# Supplementary material for: Improved RNA stability estimation indicates that transcriptional interference is frequent in diverse bacteria
Source: Commun Biol. 2023 Jul 15;6:732. doi: 10.1038/s42003-023-05097-2 (PMC10349824; doi:10.1038/s42003-023-05097-2)
Supplement: Supplementary file 2 — Supplemental Material [file 42003_2023_5097_MOESM2_ESM.pdf]

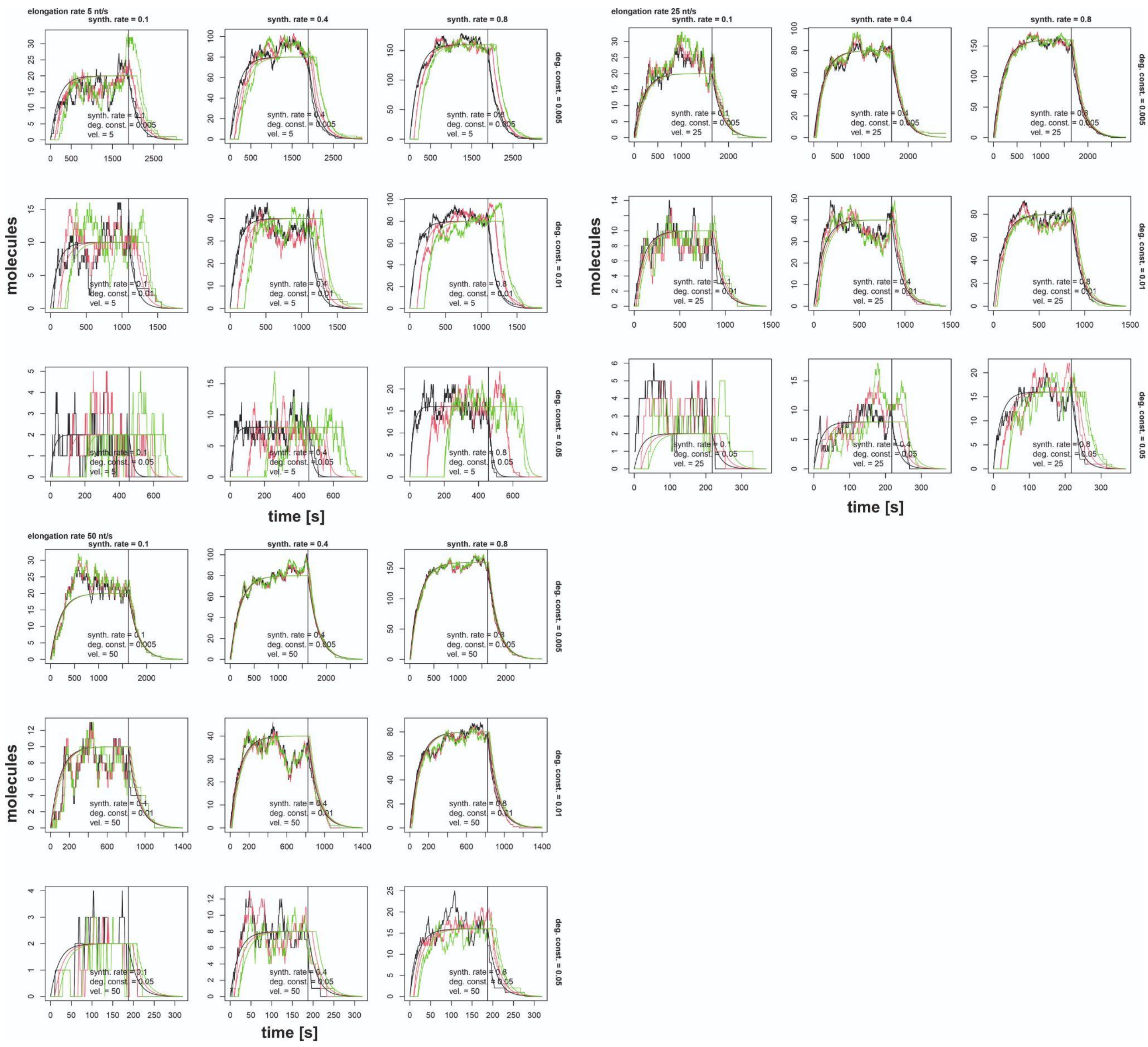

Supplementary Figure 1: Comparison of calculated and simulated (co-transcriptional decay mode) RNA counts for a wide range of parameters (27 combinations of synthesis rates [molecules/s], decay constants [1/s] and elongation rates [nt/s]) and 3 positions in a 1000nt transcript (1nt - black, 500nt - red, 1000nt - green). The time point of rifampicin addition is indicated by a vertical black line.

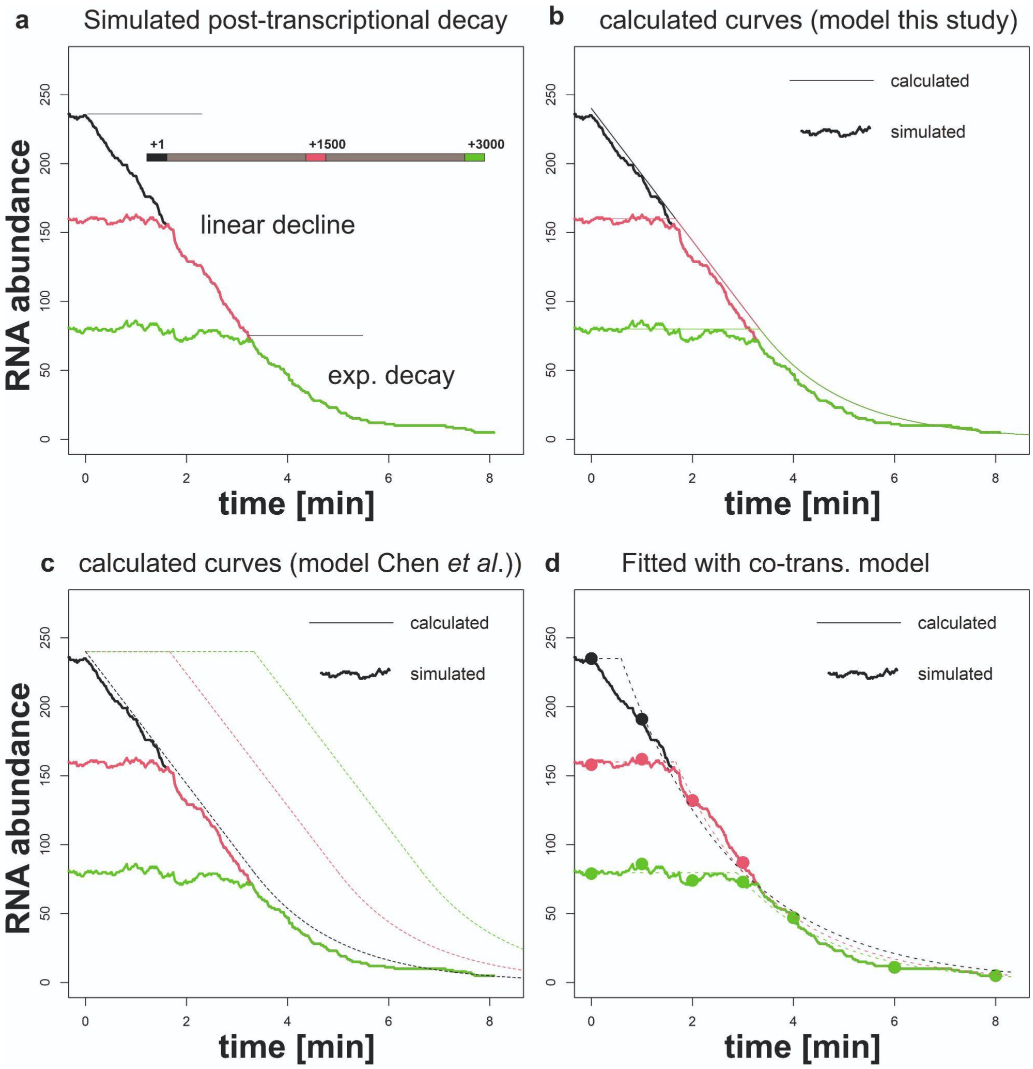

Supplementary Figure 2: **a)** Simulation of the decay curves under the assumption of **post-transcriptional decay** for three positions (1,1500, 3000 nt after TSS) in a 3000 nt transcript. The 5' end has the highest concentration and the 3' end the has the lowest concentration defined by α/λ. The curves for the linear decline and the exponential decline are the same for all positions. **b)** Simulated data and expected curves calculated with our post-transcriptional decay model (thin lines). **c)** Simulated data and the calculated curves based on the previously published model from Chen *et al.*,[7]. This model does not consider the position dependent RNA abundance differences and that the decay curves for all positions are the same. **d)** We extracted data points from the simulation for 10 time points (0, 1, 2, 3, 4, 6, 8, 10, 15, 20 min, dots) and used them for a fit with the co-transcriptional decay model (broken thin lines). Each individual curve is well fitted by the wrong model. The fitted half-life for 5' end (black) is 1.55 min and close to the half-life of 1.15 min used for the simulation.

Earlier it was proposed to distinguish the decay modes based on the decay curves[7], however we found that this is not possible, especially with the typical rifampicin time-series data. The main argument used for a global investigation of the data is that the calculated lifetime of the 5' end should be longer than the total synthesis time for the full-length transcript[7]. This is true for the "biological lifetime" of the 5' end which is the average lifetime τ fitted from the exponential decay curve plus the synthesis time of the full-length transcript. However, if the lifetime is calculated based on the model for the co-transcriptional decay (lifetime: $\tau = \frac{1}{\lambda}$, λ = decay constant) as proposed in[7], the synthesis time is not taken into account and the argument can not be used. This problem could be solved if the

post-transcriptional model is used for a fit. However, then for each bin it needs to be decided which model is most appropriate to fit the respective decay curves based on the fitting results. For the example in Supplementary Figure 2d we simulated the post-transcriptional decay. The half-life used for the simulation was 1.15 min (λ = 0.6 1/min, τ = 1.66 min). The elongation rate was 15 nt/s and the length of the transcript was 3000 nt. That means the transcription of the full-length RNA takes 3.33 min and the "biological lifetime" of the 5' end is 1.66 + 3.33 = 4.99 min. However, the calculated lifetime, based on the co-transcriptional fit of the simulated data (Supplementary Fig. 2d) is 2.24 min. In this case the synthesis time is greater than the fitted lifetime (3.33 min > 2.24 min) and the example would be miss-classified as co-transcriptional decay. On the contrary, assuming co-transcriptional decay, a transcript of 1000nt, an elongation rate of 30 nt/s and a decay constant of 0.6 1/min. The synthesis time would be 0.56 min and the lifetime would be 1.67 min. Here the lifetime is greater than the synthesis time (0.56 min < 1.67 min) and according to the above logic a post-transcriptional decay would be assumed.

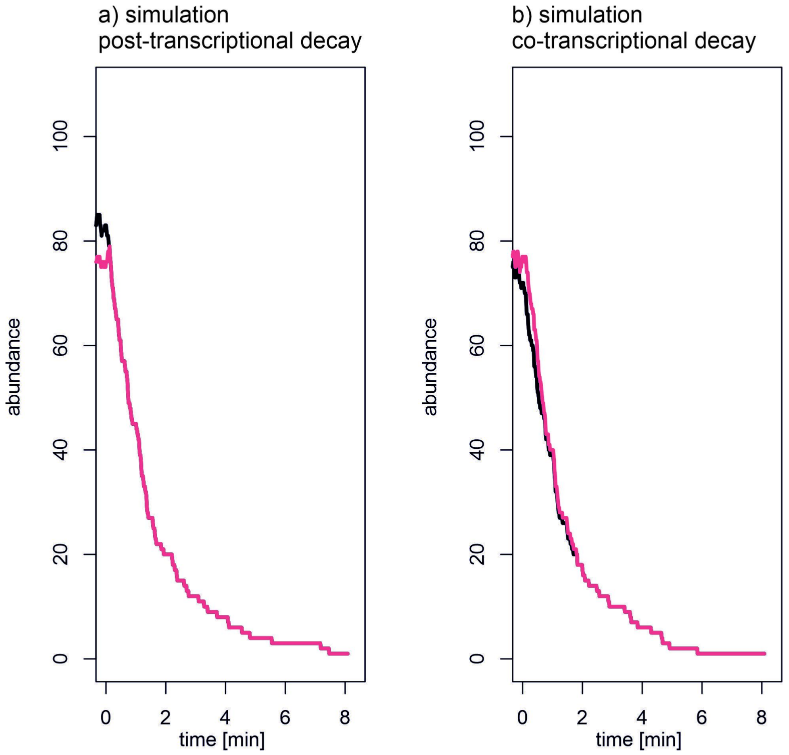

Supplementary Figure 3: Simulation of the decay curves for position 1 (black) and 500 (red) of a 500nt transcript, an elongation rate of 60 nt/s and a decay constant of 0.01 1/s. The curves for the post-transcriptional decay (a) and the co-transcriptional decay (b) are very similar and would not be distinguishable with real-life data.

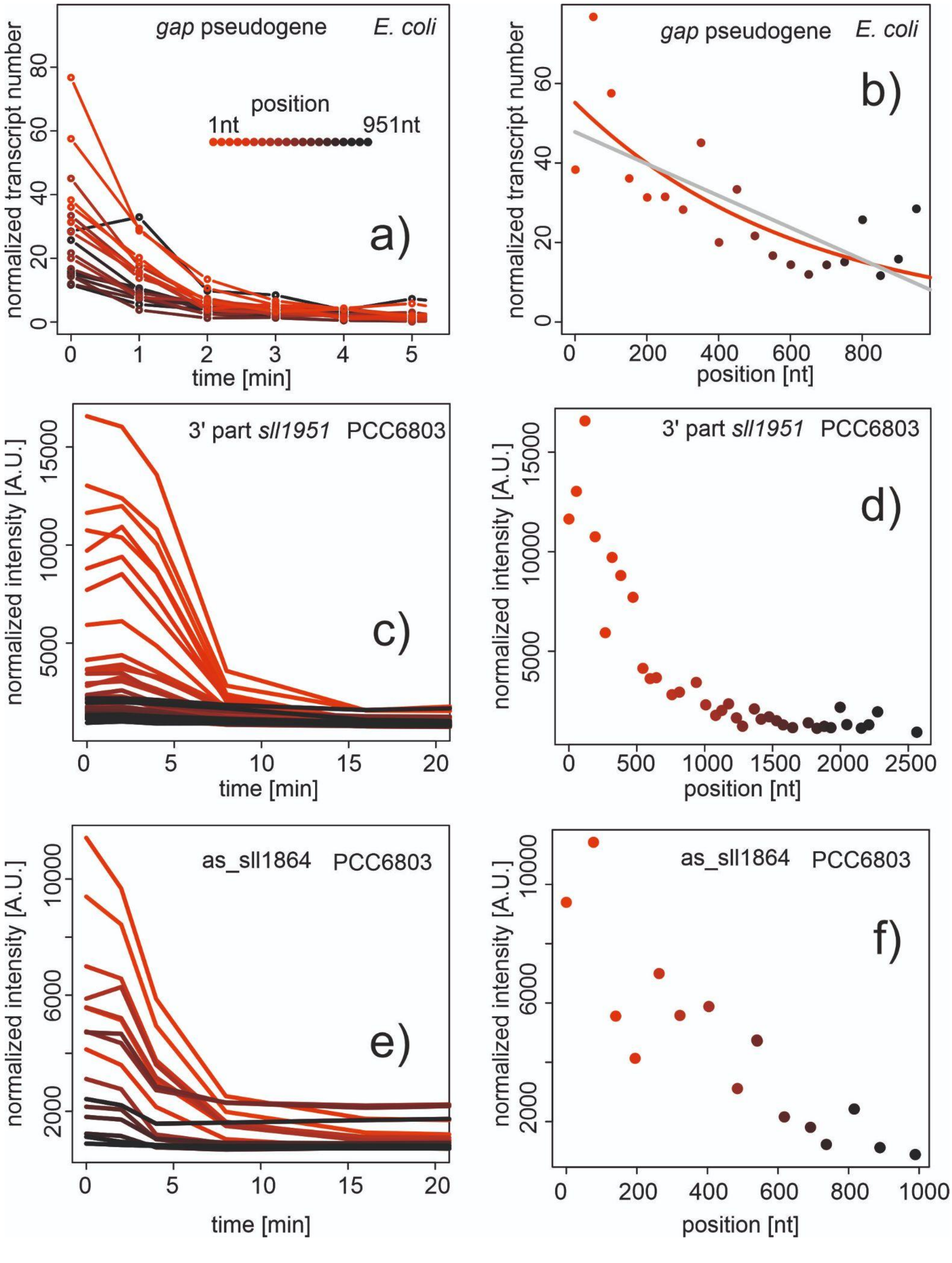

Supplementary Figure 4: (**a**) Decay curves for the bins of the *E. coli gap* pseudogene (Dar & Sorek dataset). (**b**) RNA abundances for the bins of the *gap* pseudogene before rifampicin addition (t=0). There is clearly a position-dependent abundance decline at t = 0, but this follows rather an exponential curve (red) than the linear decline (gray). Also the decay curves do not fall together as it would be expected for the post-transcriptional decay case. (**c-f**) Examples for a position-dependent abundance decline from *Synechocystis* PCC6803 that do not comply with the post-transcriptional decay mode.

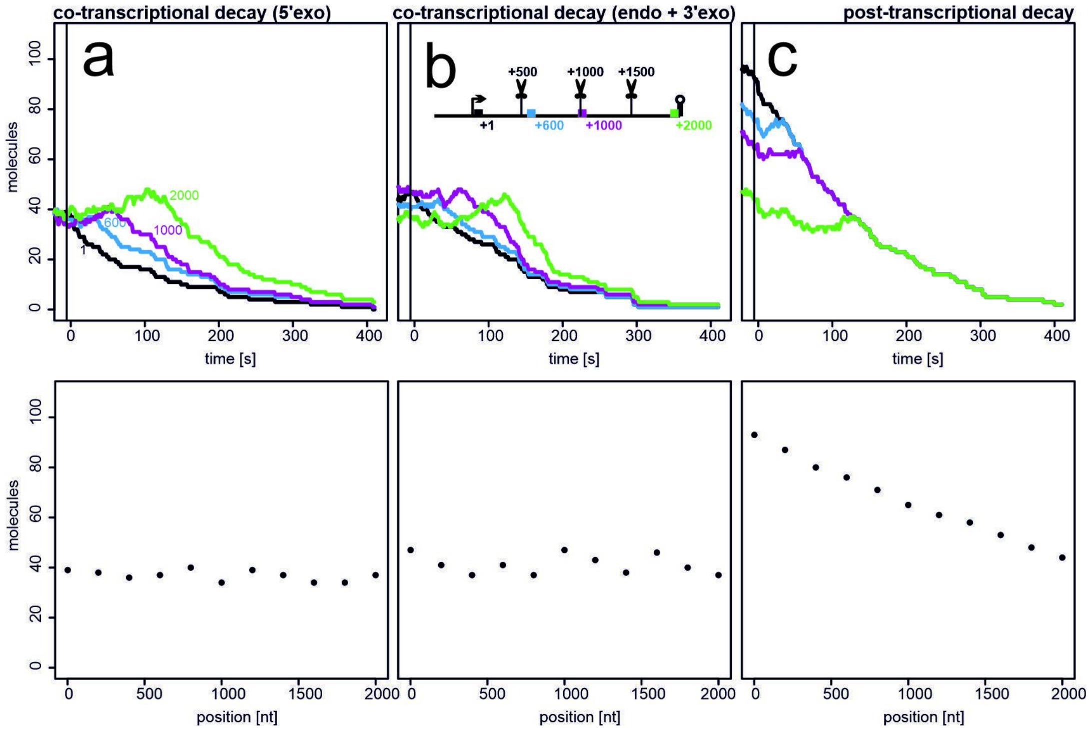

Supplementary Figure 5: Simulation of a 2000 nt transcript with a synthesis rate of 0.4 mol/s, a decay constant of 0.01 1/s and an elongation rate of 15 nt/s. Initiation of transcription was stopped at 0s which resembles addition of rifampicin. The upper series shows the decay patterns for 4 positions in the transcript after rifampicin addition. The lower series shows the RNA abundance over the whole transcript in the steady-state. **(a)** Co-transcriptional degradation of the growing transcript by 5' exonucleases and decay of the full-length transcript by 3' exonucleases. **(b)** Co-transcriptional degradation of the growing transcript only by the combined action of endonucleases and 3' exonucleases and decay of the full-length transcript by 3' exonucleases. The endonucleases can only cut at 3 defined positions (500, 1000 and 1500 nt). **(c)** only post-transcriptional decay of the full-length transcript by 3' exonucleases.

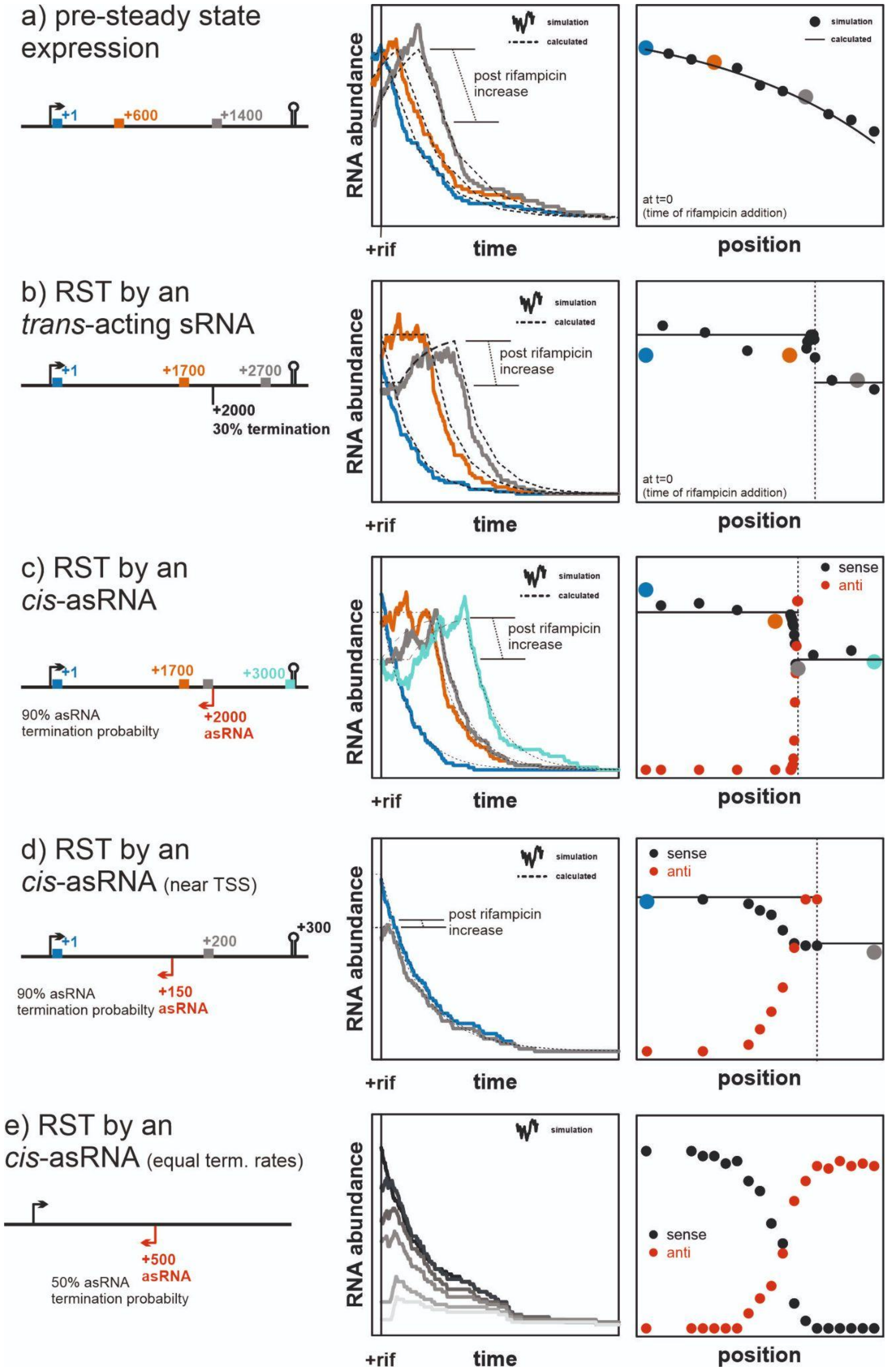

a) pre-steady state expression
+1
+600
+1400
simulation
calculated
post rifampicin increase
RNA abundance
+rif
time
at t=0
(time of rifampicin addition)
position
b) RST by an trans-acting sRNA
+1
+1700
+2700
+2000
30% termination
post rifampicin increase
c) RST by an cis-asRNA
+1
+1700
+3000
90% asRNA termination probabilty
+2000
asRNA
sense
anti
d) RST by an cis-asRNA (near TSS)
+1
+200
+300
+150
asRNA
e) RST by an cis-asRNA (equal term. rates)
+500
asRNA
50% asRNA termination probabilty

Supplementary Figure 6: For all scenarios the gene organization and the investigated probe positions are indicated in a scheme on the left side. In the middle the simulated decay curves of the indicated positions after addition of rifampicin and the calculated curves are drawn. The plot on the right side shows the simulated RNA abundances (dots) for different positions in the transcript at the time of rifampicin addition and the calculated curves (black line). **a)** Pre-steady state expression at the time of rifampicin addition. The post rifampicin abundance increase becomes stronger with a higher distance to the TSS. The positional RNA abundances at t=0 follow show a gradual decline. **b)** A fixed 30% termination probability was simulated 2000 nt after the TSS. The termination was stopped at the time of rifampicin addition to simulate that the factor responsible for the termination could not be re-synthesized any longer. In contrast to the pre-steady state case the amplitude of the increase is the same for all positions after the termination site. Depending on the distance to the termination site the increase can be delayed. The positional RNA abundances at t=0 show a sharp drop after the termination site. **c)** RST by an asRNA was simulated. If opposing RNAPs are in a range of 30 nt either the sense or the asRNA terminate. For this scenario we assumed that the sense RNA is translated and that the sense RNAP has a lower termination probability (10%) than the asRNA RNAP (90%). The asRNA has a 3 times higher synthesis rate, which leads to a final termination probability of 30% close to the asRNA TSS. The asRNA also has a 3 times higher decay constant, which results in equal maximal sense/antisense RNA counts. As in panel B, the increase is the same for all positions after the asRNA, the increase can be delayed and the positional RNA abundances at t=0 show a sharp drop after the asRNA TSS. **d)** All parameters are the same as in C), but the asRNA TSS is now much closer after the sense TSS (150 nt vs 2000 nt). If the RST appears close to the TSS, the post-rifampicin RNA abundance increase becomes very small and is not detectable in real data. Nevertheless, there is still termination and the decline is visible in the positional RNA abundances at t=0. **e)** In this example the synthesis rates and the termination probabilities for sense and antisense transcription are the same.

In the pre-steady-state case, the positional abundance-decline is gradual (Supplementary Figure 6a), while we expect a sudden, more step-like decrease in the case of RST, especially for *trans*-factors like sRNAs (Supplementary Figure 6bcd). In case of collision TI, a higher asymmetry between the termination probabilities of the interfering RNAPs leads to a sharper decline. We expect a much higher termination probability for the RNAP transcribing the un-translated *cis*-antisense RNAs (asRNAs) upon collision with the RNAP transcribing a translated mRNA[8,9]. This should result in a rapid decline of the asRNA concentration towards the 3’ end (Fig. 3, Supplementary Figure 6bcd). If both sense and asRNA are not translated, the collision termination probability should be equal and a shallower decline is expected[9] (Supplementary Figure 6e).

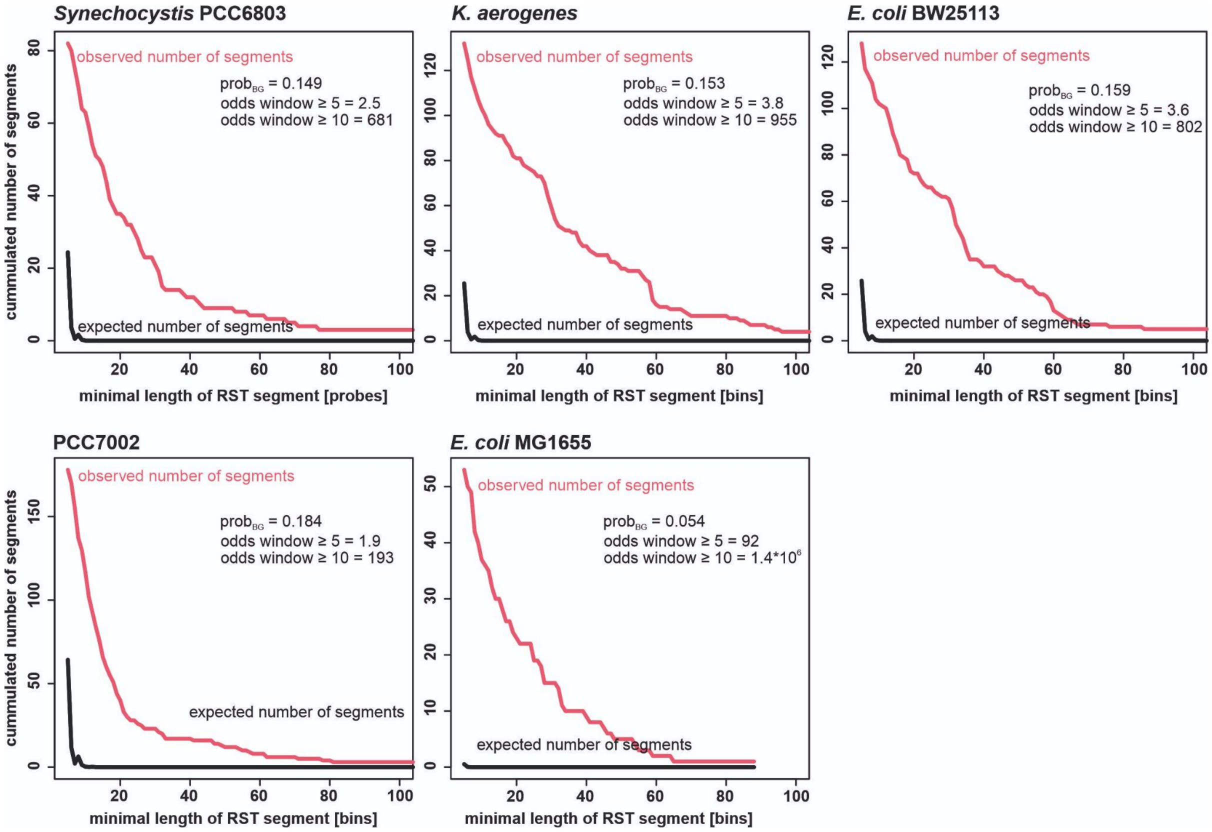

Supplementary Fig. 7: Expected (in case of random noise) and observed numbers of segments with an post-rifampicin abundancy increase. Under the rationale that post-rifampicin-increases are based on technical noise, they should be randomly distributed within all reads/probes. In contrast a systematic effect, such as TI, should affect consecutive reads/probes after an RST event. The background probability of RST bins/probes was calculated from the total number of bins/probes tagged as potential RST bins divided by the total number of bins/probes. In case of *E. coli* BW25113 7420/46803 bins are tagged as potential RST probes resulting in a background probability of $prop_{BG}$ = 0.159. Based on this background probability we calculated the probability of getting >= 0.75*n RST-bins in a sequence of n bins, i.e. the probability of getting <= 0.25*n non-RST bins, with the cumulative probability function for binomial distributions in R (pbinom) for all segment sizes from 5 to the maximal segment length of an 'rifi' detected RST segment in the respective organisms. The 0.75*n threshold was used, because by default 'rifi' requires only 75% of RST bins in a potential RST-segment prior to the actual fit. The expected numbers and probabilities of the occurrence of RST segments of length n were compared to the actual observed numbers and frequencies, showing that the observed RST segments are not explainable by random sequencing noise. Observed segments needed to fit the following requirements: 1. Segment size >= 5 bins; 2. average fitted termination rate ≥ 0.15; 3. number of individual bins with a fitted termination rate of ≥ 0.15 needed to be ≥ 75% of the total number of bins (always rounded up to the next integer to be more stringent).

Exclusion of technical artifacts for the post-rifampicin abundance increase:

Sequencing noise: To globally detect instances of potential RST we scanned the data for bins/probes that showed an RNA abundance increase of at least 7.5% after rifampicin addition. For individual probes such an increase might be due to technical noise. Especially for weakly expressed genes the post-rifampicin increase could be

due to variations in small read counts. In case of noise, the bins should be randomly distributed if they belong to independent reads. In contrast, a systematic reason, such as RST, would result in consecutive bins with an abundance increase. For that reason, we used a segmentation strategy that finds consecutive stretches of positions with a post-rifampicin abundance increase. Within these segments at least 75% of the included bins needed to show the abundance increase to be considered for RST. Using the binomial distribution function, we calculated that consecutive stretches from ≥ 5 bins to ≥ 100 bins/probes with ≥ 75% increasing elements are significantly more frequent than expected in all investigated datasets. For the *E. coli* BW25115 dataset, segments increasing over ≥ 5 bins are 3.6 times and segments ≥ 10 bins are 681 times more frequent than expected. Thus, the potential RST-segments cannot be explained by random sequencing noise (Fig. 5a, Supplementary Figure 7).

Normalization artifact: Normalization is particularly difficult for rifampicin time-series data because the key assumptions of most normalization strategies, such as that the majority of genes do not change expression and that genes expressed at higher and lower levels are symmetrically distributed, clearly do not hold. The cellular concentrations of most RNAs decrease after rifampicin addition, but for some RNA (positions) the concentration remains constant for the period of their delay. The relative proportions of these and of very stable transcripts in the total RNA pool will increase in samples of time points . If not optimally normalized, this can be misinterpreted as an abundance increase relative to . To prevent this artifact, the *E.coli*, *Klebsiella* and *Synechococcus* PCC 7002 data were normalized with external spike-in RNAs[6,10,11] and the *Synechocystis* PCC 6803 microarray data were normalized using a cyclic loess normalization strategy based on technical control

spots and the least changed genes. We also looked into the data to find indications of global normalization artifacts. Due to the typical delayed exponential decay in rifampicin experiments, a large number of bins are expected to have approximately constant abundance levels at time points t > 0 min. An abundance increase artificial caused by the normalization would affect all of these bins with constant concentrations relative to at the given later time point. However, in all datasets the bins/probes with a delay > 2 min consist of both, increase and non-increase cases (Supplementary Figure 8), which excludes a systematic normalization artifact.

Other technical artifacts: Nevertheless, for a few individual cases of post-rifampicin increases a technical artifact seems likely. For example, ribosomal RNAs in the *E. coli* BW25113 dataset showed an unusual ~20-fold increase in abundance after 2 min in one of the replicates. This might be related to a potentially uneven depletion of ribosomal RNAs during the library preparation. A similar effect is not seen for ribosomal RNAs in the *E. coli* MG1655 data.

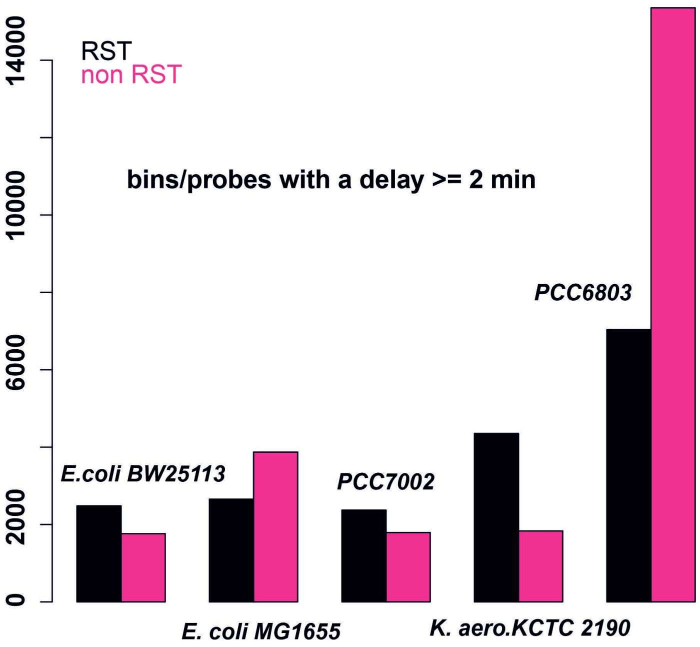

Supplementary Fig. 8: Number of initially flagged increase (RST) and non increase (non RST) bins/probes with a delay >= 2 min.

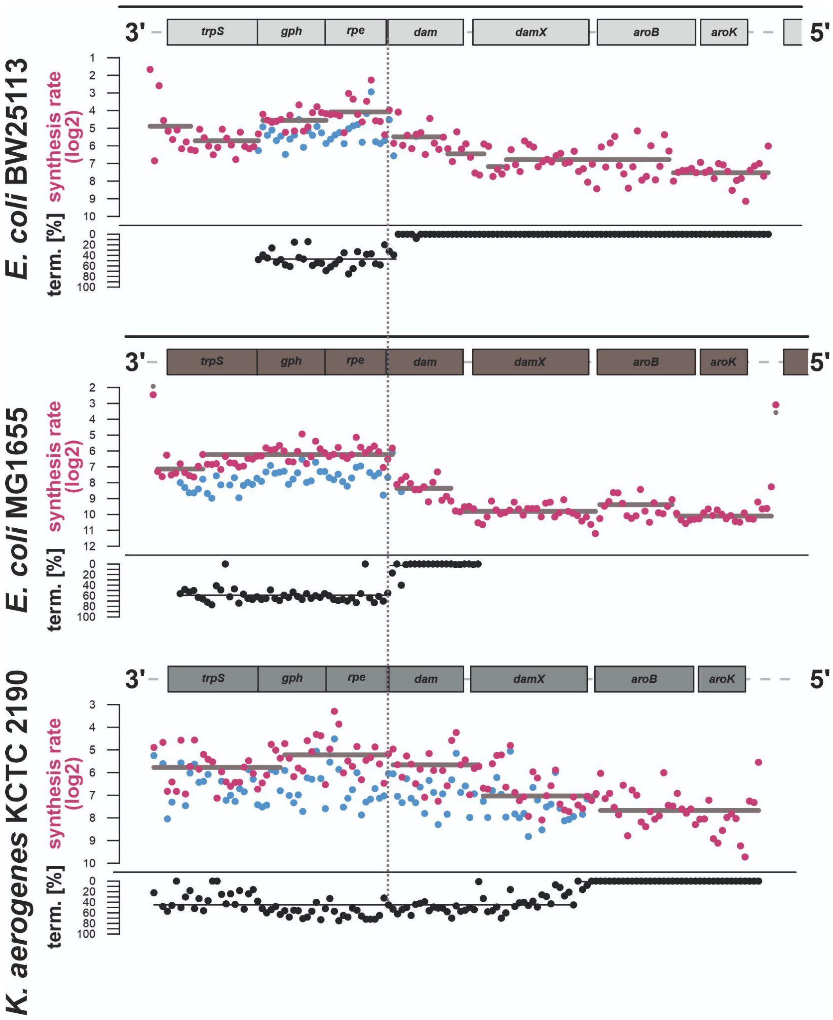

Supplementary Fig. 9: Conserved RST event between *damX/dam/rpe* in three different organisms. In all organisms there is a synthesis rate drop between *dam/rpe*. In both *E. coli* strains this is correlated with the onset of RST. In *Klebsiella* the RST starts earlier.

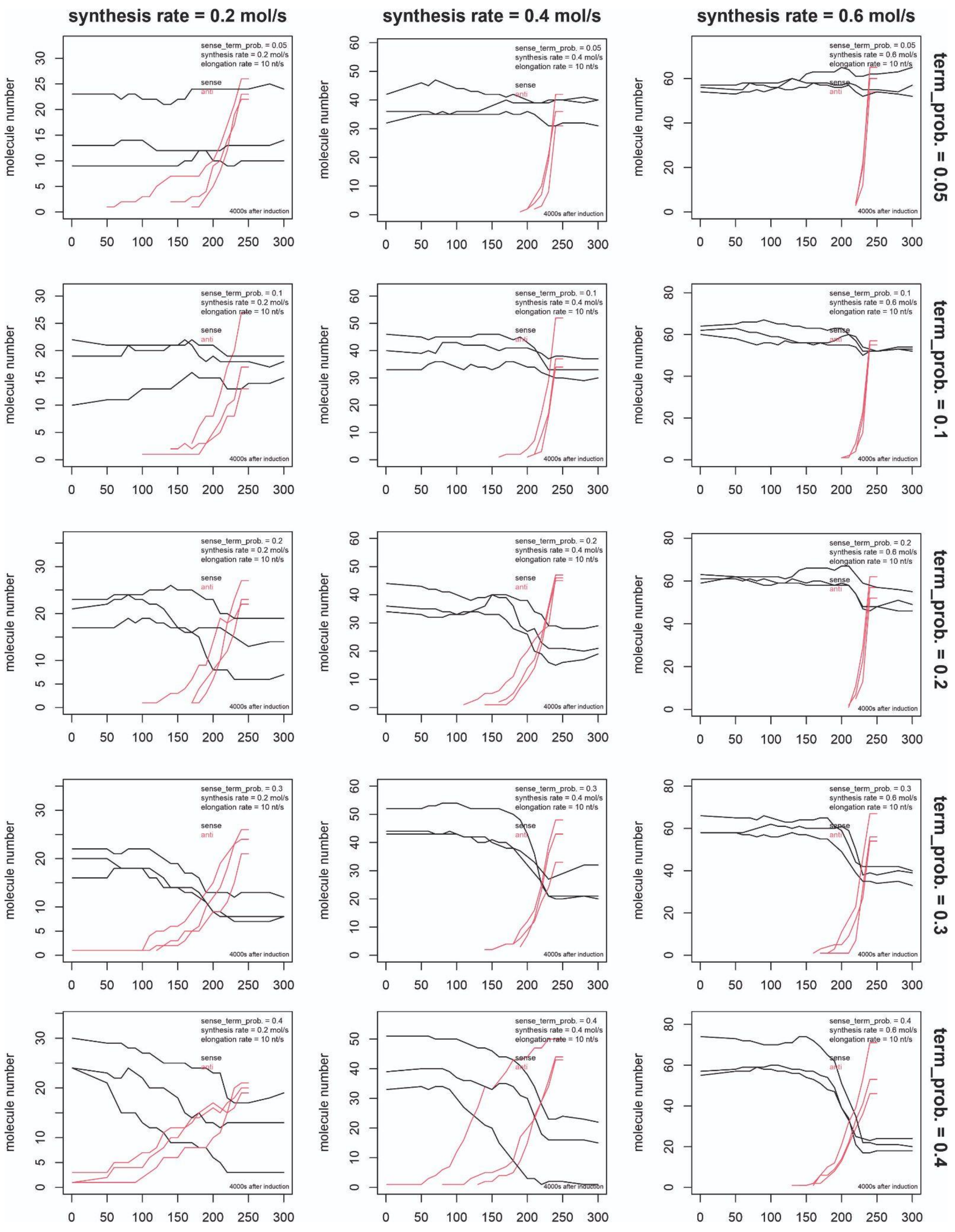

Supplementary Fig. 10: Simulation (n=3) for sense mRNA and asRNA lengths for different sense RNA termination probabilities (asRNA term. prob. = 1- sense RNA term. prob.) and synthesis rates at a fixed degradation constant of 0.01 1/s and a fixed elongation rate of 10 nt/s. The asRNA TSS is at position 250 relative to the sense RNA TSS. The length resolution is 10 nt.

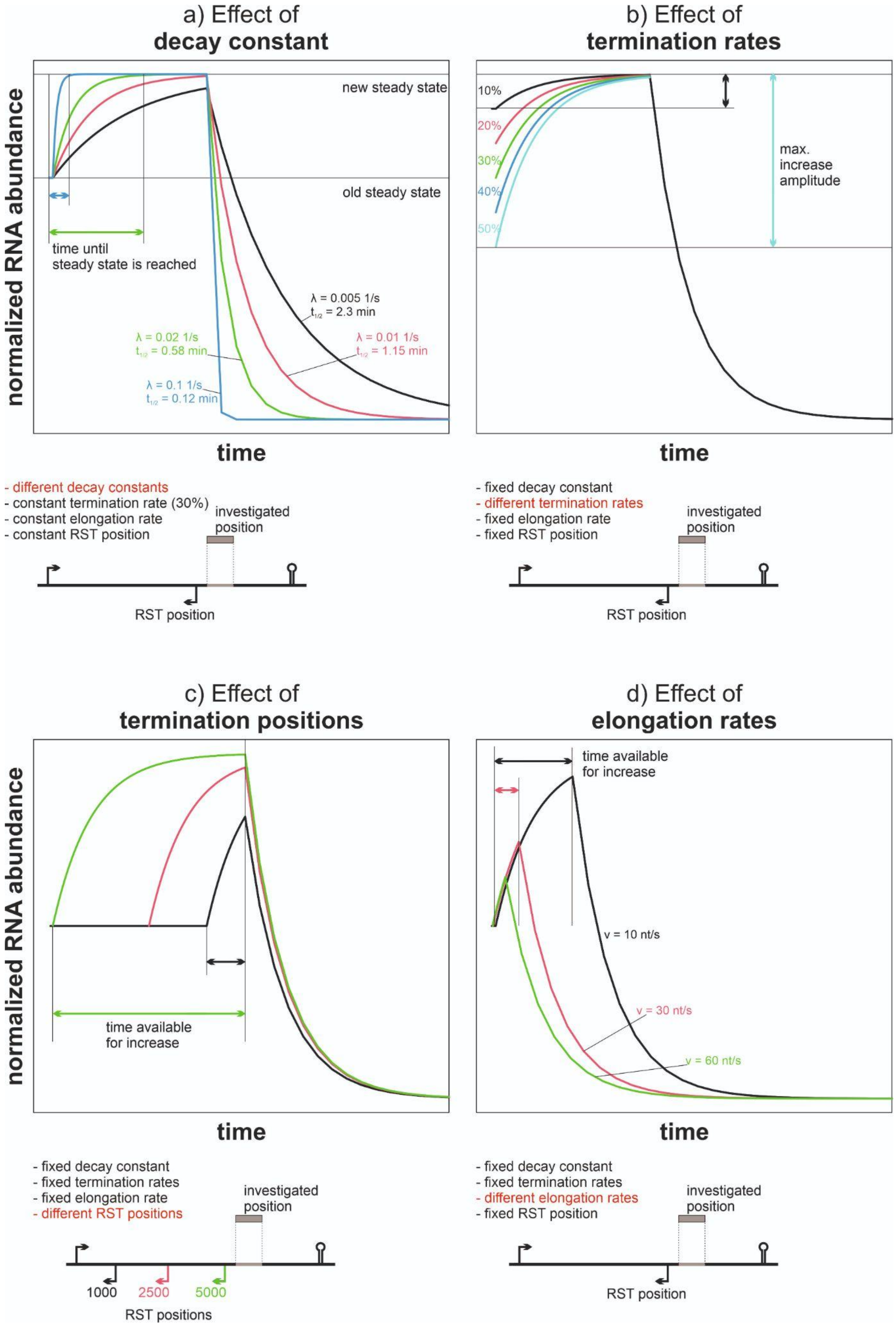

Supplementary Fig. 11: Effect of decay constant, termination rates, termination positions and elongation rates on the shape of the post-rifampicin RNA abundance increase. **a)** The steady state concentration is faster reached with a higher decay constant. **b)** A higher RST termination rate leads to a higher maximal possible intensity increase after rifampicin addition. **c)** A high distance of the RST termination site from the transcriptional start site increases the available time window to reach the new steady state. **d)** A lower elongation rate increases the available time window to reach the new steady state.

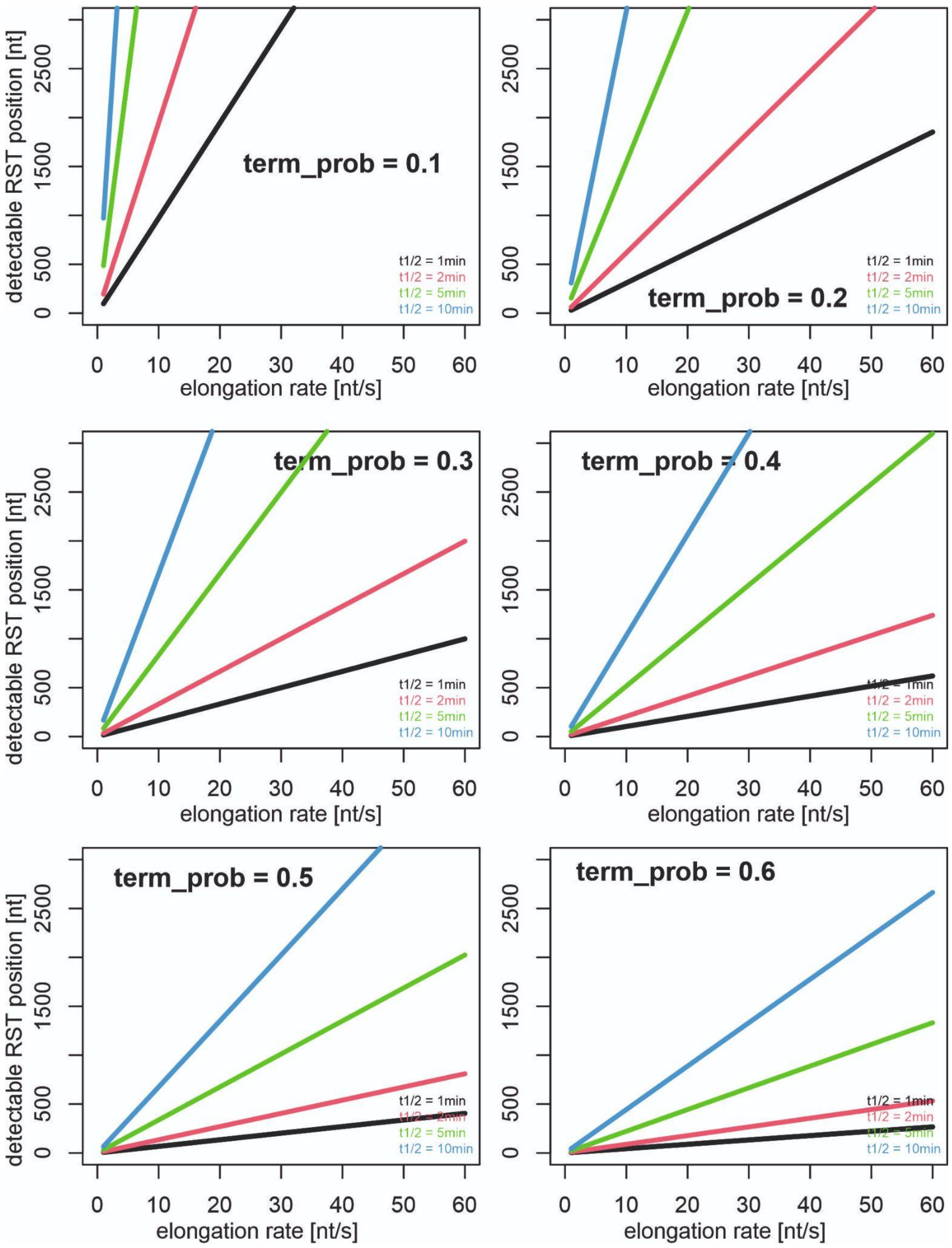

Supplementary Fig. 12: Detectability of RST instances. Minimal termination site distance from the transcriptional start site to reach an RNA abundance increase of ≥ 7.5% dependent on termination rate, elongation rate and RNA stability.

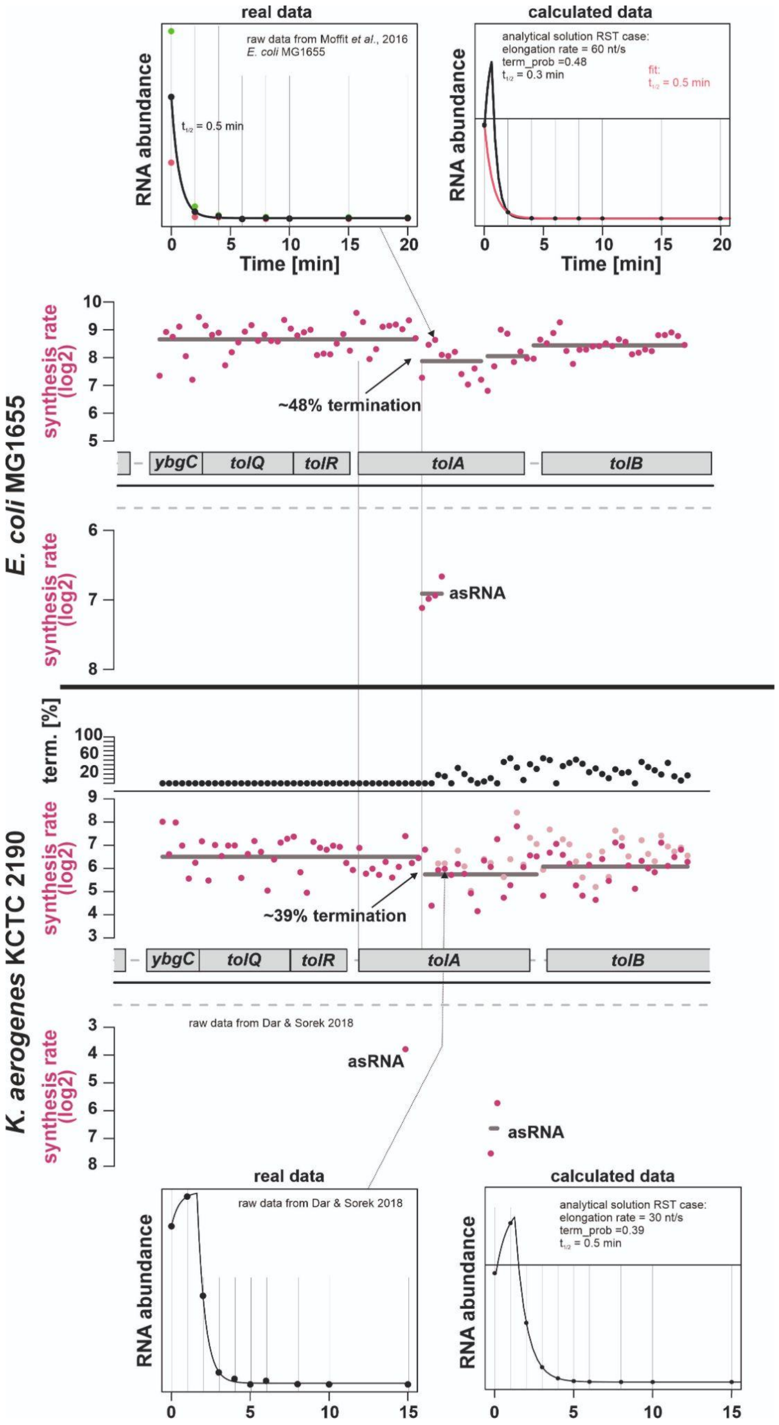
real data
raw data from Moffit et al., 2016
E. coli MG1655
RNA abundance
t1/2 = 0.5 min
Time [min]
calculated data
analytical solution RST case:
elongation rate = 60 nt/s
term_prob =0.48
t1/2 = 0.3 min
fit:
t1/2 = 0.5 min
E. coli MG1655
synthesis rate (log2)
~48% termination
ybgC
tolQ
tolR
tolA
tolB
asRNA
K. aerogenes KCTC 2190
term. [%]
~39% termination
raw data from Dar & Sorek 2018
asRNA
real data
raw data from Dar & Sorek 2018
calculated data
analytical solution RST case:
elongation rate = 30 nt/s
term_prob =0.39
t1/2 = 0.5 min

Supplementary Fig. 13: Example for a probable RST instance in *E.coli* MG1655, that is not detectable due to a relatively low time resolution. Upper part: *E. coli tol*-operon with an asRNA in the middle of *tolA*. Exactly at the position of the asRNA we detected 48% drop in the synthesis rate. However, we did not detect a post rifampicin RNA abundance increase as expected for RST. The time course for an example bin is given at the top left (the sampled timepoints 0, 2, 4, 6, 8,10, 15, 20 min are indicated by lines and dots). We calculated the expected time course for the RST case with the fitted information (top right). At the fitted high elongation rate and low half-life the post rifampicin RNA abundance increase would not be visible at the sampled time points. Note that the fitted half-life would be slightly too high (0.5 vs 0.3 min) if there would be a non detected RST in reality. Below, the same operon is displayed for *K. aerogenes*. We see an asRNA and a 39% termination at the same position in *tolA*. Here, a post rifampicin RNA abundance increase is detectable, because the 1 min time point is available.

An asRNA to the *tolA* gene perfectly correlates with a partial termination site (~48% termination), nevertheless, no RST typic increase is visible in the data (Fig 5e, Supplementary Figure 13). We calculated the expected decay curve for a potential RST of 48%. The combination of the high measured elongation rate (≥ 60 nt/s) and the low RNA stability ($t_{1/2}$ < 0.5 min) would theoretically cause a very rapid increase to ~169% of the initial steady state within 0.58 min and a decline to 8% of the initial abundance after 2 min (Fig. 5e). Thus, a hypothetical RST would not be covered by the first sampled 2 min time point and the data would be misinterpreted. Interestingly, the same asRNA and termination site is visible in the *Klebsiella* data[6], but here also the 1 min time point is sampled. Together with a lower elongation rate (~30 nt/s) the RST typic increase is detectable, which indicates that the termination in both organisms might be, at least partly, due to TI by asRNA transcription (Supplementary Figure 13).

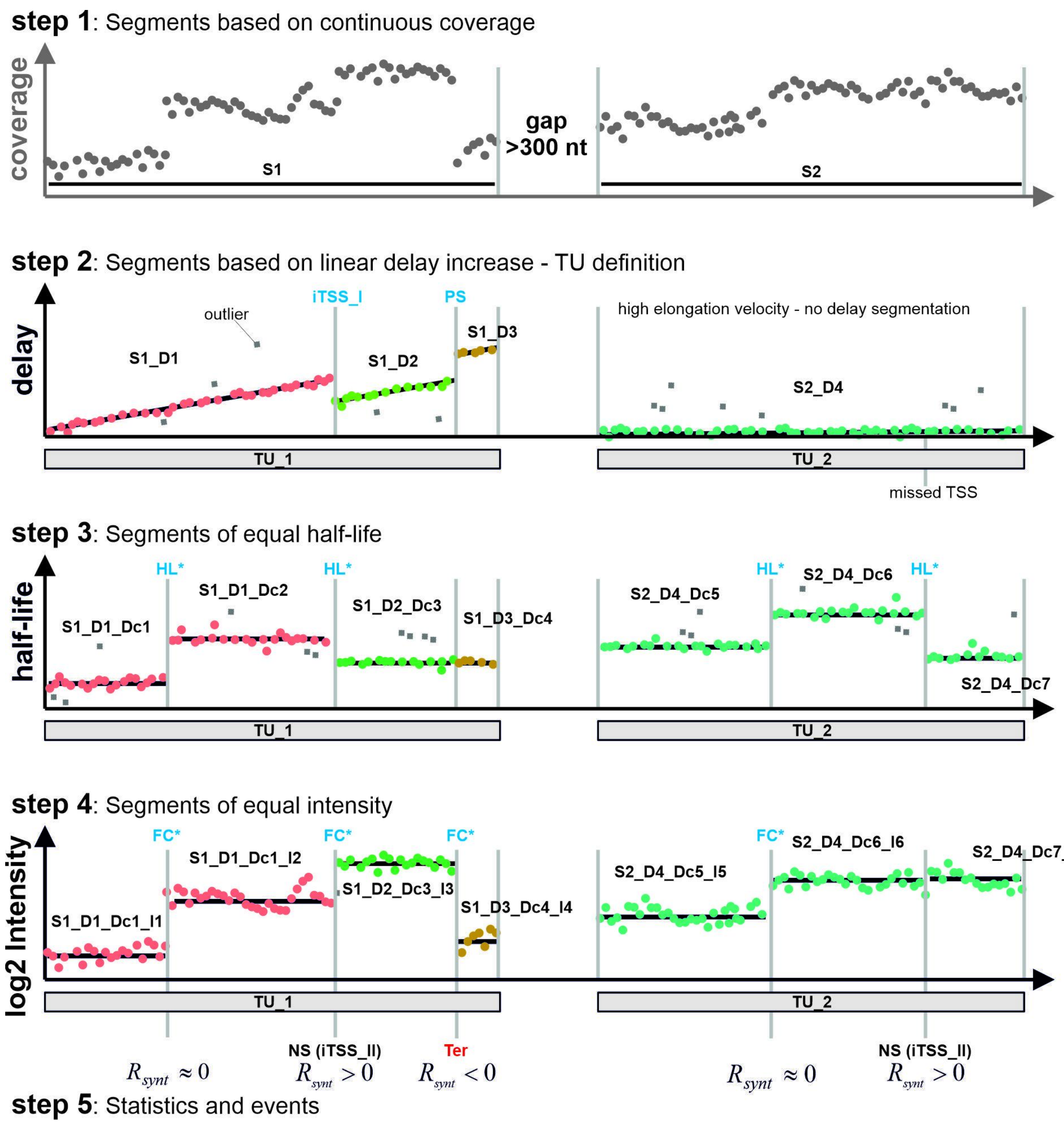

Supplementary Fig. 14: 'rifi' segmentation workflow (idealized example). **Step 1**: All regions with continuous coverage, i.e. regions with no coverage gap greater than a given threshold (default: >300nt), are taken as initial segments. This step dramatically reduces the runtime for the subsequent dynamic programming steps. Each segment is numbered progressively (S1 - Sn; n = number of segments). **Step 2**: The delay is dependent on the distance of a given position in the transcript from the transcriptional start site. Given a constant elongation rate and no internal transcriptional start site and no pausing site, the delay should increase linearly from the transcriptional start site to the end of the transcript. In this step the best set of linear fits for all bins/probes within the same coverage segment is searched by dynamic programming with the respective penalties for splits and outliers (see methods for more details). A split either represents a new TSS, a pausing site (PS) or a change of transcription velocity. The delay segments are again named progressively and inherit the respective Segment name as prefix (e.g. S1_D1, for the first delay segment or S2_D4 for the fourth delay segment). Outliers that are excluded from the segment are indicated as gray dots. Within a transcriptional unit, i.e. a transcript resulting from the continuous elongation of RNA polymerase from a TSS to the final terminator, the delay should also increase continuously. We use the delay segments for a TU estimate. A new TU is triggered if the delay of a segment starts close to 0 or if the drop in the delay between two consecutive segments is high enough. A minor delay-decrease is taken as internal start sites (iTSS_I). Note that the elongation velocity within coverage segment S2 is high, which leads to delays of ~0 for all bins of the segment. Here the segmentation based on the delay fails and a start was missed (missed TSS).

Step 3: Each delay segment is splitted into the best set of half-life segments. The segments are scored by the deviation of the individual bin/probe-wise half-lifes to the mean half-life of the segment. The segments are named progressively and inherit the names of the respective delay segment as prefixes (e.g. S1_D1_Dc3, third half-life (decay - Dc) segment). **Step 4**: Each half-life segment is splitted into the best set of RNA abundance segments based on the log2 abundance of each bin/probe. Again the splits are optimized to get the lowest deviation of the individual bin/probe-wise intensities to the mean abundance of the segment. The segments are named progressively and inherit the names of the respective delay segment as prefixes (e.g. S2_D4_Dc5_I5). **Step 5**: ‘rifi’ investigates if the consecutive segments before and after a split are significantly different from each other. Only splits within the same estimated TU are analyzed. The p-values for all splits are available in the final output. Delay: Consecutive delay segments are analyzed via ANCOVA for differences in the slope (velocity change) or via a two-sided student's t-test for pausing sites (PS) or internal start sites (iTSS_I). Half-life: Consecutive segments are tested via a two-sided student's t-test. Significant splits ($p \leq 0.05$) are indicated by HL* and indicate transcript fragments with different half-lives within the same transcriptional unit. RNA abundance: Consecutive segments are tested via a two-sided student's t-test. Significant splits ($p \leq 0.05$) are indicated by FC*. Synthesis rate based events: RNA abundance changes between two transcript segments in the same TU can be due to stability differences, differences in the synthesis rate (partial termination or internal start site) or a combination of both. We calculate the log2 foldchange of the synthesis rates (R_{synt}) between consecutive segments (see methods). $R_{synt} \approx 0$ indicates no change in the synthesis rate, i.e. the abundance change is only due to different transcript stabilities. $R_{synt} > 0$ indicates a new start (NS, iTSS_II). In the example the missed start in S2 is detected by the R_{synt} measure. $R_{synt} < 0$ indicates a partial termination (Ter). The significance of the R_{synt} value is tested via MANOVA. For further details see methods.

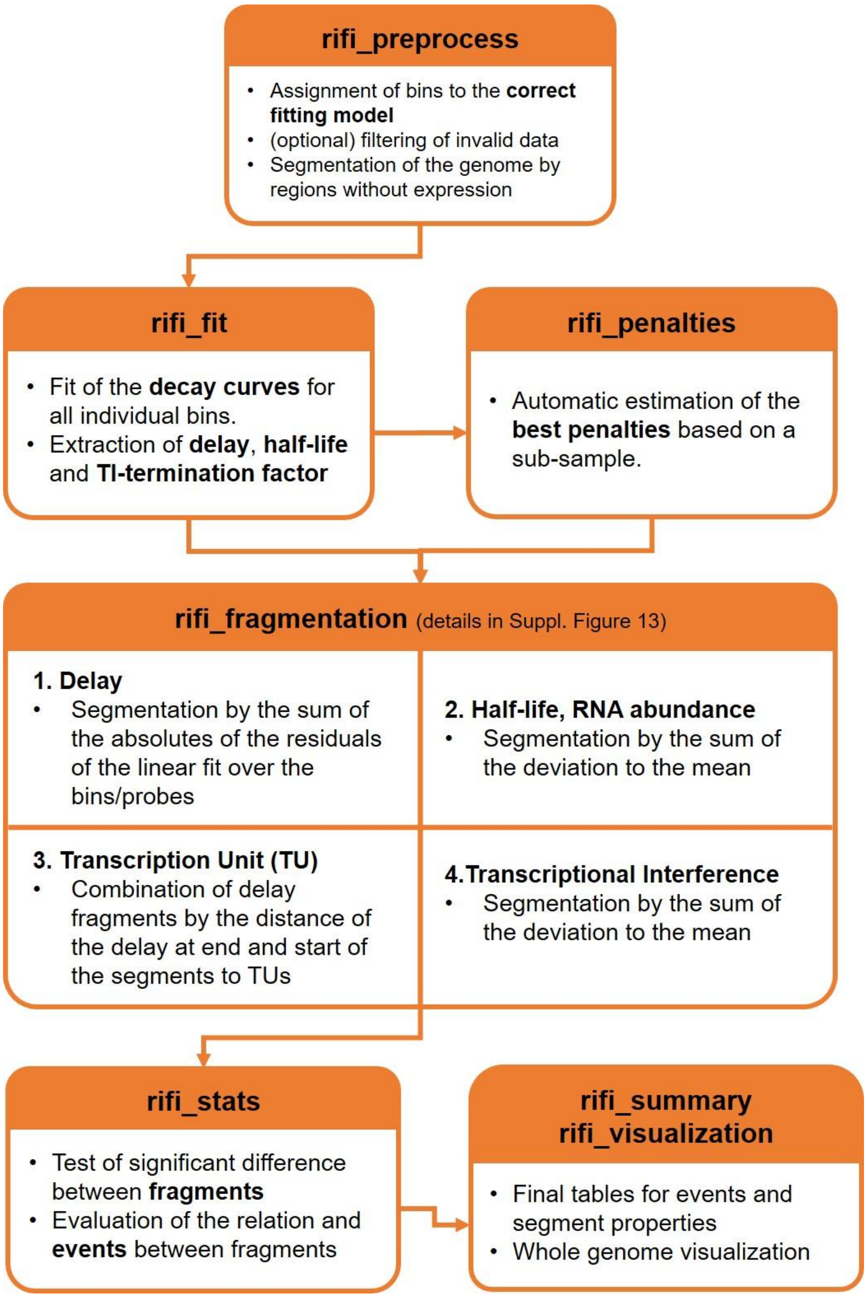

Supplementary Fig. 15: Overview workflow of the ‘rifi’ tool.

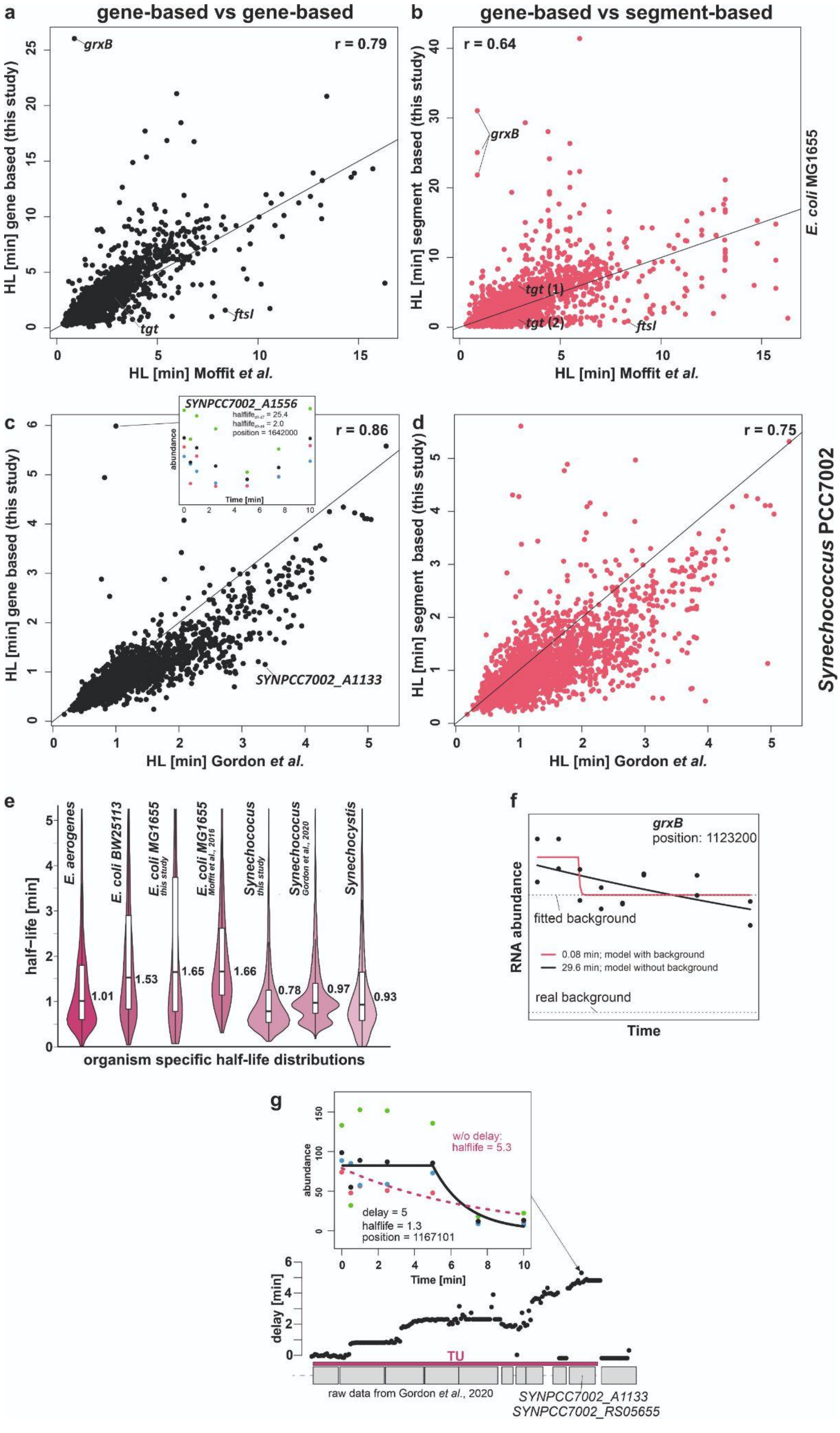
a
gene-based vs gene-based
r = 0.79
grxB
tgt
ftsI
HL [min] gene based (this study)
HL [min] Moffit et al.
b
gene-based vs segment-based
r = 0.64
grxB
tgt (1)
tgt (2)
ftsI
HL [min] segment based (this study)
HL [min] Moffit et al.
E. coli MG1655
c
SYNPCC7002_A1556
position = 1642000
abundance
Time [min]
r = 0.86
SYNPCC7002_A1133
HL [min] gene based (this study)
HL [min] Gordon et al.
d
r = 0.75
HL [min] segment based (this study)
HL [min] Gordon et al.
Synechococcus PCC7002
e
E. aerogenes
E. coli BW25113
E. coli MG1655 this study
E. coli MG1655 Moffit et al., 2016
Synechococcus this study
Synechococcus Gordon et al., 2020
Synechocystis
half-life [min]
1.01
1.53
1.65
1.66
0.78
0.97
0.93
organism specific half-life distributions
f
grxB
position: 1123200
RNA abundance
fitted background
0.08 min; model with background
29.6 min; model without background
real background
Time
g
w/o delay:
halflife = 5.3
abundance
delay = 5
halflife = 1.3
position = 1167101
Time [min]
delay [min]
TU
raw data from Gordon et al., 2020
SYNPCC7002_A1133
SYNPCC7002_RS05655

Supplementary Figure 16: Comparison of stability estimations with examples for deviations. **a)** Scatterplot showing the original half-lifes from Moffit *et al.*[10] and our results for *E.coli* MG1655 on the same data. For this plot we averaged the bin based results for each gene. **b)** Here we compared the original gene based results[10] with our segment based data. **c)** Scatterplot comparing the original *Synechococcus* PCC 7002[11] data with our gene-based results and **d)** with the fragment-based results. C) Inlay: Example for difficult input data. After the rifampicin addition the RNA abundance initially declines, but after the 4th data point the abundance increases again which leads to a U-shaped pattern. Fitting all 7 data points results in a half-life of 25.4 min while a fit based on the first 4 data points results in a half-life of 2.0 min. In the original paper[11] later times were excluded if they increased the residuals of the fit. 'rifi' allows the use of custom filter functions to deal with that data. **e)** Violin plots showing the continuous distributions of HLs for all investigated datasets based on the half-life fragments. The median half-lifes are indicated. The lower and upper hinges correspond to the first and third quartiles. For *E. coli* MG1655 and *Synechococcus* PCC 7002 also the published gene-based data are shown. The y-axis is cut at 5 min. **f)** The effect for a fit with a model with and without the consideration of a general background for a bin within the *grxB* gene. **g)** Example for a long TU in *Synechococcus* PCC 7002. Not considering the ~5 min delay for a bin in the *SYNPCC7002_A1133* transcript will lead to an artificial higher half-life of 5.3 min (broken pink line: no-delay fit; black line: delay adjusted fit).

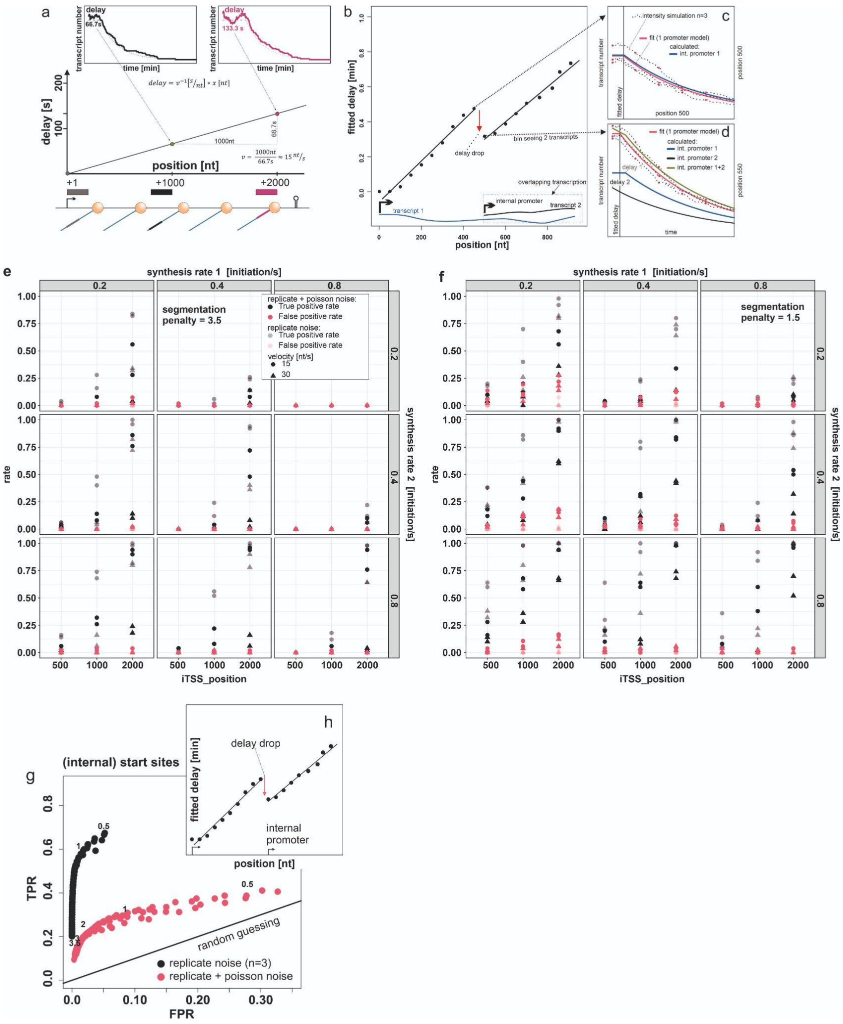

Supplementary Figure 17: Delay based TSS detection. **a)** Idealized example for a 2000 nt transcript with a constant transcription velocity over the whole transcript. The delay is linearly dependent on the distance from the transcriptional start site and the slope is the inverse of the velocity. The inlays show the decay curves of simulated data at position 1000 nt and 2000 nt with the fitted delay. **b)** Fitted delays based on stochastic simulation of an internal promoter. **c+d**) show the simulation data (dotted black lines, n=3) and data points taken for the fit (pink dots), the delayed exponential decay based on the fit with the one promoter model (pink line) and the analytical curves for the transcript starting at promoter 1 (blue), promoter 2 (black) and combined for both transcripts (green). **c**) position 500 directly before the second promoter and **d**) position 550, 50 nucleotides upstream the second promoter. Velocity and decay constants for both transcripts were 15 nt/s and 0.3 1/s. The synthesis rate at promoter one and two were 0.4 molecules/s and 0.3 molecules/s, respectively. **e+f)** In order to estimate the accuracy and sensitivity of the iTSS detection we used simulated data with different initial and secondary synthesis rates, decay constants and synthesis velocities. For each of the 108 parameter combinations (half-life: 1, 2; velocity: 15 or 30 nt/s; synthesis rate 1 +2: 0.2, 0.4 or 0.8 molecules/s; iTSS position: 500, 1000 or 2000nt) we run 50 independent simulations. The true positive rate (black symbols with and gray symbols without technical noise) is calculated based on the correctly identified pausing sites. **g)** TSS and iTSS detection: A new TSS is indicated by a drop in the delay. We used simulated data with different initial and secondary synthesis rates, decay constants and synthesis velocities. For each of the 108 parameter combinations (half-life: 1, 2; velocity: 15 or 30 nt/s; synthesis rate 1+2: 0.2, 0.4 or 0.8 molecules/s; iTSS position: 500, 1000 or 2000nt) we run 50 independent simulations. The sensitivity and specificity is dependent on the parameters for the dynamic programming. The ROC-like curve shows the performance of the approach for segmentation penalties from 4 to 0.5 and variable outlier penalties. For each penalty the average true positive rate (TPR) and false positive rate (FPR) from the simulation based on the 108 parameters was calculated. **h**) shows an example simulation.

The complicated case of internal start sites and overlapping transcription

Many attempts to map the whole transcriptome of bacterial organisms have revealed that transcription does not exclusively start at the 5' end of operons or single gene transcripts. Actually, there are plenty of internal transcriptional start sites within operons and genes[12–14]. This leads to overlapping transcription processes and transcripts initiated from different starting sites, which violates the simple delayed-co-transcriptional decay model. Fitting those cases with the simple model can lead to an "artificial" delay (Supplementary Fig 17bcd), which lies between the delay of the two independent transcription processes. In theory, the model could be adapted to two or more overlapping transcripts (see Methods). However, as displayed in Supplementary Fig 17d the fit with the wrong simple model and the solution for the two-promoter model are very close. Together with the relatively coarse sampling points, the subtle differences in the delay will not result in major differences in the residuals. Furthermore, the two-promoter model introduces up to three additional free parameters (a second synthesis rate, delay, and decay constant each). In reality a decision between models for unknown data would require accounting for these additional parameters using e.g. the Bayesian information criterion[15]. Together with the inherent noise in the data it appears unlikely that the individual parameters of two or even more overlapping transcripts are detectable. In reality, we only use the one-promoter model and a combination of the real individual transcript parameters is fitted. The simple model can not estimate the strength (synthesis rate) of an internal start site, but the drop in the delay between pre and post internal starting site positions is in principle detectable. The performance to detect internal transcriptional start sites (iTSS) was estimated based on simulated data with 108 parameter combinations. The subsequent delay fragment detection

was done with and without the addition of poisson noise to account for technical sequencing noise. The sensitivity and specificity is dependent on the stringency of the dynamical programming segmentation and hence the segmentation penalty. The ROC-like curves in (Supplementary Fig. 17g) shows that the method works in general. Every aspect that increases the duration of the delay drop, i.e. a low transcription velocity, a higher distance from the previous start and a relatively higher second synthesis rate, improves the detection of a new start (Supplementary Fig. 17ef). We tried to distinguish between weaker internal starts that do not start a new transcriptional unit (TU) and stronger starts that do trigger a new TU via our segmentation strategy. The 'rifi' internal start site detection (iTSS_I) based on the delay has only an overlap of 4 sites with the dRNA-seq starts[16], which is not better than random (p = 0.53). In contrast, the stronger delay drops used for the delay based TU-start have a significant overlap with the dRNA-seq data ($p = 1.8*10^{-9}$) (Supplementary Fig. 19b).

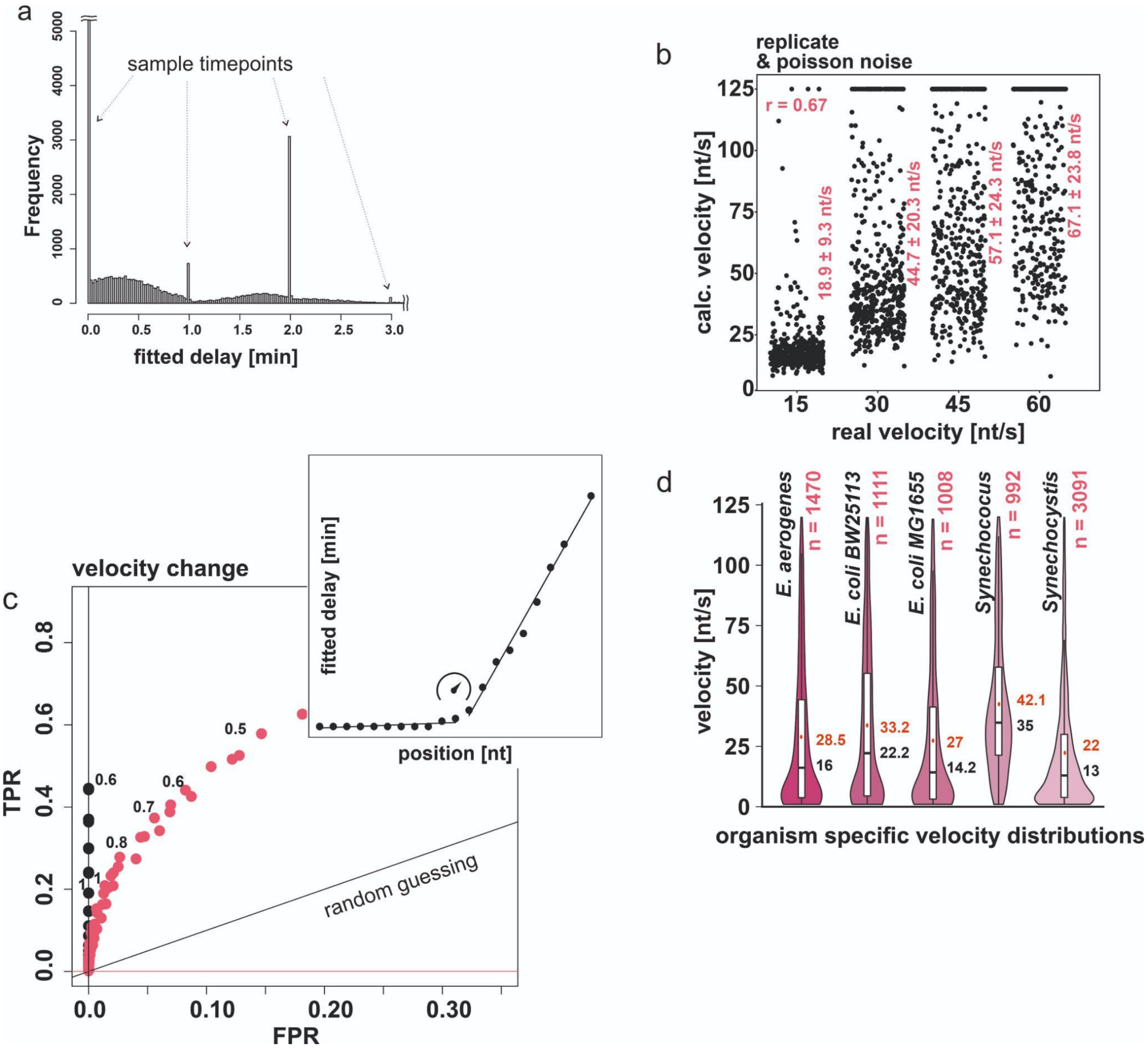

Supplementary Figure 18: **Delay based elongation rate calculation. a)** Histogram of the fitted delays for the *E. coli* BW25113[6] dataset. Sampling timepoints are over-represented. The x -and y-axes are cut and not all data points are represented. **b)** A simulation was done to assess the general accuracy of the elongation rate estimate. Data are based on the simulations with 36 parameter combinations (half-life: 1, 2 or 3.33 min; velocity: 15, 30, 45 or 60 nt/s; synthesis rate: 0.2, 0.4 or 0.8 molecules/s) and a continuous transcription without a parameter change. The jitter plots show the real input velocity used for the simulation versus the calculated velocity based on the fits. The Pearson correlation r-value and the mean fitted velocities with their standard deviation are given in the plots. At the top the number of fragments with a velocity higher than 120 nt/s and the total number of fitted segments are given. The fragments with a biologically unrealistic velocity higher than 120 nt/s were excluded from the mean, sd and correlation calculation. Poisson noise was added to the counts to simulate technical sequencing noise **c)** Evaluation of the performance of delay based event prediction based on stochastic simulation for a wide range of segmentation penalties. velocity changes: A first simulation was done to evaluate the accuracy of velocity change point estimation with 108 parameter combinations (half-life: 1, 2 or 3.33 min; velocity: 15, 30, 45 or 60 nt/s; synthesis rate: 0.2, 0.4 or 0.8 molecules/s) and a velocity change from each velocity to each other velocity at position 500. The ROC-like curve shows the segmentation performance at different breakpoint penalties from 4 to 0.5 and variable outlier penalties for the dynamic programming. The inlay shows an example for the fit and the automatic segmentation of a 1000 nt fragment with a velocity change from 60 nt/s to 15 nt/s at 500nt. **d)** Violin plots of fragment based velocities for the investigated organisms. The black and red numbers indicate the median and mean velocities, respectively. All velocities >120 nt/s (n_{ex}) were excluded from the analysis.

Chen et al.[7] used the delay to calculate the transcription elongation rate. Here, we estimate the performance of the velocity estimation and velocity change point detection based on simulation data (Supplementary Fig. 18c). The calculated velocities are clearly correlated with the actual input velocities, showing that the delay is valuable for an elongation rate estimate. Lower elongation rates are more reliable and the variability increases towards higher transcription velocities (Supplementary Fig. 18b). A comparison of the global transcription velocity distributions of the investigated organisms shows the highest mean elongation rate for Synechococcus PCC 7002 (42.1 nt/s) (Supplementary Fig. 18d). Synechocystis PCC 6803 has the lowest mean elongation rate (22 nt/s) and K. aerogenes (28.5 nt/s), E. coli MG1655 (27 nt/s) and E. coli BW25113 (33.2 nt/s) lie in between. The mean elongation rate of 27 nt/s from E. coli MG1655 is close to the previously reported 25 nt/s[7].

To test the ability to detect velocity changes during transcription we used a 1000nt transcript with a velocity change position at 500nt (for parameters see legend Supplementary Fig. 18). A simulation example is given in Supplementary Fig. 18c. The method works in general, but for the chosen transcript length and speed change position in the simulation, no segmentations were done with the segmentation penalty of 3.5 indicating an underestimation of velocity change points in the real life E. coli dataset. It needs to be considered that the segmentation for longer segments >500nt will be better.

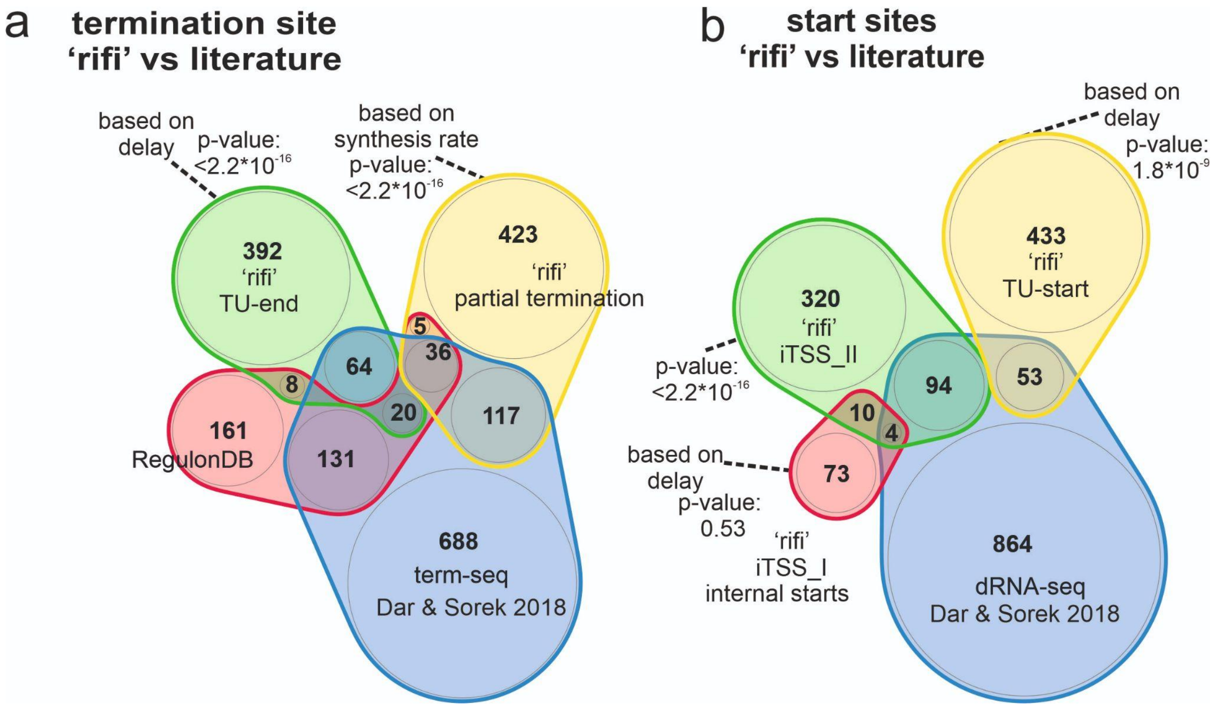

Supplementary Figure 19: Comparison of rifi events with other experimental methods. rifi detects start sites and terminators based on changes in the synthesis rate (iTSS_II, (partial) termination) or changes in the delay (start_of_TU, iTSS_I and end_of_TU). The delay based estimates are discussed in a later section. **a)** Comparison of 'rifi' terminator predictions based on the synthesis rate (all events with p-value ≤ 0.05 & >15% termination rate) or based on the delay based TU-definition with term-seq[16] and RegulonDB terminators[17]. The p-values for the overlap with the term-seq sites better than expected by chance were calculated with the exact binomial test. **b)** Comparison of 'rifi' transcriptional start site predictions based on the synthesis rate (iTSS_II, p-value ≤ 0.05), based on the delay based TU-definition (major starts) and delay based minor internal starts (iTSS_I, p-value ≤ 0.05) with a dRNAseq dataset[16]. Start sites are estimated based on synthesis rate changes between two segments (iTSS_II), too. Only 14 of the delay based and synthesis rate TSS predictions overlap. That is mostly due to the lower sensitivity within the delay based method. If the elongation rate is fast, the time resolution of the data is not good enough and there is often no detectable delay change at a start. Nevertheless, an increased synthesis rate still indicates a start. In addition to the synthesis rate based terminator estimate, each end of an TU also resembles a predicted termination site. We compared the "end of TU" terminators with the term-seq data and the RegulonDB dataset as for the synthesis rate based terminator estimates. Here, 84/484 sites overlap with term-seq terminators ($p < 2.2 \times 10^{-16}$) and 28/484 with the RegulonDB set ($p = 7.6 \times 10^{-9}$).

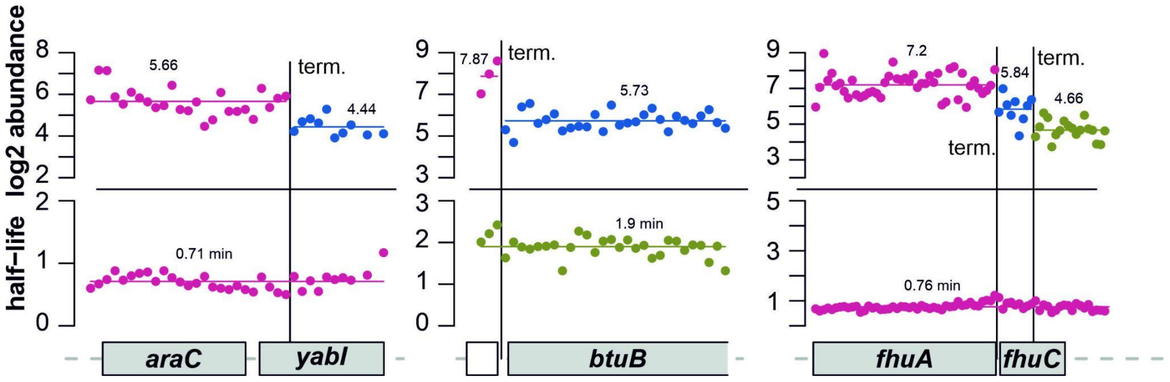

Supplementary Figure 20: Examples for high confidence terminators detected by rifi but not by term-seq[6]. The termination sites are indicated by vertical lines.

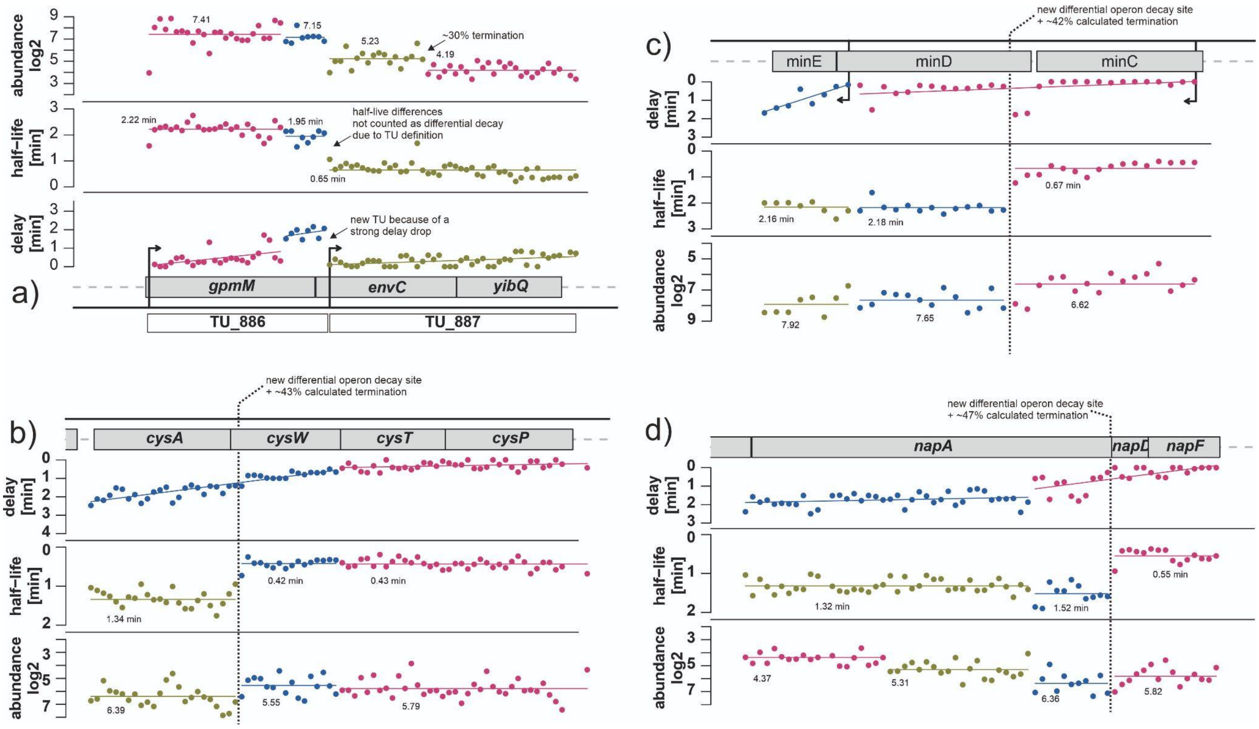

Supplementary Figure 21: Differential operon decay. **a)** Example for a previously reported differential decay site between *gpmM* and *envC* which we excluded due to the delay based TU definition. **b-d)** Examples for newly defined sites of differential operon decay.

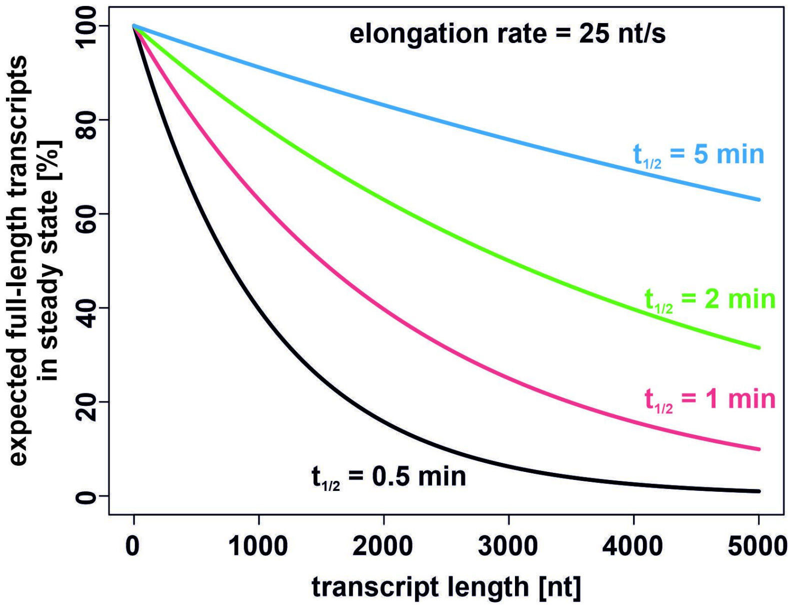

Supplementary Figure 22: Expected percentage of full-length transcripts depending on the transcript length and the RNA stability at a fixed elongation rate of 25 nt/s under the assumption of co-transcriptional decay. A lower elongation rate would increase the probability that the transcript is decayed before the transcription has concluded. $Perc_{full} = e^{-\lambda * \frac{L}{V}} * 100$. λ = decay constant [1/s], v = elongation rate [nt/s], L = total transcript length [nt].

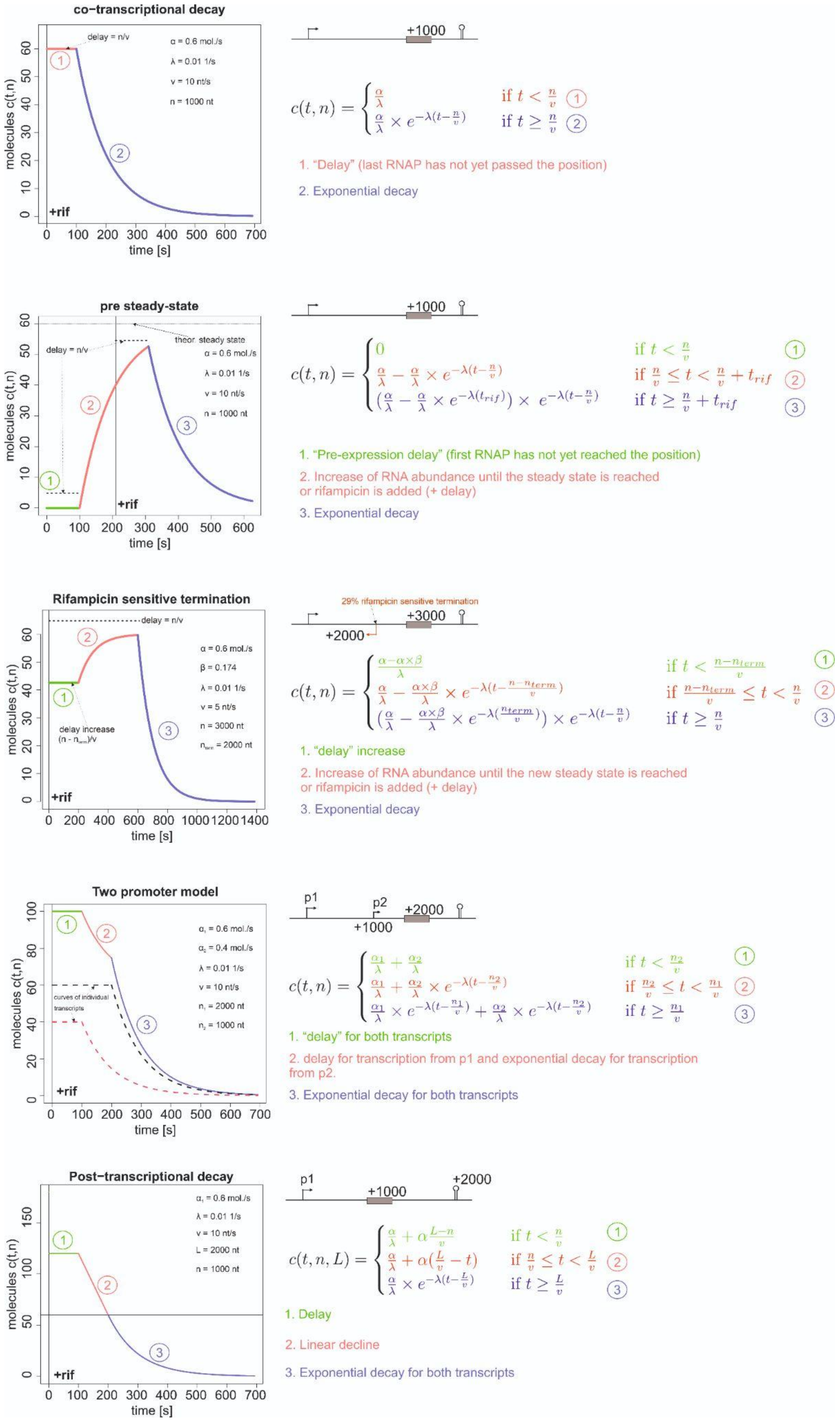

Supplementary Figure 23: Visual description of the different mathematical models.

Supplementary methods:

Overlap of terminator/start sites and statistics

Sites from other *E. coli* strains than BW25113 were mapped to the BW25113 genome by perfect string matching. 3' sites (term-seq) and 5' sites (dRNA-seq) were from[16] and pausing sites from[18,19]. We additionally used the experimentally verified terminator list from RegulonDB (RegulonDB 10.10, 02/28/2022) [17]. Pausing sites from the Larson data within a range of 50 nt were clustered to one representative site. For the terminators we allowed an overlap of +/- 2bins, i.e +/-100 nt to account for the relative coarse 'rifi' site detection. Due to the high number of sites in the Larson pausing set the overlap for pausing sites was set to +/- 1bins, i.e +/-50 nt. The p-values for the overlaps being higher than expected by chance were calculated with the exact binomial test. Based on the estimate, that roughly the whole genome is transcribed either in forward or reverse direction, we used a total transcriptome size of 4,631,470 nt. The transcriptome size was divided by the allowed overlap window size into n windows. The assumed random probability for hitting a site is the number of sites in the reference divided by the number of windows $P_{random} = \frac{\#_{sites}}{n}$.

The quasi-proportional Venn diagrams were done with the nVennR R-package [20].

Velocity sensitivity and specificity

To test the accuracy of the velocity estimate a 1000nt transcript with constant elongation rate was simulated. In total 36 parameter combinations (decay constants: 0.003465736, 0.01155 and 0.00578 1/s; initiation rates: 0.2, 0.4 and 0.8 initiations/s) including 4 different elongation rates (15, 30, 45 and 60 nt/s) were used. The simulations for each parameter set were repeated 50 times and the resulting data segmented with the penalties used for the Dar & Sorek data (split penalty 3.5, outlier

penalty 2.5). The velocities of the resulting segments were compared with the input velocities.

In order to investigate the segmentation performance upon a velocity change the simulation was done for a 1000nt RNA with a transcription velocity change at 500nt. The parameters were as above besides that all 12 possible speed changes were considered, which leads to 108 different parameter sets. A correct split needs to be in a window of +/- 2 bins and the p-value needs to be $p \leq 0.01$. There could not be more than 1 correct split for each segmentation. First the number of true positive splits (TP) for each parameter set (50 replications) was counted and divided by the number of the actual splits, i.e $TP + FN = 50$ to get the true positive rate $TPR_{parset} = \frac{TP}{50}$, yielding in total 108 parameter dependent TPR_{parset} for each penalty set. These TPR_{parset} were averaged to get the final penalty-set dependent TPR_{penset}. To calculate the final penalty-set dependent false positive rate

FPR_{penset}, again the 108 parameter dependent FPR_{parset} were averaged. The $FPR_{parset} = \frac{FP}{FP + TN}$ was calculated from the FPs, i.e. the total number of splits minus the true positives, and the true negatives (TN). For each parameter set we did 50 replications and each replication contains exactly one true positive split, thus there is no replication without a split and the number of true negatives is 50. The 108 FPR_{penset} and FPR_{penset} were used for the ROC-like curves in Fig. 5.

Internal start sites

Simulation was done as above with 108 different parameter sets (half-life: 1, 2; velocity: 15 or 30 nt/s; synthesis rate 1 + 2: 0.2, 0.4 or 0.8 molecules/s; iTSS position: 500, 1000 or 2000nt), with a data point every 100 nt instead of every 50nt.

The calculation of TPR and FPR based on different segmentation penalties was done as described above.

Supplementary References:

1. Thomason, M. K. *et al.* Global transcriptional start site mapping using differential RNA sequencing reveals novel antisense RNAs in *Escherichia coli*. *J. Bacteriol.* **197**, 18–28 (2015).
2. Van Gundy, T., Martin, E., Bono, J., Hatton, O. & Lybecker, M. C. An Antisense RNA Fine-Tunes Gene Expression of the Type II MazEF Toxin-Antitoxin System. *mBio* **13**, e03443-21 (2022).
3. Wang, X., Ji, S. C., Jeon, H. J., Lee, Y. & Lim, H. M. Two-level inhibition of *galK* expression by Spot 42: Degradation of mRNA mK2 and enhanced transcription termination before the *galK* gene. *Proc. Natl. Acad. Sci.* **112**, 7581–7586 (2015).
4. Balasubramanian, D., Ragunathan, P. T., Fei, J. & Vanderpool, C. K. A Prophage-Encoded Small RNA Controls Metabolism and Cell Division in *Escherichia coli*. *mSystems* **1**, e00021-15 (2016).
5. Melamed, S., Adams, P. P., Zhang, A., Zhang, H. & Storz, G. RNA-RNA Interactomes of ProQ and Hfq Reveal Overlapping and Competing Roles. *Mol. Cell* **77**, 411-425.e7 (2020).
6. Dar, D. & Sorek, R. Extensive reshaping of bacterial operons by programmed mRNA decay. *PLOS Genet.* **14**, e1007354 (2018).
7. Chen, H., Shiroguchi, K., Ge, H. & Xie, X. S. Genome-wide study of mRNA degradation and transcript elongation in Escherichia coli. *Mol. Syst. Biol.* **11**, 781 (2015).
8. Brophy, J. A. N. & Voigt, C. A. Antisense transcription as a tool to tune

gene expression. *Mol. Syst. Biol.* **12**, (2016).

9. Hoffmann, S. A., Hao, N., Shearwin, K. E. & Arndt, K. M. Characterizing Transcriptional Interference between Converging Genes in Bacteria. *ACS Synth. Biol.* **8**, 466–473 (2019).
10. Moffitt, J. R., Pandey, S., Boettiger, A. N., Wang, S. & Zhuang, X. Spatial organization shapes the turnover of a bacterial transcriptome. *eLife* **5**, e13065 (2016).
11. Gordon, G. C. *et al.* Genome-Wide Analysis of RNA Decay in the Cyanobacterium *Synechococcus* sp. Strain PCC 7002. *mSystems* **5**, (2020).
12. Mitschke, J. *et al.* An experimentally anchored map of transcriptional start sites in the model cyanobacterium *Synechocystis* sp. PCC6803. *Proc. Natl. Acad. Sci.* **108**, 2124–9 (2011).
13. Sharma, C. M. *et al.* The primary transcriptome of the major human pathogen *Helicobacter pylori*. *Nature* **464**, 250–255 (2010).
14. Thomason, M. K. *et al.* Global Transcriptional Start Site Mapping Using Differential RNA Sequencing Reveals Novel Antisense RNAs in *Escherichia coli*. *J. Bacteriol.* (2014) doi:10.1128/JB.02096-14.
15. Schwarz, G. Estimating the Dimension of a Model. *Ann. Stat.* **6**, 461–464 (1978).
16. Dar, D. & Sorek, R. Extensive reshaping of bacterial operons by programmed mRNA decay. *PLOS Genet.* **14**, e1007354 (2018).
17. Santos-Zavaleta, A. *et al.* RegulonDB v 10.5: tackling challenges to unify classic and high throughput knowledge of gene regulation in *E. coli* K-12. *Nucleic Acids Res.* **47**, D212–D220 (2019).
18. Larson, M. H. *et al.* A pause sequence enriched at translation start sites drives transcription dynamics in vivo. *Science* **344**, 1042–1047 (2014).

19. Vvedenskaya, I. O. *et al.* Interactions between RNA polymerase and the “core recognition element” counteract pausing. *Science* **344**, 1285–1289 (2014).

20. Pérez-Silva, J. G., Araujo-Voces, M. & Quesada, V. nVenn: generalized, quasi-proportional Venn and Euler diagrams. *Bioinformatics* **34**, 2322–2324 (2018).
