# Supplementary material for: Improved RNA stability estimation indicates that transcriptional interference is frequent in diverse bacteria
Source: Commun Biol. 2023 Jul 15;6:732. doi: 10.1038/s42003-023-05097-2 (PMC10349824; doi:10.1038/s42003-023-05097-2)
Supplement: Supplementary file 3 — Description of Additional Supplementary Files [file 42003_2023_5097_MOESM3_ESM.pdf]

Description of Additional Supplementary Files

File name: Supplementary Data 1
Description: Table of high confidence RST instances in the investigated organisms

File name: Supplementary Data 2
Description: Visualization of selected E. coli RST instances

File name: Supplementary Data 3
Description: Full genome visualizations of the ‘rifi’ results from all 4 investigated organisms

File name: Supplementary Data 4
Description: Table containing all HL-segment borders within continuous TUs for all investigated organisms

File name: Supplementary Data 5
Description: rifi R-objects containing the source data for all analyses and figures based on experimental data
