# Supplementary figures and images for: Improved RNA stability estimation indicates that transcriptional interference is frequent in diverse bacteria
Source: Commun Biol. 2023 Jul 15;6:732. doi: 10.1038/s42003-023-05097-2 (PMC10349824; doi:10.1038/s42003-023-05097-2)

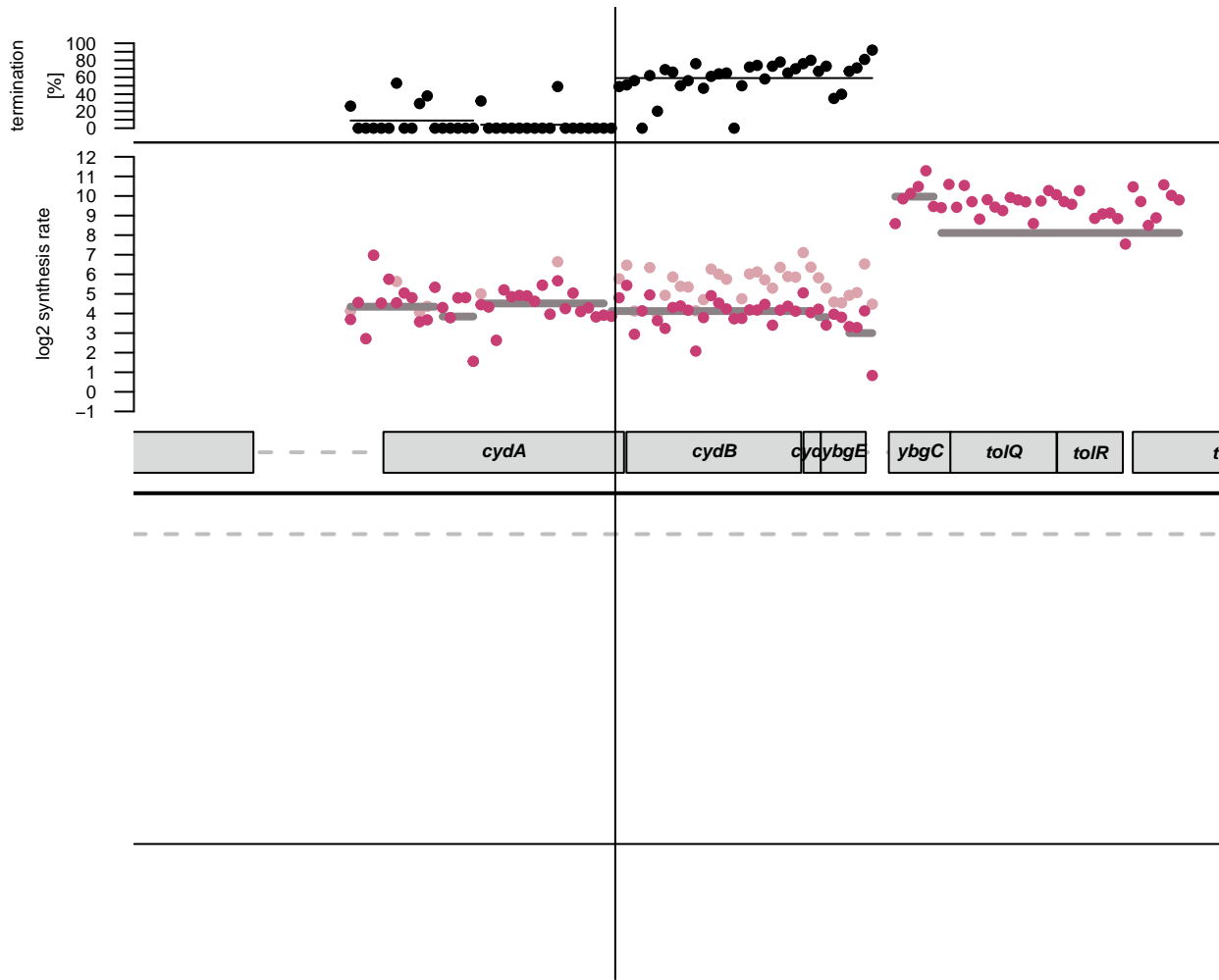

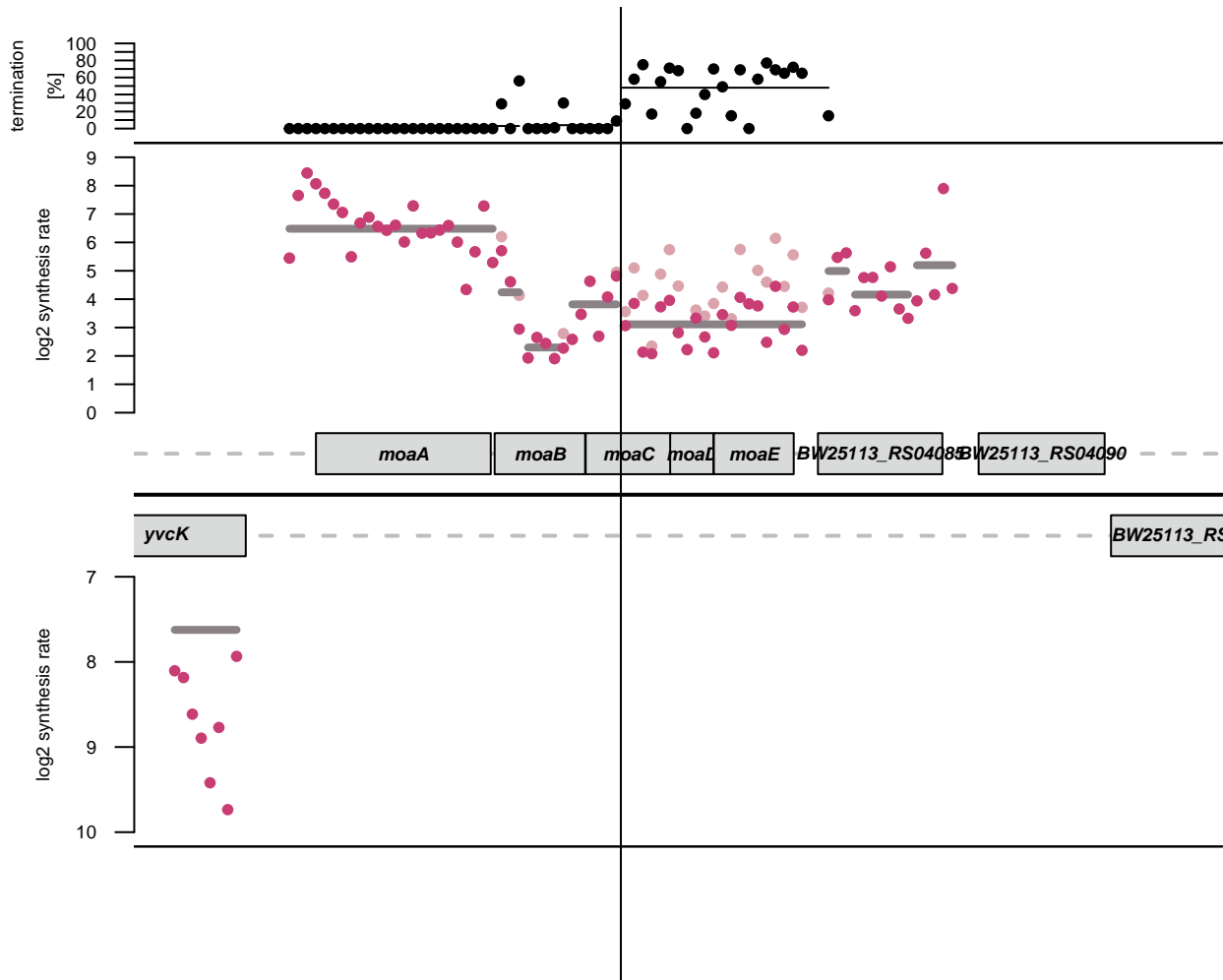

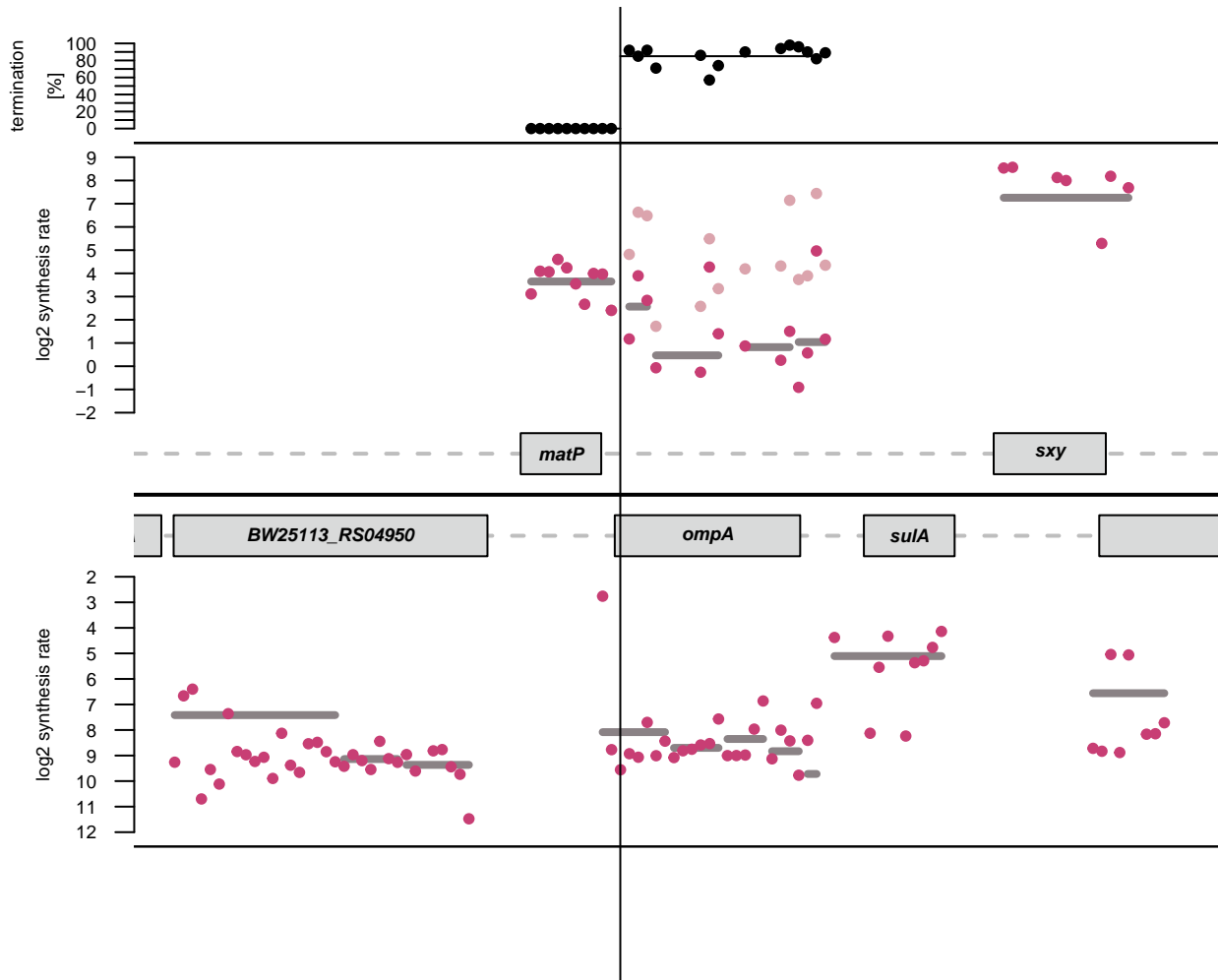

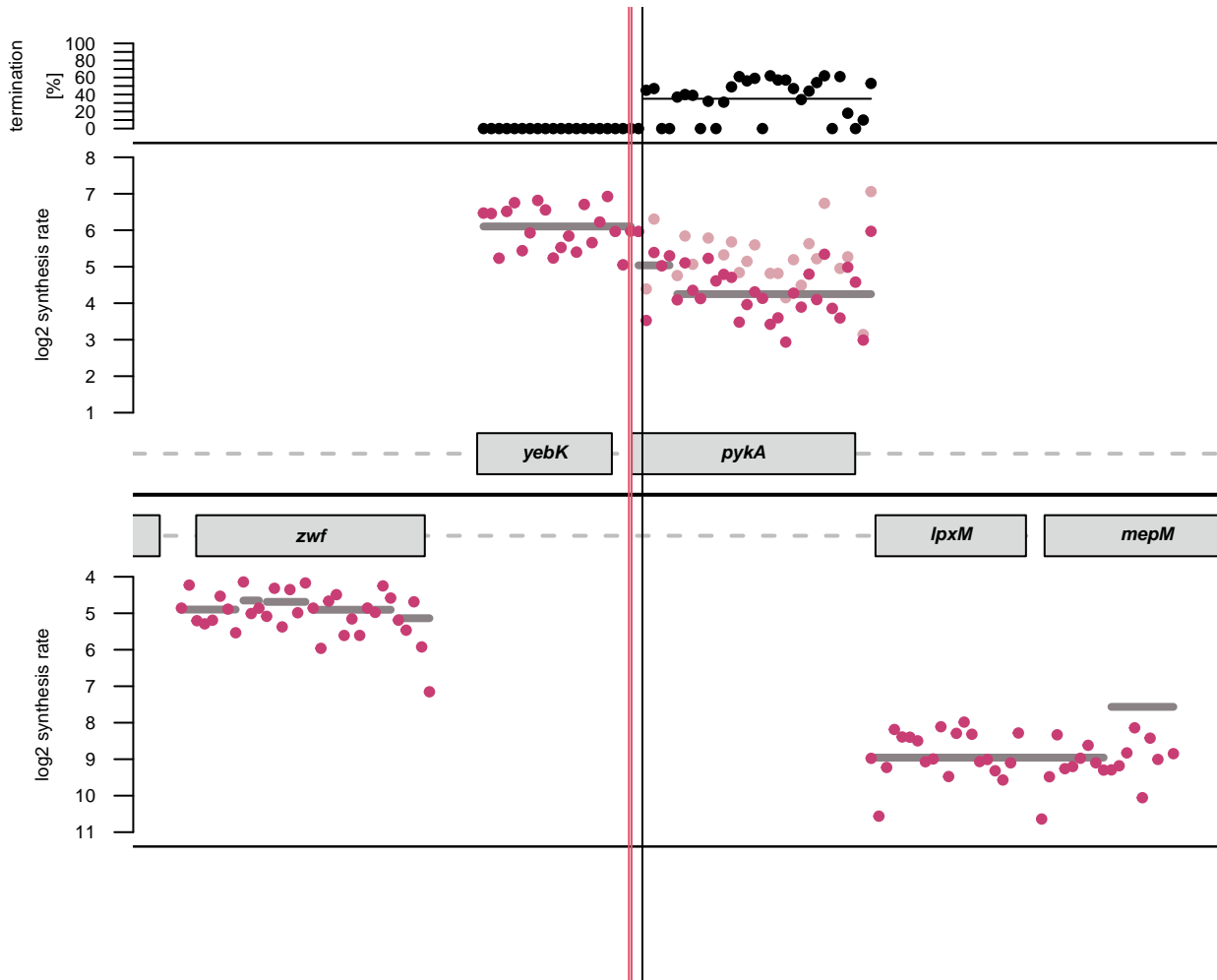

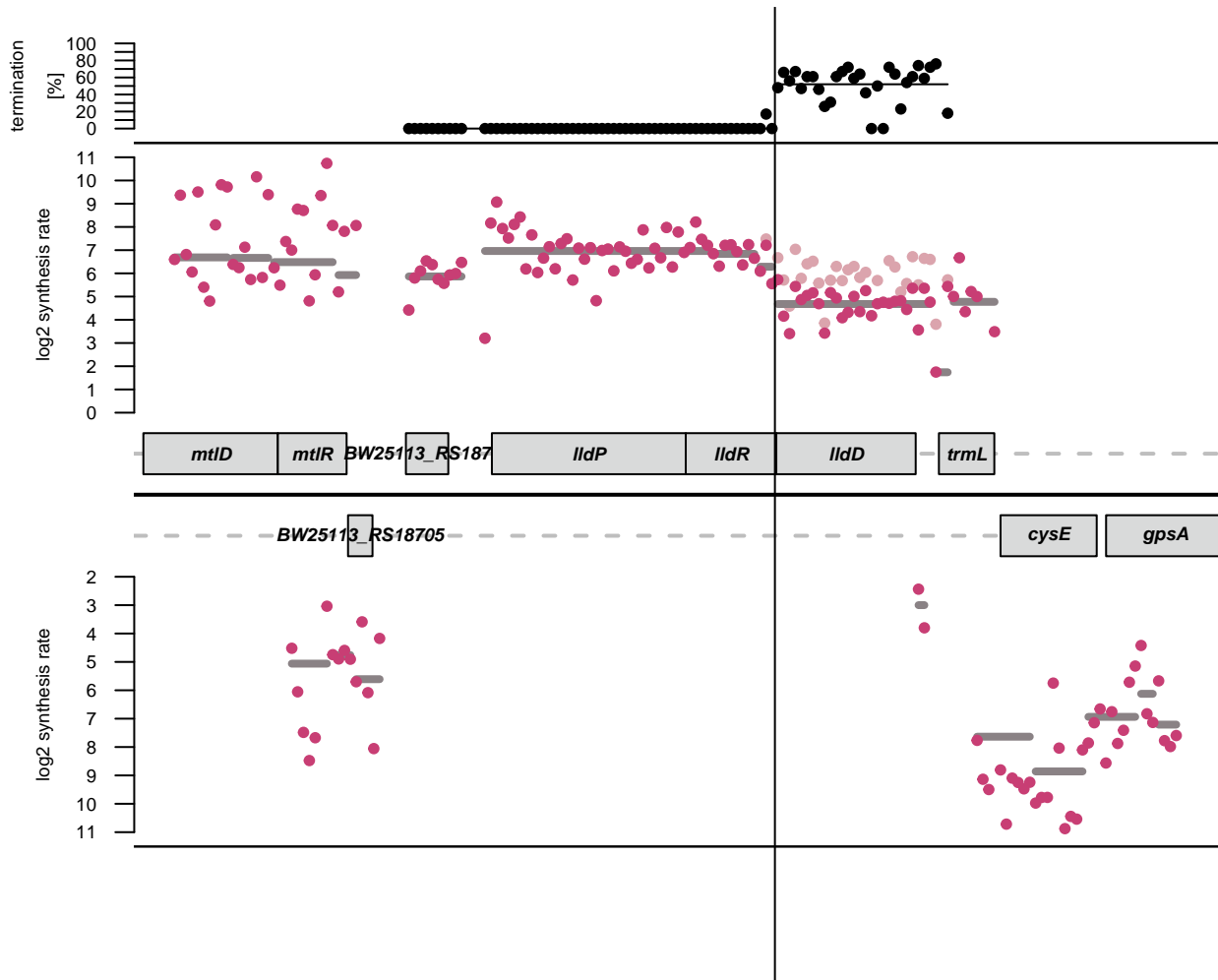

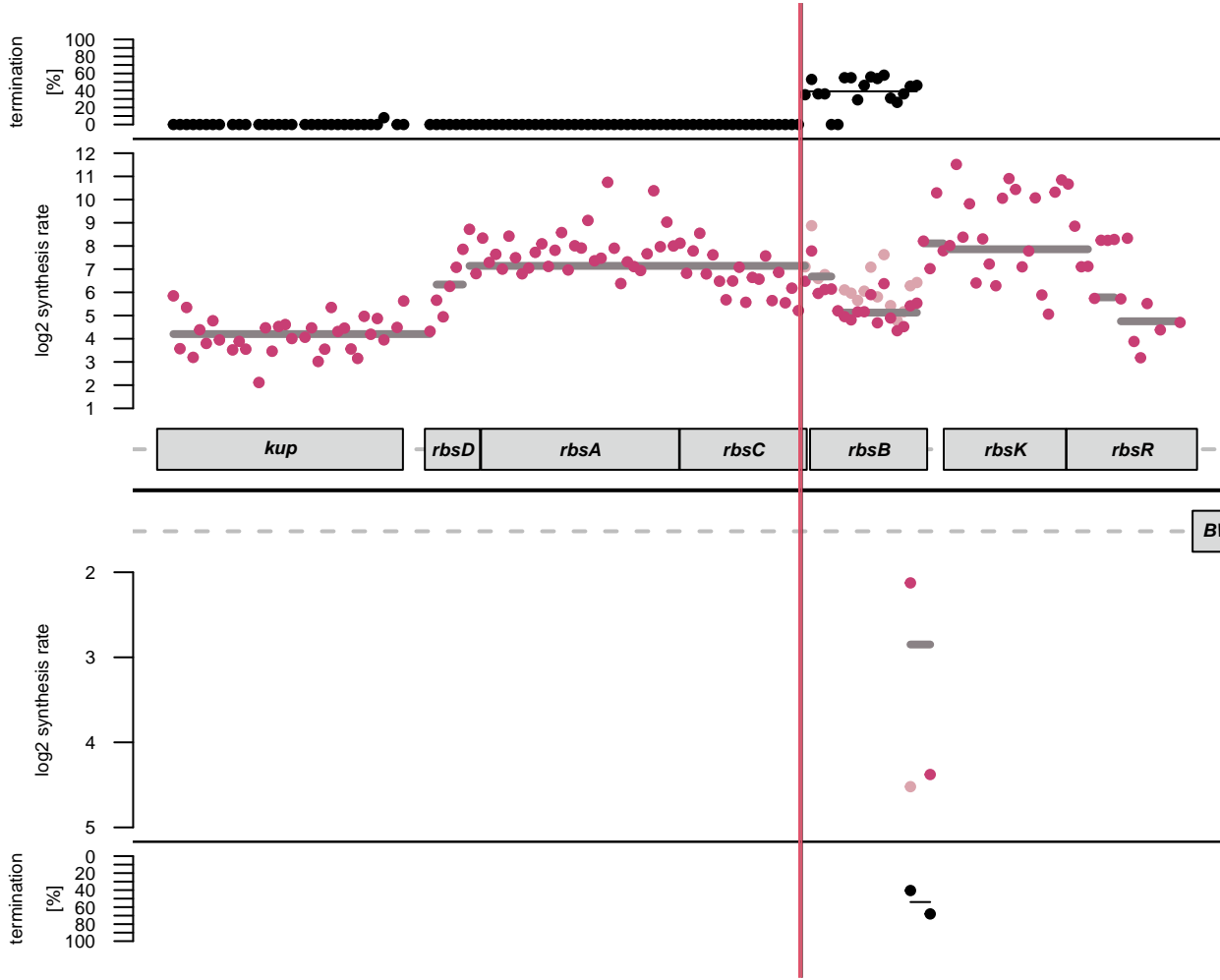

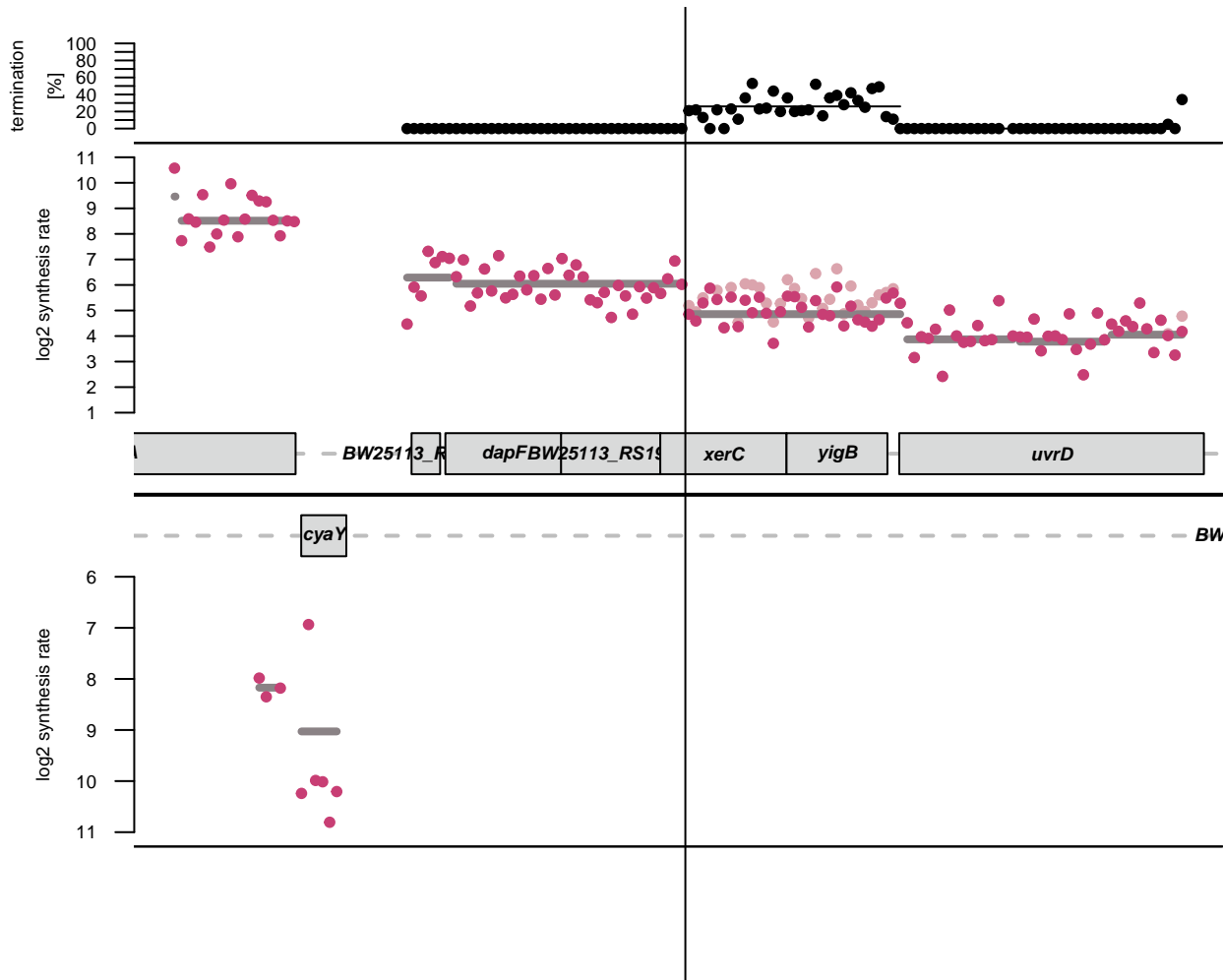

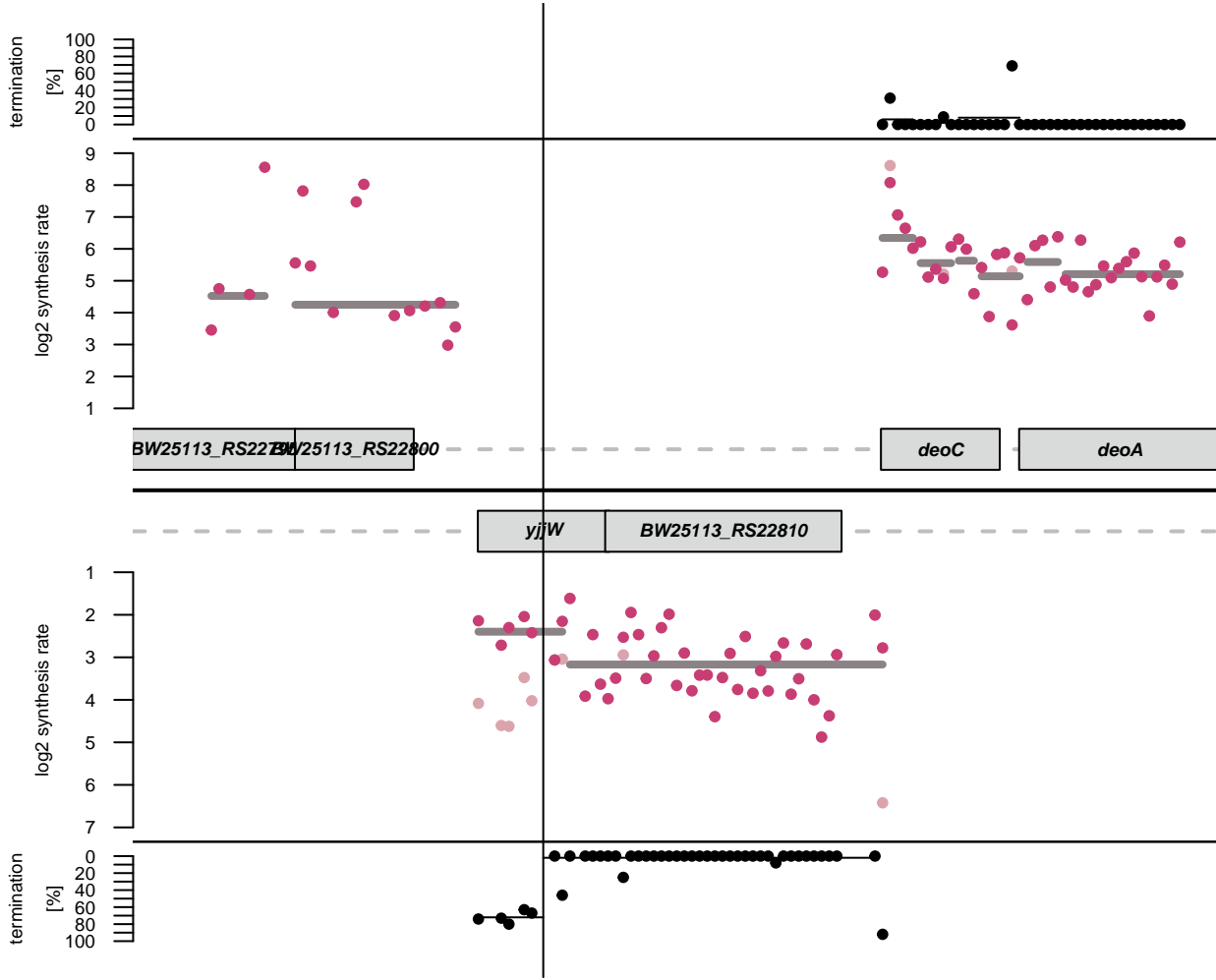

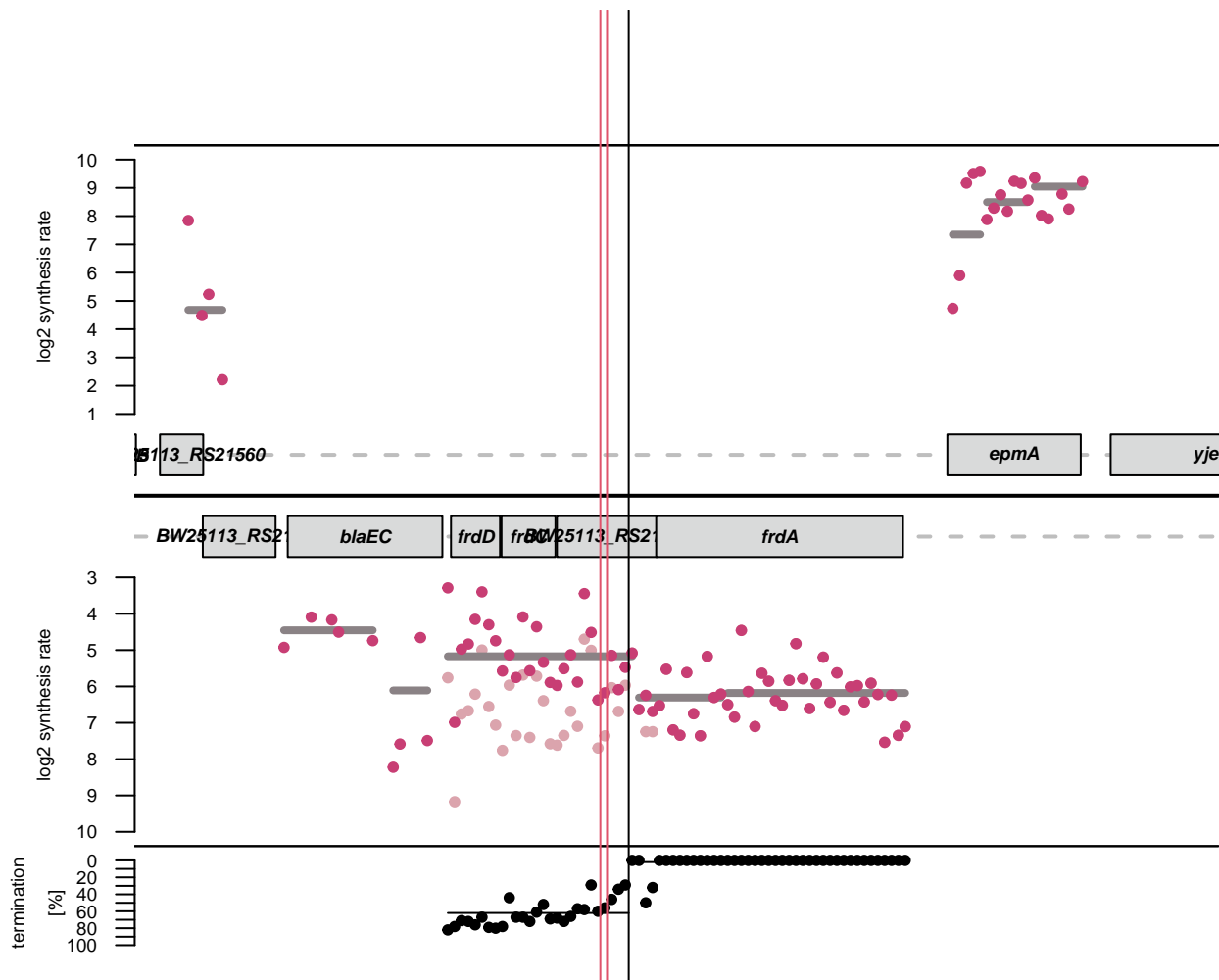

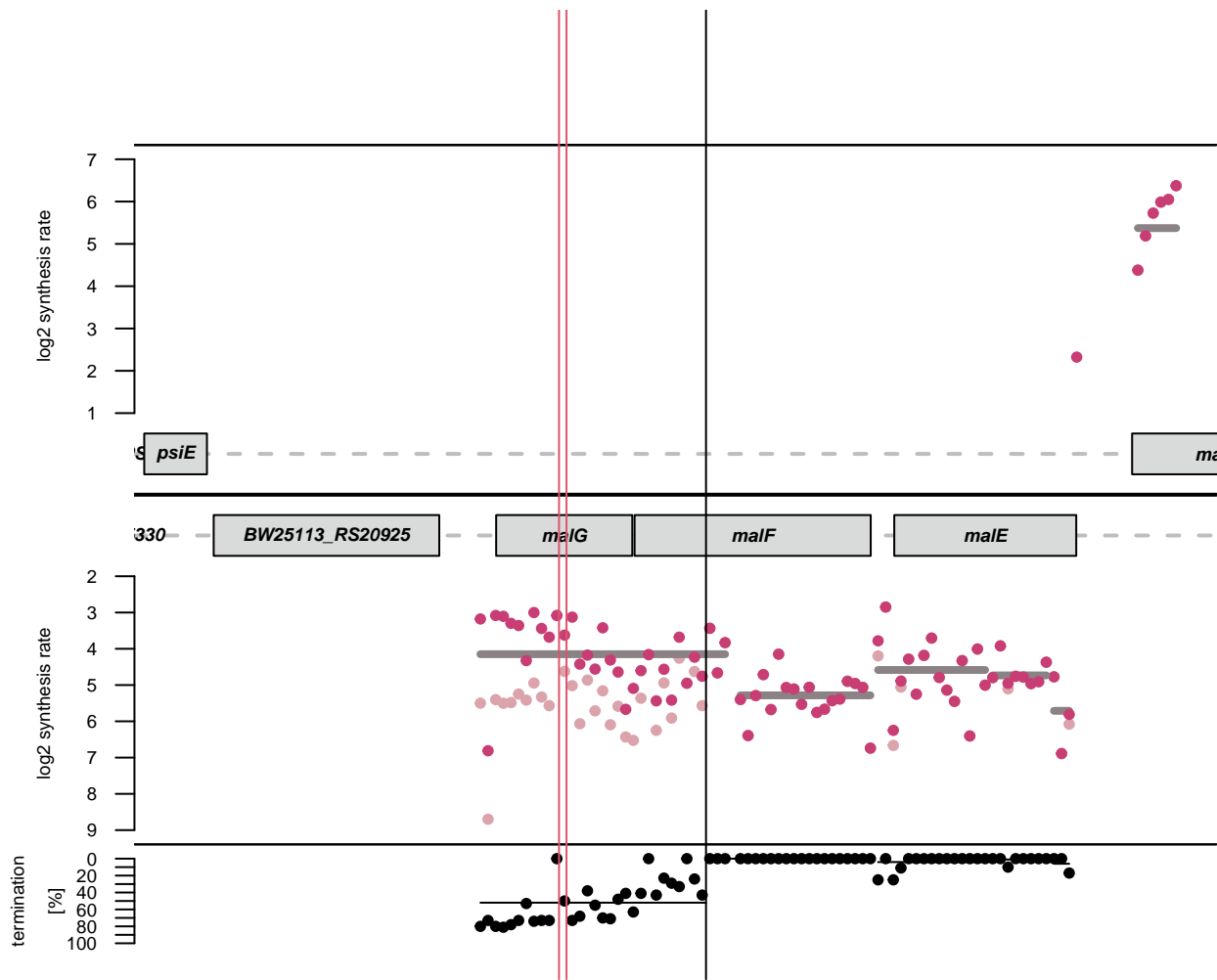

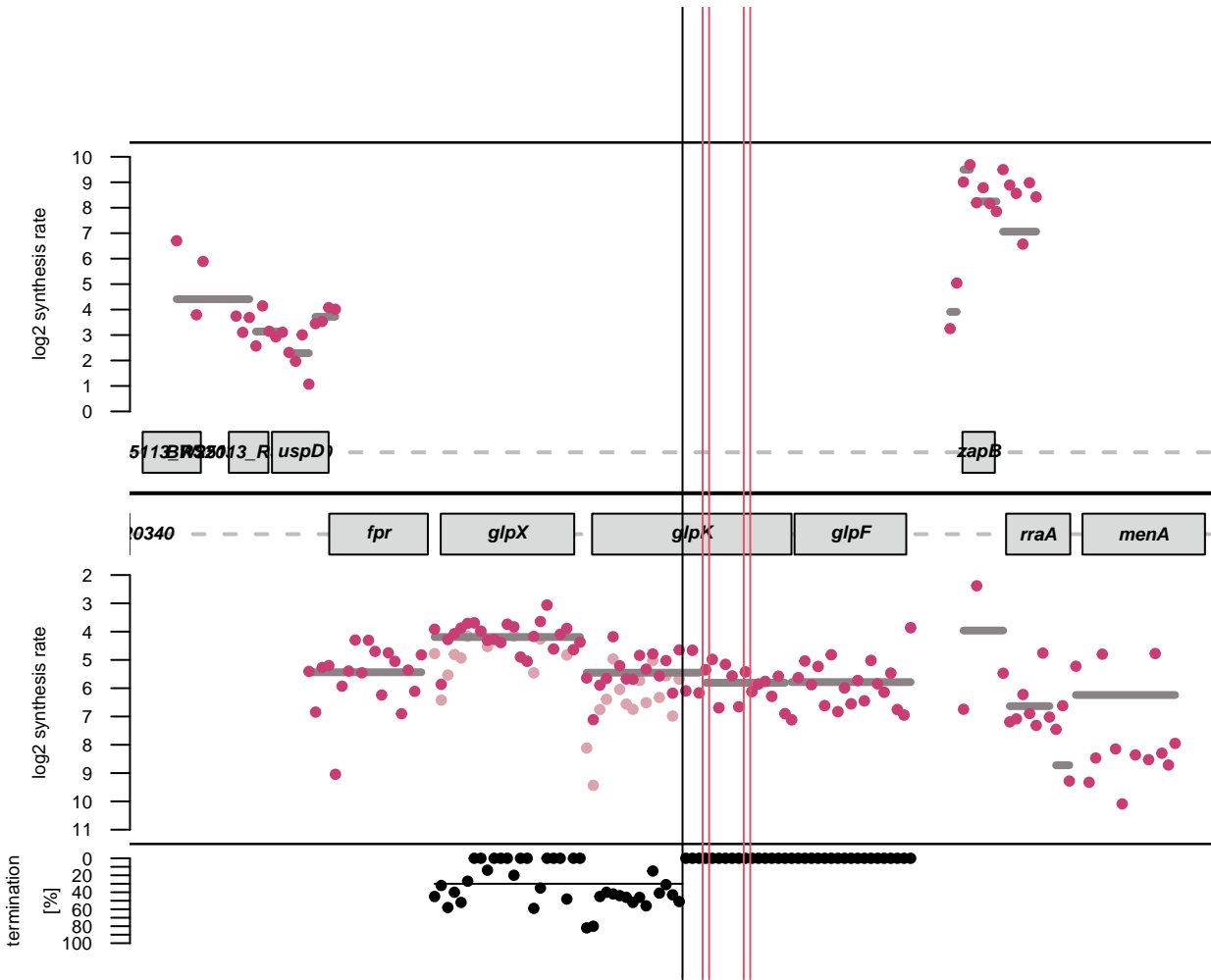

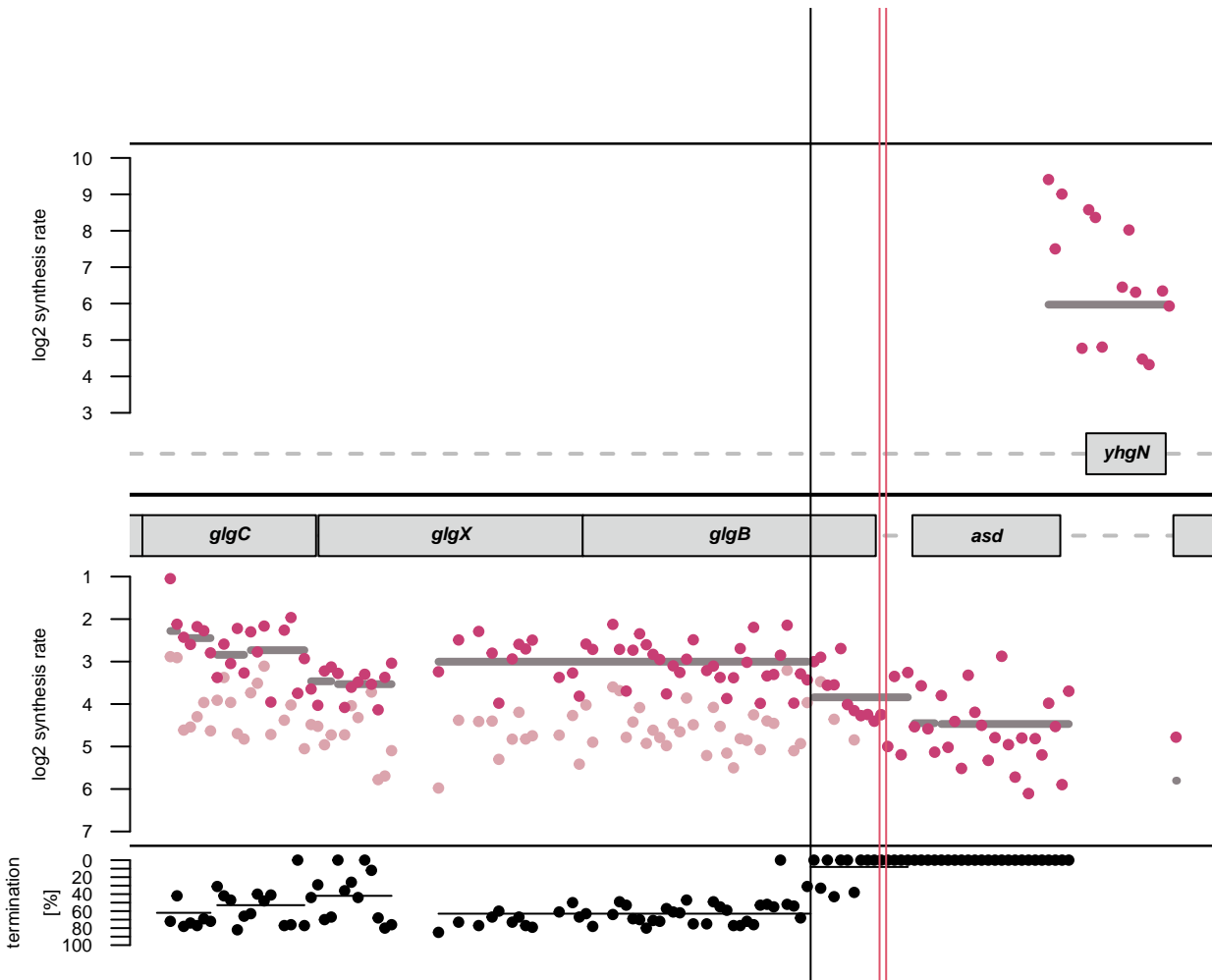

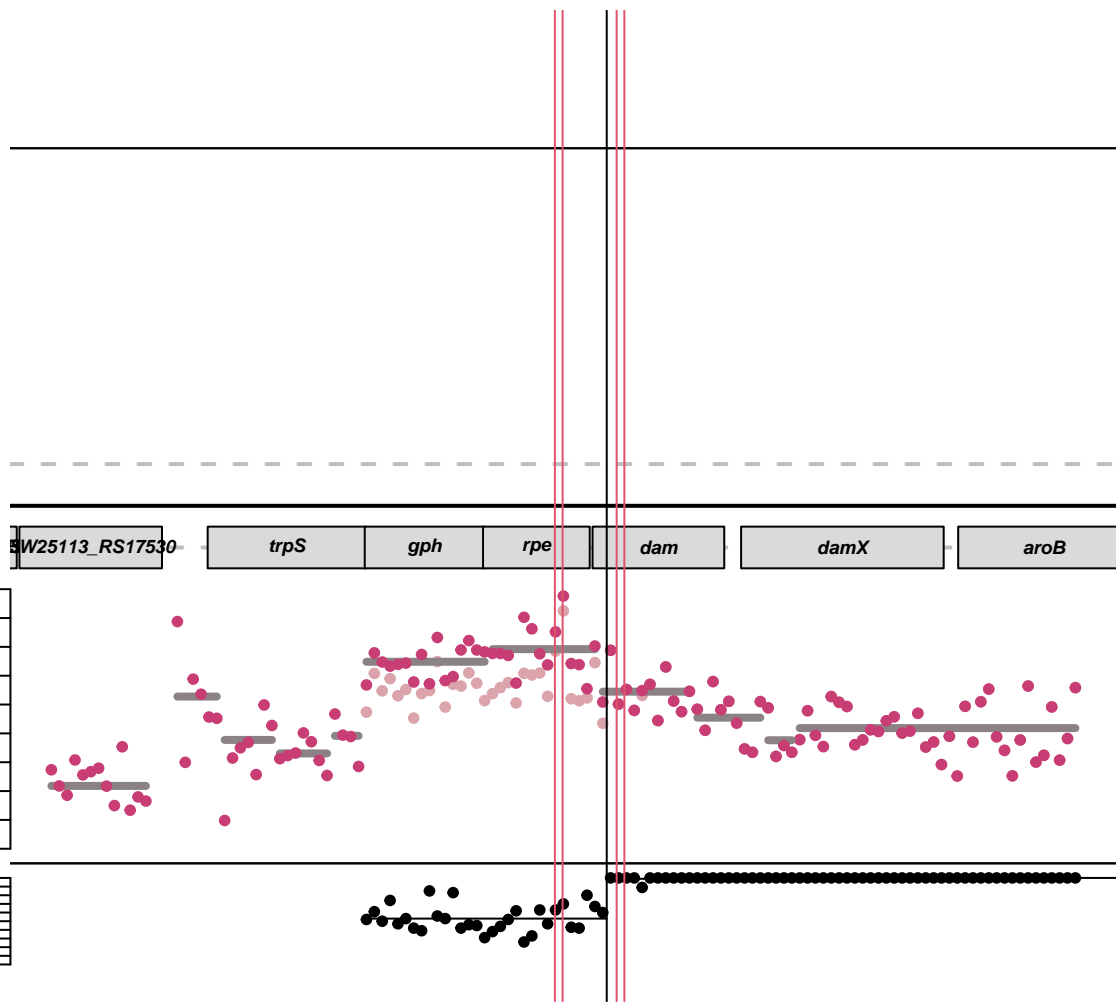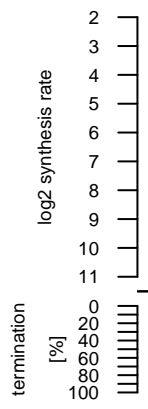

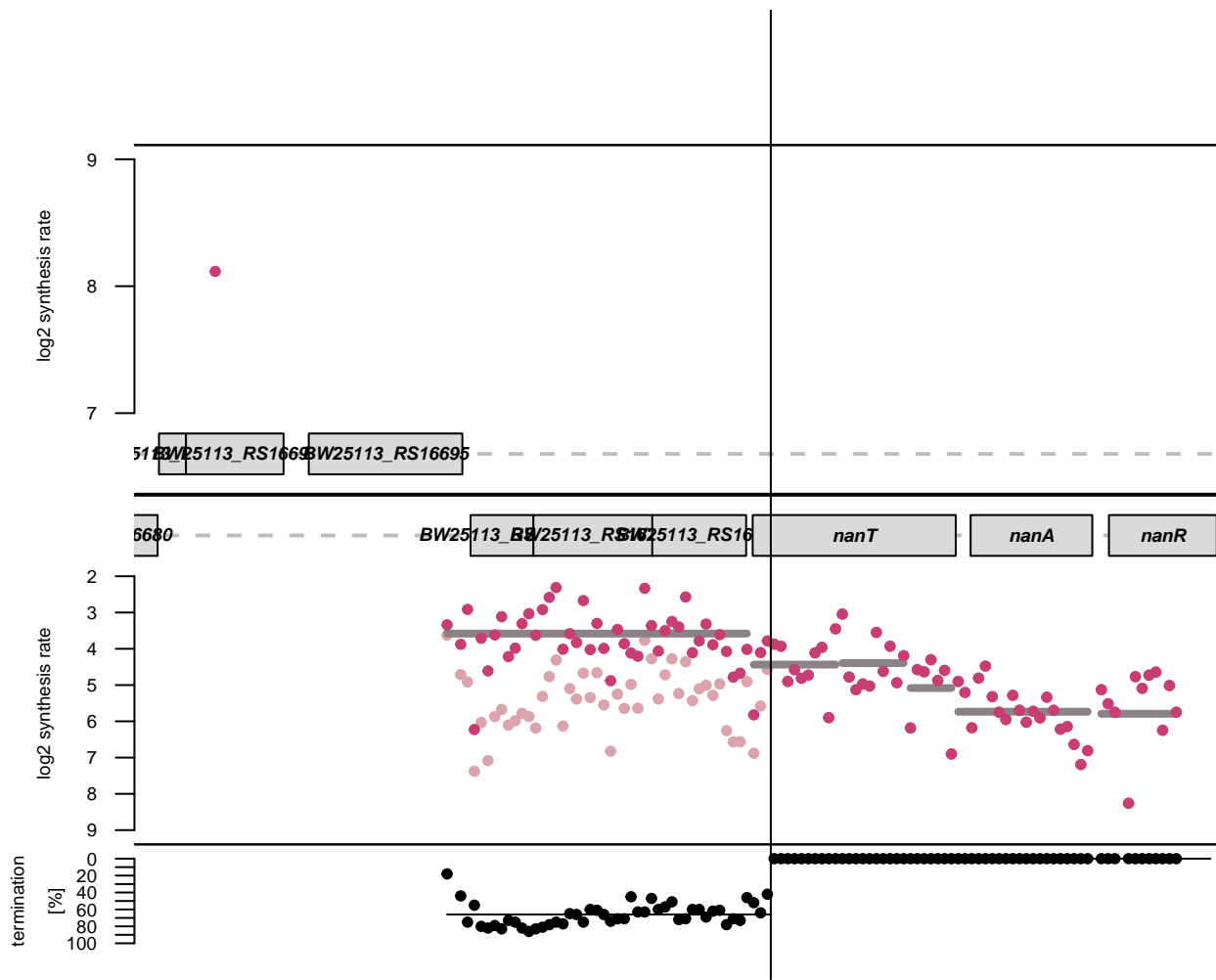

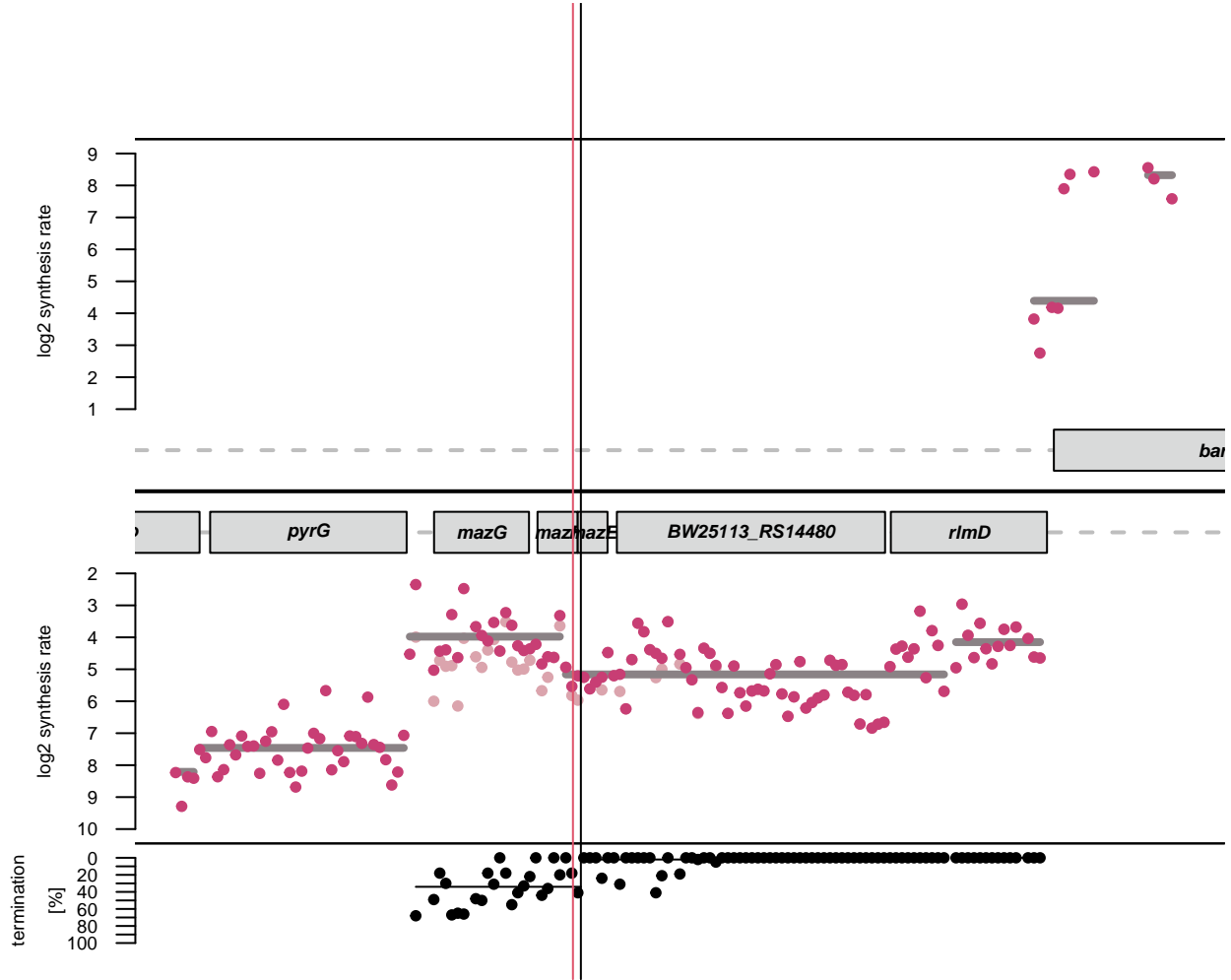

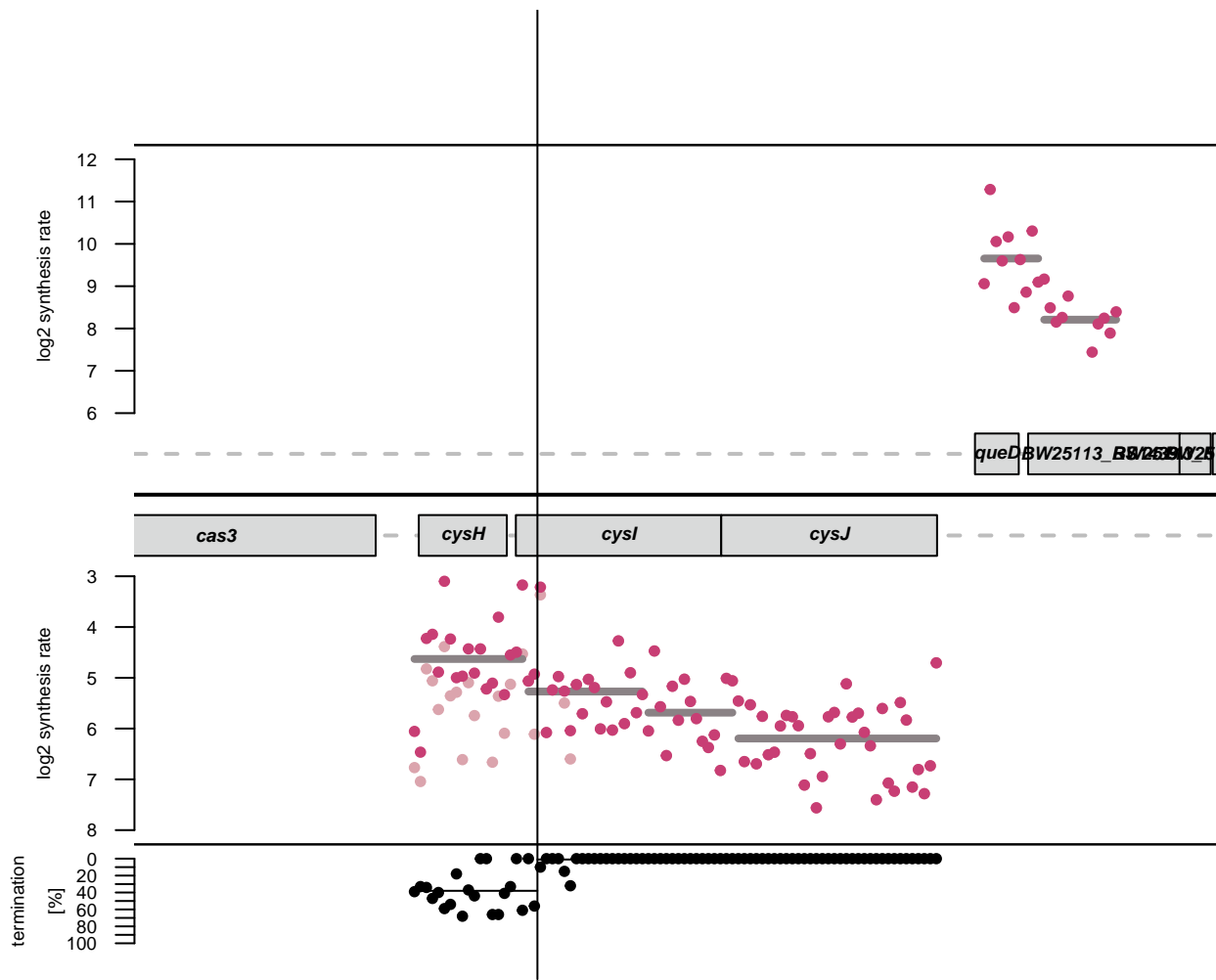

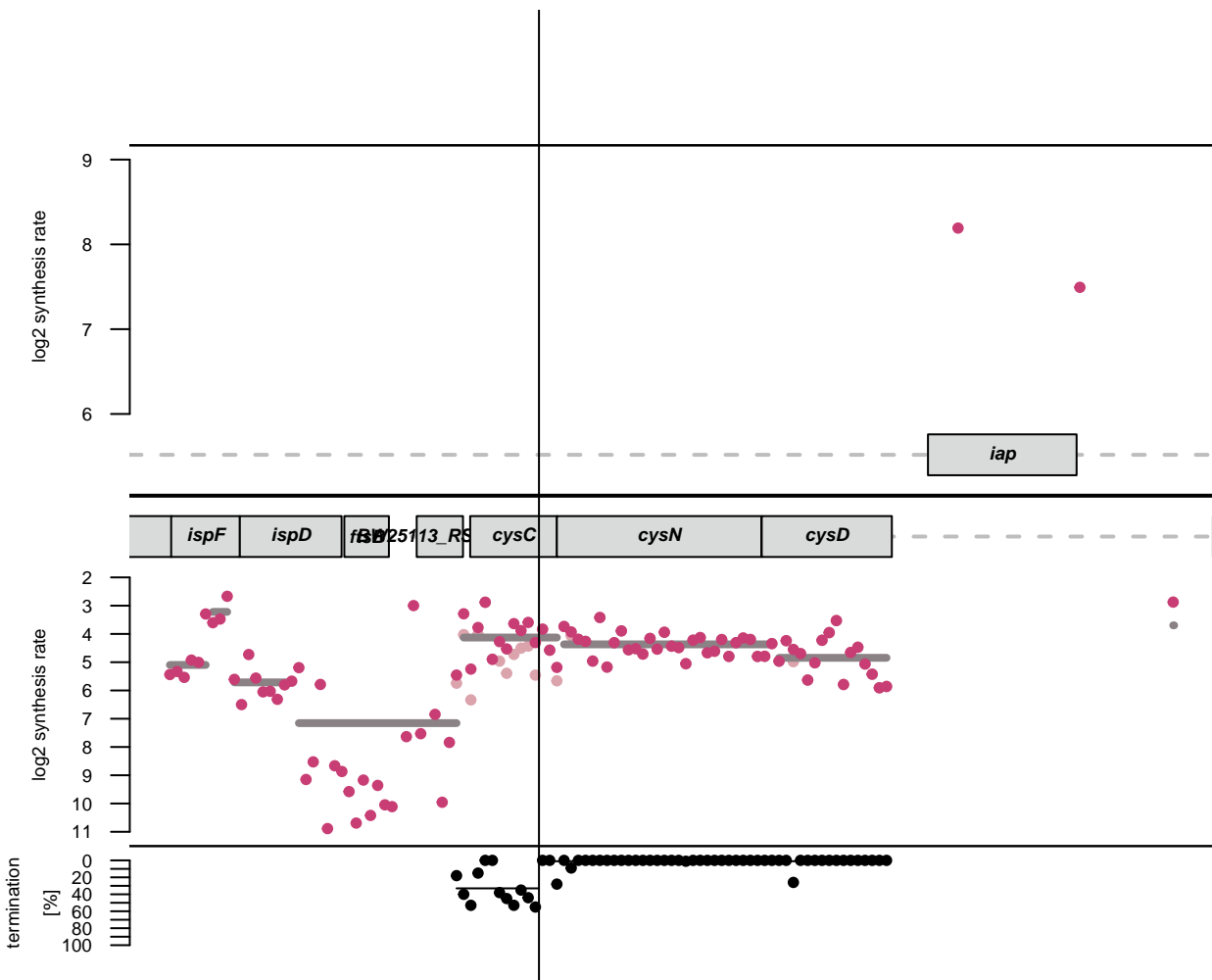

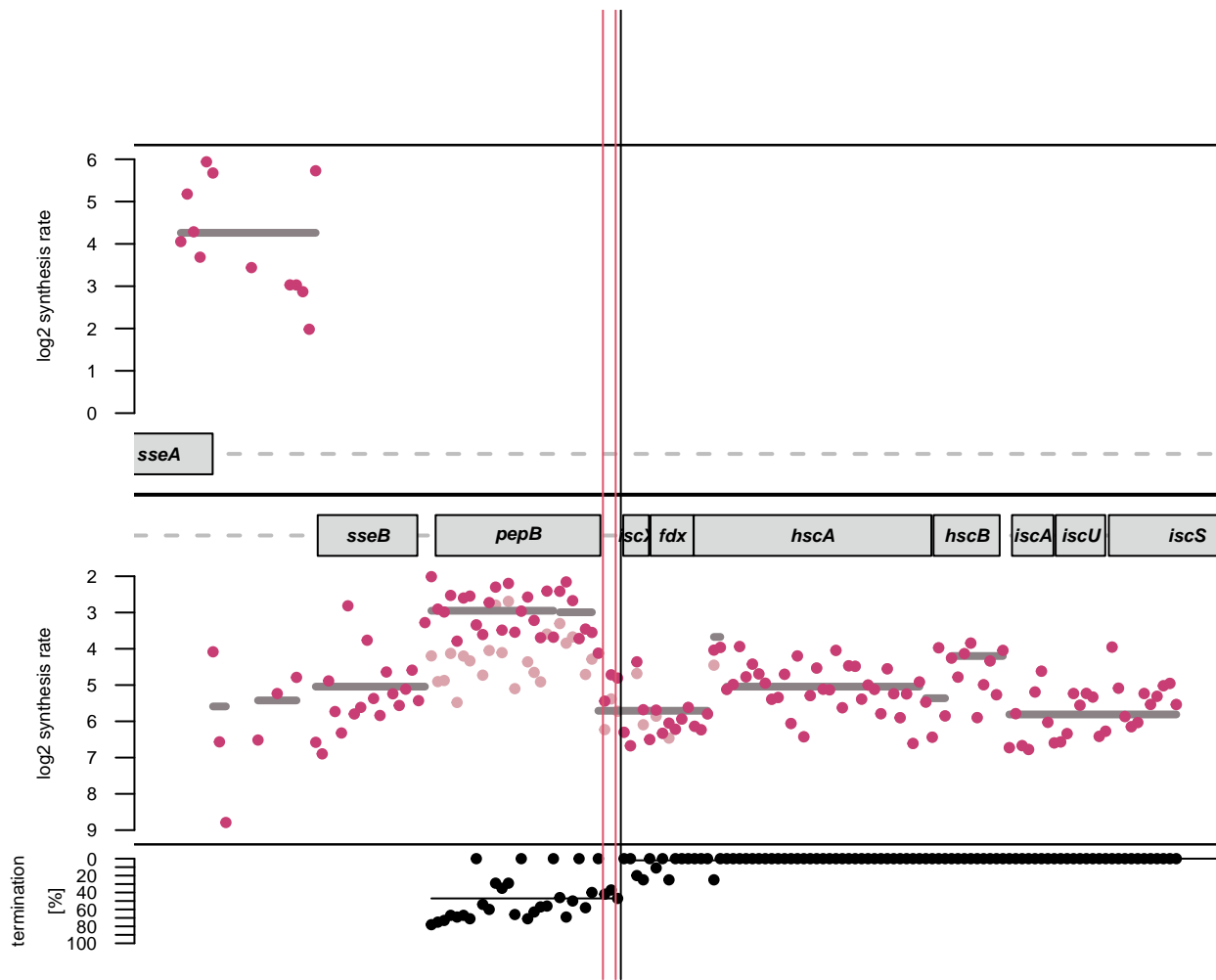

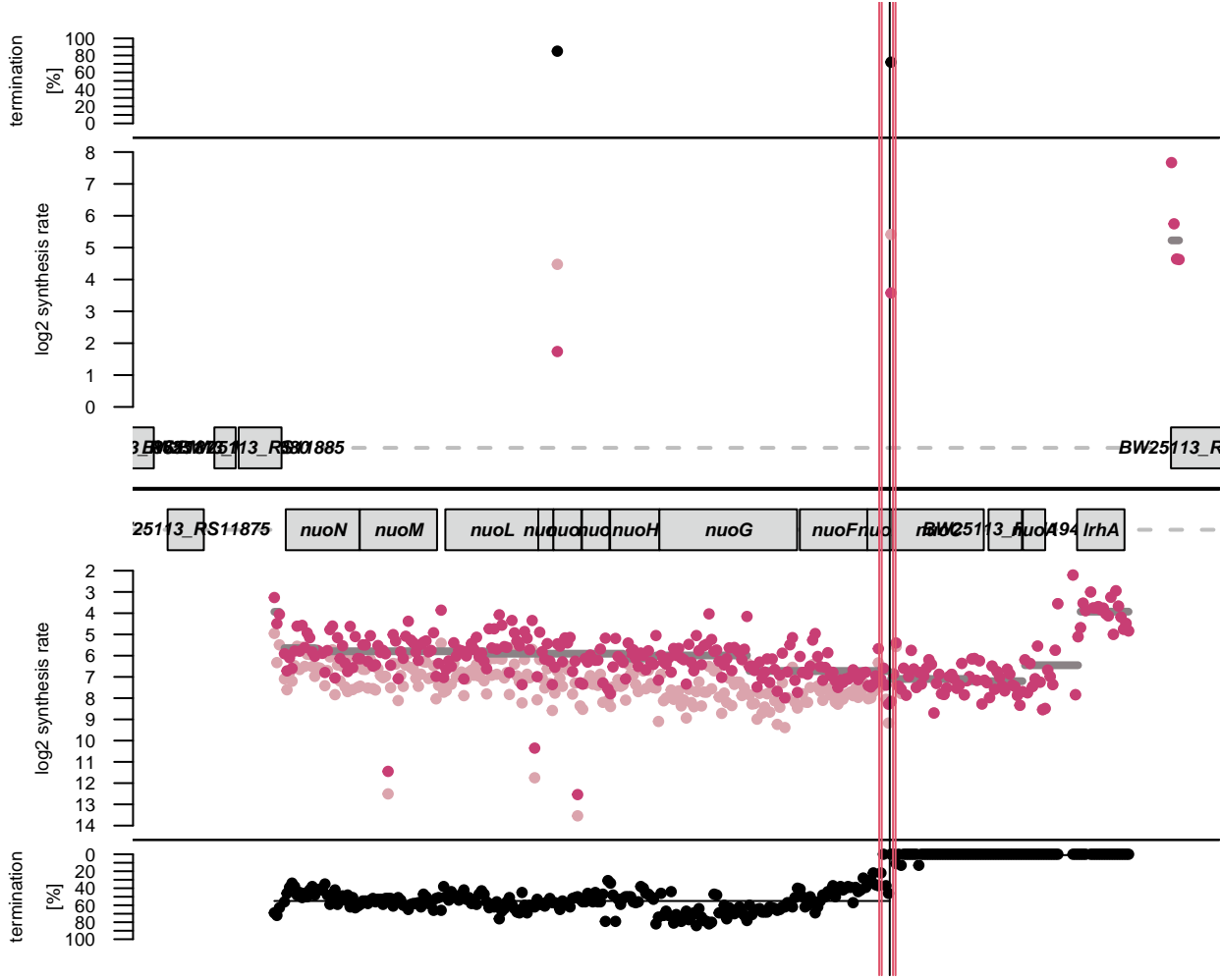

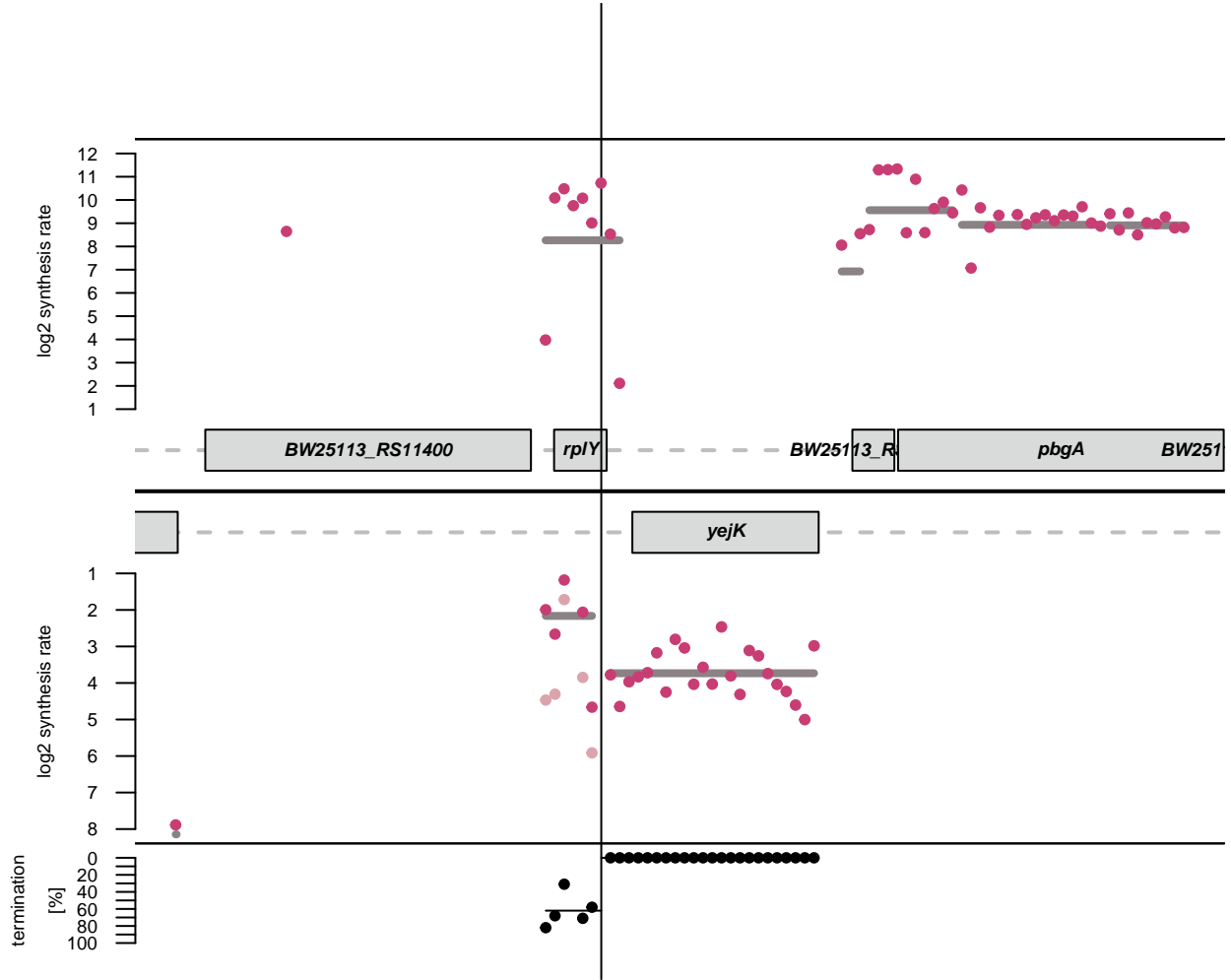

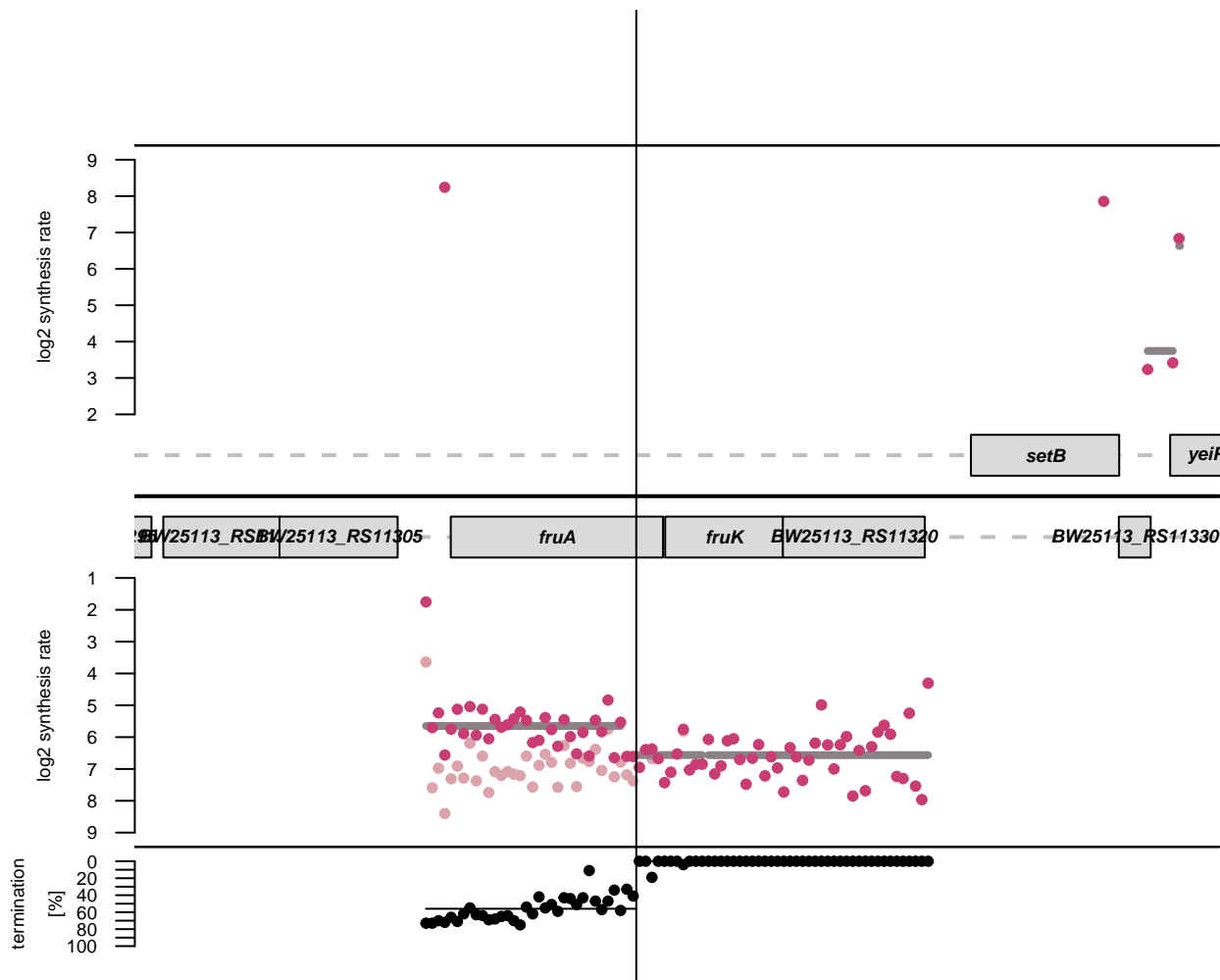

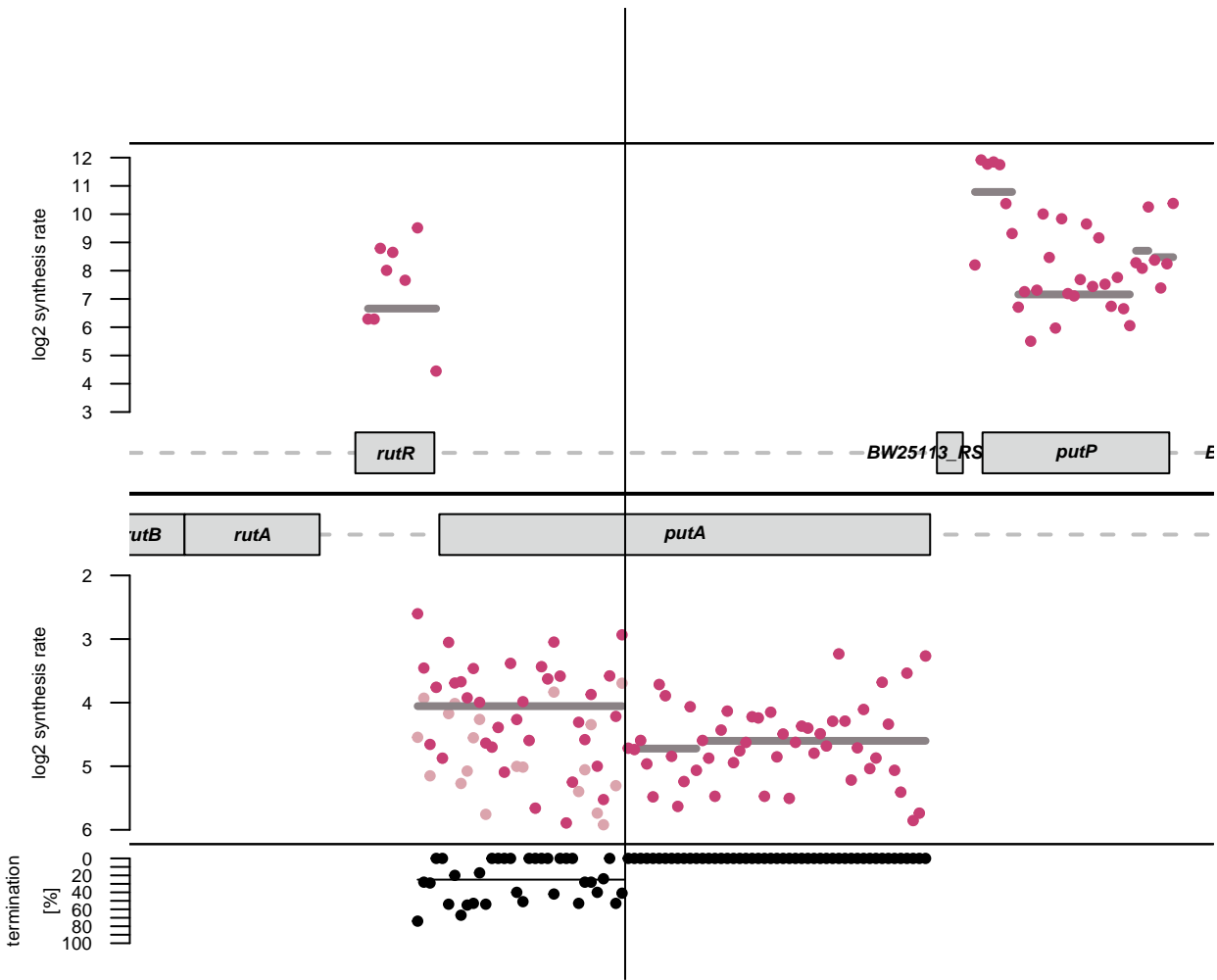

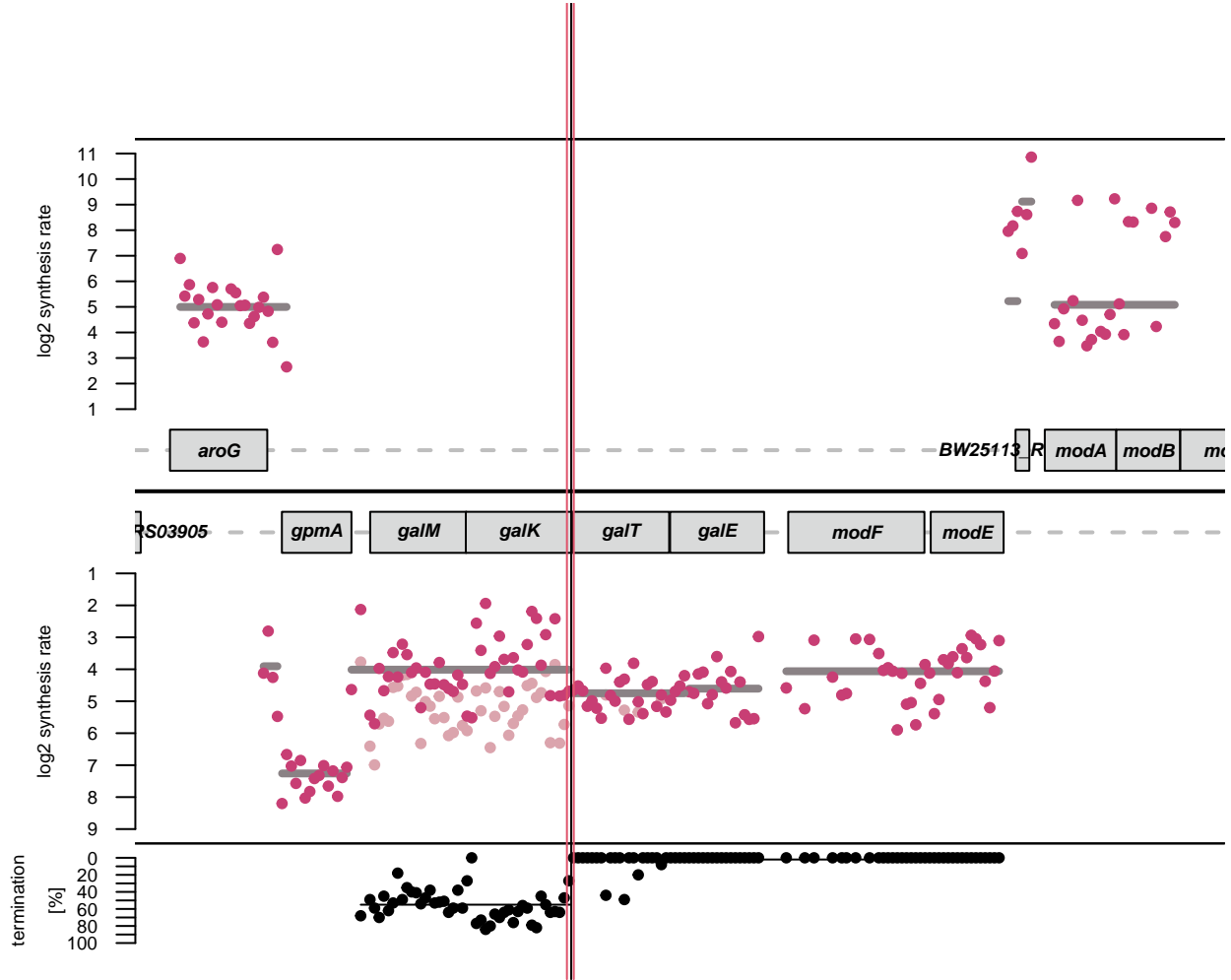

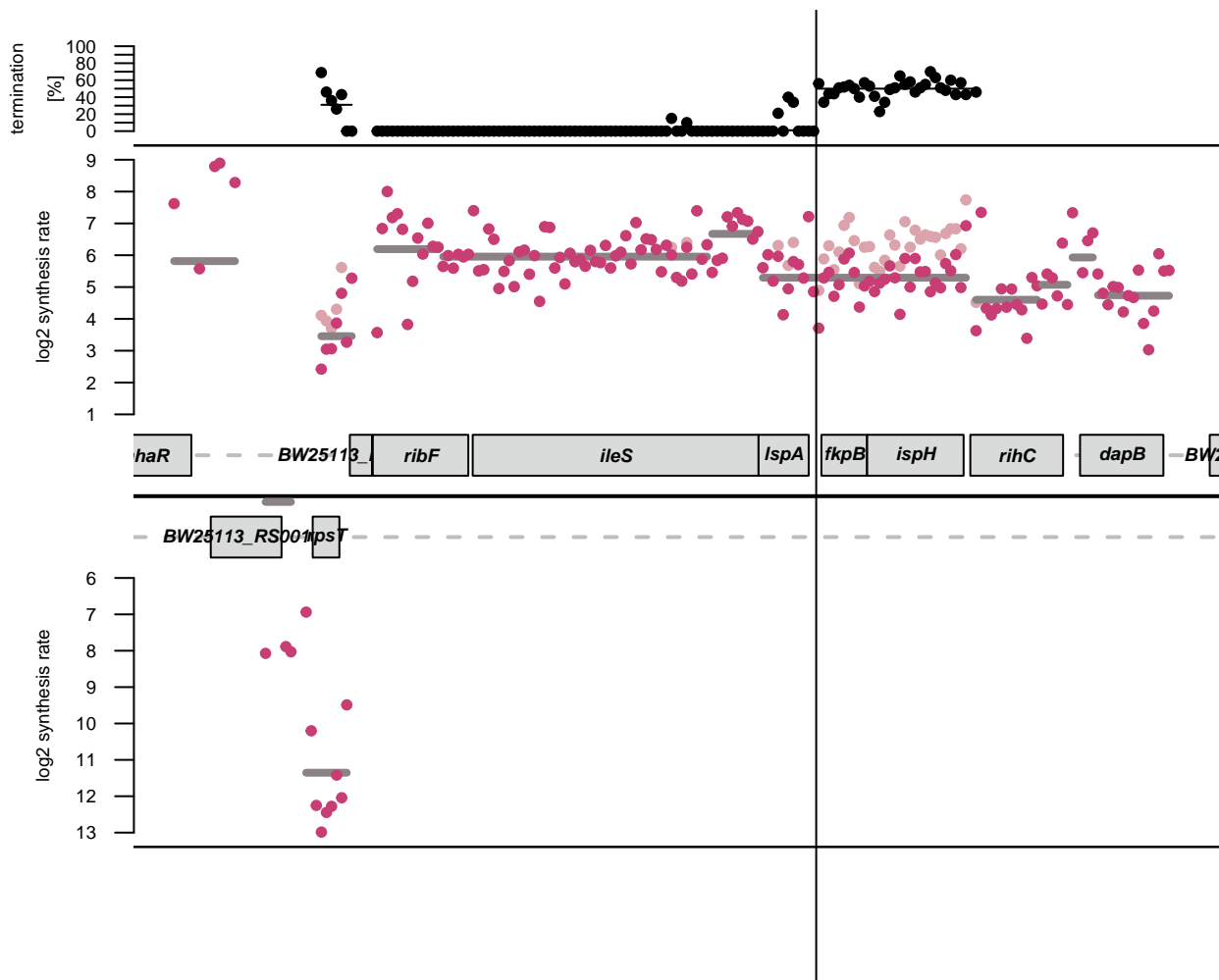

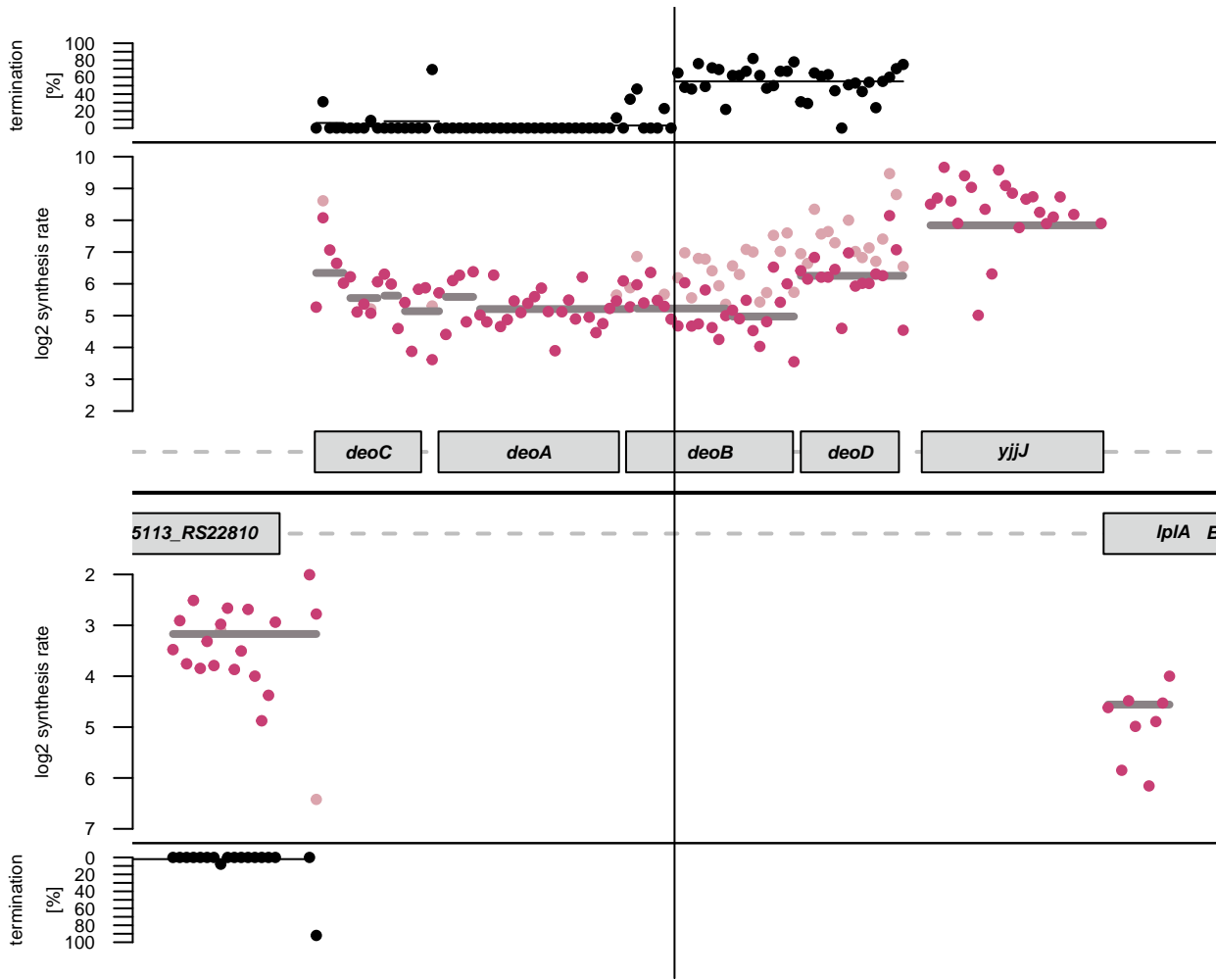

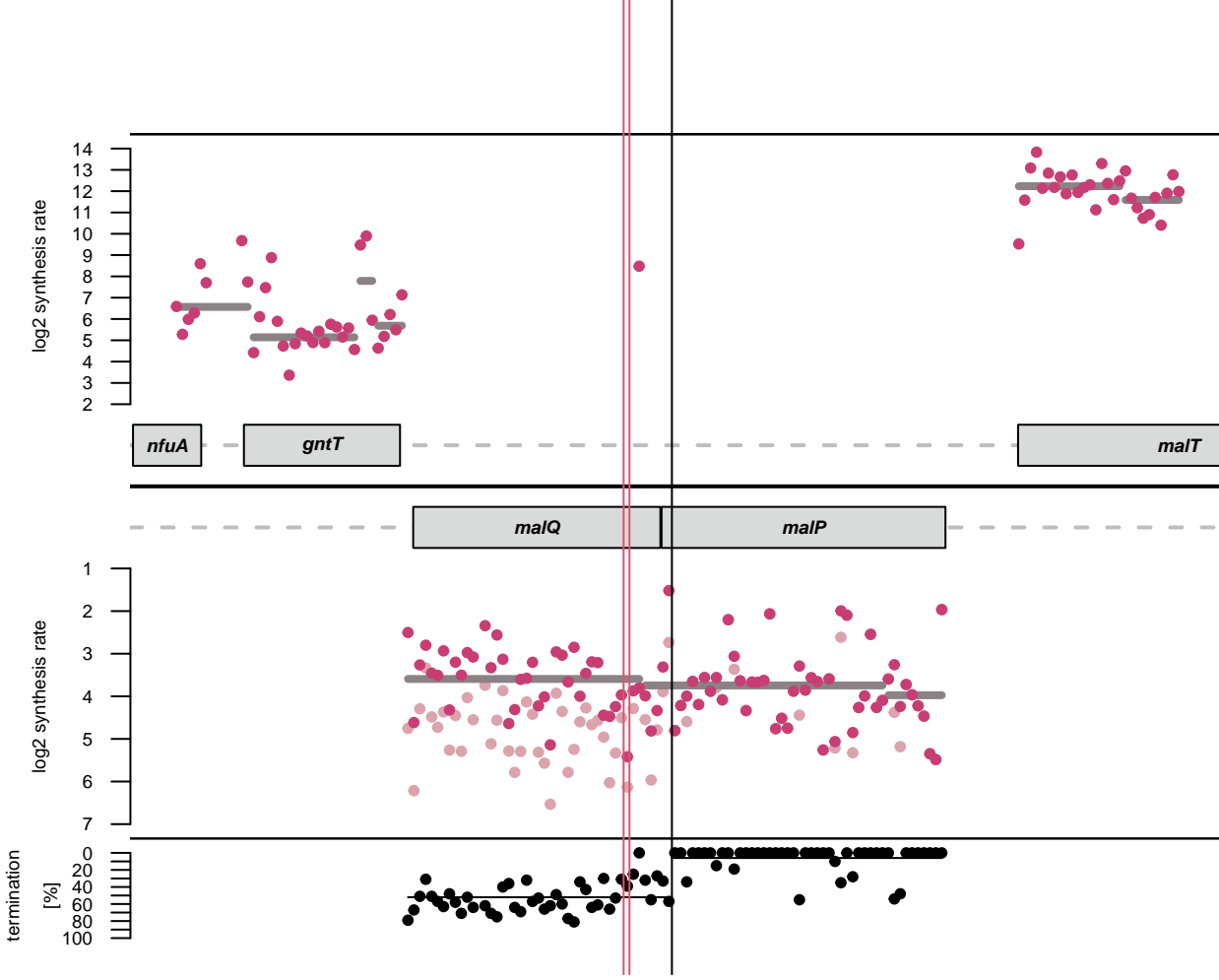

Supplement: Supplementary file 5 — Supplementary Data 2 [file 42003_2023_5097_MOESM5_ESM.pdf]
