# Supplementary material for: Improved RNA stability estimation indicates that transcriptional interference is frequent in diverse bacteria
Source: Commun Biol. 2023 Jul 15;6:732. doi: 10.1038/s42003-023-05097-2 (PMC10349824; doi:10.1038/s42003-023-05097-2)
Supplement: Supplementary file 6 — Supplementary Data 3 [file 42003_2023_5097_MOESM6_ESM.zip › E_coli_BW25113.pdf]

ID: 2−200; FC*: significant t−test of two consecutive segments;
Term: termination (4), NS: new start (2), PS: pausing site (2), iTSS_I: internal starting site (0), (*): p_value below 0.05; TI: transcription interferance.

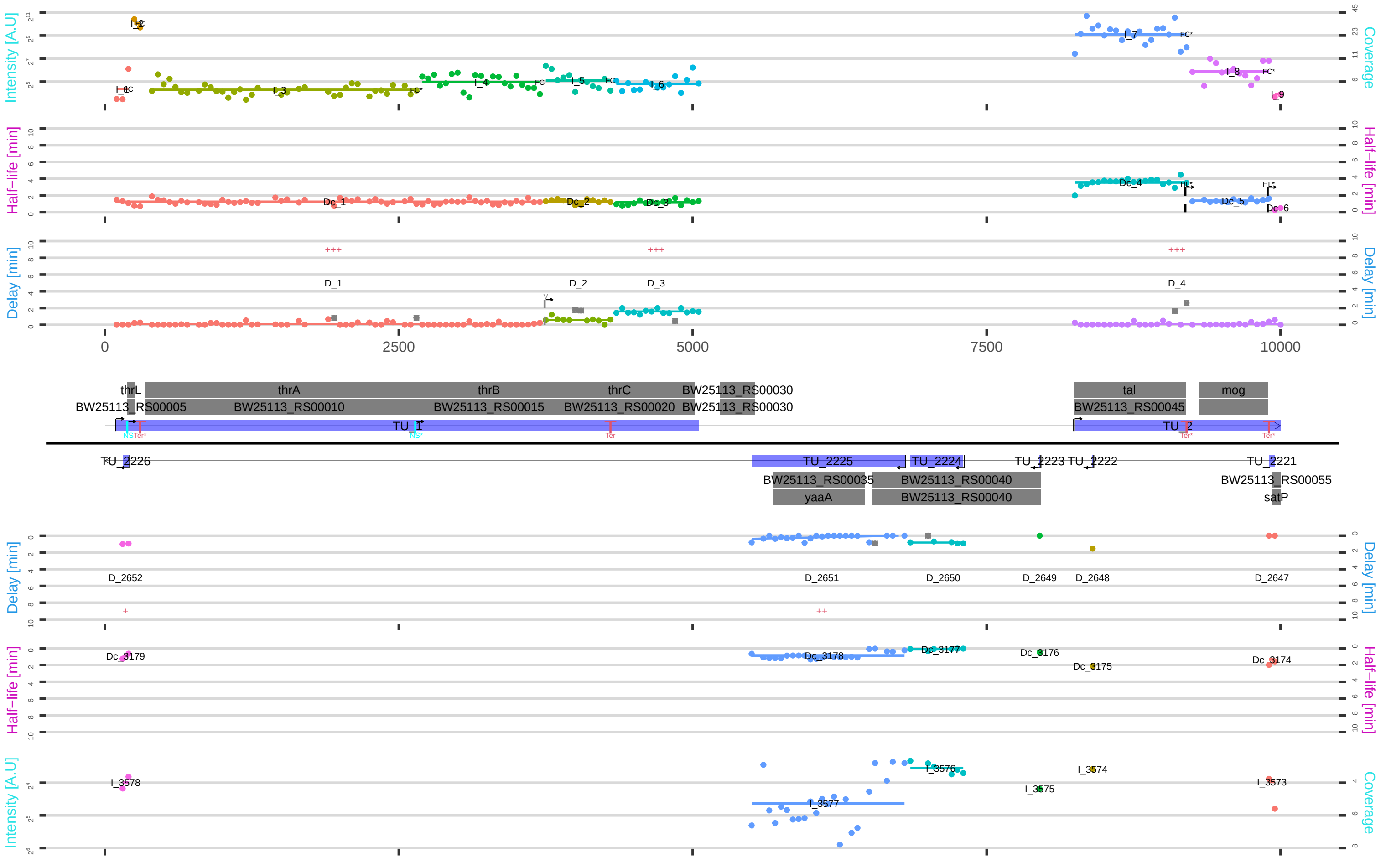

Term: termination (0), NS: new start (0), PS: pausing site (0), iTSS_I: internal starting site (0)

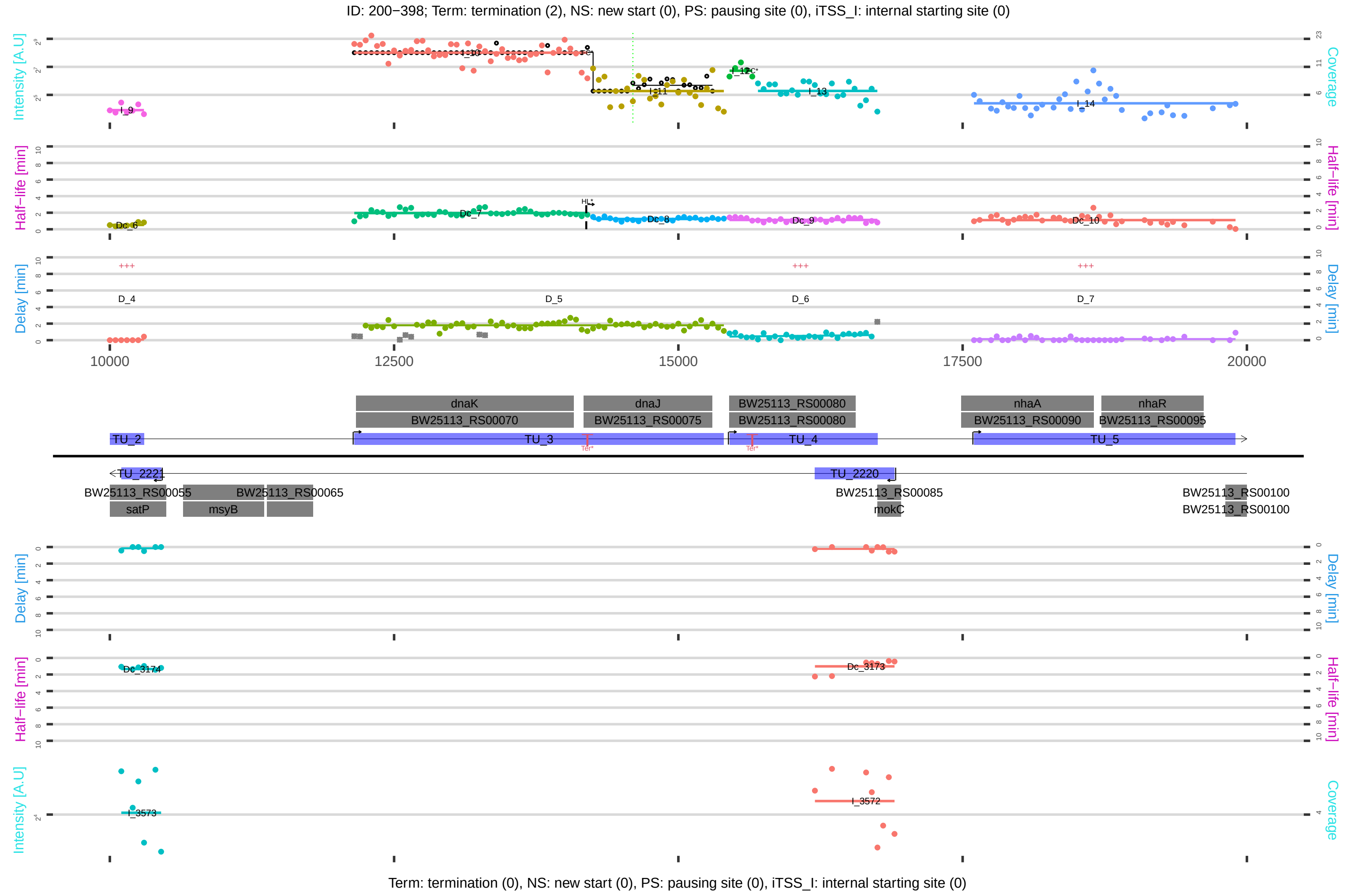
ID: 200−398; Term: termination (2), NS: new start (0), PS: pausing site (0), iTSS_I: internal starting site (0)
Intensity [A.U]
Coverage
Half−life [min]
Delay [min]
I_9
I_10
I_11
I_12
I_13
I_14
Dc_6
Dc_7
Dc_8
Dc_9
Dc_10
D_4
D_5
D_6
D_7
10000
12500
15000
17500
20000
dnaK
BW25113_RS00070
dnaJ
BW25113_RS00075
BW25113_RS00080
nhaA
BW25113_RS00090
nhaR
BW25113_RS00095
TU_2
TU_3
TU_4
TU_5
TU_2221
TU_2220
BW25113_RS00055
satP
msyB
BW25113_RS00065
BW25113_RS00085
mokC
BW25113_RS00100
Dc_3174
Dc_3173
I_3573
I_3572
Term: termination (0), NS: new start (0), PS: pausing site (0), iTSS_I: internal starting site (0)

ID: 401−600; Term: termination (2), NS: new start (1), PS: pausing site (1), iTSS_I: internal starting site (0)

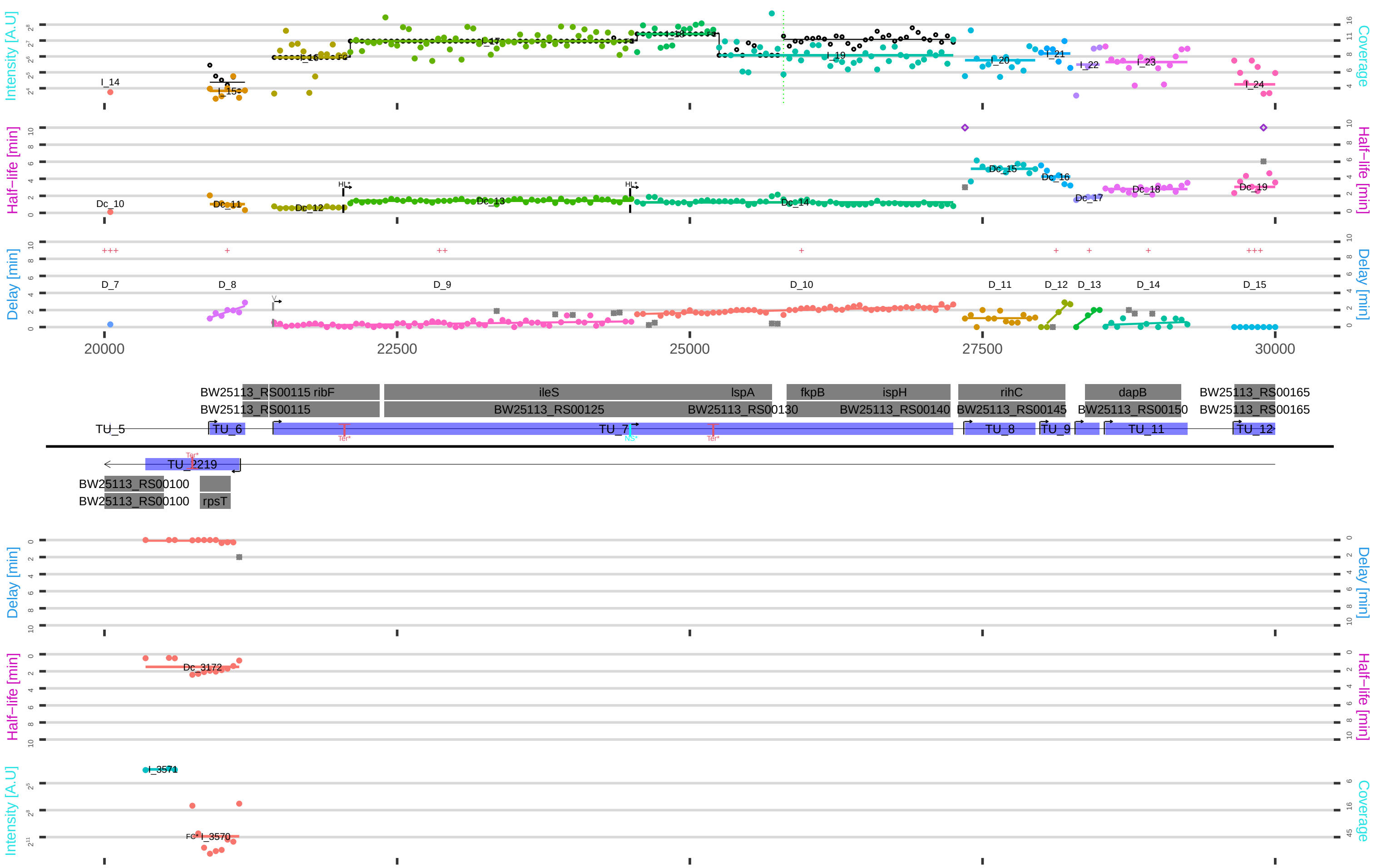

Term: termination (1), NS: new start (0), PS: pausing site (0), iTSS_I: internal starting site (0)

ID: 600−696; Term: termination (0), NS: new start (0), PS: pausing site (0), iTSS_I: internal starting site (0)

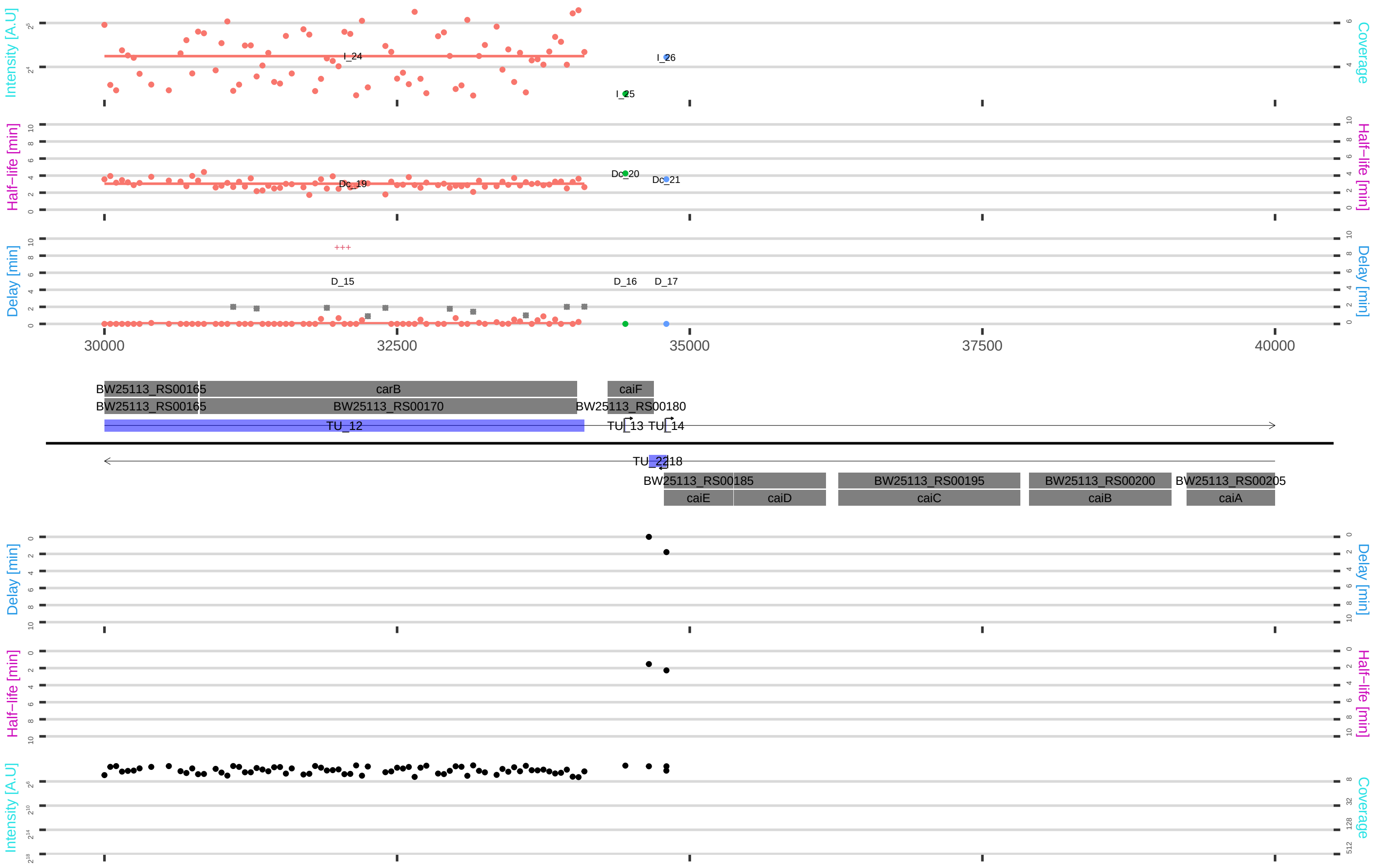

Term: termination (0), NS: new start (0), PS: pausing site (0), iTSS_I: internal starting site (0)

ID: 947−1000; Term: termination (1), NS: new start (1), PS: pausing site (0), iTSS_I: internal starting site (0)

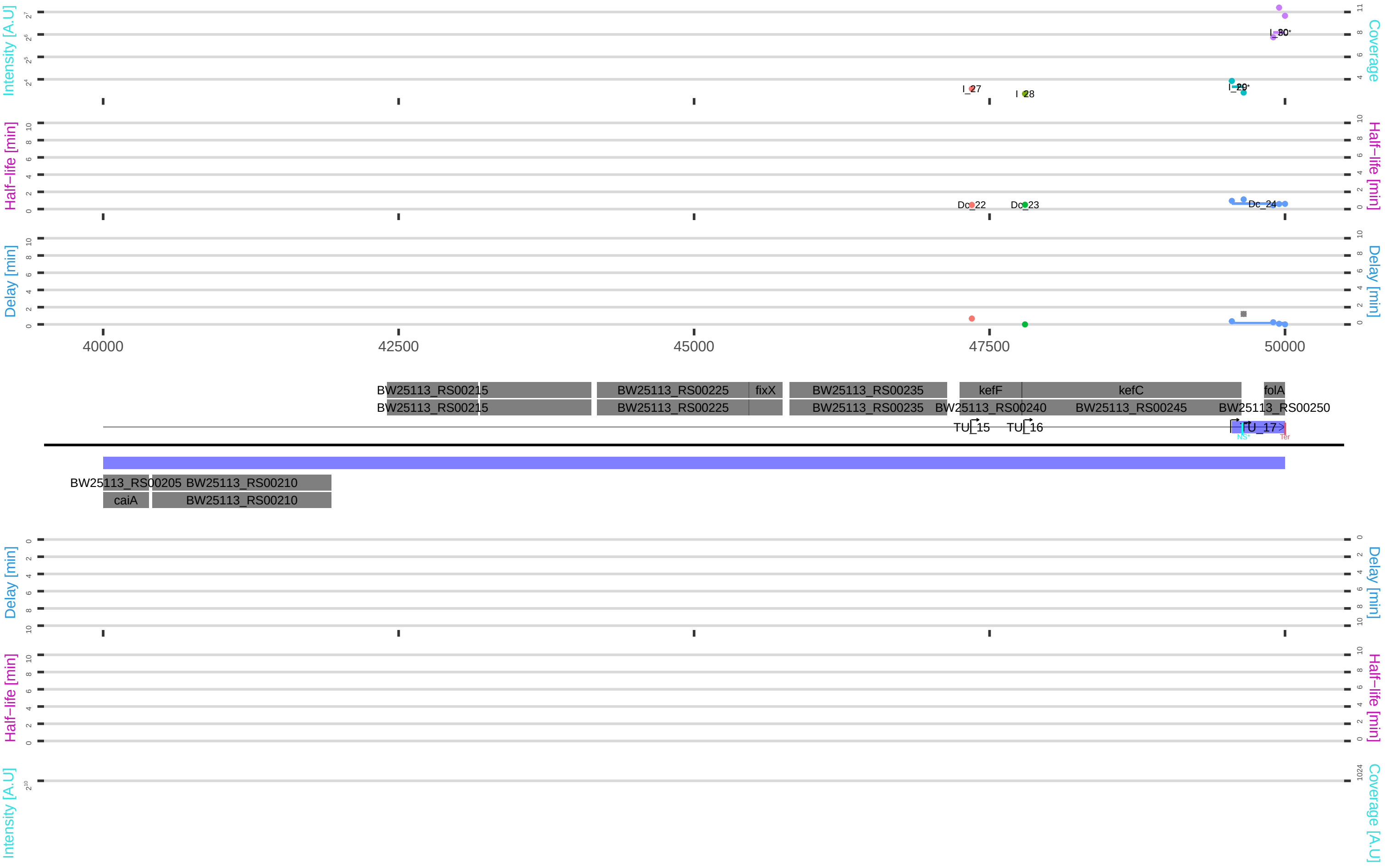

NA

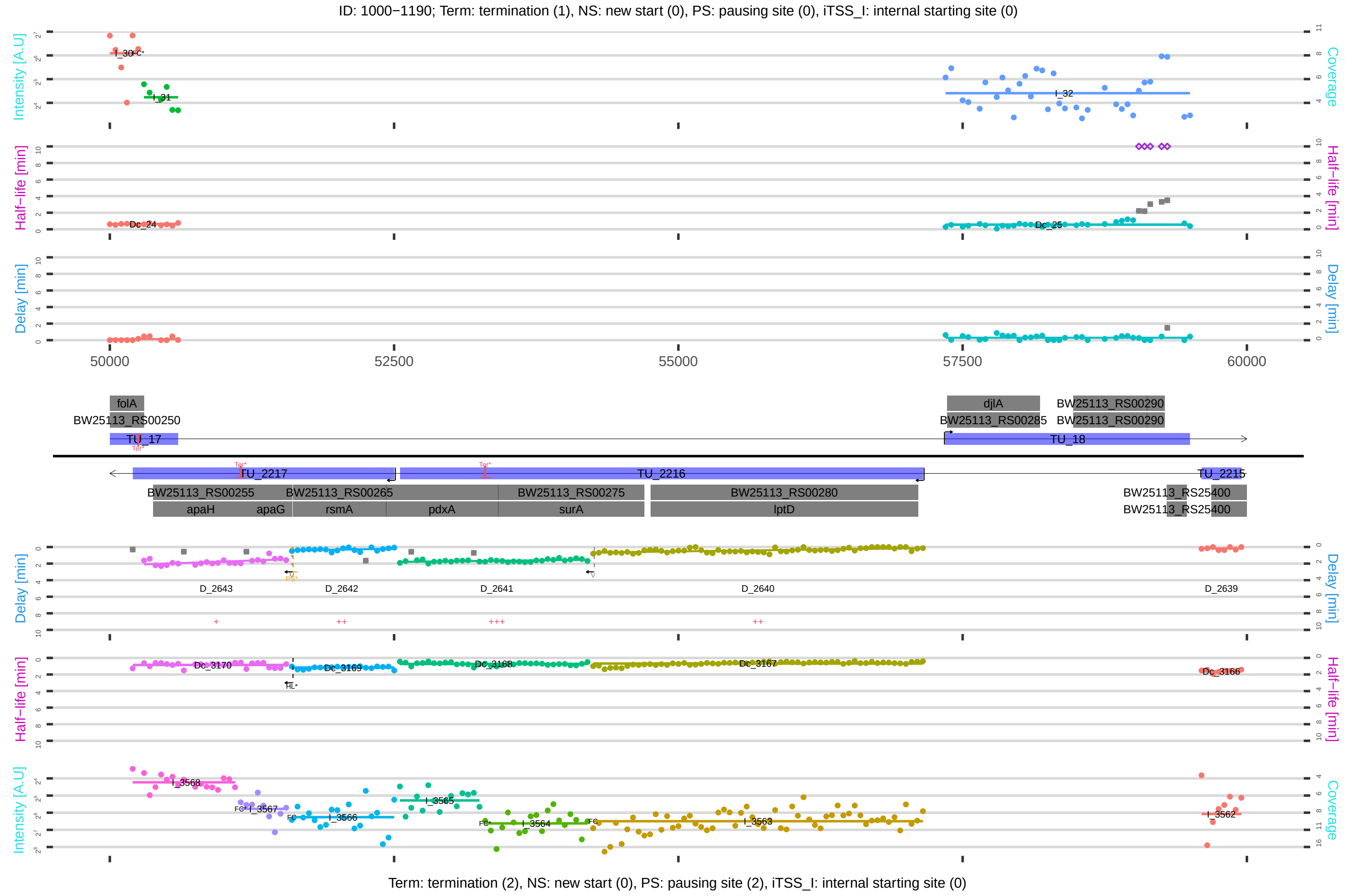

ID: 1000−1190; Term: termination (1), NS: new start (0), PS: pausing site (0), iTSS_I: internal starting site (0)
Intensity [A.U]
Coverage
Half−life [min]
Delay [min]
50000
52500
55000
57500
60000
I_30
I_31
I_32
Dc_24
Dc_25
folA
BW25113_RS00250
TU_17
djlA
BW25113_RS00285
BW25113_RS00290
TU_18
TU_2217
TU_2216
TU_2215
BW25113_RS00255
BW25113_RS00265
BW25113_RS00275
BW25113_RS00280
BW25113_RS25400
apaH
apaG
rsmA
pdxA
surA
lptD
D_2643
D_2642
D_2641
D_2640
D_2639
Dc_3170
Dc_3169
Dc_3168
Dc_3167
Dc_3166
I_3568
I_3567
I_3566
I_3565
I_3564
I_3563
I_3562
Term: termination (2), NS: new start (0), PS: pausing site (2), iTSS_I: internal starting site (0)

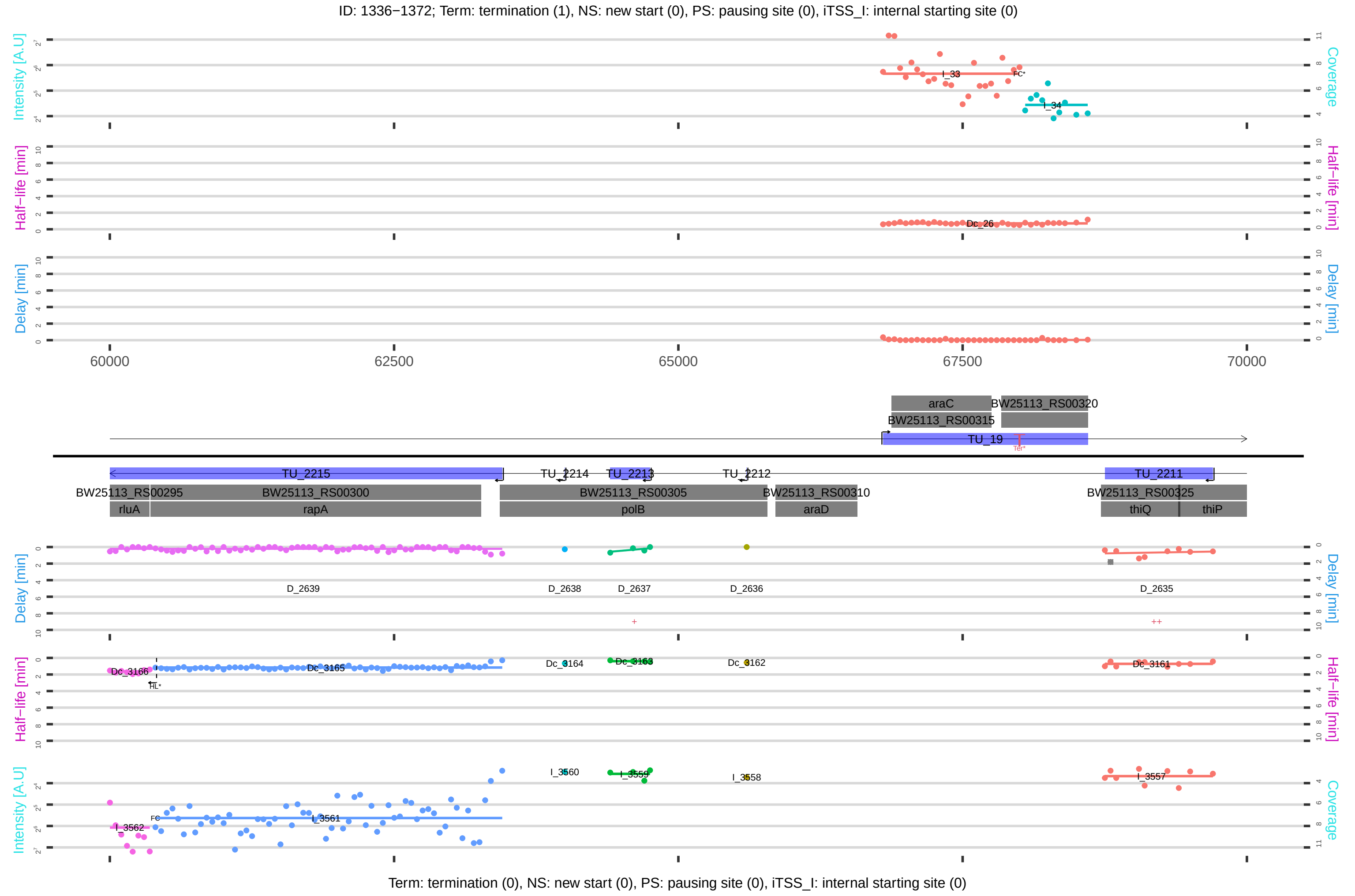
ID: 1336−1372; Term: termination (1), NS: new start (0), PS: pausing site (0), iTSS_I: internal starting site (0)
Intensity [A.U]
Coverage
I_33
FC*
I_34
Half−life [min]
Dc_26
Delay [min]
60000
62500
65000
67500
70000
araC
BW25113_RS00320
BW25113_RS00315
TU_19
Ter*
TU_2215
TU_2214
TU_2213
TU_2212
TU_2211
BW25113_RS00295
BW25113_RS00300
BW25113_RS00305
BW25113_RS00310
BW25113_RS00325
rluA
rapA
polB
araD
thiQ
thiP
D_2639
D_2638
D_2637
D_2636
D_2635
Dc_3166
HL*
Dc_3165
Dc_3164
Dc_3163
Dc_3162
Dc_3161
I_3560
I_3559
I_3558
I_3557
I_3562
FC
I_3561
Term: termination (0), NS: new start (0), PS: pausing site (0), iTSS_I: internal starting site (0)

ID: 1480−1503; Term: termination (0), NS: new start (1), PS: pausing site (0), iTSS_I: internal starting site (0)

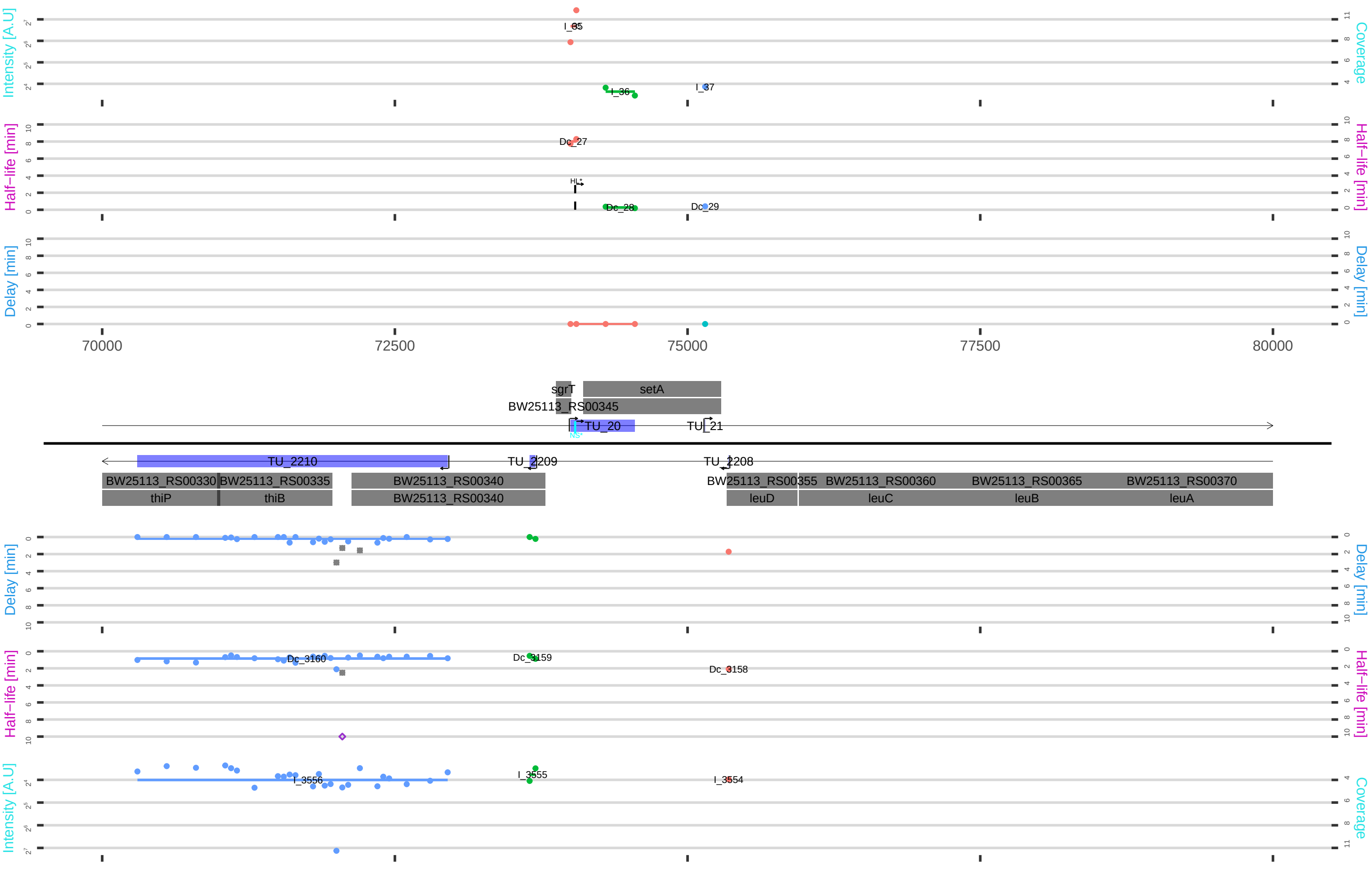

Term: termination (0), NS: new start (0), PS: pausing site (0), iTSS_I: internal starting site (0)

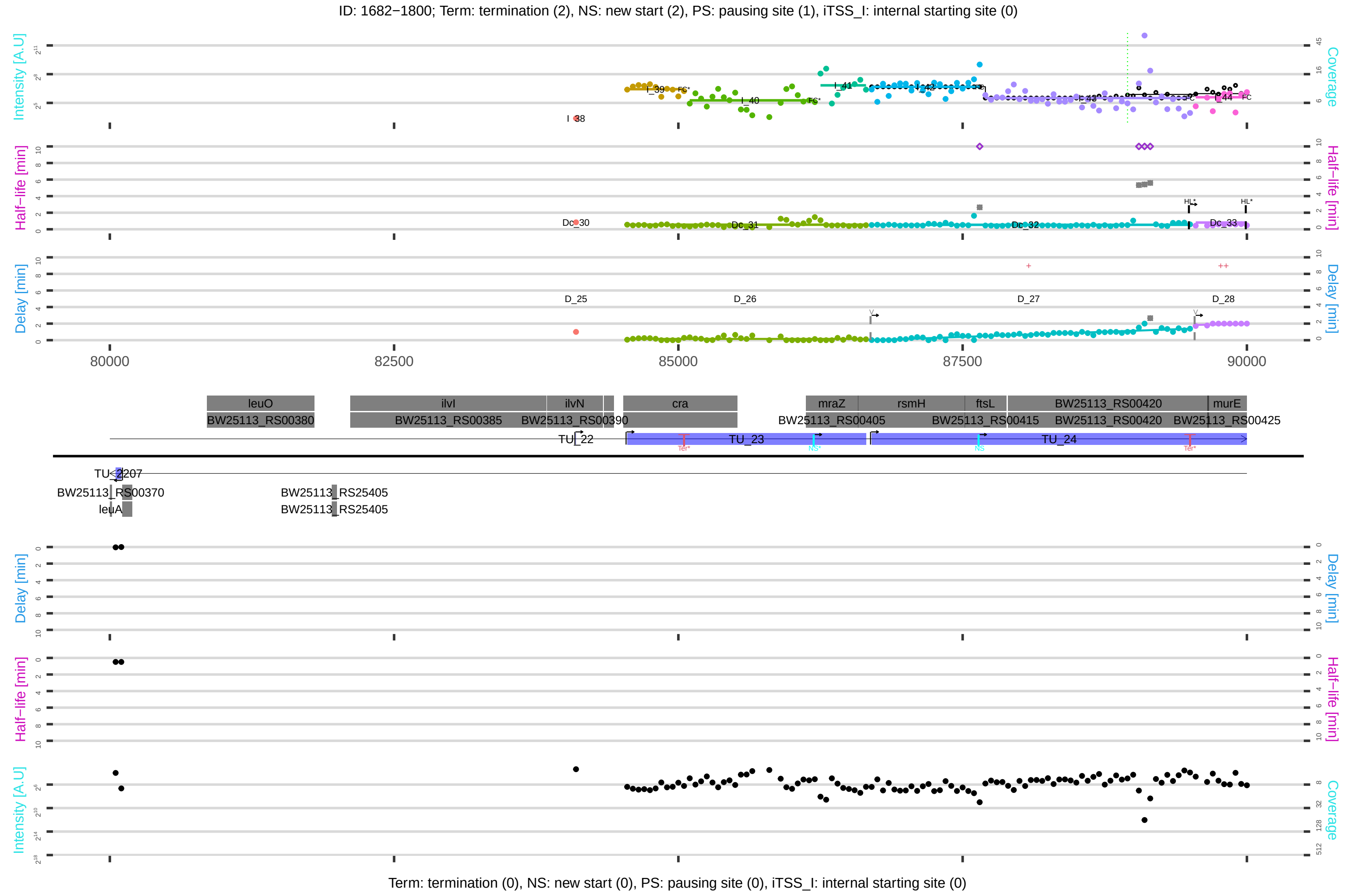
ID: 1682−1800; Term: termination (2), NS: new start (2), PS: pausing site (1), iTSS_I: internal starting site (0)
Intensity [A.U]
Coverage
Half−life [min]
Delay [min]
80000
82500
85000
87500
90000
leuO
ilvI
ilvN
cra
mraZ
rsmH
ftsL
BW25113_RS00420
murE
BW25113_RS00380
BW25113_RS00385
BW25113_RS00390
BW25113_RS00405
BW25113_RS00415
BW25113_RS00425
TU_22
TU_23
TU_24
TU_207
BW25113_RS00370
leuA
BW25113_RS25405
Term: termination (0), NS: new start (0), PS: pausing site (0), iTSS_I: internal starting site (0)

ID: 1800−2000; Term: termination (2), NS: new start (1), PS: pausing site (3), iTSS_I: internal starting site (0)

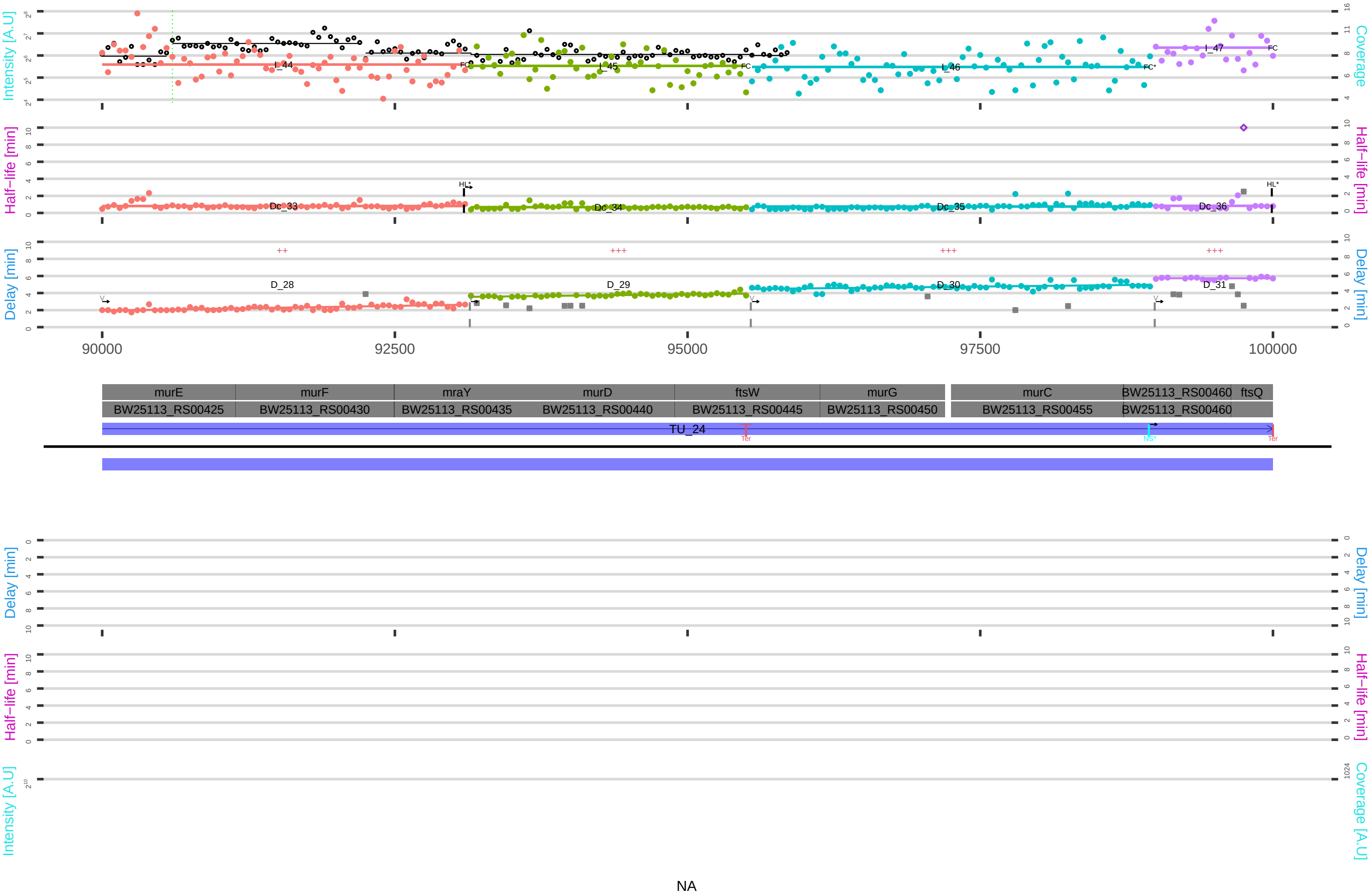

ID: 2000−2200; Term: termination (3), NS: new start (3), PS: pausing site (2), iTSS_I: internal starting site (0)

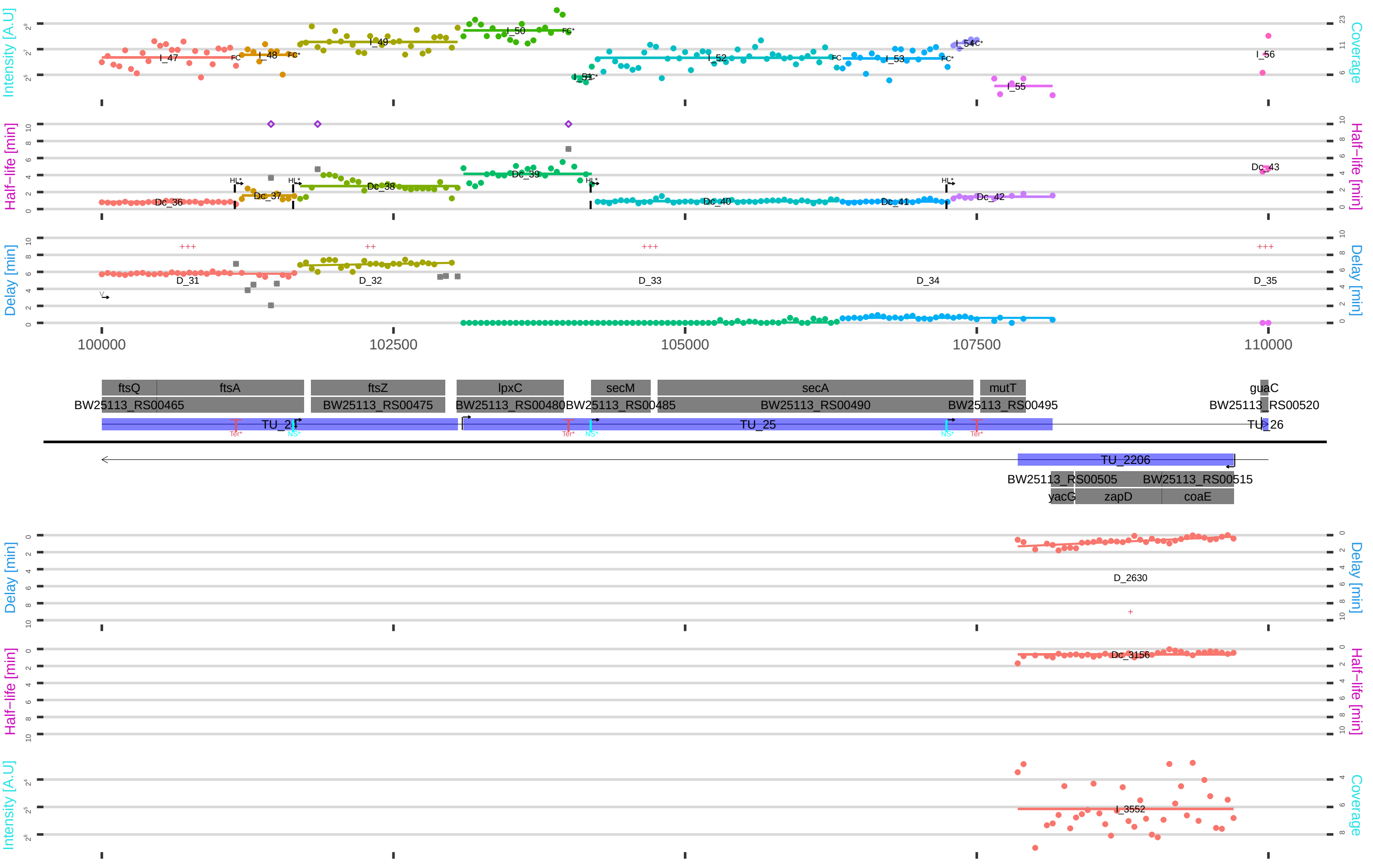

Term: termination (0), NS: new start (0), PS: pausing site (0), iTSS_I: internal starting site (0)

ID: 2200−2400; Term: termination (2), NS: new start (1), PS: pausing site (0), iTSS_I: internal starting site (0)

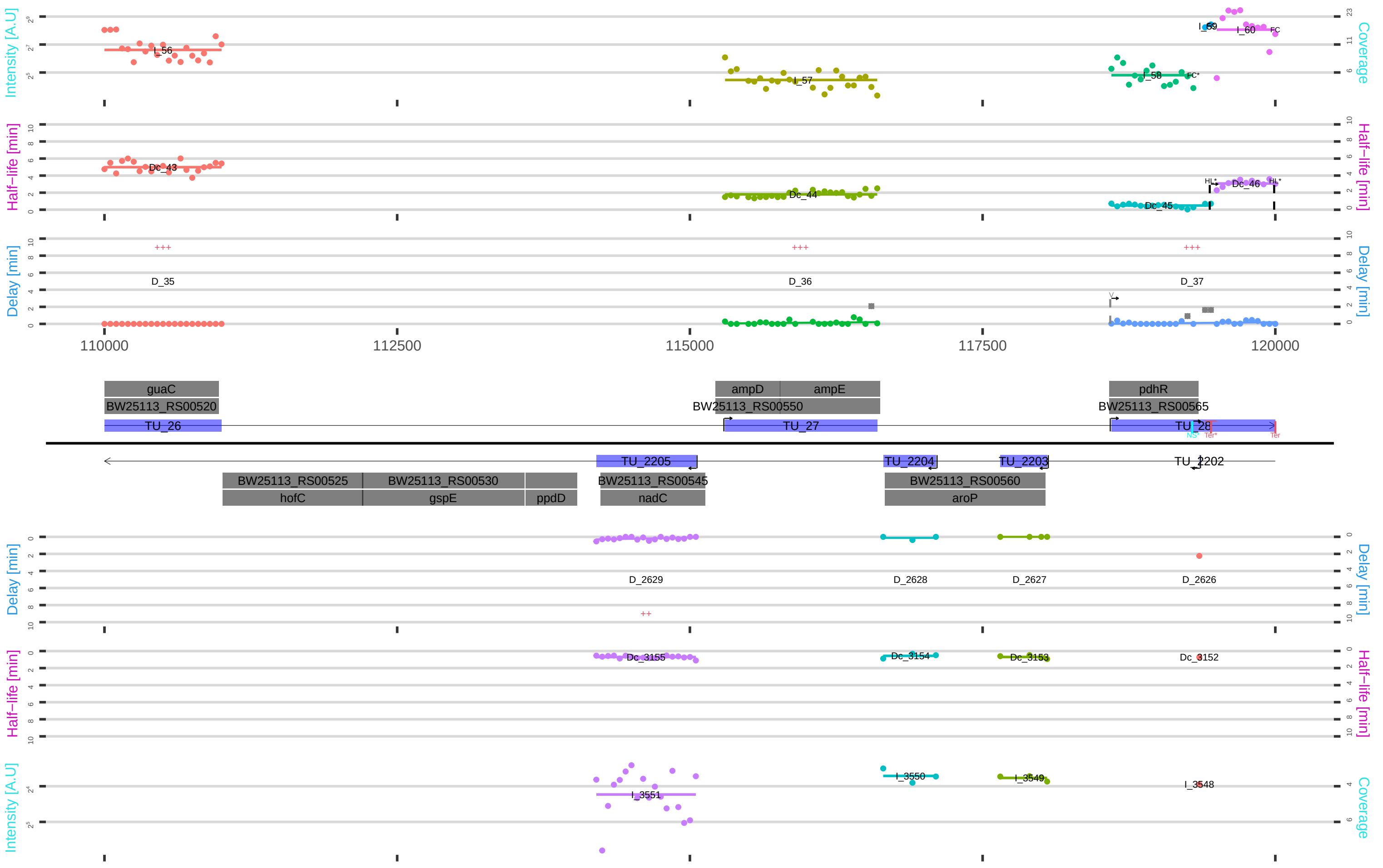

Term: termination (0), NS: new start (0), PS: pausing site (0), iTSS_I: internal starting site (0)

ID: 2400−2600; Term: termination (3), NS: new start (2), PS: pausing site (2), iTSS_I: internal starting site (0)

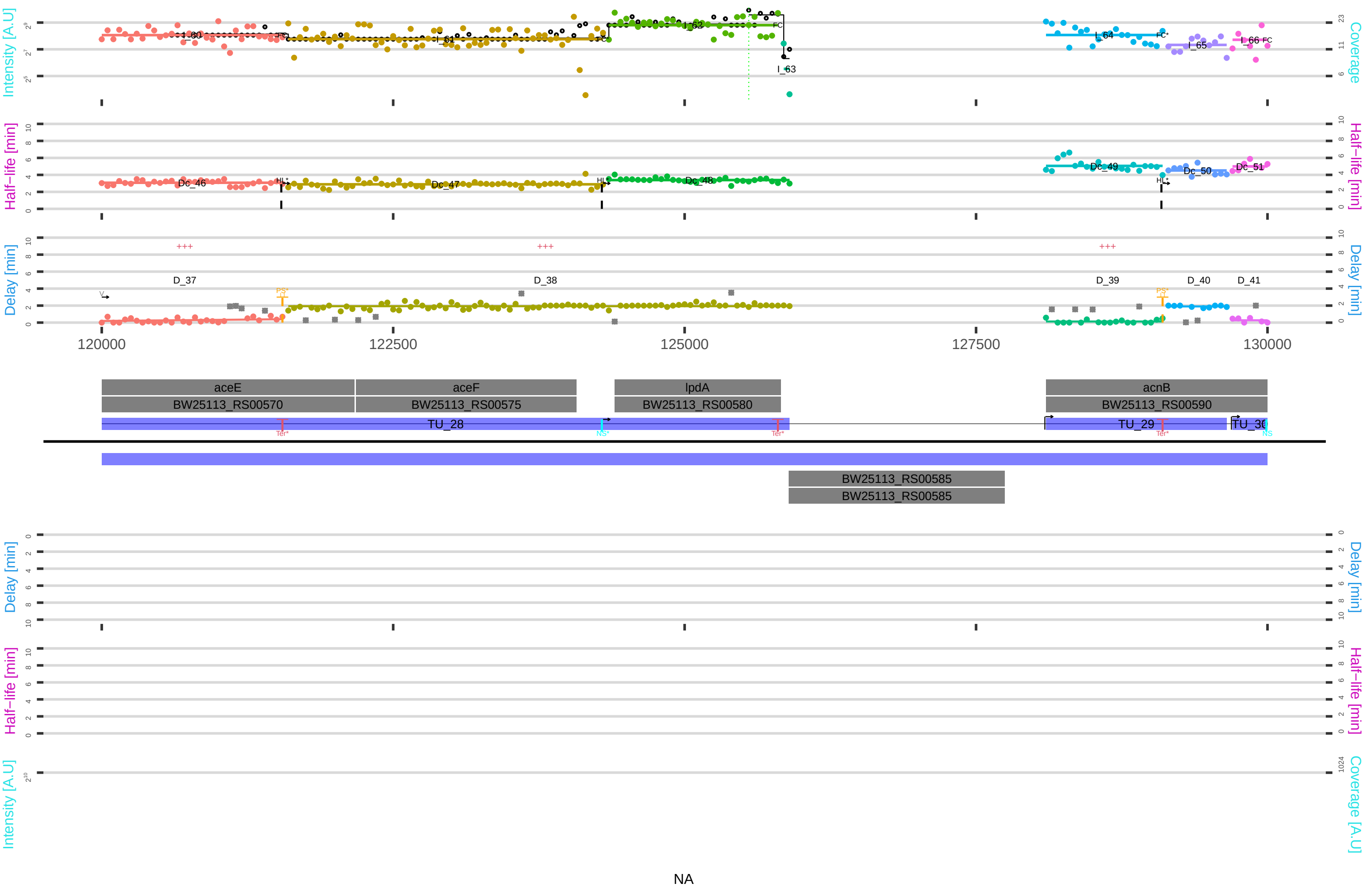

ID: 2600−2800; Term: termination (2), NS: new start (2), PS: pausing site (1), iTSS_I: internal starting site (0)

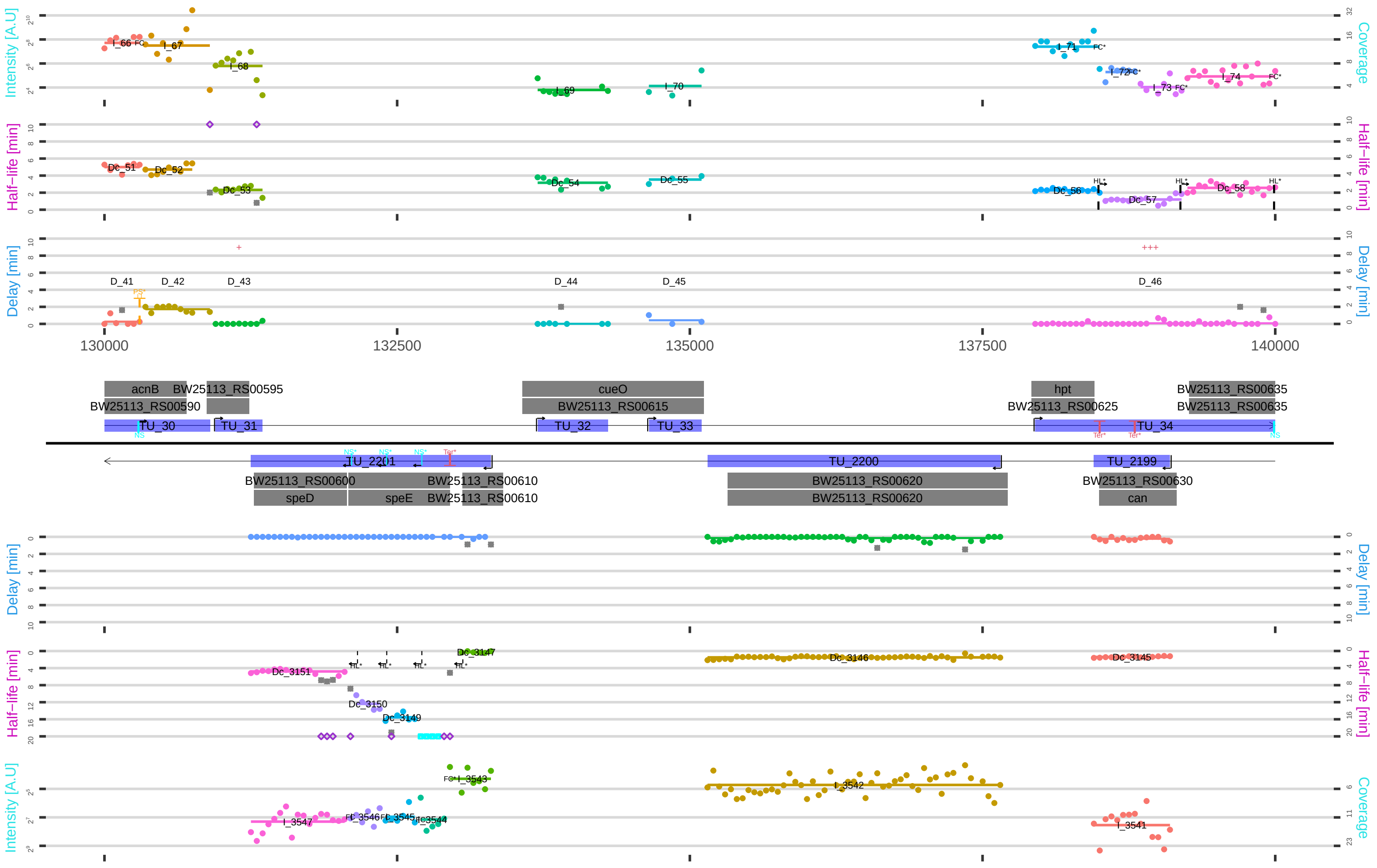

Term: termination (1), NS: new start (3), PS: pausing site (0), iTSS_I: internal starting site (0)

ID: 2800−2888; Term: termination (0), NS: new start (1), PS: pausing site (0), iTSS_I: internal starting site (0)

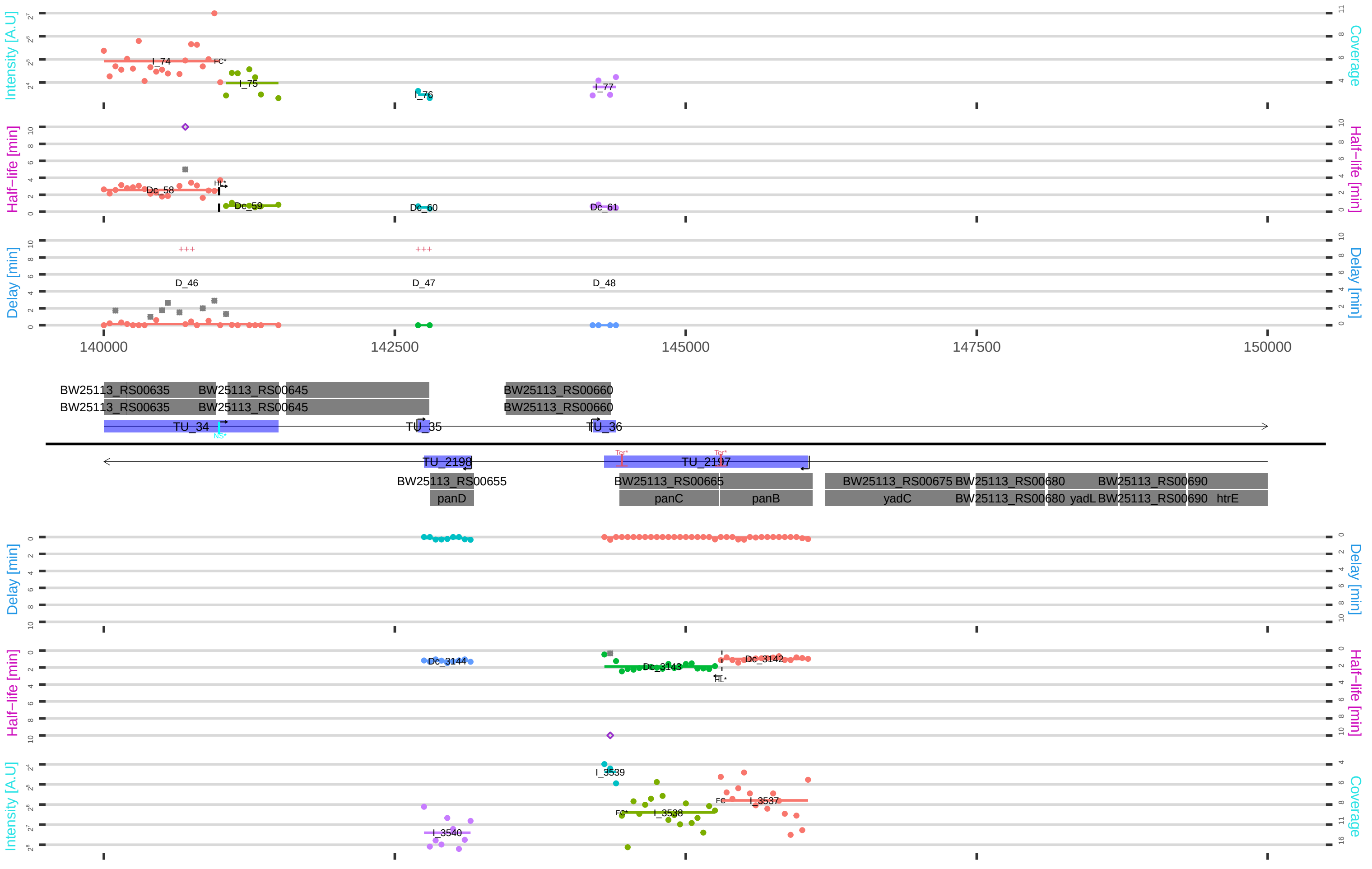

Term: termination (2), NS: new start (0), PS: pausing site (0), iTSS_I: internal starting site (0)

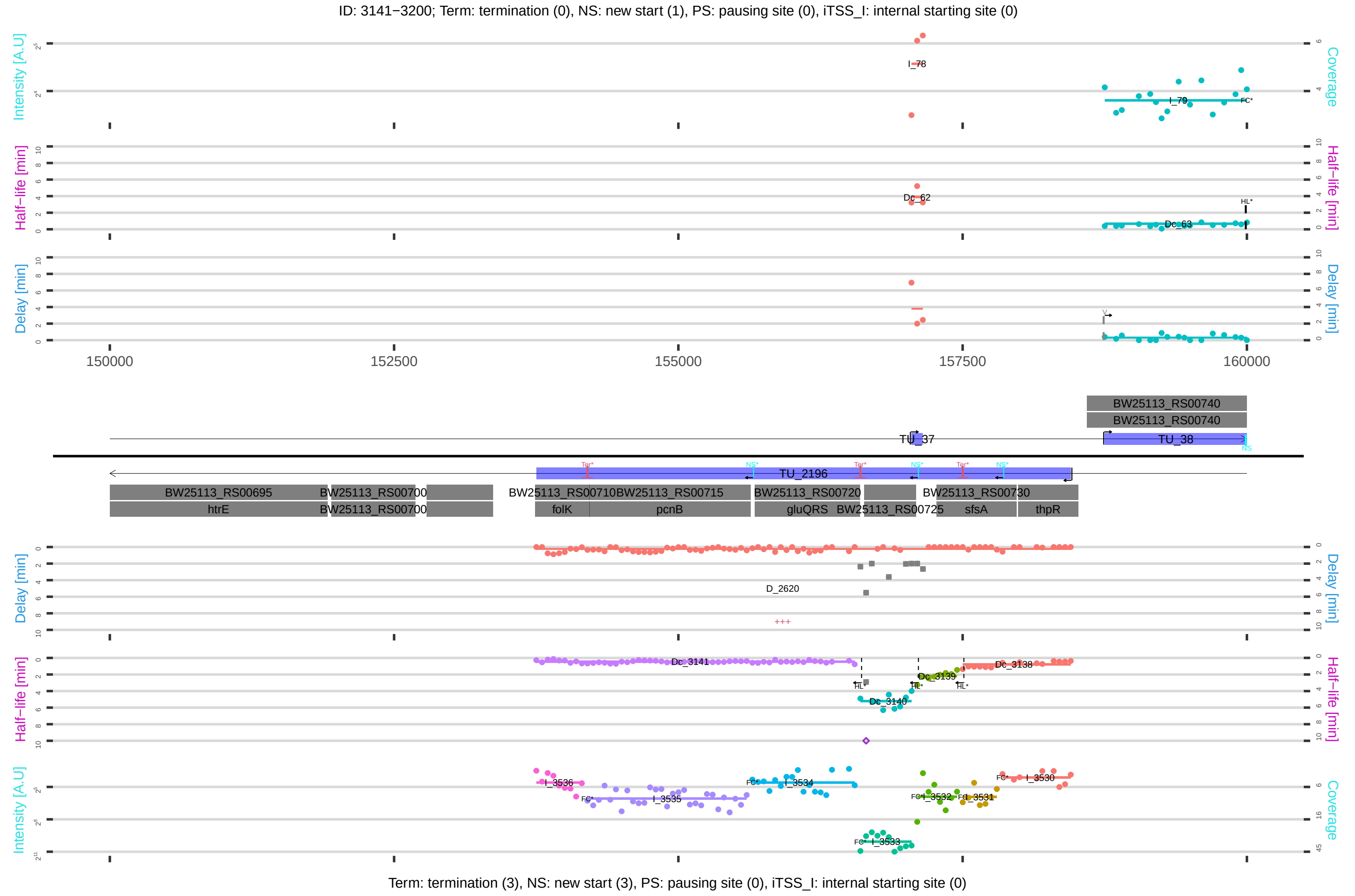

ID: 3141−3200; Term: termination (0), NS: new start (1), PS: pausing site (0), iTSS_I: internal starting site (0)
Intensity [A.U]
Coverage
Half−life [min]
Delay [min]
I_78
I_79
FC*
Dc_62
Dc_63
HL*
150000
152500
155000
157500
160000
BW25113_RS00740
BW25113_RS00740
TU_37
TU_38
NS
Ter*
NS*
TU_2196
BW25113_RS00695
BW25113_RS00700
BW25113_RS00710
BW25113_RS00715
BW25113_RS00720
BW25113_RS00730
htrE
BW25113_RS00700
folK
pcnB
gluQRS
BW25113_RS00725
sfsA
thpR
D_2620
Dc_3141
Dc_3138
Dc_3139
Dc_3140
I_3536
I_3535
I_3534
I_3533
I_3532
I_3531
I_3530
Term: termination (3), NS: new start (3), PS: pausing site (0), iTSS_I: internal starting site (0)

ID: 3200−3400; Term: termination (3), NS: new start (2), PS: pausing site (2), iTSS_I: internal starting site (0)

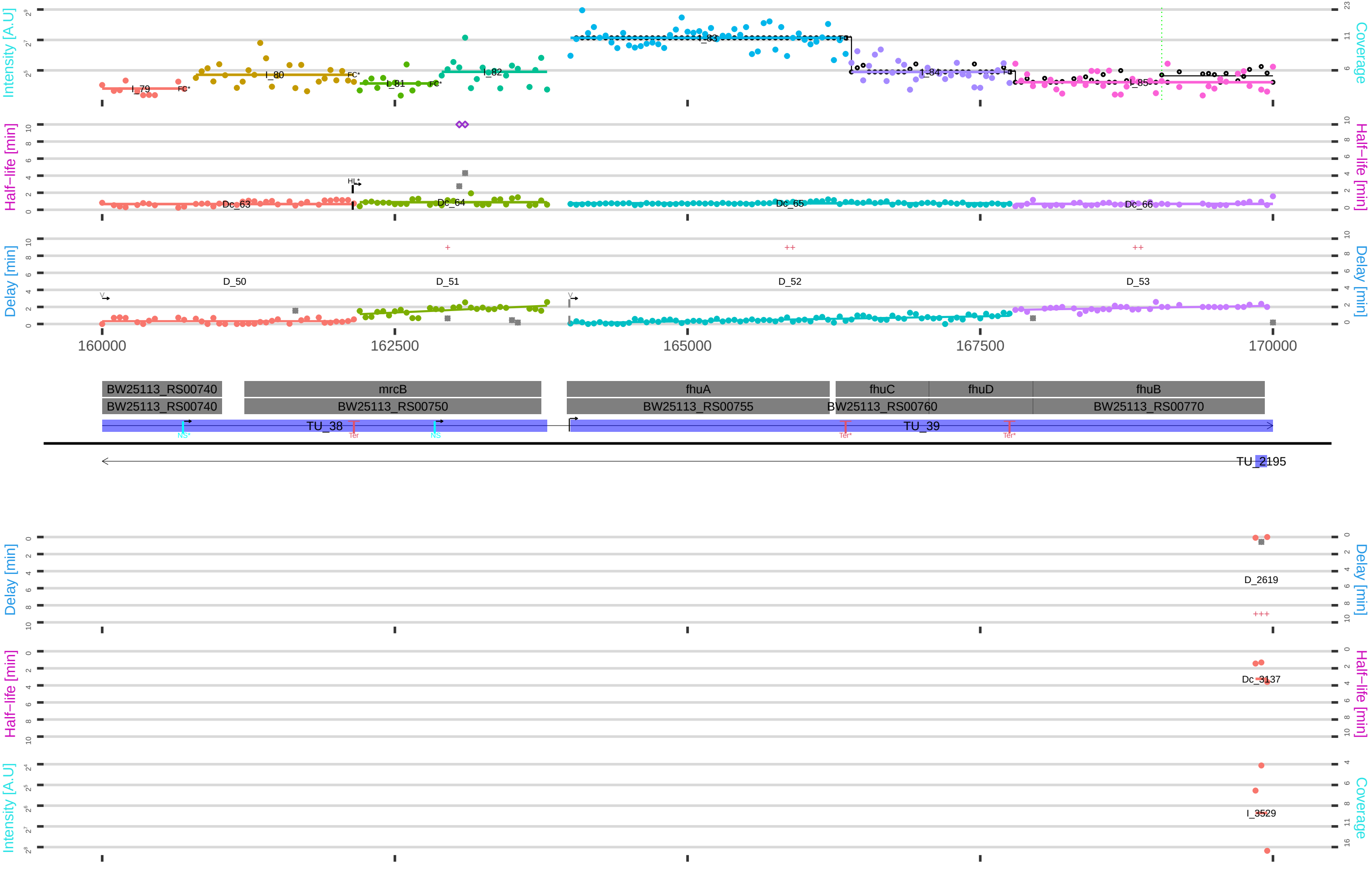

Term: termination (0), NS: new start (0), PS: pausing site (0), iTSS_I: internal starting site (0)

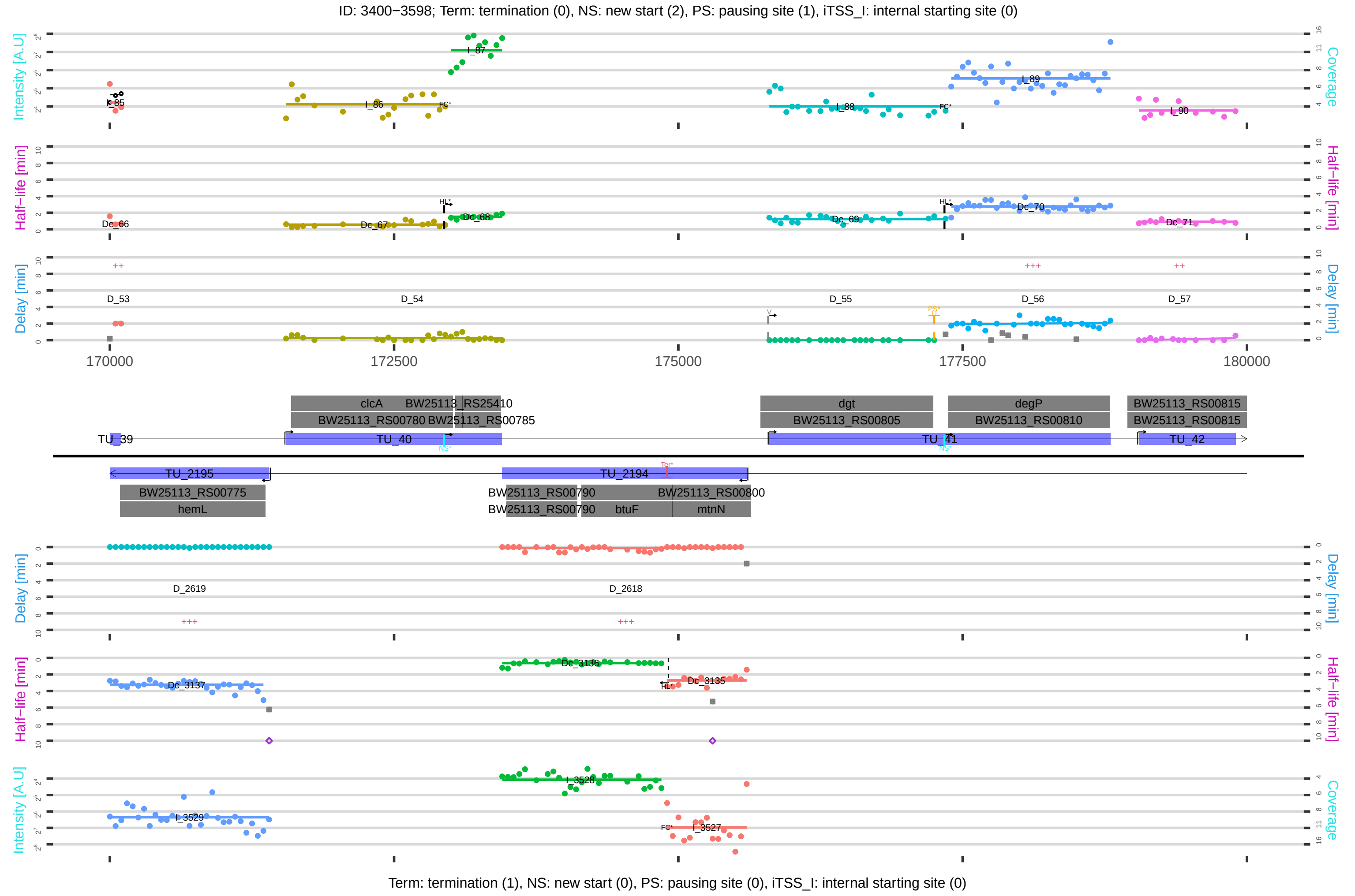

ID: 3400−3598; Term: termination (0), NS: new start (2), PS: pausing site (1), iTSS_I: internal starting site (0)
Intensity [A.U]
Coverage
Half−life [min]
Delay [min]
I_85
I_86
I_87
I_88
I_89
I_90
FC*
Dc_66
Dc_67
Dc_68
Dc_69
Dc_70
Dc_71
HL*
D_53
D_54
D_55
D_56
D_57
PS*
170000
172500
175000
177500
180000
clcA
BW25113_RS25410
BW25113_RS00780
BW25113_RS00785
dgt
BW25113_RS00805
degP
BW25113_RS00810
BW25113_RS00815
TU_39
TU_40
TU_41
TU_42
NS*
TU_2195
TU_2194
Ter*
BW25113_RS00775
hemL
BW25113_RS00790
BW25113_RS00800
btuF
mtnN
D_2619
D_2618
Dc_3137
Dc_3136
Dc_3135
I_3529
I_3528
I_3527
Term: termination (1), NS: new start (0), PS: pausing site (0), iTSS_I: internal starting site (0)

ID: 3603−3800; Term: termination (2), NS: new start (2), PS: pausing site (0), iTSS_I: internal starting site (0)

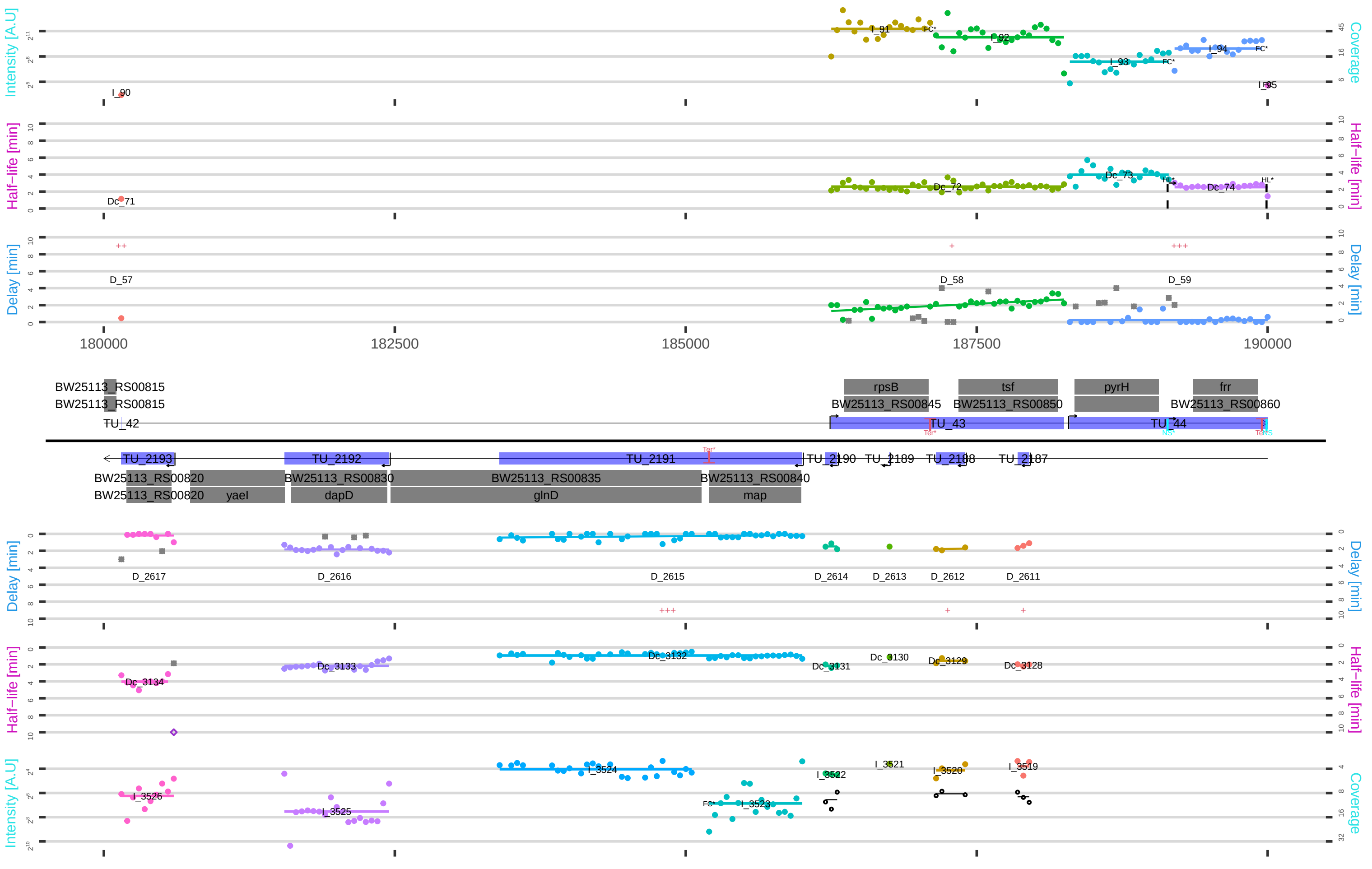

Term: termination (1), NS: new start (0), PS: pausing site (0), iTSS_I: internal starting site (0)

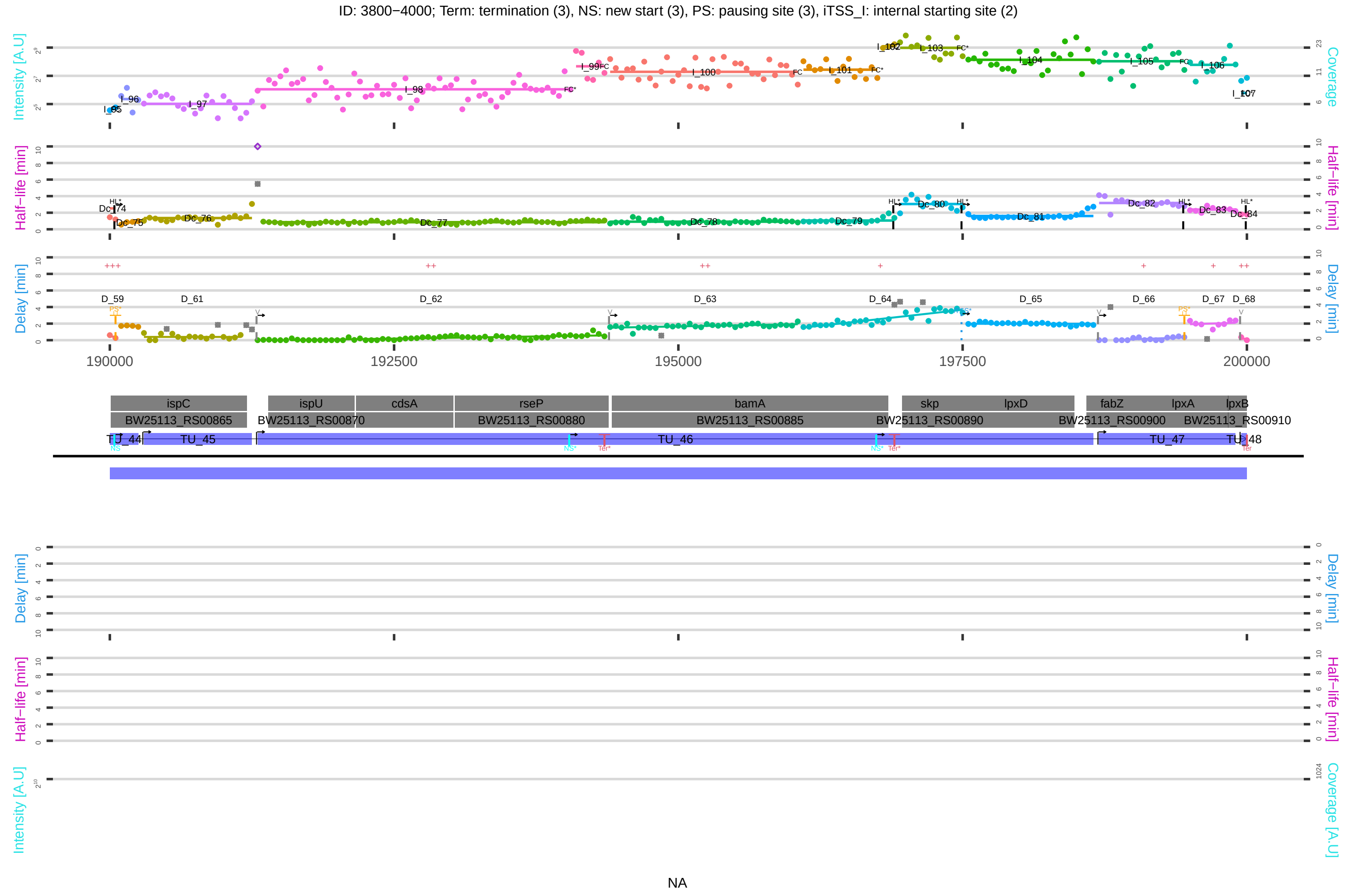
ID: 3800−4000; Term: termination (3), NS: new start (3), PS: pausing site (3), iTSS_I: internal starting site (2)
Intensity [A.U]
Coverage
Half−life [min]
Delay [min]
I_95
I_96
I_97
I_98
I_99
I_100
I_101
I_102
I_103
I_104
I_105
I_106
I_107
Dc_74
Dc_75
Dc_76
Dc_77
Dc_78
Dc_79
Dc_80
Dc_81
Dc_82
Dc_83
Dc_84
D_59
D_61
D_62
D_63
D_64
D_65
D_66
D_67
D_68
190000
192500
195000
197500
200000
ispC
ispU
cdsA
rseP
bamA
skp
lpxD
fabZ
lpxA
lpxB
BW25113_RS00865
BW25113_RS00870
BW25113_RS00880
BW25113_RS00885
BW25113_RS00890
BW25113_RS00900
BW25113_RS00910
TU_44
TU_45
TU_46
TU_47
TU_48
Delay [min]
Half−life [min]
Intensity [A.U]
Coverage [A.U]
NA

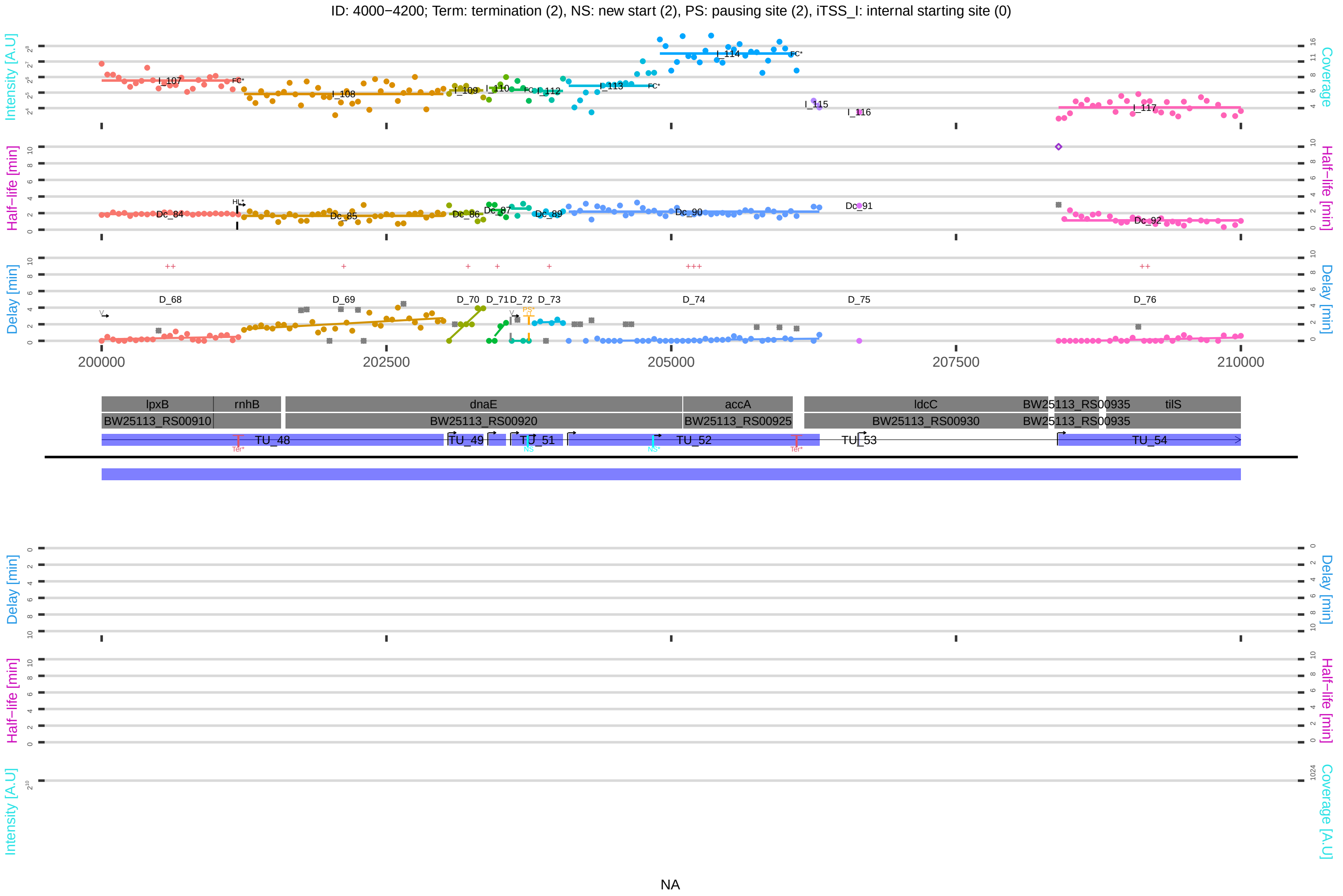
ID: 4000−4200; Term: termination (2), NS: new start (2), PS: pausing site (2), iTSS_I: internal starting site (0)
Intensity [A.U]
Coverage
Half−life [min]
Delay [min]
I_107
I_108
I_109
I_110
I_112
I_113
I_114
I_115
I_116
I_117
Dc_84
Dc_85
Dc_86
Dc_87
Dc_89
Dc_90
Dc_91
Dc_92
D_68
D_69
D_70
D_71
D_72
D_73
D_74
D_75
D_76
200000
202500
205000
207500
210000
lpxB
rnhB
dnaE
accA
ldcC
BW25113_RS00935
tilS
BW25113_RS00910
BW25113_RS00920
BW25113_RS00925
BW25113_RS00930
TU_48
TU_49
TU_51
TU_52
TU_53
TU_54
NA

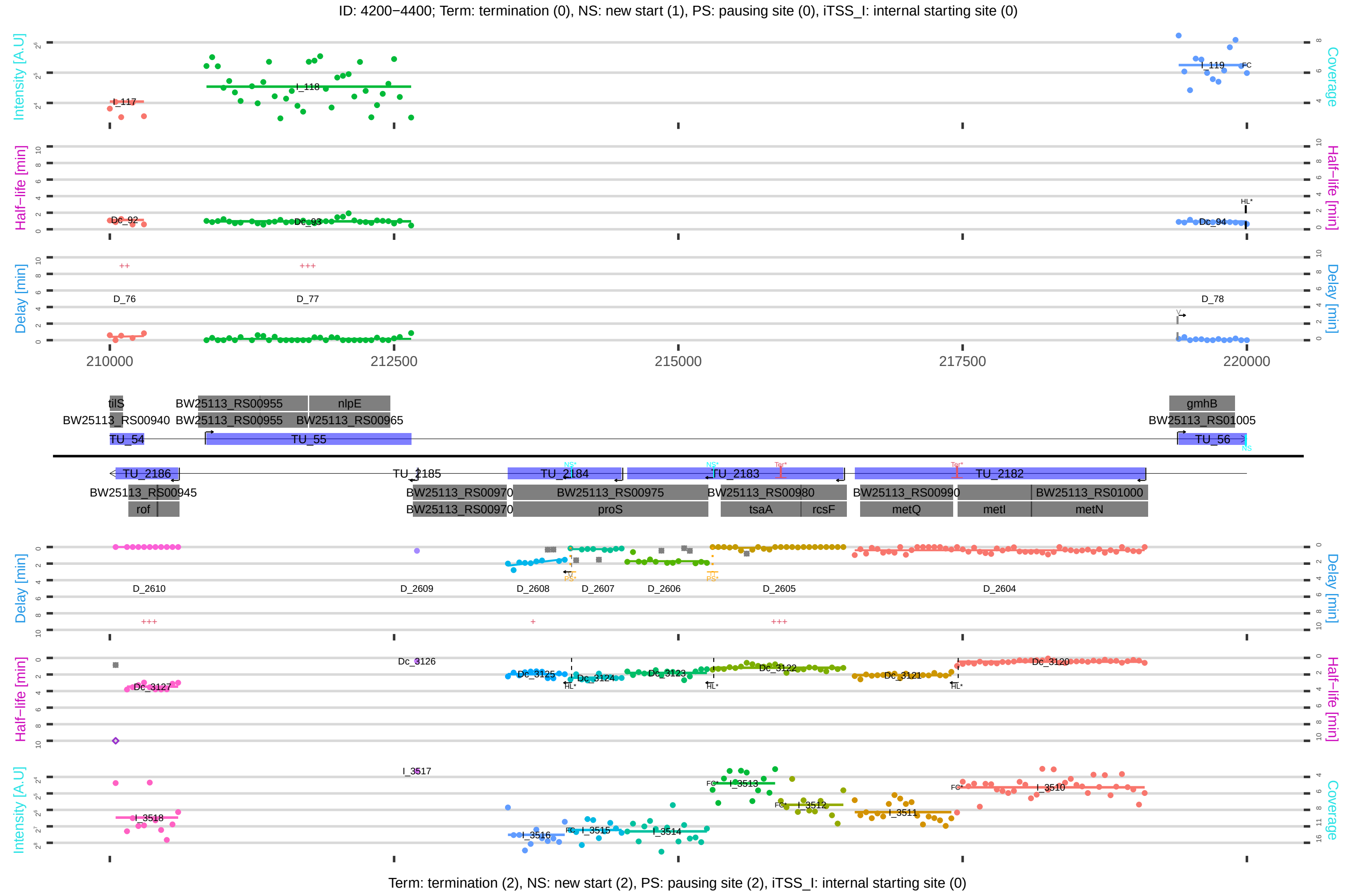

ID: 4200−4400; Term: termination (0), NS: new start (1), PS: pausing site (0), iTSS_I: internal starting site (0)
Intensity [A.U]
Coverage
Half−life [min]
Delay [min]
I_117
I_118
I_119
Dc_92
Dc_93
Dc_94
D_76
D_77
D_78
210000
212500
215000
217500
220000
tilS
BW25113_RS00940
BW25113_RS00955
nlpE
BW25113_RS00965
gmhB
BW25113_RS01005
TU_54
TU_55
TU_56
TU_2186
TU_2185
TU_2184
TU_2183
TU_2182
BW25113_RS00945
rof
BW25113_RS00970
BW25113_RS00975
proS
BW25113_RS00980
tsaA
rcsF
BW25113_RS00990
metQ
metI
BW25113_RS01000
metN
D_2610
D_2609
D_2608
D_2607
D_2606
D_2605
D_2604
Dc_3127
Dc_3126
Dc_3125
Dc_3124
Dc_3123
Dc_3122
Dc_3121
Dc_3120
I_3518
I_3517
I_3516
I_3515
I_3514
I_3513
I_3512
I_3511
I_3510
Term: termination (2), NS: new start (2), PS: pausing site (2), iTSS_I: internal starting site (0)

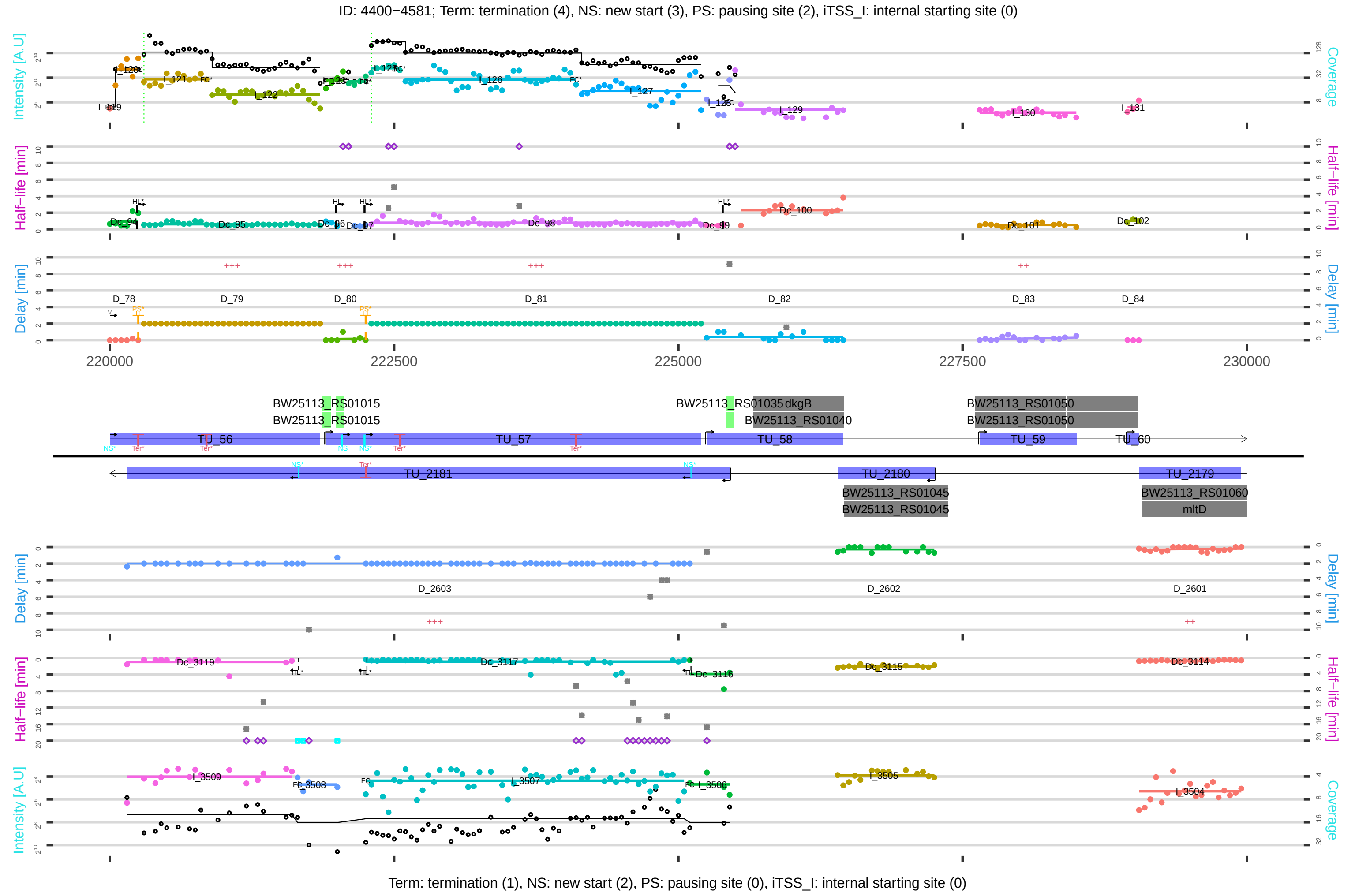

ID: 4400−4581; Term: termination (4), NS: new start (3), PS: pausing site (2), iTSS_I: internal starting site (0)
Intensity [A.U]
Coverage
Half−life [min]
Delay [min]
220000
222500
225000
227500
230000
BW25113_RS01015
BW25113_RS01015
BW25113_RS01035
dkgB
BW25113_RS01040
BW25113_RS01050
BW25113_RS01050
TU_56
TU_57
TU_58
TU_59
TU_60
TU_2181
TU_2180
TU_2179
BW25113_RS01045
BW25113_RS01045
BW25113_RS01060
mltD
D_78
D_79
D_80
D_81
D_82
D_83
D_84
D_2603
D_2602
D_2601
Term: termination (1), NS: new start (2), PS: pausing site (0), iTSS_I: internal starting site (0)

ID: 4628−4747; Term: termination (0), NS: new start (0), PS: pausing site (0), iTSS_I: internal starting site (0)

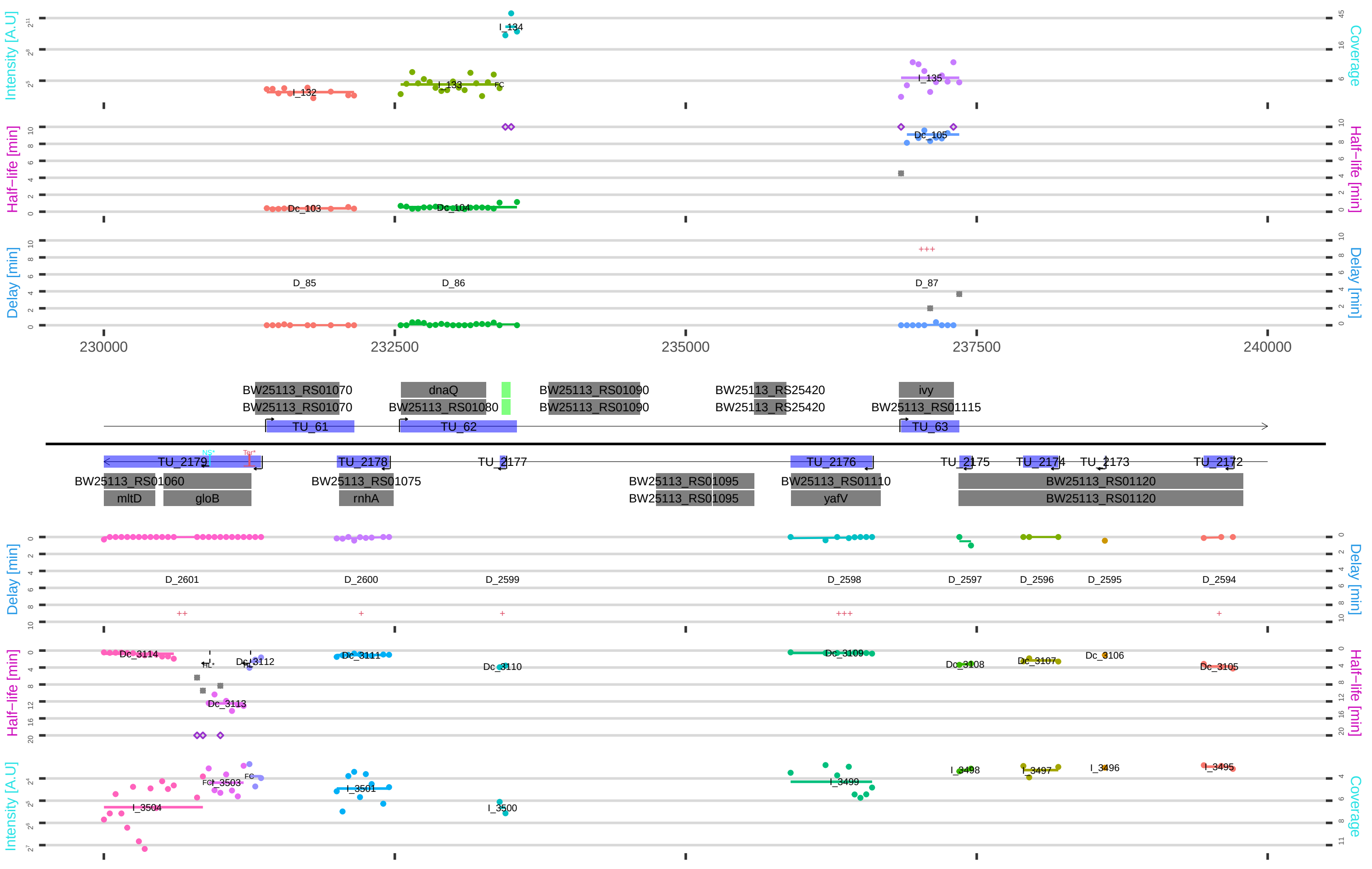

Term: termination (1), NS: new start (1), PS: pausing site (0), iTSS_I: internal starting site (0)

ID: 4802−4973; Term: termination (1), NS: new start (1), PS: pausing site (0), iTSS_I: internal starting site (0)

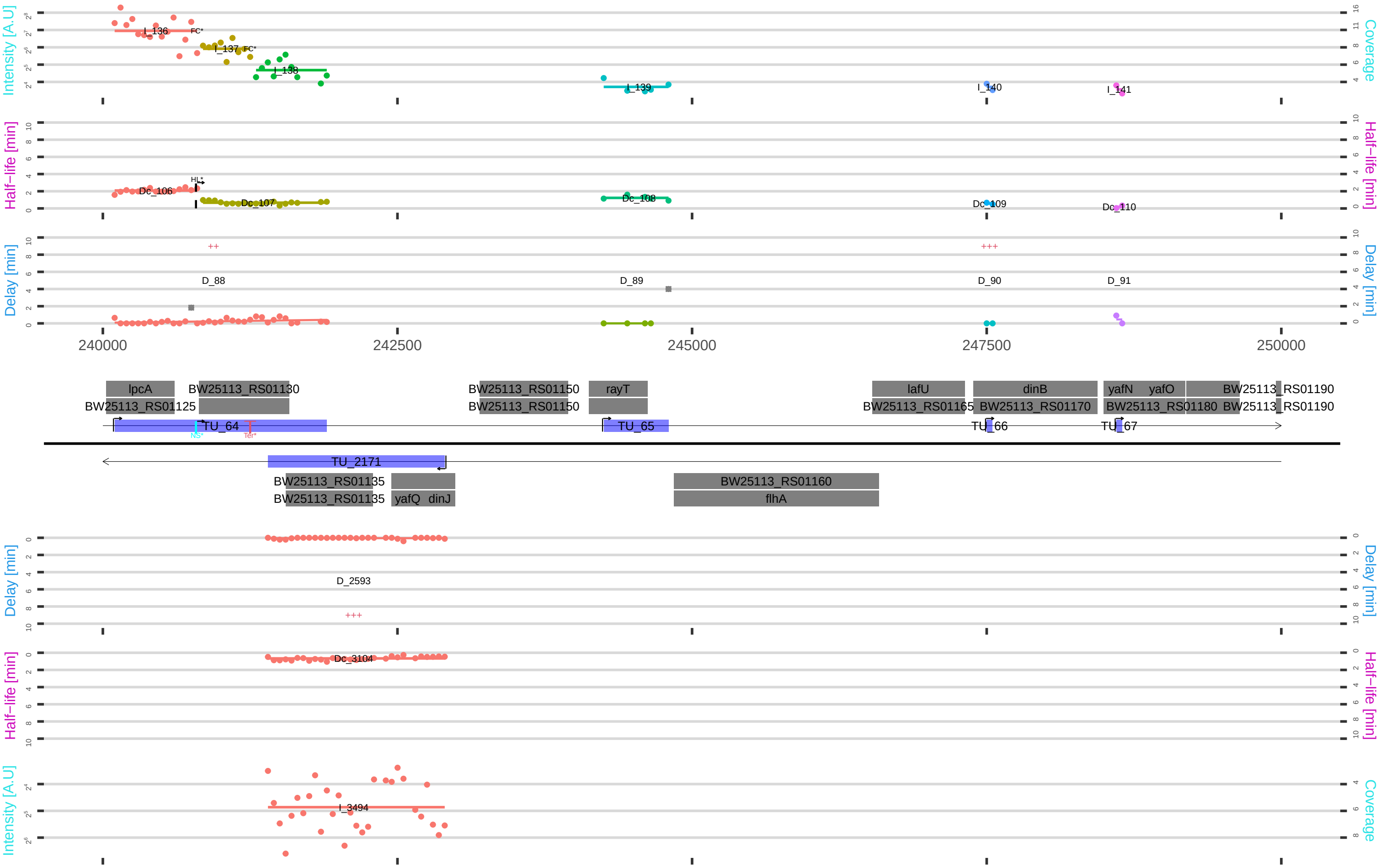

Term: termination (0), NS: new start (0), PS: pausing site (0), iTSS_I: internal starting site (0)

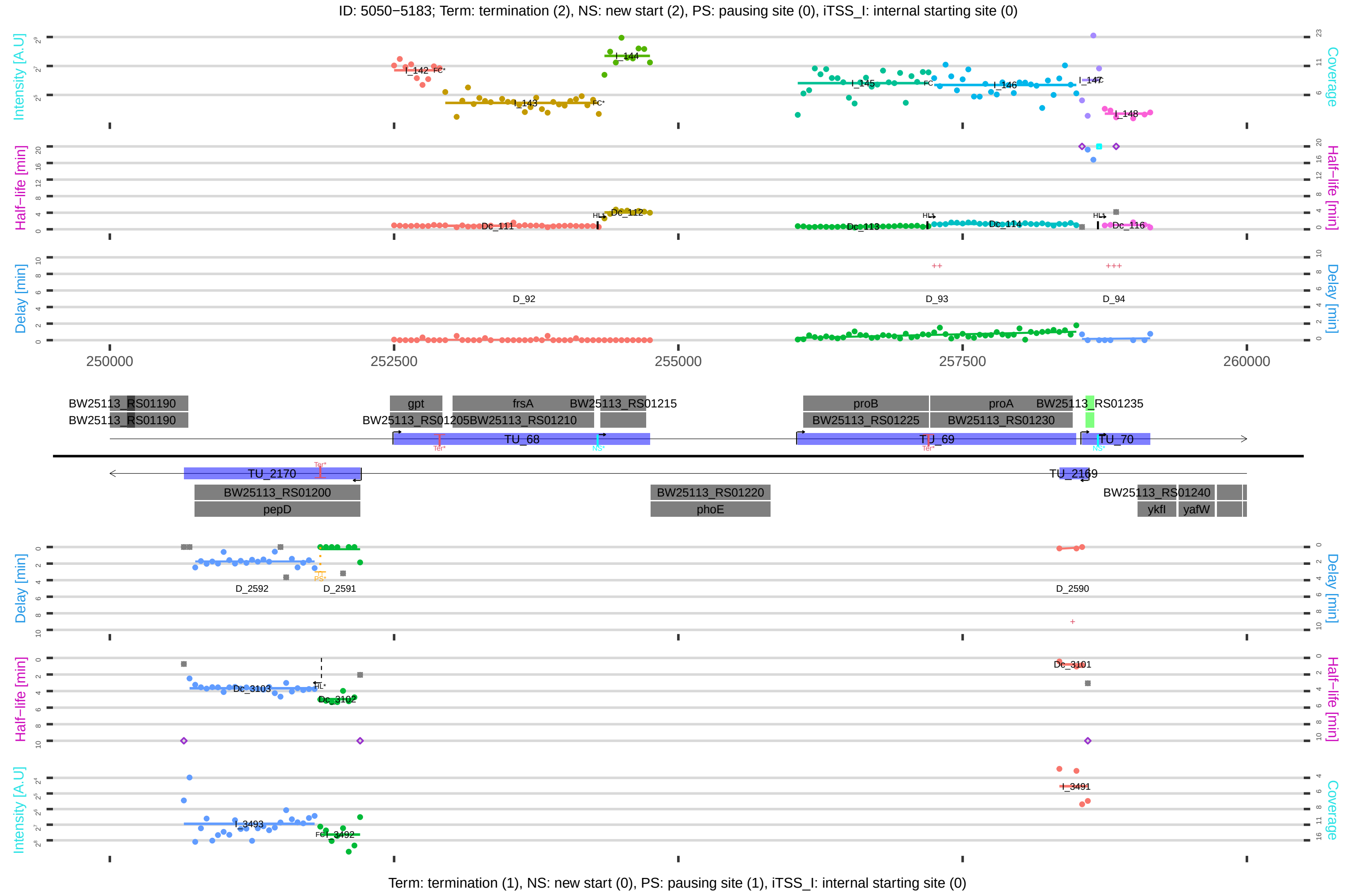
ID: 5050−5183; Term: termination (2), NS: new start (2), PS: pausing site (0), iTSS_I: internal starting site (0)
Intensity [A.U]
Coverage
Half−life [min]
Delay [min]
I_142
I_143
I_144
I_145
I_146
I_147
I_148
Dc_111
Dc_112
Dc_113
Dc_114
Dc_116
D_92
D_93
D_94
250000
252500
255000
257500
260000
BW25113_RS01190
gpt
frsA
BW25113_RS01215
BW25113_RS01205
BW25113_RS01210
proB
proA
BW25113_RS01235
BW25113_RS01225
BW25113_RS01230
TU_68
TU_69
TU_70
TU_2170
TU_2169
BW25113_RS01200
pepD
BW25113_RS01220
phoE
BW25113_RS01240
ykfI
yafW
D_2592
D_2591
D_2590
Dc_3103
Dc_3102
Dc_3101
I_3493
I_3492
I_3491
Term: termination (1), NS: new start (0), PS: pausing site (1), iTSS_I: internal starting site (0)

ID: 5213−5367; Term: termination (0), NS: new start (0), PS: pausing site (0), iTSS_I: internal starting site (0)

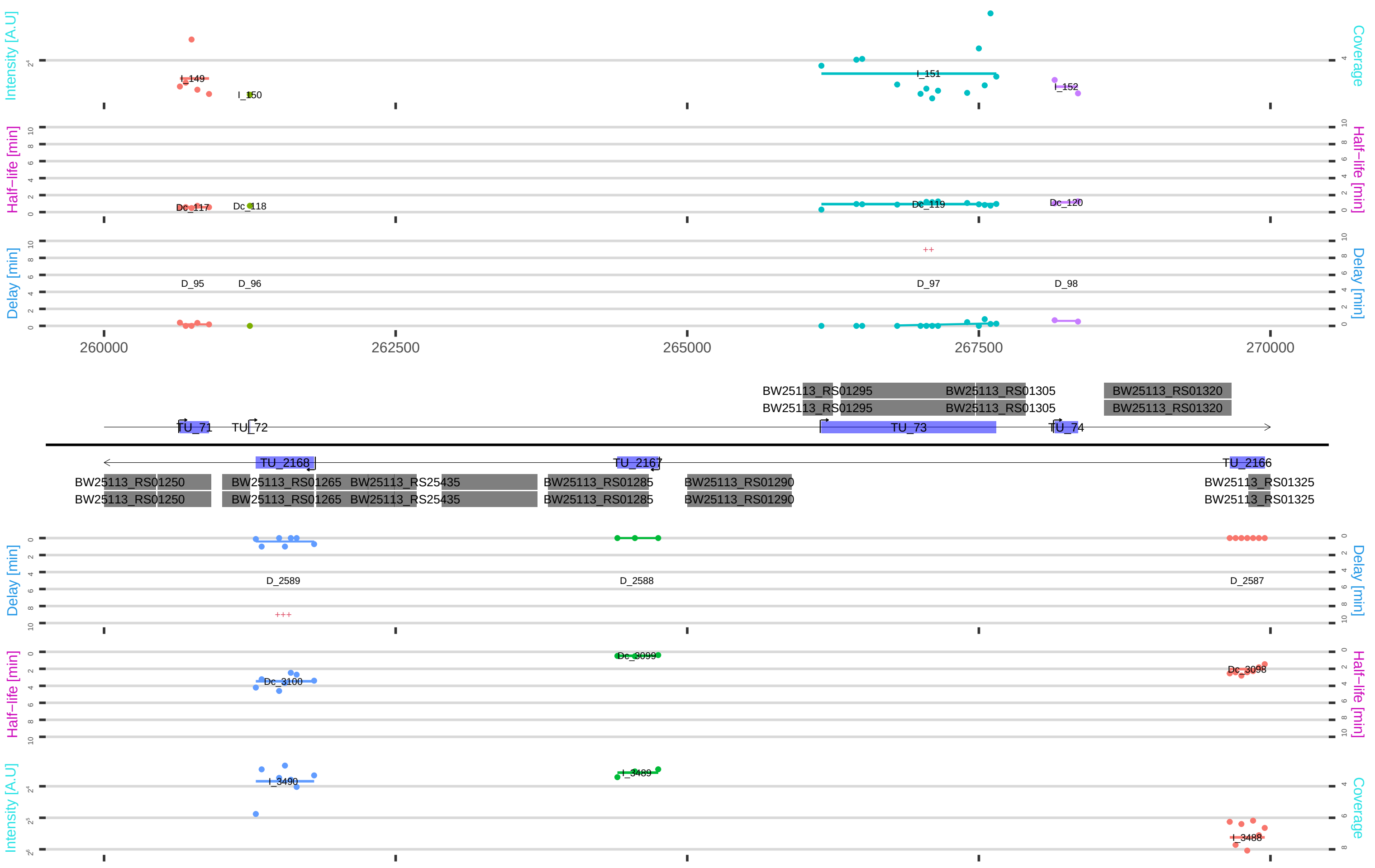

Term: termination (0), NS: new start (0), PS: pausing site (0), iTSS_I: internal starting site (0)

ID: 5450−5600; Term: termination (1), NS: new start (0), PS: pausing site (0), iTSS_I: internal starting site (0)

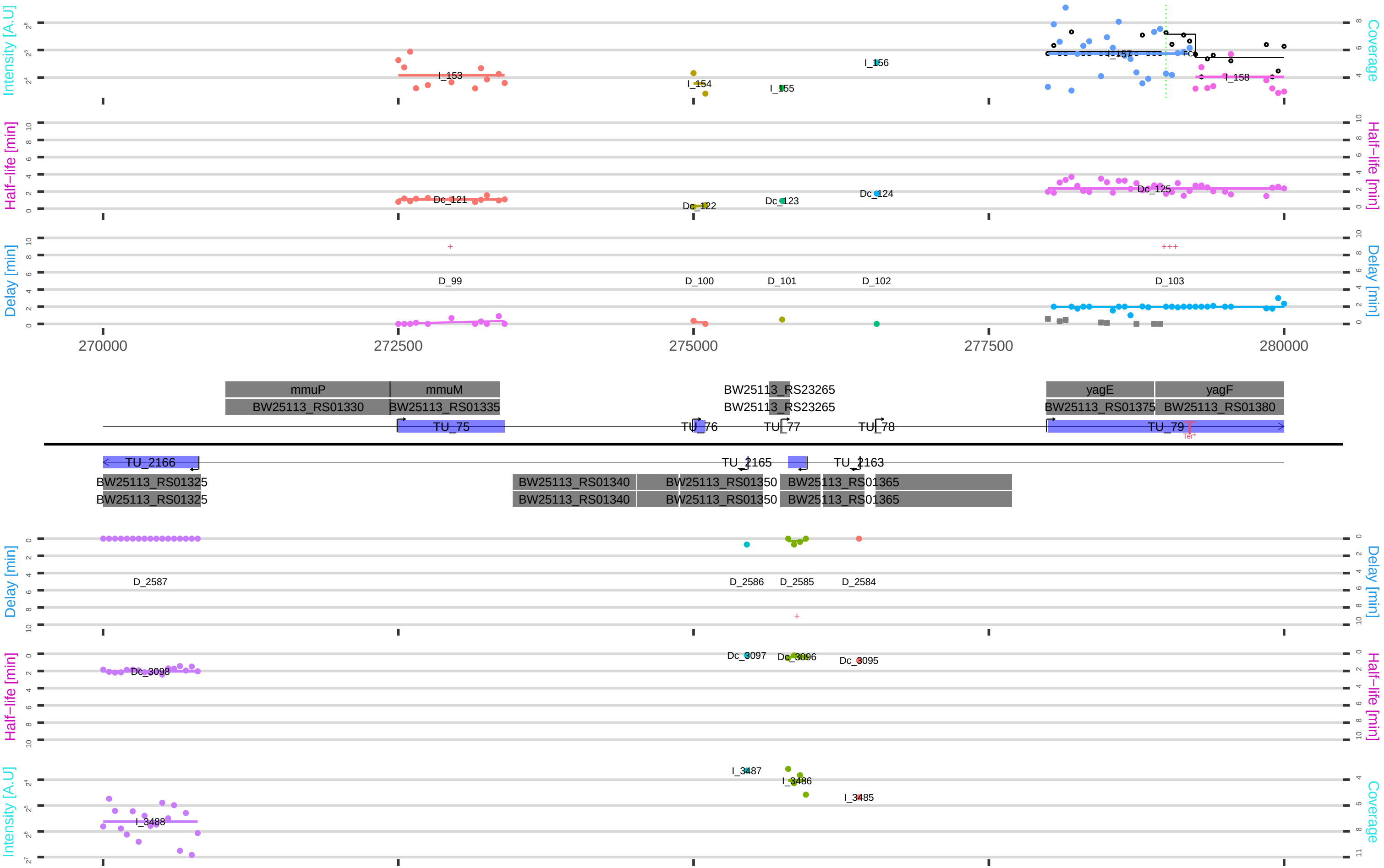

Term: termination (0), NS: new start (0), PS: pausing site (0), iTSS_I: internal starting site (0)

ID: 5600−5760; Term: termination (0), NS: new start (0), PS: pausing site (0), iTSS_I: internal starting site (0)

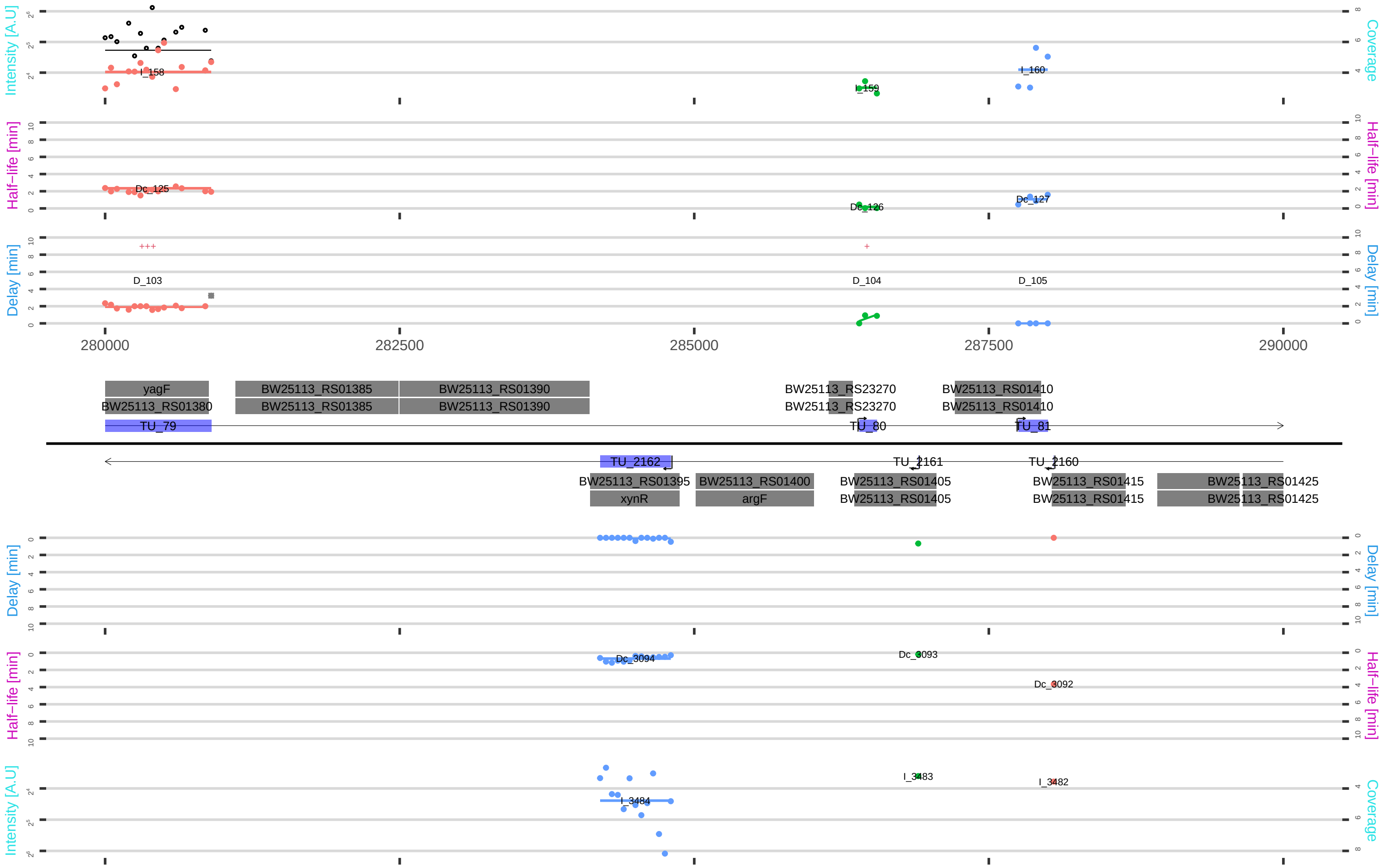

Term: termination (0), NS: new start (0), PS: pausing site (0), iTSS_I: internal starting site (0)

ID: 5843−5990; Term: termination (0), NS: new start (0), PS: pausing site (0), iTSS_I: internal starting site (0)

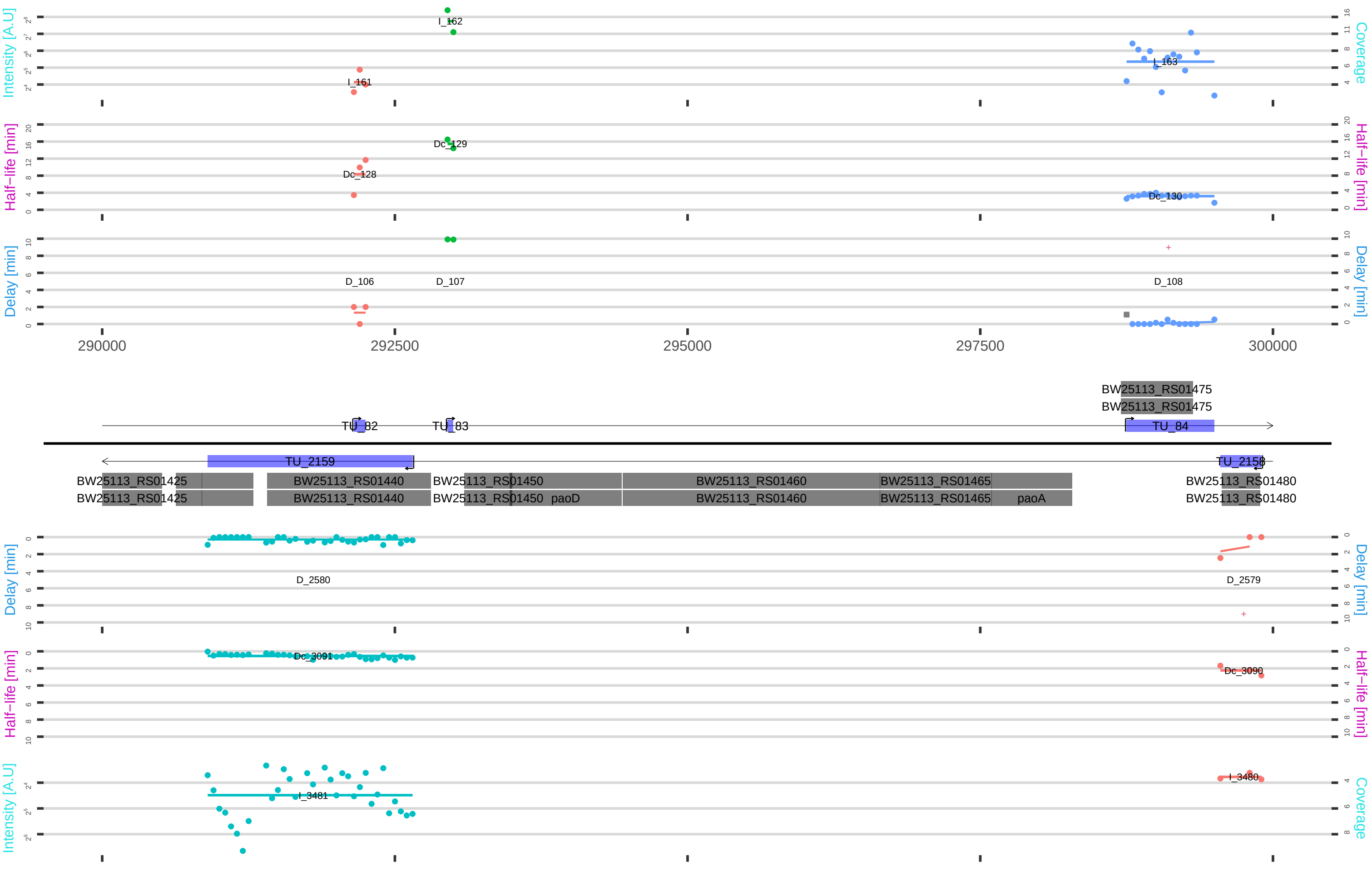

Term: termination (0), NS: new start (0), PS: pausing site (0), iTSS_I: internal starting site (0)

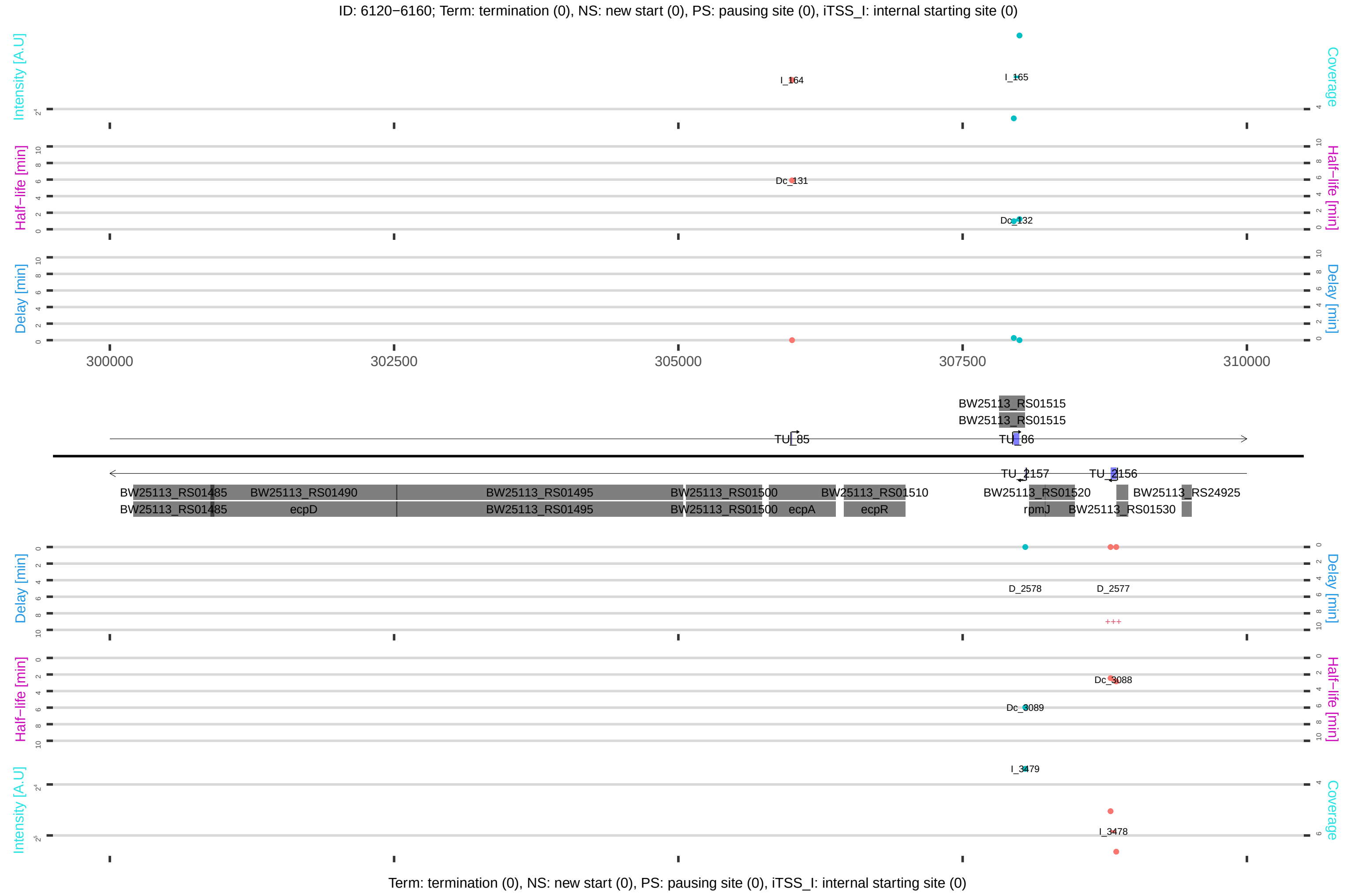
ID: 6120−6160; Term: termination (0), NS: new start (0), PS: pausing site (0), iTSS_I: internal starting site (0)
Intensity [A.U]
Coverage
I_164
I_165
Half−life [min]
Dc_131
Dc_132
Delay [min]
300000
302500
305000
307500
310000
BW25113_RS01515
BW25113_RS01515
TU_85
TU_86
TU_2157
TU_2156
BW25113_RS01485
BW25113_RS01490
BW25113_RS01495
BW25113_RS01500
BW25113_RS01510
BW25113_RS01520
BW25113_RS24925
BW25113_RS01485
ecpD
BW25113_RS01495
BW25113_RS01500
ecpA
ecpR
rpmJ
BW25113_RS01530
D_2578
D_2577
Dc_3088
Dc_3089
I_3479
I_3478
Term: termination (0), NS: new start (0), PS: pausing site (0), iTSS_I: internal starting site (0)

ID: 6222−6397; Term: termination (0), NS: new start (0), PS: pausing site (0), iTSS_I: internal starting site (0)

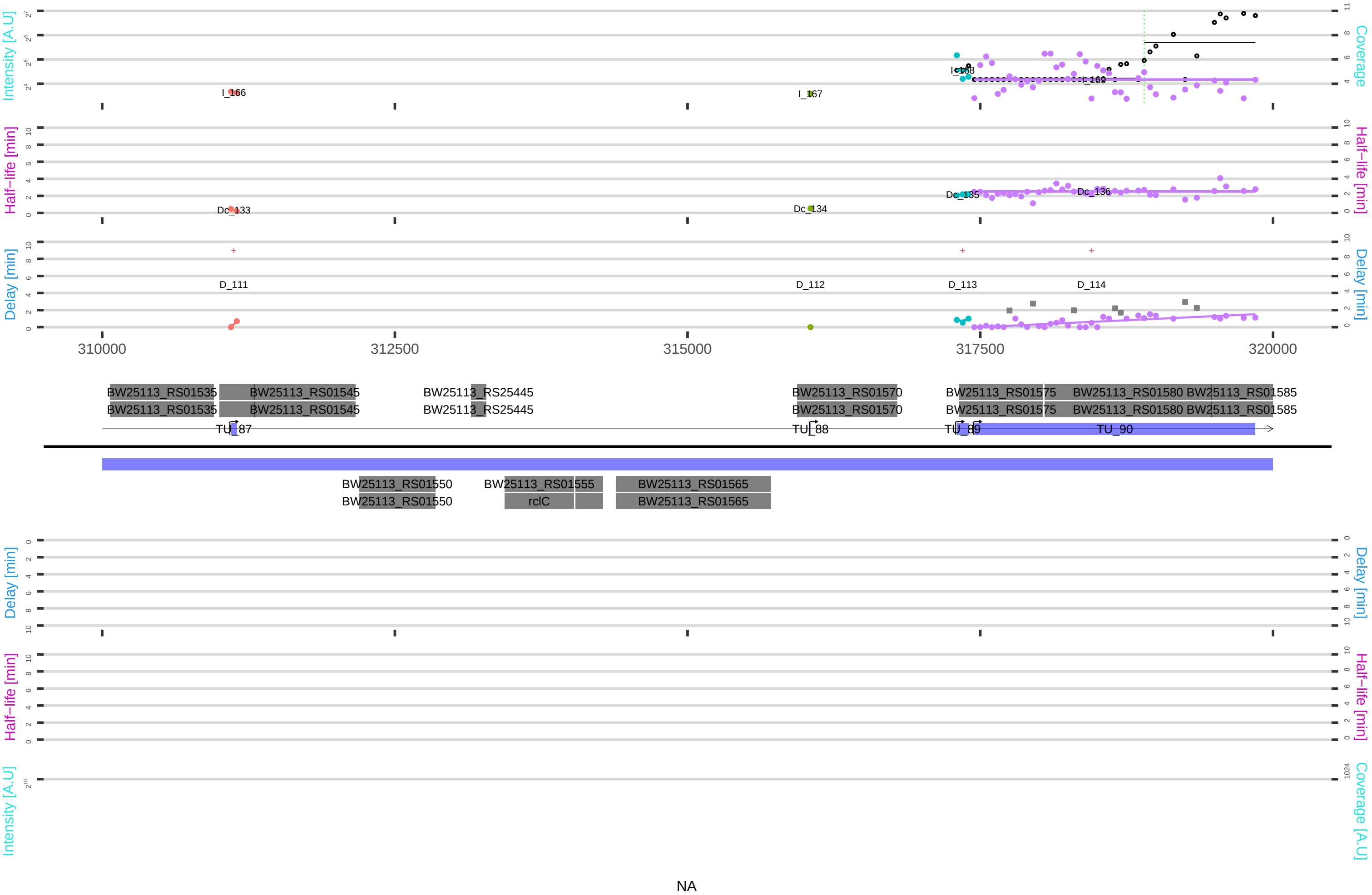

ID: 6403−6543; Term: termination (0), NS: new start (0), PS: pausing site (0), iTSS_I: internal starting site (0)

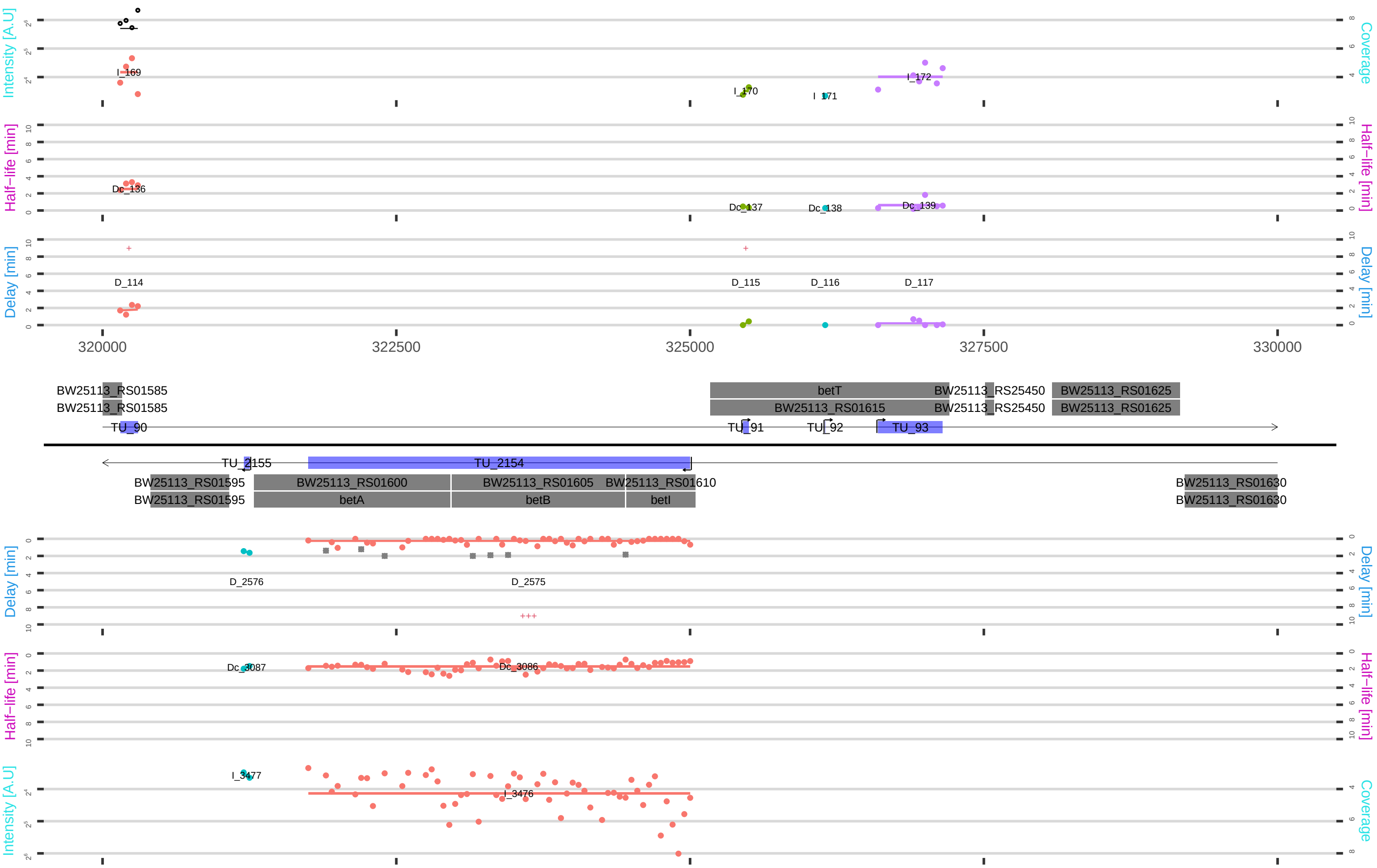

Term: termination (0), NS: new start (0), PS: pausing site (0), iTSS_I: internal starting site (0)

ID: 6740−6793; Term: termination (0), NS: new start (0), PS: pausing site (0), iTSS_I: internal starting site (0)

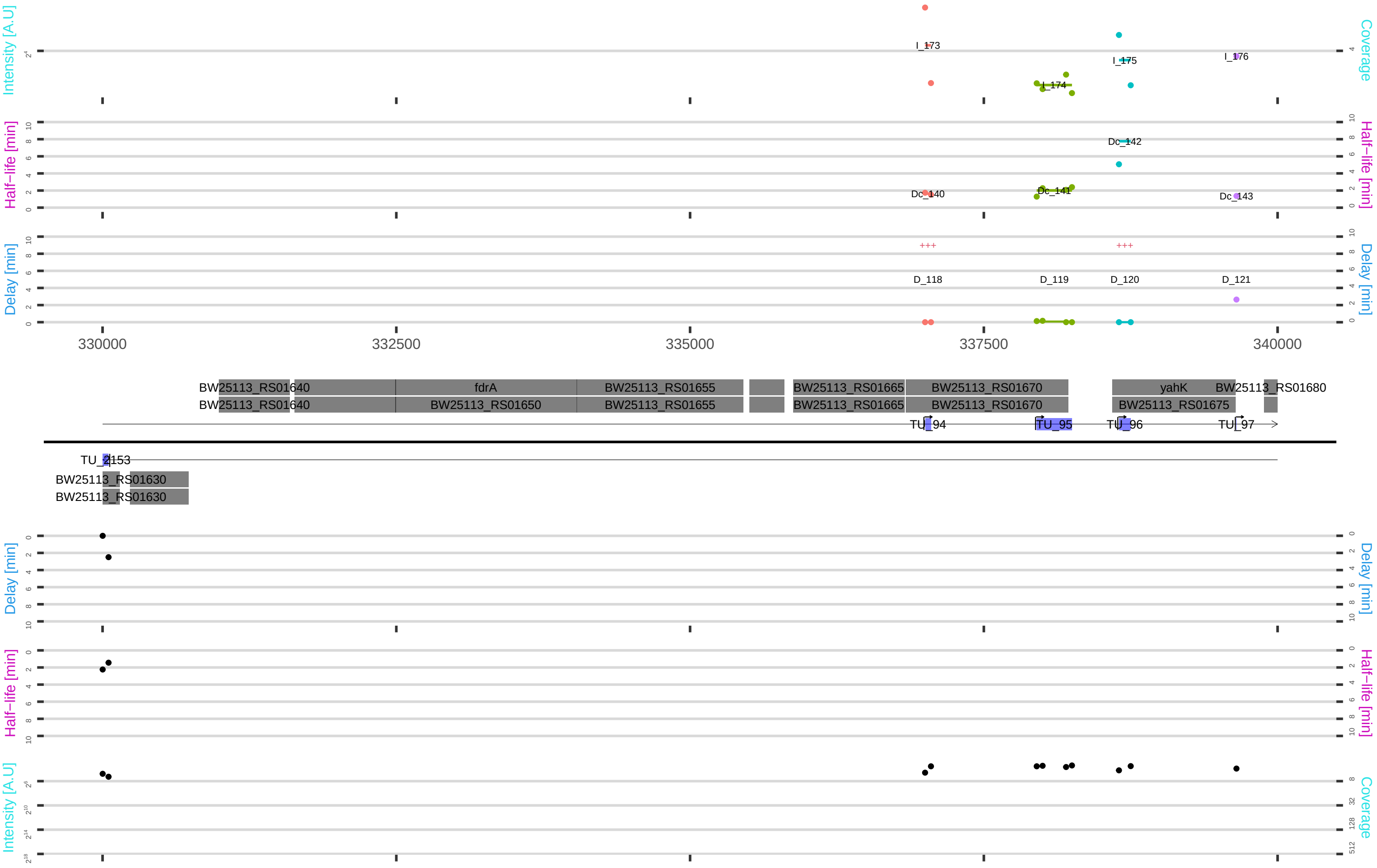

Term: termination (0), NS: new start (0), PS: pausing site (0), iTSS_I: internal starting site (0)

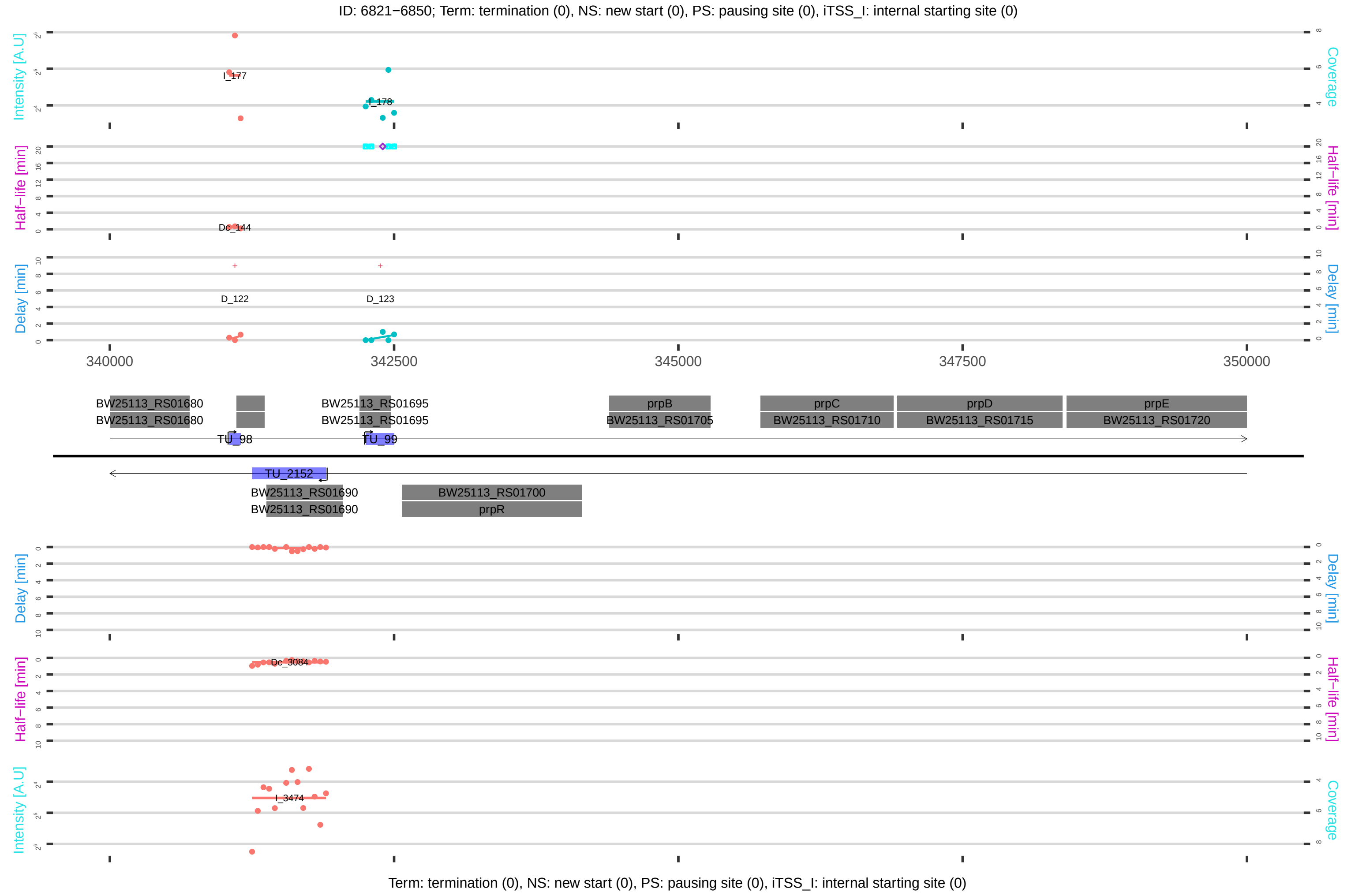
ID: 6821−6850; Term: termination (0), NS: new start (0), PS: pausing site (0), iTSS_I: internal starting site (0)
Intensity [A.U]
Coverage
I_177
I_178
Half−life [min]
Dc_144
Delay [min]
D_122
D_123
340000
342500
345000
347500
350000
BW25113_RS01680
BW25113_RS01695
prpB
BW25113_RS01705
prpC
BW25113_RS01710
prpD
BW25113_RS01715
prpE
BW25113_RS01720
TU_98
TU_99
TU_2152
BW25113_RS01690
BW25113_RS01700
prpR
Dc_3084
I_3474
Term: termination (0), NS: new start (0), PS: pausing site (0), iTSS_I: internal starting site (0)

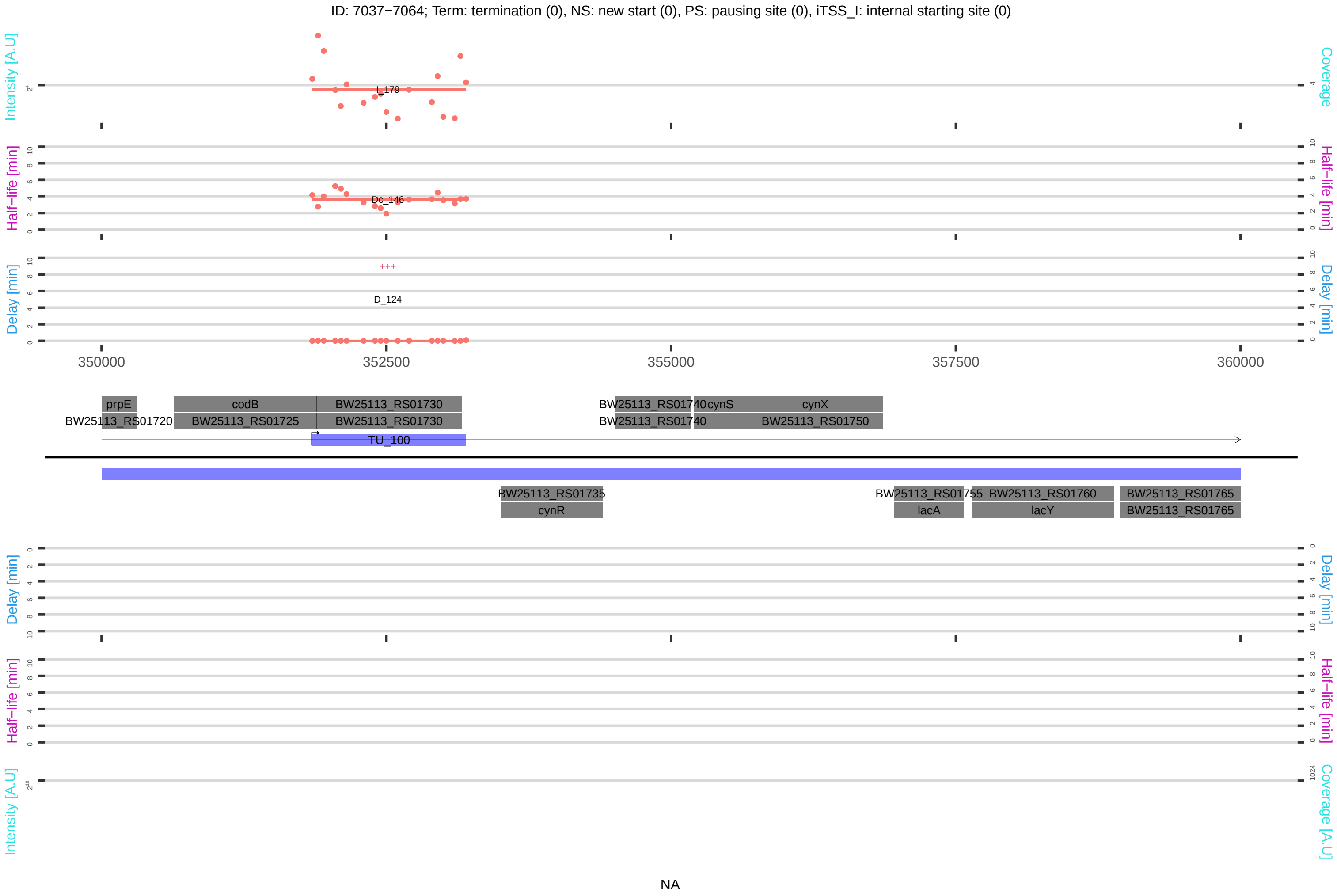
ID: 7037−7064; Term: termination (0), NS: new start (0), PS: pausing site (0), iTSS_I: internal starting site (0)
Intensity [A.U]
Coverage
I_179
Half−life [min]
Dc_146
Delay [min]
+++
D_124
350000
352500
355000
357500
360000
prpE
BW25113_RS01720
codB
BW25113_RS01725
BW25113_RS01730
BW25113_RS01740
cynS
cynX
BW25113_RS01750
TU_100
BW25113_RS01735
cynR
BW25113_RS01755
lacA
BW25113_RS01760
lacY
BW25113_RS01765
Coverage [A.U]
NA

ID: 7210−7399; Term: termination (0), NS: new start (0), PS: pausing site (0), iTSS_I: internal starting site (0)

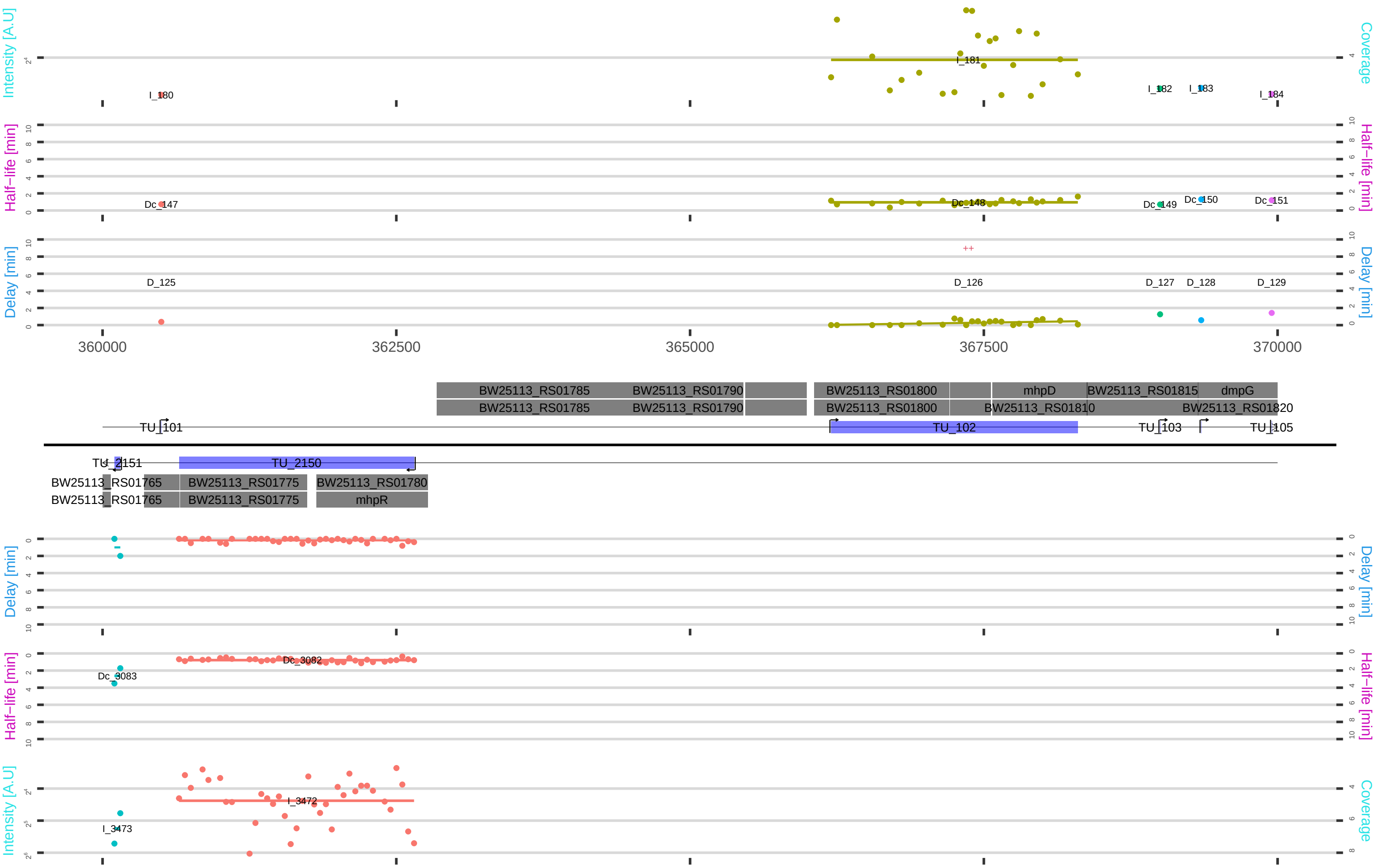

Term: termination (0), NS: new start (0), PS: pausing site (0), iTSS_I: internal starting site (0)

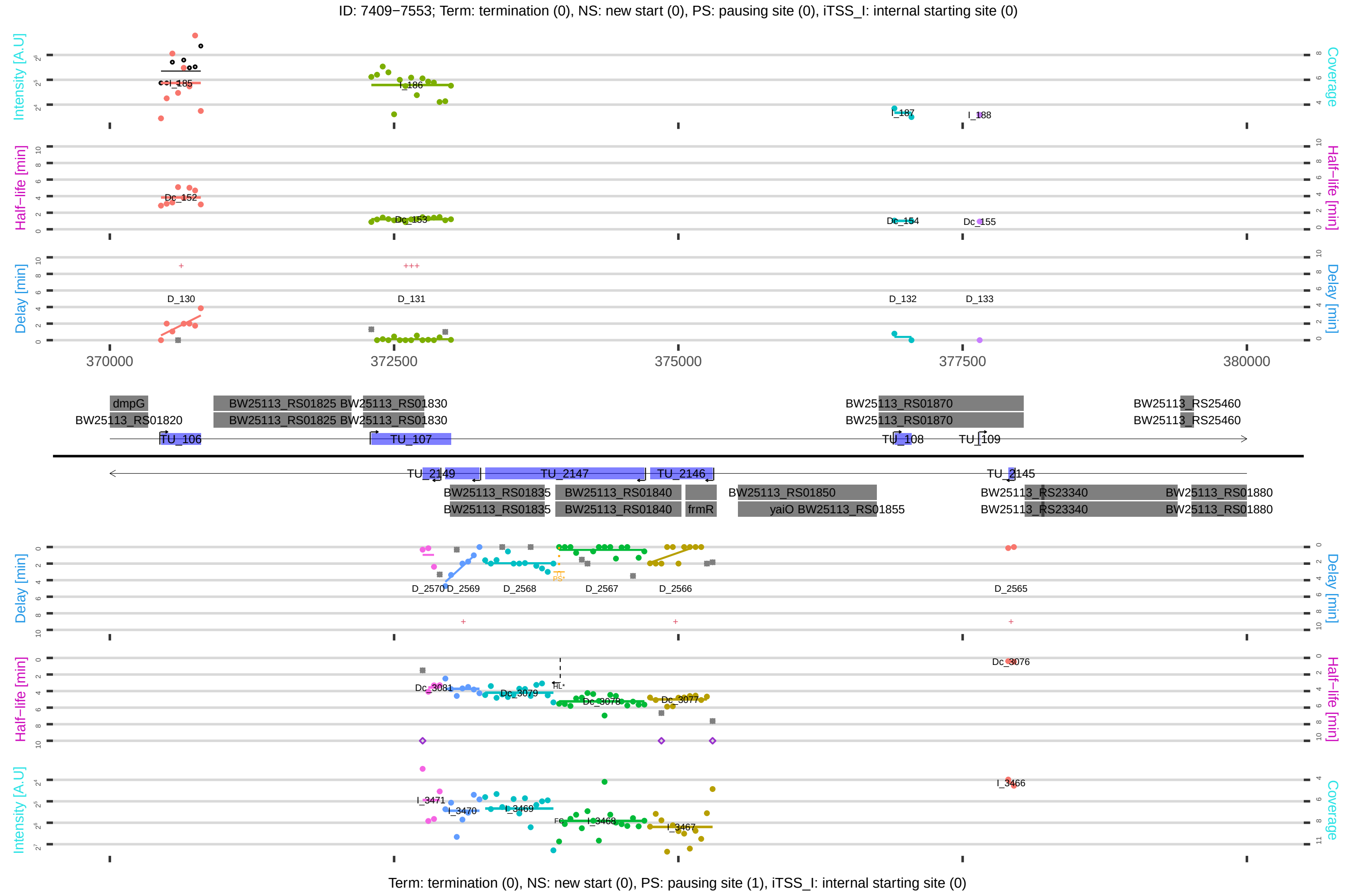

ID: 7409−7553; Term: termination (0), NS: new start (0), PS: pausing site (0), iTSS_I: internal starting site (0)
Intensity [A.U]
Coverage
Half−life [min]
Delay [min]
I_185
I_186
I_187
I_188
Dc_152
Dc_153
Dc_154
Dc_155
D_130
D_131
D_132
D_133
370000
372500
375000
377500
380000
dmpG
BW25113_RS01820
BW25113_RS01825
BW25113_RS01830
BW25113_RS01870
BW25113_RS25460
TU_106
TU_107
TU_108
TU_109
TU_2149
TU_2147
TU_2146
TU_2145
BW25113_RS01835
BW25113_RS01840
frmR
BW25113_RS01850
yaiO BW25113_RS01855
BW25113_RS23340
BW25113_RS01880
D_2570
D_2569
D_2568
D_2567
D_2566
D_2565
PS*
HL*
Dc_3081
Dc_3079
Dc_3078
Dc_3077
Dc_3076
I_3471
I_3470
I_3469
I_3468
I_3467
I_3466
FC
Term: termination (0), NS: new start (0), PS: pausing site (1), iTSS_I: internal starting site (0)

ID: 177577−177495; FC*: significant t−test of two consecutive segments; Term: termination, NS: new start, PS: pausing site,iTSS_I: internal starting site,TI: transcription interferance.

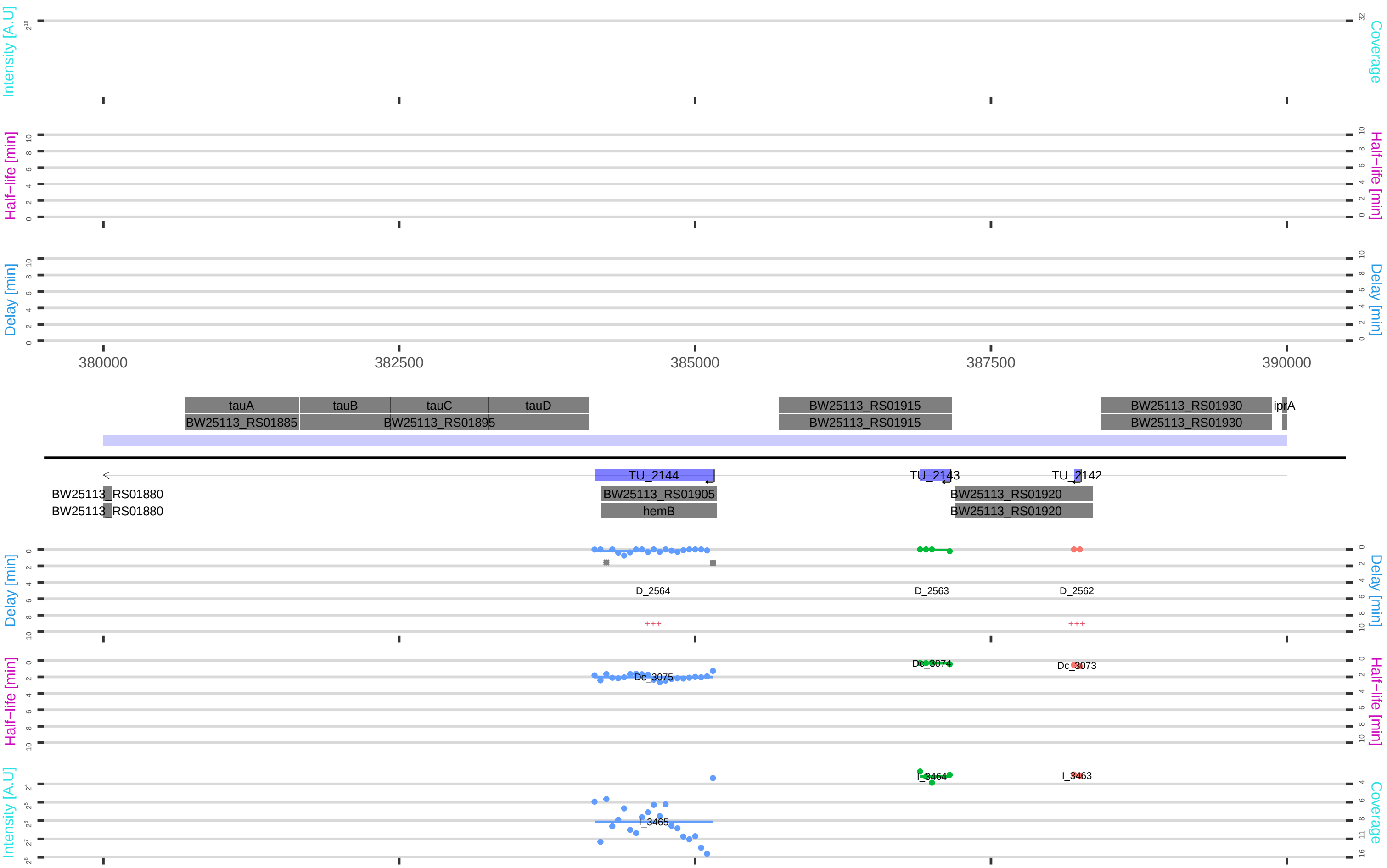

Term: termination (0), NS: new start (0), PS: pausing site (0), iTSS_I: internal starting site (0)

ID: 7840−7972; Term: termination (0), NS: new start (0), PS: pausing site (0), iTSS_I: internal starting site (0)

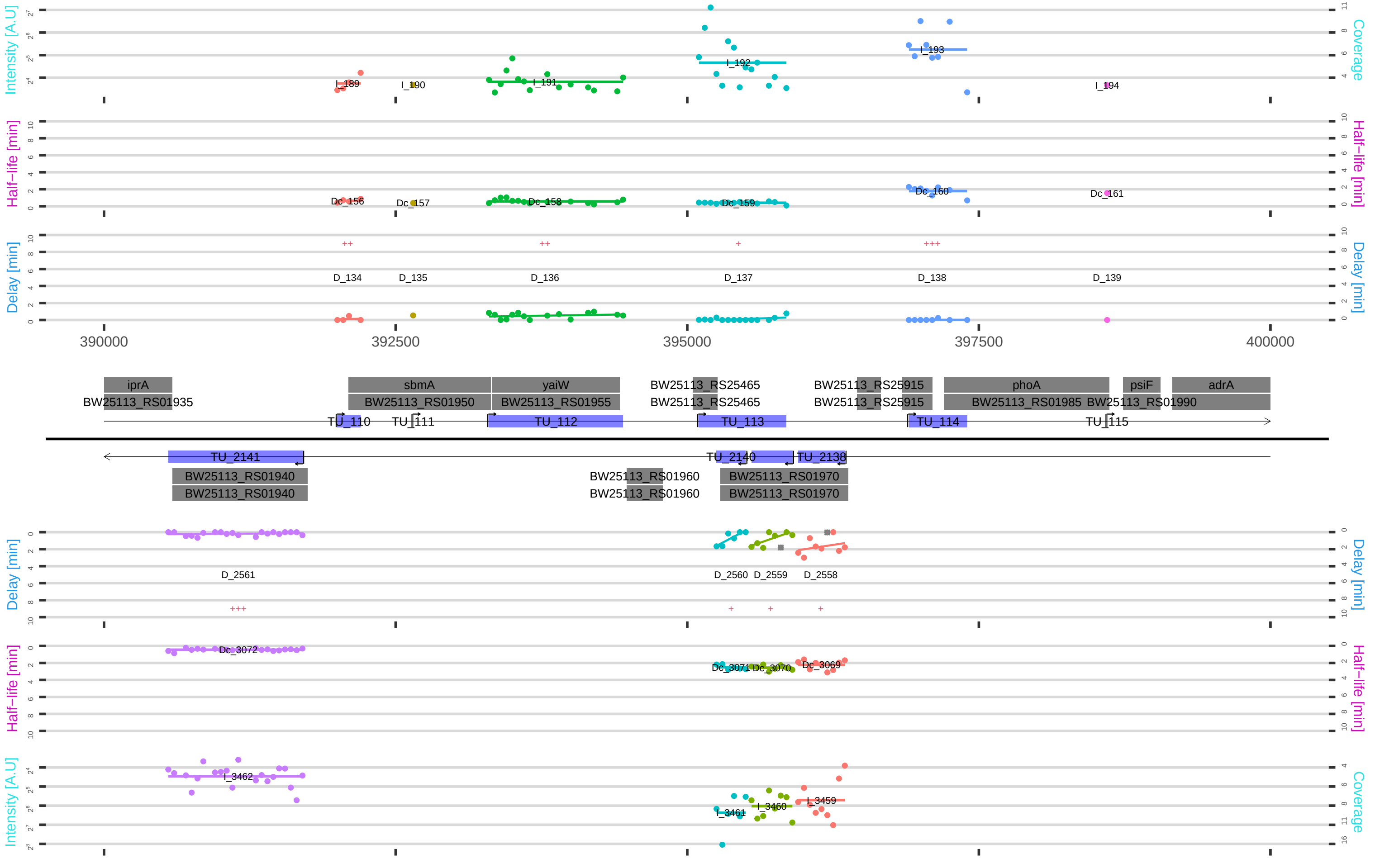

Term: termination (0), NS: new start (0), PS: pausing site (0), iTSS_I: internal starting site (0)

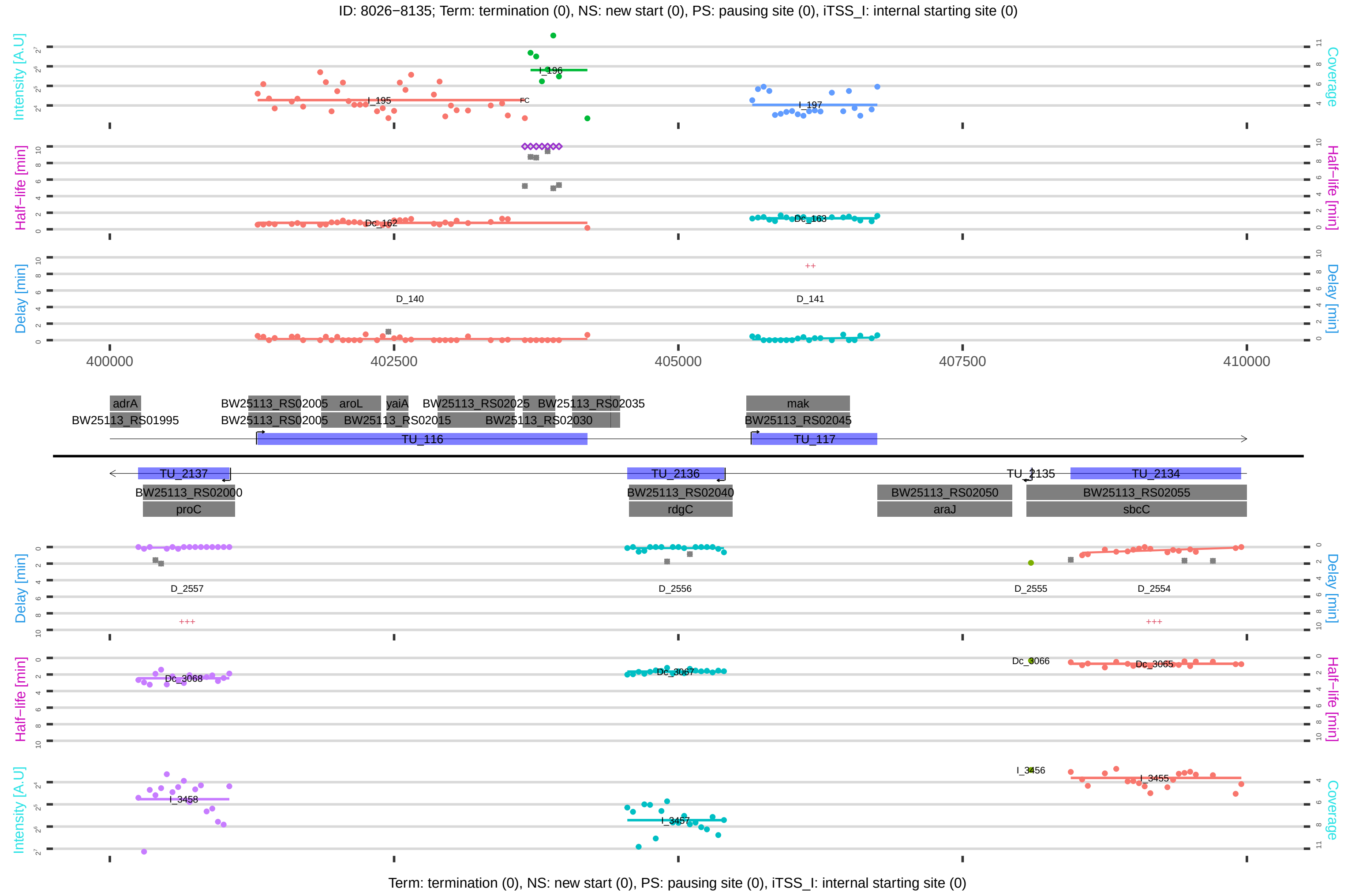

ID: 8026−8135; Term: termination (0), NS: new start (0), PS: pausing site (0), iTSS_I: internal starting site (0)
Intensity [A.U]
Coverage
Half−life [min]
Delay [min]
I_195
I_196
I_197
Dc_162
Dc_163
D_140
D_141
400000
402500
405000
407500
410000
adrA
BW25113_RS01995
BW25113_RS02005
aroL
yaiA
BW25113_RS02015
BW25113_RS02025
BW25113_RS02030
BW25113_RS02035
mak
BW25113_RS02045
TU_116
TU_117
TU_2137
TU_2136
TU_2135
TU_2134
BW25113_RS02000
proC
BW25113_RS02040
rdgC
BW25113_RS02050
araJ
BW25113_RS02055
sbcC
D_2557
D_2556
D_2555
D_2554
Dc_3068
Dc_3067
Dc_3066
Dc_3065
I_3458
I_3457
I_3456
I_3455
Term: termination (0), NS: new start (0), PS: pausing site (0), iTSS_I: internal starting site (0)

ID: 8255−8398; Term: termination (0), NS: new start (0), PS: pausing site (0), iTSS_I: internal starting site (0)

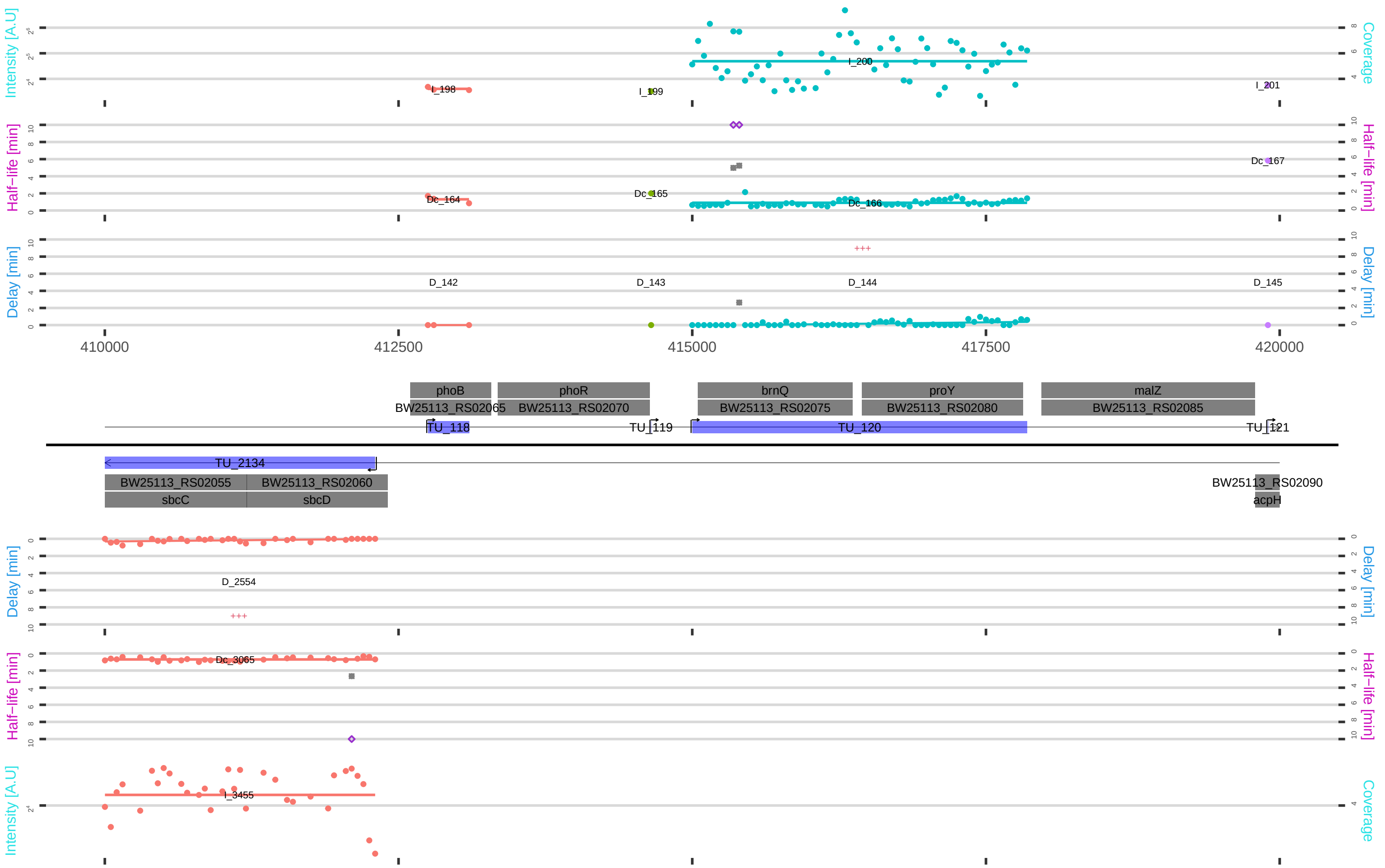

Term: termination (0), NS: new start (0), PS: pausing site (0), iTSS_I: internal starting site (0)

ID: 8411−8600; Term: termination (0), NS: new start (4), PS: pausing site (1), iTSS_I: internal starting site (0)

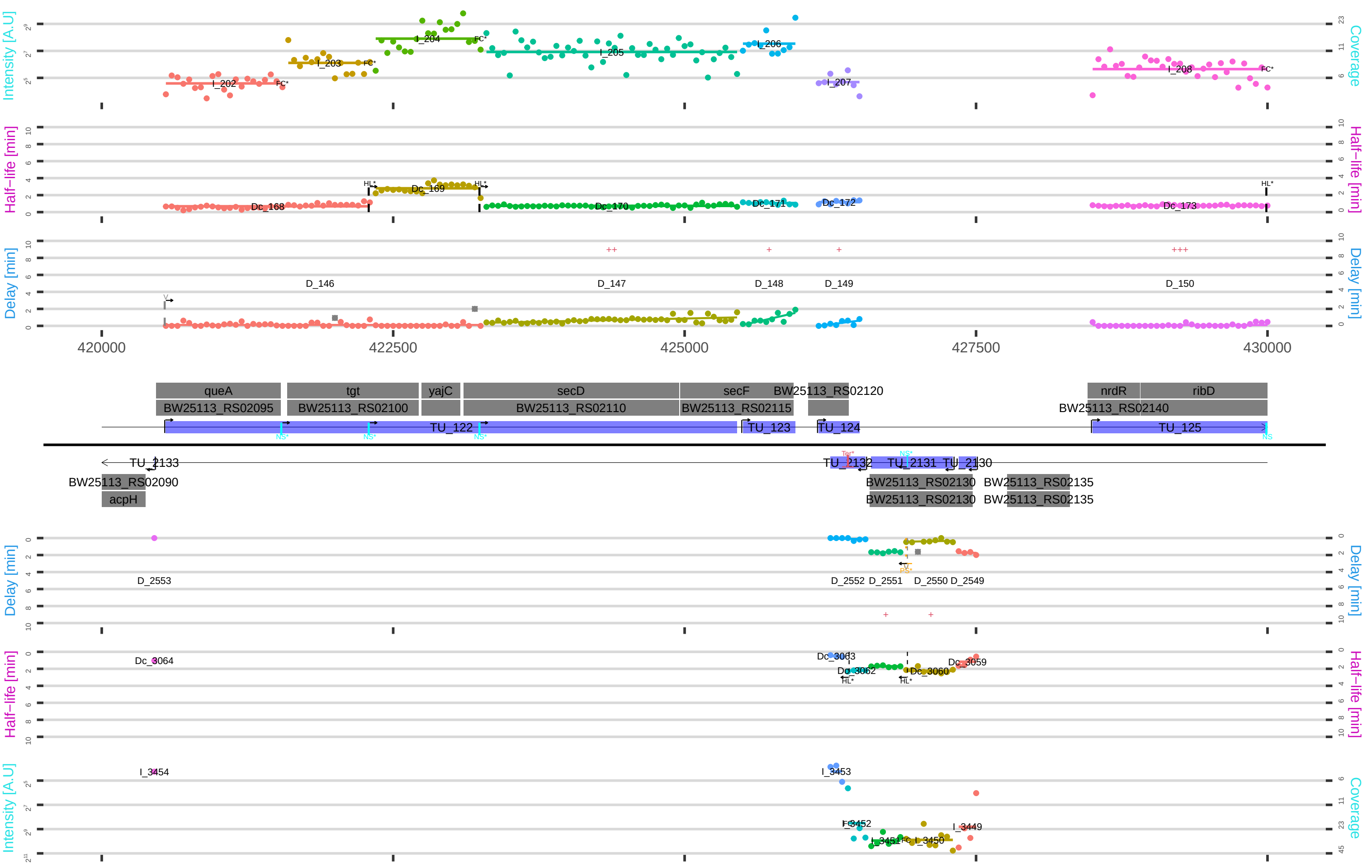

Term: termination (1), NS: new start (1), PS: pausing site (1), iTSS_I: internal starting site (0)

ID: 8600−8769; Term: termination (1), NS: new start (1), PS: pausing site (1), iTSS_I: internal starting site (0)

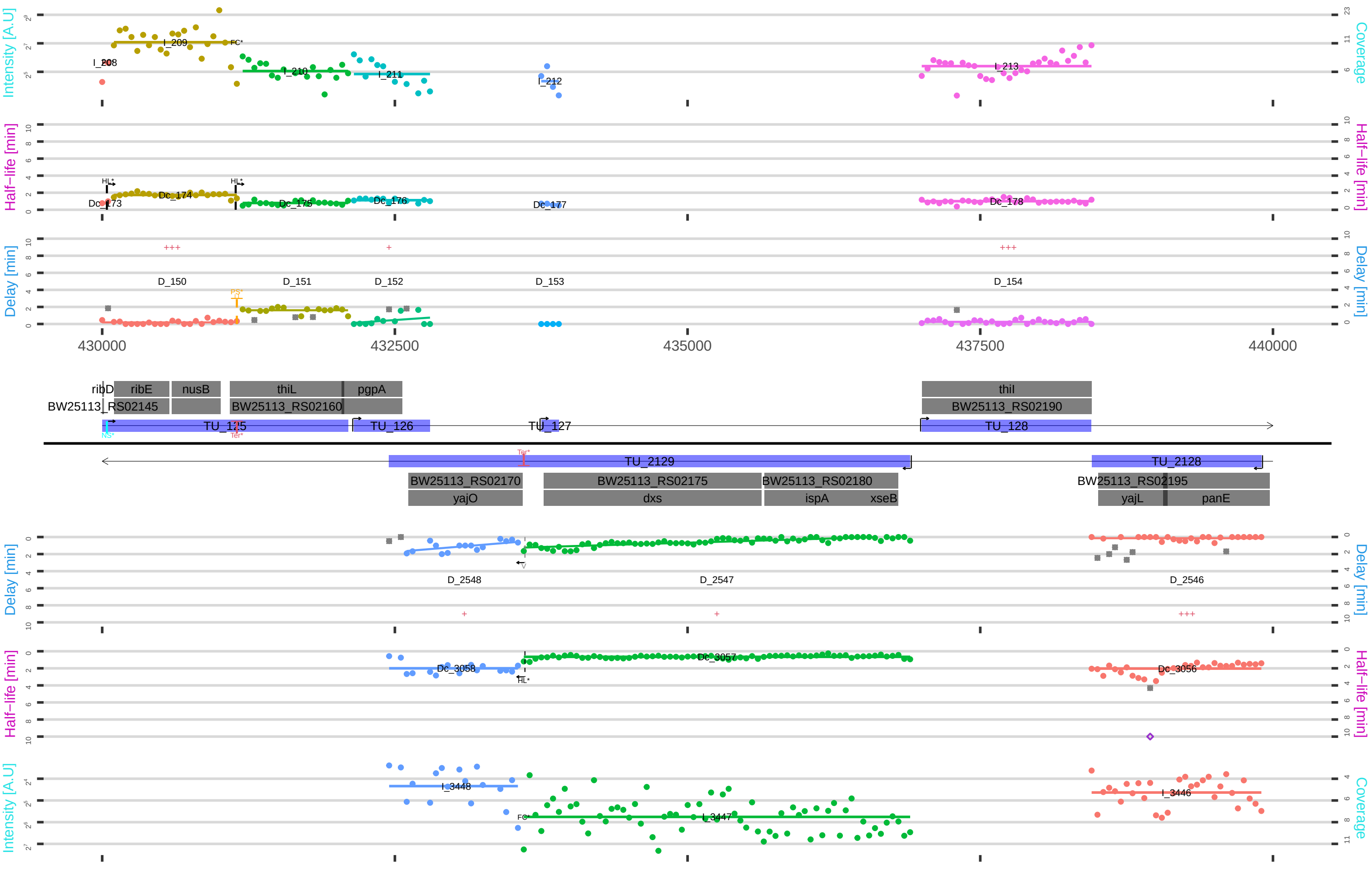

Term: termination (1), NS: new start (0), PS: pausing site (0), iTSS_I: internal starting site (1)

ID: 8803−9000; Term: termination (1), NS: new start (1), PS: pausing site (1), iTSS_I: internal starting site (0)

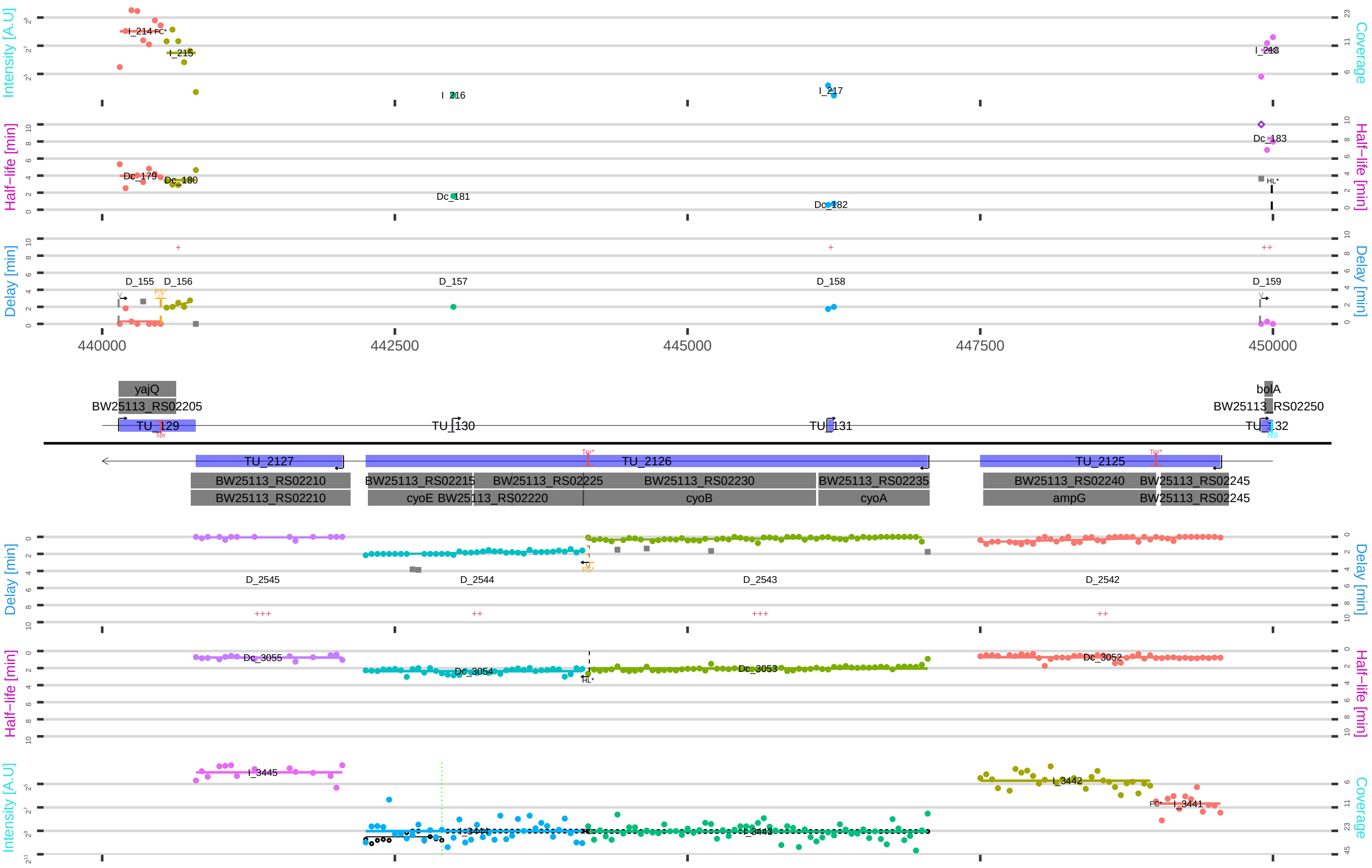

Term: termination (2), NS: new start (0), PS: pausing site (1), iTSS_I: internal starting site (0)

ID: 9000−9194; Term: termination (4), NS: new start (3), PS: pausing site (2), iTSS_I: internal starting site (0)

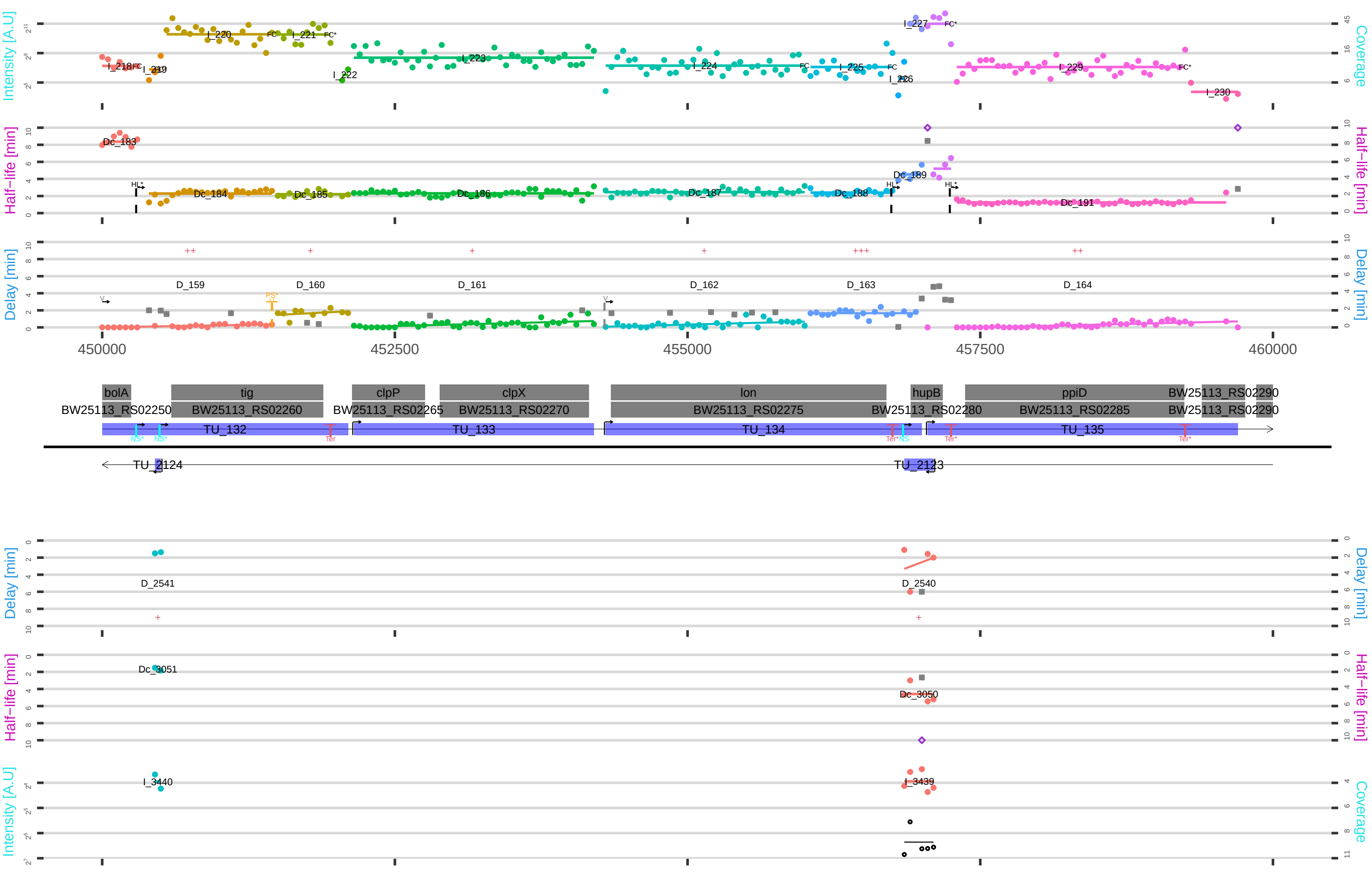

Term: termination (0), NS: new start (0), PS: pausing site (0), iTSS_I: internal starting site (0)

ID: 9260−9394; Term: termination (0), NS: new start (1), PS: pausing site (0), iTSS_I: internal starting site (0)

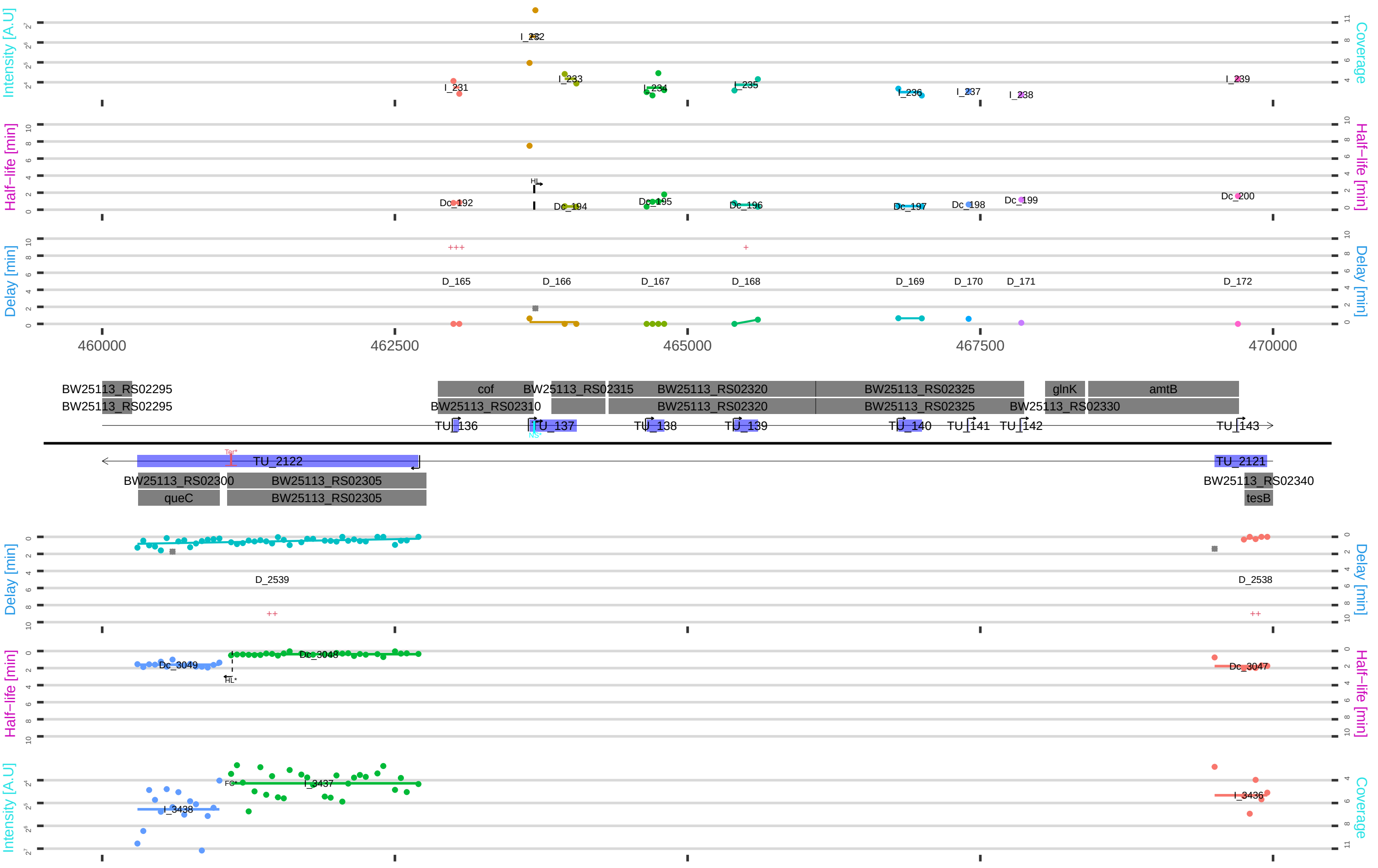

Term: termination (1), NS: new start (0), PS: pausing site (0), iTSS_I: internal starting site (0)

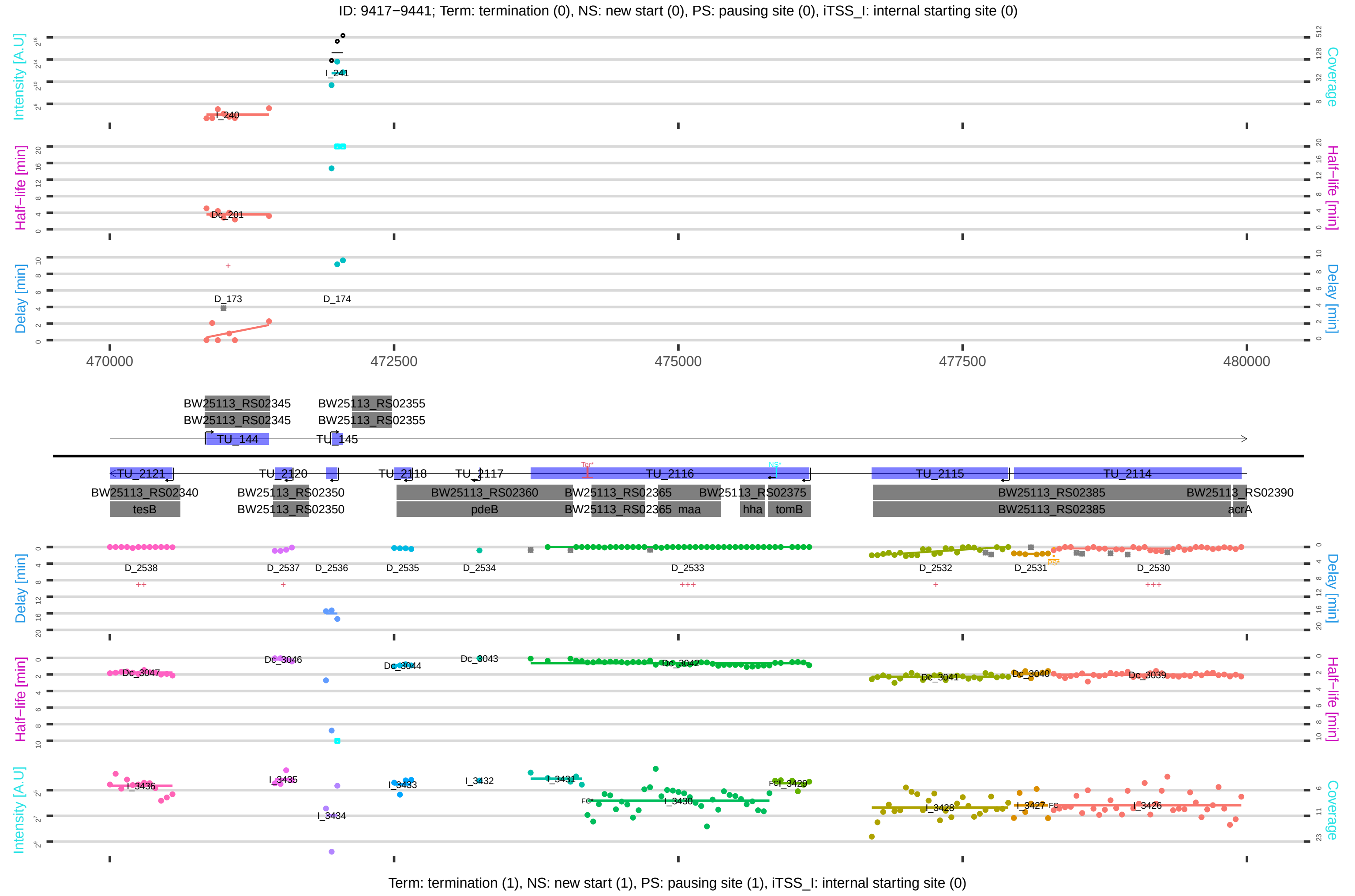

ID: 9417−9441; Term: termination (0), NS: new start (0), PS: pausing site (0), iTSS_I: internal starting site (0)
Intensity [A.U]
Coverage
Half−life [min]
Delay [min]
I_241
I_240
Dc_201
D_173
D_174
470000
472500
475000
477500
480000
BW25113_RS02345
BW25113_RS02355
TU_144
TU_145
TU_2121
TU_2120
TU_2118
TU_2117
TU_2116
TU_2115
TU_2114
BW25113_RS02340
tesB
BW25113_RS02350
BW25113_RS02360
pdeB
BW25113_RS02365
maa
BW25113_RS02375
hha
tomB
BW25113_RS02385
BW25113_RS02390
acrA
D_2538
D_2537
D_2536
D_2535
D_2534
D_2533
D_2532
D_2531
D_2530
Dc_3047
Dc_3046
Dc_3044
Dc_3043
Dc_3042
Dc_3041
Dc_3040
Dc_3039
I_3436
I_3435
I_3434
I_3433
I_3432
I_3431
I_3430
I_3429
I_3428
I_3427
I_3426
Term: termination (1), NS: new start (1), PS: pausing site (1), iTSS_I: internal starting site (0)

ID: 9626−9800; Term: termination (4), NS: new start (2), PS: pausing site (1), iTSS_I: internal starting site (0)

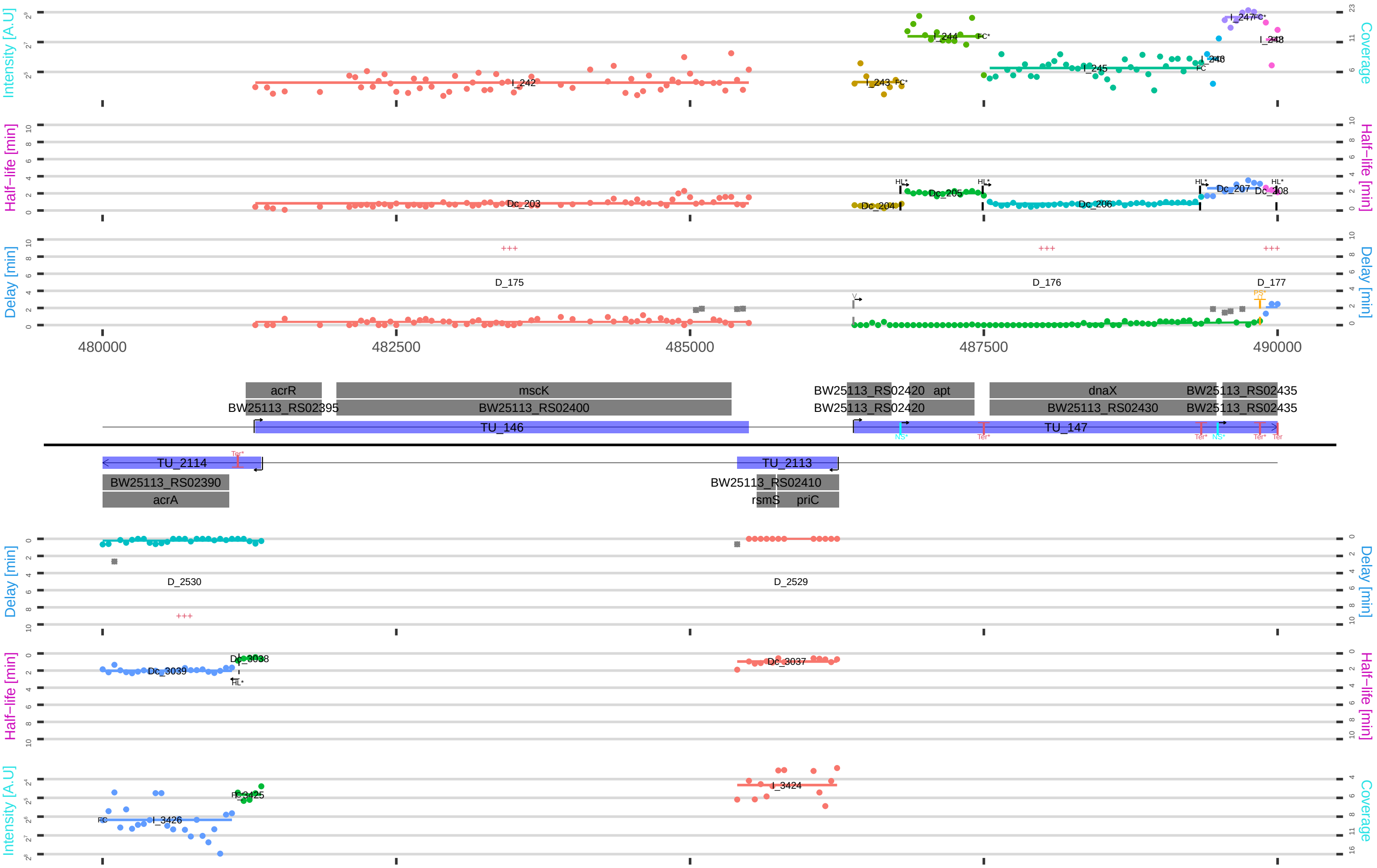

Term: termination (1), NS: new start (0), PS: pausing site (0), iTSS_I: internal starting site (0)

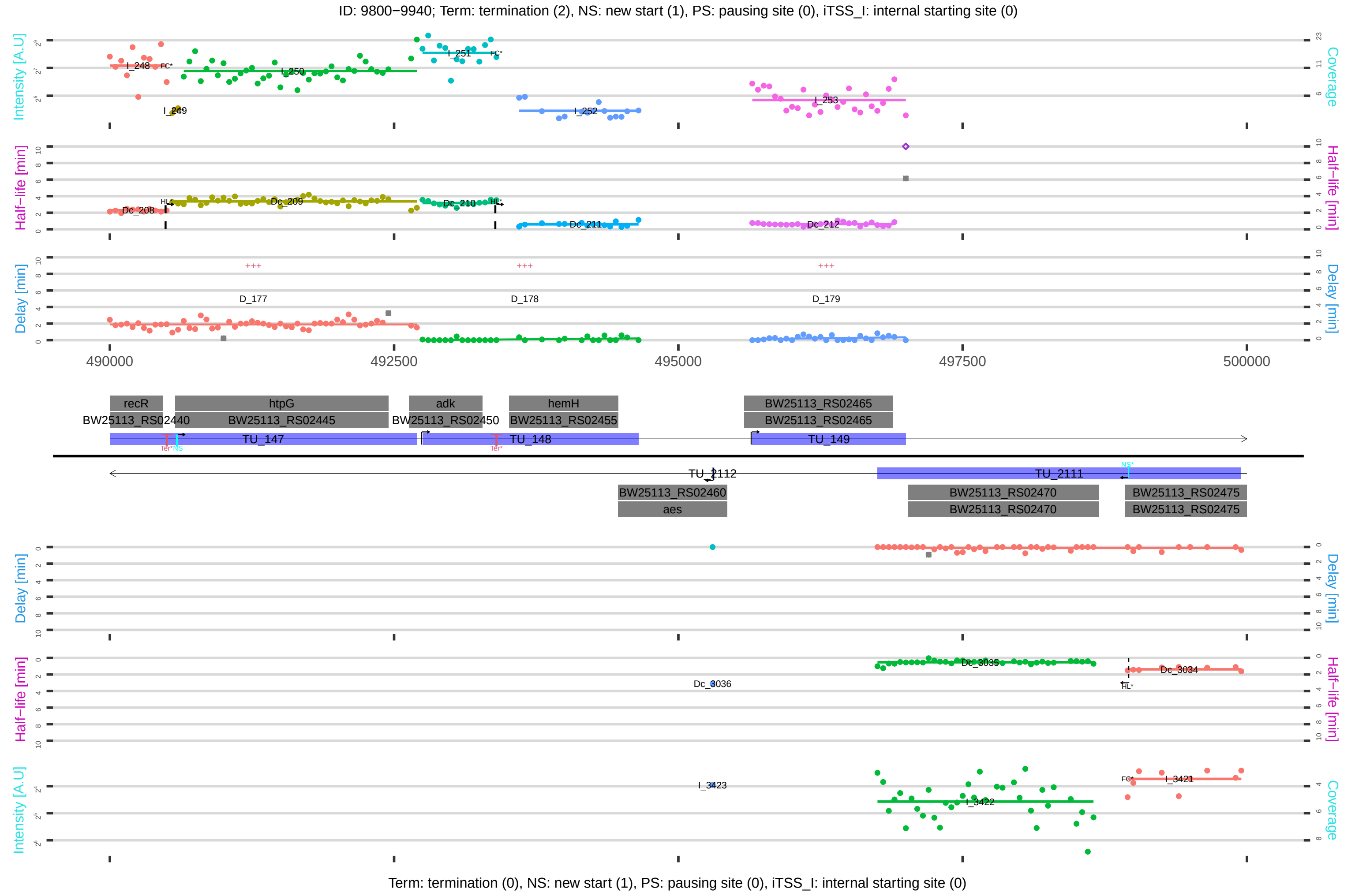

ID: 9800−9940; Term: termination (2), NS: new start (1), PS: pausing site (0), iTSS_I: internal starting site (0)
Intensity [A.U]
Coverage
Half−life [min]
Delay [min]
I_248
I_249
I_250
I_251
I_252
I_253
Dc_208
Dc_209
Dc_210
Dc_211
Dc_212
D_177
D_178
D_179
490000
492500
495000
497500
500000
recR
htpG
adk
hemH
BW25113_RS02440
BW25113_RS02445
BW25113_RS02450
BW25113_RS02455
BW25113_RS02465
TU_147
TU_148
TU_149
TU_2112
TU_2111
BW25113_RS02460
aes
BW25113_RS02470
BW25113_RS02475
Dc_3036
Dc_3035
Dc_3034
I_3423
I_3422
I_3421
Term: termination (0), NS: new start (1), PS: pausing site (0), iTSS_I: internal starting site (0)

ID: 10009−10197; Term: termination (0), NS: new start (0), PS: pausing site (0), iTSS_I: internal starting site (0)

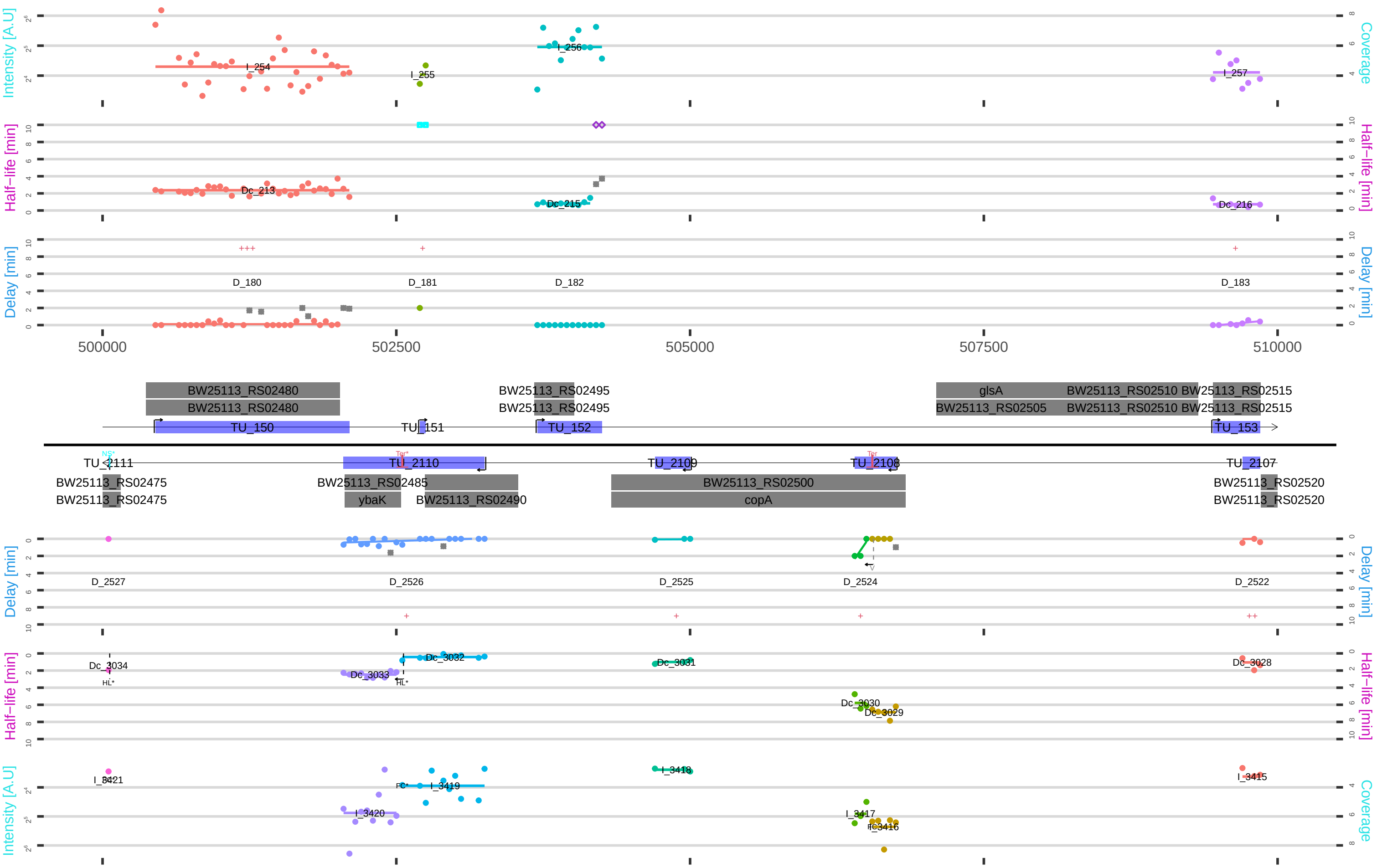

Term: termination (2), NS: new start (1), PS: pausing site (0), iTSS_I: internal starting site (1)

ID: 10229−10308; Term: termination (0), NS: new start (0), PS: pausing site (0), iTSS_I: internal starting site (0)

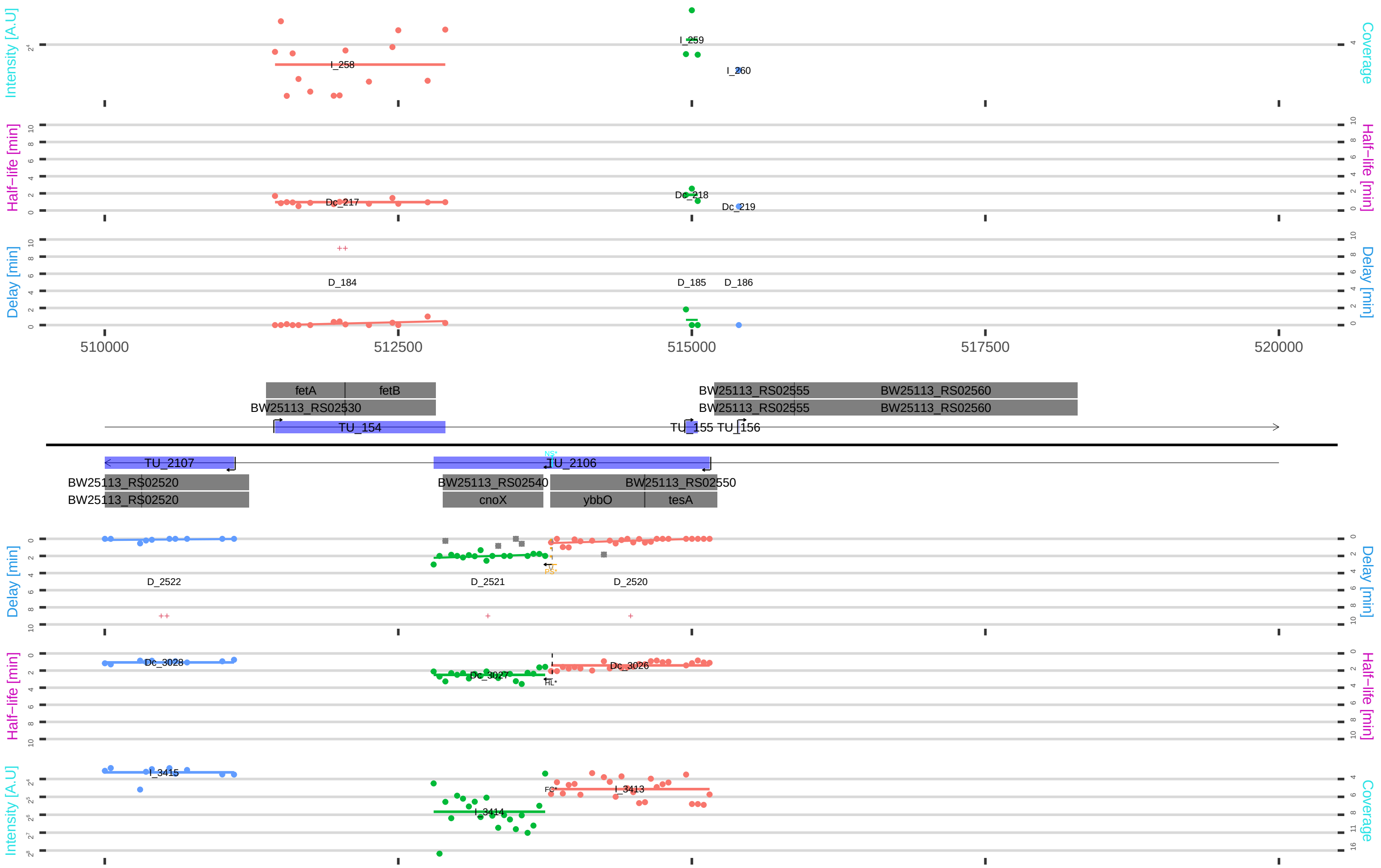

Term: termination (0), NS: new start (1), PS: pausing site (1), iTSS_I: internal starting site (0)

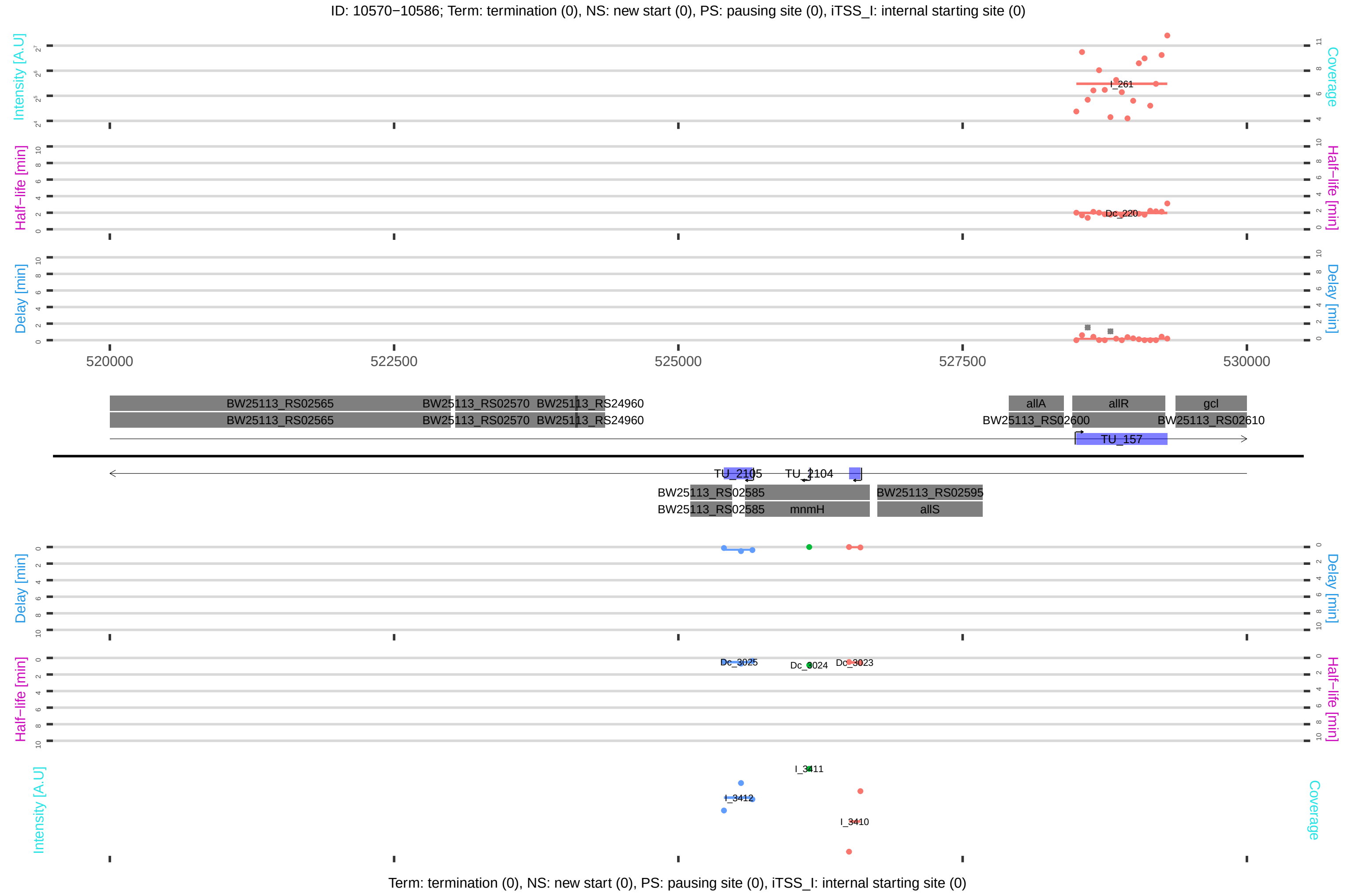

ID: 10570−10586; Term: termination (0), NS: new start (0), PS: pausing site (0), iTSS_I: internal starting site (0)
Intensity [A.U]
Coverage
I_261
Half−life [min]
Dc_220
Delay [min]
520000
522500
525000
527500
530000
BW25113_RS02565
BW25113_RS02570
BW25113_RS24960
allA
allR
gcl
BW25113_RS02600
BW25113_RS02610
TU_157
TU_2105
TU_2104
BW25113_RS02585
BW25113_RS02595
mnmH
allS
Dc_3025
Dc_3024
Dc_3023
I_3411
I_3412
I_3410
Term: termination (0), NS: new start (0), PS: pausing site (0), iTSS_I: internal starting site (0)

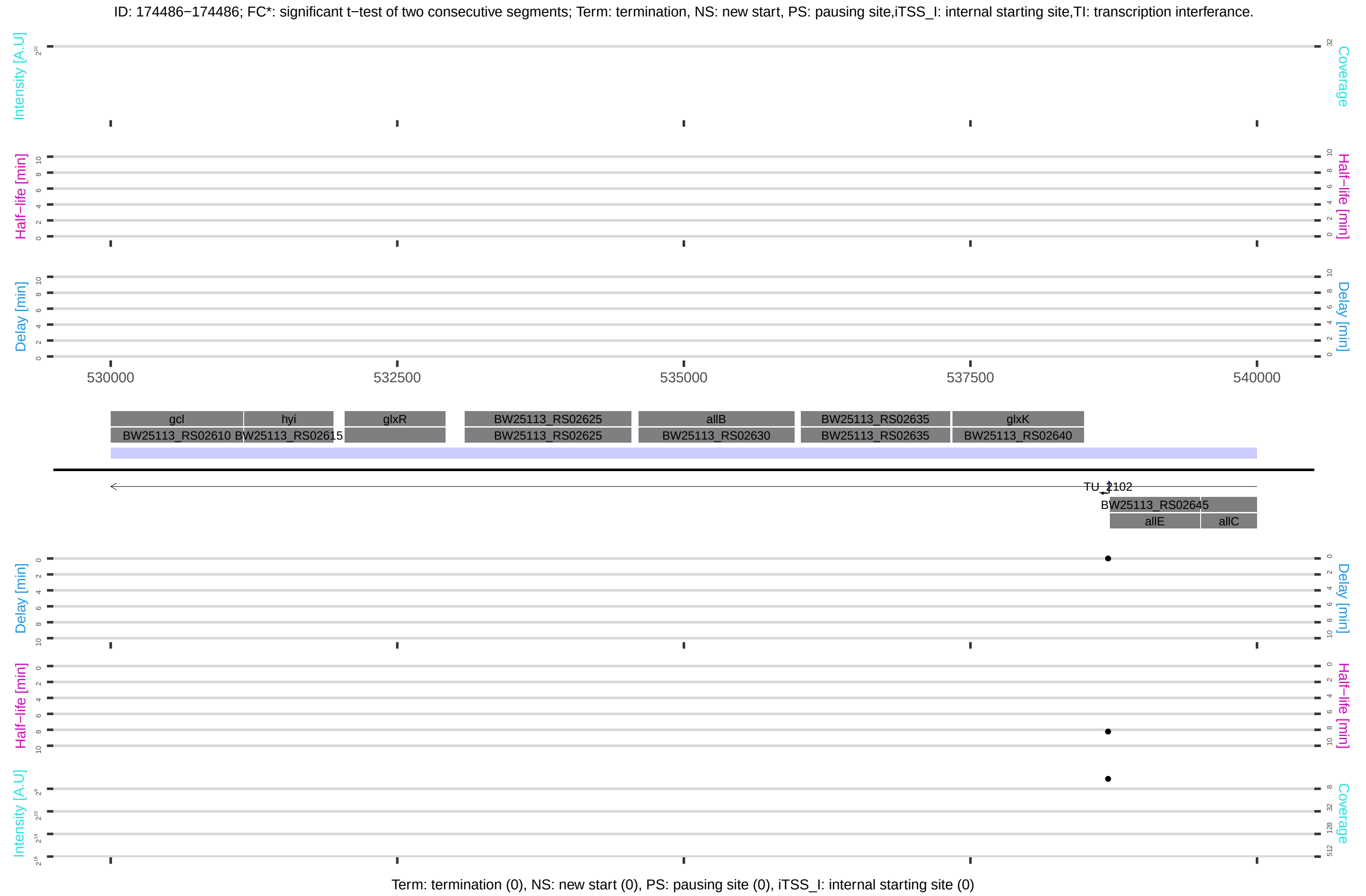
ID: 174486−174486; FC*: significant t−test of two consecutive segments; Term: termination, NS: new start, PS: pausing site,iTSS_I: internal starting site,TI: transcription interferance.
Intensity [A.U]
Coverage
Half−life [min]
Delay [min]
530000
532500
535000
537500
540000
gcl
hyi
glxR
BW25113_RS02625
allB
BW25113_RS02635
glxK
BW25113_RS02610
BW25113_RS02615
BW25113_RS02630
BW25113_RS02640
TU_2102
BW25113_RS02645
allE
allC
Term: termination (0), NS: new start (0), PS: pausing site (0), iTSS_I: internal starting site (0)

ID: 10994−10994; Term: termination (0), NS: new start (0), PS: pausing site (0), iTSS_I: internal starting site (0)

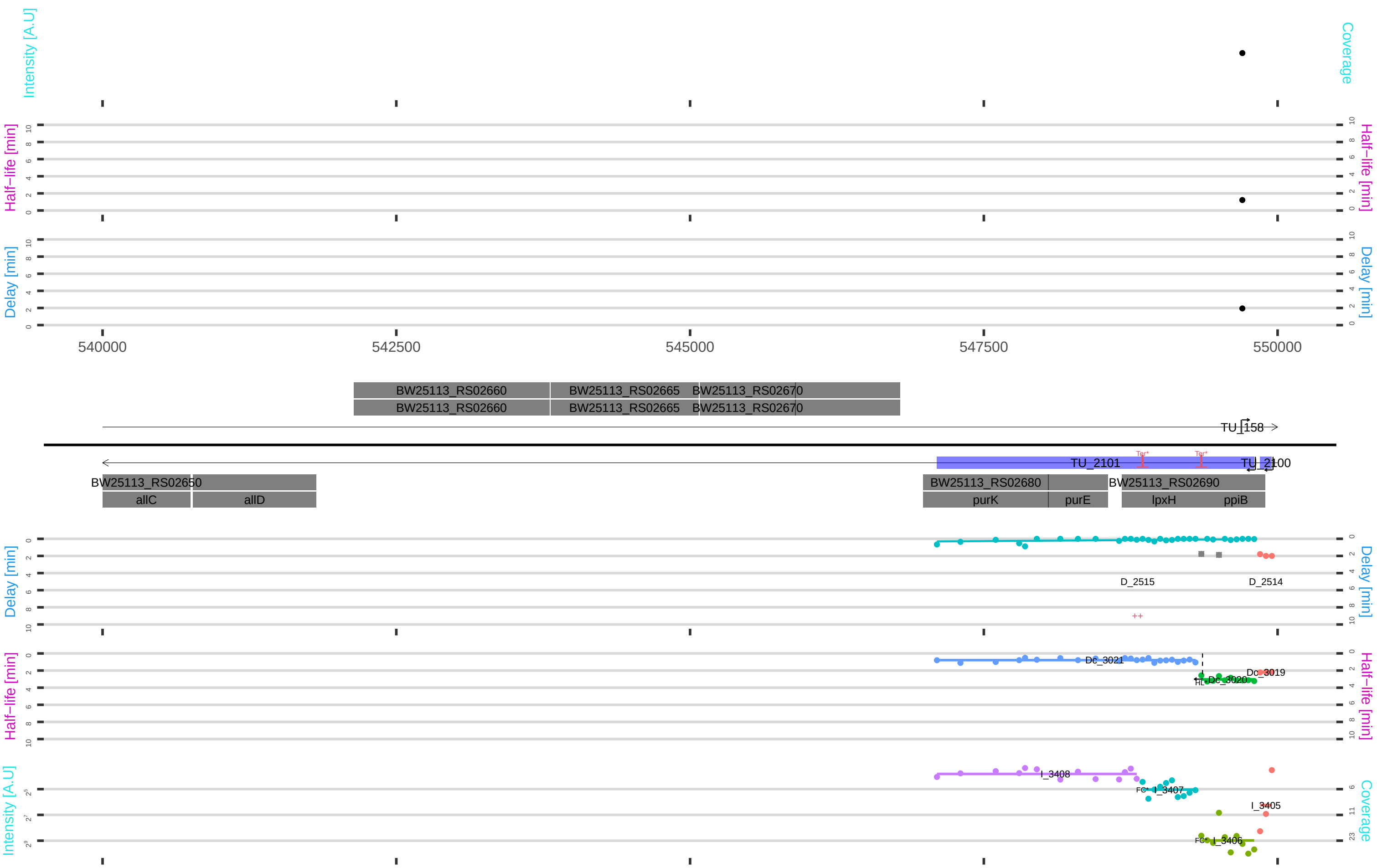

Term: termination (2), NS: new start (0), PS: pausing site (0), iTSS_I: internal starting site (0)

ID: 11002−11030; Term: termination (0), NS: new start (0), PS: pausing site (0), iTSS_I: internal starting site (0)

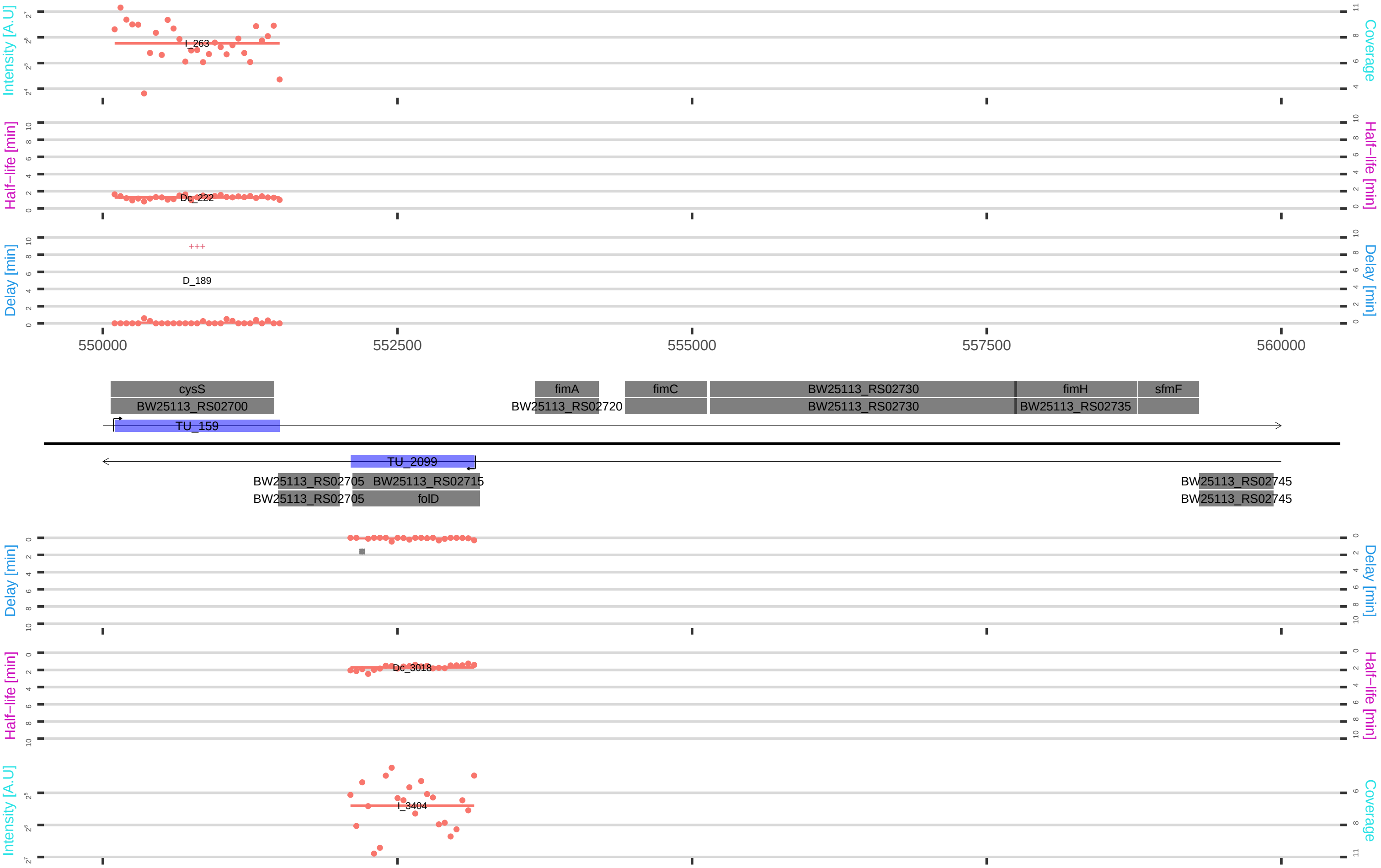

Term: termination (0), NS: new start (0), PS: pausing site (0), iTSS_I: internal starting site (0)

ID: 11205−11355; Term: termination (0), NS: new start (0), PS: pausing site (0), iTSS_I: internal starting site (0)

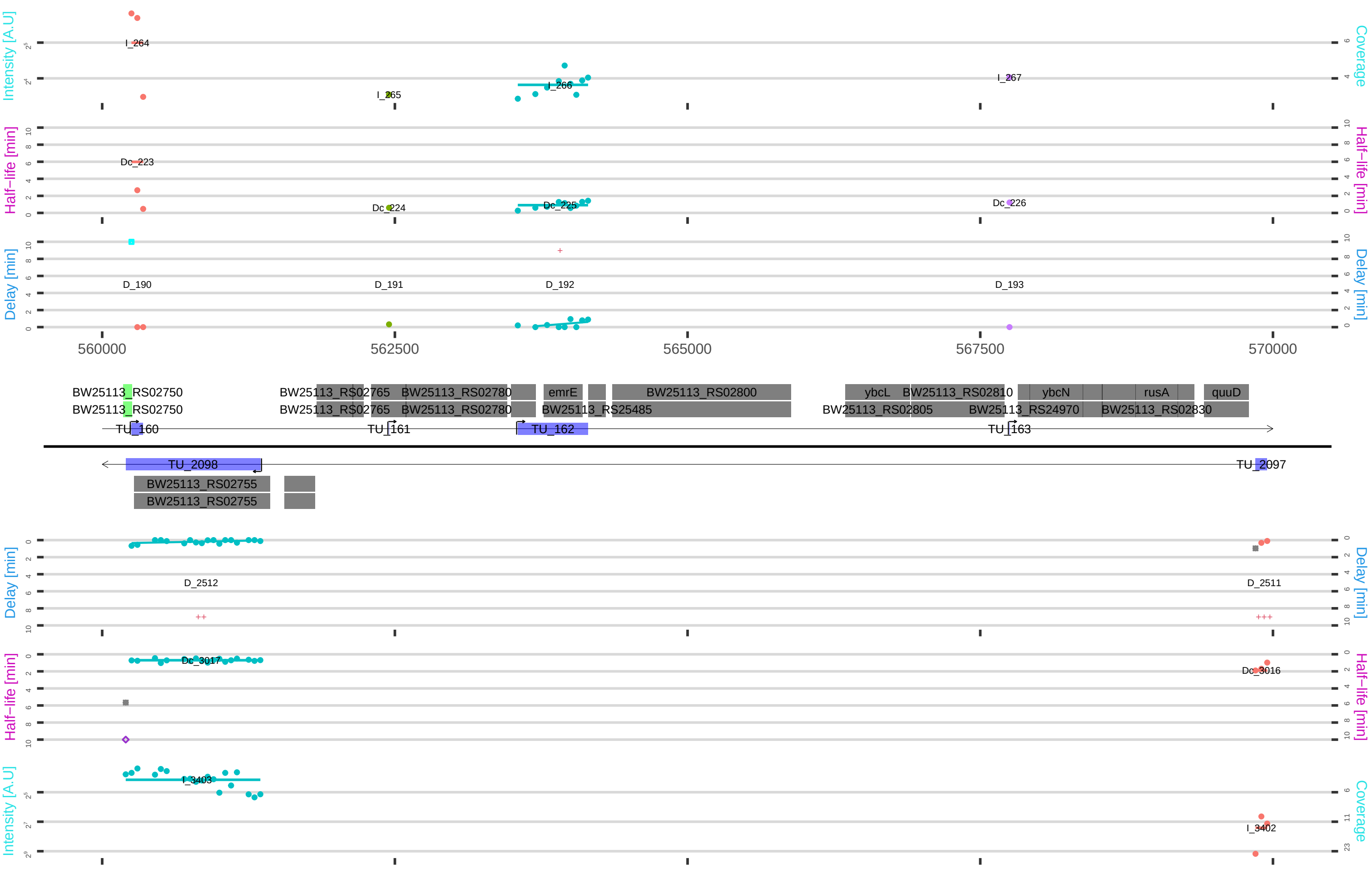

Term: termination (0), NS: new start (0), PS: pausing site (0), iTSS_I: internal starting site (0)

ID: 11506−11589; Term: termination (0), NS: new start (0), PS: pausing site (0), iTSS_I: internal starting site (0)

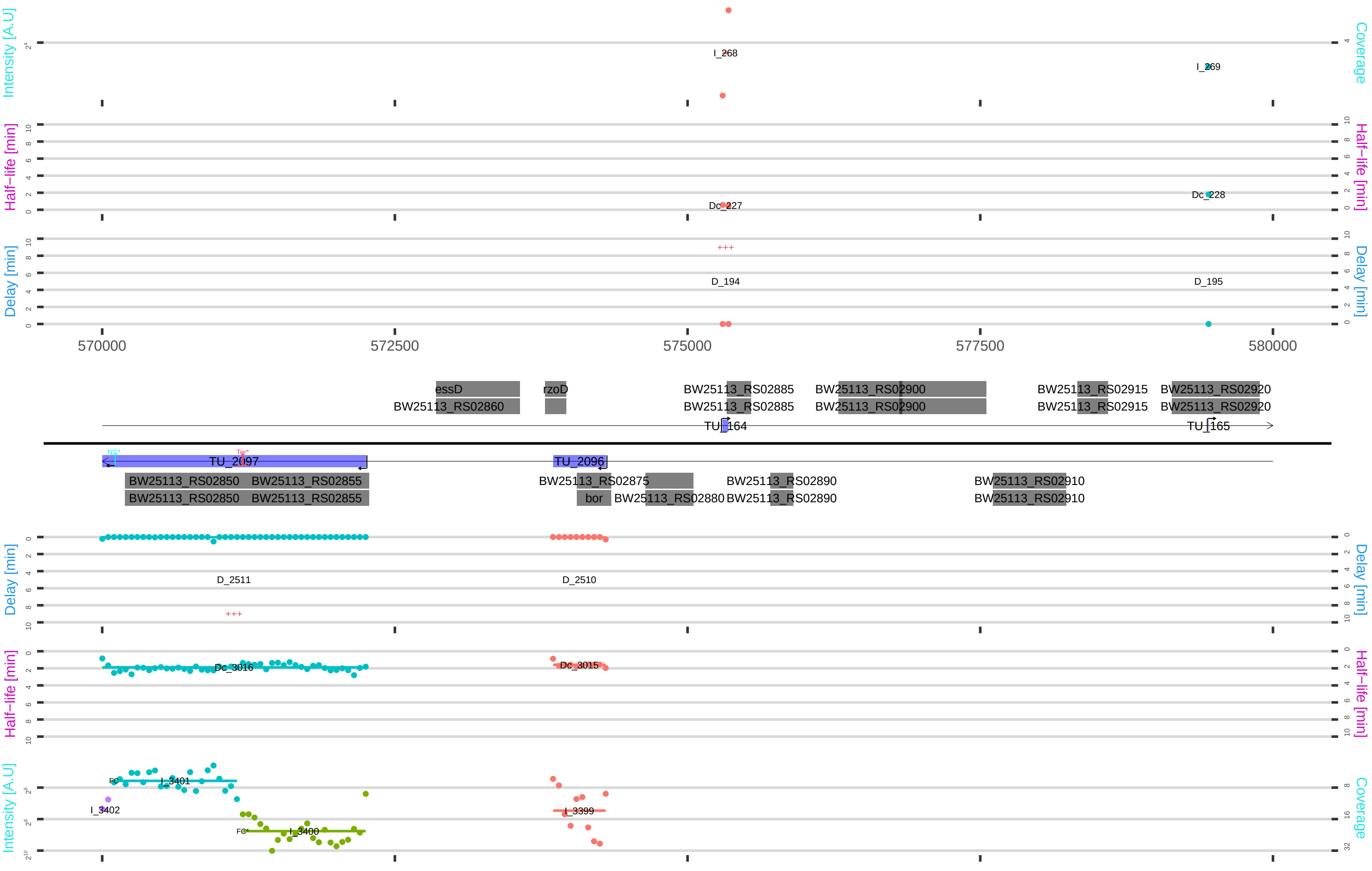

Term: termination (1), NS: new start (1), PS: pausing site (0), iTSS_I: internal starting site (0)

ID: 11615−11631; Term: termination (0), NS: new start (0), PS: pausing site (0), iTSS_I: internal starting site (0)

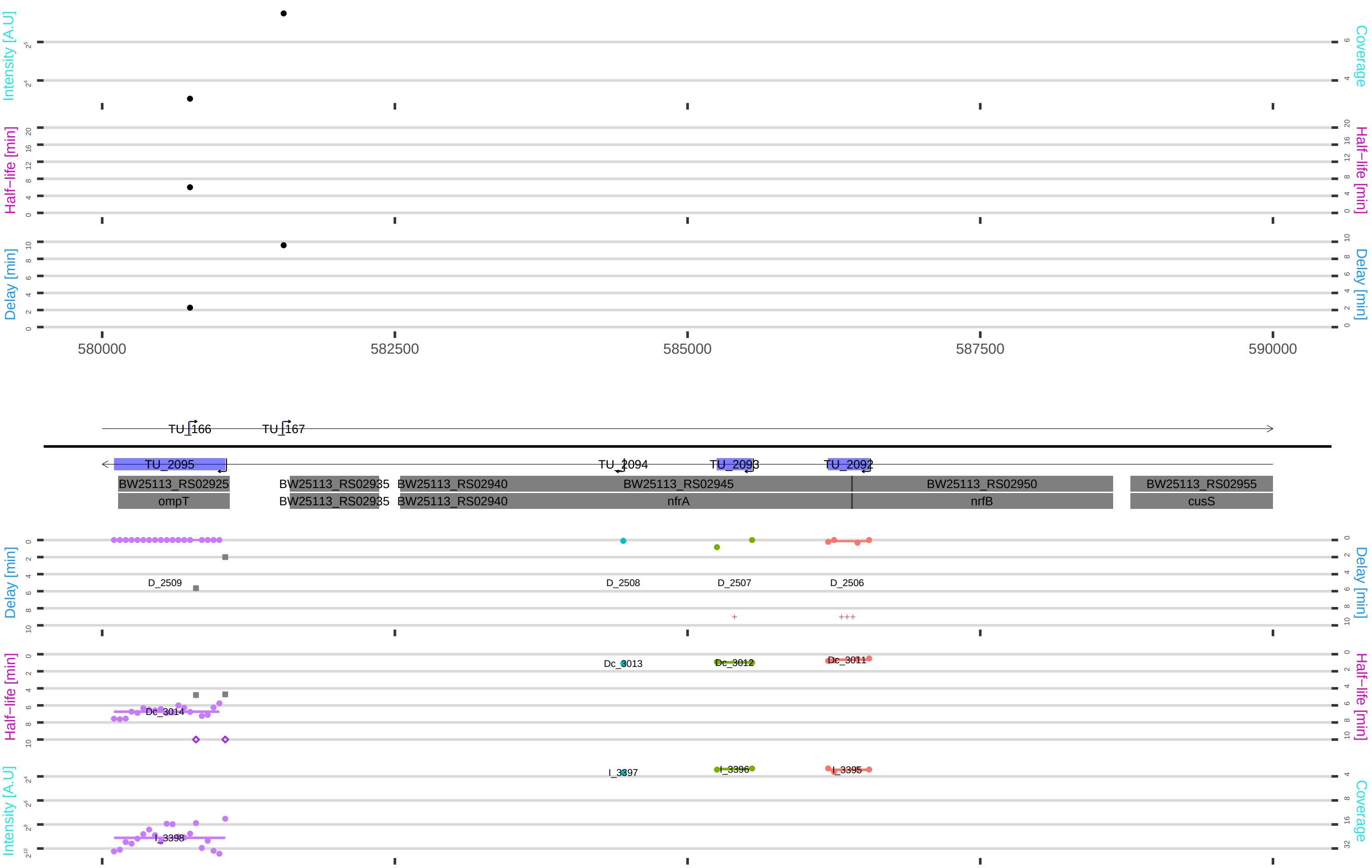

Term: termination (0), NS: new start (0), PS: pausing site (0), iTSS_I: internal starting site (0)

ID: 11950−11977; Term: termination (0), NS: new start (0), PS: pausing site (0), iTSS_I: internal starting site (0)

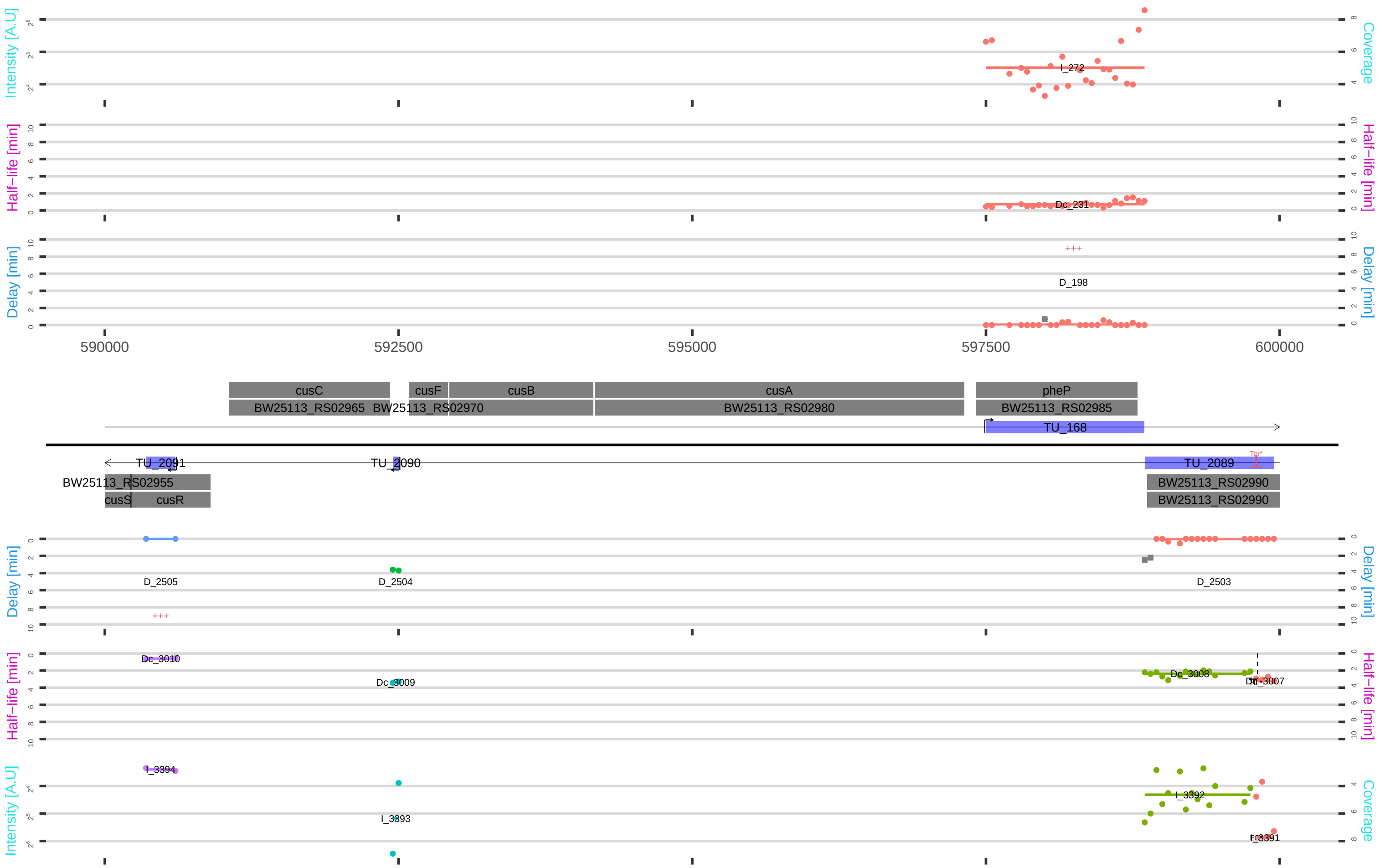

Term: termination (1), NS: new start (0), PS: pausing site (0), iTSS_I: internal starting site (0)

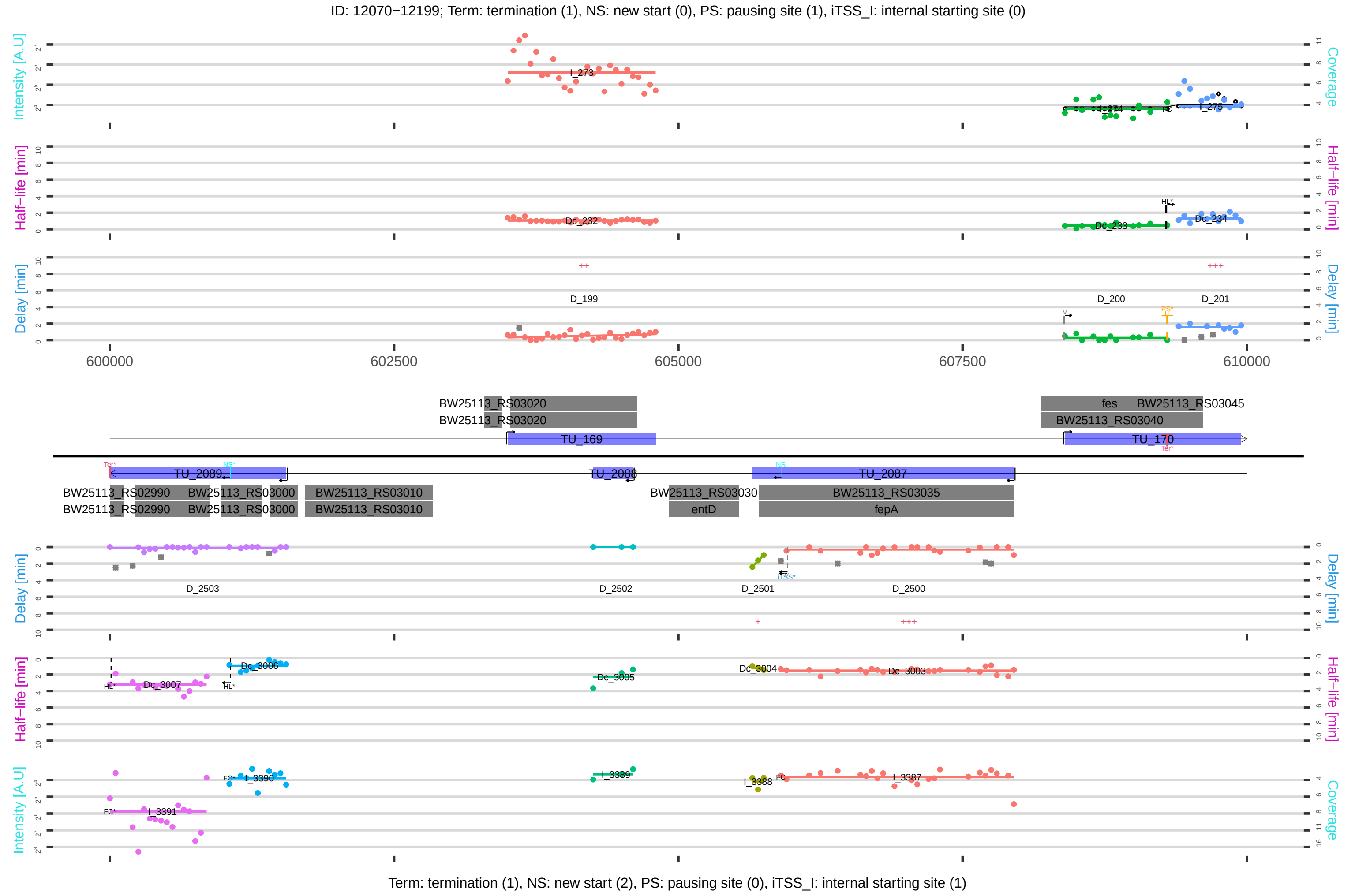
ID: 12070−12199; Term: termination (1), NS: new start (0), PS: pausing site (1), iTSS_I: internal starting site (0)
Intensity [A.U]
Coverage
Half−life [min]
Delay [min]
I_273
I_274
I_275
Dc_232
Dc_233
Dc_234
D_199
D_200
D_201
600000
602500
605000
607500
610000
BW25113_RS03020
fes
BW25113_RS03045
BW25113_RS03040
TU_169
TU_170
TU_2089
TU_2088
TU_2087
BW25113_RS02990
BW25113_RS03000
BW25113_RS03010
BW25113_RS03030
BW25113_RS03035
entD
fepA
D_2503
D_2502
D_2501
D_2500
Dc_3007
Dc_3006
Dc_3005
Dc_3004
Dc_3003
I_3391
I_3390
I_3389
I_3388
I_3387
Term: termination (1), NS: new start (2), PS: pausing site (0), iTSS_I: internal starting site (1)

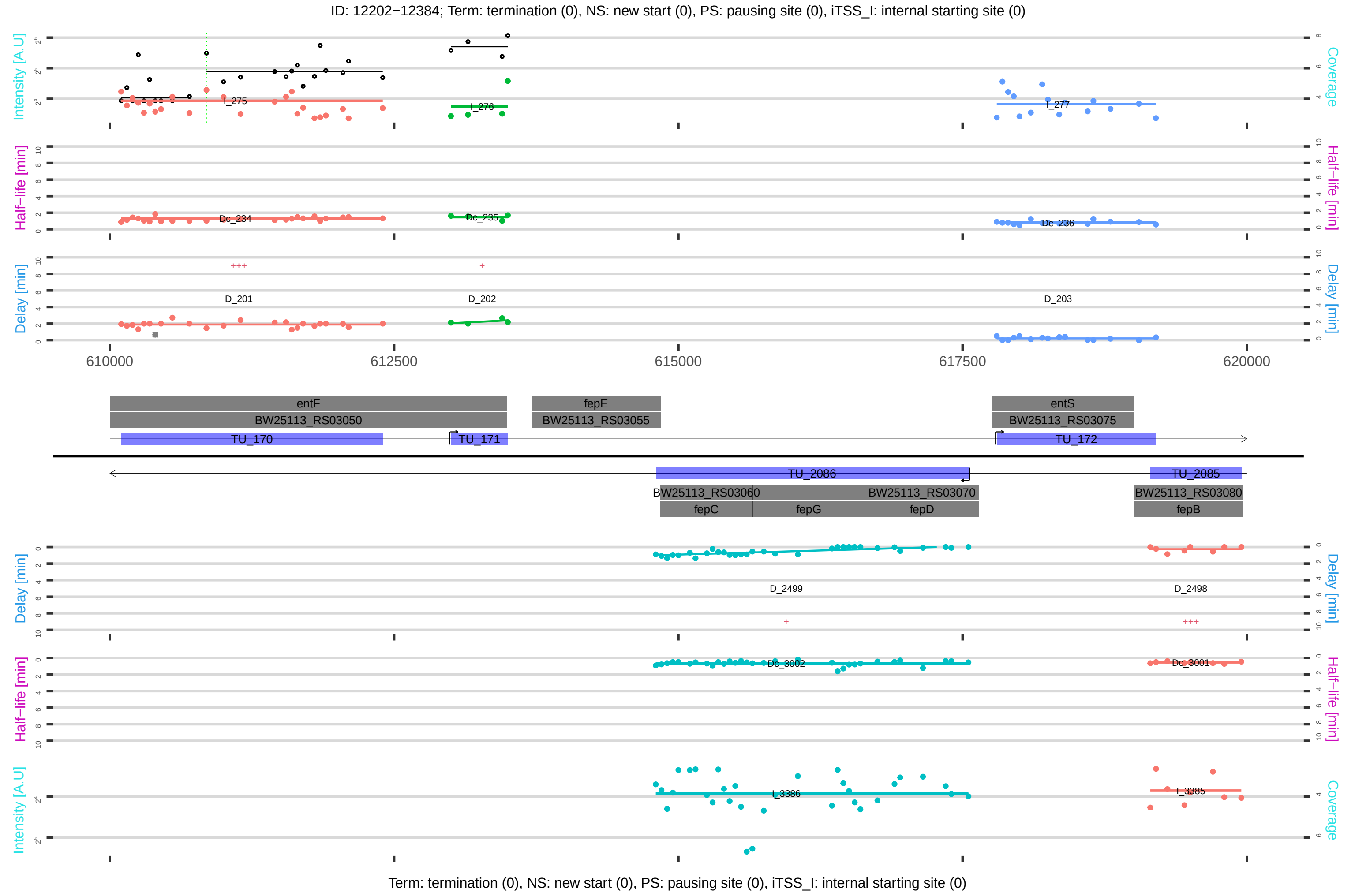

ID: 12202−12384; Term: termination (0), NS: new start (0), PS: pausing site (0), iTSS_I: internal starting site (0)
Intensity [A.U]
Coverage
I_275
I_276
I_277
Half−life [min]
Dc_234
Dc_235
Dc_236
Delay [min]
D_201
D_202
D_203
610000
612500
615000
617500
620000
entF
BW25113_RS03050
fepE
BW25113_RS03055
entS
BW25113_RS03075
TU_170
TU_171
TU_172
TU_2086
TU_2085
BW25113_RS03060
BW25113_RS03070
BW25113_RS03080
fepC
fepG
fepD
fepB
D_2499
D_2498
Dc_3002
Dc_3001
I_3386
I_3385
Term: termination (0), NS: new start (0), PS: pausing site (0), iTSS_I: internal starting site (0)

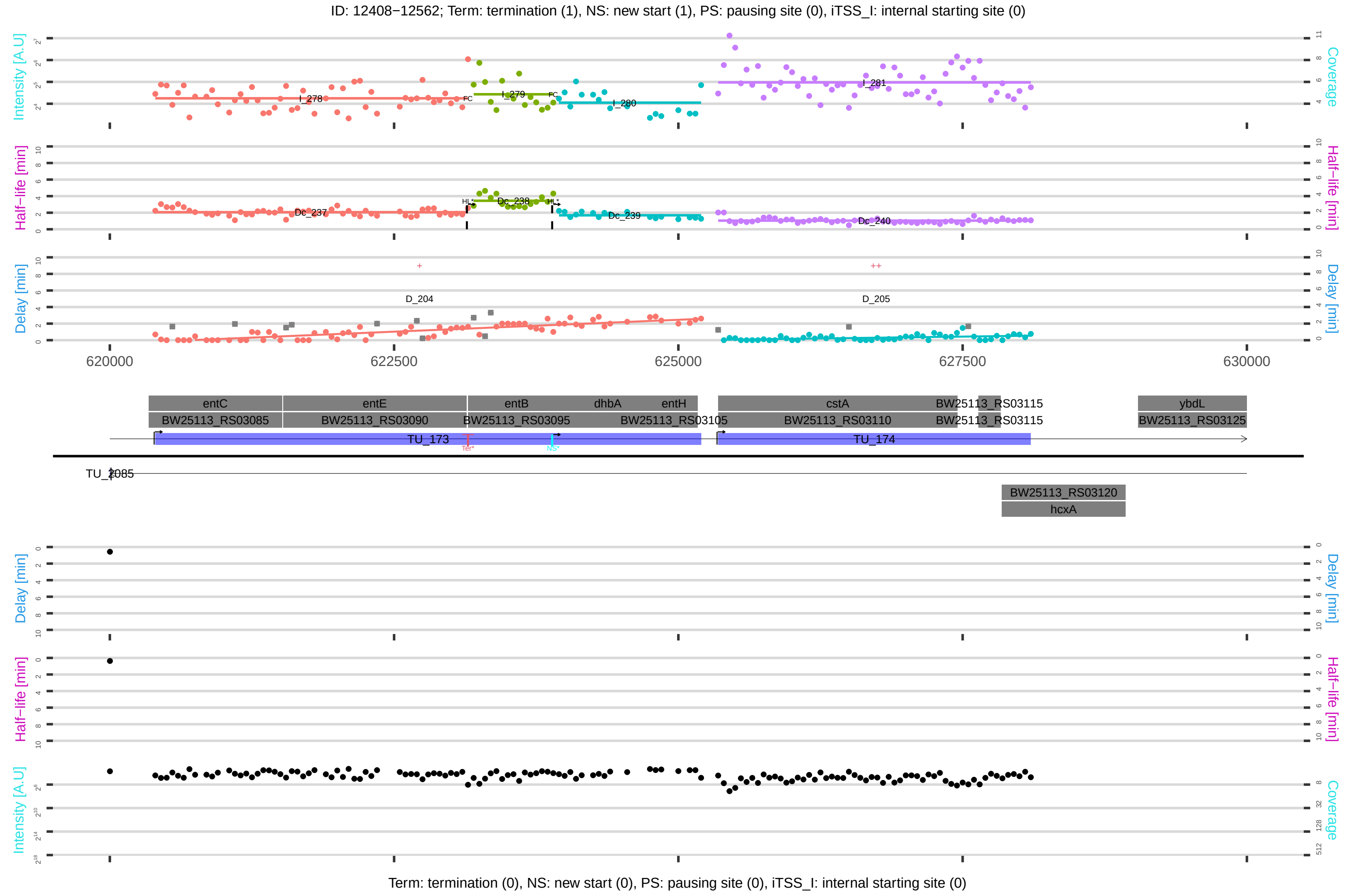
ID: 12408−12562; Term: termination (1), NS: new start (1), PS: pausing site (0), iTSS_I: internal starting site (0)
Intensity [A.U]
Coverage
Half−life [min]
Delay [min]
I_278
I_279
I_280
I_281
Dc_237
Dc_238
Dc_239
Dc_240
D_204
D_205
620000
622500
625000
627500
630000
entC
entE
entB
dhbA
entH
cstA
ybdL
BW25113_RS03085
BW25113_RS03090
BW25113_RS03095
BW25113_RS03105
BW25113_RS03110
BW25113_RS03115
BW25113_RS03125
BW25113_RS03120
hcxA
TU_173
TU_174
TU_2085
Term: termination (0), NS: new start (0), PS: pausing site (0), iTSS_I: internal starting site (0)

ID: 12683−12736; Term: termination (0), NS: new start (2), PS: pausing site (0), iTSS_I: internal starting site (0)

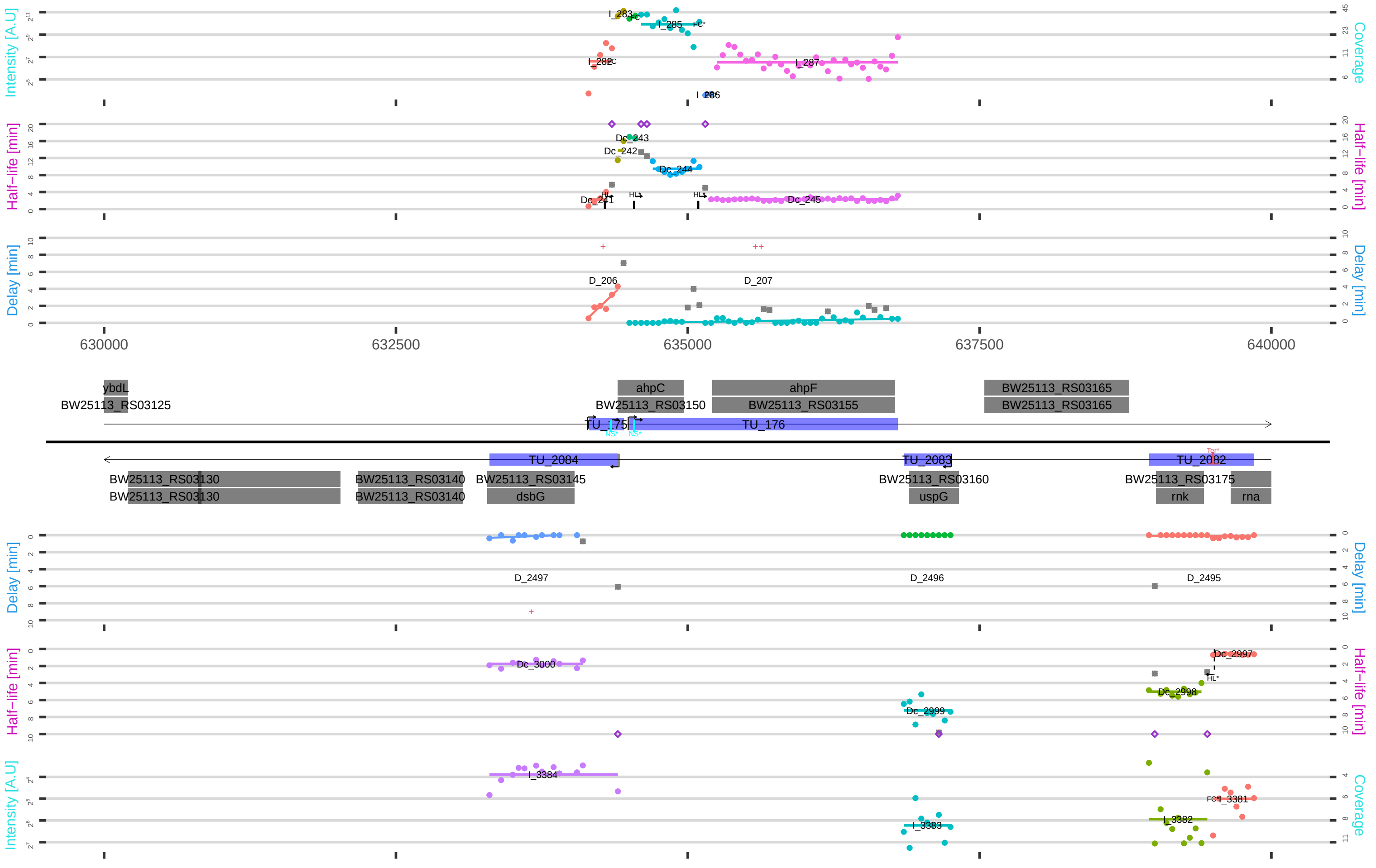

Term: termination (1), NS: new start (0), PS: pausing site (0), iTSS_I: internal starting site (0)

ID: 12964−13000; Term: termination (0), NS: new start (0), PS: pausing site (0), iTSS_I: internal starting site (0)

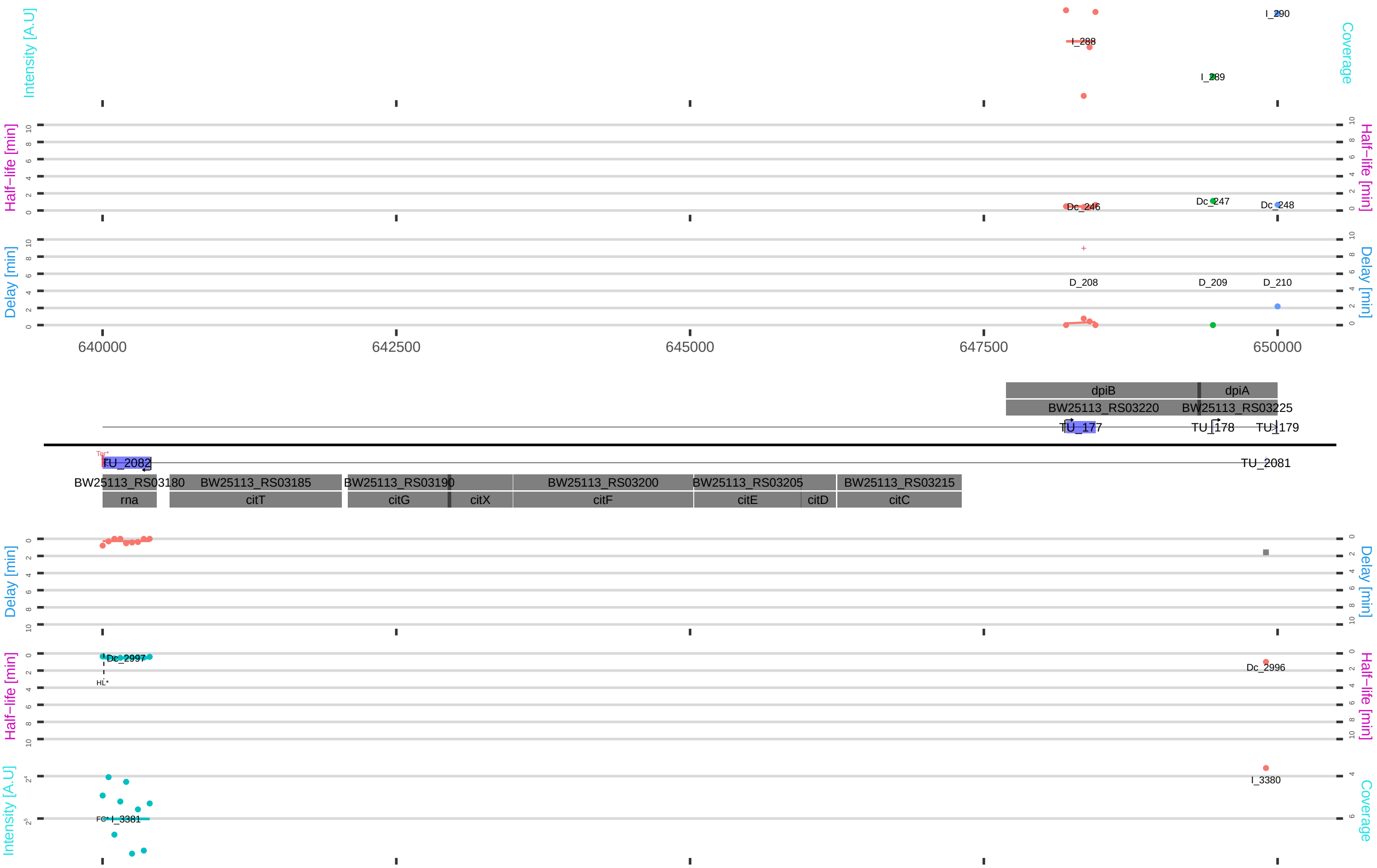

Term: termination (1), NS: new start (0), PS: pausing site (0), iTSS_I: internal starting site (0)

ID: 13000−13093; Term: termination (0), NS: new start (0), PS: pausing site (0), iTSS_I: internal starting site (0)

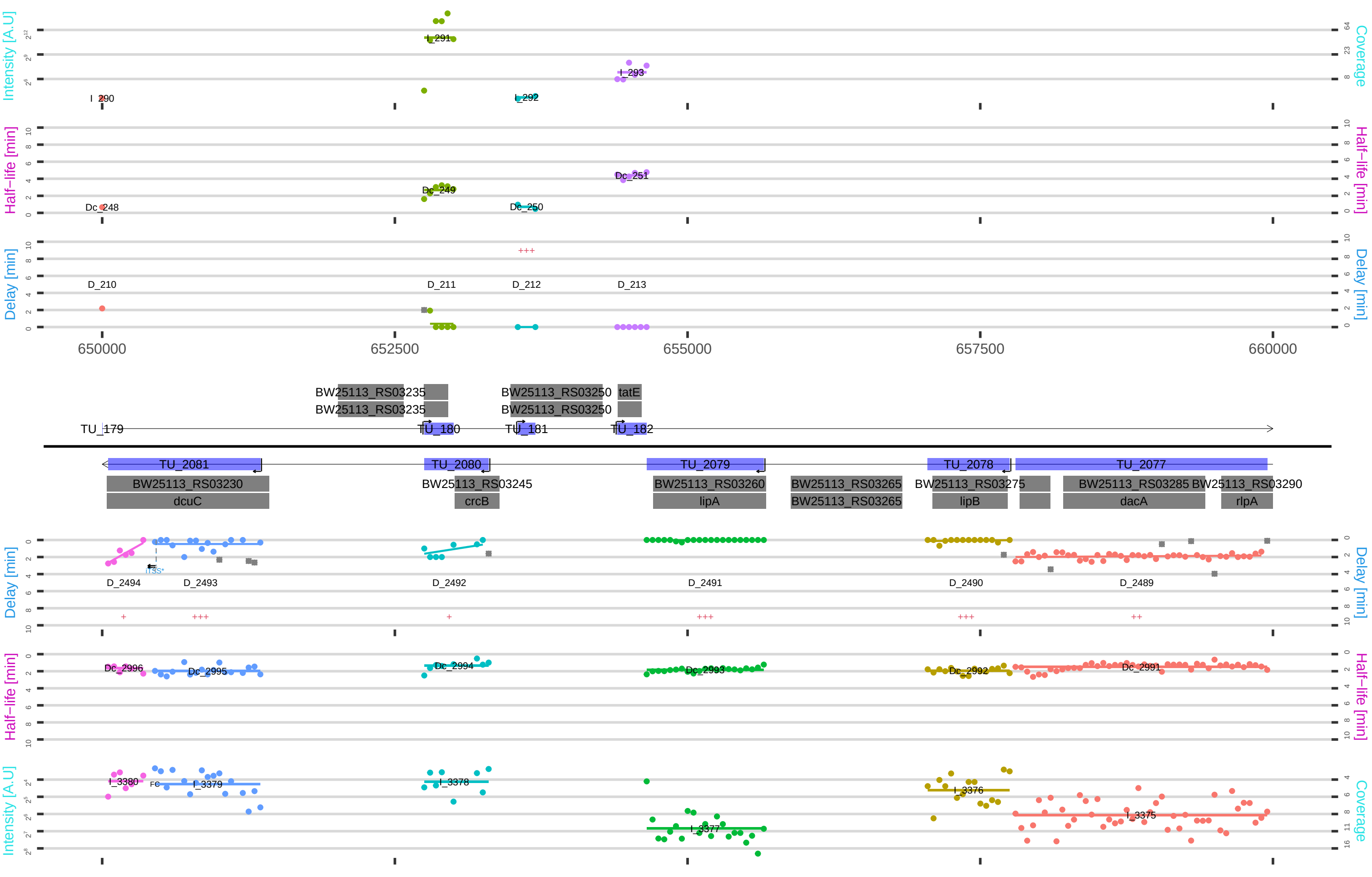

Term: termination (0), NS: new start (0), PS: pausing site (0), iTSS_I: internal starting site (1)

ID: 172060−171861; FC*: significant t−test of two consecutive segments; Term: termination, NS: new start, PS: pausing site,iTSS_I: internal starting site,TI: transcription interferance.

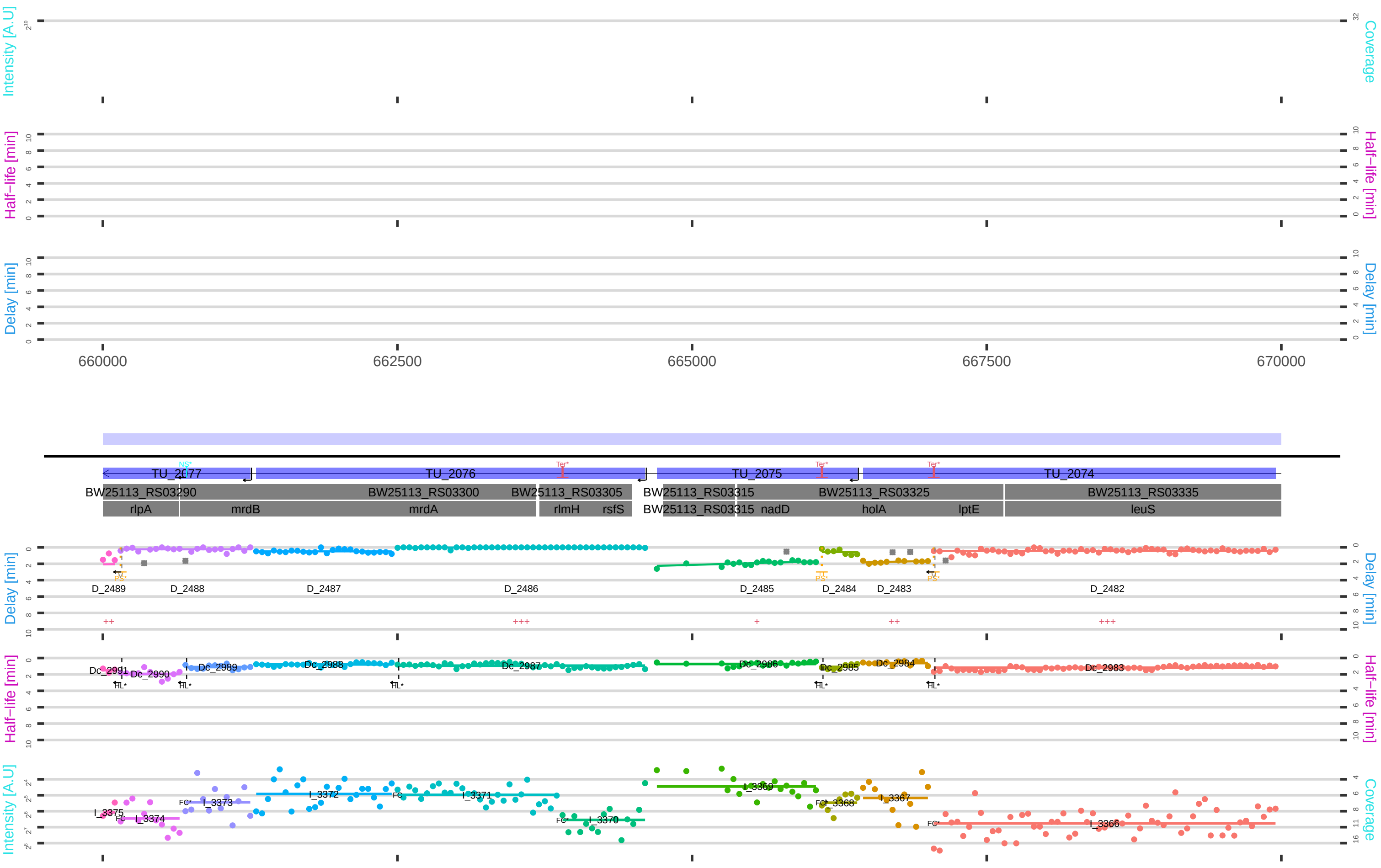

Term: termination (3), NS: new start (1), PS: pausing site (4), iTSS_I: internal starting site (0)

ID: 13410−13422; Term: termination (0), NS: new start (0), PS: pausing site (0), iTSS_I: internal starting site (0)

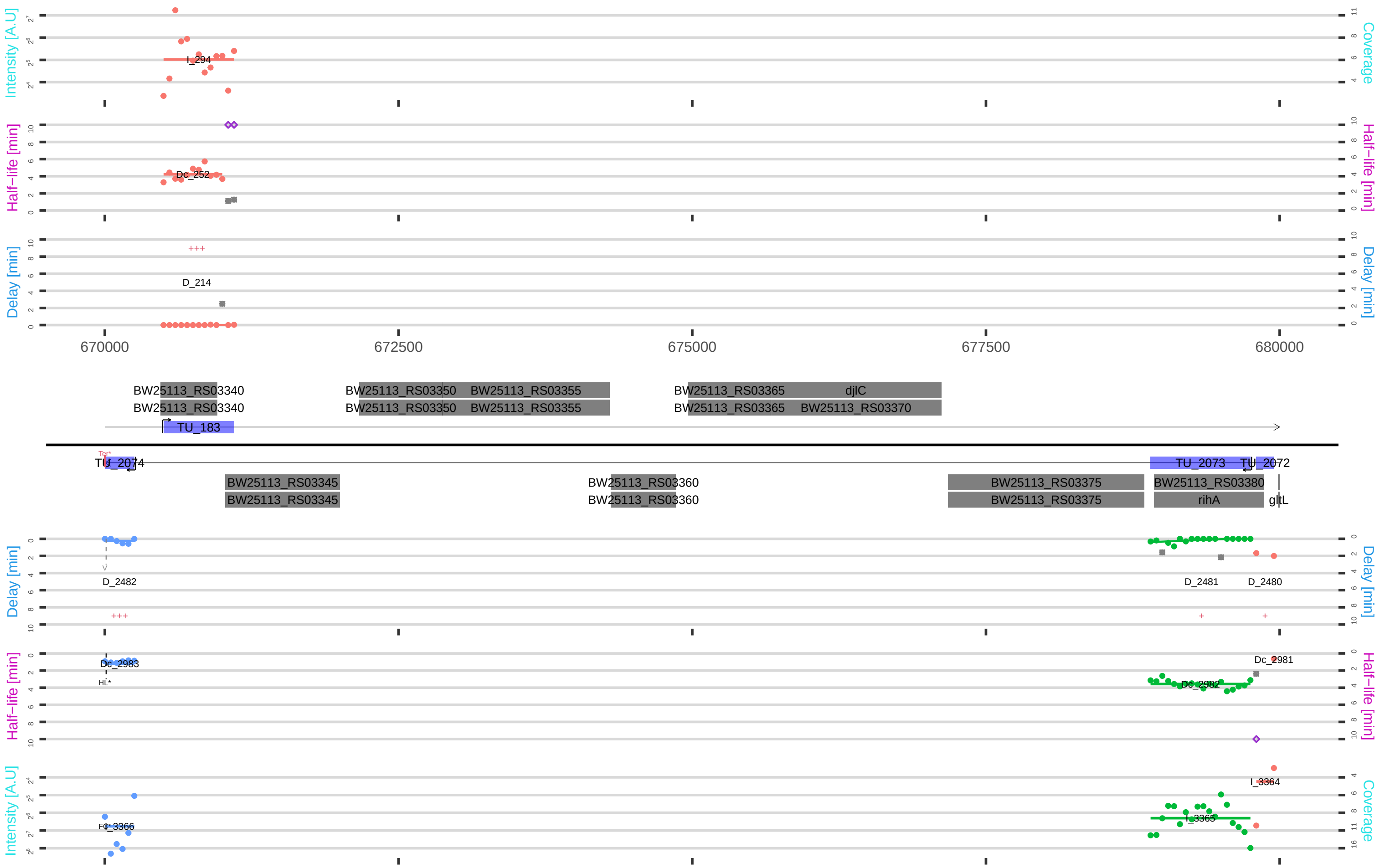

Term: termination (1), NS: new start (0), PS: pausing site (0), iTSS_I: internal starting site (0)

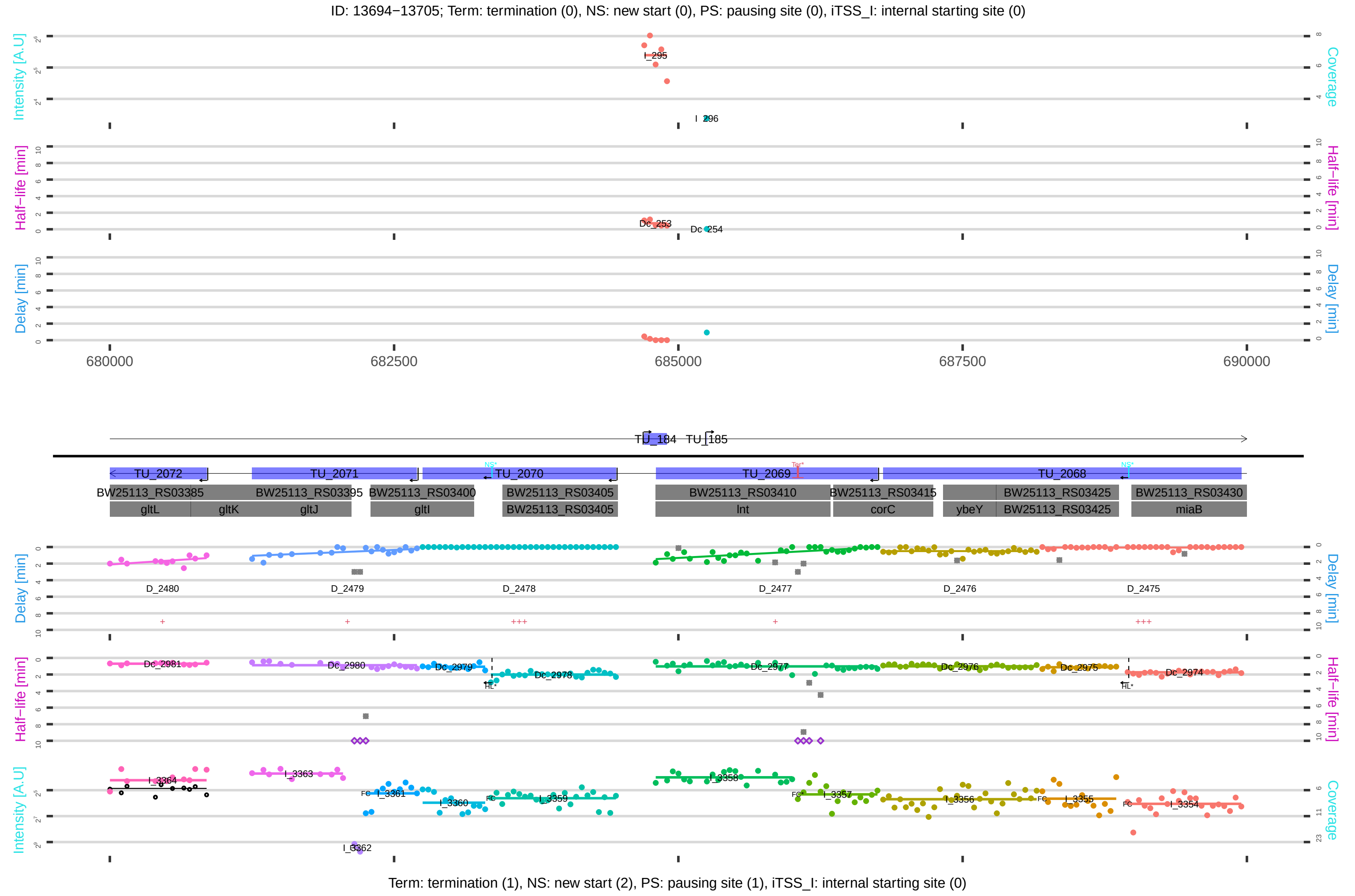
ID: 13694−13705; Term: termination (0), NS: new start (0), PS: pausing site (0), iTSS_I: internal starting site (0)
Intensity [A.U]
Coverage
Half−life [min]
Delay [min]
680000
682500
685000
687500
690000
TU_2072
TU_2071
TU_2070
TU_2069
TU_2068
BW25113_RS03385
BW25113_RS03395
BW25113_RS03400
BW25113_RS03405
BW25113_RS03410
BW25113_RS03415
BW25113_RS03425
BW25113_RS03430
gltL
gltK
gltJ
gltI
lnt
corC
ybeY
miaB
Term: termination (1), NS: new start (2), PS: pausing site (1), iTSS_I: internal starting site (0)

ID: 13813−14000; Term: termination (2), NS: new start (1), PS: pausing site (1), iTSS_I: internal starting site (0)

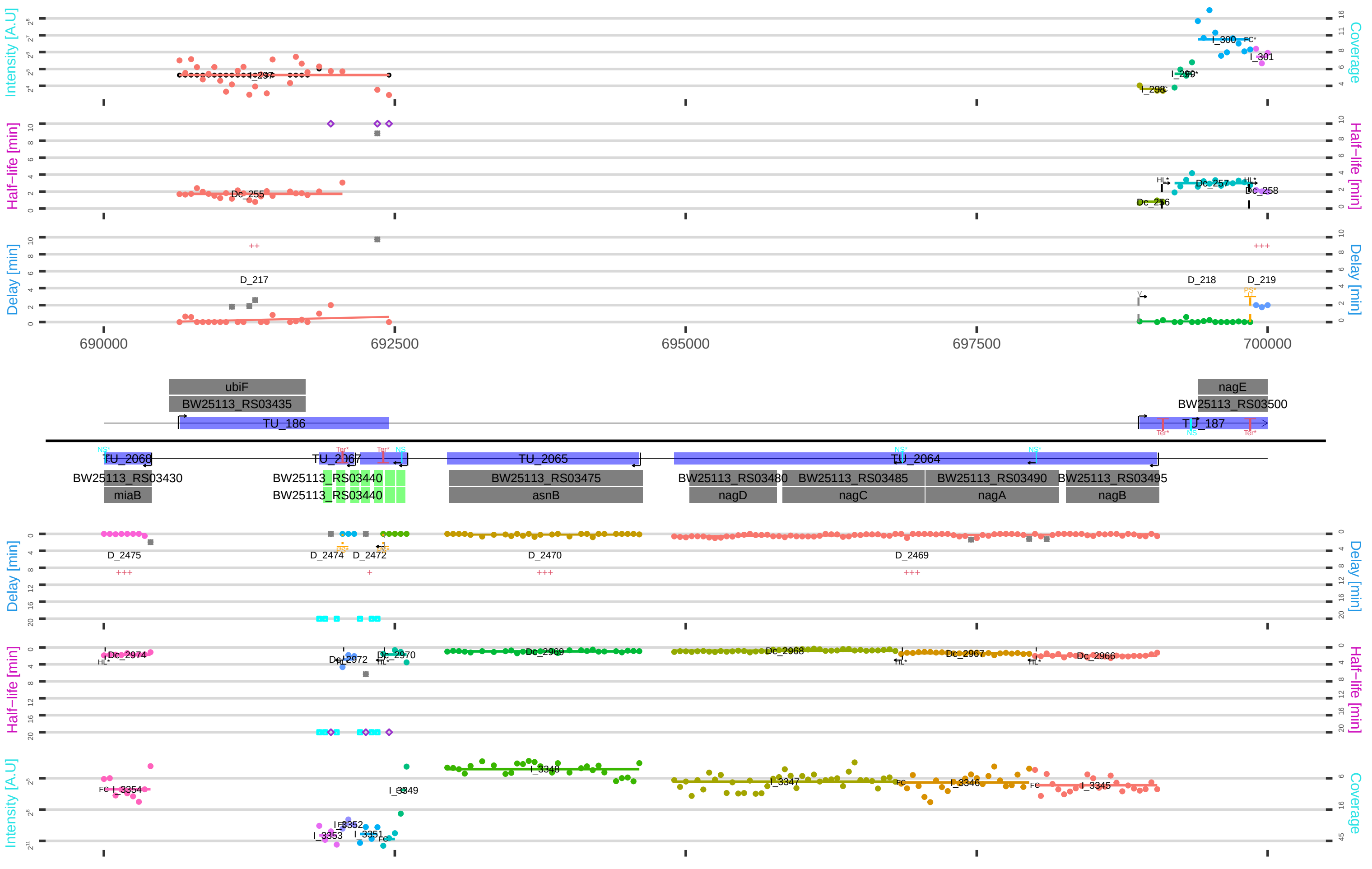

Term: termination (2), NS: new start (4), PS: pausing site (2), iTSS_I: internal starting site (0)

ID: 14000−14200; Term: termination (2), NS: new start (1), PS: pausing site (2), iTSS_I: internal starting site (0)

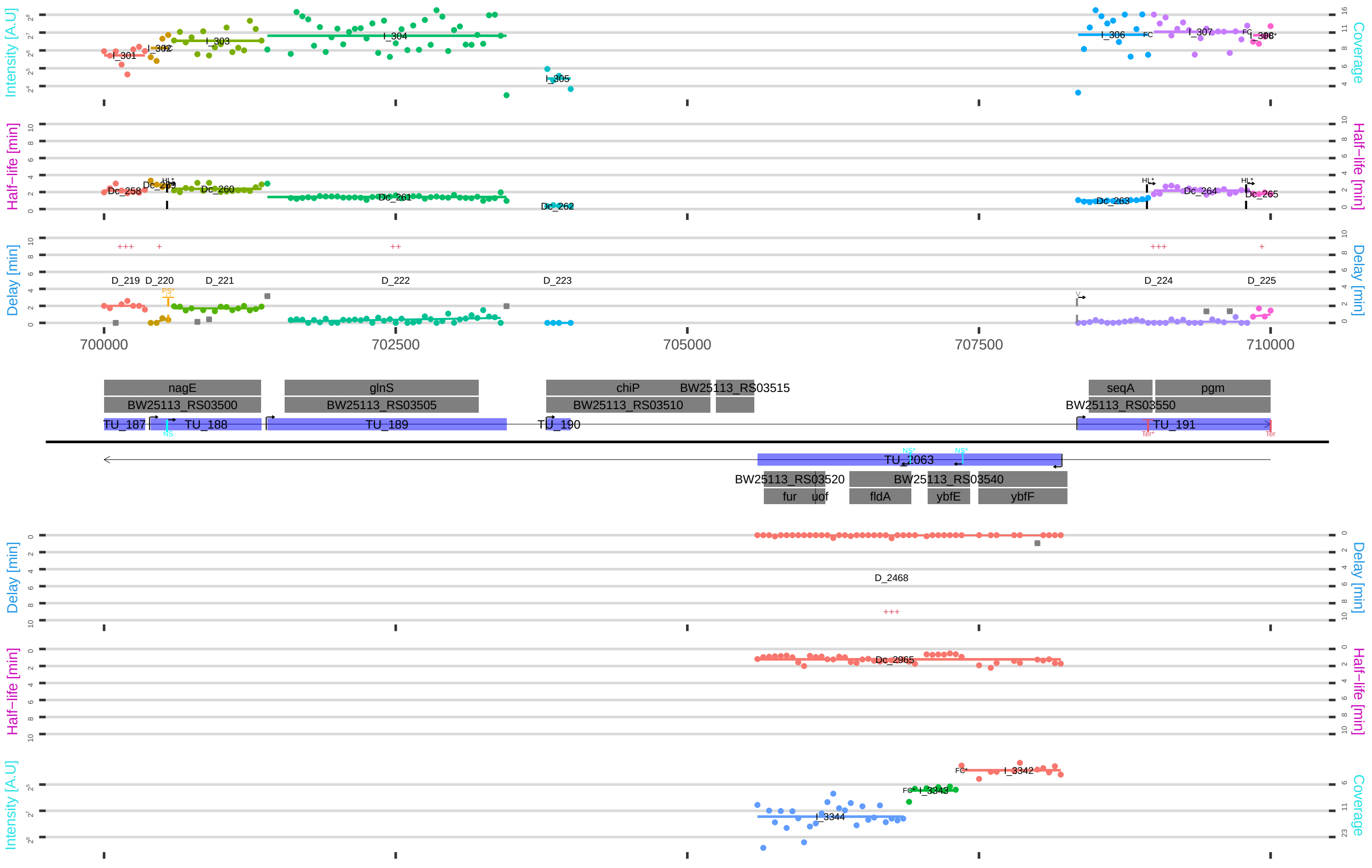

Term: termination (0), NS: new start (2), PS: pausing site (0), iTSS_I: internal starting site (0)

ID: 14200−14217; Term: termination (1), NS: new start (0), PS: pausing site (0), iTSS_I: internal starting site (0)

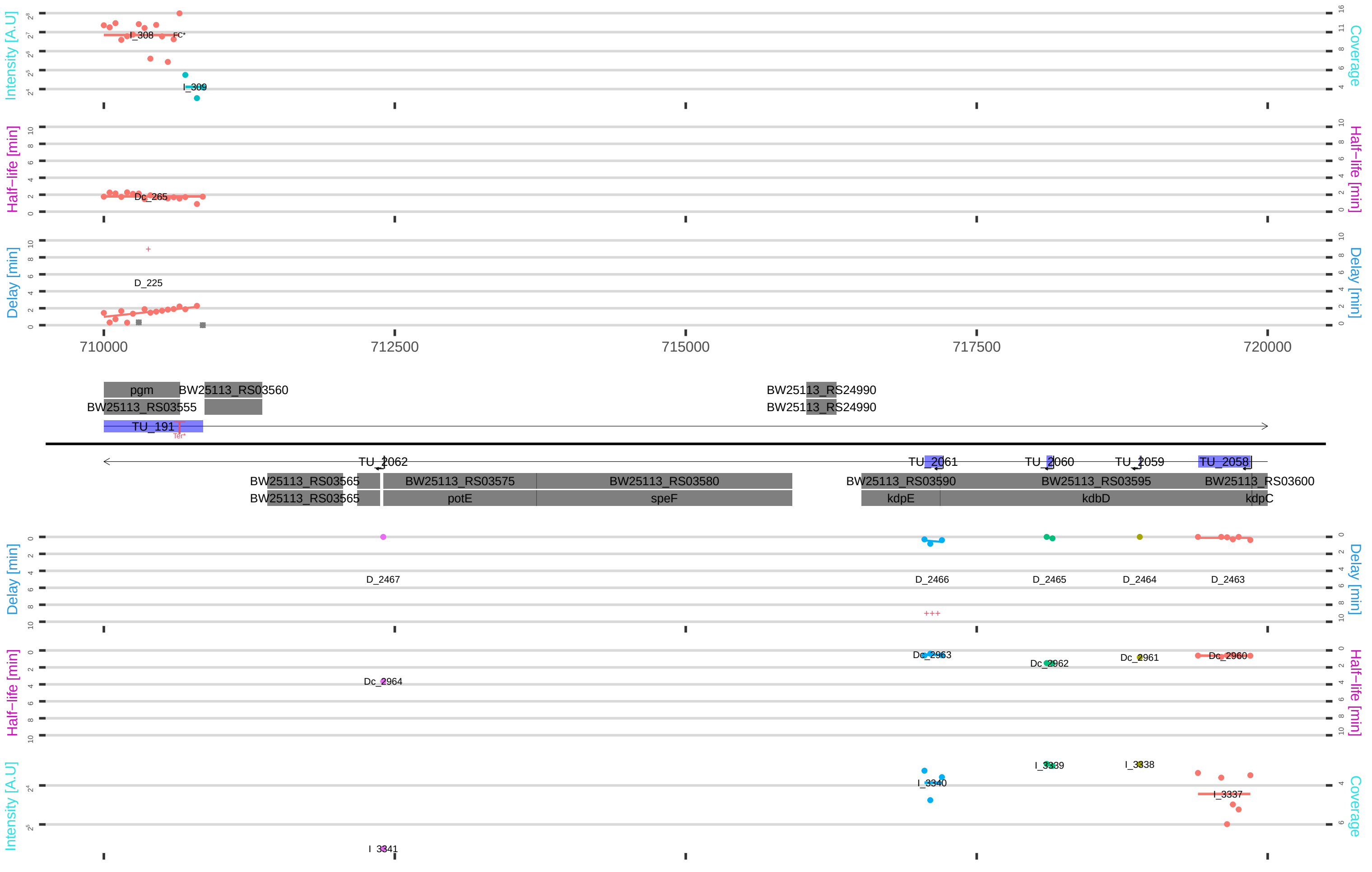

Term: termination (0), NS: new start (0), PS: pausing site (0), iTSS_I: internal starting site (0)

ID: 14493−14496; Term: termination (0), NS: new start (0), PS: pausing site (0), iTSS_I: internal starting site (0)

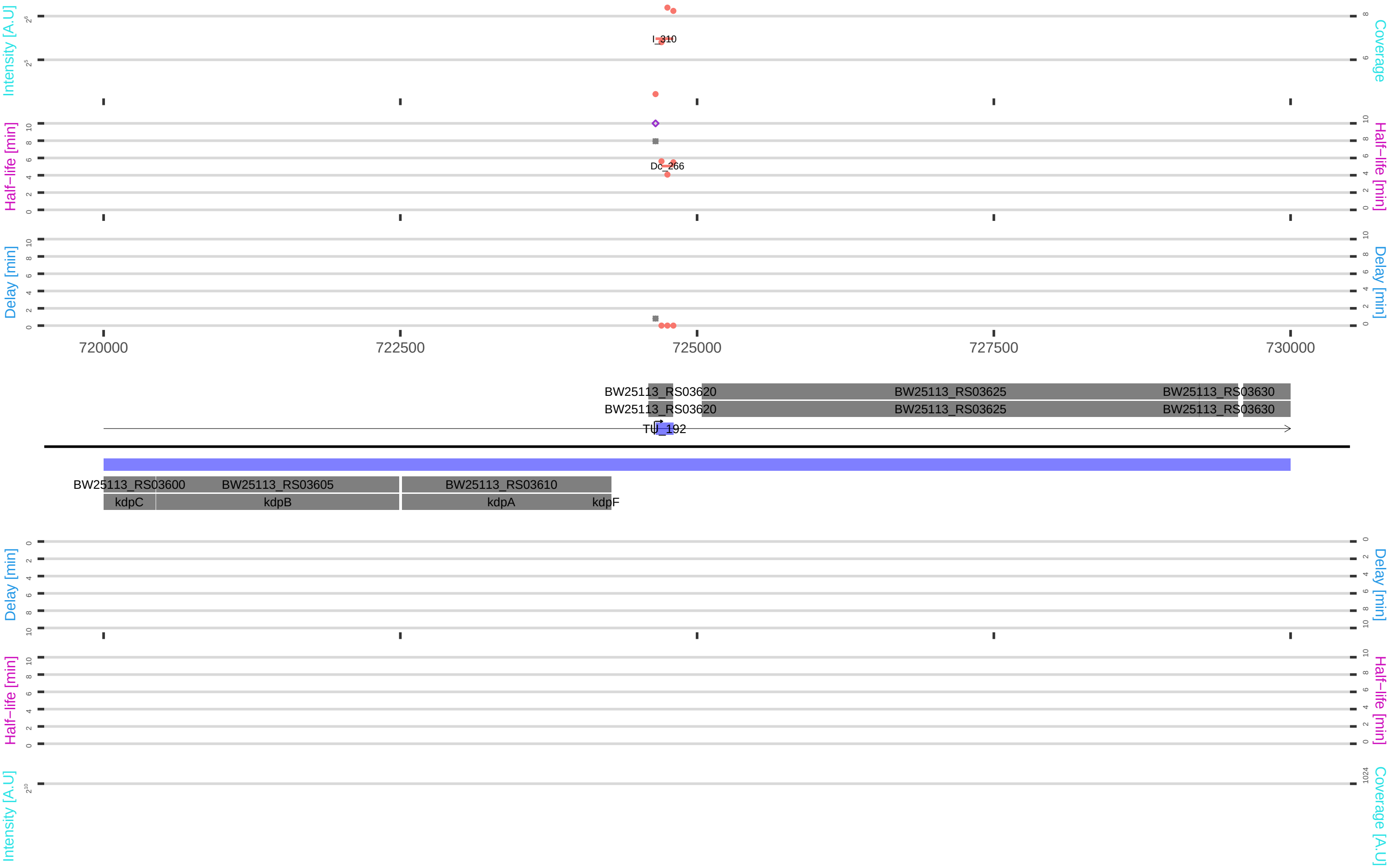

NA

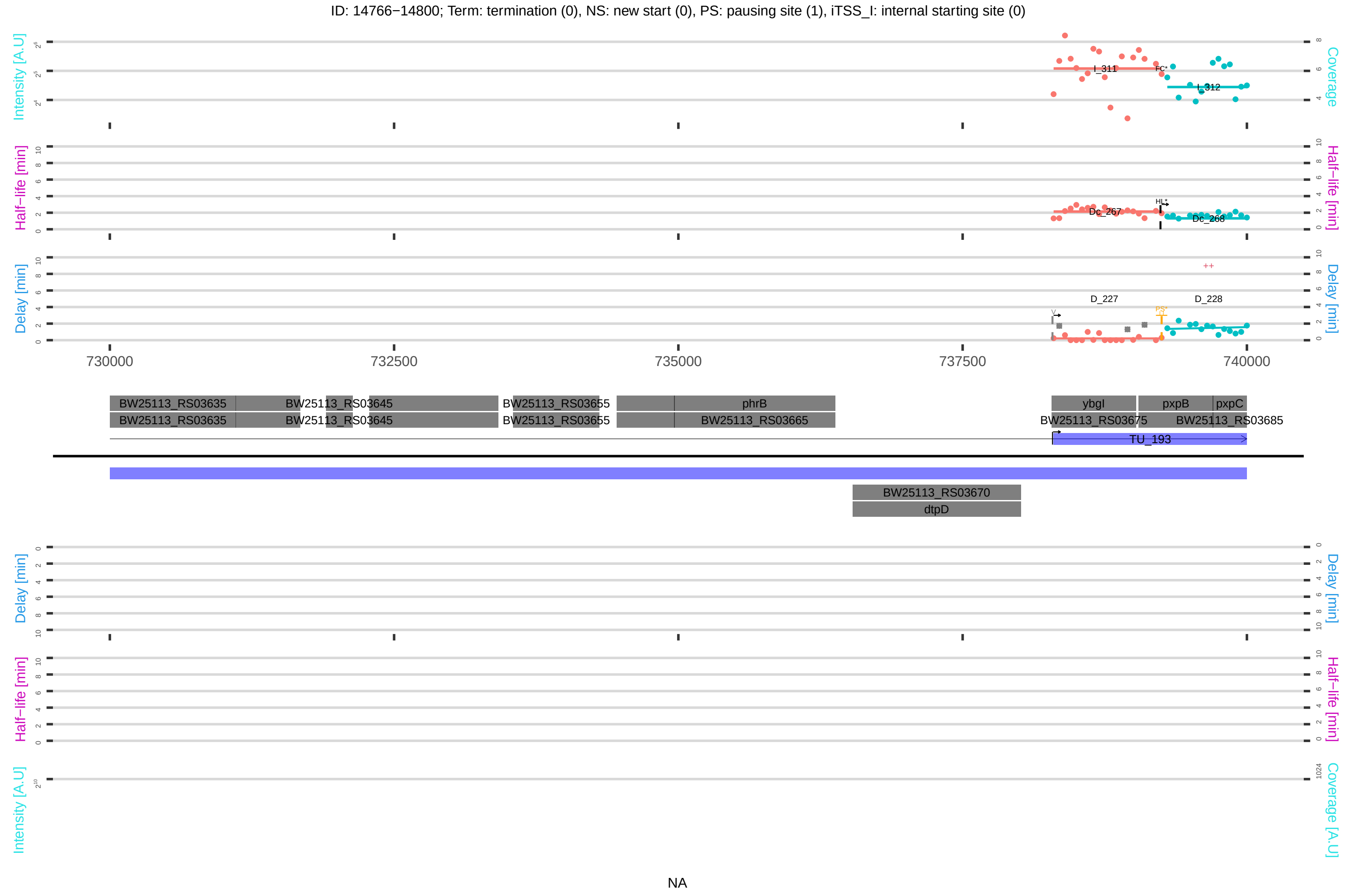

ID: 14766−14800; Term: termination (0), NS: new start (0), PS: pausing site (1), iTSS_I: internal starting site (0)
Intensity [A.U]
Coverage
I_311
FC*
I_312
Half−life [min]
HL*
Dc_267
Dc_268
Delay [min]
++
D_227
D_228
PS*
730000
732500
735000
737500
740000
BW25113_RS03635
BW25113_RS03645
BW25113_RS03655
phrB
BW25113_RS03665
ybgI
pxpB
pxpC
BW25113_RS03675
BW25113_RS03685
TU_193
BW25113_RS03670
dtpD
NA

ID: 14800−14841; Term: termination (0), NS: new start (0), PS: pausing site (0), iTSS_I: internal starting site (0)

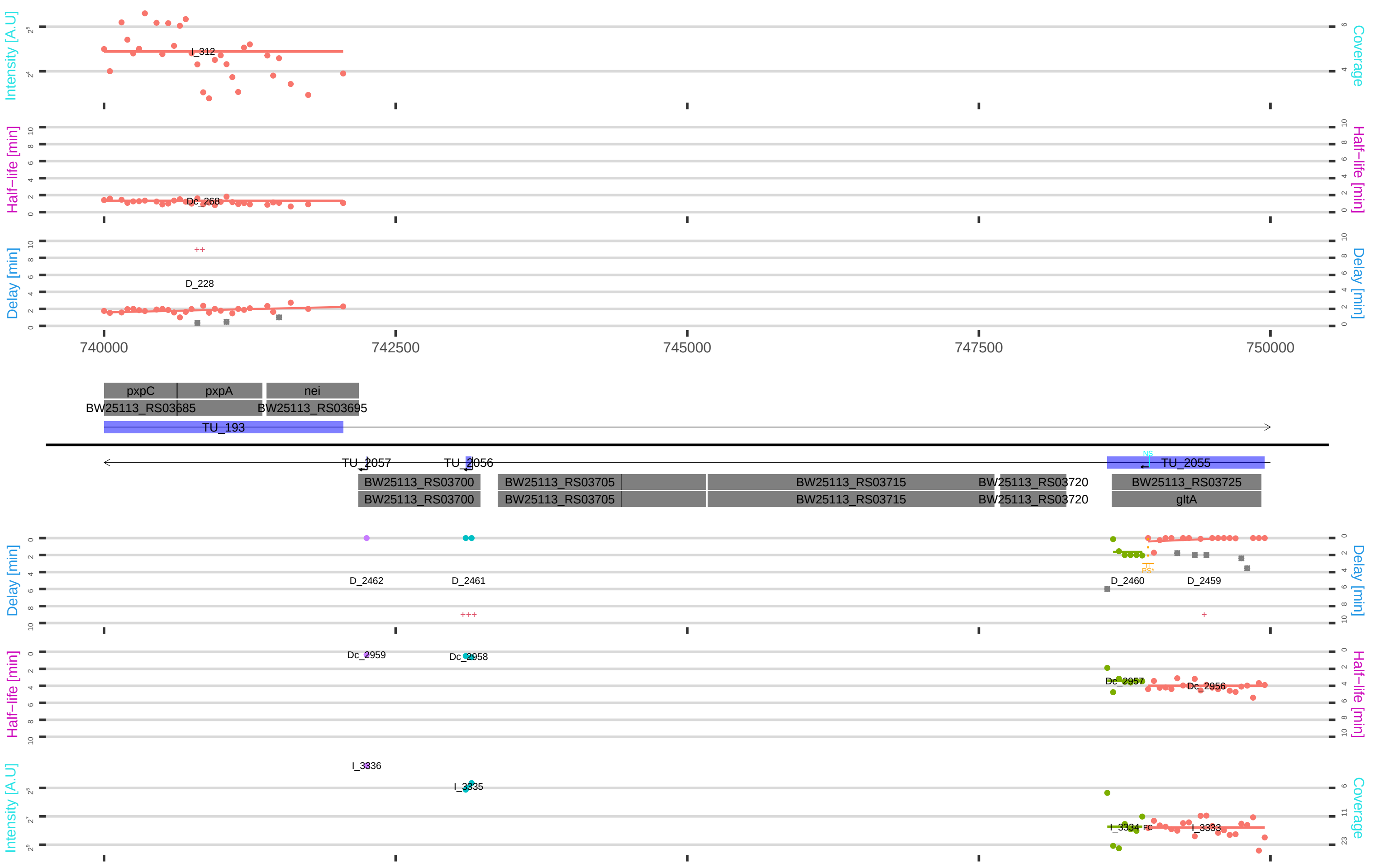

Term: termination (0), NS: new start (1), PS: pausing site (1), iTSS_I: internal starting site (0)

ID: 15010−15200; Term: termination (4), NS: new start (2), PS: pausing site (3), iTSS_I: internal starting site (1)

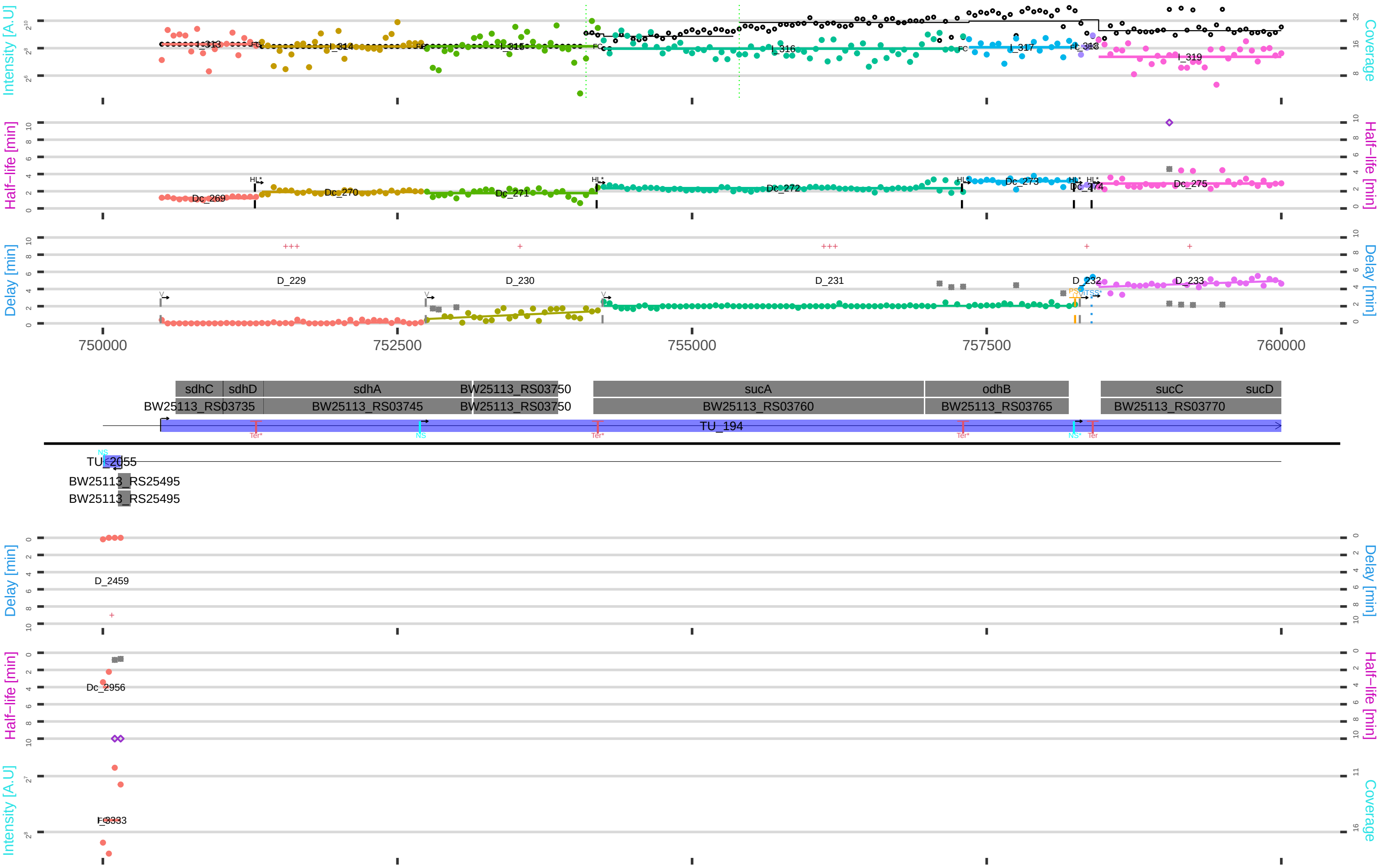

Term: termination (0), NS: new start (1), PS: pausing site (0), iTSS_I: internal starting site (0)

ID: 15200−15400; Term: termination (1), NS: new start (1), PS: pausing site (1), iTSS_I: internal starting site (1)

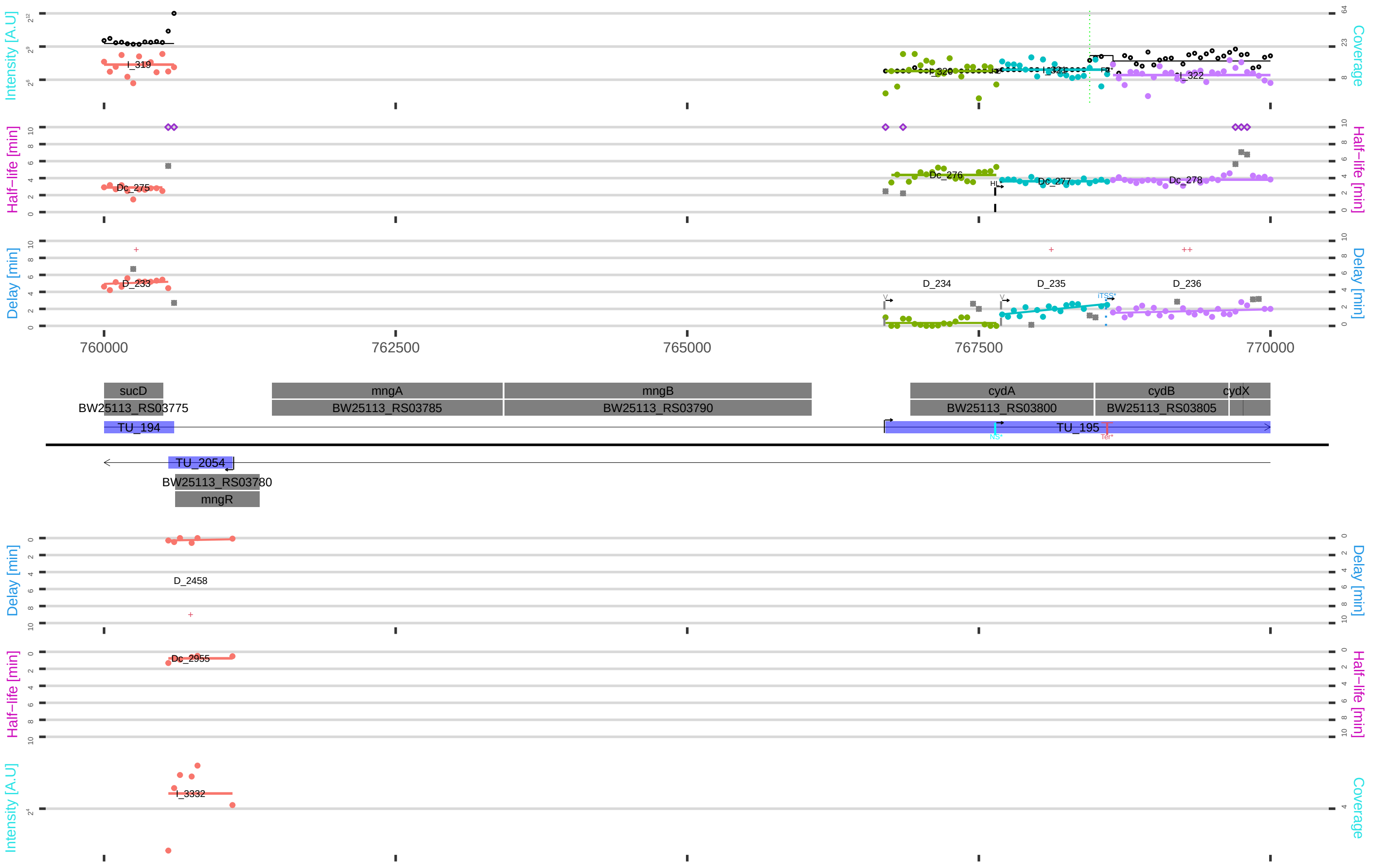

Term: termination (0), NS: new start (0), PS: pausing site (0), iTSS_I: internal starting site (0)

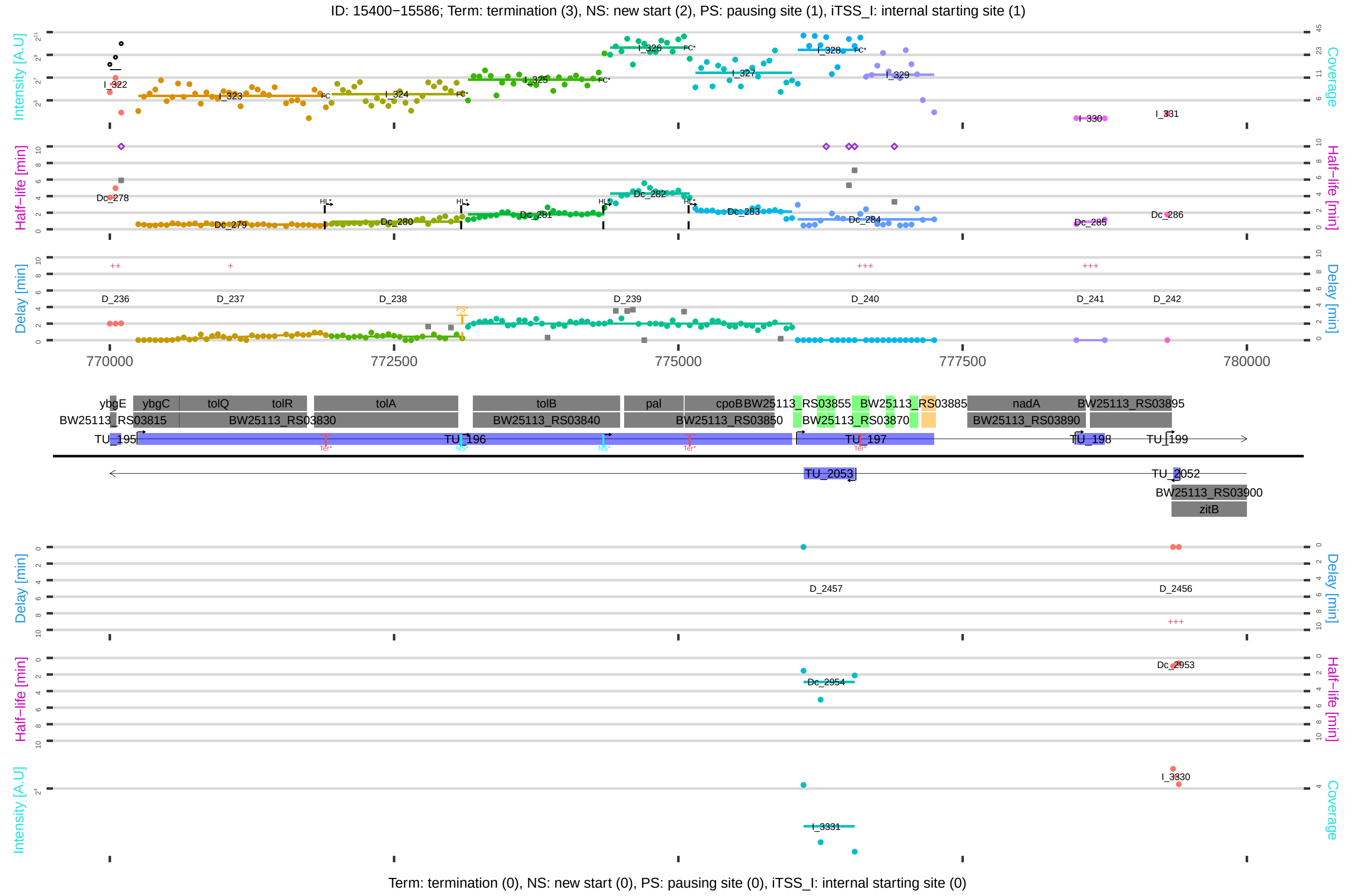
ID: 15400−15586; Term: termination (3), NS: new start (2), PS: pausing site (1), iTSS_I: internal starting site (1)
Intensity [A.U]
Coverage
Half−life [min]
Delay [min]
770000
772500
775000
777500
780000
ybgE
ybgC
tolQ
tolR
tolA
tolB
pal
cpoB
BW25113_RS03815
BW25113_RS03830
BW25113_RS03840
BW25113_RS03850
BW25113_RS03855
BW25113_RS03870
BW25113_RS03885
nadA
BW25113_RS03890
BW25113_RS03895
BW25113_RS03900
zitB
TU_195
TU_196
TU_197
TU_198
TU_199
TU_2053
TU_2052
Term: termination (0), NS: new start (0), PS: pausing site (0), iTSS_I: internal starting site (0)

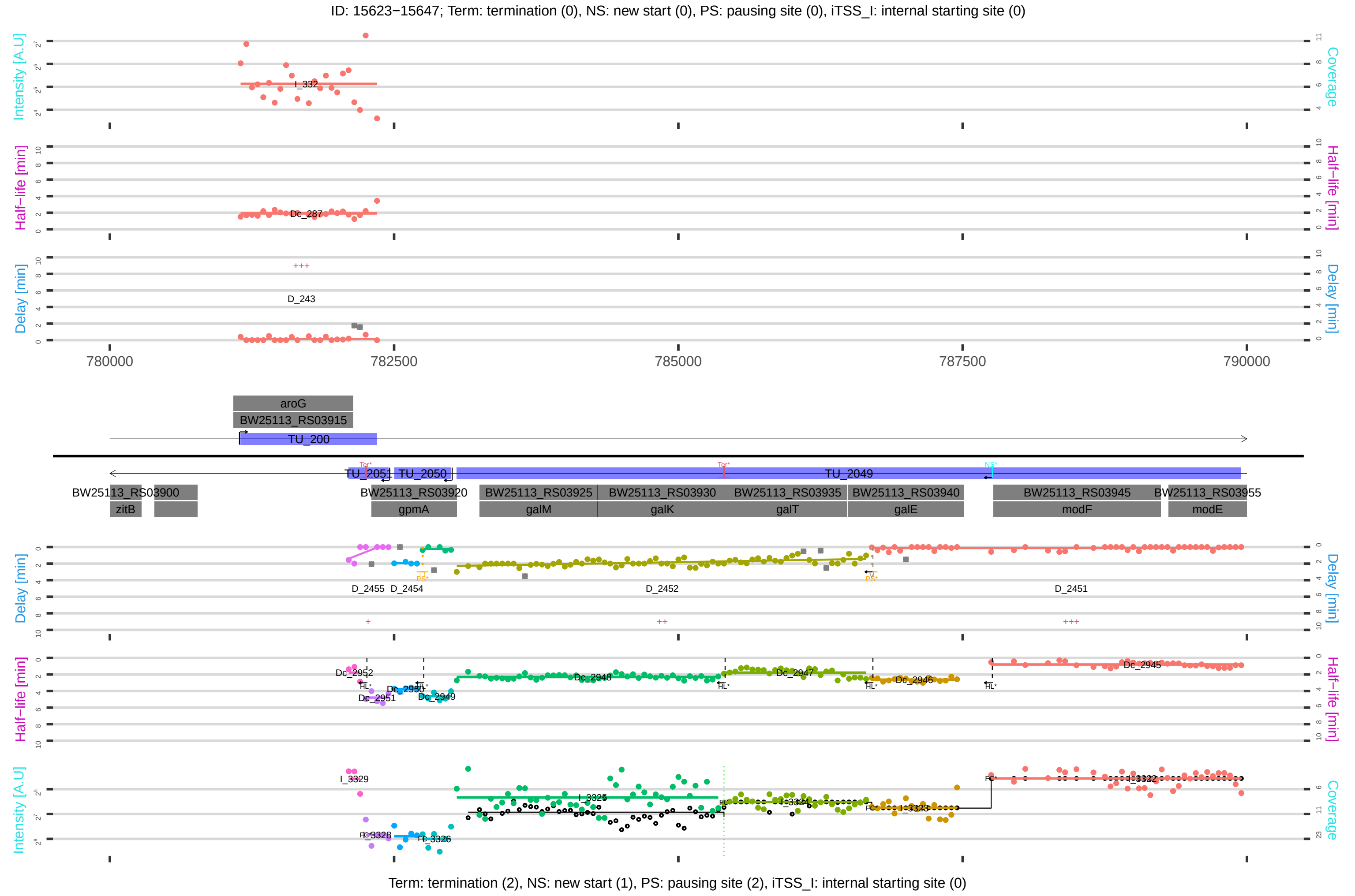

ID: 15623−15647; Term: termination (0), NS: new start (0), PS: pausing site (0), iTSS_I: internal starting site (0)
Intensity [A.U]
Coverage
I_332
Half−life [min]
Dc_287
Delay [min]
D_243
+++
780000
782500
785000
787500
790000
aroG
BW25113_RS03915
TU_200
TU_2051
TU_2050
TU_2049
BW25113_RS03900
zitB
BW25113_RS03920
gpmA
BW25113_RS03925
galM
BW25113_RS03930
galK
BW25113_RS03935
galT
BW25113_RS03940
galE
BW25113_RS03945
modF
BW25113_RS03955
modE
D_2455
D_2454
D_2452
D_2451
Dc_2952
Dc_2951
Dc_2950
Dc_2949
Dc_2948
Dc_2947
Dc_2946
Dc_2945
I_3329
I_3328
I_3326
I_3325
I_3324
I_3323
I_3322
Term: termination (2), NS: new start (1), PS: pausing site (2), iTSS_I: internal starting site (0)

ID: 15803−15901; Term: termination (2), NS: new start (2), PS: pausing site (0), iTSS_I: internal starting site (0)

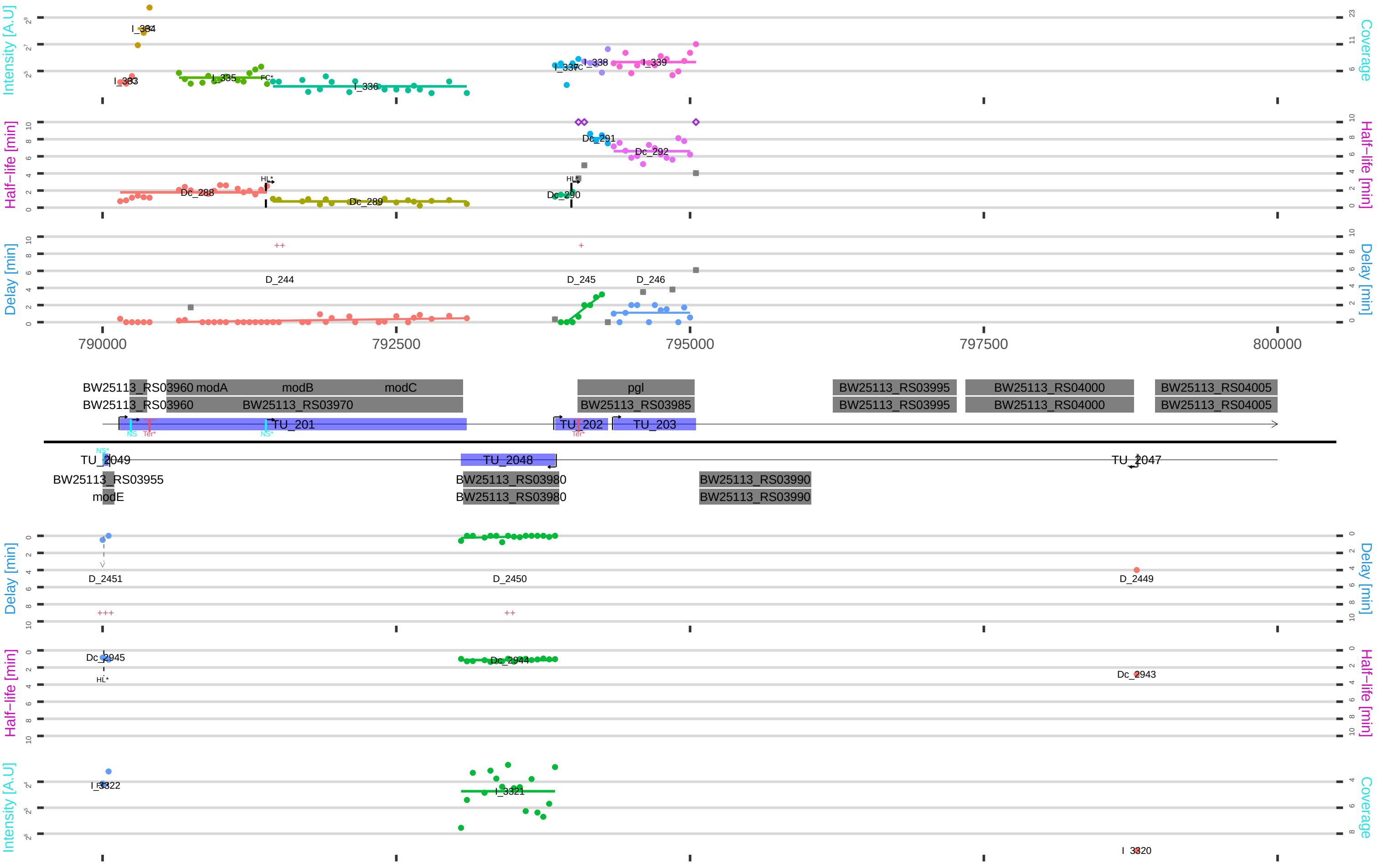

Term: termination (0), NS: new start (1), PS: pausing site (0), iTSS_I: internal starting site (0)

ID: 16181−16200; Term: termination (0), NS: new start (0), PS: pausing site (0), iTSS_I: internal starting site (0)

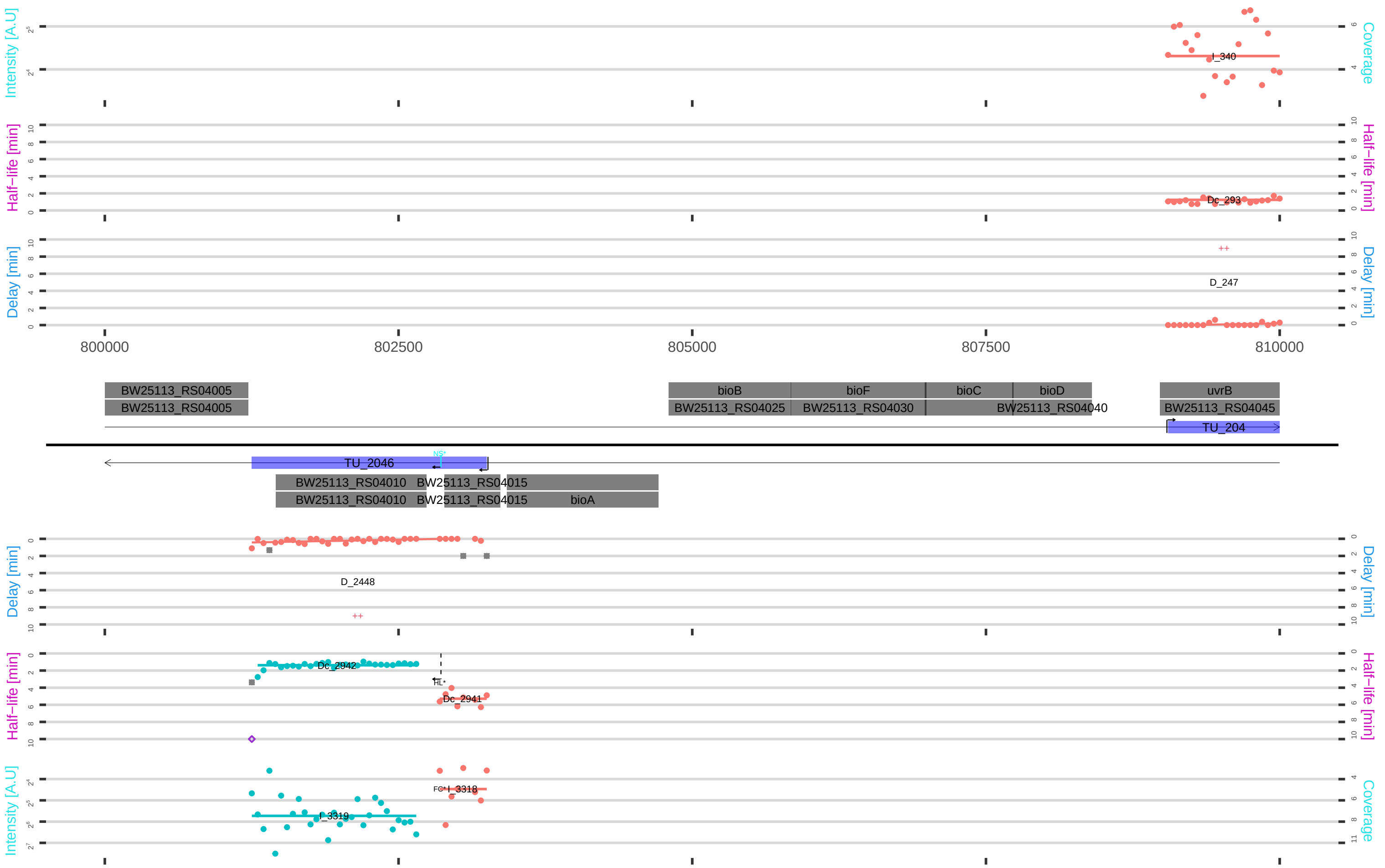

Term: termination (0), NS: new start (1), PS: pausing site (0), iTSS_I: internal starting site (0)

ID: 16200−16322; Term: termination (1), NS: new start (1), PS: pausing site (1), iTSS_I: internal starting site (0)

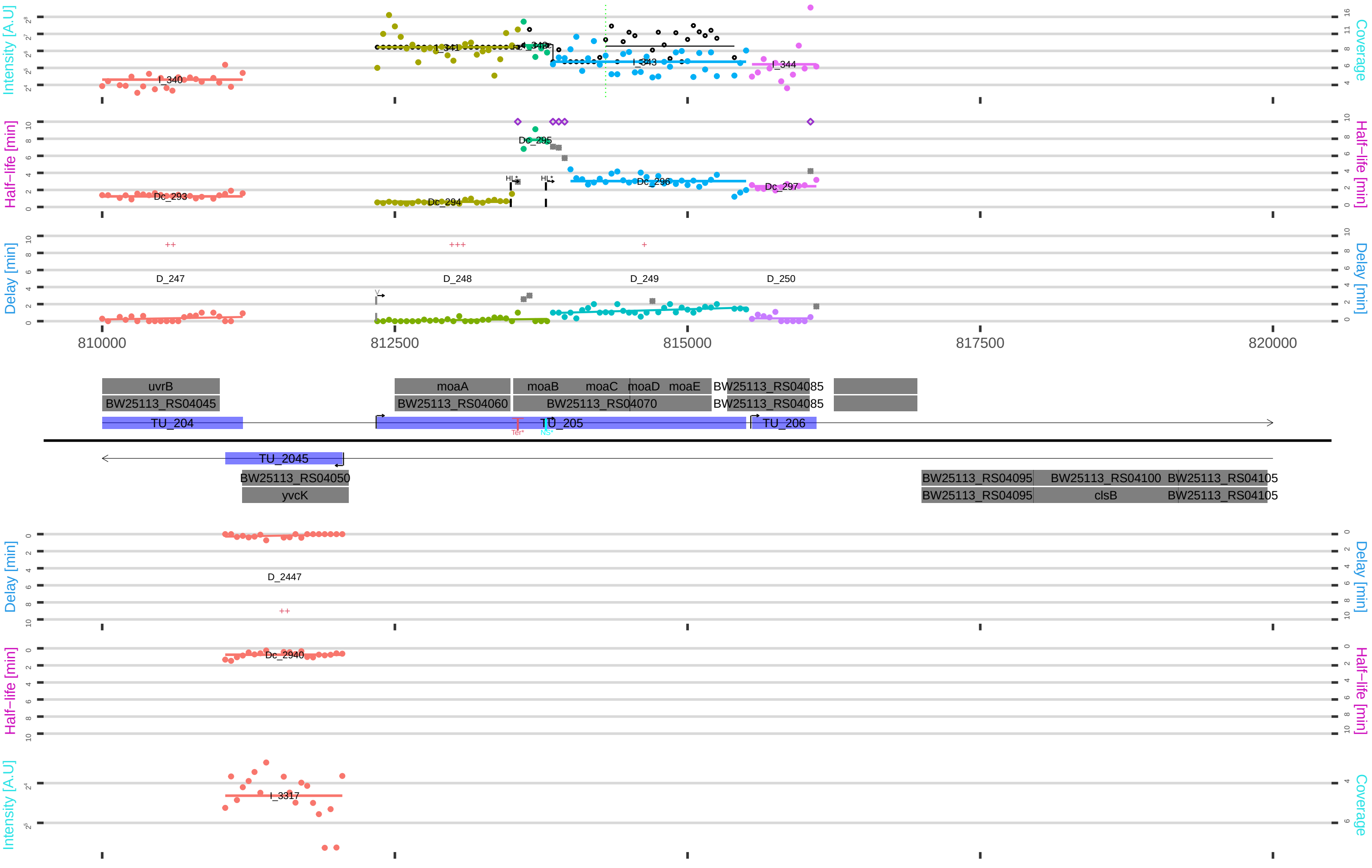

Term: termination (0), NS: new start (0), PS: pausing site (0), iTSS_I: internal starting site (0)

ID: 16403−16596; Term: termination (1), NS: new start (0), PS: pausing site (0), iTSS_I: internal starting site (0)

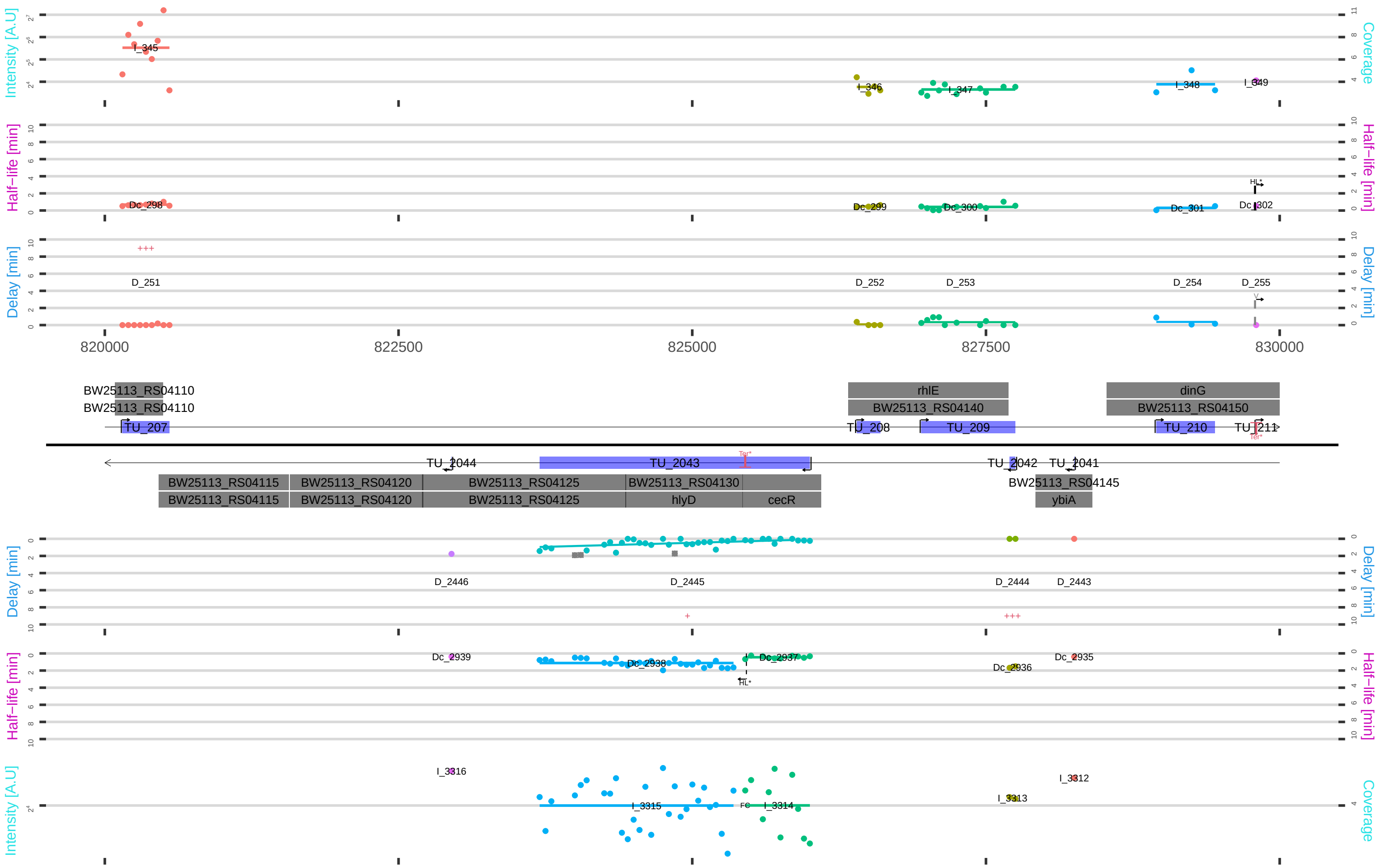

Term: termination (1), NS: new start (0), PS: pausing site (0), iTSS_I: internal starting site (0)

ID: 16601−16774; Term: termination (1), NS: new start (0), PS: pausing site (1), iTSS_I: internal starting site (0)

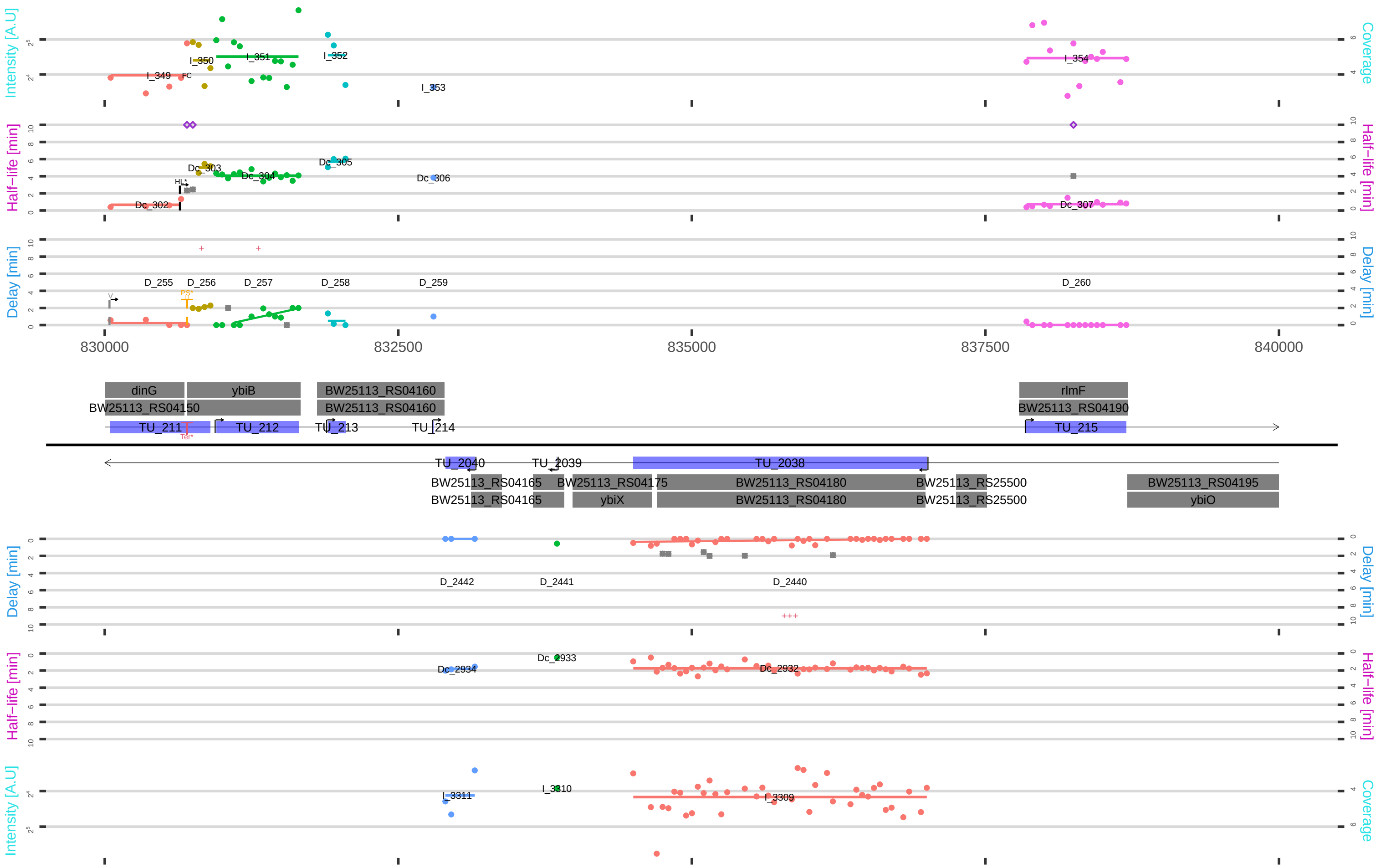

Term: termination (0), NS: new start (0), PS: pausing site (0), iTSS_I: internal starting site (0)

ID: 16915−16999; Term: termination (0), NS: new start (1), PS: pausing site (0), iTSS_I: internal starting site (0)

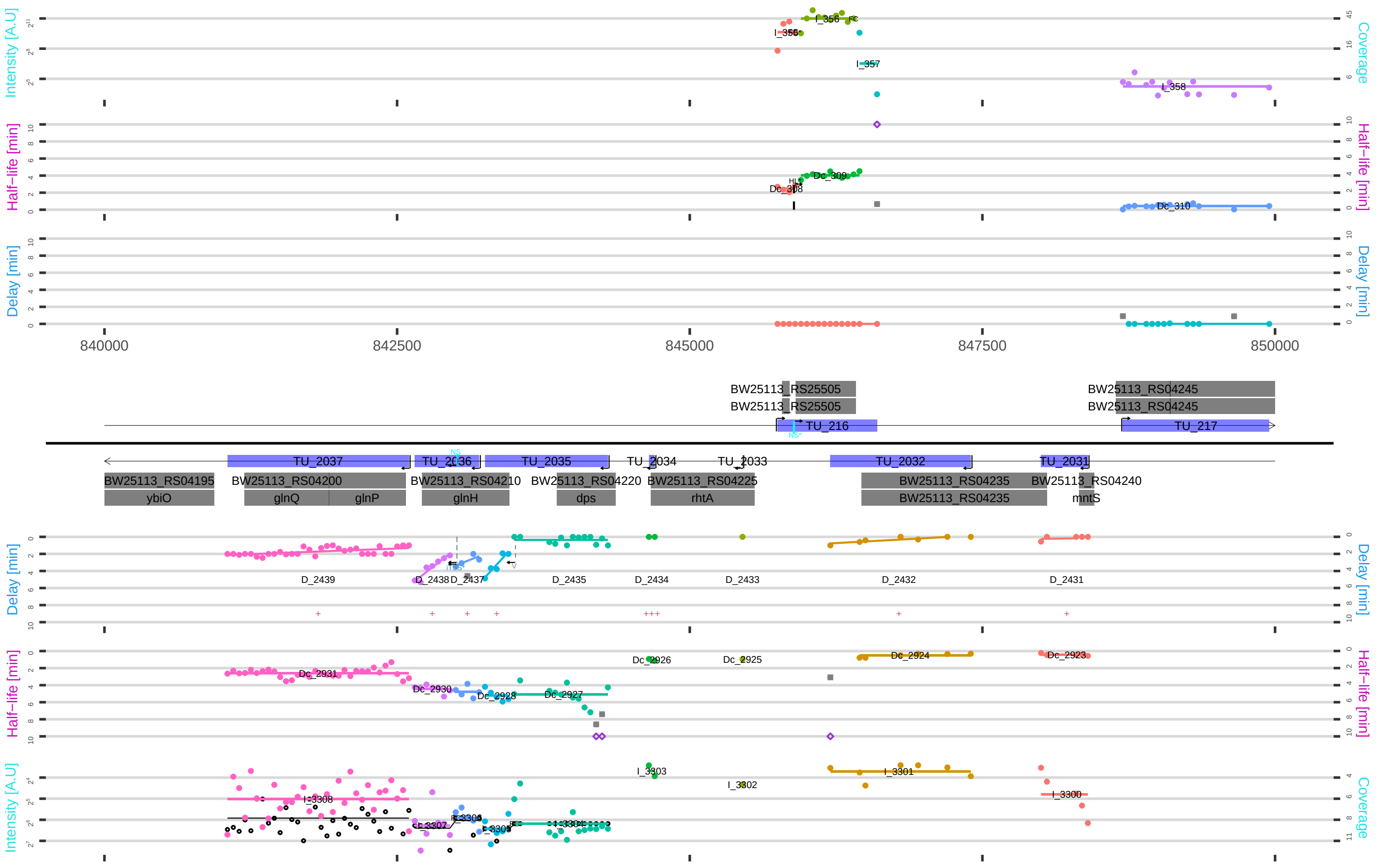

Term: termination (0), NS: new start (1), PS: pausing site (1), iTSS_I: internal starting site (1)

ID: 17030−17195; Term: termination (0), NS: new start (0), PS: pausing site (0), iTSS_I: internal starting site (0)

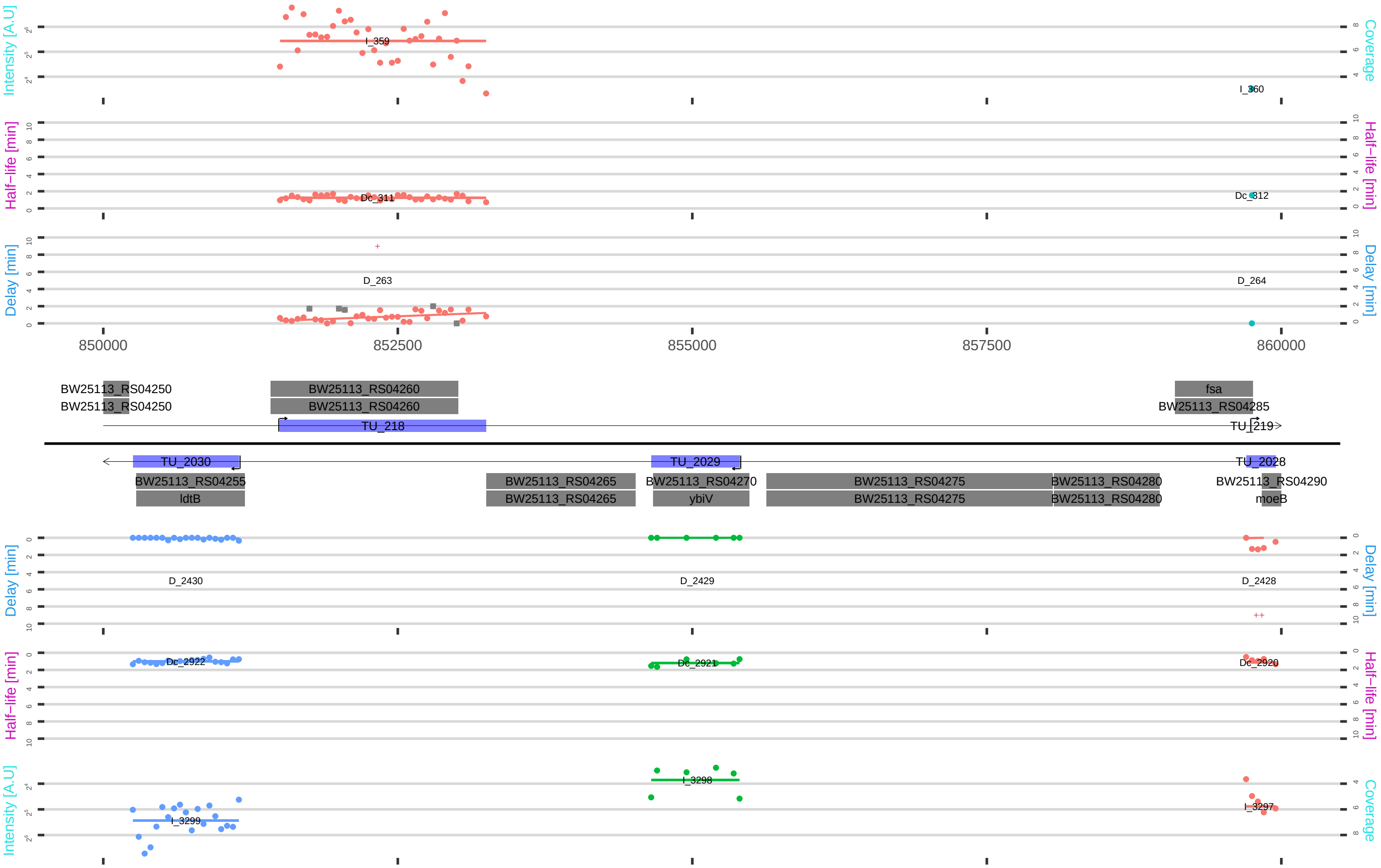

Term: termination (0), NS: new start (0), PS: pausing site (0), iTSS_I: internal starting site (0)

ID: 17241−17359; Term: termination (1), NS: new start (0), PS: pausing site (0), iTSS_I: internal starting site (0)

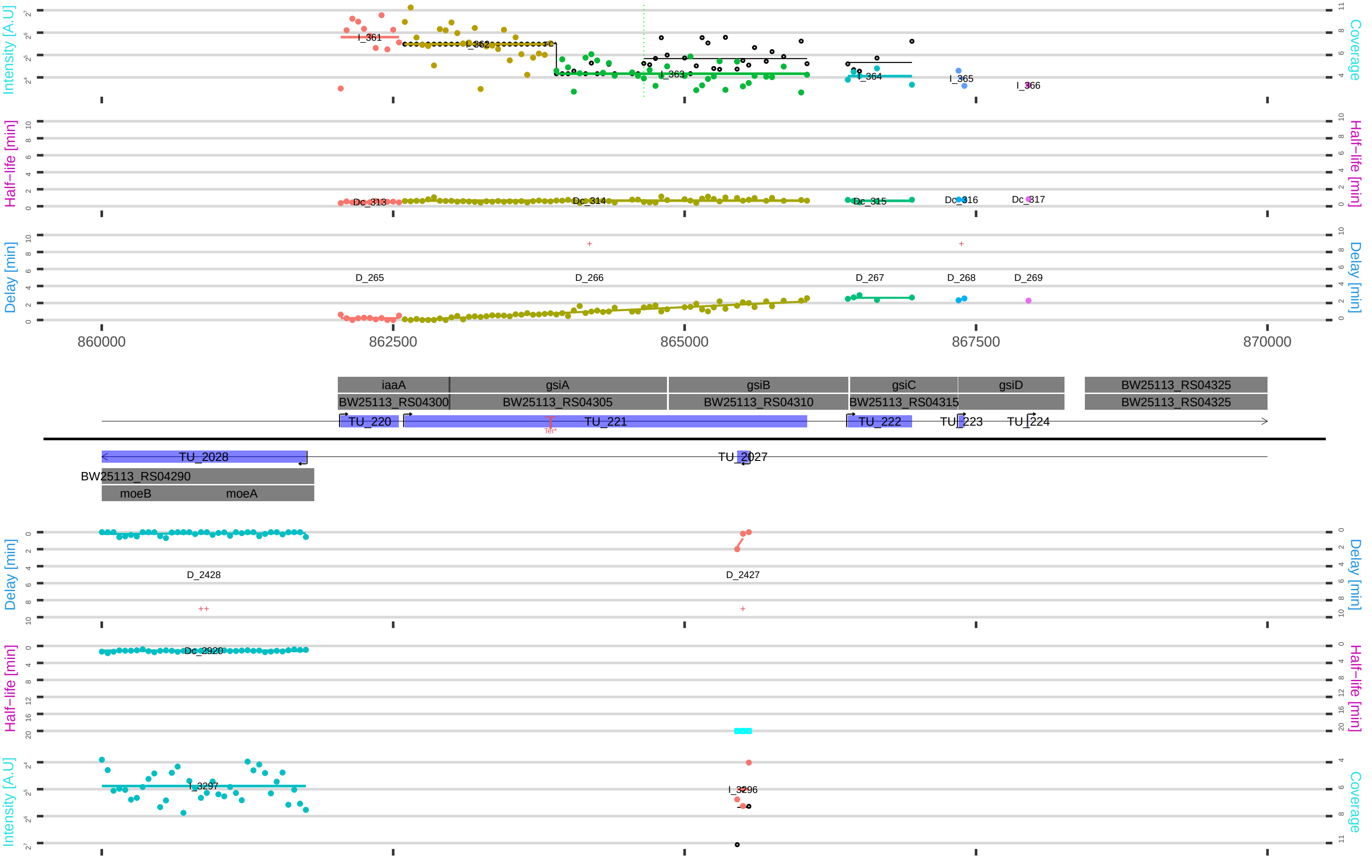

Term: termination (0), NS: new start (0), PS: pausing site (0), iTSS_I: internal starting site (0)

ID: 17411−17599; Term: termination (0), NS: new start (0), PS: pausing site (0), iTSS_I: internal starting site (0)

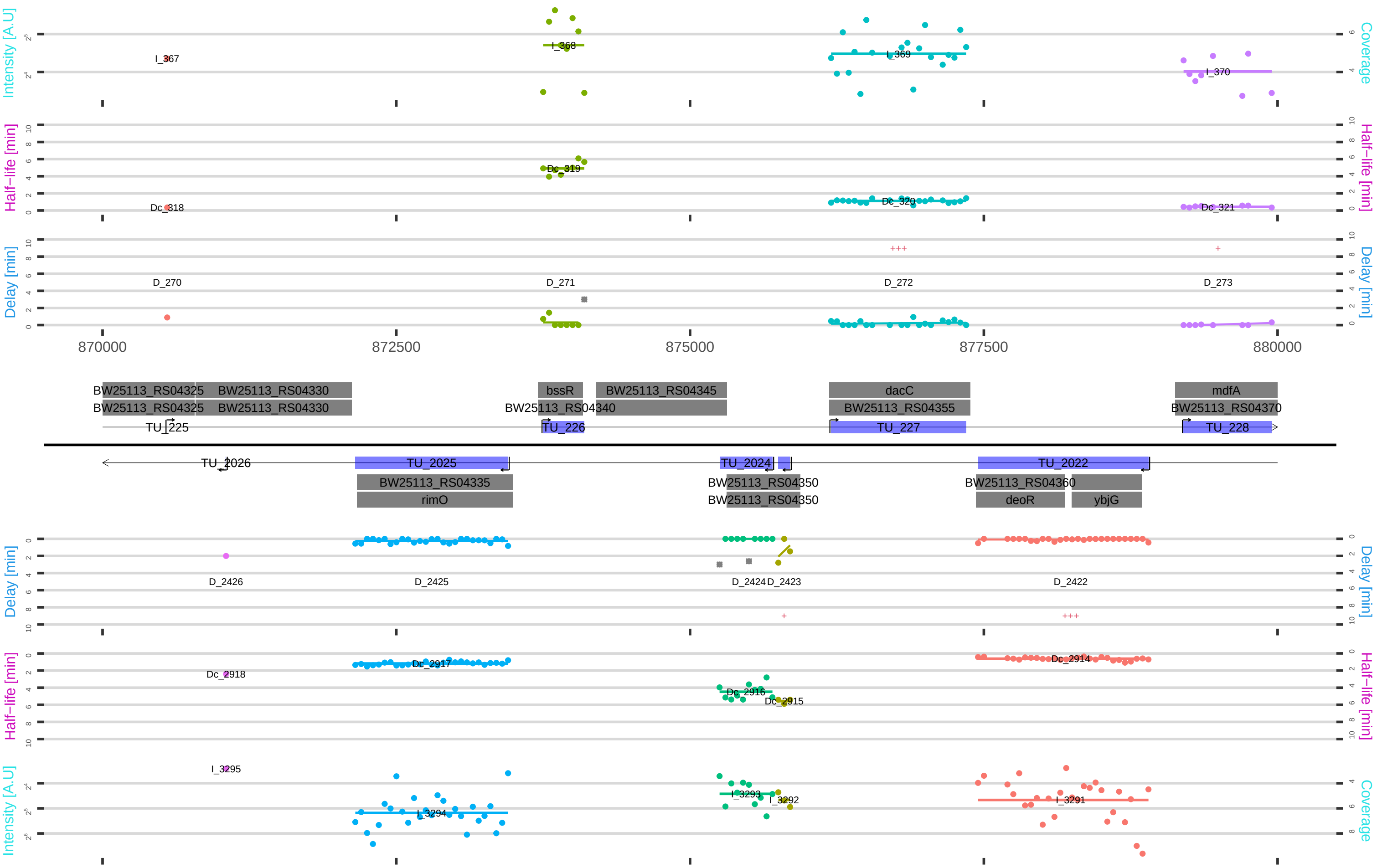

Term: termination (0), NS: new start (0), PS: pausing site (0), iTSS_I: internal starting site (0)

ID: 17603−17778; Term: termination (1), NS: new start (1), PS: pausing site (0), iTSS_I: internal starting site (0)

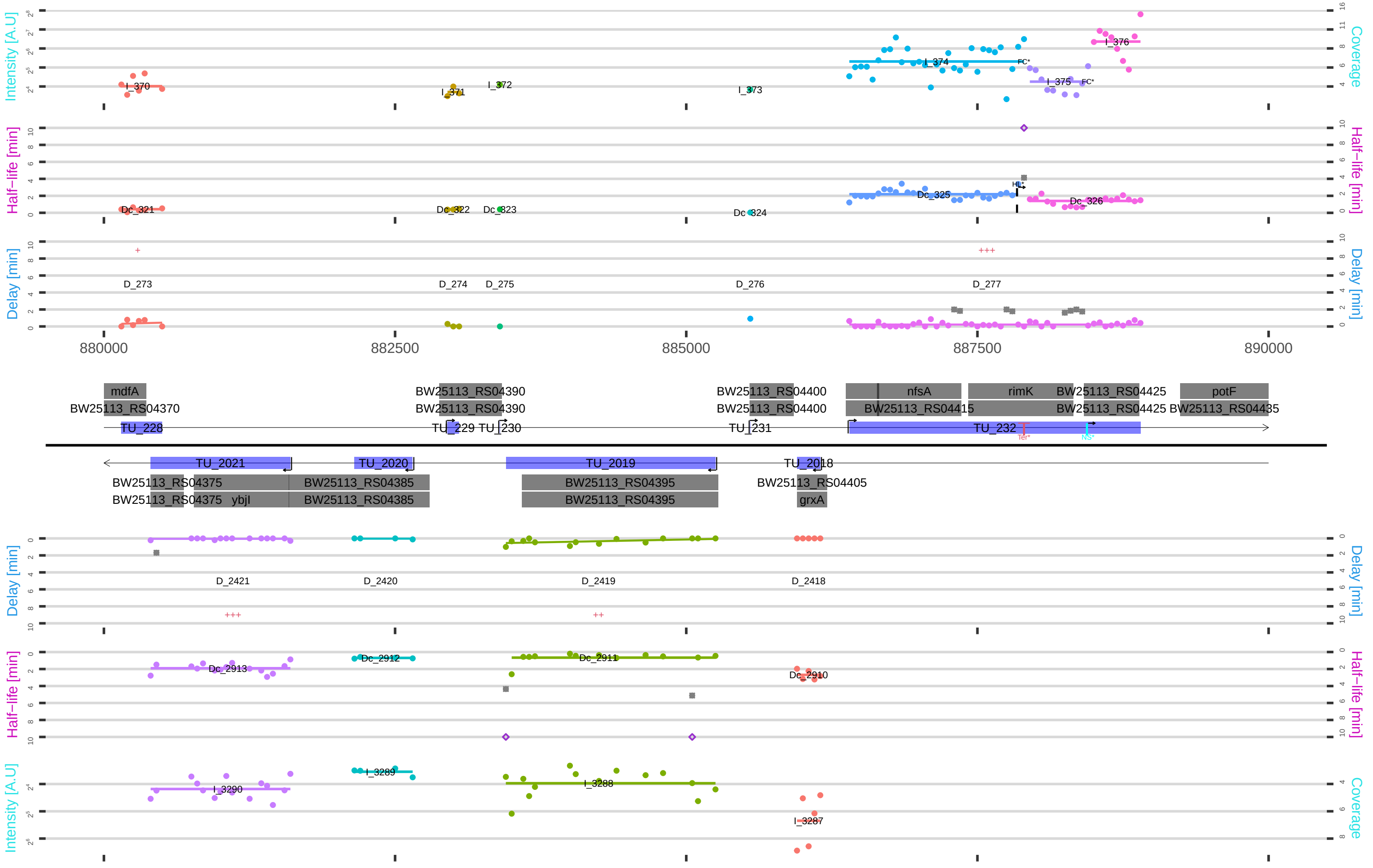

Term: termination (0), NS: new start (0), PS: pausing site (0), iTSS_I: internal starting site (0)

ID: 17875−17905; Term: termination (0), NS: new start (0), PS: pausing site (0), iTSS_I: internal starting site (0)

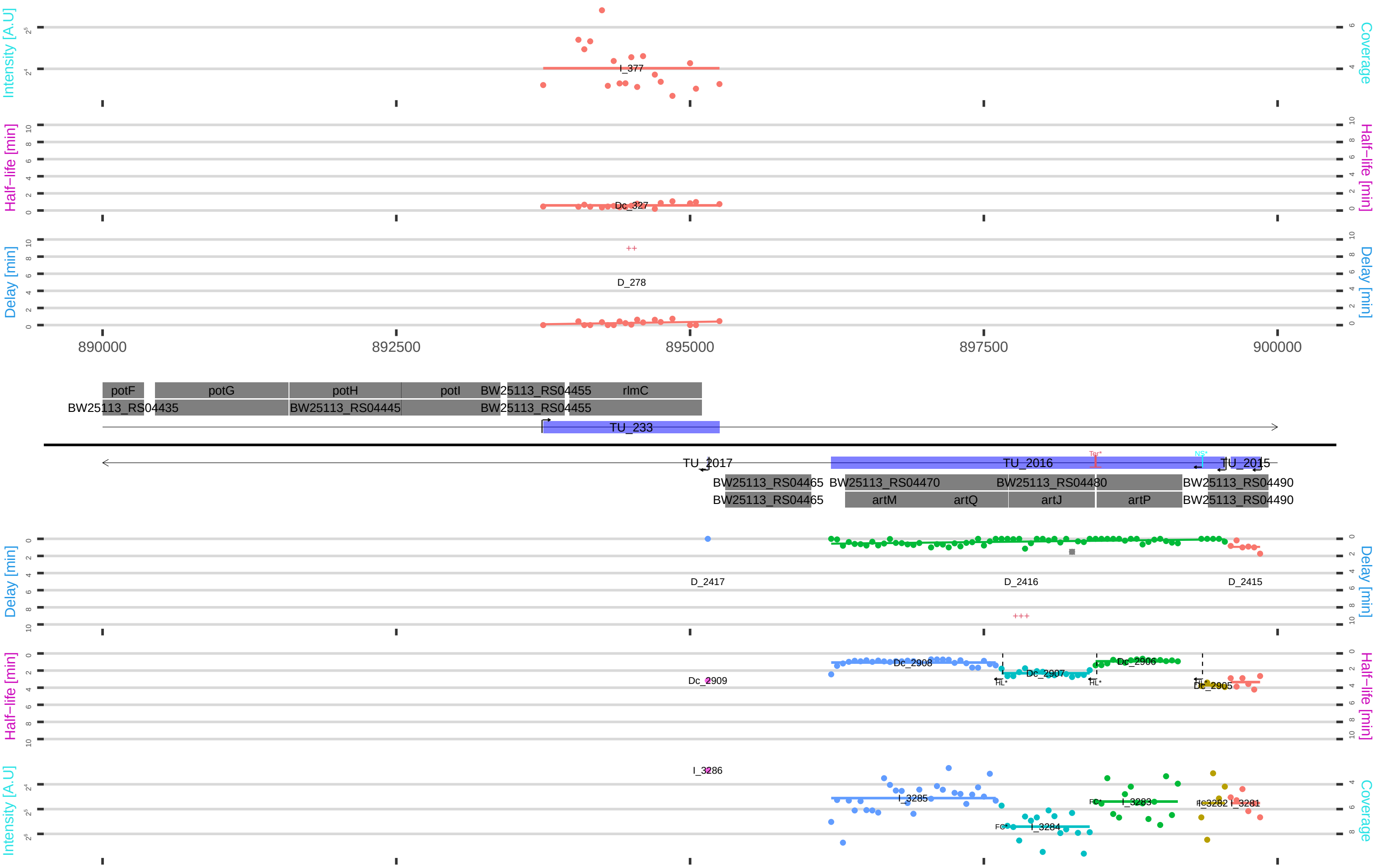

Term: termination (1), NS: new start (1), PS: pausing site (0), iTSS_I: internal starting site (0)

ID: 18001−18029; Term: termination (0), NS: new start (1), PS: pausing site (0), iTSS_I: internal starting site (0)

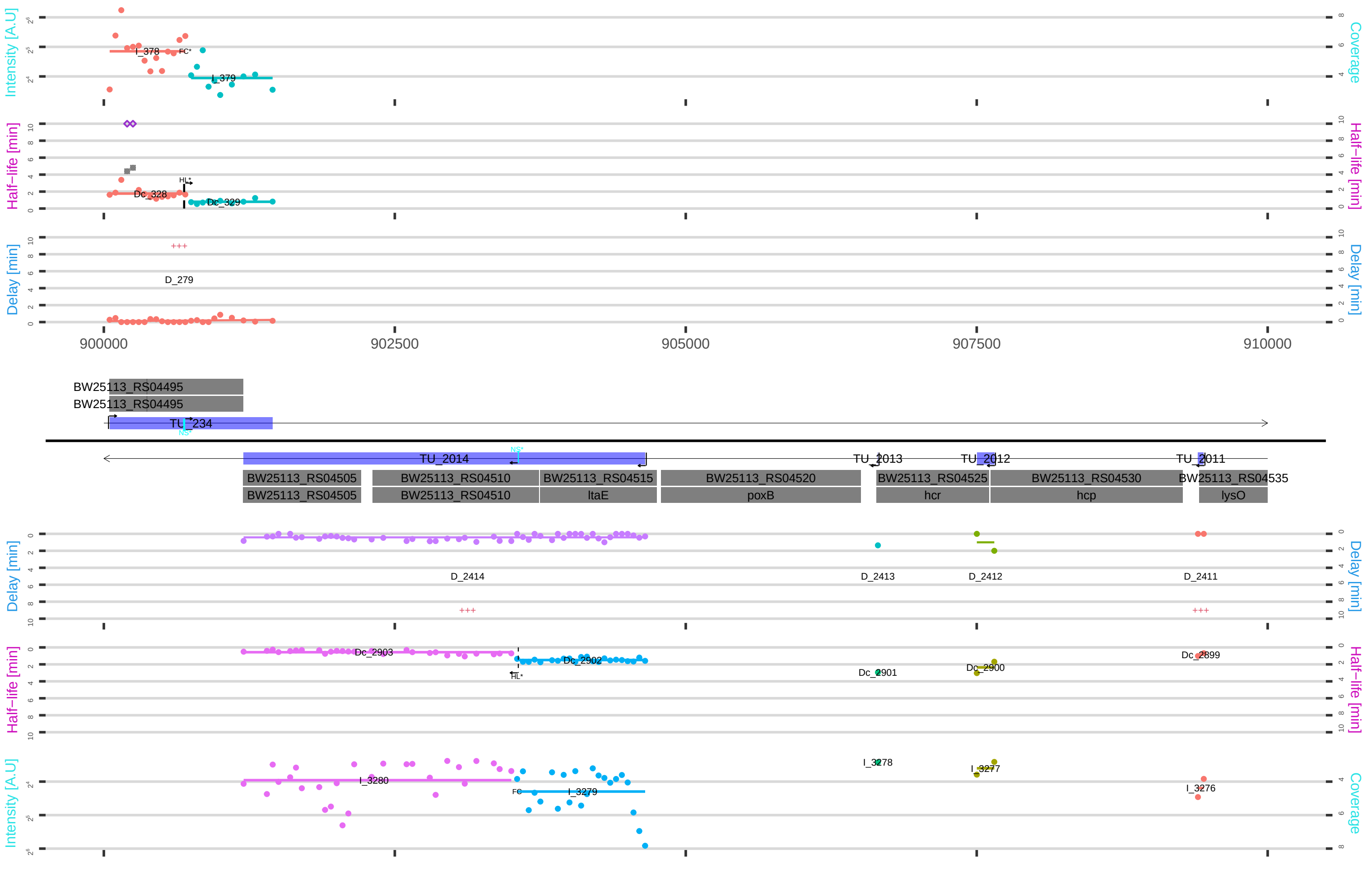

Term: termination (0), NS: new start (1), PS: pausing site (0), iTSS_I: internal starting site (0)

ID: 18212−18400; Term: termination (0), NS: new start (0), PS: pausing site (0), iTSS_I: internal starting site (0)

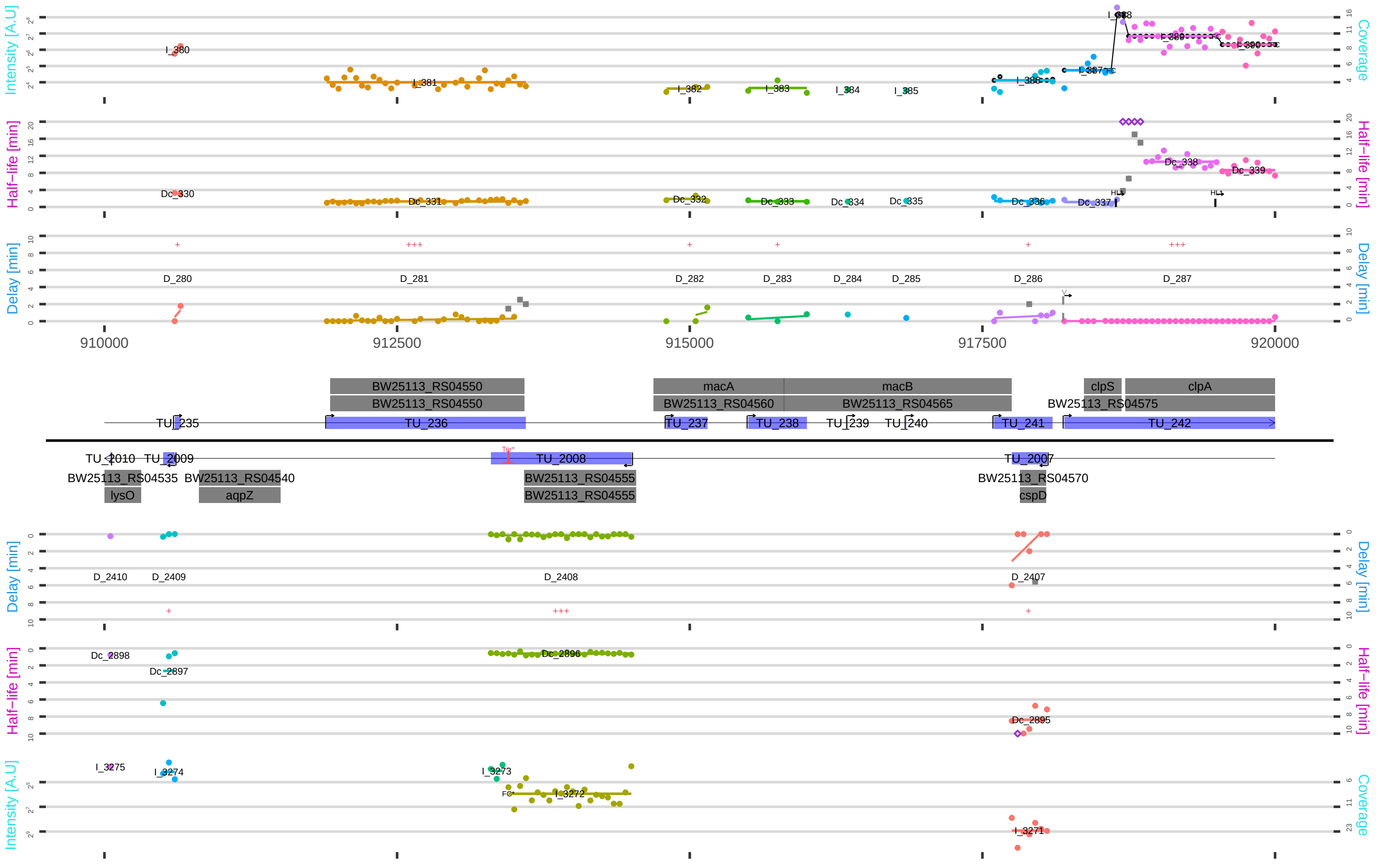

Term: termination (1), NS: new start (0), PS: pausing site (0), iTSS_I: internal starting site (0)

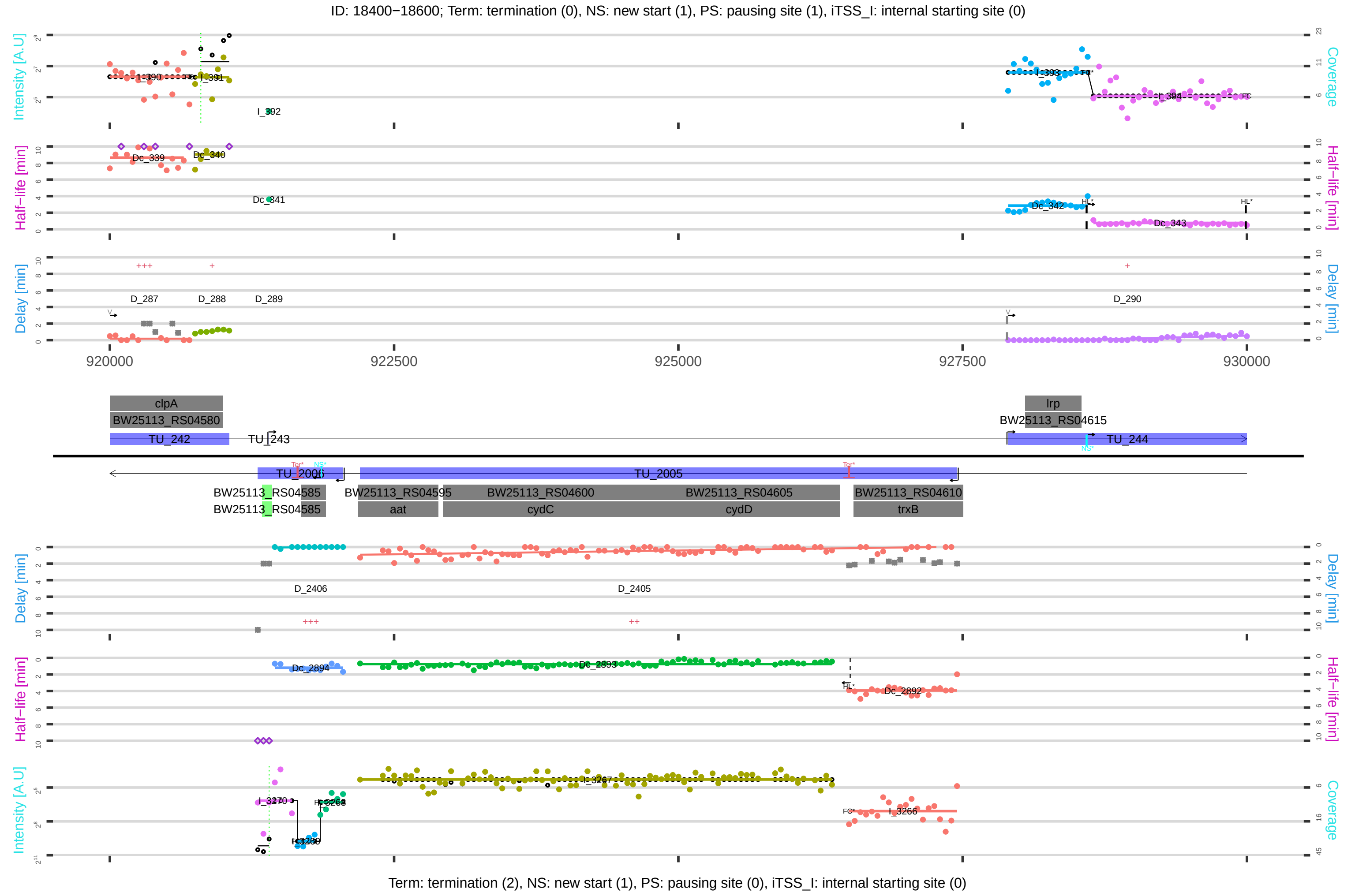

ID: 18400−18600; Term: termination (0), NS: new start (1), PS: pausing site (1), iTSS_I: internal starting site (0)
Intensity [A.U]
Coverage
Half−life [min]
Delay [min]
I_390
I_391
I_392
I_393
I_394
Dc_339
Dc_340
Dc_341
Dc_342
Dc_343
D_287
D_288
D_289
D_290
920000
922500
925000
927500
930000
clpA
BW25113_RS04580
TU_242
TU_243
lrp
BW25113_RS04615
TU_244
TU_2006
TU_2005
BW25113_RS04585
BW25113_RS04595
BW25113_RS04600
BW25113_RS04605
BW25113_RS04610
aat
cydC
cydD
trxB
D_2406
D_2405
Dc_2894
Dc_2893
Dc_2892
I_3270
I_3268
I_3269
I_3267
I_3266
Term: termination (2), NS: new start (1), PS: pausing site (0), iTSS_I: internal starting site (0)

ID: 18600−18772; Term: termination (3), NS: new start (0), PS: pausing site (1), iTSS_I: internal starting site (0)

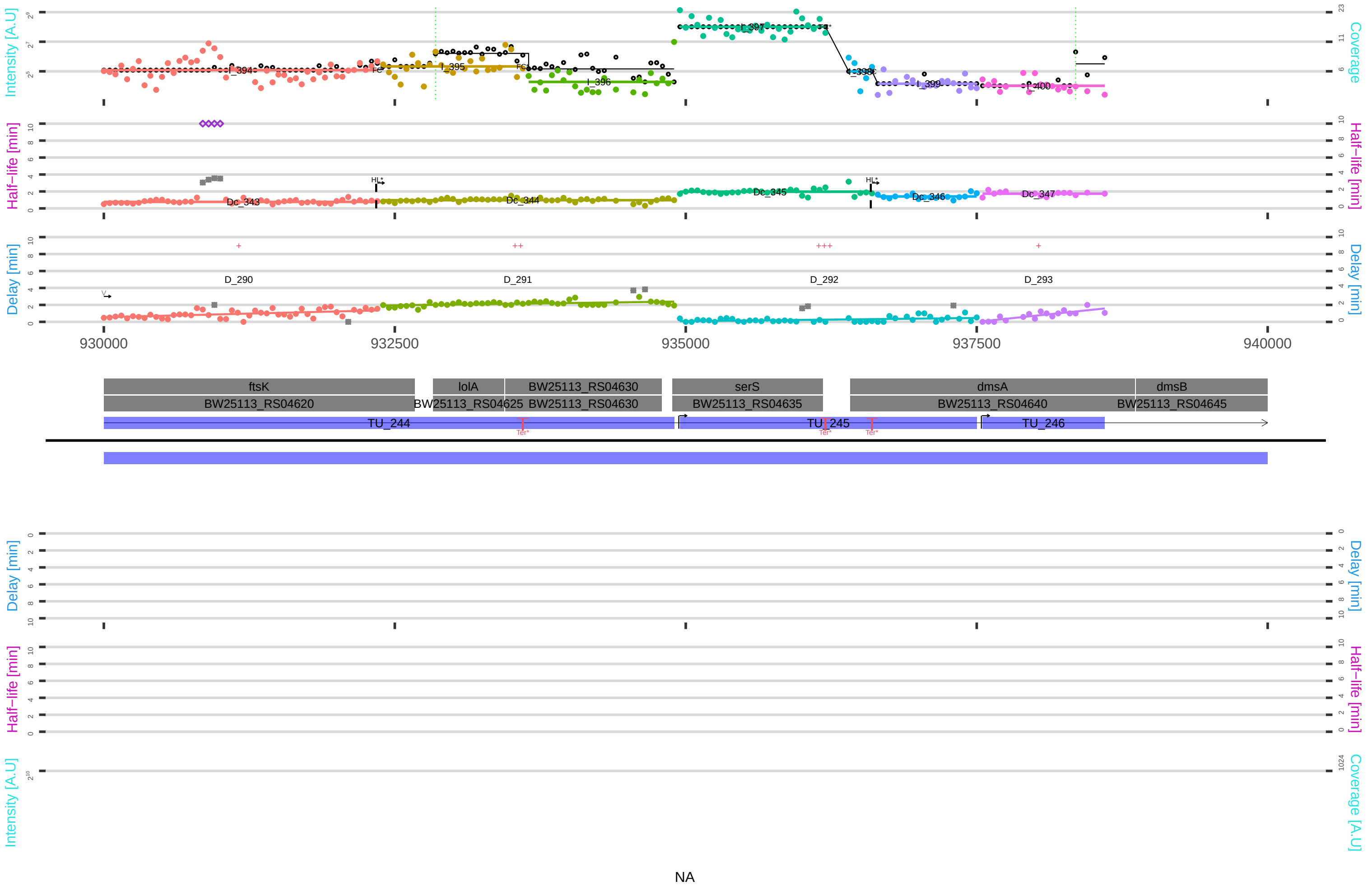

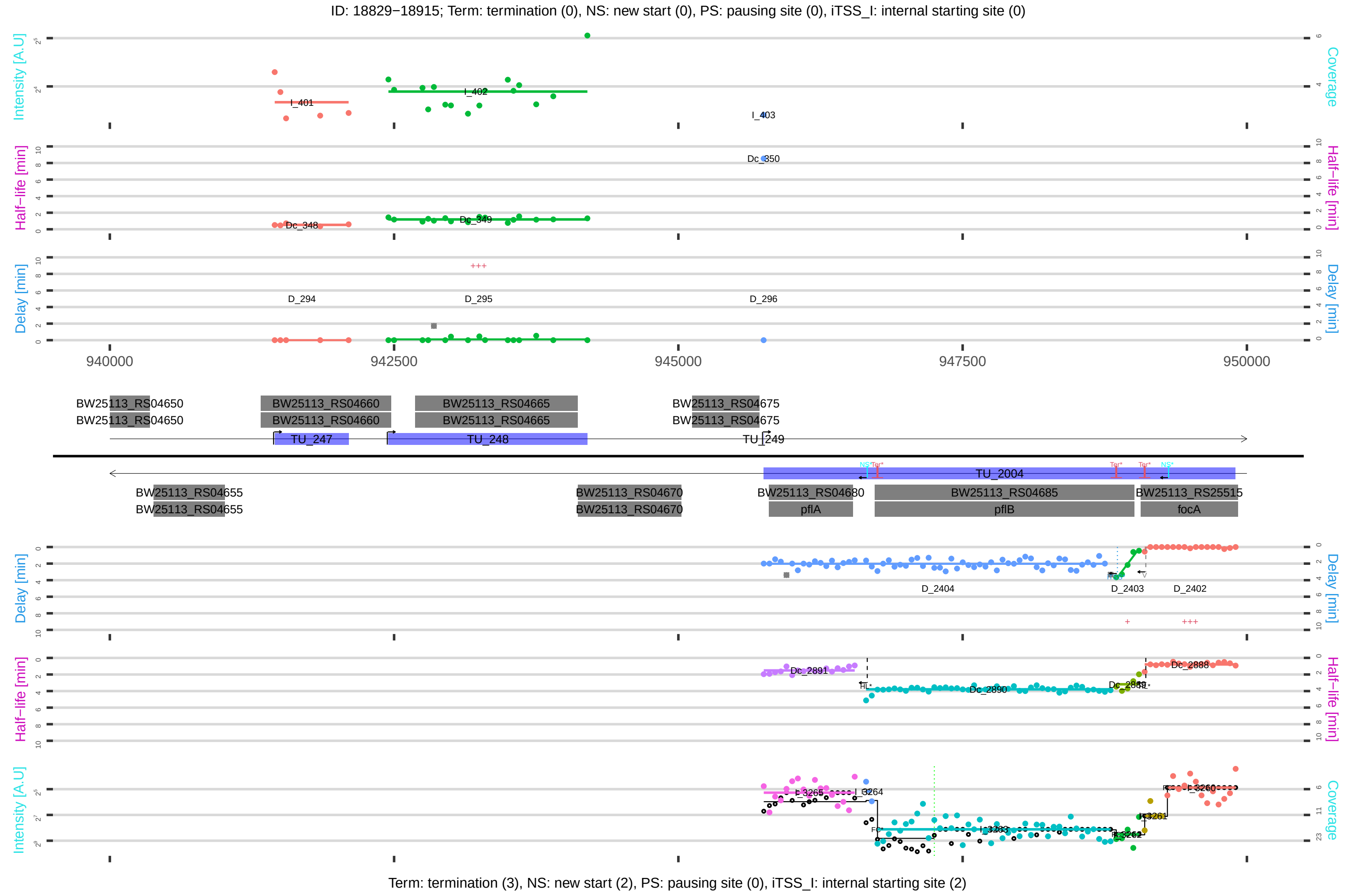

ID: 18829−18915; Term: termination (0), NS: new start (0), PS: pausing site (0), iTSS_I: internal starting site (0)
Intensity [A.U]
Coverage
Half−life [min]
Delay [min]
I_401
I_402
I_403
Dc_348
Dc_349
Dc_350
D_294
D_295
D_296
940000
942500
945000
947500
950000
BW25113_RS04650
BW25113_RS04660
BW25113_RS04665
BW25113_RS04675
TU_247
TU_248
TU_249
TU_2004
BW25113_RS04655
BW25113_RS04670
BW25113_RS04680
BW25113_RS04685
BW25113_RS25515
pflA
pflB
focA
D_2404
D_2403
D_2402
Dc_2891
Dc_2890
Dc_2889
Dc_2888
I_3265
I_3264
I_3263
I_3262
I_3261
I_3260
Term: termination (3), NS: new start (2), PS: pausing site (0), iTSS_I: internal starting site (2)

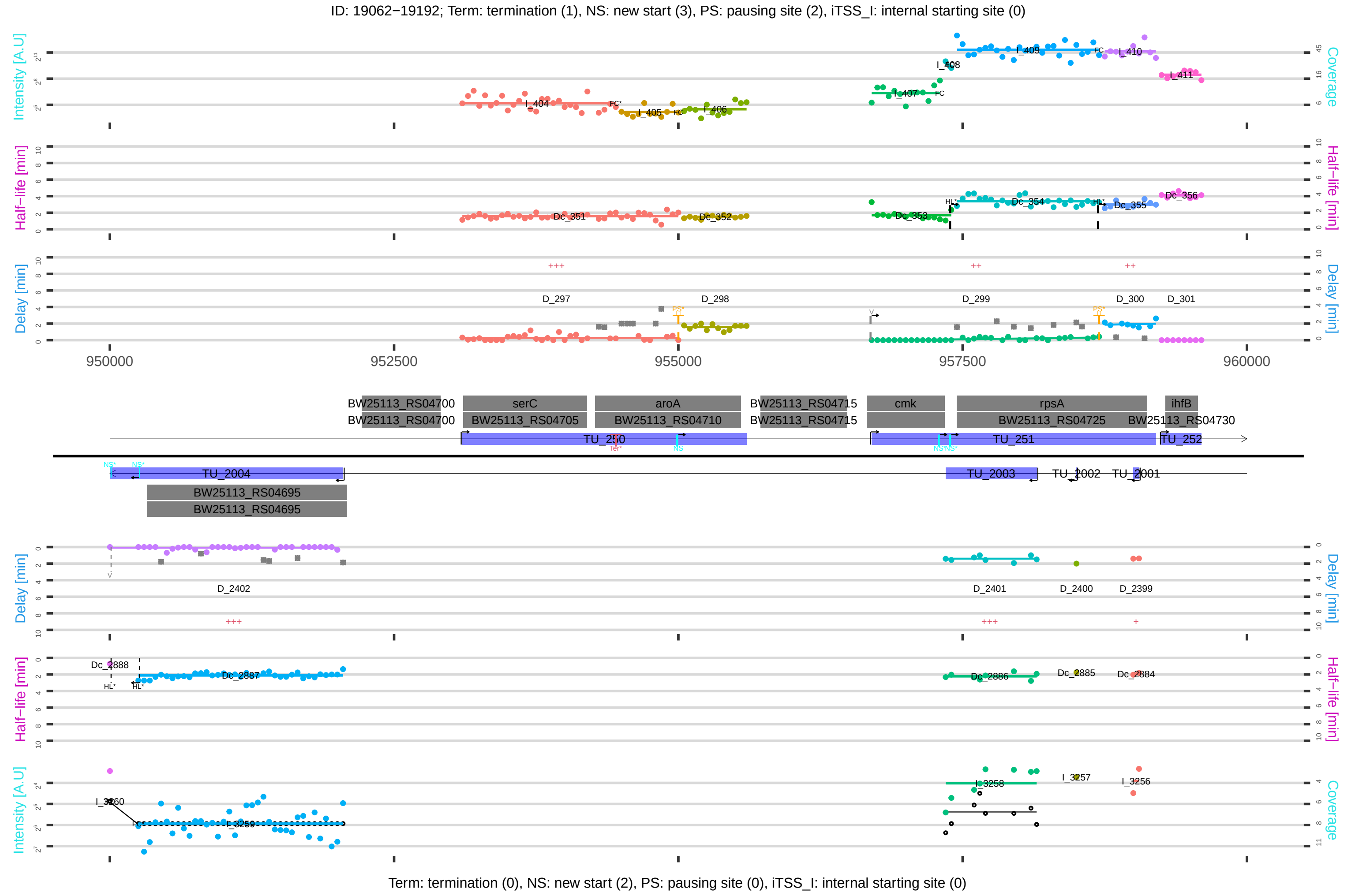

ID: 19062−19192; Term: termination (1), NS: new start (3), PS: pausing site (2), iTSS_I: internal starting site (0)
Intensity [A.U]
Coverage
Half−life [min]
Delay [min]
950000
952500
955000
957500
960000
BW25113_RS04700
serC
BW25113_RS04705
aroA
BW25113_RS04710
BW25113_RS04715
cmk
rpsA
BW25113_RS04725
ihfB
BW25113_RS04730
TU_250
TU_251
TU_252
TU_2004
BW25113_RS04695
TU_2003
TU_2002
TU_2001
D_297
D_298
D_299
D_300
D_301
D_2402
D_2401
D_2400
D_2399
Term: termination (0), NS: new start (2), PS: pausing site (0), iTSS_I: internal starting site (0)

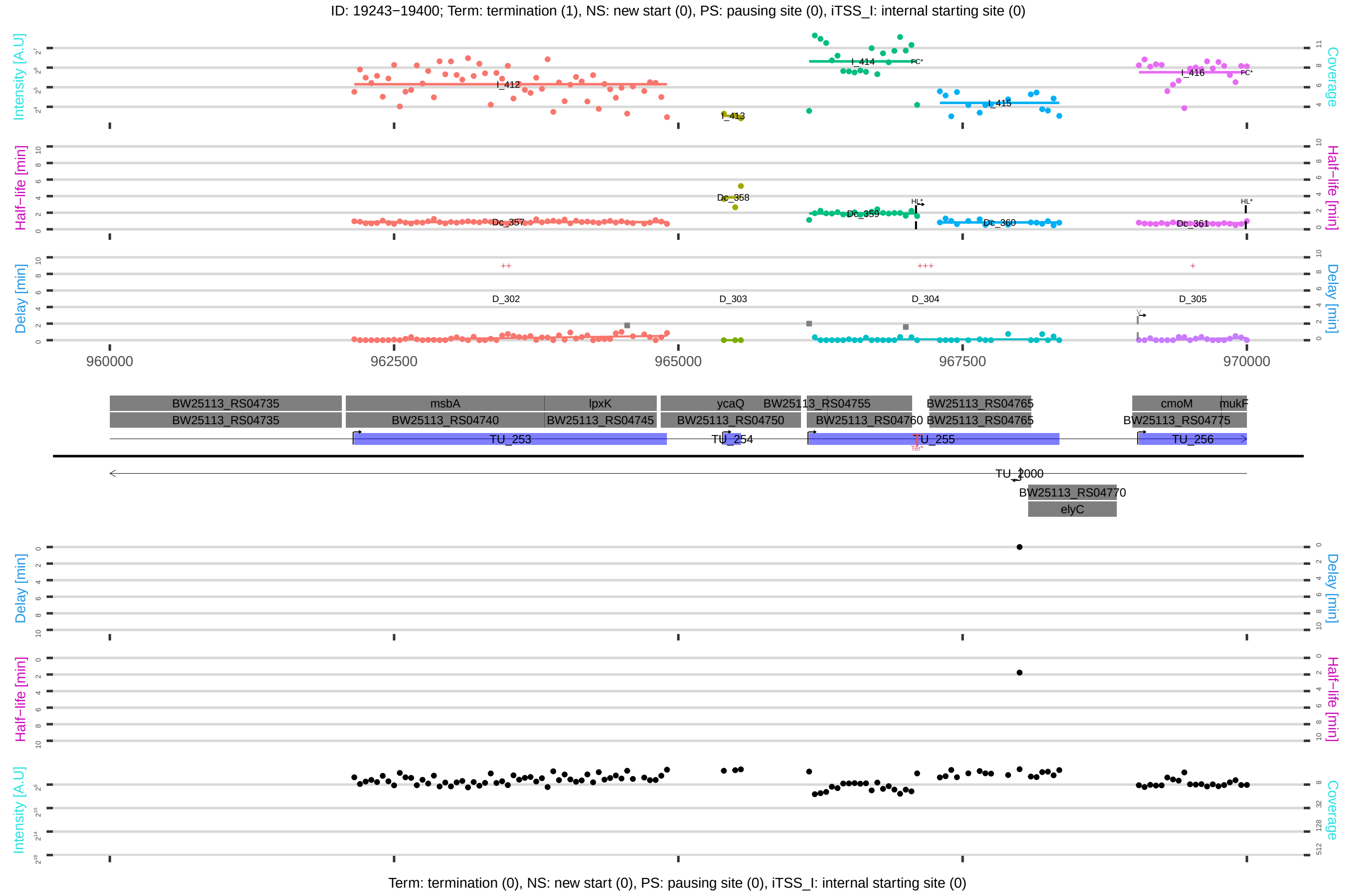

ID: 19243−19400; Term: termination (1), NS: new start (0), PS: pausing site (0), iTSS_I: internal starting site (0)
Intensity [A.U]
Coverage
Half−life [min]
Delay [min]
I_412
I_413
I_414
I_415
I_416
FC*
Dc_357
Dc_358
Dc_359
Dc_360
Dc_361
HL*
D_302
D_303
D_304
D_305
960000
962500
965000
967500
970000
BW25113_RS04735
msbA
lpxK
ycaQ
BW25113_RS04755
BW25113_RS04765
cmoM
mukF
BW25113_RS04740
BW25113_RS04745
BW25113_RS04750
BW25113_RS04760
BW25113_RS04775
TU_253
TU_254
TU_255
TU_256
Ter*
TU_2000
BW25113_RS04770
elyC
Term: termination (0), NS: new start (0), PS: pausing site (0), iTSS_I: internal starting site (0)

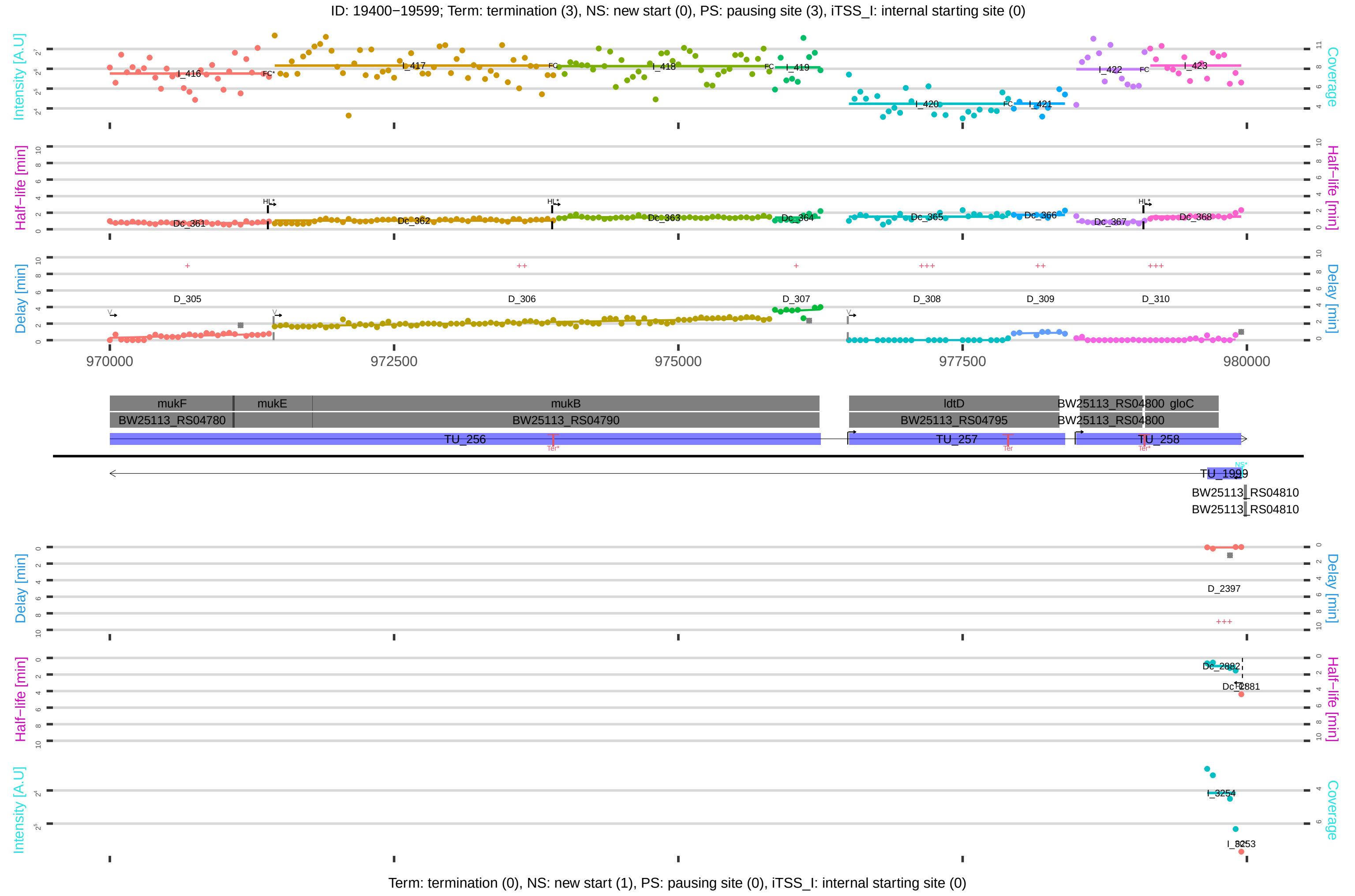

ID: 19400−19599; Term: termination (3), NS: new start (0), PS: pausing site (3), iTSS_I: internal starting site (0)
Intensity [A.U]
Coverage
Half−life [min]
Delay [min]
I_416
I_417
I_418
I_419
I_420
I_421
I_422
I_423
Dc_361
Dc_362
Dc_363
Dc_364
Dc_365
Dc_366
Dc_367
Dc_368
D_305
D_306
D_307
D_308
D_309
D_310
970000
972500
975000
977500
980000
mukF
mukE
mukB
ldtD
BW25113_RS04800 gloC
BW25113_RS04780
BW25113_RS04790
BW25113_RS04795
BW25113_RS04800
TU_256
TU_257
TU_258
TU_1999
BW25113_RS04810
BW25113_RS04810
D_2397
Dc_2882
Dc_2881
I_3254
I_3253
Term: termination (0), NS: new start (1), PS: pausing site (0), iTSS_I: internal starting site (0)

ID: 19660−19777; Term: termination (0), NS: new start (0), PS: pausing site (1), iTSS_I: internal starting site (0)

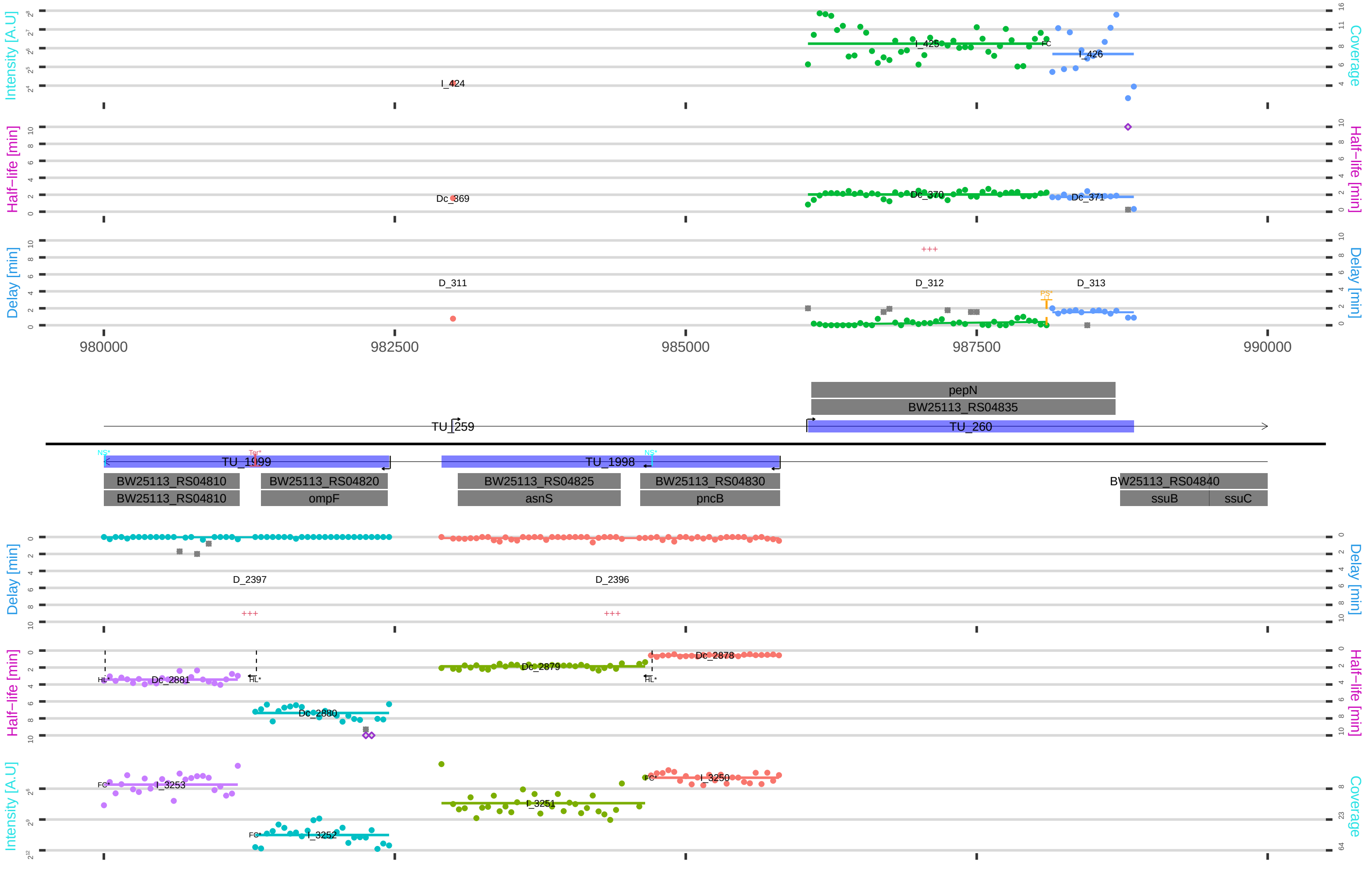

Term: termination (1), NS: new start (2), PS: pausing site (0), iTSS_I: internal starting site (0)

ID: 165264−165264; FC*: significant t−test of two consecutive segments; Term: termination, NS: new start, PS: pausing site,iTSS_I: internal starting site,TI: transcription interferance.

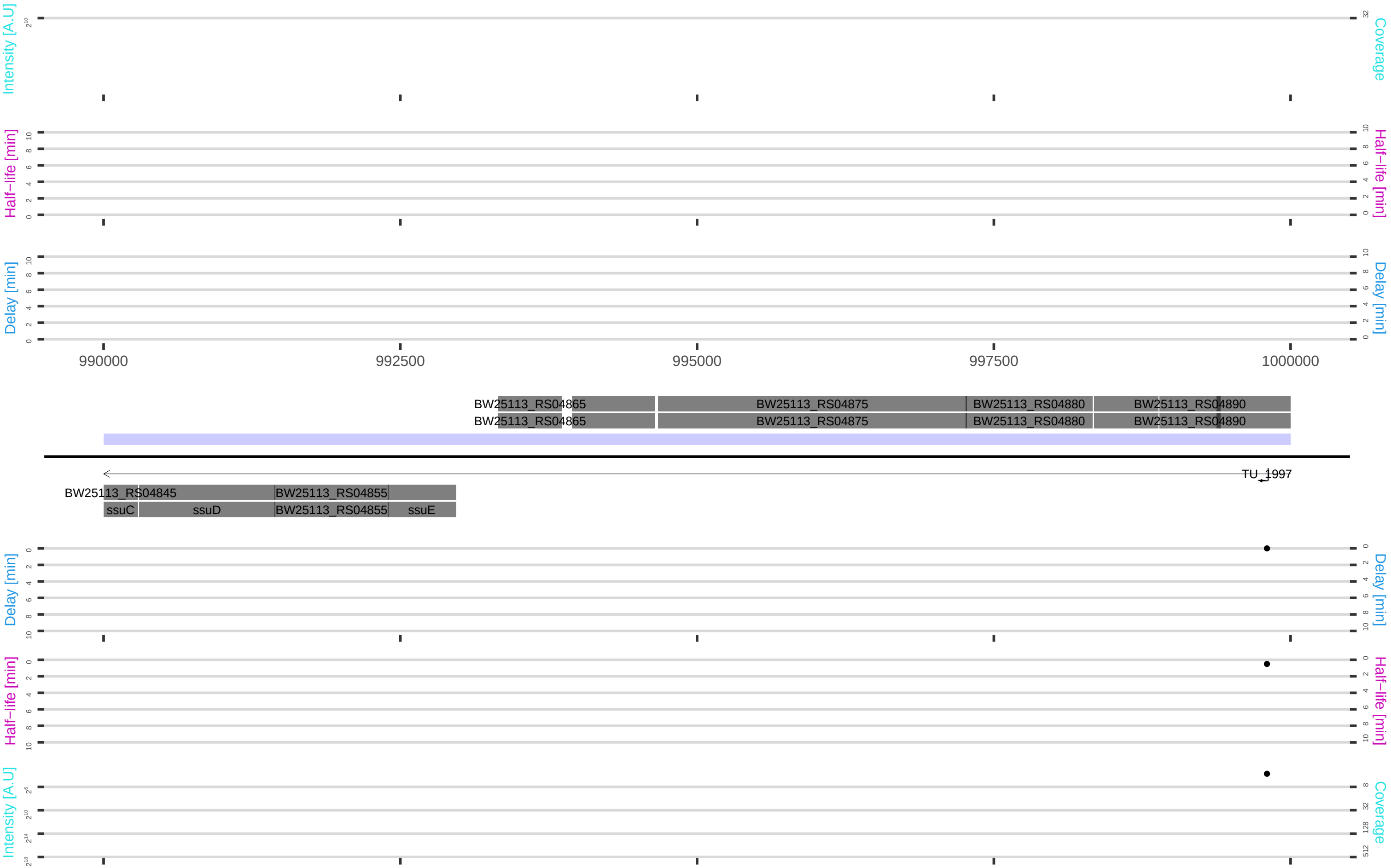

Term: termination (0), NS: new start (0), PS: pausing site (0), iTSS_I: internal starting site (0)

ID: 20006−20200; Term: termination (2), NS: new start (1), PS: pausing site (1), iTSS_I: internal starting site (0)

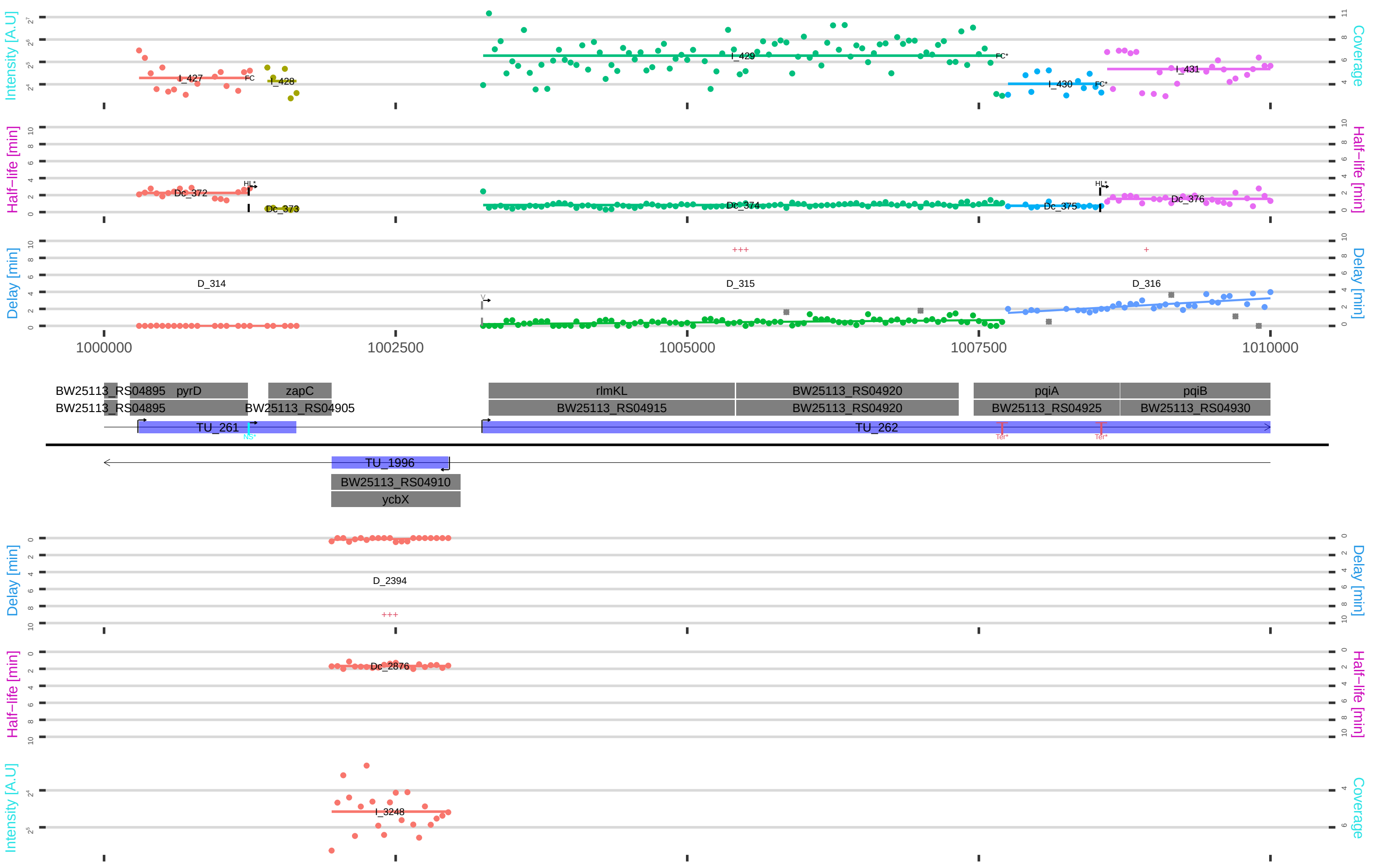

Term: termination (0), NS: new start (0), PS: pausing site (0), iTSS_I: internal starting site (0)

ID: 20200−20400; Term: termination (1), NS: new start (0), PS: pausing site (1), iTSS_I: internal starting site (0)

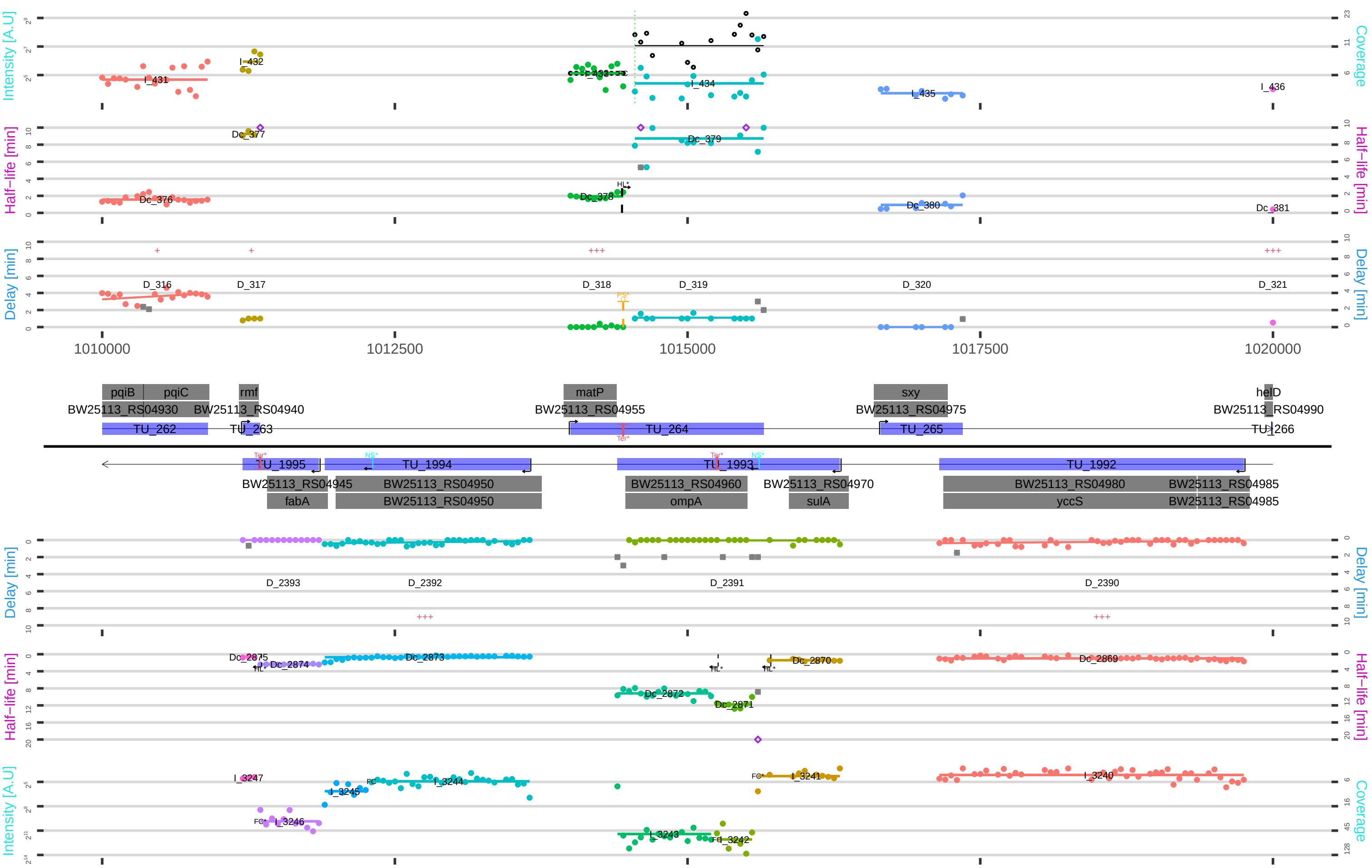

Term: termination (2), NS: new start (2), PS: pausing site (0), iTSS_I: internal starting site (0)

ID: 20400−20516; Term: termination (1), NS: new start (0), PS: pausing site (1), iTSS_I: internal starting site (0)

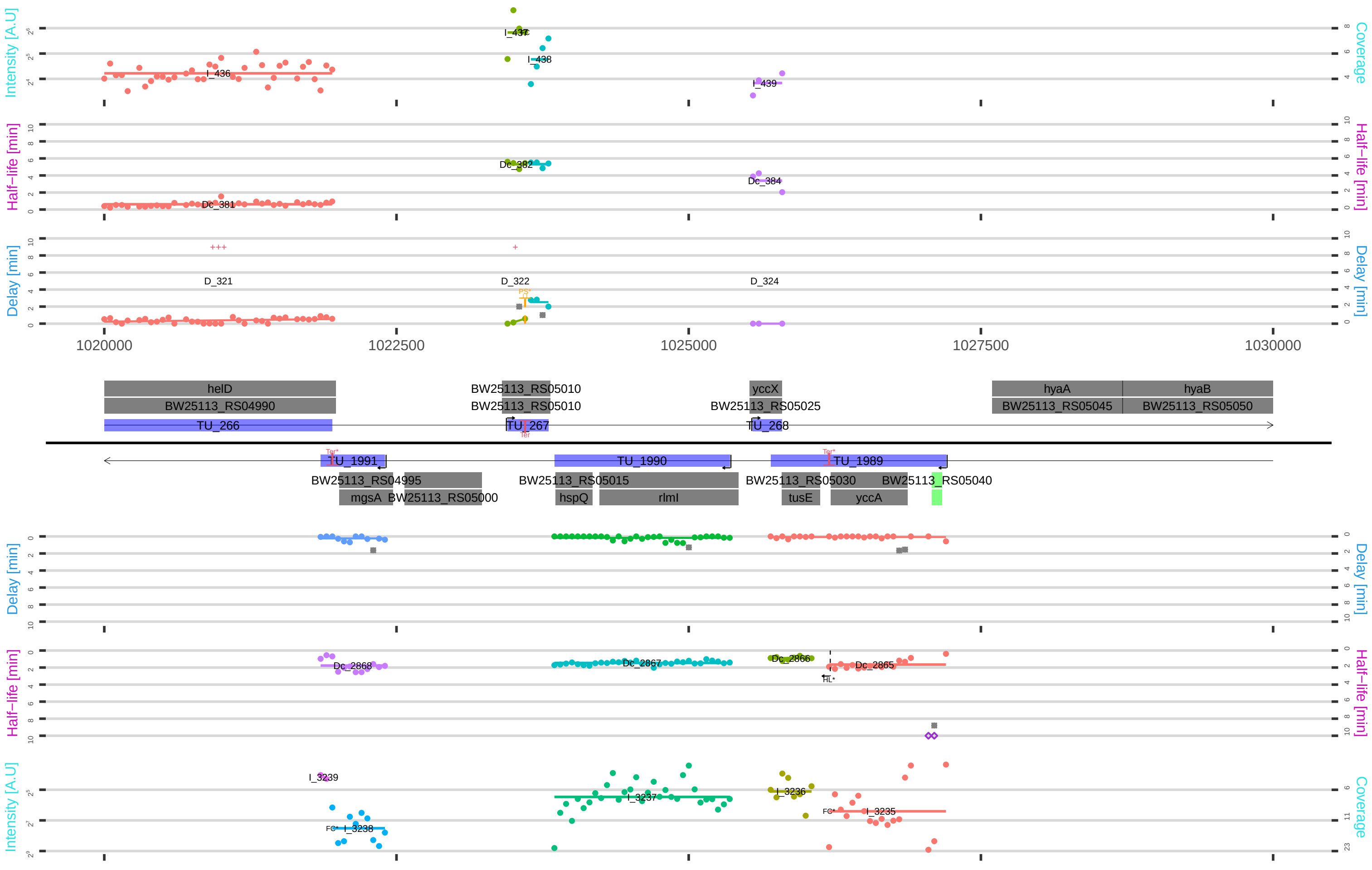

Term: termination (2), NS: new start (0), PS: pausing site (0), iTSS_I: internal starting site (0)

ID: 164512−164501; FC*: significant t−test of two consecutive segments; Term: termination, NS: new start, PS: pausing site,iTSS_I: internal starting site,TI: transcription interferance.

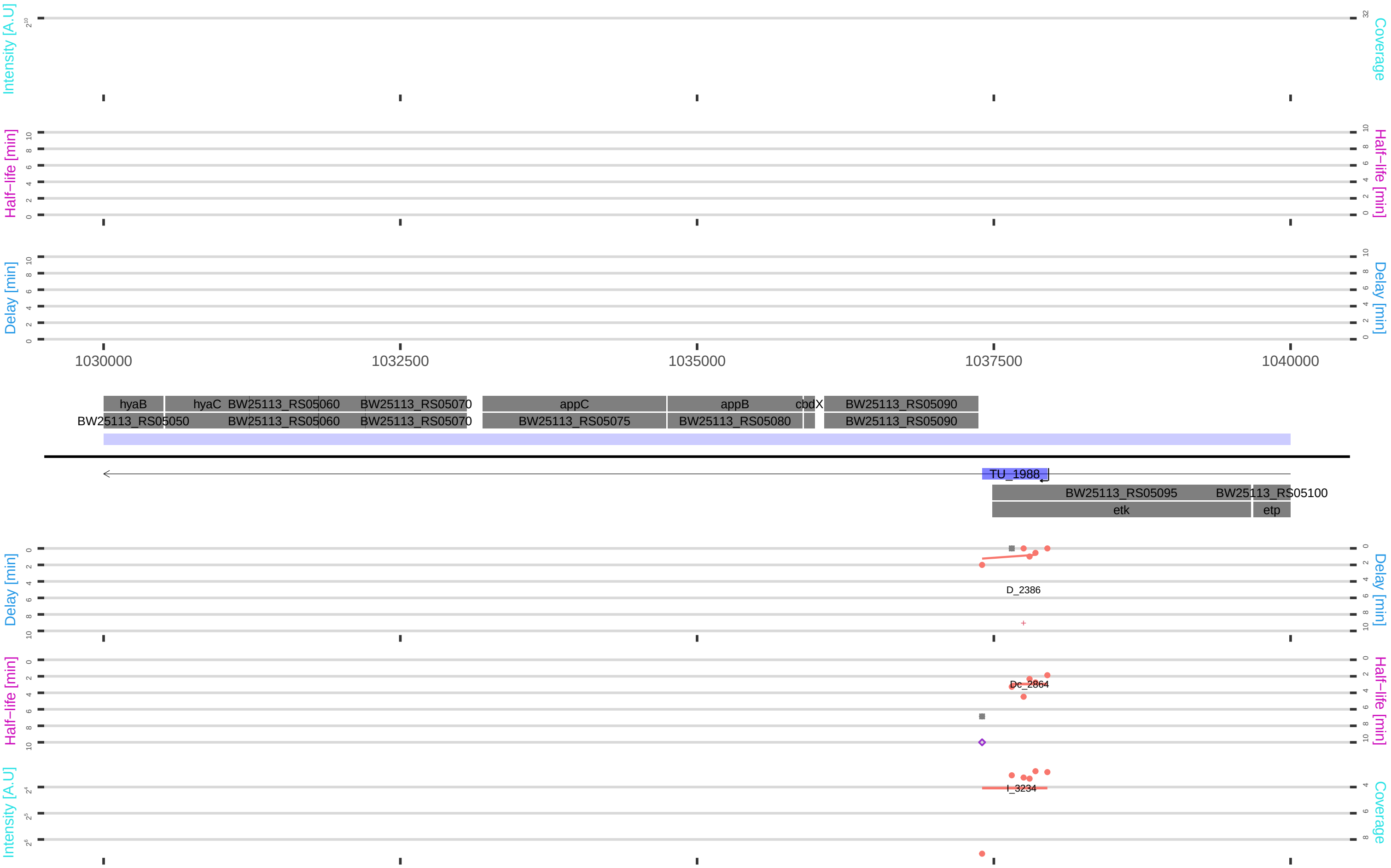

Term: termination (0), NS: new start (0), PS: pausing site (0), iTSS_I: internal starting site (0)

ID: 20908−20955; Term: termination (0), NS: new start (0), PS: pausing site (0), iTSS_I: internal starting site (0)

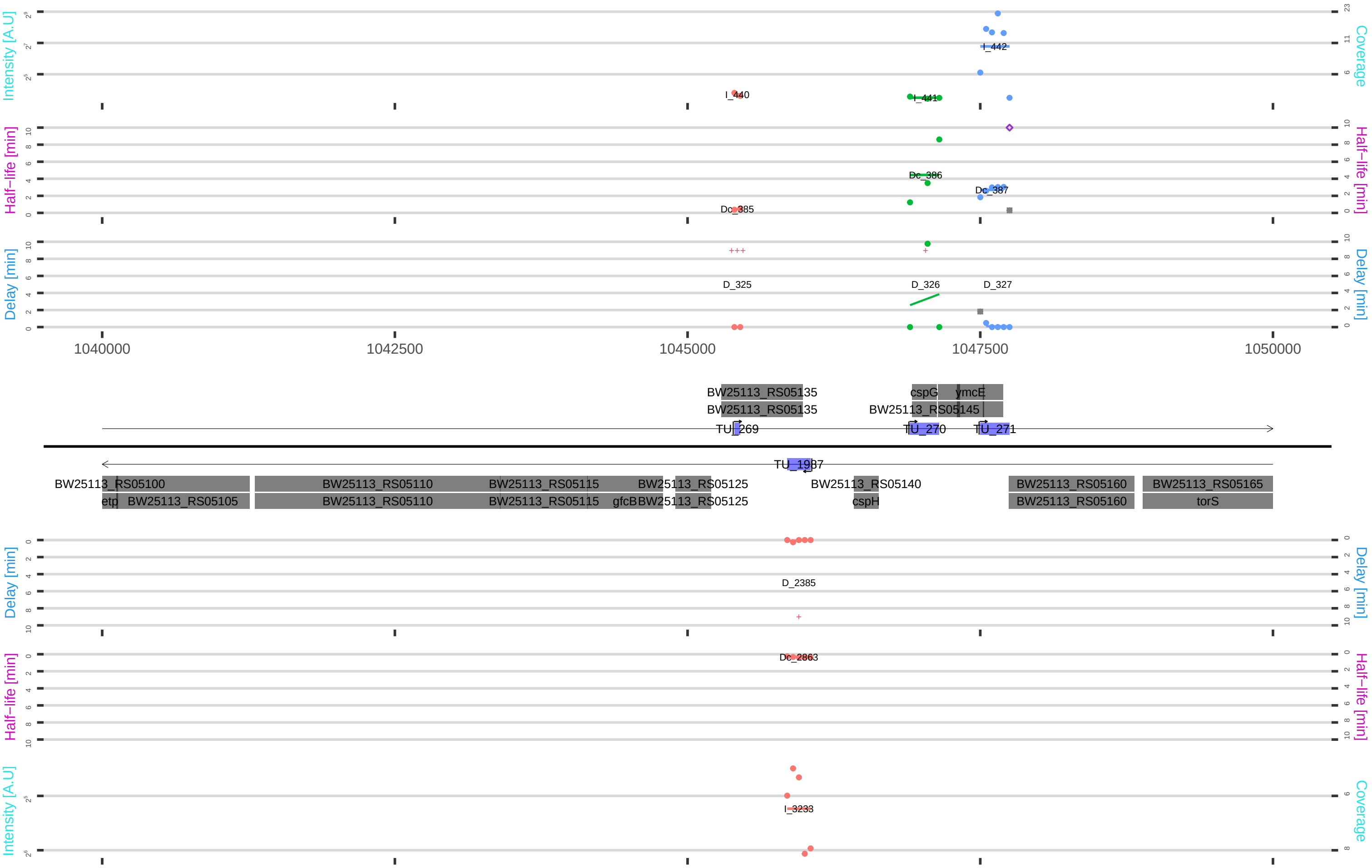

Term: termination (0), NS: new start (0), PS: pausing site (0), iTSS_I: internal starting site (0)

ID: 164197−164076; FC*: significant t−test of two consecutive segments; Term: termination, NS: new start, PS: pausing site,iTSS_I: internal starting site,TI: transcription interferance.

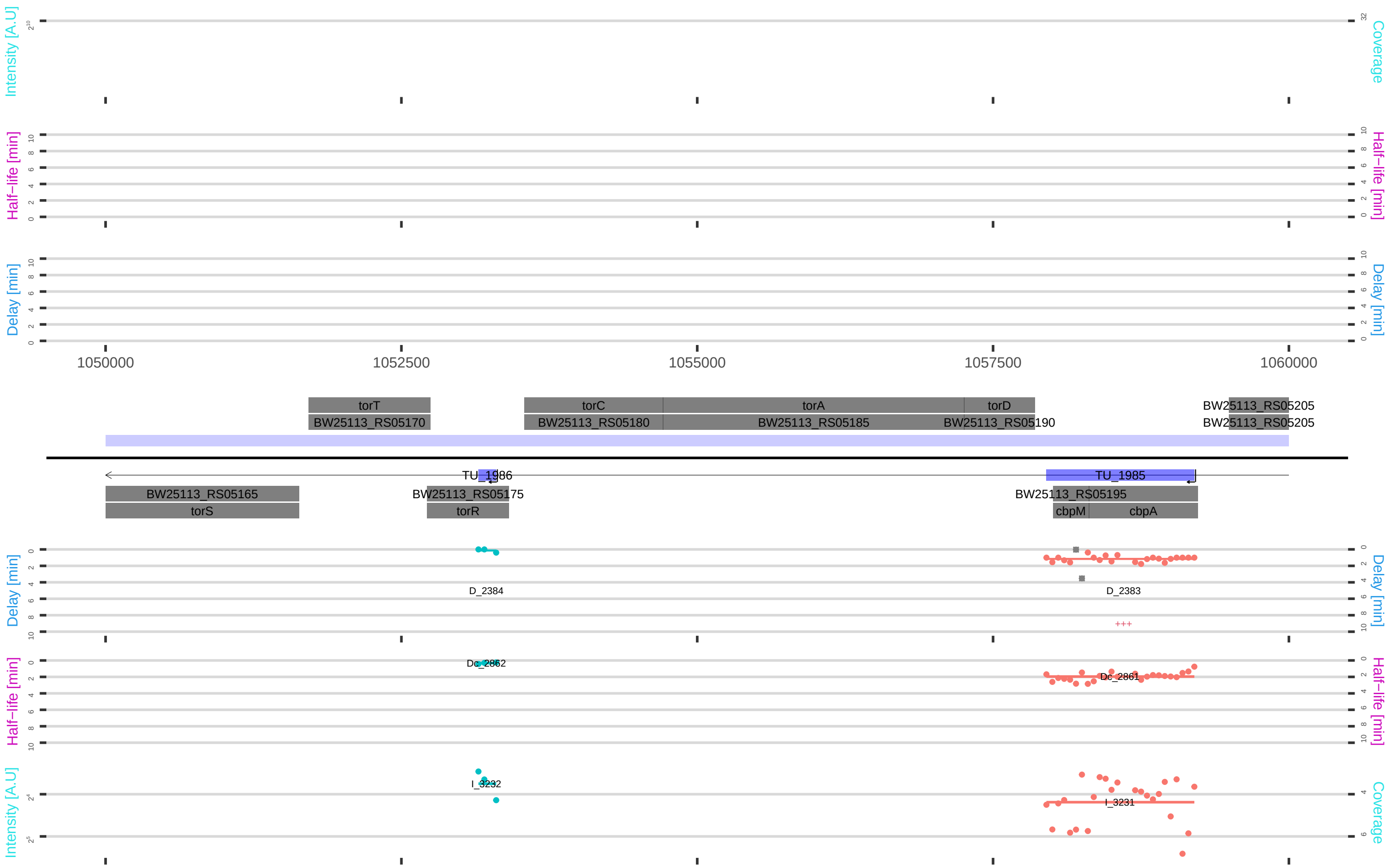

Term: termination (0), NS: new start (0), PS: pausing site (0), iTSS_I: internal starting site (0)

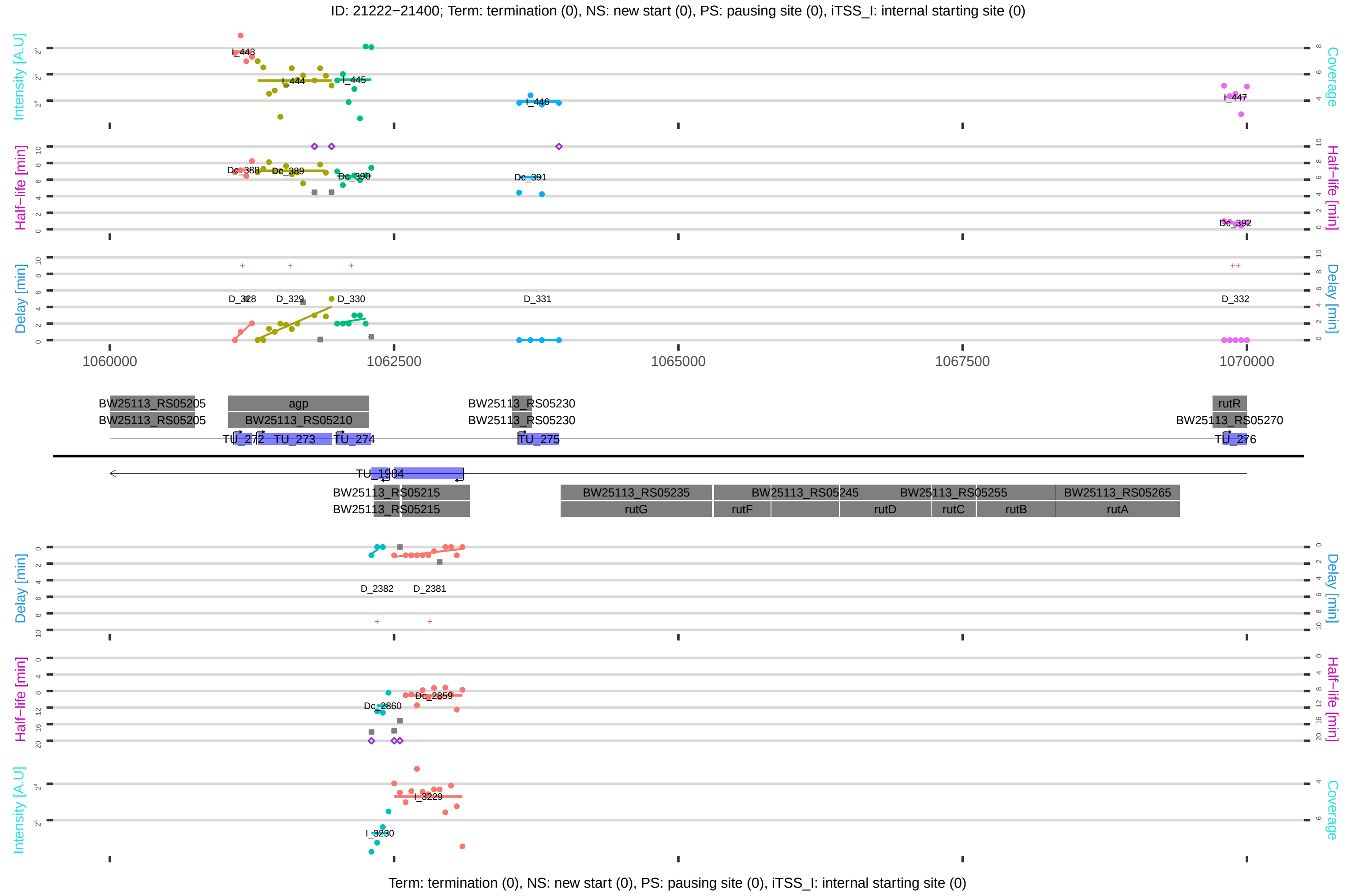
ID: 21222−21400; Term: termination (0), NS: new start (0), PS: pausing site (0), iTSS_I: internal starting site (0)
Intensity [A.U]
Coverage
I_443
I_444
I_445
I_446
I_447
Half−life [min]
Dc_388
Dc_389
Dc_390
Dc_391
Dc_392
Delay [min]
D_328
D_329
D_330
D_331
D_332
1060000
1062500
1065000
1067500
1070000
BW25113_RS05205
agp
BW25113_RS05210
BW25113_RS05230
rutR
BW25113_RS05270
TU_272
TU_273
TU_274
TU_275
TU_276
TU_1984
BW25113_RS05215
BW25113_RS05235
BW25113_RS05245
BW25113_RS05255
BW25113_RS05265
rutG
rutF
rutD
rutC
rutB
rutA
D_2382
D_2381
Dc_2860
Dc_2859
I_3229
I_3230
Term: termination (0), NS: new start (0), PS: pausing site (0), iTSS_I: internal starting site (0)

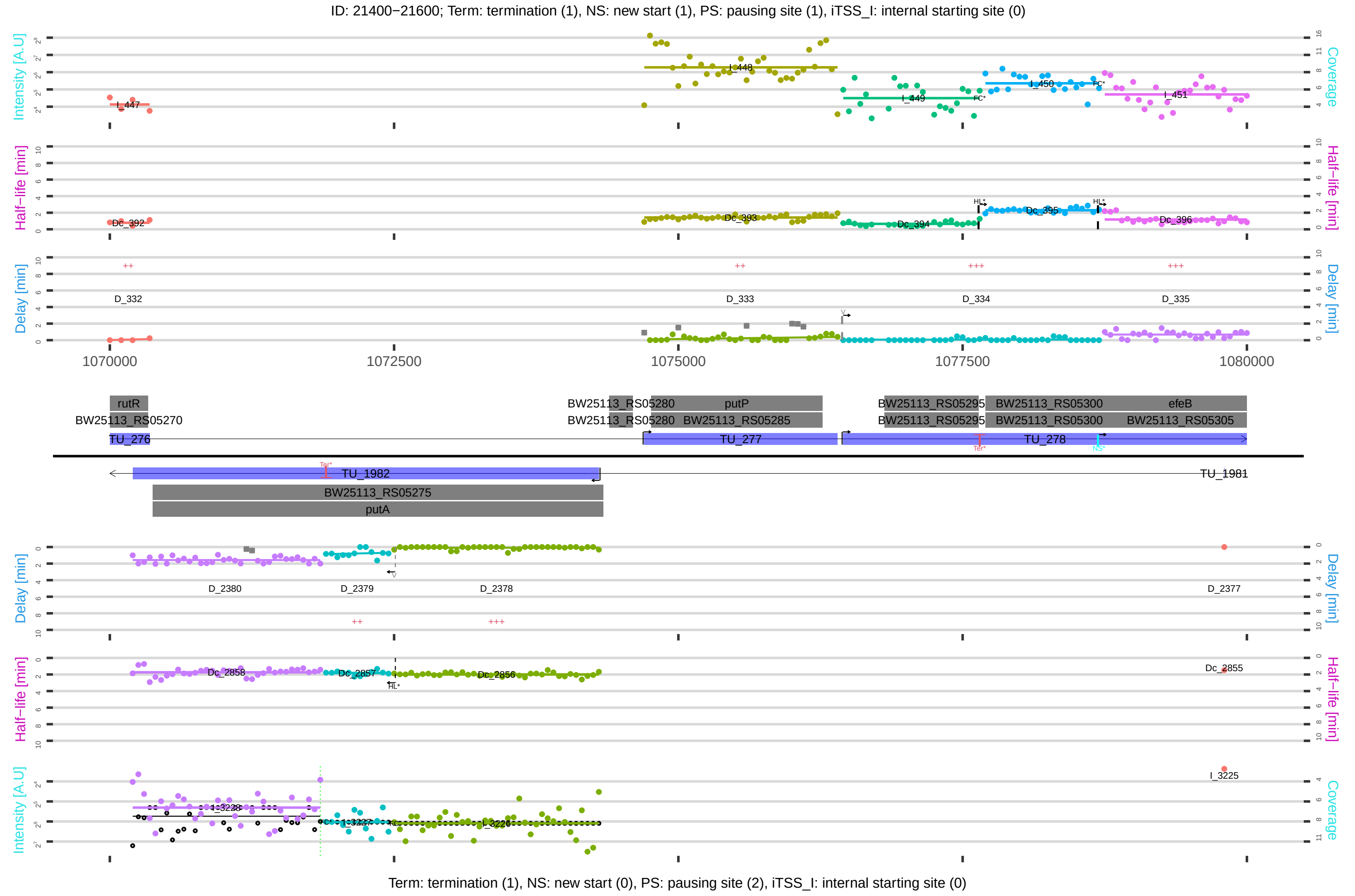
ID: 21400−21600; Term: termination (1), NS: new start (1), PS: pausing site (1), iTSS_I: internal starting site (0)
Intensity [A.U]
Coverage
Half−life [min]
Delay [min]
I_447
I_448
I_449
I_450
I_451
Dc_392
Dc_393
Dc_394
Dc_395
Dc_396
D_332
D_333
D_334
D_335
1070000
1072500
1075000
1077500
1080000
rutR
BW25113_RS05270
BW25113_RS05280
putP
BW25113_RS05285
BW25113_RS05295
BW25113_RS05300
efeB
BW25113_RS05305
TU_276
TU_277
TU_278
Ter*
NS*
TU_1982
TU_1981
BW25113_RS05275
putA
D_2380
D_2379
D_2378
D_2377
Dc_2858
Dc_2857
Dc_2856
Dc_2855
I_3228
I_3227
I_3226
I_3225
Term: termination (1), NS: new start (0), PS: pausing site (2), iTSS_I: internal starting site (0)

ID: 21600−21631; Term: termination (0), NS: new start (0), PS: pausing site (0), iTSS_I: internal starting site (0)

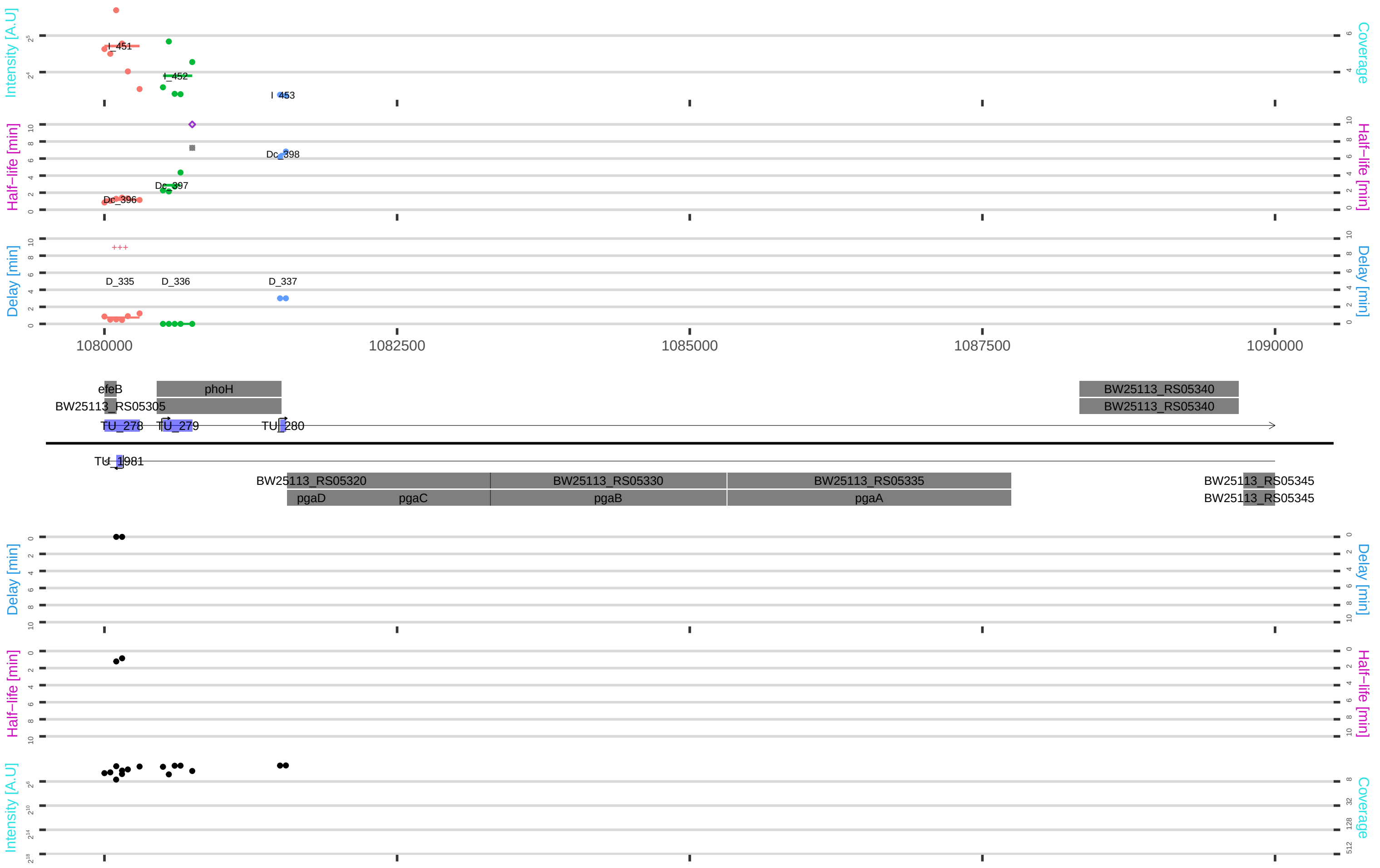

Term: termination (0), NS: new start (0), PS: pausing site (0), iTSS_I: internal starting site (0)

ID: 21862−21928; Term: termination (0), NS: new start (2), PS: pausing site (0), iTSS_I: internal starting site (0)

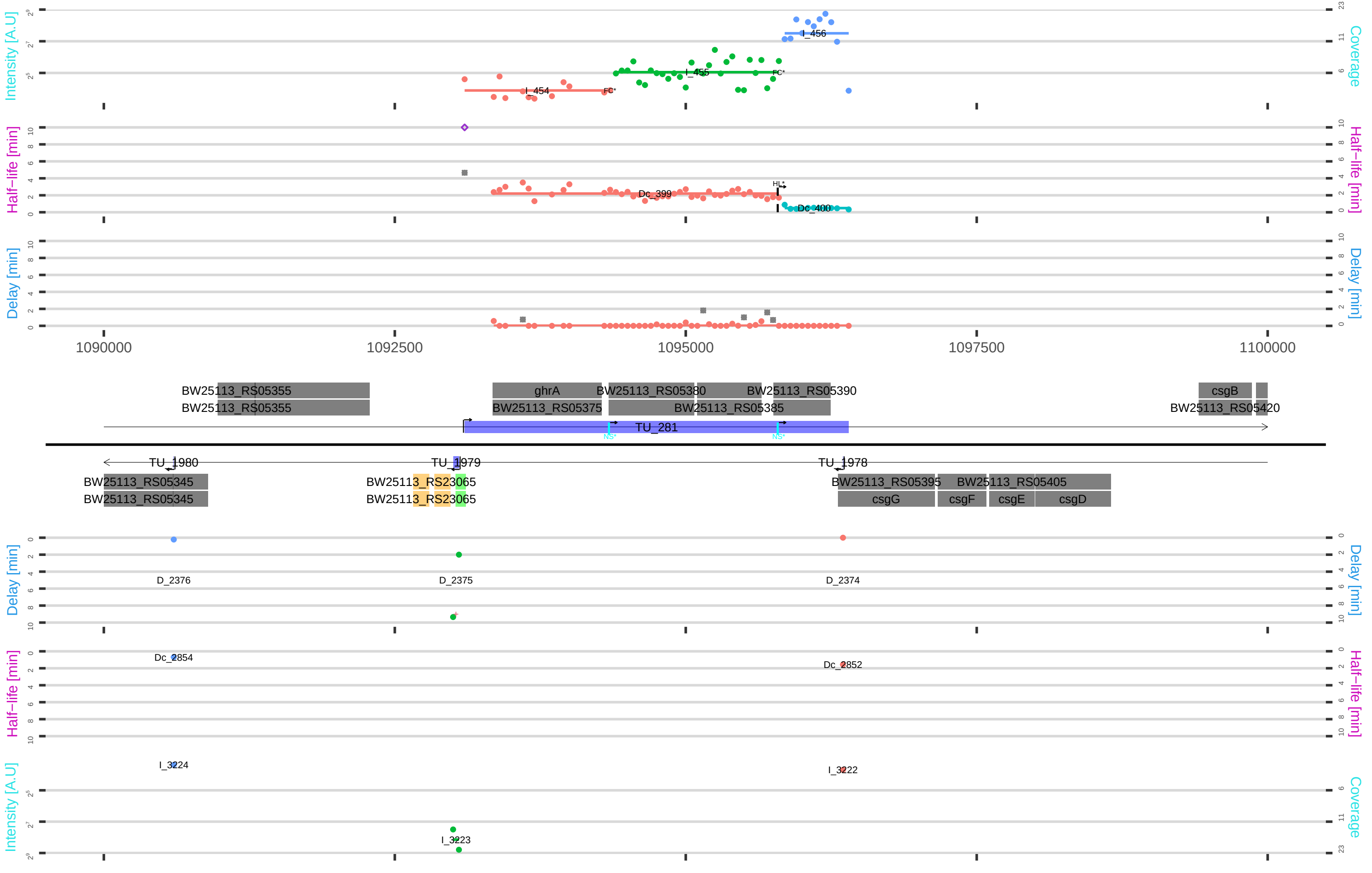

Term: termination (0), NS: new start (0), PS: pausing site (0), iTSS_I: internal starting site (0)

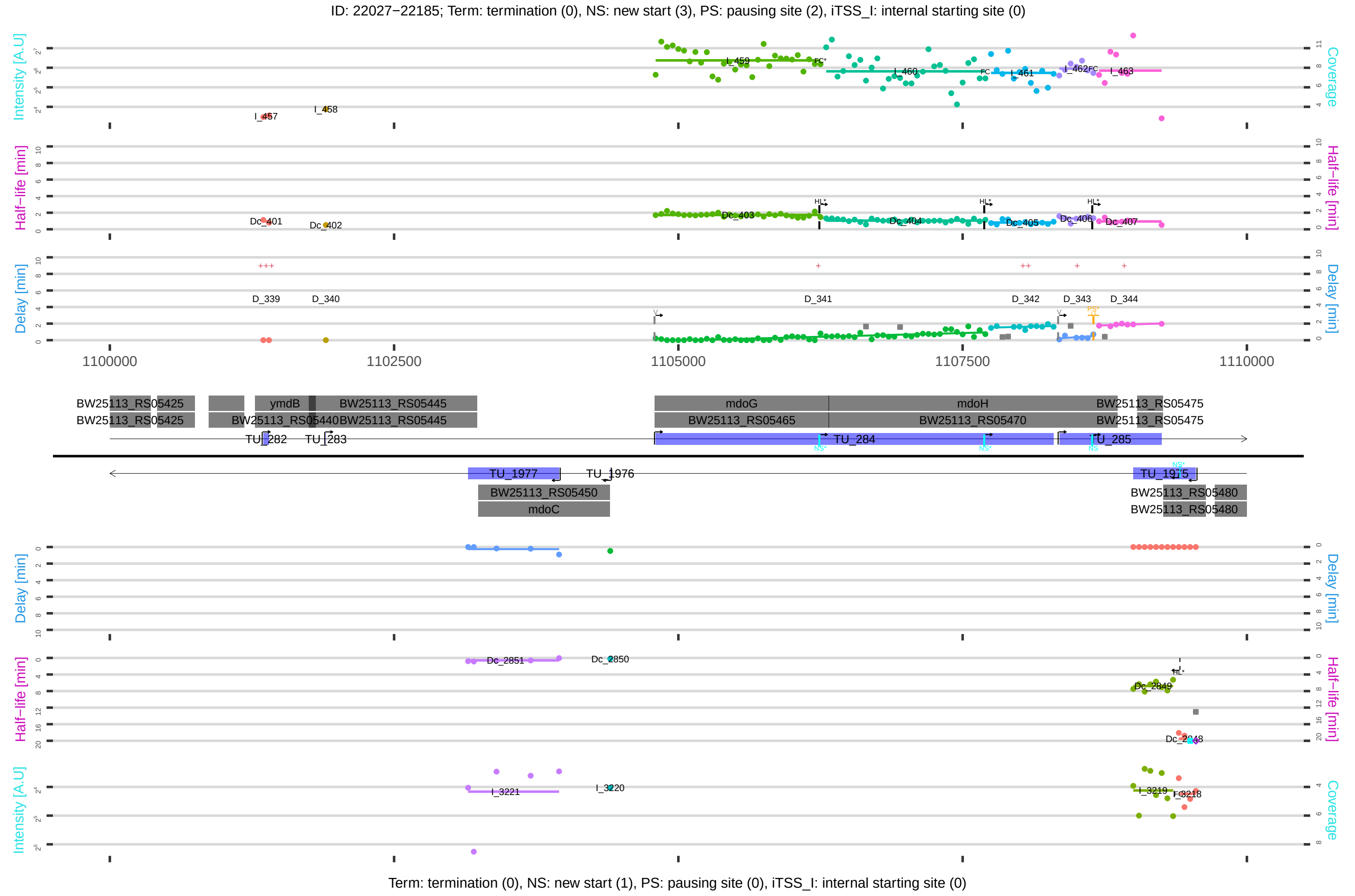

ID: 22027−22185; Term: termination (0), NS: new start (3), PS: pausing site (2), iTSS_I: internal starting site (0)
Intensity [A.U]
Coverage
Half−life [min]
Delay [min]
1100000
1102500
1105000
1107500
1110000
mdoG
mdoH
ymdB
mdoC
TU_282
TU_283
TU_284
TU_285
TU_1977
TU_1976
TU_1975
BW25113_RS05425
BW25113_RS05445
BW25113_RS05465
BW25113_RS05470
BW25113_RS05475
BW25113_RS05450
BW25113_RS05480
Term: termination (0), NS: new start (1), PS: pausing site (0), iTSS_I: internal starting site (0)

ID: 22246−22267; Term: termination (0), NS: new start (0), PS: pausing site (0), iTSS_I: internal starting site (0)

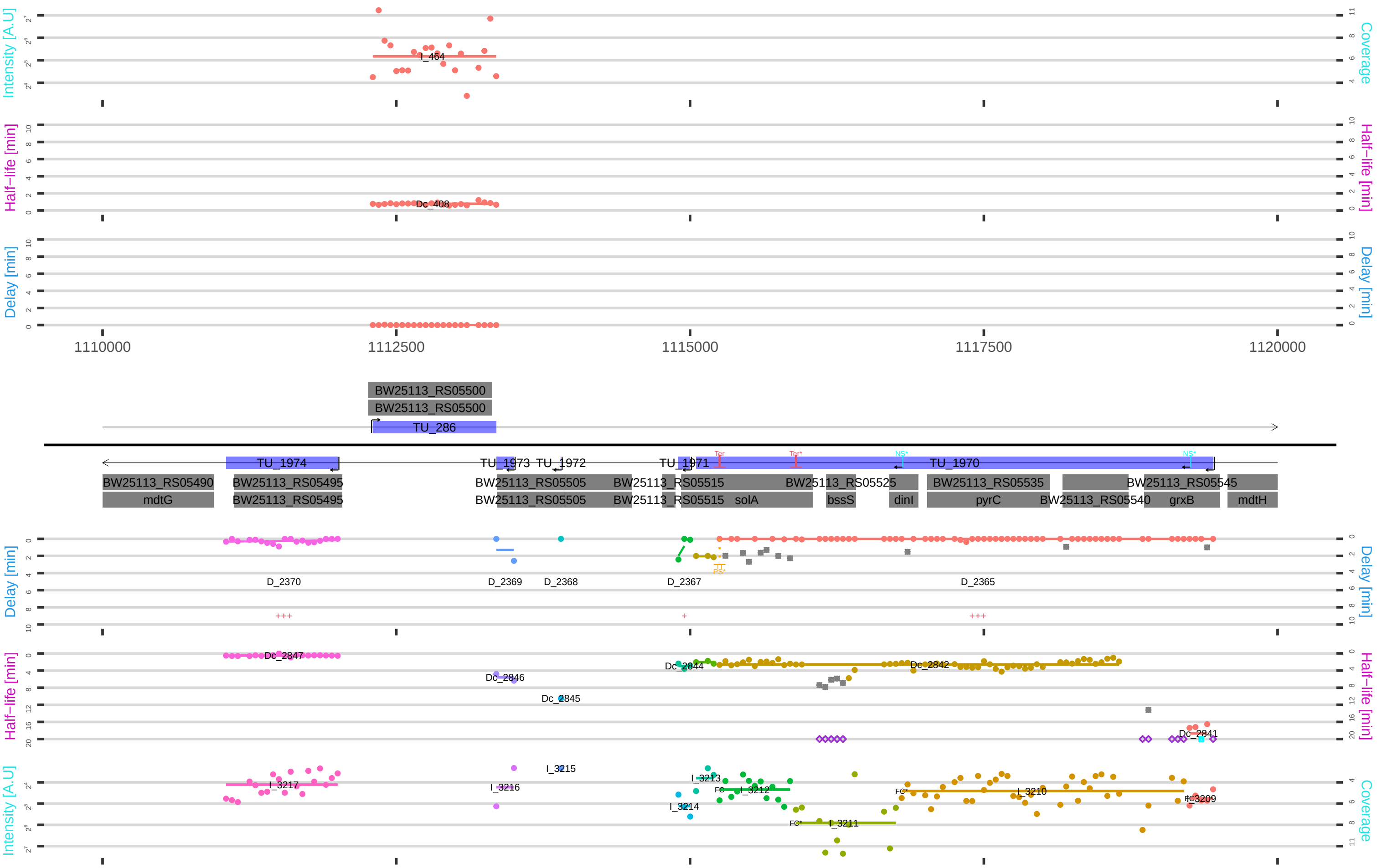

Term: termination (2), NS: new start (2), PS: pausing site (1), iTSS_I: internal starting site (0)

ID: 22421−22574; Term: termination (1), NS: new start (1), PS: pausing site (1), iTSS_I: internal starting site (0)

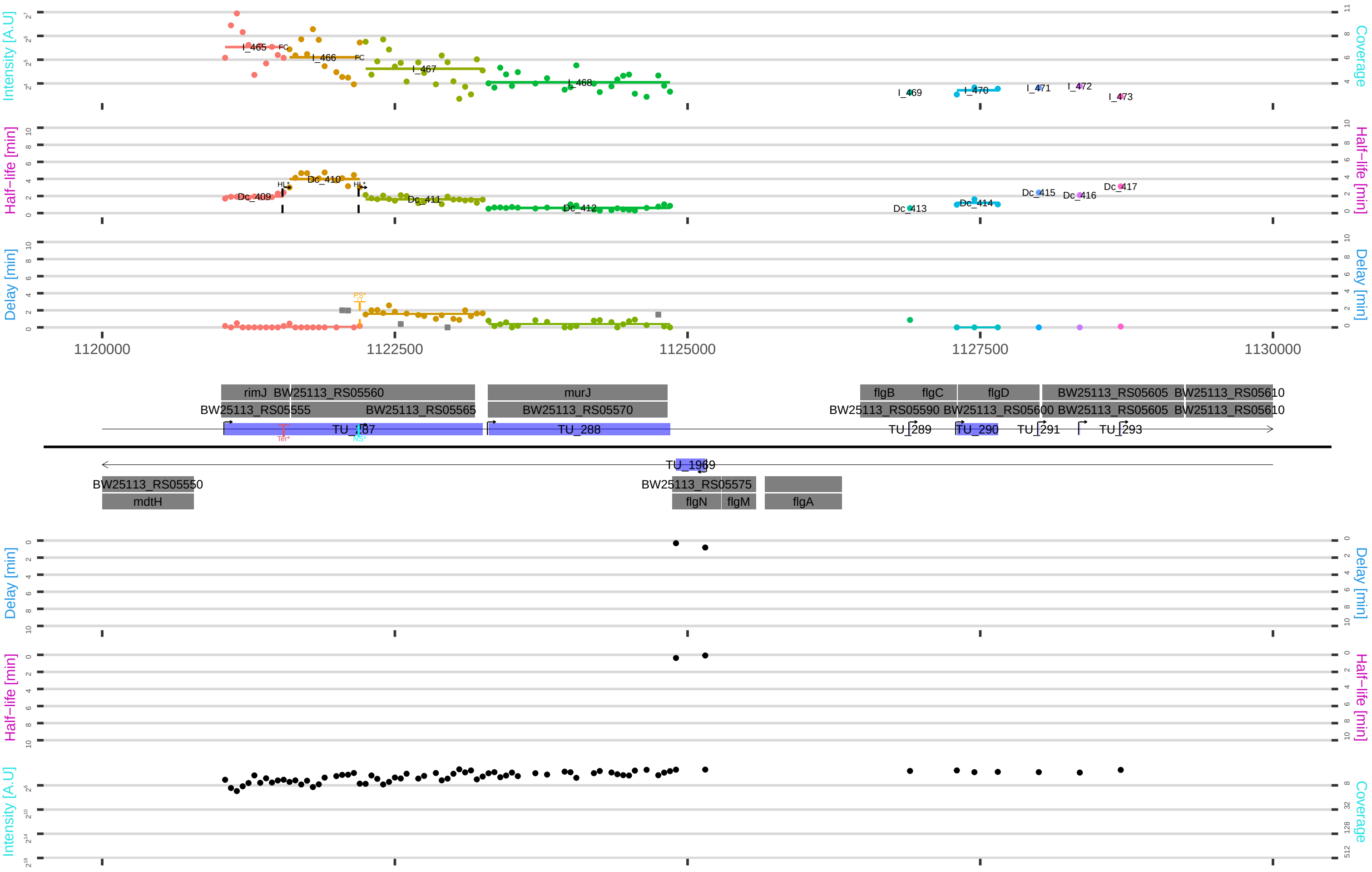

Term: termination (0), NS: new start (0), PS: pausing site (0), iTSS_I: internal starting site (0)

ID: 22619−22619; Term: termination (0), NS: new start (0), PS: pausing site (0), iTSS_I: internal starting site (0)

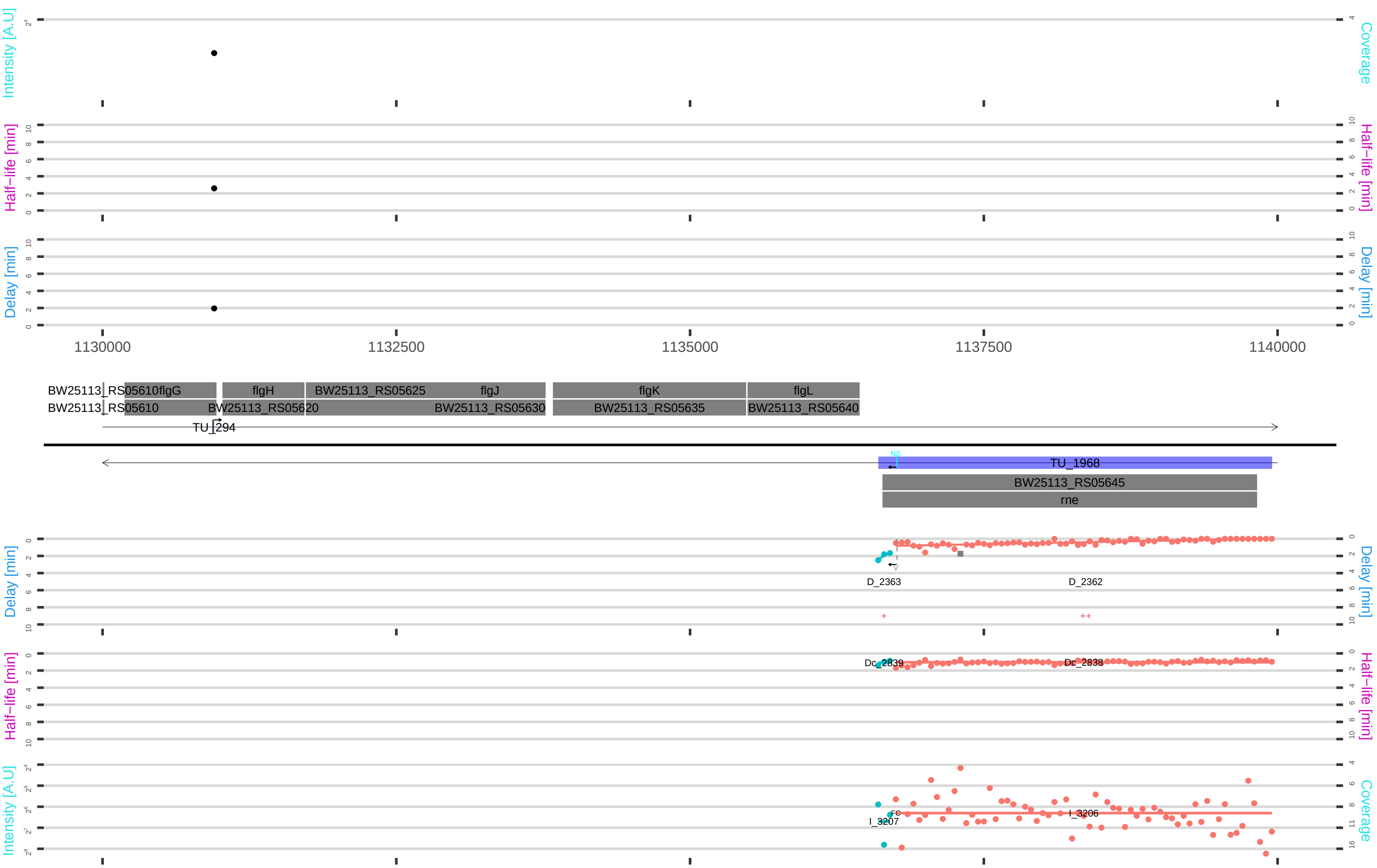

Term: termination (0), NS: new start (1), PS: pausing site (1), iTSS_I: internal starting site (0)

ID: 22810−23000; Term: termination (1), NS: new start (6), PS: pausing site (4), iTSS_I: internal starting site (0)

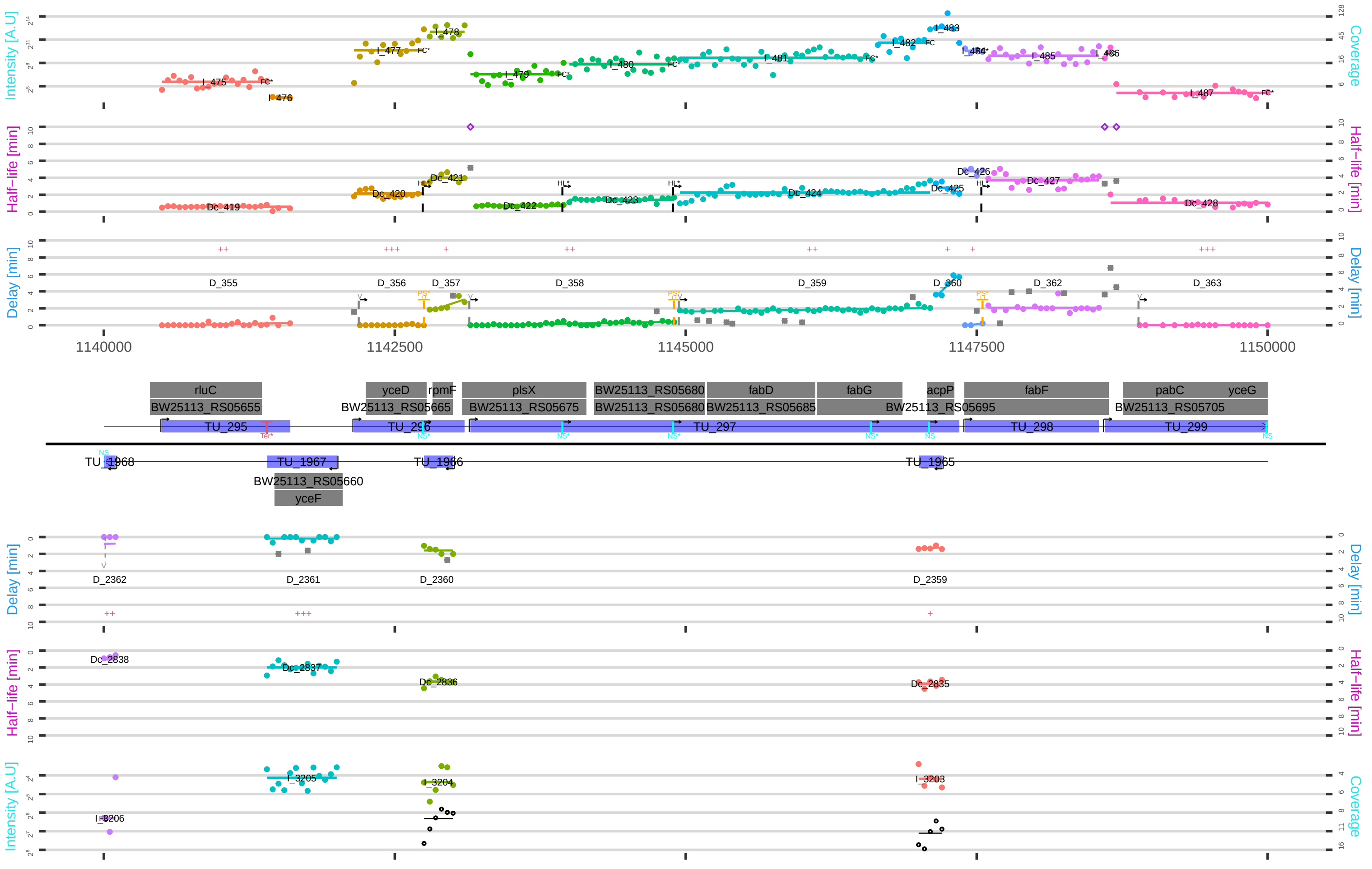

Term: termination (0), NS: new start (1), PS: pausing site (0), iTSS_I: internal starting site (0)

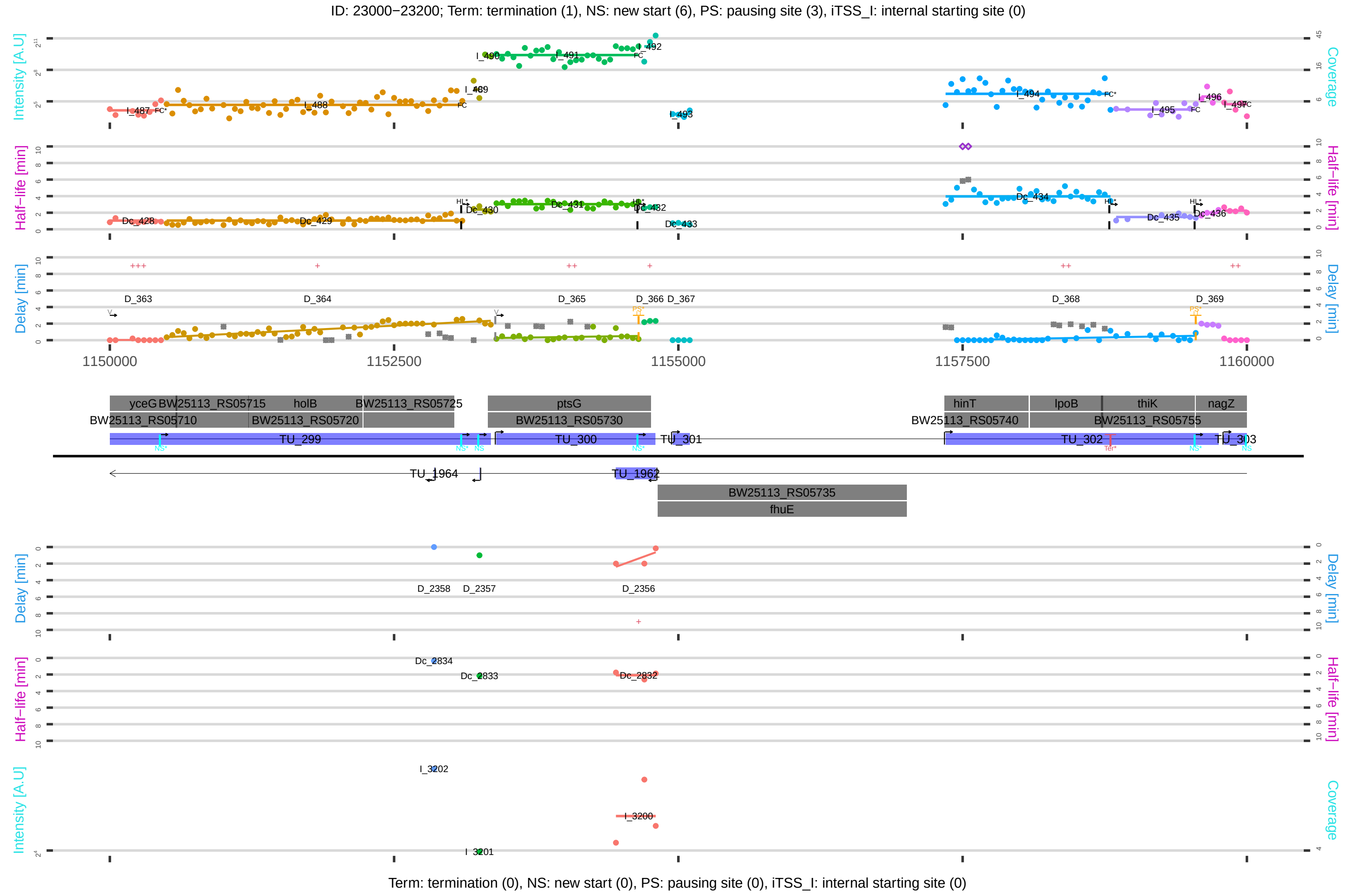
ID: 23000−23200; Term: termination (1), NS: new start (6), PS: pausing site (3), iTSS_I: internal starting site (0)
Intensity [A.U]
Coverage
Half−life [min]
Delay [min]
I_487
I_488
I_489
I_490
I_491
I_492
I_493
I_494
I_495
I_496
I_497
Dc_428
Dc_429
Dc_430
Dc_431
Dc_432
Dc_433
Dc_434
Dc_435
Dc_436
D_363
D_364
D_365
D_366
D_367
D_368
D_369
1150000
1152500
1155000
1157500
1160000
yceG
BW25113_RS05715
holB
BW25113_RS05725
BW25113_RS05710
BW25113_RS05720
ptsG
BW25113_RS05730
hinT
lpoB
thiK
nagZ
BW25113_RS05740
BW25113_RS05755
TU_299
TU_300
TU_301
TU_302
TU_303
TU_1964
TU_1962
BW25113_RS05735
fhuE
D_2358
D_2357
D_2356
Dc_2834
Dc_2833
Dc_2832
I_3202
I_3200
I_3201
Term: termination (0), NS: new start (0), PS: pausing site (0), iTSS_I: internal starting site (0)

ID: 23200−23295; Term: termination (0), NS: new start (2), PS: pausing site (1), iTSS_I: internal starting site (0)

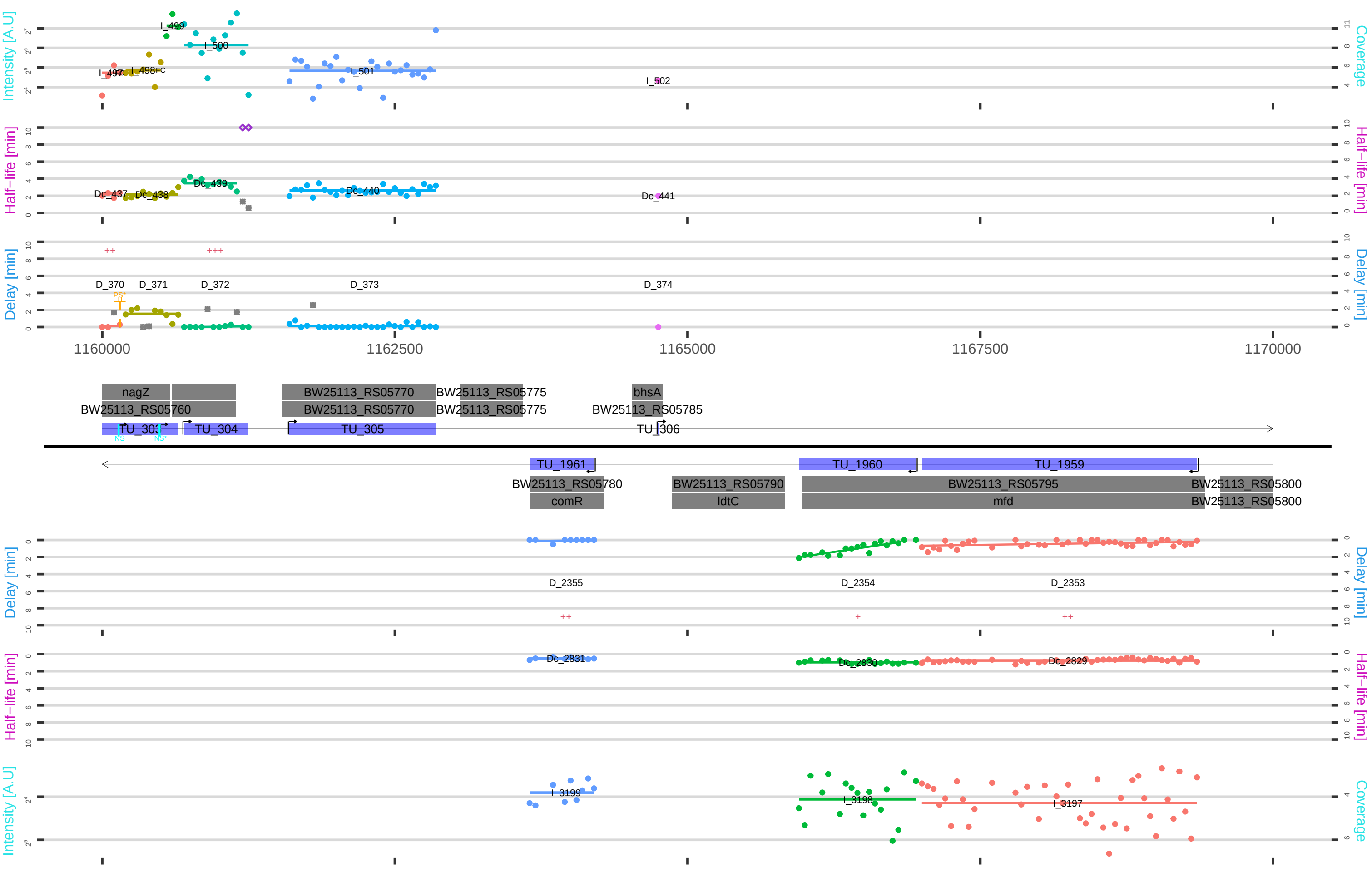

Term: termination (0), NS: new start (0), PS: pausing site (0), iTSS_I: internal starting site (0)

ID: 23419−23520; Term: termination (2), NS: new start (0), PS: pausing site (1), iTSS_I: internal starting site (0)

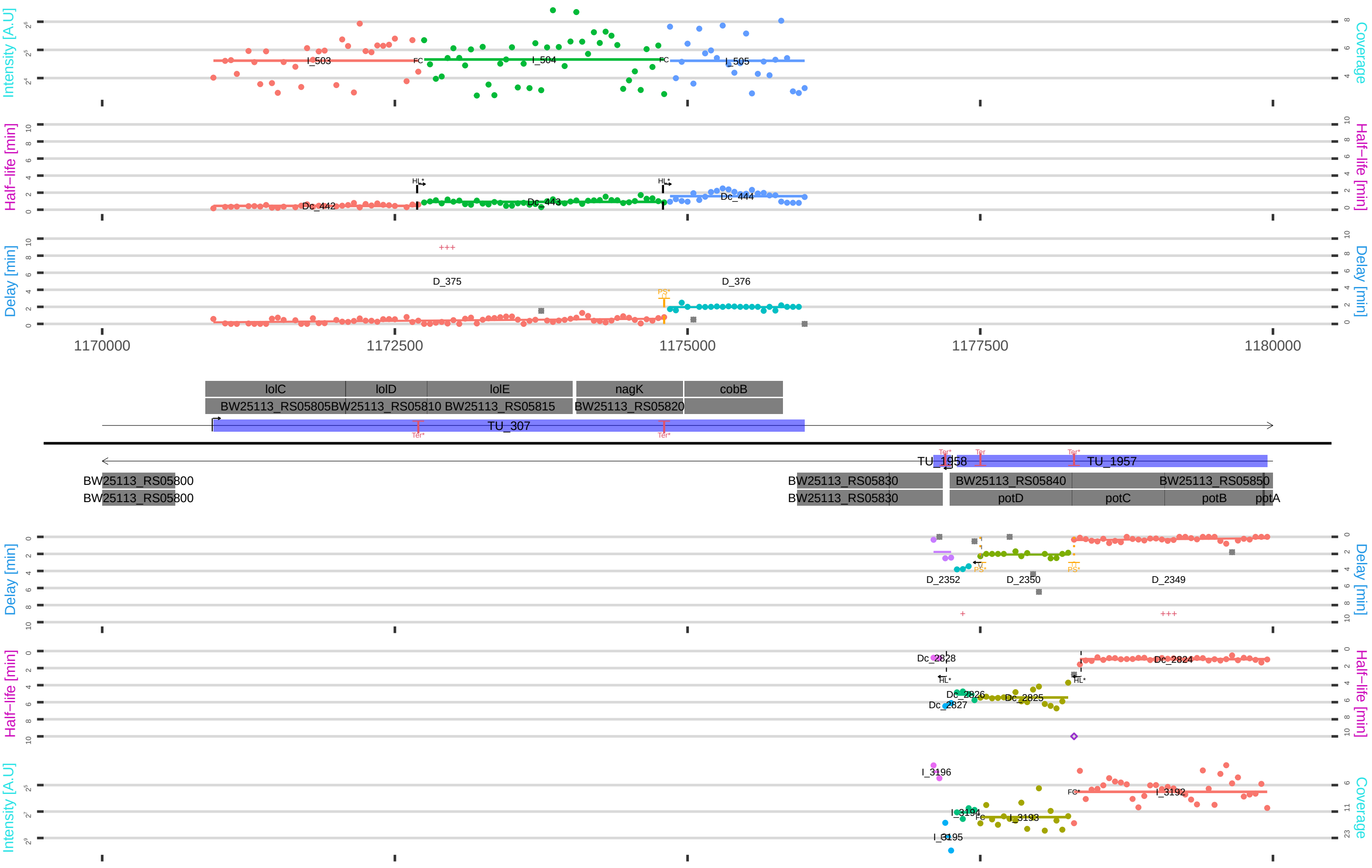

Term: termination (3), NS: new start (0), PS: pausing site (2), iTSS_I: internal starting site (0)

ID: 23626−23654; Term: termination (0), NS: new start (0), PS: pausing site (1), iTSS_I: internal starting site (1)

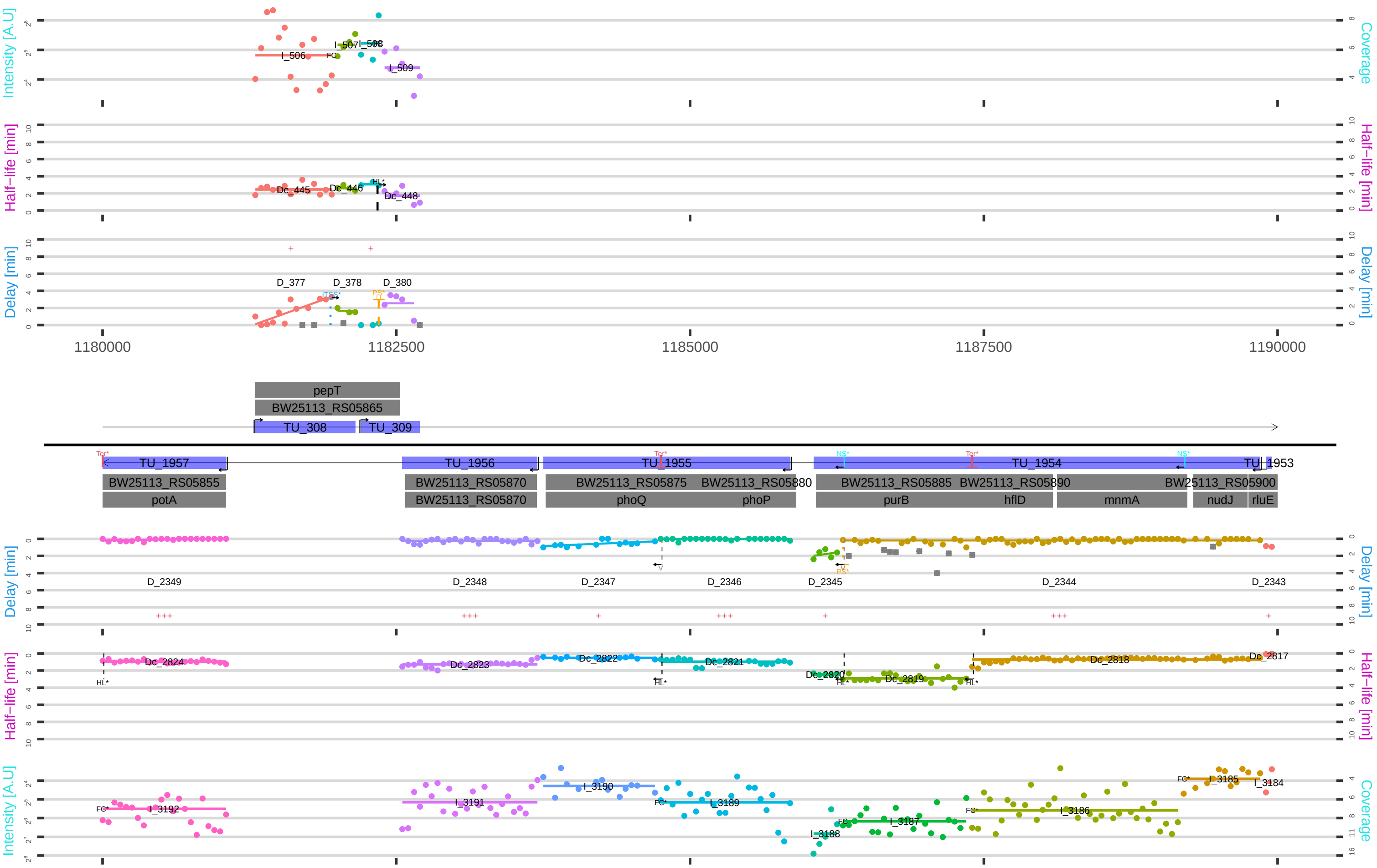

Term: termination (3), NS: new start (2), PS: pausing site (2), iTSS_I: internal starting site (0)

ID: 23811−23948; Term: termination (0), NS: new start (1), PS: pausing site (1), iTSS_I: internal starting site (0)

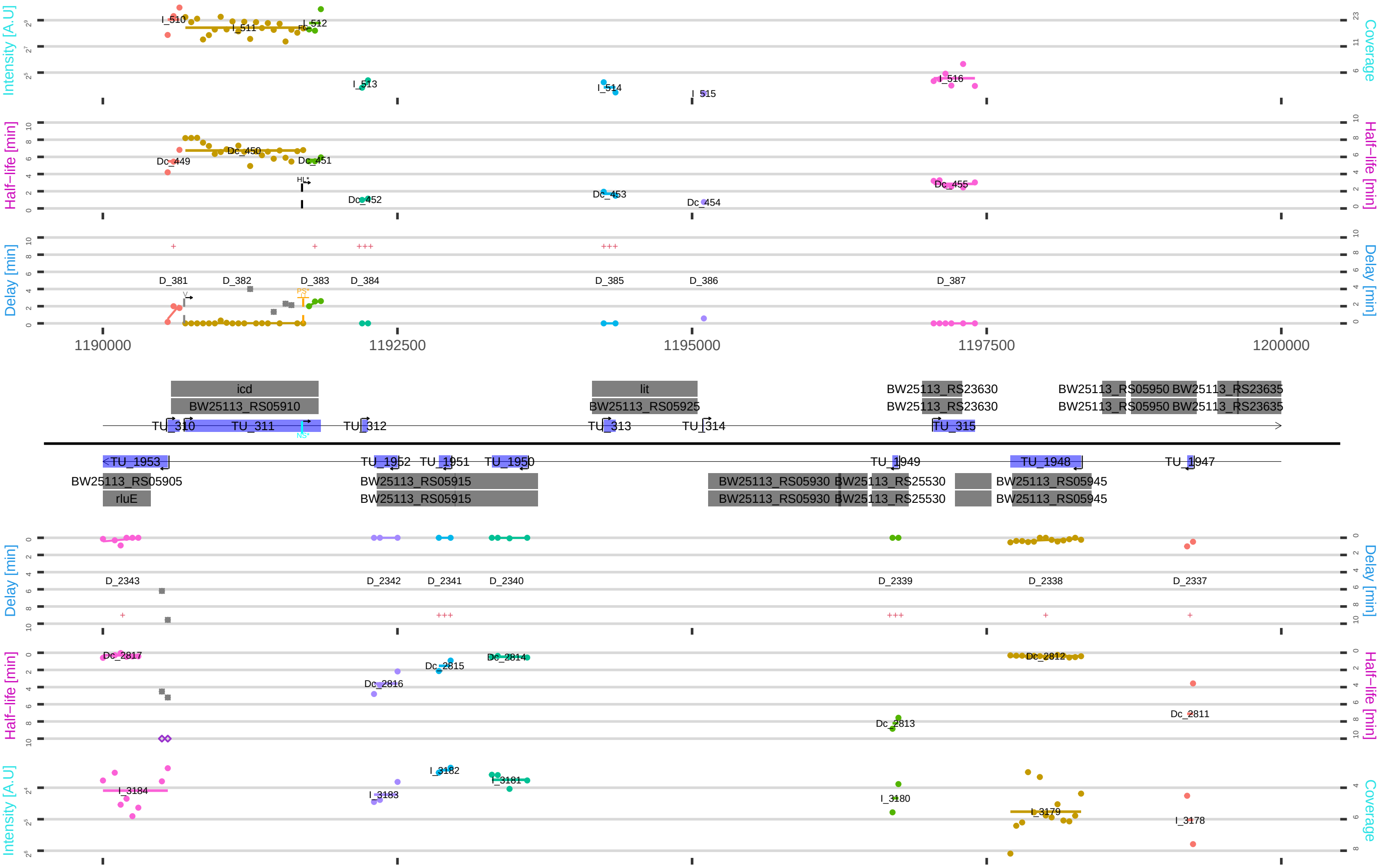

Term: termination (0), NS: new start (0), PS: pausing site (0), iTSS_I: internal starting site (0)

ID: 161093−161071; FC*: significant t−test of two consecutive segments; Term: termination, NS: new start, PS: pausing site,iTSS_I: internal starting site,TI: transcription interferance.

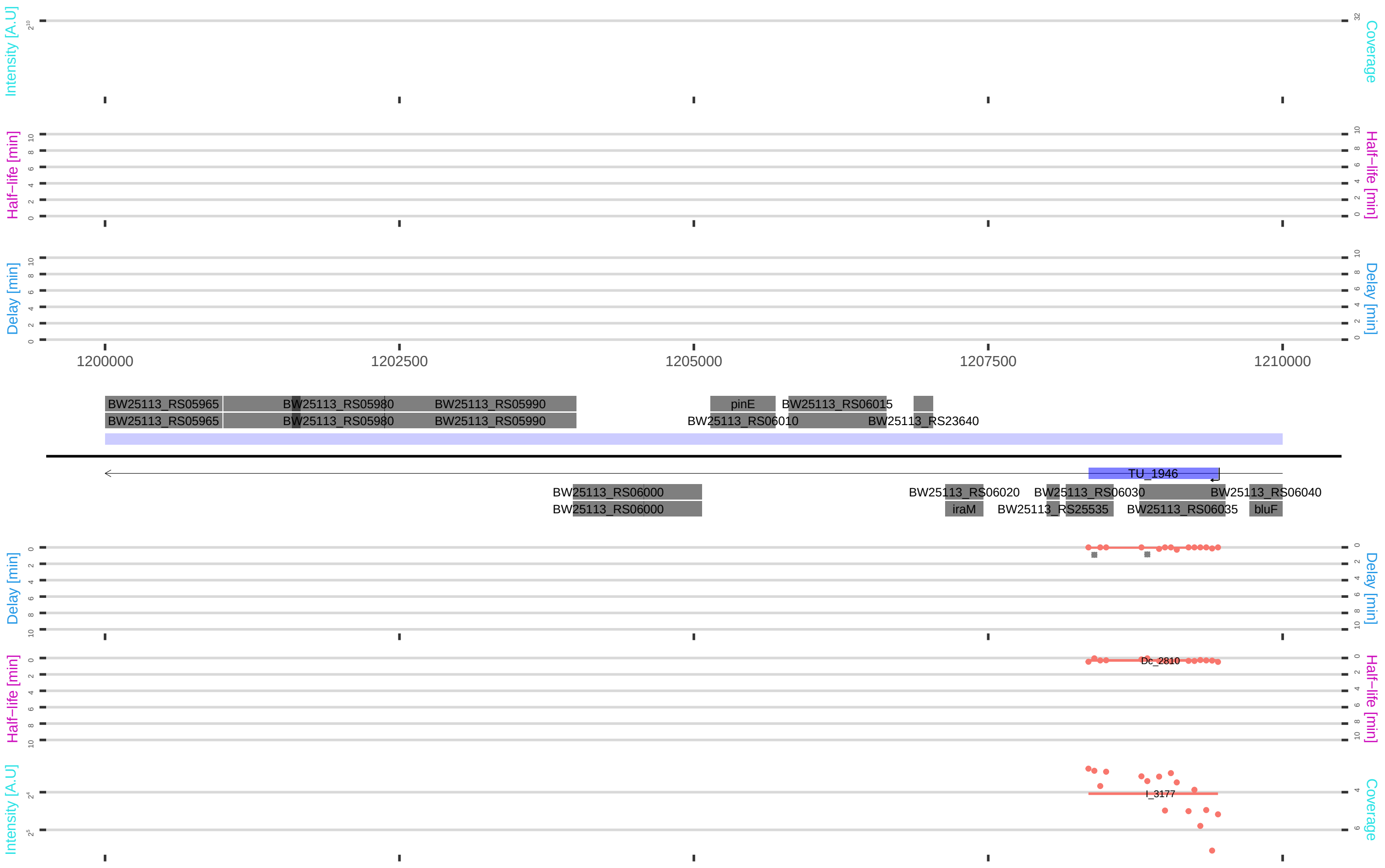

Term: termination (0), NS: new start (0), PS: pausing site (0), iTSS_I: internal starting site (0)

ID: 24291−24292; Term: termination (0), NS: new start (0), PS: pausing site (0), iTSS_I: internal starting site (0)

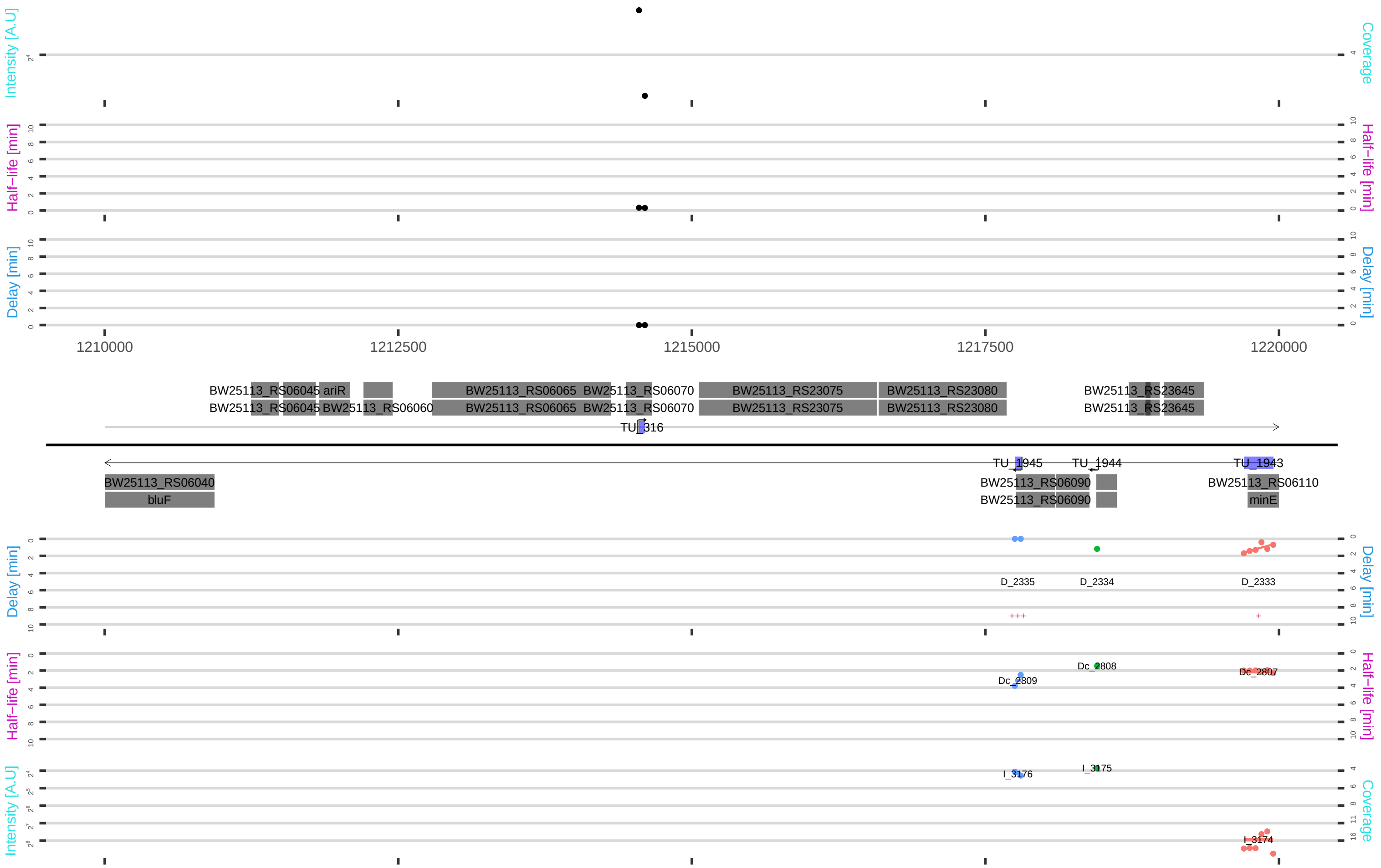

Term: termination (0), NS: new start (0), PS: pausing site (0), iTSS_I: internal starting site (0)

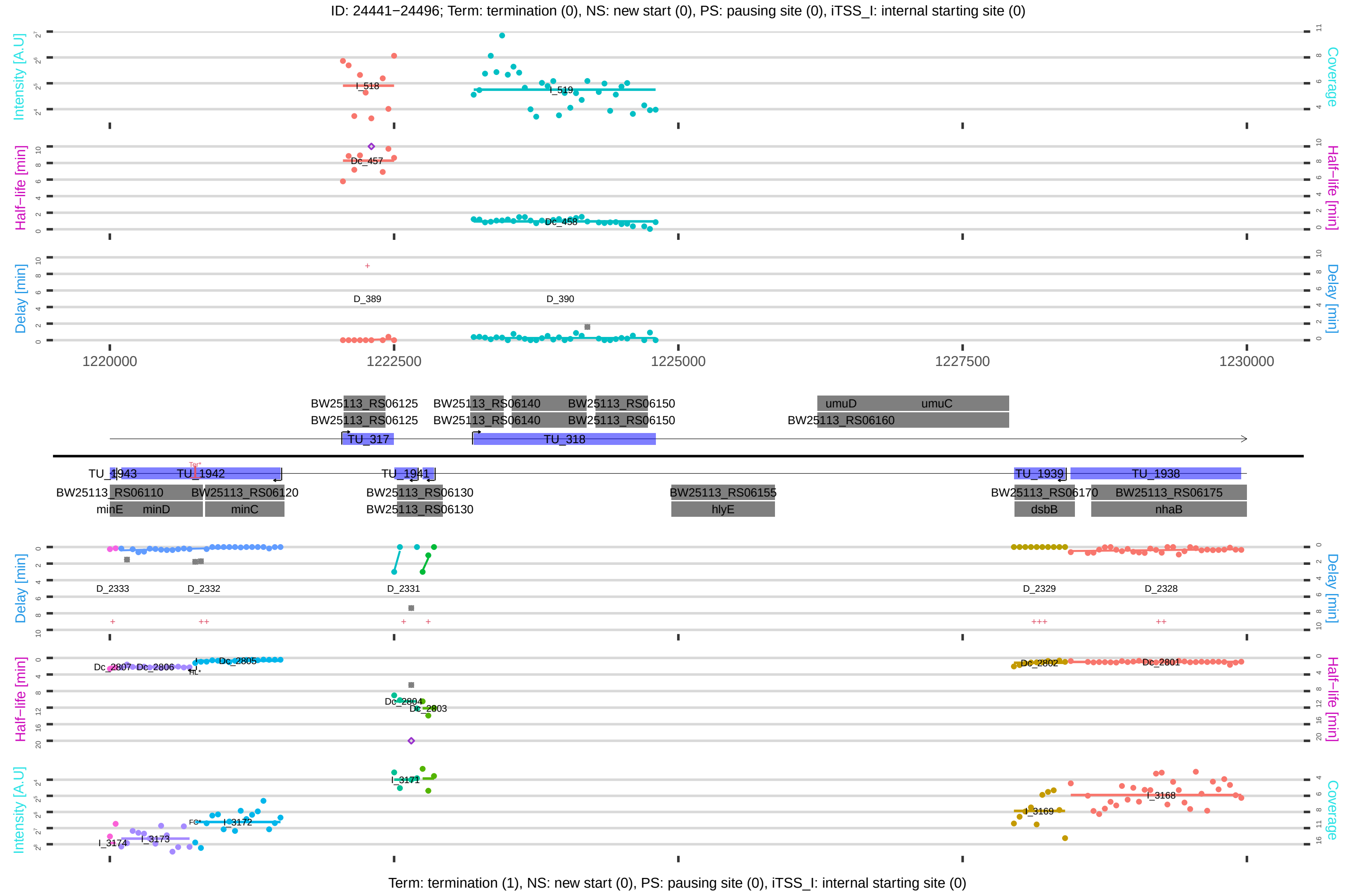

ID: 24441−24496; Term: termination (0), NS: new start (0), PS: pausing site (0), iTSS_I: internal starting site (0)
Intensity [A.U]
Coverage
I_518
I_519
Half−life [min]
Dc_457
Dc_458
Delay [min]
D_389
D_390
1220000
1222500
1225000
1227500
1230000
BW25113_RS06125
BW25113_RS06140
BW25113_RS06150
umuD
umuC
BW25113_RS06160
TU_317
TU_318
TU_1943
TU_1942
TU_1941
TU_1939
TU_1938
BW25113_RS06110
BW25113_RS06120
BW25113_RS06130
BW25113_RS06155
BW25113_RS06170
BW25113_RS06175
minE
minD
minC
hlyE
dsbB
nhaB
D_2333
D_2332
D_2331
D_2329
D_2328
Dc_2807
Dc_2806
Dc_2805
Dc_2804
Dc_2803
Dc_2802
Dc_2801
I_3174
I_3173
I_3172
I_3171
I_3169
I_3168
Term: termination (1), NS: new start (0), PS: pausing site (0), iTSS_I: internal starting site (0)

ID: 24609−24787; Term: termination (1), NS: new start (1), PS: pausing site (1), iTSS_I: internal starting site (0)

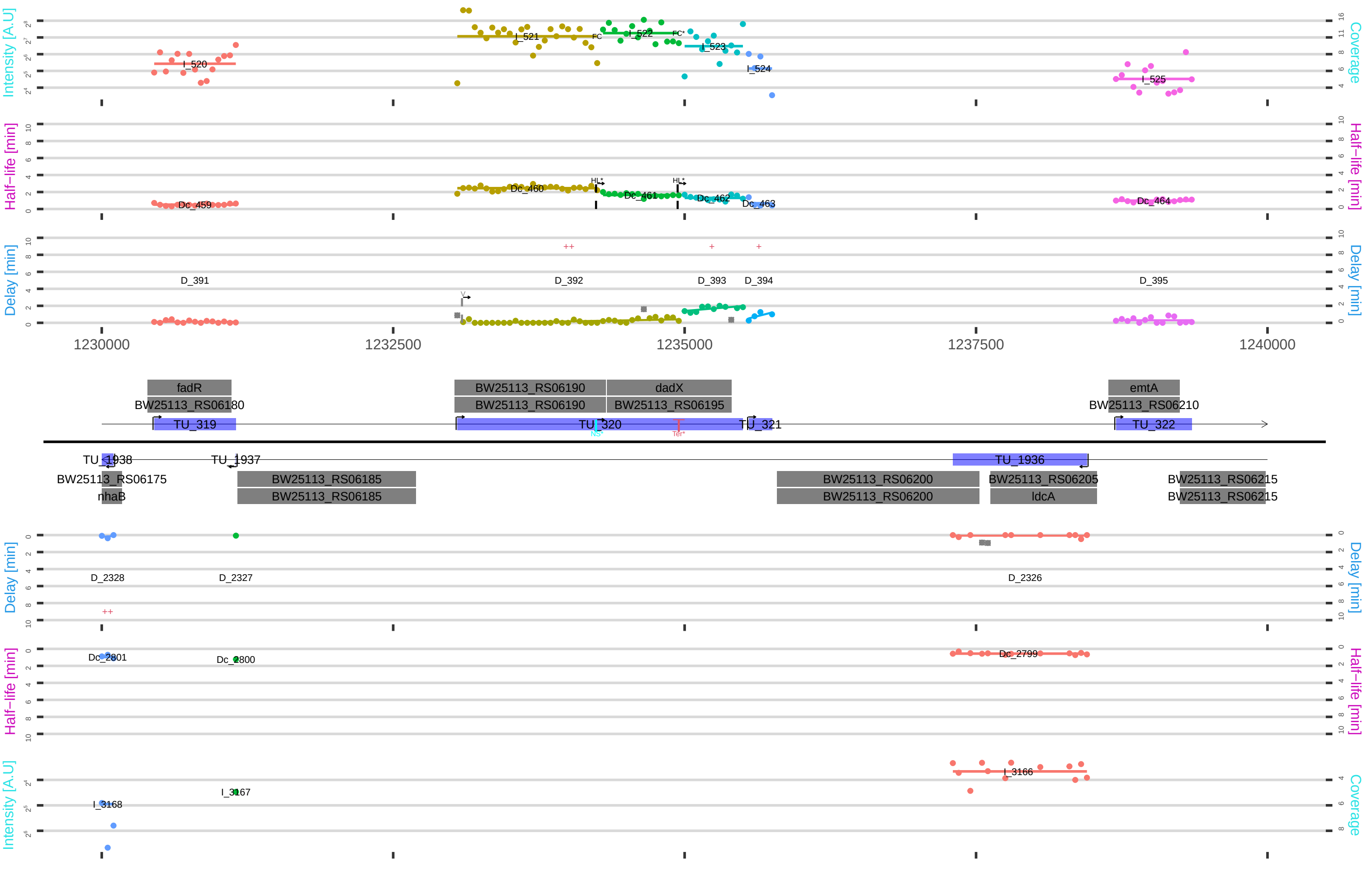

Term: termination (0), NS: new start (0), PS: pausing site (0), iTSS_I: internal starting site (0)

ID: 24933−24933; Term: termination (0), NS: new start (0), PS: pausing site (0), iTSS_I: internal starting site (0)

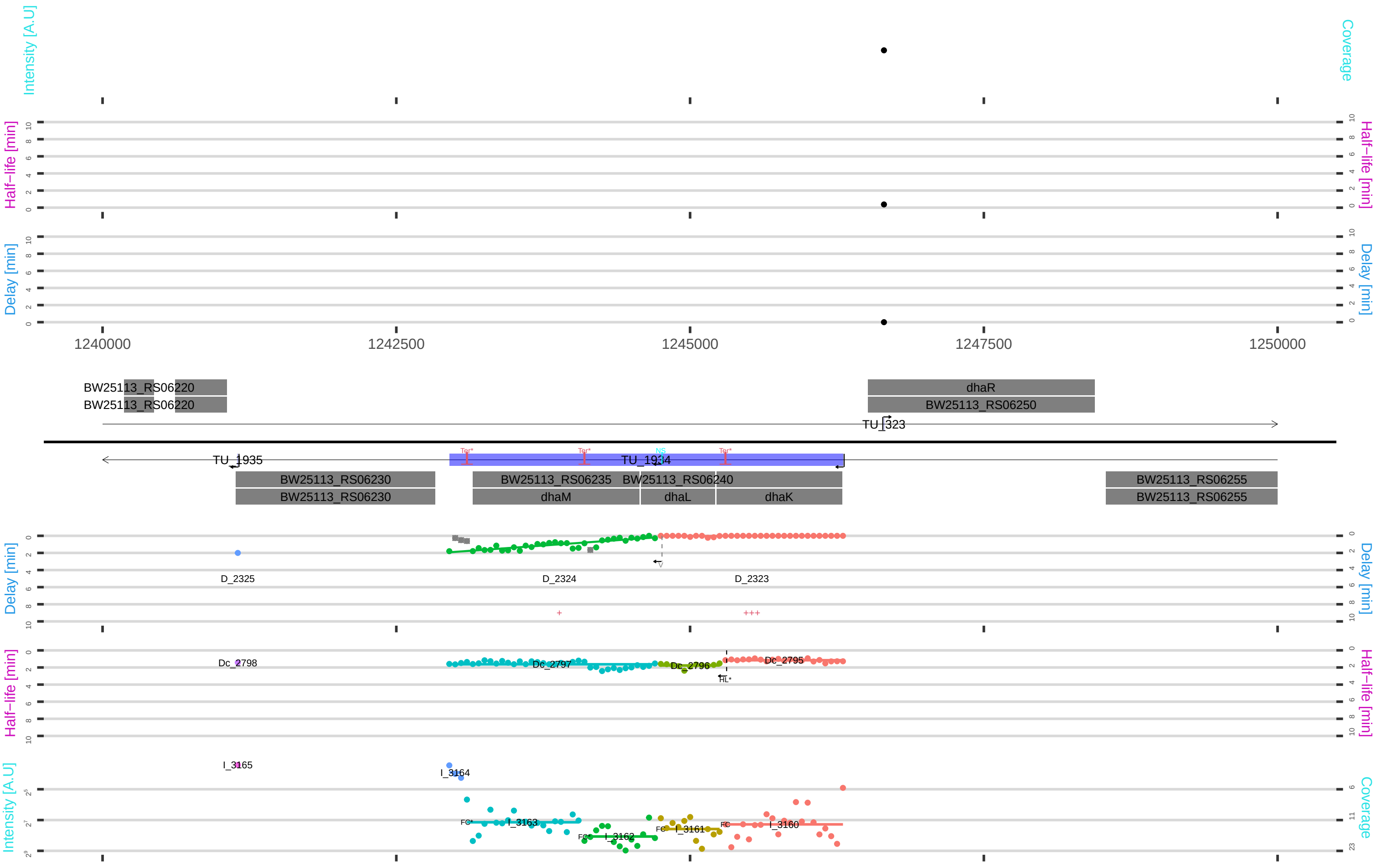

Term: termination (3), NS: new start (1), PS: pausing site (1), iTSS_I: internal starting site (0)

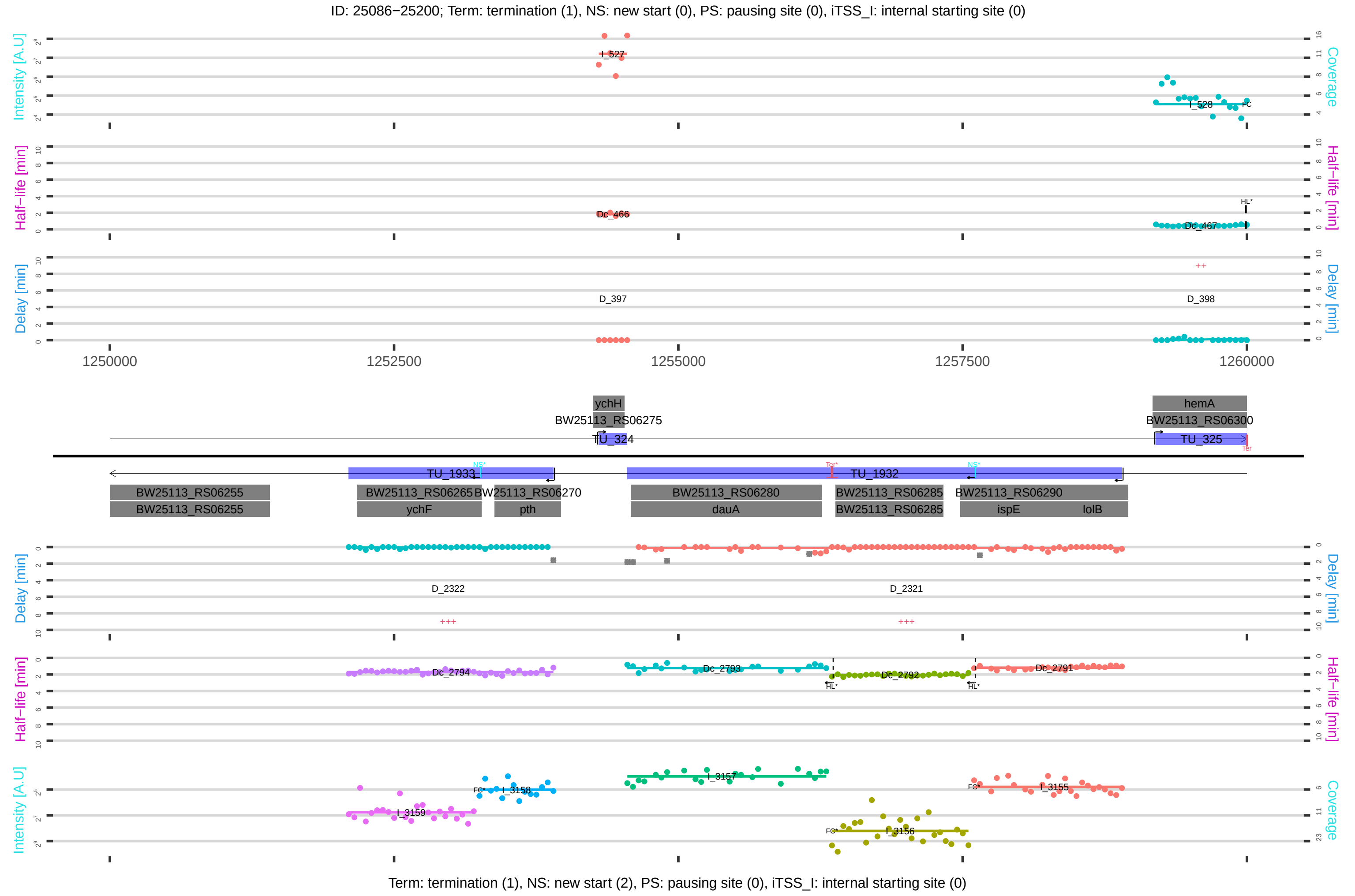
ID: 25086−25200; Term: termination (1), NS: new start (0), PS: pausing site (0), iTSS_I: internal starting site (0)
Intensity [A.U]
Coverage
Half−life [min]
Delay [min]
I_527
I_528
Dc_466
Dc_467
D_397
D_398
1250000
1252500
1255000
1257500
1260000
ychH
BW25113_RS06275
TU_324
hemA
BW25113_RS06300
TU_325
TU_1933
TU_1932
BW25113_RS06255
BW25113_RS06265
BW25113_RS06270
BW25113_RS06280
BW25113_RS06285
BW25113_RS06290
ychF
pth
dauA
ispE
lolB
D_2322
D_2321
Dc_2794
Dc_2793
Dc_2792
Dc_2791
I_3159
I_3158
I_3157
I_3156
I_3155
Term: termination (1), NS: new start (2), PS: pausing site (0), iTSS_I: internal starting site (0)

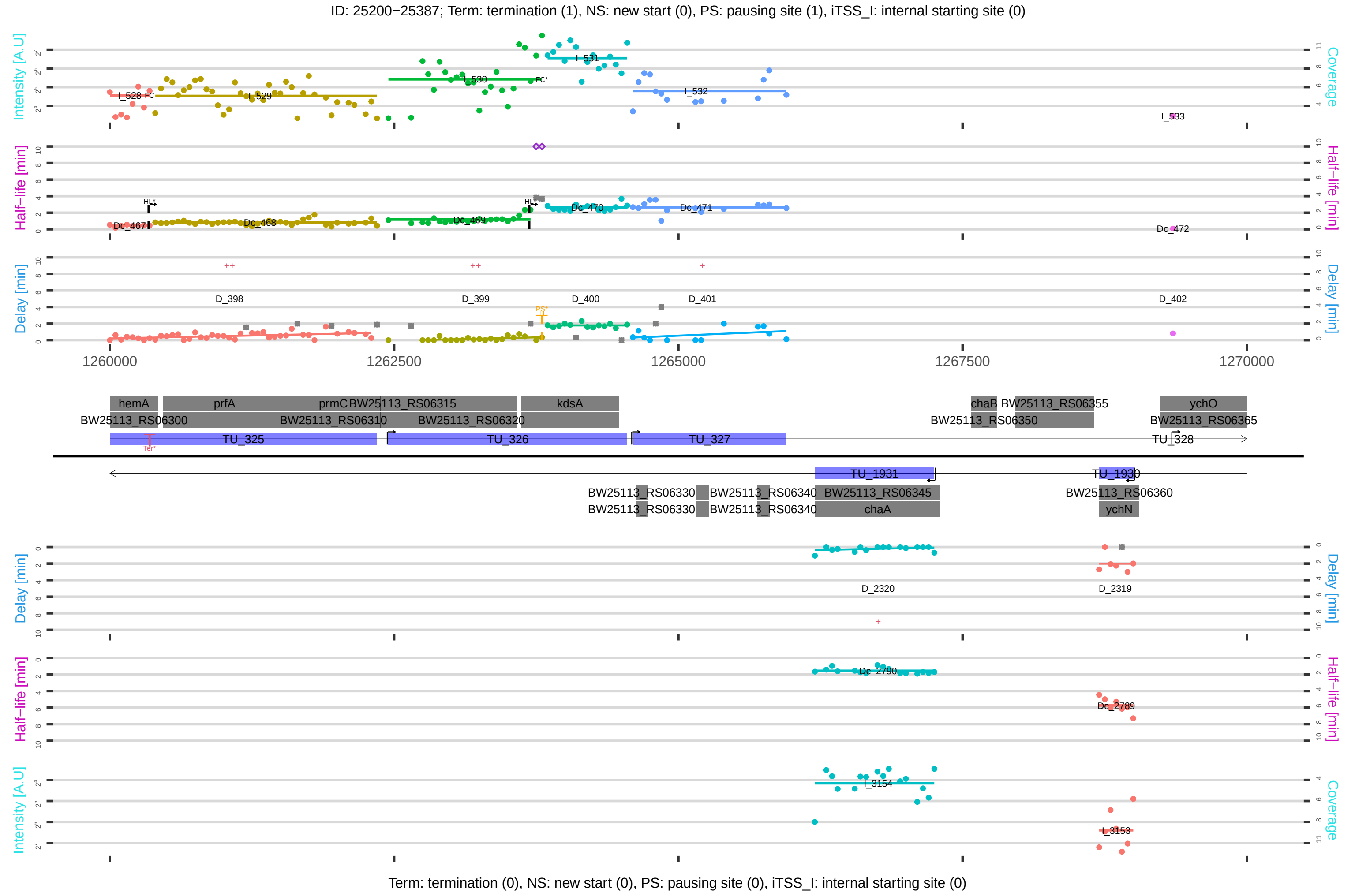

ID: 25200−25387; Term: termination (1), NS: new start (0), PS: pausing site (1), iTSS_I: internal starting site (0)
Intensity [A.U]
Coverage
Half−life [min]
Delay [min]
I_528
I_529
I_530
I_531
I_532
I_533
Dc_467
Dc_468
Dc_469
Dc_470
Dc_471
Dc_472
D_398
D_399
D_400
D_401
D_402
1260000
1262500
1265000
1267500
1270000
hemA
prfA
prmC
BW25113_RS06315
kdsA
chaB
BW25113_RS06355
ychO
BW25113_RS06300
BW25113_RS06310
BW25113_RS06320
BW25113_RS06350
BW25113_RS06365
TU_325
TU_326
TU_327
TU_328
TU_1931
TU_1930
BW25113_RS06330
BW25113_RS06340
BW25113_RS06345
BW25113_RS06360
chaA
ychN
D_2320
D_2319
Dc_2790
Dc_2789
I_3154
I_3153
Term: termination (0), NS: new start (0), PS: pausing site (0), iTSS_I: internal starting site (0)

ID: 25469−25600; Term: termination (3), NS: new start (0), PS: pausing site (1), iTSS_I: internal starting site (1)

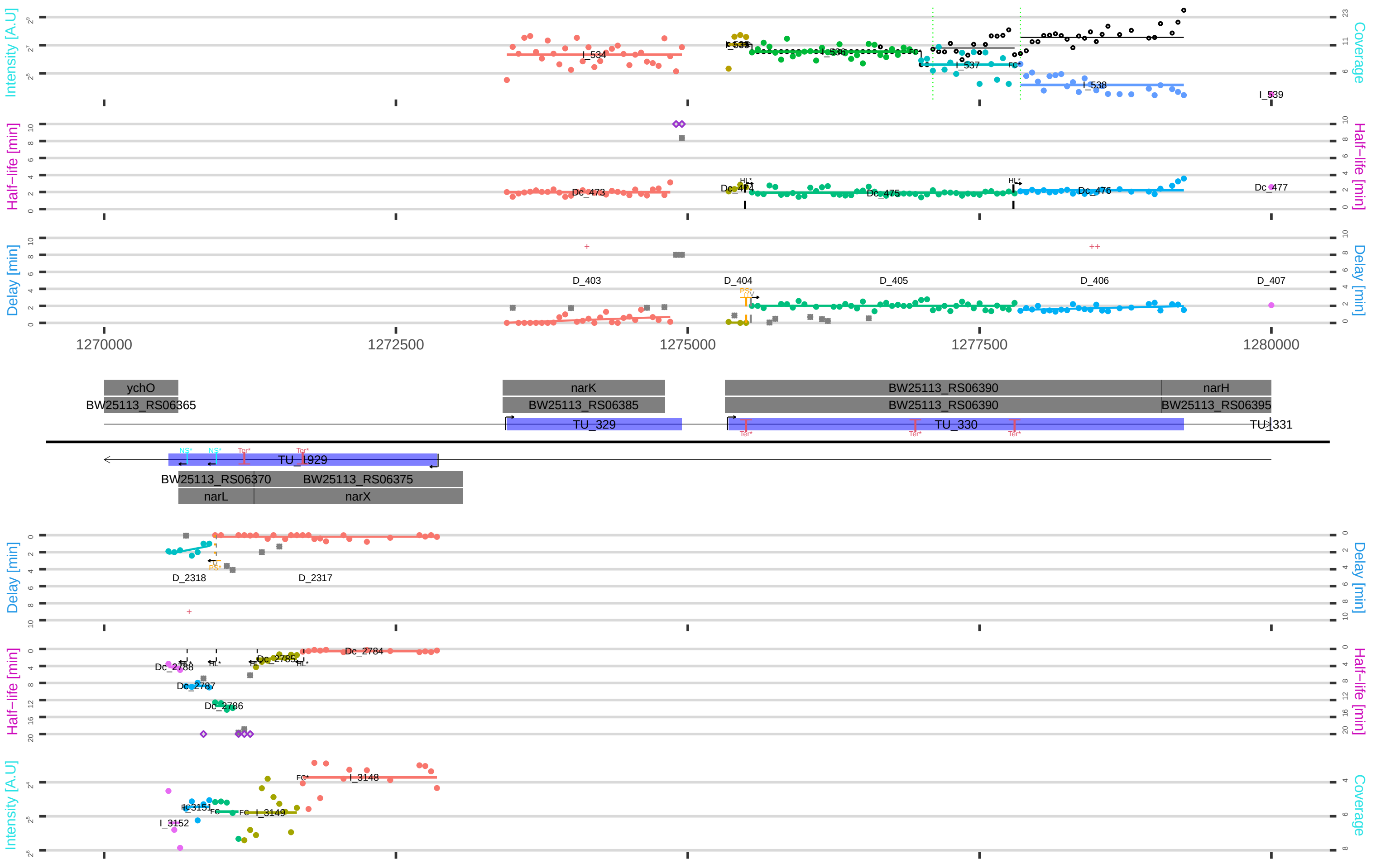

Term: termination (2), NS: new start (2), PS: pausing site (1), iTSS_I: internal starting site (0)

ID: 25600−25799; Term: termination (1), NS: new start (2), PS: pausing site (1), iTSS_I: internal starting site (0)

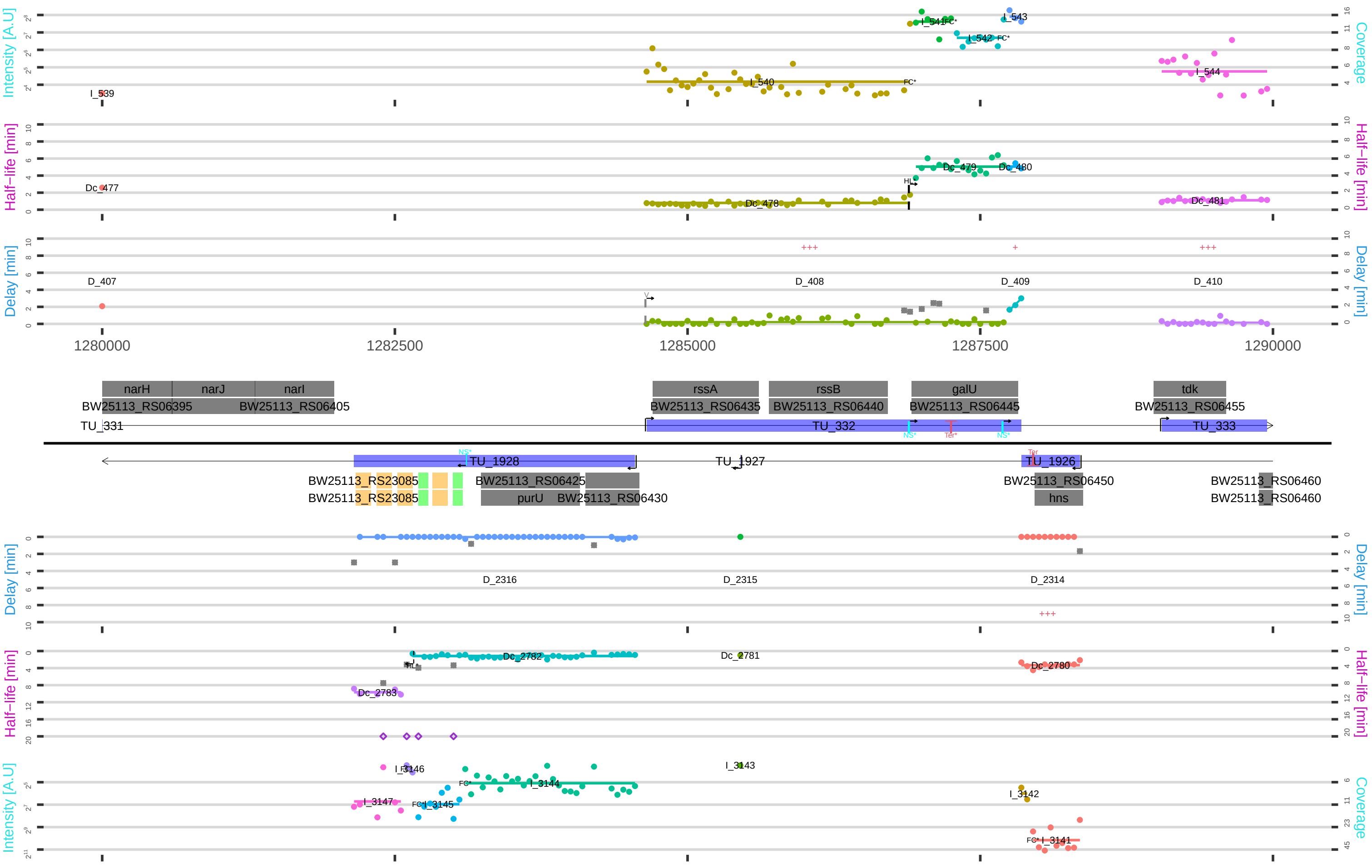

Term: termination (1), NS: new start (1), PS: pausing site (0), iTSS_I: internal starting site (0)

ID: 25880−26000; Term: termination (2), NS: new start (1), PS: pausing site (2), iTSS_I: internal starting site (0)

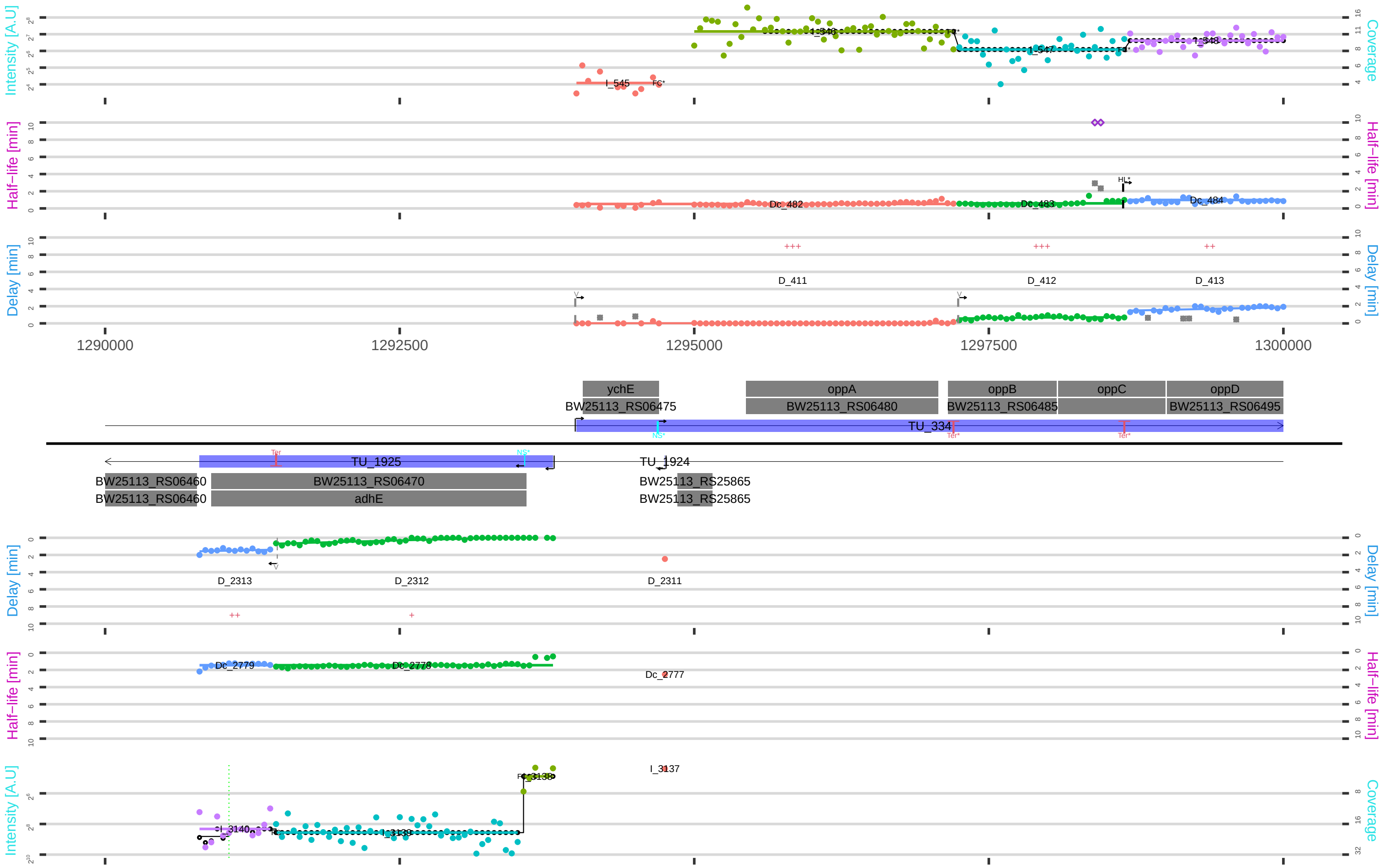

Term: termination (1), NS: new start (1), PS: pausing site (1), iTSS_I: internal starting site (0)

ID: 26000−26179; Term: termination (0), NS: new start (0), PS: pausing site (0), iTSS_I: internal starting site (0)

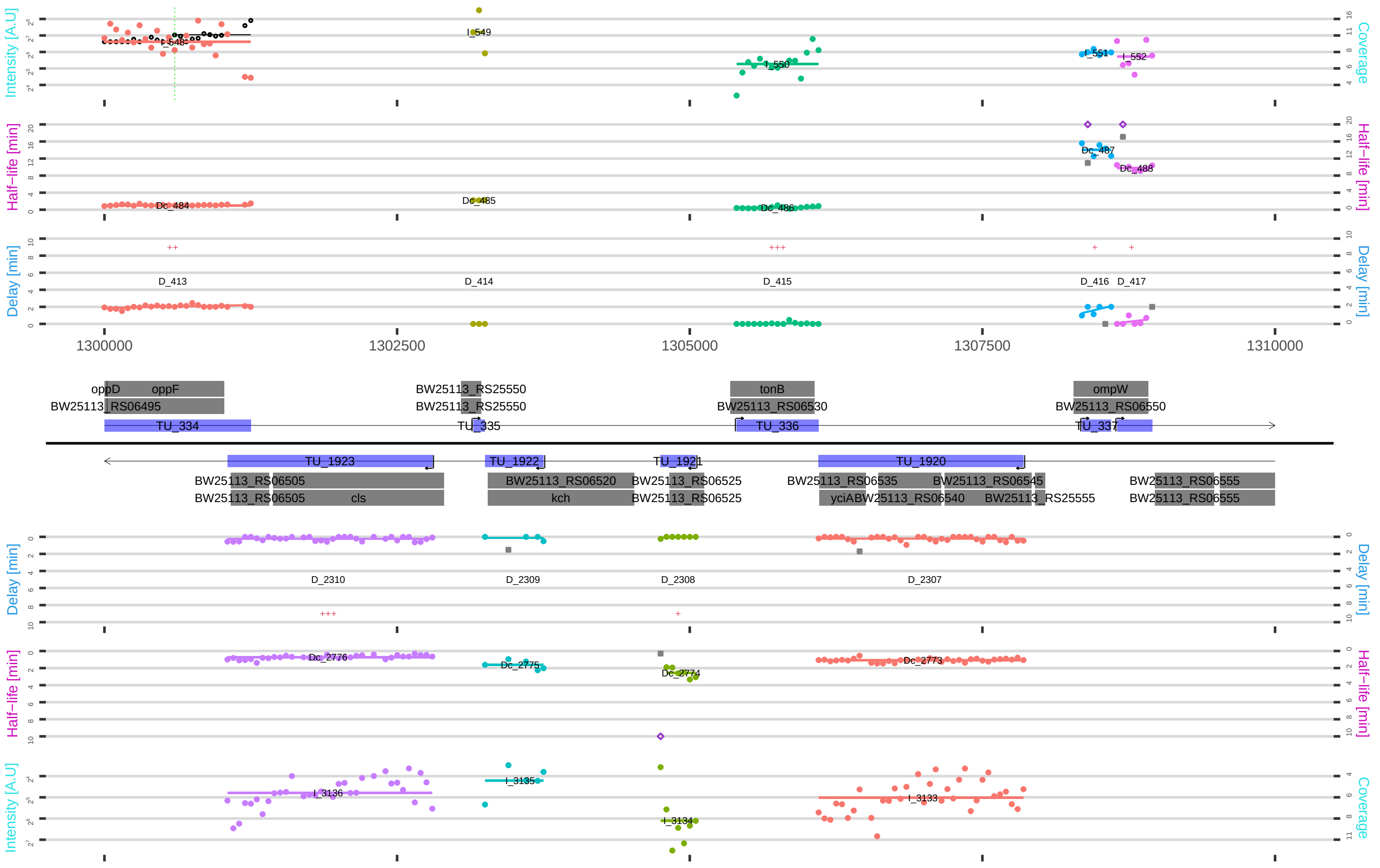

Term: termination (0), NS: new start (0), PS: pausing site (0), iTSS_I: internal starting site (0)

ID: 26352−26382; Term: termination (0), NS: new start (0), PS: pausing site (0), iTSS_I: internal starting site (0)

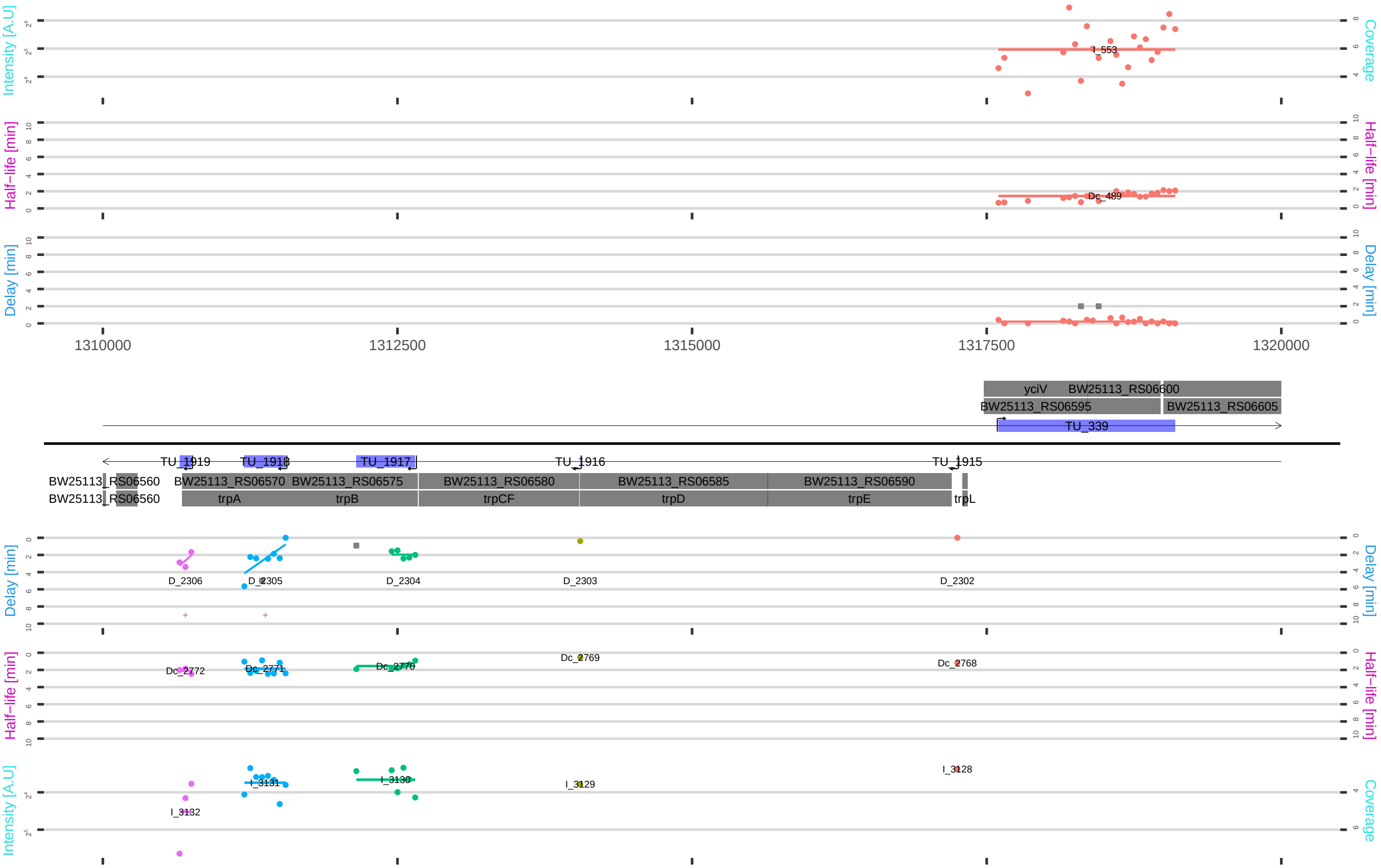

Term: termination (0), NS: new start (0), PS: pausing site (0), iTSS_I: internal starting site (0)

ID: 26421−26597; Term: termination (3), NS: new start (0), PS: pausing site (0), iTSS_I: internal starting site (0)

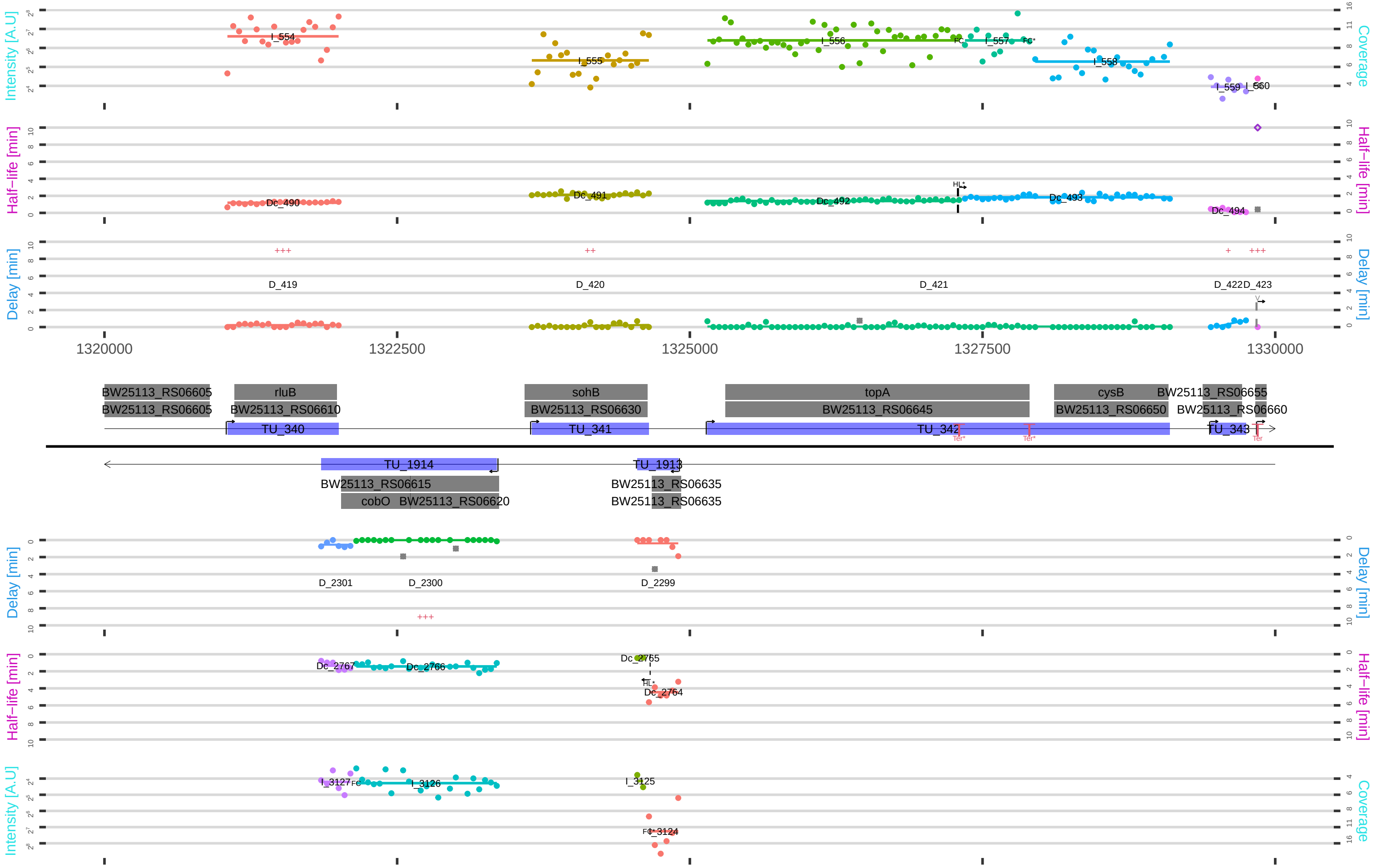

Term: termination (0), NS: new start (0), PS: pausing site (1), iTSS_I: internal starting site (0)

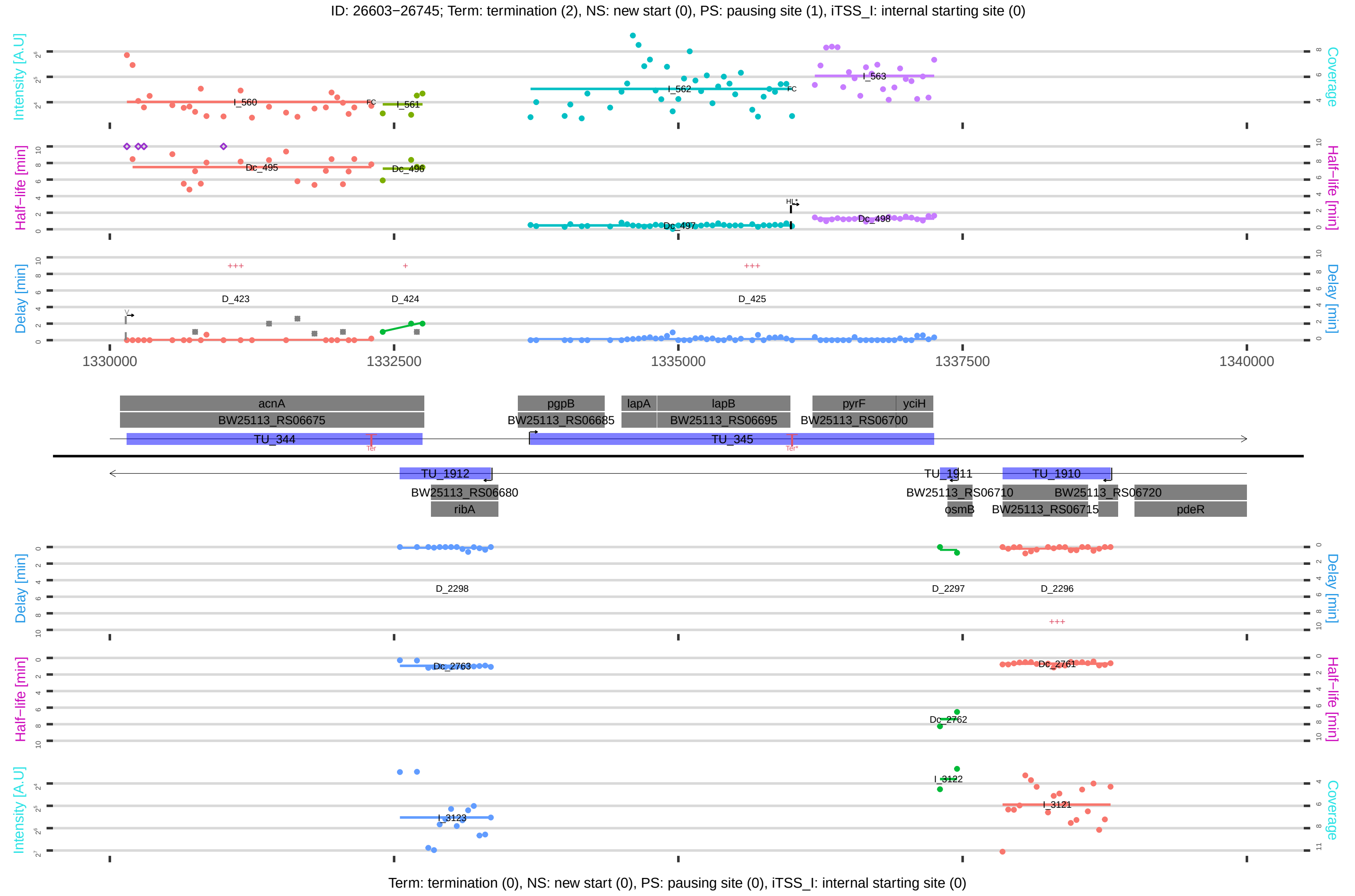

ID: 26603−26745; Term: termination (2), NS: new start (0), PS: pausing site (1), iTSS_I: internal starting site (0)
Intensity [A.U]
Coverage
Half−life [min]
Delay [min]
I_560
I_561
I_562
I_563
Dc_495
Dc_496
Dc_497
Dc_498
D_423
D_424
D_425
1330000
1332500
1335000
1337500
1340000
acnA
BW25113_RS06675
pgpB
BW25113_RS06685
lapA
lapB
BW25113_RS06695
pyrF
yciH
BW25113_RS06700
TU_344
TU_345
Ter
Ter*
TU_1912
TU_1911
TU_1910
BW25113_RS06680
ribA
BW25113_RS06710
osmB
BW25113_RS06715
BW25113_RS06720
pdeR
D_2298
D_2297
D_2296
Dc_2763
Dc_2762
Dc_2761
I_3123
I_3122
I_3121
Term: termination (0), NS: new start (0), PS: pausing site (0), iTSS_I: internal starting site (0)

ID: 26889−26889; Term: termination (0), NS: new start (0), PS: pausing site (0), iTSS_I: internal starting site (0)

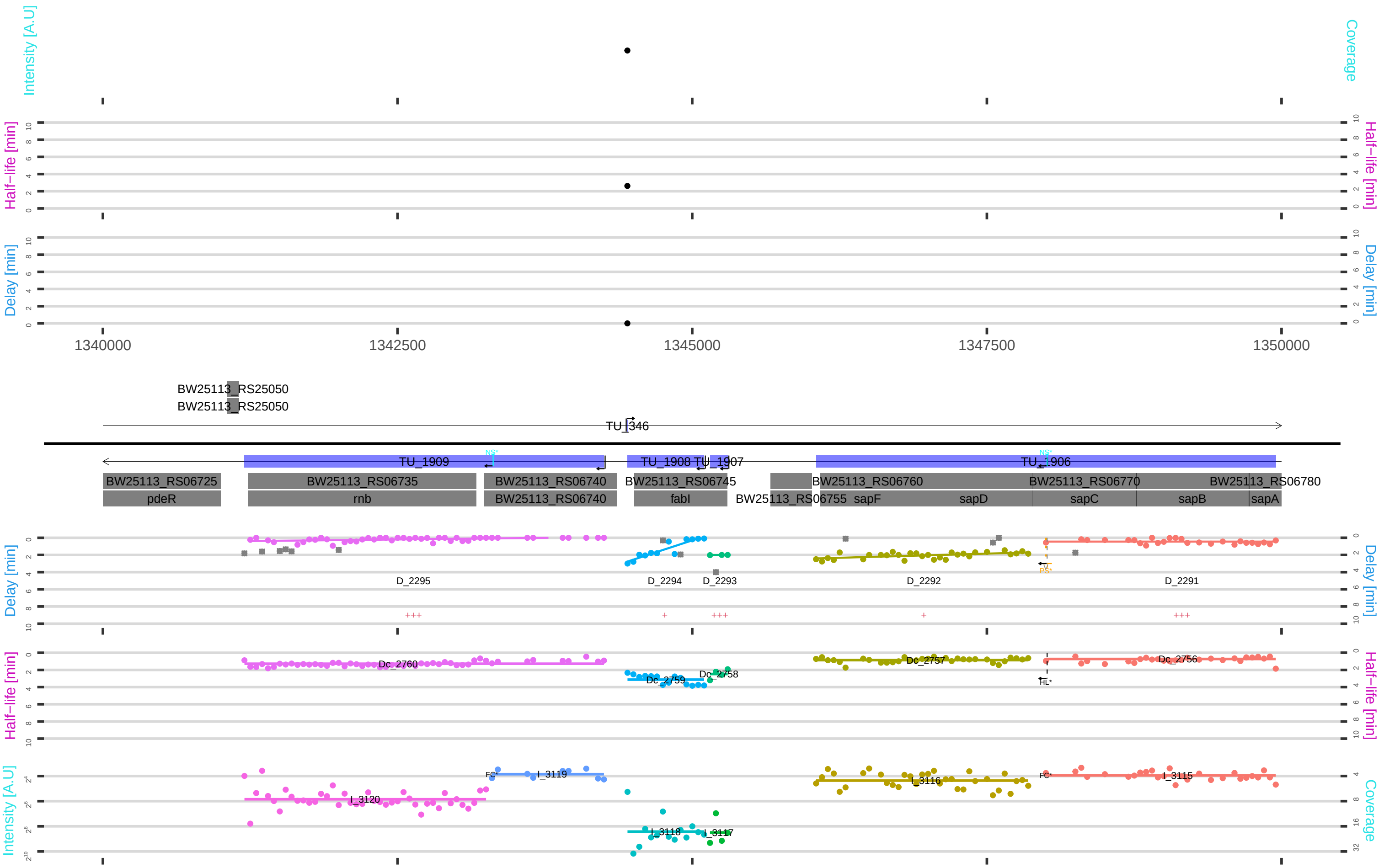

Term: termination (0), NS: new start (2), PS: pausing site (1), iTSS_I: internal starting site (0)

ID: 27121−27131; Term: termination (0), NS: new start (0), PS: pausing site (0), iTSS_I: internal starting site (0)

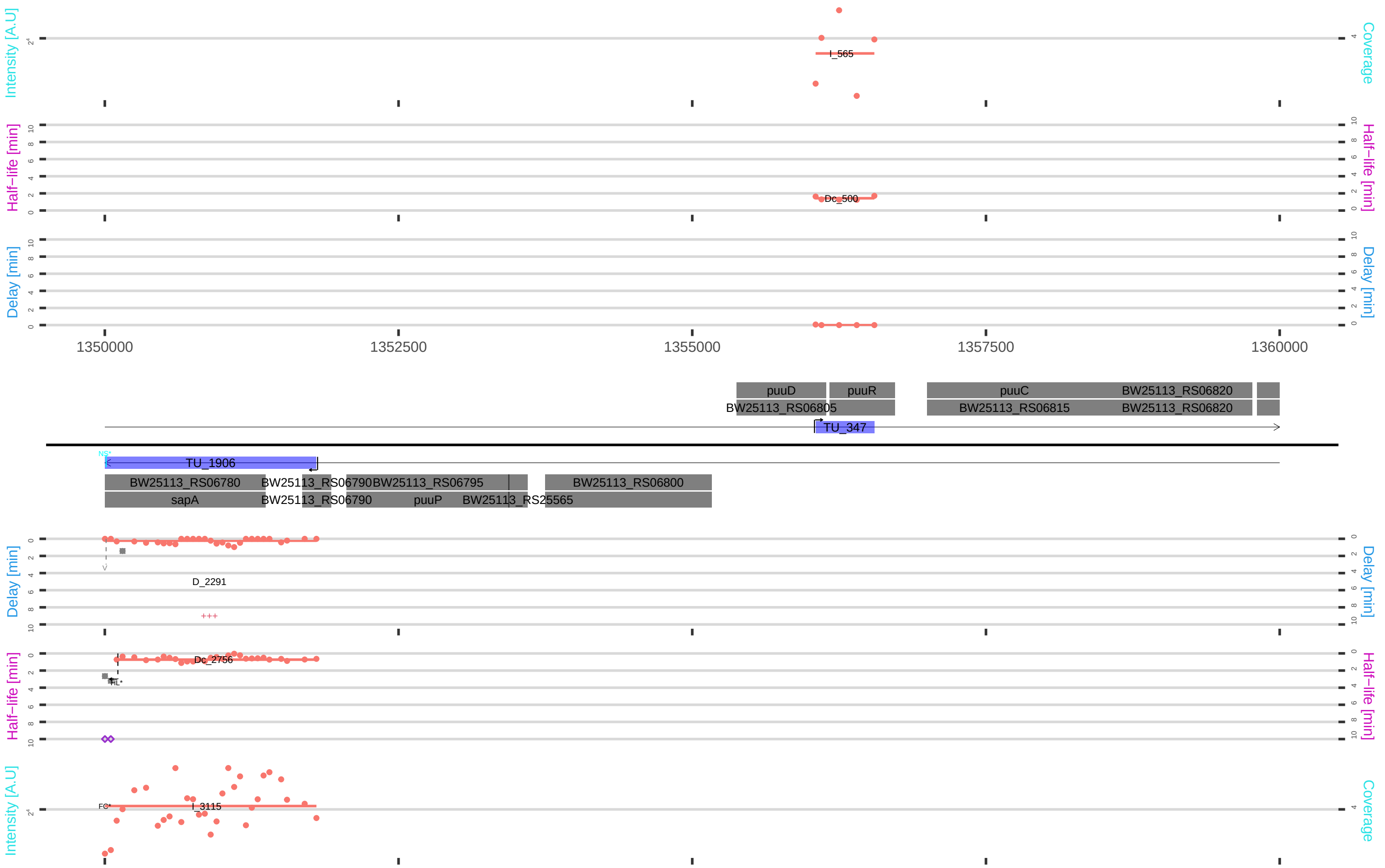

Term: termination (0), NS: new start (1), PS: pausing site (0), iTSS_I: internal starting site (0)

ID: 27248−27286; Term: termination (1), NS: new start (1), PS: pausing site (0), iTSS_I: internal starting site (0)

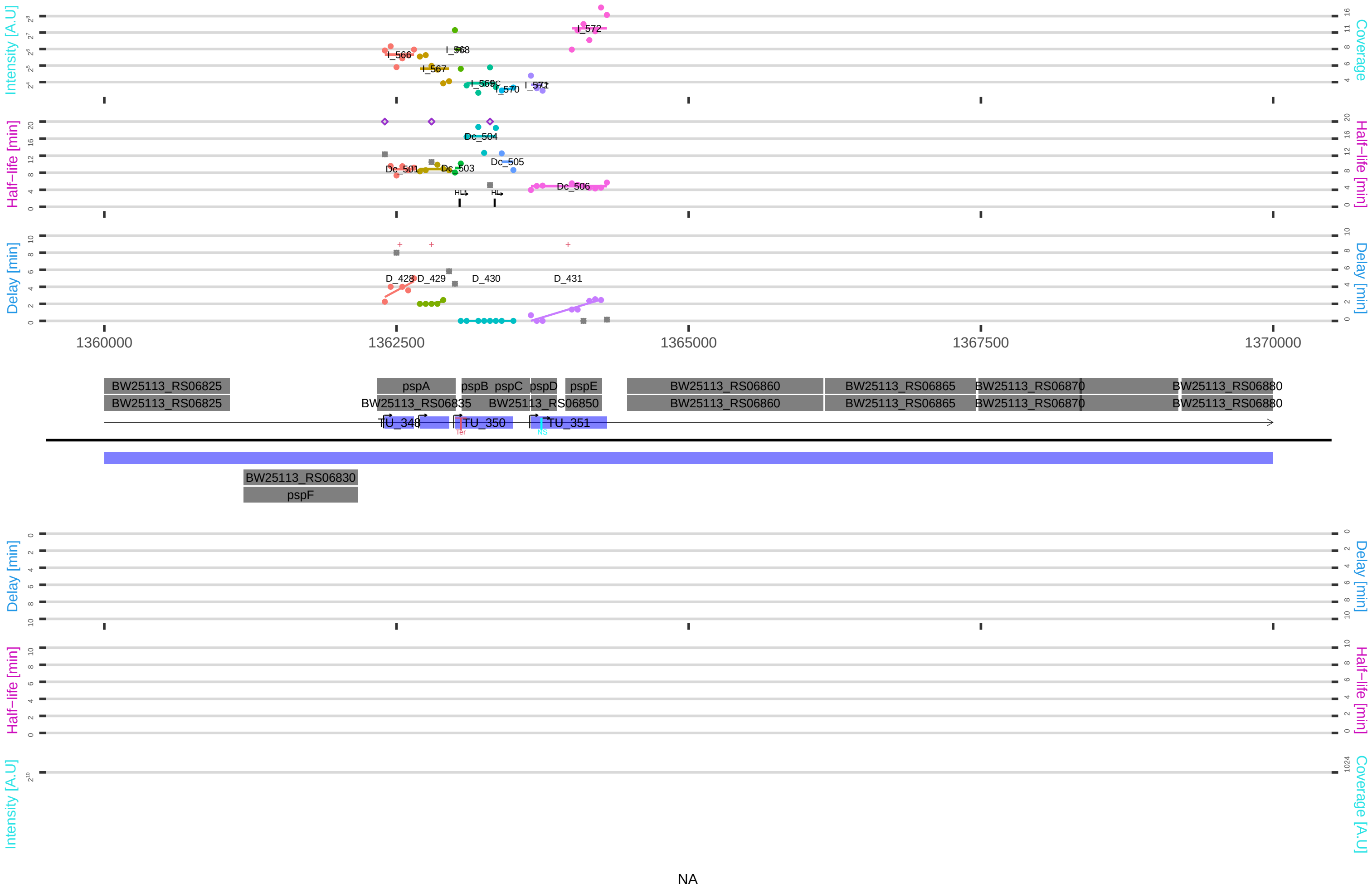

ID: 27570−27600; Term: termination (0), NS: new start (0), PS: pausing site (0), iTSS_I: internal starting site (0)

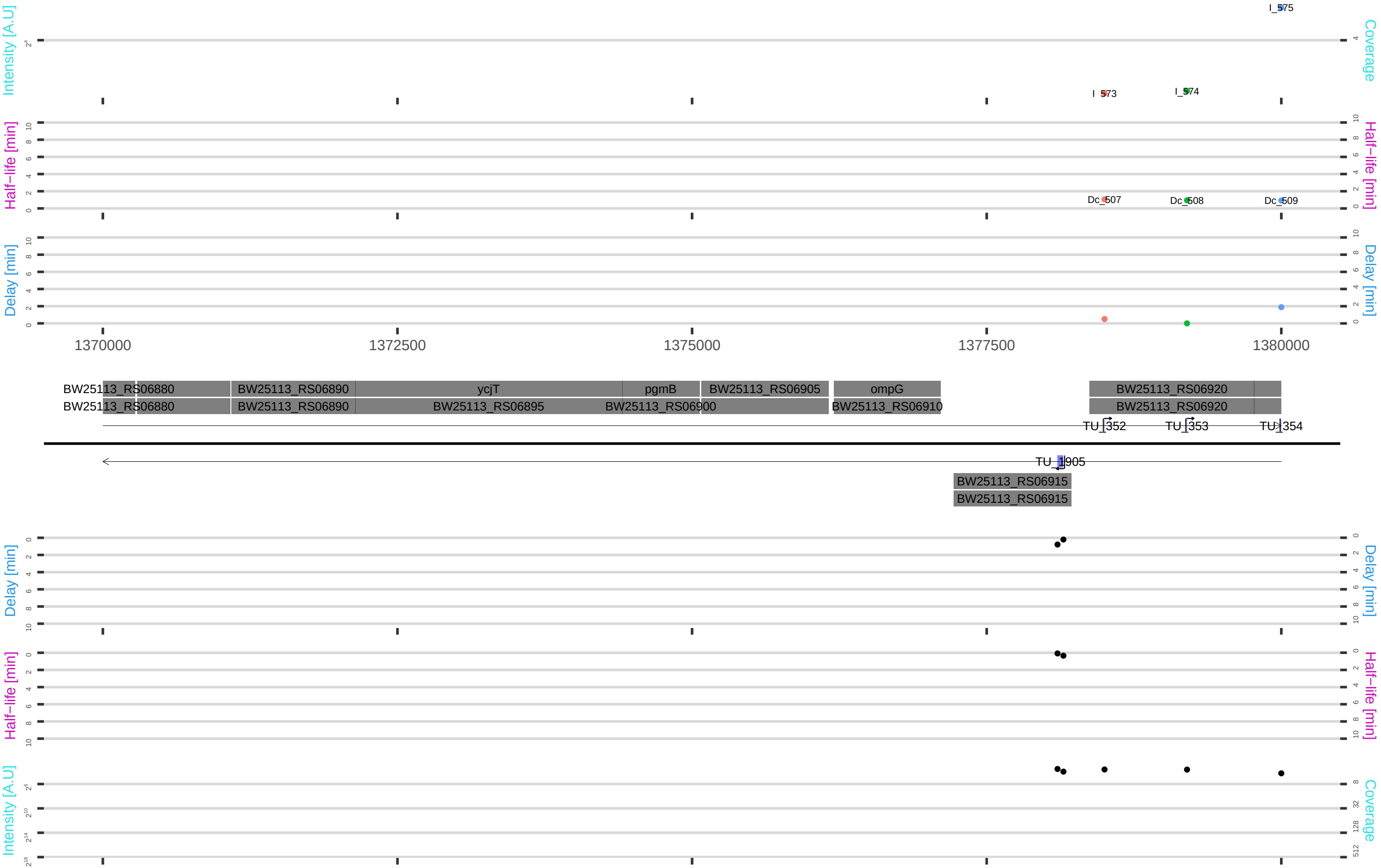

Term: termination (0), NS: new start (0), PS: pausing site (0), iTSS_I: internal starting site (0)

ID: 27600−27786; Term: termination (1), NS: new start (0), PS: pausing site (0), iTSS_I: internal starting site (0)

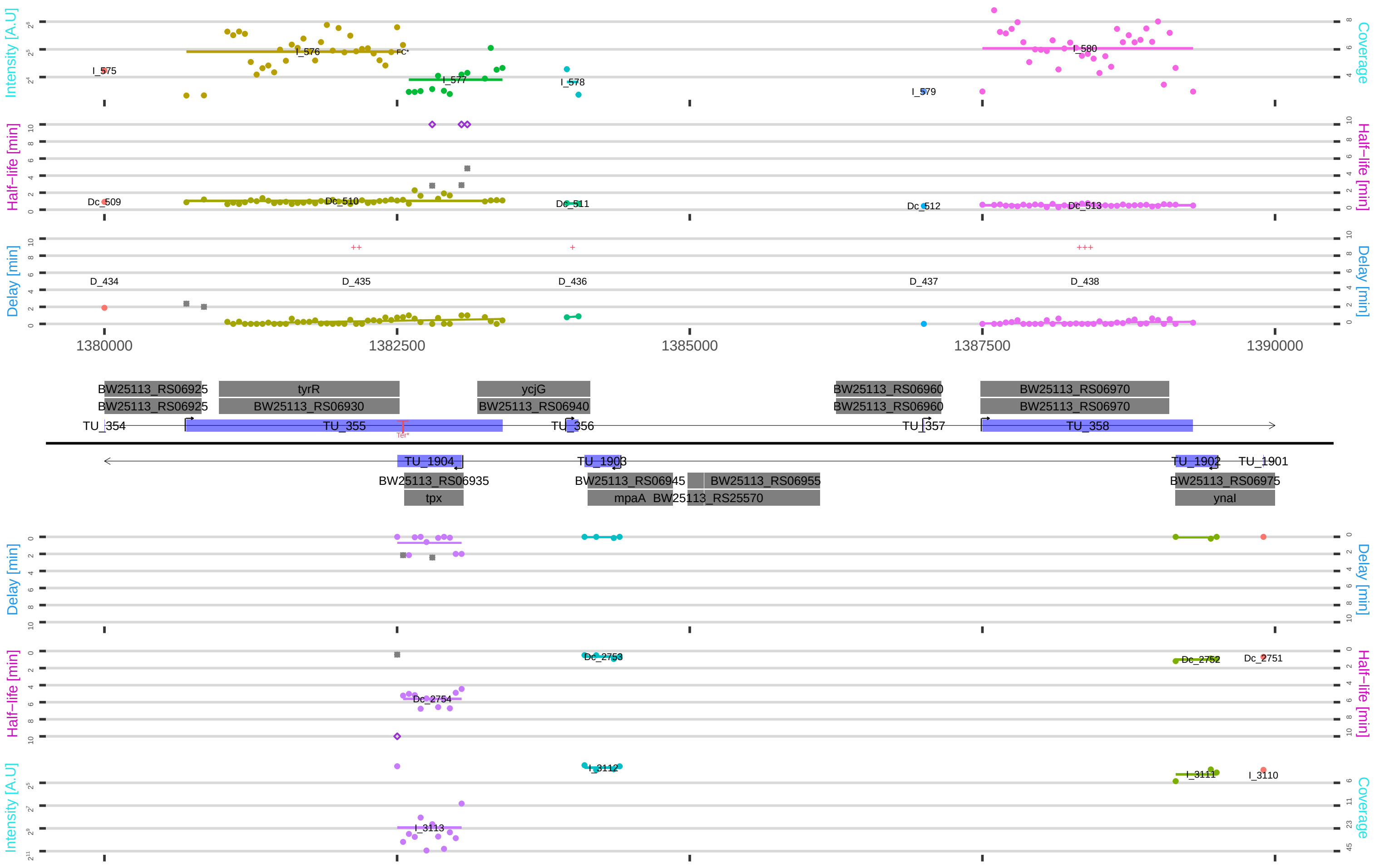

Term: termination (0), NS: new start (0), PS: pausing site (0), iTSS_I: internal starting site (0)

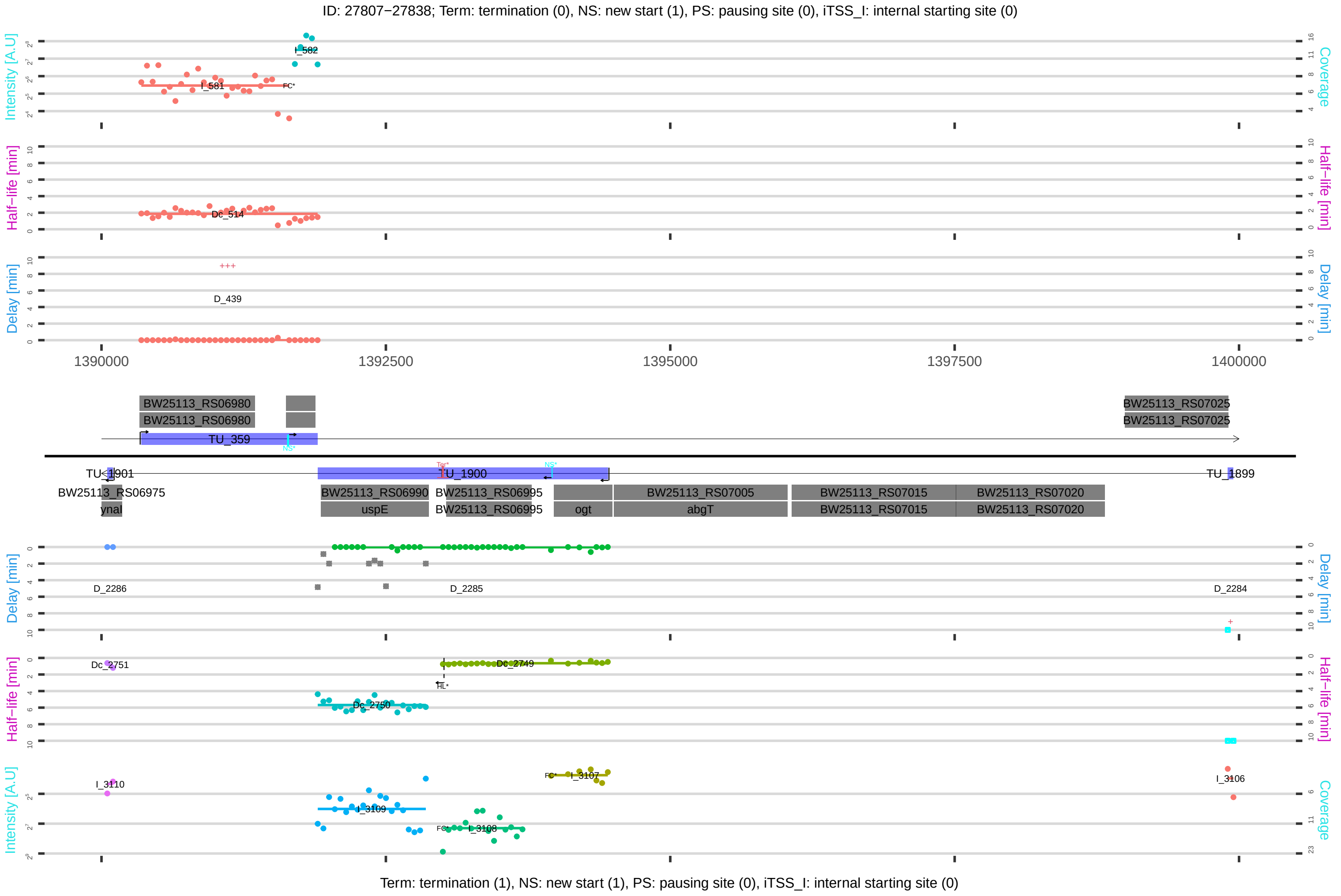

ID: 27807−27838; Term: termination (0), NS: new start (1), PS: pausing site (0), iTSS_I: internal starting site (0)
Intensity [A.U]
Coverage
Half−life [min]
Delay [min]
I_582
I_581
FC*
Dc_514
D_439
1390000
1392500
1395000
1397500
1400000
BW25113_RS06980
BW25113_RS07025
TU_359
NS*
Ter*
TU_1901
TU_1900
TU_1899
BW25113_RS06975
ynal
BW25113_RS06990
uspE
BW25113_RS06995
ogt
BW25113_RS07005
abgT
BW25113_RS07015
BW25113_RS07020
D_2286
D_2285
D_2284
Dc_2751
Dc_2749
Dc_2750
HL*
I_3110
I_3109
I_3108
I_3107
I_3106
Term: termination (1), NS: new start (1), PS: pausing site (0), iTSS_I: internal starting site (0)

ID: 28006−28096; Term: termination (0), NS: new start (0), PS: pausing site (0), iTSS_I: internal starting site (0)

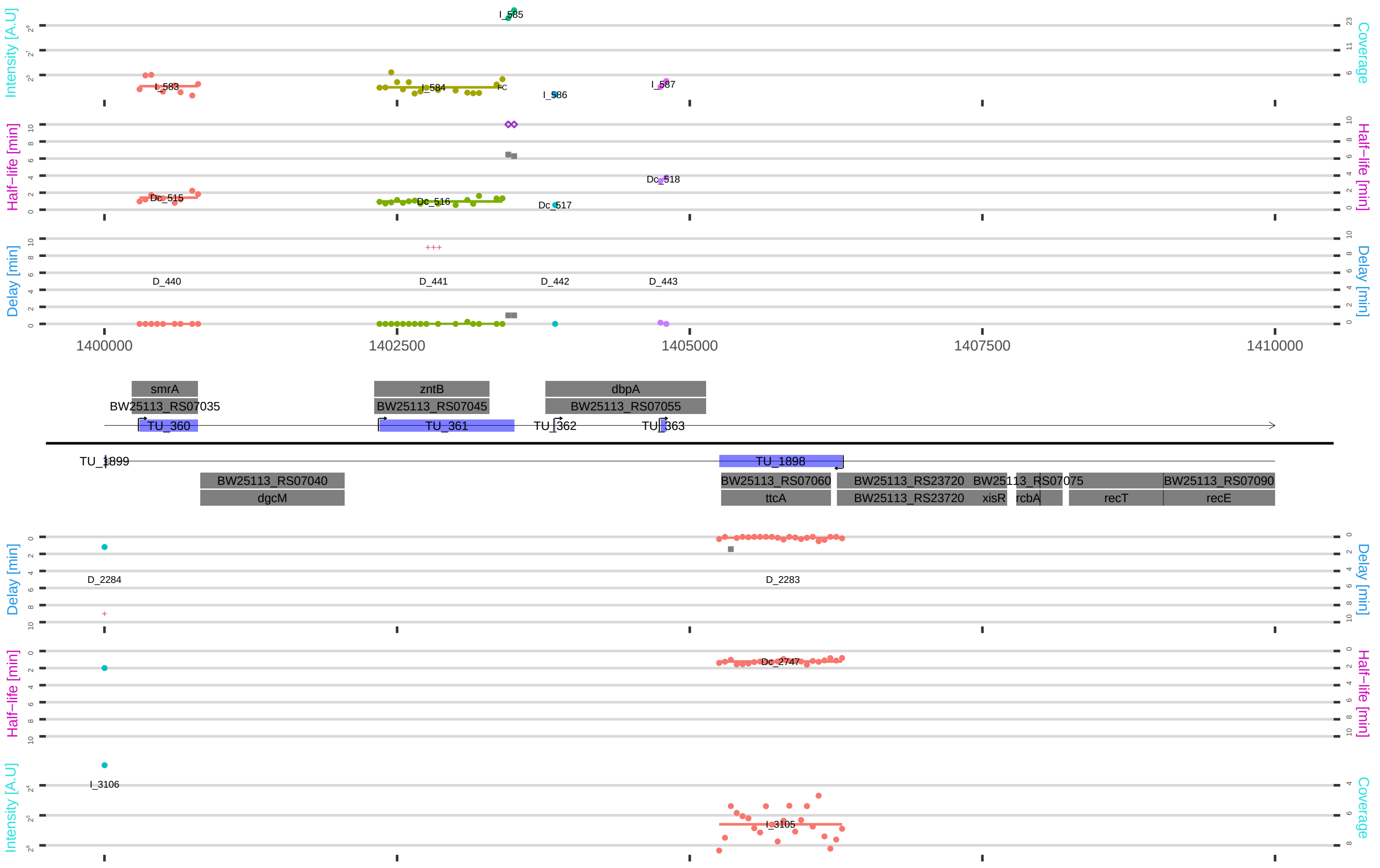

Term: termination (0), NS: new start (0), PS: pausing site (0), iTSS_I: internal starting site (0)

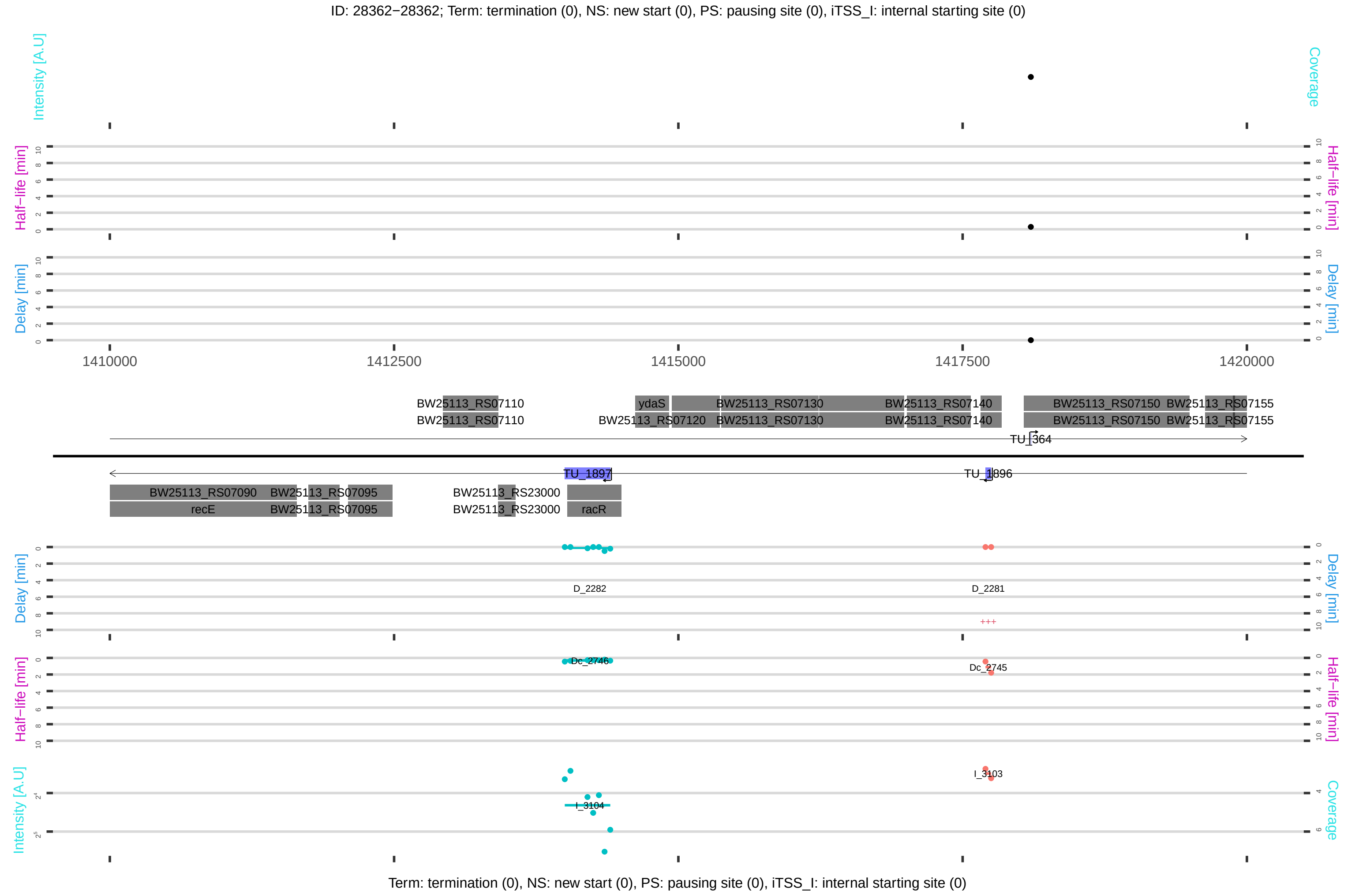
ID: 28362−28362; Term: termination (0), NS: new start (0), PS: pausing site (0), iTSS_I: internal starting site (0)
Intensity [A.U]
Coverage
Half−life [min]
Delay [min]
1410000
1412500
1415000
1417500
1420000
BW25113_RS07110
ydaS
BW25113_RS07120
BW25113_RS07130
BW25113_RS07140
BW25113_RS07150
BW25113_RS07155
TU_364
TU_1897
TU_1896
BW25113_RS07090
recE
BW25113_RS07095
BW25113_RS23000
racR
D_2282
D_2281
Dc_2746
Dc_2745
I_3104
I_3103
Term: termination (0), NS: new start (0), PS: pausing site (0), iTSS_I: internal starting site (0)

ID: 156814−156664; FC*: significant t−test of two consecutive segments; Term: termination, NS: new start, PS: pausing site,iTSS_I: internal starting site,TI: transcription interferance.

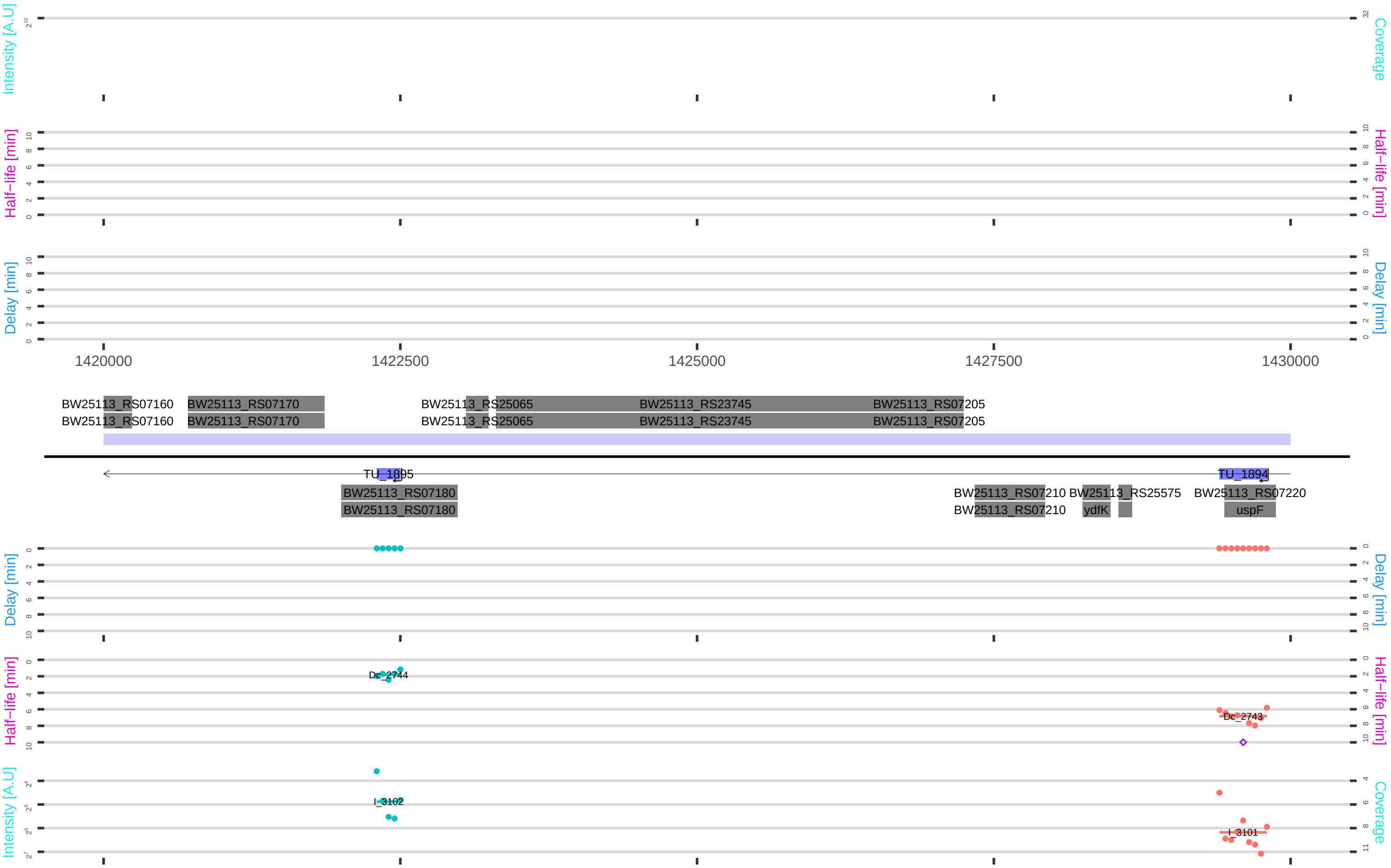

Term: termination (0), NS: new start (0), PS: pausing site (0), iTSS_I: internal starting site (0)

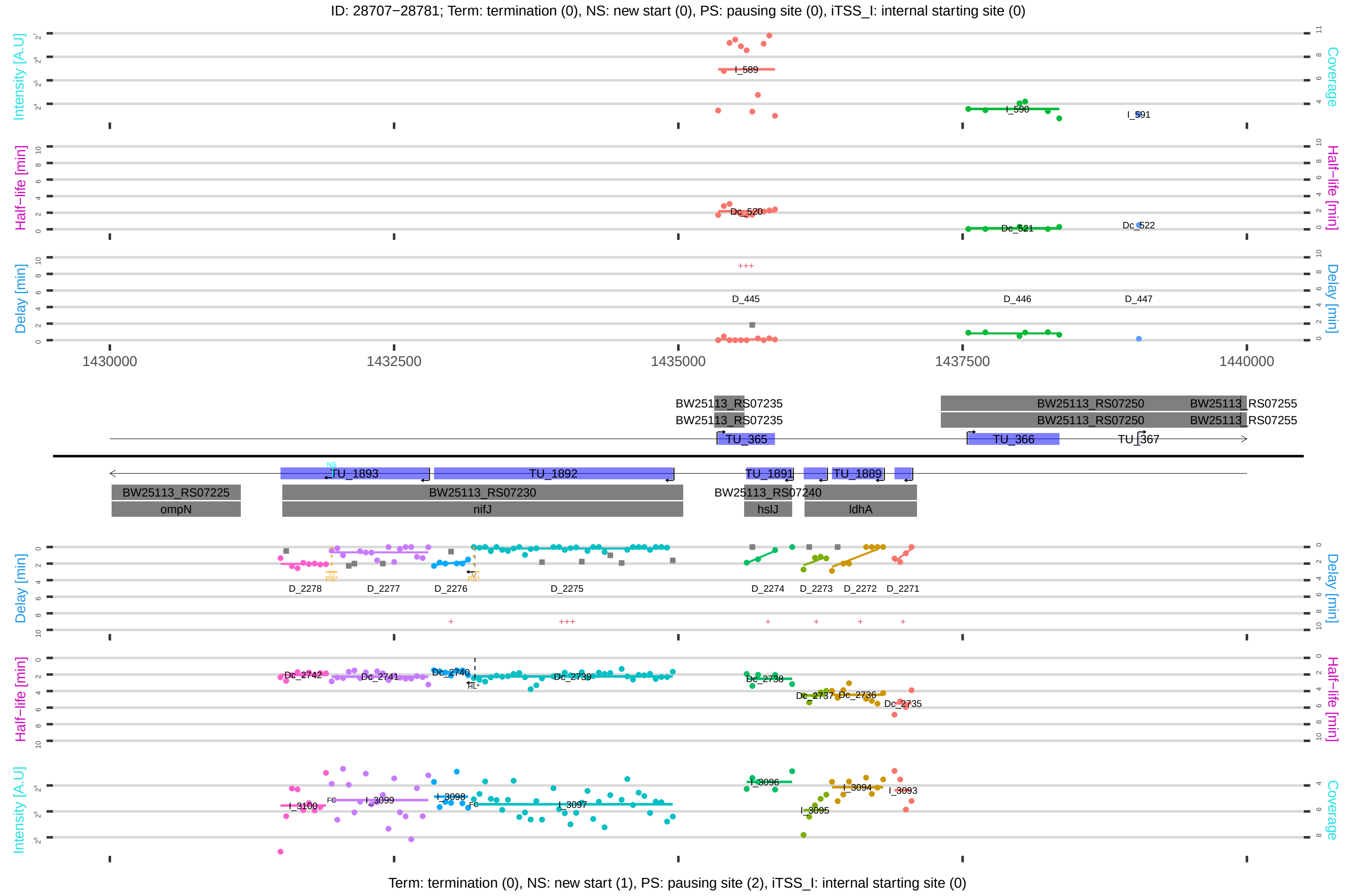

ID: 28707−28781; Term: termination (0), NS: new start (0), PS: pausing site (0), iTSS_I: internal starting site (0)
Intensity [A.U]
Coverage
I_589
I_590
I_591
Half−life [min]
Dc_520
Dc_521
Dc_522
Delay [min]
D_445
D_446
D_447
1430000
1432500
1435000
1437500
1440000
BW25113_RS07235
BW25113_RS07250
BW25113_RS07255
TU_365
TU_366
TU_367
TU_1893
TU_1892
TU_1891
TU_1889
BW25113_RS07225
ompN
BW25113_RS07230
nifJ
BW25113_RS07240
hslJ
ldhA
D_2278
D_2277
D_2276
D_2275
D_2274
D_2273
D_2272
D_2271
Dc_2742
Dc_2741
Dc_2740
Dc_2739
Dc_2738
Dc_2737
Dc_2736
Dc_2735
I_3100
I_3099
I_3098
I_3097
I_3096
I_3095
I_3094
I_3093
Term: termination (0), NS: new start (1), PS: pausing site (2), iTSS_I: internal starting site (0)

ID: 28801−28809; Term: termination (0), NS: new start (0), PS: pausing site (0), iTSS_I: internal starting site (0)

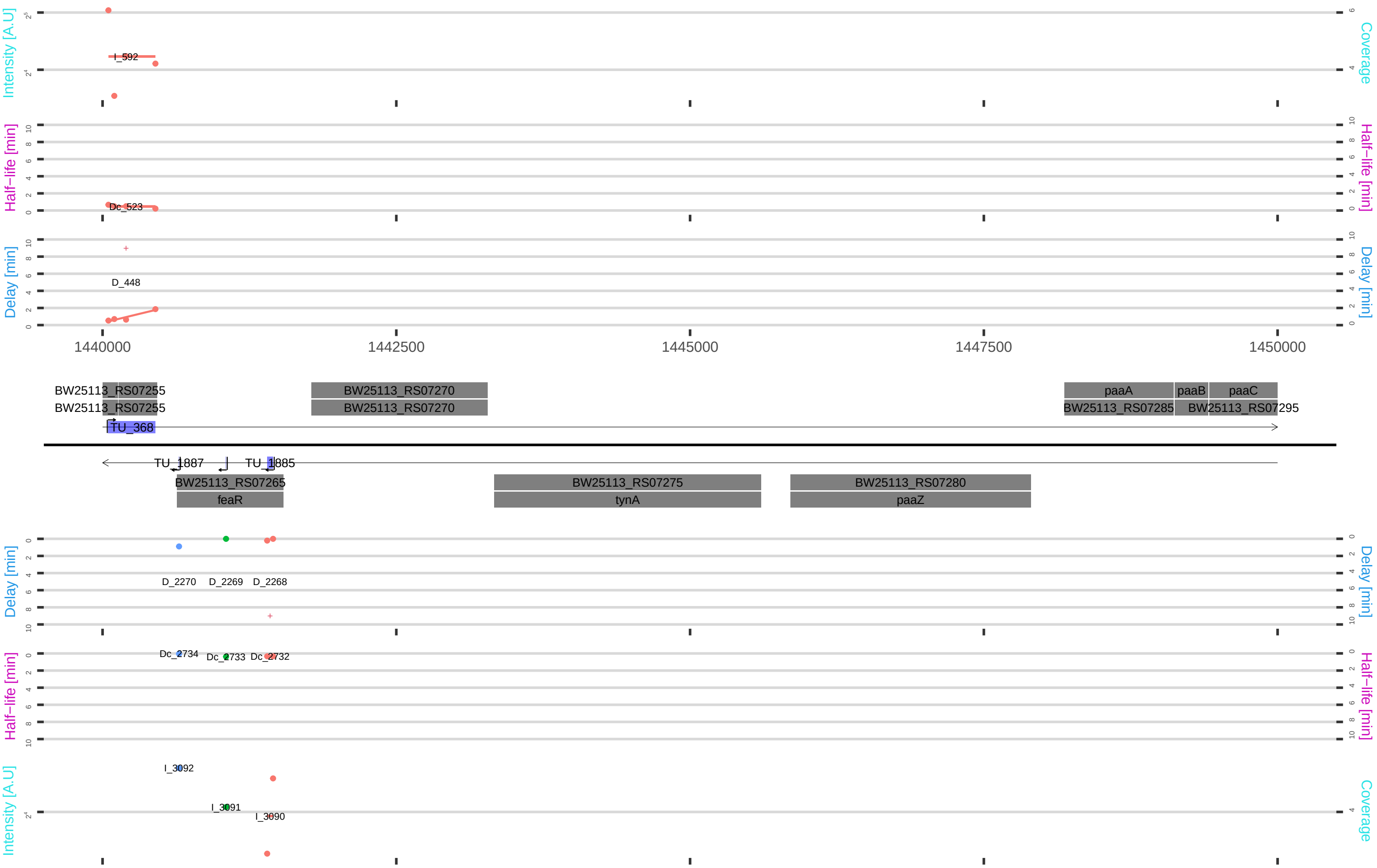

Term: termination (0), NS: new start (0), PS: pausing site (0), iTSS_I: internal starting site (0)

ID: 29001−29187; Term: termination (0), NS: new start (0), PS: pausing site (0), iTSS_I: internal starting site (0)

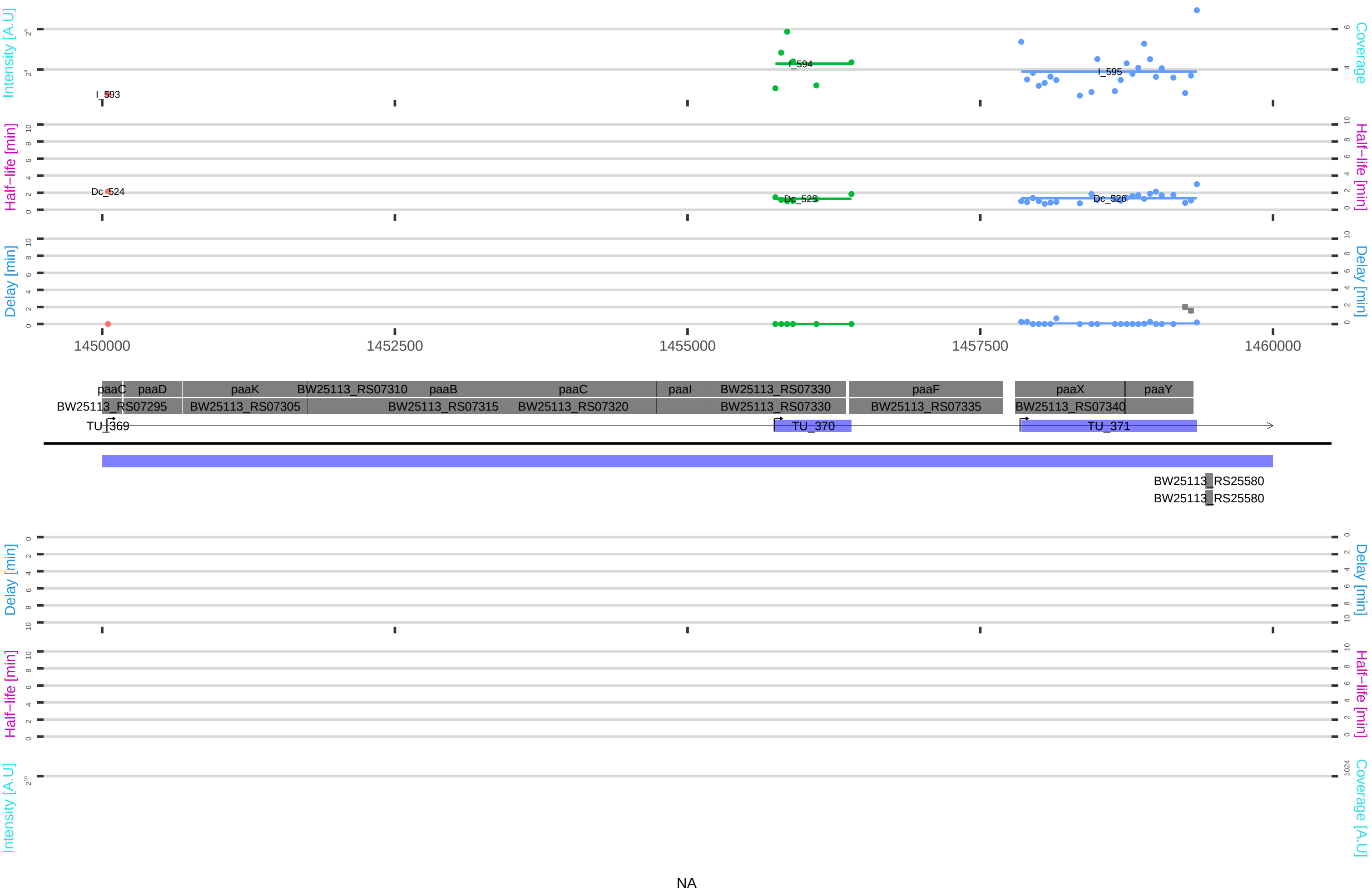

ID: 29245−29387; Term: termination (0), NS: new start (0), PS: pausing site (0), iTSS_I: internal starting site (0)

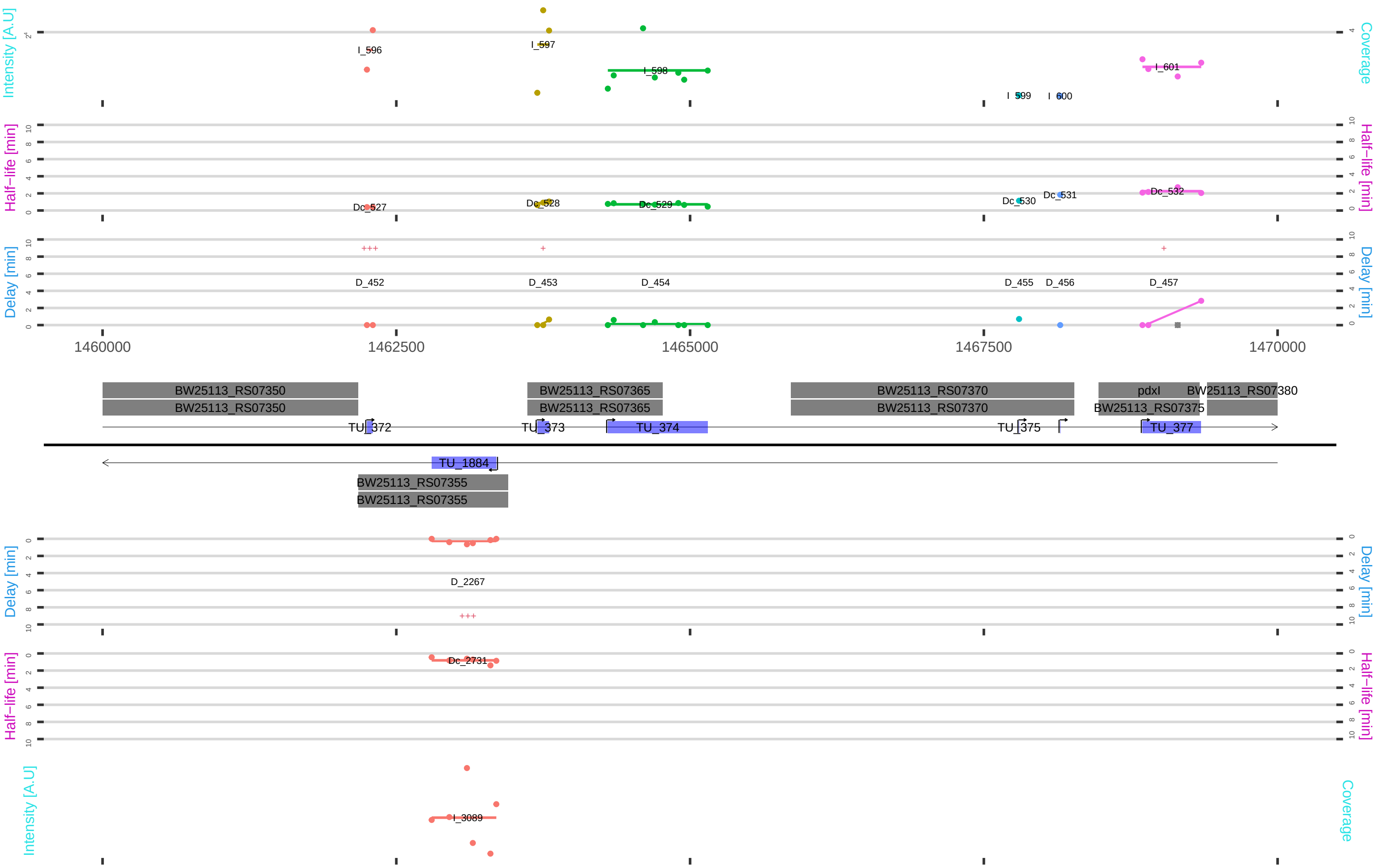

Term: termination (0), NS: new start (0), PS: pausing site (0), iTSS_I: internal starting site (0)

ID: 29547−29600; Term: termination (0), NS: new start (0), PS: pausing site (0), iTSS_I: internal starting site (0)

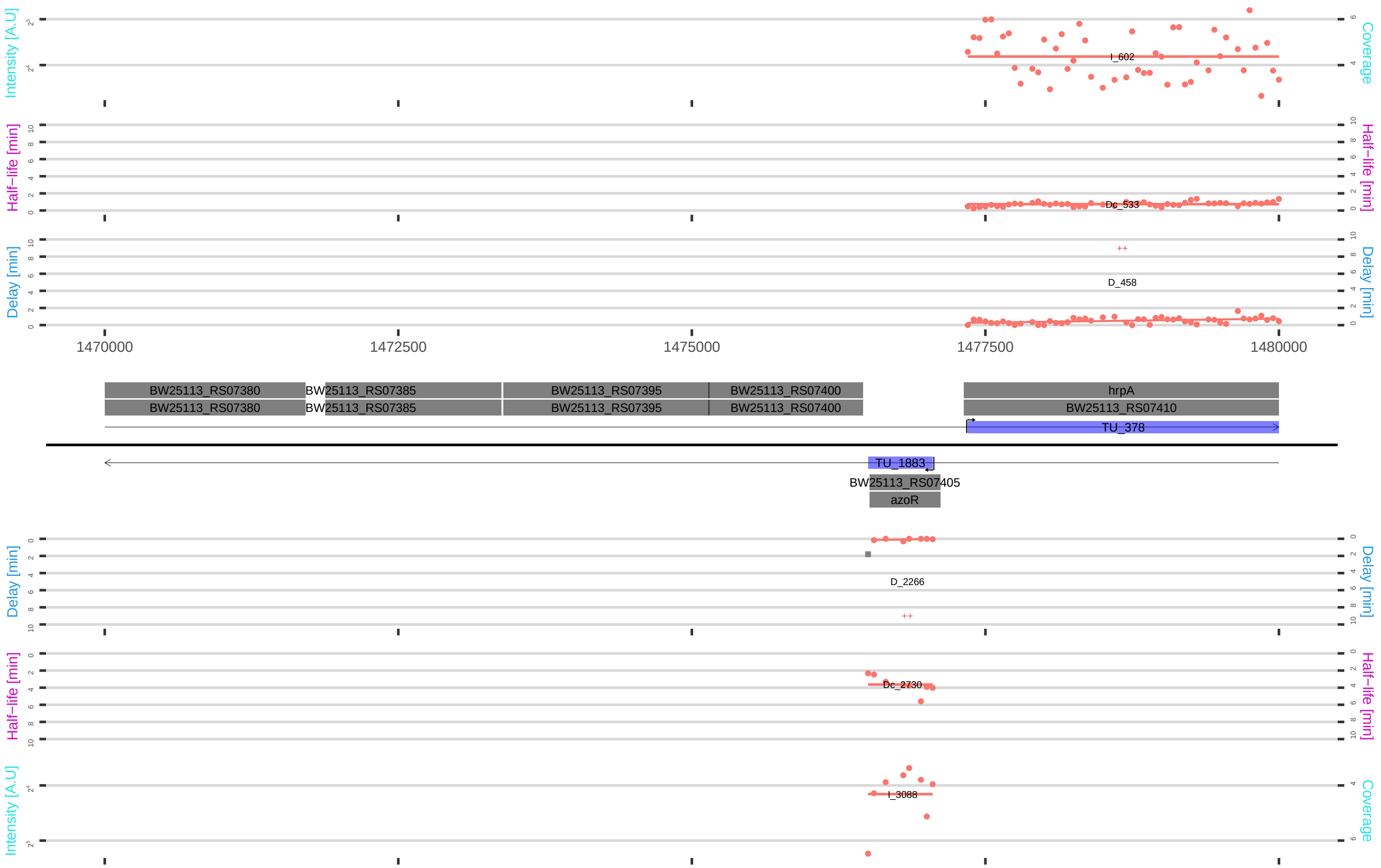

Term: termination (0), NS: new start (0), PS: pausing site (0), iTSS_I: internal starting site (0)

ID: 29600−29728; Term: termination (2), NS: new start (1), PS: pausing site (0), iTSS_I: internal starting site (0)

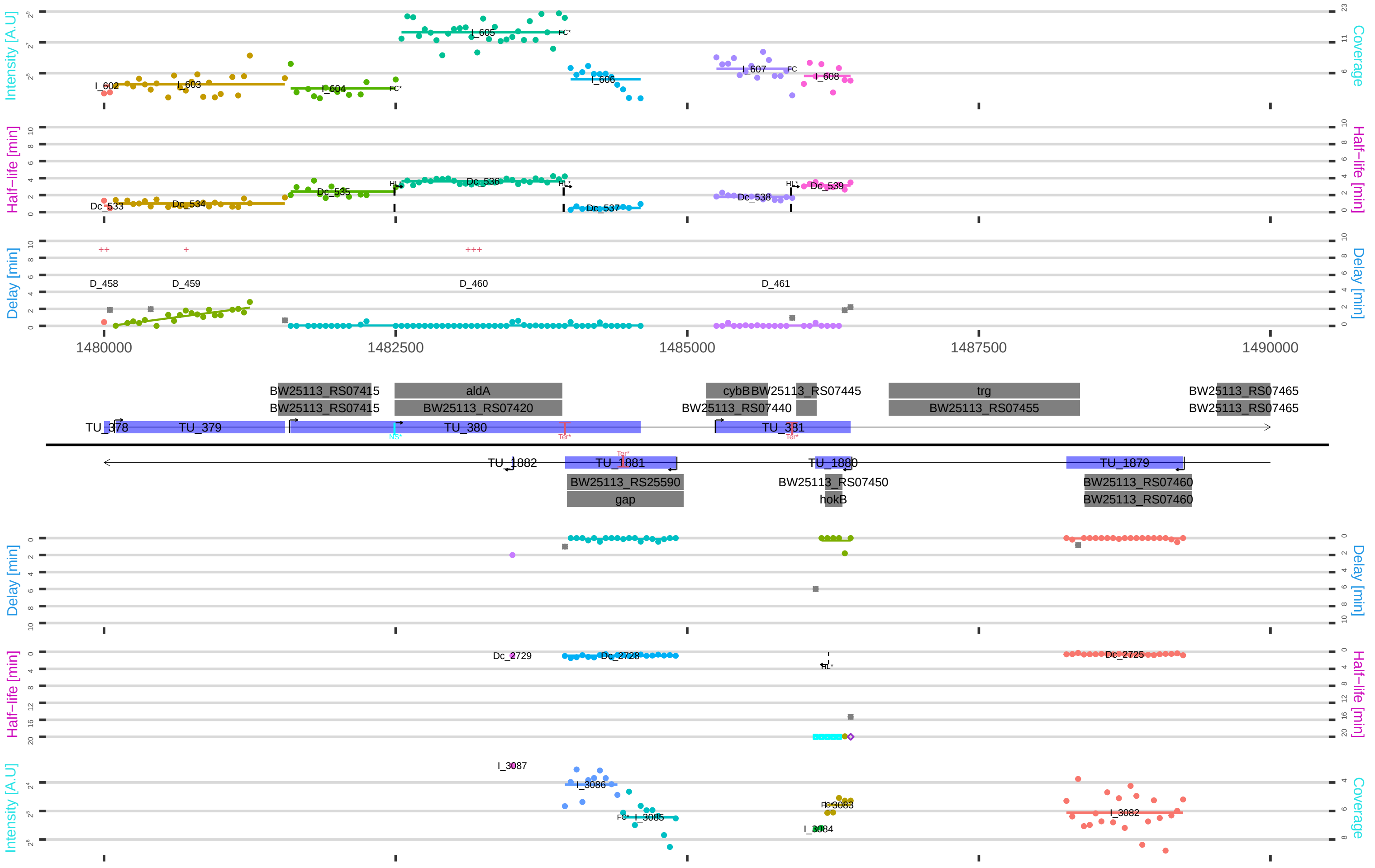

Term: termination (1), NS: new start (0), PS: pausing site (0), iTSS_I: internal starting site (0)

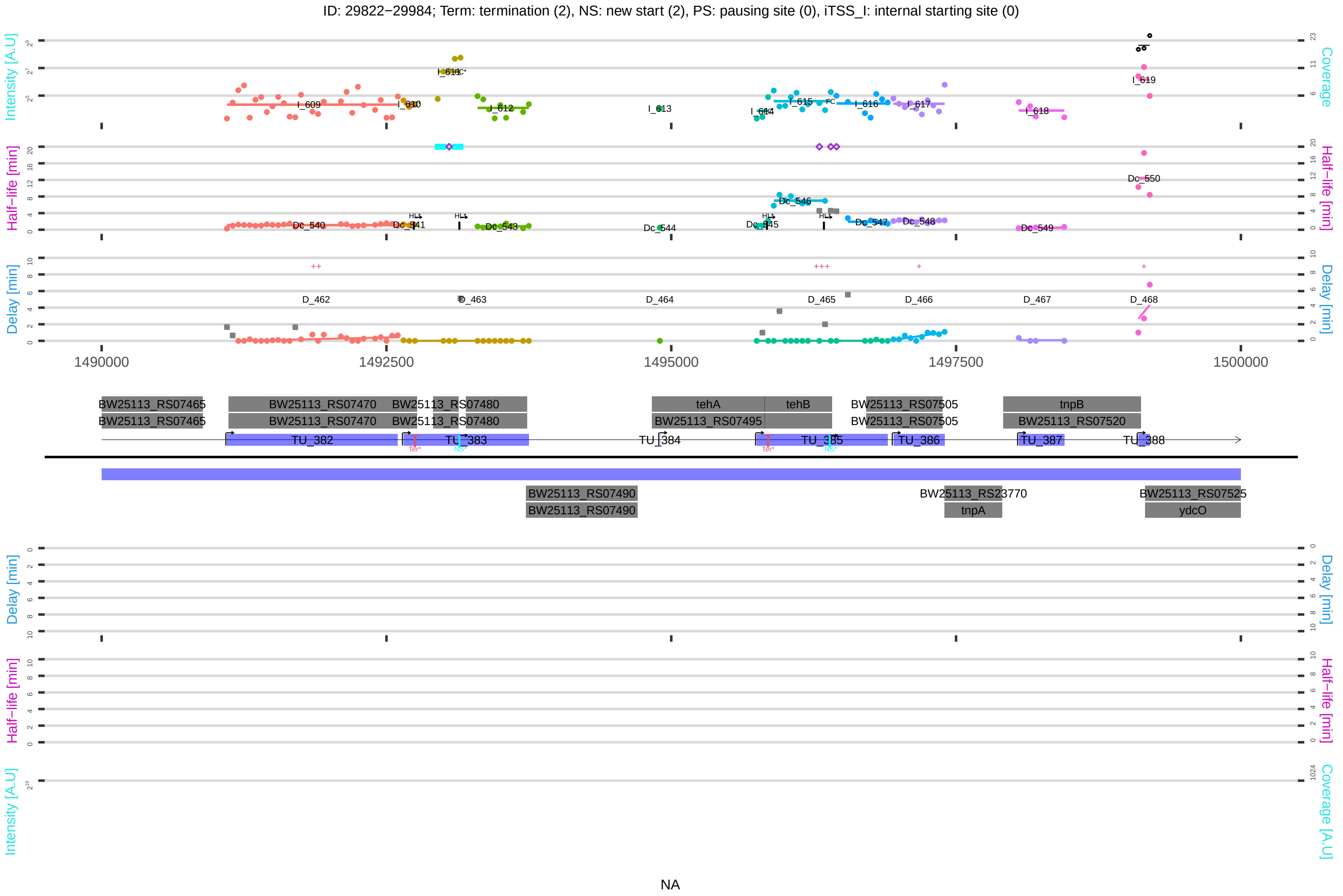
ID: 29822−29984; Term: termination (2), NS: new start (2), PS: pausing site (0), iTSS_I: internal starting site (0)
Intensity [A.U]
Coverage
Half−life [min]
Delay [min]
1490000
1492500
1495000
1497500
1500000
BW25113_RS07465
BW25113_RS07470
BW25113_RS07480
tehA
tehB
BW25113_RS07495
BW25113_RS07505
tnpB
BW25113_RS07520
TU_382
TU_383
TU_384
TU_385
TU_386
TU_387
TU_388
BW25113_RS07490
BW25113_RS23770
tnpA
BW25113_RS07525
ydcO
NA

ID: 30012−30103; Term: termination (0), NS: new start (0), PS: pausing site (0), iTSS_I: internal starting site (0)

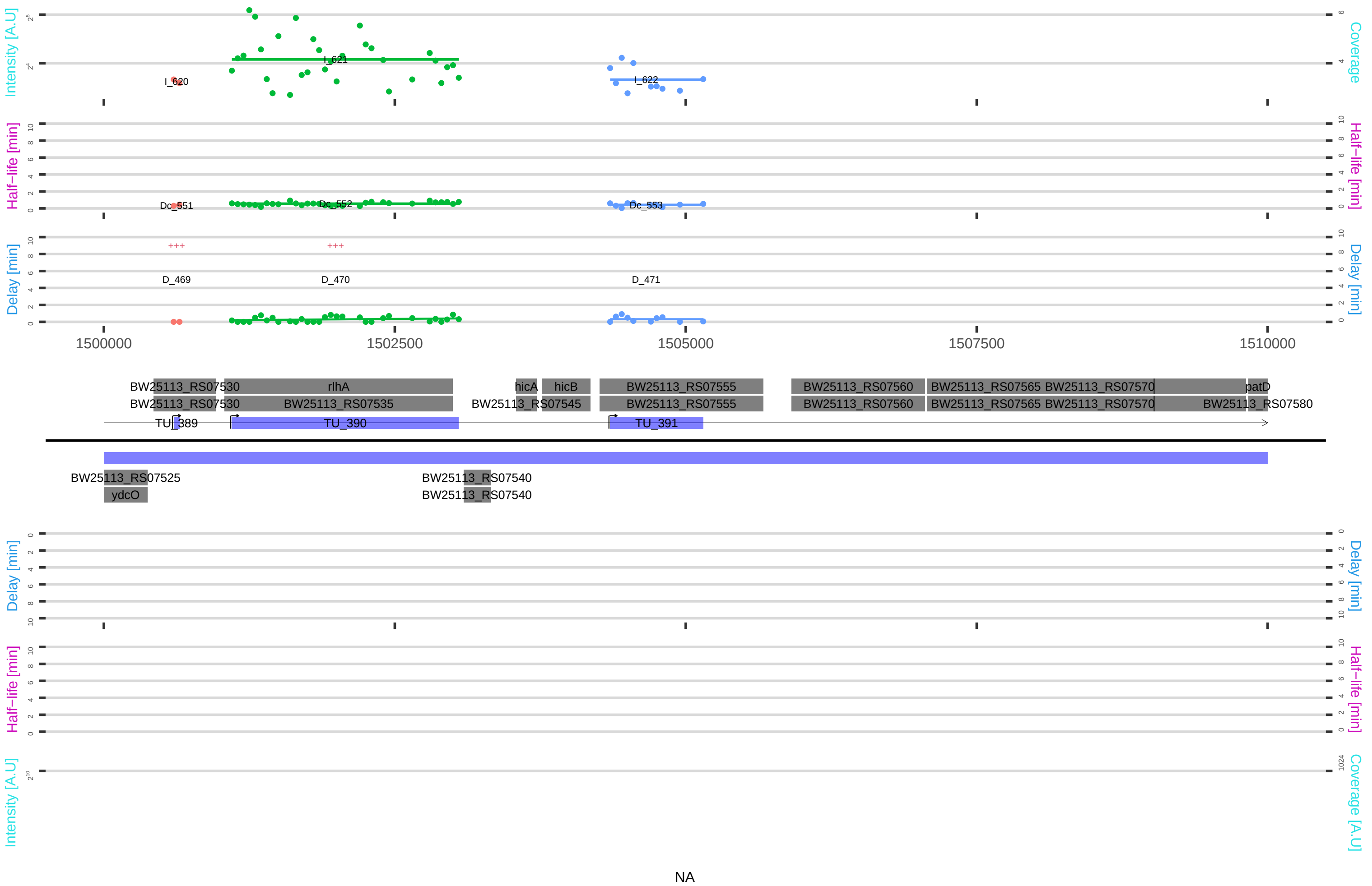

ID: 30239−30374; Term: termination (0), NS: new start (0), PS: pausing site (0), iTSS_I: internal starting site (0)

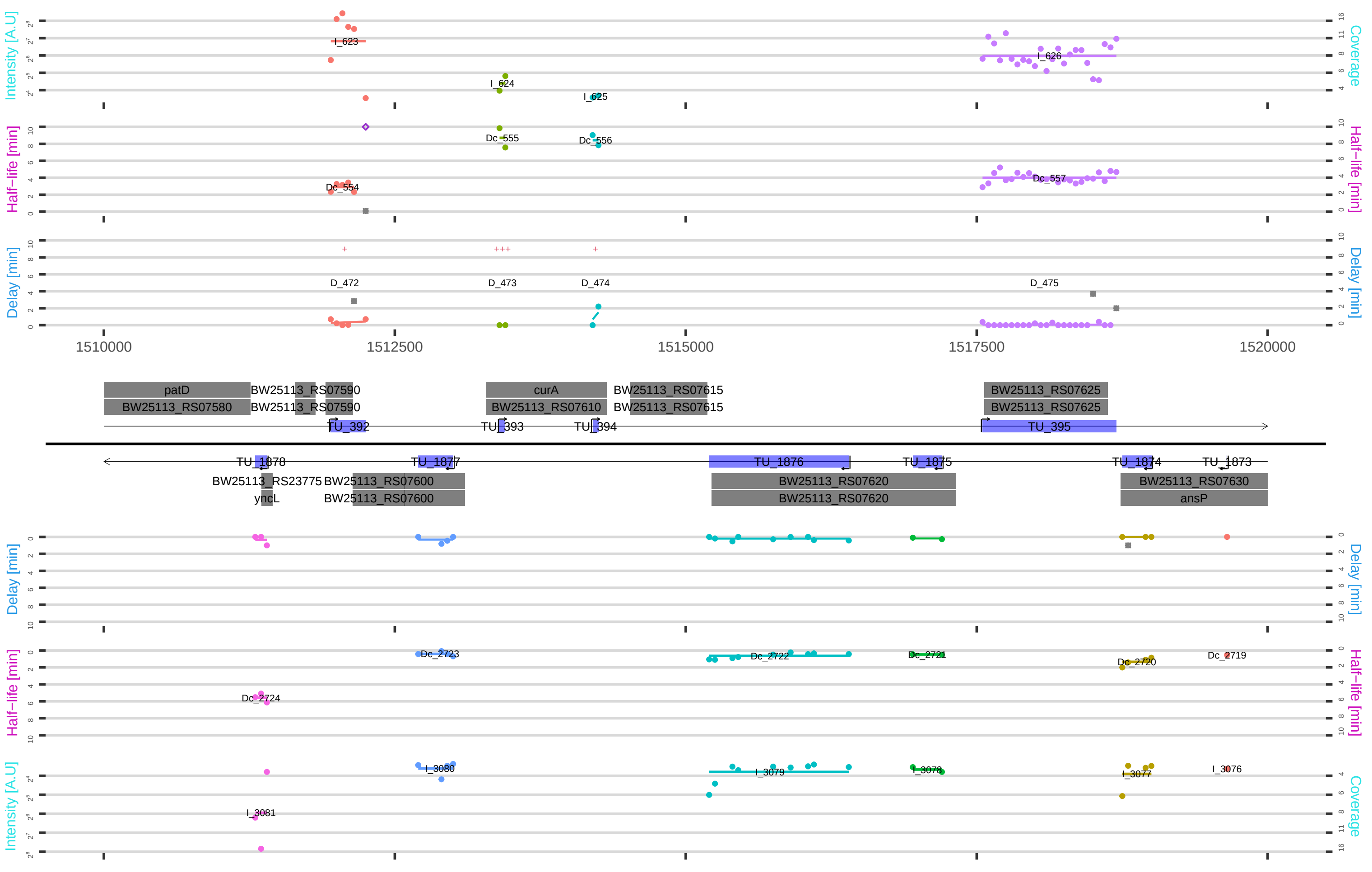

Term: termination (0), NS: new start (0), PS: pausing site (0), iTSS_I: internal starting site (0)

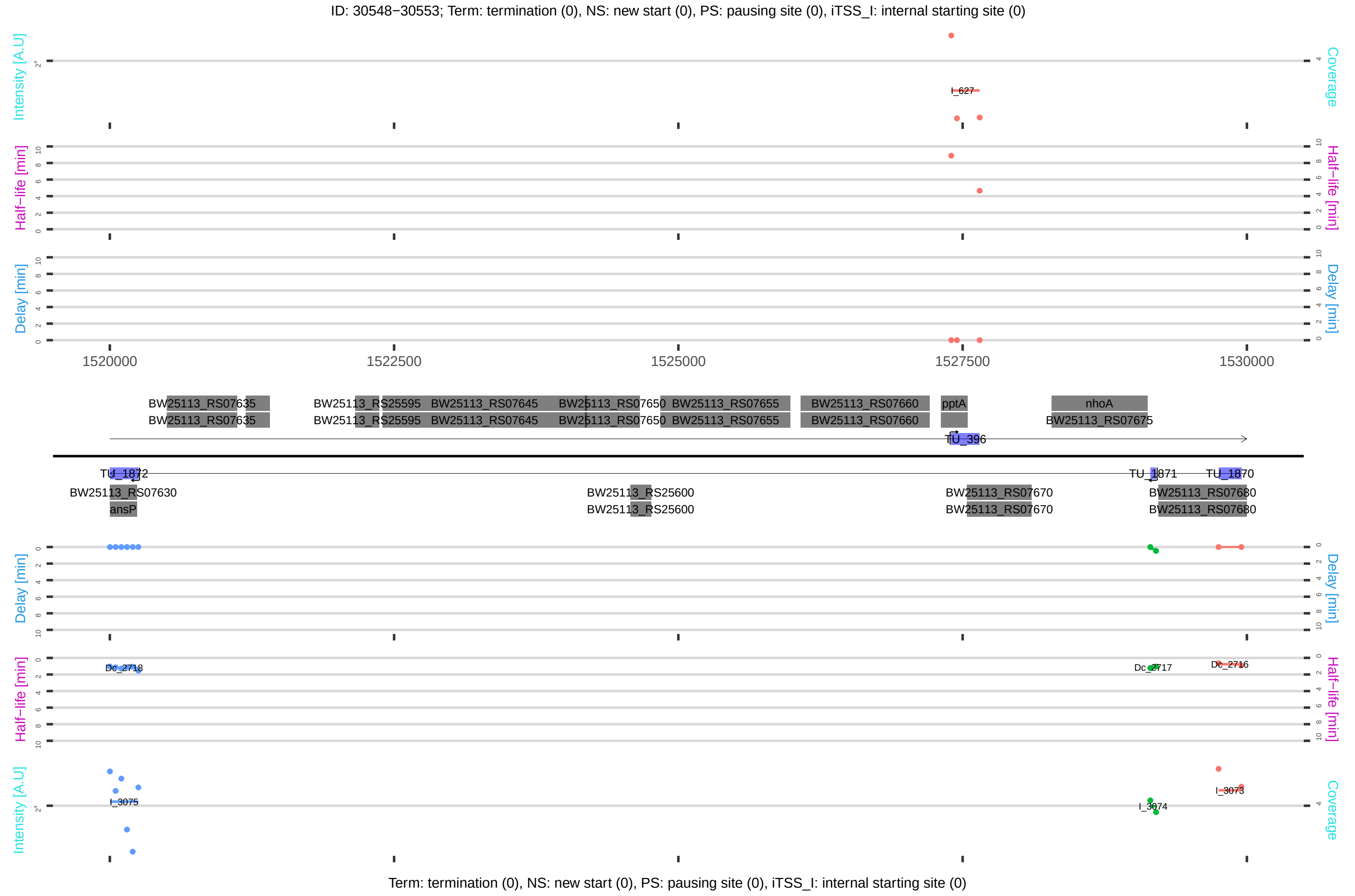
ID: 30548−30553; Term: termination (0), NS: new start (0), PS: pausing site (0), iTSS_I: internal starting site (0)
Intensity [A.U]
Coverage
Half−life [min]
Delay [min]
I_627
1520000
1522500
1525000
1527500
1530000
BW25113_RS07635
BW25113_RS25595
BW25113_RS07645
BW25113_RS07650
BW25113_RS07655
BW25113_RS07660
pptA
nhoA
BW25113_RS07675
TU_396
TU_1872
TU_1871
TU_1870
BW25113_RS07630
ansP
BW25113_RS25600
BW25113_RS07670
BW25113_RS07680
Dc_2718
Dc_2717
Dc_2716
I_3075
I_3074
I_3073
Term: termination (0), NS: new start (0), PS: pausing site (0), iTSS_I: internal starting site (0)

ID: 154660−154660; FC*: significant t−test of two consecutive segments; Term: termination, NS: new start, PS: pausing site,iTSS_I: internal starting site,TI: transcription interferance.

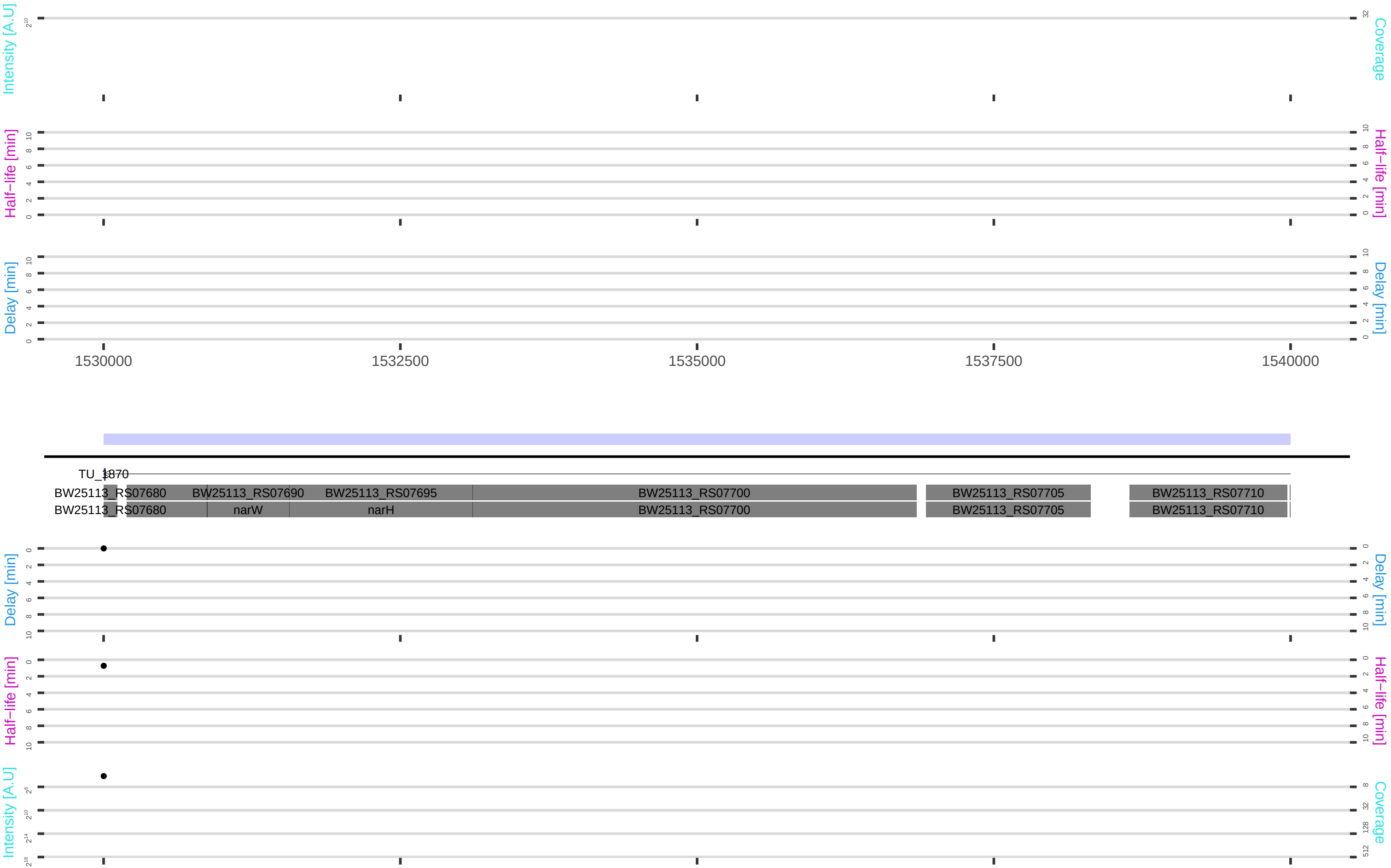

Term: termination (0), NS: new start (0), PS: pausing site (0), iTSS_I: internal starting site (0)

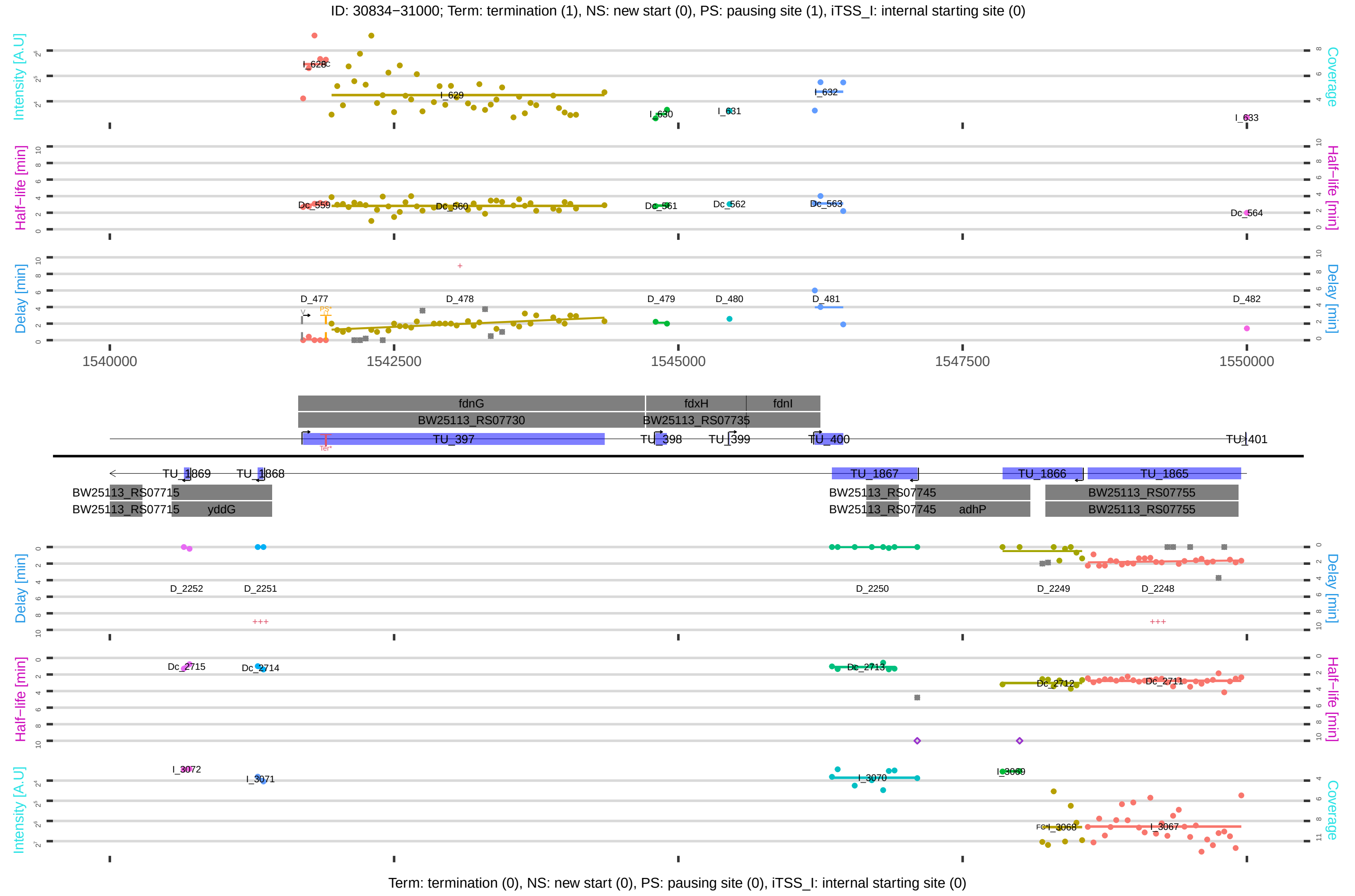
ID: 30834−31000; Term: termination (1), NS: new start (0), PS: pausing site (1), iTSS_I: internal starting site (0)
Intensity [A.U]
Coverage
Half−life [min]
Delay [min]
1540000
1542500
1545000
1547500
1550000
fdnG
fdxH
fdnI
BW25113_RS07730
BW25113_RS07735
TU_397
TU_398
TU_399
TU_400
TU_401
TU_1869
TU_1868
TU_1867
TU_1866
TU_1865
BW25113_RS07715
yddG
BW25113_RS07745
adhP
BW25113_RS07755
Term: termination (0), NS: new start (0), PS: pausing site (0), iTSS_I: internal starting site (0)

ID: 31000−31028; Term: termination (1), NS: new start (0), PS: pausing site (1), iTSS_I: internal starting site (0)

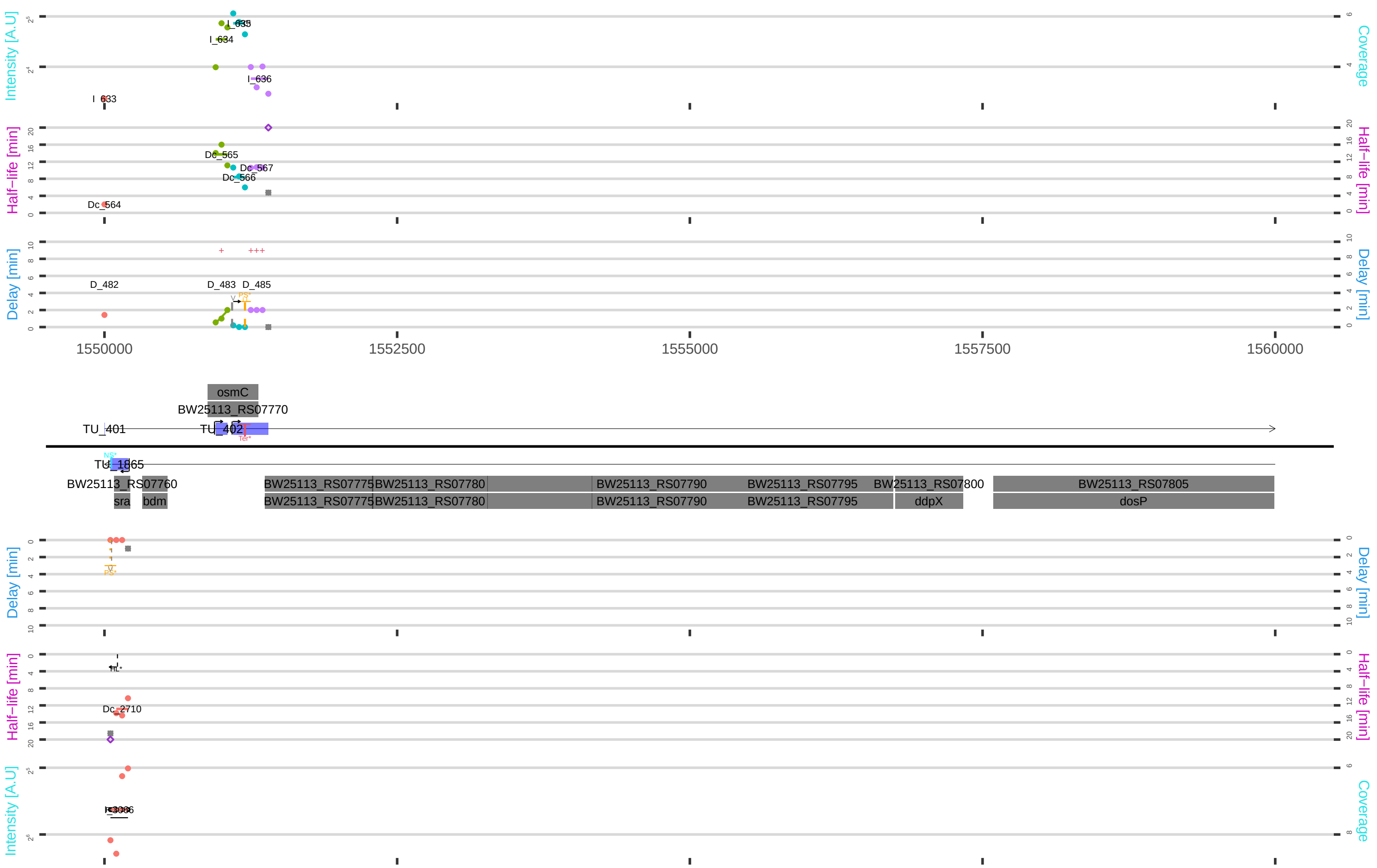

Term: termination (0), NS: new start (1), PS: pausing site (1), iTSS_I: internal starting site (0)

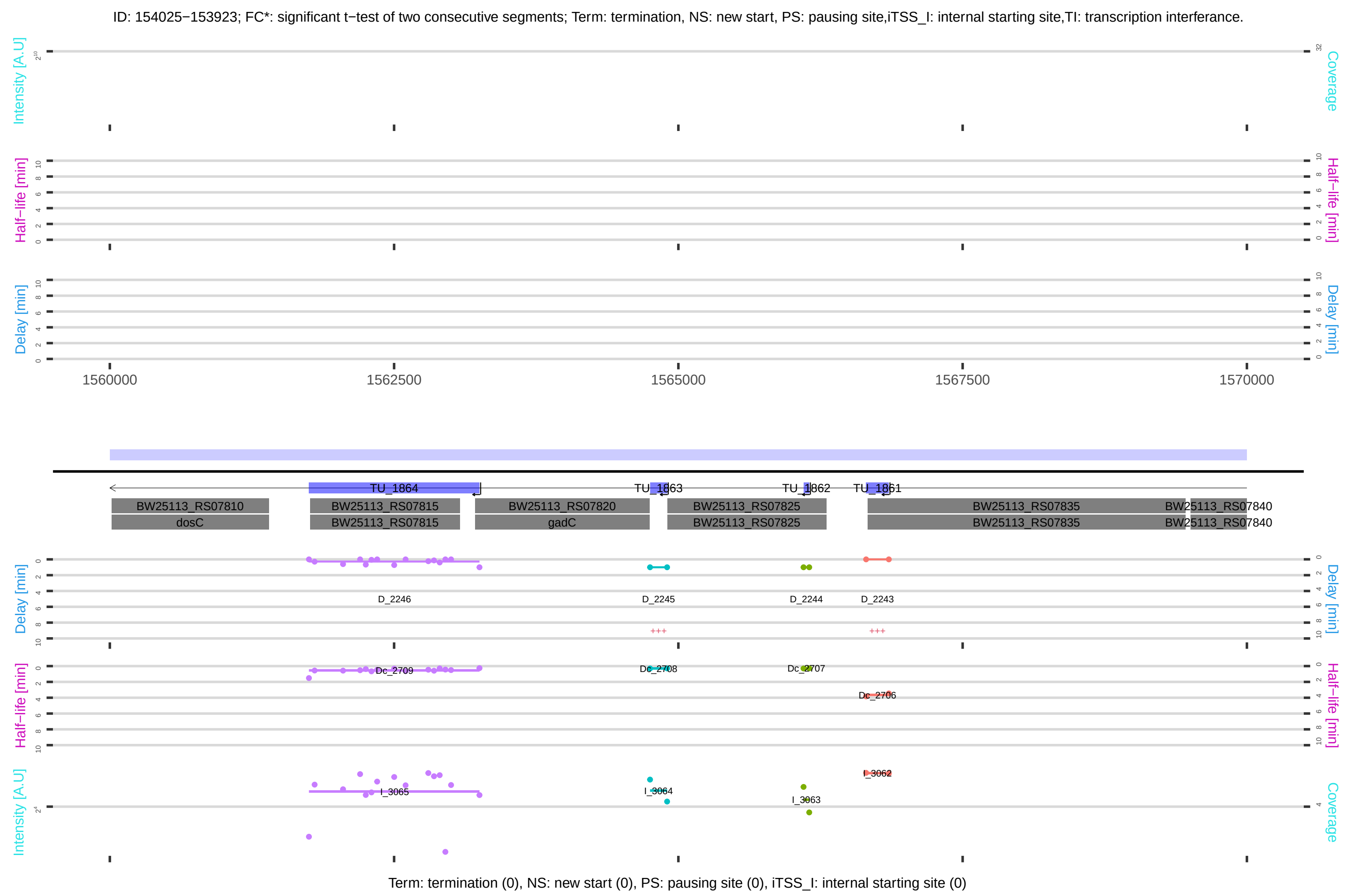
ID: 154025−153923; FC*: significant t−test of two consecutive segments; Term: termination, NS: new start, PS: pausing site,iTSS_I: internal starting site,TI: transcription interferance.
Intensity [A.U]
Coverage
Half−life [min]
Delay [min]
1560000
1562500
1565000
1567500
1570000
TU_1864
TU_1863
TU_1862
TU_1861
BW25113_RS07810
dosC
BW25113_RS07815
BW25113_RS07815
BW25113_RS07820
gadC
BW25113_RS07825
BW25113_RS07825
BW25113_RS07835
BW25113_RS07835
BW25113_RS07840
BW25113_RS07840
D_2246
D_2245
D_2244
D_2243
Dc_2709
Dc_2708
Dc_2707
Dc_2706
I_3065
I_3064
I_3063
I_3062
Term: termination (0), NS: new start (0), PS: pausing site (0), iTSS_I: internal starting site (0)

ID: 153758−153728; FC*: significant t−test of two consecutive segments; Term: termination, NS: new start, PS: pausing site,iTSS_I: internal starting site,TI: transcription interferance.

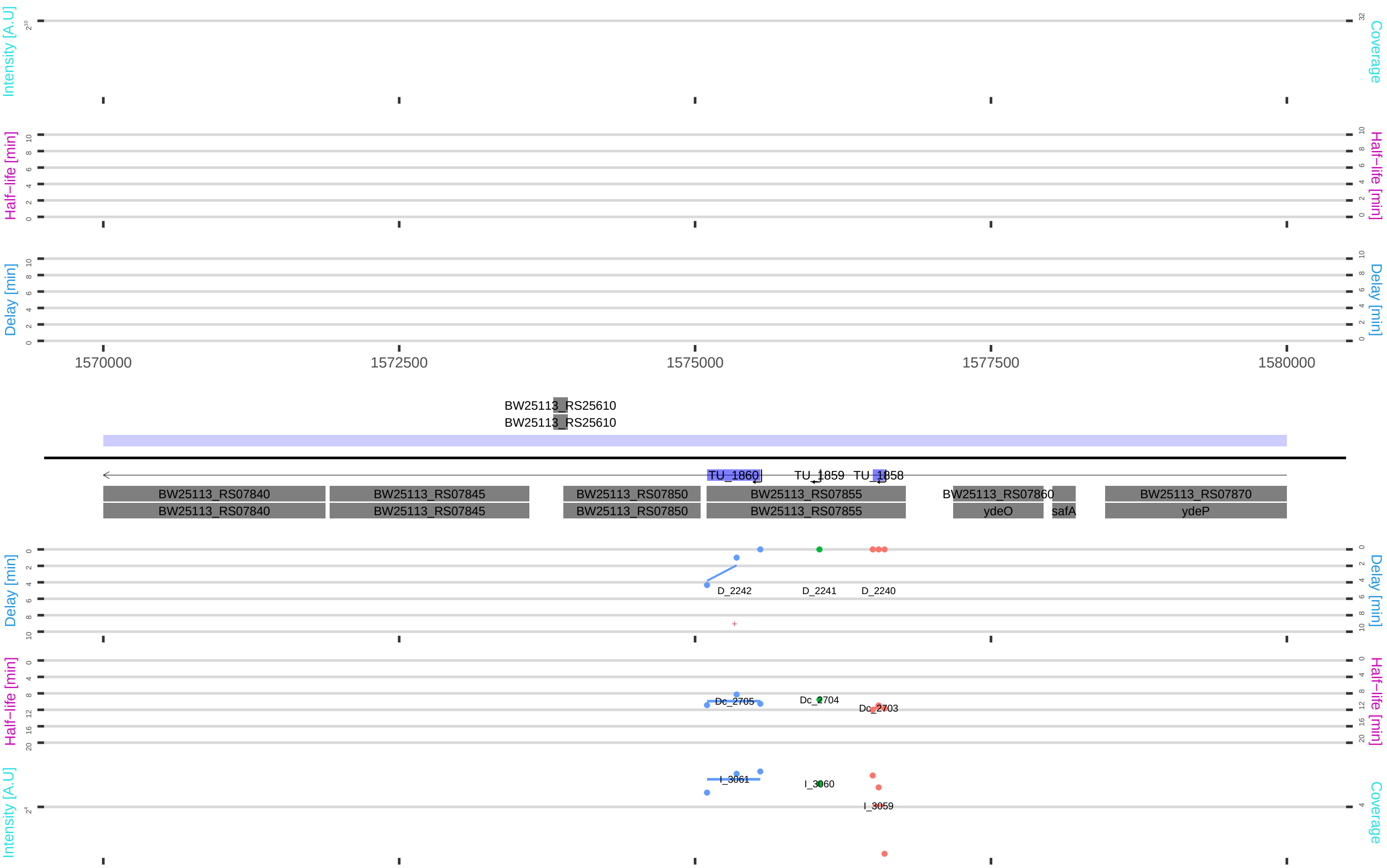

Term: termination (0), NS: new start (0), PS: pausing site (0), iTSS_I: internal starting site (0)

ID: 31737−31738; Term: termination (0), NS: new start (0), PS: pausing site (0), iTSS_I: internal starting site (0)

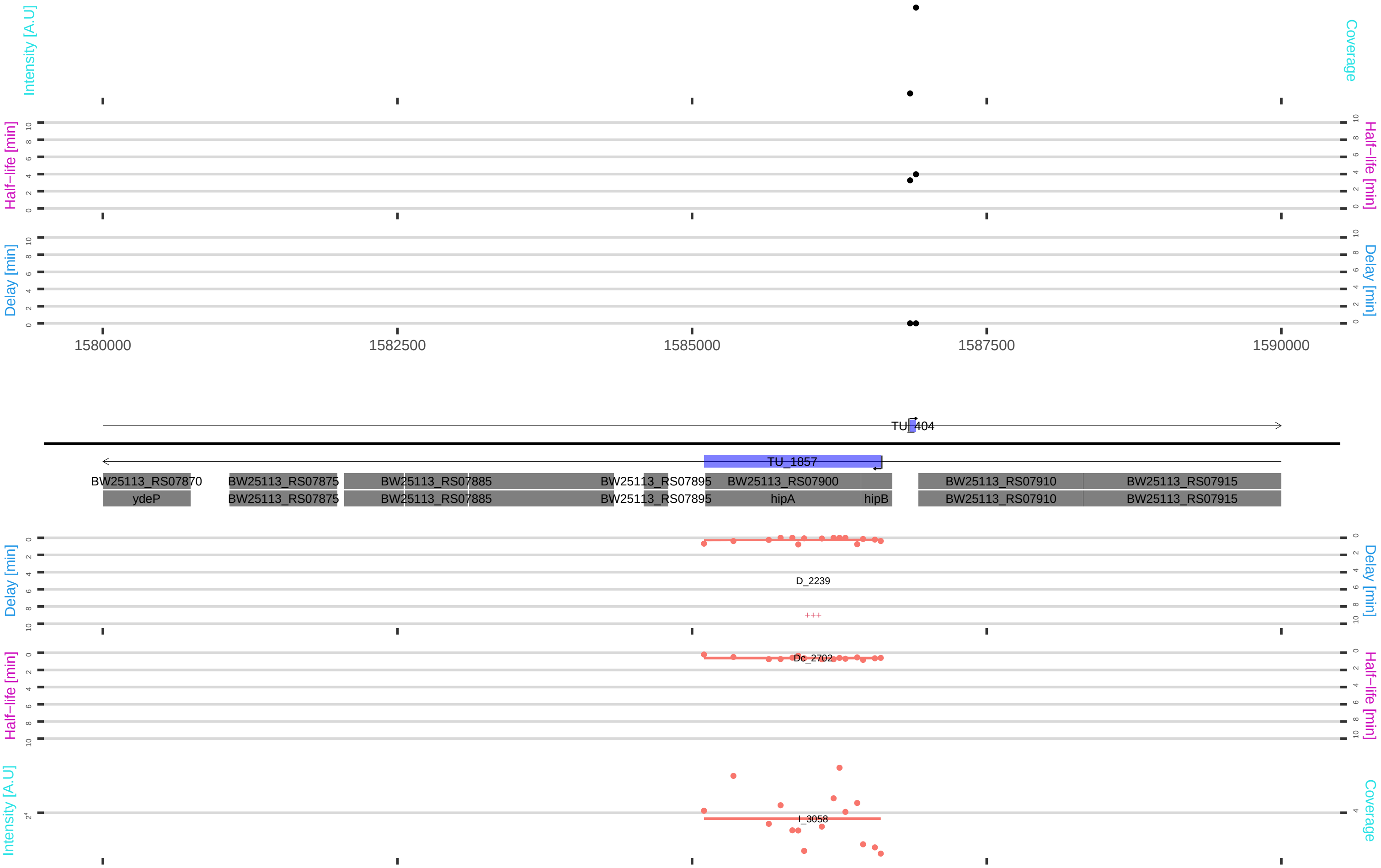

Term: termination (0), NS: new start (0), PS: pausing site (0), iTSS_I: internal starting site (0)

ID: 153375−153351; FC*: significant t−test of two consecutive segments; Term: termination, NS: new start, PS: pausing site,iTSS_I: internal starting site,TI: transcription interferance.

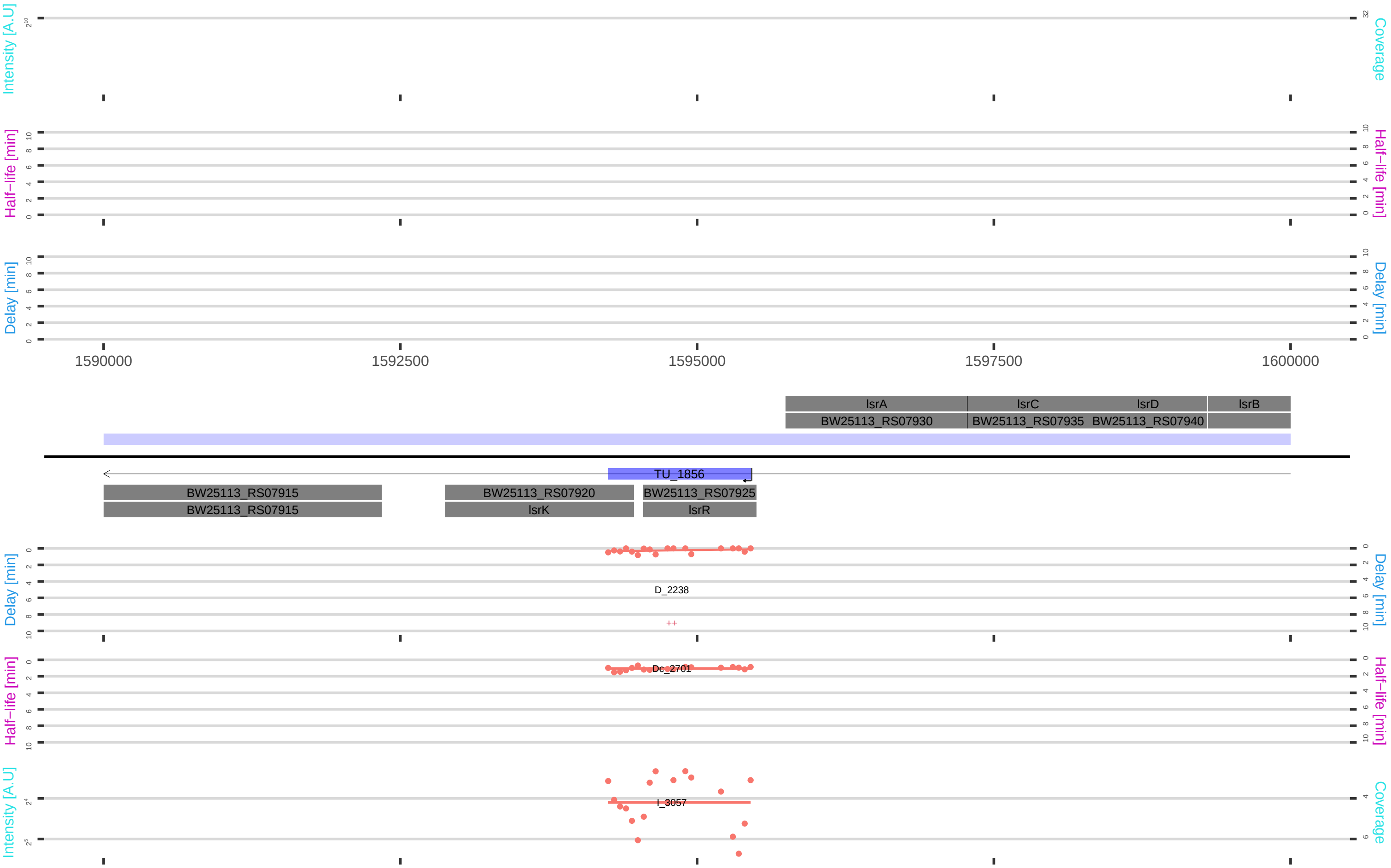

Term: termination (0), NS: new start (0), PS: pausing site (0), iTSS_I: internal starting site (0)

ID: 32126−32127; Term: termination (0), NS: new start (0), PS: pausing site (0), iTSS_I: internal starting site (0)

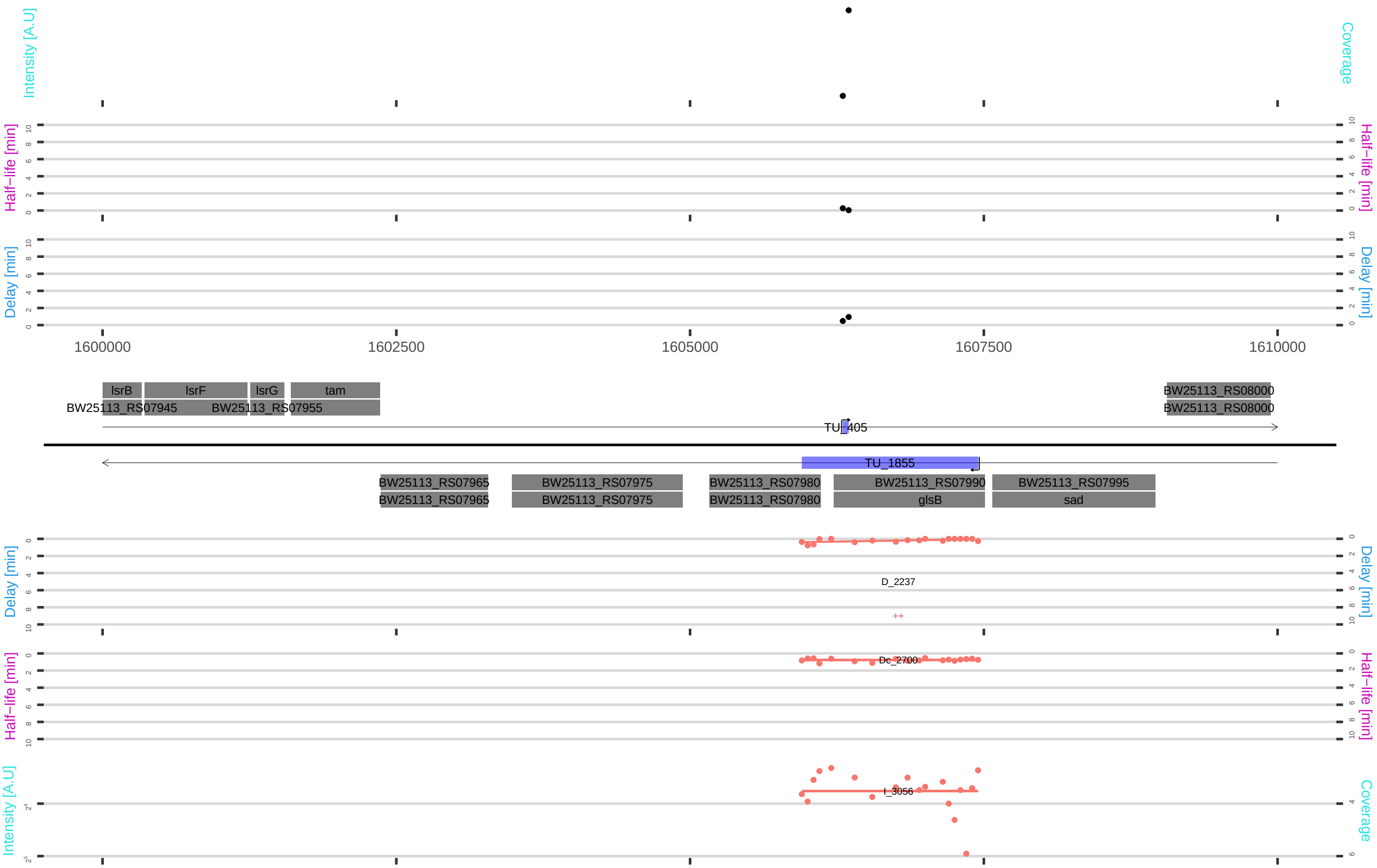

Term: termination (0), NS: new start (0), PS: pausing site (0), iTSS_I: internal starting site (0)

ID: 32275−32344; Term: termination (1), NS: new start (0), PS: pausing site (1), iTSS_I: internal starting site (0)

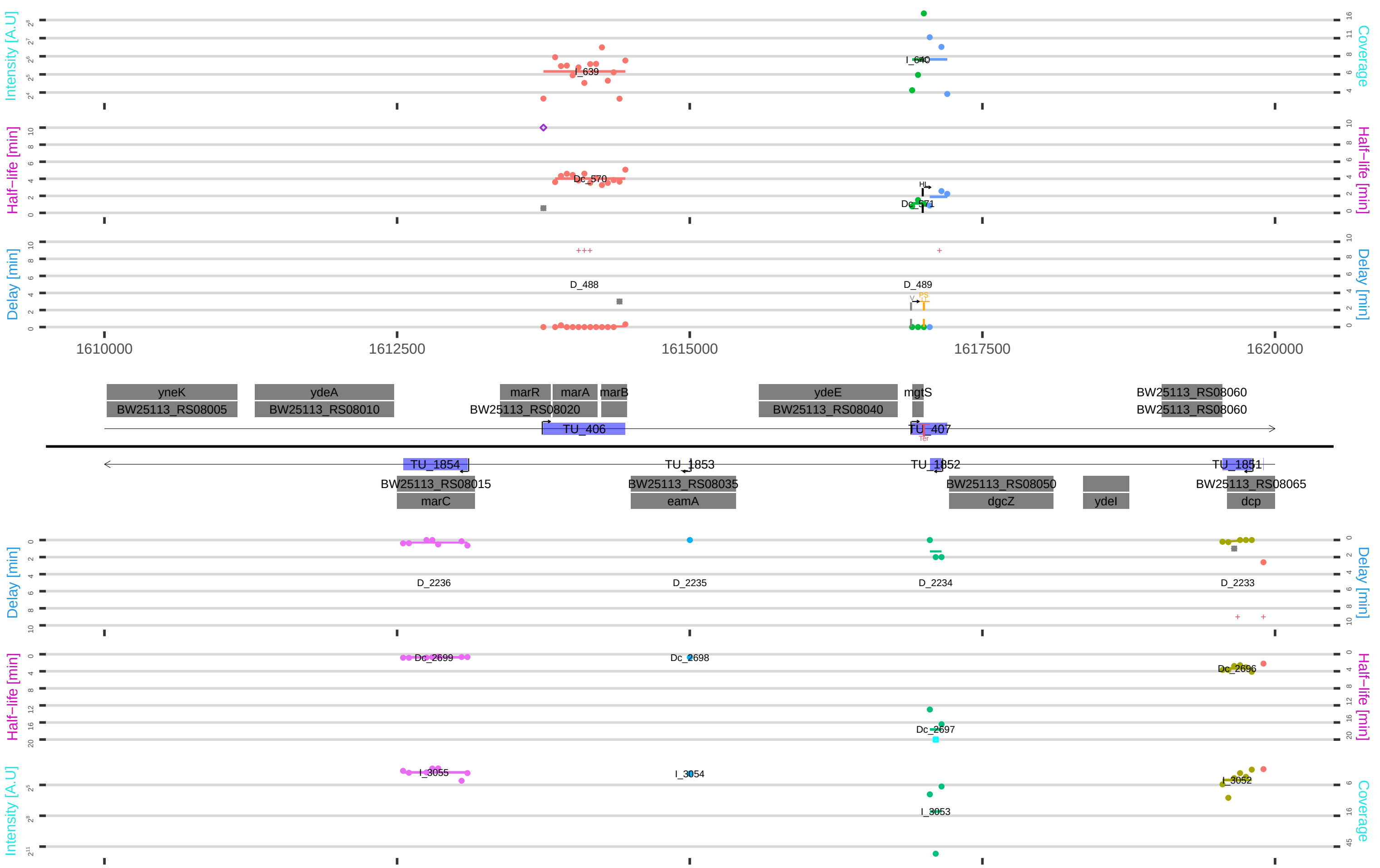

Term: termination (0), NS: new start (0), PS: pausing site (0), iTSS_I: internal starting site (0)

ID: 32437−32474; Term: termination (2), NS: new start (1), PS: pausing site (0), iTSS_I: internal starting site (0)

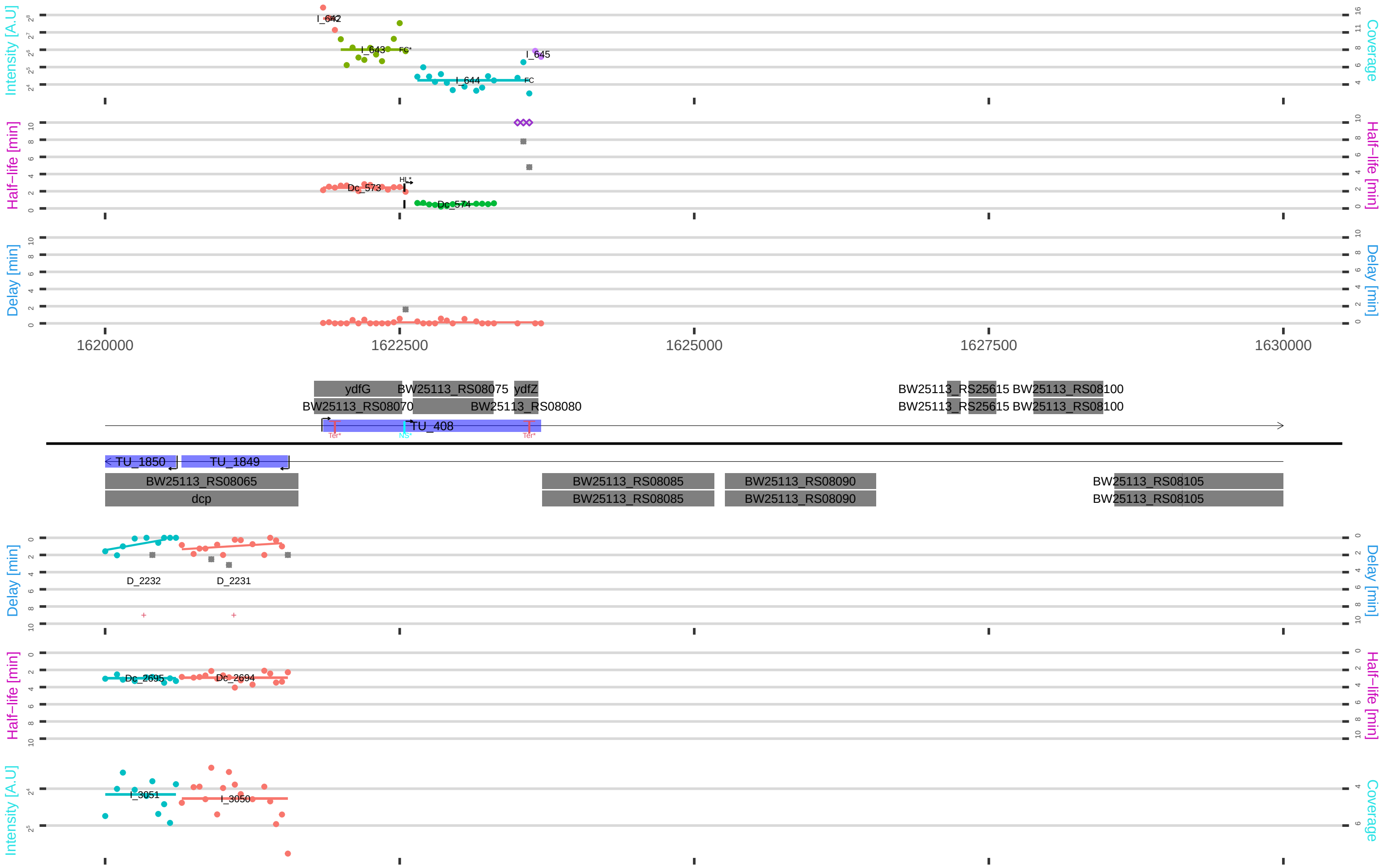

Term: termination (0), NS: new start (0), PS: pausing site (0), iTSS_I: internal starting site (0)

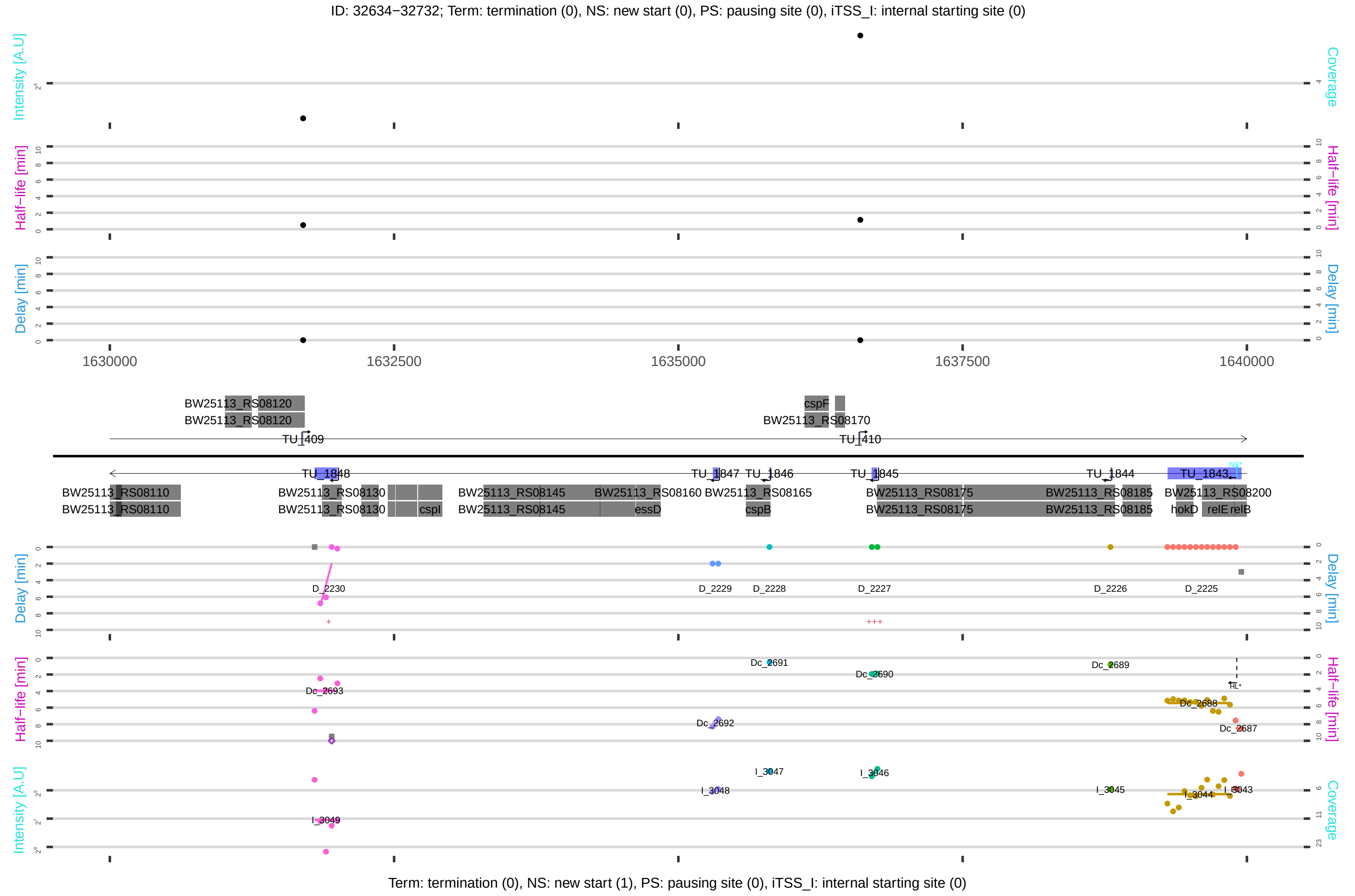

ID: 32634−32732; Term: termination (0), NS: new start (0), PS: pausing site (0), iTSS_I: internal starting site (0)
Intensity [A.U]
Coverage
Half−life [min]
Delay [min]
1630000
1632500
1635000
1637500
1640000
BW25113_RS08120
cspF
BW25113_RS08170
TU_1409
TU_1410
TU_1848
TU_1847
TU_1846
TU_1845
TU_1844
TU_1843
BW25113_RS08110
BW25113_RS08130
cspI
BW25113_RS08145
BW25113_RS08160
BW25113_RS08165
essD
cspB
BW25113_RS08175
BW25113_RS08185
BW25113_RS08200
hokD
relE
relB
D_2230
D_2229
D_2228
D_2227
D_2226
D_2225
Dc_2693
Dc_2692
Dc_2691
Dc_2690
Dc_2689
Dc_2688
Dc_2687
I_3049
I_3048
I_3047
I_3046
I_3045
I_3044
I_3043
Term: termination (0), NS: new start (1), PS: pausing site (0), iTSS_I: internal starting site (0)

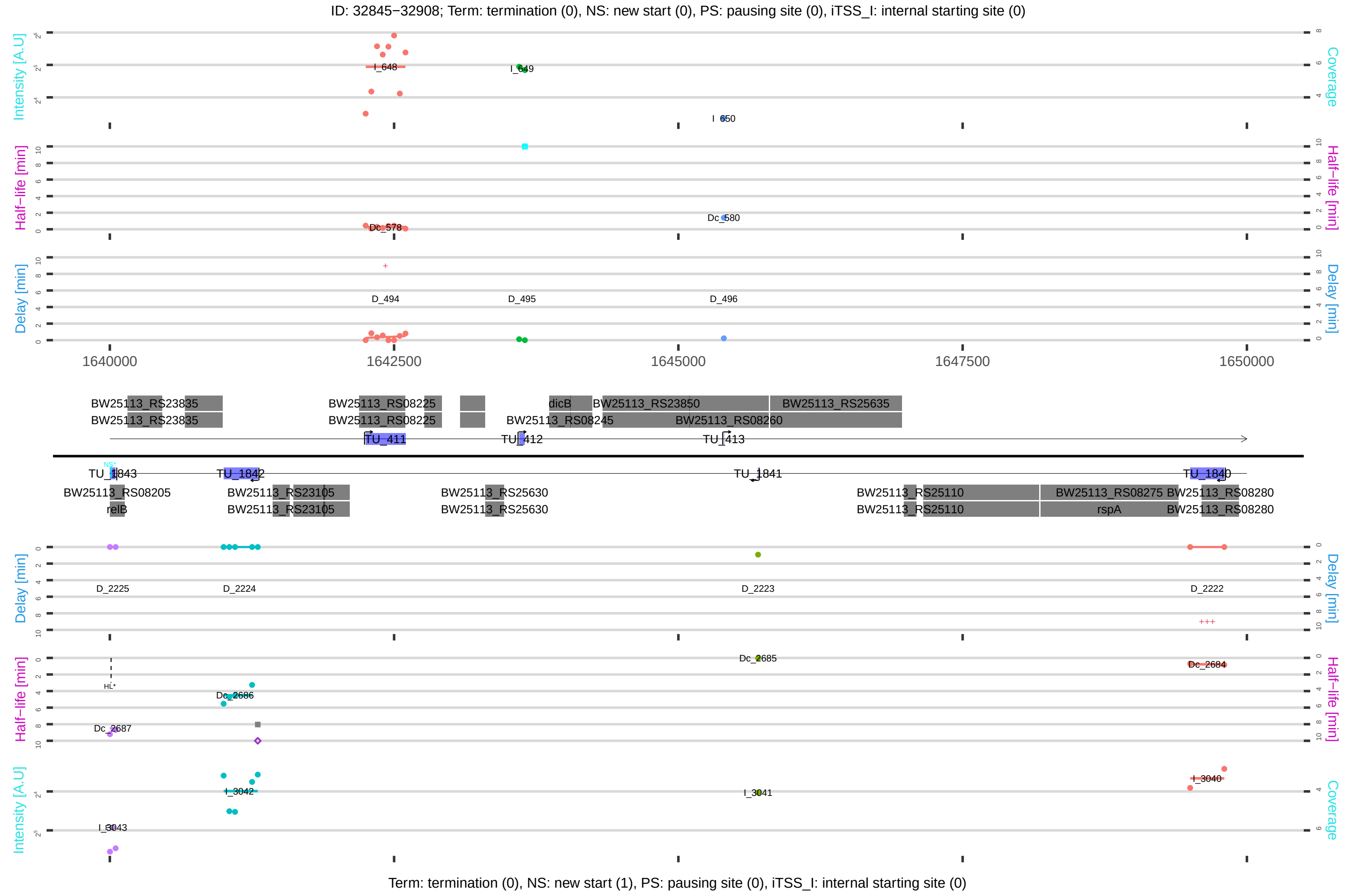

ID: 32845−32908; Term: termination (0), NS: new start (0), PS: pausing site (0), iTSS_I: internal starting site (0)
Intensity [A.U]
Coverage
Half−life [min]
Delay [min]
I_648
I_649
I_650
Dc_578
Dc_580
D_494
D_495
D_496
1640000
1642500
1645000
1647500
1650000
BW25113_RS23835
BW25113_RS08225
dicB
BW25113_RS23850
BW25113_RS25635
BW25113_RS08245
BW25113_RS08260
TU_411
TU_412
TU_413
TU_1843
TU_1842
TU_1841
TU_1840
BW25113_RS08205
relB
BW25113_RS23105
BW25113_RS25630
BW25113_RS25110
BW25113_RS08275
BW25113_RS08280
rspA
D_2225
D_2224
D_2223
D_2222
Dc_2685
Dc_2684
Dc_2686
Dc_2687
I_3040
I_3041
I_3042
I_3043
Term: termination (0), NS: new start (1), PS: pausing site (0), iTSS_I: internal starting site (0)

ID: 33001−33188; Term: termination (0), NS: new start (0), PS: pausing site (0), iTSS_I: internal starting site (0)

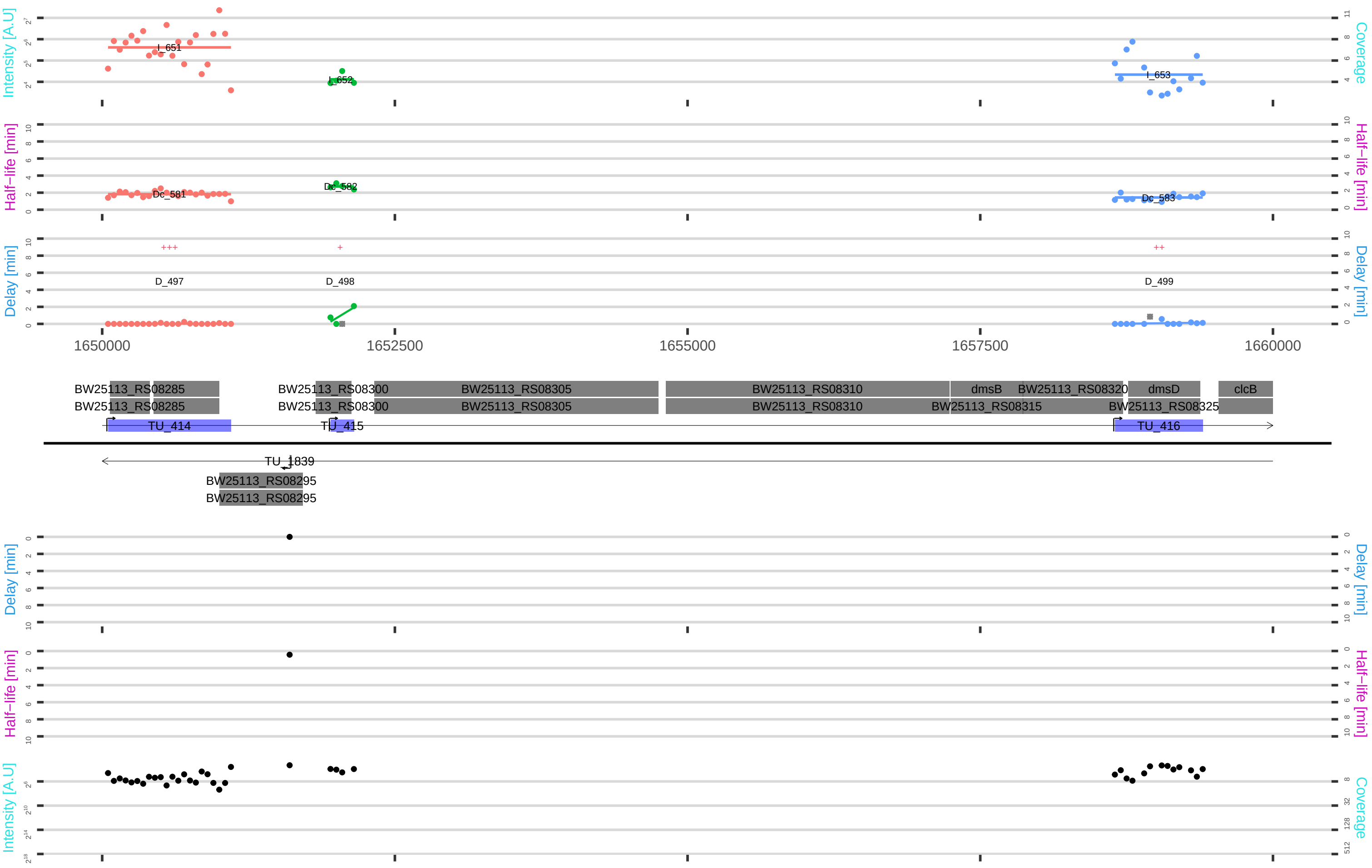

Term: termination (0), NS: new start (0), PS: pausing site (0), iTSS_I: internal starting site (0)

ID: 33300−33329; Term: termination (0), NS: new start (0), PS: pausing site (0), iTSS_I: internal starting site (0)

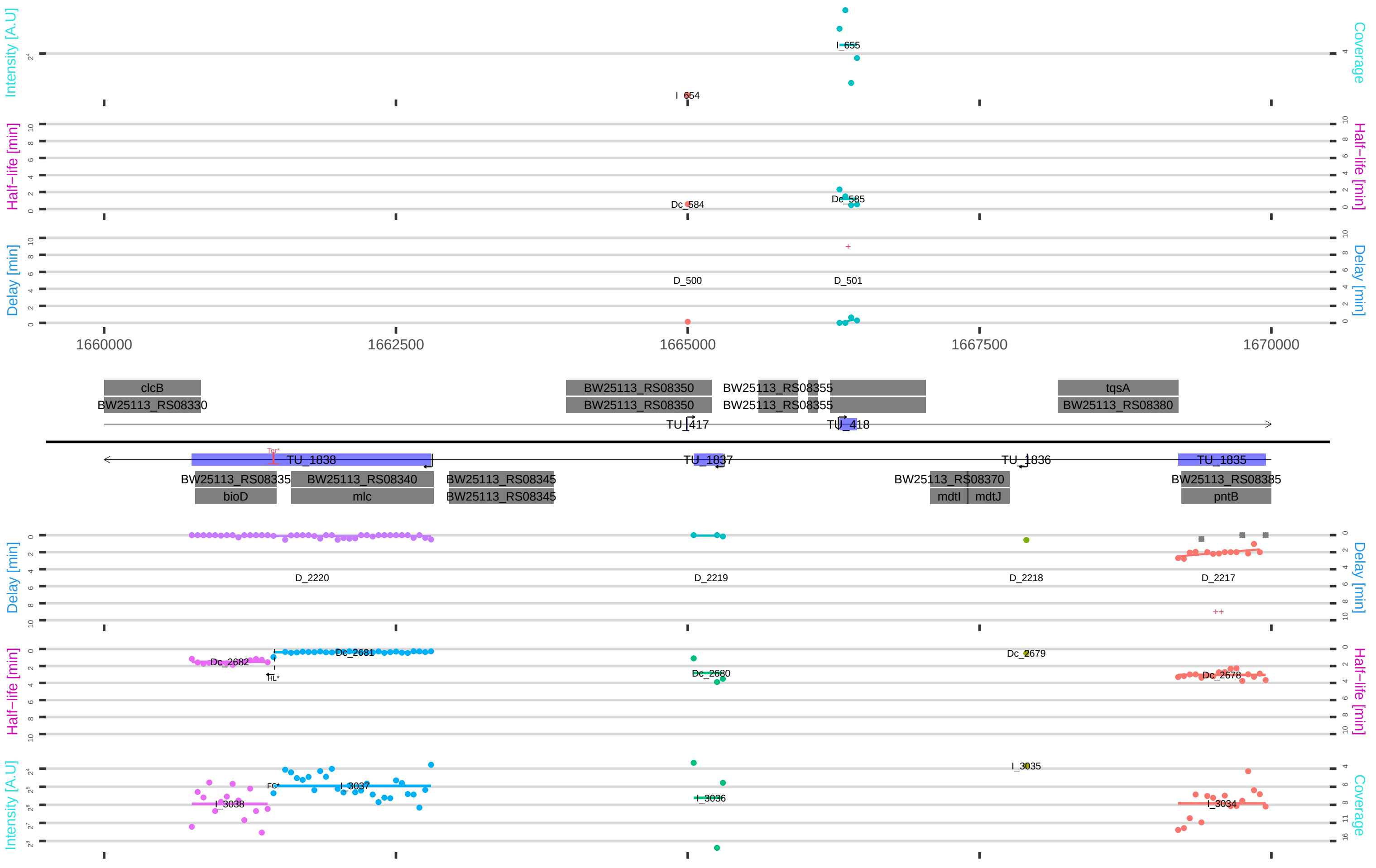

Term: termination (1), NS: new start (0), PS: pausing site (0), iTSS_I: internal starting site (0)

ID: 33454−33585; Term: termination (1), NS: new start (0), PS: pausing site (0), iTSS_I: internal starting site (0)

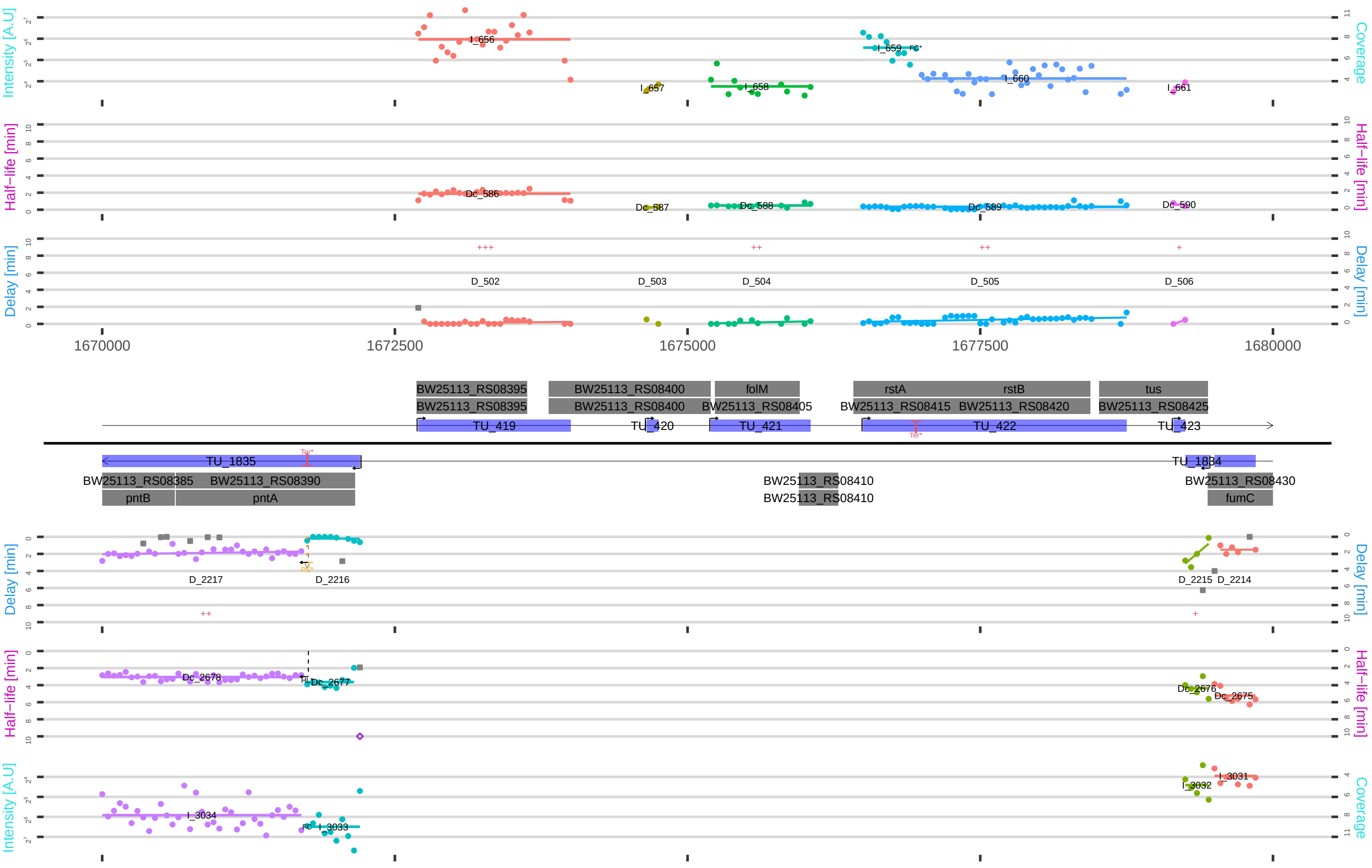

Term: termination (1), NS: new start (0), PS: pausing site (1), iTSS_I: internal starting site (0)

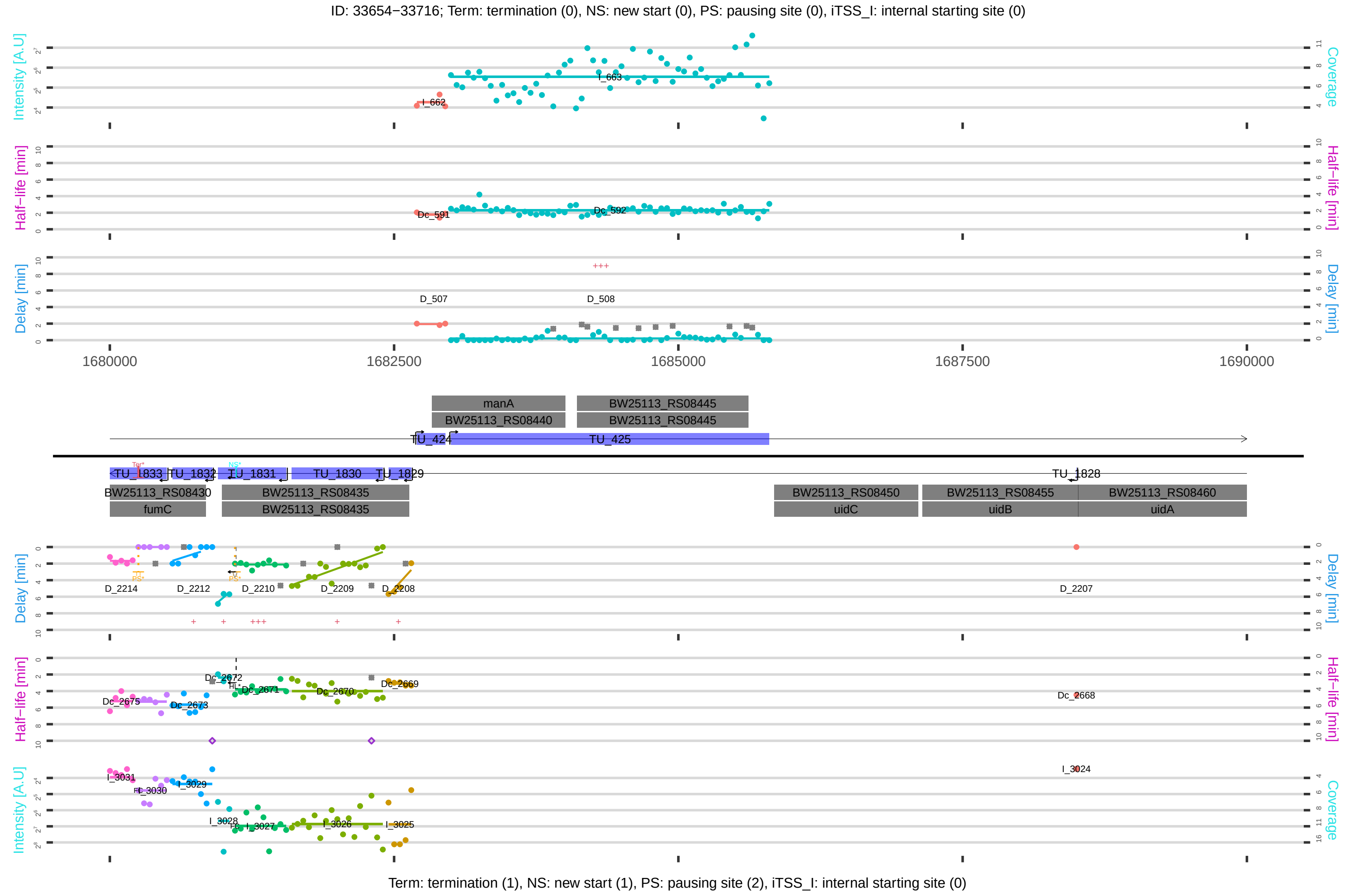

ID: 33654−33716; Term: termination (0), NS: new start (0), PS: pausing site (0), iTSS_I: internal starting site (0)
Intensity [A.U]
Coverage
I_662
I_663
Half−life [min]
Dc_591
Dc_592
Delay [min]
D_507
D_508
1680000
1682500
1685000
1687500
1690000
manA
BW25113_RS08440
BW25113_RS08445
TU_424
TU_425
TU_1833
TU_1832
TU_1831
TU_1830
TU_1829
TU_1828
BW25113_RS08430
fumC
BW25113_RS08435
BW25113_RS08450
uidC
BW25113_RS08455
uidB
BW25113_RS08460
uidA
D_2214
D_2212
D_2210
D_2209
D_2208
D_2207
Dc_2675
Dc_2673
Dc_2672
Dc_2671
Dc_2670
Dc_2669
Dc_2668
I_3031
I_3030
I_3029
I_3028
I_3027
I_3026
I_3025
I_3024
Term: termination (1), NS: new start (1), PS: pausing site (2), iTSS_I: internal starting site (0)

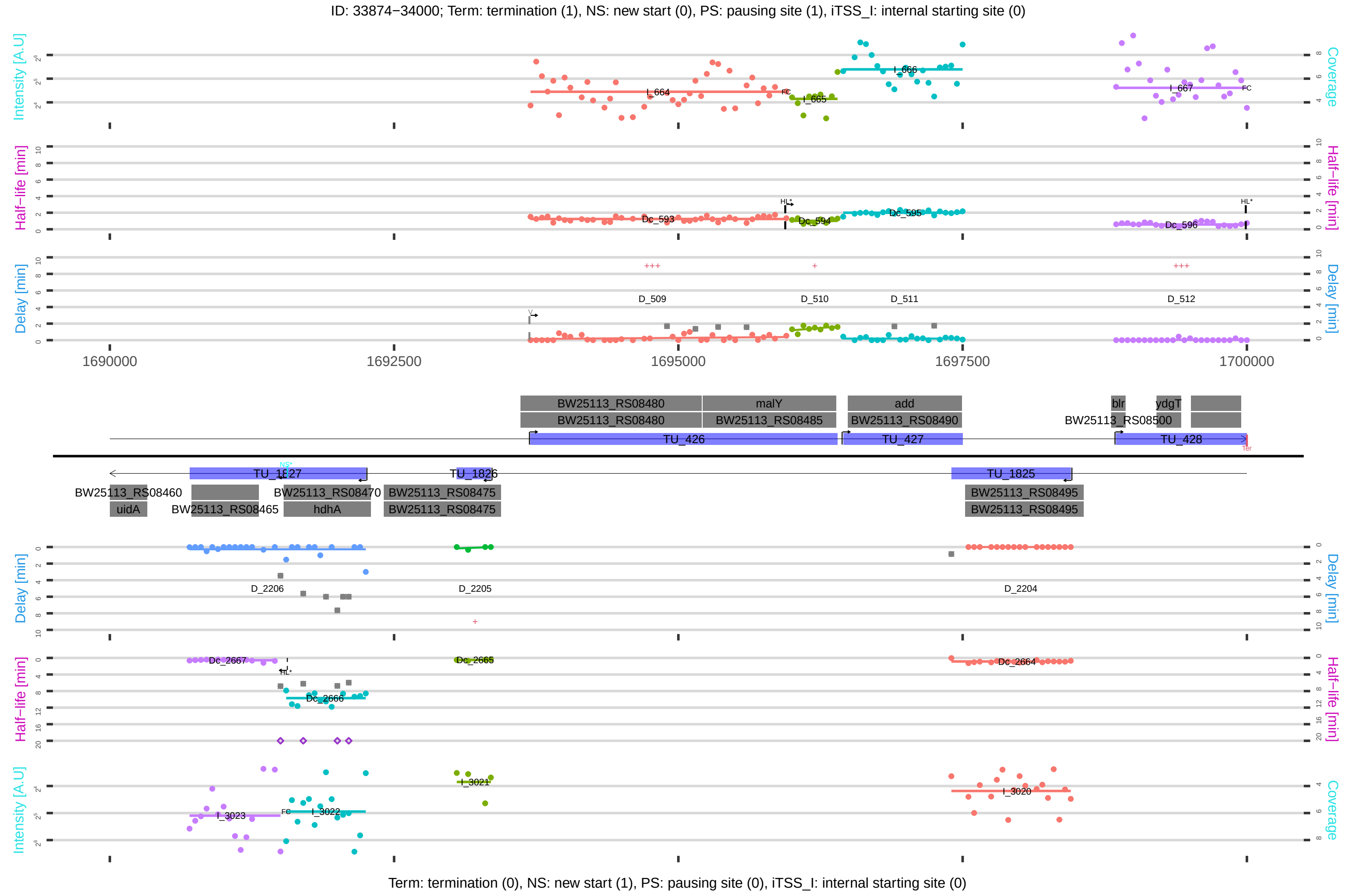

ID: 33874−34000; Term: termination (1), NS: new start (0), PS: pausing site (1), iTSS_I: internal starting site (0)
Intensity [A.U]
Coverage
Half−life [min]
Delay [min]
I_664
I_665
I_666
I_667
Dc_593
Dc_594
Dc_595
Dc_596
D_509
D_510
D_511
D_512
1690000
1692500
1695000
1697500
1700000
BW25113_RS08480
malY
BW25113_RS08485
add
BW25113_RS08490
blr
ydgT
BW25113_RS08500
TU_426
TU_427
TU_428
TU_1827
TU_1826
TU_1825
BW25113_RS08460
uidA
BW25113_RS08465
BW25113_RS08470
hdhA
BW25113_RS08475
BW25113_RS08495
D_2206
D_2205
D_2204
Dc_2667
Dc_2666
Dc_2665
Dc_2664
I_3023
I_3022
I_3021
I_3020
Term: termination (0), NS: new start (1), PS: pausing site (0), iTSS_I: internal starting site (0)

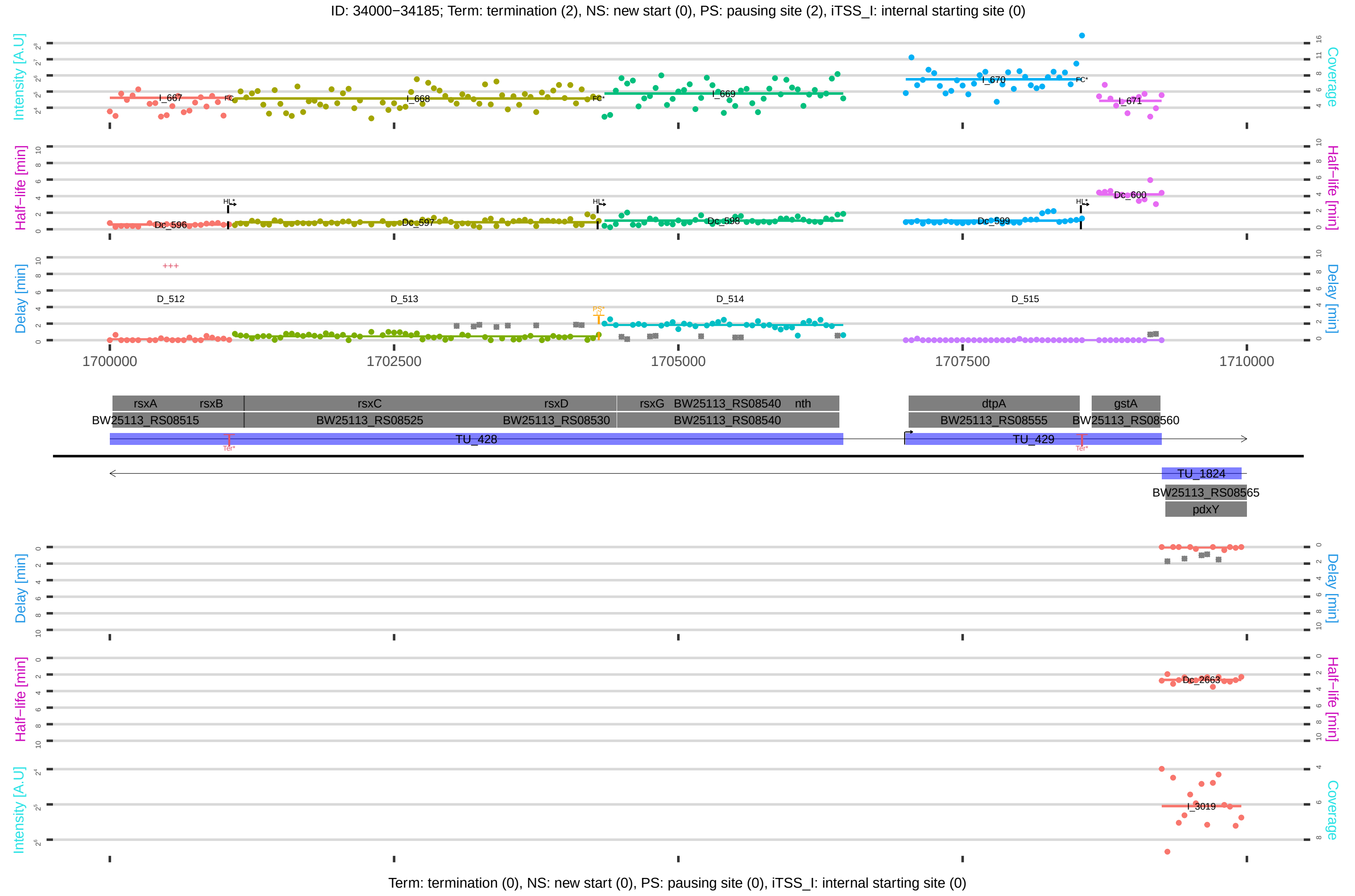

ID: 34000−34185; Term: termination (2), NS: new start (0), PS: pausing site (2), iTSS_I: internal starting site (0)
Intensity [A.U]
Coverage
I_667
I_668
I_669
I_670
I_671
Half−life [min]
Dc_596
Dc_597
Dc_598
Dc_599
Dc_600
Delay [min]
D_512
D_513
D_514
D_515
1700000
1702500
1705000
1707500
1710000
rsxA
rsxB
rsxC
rsxD
rsxG
BW25113_RS08540
nth
dtpA
gstA
BW25113_RS08515
BW25113_RS08525
BW25113_RS08530
BW25113_RS08540
BW25113_RS08555
BW25113_RS08560
TU_428
TU_429
TU_1824
BW25113_RS08565
pdxY
Dc_2663
I_3019
Term: termination (0), NS: new start (0), PS: pausing site (0), iTSS_I: internal starting site (0)

ID: 34282−34333; Term: termination (1), NS: new start (0), PS: pausing site (0), iTSS_I: internal starting site (0)

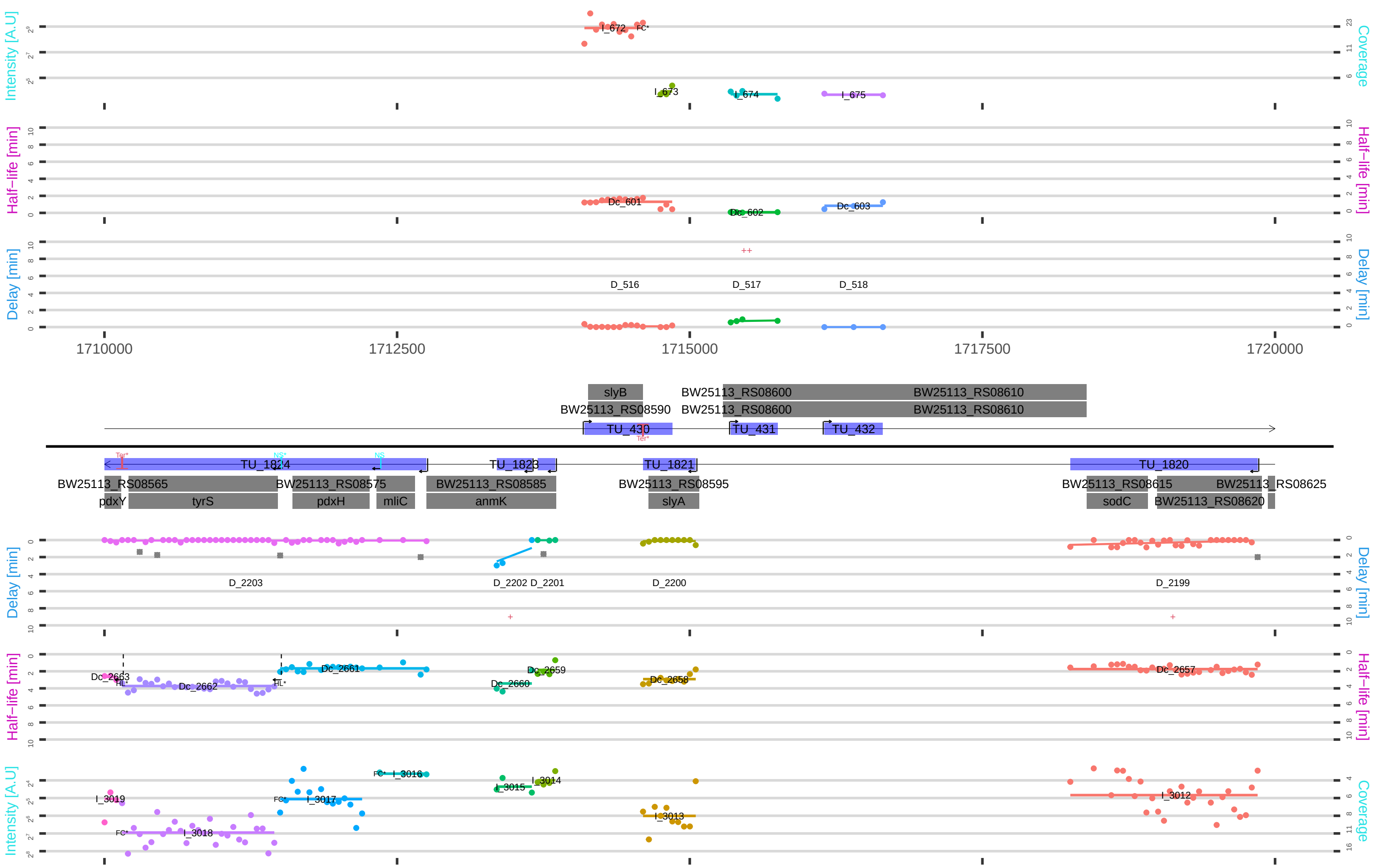

Term: termination (1), NS: new start (2), PS: pausing site (0), iTSS_I: internal starting site (0)

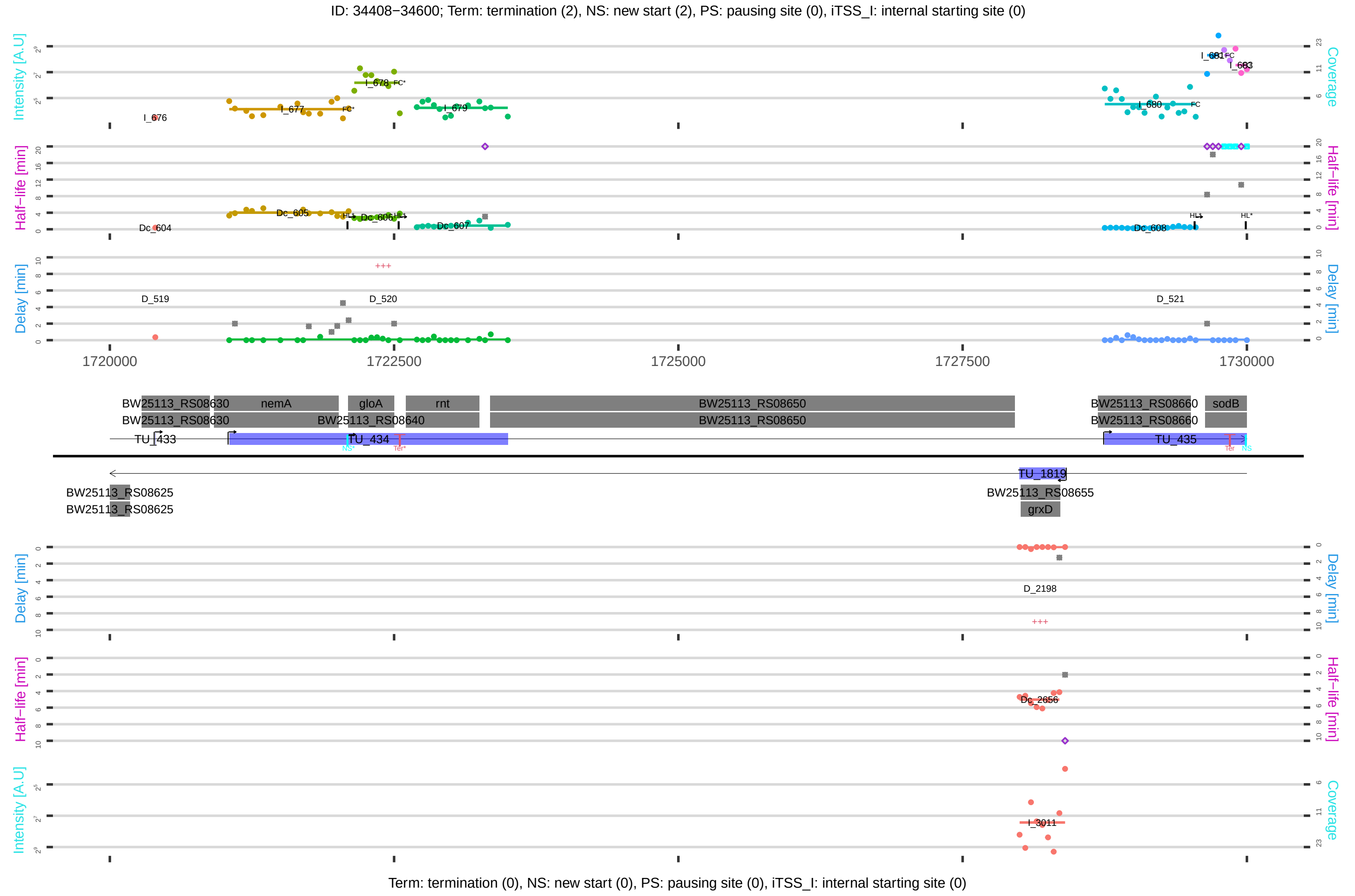

ID: 34408−34600; Term: termination (2), NS: new start (2), PS: pausing site (0), iTSS_I: internal starting site (0)
Intensity [A.U]
Coverage
I_676
I_677
I_678
I_679
I_680
I_681
I_683
Half−life [min]
Dc_604
Dc_605
Dc_606
Dc_607
Dc_608
Delay [min]
D_519
D_520
D_521
1720000
1722500
1725000
1727500
1730000
BW25113_RS08630
nemA
gloA
rnt
BW25113_RS08650
BW25113_RS08660
sodB
BW25113_RS08640
TU_433
TU_434
TU_435
TU_1819
BW25113_RS08625
BW25113_RS08655
grxD
D_2198
Dc_2656
I_3011
Term: termination (0), NS: new start (0), PS: pausing site (0), iTSS_I: internal starting site (0)

ID: 34600−34782; Term: termination (1), NS: new start (2), PS: pausing site (0), iTSS_I: internal starting site (0)

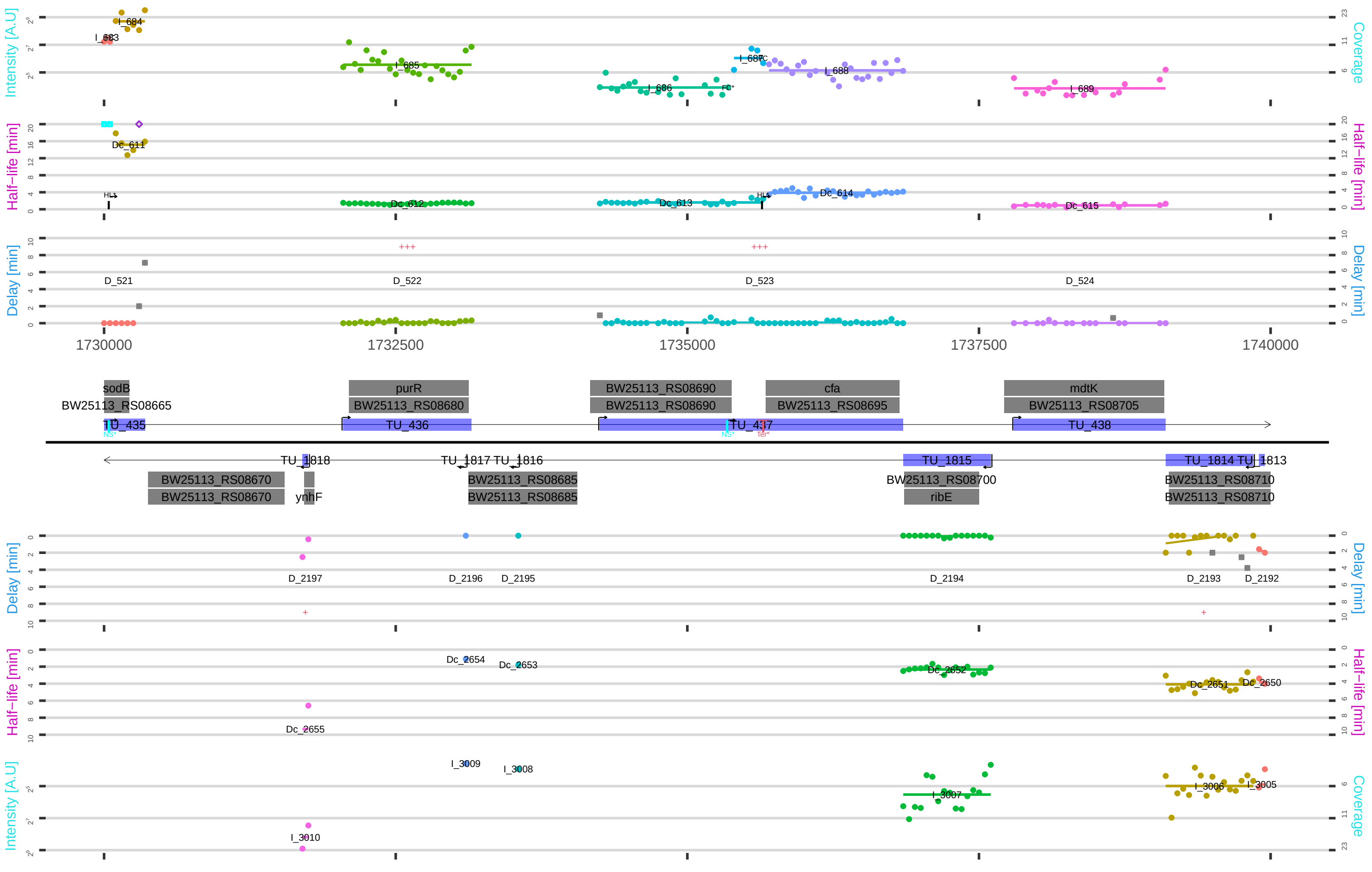

Term: termination (0), NS: new start (0), PS: pausing site (0), iTSS_I: internal starting site (0)

ID: 34815−35000; Term: termination (0), NS: new start (0), PS: pausing site (0), iTSS_I: internal starting site (0)

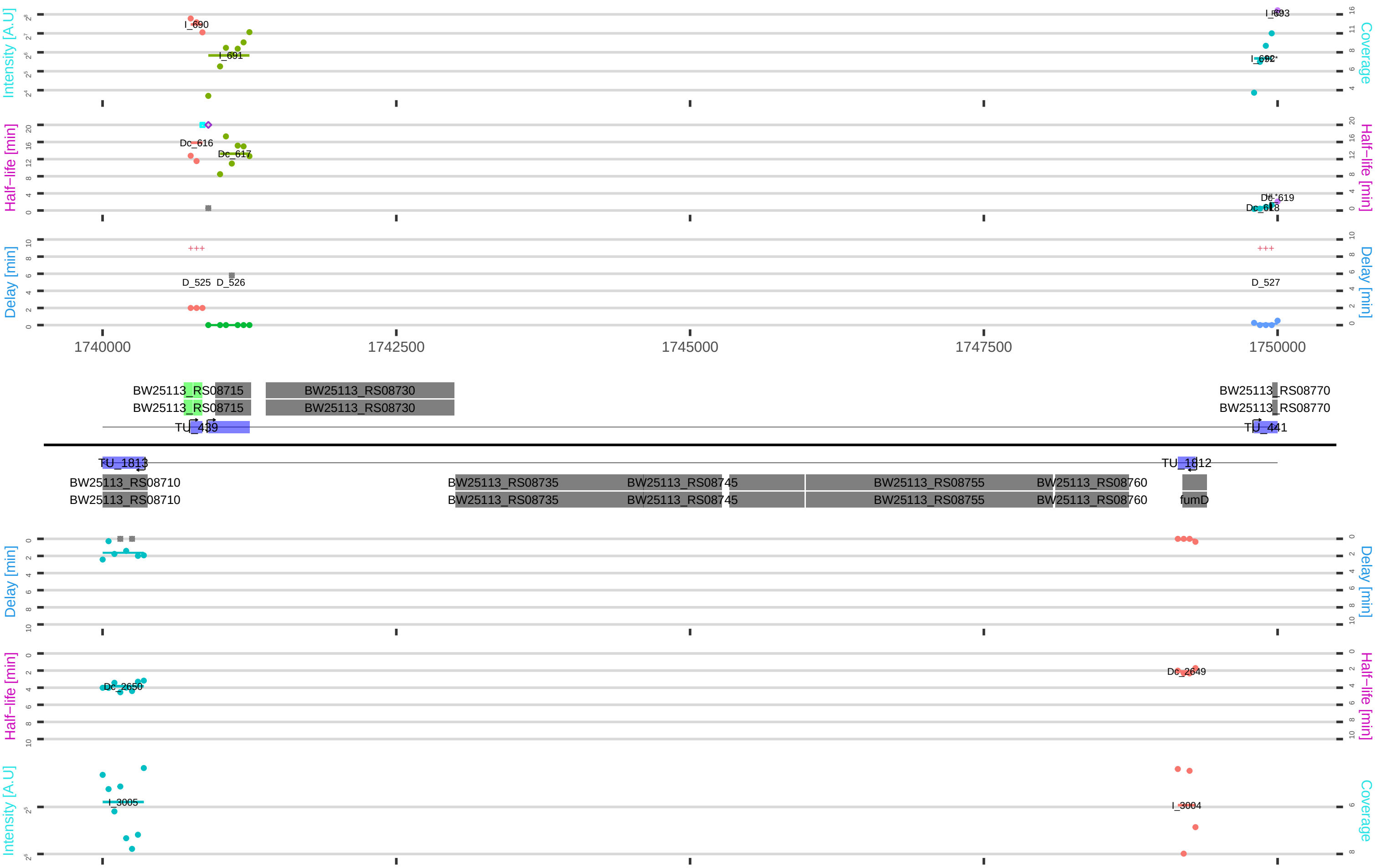

Term: termination (0), NS: new start (0), PS: pausing site (0), iTSS_I: internal starting site (0)

ID: 35000−35039; Term: termination (0), NS: new start (0), PS: pausing site (0), iTSS_I: internal starting site (0)

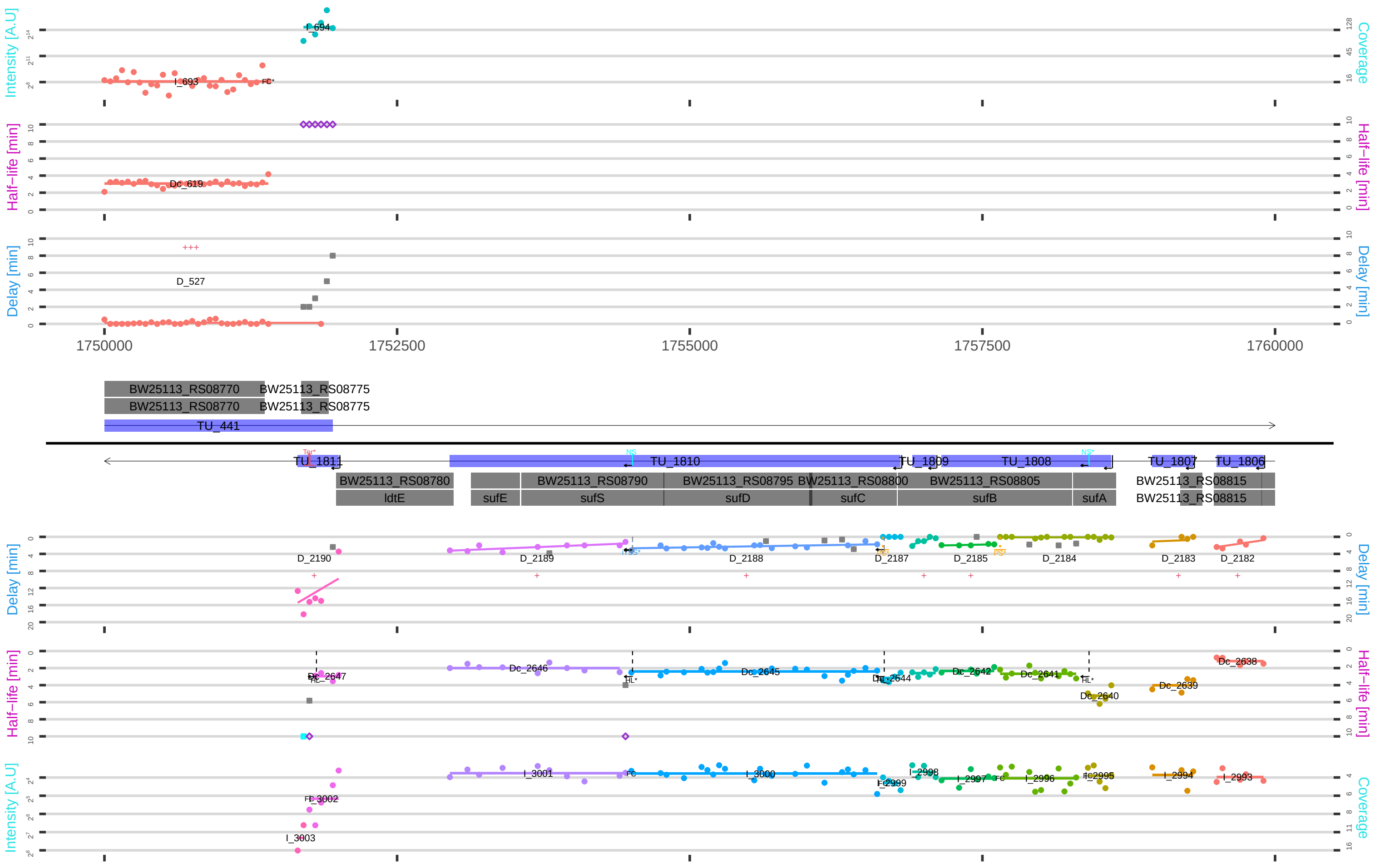

Term: termination (1), NS: new start (2), PS: pausing site (2), iTSS_I: internal starting site (1)

ID: 35267−35394; Term: termination (0), NS: new start (0), PS: pausing site (0), iTSS_I: internal starting site (0)

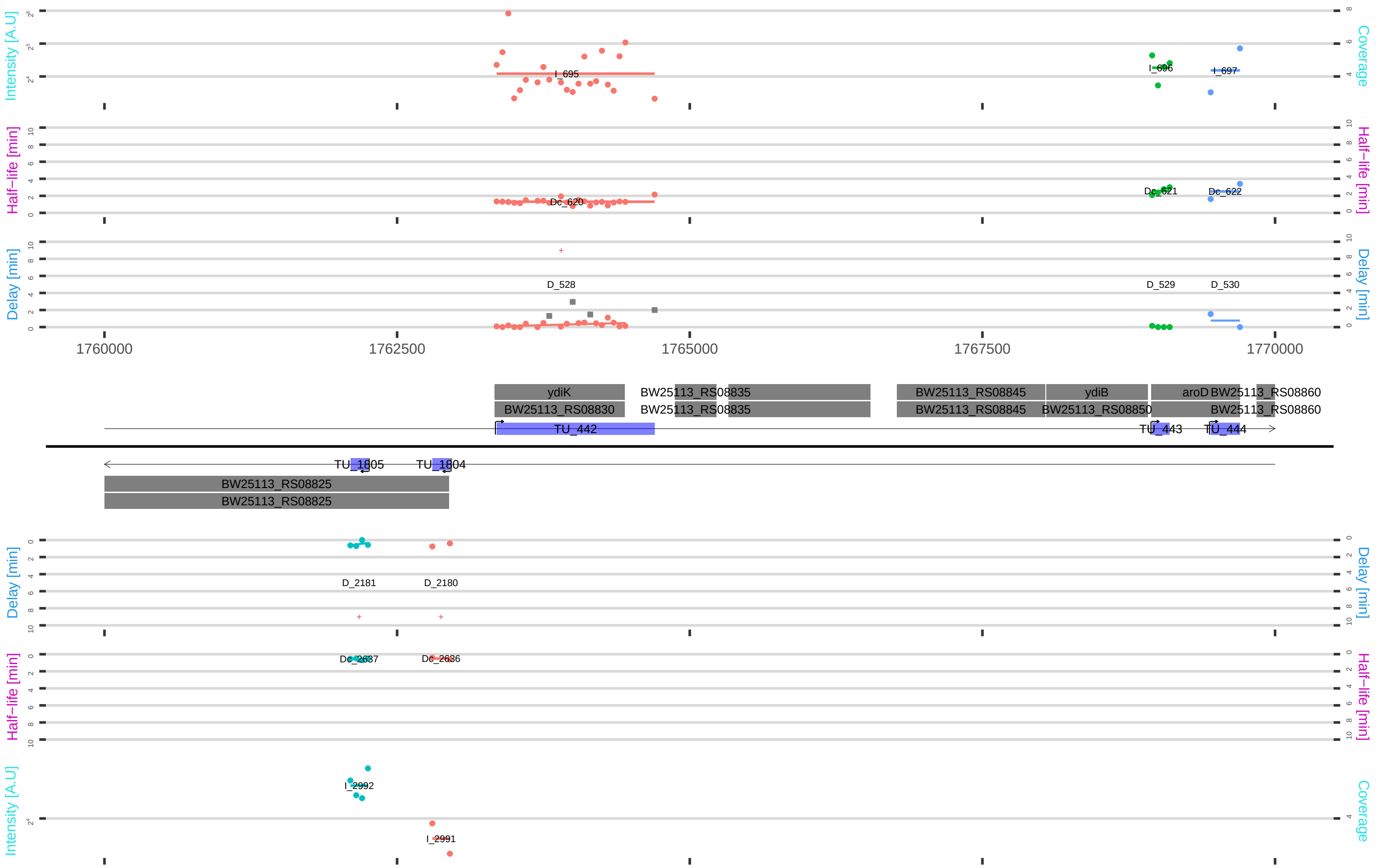

Term: termination (0), NS: new start (0), PS: pausing site (0), iTSS_I: internal starting site (0)

ID: 149680−149680; FC*: significant t−test of two consecutive segments; Term: termination, NS: new start, PS: pausing site,iTSS_I: internal starting site,TI: transcription interferance.

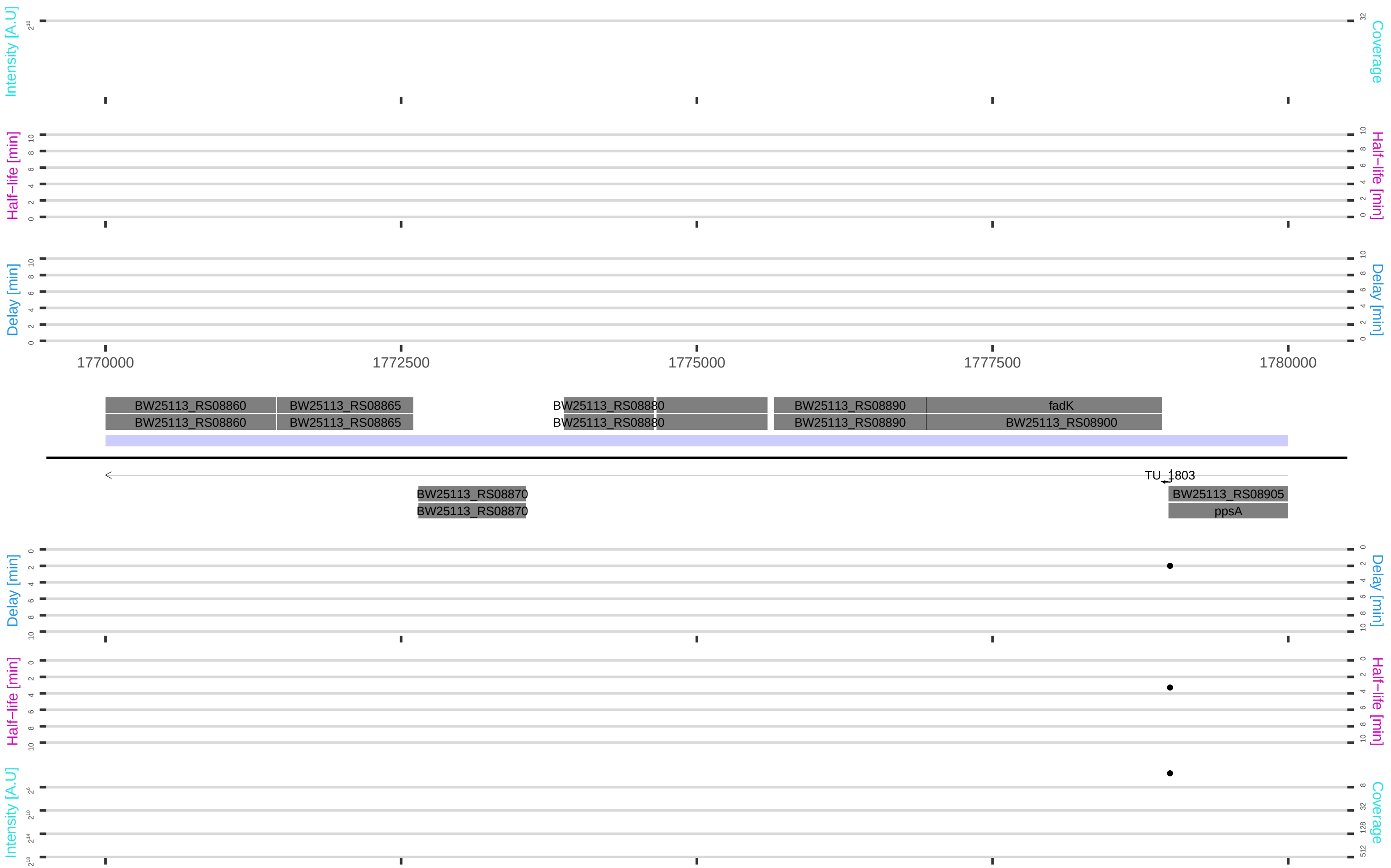

Term: termination (0), NS: new start (0), PS: pausing site (0), iTSS_I: internal starting site (0)

ID: 35636−35799; Term: termination (0), NS: new start (0), PS: pausing site (0), iTSS_I: internal starting site (0)

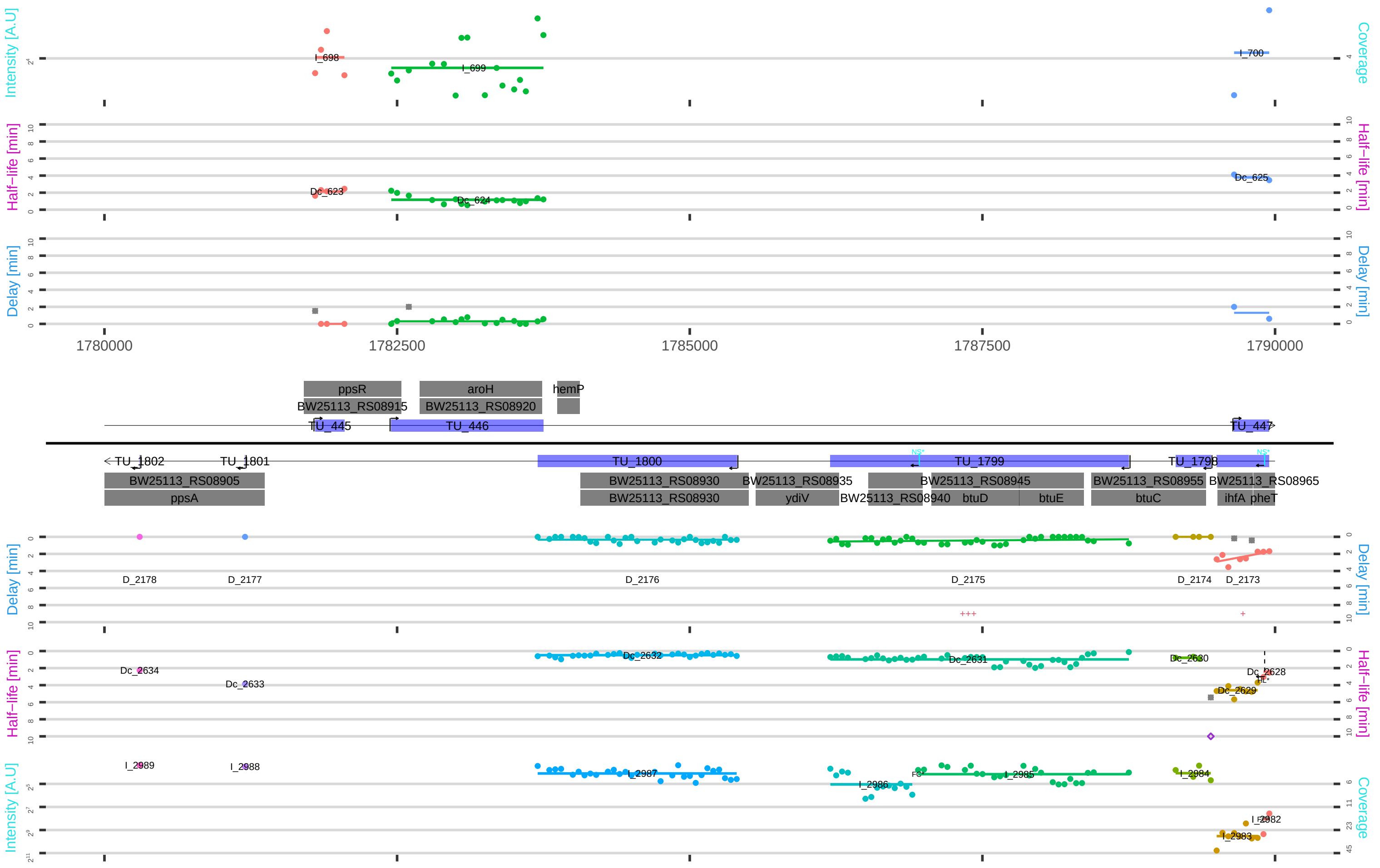

Term: termination (0), NS: new start (2), PS: pausing site (0), iTSS_I: internal starting site (0)

ID: 35877−35901; Term: termination (0), NS: new start (0), PS: pausing site (0), iTSS_I: internal starting site (0)

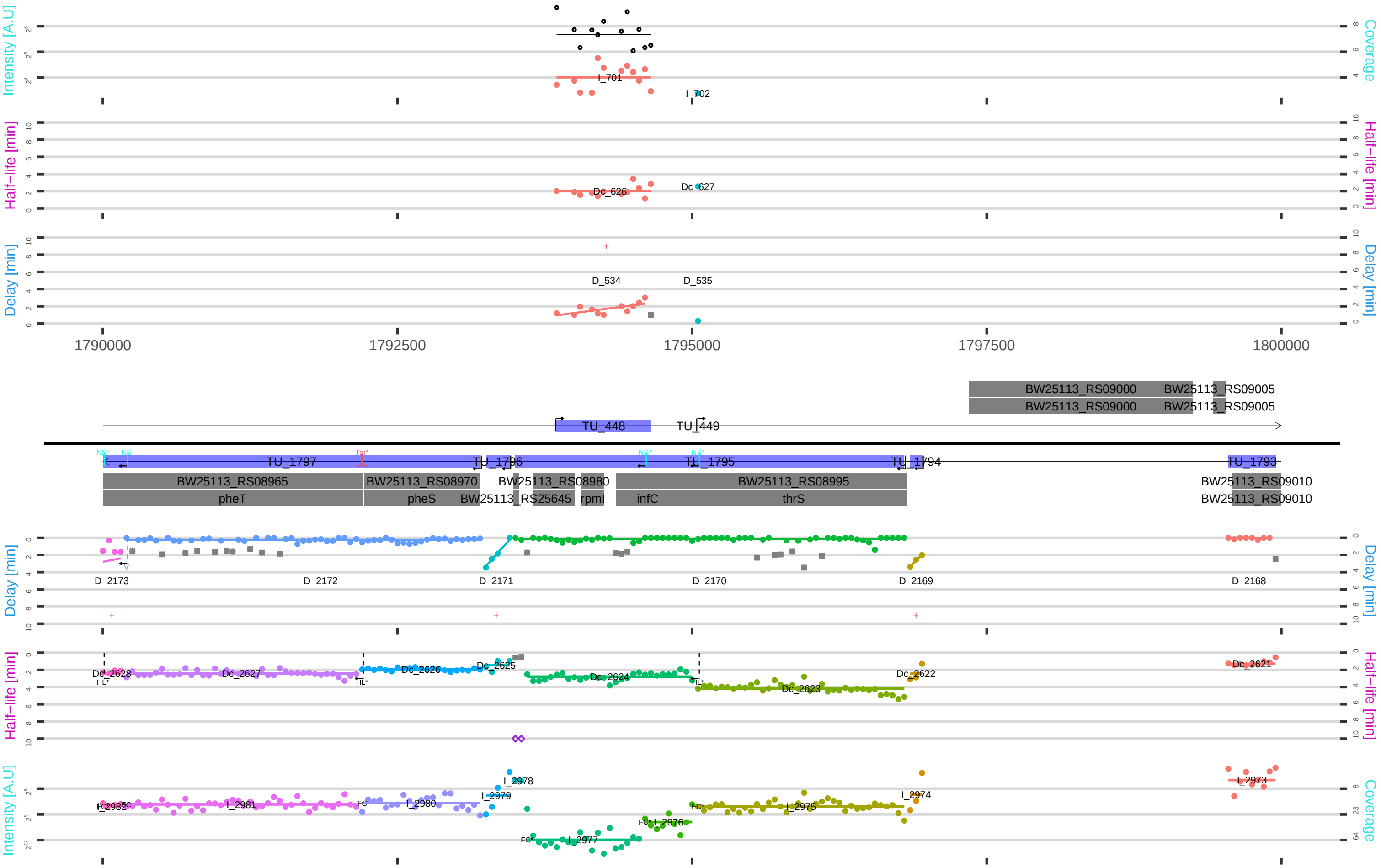

Term: termination (1), NS: new start (4), PS: pausing site (1), iTSS_I: internal starting site (0)

ID: 36014−36135; Term: termination (1), NS: new start (1), PS: pausing site (0), iTSS_I: internal starting site (0)

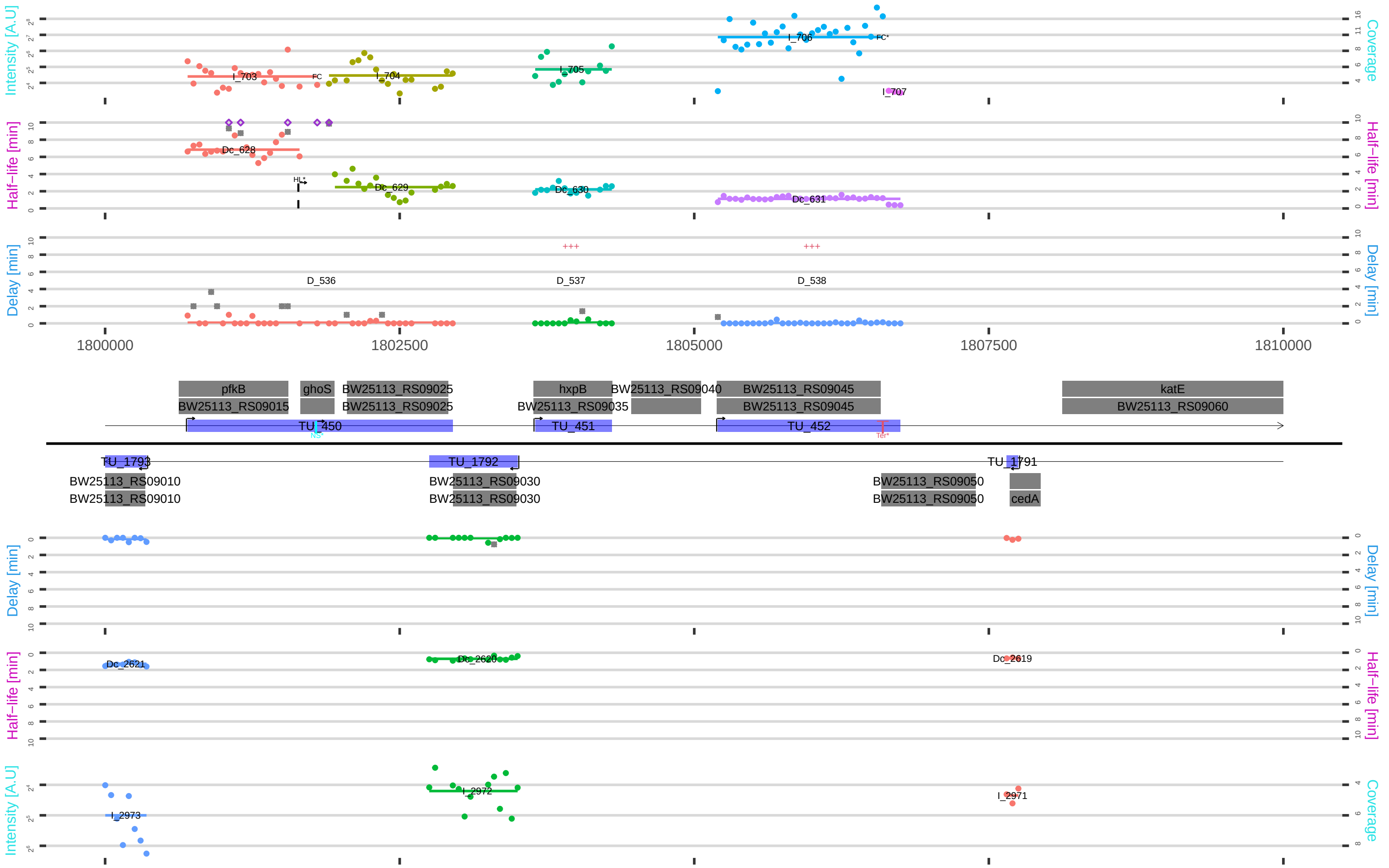

Term: termination (0), NS: new start (0), PS: pausing site (0), iTSS_I: internal starting site (0)

ID: 36336−36354; Term: termination (0), NS: new start (0), PS: pausing site (0), iTSS_I: internal starting site (0)

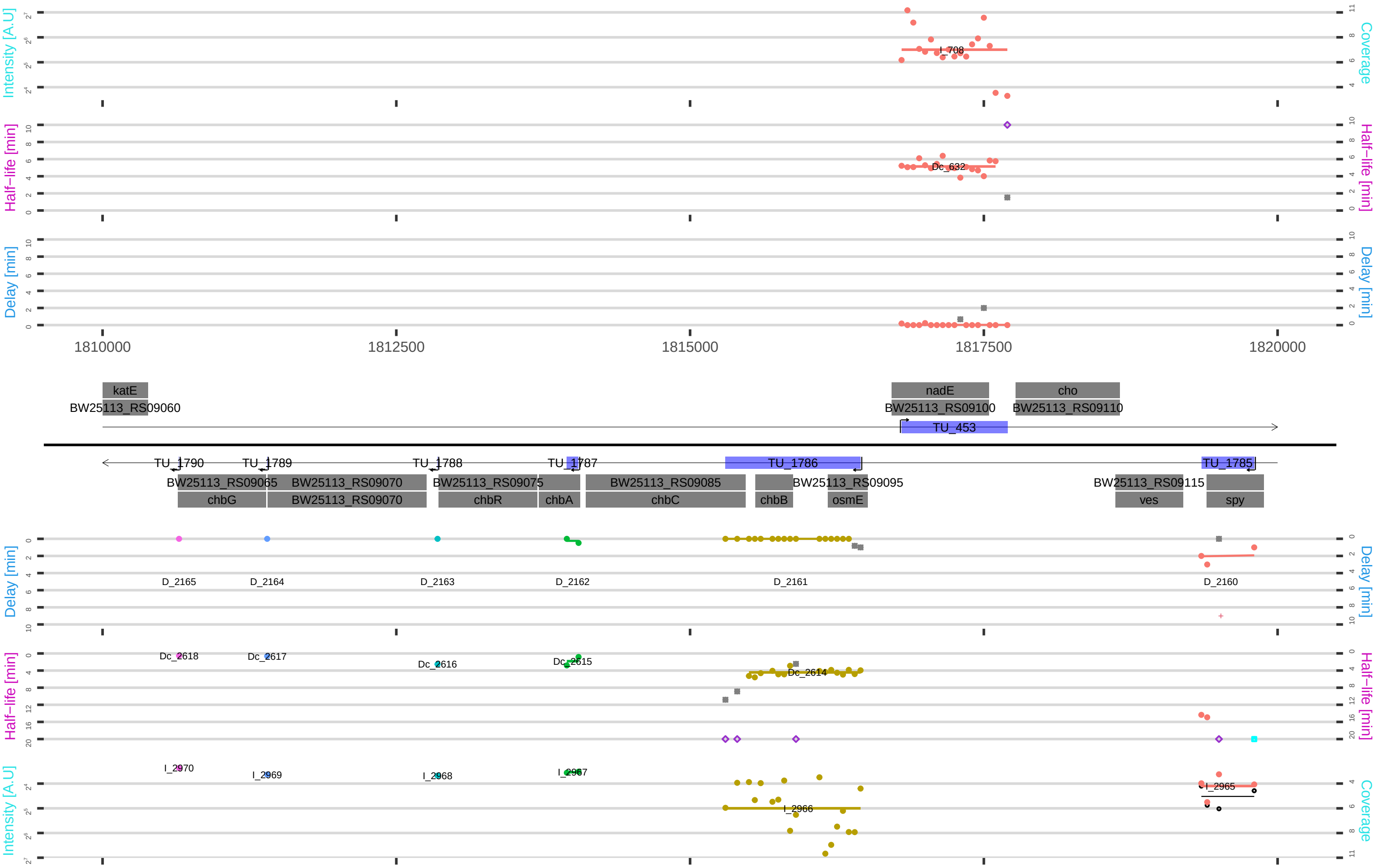

Term: termination (0), NS: new start (0), PS: pausing site (0), iTSS_I: internal starting site (0)

ID: 36535−36598; Term: termination (0), NS: new start (0), PS: pausing site (0), iTSS_I: internal starting site (0)

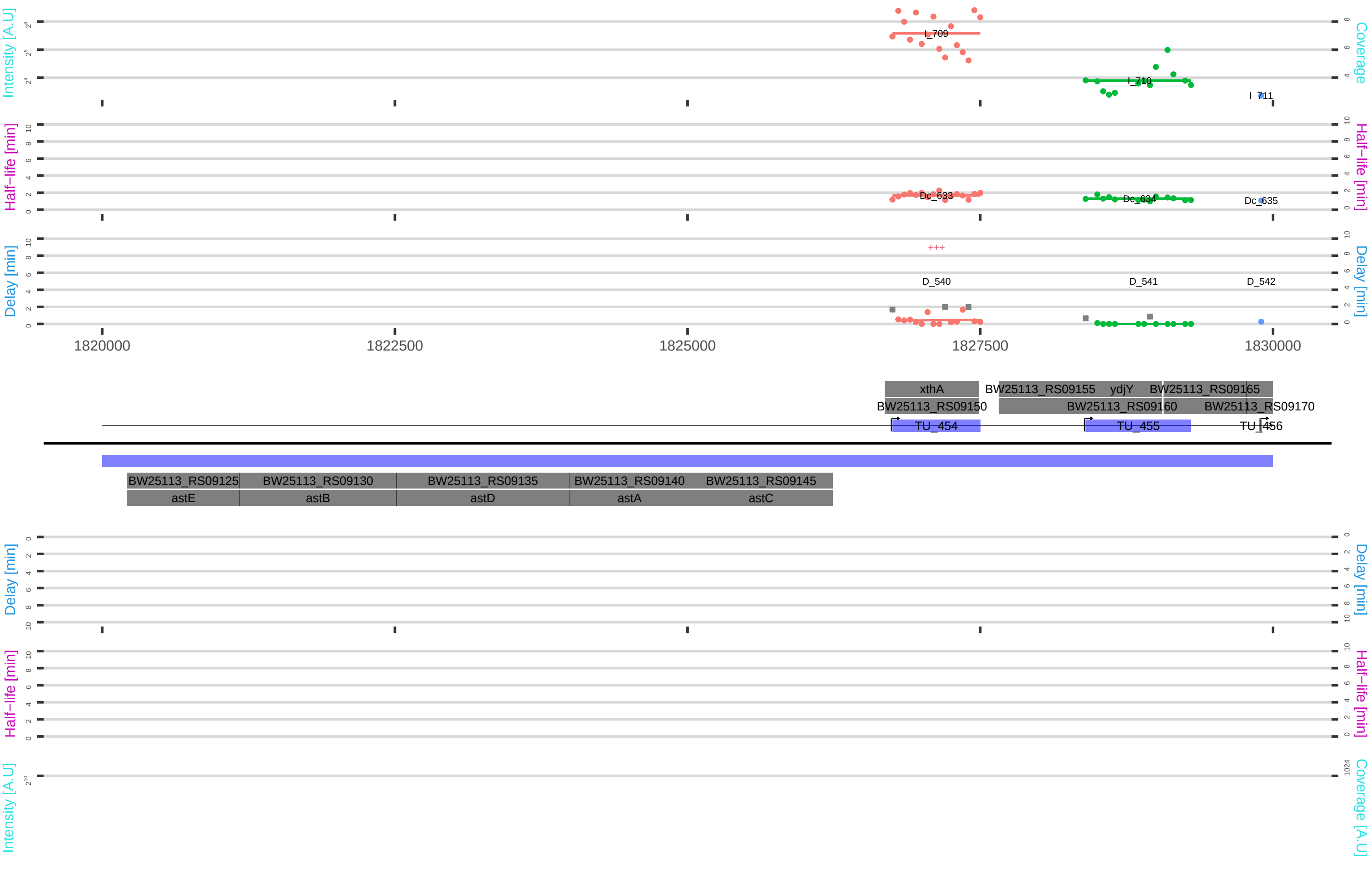

NA

ID: 36605−36760; Term: termination (0), NS: new start (0), PS: pausing site (0), iTSS_I: internal starting site (0)

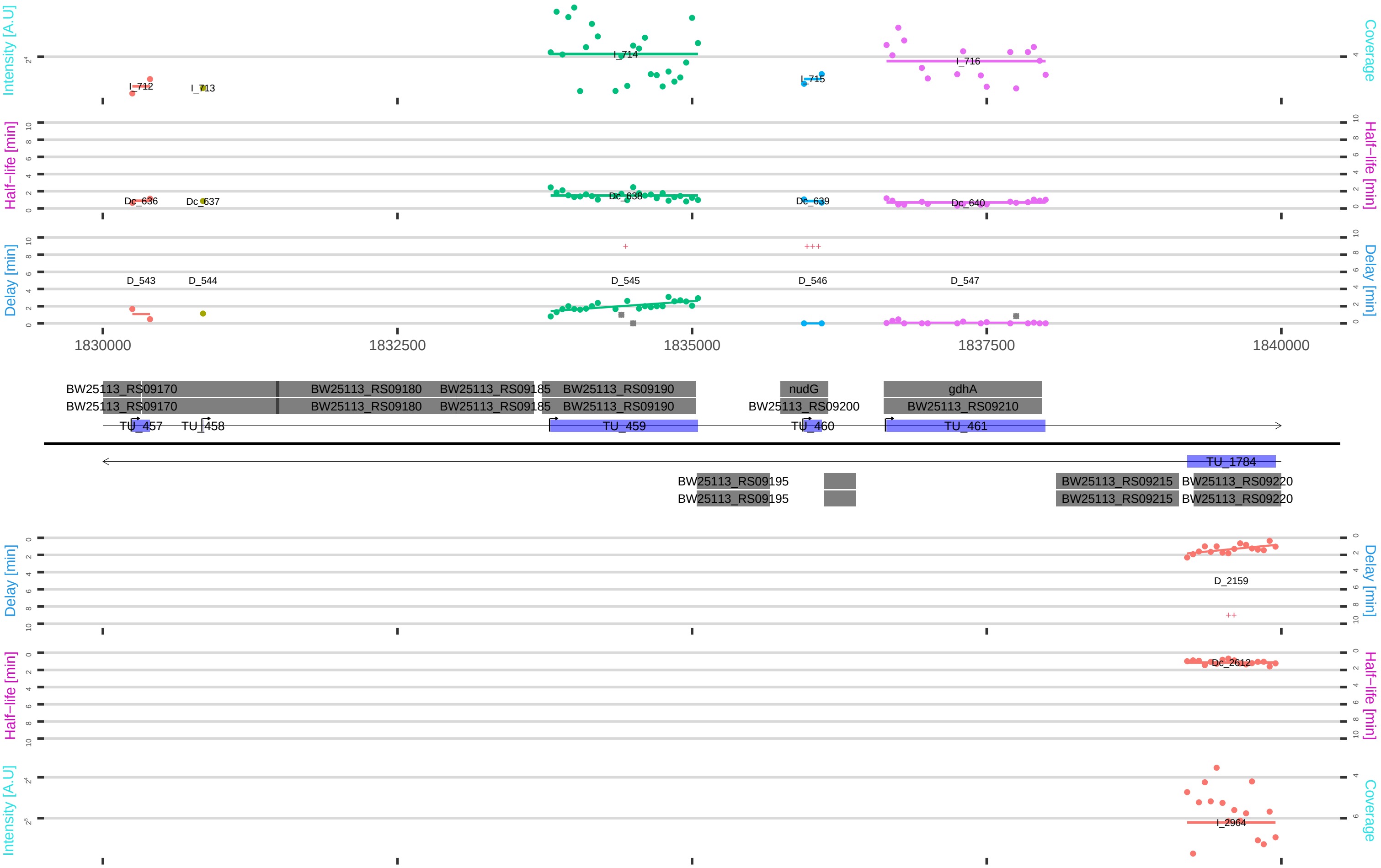

Term: termination (0), NS: new start (0), PS: pausing site (0), iTSS_I: internal starting site (0)

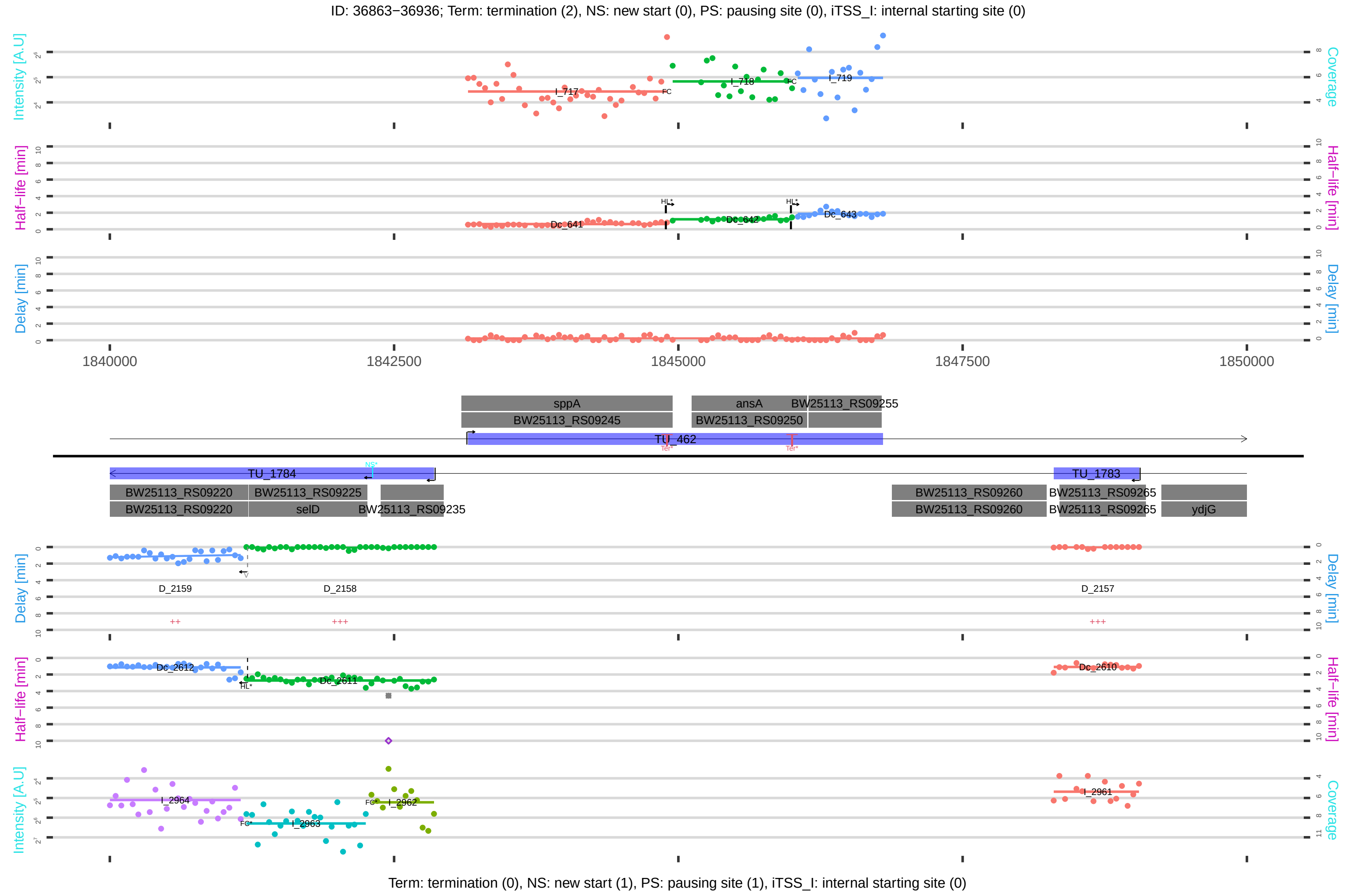
ID: 36863−36936; Term: termination (2), NS: new start (0), PS: pausing site (0), iTSS_I: internal starting site (0)
Intensity [A.U]
Coverage
Half−life [min]
Delay [min]
I_717
I_718
I_719
Dc_641
Dc_642
Dc_643
1840000
1842500
1845000
1847500
1850000
sppA
BW25113_RS09245
ansA
BW25113_RS09250
BW25113_RS09255
TU_462
TU_1784
TU_1783
BW25113_RS09220
BW25113_RS09225
selD
BW25113_RS09235
BW25113_RS09260
BW25113_RS09265
ydjG
D_2159
D_2158
D_2157
Dc_2612
Dc_2611
Dc_2610
I_2964
I_2963
I_2962
I_2961
Term: termination (0), NS: new start (1), PS: pausing site (1), iTSS_I: internal starting site (0)

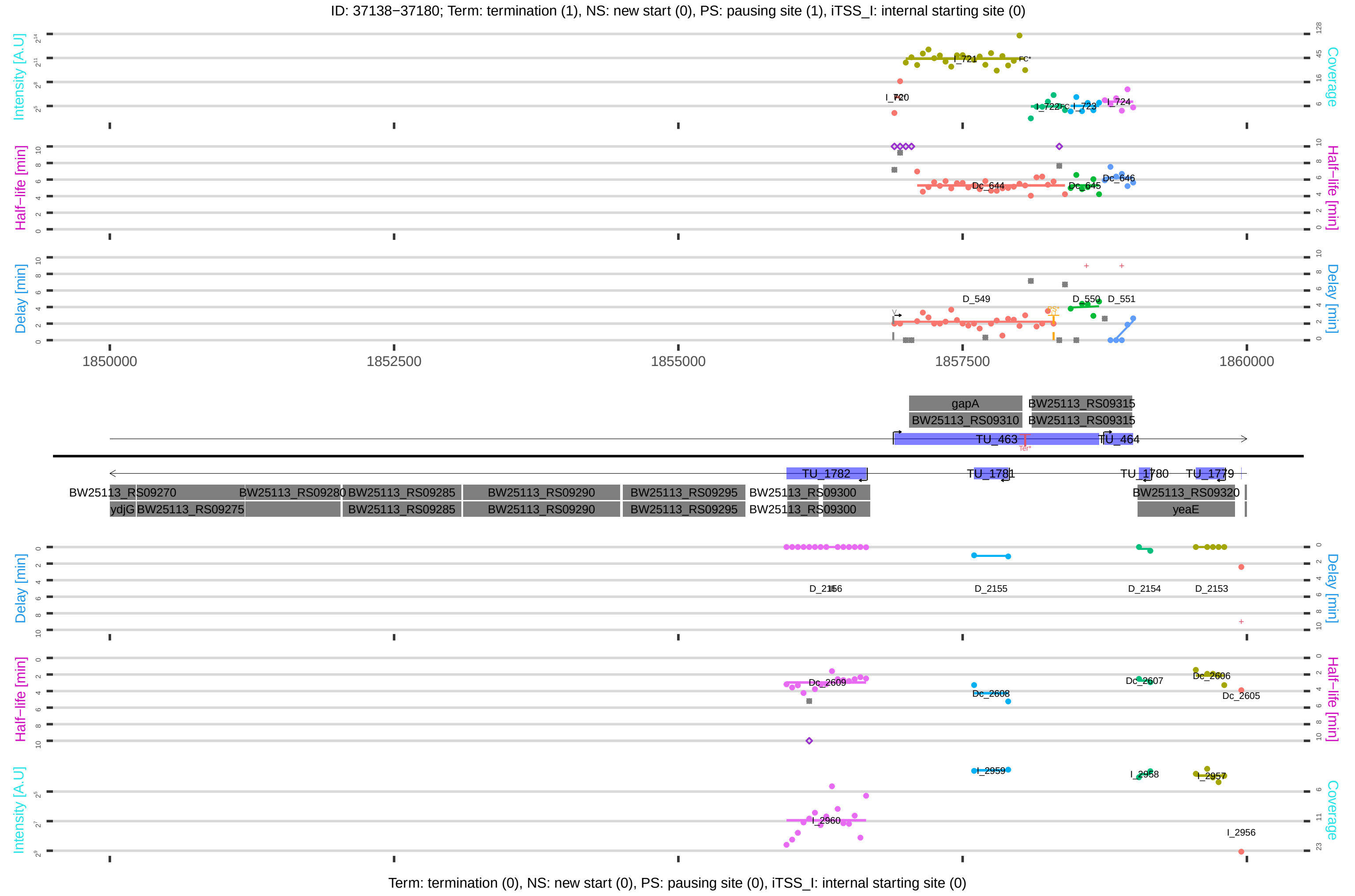

ID: 37138−37180; Term: termination (1), NS: new start (0), PS: pausing site (1), iTSS_I: internal starting site (0)
Intensity [A.U]
Coverage
Half−life [min]
Delay [min]
1850000
1852500
1855000
1857500
1860000
I_720
I_721
I_722
I_723
I_724
Dc_644
Dc_645
Dc_646
D_549
D_550
D_551
gapA
BW25113_RS09310
BW25113_RS09315
TU_463
TU_464
TU_1782
TU_1781
TU_1780
TU_1779
BW25113_RS09270
BW25113_RS09275
BW25113_RS09280
BW25113_RS09285
BW25113_RS09290
BW25113_RS09295
BW25113_RS09300
BW25113_RS09320
ydjG
yeaE
D_2156
D_2155
D_2154
D_2153
Dc_2609
Dc_2608
Dc_2607
Dc_2606
Dc_2605
I_2960
I_2959
I_2958
I_2957
I_2956
Term: termination (0), NS: new start (0), PS: pausing site (0), iTSS_I: internal starting site (0)

ID: 37227−37374; Term: termination (1), NS: new start (0), PS: pausing site (0), iTSS_I: internal starting site (0)

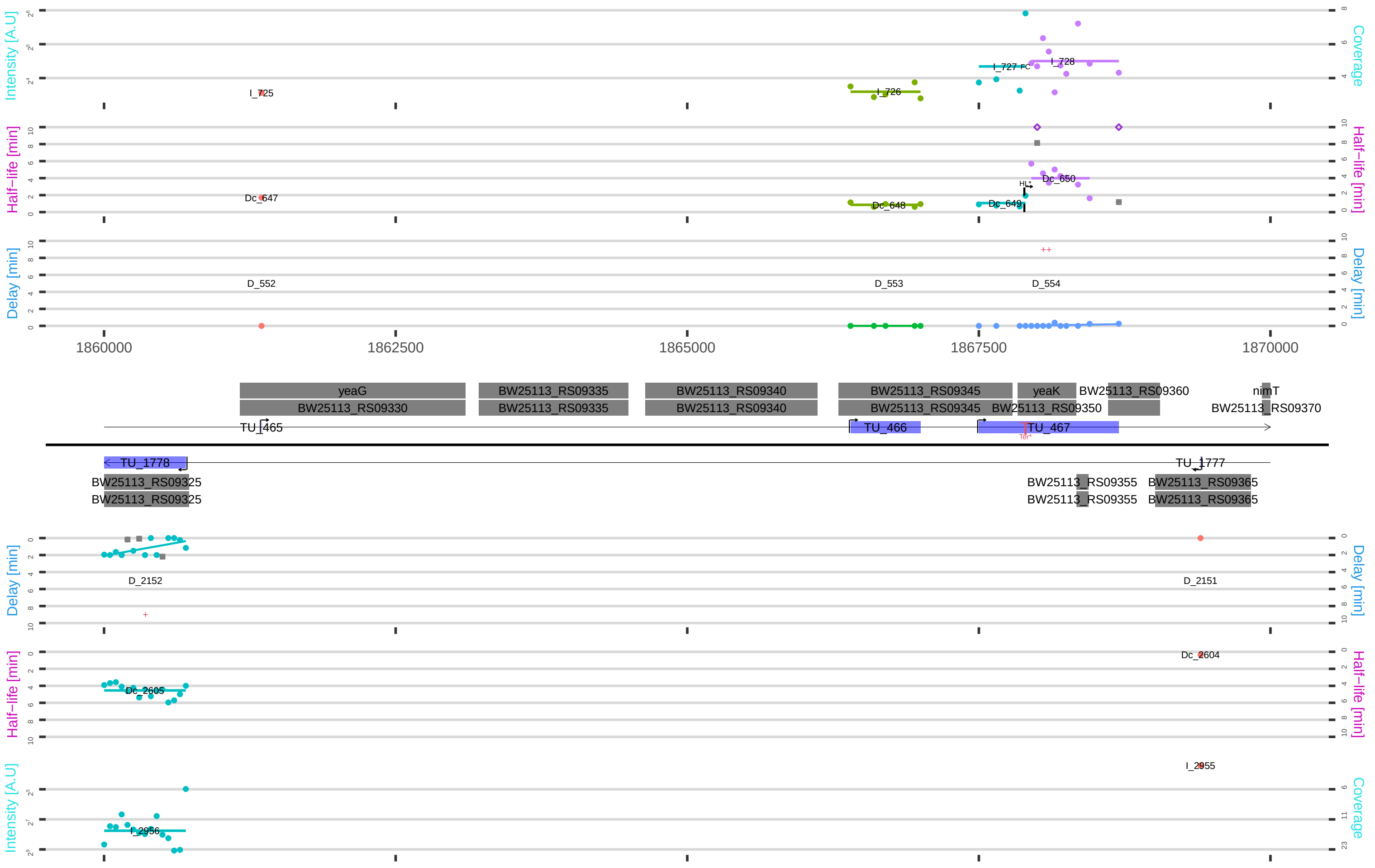

Term: termination (0), NS: new start (0), PS: pausing site (0), iTSS_I: internal starting site (0)

ID: 37425−37464; Term: termination (1), NS: new start (0), PS: pausing site (0), iTSS_I: internal starting site (0)

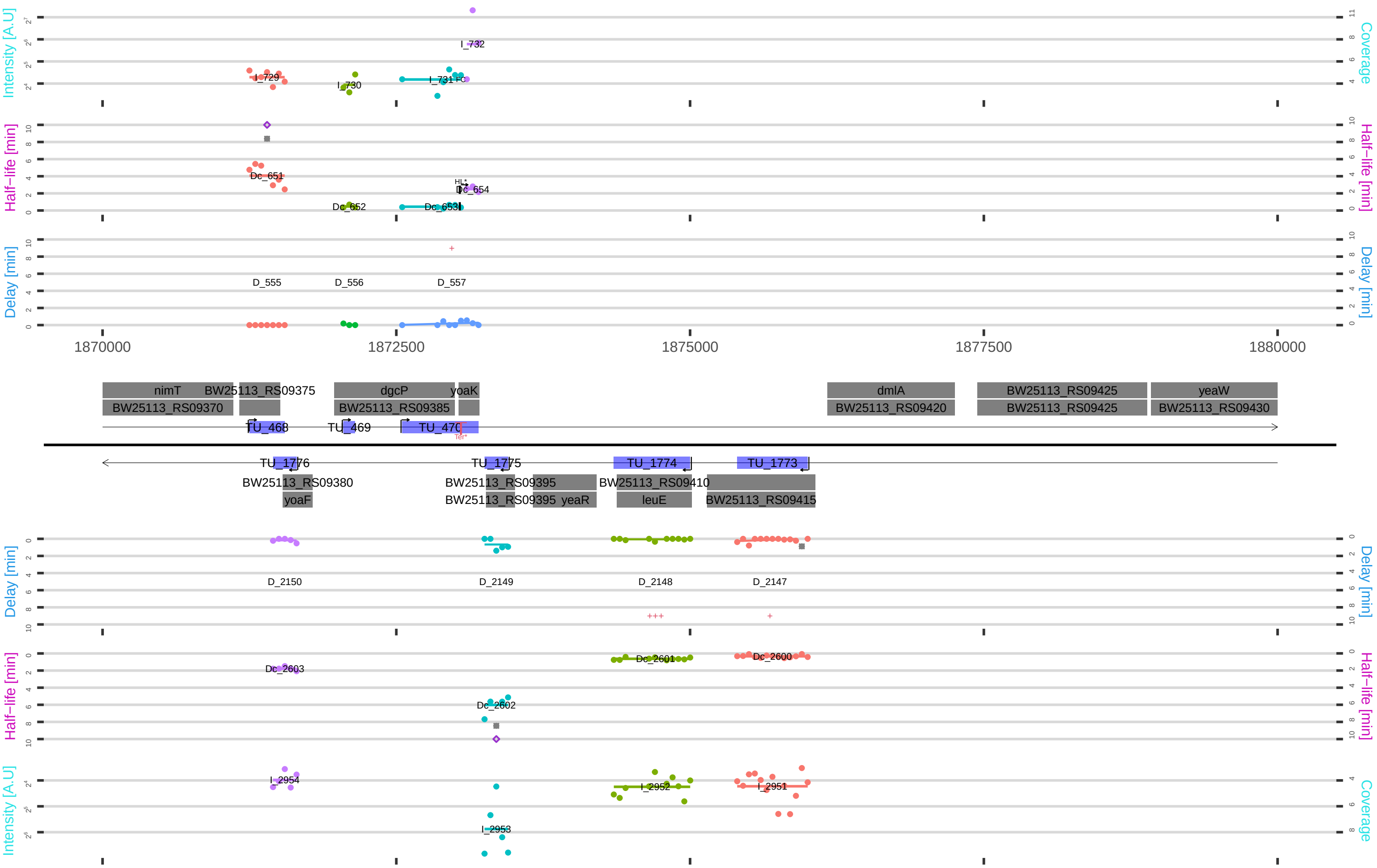

Term: termination (0), NS: new start (0), PS: pausing site (0), iTSS_I: internal starting site (0)

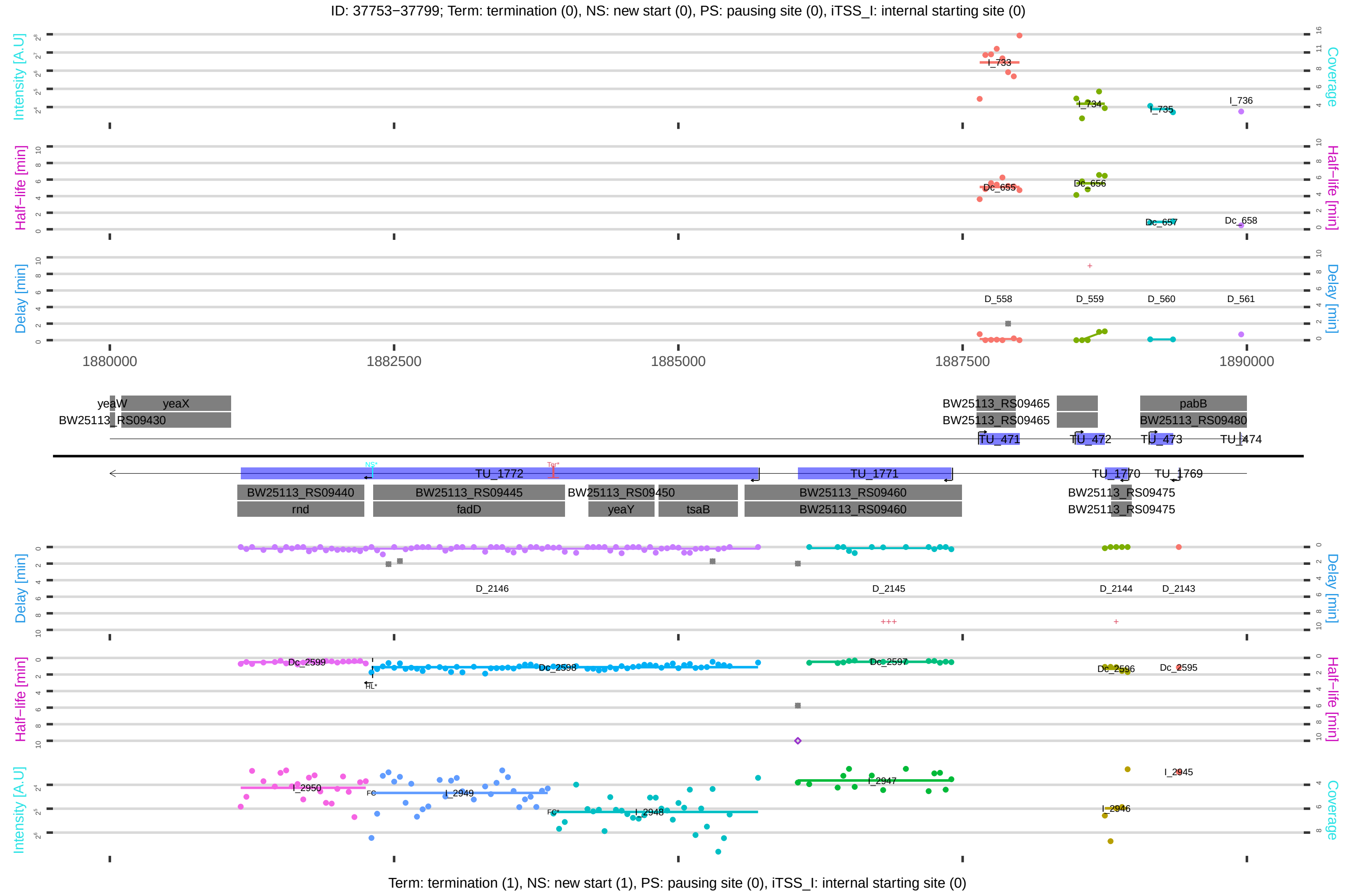

ID: 37753−37799; Term: termination (0), NS: new start (0), PS: pausing site (0), iTSS_I: internal starting site (0)
Intensity [A.U]
Coverage
I_733
I_734
I_735
I_736
Half−life [min]
Dc_655
Dc_656
Dc_657
Dc_658
Delay [min]
D_558
D_559
D_560
D_561
1880000
1882500
1885000
1887500
1890000
yeaW
yeaX
BW25113_RS09430
BW25113_RS09465
BW25113_RS09465
pabB
BW25113_RS09480
TU_471
TU_472
TU_473
TU_474
TU_1772
TU_1771
TU_1770
TU_1769
NS*
Ter*
BW25113_RS09440
BW25113_RS09445
BW25113_RS09450
BW25113_RS09460
BW25113_RS09475
rnd
fadD
yeaY
tsaB
BW25113_RS09460
BW25113_RS09475
Delay [min]
D_2146
D_2145
D_2144
D_2143
Half−life [min]
Dc_2599
Dc_2598
Dc_2597
Dc_2596
Dc_2595
HL*
Intensity [A.U]
Coverage
I_2950
I_2949
I_2948
I_2947
I_2946
I_2945
FC
FC*
Term: termination (1), NS: new start (1), PS: pausing site (0), iTSS_I: internal starting site (0)

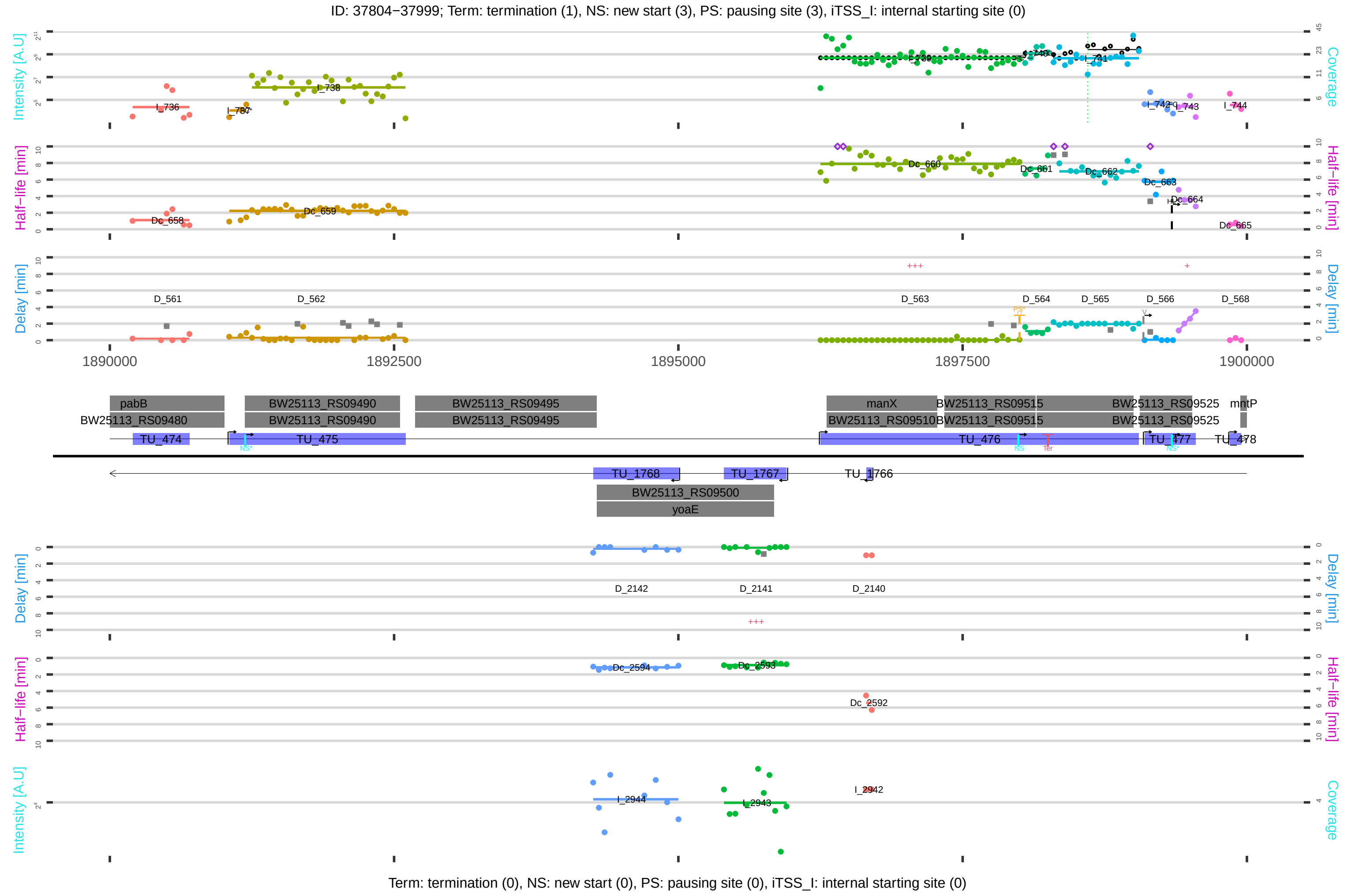
ID: 37804−37999; Term: termination (1), NS: new start (3), PS: pausing site (3), iTSS_I: internal starting site (0)
Intensity [A.U]
Coverage
Half−life [min]
Delay [min]
I_736
I_737
I_738
I_739
I_740
I_741
I_742
I_743
I_744
Dc_658
Dc_659
Dc_660
Dc_661
Dc_662
Dc_663
Dc_664
Dc_665
D_561
D_562
D_563
D_564
D_565
D_566
D_568
1890000
1892500
1895000
1897500
1900000
pabB
BW25113_RS09480
BW25113_RS09490
BW25113_RS09495
manX
BW25113_RS09510
BW25113_RS09515
BW25113_RS09525
mntP
TU_474
TU_475
TU_476
TU_477
TU_478
NS
Ter
PS
TU_1768
TU_1767
TU_1766
BW25113_RS09500
yoaE
D_2142
D_2141
D_2140
Dc_2594
Dc_2593
Dc_2592
I_2944
I_2943
I_2942
Term: termination (0), NS: new start (0), PS: pausing site (0), iTSS_I: internal starting site (0)

ID: 38001−38189; Term: termination (0), NS: new start (0), PS: pausing site (0), iTSS_I: internal starting site (0)

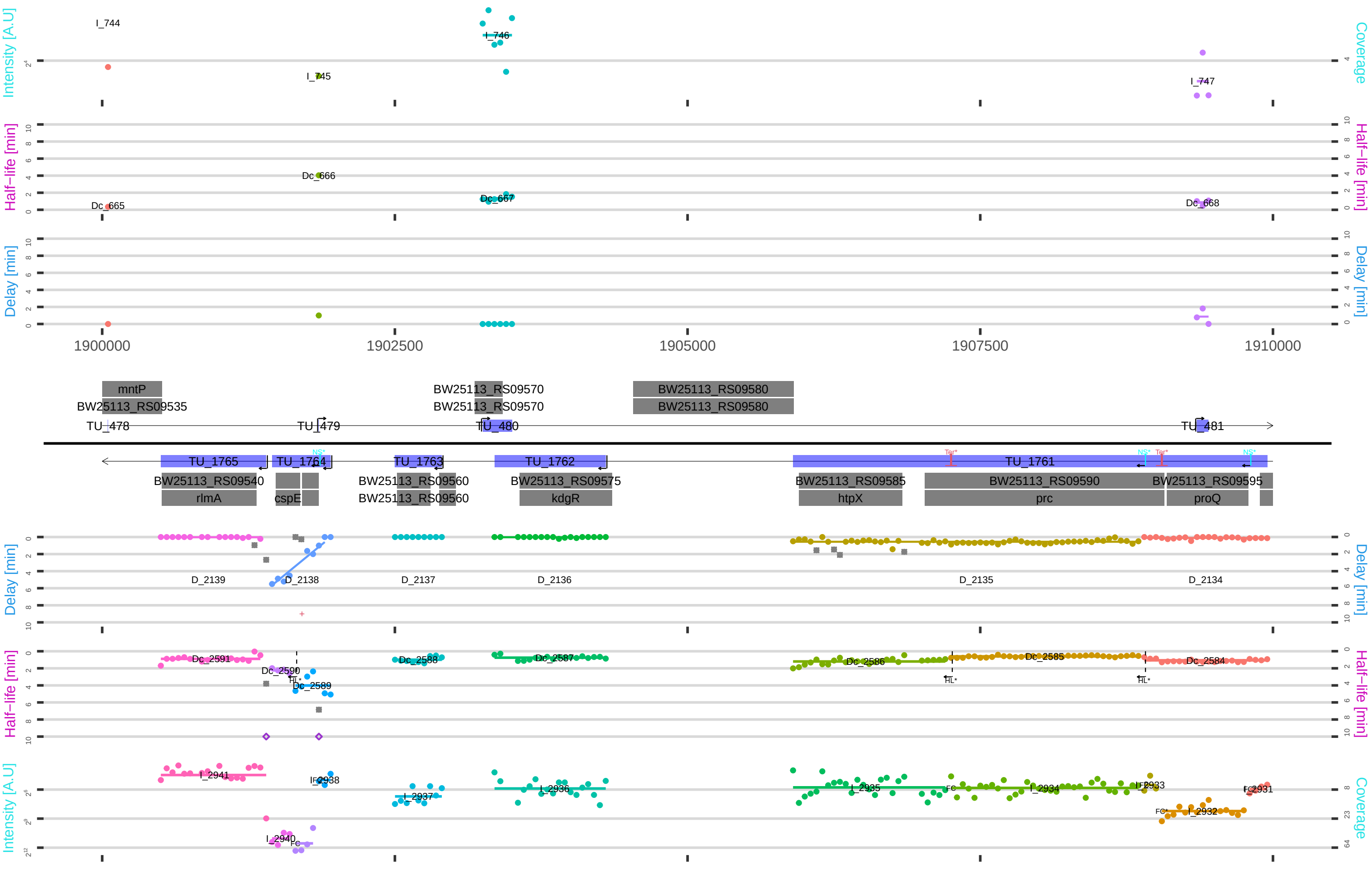

Term: termination (2), NS: new start (3), PS: pausing site (1), iTSS_I: internal starting site (0)

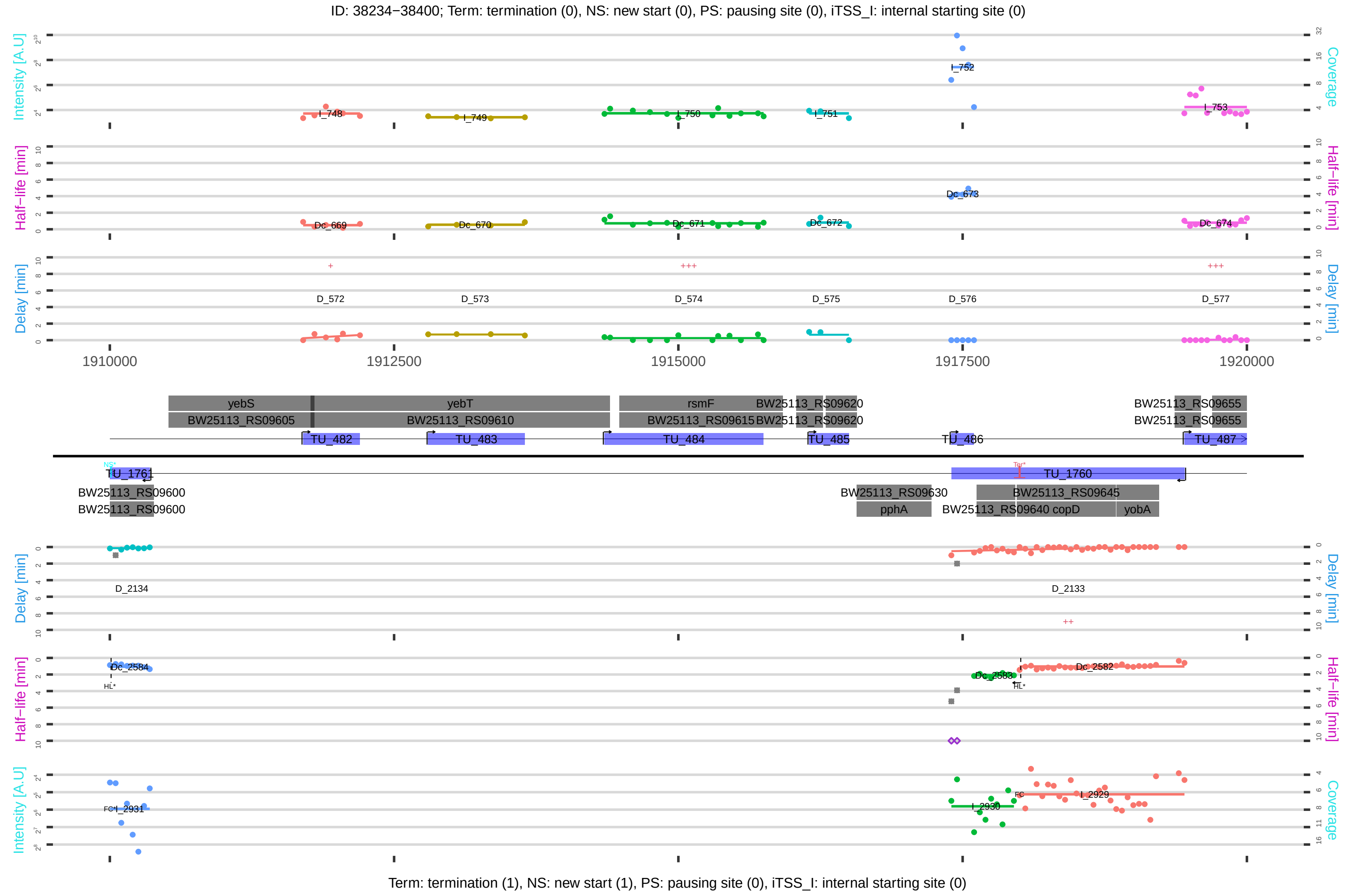

ID: 38234−38400; Term: termination (0), NS: new start (0), PS: pausing site (0), iTSS_I: internal starting site (0)
Intensity [A.U]
Coverage
Half−life [min]
Delay [min]
I_748
I_749
I_750
I_751
I_752
I_753
Dc_669
Dc_670
Dc_671
Dc_672
Dc_673
Dc_674
D_572
D_573
D_574
D_575
D_576
D_577
1910000
1912500
1915000
1917500
1920000
yebS
yebT
rsmF
BW25113_RS09605
BW25113_RS09610
BW25113_RS09615
BW25113_RS09620
BW25113_RS09655
TU_482
TU_483
TU_484
TU_485
TU_486
TU_487
TU_1761
TU_1760
BW25113_RS09600
BW25113_RS09630
BW25113_RS09645
BW25113_RS09640 copD
pphA
yobA
D_2134
D_2133
Dc_2584
Dc_2583
Dc_2582
I_2931
I_2930
I_2929
Term: termination (1), NS: new start (1), PS: pausing site (0), iTSS_I: internal starting site (0)

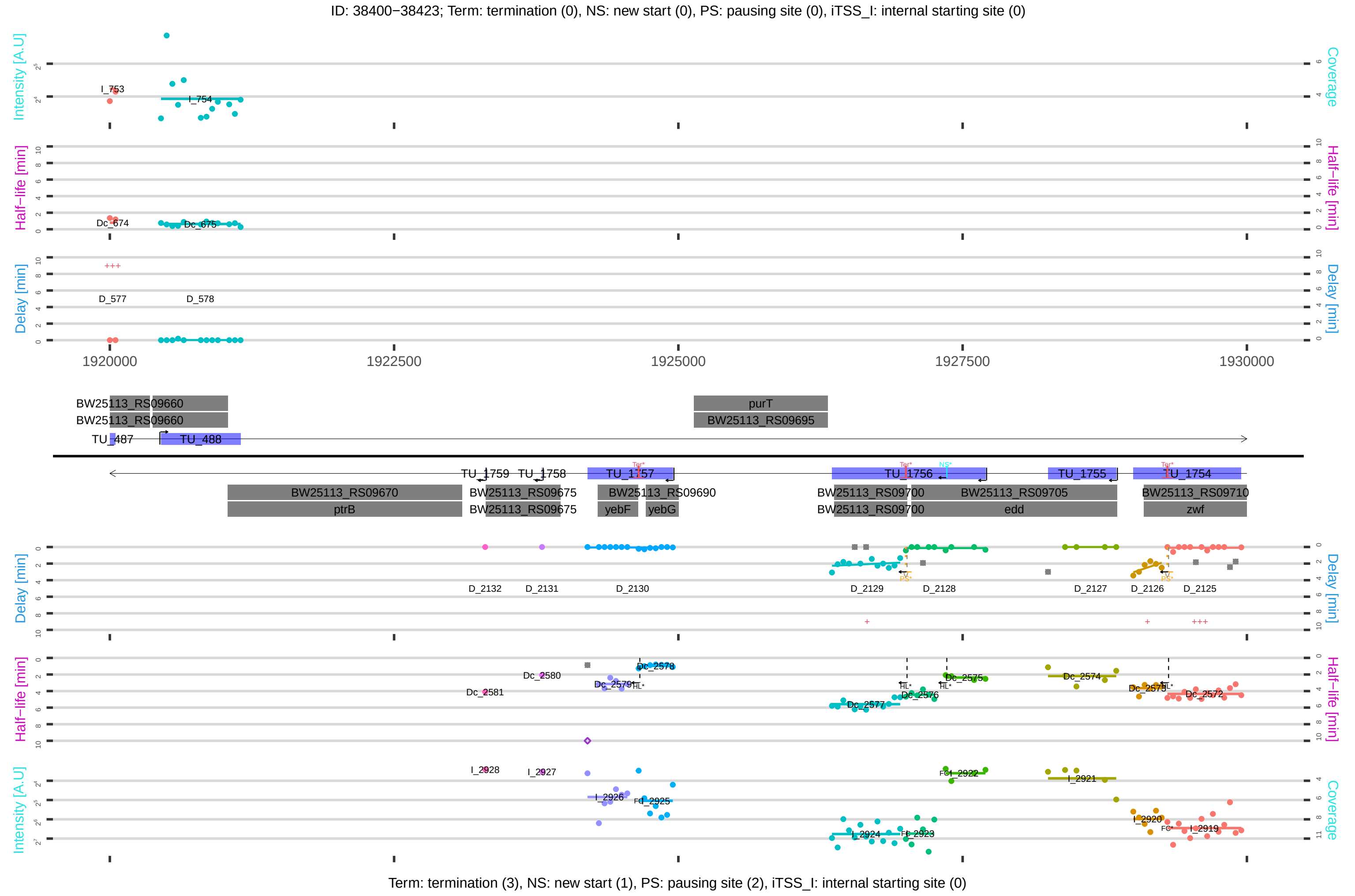
ID: 38400−38423; Term: termination (0), NS: new start (0), PS: pausing site (0), iTSS_I: internal starting site (0)
Intensity [A.U]
Coverage
Half−life [min]
Delay [min]
1920000
1922500
1925000
1927500
1930000
BW25113_RS09660
TU_487
TU_488
purT
BW25113_RS09695
TU_1759
TU_1758
TU_1757
TU_1756
TU_1755
TU_1754
BW25113_RS09670
ptrB
BW25113_RS09675
BW25113_RS09690
yebF
yebG
BW25113_RS09700
BW25113_RS09705
edd
BW25113_RS09710
zwf
Term: termination (3), NS: new start (1), PS: pausing site (2), iTSS_I: internal starting site (0)

ID: 38619−38763; Term: termination (1), NS: new start (0), PS: pausing site (1), iTSS_I: internal starting site (0)

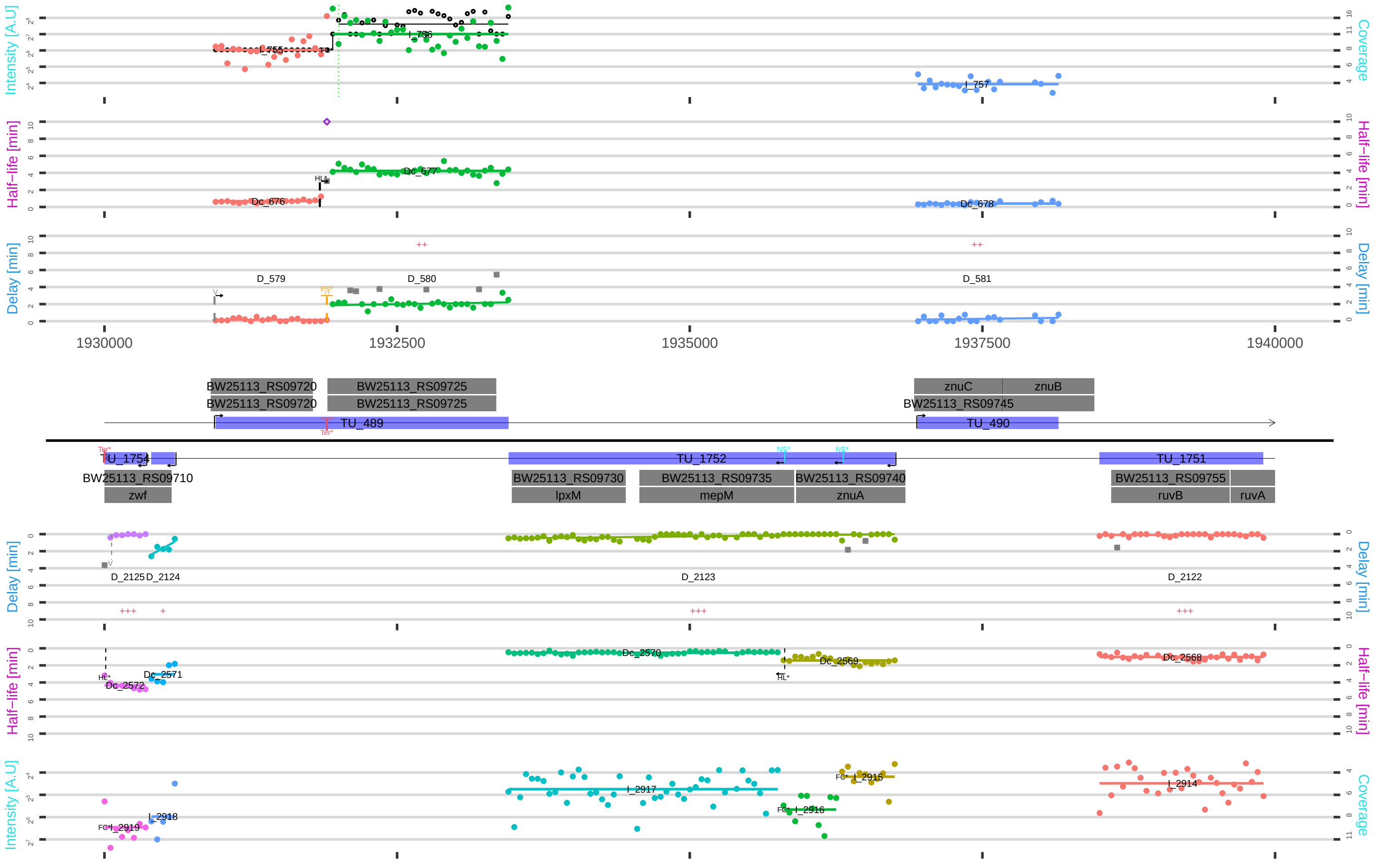

Term: termination (1), NS: new start (2), PS: pausing site (0), iTSS_I: internal starting site (0)

ID: 38903−38977; Term: termination (0), NS: new start (0), PS: pausing site (0), iTSS_I: internal starting site (0)

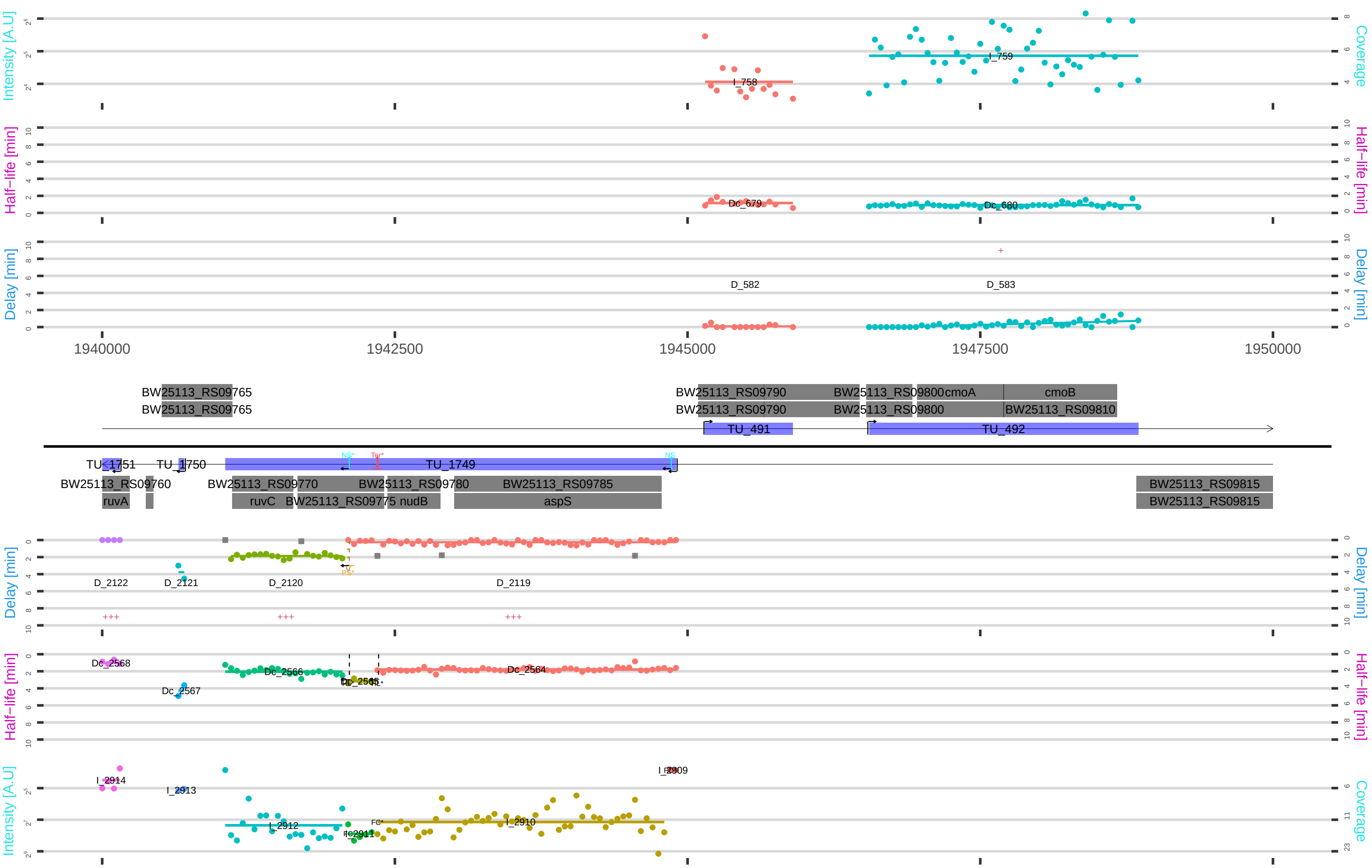

Term: termination (1), NS: new start (2), PS: pausing site (1), iTSS_I: internal starting site (0)

ID: 39087−39122; Term: termination (0), NS: new start (0), PS: pausing site (0), iTSS_I: internal starting site (0)

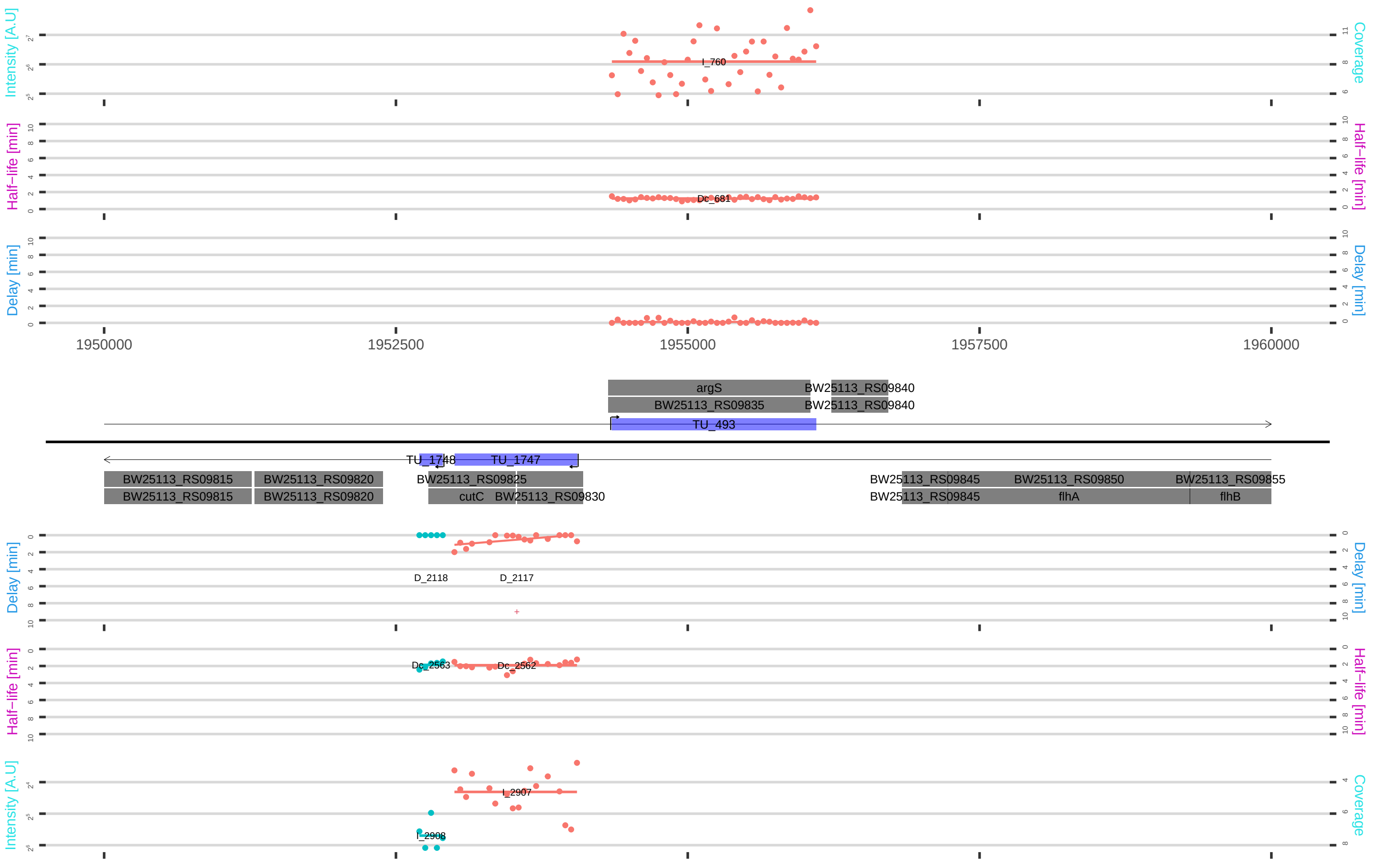

Term: termination (0), NS: new start (0), PS: pausing site (0), iTSS_I: internal starting site (0)

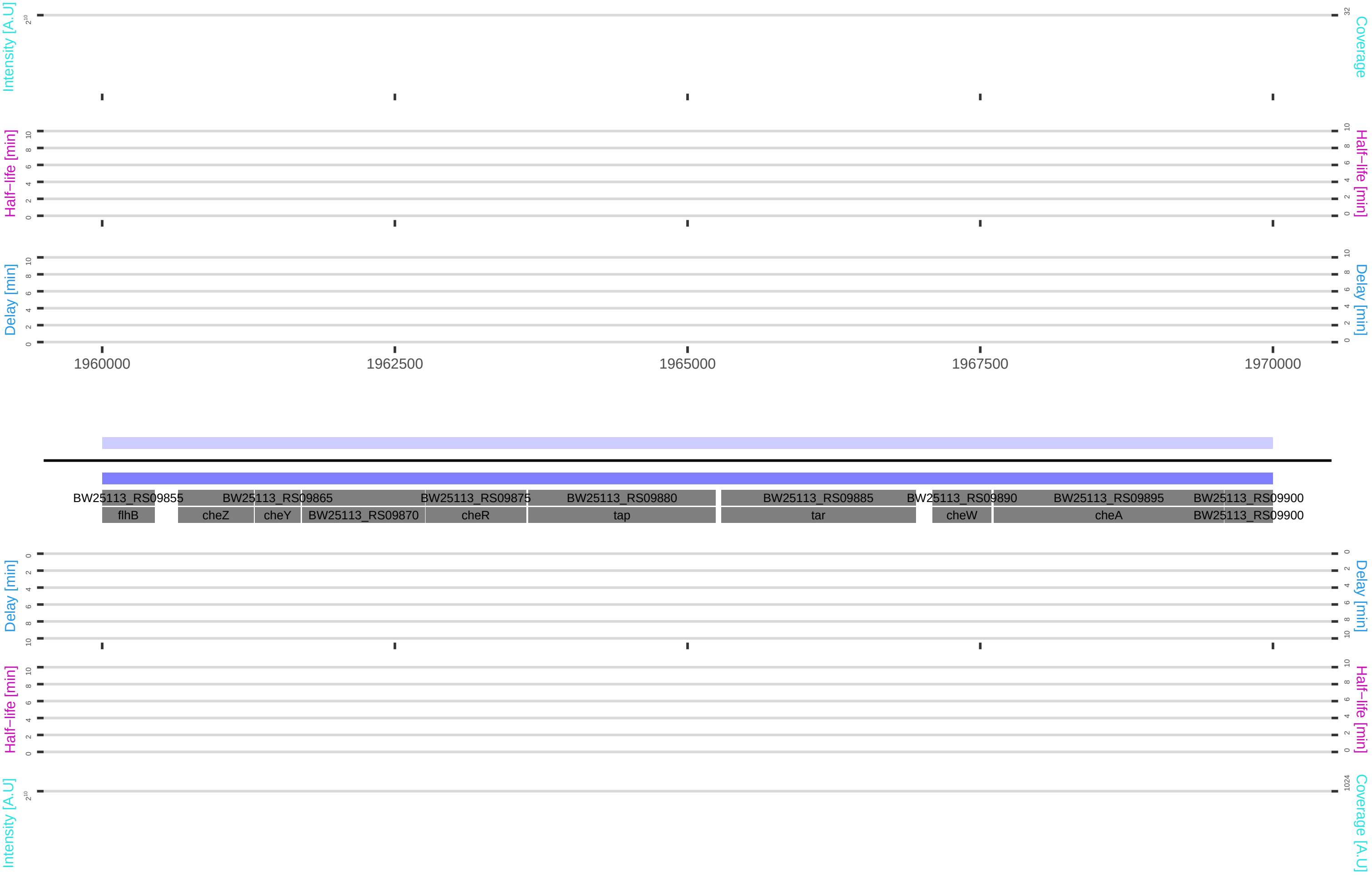

Intensity [A.U]
Coverage
Half−life [min]
Delay [min]
1960000
1962500
1965000
1967500
1970000
BW25113_RS09855
BW25113_RS09865
BW25113_RS09875
BW25113_RS09880
BW25113_RS09885
BW25113_RS09890
BW25113_RS09895
BW25113_RS09900
flhB
cheZ
cheY
BW25113_RS09870
cheR
tap
tar
cheW
cheA
BW25113_RS09900
Delay [min]
Half−life [min]
Intensity [A.U]
Coverage [A.U]

NA

ID: 39466−39553; Term: termination (0), NS: new start (0), PS: pausing site (0), iTSS_I: internal starting site (0)

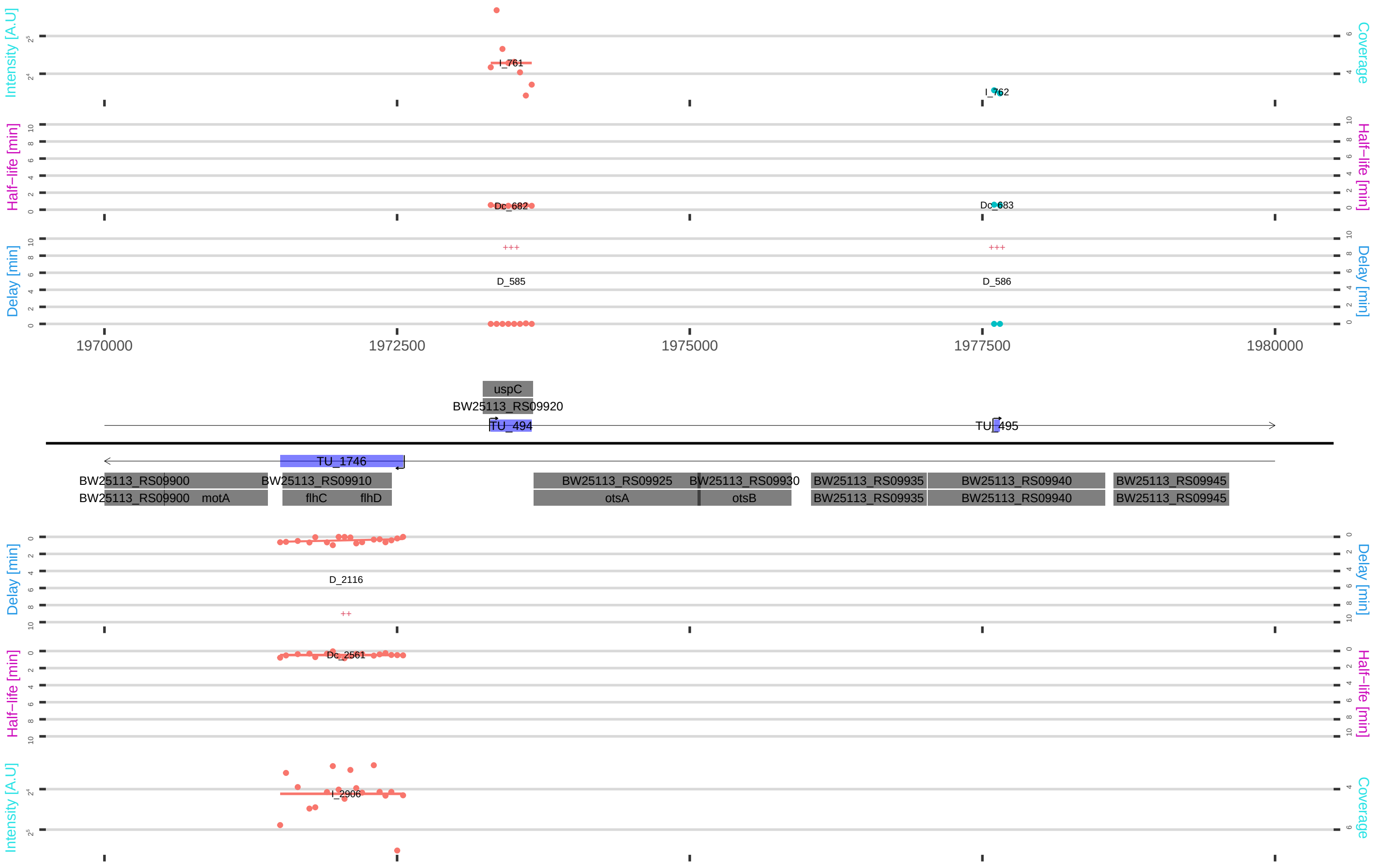

Term: termination (0), NS: new start (0), PS: pausing site (0), iTSS_I: internal starting site (0)

ID: 39608−39791; Term: termination (0), NS: new start (0), PS: pausing site (0), iTSS_I: internal starting site (0)

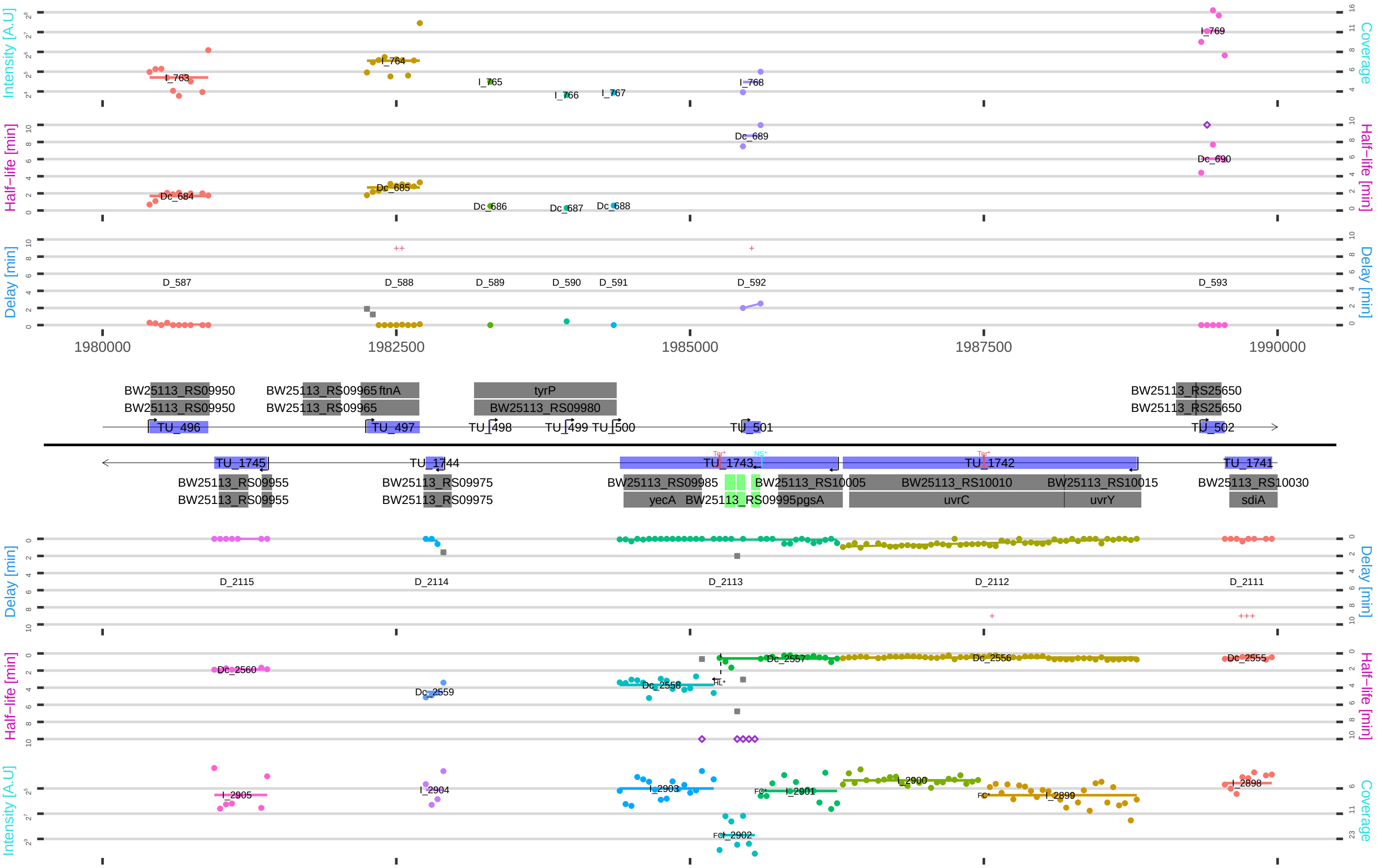

Term: termination (2), NS: new start (1), PS: pausing site (0), iTSS_I: internal starting site (0)

ID: 39810−39811; Term: termination (0), NS: new start (0), PS: pausing site (0), iTSS_I: internal starting site (0)

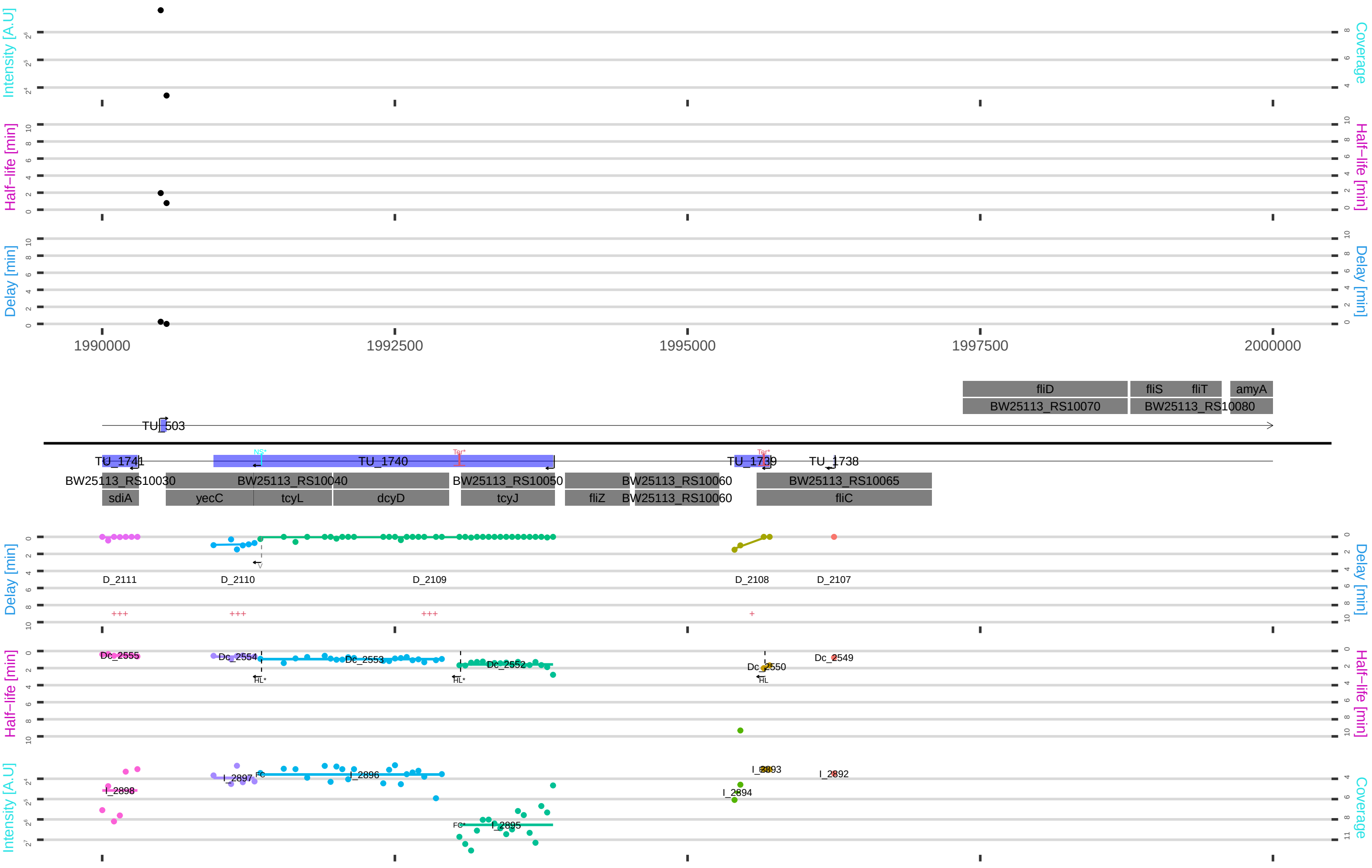

Term: termination (2), NS: new start (1), PS: pausing site (1), iTSS_I: internal starting site (0)

ID: 40018−40068; Term: termination (1), NS: new start (0), PS: pausing site (1), iTSS_I: internal starting site (0)

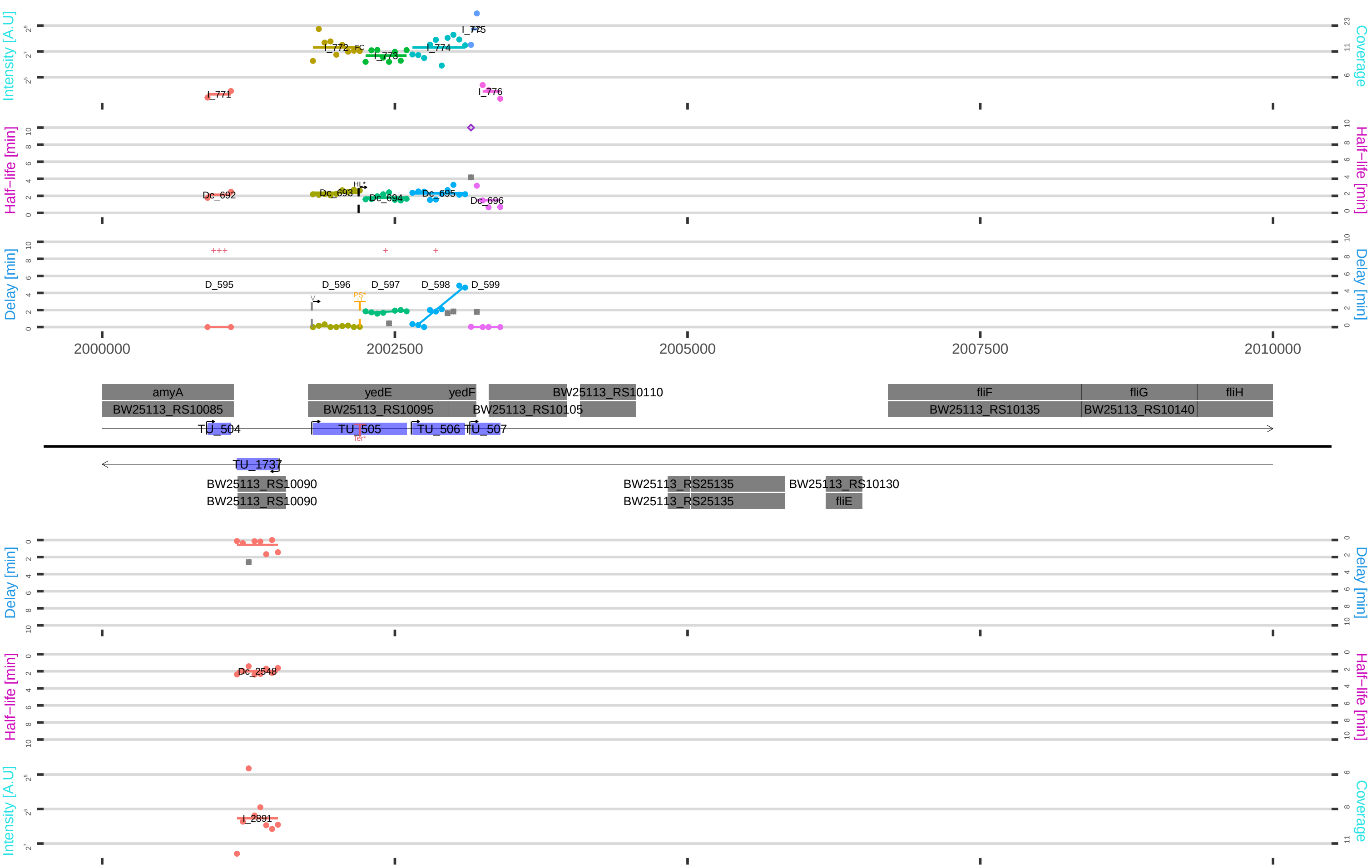

Term: termination (0), NS: new start (0), PS: pausing site (0), iTSS_I: internal starting site (0)

ID: 144898−144863; FC*: significant t−test of two consecutive segments; Term: termination, NS: new start, PS: pausing site,iTSS_I: internal starting site,TI: transcription interferance.

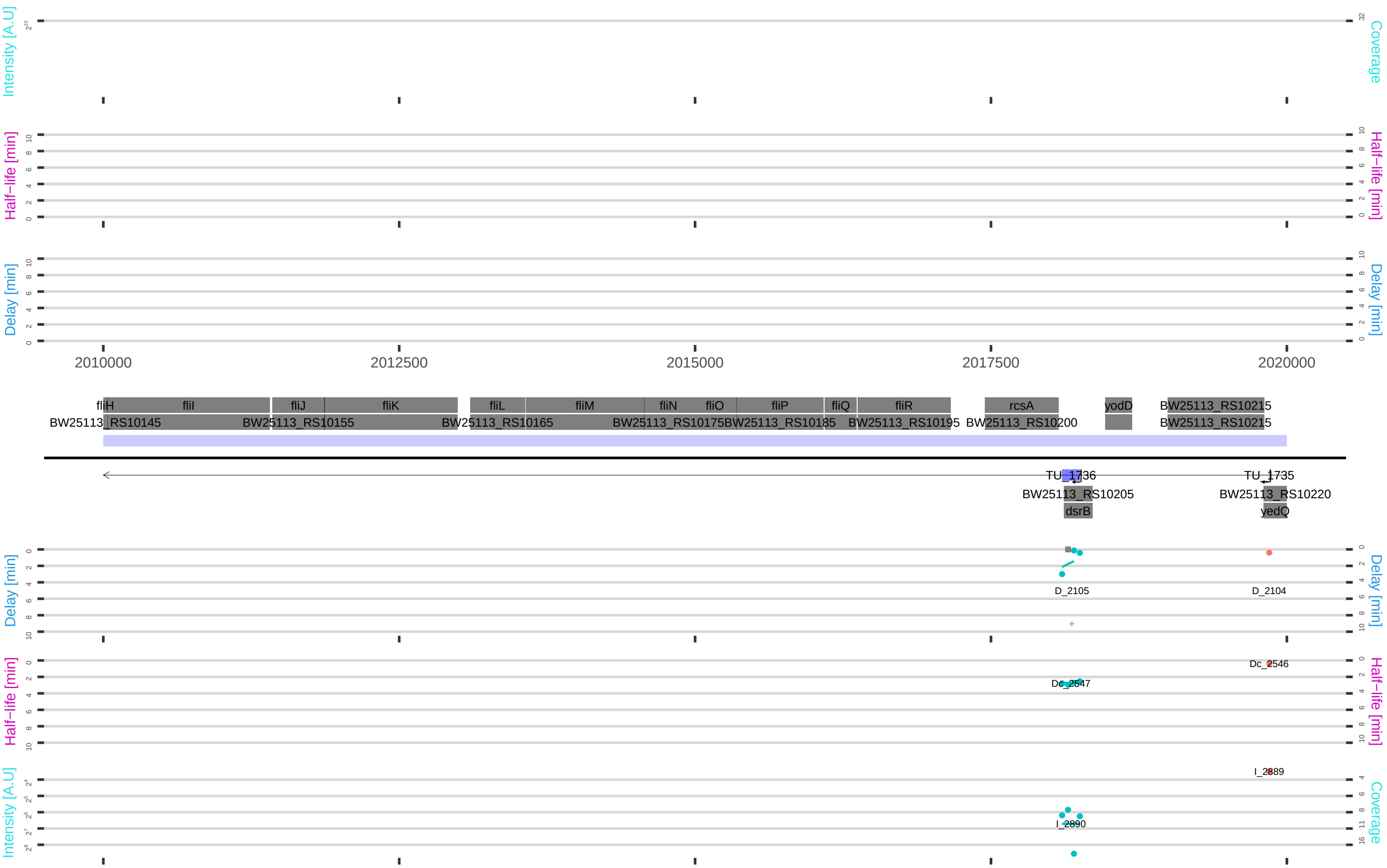

Term: termination (0), NS: new start (0), PS: pausing site (0), iTSS_I: internal starting site (0)

ID: 40587−40599; Term: termination (1), NS: new start (2), PS: pausing site (0), iTSS_I: internal starting site (0)

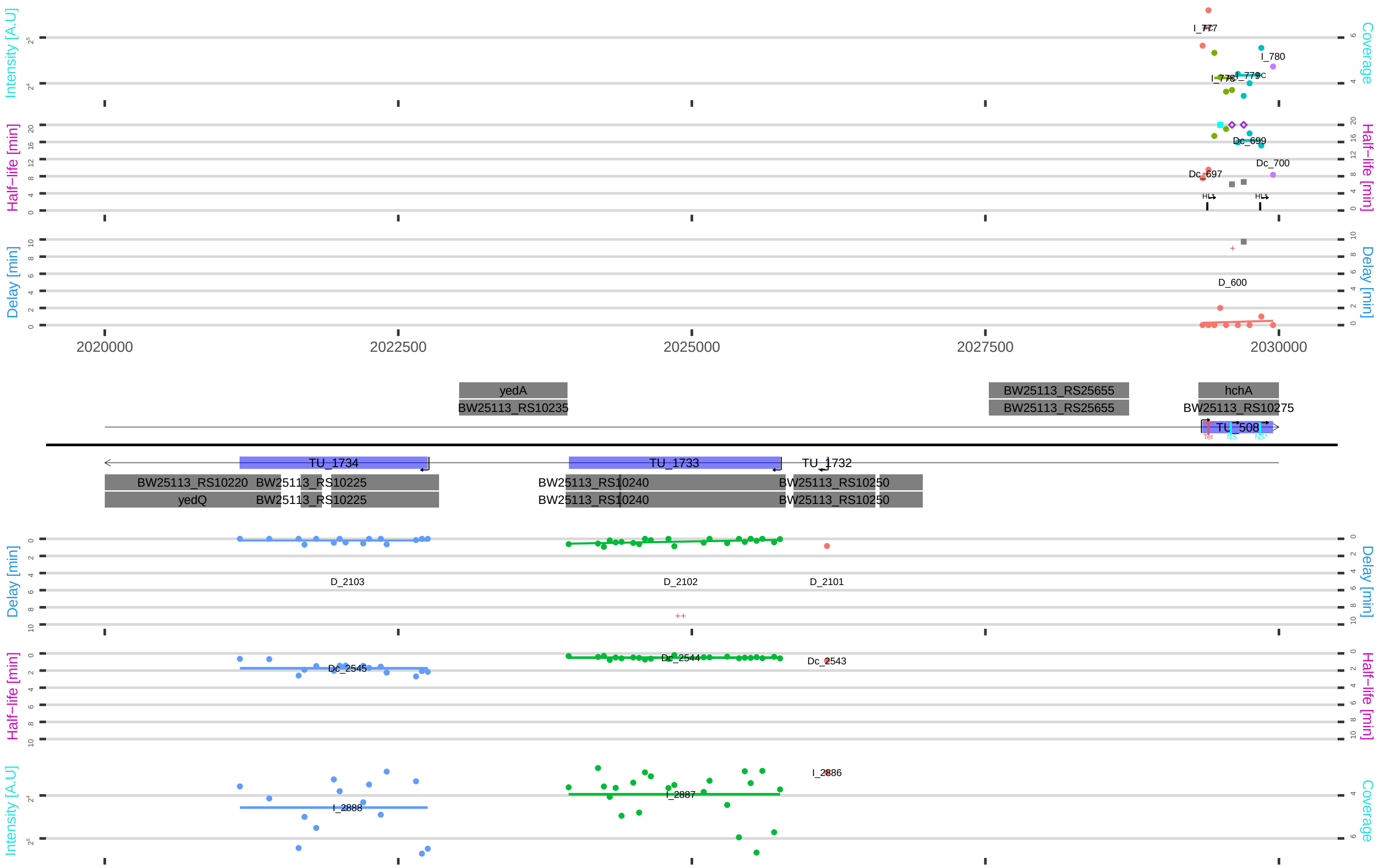

Term: termination (0), NS: new start (0), PS: pausing site (0), iTSS_I: internal starting site (0)

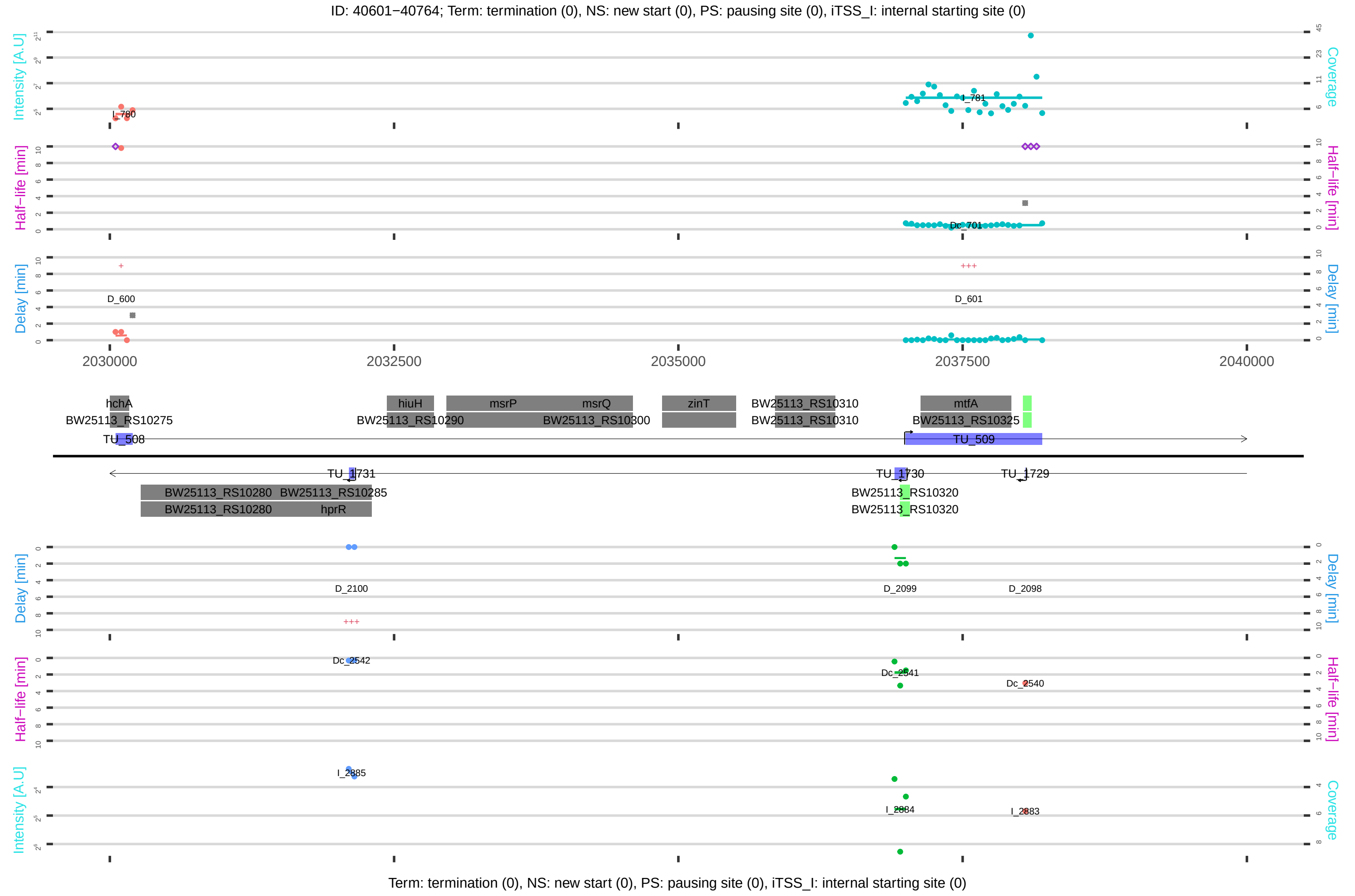

ID: 40601−40764; Term: termination (0), NS: new start (0), PS: pausing site (0), iTSS_I: internal starting site (0)
Intensity [A.U]
Coverage
Half−life [min]
Delay [min]
I_780
I_781
Dc_701
D_600
D_601
2030000
2032500
2035000
2037500
2040000
hchA
BW25113_RS10275
hiuH
BW25113_RS10290
msrP
msrQ
BW25113_RS10300
zinT
BW25113_RS10310
mtfA
BW25113_RS10325
TU_508
TU_509
TU_1731
TU_1730
TU_1729
BW25113_RS10280
BW25113_RS10285
hprR
BW25113_RS10320
D_2100
D_2099
D_2098
Dc_2542
Dc_2541
Dc_2540
I_2885
I_2884
I_2883
Term: termination (0), NS: new start (0), PS: pausing site (0), iTSS_I: internal starting site (0)

ID: 40943−41000; Term: termination (1), NS: new start (1), PS: pausing site (0), iTSS_I: internal starting site (0)

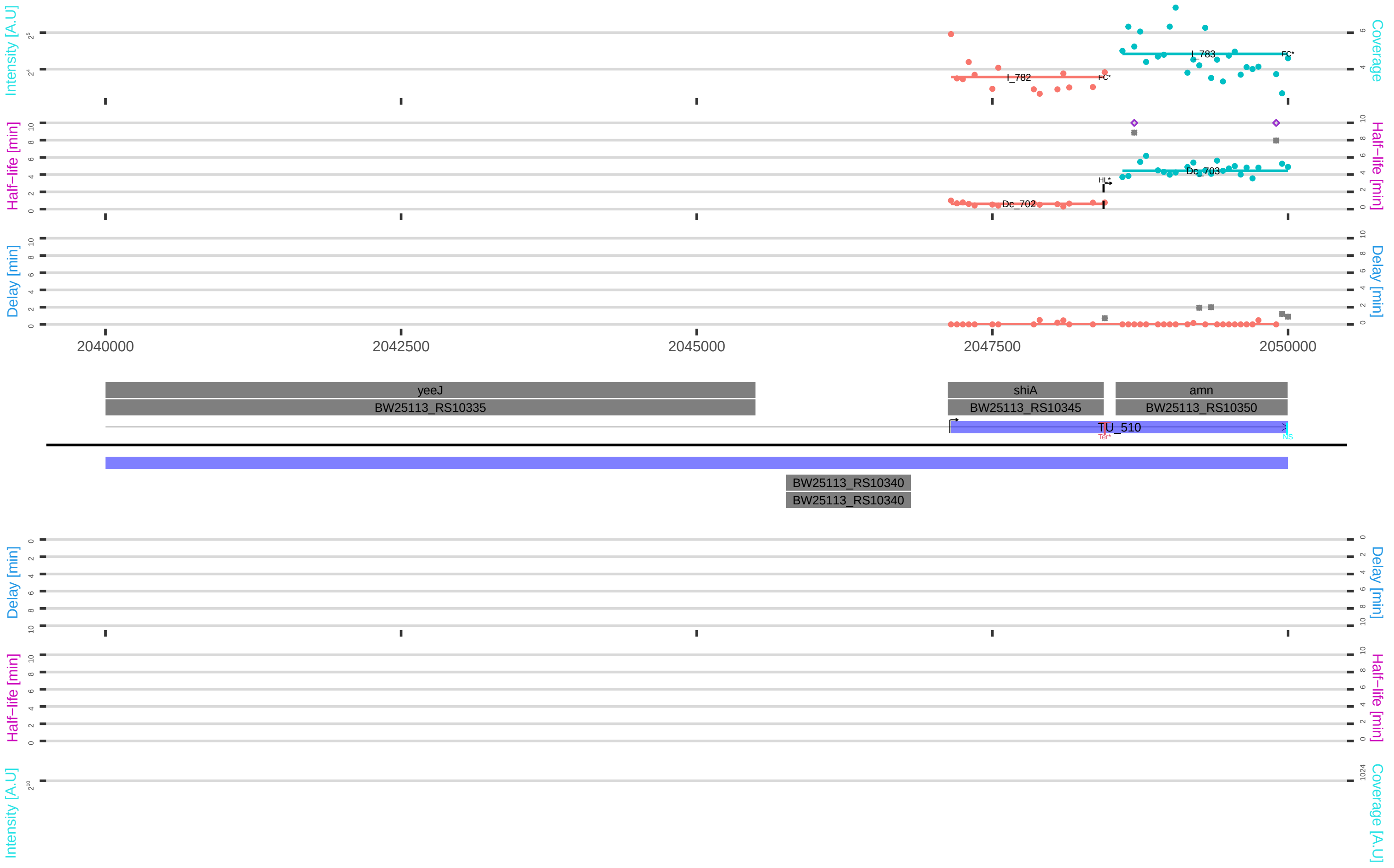

NA

ID: 41000−41117; Term: termination (0), NS: new start (1), PS: pausing site (0), iTSS_I: internal starting site (0)

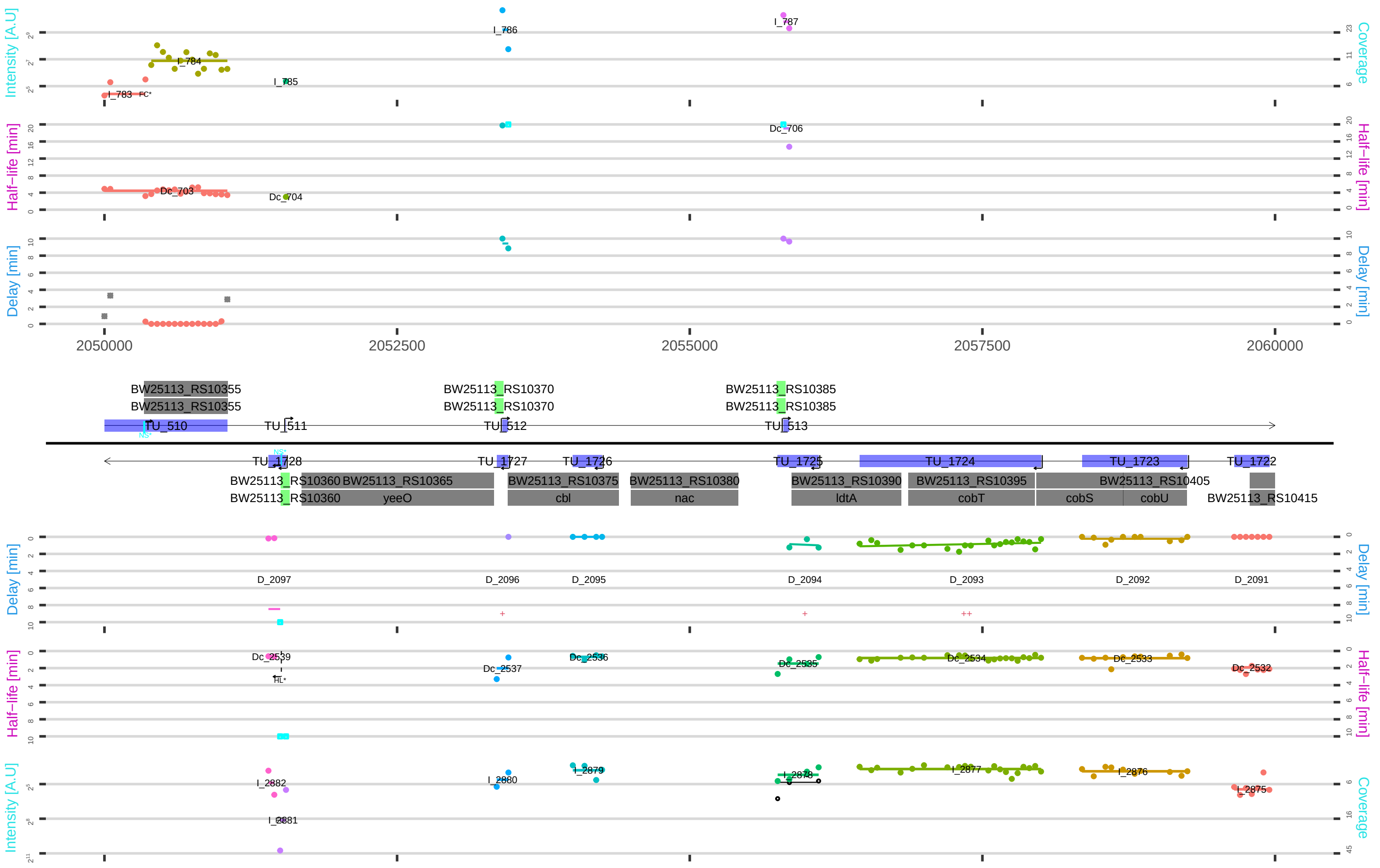

Term: termination (0), NS: new start (1), PS: pausing site (0), iTSS_I: internal starting site (0)

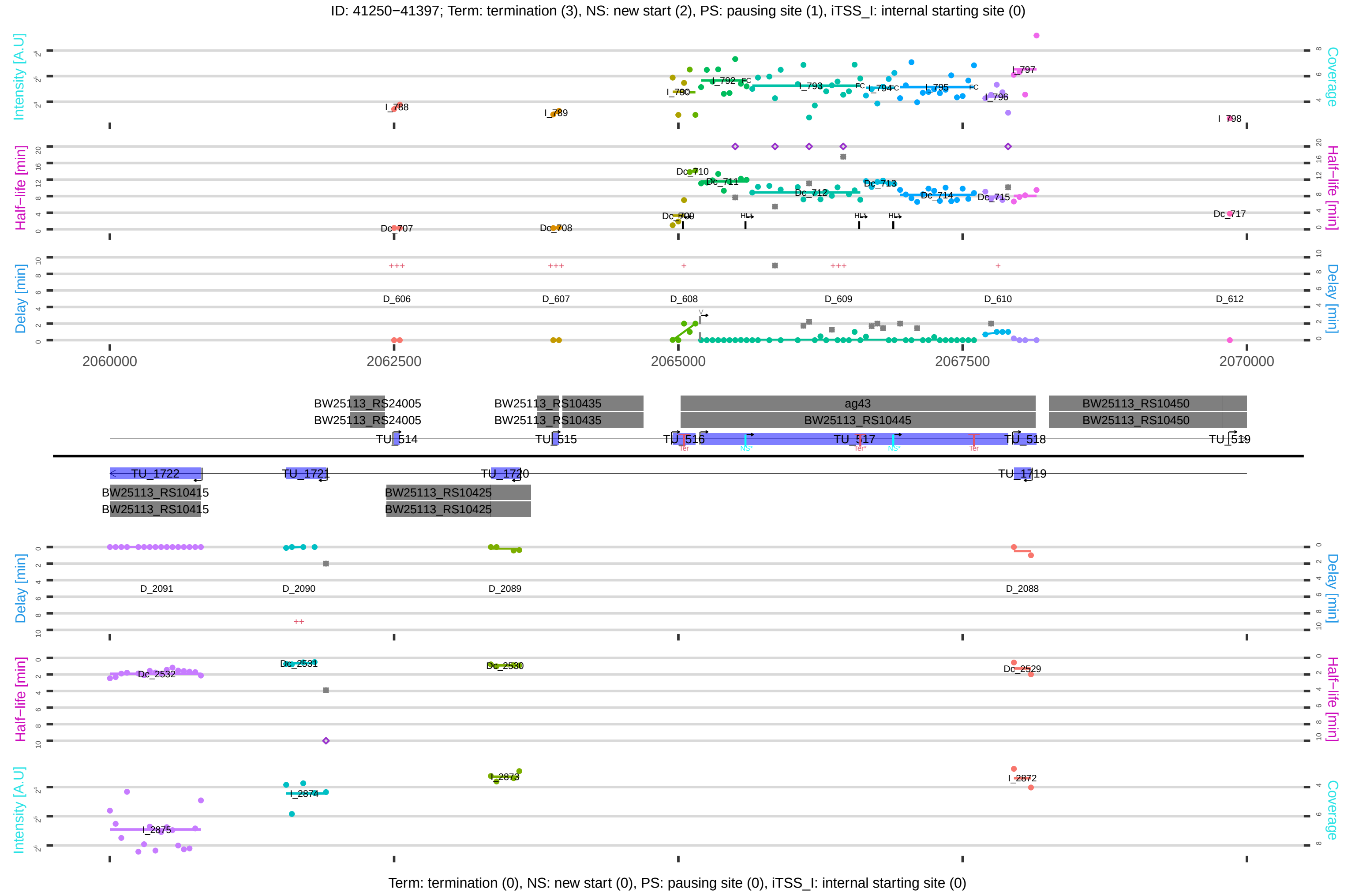
ID: 41250−41397; Term: termination (3), NS: new start (2), PS: pausing site (1), iTSS_I: internal starting site (0)
Intensity [A.U]
Coverage
Half−life [min]
Delay [min]
I_788
I_789
I_790
I_792
I_793
I_794
I_795
I_796
I_797
I_798
Dc_707
Dc_708
Dc_709
Dc_710
Dc_711
Dc_712
Dc_713
Dc_714
Dc_715
Dc_717
D_606
D_607
D_608
D_609
D_610
D_612
2060000
2062500
2065000
2067500
2070000
BW25113_RS24005
BW25113_RS10435
ag43
BW25113_RS10445
BW25113_RS10450
TU_514
TU_515
TU_516
TU_517
TU_518
TU_519
TU_1722
TU_1721
TU_1720
TU_1719
BW25113_RS10415
BW25113_RS10425
D_2091
D_2090
D_2089
D_2088
Dc_2532
Dc_2531
Dc_2530
Dc_2529
I_2875
I_2874
I_2873
I_2872
Term: termination (0), NS: new start (0), PS: pausing site (0), iTSS_I: internal starting site (0)

ID: 41435−41553; Term: termination (0), NS: new start (0), PS: pausing site (0), iTSS_I: internal starting site (0)

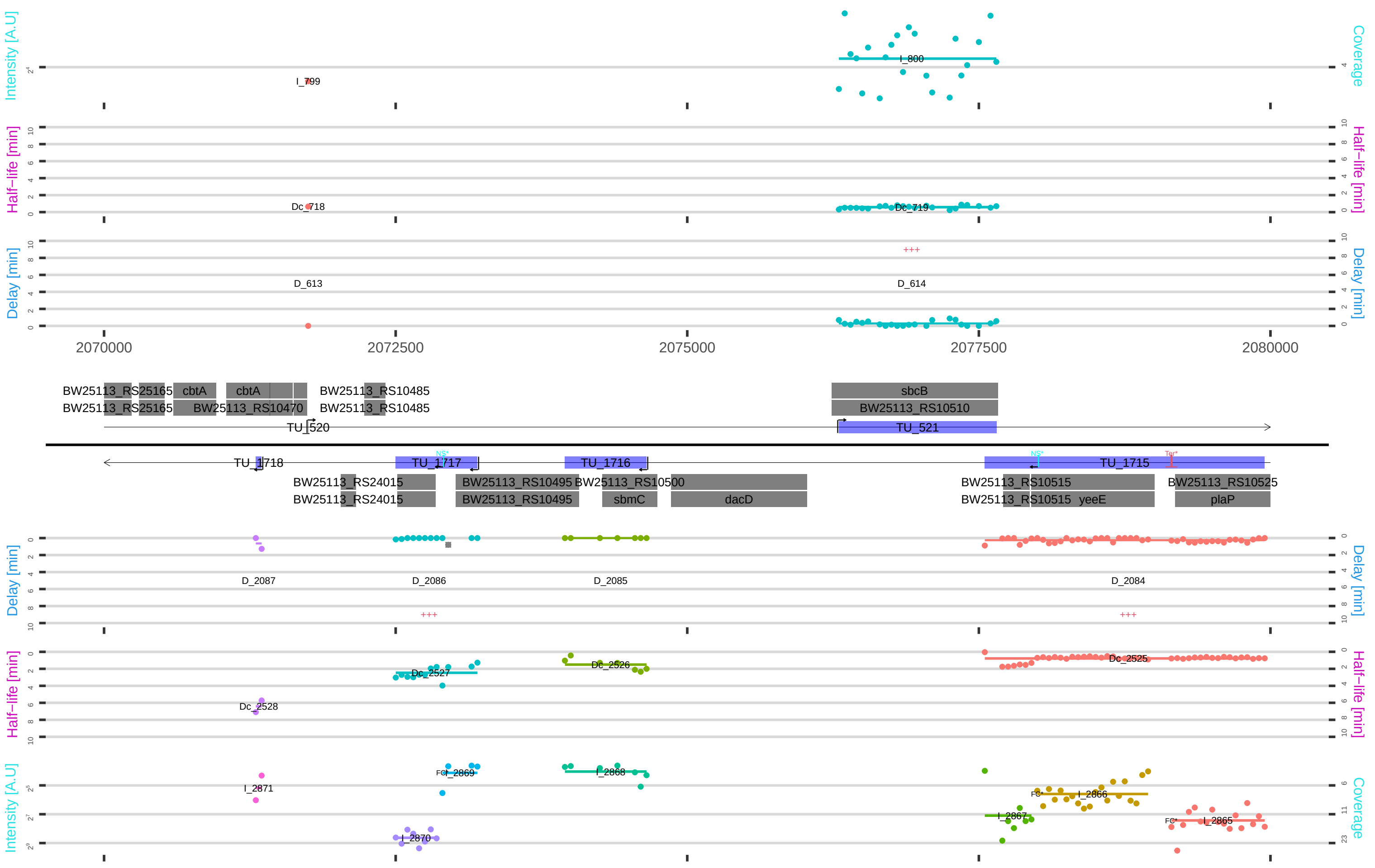

Term: termination (1), NS: new start (2), PS: pausing site (0), iTSS_I: internal starting site (0)

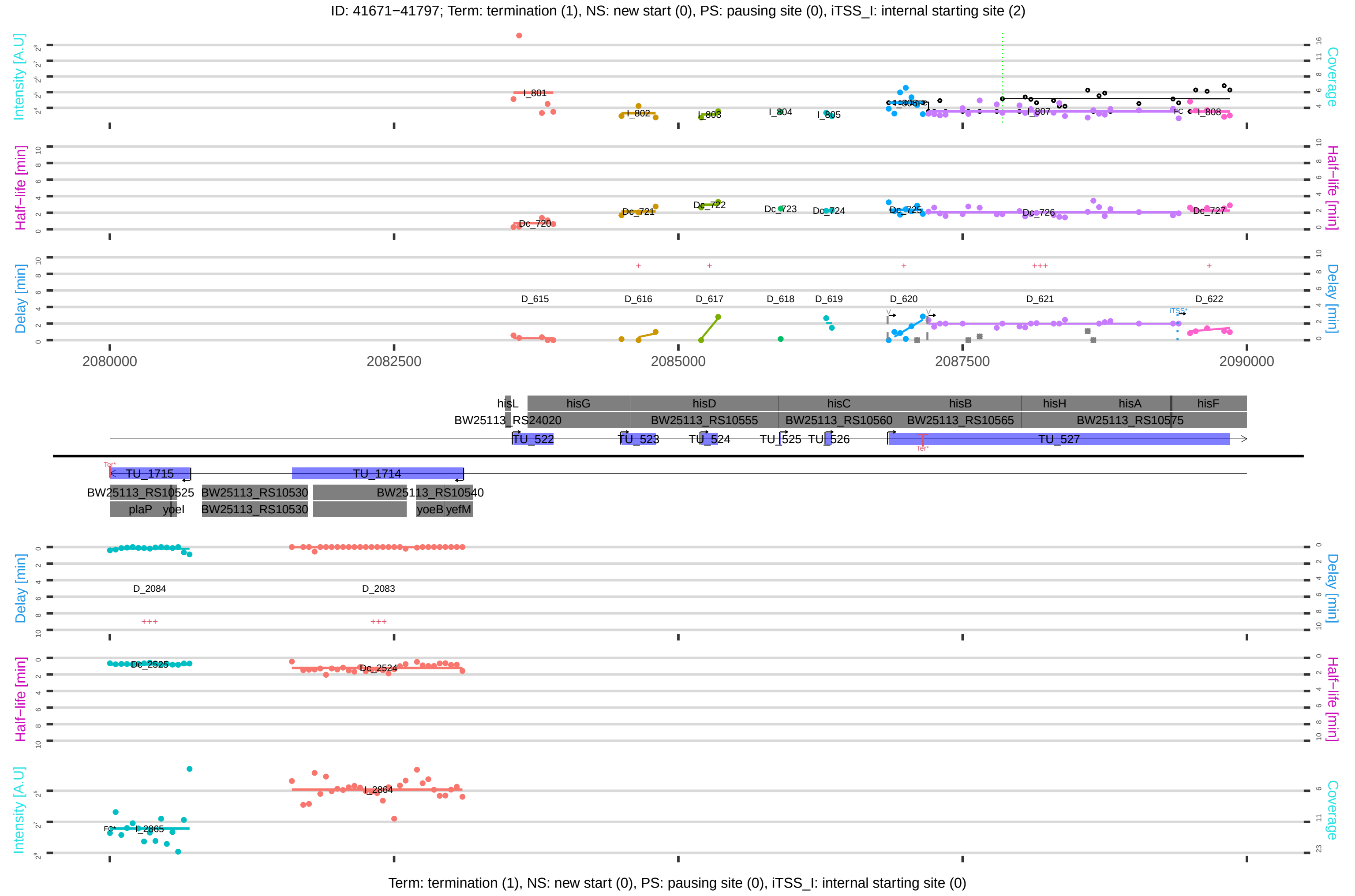

ID: 41671−41797; Term: termination (1), NS: new start (0), PS: pausing site (0), iTSS_I: internal starting site (2)
Intensity [A.U]
Coverage
Half−life [min]
Delay [min]
I_801
I_802
I_803
I_804
I_805
I_807
I_808
Dc_720
Dc_721
Dc_722
Dc_723
Dc_724
Dc_725
Dc_726
Dc_727
D_615
D_616
D_617
D_618
D_619
D_620
D_621
D_622
2080000
2082500
2085000
2087500
2090000
hisL
hisG
hisD
hisC
hisB
hisH
hisA
hisF
BW25113_RS24020
BW25113_RS10555
BW25113_RS10560
BW25113_RS10565
BW25113_RS10575
TU_522
TU_523
TU_524
TU_525
TU_526
TU_527
TU_1715
TU_1714
BW25113_RS10525
BW25113_RS10530
BW25113_RS10540
plaP
yoeI
yoeB
yefM
D_2084
D_2083
Dc_2525
Dc_2524
I_2864
I_2865
Term: termination (1), NS: new start (0), PS: pausing site (0), iTSS_I: internal starting site (0)

ID: 41801−41815; Term: termination (0), NS: new start (0), PS: pausing site (0), iTSS_I: internal starting site (0)

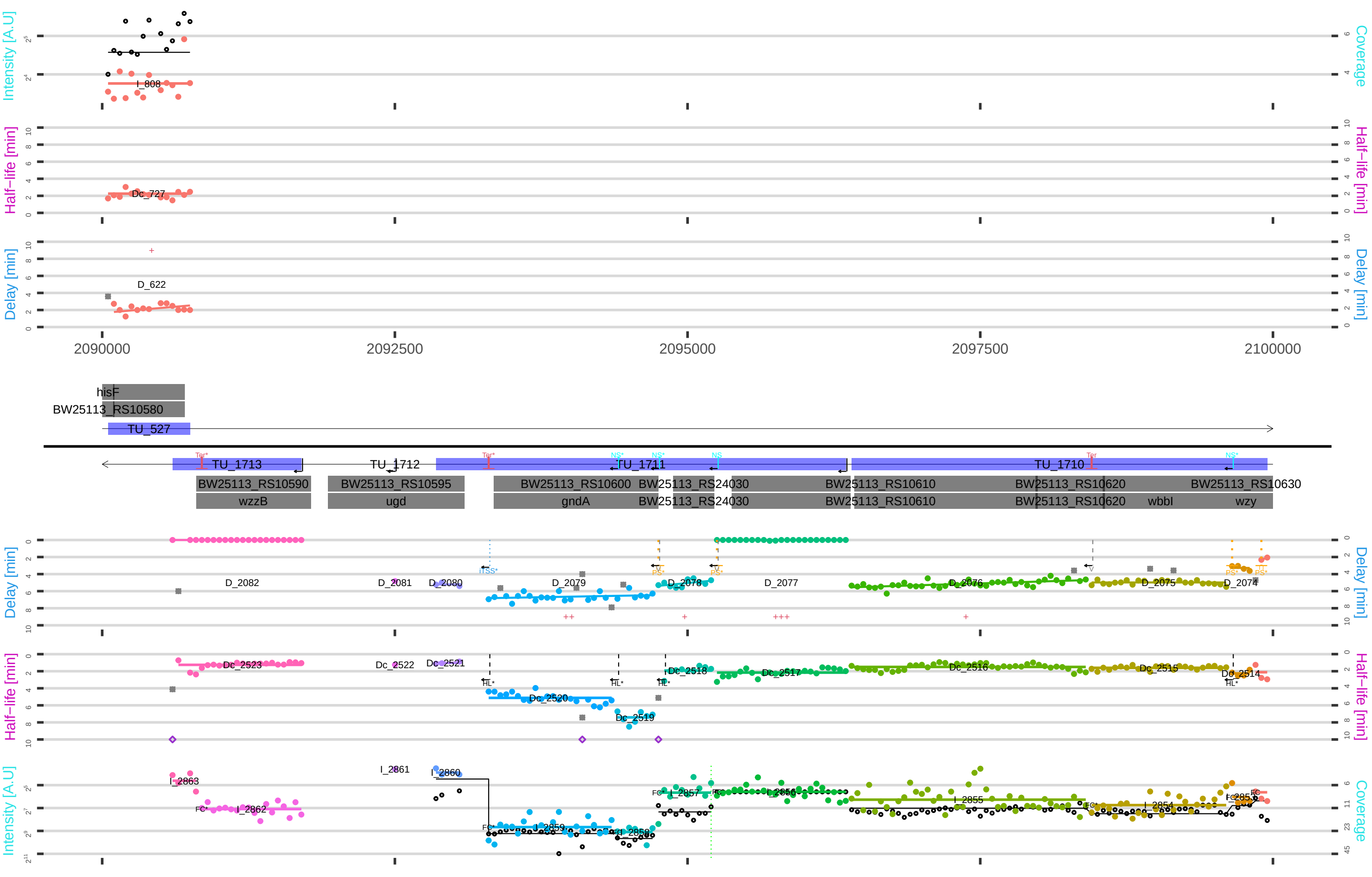

Term: termination (3), NS: new start (4), PS: pausing site (4), iTSS_I: internal starting site (2)

ID: 143260−143104; FC*: significant t−test of two consecutive segments; Term: termination, NS: new start, PS: pausing site,iTSS_I: internal starting site,TI: transcription interferance.

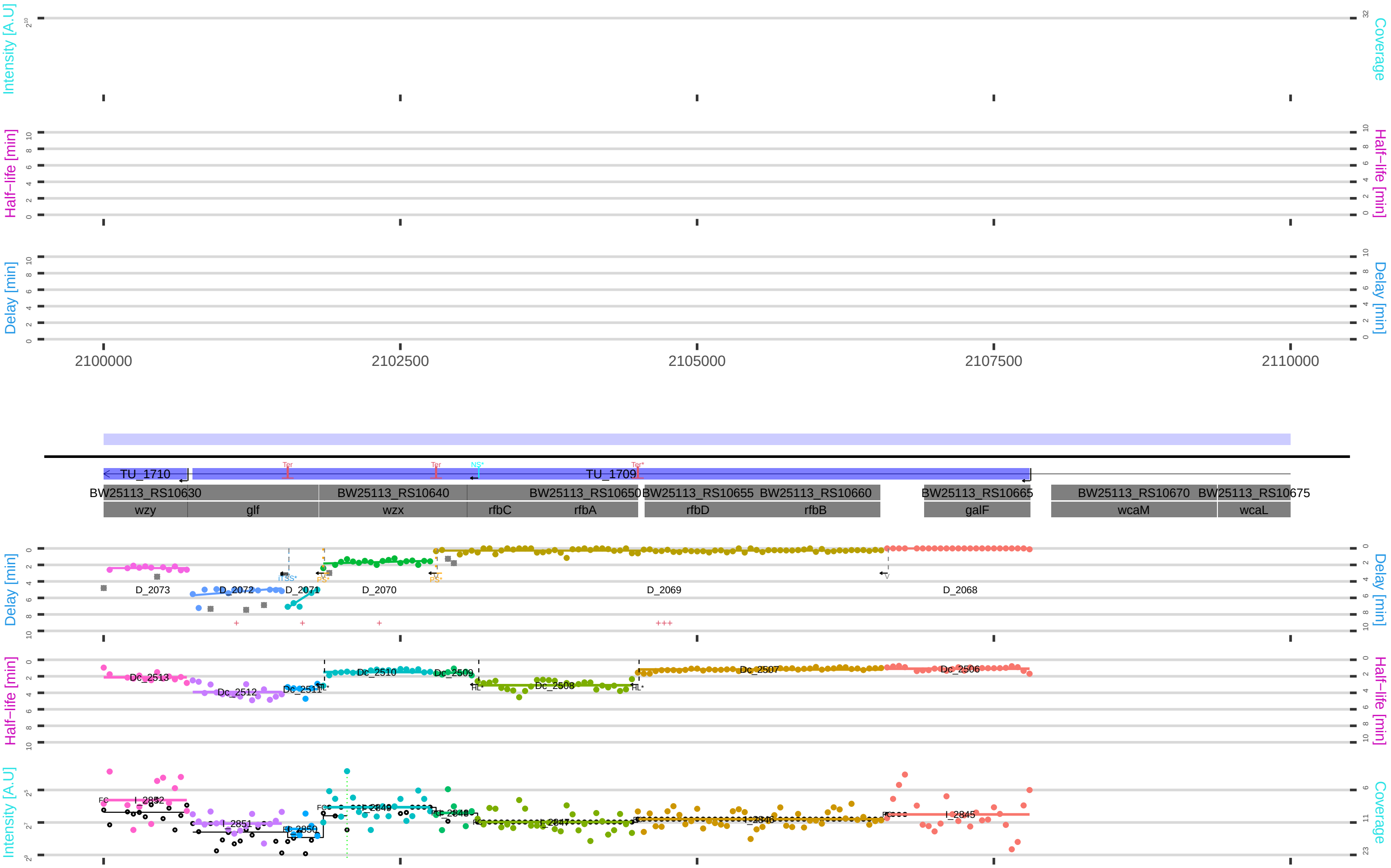

Term: termination (3), NS: new start (1), PS: pausing site (3), iTSS_I: internal starting site (1)

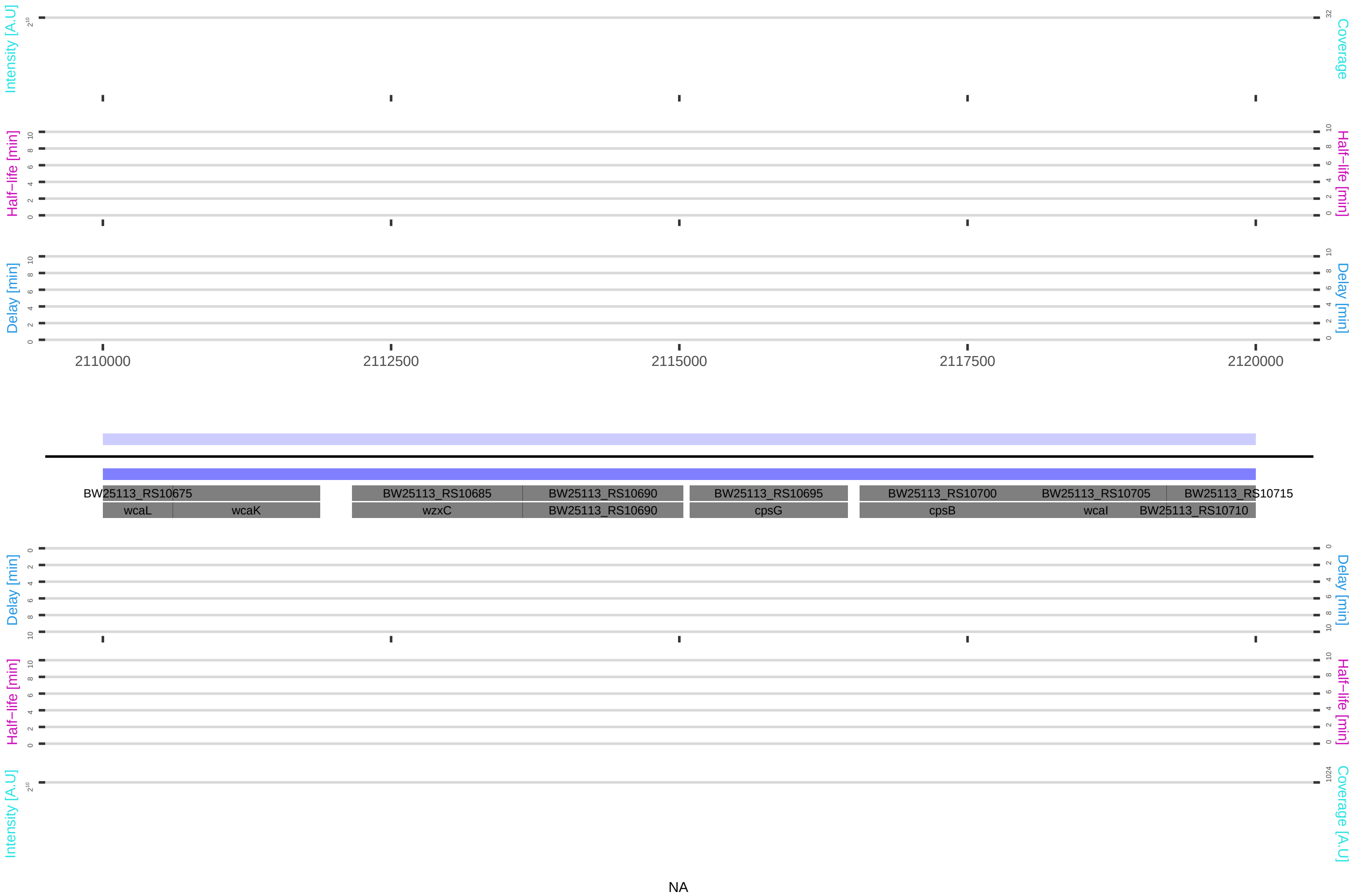

Intensity [A.U]
Coverage
Half−life [min]
Delay [min]
2110000
2112500
2115000
2117500
2120000
BW25113_RS10675
wcaL
wcaK
BW25113_RS10685
wzxC
BW25113_RS10690
BW25113_RS10690
BW25113_RS10695
cpsG
BW25113_RS10700
cpsB
BW25113_RS10705
wcaI
BW25113_RS10715
BW25113_RS10710
Coverage [A.U]
NA

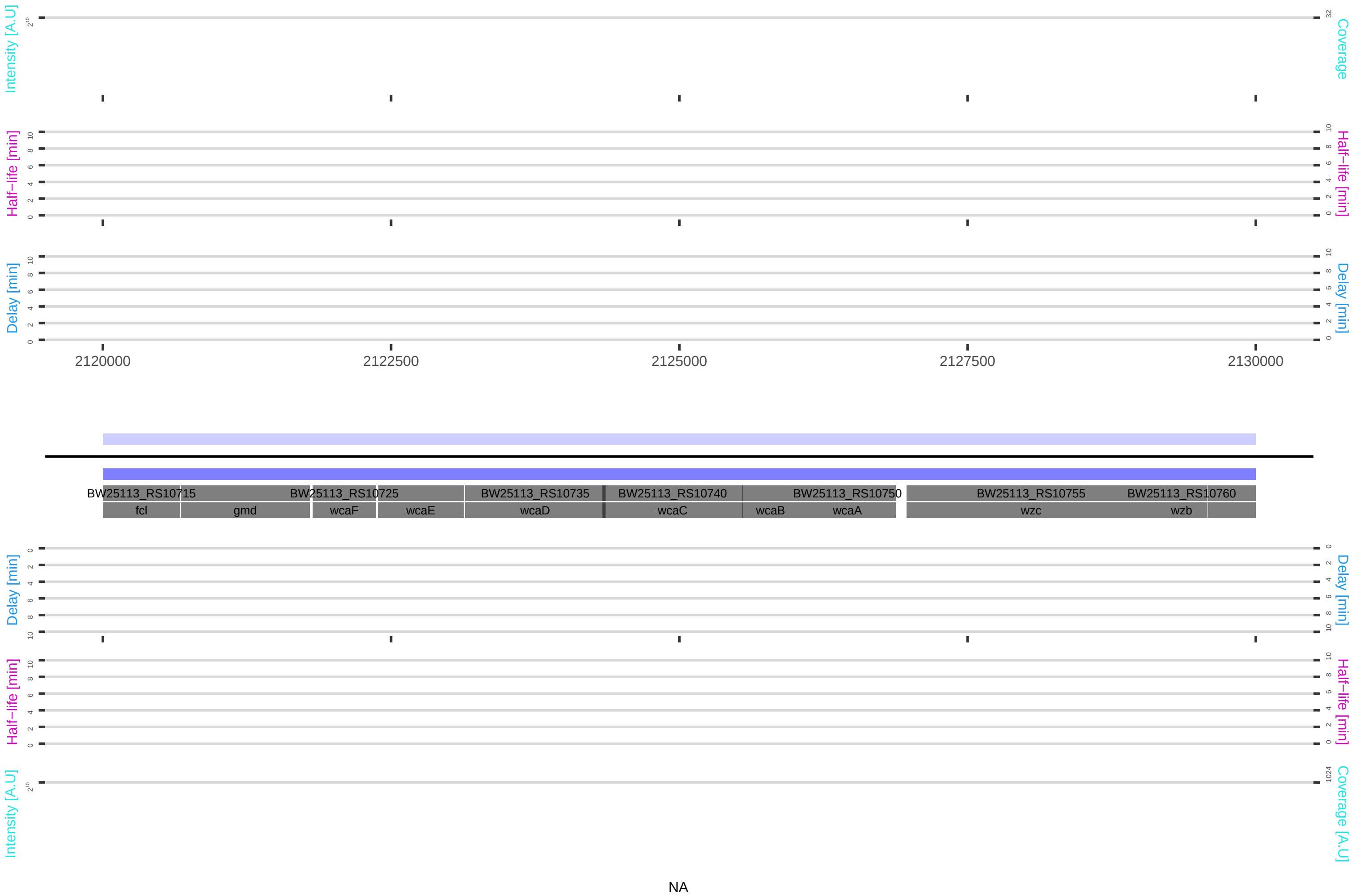

Intensity [A.U]
Coverage
Half−life [min]
Delay [min]
2120000
2122500
2125000
2127500
2130000
BW25113_RS10715
BW25113_RS10725
BW25113_RS10735
BW25113_RS10740
BW25113_RS10750
BW25113_RS10755
BW25113_RS10760
fcl
gmd
wcaF
wcaE
wcaD
wcaC
wcaB
wcaA
wzc
wzb
Coverage [A.U]
NA

ID: 42630−42661; Term: termination (0), NS: new start (0), PS: pausing site (0), iTSS_I: internal starting site (0)

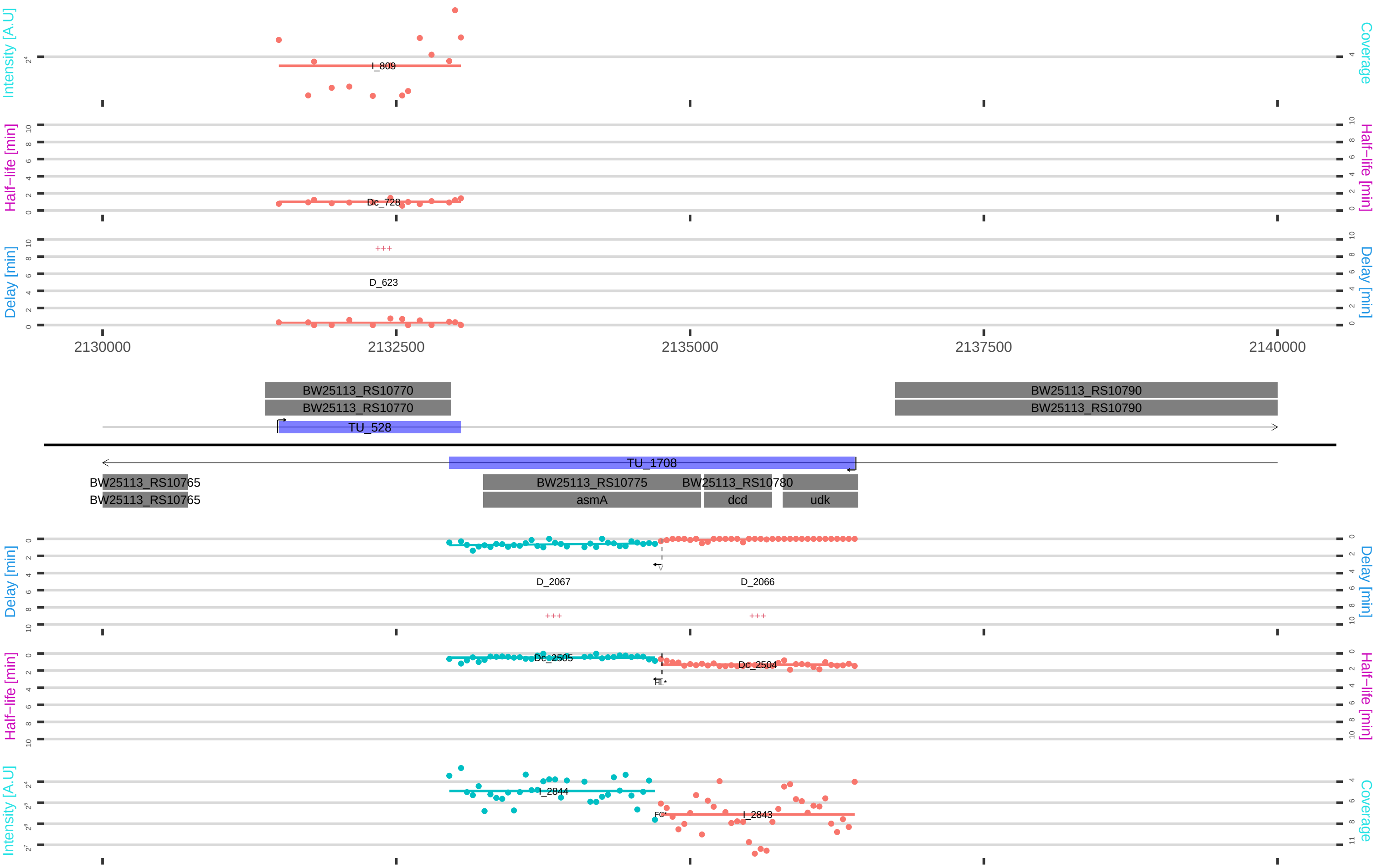

Term: termination (0), NS: new start (0), PS: pausing site (1), iTSS_I: internal starting site (0)

ID: 42824−42945; Term: termination (0), NS: new start (0), PS: pausing site (0), iTSS_I: internal starting site (0)

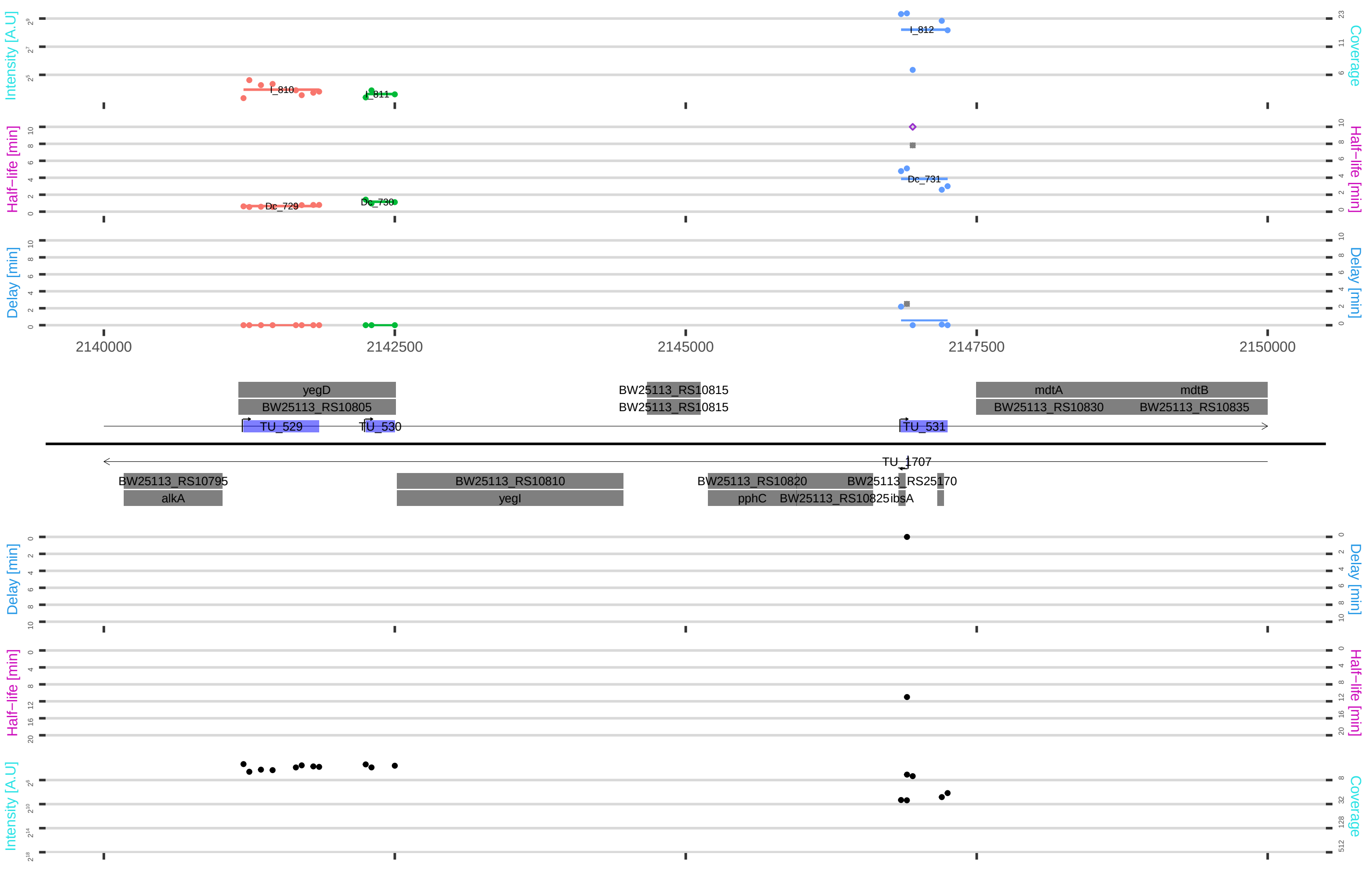

Term: termination (0), NS: new start (0), PS: pausing site (0), iTSS_I: internal starting site (0)

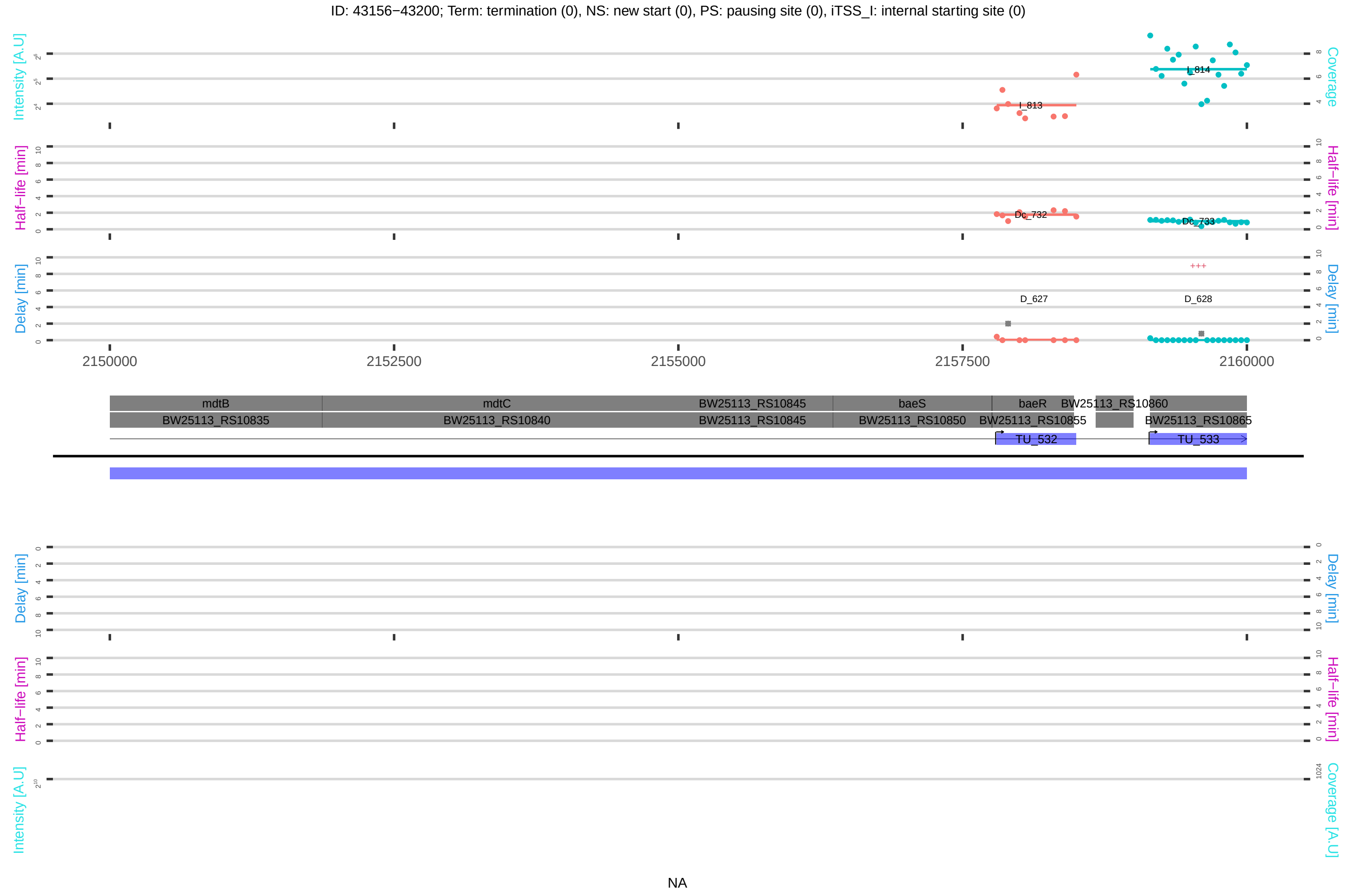

ID: 43156−43200; Term: termination (0), NS: new start (0), PS: pausing site (0), iTSS_I: internal starting site (0)
Intensity [A.U]
Coverage
Half−life [min]
Delay [min]
I_813
I_814
Dc_732
Dc_733
D_627
D_628
2150000
2152500
2155000
2157500
2160000
mdtB
mdtC
BW25113_RS10845
baeS
baeR
BW25113_RS10860
BW25113_RS10835
BW25113_RS10840
BW25113_RS10850
BW25113_RS10855
BW25113_RS10865
TU_532
TU_533
Coverage [A.U]
NA

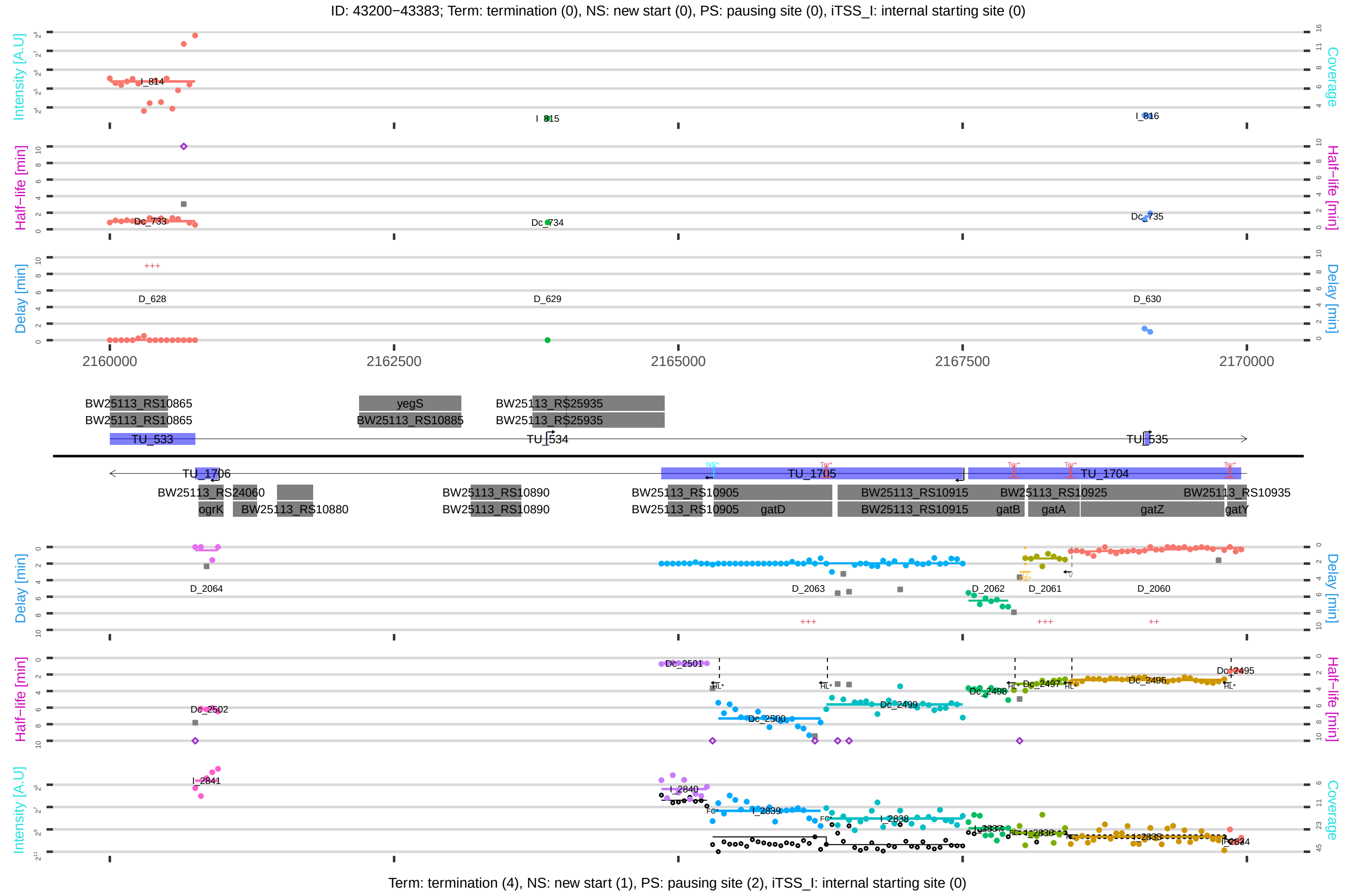
ID: 43200−43383; Term: termination (0), NS: new start (0), PS: pausing site (0), iTSS_I: internal starting site (0)
Intensity [A.U]
Coverage
Half−life [min]
Delay [min]
I_814
I_815
I_816
Dc_733
Dc_734
Dc_735
D_628
D_629
D_630
2160000
2162500
2165000
2167500
2170000
BW25113_RS10865
yegS
BW25113_RS10885
BW25113_RS25935
TU_533
TU_534
TU_535
TU_1706
TU_1705
TU_1704
BW25113_RS24060
ogrK
BW25113_RS10880
BW25113_RS10890
BW25113_RS10905
gatD
BW25113_RS10915
gatB
BW25113_RS10925
gatA
gatZ
BW25113_RS10935
gatY
D_2064
D_2063
D_2062
D_2061
D_2060
Dc_2501
Dc_2502
Dc_2500
Dc_2499
Dc_2498
Dc_2497
Dc_2496
Dc_2495
I_2841
I_2840
I_2839
I_2838
I_2837
I_2836
I_2835
I_2834
Term: termination (4), NS: new start (1), PS: pausing site (2), iTSS_I: internal starting site (0)

ID: 43414−43473; Term: termination (0), NS: new start (0), PS: pausing site (0), iTSS_I: internal starting site (0)

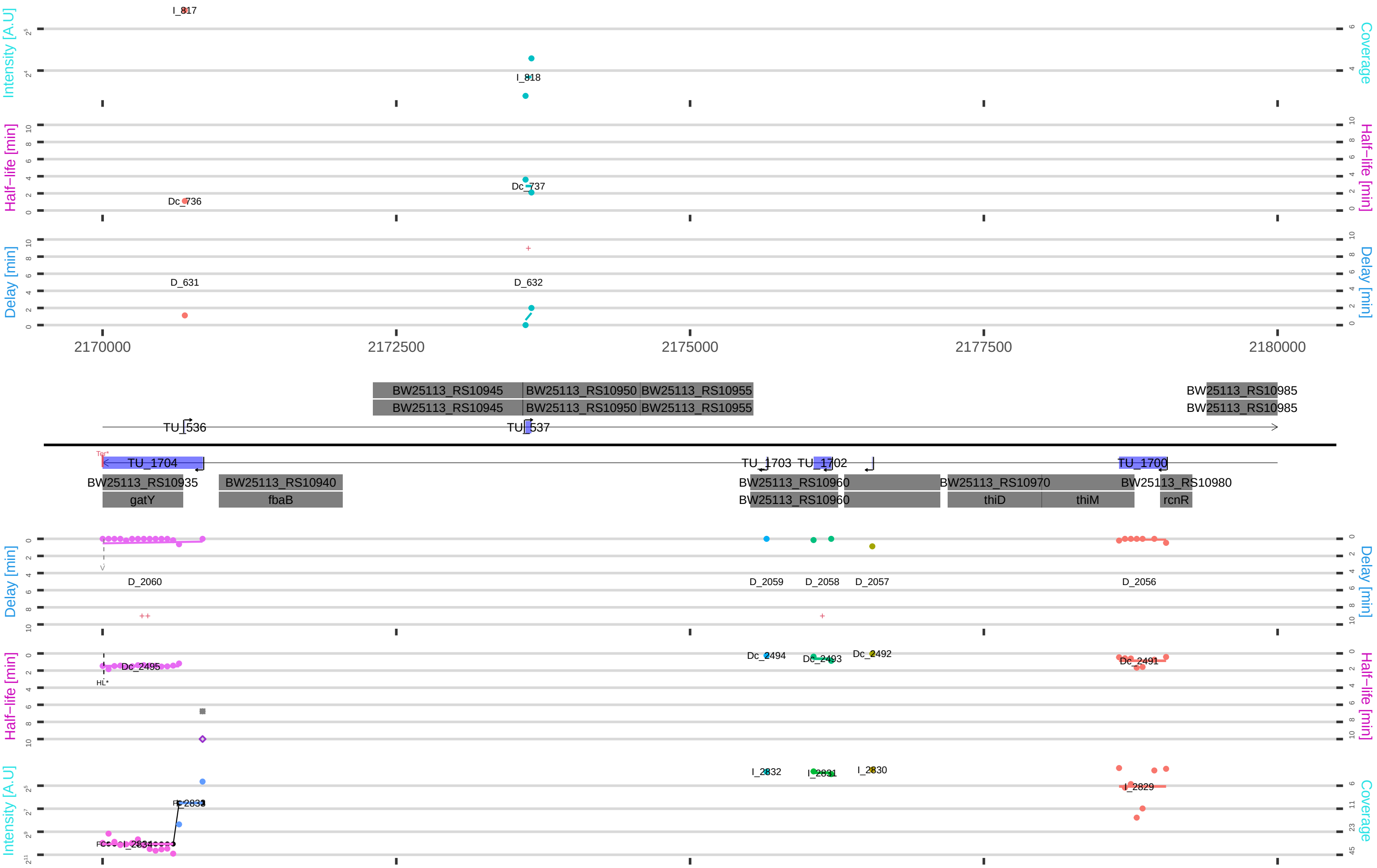

Term: termination (1), NS: new start (0), PS: pausing site (0), iTSS_I: internal starting site (0)

ID: 43609−43797; Term: termination (0), NS: new start (0), PS: pausing site (0), iTSS_I: internal starting site (0)

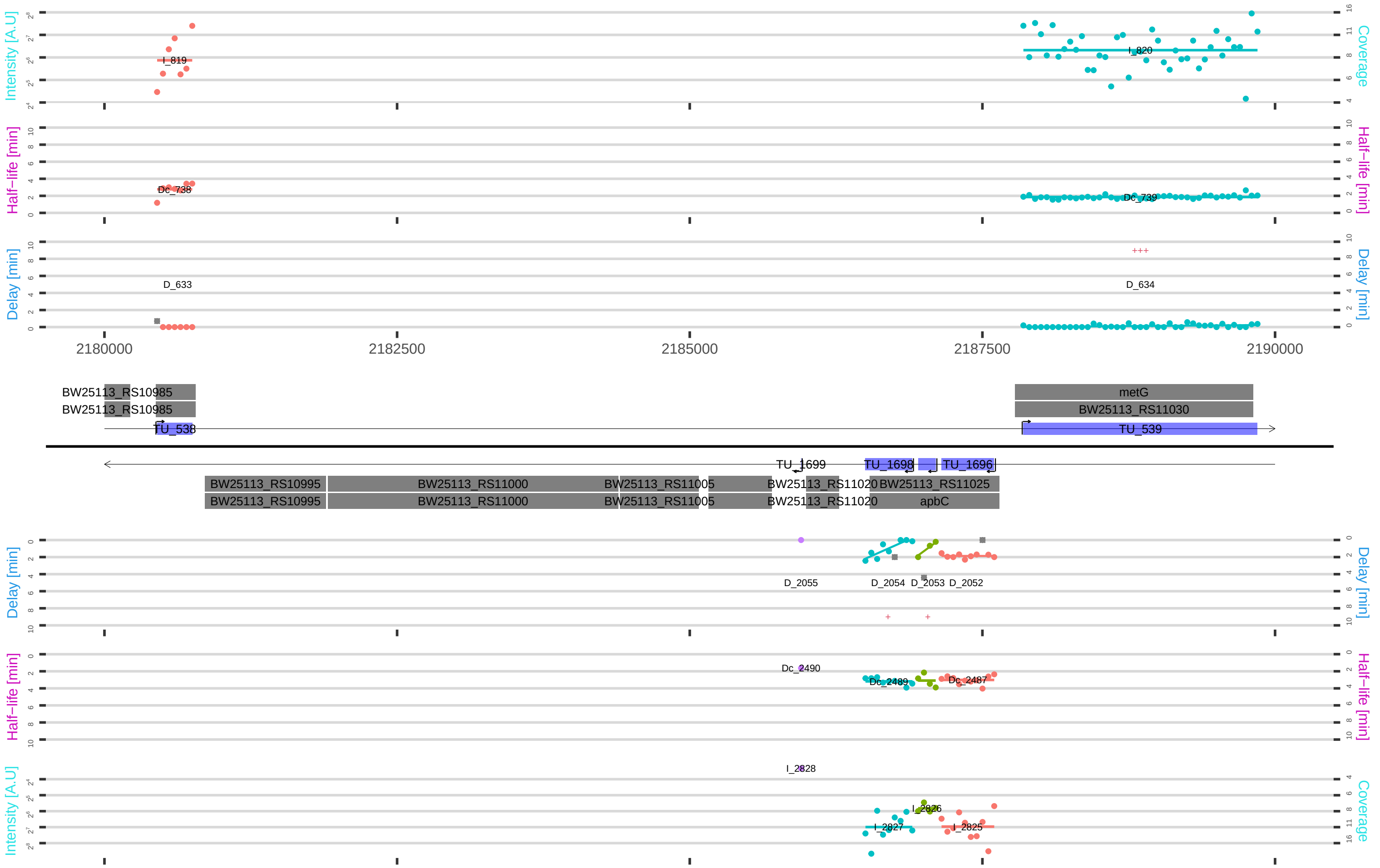

Term: termination (0), NS: new start (0), PS: pausing site (0), iTSS_I: internal starting site (0)

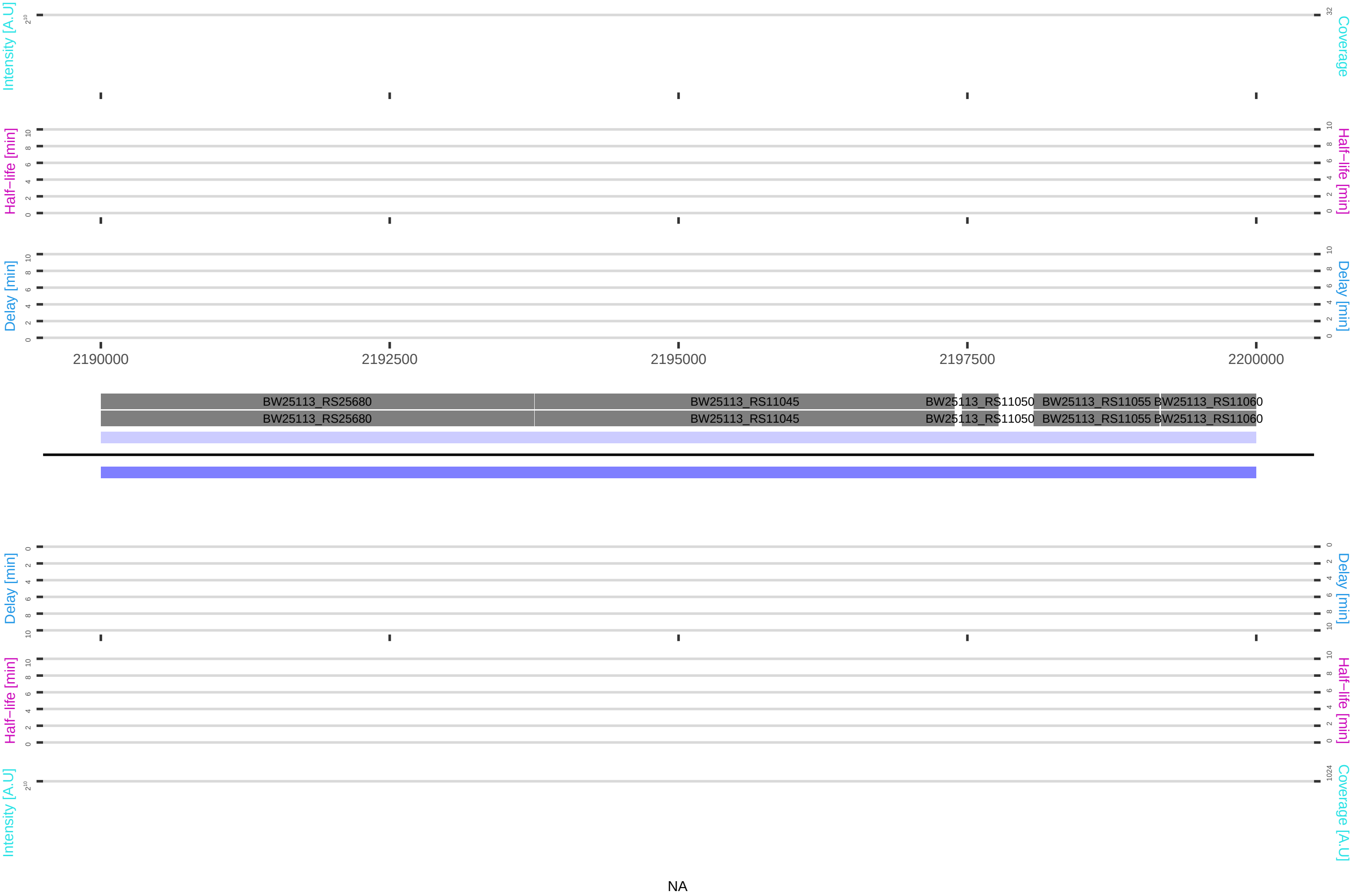

Intensity [A.U]
Coverage
Half−life [min]
Delay [min]
2190000
2192500
2195000
2197500
2200000
BW25113_RS25680
BW25113_RS11045
BW25113_RS11050
BW25113_RS11055
BW25113_RS11060
Coverage [A.U]
NA

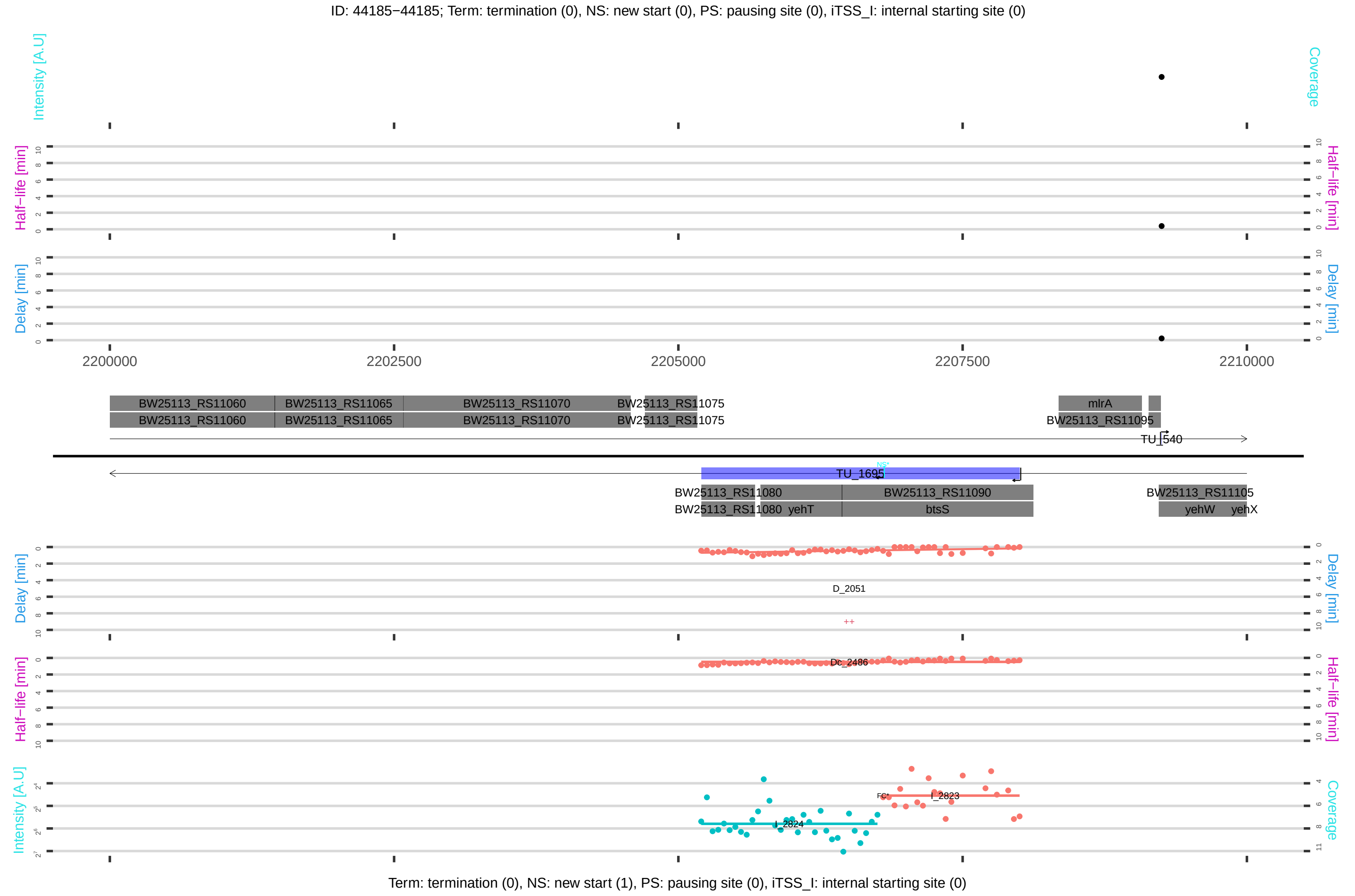

ID: 44185−44185; Term: termination (0), NS: new start (0), PS: pausing site (0), iTSS_I: internal starting site (0)
Intensity [A.U]
Coverage
Half−life [min]
Delay [min]
2200000
2202500
2205000
2207500
2210000
BW25113_RS11060
BW25113_RS11065
BW25113_RS11070
BW25113_RS11075
mlrA
BW25113_RS11095
TU_540
TU_1695
NS*
BW25113_RS11080
BW25113_RS11090
BW25113_RS11105
yehT
btsS
yehW
yehX
D_2051
Dc_2486
I_2824
I_2823
FC*
Term: termination (0), NS: new start (1), PS: pausing site (0), iTSS_I: internal starting site (0)

ID: 44315−44399; Term: termination (1), NS: new start (0), PS: pausing site (1), iTSS_I: internal starting site (0)

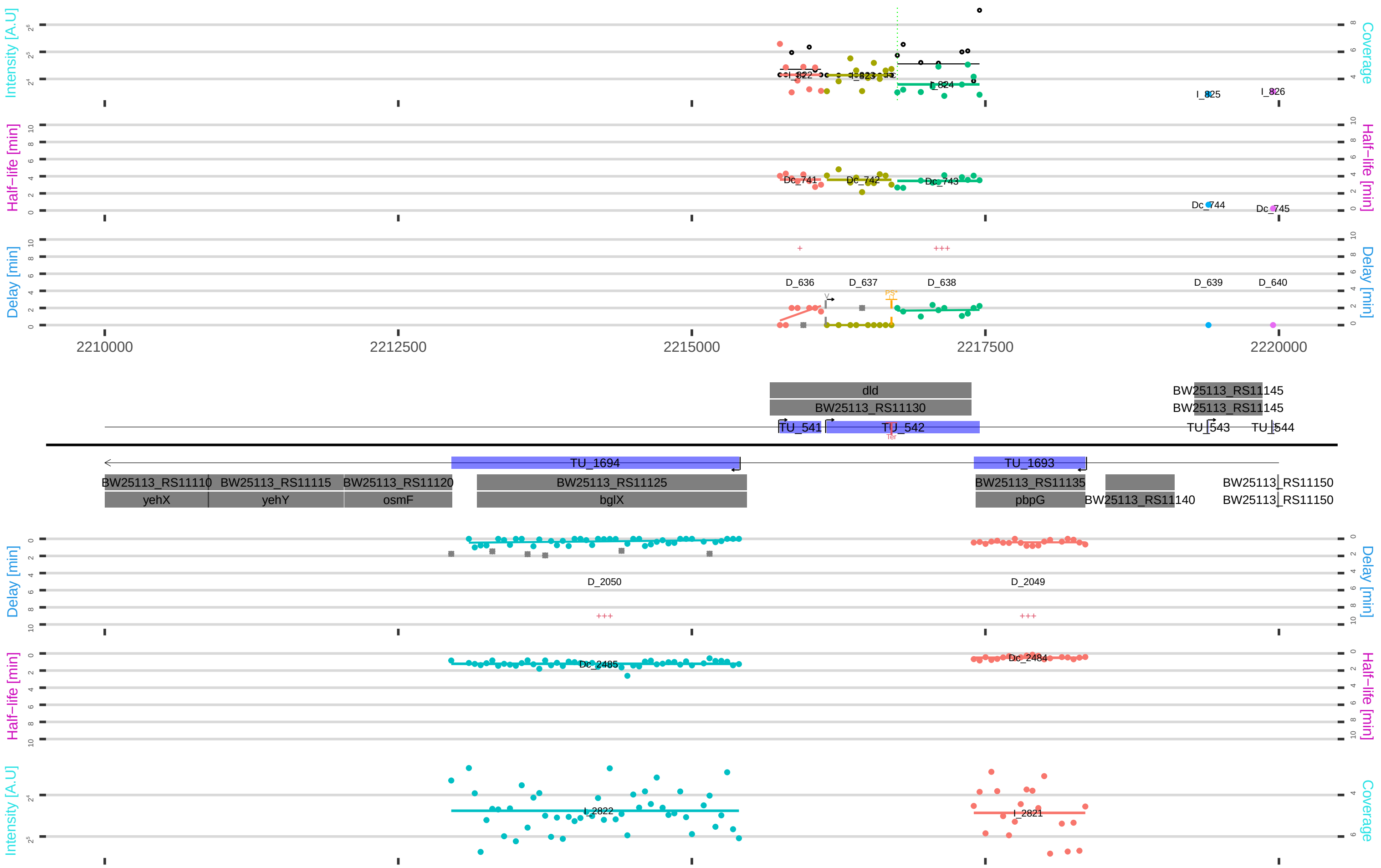

Term: termination (0), NS: new start (0), PS: pausing site (0), iTSS_I: internal starting site (0)

ID: 44507−44600; Term: termination (4), NS: new start (0), PS: pausing site (1), iTSS_I: internal starting site (0)

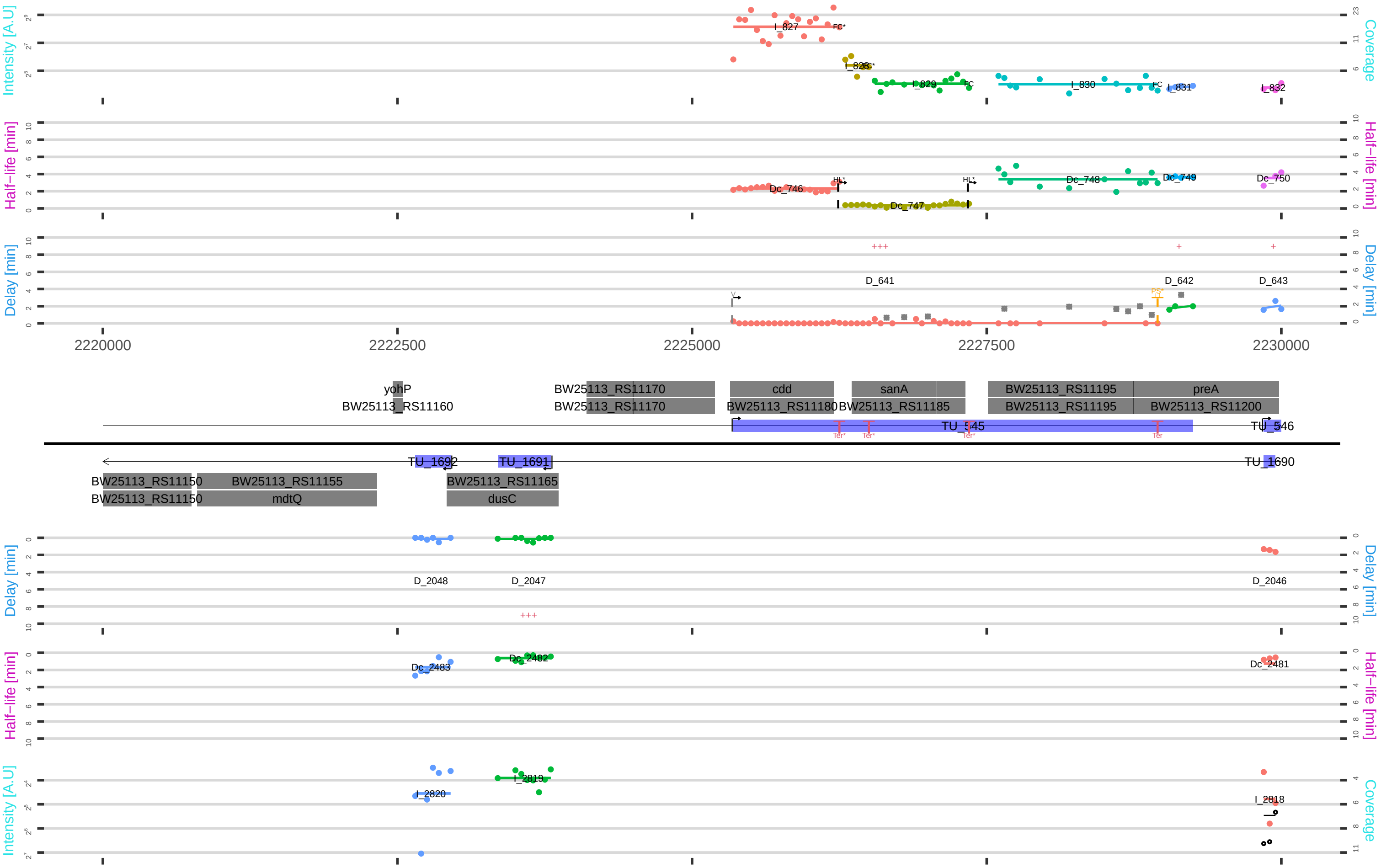

Term: termination (0), NS: new start (0), PS: pausing site (0), iTSS_I: internal starting site (0)

ID: 44600−44763; Term: termination (0), NS: new start (0), PS: pausing site (0), iTSS_I: internal starting site (0)

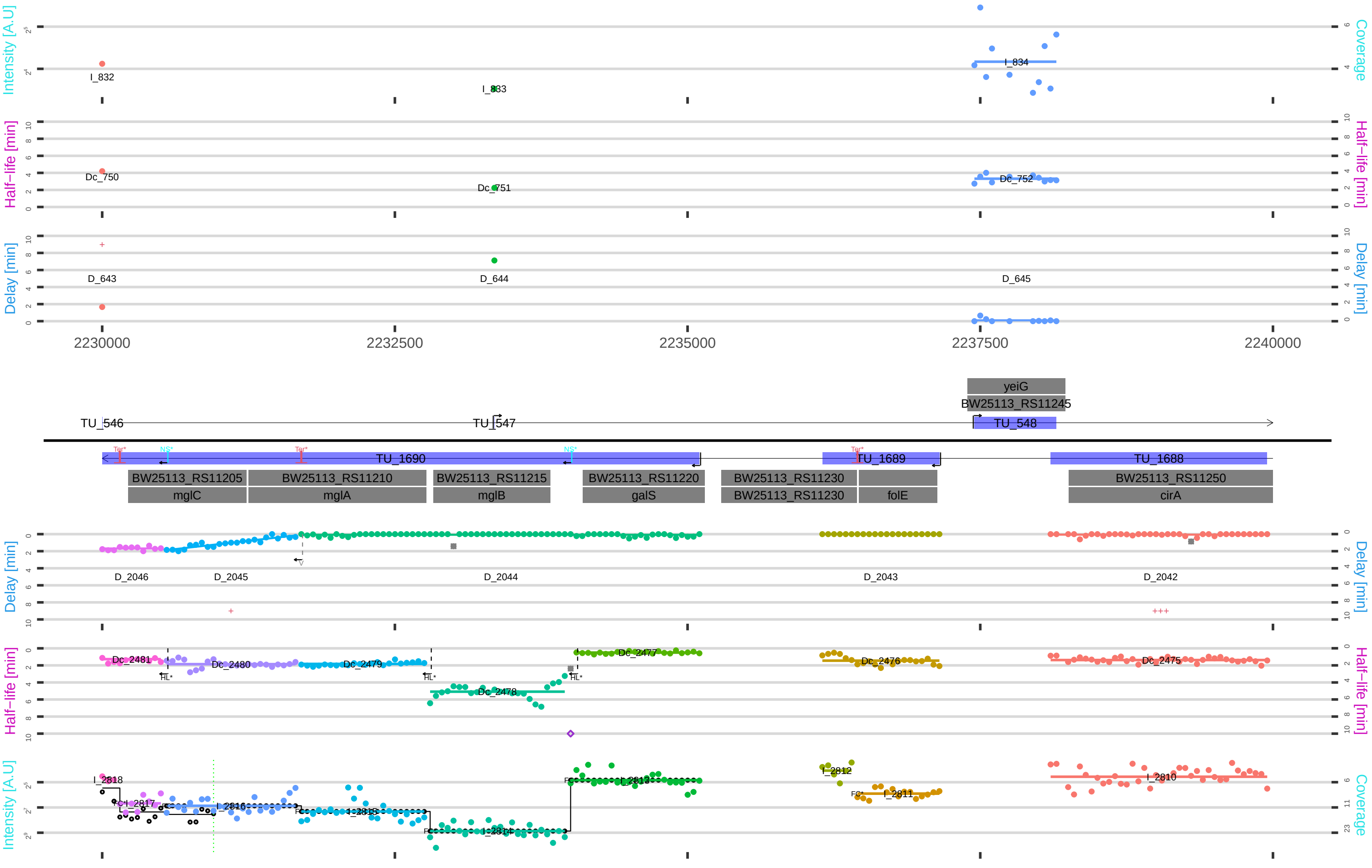

Term: termination (3), NS: new start (2), PS: pausing site (1), iTSS_I: internal starting site (1)

ID: 44886−44925; Term: termination (0), NS: new start (1), PS: pausing site (0), iTSS_I: internal starting site (0)

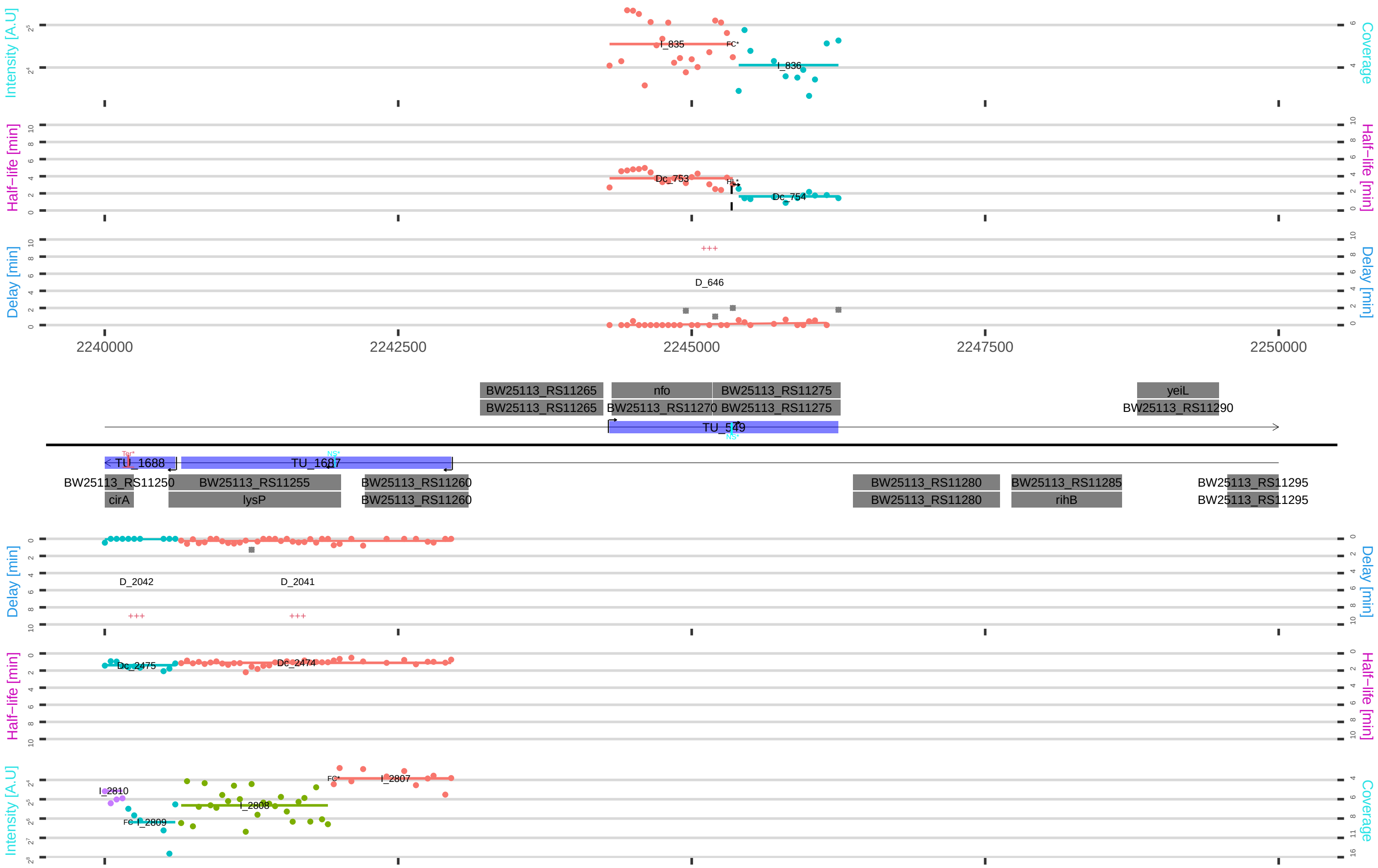

Term: termination (1), NS: new start (1), PS: pausing site (0), iTSS_I: internal starting site (0)

ID: 45063−45200; Term: termination (1), NS: new start (2), PS: pausing site (0), iTSS_I: internal starting site (0)

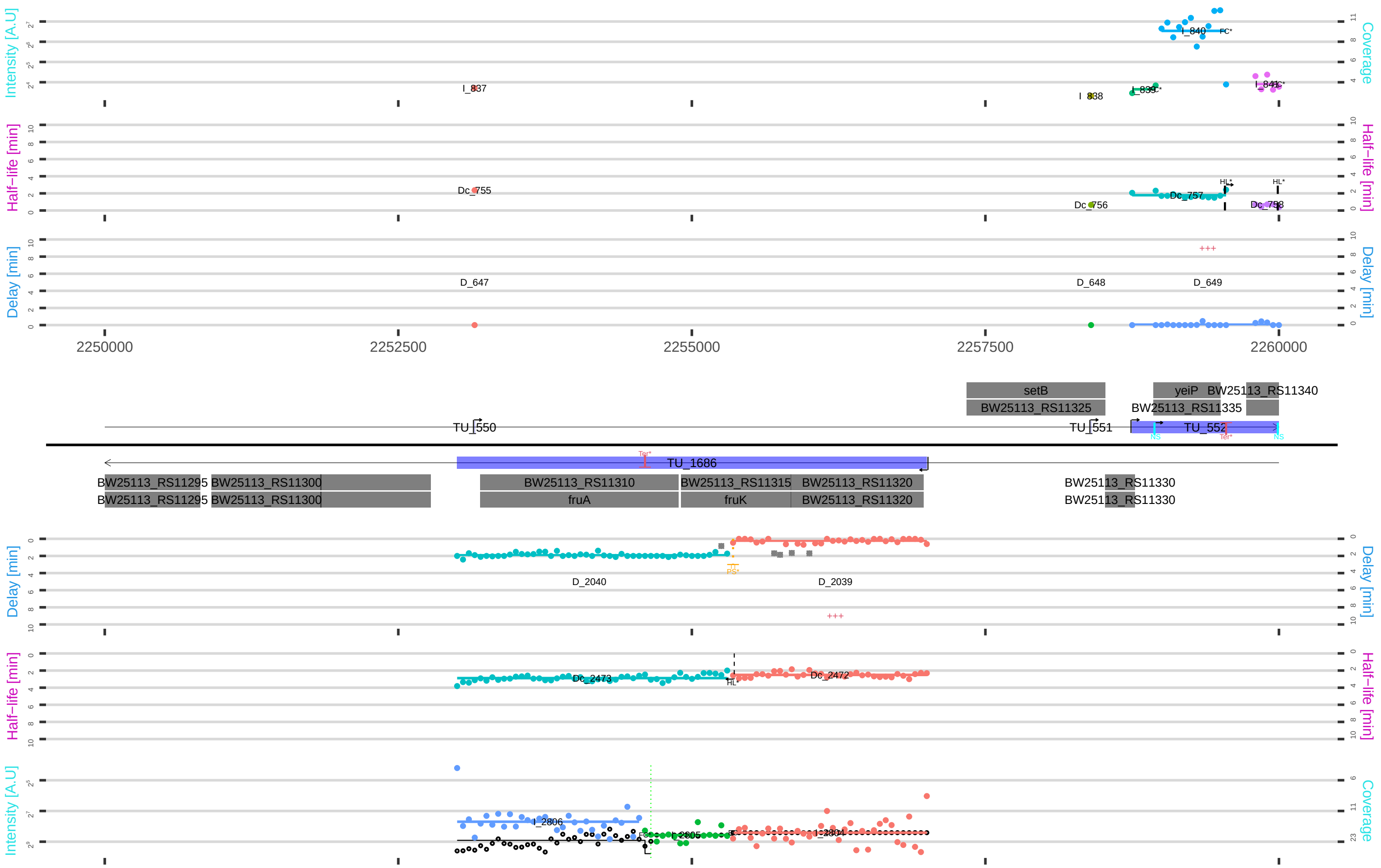

Term: termination (1), NS: new start (0), PS: pausing site (1), iTSS_I: internal starting site (0)

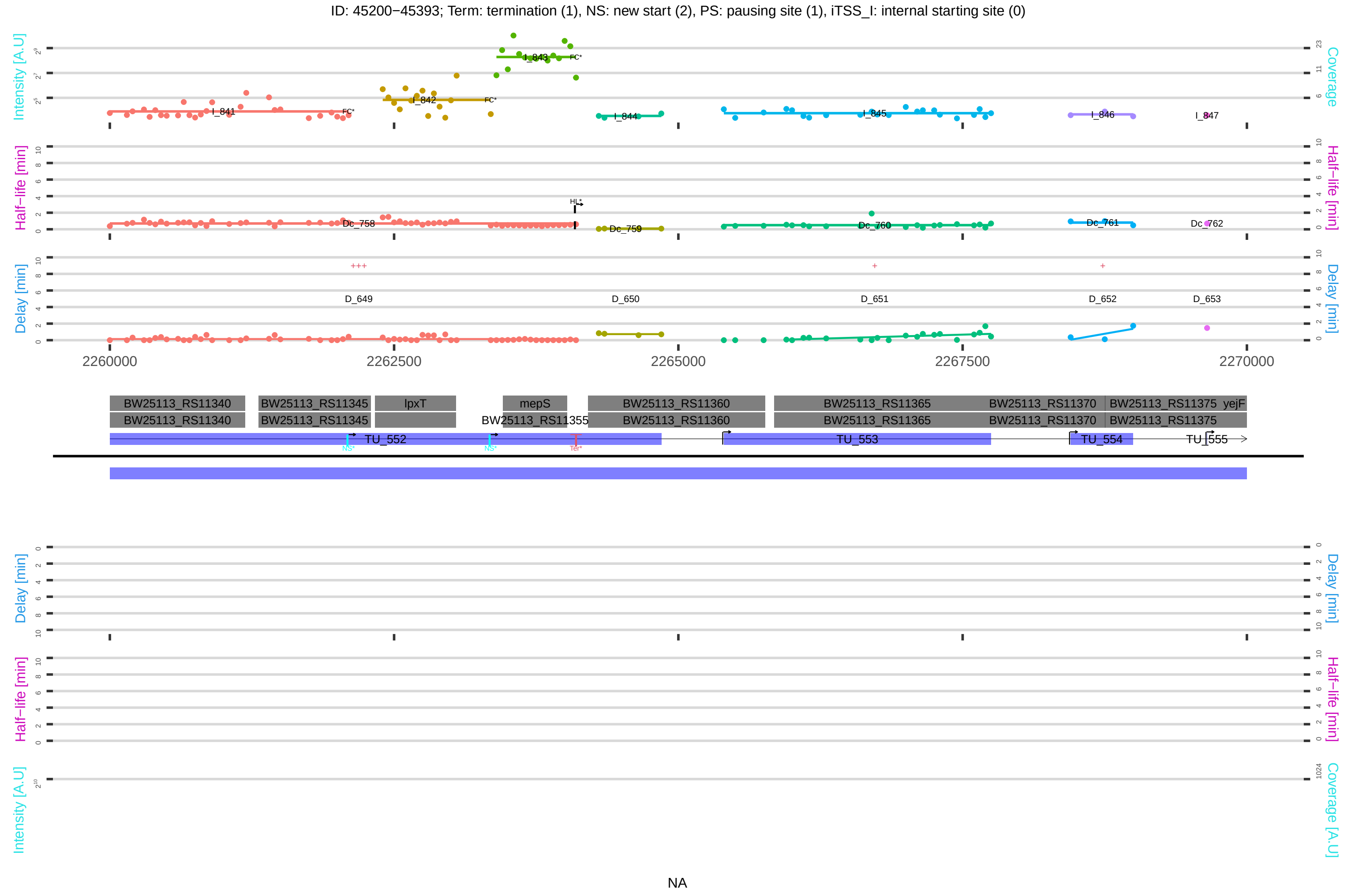

ID: 45200−45393; Term: termination (1), NS: new start (2), PS: pausing site (1), iTSS_I: internal starting site (0)
Intensity [A.U]
Coverage
Half−life [min]
Delay [min]
I_841
I_842
I_843
I_844
I_845
I_846
I_847
Dc_758
Dc_759
Dc_760
Dc_761
Dc_762
D_649
D_650
D_651
D_652
D_653
2260000
2262500
2265000
2267500
2270000
BW25113_RS11340
BW25113_RS11345
lpxT
mepS
BW25113_RS11355
BW25113_RS11360
BW25113_RS11365
BW25113_RS11370
BW25113_RS11375
yejF
TU_552
TU_553
TU_554
TU_555
NA

ID: 45409−45596; Term: termination (1), NS: new start (1), PS: pausing site (0), iTSS_I: internal starting site (0)

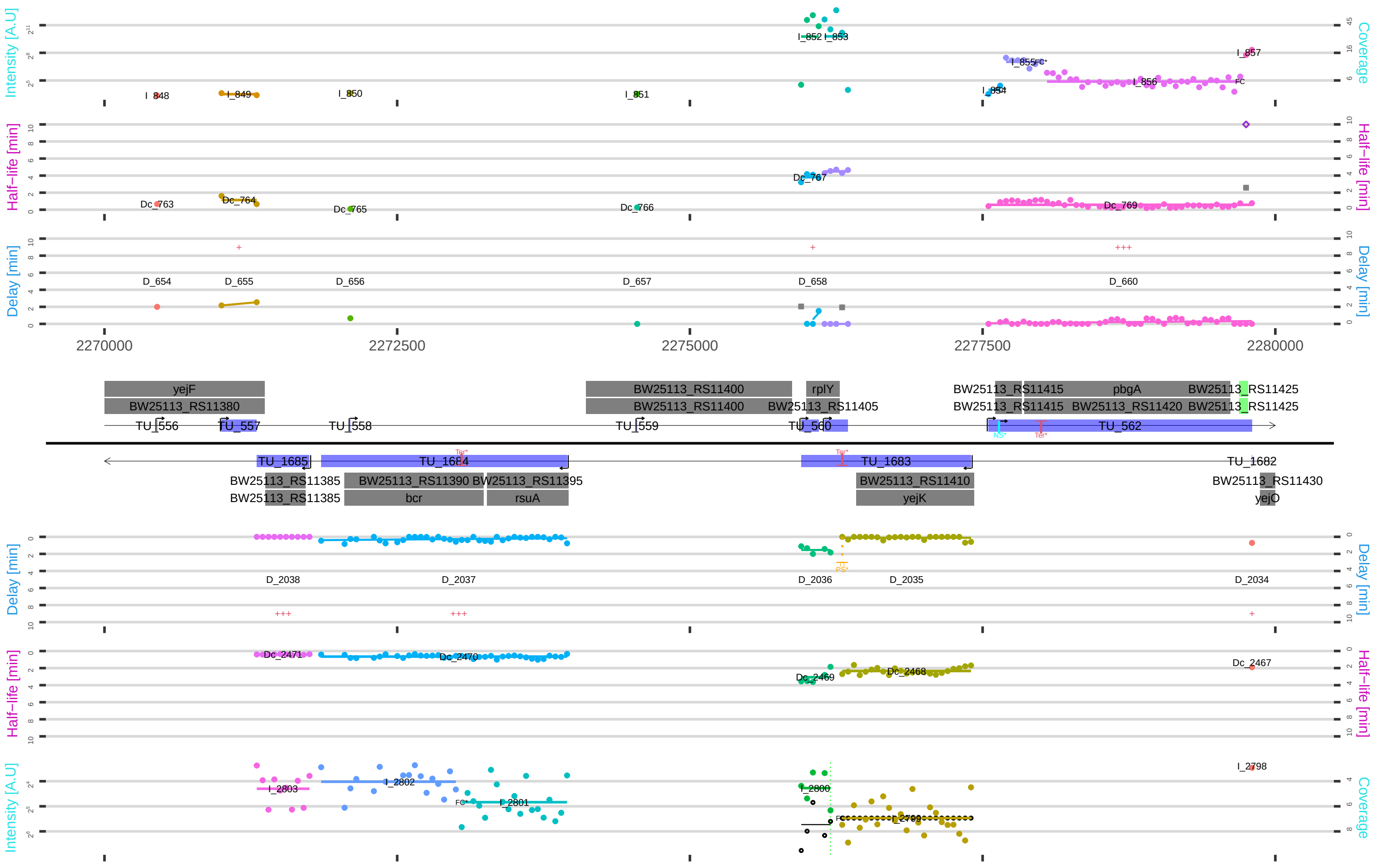

Term: termination (2), NS: new start (0), PS: pausing site (1), iTSS_I: internal starting site (0)

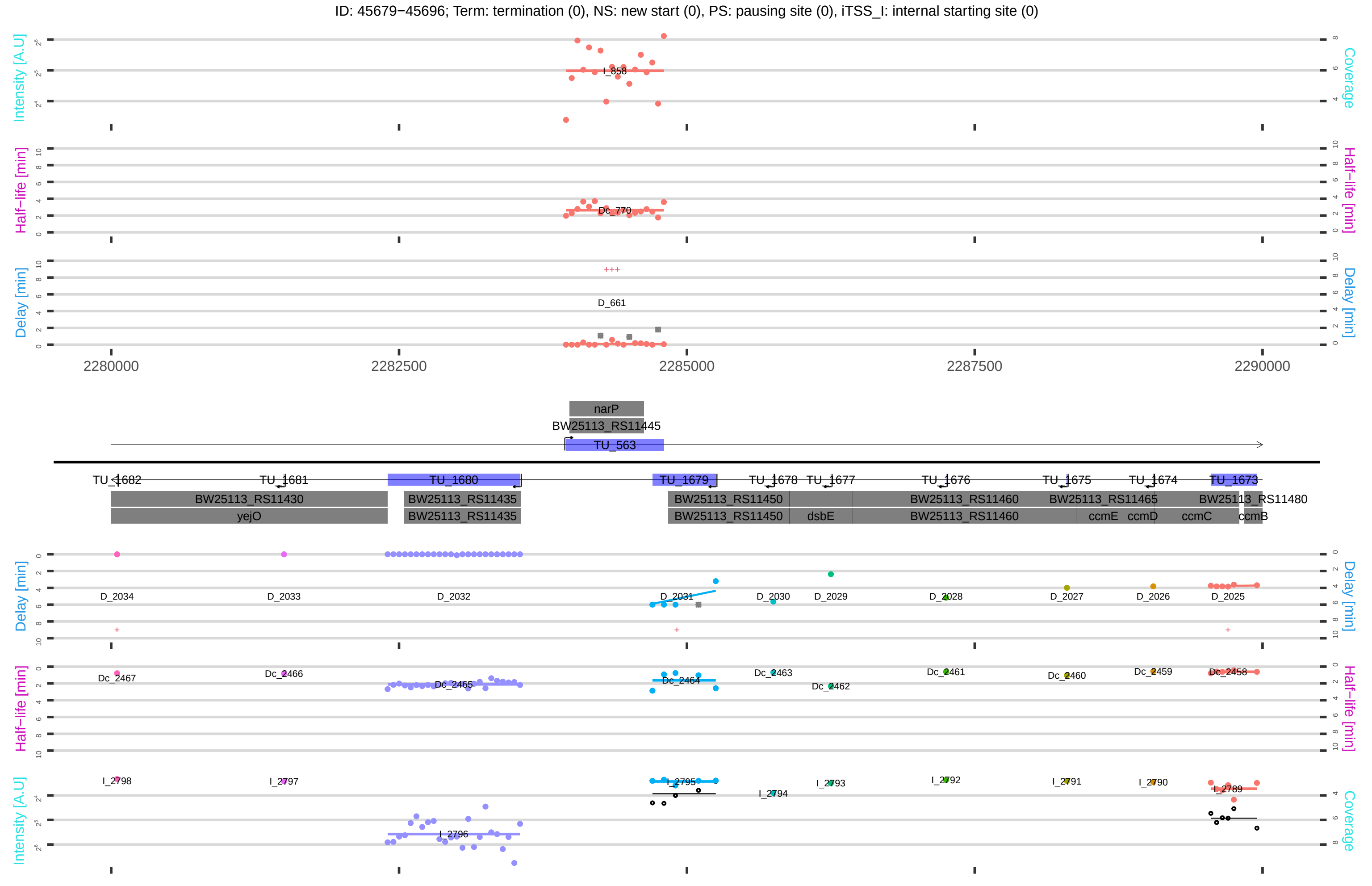

Term: termination (0), NS: new start (0), PS: pausing site (0), iTSS_I: internal starting site (0)

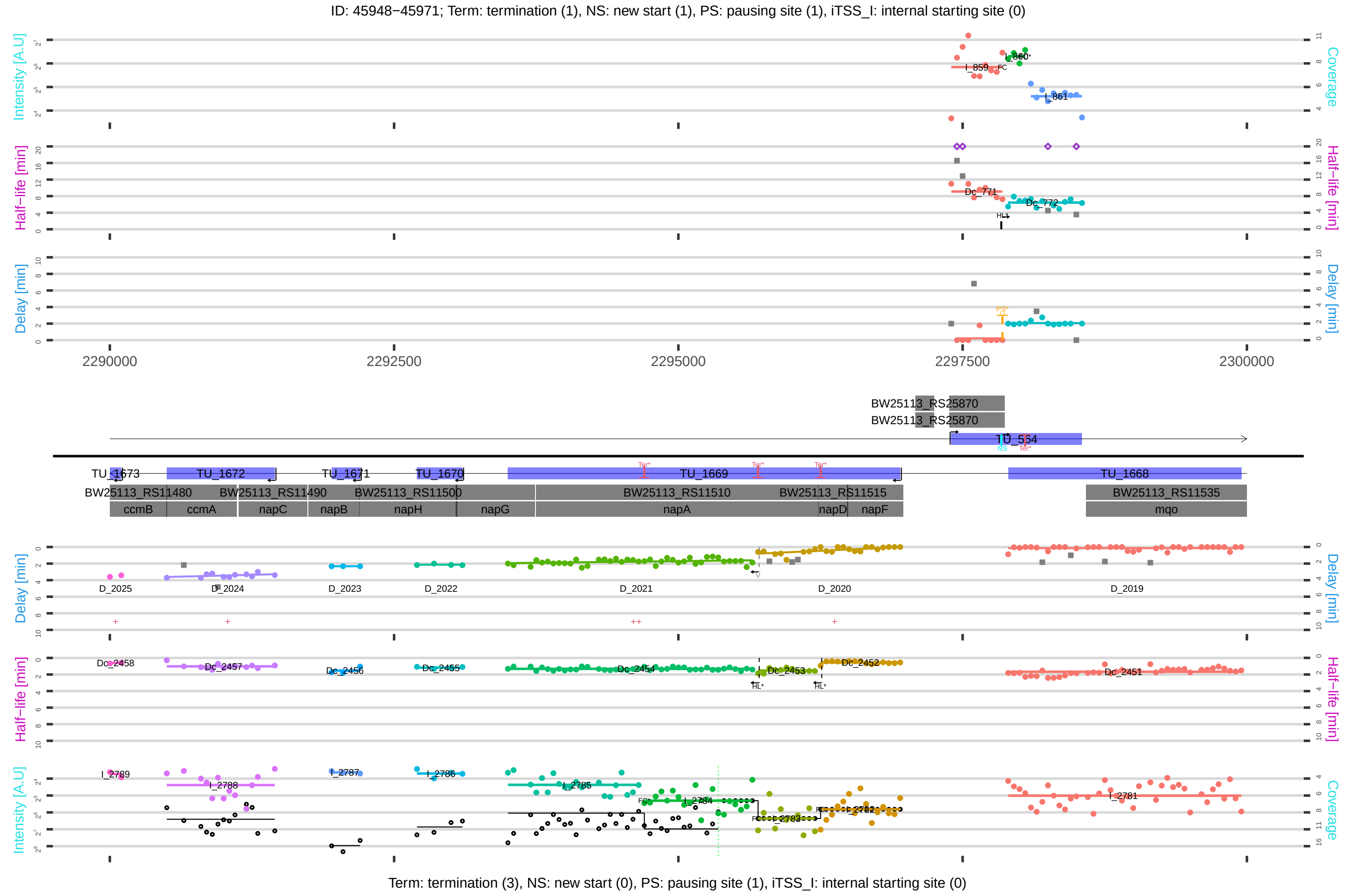
ID: 45948−45971; Term: termination (1), NS: new start (1), PS: pausing site (1), iTSS_I: internal starting site (0)
Intensity [A.U]
Coverage
Half−life [min]
Delay [min]
2290000
2292500
2295000
2297500
2300000
BW25113_RS25870
TU_564
TU_1673
TU_1672
TU_1671
TU_1670
TU_1669
TU_1668
BW25113_RS11480
BW25113_RS11490
BW25113_RS11500
BW25113_RS11510
BW25113_RS11515
BW25113_RS11535
ccmB
ccmA
napC
napB
napH
napG
napA
napD
napF
mqo
D_2025
D_2024
D_2023
D_2022
D_2021
D_2020
D_2019
Dc_2458
Dc_2457
Dc_2456
Dc_2455
Dc_2454
Dc_2453
Dc_2452
Dc_2451
I_2789
I_2788
I_2787
I_2786
I_2785
I_2784
I_2783
I_2782
I_2781
Term: termination (3), NS: new start (0), PS: pausing site (1), iTSS_I: internal starting site (0)

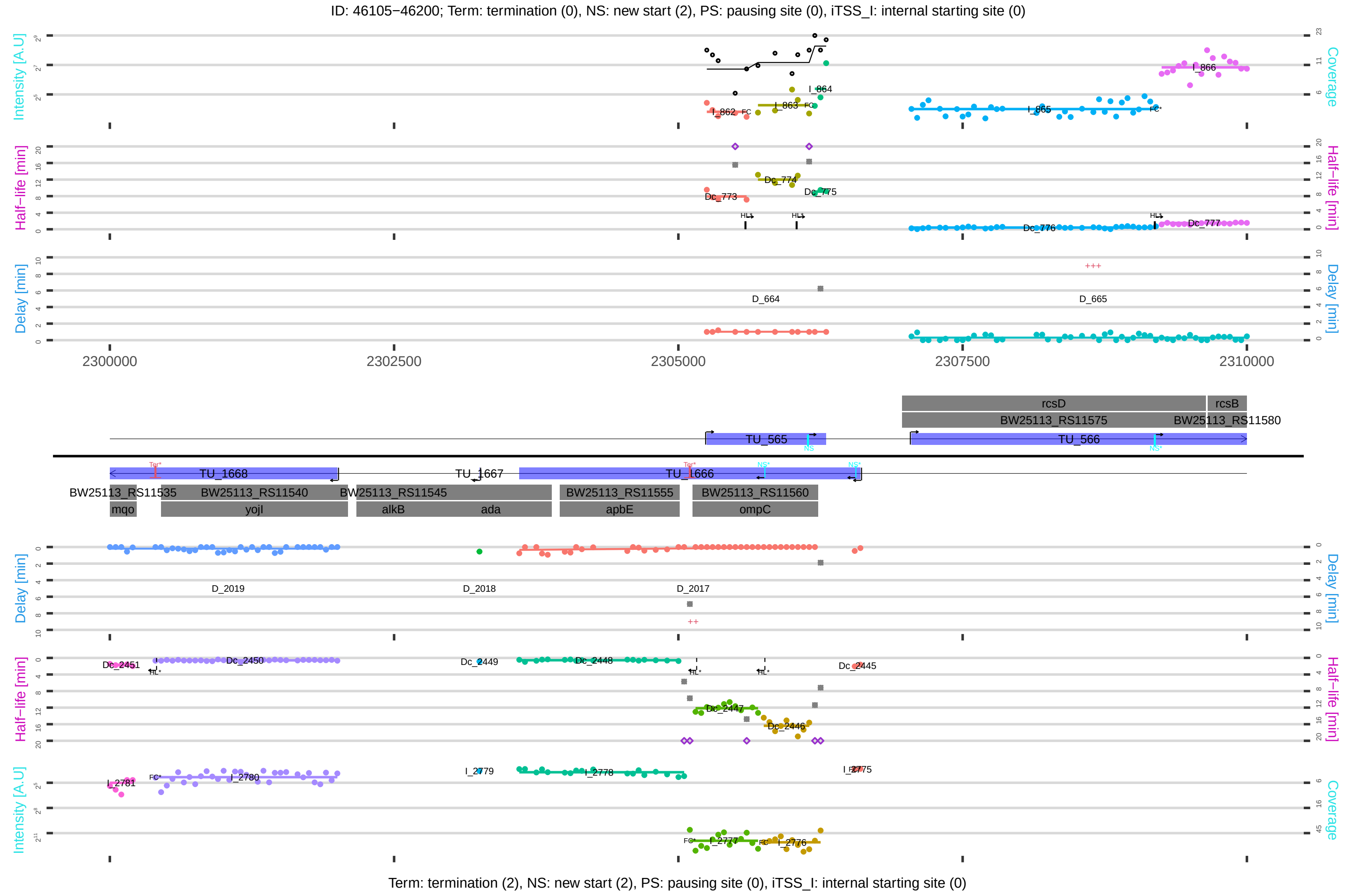

ID: 46105−46200; Term: termination (0), NS: new start (2), PS: pausing site (0), iTSS_I: internal starting site (0)
Intensity [A.U]
Coverage
Half−life [min]
Delay [min]
I_862
I_863
I_864
I_865
I_866
Dc_773
Dc_774
Dc_775
Dc_776
Dc_777
D_664
D_665
2300000
2302500
2305000
2307500
2310000
rcsD
rcsB
BW25113_RS11575
BW25113_RS11580
TU_565
TU_566
TU_1668
TU_1667
TU_1666
BW25113_RS11535
BW25113_RS11540
BW25113_RS11545
BW25113_RS11555
BW25113_RS11560
mqo
yojI
alkB
ada
apbE
ompC
D_2019
D_2018
D_2017
Dc_2451
Dc_2450
Dc_2449
Dc_2448
Dc_2447
Dc_2446
Dc_2445
I_2781
I_2780
I_2779
I_2778
I_2777
I_2776
I_2775
Term: termination (2), NS: new start (2), PS: pausing site (0), iTSS_I: internal starting site (0)

ID: 46200−46312; Term: termination (0), NS: new start (0), PS: pausing site (0), iTSS_I: internal starting site (0)

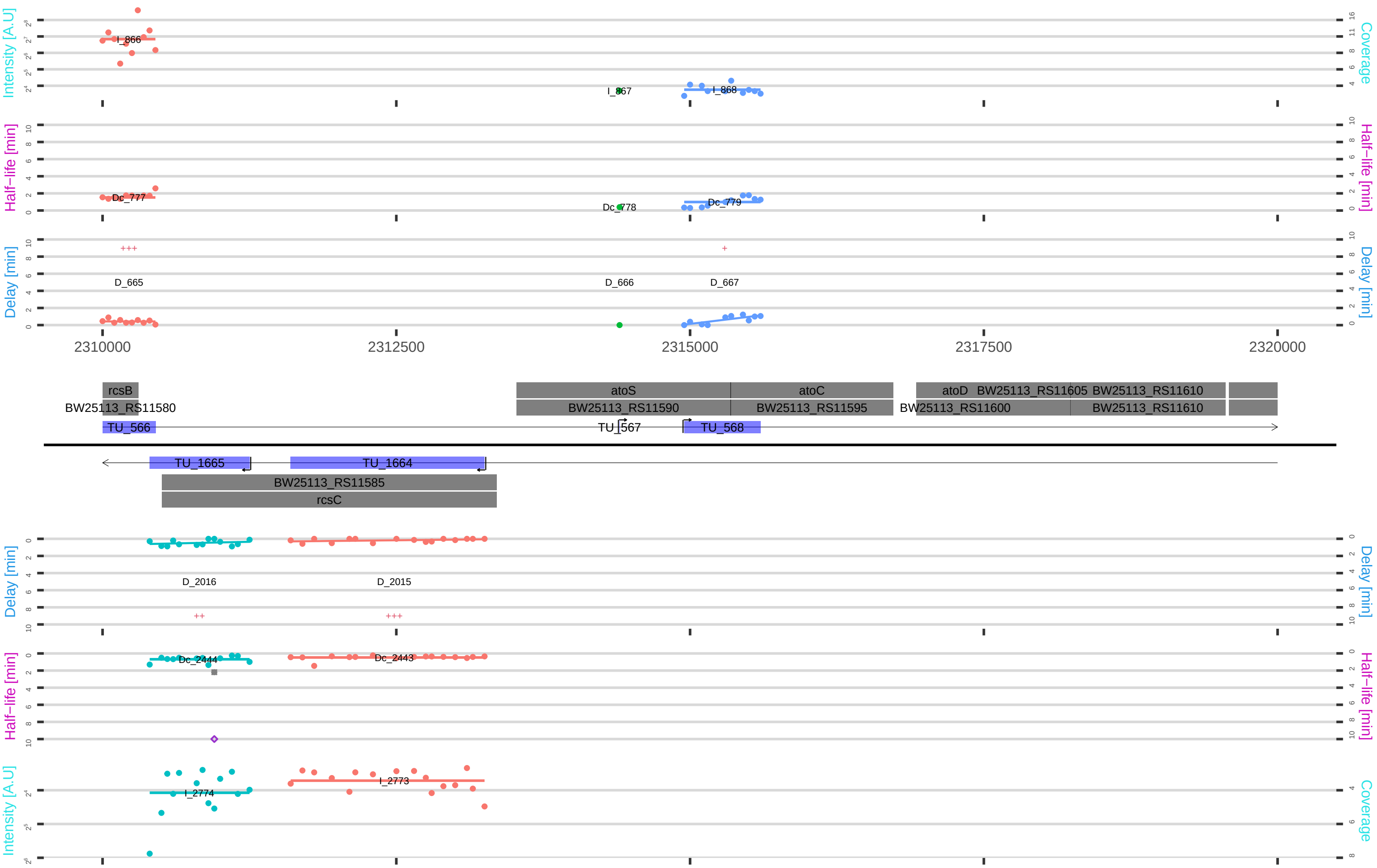

Term: termination (0), NS: new start (0), PS: pausing site (0), iTSS_I: internal starting site (0)

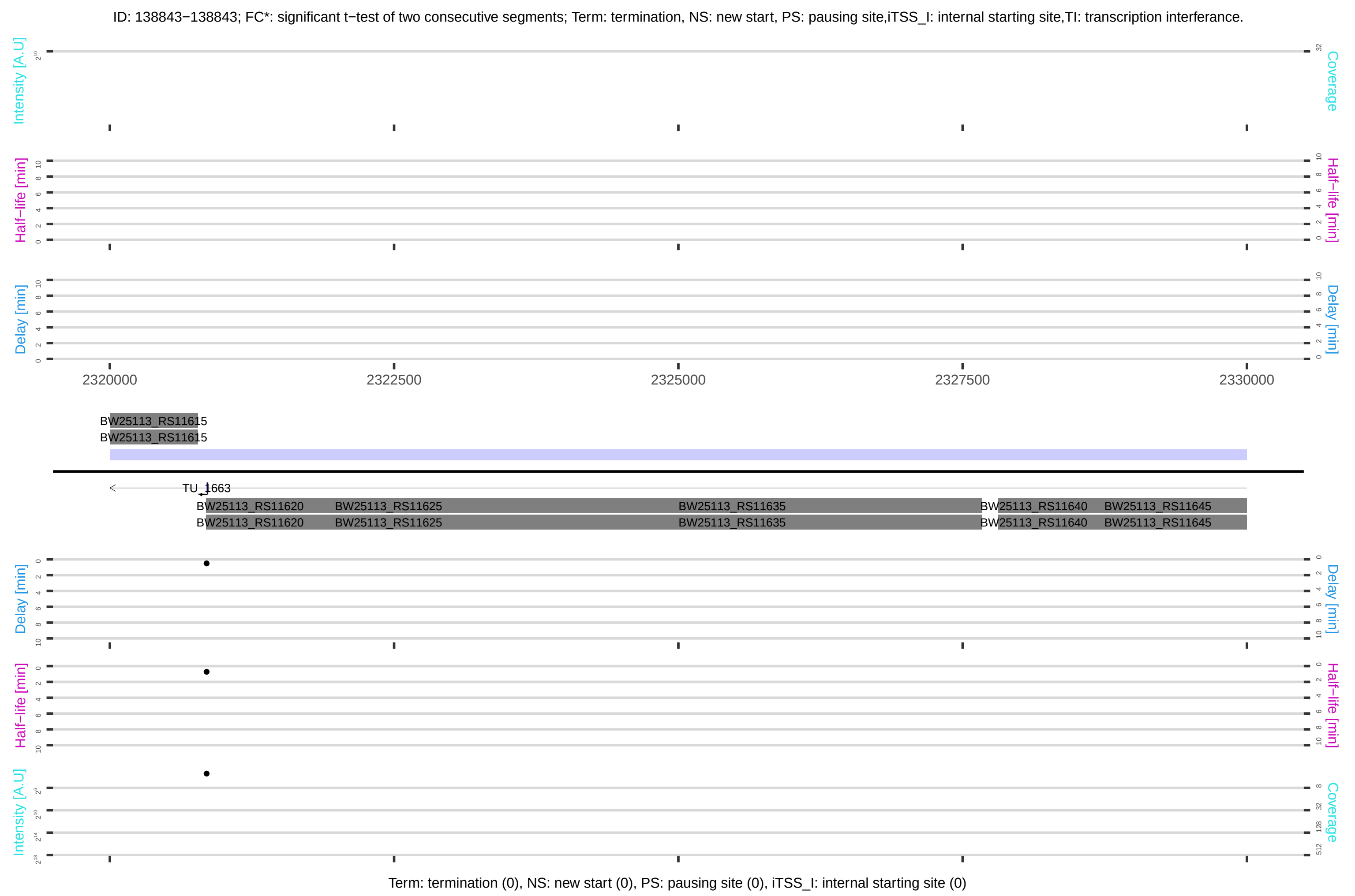
ID: 138843−138843; FC*: significant t−test of two consecutive segments; Term: termination, NS: new start, PS: pausing site,iTSS_I: internal starting site,TI: transcription interferance.
Intensity [A.U]
Coverage
Half−life [min]
Delay [min]
2320000
2322500
2325000
2327500
2330000
BW25113_RS11615
TU_1663
BW25113_RS11620
BW25113_RS11625
BW25113_RS11635
BW25113_RS11640
BW25113_RS11645
Term: termination (0), NS: new start (0), PS: pausing site (0), iTSS_I: internal starting site (0)

ID: 46662−46800; Term: termination (1), NS: new start (0), PS: pausing site (0), iTSS_I: internal starting site (0)

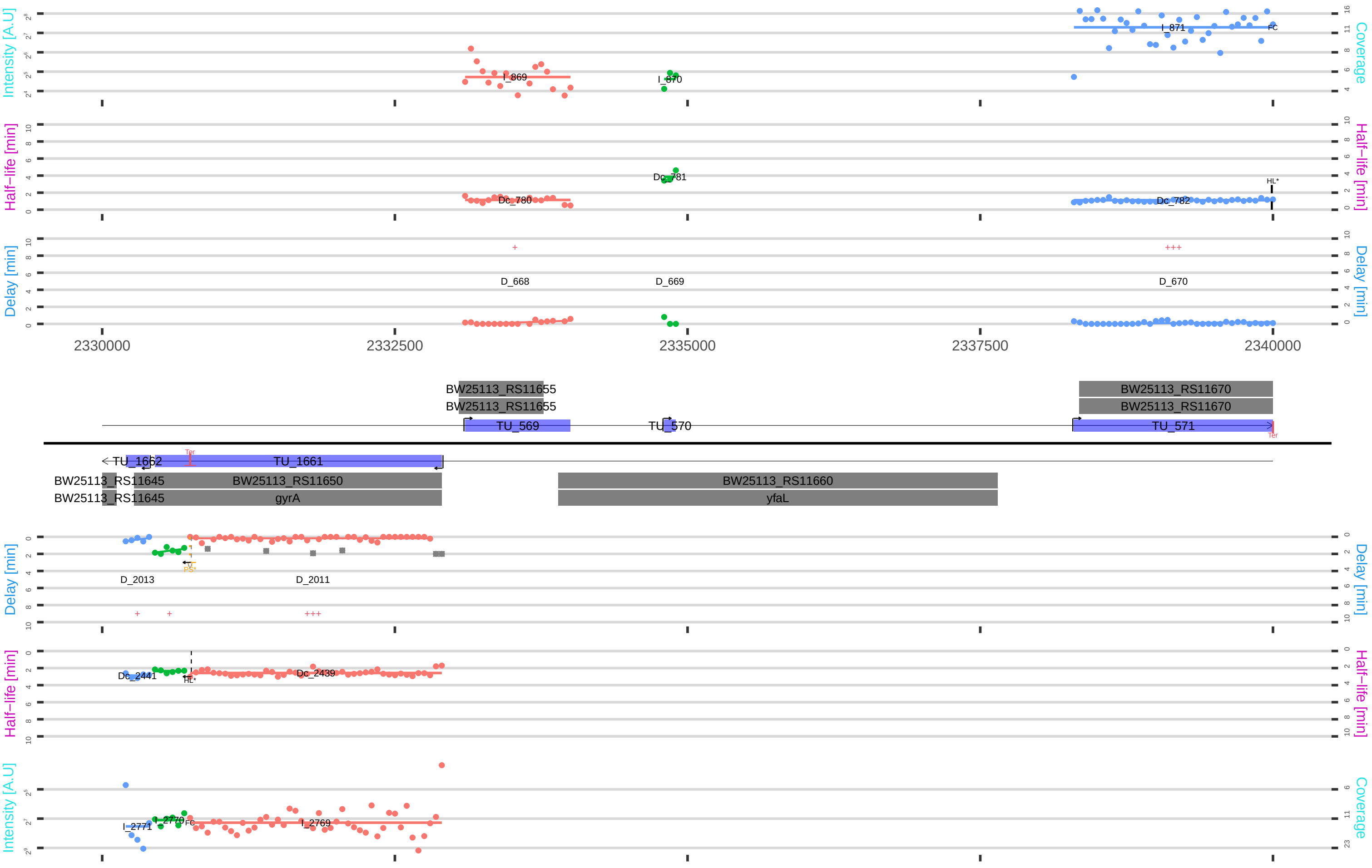

Term: termination (1), NS: new start (0), PS: pausing site (1), iTSS_I: internal starting site (0)

ID: 46800−46998; Term: termination (4), NS: new start (1), PS: pausing site (1), iTSS_I: internal starting site (3)

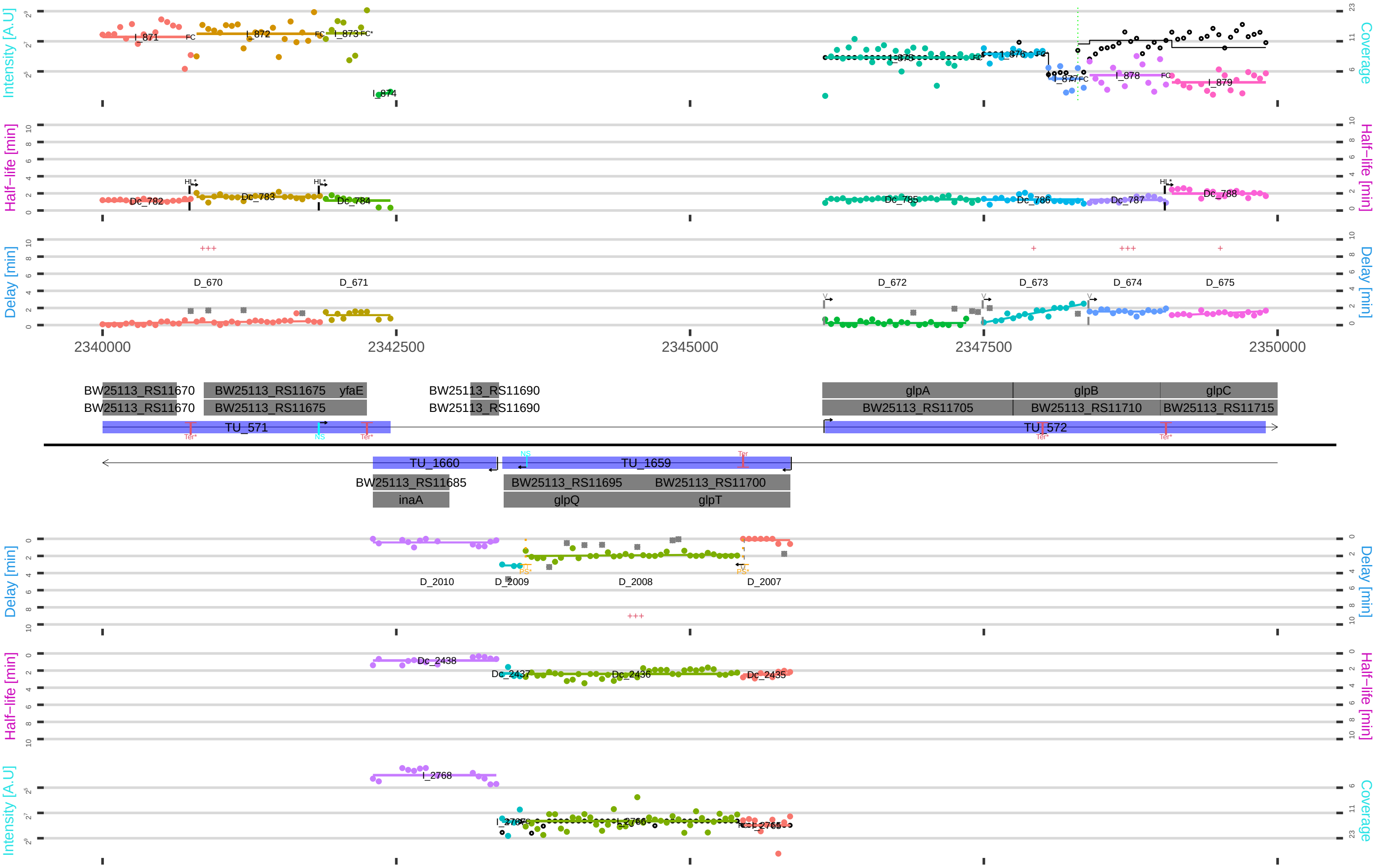

Term: termination (1), NS: new start (1), PS: pausing site (2), iTSS_I: internal starting site (0)

ID: 47001−47169; Term: termination (0), NS: new start (0), PS: pausing site (0), iTSS_I: internal starting site (0)

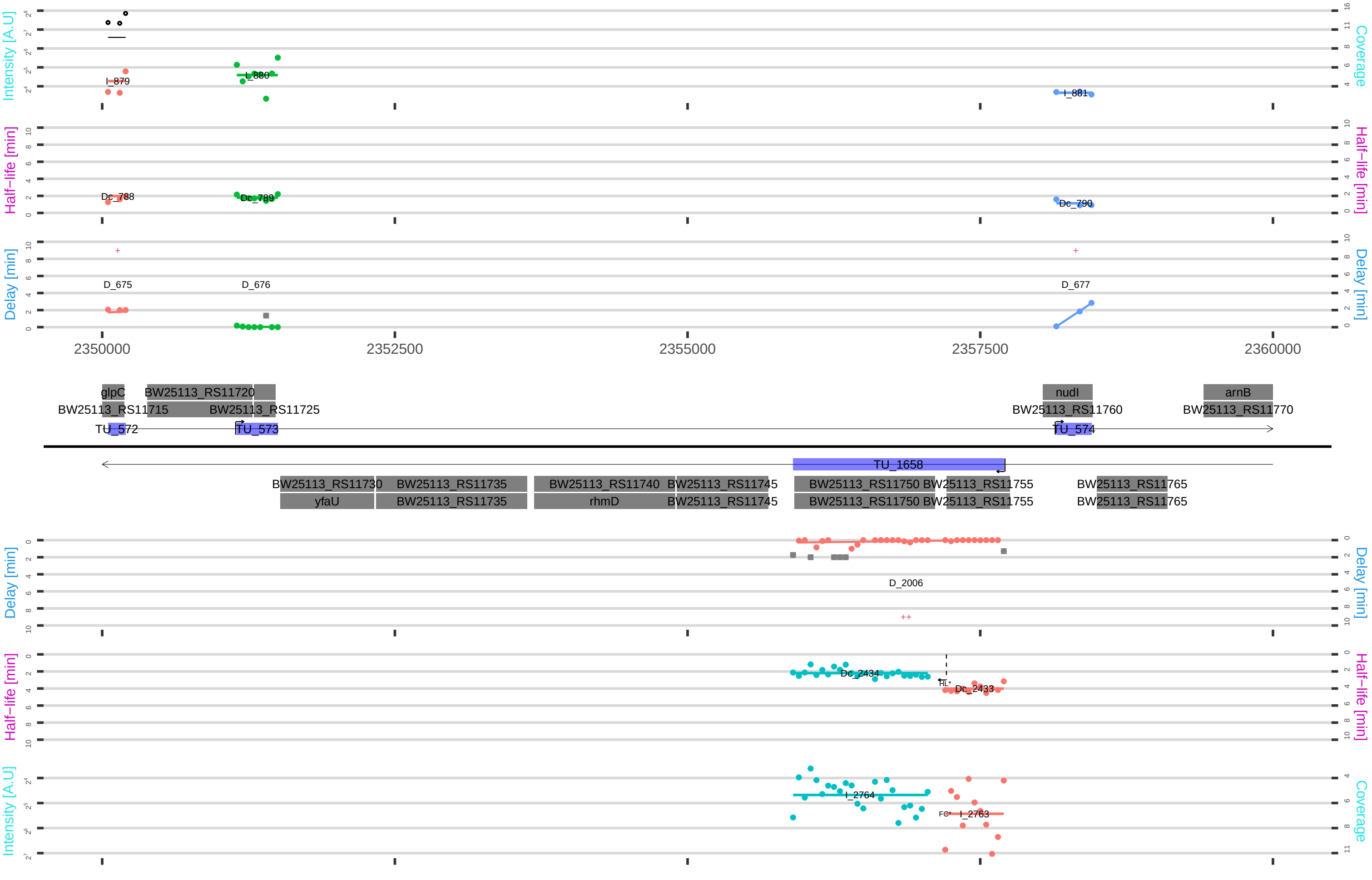

Term: termination (0), NS: new start (0), PS: pausing site (0), iTSS_I: internal starting site (0)

ID: 138000−137861; FC*: significant t−test of two consecutive segments; Term: termination, NS: new start, PS: pausing site,iTSS_I: internal starting site,TI: transcription interferance.

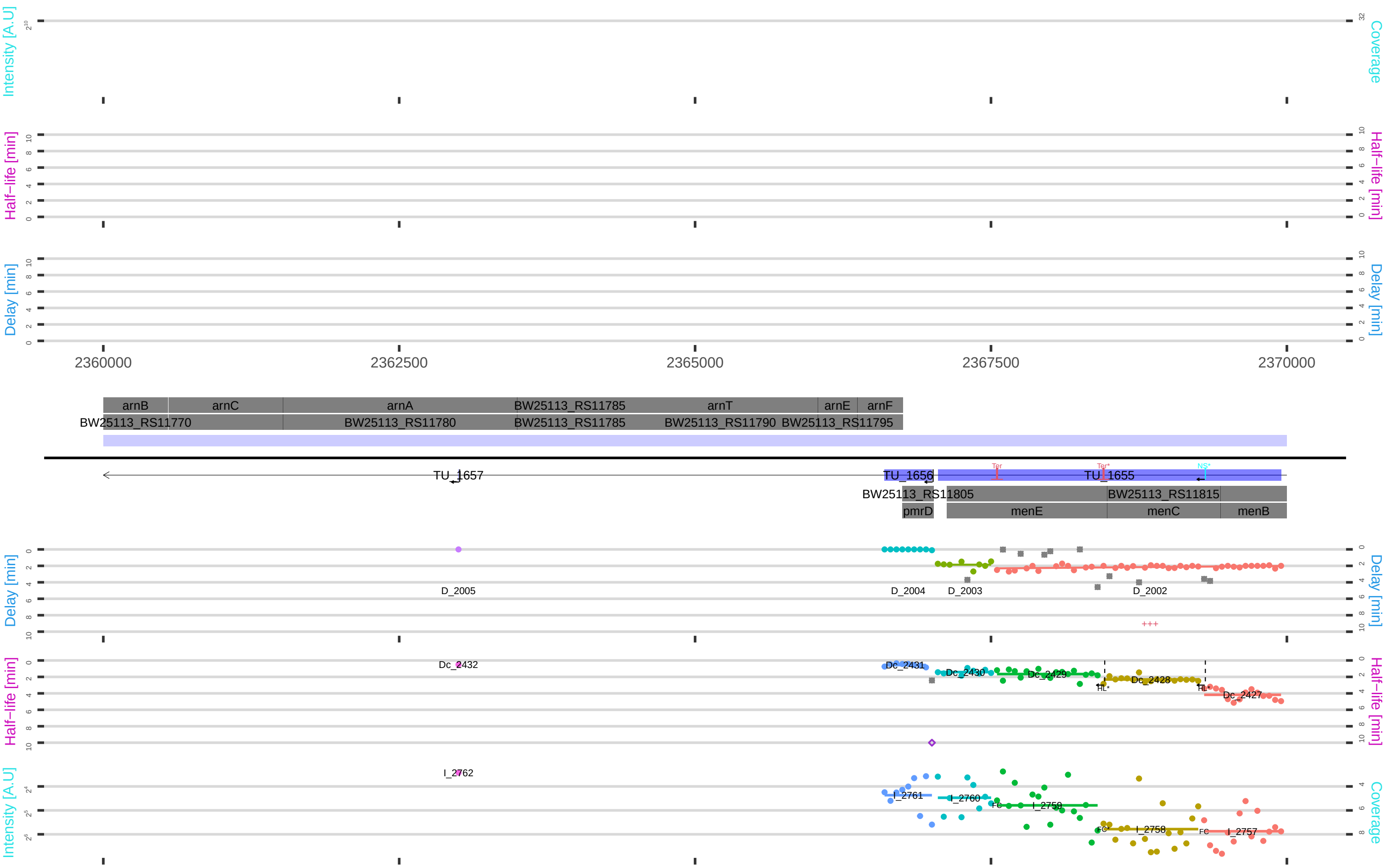

Term: termination (2), NS: new start (1), PS: pausing site (0), iTSS_I: internal starting site (1)

ID: 47504−47580; Term: termination (0), NS: new start (0), PS: pausing site (0), iTSS_I: internal starting site (0)

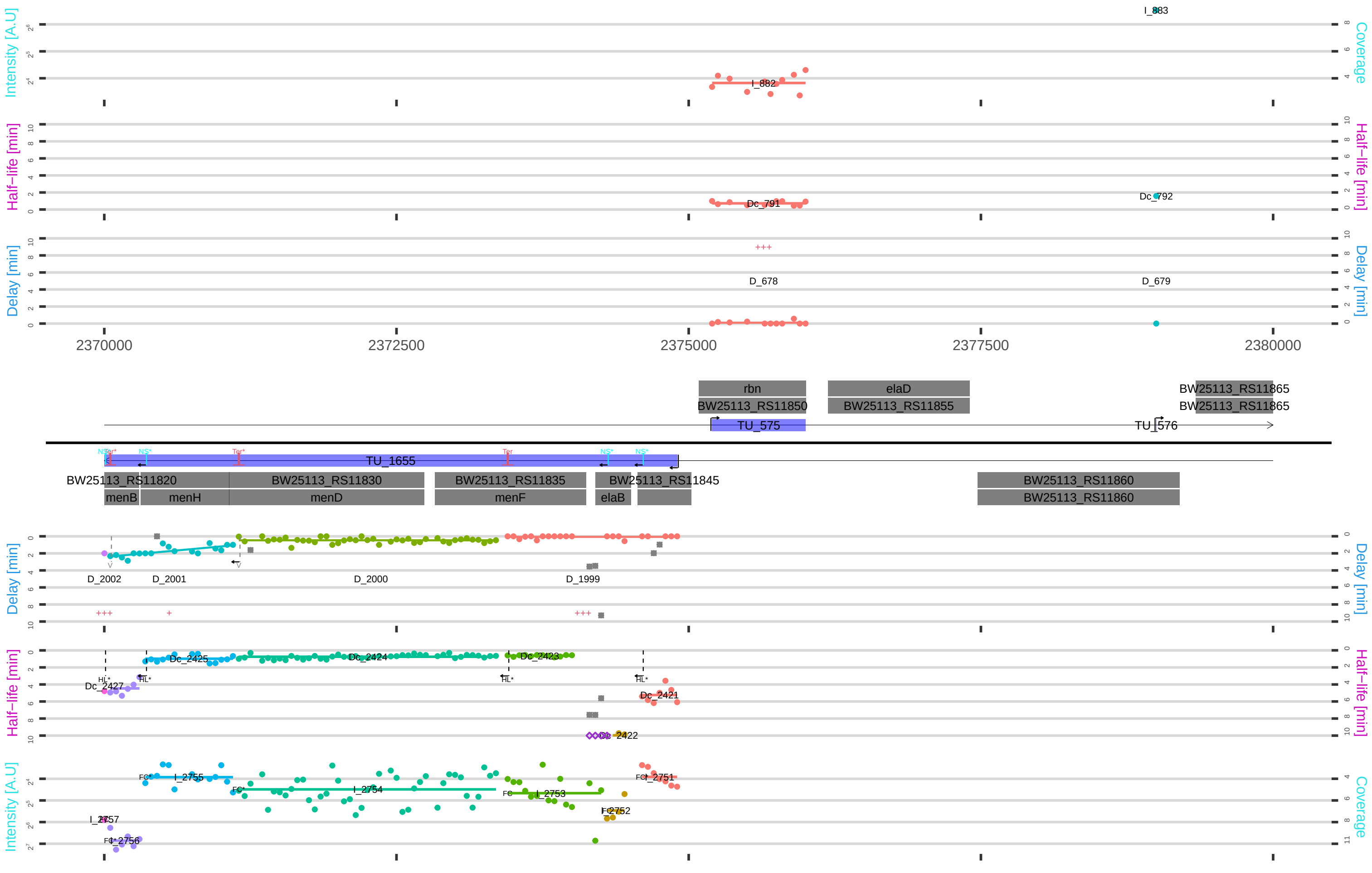

Term: termination (3), NS: new start (4), PS: pausing site (2), iTSS_I: internal starting site (1)

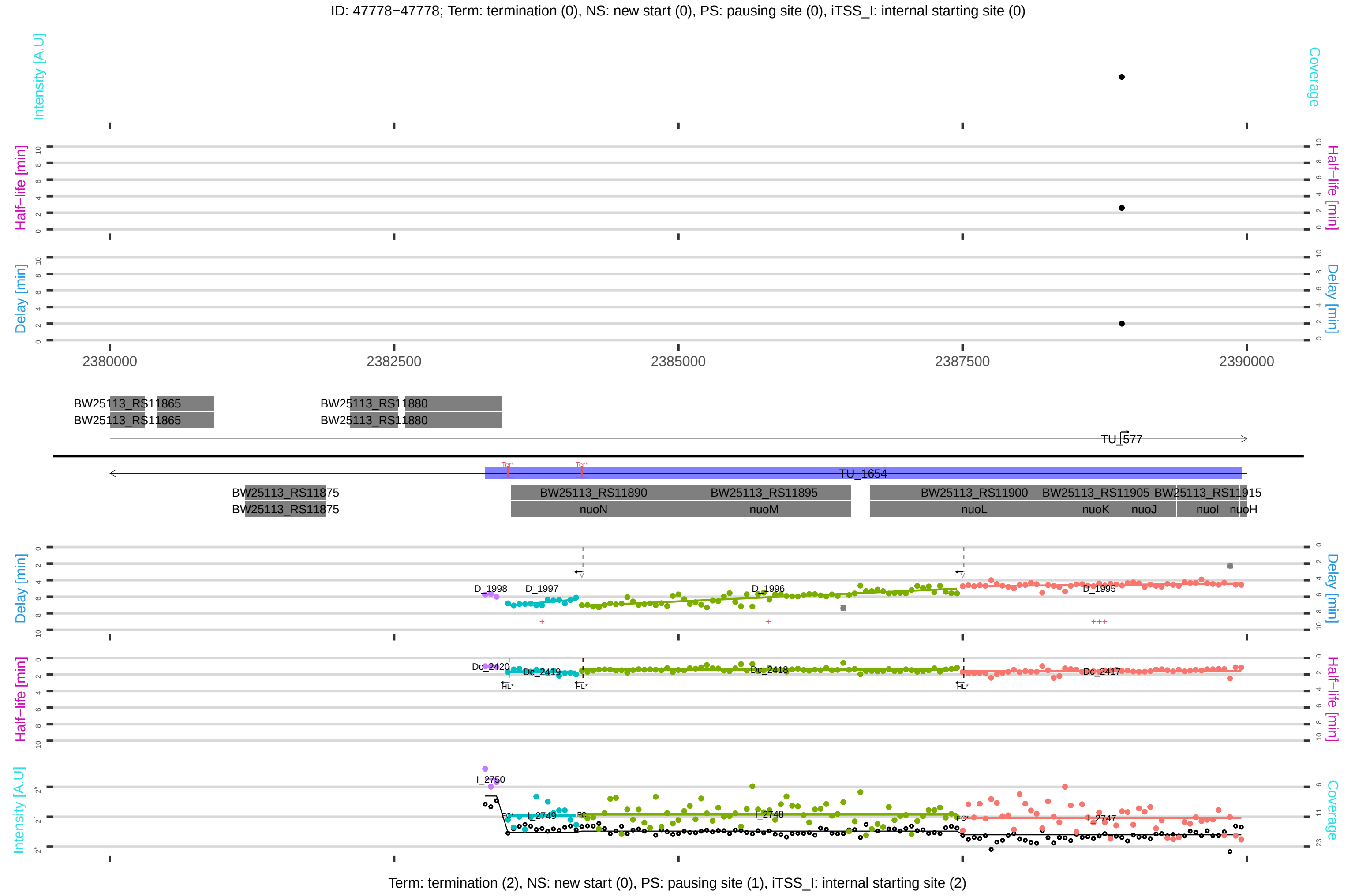
ID: 47778−47778; Term: termination (0), NS: new start (0), PS: pausing site (0), iTSS_I: internal starting site (0)
Intensity [A.U]
Coverage
Half−life [min]
Delay [min]
2380000
2382500
2385000
2387500
2390000
BW25113_RS11865
BW25113_RS11880
TU_1577
TU_1654
BW25113_RS11875
BW25113_RS11890
BW25113_RS11895
BW25113_RS11900
BW25113_RS11905
BW25113_RS11915
nuoN
nuoM
nuoL
nuoK
nuoJ
nuoI
nuoH
D_1998
D_1997
D_1996
D_1995
Dc_2420
Dc_2419
Dc_2418
Dc_2417
I_2750
I_2749
I_2748
I_2747
Term: termination (2), NS: new start (0), PS: pausing site (1), iTSS_I: internal starting site (2)

ID: 47910−47910; Term: termination (0), NS: new start (0), PS: pausing site (0), iTSS_I: internal starting site (0)

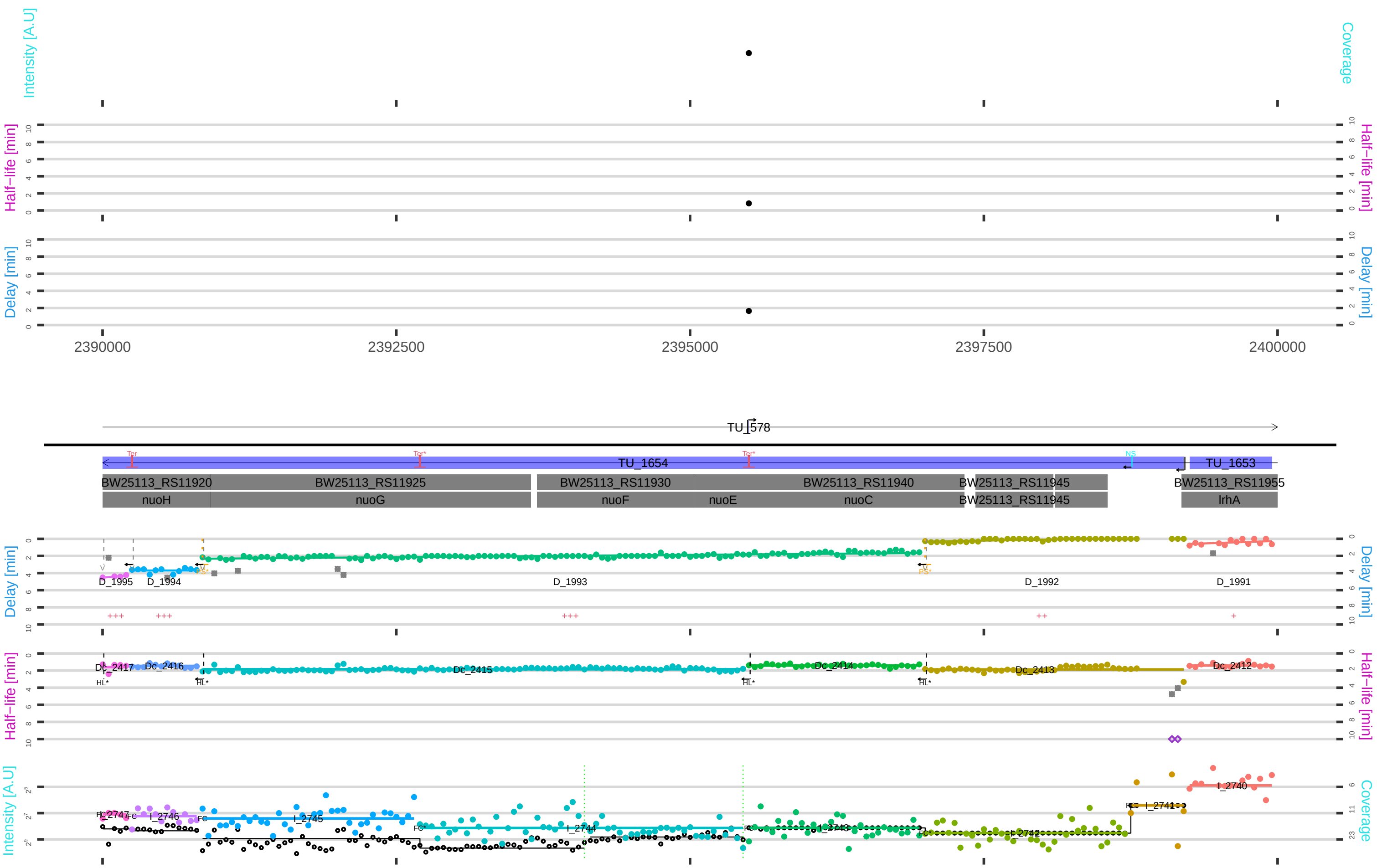

Term: termination (3), NS: new start (1), PS: pausing site (3), iTSS_I: internal starting site (0)

ID: 48021−48200; Term: termination (2), NS: new start (0), PS: pausing site (1), iTSS_I: internal starting site (0)

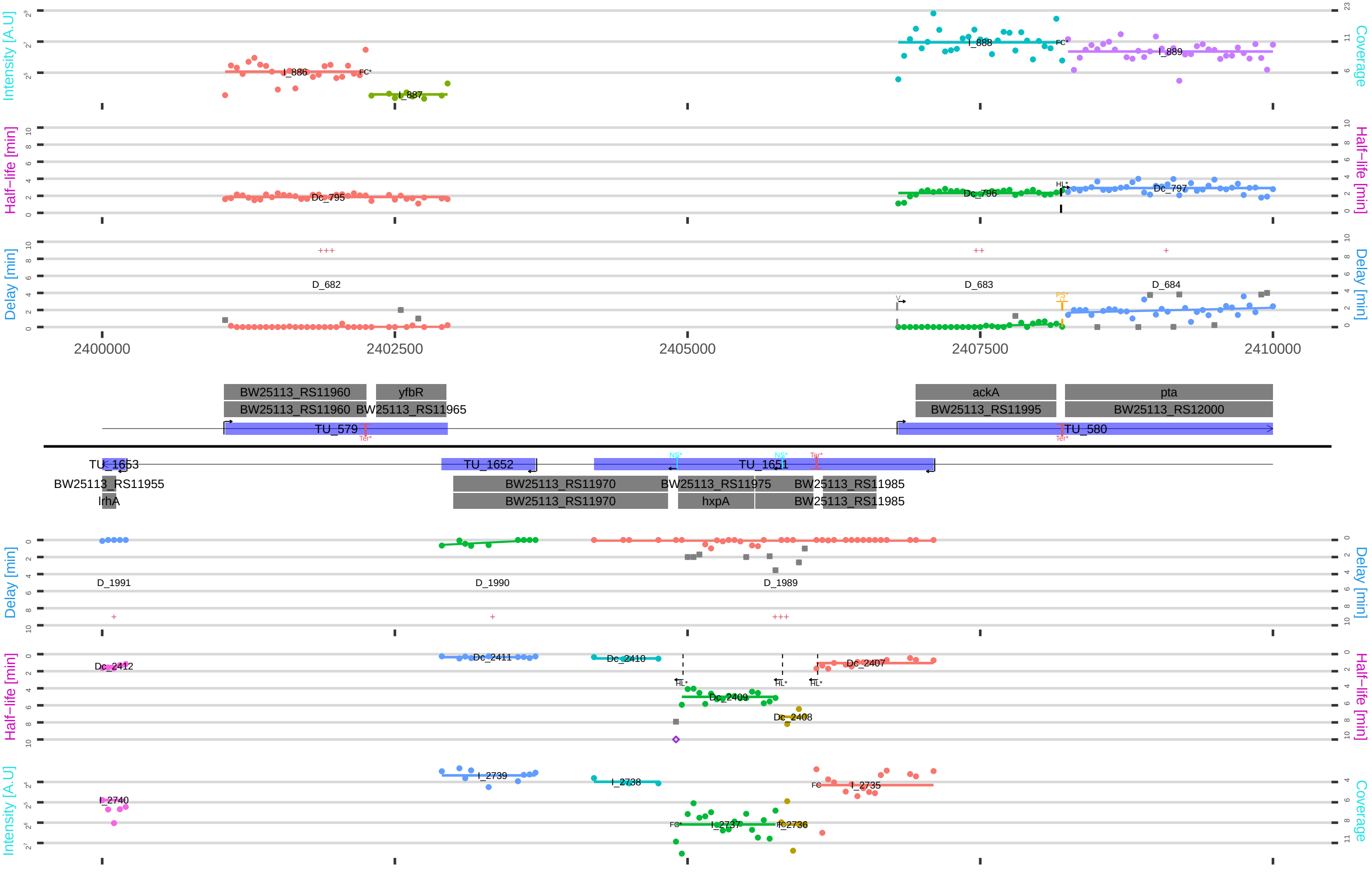

Term: termination (1), NS: new start (2), PS: pausing site (0), iTSS_I: internal starting site (0)

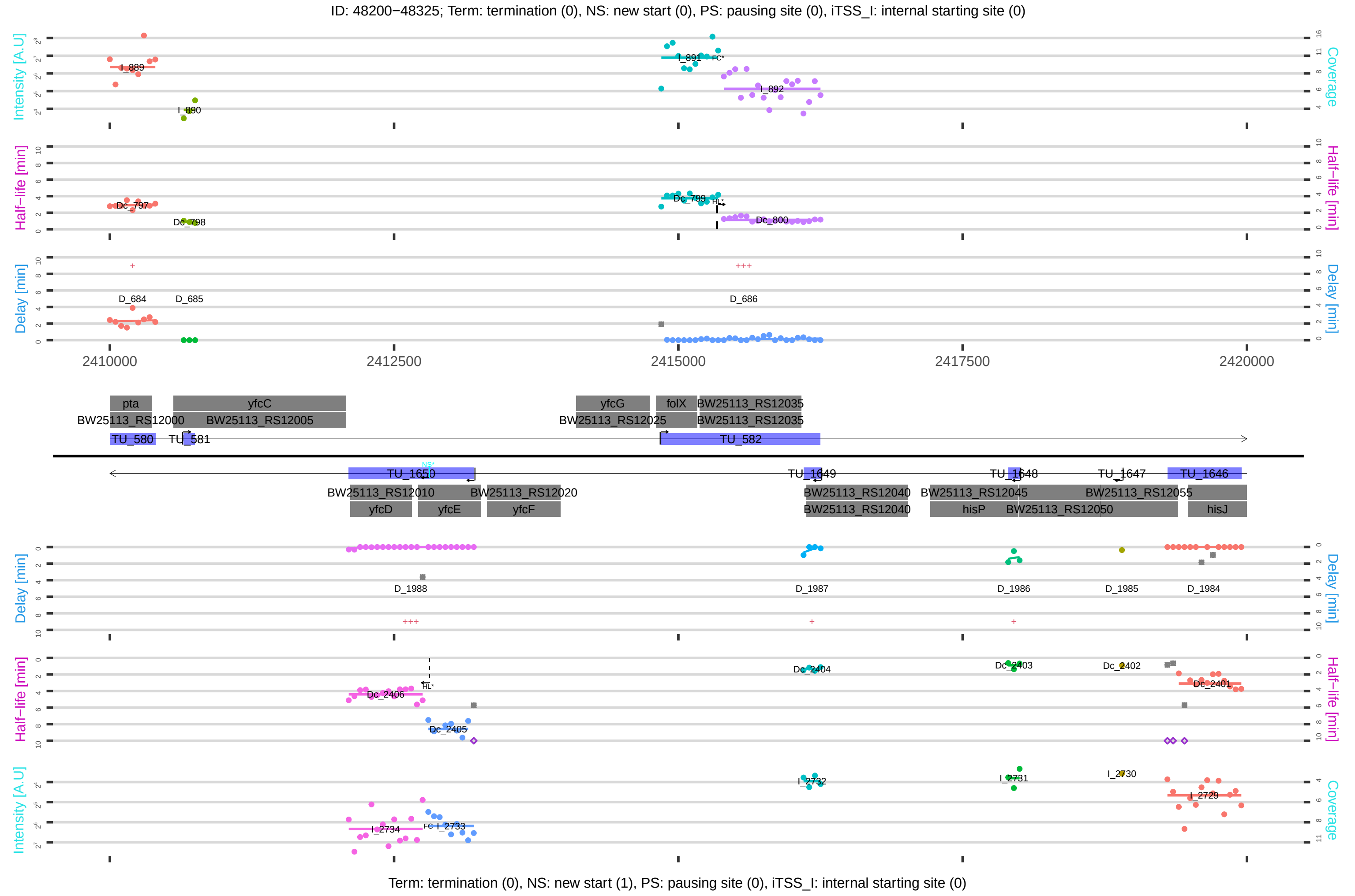
ID: 48200−48325; Term: termination (0), NS: new start (0), PS: pausing site (0), iTSS_I: internal starting site (0)
Intensity [A.U]
Coverage
Half−life [min]
Delay [min]
I_889
I_890
I_891
I_892
FC*
Dc_797
Dc_798
Dc_799
Dc_800
HL*
D_684
D_685
D_686
2410000
2412500
2415000
2417500
2420000
pta
yfcC
yfcG
folX
BW25113_RS12035
BW25113_RS12000
BW25113_RS12005
BW25113_RS12025
TU_580
TU_581
TU_582
NS*
TU_1650
TU_1649
TU_1648
TU_1647
TU_1646
BW25113_RS12010
BW25113_RS12020
BW25113_RS12040
BW25113_RS12045
BW25113_RS12055
yfcD
yfcE
yfcF
hisP
BW25113_RS12050
hisJ
D_1988
D_1987
D_1986
D_1985
D_1984
Dc_2406
Dc_2405
Dc_2404
Dc_2403
Dc_2402
Dc_2401
I_2734
I_2733
I_2732
I_2731
I_2730
I_2729
FC
Term: termination (0), NS: new start (1), PS: pausing site (0), iTSS_I: internal starting site (0)

ID: 136860−136661; FC*: significant t−test of two consecutive segments; Term: termination, NS: new start, PS: pausing site,iTSS_I: internal starting site,TI: transcription interferance.

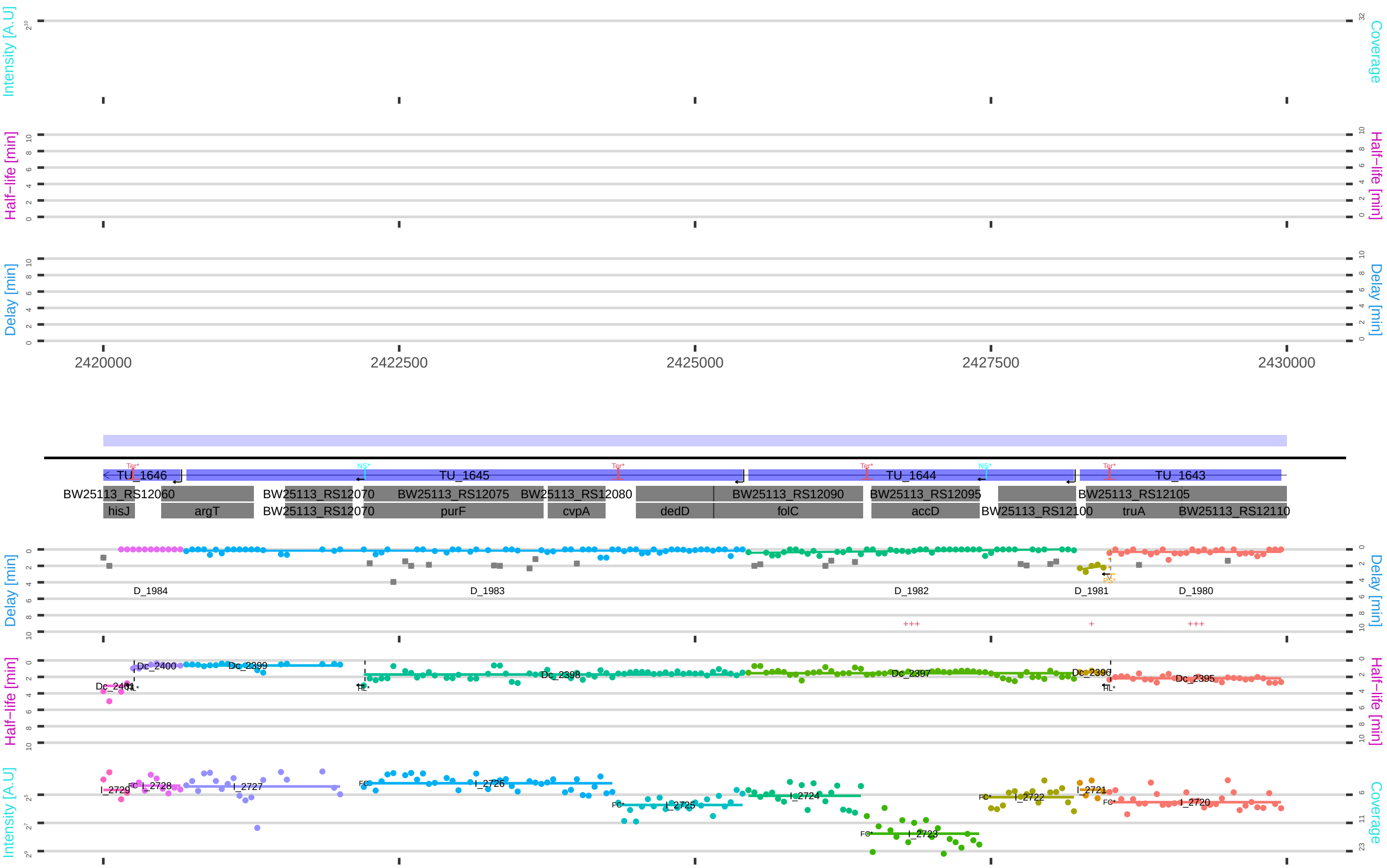

Term: termination (4), NS: new start (2), PS: pausing site (1), iTSS_I: internal starting site (0)

ID: 48630−48745; Term: termination (0), NS: new start (0), PS: pausing site (0), iTSS_I: internal starting site (0)

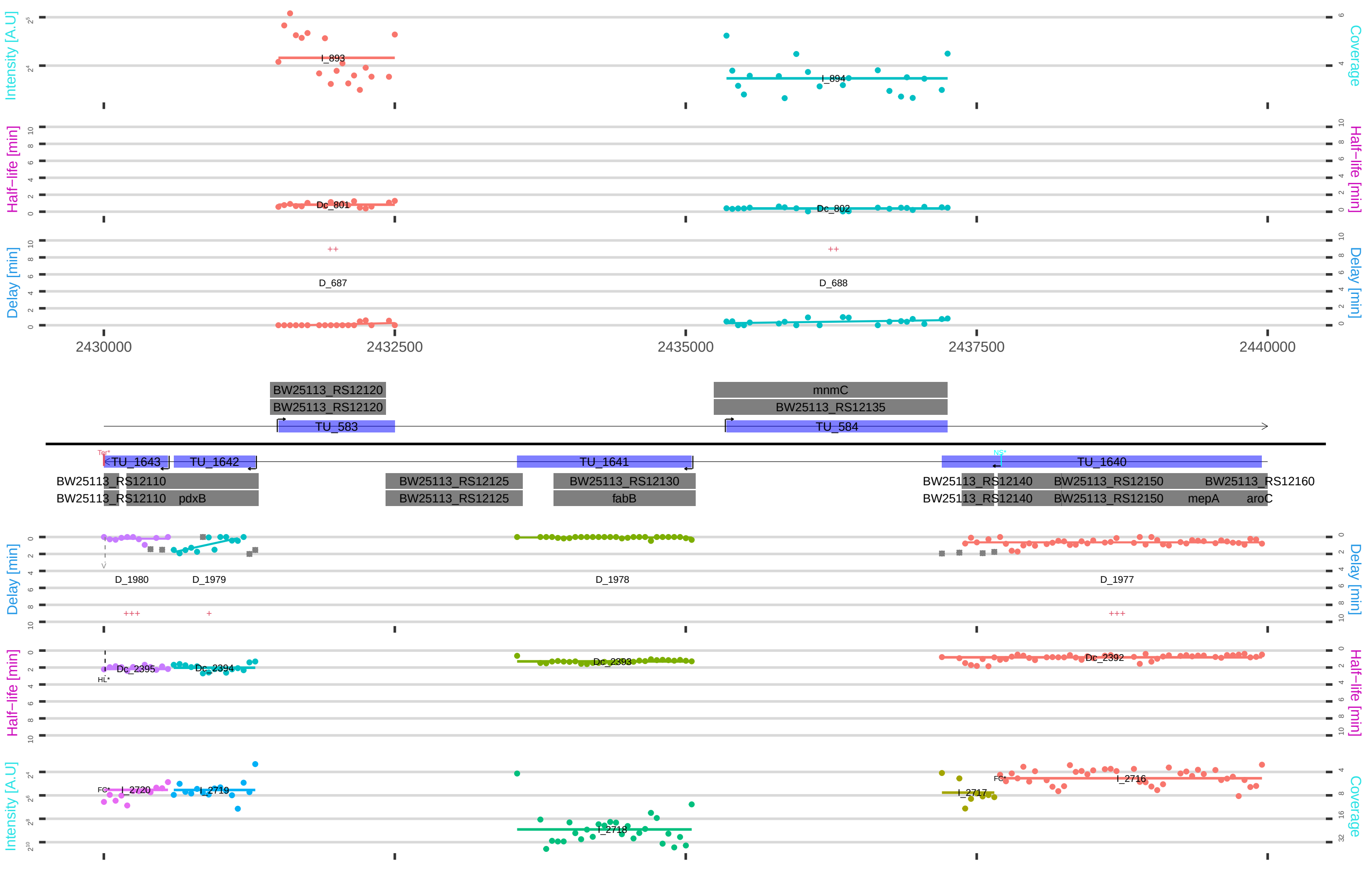

Term: termination (1), NS: new start (1), PS: pausing site (0), iTSS_I: internal starting site (0)

ID: 48843−48858; Term: termination (0), NS: new start (0), PS: pausing site (0), iTSS_I: internal starting site (0)

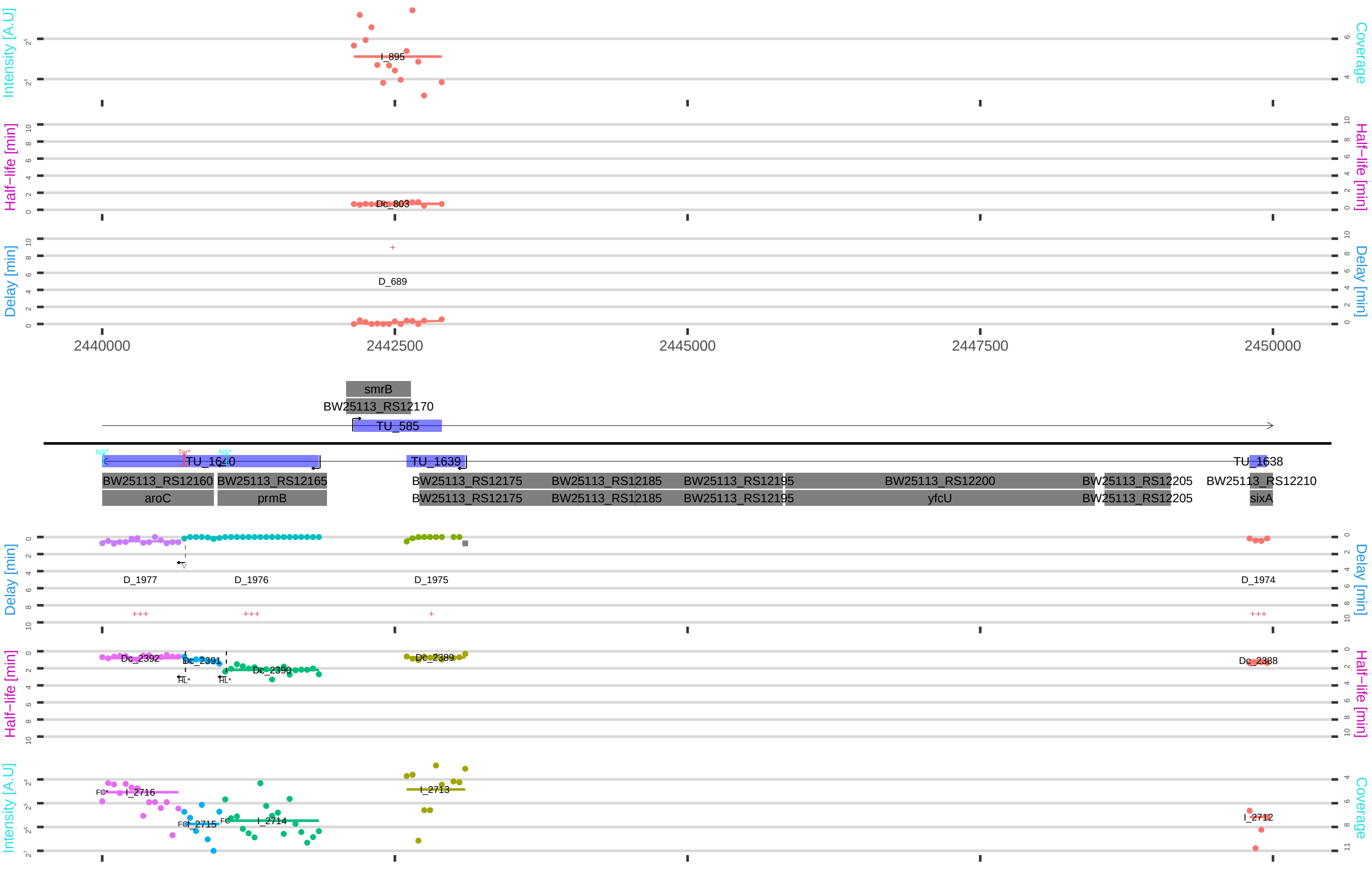

Term: termination (1), NS: new start (2), PS: pausing site (1), iTSS_I: internal starting site (0)

ID: 49096−49198; Term: termination (0), NS: new start (1), PS: pausing site (0), iTSS_I: internal starting site (0)

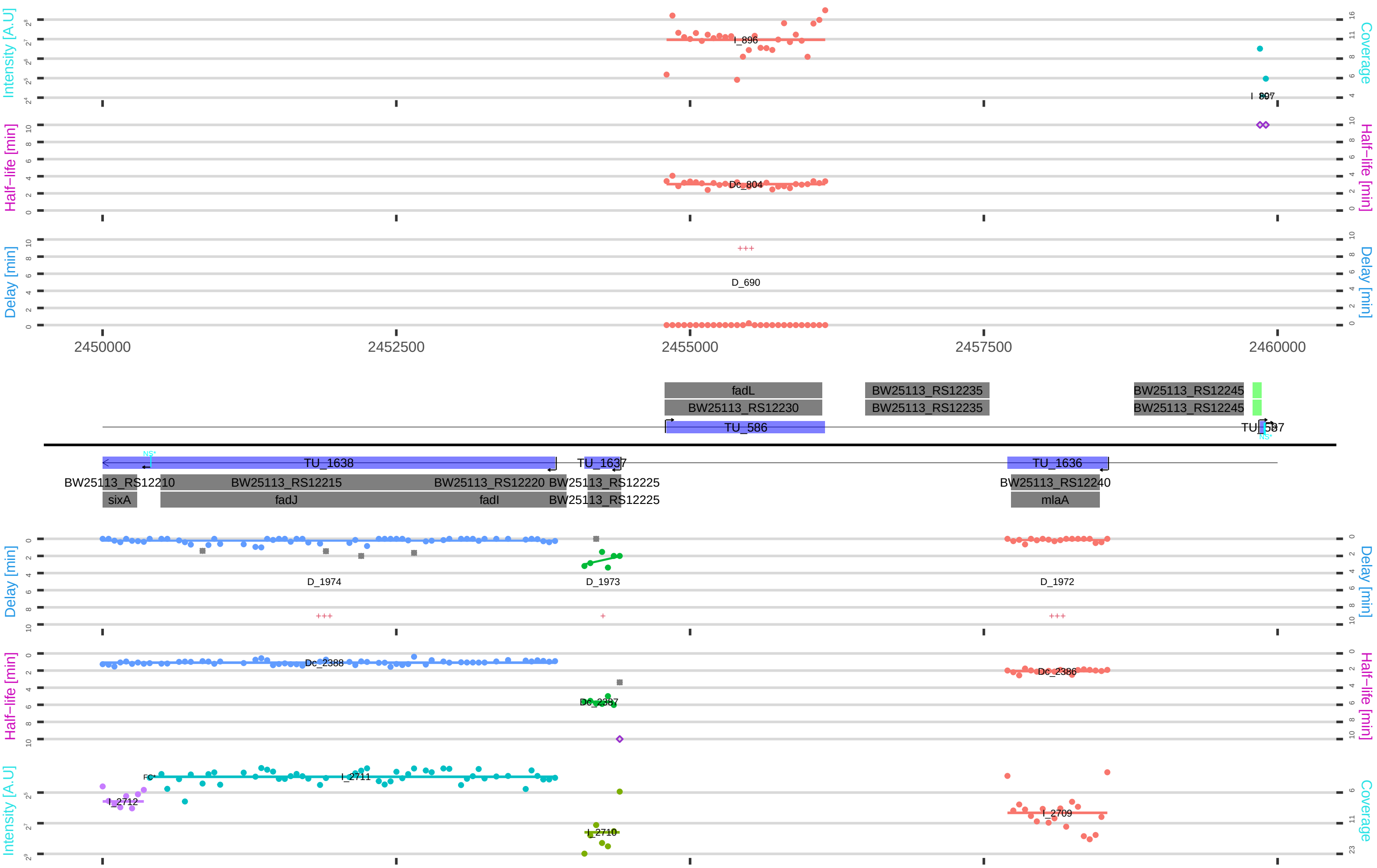

Term: termination (0), NS: new start (1), PS: pausing site (0), iTSS_I: internal starting site (0)

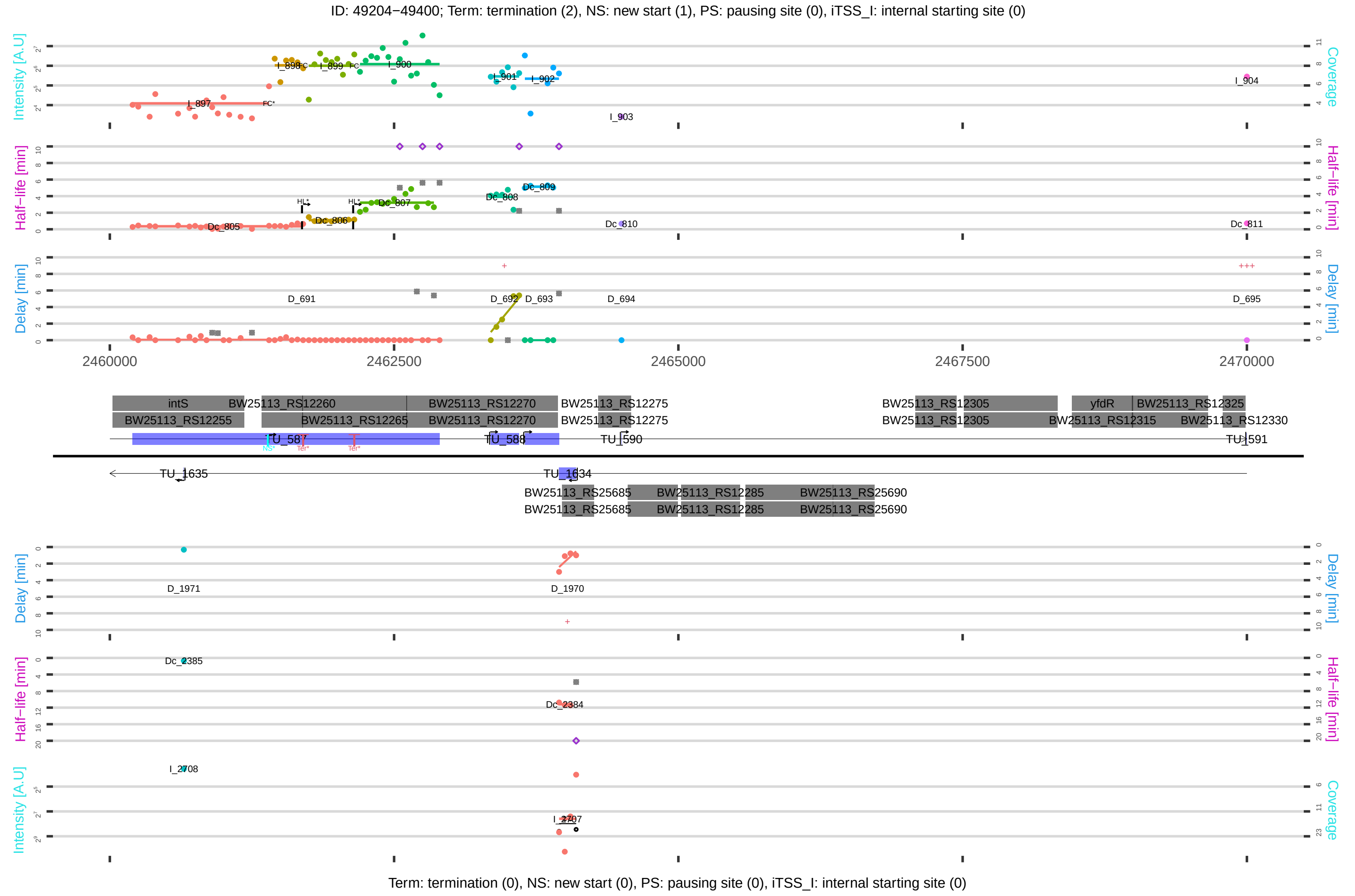

ID: 49204−49400; Term: termination (2), NS: new start (1), PS: pausing site (0), iTSS_I: internal starting site (0)
Intensity [A.U]
Coverage
Half−life [min]
Delay [min]
I_897
I_898
I_899
I_900
I_901
I_902
I_903
I_904
Dc_805
Dc_806
Dc_807
Dc_808
Dc_809
Dc_810
Dc_811
D_691
D_692
D_693
D_694
D_695
2460000
2462500
2465000
2467500
2470000
intS
BW25113_RS12255
BW25113_RS12260
BW25113_RS12265
BW25113_RS12270
BW25113_RS12275
BW25113_RS12305
yfdR
BW25113_RS12315
BW25113_RS12325
BW25113_RS12330
TU_587
TU_588
TU_590
TU_591
NS*
Ter*
TU_1635
TU_1634
BW25113_RS25685
BW25113_RS12285
BW25113_RS25690
D_1971
D_1970
Dc_2385
Dc_2384
I_2708
I_2707
Term: termination (0), NS: new start (0), PS: pausing site (0), iTSS_I: internal starting site (0)

ID: 49400−49561; Term: termination (2), NS: new start (0), PS: pausing site (1), iTSS_I: internal starting site (0)

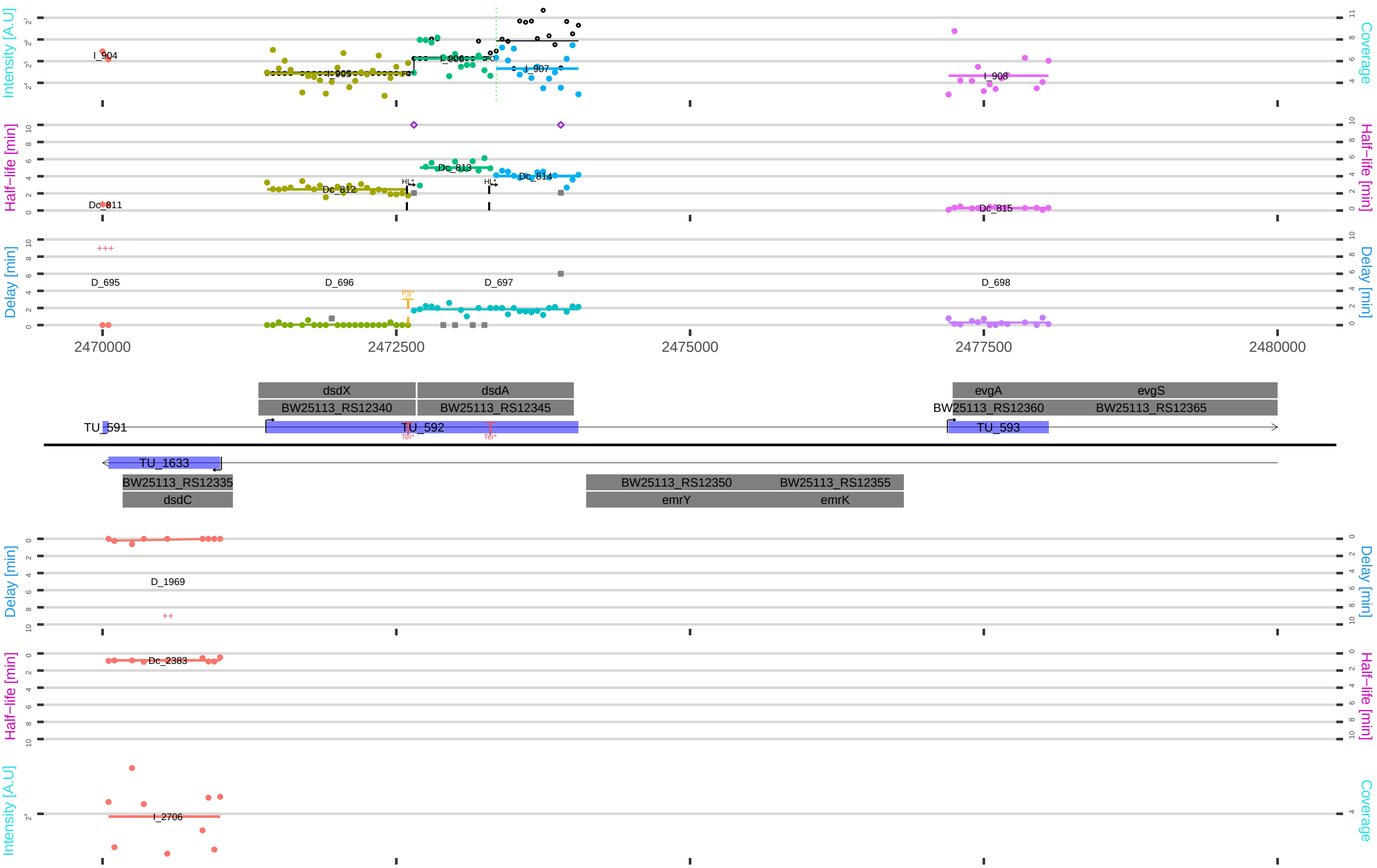

Term: termination (0), NS: new start (0), PS: pausing site (0), iTSS_I: internal starting site (0)

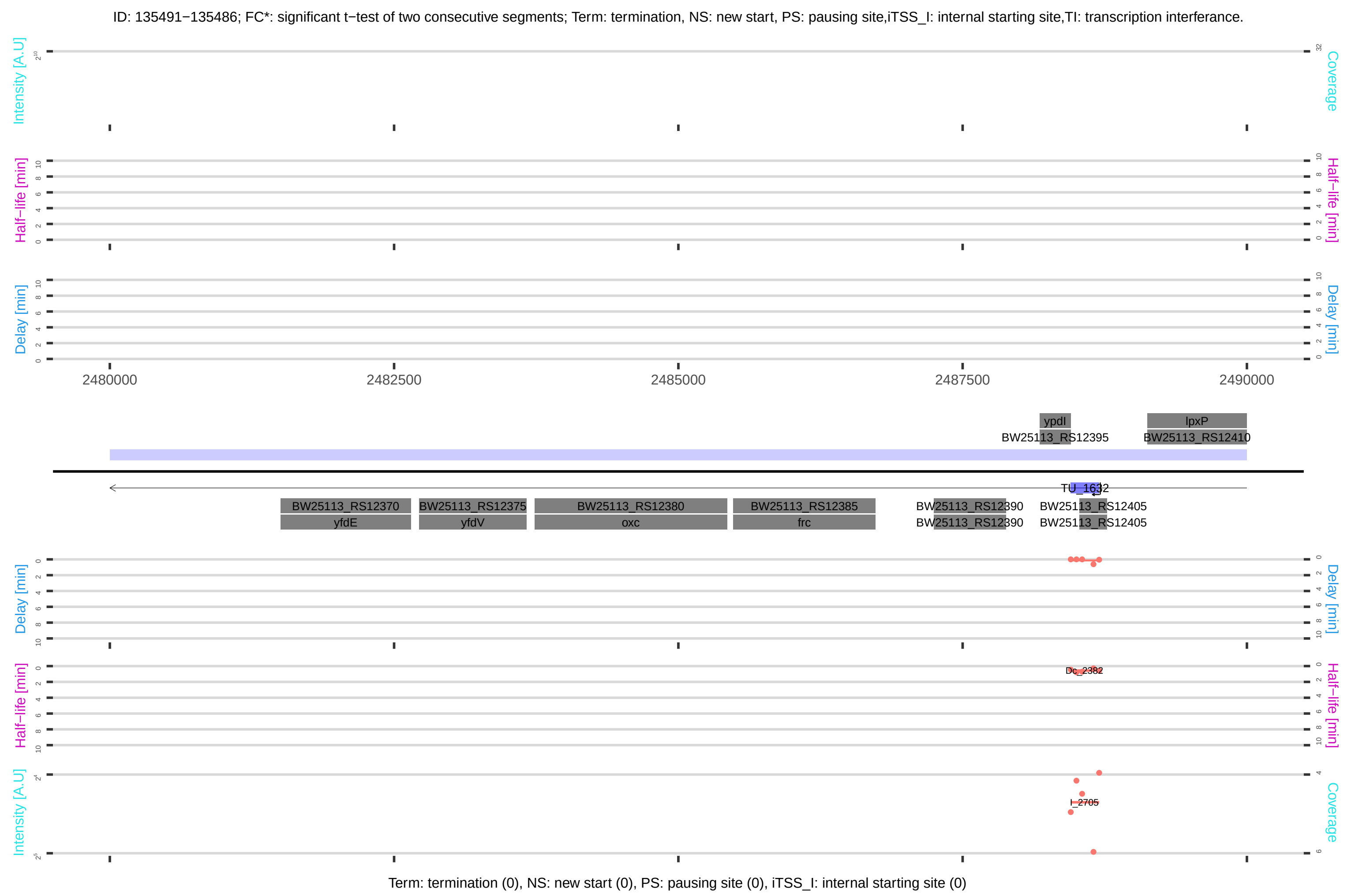
ID: 135491−135486; FC*: significant t−test of two consecutive segments; Term: termination, NS: new start, PS: pausing site,iTSS_I: internal starting site,TI: transcription interferance.
Intensity [A.U]
Coverage
Half−life [min]
Delay [min]
2480000
2482500
2485000
2487500
2490000
ypdI
BW25113_RS12395
lpxP
BW25113_RS12410
TU_1632
BW25113_RS12370
yfdE
BW25113_RS12375
yfdV
BW25113_RS12380
oxc
BW25113_RS12385
frc
BW25113_RS12390
BW25113_RS12405
Dc_2382
I_2705
Term: termination (0), NS: new start (0), PS: pausing site (0), iTSS_I: internal starting site (0)

ID: 49807−49901; Term: termination (0), NS: new start (0), PS: pausing site (0), iTSS_I: internal starting site (0)

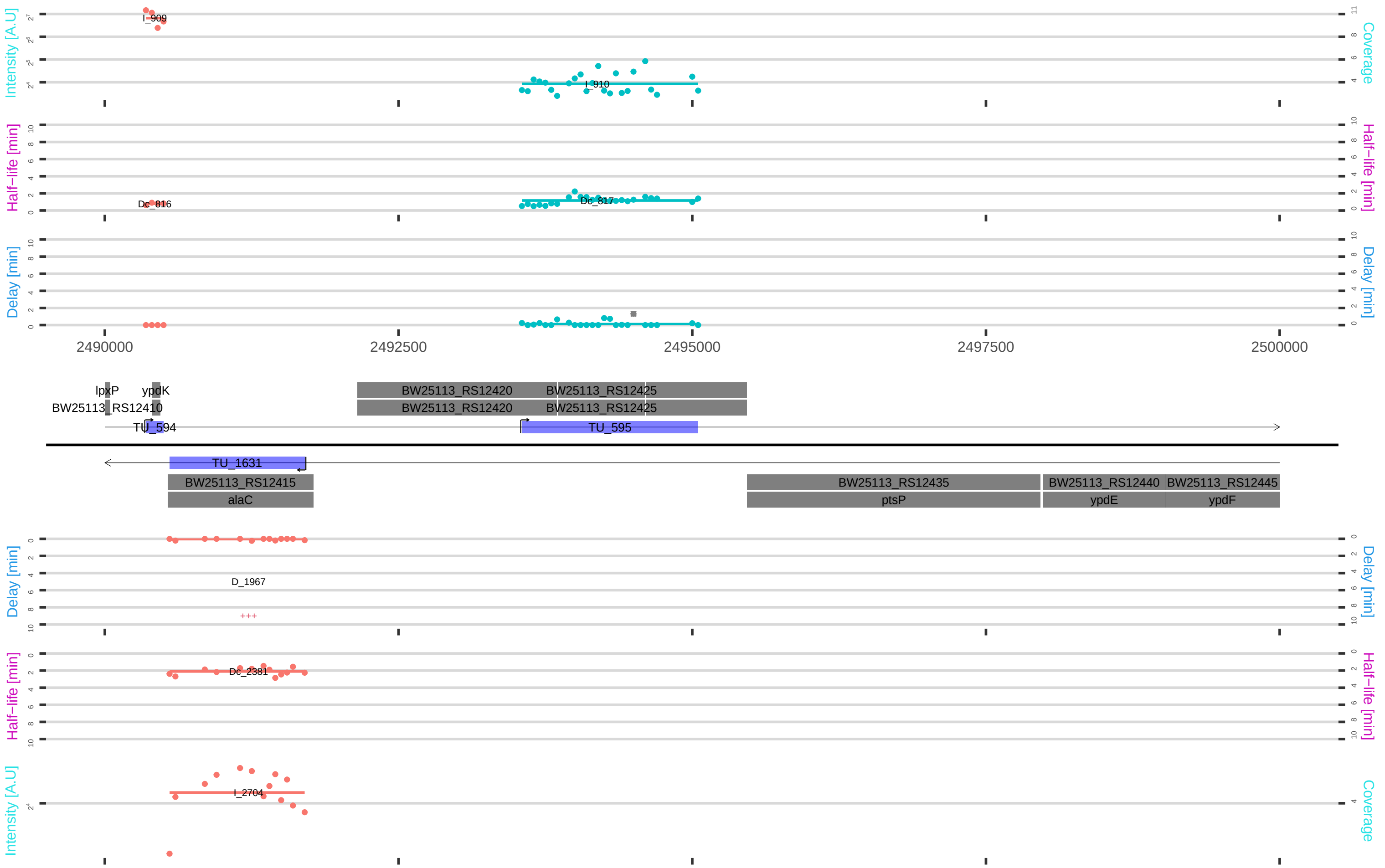

Term: termination (0), NS: new start (0), PS: pausing site (0), iTSS_I: internal starting site (0)

ID: 50091−50183; Term: termination (1), NS: new start (1), PS: pausing site (0), iTSS_I: internal starting site (0)

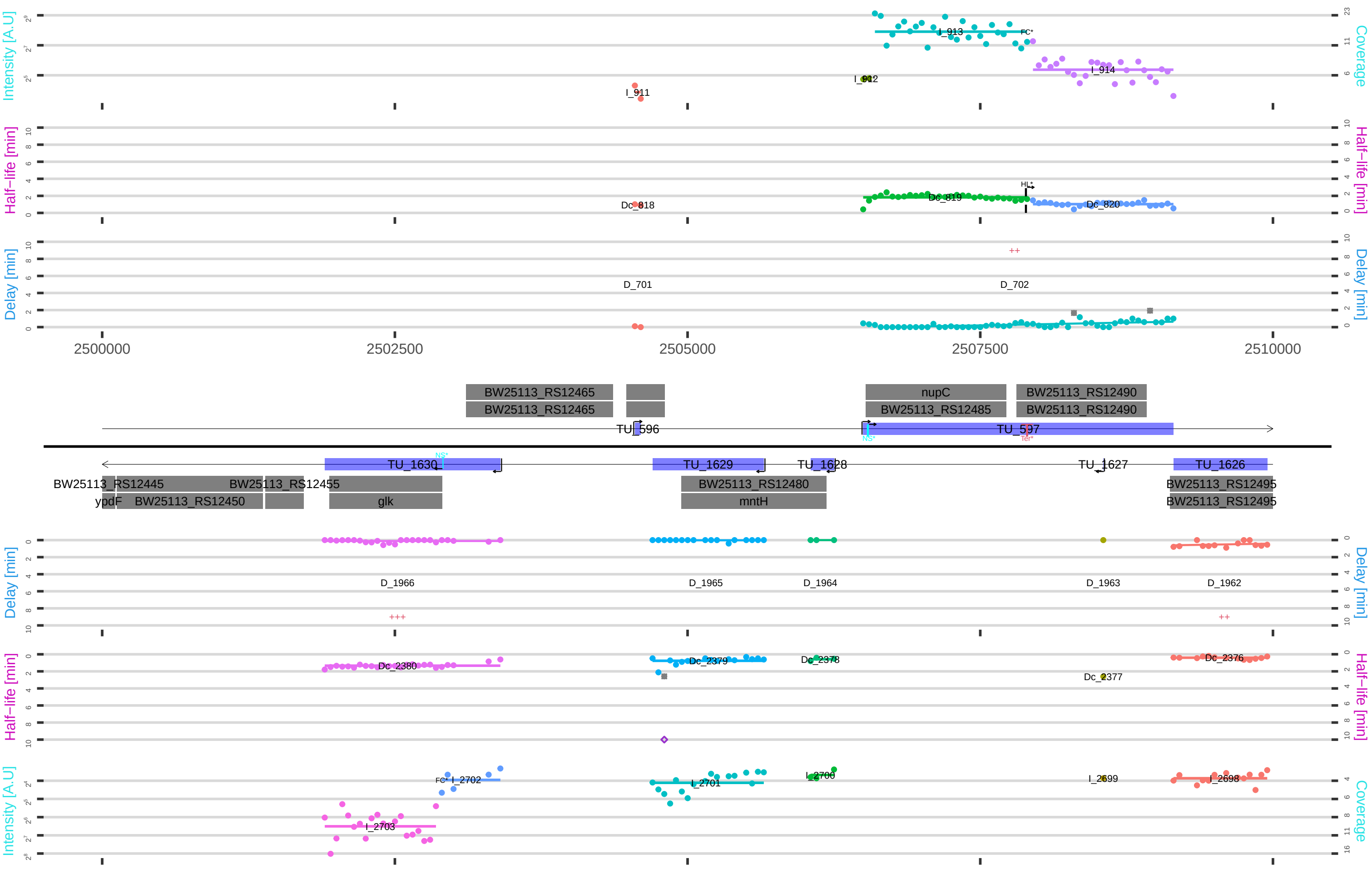

Term: termination (0), NS: new start (1), PS: pausing site (0), iTSS_I: internal starting site (0)

ID: 50232−50389; Term: termination (0), NS: new start (0), PS: pausing site (0), iTSS_I: internal starting site (0)

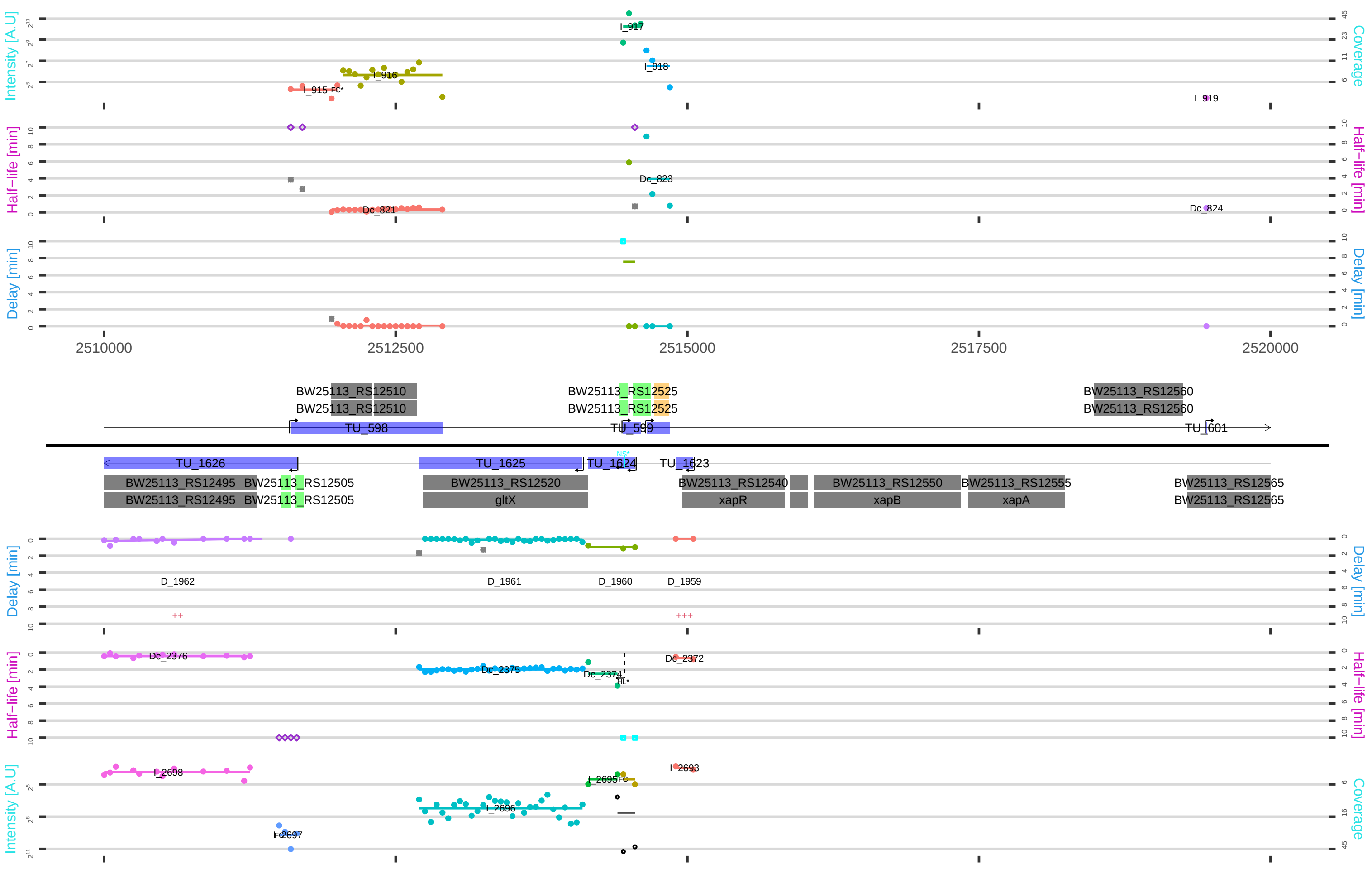

Term: termination (0), NS: new start (1), PS: pausing site (0), iTSS_I: internal starting site (0)

ID: 50408−50595; Term: termination (1), NS: new start (3), PS: pausing site (0), iTSS_I: internal starting site (0)

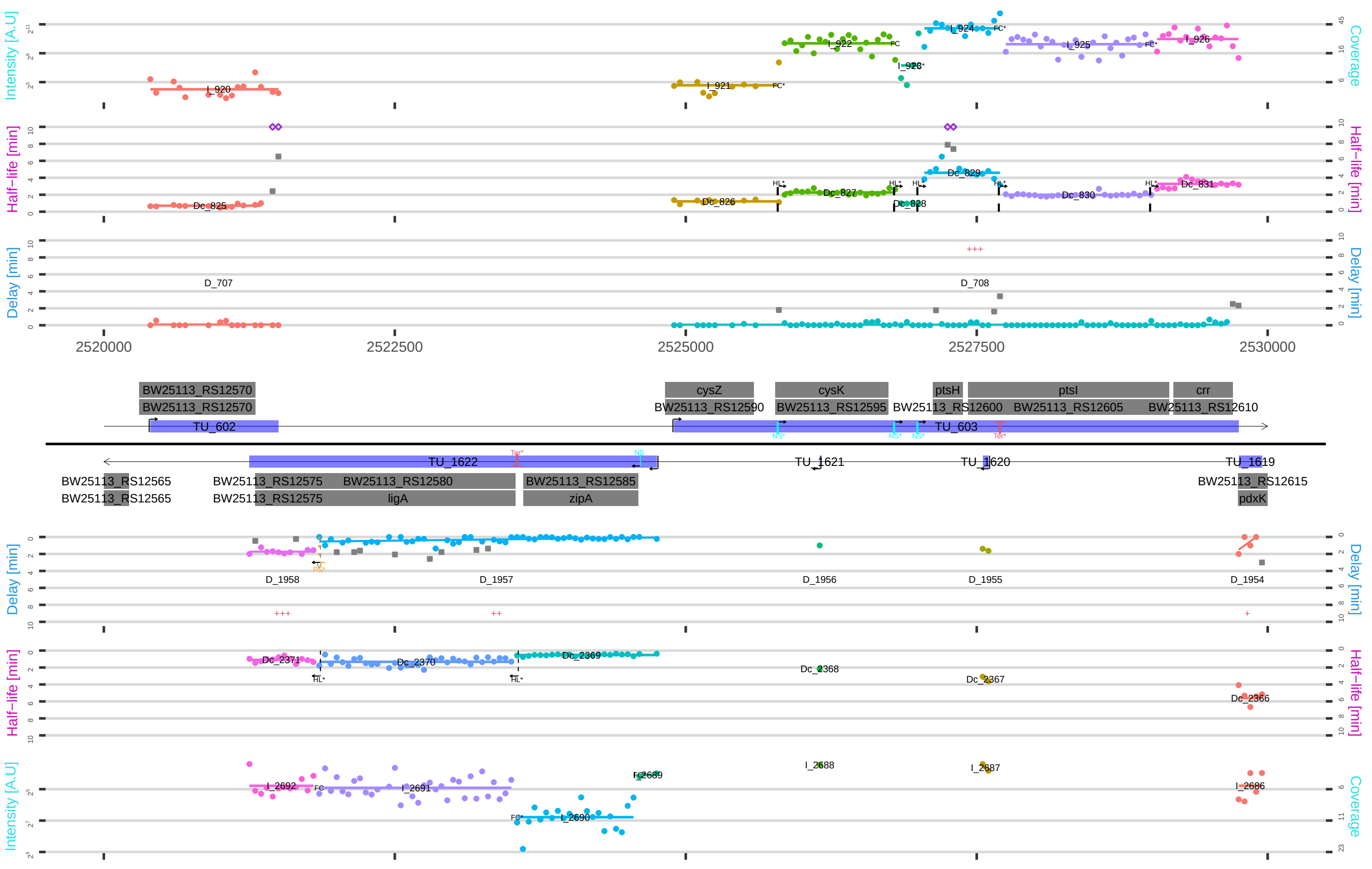

Term: termination (1), NS: new start (1), PS: pausing site (1), iTSS_I: internal starting site (0)

ID: 50684−50800; Term: termination (0), NS: new start (1), PS: pausing site (0), iTSS_I: internal starting site (0)

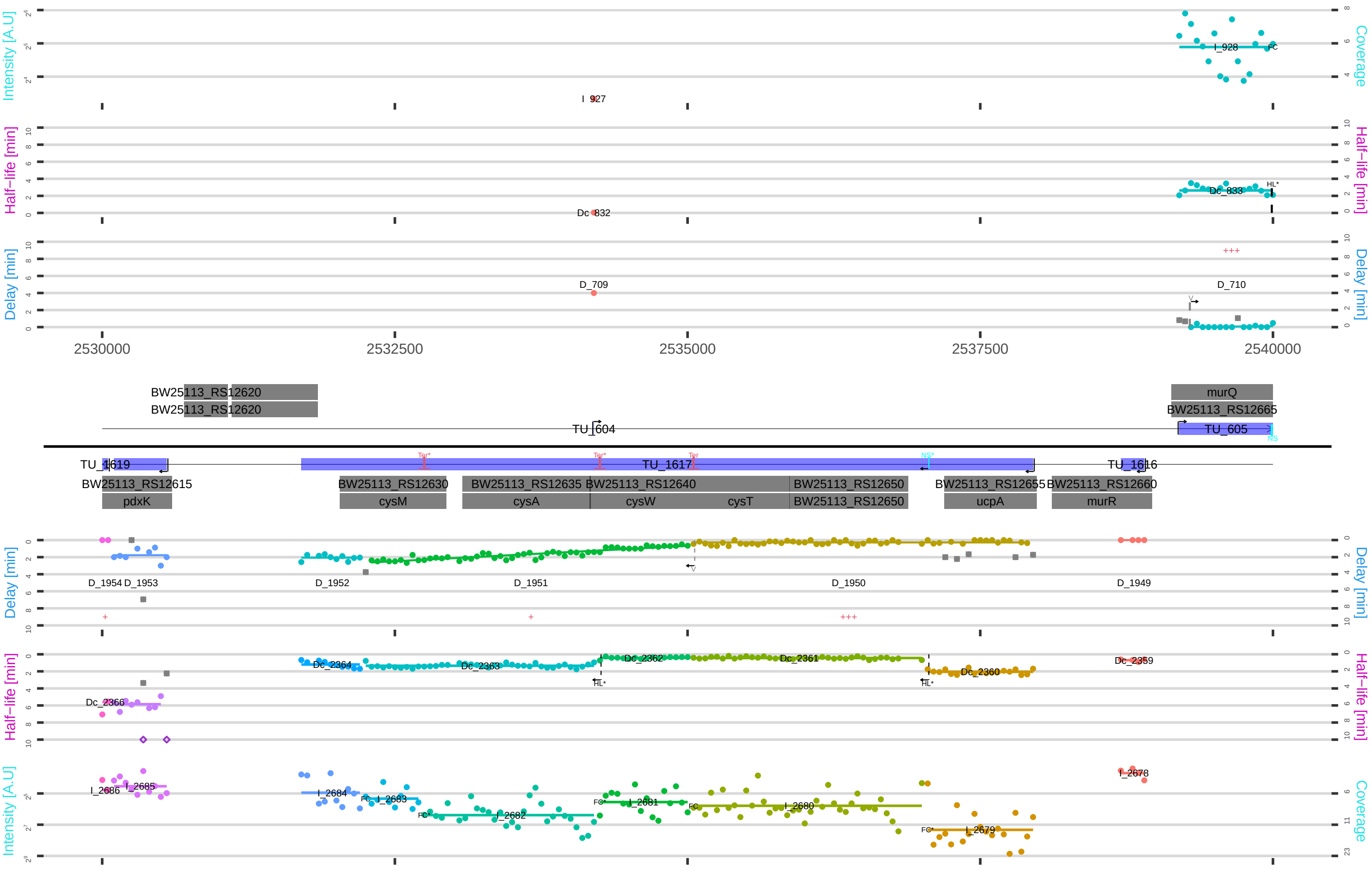

Term: termination (3), NS: new start (1), PS: pausing site (1), iTSS_I: internal starting site (1)

ID: 50800−50949; Term: termination (2), NS: new start (1), PS: pausing site (1), iTSS_I: internal starting site (0)

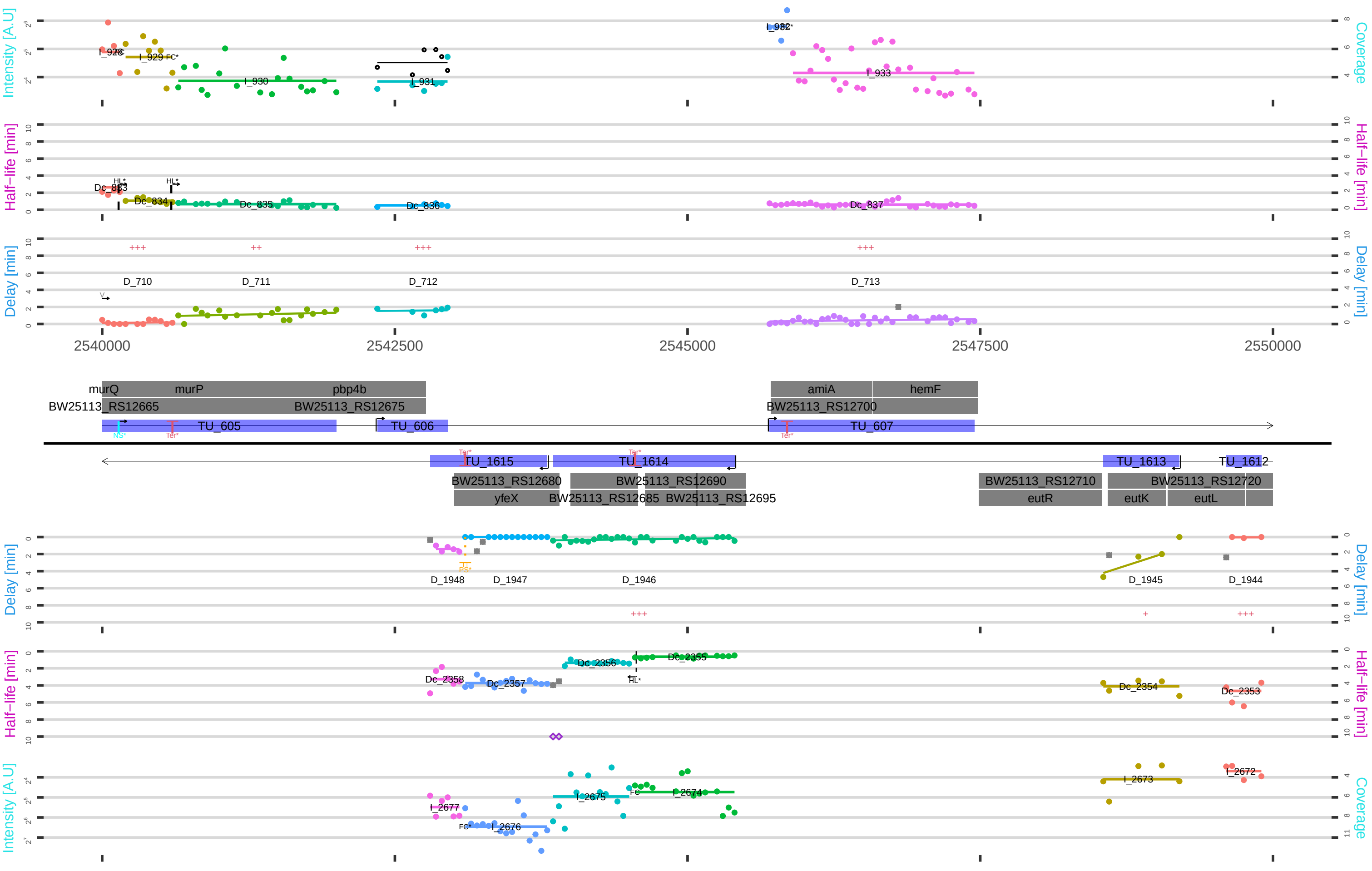

Term: termination (2), NS: new start (0), PS: pausing site (1), iTSS_I: internal starting site (0)

ID: 51015−51175; Term: termination (1), NS: new start (0), PS: pausing site (0), iTSS_I: internal starting site (0)

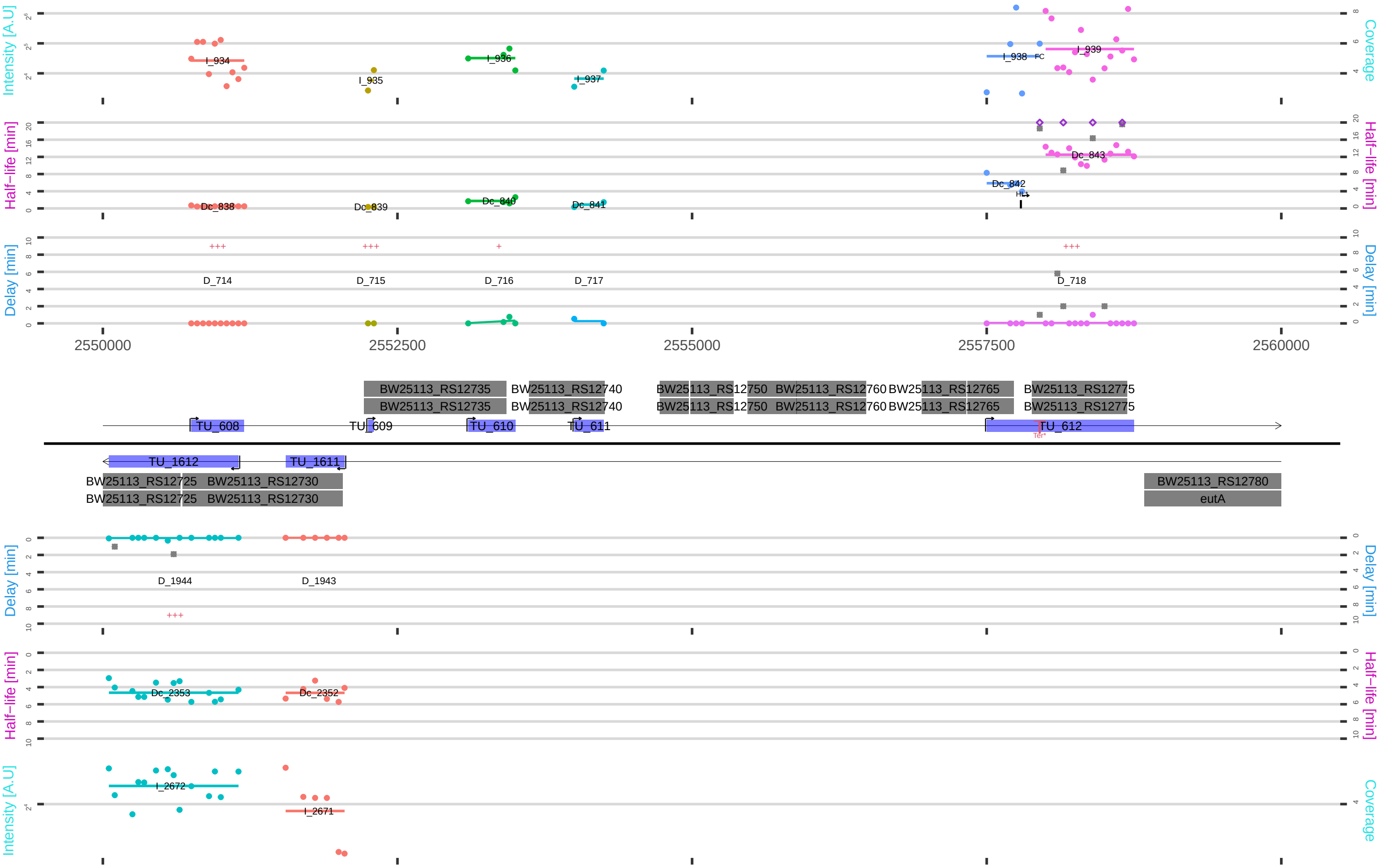

Term: termination (0), NS: new start (0), PS: pausing site (0), iTSS_I: internal starting site (0)

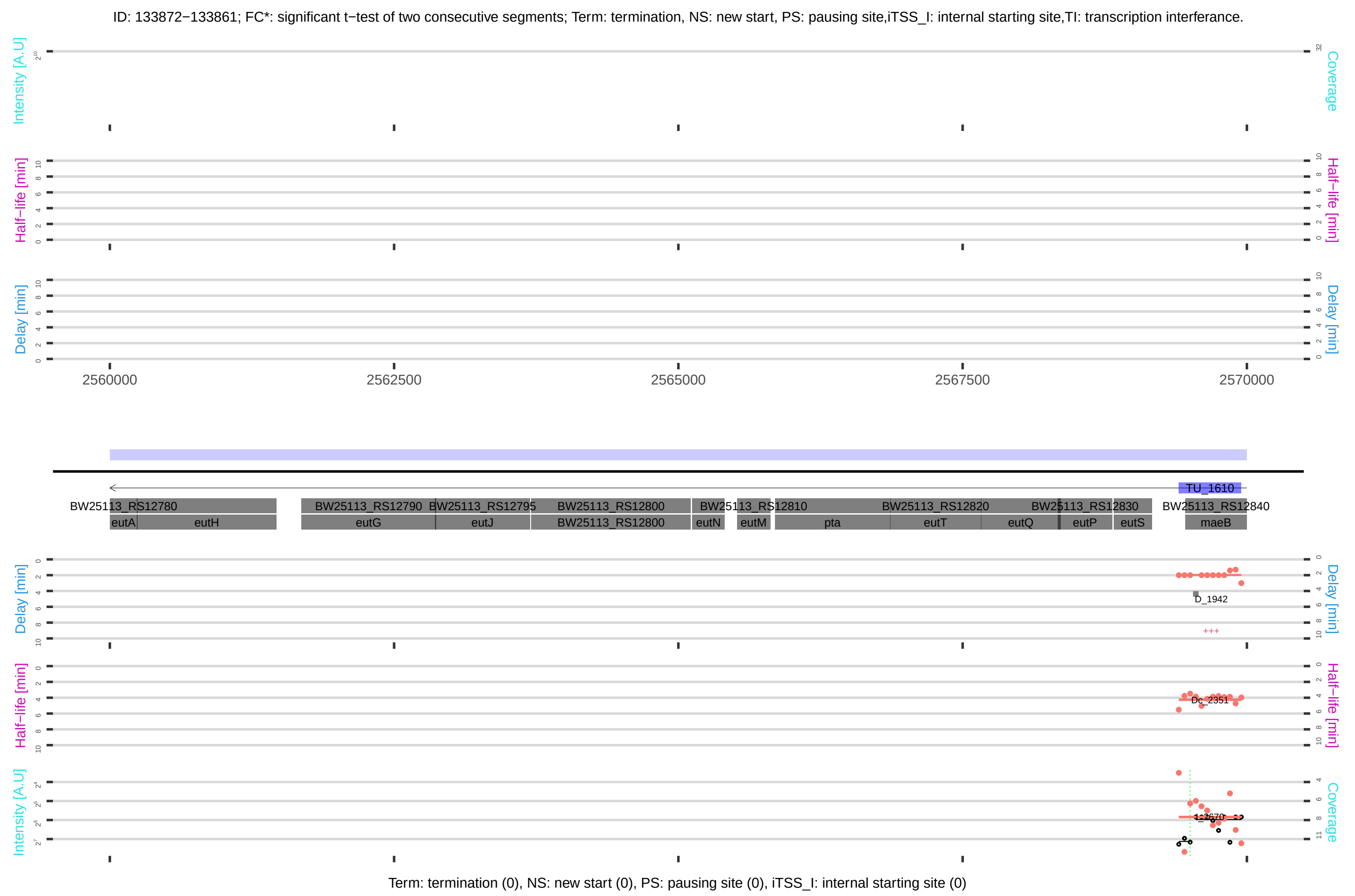
ID: 133872−133861; FC*: significant t−test of two consecutive segments; Term: termination, NS: new start, PS: pausing site,iTSS_I: internal starting site,TI: transcription interferance.
Intensity [A.U]
Coverage
Half−life [min]
Delay [min]
2560000
2562500
2565000
2567500
2570000
TU_1610
BW25113_RS12780
BW25113_RS12790
BW25113_RS12795
BW25113_RS12800
BW25113_RS12810
BW25113_RS12820
BW25113_RS12830
BW25113_RS12840
eutA
eutH
eutG
eutJ
BW25113_RS12800
eutN
eutM
pta
eutT
eutQ
eutP
eutS
maeB
D_1942
Dc_2351
Term: termination (0), NS: new start (0), PS: pausing site (0), iTSS_I: internal starting site (0)

ID: 51442−51582; Term: termination (0), NS: new start (0), PS: pausing site (0), iTSS_I: internal starting site (0)

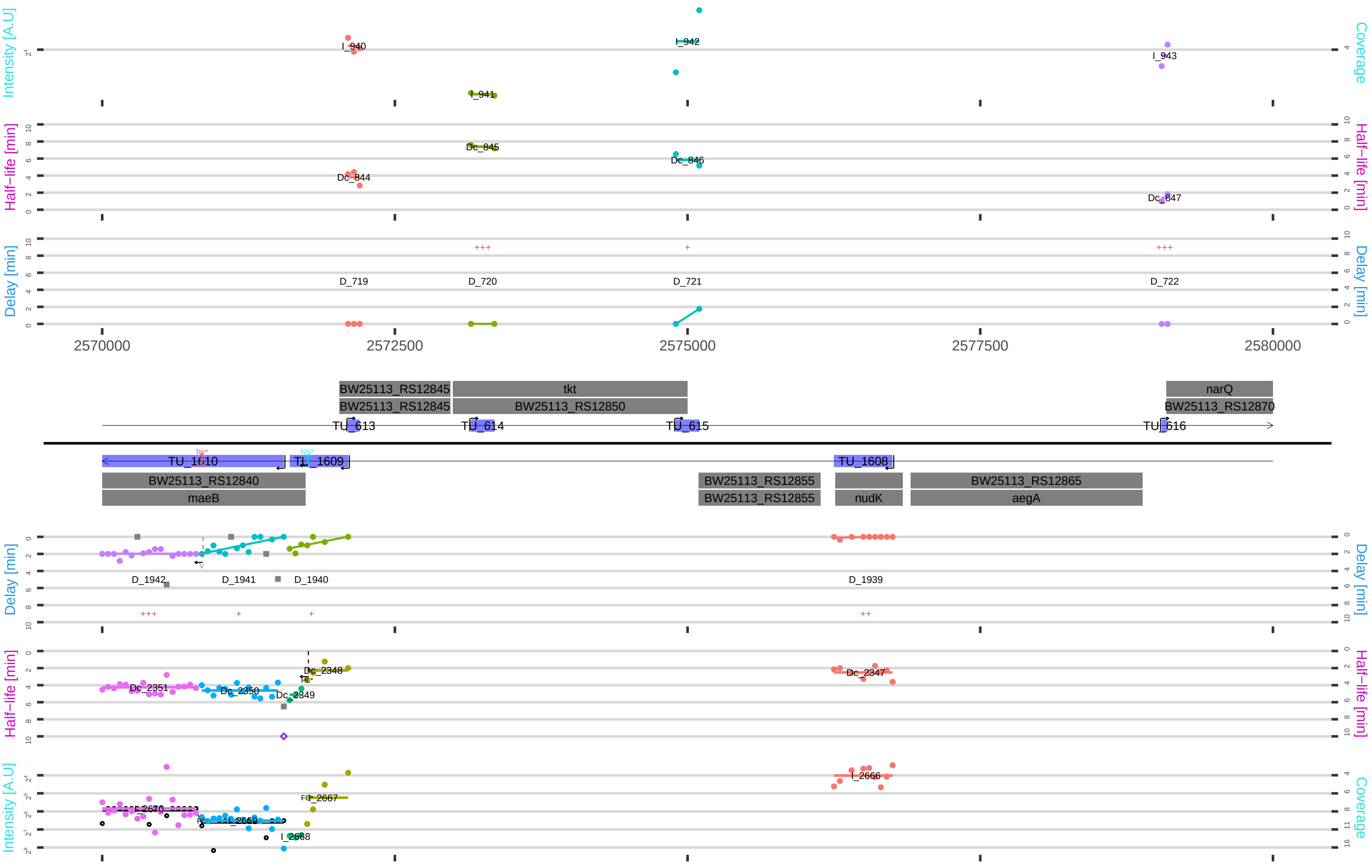

Term: termination (1), NS: new start (1), PS: pausing site (0), iTSS_I: internal starting site (1)

ID: 51664−51727; Term: termination (0), NS: new start (1), PS: pausing site (0), iTSS_I: internal starting site (0)

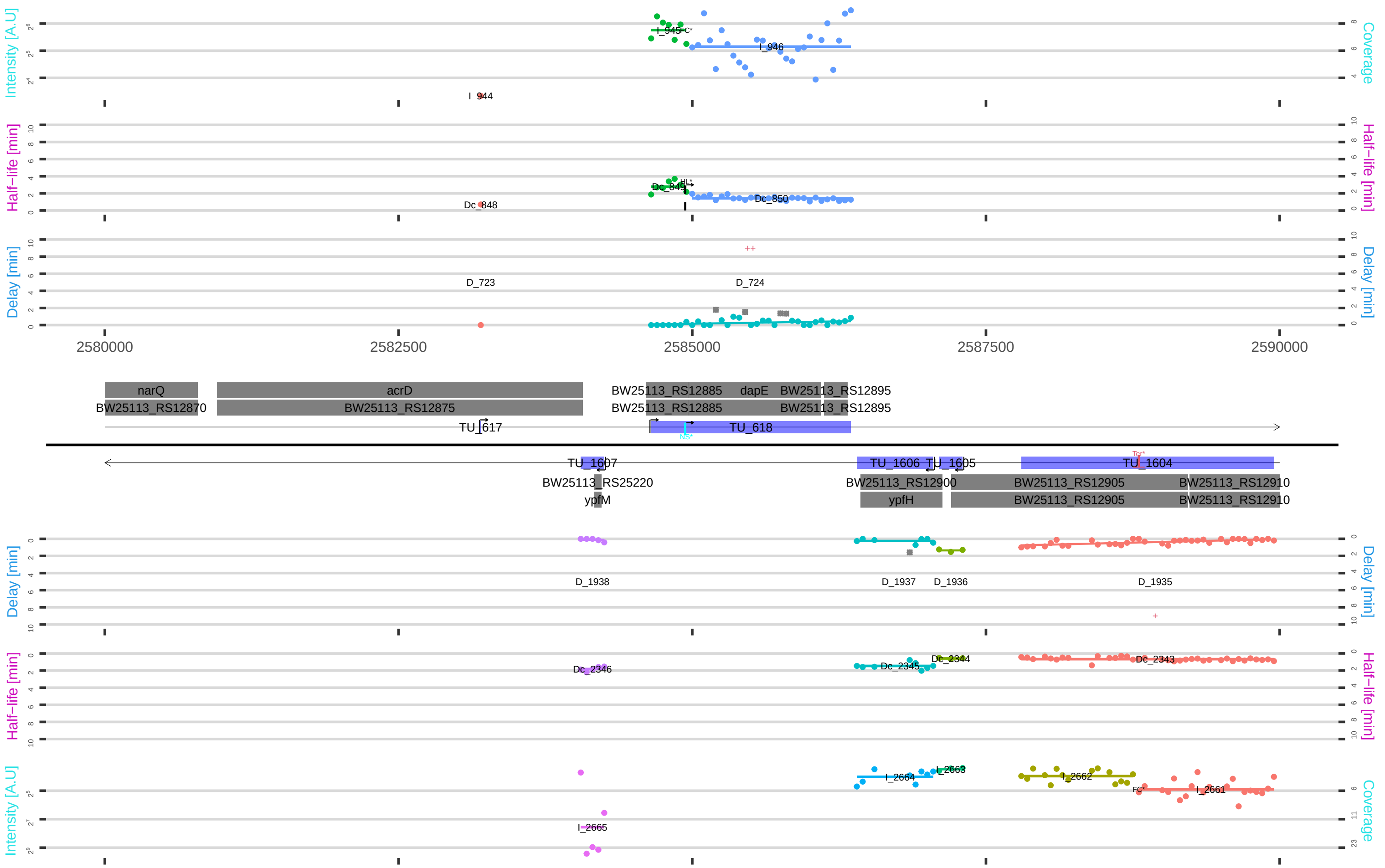

Term: termination (1), NS: new start (0), PS: pausing site (0), iTSS_I: internal starting site (0)

ID: 51865−51887; Term: termination (0), NS: new start (2), PS: pausing site (1), iTSS_I: internal starting site (0)

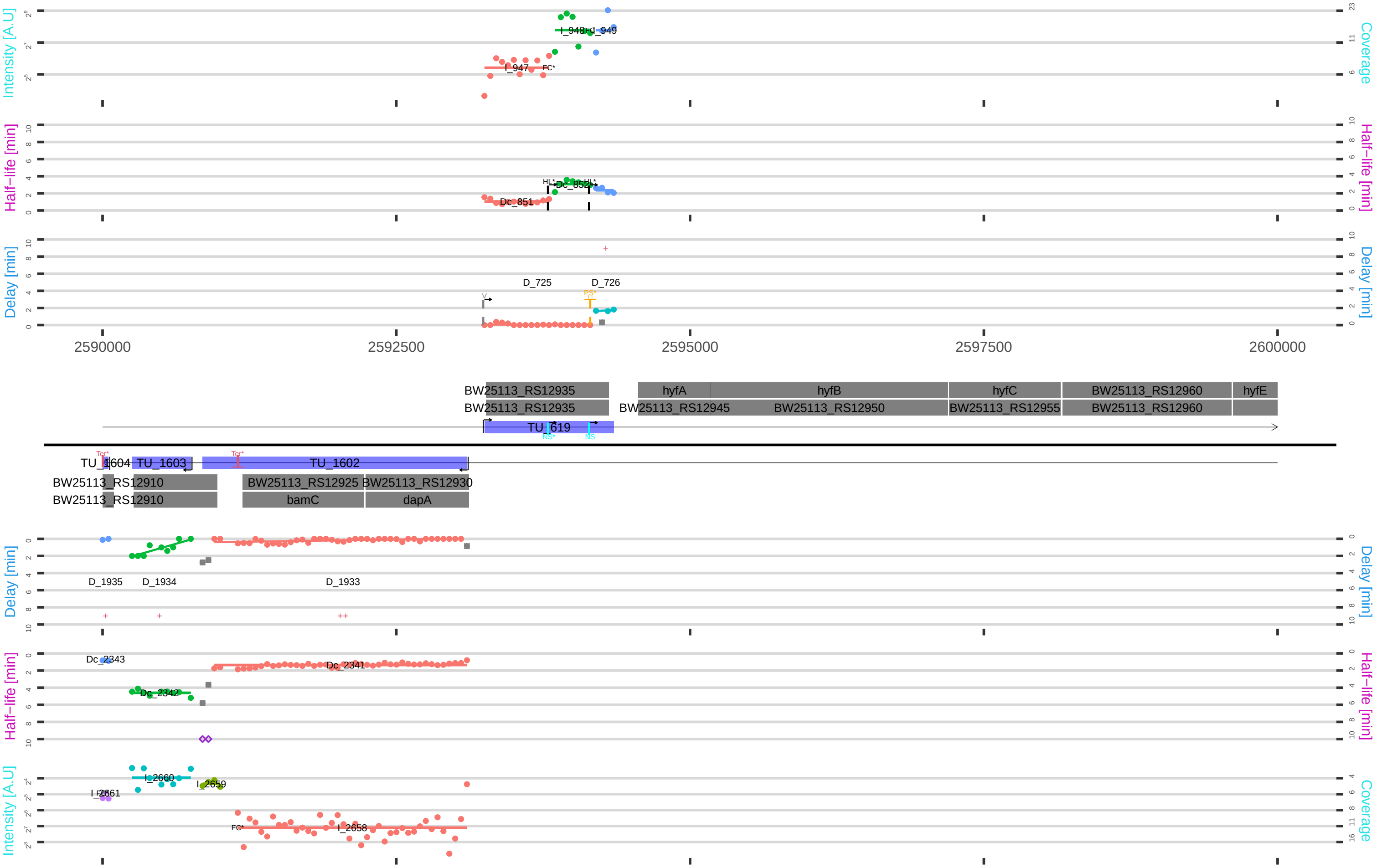

Term: termination (2), NS: new start (0), PS: pausing site (0), iTSS_I: internal starting site (0)

ID: 52189−52200; Term: termination (0), NS: new start (1), PS: pausing site (0), iTSS_I: internal starting site (0)

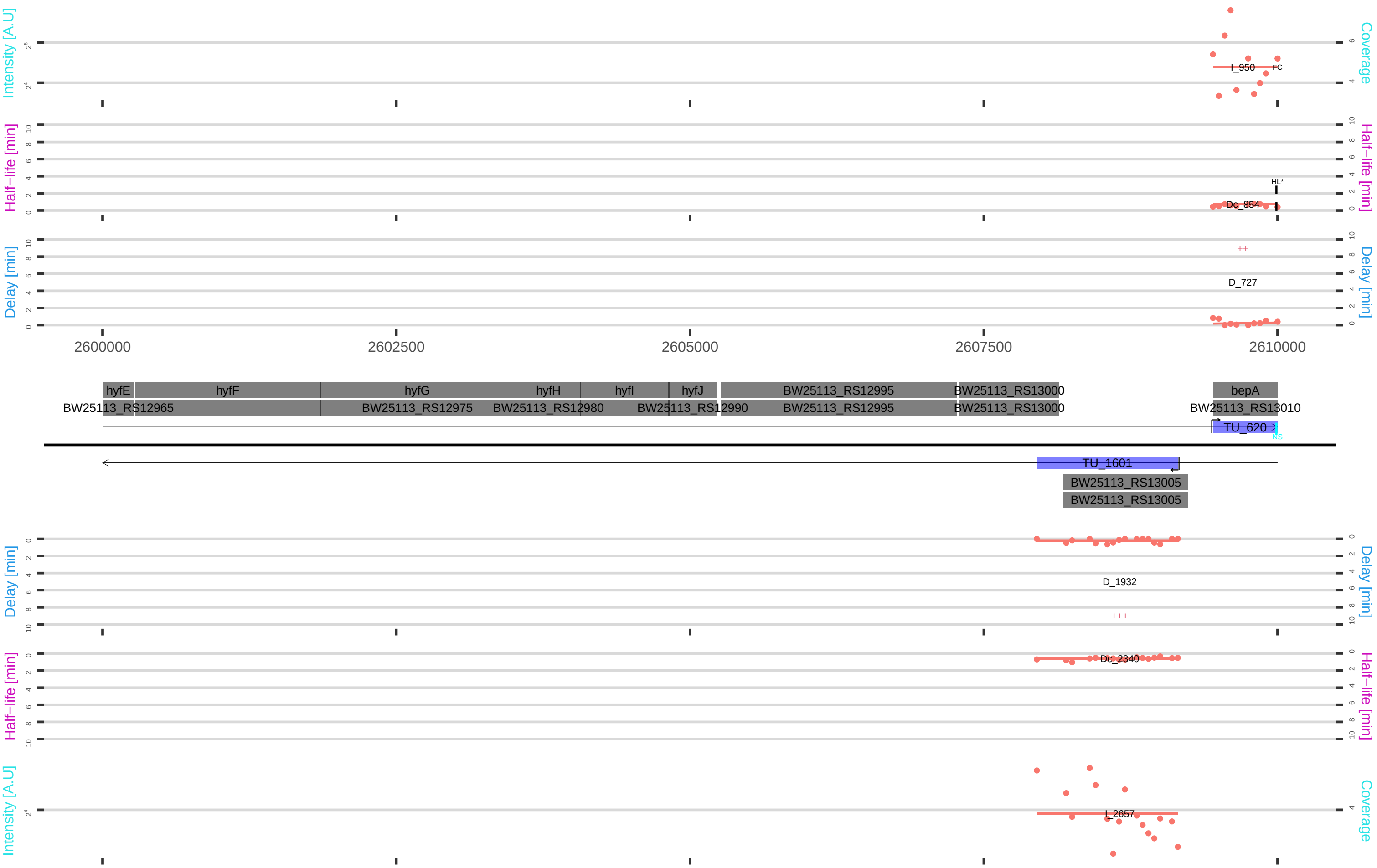

Term: termination (0), NS: new start (0), PS: pausing site (0), iTSS_I: internal starting site (0)

ID: 52200−52400; Term: termination (1), NS: new start (1), PS: pausing site (0), iTSS_I: internal starting site (0)

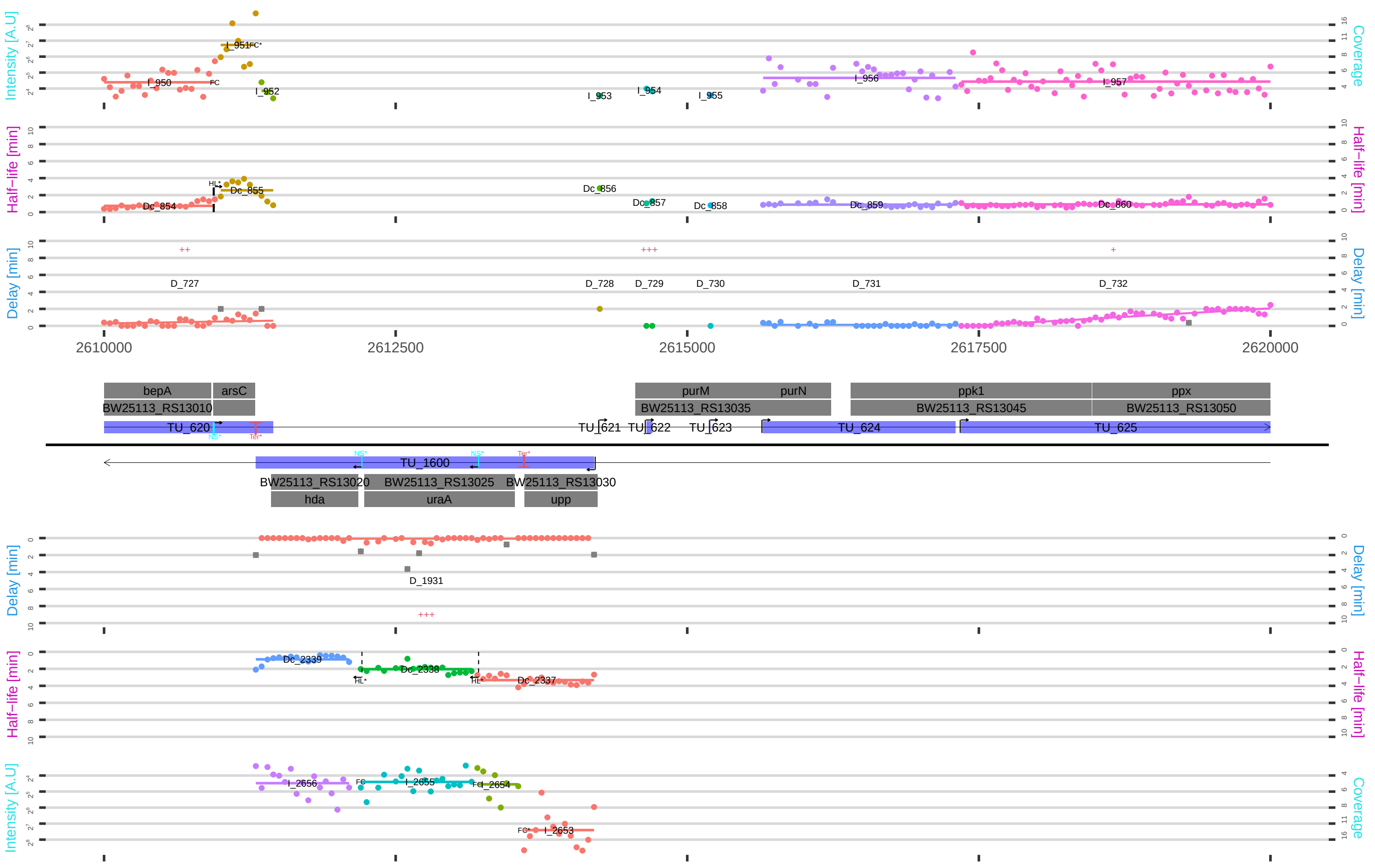

Term: termination (1), NS: new start (2), PS: pausing site (0), iTSS_I: internal starting site (0)

ID: 52400−52584; Term: termination (0), NS: new start (0), PS: pausing site (0), iTSS_I: internal starting site (0)

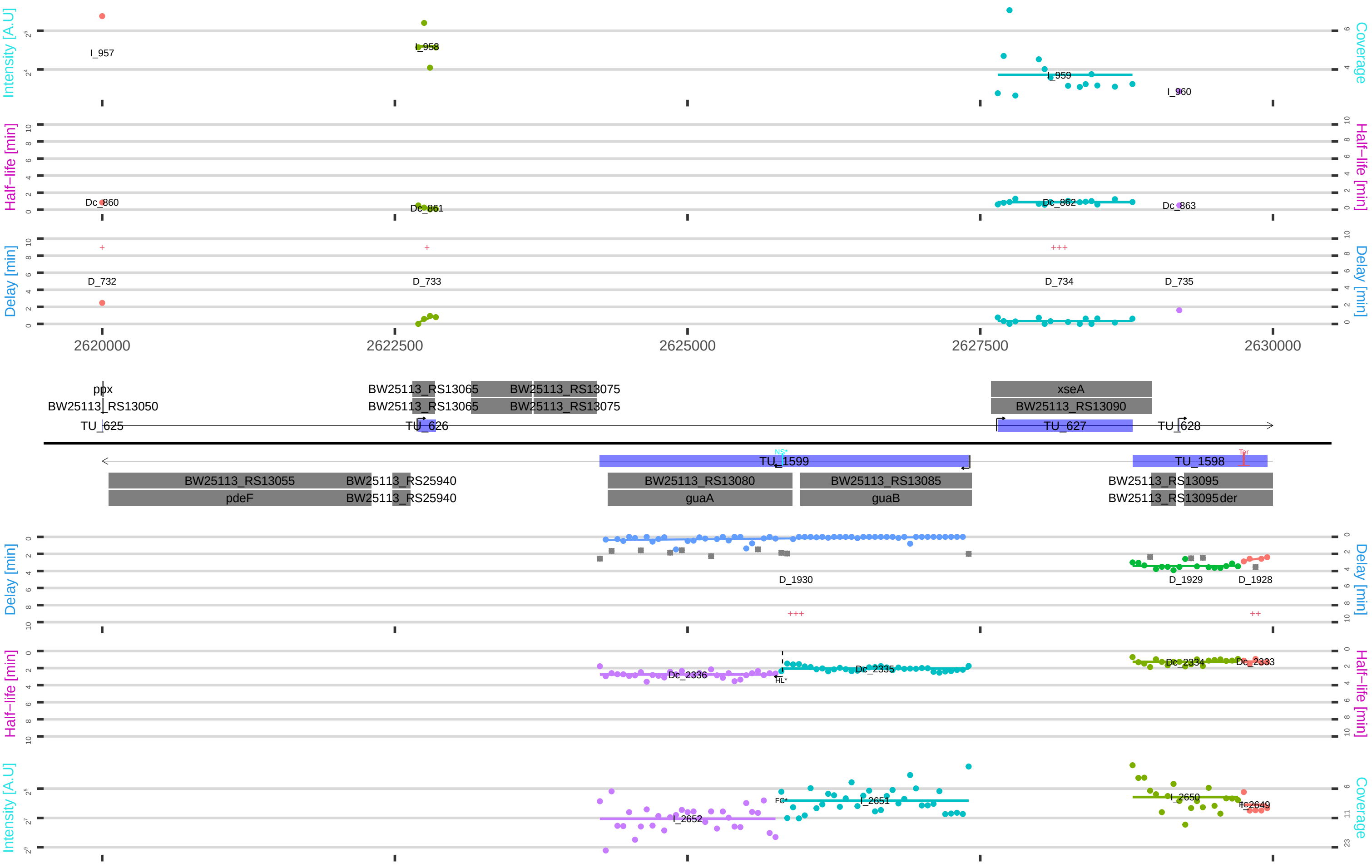

Term: termination (1), NS: new start (1), PS: pausing site (1), iTSS_I: internal starting site (0)

ID: 132660−132496; FC*: significant t−test of two consecutive segments; Term: termination, NS: new start, PS: pausing site,iTSS_I: internal starting site,TI: transcription interferance.

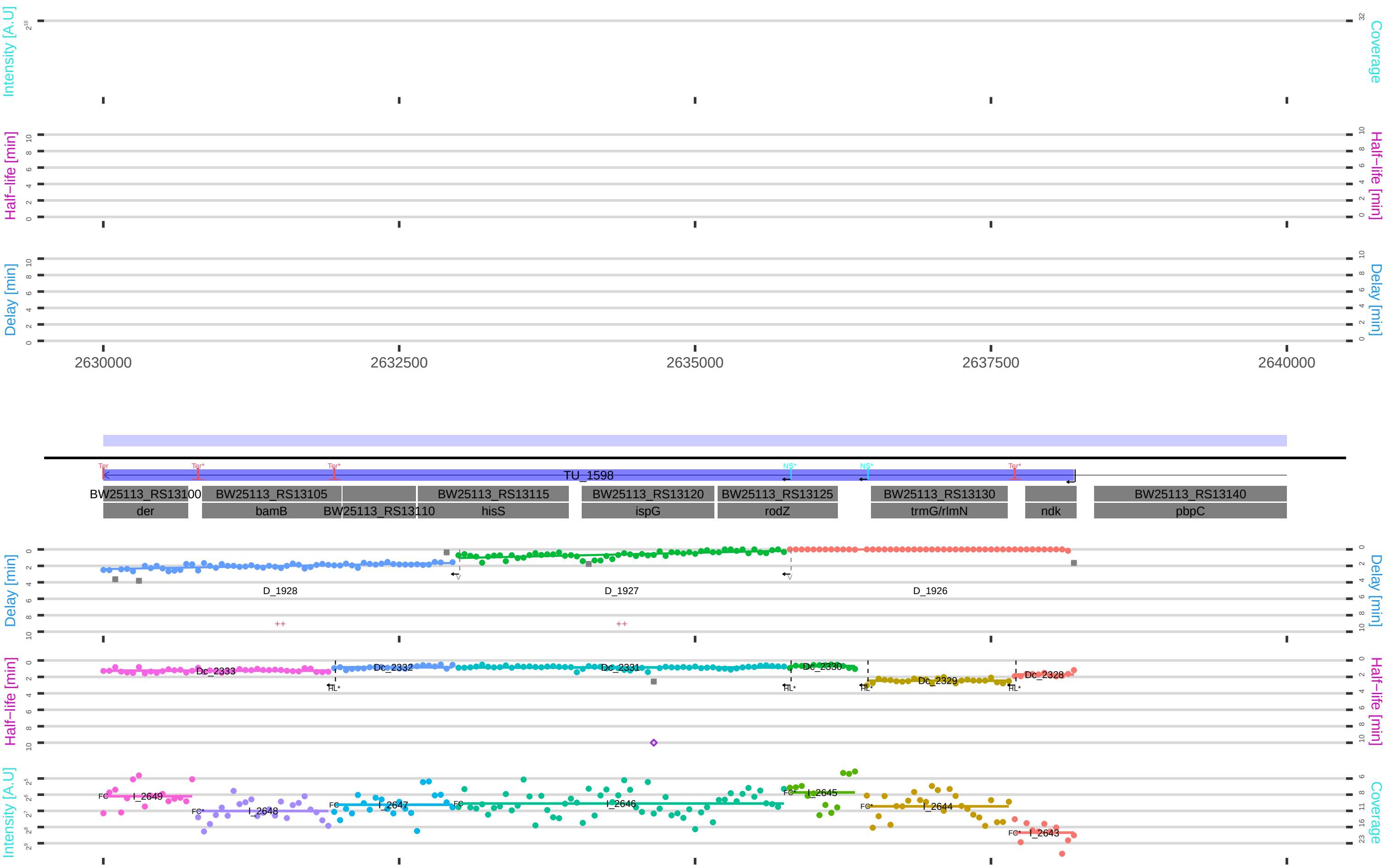

Term: termination (4), NS: new start (2), PS: pausing site (2), iTSS_I: internal starting site (0)

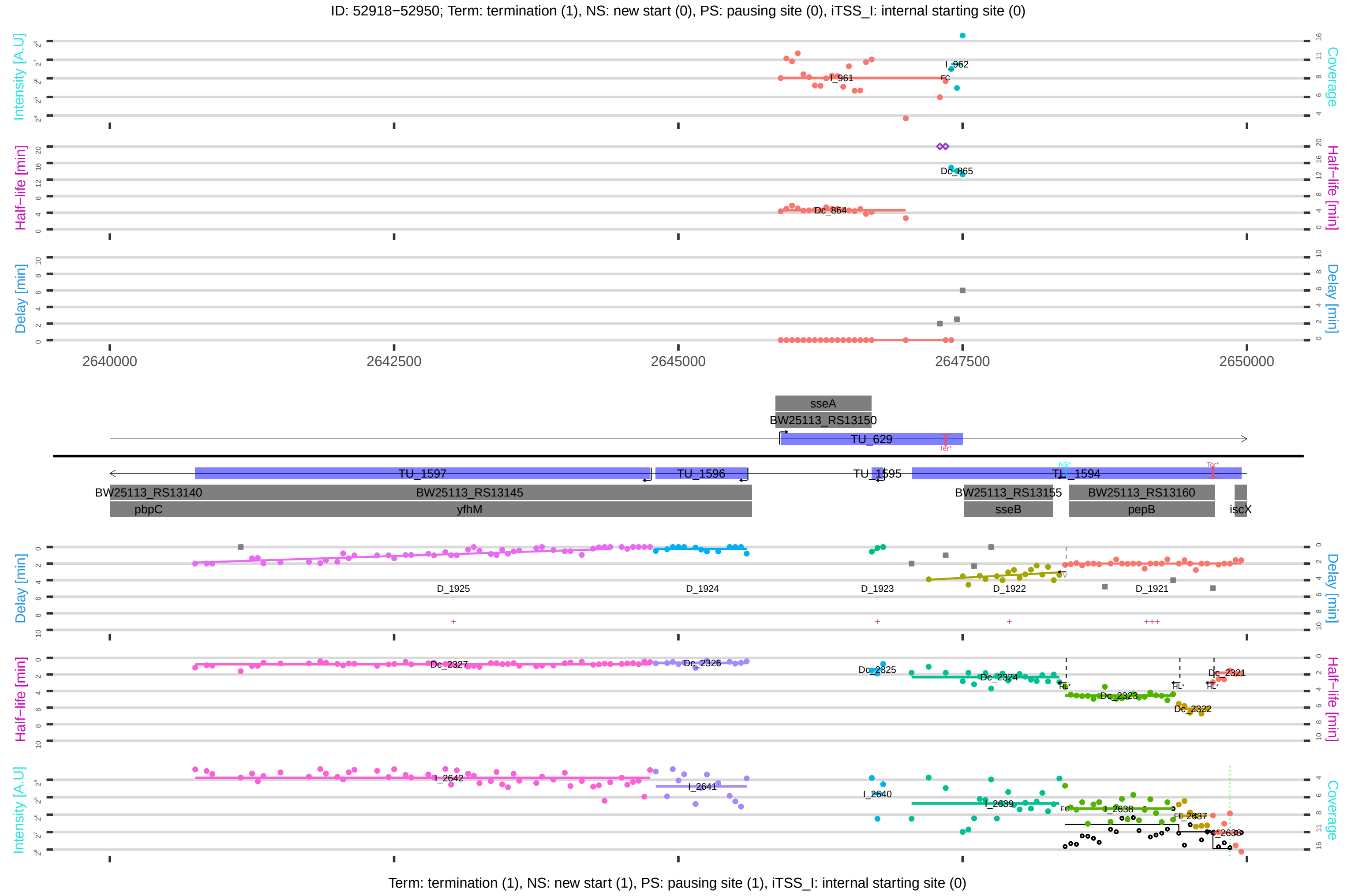

ID: 52918−52950; Term: termination (1), NS: new start (0), PS: pausing site (0), iTSS_I: internal starting site (0)
Intensity [A.U]
Coverage
Half−life [min]
Delay [min]
2640000
2642500
2645000
2647500
2650000
I_962
I_961
Dc_865
Dc_864
sseA
BW25113_RS13150
TU_629
TU_1597
TU_1596
TU_1595
TU_1594
BW25113_RS13140
BW25113_RS13145
BW25113_RS13155
BW25113_RS13160
pbpC
yfhM
sseB
pepB
iscX
D_1925
D_1924
D_1923
D_1922
D_1921
Dc_2327
Dc_2326
Dc_2325
Dc_2324
Dc_2323
Dc_2322
Dc_2321
I_2642
I_2641
I_2640
I_2639
I_2638
I_2637
I_2636
Term: termination (1), NS: new start (1), PS: pausing site (1), iTSS_I: internal starting site (0)

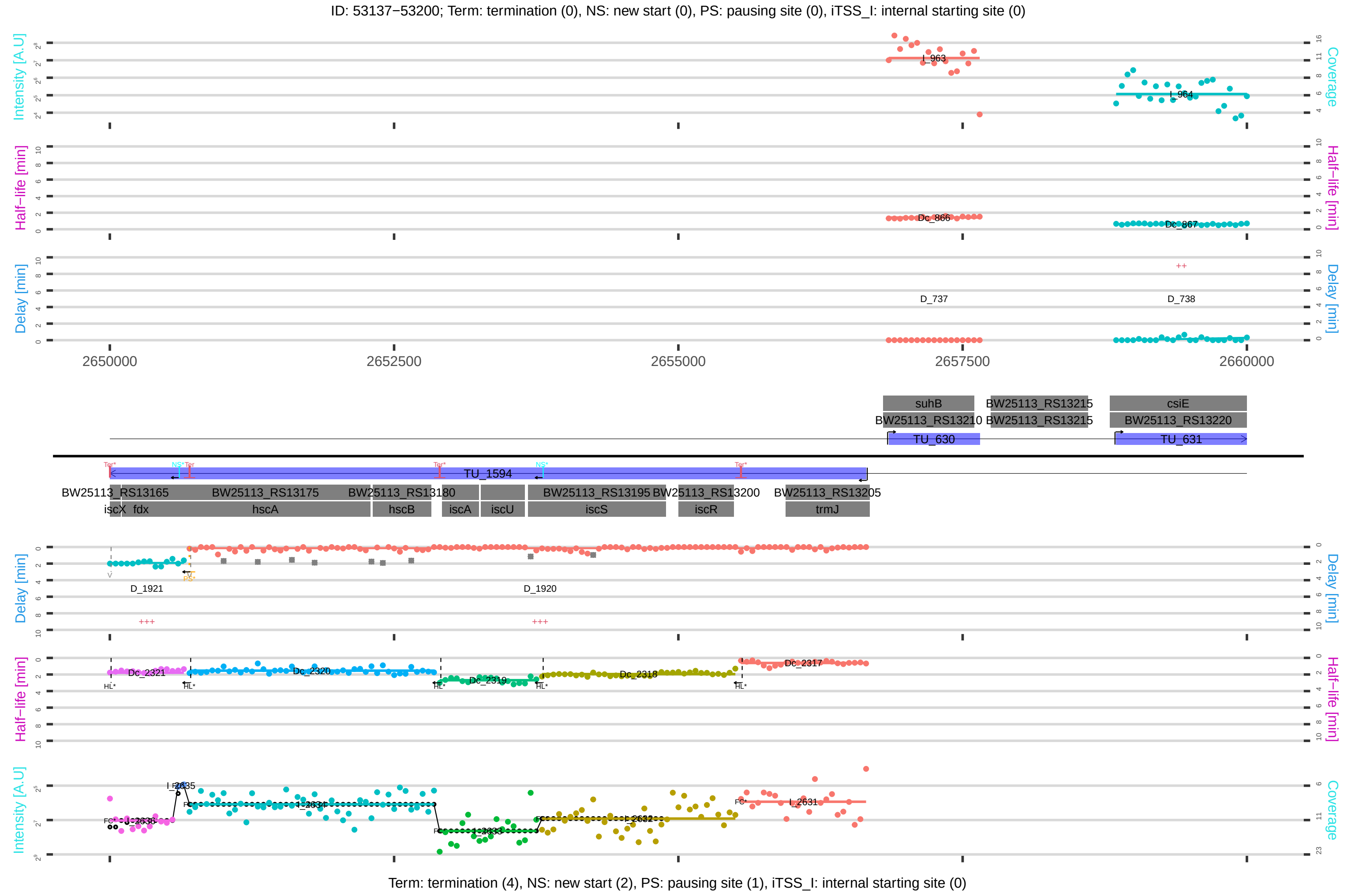

ID: 53137−53200; Term: termination (0), NS: new start (0), PS: pausing site (0), iTSS_I: internal starting site (0)
Intensity [A.U]
Coverage
Half−life [min]
Delay [min]
I_963
I_964
Dc_866
Dc_867
D_737
D_738
2650000
2652500
2655000
2657500
2660000
suhB
BW25113_RS13215
csiE
BW25113_RS13210
BW25113_RS13220
TU_630
TU_631
TU_1594
BW25113_RS13165
BW25113_RS13175
BW25113_RS13180
BW25113_RS13195
BW25113_RS13200
BW25113_RS13205
iscX
fdx
hscA
hscB
iscA
iscU
iscS
iscR
trmJ
D_1921
D_1920
Dc_2321
Dc_2320
Dc_2319
Dc_2318
Dc_2317
I_2635
I_2634
I_2633
I_2632
I_2631
I_2636
Term: termination (4), NS: new start (2), PS: pausing site (1), iTSS_I: internal starting site (0)

ID: 53200−53337; Term: termination (0), NS: new start (0), PS: pausing site (0), iTSS_I: internal starting site (0)

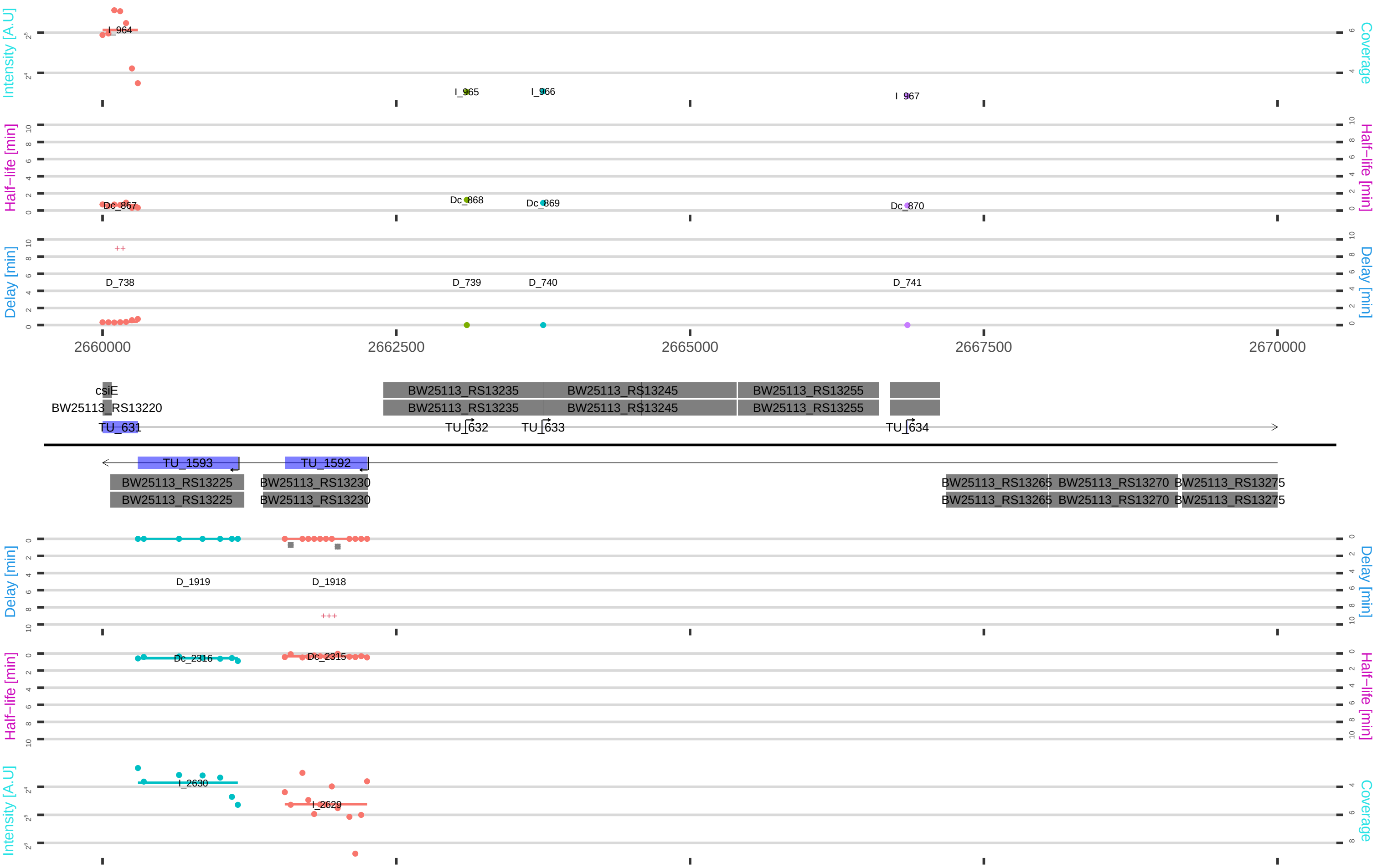

Term: termination (0), NS: new start (0), PS: pausing site (0), iTSS_I: internal starting site (0)

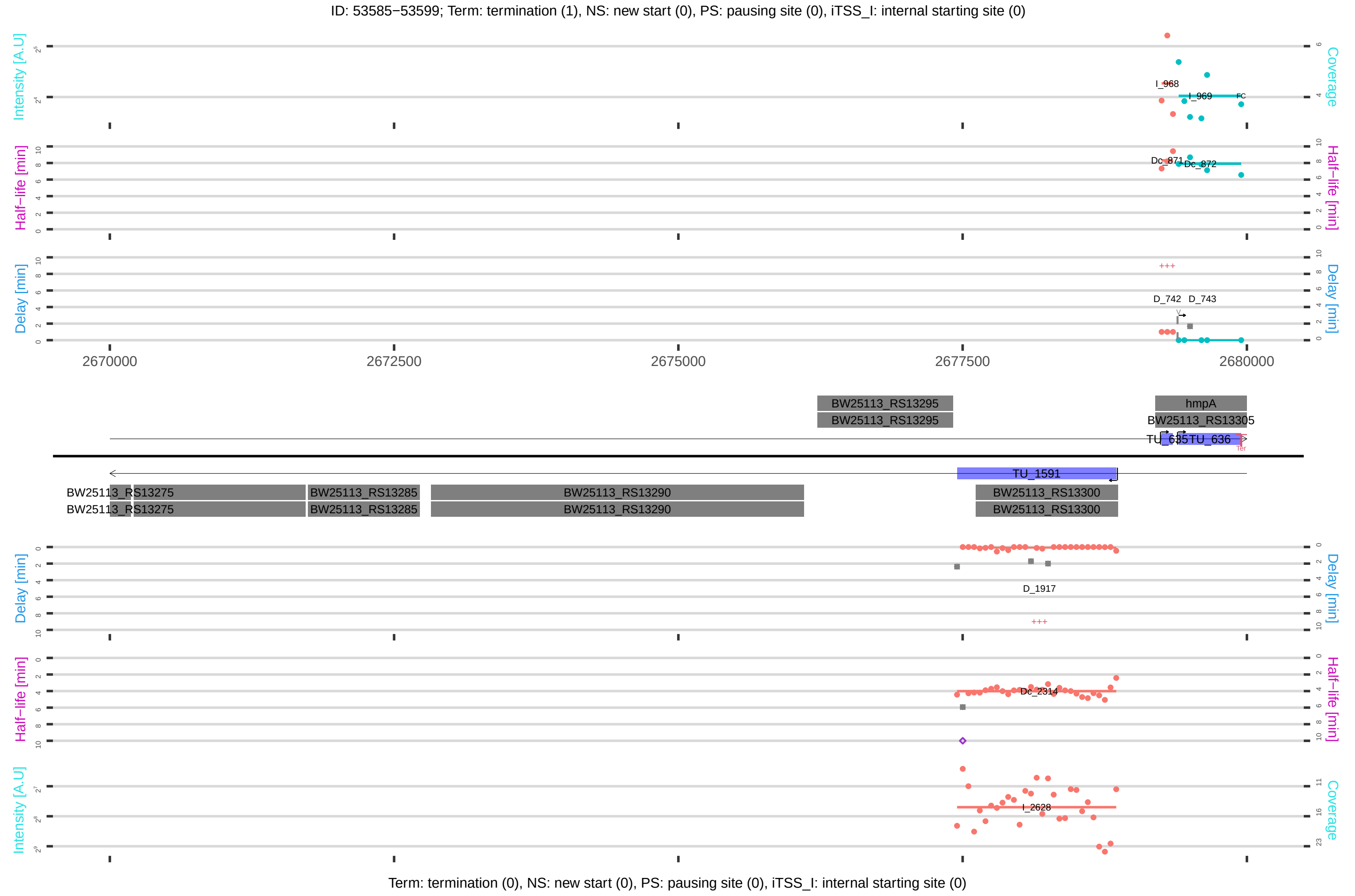

ID: 53585−53599; Term: termination (1), NS: new start (0), PS: pausing site (0), iTSS_I: internal starting site (0)
Intensity [A.U]
Coverage
I_968
I_969
FC
Half−life [min]
Dc_871
Dc_872
Delay [min]
D_742
D_743
2670000
2672500
2675000
2677500
2680000
BW25113_RS13295
hmpA
BW25113_RS13305
TU_635
TU_636
Ter
TU_1591
BW25113_RS13275
BW25113_RS13285
BW25113_RS13290
BW25113_RS13300
D_1917
Dc_2314
I_2628
Term: termination (0), NS: new start (0), PS: pausing site (0), iTSS_I: internal starting site (0)

ID: 53601−53694; Term: termination (1), NS: new start (0), PS: pausing site (1), iTSS_I: internal starting site (0)

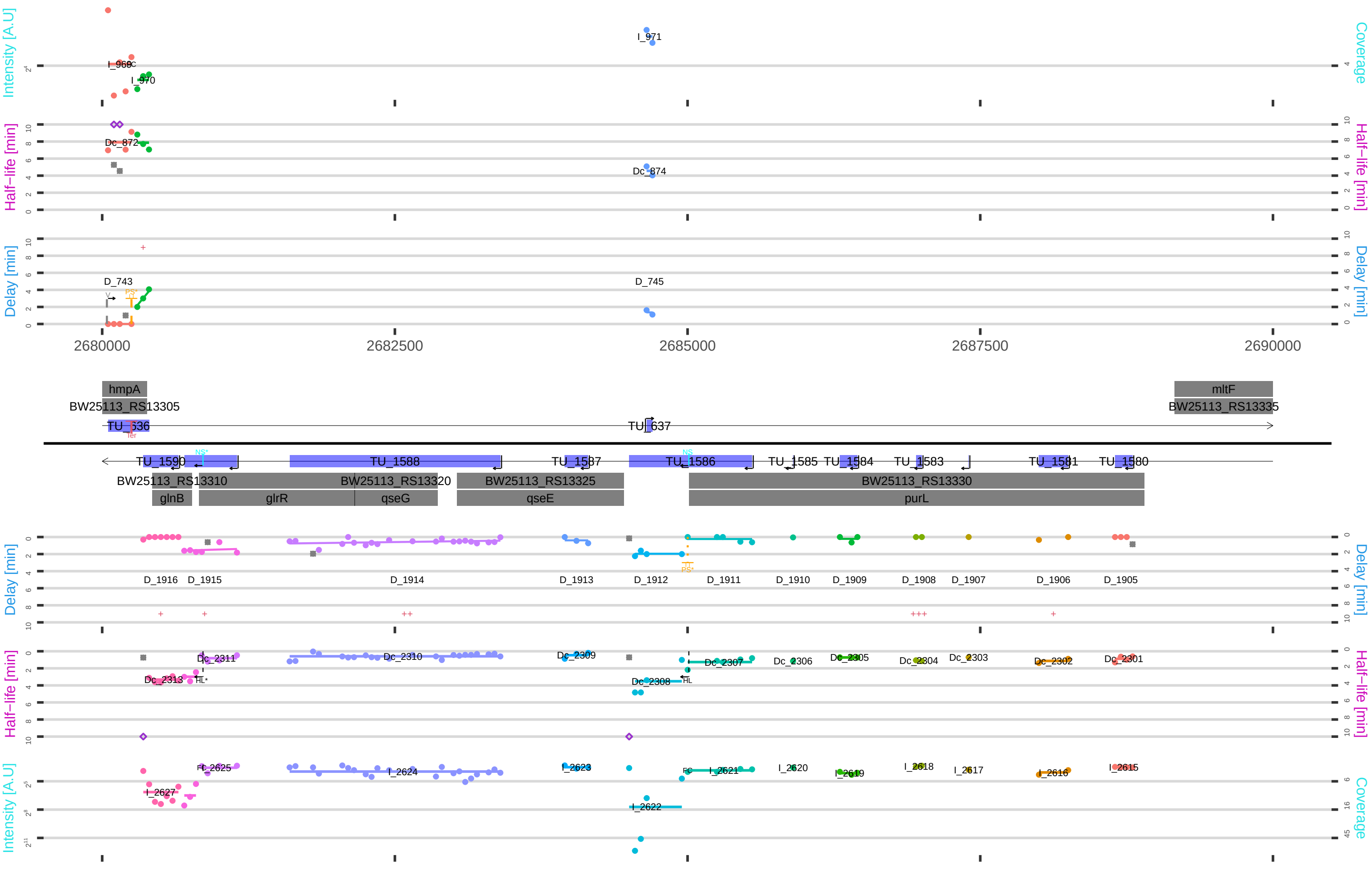

Term: termination (0), NS: new start (2), PS: pausing site (1), iTSS_I: internal starting site (0)

ID: 53801−53866; Term: termination (0), NS: new start (0), PS: pausing site (0), iTSS_I: internal starting site (0)

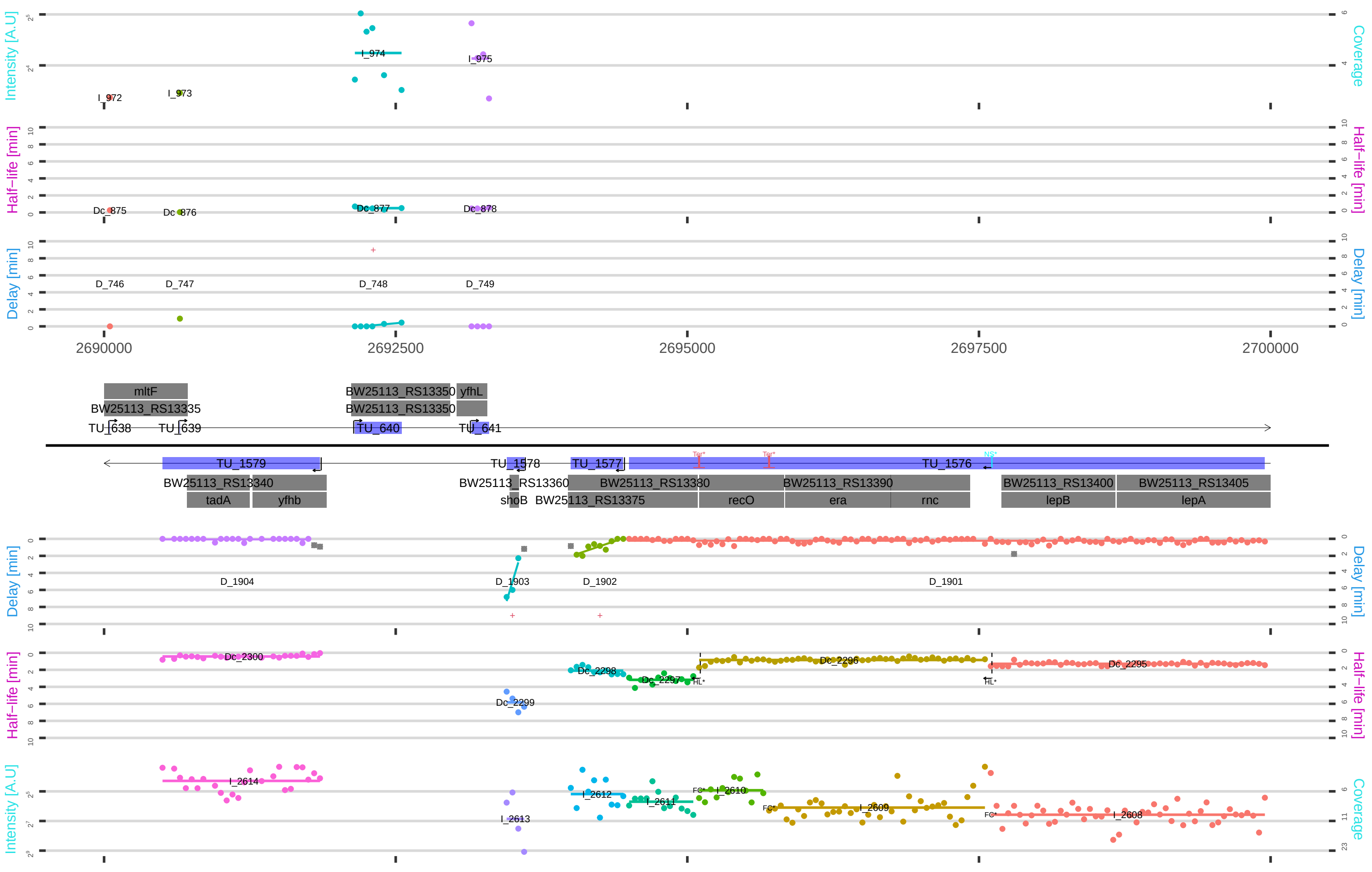

Term: termination (2), NS: new start (1), PS: pausing site (0), iTSS_I: internal starting site (0)

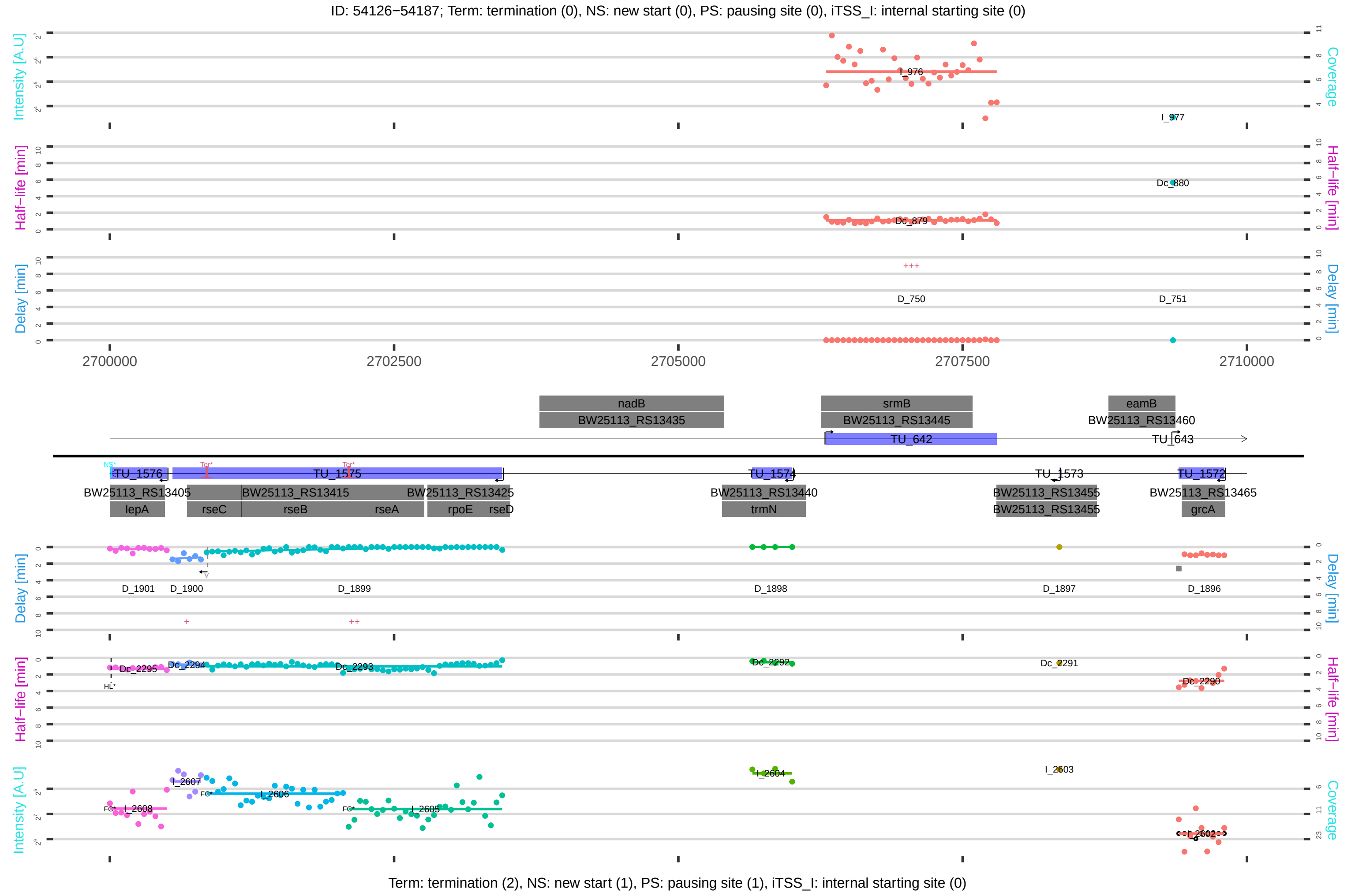
ID: 54126−54187; Term: termination (0), NS: new start (0), PS: pausing site (0), iTSS_I: internal starting site (0)
Intensity [A.U]
Coverage
Half−life [min]
Delay [min]
I_976
I_977
Dc_879
Dc_880
D_750
D_751
2700000
2702500
2705000
2707500
2710000
nadB
BW25113_RS13435
srmB
BW25113_RS13445
eamB
BW25113_RS13460
TU_642
TU_643
TU_1576
TU_1575
TU_1574
TU_1573
TU_1572
BW25113_RS13405
BW25113_RS13415
BW25113_RS13425
BW25113_RS13440
BW25113_RS13455
BW25113_RS13465
lepA
rseC
rseB
rseA
rpoE
rseD
trmN
grcA
D_1901
D_1900
D_1899
D_1898
D_1897
D_1896
Dc_2295
Dc_2294
Dc_2293
Dc_2292
Dc_2291
Dc_2290
I_2608
I_2607
I_2606
I_2605
I_2604
I_2603
I_2602
Term: termination (2), NS: new start (1), PS: pausing site (1), iTSS_I: internal starting site (0)

ID: 54204−54400; Term: termination (1), NS: new start (1), PS: pausing site (0), iTSS_I: internal starting site (0)

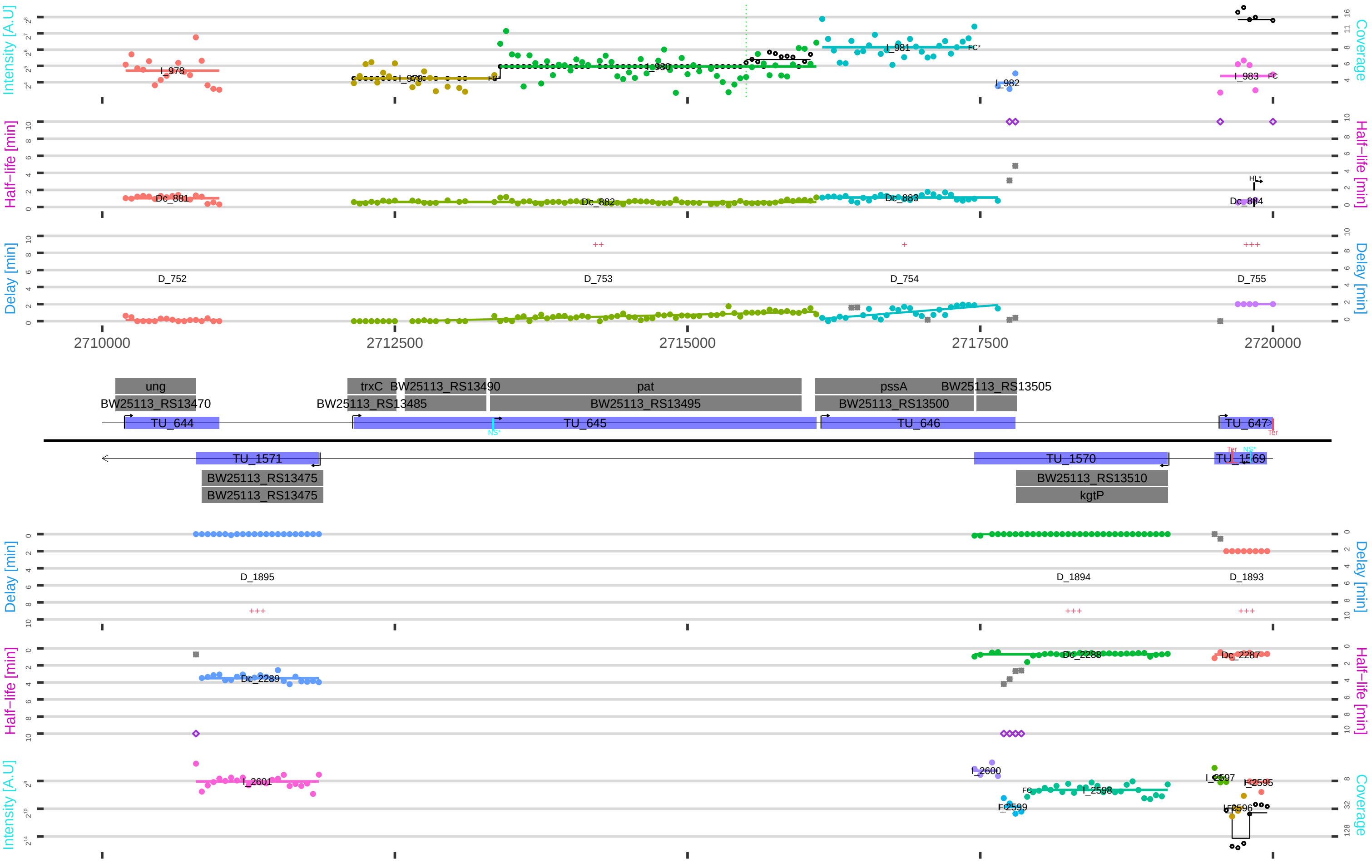

Term: termination (1), NS: new start (1), PS: pausing site (0), iTSS_I: internal starting site (0)

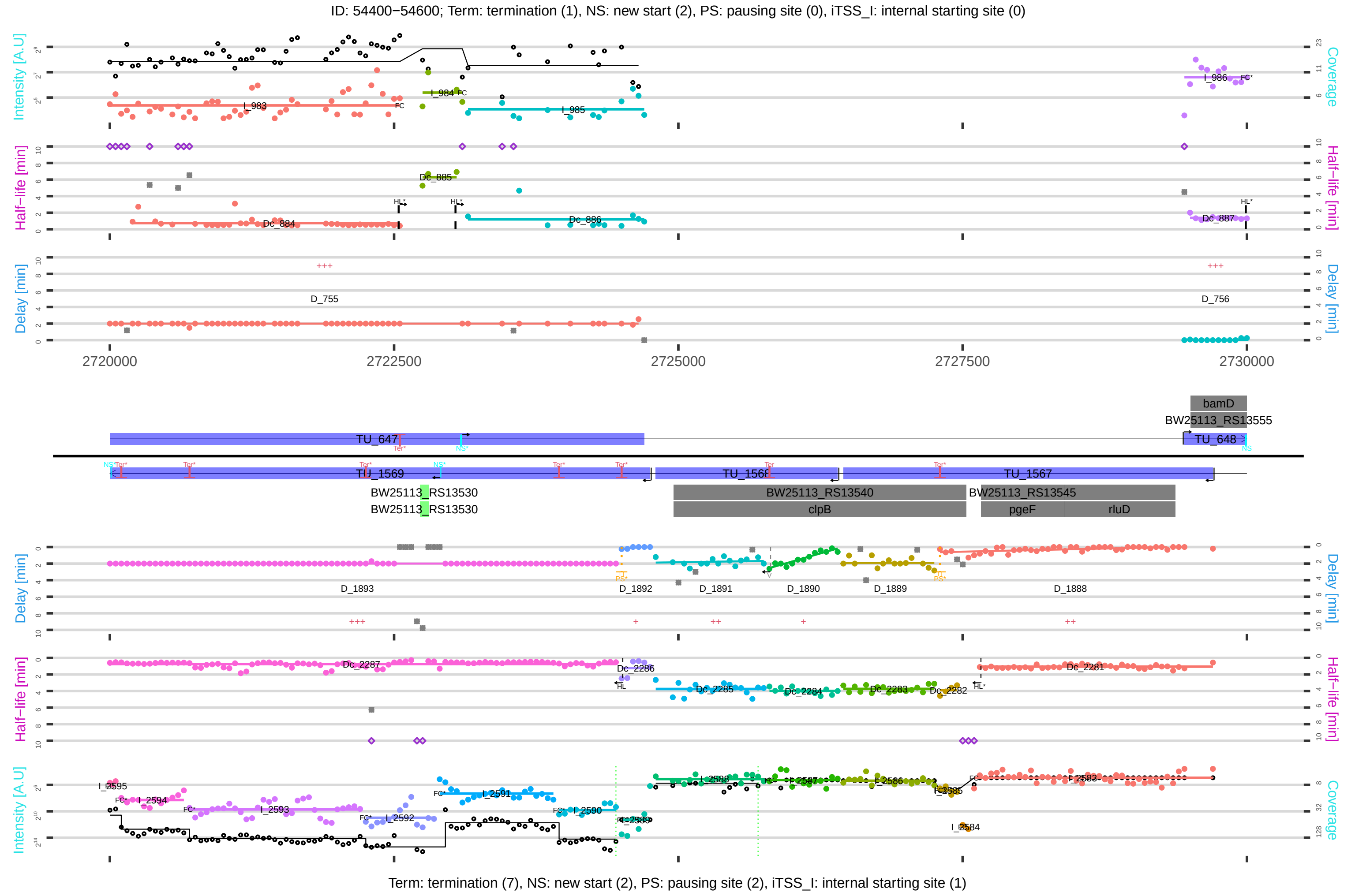
ID: 54400−54600; Term: termination (1), NS: new start (2), PS: pausing site (0), iTSS_I: internal starting site (0)
Intensity [A.U]
Coverage
Half−life [min]
Delay [min]
2720000
2722500
2725000
2727500
2730000
I_983
I_984
I_985
I_986
Dc_884
Dc_885
Dc_886
Dc_887
D_755
D_756
bamD
BW25113_RS13555
TU_647
TU_648
TU_1569
TU_1568
TU_1567
BW25113_RS13530
BW25113_RS13540
BW25113_RS13545
clpB
pgeF
rluD
D_1893
D_1892
D_1891
D_1890
D_1889
D_1888
Dc_2287
Dc_2286
Dc_2285
Dc_2284
Dc_2283
Dc_2282
Dc_2281
I_2595
I_2594
I_2593
I_2592
I_2591
I_2590
I_2589
I_2588
I_2587
I_2586
I_2585
I_2584
I_2583
Term: termination (7), NS: new start (2), PS: pausing site (2), iTSS_I: internal starting site (1)

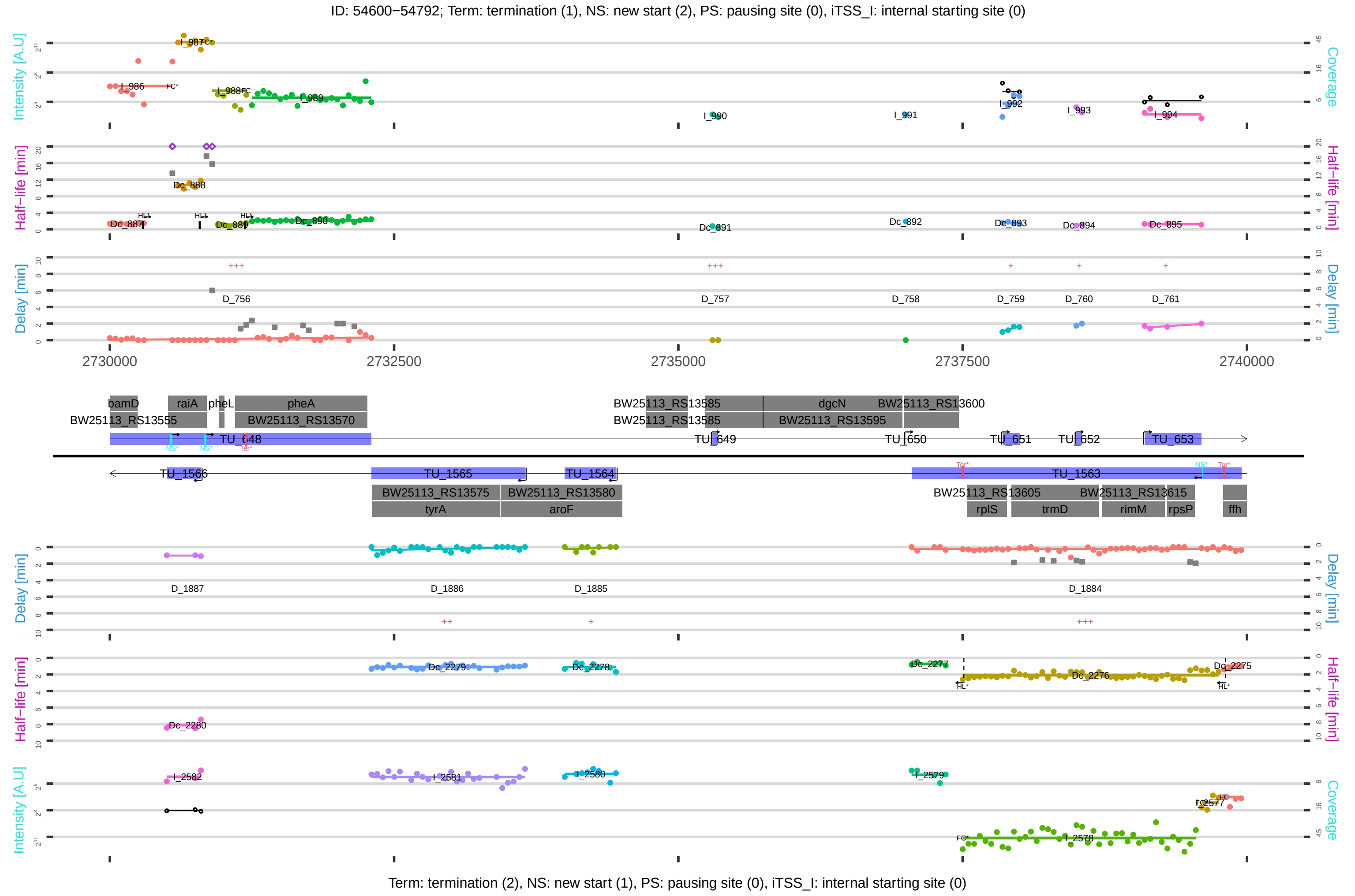

ID: 54600−54792; Term: termination (1), NS: new start (2), PS: pausing site (0), iTSS_I: internal starting site (0)
Intensity [A.U]
Coverage
Half−life [min]
Delay [min]
2730000
2732500
2735000
2737500
2740000
bamD
BW25113_RS13555
raiA
pheL
pheA
BW25113_RS13570
BW25113_RS13585
dgcN
BW25113_RS13595
BW25113_RS13600
TU_648
TU_649
TU_650
TU_651
TU_652
TU_653
TU_1566
TU_1565
TU_1564
TU_1563
BW25113_RS13575
BW25113_RS13580
tyrA
aroF
BW25113_RS13605
BW25113_RS13615
rplS
trmD
rimM
rpsP
ffh
D_756
D_757
D_758
D_759
D_760
D_761
D_1887
D_1886
D_1885
D_1884
Dc_887
Dc_888
Dc_889
Dc_890
Dc_891
Dc_892
Dc_893
Dc_894
Dc_895
Dc_2280
Dc_2279
Dc_2278
Dc_2277
Dc_2276
Dc_2275
I_986
I_987
I_988
I_989
I_990
I_991
I_992
I_993
I_994
I_2582
I_2581
I_2580
I_2579
I_2578
I_2577
Term: termination (2), NS: new start (1), PS: pausing site (0), iTSS_I: internal starting site (0)

ID: 54828−55000; Term: termination (0), NS: new start (3), PS: pausing site (1), iTSS_I: internal starting site (0)

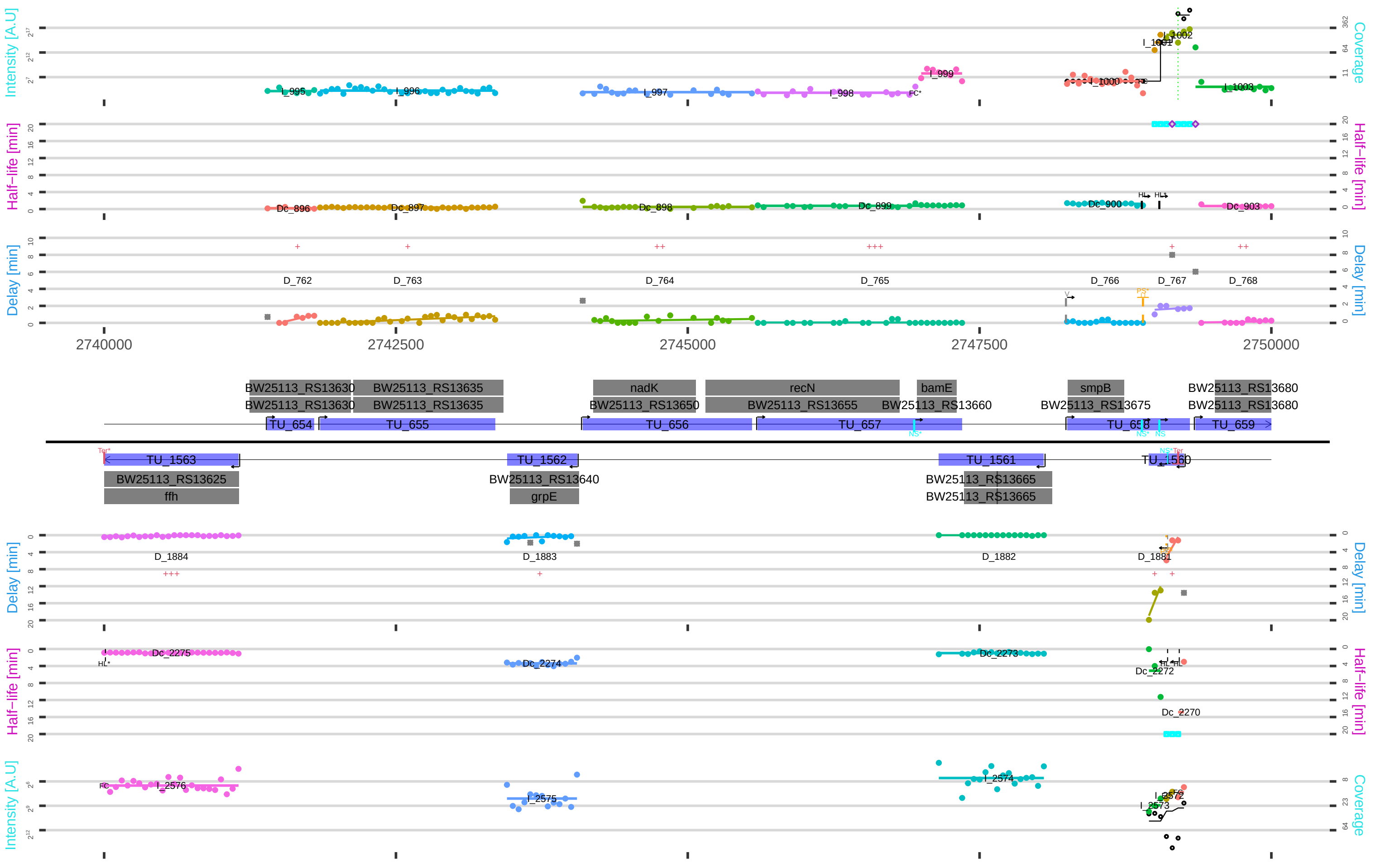

Term: termination (2), NS: new start (1), PS: pausing site (1), iTSS_I: internal starting site (0)

ID: 55000−55200; Term: termination (0), NS: new start (0), PS: pausing site (0), iTSS_I: internal starting site (0)

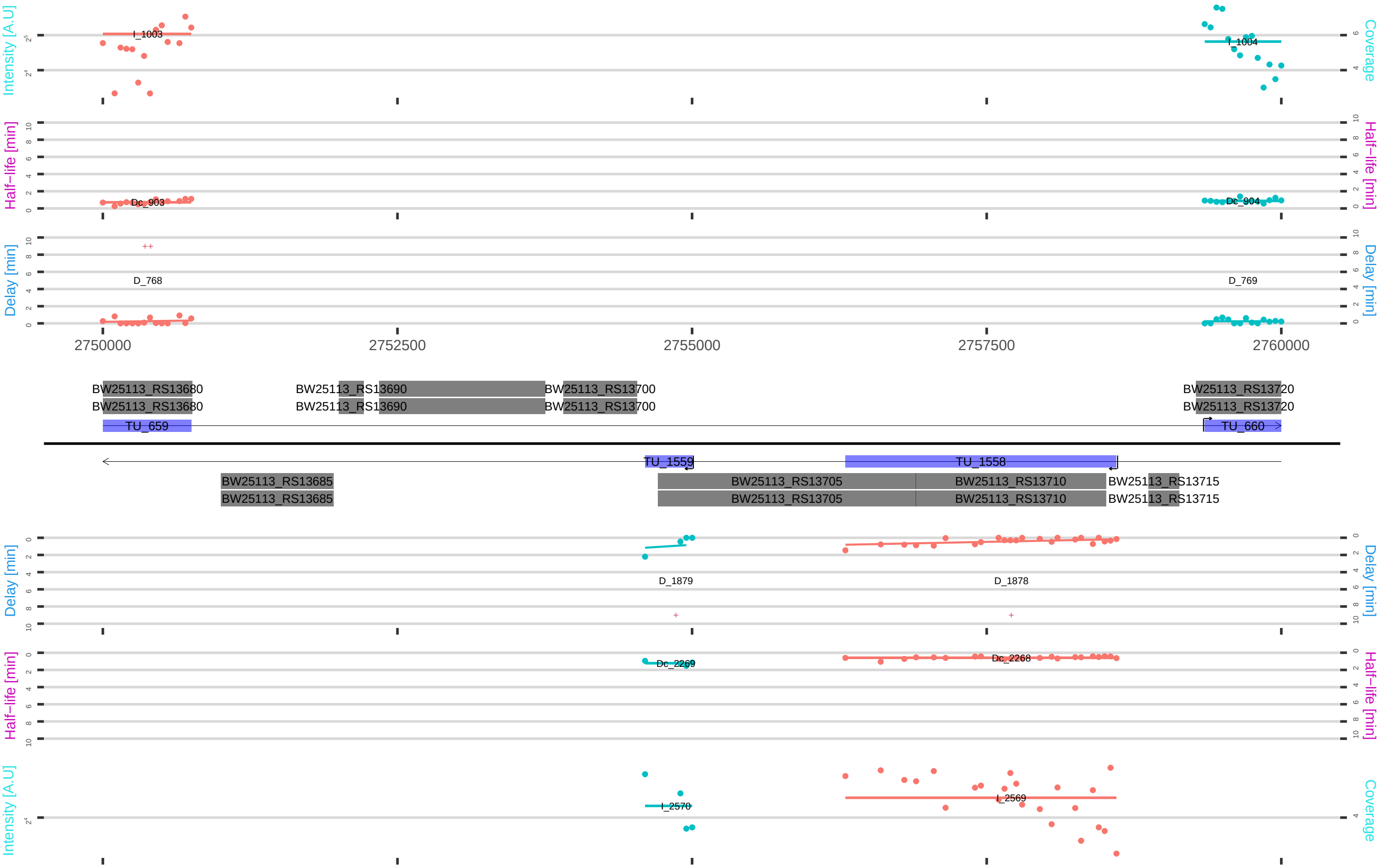

Term: termination (0), NS: new start (0), PS: pausing site (0), iTSS_I: internal starting site (0)

ID: 55200−55308; Term: termination (0), NS: new start (0), PS: pausing site (0), iTSS_I: internal starting site (0)

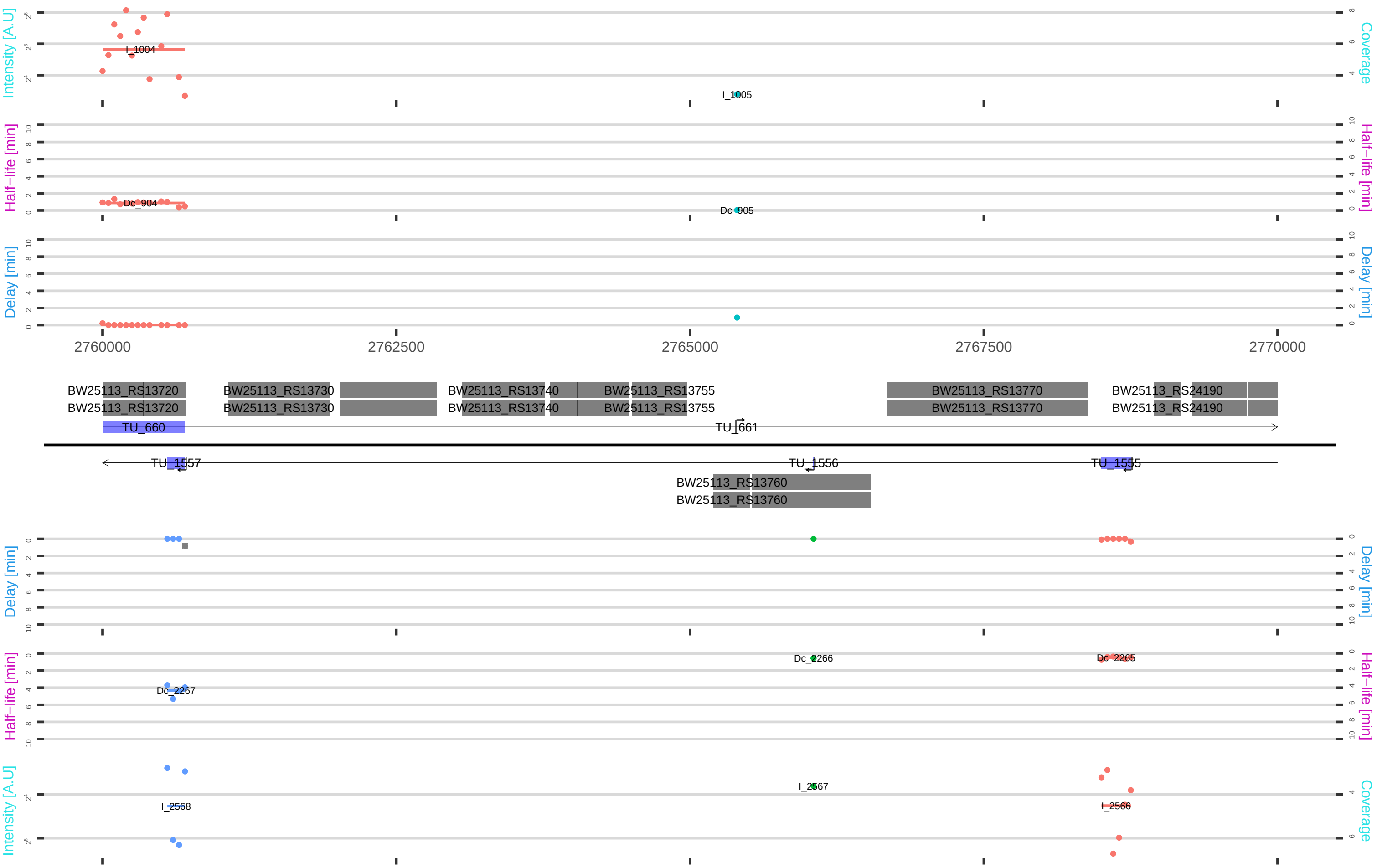

Term: termination (0), NS: new start (0), PS: pausing site (0), iTSS_I: internal starting site (0)

ID: 55428−55429; Term: termination (0), NS: new start (0), PS: pausing site (0), iTSS_I: internal starting site (0)

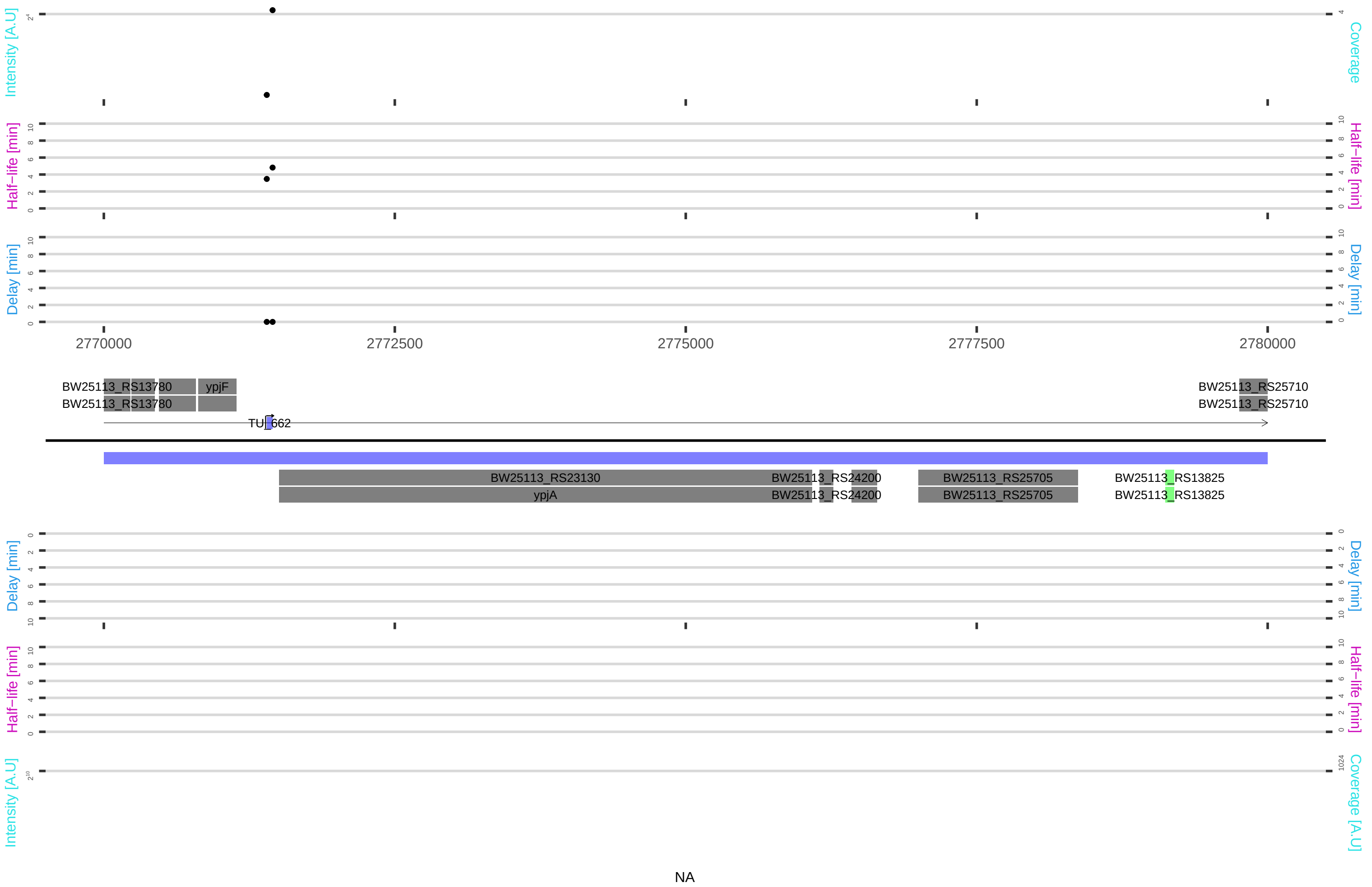

ID: 129467−129461; FC*: significant t−test of two consecutive segments; Term: termination, NS: new start, PS: pausing site,iTSS_I: internal starting site,TI: transcription interferance.

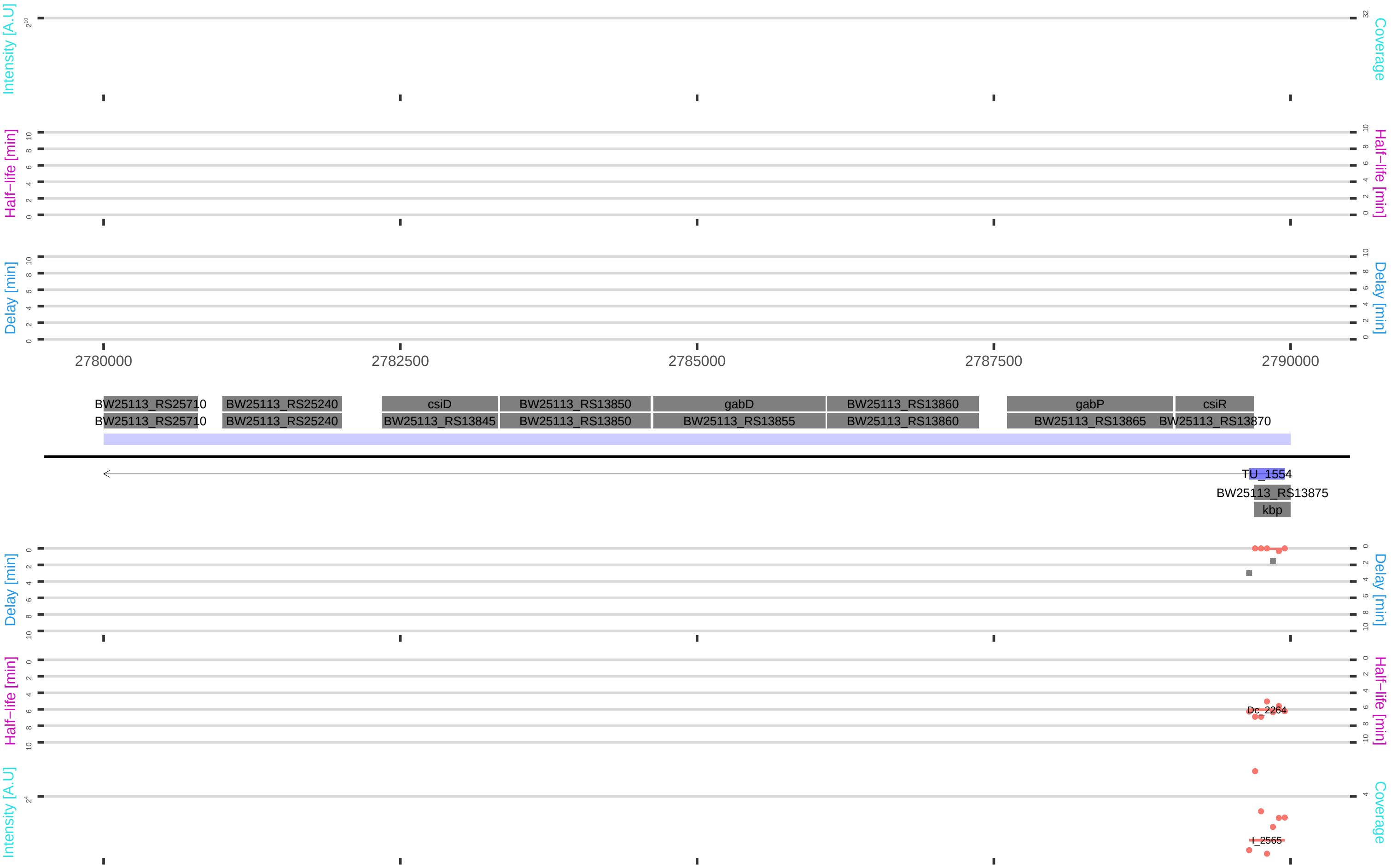

Term: termination (0), NS: new start (0), PS: pausing site (0), iTSS_I: internal starting site (0)

ID: 55822−55999; Term: termination (1), NS: new start (1), PS: pausing site (0), iTSS_I: internal starting site (0)

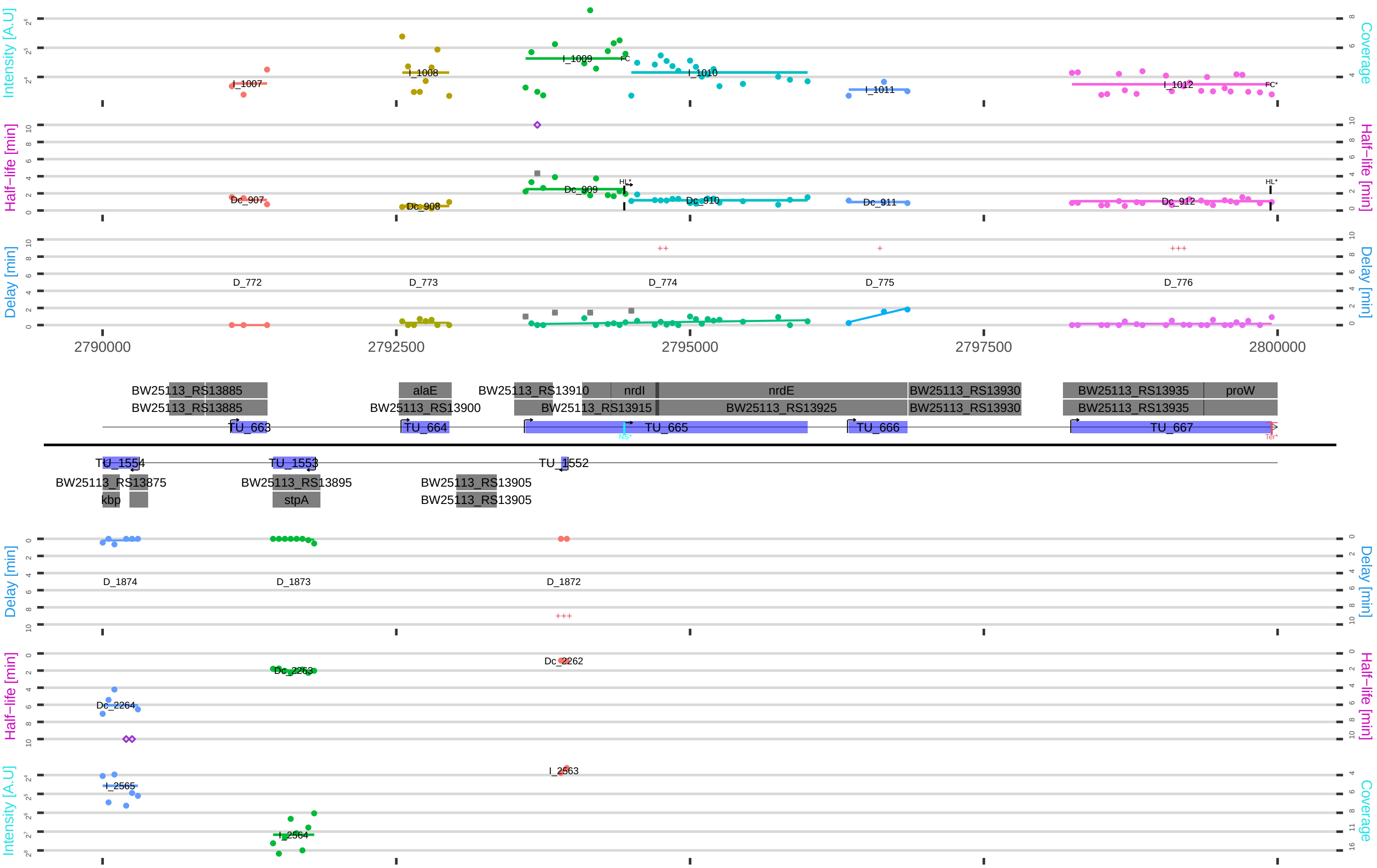

Term: termination (0), NS: new start (0), PS: pausing site (0), iTSS_I: internal starting site (0)

ID: 56001−56150; Term: termination (3), NS: new start (2), PS: pausing site (1), iTSS_I: internal starting site (3)

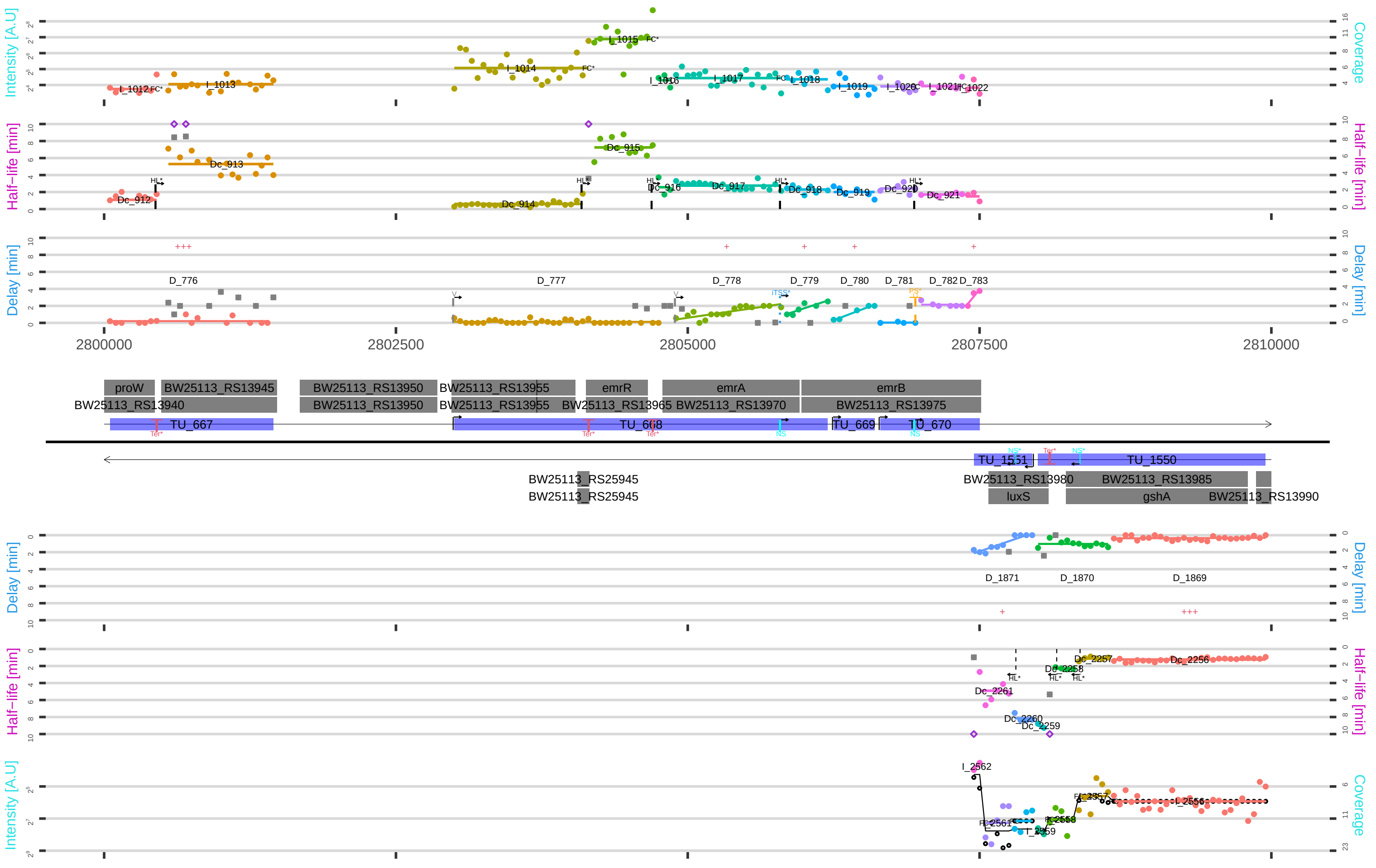

Term: termination (1), NS: new start (2), PS: pausing site (1), iTSS_I: internal starting site (0)

ID: 56239−56400; Term: termination (0), NS: new start (1), PS: pausing site (0), iTSS_I: internal starting site (0)

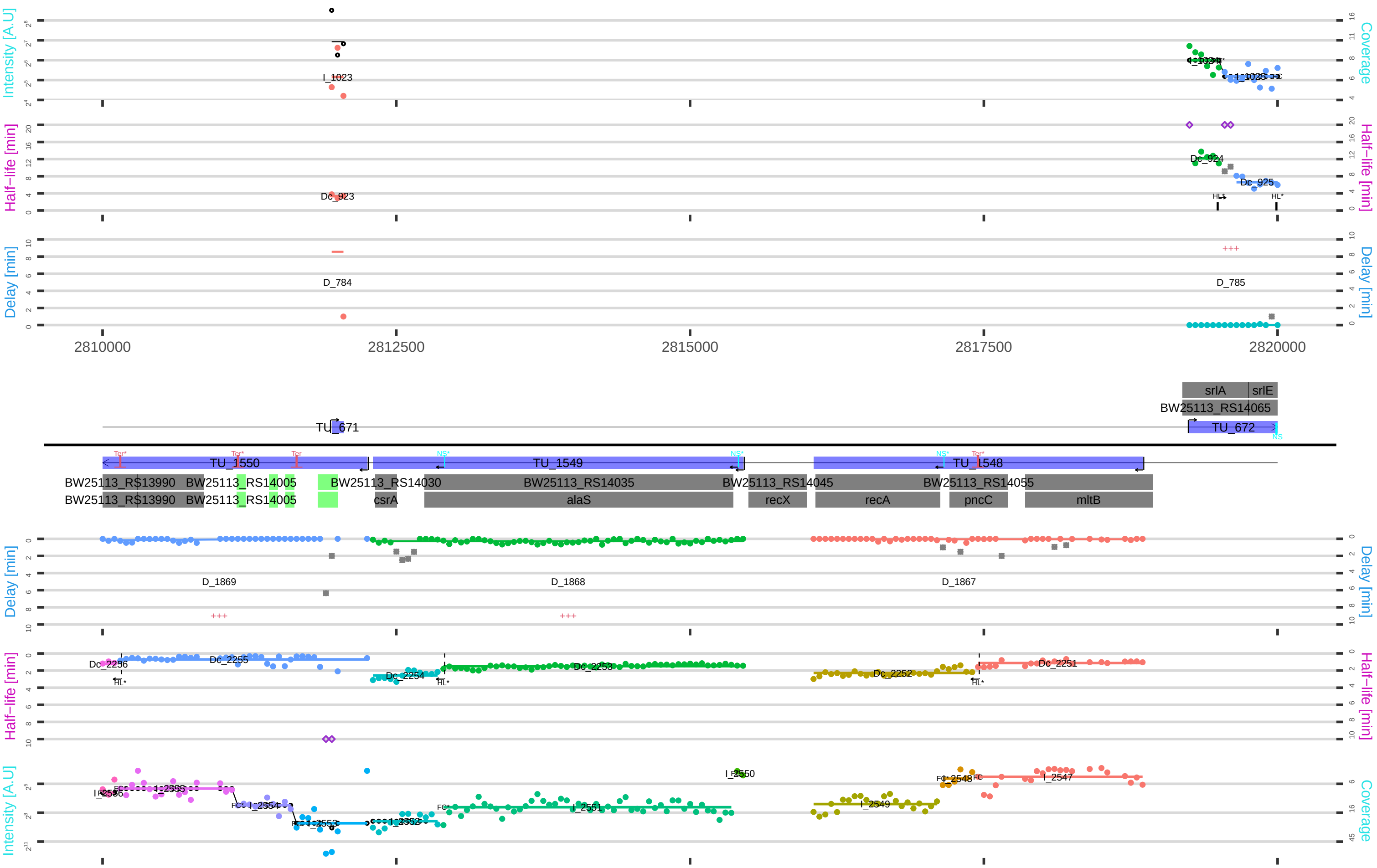

Term: termination (4), NS: new start (3), PS: pausing site (0), iTSS_I: internal starting site (0)

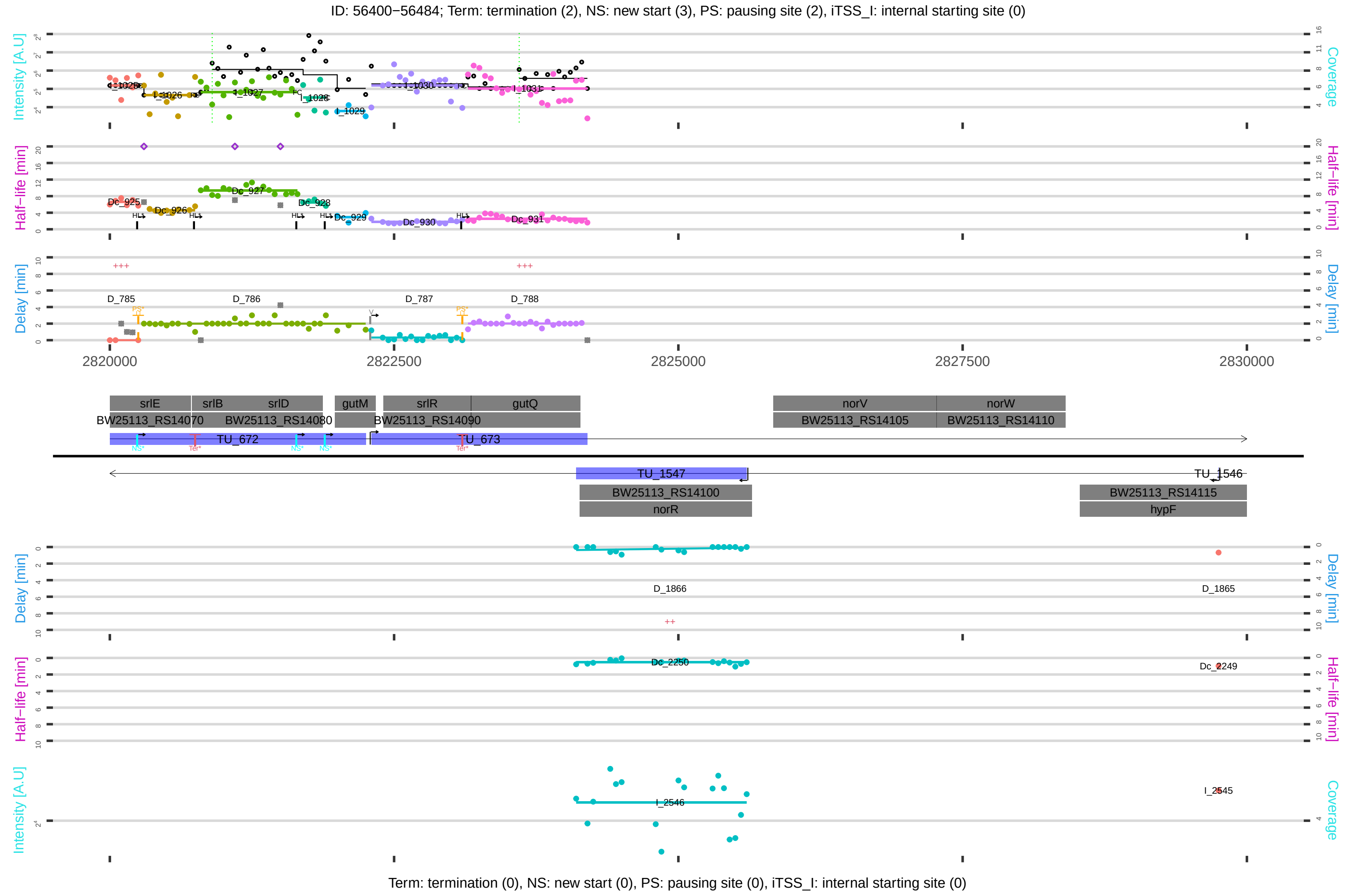

ID: 56400−56484; Term: termination (2), NS: new start (3), PS: pausing site (2), iTSS_I: internal starting site (0)
Intensity [A.U]
Coverage
Half−life [min]
Delay [min]
I_1025
I_1026
I_1027
I_1028
I_1029
I_1030
I_1031
Dc_925
Dc_926
Dc_927
Dc_928
Dc_929
Dc_930
Dc_931
D_785
D_786
D_787
D_788
2820000
2822500
2825000
2827500
2830000
srlE
srlB
srlD
gutM
srlR
gutQ
norV
norW
BW25113_RS14070
BW25113_RS14080
BW25113_RS14090
BW25113_RS14105
BW25113_RS14110
TU_672
TU_673
TU_1547
TU_1546
BW25113_RS14100
norR
BW25113_RS14115
hypF
D_1866
D_1865
Dc_2250
Dc_2249
I_2546
I_2545
Term: termination (0), NS: new start (0), PS: pausing site (0), iTSS_I: internal starting site (0)

ID: 128655−128533; FC*: significant t−test of two consecutive segments; Term: termination, NS: new start, PS: pausing site,iTSS_I: internal starting site,TI: transcription interferance.

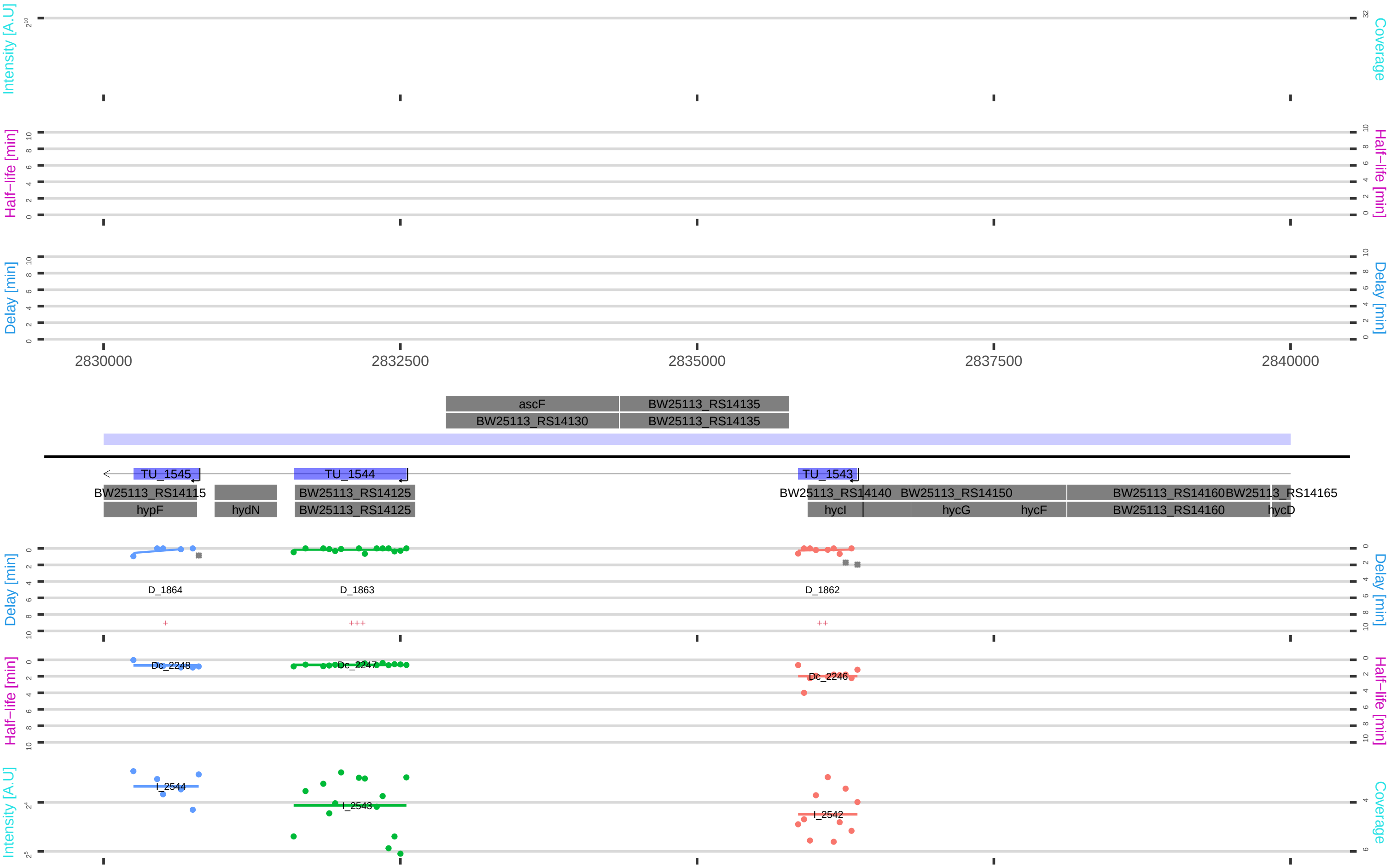

Term: termination (0), NS: new start (0), PS: pausing site (0), iTSS_I: internal starting site (0)

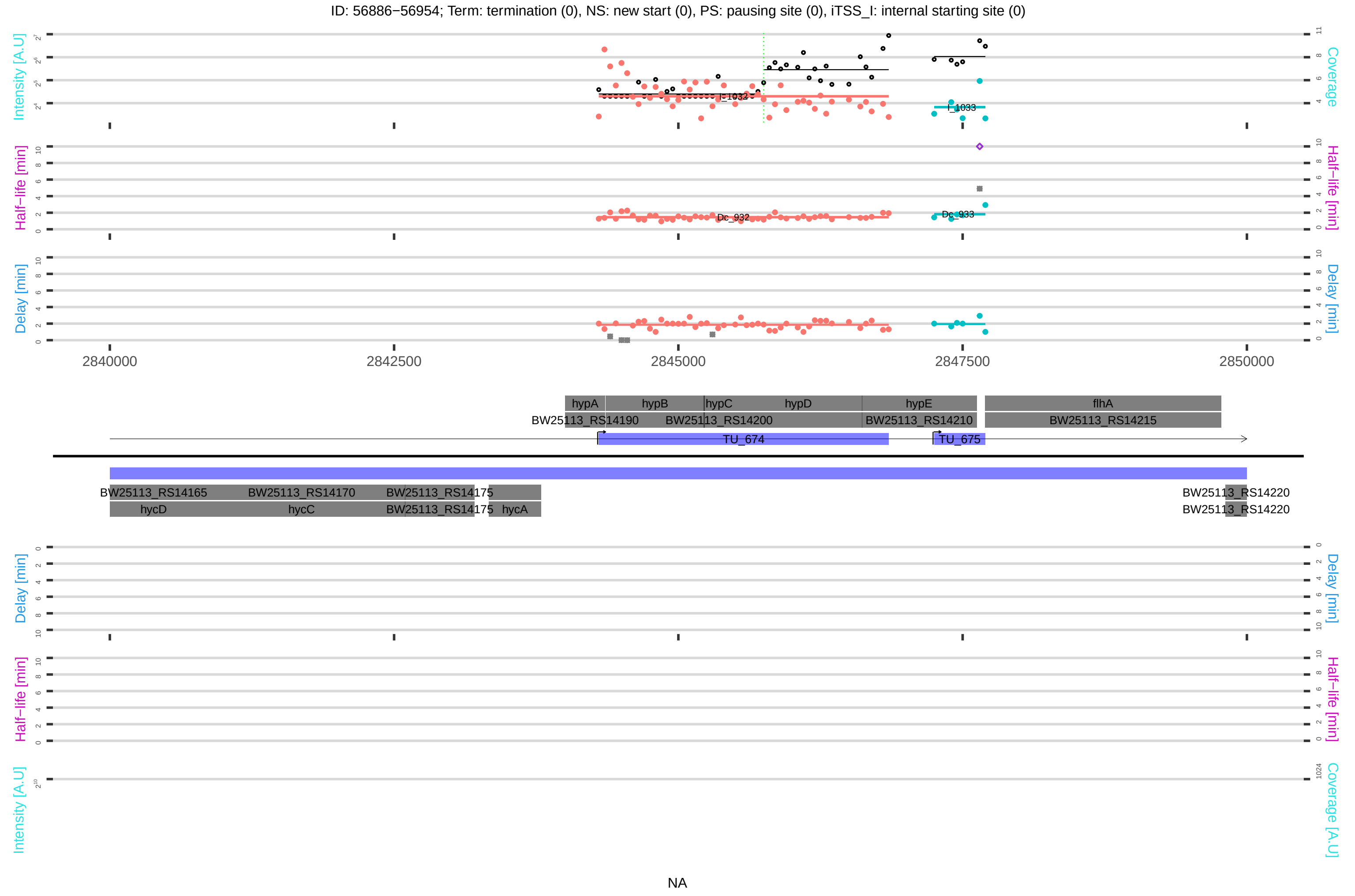

ID: 56886−56954; Term: termination (0), NS: new start (0), PS: pausing site (0), iTSS_I: internal starting site (0)
Intensity [A.U]
Coverage
I_1032
I_1033
Half−life [min]
Dc_932
Dc_933
Delay [min]
2840000
2842500
2845000
2847500
2850000
hypA
hypB
hypC
hypD
hypE
flhA
BW25113_RS14190
BW25113_RS14200
BW25113_RS14210
BW25113_RS14215
TU_674
TU_675
BW25113_RS14165
BW25113_RS14170
BW25113_RS14175
BW25113_RS14220
hycD
hycC
hycA
NA

ID: 57009−57062; Term: termination (0), NS: new start (0), PS: pausing site (0), iTSS_I: internal starting site (0)

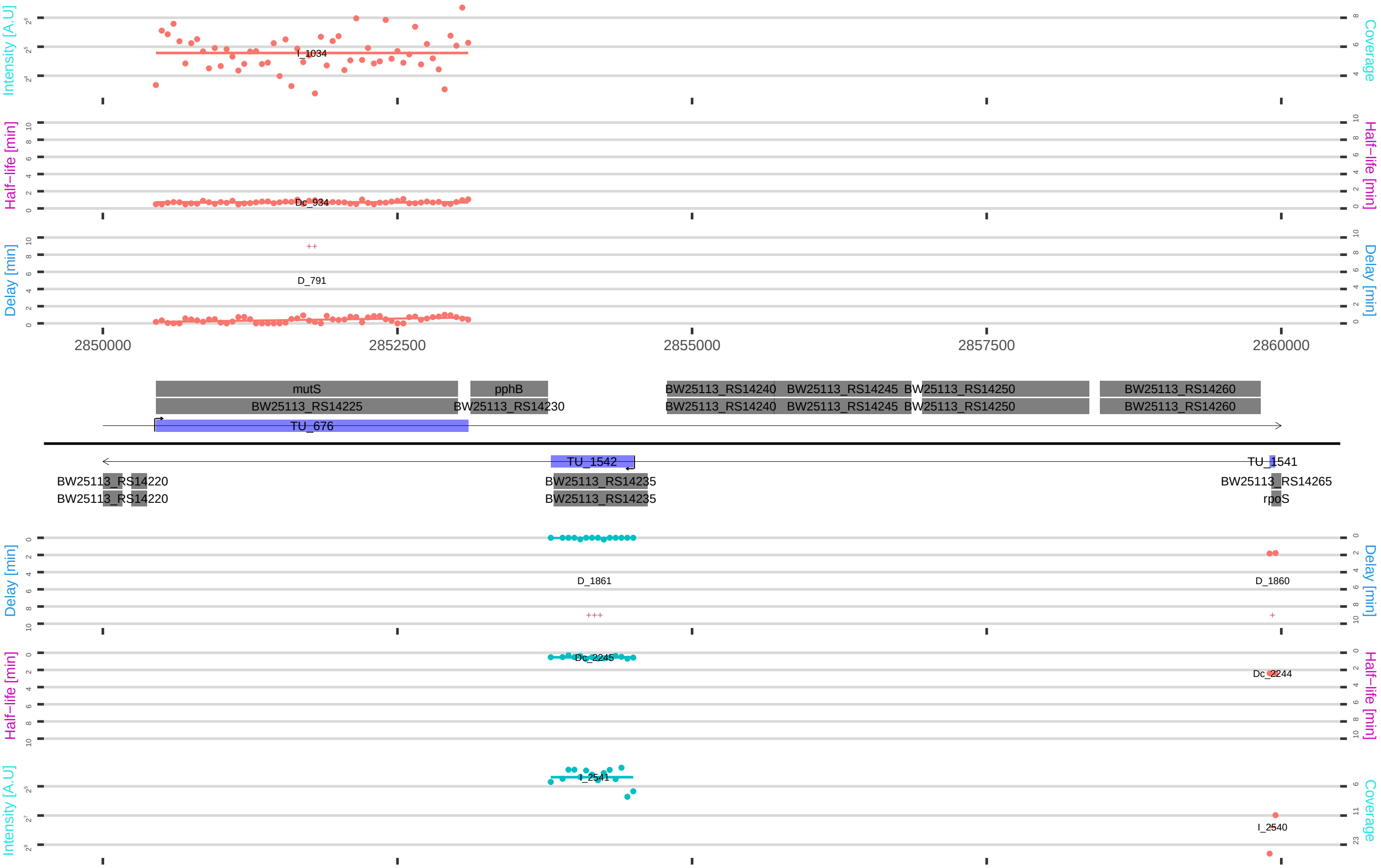

Term: termination (0), NS: new start (0), PS: pausing site (0), iTSS_I: internal starting site (0)

ID: 57229−57229; Term: termination (0), NS: new start (0), PS: pausing site (0), iTSS_I: internal starting site (0)

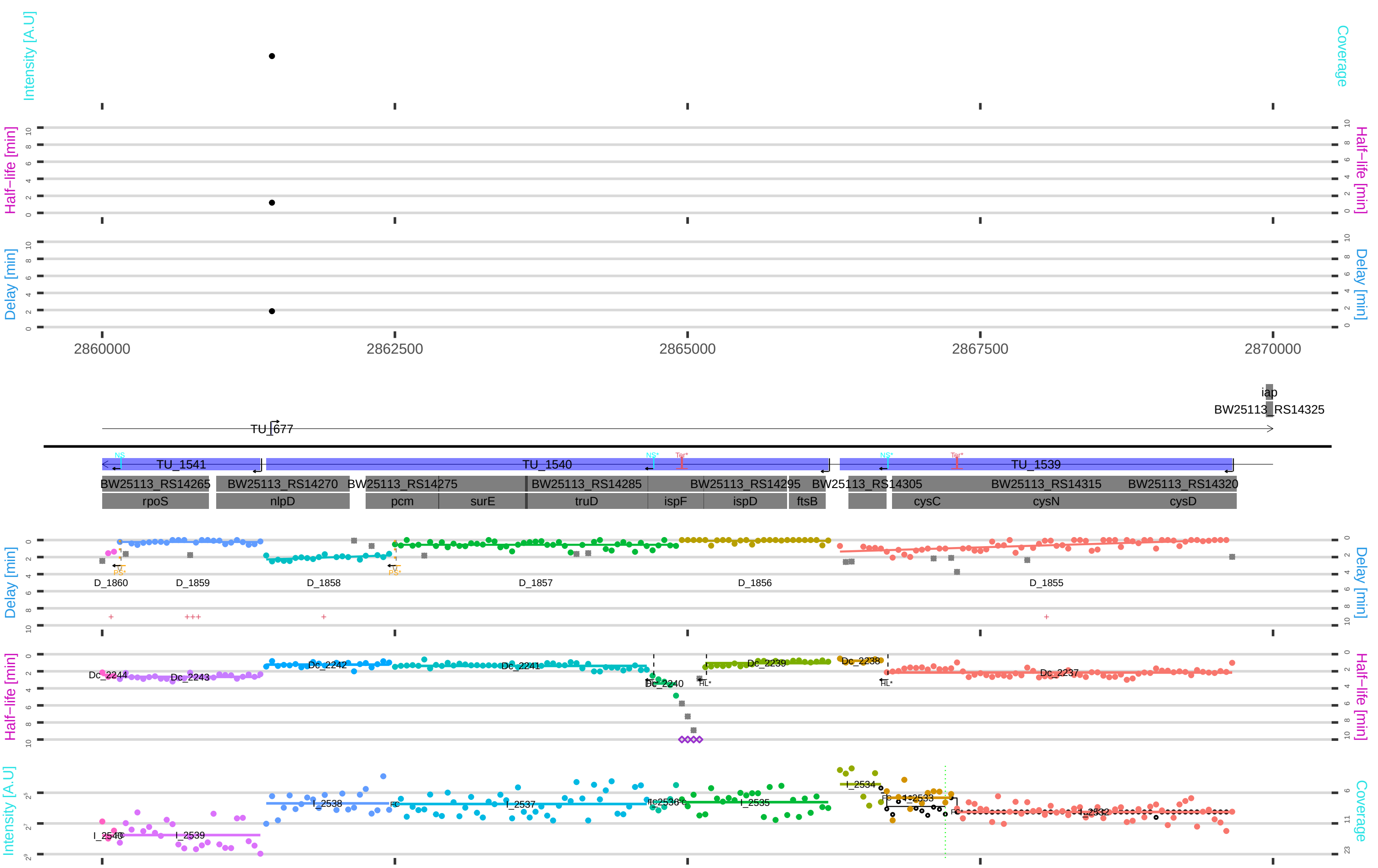

Term: termination (2), NS: new start (3), PS: pausing site (3), iTSS_I: internal starting site (0)

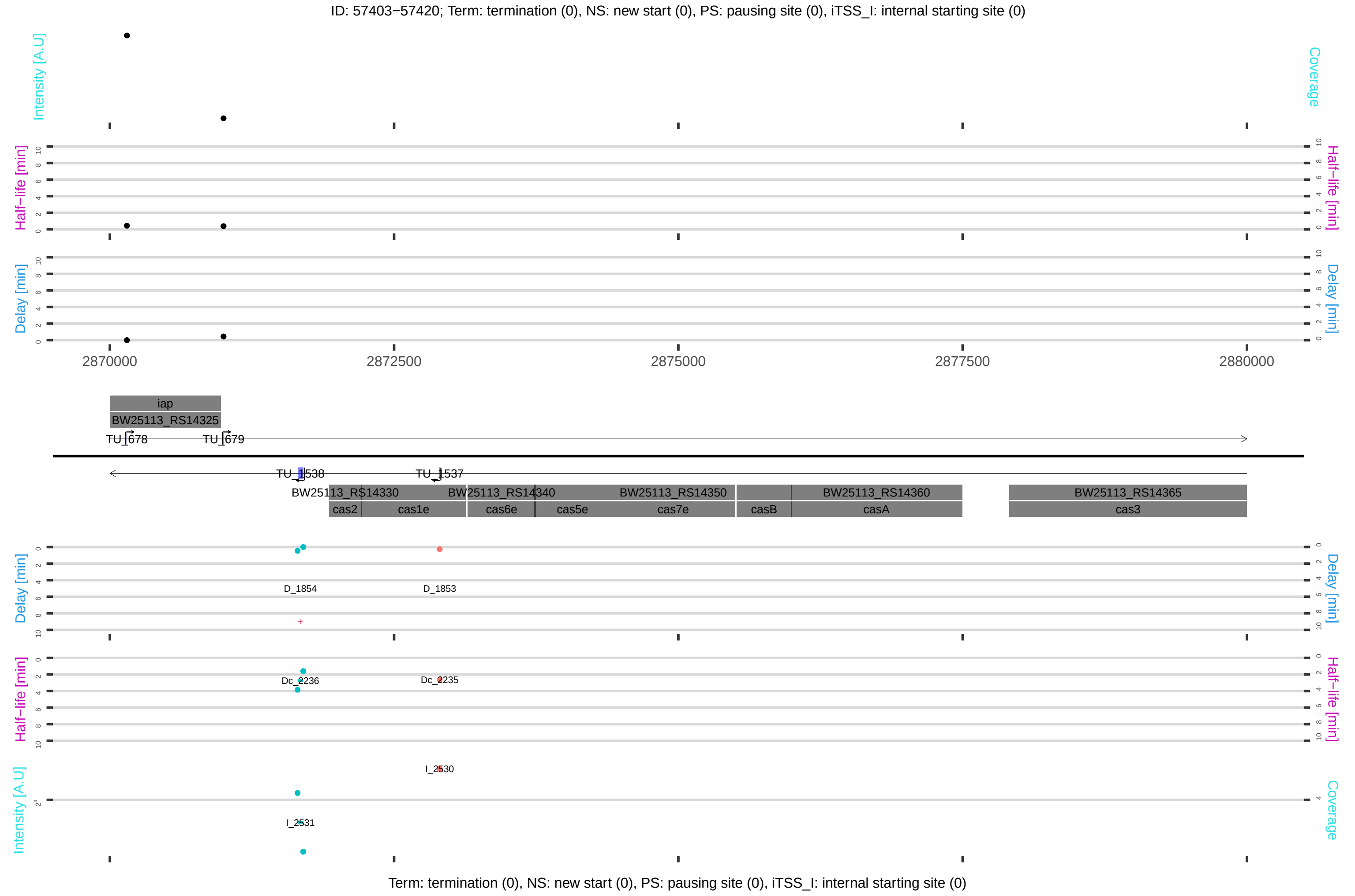

ID: 57403−57420; Term: termination (0), NS: new start (0), PS: pausing site (0), iTSS_I: internal starting site (0)
Intensity [A.U]
Coverage
Half−life [min]
Delay [min]
2870000
2872500
2875000
2877500
2880000
iap
BW25113_RS14325
TU_678
TU_679
TU_1538
TU_1537
BW25113_RS14330
BW25113_RS14340
BW25113_RS14350
BW25113_RS14360
BW25113_RS14365
cas2
cas1e
cas6e
cas5e
cas7e
casB
casA
cas3
D_1854
D_1853
Dc_2236
Dc_2235
I_2530
I_2531
Term: termination (0), NS: new start (0), PS: pausing site (0), iTSS_I: internal starting site (0)

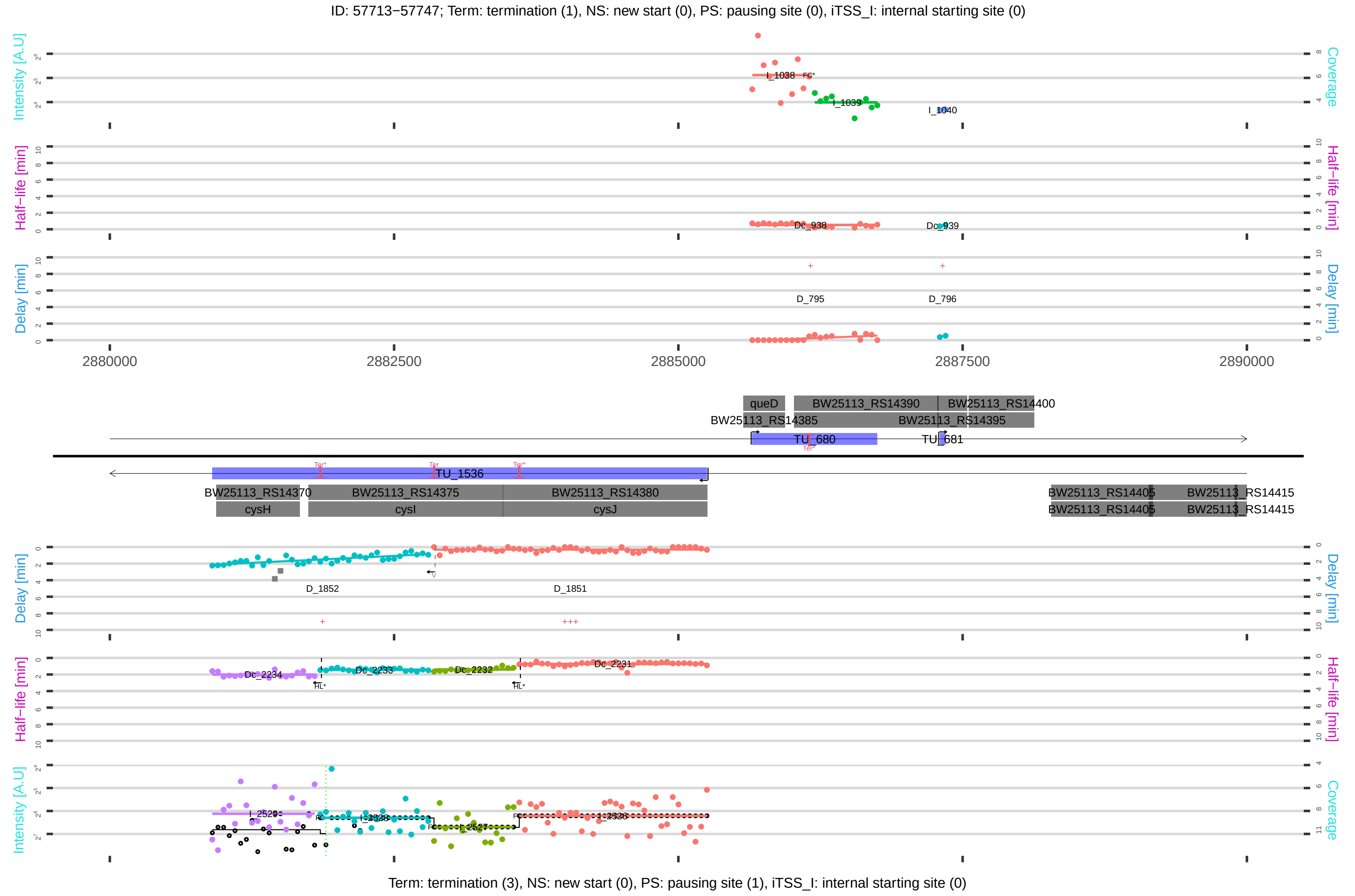

ID: 57713−57747; Term: termination (1), NS: new start (0), PS: pausing site (0), iTSS_I: internal starting site (0)
Intensity [A.U]
Coverage
I_1038
I_1039
I_1040
Half−life [min]
Dc_938
Dc_939
Delay [min]
D_795
D_796
2880000
2882500
2885000
2887500
2890000
queD
BW25113_RS14390
BW25113_RS14400
BW25113_RS14385
BW25113_RS14395
TU_680
TU_681
TU_1536
BW25113_RS14370
BW25113_RS14375
BW25113_RS14380
cysH
cysI
cysJ
BW25113_RS14405
BW25113_RS14415
D_1852
D_1851
Dc_2234
Dc_2233
Dc_2232
Dc_2231
Term: termination (3), NS: new start (0), PS: pausing site (1), iTSS_I: internal starting site (0)

ID: 127312−127261; FC*: significant t−test of two consecutive segments; Term: termination, NS: new start, PS: pausing site,iTSS_I: internal starting site,TI: transcription interferance.

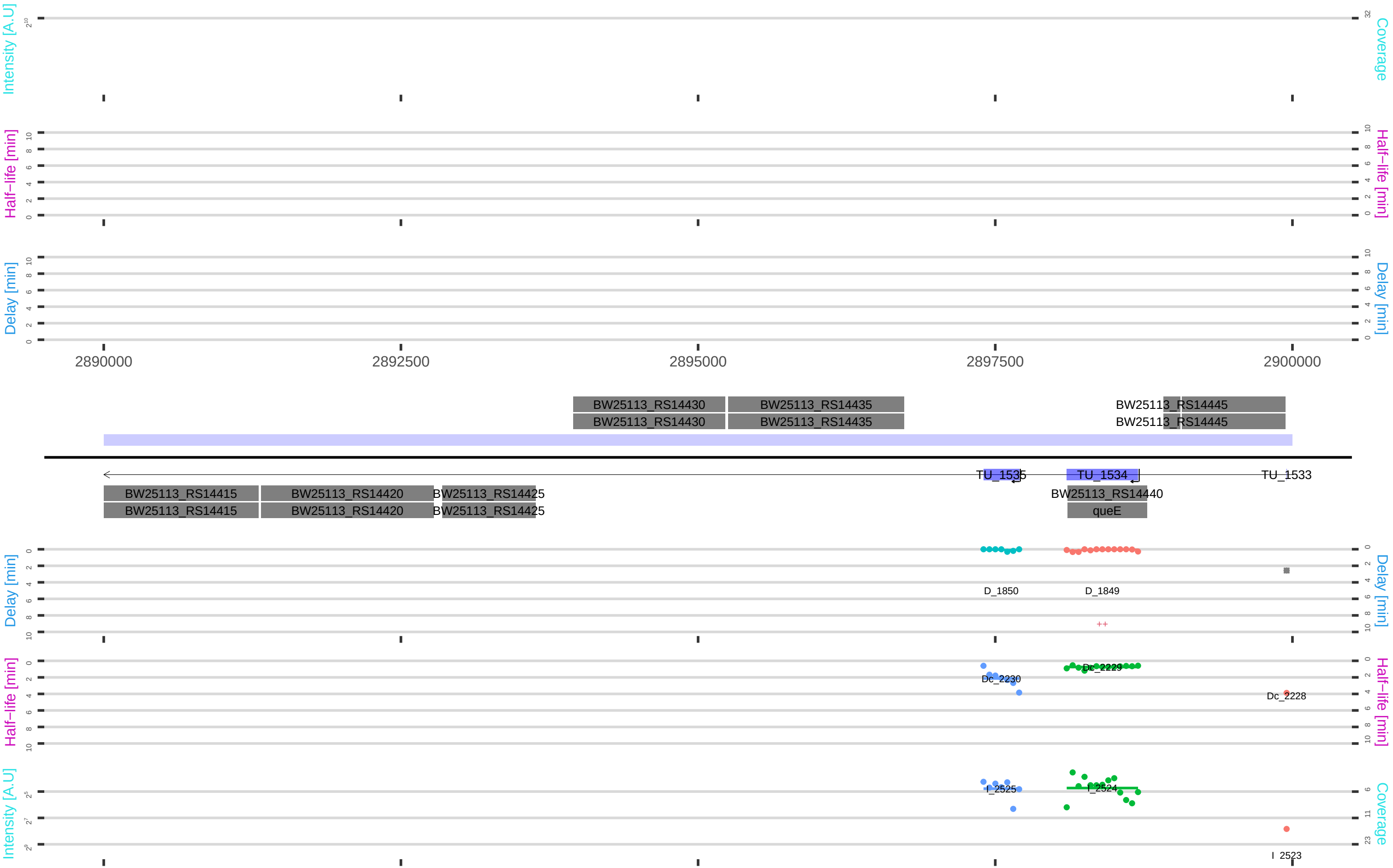

Term: termination (0), NS: new start (0), PS: pausing site (0), iTSS_I: internal starting site (0)

ID: 58017−58197; Term: termination (0), NS: new start (1), PS: pausing site (0), iTSS_I: internal starting site (0)

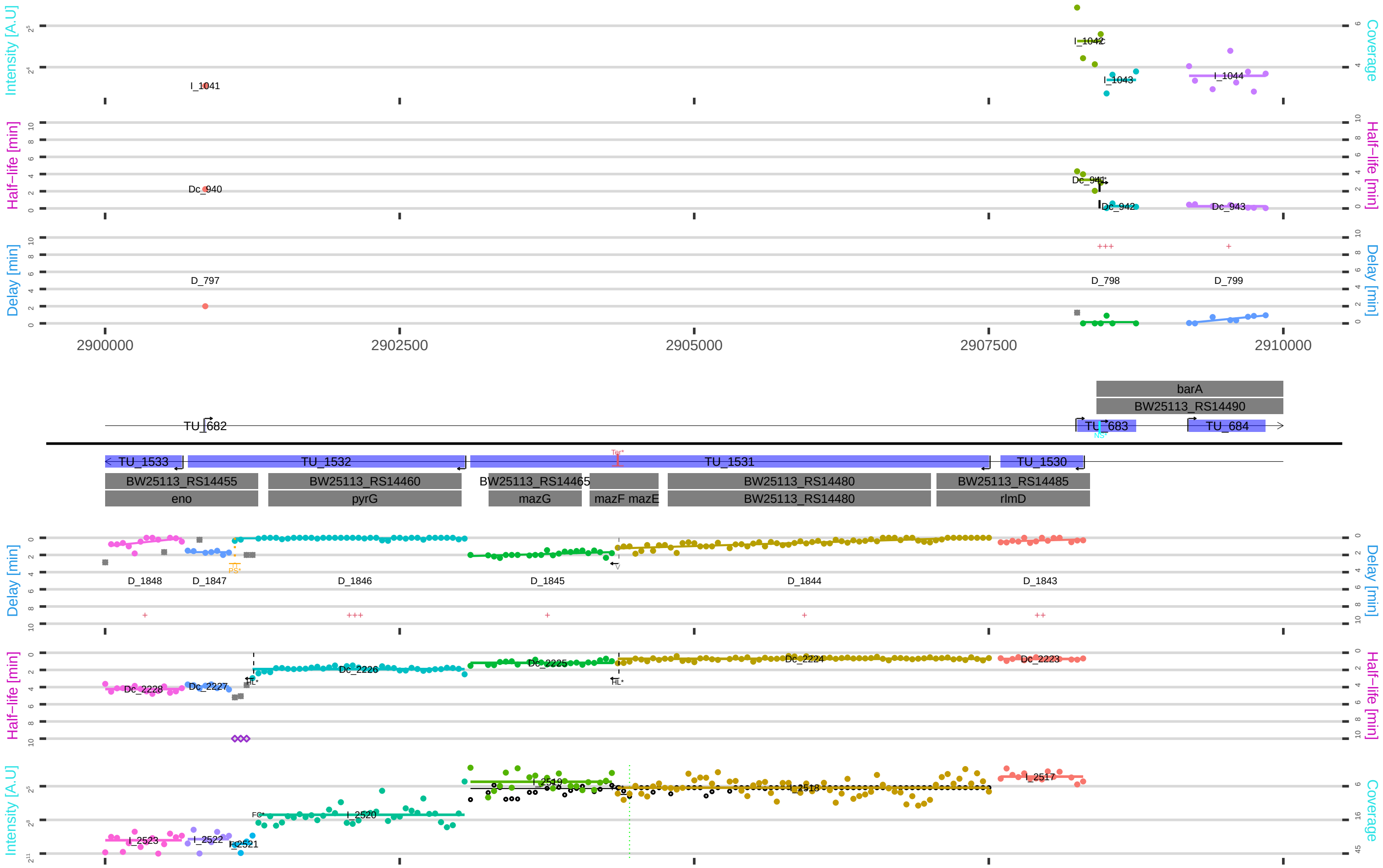

Term: termination (1), NS: new start (0), PS: pausing site (2), iTSS_I: internal starting site (0)

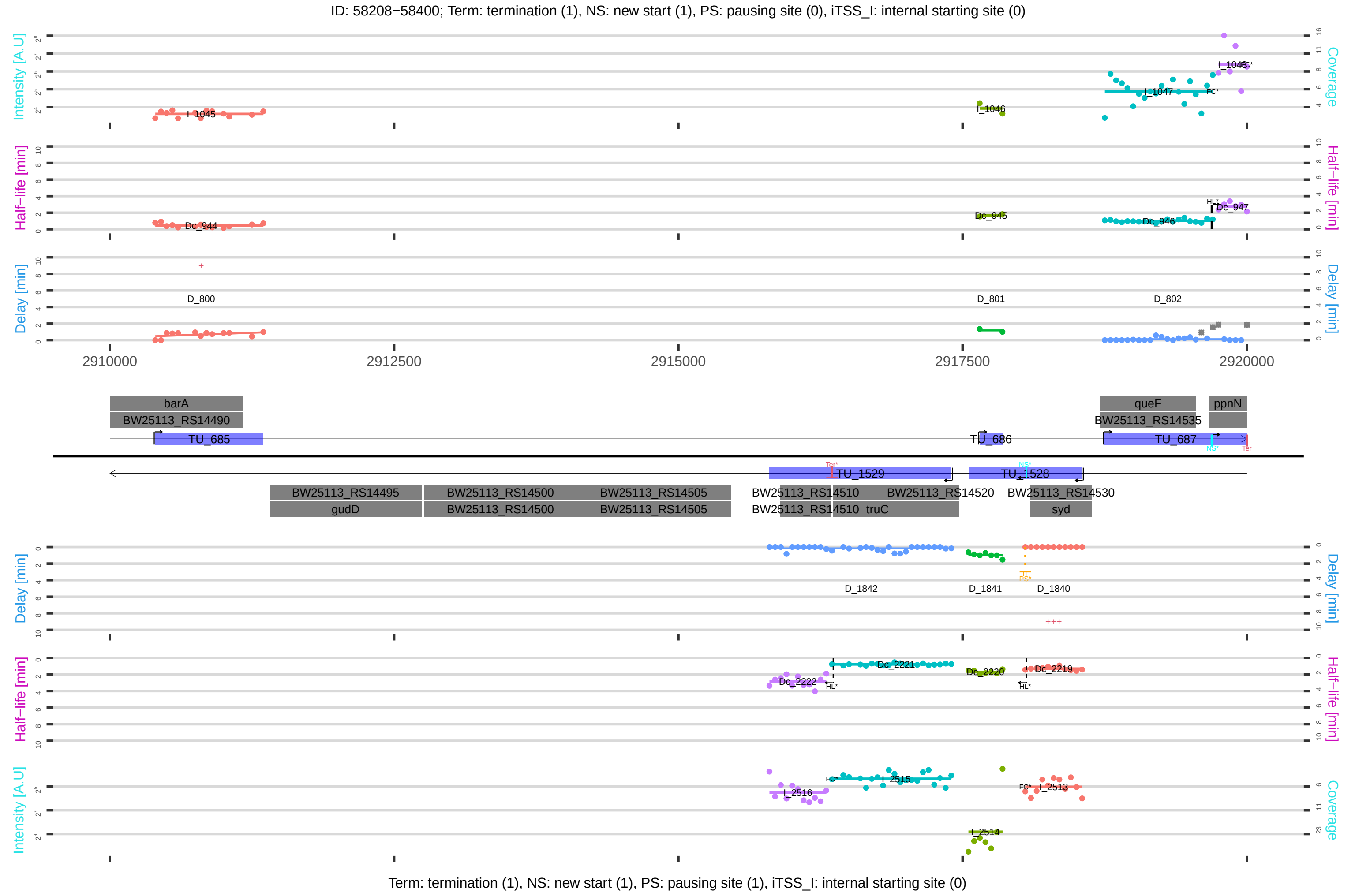
ID: 58208−58400; Term: termination (1), NS: new start (1), PS: pausing site (0), iTSS_I: internal starting site (0)
Intensity [A.U]
Coverage
Half−life [min]
Delay [min]
I_1045
I_1046
I_1047
I_1048
Dc_944
Dc_945
Dc_946
Dc_947
D_800
D_801
D_802
2910000
2912500
2915000
2917500
2920000
barA
BW25113_RS14490
TU_685
TU_686
queF
BW25113_RS14535
ppnN
TU_687
NS*
Ter
Ter*
TU_1529
TU_1528
BW25113_RS14495
gudD
BW25113_RS14500
BW25113_RS14505
BW25113_RS14510
BW25113_RS14520
truC
BW25113_RS14530
syd
D_1842
D_1841
D_1840
PS*
Dc_2222
Dc_2221
Dc_2220
Dc_2219
HL*
FC*
I_2516
I_2515
I_2514
I_2513
Term: termination (1), NS: new start (1), PS: pausing site (1), iTSS_I: internal starting site (0)

ID: 58400−58600; Term: termination (2), NS: new start (1), PS: pausing site (3), iTSS_I: internal starting site (0)

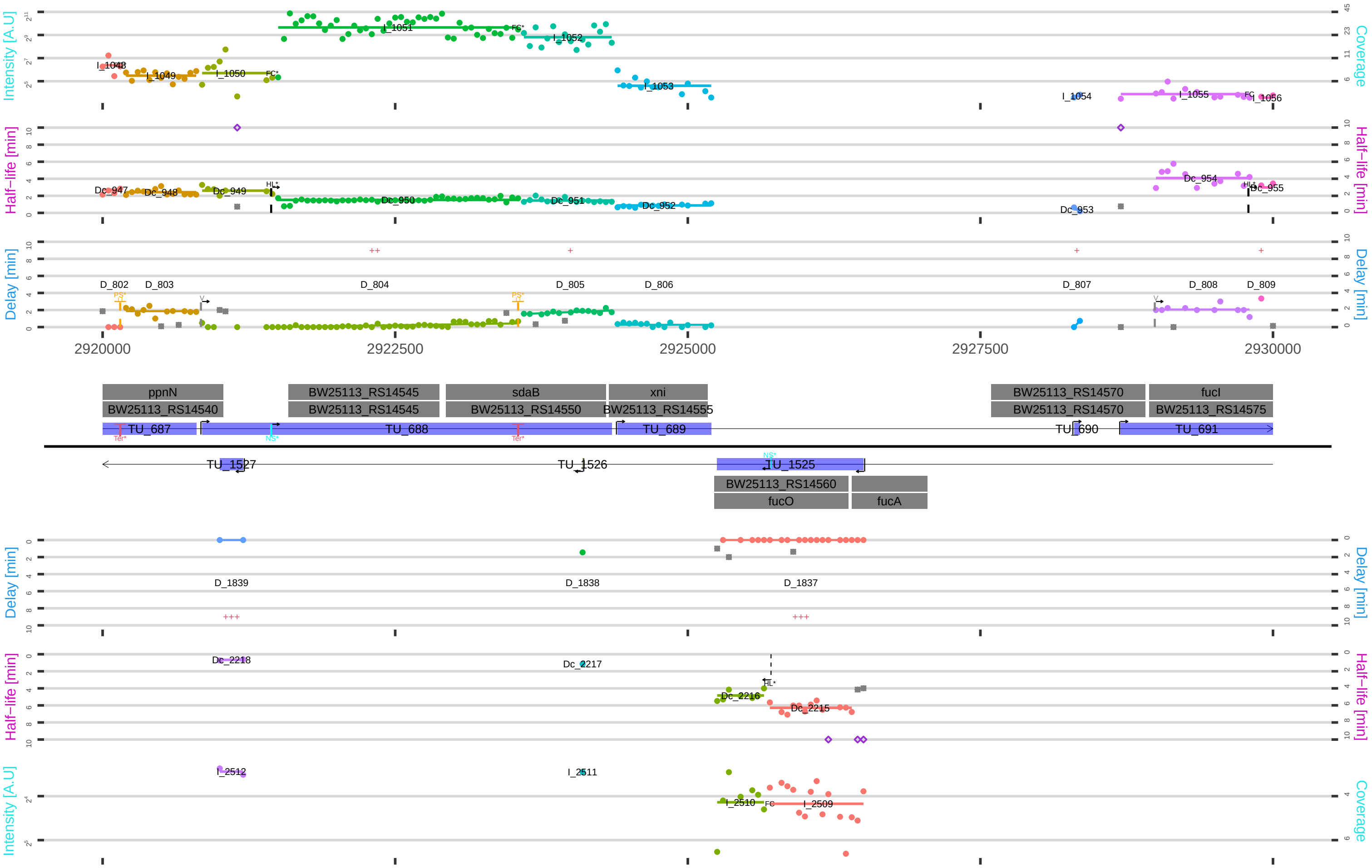

Term: termination (0), NS: new start (1), PS: pausing site (0), iTSS_I: internal starting site (0)

ID: 58600−58769; Term: termination (1), NS: new start (1), PS: pausing site (0), iTSS_I: internal starting site (0)

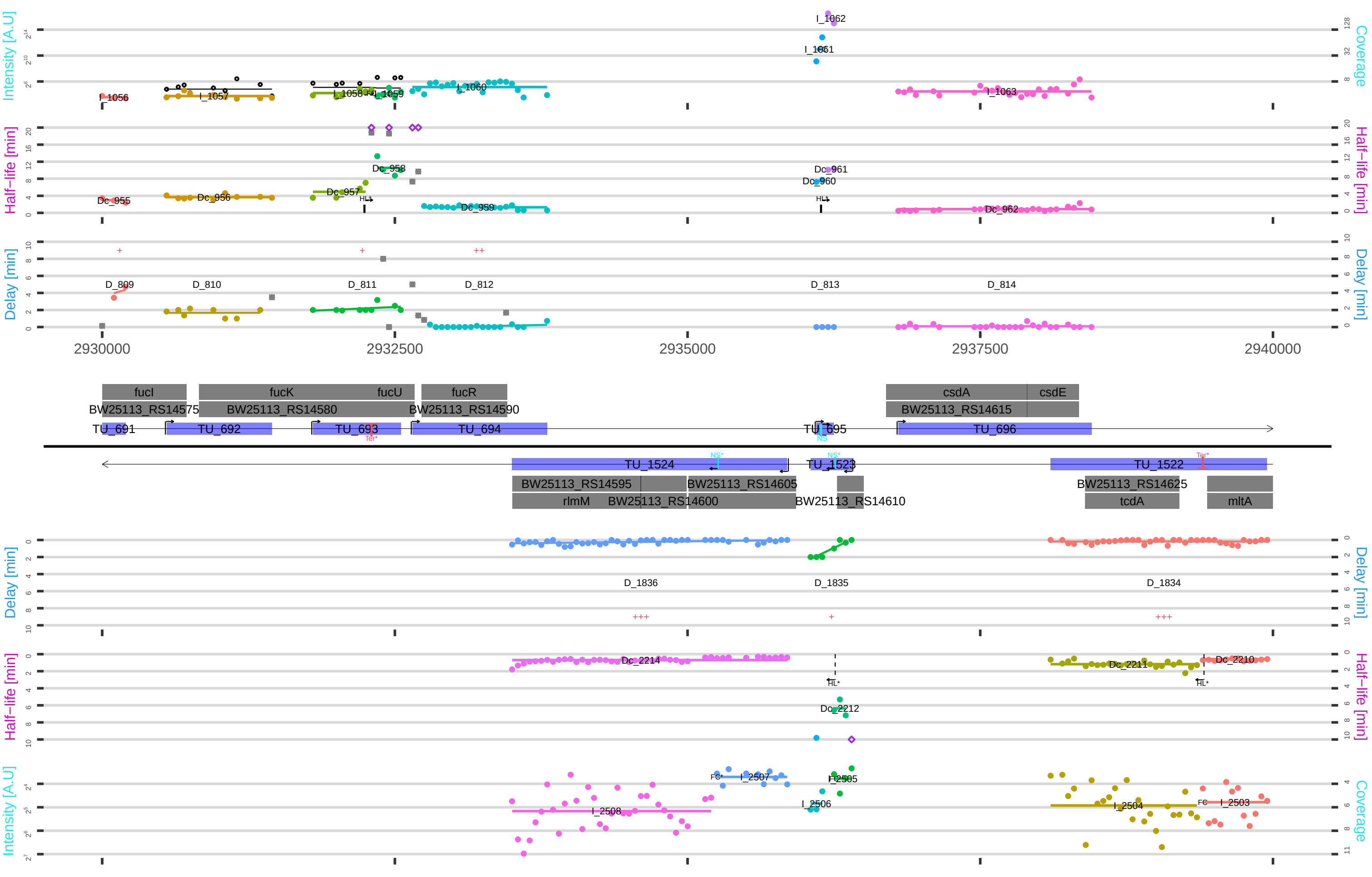

Term: termination (1), NS: new start (2), PS: pausing site (0), iTSS_I: internal starting site (0)

ID: 58814−58821; Term: termination (0), NS: new start (0), PS: pausing site (1), iTSS_I: internal starting site (0)

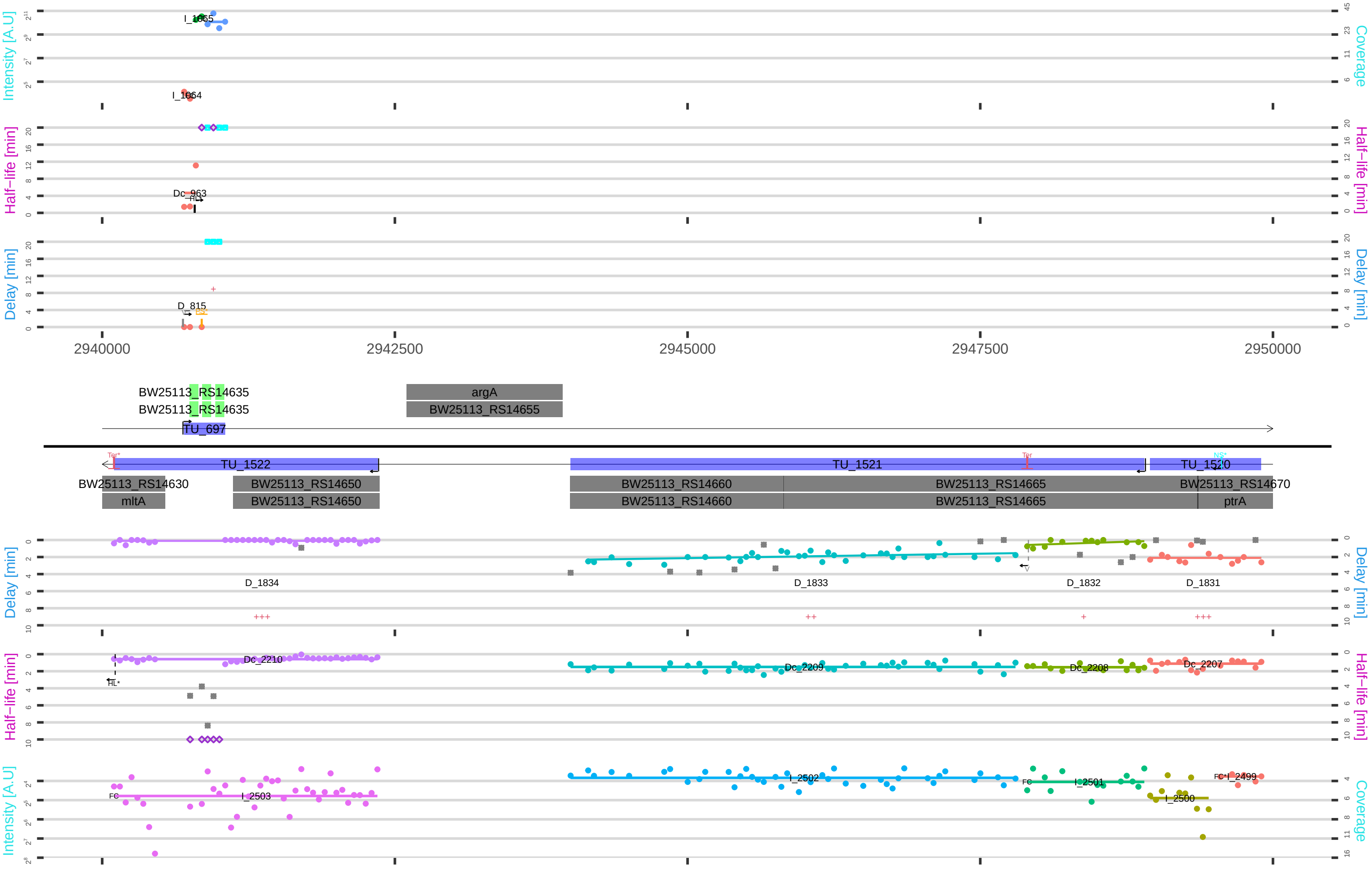

Term: termination (2), NS: new start (1), PS: pausing site (1), iTSS_I: internal starting site (0)

ID: 126257−126061; FC*: significant t−test of two consecutive segments; Term: termination, NS: new start, PS: pausing site,iTSS_I: internal starting site,TI: transcription interferance.

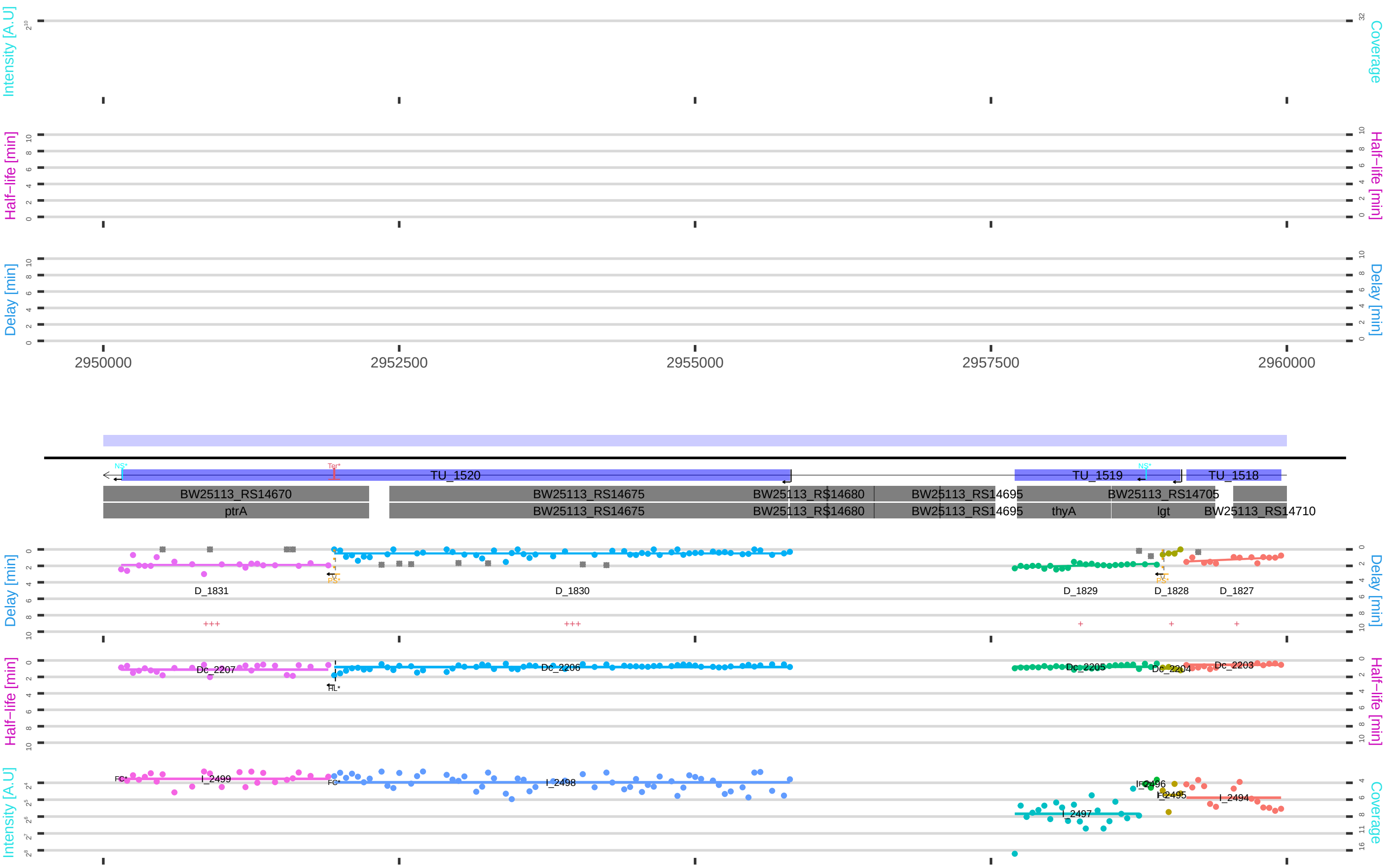

Term: termination (1), NS: new start (2), PS: pausing site (2), iTSS_I: internal starting site (0)

ID: 59263−59323; Term: termination (0), NS: new start (1), PS: pausing site (2), iTSS_I: internal starting site (0)

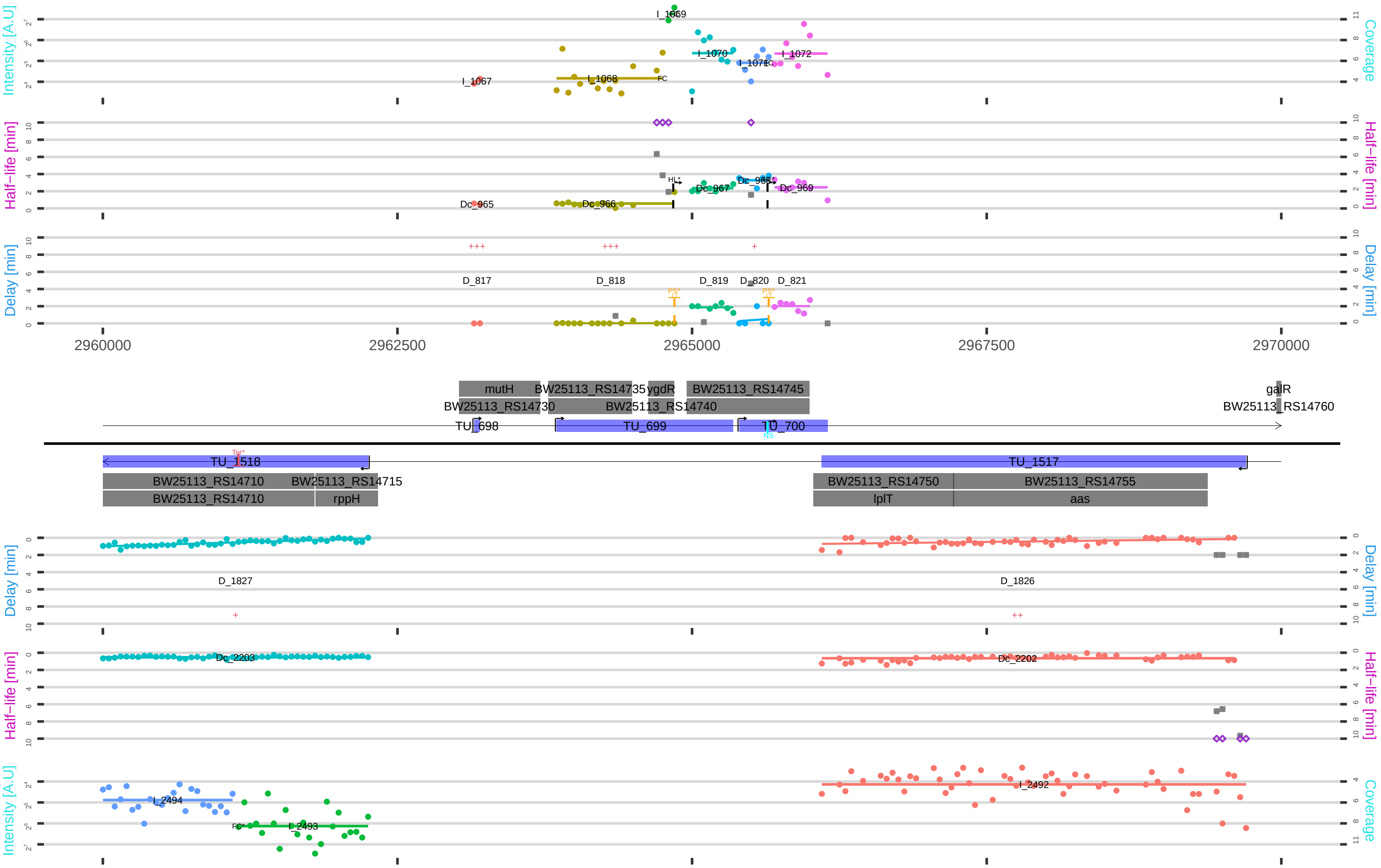

Term: termination (1), NS: new start (0), PS: pausing site (0), iTSS_I: internal starting site (0)

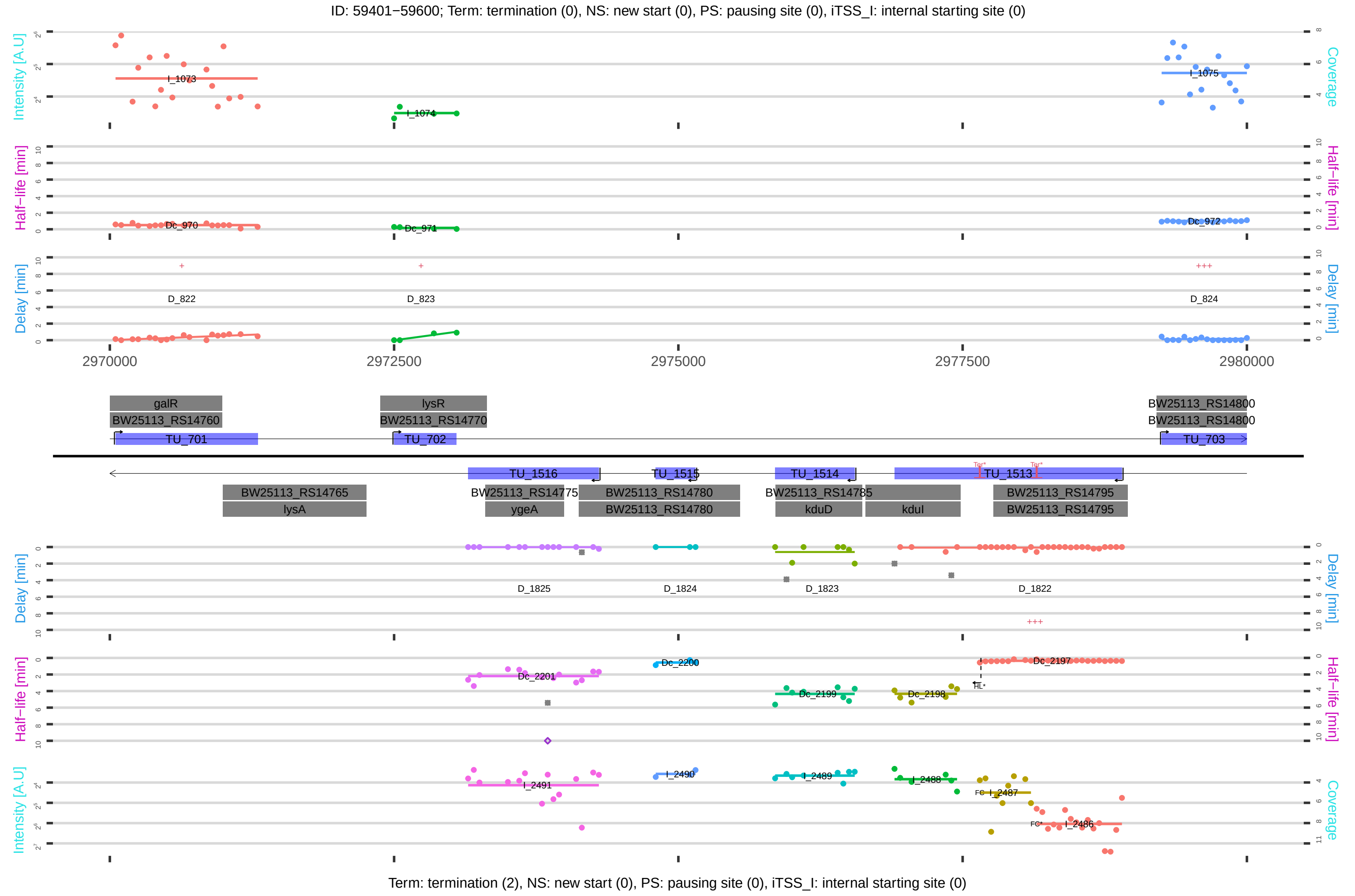
ID: 59401−59600; Term: termination (0), NS: new start (0), PS: pausing site (0), iTSS_I: internal starting site (0)
Intensity [A.U]
Coverage
Half−life [min]
Delay [min]
I_1073
I_1074
I_1075
Dc_970
Dc_971
Dc_972
D_822
D_823
D_824
2970000
2972500
2975000
2977500
2980000
galR
BW25113_RS14760
lysR
BW25113_RS14770
BW25113_RS14800
TU_701
TU_702
TU_703
TU_1516
TU_1515
TU_1514
TU_1513
Ter*
BW25113_RS14765
lysA
BW25113_RS14775
ygeA
BW25113_RS14780
BW25113_RS14785
kduD
kduI
BW25113_RS14795
D_1825
D_1824
D_1823
D_1822
Dc_2200
Dc_2201
Dc_2199
Dc_2198
Dc_2197
HL*
I_2490
I_2491
I_2489
I_2488
I_2487
I_2486
FC
FC*
Term: termination (2), NS: new start (0), PS: pausing site (0), iTSS_I: internal starting site (0)

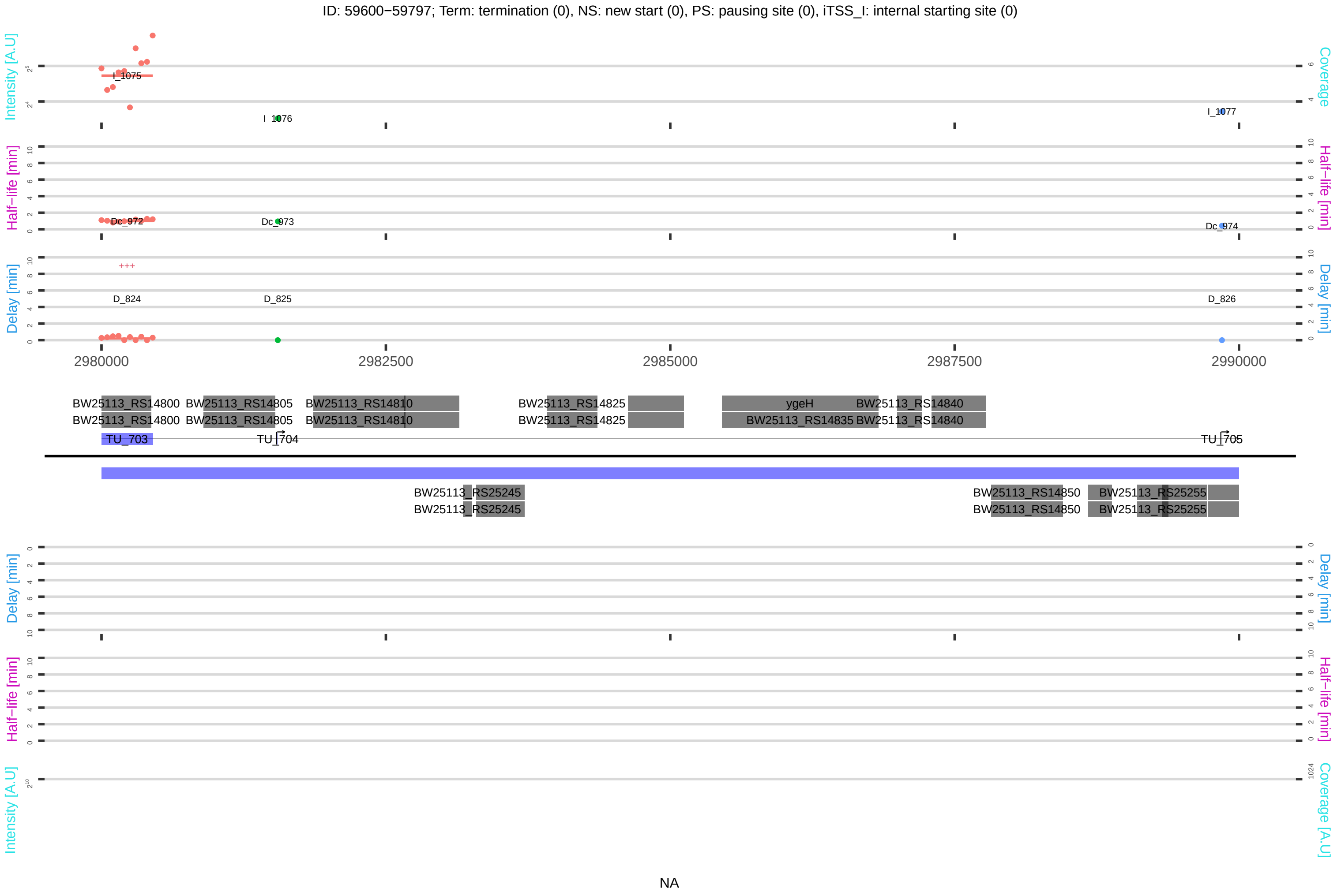

ID: 59600−59797; Term: termination (0), NS: new start (0), PS: pausing site (0), iTSS_I: internal starting site (0)
Intensity [A.U]
Coverage
I_1075
I_1076
I_1077
Half−life [min]
Dc_972
Dc_973
Dc_974
Delay [min]
D_824
D_825
D_826
2980000
2982500
2985000
2987500
2990000
BW25113_RS14800
BW25113_RS14805
BW25113_RS14810
BW25113_RS14825
ygeH
BW25113_RS14835
BW25113_RS14840
TU_703
TU_704
TU_705
BW25113_RS25245
BW25113_RS14850
BW25113_RS25255
Coverage [A.U]
NA

ID: 59848−59875; Term: termination (0), NS: new start (0), PS: pausing site (0), iTSS_I: internal starting site (0)

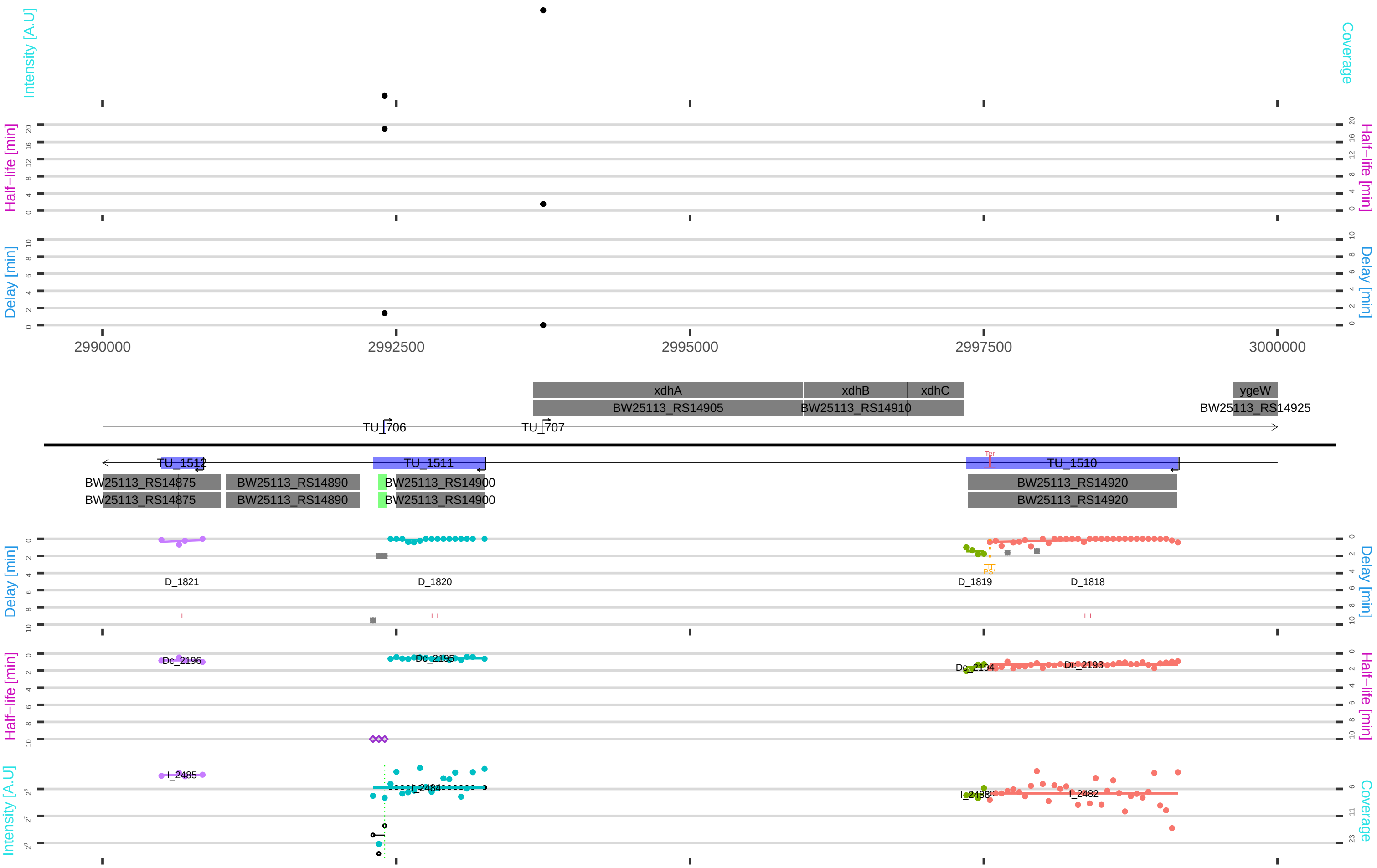

Term: termination (1), NS: new start (0), PS: pausing site (1), iTSS_I: internal starting site (0)

ID: 60115−60172; Term: termination (0), NS: new start (0), PS: pausing site (0), iTSS_I: internal starting site (0)

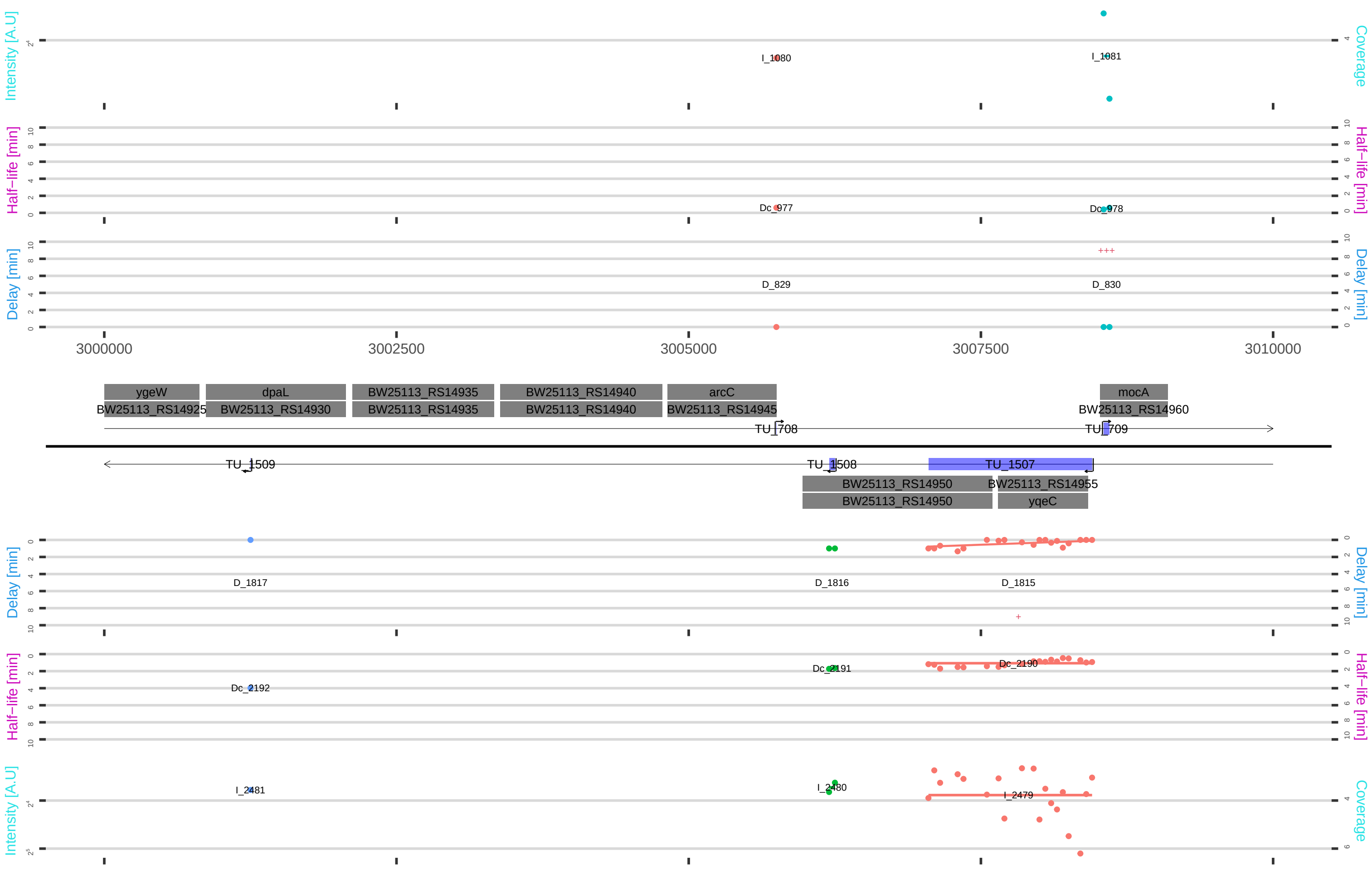

Term: termination (0), NS: new start (0), PS: pausing site (0), iTSS_I: internal starting site (0)

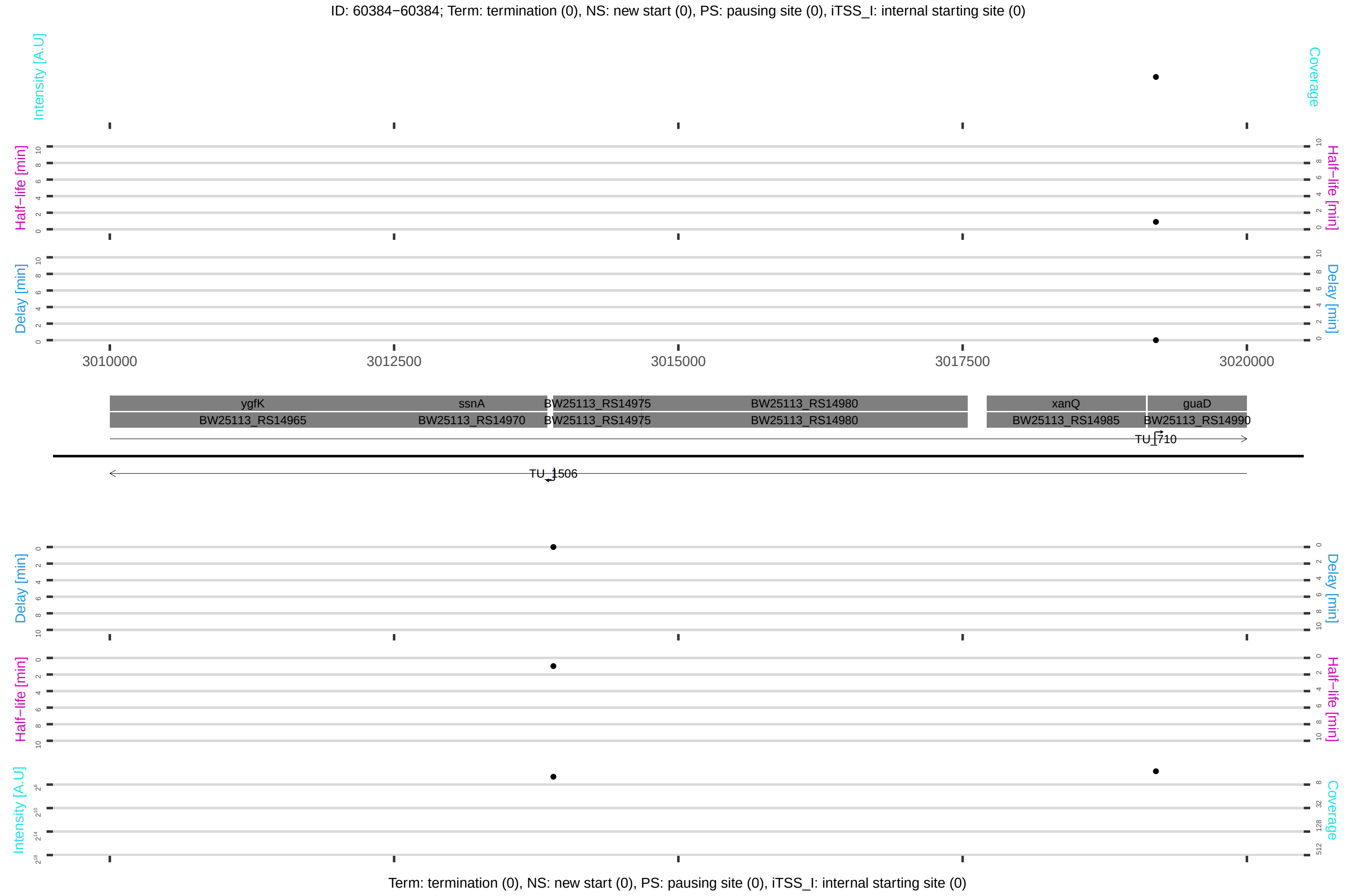

ID: 60384−60384; Term: termination (0), NS: new start (0), PS: pausing site (0), iTSS_I: internal starting site (0)
Intensity [A.U]
Coverage
Half−life [min]
Delay [min]
3010000
3012500
3015000
3017500
3020000
ygfK
ssnA
BW25113_RS14975
BW25113_RS14980
xanQ
guaD
BW25113_RS14965
BW25113_RS14970
BW25113_RS14985
BW25113_RS14990
TU_710
TU_1506
Term: termination (0), NS: new start (0), PS: pausing site (0), iTSS_I: internal starting site (0)

ID: 60531−60539; Term: termination (0), NS: new start (0), PS: pausing site (0), iTSS_I: internal starting site (0)

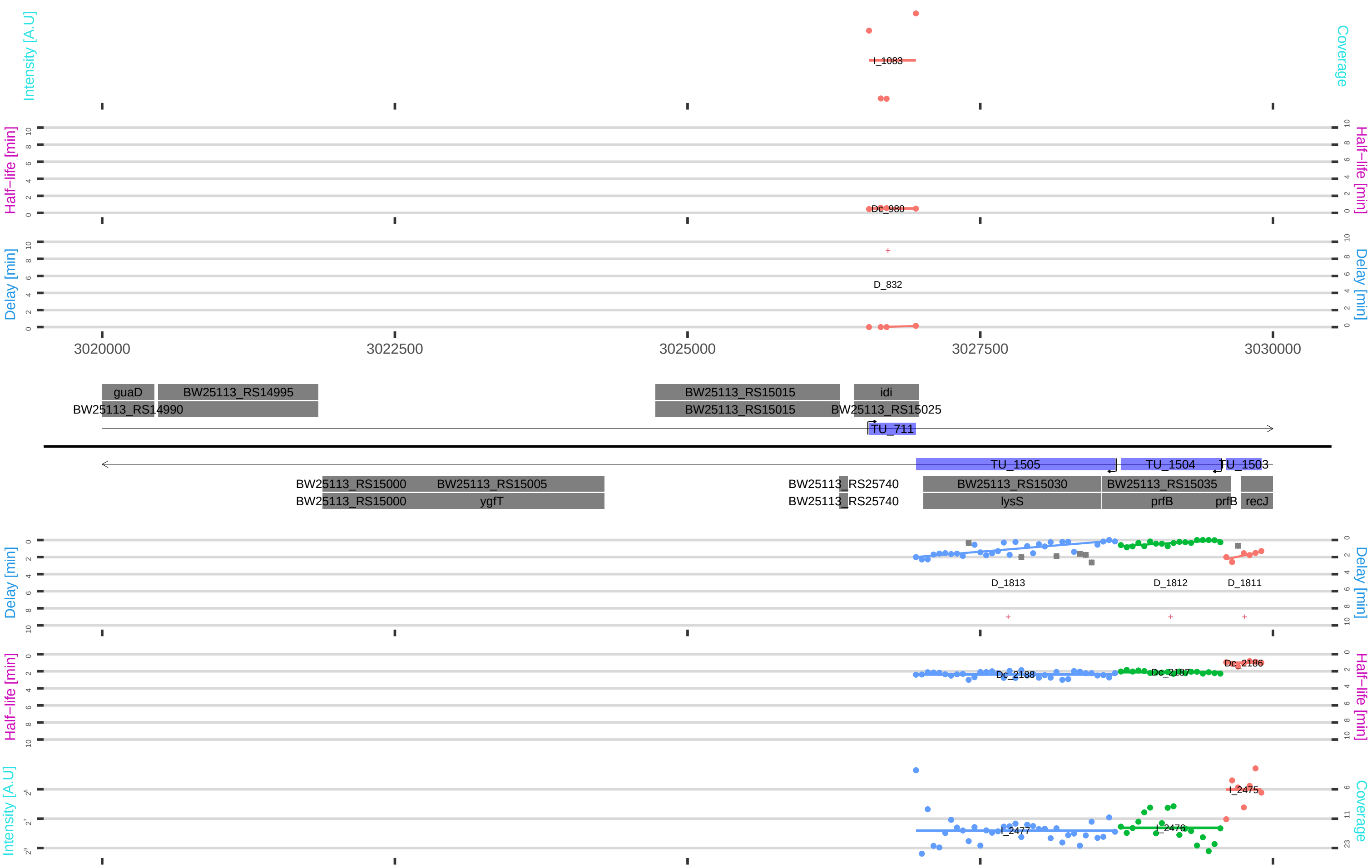

Term: termination (0), NS: new start (0), PS: pausing site (0), iTSS_I: internal starting site (0)

ID: 60665−60770; Term: termination (0), NS: new start (1), PS: pausing site (1), iTSS_I: internal starting site (0)

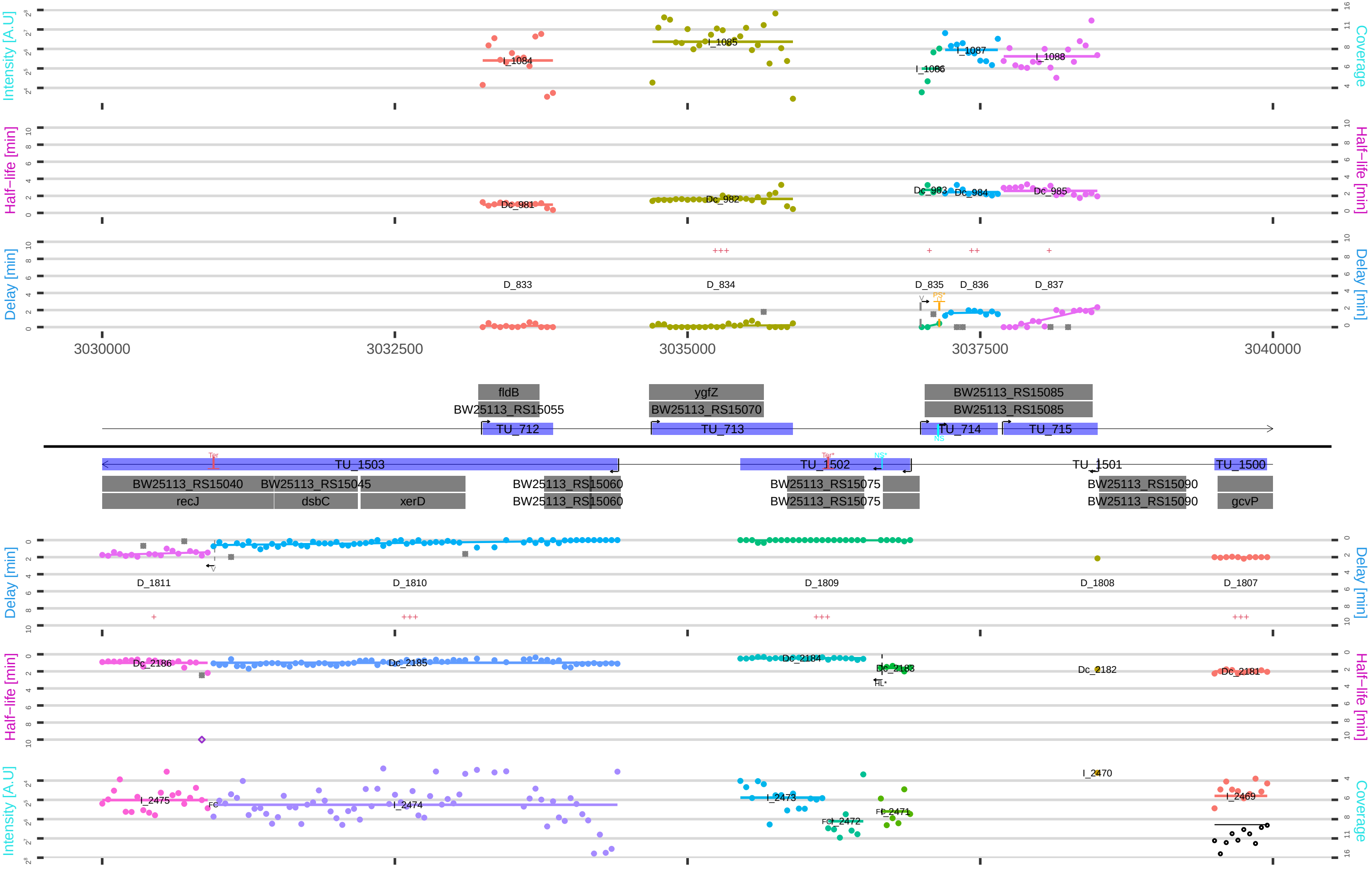

Term: termination (2), NS: new start (1), PS: pausing site (1), iTSS_I: internal starting site (0)

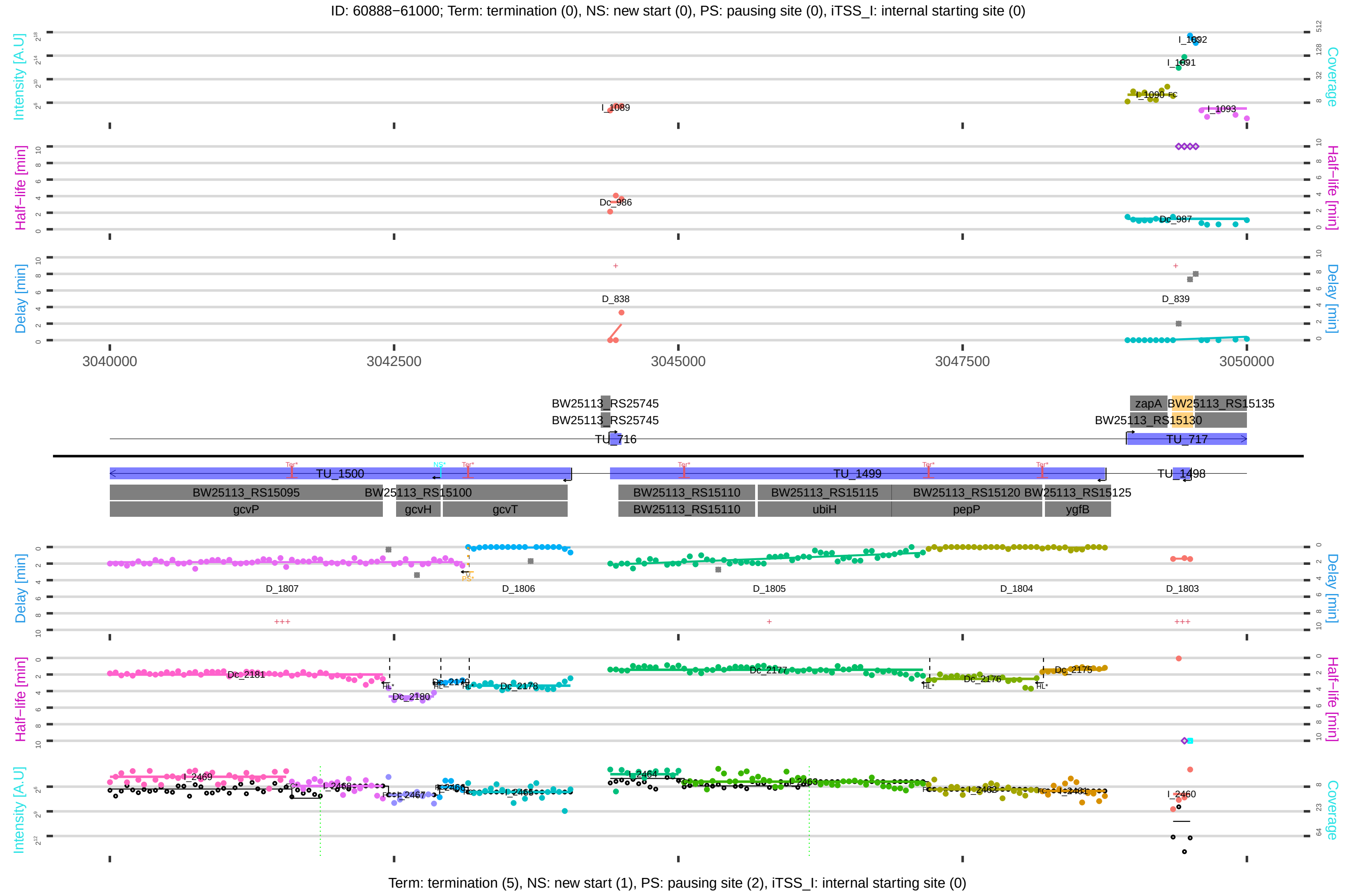
ID: 60888−61000; Term: termination (0), NS: new start (0), PS: pausing site (0), iTSS_I: internal starting site (0)
Intensity [A.U]
Coverage
Half−life [min]
Delay [min]
3040000
3042500
3045000
3047500
3050000
I_1092
I_1091
I_1090
I_1089
I_1093
Dc_986
Dc_987
D_838
D_839
BW25113_RS25745
zapA
BW25113_RS15135
BW25113_RS15130
TU_716
TU_717
TU_1500
TU_1499
TU_1498
BW25113_RS15095
BW25113_RS15100
BW25113_RS15110
BW25113_RS15115
BW25113_RS15120
BW25113_RS15125
gcvP
gcvH
gcvT
ubiH
pepP
ygfB
D_1807
D_1806
D_1805
D_1804
D_1803
Dc_2181
Dc_2180
Dc_2179
Dc_2178
Dc_2177
Dc_2176
Dc_2175
I_2469
I_2468
I_2467
I_2466
I_2465
I_2464
I_2463
I_2462
I_2461
I_2460
Term: termination (5), NS: new start (1), PS: pausing site (2), iTSS_I: internal starting site (0)

ID: 61000−61081; Term: termination (0), NS: new start (0), PS: pausing site (0), iTSS_I: internal starting site (0)

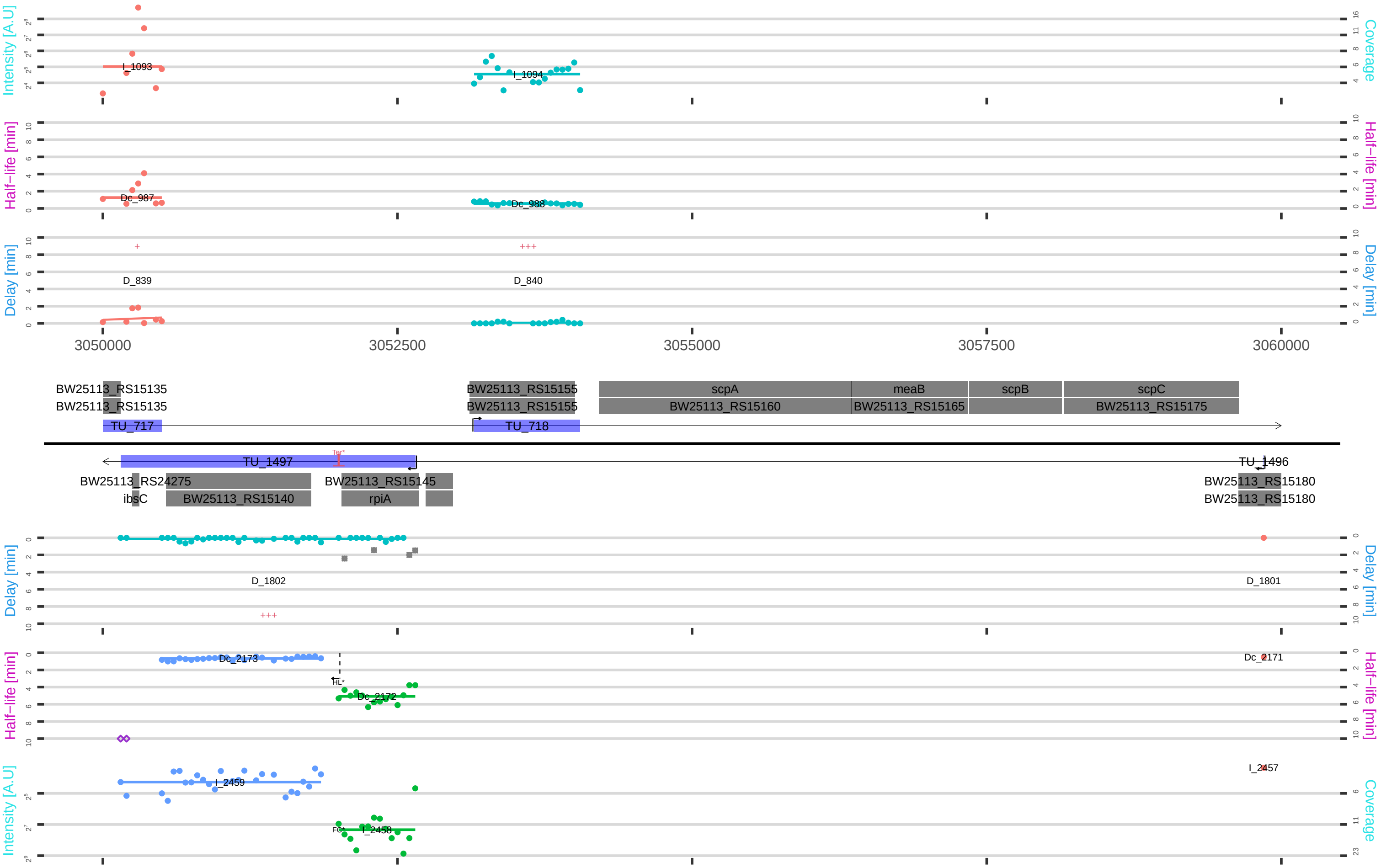

Term: termination (1), NS: new start (0), PS: pausing site (0), iTSS_I: internal starting site (0)

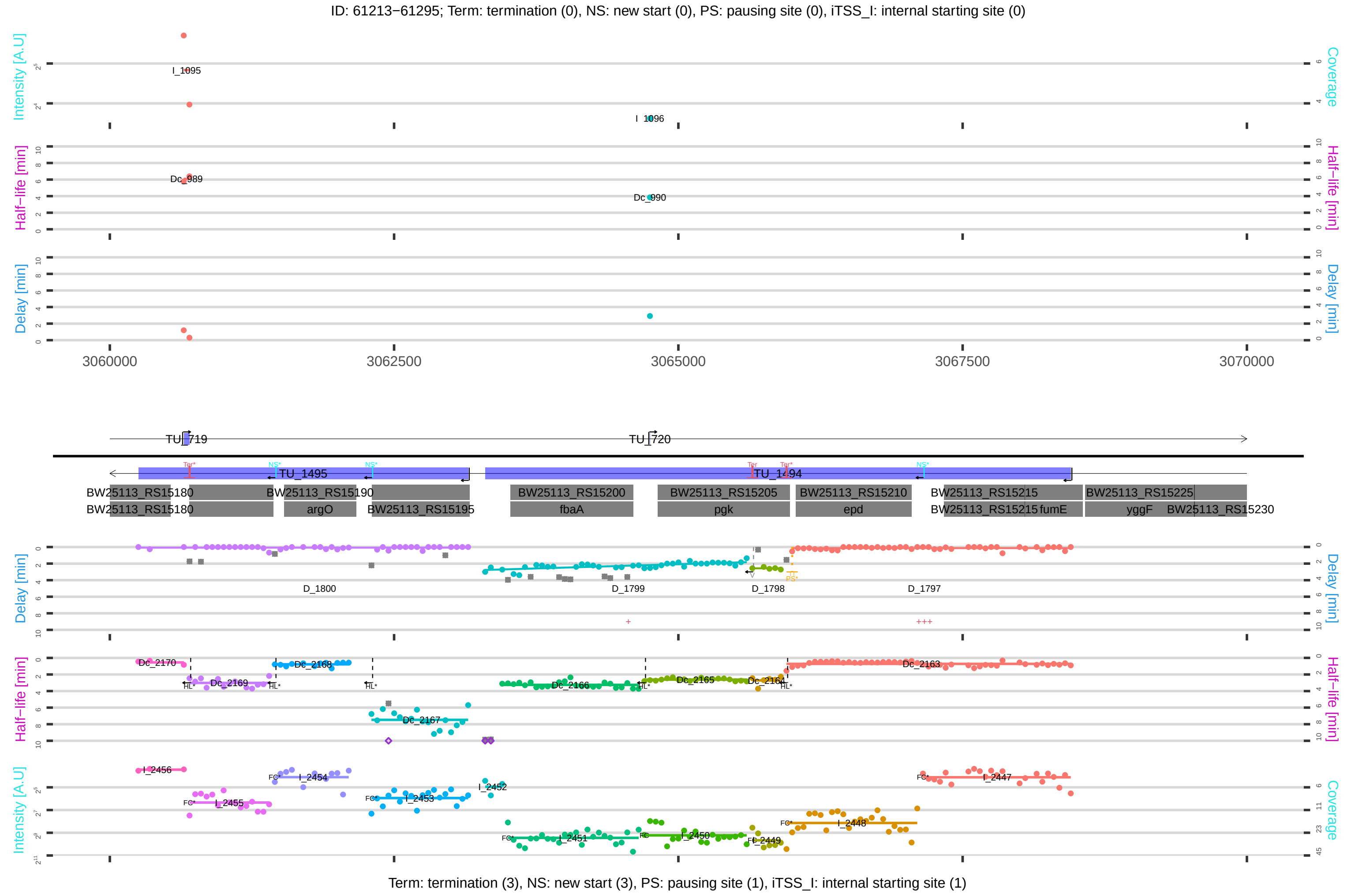
ID: 61213−61295; Term: termination (0), NS: new start (0), PS: pausing site (0), iTSS_I: internal starting site (0)
Intensity [A.U]
Coverage
Half−life [min]
Delay [min]
I_1095
I_1096
Dc_989
Dc_990
3060000
3062500
3065000
3067500
3070000
TU_719
TU_720
TU_1495
TU_1494
BW25113_RS15180
BW25113_RS15190
argO
BW25113_RS15195
BW25113_RS15200
fbaA
BW25113_RS15205
pgk
BW25113_RS15210
epd
BW25113_RS15215
fumE
BW25113_RS15225
yggF
BW25113_RS15230
D_1800
D_1799
D_1798
D_1797
Dc_2170
Dc_2169
Dc_2168
Dc_2167
Dc_2166
Dc_2165
Dc_2164
Dc_2163
I_2456
I_2455
I_2454
I_2453
I_2452
I_2451
I_2450
I_2449
I_2448
I_2447
Term: termination (3), NS: new start (3), PS: pausing site (1), iTSS_I: internal starting site (1)

ID: 61506−61600; Term: termination (0), NS: new start (0), PS: pausing site (0), iTSS_I: internal starting site (0)

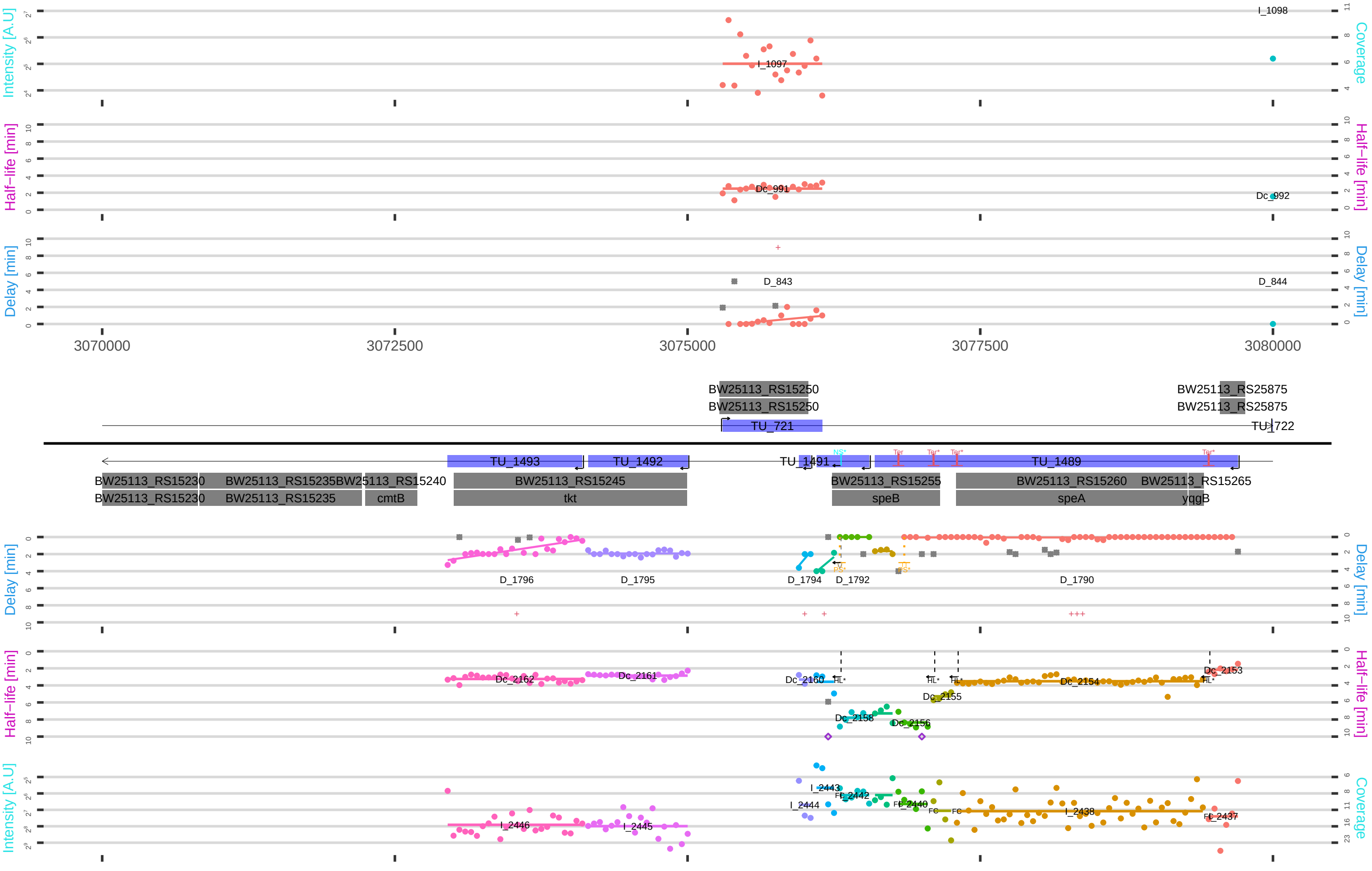

Term: termination (4), NS: new start (1), PS: pausing site (2), iTSS_I: internal starting site (0)

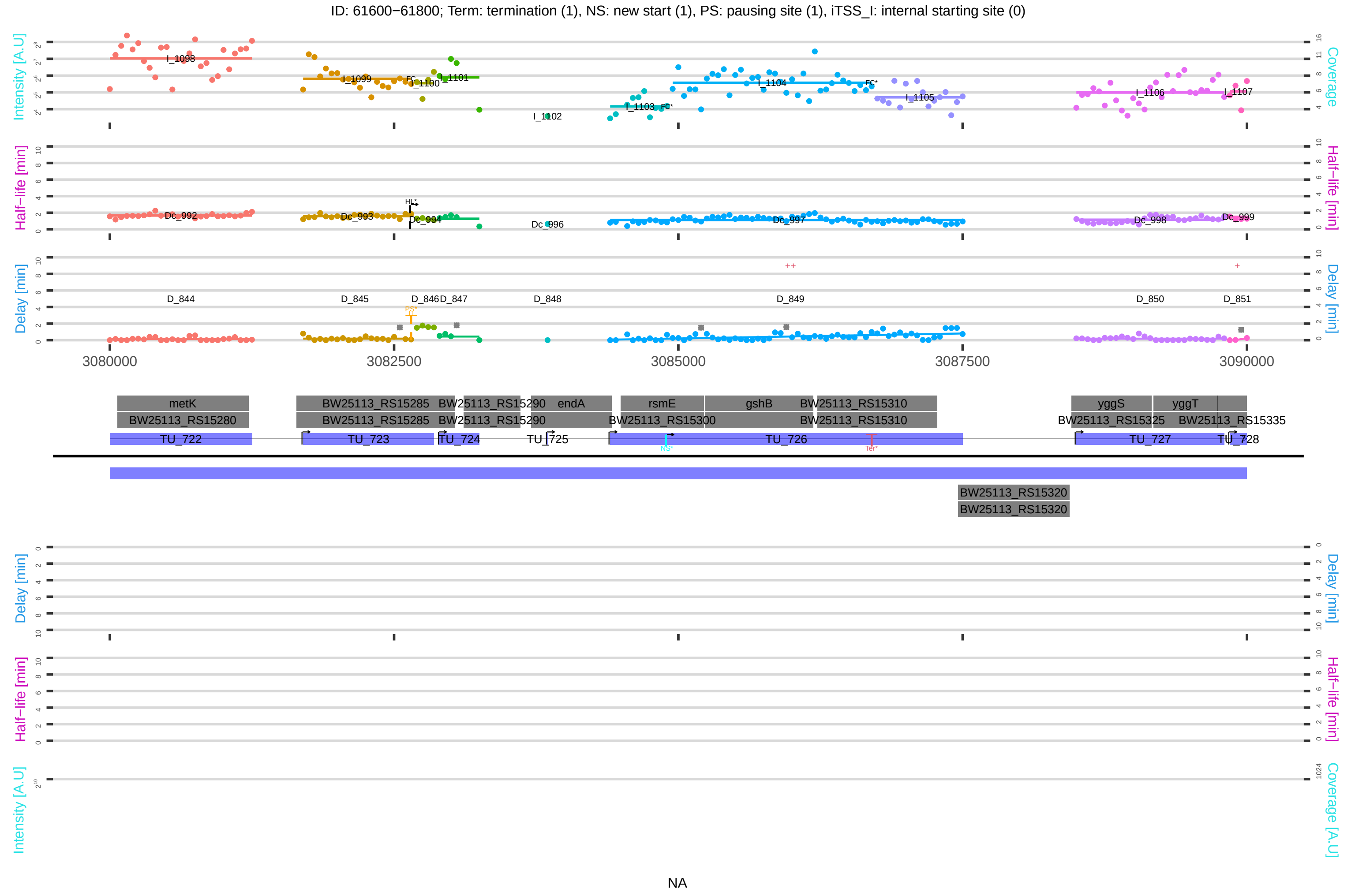
ID: 61600−61800; Term: termination (1), NS: new start (1), PS: pausing site (1), iTSS_I: internal starting site (0)
Intensity [A.U]
Coverage
Half−life [min]
Delay [min]
I_1098
I_1099
I_1100
I_1101
I_1102
I_1103
I_1104
I_1105
I_1106
I_1107
Dc_992
Dc_993
Dc_994
Dc_996
Dc_997
Dc_998
Dc_999
D_844
D_845
D_846
D_847
D_848
D_849
D_850
D_851
3080000
3082500
3085000
3087500
3090000
metK
BW25113_RS15280
BW25113_RS15285
BW25113_RS15290
endA
rsmE
gshB
BW25113_RS15300
BW25113_RS15310
yggS
yggT
BW25113_RS15325
BW25113_RS15335
TU_722
TU_723
TU_724
TU_725
TU_726
TU_727
TU_728
BW25113_RS15320
NA

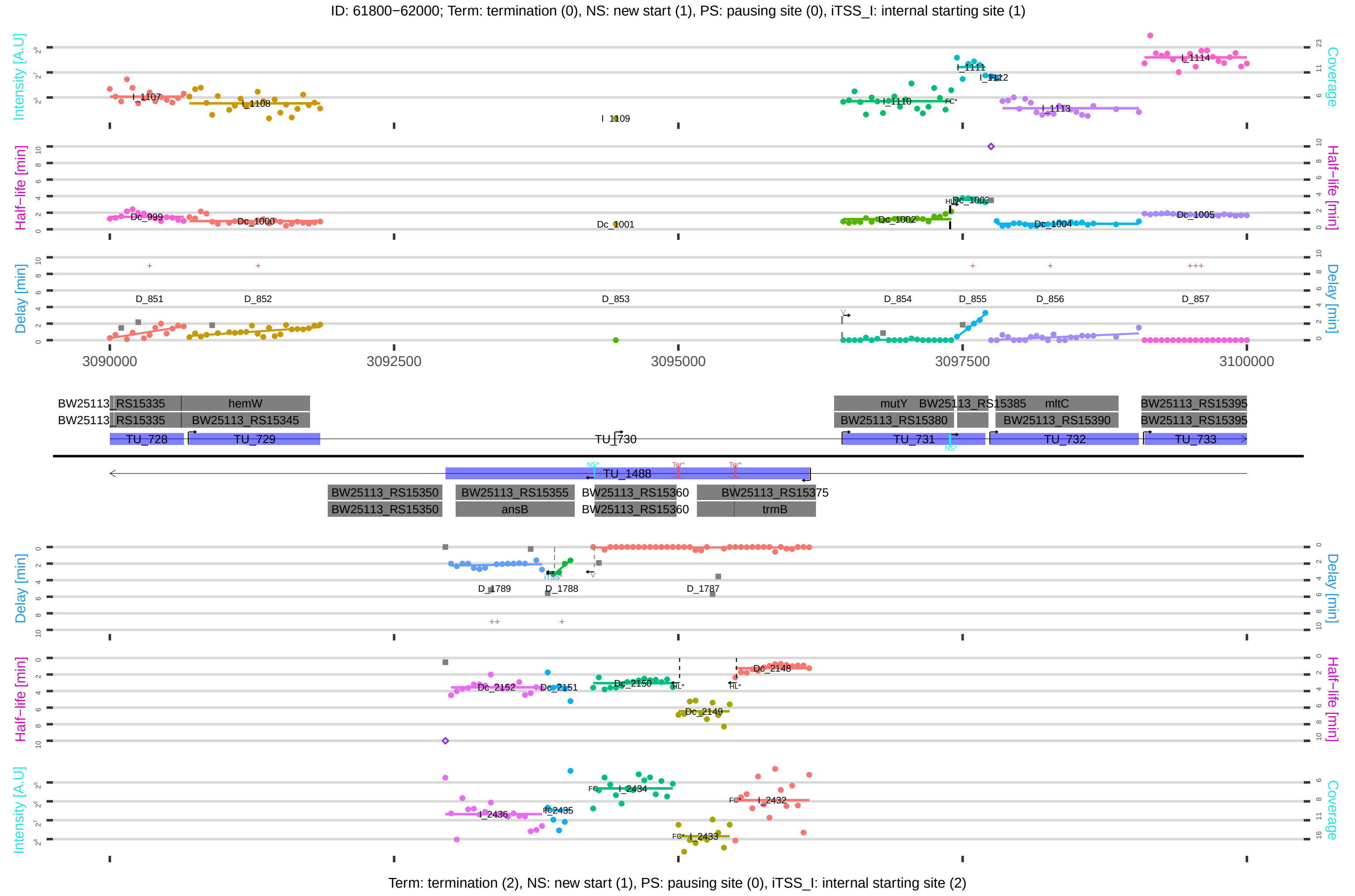

ID: 61800−62000; Term: termination (0), NS: new start (1), PS: pausing site (0), iTSS_I: internal starting site (1)
Intensity [A.U]
Coverage
Half−life [min]
Delay [min]
I_1107
I_1108
I_1109
I_1110
I_1111
I_1112
I_1113
I_1114
Dc_999
Dc_1000
Dc_1001
Dc_1002
Dc_1003
Dc_1004
Dc_1005
D_851
D_852
D_853
D_854
D_855
D_856
D_857
3090000
3092500
3095000
3097500
3100000
BW25113_RS15335
hemW
BW25113_RS15345
mutY
BW25113_RS15385
mltC
BW25113_RS15380
BW25113_RS15390
BW25113_RS15395
TU_728
TU_729
TU_730
TU_731
TU_732
TU_733
TU_1488
BW25113_RS15350
BW25113_RS15355
ansB
BW25113_RS15360
BW25113_RS15375
trmB
D_1789
D_1788
D_1787
Dc_2152
Dc_2151
Dc_2150
Dc_2149
Dc_2148
I_2436
I_2435
I_2434
I_2433
I_2432
Term: termination (2), NS: new start (1), PS: pausing site (0), iTSS_I: internal starting site (2)

ID: 62000−62105; Term: termination (0), NS: new start (0), PS: pausing site (0), iTSS_I: internal starting site (0)

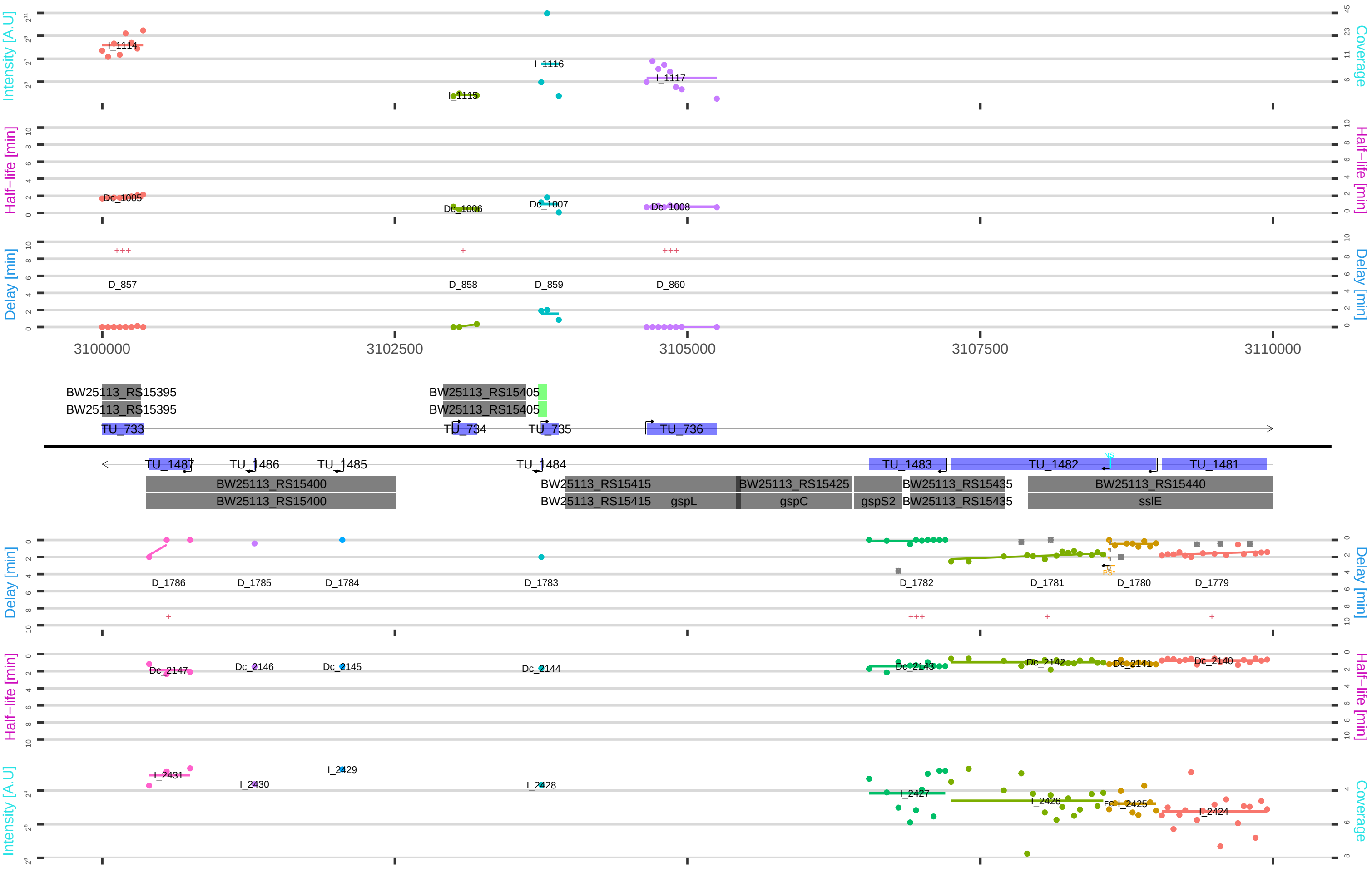

Term: termination (0), NS: new start (1), PS: pausing site (1), iTSS_I: internal starting site (0)

ID: 123060−122913; FC*: significant t−test of two consecutive segments; Term: termination, NS: new start, PS: pausing site,iTSS_I: internal starting site,TI: transcription interferance.

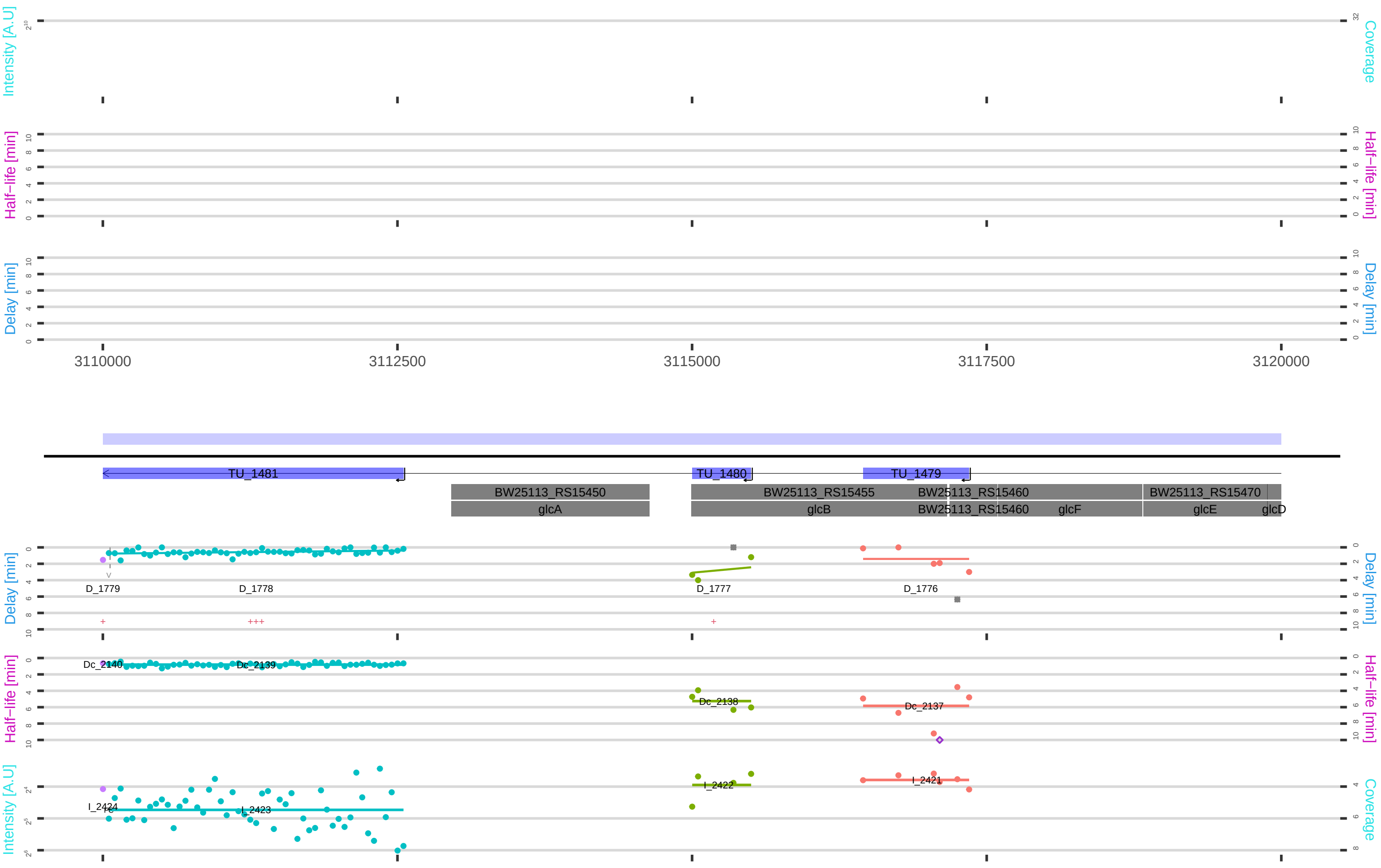

Term: termination (0), NS: new start (0), PS: pausing site (1), iTSS_I: internal starting site (0)

ID: 62433−62497; Term: termination (0), NS: new start (0), PS: pausing site (0), iTSS_I: internal starting site (0)

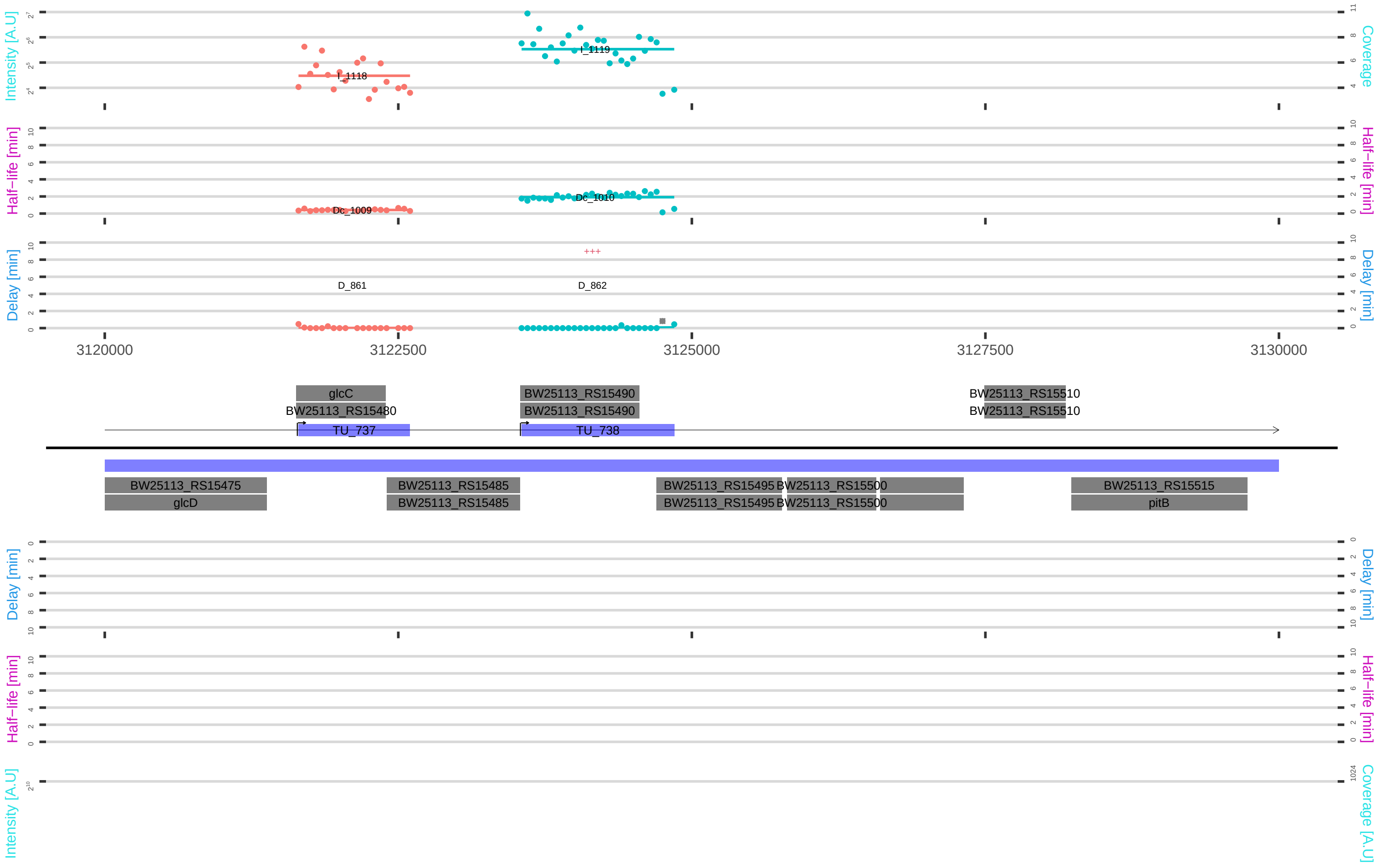

NA

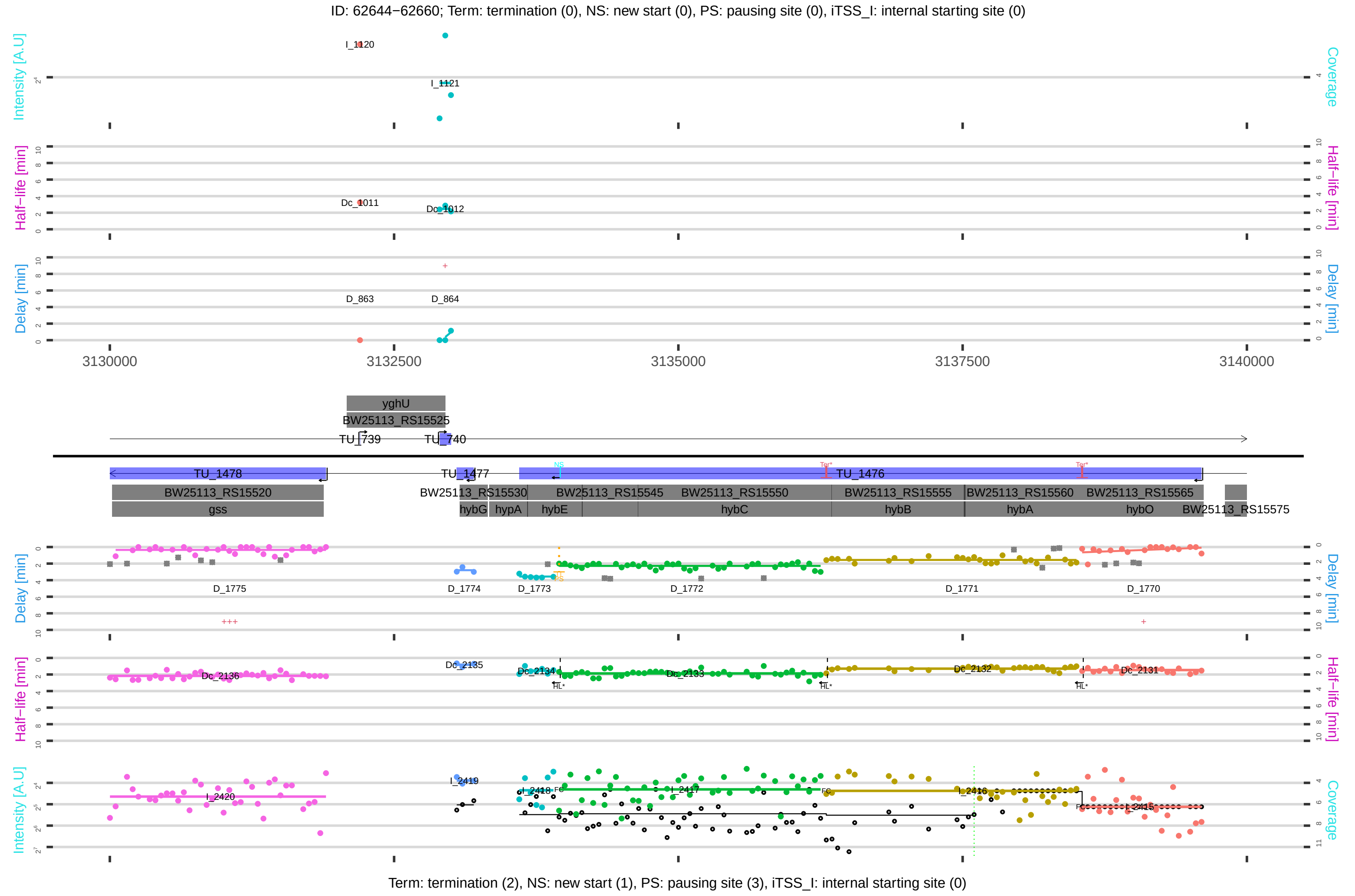
ID: 62644−62660; Term: termination (0), NS: new start (0), PS: pausing site (0), iTSS_I: internal starting site (0)
Intensity [A.U]
Coverage
Half−life [min]
Delay [min]
I_1120
I_1121
Dc_1011
Dc_1012
D_863
D_864
3130000
3132500
3135000
3137500
3140000
yghU
BW25113_RS15525
TU_739
TU_740
TU_1478
TU_1477
TU_1476
BW25113_RS15520
gss
BW25113_RS15530
hybG
hypA
hybE
BW25113_RS15545
BW25113_RS15550
hybC
BW25113_RS15555
hybB
BW25113_RS15560
hybA
BW25113_RS15565
hybO
BW25113_RS15575
D_1775
D_1774
D_1773
D_1772
D_1771
D_1770
Dc_2136
Dc_2135
Dc_2134
Dc_2133
Dc_2132
Dc_2131
I_2420
I_2419
I_2418
I_2417
I_2416
I_2415
Term: termination (2), NS: new start (1), PS: pausing site (3), iTSS_I: internal starting site (0)

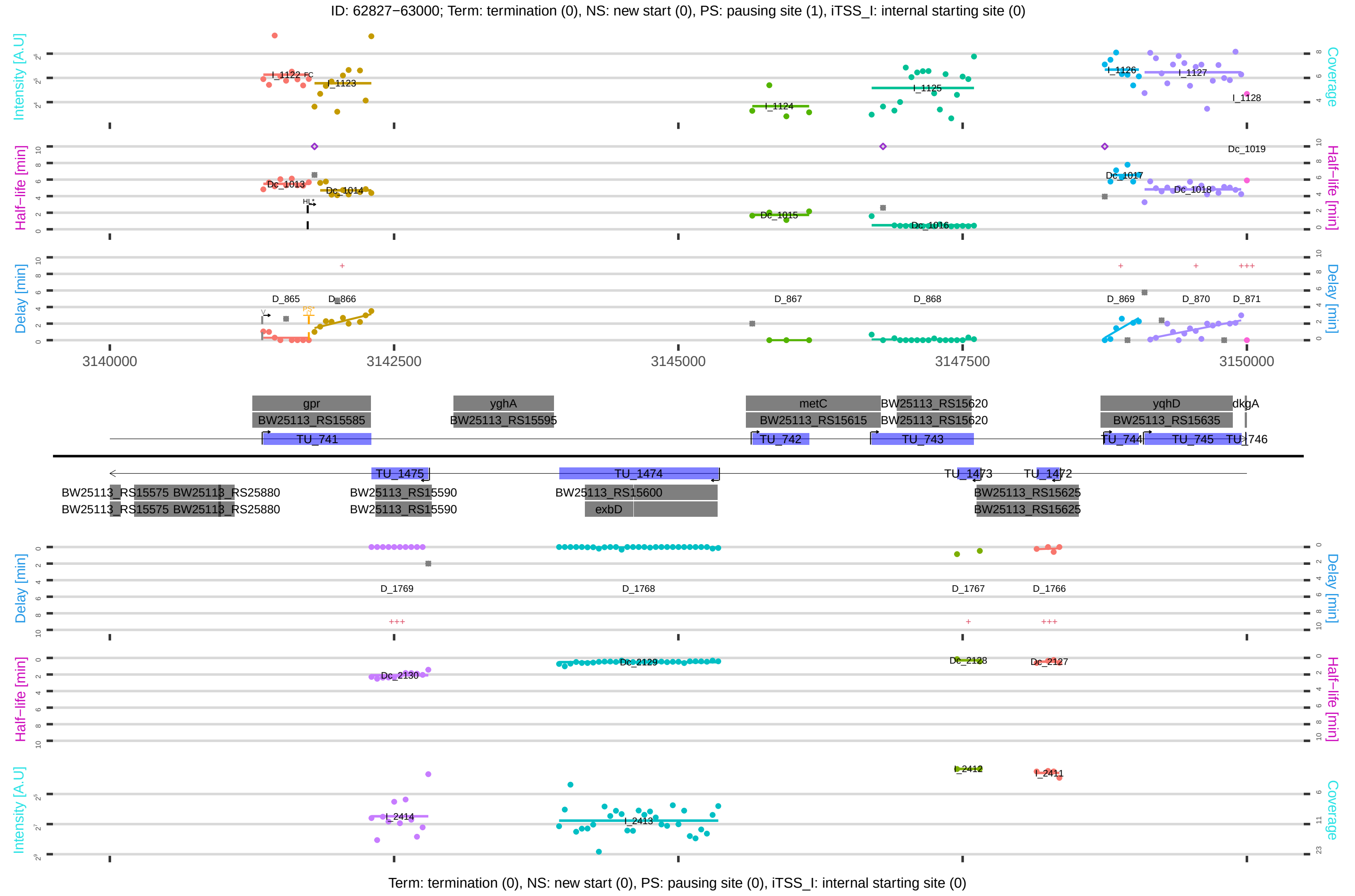
ID: 62827−63000; Term: termination (0), NS: new start (0), PS: pausing site (1), iTSS_I: internal starting site (0)
Intensity [A.U]
Coverage
Half−life [min]
Delay [min]
I_1122 FC
I_1123
I_1124
I_1125
I_1126
I_1127
I_1128
Dc_1013
Dc_1014
Dc_1015
Dc_1016
Dc_1017
Dc_1018
Dc_1019
D_865
D_866
D_867
D_868
D_869
D_870
D_871
3140000
3142500
3145000
3147500
3150000
gpr
BW25113_RS15585
yghA
BW25113_RS15595
metC
BW25113_RS15615
BW25113_RS15620
yqhD
BW25113_RS15635
dkgA
TU_741
TU_742
TU_743
TU_744
TU_745
TU_746
TU_1475
TU_1474
TU_1473
TU_1472
BW25113_RS15575
BW25113_RS25880
BW25113_RS15590
BW25113_RS15600
exbD
BW25113_RS15625
D_1769
D_1768
D_1767
D_1766
Dc_2130
Dc_2129
Dc_2128
Dc_2127
I_2414
I_2413
I_2412
I_2411
Term: termination (0), NS: new start (0), PS: pausing site (0), iTSS_I: internal starting site (0)

ID: 63000−63016; Term: termination (0), NS: new start (0), PS: pausing site (0), iTSS_I: internal starting site (0)

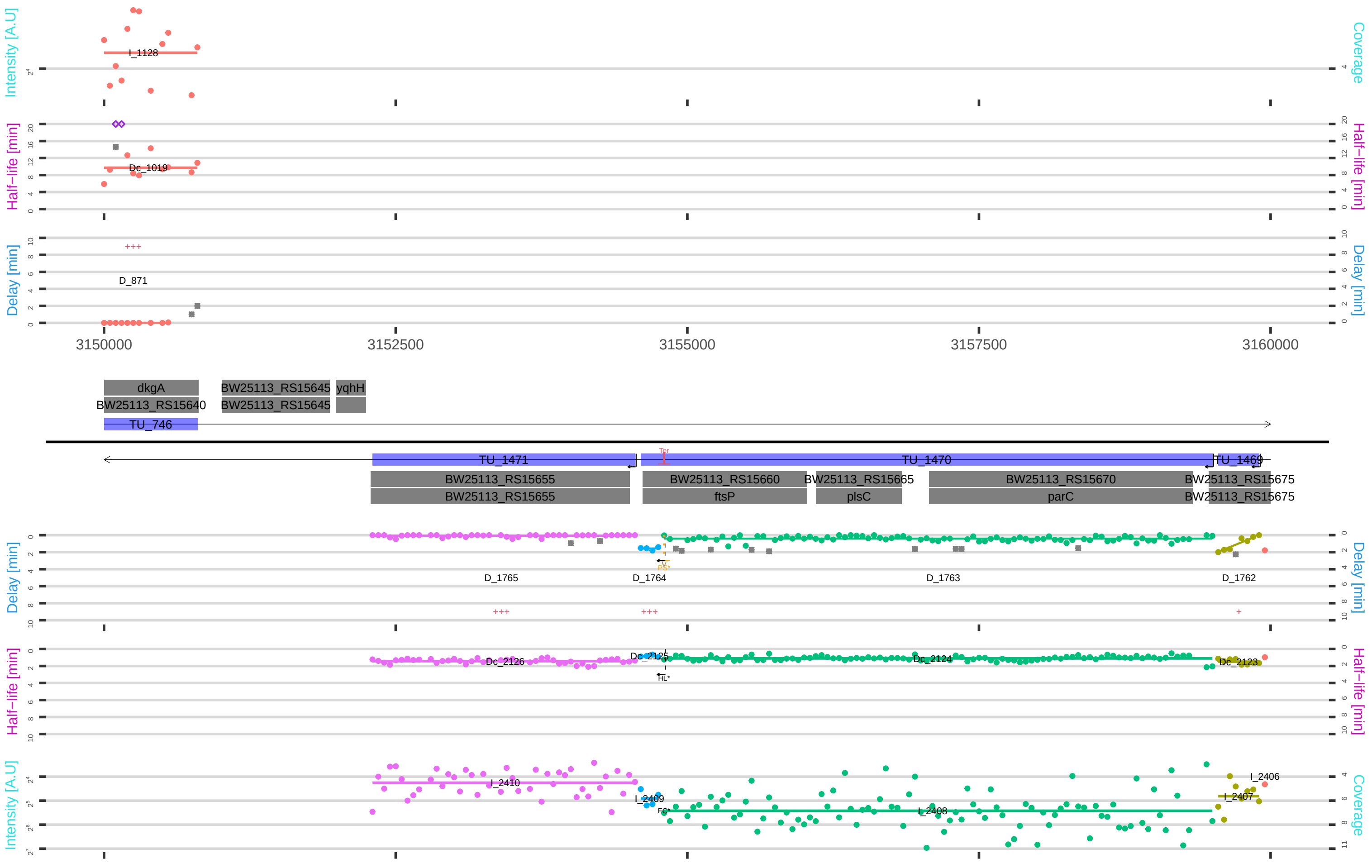

Term: termination (1), NS: new start (0), PS: pausing site (1), iTSS_I: internal starting site (0)

ID: 63265−63337; Term: termination (0), NS: new start (1), PS: pausing site (0), iTSS_I: internal starting site (0)

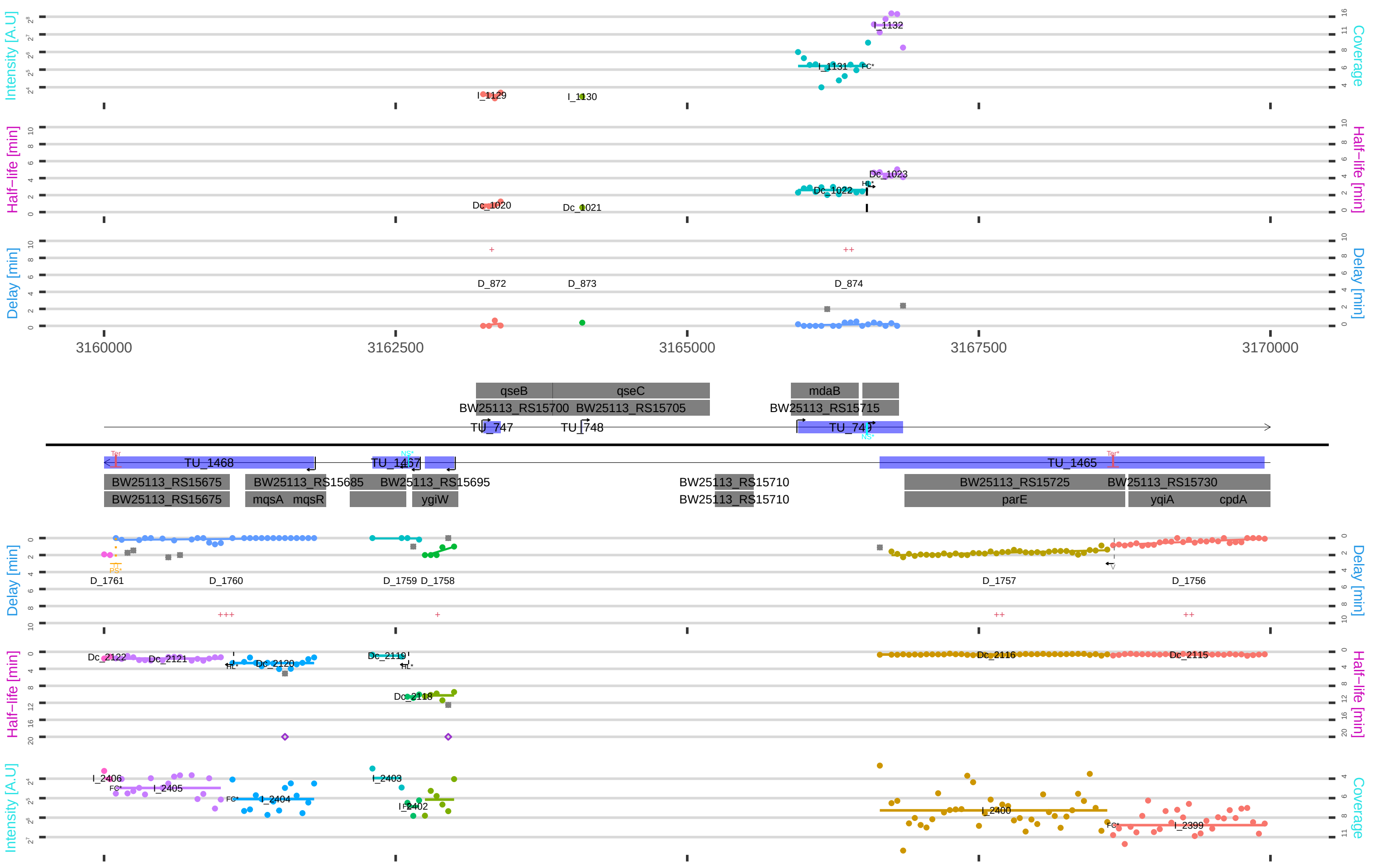

Term: termination (2), NS: new start (1), PS: pausing site (2), iTSS_I: internal starting site (0)

ID: 63426−63600; Term: termination (3), NS: new start (2), PS: pausing site (1), iTSS_I: internal starting site (0)

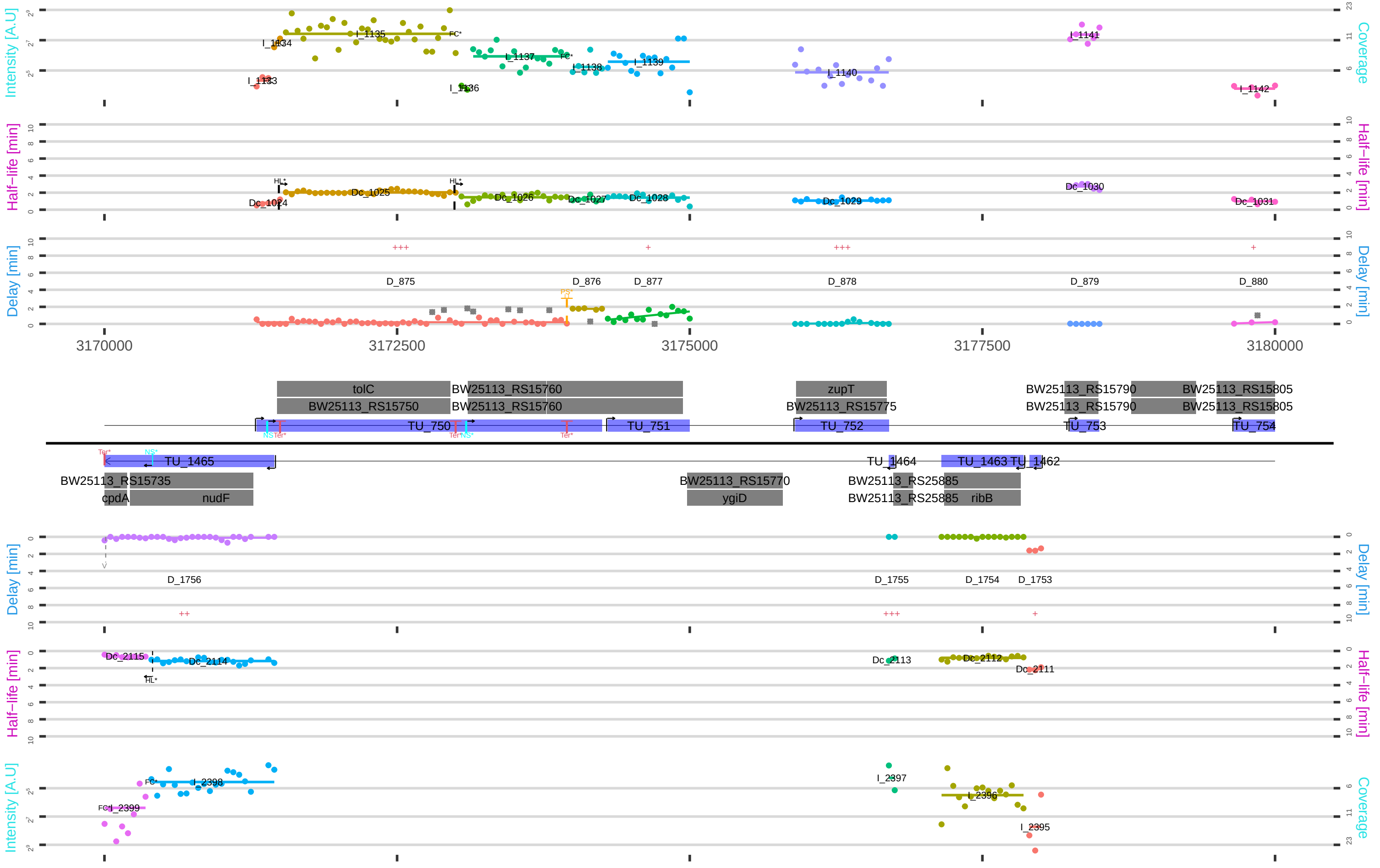

Term: termination (1), NS: new start (1), PS: pausing site (0), iTSS_I: internal starting site (0)

ID: 63600−63600; Term: termination (0), NS: new start (0), PS: pausing site (0), iTSS_I: internal starting site (0)

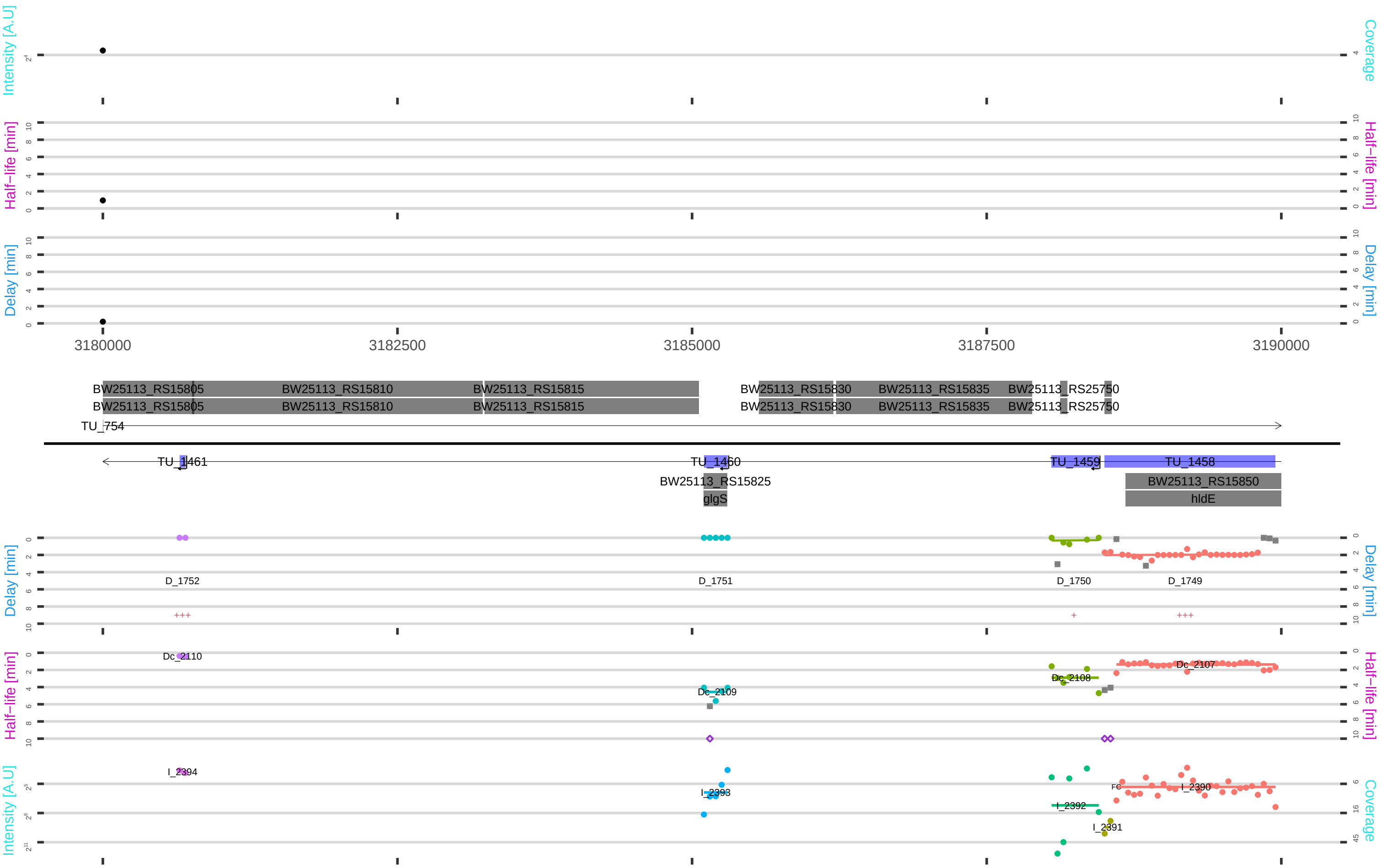

Term: termination (0), NS: new start (0), PS: pausing site (0), iTSS_I: internal starting site (0)

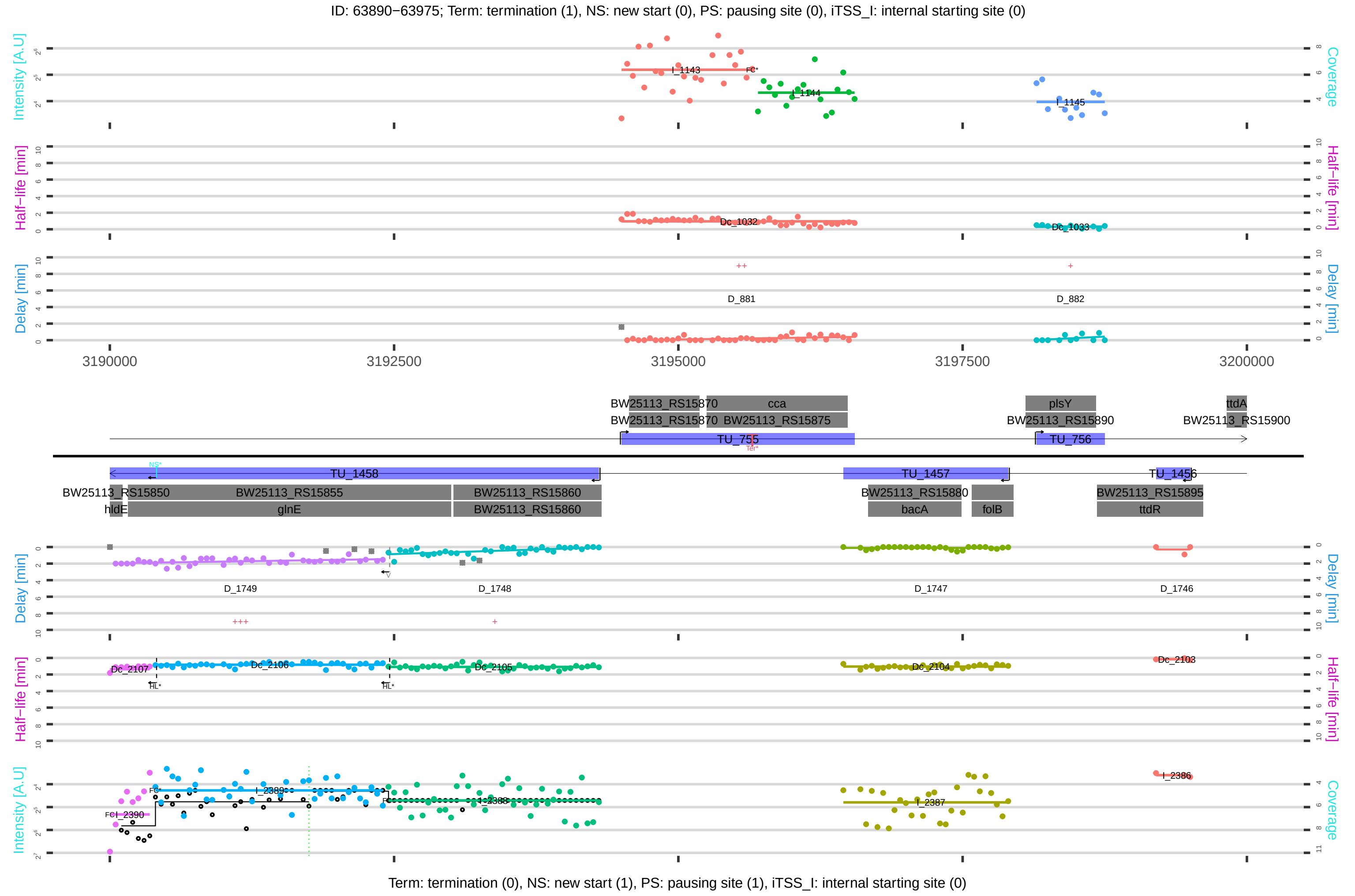

ID: 63890−63975; Term: termination (1), NS: new start (0), PS: pausing site (0), iTSS_I: internal starting site (0)
Intensity [A.U]
Coverage
Half−life [min]
Delay [min]
I_1143
I_1144
I_1145
Dc_1032
Dc_1033
D_881
D_882
3190000
3192500
3195000
3197500
3200000
BW25113_RS15870
cca
BW25113_RS15875
plsY
BW25113_RS15890
ttdA
BW25113_RS15900
TU_755
TU_756
TU_1458
TU_1457
TU_1456
BW25113_RS15850
BW25113_RS15855
BW25113_RS15860
BW25113_RS15880
BW25113_RS15895
hldE
glnE
bacA
folB
ttdR
D_1749
D_1748
D_1747
D_1746
Dc_2107
Dc_2106
Dc_2105
Dc_2104
Dc_2103
I_2390
I_2389
I_2388
I_2387
I_2386
Term: termination (0), NS: new start (1), PS: pausing site (1), iTSS_I: internal starting site (0)

ID: 64082−64181; Term: termination (3), NS: new start (1), PS: pausing site (2), iTSS_I: internal starting site (1)

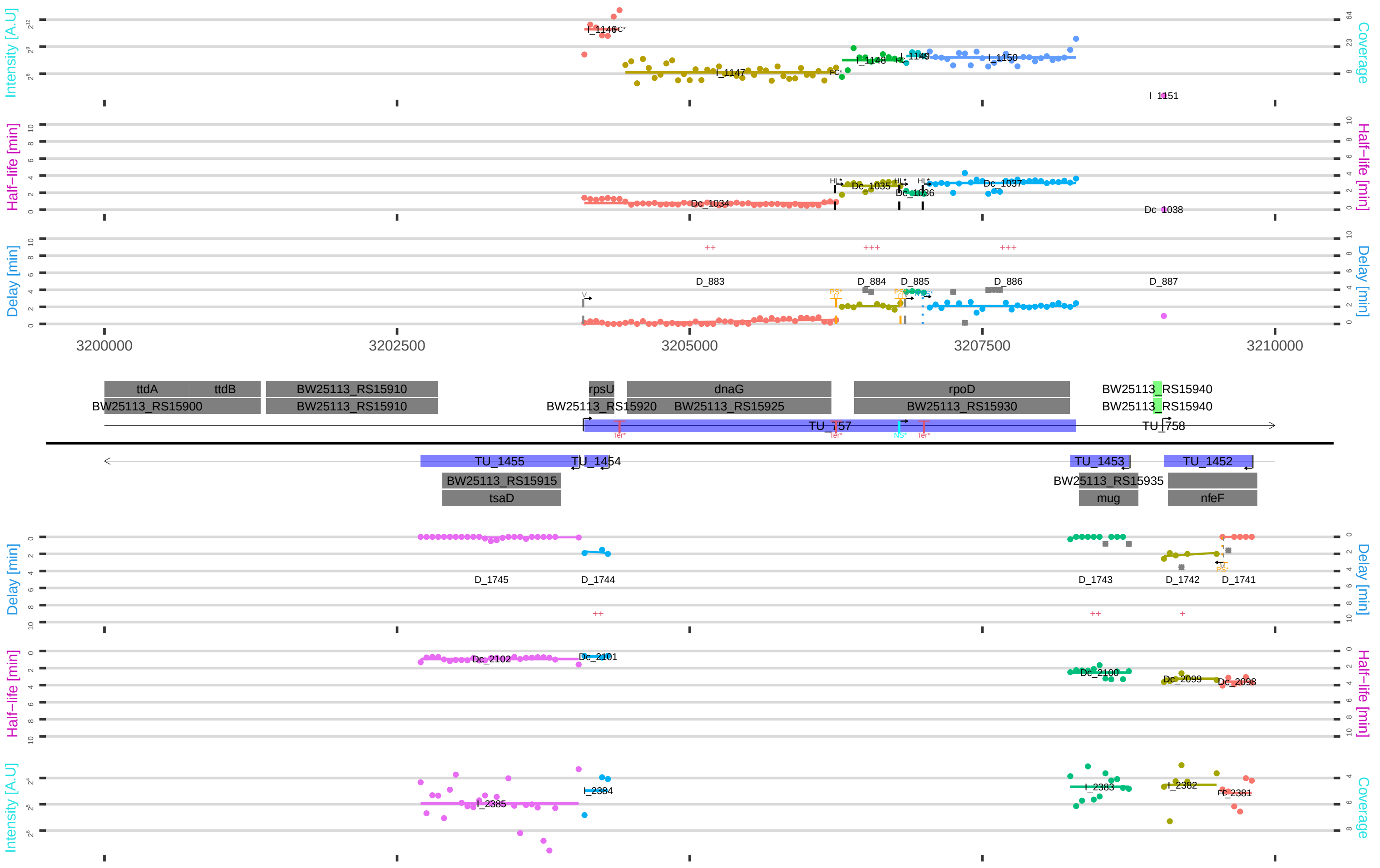

Term: termination (0), NS: new start (0), PS: pausing site (1), iTSS_I: internal starting site (0)

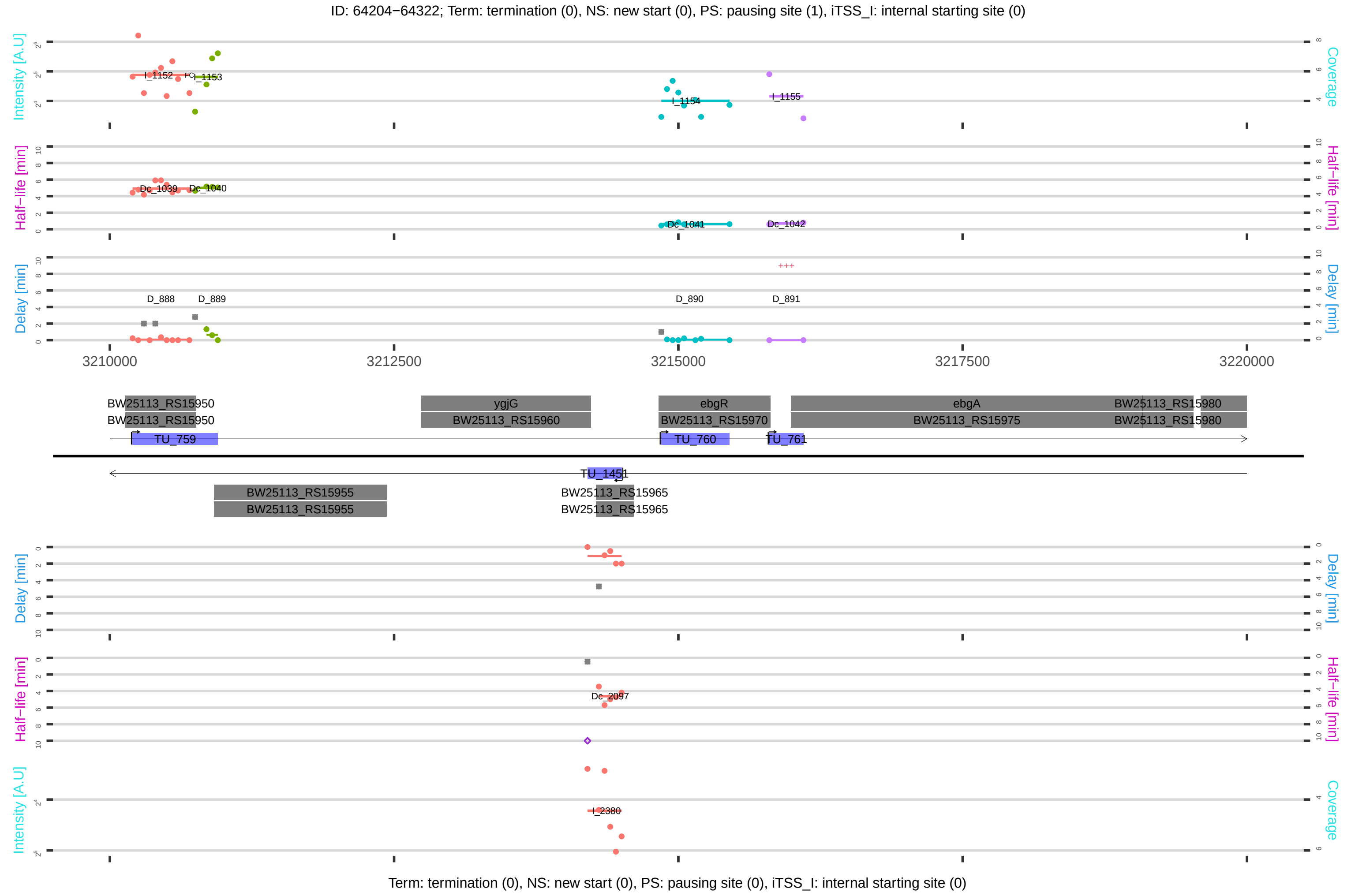
ID: 64204−64322; Term: termination (0), NS: new start (0), PS: pausing site (1), iTSS_I: internal starting site (0)
Intensity [A.U]
Coverage
I_1152
I_1153
I_1154
I_1155
Half−life [min]
Dc_1039
Dc_1040
Dc_1041
Dc_1042
Delay [min]
D_888
D_889
D_890
D_891
+++
3210000
3212500
3215000
3217500
3220000
BW25113_RS15950
ygjG
BW25113_RS15960
ebgR
BW25113_RS15970
ebgA
BW25113_RS15975
BW25113_RS15980
TU_759
TU_760
TU_761
TU_1451
BW25113_RS15955
BW25113_RS15965
Dc_2097
I_2380
Term: termination (0), NS: new start (0), PS: pausing site (0), iTSS_I: internal starting site (0)

ID: 64421−64588; Term: termination (0), NS: new start (0), PS: pausing site (0), iTSS_I: internal starting site (0)

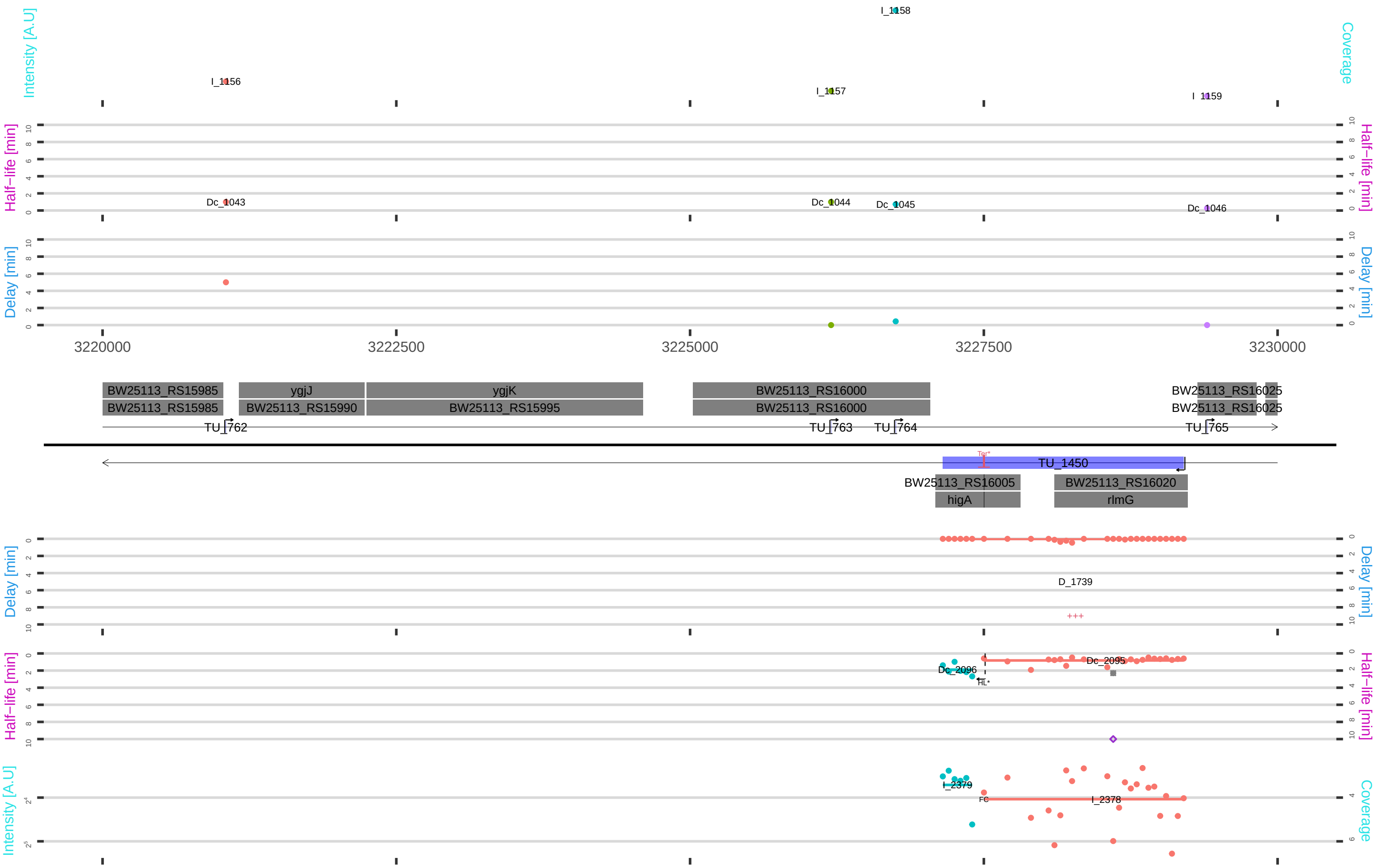

Term: termination (1), NS: new start (0), PS: pausing site (0), iTSS_I: internal starting site (0)

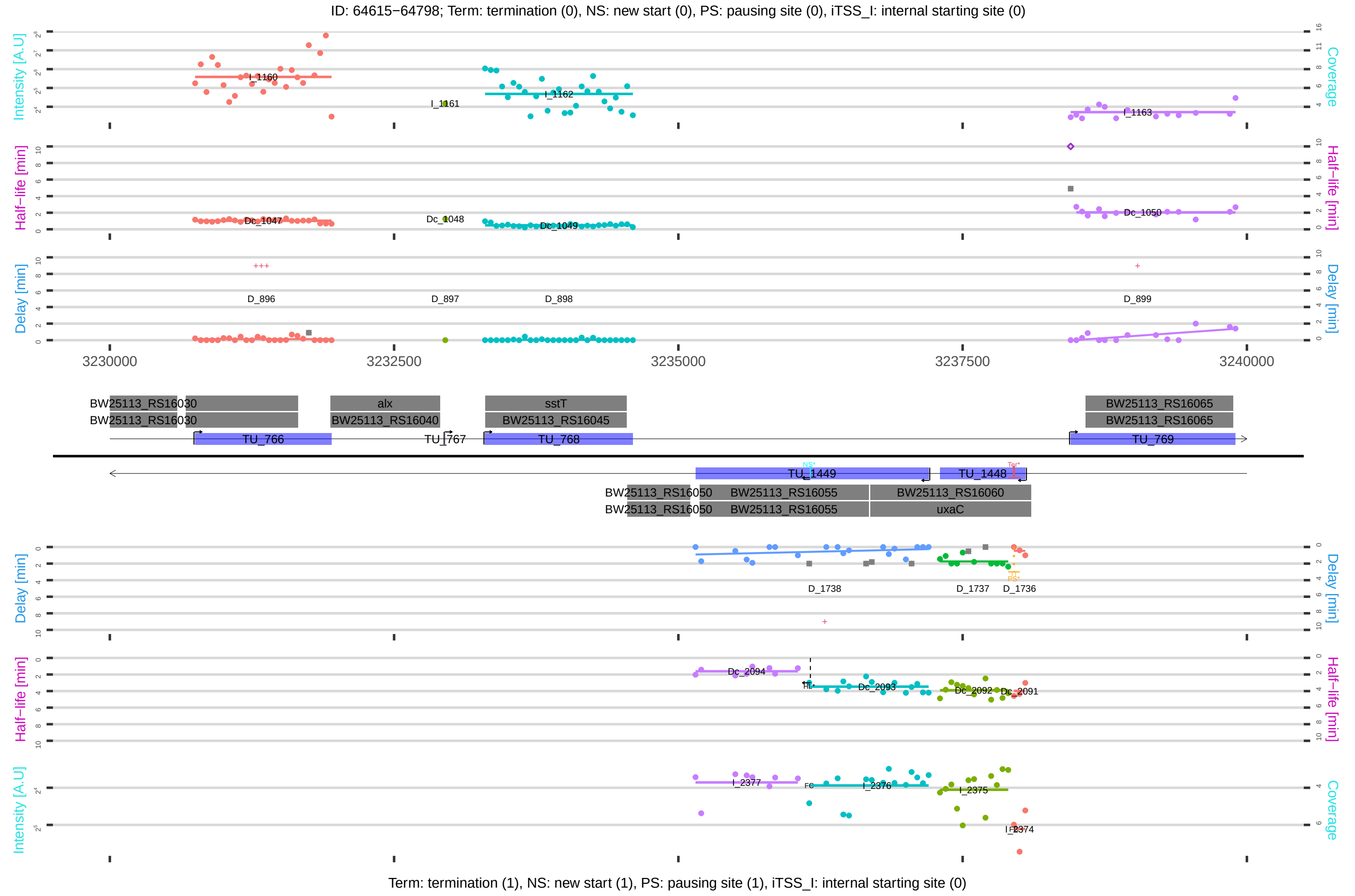

ID: 64615−64798; Term: termination (0), NS: new start (0), PS: pausing site (0), iTSS_I: internal starting site (0)
Intensity [A.U]
Coverage
Half−life [min]
Delay [min]
I_1160
I_1161
I_1162
I_1163
Dc_1047
Dc_1048
Dc_1049
Dc_1050
D_896
D_897
D_898
D_899
3230000
3232500
3235000
3237500
3240000
BW25113_RS16030
alx
BW25113_RS16040
sstT
BW25113_RS16045
BW25113_RS16065
TU_766
TU_767
TU_768
TU_769
TU_1449
TU_1448
BW25113_RS16050
BW25113_RS16055
BW25113_RS16060
uxaC
D_1738
D_1737
D_1736
Dc_2094
Dc_2093
Dc_2092
Dc_2091
I_2377
I_2376
I_2375
I_2374
Term: termination (1), NS: new start (1), PS: pausing site (1), iTSS_I: internal starting site (0)

ID: 64801−64975; Term: termination (4), NS: new start (1), PS: pausing site (1), iTSS_I: internal starting site (0)

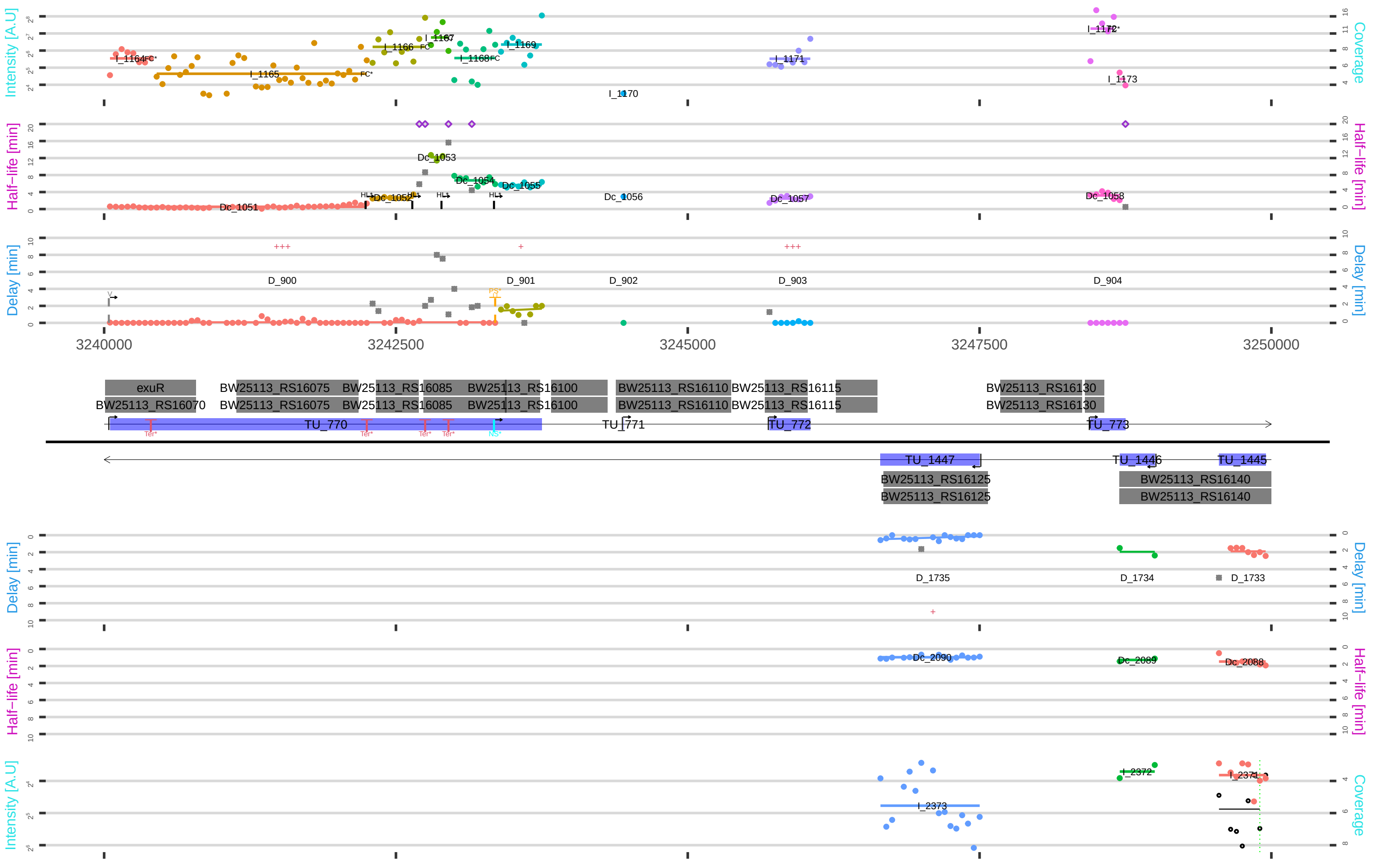

Term: termination (0), NS: new start (0), PS: pausing site (0), iTSS_I: internal starting site (0)

ID: 120260−120061; FC*: significant t−test of two consecutive segments; Term: termination, NS: new start, PS: pausing site,iTSS_I: internal starting site,TI: transcription interferance.

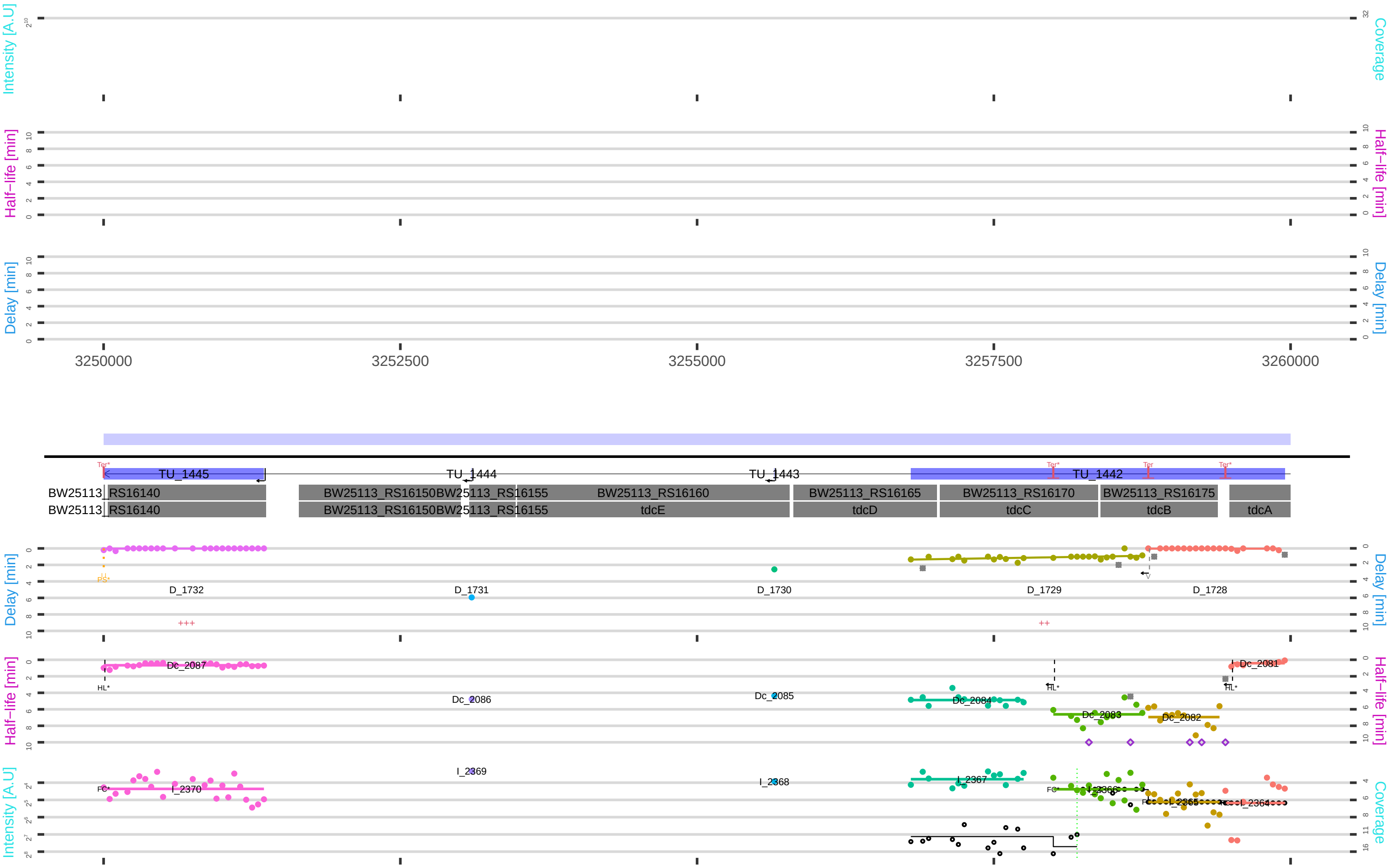

Term: termination (4), NS: new start (0), PS: pausing site (2), iTSS_I: internal starting site (0)

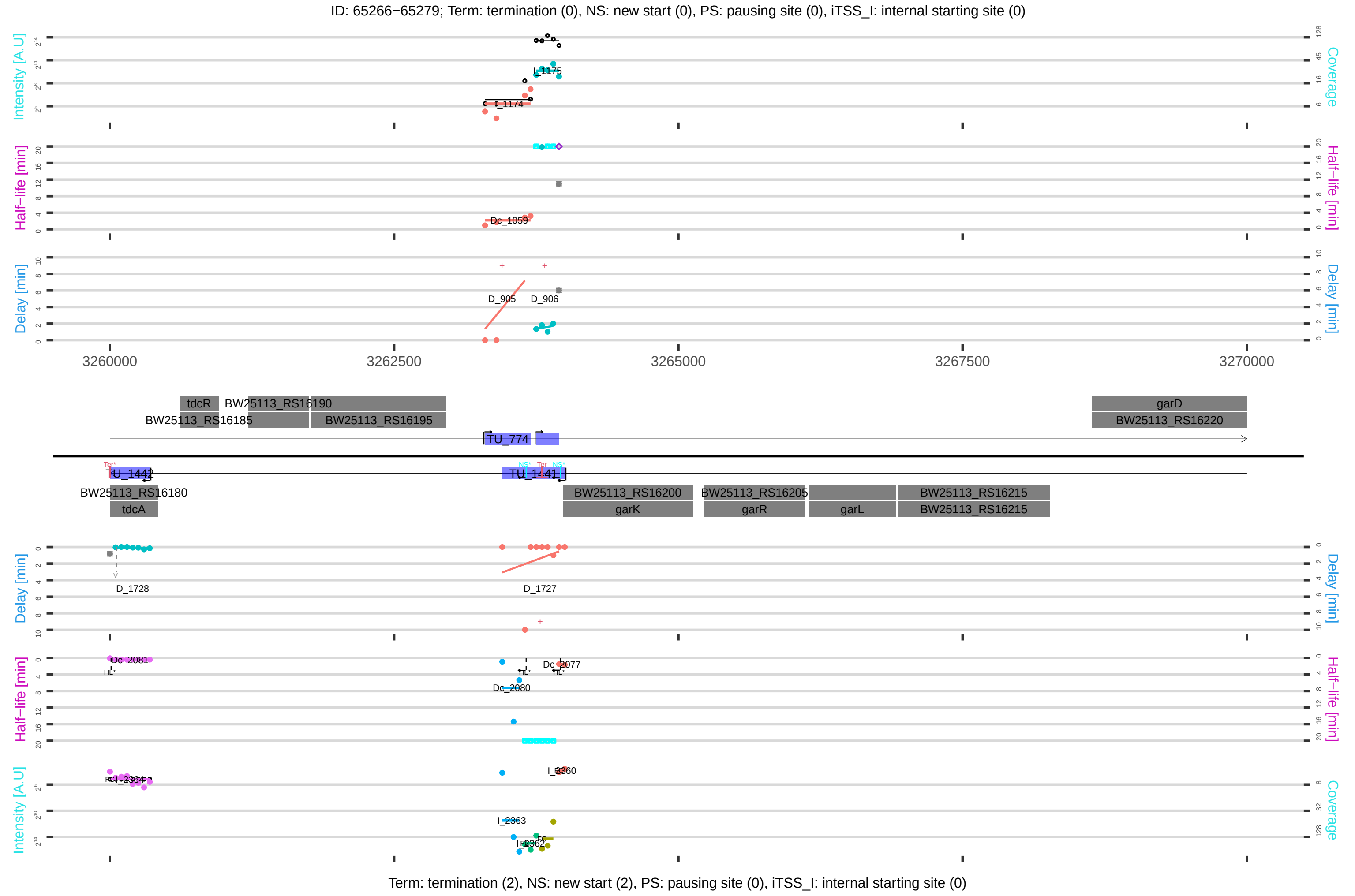

ID: 65266−65279; Term: termination (0), NS: new start (0), PS: pausing site (0), iTSS_I: internal starting site (0)
Intensity [A.U]
Coverage
Half−life [min]
Delay [min]
I_1175
I_1174
Dc_1059
D_905
D_906
3260000
3262500
3265000
3267500
3270000
tdcR
BW25113_RS16190
BW25113_RS16185
BW25113_RS16195
garD
BW25113_RS16220
TU_774
TU_1442
TU_1441
BW25113_RS16180
tdcA
BW25113_RS16200
garK
BW25113_RS16205
garR
garL
BW25113_RS16215
BW25113_RS16215
D_1728
D_1727
Dc_2081
Dc_2077
Dc_2080
I_2360
I_2363
I_2362
Term: termination (2), NS: new start (2), PS: pausing site (0), iTSS_I: internal starting site (0)

ID: 65404−65424; Term: termination (0), NS: new start (0), PS: pausing site (0), iTSS_I: internal starting site (0)

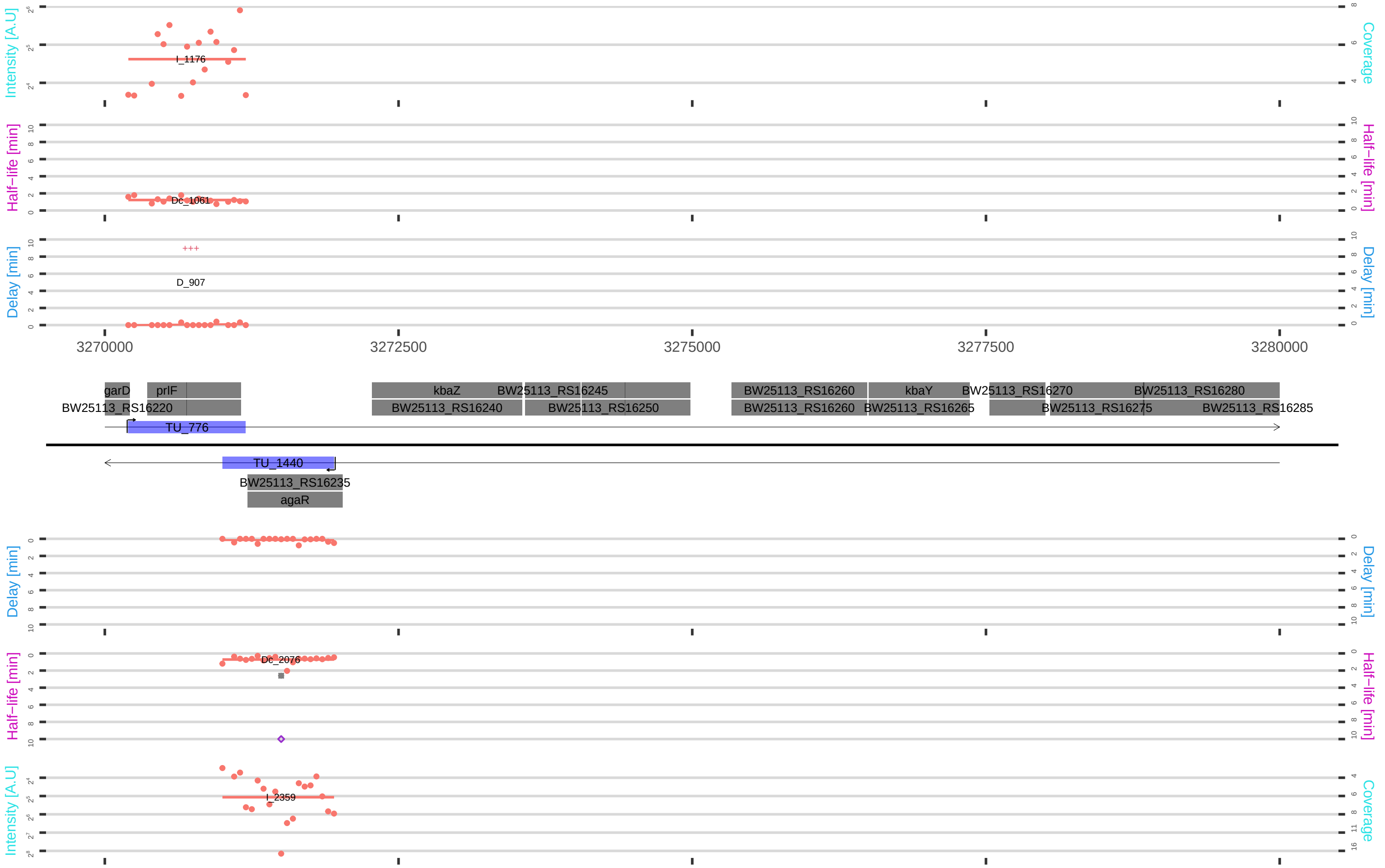

Term: termination (0), NS: new start (0), PS: pausing site (0), iTSS_I: internal starting site (0)

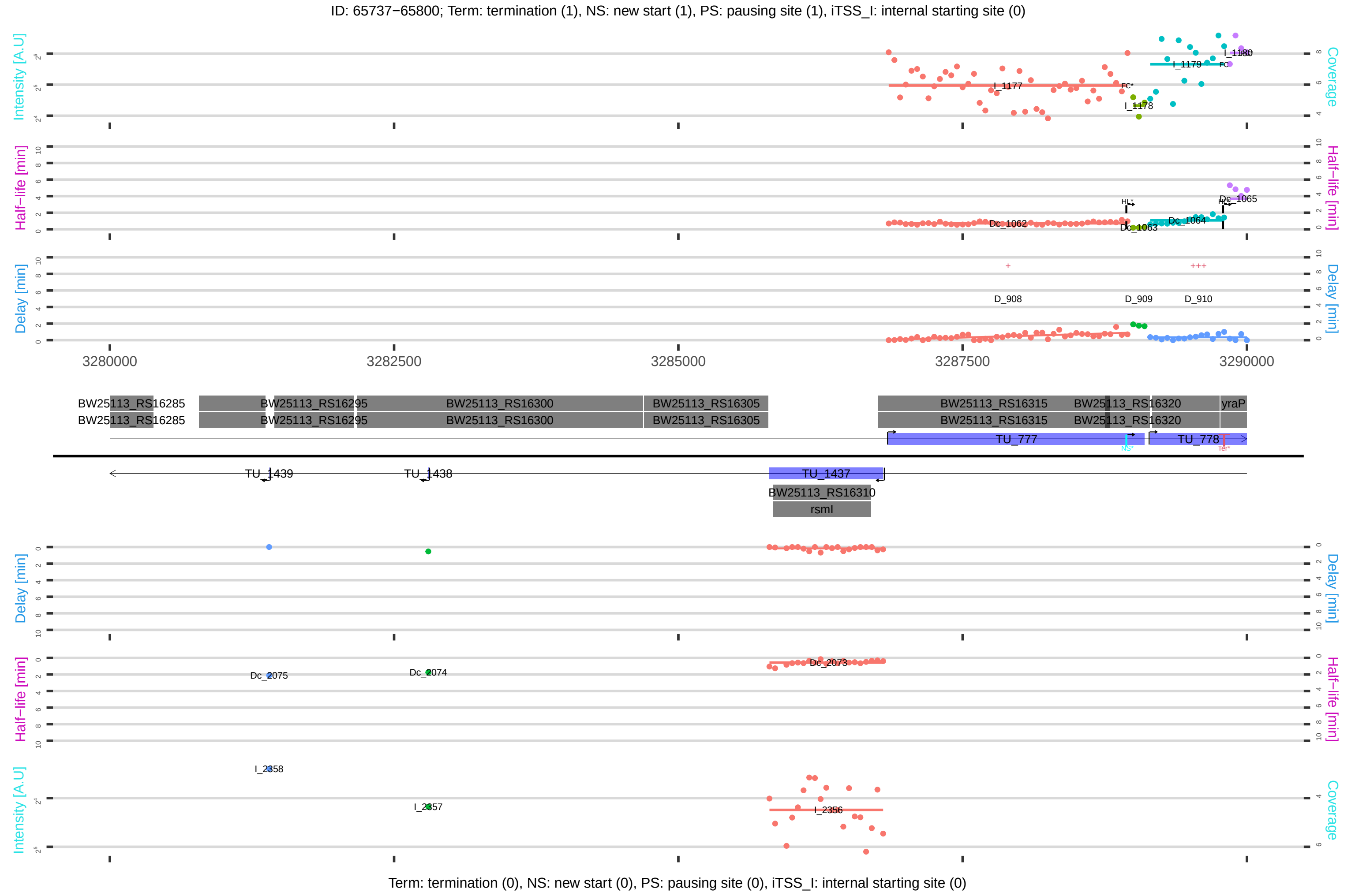

ID: 65737−65800; Term: termination (1), NS: new start (1), PS: pausing site (1), iTSS_I: internal starting site (0)
Intensity [A.U]
Coverage
Half−life [min]
Delay [min]
I_1177
I_1178
I_1179
I_1180
FC*
Dc_1062
Dc_1063
Dc_1064
Dc_1065
HL*
D_908
D_909
D_910
3280000
3282500
3285000
3287500
3290000
BW25113_RS16285
BW25113_RS16295
BW25113_RS16300
BW25113_RS16305
BW25113_RS16315
BW25113_RS16320
yraP
TU_777
TU_778
NS*
Ter*
TU_1439
TU_1438
TU_1437
BW25113_RS16310
rsmI
Dc_2075
Dc_2074
Dc_2073
I_2358
I_2357
I_2356
Term: termination (0), NS: new start (0), PS: pausing site (0), iTSS_I: internal starting site (0)

ID: 65800−65963; Term: termination (0), NS: new start (0), PS: pausing site (0), iTSS_I: internal starting site (0)

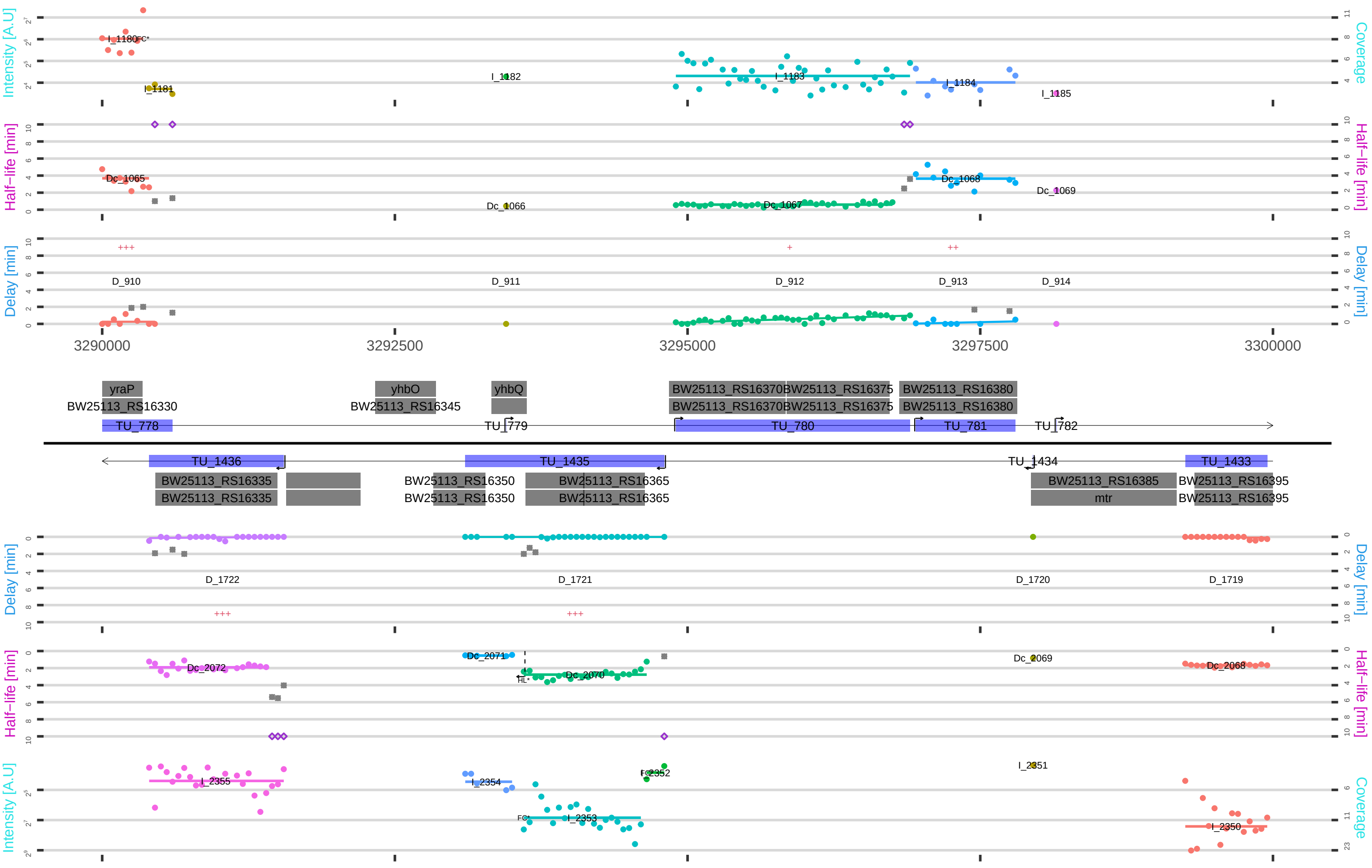

Term: termination (0), NS: new start (0), PS: pausing site (0), iTSS_I: internal starting site (0)

ID: 66092−66183; Term: termination (0), NS: new start (0), PS: pausing site (0), iTSS_I: internal starting site (0)

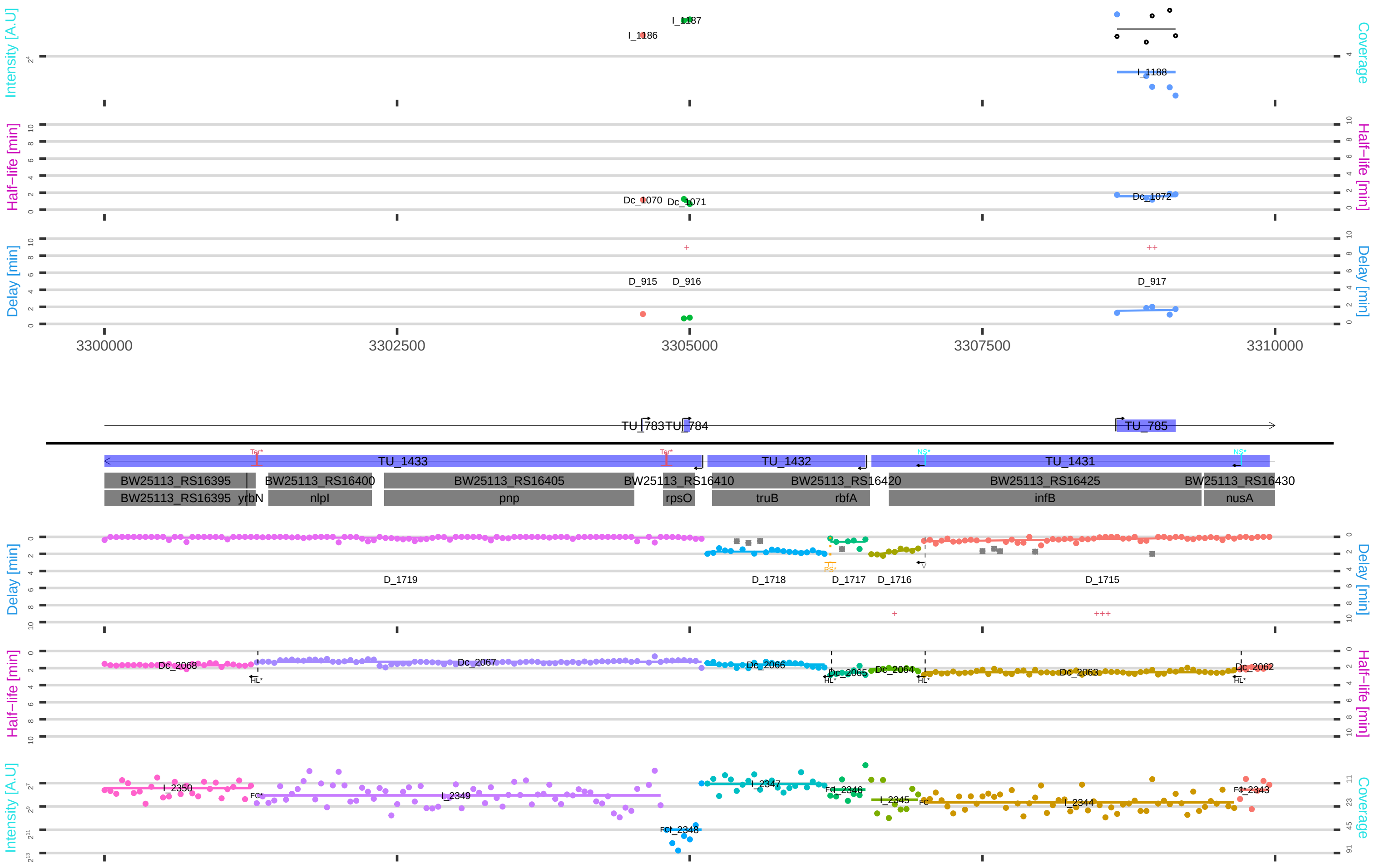

Term: termination (2), NS: new start (2), PS: pausing site (2), iTSS_I: internal starting site (0)

ID: 66233−66267; Term: termination (0), NS: new start (0), PS: pausing site (0), iTSS_I: internal starting site (0)

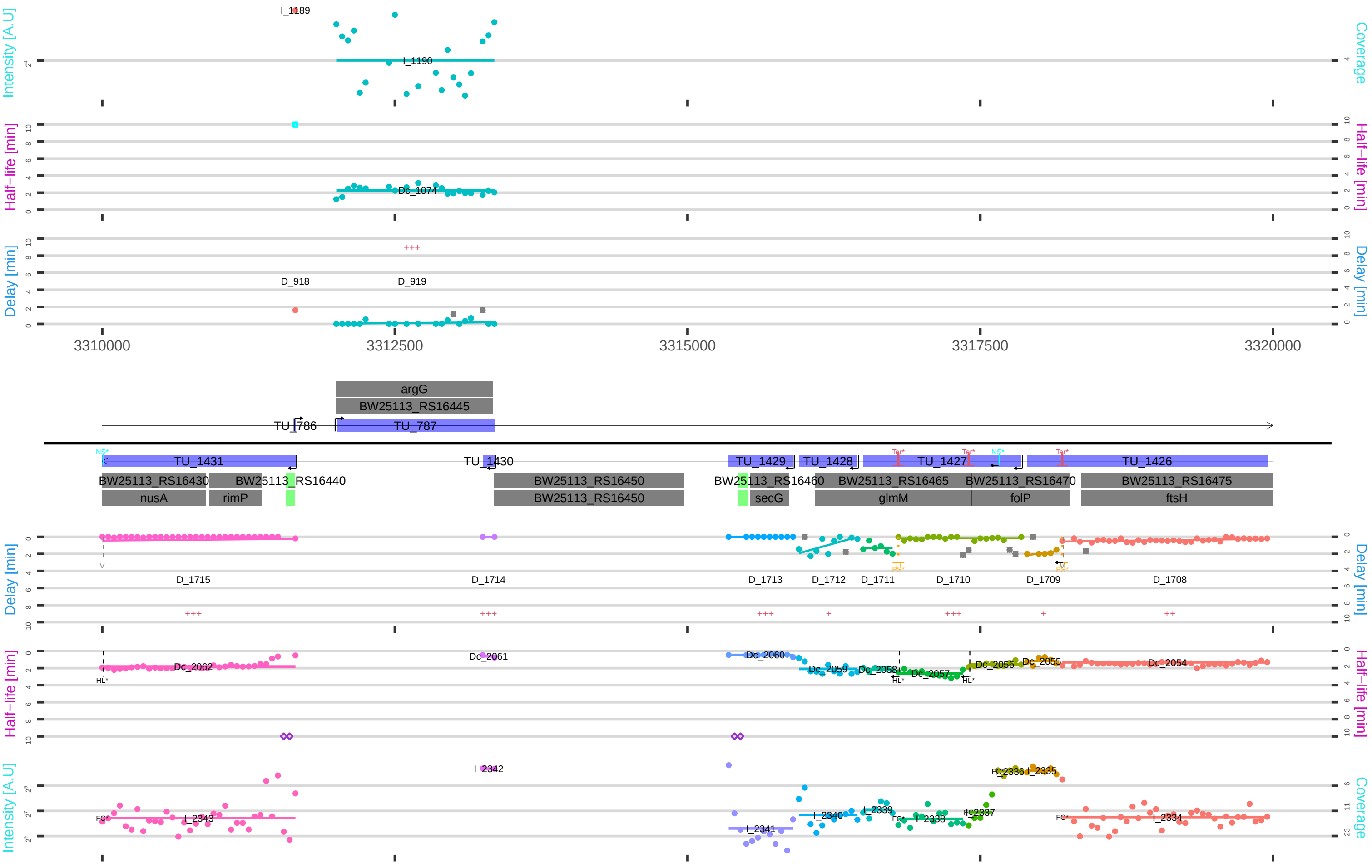

Term: termination (3), NS: new start (2), PS: pausing site (2), iTSS_I: internal starting site (0)

ID: 66423−66571; Term: termination (1), NS: new start (0), PS: pausing site (0), iTSS_I: internal starting site (0)

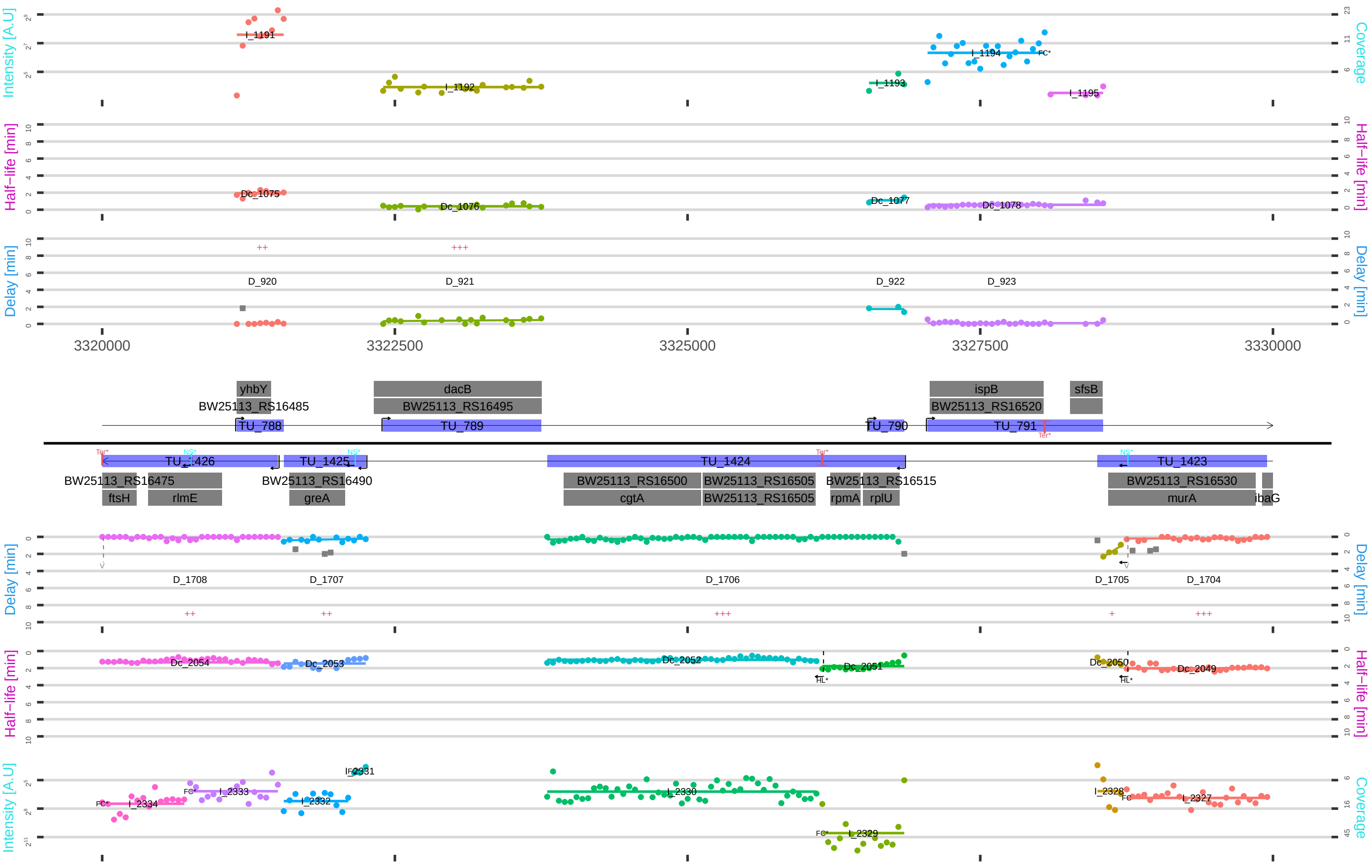

Term: termination (2), NS: new start (3), PS: pausing site (1), iTSS_I: internal starting site (0)

ID: 66674−66800; Term: termination (2), NS: new start (2), PS: pausing site (2), iTSS_I: internal starting site (0)

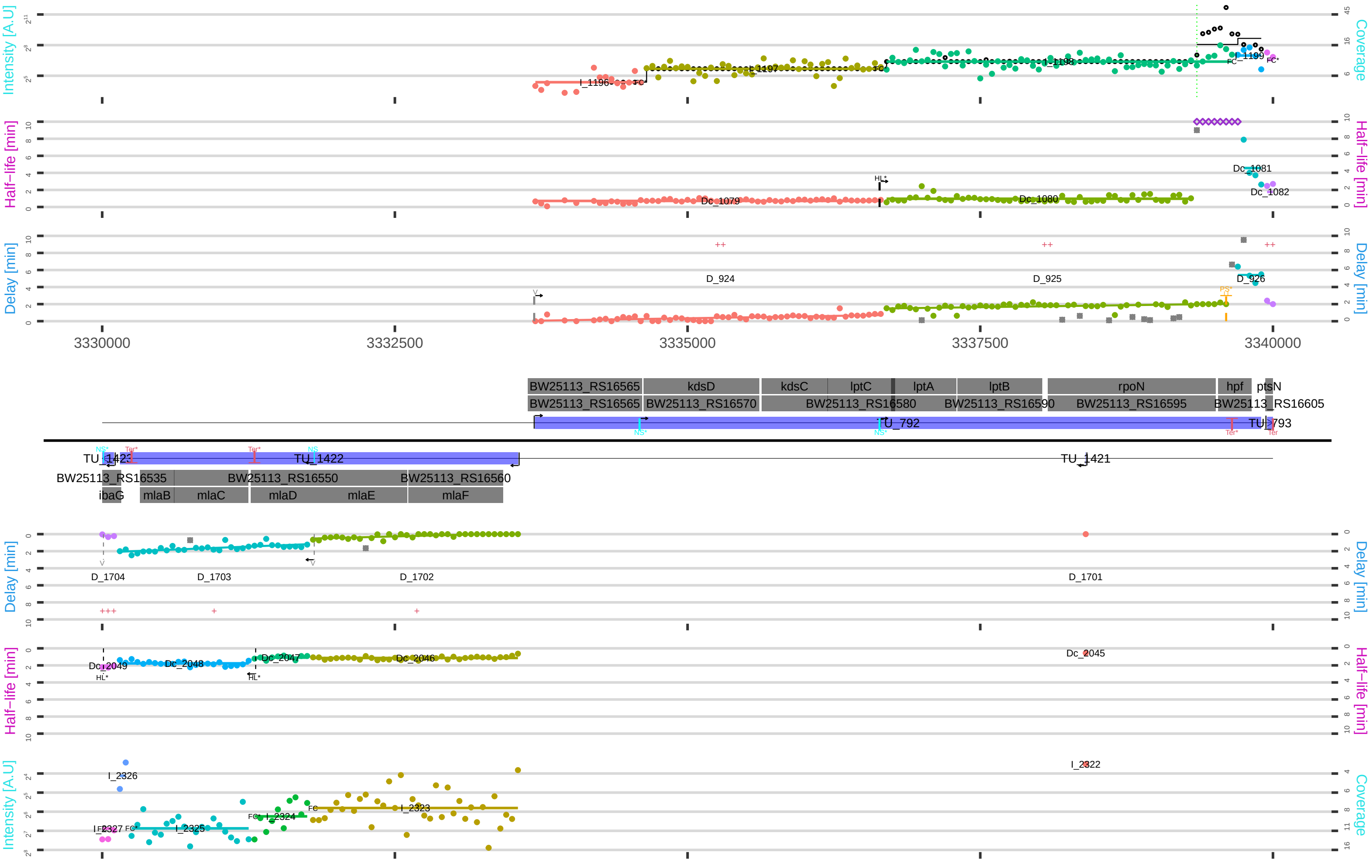

Term: termination (2), NS: new start (2), PS: pausing site (1), iTSS_I: internal starting site (0)

ID: 66800−67000; Term: termination (2), NS: new start (0), PS: pausing site (0), iTSS_I: internal starting site (0)

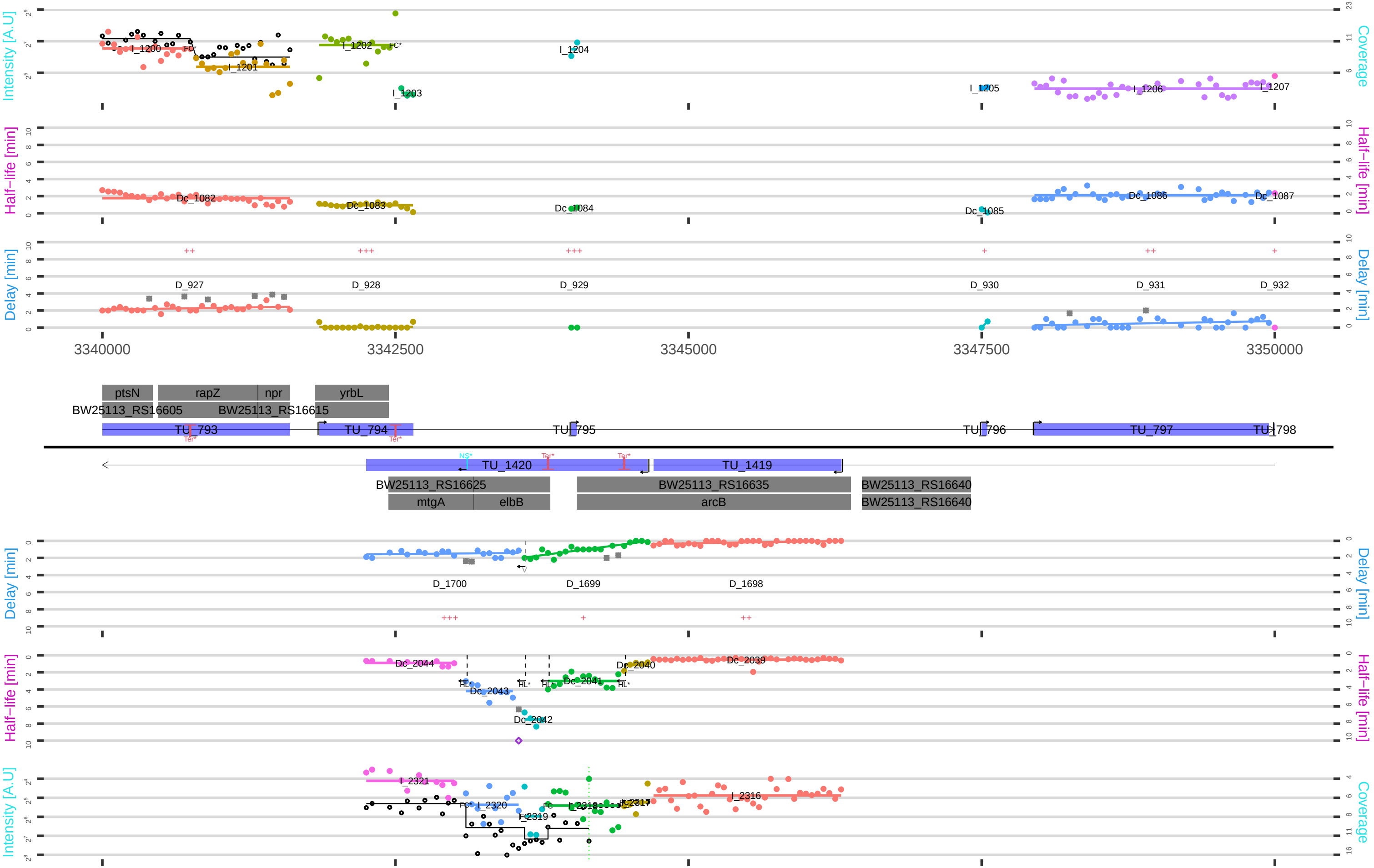

Term: termination (2), NS: new start (1), PS: pausing site (0), iTSS_I: internal starting site (1)

ID: 67000−67107; Term: termination (0), NS: new start (2), PS: pausing site (2), iTSS_I: internal starting site (0)

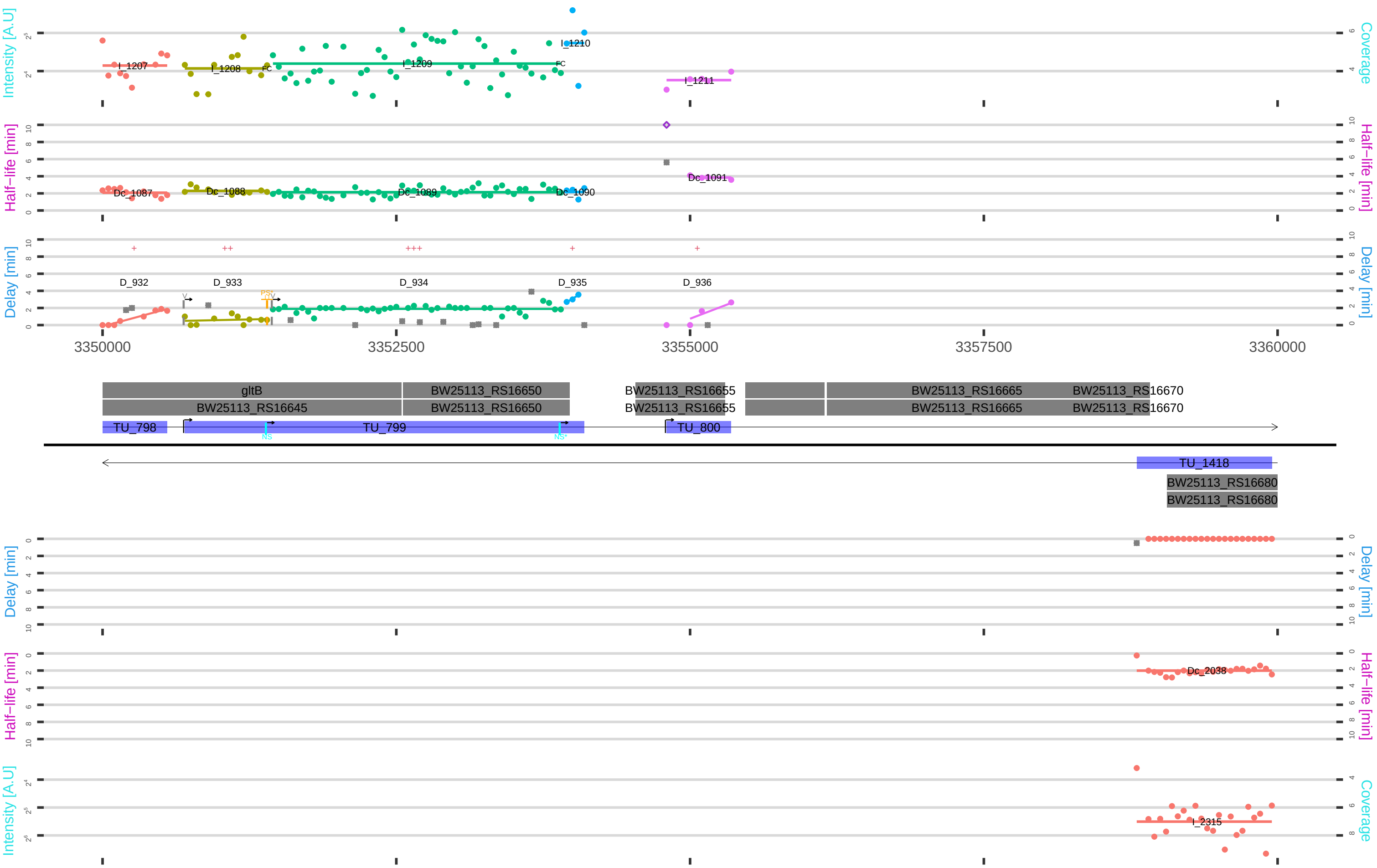

Term: termination (0), NS: new start (0), PS: pausing site (0), iTSS_I: internal starting site (0)

ID: 67210−67210; Term: termination (0), NS: new start (0), PS: pausing site (0), iTSS_I: internal starting site (0)

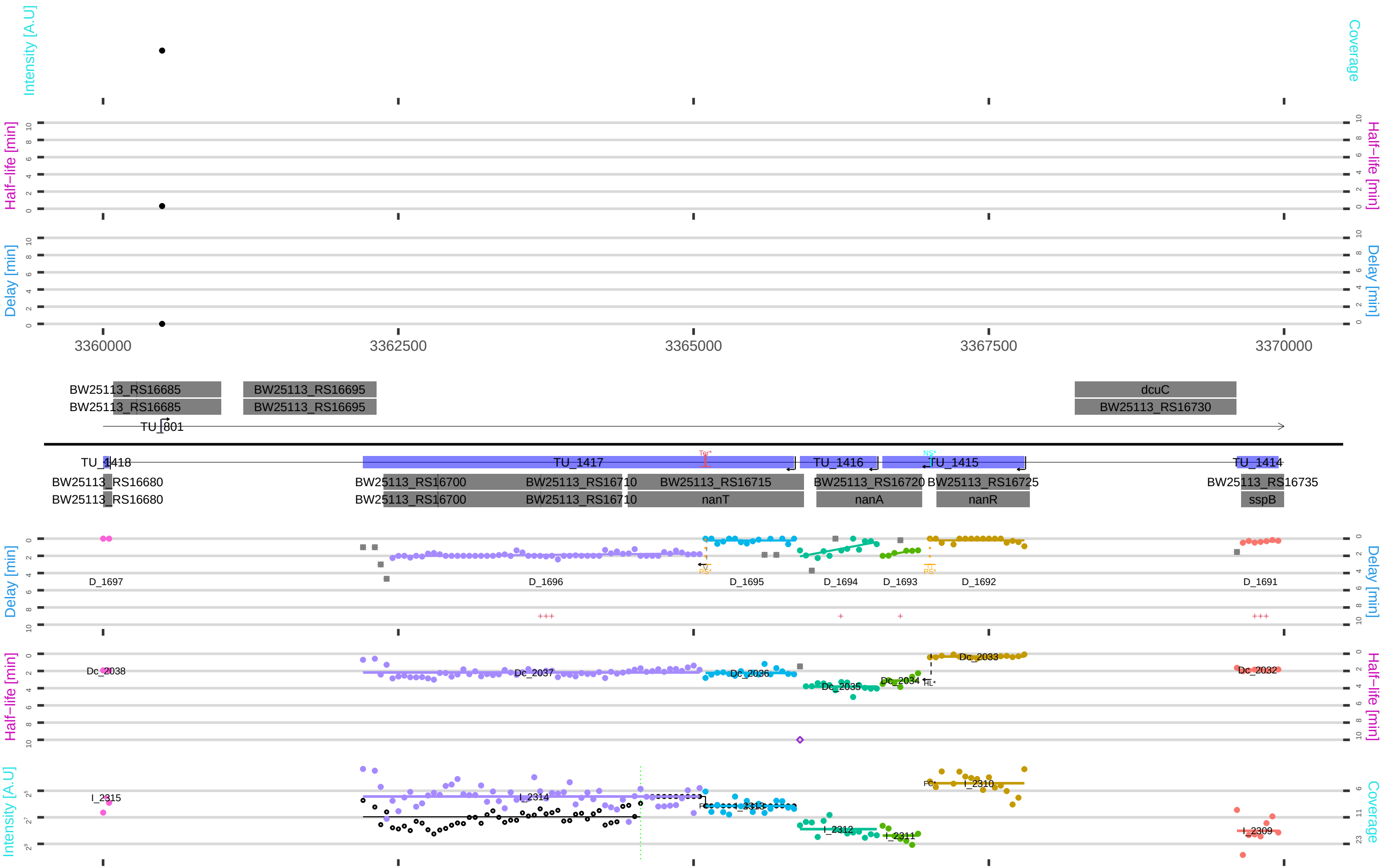

Term: termination (1), NS: new start (1), PS: pausing site (2), iTSS_I: internal starting site (0)

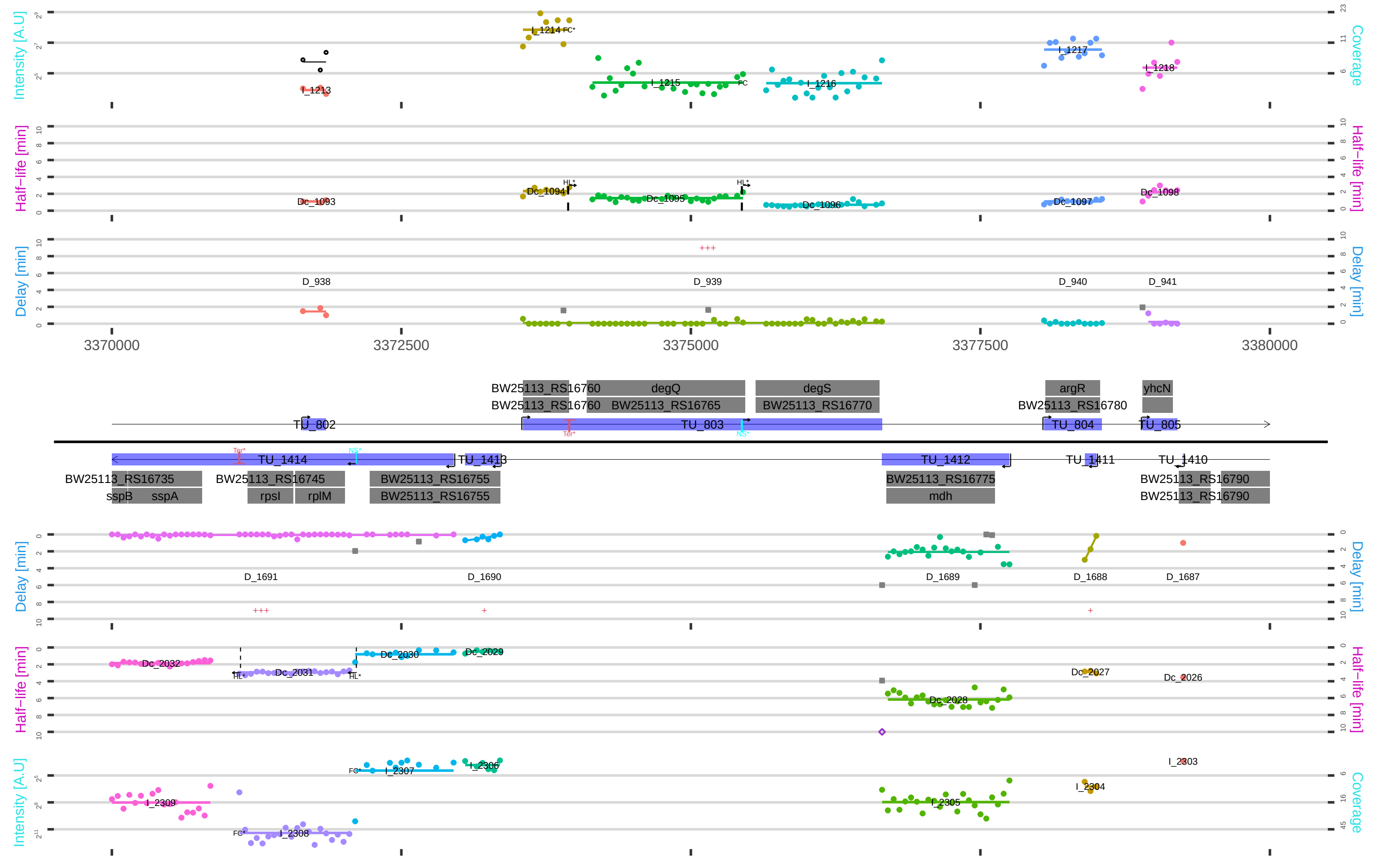

Term: termination (1), NS: new start (1), PS: pausing site (0), iTSS_I: internal starting site (0)

ID: 67649−67759; Term: termination (0), NS: new start (0), PS: pausing site (0), iTSS_I: internal starting site (0)

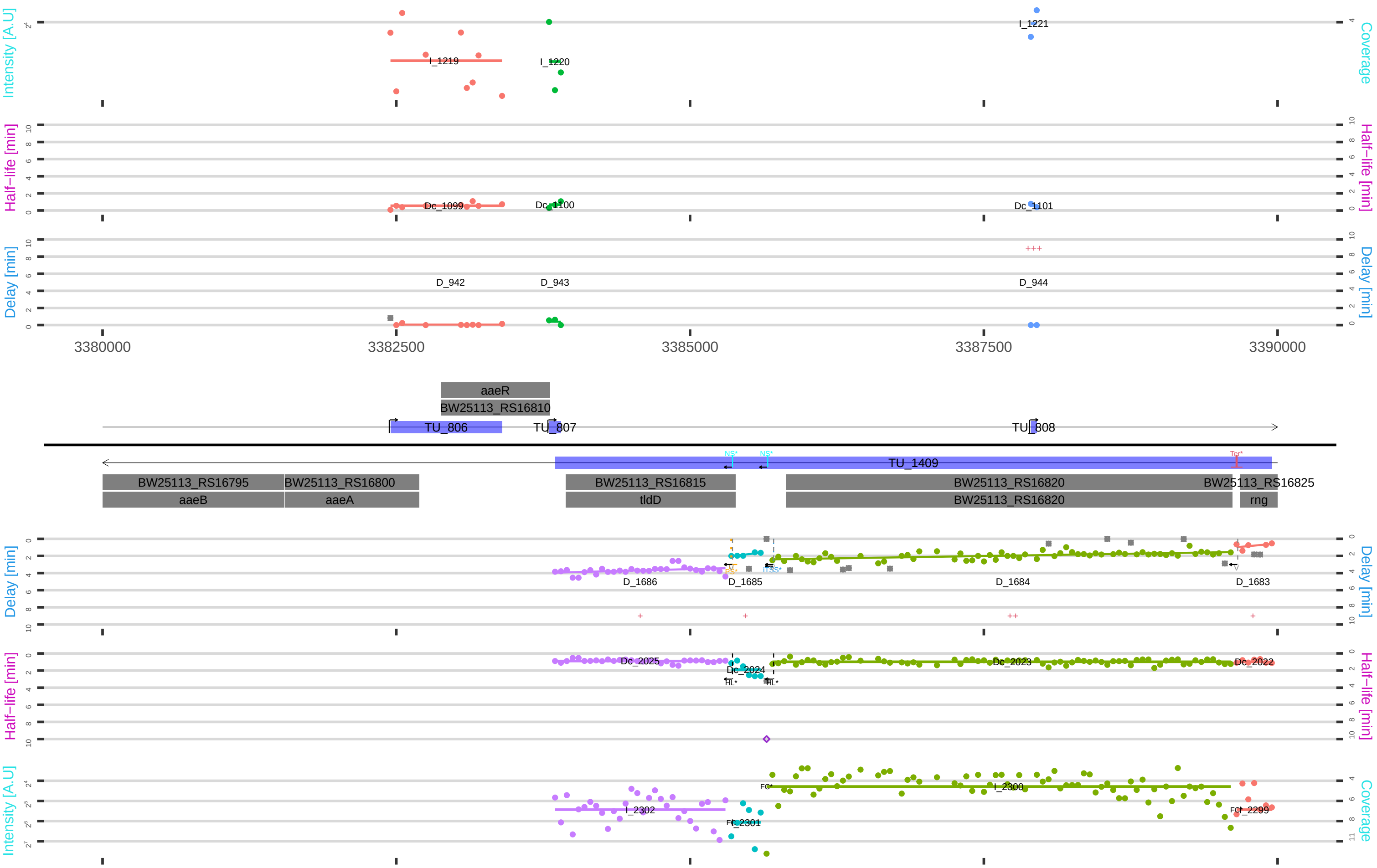

Term: termination (1), NS: new start (2), PS: pausing site (2), iTSS_I: internal starting site (1)

ID: 67937−68000; Term: termination (1), NS: new start (2), PS: pausing site (1), iTSS_I: internal starting site (0)

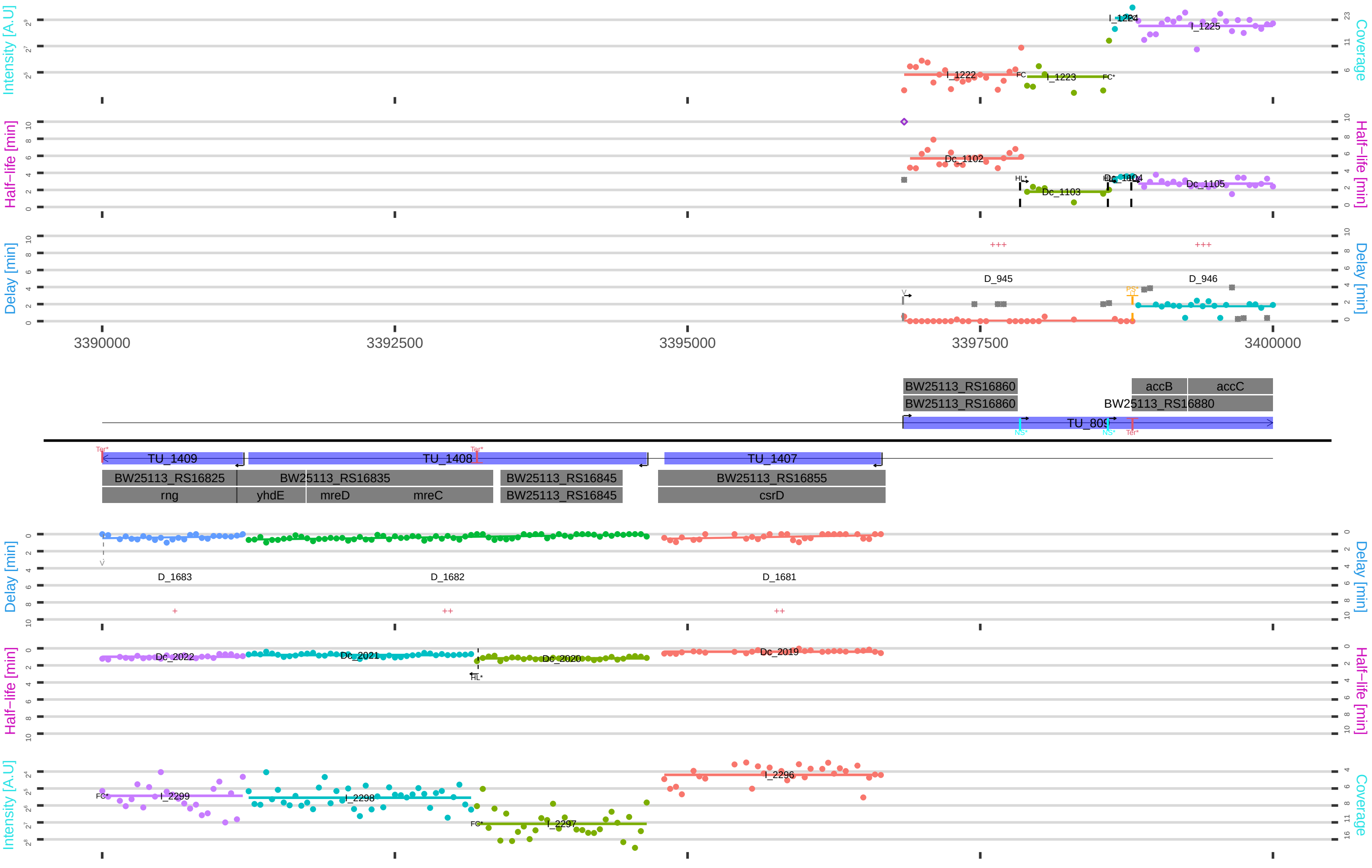

Term: termination (2), NS: new start (0), PS: pausing site (0), iTSS_I: internal starting site (0)

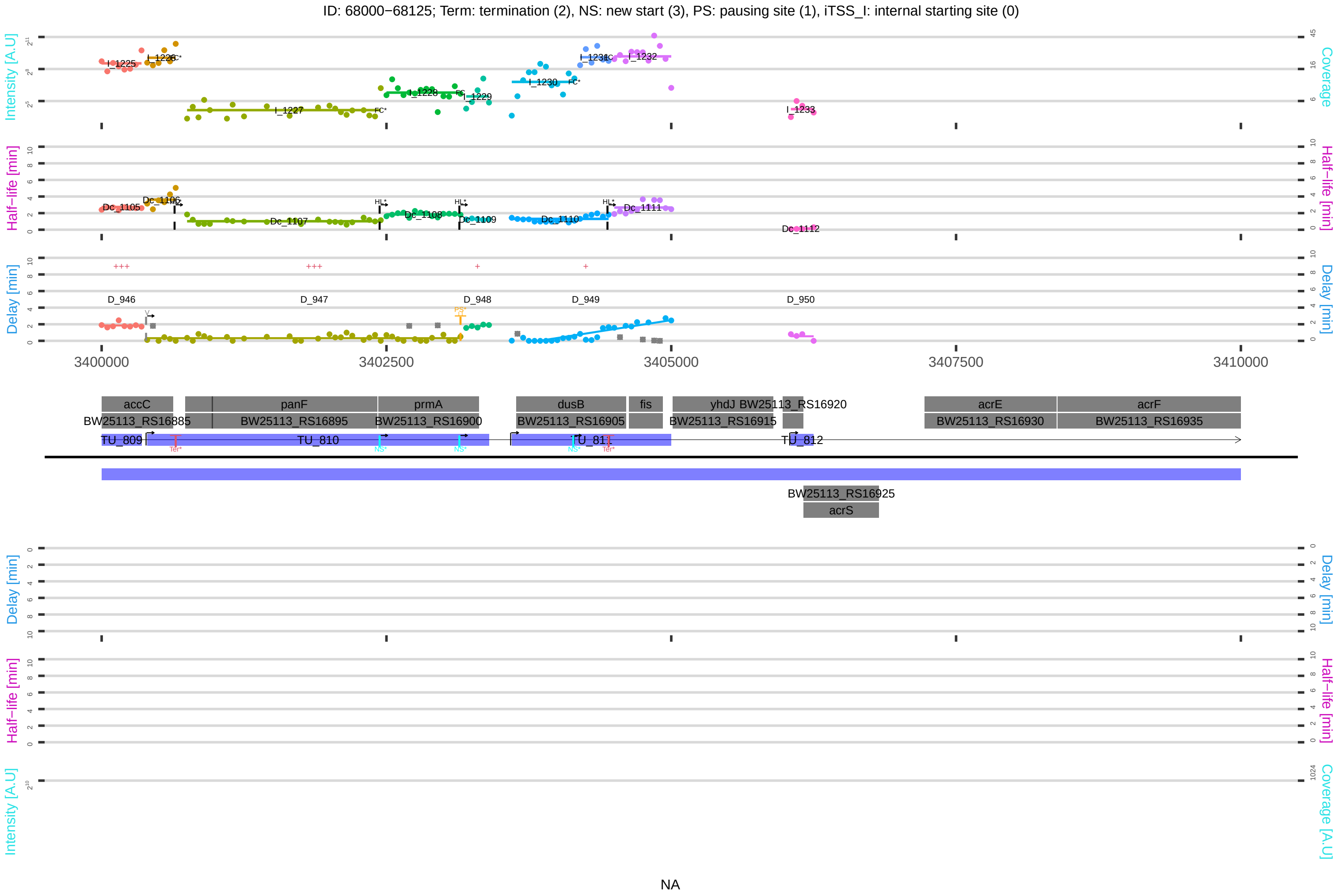

ID: 68000−68125; Term: termination (2), NS: new start (3), PS: pausing site (1), iTSS_I: internal starting site (0)
Intensity [A.U]
Coverage
Half−life [min]
Delay [min]
3400000
3402500
3405000
3407500
3410000
accC
BW25113_RS16885
panF
BW25113_RS16895
prmA
BW25113_RS16900
dusB
BW25113_RS16905
fis
yhdJ BW25113_RS16920
BW25113_RS16915
acrE
BW25113_RS16930
acrF
BW25113_RS16935
TU_809
TU_810
TU_811
TU_812
BW25113_RS16925
acrS
Coverage [A.U]
NA

ID: 68230−68400; Term: termination (1), NS: new start (1), PS: pausing site (0), iTSS_I: internal starting site (0)

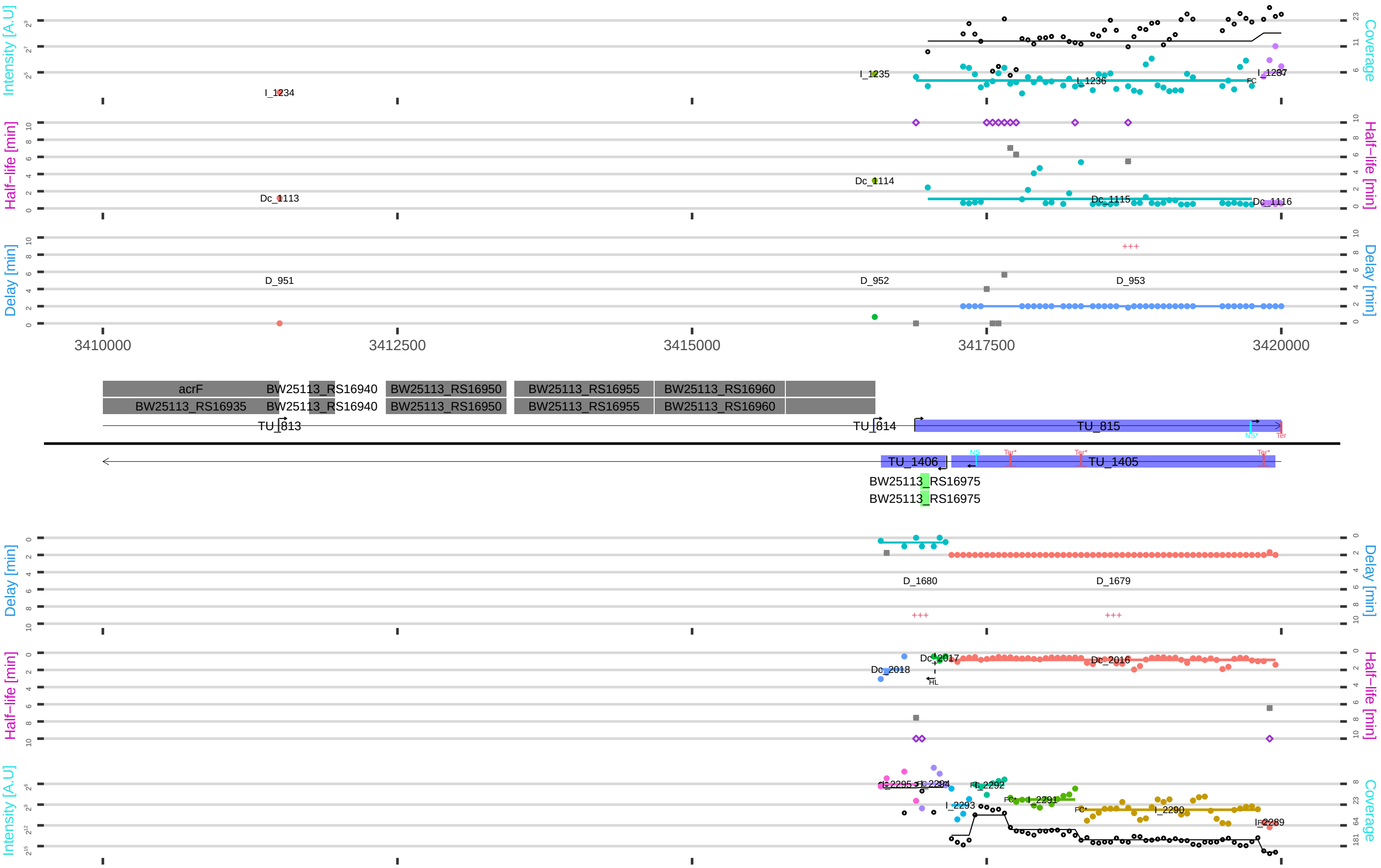

Term: termination (3), NS: new start (1), PS: pausing site (0), iTSS_I: internal starting site (0)

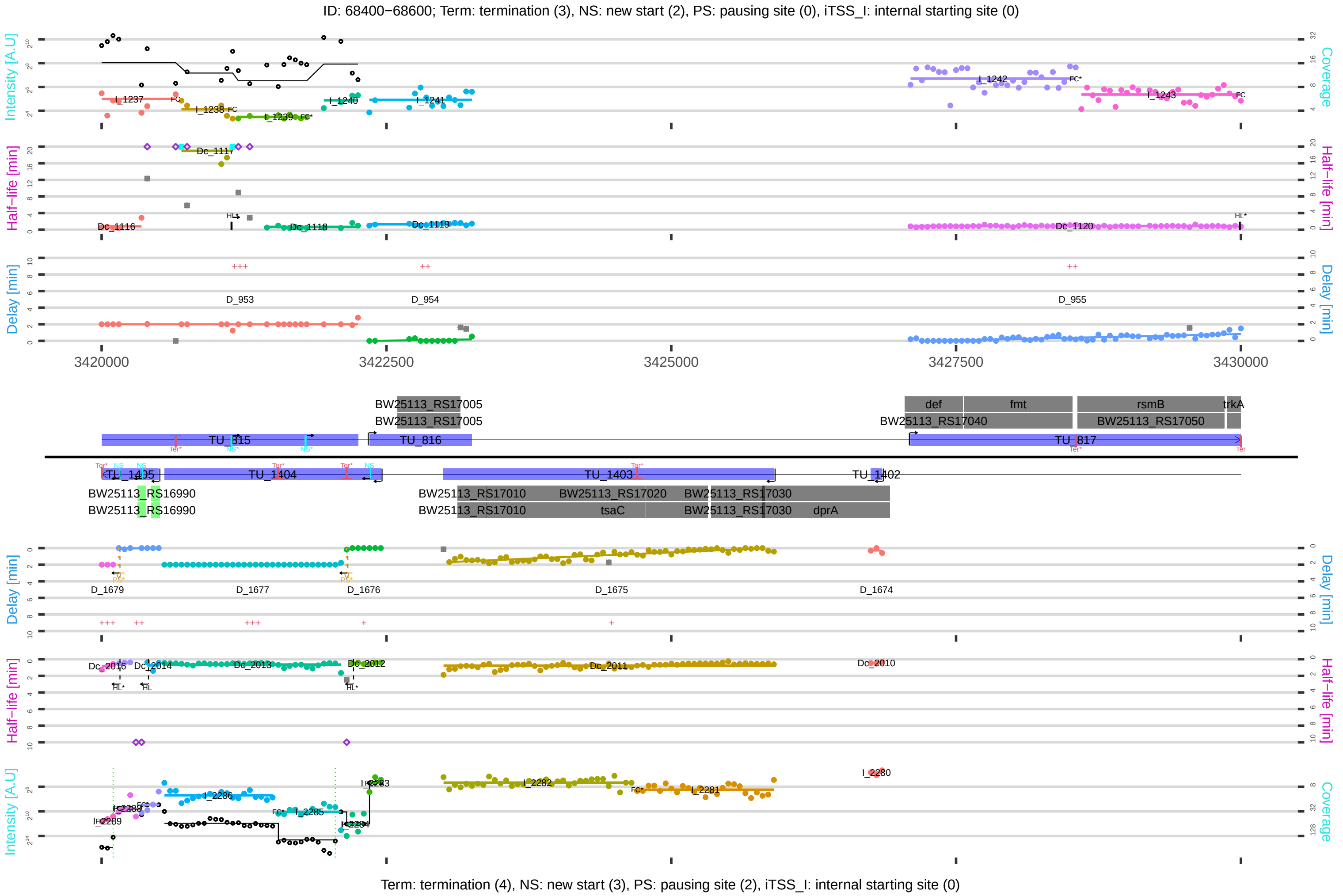

ID: 68400−68600; Term: termination (3), NS: new start (2), PS: pausing site (0), iTSS_I: internal starting site (0)
Intensity [A.U]
Coverage
Half−life [min]
Delay [min]
3420000
3422500
3425000
3427500
3430000
I_1237
I_1238
I_1239
I_1240
I_1241
I_1242
I_1243
Dc_1116
Dc_1117
Dc_1118
Dc_1119
Dc_1120
D_953
D_954
D_955
BW25113_RS17005
def
fmt
rsmB
trkA
BW25113_RS17040
BW25113_RS17050
TU_815
TU_816
TU_817
TU_1405
TU_1404
TU_1403
TU_1402
BW25113_RS16990
BW25113_RS17010
BW25113_RS17020
BW25113_RS17030
tsaC
dprA
D_1679
D_1677
D_1676
D_1675
D_1674
Dc_2016
Dc_2014
Dc_2013
Dc_2012
Dc_2011
Dc_2010
I_2289
I_2288
I_2286
I_2285
I_2284
I_2283
I_2282
I_2281
I_2280
Term: termination (4), NS: new start (3), PS: pausing site (2), iTSS_I: internal starting site (0)

ID: 68600−68800; Term: termination (3), NS: new start (2), PS: pausing site (1), iTSS_I: internal starting site (3)

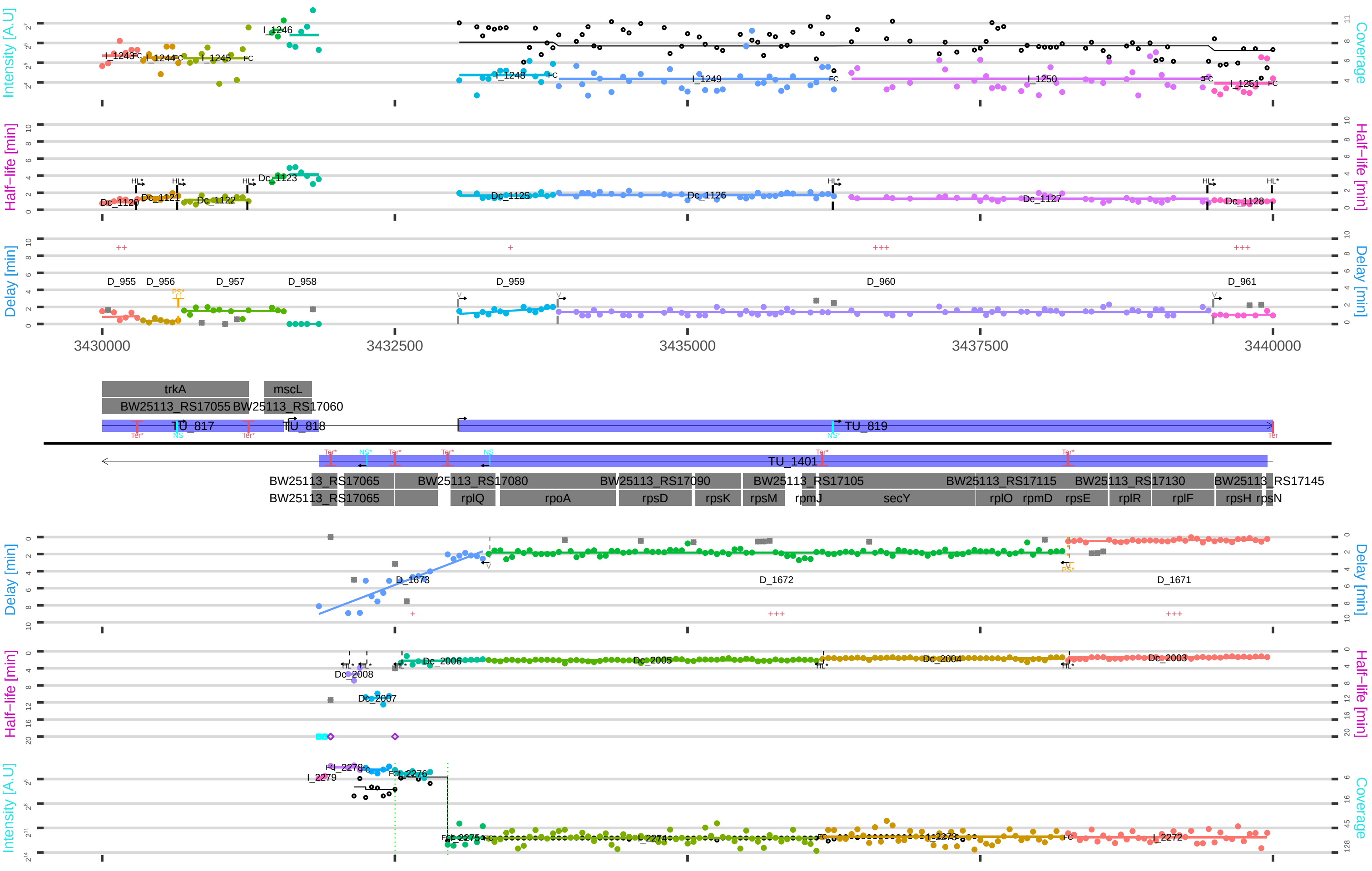

Term: termination (5), NS: new start (2), PS: pausing site (1), iTSS_I: internal starting site (1)

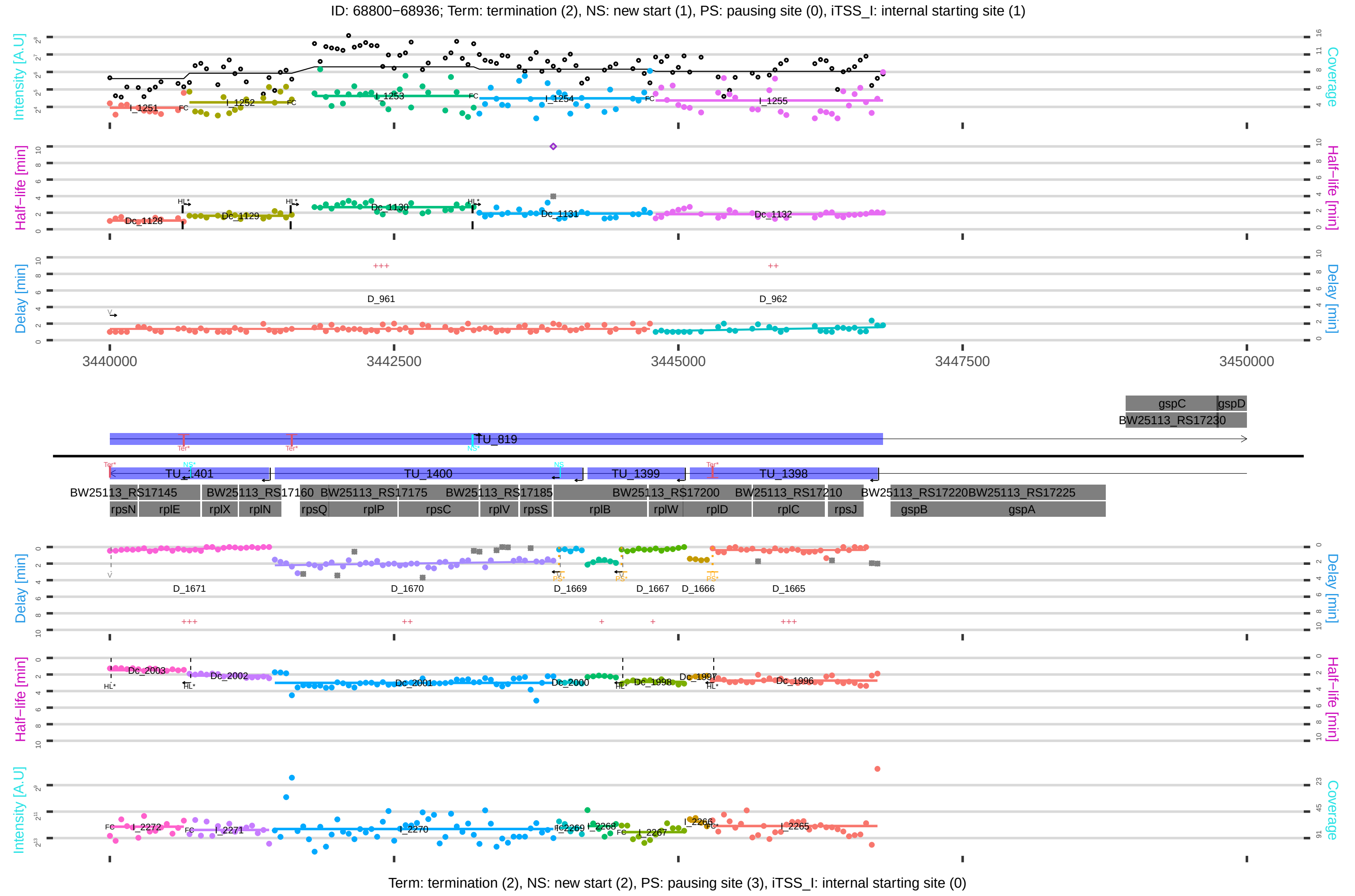
ID: 68800−68936; Term: termination (2), NS: new start (1), PS: pausing site (0), iTSS_I: internal starting site (1)
Intensity [A.U]
Coverage
Half−life [min]
Delay [min]
I_1251
I_1252
I_1253
I_1254
I_1255
Dc_1128
Dc_1129
Dc_1130
Dc_1131
Dc_1132
D_961
D_962
3440000
3442500
3445000
3447500
3450000
gspC
gspD
BW25113_RS17230
TU_819
TU_1401
TU_1400
TU_1399
TU_1398
BW25113_RS17145
BW25113_RS17160
BW25113_RS17175
BW25113_RS17185
BW25113_RS17200
BW25113_RS17210
BW25113_RS17220
BW25113_RS17225
rpsN
rplE
rplX
rplN
rpsQ
rplP
rpsC
rplV
rpsS
rplB
rplW
rplD
rplC
rpsJ
gspB
gspA
D_1671
D_1670
D_1669
D_1667
D_1666
D_1665
Dc_2003
Dc_2002
Dc_2001
Dc_2000
Dc_1998
Dc_1997
Dc_1996
I_2272
I_2271
I_2270
I_2269
I_2268
I_2267
I_2266
I_2265
Term: termination (2), NS: new start (2), PS: pausing site (3), iTSS_I: internal starting site (0)

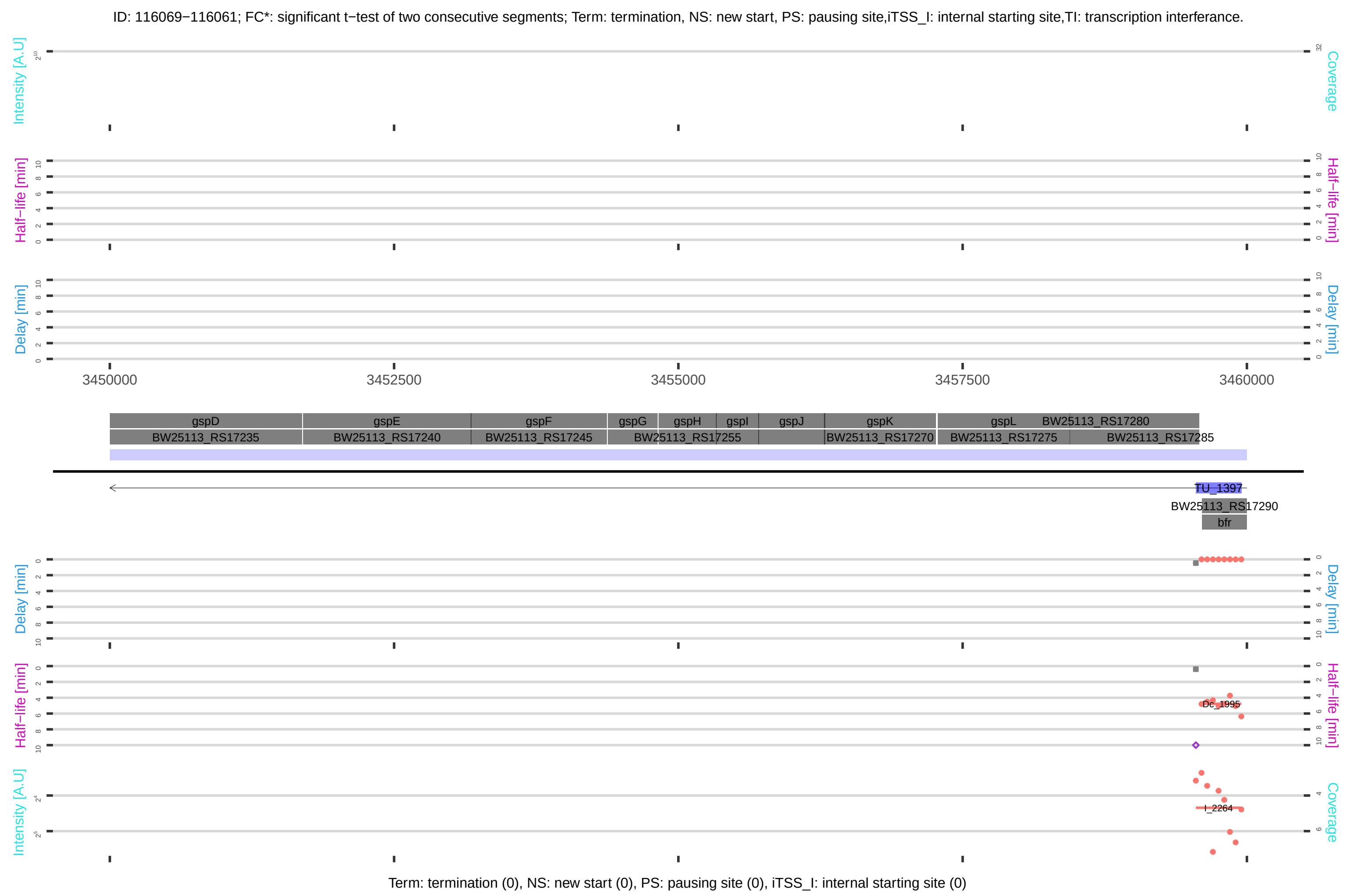
ID: 116069−116061; FC*: significant t−test of two consecutive segments; Term: termination, NS: new start, PS: pausing site,iTSS_I: internal starting site,TI: transcription interferance.
Intensity [A.U]
Coverage
Half−life [min]
Delay [min]
3450000
3452500
3455000
3457500
3460000
gspD
gspE
gspF
gspG
gspH
gspI
gspJ
gspK
gspL
BW25113_RS17280
BW25113_RS17235
BW25113_RS17240
BW25113_RS17245
BW25113_RS17255
BW25113_RS17270
BW25113_RS17275
BW25113_RS17285
TU_1397
BW25113_RS17290
bfr
Dc_1995
I_2264
Term: termination (0), NS: new start (0), PS: pausing site (0), iTSS_I: internal starting site (0)

ID: 69273−69359; Term: termination (0), NS: new start (0), PS: pausing site (0), iTSS_I: internal starting site (0)

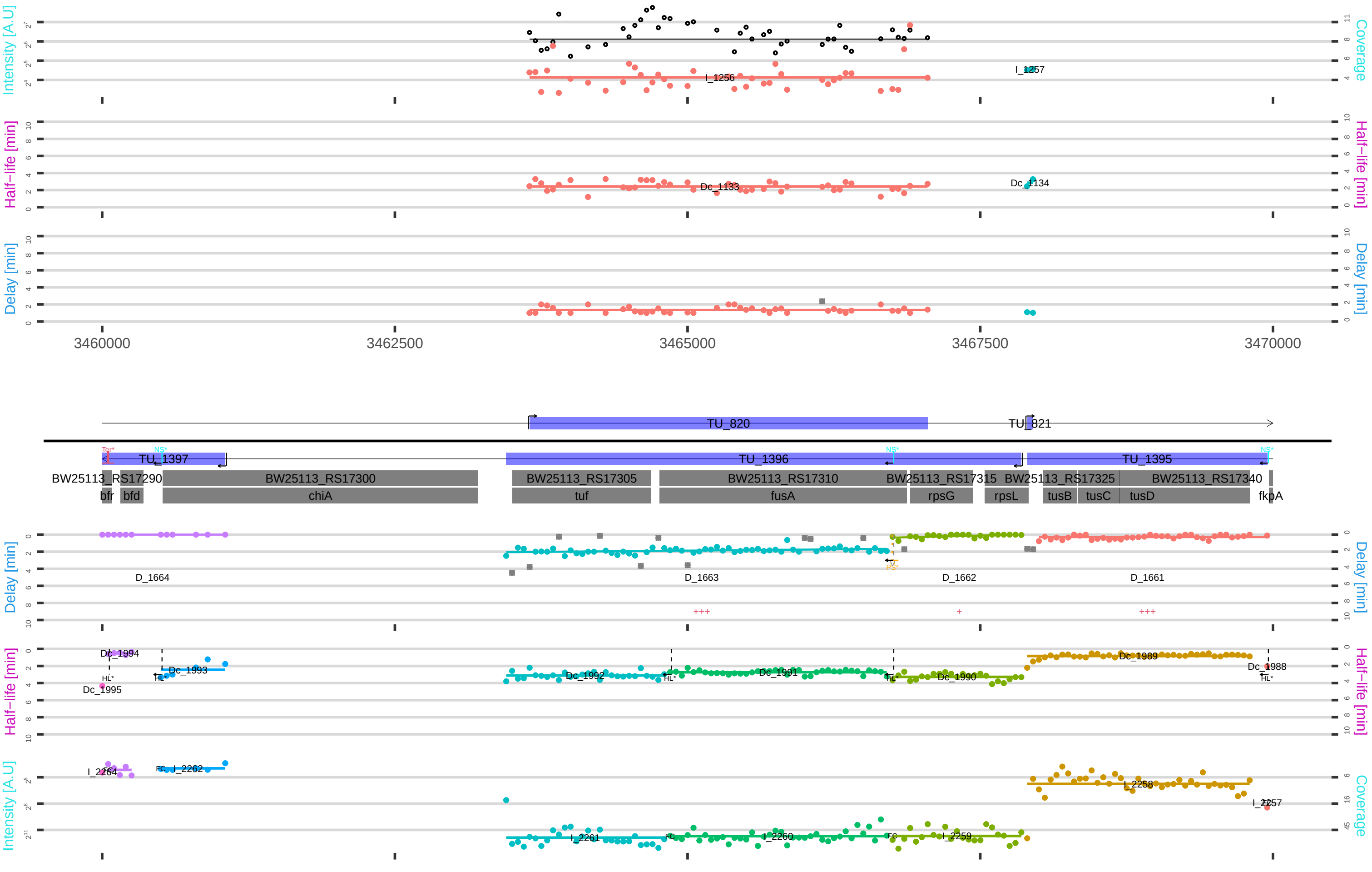

Term: termination (1), NS: new start (3), PS: pausing site (1), iTSS_I: internal starting site (0)

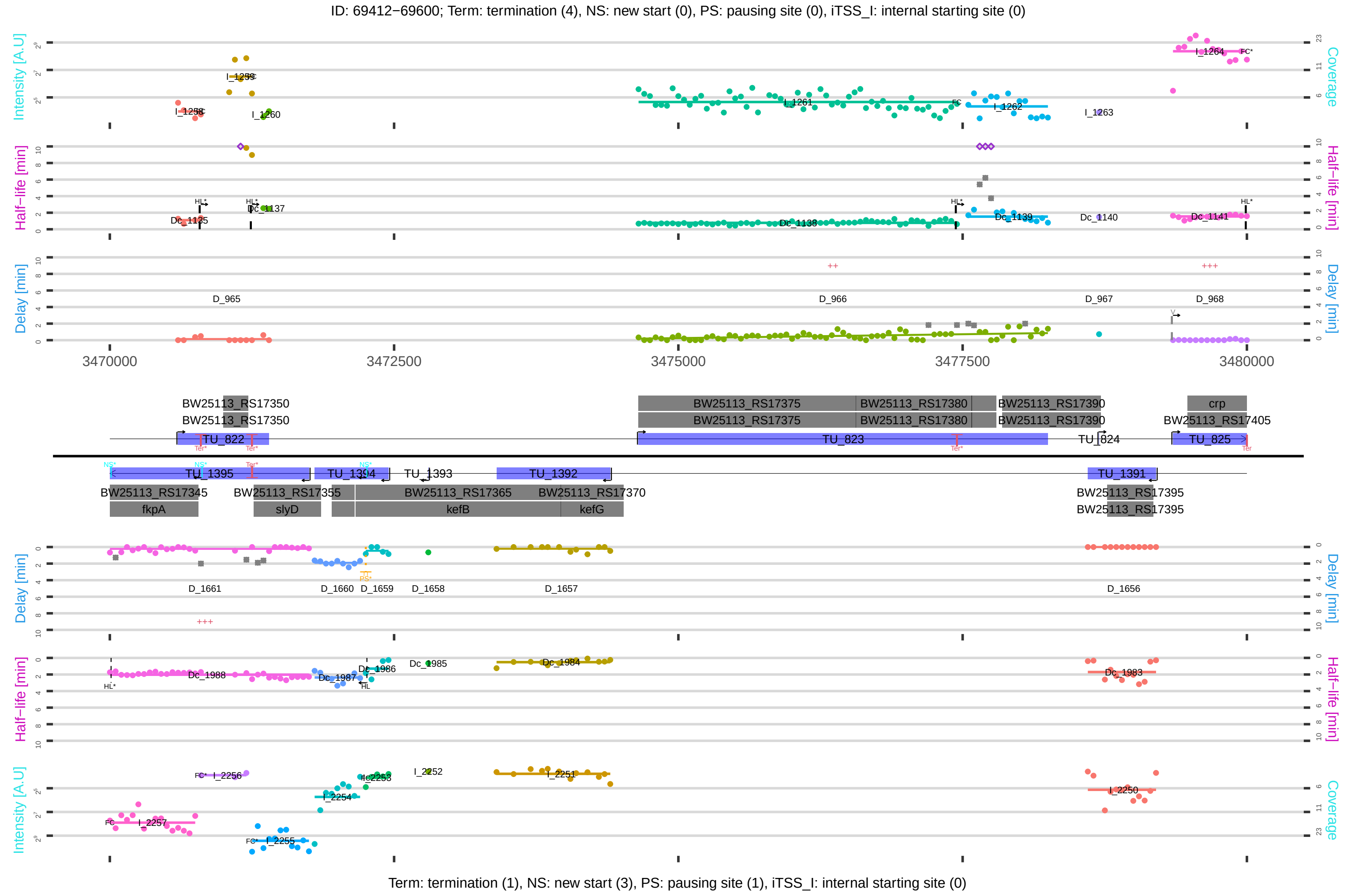

ID: 69412−69600; Term: termination (4), NS: new start (0), PS: pausing site (0), iTSS_I: internal starting site (0)
Intensity [A.U]
Coverage
Half−life [min]
Delay [min]
I_1258
I_1259
I_1260
I_1261
I_1262
I_1263
I_1264
Dc_1135
Dc_1137
Dc_1138
Dc_1139
Dc_1140
Dc_1141
D_965
D_966
D_967
D_968
3470000
3472500
3475000
3477500
3480000
BW25113_RS17350
BW25113_RS17375
BW25113_RS17380
BW25113_RS17390
crp
BW25113_RS17405
TU_822
TU_823
TU_824
TU_825
TU_1395
TU_1394
TU_1393
TU_1392
TU_1391
BW25113_RS17345
BW25113_RS17355
BW25113_RS17365
BW25113_RS17370
BW25113_RS17395
fkpA
slyD
kefB
kefG
D_1661
D_1660
D_1659
D_1658
D_1657
D_1656
Dc_1988
Dc_1987
Dc_1986
Dc_1985
Dc_1984
Dc_1983
I_2257
I_2256
I_2255
I_2254
I_2253
I_2252
I_2251
I_2250
Term: termination (1), NS: new start (3), PS: pausing site (1), iTSS_I: internal starting site (0)

ID: 69600−69800; Term: termination (7), NS: new start (1), PS: pausing site (3), iTSS_I: internal starting site (0)

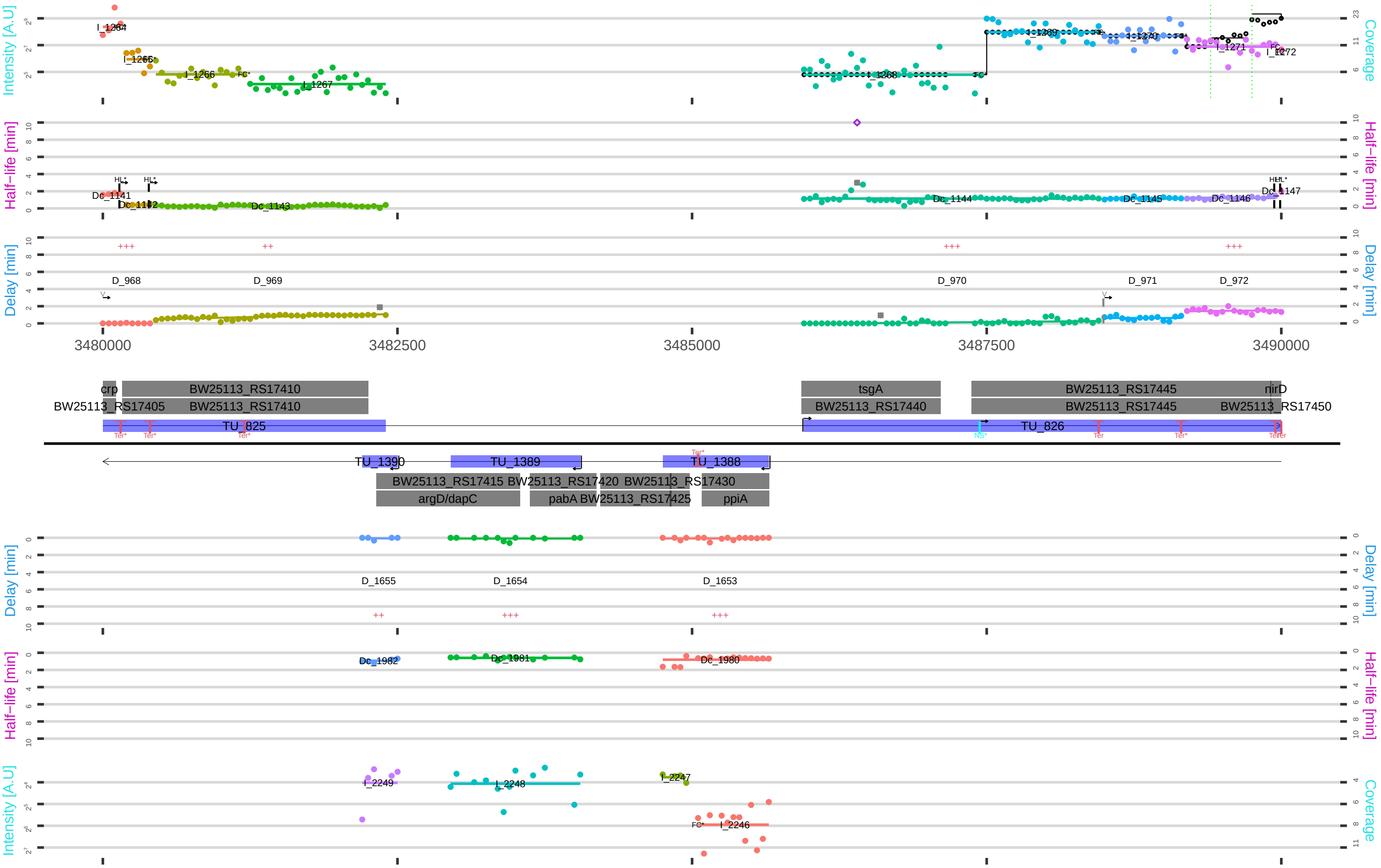

Term: termination (1), NS: new start (0), PS: pausing site (0), iTSS_I: internal starting site (0)

ID: 69800−69963; Term: termination (2), NS: new start (1), PS: pausing site (1), iTSS_I: internal starting site (0)

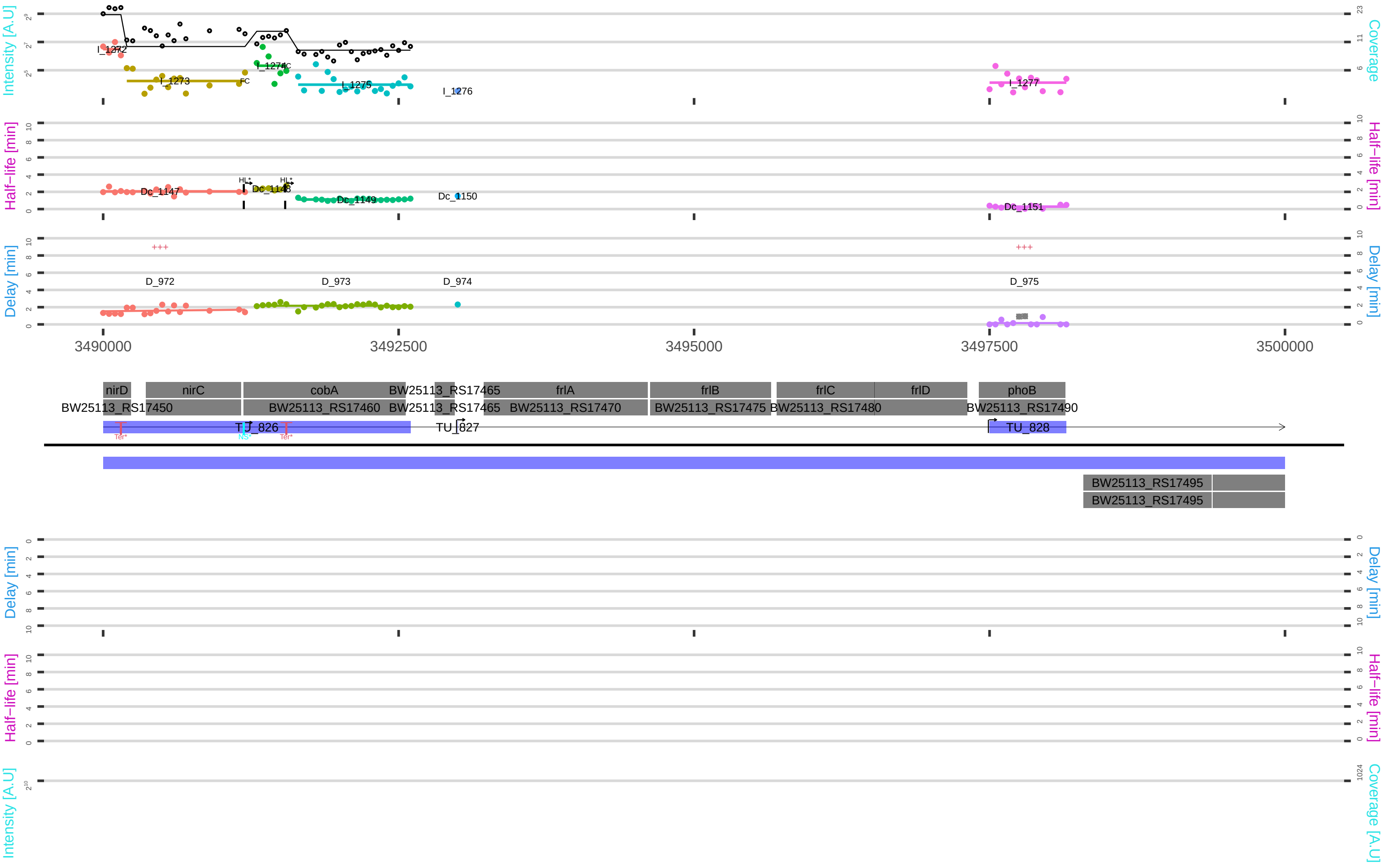

NA

ID: 115172−115061; FC*: significant t−test of two consecutive segments; Term: termination, NS: new start, PS: pausing site,iTSS_I: internal starting site,TI: transcription interferance.

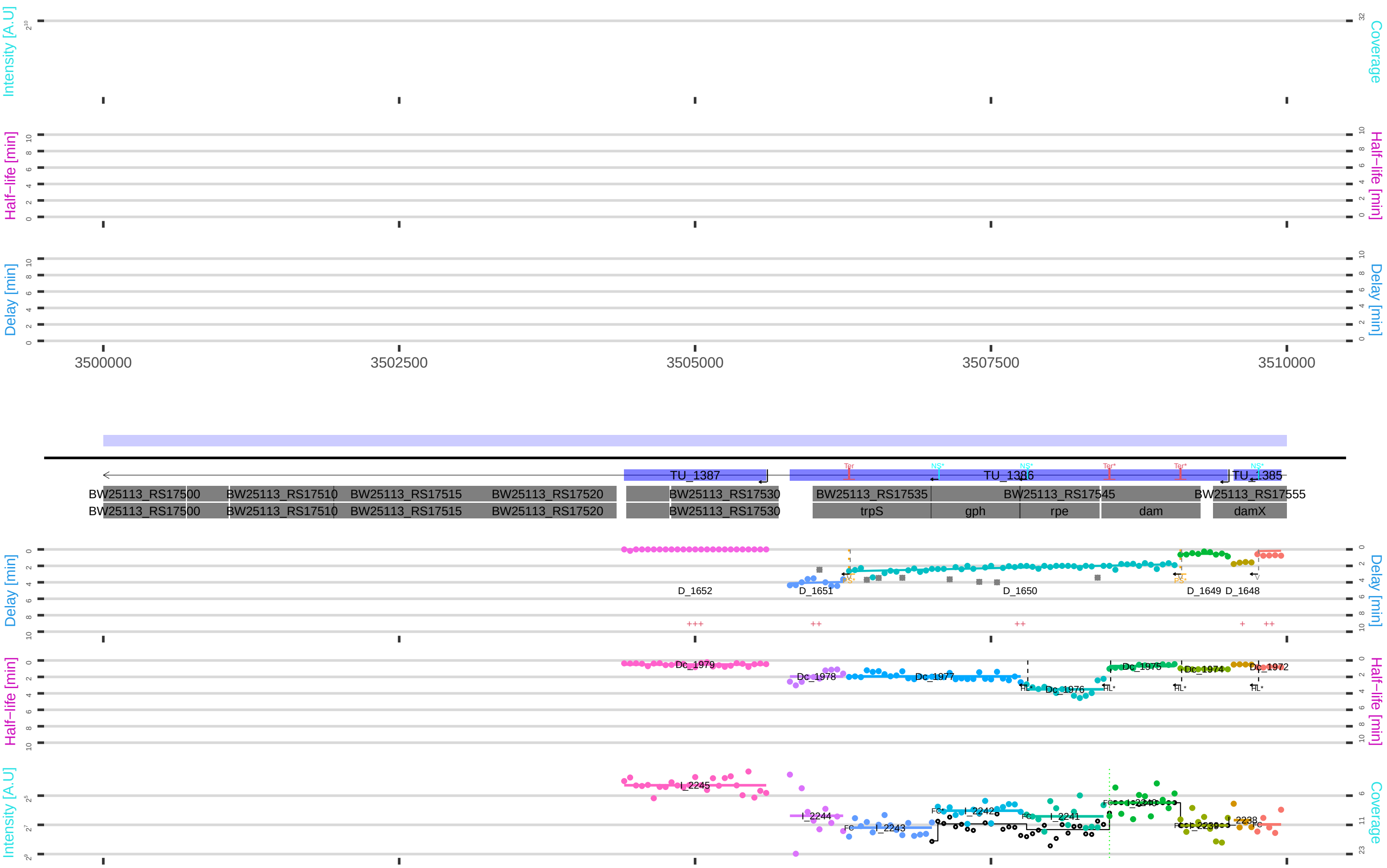

Term: termination (3), NS: new start (3), PS: pausing site (3), iTSS_I: internal starting site (0)

ID: 70326−70400; Term: termination (1), NS: new start (0), PS: pausing site (0), iTSS_I: internal starting site (0)

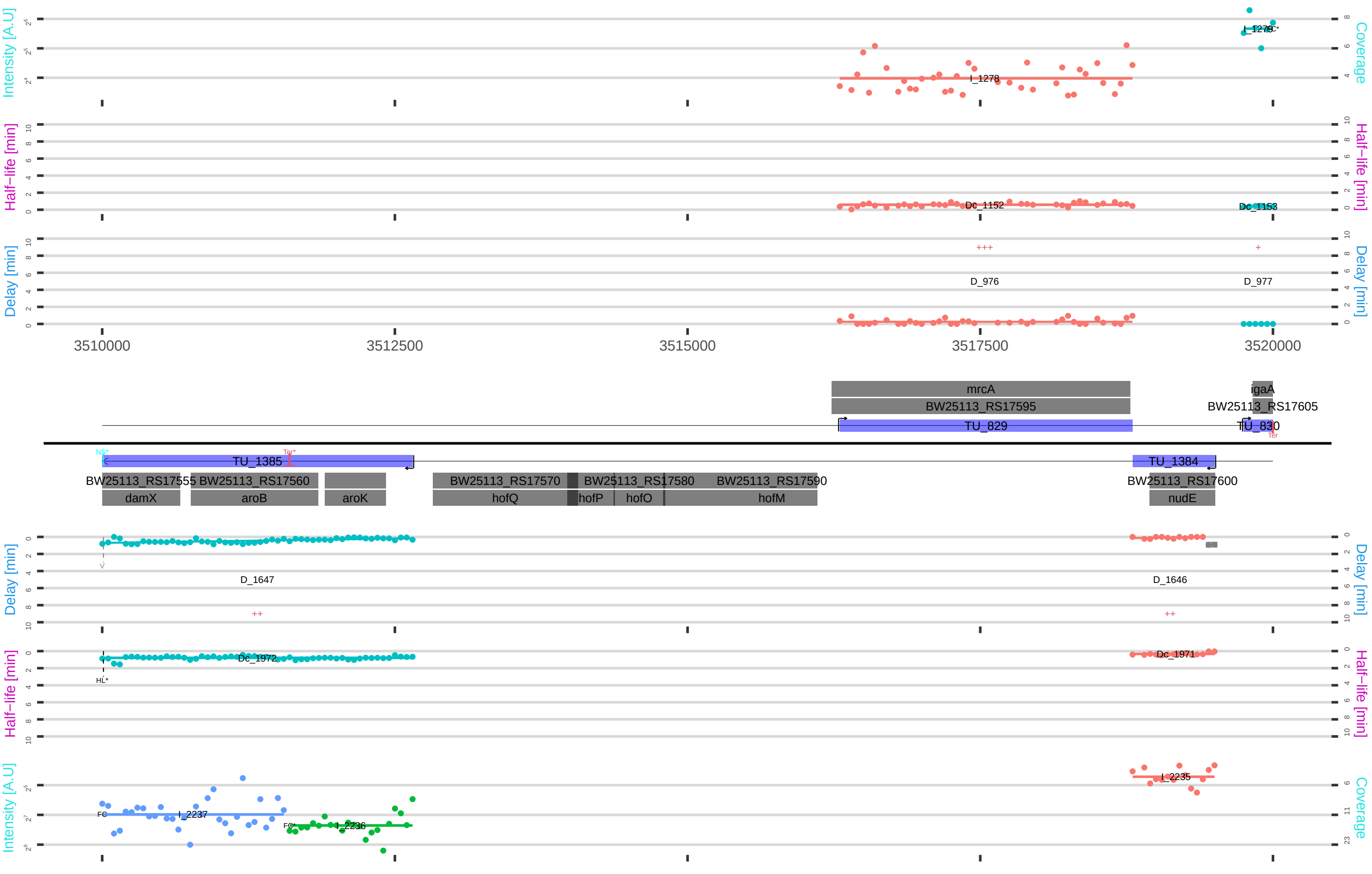

Term: termination (1), NS: new start (1), PS: pausing site (0), iTSS_I: internal starting site (0)

ID: 70400−70600; Term: termination (3), NS: new start (1), PS: pausing site (1), iTSS_I: internal starting site (0)

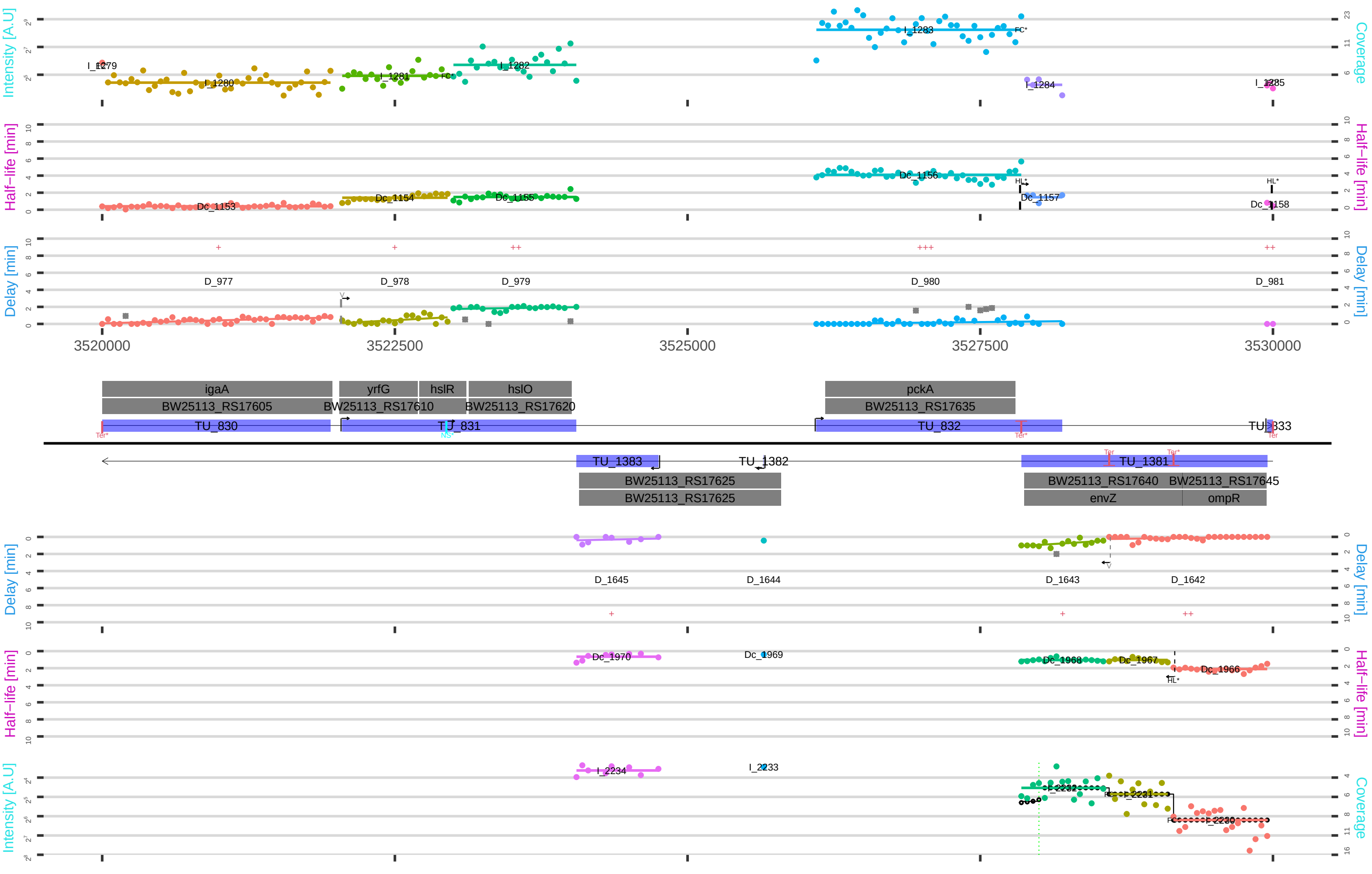

Term: termination (2), NS: new start (0), PS: pausing site (1), iTSS_I: internal starting site (0)

ID: 70600−70800; Term: termination (3), NS: new start (0), PS: pausing site (1), iTSS_I: internal starting site (0)

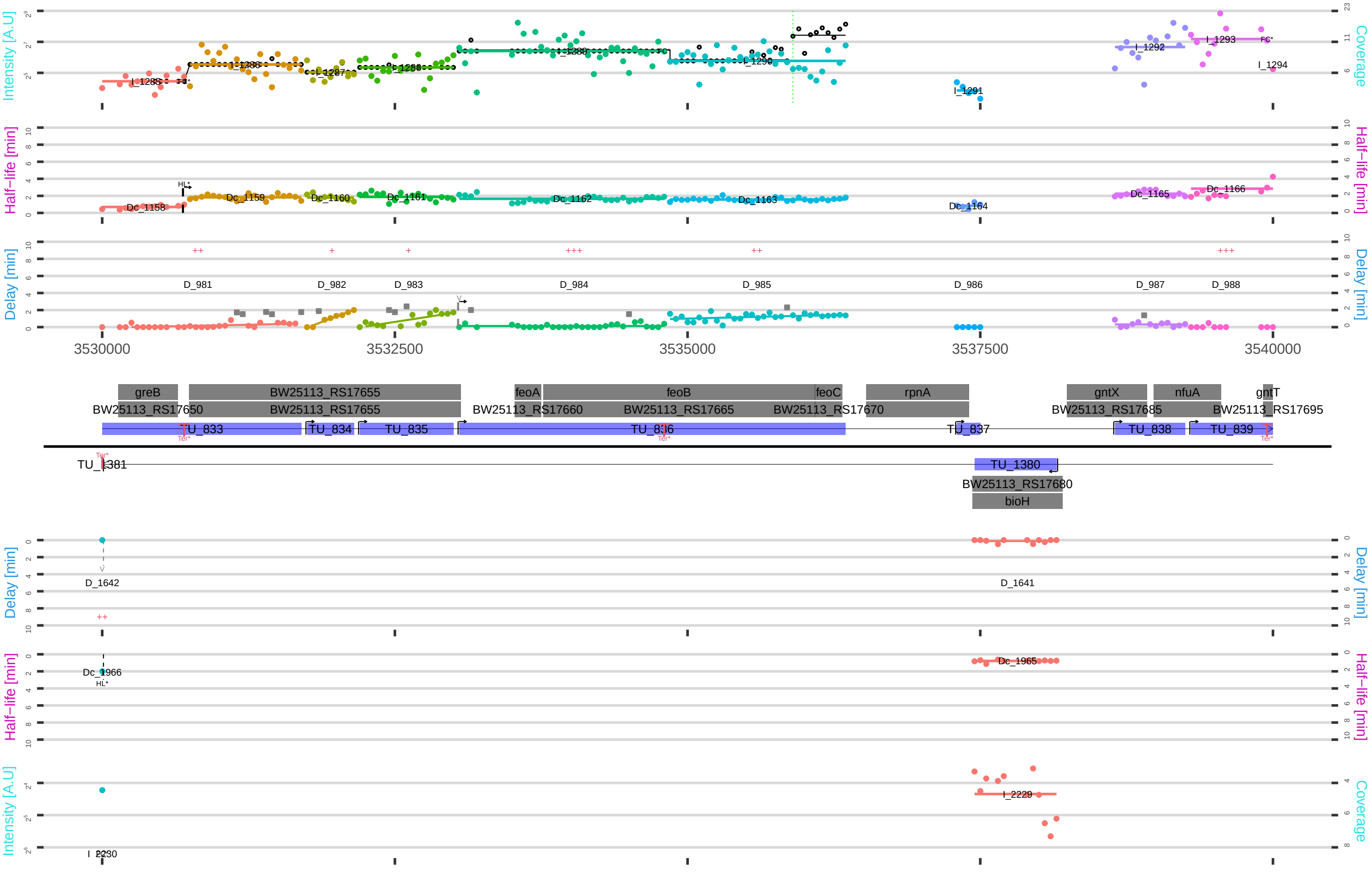

Term: termination (1), NS: new start (0), PS: pausing site (0), iTSS_I: internal starting site (0)

ID: 70800−70986; Term: termination (1), NS: new start (0), PS: pausing site (0), iTSS_I: internal starting site (0)

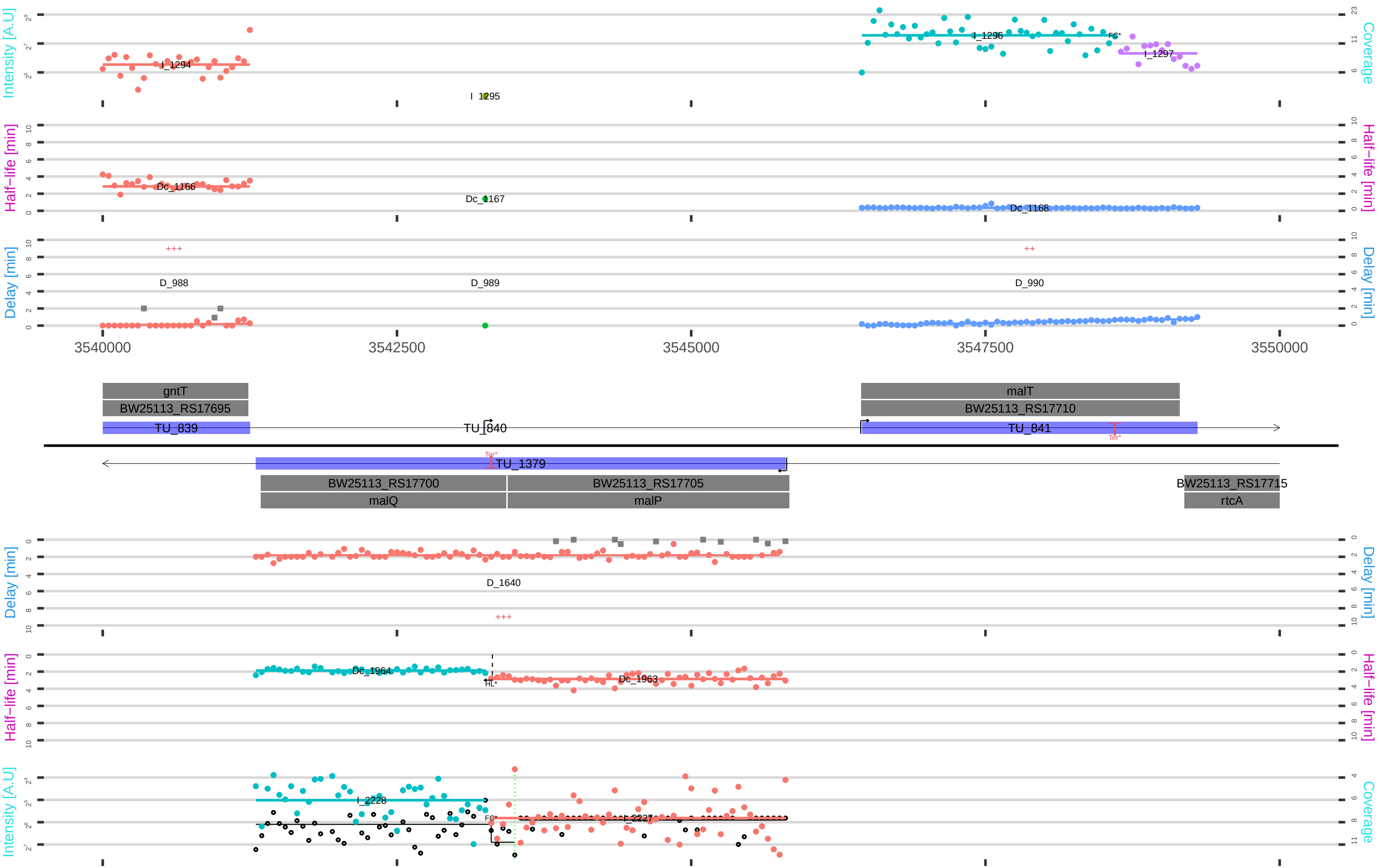

Term: termination (1), NS: new start (0), PS: pausing site (0), iTSS_I: internal starting site (0)

ID: 71108−71149; Term: termination (1), NS: new start (0), PS: pausing site (0), iTSS_I: internal starting site (0)

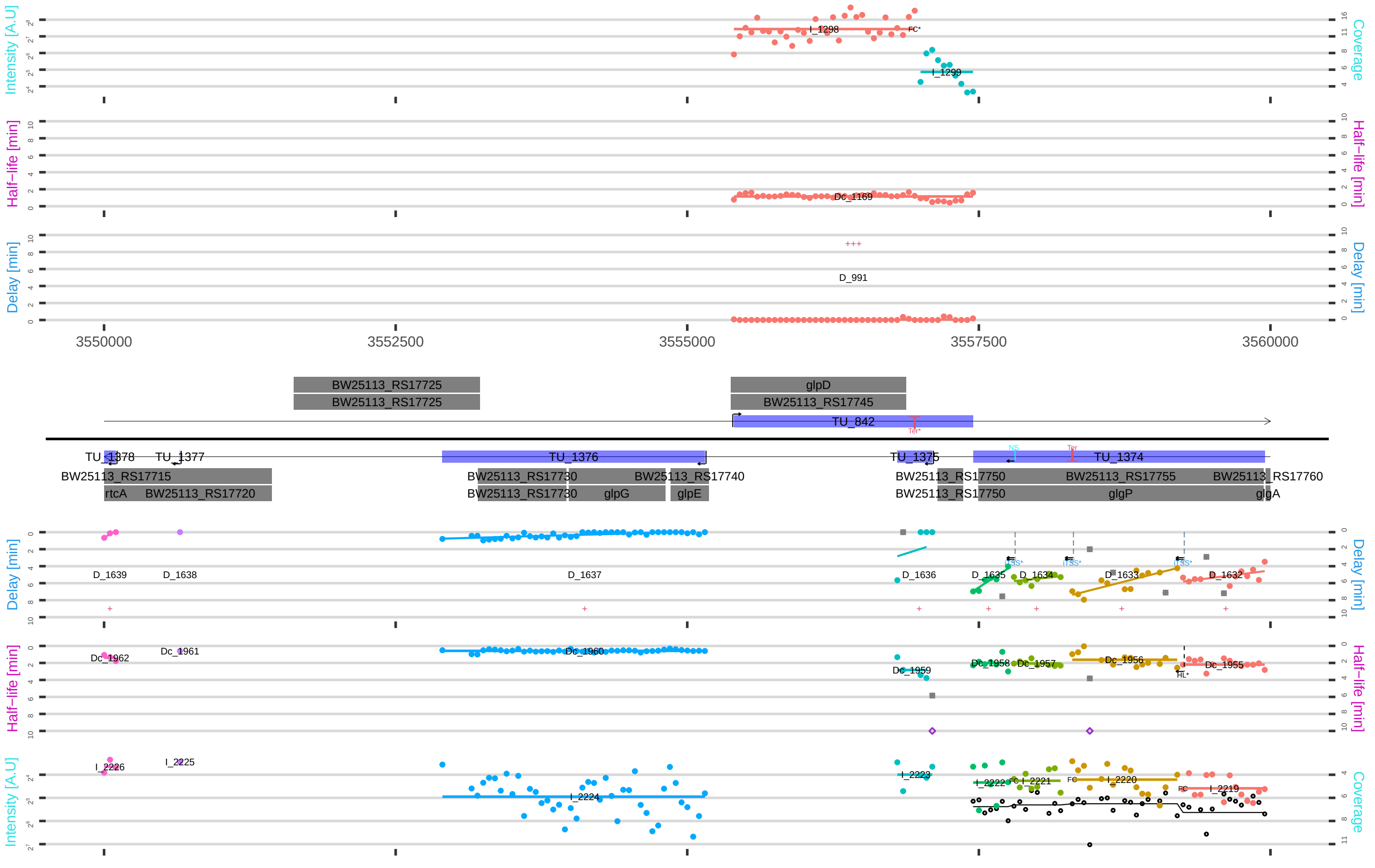

Term: termination (1), NS: new start (1), PS: pausing site (0), iTSS_I: internal starting site (3)

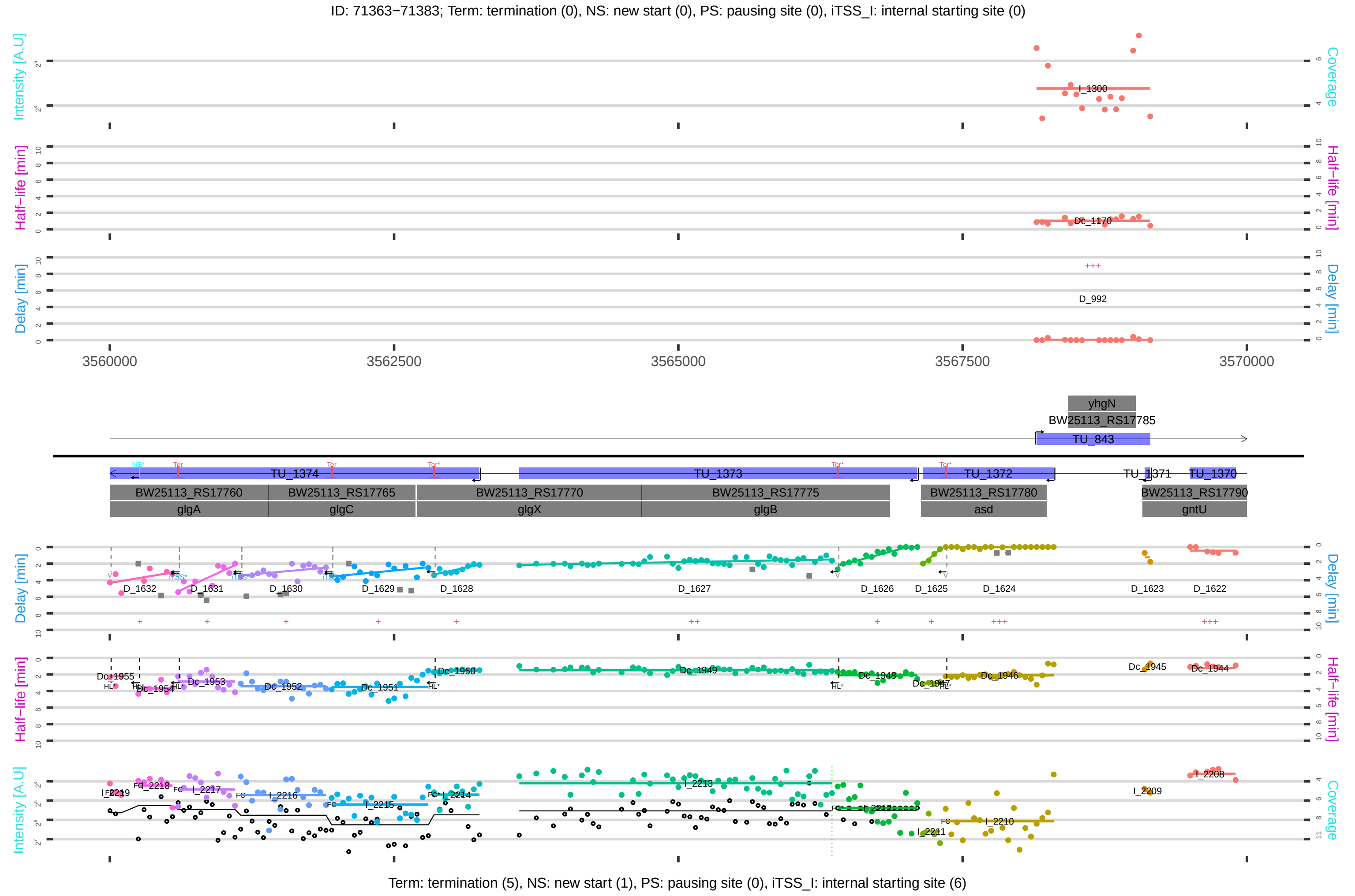
ID: 71363−71383; Term: termination (0), NS: new start (0), PS: pausing site (0), iTSS_I: internal starting site (0)
Intensity [A.U]
Coverage
I_1300
Half−life [min]
Dc_1170
Delay [min]
D_992
3560000
3562500
3565000
3567500
3570000
yhgN
BW25113_RS17785
TU_843
TU_1374
TU_1373
TU_1372
TU_1371
TU_1370
BW25113_RS17760
BW25113_RS17765
BW25113_RS17770
BW25113_RS17775
BW25113_RS17780
BW25113_RS17790
glgA
glgC
glgX
glgB
asd
gntU
D_1632
D_1631
D_1630
D_1629
D_1628
D_1627
D_1626
D_1625
D_1624
D_1623
D_1622
Dc_1955
Dc_1954
Dc_1953
Dc_1952
Dc_1951
Dc_1950
Dc_1949
Dc_1948
Dc_1947
Dc_1946
Dc_1945
Dc_1944
I_2219
I_2218
I_2217
I_2216
I_2215
I_2214
I_2213
I_2212
I_2211
I_2210
I_2209
I_2208
Term: termination (5), NS: new start (1), PS: pausing site (0), iTSS_I: internal starting site (6)

ID: 71445−71500; Term: termination (0), NS: new start (1), PS: pausing site (0), iTSS_I: internal starting site (0)

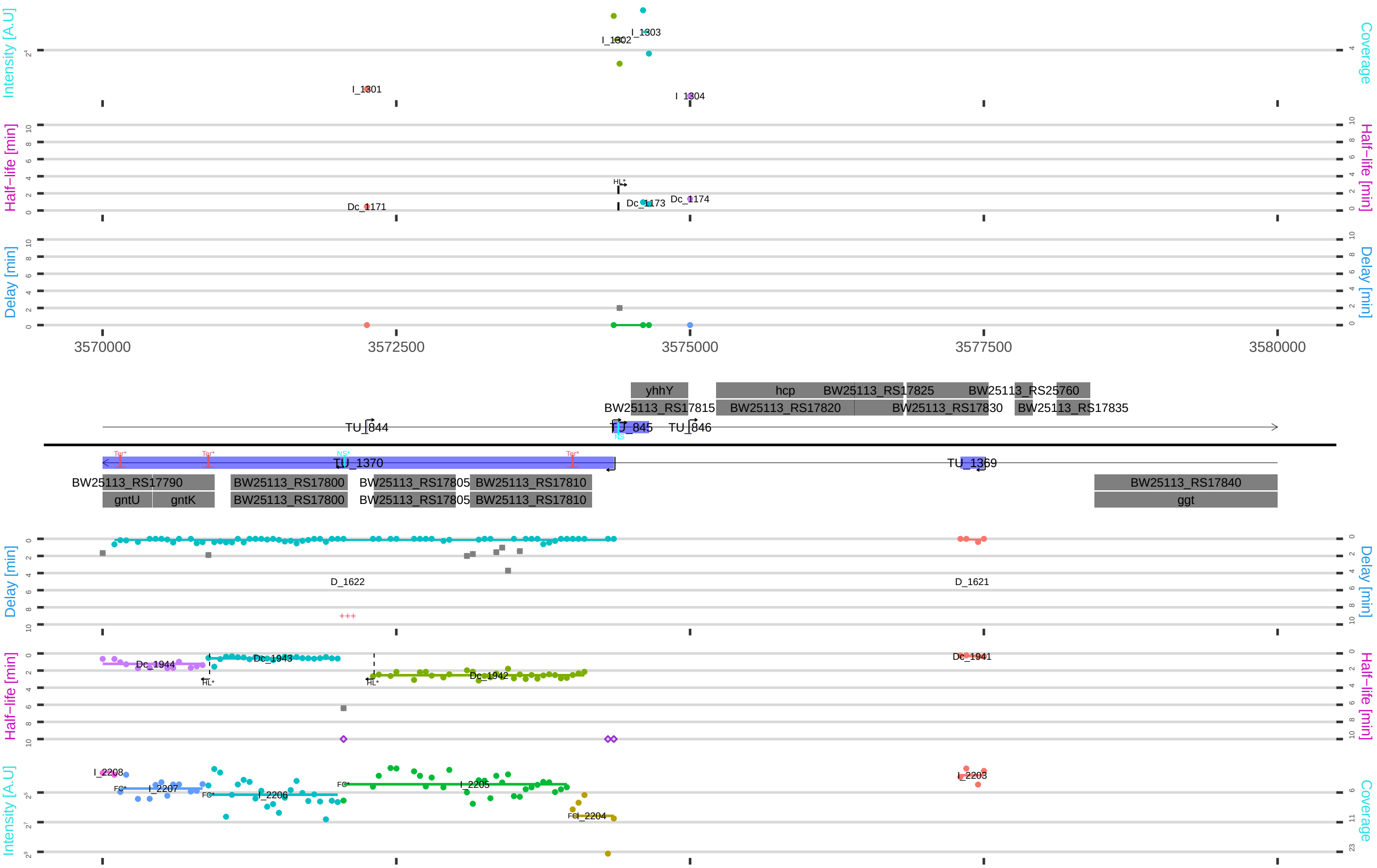

Term: termination (3), NS: new start (1), PS: pausing site (0), iTSS_I: internal starting site (0)

ID: 71608−71720; Term: termination (0), NS: new start (0), PS: pausing site (0), iTSS_I: internal starting site (0)

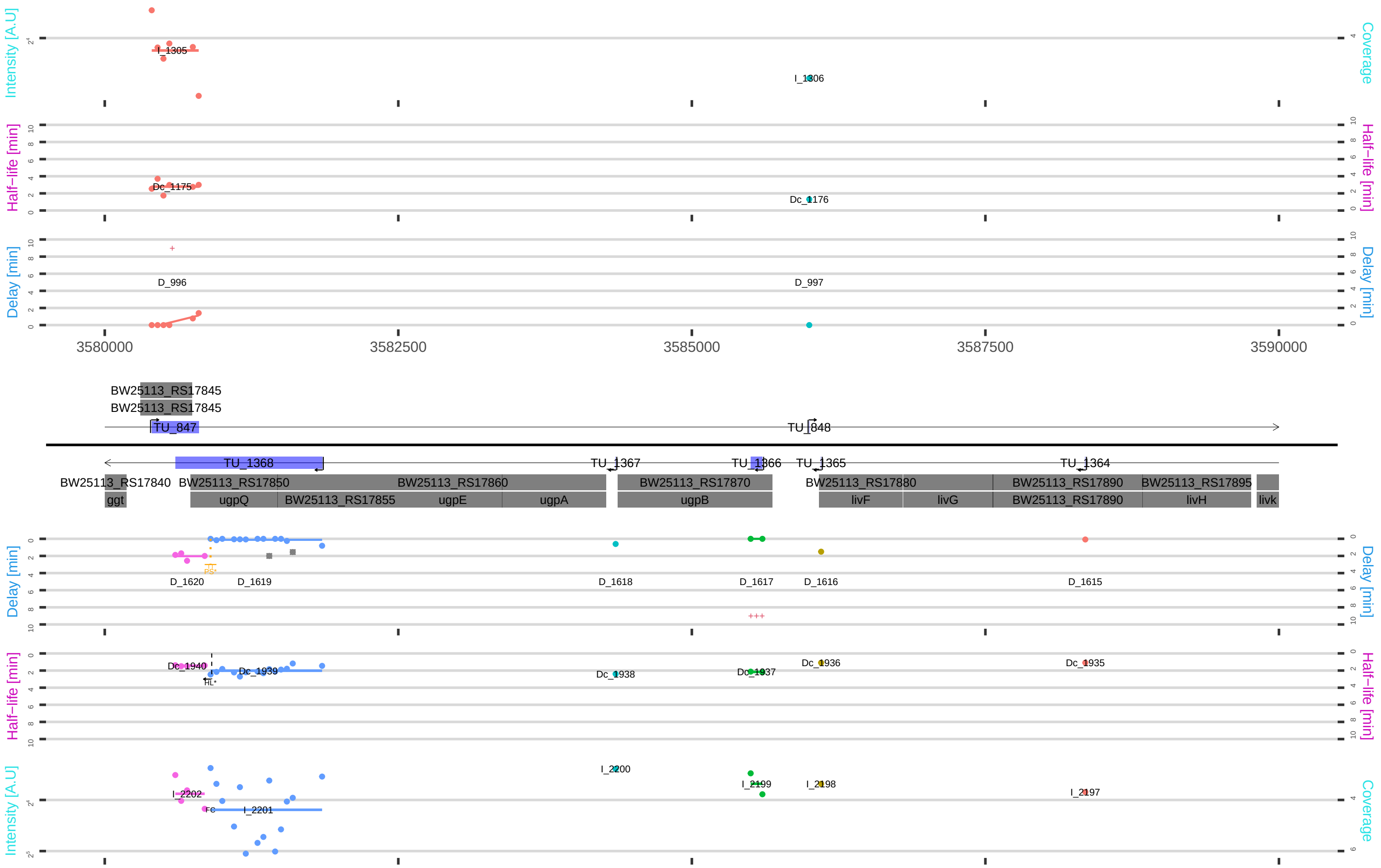

Term: termination (0), NS: new start (0), PS: pausing site (1), iTSS_I: internal starting site (0)

ID: 71828−72000; Term: termination (1), NS: new start (0), PS: pausing site (0), iTSS_I: internal starting site (0)

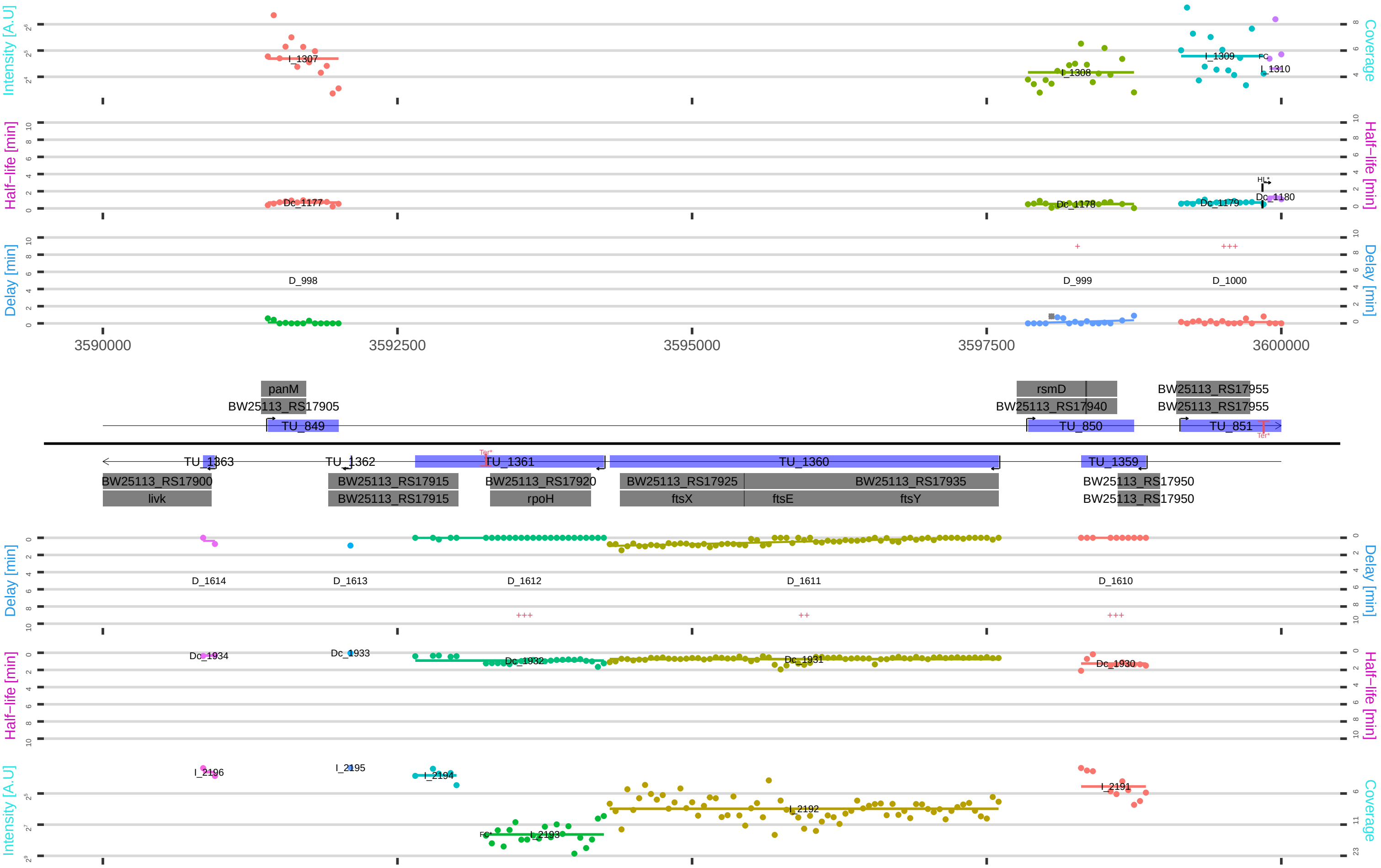

Term: termination (1), NS: new start (0), PS: pausing site (0), iTSS_I: internal starting site (0)

ID: 72000−72196; Term: termination (1), NS: new start (1), PS: pausing site (0), iTSS_I: internal starting site (0)

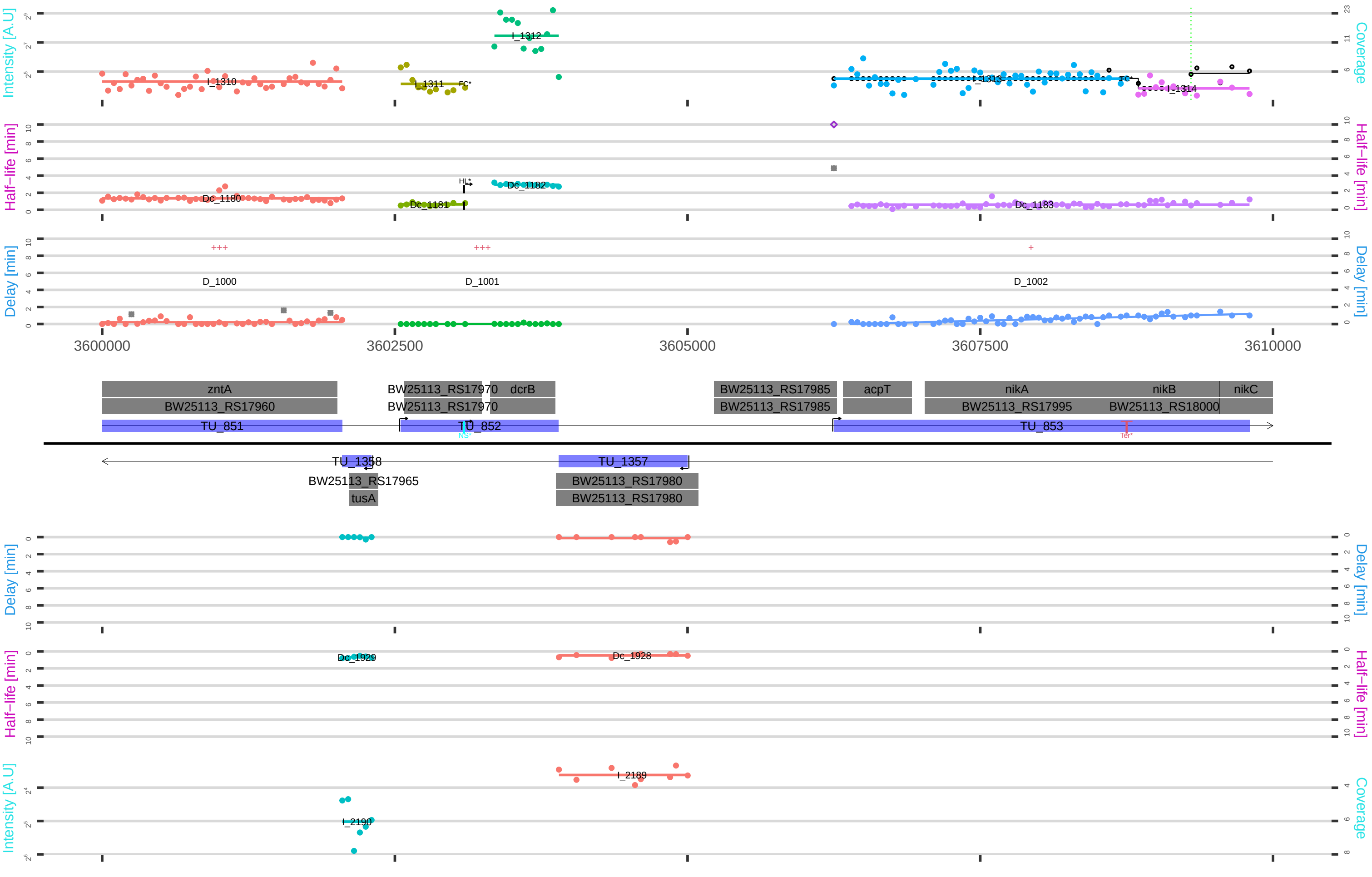

Term: termination (0), NS: new start (0), PS: pausing site (0), iTSS_I: internal starting site (0)

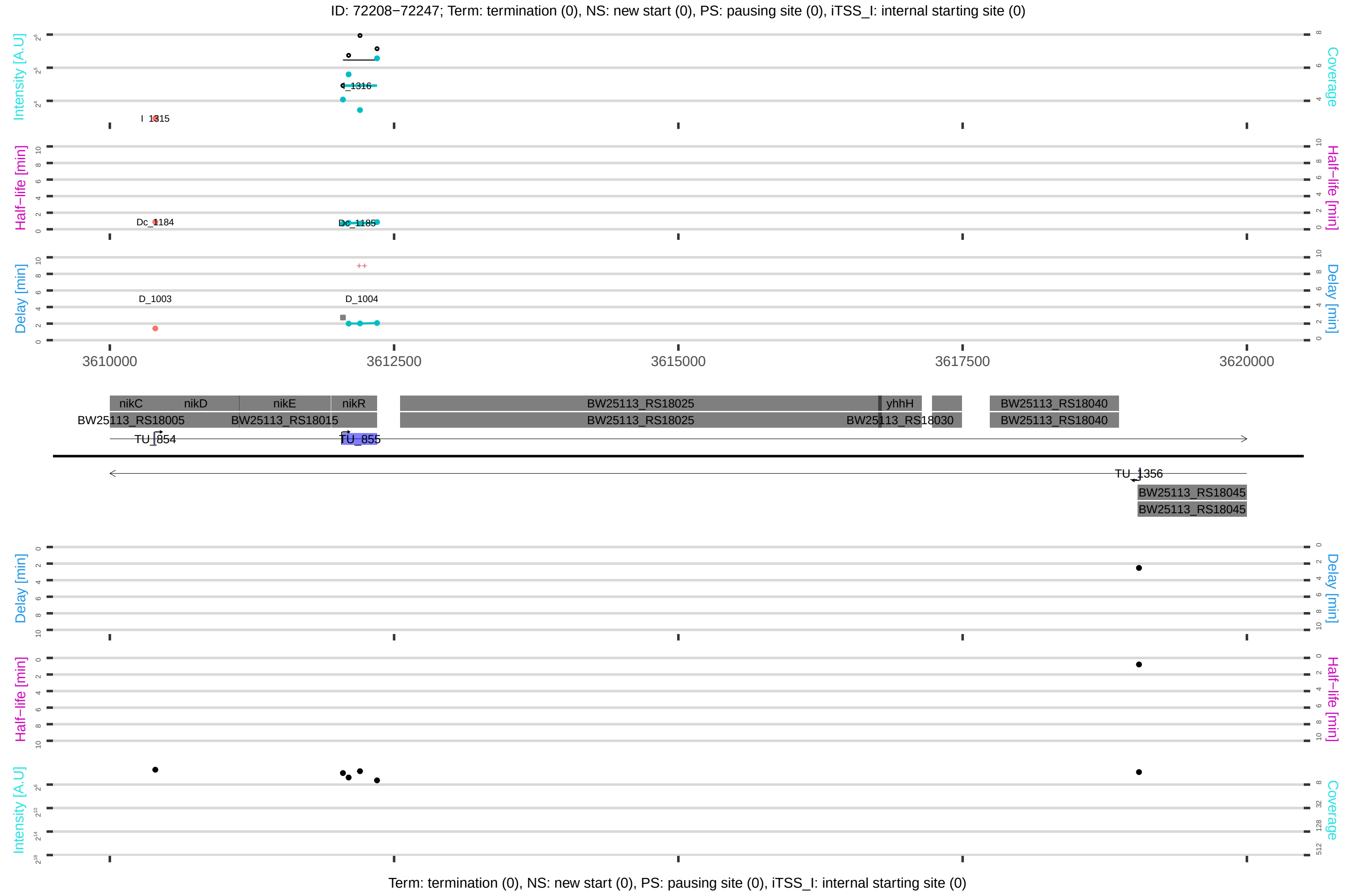
ID: 72208−72247; Term: termination (0), NS: new start (0), PS: pausing site (0), iTSS_I: internal starting site (0)
Intensity [A.U]
Coverage
Half−life [min]
Delay [min]
I_1315
I_1316
Dc_1184
Dc_1185
D_1003
D_1004
3610000
3612500
3615000
3617500
3620000
nikC
nikD
nikE
nikR
BW25113_RS18005
BW25113_RS18015
BW25113_RS18025
yhhH
BW25113_RS18030
BW25113_RS18040
TU_854
TU_855
TU_1356
BW25113_RS18045
Term: termination (0), NS: new start (0), PS: pausing site (0), iTSS_I: internal starting site (0)

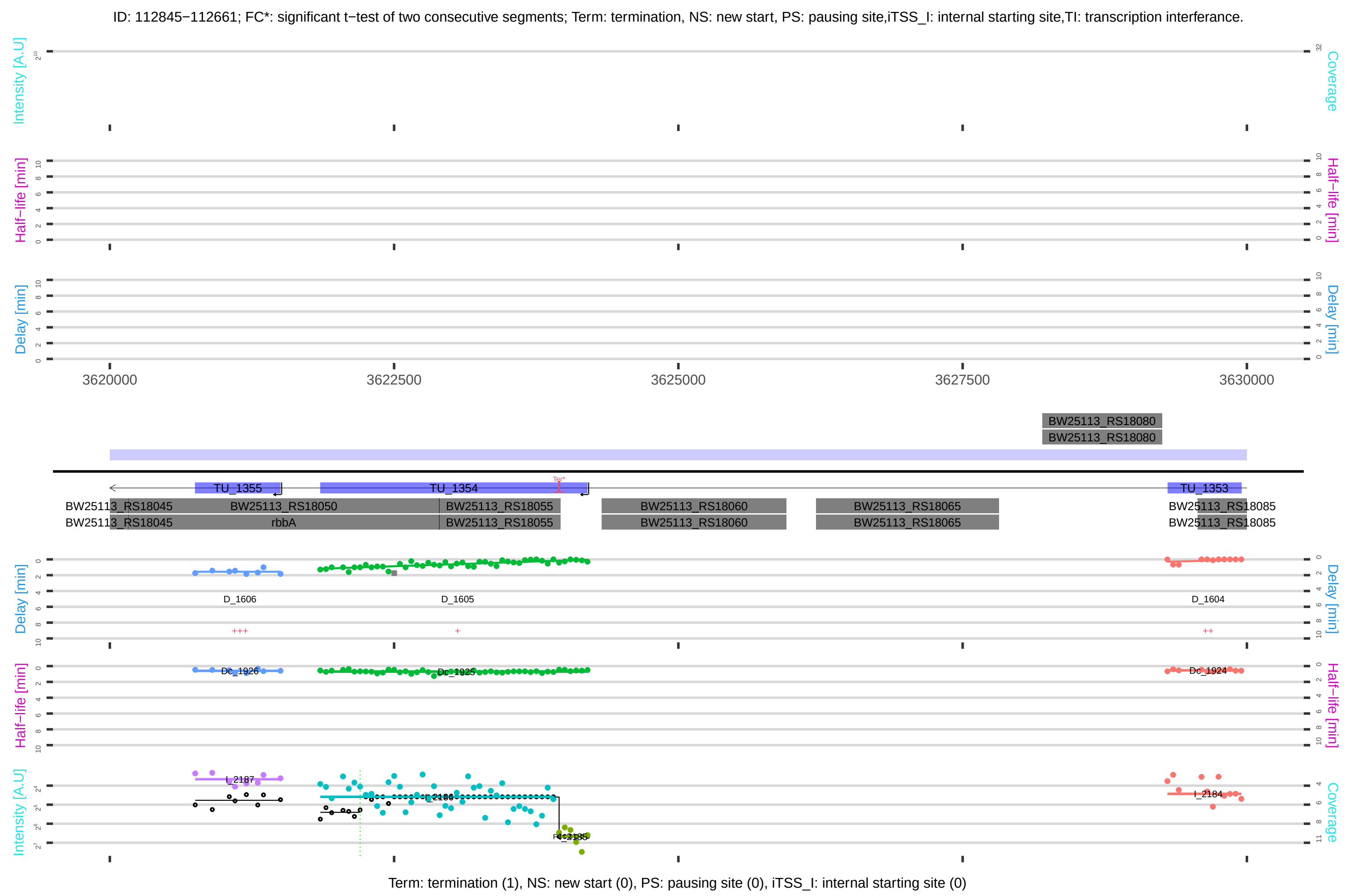
ID: 112845−112661; FC*: significant t−test of two consecutive segments; Term: termination, NS: new start, PS: pausing site,iTSS_I: internal starting site,TI: transcription interferance.
Intensity [A.U]
Coverage
Half−life [min]
Delay [min]
3620000
3622500
3625000
3627500
3630000
BW25113_RS18080
TU_1355
TU_1354
TU_1353
Ter*
BW25113_RS18045
BW25113_RS18050
rbbA
BW25113_RS18055
BW25113_RS18060
BW25113_RS18065
BW25113_RS18085
D_1606
D_1605
D_1604
Dc_1926
Dc_1925
Dc_1924
I_2187
I_2186
I_2184
FC*2185
Term: termination (1), NS: new start (0), PS: pausing site (0), iTSS_I: internal starting site (0)

ID: 72620−72800; Term: termination (3), NS: new start (1), PS: pausing site (1), iTSS_I: internal starting site (0)

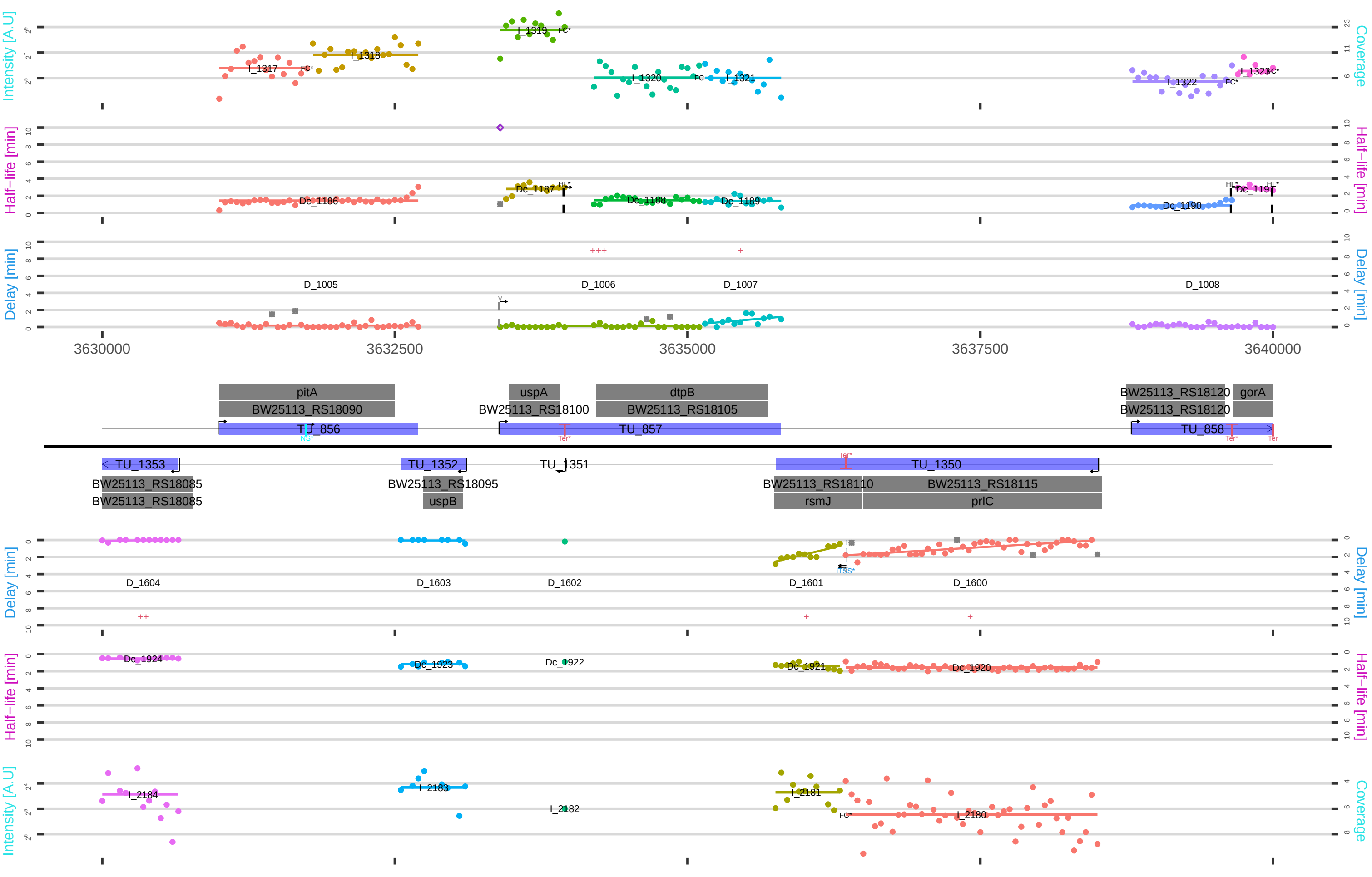

Term: termination (1), NS: new start (0), PS: pausing site (0), iTSS_I: internal starting site (1)

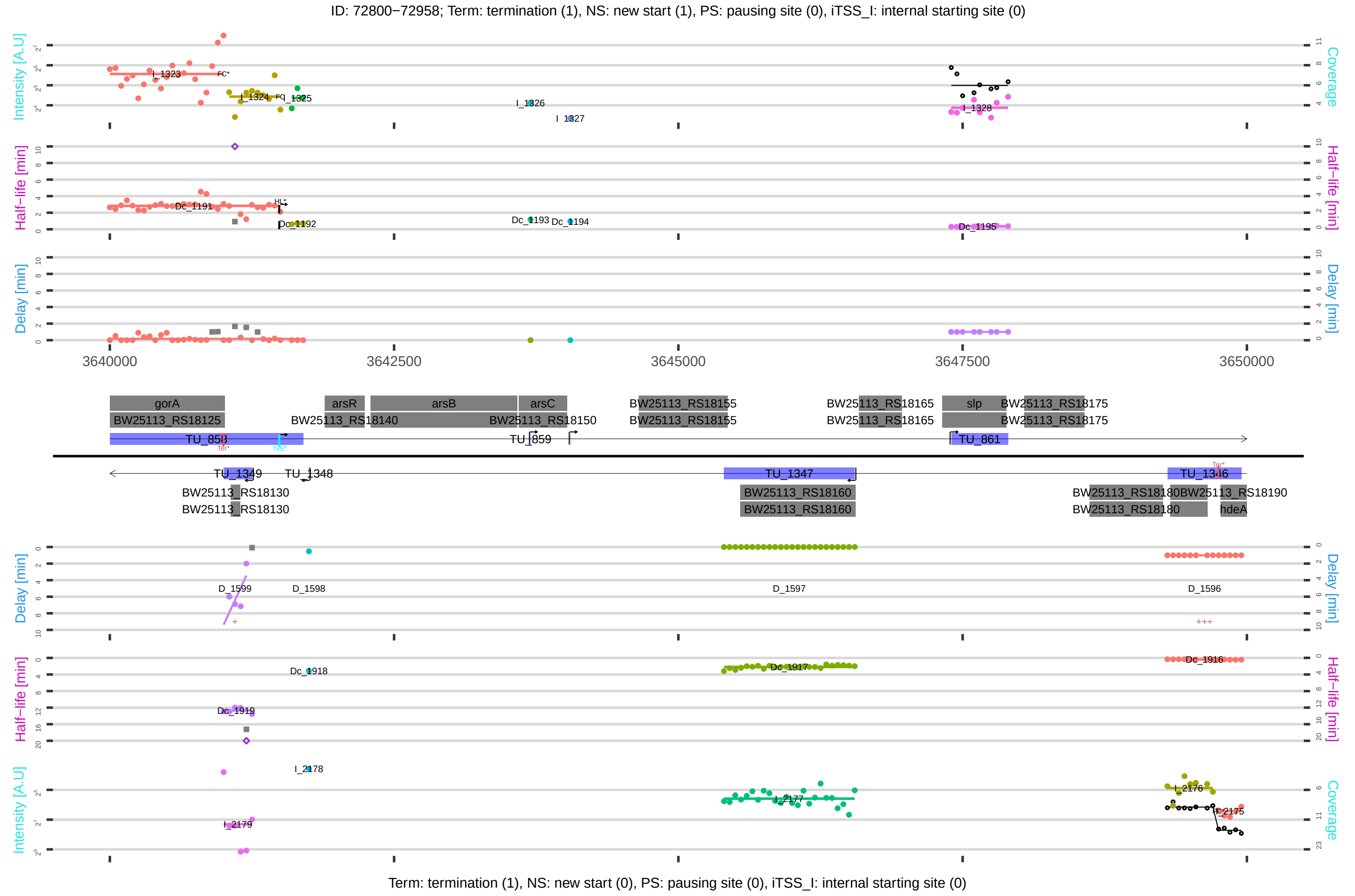
ID: 72800−72958; Term: termination (1), NS: new start (1), PS: pausing site (0), iTSS_I: internal starting site (0)
Intensity [A.U]
Coverage
Half−life [min]
Delay [min]
I_1323
I_1324
I_1325
I_1326
I_1327
I_1328
Dc_1191
Dc_1192
Dc_1193
Dc_1194
Dc_1195
3640000
3642500
3645000
3647500
3650000
gorA
BW25113_RS18125
arsR
arsB
arsC
BW25113_RS18140
BW25113_RS18150
BW25113_RS18155
BW25113_RS18165
slp
BW25113_RS18175
TU_858
TU_859
TU_861
TU_1349
TU_1348
TU_1347
TU_1346
BW25113_RS18130
BW25113_RS18160
BW25113_RS18180
BW25113_RS18190
hdeA
D_1599
D_1598
D_1597
D_1596
Dc_1918
Dc_1917
Dc_1916
Dc_1919
I_2178
I_2177
I_2176
I_2175
I_2179
Term: termination (1), NS: new start (0), PS: pausing site (0), iTSS_I: internal starting site (0)

ID: 73019−73167; Term: termination (0), NS: new start (0), PS: pausing site (0), iTSS_I: internal starting site (0)

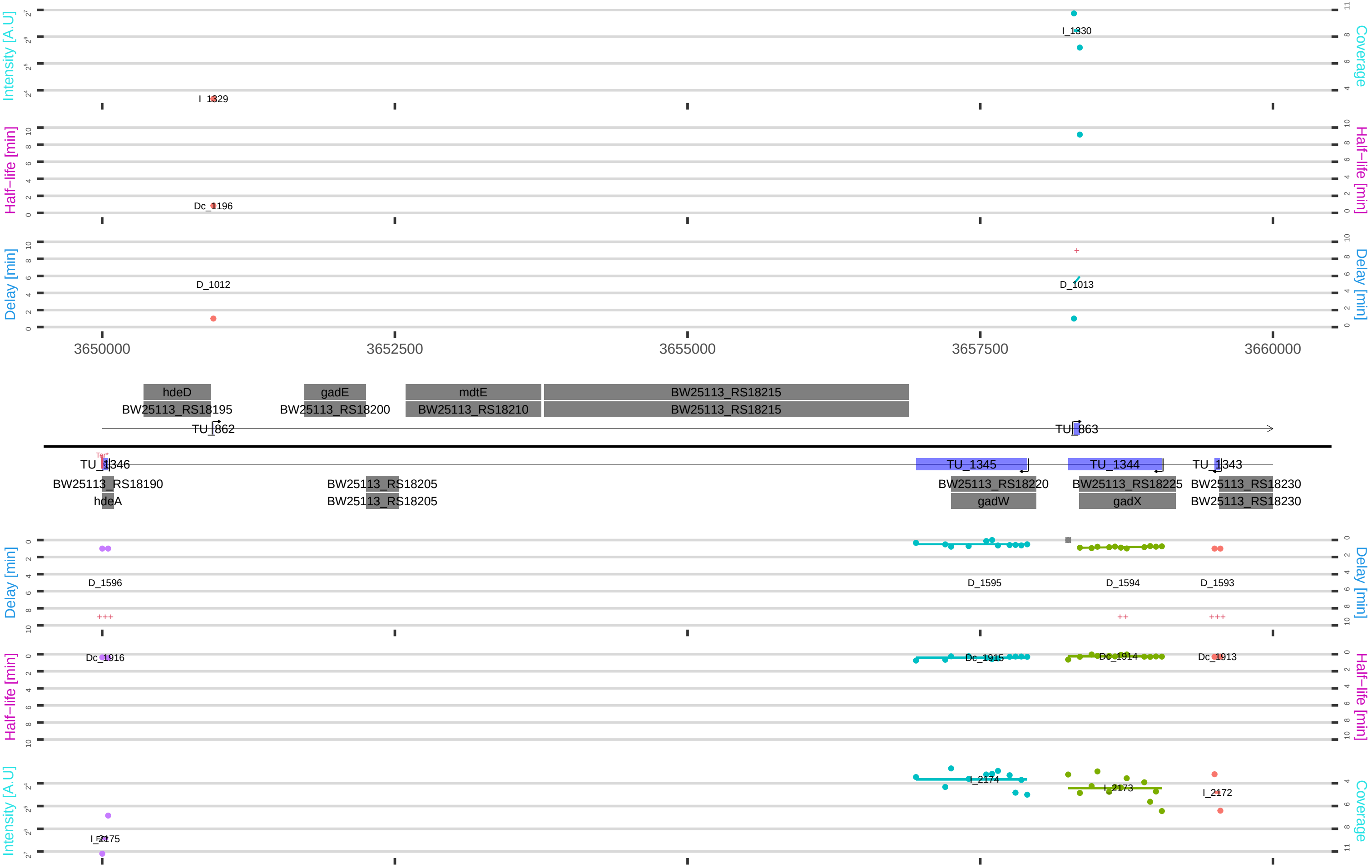

Term: termination (1), NS: new start (0), PS: pausing site (0), iTSS_I: internal starting site (0)

ID: 73318−73354; Term: termination (0), NS: new start (0), PS: pausing site (0), iTSS_I: internal starting site (0)

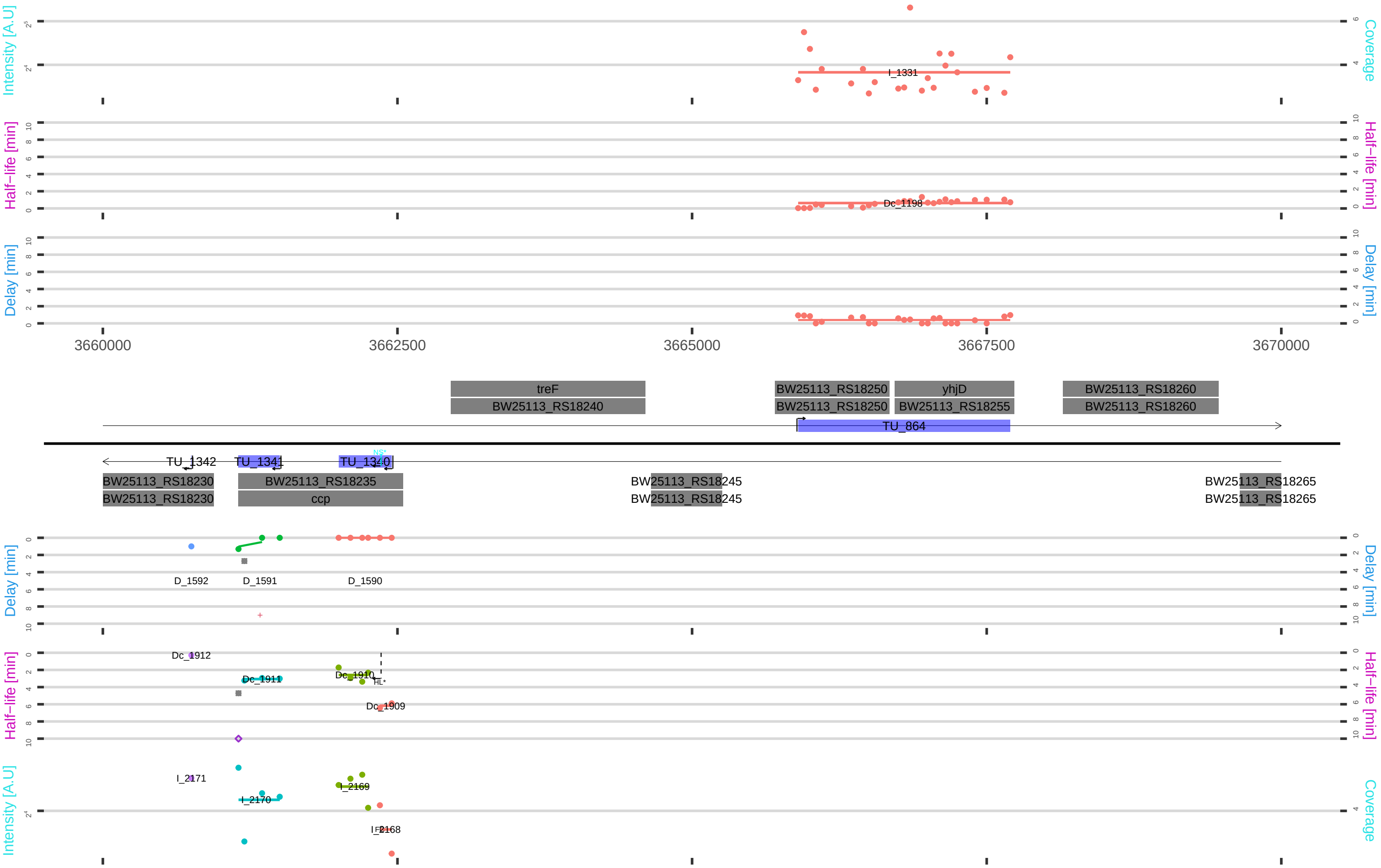

Term: termination (0), NS: new start (1), PS: pausing site (0), iTSS_I: internal starting site (0)

ID: 73457−73510; Term: termination (0), NS: new start (0), PS: pausing site (0), iTSS_I: internal starting site (0)

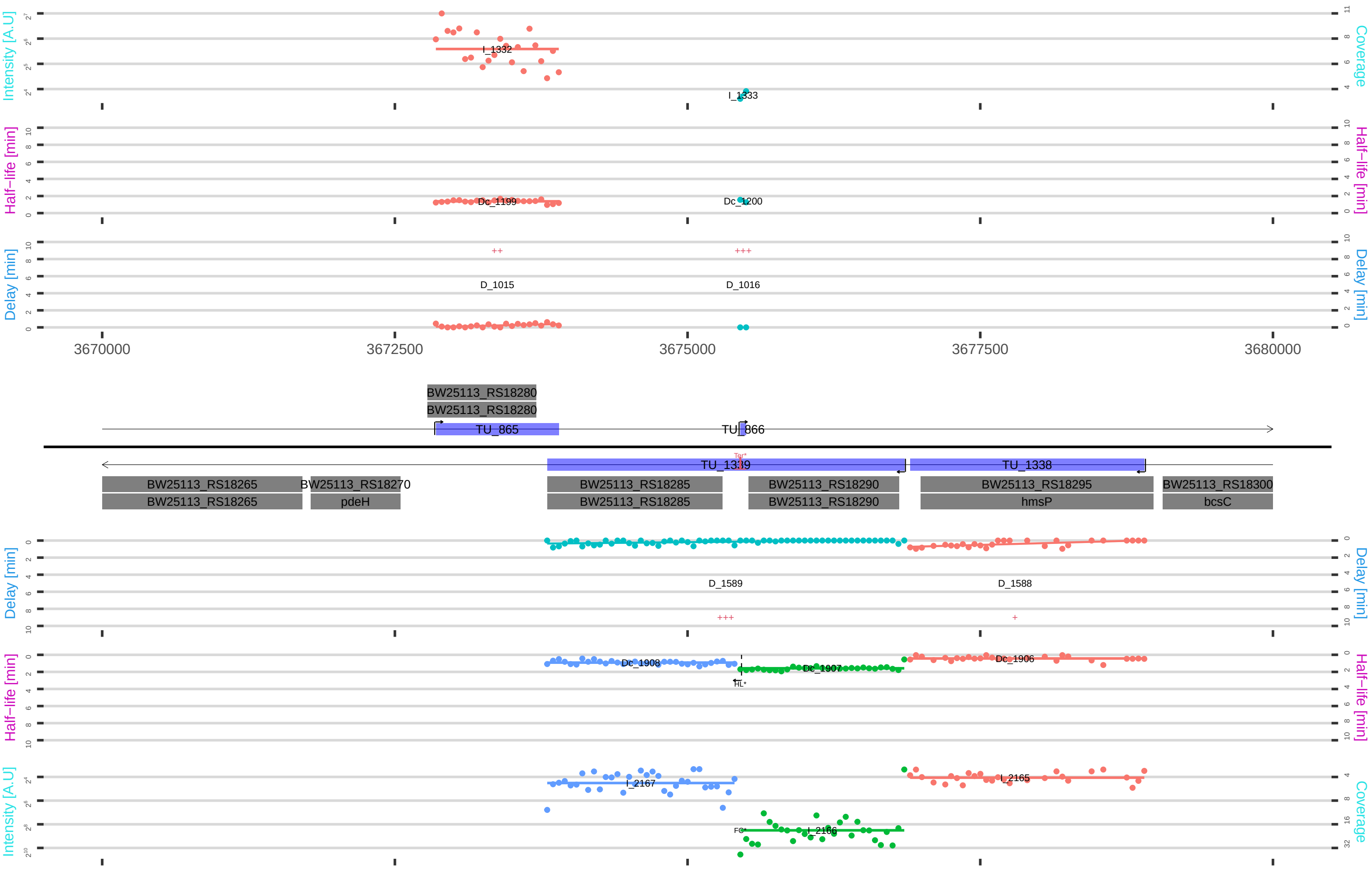

Term: termination (1), NS: new start (0), PS: pausing site (0), iTSS_I: internal starting site (0)

ID: 73797−73800; Term: termination (0), NS: new start (0), PS: pausing site (0), iTSS_I: internal starting site (0)

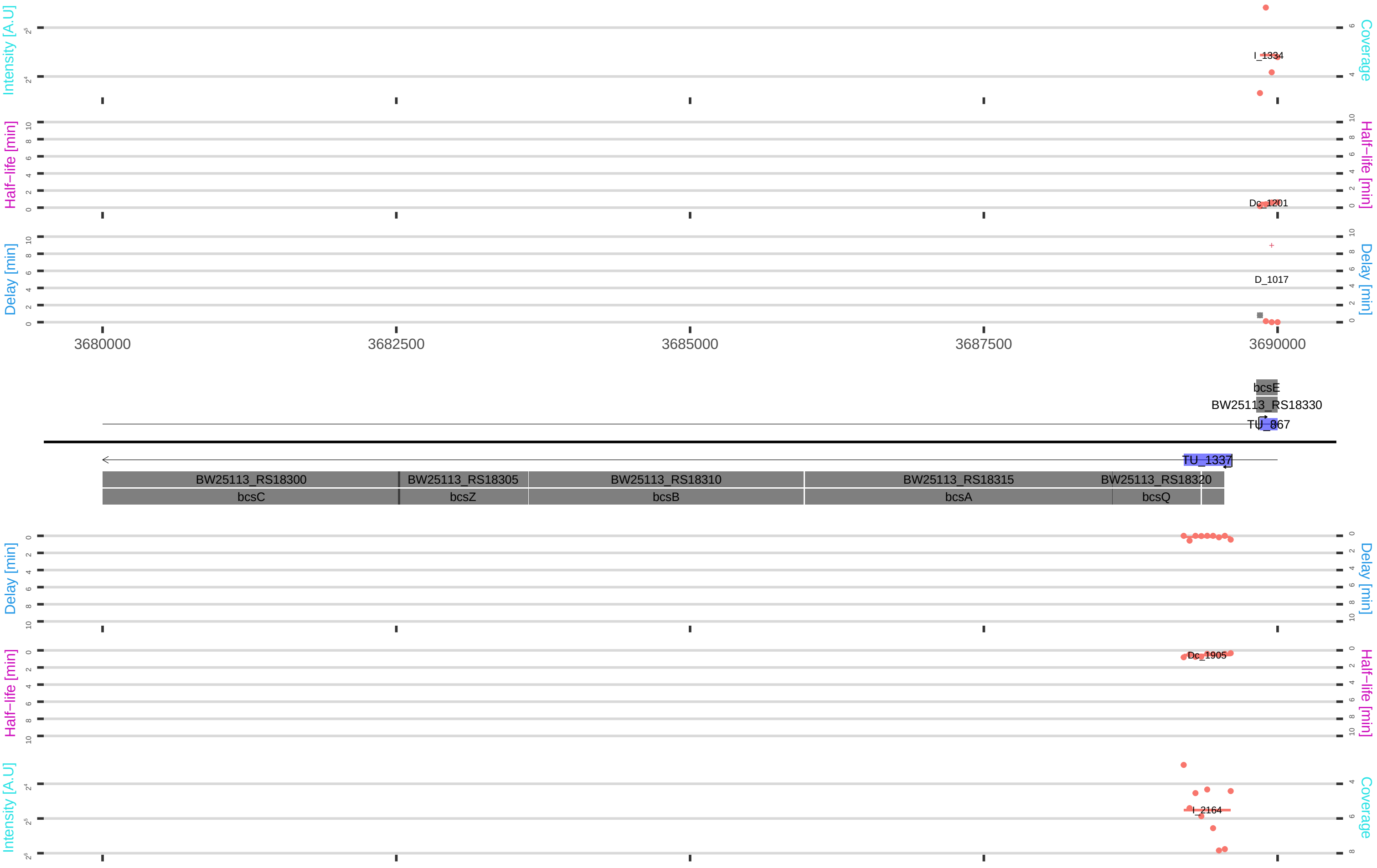

Term: termination (0), NS: new start (0), PS: pausing site (0), iTSS_I: internal starting site (0)

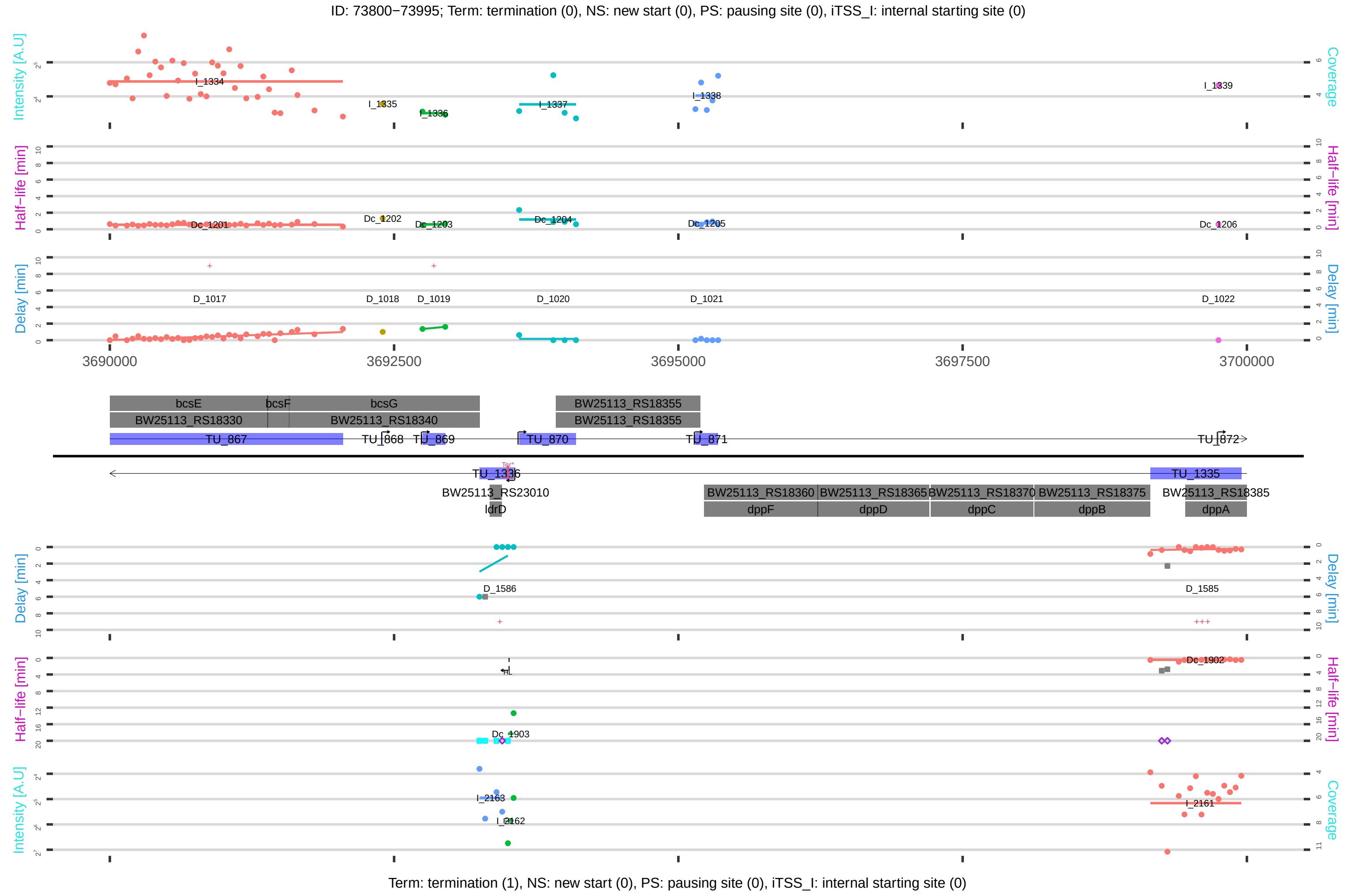

ID: 73800−73995; Term: termination (0), NS: new start (0), PS: pausing site (0), iTSS_I: internal starting site (0)
Intensity [A.U]
Coverage
Half−life [min]
Delay [min]
I_1334
I_1335
I_1336
I_1337
I_1338
I_1339
Dc_1201
Dc_1202
Dc_1203
Dc_1204
Dc_1205
Dc_1206
D_1017
D_1018
D_1019
D_1020
D_1021
D_1022
3690000
3692500
3695000
3697500
3700000
bcsE
bcsF
bcsG
BW25113_RS18355
BW25113_RS18330
BW25113_RS18340
TU_867
TU_868
TU_869
TU_870
TU_871
TU_872
TU_1336
TU_1335
BW25113_RS23010
ldrD
BW25113_RS18360
BW25113_RS18365
BW25113_RS18370
BW25113_RS18375
BW25113_RS18385
dppF
dppD
dppC
dppB
dppA
D_1586
D_1585
Dc_1902
Dc_1903
I_2163
I_2162
I_2161
Term: termination (1), NS: new start (0), PS: pausing site (0), iTSS_I: internal starting site (0)

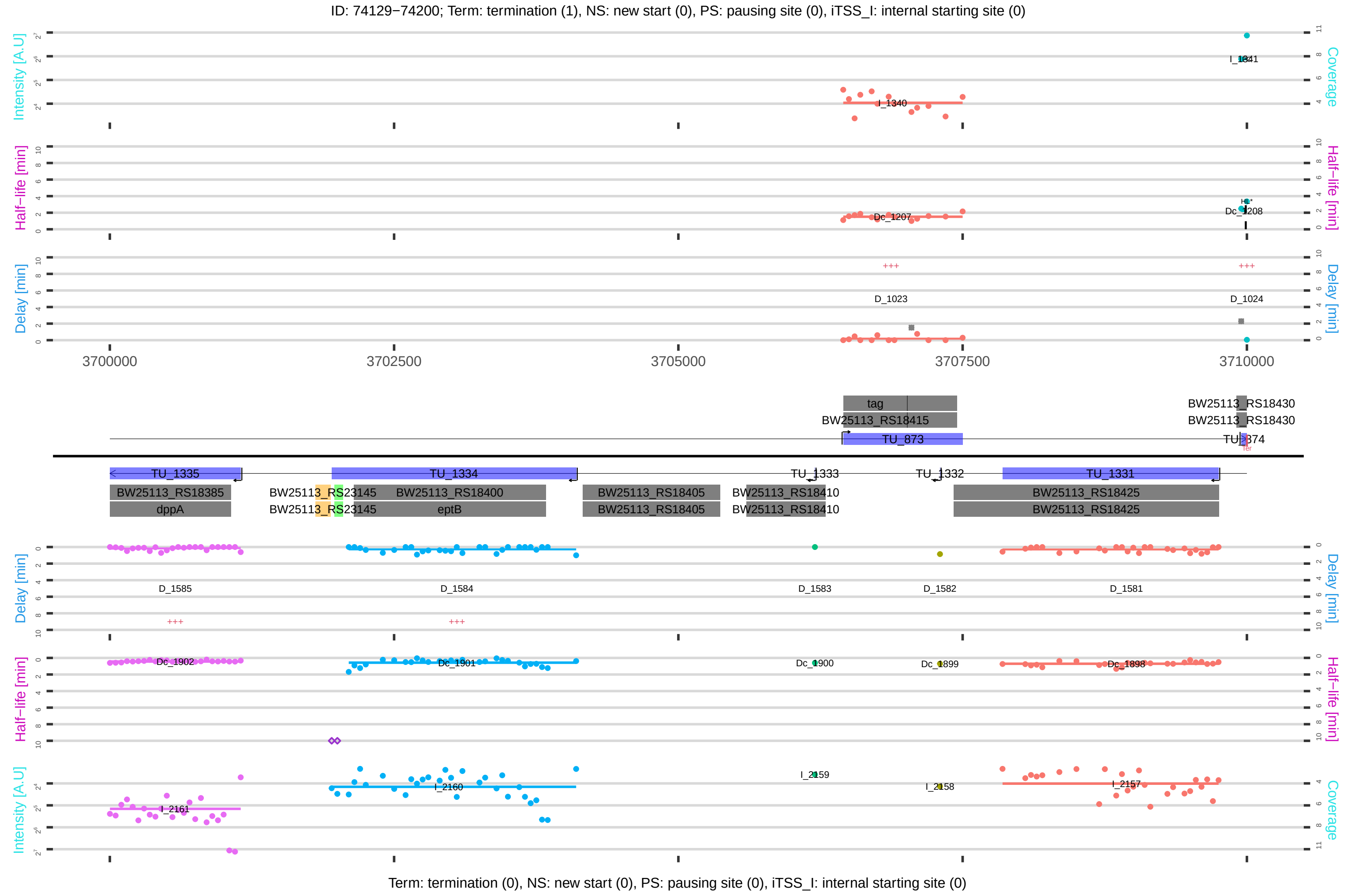

ID: 74129−74200; Term: termination (1), NS: new start (0), PS: pausing site (0), iTSS_I: internal starting site (0)
Intensity [A.U]
Coverage
Half−life [min]
Delay [min]
I_1340
I_1341
Dc_1207
Dc_1208
D_1023
D_1024
+++
3700000
3702500
3705000
3707500
3710000
tag
BW25113_RS18415
BW25113_RS18430
TU_873
TU_874
TU_1335
TU_1334
TU_1333
TU_1332
TU_1331
BW25113_RS18385
dppA
BW25113_RS23145
BW25113_RS18400
eptB
BW25113_RS18405
BW25113_RS18410
BW25113_RS18425
D_1585
D_1584
D_1583
D_1582
D_1581
Dc_1902
Dc_1901
Dc_1900
Dc_1899
Dc_1898
I_2161
I_2160
I_2159
I_2158
I_2157
Term: termination (0), NS: new start (0), PS: pausing site (0), iTSS_I: internal starting site (0)

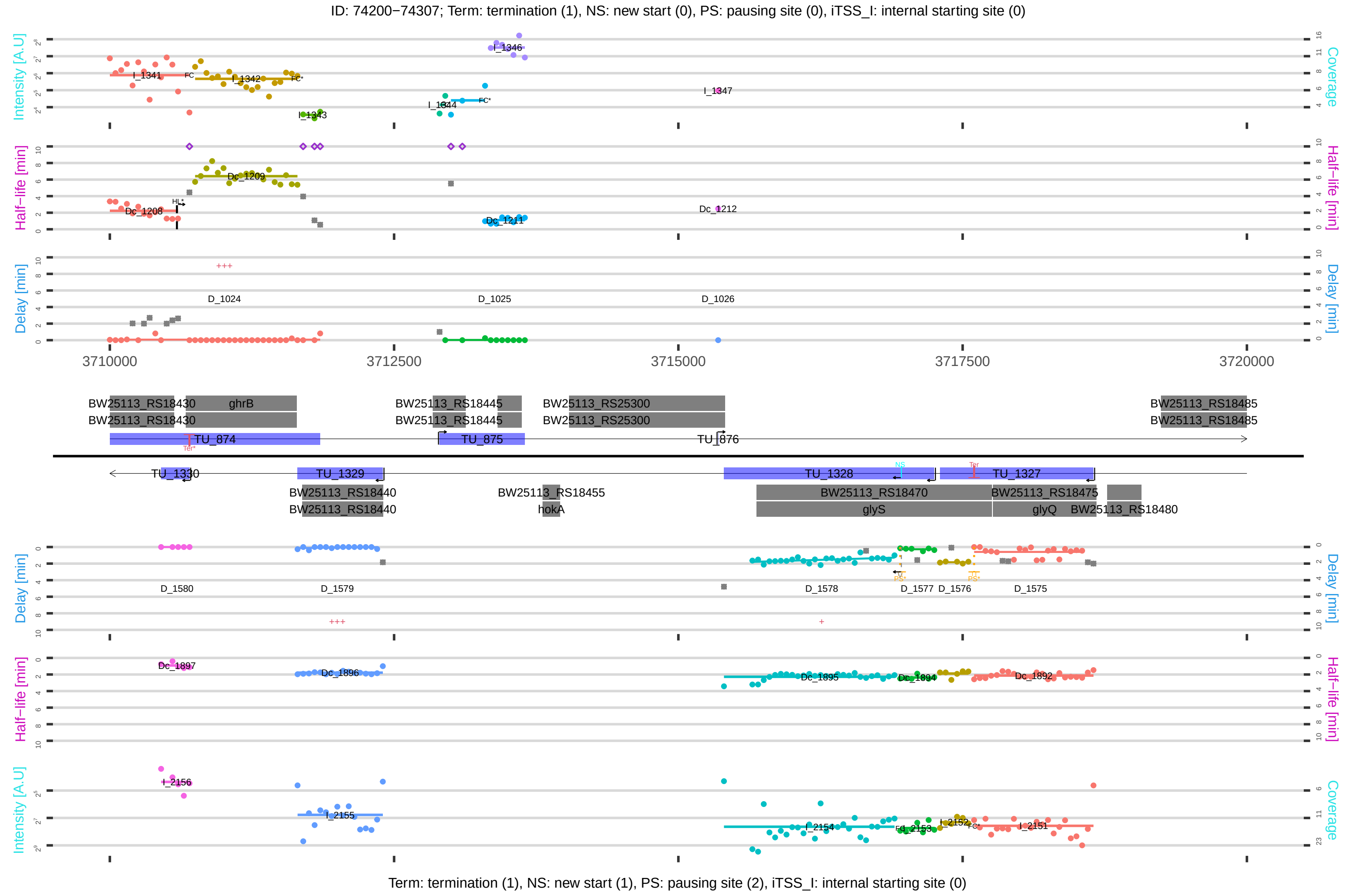

ID: 74200−74307; Term: termination (1), NS: new start (0), PS: pausing site (0), iTSS_I: internal starting site (0)
Intensity [A.U]
Coverage
Half−life [min]
Delay [min]
I_1341
I_1342
I_1343
I_1344
I_1346
I_1347
Dc_1208
Dc_1209
Dc_1211
Dc_1212
D_1024
D_1025
D_1026
3710000
3712500
3715000
3717500
3720000
BW25113_RS18430
ghrB
BW25113_RS18445
BW25113_RS25300
BW25113_RS18485
TU_874
TU_875
TU_876
TU_1330
TU_1329
TU_1328
TU_1327
BW25113_RS18440
BW25113_RS18455
hokA
BW25113_RS18470
glyS
BW25113_RS18475
glyQ
BW25113_RS18480
D_1580
D_1579
D_1578
D_1577
D_1576
D_1575
Dc_1897
Dc_1896
Dc_1895
Dc_1894
Dc_1892
I_2156
I_2155
I_2154
I_2153
I_2152
I_2151
Term: termination (1), NS: new start (1), PS: pausing site (2), iTSS_I: internal starting site (0)

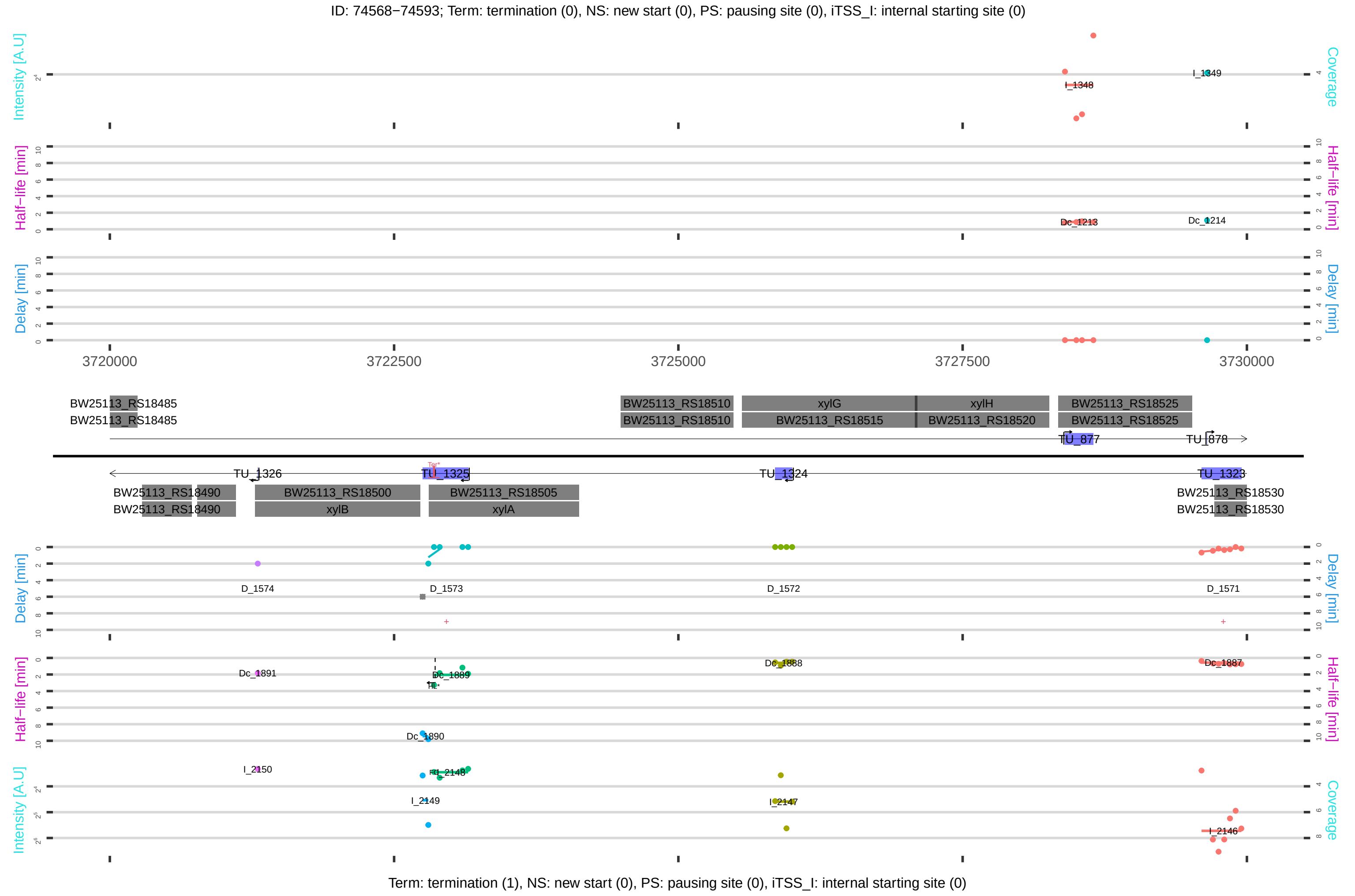

ID: 74568−74593; Term: termination (0), NS: new start (0), PS: pausing site (0), iTSS_I: internal starting site (0)
Intensity [A.U]
Coverage
I_1348
I_1349
Half−life [min]
Dc_1213
Dc_1214
Delay [min]
3720000
3722500
3725000
3727500
3730000
BW25113_RS18485
BW25113_RS18510
xylG
BW25113_RS18515
xylH
BW25113_RS18520
BW25113_RS18525
TU_877
TU_878
TU_1326
TU_1325
TU_1324
TU_1323
BW25113_RS18490
BW25113_RS18500
xylB
BW25113_RS18505
xylA
BW25113_RS18530
D_1574
D_1573
D_1572
D_1571
Dc_1891
Dc_1889
Dc_1888
Dc_1887
Dc_1890
I_2150
I_2148
I_2149
I_2147
I_2146
Term: termination (1), NS: new start (0), PS: pausing site (0), iTSS_I: internal starting site (0)

ID: 74663−74689; Term: termination (0), NS: new start (0), PS: pausing site (0), iTSS_I: internal starting site (0)

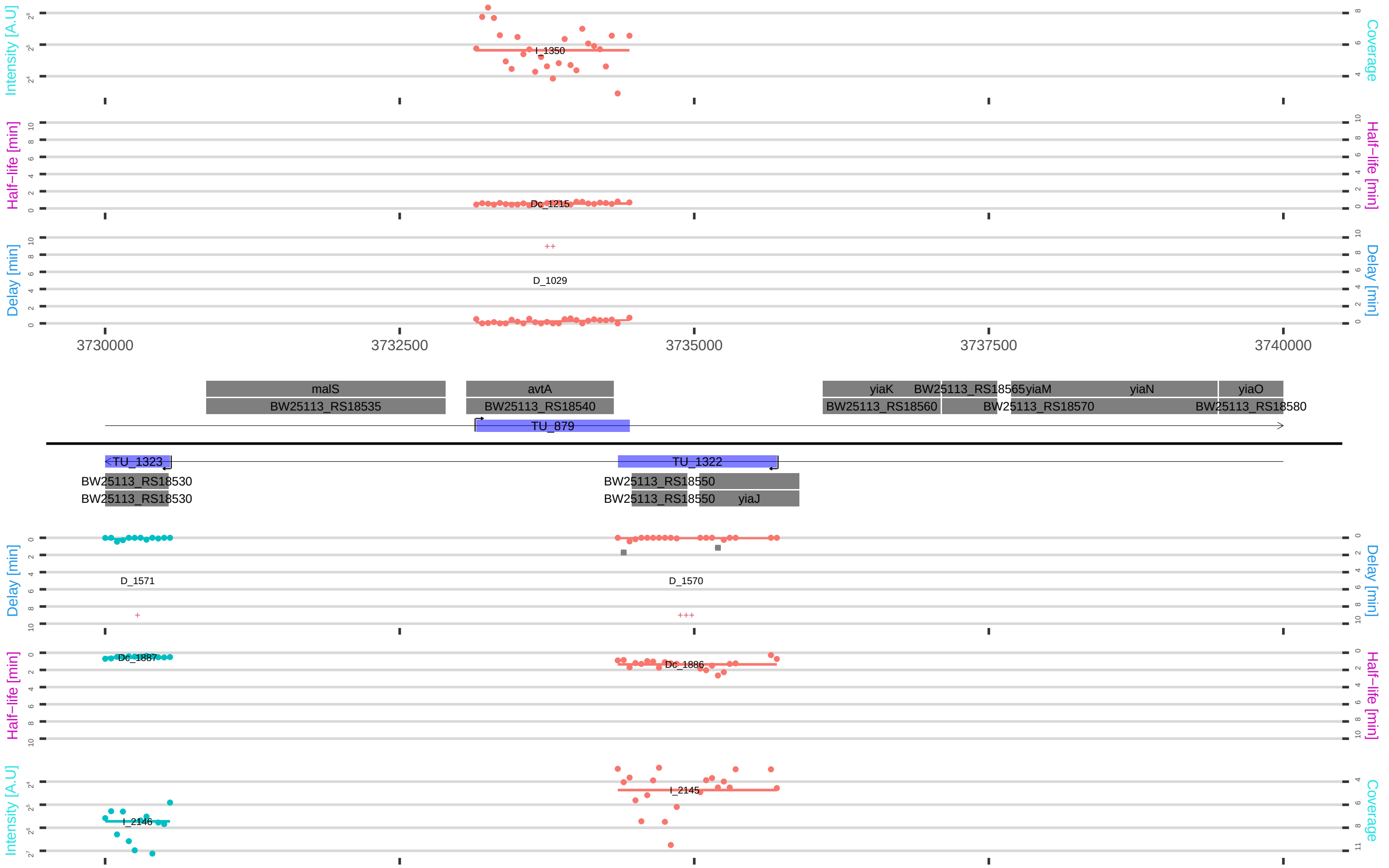

Term: termination (0), NS: new start (0), PS: pausing site (0), iTSS_I: internal starting site (0)

ID: 74922−74922; Term: termination (0), NS: new start (0), PS: pausing site (0), iTSS_I: internal starting site (0)

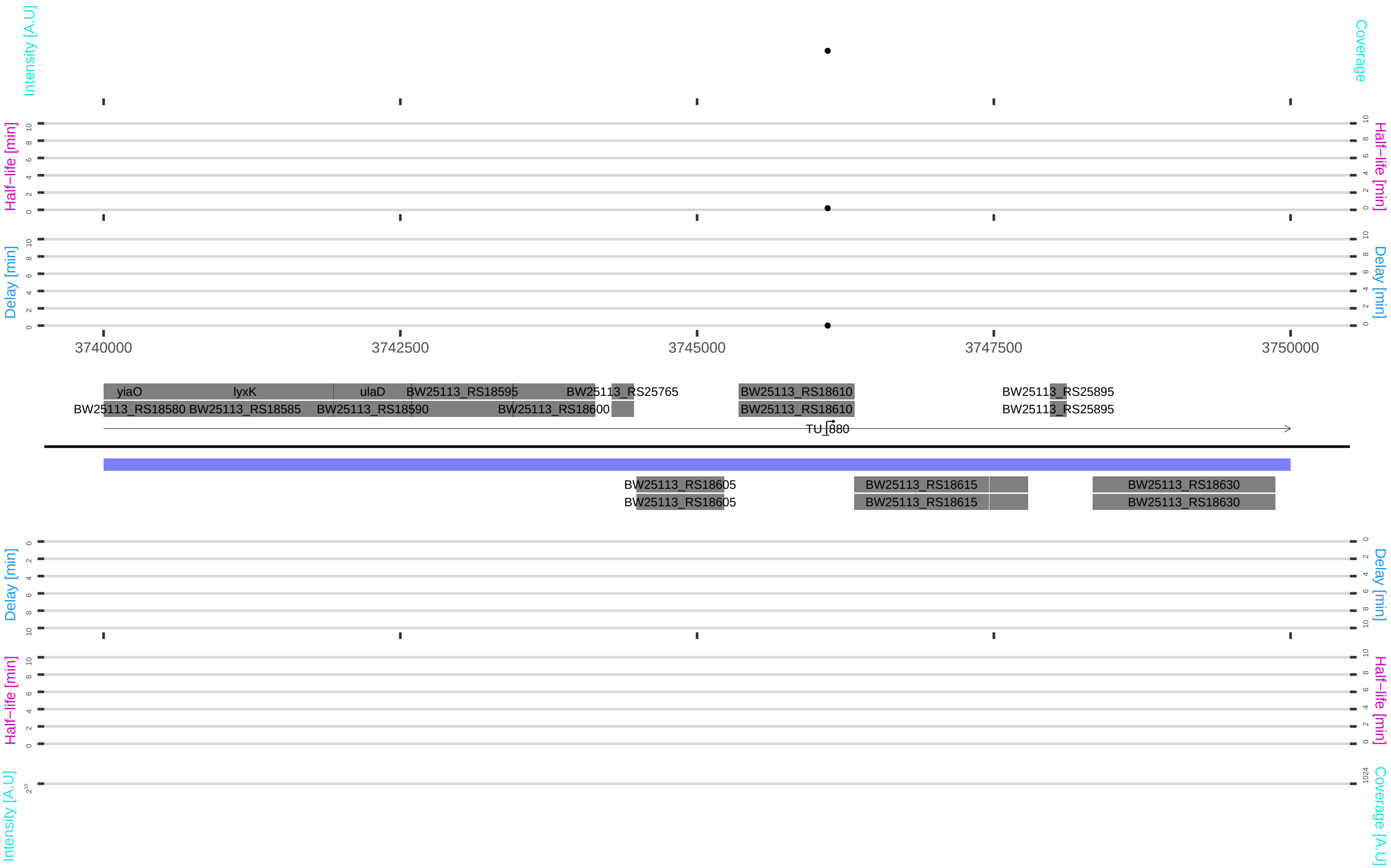

NA

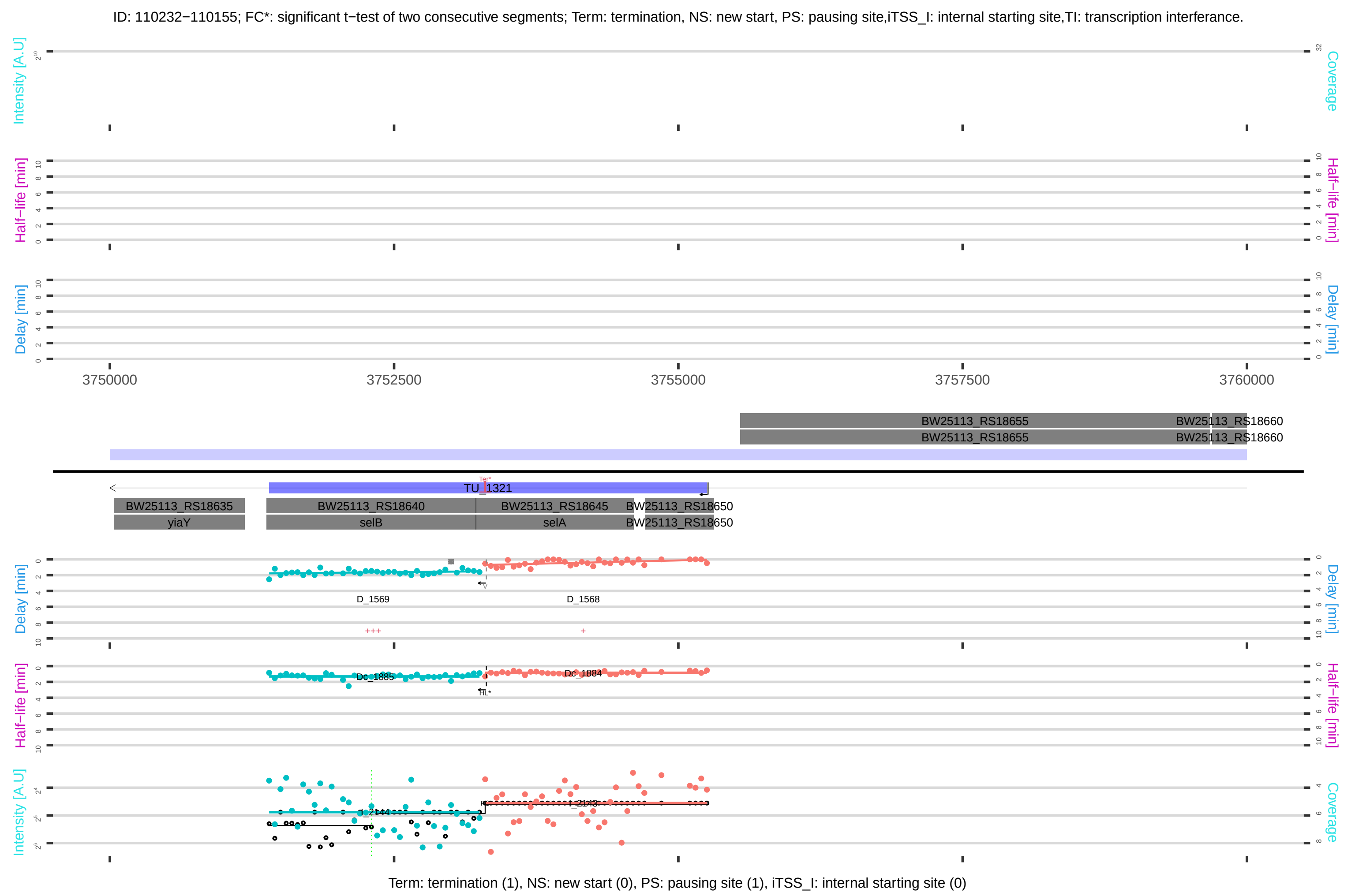

ID: 110232−110155; FC*: significant t−test of two consecutive segments; Term: termination, NS: new start, PS: pausing site,iTSS_I: internal starting site,TI: transcription interferance.
Intensity [A.U]
Coverage
Half−life [min]
Delay [min]
3750000
3752500
3755000
3757500
3760000
BW25113_RS18655
BW25113_RS18660
TU_1321
Ter*
BW25113_RS18635
yiaY
BW25113_RS18640
selB
BW25113_RS18645
selA
BW25113_RS18650
D_1569
D_1568
Dc_1885
Dc_1884
HL*
t_2144
t_2143
Term: termination (1), NS: new start (0), PS: pausing site (1), iTSS_I: internal starting site (0)

ID: 75208−75392; Term: termination (1), NS: new start (1), PS: pausing site (1), iTSS_I: internal starting site (0)

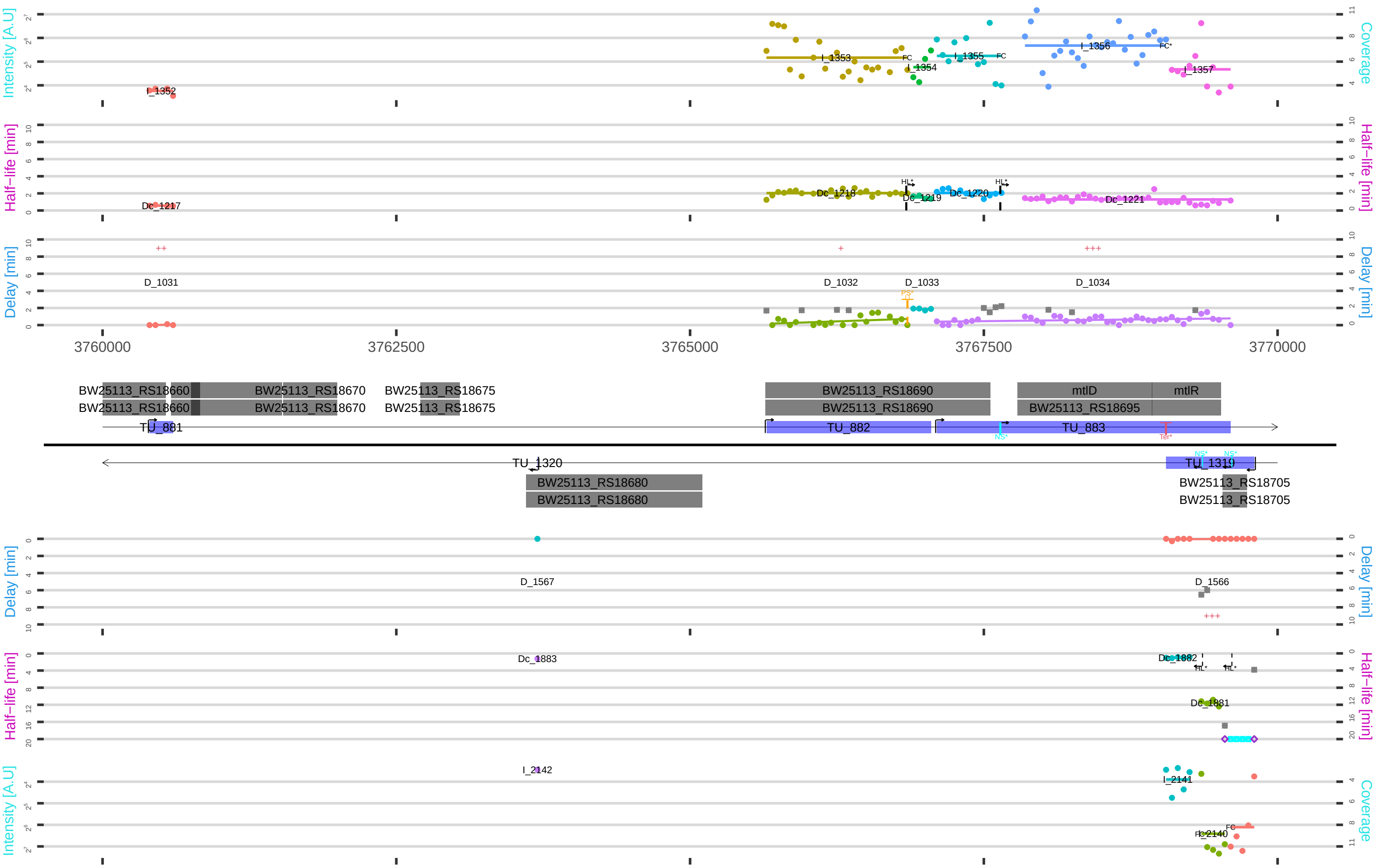

Term: termination (0), NS: new start (2), PS: pausing site (0), iTSS_I: internal starting site (0)

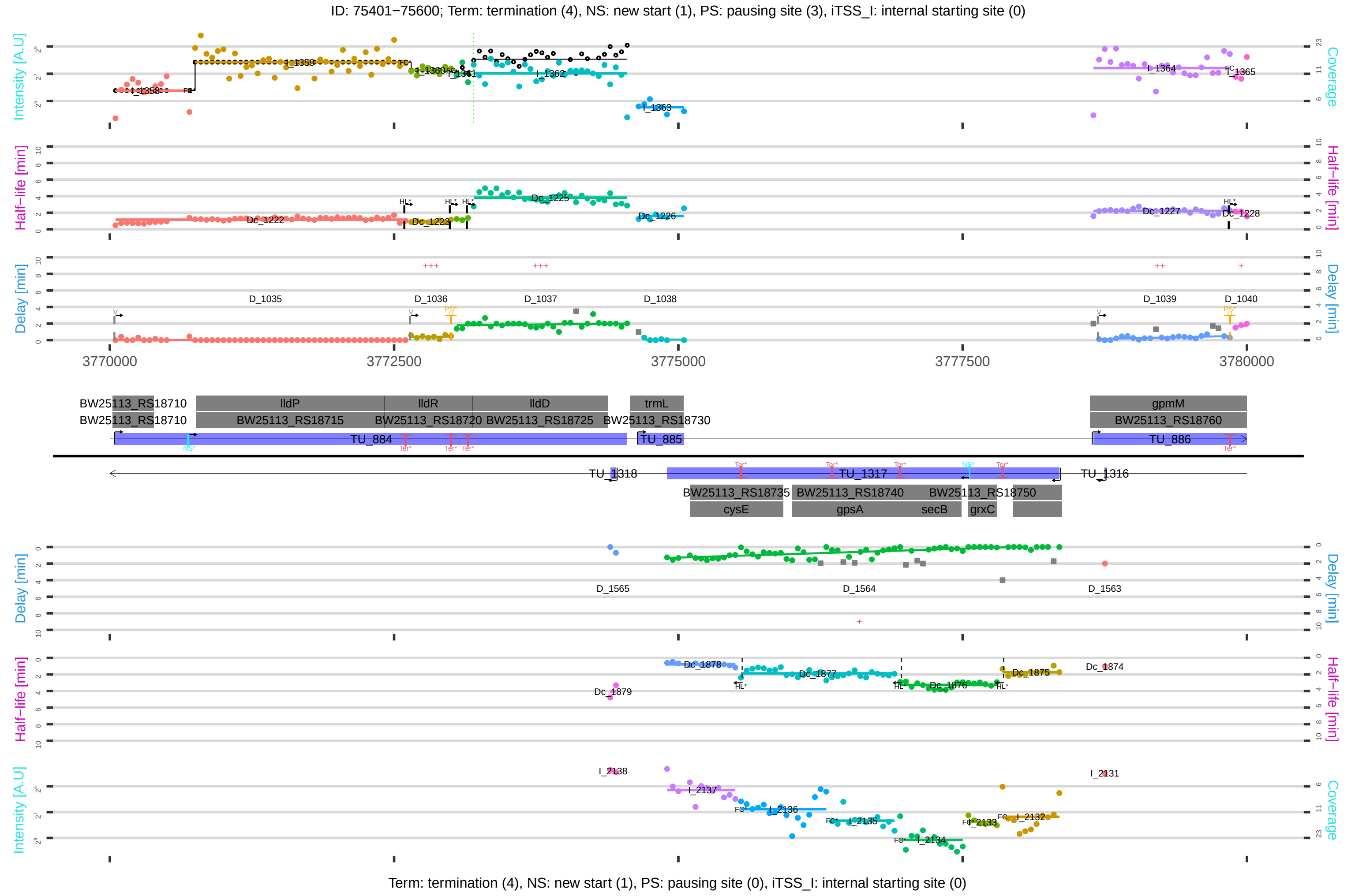

ID: 75401−75600; Term: termination (4), NS: new start (1), PS: pausing site (3), iTSS_I: internal starting site (0)
Intensity [A.U]
Coverage
Half−life [min]
Delay [min]
I_1358
I_1359
I_1360
I_1361
I_1362
I_1363
I_1364
I_1365
Dc_1222
Dc_1223
Dc_1225
Dc_1226
Dc_1227
Dc_1228
D_1035
D_1036
D_1037
D_1038
D_1039
D_1040
3770000
3772500
3775000
3777500
3780000
BW25113_RS18710
lldP
BW25113_RS18715
lldR
BW25113_RS18720
lldD
BW25113_RS18725
trmL
BW25113_RS18730
gpmM
BW25113_RS18760
TU_884
TU_885
TU_886
TU_1318
TU_1317
TU_1316
BW25113_RS18735
BW25113_RS18740
BW25113_RS18750
cysE
gpsA
secB
grxC
D_1565
D_1564
D_1563
Dc_1879
Dc_1878
Dc_1877
Dc_1876
Dc_1875
Dc_1874
I_2138
I_2137
I_2136
I_2135
I_2134
I_2133
I_2132
I_2131
Term: termination (4), NS: new start (1), PS: pausing site (0), iTSS_I: internal starting site (0)

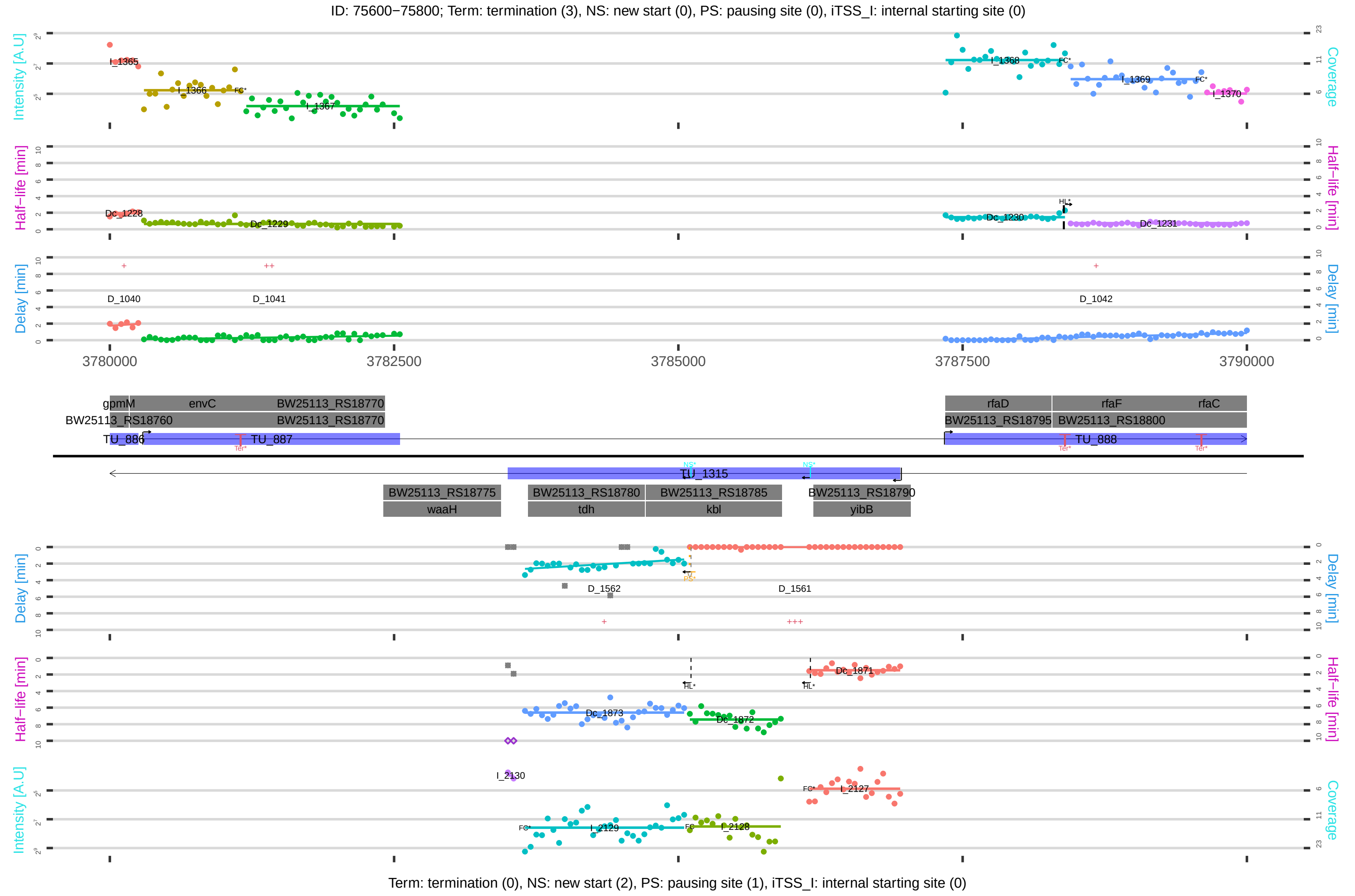

ID: 75600−75800; Term: termination (3), NS: new start (0), PS: pausing site (0), iTSS_I: internal starting site (0)
Intensity [A.U]
Coverage
Half−life [min]
Delay [min]
I_1365
I_1366
I_1367
I_1368
I_1369
I_1370
Dc_1228
Dc_1229
Dc_1230
Dc_1231
D_1040
D_1041
D_1042
3780000
3782500
3785000
3787500
3790000
gpmM
envC
BW25113_RS18770
BW25113_RS18760
rfaD
rfaF
rfaC
BW25113_RS18795
BW25113_RS18800
TU_886
TU_887
TU_888
TU_1315
BW25113_RS18775
BW25113_RS18780
BW25113_RS18785
BW25113_RS18790
waaH
tdh
kbl
yibB
D_1562
D_1561
Dc_1873
Dc_1872
Dc_1871
I_2130
I_2129
I_2128
I_2127
Term: termination (0), NS: new start (2), PS: pausing site (1), iTSS_I: internal starting site (0)

ID: 75800−75832; Term: termination (1), NS: new start (0), PS: pausing site (0), iTSS_I: internal starting site (0)

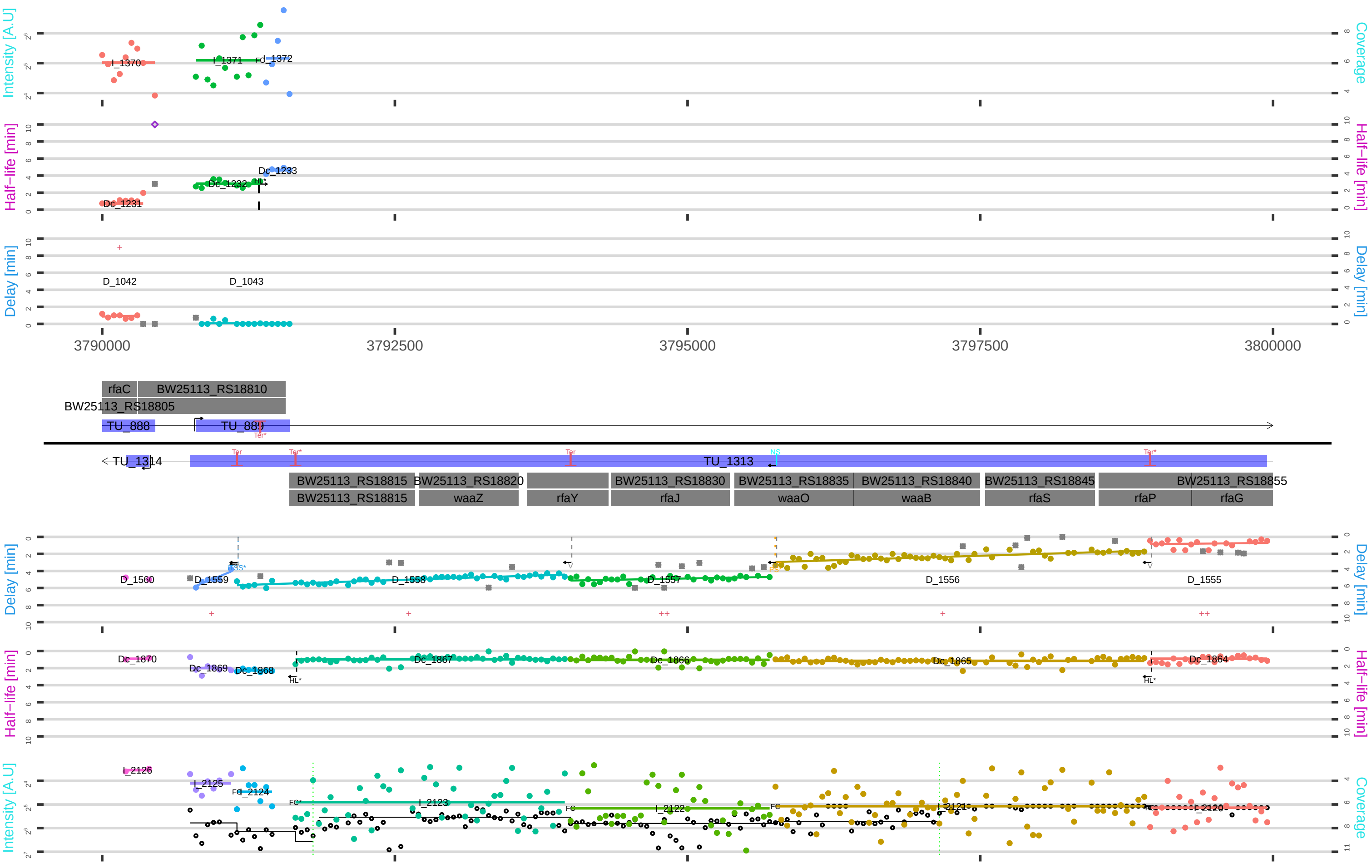

Term: termination (4), NS: new start (1), PS: pausing site (2), iTSS_I: internal starting site (2)

ID: 76040−76169; Term: termination (2), NS: new start (2), PS: pausing site (1), iTSS_I: internal starting site (0)

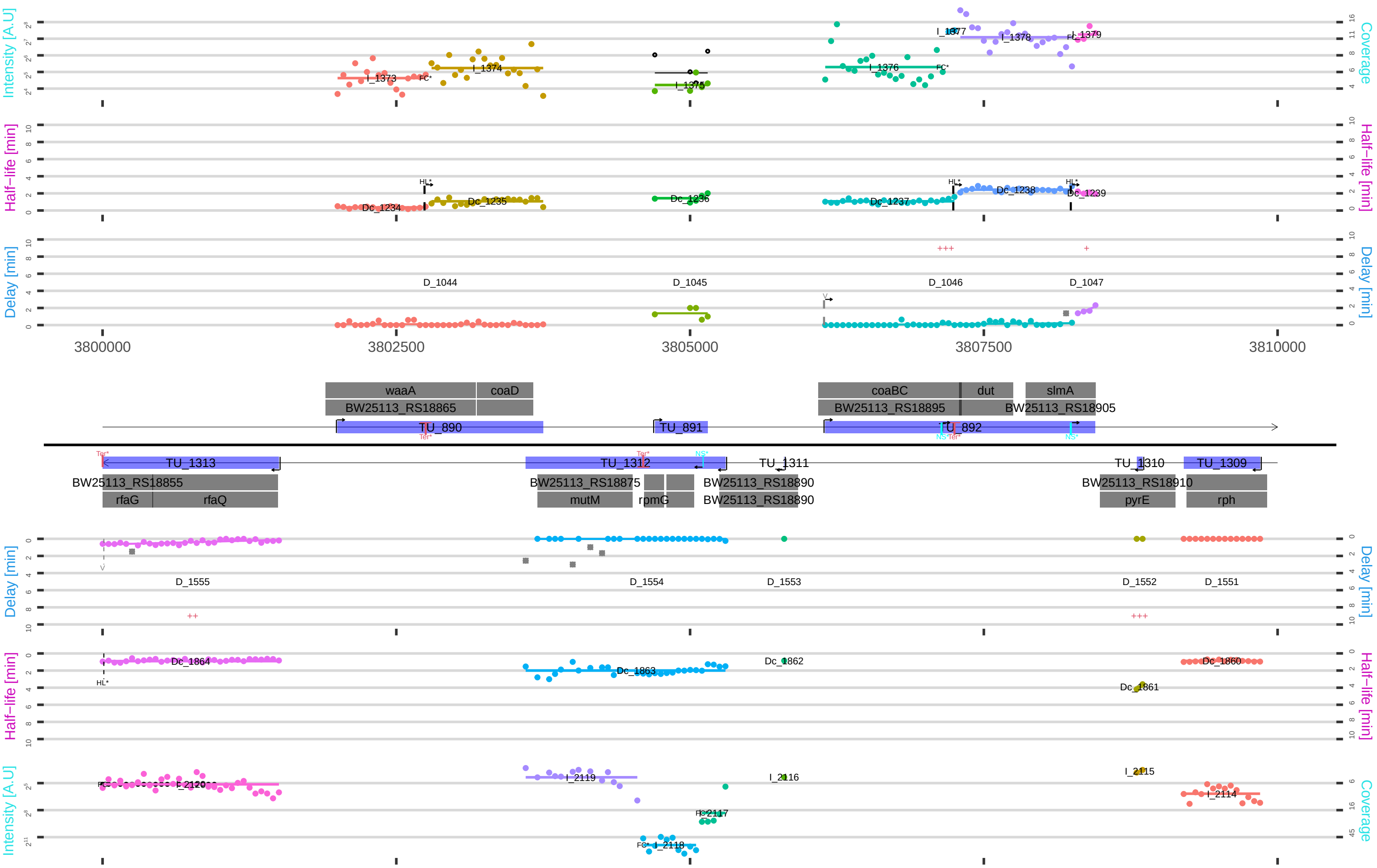

Term: termination (2), NS: new start (1), PS: pausing site (0), iTSS_I: internal starting site (0)

ID: 76202−76400; Term: termination (4), NS: new start (1), PS: pausing site (2), iTSS_I: internal starting site (0)

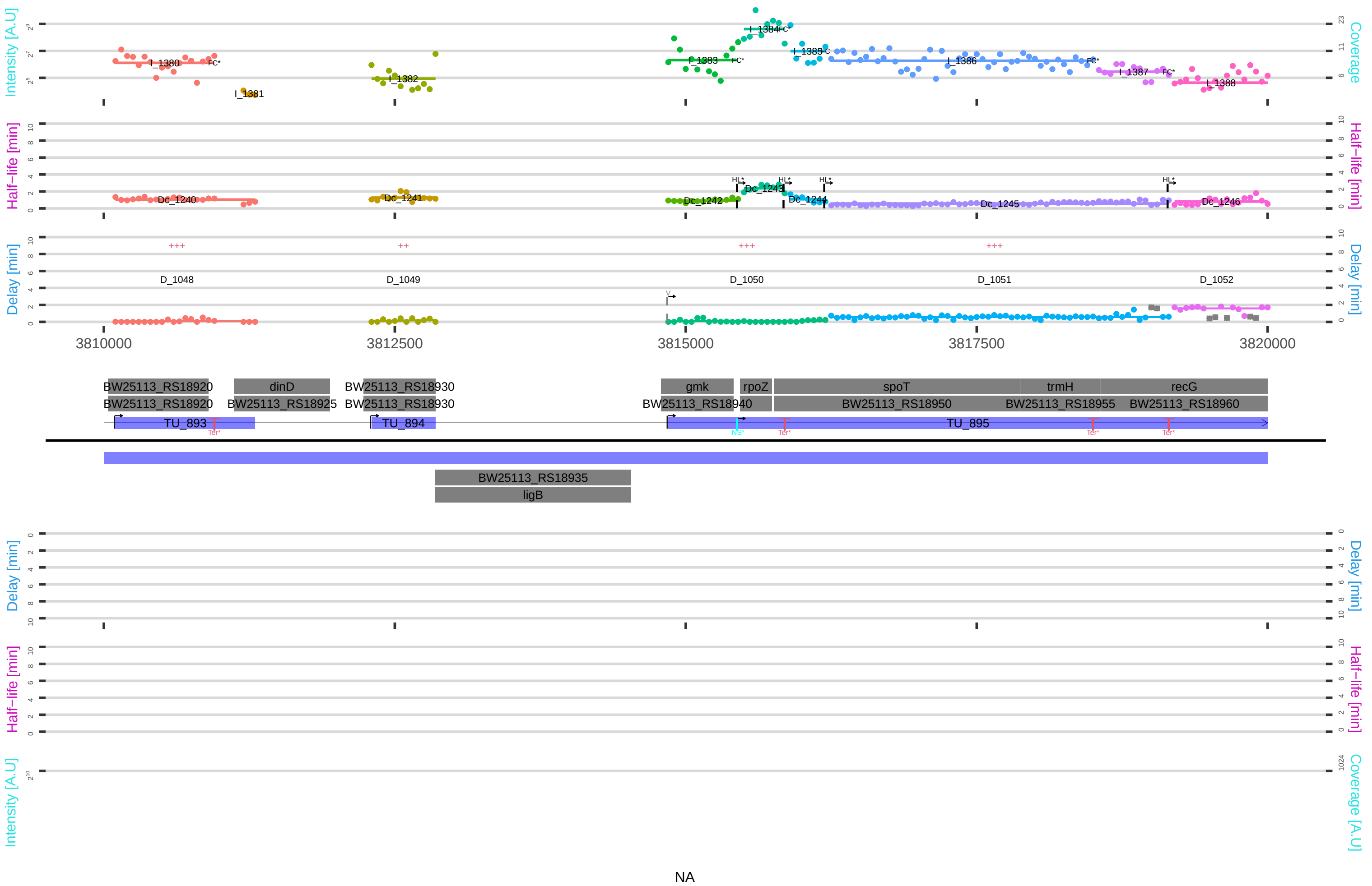

ID: 76400−76595; Term: termination (0), NS: new start (1), PS: pausing site (0), iTSS_I: internal starting site (0)

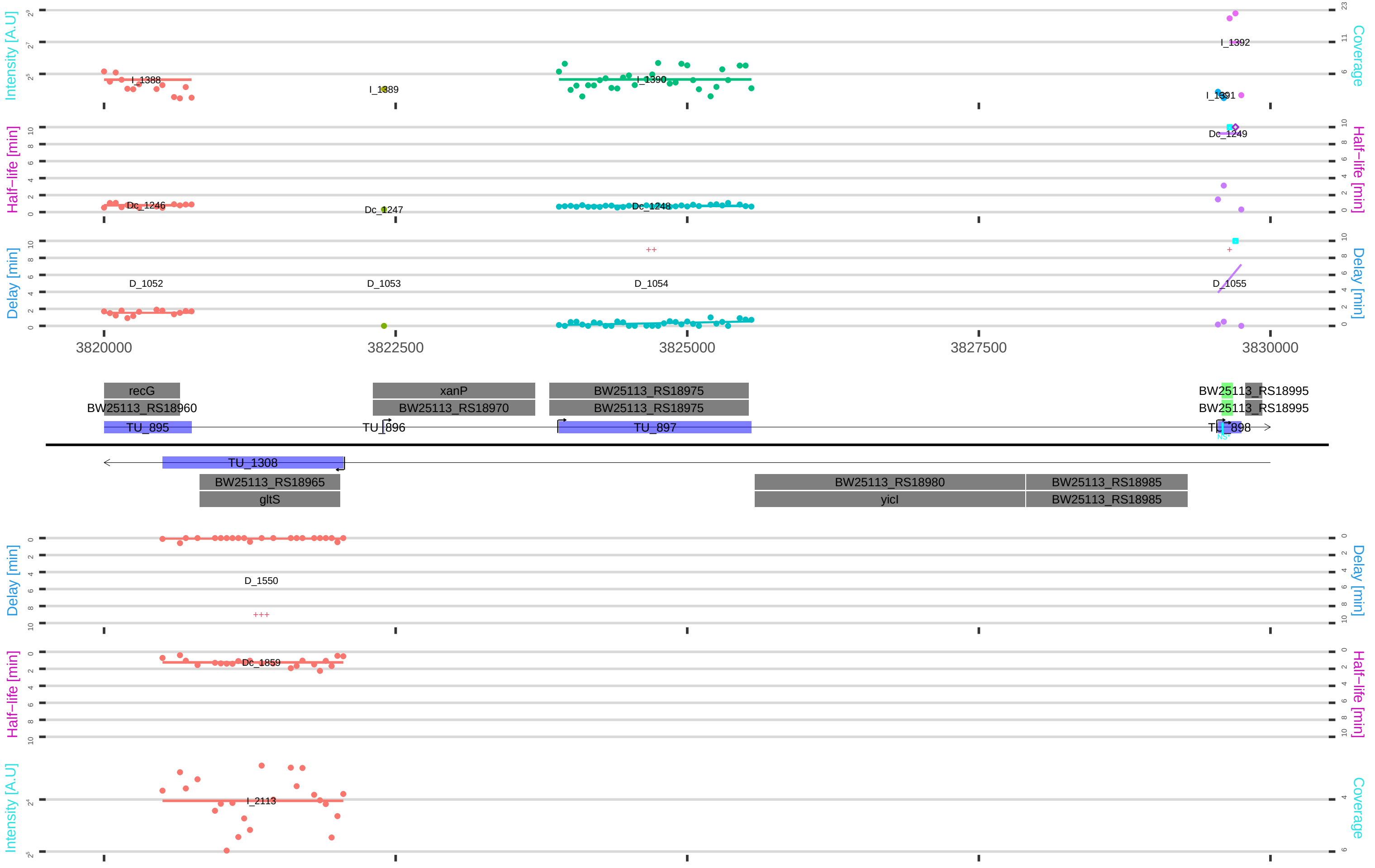

Term: termination (0), NS: new start (0), PS: pausing site (0), iTSS_I: internal starting site (0)

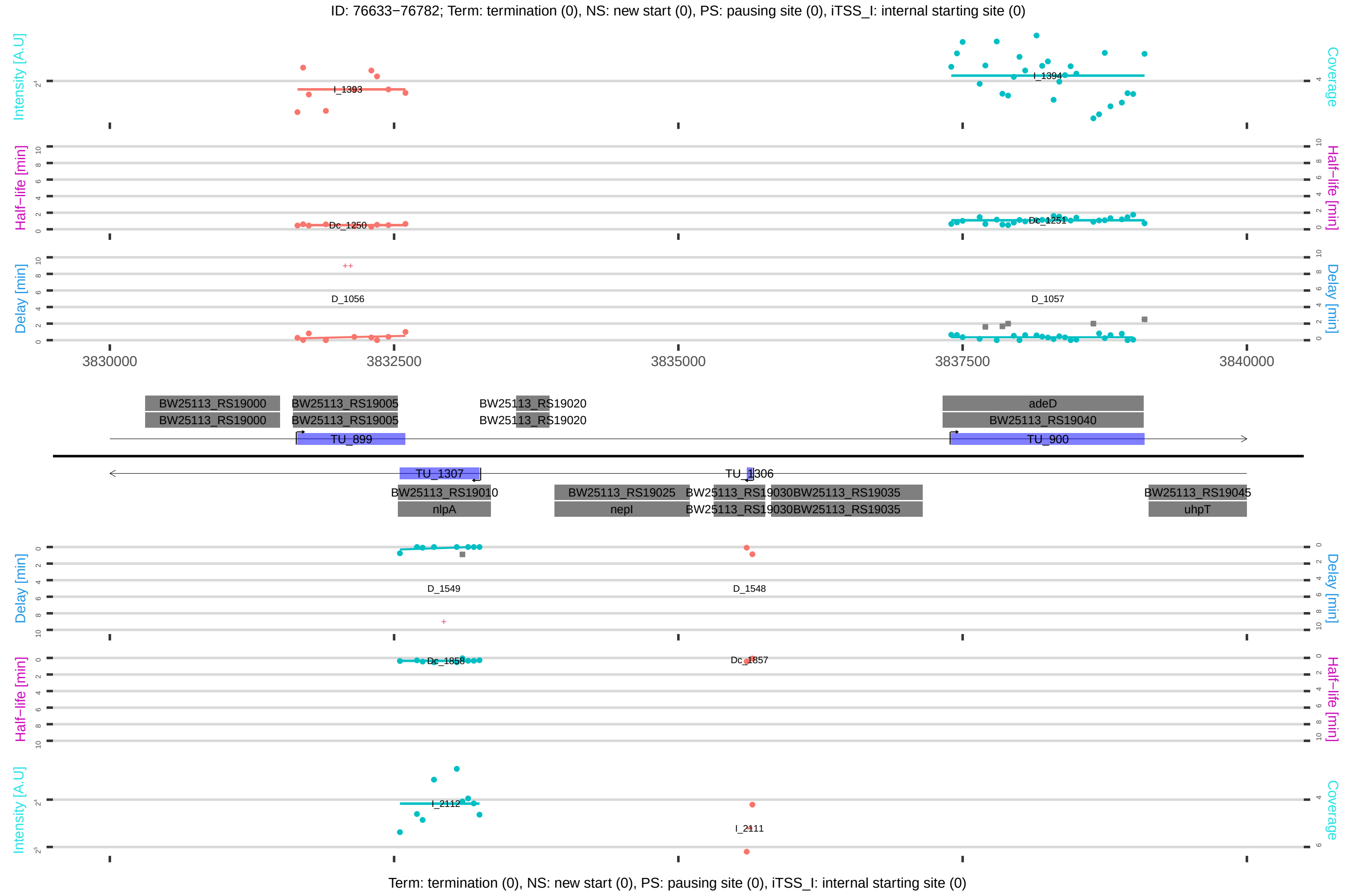

ID: 76633−76782; Term: termination (0), NS: new start (0), PS: pausing site (0), iTSS_I: internal starting site (0)
Intensity [A.U]
Coverage
I_1393
I_1394
Half−life [min]
Dc_1250
Dc_1251
Delay [min]
D_1056
D_1057
3830000
3832500
3835000
3837500
3840000
BW25113_RS19000
BW25113_RS19005
BW25113_RS19020
adeD
BW25113_RS19040
TU_899
TU_900
TU_1307
TU_1306
BW25113_RS19010
nlpA
BW25113_RS19025
nepI
BW25113_RS19030
BW25113_RS19035
BW25113_RS19045
uhpT
D_1549
D_1548
Dc_1858
Dc_1857
I_2112
I_2111
Term: termination (0), NS: new start (0), PS: pausing site (0), iTSS_I: internal starting site (0)

ID: 76936−76975; Term: termination (1), NS: new start (0), PS: pausing site (0), iTSS_I: internal starting site (0)

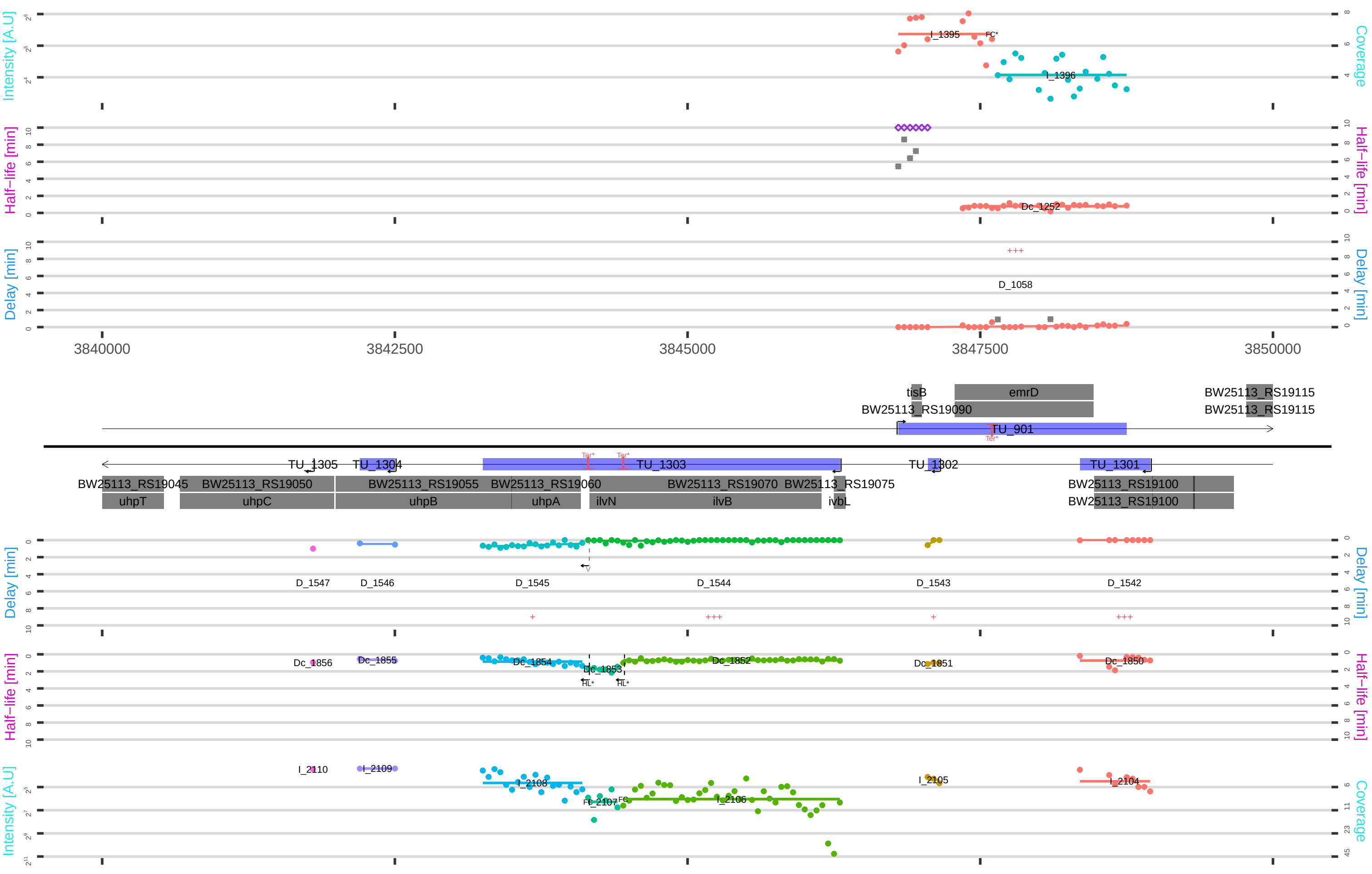

Term: termination (2), NS: new start (0), PS: pausing site (1), iTSS_I: internal starting site (0)

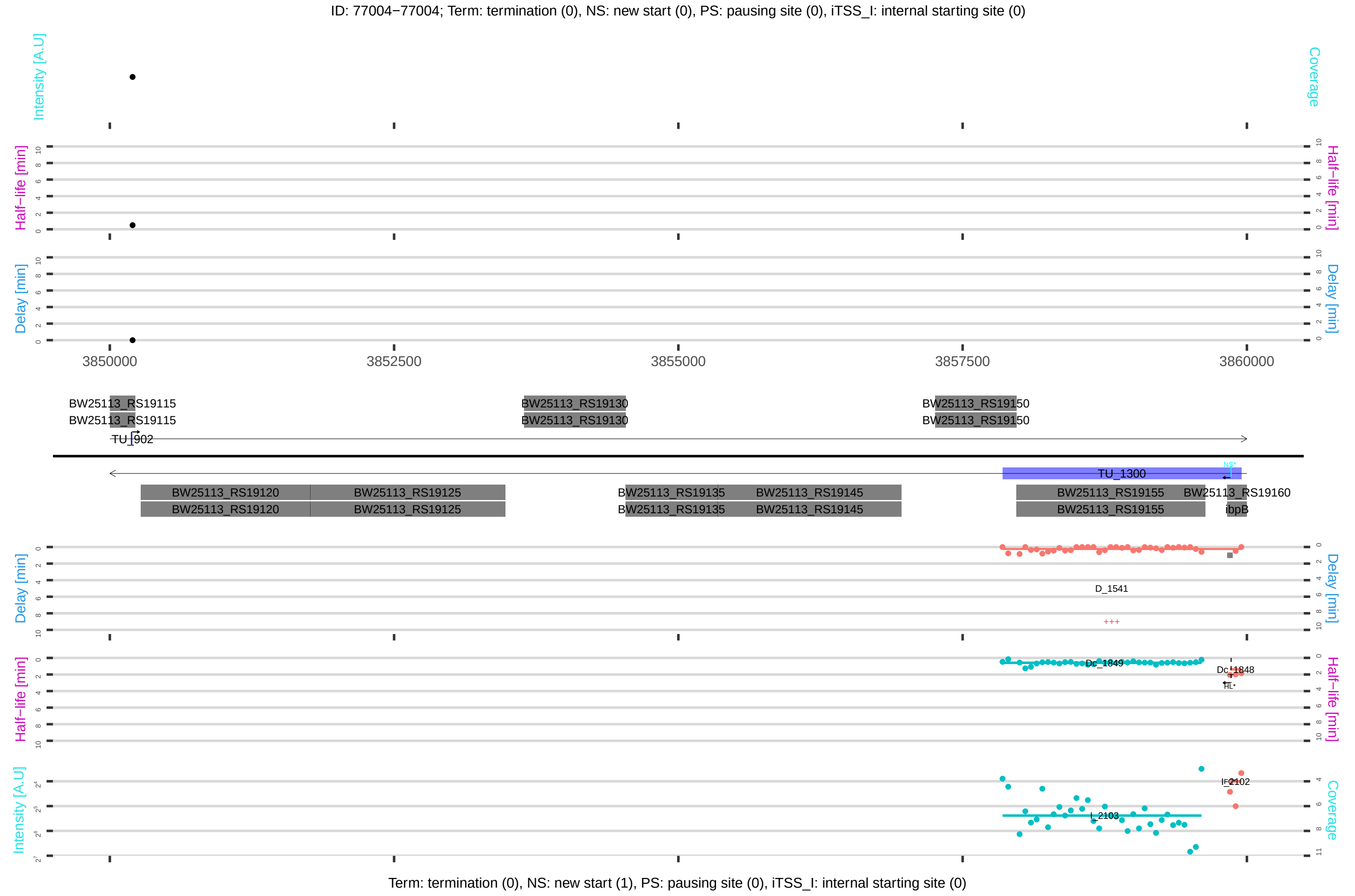

ID: 77004−77004; Term: termination (0), NS: new start (0), PS: pausing site (0), iTSS_I: internal starting site (0)
Intensity [A.U]
Coverage
Half−life [min]
Delay [min]
3850000
3852500
3855000
3857500
3860000
BW25113_RS19115
BW25113_RS19130
BW25113_RS19150
TU_902
TU_1300
BW25113_RS19120
BW25113_RS19125
BW25113_RS19135
BW25113_RS19145
BW25113_RS19155
BW25113_RS19160
ibpB
D_1541
Dc_1849
Dc_1848
I_2103
I_2102
Term: termination (0), NS: new start (1), PS: pausing site (0), iTSS_I: internal starting site (0)

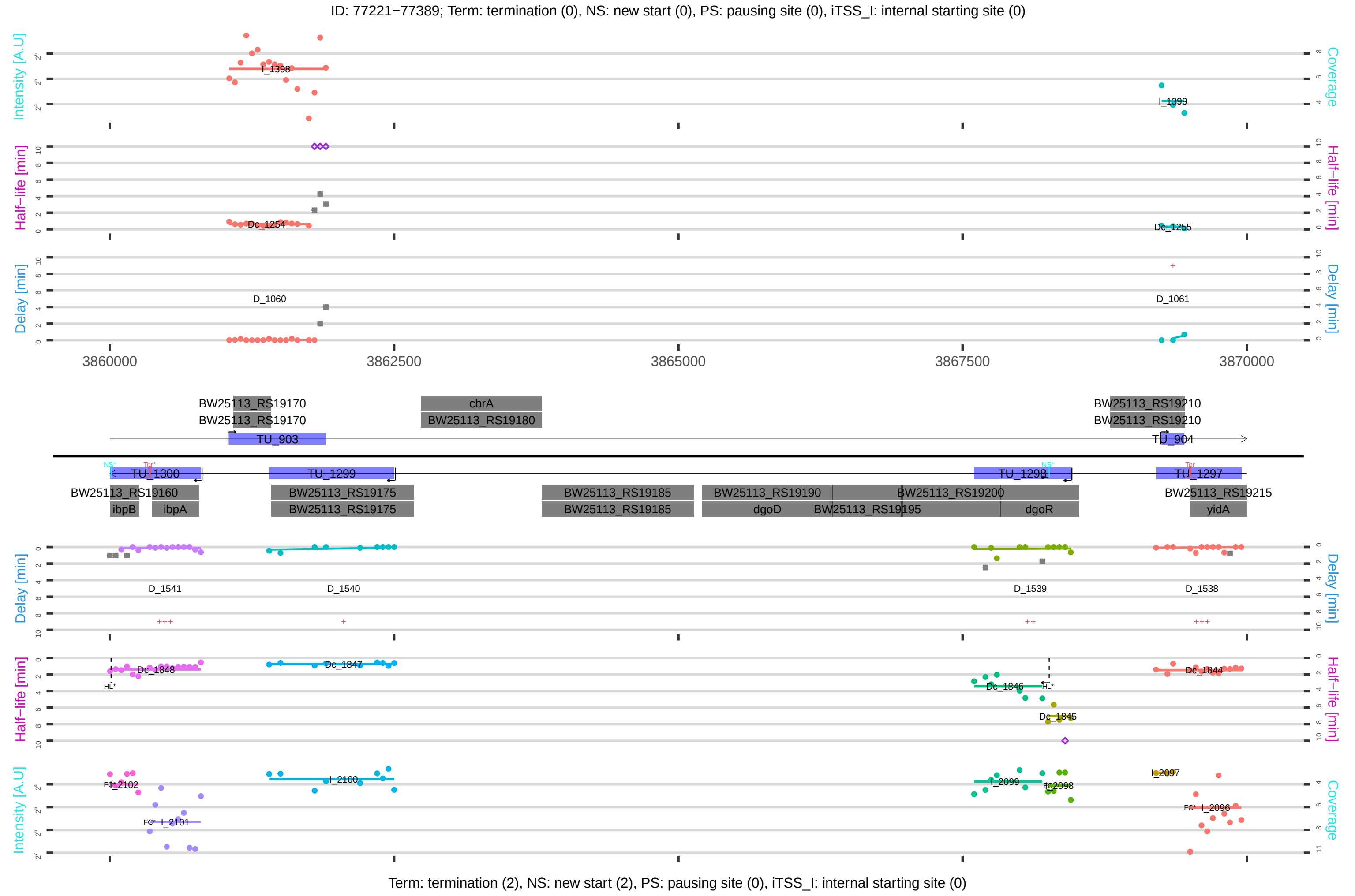

ID: 77221−77389; Term: termination (0), NS: new start (0), PS: pausing site (0), iTSS_I: internal starting site (0)
Intensity [A.U]
Coverage
I_1398
I_1399
Half−life [min]
Dc_1254
Dc_1255
Delay [min]
D_1060
D_1061
3860000
3862500
3865000
3867500
3870000
BW25113_RS19170
cbrA
BW25113_RS19180
BW25113_RS19210
TU_903
TU_904
TU_1300
TU_1299
TU_1298
TU_1297
BW25113_RS19160
ibpB
ibpA
BW25113_RS19175
BW25113_RS19185
BW25113_RS19190
dgoD
BW25113_RS19195
BW25113_RS19200
dgoR
BW25113_RS19215
yidA
D_1541
D_1540
D_1539
D_1538
Dc_1848
Dc_1847
Dc_1846
Dc_1845
Dc_1844
I_2102
I_2101
I_2100
I_2099
I_2098
I_2097
I_2096
Term: termination (2), NS: new start (2), PS: pausing site (0), iTSS_I: internal starting site (0)

ID: 77551−77600; Term: termination (1), NS: new start (0), PS: pausing site (0), iTSS_I: internal starting site (0)

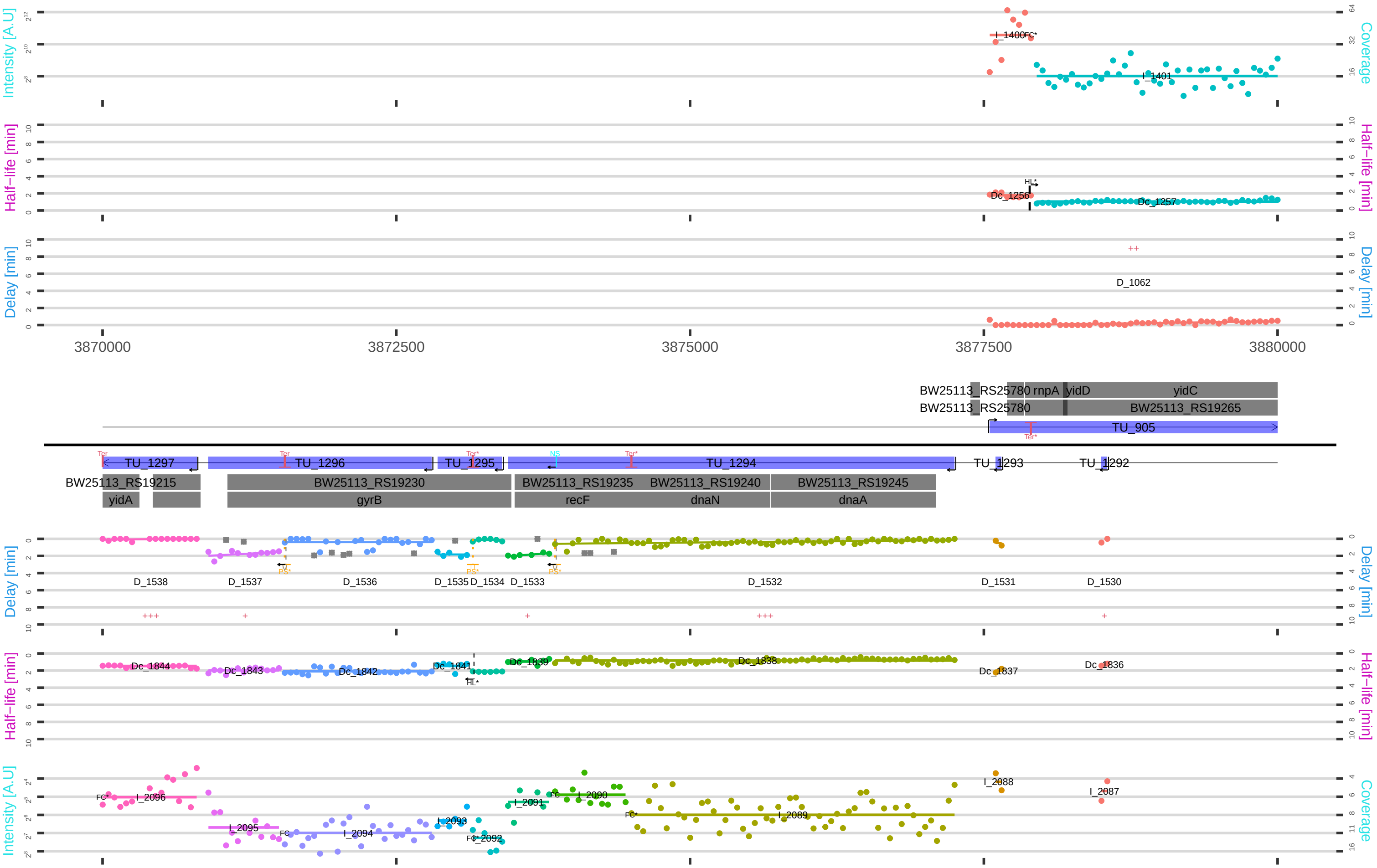

Term: termination (4), NS: new start (1), PS: pausing site (3), iTSS_I: internal starting site (0)

ID: 77600−77772; Term: termination (4), NS: new start (2), PS: pausing site (3), iTSS_I: internal starting site (0)

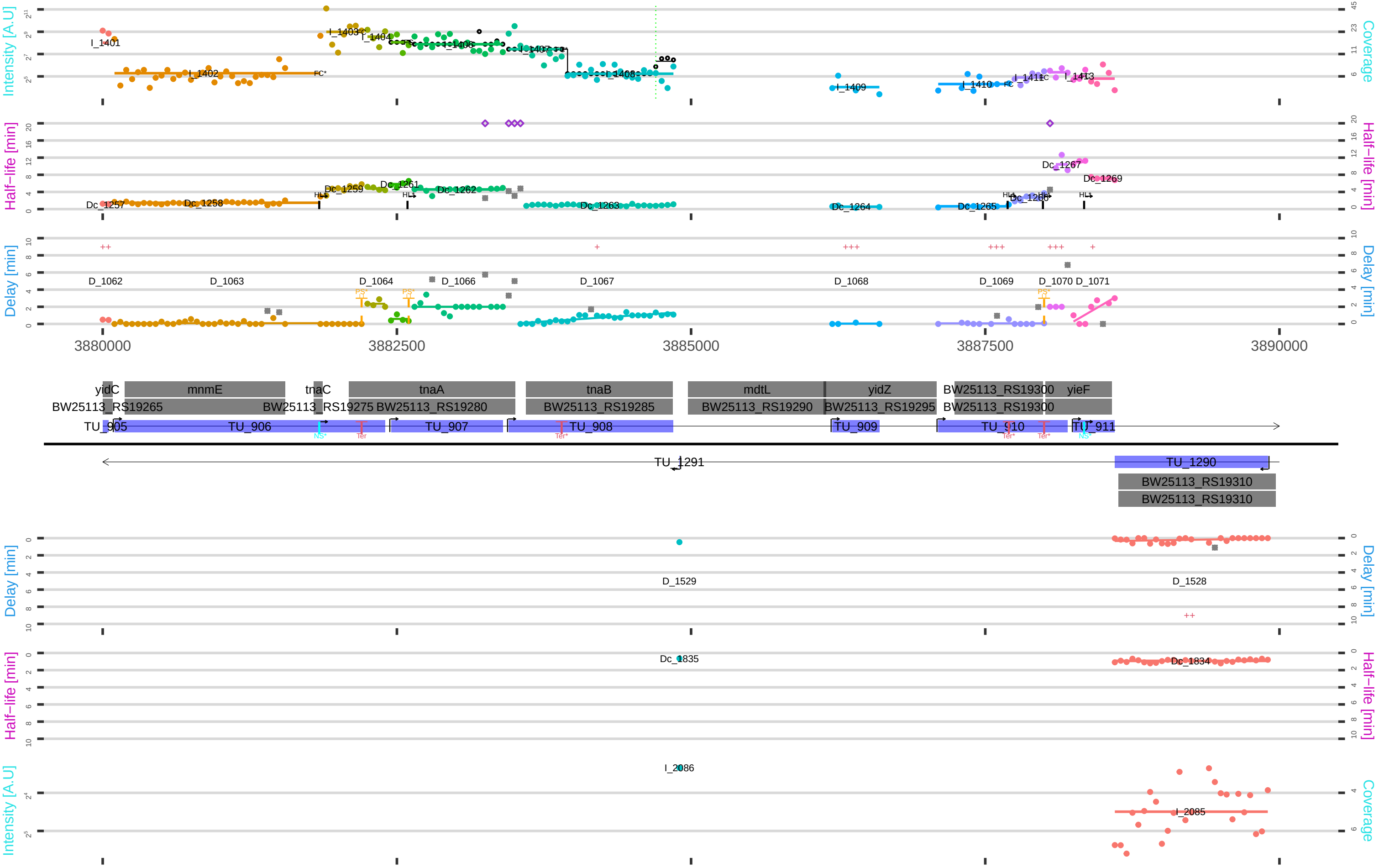

Term: termination (0), NS: new start (0), PS: pausing site (0), iTSS_I: internal starting site (0)

ID: 77803−77961; Term: termination (0), NS: new start (0), PS: pausing site (0), iTSS_I: internal starting site (0)

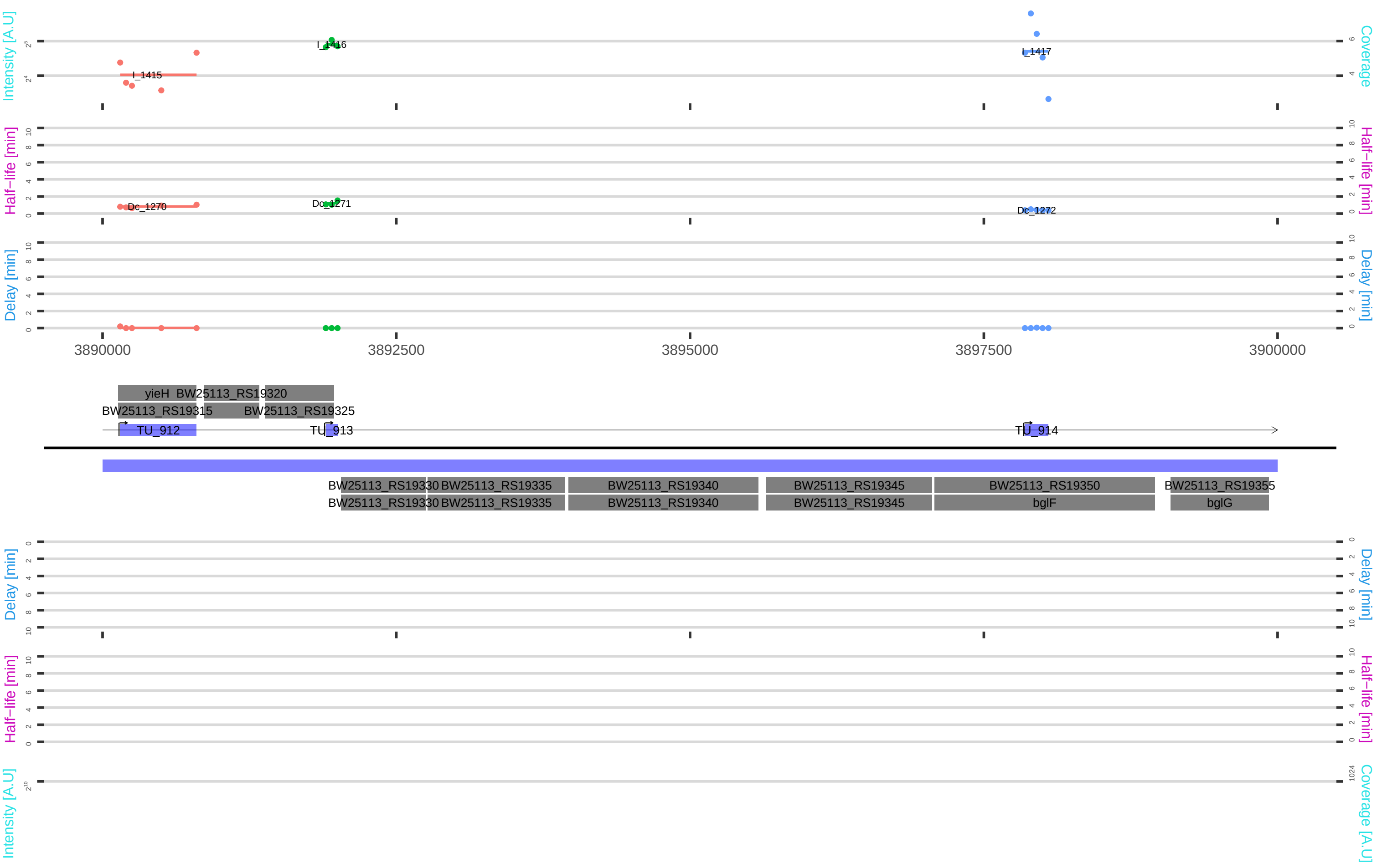

NA

ID: 78103−78182; Term: termination (0), NS: new start (0), PS: pausing site (0), iTSS_I: internal starting site (0)

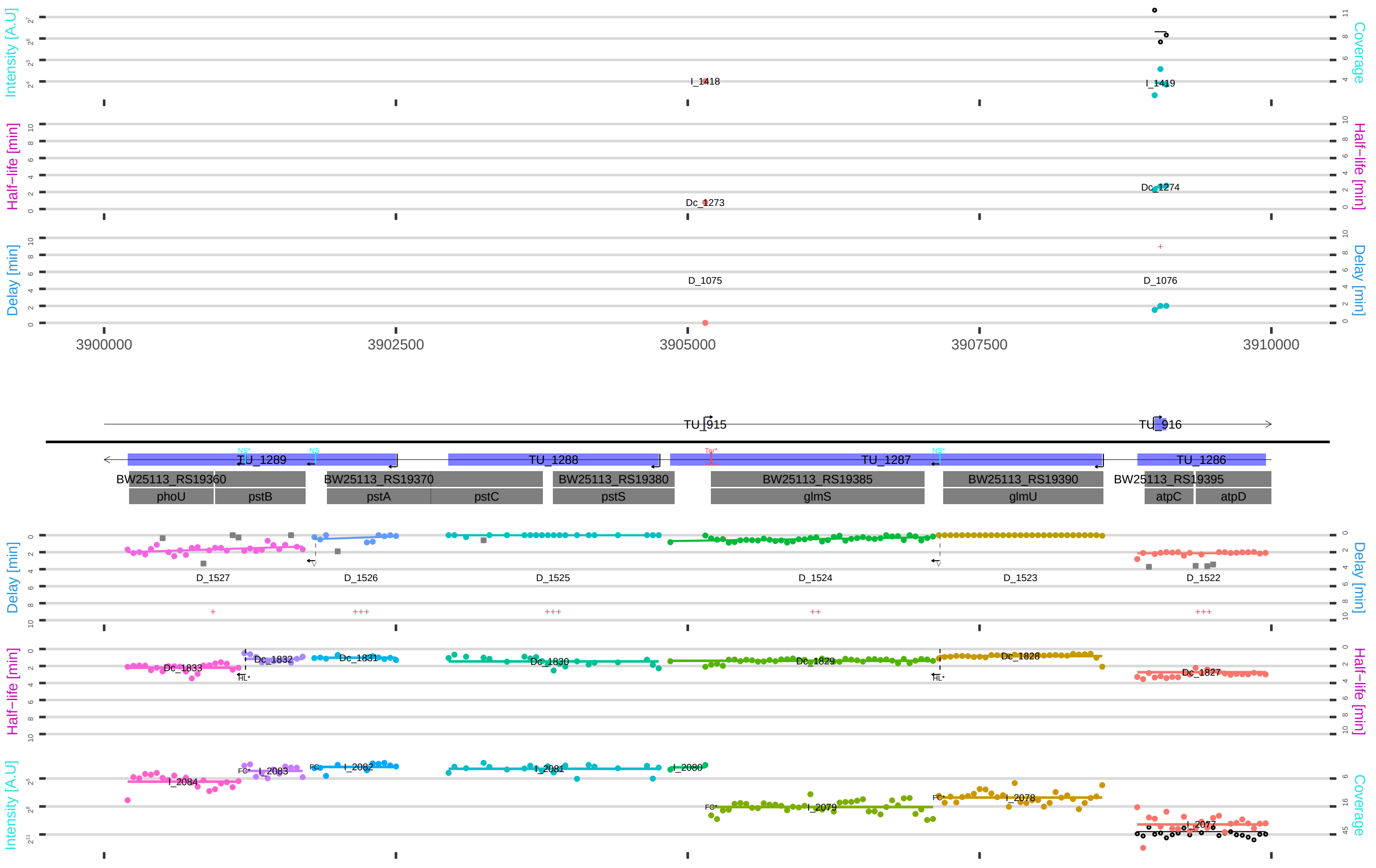

Term: termination (1), NS: new start (3), PS: pausing site (2), iTSS_I: internal starting site (0)

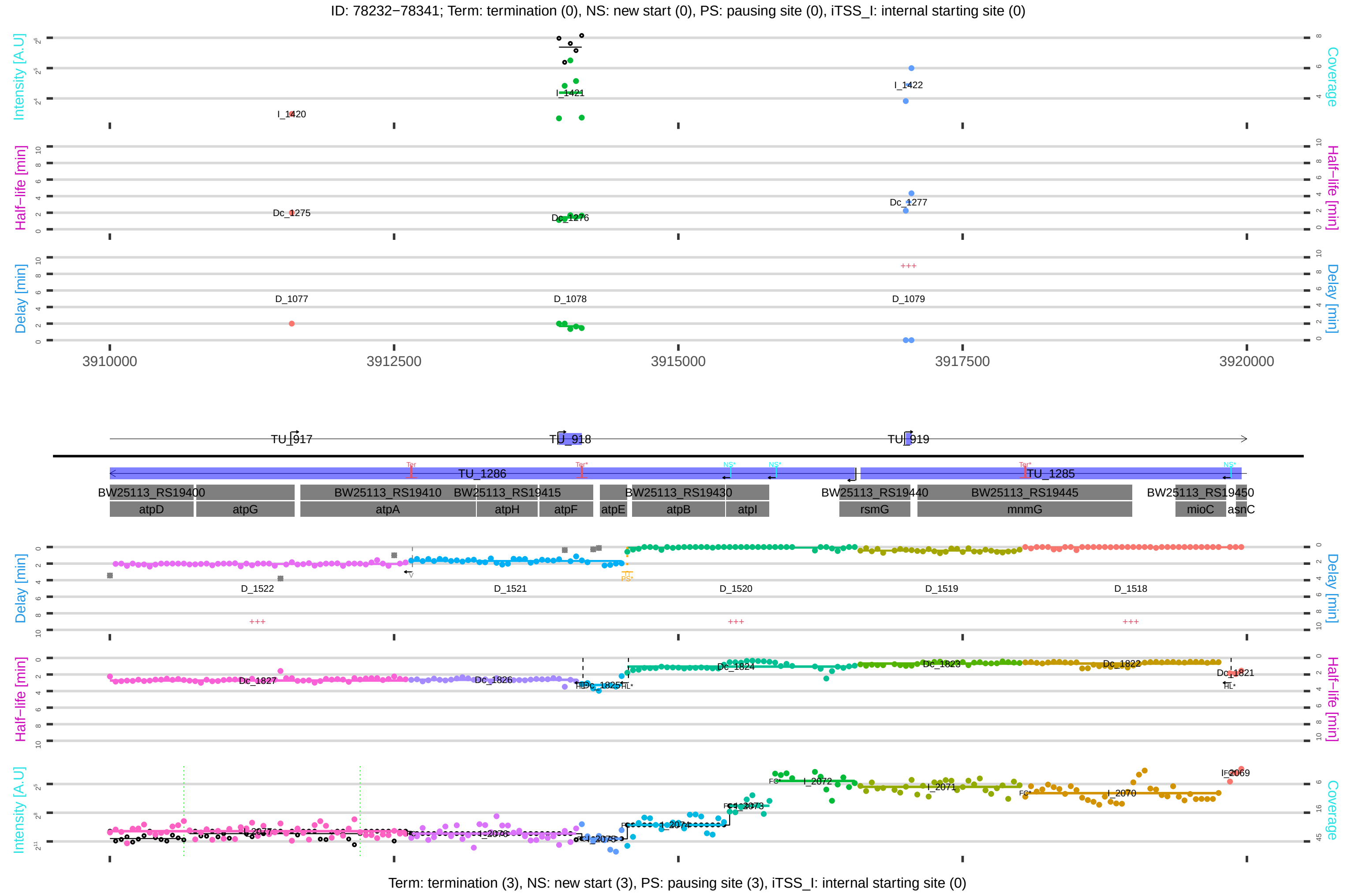
ID: 78232−78341; Term: termination (0), NS: new start (0), PS: pausing site (0), iTSS_I: internal starting site (0)
Intensity [A.U]
Coverage
I_1420
I_1421
I_1422
Half−life [min]
Dc_1275
Dc_1276
Dc_1277
Delay [min]
D_1077
D_1078
D_1079
3910000
3912500
3915000
3917500
3920000
TU_917
TU_918
TU_919
TU_1286
TU_1285
BW25113_RS19400
BW25113_RS19410
BW25113_RS19415
BW25113_RS19430
BW25113_RS19440
BW25113_RS19445
BW25113_RS19450
atpD
atpG
atpA
atpH
atpF
atpE
atpB
atpI
rsmG
mnmG
mioC
asnC
D_1522
D_1521
D_1520
D_1519
D_1518
Dc_1827
Dc_1826
Dc_1825
Dc_1824
Dc_1823
Dc_1822
Dc_1821
I_2077
I_2076
I_2075
I_2074
I_2073
I_2072
I_2071
I_2070
I_2069
Term: termination (3), NS: new start (3), PS: pausing site (3), iTSS_I: internal starting site (0)

ID: 78411−78600; Term: termination (3), NS: new start (2), PS: pausing site (1), iTSS_I: internal starting site (0)

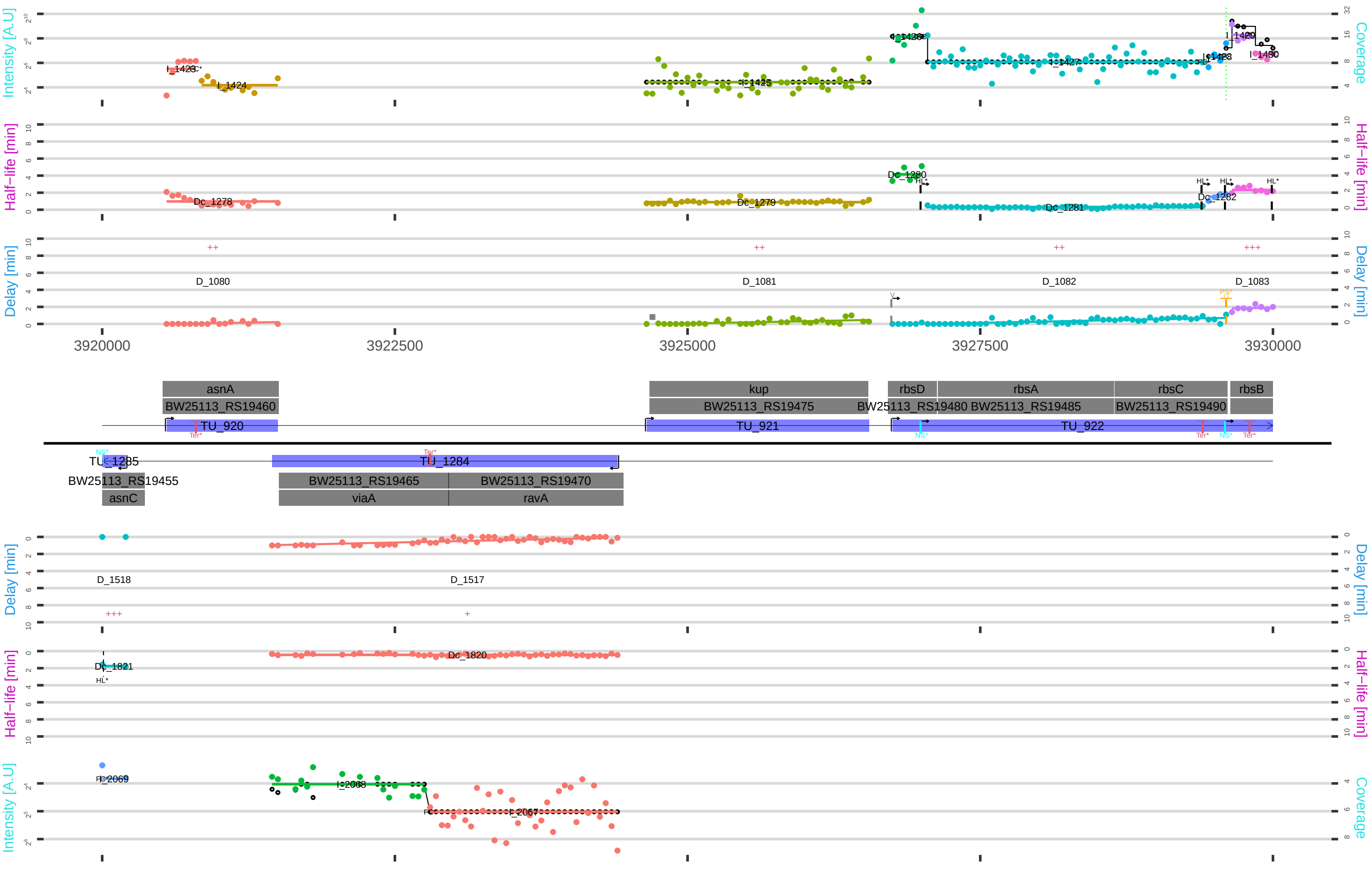

Term: termination (1), NS: new start (1), PS: pausing site (0), iTSS_I: internal starting site (0)

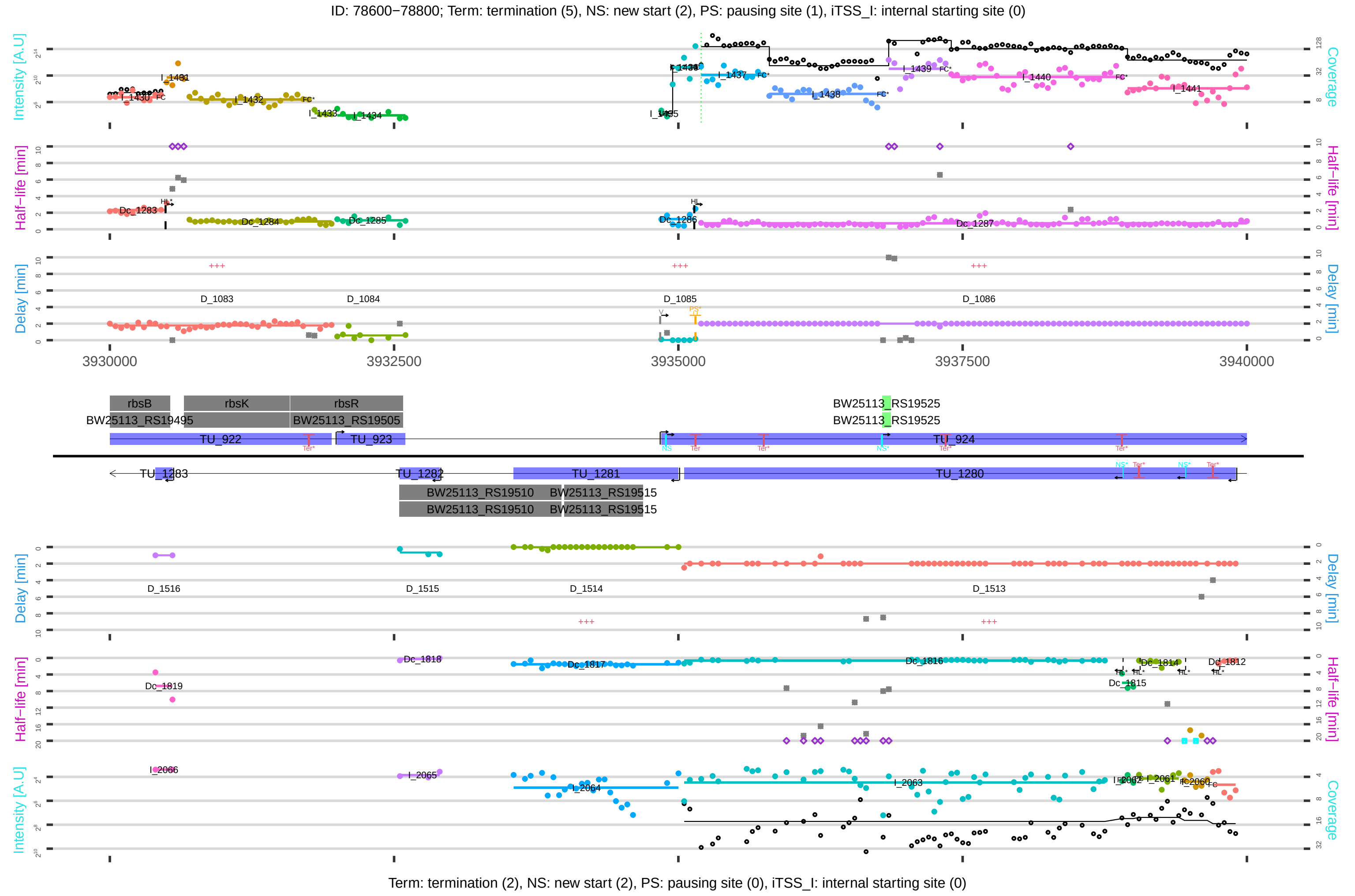
ID: 78600−78800; Term: termination (5), NS: new start (2), PS: pausing site (1), iTSS_I: internal starting site (0)
Intensity [A.U]
Coverage
Half−life [min]
Delay [min]
3930000
3932500
3935000
3937500
3940000
rbsB
rbsK
rbsR
BW25113_RS19495
BW25113_RS19505
BW25113_RS19525
TU_922
TU_923
TU_924
TU_1283
TU_1282
TU_1281
TU_1280
BW25113_RS19510
BW25113_RS19515
D_1083
D_1084
D_1085
D_1086
D_1516
D_1515
D_1514
D_1513
Term: termination (2), NS: new start (2), PS: pausing site (0), iTSS_I: internal starting site (0)

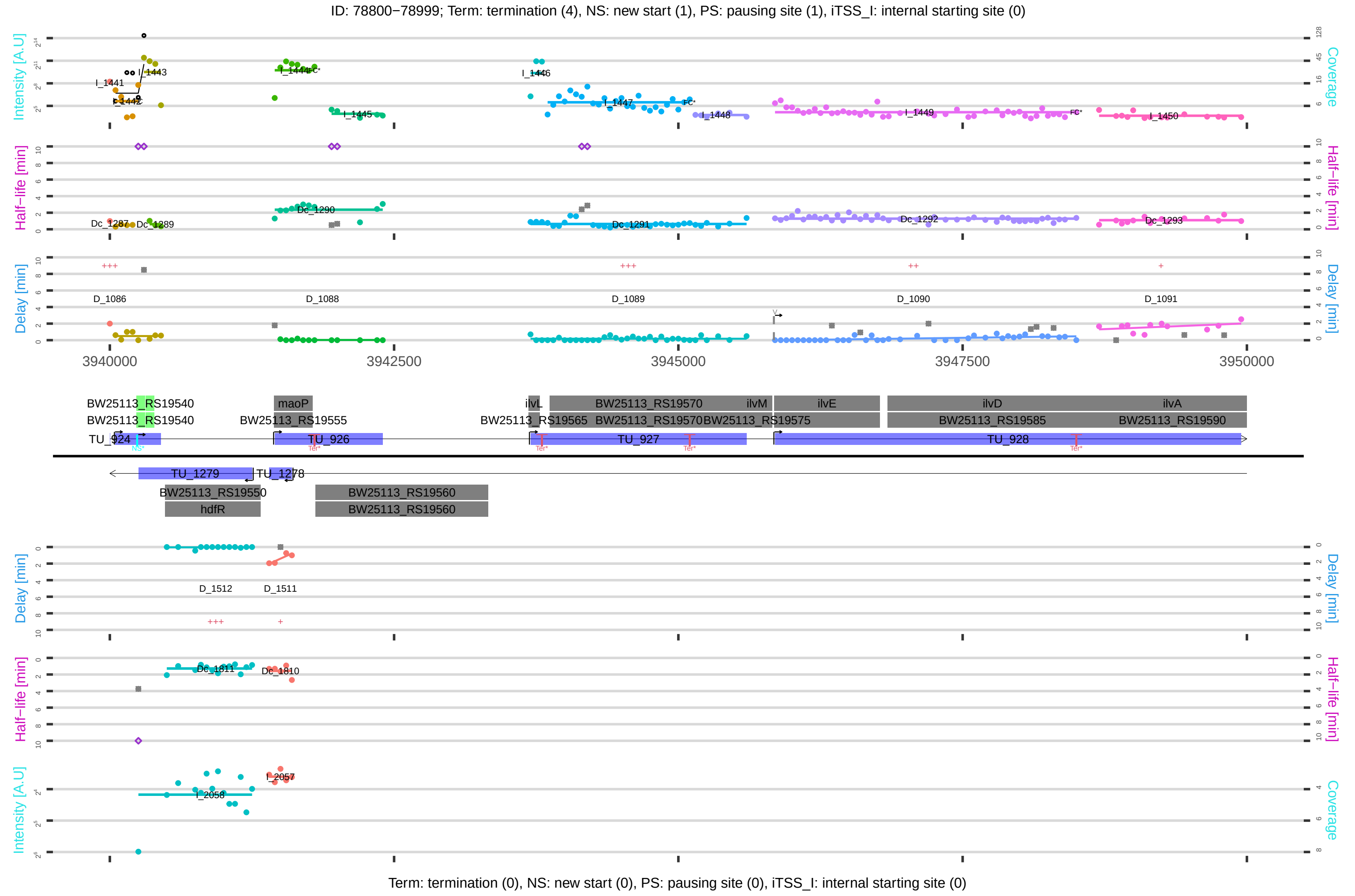

ID: 78800−78999; Term: termination (4), NS: new start (1), PS: pausing site (1), iTSS_I: internal starting site (0)
Intensity [A.U]
Coverage
Half−life [min]
Delay [min]
I_1441
I_1442
I_1443
I_1444
I_1445
I_1446
I_1447
I_1448
I_1449
I_1450
Dc_1287
Dc_1289
Dc_1290
Dc_1291
Dc_1292
Dc_1293
D_1086
D_1088
D_1089
D_1090
D_1091
3940000
3942500
3945000
3947500
3950000
BW25113_RS19540
maoP
BW25113_RS19555
ilvL
BW25113_RS19565
BW25113_RS19570
ilvM
BW25113_RS19575
ilvE
ilvD
BW25113_RS19585
ilvA
BW25113_RS19590
TU_924
TU_926
TU_927
TU_928
NS*
Ter*
TU_1279
TU_1278
BW25113_RS19550
hdfR
BW25113_RS19560
D_1512
D_1511
Dc_1811
Dc_1810
I_2057
I_2058
Term: termination (0), NS: new start (0), PS: pausing site (0), iTSS_I: internal starting site (0)

ID: 79004−79200; Term: termination (2), NS: new start (1), PS: pausing site (0), iTSS_I: internal starting site (0)

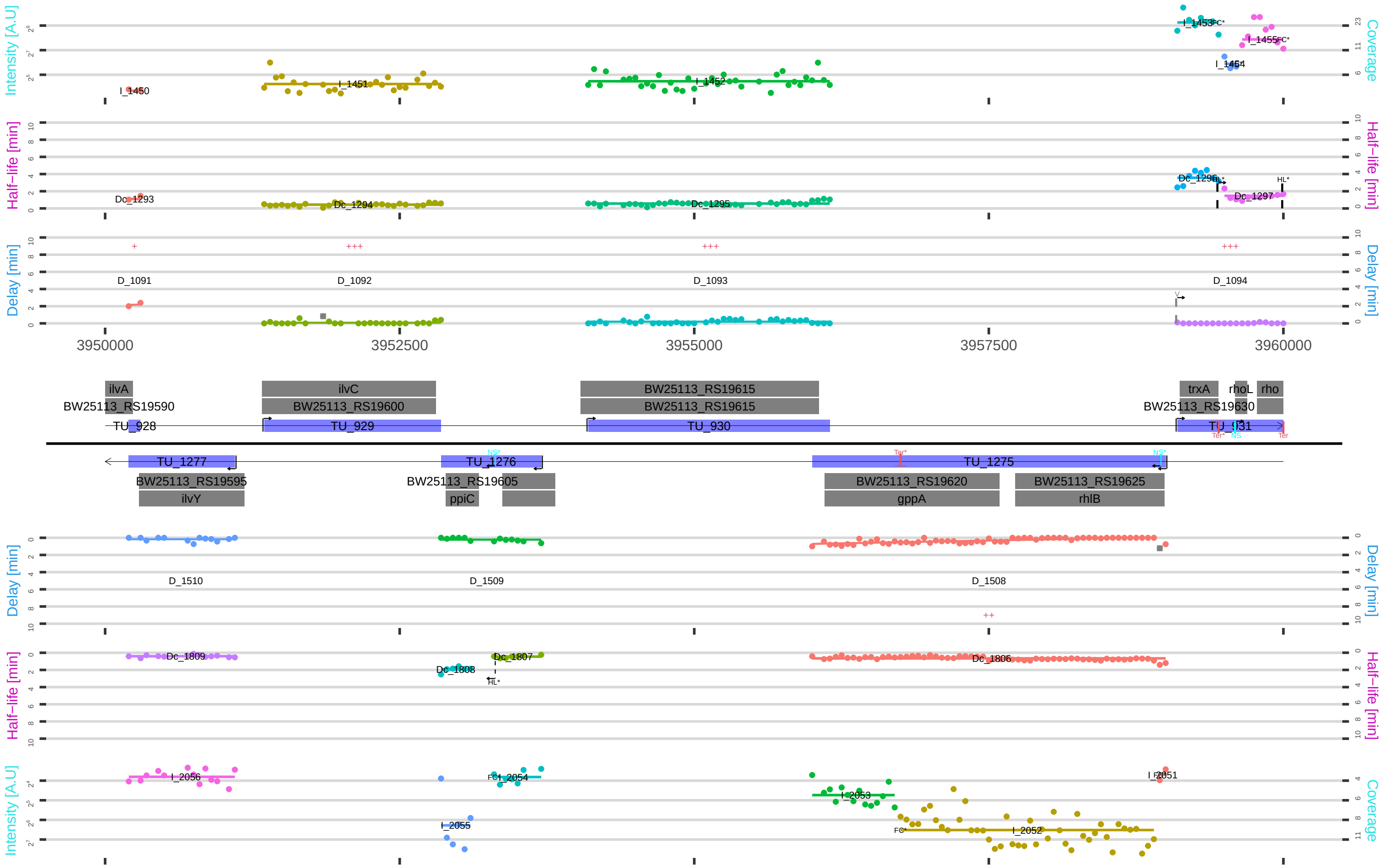

Term: termination (1), NS: new start (2), PS: pausing site (0), iTSS_I: internal starting site (0)

ID: 79200−79400; Term: termination (4), NS: new start (0), PS: pausing site (2), iTSS_I: internal starting site (0)

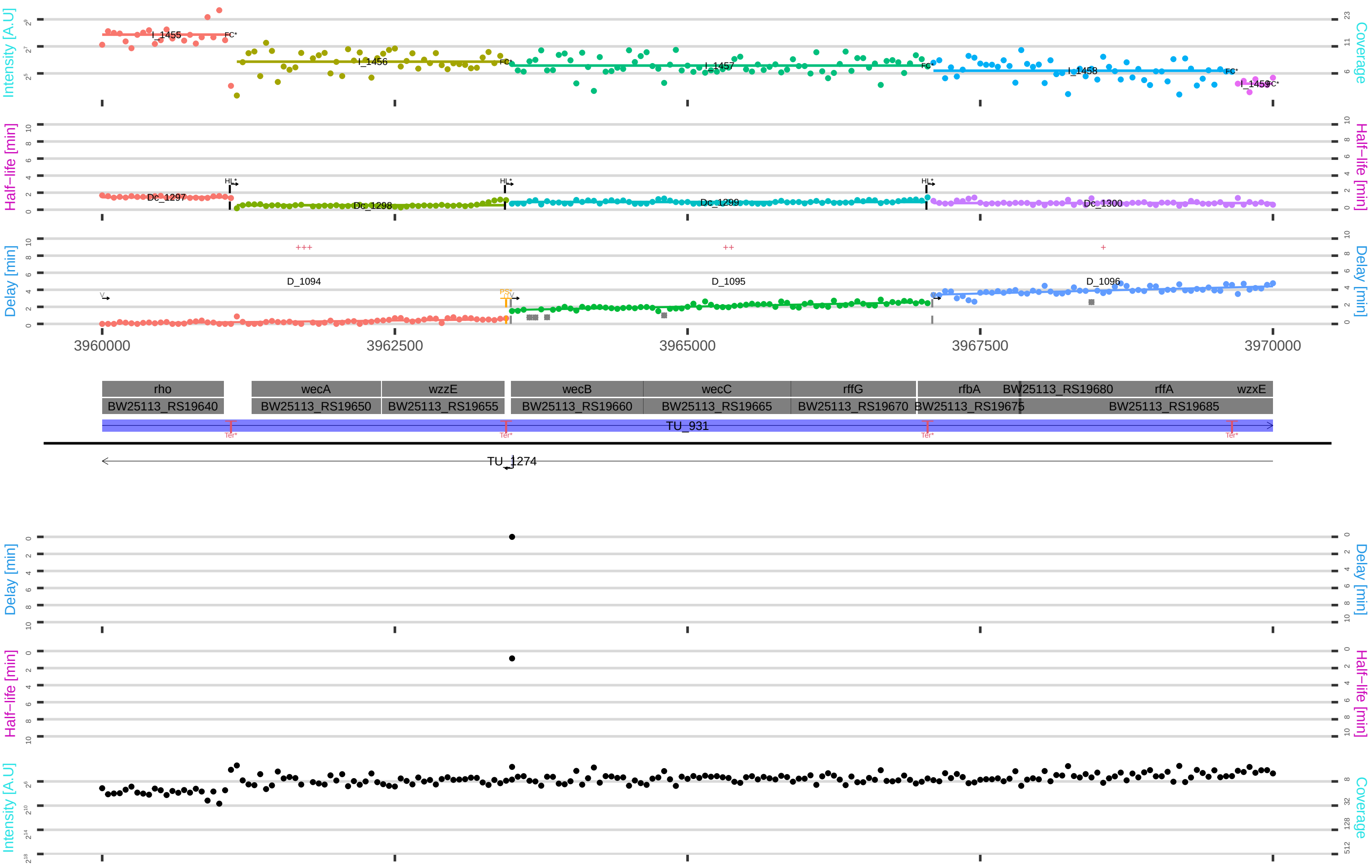

Term: termination (0), NS: new start (0), PS: pausing site (0), iTSS_I: internal starting site (0)

ID: 79400−79600; Term: termination (1), NS: new start (1), PS: pausing site (0), iTSS_I: internal starting site (2)

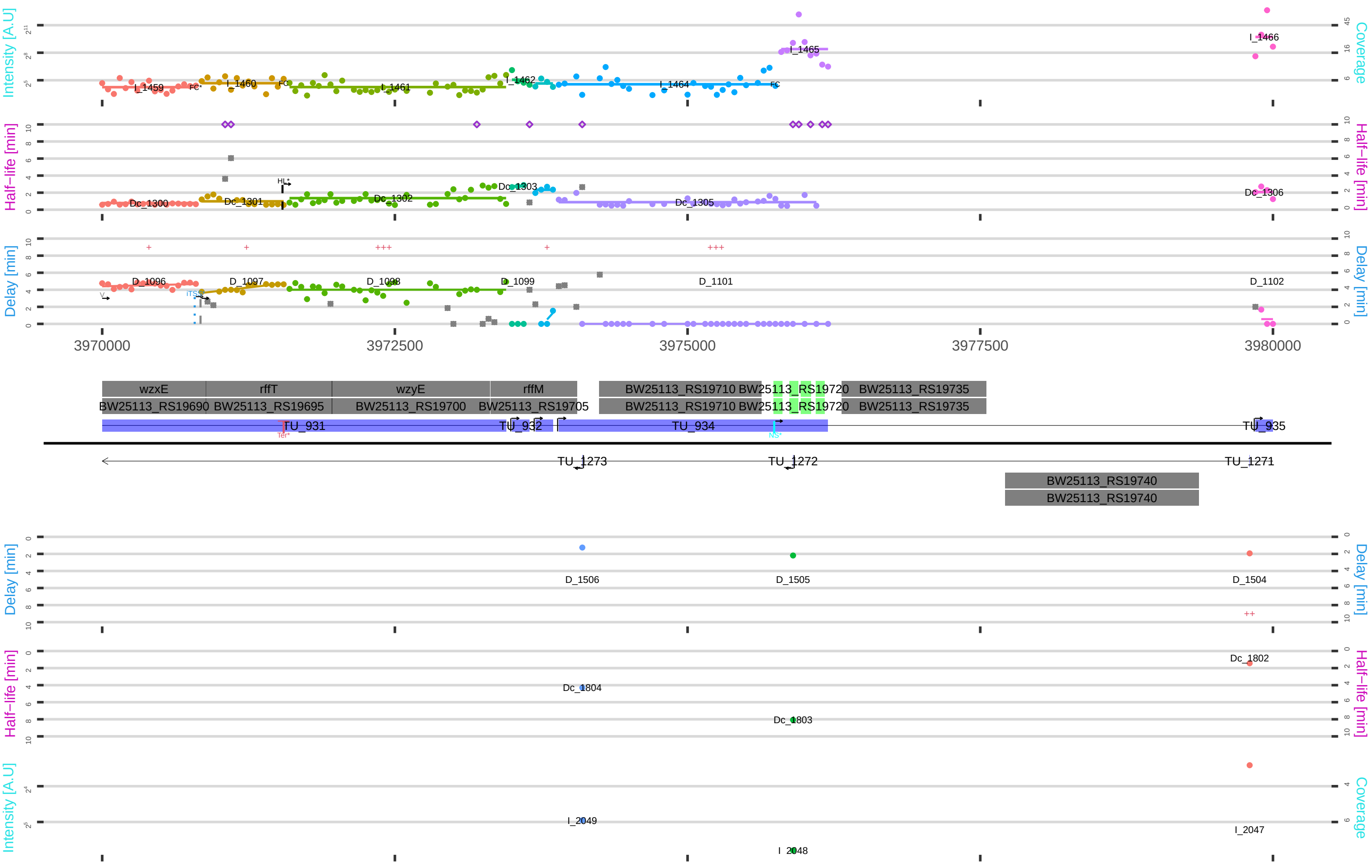

Term: termination (0), NS: new start (0), PS: pausing site (0), iTSS_I: internal starting site (0)

ID: 79600−79800; Term: termination (2), NS: new start (0), PS: pausing site (0), iTSS_I: internal starting site (0)

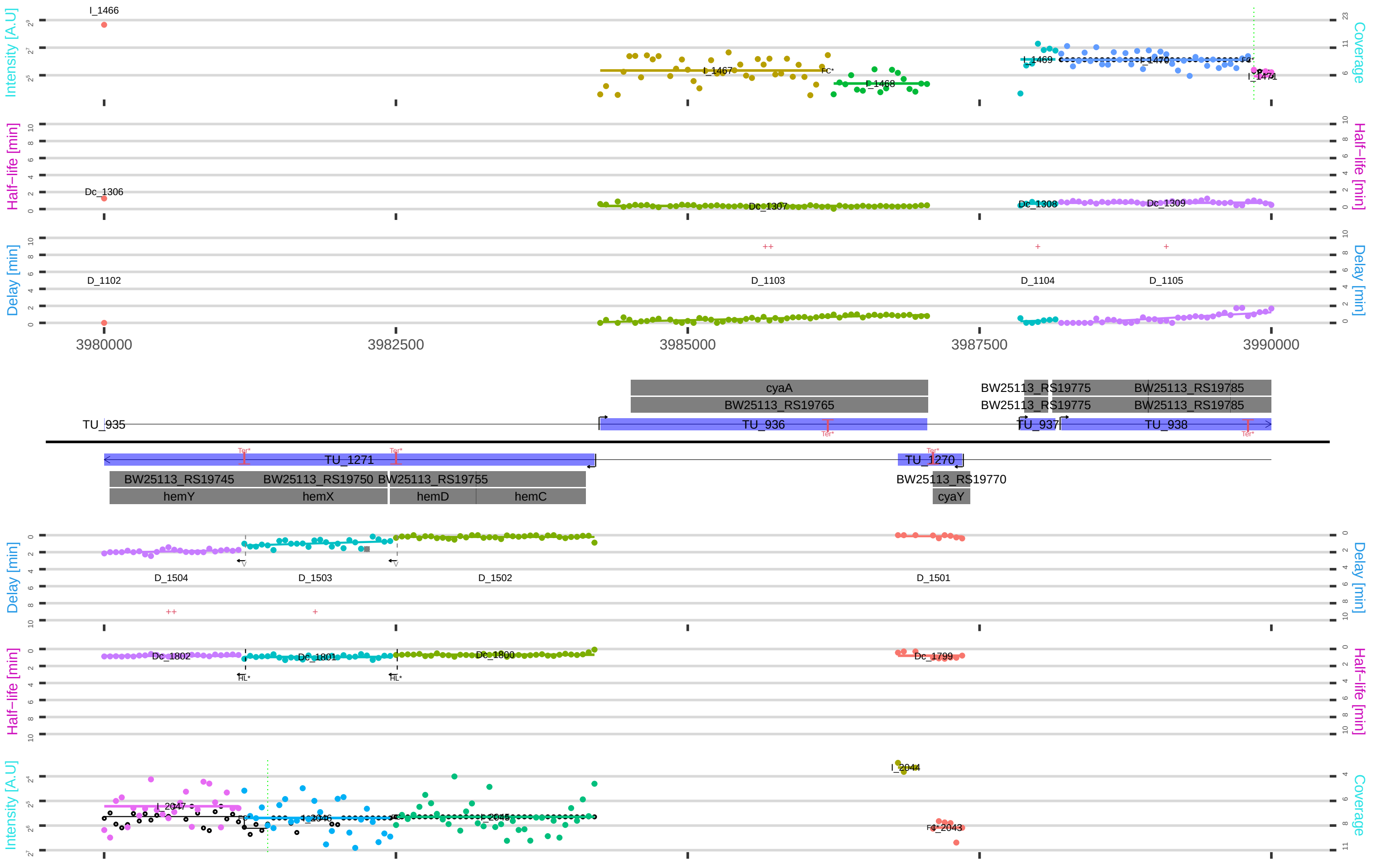

Term: termination (3), NS: new start (0), PS: pausing site (2), iTSS_I: internal starting site (0)

ID: 79800−80000; Term: termination (2), NS: new start (1), PS: pausing site (1), iTSS_I: internal starting site (0)

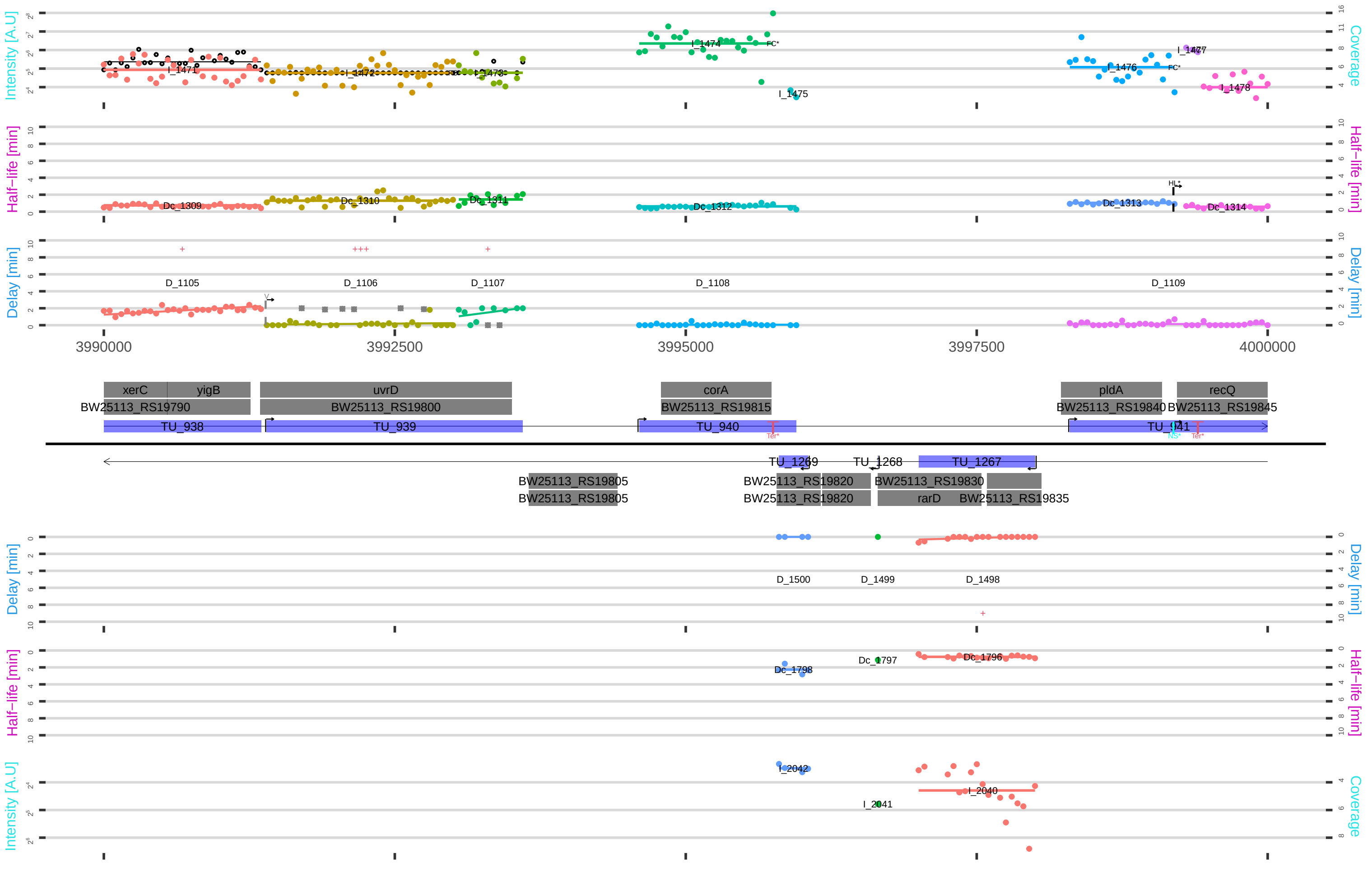

Term: termination (0), NS: new start (0), PS: pausing site (0), iTSS_I: internal starting site (0)

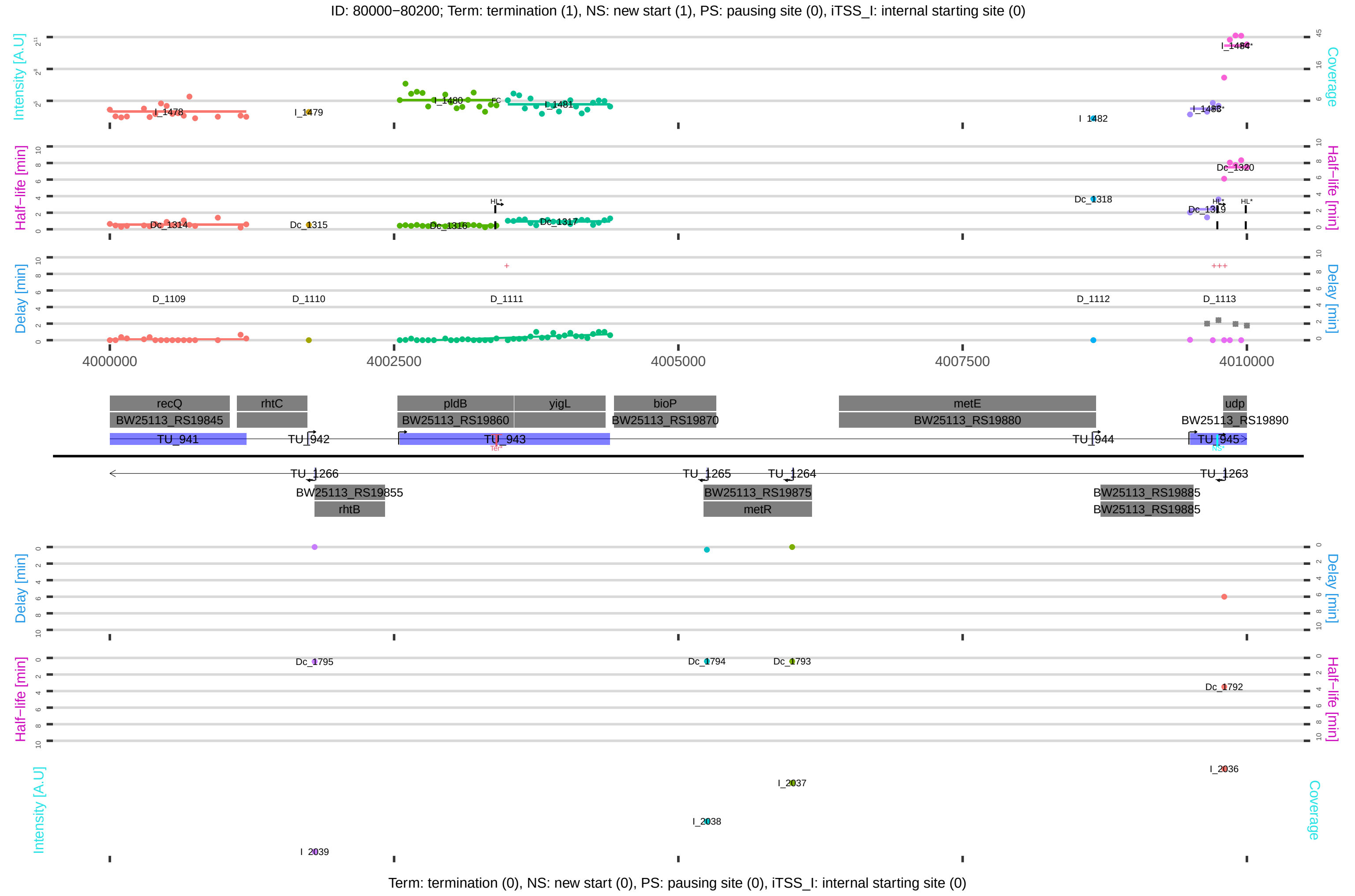

ID: 80000−80200; Term: termination (1), NS: new start (1), PS: pausing site (0), iTSS_I: internal starting site (0)
Intensity [A.U]
Coverage
Half−life [min]
Delay [min]
I_1478
I_1479
I_1480
I_1481
I_1482
I_1483*
I_1484*
Dc_1314
Dc_1315
Dc_1316
Dc_1317
Dc_1318
Dc_1319
Dc_1320
D_1109
D_1110
D_1111
D_1112
D_1113
4000000
4002500
4005000
4007500
4010000
recQ
rhtC
pldB
yigL
bioP
metE
udp
BW25113_RS19845
BW25113_RS19860
BW25113_RS19870
BW25113_RS19880
BW25113_RS19890
TU_941
TU_942
TU_943
TU_944
TU_945
TU_1266
TU_1265
TU_1264
TU_1263
BW25113_RS19855
rhtB
BW25113_RS19875
metR
BW25113_RS19885
BW25113_RS19885
Dc_1795
Dc_1794
Dc_1793
Dc_1792
I_2036
I_2037
I_2038
I_2039
Term: termination (0), NS: new start (0), PS: pausing site (0), iTSS_I: internal starting site (0)

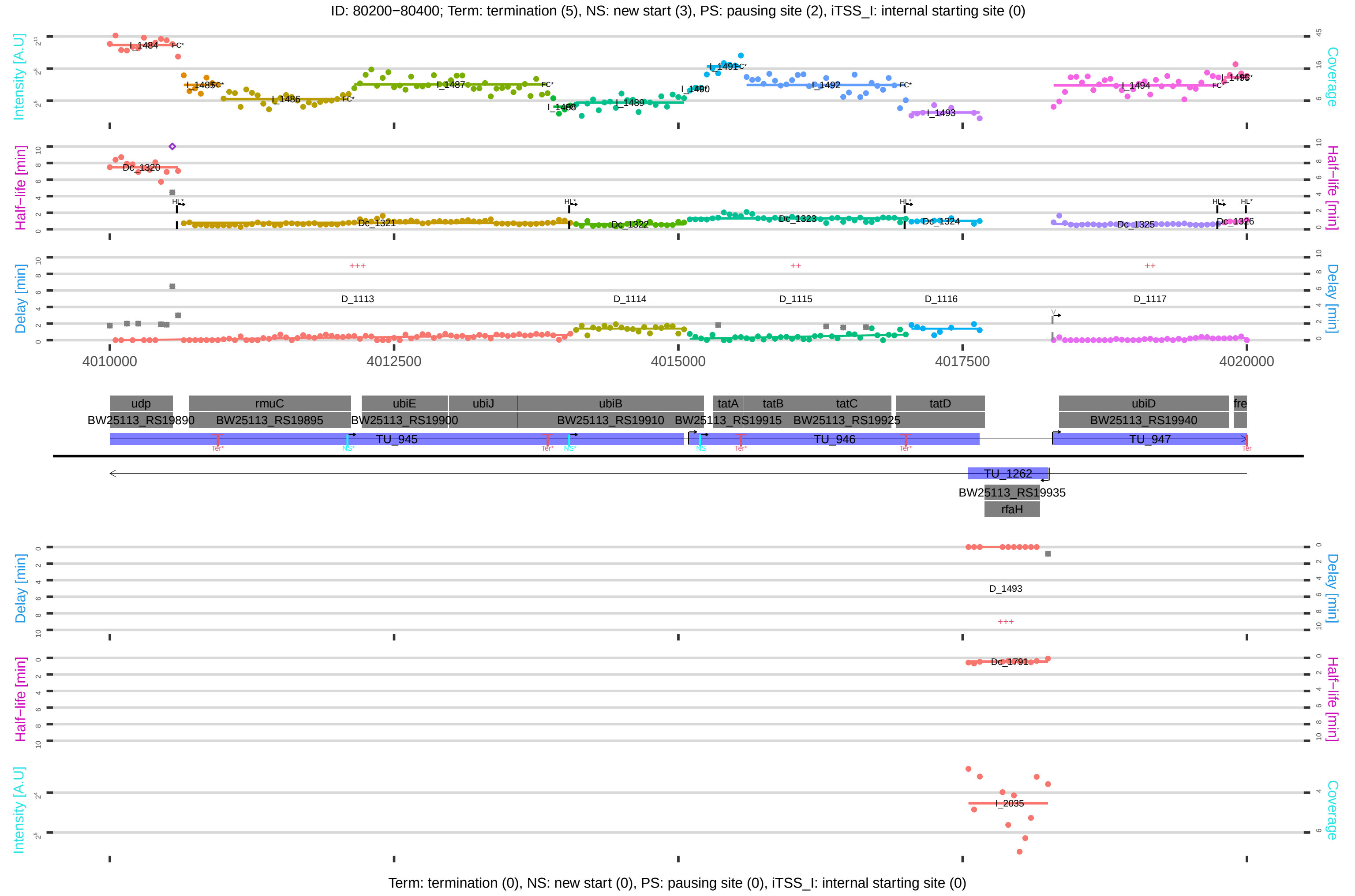

ID: 80200−80400; Term: termination (5), NS: new start (3), PS: pausing site (2), iTSS_I: internal starting site (0)
Intensity [A.U]
Coverage
Half−life [min]
Delay [min]
4010000
4012500
4015000
4017500
4020000
udp
rmuC
ubiE
ubiJ
ubiB
tatA
tatB
tatC
tatD
ubiD
BW25113_RS19890
BW25113_RS19895
BW25113_RS19900
BW25113_RS19910
BW25113_RS19915
BW25113_RS19925
BW25113_RS19940
TU_945
TU_946
TU_947
TU_1262
BW25113_RS19935
rfaH
D_1113
D_1114
D_1115
D_1116
D_1117
D_1493
Dc_1791
I_2035
Term: termination (0), NS: new start (0), PS: pausing site (0), iTSS_I: internal starting site (0)

ID: 80400−80600; Term: termination (4), NS: new start (1), PS: pausing site (2), iTSS_I: internal starting site (0)

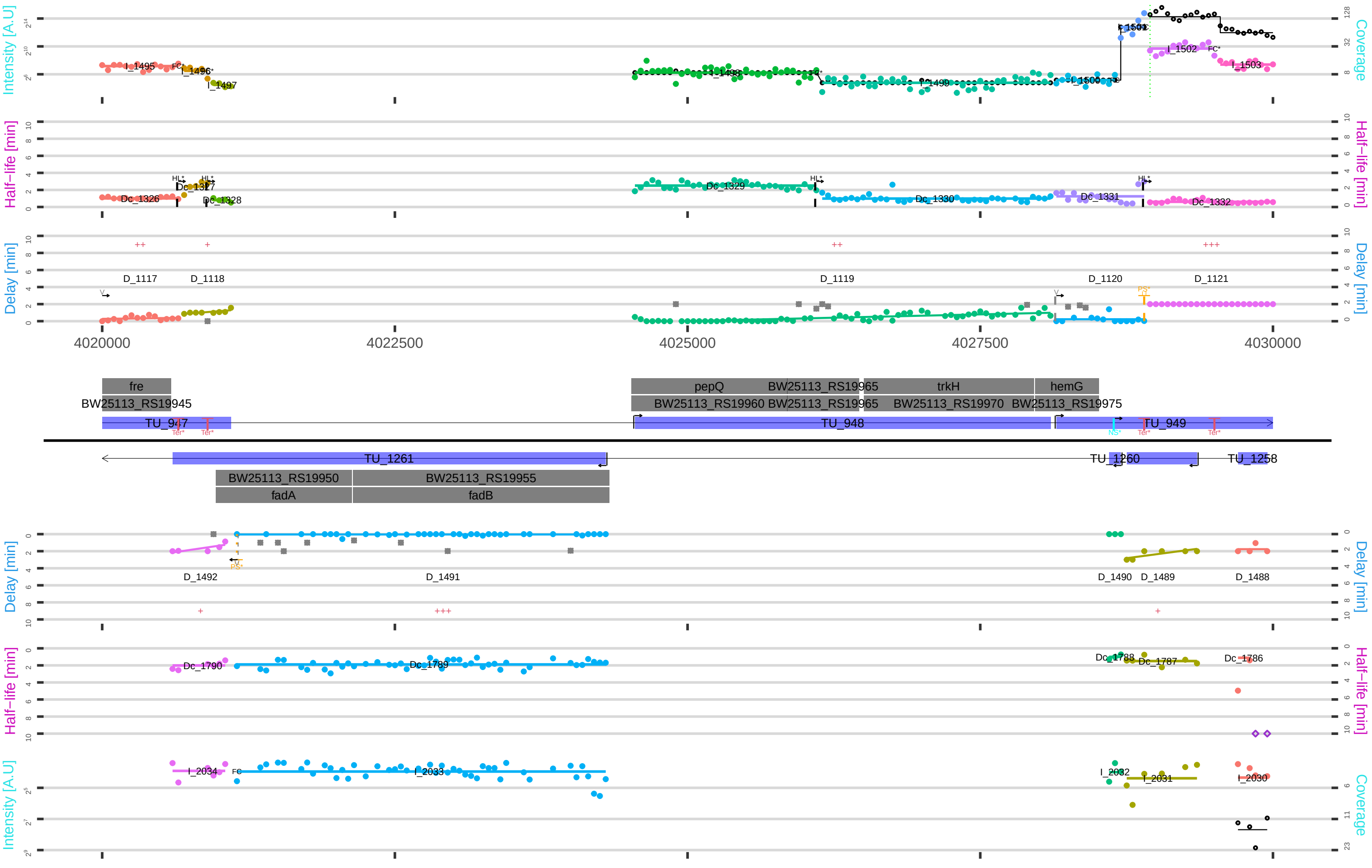

Term: termination (0), NS: new start (0), PS: pausing site (1), iTSS_I: internal starting site (0)

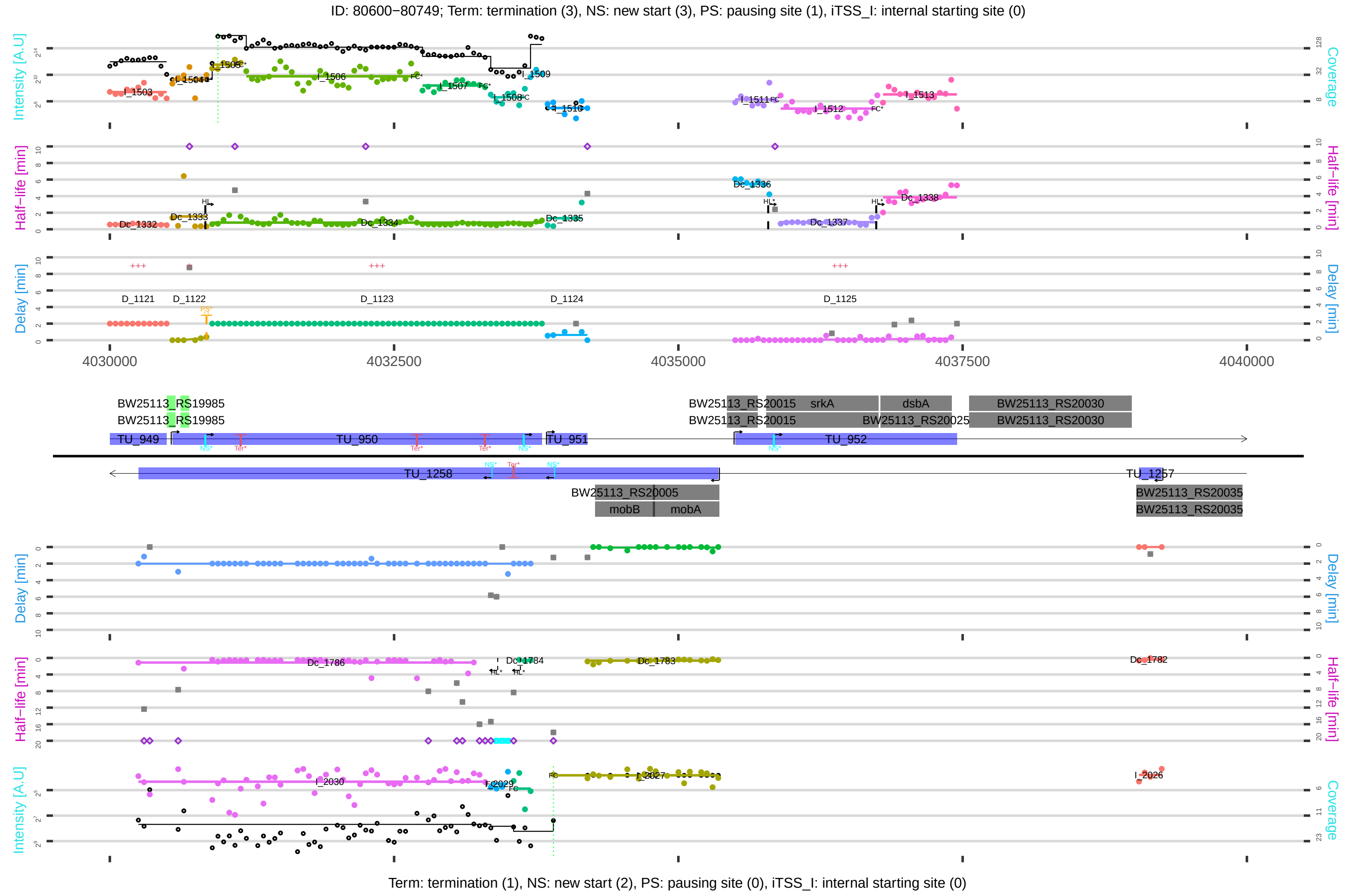
ID: 80600−80749; Term: termination (3), NS: new start (3), PS: pausing site (1), iTSS_I: internal starting site (0)
Intensity [A.U]
Coverage
Half−life [min]
Delay [min]
I_1503
I_1504
I_1505
I_1506
I_1507
I_1508
I_1509
I_1510
I_1511
I_1512
I_1513
Dc_1332
Dc_1333
Dc_1334
Dc_1335
Dc_1336
Dc_1337
Dc_1338
D_1121
D_1122
D_1123
D_1124
D_1125
4030000
4032500
4035000
4037500
4040000
BW25113_RS19985
BW25113_RS20015
srkA
dsbA
BW25113_RS20025
BW25113_RS20030
TU_949
TU_950
TU_951
TU_952
TU_1258
TU_1257
BW25113_RS20005
mobB
mobA
BW25113_RS20035
Dc_1786
Dc_1784
Dc_1783
Dc_1782
I_2030
I_2029
I_2027
I_2026
Term: termination (1), NS: new start (2), PS: pausing site (0), iTSS_I: internal starting site (0)

ID: 80808−80940; Term: termination (2), NS: new start (1), PS: pausing site (1), iTSS_I: internal starting site (0)

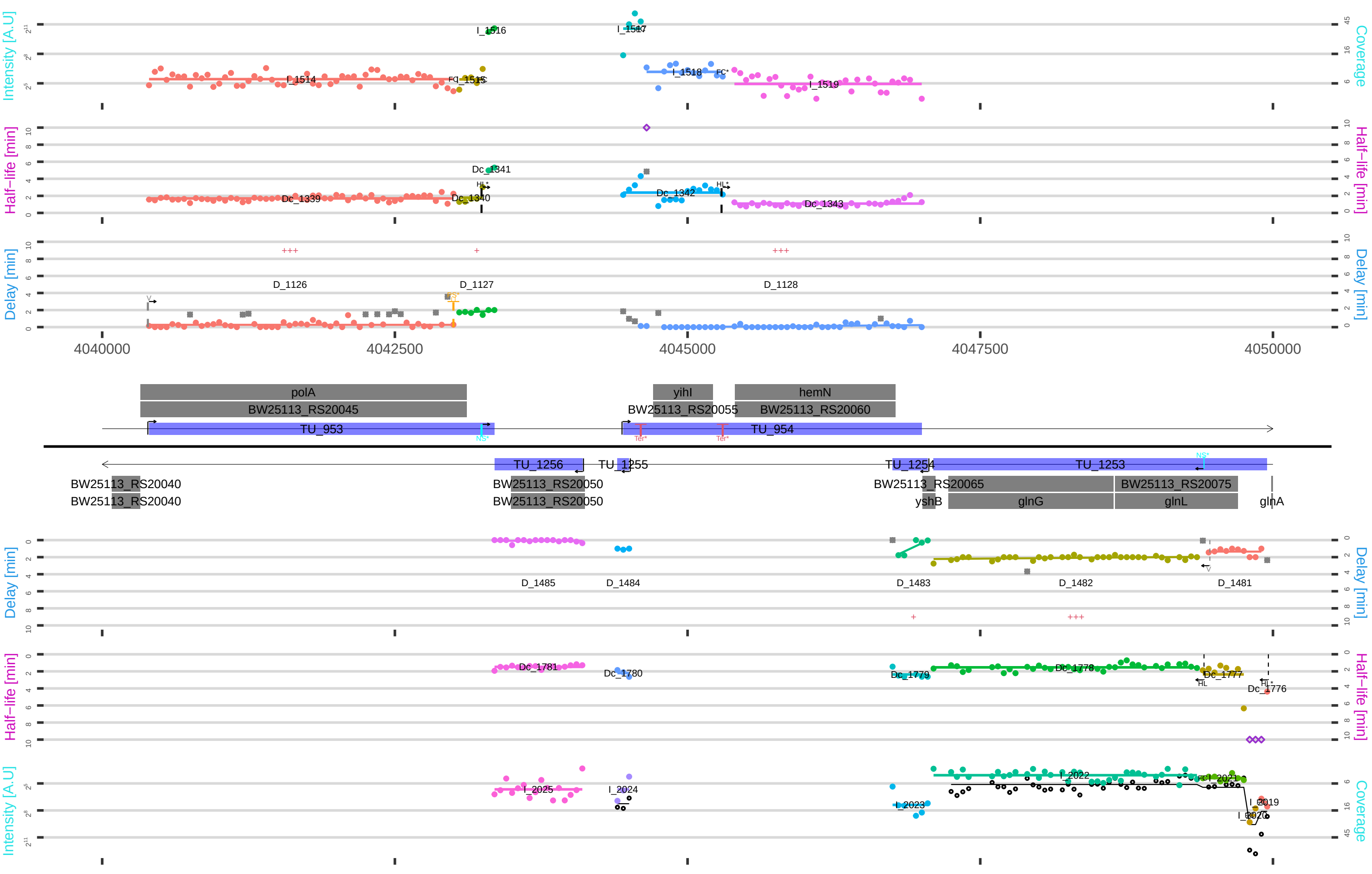

Term: termination (0), NS: new start (1), PS: pausing site (1), iTSS_I: internal starting site (0)

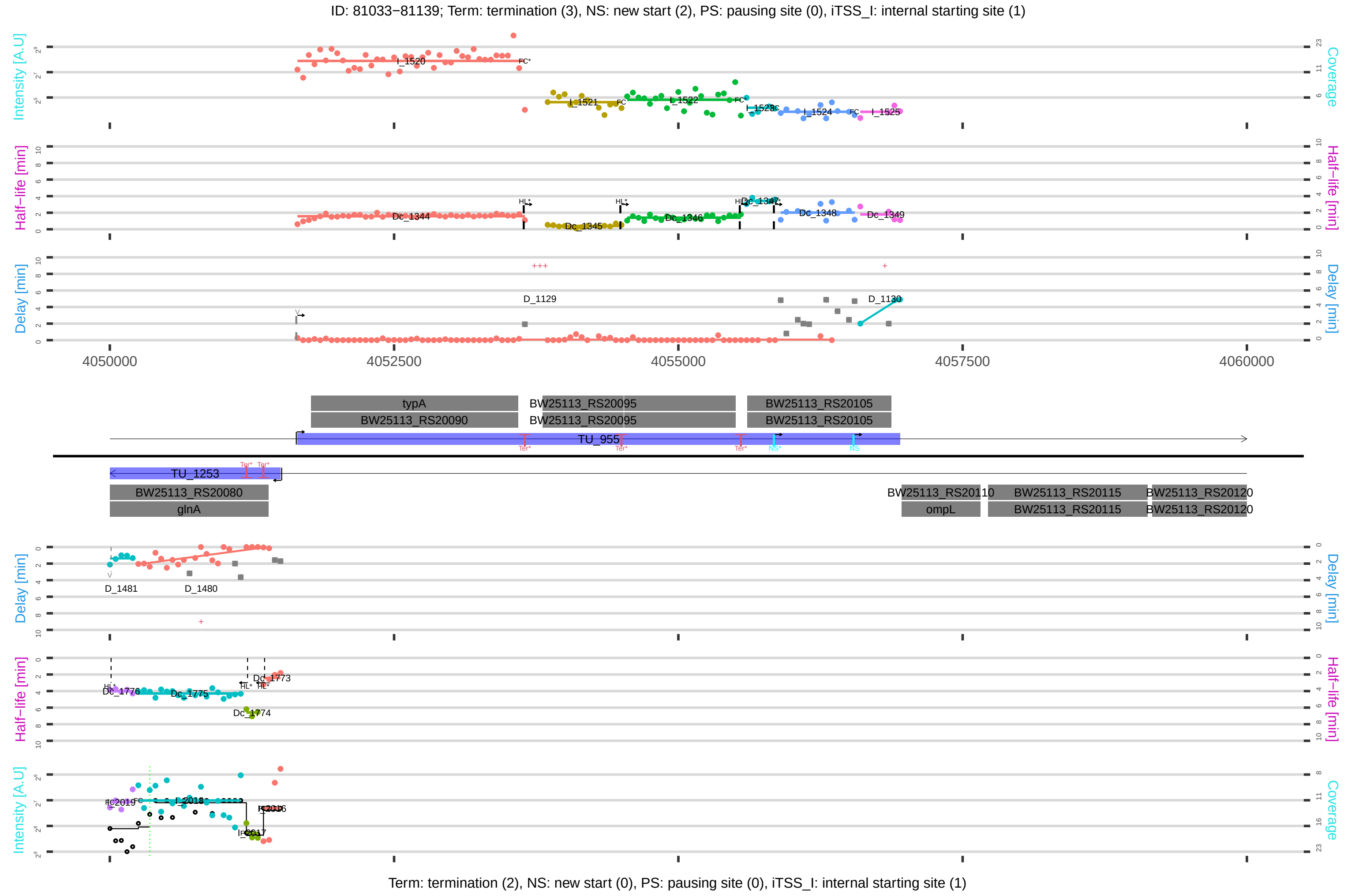

ID: 81033−81139; Term: termination (3), NS: new start (2), PS: pausing site (0), iTSS_I: internal starting site (1)
Intensity [A.U]
Coverage
Half−life [min]
Delay [min]
I_1520
I_1521
I_1522
I_1523
I_1524
I_1525
Dc_1344
Dc_1345
Dc_1346
Dc_1347
Dc_1348
Dc_1349
D_1129
D_1130
4050000
4052500
4055000
4057500
4060000
typA
BW25113_RS20090
BW25113_RS20095
BW25113_RS20105
TU_955
TU_1253
BW25113_RS20080
glnA
BW25113_RS20110
ompL
BW25113_RS20115
BW25113_RS20120
D_1481
D_1480
Dc_1776
Dc_1775
Dc_1774
Dc_1773
Term: termination (2), NS: new start (0), PS: pausing site (0), iTSS_I: internal starting site (1)

ID: 81362−81400; Term: termination (1), NS: new start (1), PS: pausing site (0), iTSS_I: internal starting site (0)

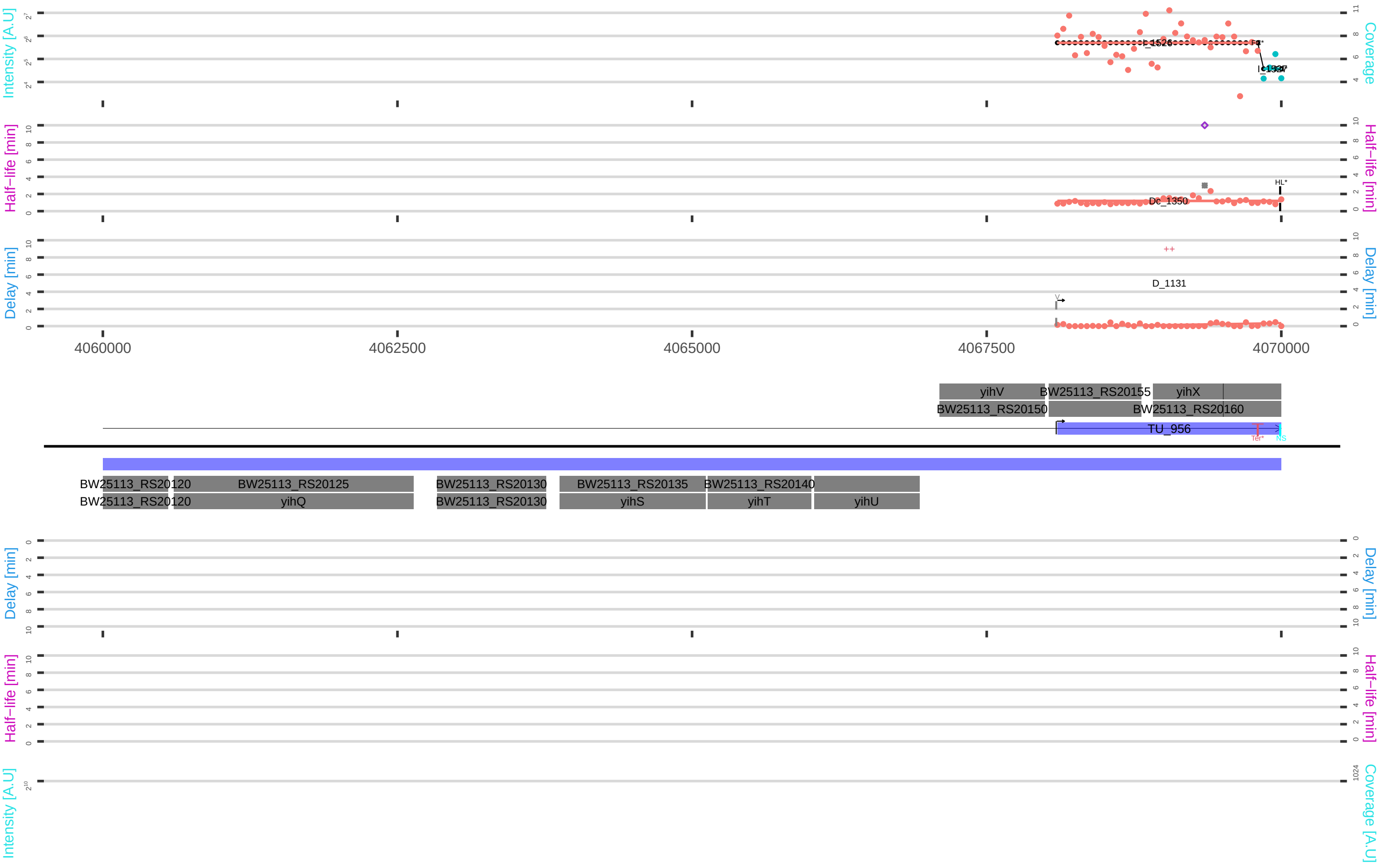

NA

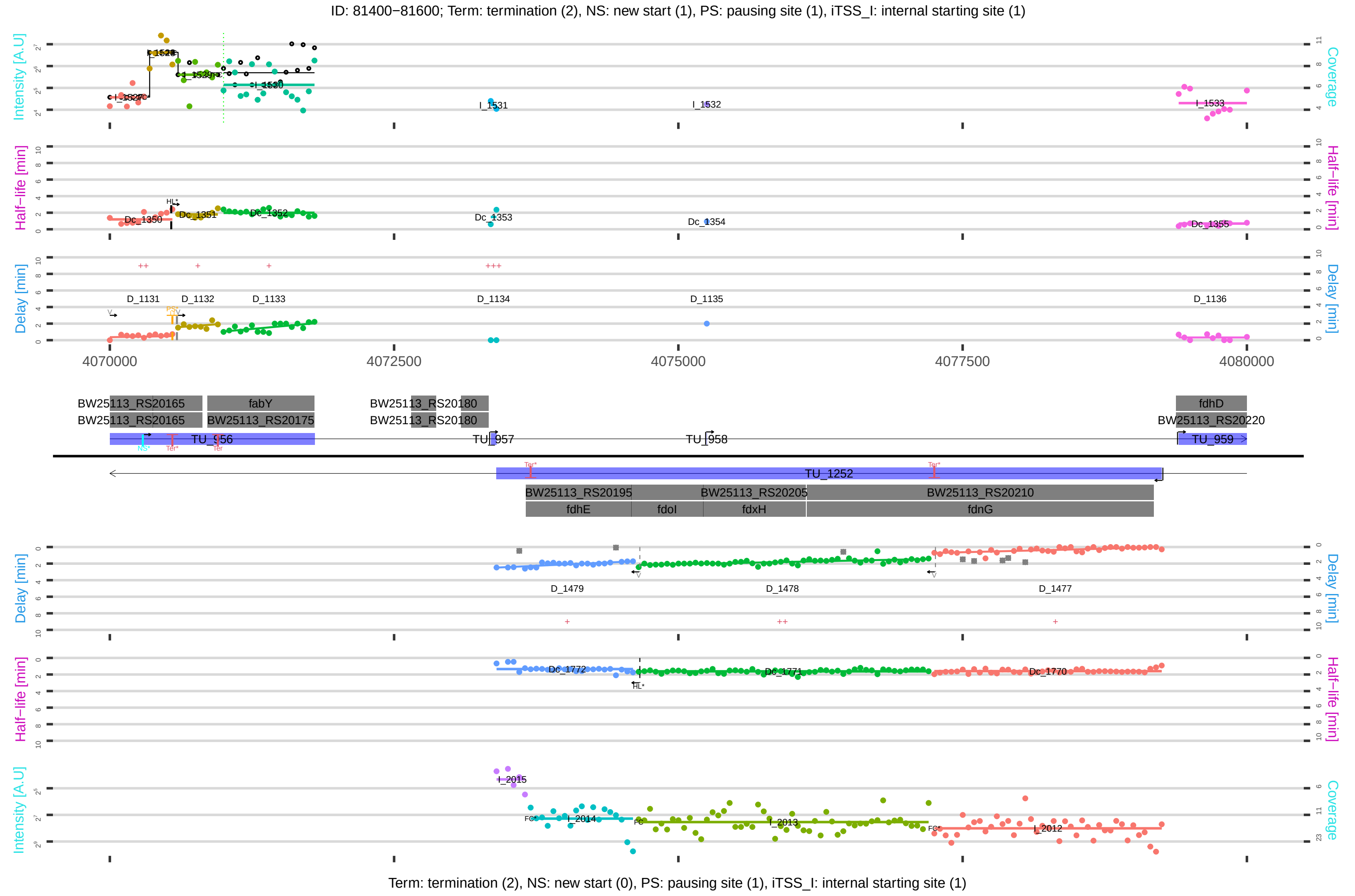

ID: 81400−81600; Term: termination (2), NS: new start (1), PS: pausing site (1), iTSS_I: internal starting site (1)
Intensity [A.U]
Coverage
Half−life [min]
Delay [min]
I_1527
I_1528
I_1529
I_1530
I_1531
I_1532
I_1533
Dc_1350
Dc_1351
Dc_1352
Dc_1353
Dc_1354
Dc_1355
D_1131
D_1132
D_1133
D_1134
D_1135
D_1136
4070000
4072500
4075000
4077500
4080000
BW25113_RS20165
fabY
BW25113_RS20175
BW25113_RS20180
fdhD
BW25113_RS20220
TU_956
TU_957
TU_958
TU_959
TU_1252
BW25113_RS20195
BW25113_RS20205
BW25113_RS20210
fdhE
fdoI
fdxH
fdnG
D_1479
D_1478
D_1477
Dc_1772
Dc_1771
Dc_1770
I_2015
I_2014
I_2013
I_2012
Term: termination (2), NS: new start (0), PS: pausing site (1), iTSS_I: internal starting site (1)

ID: 81600−81730; Term: termination (0), NS: new start (0), PS: pausing site (0), iTSS_I: internal starting site (0)

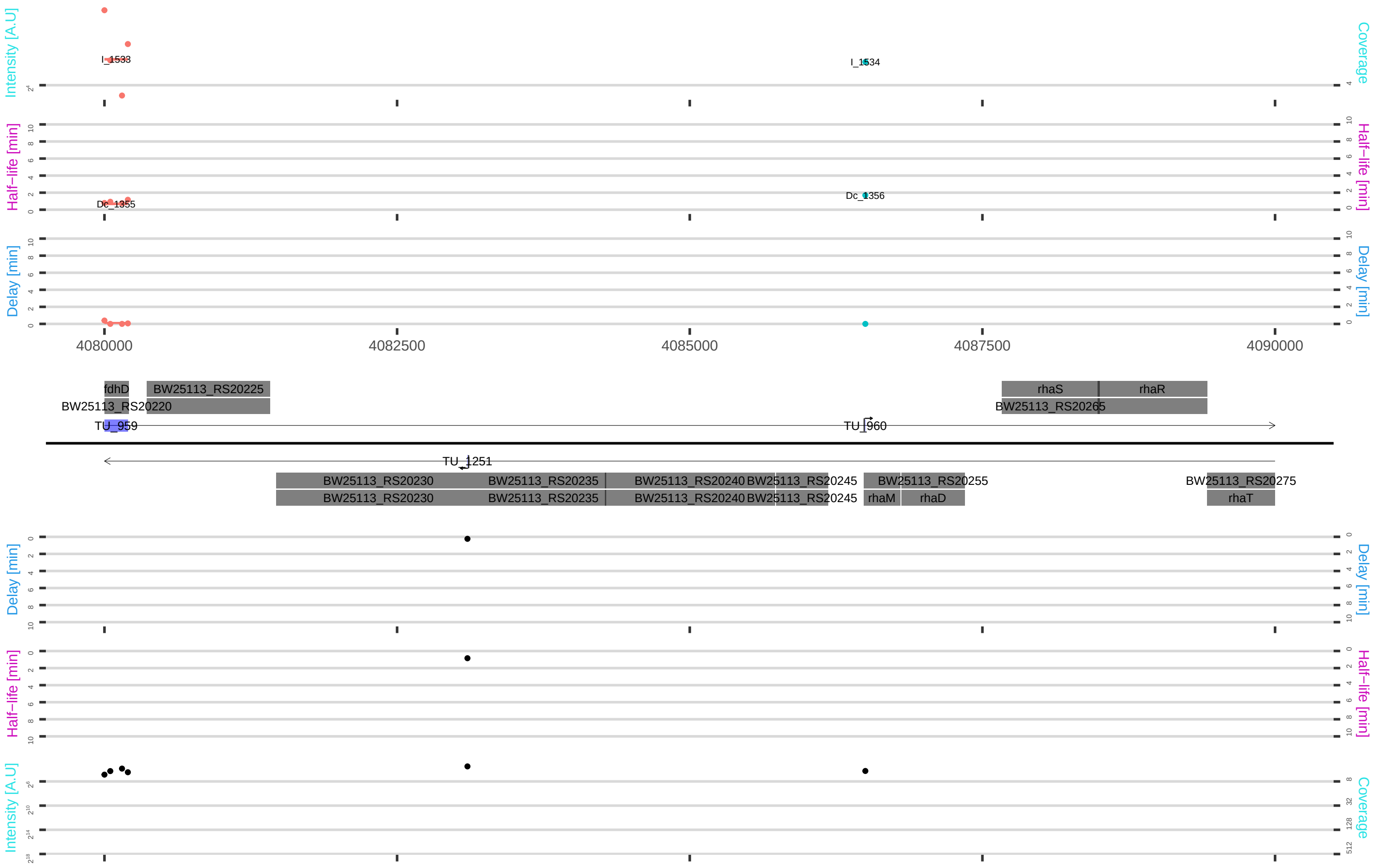

Term: termination (0), NS: new start (0), PS: pausing site (0), iTSS_I: internal starting site (0)

ID: 81815−82000; Term: termination (0), NS: new start (1), PS: pausing site (0), iTSS_I: internal starting site (0)

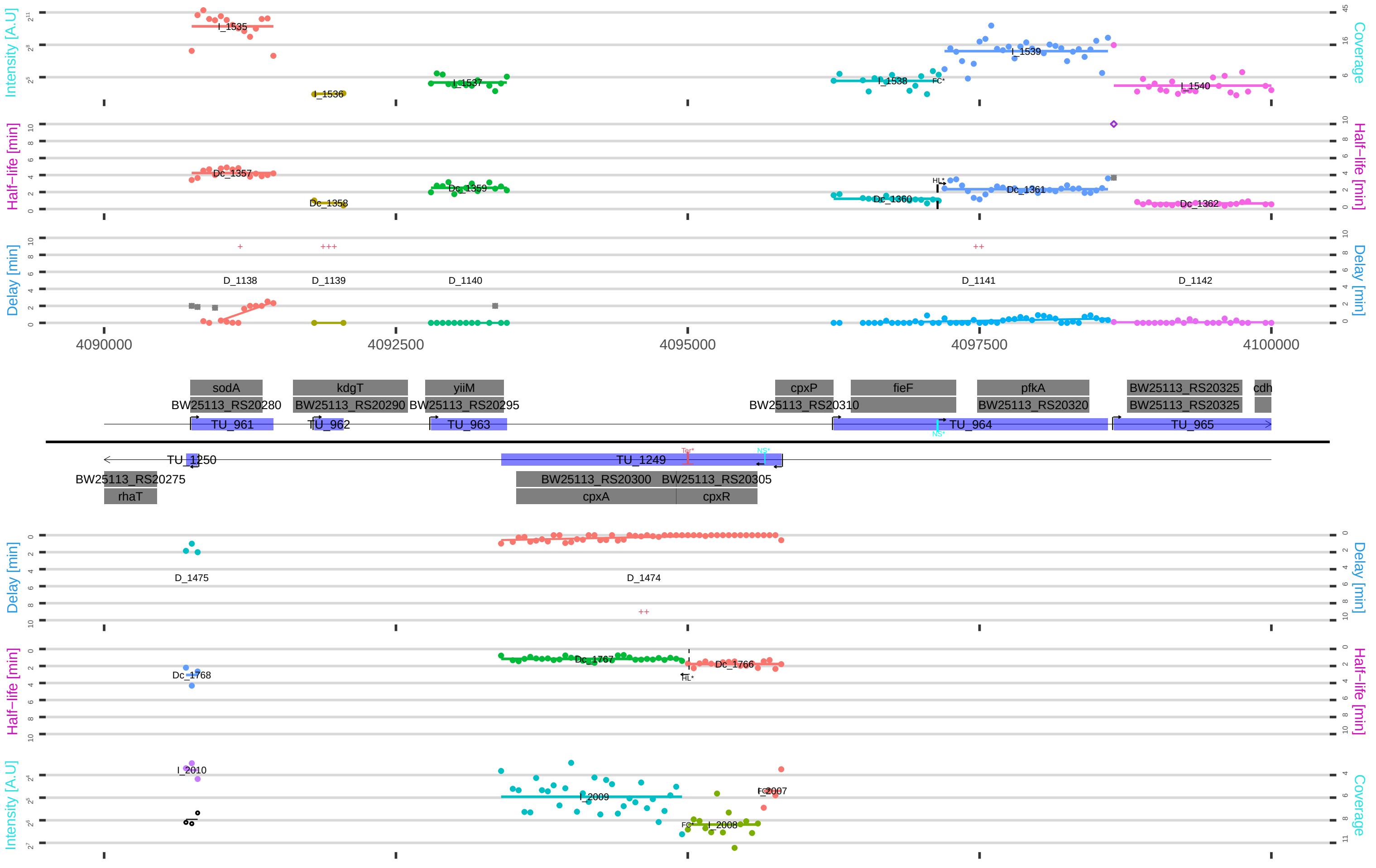

Term: termination (1), NS: new start (1), PS: pausing site (0), iTSS_I: internal starting site (0)

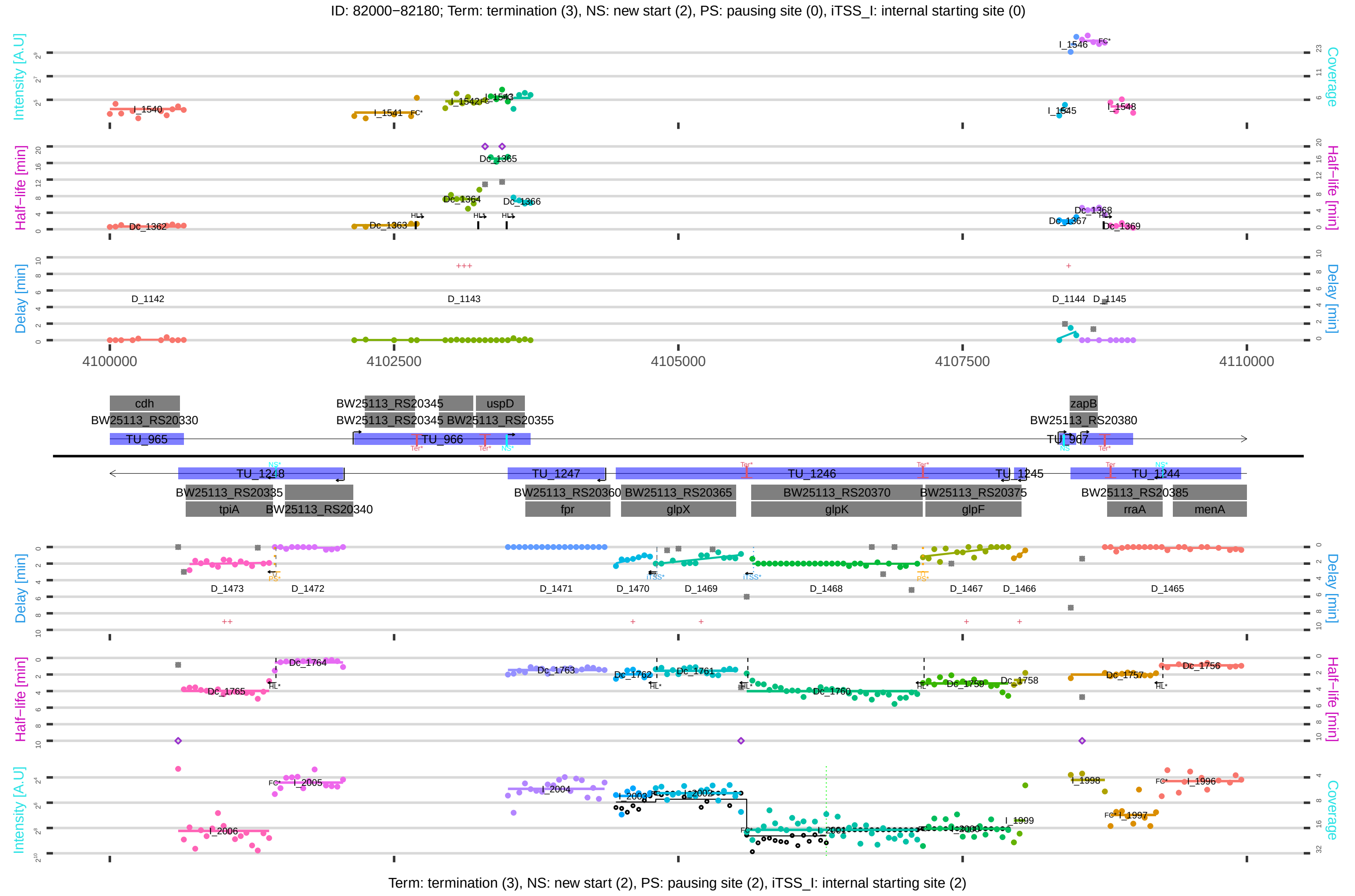
ID: 82000−82180; Term: termination (3), NS: new start (2), PS: pausing site (0), iTSS_I: internal starting site (0)
Intensity [A.U]
Coverage
Half−life [min]
Delay [min]
4100000
4102500
4105000
4107500
4110000
cdh
BW25113_RS20330
TU_965
BW25113_RS20345
uspD
BW25113_RS20355
TU_966
zapB
BW25113_RS20380
TU_967
TU_1248
BW25113_RS20335
tpiA
BW25113_RS20340
TU_1247
BW25113_RS20360
fpr
TU_1246
BW25113_RS20365
glpX
BW25113_RS20370
glpK
BW25113_RS20375
glpF
TU_1245
TU_1244
BW25113_RS20385
rraA
menA
Term: termination (3), NS: new start (2), PS: pausing site (2), iTSS_I: internal starting site (2)

ID: 82305−82400; Term: termination (1), NS: new start (1), PS: pausing site (0), iTSS_I: internal starting site (0)

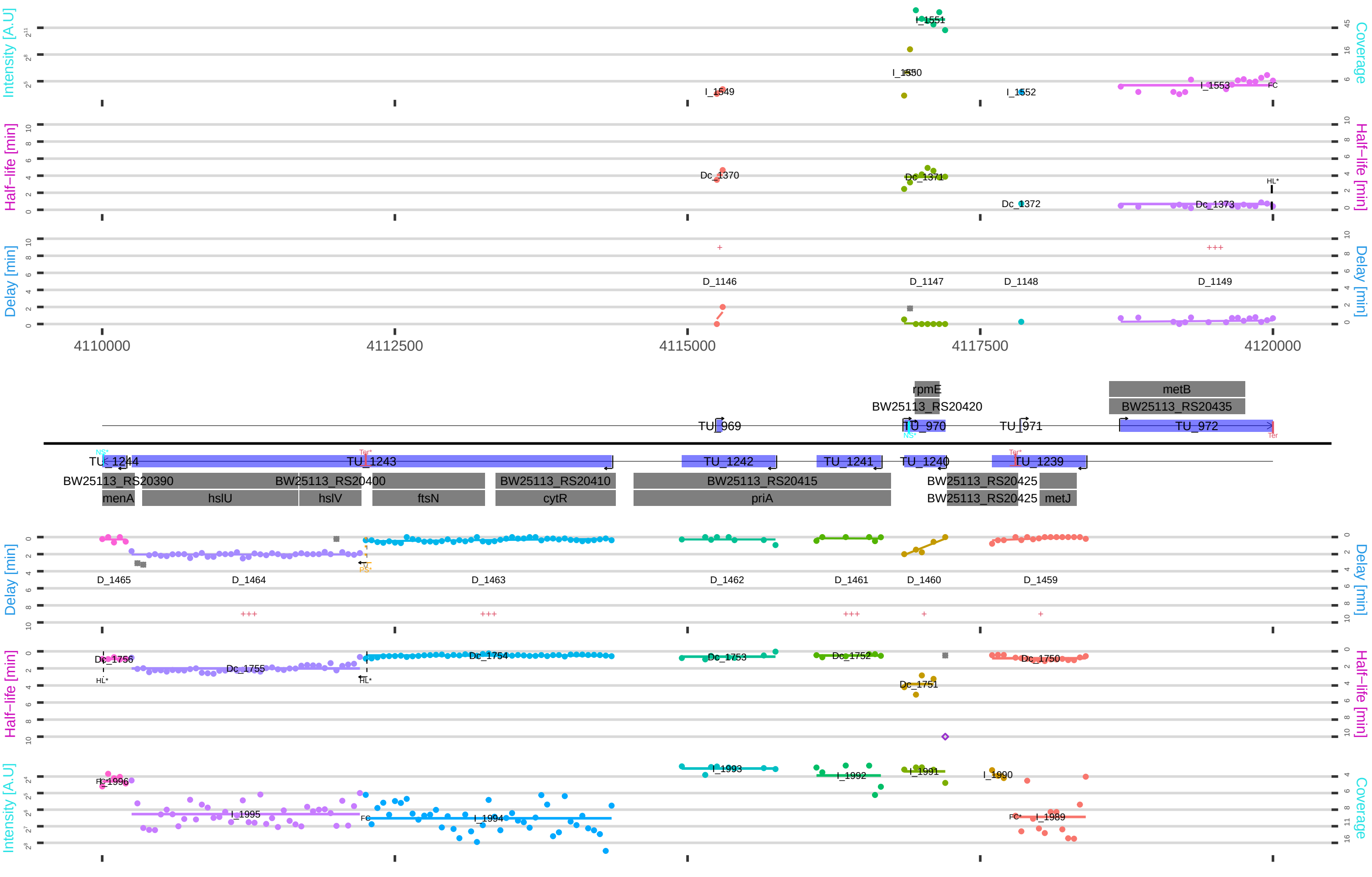

Term: termination (2), NS: new start (1), PS: pausing site (1), iTSS_I: internal starting site (0)

ID: 82400−82524; Term: termination (2), NS: new start (2), PS: pausing site (1), iTSS_I: internal starting site (0)

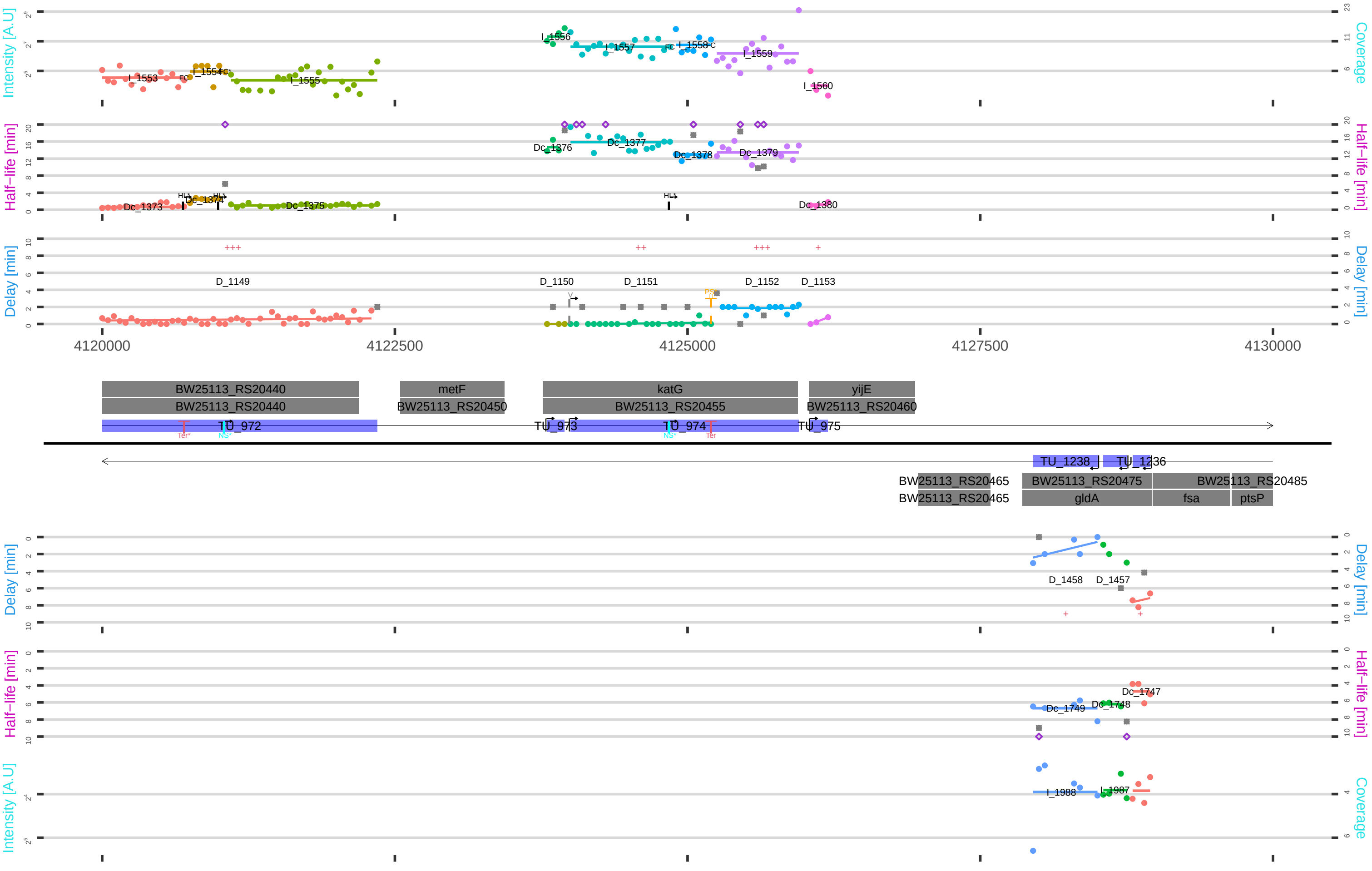

Term: termination (0), NS: new start (0), PS: pausing site (0), iTSS_I: internal starting site (0)

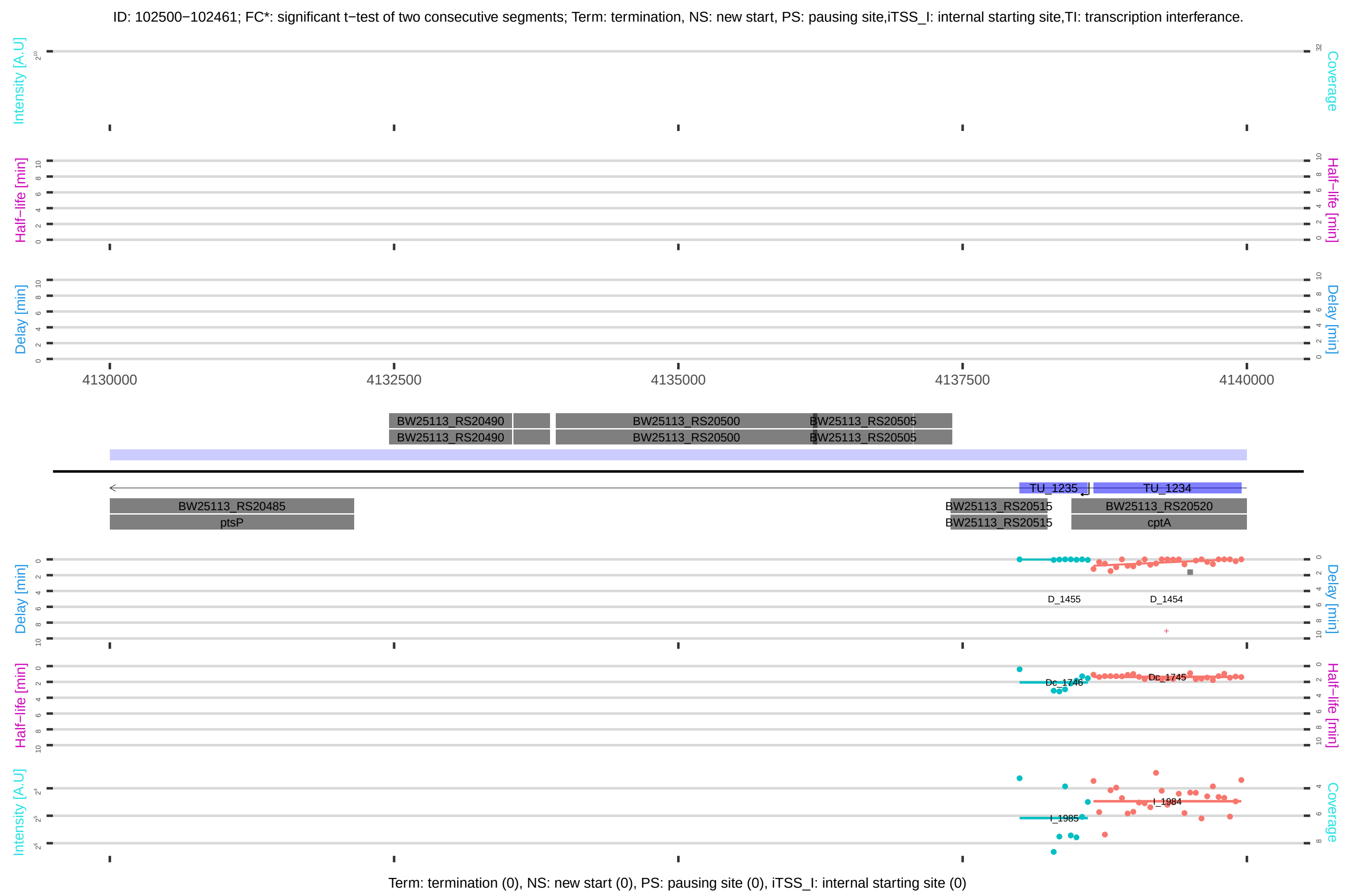

ID: 102500−102461; FC*: significant t−test of two consecutive segments; Term: termination, NS: new start, PS: pausing site,iTSS_I: internal starting site,TI: transcription interferance.
Intensity [A.U]
Coverage
Half−life [min]
Delay [min]
4130000
4132500
4135000
4137500
4140000
BW25113_RS20490
BW25113_RS20500
BW25113_RS20505
TU_1235
TU_1234
BW25113_RS20485
ptsP
BW25113_RS20515
BW25113_RS20520
cptA
D_1455
D_1454
Dc_1746
Dc_1745
I_1985
I_1984
Term: termination (0), NS: new start (0), PS: pausing site (0), iTSS_I: internal starting site (0)

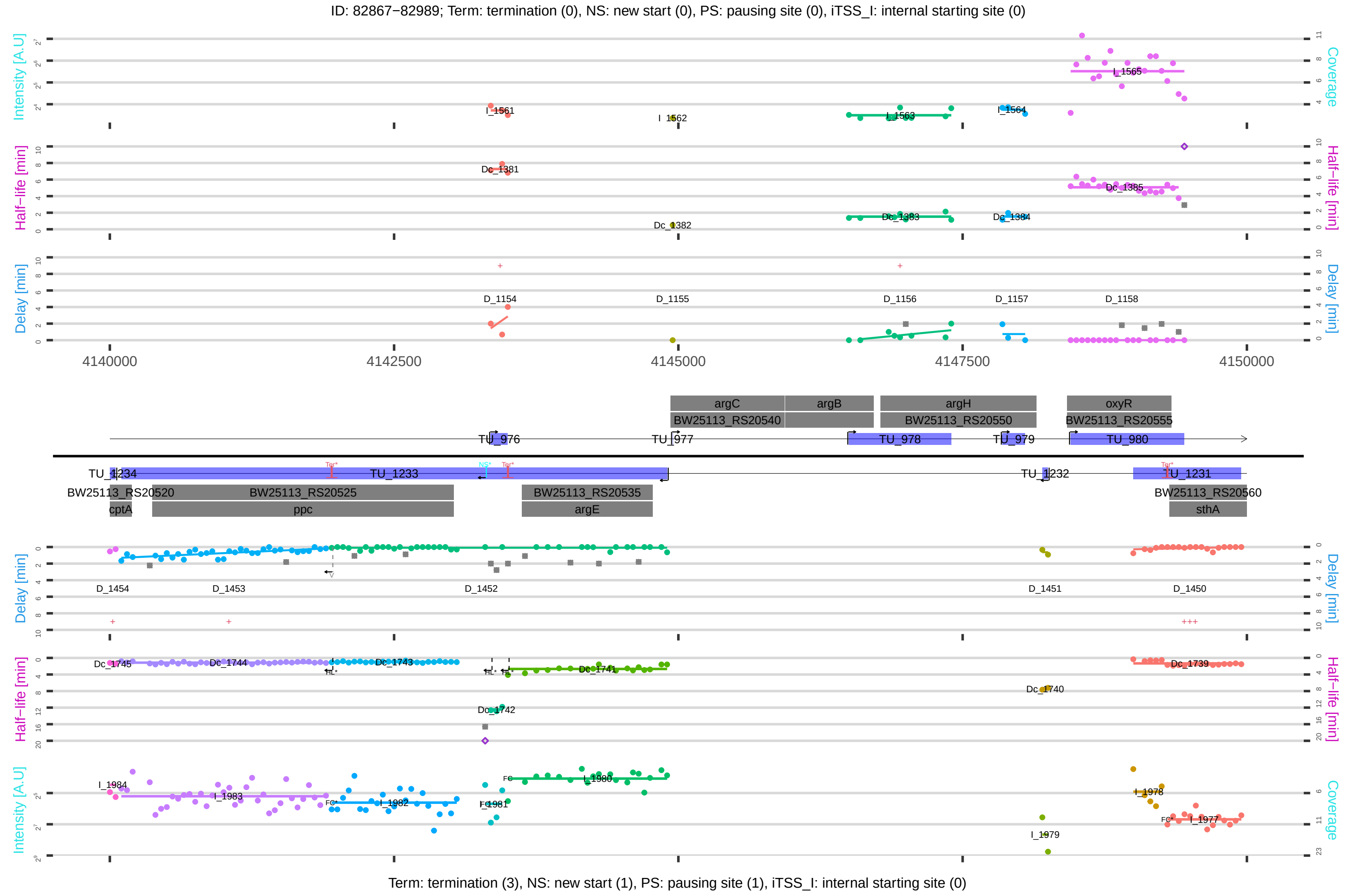

ID: 82867−82989; Term: termination (0), NS: new start (0), PS: pausing site (0), iTSS_I: internal starting site (0)
Intensity [A.U]
Coverage
Half−life [min]
Delay [min]
I_1561
I_1562
I_1563
I_1564
I_1565
Dc_1381
Dc_1382
Dc_1383
Dc_1384
Dc_1385
D_1154
D_1155
D_1156
D_1157
D_1158
4140000
4142500
4145000
4147500
4150000
argC
argB
argH
oxyR
BW25113_RS20540
BW25113_RS20550
BW25113_RS20555
TU_976
TU_977
TU_978
TU_979
TU_980
TU_1234
TU_1233
TU_1232
TU_1231
BW25113_RS20520
BW25113_RS20525
BW25113_RS20535
BW25113_RS20560
cptA
ppc
argE
sthA
D_1454
D_1453
D_1452
D_1451
D_1450
Dc_1745
Dc_1744
Dc_1743
Dc_1742
Dc_1741
Dc_1740
Dc_1739
I_1984
I_1983
I_1982
I_1981
I_1980
I_1979
I_1978
I_1977
Term: termination (3), NS: new start (1), PS: pausing site (1), iTSS_I: internal starting site (0)

ID: 83022−83200; Term: termination (7), NS: new start (4), PS: pausing site (1), iTSS_I: internal starting site (1)

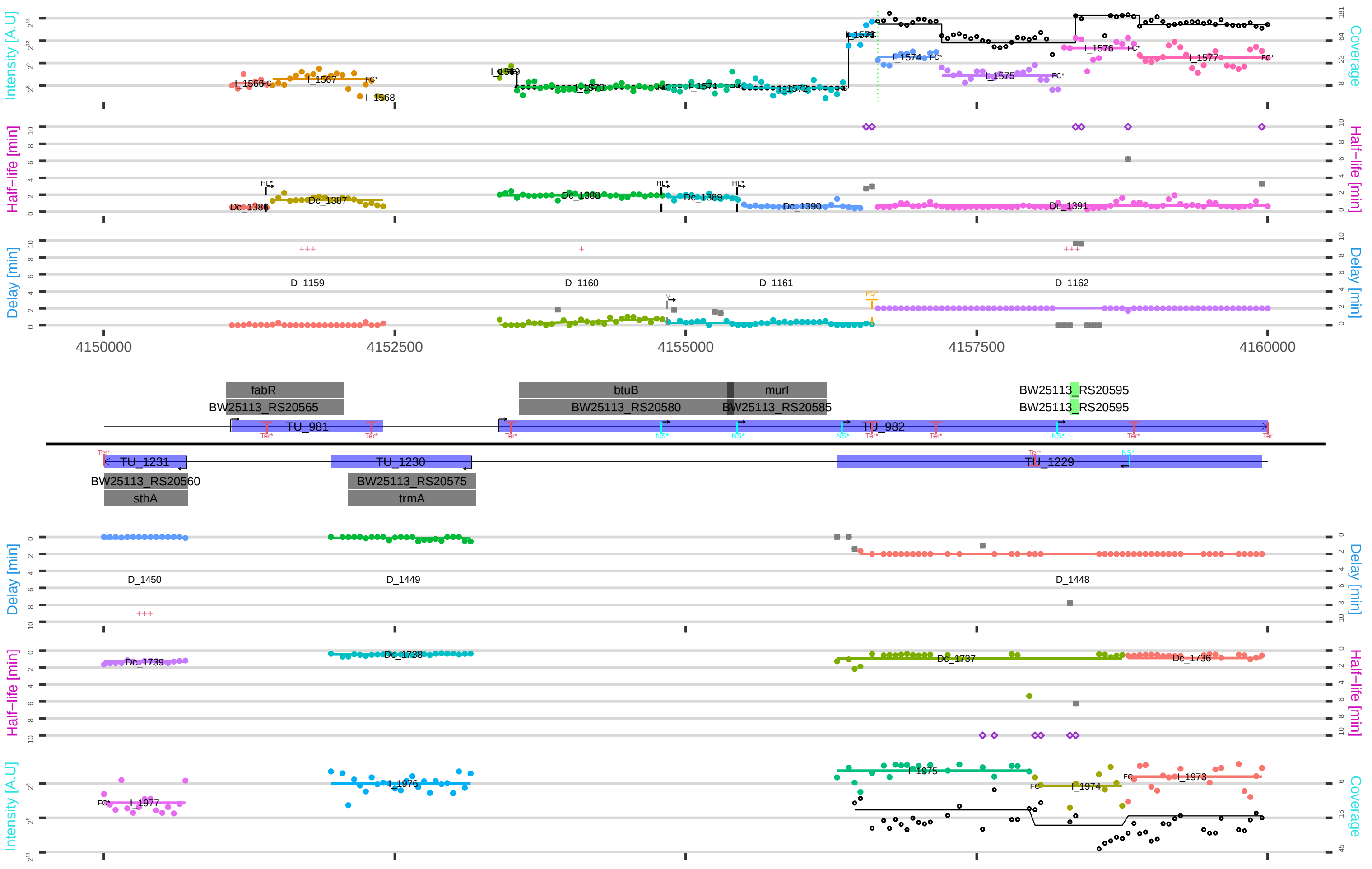

Term: termination (2), NS: new start (1), PS: pausing site (0), iTSS_I: internal starting site (0)

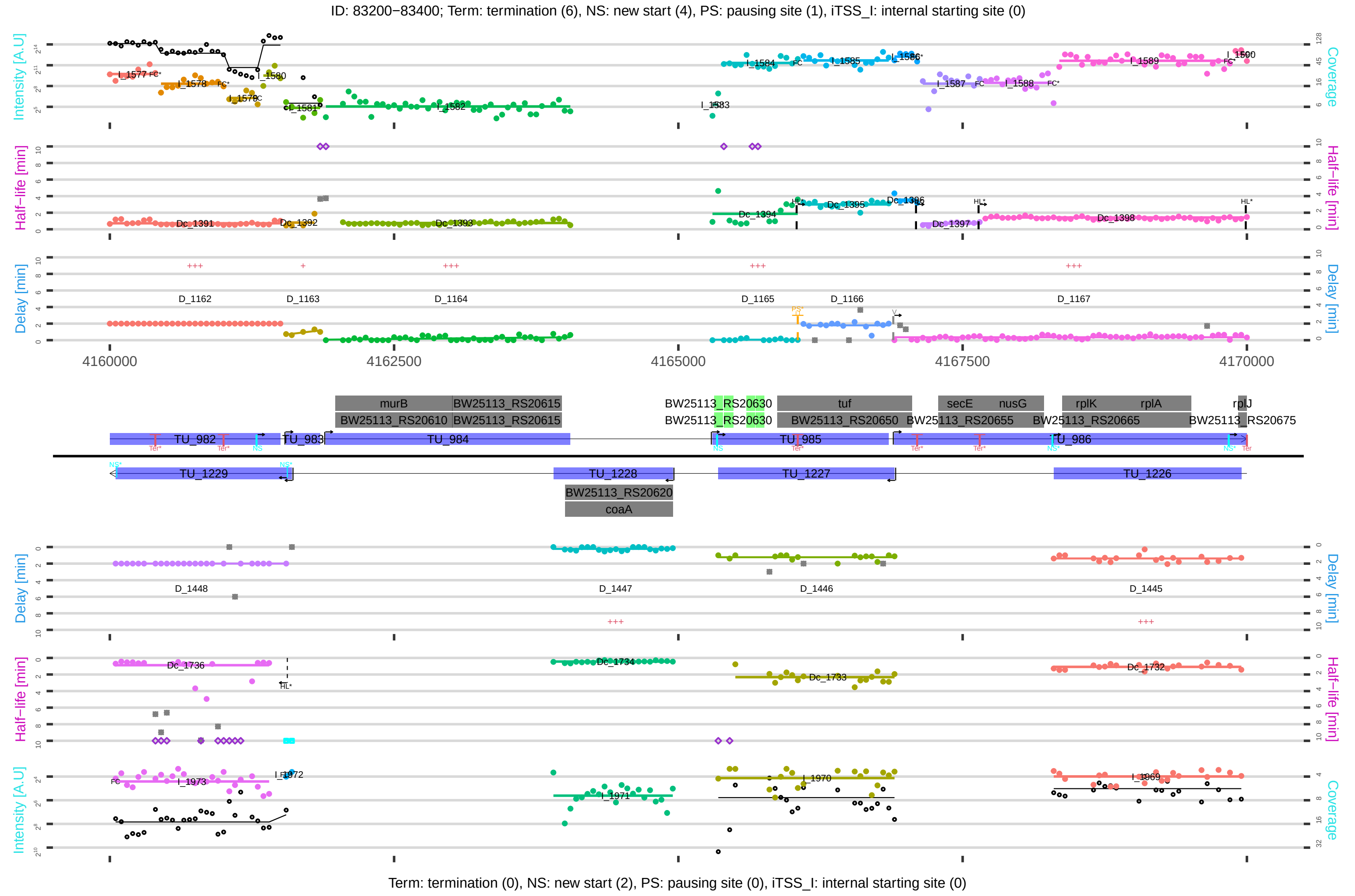

ID: 83200−83400; Term: termination (6), NS: new start (4), PS: pausing site (1), iTSS_I: internal starting site (0)
Intensity [A.U]
Coverage
Half−life [min]
Delay [min]
I_1577
I_1578
I_1579
I_1580
I_1581
I_1582
I_1583
I_1584
I_1585
I_1586
I_1587
I_1588
I_1589
I_1590
Dc_1391
Dc_1392
Dc_1393
Dc_1394
Dc_1395
Dc_1396
Dc_1397
Dc_1398
D_1162
D_1163
D_1164
D_1165
D_1166
D_1167
4160000
4162500
4165000
4167500
4170000
murB
BW25113_RS20615
BW25113_RS20610
BW25113_RS20630
tuf
BW25113_RS20650
secE
nusG
BW25113_RS20655
rplK
rplA
BW25113_RS20665
rplJ
BW25113_RS20675
TU_982
TU_983
TU_984
TU_985
TU_986
TU_1229
TU_1228
TU_1227
TU_1226
BW25113_RS20620
coaA
D_1448
D_1447
D_1446
D_1445
Dc_1736
Dc_1734
Dc_1733
Dc_1732
I_1973
I_1972
I_1971
I_1970
I_1969
Term: termination (0), NS: new start (2), PS: pausing site (0), iTSS_I: internal starting site (0)

ID: 83400−83591; Term: termination (2), NS: new start (1), PS: pausing site (2), iTSS_I: internal starting site (0)

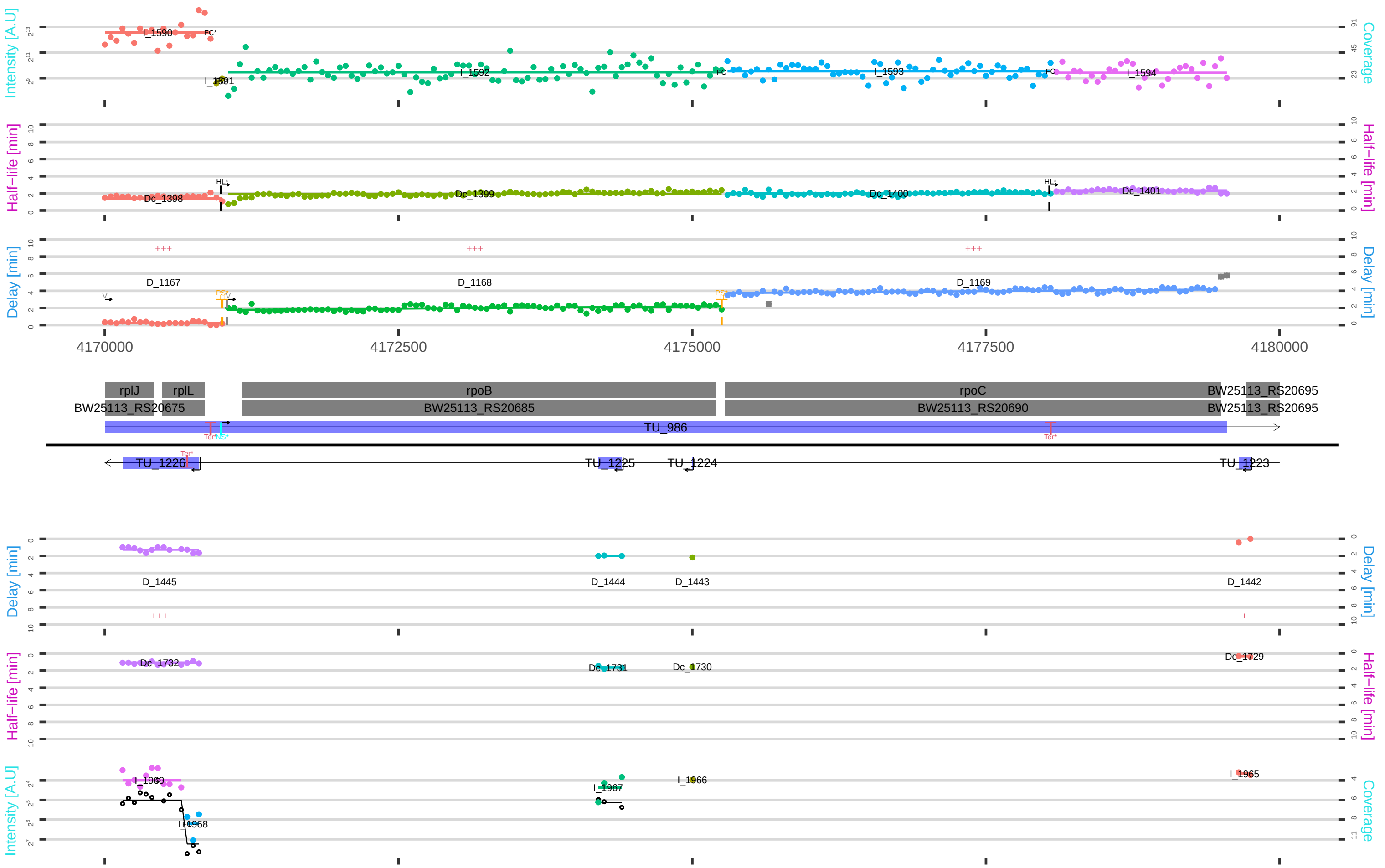

Term: termination (1), NS: new start (0), PS: pausing site (0), iTSS_I: internal starting site (0)

ID: 83605−83800; Term: termination (1), NS: new start (2), PS: pausing site (0), iTSS_I: internal starting site (0)

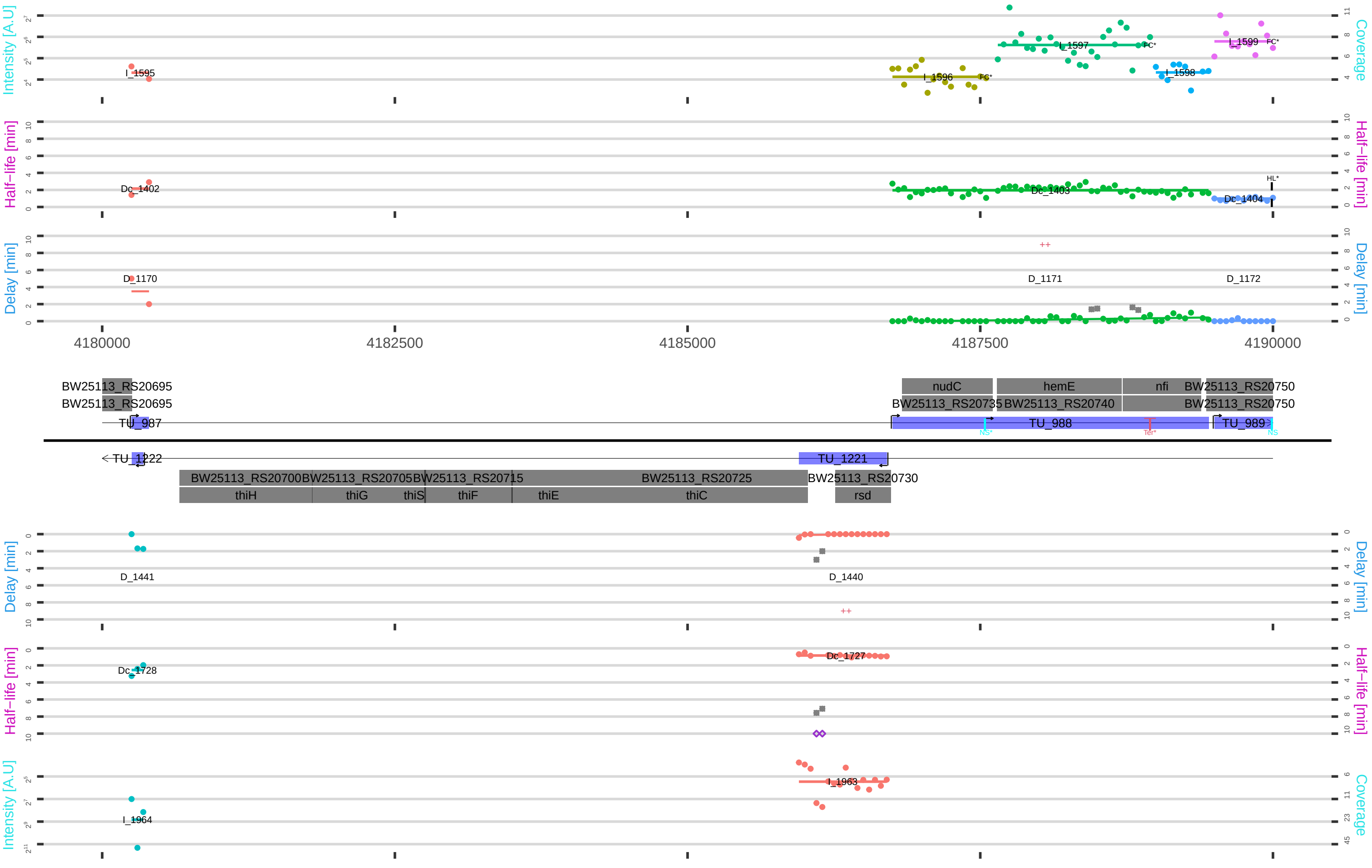

Term: termination (0), NS: new start (0), PS: pausing site (0), iTSS_I: internal starting site (0)

ID: 83800−84000; Term: termination (5), NS: new start (2), PS: pausing site (1), iTSS_I: internal starting site (0)

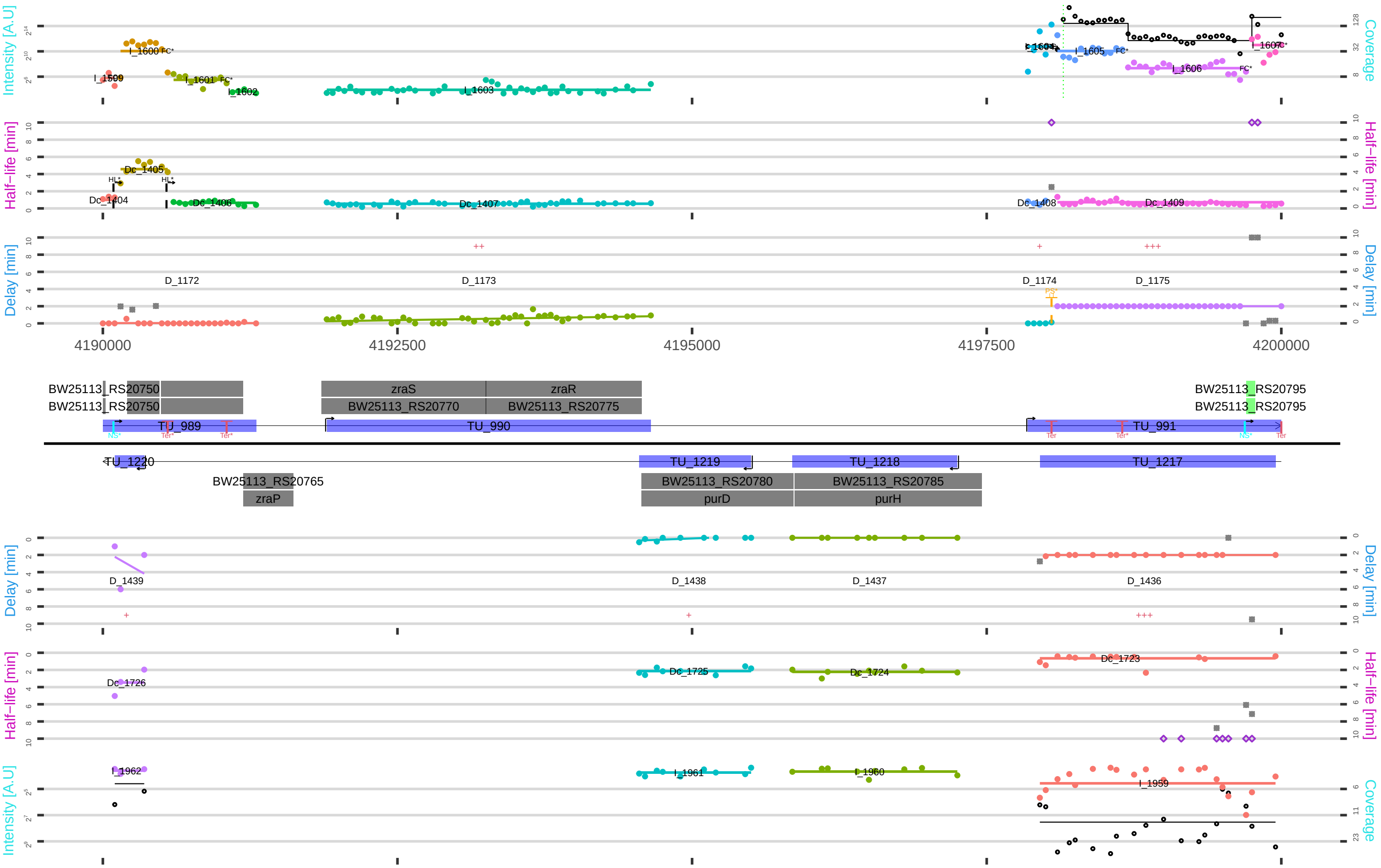

Term: termination (0), NS: new start (0), PS: pausing site (0), iTSS_I: internal starting site (0)

ID: 84000−84179; Term: termination (4), NS: new start (4), PS: pausing site (1), iTSS_I: internal starting site (0)

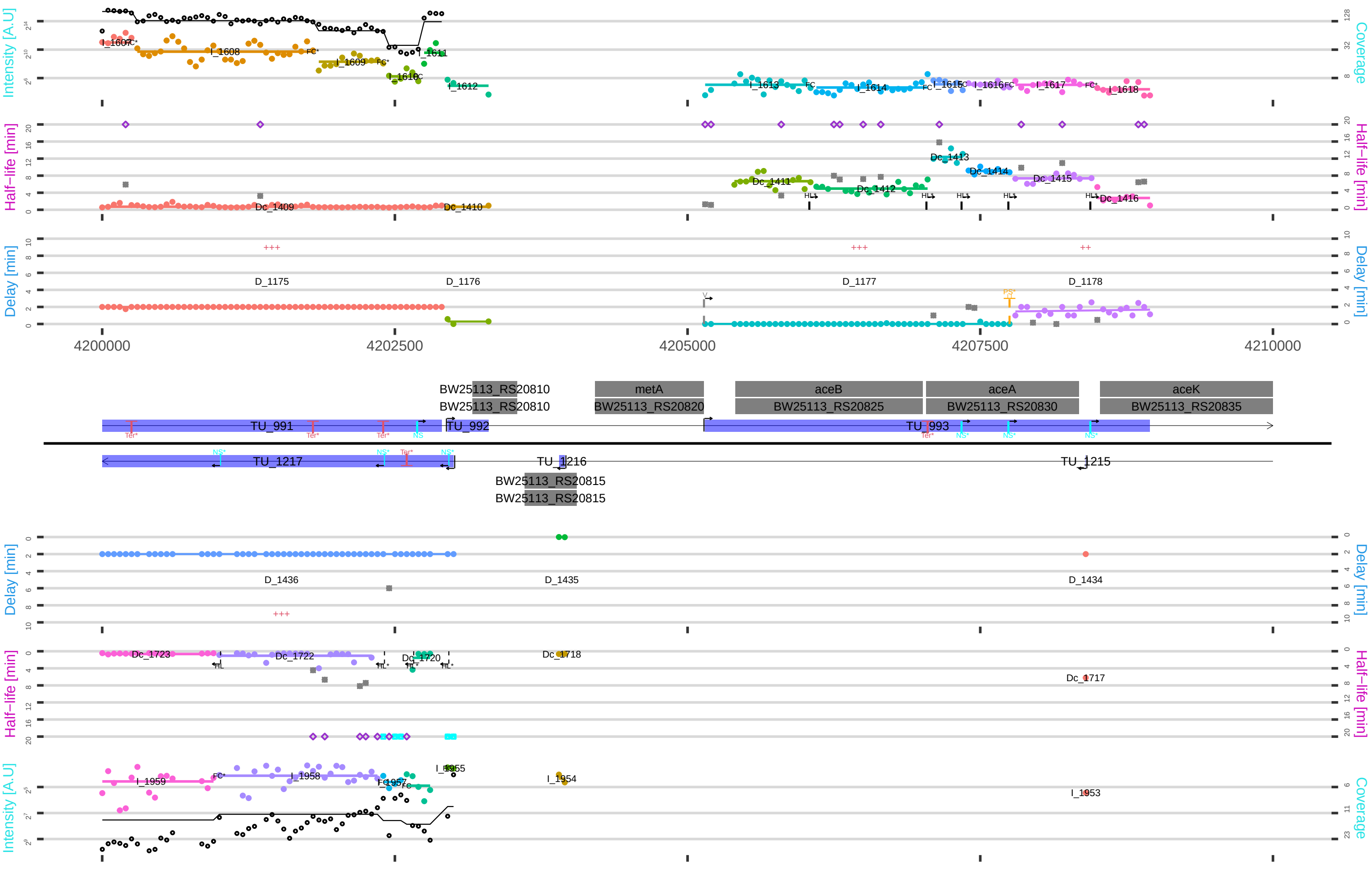

Term: termination (1), NS: new start (3), PS: pausing site (0), iTSS_I: internal starting site (0)

ID: 84276−84383; Term: termination (0), NS: new start (0), PS: pausing site (0), iTSS_I: internal starting site (0)

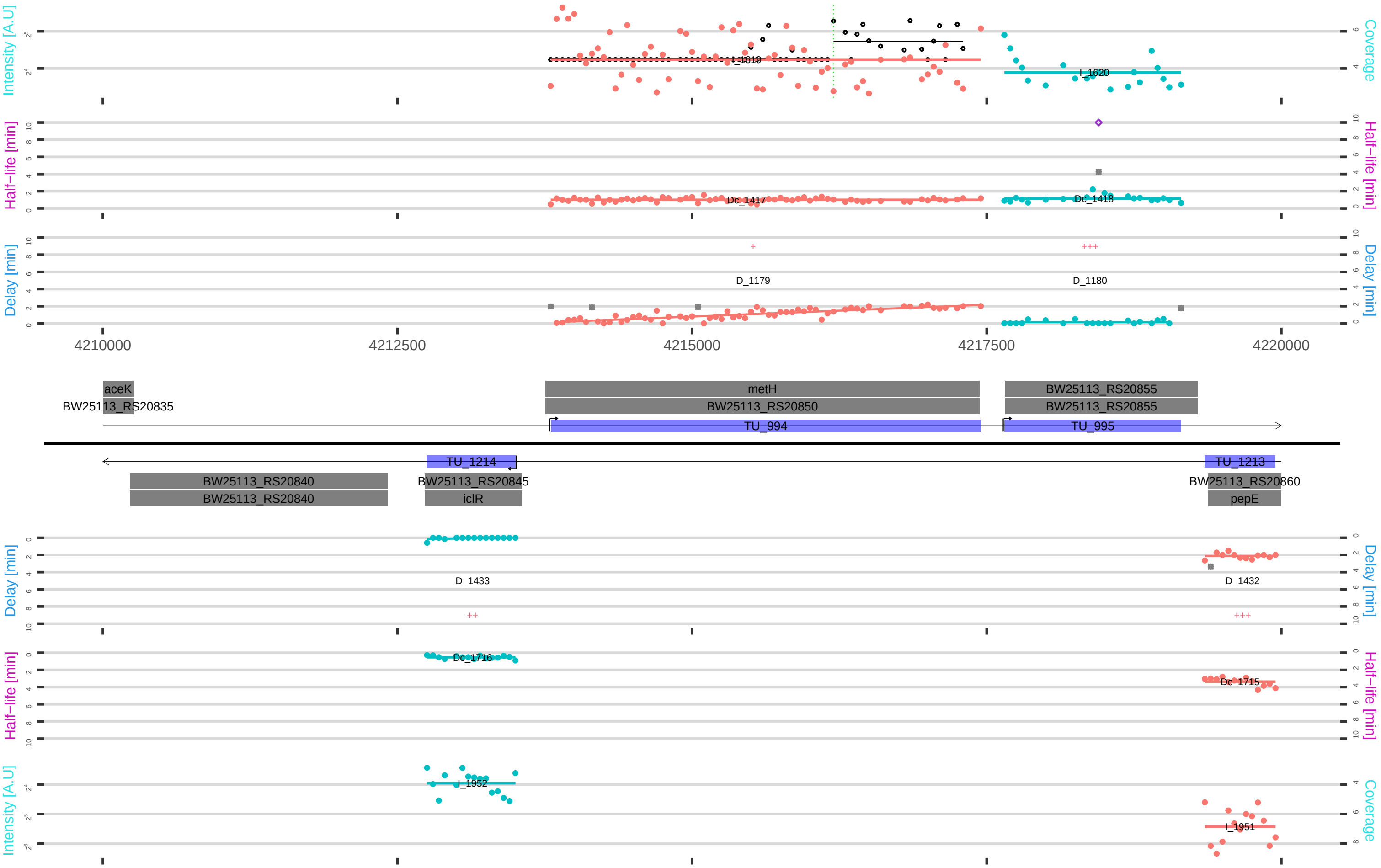

Term: termination (0), NS: new start (0), PS: pausing site (0), iTSS_I: internal starting site (0)

ID: 84407−84510; Term: termination (0), NS: new start (0), PS: pausing site (0), iTSS_I: internal starting site (0)

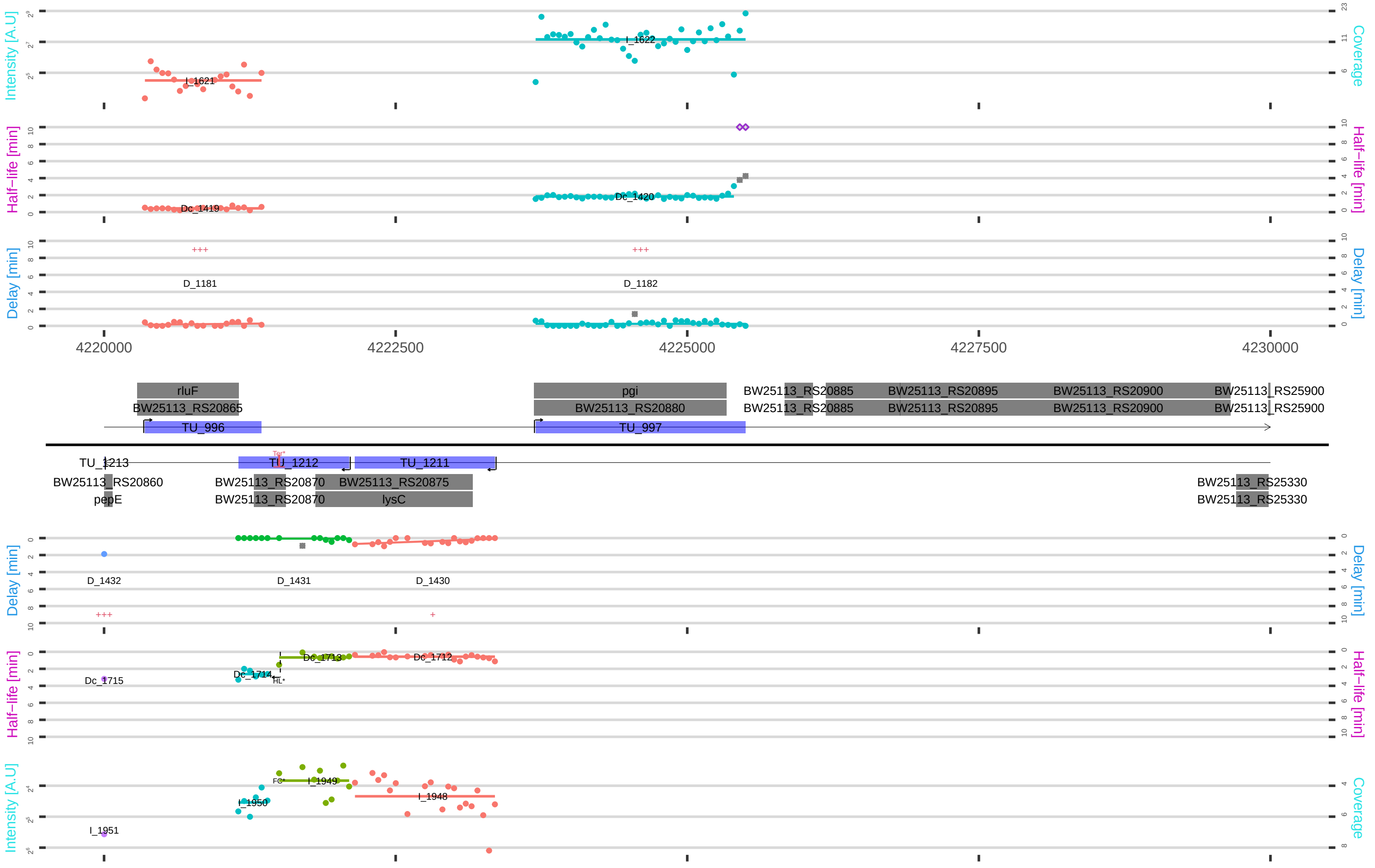

Term: termination (1), NS: new start (0), PS: pausing site (0), iTSS_I: internal starting site (0)

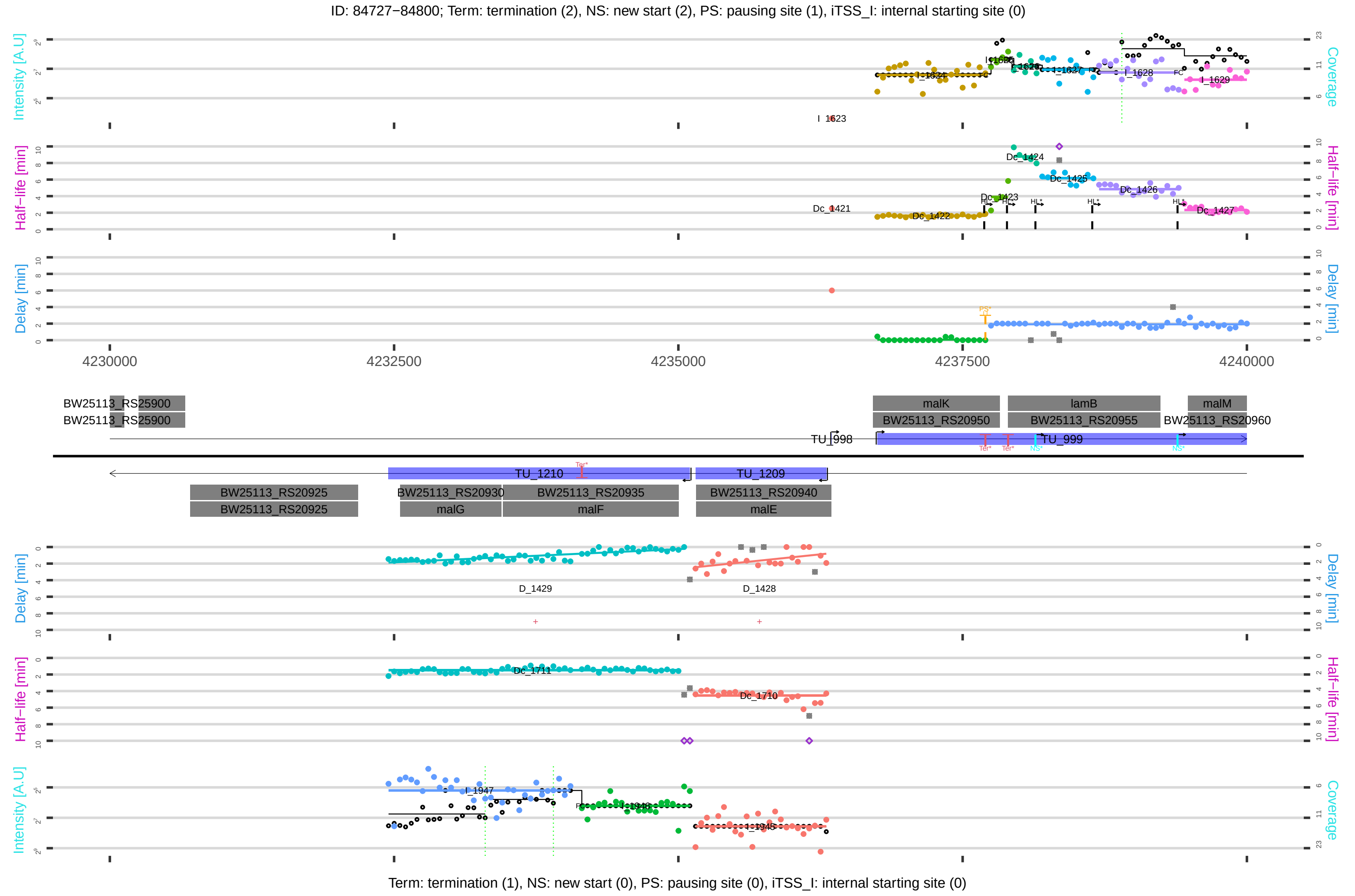
ID: 84727−84800; Term: termination (2), NS: new start (2), PS: pausing site (1), iTSS_I: internal starting site (0)
Intensity [A.U]
Coverage
Half−life [min]
Delay [min]
4230000
4232500
4235000
4237500
4240000
BW25113_RS25900
malK
BW25113_RS20950
lamB
BW25113_RS20955
malM
BW25113_RS20960
TU_998
TU_999
TU_1210
TU_1209
BW25113_RS20925
BW25113_RS20930
BW25113_RS20935
BW25113_RS20940
malG
malF
malE
D_1429
D_1428
Dc_1711
Dc_1710
Term: termination (1), NS: new start (0), PS: pausing site (0), iTSS_I: internal starting site (0)

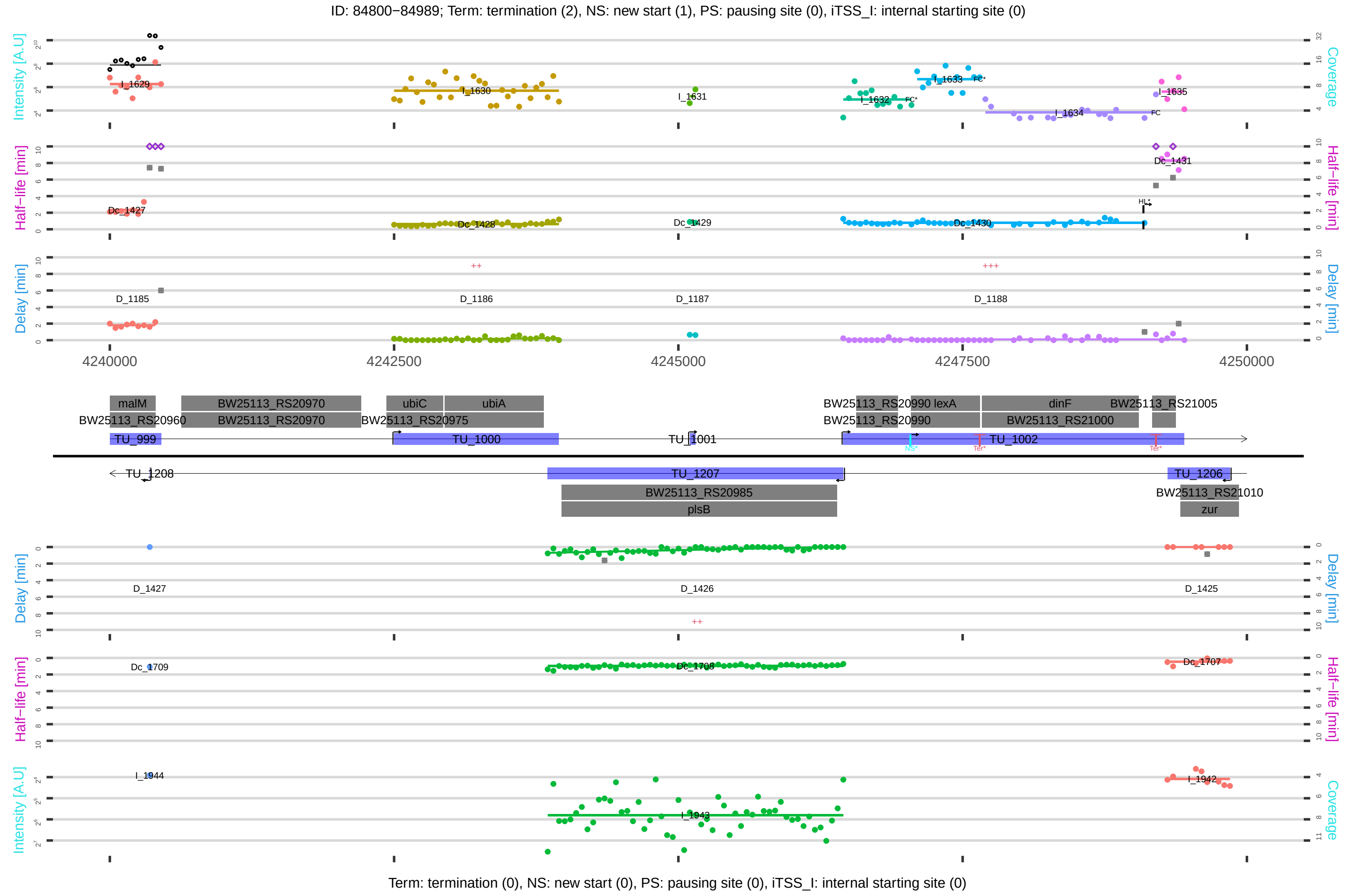

ID: 84800−84989; Term: termination (2), NS: new start (1), PS: pausing site (0), iTSS_I: internal starting site (0)
Intensity [A.U]
Coverage
I_1629
I_1630
I_1631
I_1632
I_1633
I_1634
I_1635
Half−life [min]
Dc_1427
Dc_1428
Dc_1429
Dc_1430
Dc_1431
Delay [min]
D_1185
D_1186
D_1187
D_1188
4240000
4242500
4245000
4247500
4250000
malM
BW25113_RS20960
BW25113_RS20970
ubiC
ubiA
BW25113_RS20975
BW25113_RS20990
lexA
dinF
BW25113_RS21000
BW25113_RS21005
TU_999
TU_1000
TU_1001
TU_1002
TU_1208
TU_1207
TU_1206
BW25113_RS20985
plsB
BW25113_RS21010
zur
D_1427
D_1426
D_1425
Dc_1709
Dc_1708
Dc_1707
I_1944
I_1943
I_1942
Term: termination (0), NS: new start (0), PS: pausing site (0), iTSS_I: internal starting site (0)

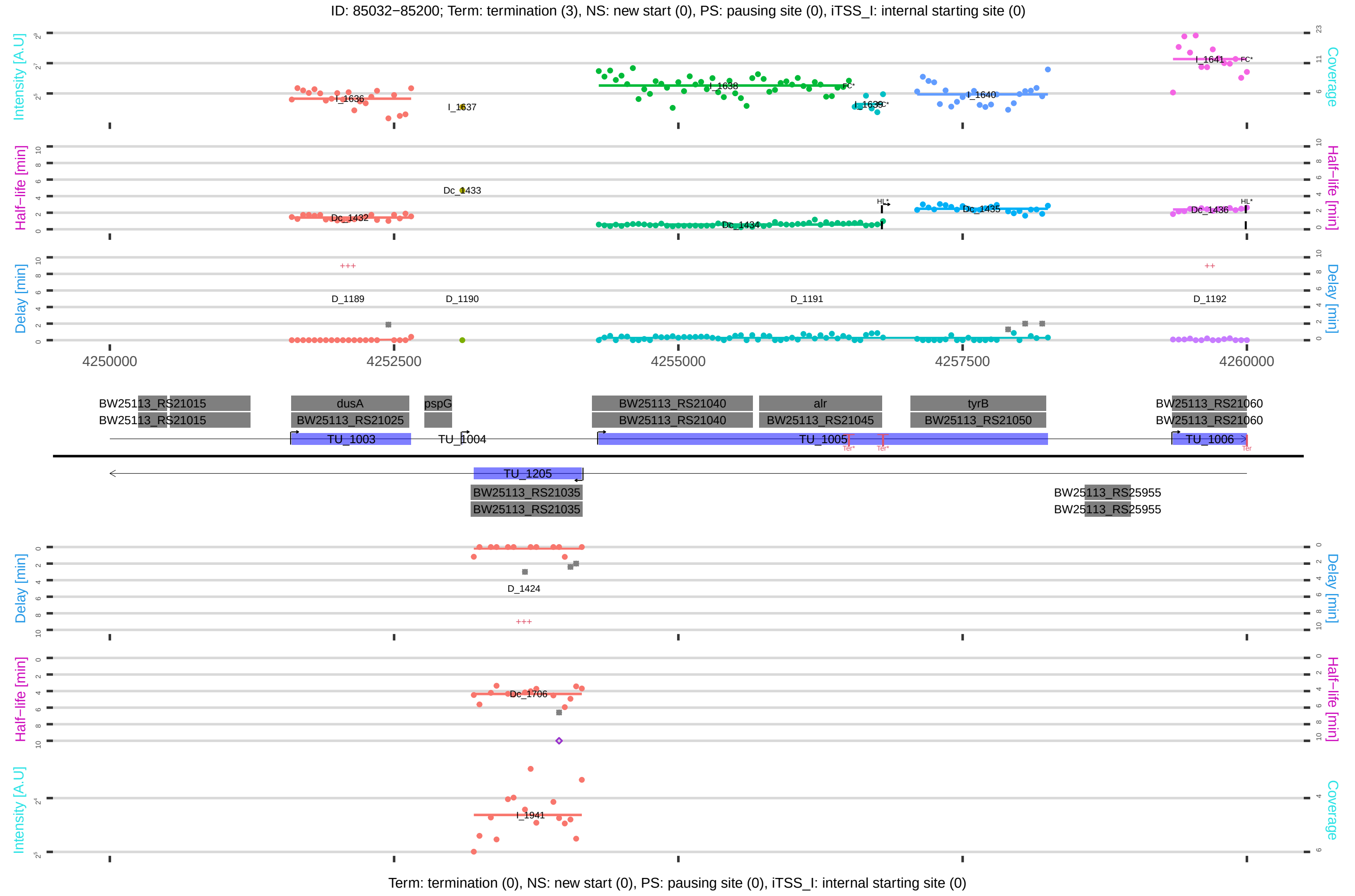

ID: 85032−85200; Term: termination (3), NS: new start (0), PS: pausing site (0), iTSS_I: internal starting site (0)
Intensity [A.U]
Coverage
Half−life [min]
Delay [min]
I_1636
I_1637
I_1638
I_1639
I_1640
I_1641
Dc_1432
Dc_1433
Dc_1434
Dc_1435
Dc_1436
D_1189
D_1190
D_1191
D_1192
4250000
4252500
4255000
4257500
4260000
BW25113_RS21015
dusA
BW25113_RS21025
pspG
BW25113_RS21040
alr
BW25113_RS21045
tyrB
BW25113_RS21050
BW25113_RS21060
TU_1003
TU_1004
TU_1005
TU_1006
TU_1205
BW25113_RS21035
BW25113_RS25955
D_1424
Dc_1706
I_1941
Term: termination (0), NS: new start (0), PS: pausing site (0), iTSS_I: internal starting site (0)

ID: 85200−85400; Term: termination (1), NS: new start (0), PS: pausing site (0), iTSS_I: internal starting site (0)

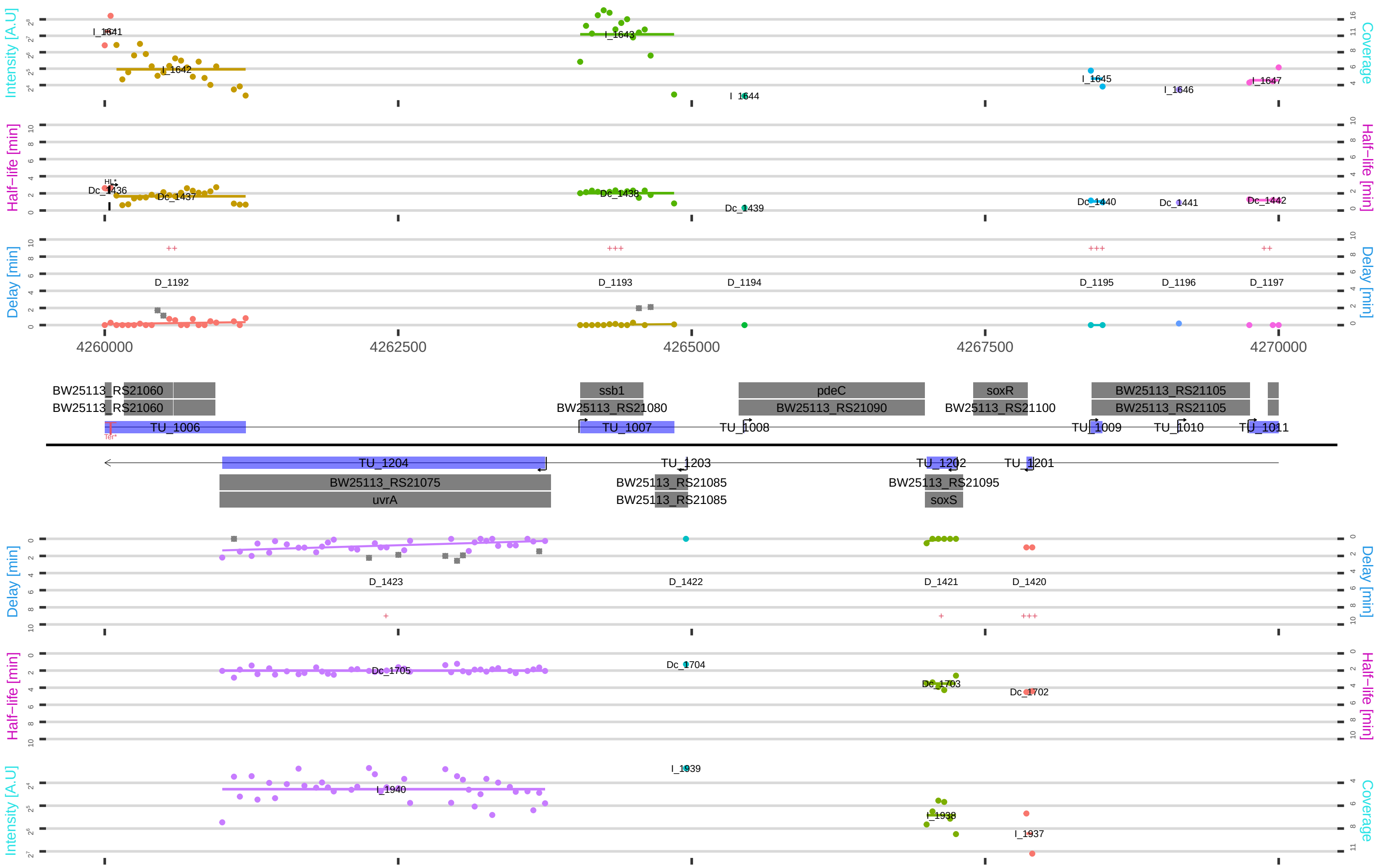

Term: termination (0), NS: new start (0), PS: pausing site (0), iTSS_I: internal starting site (0)

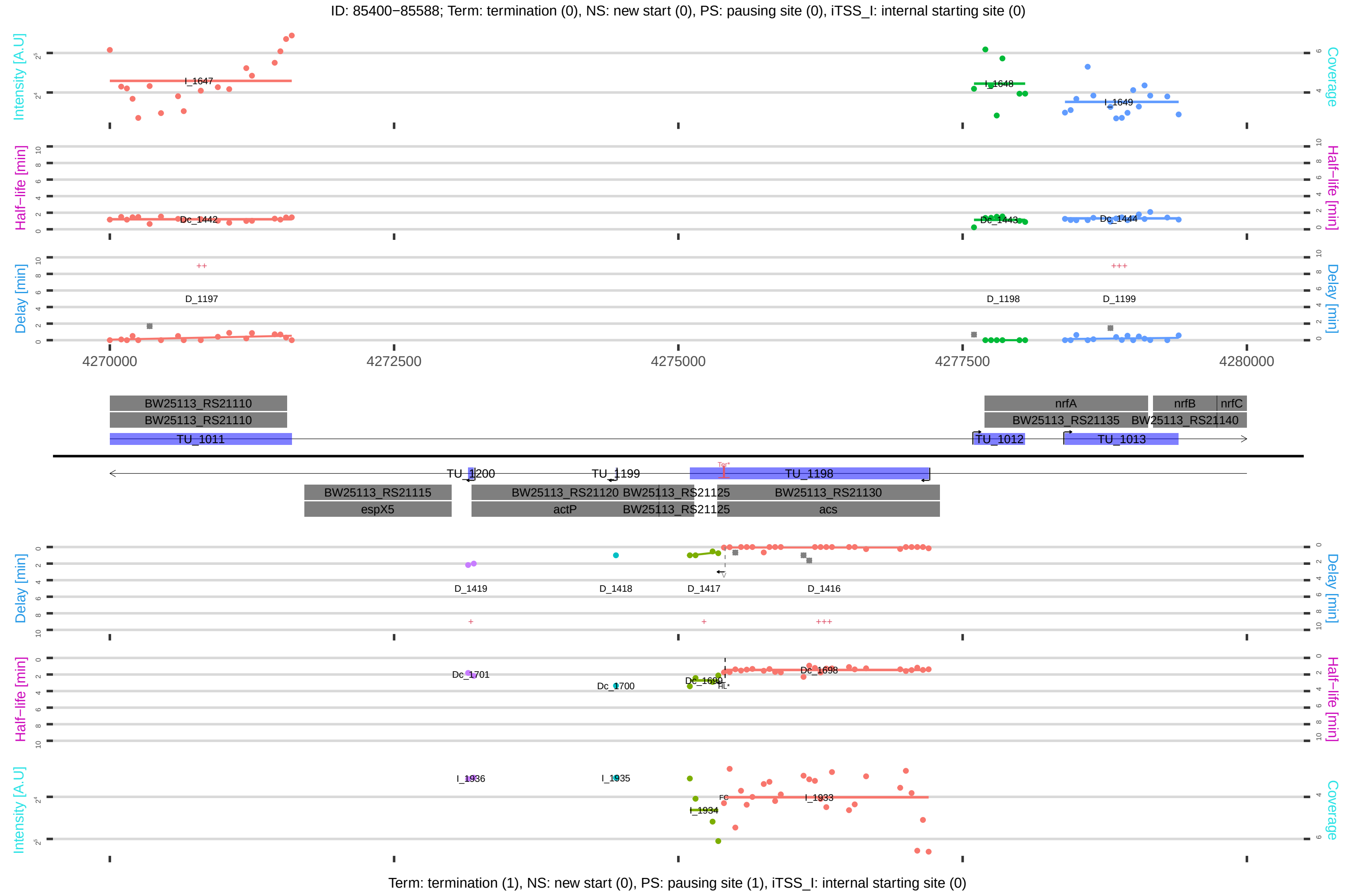

ID: 85400−85588; Term: termination (0), NS: new start (0), PS: pausing site (0), iTSS_I: internal starting site (0)
Intensity [A.U]
Coverage
Half−life [min]
Delay [min]
I_1647
I_1648
I_1649
Dc_1442
Dc_1443
Dc_1444
D_1197
D_1198
D_1199
4270000
4272500
4275000
4277500
4280000
BW25113_RS21110
nrfA
nrfB
nrfC
BW25113_RS21135
BW25113_RS21140
TU_1011
TU_1012
TU_1013
TU_1200
TU_1199
TU_1198
BW25113_RS21115
espX5
BW25113_RS21120
actP
BW25113_RS21125
BW25113_RS21130
acs
D_1419
D_1418
D_1417
D_1416
Dc_1701
Dc_1700
Dc_1699
Dc_1698
I_1936
I_1935
I_1934
I_1933
Term: termination (1), NS: new start (0), PS: pausing site (1), iTSS_I: internal starting site (0)

ID: 85688−85724; Term: termination (0), NS: new start (0), PS: pausing site (0), iTSS_I: internal starting site (0)

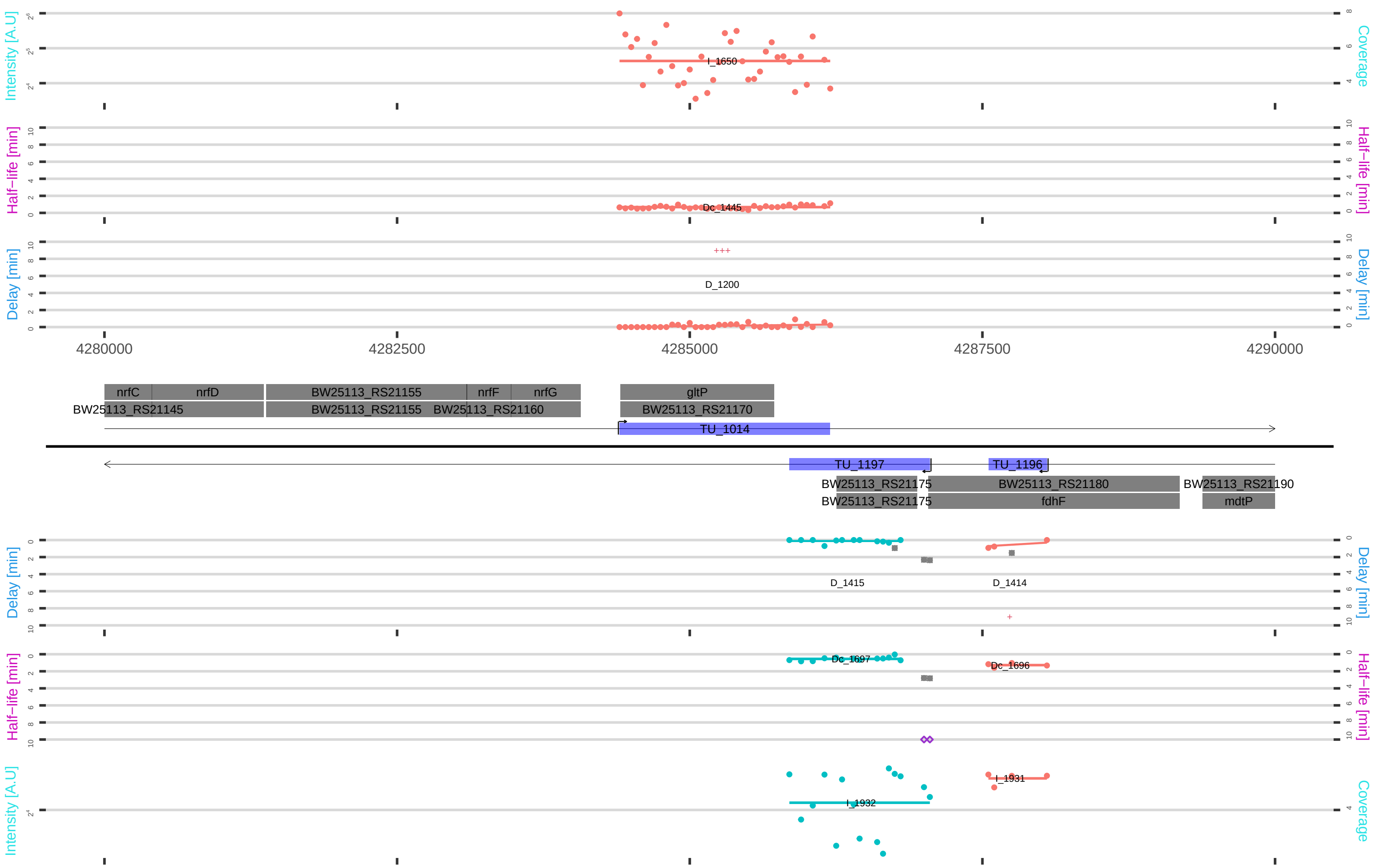

Term: termination (0), NS: new start (0), PS: pausing site (0), iTSS_I: internal starting site (0)

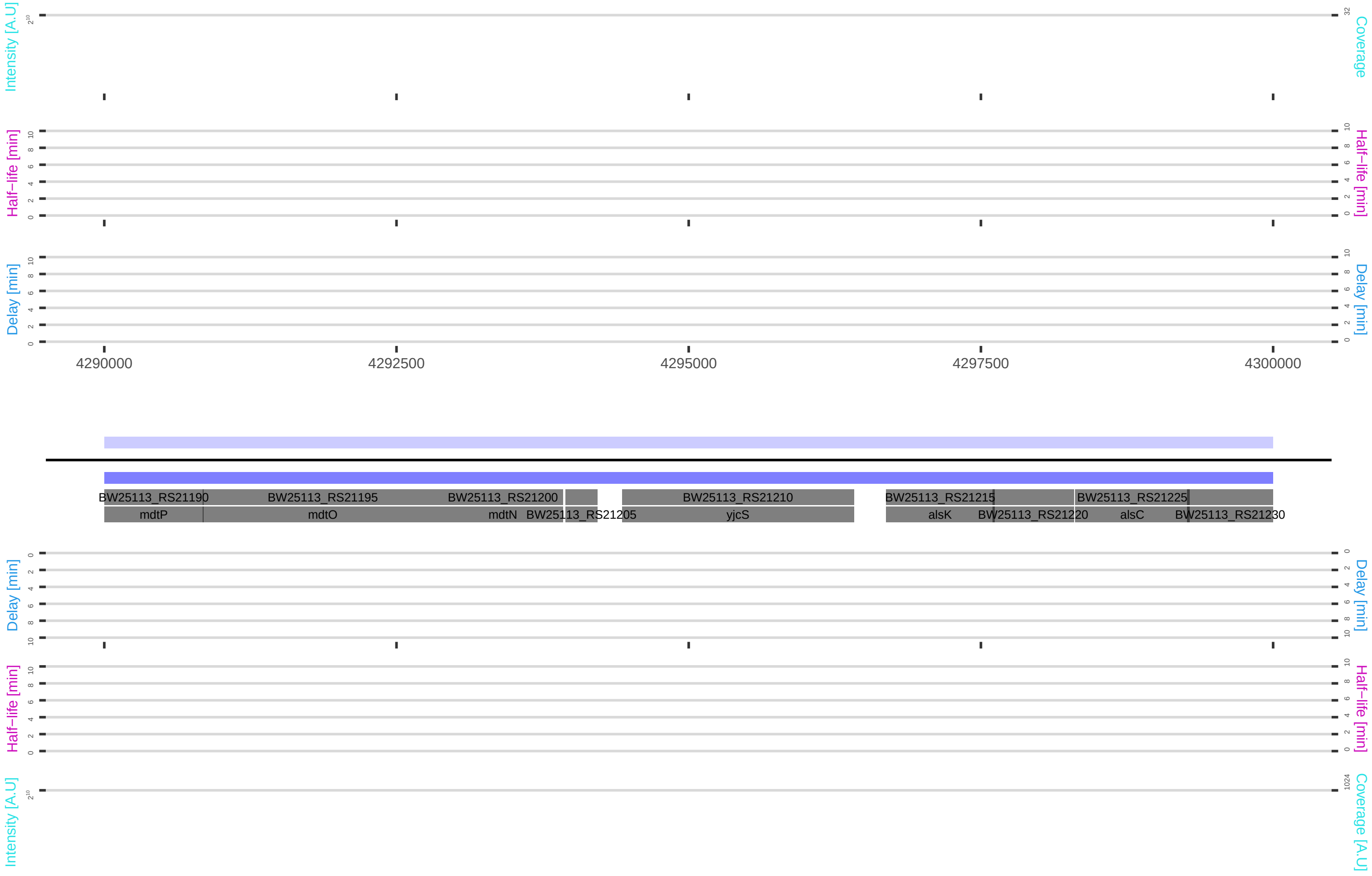
Intensity [A.U]
Coverage
Half−life [min]
Delay [min]
4290000
4292500
4295000
4297500
4300000
BW25113_RS21190
BW25113_RS21195
BW25113_RS21200
BW25113_RS21210
BW25113_RS21215
BW25113_RS21225
mdtP
mdtO
mdtN
BW25113_RS21205
yjcS
alsK
BW25113_RS21220
alsC
BW25113_RS21230
Delay [min]
Half−life [min]
Intensity [A.U]
Coverage [A.U]
NA

ID: 86065−86149; Term: termination (0), NS: new start (0), PS: pausing site (0), iTSS_I: internal starting site (0)

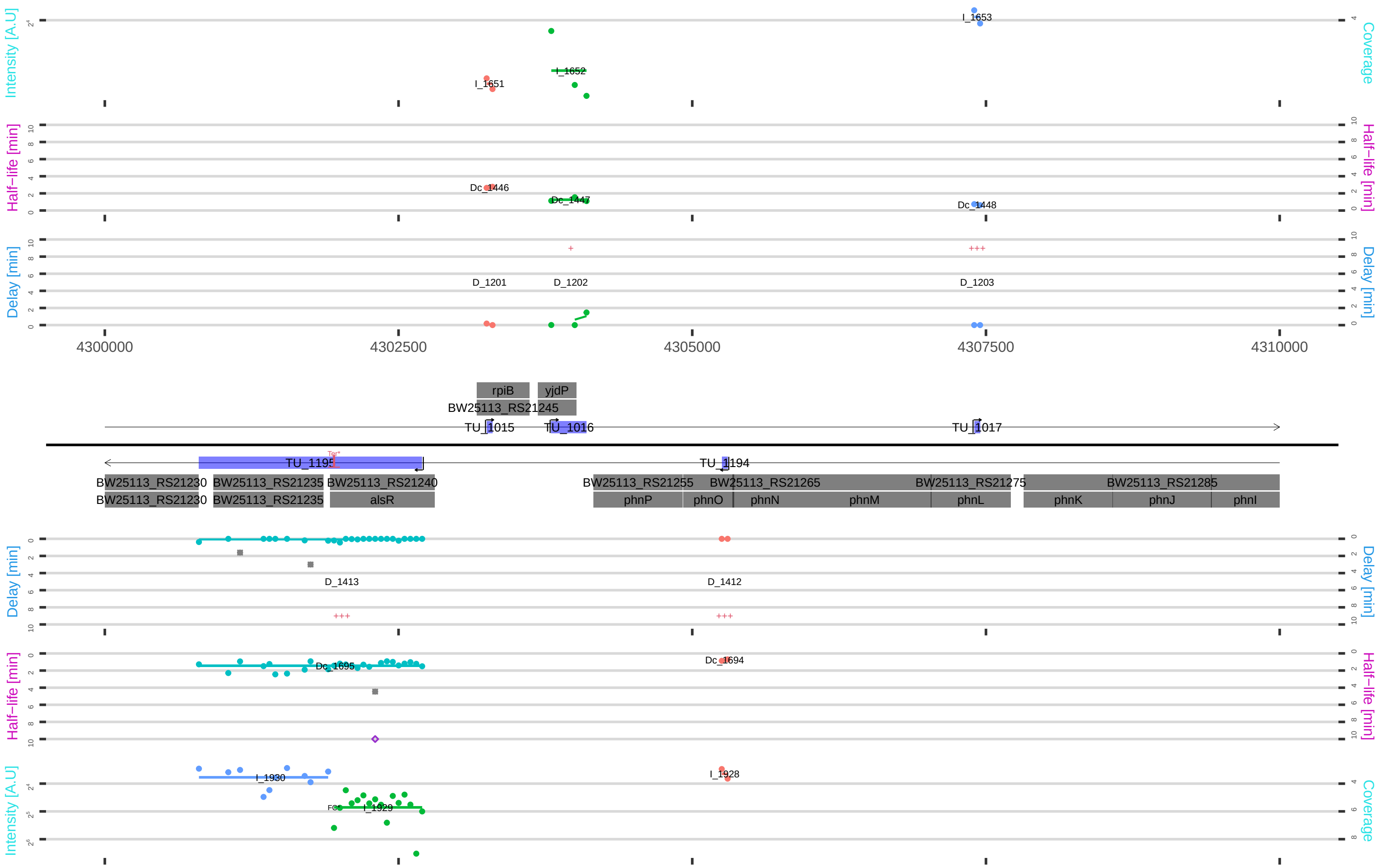

Term: termination (1), NS: new start (0), PS: pausing site (0), iTSS_I: internal starting site (0)

ID: 98946−98929; FC*: significant t−test of two consecutive segments; Term: termination, NS: new start, PS: pausing site,iTSS_I: internal starting site,TI: transcription interferance.

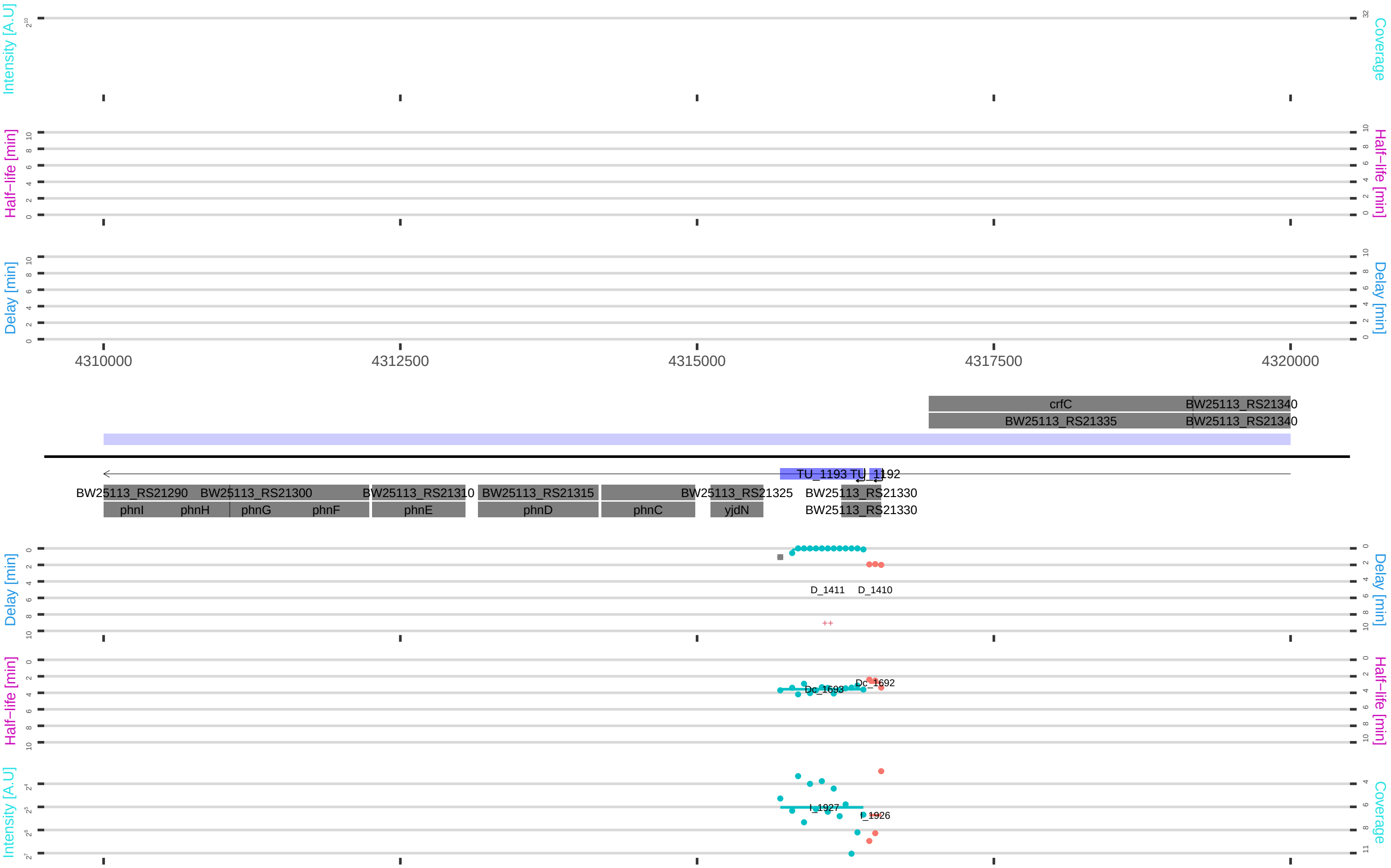

Term: termination (0), NS: new start (0), PS: pausing site (0), iTSS_I: internal starting site (0)

ID: 86406−86439; Term: termination (0), NS: new start (0), PS: pausing site (0), iTSS_I: internal starting site (0)

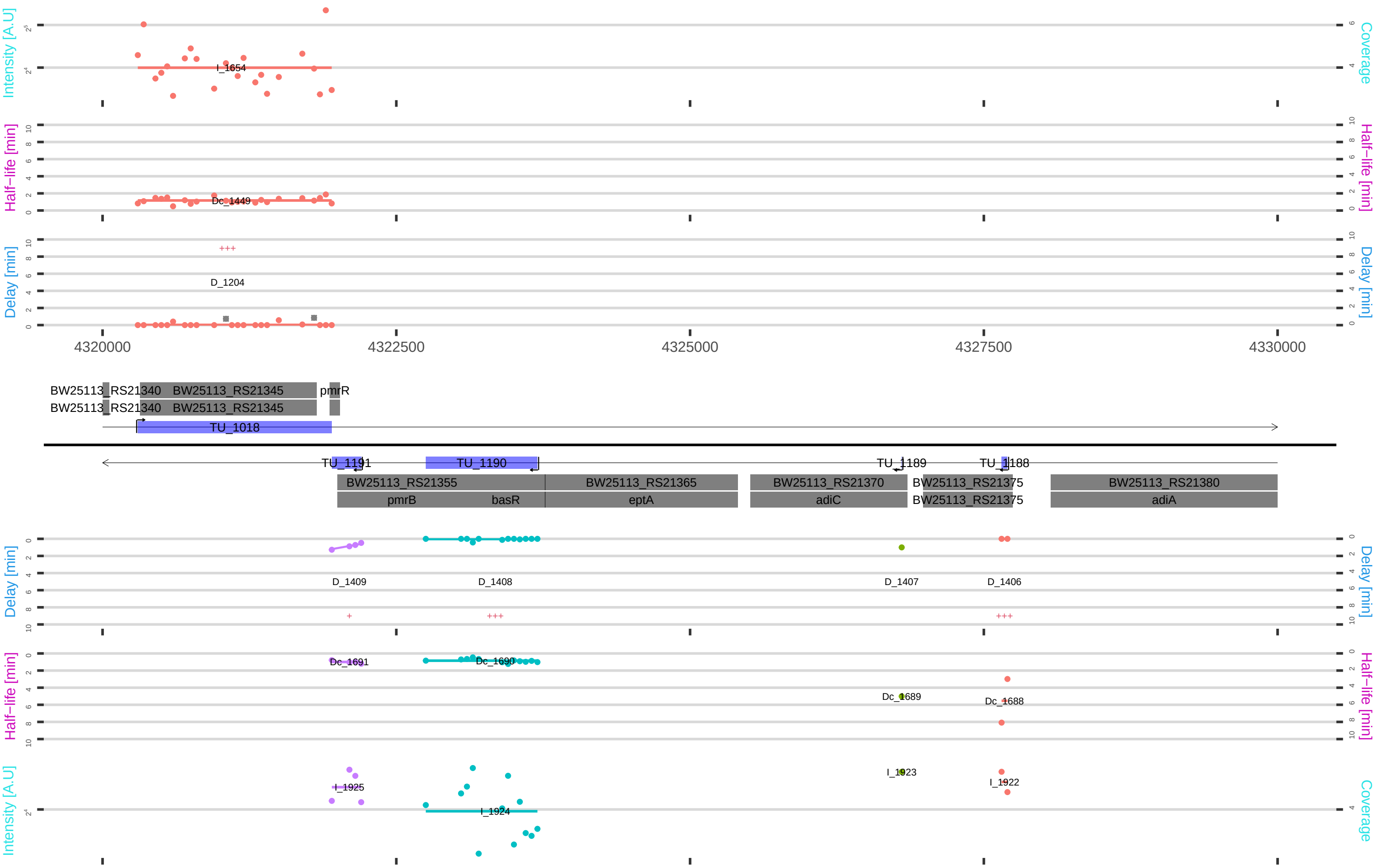

Term: termination (0), NS: new start (0), PS: pausing site (0), iTSS_I: internal starting site (0)

ID: 86780−86780; Term: termination (0), NS: new start (0), PS: pausing site (0), iTSS_I: internal starting site (0)

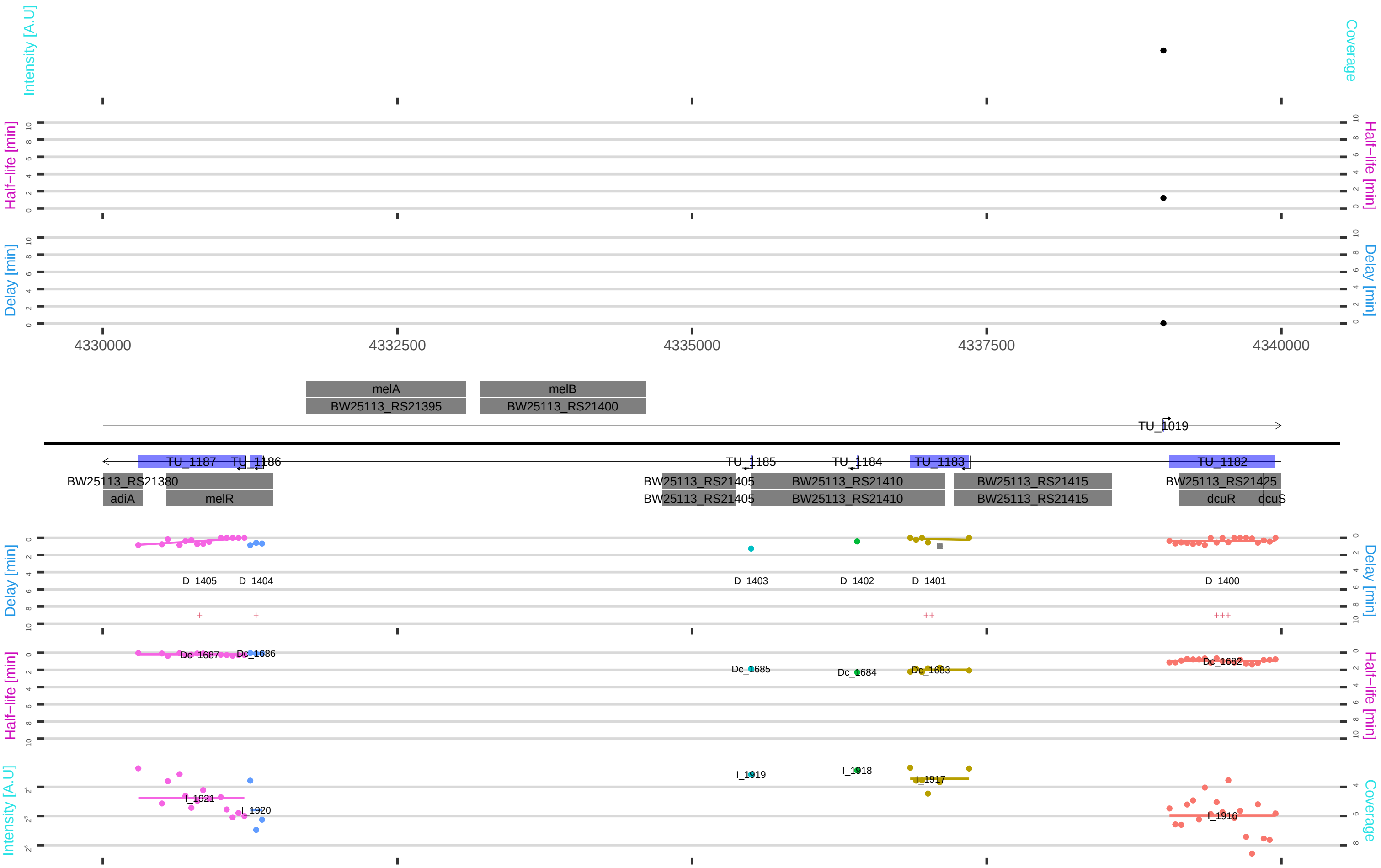

Term: termination (0), NS: new start (0), PS: pausing site (0), iTSS_I: internal starting site (0)

ID: 86844−86859; Term: termination (0), NS: new start (0), PS: pausing site (0), iTSS_I: internal starting site (0)

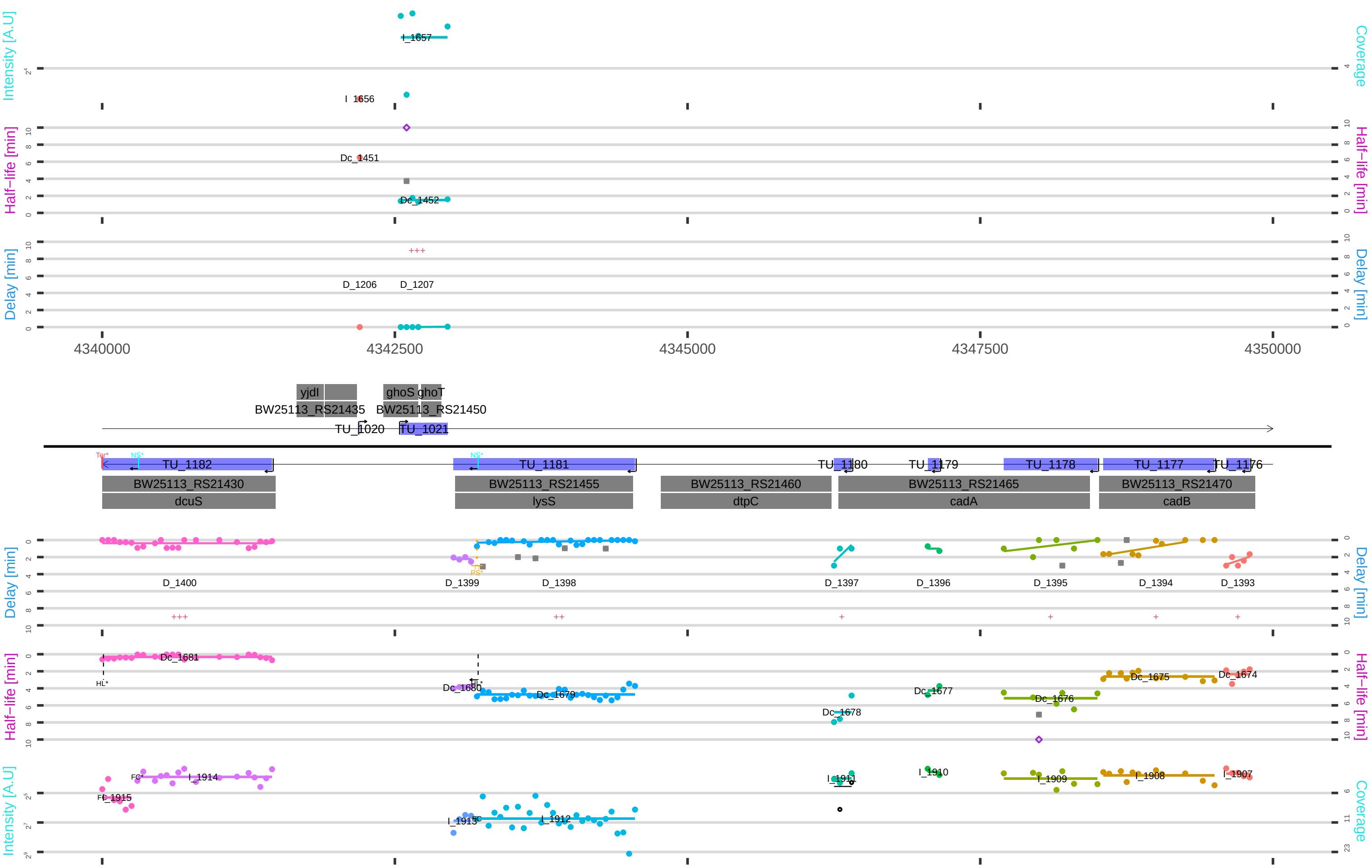

Term: termination (1), NS: new start (2), PS: pausing site (1), iTSS_I: internal starting site (0)

ID: 87156−87179; Term: termination (0), NS: new start (0), PS: pausing site (0), iTSS_I: internal starting site (0)

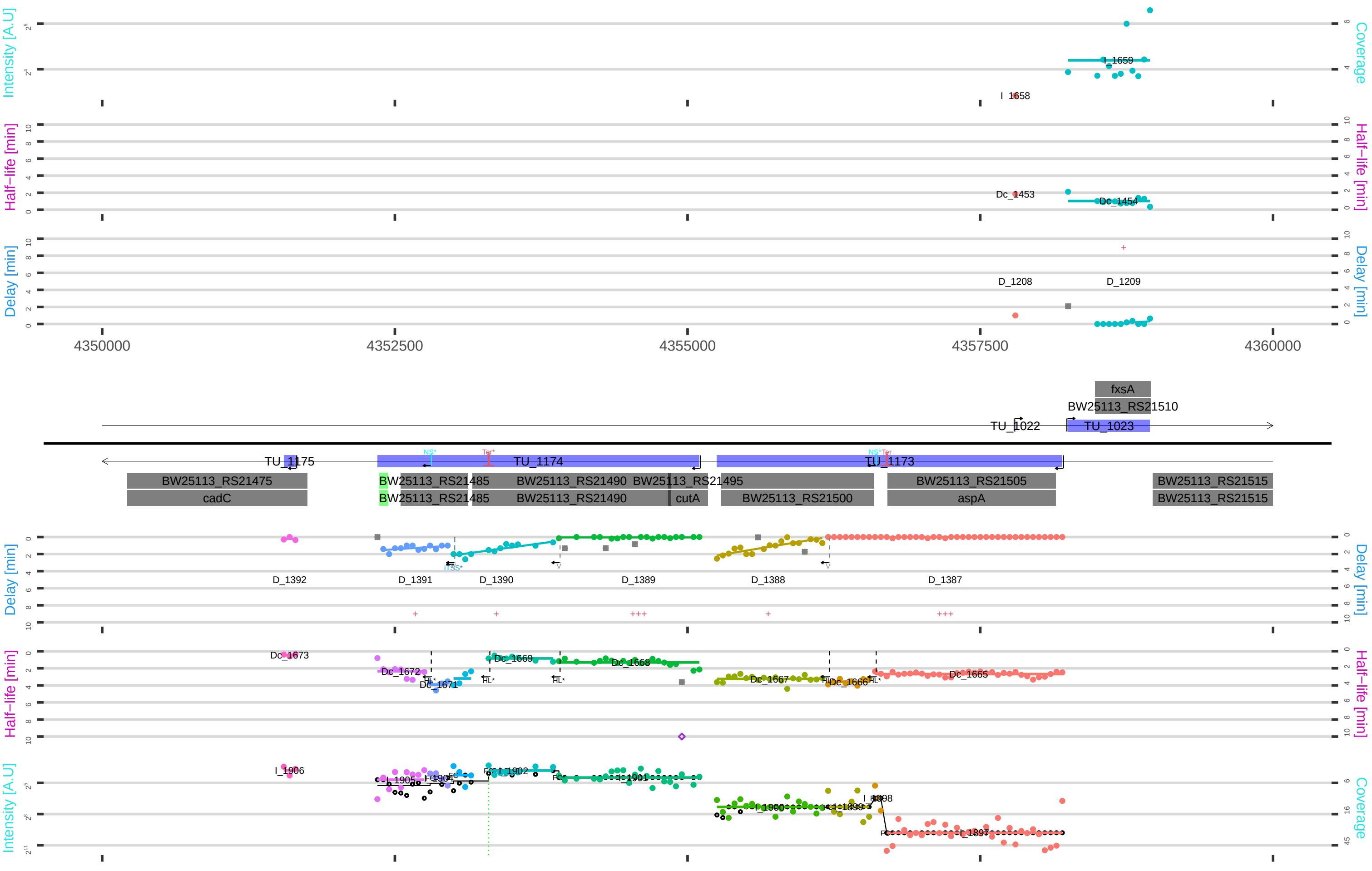

Term: termination (2), NS: new start (2), PS: pausing site (1), iTSS_I: internal starting site (2)

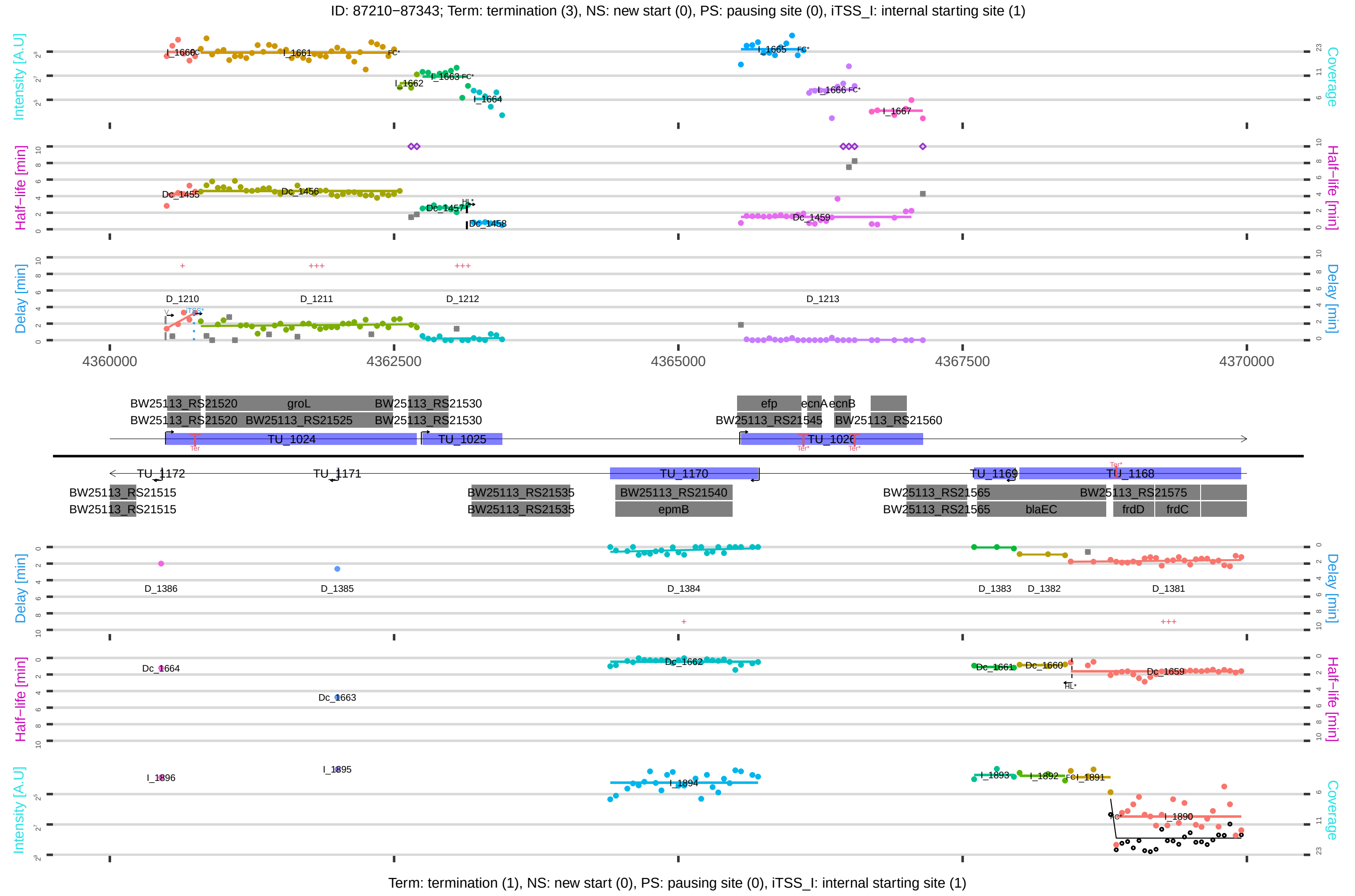

ID: 87210−87343; Term: termination (3), NS: new start (0), PS: pausing site (0), iTSS_I: internal starting site (1)
Intensity [A.U]
Coverage
Half−life [min]
Delay [min]
4360000
4362500
4365000
4367500
4370000
BW25113_RS21520
groL
BW25113_RS21525
BW25113_RS21530
efp
ecnA
ecnB
BW25113_RS21545
BW25113_RS21560
TU_1024
TU_1025
TU_1026
TU_1172
TU_1171
TU_1170
TU_1169
TU_1168
BW25113_RS21515
BW25113_RS21535
BW25113_RS21540
epmB
BW25113_RS21565
blaEC
BW25113_RS21575
frdD
frdC
Term: termination (1), NS: new start (0), PS: pausing site (0), iTSS_I: internal starting site (1)

ID: 87450−87514; Term: termination (0), NS: new start (0), PS: pausing site (0), iTSS_I: internal starting site (0)

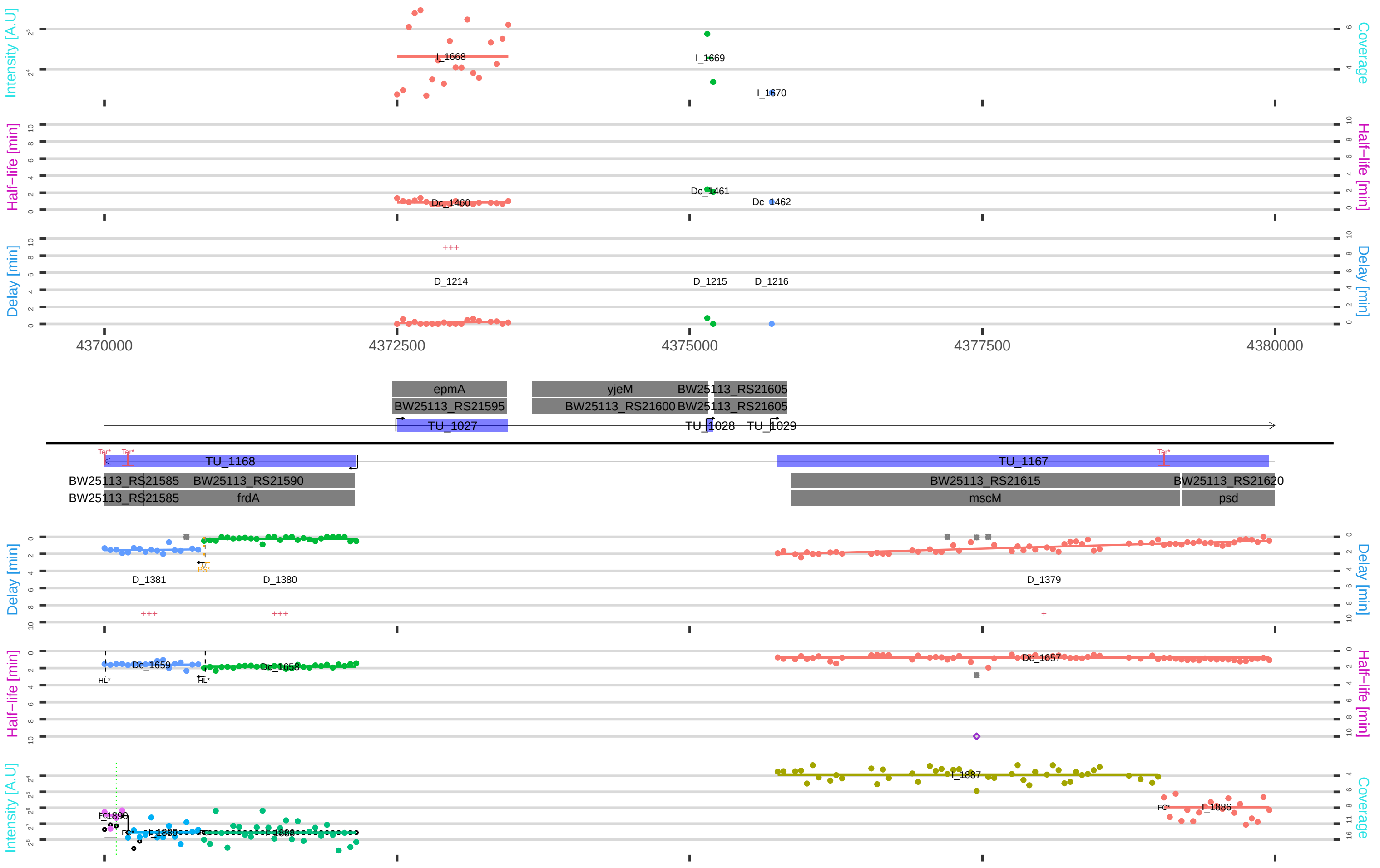

Term: termination (3), NS: new start (0), PS: pausing site (1), iTSS_I: internal starting site (0)

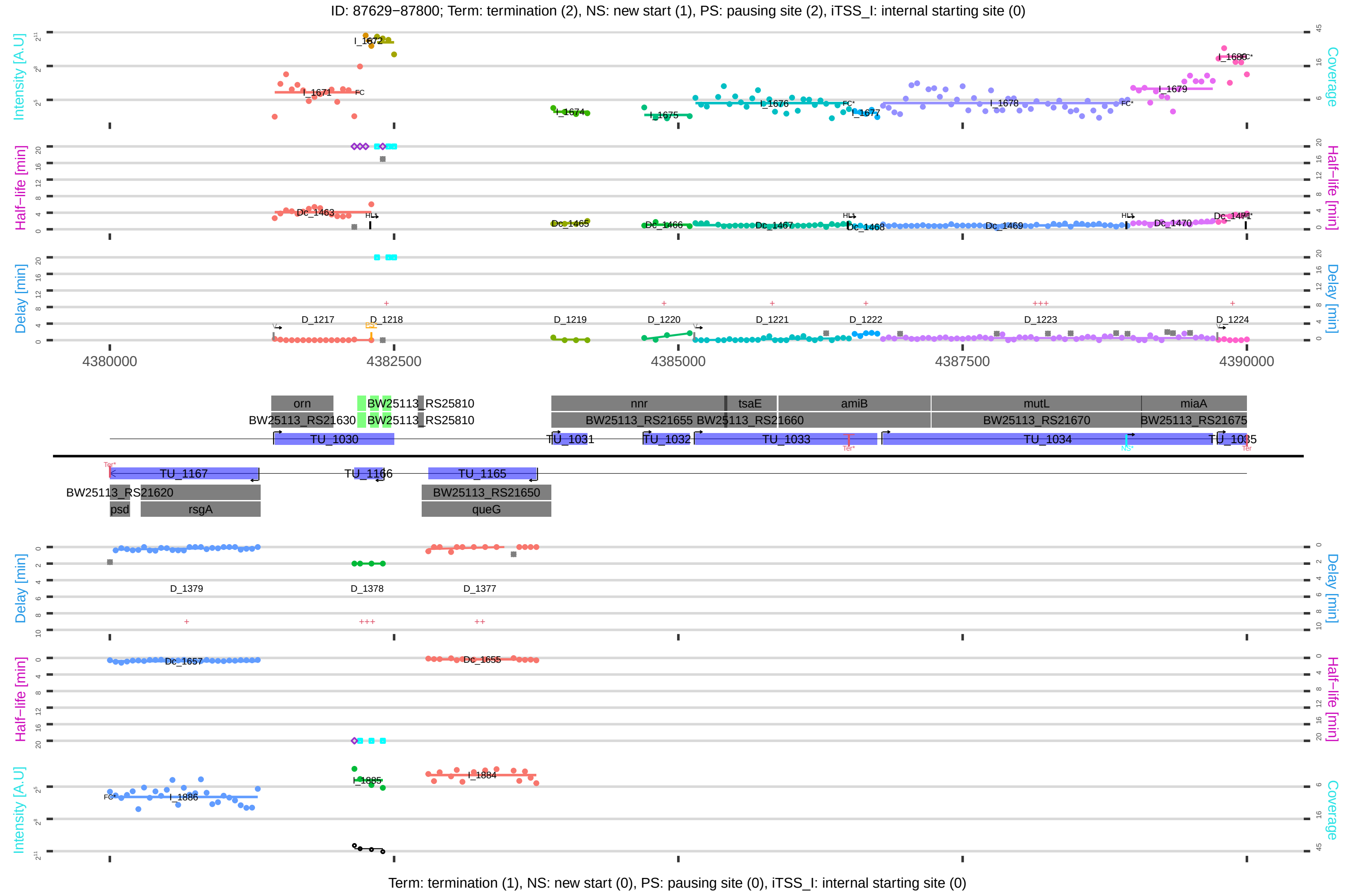
ID: 87629−87800; Term: termination (2), NS: new start (1), PS: pausing site (2), iTSS_I: internal starting site (0)
Intensity [A.U]
Coverage
Half−life [min]
Delay [min]
I_1671
I_1672
I_1674
I_1675
I_1676
I_1677
I_1678
I_1679
I_1680
Dc_1463
Dc_1465
Dc_1466
Dc_1467
Dc_1468
Dc_1469
Dc_1470
Dc_1471
D_1217
D_1218
D_1219
D_1220
D_1221
D_1222
D_1223
D_1224
4380000
4382500
4385000
4387500
4390000
orn
BW25113_RS21630
BW25113_RS25810
nnr
BW25113_RS21655
tsaE
BW25113_RS21660
amiB
mutL
BW25113_RS21670
miaA
BW25113_RS21675
TU_1030
TU_1031
TU_1032
TU_1033
TU_1034
TU_1035
Ter*
NS*
Ter
TU_1167
TU_1166
TU_1165
BW25113_RS21620
psd
rsgA
BW25113_RS21650
queG
D_1379
D_1378
D_1377
Dc_1657
Dc_1655
I_1885
I_1884
I_1886
Term: termination (1), NS: new start (0), PS: pausing site (0), iTSS_I: internal starting site (0)

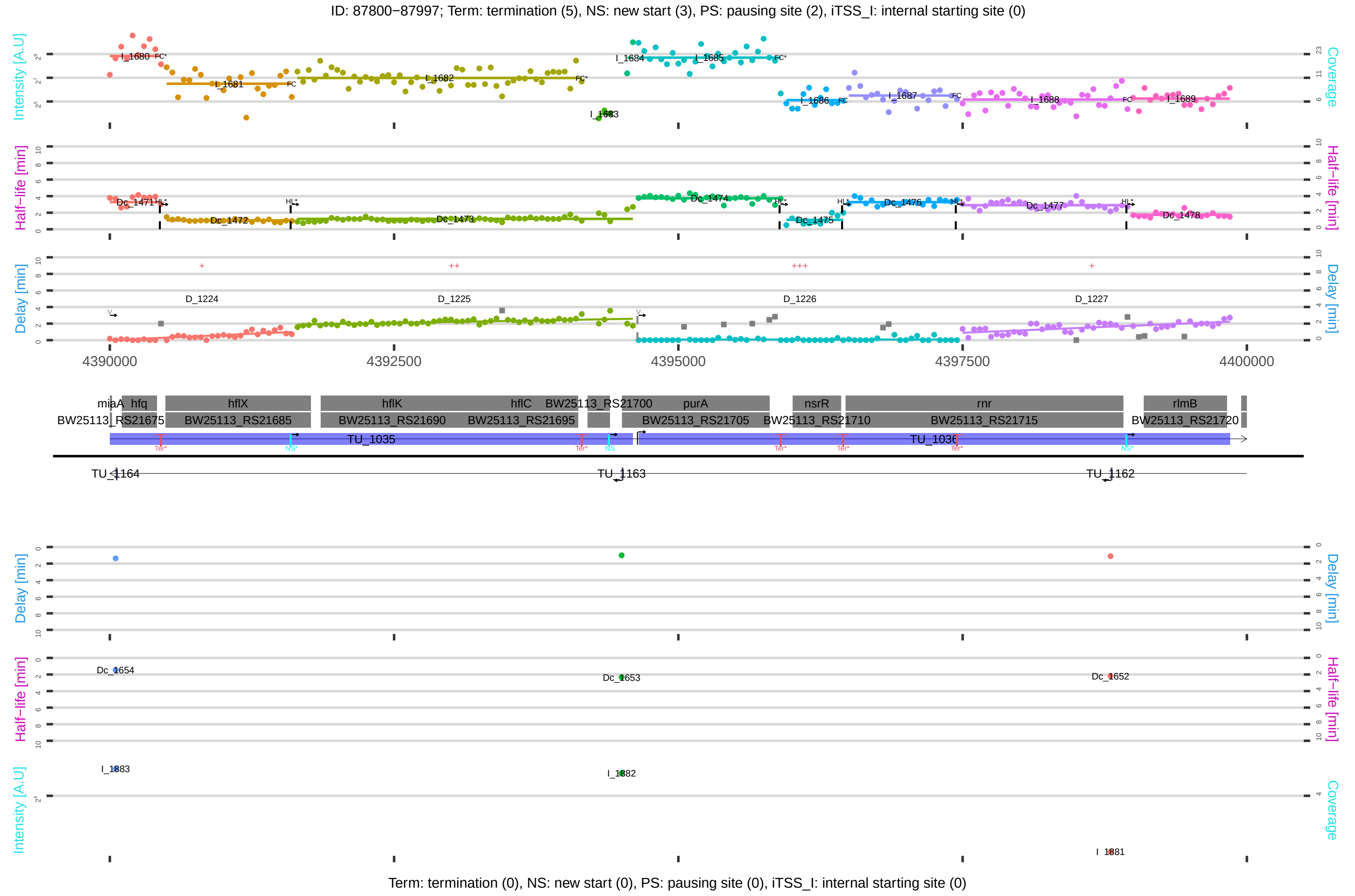

ID: 87800−87997; Term: termination (5), NS: new start (3), PS: pausing site (2), iTSS_I: internal starting site (0)
Intensity [A.U]
Coverage
Half−life [min]
Delay [min]
4390000
4392500
4395000
4397500
4400000
miaA hfq hflX hflK hflC BW25113_RS21700 purA nsrR rnr rlmB
BW25113_RS21675 BW25113_RS21685 BW25113_RS21690 BW25113_RS21695 BW25113_RS21705 BW25113_RS21710 BW25113_RS21715 BW25113_RS21720
TU_1035
TU_1036
TU_1164
TU_1163
TU_1162
D_1224
D_1225
D_1226
D_1227
Term: termination (0), NS: new start (0), PS: pausing site (0), iTSS_I: internal starting site (0)

ID: 88137−88143; Term: termination (0), NS: new start (0), PS: pausing site (0), iTSS_I: internal starting site (0)

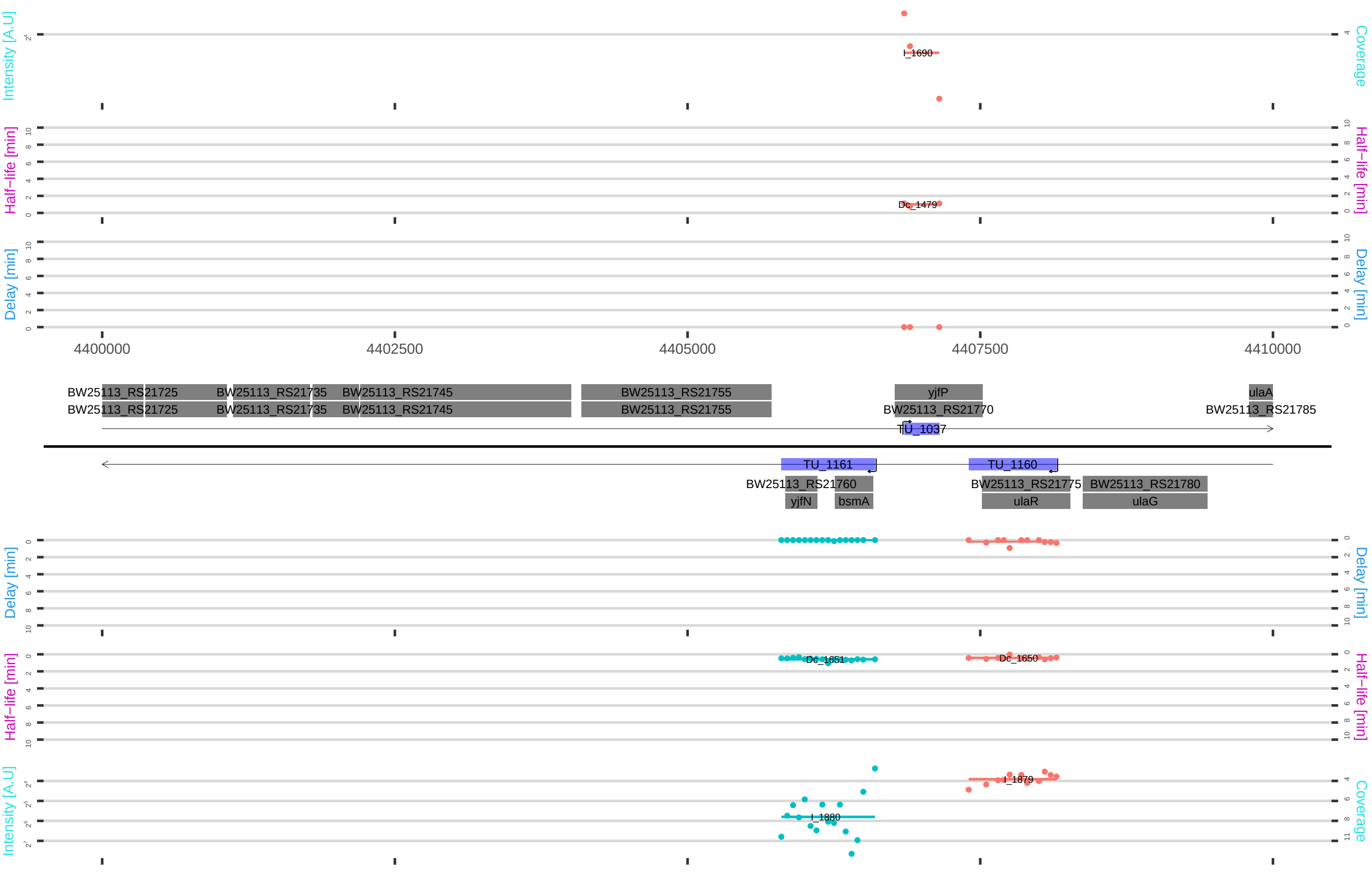

Term: termination (0), NS: new start (0), PS: pausing site (0), iTSS_I: internal starting site (0)

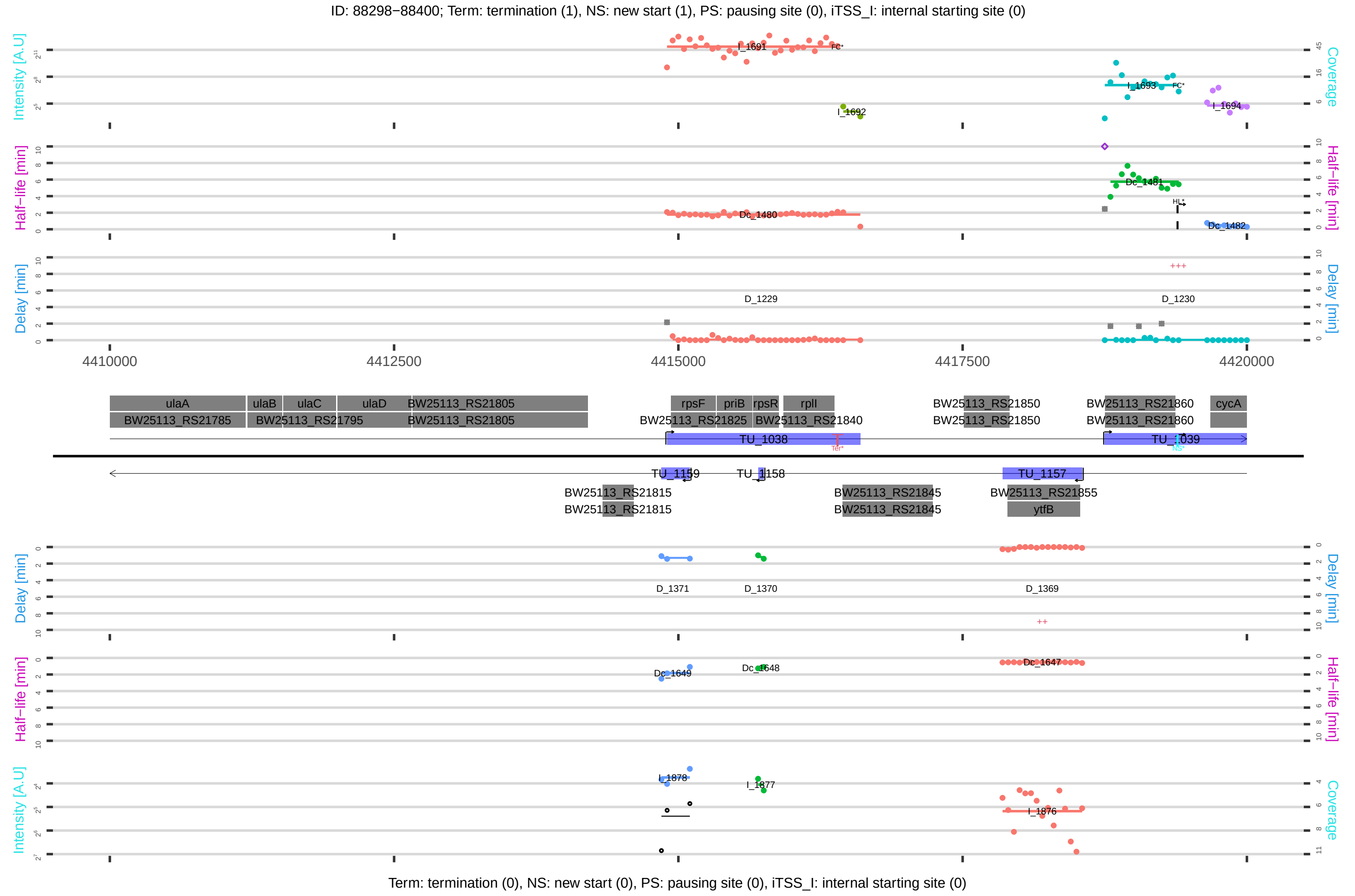

ID: 88298−88400; Term: termination (1), NS: new start (1), PS: pausing site (0), iTSS_I: internal starting site (0)
Intensity [A.U]
Coverage
Half−life [min]
Delay [min]
I_1691
I_1692
I_1693
I_1694
Dc_1480
Dc_1481
Dc_1482
D_1229
D_1230
4410000
4412500
4415000
4417500
4420000
ulaA
ulaB
ulaC
ulaD
BW25113_RS21785
BW25113_RS21795
BW25113_RS21805
rpsF
priB
rpsR
rplI
BW25113_RS21825
BW25113_RS21840
BW25113_RS21850
BW25113_RS21860
cycA
TU_1038
TU_1039
TU_1159
TU_1158
TU_1157
BW25113_RS21815
BW25113_RS21845
BW25113_RS21855
ytfB
D_1371
D_1370
D_1369
Dc_1649
Dc_1648
Dc_1647
I_1878
I_1877
I_1876
Term: termination (0), NS: new start (0), PS: pausing site (0), iTSS_I: internal starting site (0)

ID: 88400−88593; Term: termination (1), NS: new start (0), PS: pausing site (0), iTSS_I: internal starting site (0)

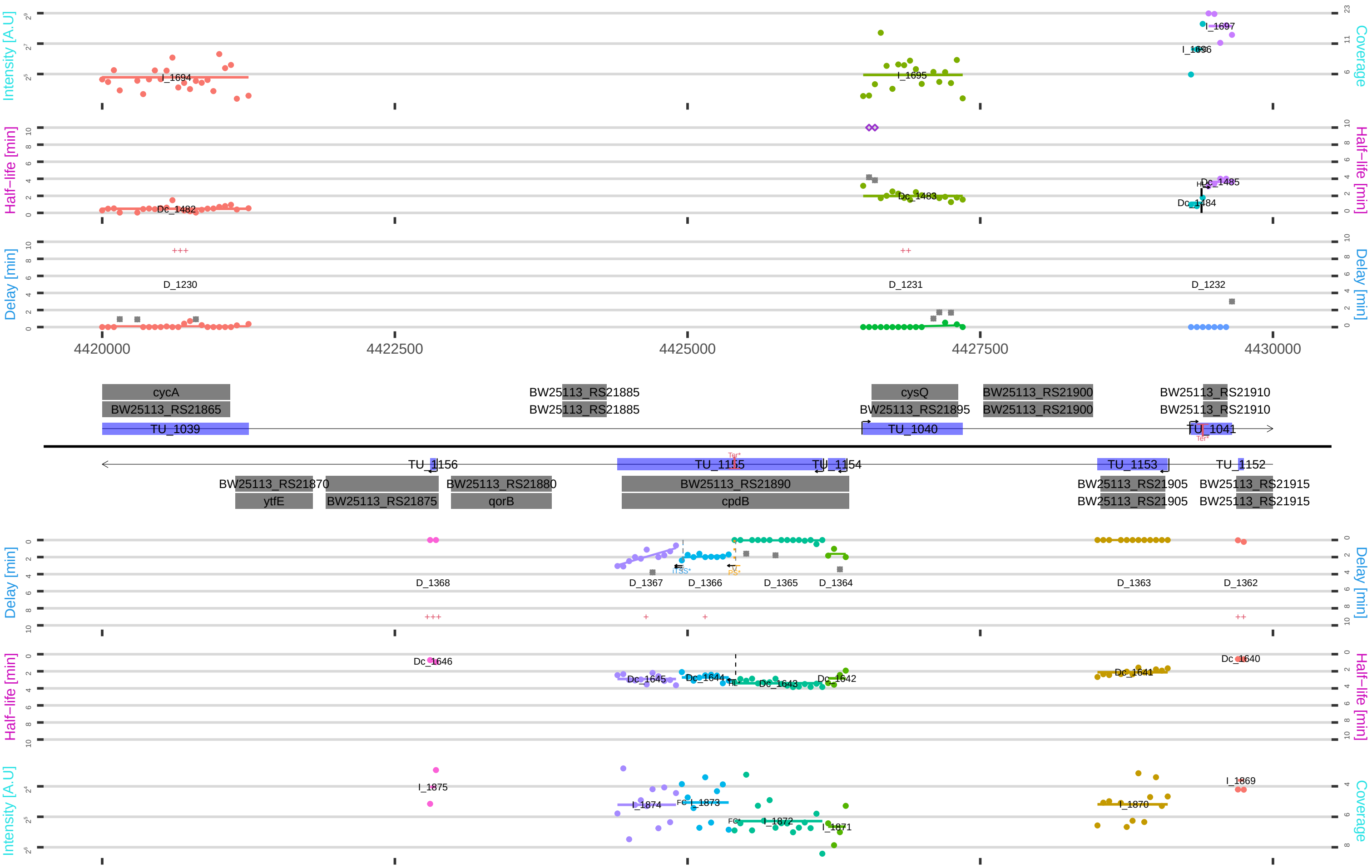

Term: termination (1), NS: new start (0), PS: pausing site (1), iTSS_I: internal starting site (1)

ID: 88644−88800; Term: termination (1), NS: new start (0), PS: pausing site (0), iTSS_I: internal starting site (0)

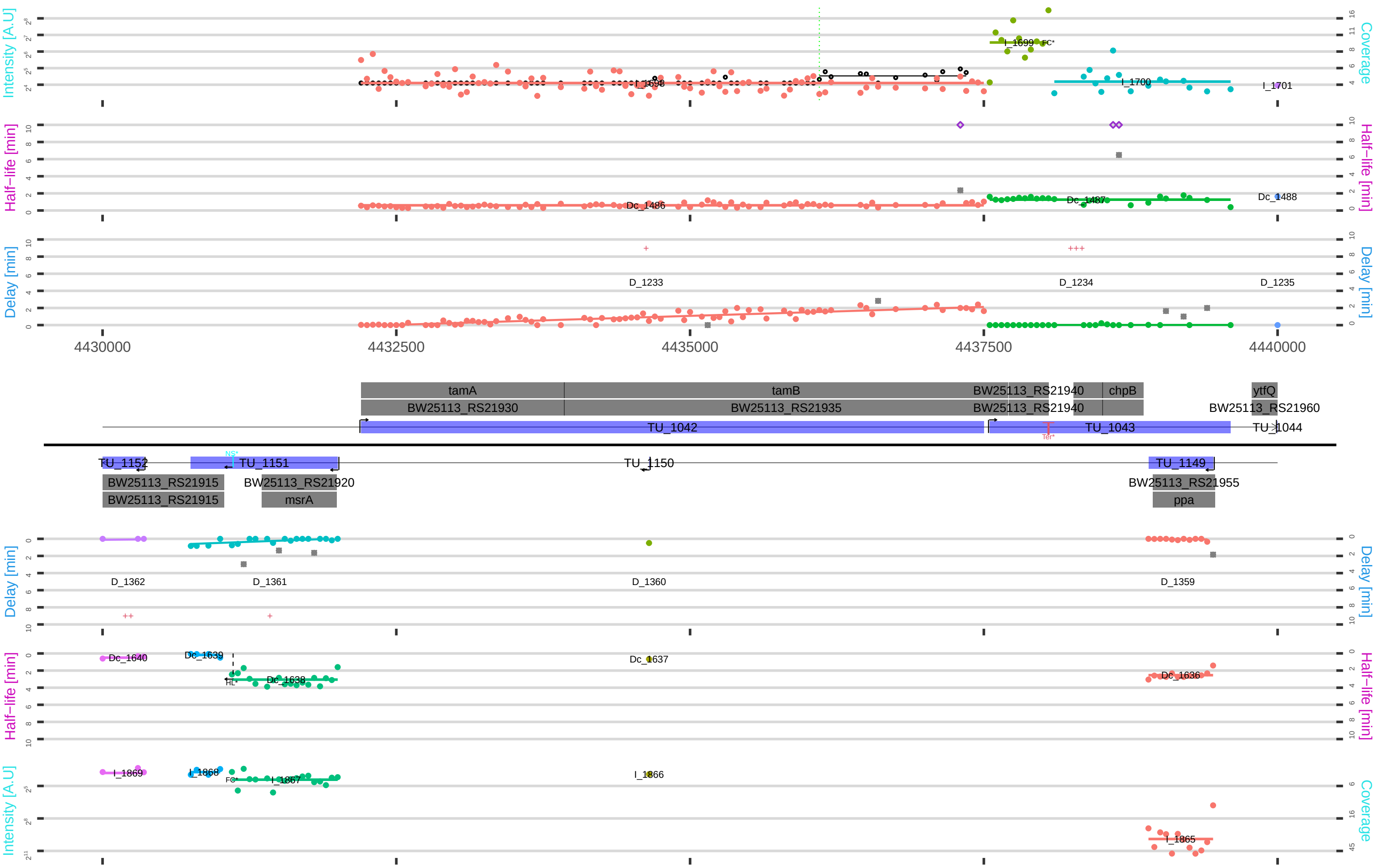

Term: termination (0), NS: new start (1), PS: pausing site (0), iTSS_I: internal starting site (0)

ID: 88800−88994; Term: termination (0), NS: new start (0), PS: pausing site (0), iTSS_I: internal starting site (0)

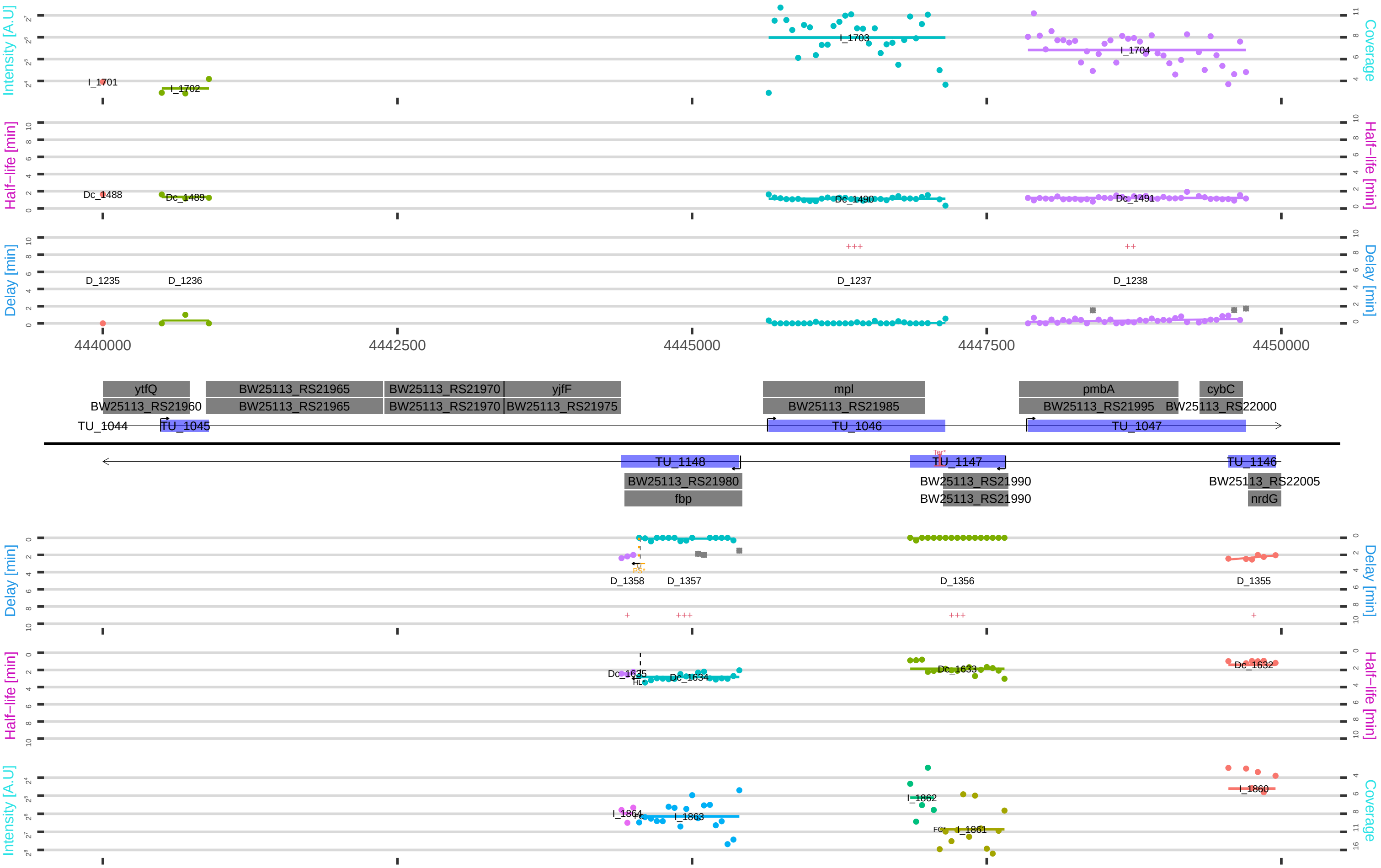

Term: termination (1), NS: new start (0), PS: pausing site (1), iTSS_I: internal starting site (0)

ID: 89145−89200; Term: termination (2), NS: new start (1), PS: pausing site (0), iTSS_I: internal starting site (0)

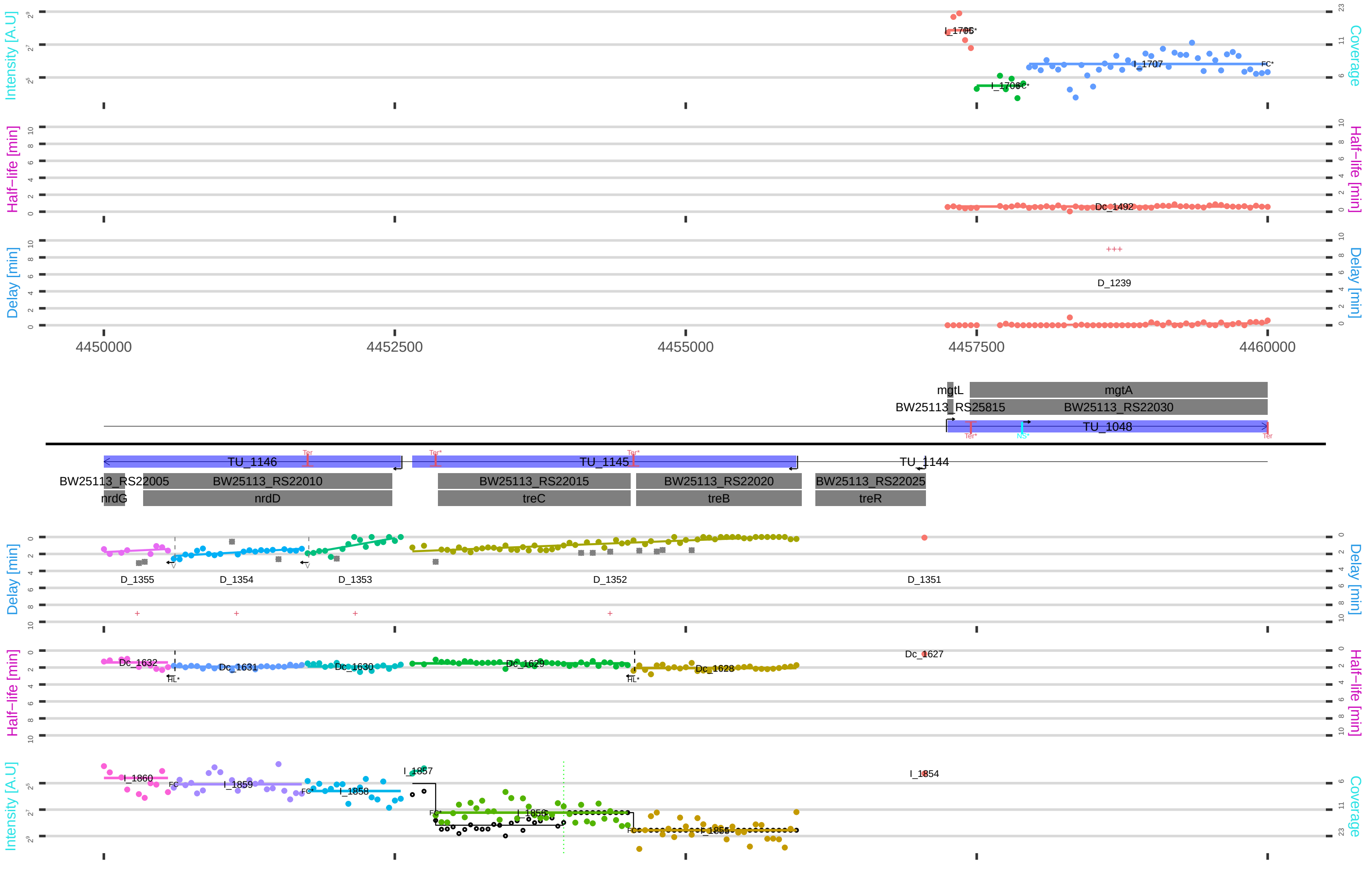

Term: termination (3), NS: new start (0), PS: pausing site (0), iTSS_I: internal starting site (2)

ID: 89200−89377; Term: termination (3), NS: new start (1), PS: pausing site (0), iTSS_I: internal starting site (0)

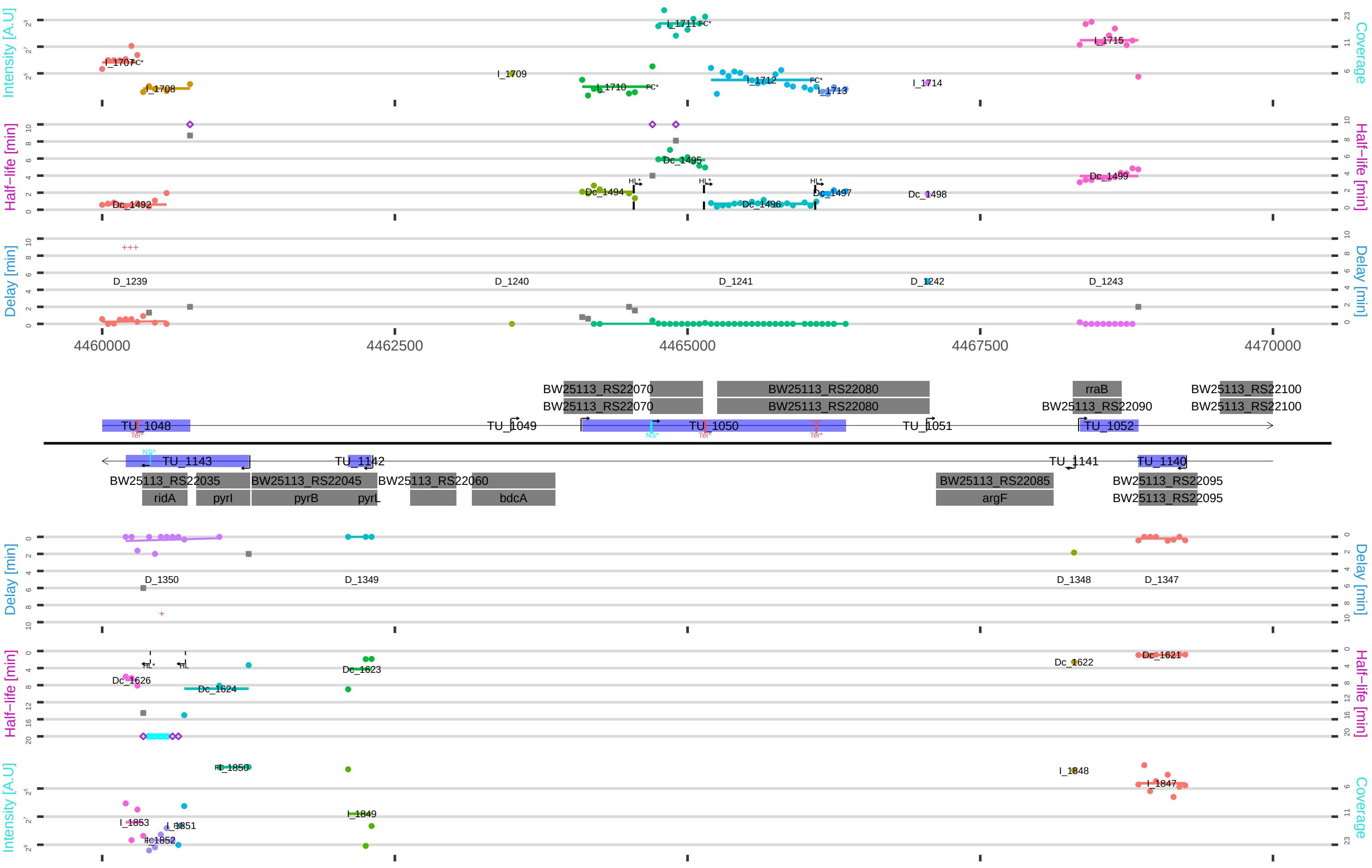

Term: termination (0), NS: new start (1), PS: pausing site (0), iTSS_I: internal starting site (0)

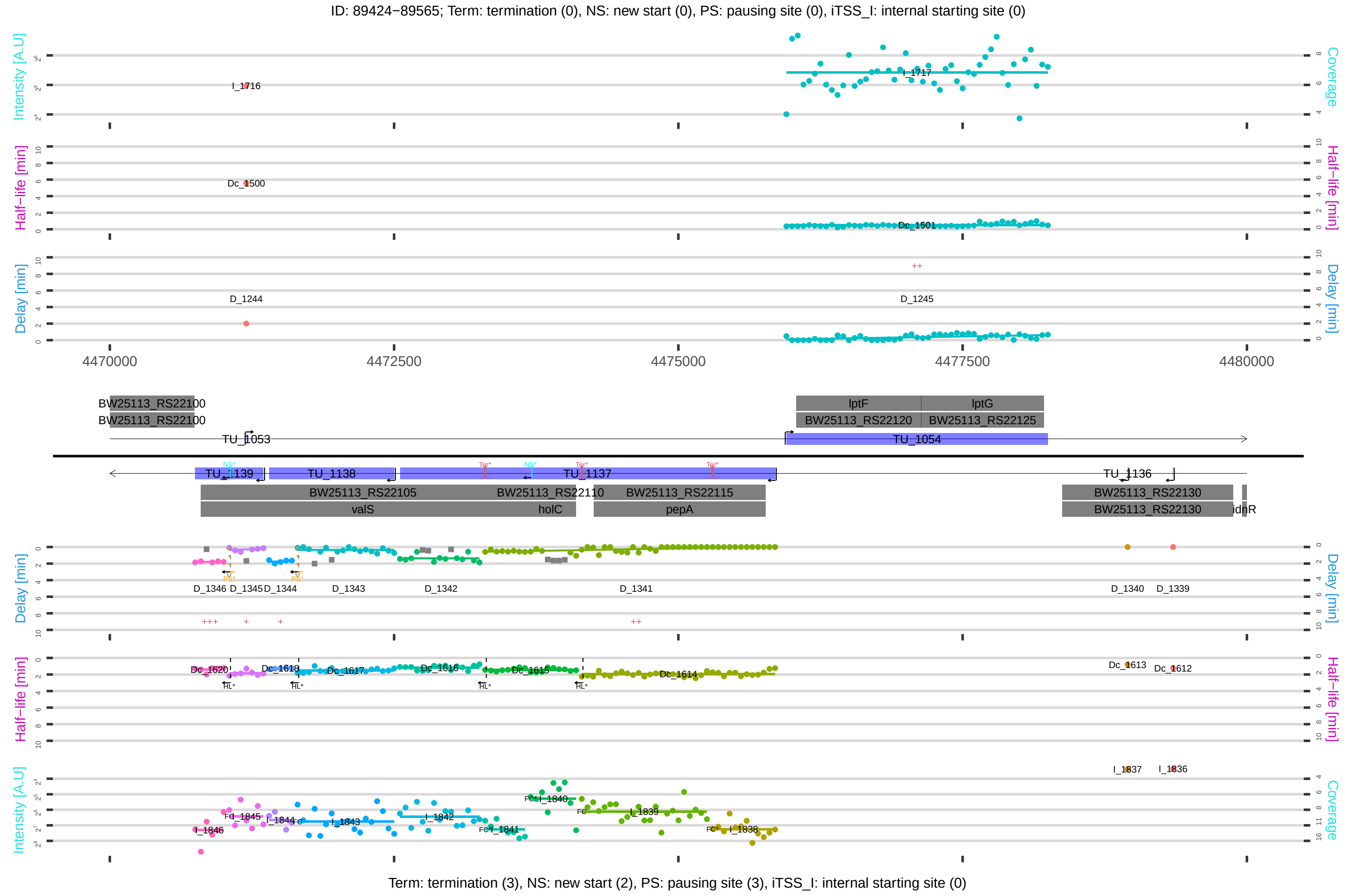
ID: 89424−89565; Term: termination (0), NS: new start (0), PS: pausing site (0), iTSS_I: internal starting site (0)
Intensity [A.U]
Coverage
Half−life [min]
Delay [min]
I_1716
I_1717
Dc_1500
Dc_1501
D_1244
D_1245
4470000
4472500
4475000
4477500
4480000
BW25113_RS22100
lptF
lptG
BW25113_RS22120
BW25113_RS22125
TU_1053
TU_1054
TU_1139
TU_1138
TU_1137
TU_1136
BW25113_RS22105
BW25113_RS22110
BW25113_RS22115
BW25113_RS22130
valS
holC
pepA
idnR
D_1346
D_1345
D_1344
D_1343
D_1342
D_1341
D_1340
D_1339
Dc_1620
Dc_1618
Dc_1617
Dc_1616
Dc_1615
Dc_1614
Dc_1613
Dc_1612
I_1846
I_1845
I_1844
I_1843
I_1842
I_1841
I_1840
I_1839
I_1838
I_1837
I_1836
Term: termination (3), NS: new start (2), PS: pausing site (3), iTSS_I: internal starting site (0)

ID: 89692−89771; Term: termination (0), NS: new start (0), PS: pausing site (0), iTSS_I: internal starting site (0)

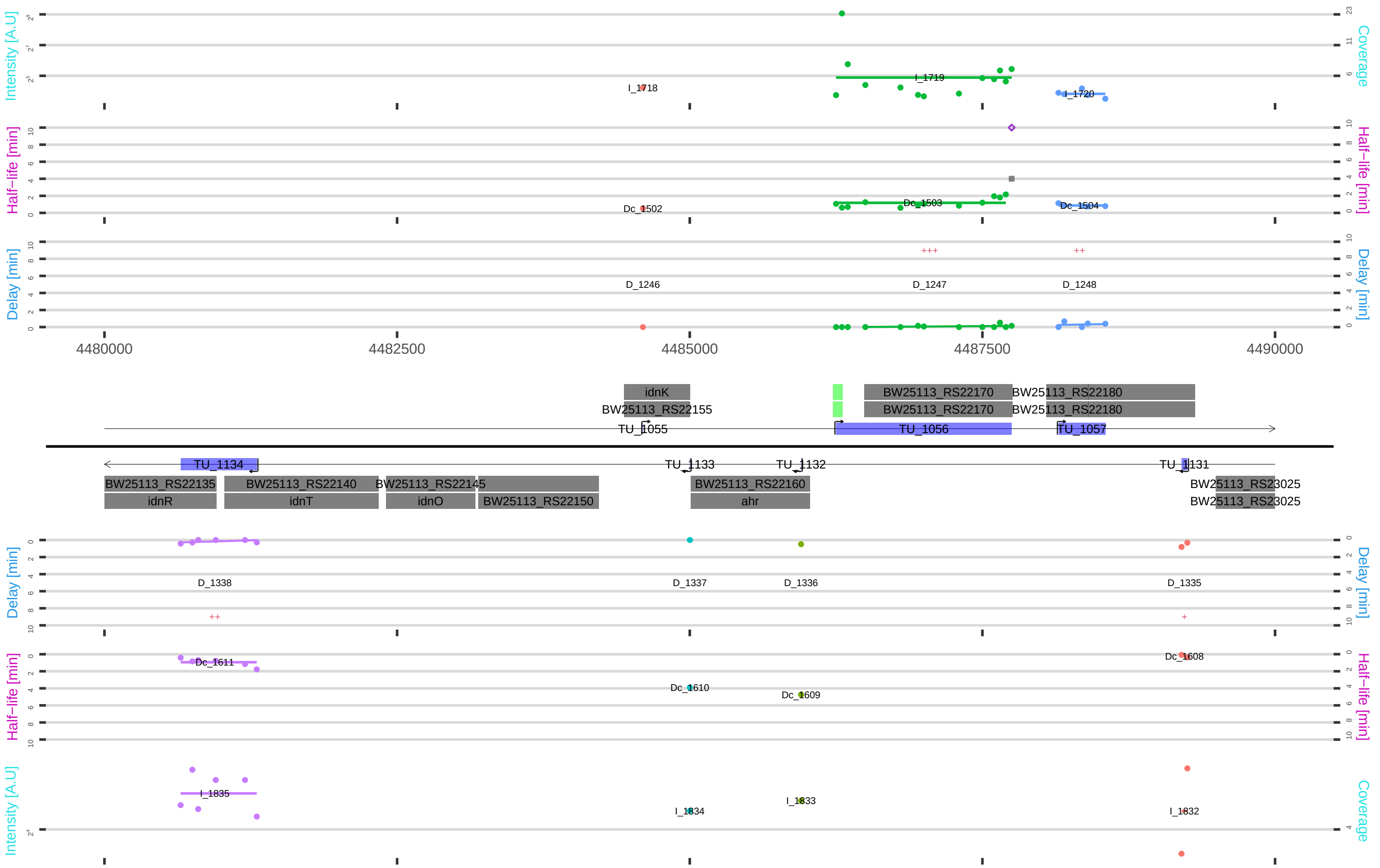

Term: termination (0), NS: new start (0), PS: pausing site (0), iTSS_I: internal starting site (0)

ID: 89835−89942; Term: termination (0), NS: new start (1), PS: pausing site (1), iTSS_I: internal starting site (0)

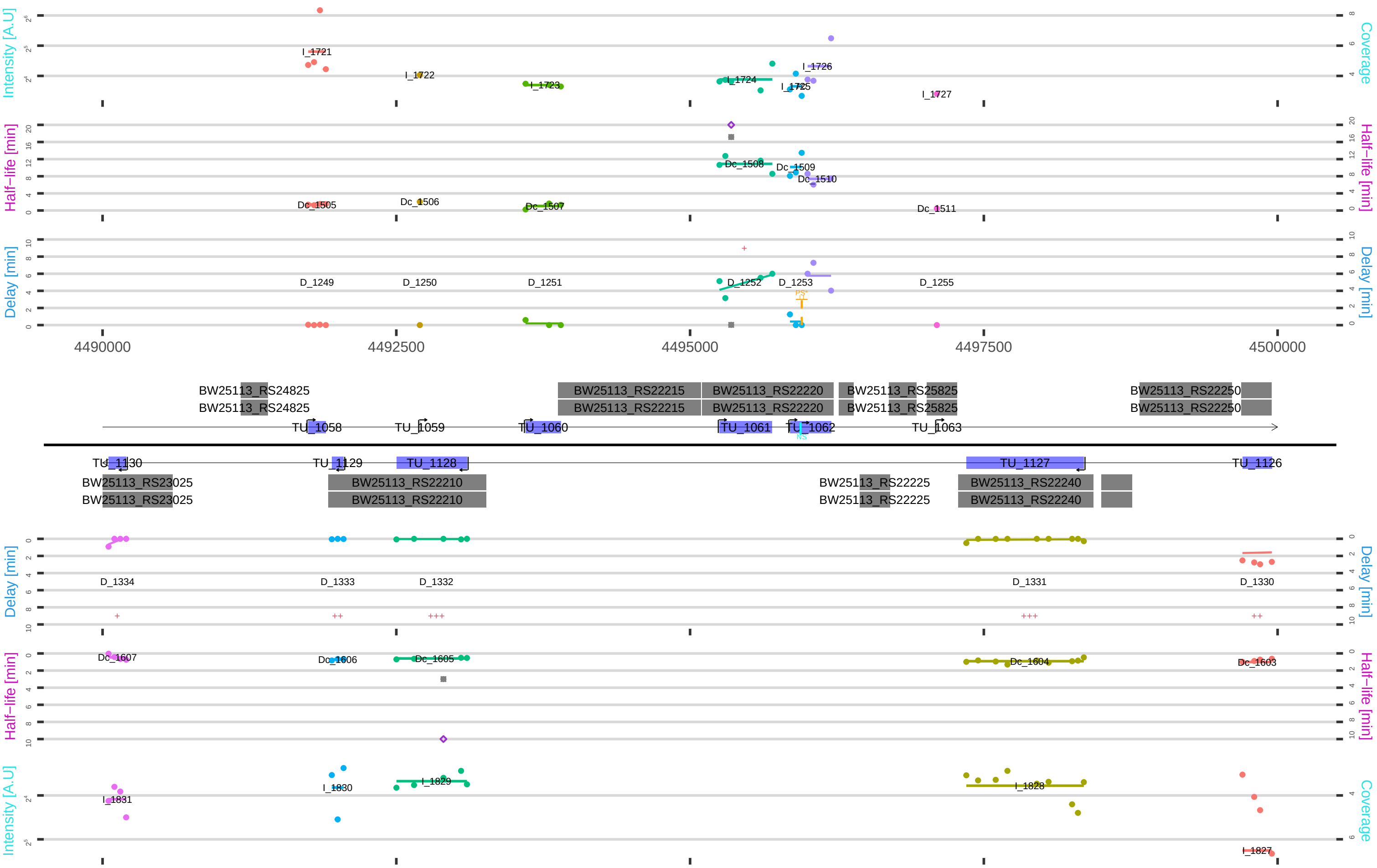

Term: termination (0), NS: new start (0), PS: pausing site (0), iTSS_I: internal starting site (0)

ID: 90166−90182; Term: termination (0), NS: new start (0), PS: pausing site (0), iTSS_I: internal starting site (0)

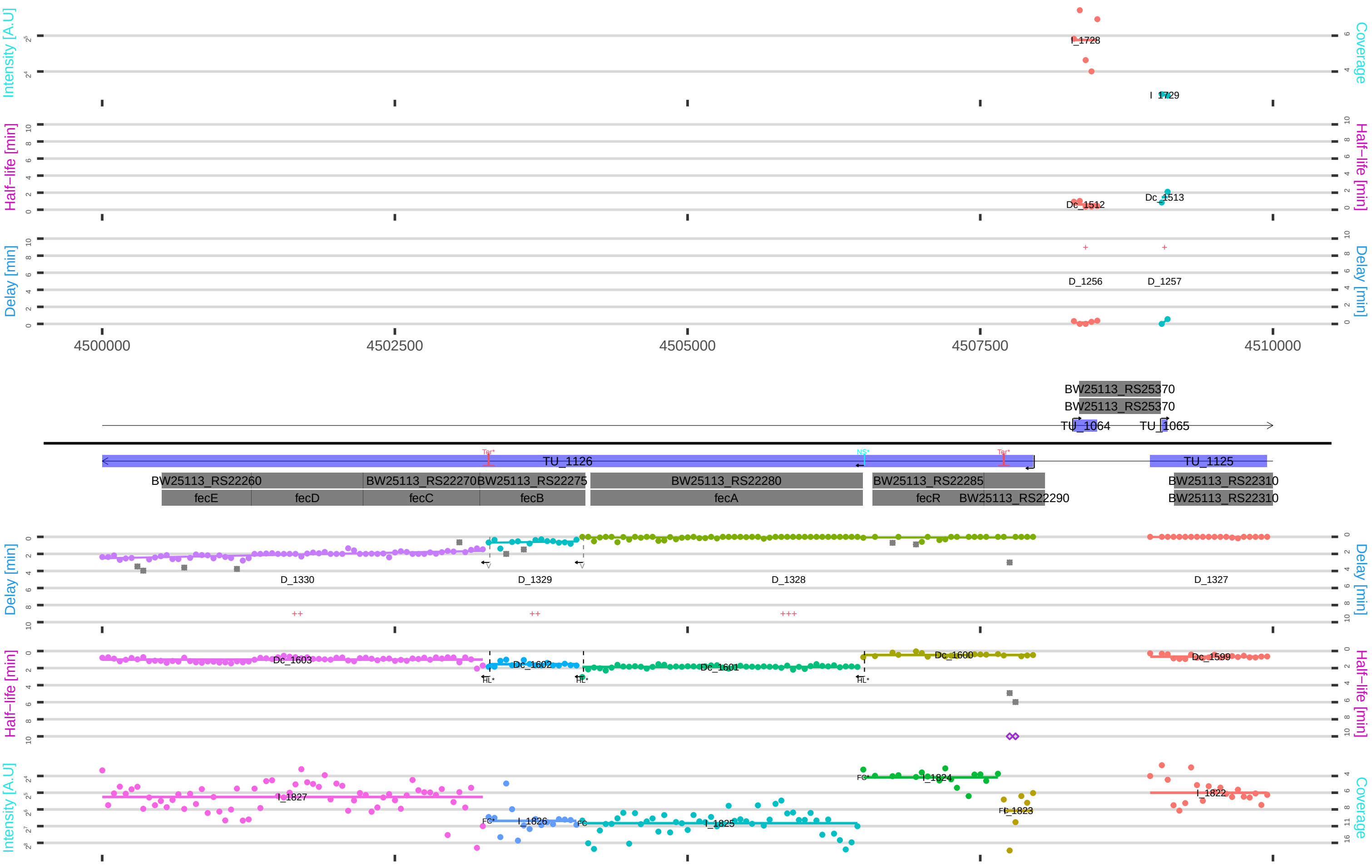

Term: termination (2), NS: new start (1), PS: pausing site (2), iTSS_I: internal starting site (0)

ID: 90358−90361; Term: termination (0), NS: new start (0), PS: pausing site (0), iTSS_I: internal starting site (0)

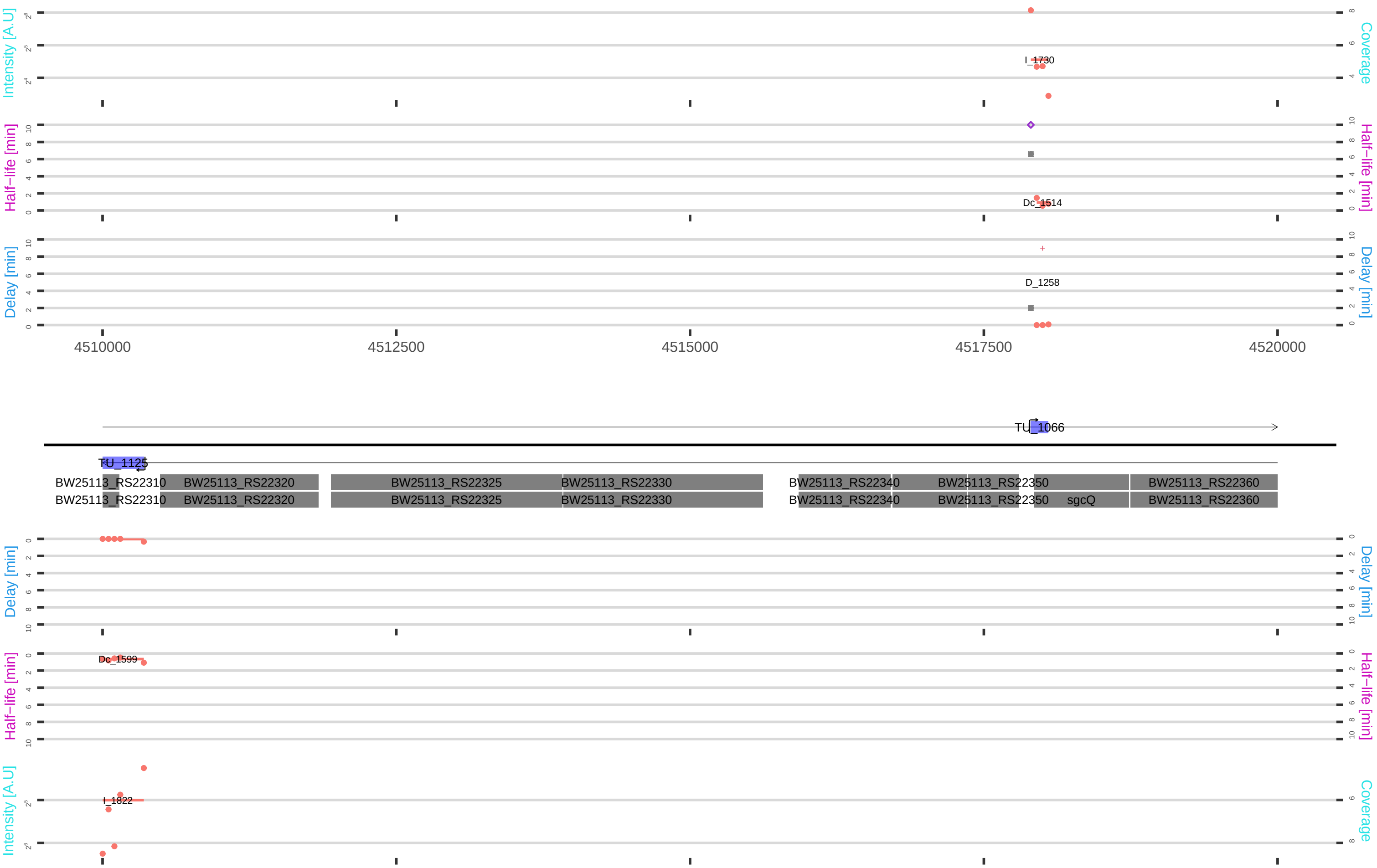

Term: termination (0), NS: new start (0), PS: pausing site (0), iTSS_I: internal starting site (0)

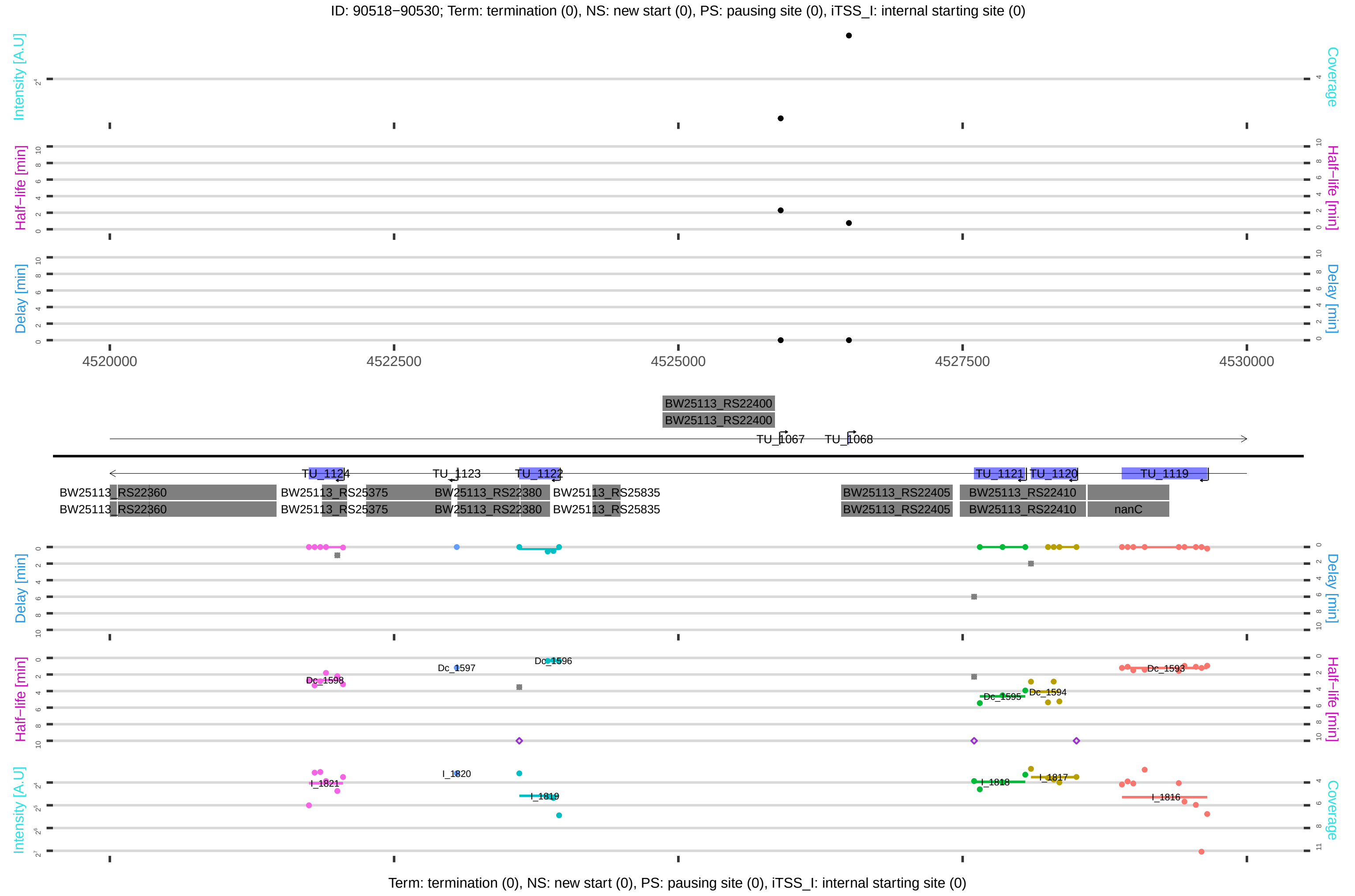
ID: 90518−90530; Term: termination (0), NS: new start (0), PS: pausing site (0), iTSS_I: internal starting site (0)
Intensity [A.U]
Coverage
Half−life [min]
Delay [min]
4520000
4522500
4525000
4527500
4530000
BW25113_RS22400
TU_1067
TU_1068
TU_1124
TU_1123
TU_1122
TU_1121
TU_1120
TU_1119
BW25113_RS22360
BW25113_RS25375
BW25113_RS22380
BW25113_RS25835
BW25113_RS22405
BW25113_RS22410
nanC
Dc_1598
Dc_1597
Dc_1596
Dc_1595
Dc_1594
Dc_1593
I_1821
I_1820
I_1819
I_1818
I_1817
I_1816
Term: termination (0), NS: new start (0), PS: pausing site (0), iTSS_I: internal starting site (0)

ID: 90613−90722; Term: termination (1), NS: new start (2), PS: pausing site (0), iTSS_I: internal starting site (0)

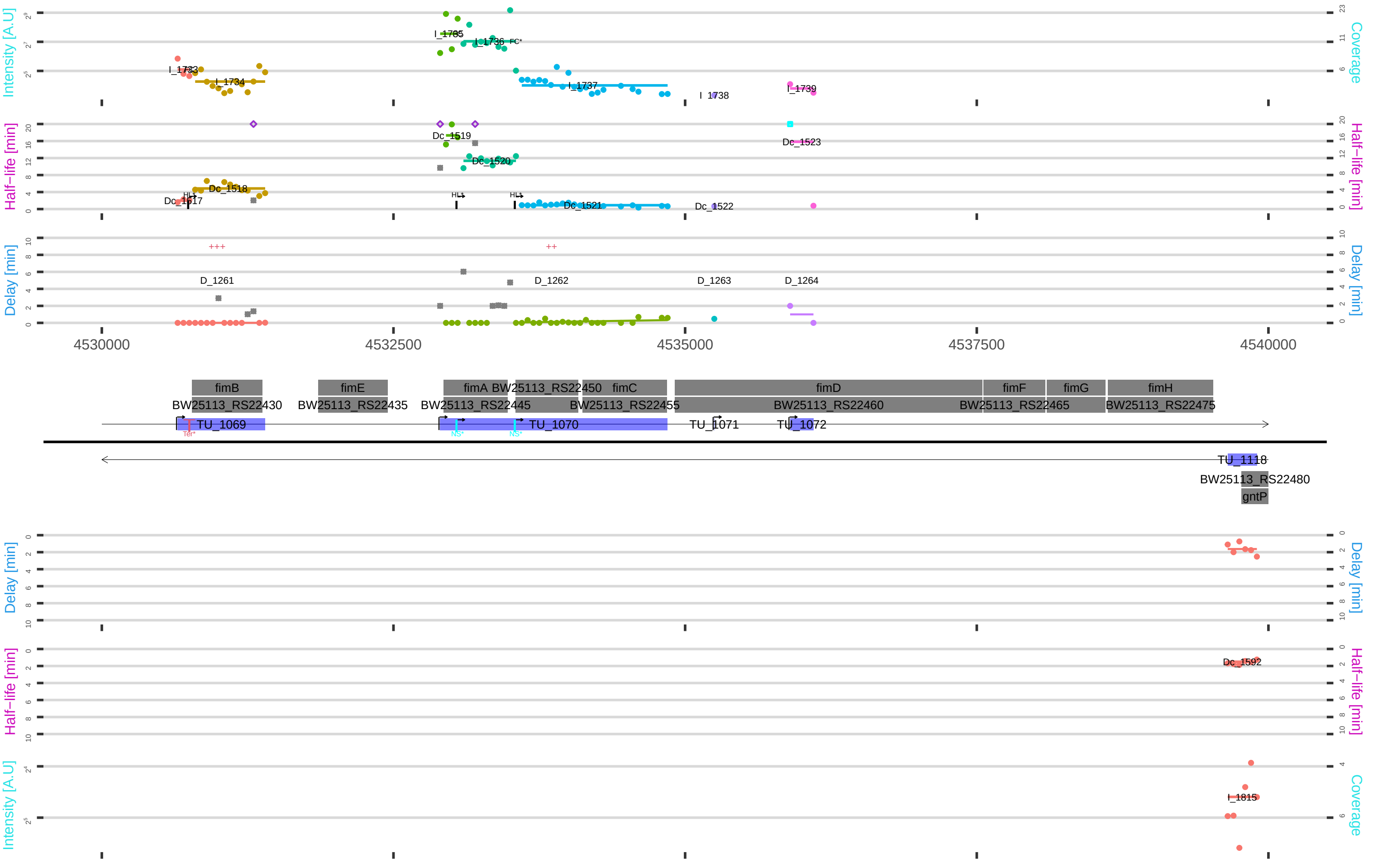

Term: termination (0), NS: new start (0), PS: pausing site (0), iTSS_I: internal starting site (0)

ID: 90829−90943; Term: termination (1), NS: new start (0), PS: pausing site (1), iTSS_I: internal starting site (0)

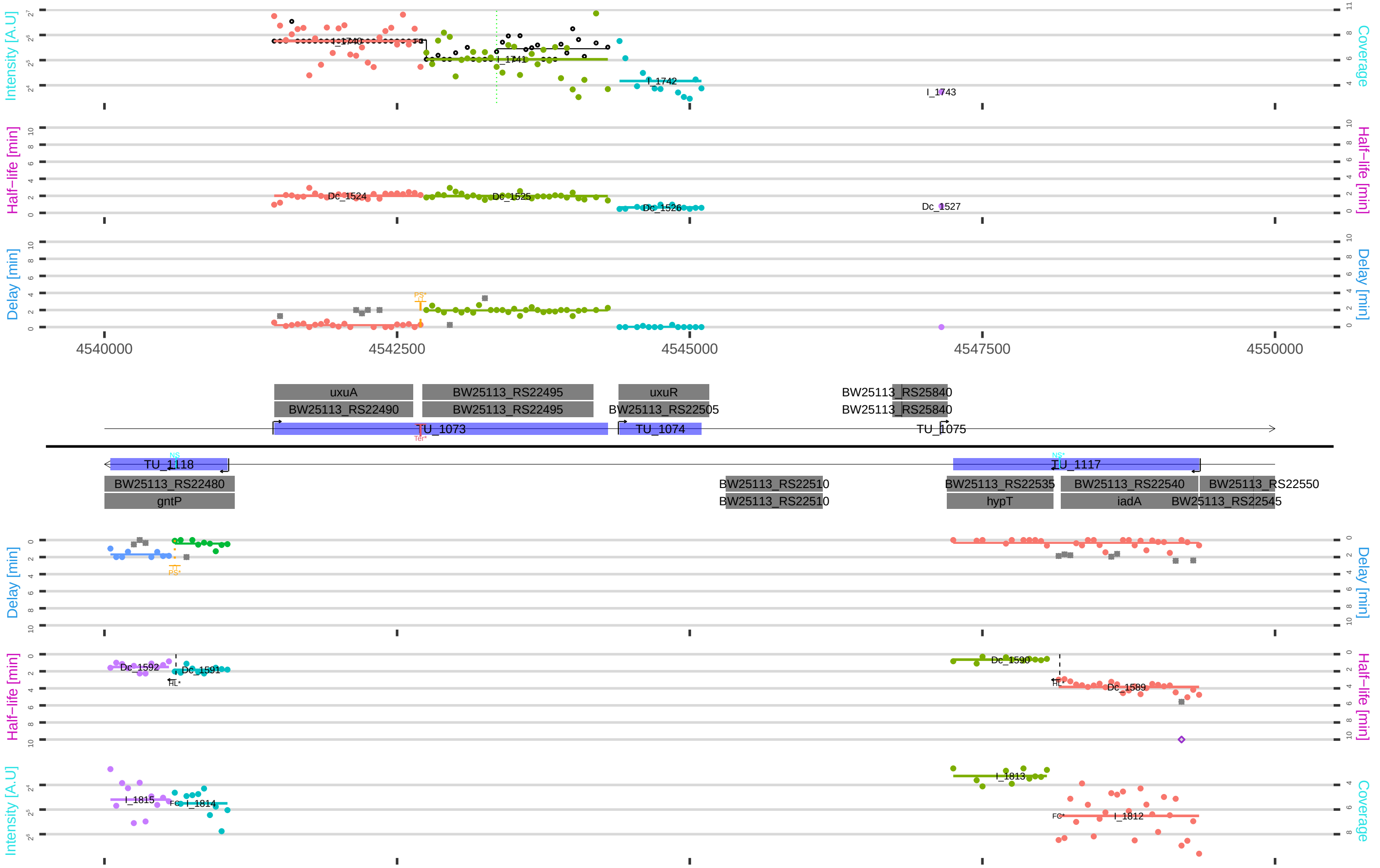

Term: termination (0), NS: new start (2), PS: pausing site (1), iTSS_I: internal starting site (0)

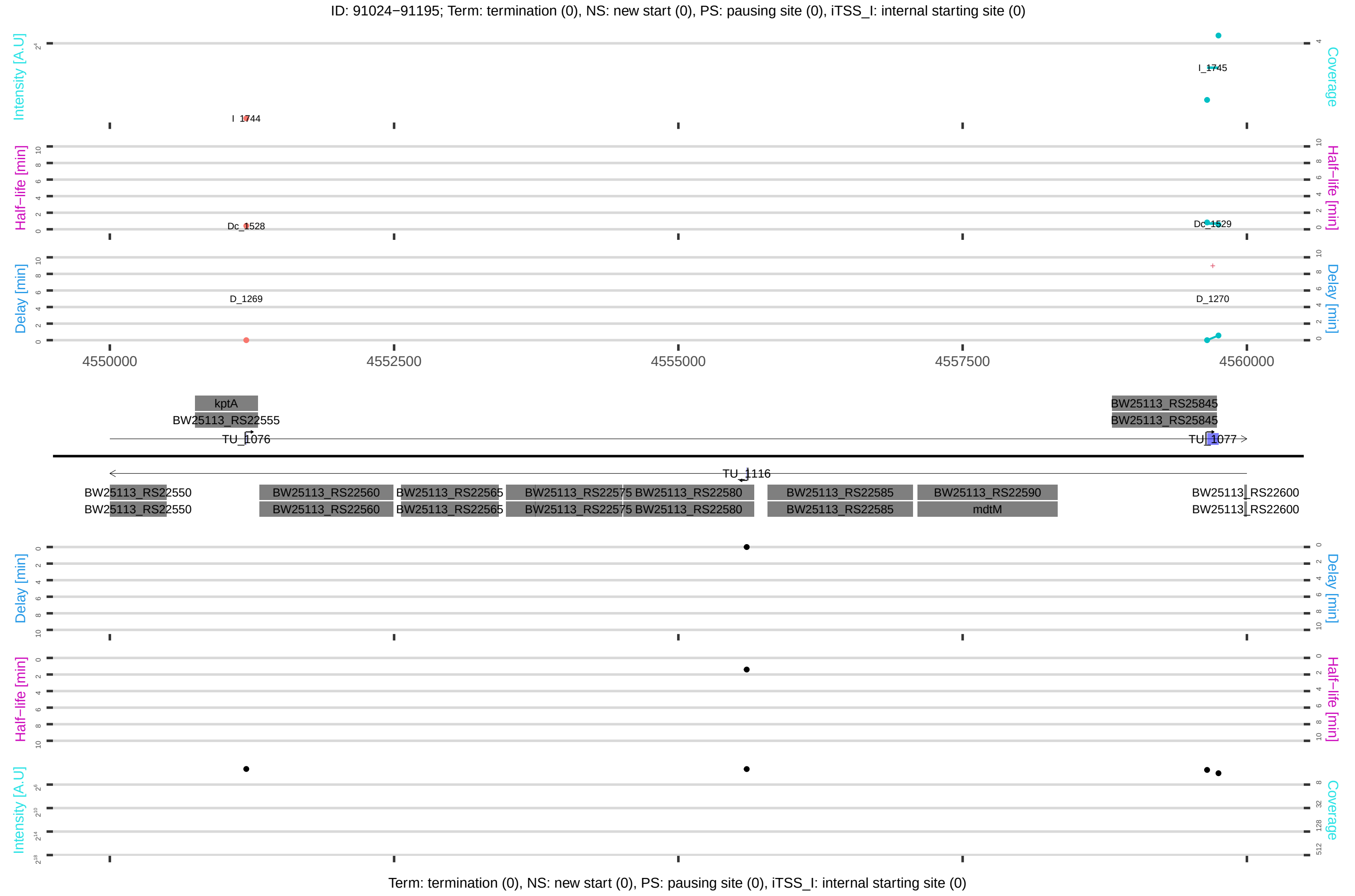
ID: 91024−91195; Term: termination (0), NS: new start (0), PS: pausing site (0), iTSS_I: internal starting site (0)
Intensity [A.U]
Coverage
I_1745
I_1744
Half−life [min]
Dc_1528
Dc_1529
Delay [min]
D_1269
D_1270
4550000
4552500
4555000
4557500
4560000
kptA
BW25113_RS22555
TU_1076
BW25113_RS25845
TU_1077
TU_1116
BW25113_RS22550
BW25113_RS22560
BW25113_RS22565
BW25113_RS22575
BW25113_RS22580
BW25113_RS22585
BW25113_RS22590
mdtM
BW25113_RS22600
Term: termination (0), NS: new start (0), PS: pausing site (0), iTSS_I: internal starting site (0)

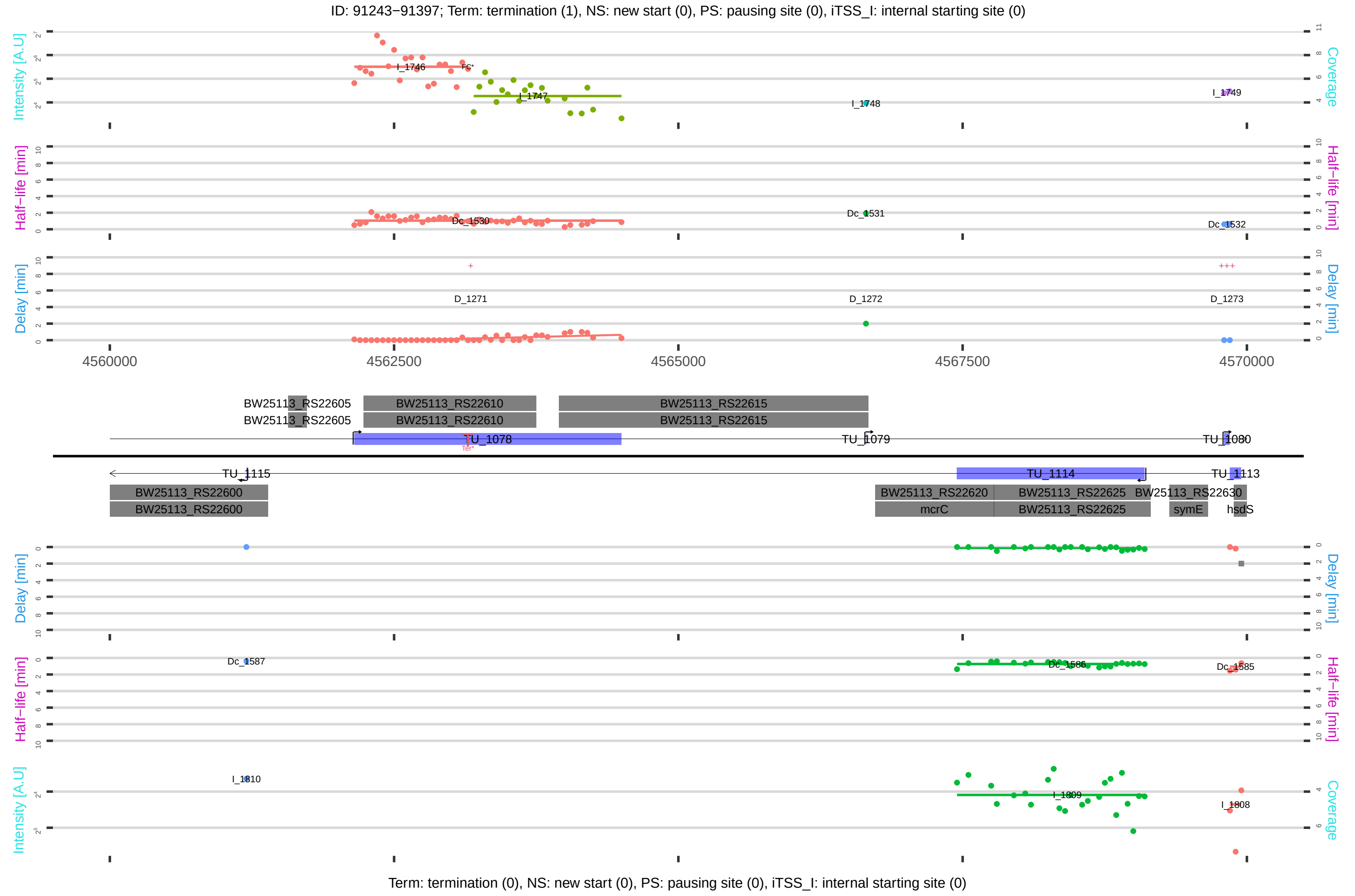

ID: 91243−91397; Term: termination (1), NS: new start (0), PS: pausing site (0), iTSS_I: internal starting site (0)
Intensity [A.U]
Coverage
I_1746
Fc*
I_1747
I_1748
I_1749
Half−life [min]
Dc_1530
Dc_1531
Dc_1532
Delay [min]
D_1271
D_1272
D_1273
4560000
4562500
4565000
4567500
4570000
BW25113_RS22605
BW25113_RS22610
BW25113_RS22615
TU_1078
Ter*
TU_1079
TU_1080
TU_1115
TU_1114
TU_1113
BW25113_RS22600
BW25113_RS22620
mcrC
BW25113_RS22625
BW25113_RS22630
symE
hsdS
Dc_1587
Dc_1586
Dc_1585
I_1810
I_1809
I_1808
Term: termination (0), NS: new start (0), PS: pausing site (0), iTSS_I: internal starting site (0)

ID: 91536−91555; Term: termination (0), NS: new start (0), PS: pausing site (0), iTSS_I: internal starting site (0)

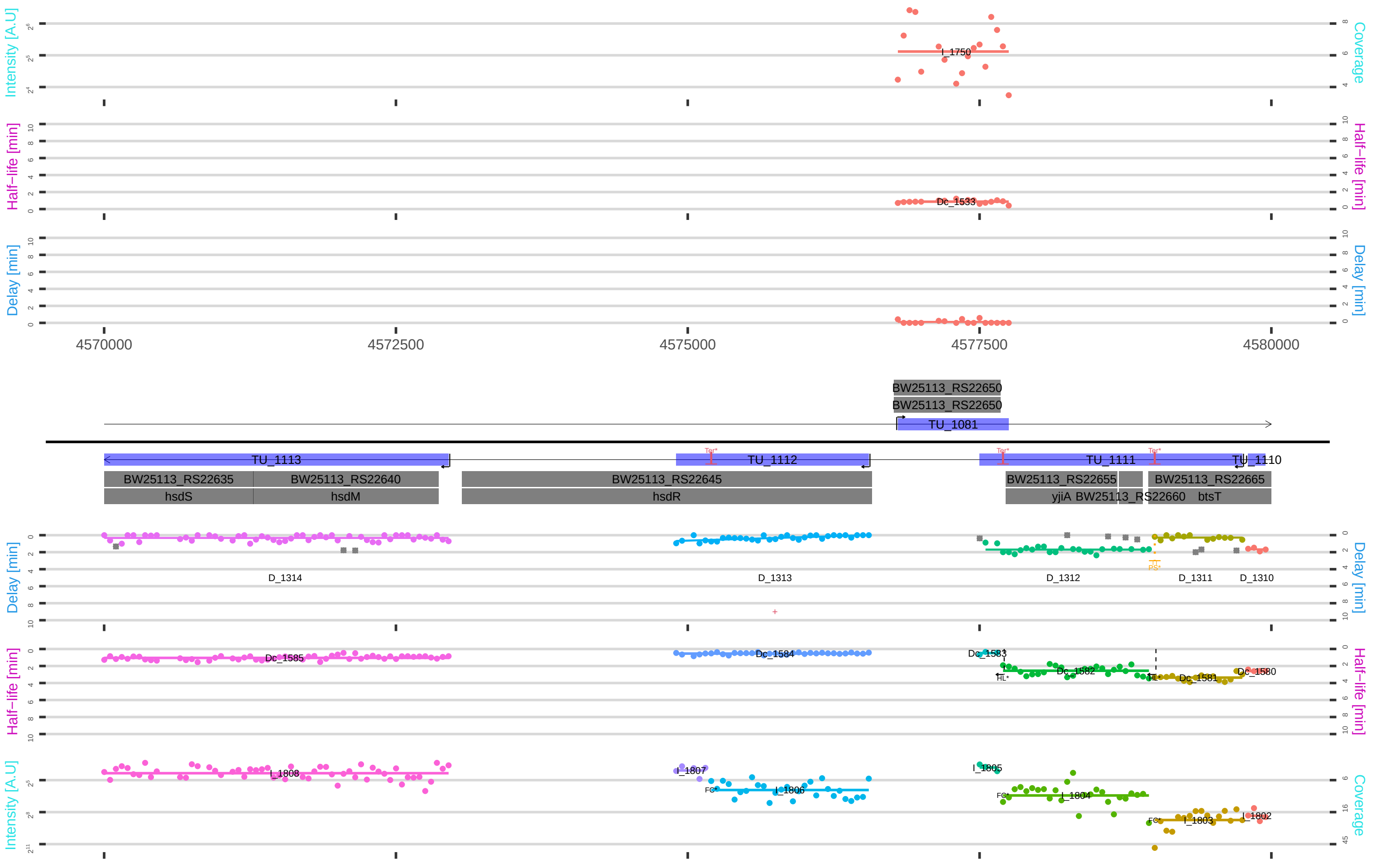

Term: termination (3), NS: new start (0), PS: pausing site (1), iTSS_I: internal starting site (0)

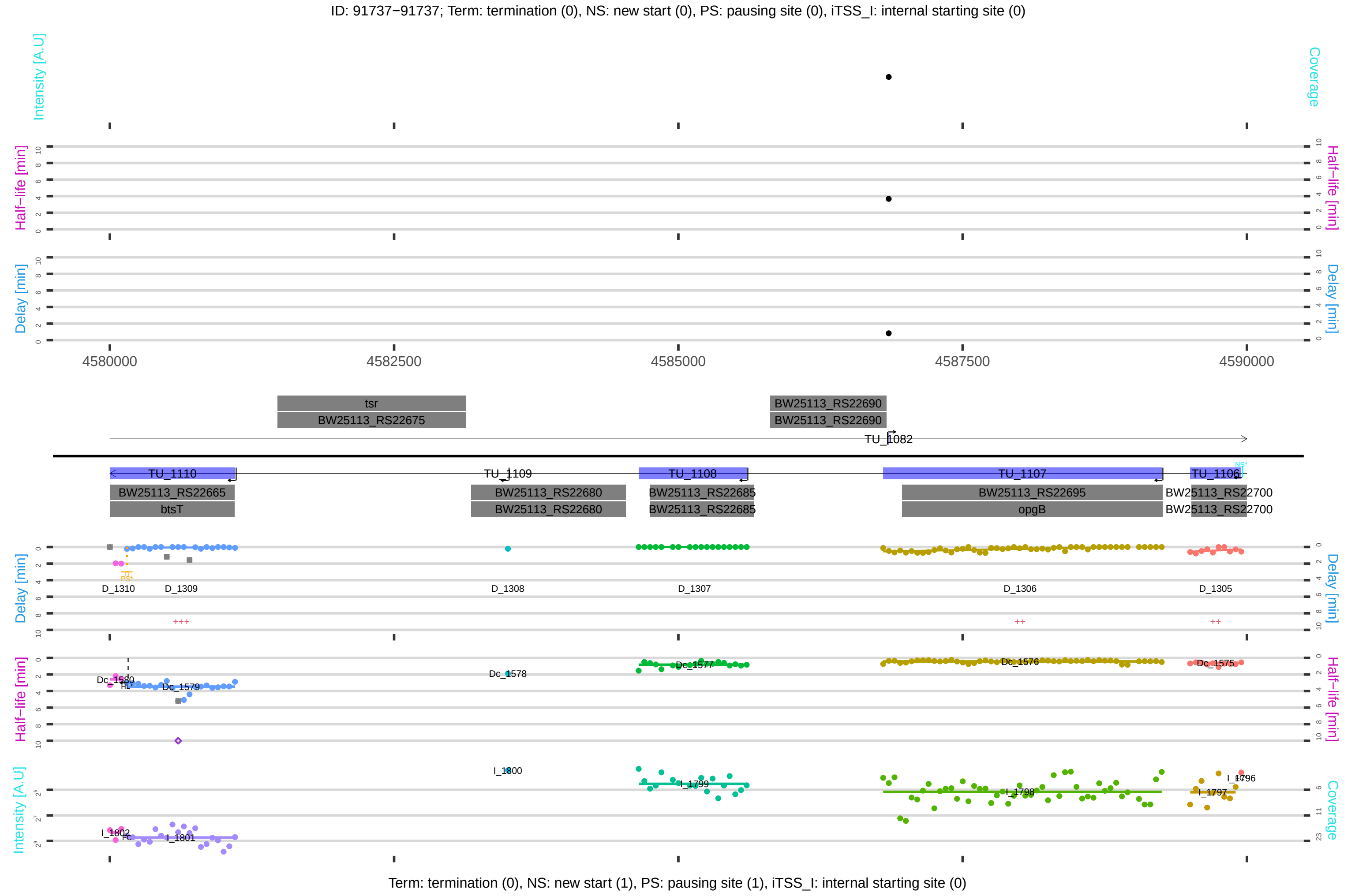

ID: 91737−91737; Term: termination (0), NS: new start (0), PS: pausing site (0), iTSS_I: internal starting site (0)
Intensity [A.U]
Coverage
Half−life [min]
Delay [min]
4580000
4582500
4585000
4587500
4590000
tsr
BW25113_RS22675
BW25113_RS22690
TU_1082
TU_1110
TU_1109
TU_1108
TU_1107
TU_1106
BW25113_RS22665
btsT
BW25113_RS22680
BW25113_RS22685
BW25113_RS22695
opgB
BW25113_RS22700
D_1310
D_1309
D_1308
D_1307
D_1306
D_1305
Dc_1580
Dc_1579
Dc_1578
Dc_1577
Dc_1576
Dc_1575
I_1802
I_1801
I_1800
I_1799
I_1798
I_1797
I_1796
Term: termination (0), NS: new start (1), PS: pausing site (1), iTSS_I: internal starting site (0)

ID: 91918−92000; Term: termination (2), NS: new start (1), PS: pausing site (0), iTSS_I: internal starting site (0)

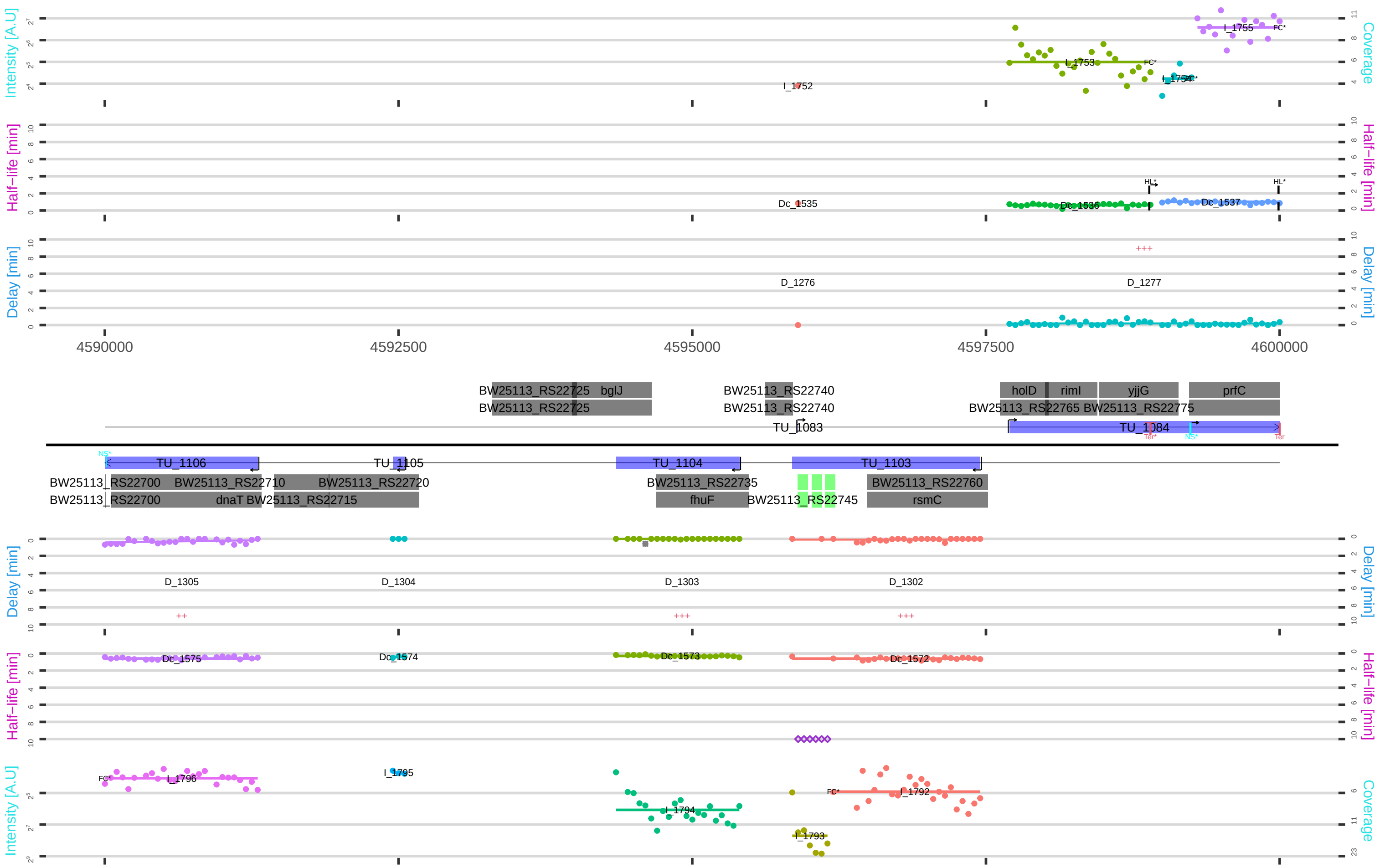

Term: termination (0), NS: new start (1), PS: pausing site (0), iTSS_I: internal starting site (0)

ID: 92000−92200; Term: termination (5), NS: new start (2), PS: pausing site (3), iTSS_I: internal starting site (0)

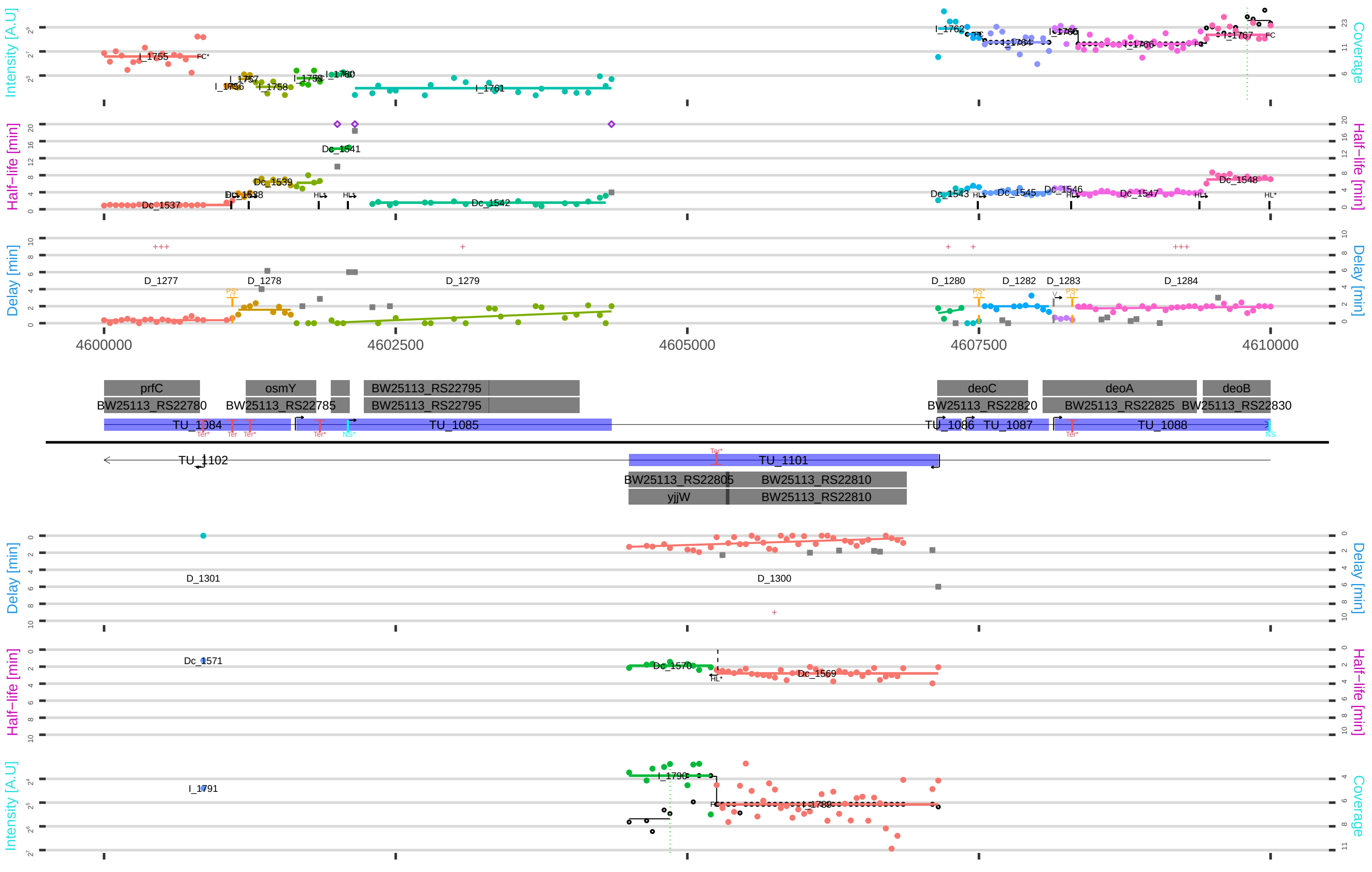

Term: termination (1), NS: new start (0), PS: pausing site (0), iTSS_I: internal starting site (0)

ID: 92200−92373; Term: termination (1), NS: new start (2), PS: pausing site (1), iTSS_I: internal starting site (0)

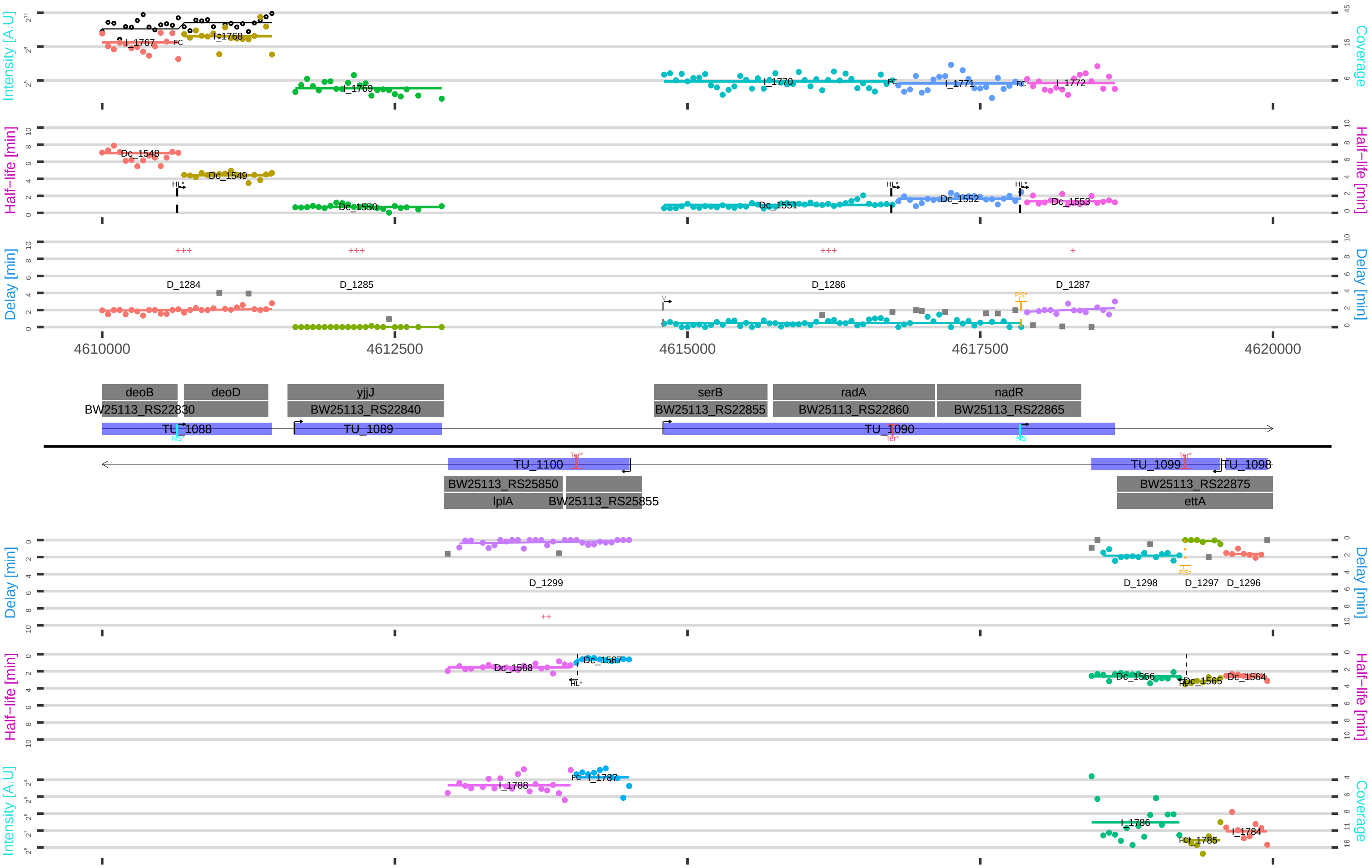

Term: termination (2), NS: new start (0), PS: pausing site (1), iTSS_I: internal starting site (0)

ID: 92413−92542; Term: termination (1), NS: new start (0), PS: pausing site (0), iTSS_I: internal starting site (0)

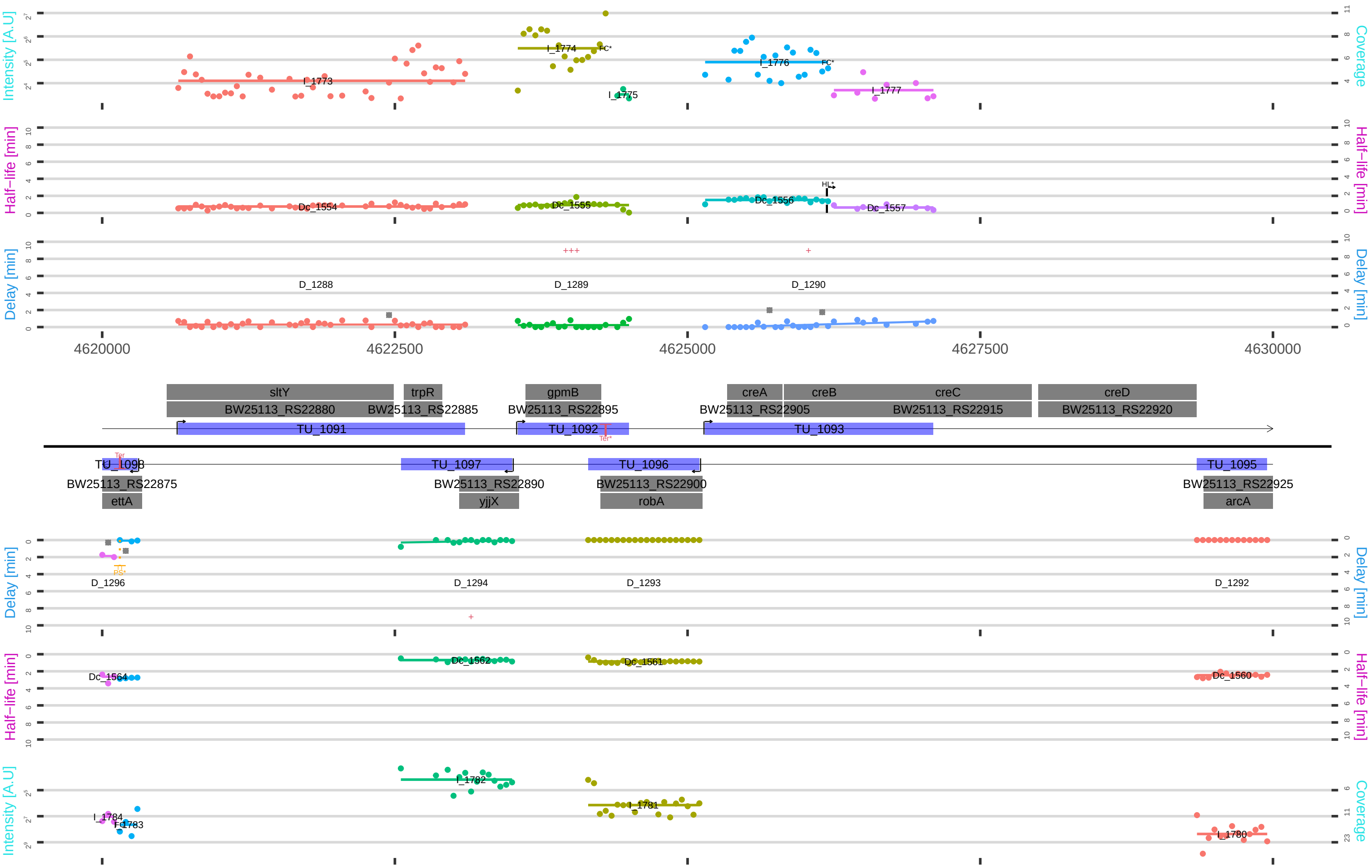

Term: termination (1), NS: new start (0), PS: pausing site (1), iTSS_I: internal starting site (0)

ID: 92613−92628; Term: termination (0), NS: new start (0), PS: pausing site (0), iTSS_I: internal starting site (0)

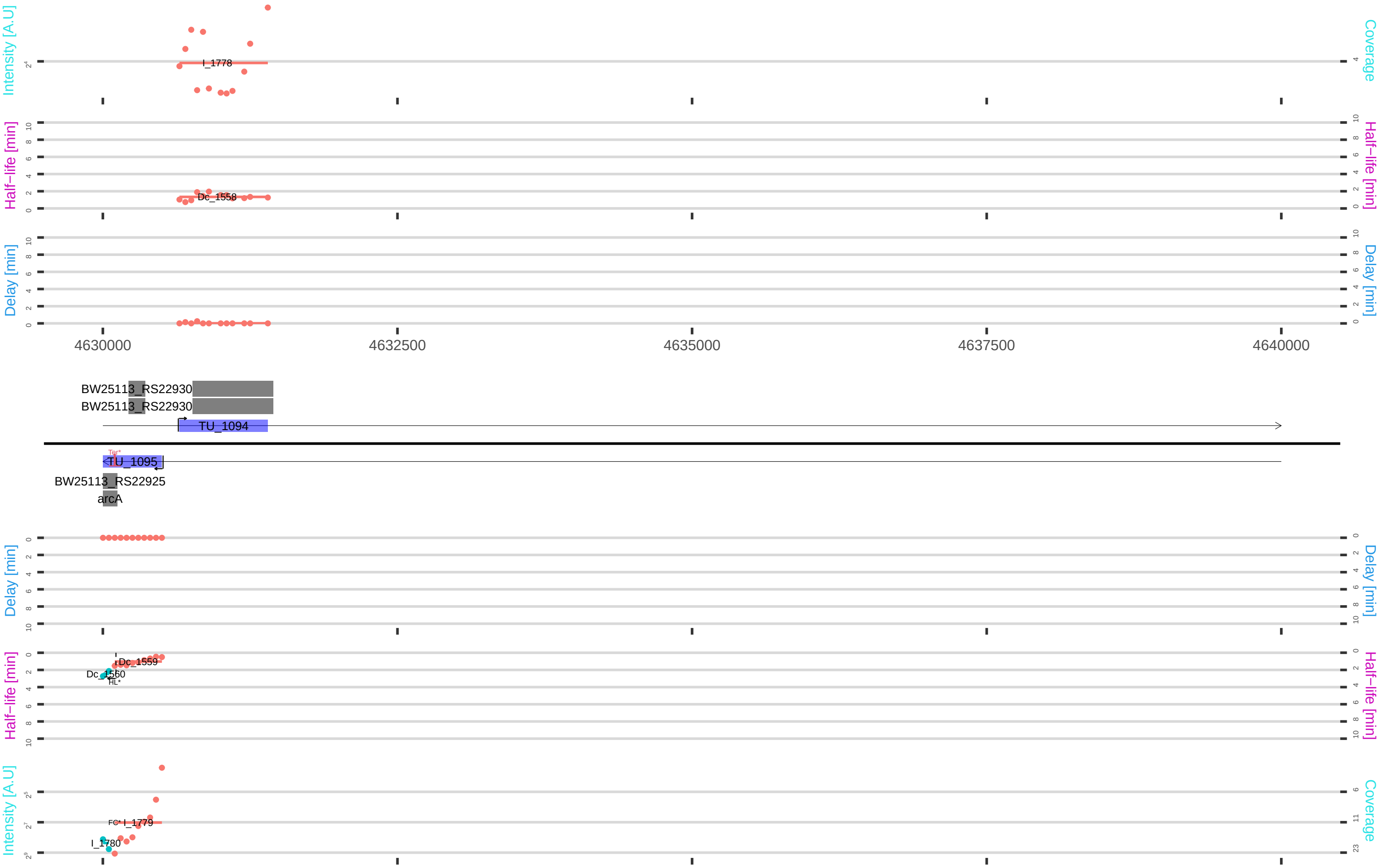

Term: termination (1), NS: new start (0), PS: pausing site (0), iTSS_I: internal starting site (0)
