# Supplementary material for: Improved RNA stability estimation indicates that transcriptional interference is frequent in diverse bacteria
Source: Commun Biol. 2023 Jul 15;6:732. doi: 10.1038/s42003-023-05097-2 (PMC10349824; doi:10.1038/s42003-023-05097-2)
Supplement: Supplementary file 6 — Supplementary Data 3 [file 42003_2023_5097_MOESM6_ESM.zip › E_coli_MG1655.pdf]

ID: 1−200; FC*: significant t−test of two consecutive segments;
Term: termination (4), NS: new start (3), PS: pausing site (2), iTSS_I: internal starting site (0), (*): p_value below 0.05; TI: transcription interferance.

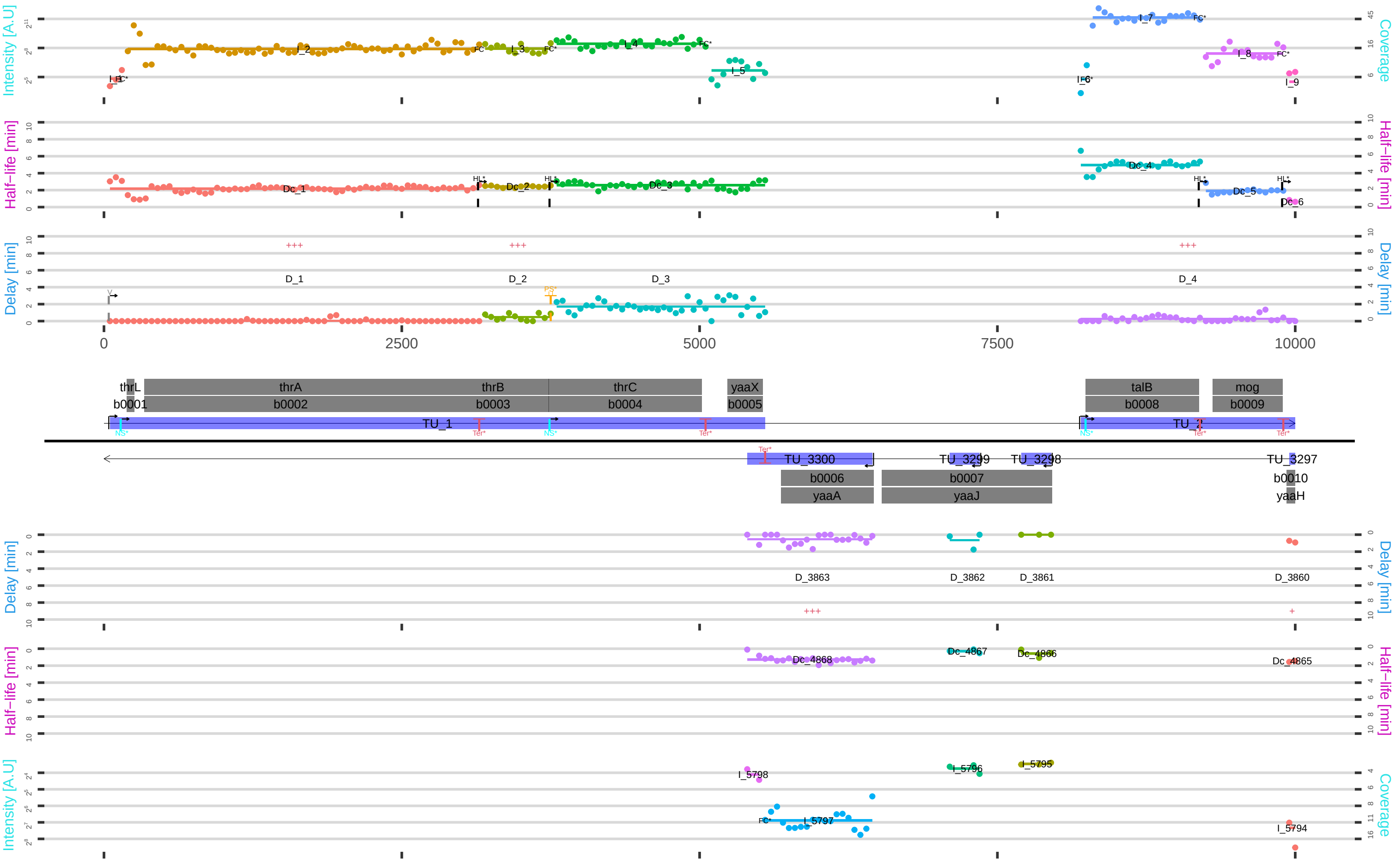

Term: termination (1), NS: new start (0), PS: pausing site (0), iTSS_I: internal starting site (0)

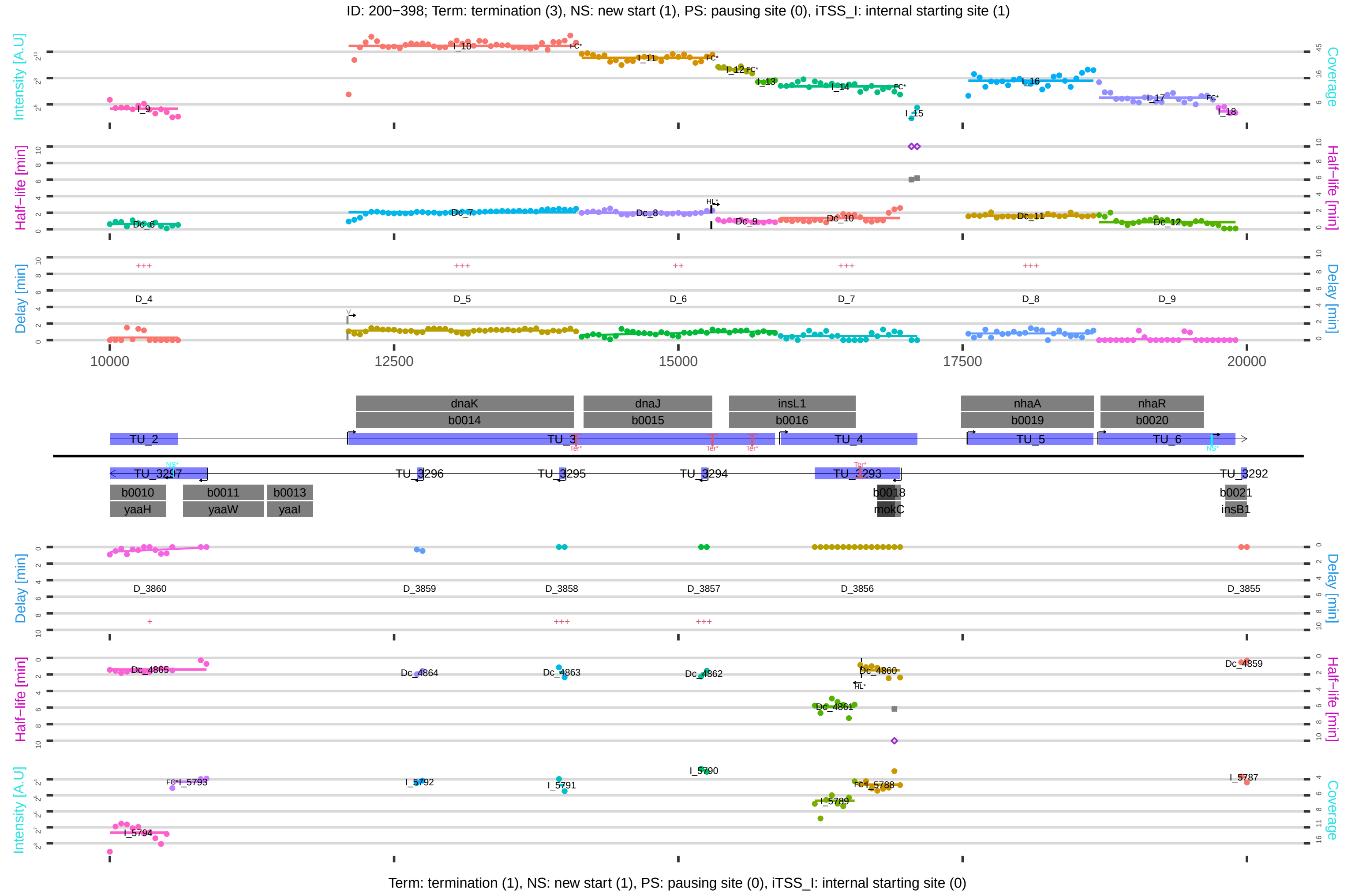
ID: 200−398; Term: termination (3), NS: new start (1), PS: pausing site (0), iTSS_I: internal starting site (1)
Intensity [A.U]
Coverage
Half−life [min]
Delay [min]
I_9
I_10
I_11
I_12
I_13
I_14
I_15
I_16
I_17
I_18
Dc_6
Dc_7
Dc_8
Dc_9
Dc_10
Dc_11
Dc_12
D_4
D_5
D_6
D_7
D_8
D_9
10000
12500
15000
17500
20000
dnaK
b0014
dnaJ
b0015
insL1
b0016
nhaA
b0019
nhaR
b0020
TU_2
TU_3
TU_4
TU_5
TU_6
TU_3297
TU_3296
TU_3295
TU_3294
TU_3293
TU_3292
b0010
yaaH
b0011
yaaW
b0013
yaaI
b0018
mokC
b0021
insB1
D_3860
D_3859
D_3858
D_3857
D_3856
D_3855
Dc_4865
Dc_4864
Dc_4863
Dc_4862
Dc_4861
Dc_4860
Dc_4859
I_5794
I_5793
I_5792
I_5791
I_5790
I_5789
I_5788
I_5787
Term: termination (1), NS: new start (1), PS: pausing site (0), iTSS_I: internal starting site (0)

ID: 401−600; Term: termination (1), NS: new start (5), PS: pausing site (1), iTSS_I: internal starting site (1)

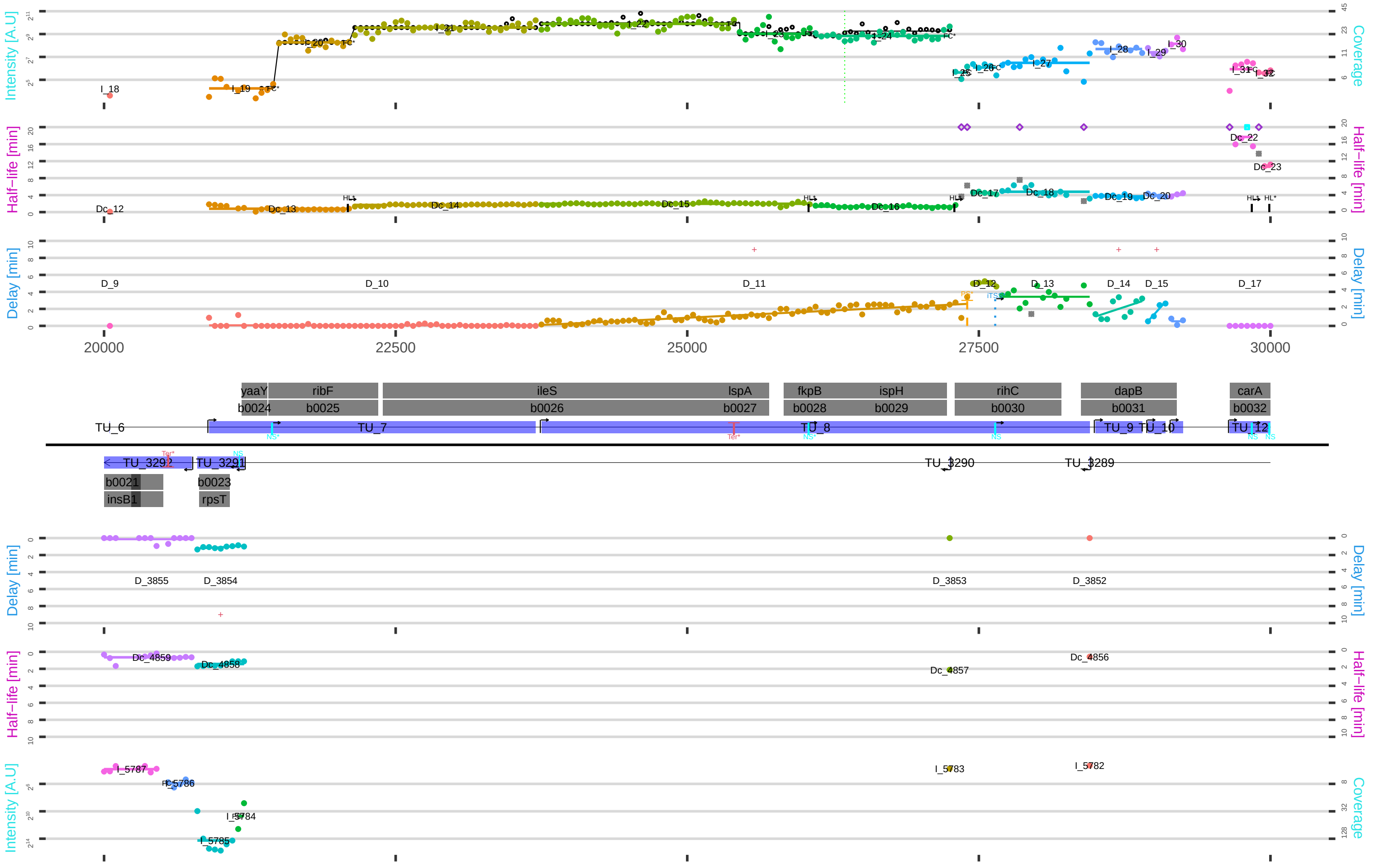

Term: termination (1), NS: new start (1), PS: pausing site (0), iTSS_I: internal starting site (0)

ID: 600−764; Term: termination (2), NS: new start (3), PS: pausing site (0), iTSS_I: internal starting site (0)

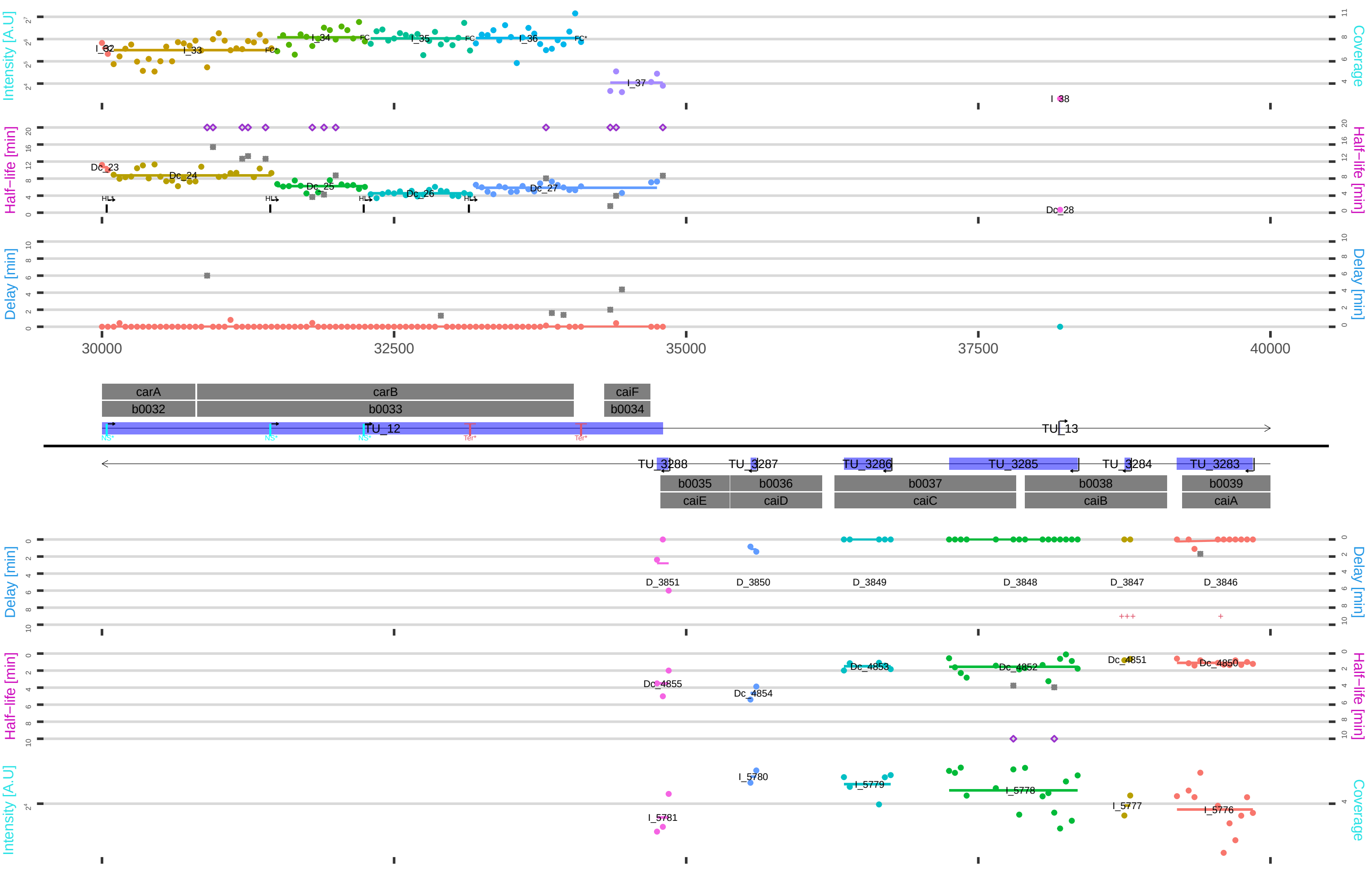

Term: termination (0), NS: new start (0), PS: pausing site (0), iTSS_I: internal starting site (0)

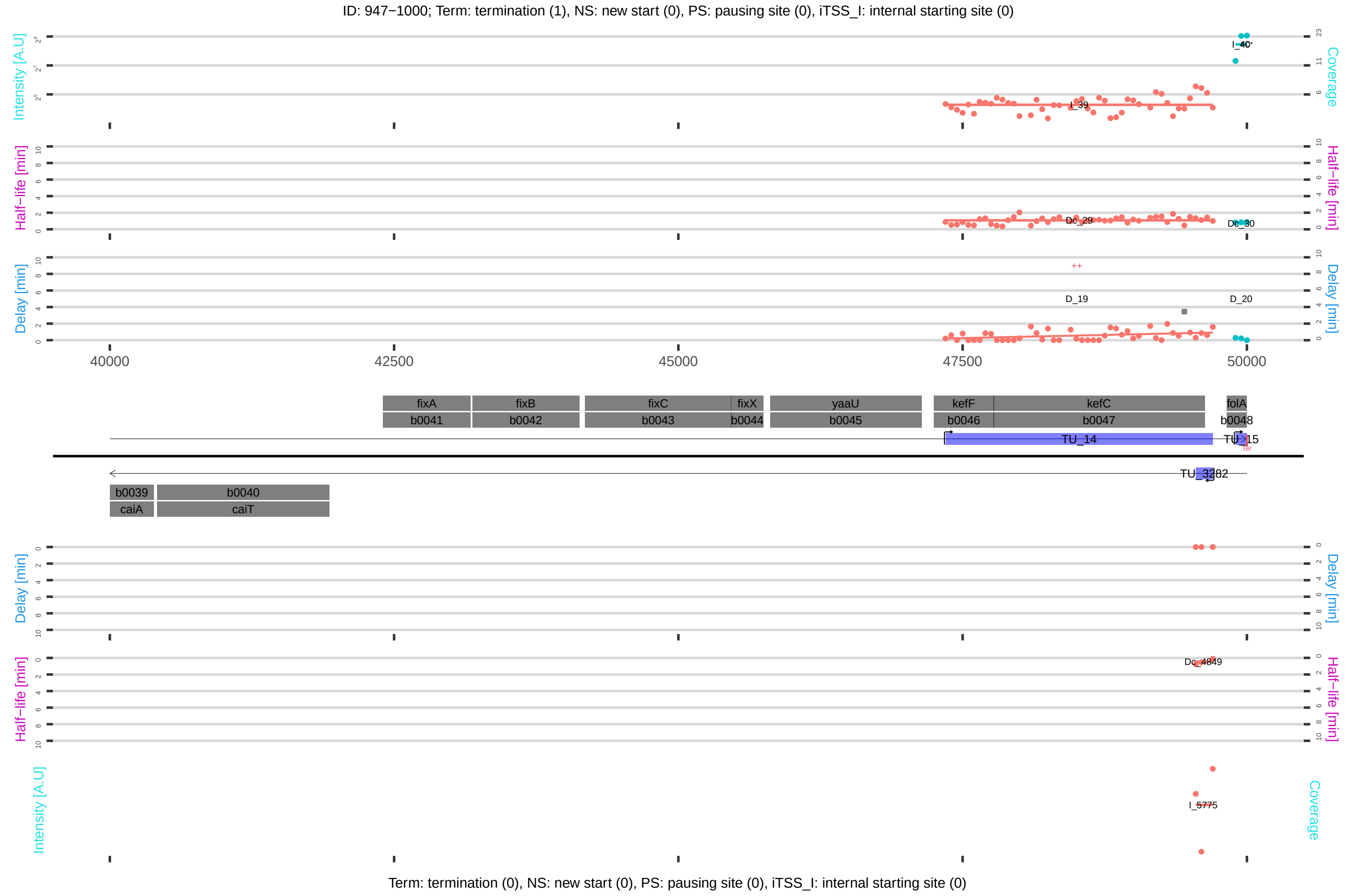

ID: 947−1000; Term: termination (1), NS: new start (0), PS: pausing site (0), iTSS_I: internal starting site (0)
Intensity [A.U]
Coverage
I_40
I_39
Half−life [min]
Dc_29
Dc_30
Delay [min]
D_19
D_20
40000
42500
45000
47500
50000
fixA
fixB
fixC
fixX
yaaU
kefF
kefC
folA
b0041
b0042
b0043
b0044
b0045
b0046
b0047
b0048
TU_14
TU_15
Ter
TU_3282
b0039
b0040
caiA
caiT
Dc_4849
I_5775
Term: termination (0), NS: new start (0), PS: pausing site (0), iTSS_I: internal starting site (0)

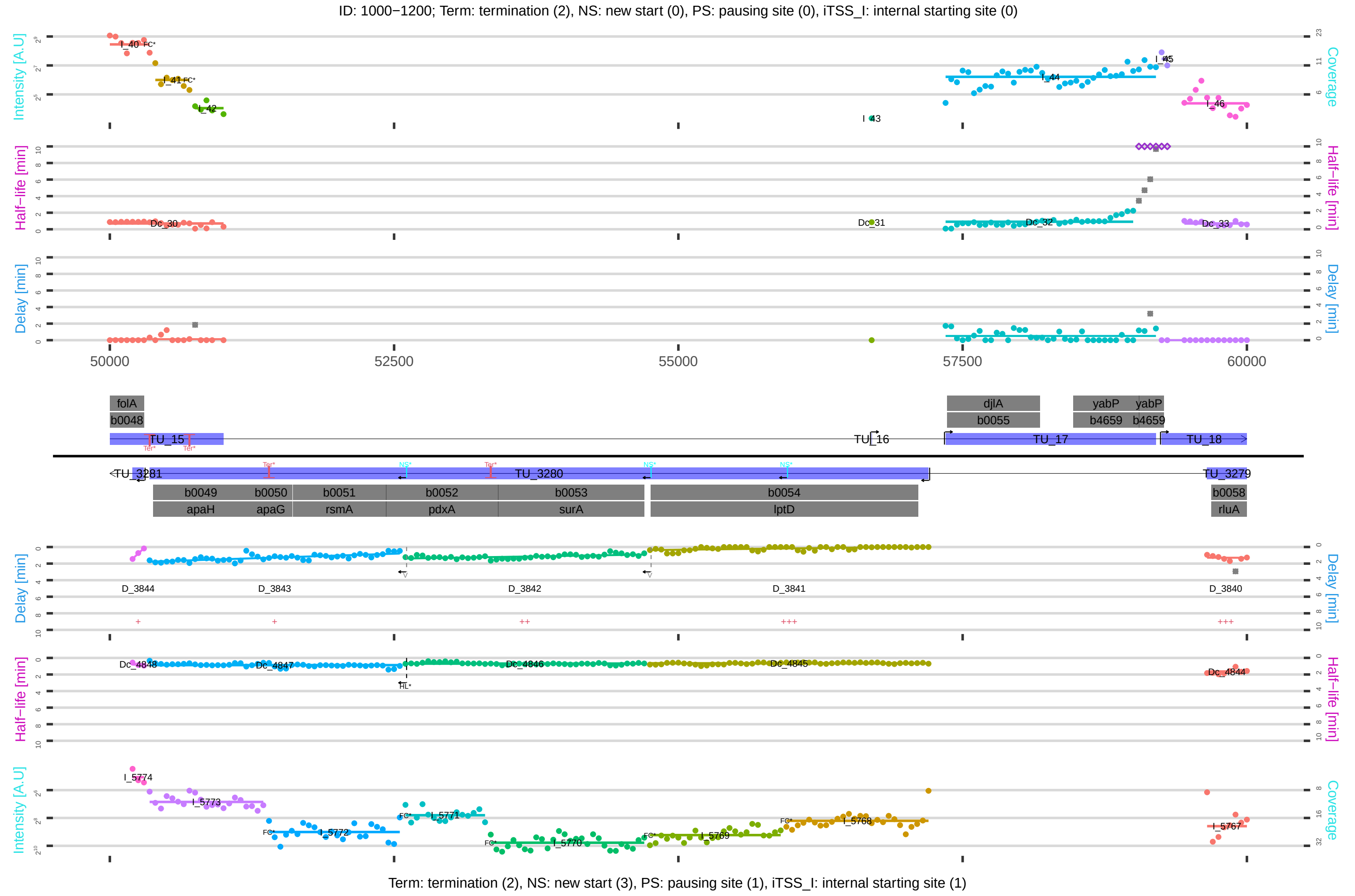

ID: 1000−1200; Term: termination (2), NS: new start (0), PS: pausing site (0), iTSS_I: internal starting site (0)
Intensity [A.U]
Coverage
Half−life [min]
Delay [min]
I_40
I_41
I_42
I_43
I_44
I_45
I_46
Dc_30
Dc_31
Dc_32
Dc_33
50000
52500
55000
57500
60000
folA
b0048
djlA
b0055
yabP
b4659
TU_15
TU_16
TU_17
TU_18
TU_3281
TU_3280
TU_3279
b0049
b0050
b0051
b0052
b0053
b0054
b0058
apaH
apaG
rsmA
pdxA
surA
lptD
rluA
D_3844
D_3843
D_3842
D_3841
D_3840
Dc_4848
Dc_4847
Dc_4846
Dc_4845
Dc_4844
I_5774
I_5773
I_5772
I_5771
I_5770
I_5769
I_5768
I_5767
Term: termination (2), NS: new start (3), PS: pausing site (1), iTSS_I: internal starting site (1)

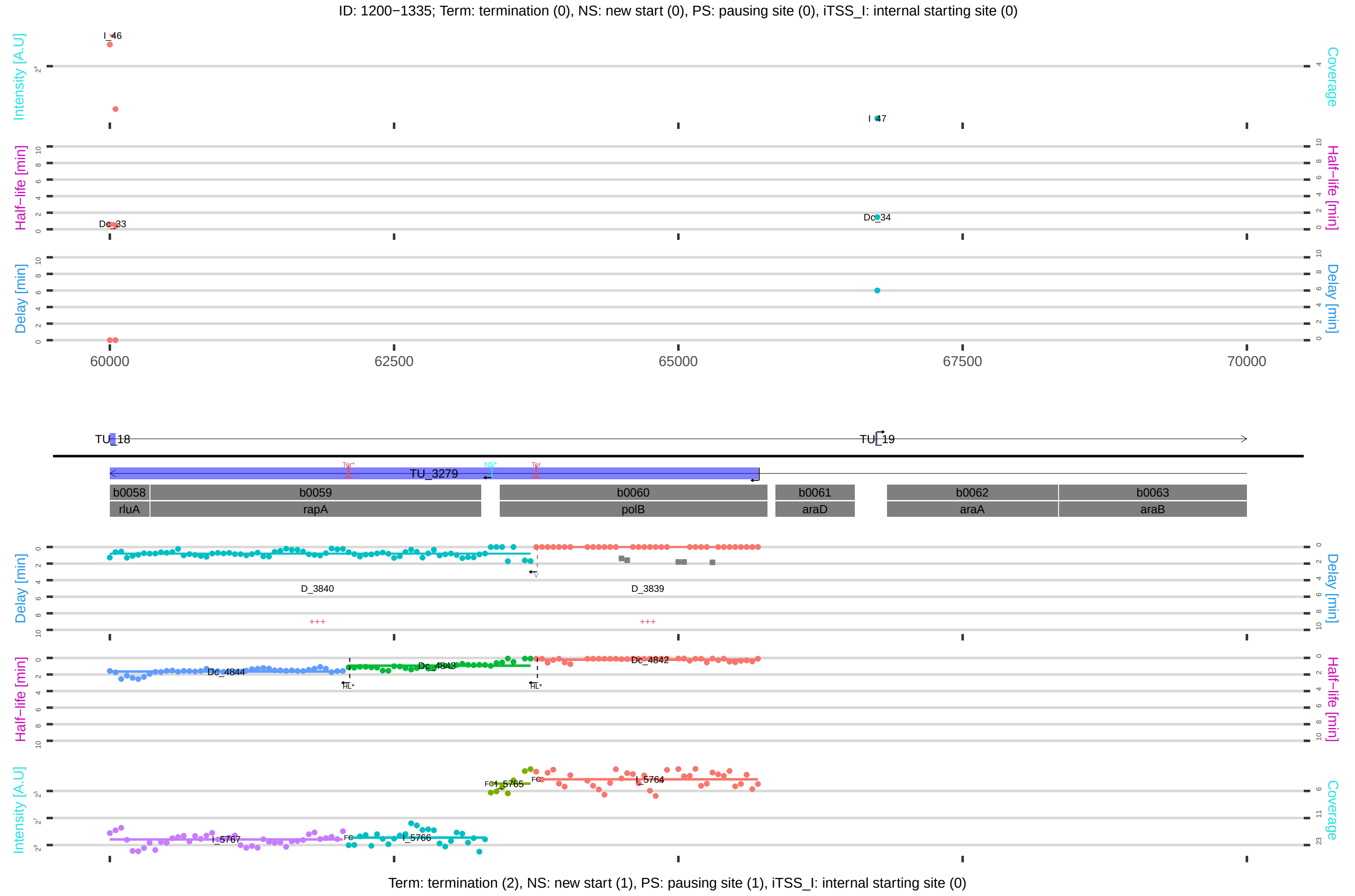
ID: 1200−1335; Term: termination (0), NS: new start (0), PS: pausing site (0), iTSS_I: internal starting site (0)
Intensity [A.U]
Coverage
Half−life [min]
Delay [min]
60000
62500
65000
67500
70000
TU_18
TU_19
TU_3279
b0058
rluA
b0059
rapA
b0060
polB
b0061
araD
b0062
araA
b0063
araB
D_3840
D_3839
Dc_4844
Dc_4843
Dc_4842
I_5767
I_5766
I_5765
I_5764
Term: termination (2), NS: new start (1), PS: pausing site (1), iTSS_I: internal starting site (0)

ID: 1406−1597; Term: termination (0), NS: new start (0), PS: pausing site (0), iTSS_I: internal starting site (0)

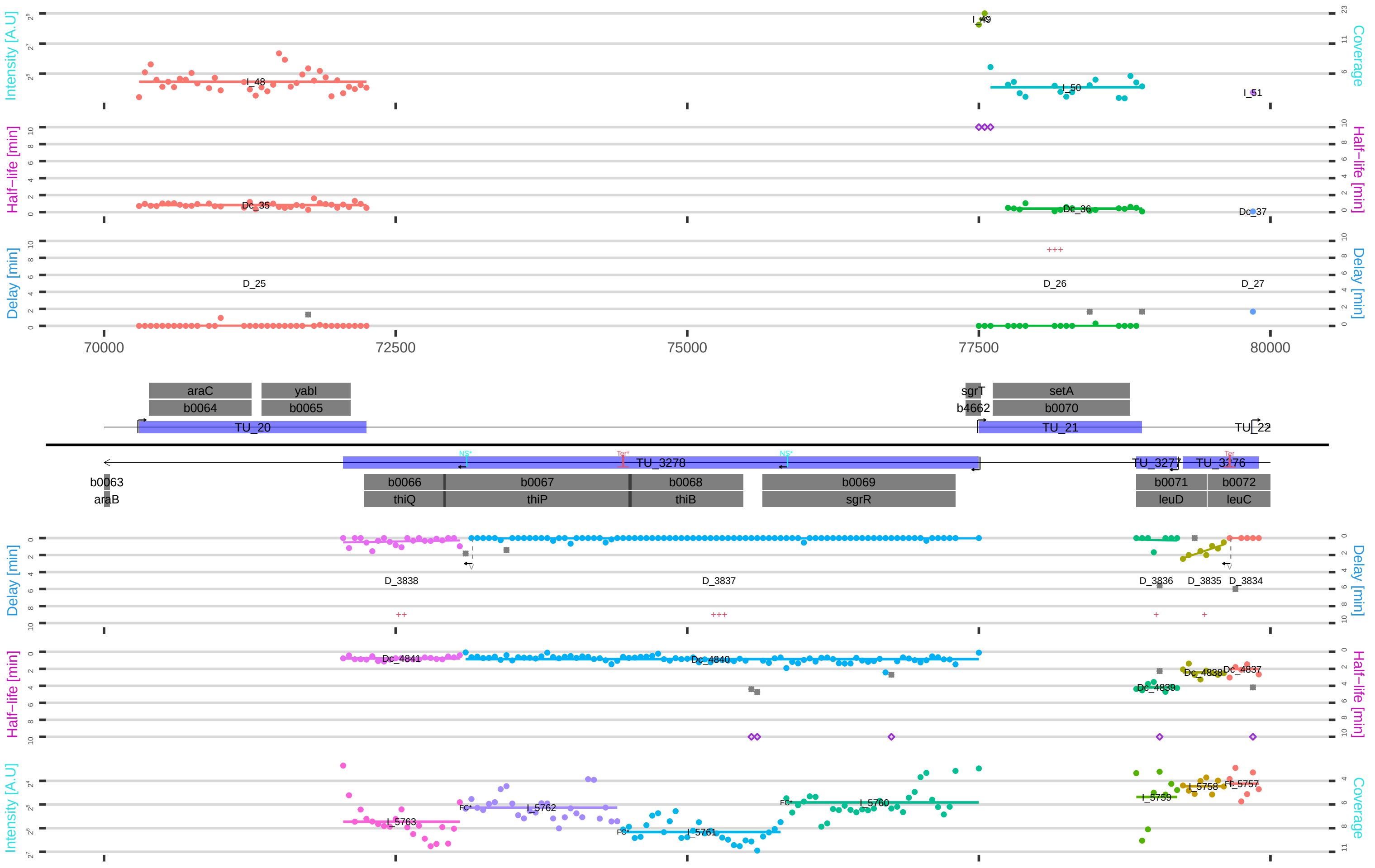

Term: termination (2), NS: new start (2), PS: pausing site (2), iTSS_I: internal starting site (0)

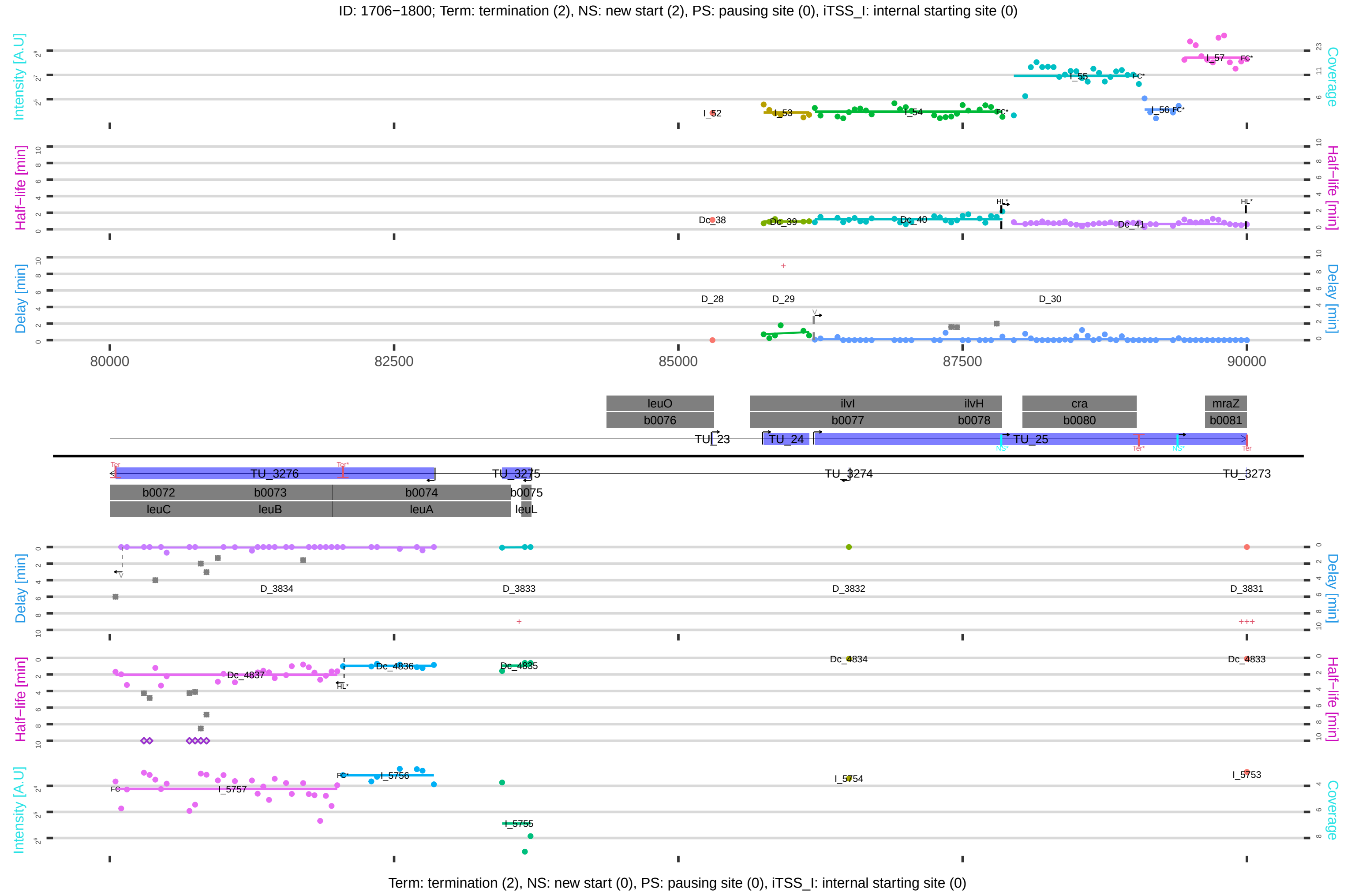

ID: 1706−1800; Term: termination (2), NS: new start (2), PS: pausing site (0), iTSS_I: internal starting site (0)
Intensity [A.U]
Coverage
Half−life [min]
Delay [min]
I_52
I_53
I_54
I_55
I_56
I_57
FC*
Dc_38
Dc_39
Dc_40
Dc_41
HL*
D_28
D_29
D_30
80000
82500
85000
87500
90000
leuO
b0076
ilvI
b0077
ilvH
b0078
cra
b0080
mraZ
b0081
TU_23
TU_24
TU_25
NS*
Ter*
Ter
TU_3276
TU_3275
TU_3274
TU_3273
b0072
b0073
b0074
b0075
leuC
leuB
leuA
leuL
D_3834
D_3833
D_3832
D_3831
Dc_4837
Dc_4836
Dc_4835
Dc_4834
Dc_4833
I_5757
I_5756
I_5755
I_5754
I_5753
FC
Term: termination (2), NS: new start (0), PS: pausing site (0), iTSS_I: internal starting site (0)

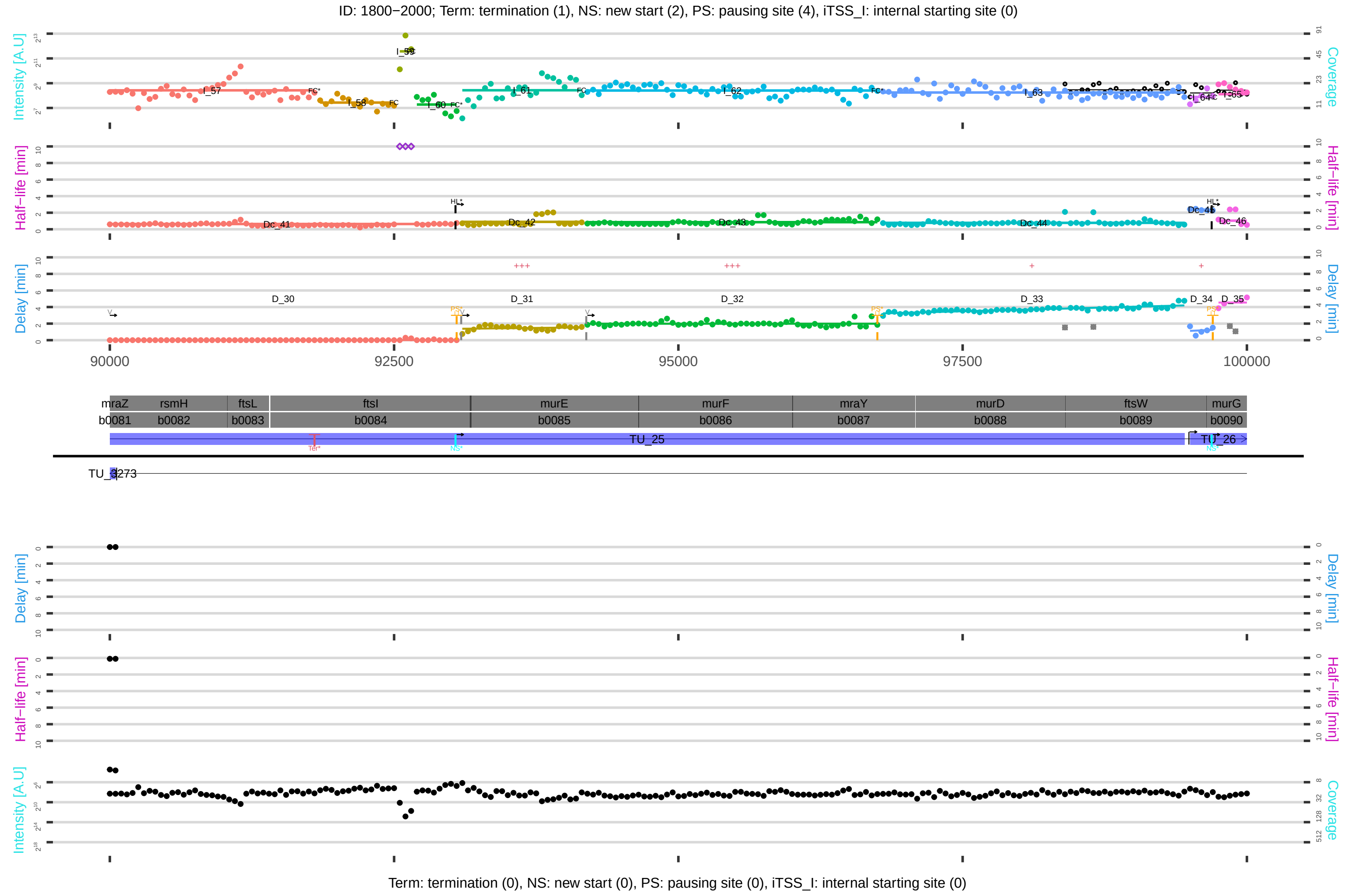

ID: 1800−2000; Term: termination (1), NS: new start (2), PS: pausing site (4), iTSS_I: internal starting site (0)
Intensity [A.U]
Coverage
Half−life [min]
Delay [min]
I_57
I_58
I_59
I_60
I_61
I_62
I_63
I_64
I_65
Dc_41
Dc_42
Dc_43
Dc_44
Dc_45
Dc_46
D_30
D_31
D_32
D_33
D_34
D_35
90000
92500
95000
97500
100000
mraZ
rsmH
ftsL
ftsI
murE
murF
mraY
murD
ftsW
murG
b0081
b0082
b0083
b0084
b0085
b0086
b0087
b0088
b0089
b0090
TU_25
TU_26
TU_3273
Term: termination (0), NS: new start (0), PS: pausing site (0), iTSS_I: internal starting site (0)

ID: 2000−2200; Term: termination (4), NS: new start (6), PS: pausing site (2), iTSS_I: internal starting site (2)

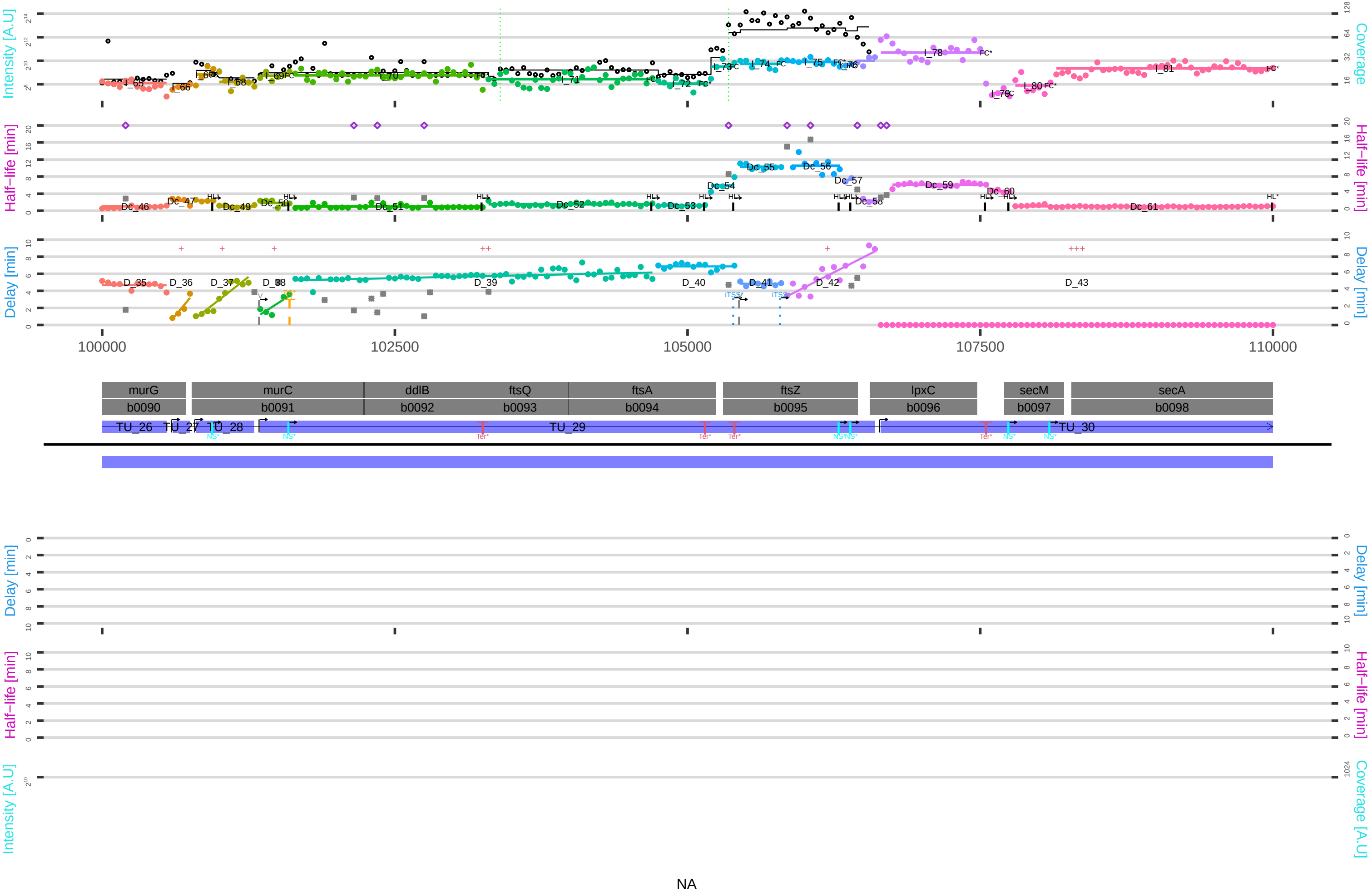

ID: 2200−2400; Term: termination (3), NS: new start (0), PS: pausing site (1), iTSS_I: internal starting site (0)

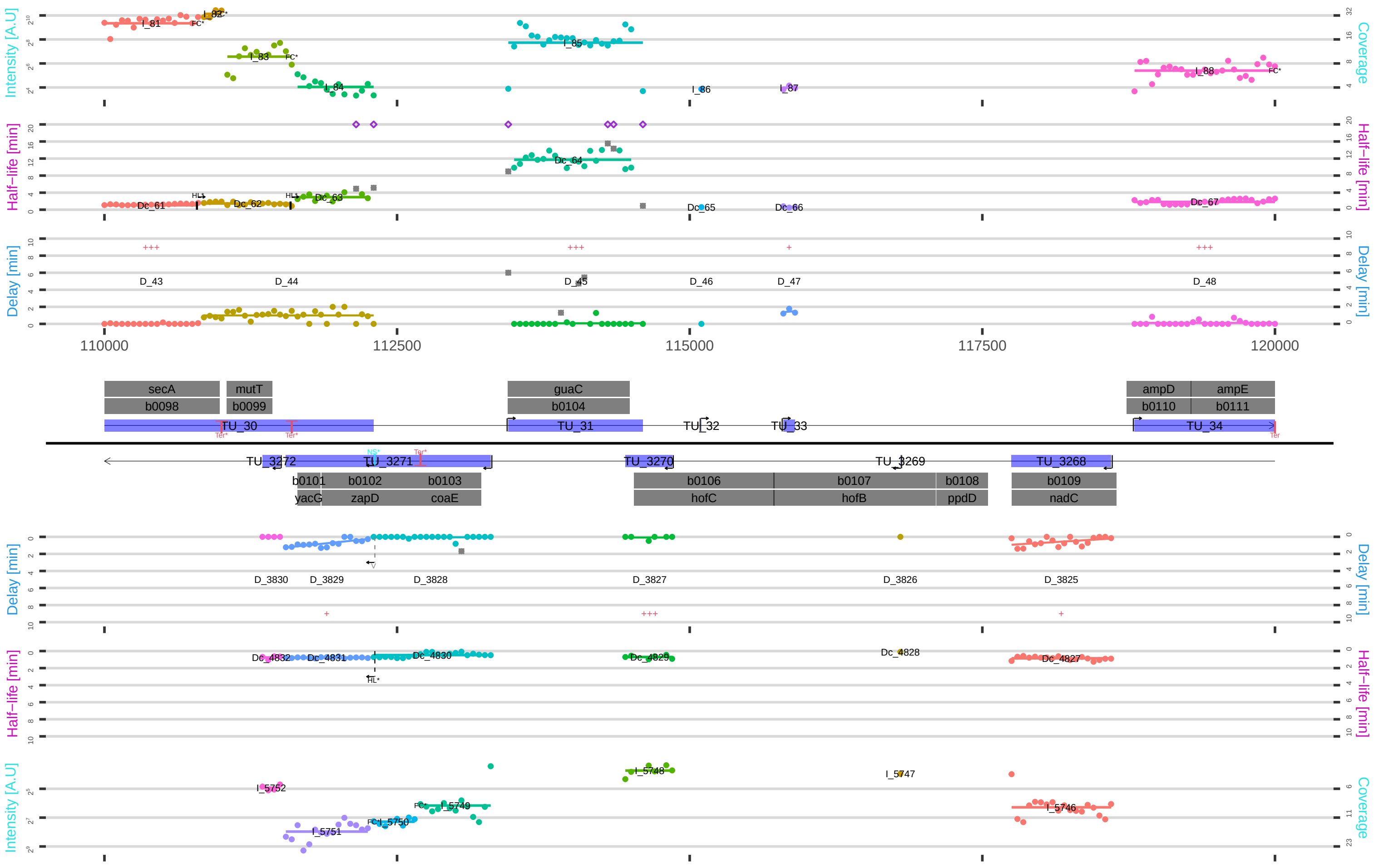

Term: termination (1), NS: new start (1), PS: pausing site (1), iTSS_I: internal starting site (0)

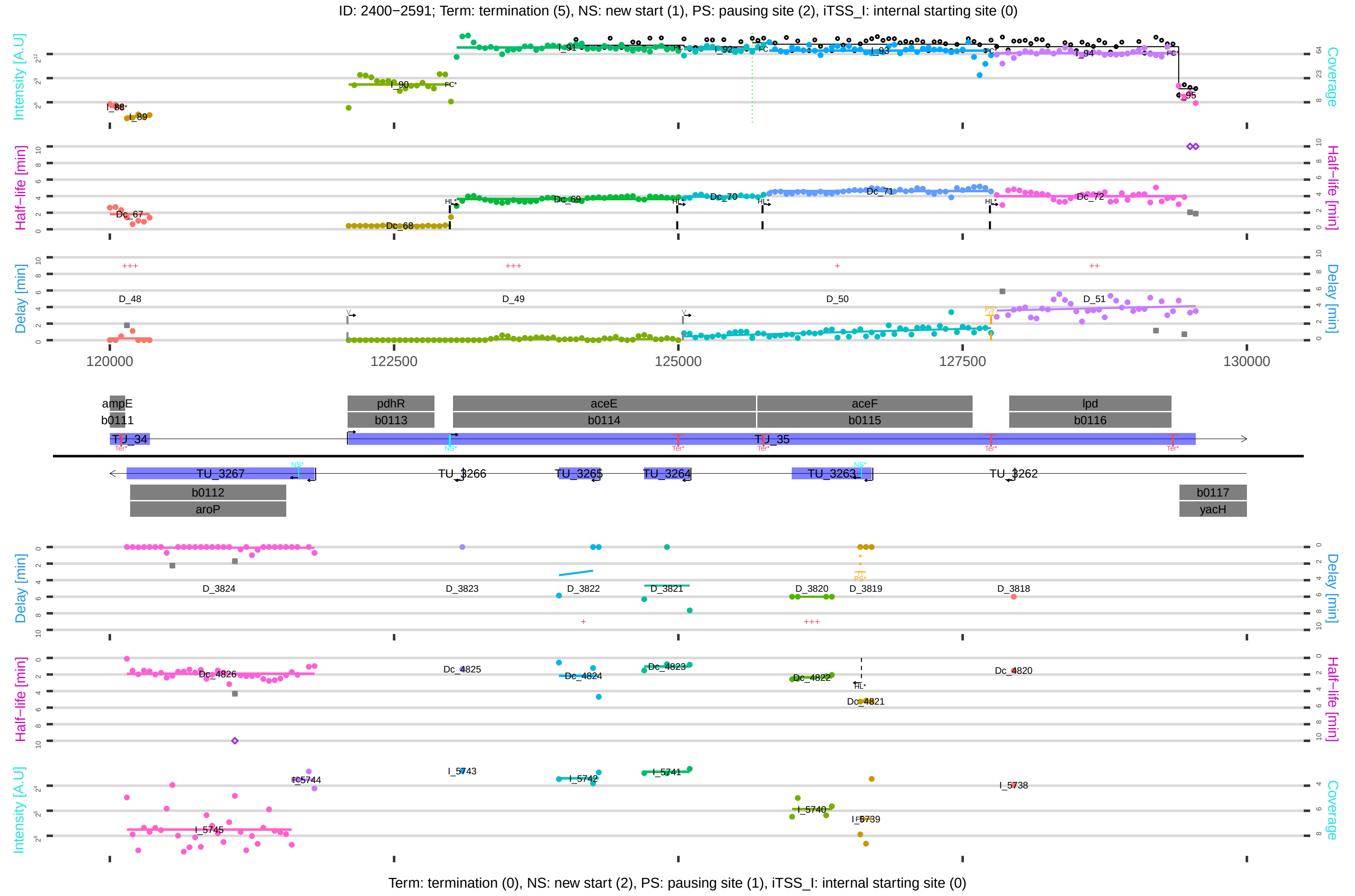
ID: 2400−2591; Term: termination (5), NS: new start (1), PS: pausing site (2), iTSS_I: internal starting site (0)
Intensity [A.U]
Coverage
Half−life [min]
Delay [min]
120000
122500
125000
127500
130000
ampE
b0111
pdhR
b0113
aceE
b0114
aceF
b0115
lpd
b0116
TU_34
TU_35
TU_3267
TU_3266
TU_3265
TU_3264
TU_3263
TU_3262
b0112
aroP
b0117
yacH
D_48
D_49
D_50
D_51
D_3824
D_3823
D_3822
D_3821
D_3820
D_3819
D_3818
Dc_67
Dc_68
Dc_69
Dc_70
Dc_71
Dc_72
Dc_4826
Dc_4825
Dc_4824
Dc_4823
Dc_4822
Dc_4821
Dc_4820
I_90
I_91
I_92
I_93
I_94
I_95
I_5745
I_5743
I_5742
I_5741
I_5740
I_5738
Term: termination (0), NS: new start (2), PS: pausing site (1), iTSS_I: internal starting site (0)

ID: 2629−2774; Term: termination (3), NS: new start (3), PS: pausing site (2), iTSS_I: internal starting site (0)

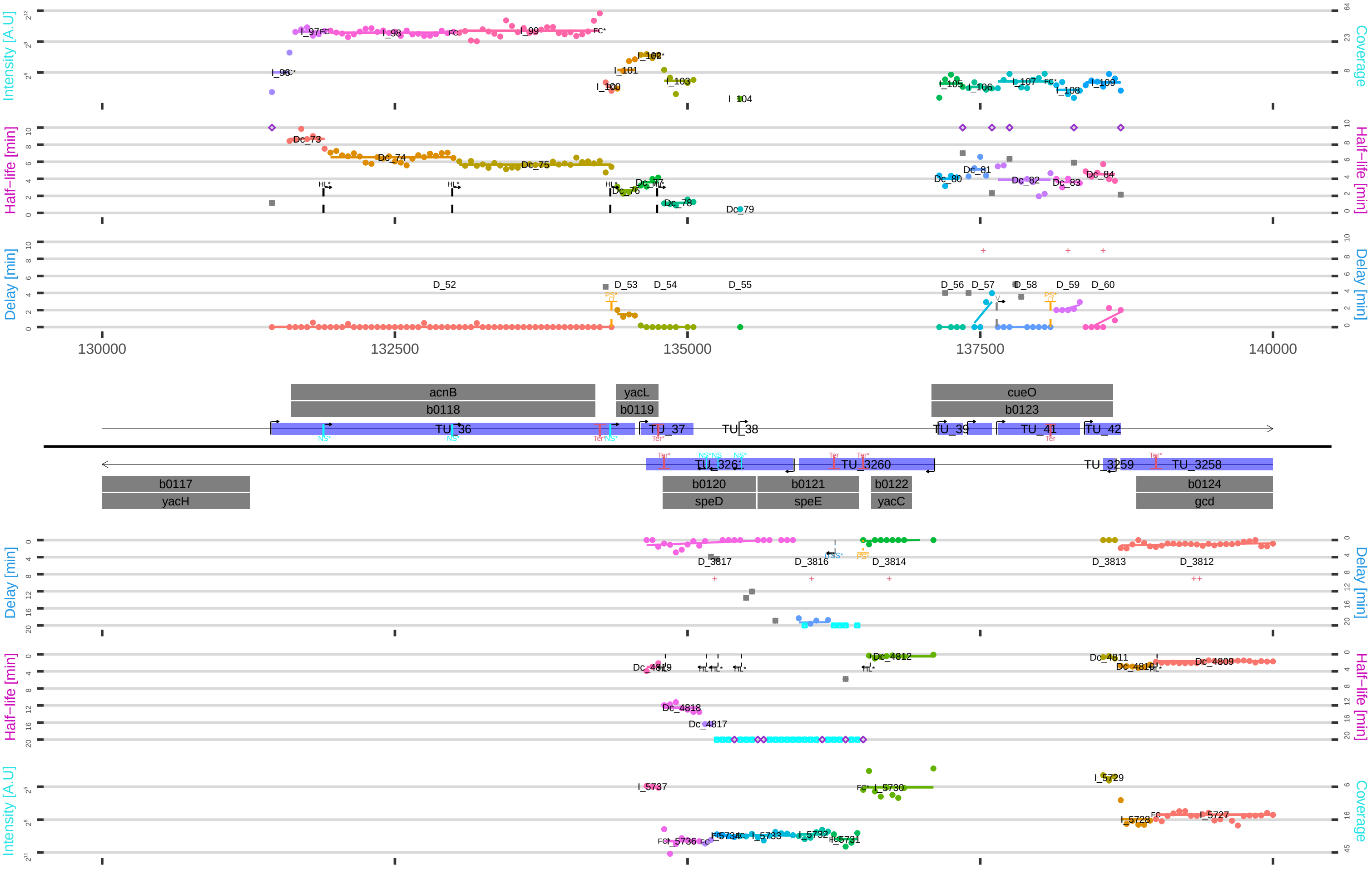

Term: termination (4), NS: new start (3), PS: pausing site (1), iTSS_I: internal starting site (1)

ID: 2829−2958; Term: termination (3), NS: new start (1), PS: pausing site (0), iTSS_I: internal starting site (0)

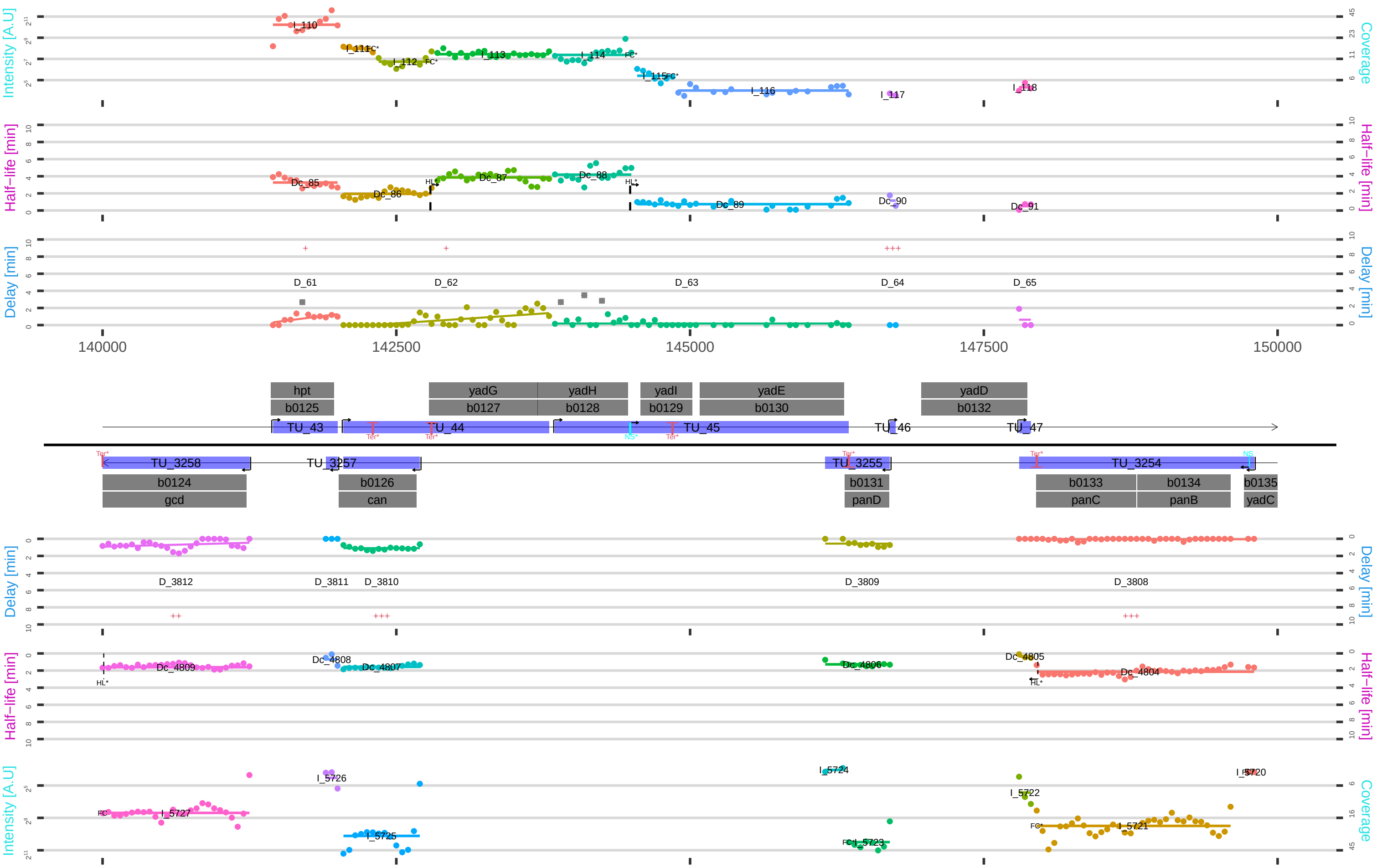

Term: termination (3), NS: new start (1), PS: pausing site (0), iTSS_I: internal starting site (0)

ID: 3042−3042; Term: termination (0), NS: new start (0), PS: pausing site (0), iTSS_I: internal starting site (0)

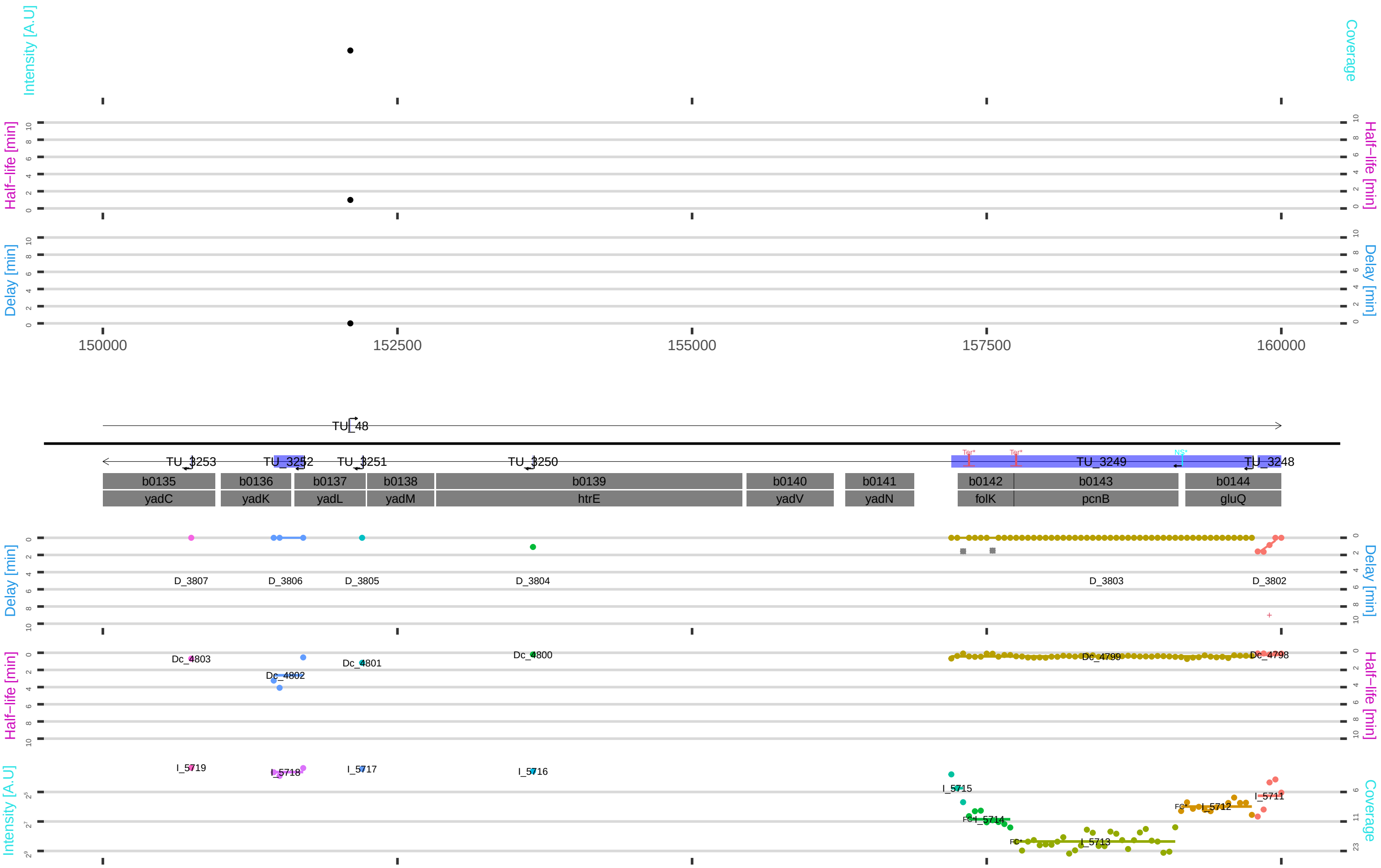

Term: termination (2), NS: new start (1), PS: pausing site (0), iTSS_I: internal starting site (0)

ID: 3227−3400; Term: termination (2), NS: new start (3), PS: pausing site (1), iTSS_I: internal starting site (0)

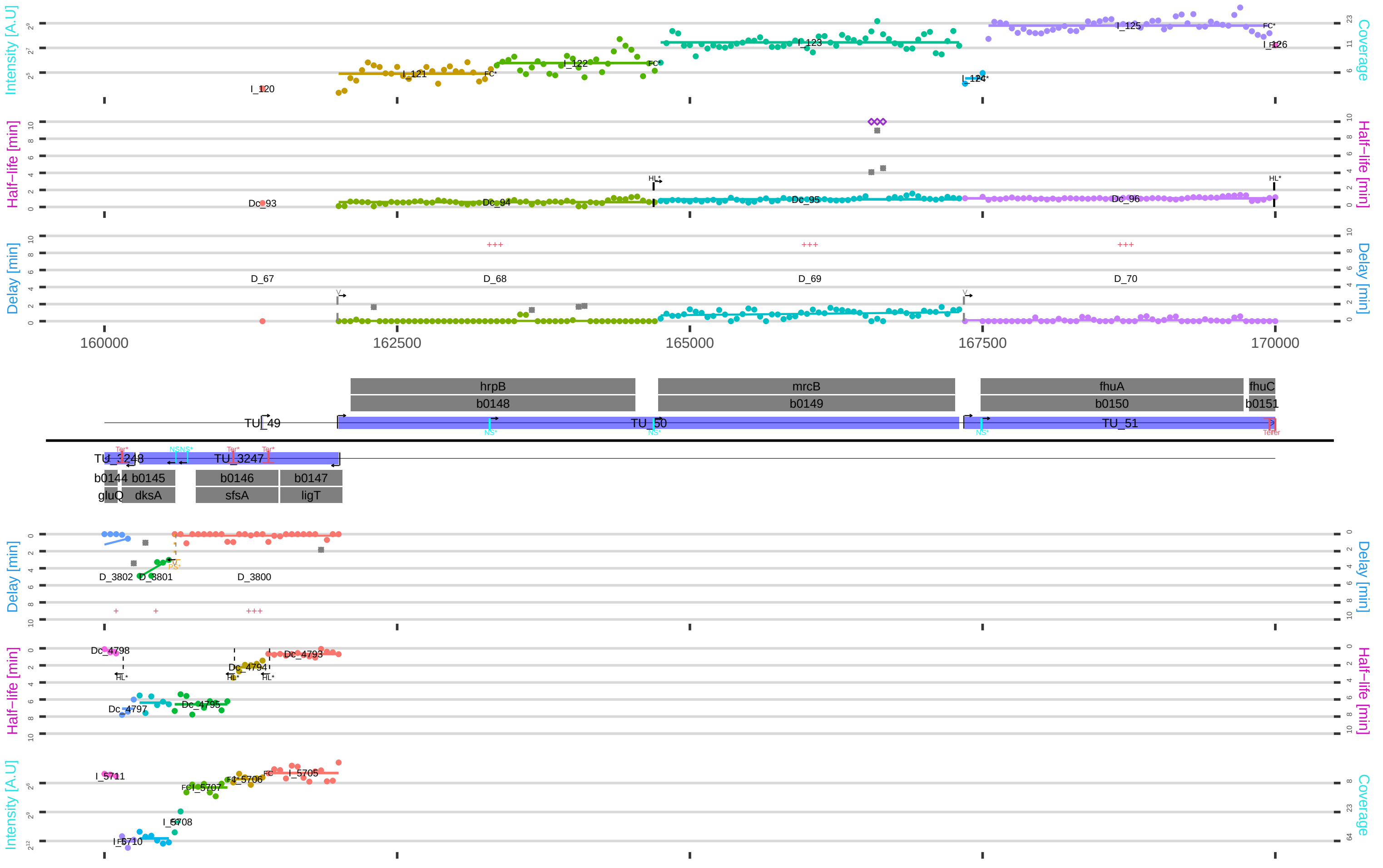

Term: termination (3), NS: new start (2), PS: pausing site (1), iTSS_I: internal starting site (0)

ID: 3400−3599; Term: termination (3), NS: new start (2), PS: pausing site (1), iTSS_I: internal starting site (0)

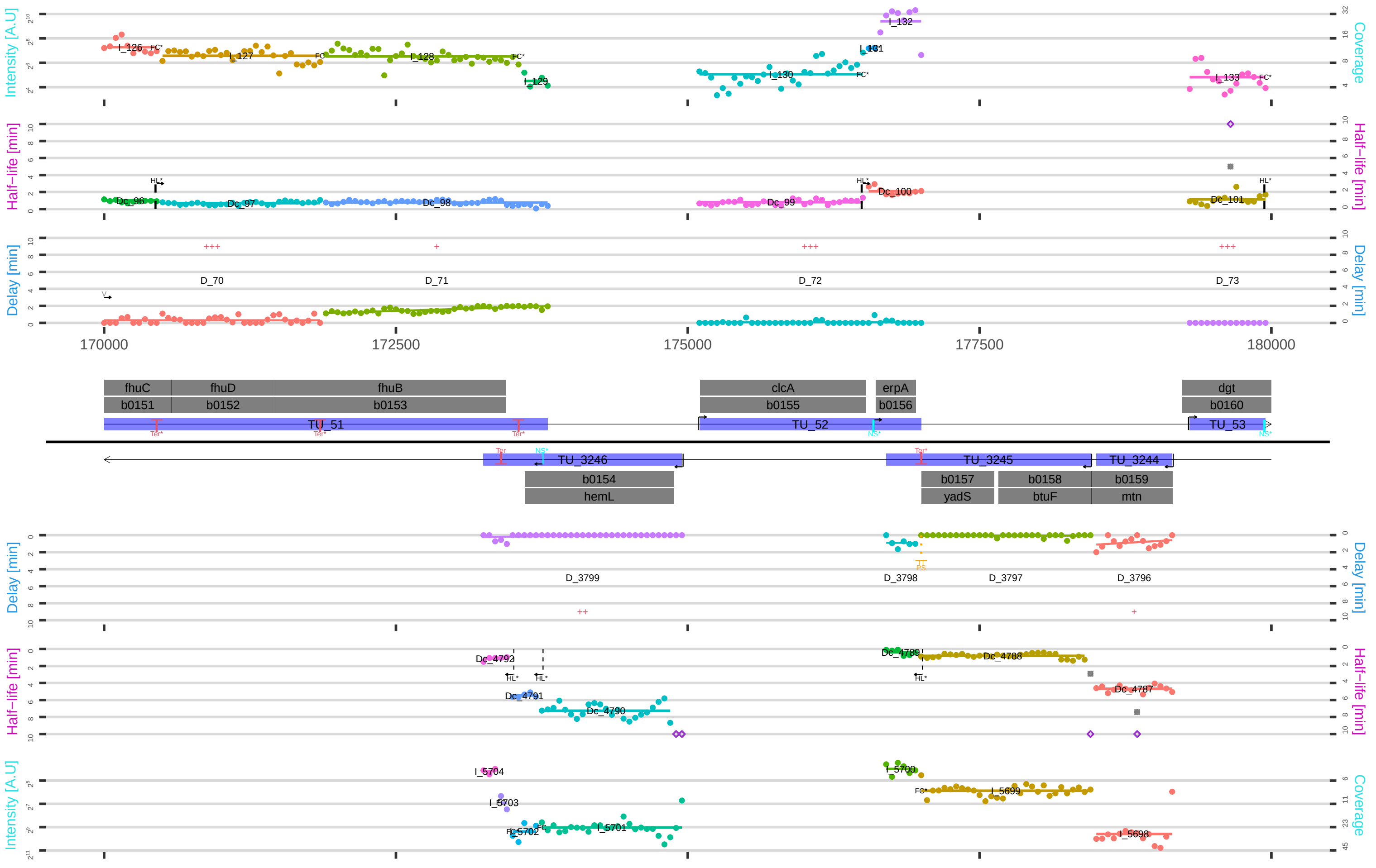

Term: termination (2), NS: new start (1), PS: pausing site (1), iTSS_I: internal starting site (0)

ID: 3601−3800; Term: termination (0), NS: new start (2), PS: pausing site (3), iTSS_I: internal starting site (0)

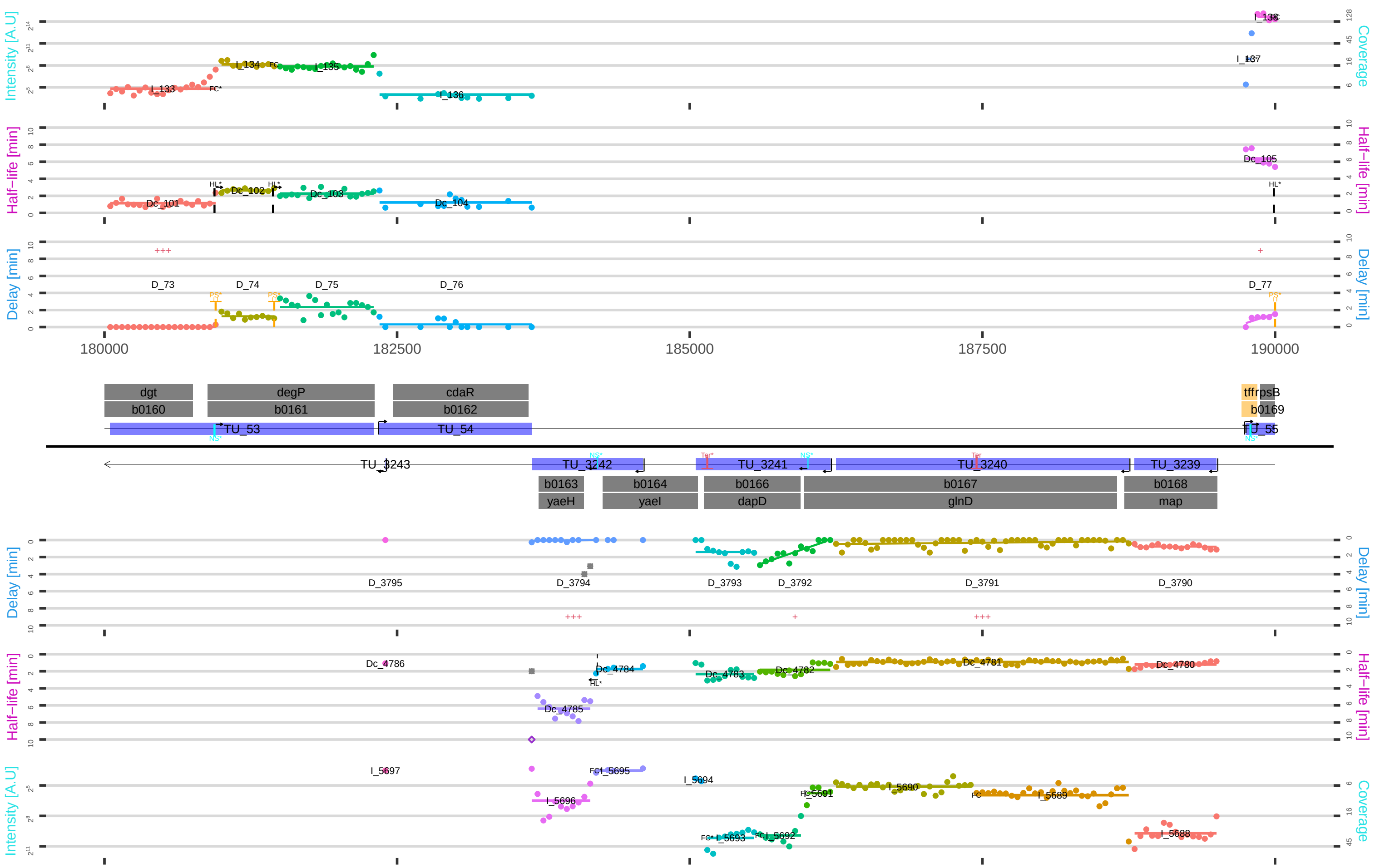

Term: termination (2), NS: new start (2), PS: pausing site (0), iTSS_I: internal starting site (1)

ID: 3800−4000; Term: termination (3), NS: new start (3), PS: pausing site (5), iTSS_I: internal starting site (0)

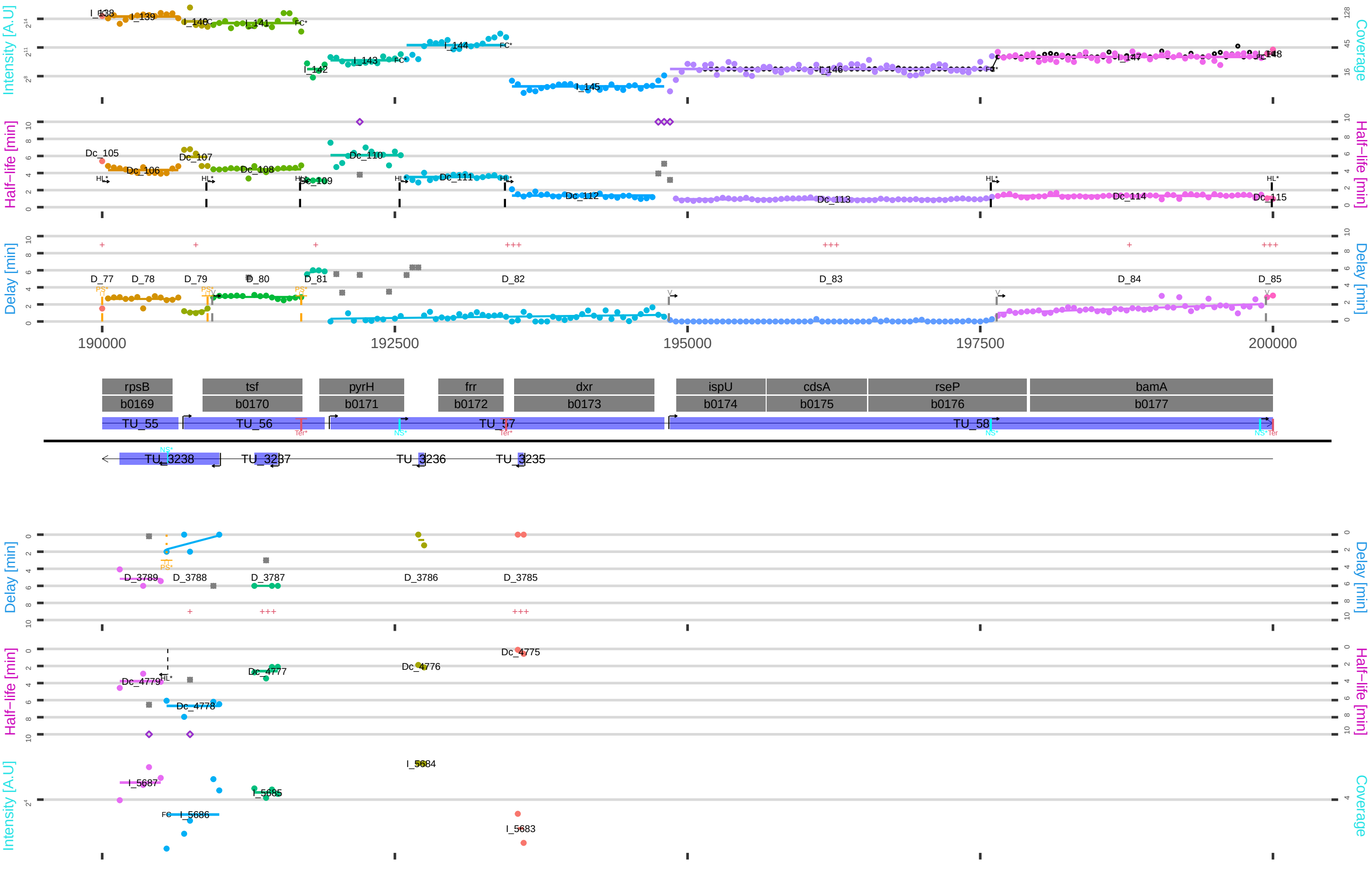

Term: termination (0), NS: new start (1), PS: pausing site (1), iTSS_I: internal starting site (0)

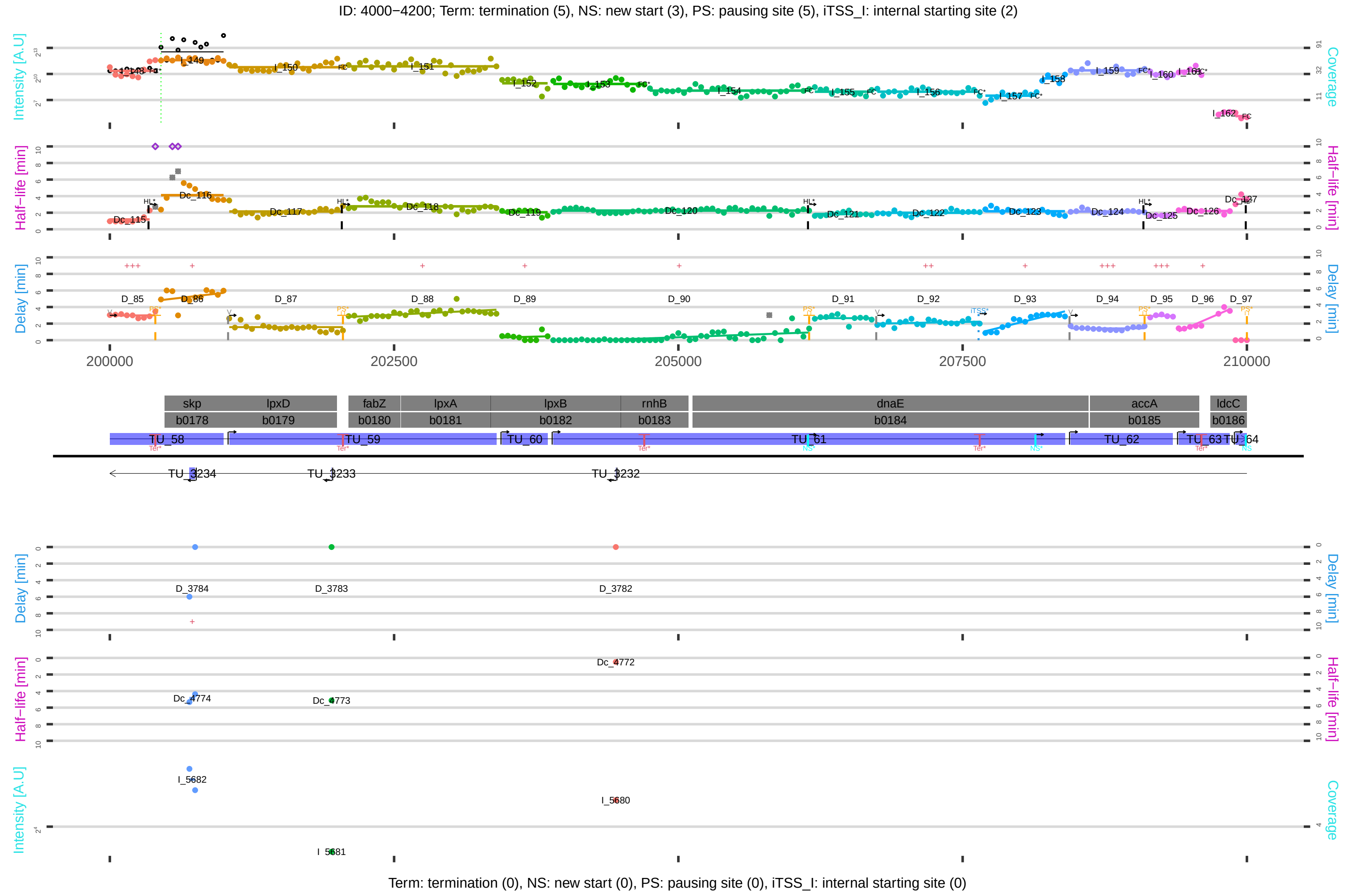

ID: 4000−4200; Term: termination (5), NS: new start (3), PS: pausing site (5), iTSS_I: internal starting site (2)
Intensity [A.U]
Coverage
Half−life [min]
Delay [min]
200000
202500
205000
207500
210000
skp
lpxD
fabZ
lpxA
lpxB
rnhB
dnaE
accA
ldcC
b0178
b0179
b0180
b0181
b0182
b0183
b0184
b0185
b0186
TU_58
TU_59
TU_60
TU_61
TU_62
TU_63
TU_64
TU_3234
TU_3233
TU_3232
D_3784
D_3783
D_3782
Dc_4774
Dc_4773
Dc_4772
I_5682
I_5681
I_5680
Term: termination (0), NS: new start (0), PS: pausing site (0), iTSS_I: internal starting site (0)

ID: 4200−4361; Term: termination (0), NS: new start (5), PS: pausing site (1), iTSS_I: internal starting site (2)

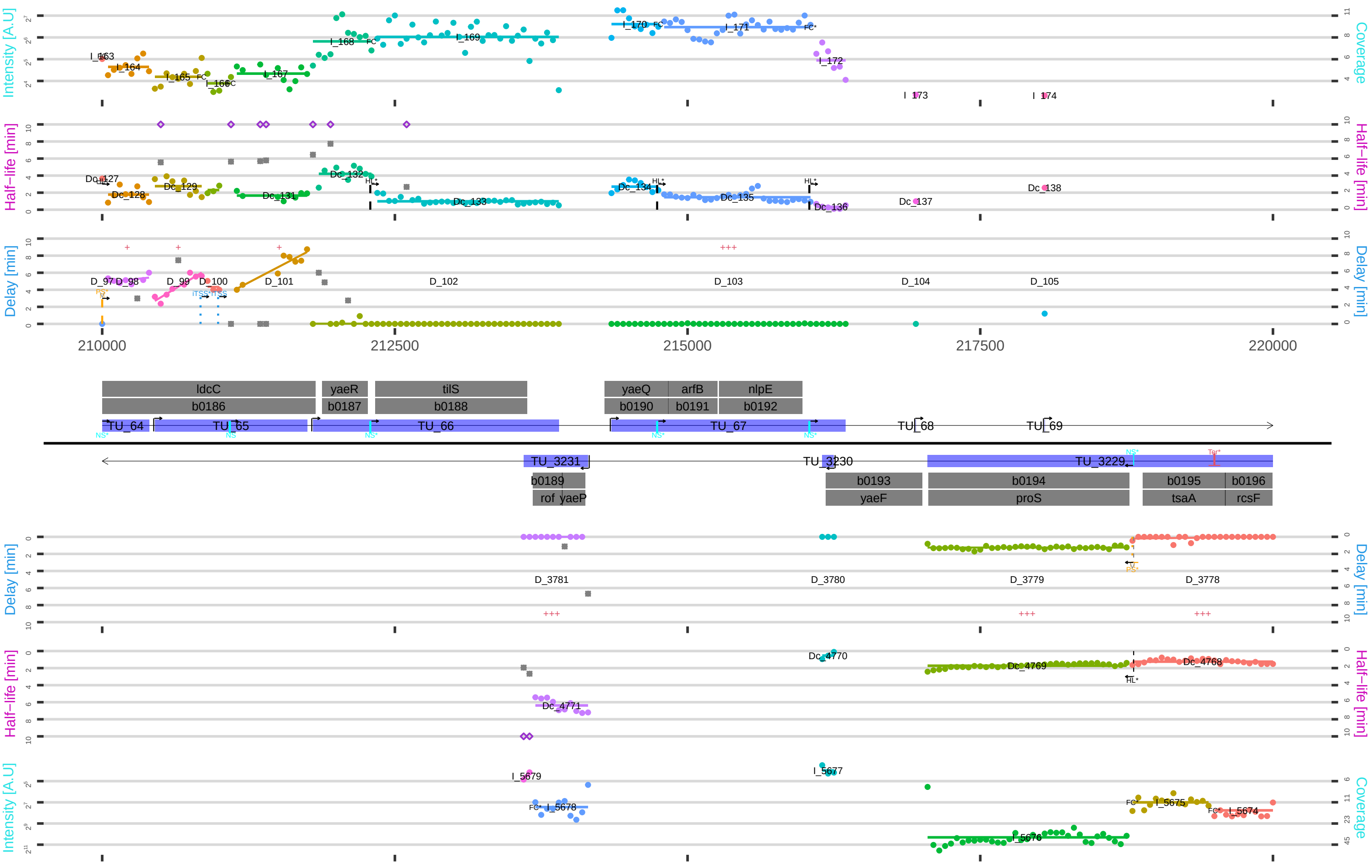

Term: termination (1), NS: new start (1), PS: pausing site (1), iTSS_I: internal starting site (0)

ID: 4440−4600; Term: termination (4), NS: new start (4), PS: pausing site (0), iTSS_I: internal starting site (0)

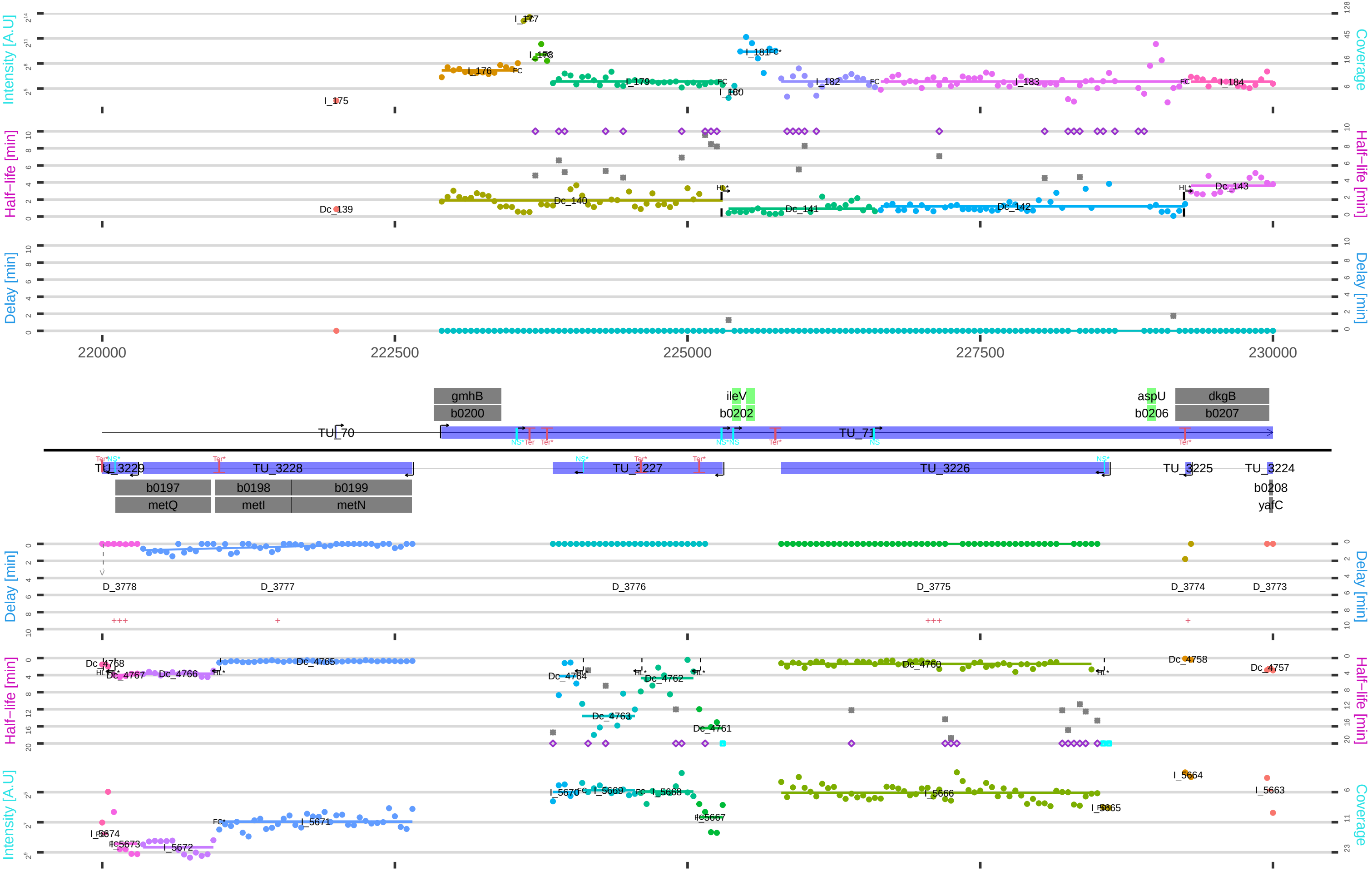

Term: termination (4), NS: new start (3), PS: pausing site (0), iTSS_I: internal starting site (0)

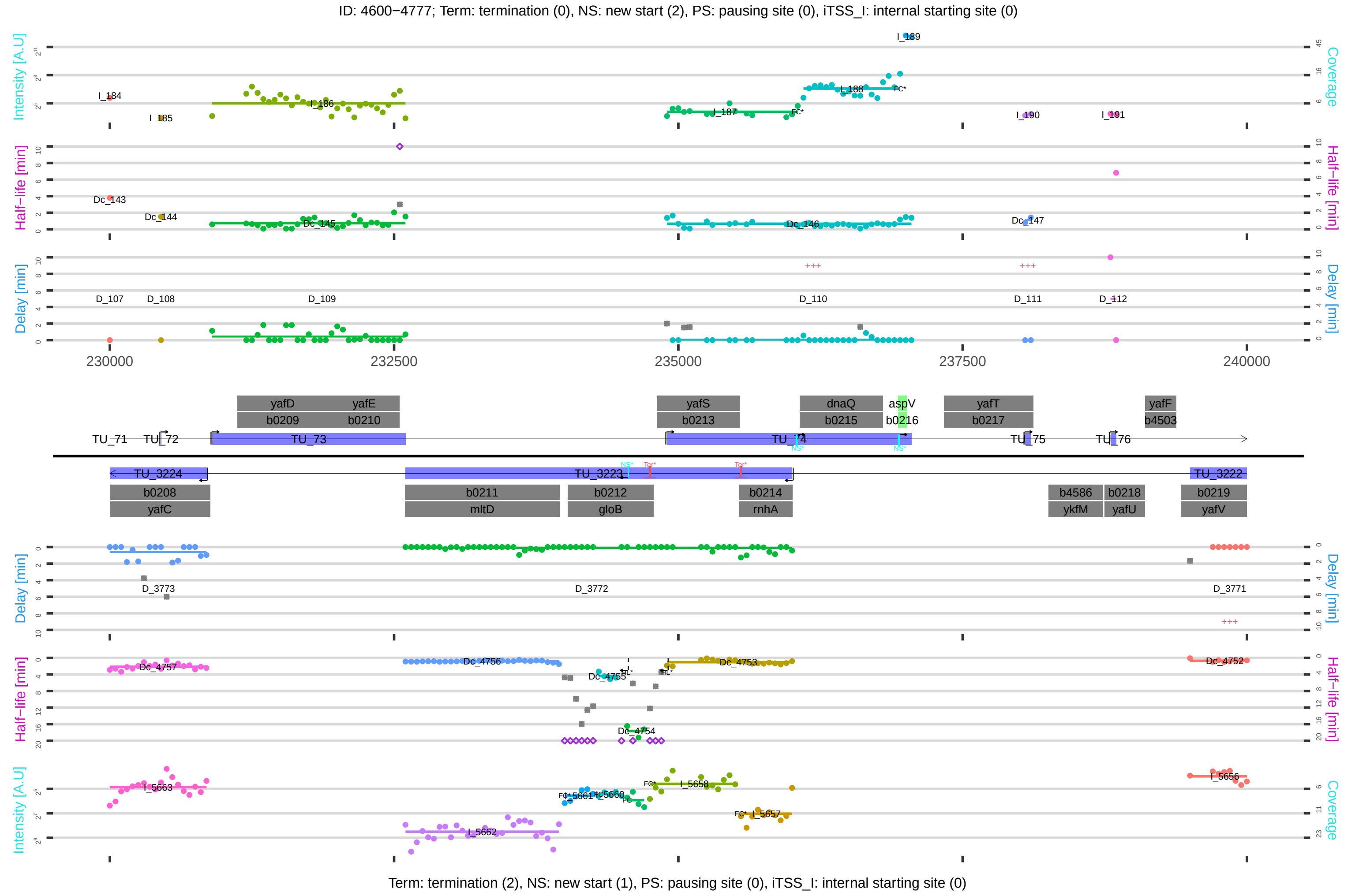

ID: 4600−4777; Term: termination (0), NS: new start (2), PS: pausing site (0), iTSS_I: internal starting site (0)
Intensity [A.U]
Coverage
Half−life [min]
Delay [min]
I_184
I_185
I_186
I_187
I_188
I_189
I_190
I_191
Dc_143
Dc_144
Dc_145
Dc_146
Dc_147
D_107
D_108
D_109
D_110
D_111
D_112
230000
232500
235000
237500
240000
yafD
yafE
b0209
b0210
yafS
b0213
dnaQ
b0215
aspV
b0216
yafT
b0217
yafF
b4503
TU_71
TU_72
TU_73
TU_74
TU_75
TU_76
TU_3224
TU_3223
TU_3222
b0208
yafC
b0211
mltD
b0212
gloB
b0214
rnhA
b4586
ykfM
b0218
yafU
b0219
yafV
D_3773
D_3772
D_3771
Dc_4757
Dc_4756
Dc_4755
Dc_4754
Dc_4753
Dc_4752
I_5663
I_5662
I_5661
I_5660
I_5658
I_5657
I_5656
Term: termination (2), NS: new start (1), PS: pausing site (0), iTSS_I: internal starting site (0)

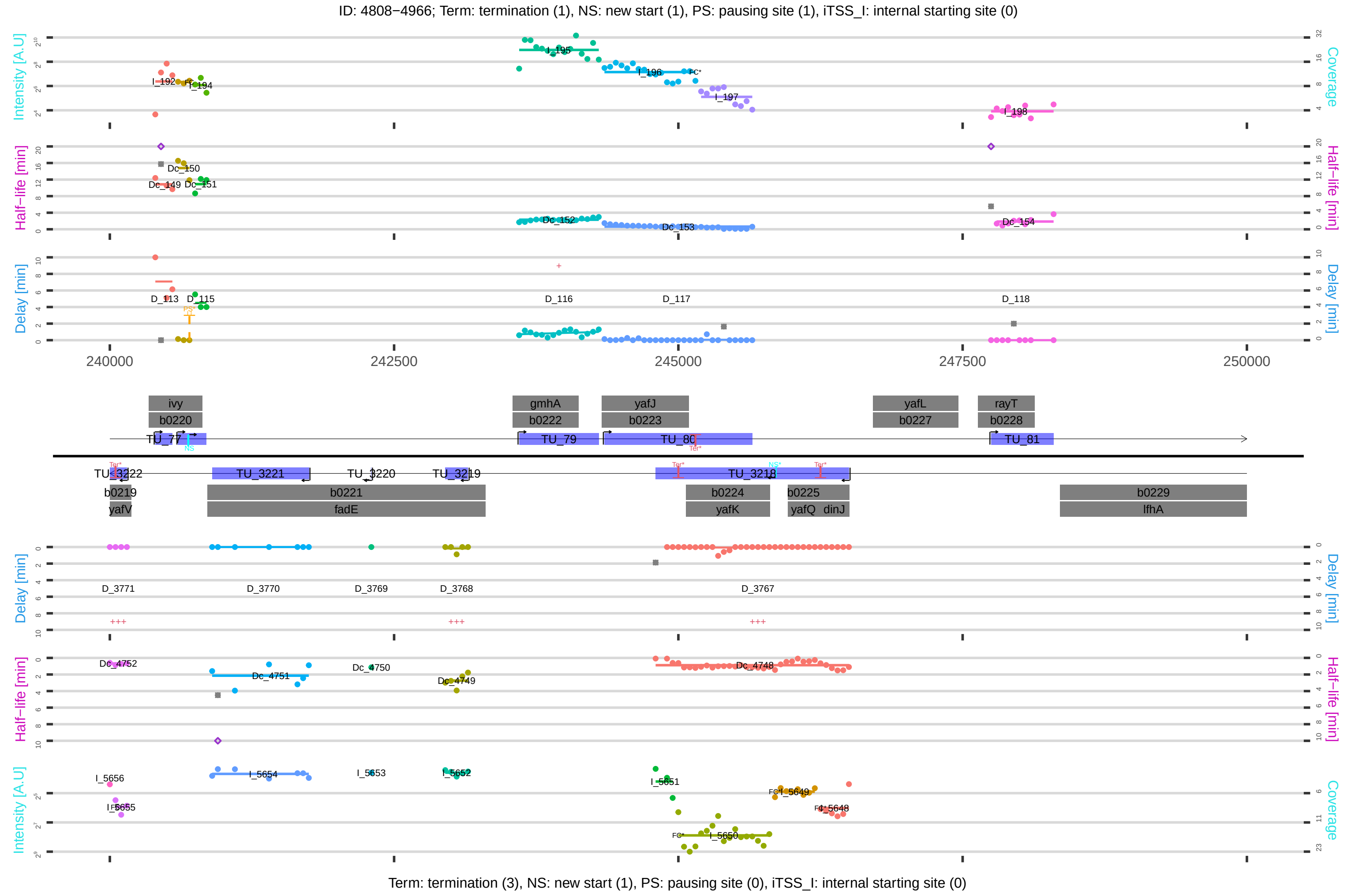

ID: 4808−4966; Term: termination (1), NS: new start (1), PS: pausing site (1), iTSS_I: internal starting site (0)
Intensity [A.U]
Coverage
Half−life [min]
Delay [min]
240000
242500
245000
247500
250000
ivy
b0220
gmhA
b0222
yafJ
b0223
yafL
b0227
rayT
b0228
TU_77
TU_79
TU_80
TU_81
TU_3222
TU_3221
TU_3220
TU_3219
TU_3218
b0219
yafV
b0221
fadE
b0224
yafK
b0225
yafQ dinJ
b0229
lfhA
Term: termination (3), NS: new start (1), PS: pausing site (0), iTSS_I: internal starting site (0)

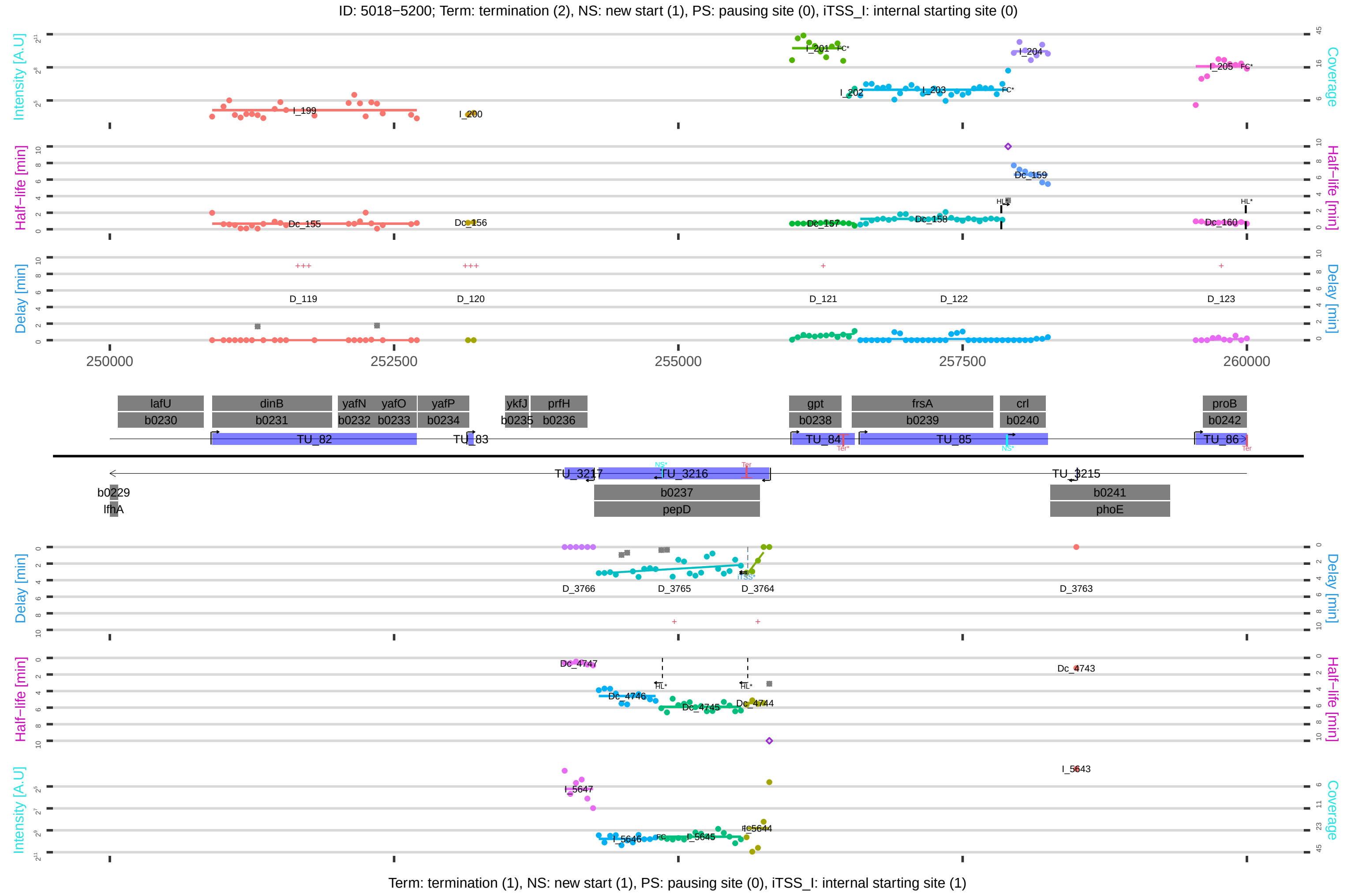

ID: 5018−5200; Term: termination (2), NS: new start (1), PS: pausing site (0), iTSS_I: internal starting site (0)
Intensity [A.U]
Coverage
Half−life [min]
Delay [min]
I_199
I_200
I_201
I_202
I_203
I_204
I_205
Dc_155
Dc_156
Dc_157
Dc_158
Dc_159
Dc_160
D_119
D_120
D_121
D_122
D_123
250000
252500
255000
257500
260000
lafU
b0230
dinB
b0231
yafN
yafO
b0232
b0233
yafP
b0234
ykfJ
b0235
prfH
b0236
gpt
b0238
frsA
b0239
crl
b0240
proB
b0242
TU_82
TU_83
TU_84
TU_85
TU_86
TU_3217
TU_3216
TU_3215
b0229
lfhA
b0237
pepD
b0241
phoE
D_3766
D_3765
D_3764
D_3763
Dc_4747
Dc_4746
Dc_4745
Dc_4744
Dc_4743
I_5647
I_5646
I_5645
I_5644
I_5643
Term: termination (1), NS: new start (1), PS: pausing site (0), iTSS_I: internal starting site (1)

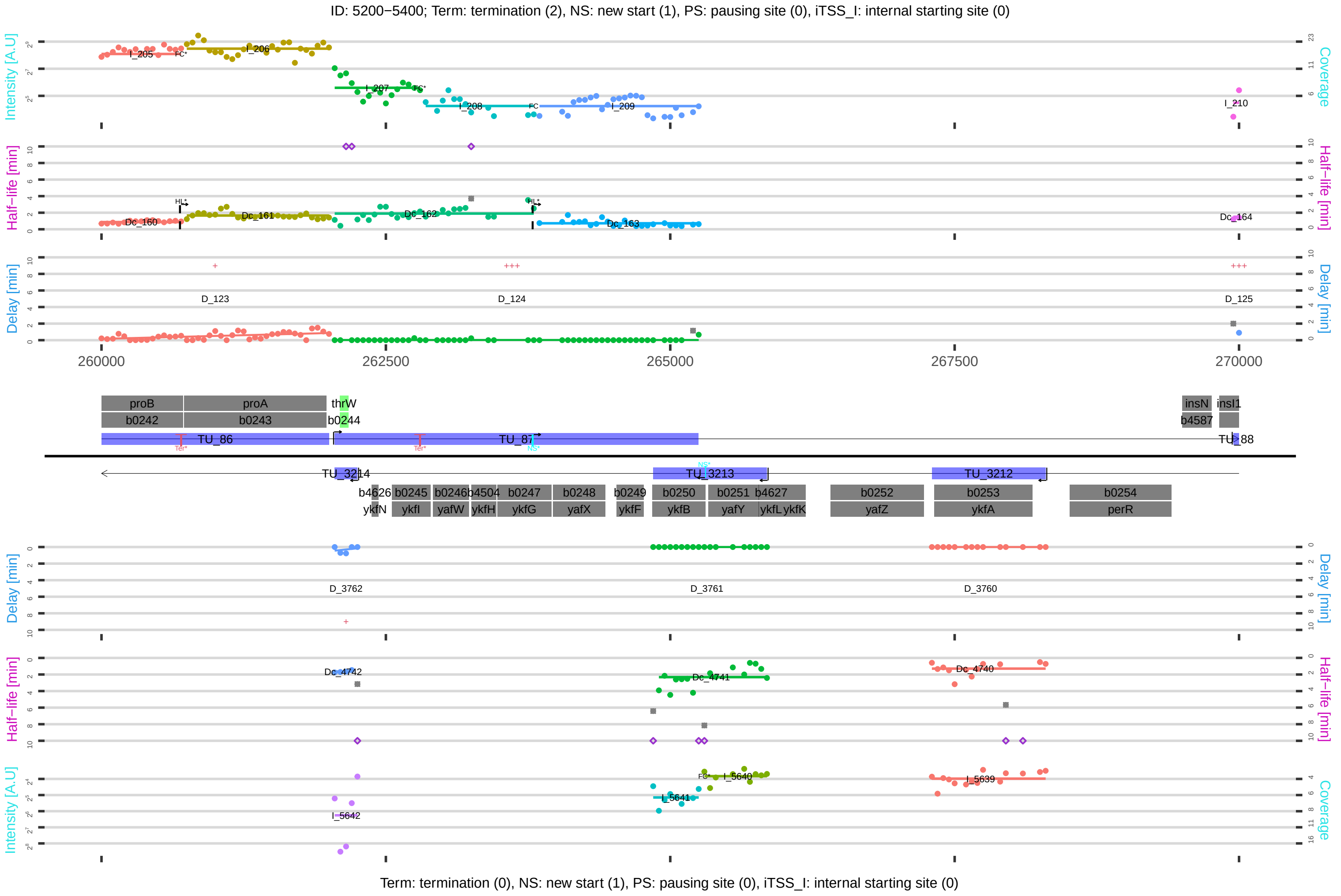

ID: 5200−5400; Term: termination (2), NS: new start (1), PS: pausing site (0), iTSS_I: internal starting site (0)
Intensity [A.U]
Coverage
Half−life [min]
Delay [min]
I_205
I_206
I_207
I_208
I_209
I_210
Dc_160
Dc_161
Dc_162
Dc_163
Dc_164
D_123
D_124
D_125
260000
262500
265000
267500
270000
proB
proA
thrW
insN
insI1
b0242
b0243
b0244
b4587
TU_86
TU_87
TU_88
TU_3214
TU_3213
TU_3212
b4626
b0245
b0246
b4504
b0247
b0248
b0249
b0250
b0251
b4627
b0252
b0253
b0254
ykfN
ykfI
yafW
ykfH
ykfG
yafX
ykfF
ykfB
yafY
ykfL
ykfK
yafZ
ykfA
perR
D_3762
D_3761
D_3760
Dc_4742
Dc_4741
Dc_4740
I_5642
I_5641
I_5640
I_5639
Term: termination (0), NS: new start (1), PS: pausing site (0), iTSS_I: internal starting site (0)

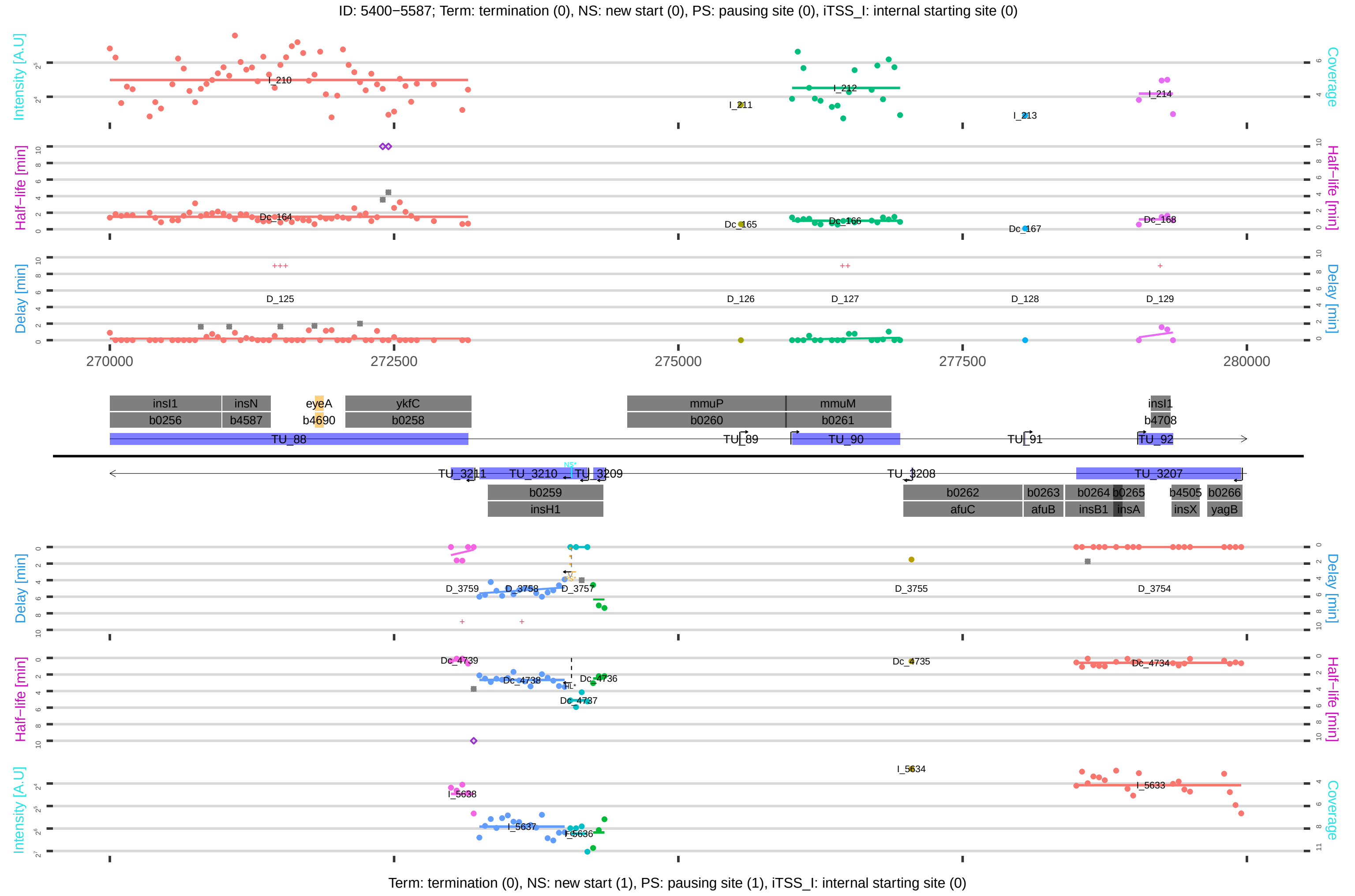

ID: 5400−5587; Term: termination (0), NS: new start (0), PS: pausing site (0), iTSS_I: internal starting site (0)
Intensity [A.U]
Coverage
Half−life [min]
Delay [min]
I_210
I_211
I_212
I_213
I_214
Dc_164
Dc_165
Dc_166
Dc_167
Dc_168
D_125
D_126
D_127
D_128
D_129
270000
272500
275000
277500
280000
insI1
insN
eyeA
ykfC
mmuP
mmuM
b0256
b4587
b4690
b0258
b0260
b0261
b4708
TU_88
TU_89
TU_90
TU_91
TU_92
TU_3211
TU_3210
TU_3209
TU_3208
TU_3207
b0259
insH1
b0262
b0263
b0264
b0265
b4505
b0266
afuC
afuB
insB1
insA
insX
yagB
D_3759
D_3758
D_3757
D_3755
D_3754
Dc_4739
Dc_4738
Dc_4737
Dc_4736
Dc_4735
Dc_4734
I_5638
I_5637
I_5636
I_5634
I_5633
Term: termination (0), NS: new start (1), PS: pausing site (1), iTSS_I: internal starting site (0)

ID: 5632−5798; Term: termination (1), NS: new start (0), PS: pausing site (1), iTSS_I: internal starting site (0)

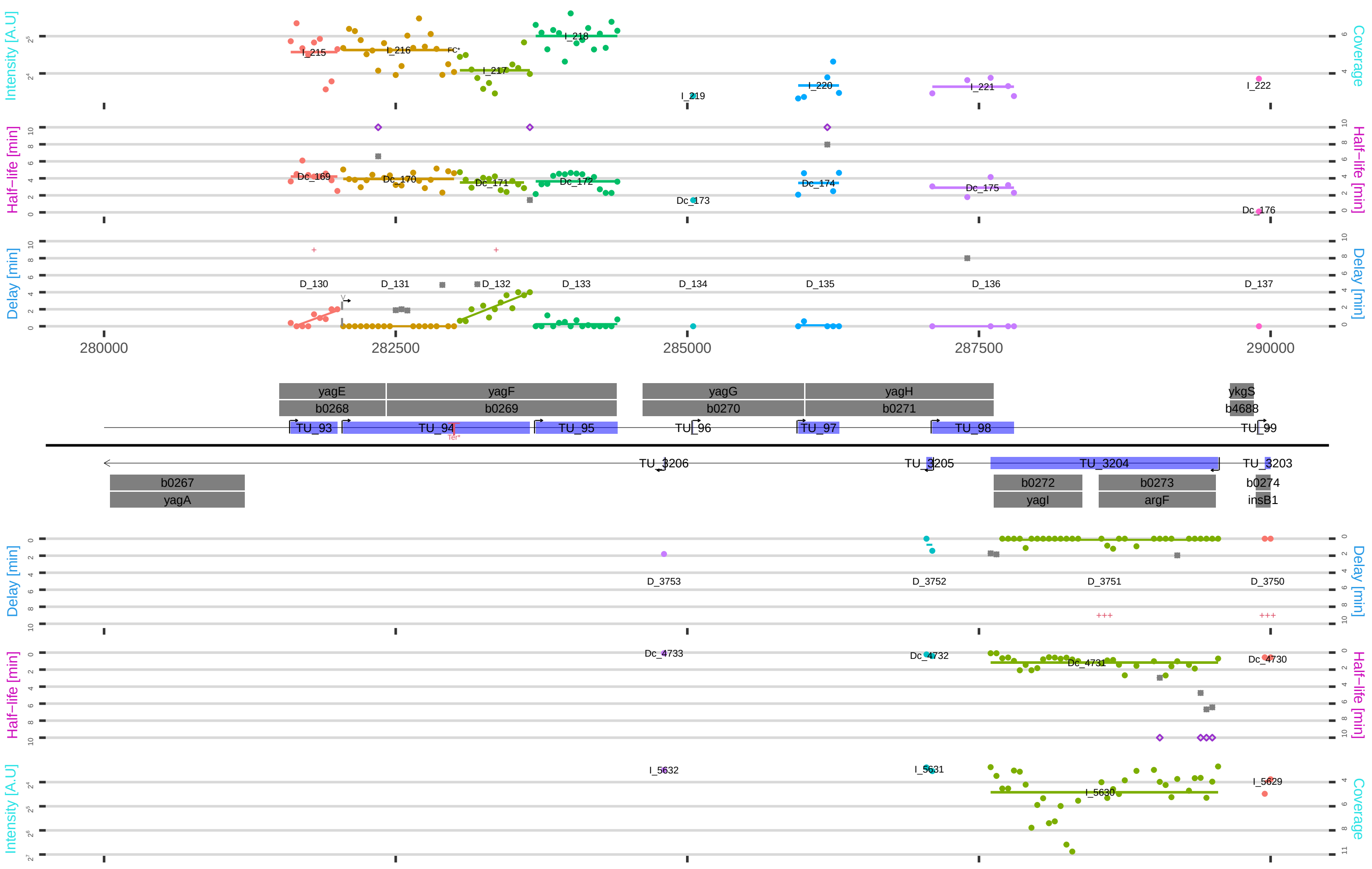

Term: termination (0), NS: new start (0), PS: pausing site (0), iTSS_I: internal starting site (0)

ID: 5802−5949; Term: termination (1), NS: new start (0), PS: pausing site (1), iTSS_I: internal starting site (0)

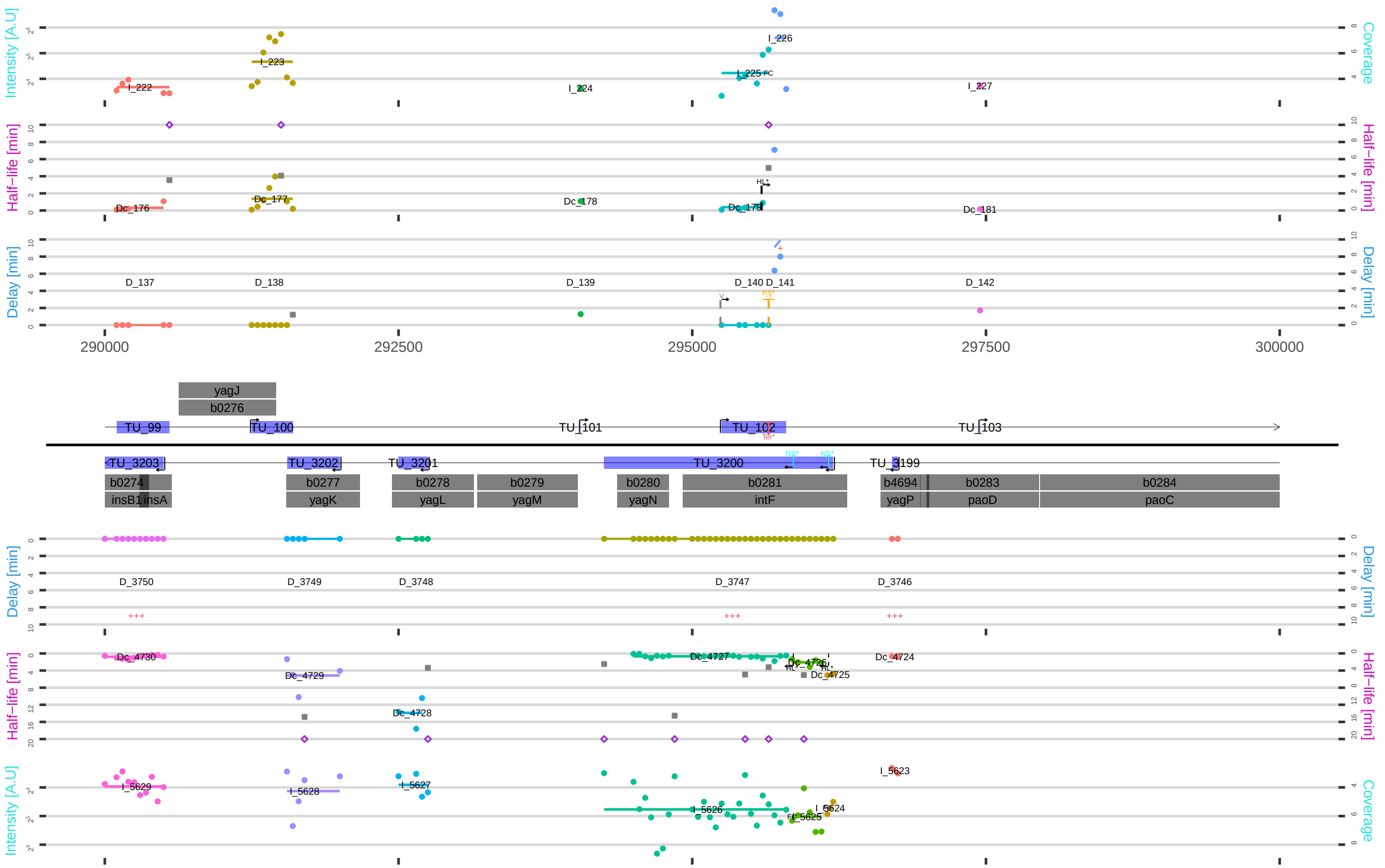

Term: termination (0), NS: new start (2), PS: pausing site (0), iTSS_I: internal starting site (0)

ID: 6046−6195; Term: termination (0), NS: new start (0), PS: pausing site (0), iTSS_I: internal starting site (0)

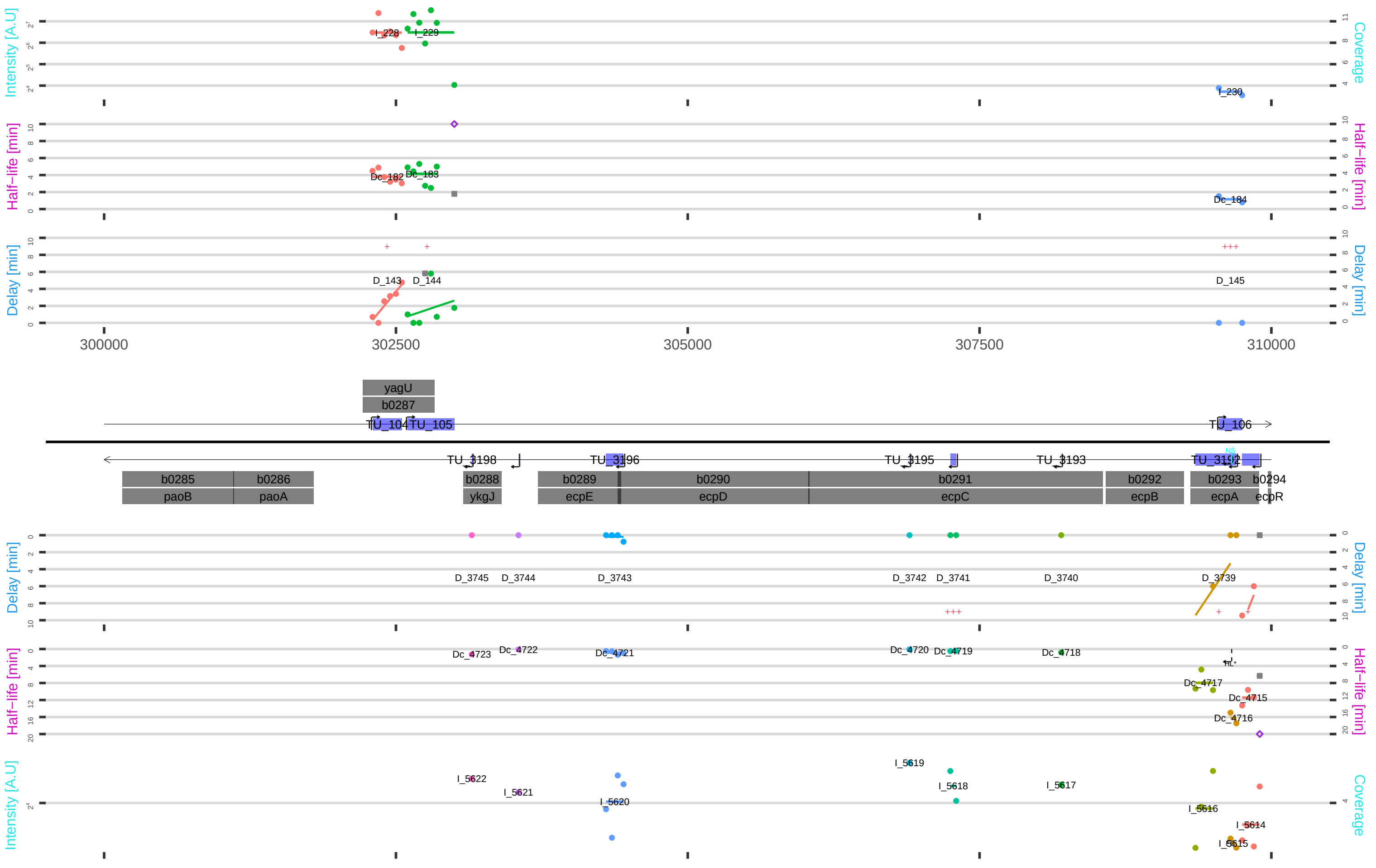

Term: termination (0), NS: new start (1), PS: pausing site (0), iTSS_I: internal starting site (0)

ID: 6229−6399; Term: termination (0), NS: new start (0), PS: pausing site (0), iTSS_I: internal starting site (0)

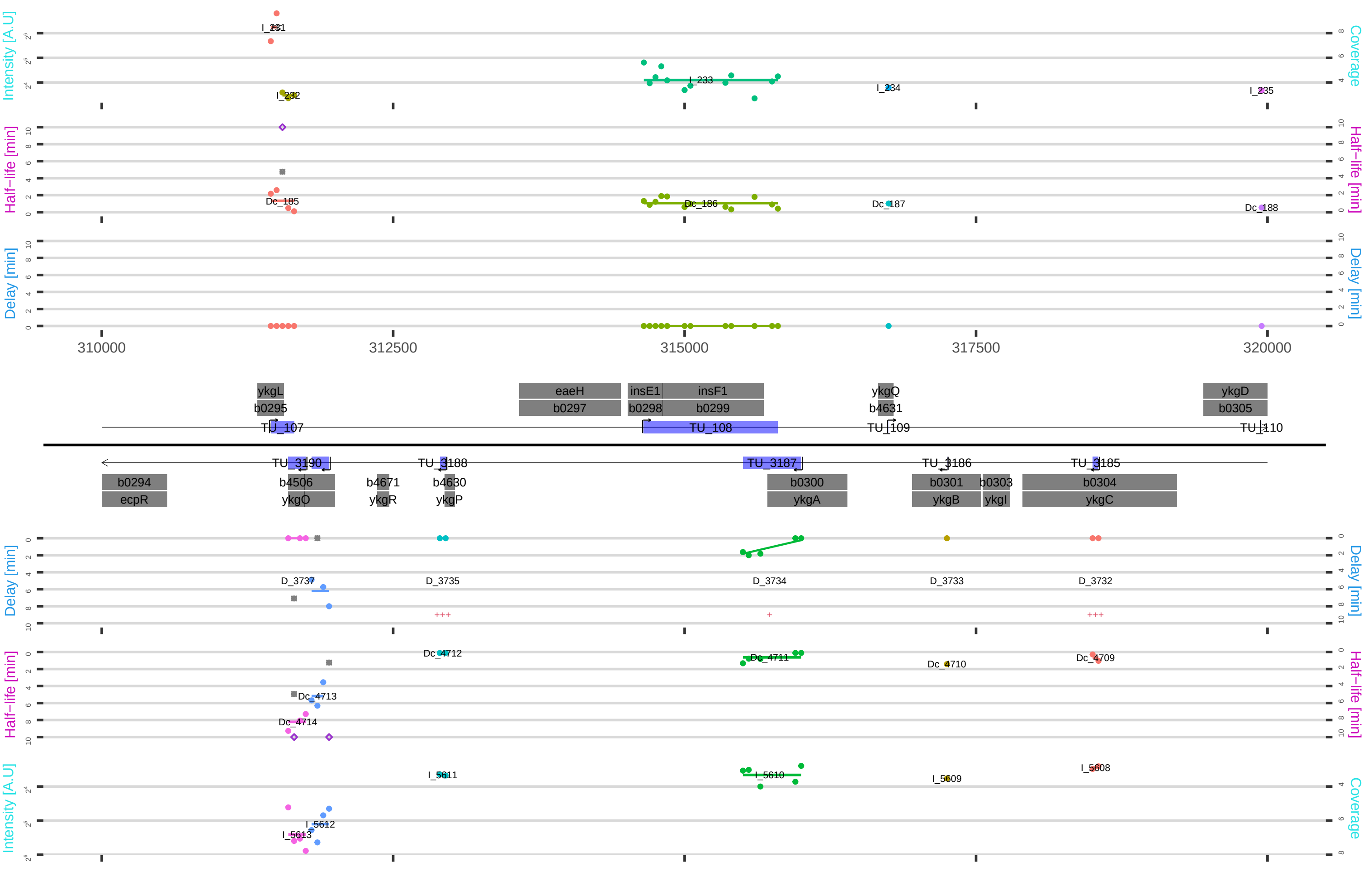

Term: termination (0), NS: new start (0), PS: pausing site (0), iTSS_I: internal starting site (0)

ID: 6418−6600; Term: termination (0), NS: new start (1), PS: pausing site (1), iTSS_I: internal starting site (0)

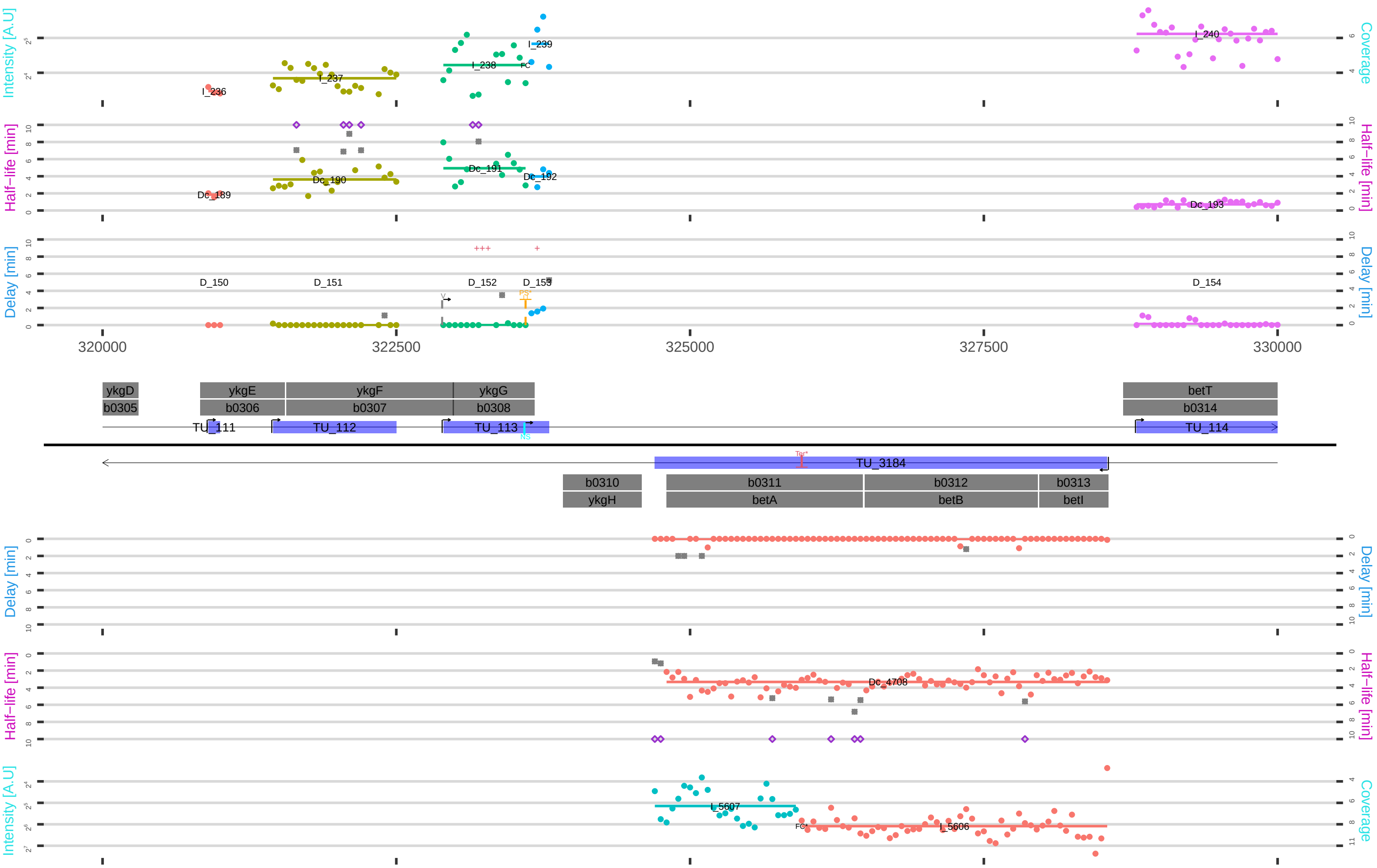

Term: termination (1), NS: new start (0), PS: pausing site (0), iTSS_I: internal starting site (0)

ID: 6600−6697; Term: termination (0), NS: new start (0), PS: pausing site (0), iTSS_I: internal starting site (0)

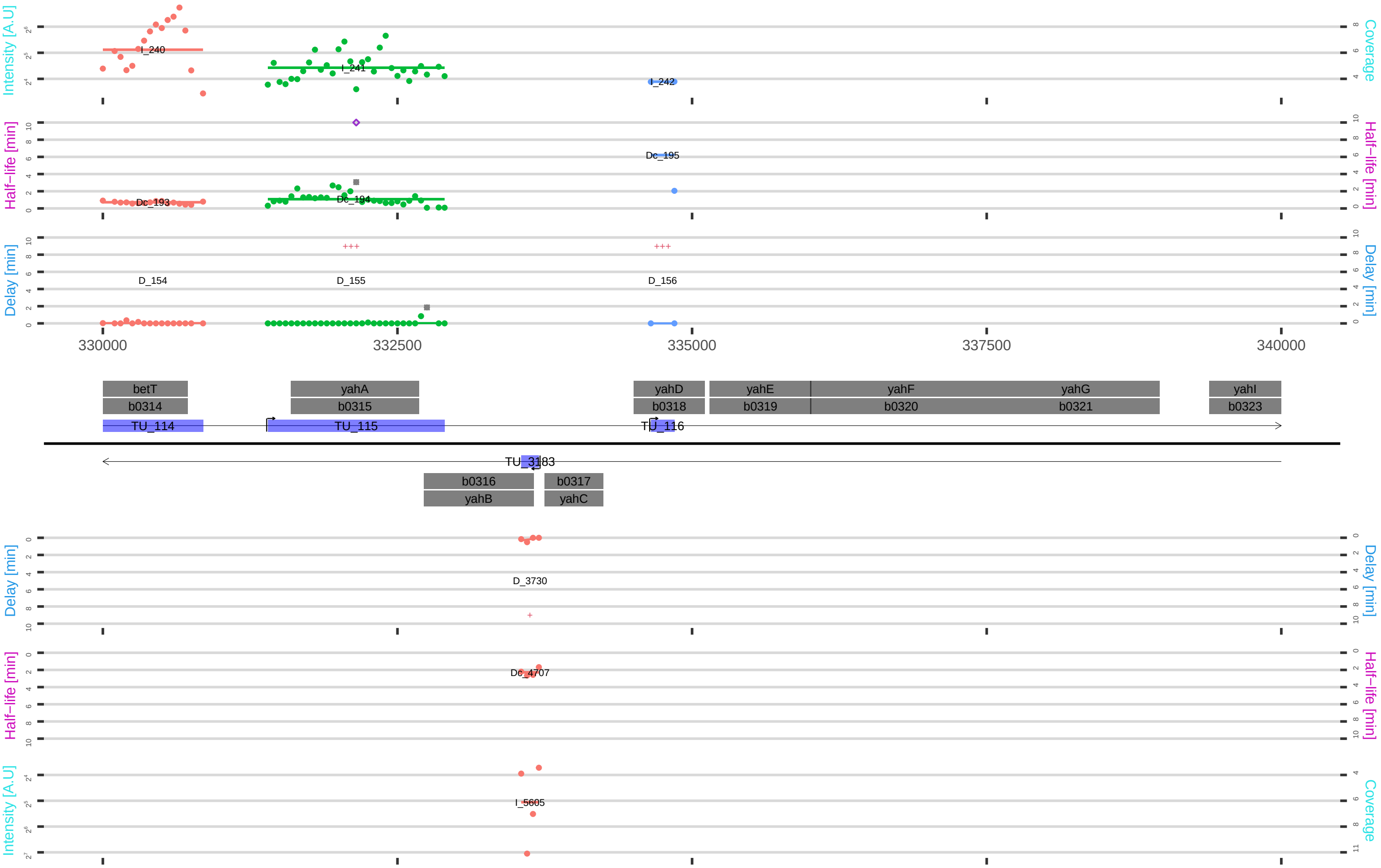

Term: termination (0), NS: new start (0), PS: pausing site (0), iTSS_I: internal starting site (0)

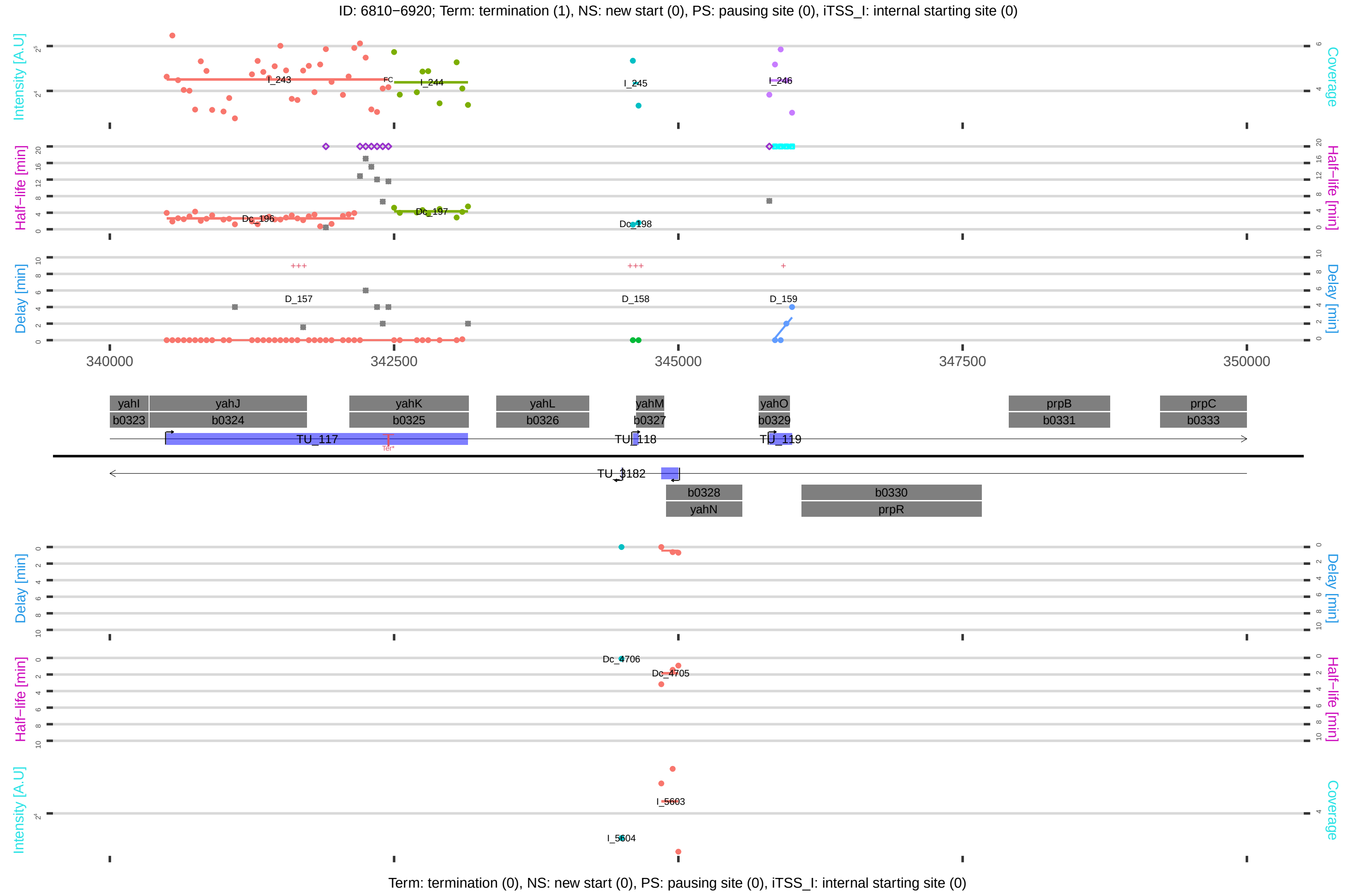

ID: 6810−6920; Term: termination (1), NS: new start (0), PS: pausing site (0), iTSS_I: internal starting site (0)
Intensity [A.U]
Coverage
I_243
I_244
I_245
I_246
Half−life [min]
Dc_196
Dc_197
Dc_198
Delay [min]
D_157
D_158
D_159
340000
342500
345000
347500
350000
yahI
b0323
yahJ
b0324
yahK
b0325
yahL
b0326
yahM
b0327
yahO
b0329
prpB
b0331
prpC
b0333
TU_117
TU_118
TU_119
TU_3182
b0328
yahN
b0330
prpR
Dc_4706
Dc_4705
I_5603
I_5604
Term: termination (0), NS: new start (0), PS: pausing site (0), iTSS_I: internal starting site (0)

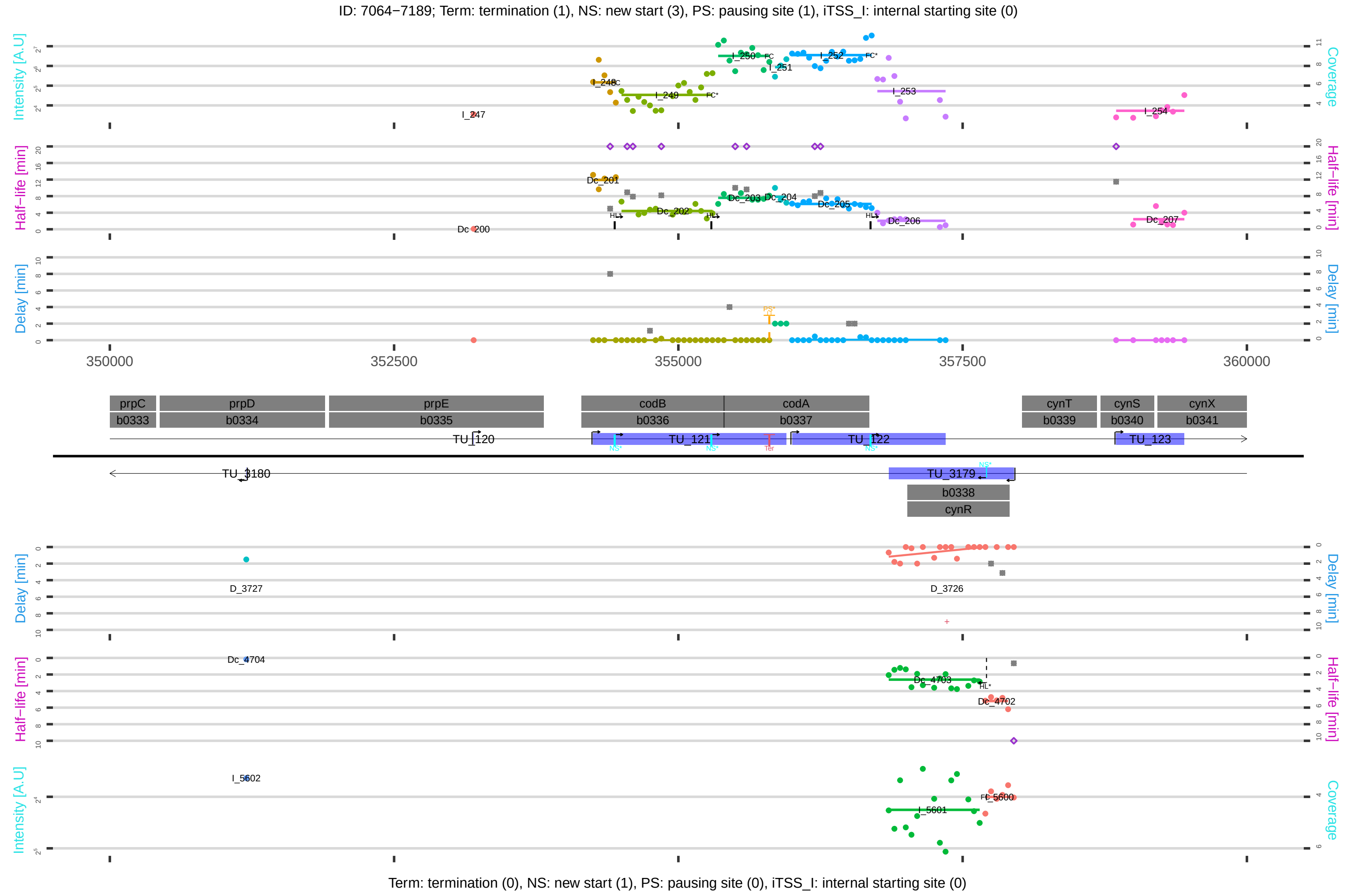

ID: 7064−7189; Term: termination (1), NS: new start (3), PS: pausing site (1), iTSS_I: internal starting site (0)
Intensity [A.U]
Coverage
Half−life [min]
Delay [min]
I_247
I_248
I_249
I_250
I_251
I_252
I_253
I_254
Dc_200
Dc_201
Dc_202
Dc_203
Dc_204
Dc_205
Dc_206
Dc_207
350000
352500
355000
357500
360000
prpC
b0333
prpD
b0334
prpE
b0335
codB
b0336
codA
b0337
cynT
b0339
cynS
b0340
cynX
b0341
TU_120
TU_121
TU_122
TU_123
TU_3180
TU_3179
b0338
cynR
D_3727
D_3726
Dc_4704
Dc_4703
Dc_4702
I_5602
I_5601
FC_5600
Term: termination (0), NS: new start (1), PS: pausing site (0), iTSS_I: internal starting site (0)

ID: 7204−7204; Term: termination (0), NS: new start (0), PS: pausing site (0), iTSS_I: internal starting site (0)

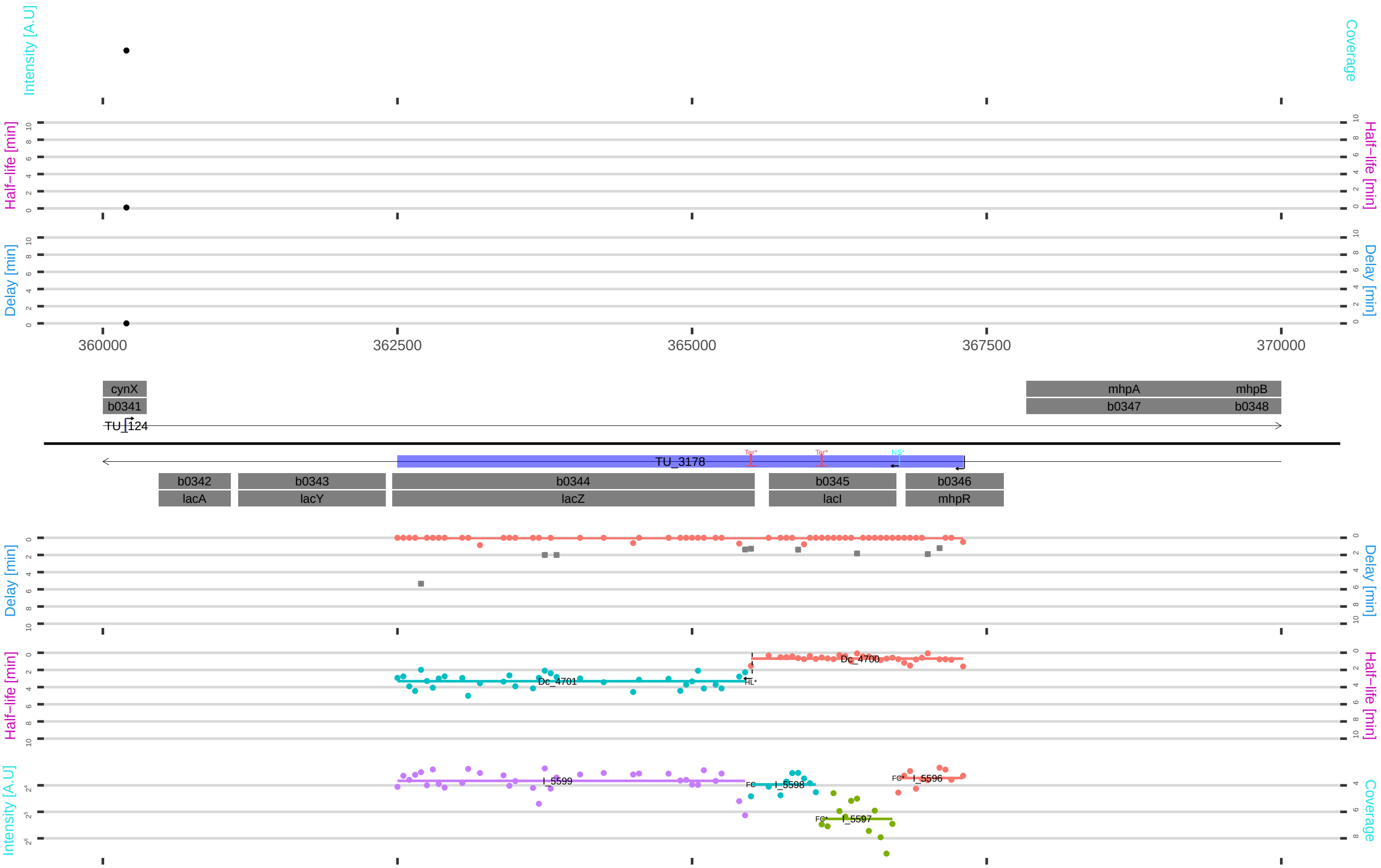

Term: termination (2), NS: new start (1), PS: pausing site (0), iTSS_I: internal starting site (0)

ID: 7484−7552; Term: termination (2), NS: new start (1), PS: pausing site (0), iTSS_I: internal starting site (0)

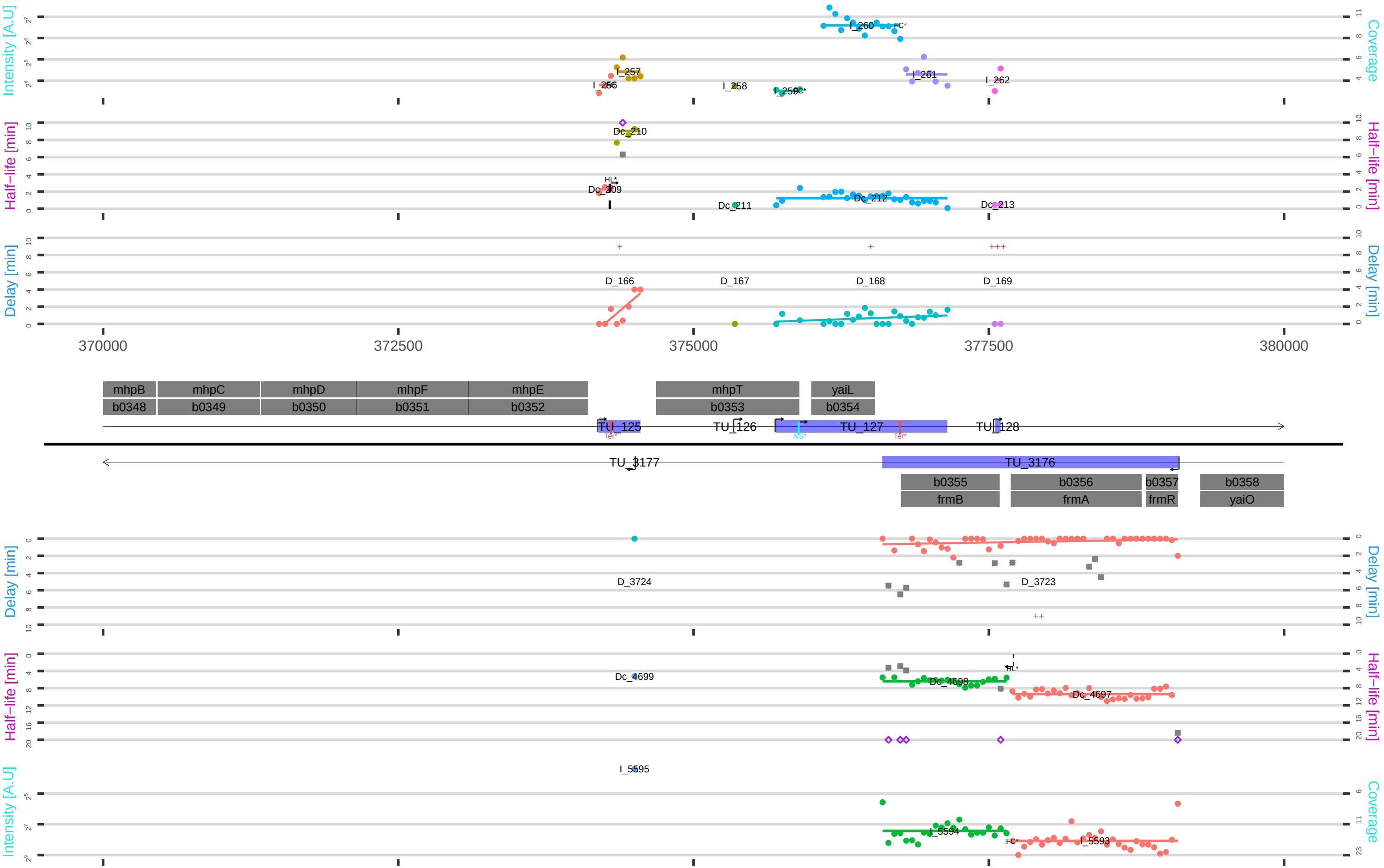

Term: termination (0), NS: new start (0), PS: pausing site (0), iTSS_I: internal starting site (0)

ID: 7613−7773; Term: termination (0), NS: new start (0), PS: pausing site (0), iTSS_I: internal starting site (0)

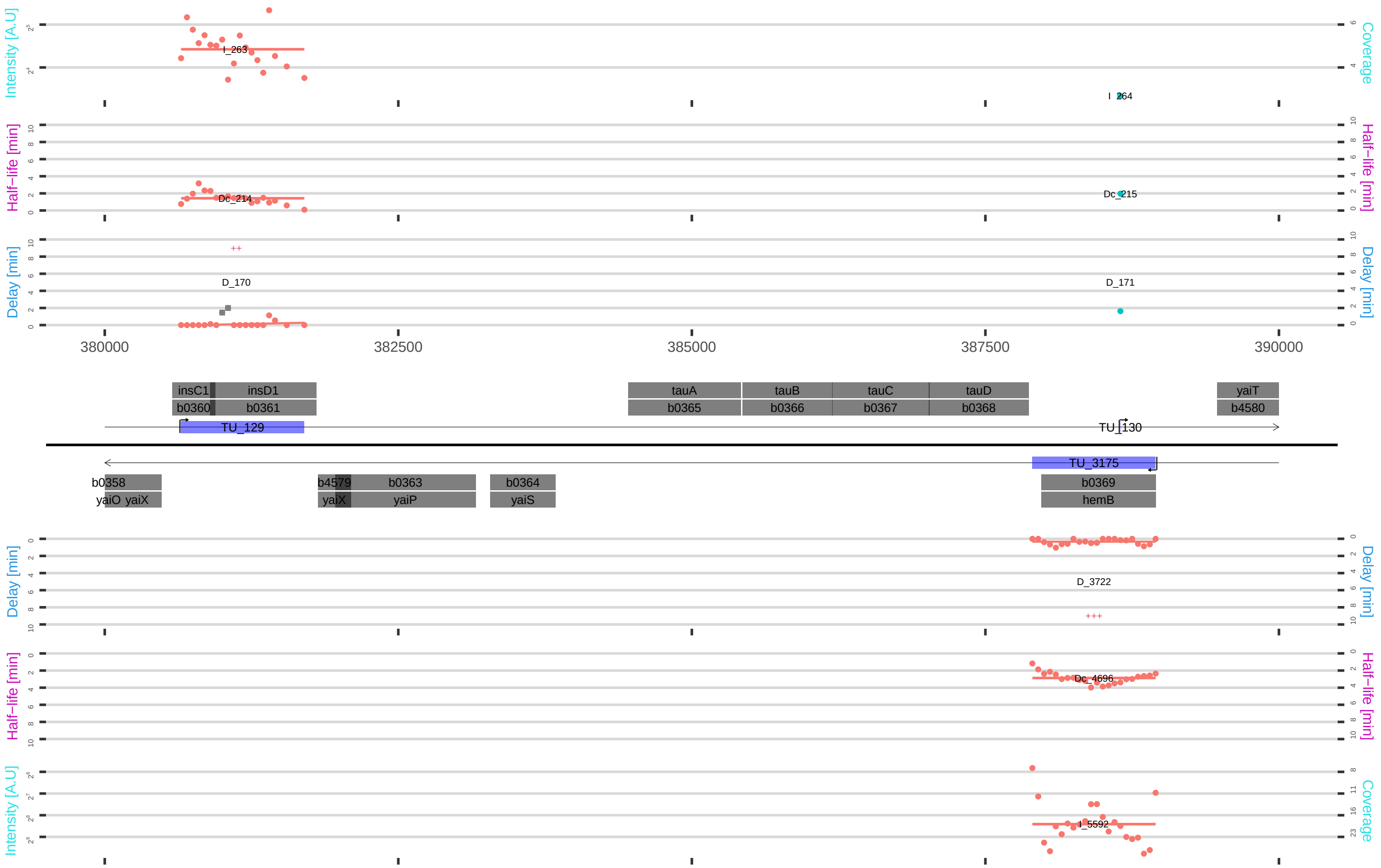

Term: termination (0), NS: new start (0), PS: pausing site (0), iTSS_I: internal starting site (0)

ID: 7818−7983; Term: termination (0), NS: new start (0), PS: pausing site (0), iTSS_I: internal starting site (0)

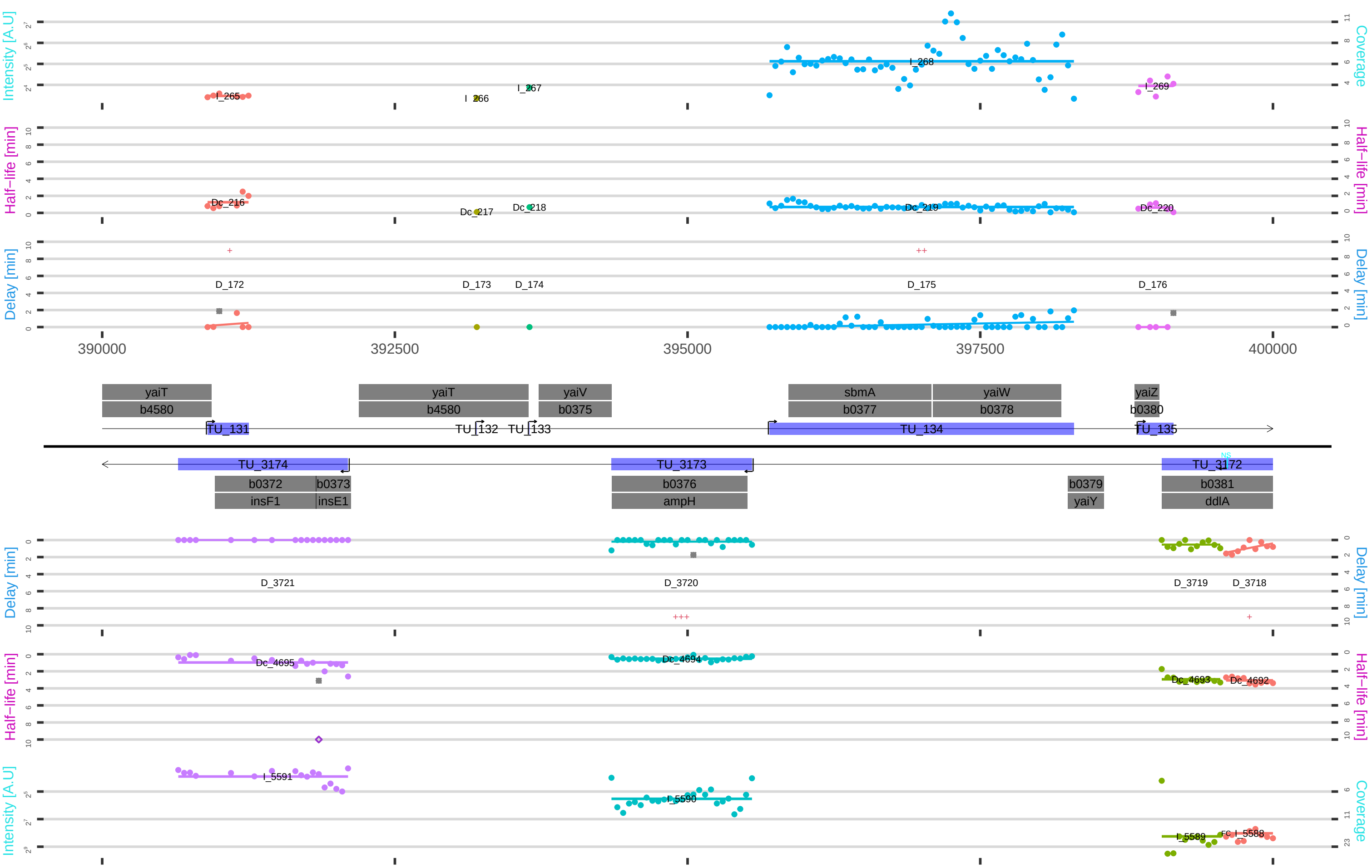

Term: termination (0), NS: new start (1), PS: pausing site (0), iTSS_I: internal starting site (1)

ID: 8014−8200; Term: termination (1), NS: new start (1), PS: pausing site (0), iTSS_I: internal starting site (0)

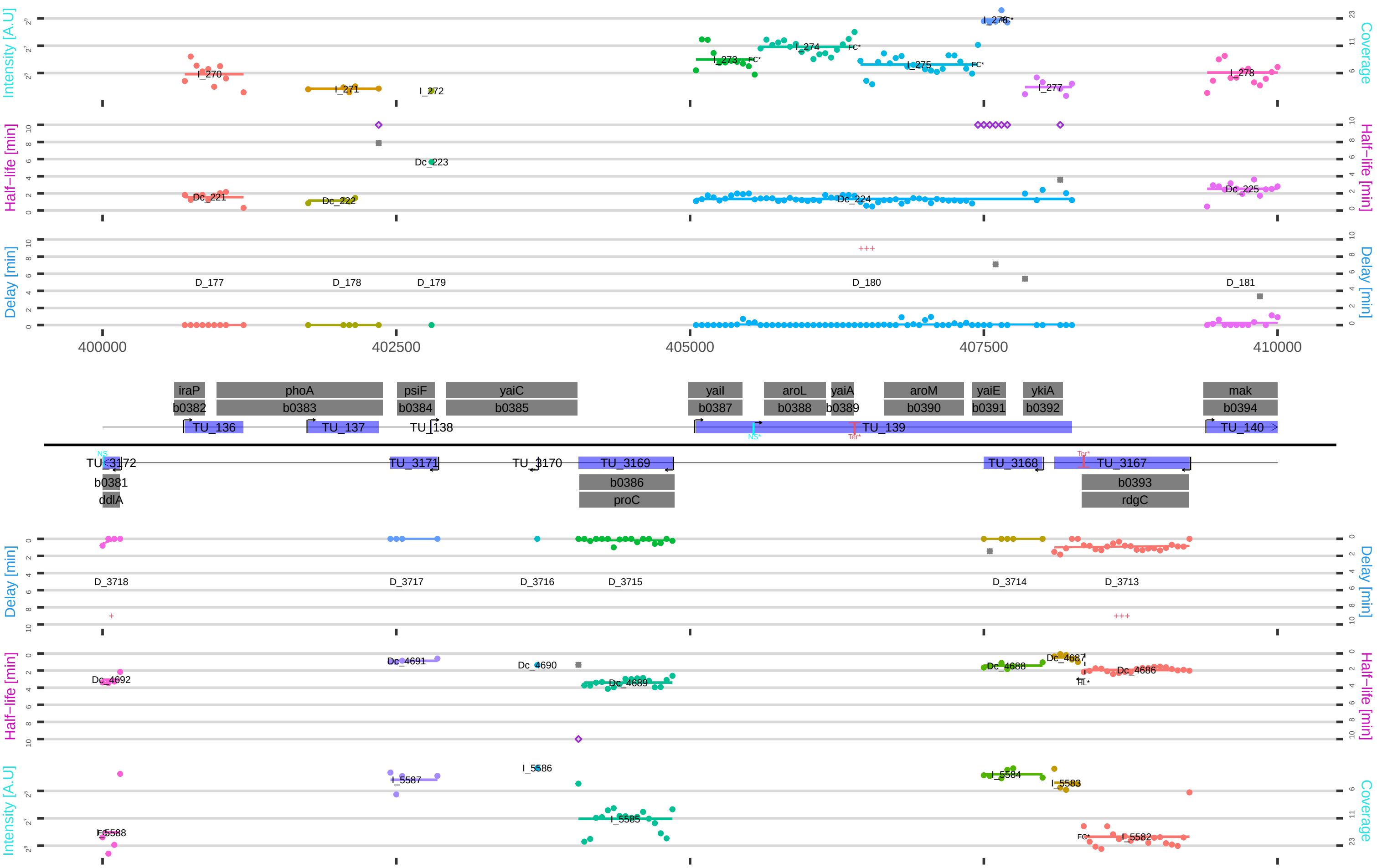

Term: termination (1), NS: new start (1), PS: pausing site (0), iTSS_I: internal starting site (0)

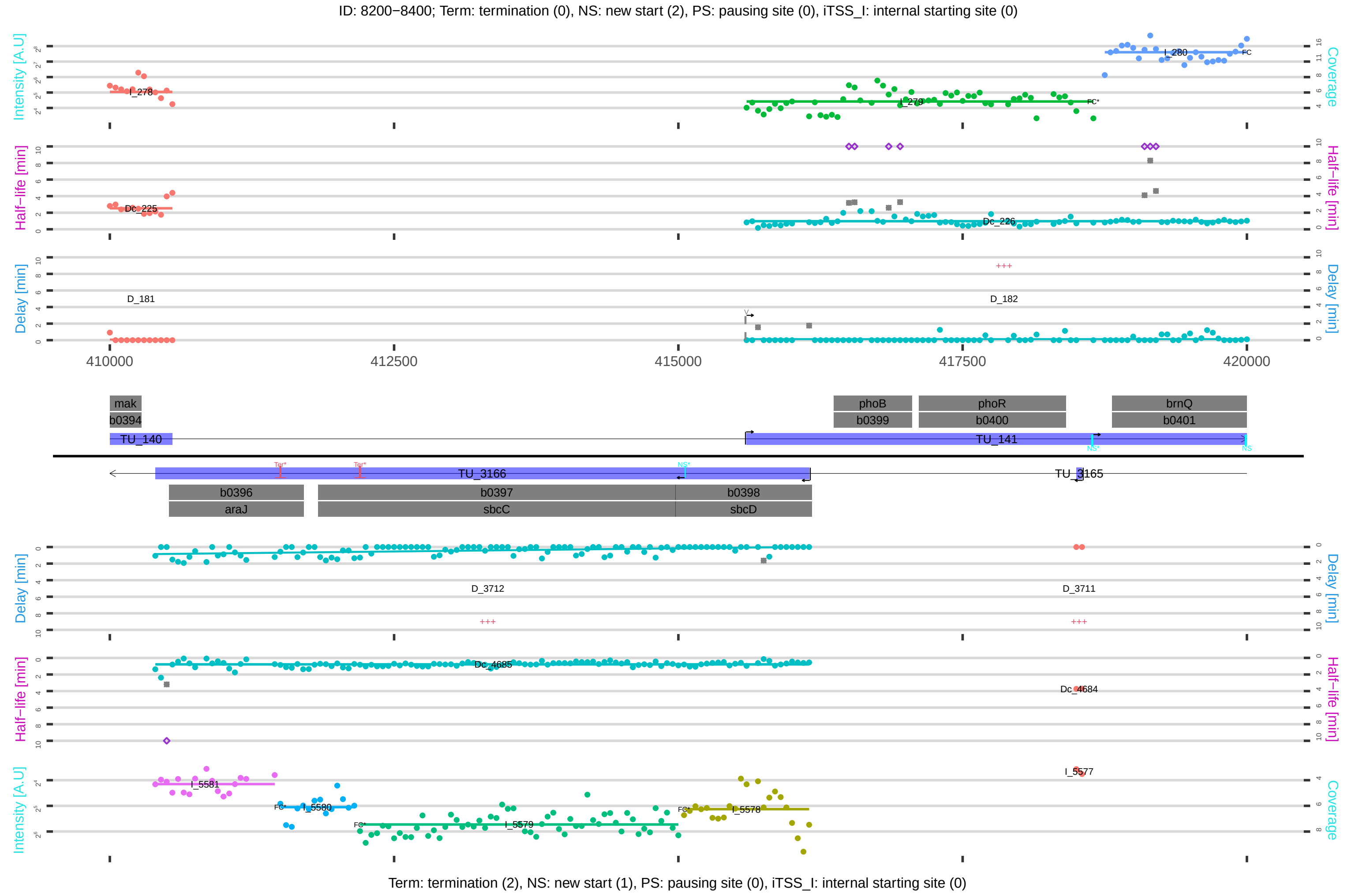

ID: 8200−8400; Term: termination (0), NS: new start (2), PS: pausing site (0), iTSS_I: internal starting site (0)
Intensity [A.U]
Coverage
Half−life [min]
Delay [min]
I_278
I_279
I_280
Dc_225
Dc_226
D_181
D_182
410000
412500
415000
417500
420000
mak
b0394
phoB
b0399
phoR
b0400
brnQ
b0401
TU_140
TU_141
TU_3166
TU_3165
b0396
araJ
b0397
sbcC
b0398
sbcD
D_3712
D_3711
Dc_4685
Dc_4684
I_5581
I_5580
I_5579
I_5578
I_5577
Term: termination (2), NS: new start (1), PS: pausing site (0), iTSS_I: internal starting site (0)

ID: 8400−8600; Term: termination (4), NS: new start (5), PS: pausing site (3), iTSS_I: internal starting site (0)

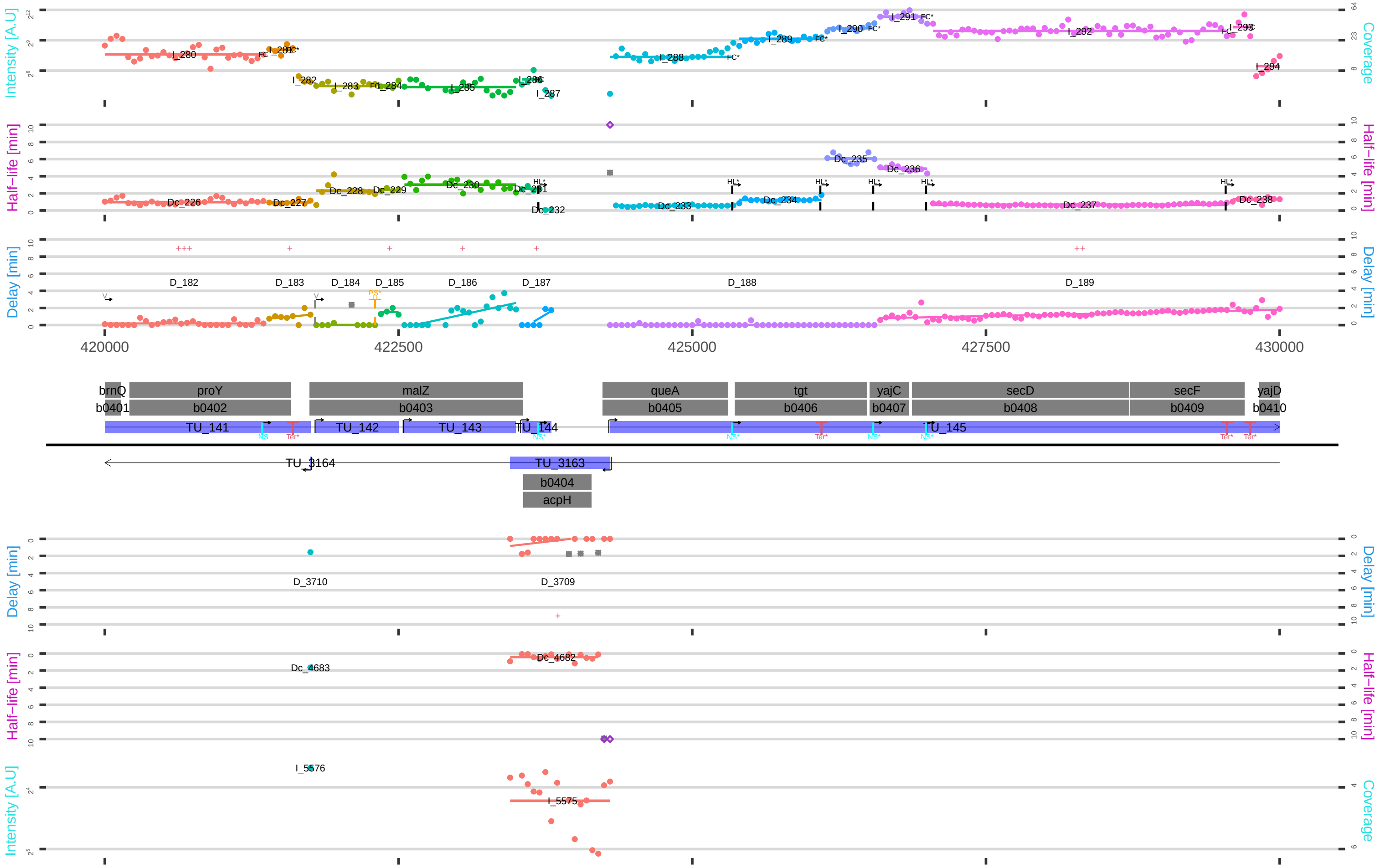

Term: termination (0), NS: new start (0), PS: pausing site (0), iTSS_I: internal starting site (0)

ID: 8600−8754; Term: termination (2), NS: new start (1), PS: pausing site (0), iTSS_I: internal starting site (0)

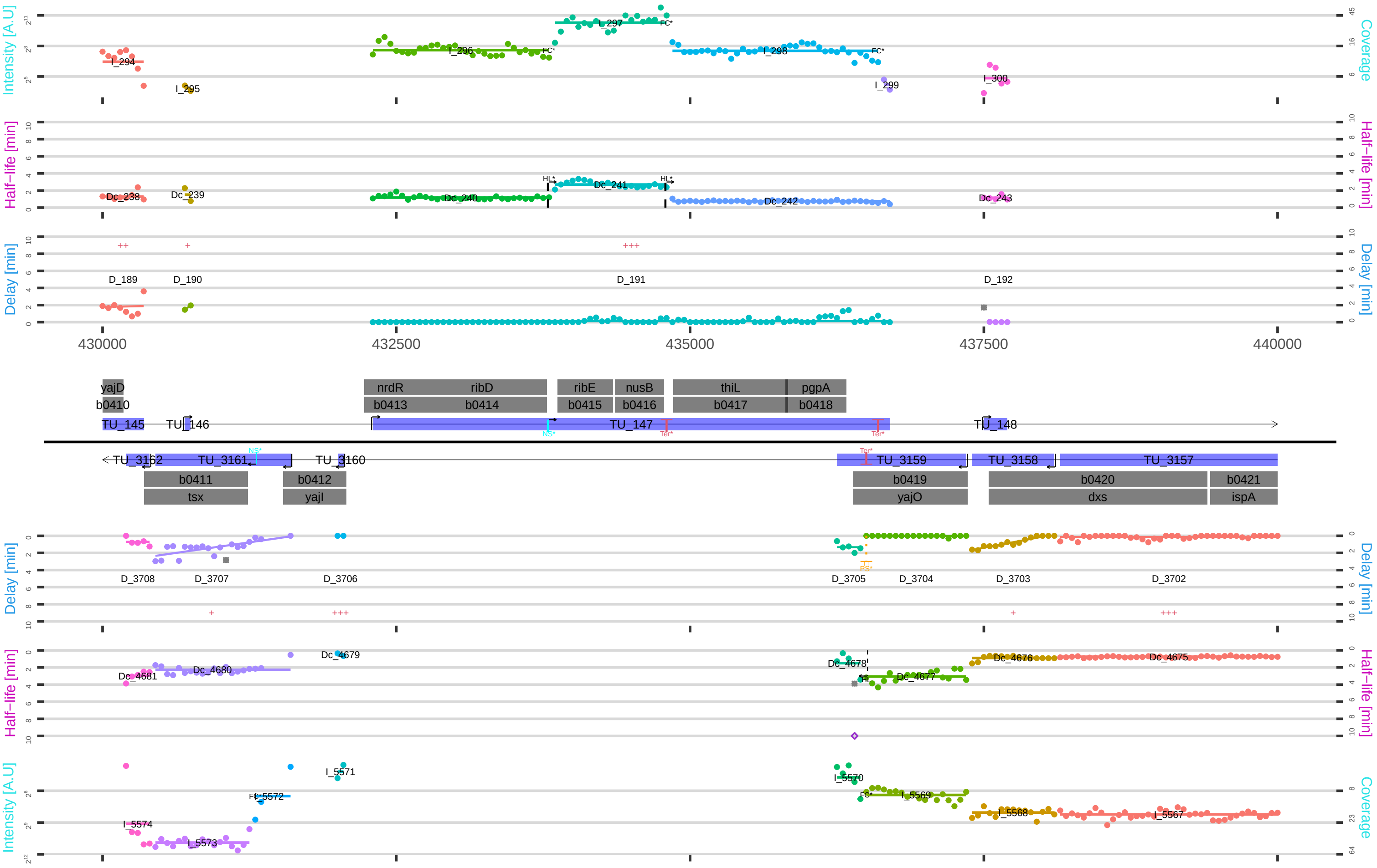

Term: termination (1), NS: new start (1), PS: pausing site (1), iTSS_I: internal starting site (0)

ID: 8810−8999; Term: termination (1), NS: new start (1), PS: pausing site (0), iTSS_I: internal starting site (0)

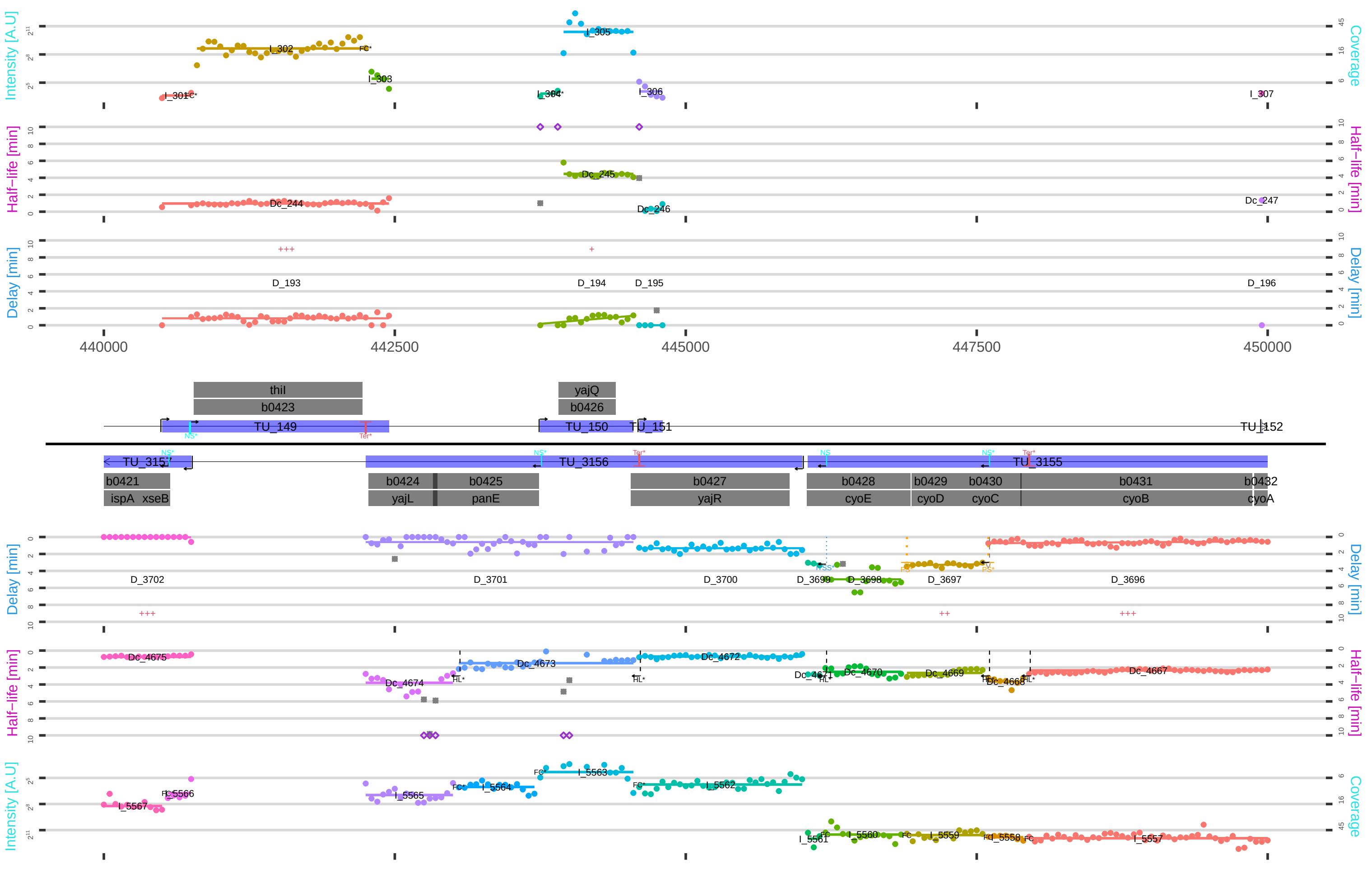

Term: termination (2), NS: new start (4), PS: pausing site (2), iTSS_I: internal starting site (2)

ID: 9069−9200; Term: termination (1), NS: new start (4), PS: pausing site (1), iTSS_I: internal starting site (0)

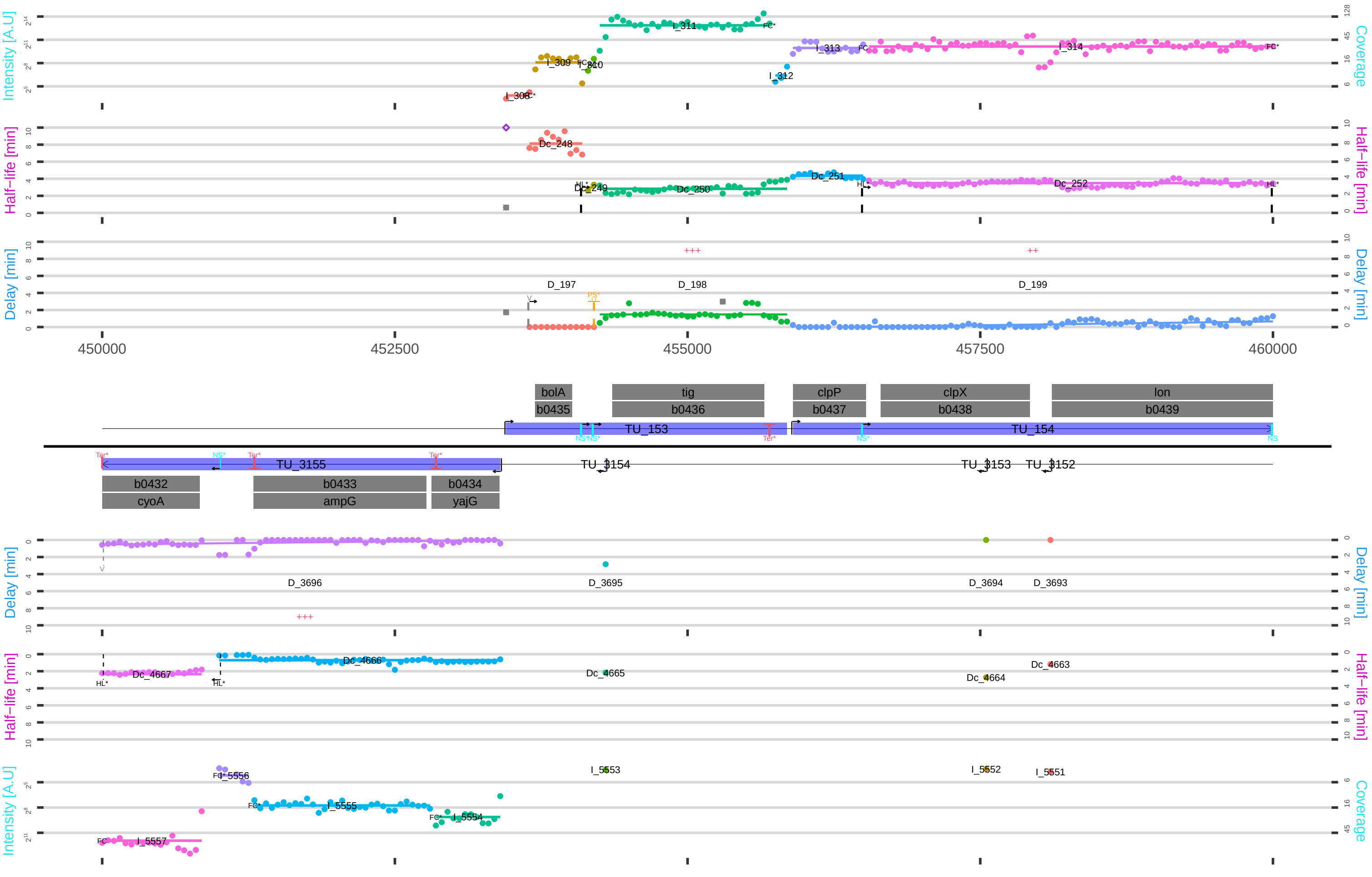

Term: termination (3), NS: new start (1), PS: pausing site (0), iTSS_I: internal starting site (0)

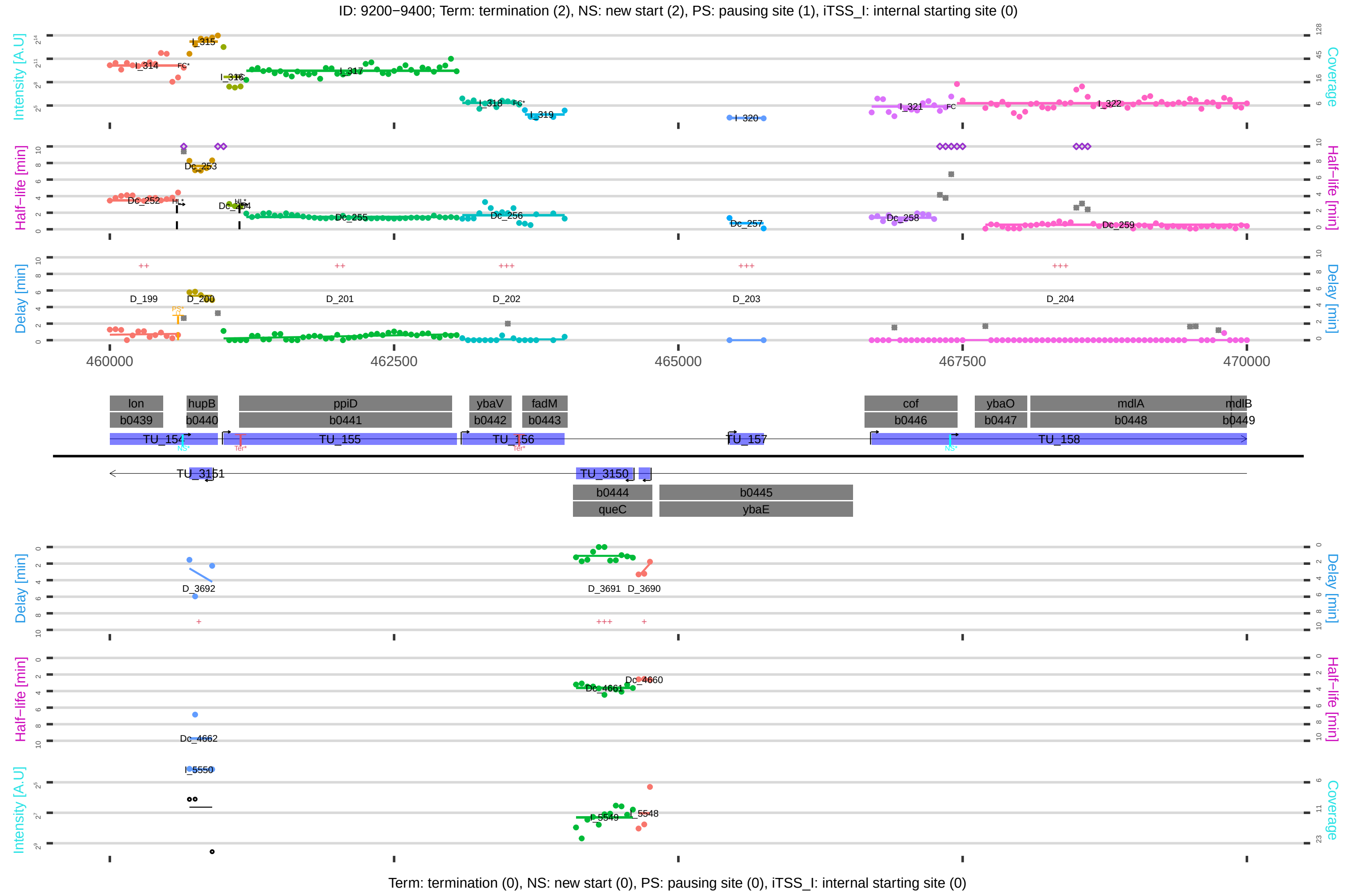

ID: 9200−9400; Term: termination (2), NS: new start (2), PS: pausing site (1), iTSS_I: internal starting site (0)
Intensity [A.U]
Coverage
Half−life [min]
Delay [min]
460000
462500
465000
467500
470000
lon
b0439
hupB
b0440
ppiD
b0441
ybaV
b0442
fadM
b0443
cof
b0446
ybaO
b0447
mdlA
b0448
mdlB
b0449
TU_154
TU_155
TU_156
TU_157
TU_158
TU_3151
TU_3150
b0444
queC
b0445
ybaE
D_3692
D_3691
D_3690
Dc_4660
Dc_4661
Dc_4662
I_5550
I_5549
I_5548
Term: termination (0), NS: new start (0), PS: pausing site (0), iTSS_I: internal starting site (0)

ID: 9400−9525; Term: termination (0), NS: new start (0), PS: pausing site (0), iTSS_I: internal starting site (0)

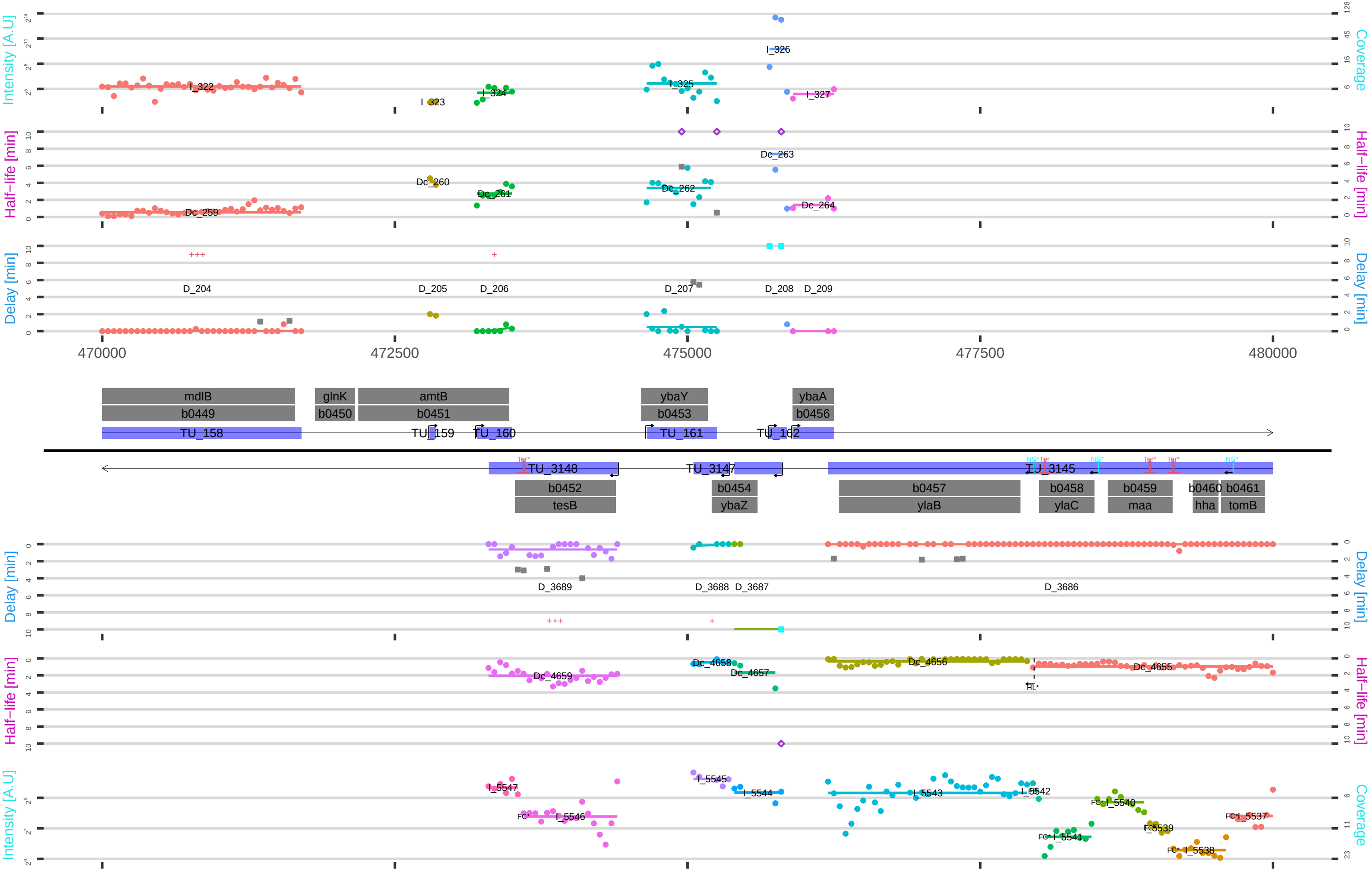

Term: termination (4), NS: new start (3), PS: pausing site (0), iTSS_I: internal starting site (0)

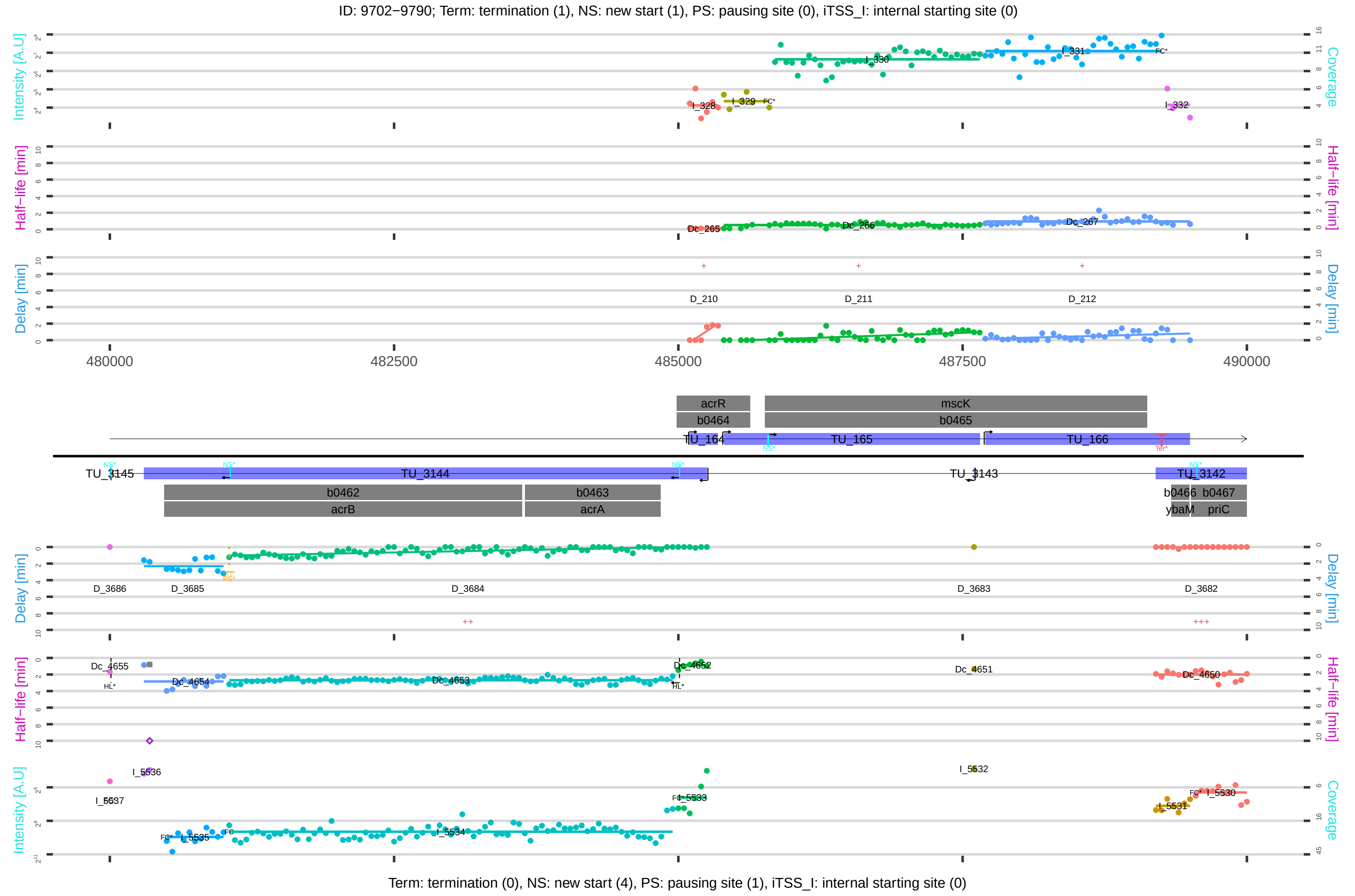

ID: 9702−9790; Term: termination (1), NS: new start (1), PS: pausing site (0), iTSS_I: internal starting site (0)
Intensity [A.U]
Coverage
Half−life [min]
Delay [min]
I_328
I_329
I_330
I_331
I_332
Dc_265
Dc_266
Dc_267
D_210
D_211
D_212
480000
482500
485000
487500
490000
acrR
b0464
mscK
b0465
TU_164
TU_165
TU_166
TU_3145
TU_3144
TU_3143
TU_3142
b0462
acrB
b0463
acrA
b0466
ybaM
b0467
priC
D_3686
D_3685
D_3684
D_3683
D_3682
Dc_4655
Dc_4654
Dc_4653
Dc_4652
Dc_4651
Dc_4650
I_5537
I_5536
I_5535
I_5534
I_5533
I_5532
I_5531
I_5530
Term: termination (0), NS: new start (4), PS: pausing site (1), iTSS_I: internal starting site (0)

ID: 9804−10000; Term: termination (3), NS: new start (5), PS: pausing site (2), iTSS_I: internal starting site (0)

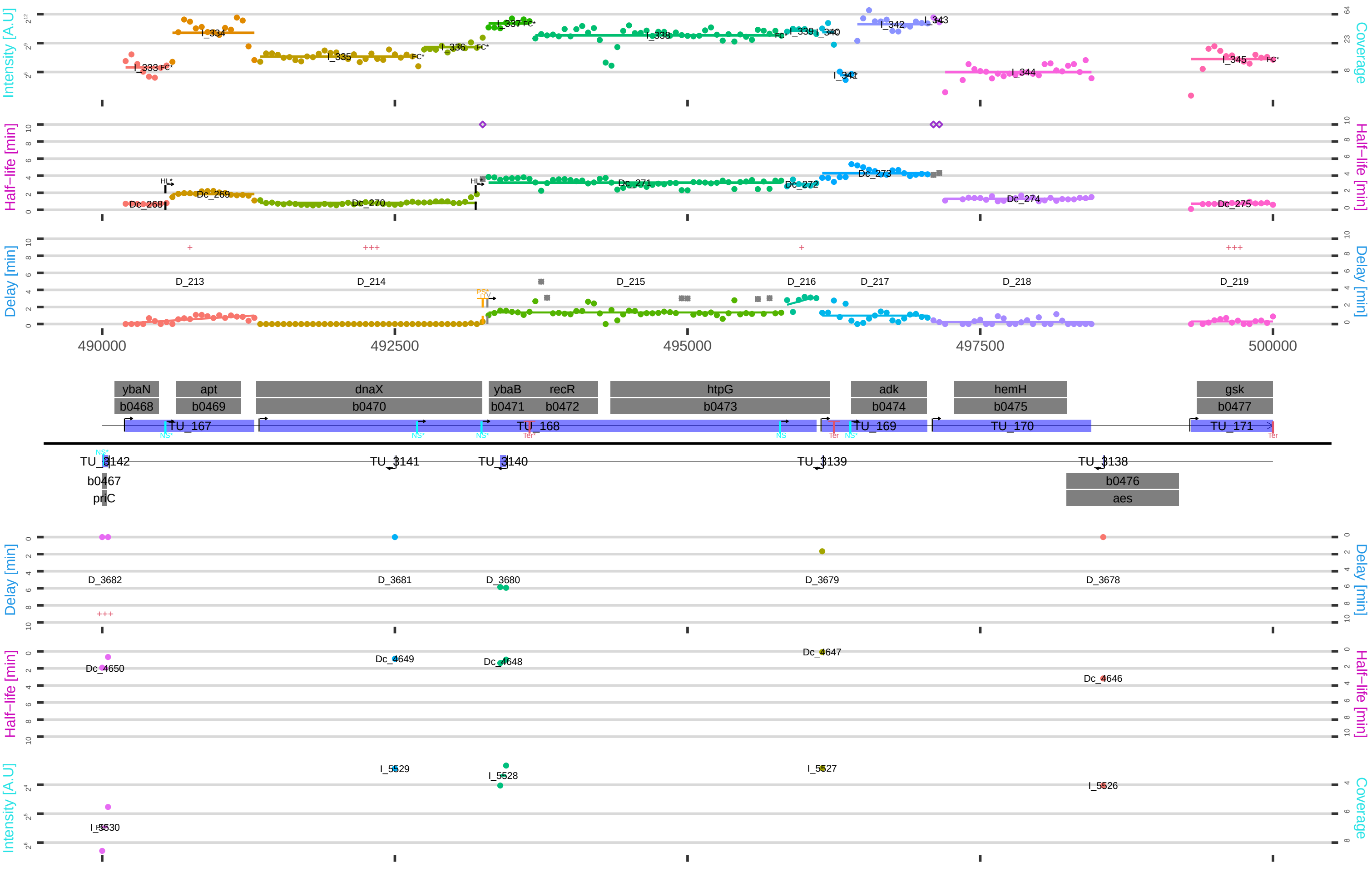

Term: termination (0), NS: new start (1), PS: pausing site (0), iTSS_I: internal starting site (0)

ID: 10000−10164; Term: termination (1), NS: new start (1), PS: pausing site (0), iTSS_I: internal starting site (0)

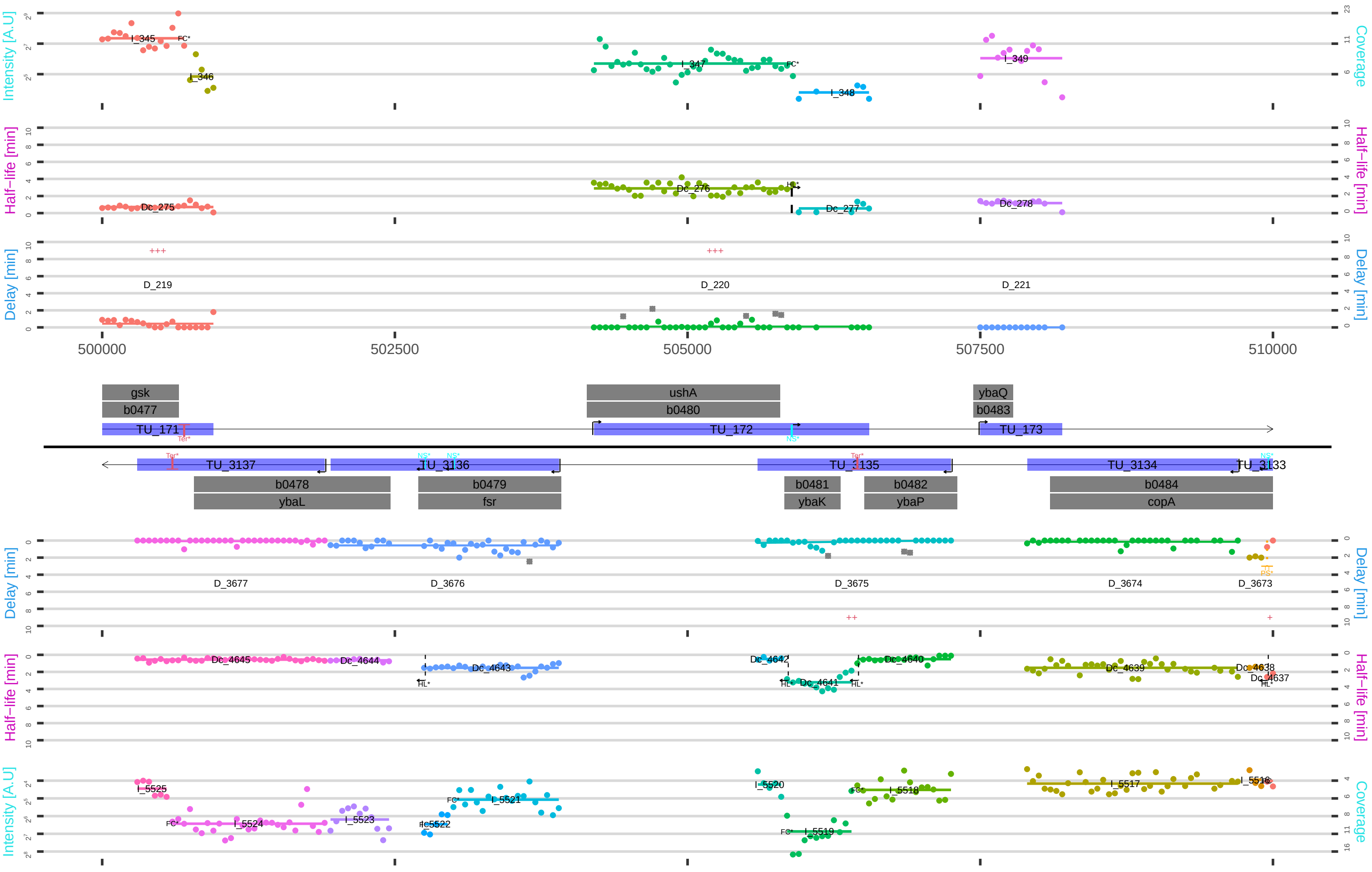

Term: termination (2), NS: new start (3), PS: pausing site (1), iTSS_I: internal starting site (0)

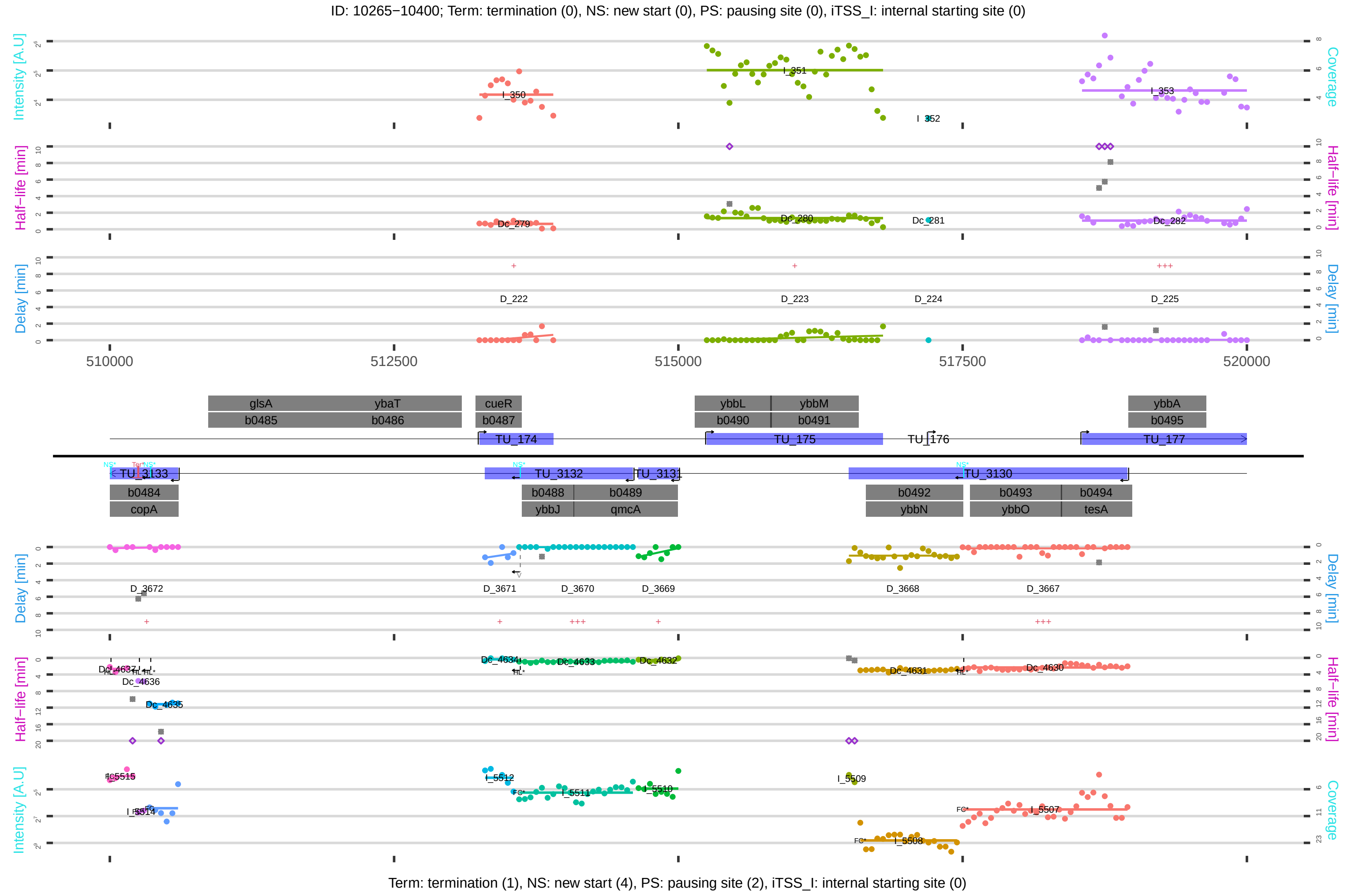
ID: 10265−10400; Term: termination (0), NS: new start (0), PS: pausing site (0), iTSS_I: internal starting site (0)
Intensity [A.U]
Coverage
Half−life [min]
Delay [min]
I_350
I_351
I_352
I_353
Dc_279
Dc_280
Dc_281
Dc_282
D_222
D_223
D_224
D_225
510000
512500
515000
517500
520000
glsA
ybaT
cueR
ybbL
ybbM
ybbA
b0485
b0486
b0487
b0490
b0491
b0495
TU_174
TU_175
TU_176
TU_177
TU_3133
TU_3132
TU_3131
TU_3130
b0484
b0488
b0489
b0492
b0493
b0494
copA
ybbJ
qmcA
ybbN
ybbO
tesA
D_3672
D_3671
D_3670
D_3669
D_3668
D_3667
Dc_4637
Dc_4636
Dc_4635
Dc_4634
Dc_4633
Dc_4632
Dc_4631
Dc_4630
I_5515
I_5514
I_5512
I_5511
I_5510
I_5509
I_5508
I_5507
Term: termination (1), NS: new start (4), PS: pausing site (2), iTSS_I: internal starting site (0)

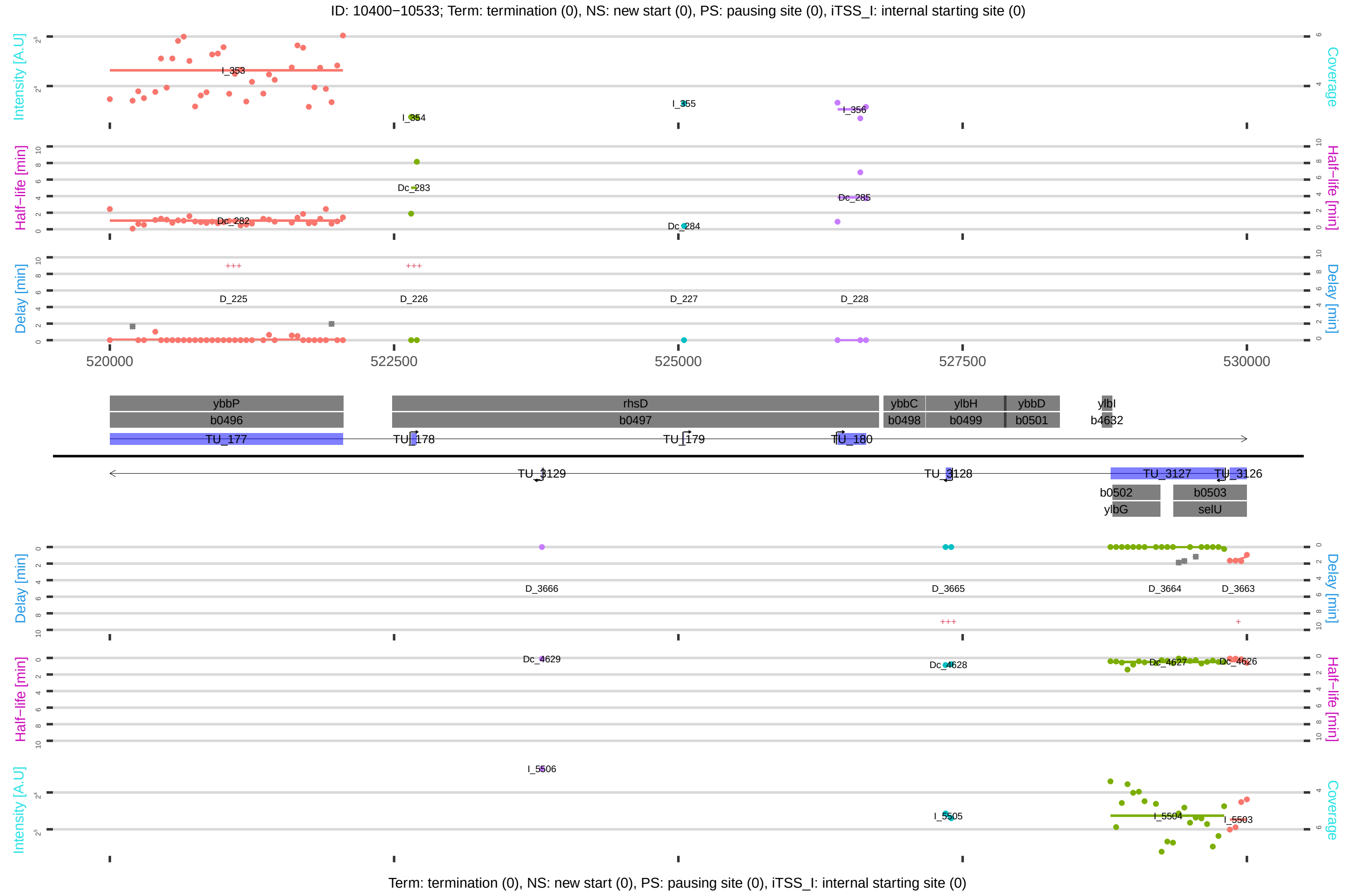

ID: 10400−10533; Term: termination (0), NS: new start (0), PS: pausing site (0), iTSS_I: internal starting site (0)
Intensity [A.U]
Coverage
I_353
I_354
I_355
I_356
Half−life [min]
Dc_282
Dc_283
Dc_284
Dc_285
Delay [min]
D_225
D_226
D_227
D_228
520000
522500
525000
527500
530000
ybbP
b0496
rhsD
b0497
ybbC
b0498
ylbH
b0499
ybbD
b0501
ylbI
b4632
TU_177
TU_178
TU_179
TU_180
TU_3129
TU_3128
TU_3127
TU_3126
b0502
ylbG
b0503
selU
D_3666
D_3665
D_3664
D_3663
Dc_4629
Dc_4628
Dc_4627
Dc_4626
I_5506
I_5505
I_5504
I_5503
Term: termination (0), NS: new start (0), PS: pausing site (0), iTSS_I: internal starting site (0)

ID: 10646−10795; Term: termination (0), NS: new start (0), PS: pausing site (0), iTSS_I: internal starting site (0)

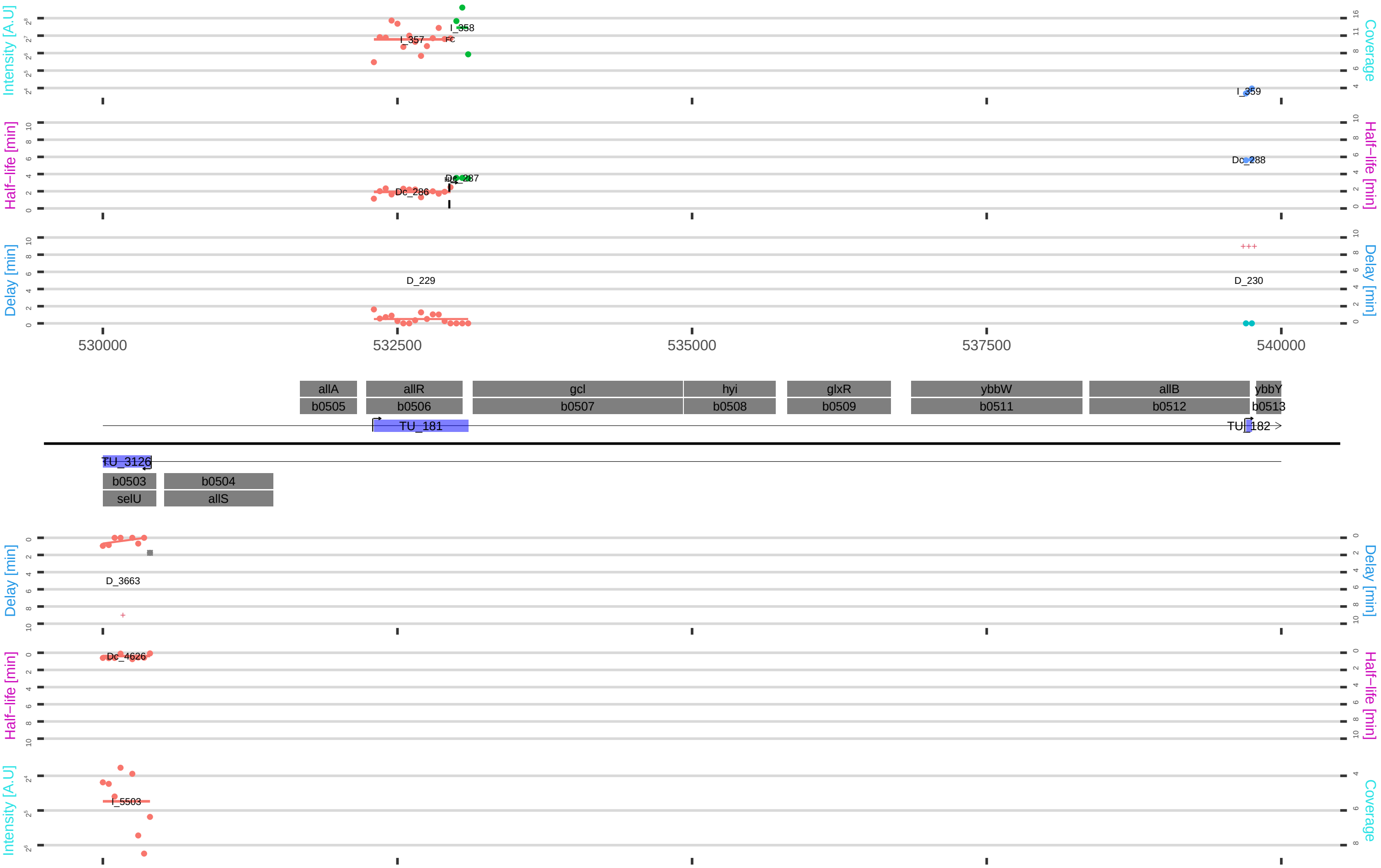

Term: termination (0), NS: new start (0), PS: pausing site (0), iTSS_I: internal starting site (0)

ID: 10892−10954; Term: termination (0), NS: new start (0), PS: pausing site (0), iTSS_I: internal starting site (0)

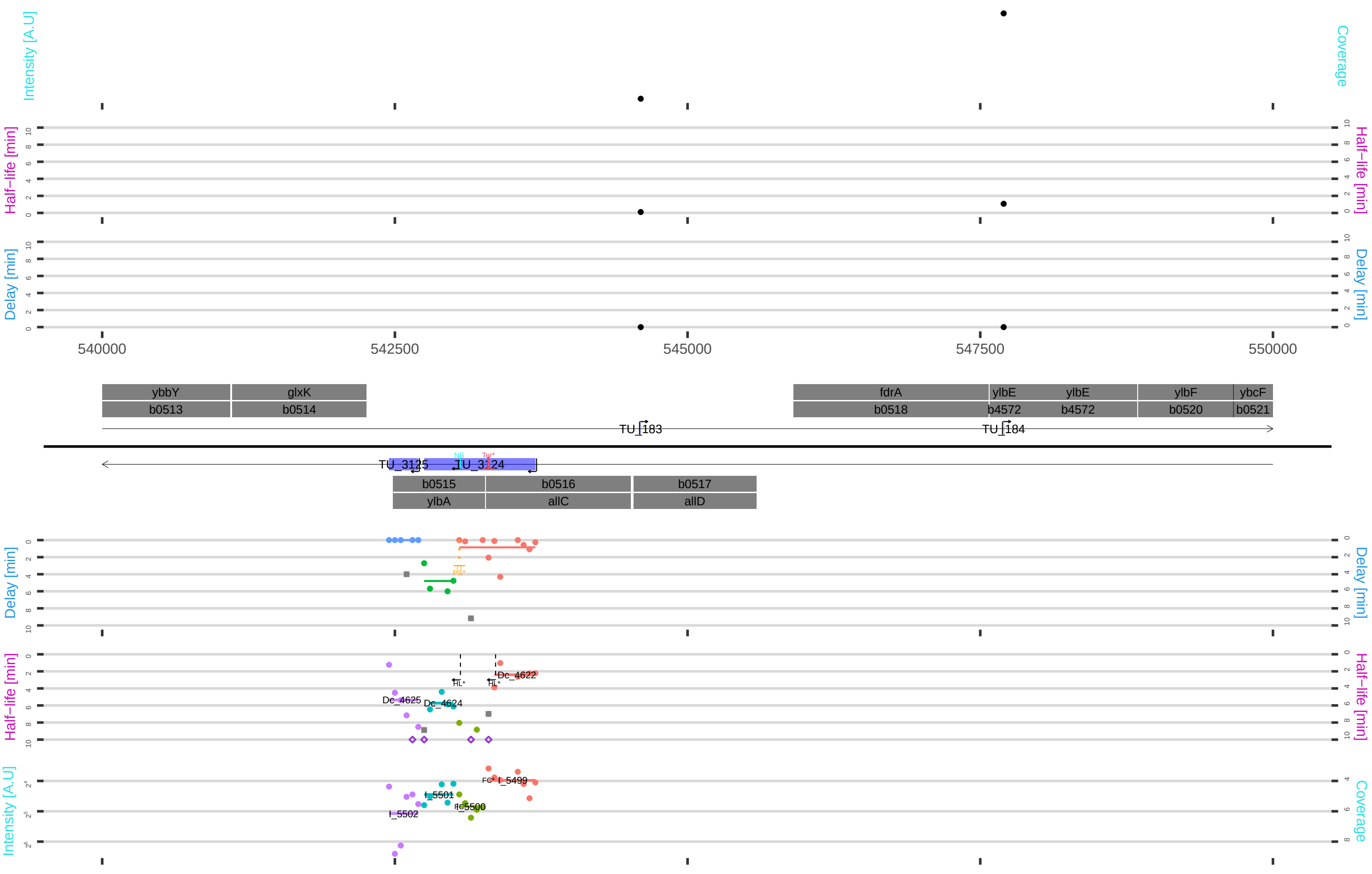

Term: termination (1), NS: new start (1), PS: pausing site (1), iTSS_I: internal starting site (0)

ID: 11078−11109; Term: termination (1), NS: new start (0), PS: pausing site (0), iTSS_I: internal starting site (0)

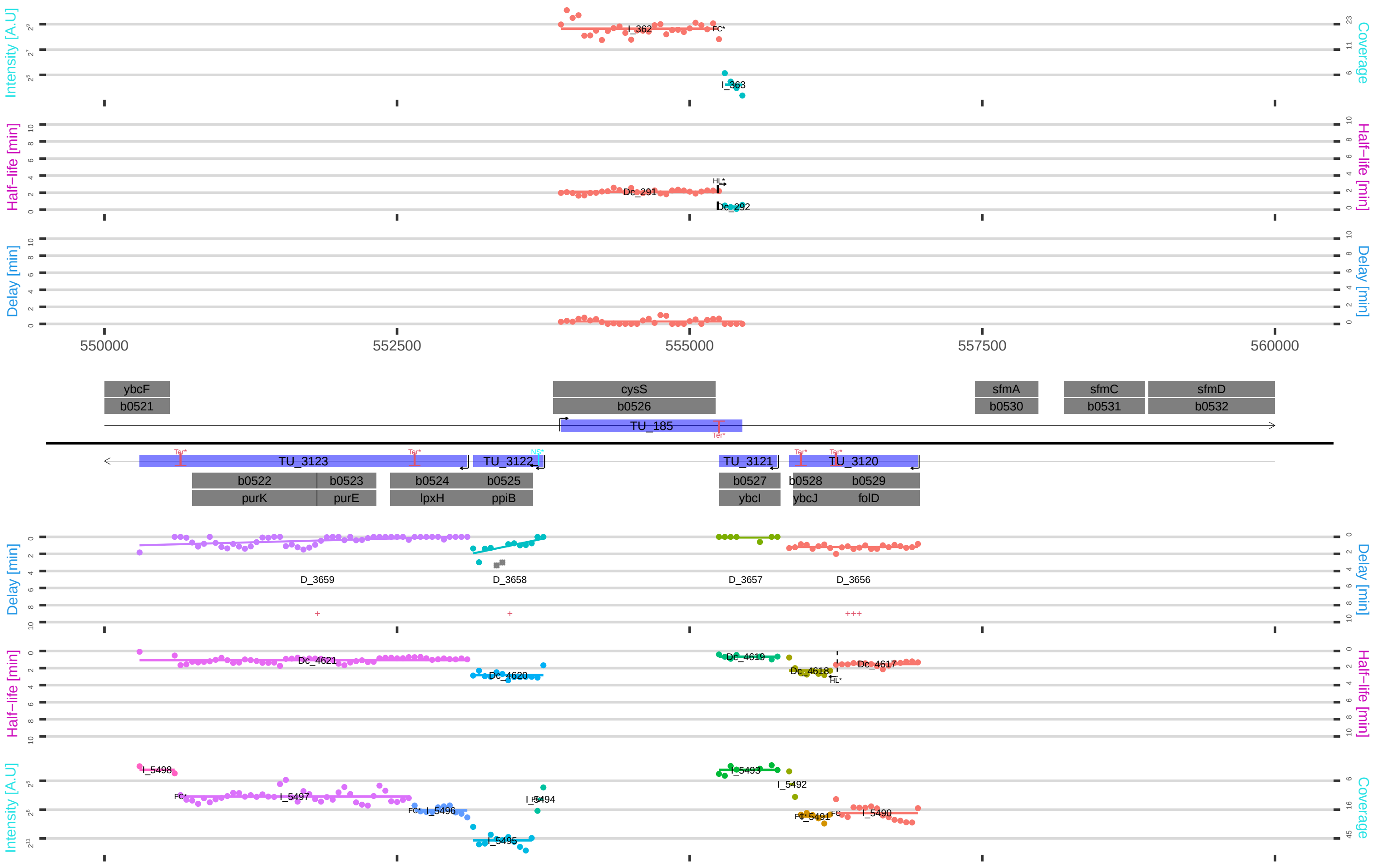

Term: termination (4), NS: new start (1), PS: pausing site (0), iTSS_I: internal starting site (0)

ID: 11275−11387; Term: termination (0), NS: new start (1), PS: pausing site (0), iTSS_I: internal starting site (0)

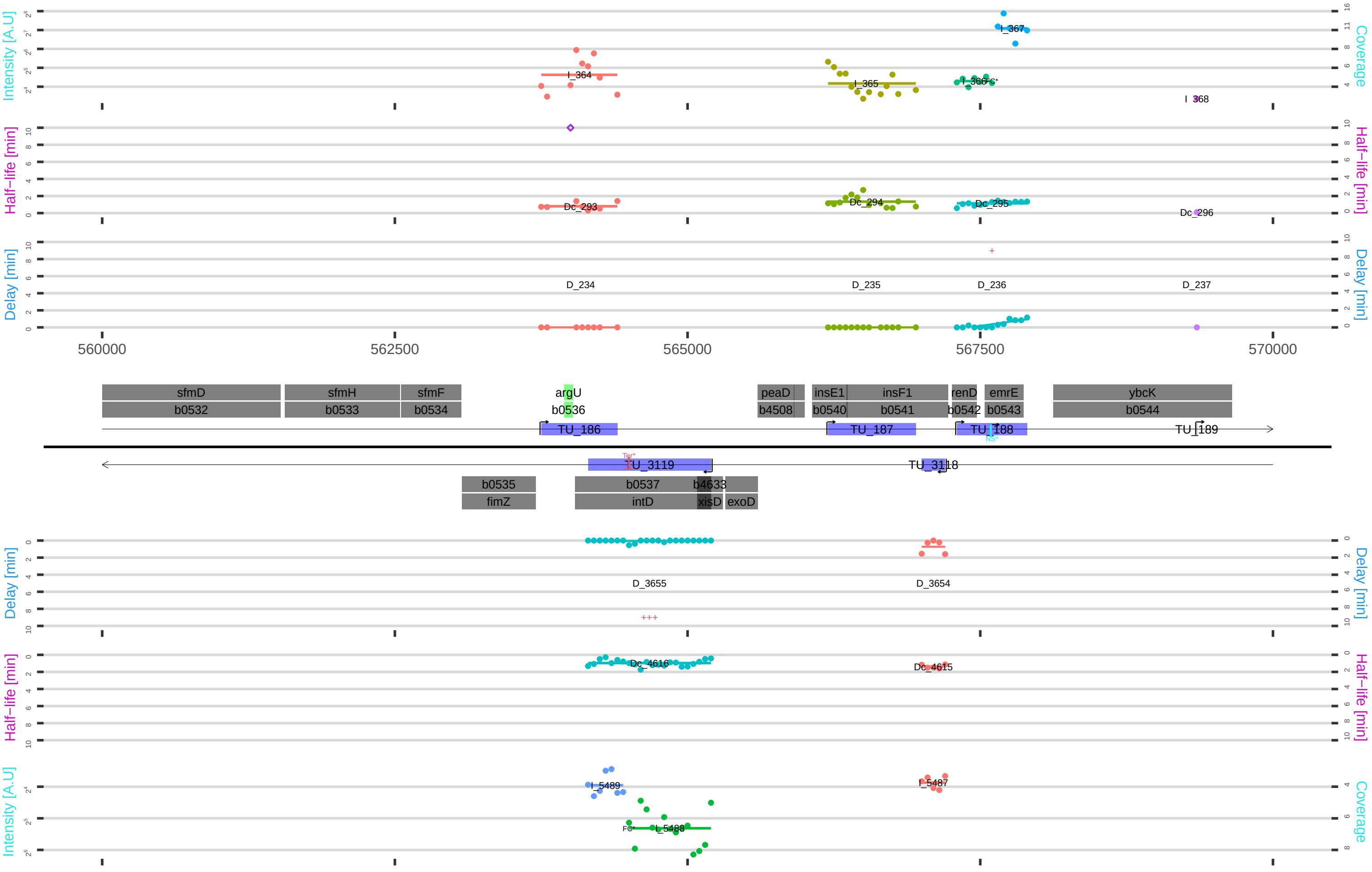

Term: termination (1), NS: new start (0), PS: pausing site (0), iTSS_I: internal starting site (0)

ID: 11402−11587; Term: termination (0), NS: new start (0), PS: pausing site (0), iTSS_I: internal starting site (0)

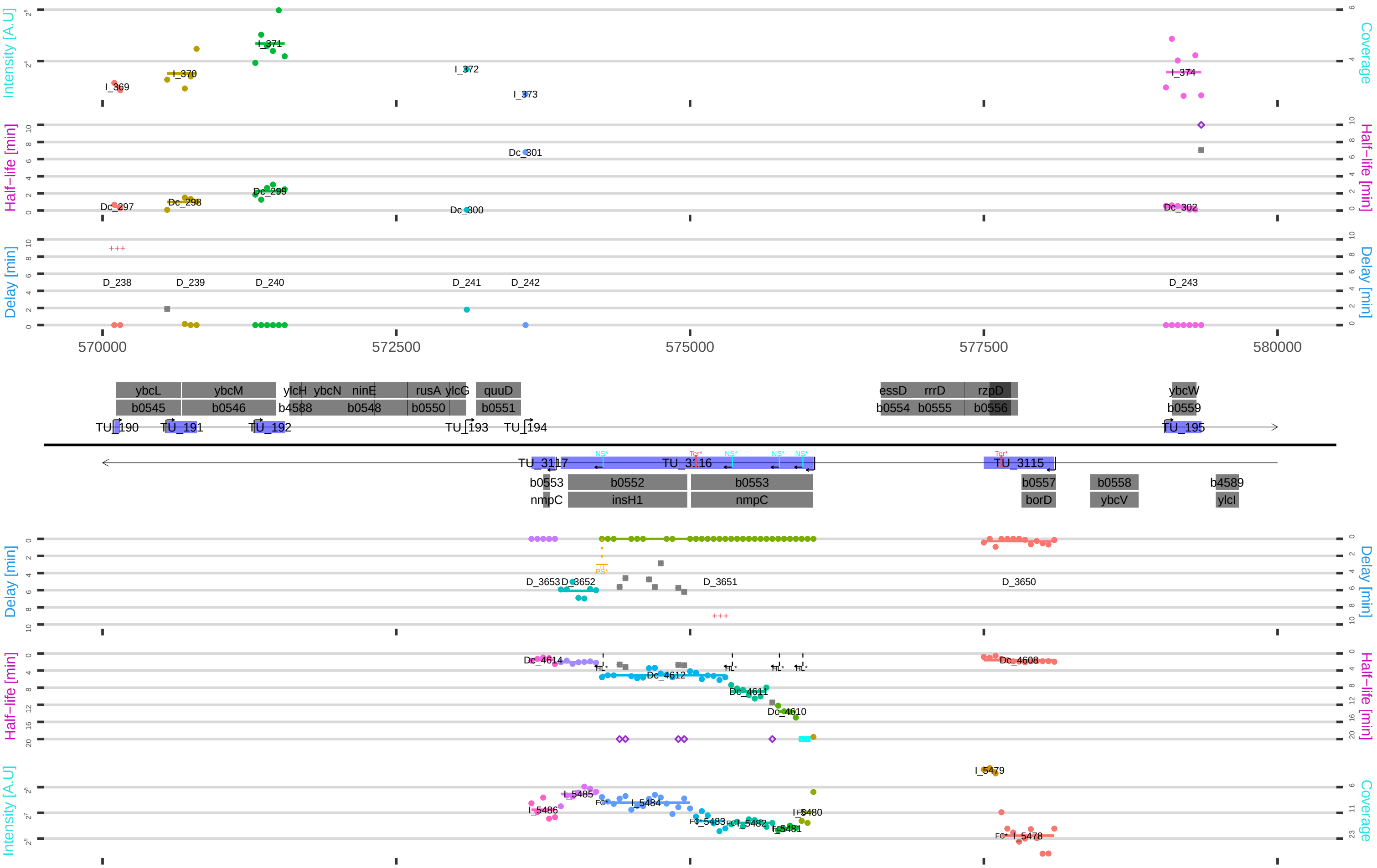

Term: termination (2), NS: new start (4), PS: pausing site (1), iTSS_I: internal starting site (0)

ID: 11606−11706; Term: termination (0), NS: new start (0), PS: pausing site (0), iTSS_I: internal starting site (0)

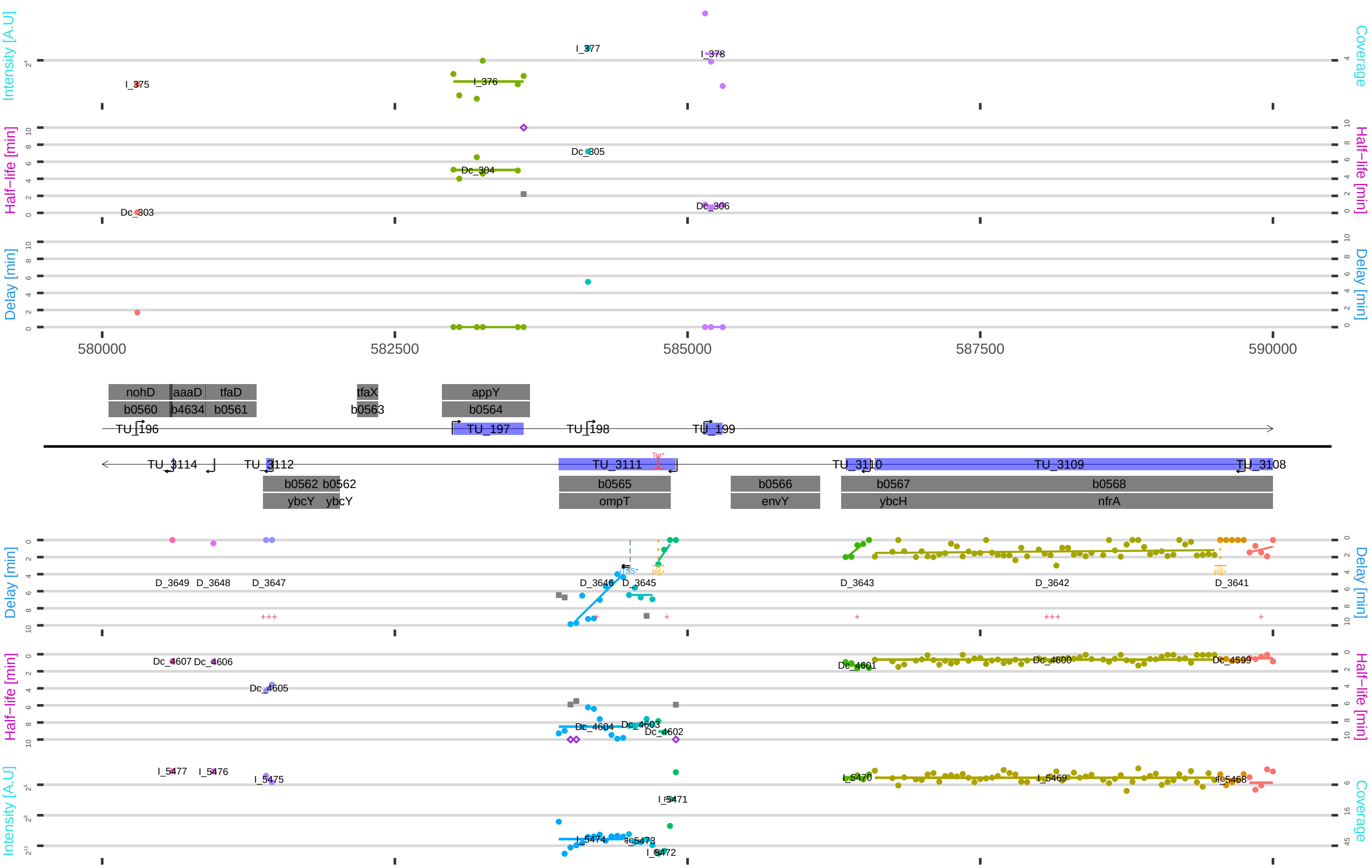

Term: termination (1), NS: new start (0), PS: pausing site (2), iTSS_I: internal starting site (1)

ID: 11941−11981; Term: termination (0), NS: new start (0), PS: pausing site (0), iTSS_I: internal starting site (0)

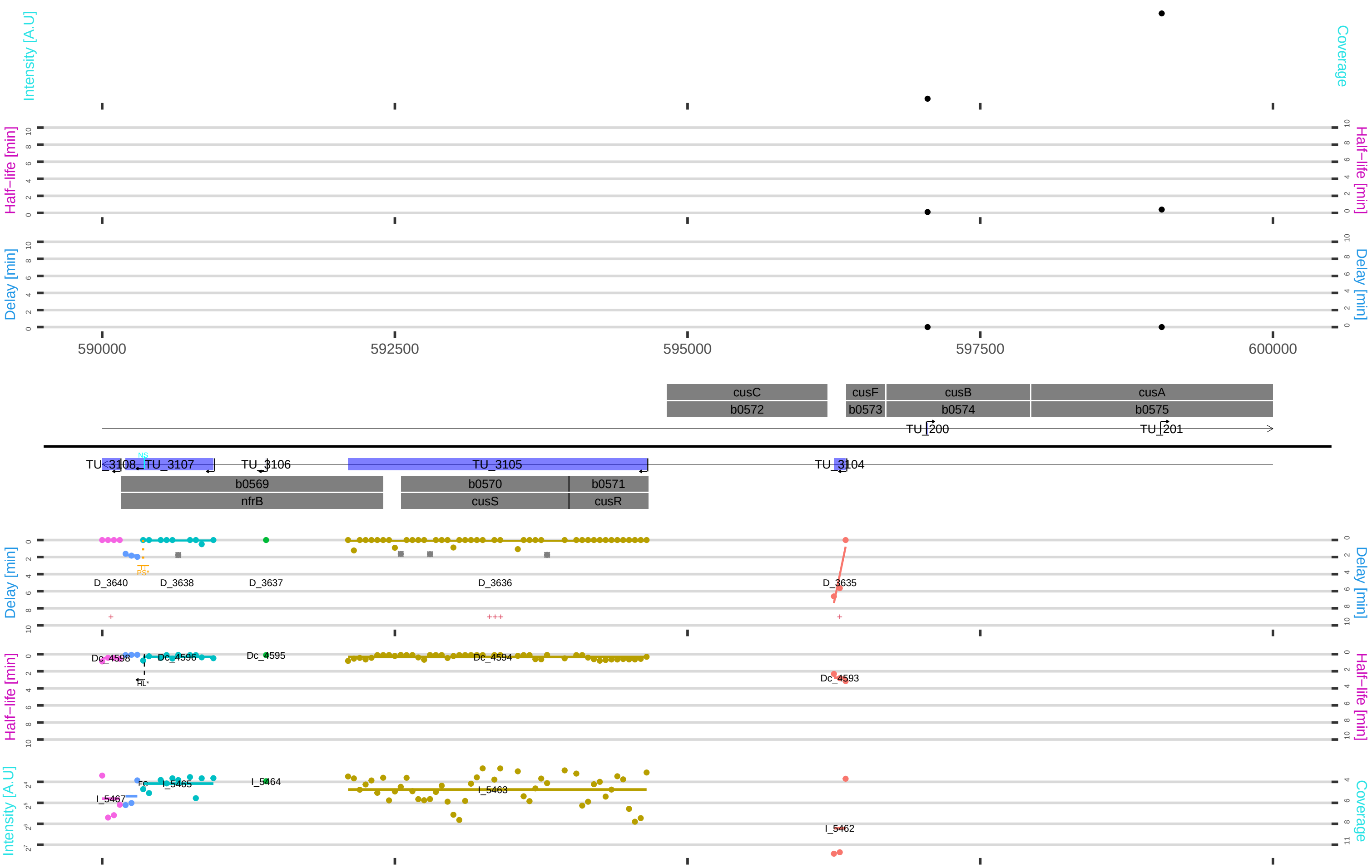

Term: termination (0), NS: new start (1), PS: pausing site (1), iTSS_I: internal starting site (0)

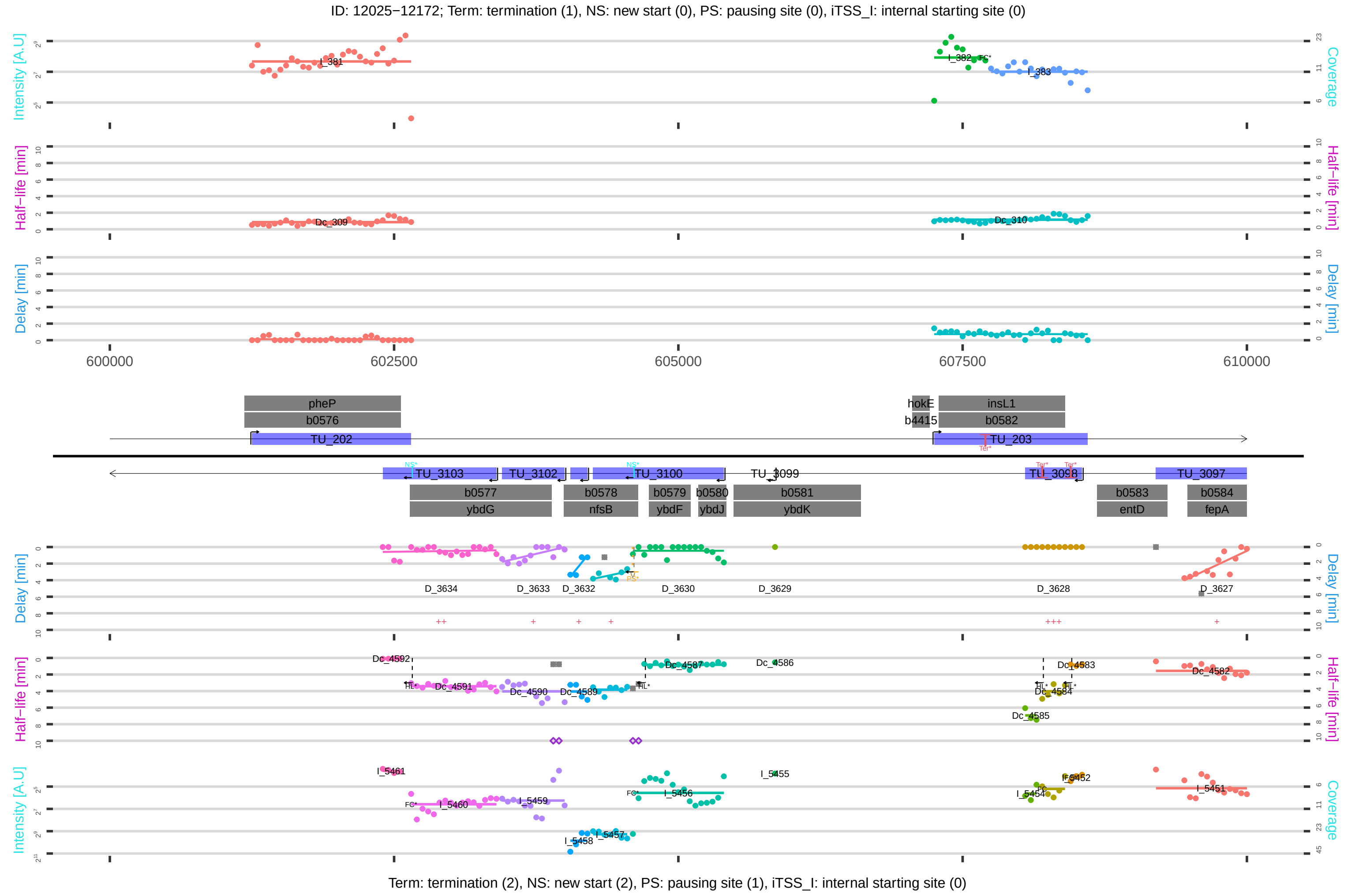
ID: 12025−12172; Term: termination (1), NS: new start (0), PS: pausing site (0), iTSS_I: internal starting site (0)
Intensity [A.U]
Coverage
Half−life [min]
Delay [min]
I_381
I_382
FC*
I_383
Dc_309
Dc_310
600000
602500
605000
607500
610000
pheP
b0576
TU_202
hokE
b4415
insL1
b0582
TU_203
Ter*
NS*
TU_3103
TU_3102
TU_3100
TU_3099
TU_3098
TU_3097
b0577
ybdG
b0578
nfsB
b0579
ybdF
b0580
ybdJ
b0581
ybdK
b0583
entD
b0584
fepA
PS*
D_3634
D_3633
D_3632
D_3630
D_3629
D_3628
D_3627
Dc_4592
Dc_4591
Dc_4590
Dc_4589
Dc_4587
Dc_4586
Dc_4583
Dc_4584
Dc_4585
Dc_4582
TL*
I_5461
I_5460
I_5459
I_5458
I_5457
I_5456
I_5455
I_5454
I_5452
I_5451
Term: termination (2), NS: new start (2), PS: pausing site (1), iTSS_I: internal starting site (0)

ID: 12241−12345; Term: termination (2), NS: new start (0), PS: pausing site (1), iTSS_I: internal starting site (0)

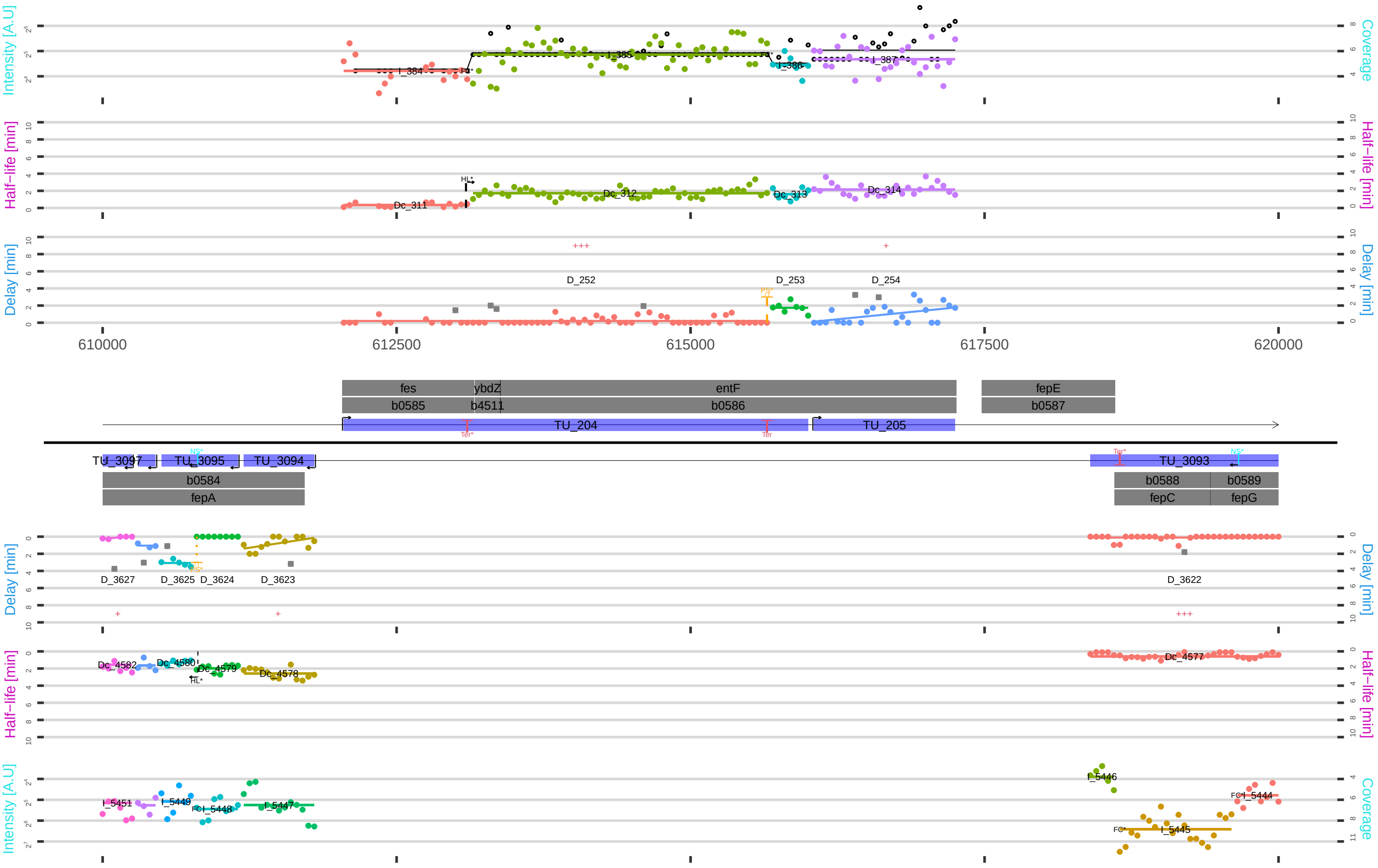

Term: termination (1), NS: new start (2), PS: pausing site (1), iTSS_I: internal starting site (0)

ID: 12431−12600; Term: termination (2), NS: new start (2), PS: pausing site (1), iTSS_I: internal starting site (0)

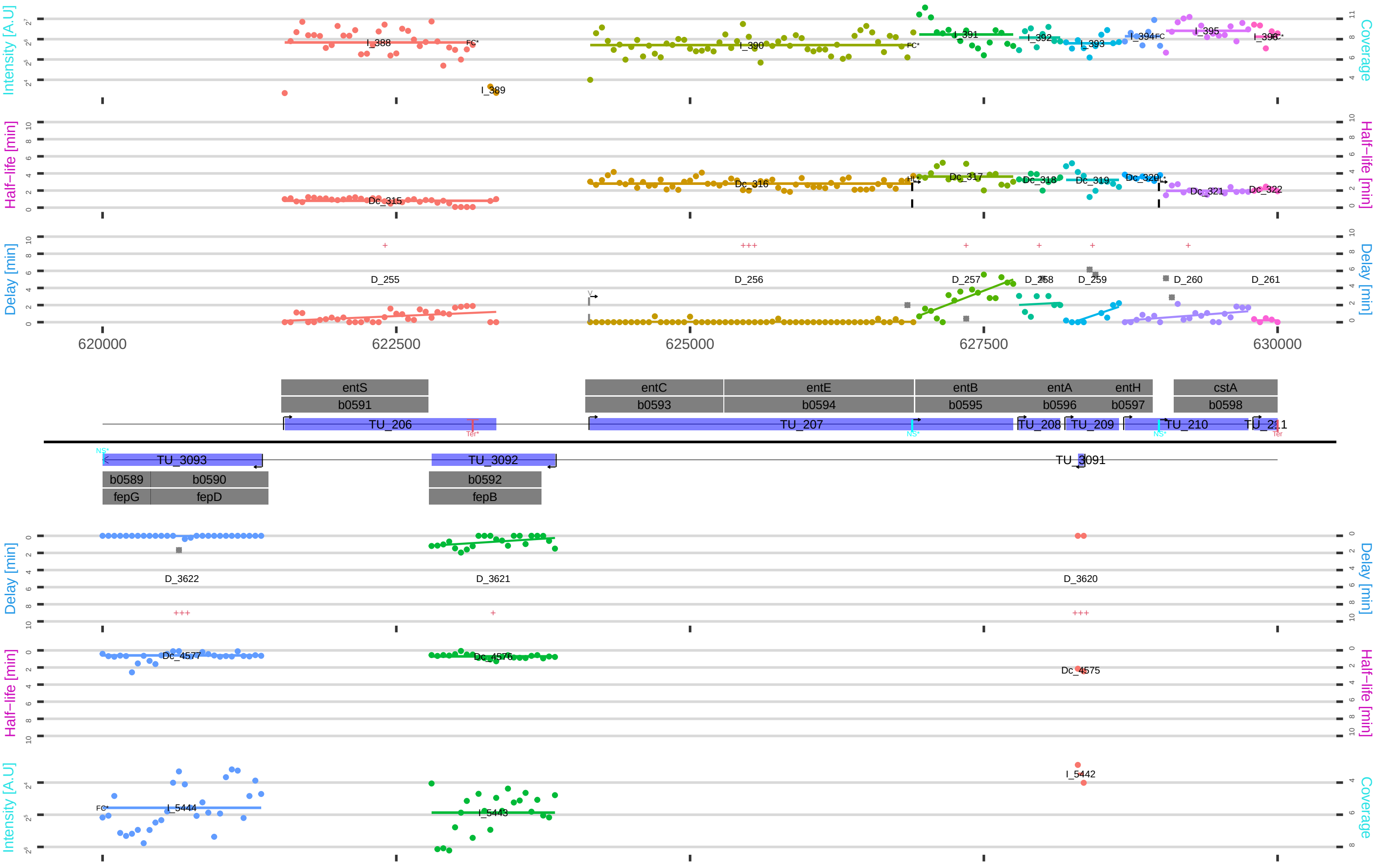

Term: termination (0), NS: new start (1), PS: pausing site (0), iTSS_I: internal starting site (0)

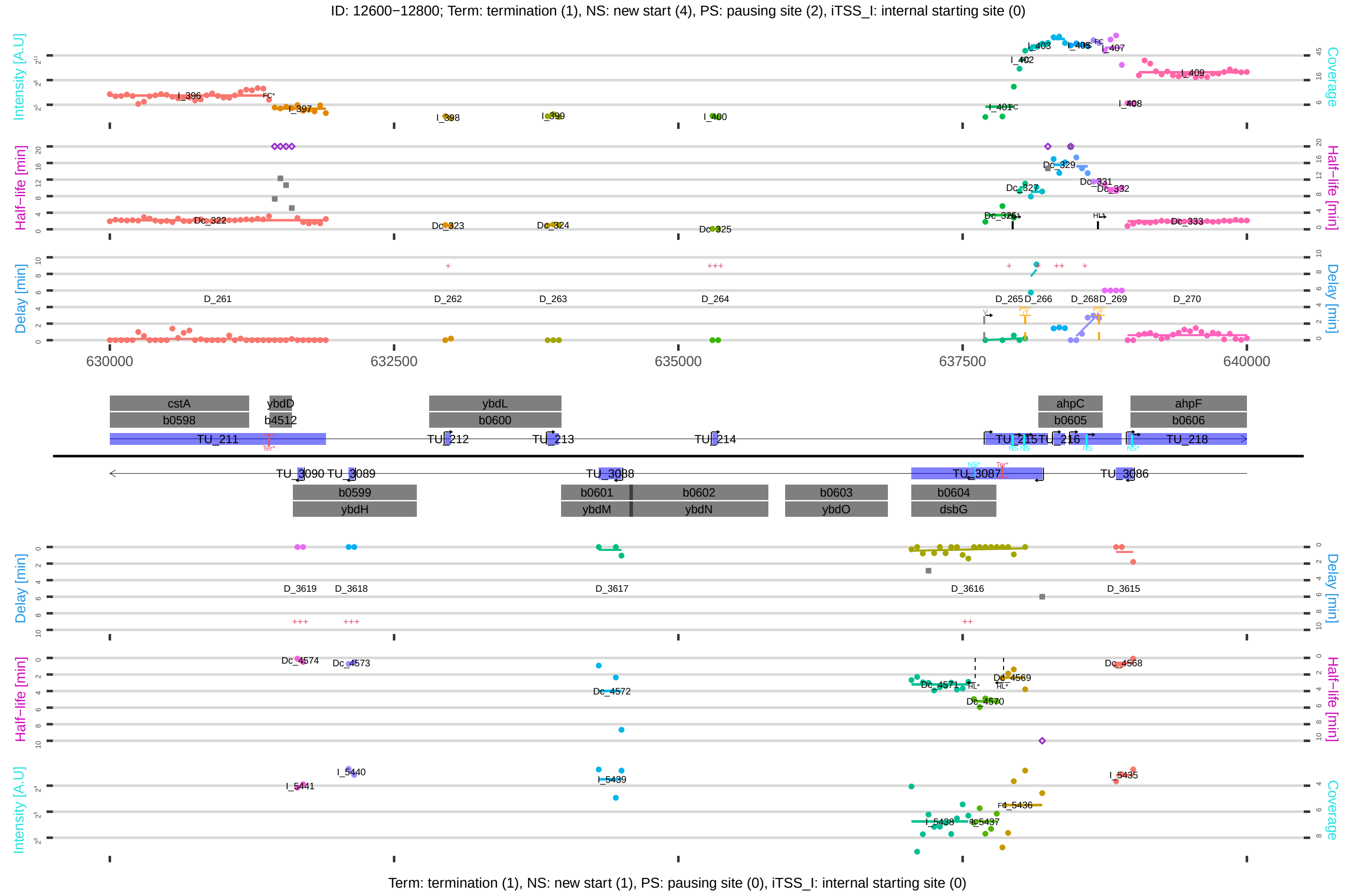
ID: 12600−12800; Term: termination (1), NS: new start (4), PS: pausing site (2), iTSS_I: internal starting site (0)
Intensity [A.U]
Coverage
Half−life [min]
Delay [min]
630000
632500
635000
637500
640000
cstA
b0598
ybdD
b4512
ybdL
b0600
ahpC
b0605
ahpF
b0606
TU_211
TU_212
TU_213
TU_214
TU_215
TU_216
TU_218
TU_3090
TU_3089
TU_3088
TU_3087
TU_3086
b0599
ybdH
b0601
ybdM
b0602
ybdN
b0603
ybdO
b0604
dsbG
Term: termination (1), NS: new start (1), PS: pausing site (0), iTSS_I: internal starting site (0)

ID: 12800−12944; Term: termination (0), NS: new start (0), PS: pausing site (0), iTSS_I: internal starting site (0)

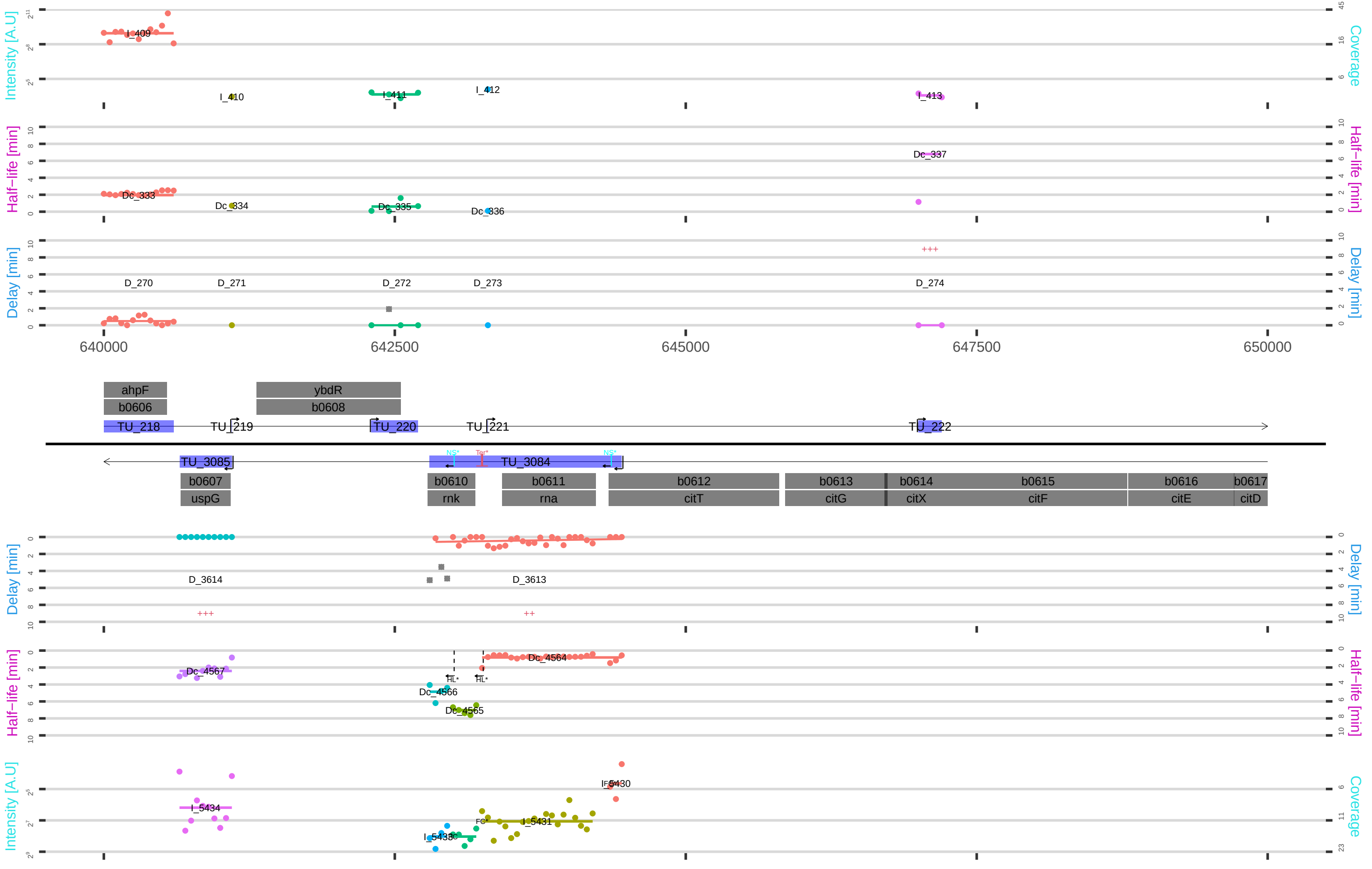

Term: termination (1), NS: new start (2), PS: pausing site (0), iTSS_I: internal starting site (0)

ID: 13039−13169; Term: termination (2), NS: new start (3), PS: pausing site (0), iTSS_I: internal starting site (0)

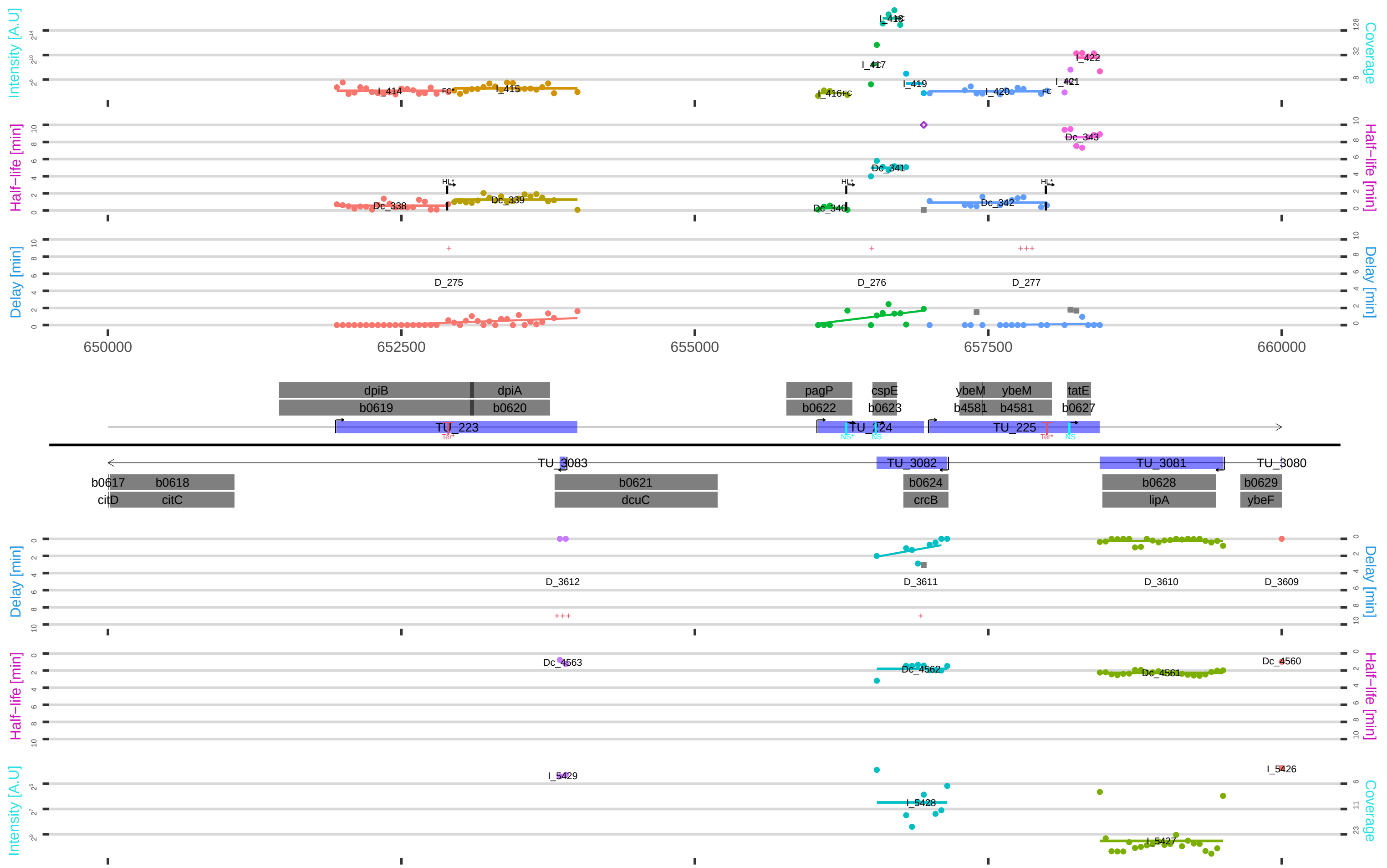

Term: termination (0), NS: new start (0), PS: pausing site (0), iTSS_I: internal starting site (0)

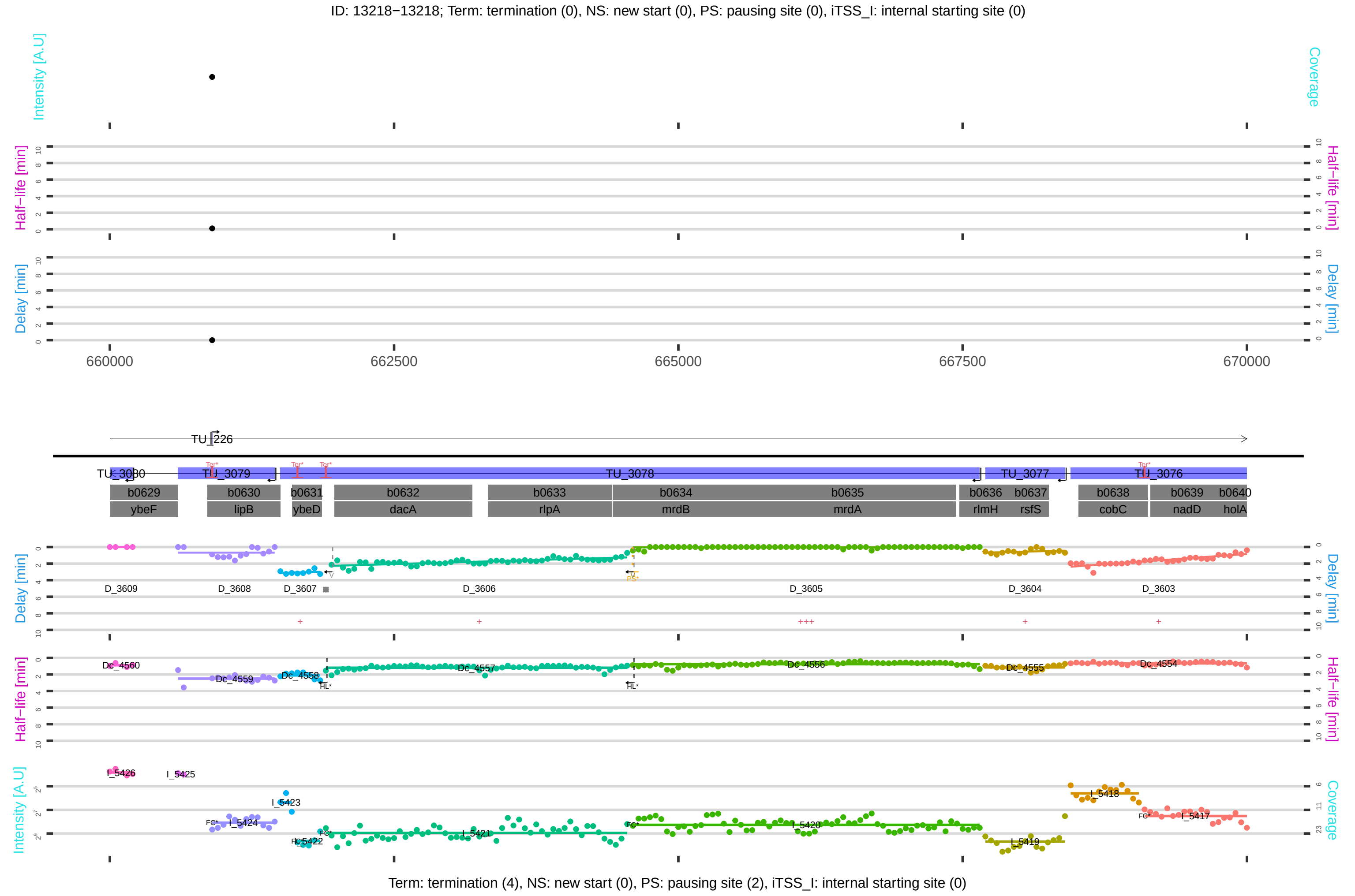
ID: 13218−13218; Term: termination (0), NS: new start (0), PS: pausing site (0), iTSS_I: internal starting site (0)
Intensity [A.U]
Coverage
Half−life [min]
Delay [min]
660000
662500
665000
667500
670000
TU_226
TU_3080
TU_3079
TU_3078
TU_3077
TU_3076
b0629 ybeF
b0630 lipB
b0631 ybeD
b0632 dacA
b0633 rlpA
b0634 mrdB
b0635 mrdA
b0636 rlmH
b0637 rsfS
b0638 cobC
b0639 nadD
b0640 holA
D_3609
D_3608
D_3607
D_3606
D_3605
D_3604
D_3603
Dc_4560
Dc_4559
Dc_4558
Dc_4557
Dc_4556
Dc_4555
Dc_4554
I_5426
I_5425
I_5424
I_5423
I_5422
I_5421
I_5420
I_5419
I_5418
I_5417
Term: termination (4), NS: new start (0), PS: pausing site (2), iTSS_I: internal starting site (0)

ID: 13408−13600; Term: termination (0), NS: new start (1), PS: pausing site (0), iTSS_I: internal starting site (0)

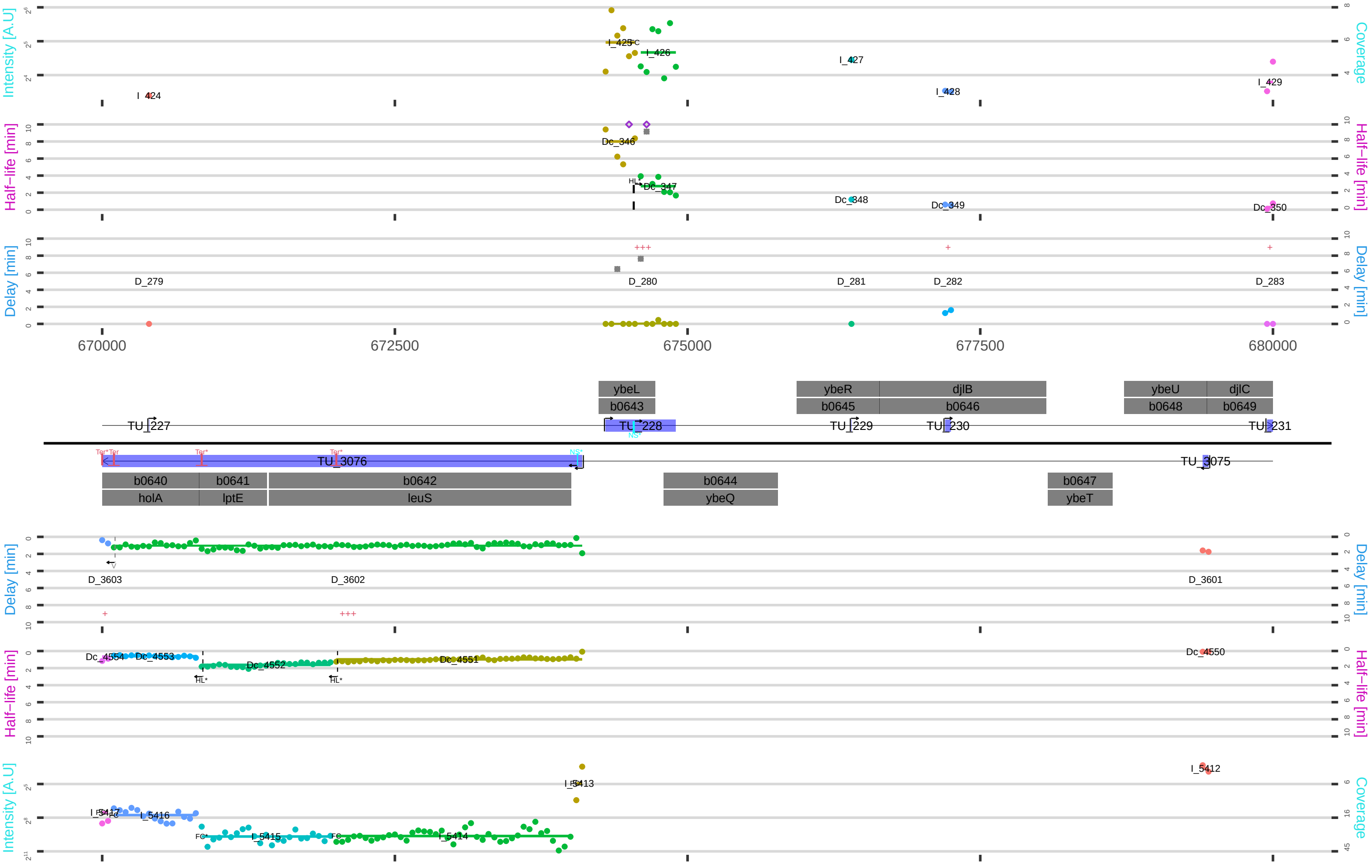

Term: termination (4), NS: new start (1), PS: pausing site (0), iTSS_I: internal starting site (1)

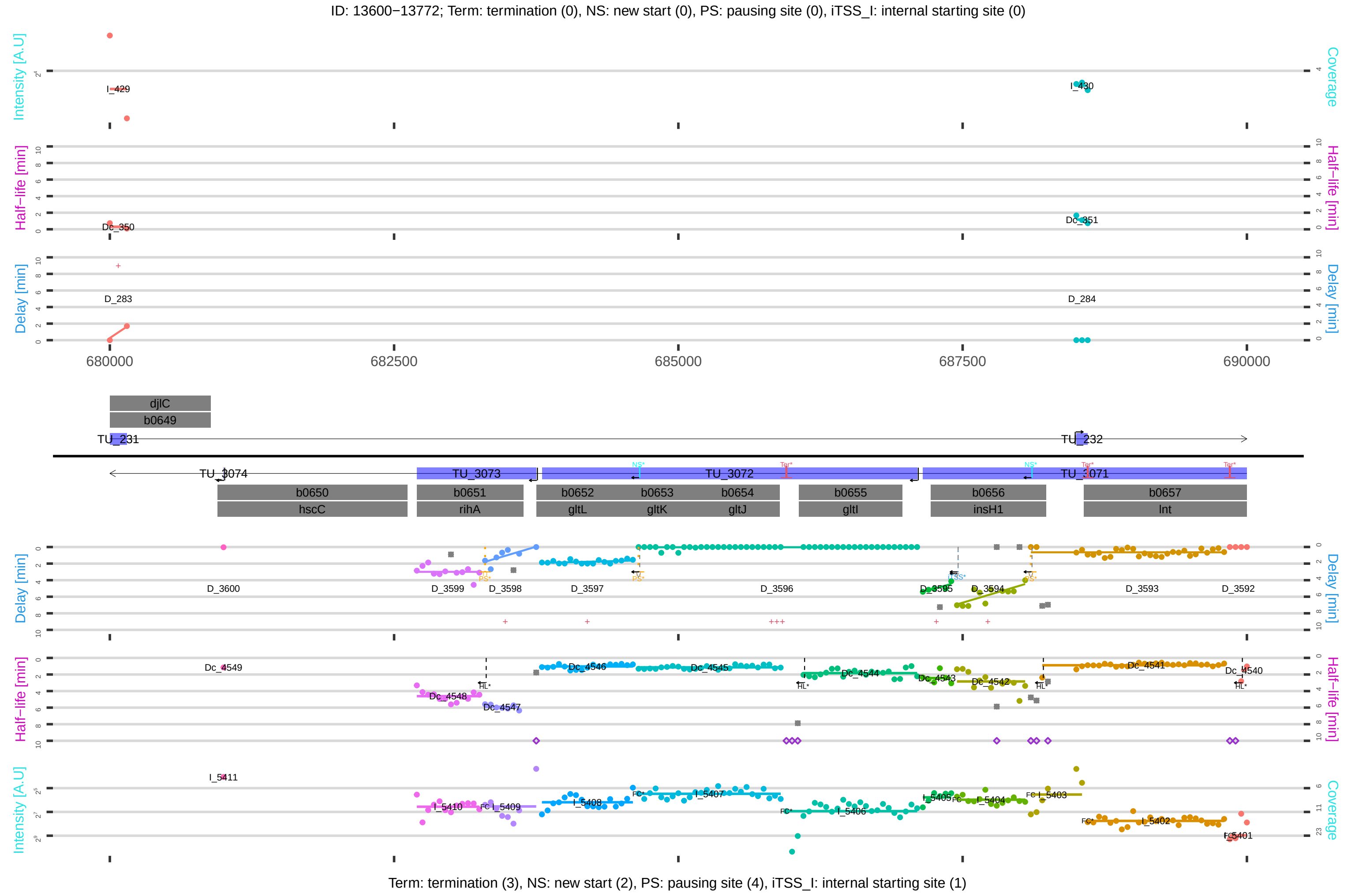

ID: 13600−13772; Term: termination (0), NS: new start (0), PS: pausing site (0), iTSS_I: internal starting site (0)
Intensity [A.U]
Coverage
I_429
I_430
Half−life [min]
Dc_350
Dc_351
Delay [min]
D_283
D_284
680000
682500
685000
687500
690000
djlC
b0649
TU_231
TU_232
TU_3074
TU_3073
TU_3072
TU_3071
b0650
hscC
b0651
rihA
b0652
gltL
b0653
gltK
b0654
gltJ
b0655
gltI
b0656
insH1
b0657
lnt
D_3600
D_3599
D_3598
D_3597
D_3596
D_3595
D_3594
D_3593
D_3592
Dc_4549
Dc_4548
Dc_4547
Dc_4546
Dc_4545
Dc_4544
Dc_4543
Dc_4542
Dc_4541
Dc_4540
I_5411
I_5410
I_5409
I_5408
I_5407
I_5406
I_5405
I_5404
I_5403
I_5402
I_5401
Term: termination (3), NS: new start (2), PS: pausing site (4), iTSS_I: internal starting site (1)

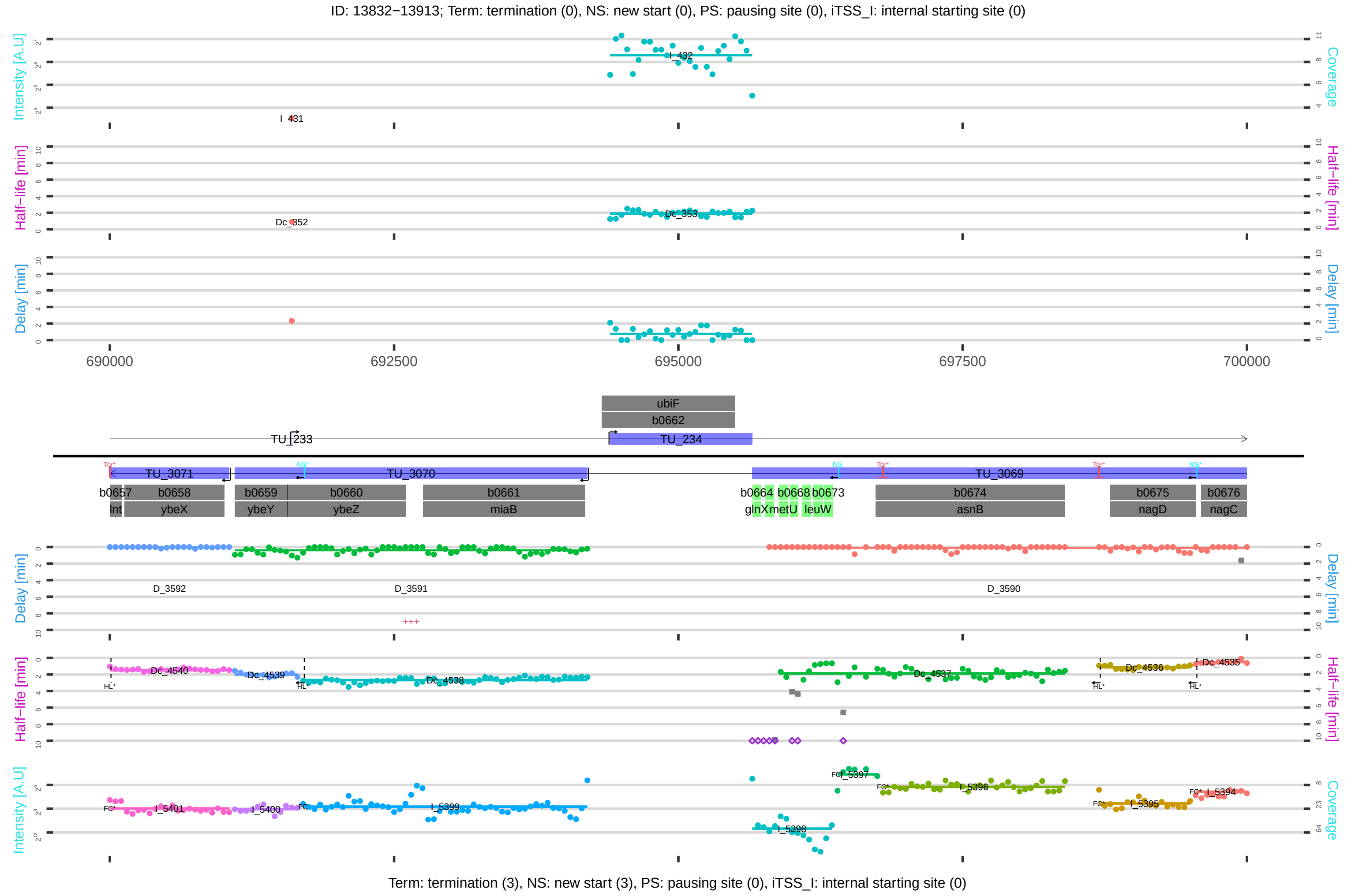
ID: 13832−13913; Term: termination (0), NS: new start (0), PS: pausing site (0), iTSS_I: internal starting site (0)
Intensity [A.U]
Coverage
I_432
I_431
Half−life [min]
Dc_353
Dc_352
Delay [min]
690000
692500
695000
697500
700000
ubiF
b0662
TU_233
TU_234
TU_3071
TU_3070
TU_3069
b0657
b0658
b0659
b0660
b0661
b0664
b0668
b0673
b0674
b0675
b0676
Int
ybeX
ybeY
ybeZ
miaB
glnX
metU
leuW
asnB
nagD
nagC
D_3592
D_3591
D_3590
Dc_4540
Dc_4539
Dc_4538
Dc_4537
Dc_4536
Dc_4535
I_5401
I_5400
I_5399
I_5398
I_5397
I_5396
I_5395
I_5394
Term: termination (3), NS: new start (3), PS: pausing site (0), iTSS_I: internal starting site (0)

ID: 14049−14186; Term: termination (0), NS: new start (2), PS: pausing site (1), iTSS_I: internal starting site (0)

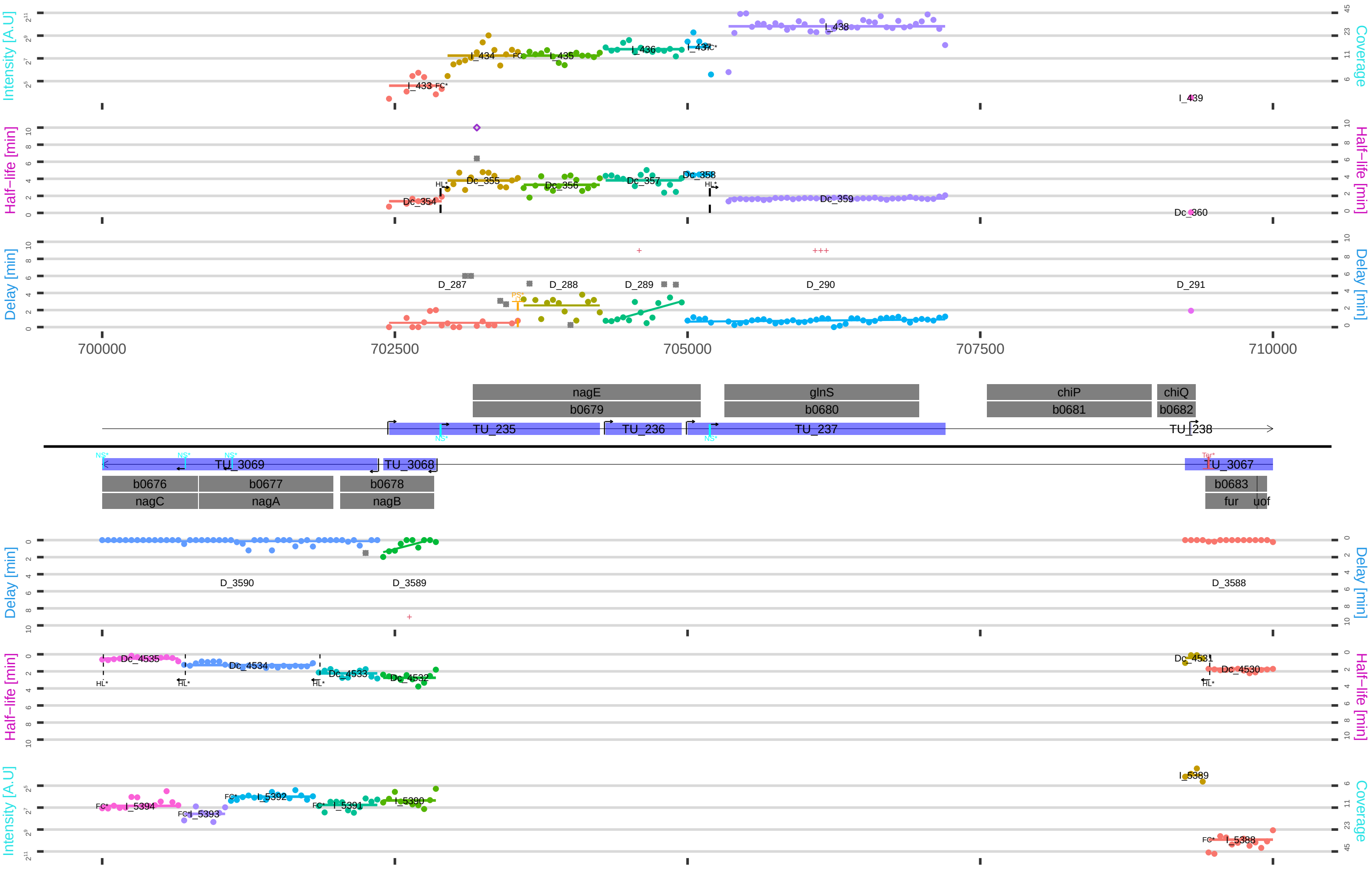

Term: termination (1), NS: new start (3), PS: pausing site (0), iTSS_I: internal starting site (0)

ID: 14202−14386; Term: termination (2), NS: new start (1), PS: pausing site (0), iTSS_I: internal starting site (0)

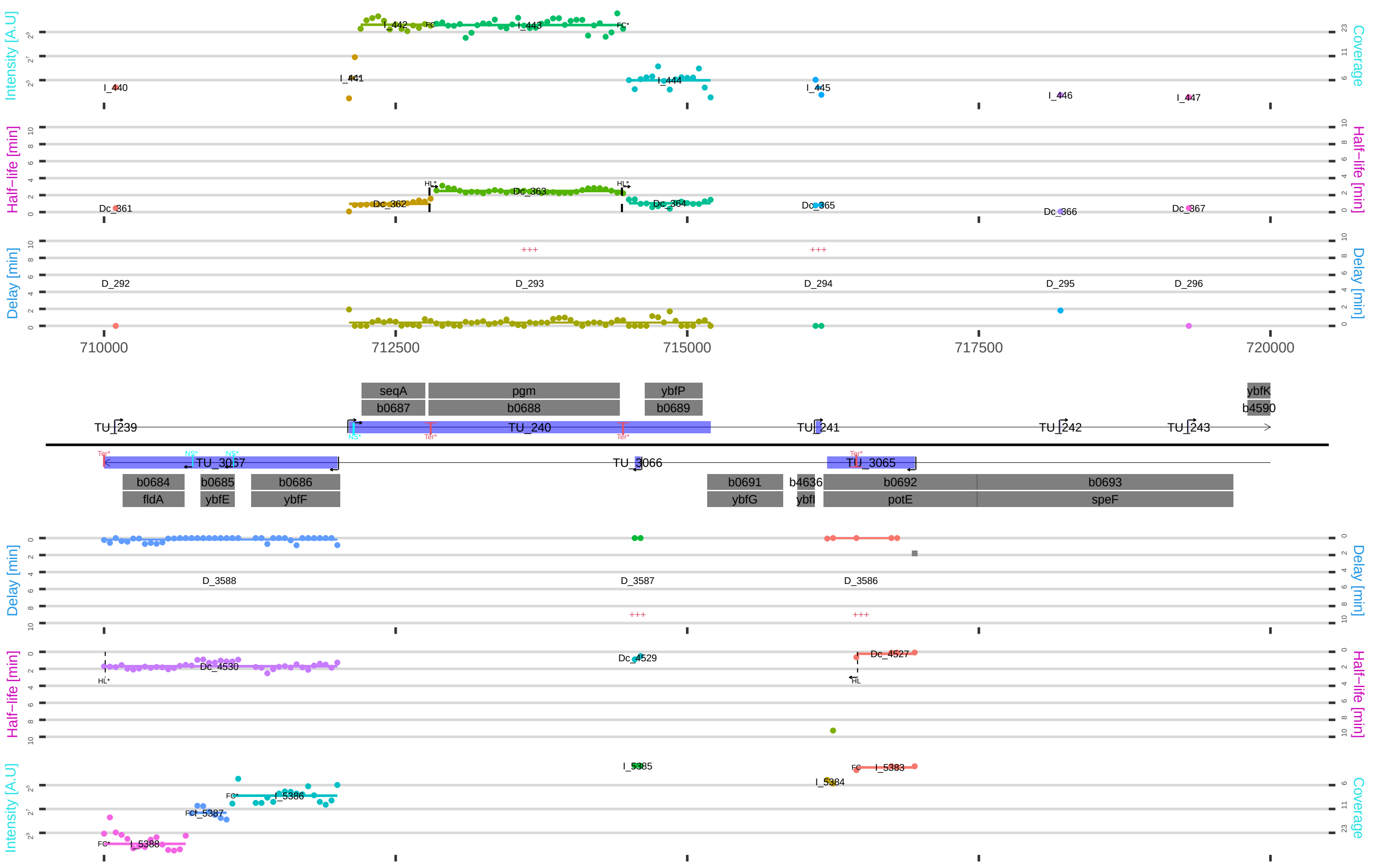

Term: termination (2), NS: new start (2), PS: pausing site (0), iTSS_I: internal starting site (0)

ID: 14569−14588; Term: termination (0), NS: new start (0), PS: pausing site (0), iTSS_I: internal starting site (0)

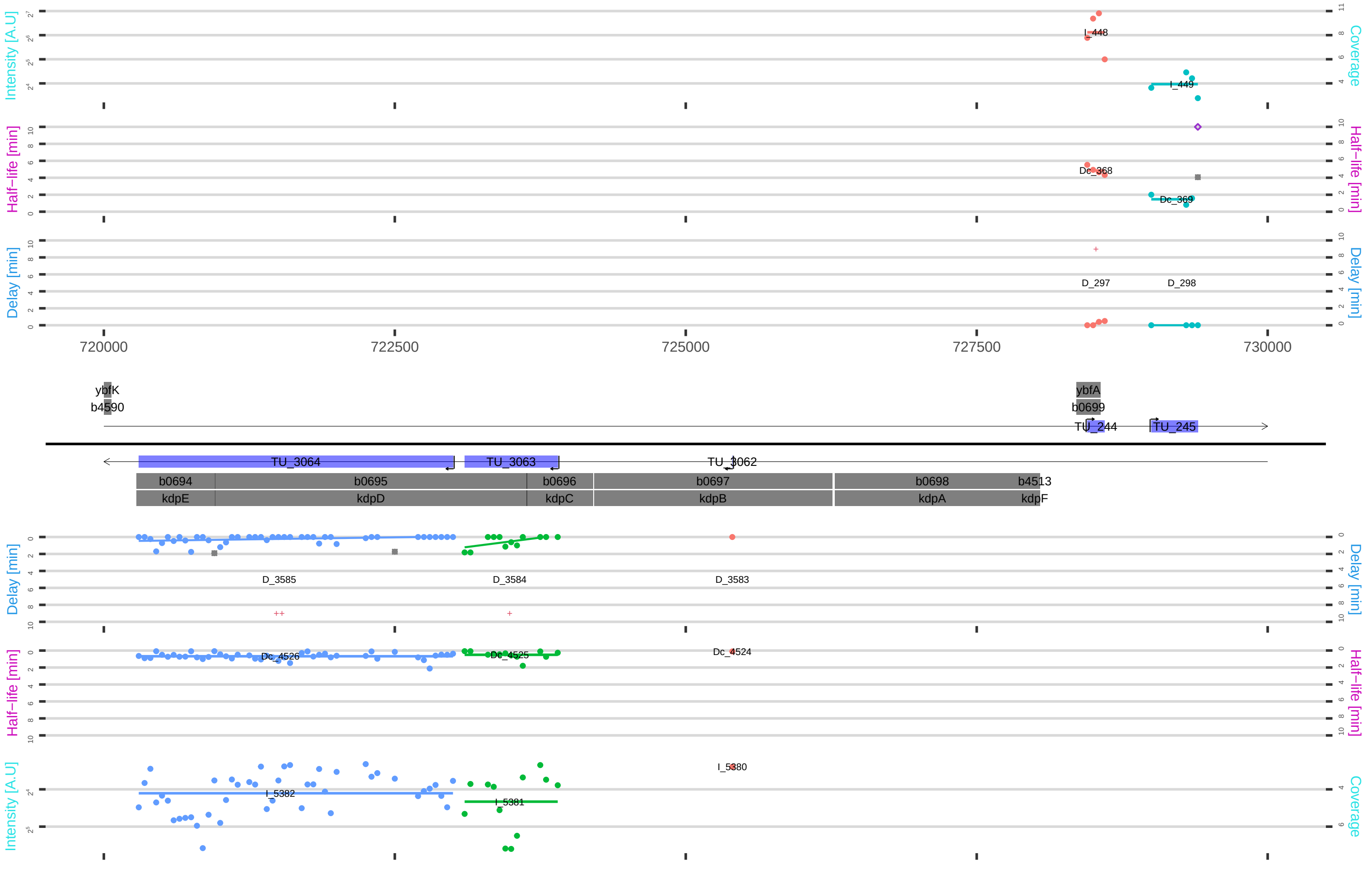

Term: termination (0), NS: new start (0), PS: pausing site (0), iTSS_I: internal starting site (0)

ID: 14608−14799; Term: termination (0), NS: new start (0), PS: pausing site (0), iTSS_I: internal starting site (0)

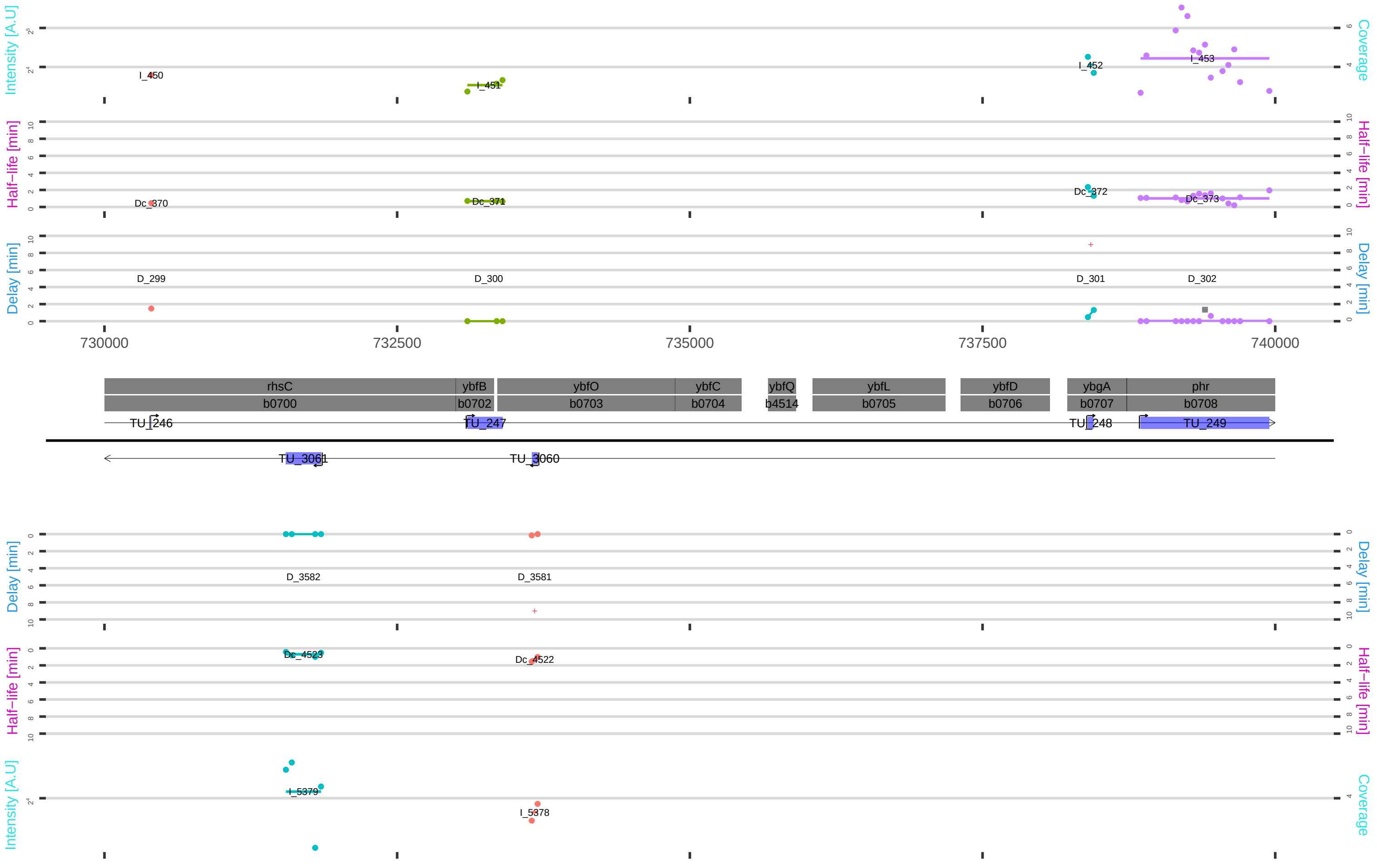

Term: termination (0), NS: new start (0), PS: pausing site (0), iTSS_I: internal starting site (0)

ID: 14801−14975; Term: termination (2), NS: new start (2), PS: pausing site (0), iTSS_I: internal starting site (0)

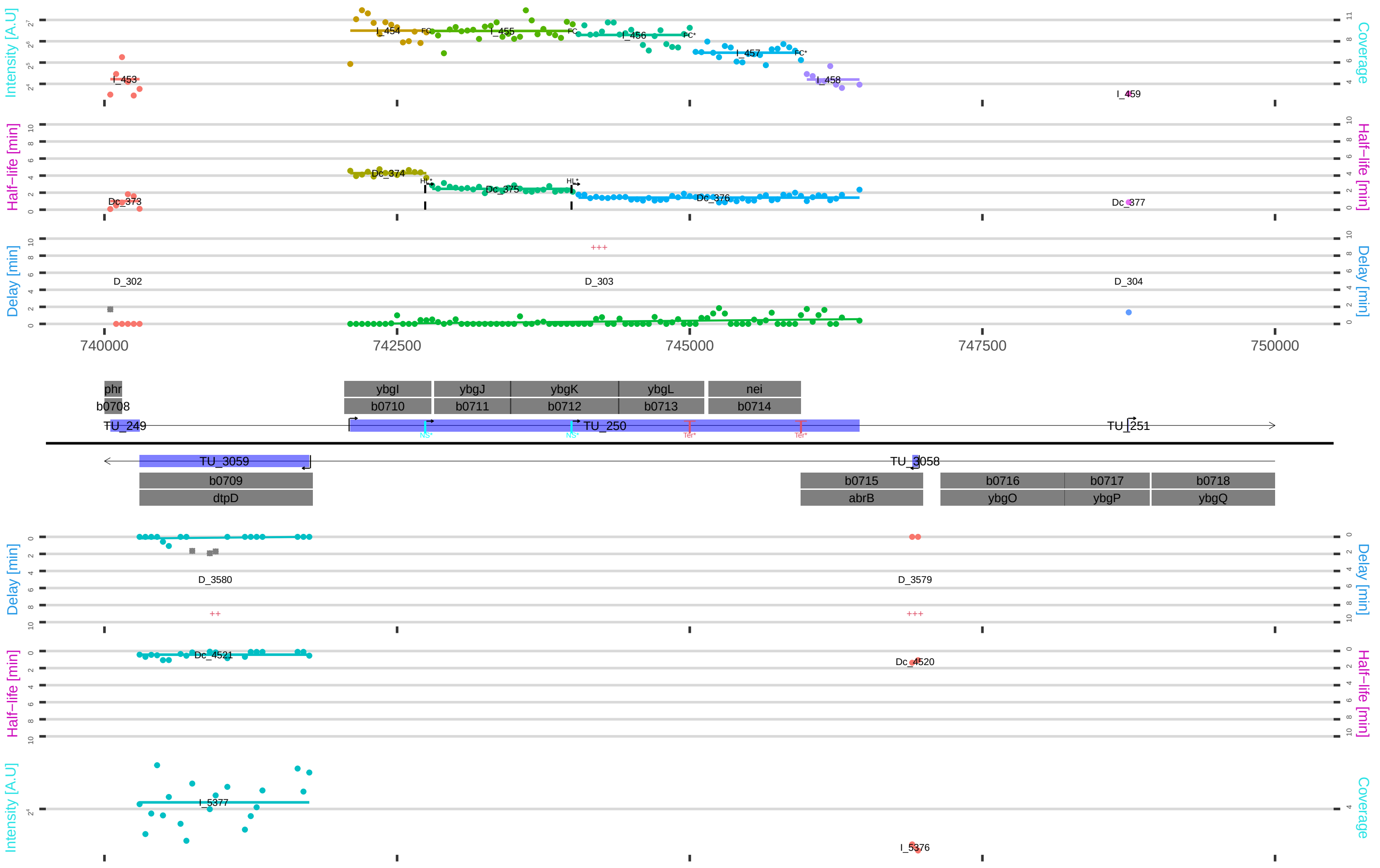

Term: termination (0), NS: new start (0), PS: pausing site (0), iTSS_I: internal starting site (0)

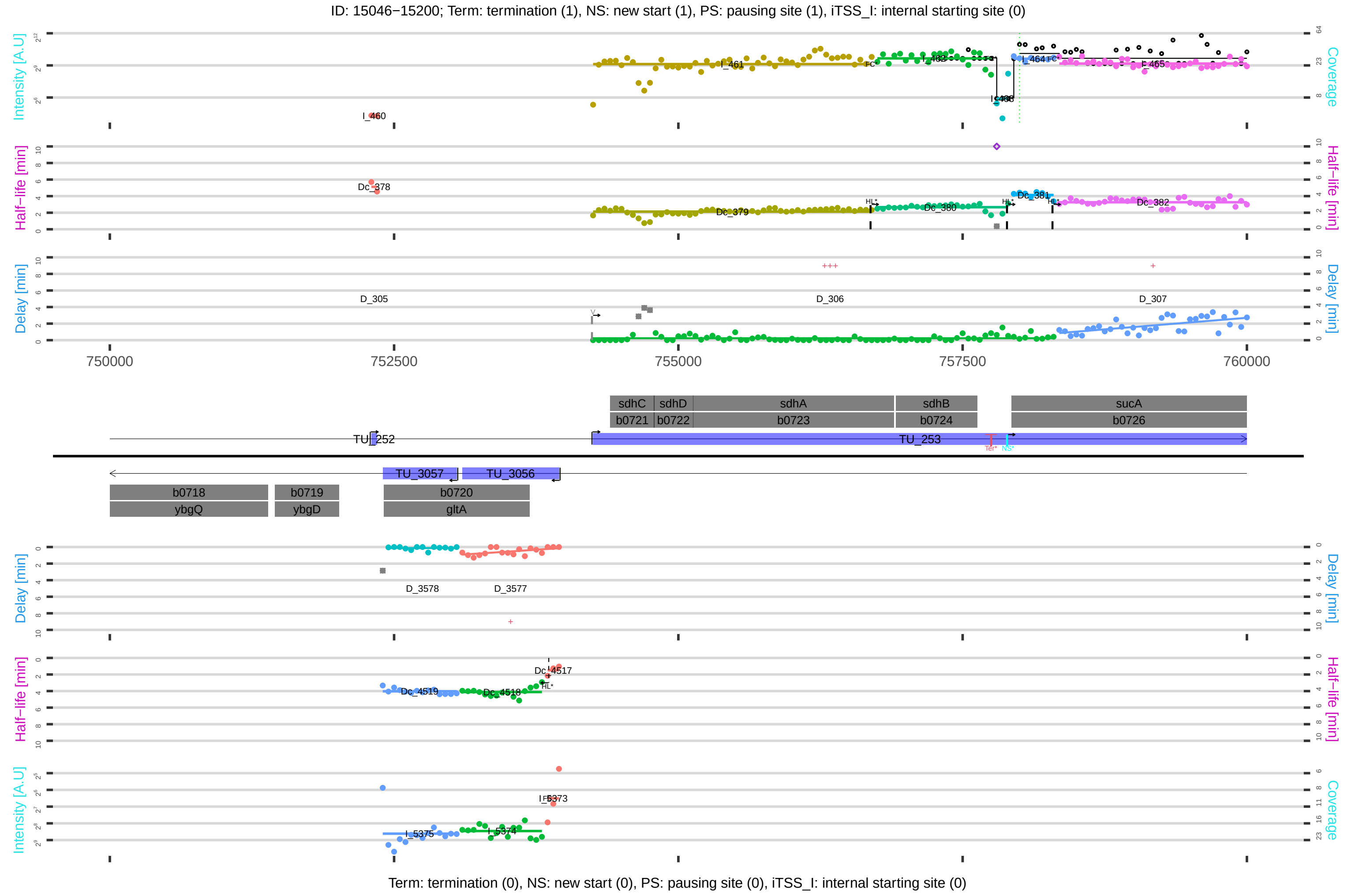

ID: 15046−15200; Term: termination (1), NS: new start (1), PS: pausing site (1), iTSS_I: internal starting site (0)
Intensity [A.U]
Coverage
Half−life [min]
Delay [min]
I_460
I_461
I_462
I_463
I_464
I_465
Dc_378
Dc_379
Dc_380
Dc_381
Dc_382
D_305
D_306
D_307
750000
752500
755000
757500
760000
sdhC
sdhD
sdhA
sdhB
sucA
b0721
b0722
b0723
b0724
b0726
TU_252
TU_253
Ter*
NS*
TU_3057
TU_3056
b0718
b0719
b0720
ybgQ
ybgD
gltA
D_3578
D_3577
Dc_4517
Dc_4519
Dc_4518
I_5373
I_5375
I_5374
Term: termination (0), NS: new start (0), PS: pausing site (0), iTSS_I: internal starting site (0)

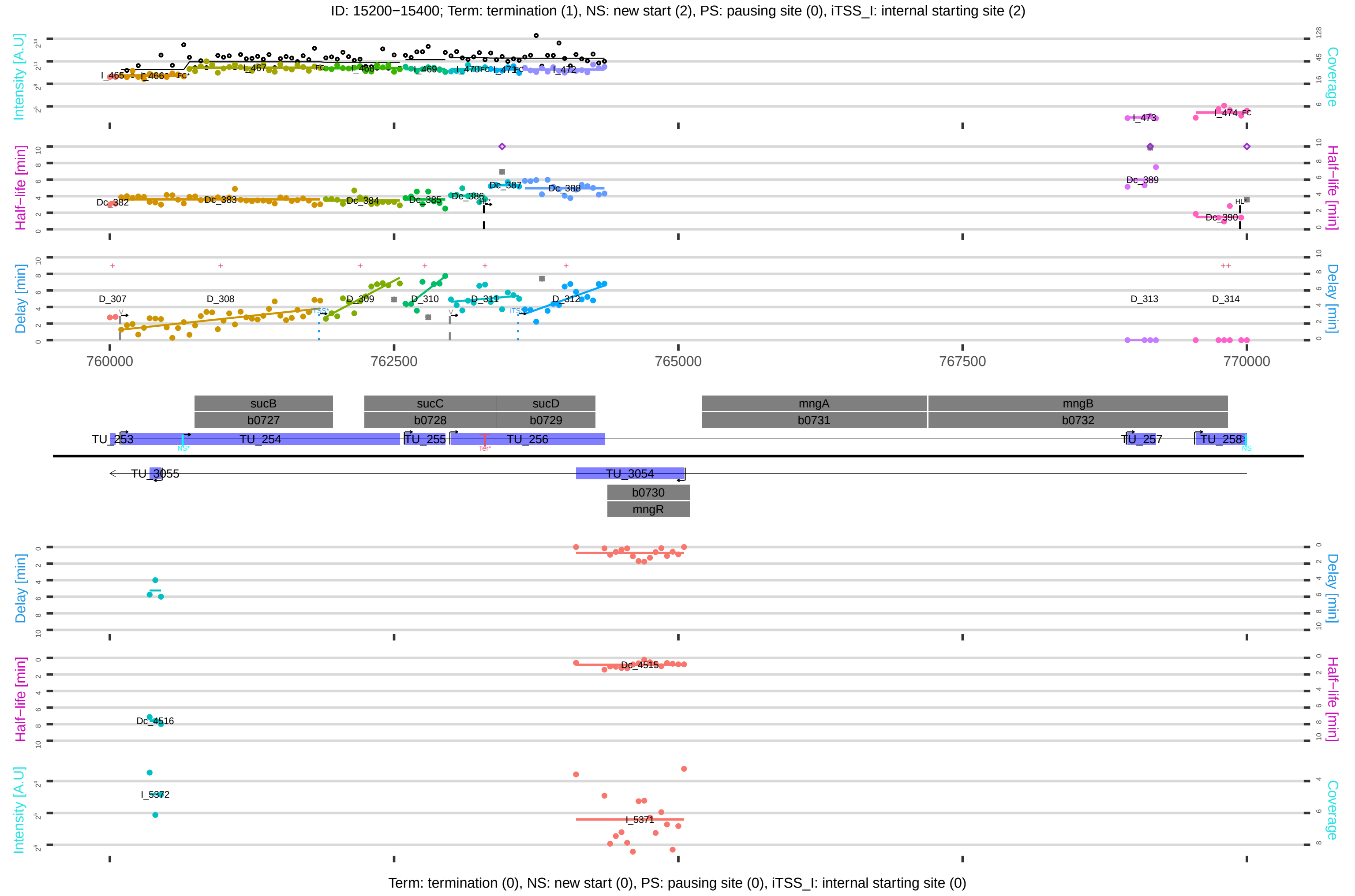
ID: 15200−15400; Term: termination (1), NS: new start (2), PS: pausing site (0), iTSS_I: internal starting site (2)
Intensity [A.U]
Coverage
Half−life [min]
Delay [min]
760000
762500
765000
767500
770000
sucB
b0727
sucC
b0728
sucD
b0729
mngA
b0731
mngB
b0732
TU_253
TU_254
TU_255
TU_256
TU_257
TU_258
TU_3055
TU_3054
b0730
mngR
Dc_4515
Dc_4516
I_5372
I_5371
Term: termination (0), NS: new start (0), PS: pausing site (0), iTSS_I: internal starting site (0)

ID: 15400−15600; Term: termination (5), NS: new start (5), PS: pausing site (3), iTSS_I: internal starting site (2)

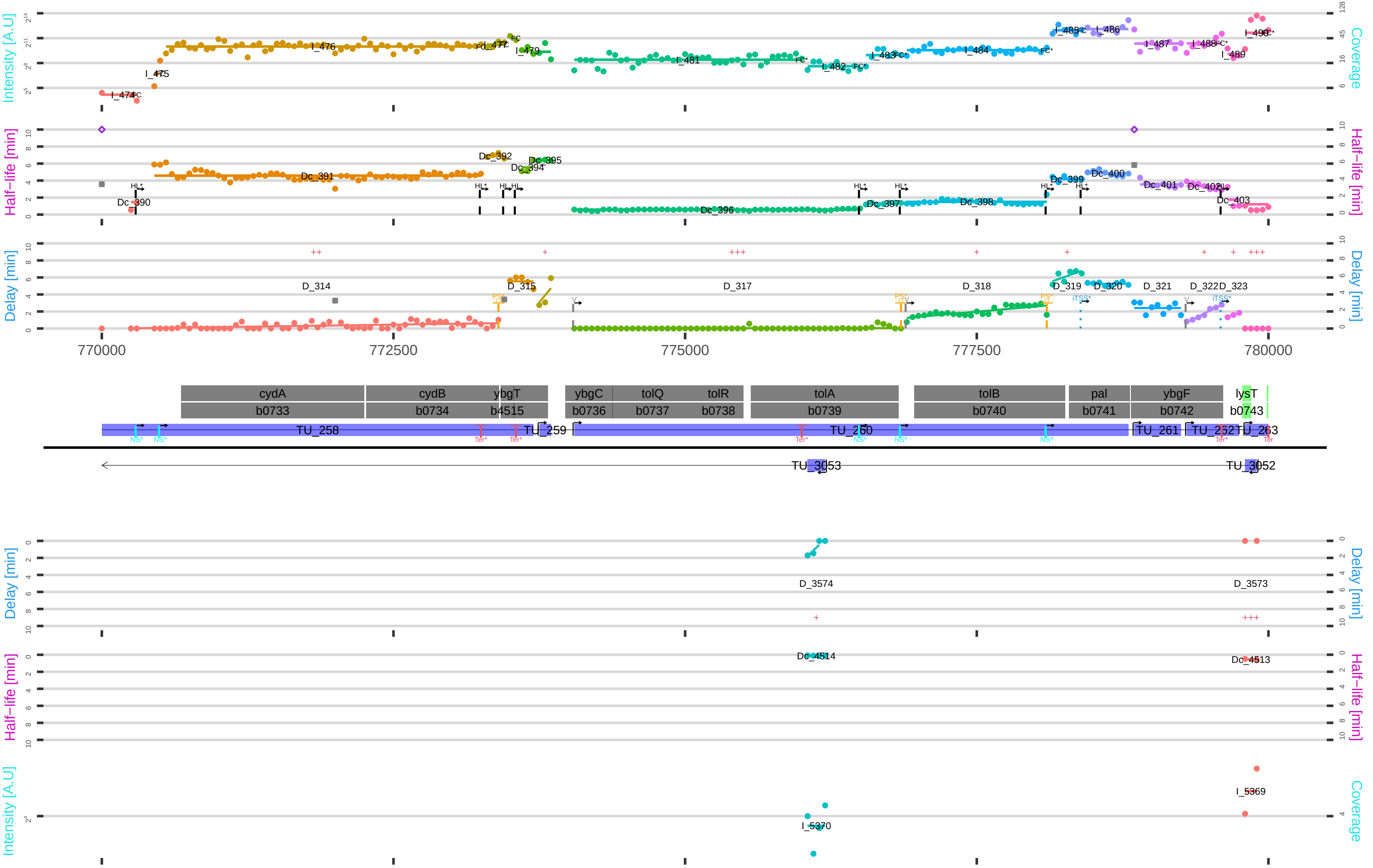

Term: termination (0), NS: new start (0), PS: pausing site (0), iTSS_I: internal starting site (0)

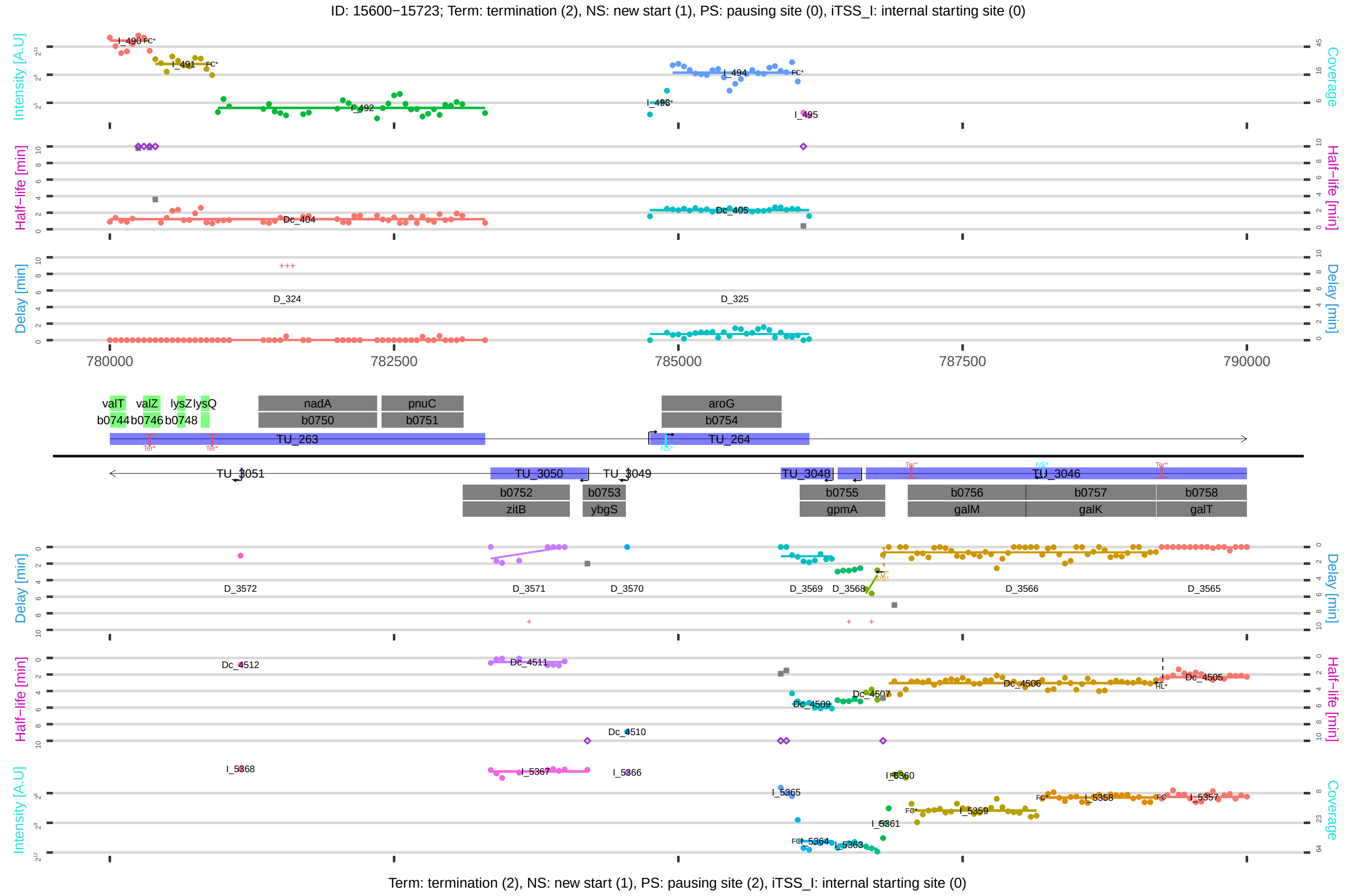

ID: 15600−15723; Term: termination (2), NS: new start (1), PS: pausing site (0), iTSS_I: internal starting site (0)
Intensity [A.U]
Coverage
Half−life [min]
Delay [min]
780000
782500
785000
787500
790000
valT
valZ
lysZ
lysQ
b0744
b0746
b0748
nadA
b0750
pnuC
b0751
aroG
b0754
TU_263
TU_264
TU_3051
TU_3050
TU_3049
TU_3048
TU_3046
b0752
zitB
b0753
ybgS
b0755
gpmA
b0756
galM
b0757
galK
b0758
galT
Term: termination (2), NS: new start (1), PS: pausing site (2), iTSS_I: internal starting site (0)

ID: 15878−15984; Term: termination (2), NS: new start (2), PS: pausing site (0), iTSS_I: internal starting site (1)

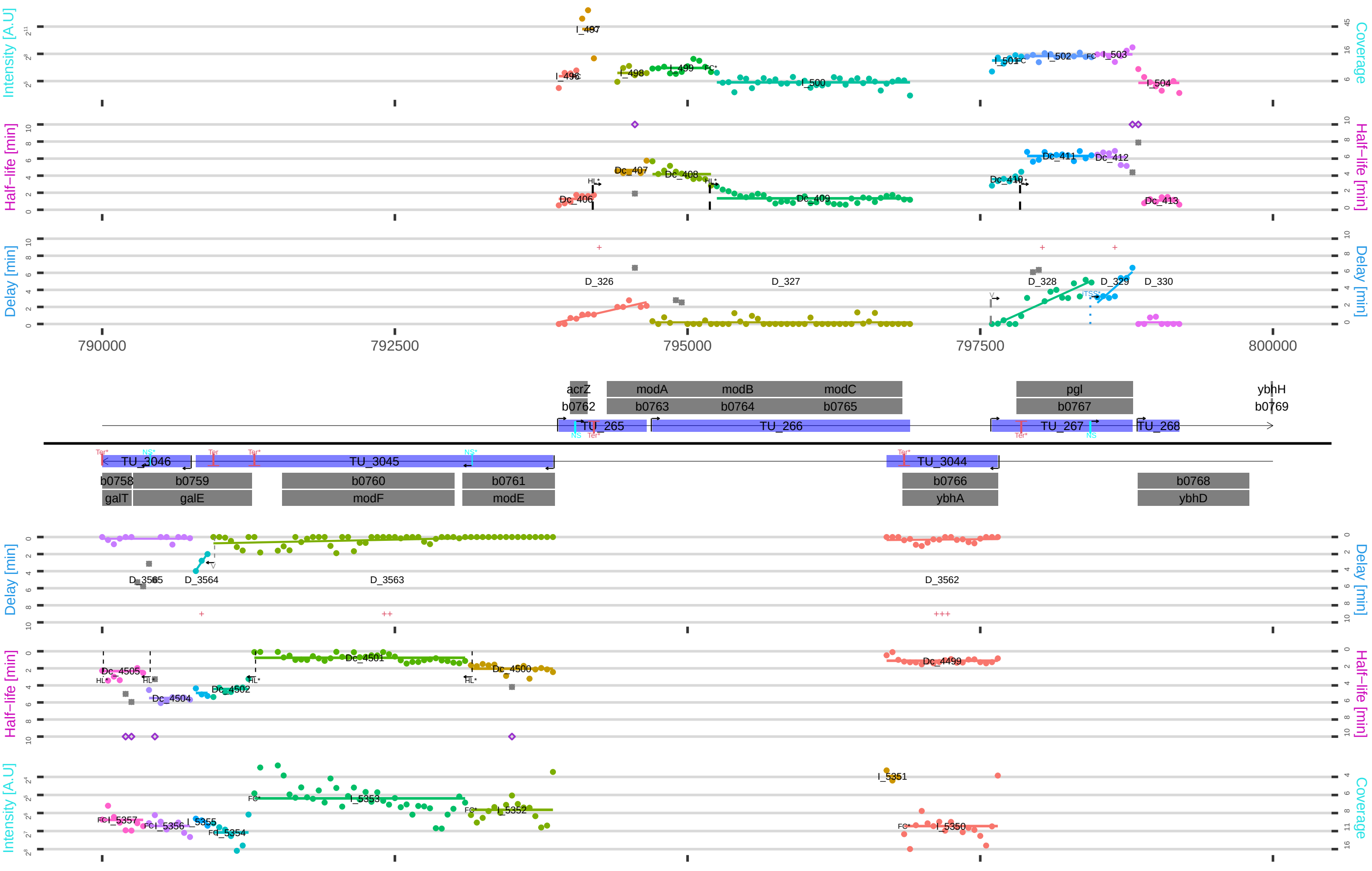

Term: termination (4), NS: new start (2), PS: pausing site (1), iTSS_I: internal starting site (0)

ID: 16029−16192; Term: termination (0), NS: new start (0), PS: pausing site (0), iTSS_I: internal starting site (0)

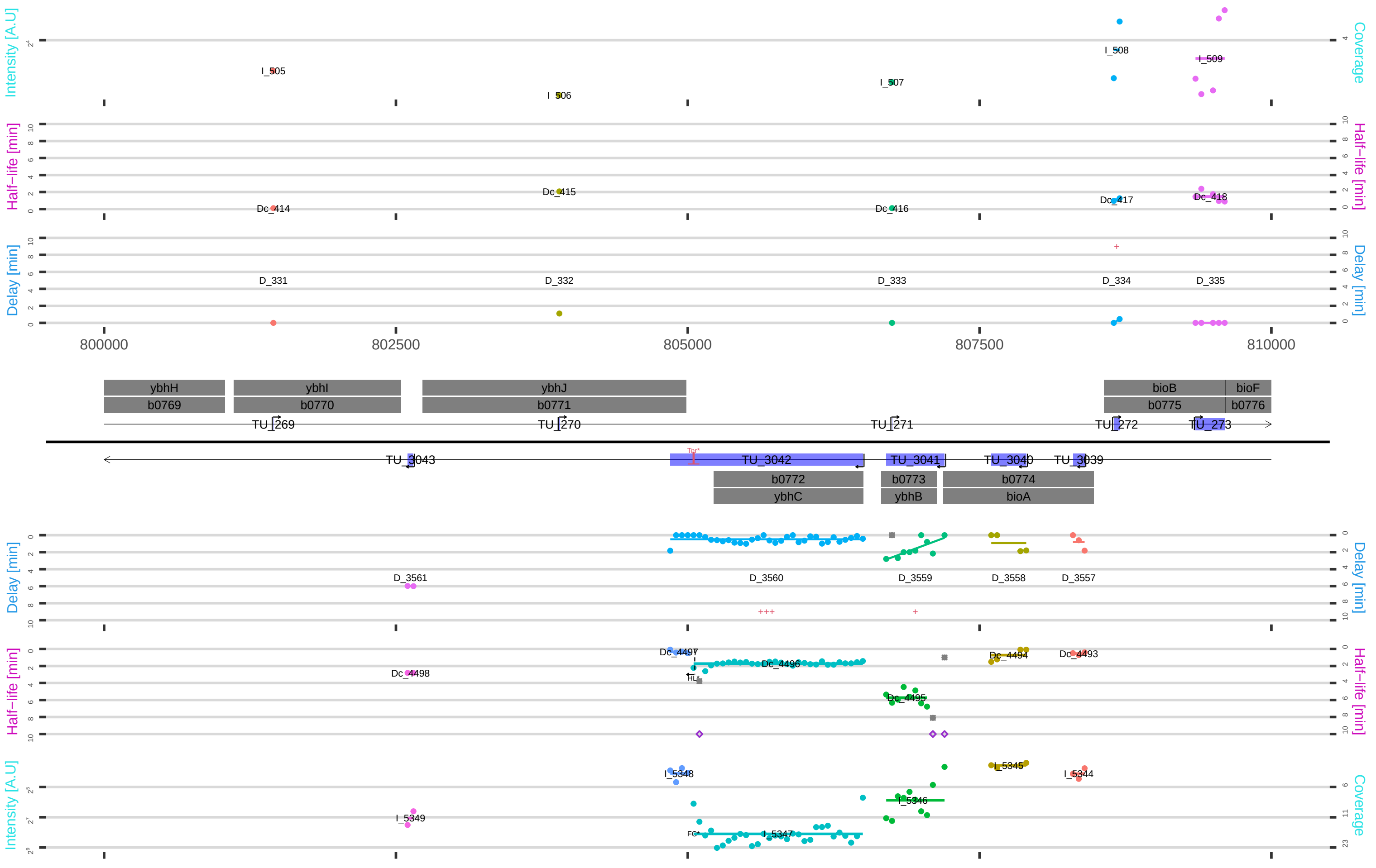

Term: termination (1), NS: new start (0), PS: pausing site (0), iTSS_I: internal starting site (0)

ID: 16214−16397; Term: termination (2), NS: new start (2), PS: pausing site (1), iTSS_I: internal starting site (0)

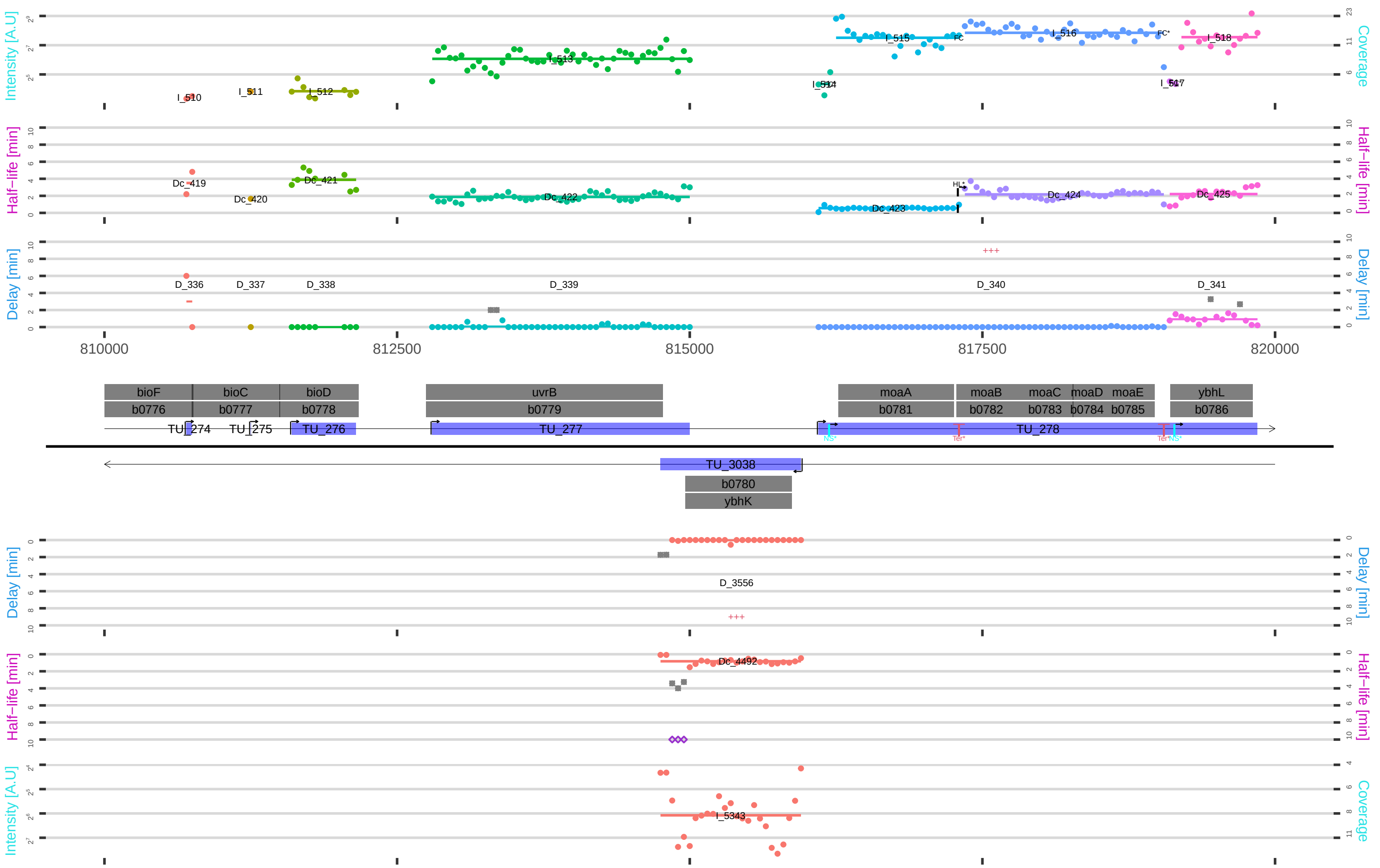

Term: termination (0), NS: new start (0), PS: pausing site (0), iTSS_I: internal starting site (0)

ID: 16417−16486; Term: termination (0), NS: new start (0), PS: pausing site (0), iTSS_I: internal starting site (0)

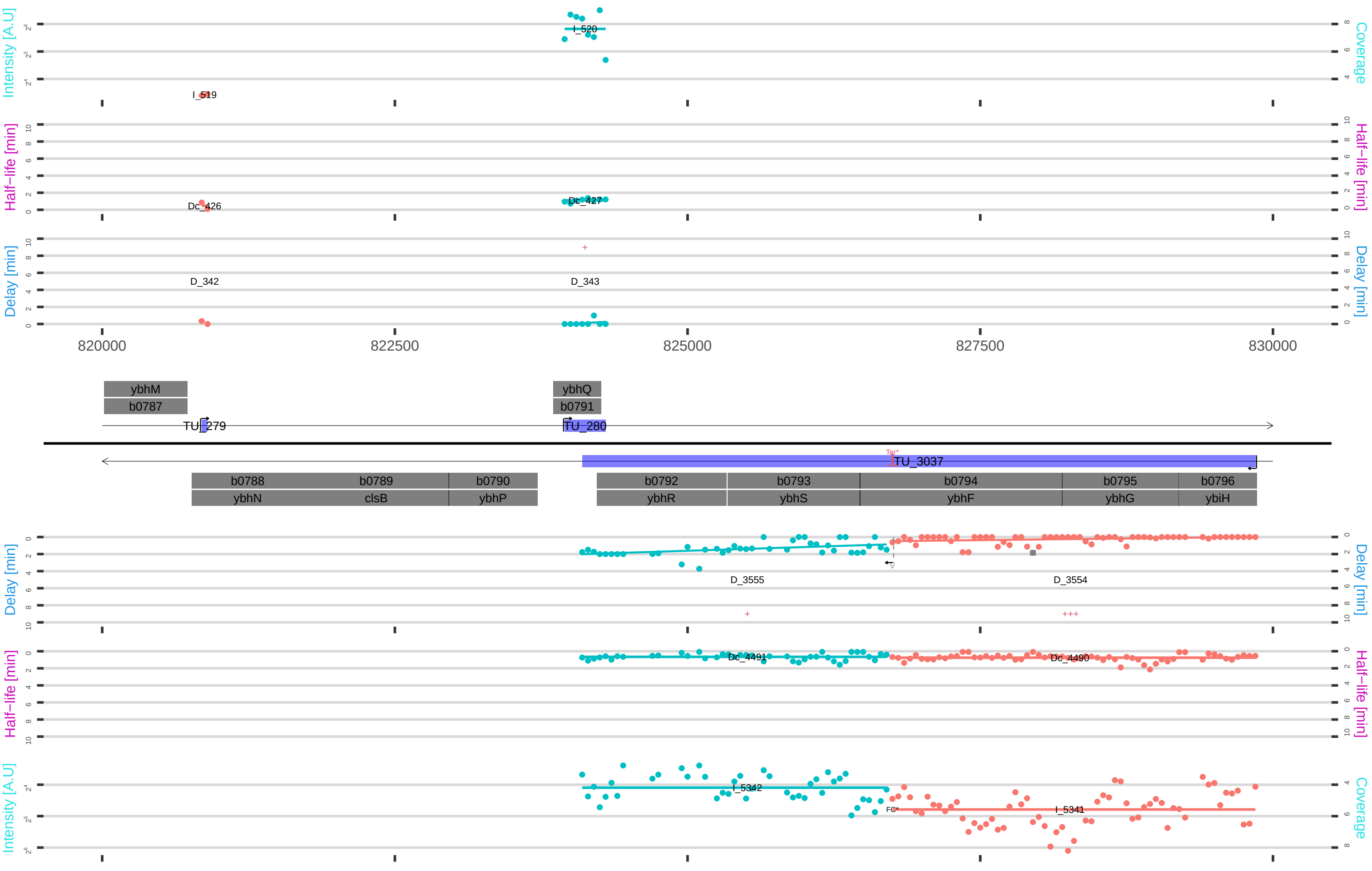

Term: termination (1), NS: new start (0), PS: pausing site (1), iTSS_I: internal starting site (0)

ID: 16602−16763; Term: termination (4), NS: new start (0), PS: pausing site (1), iTSS_I: internal starting site (0)

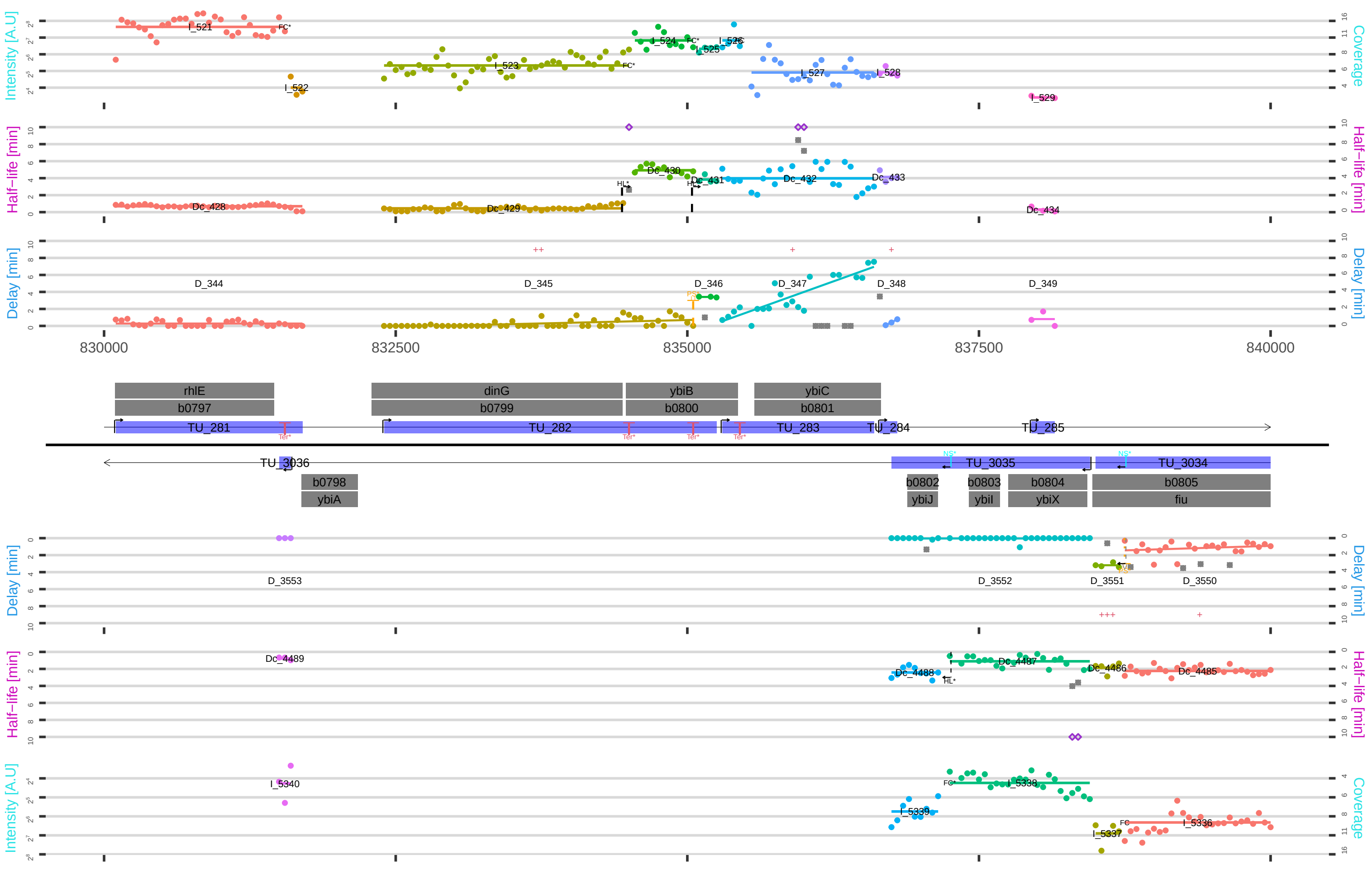

Term: termination (0), NS: new start (2), PS: pausing site (1), iTSS_I: internal starting site (0)

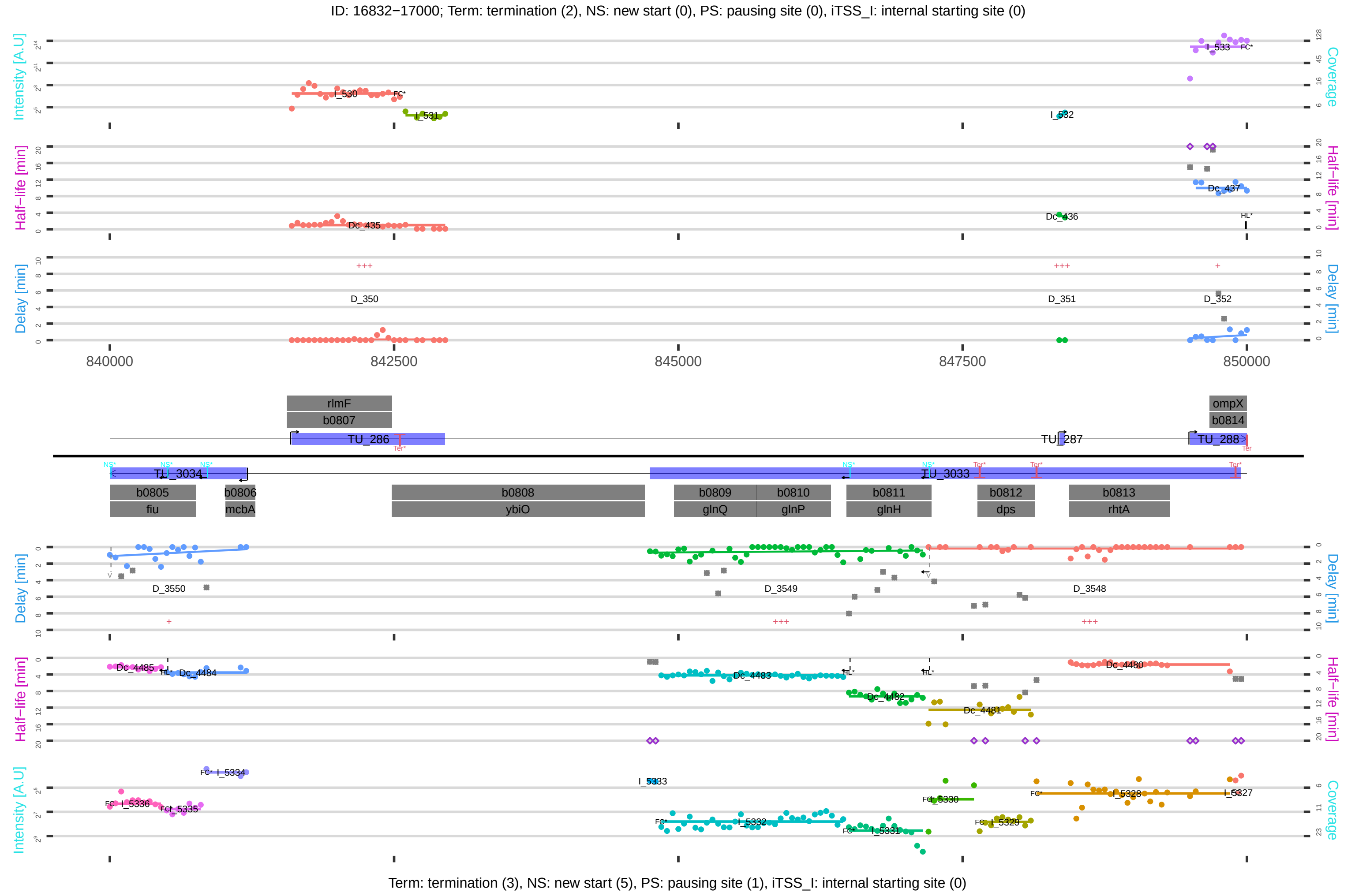

ID: 16832−17000; Term: termination (2), NS: new start (0), PS: pausing site (0), iTSS_I: internal starting site (0)
Intensity [A.U]
Coverage
Half−life [min]
Delay [min]
I_530
I_531
I_532
I_533
Dc_435
Dc_436
Dc_437
D_350
D_351
D_352
840000
842500
845000
847500
850000
rlmF
b0807
TU_286
TU_287
TU_288
ompX
b0814
TU_3034
TU_3033
b0805
fiu
b0806
mcbA
b0808
ybiO
b0809
glnQ
b0810
glnP
b0811
glnH
b0812
dps
b0813
rhtA
D_3550
D_3549
D_3548
Dc_4485
Dc_4484
Dc_4483
Dc_4482
Dc_4481
Dc_4480
I_5336
I_5335
I_5334
I_5333
I_5332
I_5331
I_5330
I_5329
I_5328
I_5327
Term: termination (3), NS: new start (5), PS: pausing site (1), iTSS_I: internal starting site (0)

ID: 17000−17186; Term: termination (3), NS: new start (0), PS: pausing site (0), iTSS_I: internal starting site (0)

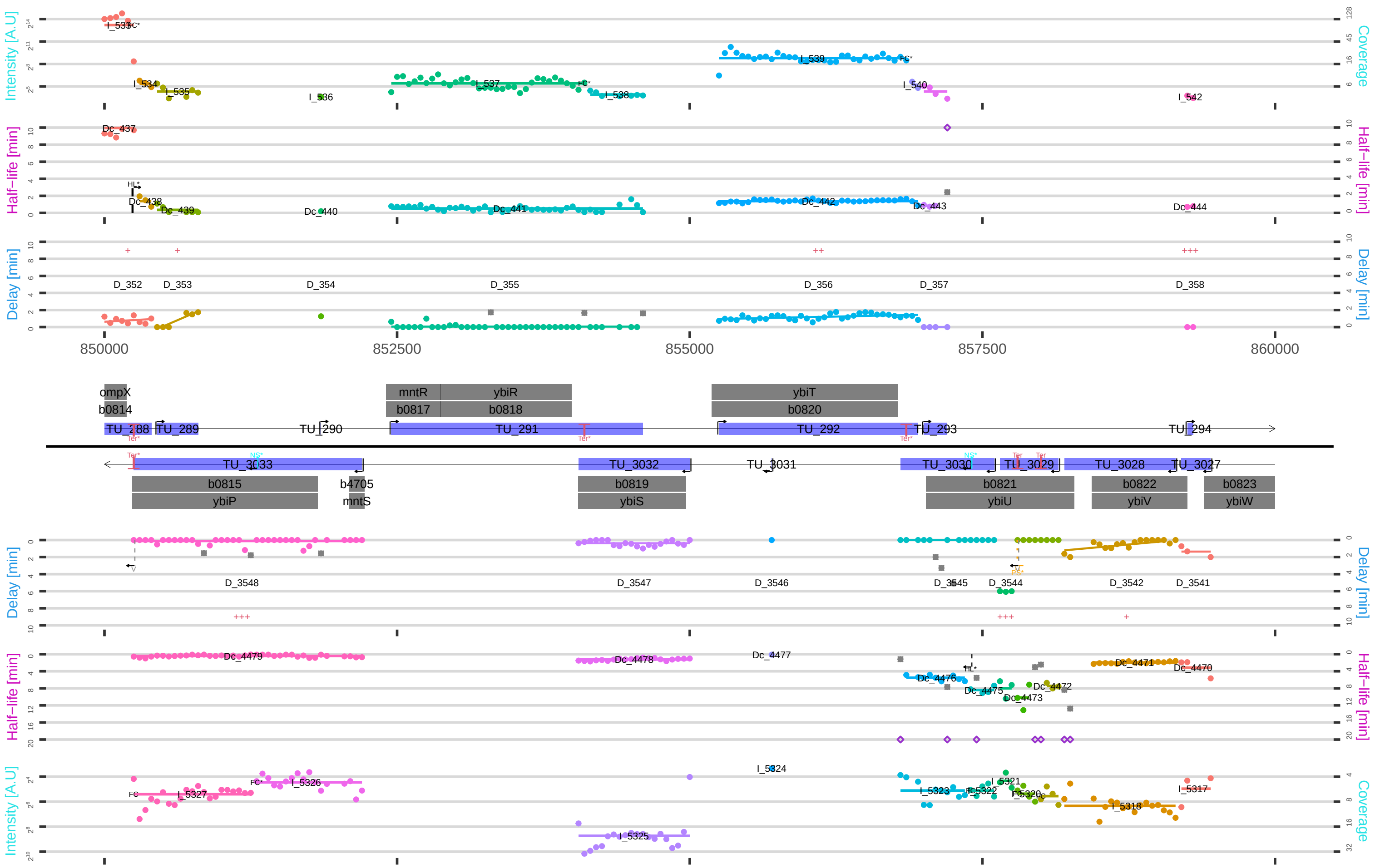

Term: termination (3), NS: new start (2), PS: pausing site (1), iTSS_I: internal starting site (0)

ID: 17260−17400; Term: termination (2), NS: new start (0), PS: pausing site (1), iTSS_I: internal starting site (0)

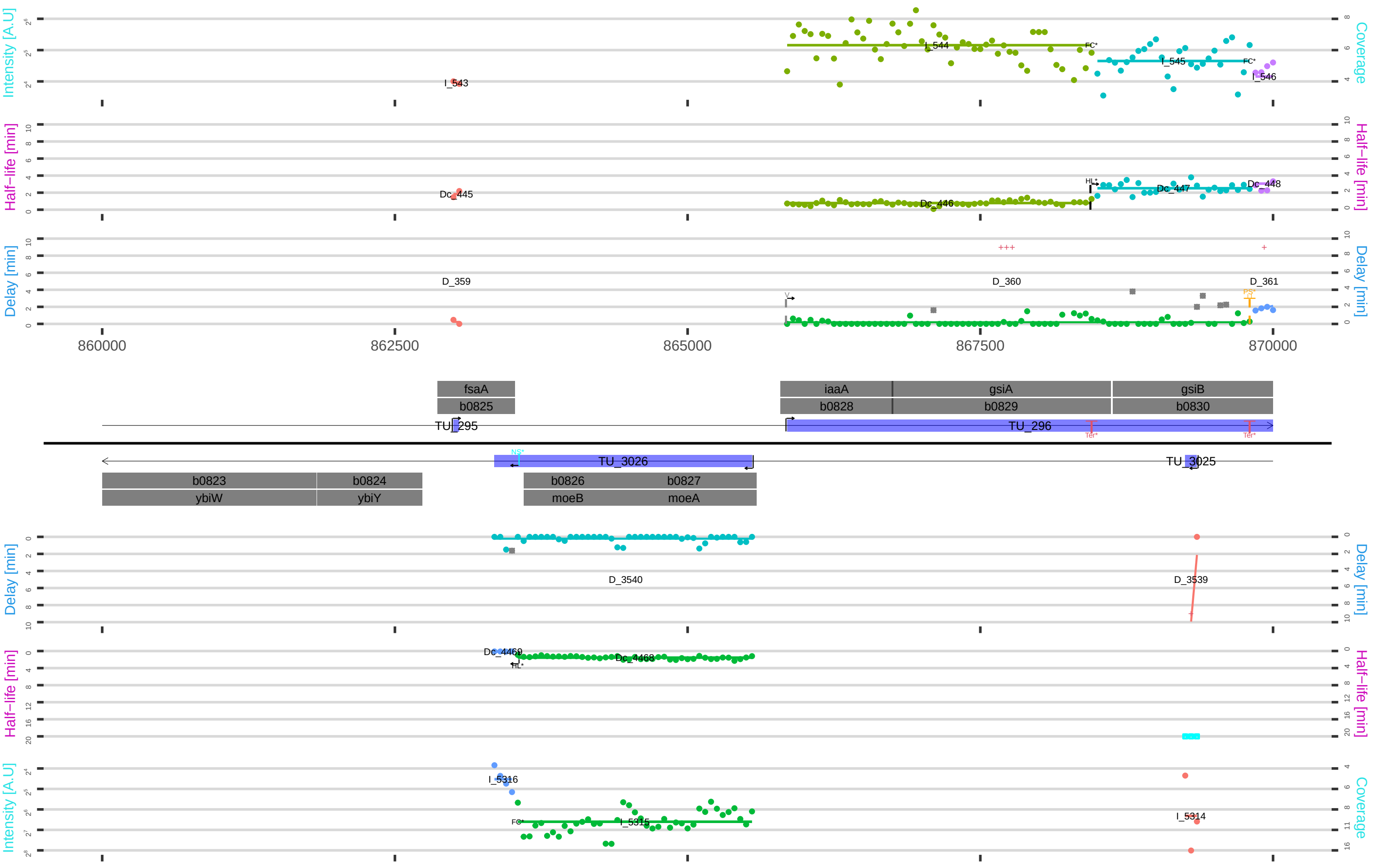

Term: termination (0), NS: new start (1), PS: pausing site (0), iTSS_I: internal starting site (0)

ID: 17400−17600; Term: termination (0), NS: new start (1), PS: pausing site (0), iTSS_I: internal starting site (1)

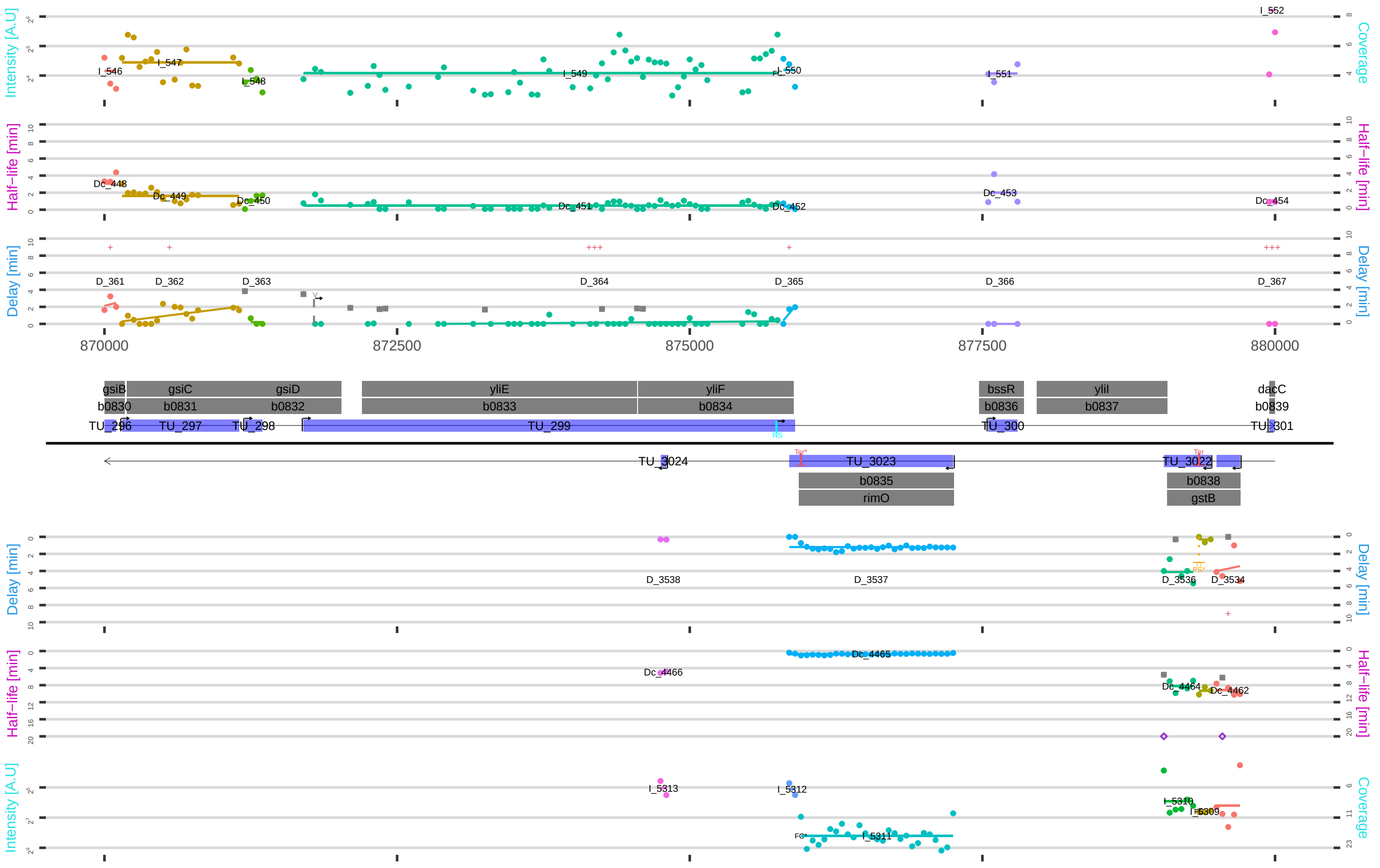

Term: termination (2), NS: new start (0), PS: pausing site (1), iTSS_I: internal starting site (0)

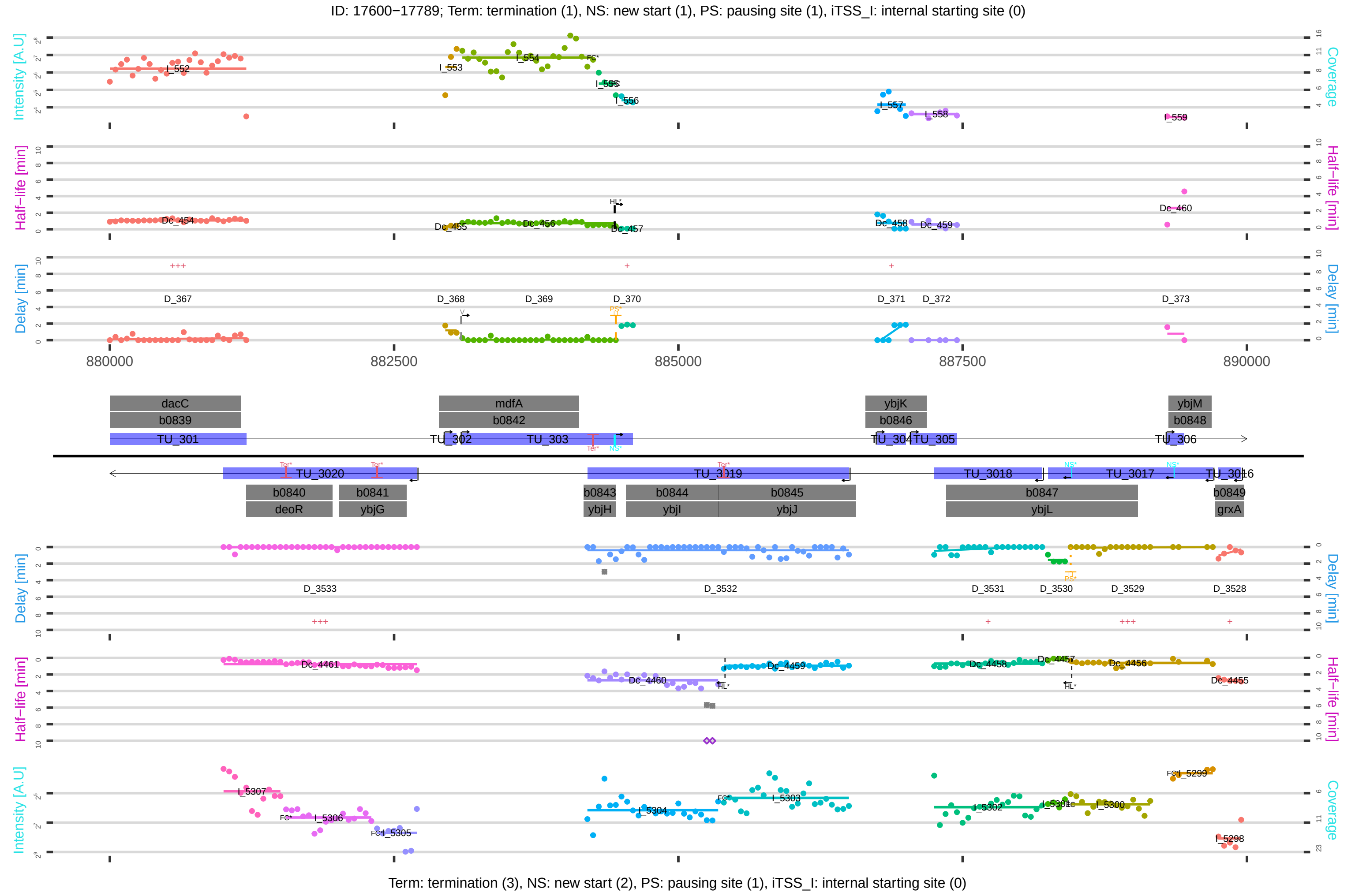
ID: 17600−17789; Term: termination (1), NS: new start (1), PS: pausing site (1), iTSS_I: internal starting site (0)
Intensity [A.U]
Coverage
Half−life [min]
Delay [min]
880000
882500
885000
887500
890000
dacC
b0839
TU_301
mdfA
b0842
TU_302
TU_303
ybjK
b0846
TU_304
TU_305
ybjM
b0848
TU_306
TU_3020
b0840
deoR
b0841
ybjG
TU_3019
b0843
ybjH
b0844
ybjI
b0845
ybjJ
TU_3018
TU_3017
TU_3016
b0847
ybjL
b0849
grxA
D_3533
D_3532
D_3531
D_3530
D_3529
D_3528
Dc_4461
Dc_4460
Dc_4459
Dc_4458
Dc_4457
Dc_4456
Dc_4455
I_5307
I_5306
I_5305
I_5304
I_5303
I_5302
I_5301
I_5300
I_5299
I_5298
Term: termination (3), NS: new start (2), PS: pausing site (1), iTSS_I: internal starting site (0)

ID: 17803−17986; Term: termination (1), NS: new start (3), PS: pausing site (0), iTSS_I: internal starting site (0)

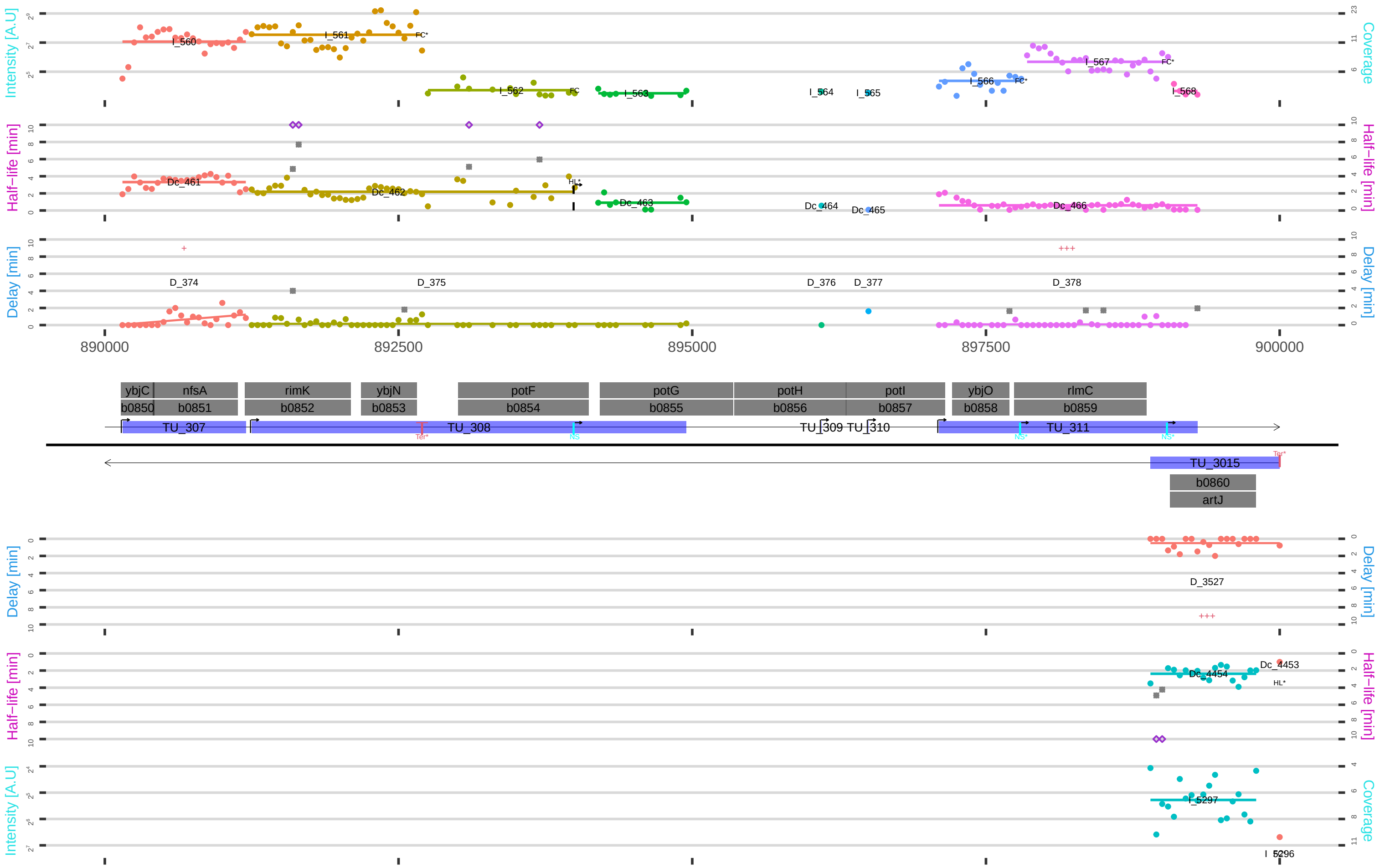

Term: termination (1), NS: new start (0), PS: pausing site (0), iTSS_I: internal starting site (0)

ID: 18077−18117; Term: termination (1), NS: new start (1), PS: pausing site (0), iTSS_I: internal starting site (0)

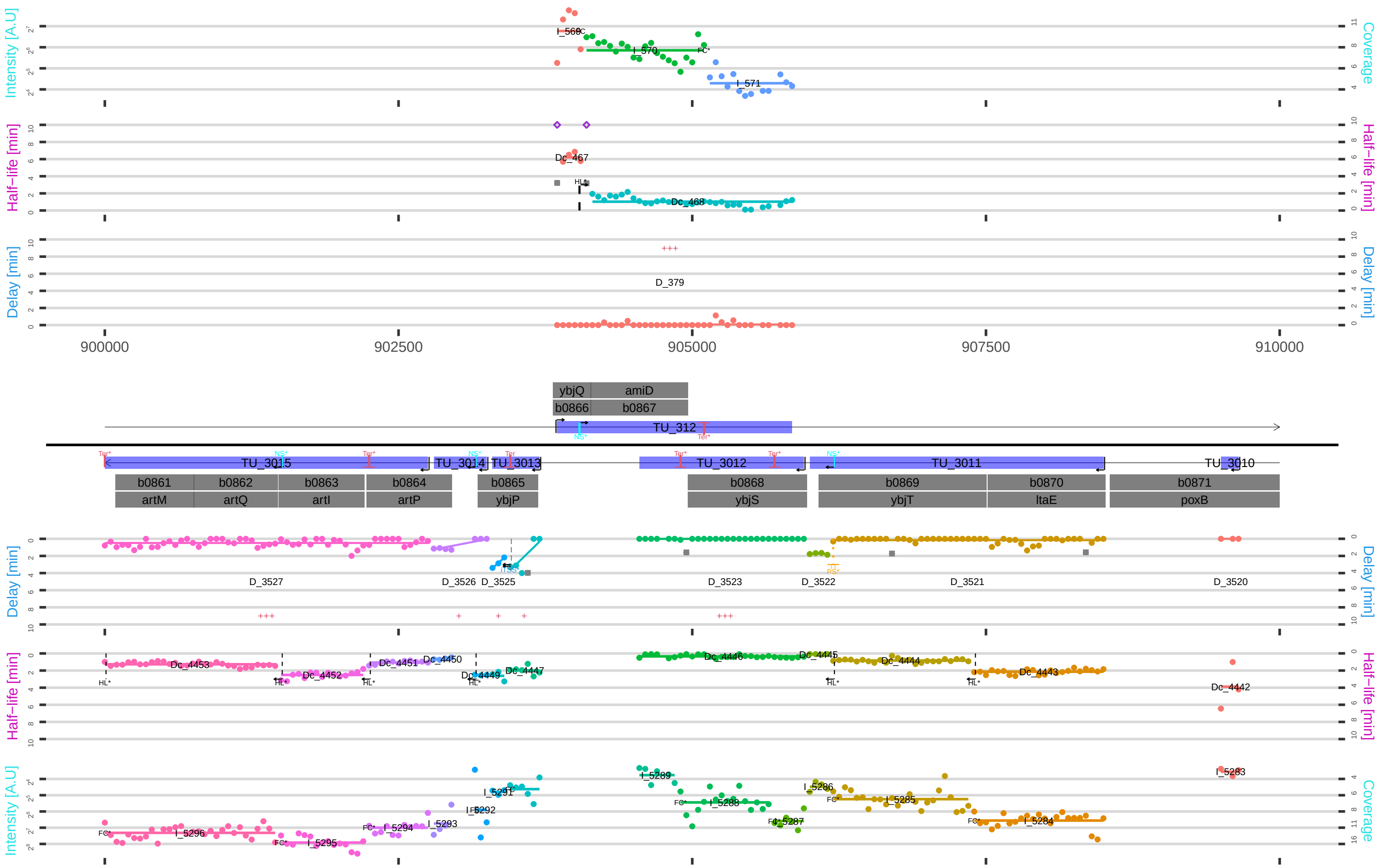

Term: termination (5), NS: new start (3), PS: pausing site (1), iTSS_I: internal starting site (1)

ID: 18285−18400; Term: termination (1), NS: new start (1), PS: pausing site (0), iTSS_I: internal starting site (0)

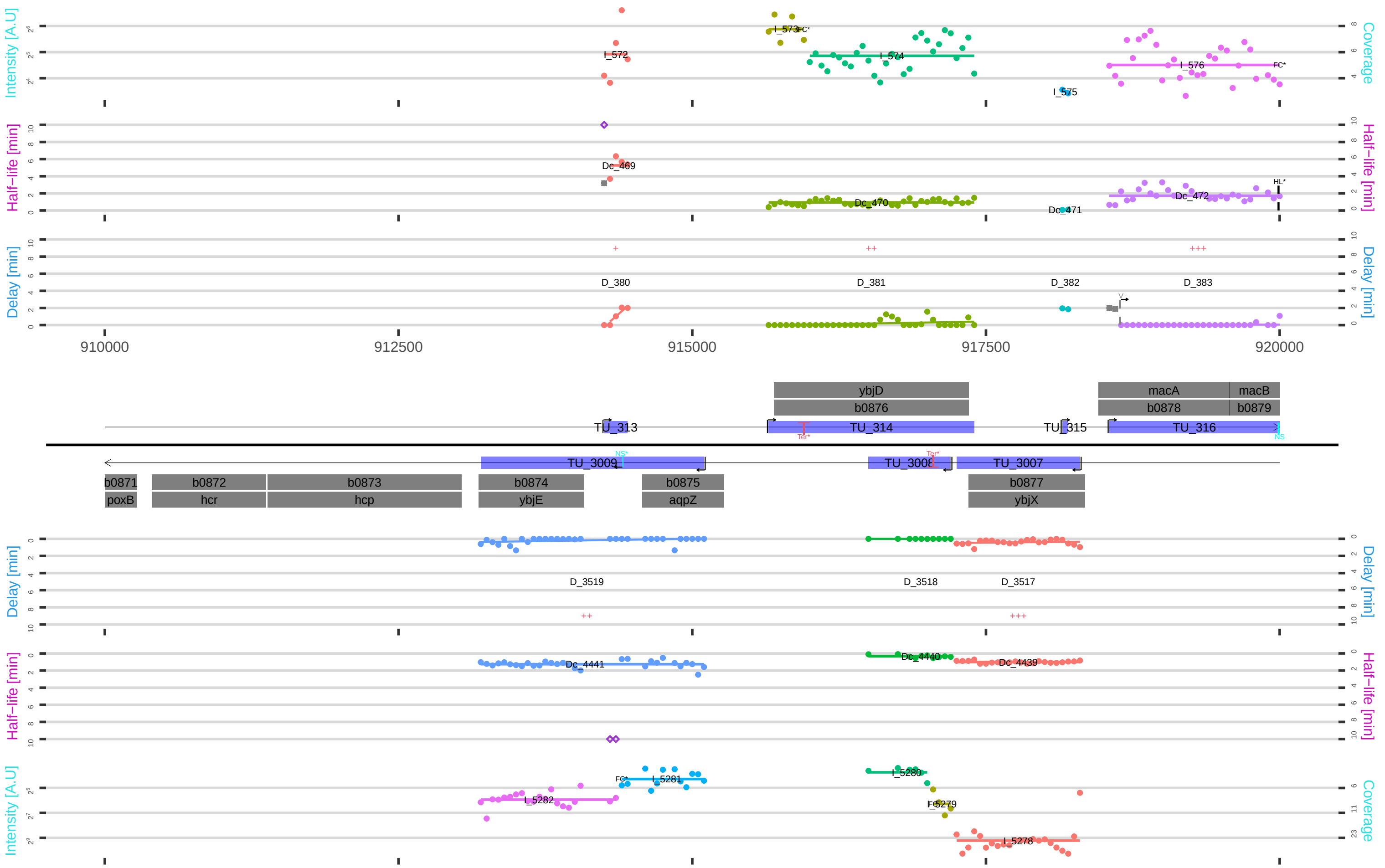

Term: termination (1), NS: new start (1), PS: pausing site (0), iTSS_I: internal starting site (0)

ID: 18400−18513; Term: termination (2), NS: new start (4), PS: pausing site (1), iTSS_I: internal starting site (0)

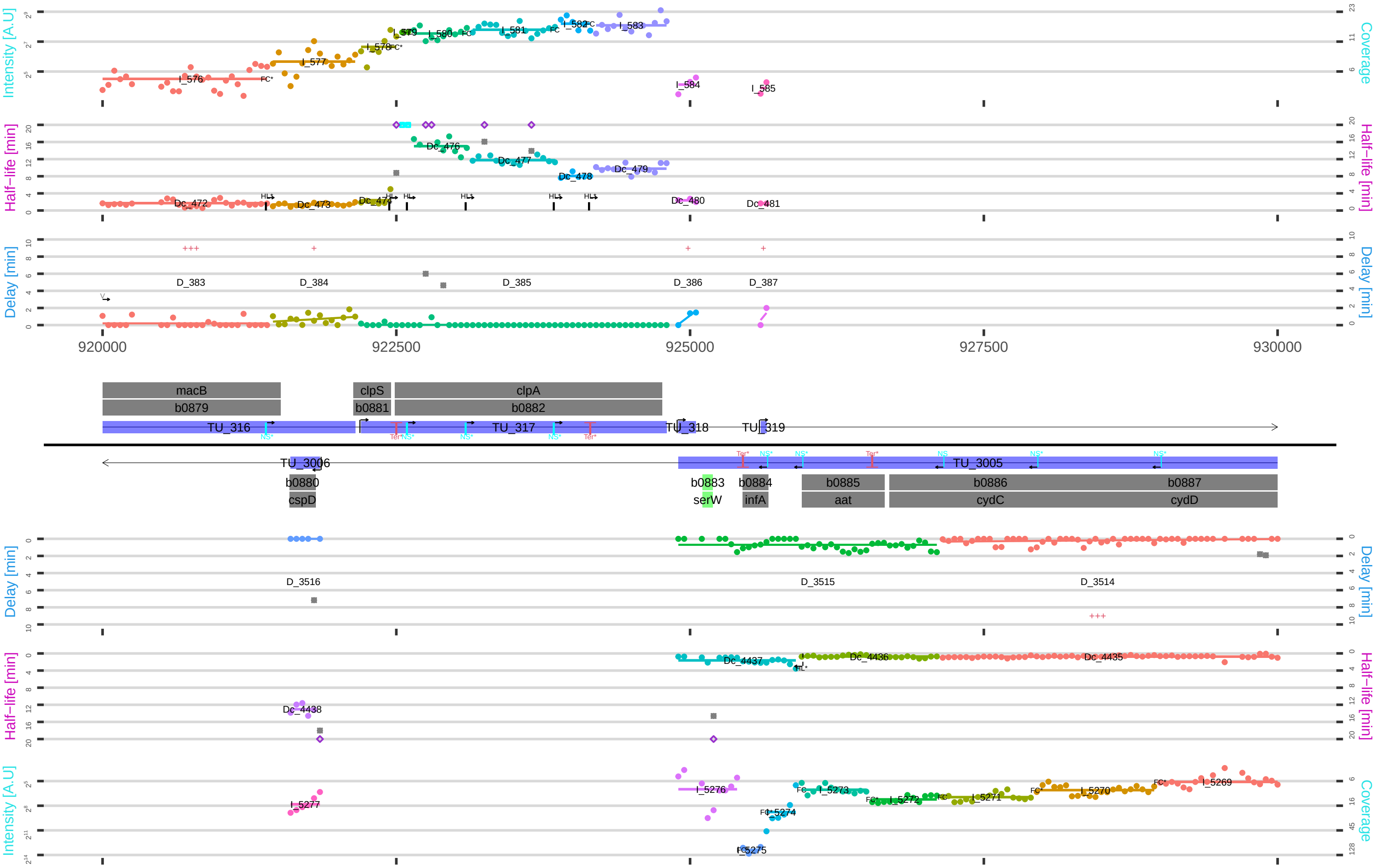

Term: termination (2), NS: new start (5), PS: pausing site (1), iTSS_I: internal starting site (0)

ID: 18633−18800; Term: termination (2), NS: new start (2), PS: pausing site (0), iTSS_I: internal starting site (2)

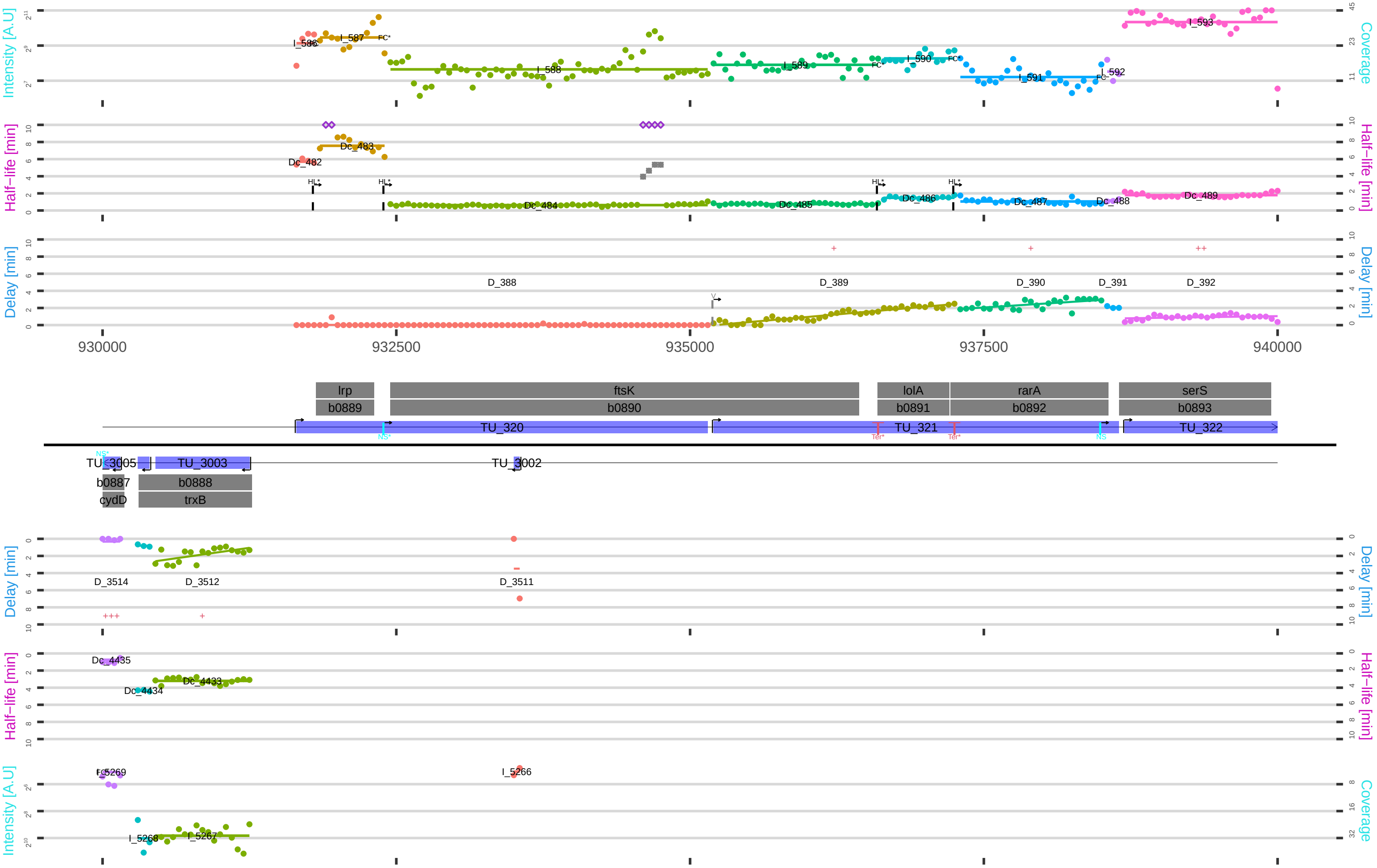

Term: termination (0), NS: new start (1), PS: pausing site (0), iTSS_I: internal starting site (0)

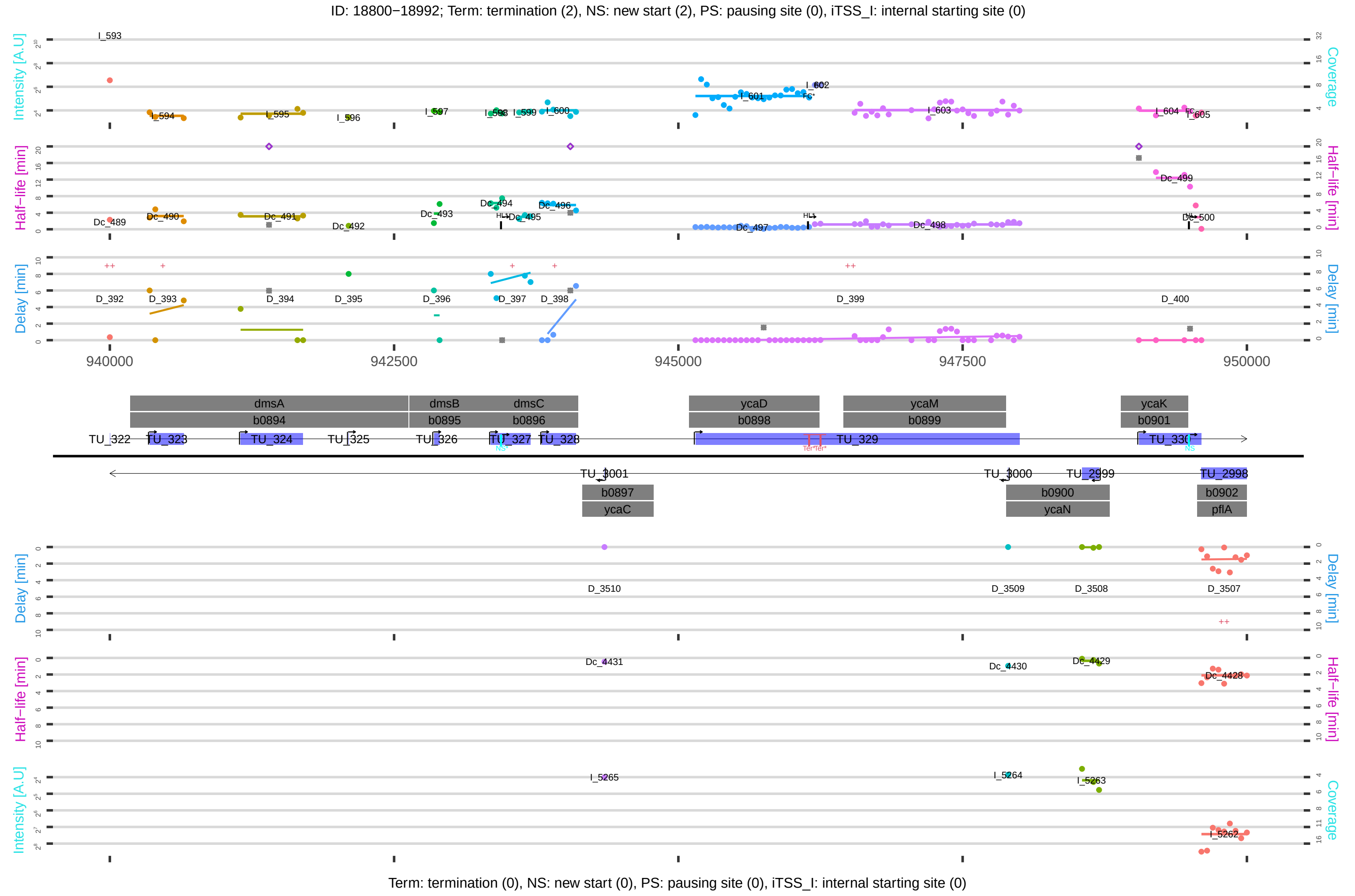
ID: 18800−18992; Term: termination (2), NS: new start (2), PS: pausing site (0), iTSS_I: internal starting site (0)
Intensity [A.U]
Coverage
Half−life [min]
Delay [min]
940000
942500
945000
947500
950000
dmsA
b0894
dmsB
b0895
dmsC
b0896
ycaD
b0898
ycaM
b0899
ycaK
b0901
TU_322
TU_323
TU_324
TU_325
TU_326
TU_327
TU_328
TU_329
TU_330
TU_3001
TU_3000
TU_2999
TU_2998
b0897
ycaC
b0900
ycaN
b0902
pflA
D_3510
D_3509
D_3508
D_3507
Dc_4431
Dc_4430
Dc_4429
Dc_4428
I_5265
I_5264
I_5263
I_5262
Term: termination (0), NS: new start (0), PS: pausing site (0), iTSS_I: internal starting site (0)

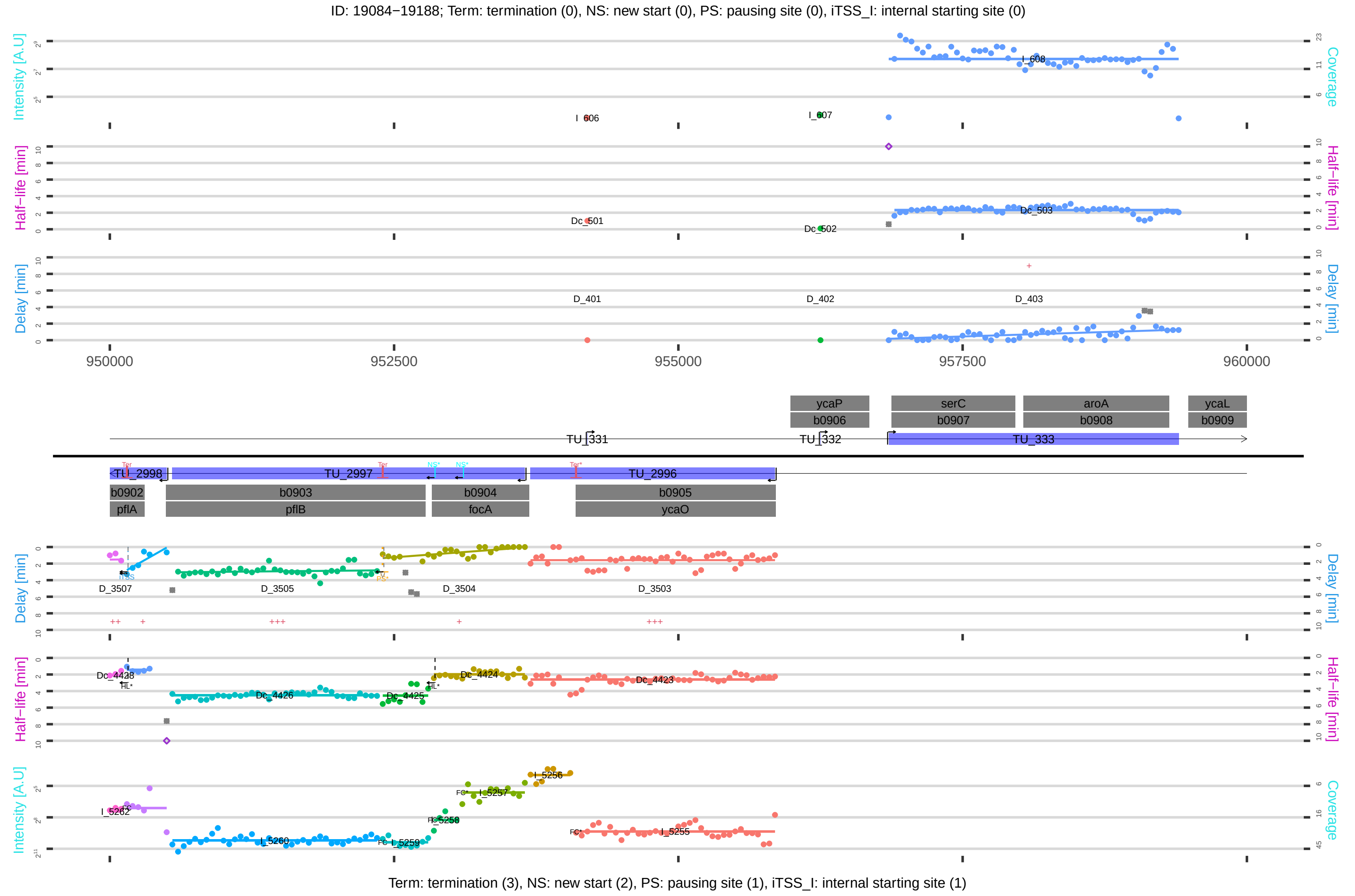
ID: 19084−19188; Term: termination (0), NS: new start (0), PS: pausing site (0), iTSS_I: internal starting site (0)
Intensity [A.U]
Coverage
Half−life [min]
Delay [min]
I_606
I_607
I_608
Dc_501
Dc_502
Dc_503
D_401
D_402
D_403
950000
952500
955000
957500
960000
ycaP
b0906
serC
b0907
aroA
b0908
ycaL
b0909
TU_331
TU_332
TU_333
TU_2998
TU_2997
TU_2996
b0902
pflA
b0903
pflB
b0904
focA
b0905
ycaO
D_3507
D_3505
D_3504
D_3503
Dc_4428
Dc_4426
Dc_4425
Dc_4424
Dc_4423
I_5262
I_5260
I_5259
I_5258
I_5257
I_5256
I_5255
Term: termination (3), NS: new start (2), PS: pausing site (1), iTSS_I: internal starting site (1)

ID: 19204−19400; Term: termination (2), NS: new start (2), PS: pausing site (2), iTSS_I: internal starting site (0)

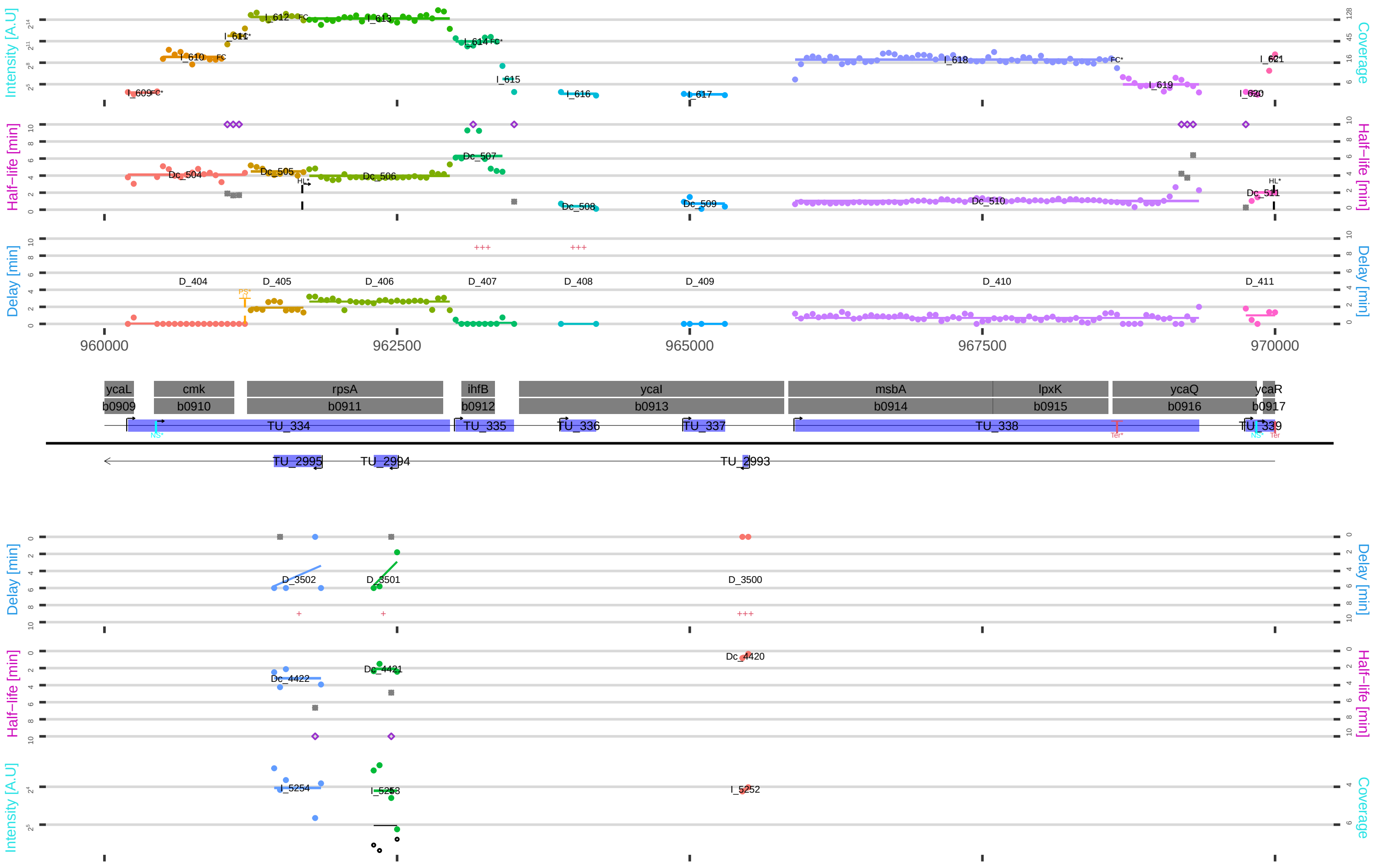

Term: termination (0), NS: new start (0), PS: pausing site (0), iTSS_I: internal starting site (0)

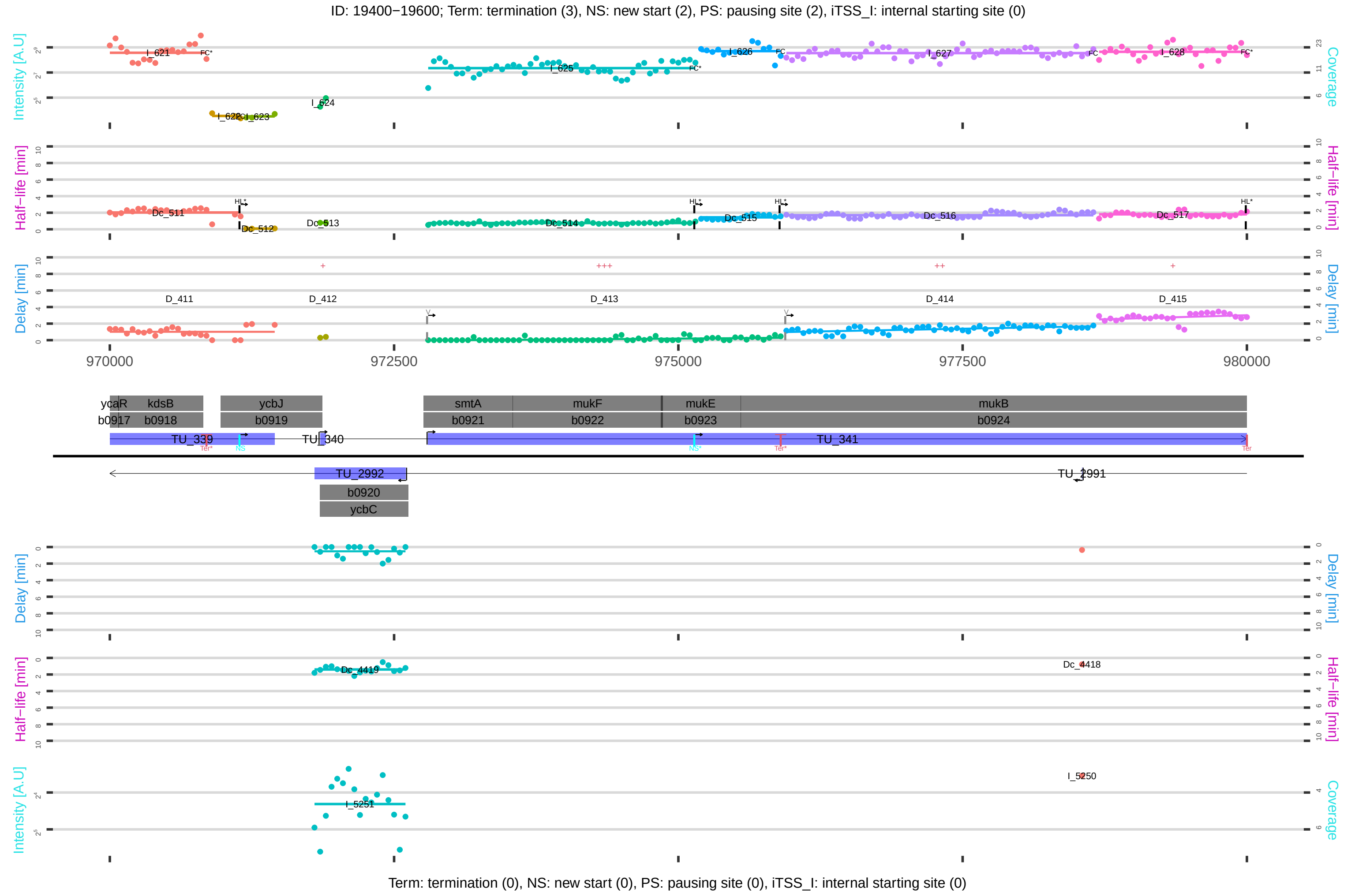
ID: 19400−19600; Term: termination (3), NS: new start (2), PS: pausing site (2), iTSS_I: internal starting site (0)
Intensity [A.U]
Coverage
Half−life [min]
Delay [min]
I_621
I_622
I_623
I_624
I_625
I_626
I_627
I_628
FC*
FC
Dc_511
Dc_512
Dc_513
Dc_514
Dc_515
Dc_516
Dc_517
HL*
D_411
D_412
D_413
D_414
D_415
970000
972500
975000
977500
980000
ycaR
kdsB
ycbJ
smtA
mukF
mukE
mukB
b0917
b0918
b0919
b0921
b0922
b0923
b0924
TU_339
TU_340
TU_341
Ter*
NS
NS*
Ter
TU_2992
TU_2991
b0920
ycbC
Dc_4419
Dc_4418
I_5251
I_5250
Term: termination (0), NS: new start (0), PS: pausing site (0), iTSS_I: internal starting site (0)

ID: 19600−19800; Term: termination (3), NS: new start (4), PS: pausing site (1), iTSS_I: internal starting site (1)

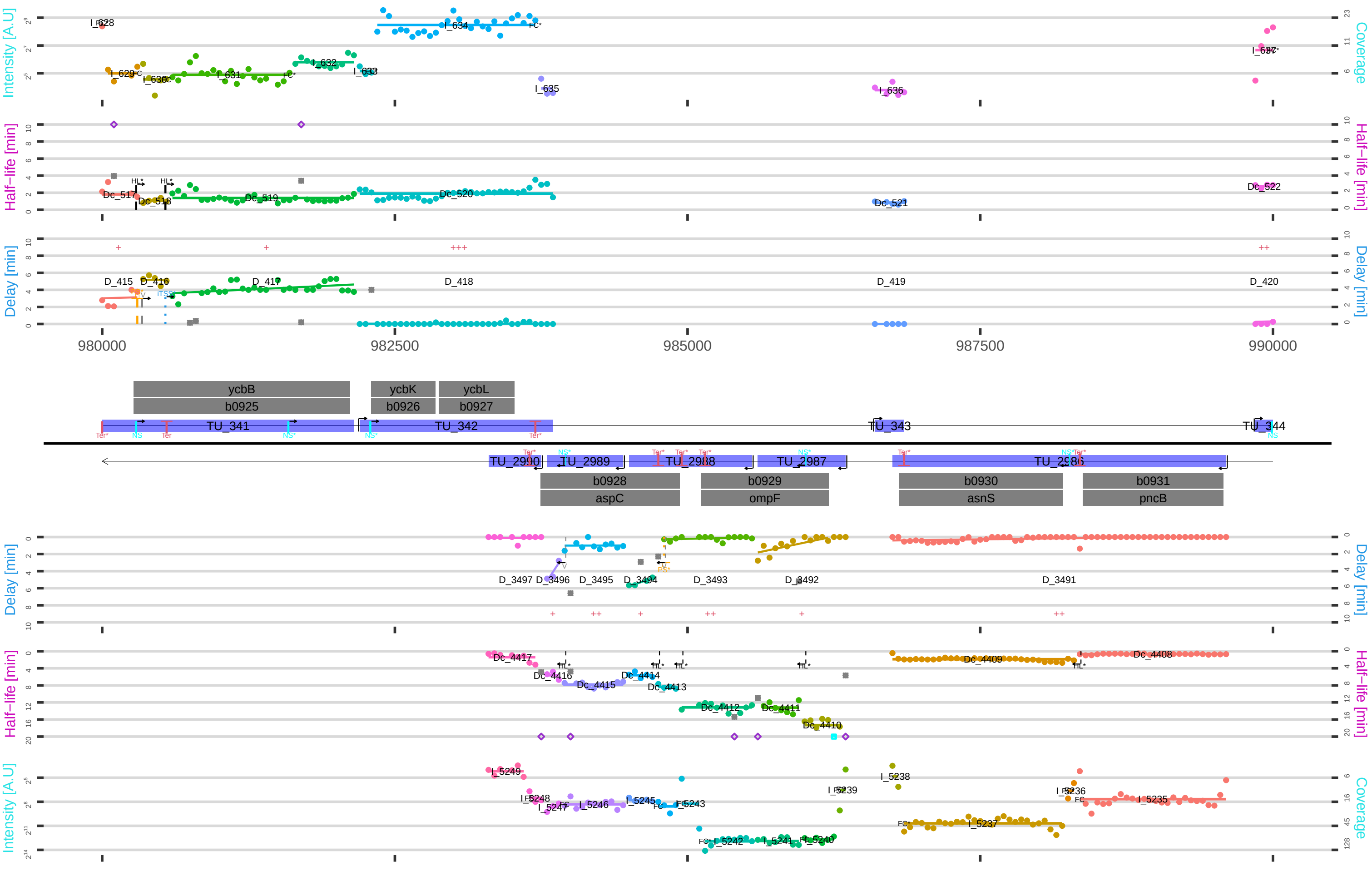

Term: termination (6), NS: new start (3), PS: pausing site (2), iTSS_I: internal starting site (0)

ID: 19800−19852; Term: termination (0), NS: new start (1), PS: pausing site (0), iTSS_I: internal starting site (0)

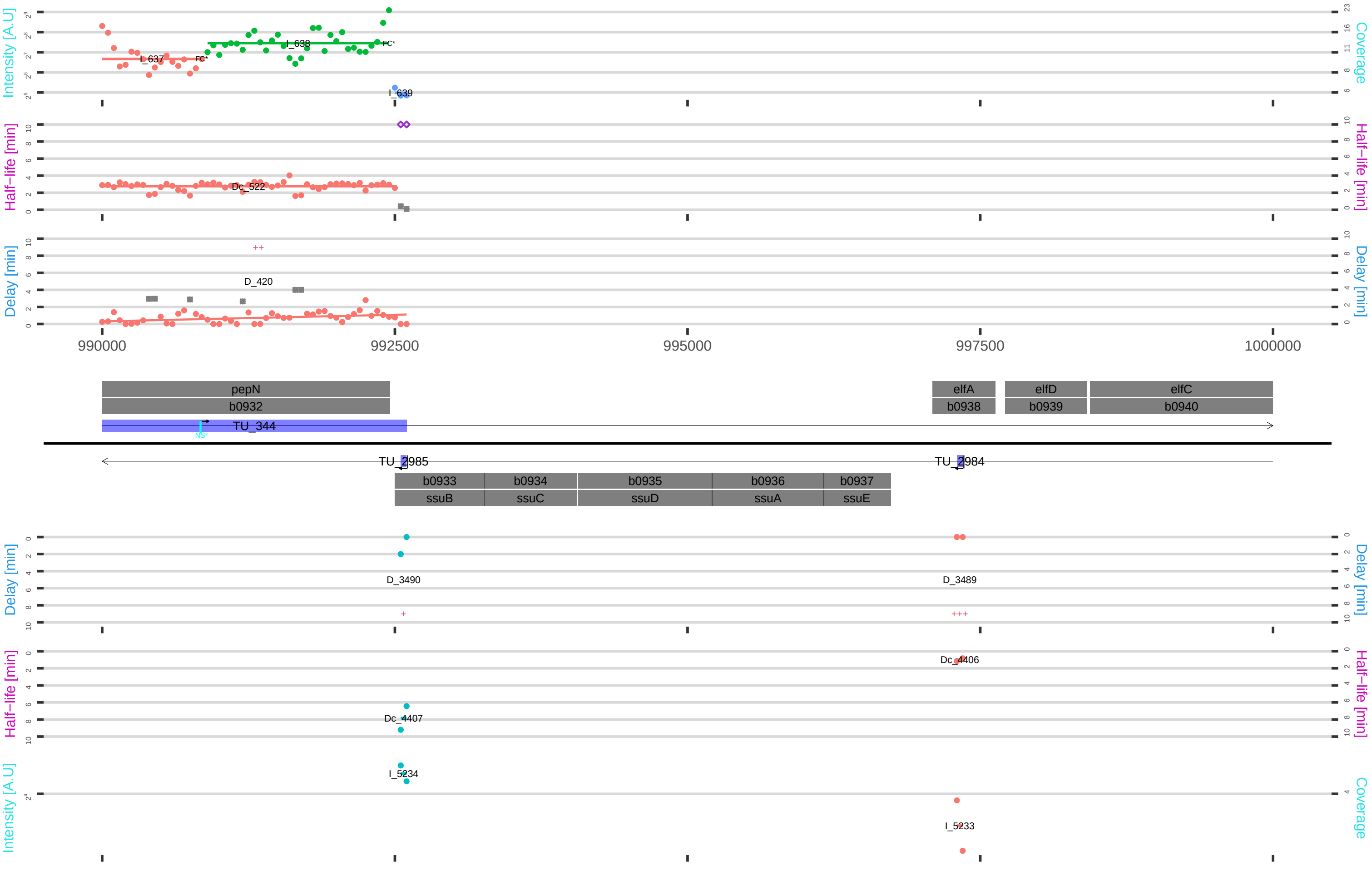

Term: termination (0), NS: new start (0), PS: pausing site (0), iTSS_I: internal starting site (0)

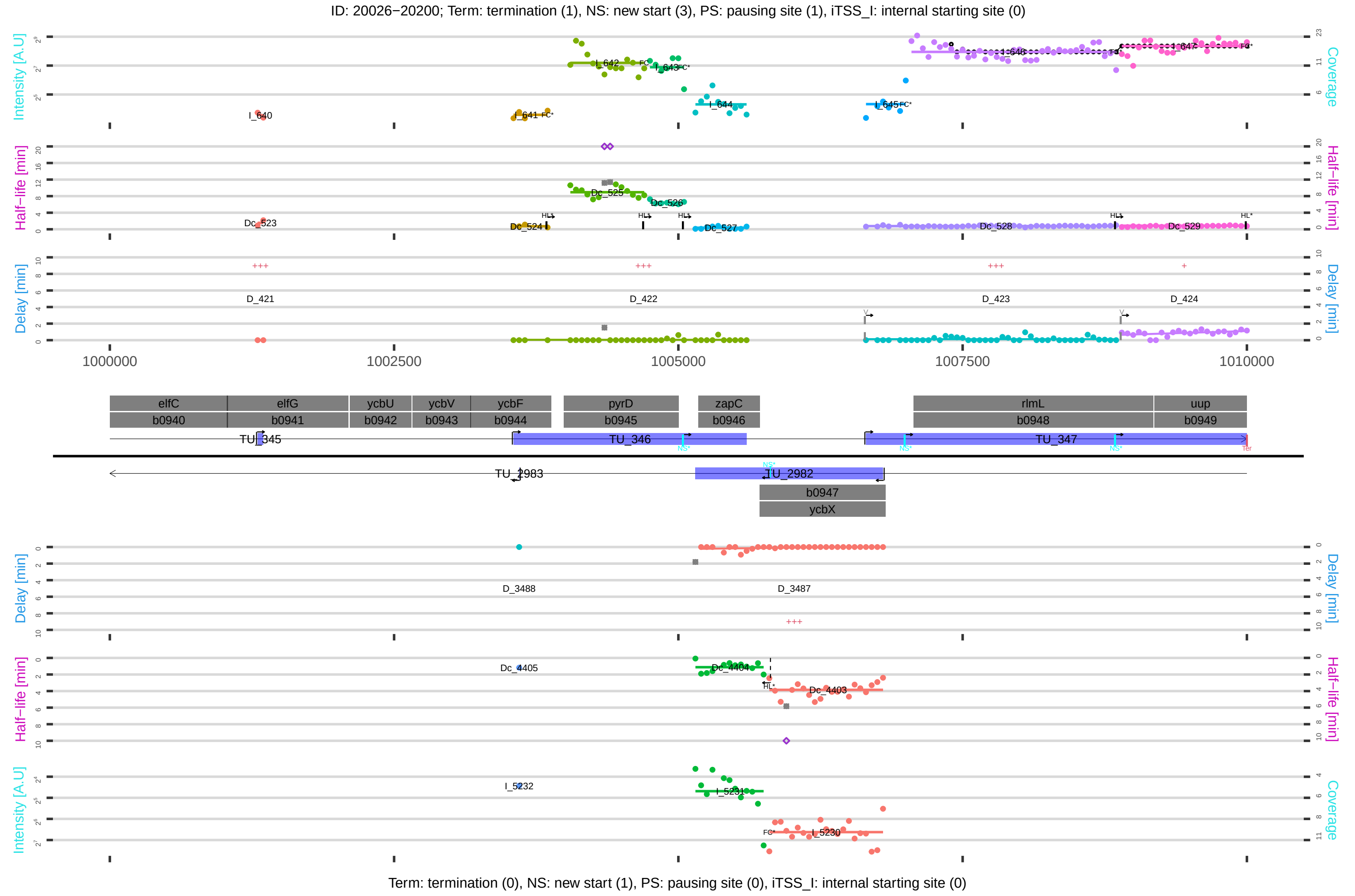

ID: 20026−20200; Term: termination (1), NS: new start (3), PS: pausing site (1), iTSS_I: internal starting site (0)
Intensity [A.U]
Coverage
Half−life [min]
Delay [min]
I_640
I_641
I_642
I_643
I_644
I_645
I_646
I_647
Dc_523
Dc_524
Dc_525
Dc_526
Dc_527
Dc_528
Dc_529
D_421
D_422
D_423
D_424
1000000
1002500
1005000
1007500
1010000
elfC
b0940
elfG
b0941
ycbU
b0942
ycbV
b0943
ycbF
b0944
pyrD
b0945
zapC
b0946
rlmL
b0948
uup
b0949
TU_345
TU_346
TU_347
TU_2983
TU_2982
b0947
ycbX
D_3488
D_3487
Dc_4405
Dc_4404
Dc_4403
I_5232
I_5231
I_5230
Term: termination (0), NS: new start (1), PS: pausing site (0), iTSS_I: internal starting site (0)

ID: 20200−20388; Term: termination (4), NS: new start (2), PS: pausing site (1), iTSS_I: internal starting site (1)

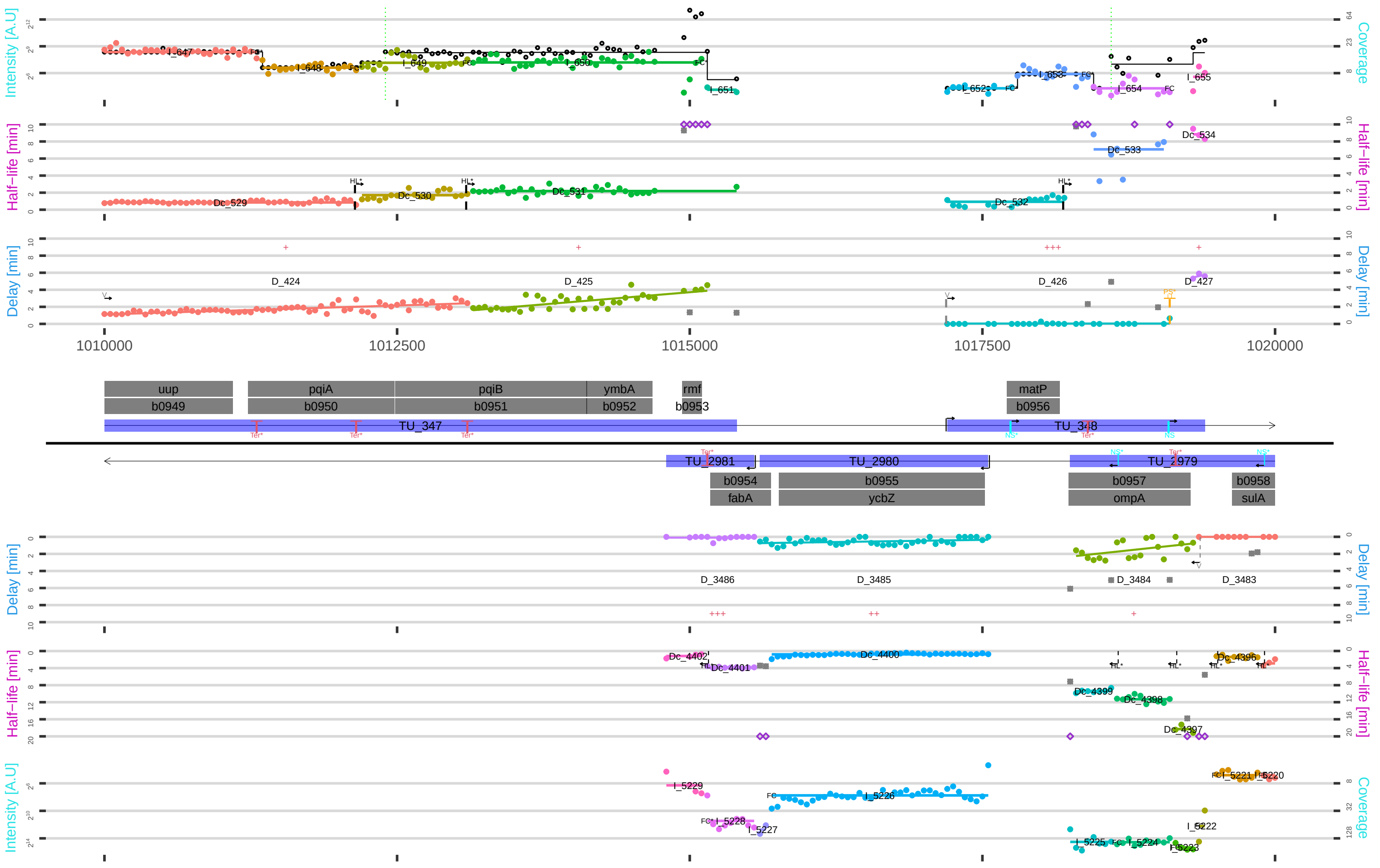

Term: termination (2), NS: new start (2), PS: pausing site (1), iTSS_I: internal starting site (0)

ID: 20410−20596; Term: termination (1), NS: new start (0), PS: pausing site (0), iTSS_I: internal starting site (0)

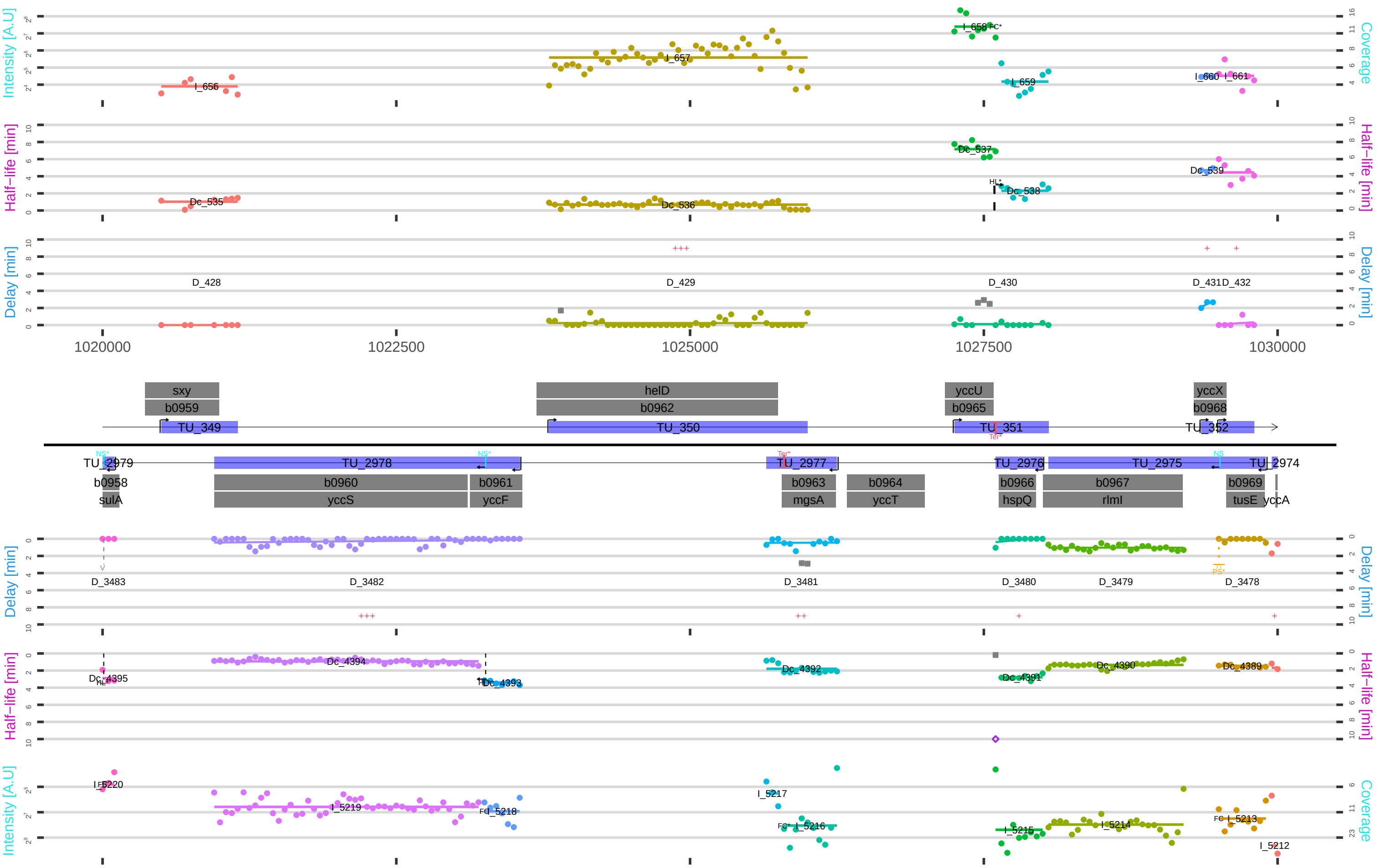

Term: termination (1), NS: new start (3), PS: pausing site (1), iTSS_I: internal starting site (0)

ID: 20741−20797; Term: termination (0), NS: new start (1), PS: pausing site (0), iTSS_I: internal starting site (0)

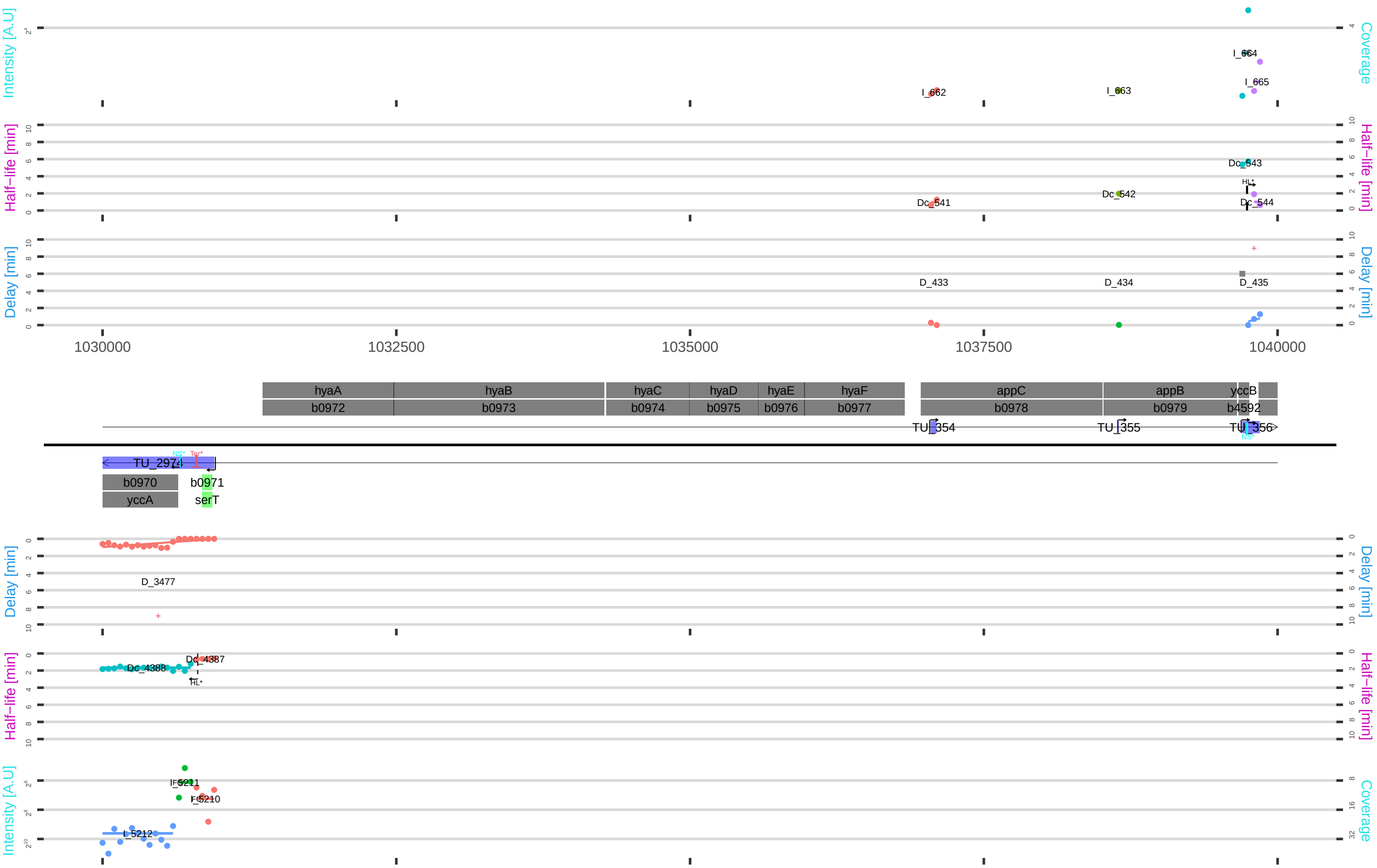

Term: termination (1), NS: new start (1), PS: pausing site (0), iTSS_I: internal starting site (0)

ID: 20801−20991; Term: termination (0), NS: new start (0), PS: pausing site (0), iTSS_I: internal starting site (0)

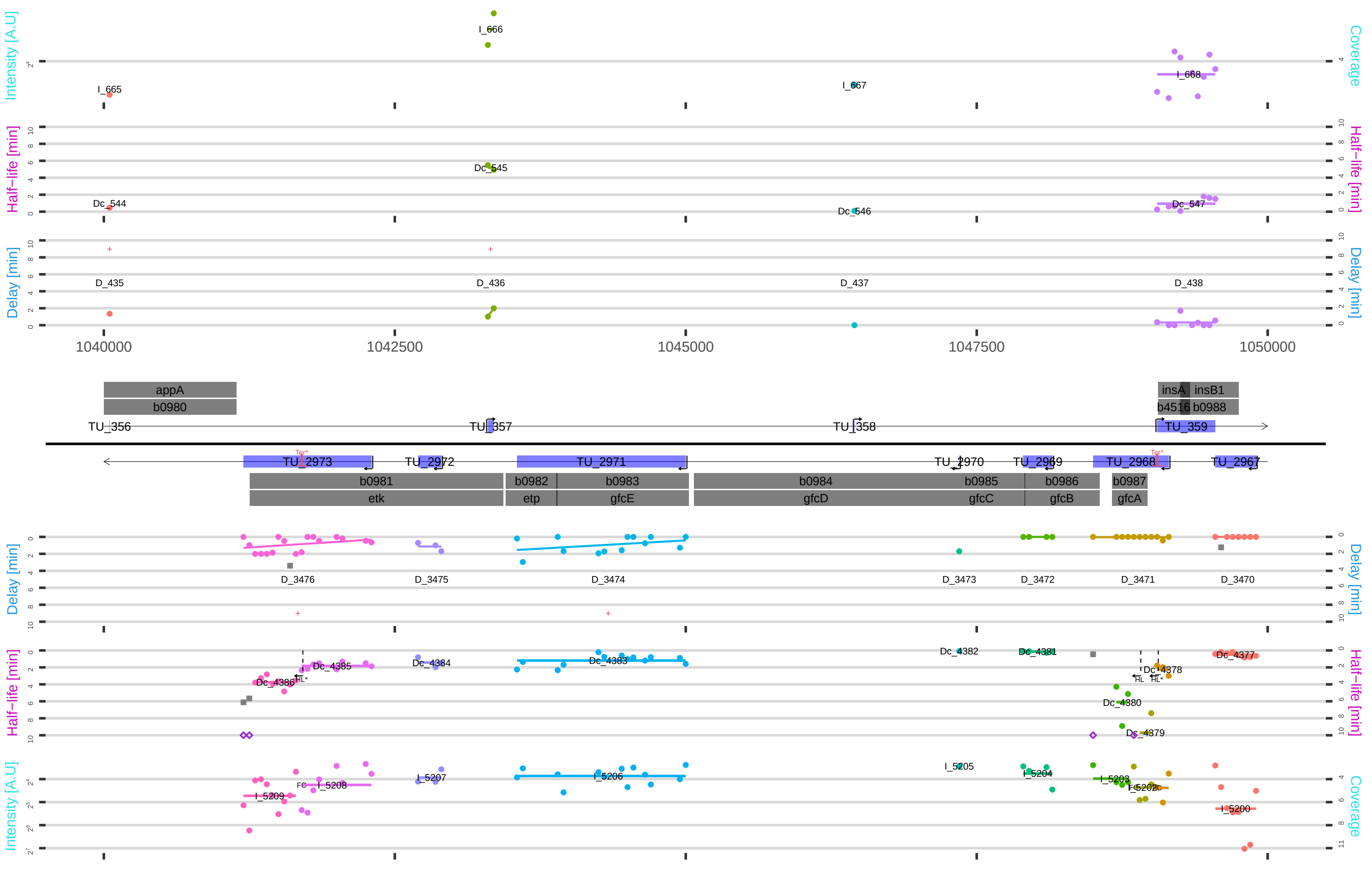

Term: termination (2), NS: new start (0), PS: pausing site (0), iTSS_I: internal starting site (0)

ID: 21001−21077; Term: termination (2), NS: new start (1), PS: pausing site (0), iTSS_I: internal starting site (0)

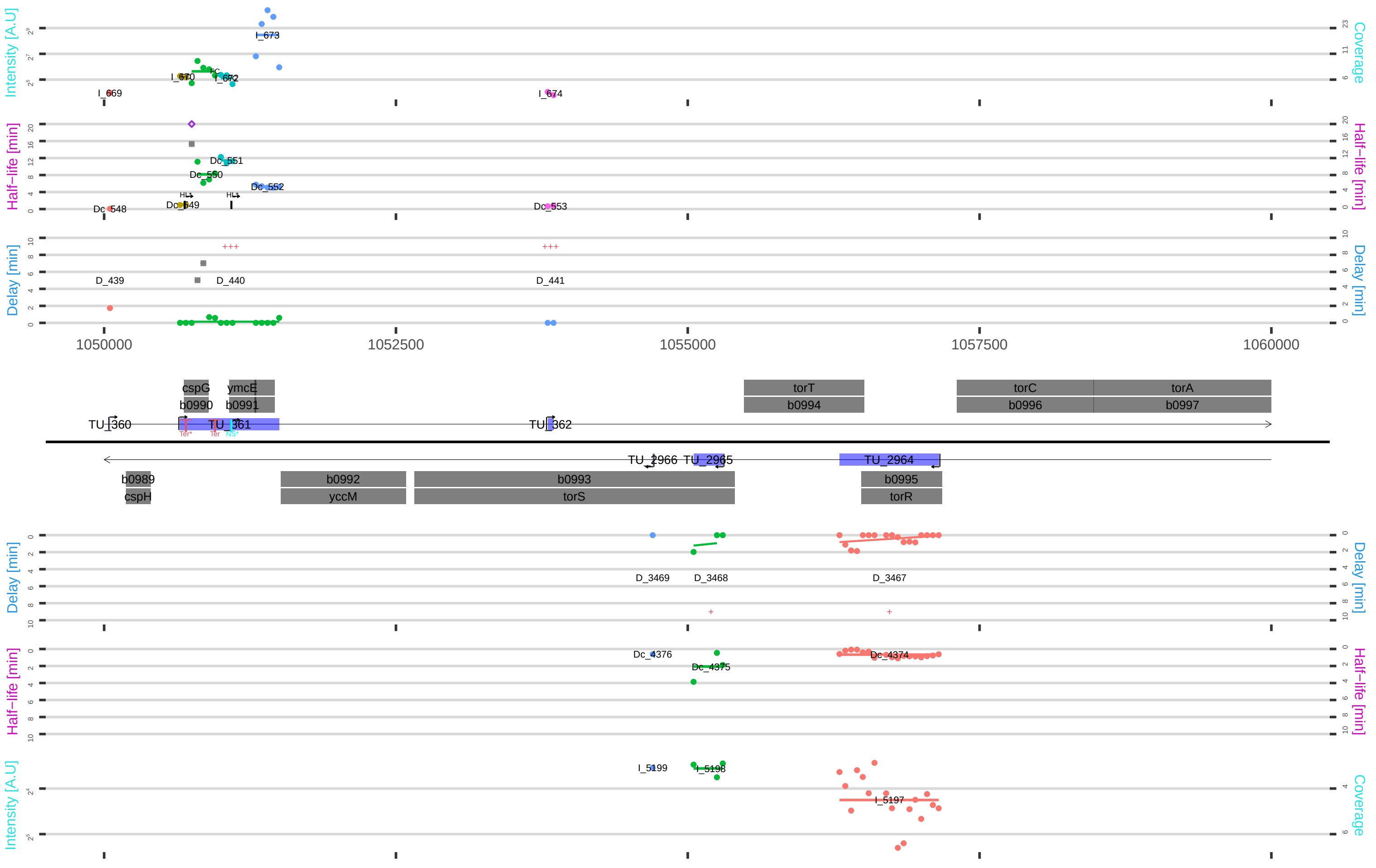

Term: termination (0), NS: new start (0), PS: pausing site (0), iTSS_I: internal starting site (0)

ID: 21220−21400; Term: termination (2), NS: new start (1), PS: pausing site (0), iTSS_I: internal starting site (0)

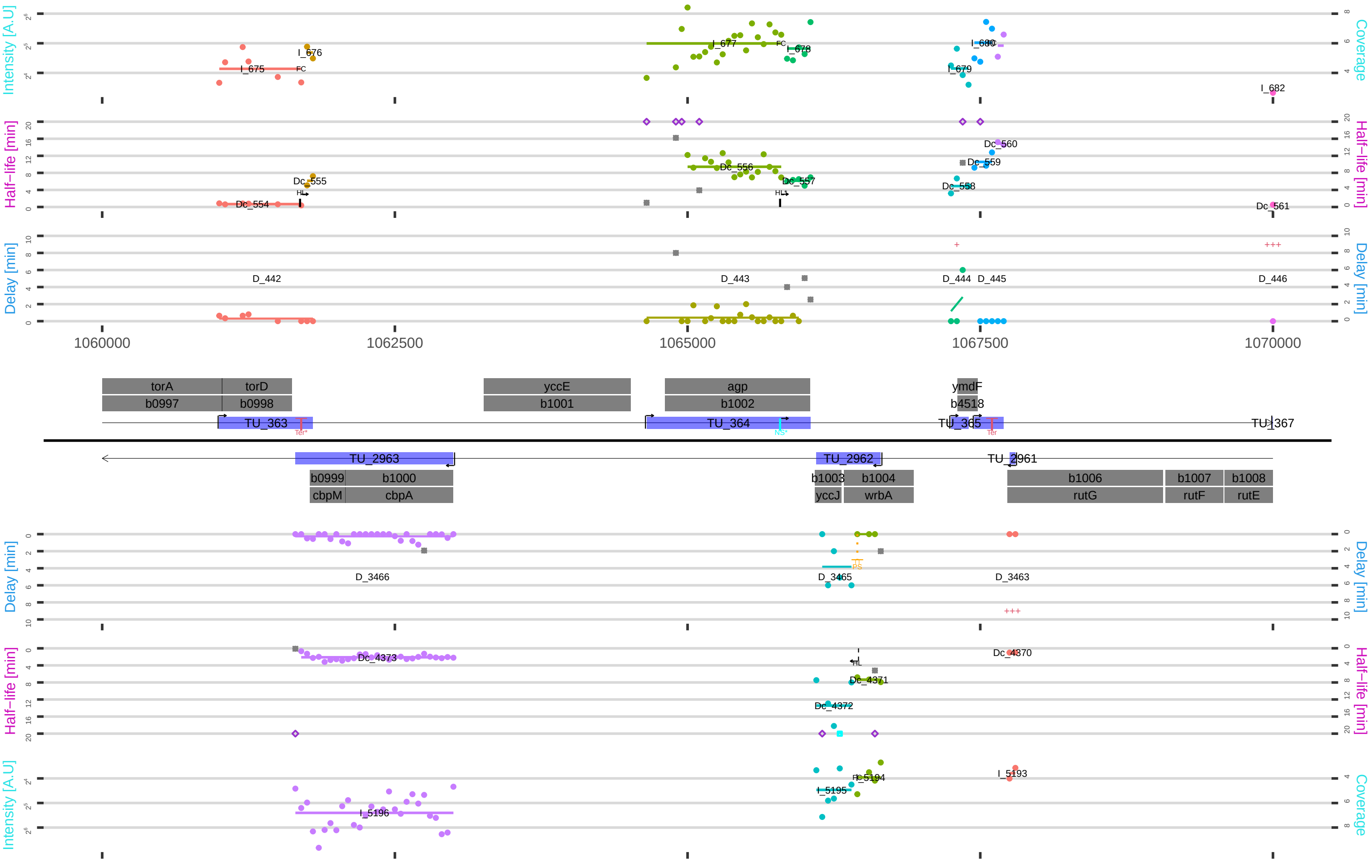

Term: termination (0), NS: new start (0), PS: pausing site (1), iTSS_I: internal starting site (0)

ID: 21400−21600; Term: termination (0), NS: new start (3), PS: pausing site (0), iTSS_I: internal starting site (0)

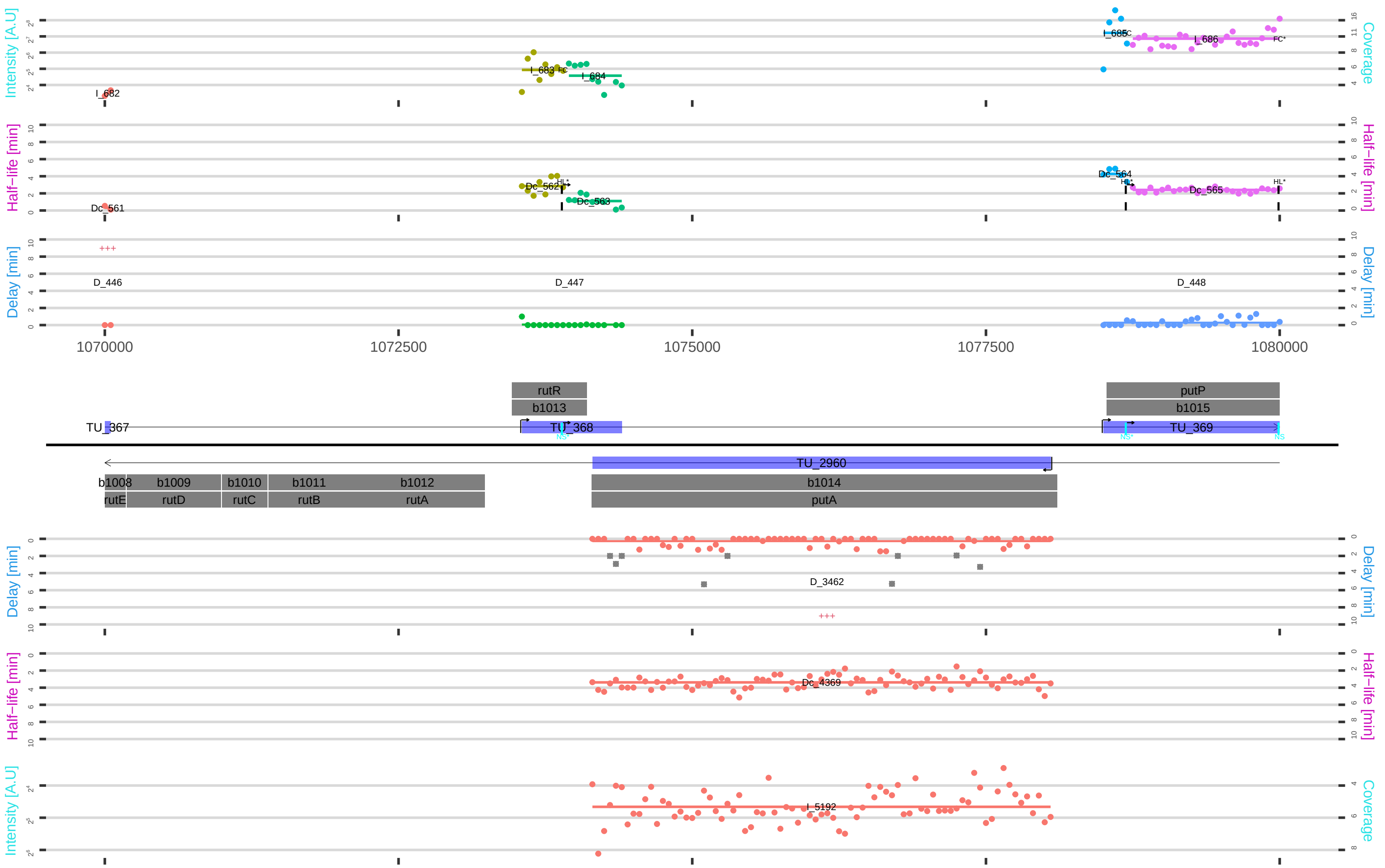

Term: termination (0), NS: new start (0), PS: pausing site (0), iTSS_I: internal starting site (0)

ID: 21600−21791; Term: termination (2), NS: new start (2), PS: pausing site (0), iTSS_I: internal starting site (0)

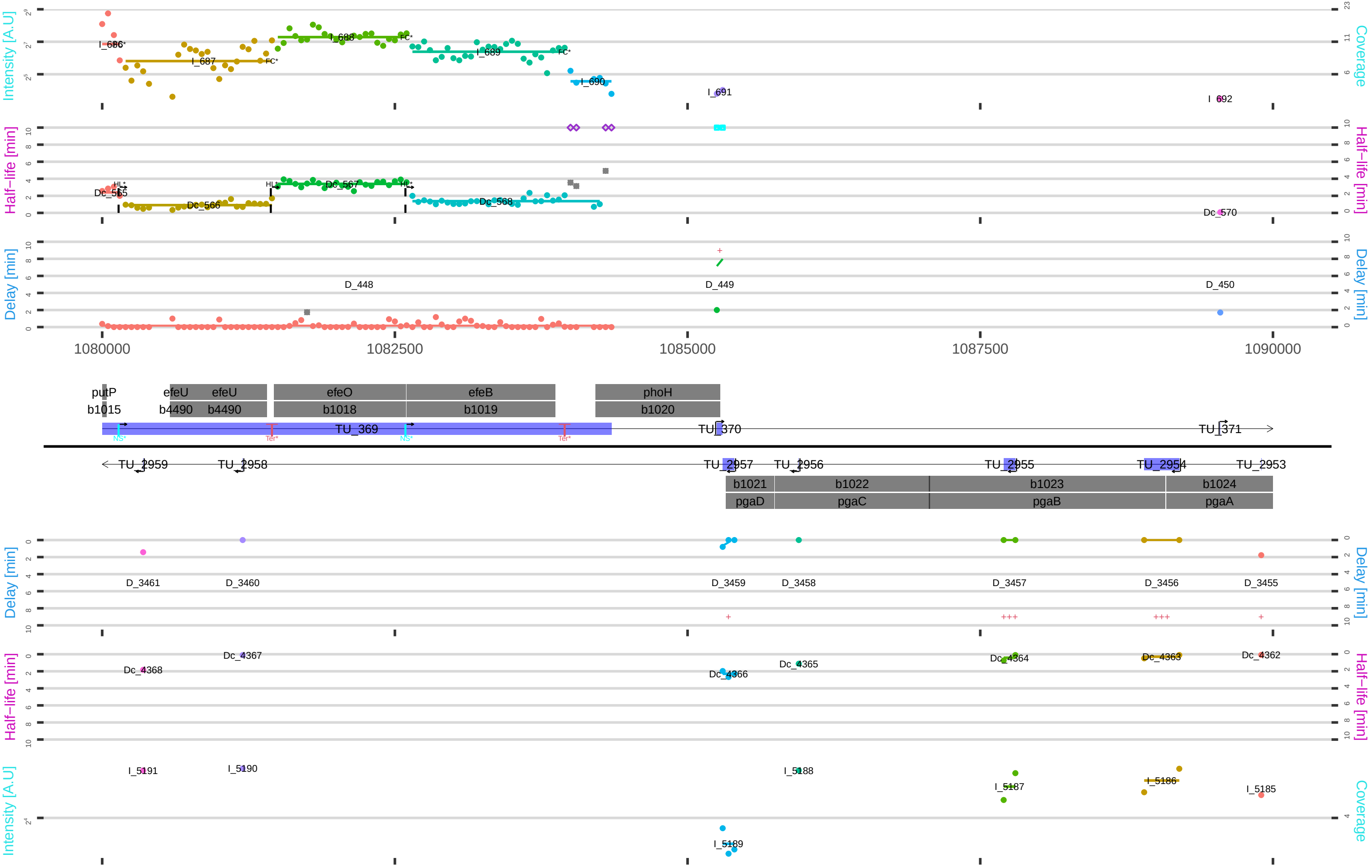

Term: termination (0), NS: new start (0), PS: pausing site (0), iTSS_I: internal starting site (0)

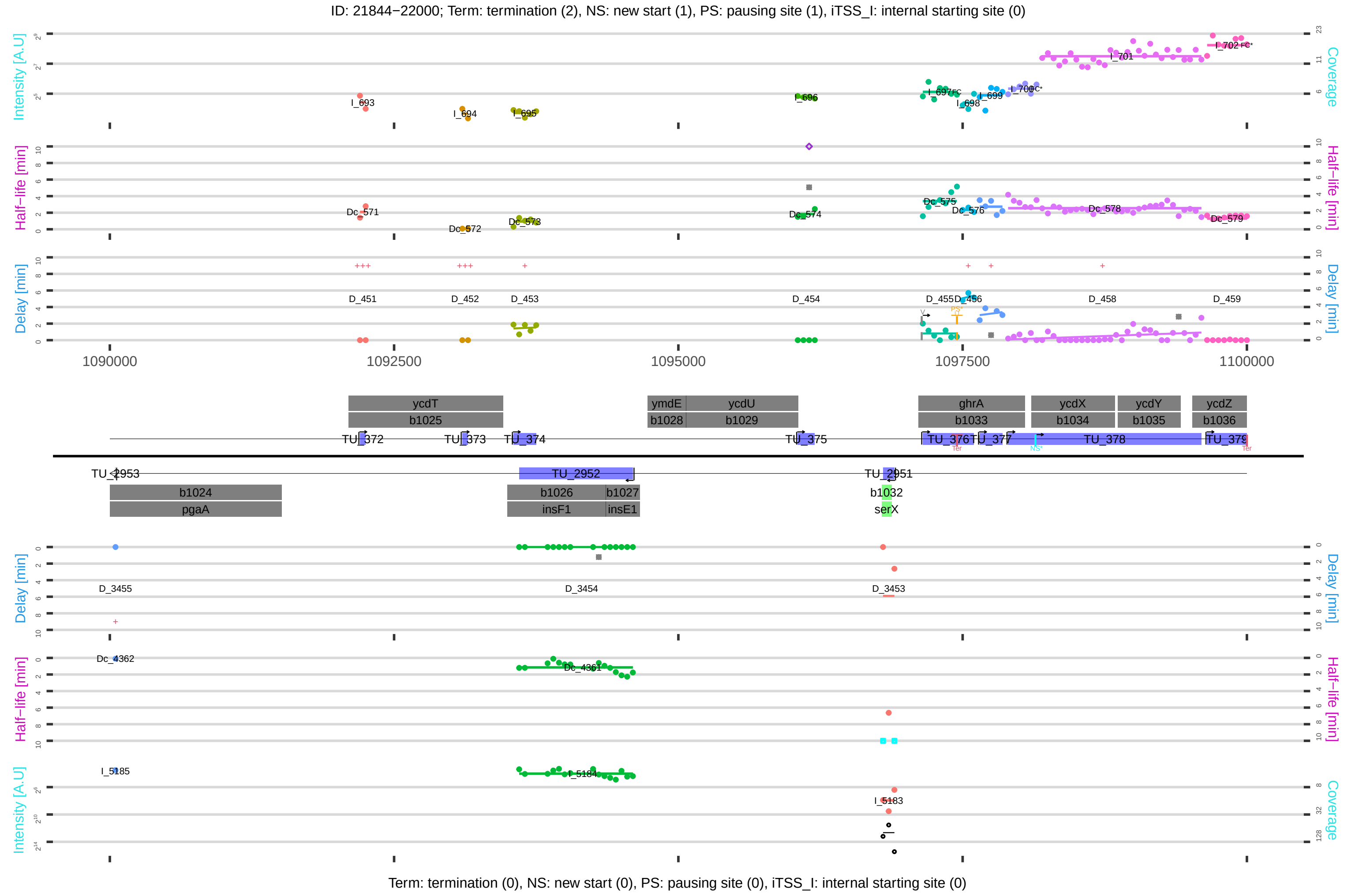

ID: 21844−22000; Term: termination (2), NS: new start (1), PS: pausing site (1), iTSS_I: internal starting site (0)
Intensity [A.U]
Coverage
Half−life [min]
Delay [min]
1090000
1092500
1095000
1097500
1100000
ycdT
b1025
ymdE
b1028
ycdU
b1029
ghrA
b1033
ycdX
b1034
ycdY
b1035
ycdZ
b1036
b1024
pgaA
b1026
insF1
b1027
insE1
b1032
serX
TU_372
TU_373
TU_374
TU_375
TU_376
TU_377
TU_378
TU_379
TU_2953
TU_2952
TU_2951
Term: termination (0), NS: new start (0), PS: pausing site (0), iTSS_I: internal starting site (0)

ID: 22000−22200; Term: termination (1), NS: new start (3), PS: pausing site (0), iTSS_I: internal starting site (0)

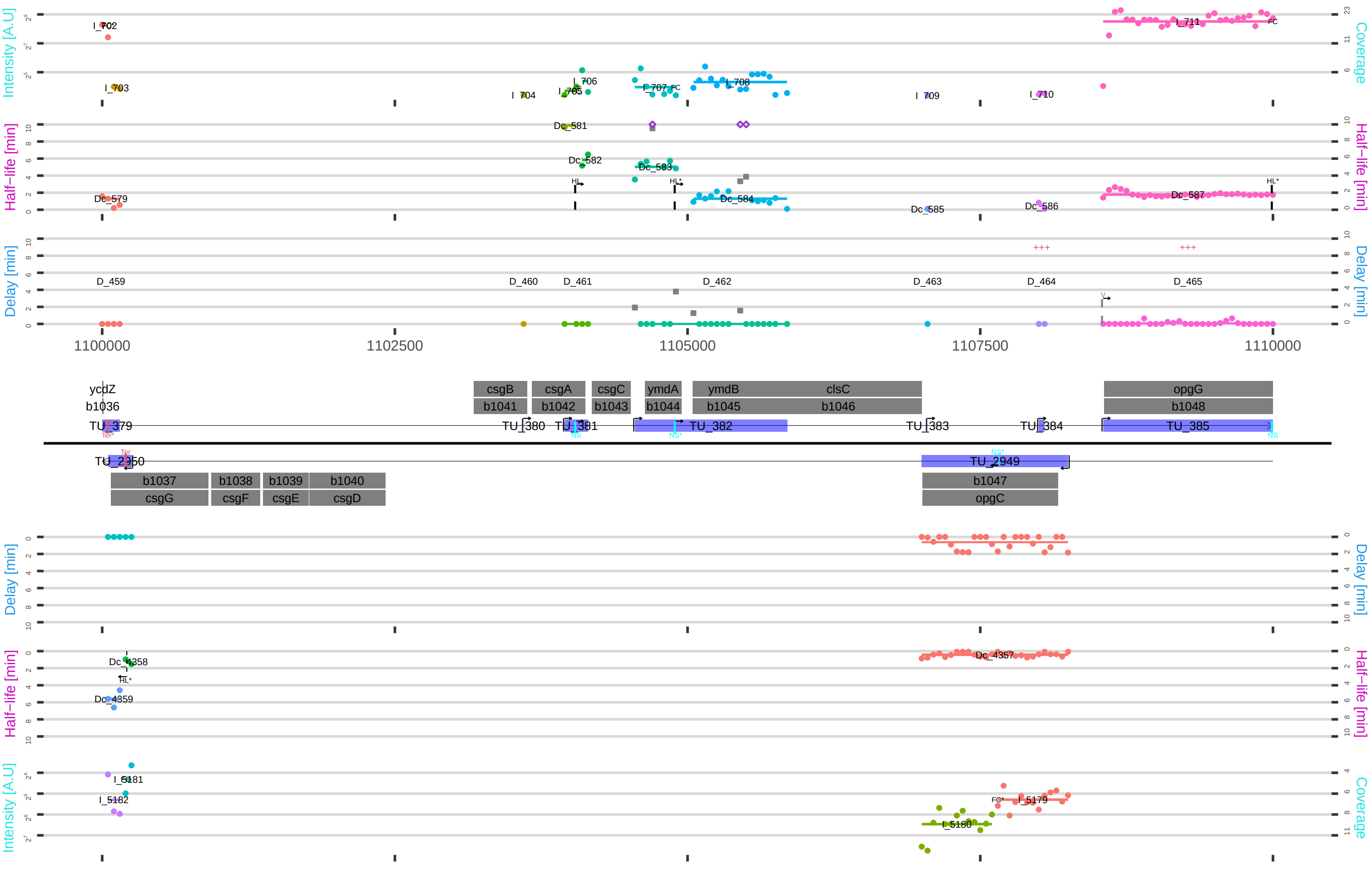

Term: termination (1), NS: new start (1), PS: pausing site (0), iTSS_I: internal starting site (0)

ID: 22200−22343; Term: termination (1), NS: new start (2), PS: pausing site (1), iTSS_I: internal starting site (0)

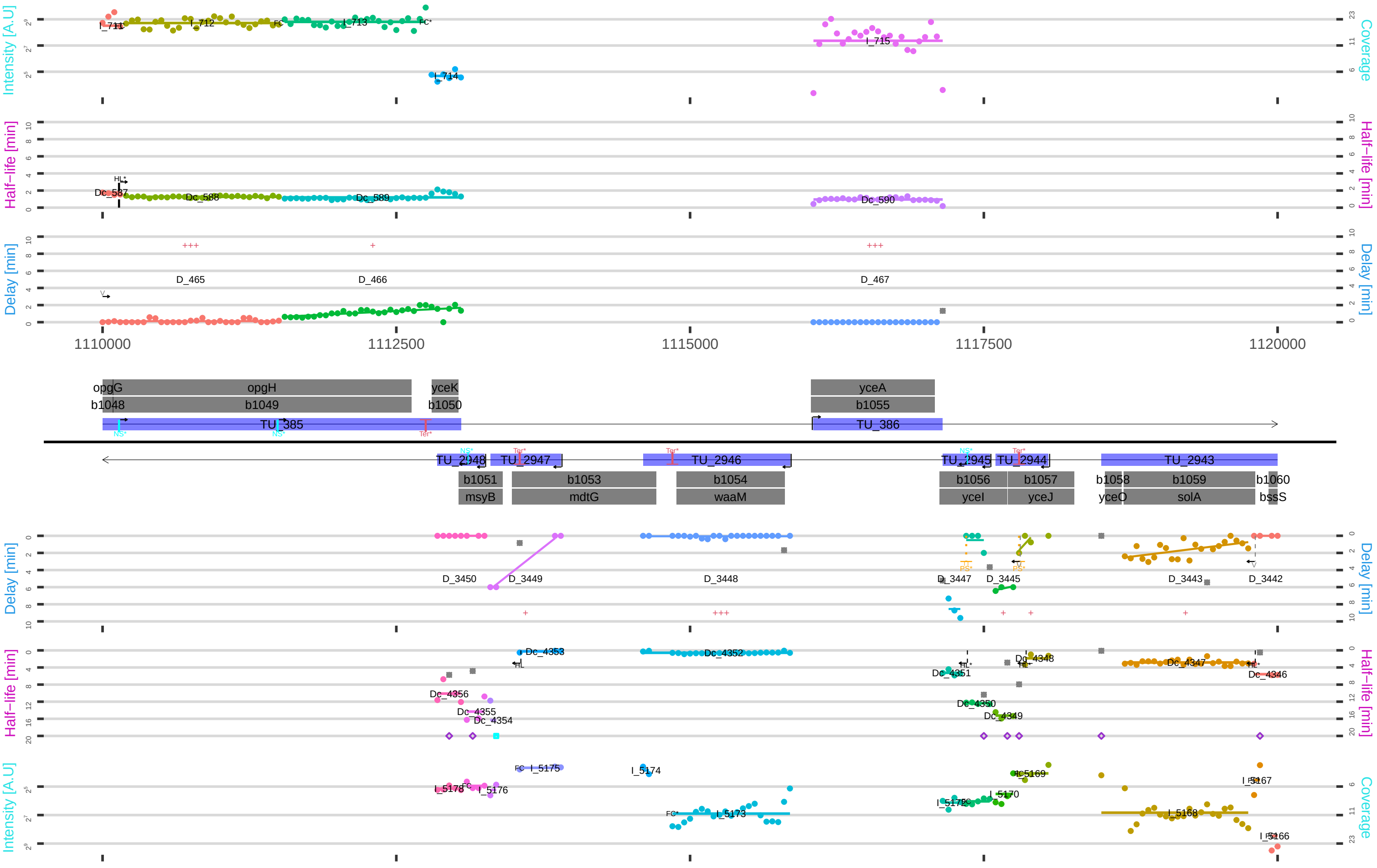

Term: termination (3), NS: new start (2), PS: pausing site (3), iTSS_I: internal starting site (0)

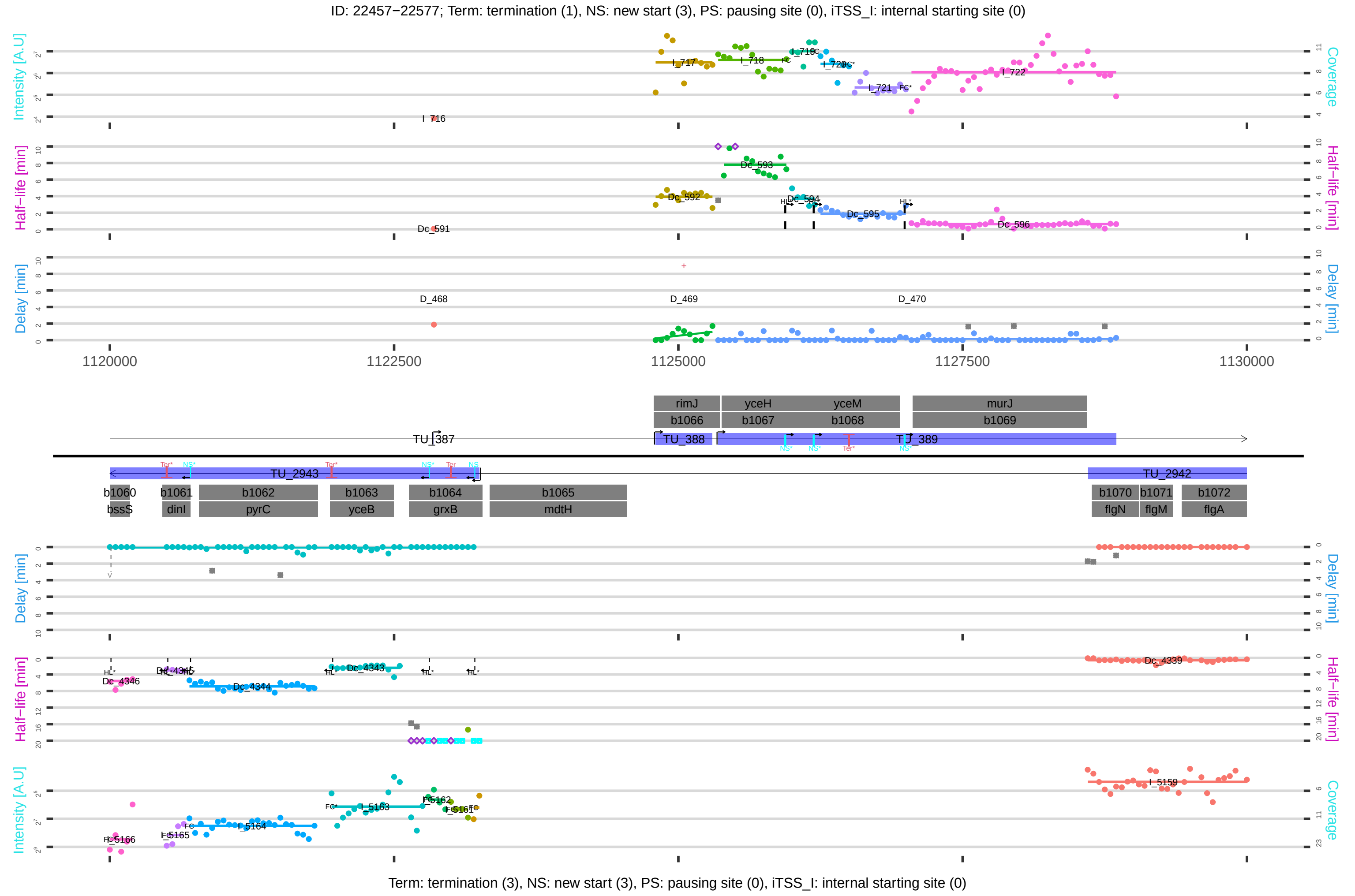

ID: 22457−22577; Term: termination (1), NS: new start (3), PS: pausing site (0), iTSS_I: internal starting site (0)
Intensity [A.U]
Coverage
Half−life [min]
Delay [min]
I_716
I_717
I_718
I_719
I_720
I_721
I_722
Dc_591
Dc_592
Dc_593
Dc_594
Dc_595
Dc_596
D_468
D_469
D_470
1120000
1122500
1125000
1127500
1130000
rimJ
yceH
yceM
murJ
b1066
b1067
b1068
b1069
TU_387
TU_388
TU_389
TU_2943
TU_2942
b1060
b1061
b1062
b1063
b1064
b1065
b1070
b1071
b1072
bssS
dinI
pyrC
yceB
grxB
mdtH
flgN
flgM
flgA
Dc_4346
Dc_4345
Dc_4344
Dc_4343
Dc_4339
I_5166
I_5165
I_5164
I_5163
I_5162
I_5161
I_5159
Term: termination (3), NS: new start (3), PS: pausing site (0), iTSS_I: internal starting site (0)

ID: 22606−22800; Term: termination (7), NS: new start (3), PS: pausing site (1), iTSS_I: internal starting site (4)

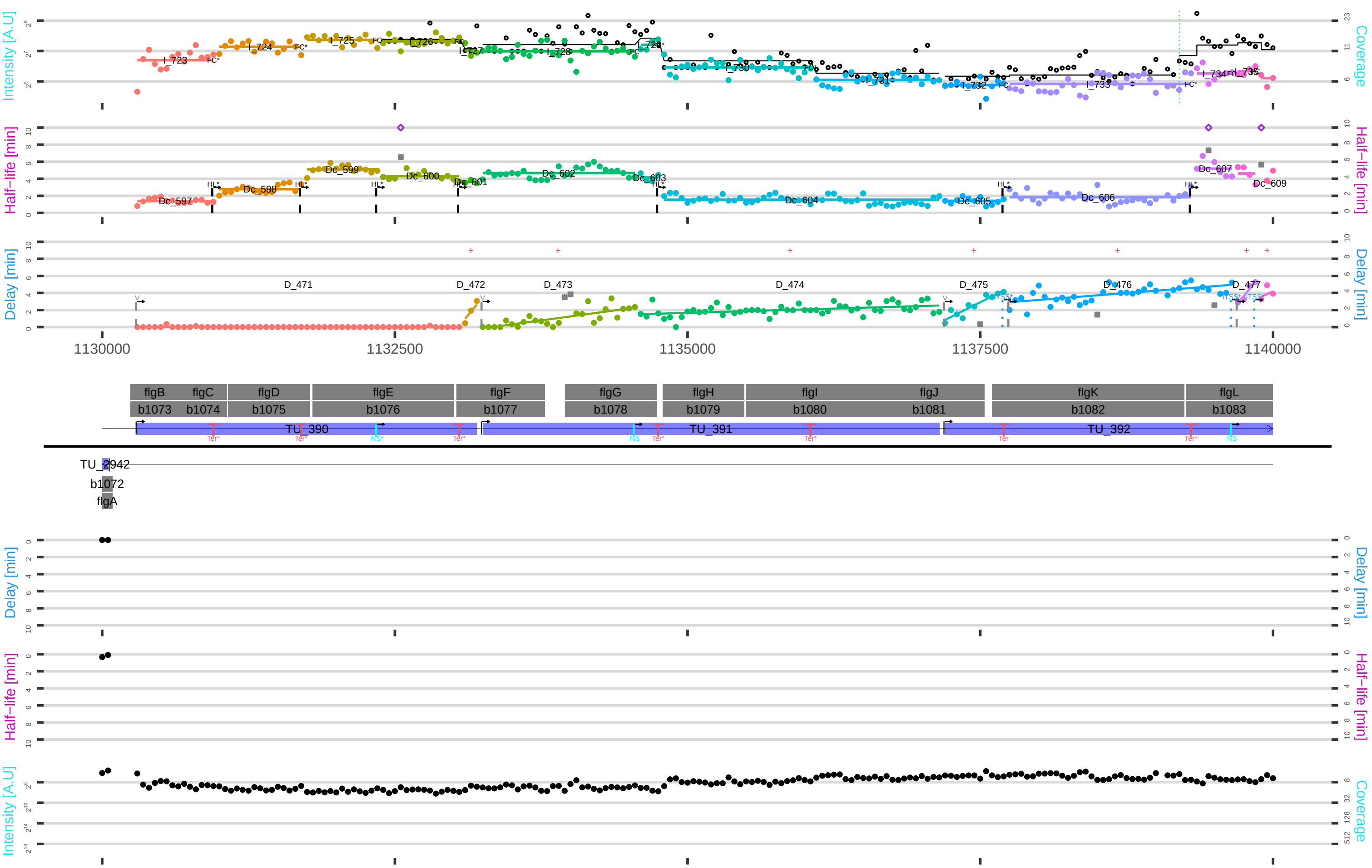

Term: termination (0), NS: new start (0), PS: pausing site (0), iTSS_I: internal starting site (0)

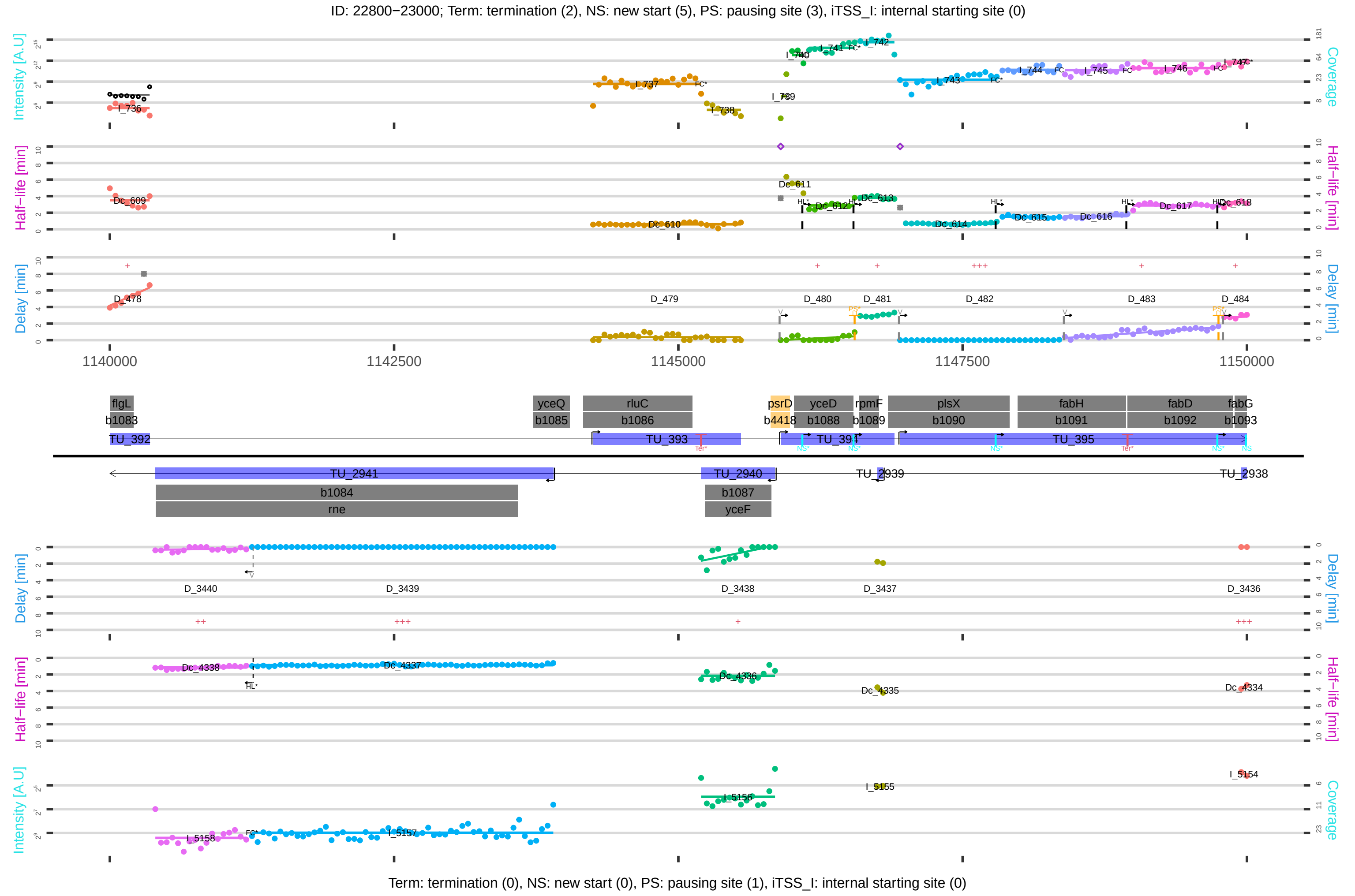

ID: 22800−23000; Term: termination (2), NS: new start (5), PS: pausing site (3), iTSS_I: internal starting site (0)
Intensity [A.U]
Coverage
Half−life [min]
Delay [min]
I_736
I_737
I_738
I_739
I_740
I_741
I_742
I_743
I_744
I_745
I_746
I_747
Dc_609
Dc_610
Dc_611
Dc_612
Dc_613
Dc_614
Dc_615
Dc_616
Dc_617
Dc_618
D_478
D_479
D_480
D_481
D_482
D_483
D_484
1140000
1142500
1145000
1147500
1150000
flgL
b1083
yceQ
b1085
rluC
b1086
psrD
b4418
yceD
b1088
rpmF
b1089
plsX
b1090
fabH
b1091
fabD
b1092
fabG
b1093
TU_392
TU_393
TU_394
TU_395
TU_2941
TU_2940
TU_2939
TU_2938
b1084
rne
b1087
yceF
D_3440
D_3439
D_3438
D_3437
D_3436
Dc_4338
Dc_4337
Dc_4336
Dc_4335
Dc_4334
I_5154
I_5155
I_5156
I_5157
I_5158
Term: termination (0), NS: new start (0), PS: pausing site (1), iTSS_I: internal starting site (0)

ID: 23000−23185; Term: termination (3), NS: new start (6), PS: pausing site (4), iTSS_I: internal starting site (2)

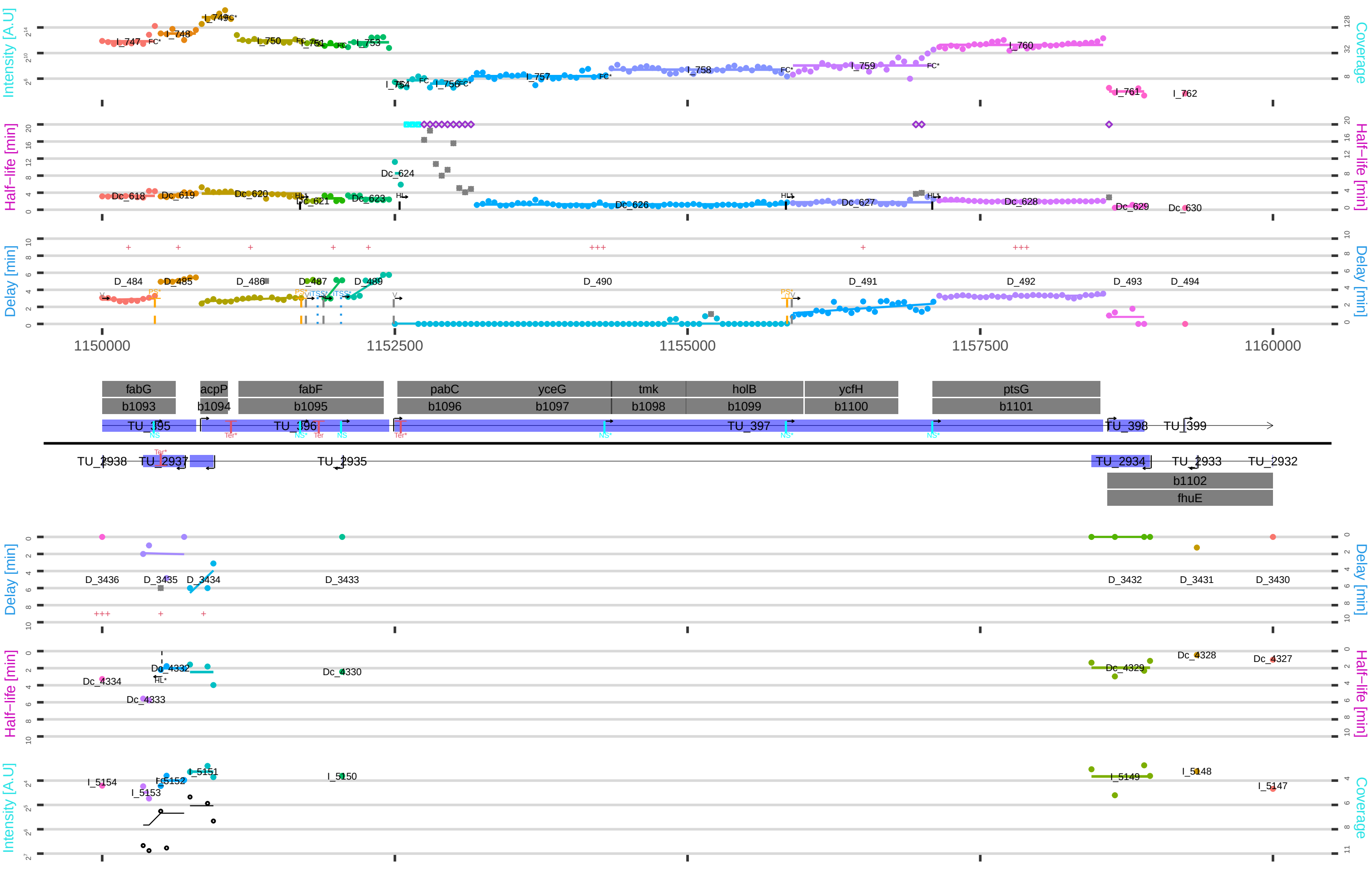

Term: termination (1), NS: new start (0), PS: pausing site (0), iTSS_I: internal starting site (0)

ID: 23222−23372; Term: termination (5), NS: new start (2), PS: pausing site (3), iTSS_I: internal starting site (0)

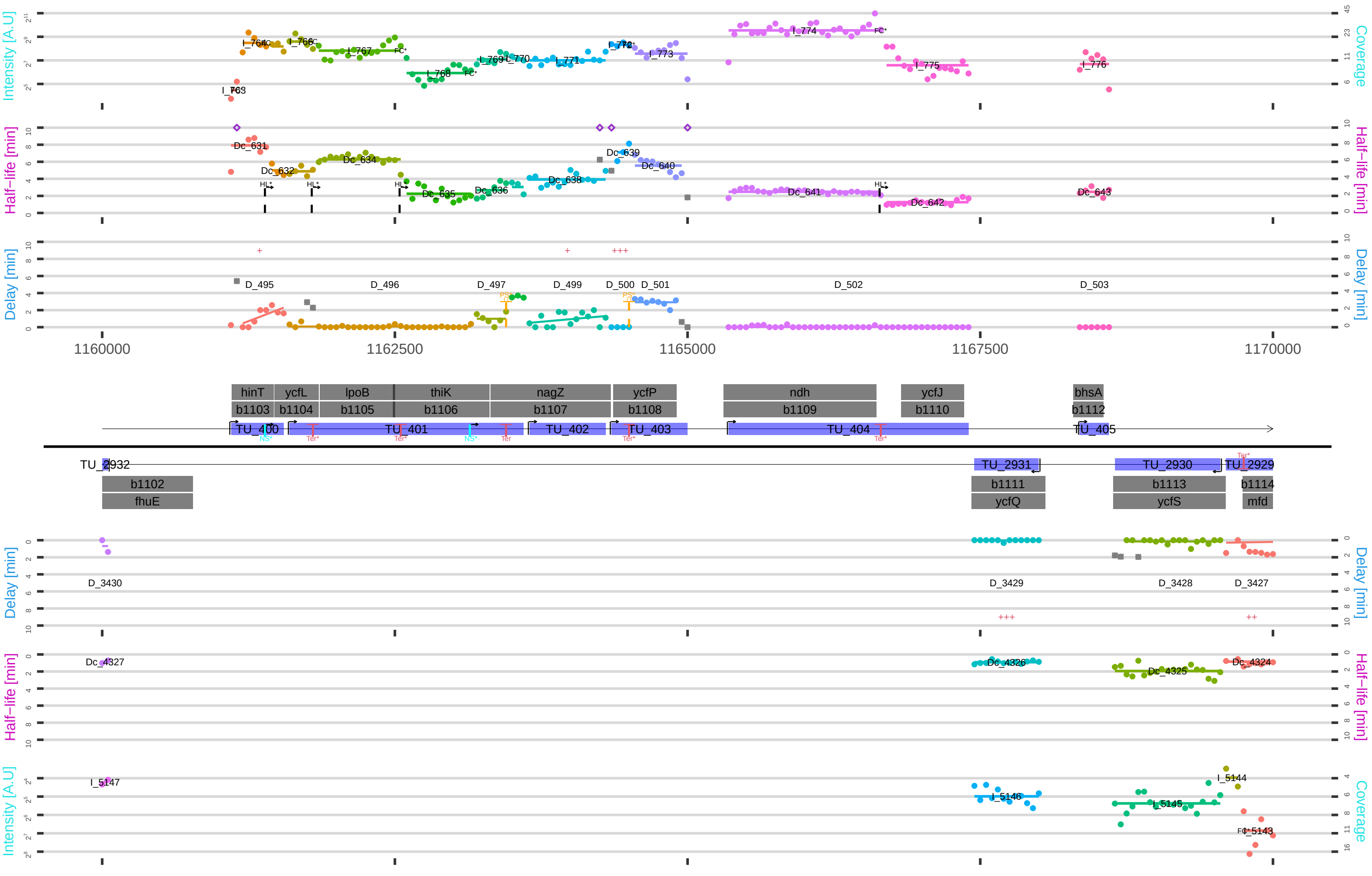

Term: termination (1), NS: new start (0), PS: pausing site (0), iTSS_I: internal starting site (0)

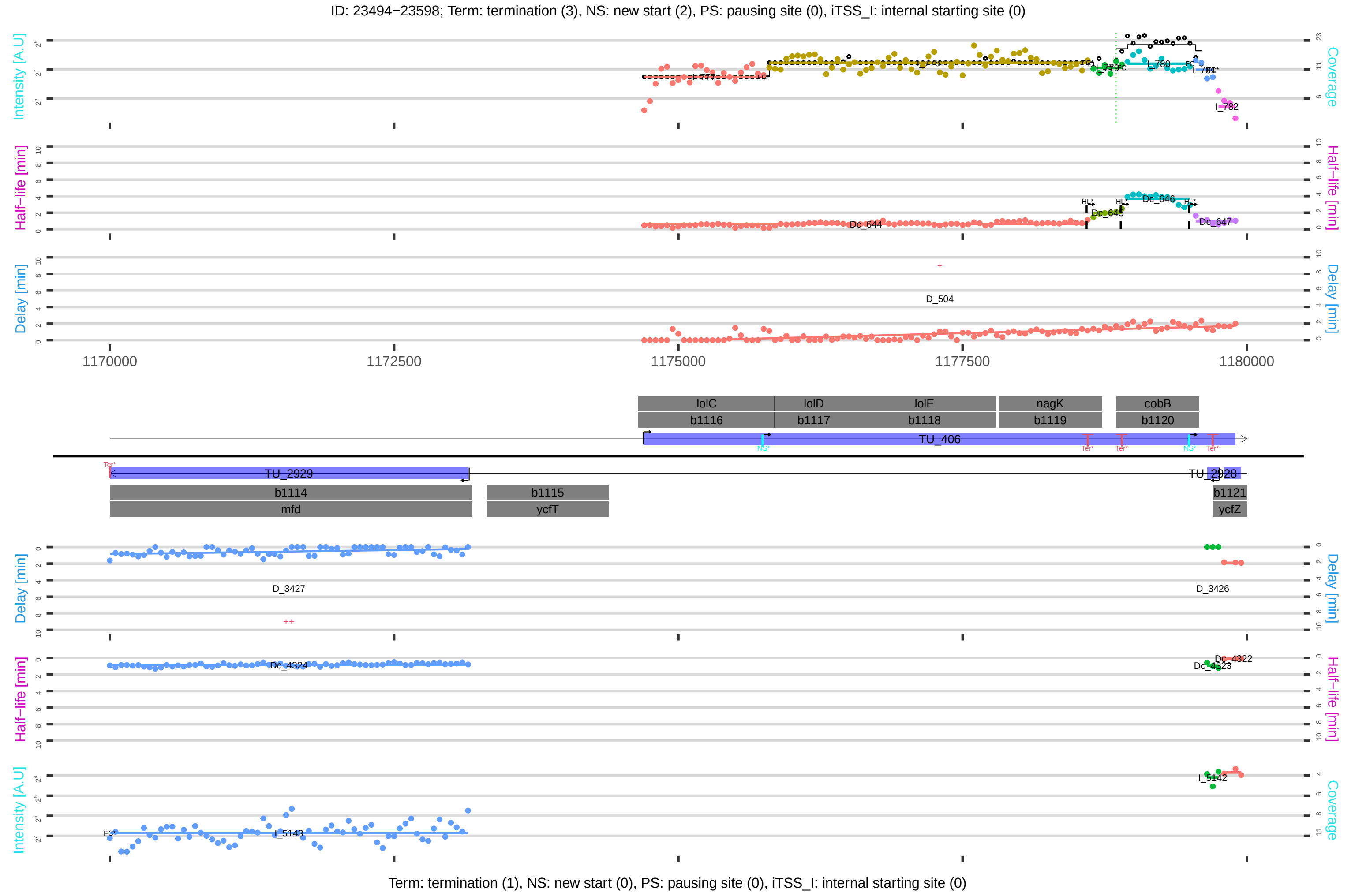

ID: 23494−23598; Term: termination (3), NS: new start (2), PS: pausing site (0), iTSS_I: internal starting site (0)
Intensity [A.U]
Coverage
Half−life [min]
Delay [min]
I_777
I_778
I_779
I_780
I_781
I_782
Dc_644
Dc_645
Dc_646
Dc_647
D_504
1170000
1172500
1175000
1177500
1180000
lolC
lolD
lolE
nagK
cobB
b1116
b1117
b1118
b1119
b1120
TU_406
TU_2929
TU_2928
b1114
b1115
b1121
mfd
ycfT
ycfZ
D_3427
D_3426
Dc_4324
Dc_4322
Dc_4323
I_5142
I_5143
Term: termination (1), NS: new start (0), PS: pausing site (0), iTSS_I: internal starting site (0)

ID: 23602−23729; Term: termination (0), NS: new start (0), PS: pausing site (0), iTSS_I: internal starting site (0)

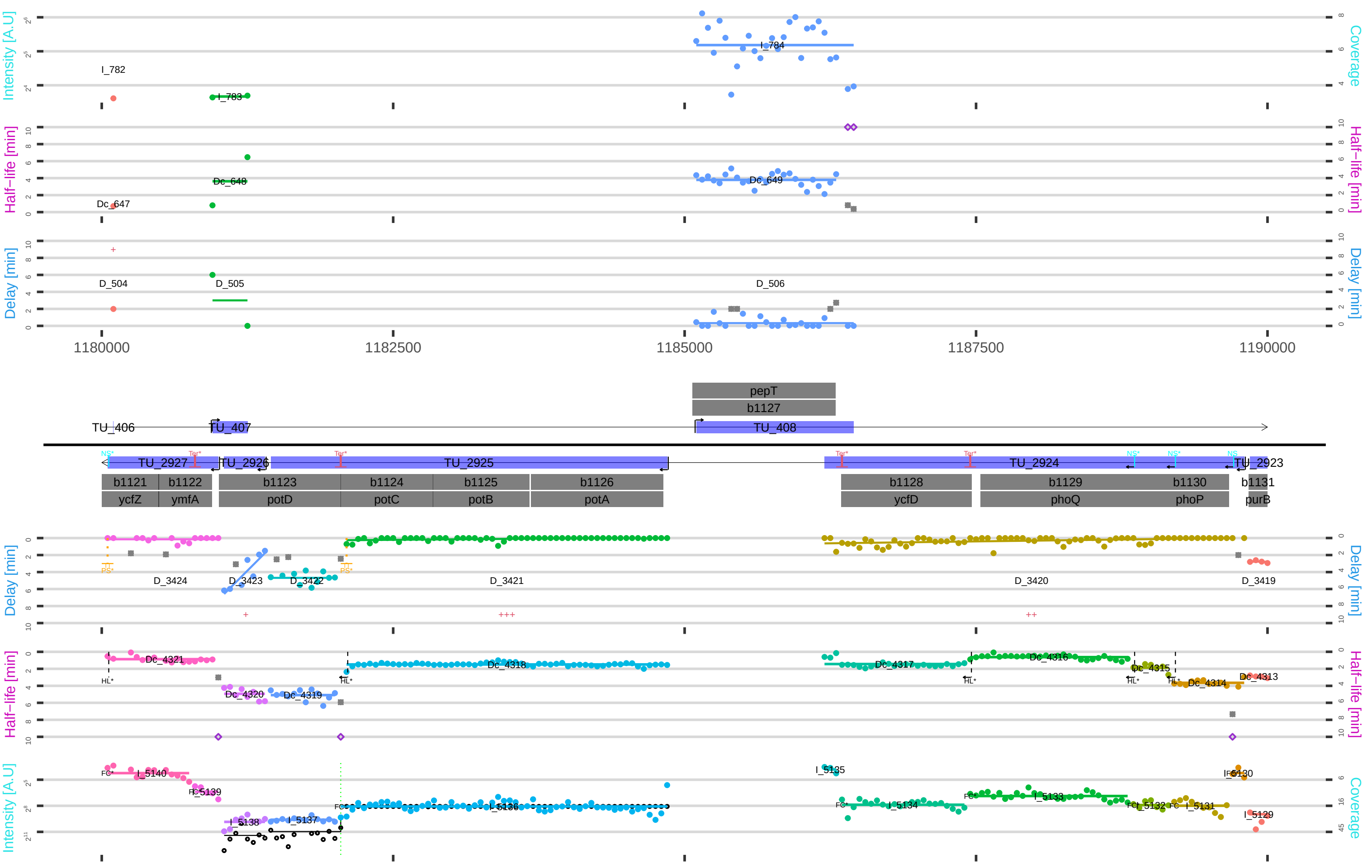

Term: termination (4), NS: new start (4), PS: pausing site (2), iTSS_I: internal starting site (0)

ID: 23886−23978; Term: termination (2), NS: new start (3), PS: pausing site (1), iTSS_I: internal starting site (1)

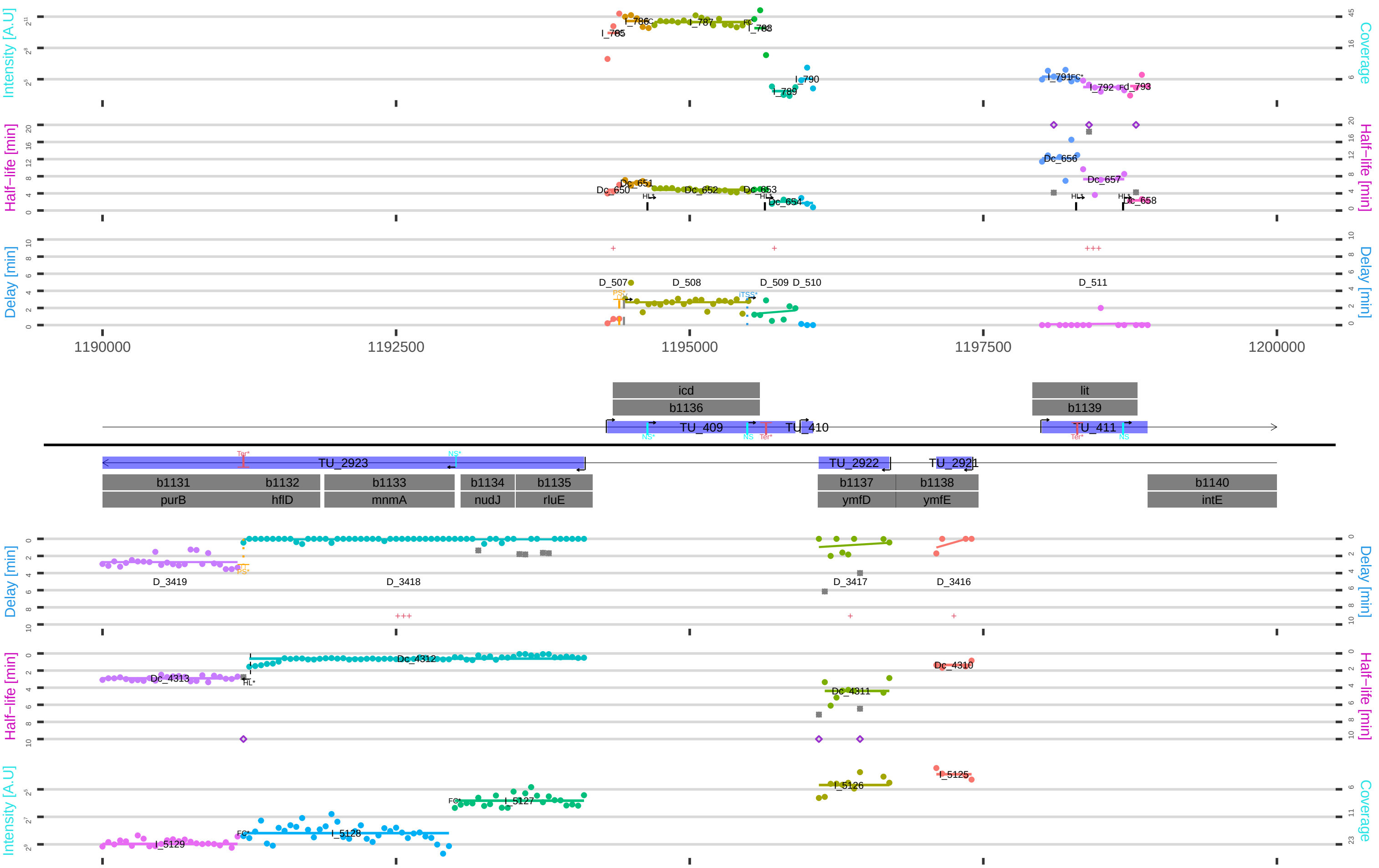

Term: termination (1), NS: new start (1), PS: pausing site (1), iTSS_I: internal starting site (0)

ID: 24015−24200; Term: termination (1), NS: new start (0), PS: pausing site (1), iTSS_I: internal starting site (0)

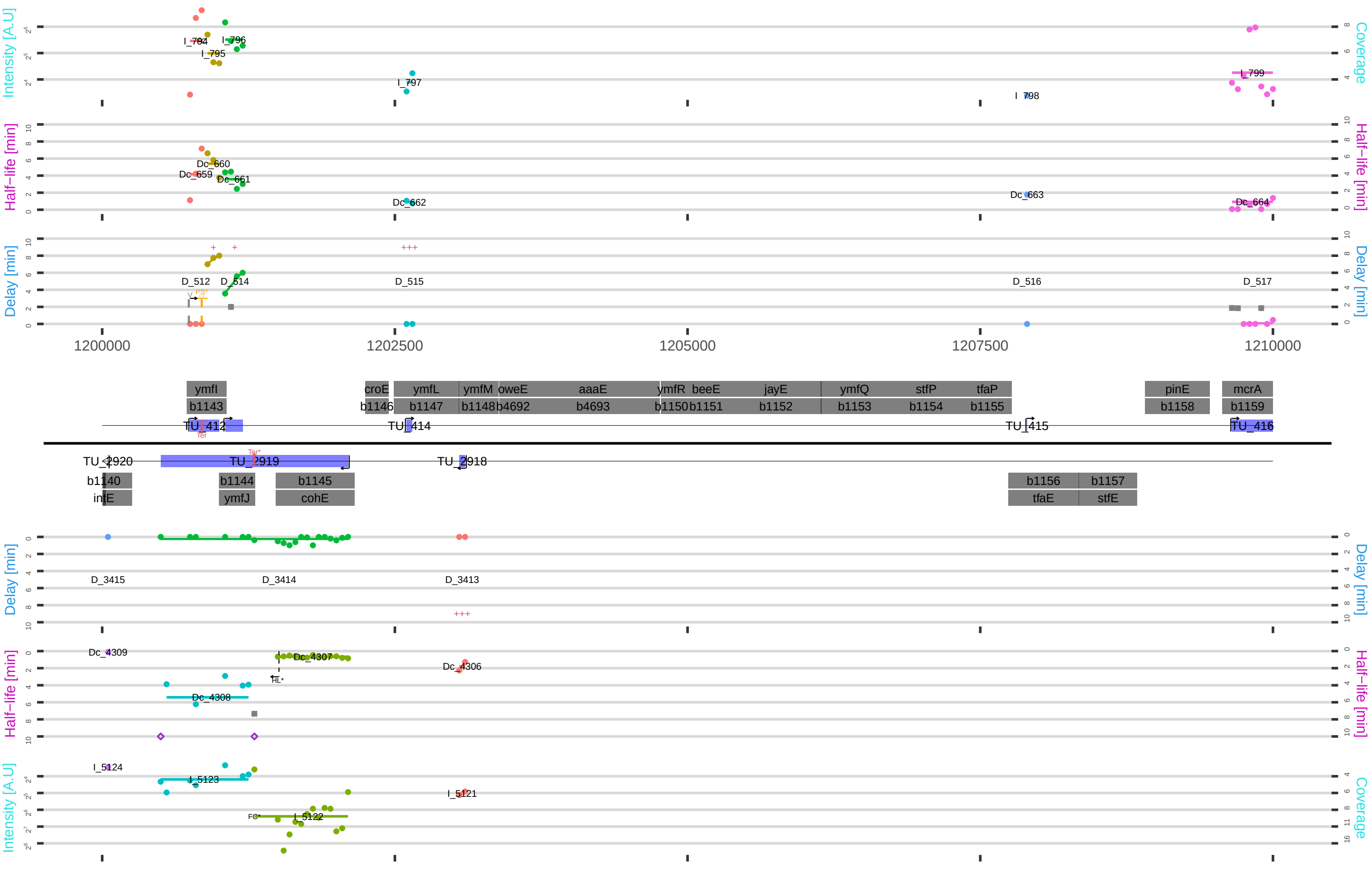

Term: termination (1), NS: new start (0), PS: pausing site (0), iTSS_I: internal starting site (0)

ID: 24200−24370; Term: termination (0), NS: new start (0), PS: pausing site (0), iTSS_I: internal starting site (0)

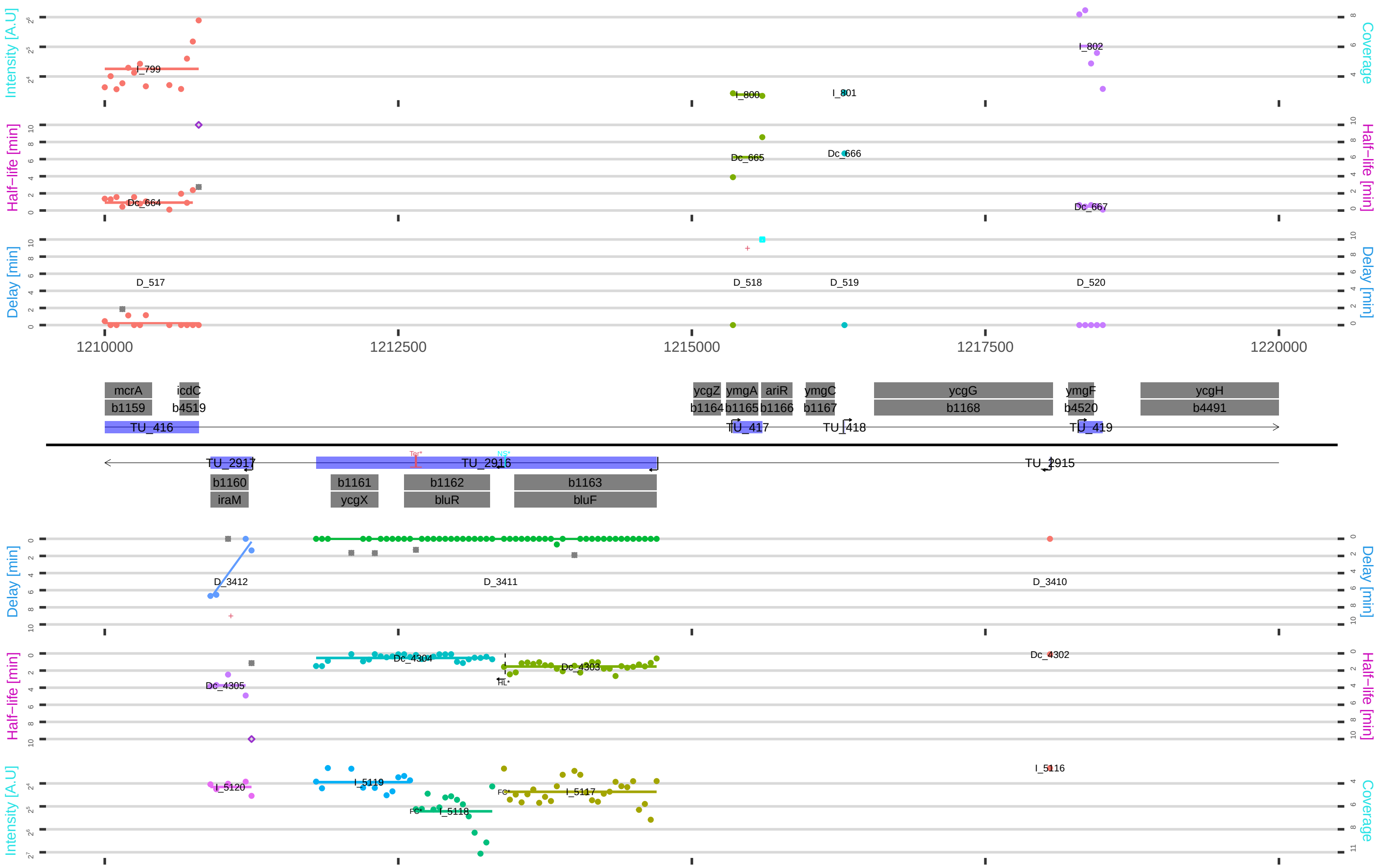

Term: termination (1), NS: new start (1), PS: pausing site (0), iTSS_I: internal starting site (0)

ID: 24419−24573; Term: termination (1), NS: new start (2), PS: pausing site (1), iTSS_I: internal starting site (0)

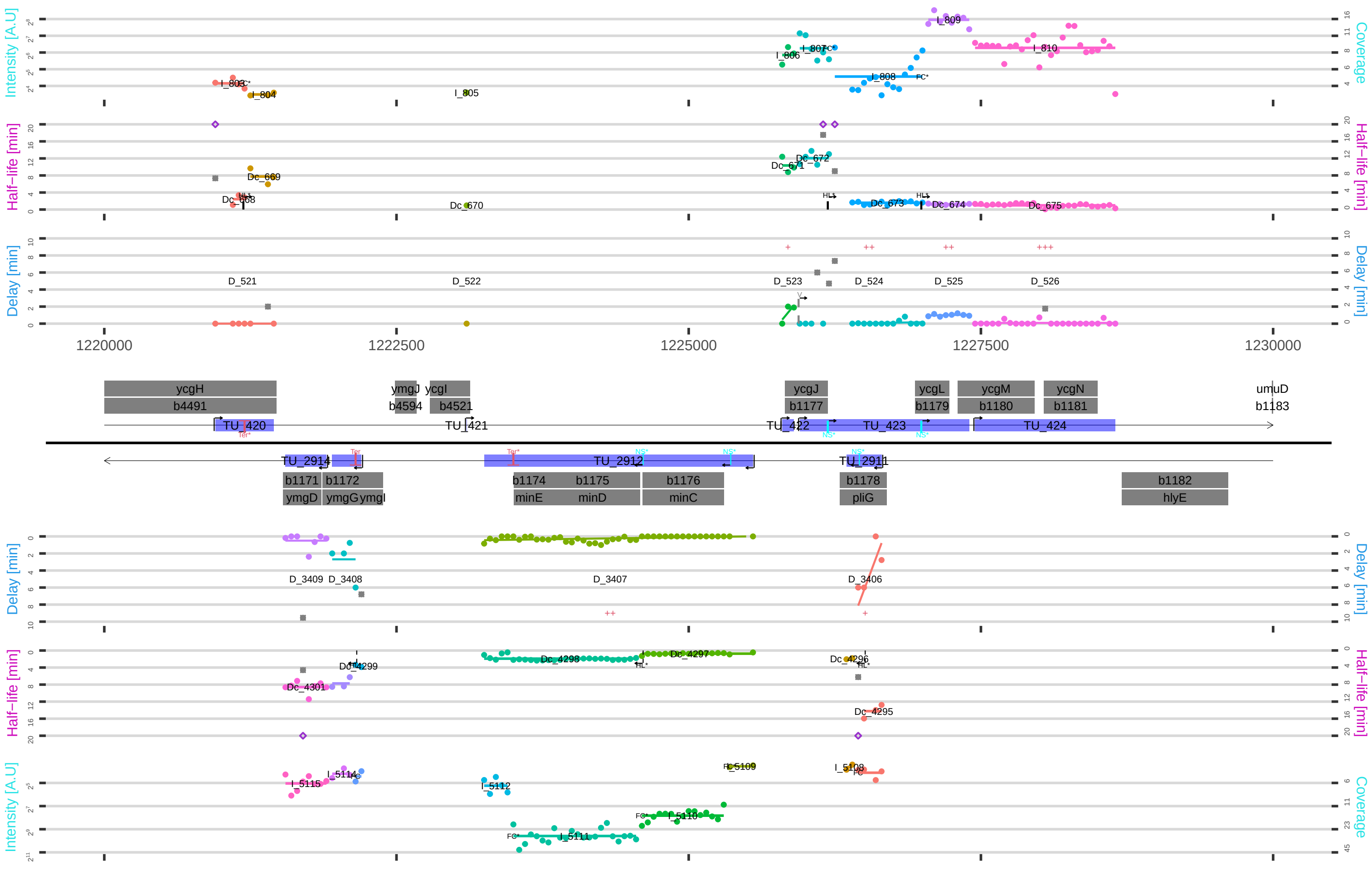

Term: termination (2), NS: new start (3), PS: pausing site (0), iTSS_I: internal starting site (0)

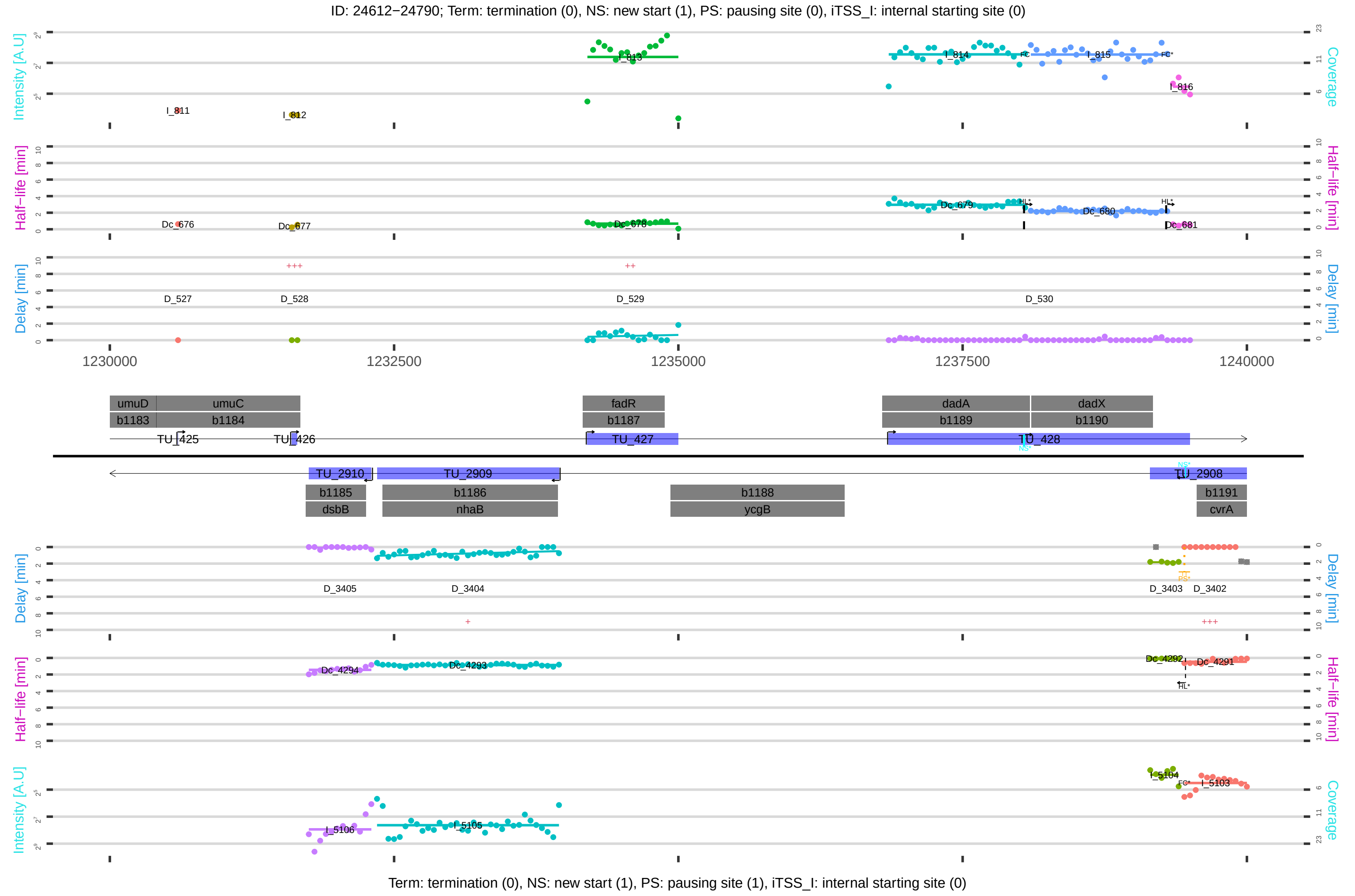

ID: 24612−24790; Term: termination (0), NS: new start (1), PS: pausing site (0), iTSS_I: internal starting site (0)
Intensity [A.U]
Coverage
Half−life [min]
Delay [min]
I_811
I_812
I_813
I_814
I_815
I_816
Dc_676
Dc_677
Dc_678
Dc_679
Dc_680
Dc_681
D_527
D_528
D_529
D_530
1230000
1232500
1235000
1237500
1240000
umuD
umuC
b1183
b1184
fadR
b1187
dadA
dadX
b1189
b1190
TU_425
TU_426
TU_427
TU_428
TU_2910
TU_2909
TU_2908
b1185
dsbB
b1186
nhaB
b1188
ycgB
b1191
cvrA
D_3405
D_3404
D_3403
D_3402
Dc_4294
Dc_4293
Dc_4292
Dc_4291
I_5106
I_5105
I_5104
I_5103
Term: termination (0), NS: new start (1), PS: pausing site (1), iTSS_I: internal starting site (0)

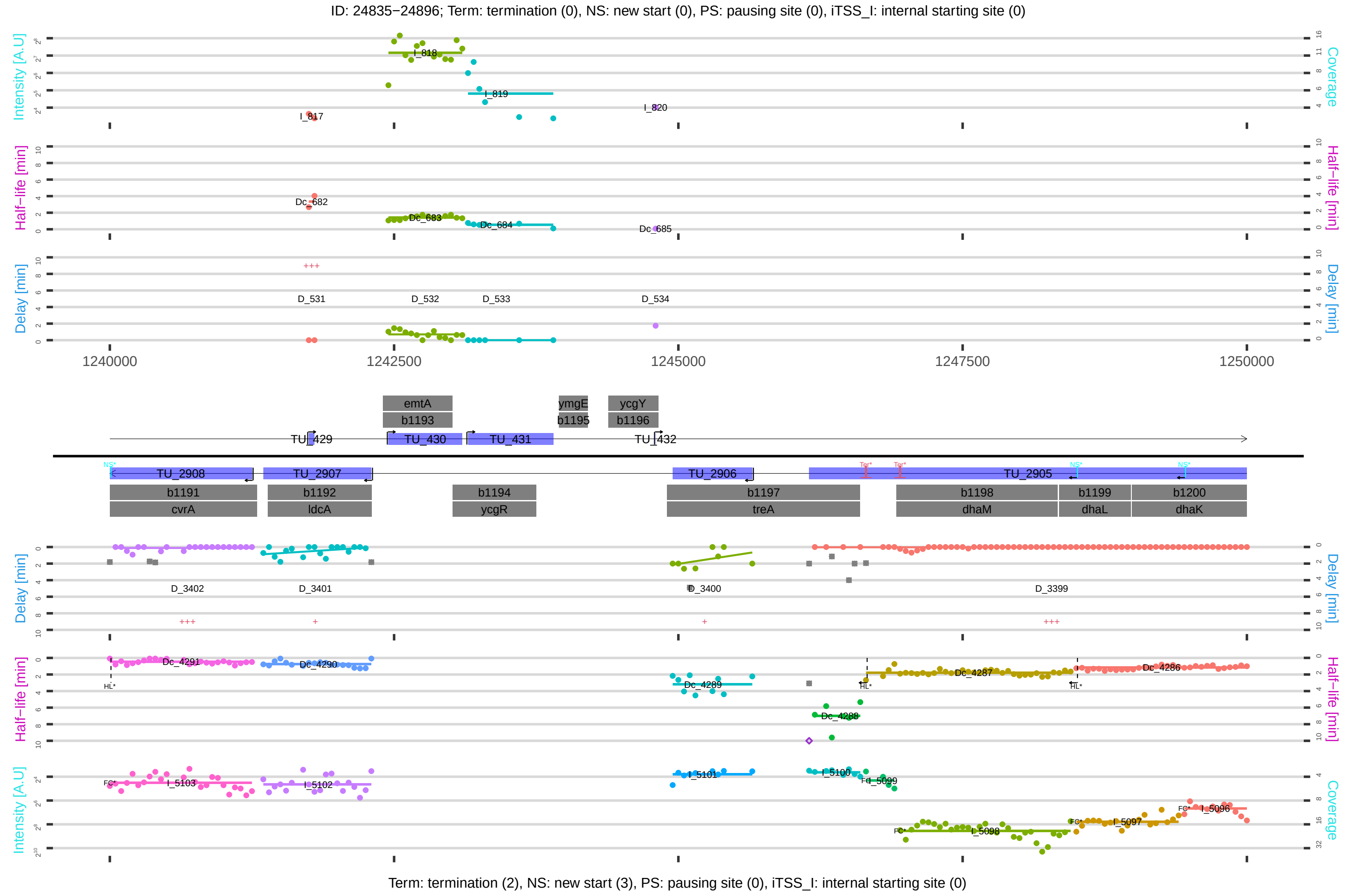
ID: 24835−24896; Term: termination (0), NS: new start (0), PS: pausing site (0), iTSS_I: internal starting site (0)
Intensity [A.U]
Coverage
I_817
I_818
I_819
I_820
Half−life [min]
Dc_682
Dc_683
Dc_684
Dc_685
Delay [min]
D_531
D_532
D_533
D_534
1240000
1242500
1245000
1247500
1250000
emtA
b1193
ymgE
b1195
ycgY
b1196
TU_429
TU_430
TU_431
TU_432
TU_2908
TU_2907
TU_2906
TU_2905
b1191
cvrA
b1192
ldcA
b1194
ycgR
b1197
treA
b1198
dhaM
b1199
dhaL
b1200
dhaK
D_3402
D_3401
D_3400
D_3399
Dc_4291
Dc_4290
Dc_4289
Dc_4288
Dc_4287
Dc_4286
I_5103
I_5102
I_5101
I_5100
I_5099
I_5098
I_5097
I_5096
Term: termination (2), NS: new start (3), PS: pausing site (0), iTSS_I: internal starting site (0)

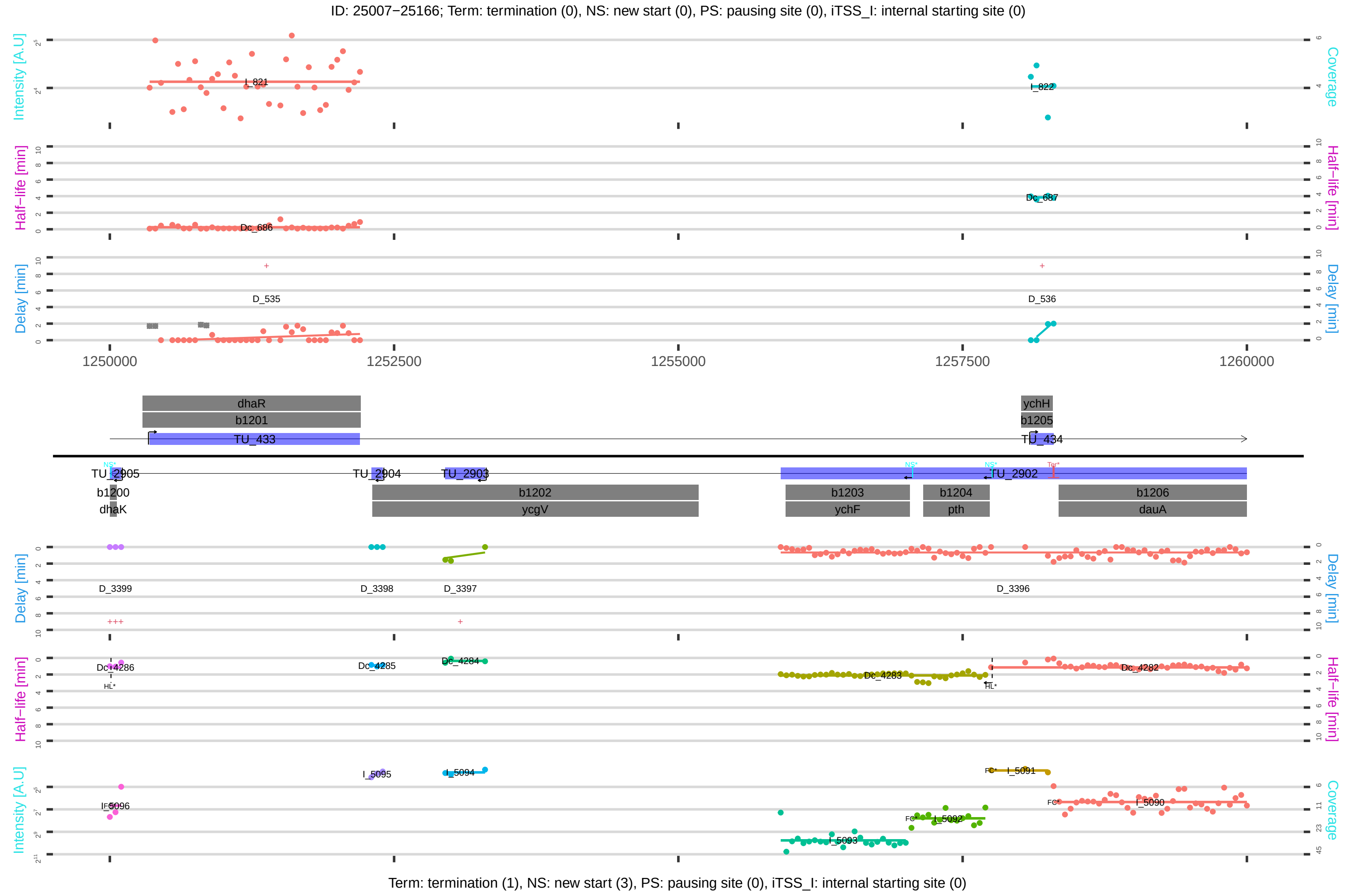

ID: 25007−25166; Term: termination (0), NS: new start (0), PS: pausing site (0), iTSS_I: internal starting site (0)
Intensity [A.U]
Coverage
I_821
I_822
Half−life [min]
Dc_686
Dc_687
Delay [min]
D_535
D_536
1250000
1252500
1255000
1257500
1260000
dhaR
b1201
TU_433
ychH
b1205
TU_434
TU_2905
b1200
dhaK
TU_2904
TU_2903
b1202
ycgV
TU_2902
b1203
ychF
b1204
pth
b1206
dauA
D_3399
D_3398
D_3397
D_3396
Dc_4286
Dc_4285
Dc_4284
Dc_4283
Dc_4282
I_5096
I_5095
I_5094
I_5093
I_5092
I_5091
I_5090
Term: termination (1), NS: new start (3), PS: pausing site (0), iTSS_I: internal starting site (0)

ID: 25260−25400; Term: termination (3), NS: new start (2), PS: pausing site (1), iTSS_I: internal starting site (1)

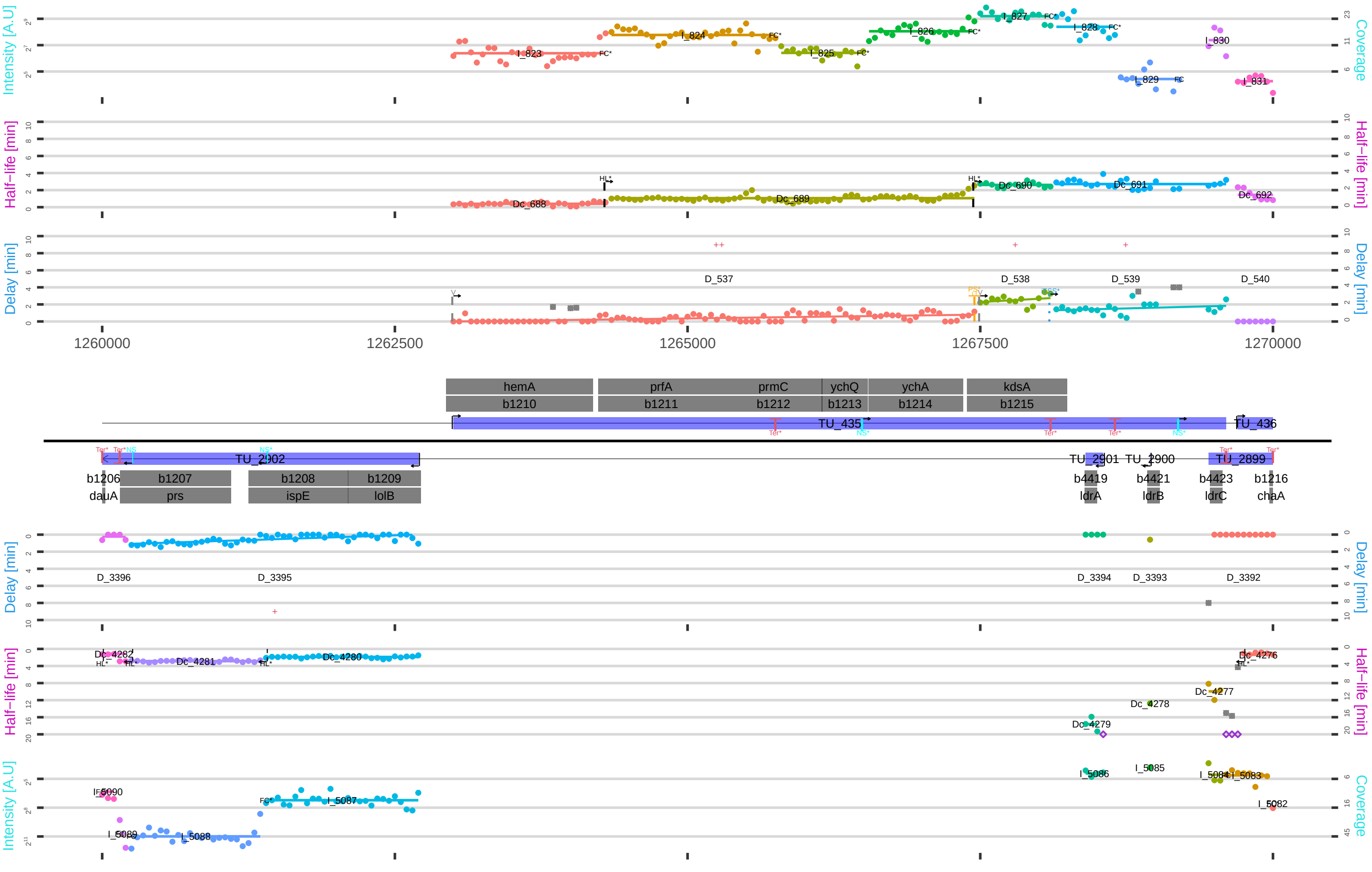

Term: termination (4), NS: new start (2), PS: pausing site (0), iTSS_I: internal starting site (1)

ID: 25400−25574; Term: termination (0), NS: new start (0), PS: pausing site (0), iTSS_I: internal starting site (0)

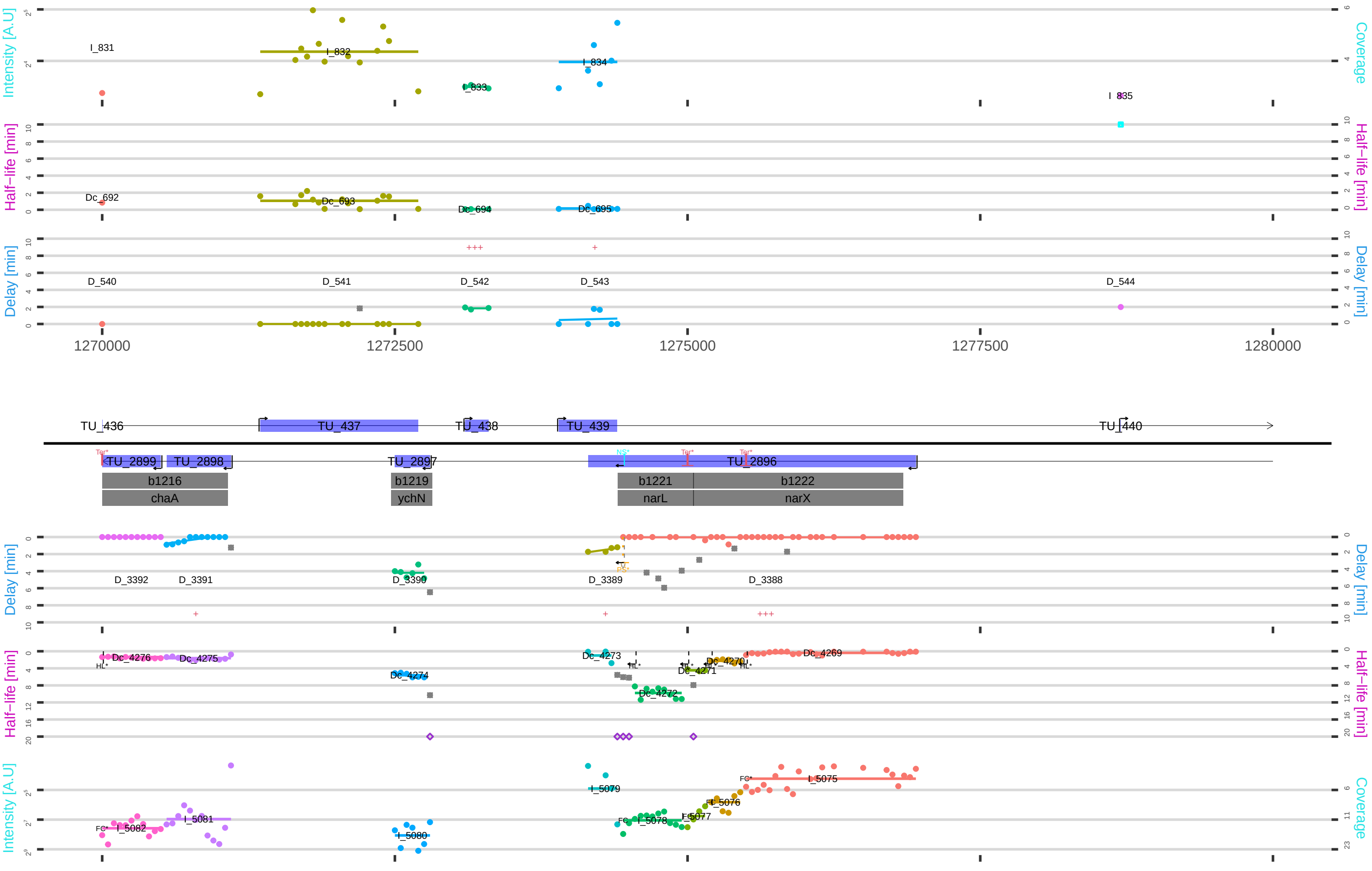

Term: termination (3), NS: new start (1), PS: pausing site (1), iTSS_I: internal starting site (0)

ID: 25667−25800; Term: termination (1), NS: new start (0), PS: pausing site (0), iTSS_I: internal starting site (0)

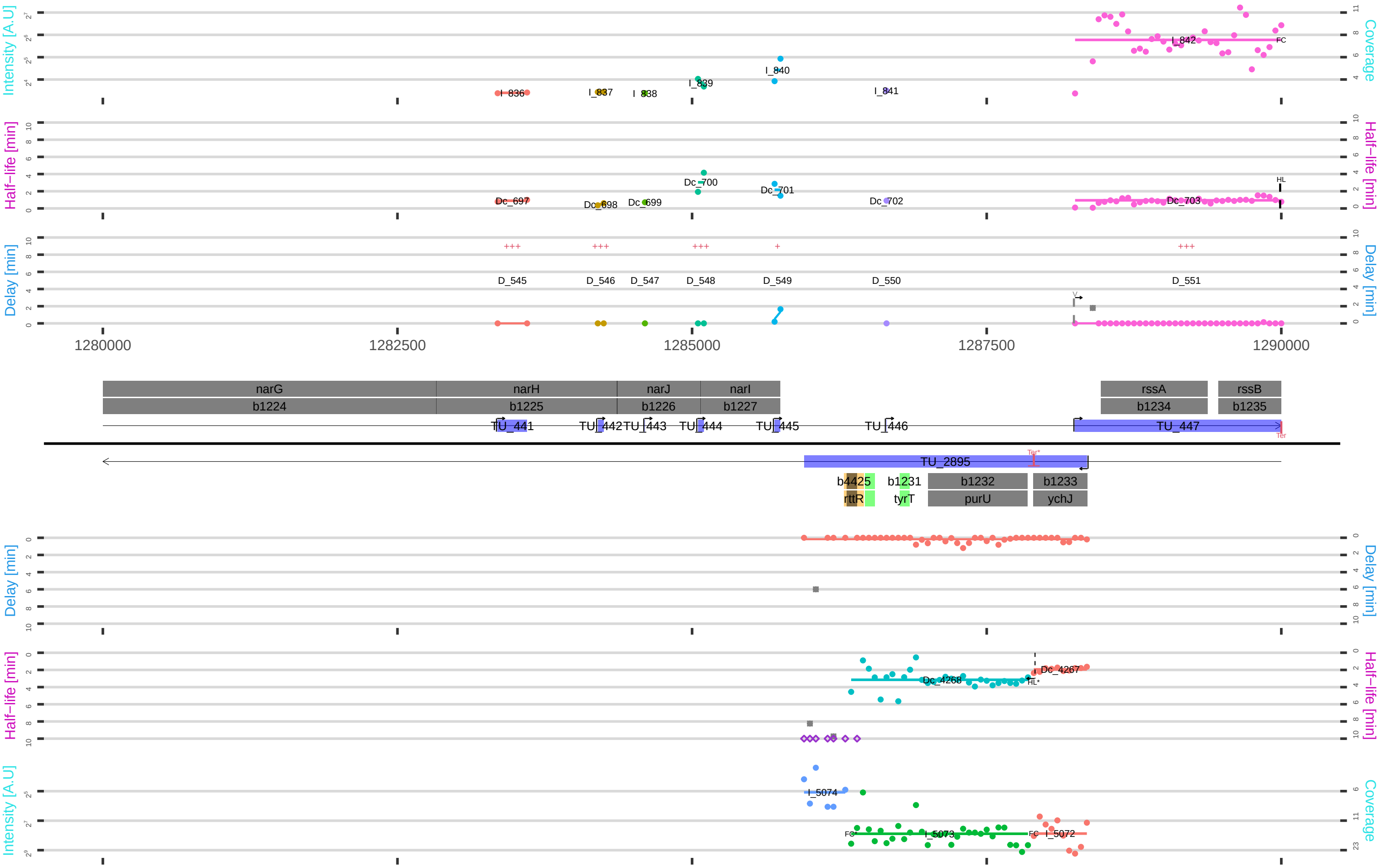

Term: termination (1), NS: new start (0), PS: pausing site (0), iTSS_I: internal starting site (0)

ID: 25800−26000; Term: termination (7), NS: new start (4), PS: pausing site (1), iTSS_I: internal starting site (0)

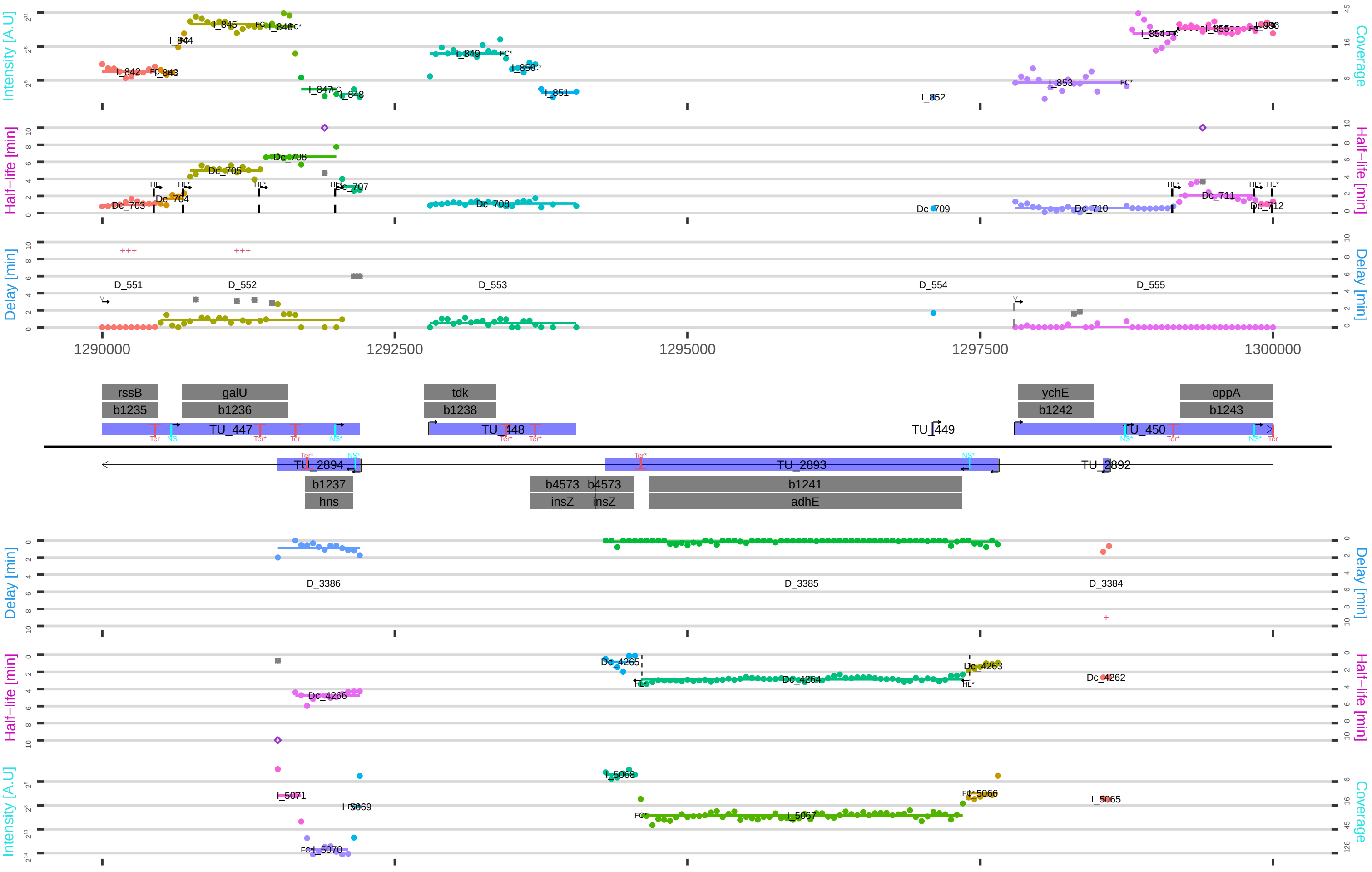

Term: termination (2), NS: new start (2), PS: pausing site (0), iTSS_I: internal starting site (0)

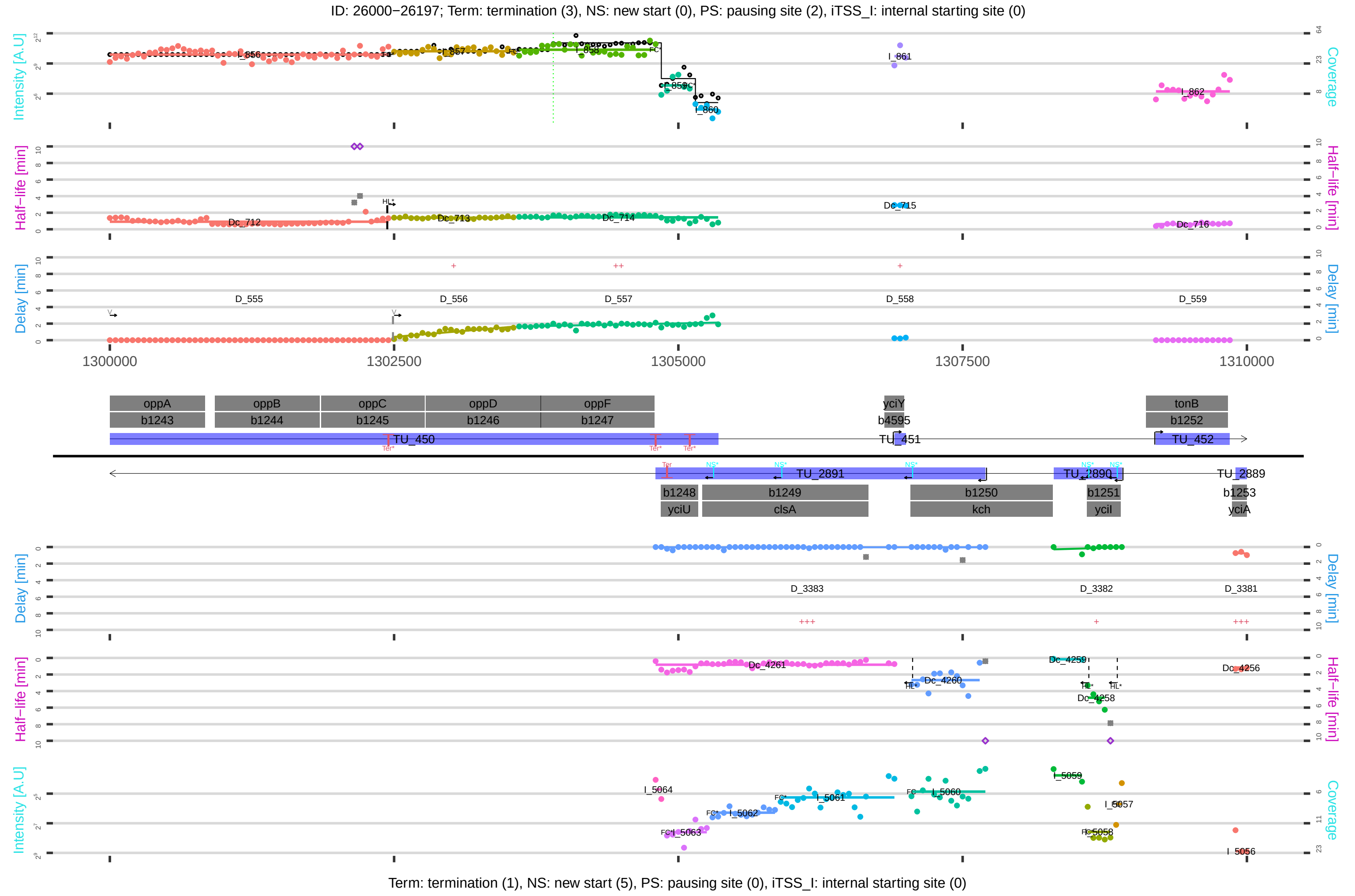

ID: 26000−26197; Term: termination (3), NS: new start (0), PS: pausing site (2), iTSS_I: internal starting site (0)
Intensity [A.U]
Coverage
Half−life [min]
Delay [min]
I_856
I_857
I_858
I_859
I_860
I_861
I_862
Dc_712
Dc_713
Dc_714
Dc_715
Dc_716
D_555
D_556
D_557
D_558
D_559
1300000
1302500
1305000
1307500
1310000
oppA
b1243
oppB
b1244
oppC
b1245
oppD
b1246
oppF
b1247
yciY
b4595
tonB
b1252
TU_450
TU_451
TU_452
TU_2891
TU_2890
TU_2889
b1248
yciU
b1249
clsA
b1250
kch
b1251
yciI
b1253
yciA
D_3383
D_3382
D_3381
Dc_4261
Dc_4260
Dc_4259
Dc_4258
Dc_4256
I_5064
I_5063
I_5062
I_5061
I_5060
I_5059
I_5058
I_5057
I_5056
Term: termination (1), NS: new start (5), PS: pausing site (0), iTSS_I: internal starting site (0)

ID: 26293−26294; Term: termination (0), NS: new start (0), PS: pausing site (0), iTSS_I: internal starting site (0)

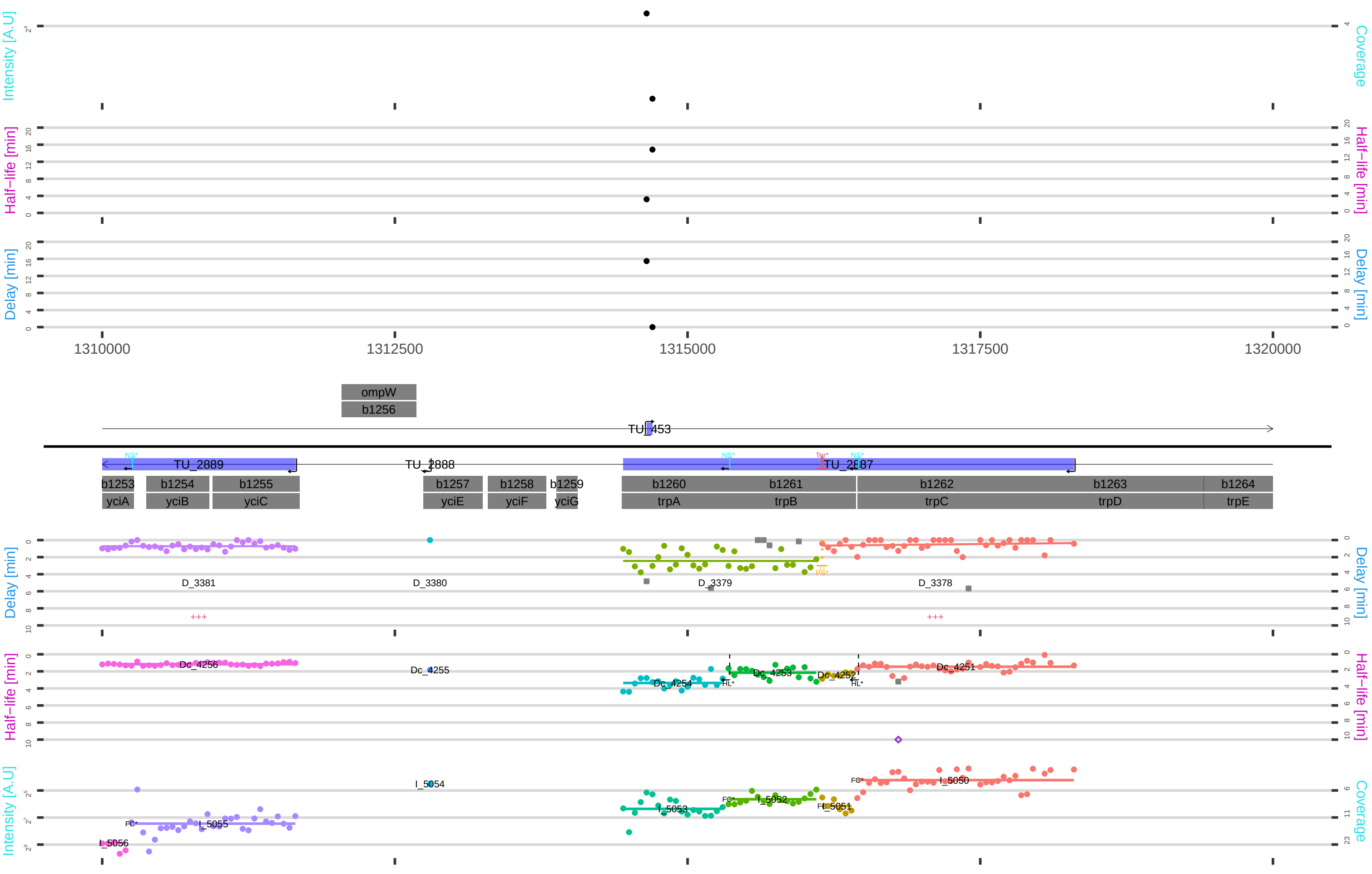

Term: termination (1), NS: new start (3), PS: pausing site (1), iTSS_I: internal starting site (0)

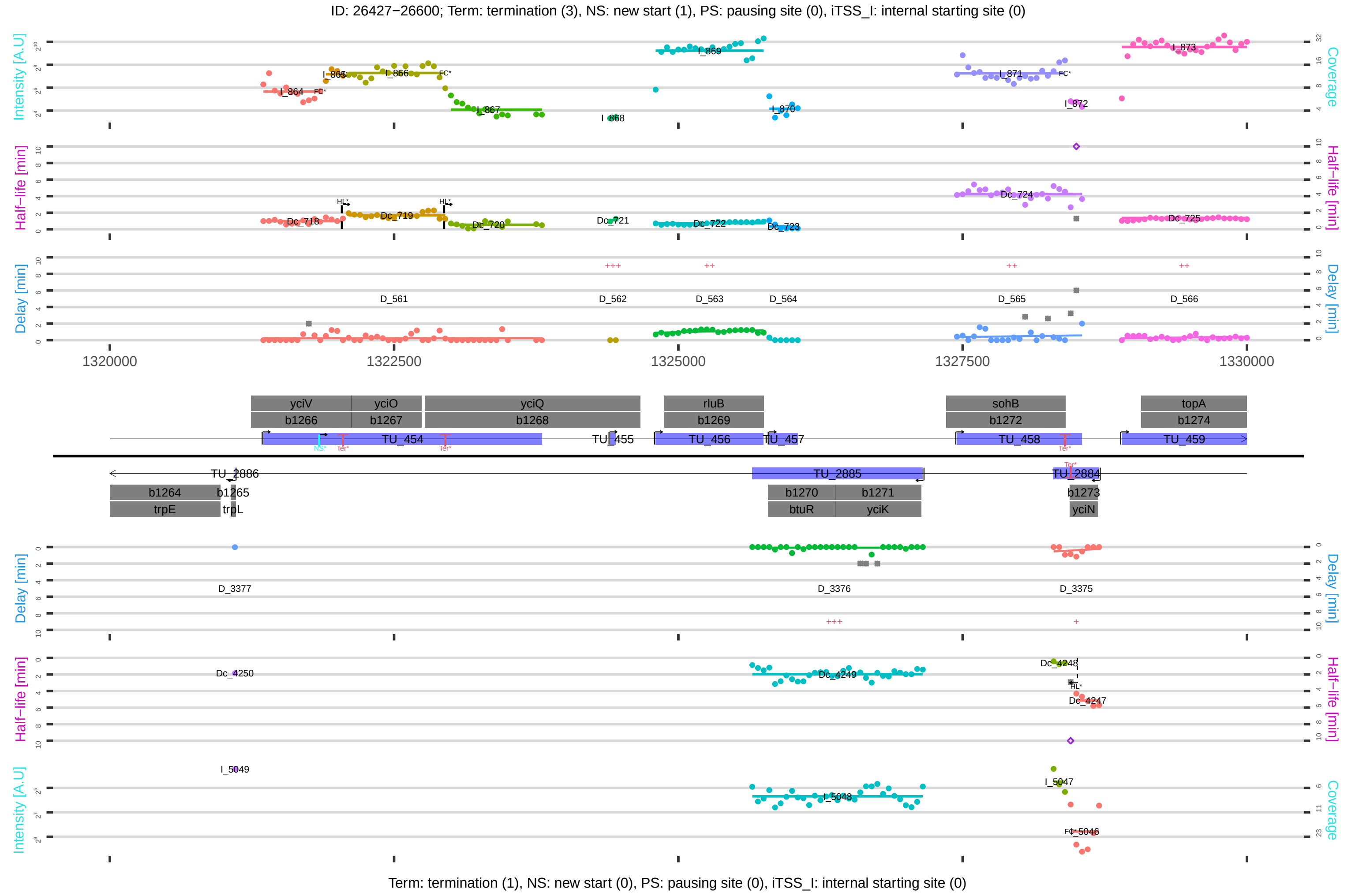

ID: 26427−26600; Term: termination (3), NS: new start (1), PS: pausing site (0), iTSS_I: internal starting site (0)
Intensity [A.U]
Coverage
Half−life [min]
Delay [min]
1320000
1322500
1325000
1327500
1330000
yciV
yciO
yciQ
rluB
sohB
topA
b1266
b1267
b1268
b1269
b1272
b1274
TU_454
TU_455
TU_456
TU_457
TU_458
TU_459
TU_2886
TU_2885
TU_2884
b1264
b1265
b1270
b1271
b1273
trpE
trpL
btuR
yciK
yciN
D_3377
D_3376
D_3375
Term: termination (1), NS: new start (0), PS: pausing site (0), iTSS_I: internal starting site (0)

ID: 26600−26800; Term: termination (4), NS: new start (5), PS: pausing site (1), iTSS_I: internal starting site (0)

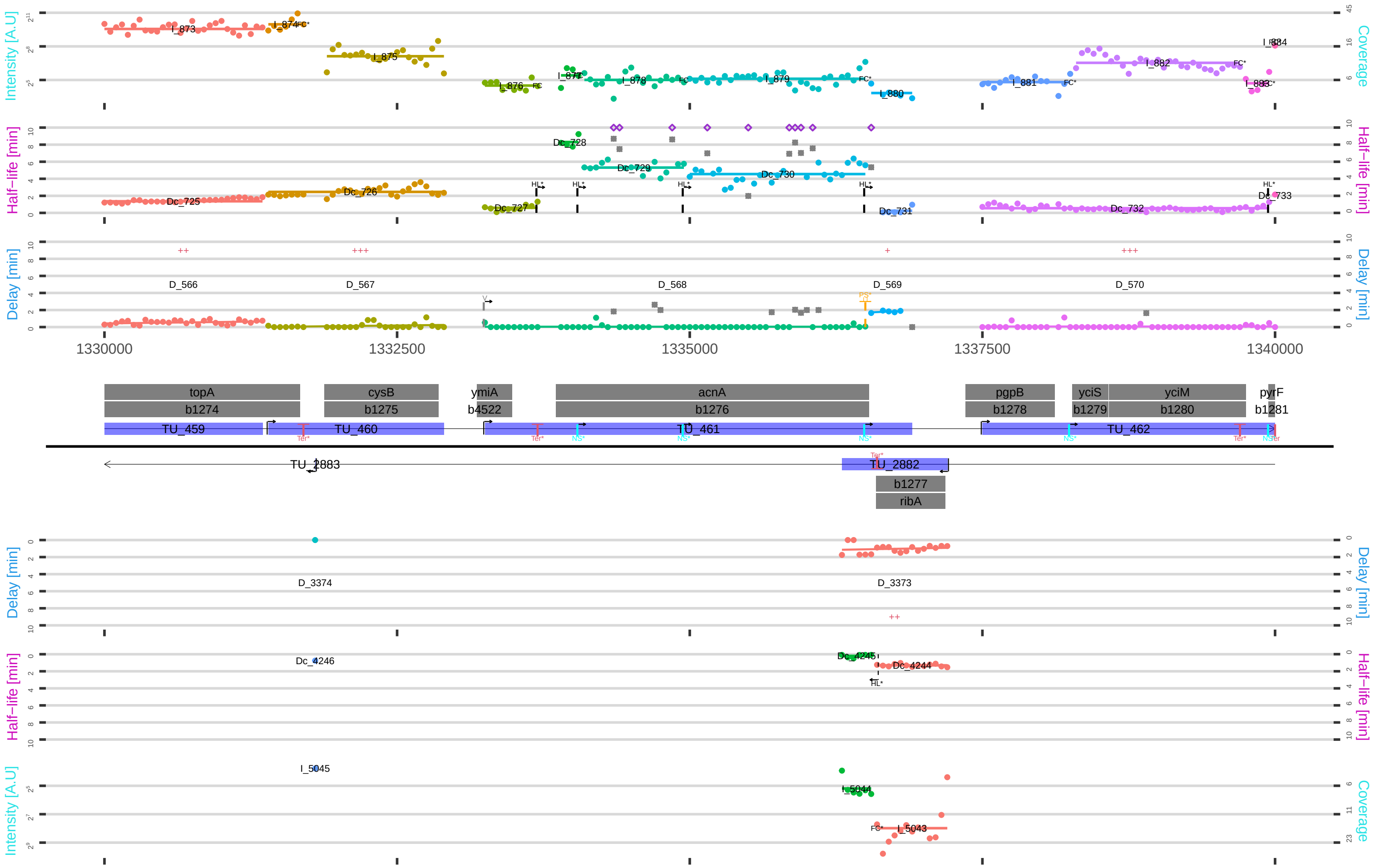

Term: termination (1), NS: new start (0), PS: pausing site (0), iTSS_I: internal starting site (0)

ID: 26800−26823; Term: termination (1), NS: new start (0), PS: pausing site (0), iTSS_I: internal starting site (0)

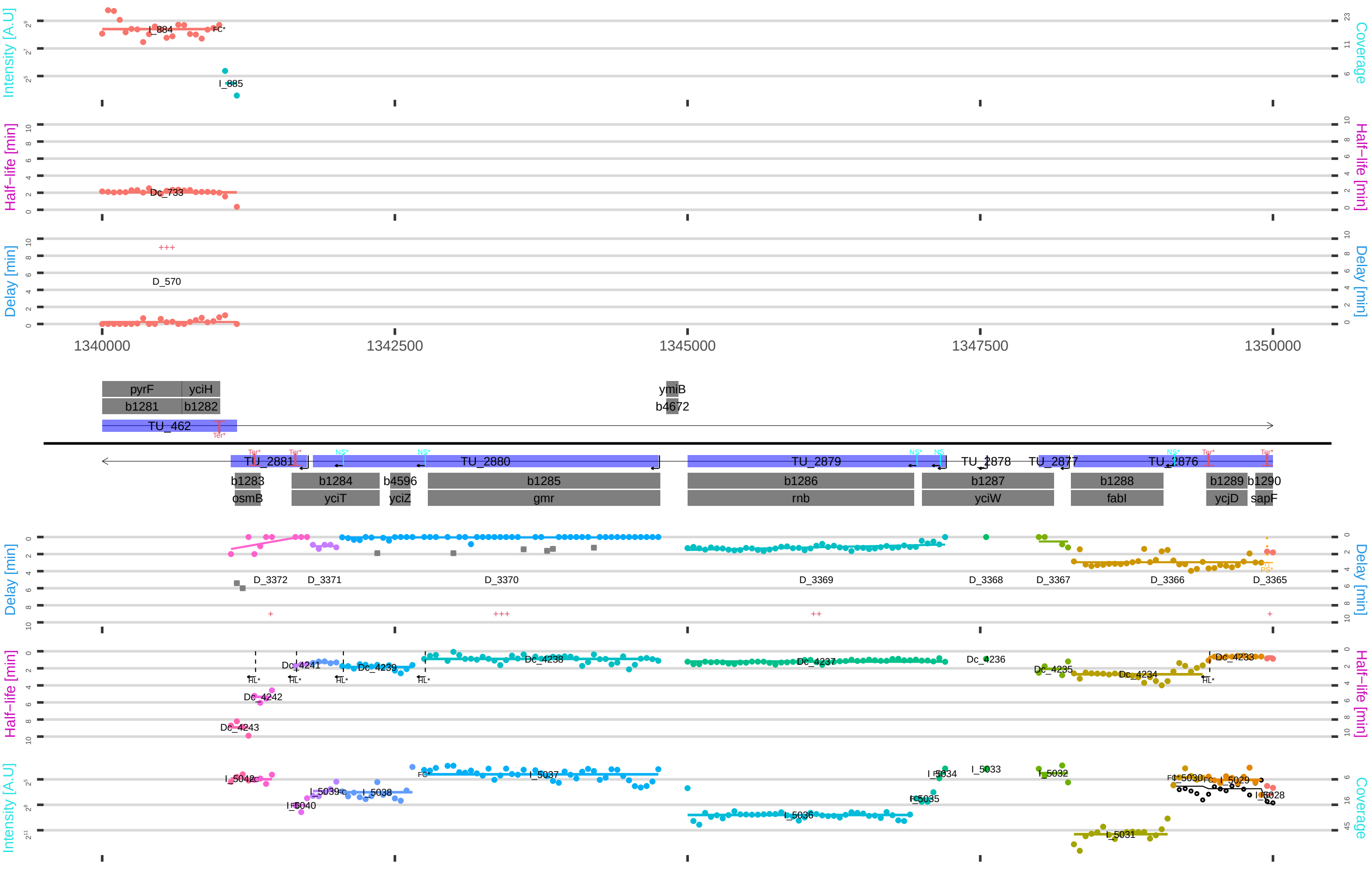

Term: termination (4), NS: new start (5), PS: pausing site (2), iTSS_I: internal starting site (0)

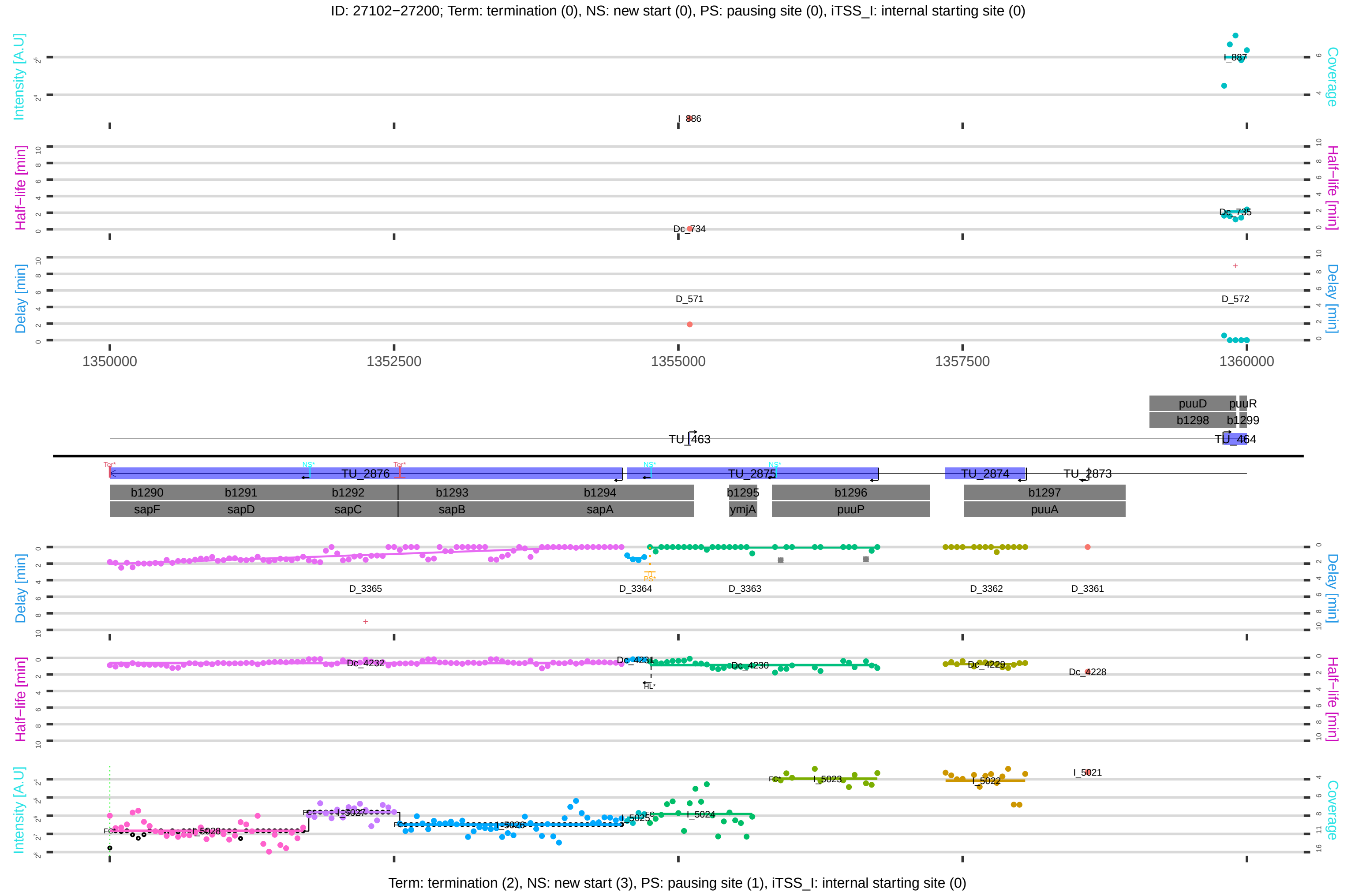
ID: 27102−27200; Term: termination (0), NS: new start (0), PS: pausing site (0), iTSS_I: internal starting site (0)
Intensity [A.U]
Coverage
I_887
I_886
Half−life [min]
Dc_735
Dc_734
Delay [min]
D_571
D_572
1350000
1352500
1355000
1357500
1360000
puuD
puuR
b1298
b1299
TU_463
TU_464
TU_2876
TU_2875
TU_2874
TU_2873
b1290
b1291
b1292
b1293
b1294
b1295
b1296
b1297
sapF
sapD
sapC
sapB
sapA
ymjA
puuP
puuA
D_3365
D_3364
D_3363
D_3362
D_3361
Dc_4232
Dc_4231
Dc_4230
Dc_4229
Dc_4228
I_5028
I_5027
I_5026
I_5025
I_5024
I_5023
I_5022
I_5021
Term: termination (2), NS: new start (3), PS: pausing site (1), iTSS_I: internal starting site (0)

ID: 27200−27383; Term: termination (1), NS: new start (4), PS: pausing site (2), iTSS_I: internal starting site (0)

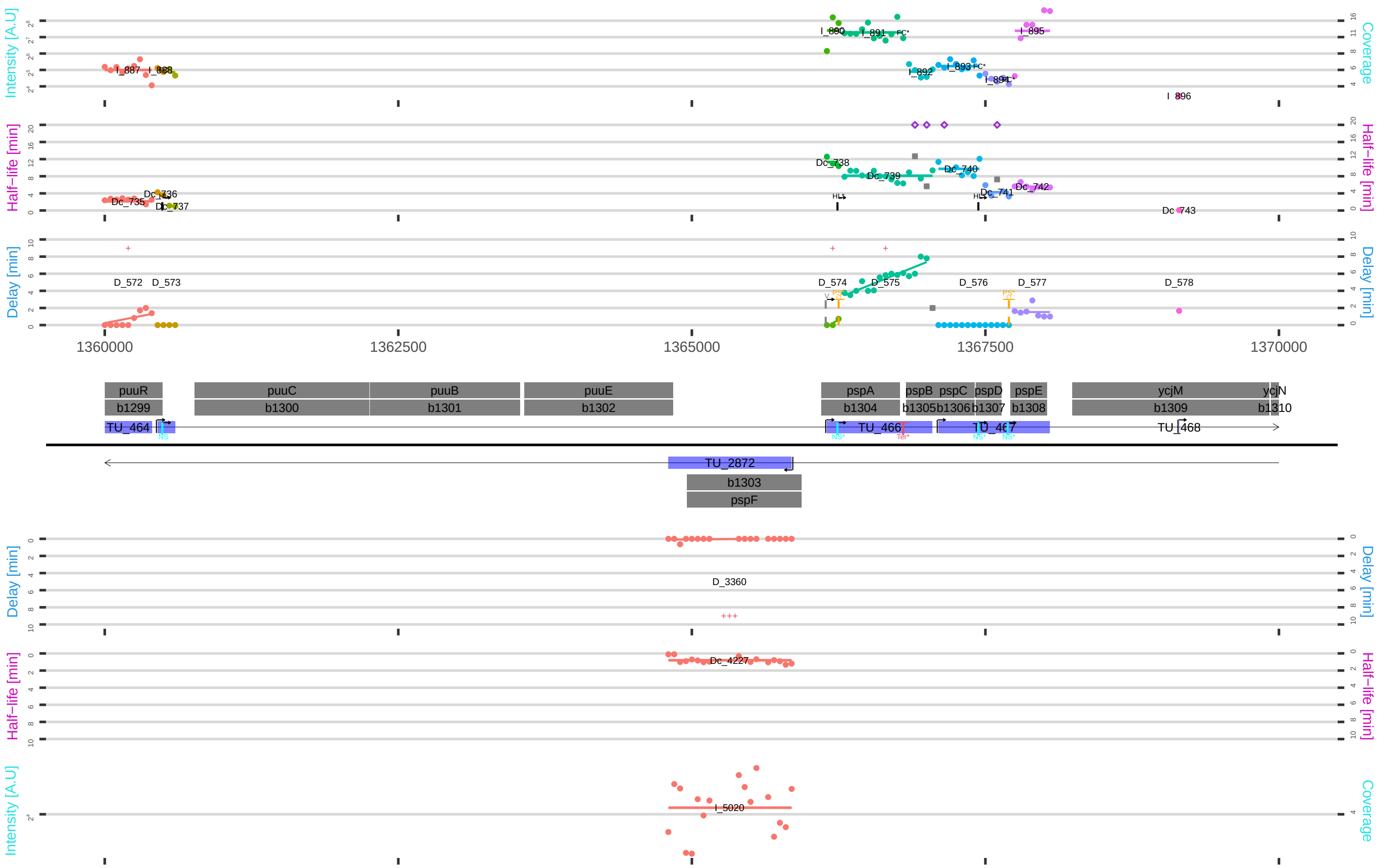

Term: termination (0), NS: new start (0), PS: pausing site (0), iTSS_I: internal starting site (0)

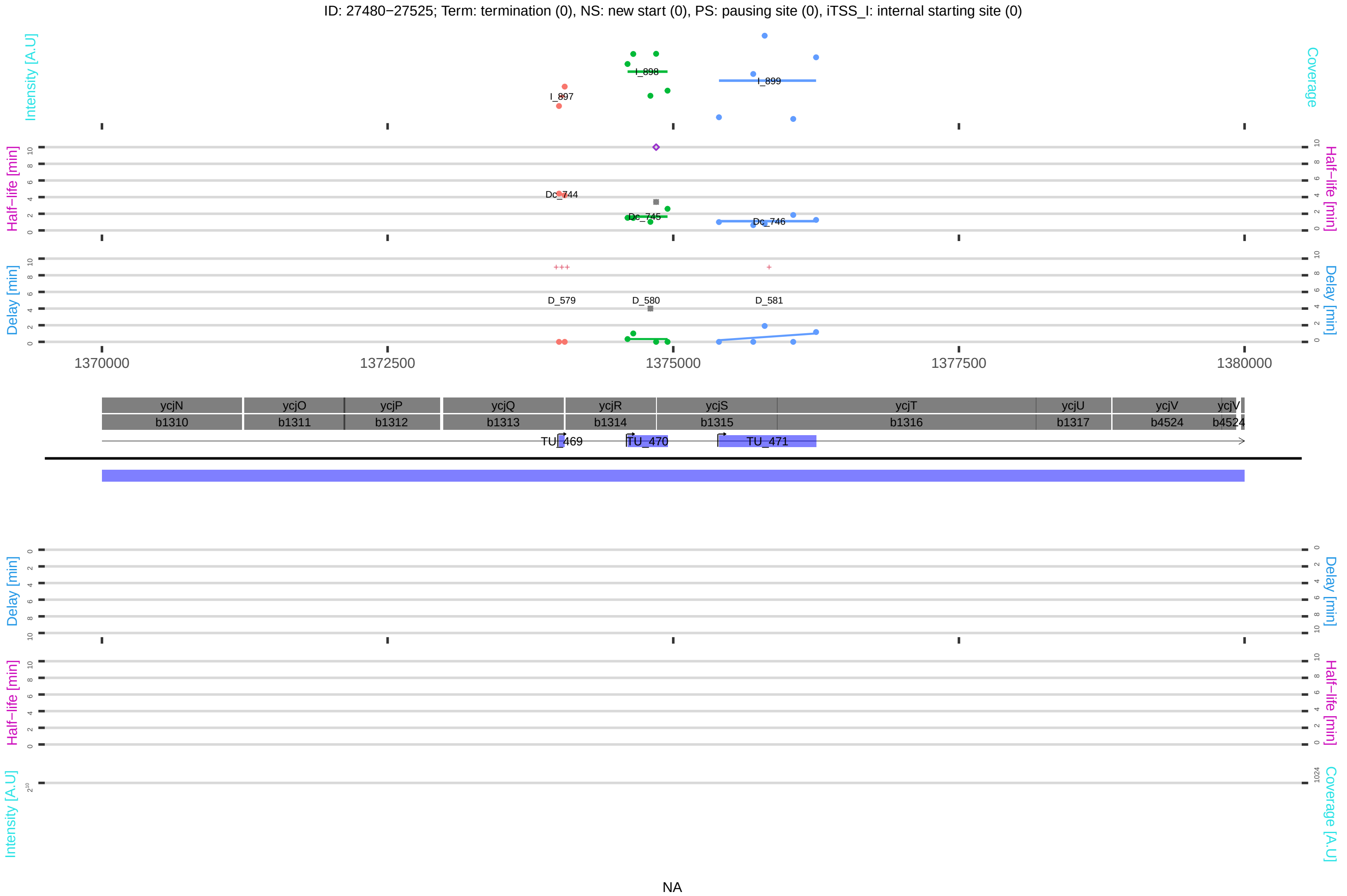

ID: 27480−27525; Term: termination (0), NS: new start (0), PS: pausing site (0), iTSS_I: internal starting site (0)
Intensity [A.U]
Coverage
I_897
I_898
I_899
Half−life [min]
Dc_744
Dc_745
Dc_746
Delay [min]
D_579
D_580
D_581
1370000
1372500
1375000
1377500
1380000
ycjN
ycjO
ycjP
ycjQ
ycjR
ycjS
ycjT
ycjU
ycjV
ycjV
b1310
b1311
b1312
b1313
b1314
b1315
b1316
b1317
b4524
b4524
TU_469
TU_470
TU_471
Delay [min]
Half−life [min]
Intensity [A.U]
Coverage [A.U]
NA

ID: 27644−27798; Term: termination (3), NS: new start (2), PS: pausing site (0), iTSS_I: internal starting site (0)

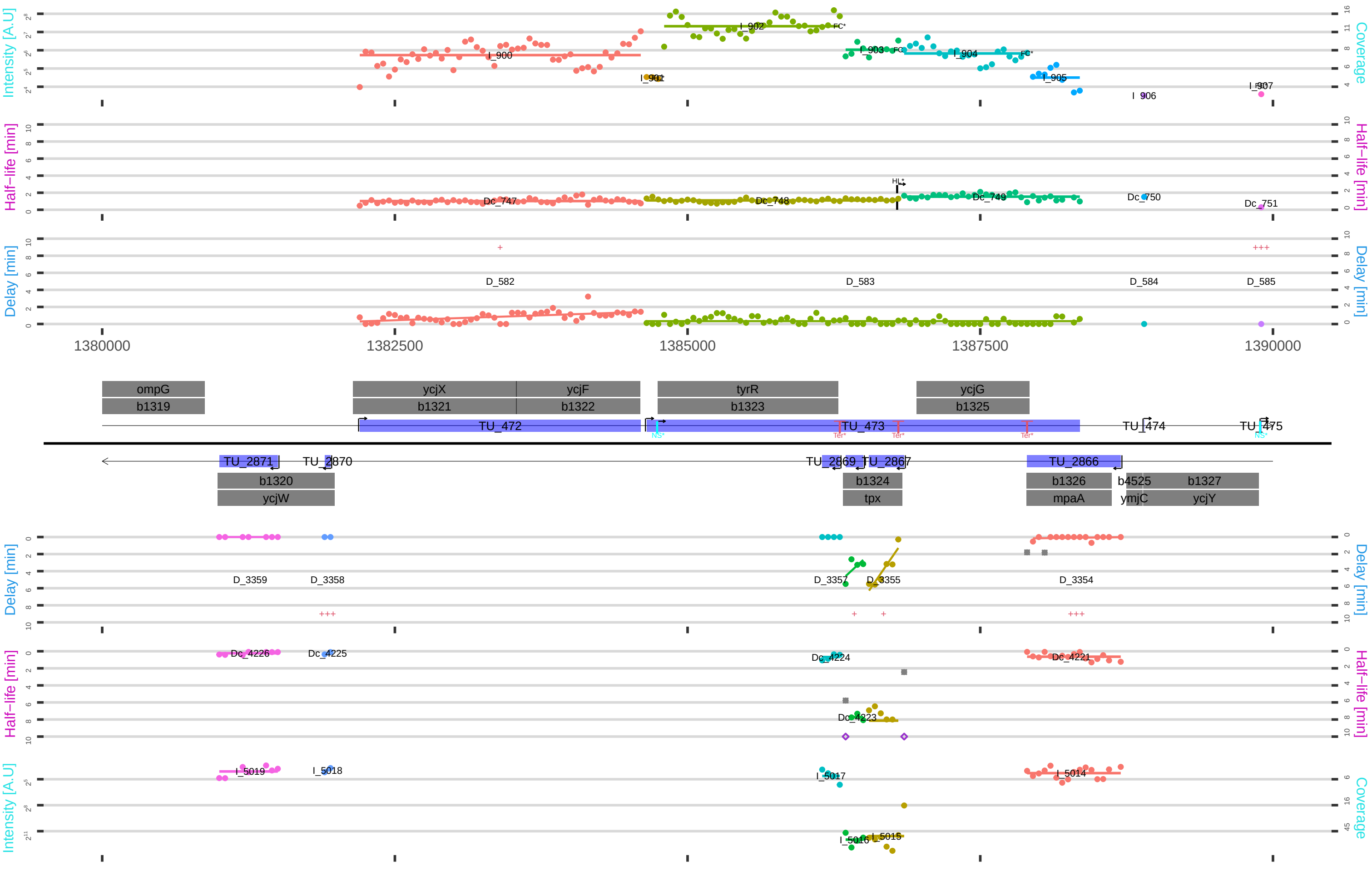

Term: termination (0), NS: new start (0), PS: pausing site (0), iTSS_I: internal starting site (0)

ID: 27801−27918; Term: termination (1), NS: new start (3), PS: pausing site (0), iTSS_I: internal starting site (1)

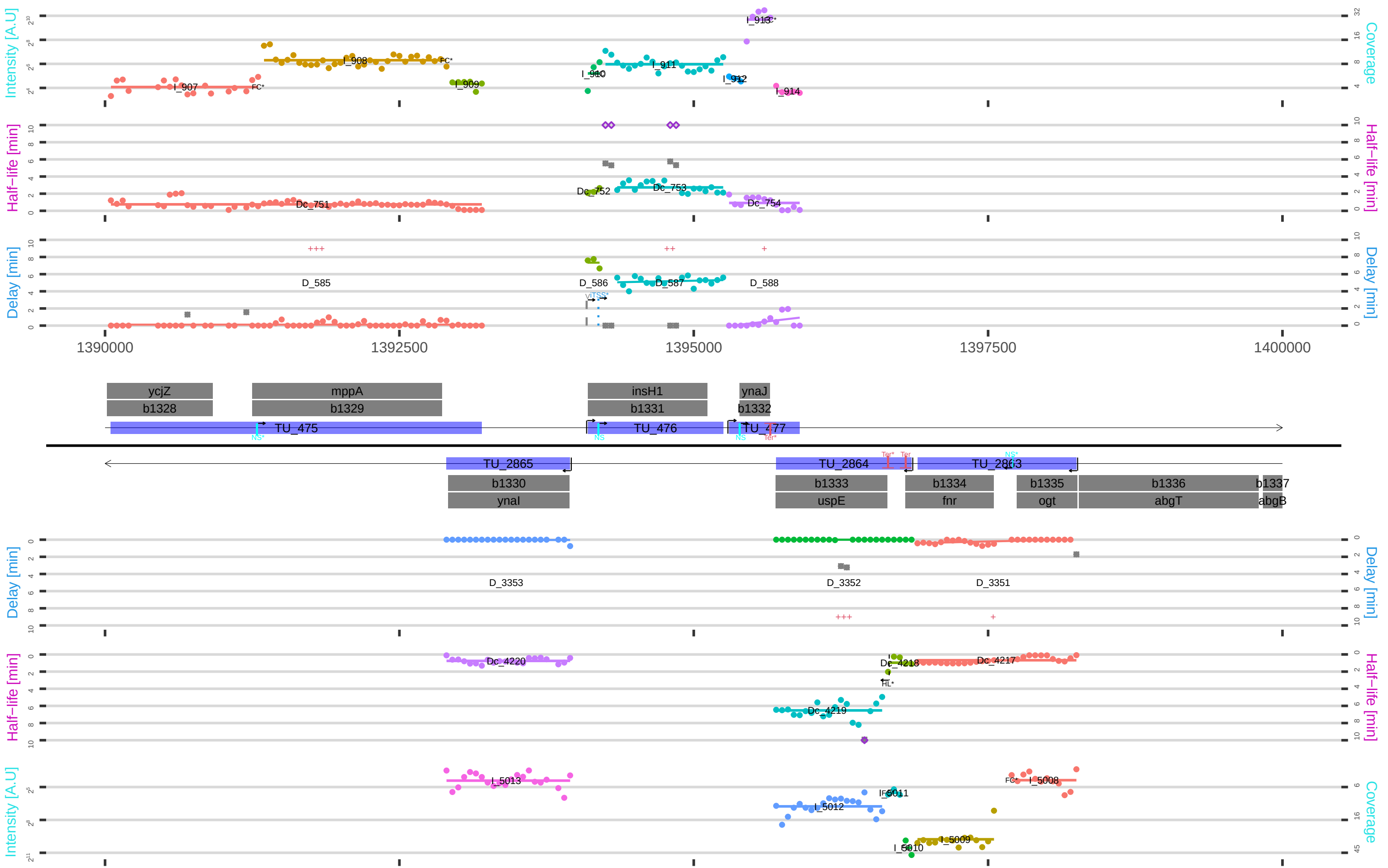

Term: termination (2), NS: new start (1), PS: pausing site (0), iTSS_I: internal starting site (0)

ID: 28062−28184; Term: termination (1), NS: new start (1), PS: pausing site (0), iTSS_I: internal starting site (0)

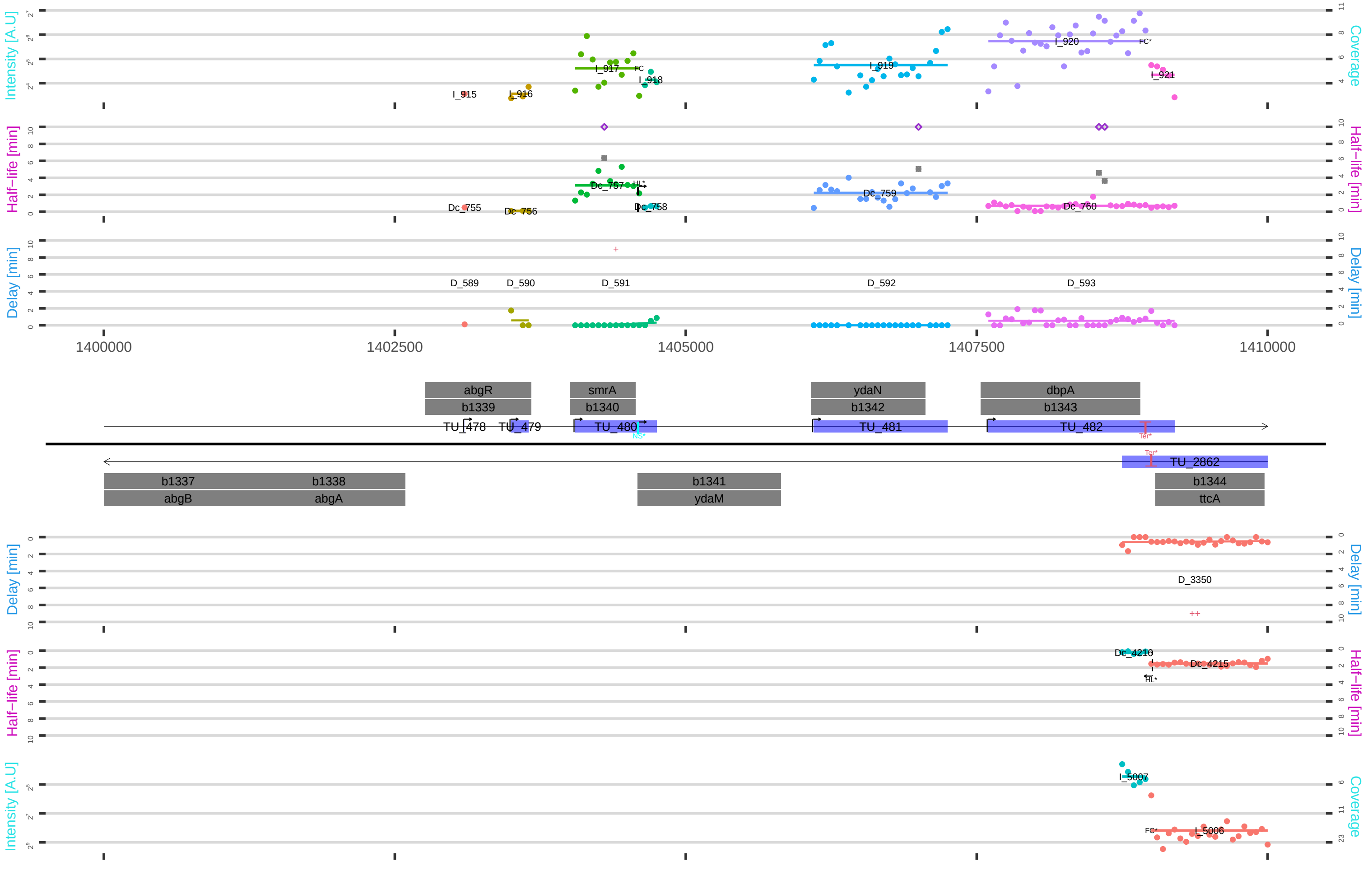

Term: termination (1), NS: new start (0), PS: pausing site (0), iTSS_I: internal starting site (0)

ID: 28308−28399; Term: termination (0), NS: new start (0), PS: pausing site (0), iTSS_I: internal starting site (0)

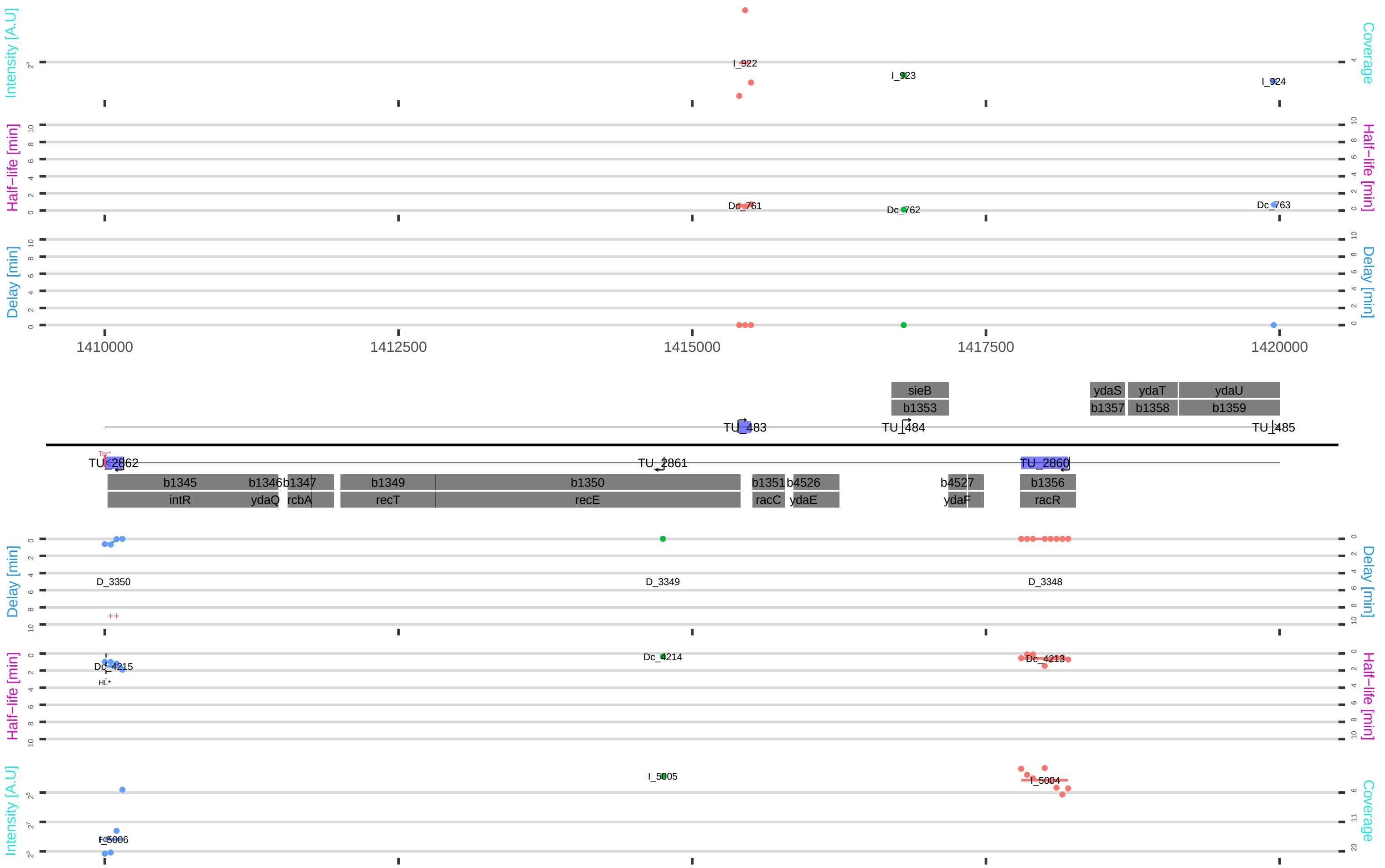

Term: termination (1), NS: new start (0), PS: pausing site (0), iTSS_I: internal starting site (0)

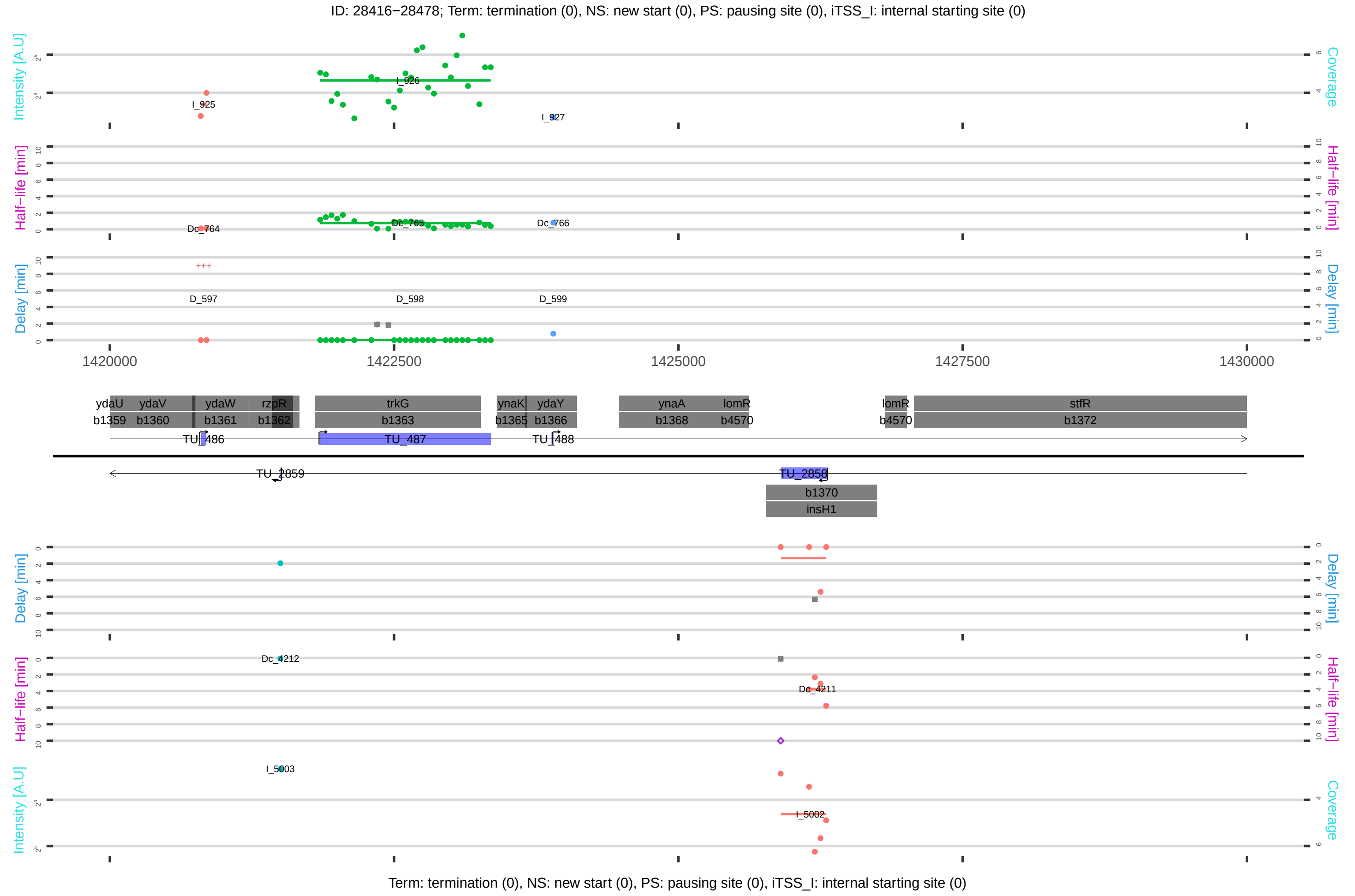
ID: 28416−28478; Term: termination (0), NS: new start (0), PS: pausing site (0), iTSS_I: internal starting site (0)
Intensity [A.U]
Coverage
I_925
I_926
I_927
Half−life [min]
Dc_764
Dc_765
Dc_766
Delay [min]
D_597
D_598
D_599
1420000
1422500
1425000
1427500
1430000
ydaU
ydaV
ydaW
rzpR
trkG
ynaK
ydaY
ynaA
lomR
stfR
b1359
b1360
b1361
b1362
b1363
b1365
b1366
b1368
b4570
b1372
TU_486
TU_487
TU_488
TU_2859
TU_2858
b1370
insH1
Dc_4212
Dc_4211
I_5003
I_5002
Term: termination (0), NS: new start (0), PS: pausing site (0), iTSS_I: internal starting site (0)

ID: 28654−28792; Term: termination (0), NS: new start (0), PS: pausing site (0), iTSS_I: internal starting site (0)

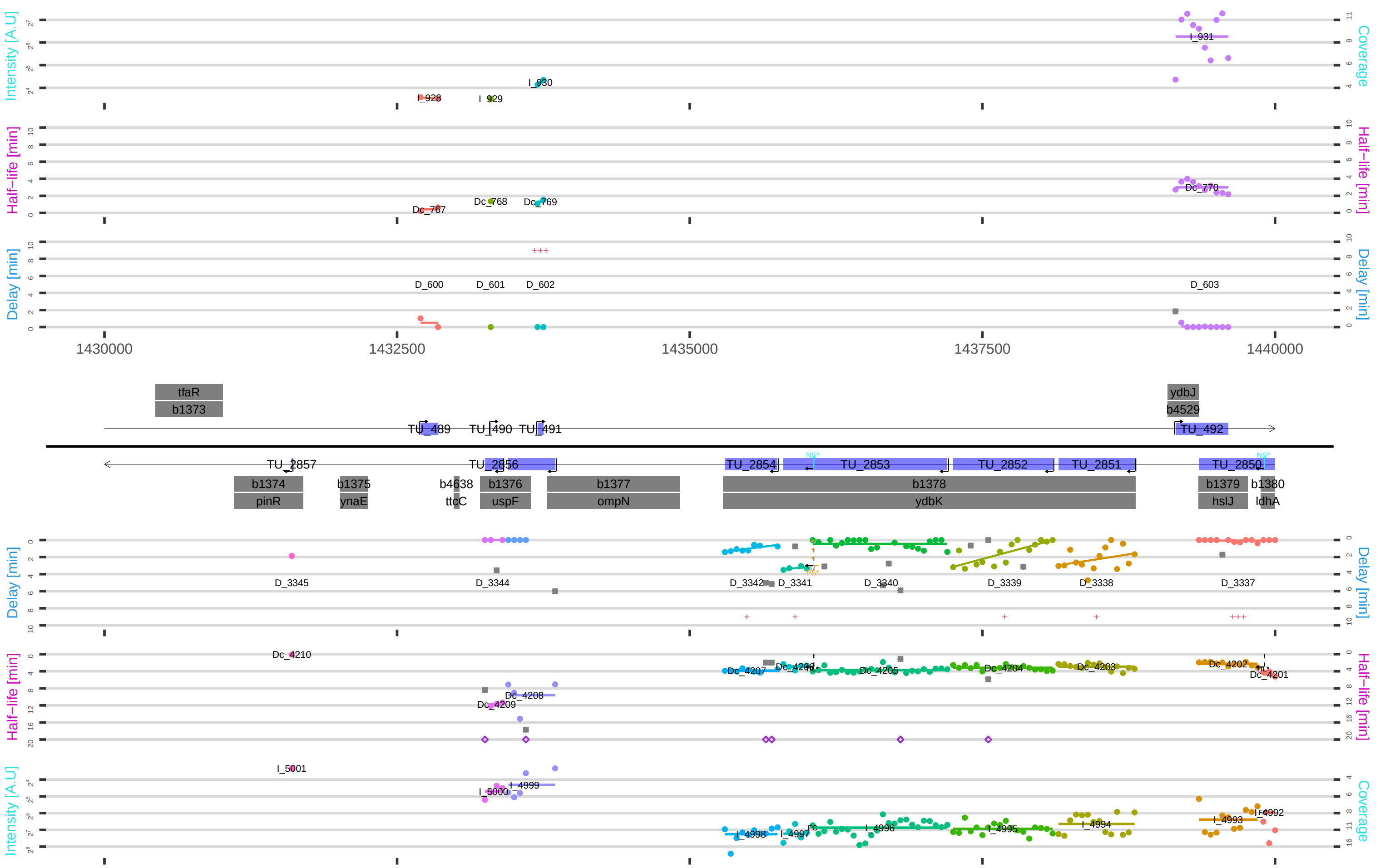

Term: termination (0), NS: new start (2), PS: pausing site (1), iTSS_I: internal starting site (0)

ID: 28824−28991; Term: termination (1), NS: new start (1), PS: pausing site (1), iTSS_I: internal starting site (0)

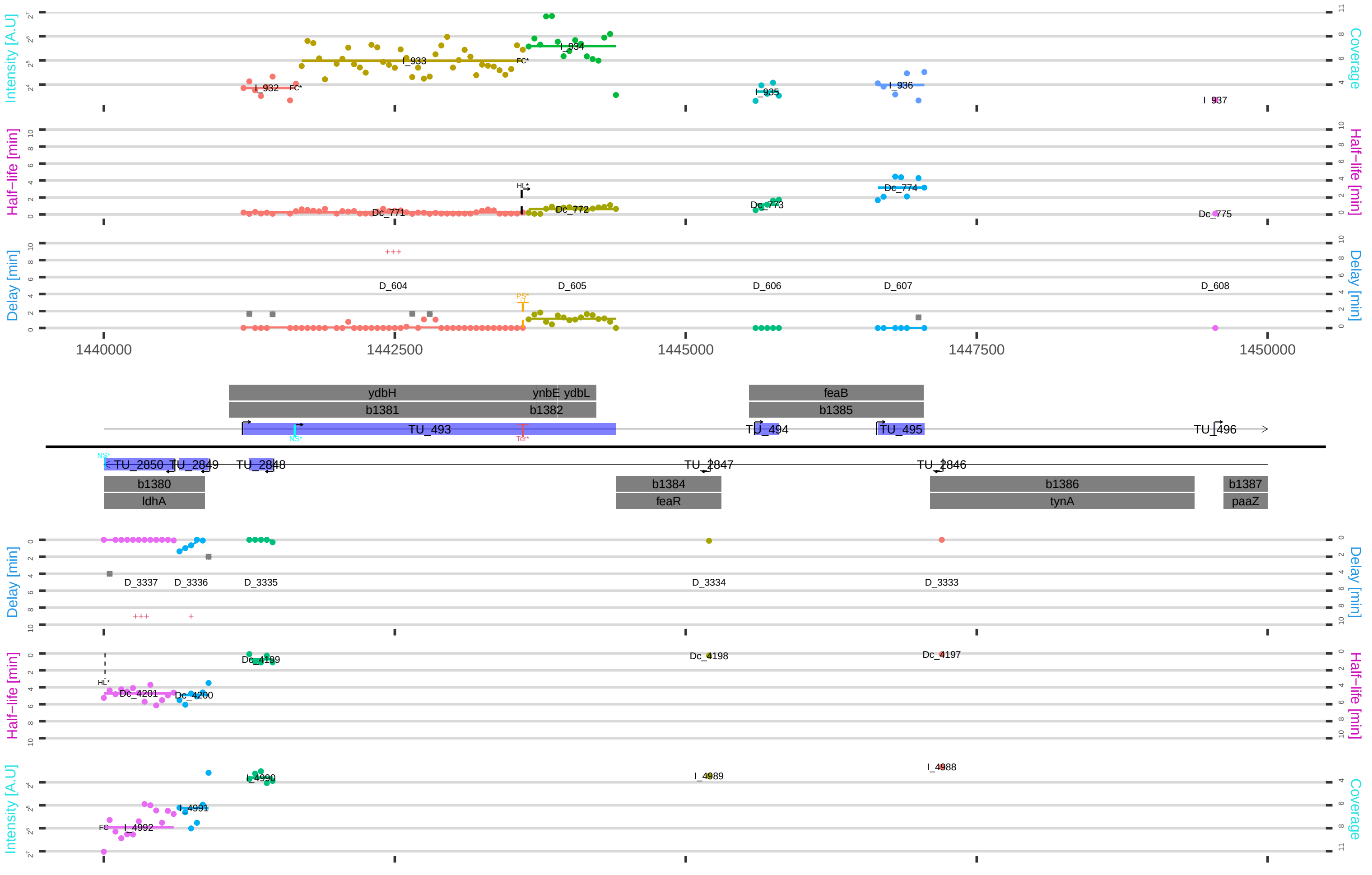

Term: termination (0), NS: new start (1), PS: pausing site (0), iTSS_I: internal starting site (0)

ID: 29085−29197; Term: termination (0), NS: new start (0), PS: pausing site (0), iTSS_I: internal starting site (0)

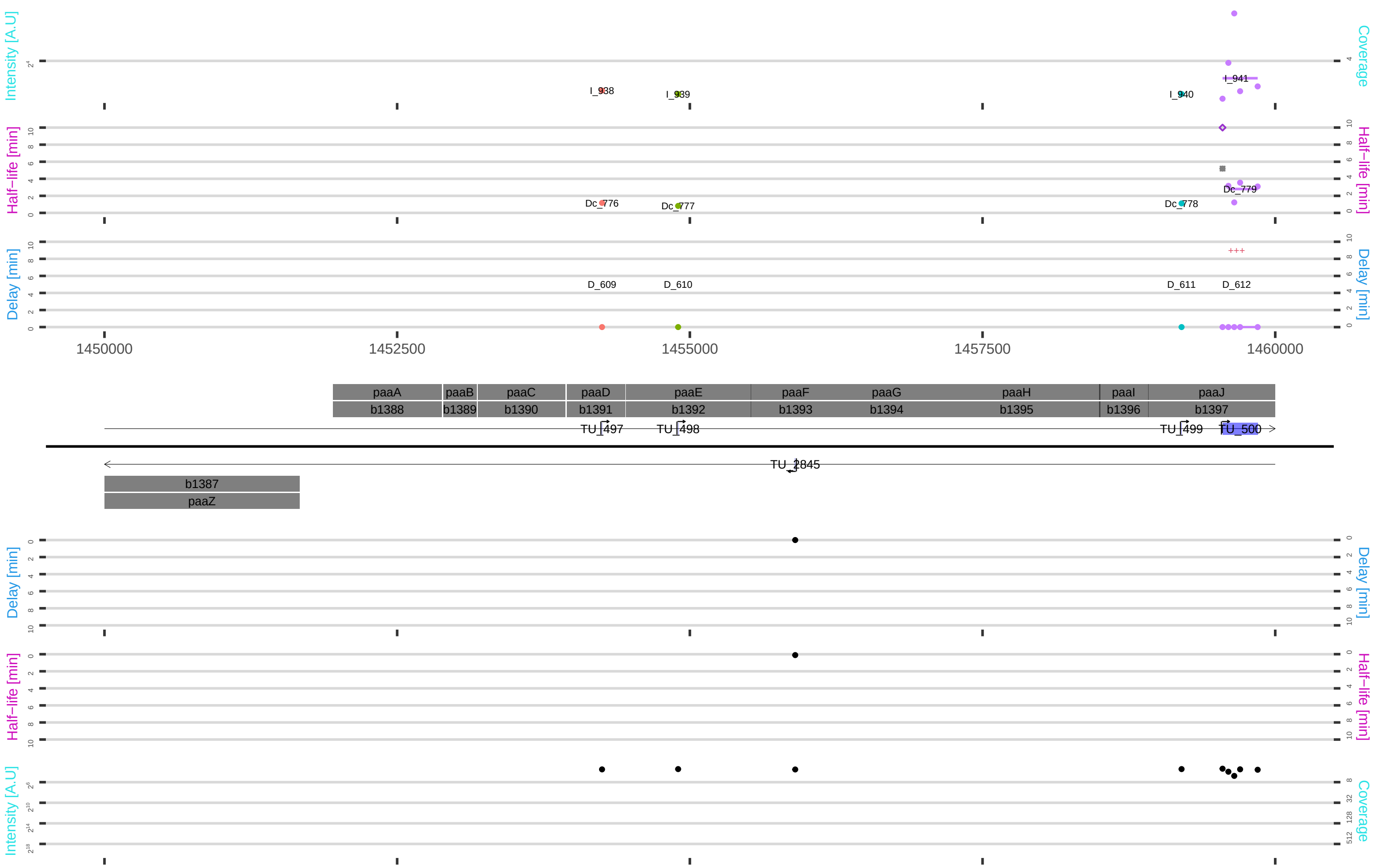

Term: termination (0), NS: new start (0), PS: pausing site (0), iTSS_I: internal starting site (0)

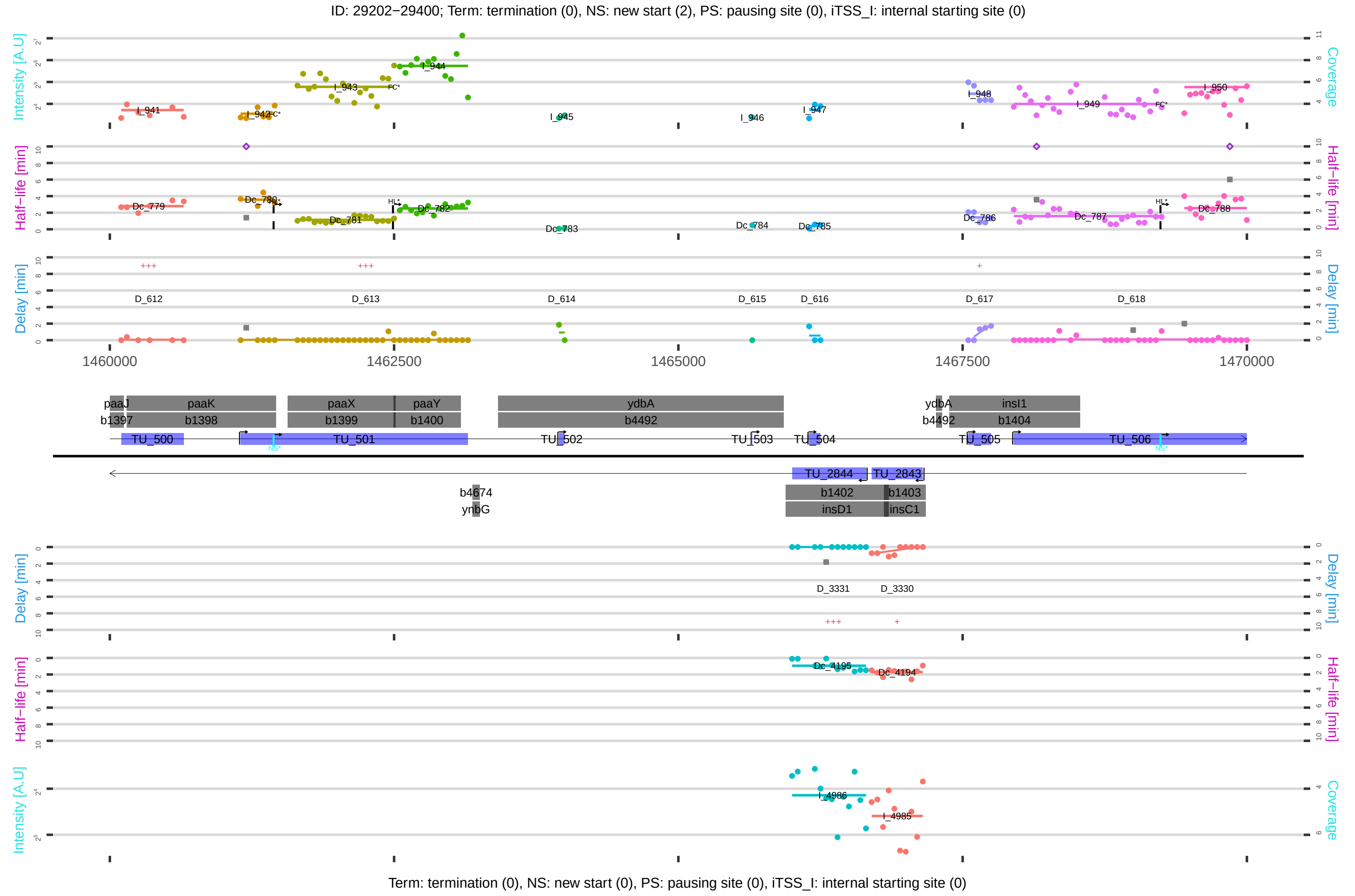

ID: 29202−29400; Term: termination (0), NS: new start (2), PS: pausing site (0), iTSS_I: internal starting site (0)
Intensity [A.U]
Coverage
Half−life [min]
Delay [min]
I_941
I_942
I_943
I_944
I_945
I_946
I_947
I_948
I_949
I_950
Dc_779
Dc_780
Dc_781
Dc_782
Dc_783
Dc_784
Dc_785
Dc_786
Dc_787
Dc_788
D_612
D_613
D_614
D_615
D_616
D_617
D_618
1460000
1462500
1465000
1467500
1470000
paaJ
paaK
paaX
paaY
ydbA
ydbA
insI1
b1397
b1398
b1399
b1400
b4492
b4492
b1404
TU_500
TU_501
TU_502
TU_503
TU_504
TU_505
TU_506
TU_2844
TU_2843
b4674
ynbG
b1402
b1403
insD1
insC1
D_3331
D_3330
Dc_4195
Dc_4194
I_4986
I_4985
Term: termination (0), NS: new start (0), PS: pausing site (0), iTSS_I: internal starting site (0)

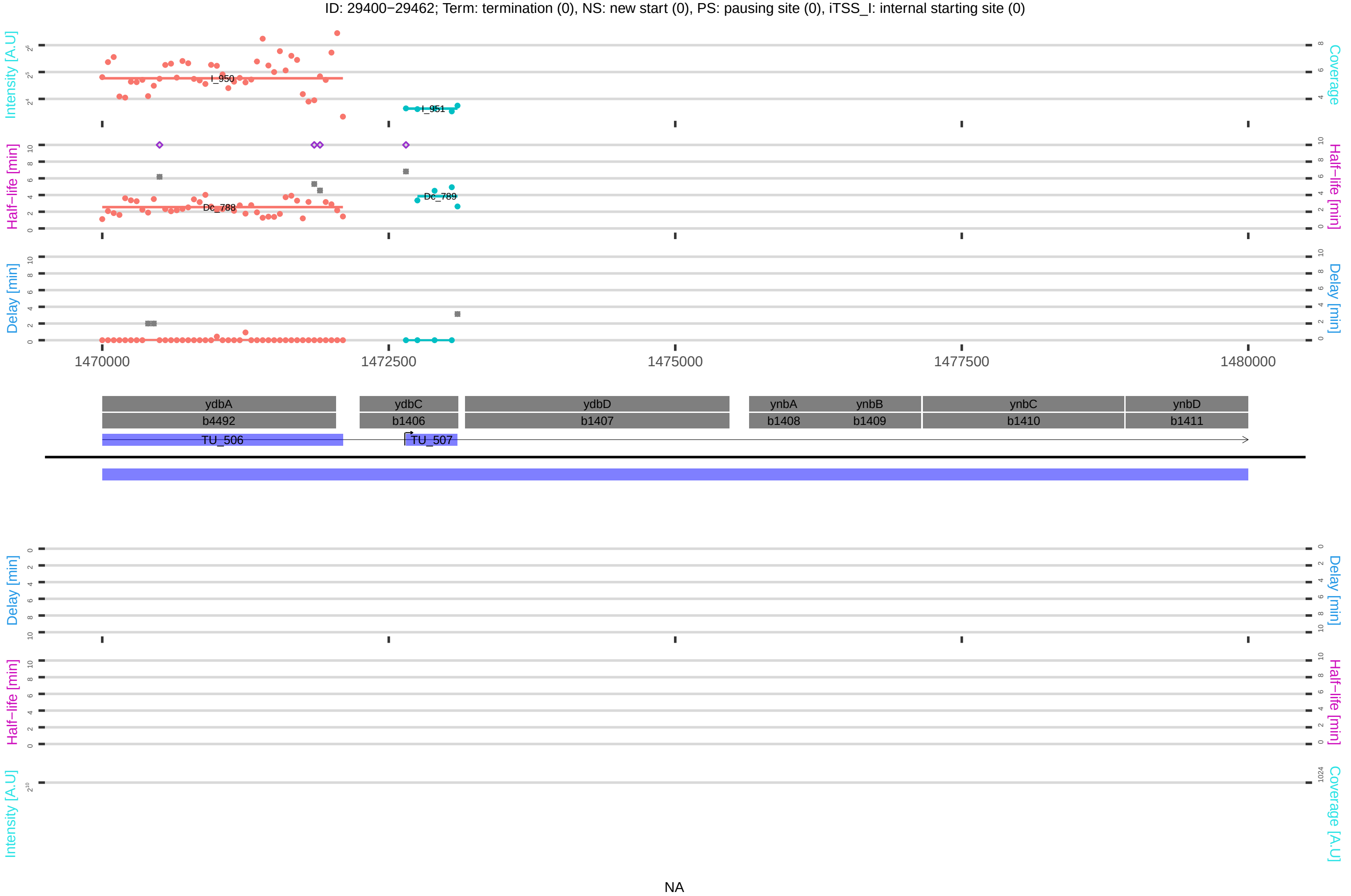
ID: 29400−29462; Term: termination (0), NS: new start (0), PS: pausing site (0), iTSS_I: internal starting site (0)
Intensity [A.U]
Coverage
I_950
I_951
Half−life [min]
Dc_788
Dc_789
Delay [min]
1470000
1472500
1475000
1477500
1480000
ydbA
b4492
ydbC
b1406
ydbD
b1407
ynbA
b1408
ynbB
b1409
ynbC
b1410
ynbD
b1411
TU_506
TU_507
Delay [min]
Half−life [min]
Intensity [A.U]
Coverage [A.U]
NA

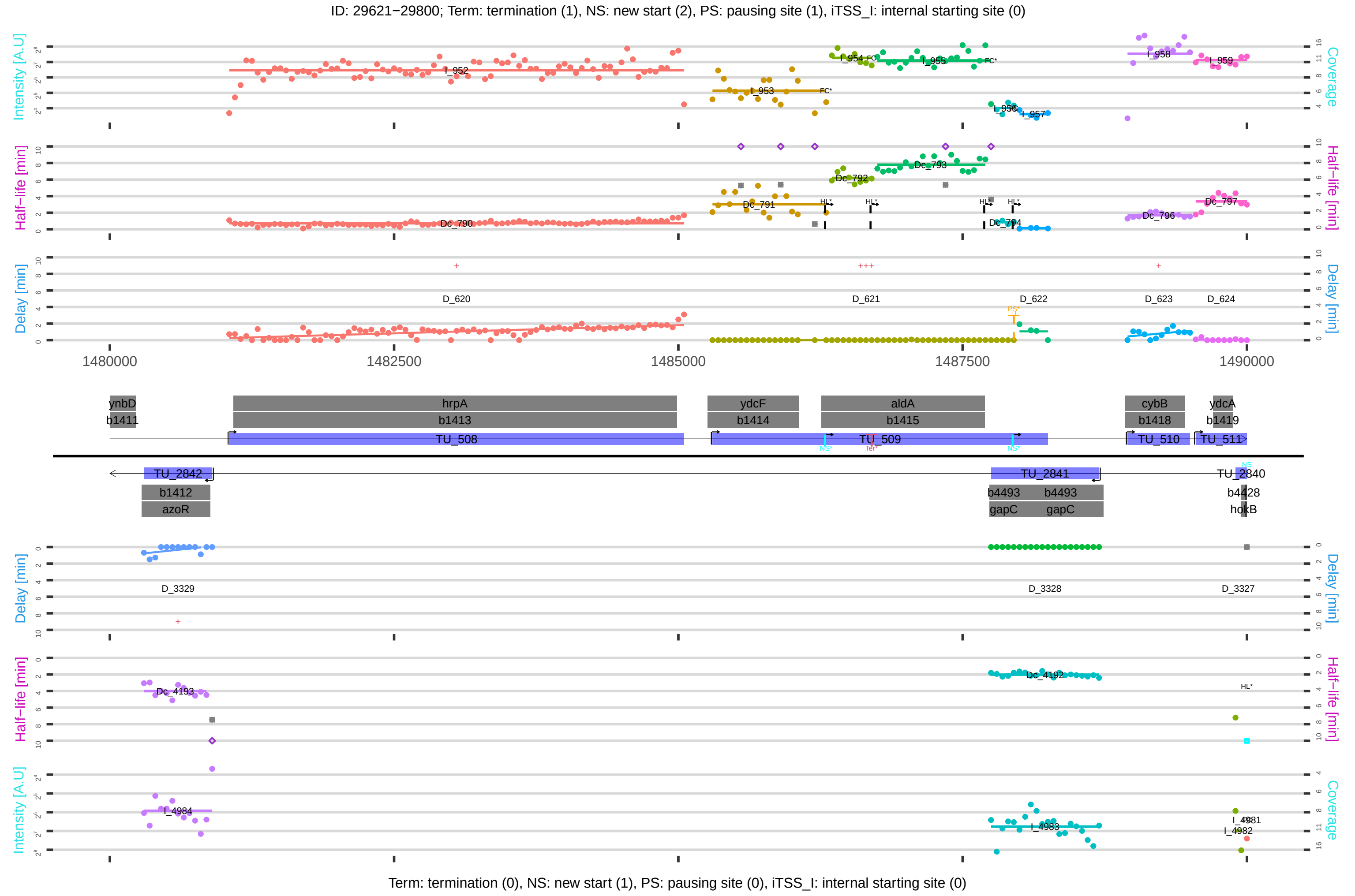

ID: 29621−29800; Term: termination (1), NS: new start (2), PS: pausing site (1), iTSS_I: internal starting site (0)
Intensity [A.U]
Coverage
Half−life [min]
Delay [min]
I_952
I_953
I_954
I_955
I_956
I_957
I_958
I_959
Dc_790
Dc_791
Dc_792
Dc_793
Dc_794
Dc_796
Dc_797
D_620
D_621
D_622
D_623
D_624
1480000
1482500
1485000
1487500
1490000
ynbD
b1411
hrpA
b1413
ydcF
b1414
aldA
b1415
cybB
b1418
ydcA
b1419
TU_508
TU_509
TU_510
TU_511
TU_2842
TU_2841
TU_2840
b1412
azoR
b4493
gapC
b4428
hokB
D_3329
D_3328
D_3327
Dc_4193
Dc_4192
I_4984
I_4983
I_4981
I_4982
Term: termination (0), NS: new start (1), PS: pausing site (0), iTSS_I: internal starting site (0)

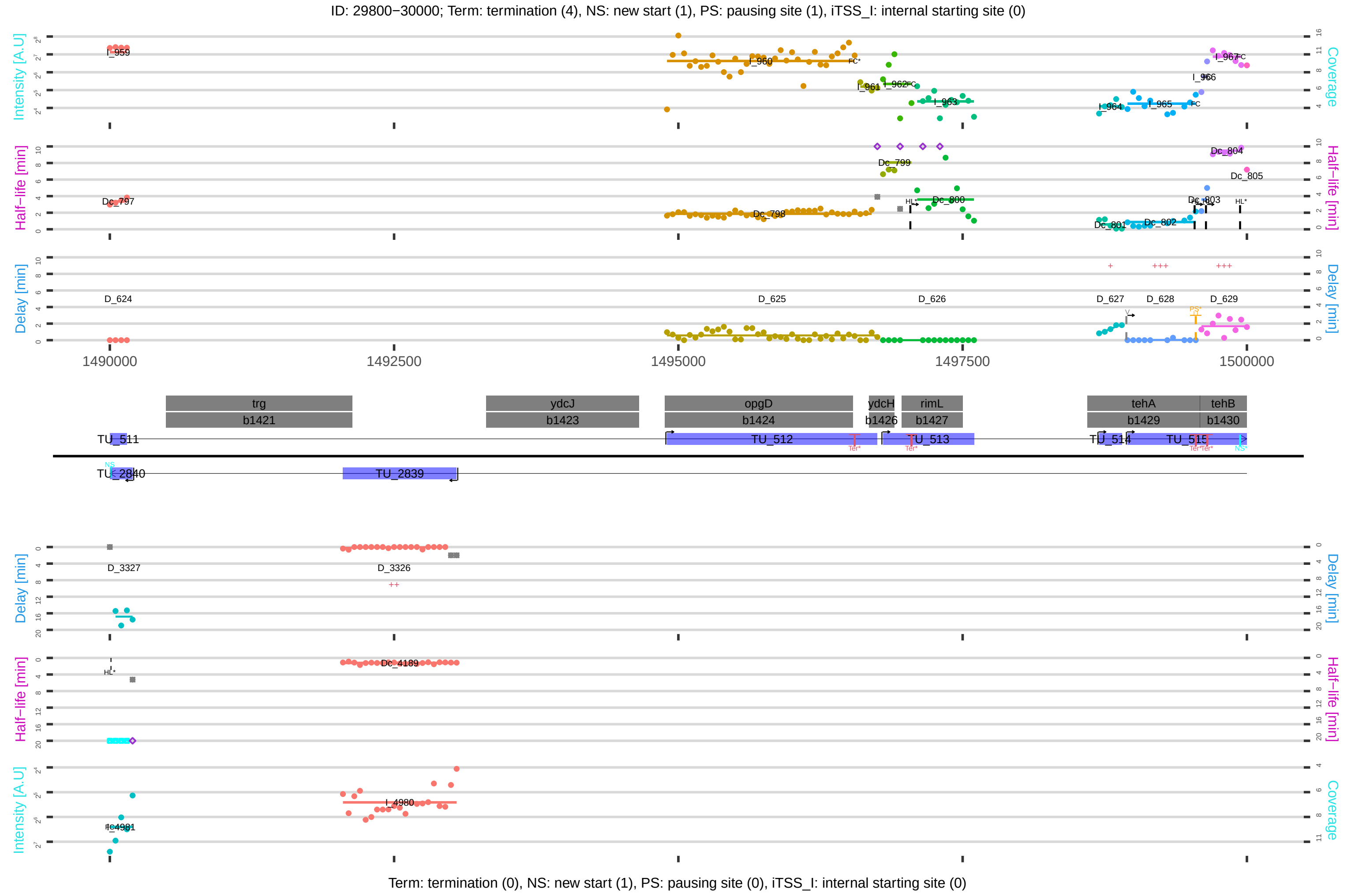
ID: 29800−30000; Term: termination (4), NS: new start (1), PS: pausing site (1), iTSS_I: internal starting site (0)
Intensity [A.U]
Coverage
Half−life [min]
Delay [min]
1490000
1492500
1495000
1497500
1500000
trg
b1421
ydcJ
b1423
opgD
b1424
ydcH
b1426
rimL
b1427
tehA
b1429
tehB
b1430
TU_511
TU_512
TU_513
TU_514
TU_515
TU_2840
TU_2839
Term: termination (0), NS: new start (1), PS: pausing site (0), iTSS_I: internal starting site (0)

ID: 30000−30196; Term: termination (2), NS: new start (3), PS: pausing site (0), iTSS_I: internal starting site (0)

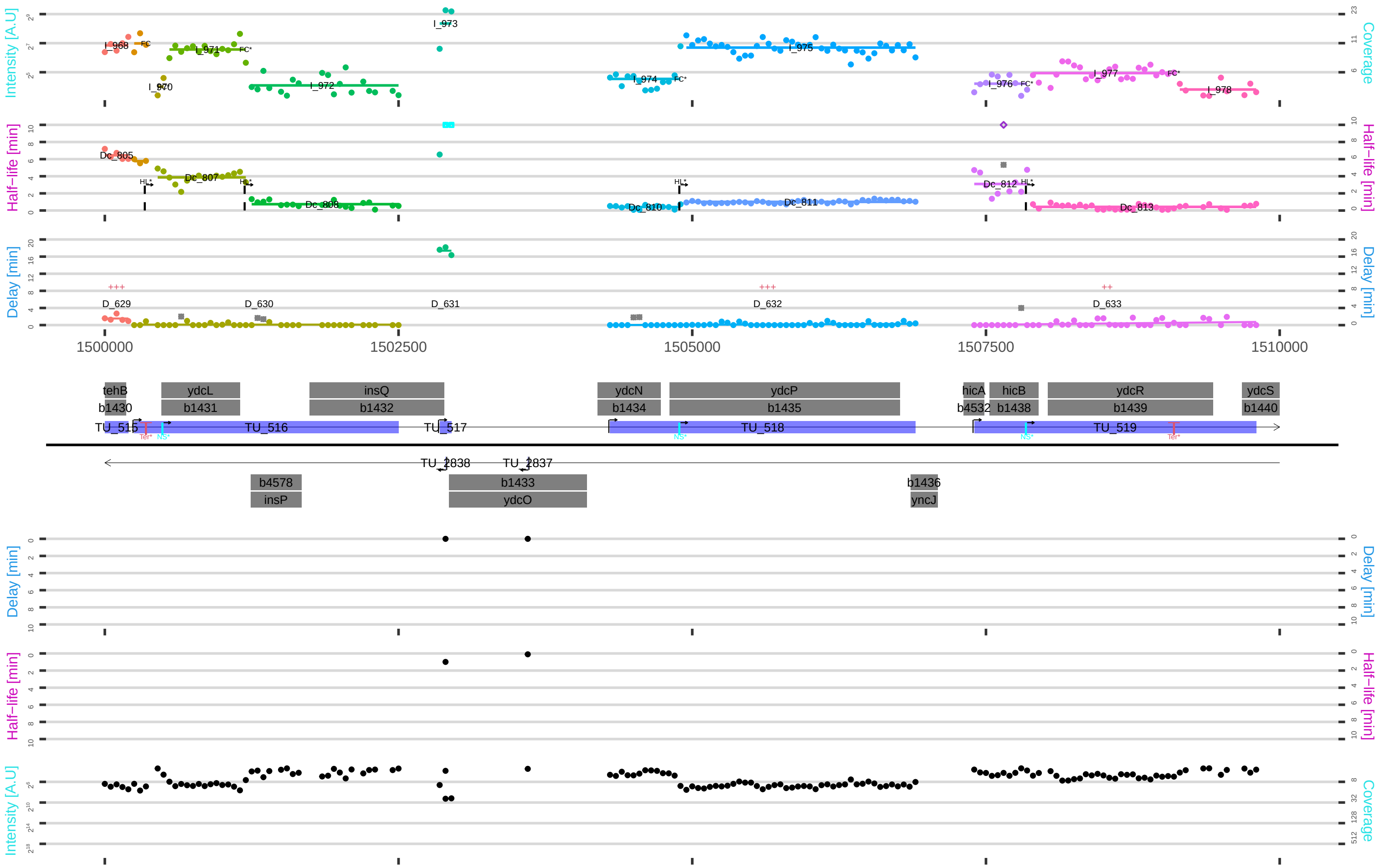

Term: termination (0), NS: new start (0), PS: pausing site (0), iTSS_I: internal starting site (0)

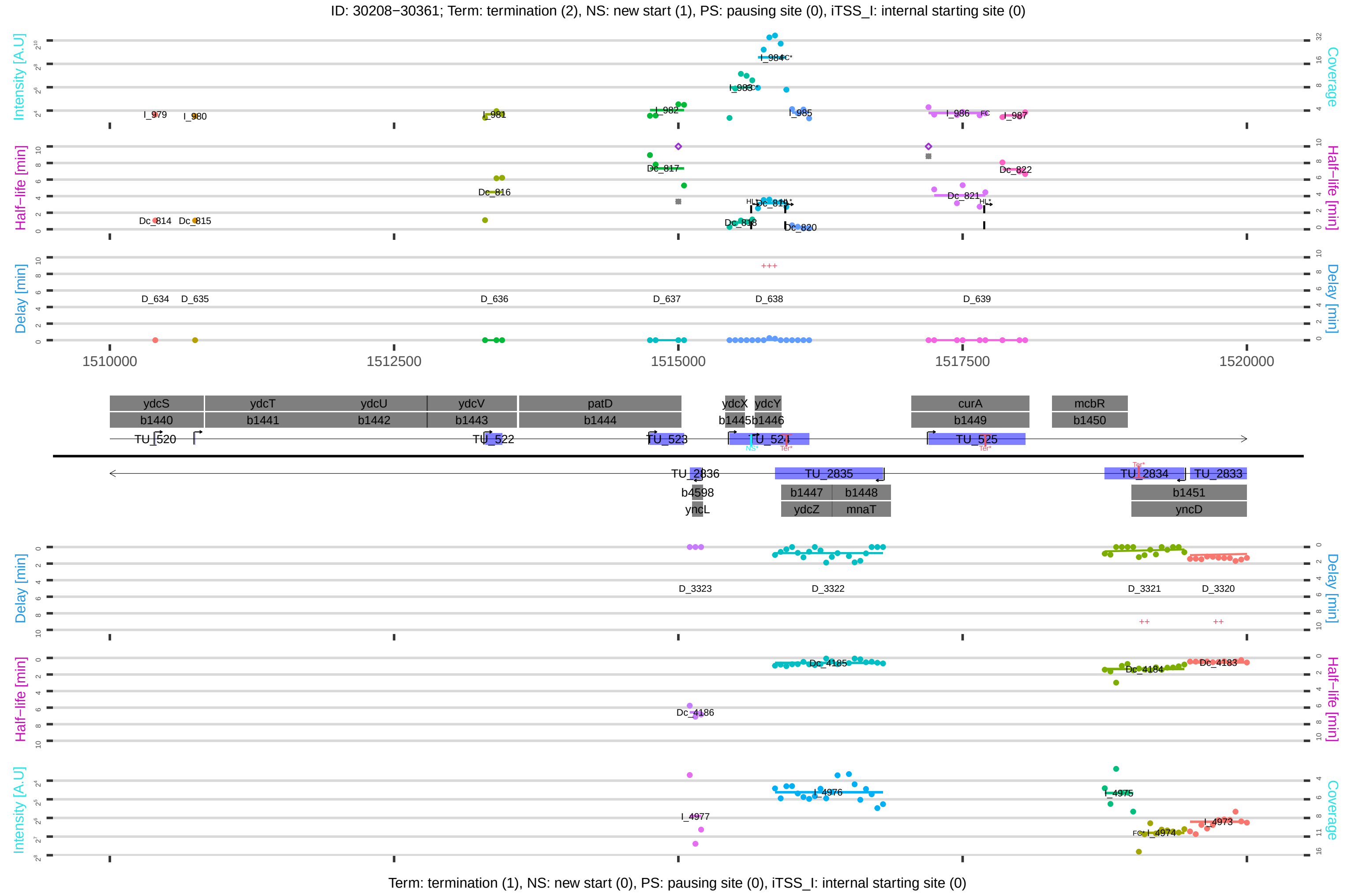

ID: 30208−30361; Term: termination (2), NS: new start (1), PS: pausing site (0), iTSS_I: internal starting site (0)
Intensity [A.U]
Coverage
Half−life [min]
Delay [min]
I_979
I_980
I_981
I_982
I_983
I_984
I_985
I_986
I_987
Dc_814
Dc_815
Dc_816
Dc_817
Dc_818
Dc_819
Dc_820
Dc_821
Dc_822
D_634
D_635
D_636
D_637
D_638
D_639
1510000
1512500
1515000
1517500
1520000
ydcS
b1440
ydcT
b1441
ydcU
b1442
ydcV
b1443
patD
b1444
ydcX
b1445
ydcY
b1446
curA
b1449
mcbR
b1450
TU_520
TU_522
TU_523
TU_524
TU_525
NS*
Ter*
TU_2836
TU_2835
TU_2834
TU_2833
b4598
yncL
b1447
ydcZ
b1448
mnaT
b1451
yncD
D_3323
D_3322
D_3321
D_3320
Dc_4186
Dc_4185
Dc_4184
Dc_4183
I_4977
I_4976
I_4975
I_4974
I_4973
Term: termination (1), NS: new start (0), PS: pausing site (0), iTSS_I: internal starting site (0)

ID: 30426−30483; Term: termination (0), NS: new start (1), PS: pausing site (0), iTSS_I: internal starting site (0)

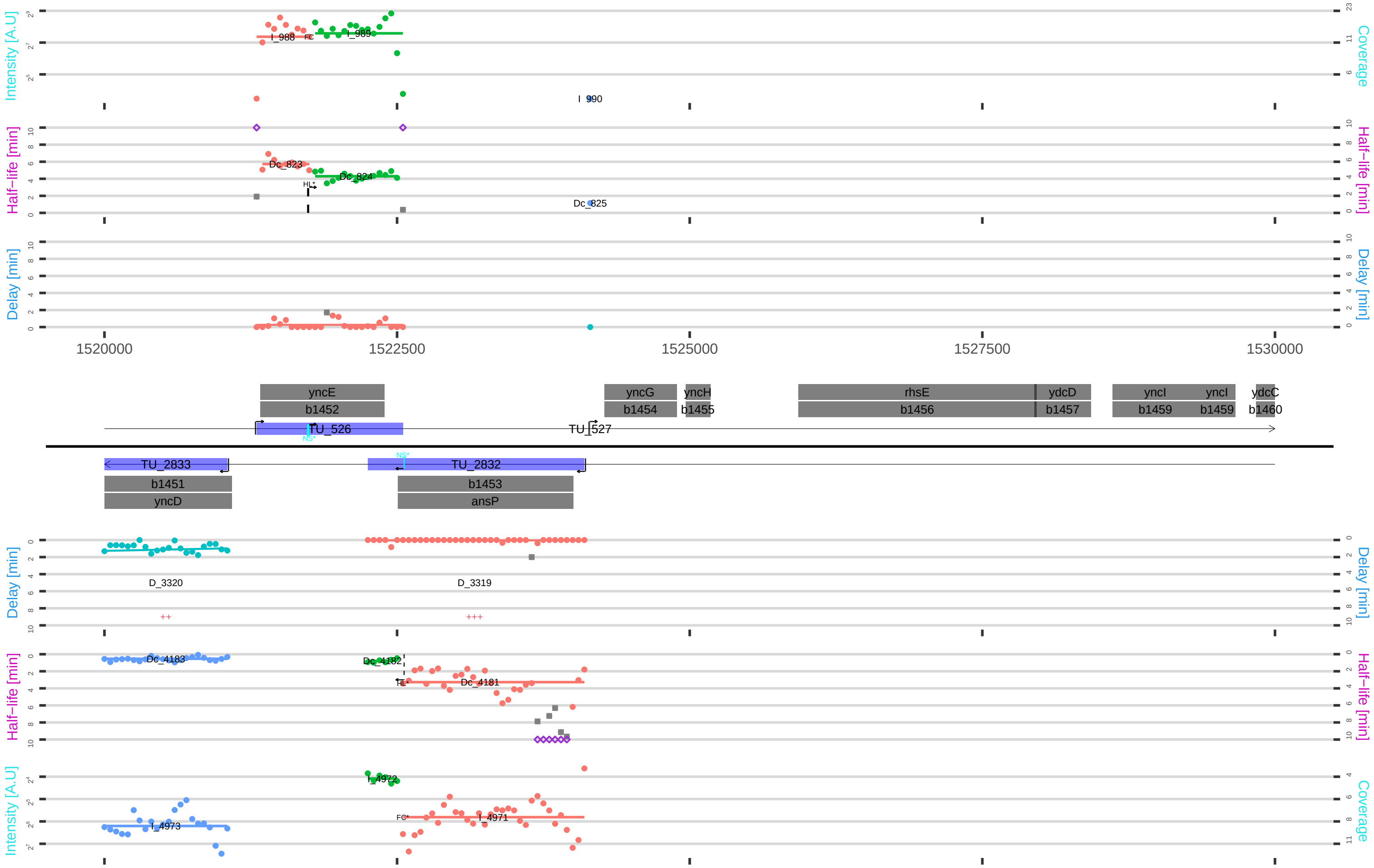

Term: termination (0), NS: new start (1), PS: pausing site (0), iTSS_I: internal starting site (0)

ID: 30623−30644; Term: termination (0), NS: new start (0), PS: pausing site (0), iTSS_I: internal starting site (0)

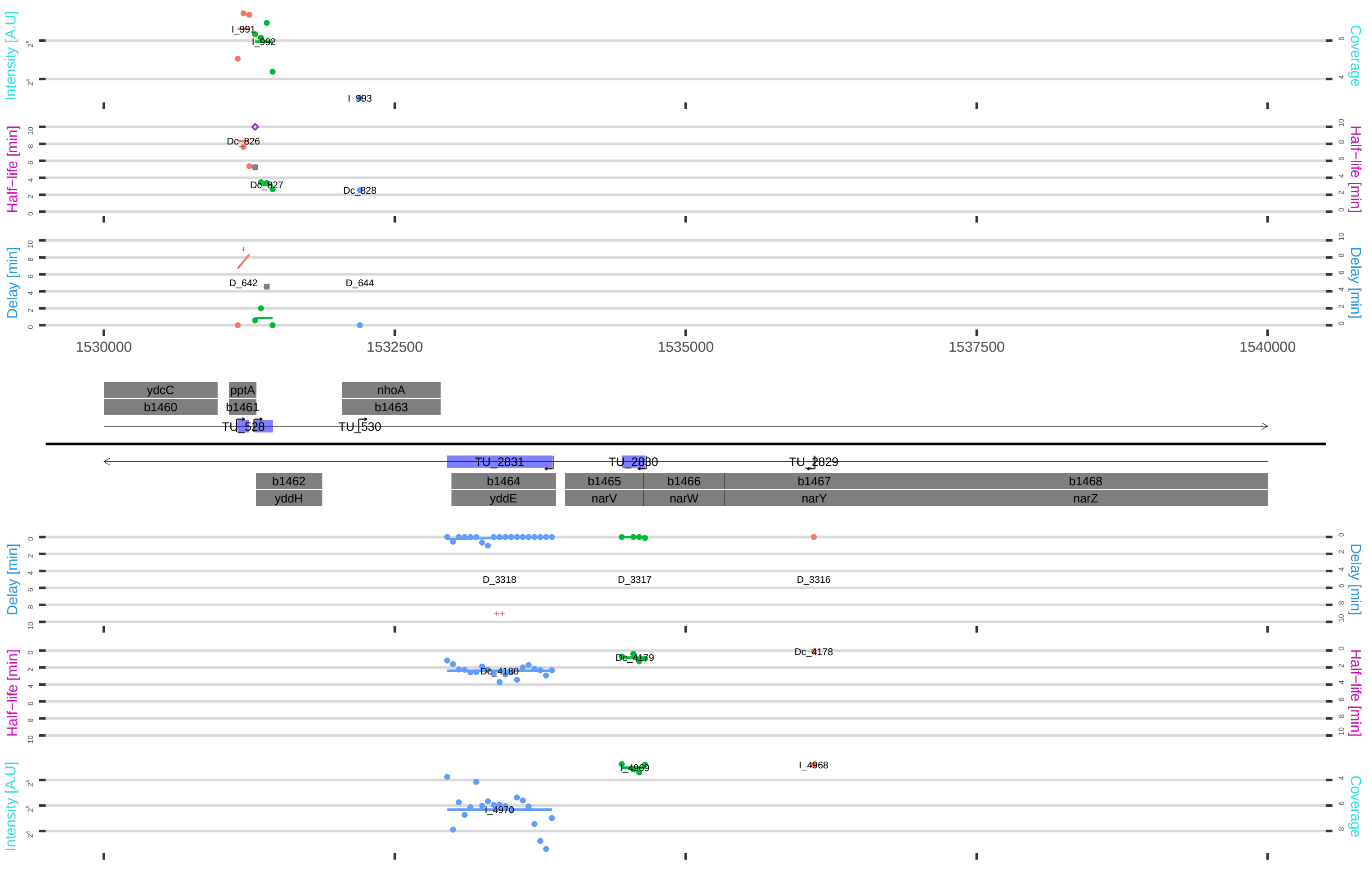

Term: termination (0), NS: new start (0), PS: pausing site (0), iTSS_I: internal starting site (0)

ID: 30911−31000; Term: termination (0), NS: new start (0), PS: pausing site (0), iTSS_I: internal starting site (0)

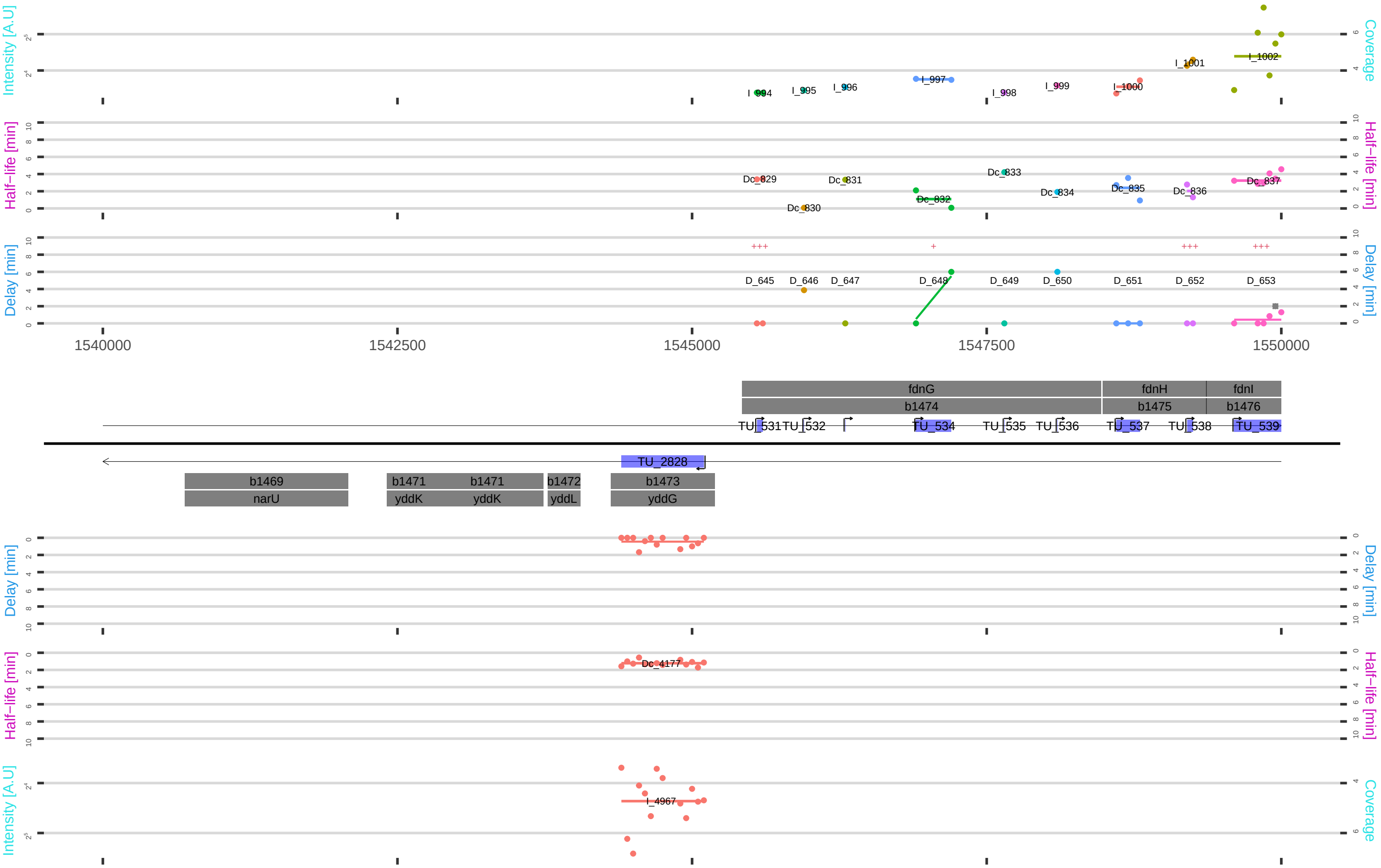

Term: termination (0), NS: new start (0), PS: pausing site (0), iTSS_I: internal starting site (0)

ID: 31000−31114; Term: termination (0), NS: new start (2), PS: pausing site (0), iTSS_I: internal starting site (0)

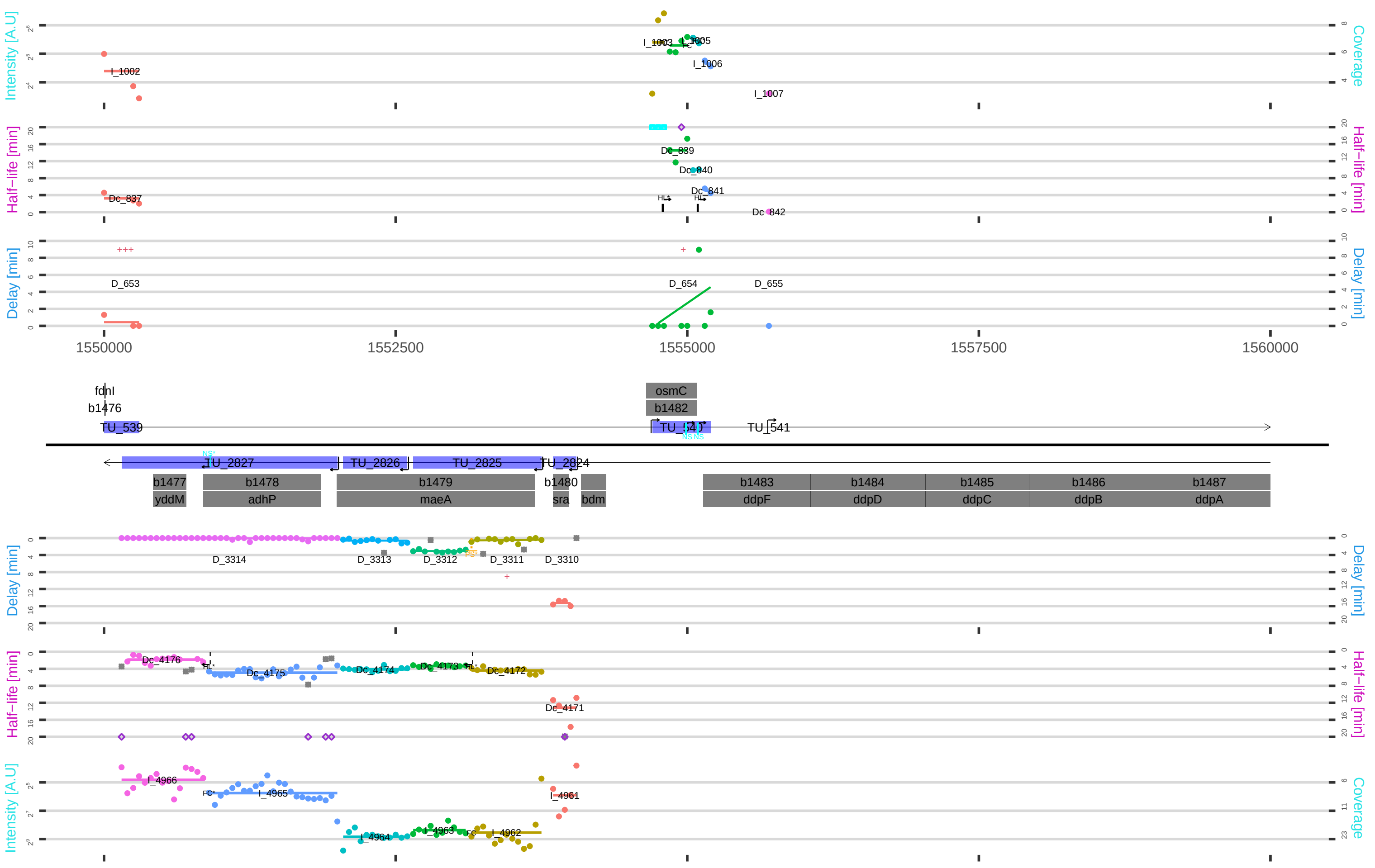

Term: termination (0), NS: new start (1), PS: pausing site (1), iTSS_I: internal starting site (0)

ID: 31343−31343; Term: termination (0), NS: new start (0), PS: pausing site (0), iTSS_I: internal starting site (0)

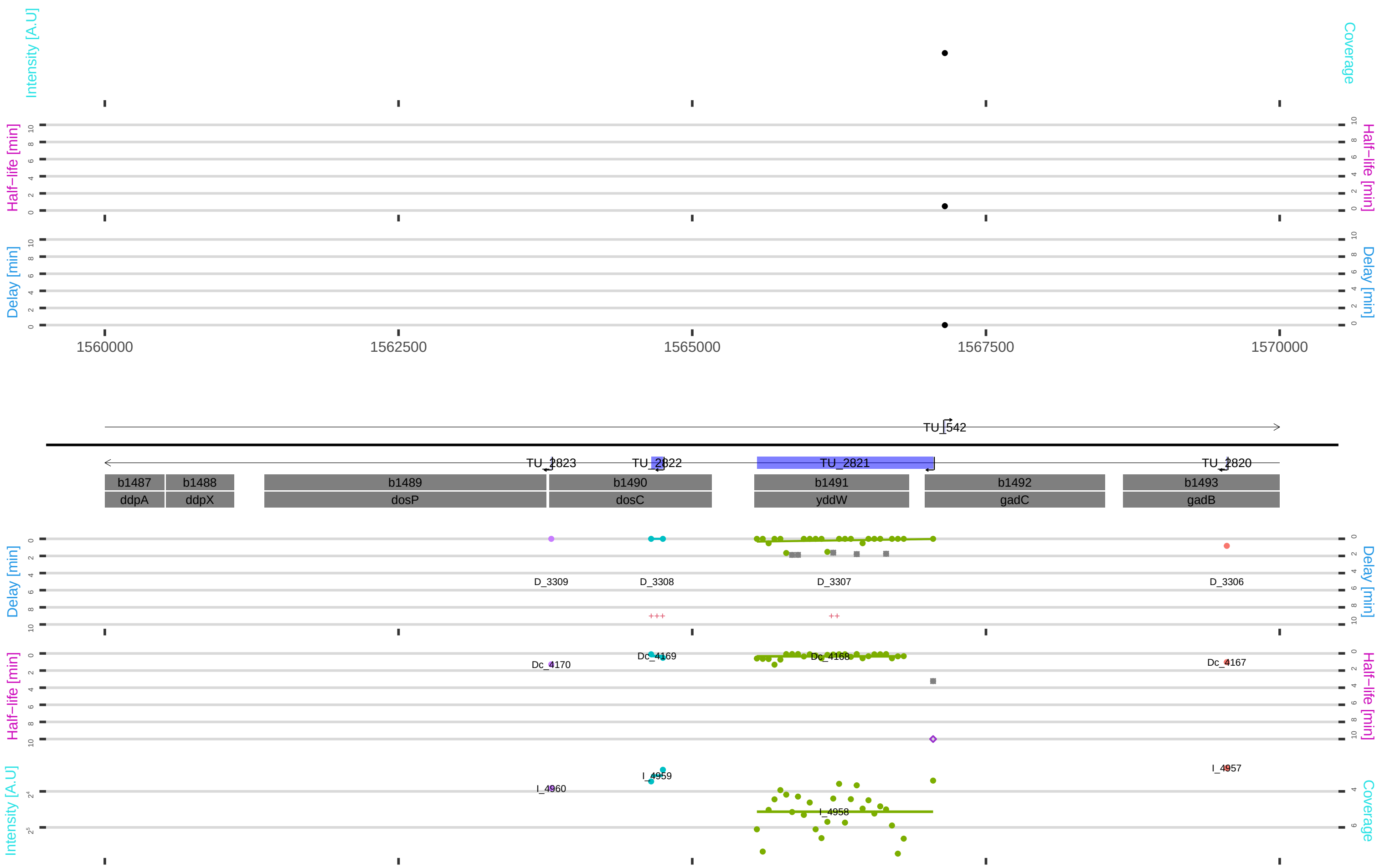

Term: termination (0), NS: new start (0), PS: pausing site (0), iTSS_I: internal starting site (0)

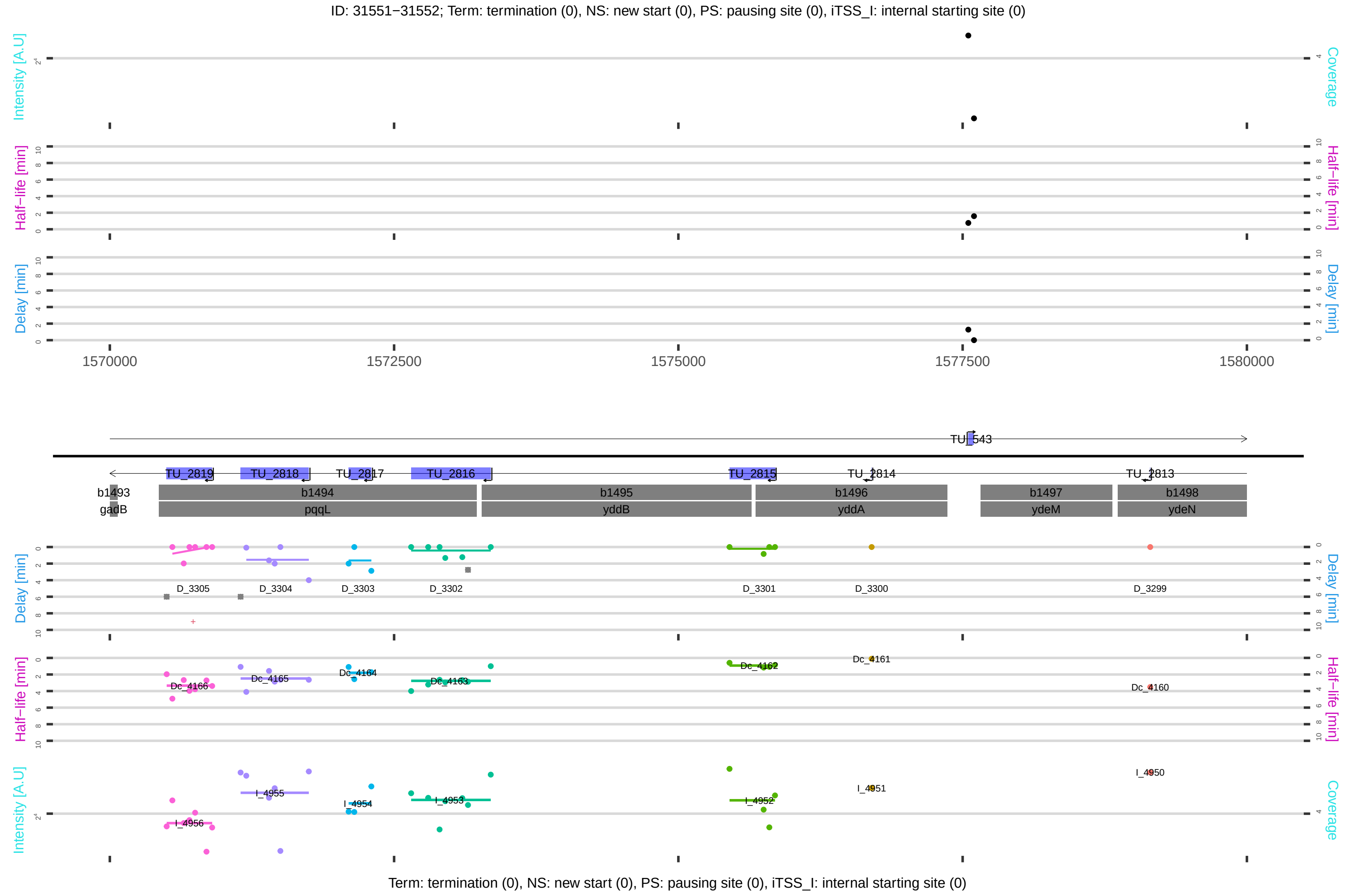
ID: 31551−31552; Term: termination (0), NS: new start (0), PS: pausing site (0), iTSS_I: internal starting site (0)
Intensity [A.U]
Coverage
Half−life [min]
Delay [min]
1570000
1572500
1575000
1577500
1580000
TU_543
TU_2819
TU_2818
TU_2817
TU_2816
TU_2815
TU_2814
TU_2813
b1493
gadB
b1494
pqqL
b1495
yddB
b1496
yddA
b1497
ydeM
b1498
ydeN
D_3305
D_3304
D_3303
D_3302
D_3301
D_3300
D_3299
Dc_4166
Dc_4165
Dc_4164
Dc_4163
Dc_4162
Dc_4161
Dc_4160
I_4956
I_4955
I_4954
I_4953
I_4952
I_4951
I_4950
Term: termination (0), NS: new start (0), PS: pausing site (0), iTSS_I: internal starting site (0)

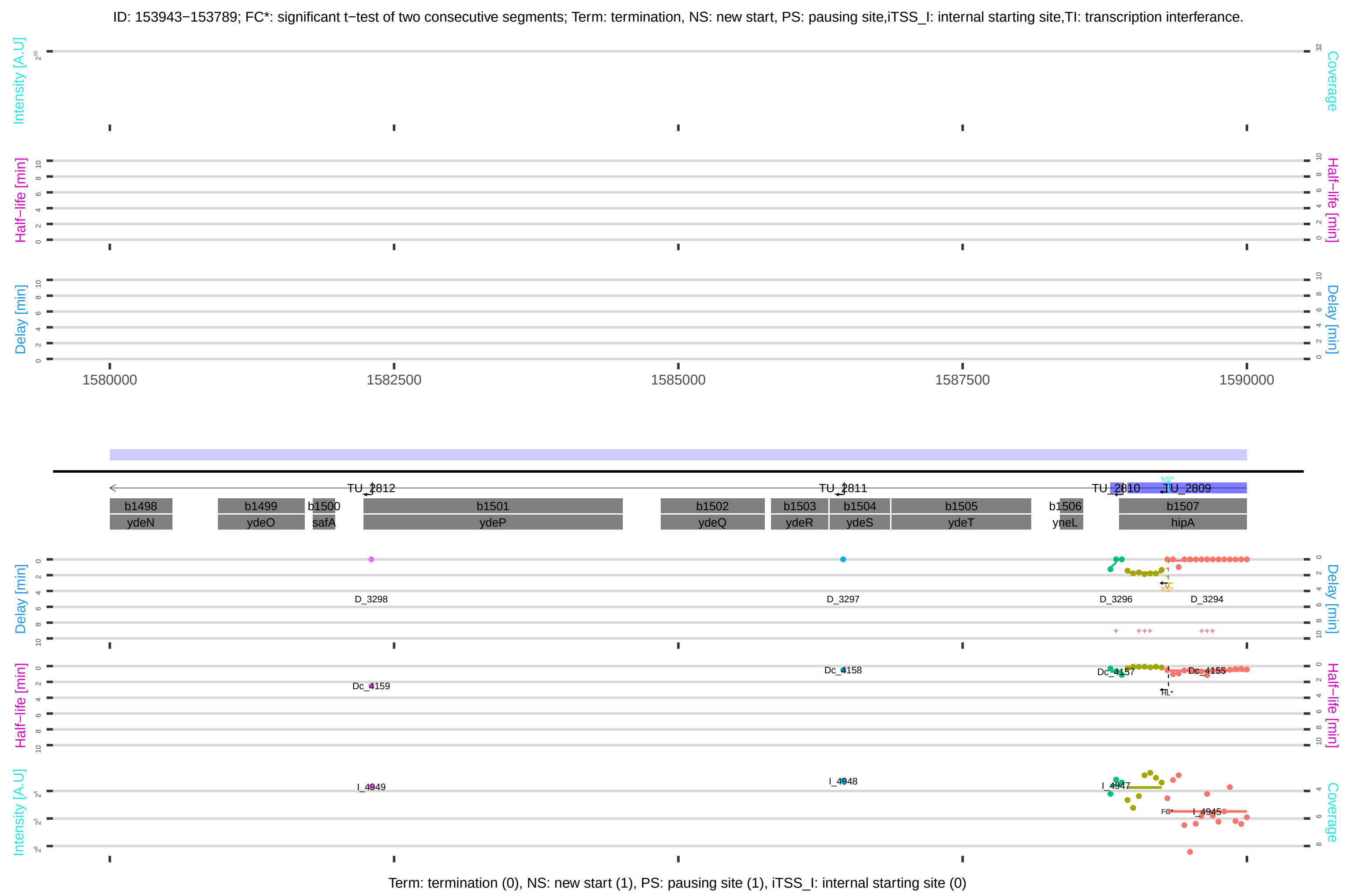

ID: 153943−153789; FC*: significant t−test of two consecutive segments; Term: termination, NS: new start, PS: pausing site,iTSS_I: internal starting site,TI: transcription interferance.
Intensity [A.U]
Coverage
Half−life [min]
Delay [min]
1580000
1582500
1585000
1587500
1590000
TU_2812
TU_2811
TU_2810
TU_2809
NS*
b1498
ydeN
b1499
ydeO
b1500
safA
b1501
ydeP
b1502
ydeQ
b1503
ydeR
b1504
ydeS
b1505
ydeT
b1506
yneL
b1507
hipA
D_3298
D_3297
D_3296
D_3294
PS*
Dc_4159
Dc_4158
Dc_4157
Dc_4155
HL*
I_4949
I_4948
I_4947
I_4945
FC*
Term: termination (0), NS: new start (1), PS: pausing site (1), iTSS_I: internal starting site (0)

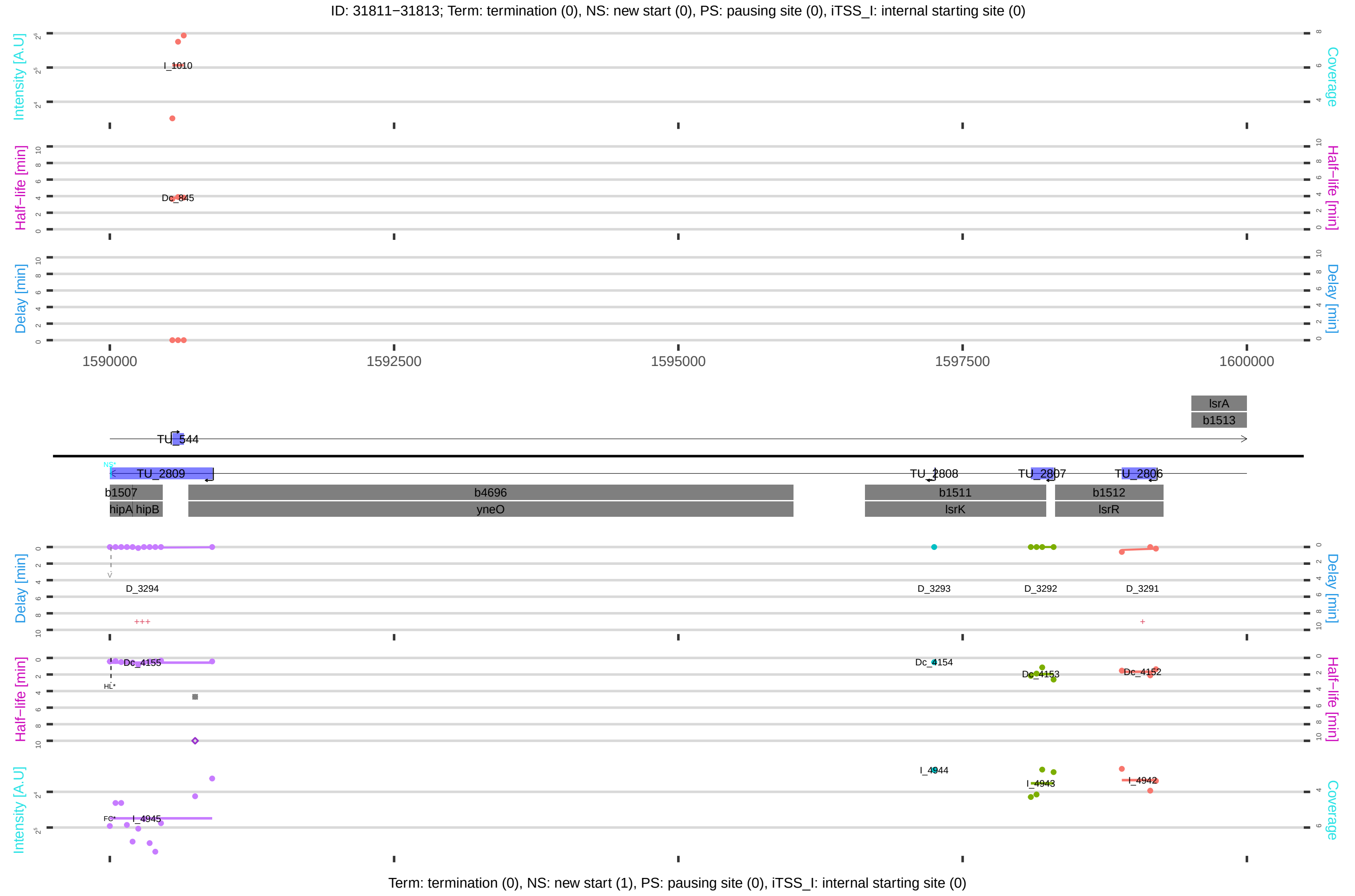
ID: 31811−31813; Term: termination (0), NS: new start (0), PS: pausing site (0), iTSS_I: internal starting site (0)
Intensity [A.U]
Coverage
I_1010
Half−life [min]
Dc_845
Delay [min]
1590000
1592500
1595000
1597500
1600000
lsrA
b1513
TU_544
NS*
TU_2809
TU_2808
TU_2807
TU_2806
b1507
hipA
hipB
b4696
yneO
b1511
lsrK
b1512
lsrR
D_3294
D_3293
D_3292
D_3291
Dc_4155
HL*
Dc_4154
Dc_4153
Dc_4152
I_4944
I_4943
I_4942
FC*
I_4945
Term: termination (0), NS: new start (1), PS: pausing site (0), iTSS_I: internal starting site (0)

ID: 32101−32110; Term: termination (0), NS: new start (1), PS: pausing site (0), iTSS_I: internal starting site (0)

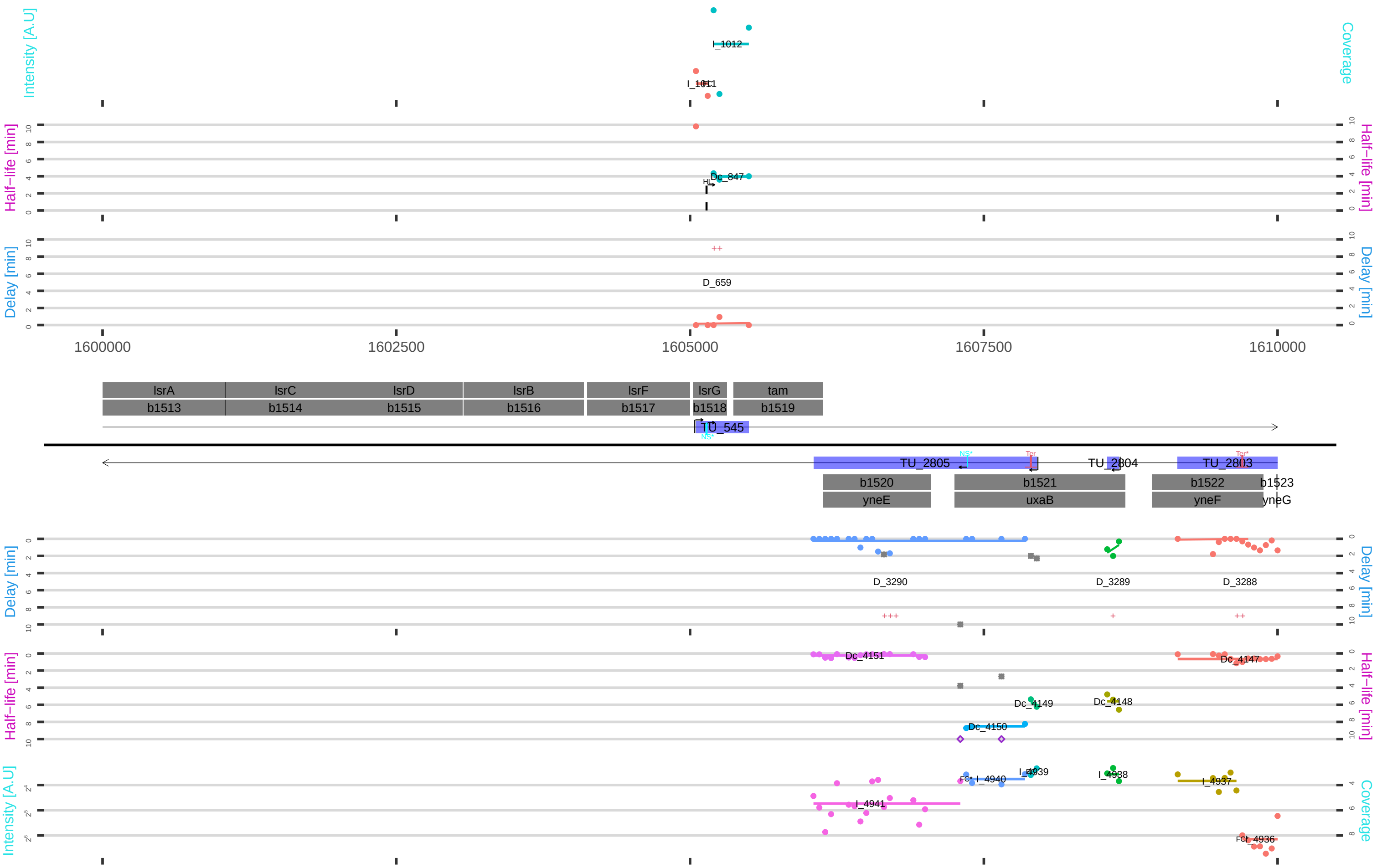

Term: termination (2), NS: new start (1), PS: pausing site (0), iTSS_I: internal starting site (0)

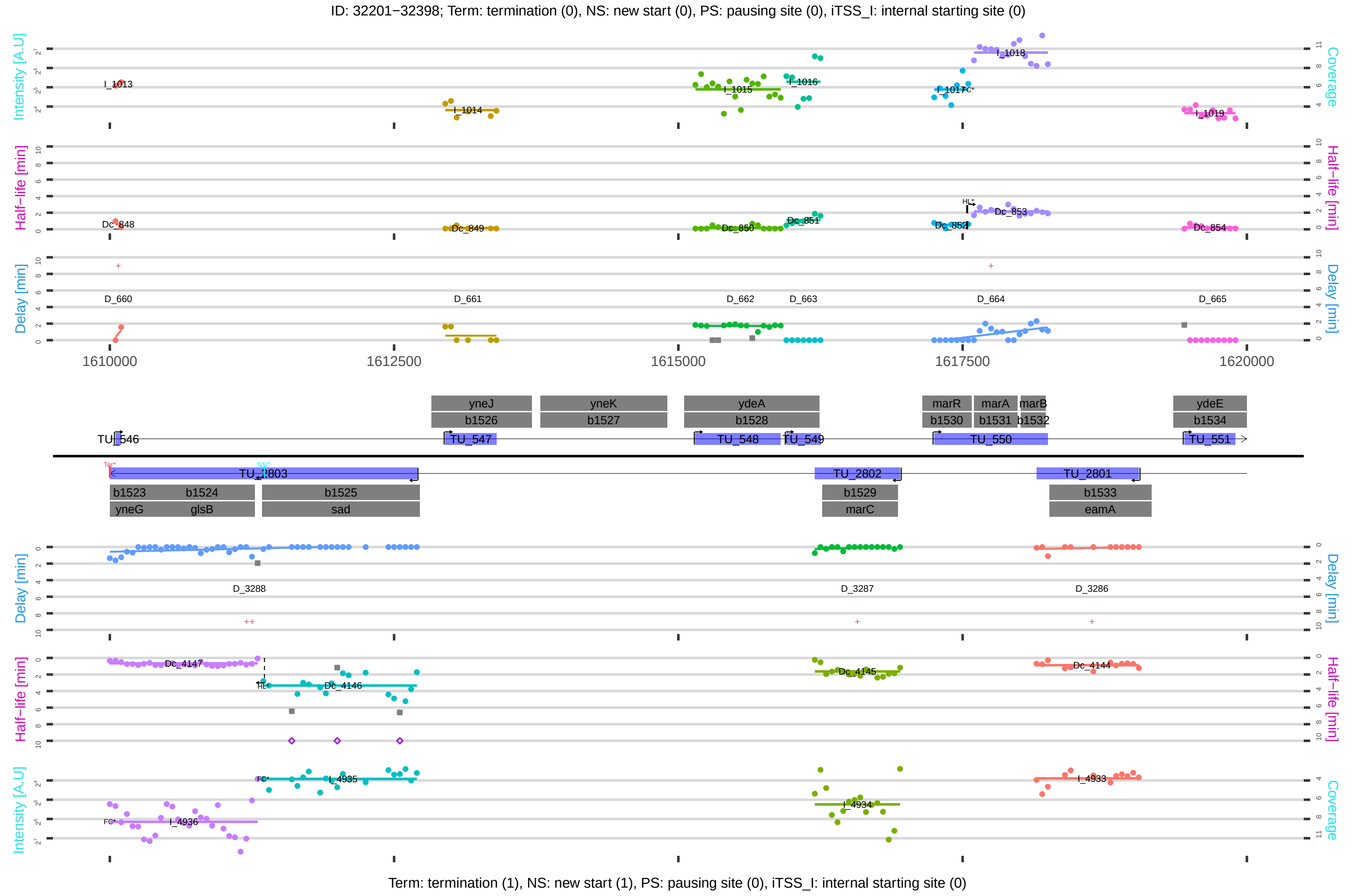
ID: 32201−32398; Term: termination (0), NS: new start (0), PS: pausing site (0), iTSS_I: internal starting site (0)
Intensity [A.U]
Coverage
Half−life [min]
Delay [min]
I_1013
I_1014
I_1015
I_1016
I_1017
I_1018
I_1019
Dc_848
Dc_849
Dc_850
Dc_851
Dc_852
Dc_853
Dc_854
D_660
D_661
D_662
D_663
D_664
D_665
1610000
1612500
1615000
1617500
1620000
yneJ
b1526
yneK
b1527
ydeA
b1528
marR
b1530
marA
b1531
marB
b1532
ydeE
b1534
TU_546
TU_547
TU_548
TU_549
TU_550
TU_551
TU_2803
TU_2802
TU_2801
b1523
b1524
b1525
b1529
b1533
yneG
glsB
sad
marC
eamA
D_3288
D_3287
D_3286
Dc_4147
Dc_4146
Dc_4145
Dc_4144
I_4936
I_4935
I_4934
I_4933
Term: termination (1), NS: new start (1), PS: pausing site (0), iTSS_I: internal starting site (0)

ID: 32407−32549; Term: termination (2), NS: new start (1), PS: pausing site (0), iTSS_I: internal starting site (0)

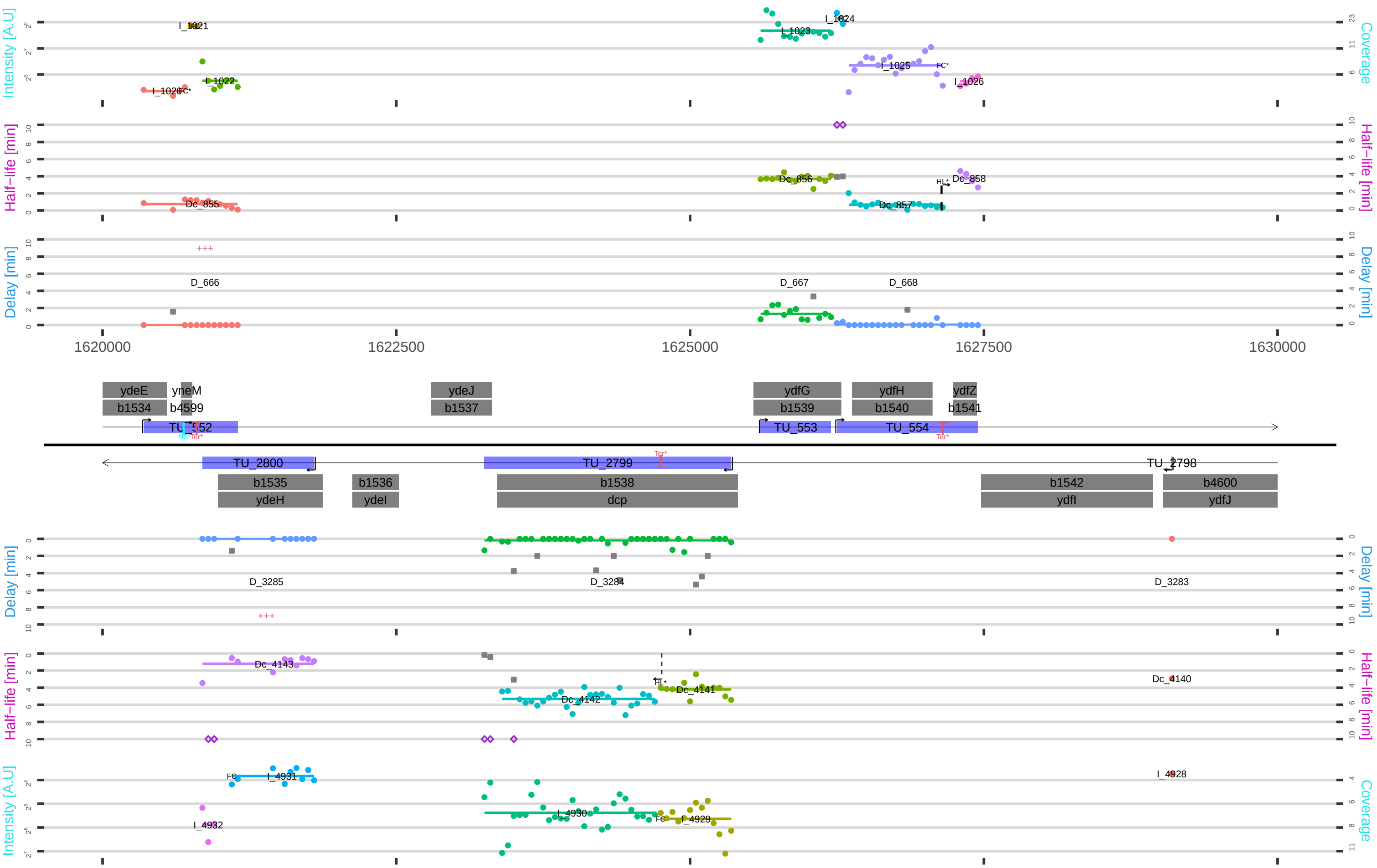

Term: termination (1), NS: new start (0), PS: pausing site (0), iTSS_I: internal starting site (0)

ID: 32703−32800; Term: termination (0), NS: new start (0), PS: pausing site (0), iTSS_I: internal starting site (0)

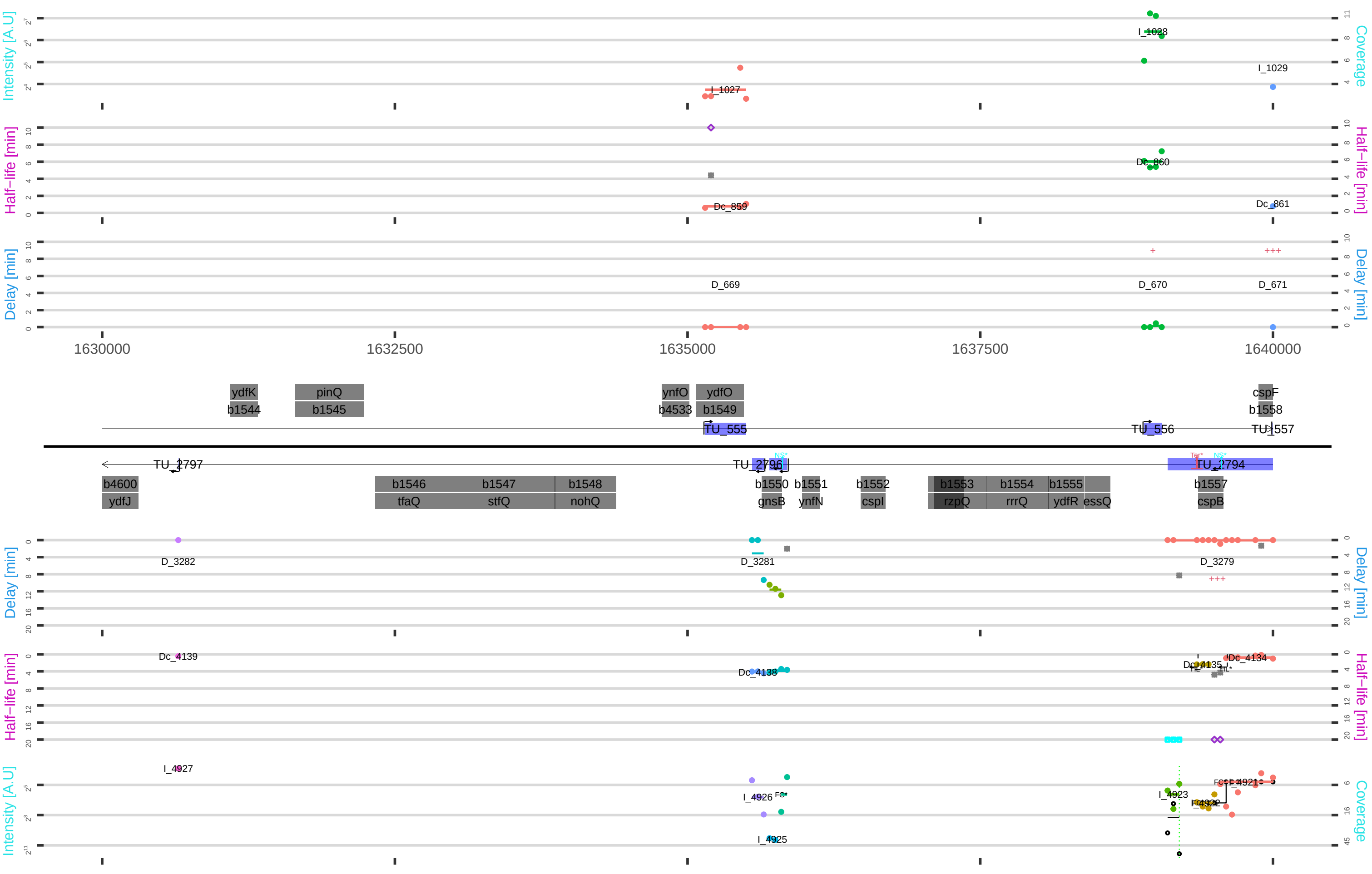

Term: termination (1), NS: new start (2), PS: pausing site (0), iTSS_I: internal starting site (0)

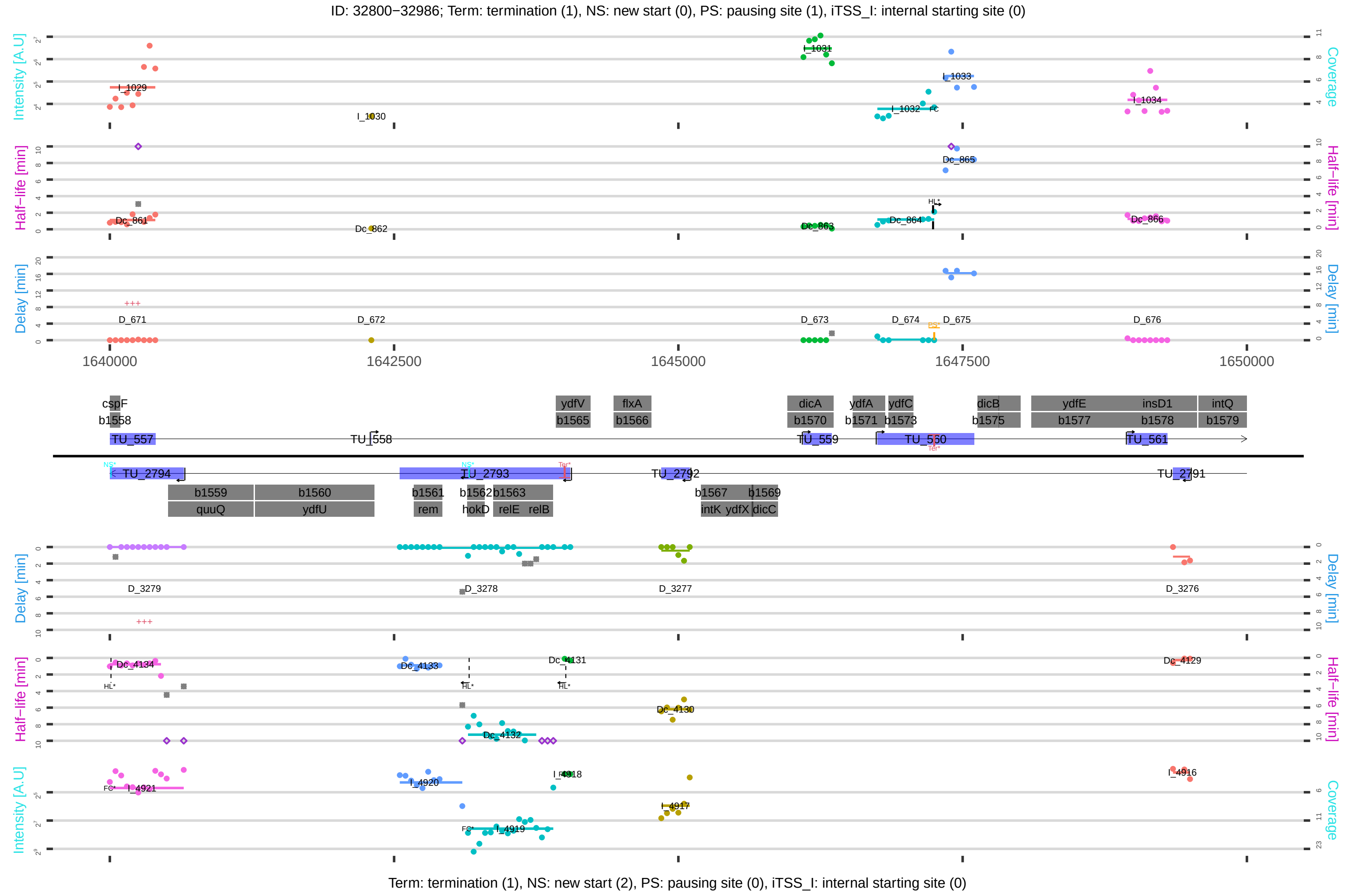
ID: 32800−32986; Term: termination (1), NS: new start (0), PS: pausing site (1), iTSS_I: internal starting site (0)
Intensity [A.U]
Coverage
I_1029
I_1030
I_1031
I_1032
I_1033
I_1034
Half−life [min]
Dc_861
Dc_862
Dc_863
Dc_864
Dc_865
Dc_866
Delay [min]
D_671
D_672
D_673
D_674
D_675
D_676
1640000
1642500
1645000
1647500
1650000
cspF
b1558
ydfV
b1565
flxA
b1566
dicA
b1570
ydfA
b1571
ydfC
b1573
dicB
b1575
ydfE
b1577
insD1
b1578
intQ
b1579
TU_557
TU_558
TU_559
TU_560
TU_561
TU_2794
TU_2793
TU_2792
TU_2791
b1559
quuQ
b1560
ydfU
b1561
rem
b1562
hokD
b1563
relE
relB
b1567
intK
ydfX
b1569
dicC
D_3279
D_3278
D_3277
D_3276
Dc_4134
Dc_4133
Dc_4132
Dc_4131
Dc_4130
Dc_4129
I_4921
I_4920
I_4919
I_4918
I_4917
I_4916
Term: termination (1), NS: new start (2), PS: pausing site (0), iTSS_I: internal starting site (0)

ID: 33077−33200; Term: termination (1), NS: new start (0), PS: pausing site (0), iTSS_I: internal starting site (0)

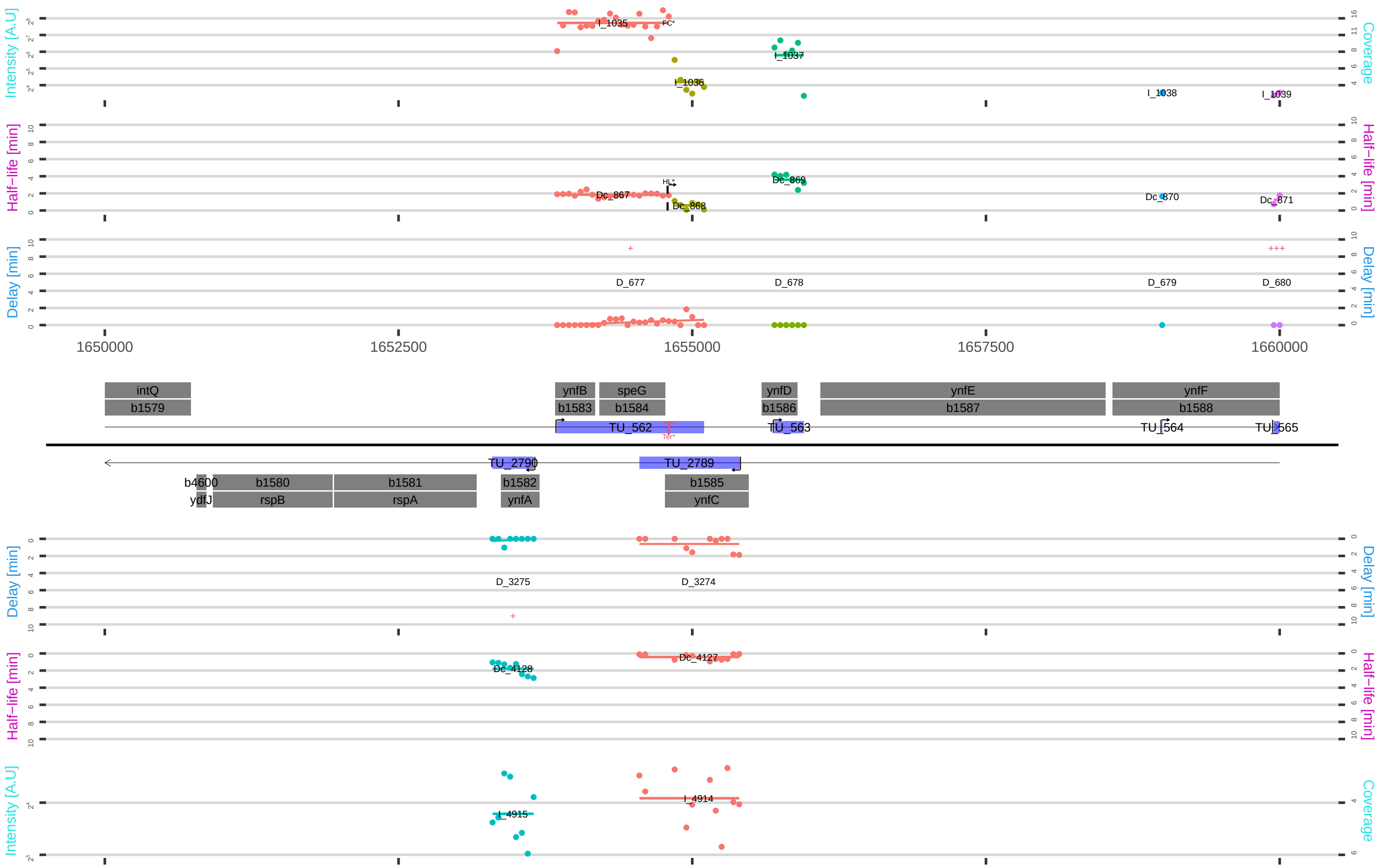

Term: termination (0), NS: new start (0), PS: pausing site (0), iTSS_I: internal starting site (0)

ID: 33200−33391; Term: termination (1), NS: new start (1), PS: pausing site (0), iTSS_I: internal starting site (0)

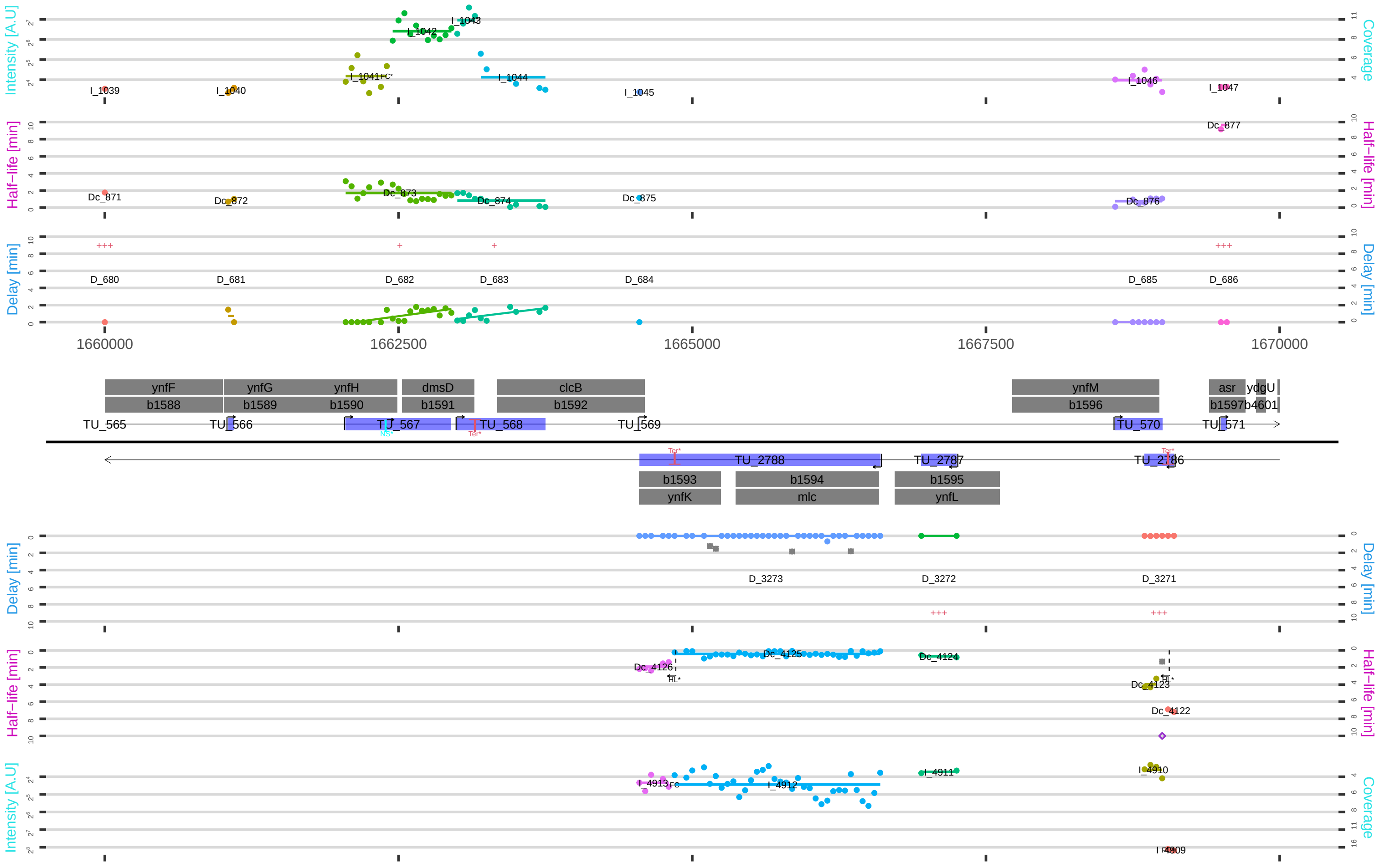

Term: termination (2), NS: new start (0), PS: pausing site (0), iTSS_I: internal starting site (0)

ID: 33401−33600; Term: termination (0), NS: new start (2), PS: pausing site (0), iTSS_I: internal starting site (1)

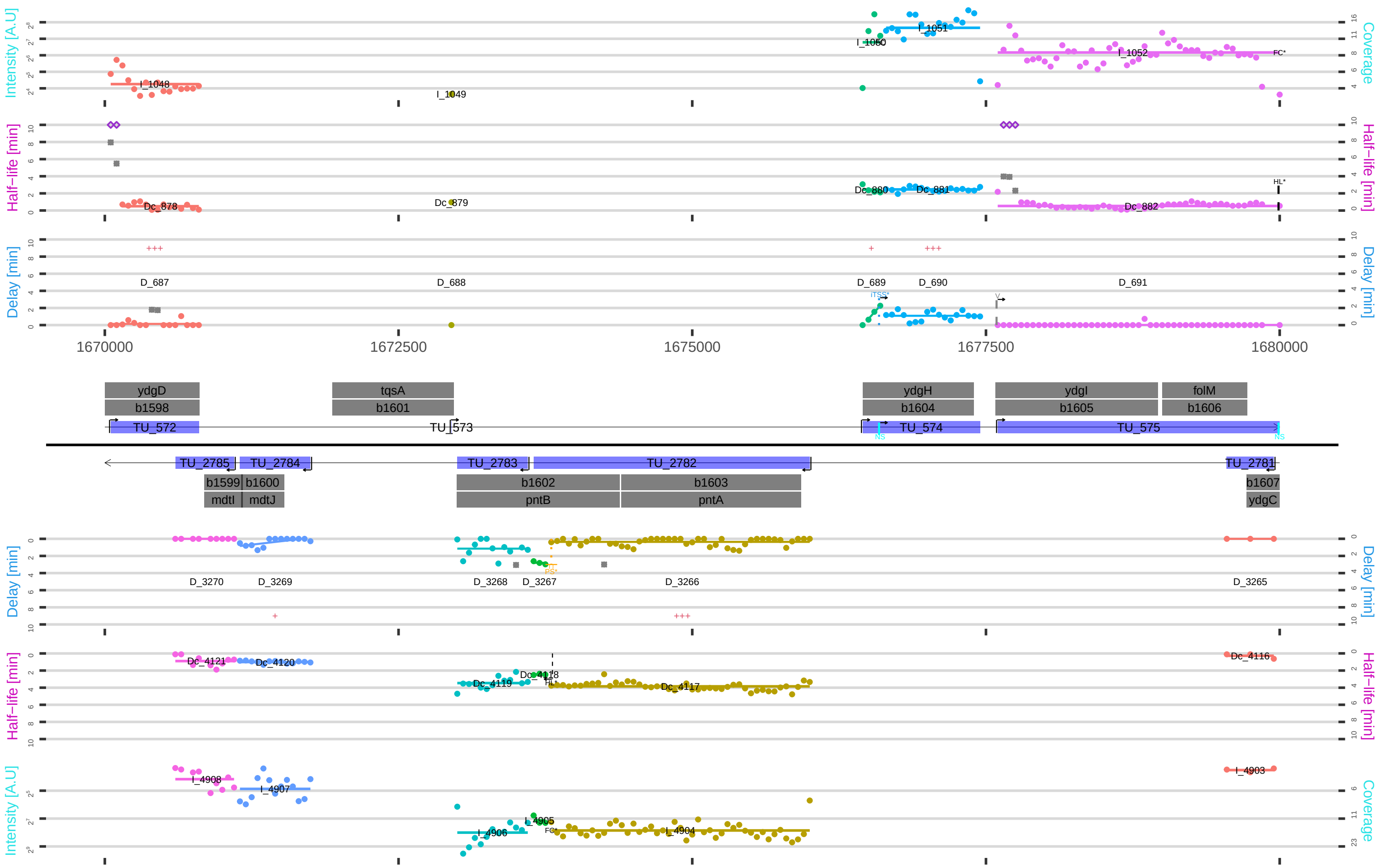

Term: termination (0), NS: new start (0), PS: pausing site (1), iTSS_I: internal starting site (0)

ID: 33600−33792; Term: termination (2), NS: new start (2), PS: pausing site (1), iTSS_I: internal starting site (0)

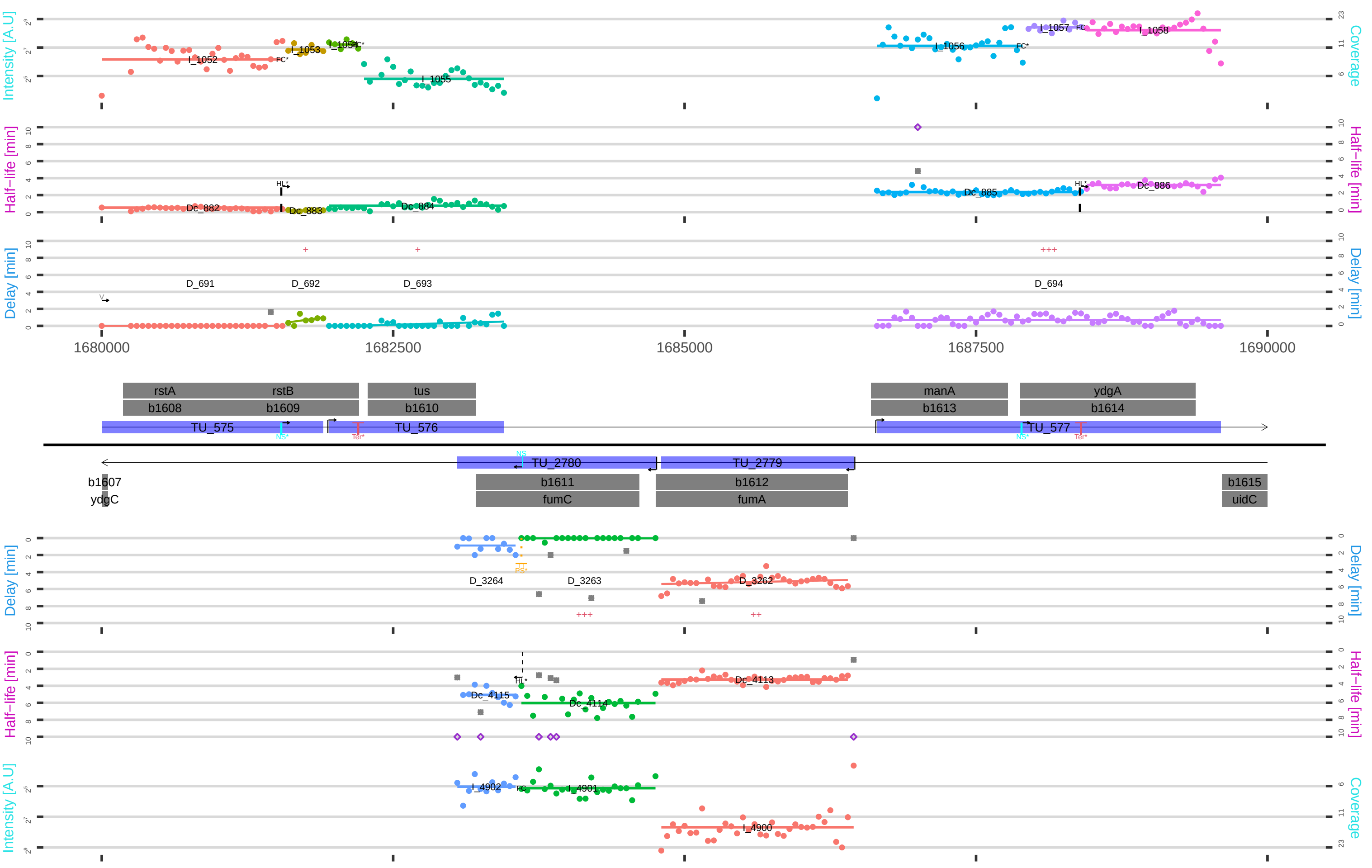

Term: termination (0), NS: new start (1), PS: pausing site (1), iTSS_I: internal starting site (0)

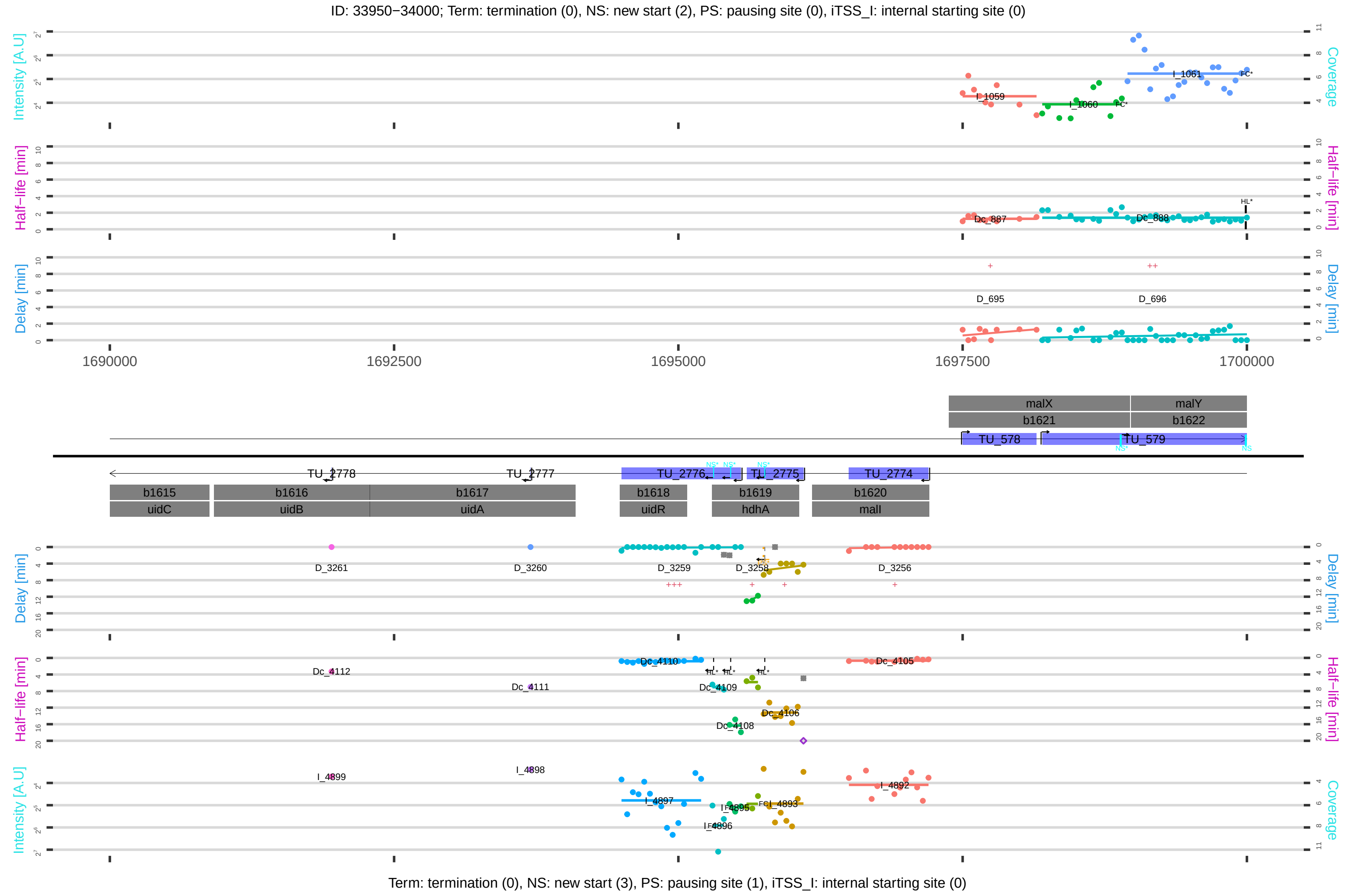
ID: 33950−34000; Term: termination (0), NS: new start (2), PS: pausing site (0), iTSS_I: internal starting site (0)
Intensity [A.U]
Coverage
Half−life [min]
Delay [min]
I_1059
I_1060
I_1061
Dc_887
Dc_888
D_695
D_696
1690000
1692500
1695000
1697500
1700000
malX
malY
b1621
b1622
TU_578
TU_579
TU_2778
TU_2777
TU_2776
TU_2775
TU_2774
b1615
b1616
b1617
b1618
b1619
b1620
uidC
uidB
uidA
uidR
hdhA
malI
D_3261
D_3260
D_3259
D_3258
D_3256
Dc_4112
Dc_4111
Dc_4110
Dc_4109
Dc_4108
Dc_4106
Dc_4105
I_4899
I_4898
I_4897
I_4896
I_4895
I_4893
I_4892
Term: termination (0), NS: new start (3), PS: pausing site (1), iTSS_I: internal starting site (0)

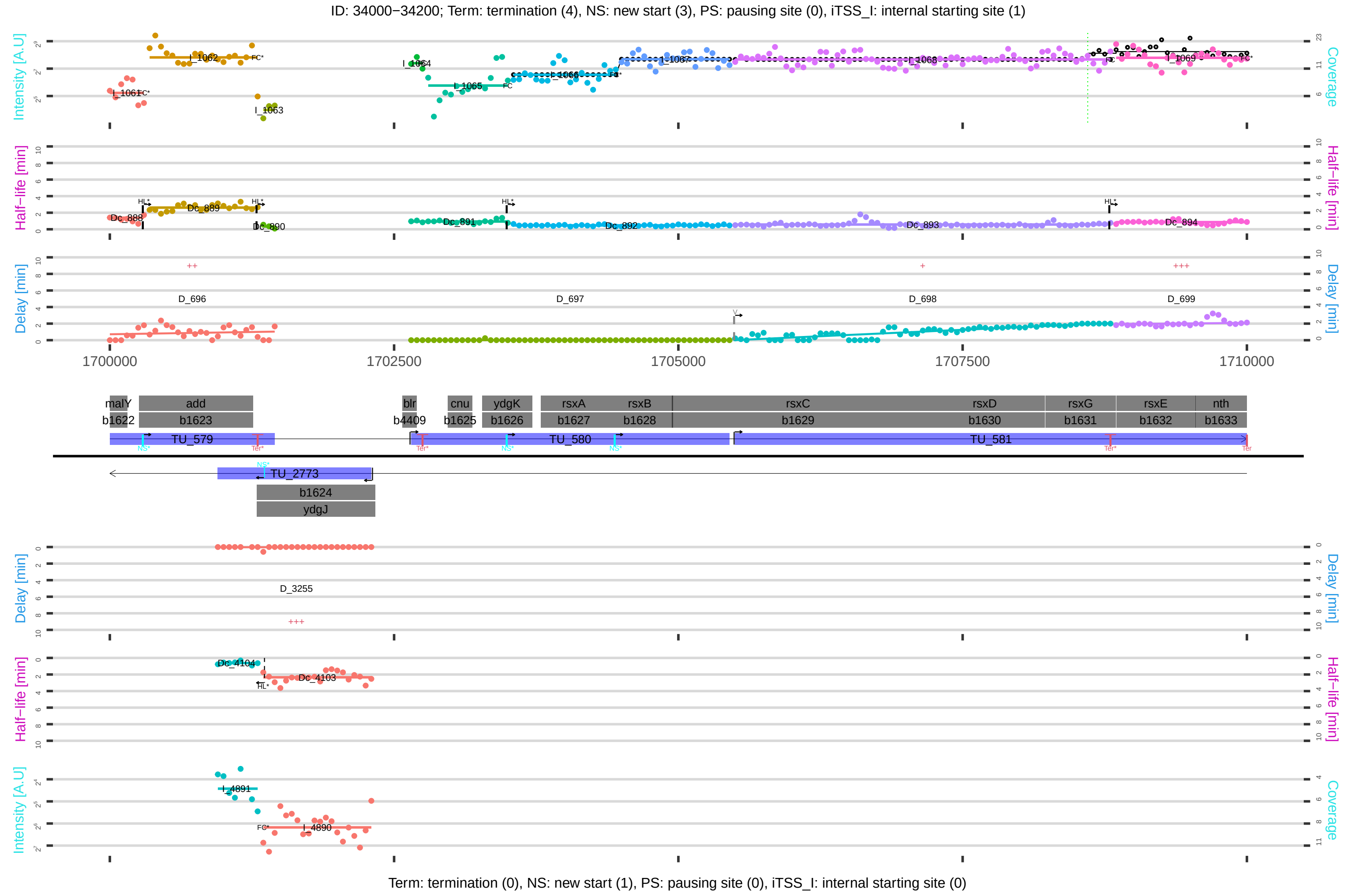

ID: 34000−34200; Term: termination (4), NS: new start (3), PS: pausing site (0), iTSS_I: internal starting site (1)
Intensity [A.U]
Coverage
Half−life [min]
Delay [min]
I_1061
I_1062
I_1063
I_1064
I_1065
I_1066
I_1067
I_1068
I_1069
Dc_888
Dc_889
Dc_890
Dc_891
Dc_892
Dc_893
Dc_894
D_696
D_697
D_698
D_699
1700000
1702500
1705000
1707500
1710000
malY
b1622
add
b1623
blr
b4409
cnu
b1625
ydgK
b1626
rsxA
b1627
rsxB
b1628
rsxC
b1629
rsxD
b1630
rsxG
b1631
rsxE
b1632
nth
b1633
TU_579
TU_580
TU_581
TU_2773
b1624
ydgJ
D_3255
Dc_4104
Dc_4103
I_4891
I_4890
Term: termination (0), NS: new start (1), PS: pausing site (0), iTSS_I: internal starting site (0)

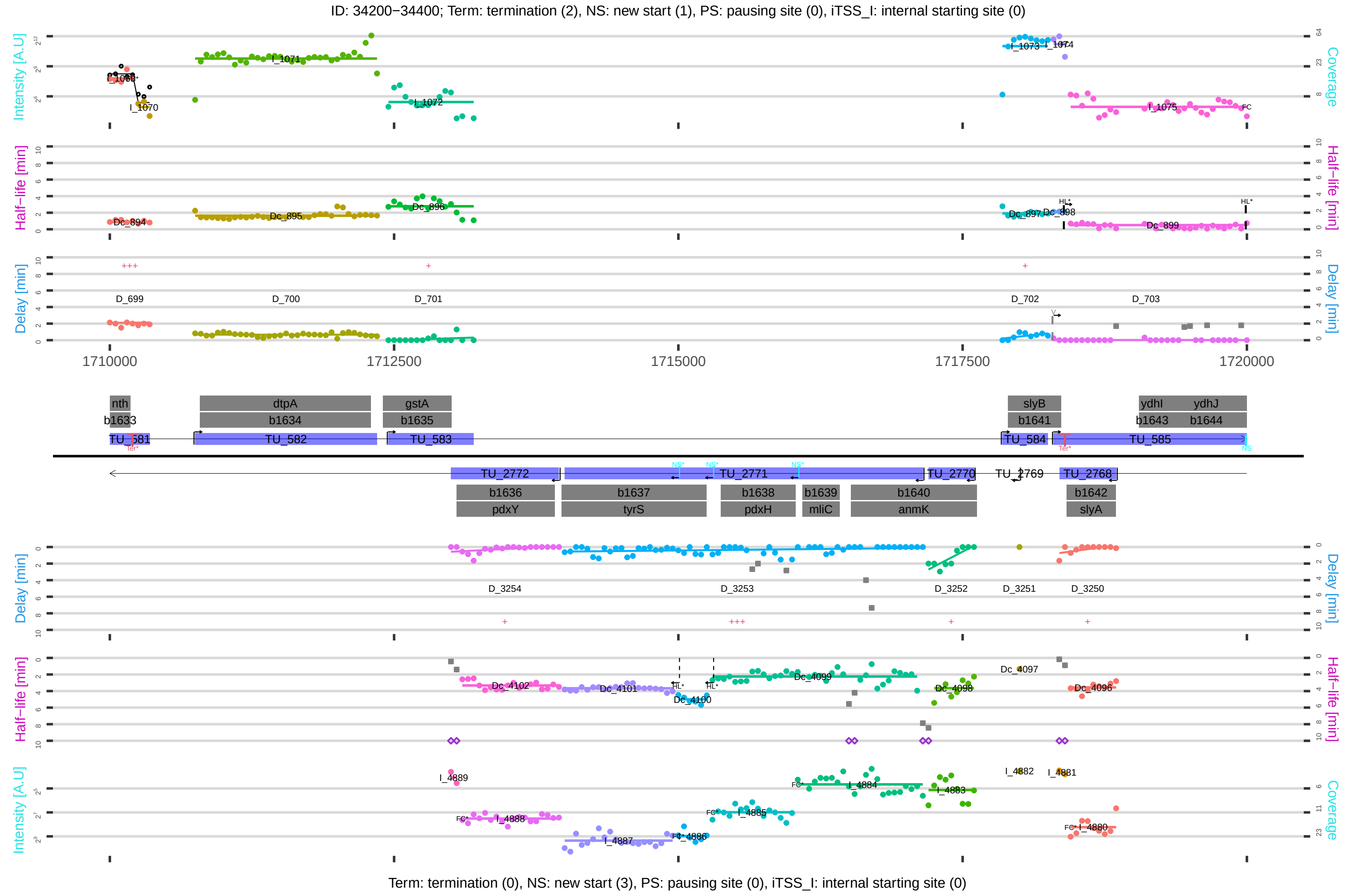
ID: 34200−34400; Term: termination (2), NS: new start (1), PS: pausing site (0), iTSS_I: internal starting site (0)
Intensity [A.U]
Coverage
Half−life [min]
Delay [min]
I_1069*
I_1070
I_1071
I_1072
I_1073
I_1074
I_1075
FC
Dc_894
Dc_895
Dc_896
Dc_897
Dc_898
Dc_899
HL*
D_699
D_700
D_701
D_702
D_703
1710000
1712500
1715000
1717500
1720000
nth
b1633
dtpA
b1634
gstA
b1635
slyB
b1641
ydhI
b1643
ydhJ
b1644
TU_581
TU_582
TU_583
TU_584
TU_585
Ter*
NS
TU_2772
TU_2771
TU_2770
TU_2769
TU_2768
NS*
b1636
pdxY
b1637
tyrS
b1638
pdxH
b1639
mliC
b1640
anmK
b1642
slyA
D_3254
D_3253
D_3252
D_3251
D_3250
Dc_4102
Dc_4101
Dc_4100
Dc_4099
Dc_4098
Dc_4097
Dc_4096
I_4889
I_4888
I_4887
I_4886
I_4885
I_4884
I_4883
I_4882
I_4881
I_4880
FC*
Term: termination (0), NS: new start (3), PS: pausing site (0), iTSS_I: internal starting site (0)

ID: 34400−34600; Term: termination (3), NS: new start (1), PS: pausing site (2), iTSS_I: internal starting site (0)

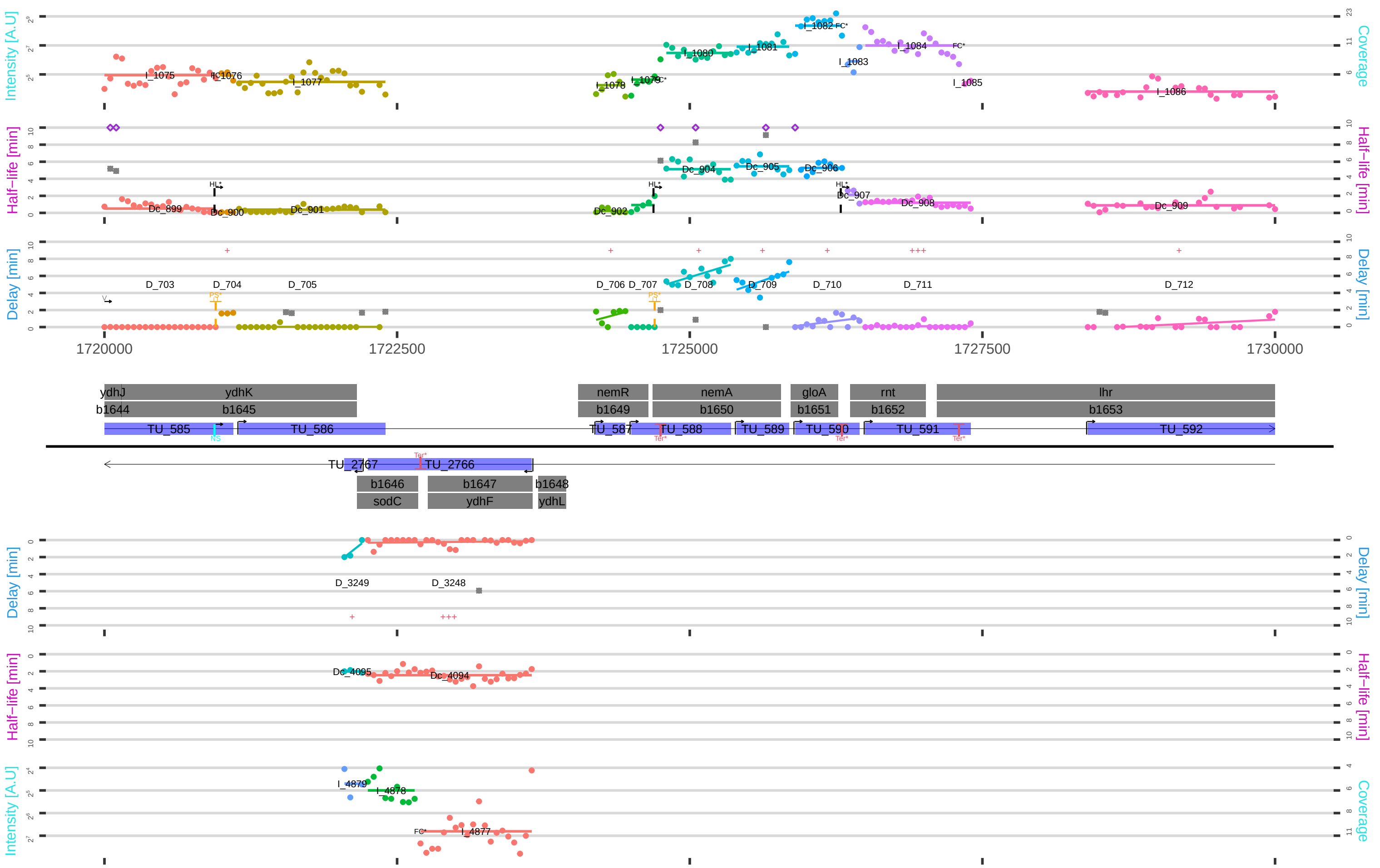

Term: termination (1), NS: new start (0), PS: pausing site (0), iTSS_I: internal starting site (0)

ID: 34600−34800; Term: termination (1), NS: new start (2), PS: pausing site (1), iTSS_I: internal starting site (0)

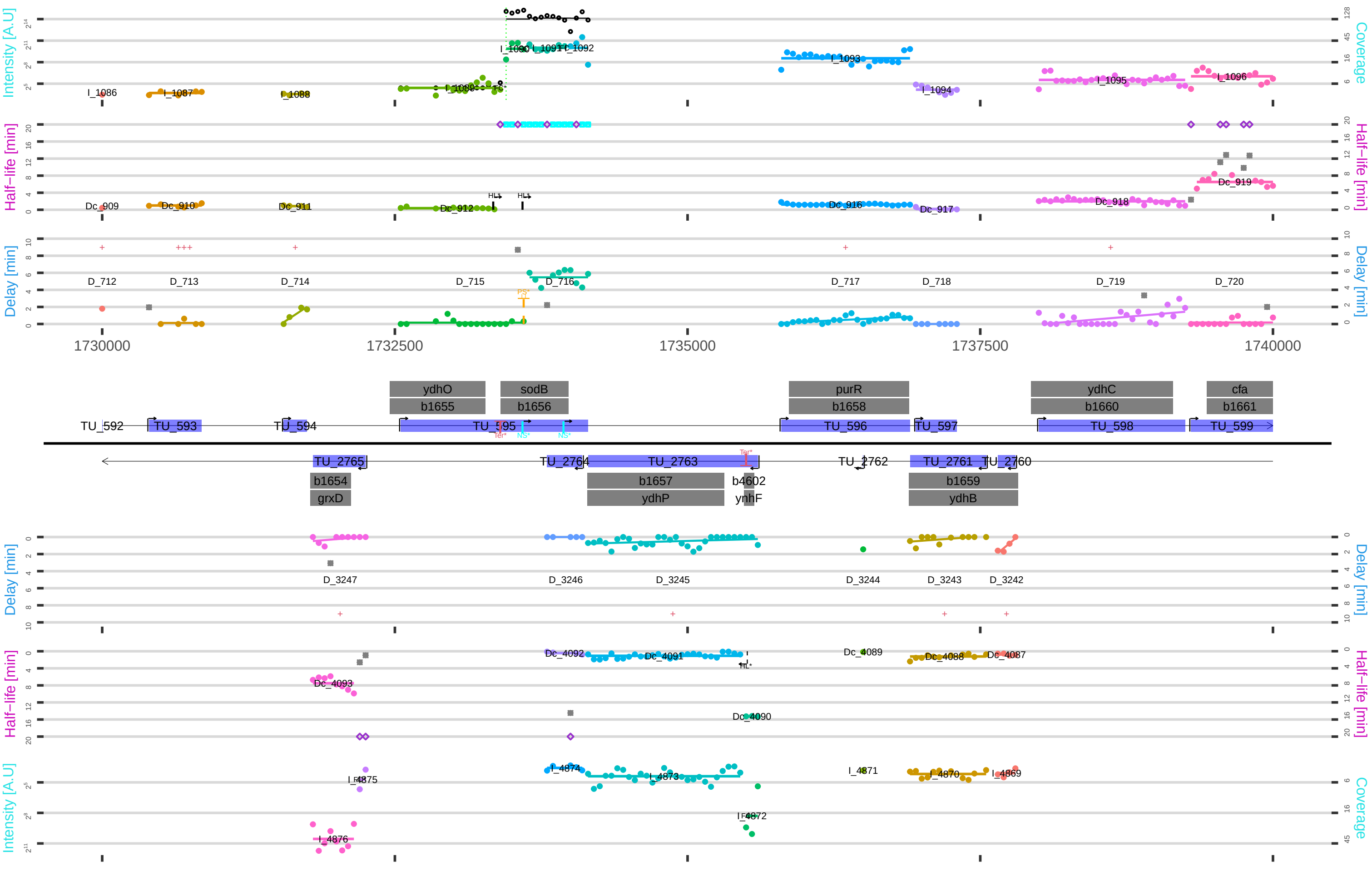

Term: termination (1), NS: new start (0), PS: pausing site (0), iTSS_I: internal starting site (0)

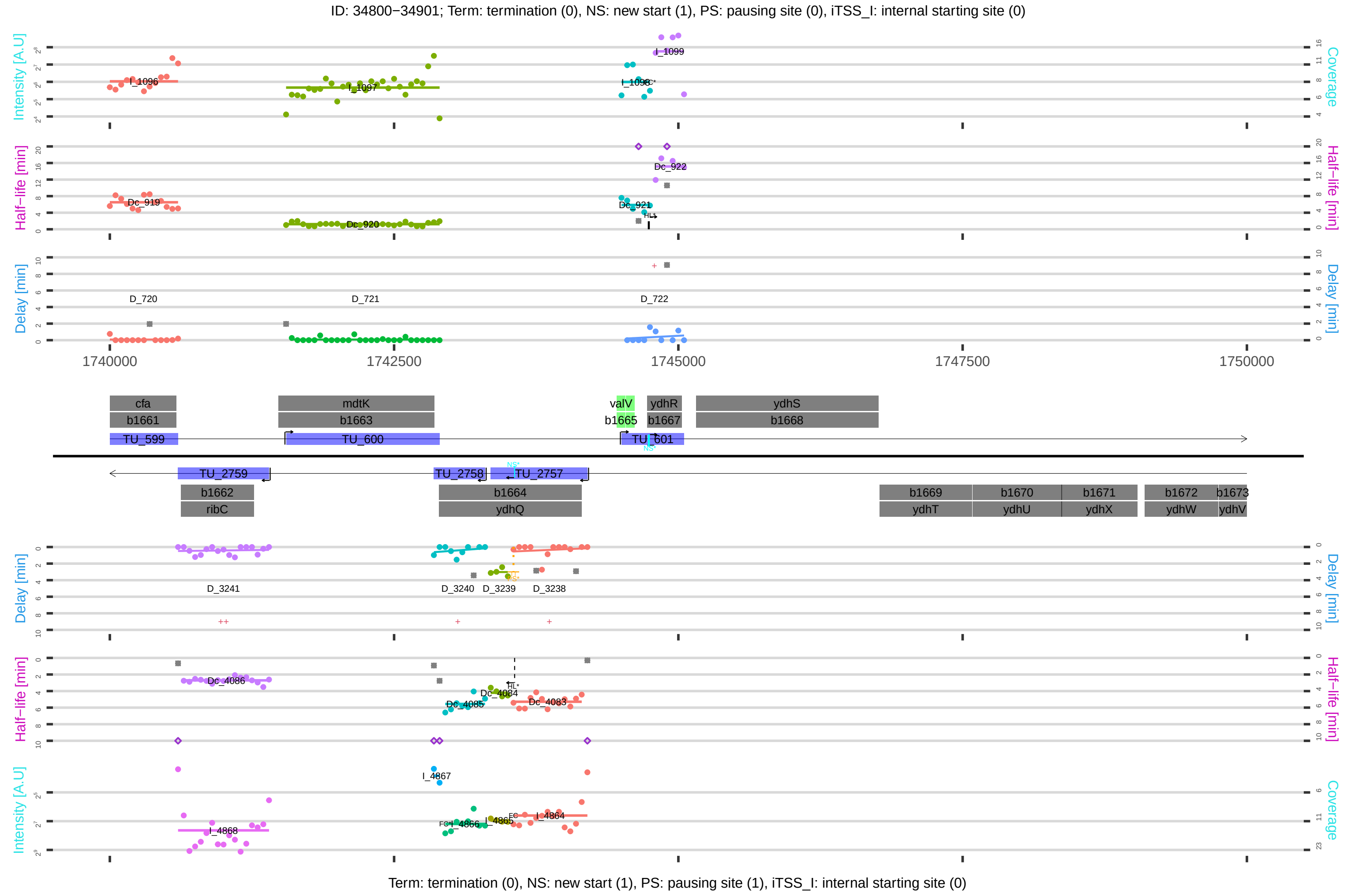

ID: 34800−34901; Term: termination (0), NS: new start (1), PS: pausing site (0), iTSS_I: internal starting site (0)
Intensity [A.U]
Coverage
Half−life [min]
Delay [min]
I_1096
I_1097
I_1098
I_1099
Dc_919
Dc_920
Dc_921
Dc_922
D_720
D_721
D_722
1740000
1742500
1745000
1747500
1750000
cfa
b1661
mdtK
b1663
valV
b1665
ydhR
b1667
ydhS
b1668
TU_599
TU_600
TU_601
TU_2759
TU_2758
TU_2757
b1662
ribC
b1664
ydhQ
b1669
ydhT
b1670
ydhU
b1671
ydhX
b1672
ydhW
b1673
ydhV
D_3241
D_3240
D_3239
D_3238
Dc_4086
Dc_4085
Dc_4084
Dc_4083
I_4868
I_4867
I_4866
I_4865
I_4864
Term: termination (0), NS: new start (1), PS: pausing site (1), iTSS_I: internal starting site (0)

ID: 35069−35122; Term: termination (1), NS: new start (2), PS: pausing site (1), iTSS_I: internal starting site (0)

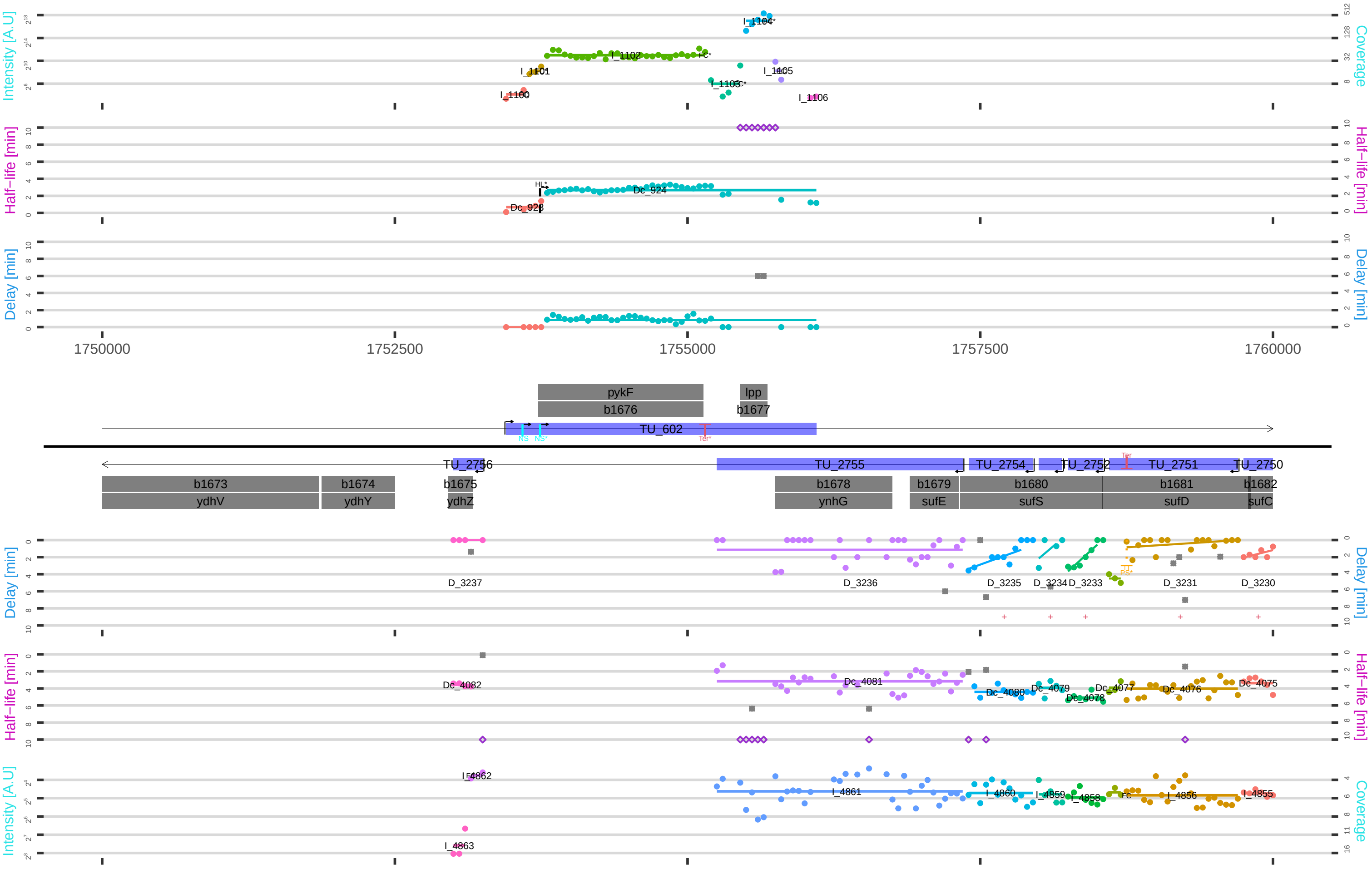

Term: termination (1), NS: new start (0), PS: pausing site (1), iTSS_I: internal starting site (0)

ID: 35336−35369; Term: termination (1), NS: new start (1), PS: pausing site (0), iTSS_I: internal starting site (0)

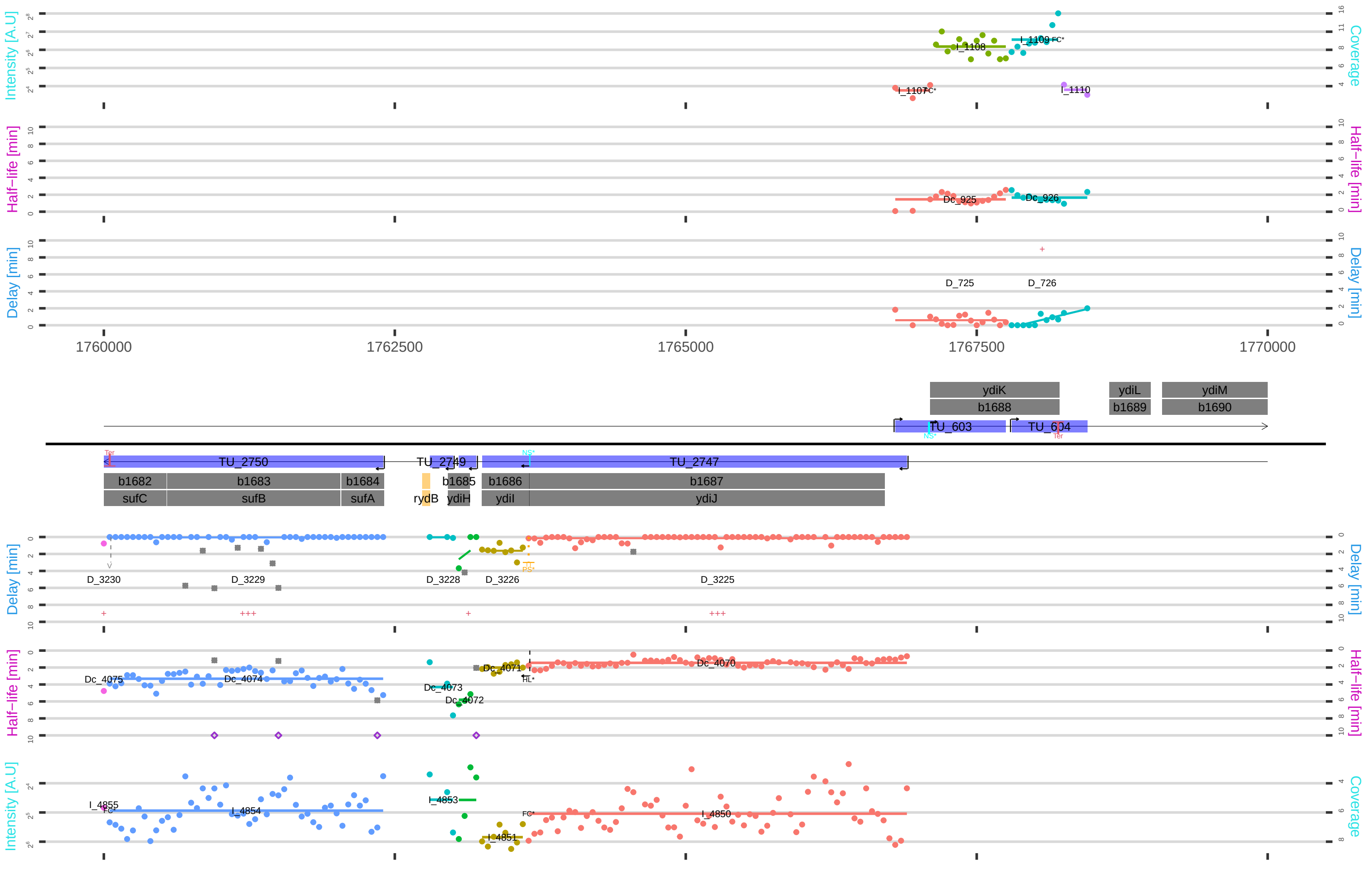

Term: termination (1), NS: new start (1), PS: pausing site (2), iTSS_I: internal starting site (0)

ID: 35454−35600; Term: termination (0), NS: new start (0), PS: pausing site (0), iTSS_I: internal starting site (0)

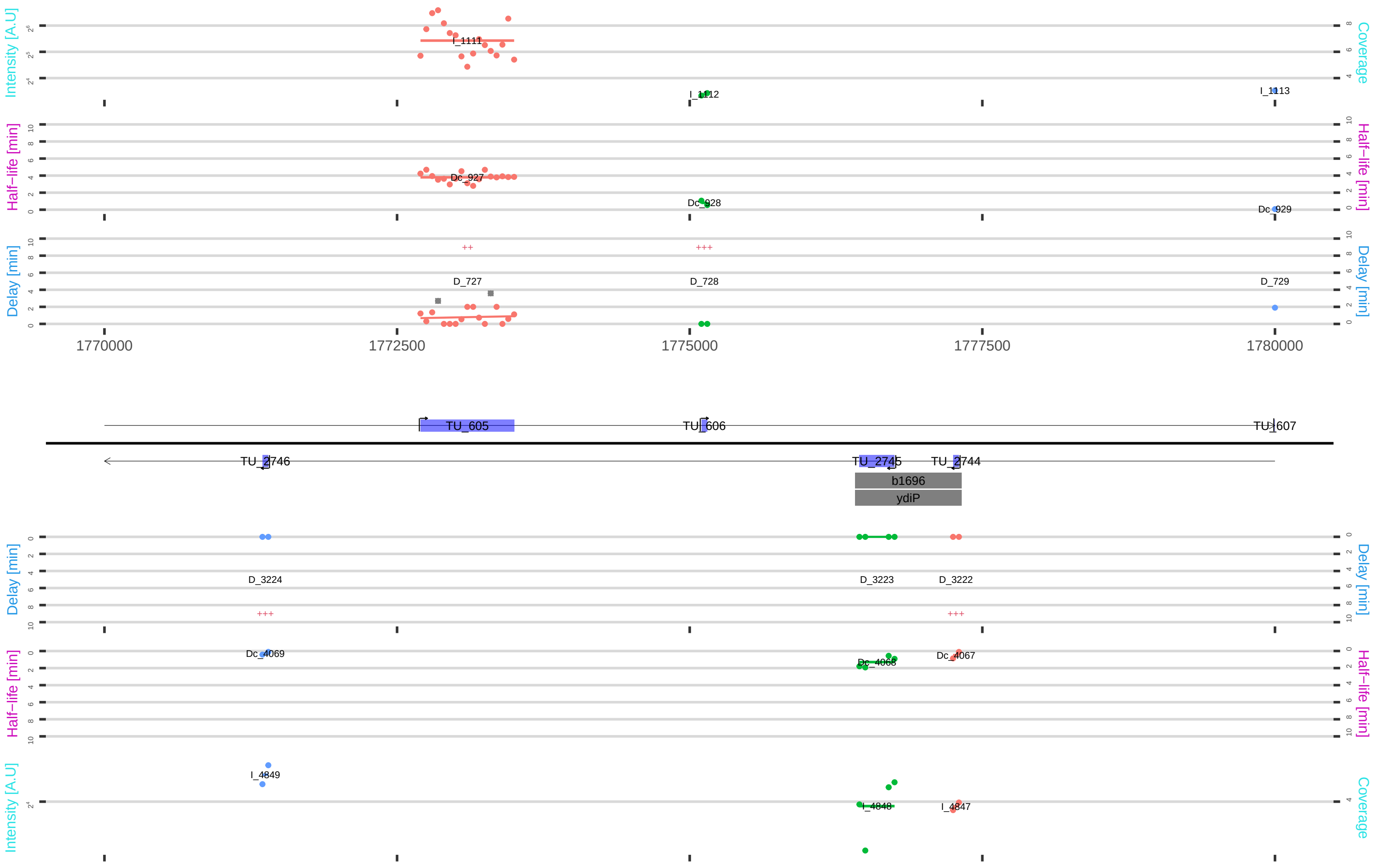

Term: termination (0), NS: new start (0), PS: pausing site (0), iTSS_I: internal starting site (0)

ID: 35600−35758; Term: termination (1), NS: new start (1), PS: pausing site (0), iTSS_I: internal starting site (0)

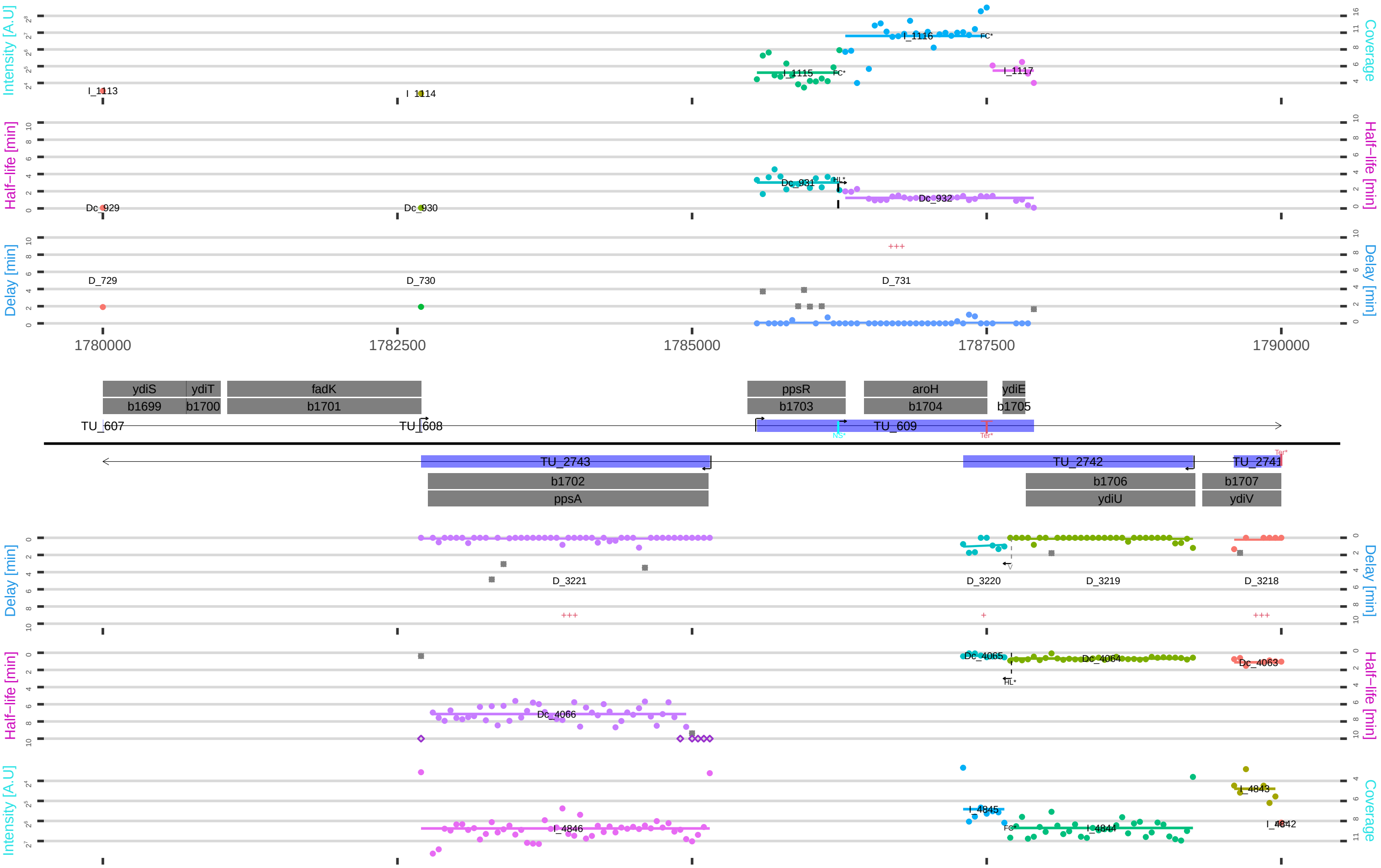

Term: termination (1), NS: new start (0), PS: pausing site (1), iTSS_I: internal starting site (0)

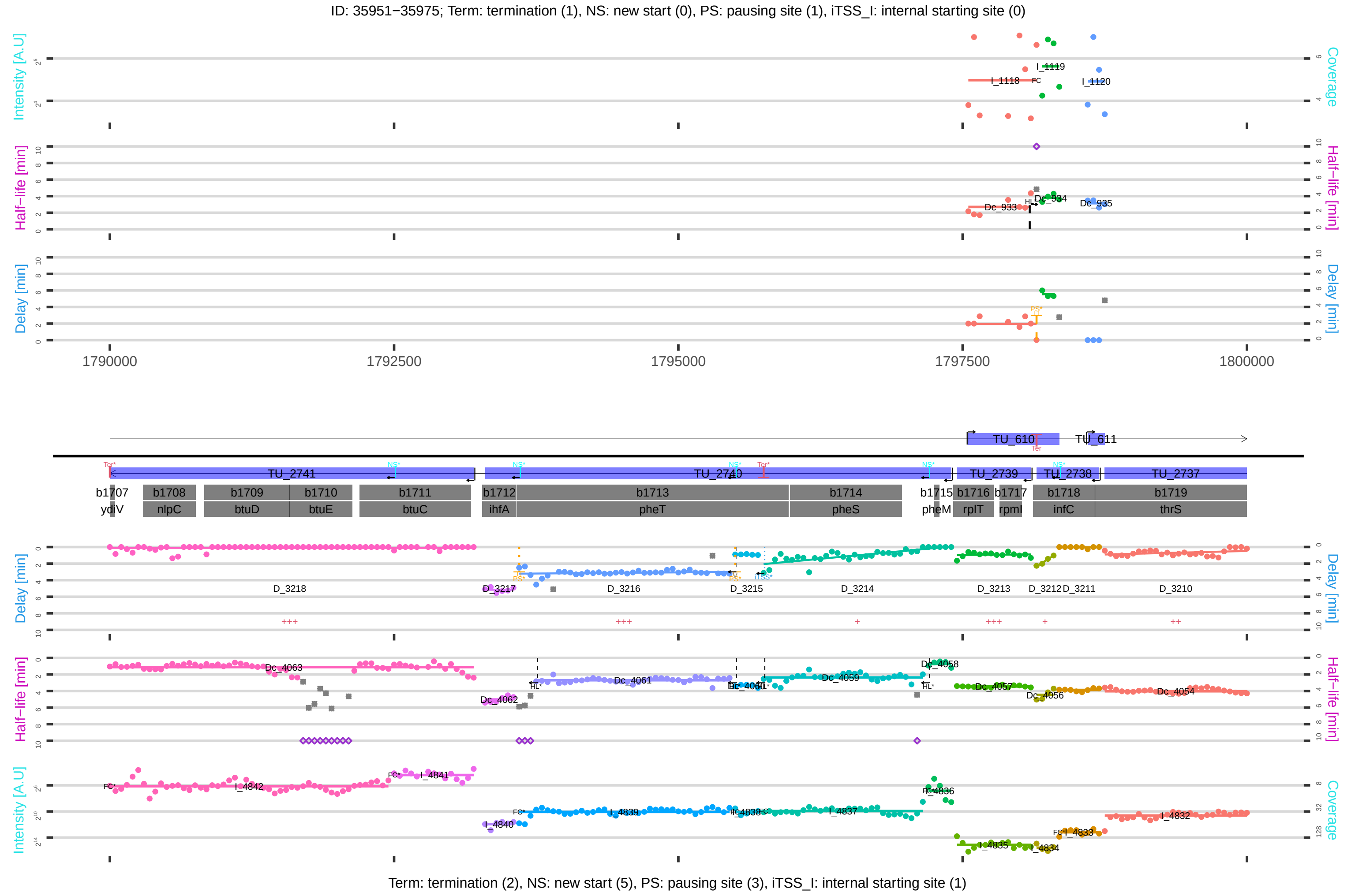

ID: 35951−35975; Term: termination (1), NS: new start (0), PS: pausing site (1), iTSS_I: internal starting site (0)
Intensity [A.U]
Coverage
Half−life [min]
Delay [min]
I_1118
I_1119
I_1120
Dc_933
Dc_934
Dc_935
1790000
1792500
1795000
1797500
1800000
TU_610
TU_611
TU_2741
TU_2740
TU_2739
TU_2738
TU_2737
b1707
b1708
b1709
b1710
b1711
b1712
b1713
b1714
b1715
b1716
b1717
b1718
b1719
ydiV
nlpC
btuD
btuE
btuC
ihfA
pheT
pheS
pheM
rplT
rpmI
infC
thrS
D_3218
D_3217
D_3216
D_3215
D_3214
D_3213
D_3212
D_3211
D_3210
Dc_4063
Dc_4062
Dc_4061
Dc_4060
Dc_4059
Dc_4058
Dc_4057
Dc_4056
Dc_4054
I_4842
I_4841
I_4840
I_4839
I_4838
I_4837
I_4836
I_4835
I_4834
I_4833
I_4832
Term: termination (2), NS: new start (5), PS: pausing site (3), iTSS_I: internal starting site (1)

ID: 36066−36200; Term: termination (1), NS: new start (3), PS: pausing site (0), iTSS_I: internal starting site (0)

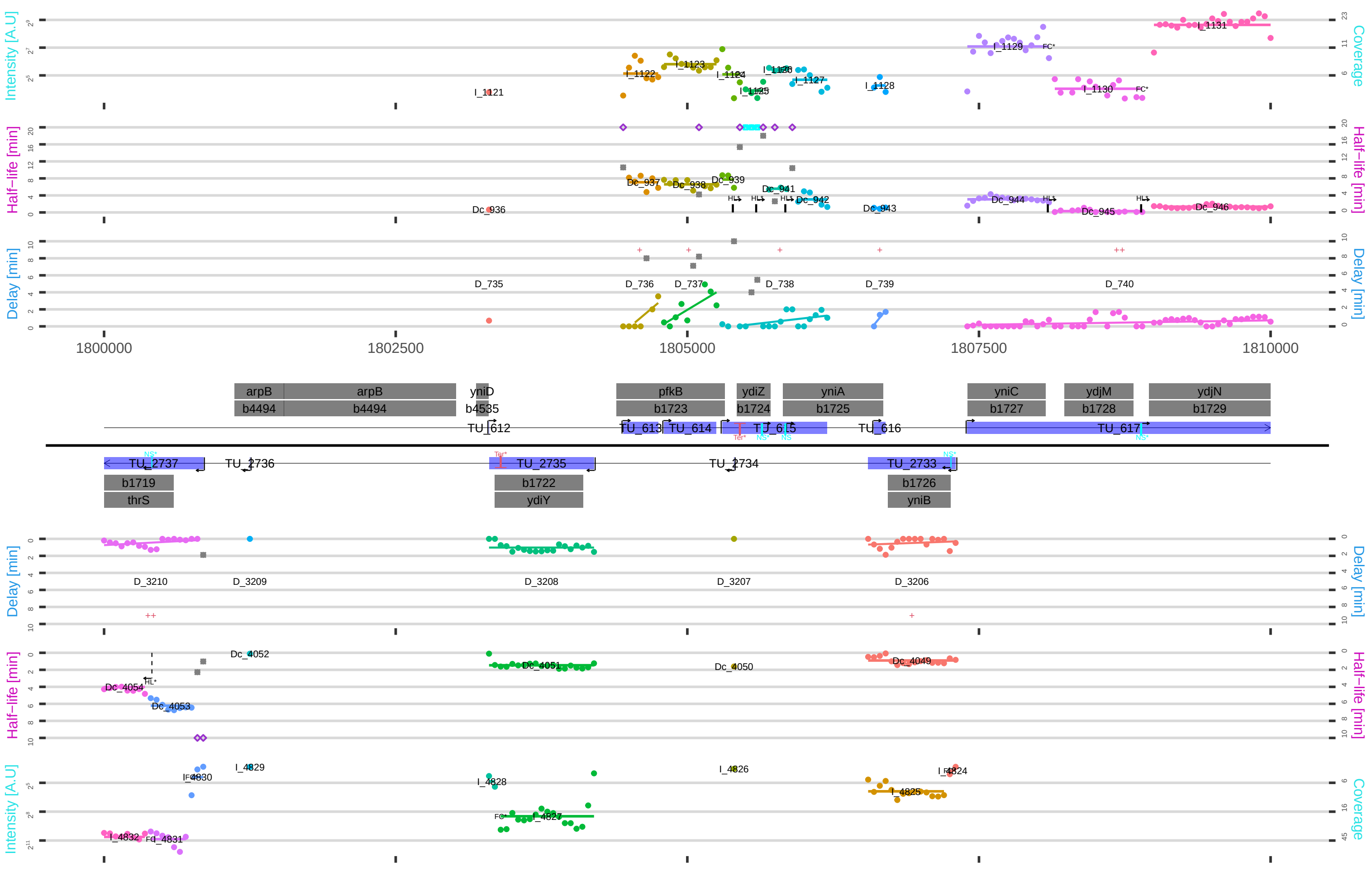

Term: termination (1), NS: new start (2), PS: pausing site (0), iTSS_I: internal starting site (0)

ID: 36200−36241; Term: termination (0), NS: new start (0), PS: pausing site (0), iTSS_I: internal starting site (0)

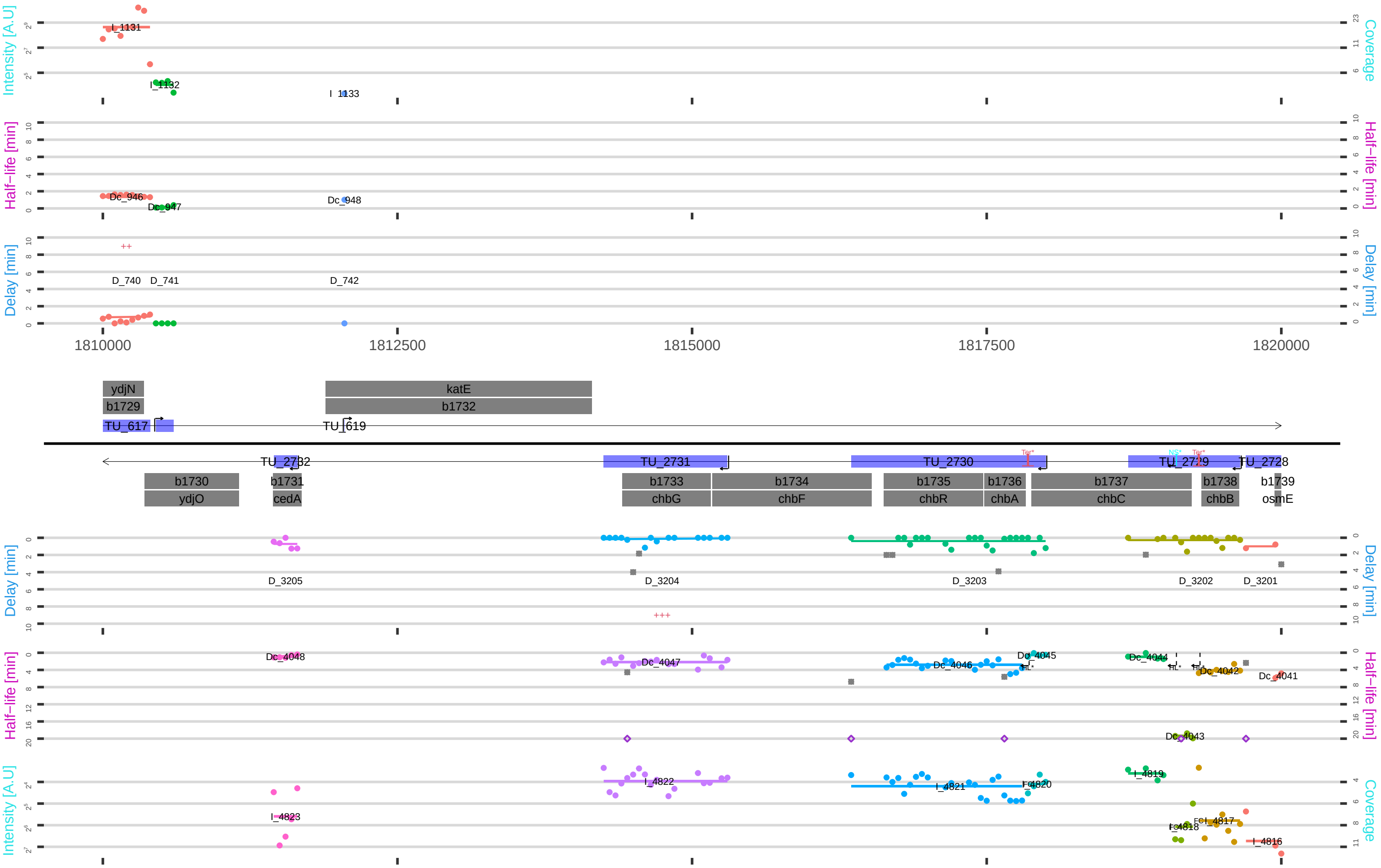

Term: termination (2), NS: new start (1), PS: pausing site (0), iTSS_I: internal starting site (0)

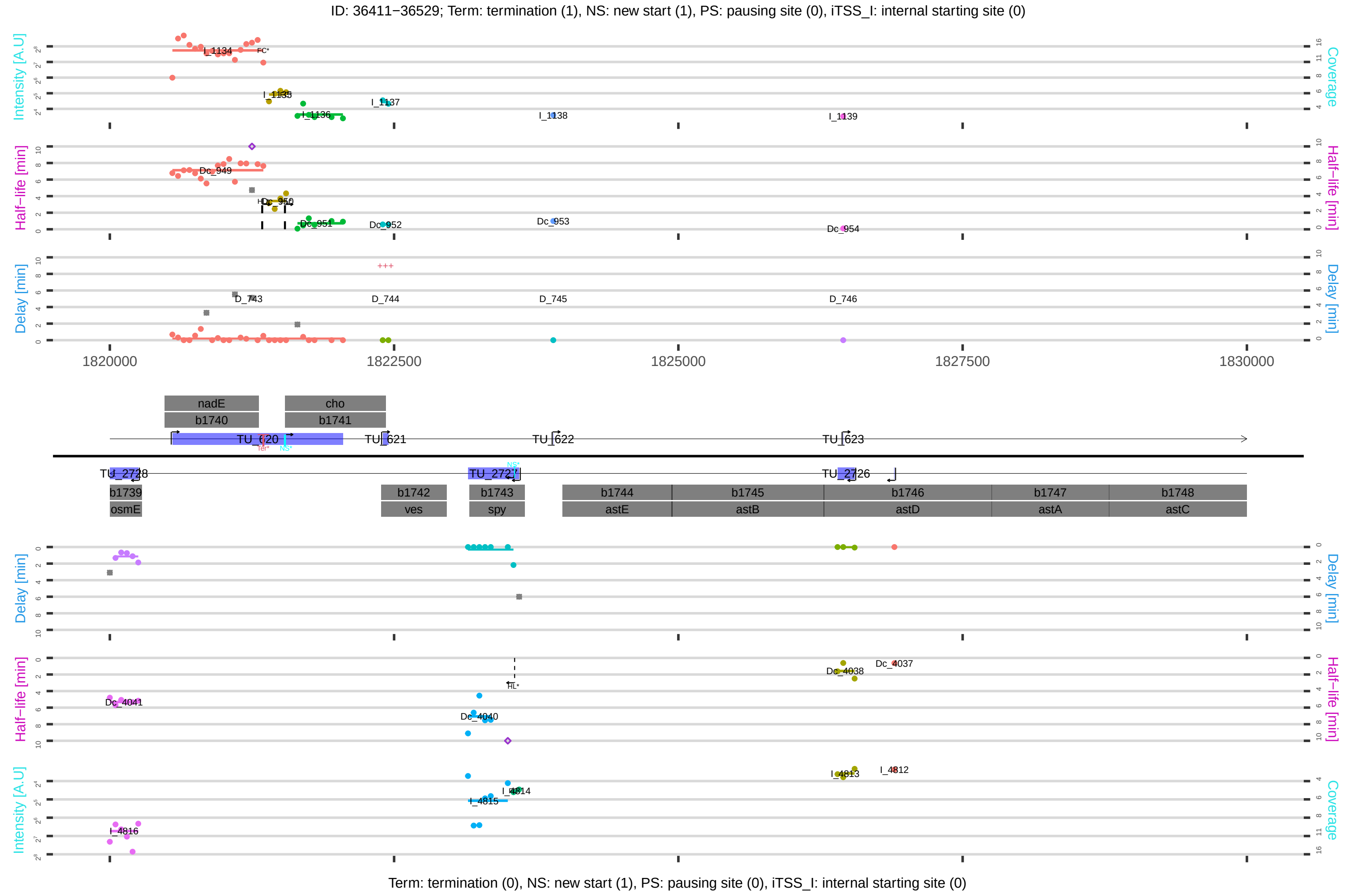
ID: 36411−36529; Term: termination (1), NS: new start (1), PS: pausing site (0), iTSS_I: internal starting site (0)
Intensity [A.U]
Coverage
Half−life [min]
Delay [min]
I_1134
I_1135
I_1136
I_1137
I_1138
I_1139
Dc_949
Dc_950
Dc_951
Dc_952
Dc_953
Dc_954
D_743
D_744
D_745
D_746
1820000
1822500
1825000
1827500
1830000
nadE
b1740
cho
b1741
TU_620
TU_621
TU_622
TU_623
TU_2728
TU_2727
TU_2726
b1739
osmE
b1742
ves
b1743
spy
b1744
astE
b1745
astB
b1746
astD
b1747
astA
b1748
astC
Dc_4041
Dc_4040
Dc_4038
Dc_4037
I_4816
I_4815
I_4814
I_4813
I_4812
Term: termination (0), NS: new start (1), PS: pausing site (0), iTSS_I: internal starting site (0)

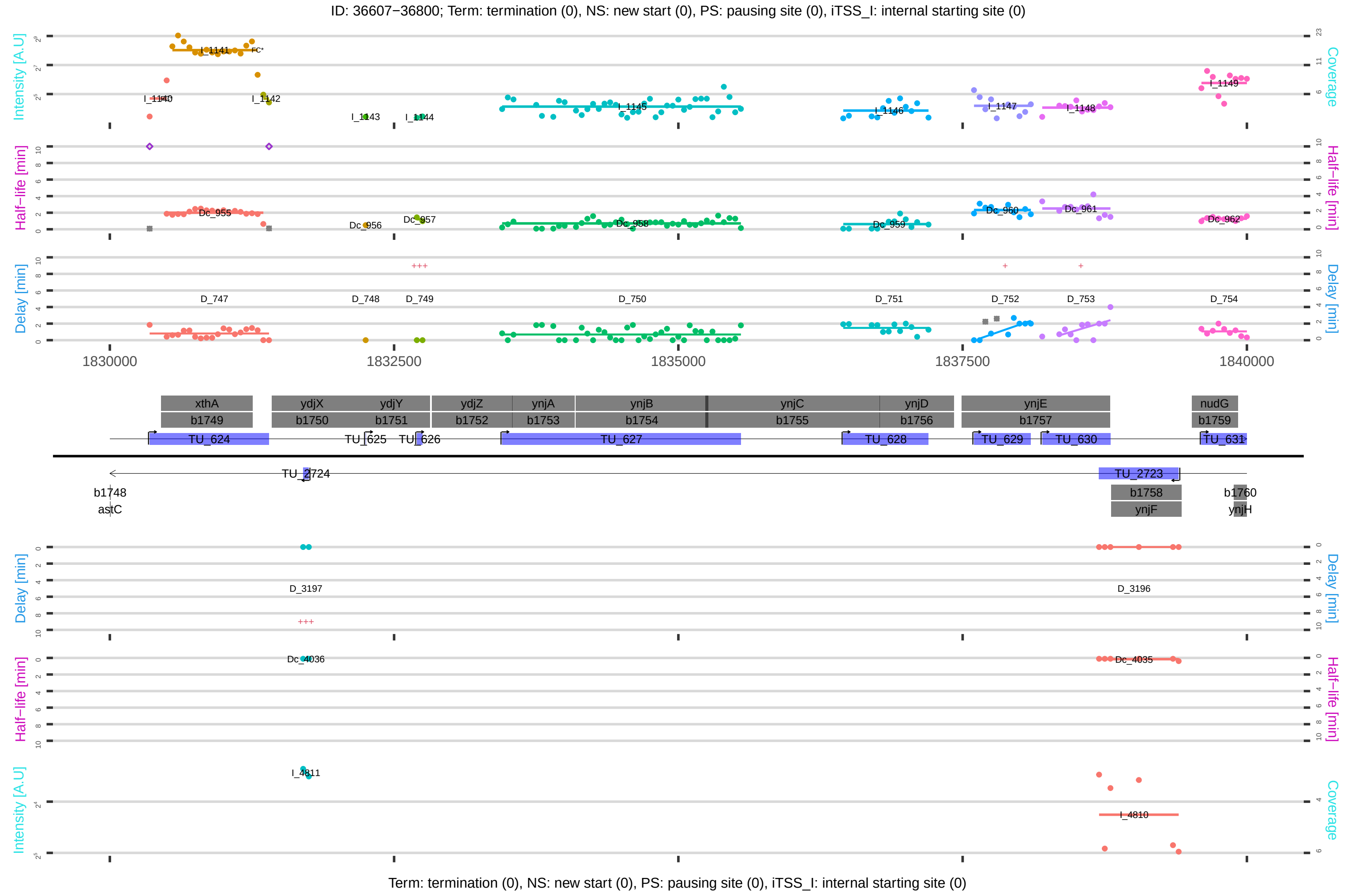
ID: 36607−36800; Term: termination (0), NS: new start (0), PS: pausing site (0), iTSS_I: internal starting site (0)
Intensity [A.U]
Coverage
Half−life [min]
Delay [min]
I_1140
I_1141
FC*
I_1142
I_1143
I_1144
I_1145
I_1146
I_1147
I_1148
I_1149
Dc_955
Dc_956
Dc_957
Dc_958
Dc_959
Dc_960
Dc_961
Dc_962
D_747
D_748
D_749
D_750
D_751
D_752
D_753
D_754
1830000
1832500
1835000
1837500
1840000
xthA
b1749
ydjX
b1750
ydjY
b1751
ydjZ
b1752
ynjA
b1753
ynjB
b1754
ynjC
b1755
ynjD
b1756
ynjE
b1757
nudG
b1759
TU_624
TU_625
TU_626
TU_627
TU_628
TU_629
TU_630
TU_631
TU_2724
TU_2723
b1748
astC
b1758
ynjF
b1760
ynjH
D_3197
D_3196
Dc_4036
Dc_4035
I_4811
I_4810
Term: termination (0), NS: new start (0), PS: pausing site (0), iTSS_I: internal starting site (0)

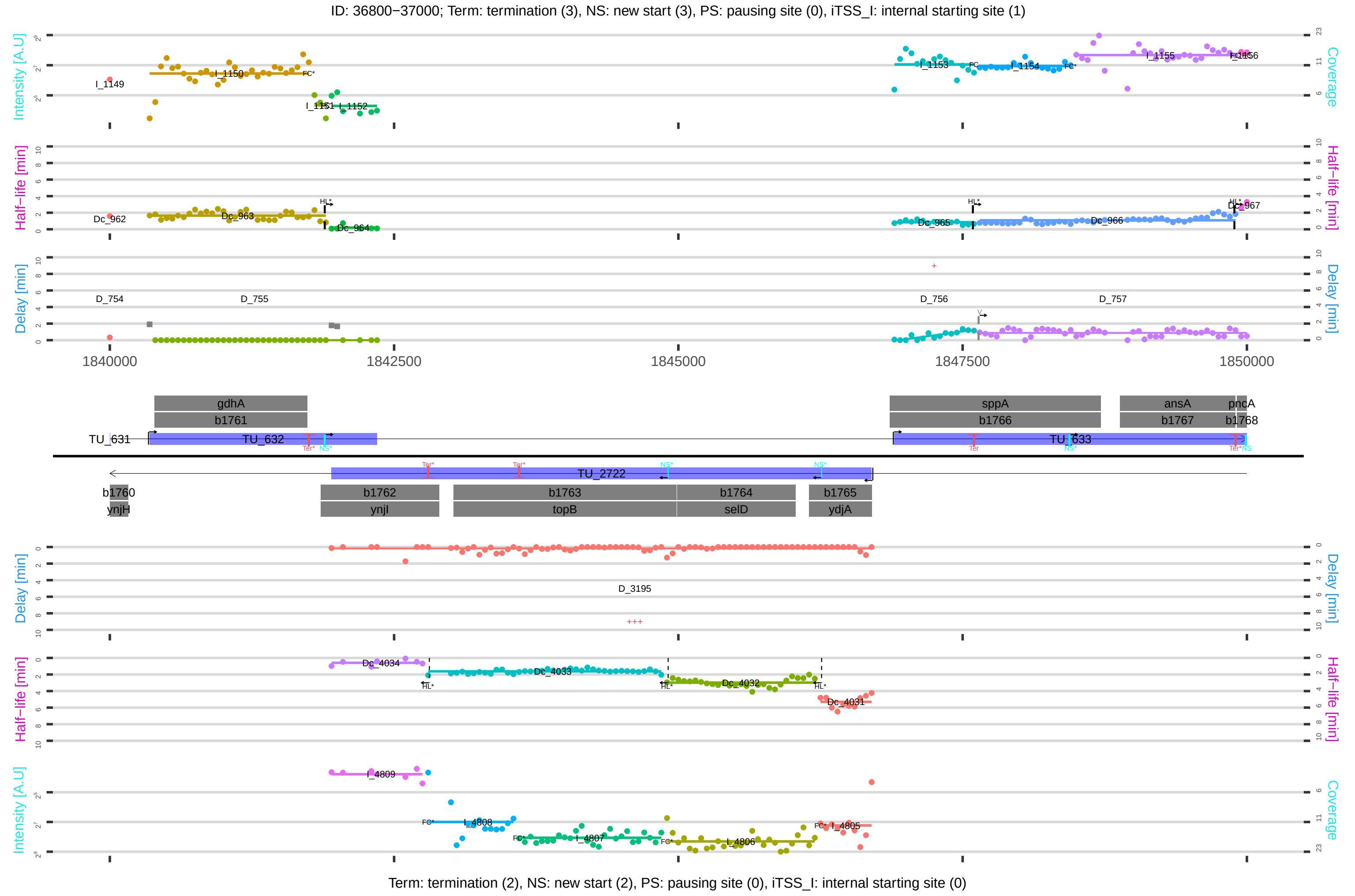

ID: 36800−37000; Term: termination (3), NS: new start (3), PS: pausing site (0), iTSS_I: internal starting site (1)
Intensity [A.U]
Coverage
Half−life [min]
Delay [min]
I_1149
I_1150
I_1151
I_1152
I_1153
I_1154
I_1155
I_1156
Dc_962
Dc_963
Dc_964
Dc_965
Dc_966
Dc_967
D_754
D_755
D_756
D_757
1840000
1842500
1845000
1847500
1850000
gdhA
b1761
sppA
b1766
ansA
b1767
pncA
b1768
TU_631
TU_632
TU_633
TU_2722
b1760
ynjH
b1762
ynjI
b1763
topB
b1764
selD
b1765
ydjA
D_3195
Dc_4034
Dc_4033
Dc_4032
Dc_4031
I_4809
I_4808
I_4807
I_4806
I_4805
Term: termination (2), NS: new start (2), PS: pausing site (0), iTSS_I: internal starting site (0)

ID: 37000−37015; Term: termination (0), NS: new start (1), PS: pausing site (0), iTSS_I: internal starting site (1)

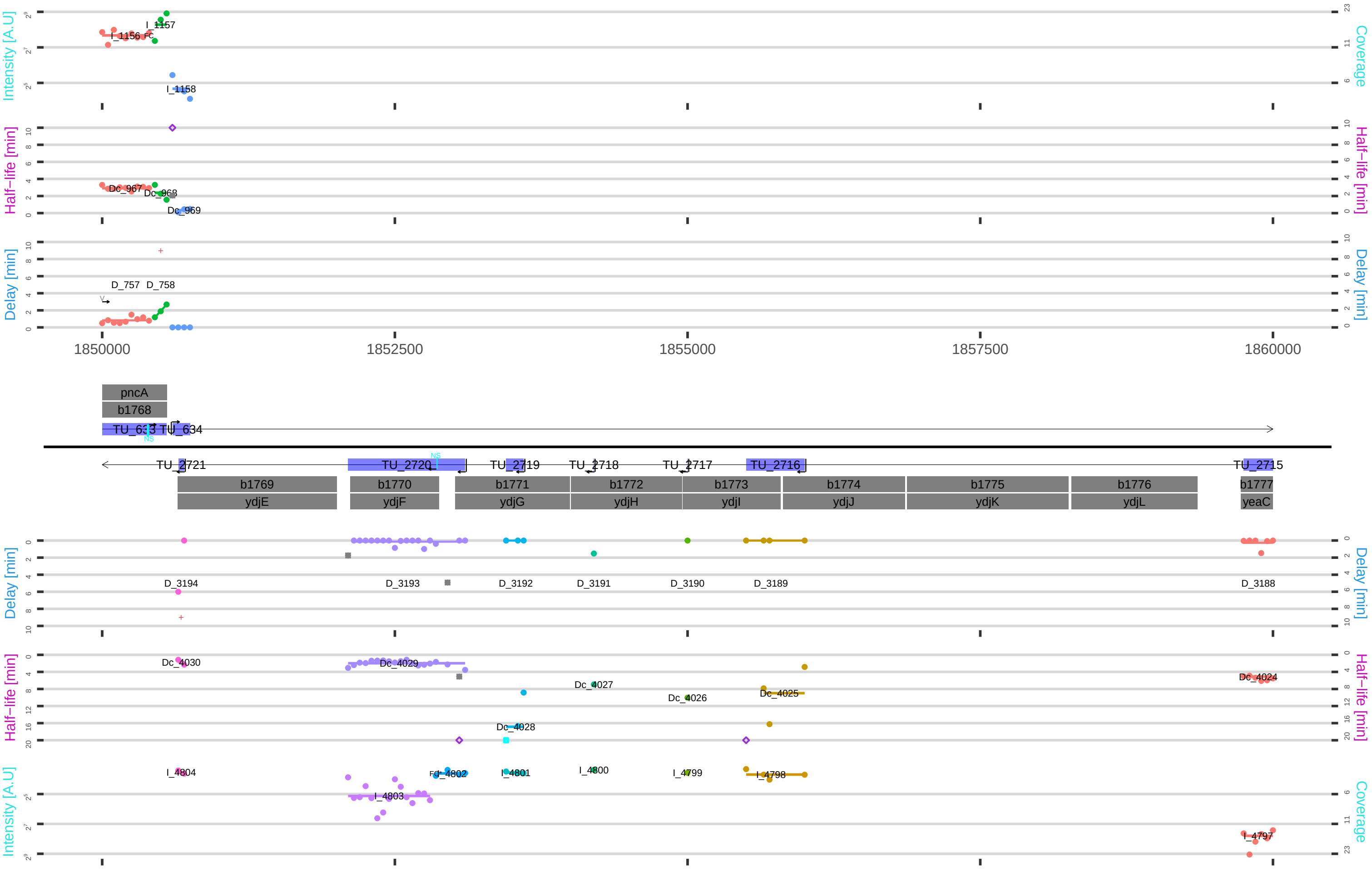

Term: termination (0), NS: new start (1), PS: pausing site (0), iTSS_I: internal starting site (0)

ID: 37213−37337; Term: termination (1), NS: new start (0), PS: pausing site (1), iTSS_I: internal starting site (0)

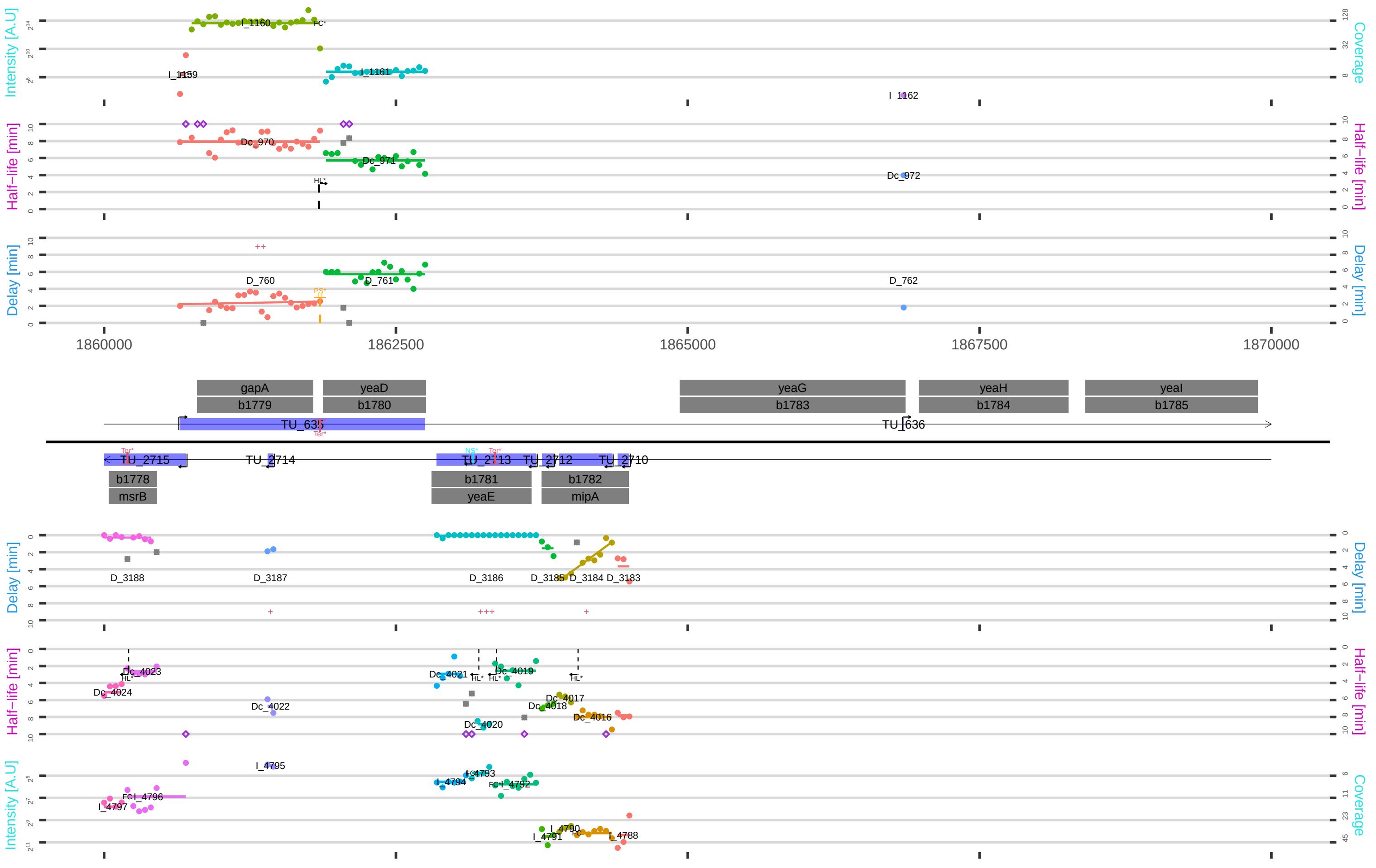

Term: termination (2), NS: new start (1), PS: pausing site (0), iTSS_I: internal starting site (0)

ID: 37402−37540; Term: termination (2), NS: new start (3), PS: pausing site (2), iTSS_I: internal starting site (0)

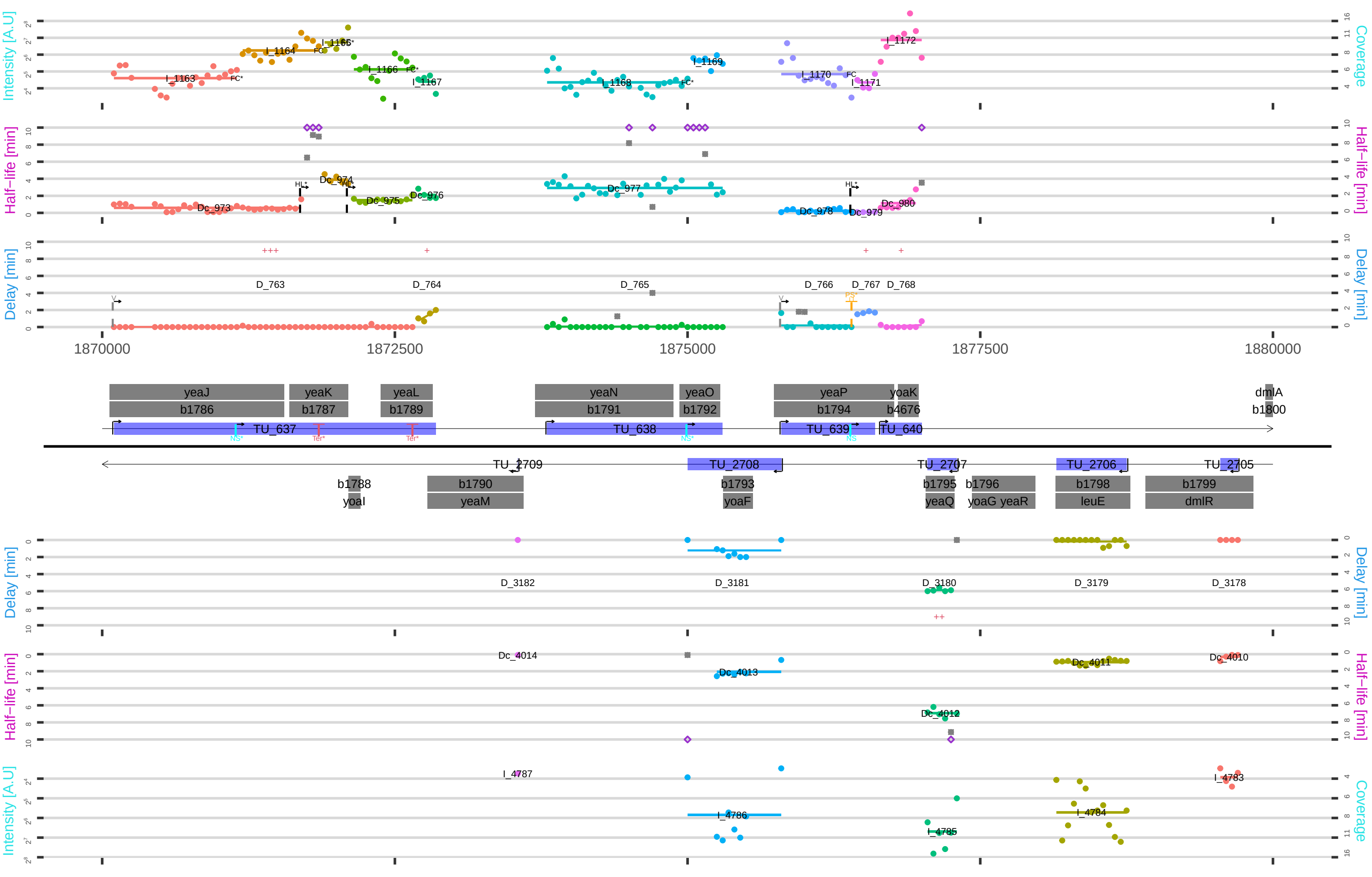

Term: termination (0), NS: new start (0), PS: pausing site (0), iTSS_I: internal starting site (0)

ID: 37620−37696; Term: termination (0), NS: new start (0), PS: pausing site (0), iTSS_I: internal starting site (0)

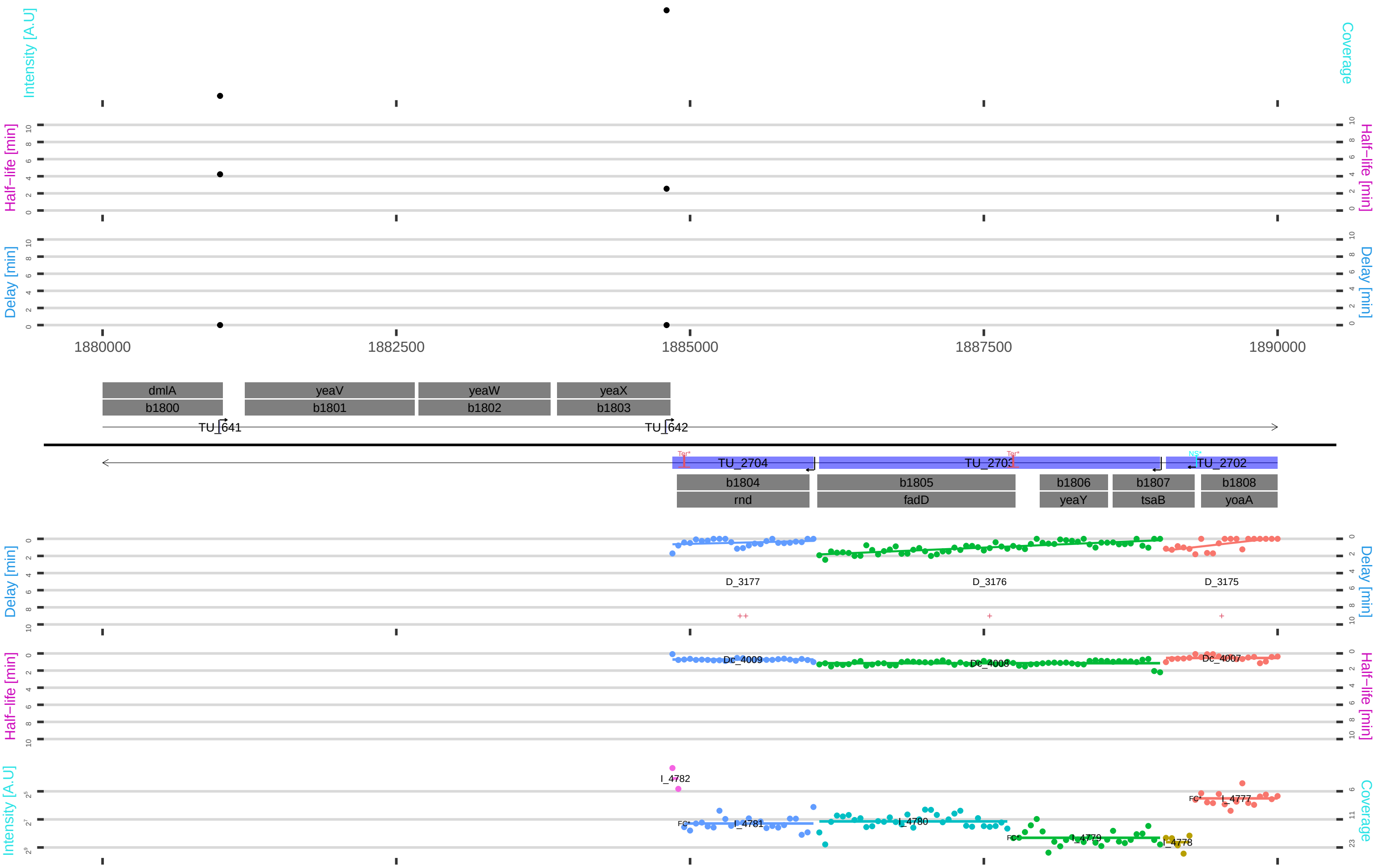

Term: termination (2), NS: new start (1), PS: pausing site (0), iTSS_I: internal starting site (0)

ID: 37829−37928; Term: termination (2), NS: new start (2), PS: pausing site (1), iTSS_I: internal starting site (0)

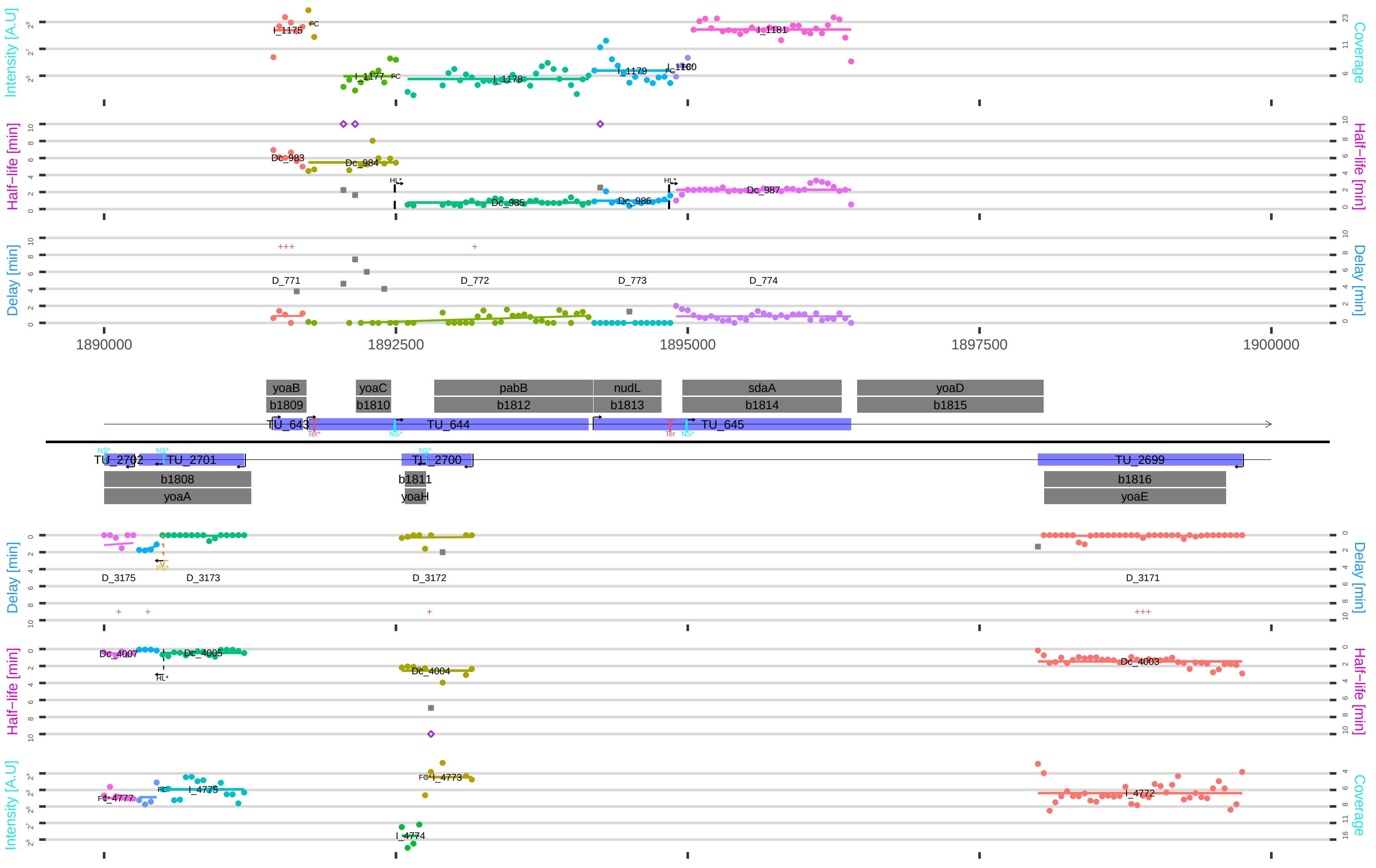

Term: termination (0), NS: new start (3), PS: pausing site (1), iTSS_I: internal starting site (0)

ID: 38001−38194; Term: termination (1), NS: new start (4), PS: pausing site (1), iTSS_I: internal starting site (0)

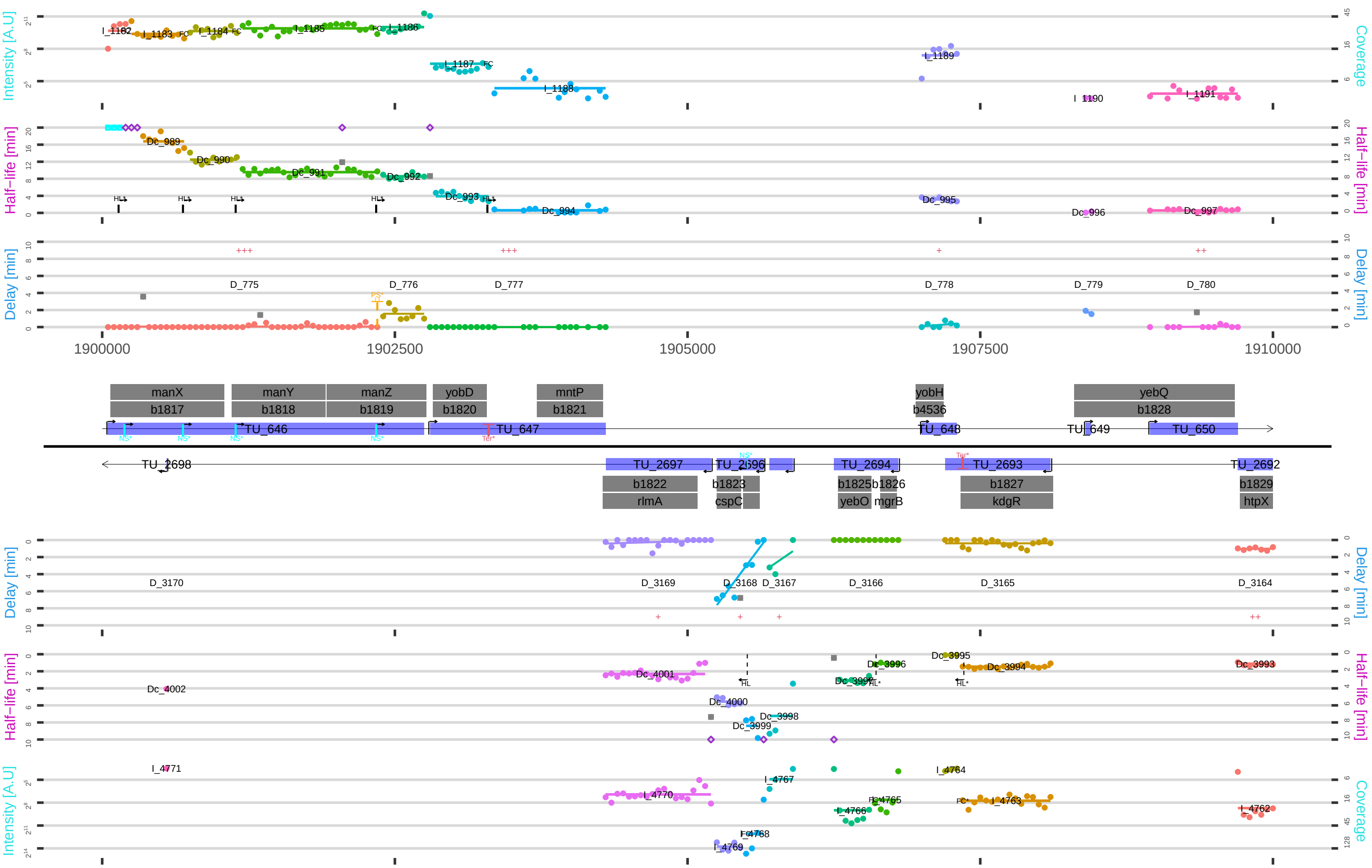

Term: termination (1), NS: new start (1), PS: pausing site (0), iTSS_I: internal starting site (0)

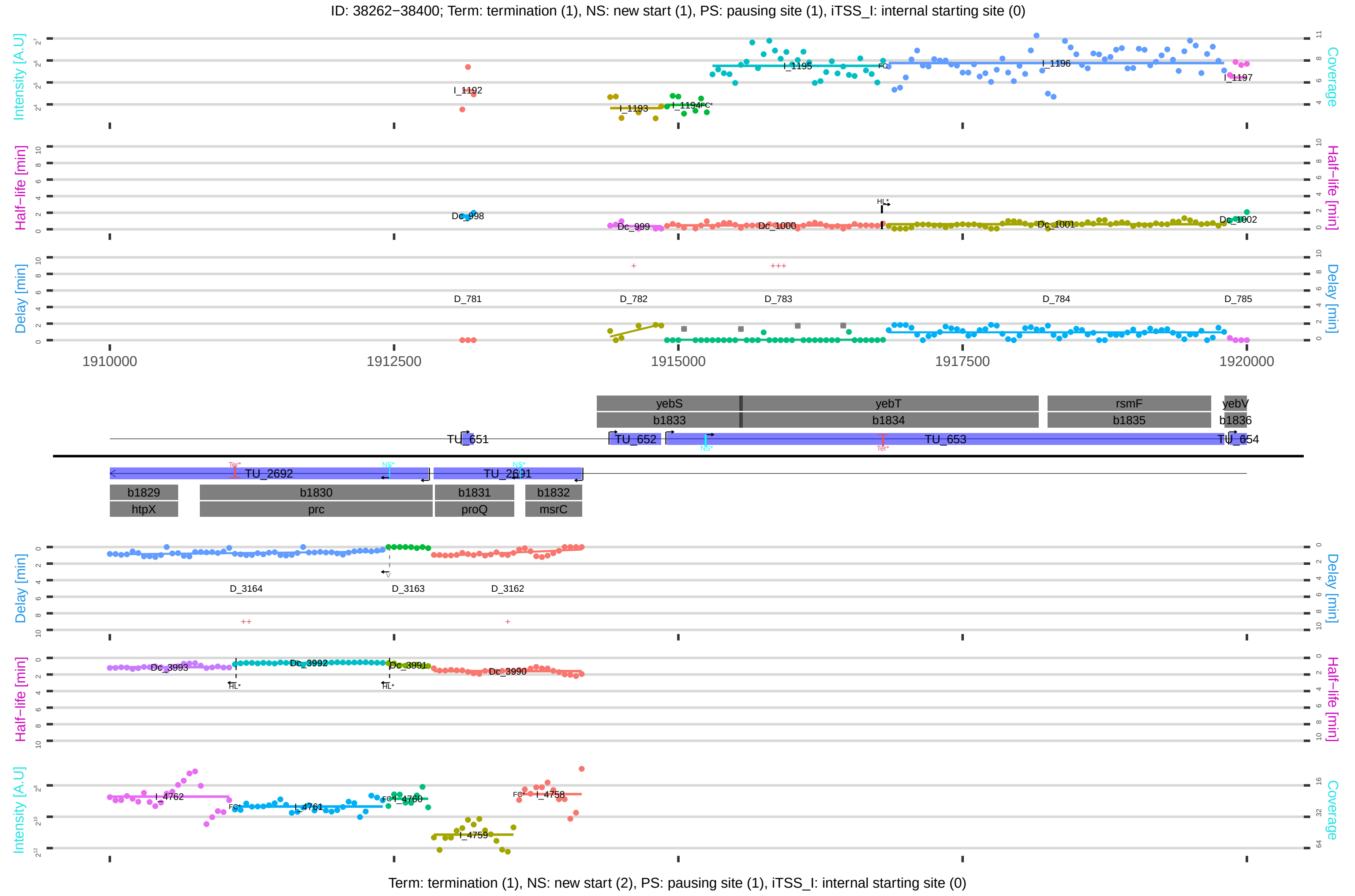

ID: 38262−38400; Term: termination (1), NS: new start (1), PS: pausing site (1), iTSS_I: internal starting site (0)
Intensity [A.U]
Coverage
Half−life [min]
Delay [min]
I_1192
I_1193
I_1194
I_1195
I_1196
I_1197
Dc_998
Dc_999
Dc_1000
Dc_1001
Dc_1002
D_781
D_782
D_783
D_784
D_785
1910000
1912500
1915000
1917500
1920000
yebS
b1833
yebT
b1834
rsmF
b1835
yebV
b1836
TU_651
TU_652
TU_653
TU_654
TU_2692
TU_2691
b1829
htpX
b1830
prc
b1831
proQ
b1832
msrC
D_3164
D_3163
D_3162
Dc_3993
Dc_3992
Dc_3991
Dc_3990
I_4762
I_4761
I_4760
I_4759
I_4758
Term: termination (1), NS: new start (2), PS: pausing site (1), iTSS_I: internal starting site (0)

ID: 38400−38600; Term: termination (2), NS: new start (2), PS: pausing site (1), iTSS_I: internal starting site (1)

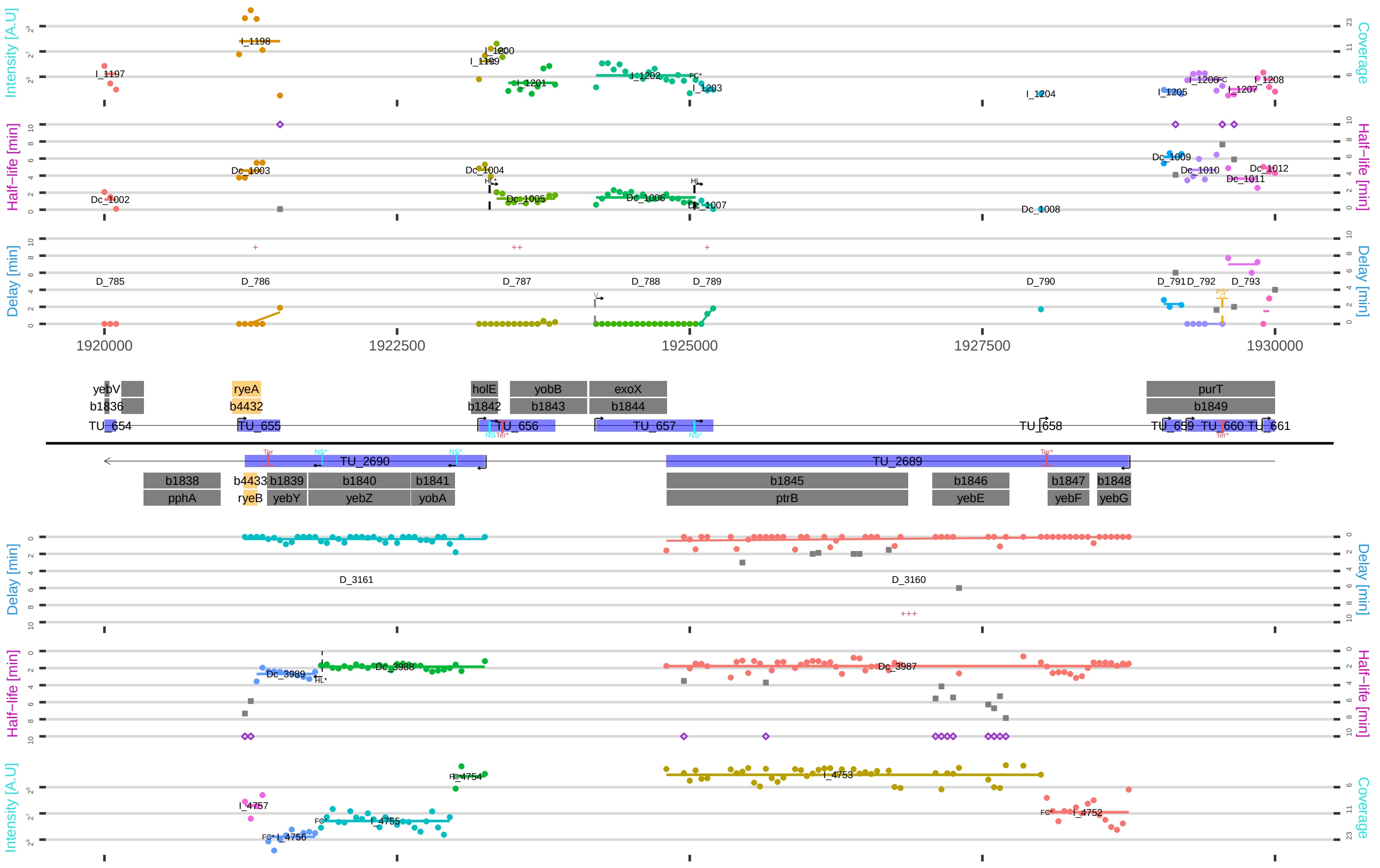

Term: termination (2), NS: new start (2), PS: pausing site (0), iTSS_I: internal starting site (0)

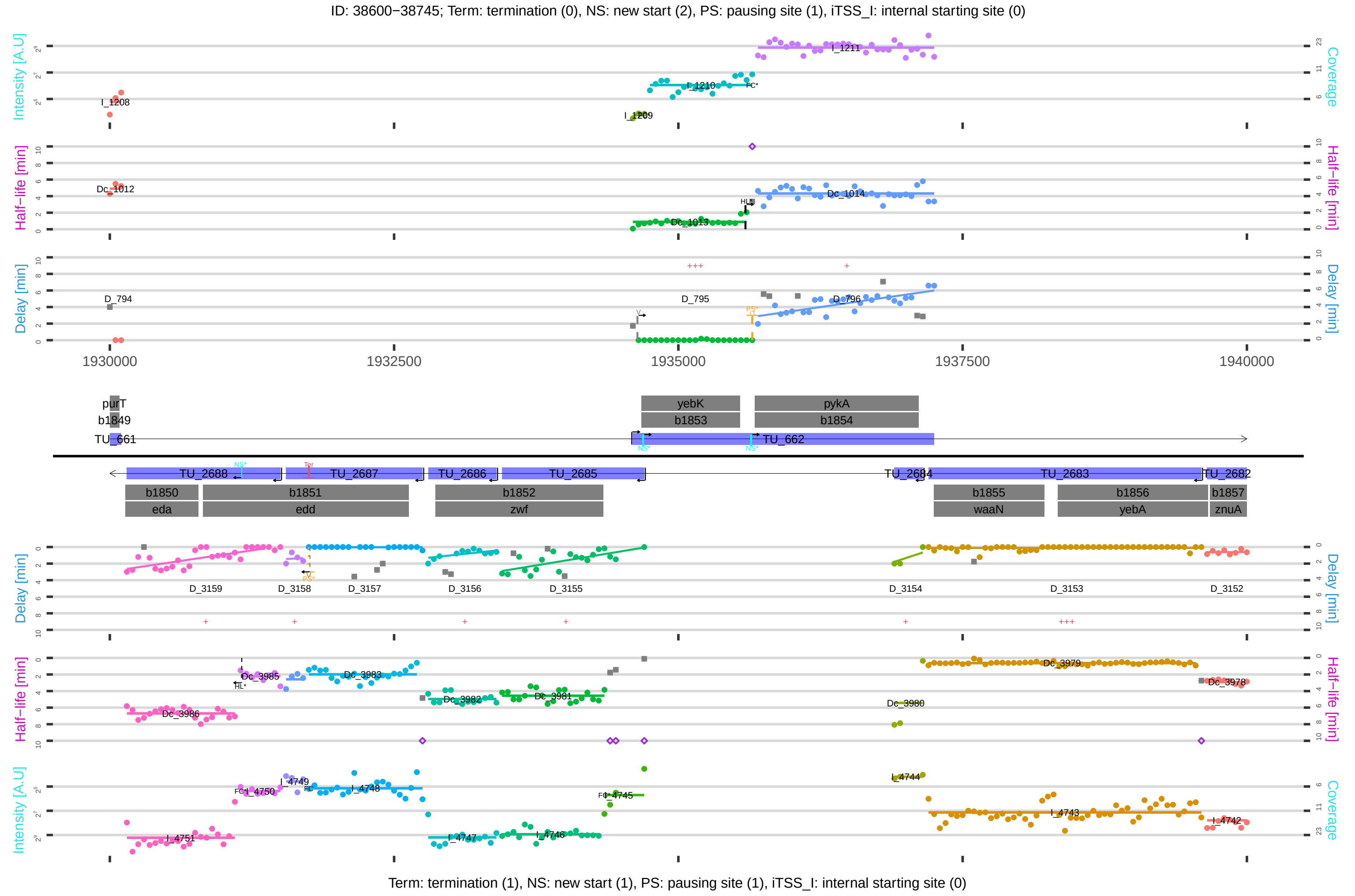

ID: 38600−38745; Term: termination (0), NS: new start (2), PS: pausing site (1), iTSS_I: internal starting site (0)
Intensity [A.U]
Coverage
Half−life [min]
Delay [min]
I_1208
I_1209
I_1210
I_1211
Dc_1012
Dc_1013
Dc_1014
D_794
D_795
D_796
1930000
1932500
1935000
1937500
1940000
purT
b1849
TU_661
yebK
b1853
pykA
b1854
TU_662
TU_2688
TU_2687
TU_2686
TU_2685
TU_2684
TU_2683
TU_2682
b1850
eda
b1851
edd
b1852
zwf
b1855
waaN
b1856
yebA
b1857
znuA
D_3159
D_3158
D_3157
D_3156
D_3155
D_3154
D_3153
D_3152
Dc_3986
Dc_3985
Dc_3983
Dc_3982
Dc_3981
Dc_3980
Dc_3979
Dc_3978
I_4751
I_4750
I_4749
I_4748
I_4747
I_4746
I_4745
I_4744
I_4743
I_4742
Term: termination (1), NS: new start (1), PS: pausing site (1), iTSS_I: internal starting site (0)

ID: 38815−39000; Term: termination (0), NS: new start (2), PS: pausing site (0), iTSS_I: internal starting site (0)

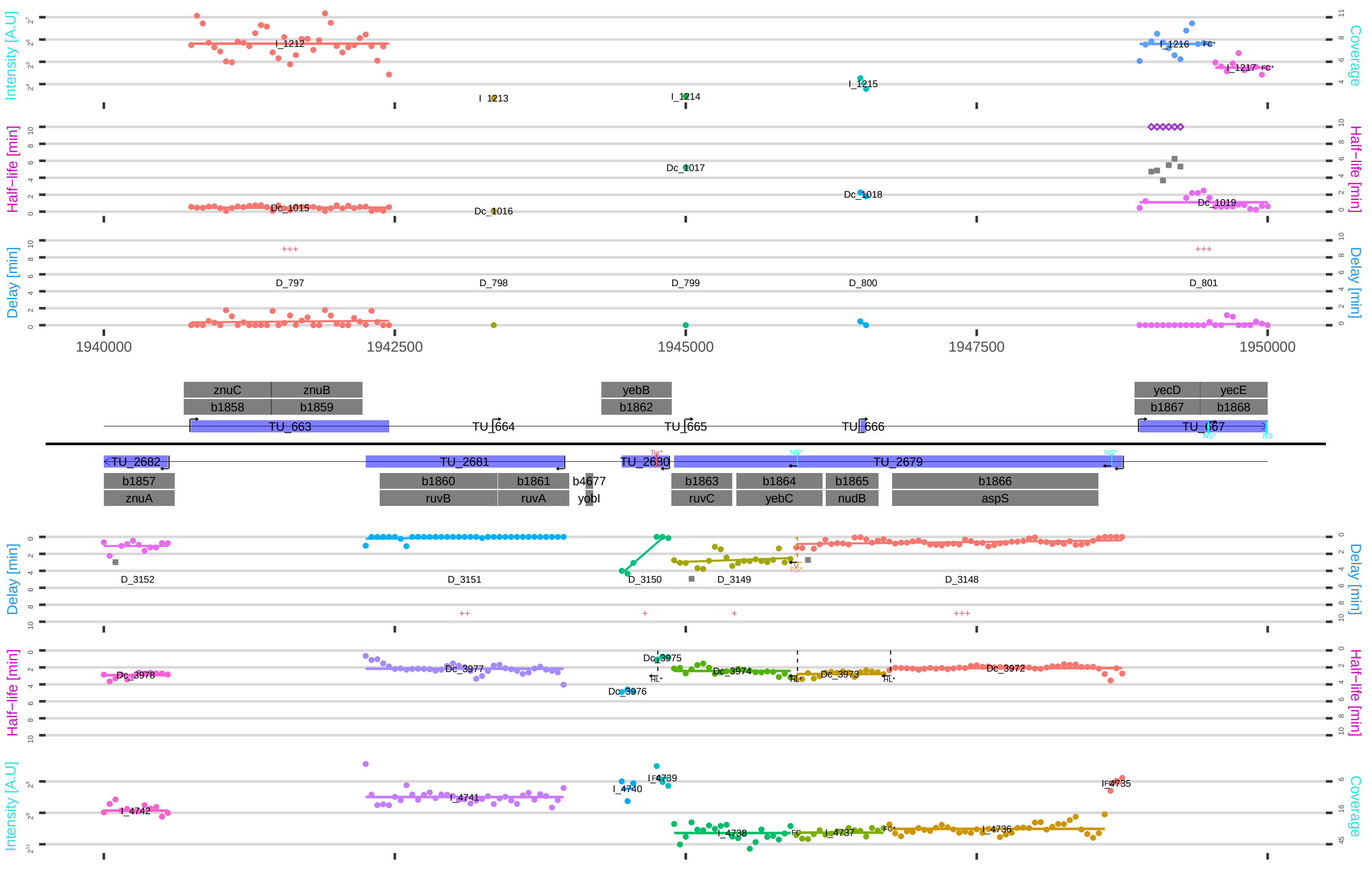

Term: termination (1), NS: new start (2), PS: pausing site (1), iTSS_I: internal starting site (0)

ID: 39000−39200; Term: termination (1), NS: new start (1), PS: pausing site (0), iTSS_I: internal starting site (0)

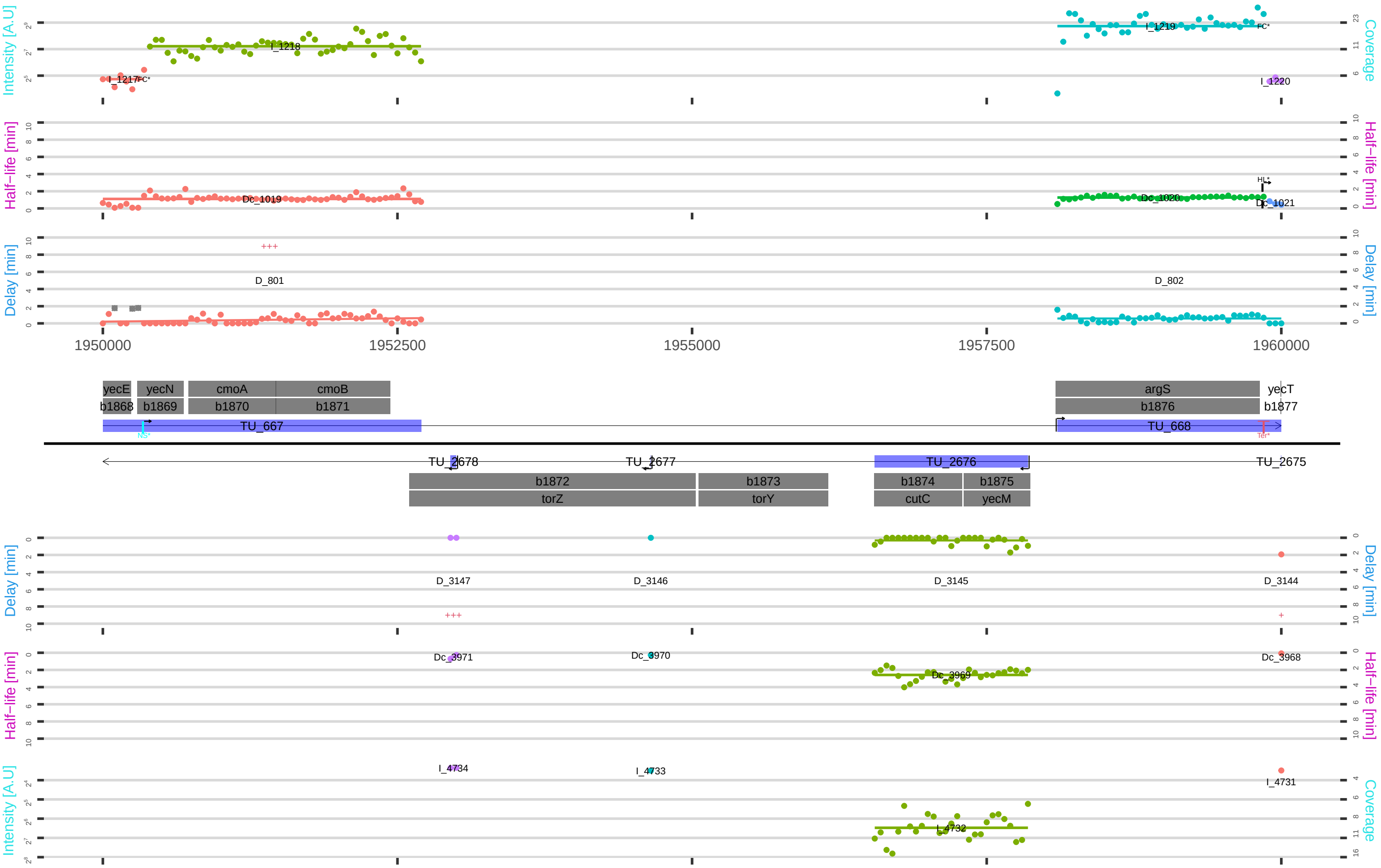

Term: termination (0), NS: new start (0), PS: pausing site (0), iTSS_I: internal starting site (0)

ID: 39200−39213; Term: termination (0), NS: new start (0), PS: pausing site (0), iTSS_I: internal starting site (0)

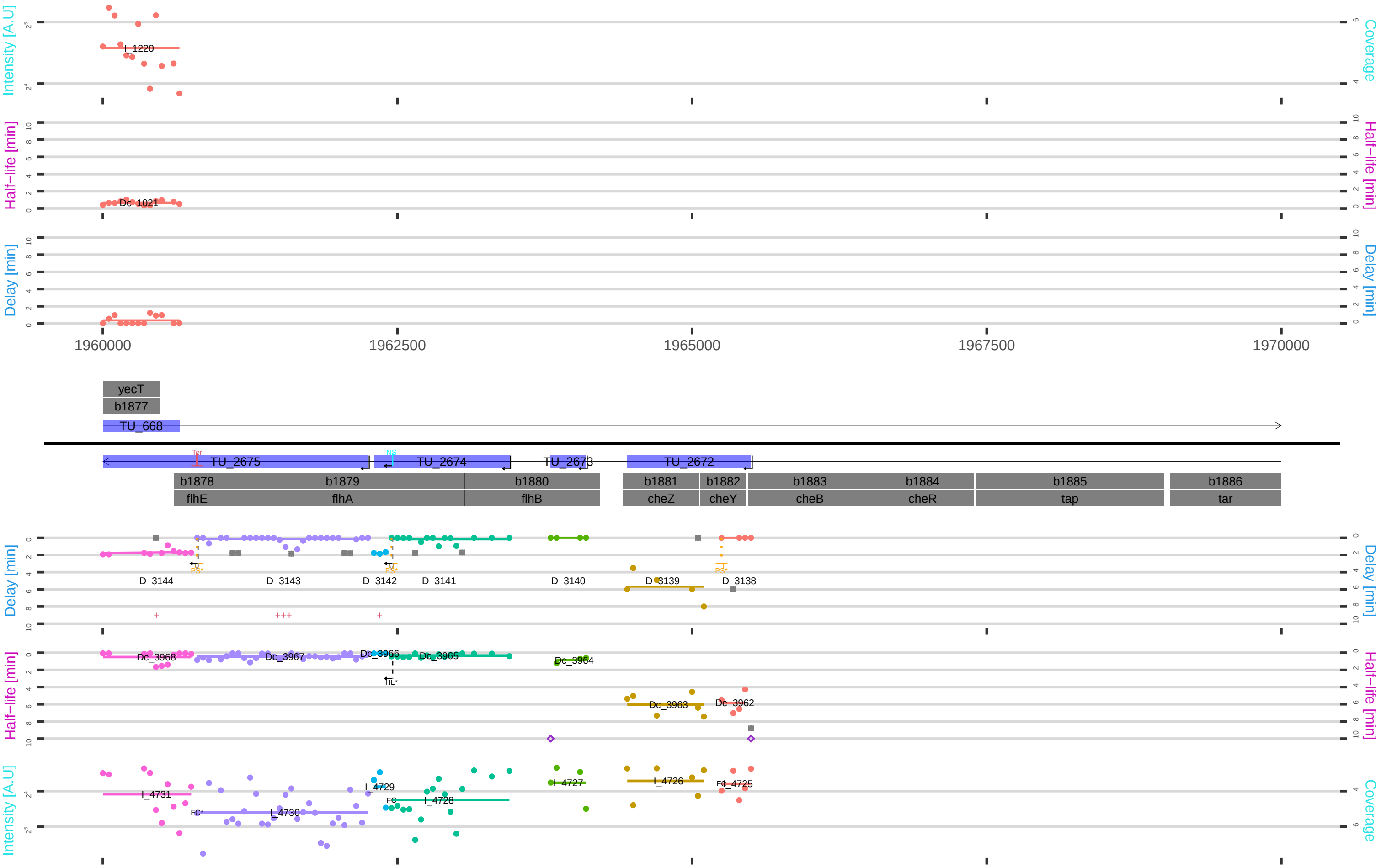

Term: termination (1), NS: new start (1), PS: pausing site (3), iTSS_I: internal starting site (0)

ID: 39533−39536; Term: termination (0), NS: new start (0), PS: pausing site (0), iTSS_I: internal starting site (0)

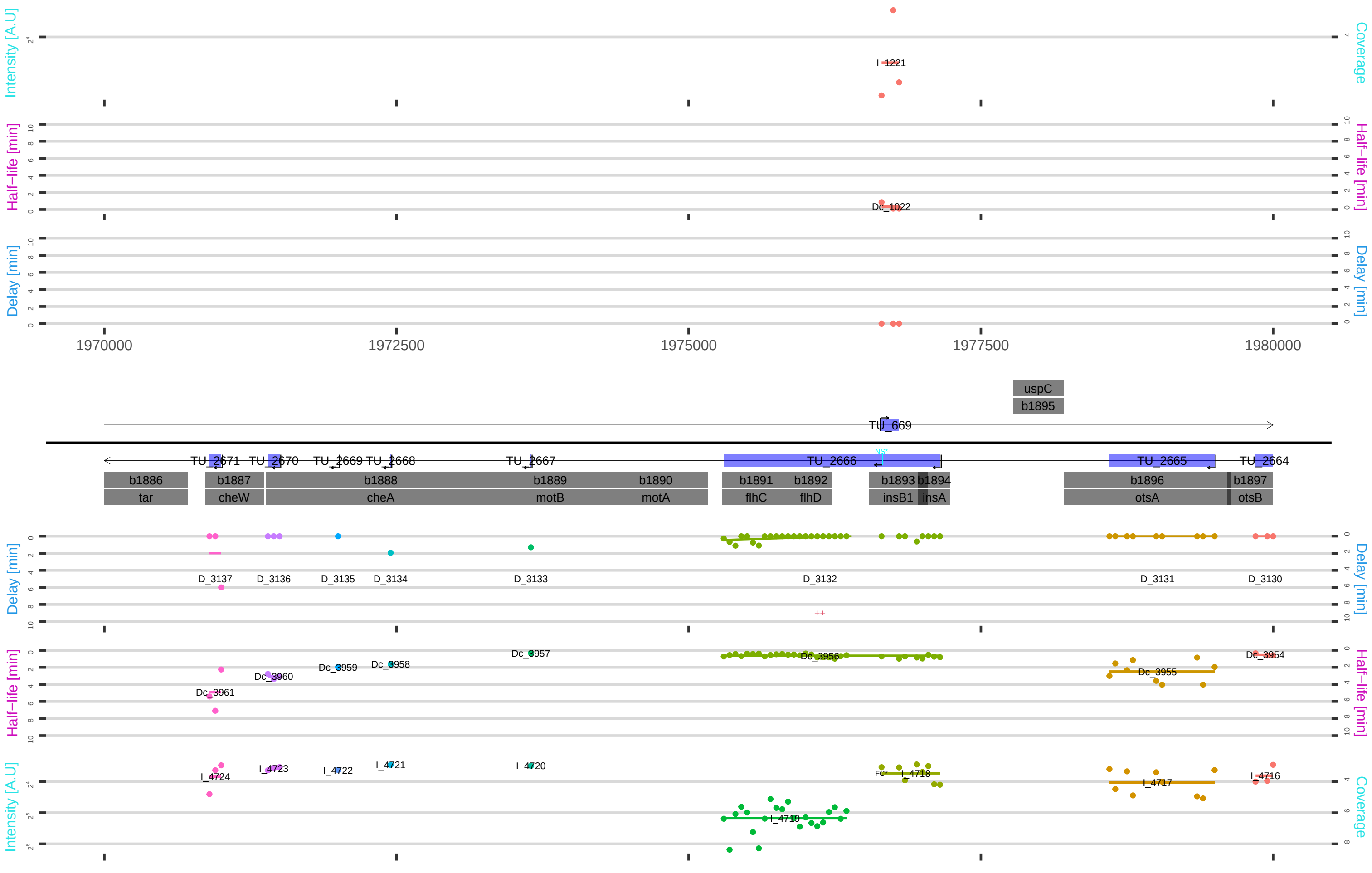

Term: termination (0), NS: new start (1), PS: pausing site (0), iTSS_I: internal starting site (0)

ID: 39644−39782; Term: termination (0), NS: new start (1), PS: pausing site (1), iTSS_I: internal starting site (0)

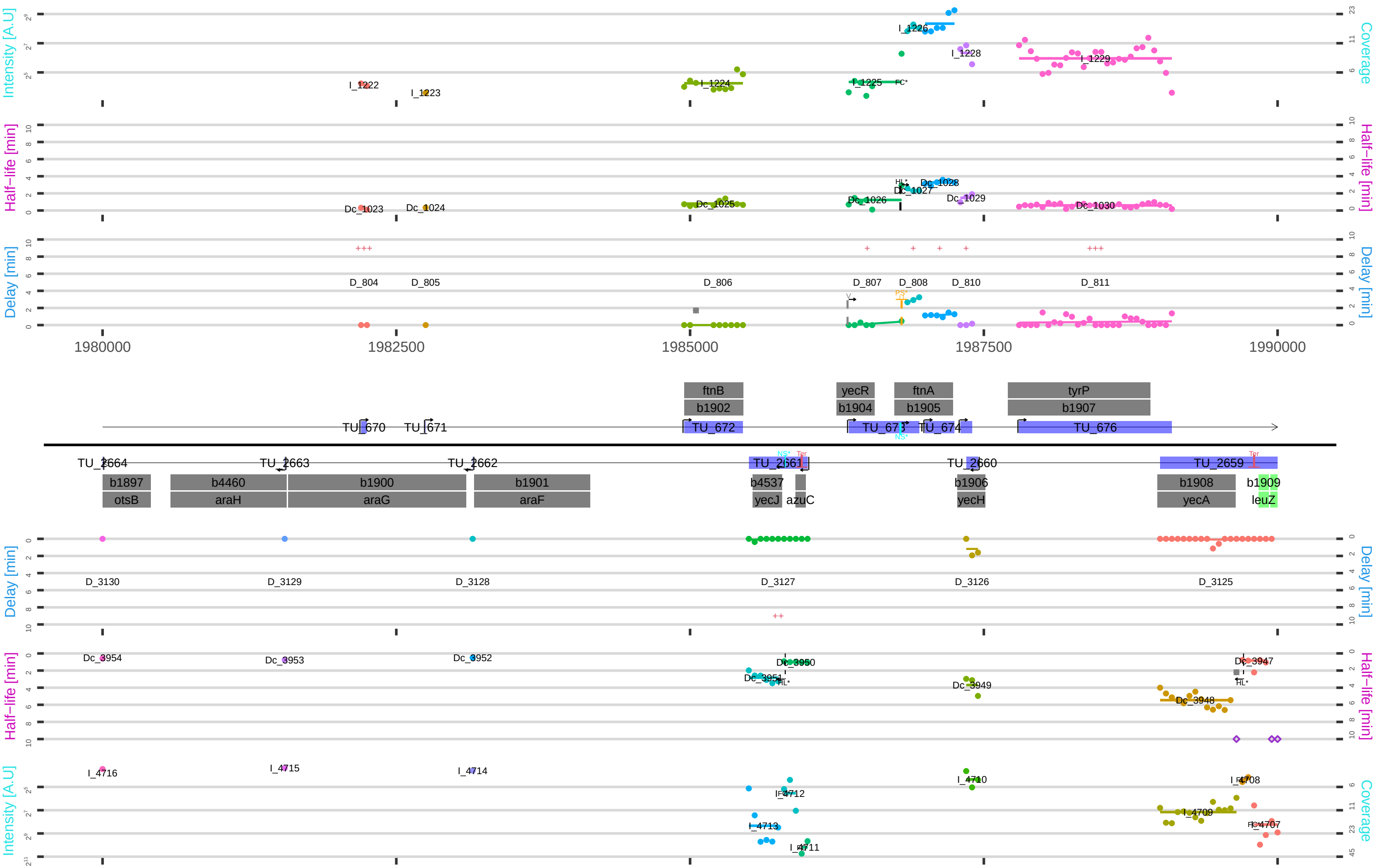

Term: termination (2), NS: new start (1), PS: pausing site (0), iTSS_I: internal starting site (0)

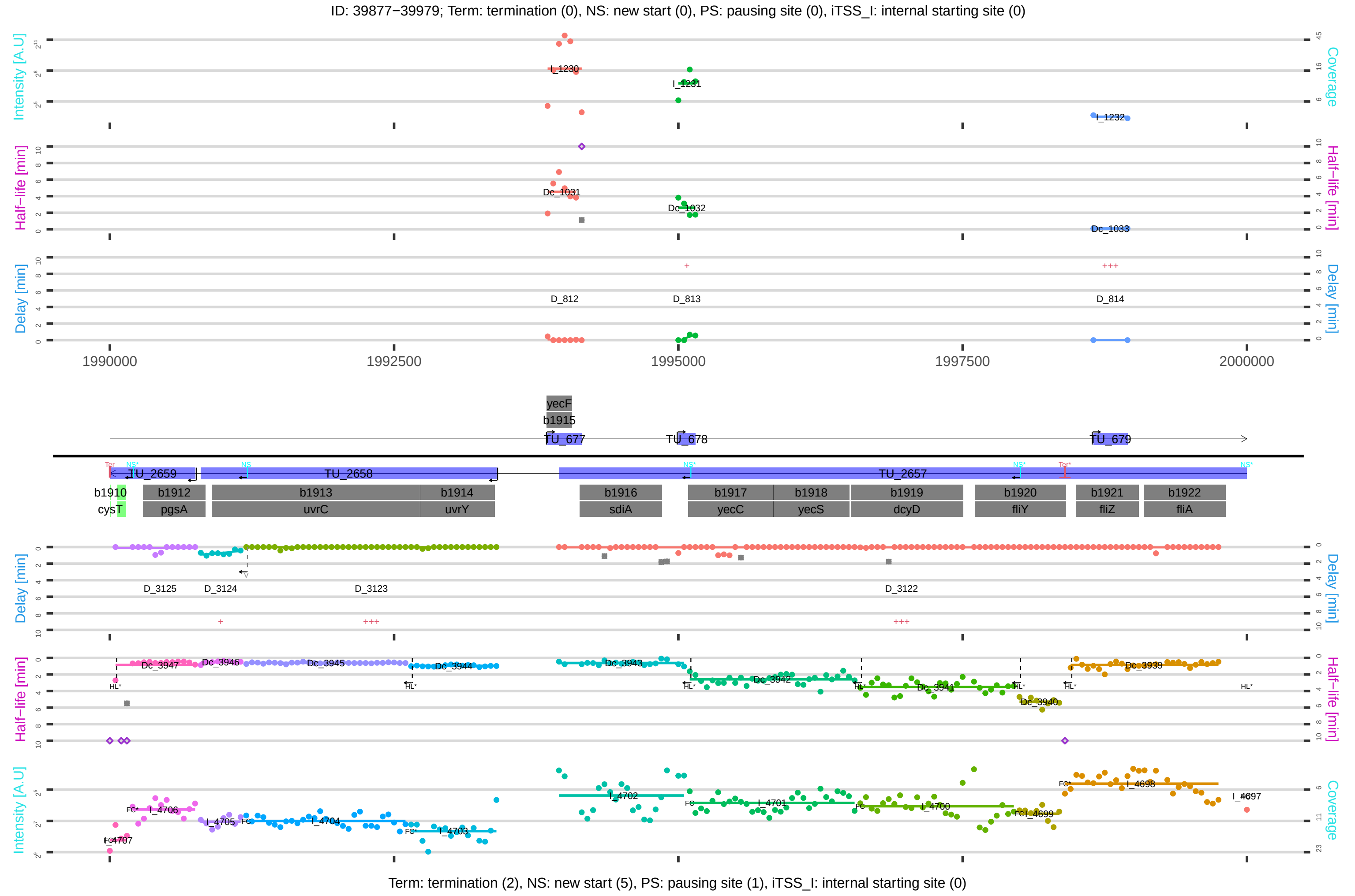
ID: 39877−39979; Term: termination (0), NS: new start (0), PS: pausing site (0), iTSS_I: internal starting site (0)
Intensity [A.U]
Coverage
I_1230
I_1231
I_1232
Half−life [min]
Dc_1031
Dc_1032
Dc_1033
Delay [min]
D_812
D_813
D_814
1990000
1992500
1995000
1997500
2000000
yecF
b1915
TU_677
TU_678
TU_679
TU_2659
TU_2658
TU_2657
b1910
cysT
b1912
pgsA
b1913
uvrC
b1914
uvrY
b1916
sdiA
b1917
yecC
b1918
yecS
b1919
dcyD
b1920
fliY
b1921
fliZ
b1922
fliA
D_3125
D_3124
D_3123
D_3122
Dc_3947
Dc_3946
Dc_3945
Dc_3944
Dc_3943
Dc_3942
Dc_3941
Dc_3940
Dc_3939
I_4707
I_4706
I_4705
I_4704
I_4703
I_4702
I_4701
I_4700
I_4699
I_4698
I_4697
Term: termination (2), NS: new start (5), PS: pausing site (1), iTSS_I: internal starting site (0)

ID: 40009−40156; Term: termination (0), NS: new start (0), PS: pausing site (0), iTSS_I: internal starting site (0)

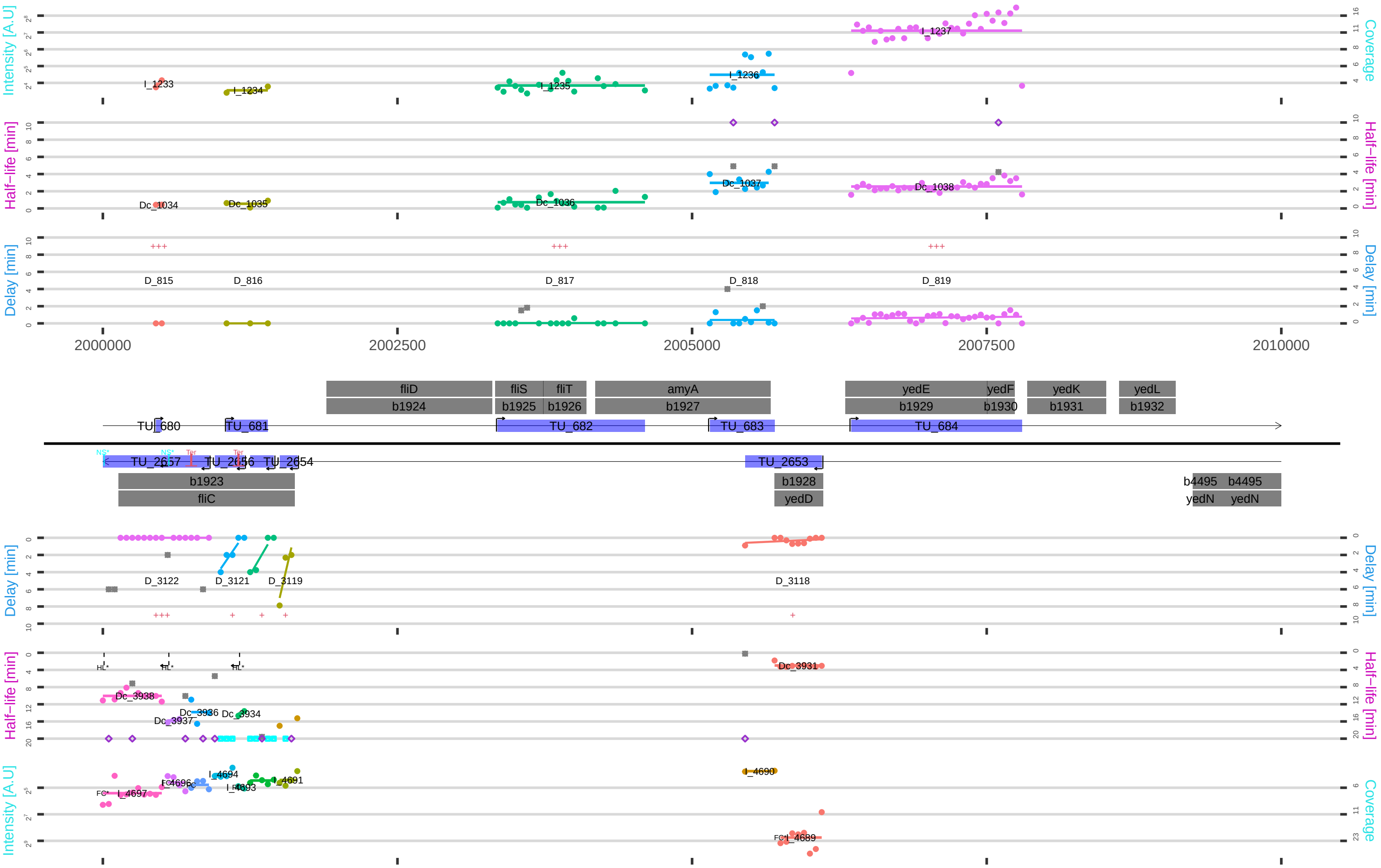

Term: termination (2), NS: new start (2), PS: pausing site (0), iTSS_I: internal starting site (0)

ID: 40210−40400; Term: termination (3), NS: new start (2), PS: pausing site (3), iTSS_I: internal starting site (0)

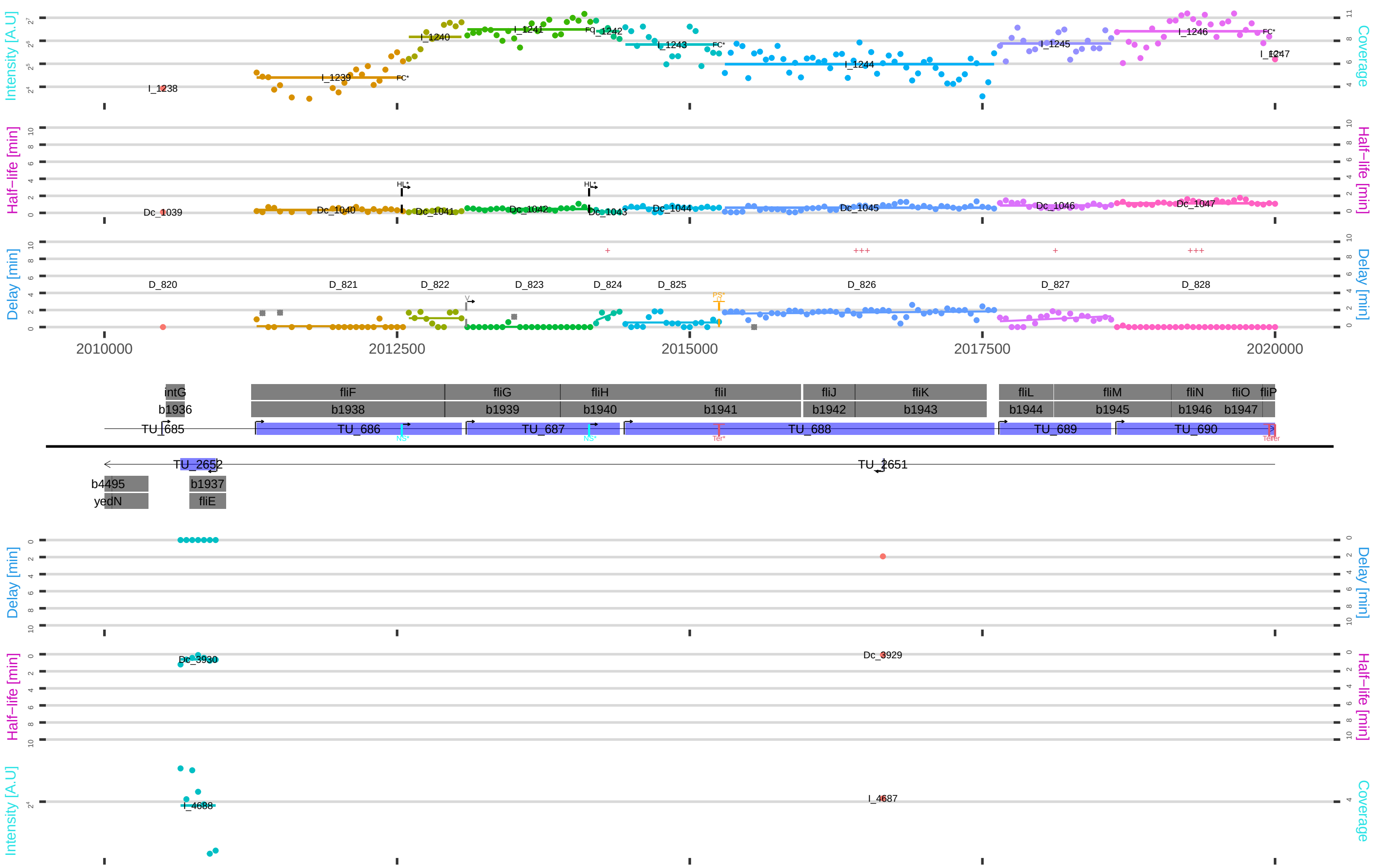

Term: termination (0), NS: new start (0), PS: pausing site (0), iTSS_I: internal starting site (0)

ID: 40400−40570; Term: termination (1), NS: new start (0), PS: pausing site (0), iTSS_I: internal starting site (0)

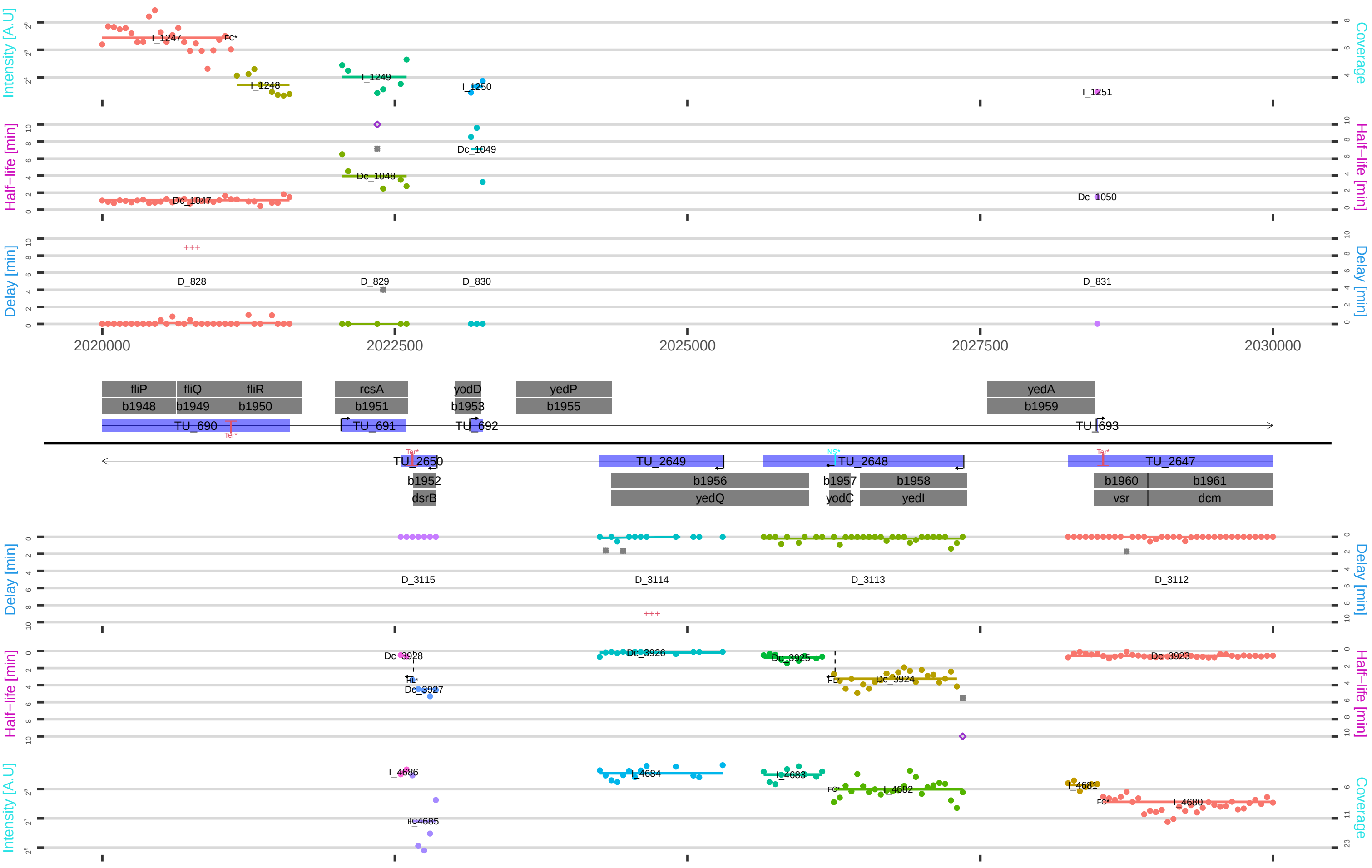

Term: termination (2), NS: new start (1), PS: pausing site (0), iTSS_I: internal starting site (0)

ID: 40655−40797; Term: termination (1), NS: new start (0), PS: pausing site (0), iTSS_I: internal starting site (0)

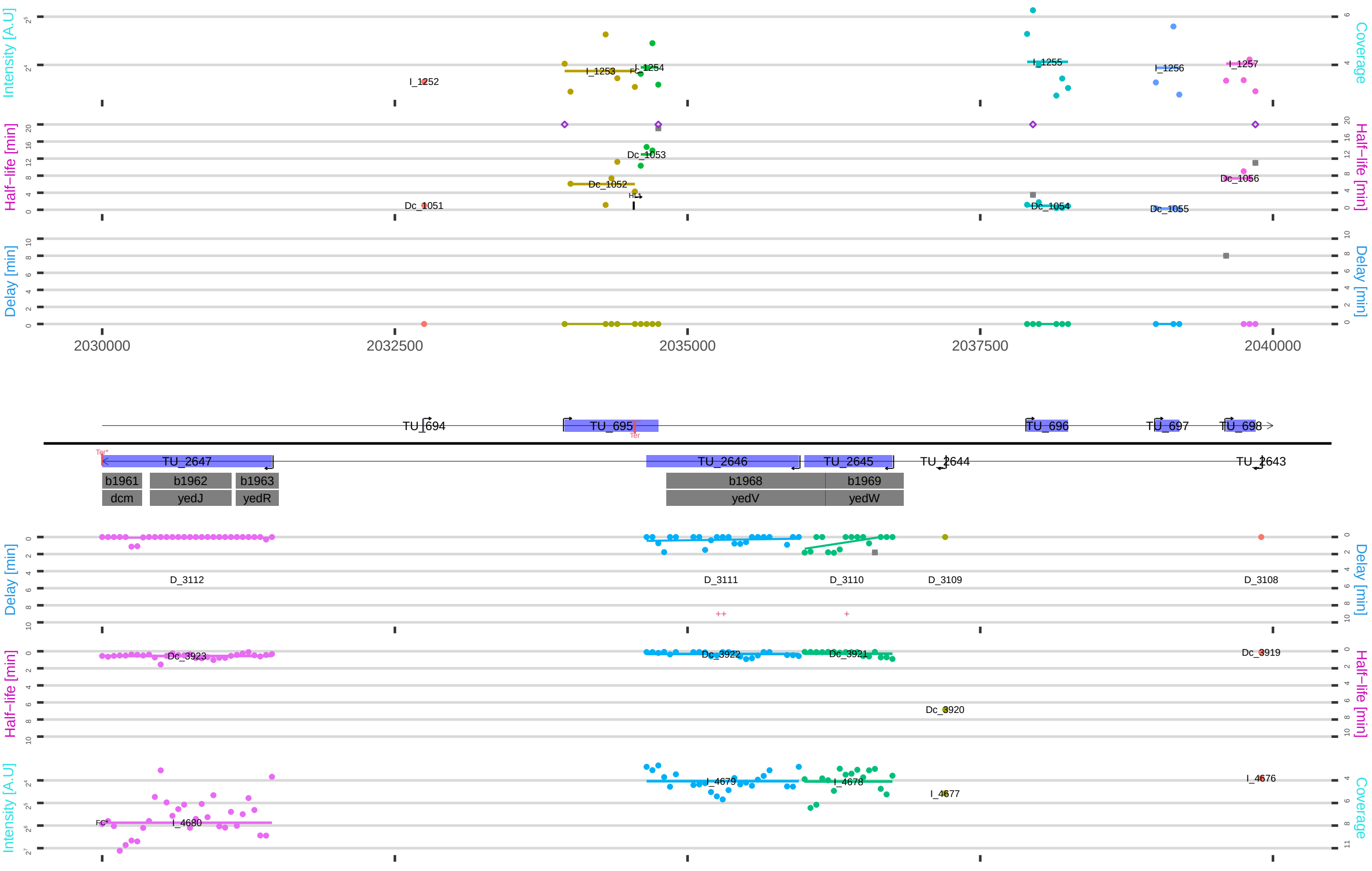

Term: termination (1), NS: new start (0), PS: pausing site (0), iTSS_I: internal starting site (0)

ID: 40802−41000; Term: termination (1), NS: new start (0), PS: pausing site (0), iTSS_I: internal starting site (0)

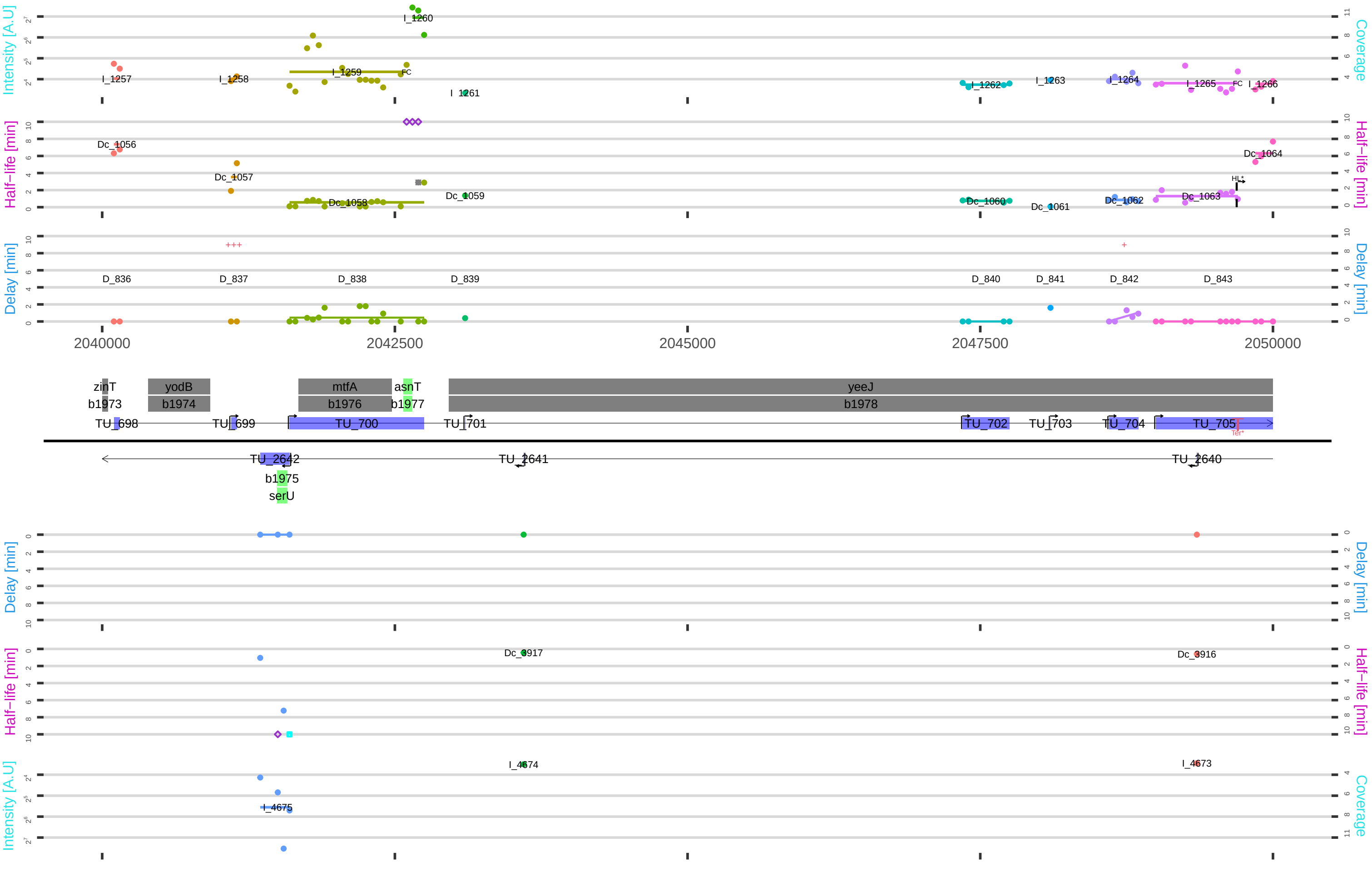

Term: termination (0), NS: new start (0), PS: pausing site (0), iTSS_I: internal starting site (0)

ID: 41000−41161; Term: termination (1), NS: new start (4), PS: pausing site (1), iTSS_I: internal starting site (0)

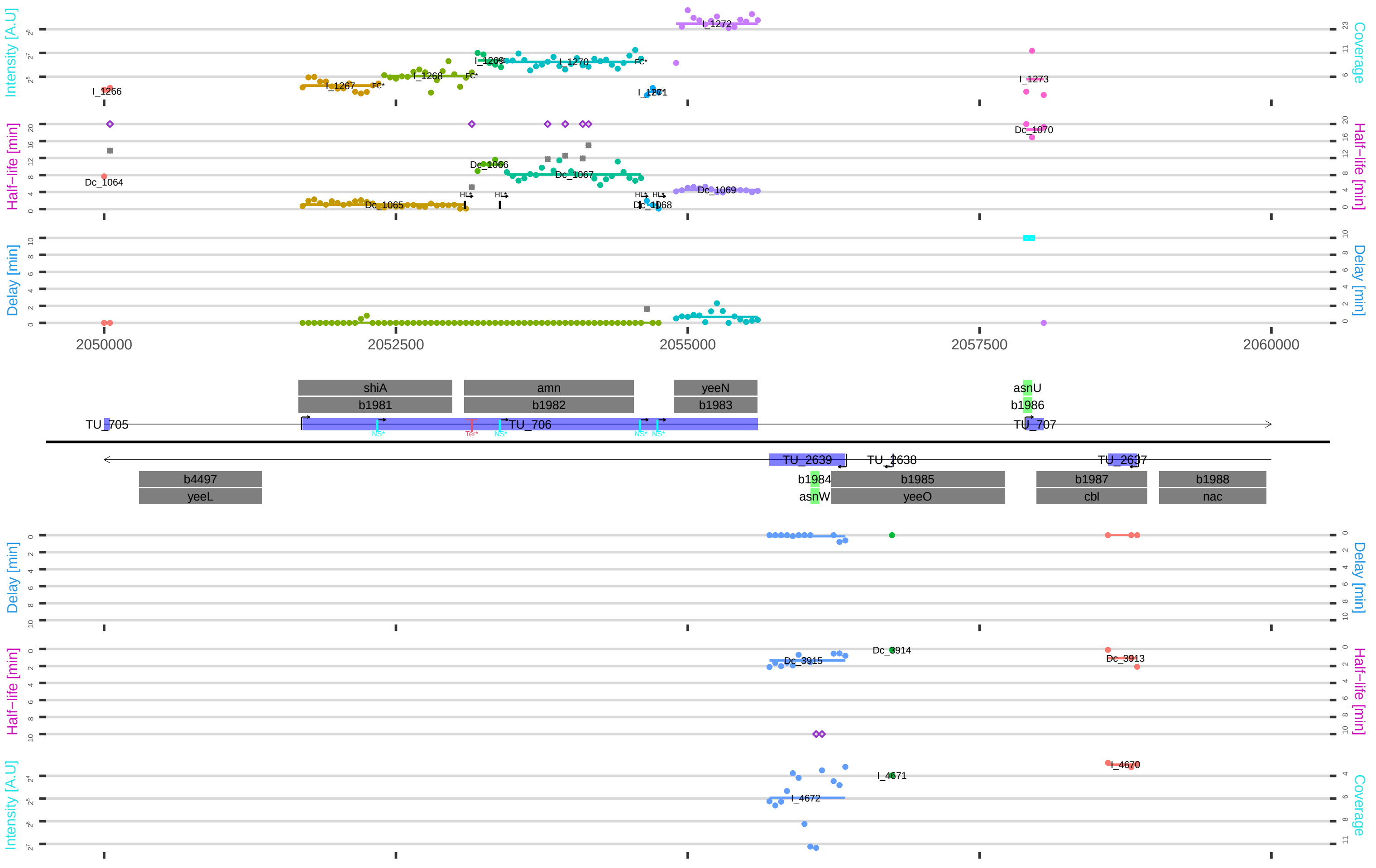

Term: termination (0), NS: new start (0), PS: pausing site (0), iTSS_I: internal starting site (0)

ID: 41206−41400; Term: termination (0), NS: new start (2), PS: pausing site (1), iTSS_I: internal starting site (0)

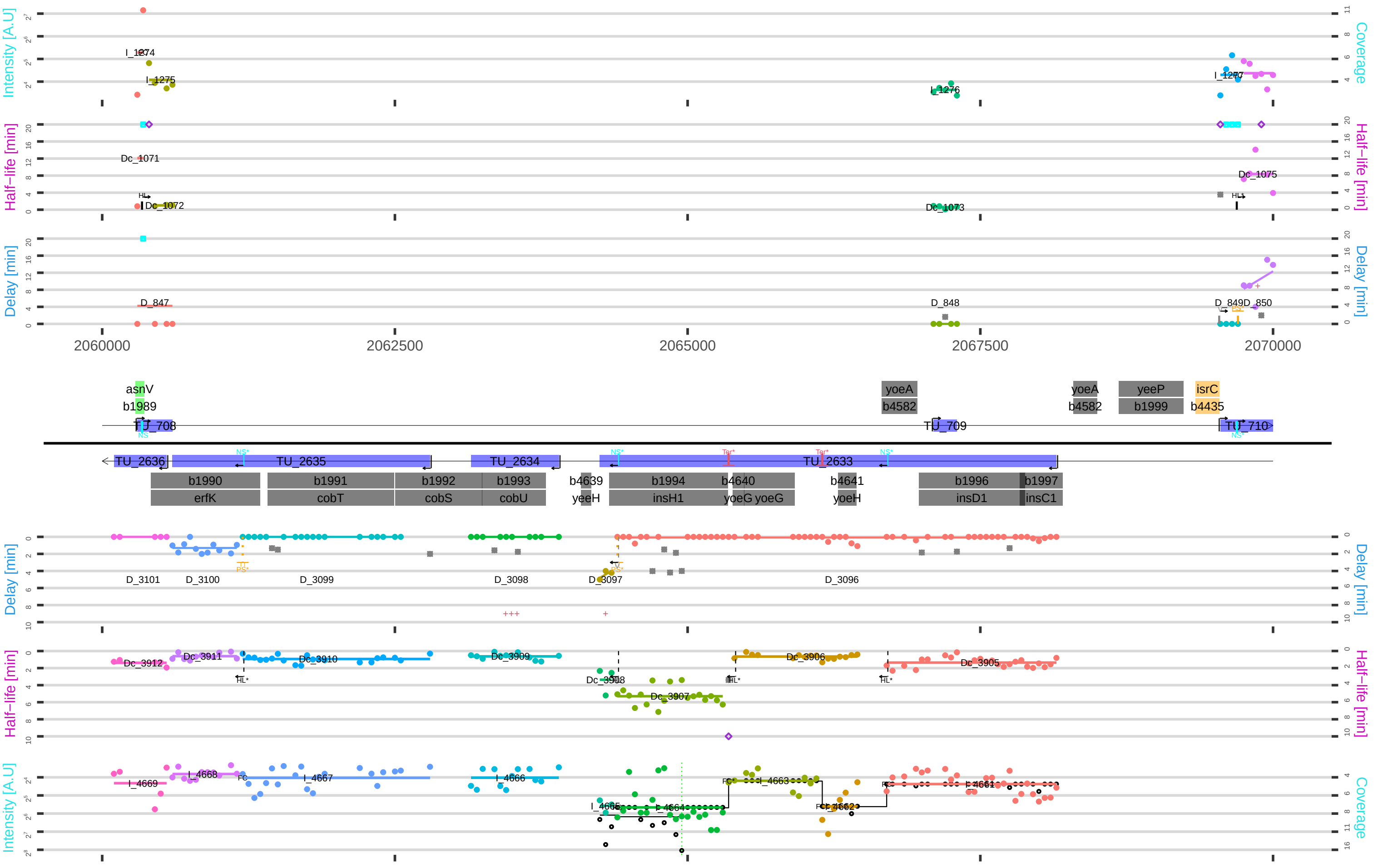

Term: termination (2), NS: new start (3), PS: pausing site (2), iTSS_I: internal starting site (0)

ID: 41400−41526; Term: termination (1), NS: new start (3), PS: pausing site (3), iTSS_I: internal starting site (0)

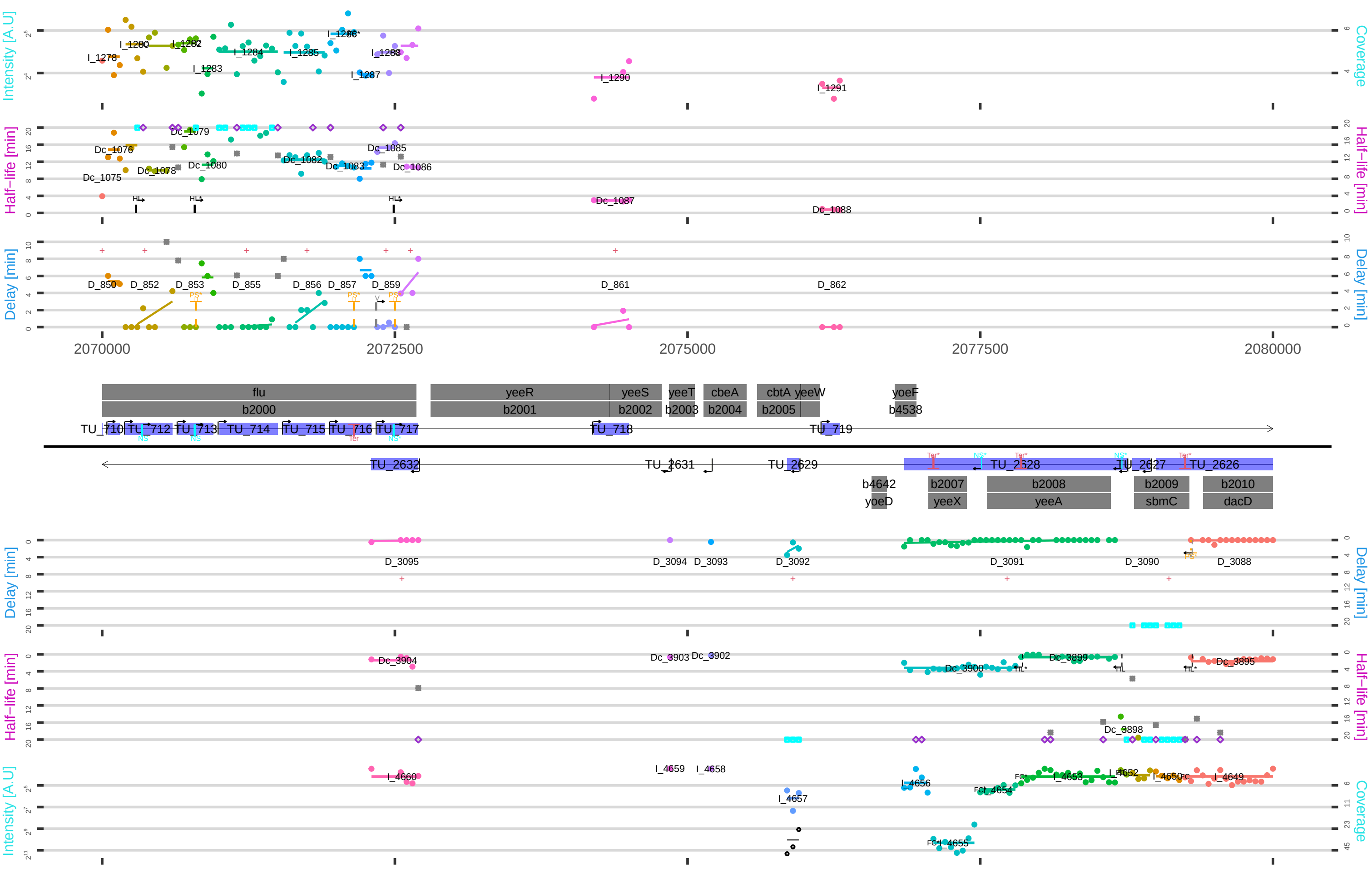

Term: termination (3), NS: new start (2), PS: pausing site (1), iTSS_I: internal starting site (0)

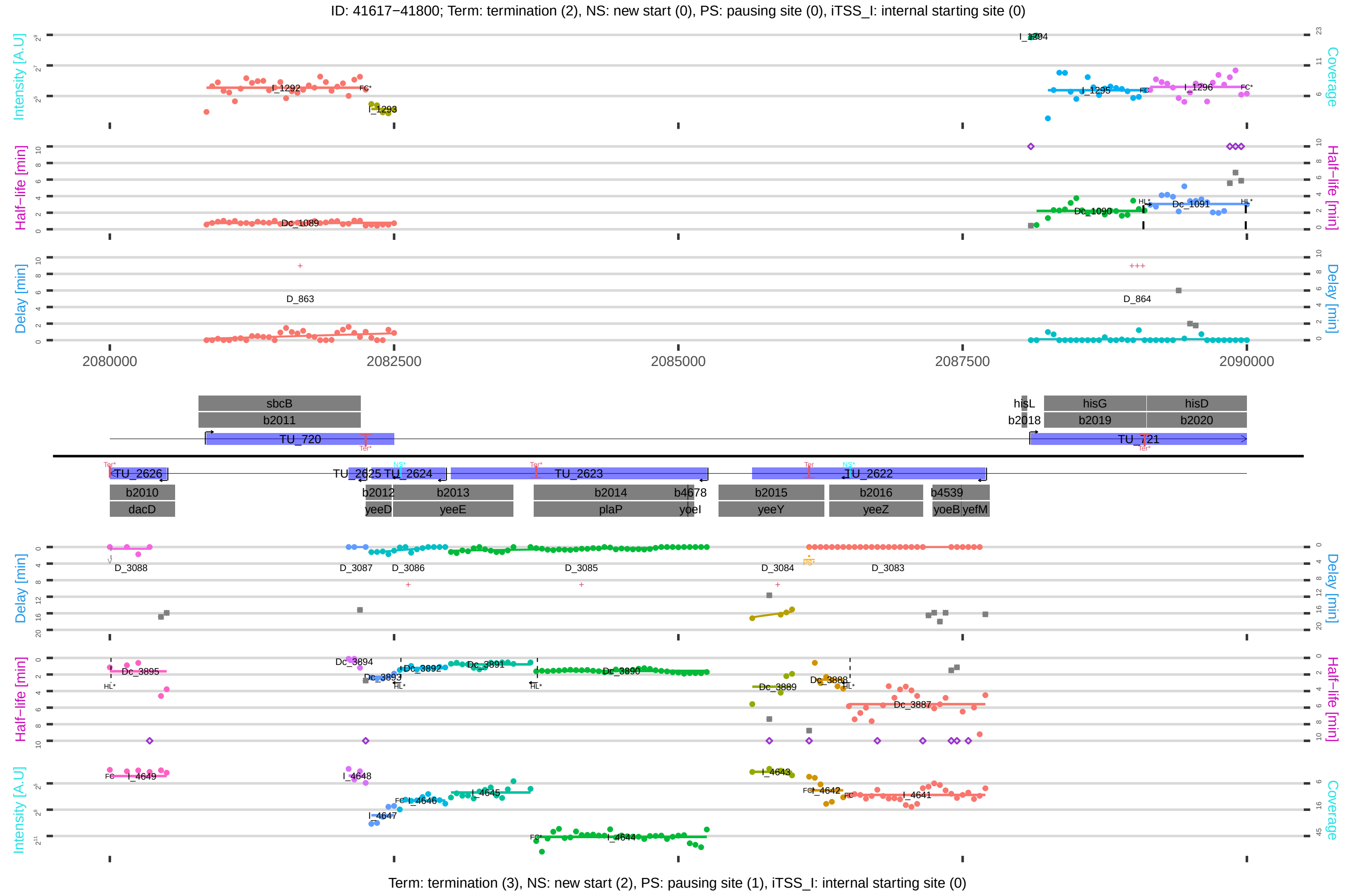

ID: 41617−41800; Term: termination (2), NS: new start (0), PS: pausing site (0), iTSS_I: internal starting site (0)
Intensity [A.U]
Coverage
Half−life [min]
Delay [min]
I_1292
I_1293
I_1294
I_1295
I_1296
Dc_1089
Dc_1090
Dc_1091
D_863
D_864
2080000
2082500
2085000
2087500
2090000
sbcB
b2011
TU_720
hisL
b2018
hisG
b2019
hisD
b2020
TU_721
TU_2626
TU_2625
TU_2624
TU_2623
TU_2622
b2010
dacD
b2012
yeeD
b2013
yeeE
b2014
plaP
b4678
yoeI
b2015
yeeY
b2016
yeeZ
b4539
yoeB
yefM
D_3088
D_3087
D_3086
D_3085
D_3084
D_3083
Dc_3895
Dc_3894
Dc_3893
Dc_3892
Dc_3891
Dc_3890
Dc_3889
Dc_3888
Dc_3887
I_4649
I_4648
I_4647
I_4646
I_4645
I_4644
I_4643
I_4642
I_4641
Term: termination (3), NS: new start (2), PS: pausing site (1), iTSS_I: internal starting site (0)

ID: 41800−41951; Term: termination (0), NS: new start (2), PS: pausing site (2), iTSS_I: internal starting site (0)

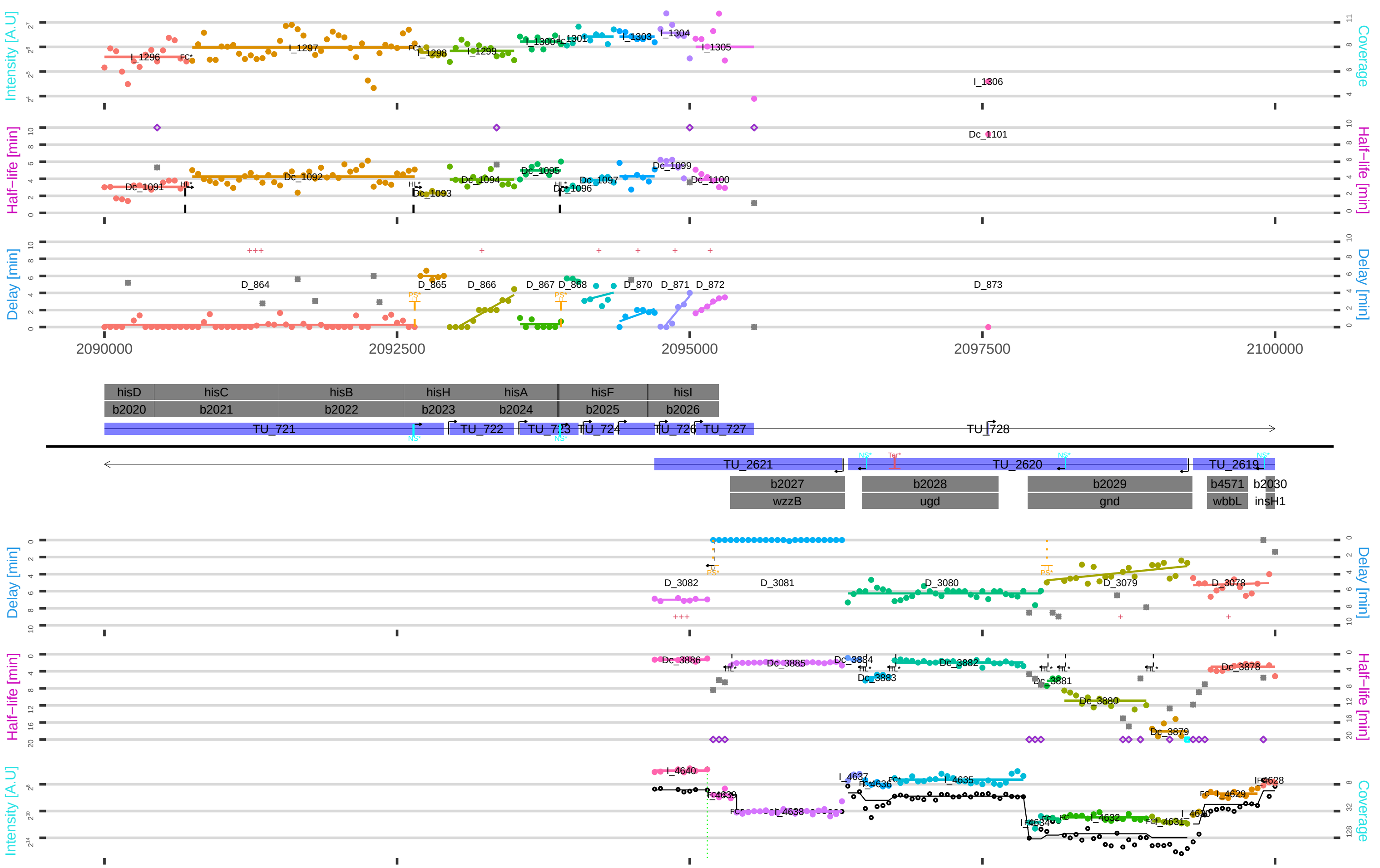

Term: termination (1), NS: new start (3), PS: pausing site (2), iTSS_I: internal starting site (0)

ID: 143589−143389; FC*: significant t−test of two consecutive segments; Term: termination, NS: new start, PS: pausing site,iTSS_I: internal starting site,TI: transcription interferance.

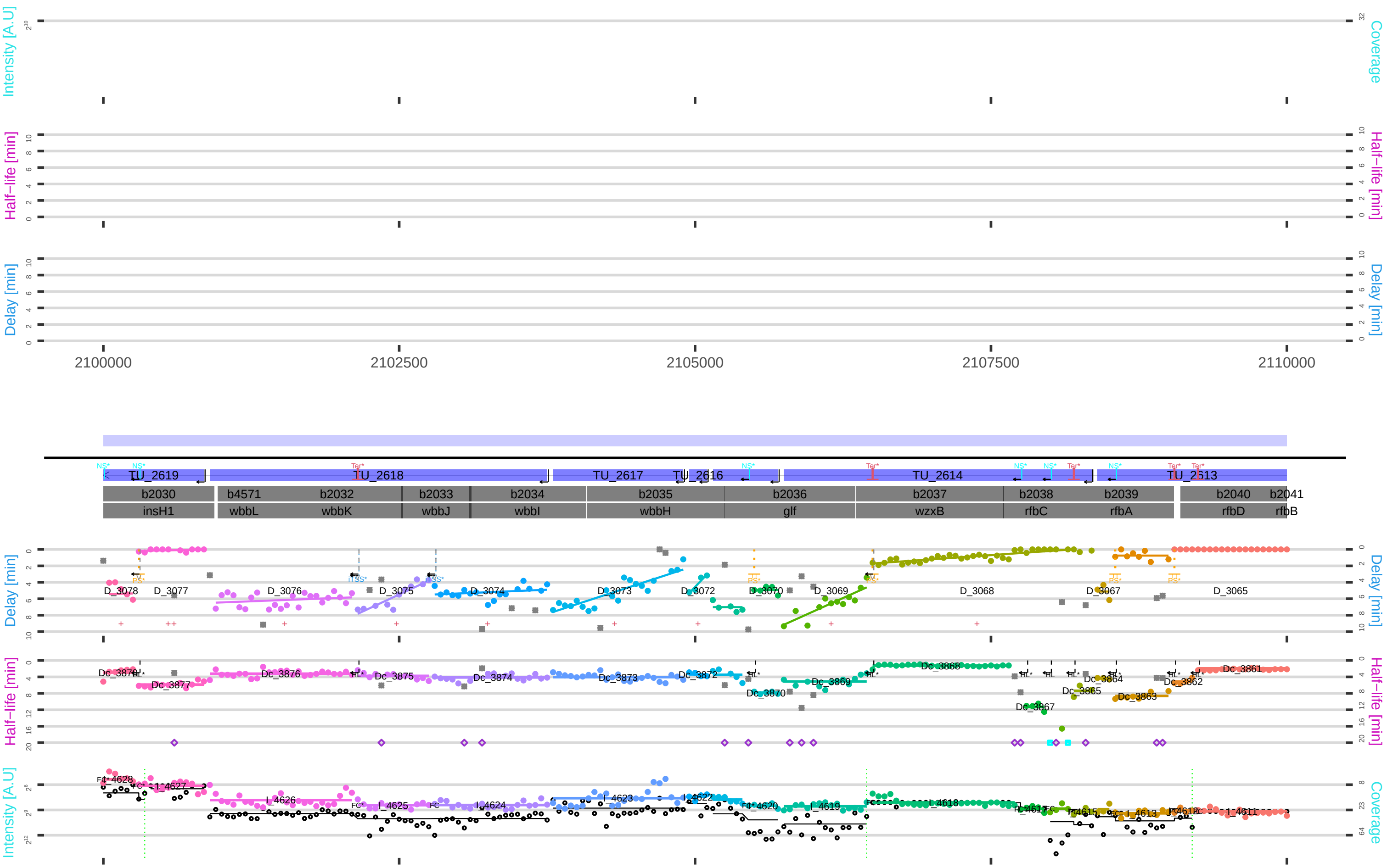

Term: termination (5), NS: new start (6), PS: pausing site (5), iTSS_I: internal starting site (2)

ID: 143389−143222; FC*: significant t−test of two consecutive segments; Term: termination, NS: new start, PS: pausing site,iTSS_I: internal starting site,TI: transcription interferance.

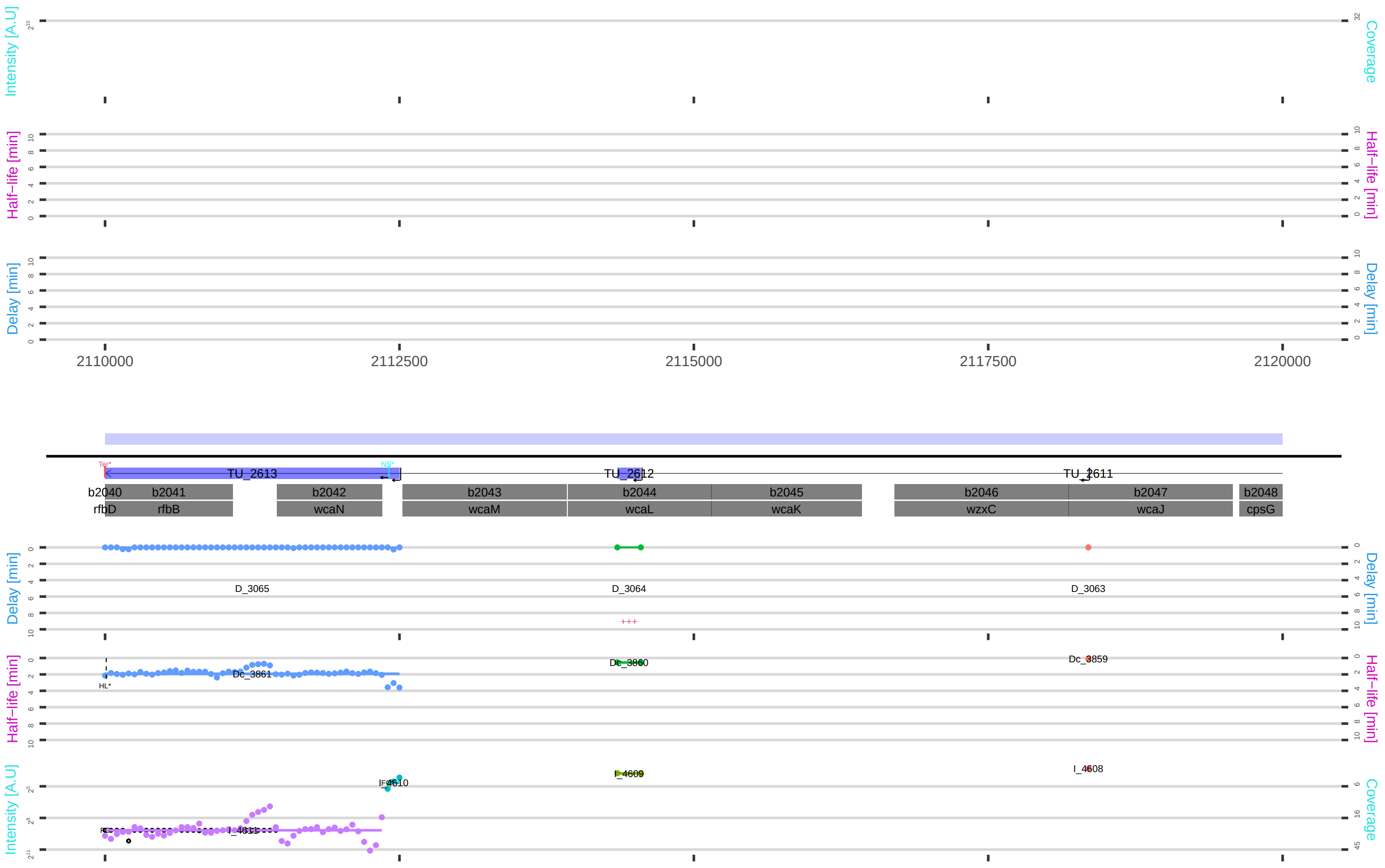

Term: termination (1), NS: new start (1), PS: pausing site (0), iTSS_I: internal starting site (0)

ID: 42517−42517; Term: termination (0), NS: new start (0), PS: pausing site (0), iTSS_I: internal starting site (0)

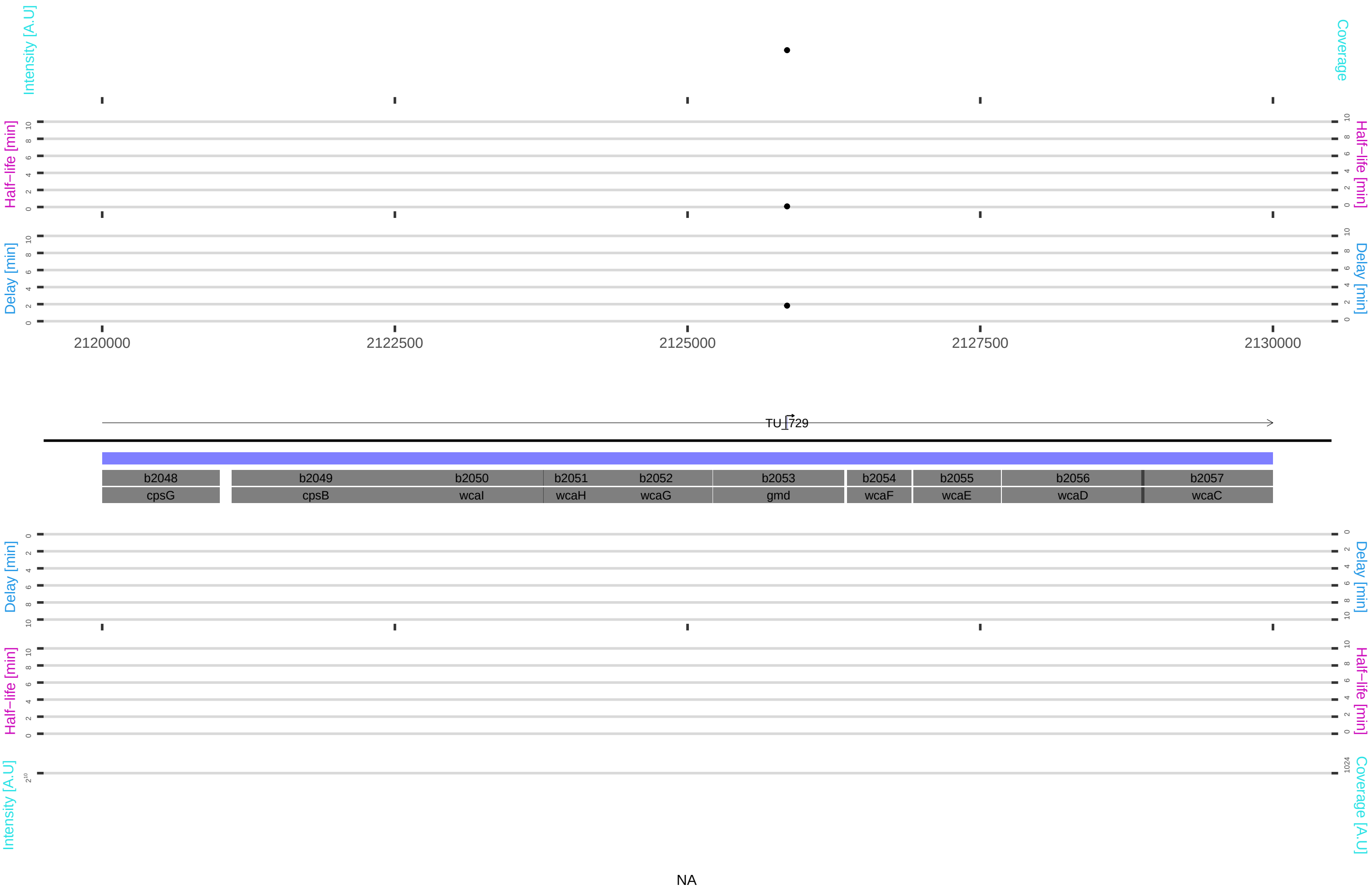

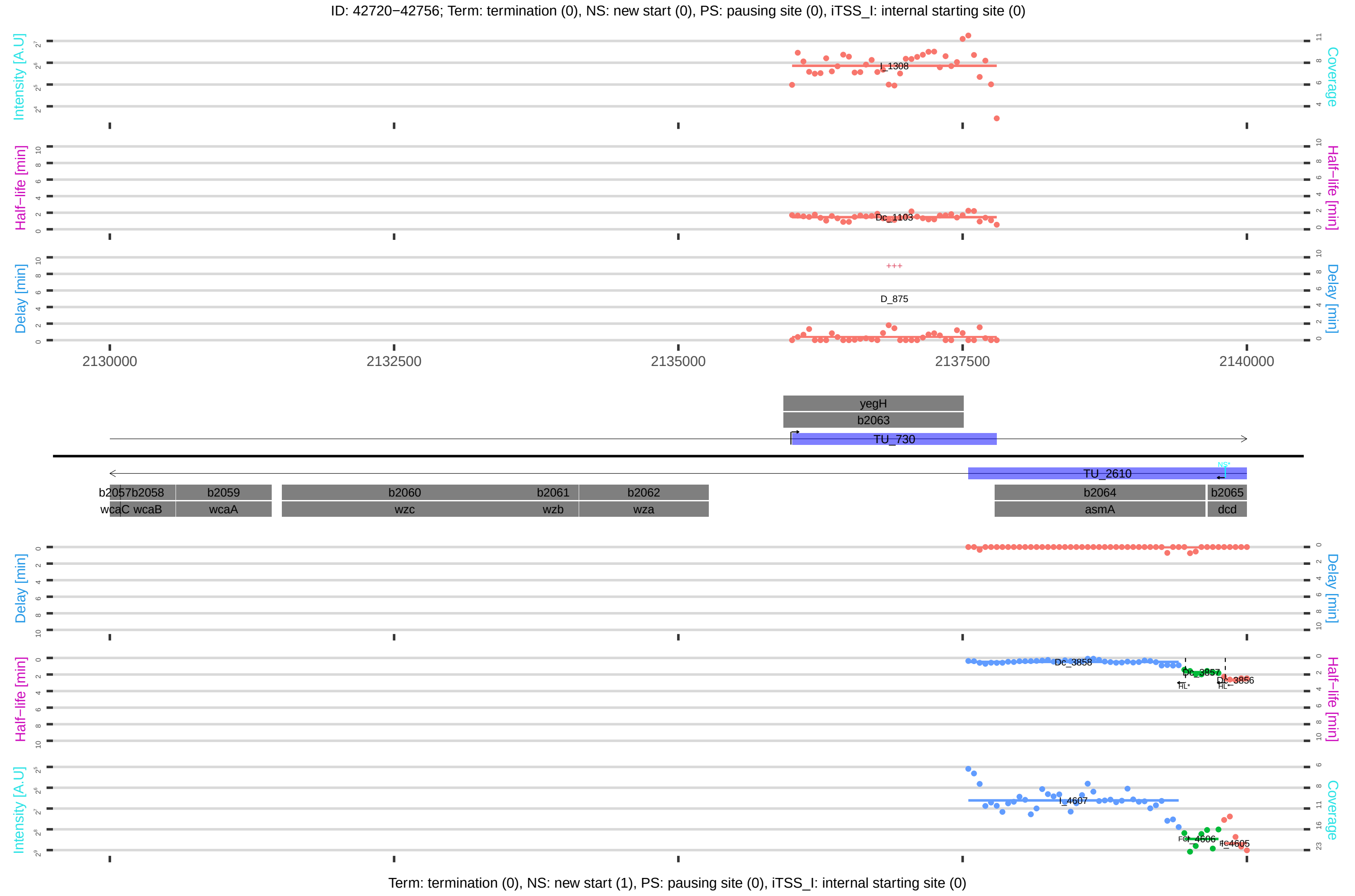
ID: 42720−42756; Term: termination (0), NS: new start (0), PS: pausing site (0), iTSS_I: internal starting site (0)
Intensity [A.U]
Coverage
I_1308
Half−life [min]
Dc_1103
Delay [min]
+++
D_875
2130000
2132500
2135000
2137500
2140000
yegH
b2063
TU_730
TU_2610
NS*
b2057
b2058
b2059
b2060
b2061
b2062
b2064
b2065
wcaC
wcaB
wcaA
wzc
wzb
wza
asmA
dcd
Dc_3858
Dc_3857
Dc_3856
HL*
I_4607
I_4606
I_4605
FC*
Term: termination (0), NS: new start (1), PS: pausing site (0), iTSS_I: internal starting site (0)

ID: 42832−42949; Term: termination (1), NS: new start (0), PS: pausing site (0), iTSS_I: internal starting site (0)

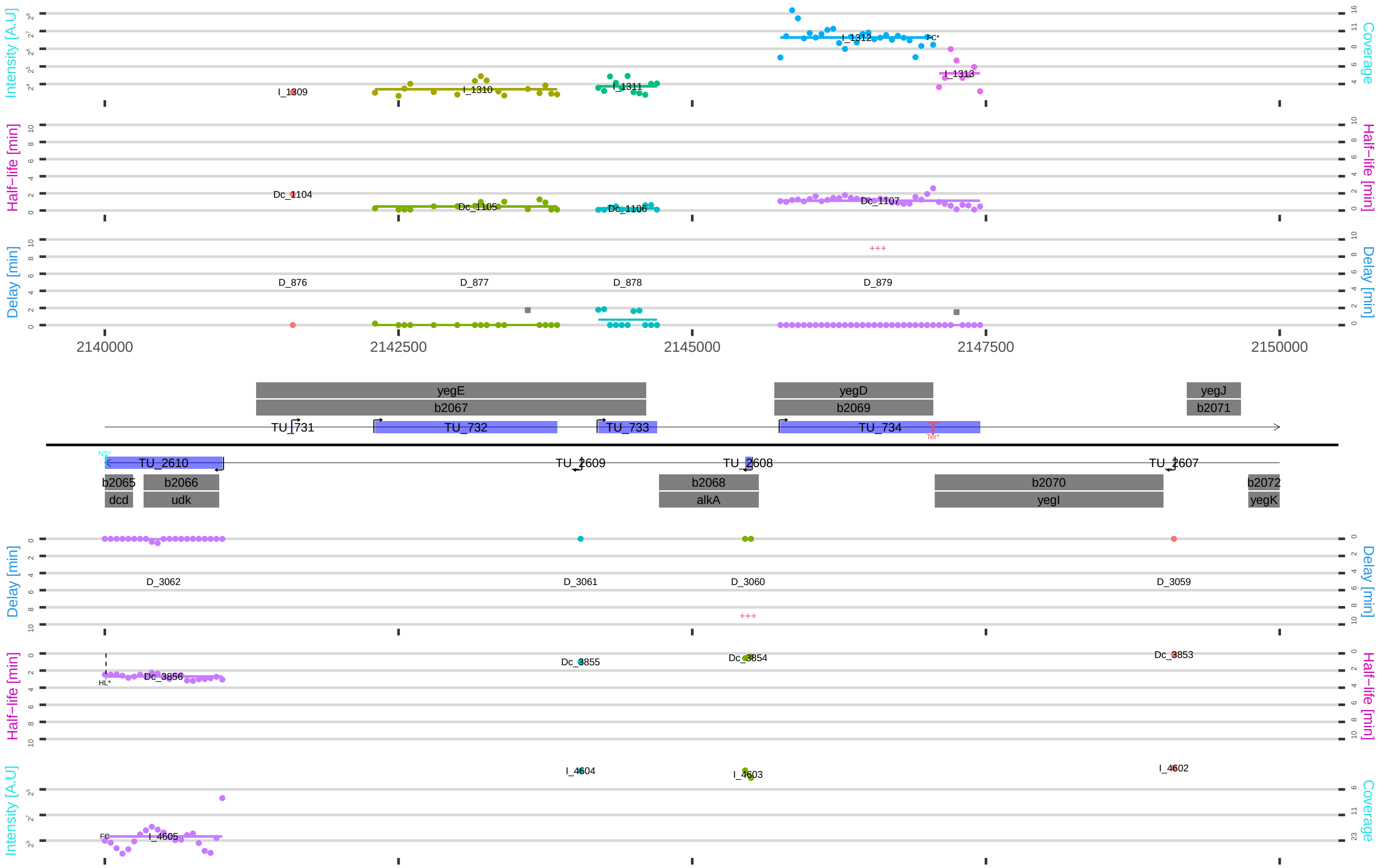

Term: termination (0), NS: new start (1), PS: pausing site (0), iTSS_I: internal starting site (0)

ID: 43028−43195; Term: termination (0), NS: new start (1), PS: pausing site (0), iTSS_I: internal starting site (0)

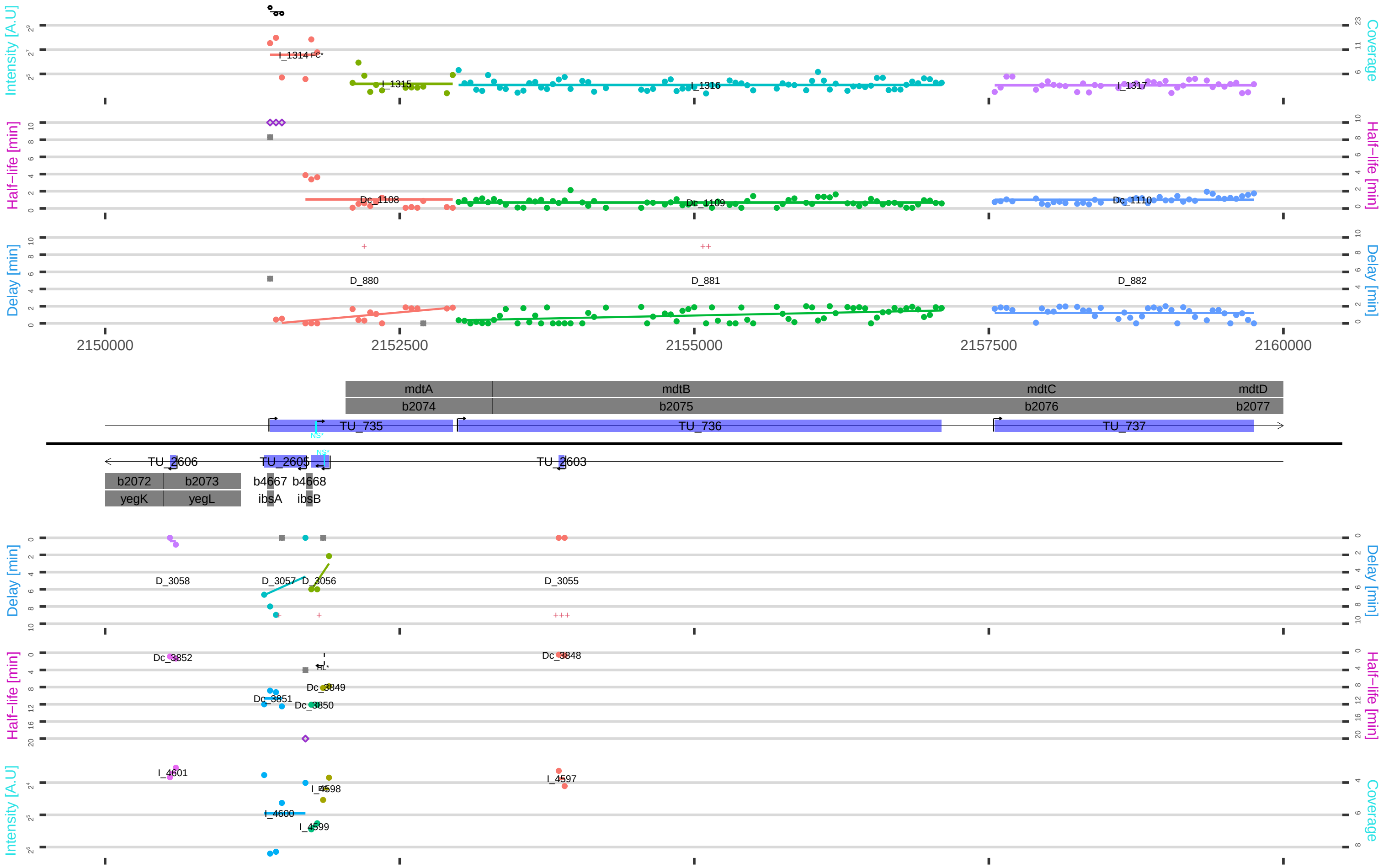

Term: termination (0), NS: new start (1), PS: pausing site (0), iTSS_I: internal starting site (0)

ID: 43203−43391; Term: termination (0), NS: new start (2), PS: pausing site (0), iTSS_I: internal starting site (0)

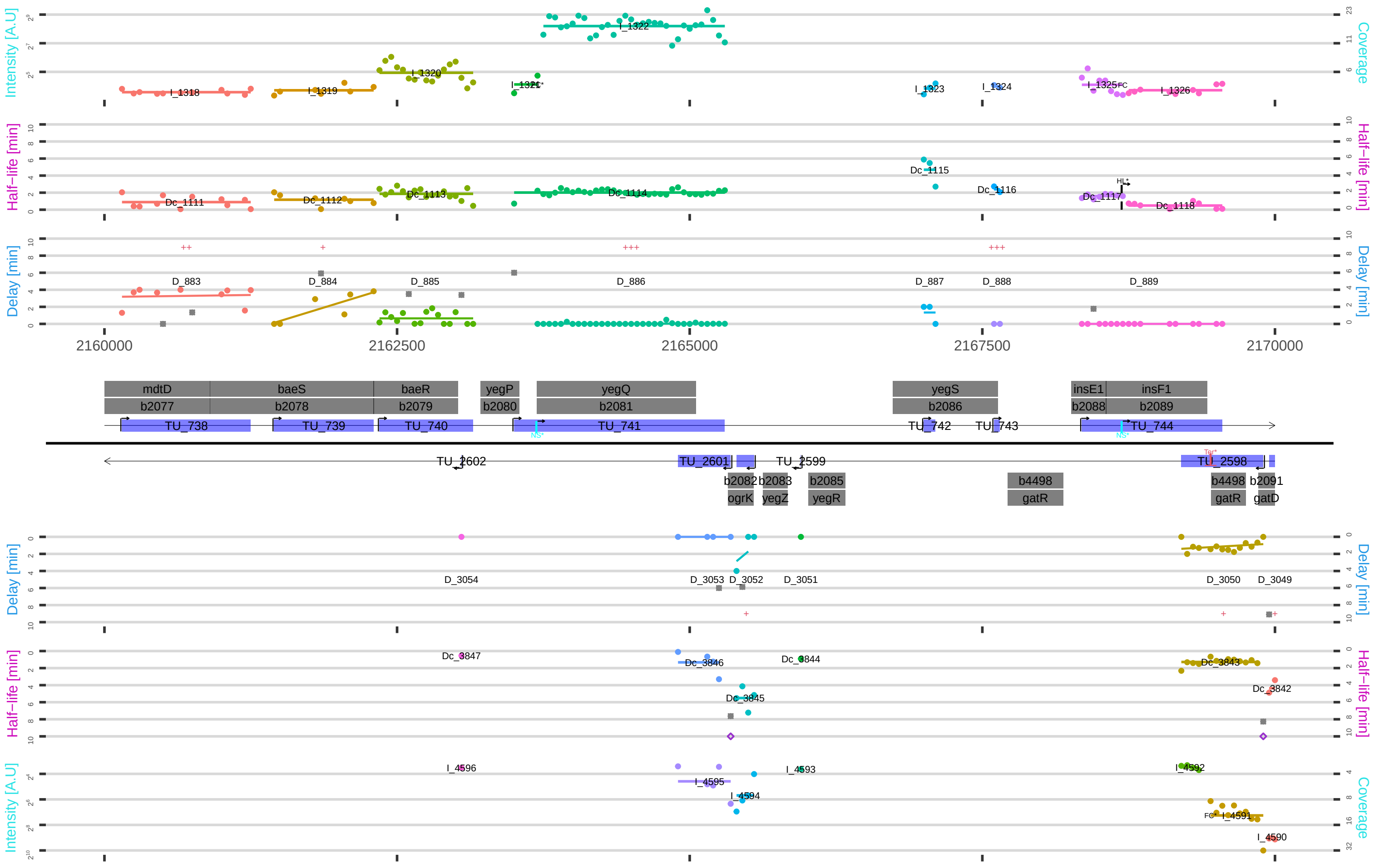

Term: termination (1), NS: new start (0), PS: pausing site (0), iTSS_I: internal starting site (0)

ID: 43449−43600; Term: termination (1), NS: new start (0), PS: pausing site (1), iTSS_I: internal starting site (0)

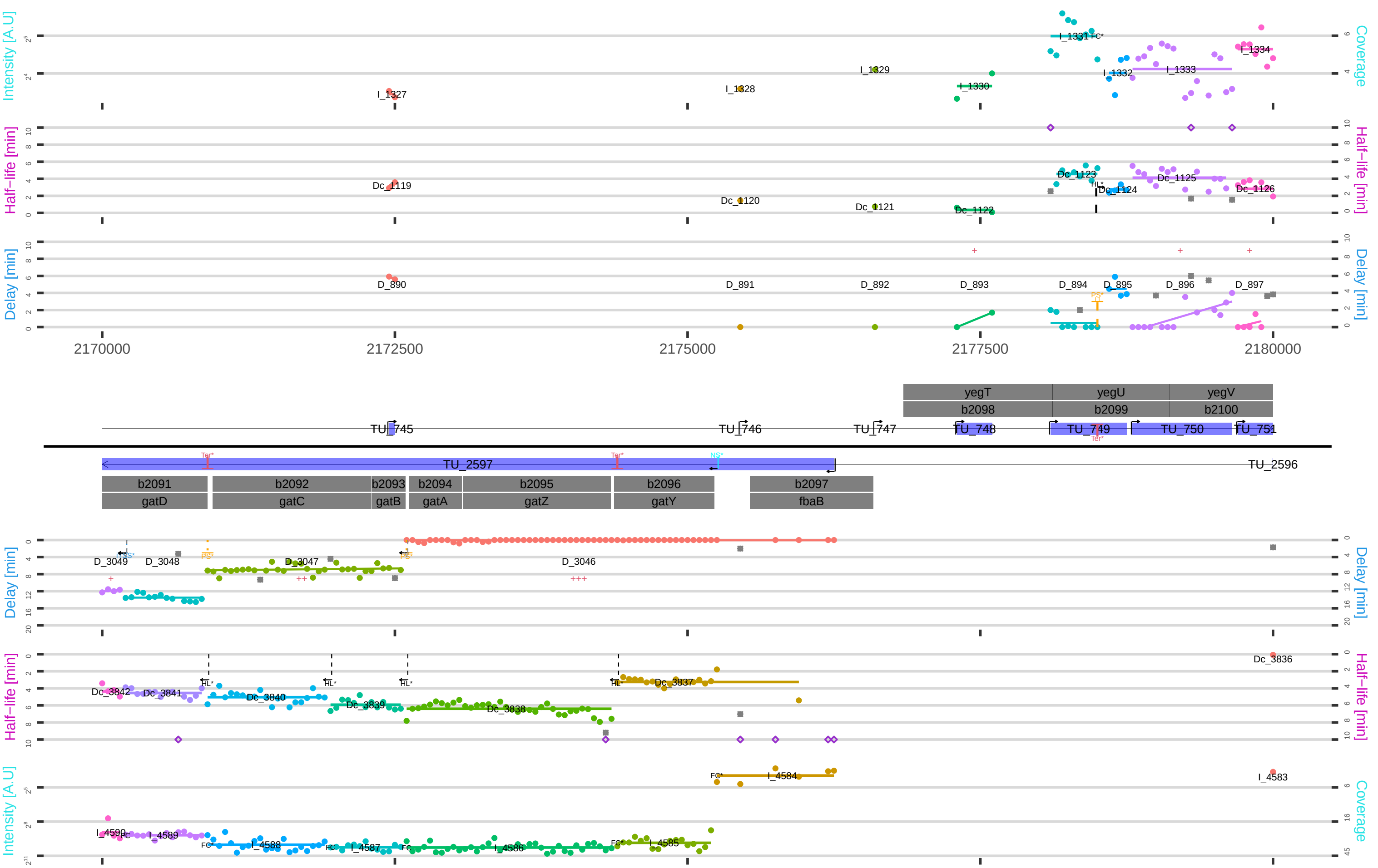

Term: termination (2), NS: new start (1), PS: pausing site (2), iTSS_I: internal starting site (1)

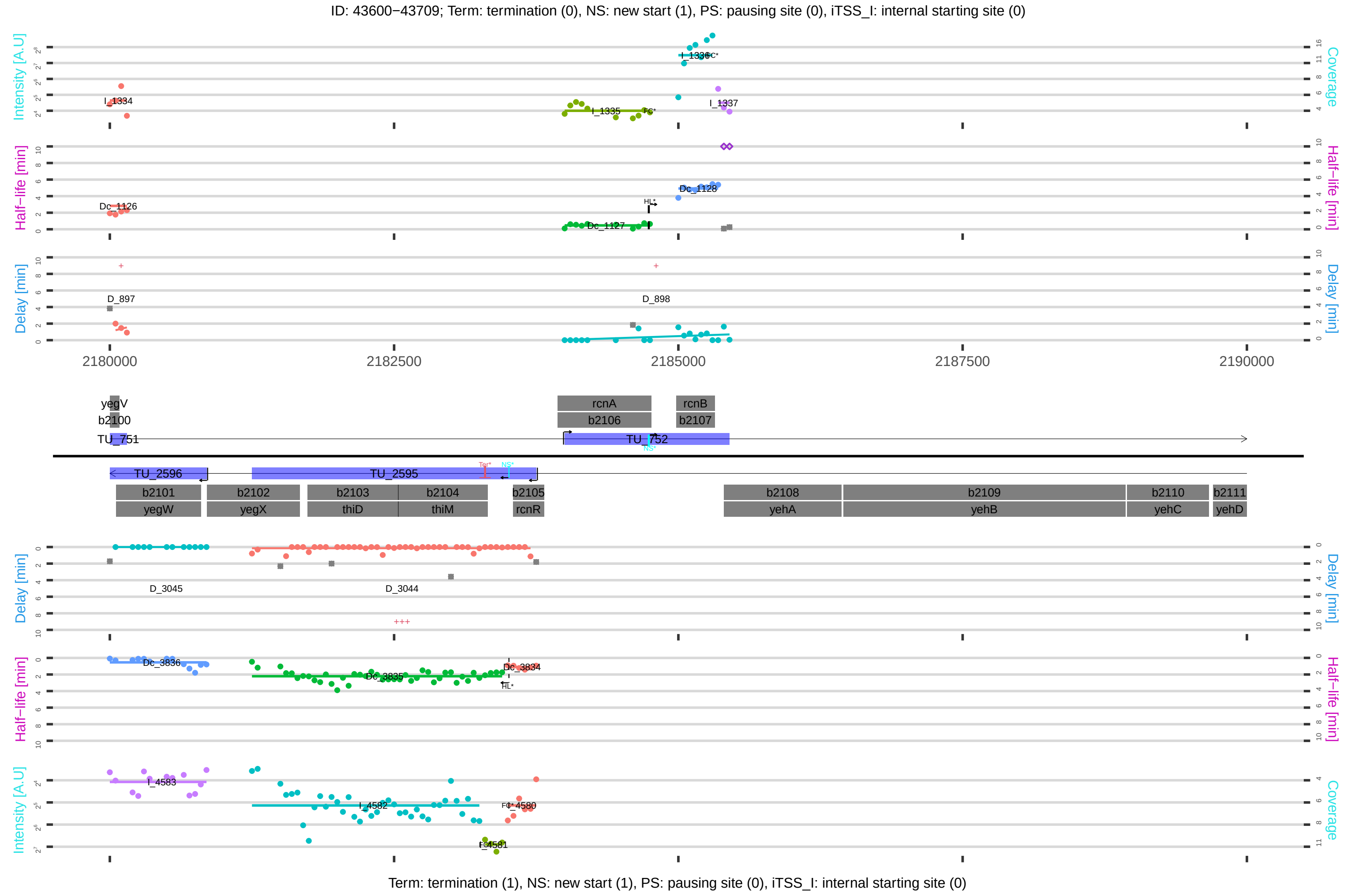
ID: 43600−43709; Term: termination (0), NS: new start (1), PS: pausing site (0), iTSS_I: internal starting site (0)
Intensity [A.U]
Coverage
Half−life [min]
Delay [min]
I_1334
I_1335
I_1336
I_1337
Dc_1126
Dc_1127
Dc_1128
D_897
D_898
2180000
2182500
2185000
2187500
2190000
yegV
b2100
TU_751
rcnA
b2106
rcnB
b2107
TU_752
TU_2596
TU_2595
b2101
yegW
b2102
yegX
b2103
thiD
b2104
thiM
b2105
rcnR
b2108
yehA
b2109
yehB
b2110
yehC
b2111
yehD
D_3045
D_3044
Dc_3836
Dc_3835
Dc_3834
I_4583
I_4582
I_4580
I_4581
Term: termination (1), NS: new start (1), PS: pausing site (0), iTSS_I: internal starting site (0)

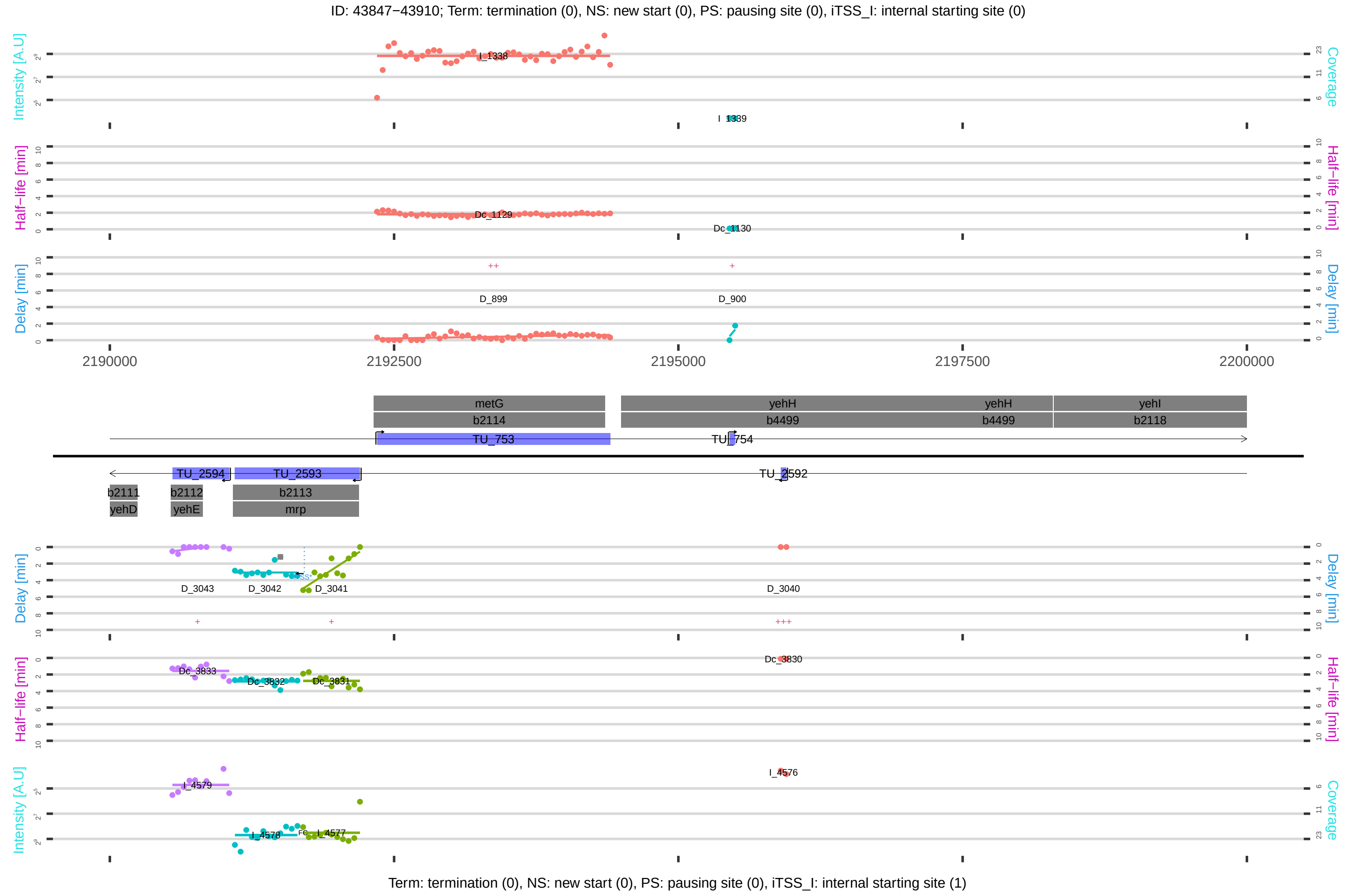
ID: 43847−43910; Term: termination (0), NS: new start (0), PS: pausing site (0), iTSS_I: internal starting site (0)
Intensity [A.U]
Coverage
I_1338
I_1339
Half−life [min]
Dc_1129
Dc_1130
Delay [min]
D_899
D_900
2190000
2192500
2195000
2197500
2200000
metG
b2114
yehH
b4499
yehH
b4499
yehI
b2118
TU_753
TU_754
TU_2594
TU_2593
TU_2592
b2111
yehD
b2112
yehE
b2113
mrp
D_3043
D_3042
D_3041
D_3040
Dc_3833
Dc_3832
Dc_3831
Dc_3830
I_4579
I_4578
I_4577
I_4576
Term: termination (0), NS: new start (0), PS: pausing site (0), iTSS_I: internal starting site (1)

ID: 44059−44194; Term: termination (0), NS: new start (0), PS: pausing site (0), iTSS_I: internal starting site (0)

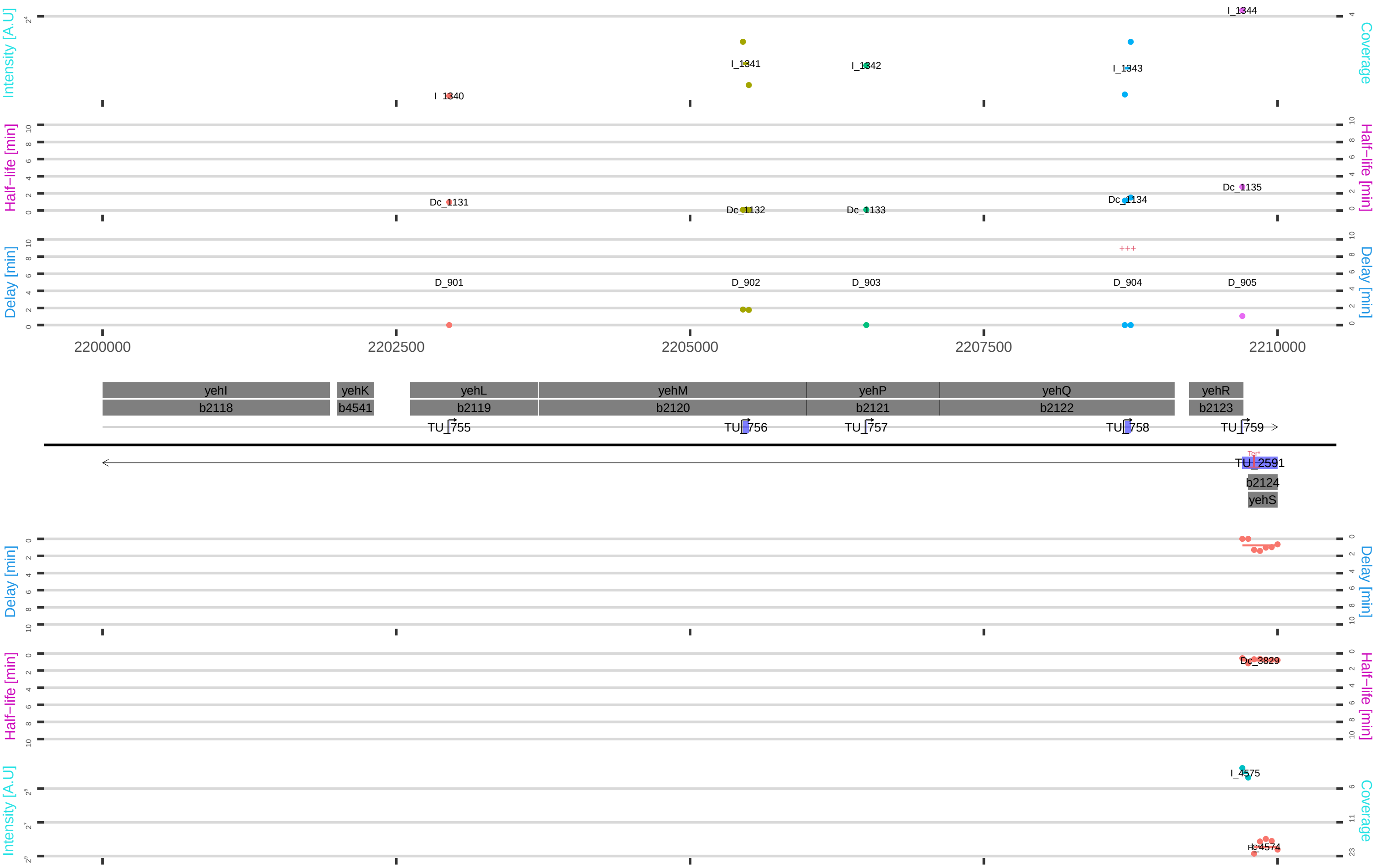

Term: termination (1), NS: new start (0), PS: pausing site (0), iTSS_I: internal starting site (0)

ID: 44275−44278; Term: termination (0), NS: new start (0), PS: pausing site (0), iTSS_I: internal starting site (0)

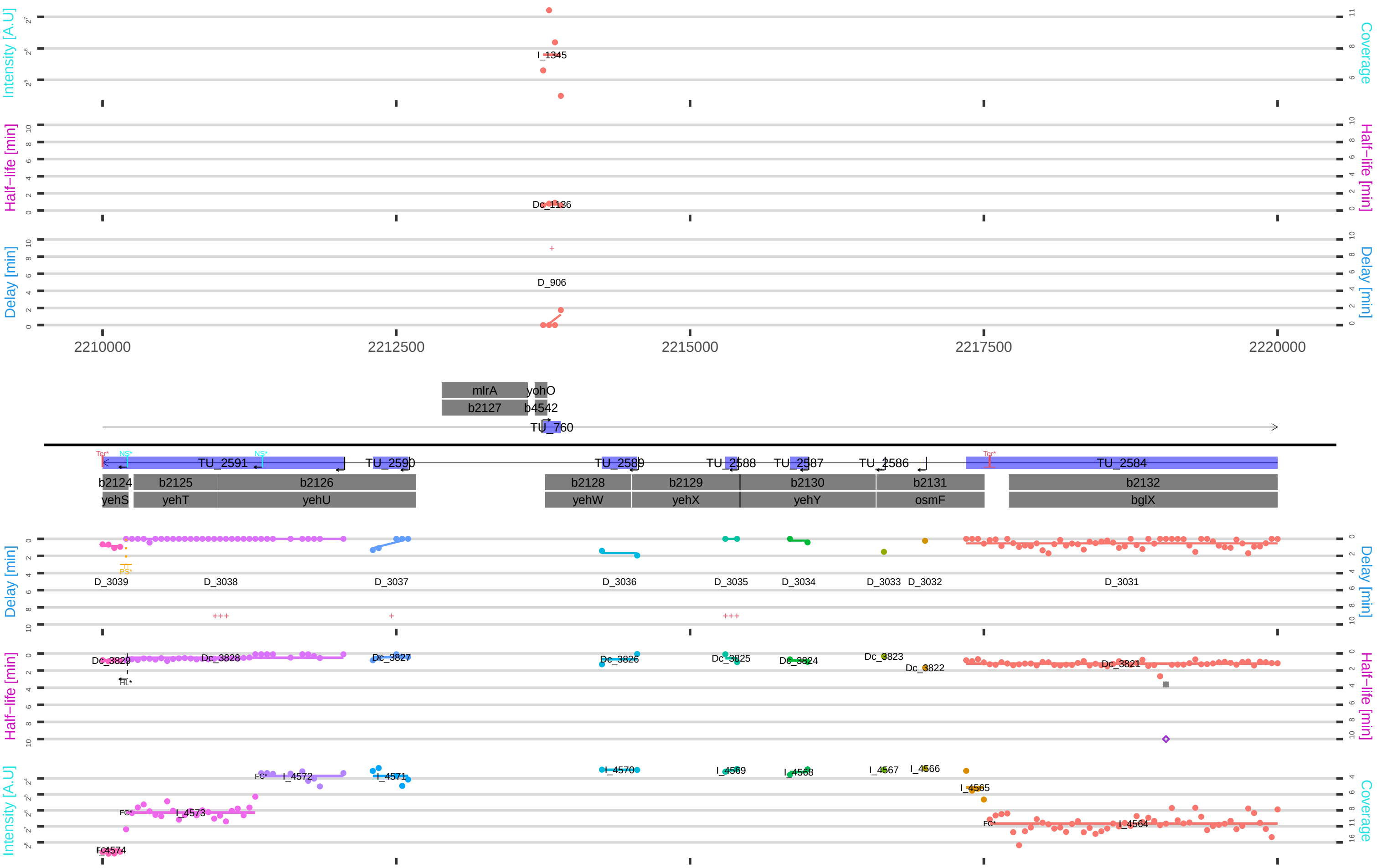

Term: termination (2), NS: new start (2), PS: pausing site (1), iTSS_I: internal starting site (0)

ID: 44404−44600; Term: termination (1), NS: new start (2), PS: pausing site (2), iTSS_I: internal starting site (0)

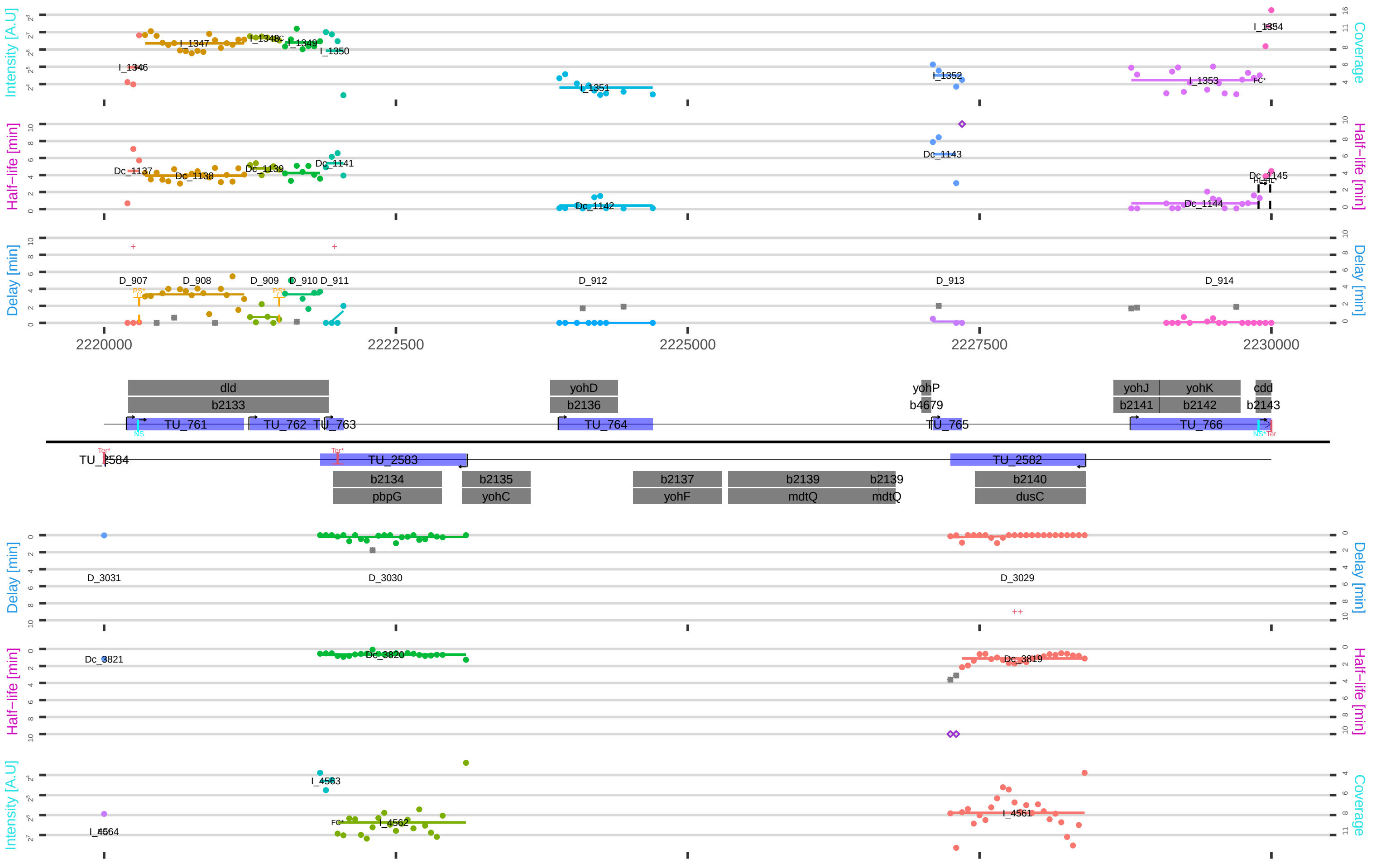

Term: termination (2), NS: new start (0), PS: pausing site (0), iTSS_I: internal starting site (0)

ID: 44600−44730; Term: termination (2), NS: new start (1), PS: pausing site (1), iTSS_I: internal starting site (0)

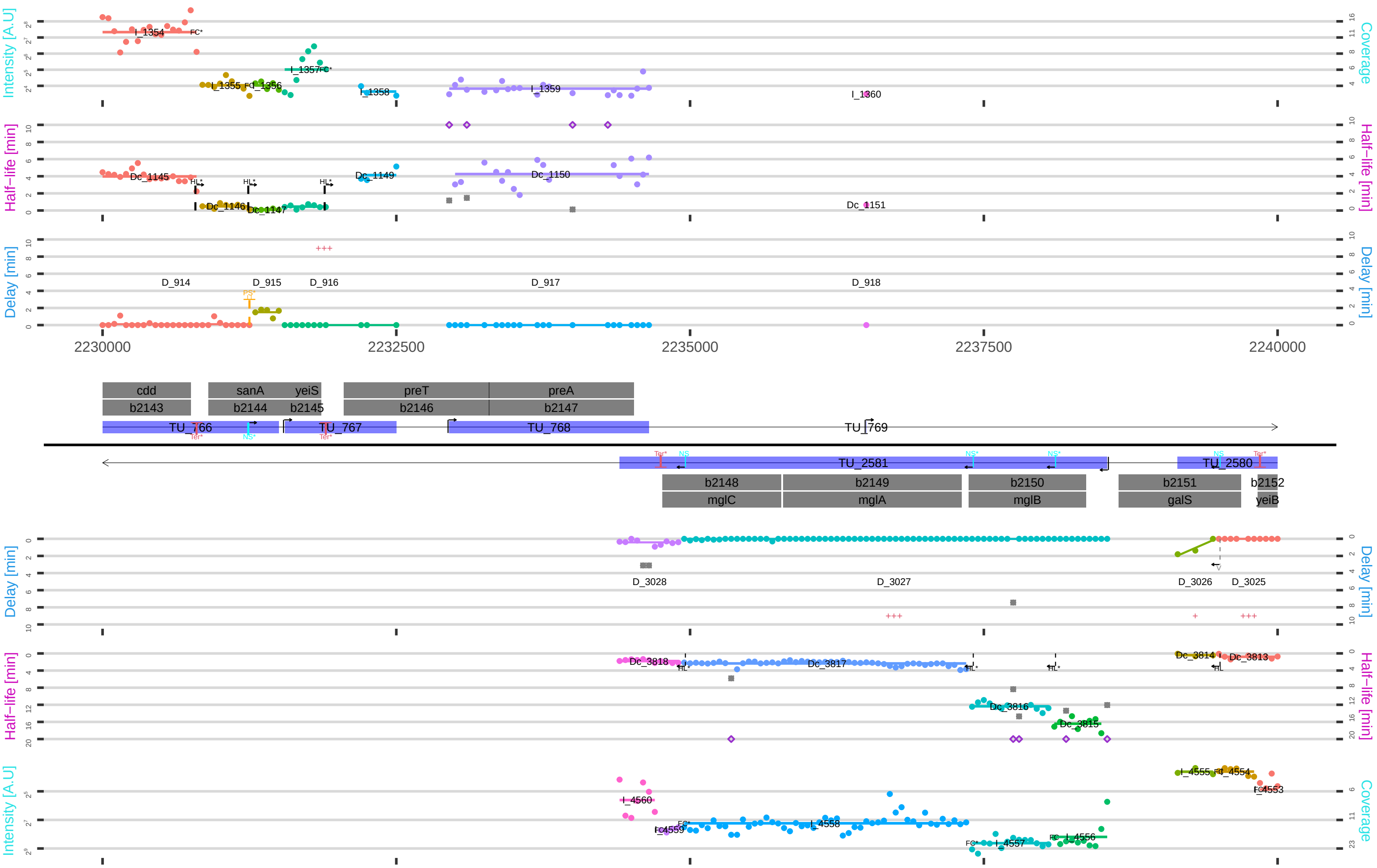

Term: termination (2), NS: new start (4), PS: pausing site (1), iTSS_I: internal starting site (1)

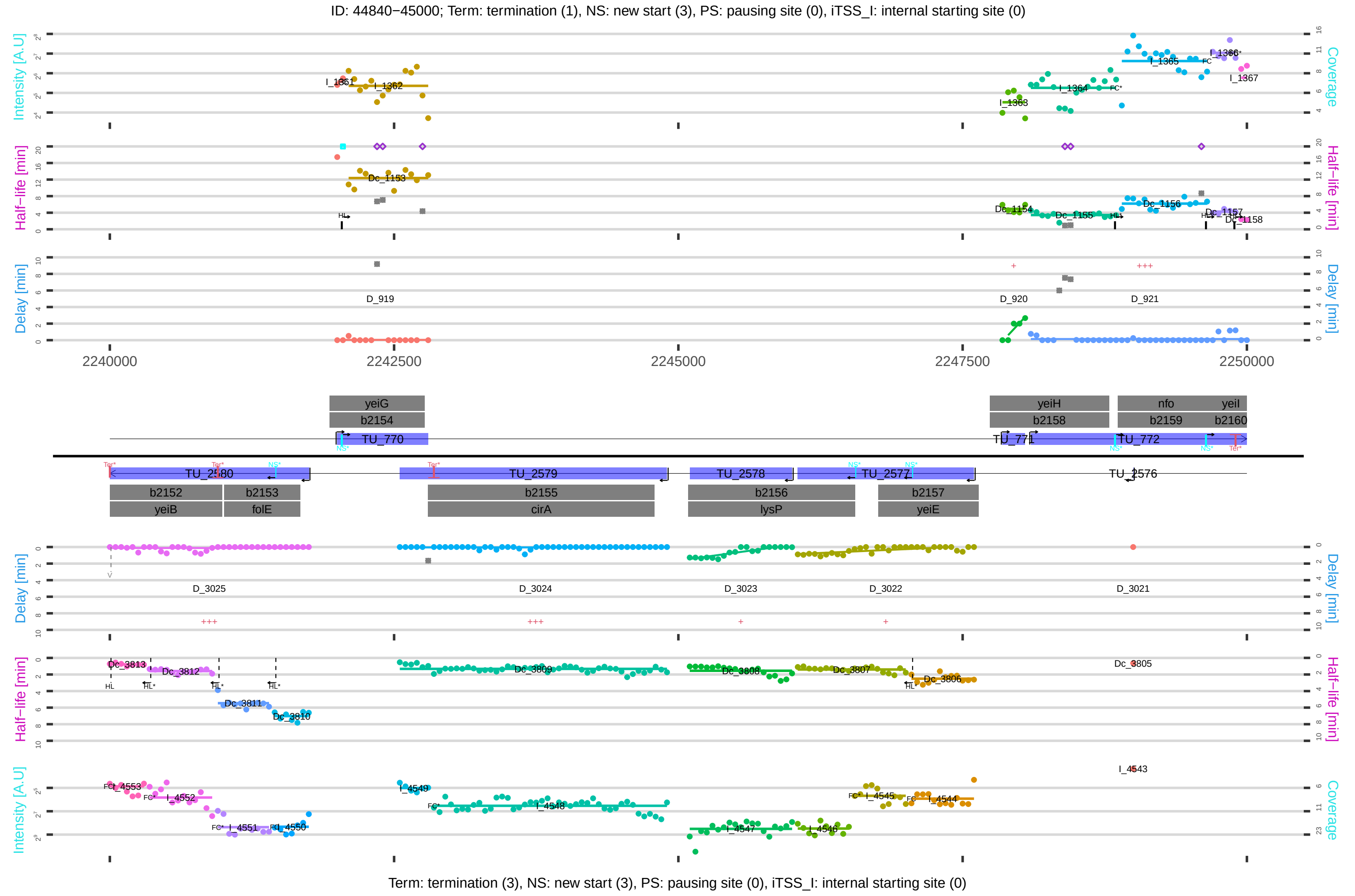

ID: 44840−45000; Term: termination (1), NS: new start (3), PS: pausing site (0), iTSS_I: internal starting site (0)
Intensity [A.U]
Coverage
Half−life [min]
Delay [min]
2240000
2242500
2245000
2247500
2250000
yeiG
b2154
TU_770
yeiH
b2158
nfo
b2159
yeiI
b2160
TU_771
TU_772
TU_2580
TU_2579
TU_2578
TU_2577
TU_2576
b2152
yeiB
b2153
folE
b2155
cirA
b2156
lysP
b2157
yeiE
D_919
D_920
D_921
D_3025
D_3024
D_3023
D_3022
D_3021
Dc_1153
Dc_1154
Dc_1155
Dc_1156
Dc_1157
Dc_1158
Dc_3813
Dc_3812
Dc_3811
Dc_3810
Dc_3809
Dc_3808
Dc_3807
Dc_3806
Dc_3805
I_1361
I_1362
I_1363
I_1364
I_1365
I_1366
I_1367
I_4553
I_4552
I_4551
I_4550
I_4549
I_4548
I_4547
I_4546
I_4545
I_4544
I_4543
Term: termination (3), NS: new start (3), PS: pausing site (0), iTSS_I: internal starting site (0)

ID: 45000−45130; Term: termination (0), NS: new start (0), PS: pausing site (0), iTSS_I: internal starting site (0)

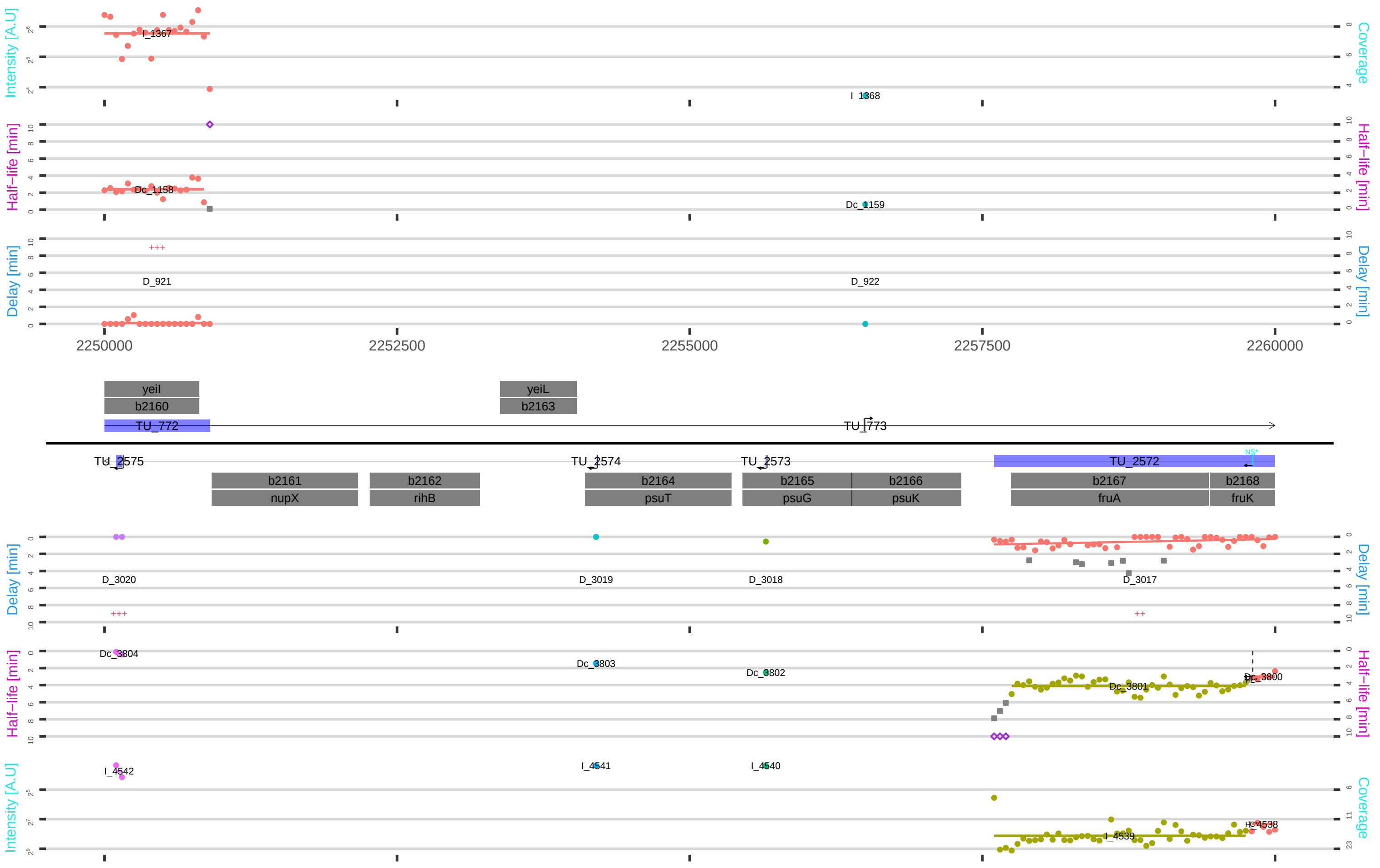

Term: termination (0), NS: new start (1), PS: pausing site (0), iTSS_I: internal starting site (0)

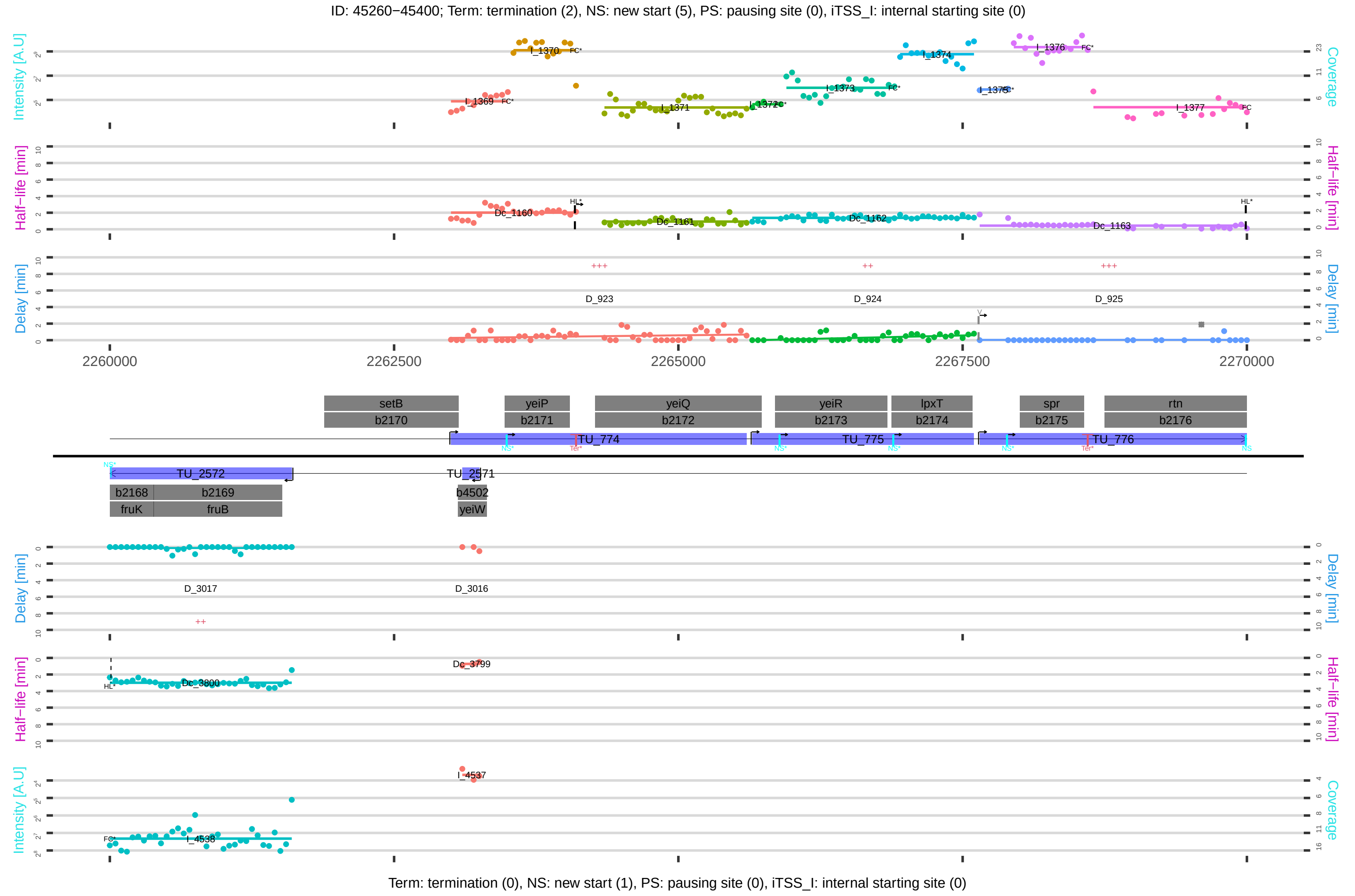
ID: 45260−45400; Term: termination (2), NS: new start (5), PS: pausing site (0), iTSS_I: internal starting site (0)
Intensity [A.U]
Coverage
Half−life [min]
Delay [min]
I_1369
I_1370
I_1371
I_1372
I_1373
I_1374
I_1375
I_1376
I_1377
Dc_1160
Dc_1161
Dc_1162
Dc_1163
D_923
D_924
D_925
2260000
2262500
2265000
2267500
2270000
setB
b2170
yeiP
b2171
yeiQ
b2172
yeiR
b2173
lpxT
b2174
spr
b2175
rtn
b2176
TU_774
TU_775
TU_776
TU_2572
TU_2571
b2168
b2169
fruK
fruB
b4502
yeiW
D_3017
D_3016
Dc_3799
Dc_3800
I_4537
I_4538
Term: termination (0), NS: new start (1), PS: pausing site (0), iTSS_I: internal starting site (0)

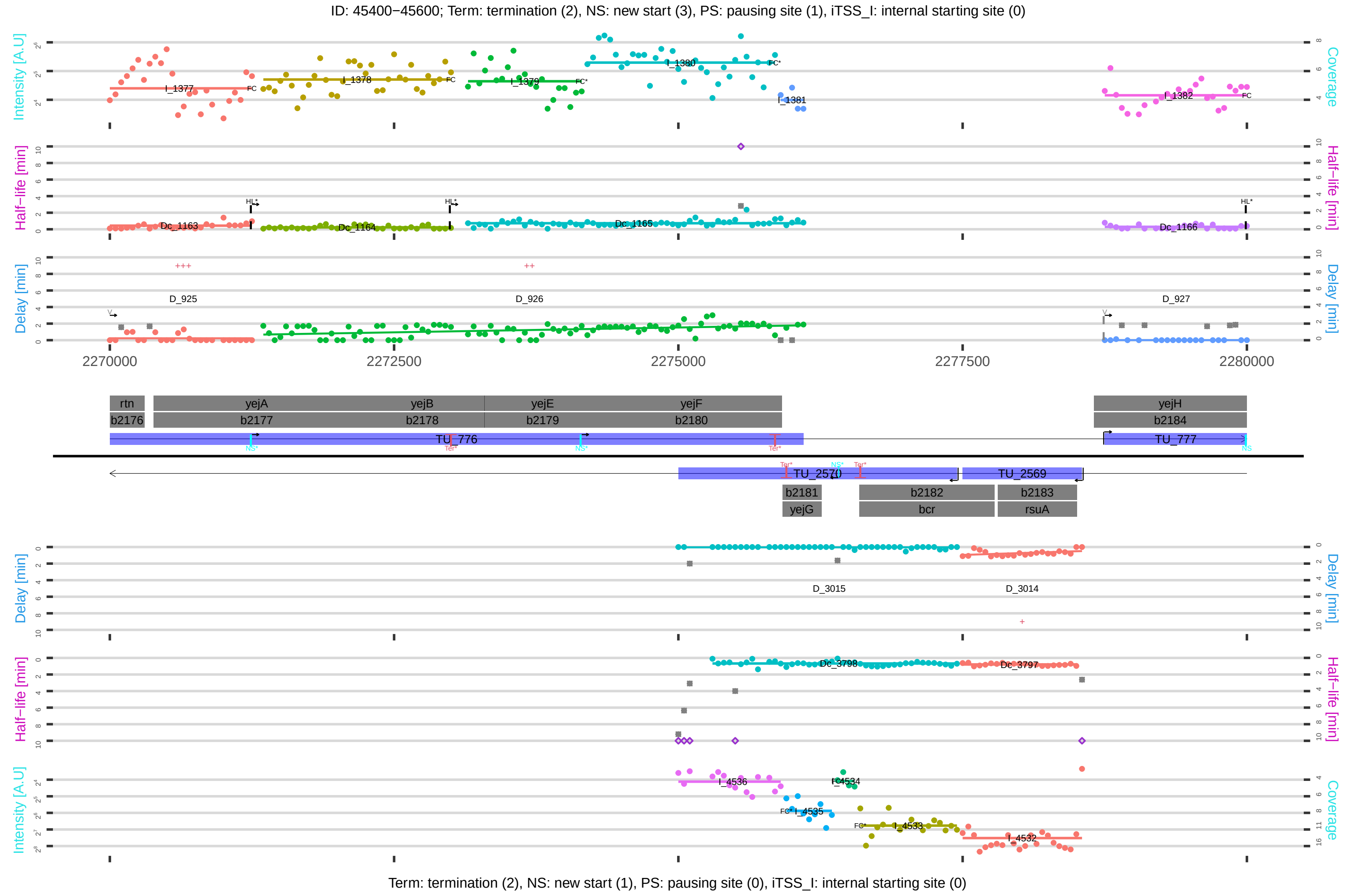
ID: 45400−45600; Term: termination (2), NS: new start (3), PS: pausing site (1), iTSS_I: internal starting site (0)
Intensity [A.U]
Coverage
Half−life [min]
Delay [min]
I_1377
I_1378
I_1379
I_1380
I_1381
I_1382
Dc_1163
Dc_1164
Dc_1165
Dc_1166
D_925
D_926
D_927
2270000
2272500
2275000
2277500
2280000
rtn
b2176
yejA
b2177
yejB
b2178
yejE
b2179
yejF
b2180
yejH
b2184
TU_776
TU_777
TU_2570
TU_2569
b2181
yejG
b2182
bcr
b2183
rsuA
D_3015
D_3014
Dc_3798
Dc_3797
I_4536
I_4534
I_4535
I_4533
I_4532
Term: termination (2), NS: new start (1), PS: pausing site (0), iTSS_I: internal starting site (0)

ID: 45600−45792; Term: termination (1), NS: new start (4), PS: pausing site (2), iTSS_I: internal starting site (0)

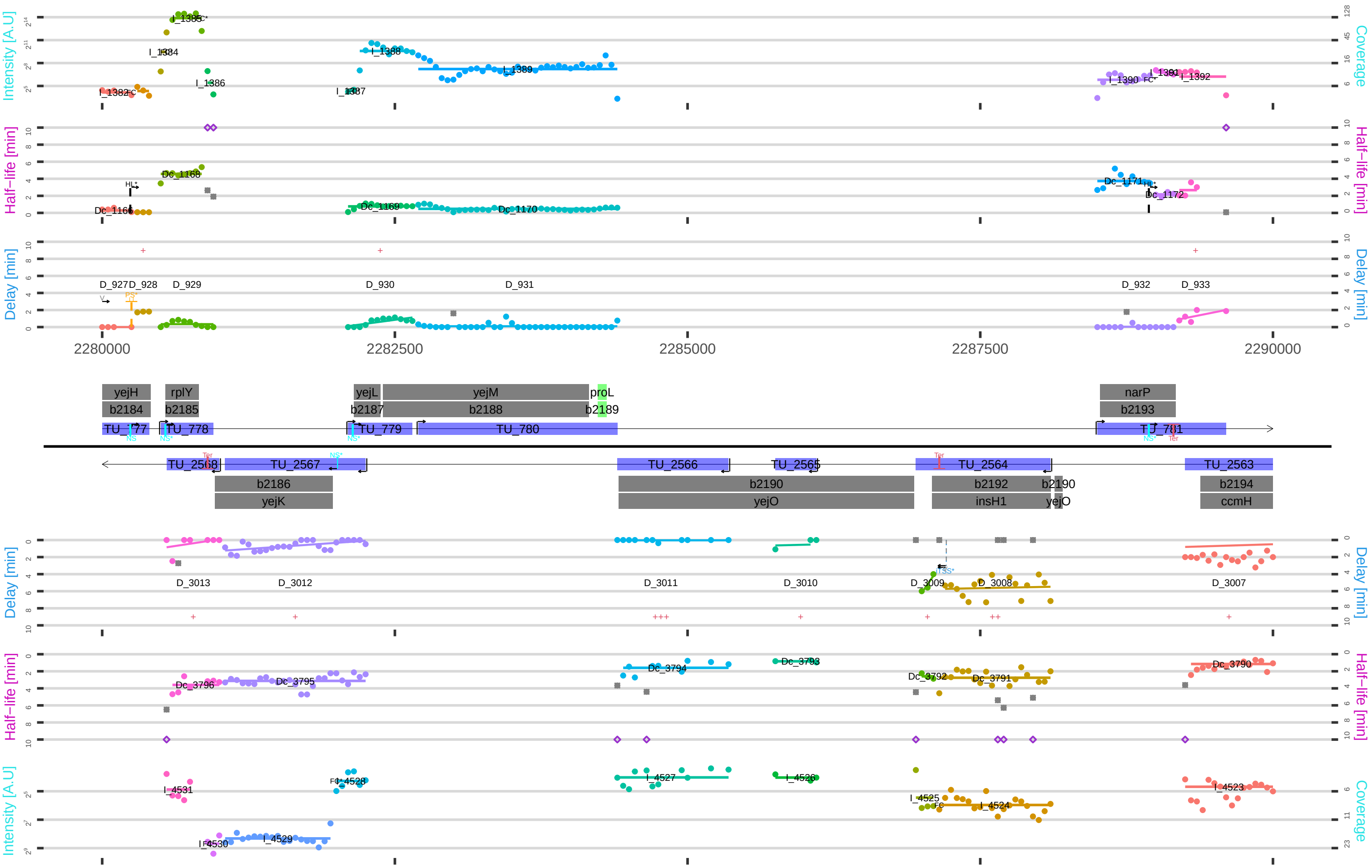

Term: termination (2), NS: new start (1), PS: pausing site (0), iTSS_I: internal starting site (1)

ID: 139789−139589; FC*: significant t−test of two consecutive segments; Term: termination, NS: new start, PS: pausing site,iTSS_I: internal starting site,TI: transcription interferance.

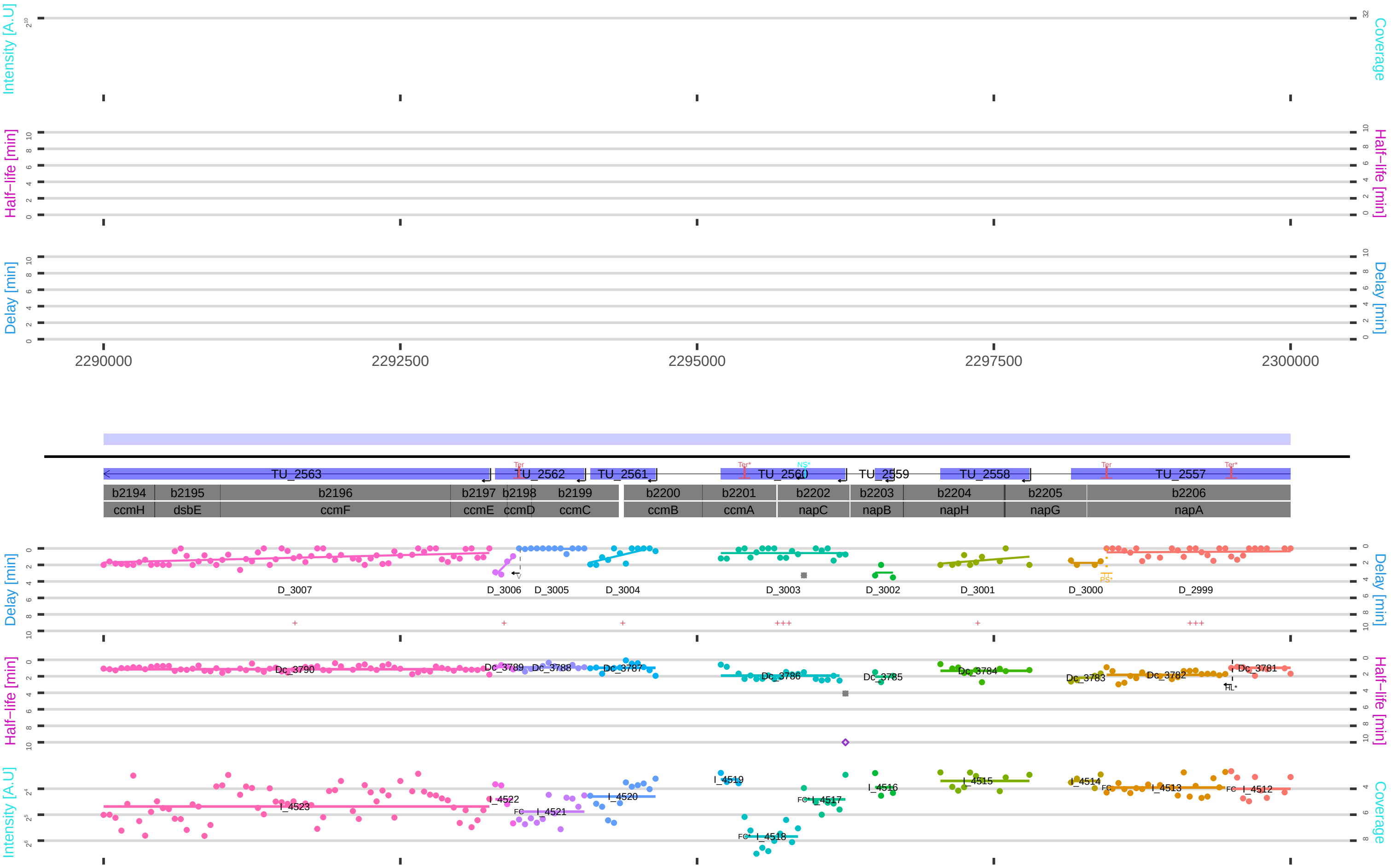

Term: termination (4), NS: new start (1), PS: pausing site (2), iTSS_I: internal starting site (0)

ID: 46039−46062; Term: termination (0), NS: new start (1), PS: pausing site (0), iTSS_I: internal starting site (0)

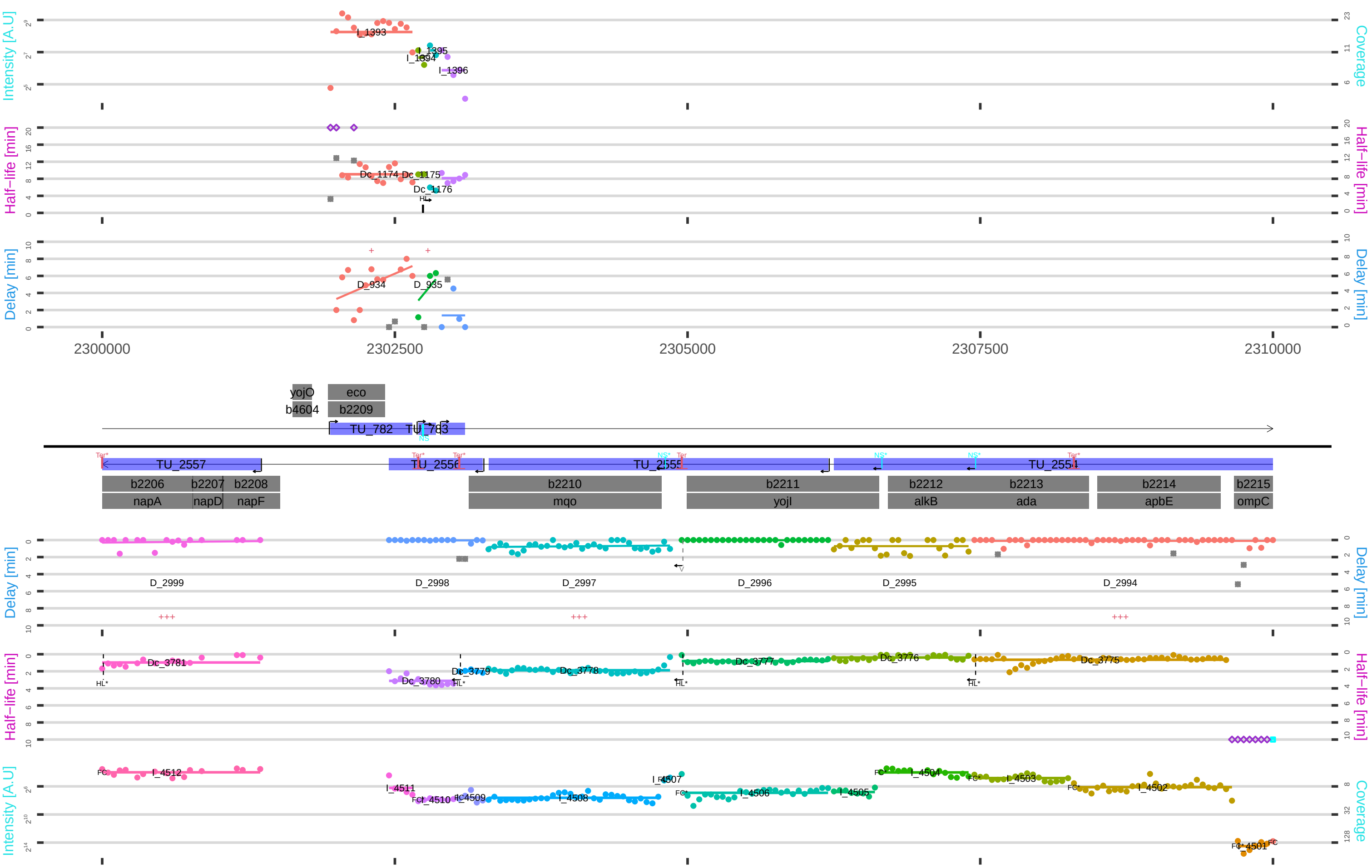

Term: termination (5), NS: new start (3), PS: pausing site (2), iTSS_I: internal starting site (0)

ID: 46206−46397; Term: termination (0), NS: new start (2), PS: pausing site (0), iTSS_I: internal starting site (0)

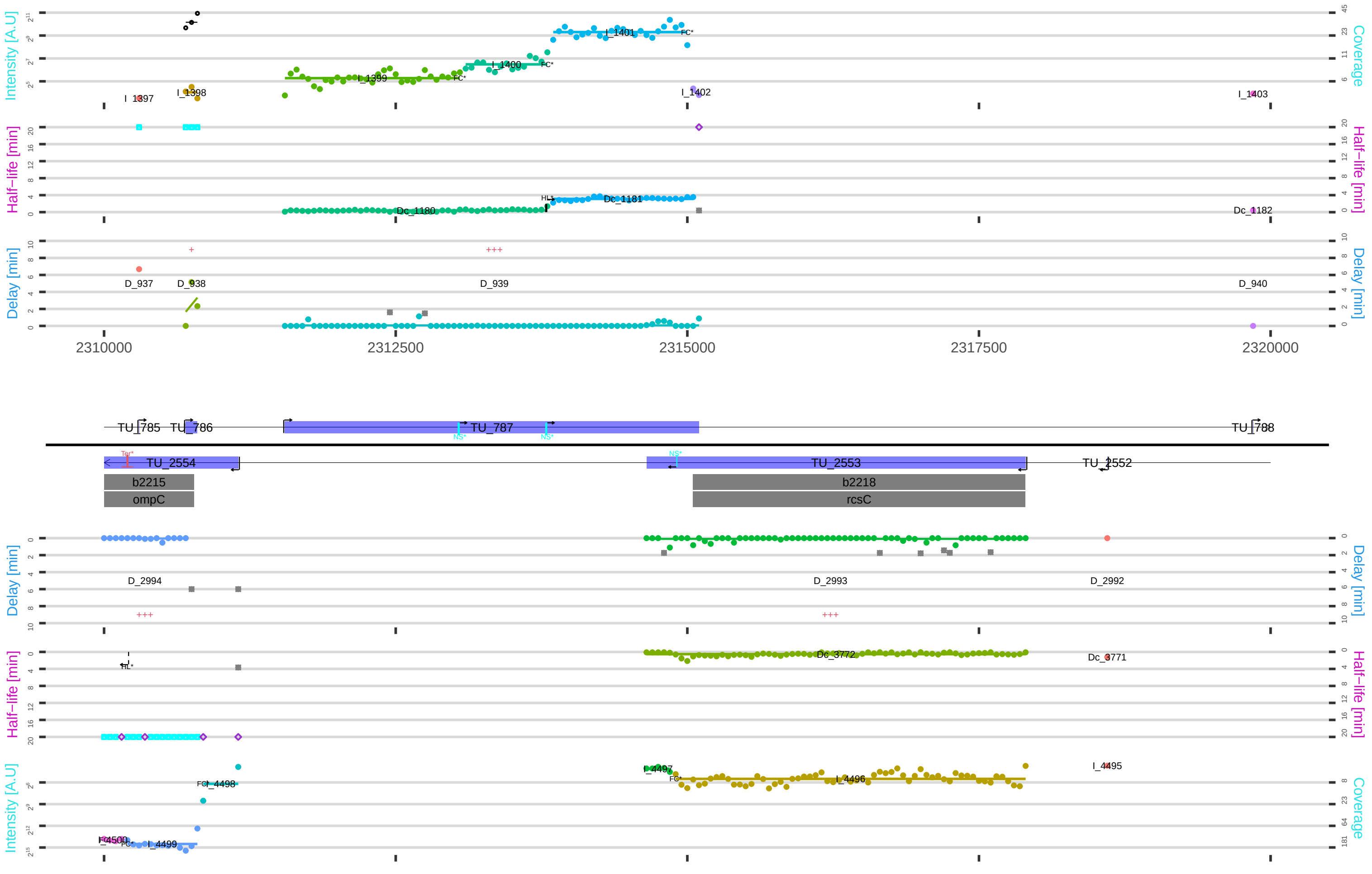

Term: termination (1), NS: new start (1), PS: pausing site (0), iTSS_I: internal starting site (0)

ID: 46535−46535; Term: termination (0), NS: new start (0), PS: pausing site (0), iTSS_I: internal starting site (0)

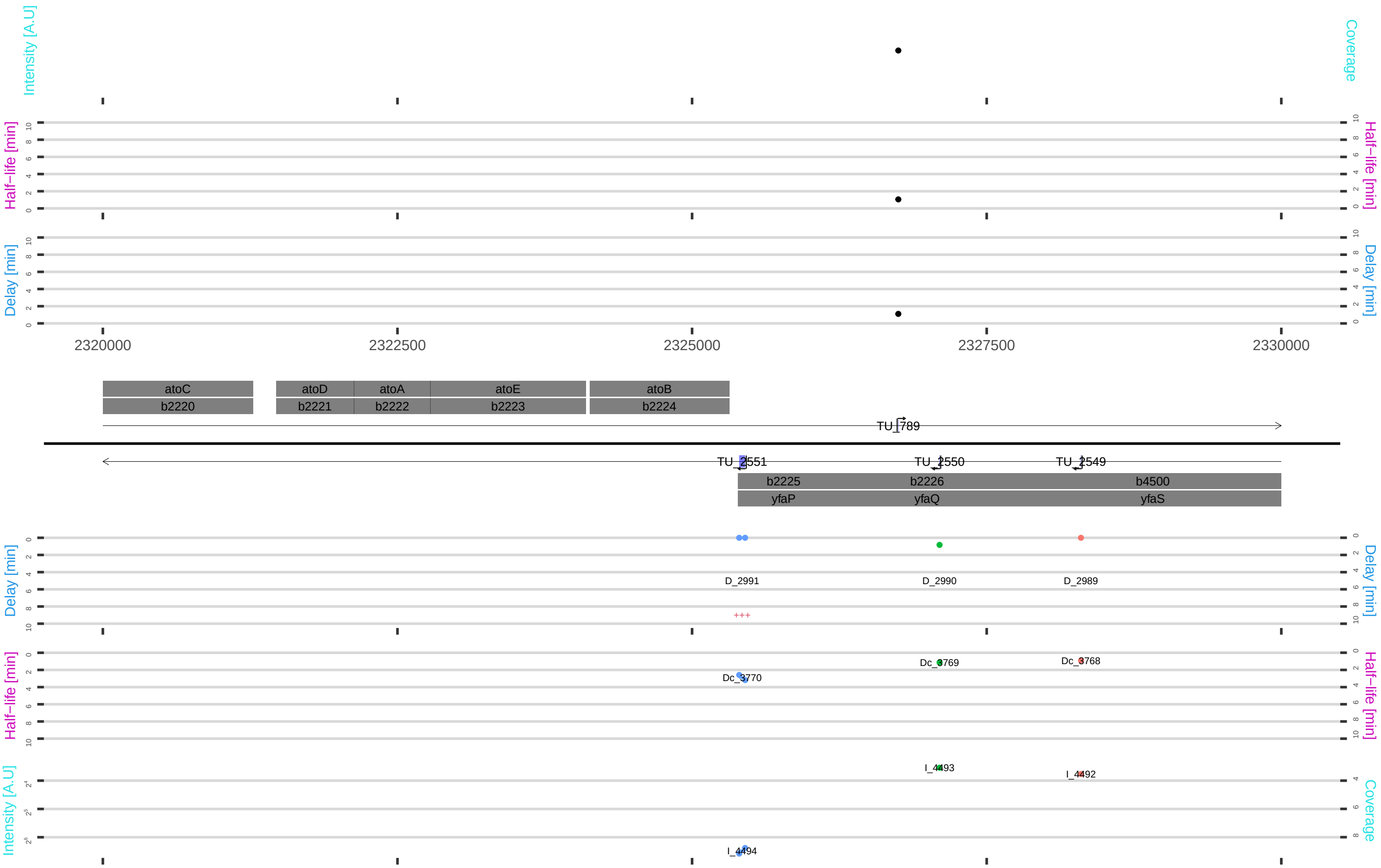

Term: termination (0), NS: new start (0), PS: pausing site (0), iTSS_I: internal starting site (0)

ID: 46753−46789; Term: termination (0), NS: new start (1), PS: pausing site (0), iTSS_I: internal starting site (0)

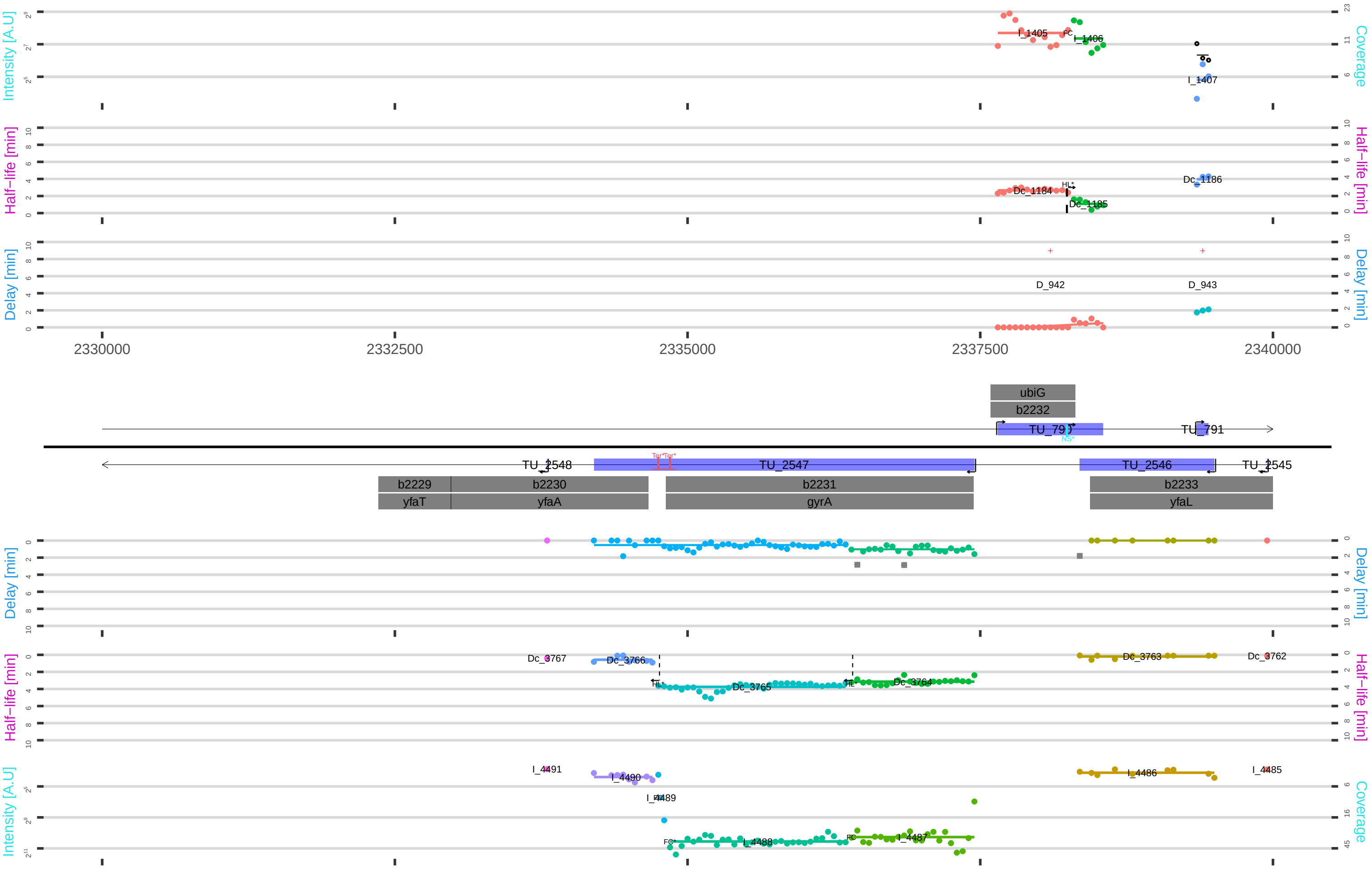

Term: termination (2), NS: new start (0), PS: pausing site (0), iTSS_I: internal starting site (1)

ID: 46857−46962; Term: termination (1), NS: new start (0), PS: pausing site (0), iTSS_I: internal starting site (0)

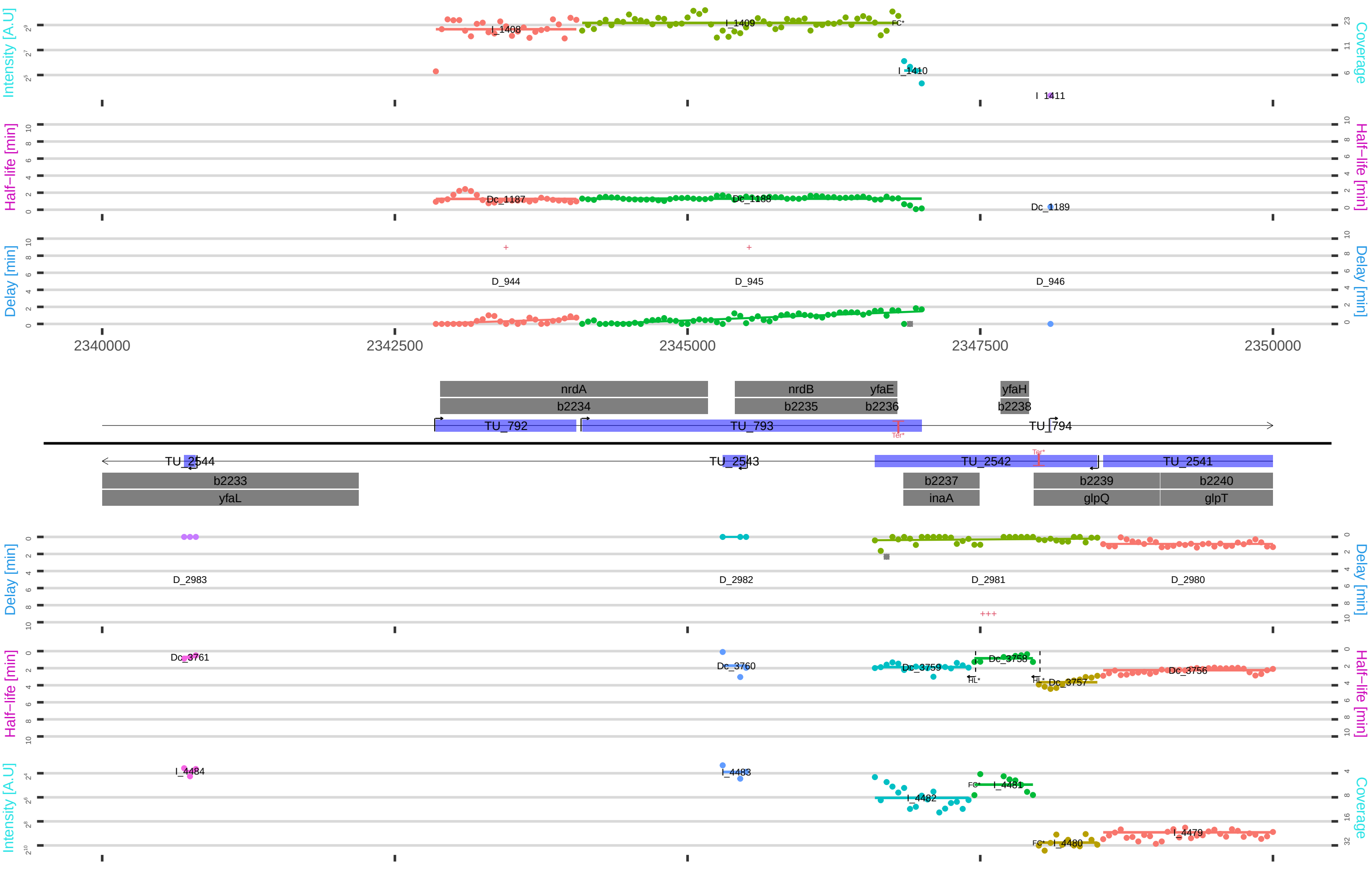

Term: termination (1), NS: new start (0), PS: pausing site (0), iTSS_I: internal starting site (0)

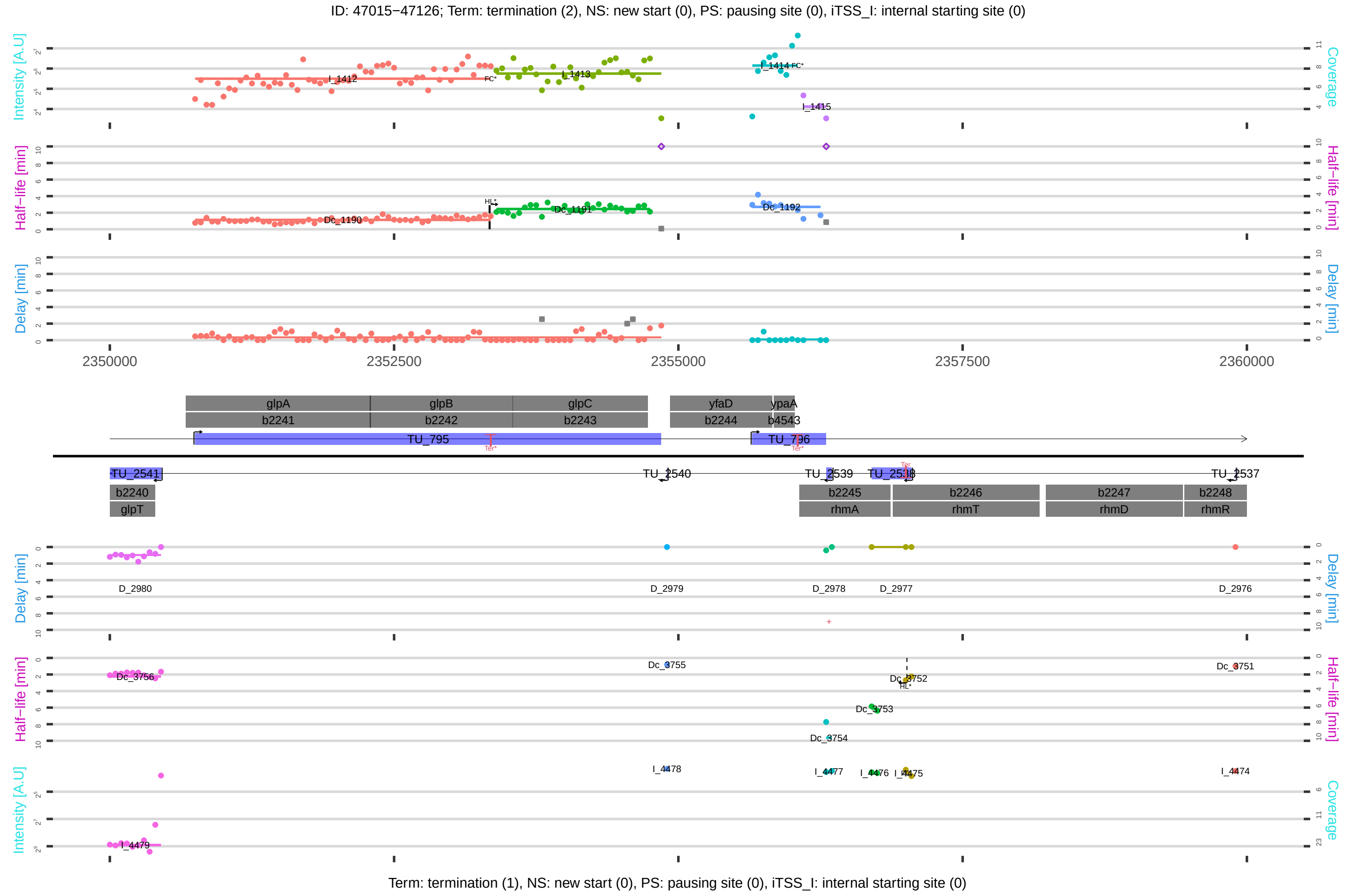
ID: 47015−47126; Term: termination (2), NS: new start (0), PS: pausing site (0), iTSS_I: internal starting site (0)
Intensity [A.U]
Coverage
Half−life [min]
Delay [min]
I_1412
I_1413
I_1414
I_1415
Dc_1190
Dc_1191
Dc_1192
2350000
2352500
2355000
2357500
2360000
glpA
glpB
glpC
yfaD
ypaA
b2241
b2242
b2243
b2244
b4543
TU_795
TU_796
TU_2541
TU_2540
TU_2539
TU_2538
TU_2537
b2240
b2245
b2246
b2247
b2248
glpT
rhmA
rhmT
rhmD
rhmR
D_2980
D_2979
D_2978
D_2977
D_2976
Dc_3756
Dc_3755
Dc_3754
Dc_3753
Dc_3752
Dc_3751
I_4479
I_4478
I_4477
I_4476
I_4475
I_4474
Term: termination (1), NS: new start (0), PS: pausing site (0), iTSS_I: internal starting site (0)

ID: 47253−47370; Term: termination (0), NS: new start (0), PS: pausing site (0), iTSS_I: internal starting site (0)

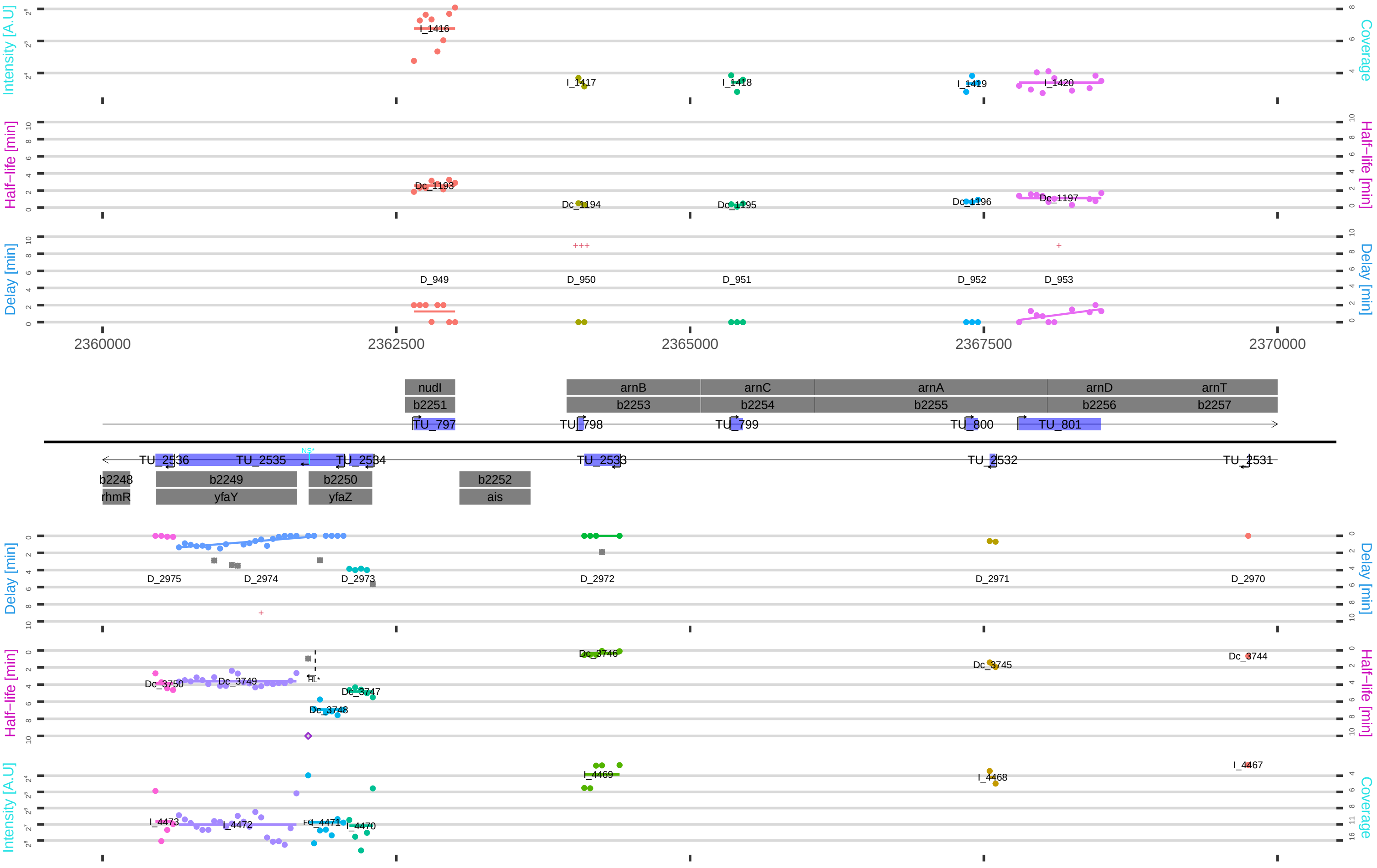

Term: termination (0), NS: new start (1), PS: pausing site (0), iTSS_I: internal starting site (0)

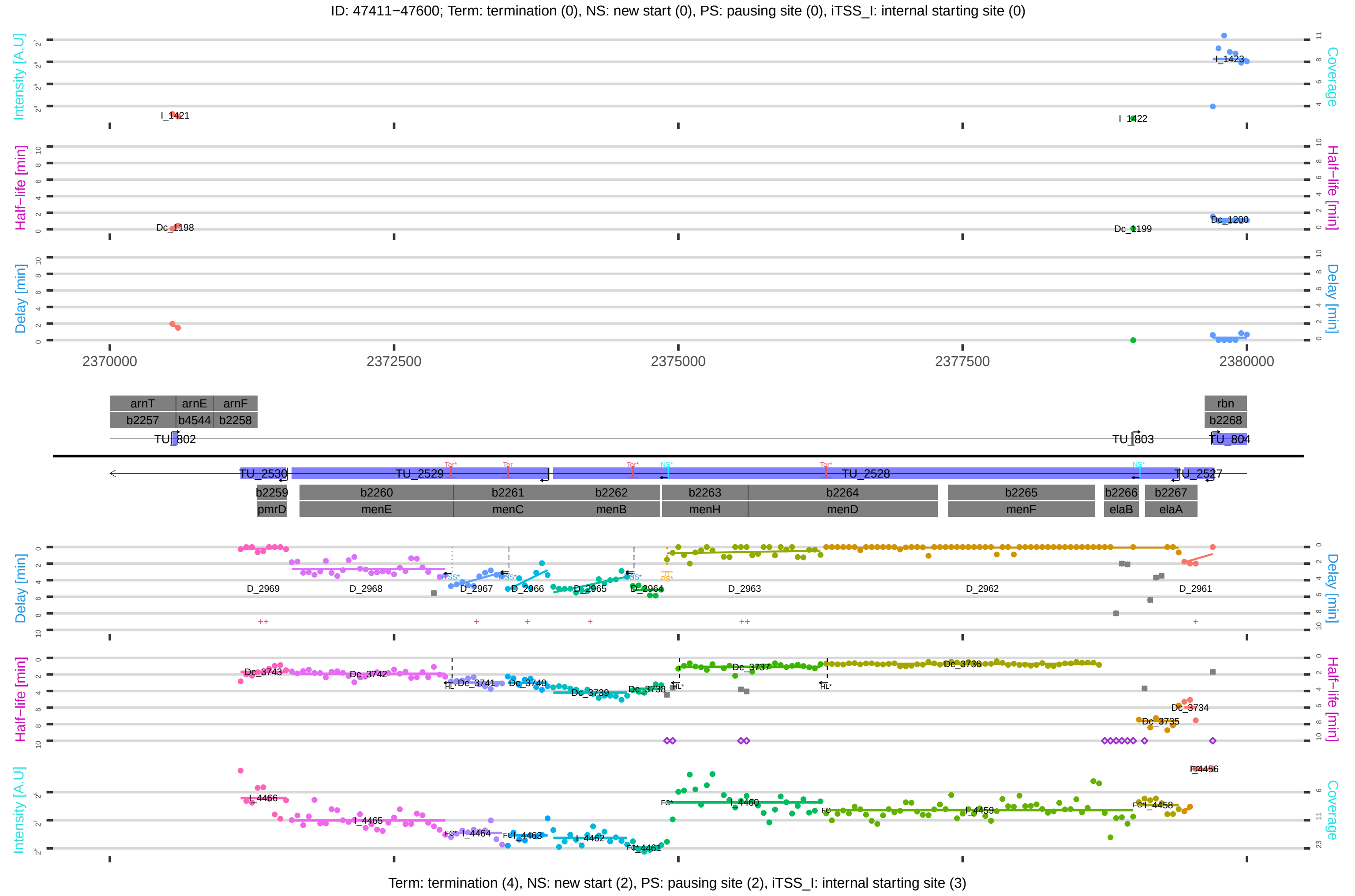

ID: 47411−47600; Term: termination (0), NS: new start (0), PS: pausing site (0), iTSS_I: internal starting site (0)
Intensity [A.U]
Coverage
Half−life [min]
Delay [min]
I_1421
I_1422
I_1423
Dc_1198
Dc_1199
Dc_1200
2370000
2372500
2375000
2377500
2380000
arnT
arnE
arnF
b2257
b4544
b2258
rbn
b2268
TU_802
TU_803
TU_804
TU_2530
TU_2529
TU_2528
TU_2527
b2259
pmrD
b2260
menE
b2261
menC
b2262
menB
b2263
menH
b2264
menD
b2265
menF
b2266
elaB
b2267
elaA
D_2969
D_2968
D_2967
D_2966
D_2965
D_2964
D_2963
D_2962
D_2961
Dc_3743
Dc_3742
Dc_3741
Dc_3740
Dc_3739
Dc_3738
Dc_3737
Dc_3736
Dc_3735
Dc_3734
I_4466
I_4465
I_4464
I_4463
I_4462
I_4461
I_4460
I_4459
I_4458
I_4456
Term: termination (4), NS: new start (2), PS: pausing site (2), iTSS_I: internal starting site (3)

ID: 47600−47760; Term: termination (0), NS: new start (1), PS: pausing site (0), iTSS_I: internal starting site (0)

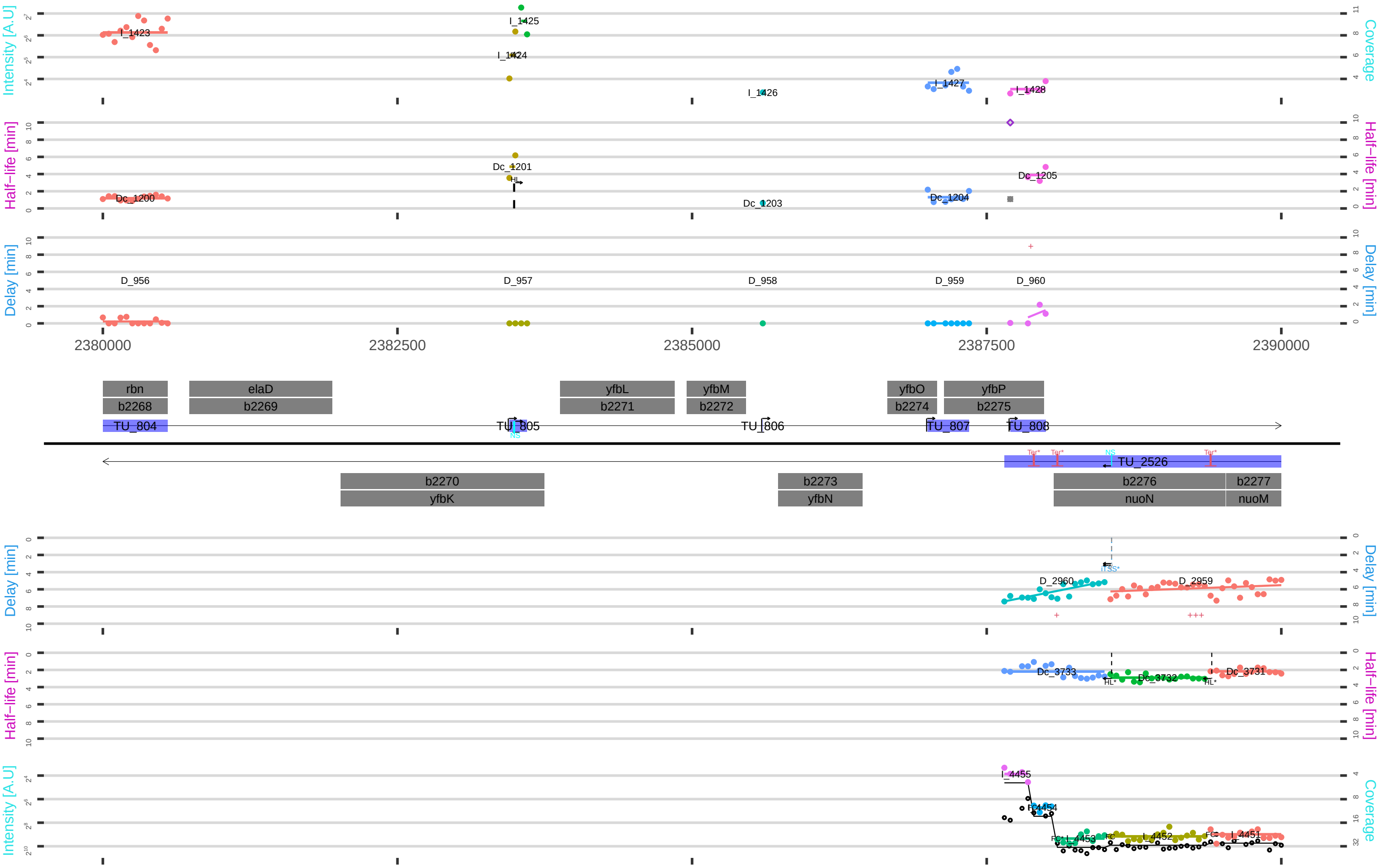

Term: termination (3), NS: new start (1), PS: pausing site (0), iTSS_I: internal starting site (1)

ID: 137789−137589; FC*: significant t−test of two consecutive segments; Term: termination, NS: new start, PS: pausing site,iTSS_I: internal starting site,TI: transcription interferance.

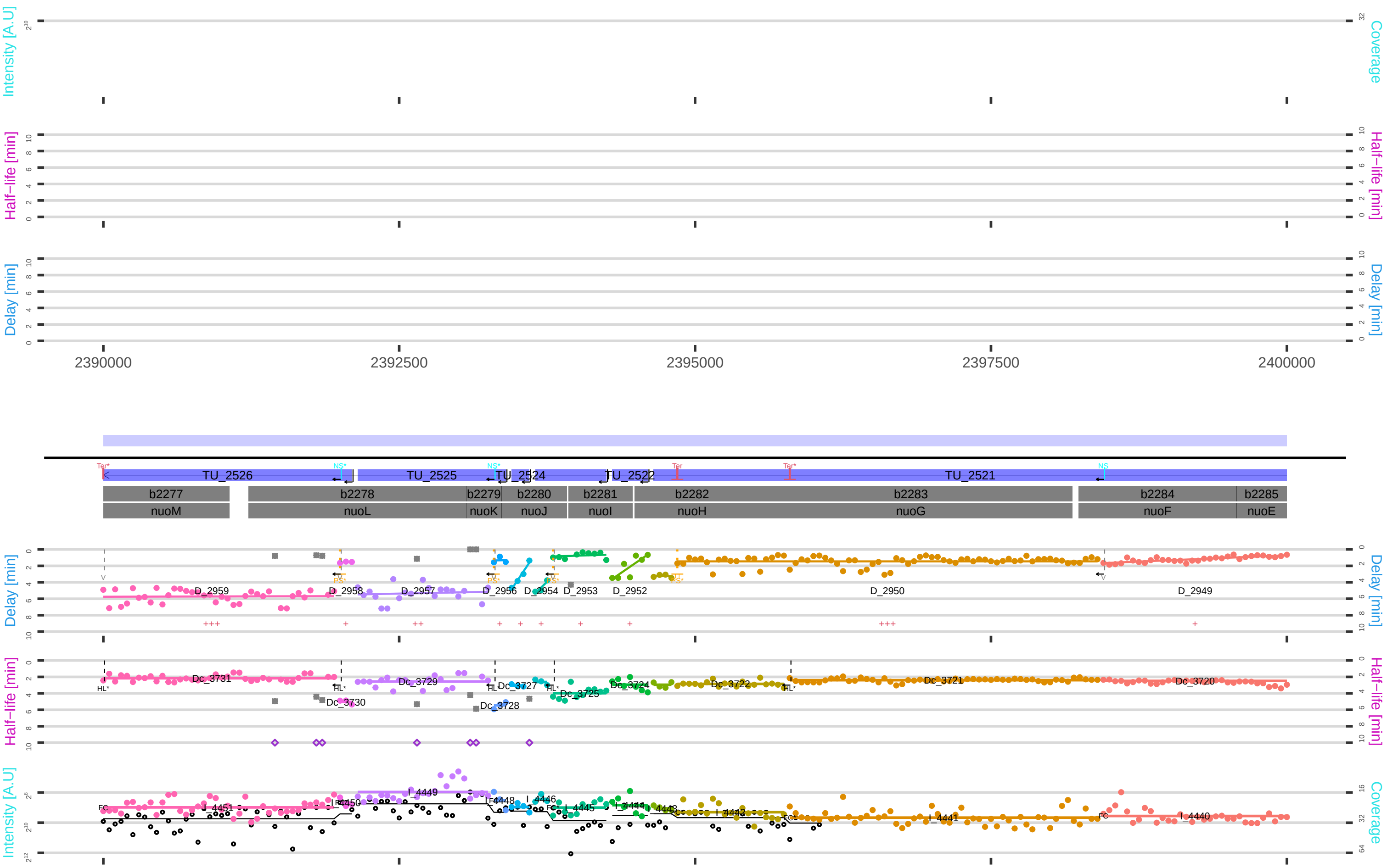

Term: termination (3), NS: new start (3), PS: pausing site (4), iTSS_I: internal starting site (1)

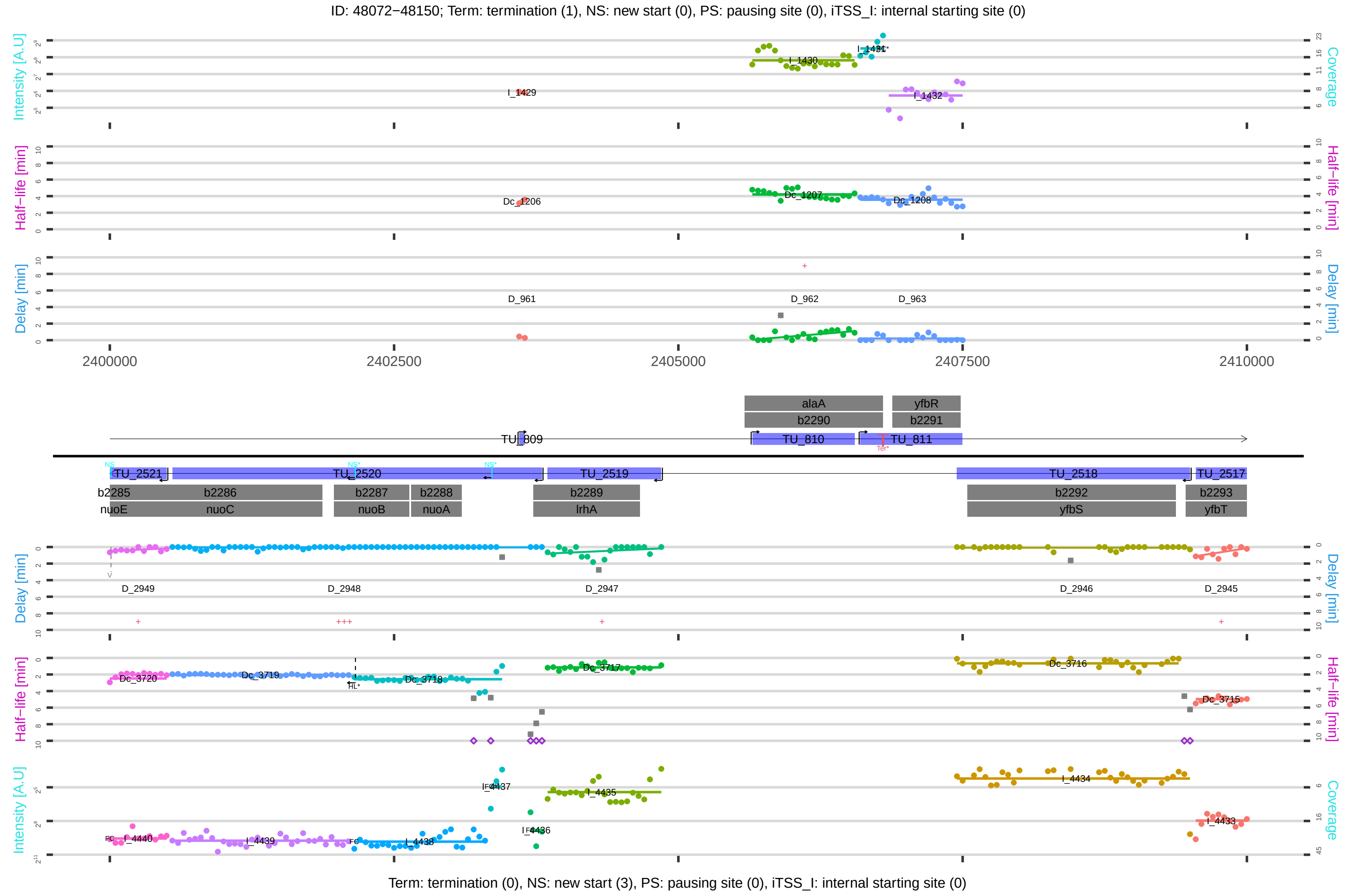

ID: 48072−48150; Term: termination (1), NS: new start (0), PS: pausing site (0), iTSS_I: internal starting site (0)
Intensity [A.U]
Coverage
Half−life [min]
Delay [min]
I_1429
I_1430
I_1431
I_1432
Dc_1206
Dc_1207
Dc_1208
D_961
D_962
D_963
2400000
2402500
2405000
2407500
2410000
alaA
b2290
yfbR
b2291
TU_809
TU_810
TU_811
Ter*
TU_2521
TU_2520
TU_2519
TU_2518
TU_2517
NS
NS*
b2285
b2286
b2287
b2288
b2289
b2292
b2293
nuoE
nuoC
nuoB
nuoA
lrhA
yfbS
yfbT
D_2949
D_2948
D_2947
D_2946
D_2945
Dc_3720
Dc_3719
Dc_3718
Dc_3717
Dc_3716
Dc_3715
HL*
I_4440
I_4439
I_4438
I_4437
I_4436
I_4435
I_4434
I_4433
Term: termination (0), NS: new start (3), PS: pausing site (0), iTSS_I: internal starting site (0)

ID: 48227−48400; Term: termination (2), NS: new start (3), PS: pausing site (1), iTSS_I: internal starting site (1)

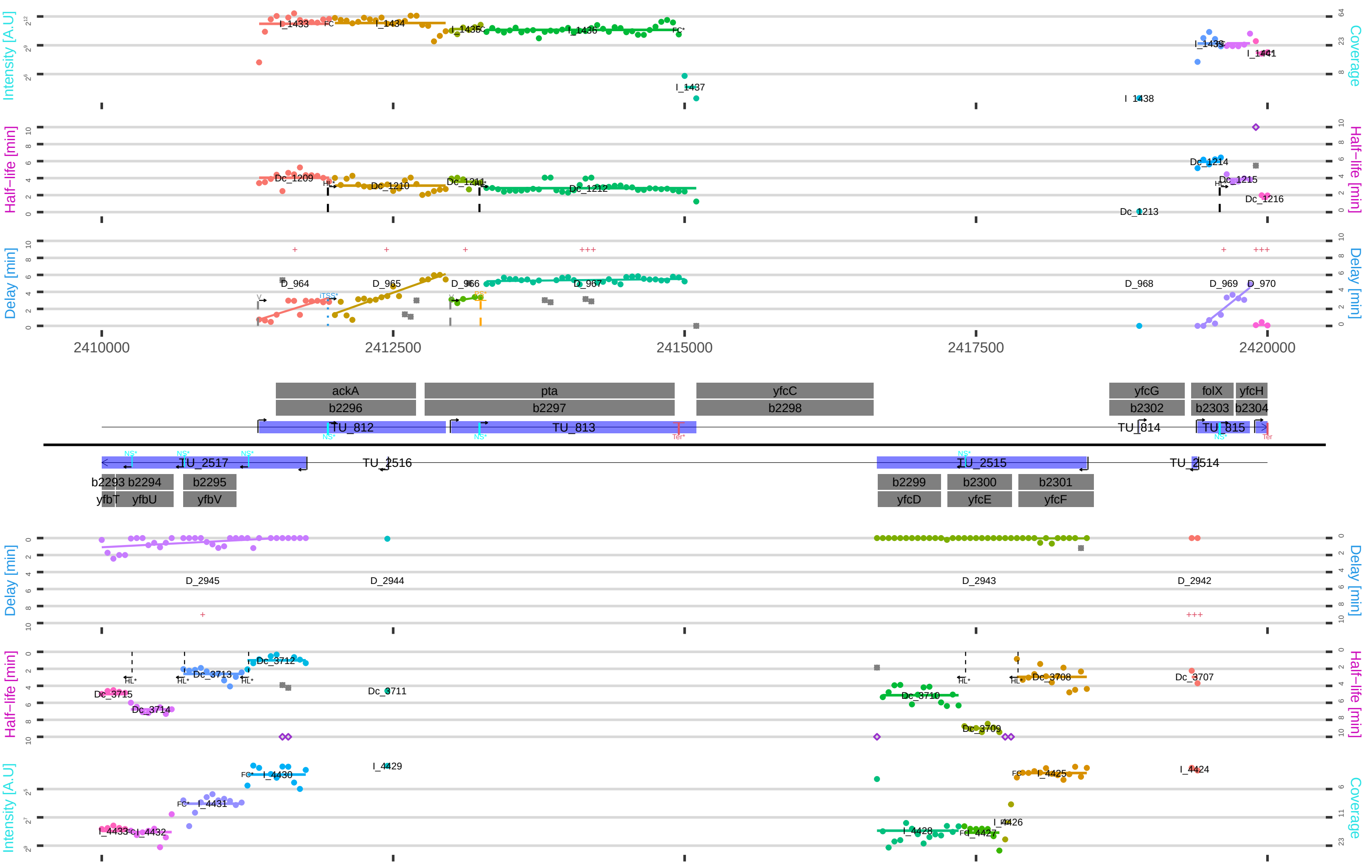

Term: termination (0), NS: new start (4), PS: pausing site (0), iTSS_I: internal starting site (0)

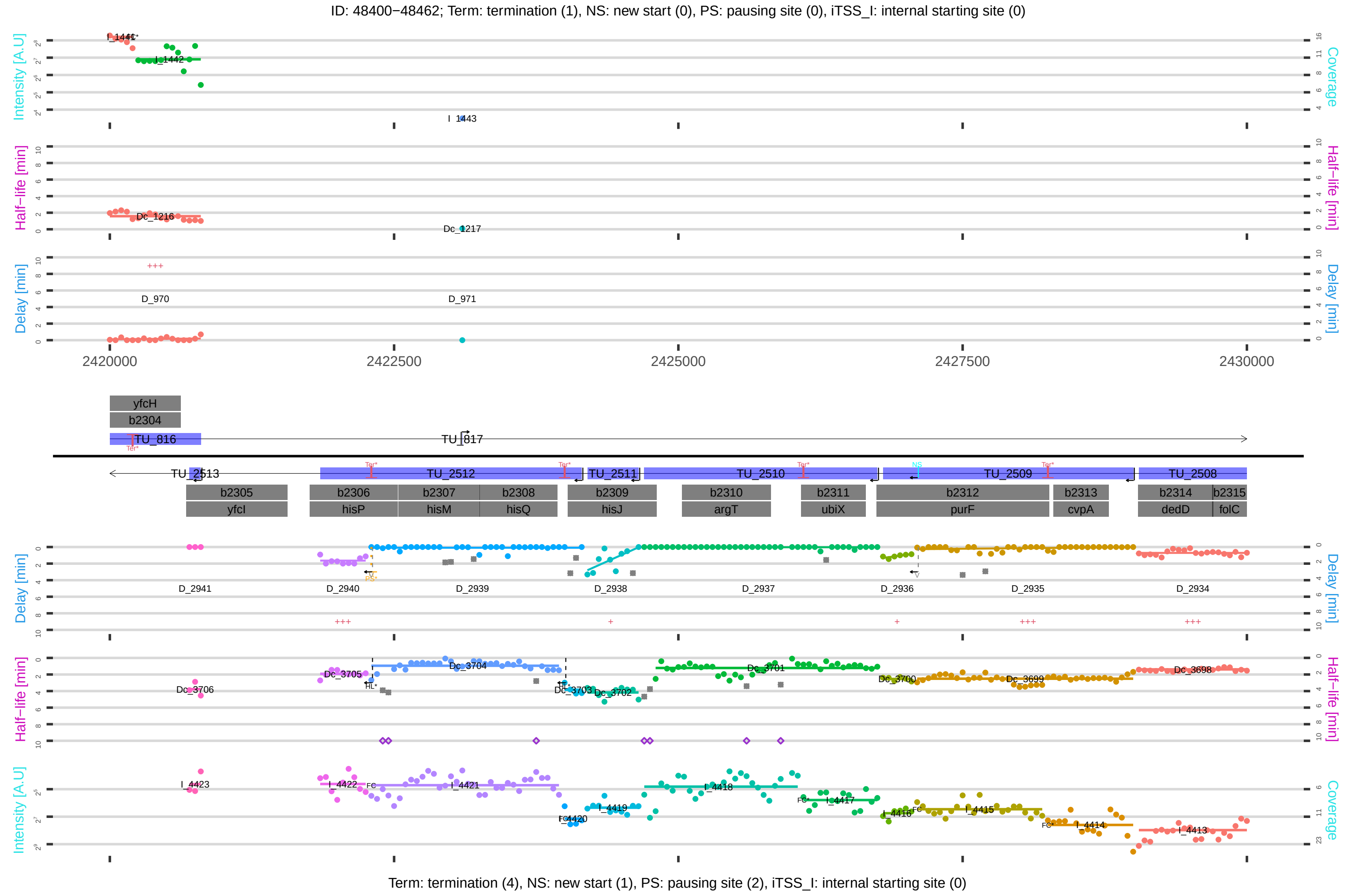

Term: termination (4), NS: new start (1), PS: pausing site (2), iTSS_I: internal starting site (0)

ID: 48721−48800; Term: termination (1), NS: new start (0), PS: pausing site (0), iTSS_I: internal starting site (0)

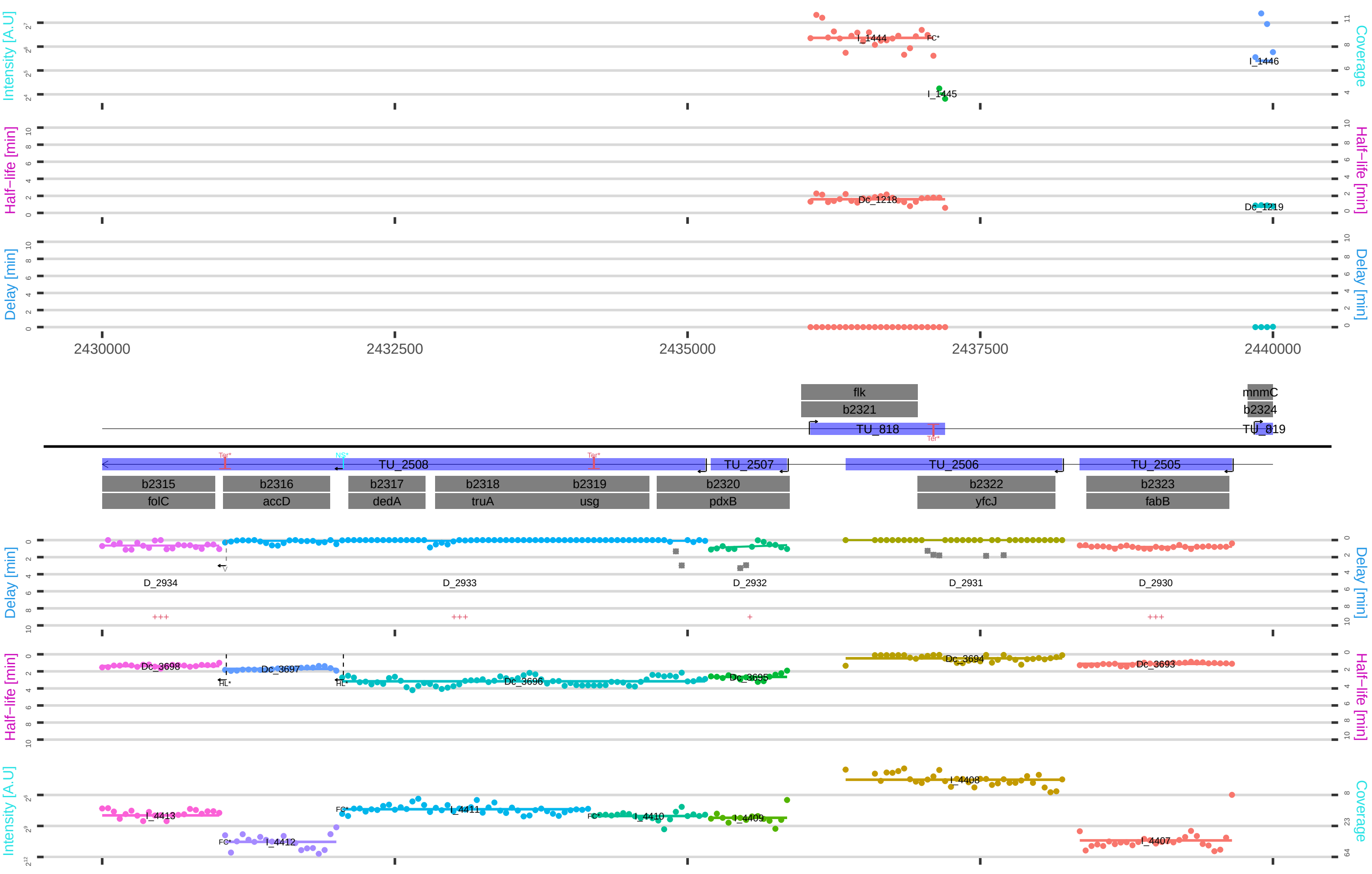

Term: termination (2), NS: new start (1), PS: pausing site (1), iTSS_I: internal starting site (0)

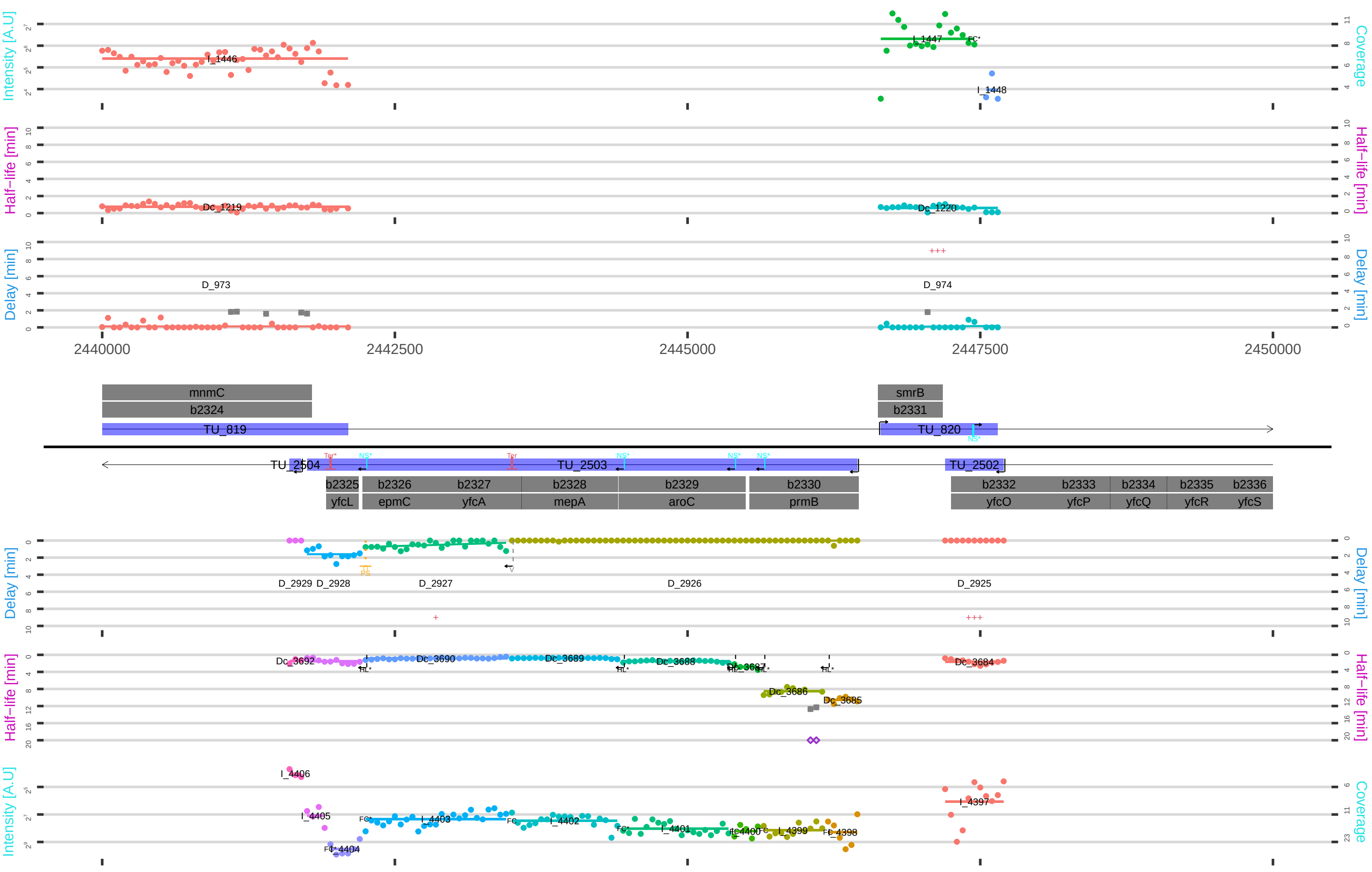

Term: termination (2), NS: new start (4), PS: pausing site (2), iTSS_I: internal starting site (0)

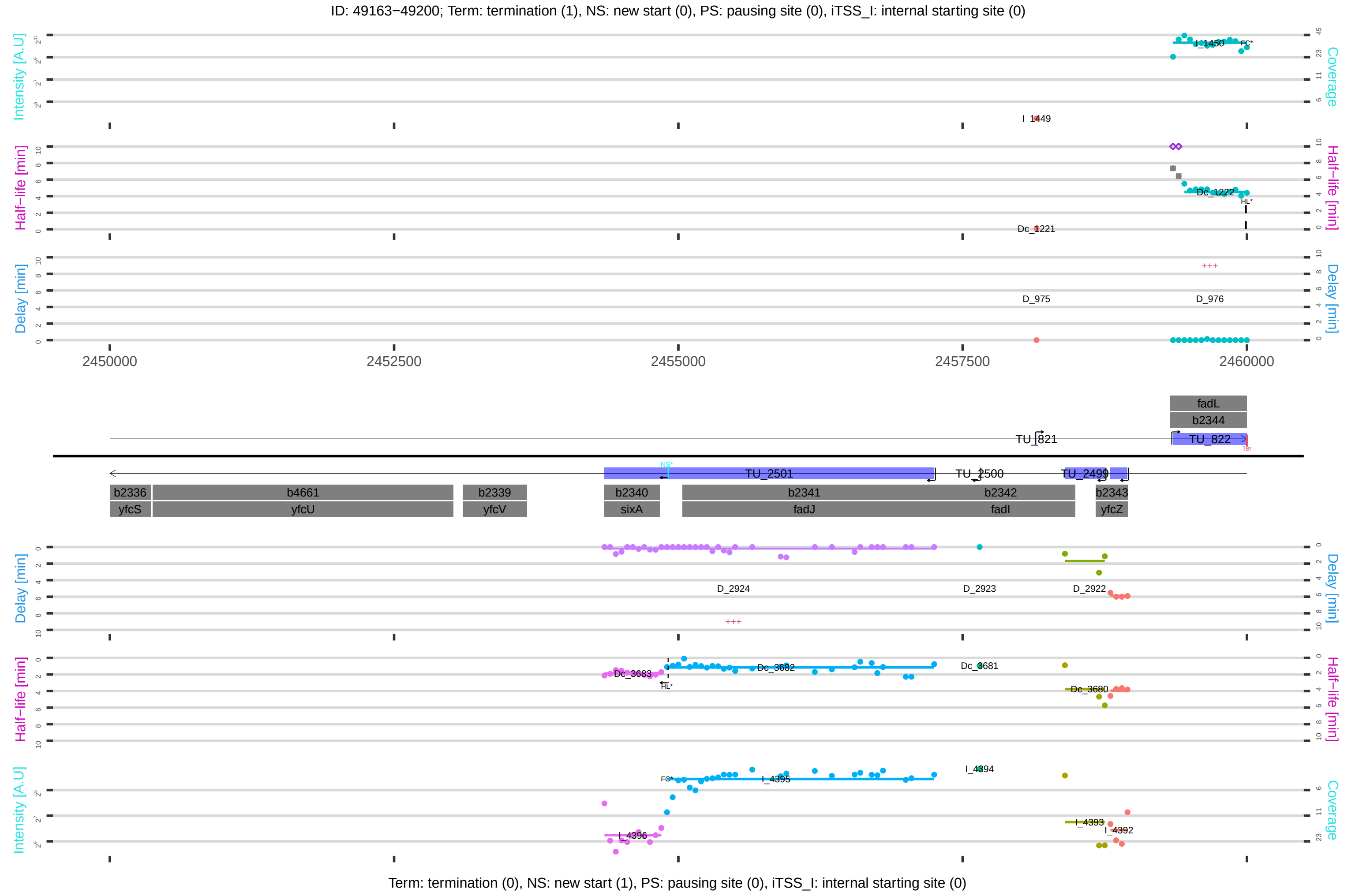
ID: 49163−49200; Term: termination (1), NS: new start (0), PS: pausing site (0), iTSS_I: internal starting site (0)
Intensity [A.U]
Coverage
Half−life [min]
Delay [min]
I_1450
I_1449
Dc_1222
Dc_1221
D_975
D_976
2450000
2452500
2455000
2457500
2460000
fadL
b2344
TU_821
TU_822
TU_2501
TU_2500
TU_2499
b2336
b4661
b2339
b2340
b2341
b2342
b2343
yfcS
yfcU
yfcV
sixA
fadJ
fadI
yfcZ
D_2924
D_2923
D_2922
Dc_3683
Dc_3682
Dc_3681
Dc_3680
I_4396
I_4395
I_4394
I_4393
I_4392
Term: termination (0), NS: new start (1), PS: pausing site (0), iTSS_I: internal starting site (0)

ID: 49200−49383; Term: termination (5), NS: new start (2), PS: pausing site (0), iTSS_I: internal starting site (0)

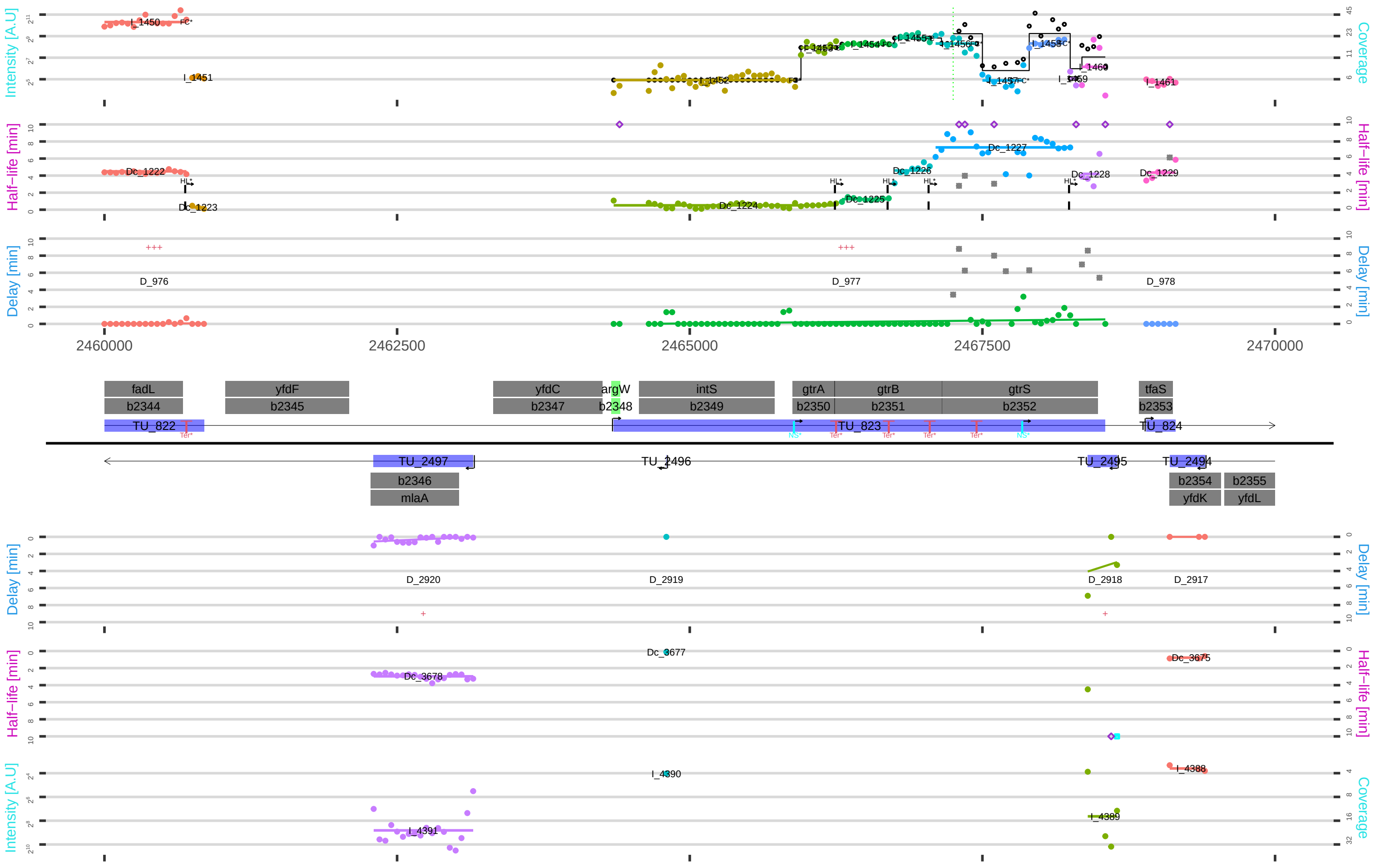

Term: termination (0), NS: new start (0), PS: pausing site (0), iTSS_I: internal starting site (0)

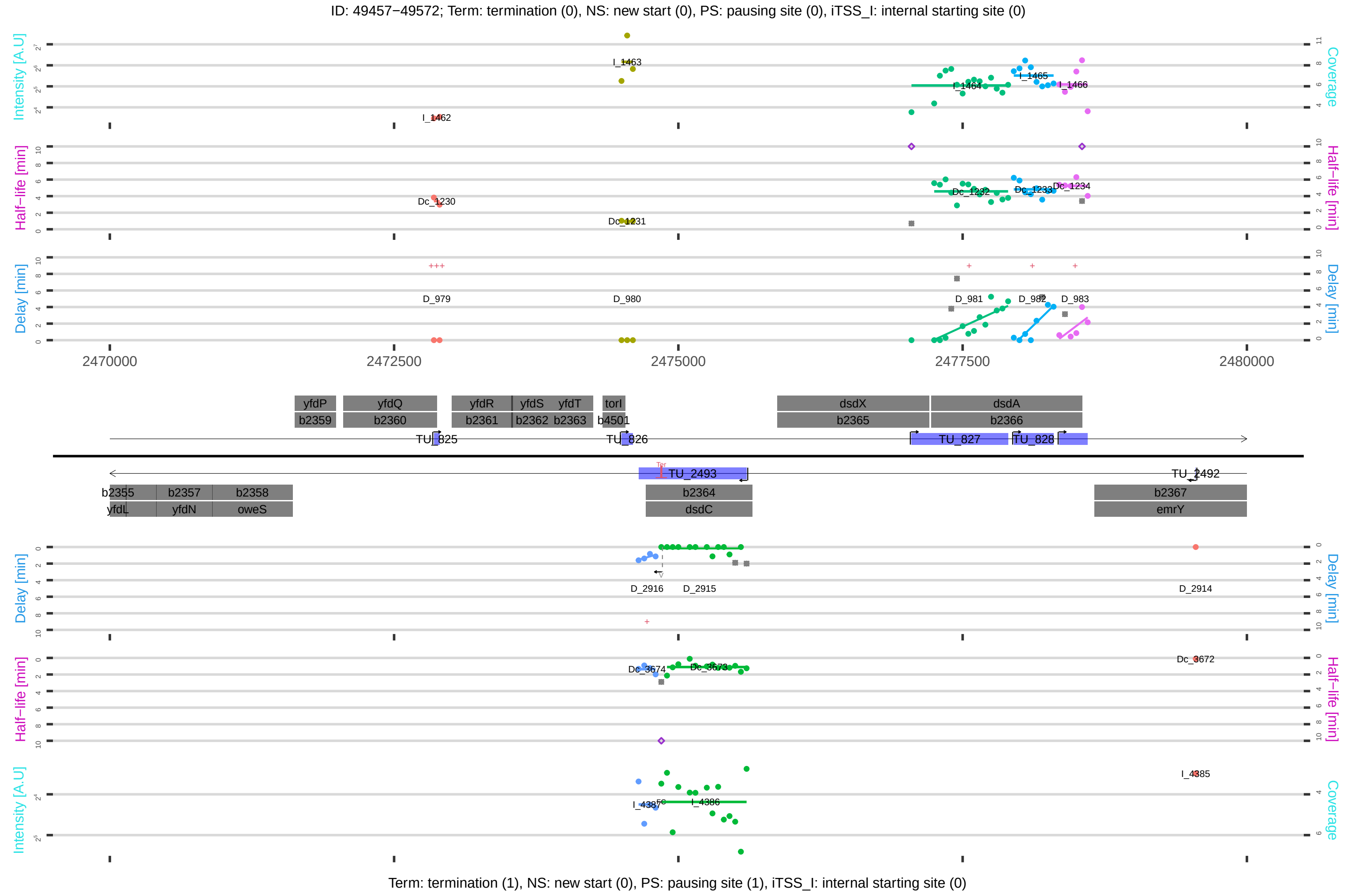
ID: 49457−49572; Term: termination (0), NS: new start (0), PS: pausing site (0), iTSS_I: internal starting site (0)
Intensity [A.U]
Coverage
Half−life [min]
Delay [min]
I_1462
I_1463
I_1464
I_1465
I_1466
Dc_1230
Dc_1231
Dc_1232
Dc_1233
Dc_1234
D_979
D_980
D_981
D_982
D_983
2470000
2472500
2475000
2477500
2480000
yfdP
b2359
yfdQ
b2360
yfdR
b2361
yfdS
b2362
yfdT
b2363
torI
b4501
dsdX
b2365
dsdA
b2366
TU_825
TU_826
TU_827
TU_828
TU_2493
TU_2492
Ter
b2355
yfdL
b2357
yfdN
b2358
oweS
b2364
dsdC
b2367
emrY
D_2916
D_2915
D_2914
Dc_3674
Dc_3673
Dc_3672
I_4387
I_4386
I_4385
Term: termination (1), NS: new start (0), PS: pausing site (1), iTSS_I: internal starting site (0)

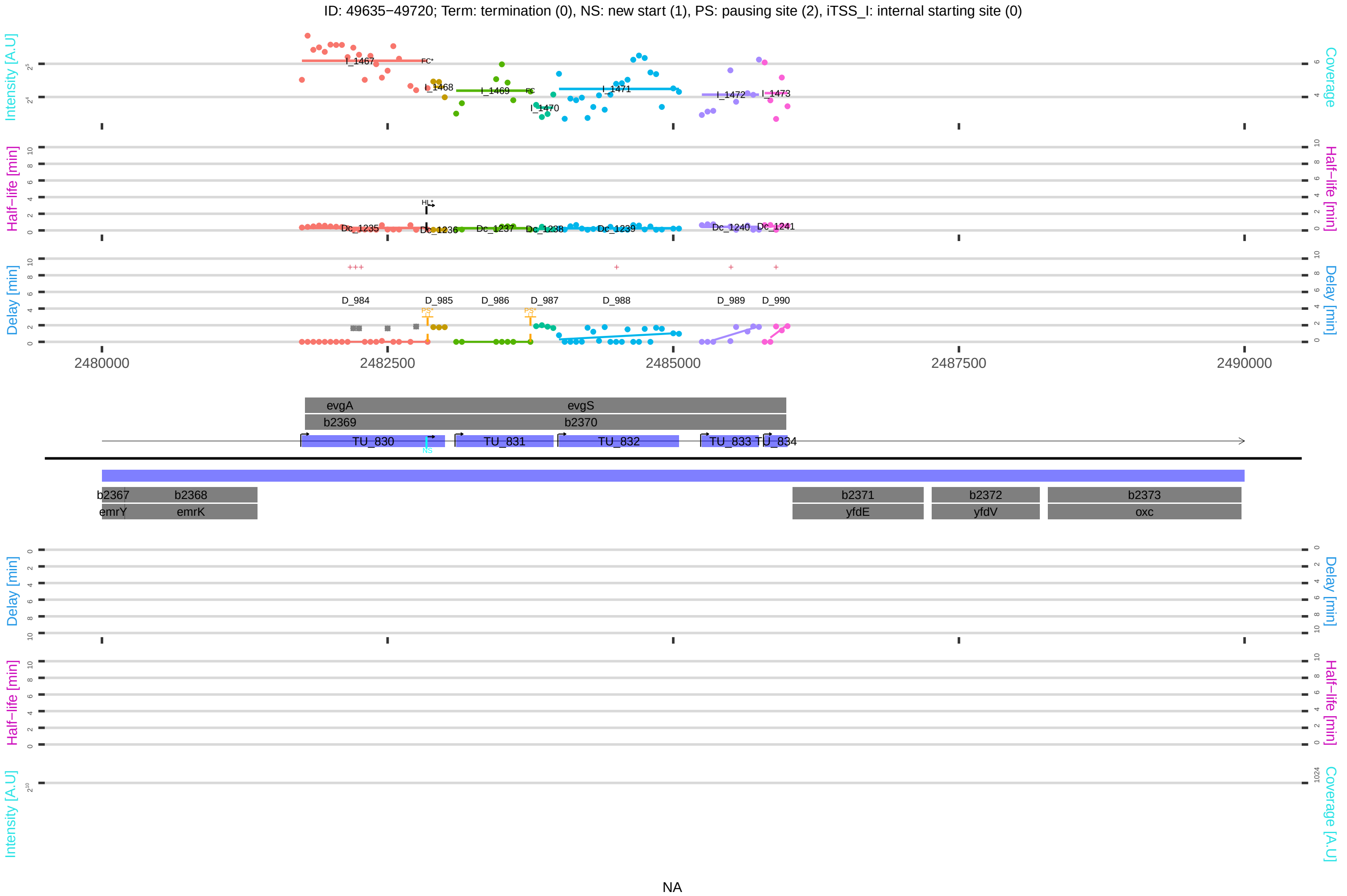
ID: 49635−49720; Term: termination (0), NS: new start (1), PS: pausing site (2), iTSS_I: internal starting site (0)
Intensity [A.U]
Coverage
I_1467
I_1468
I_1469
I_1470
I_1471
I_1472
I_1473
Half−life [min]
Dc_1235
Dc_1236
Dc_1237
Dc_1238
Dc_1239
Dc_1240
Dc_1241
Delay [min]
D_984
D_985
D_986
D_987
D_988
D_989
D_990
2480000
2482500
2485000
2487500
2490000
evgA
evgS
b2369
b2370
TU_830
TU_831
TU_832
TU_833
TU_834
NS
b2367
b2368
emrY
emrK
b2371
b2372
b2373
yfdE
yfdV
oxc
NA

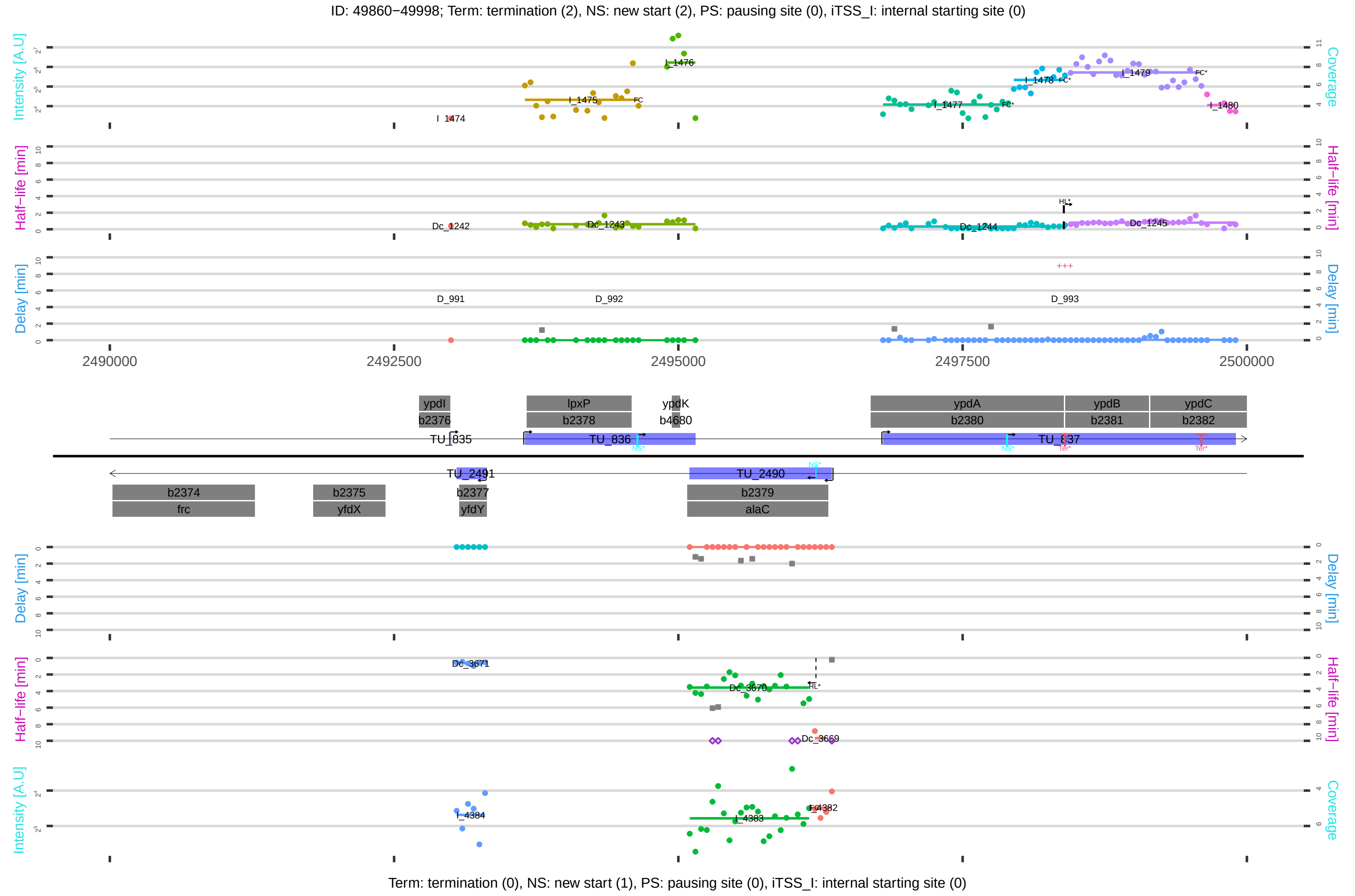
ID: 49860−49998; Term: termination (2), NS: new start (2), PS: pausing site (0), iTSS_I: internal starting site (0)
Intensity [A.U]
Coverage
Half−life [min]
Delay [min]
I_1474
I_1475
I_1476
I_1477
I_1478
I_1479
I_1480
Dc_1242
Dc_1243
Dc_1244
Dc_1245
D_991
D_992
D_993
2490000
2492500
2495000
2497500
2500000
ypdI
b2376
lpxP
b2378
ypdK
b4680
ypdA
b2380
ypdB
b2381
ypdC
b2382
TU_835
TU_836
TU_837
TU_2491
TU_2490
b2374
frc
b2375
yfdX
b2377
yfdY
b2379
alaC
Dc_3671
Dc_3670
Dc_3669
I_4384
I_4383
I_4382
Term: termination (0), NS: new start (1), PS: pausing site (0), iTSS_I: internal starting site (0)

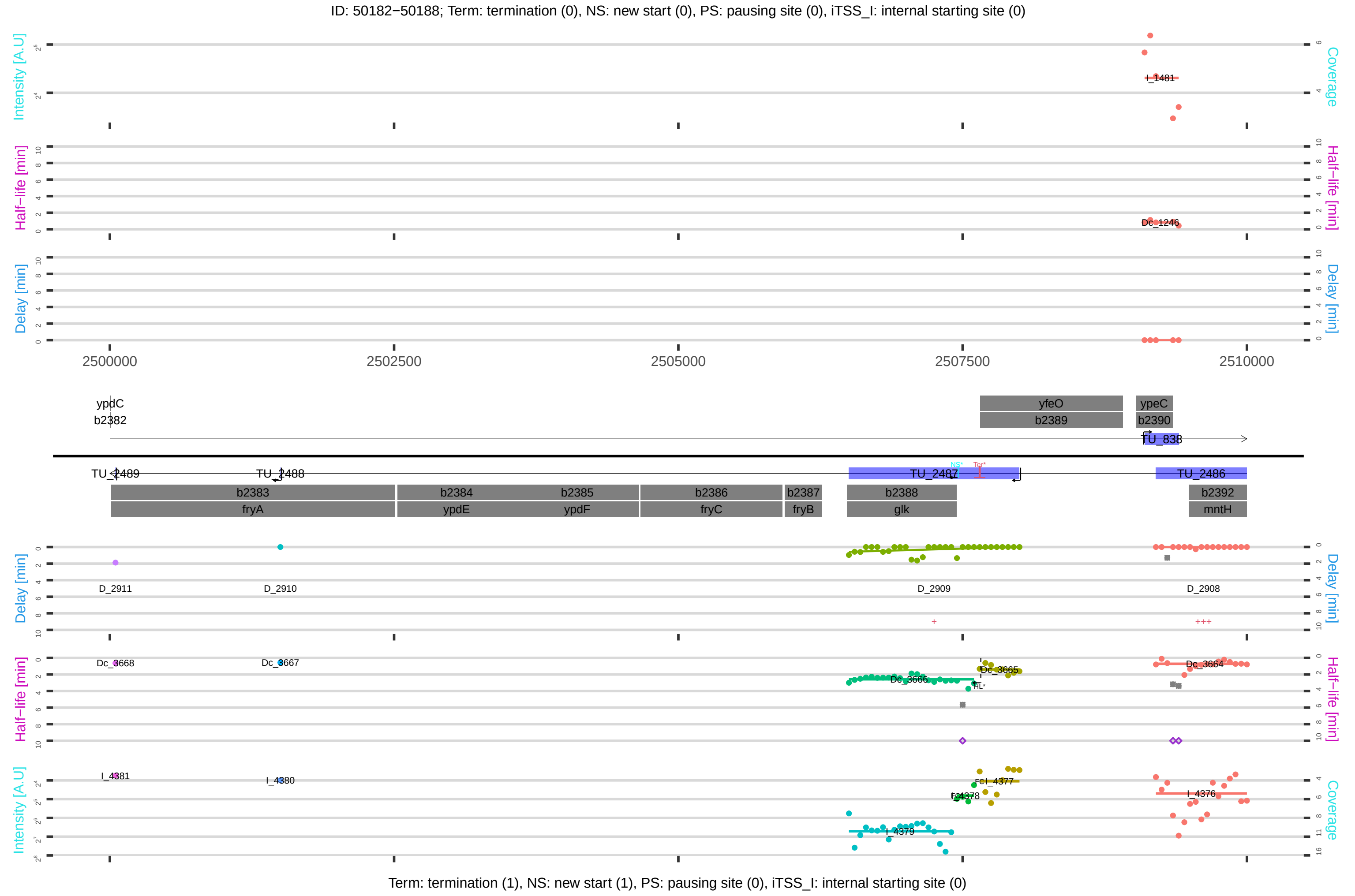

ID: 50182−50188; Term: termination (0), NS: new start (0), PS: pausing site (0), iTSS_I: internal starting site (0)
Intensity [A.U]
Coverage
I_1481
Half−life [min]
Dc_1246
Delay [min]
2500000
2502500
2505000
2507500
2510000
ypdC
b2382
yfeO
b2389
ypeC
b2390
TU_838
TU_2489
TU_2488
TU_2487
TU_2486
b2383
fryA
b2384
ypdE
b2385
ypdF
b2386
fryC
b2387
fryB
b2388
glk
b2392
mntH
D_2911
D_2910
D_2909
D_2908
Dc_3668
Dc_3667
Dc_3666
Dc_3665
Dc_3664
I_4381
I_4380
I_4379
I_4377
I_4378
I_4376
Term: termination (1), NS: new start (1), PS: pausing site (0), iTSS_I: internal starting site (0)

ID: 50219−50390; Term: termination (2), NS: new start (2), PS: pausing site (0), iTSS_I: internal starting site (0)

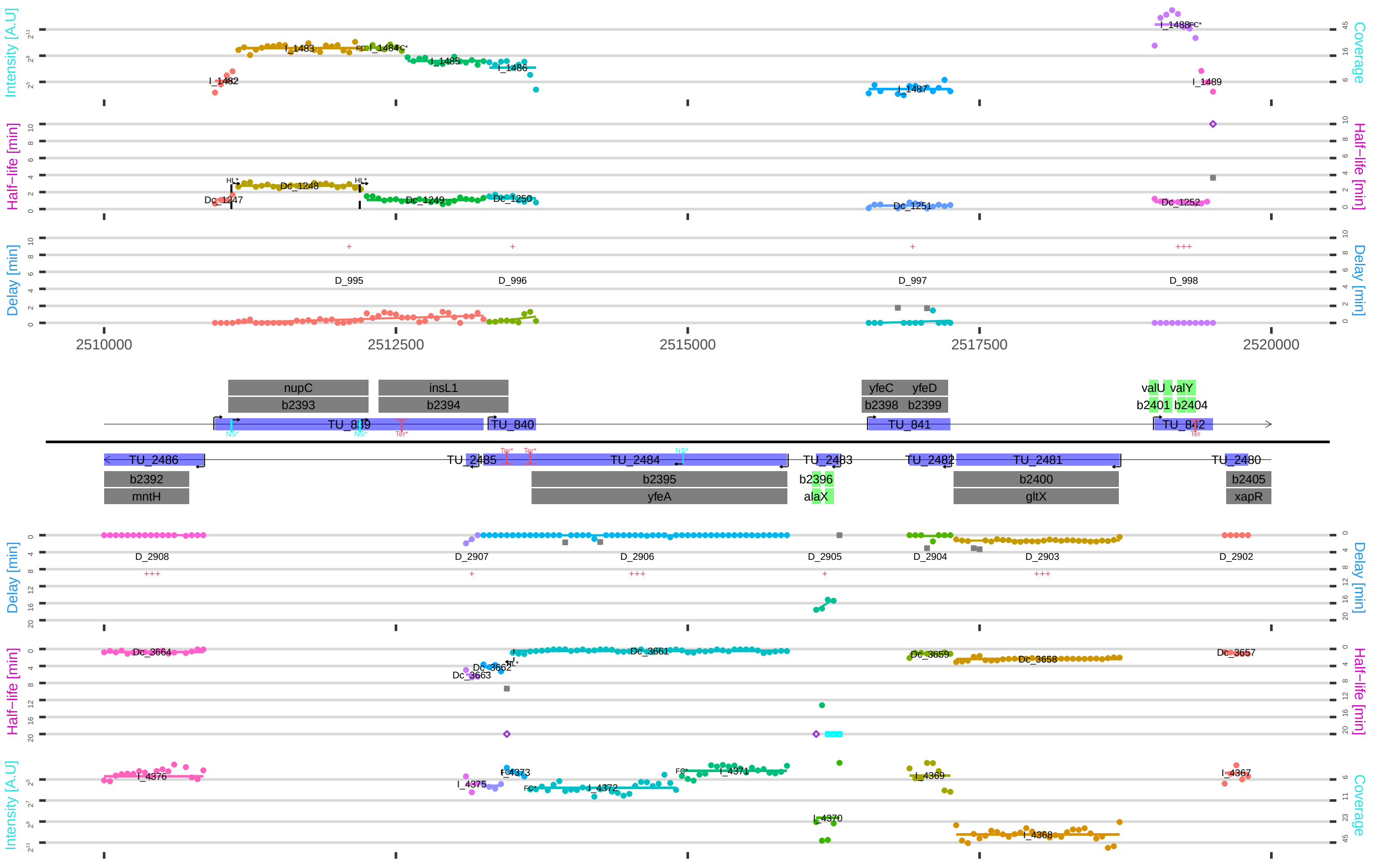

Term: termination (2), NS: new start (1), PS: pausing site (0), iTSS_I: internal starting site (0)

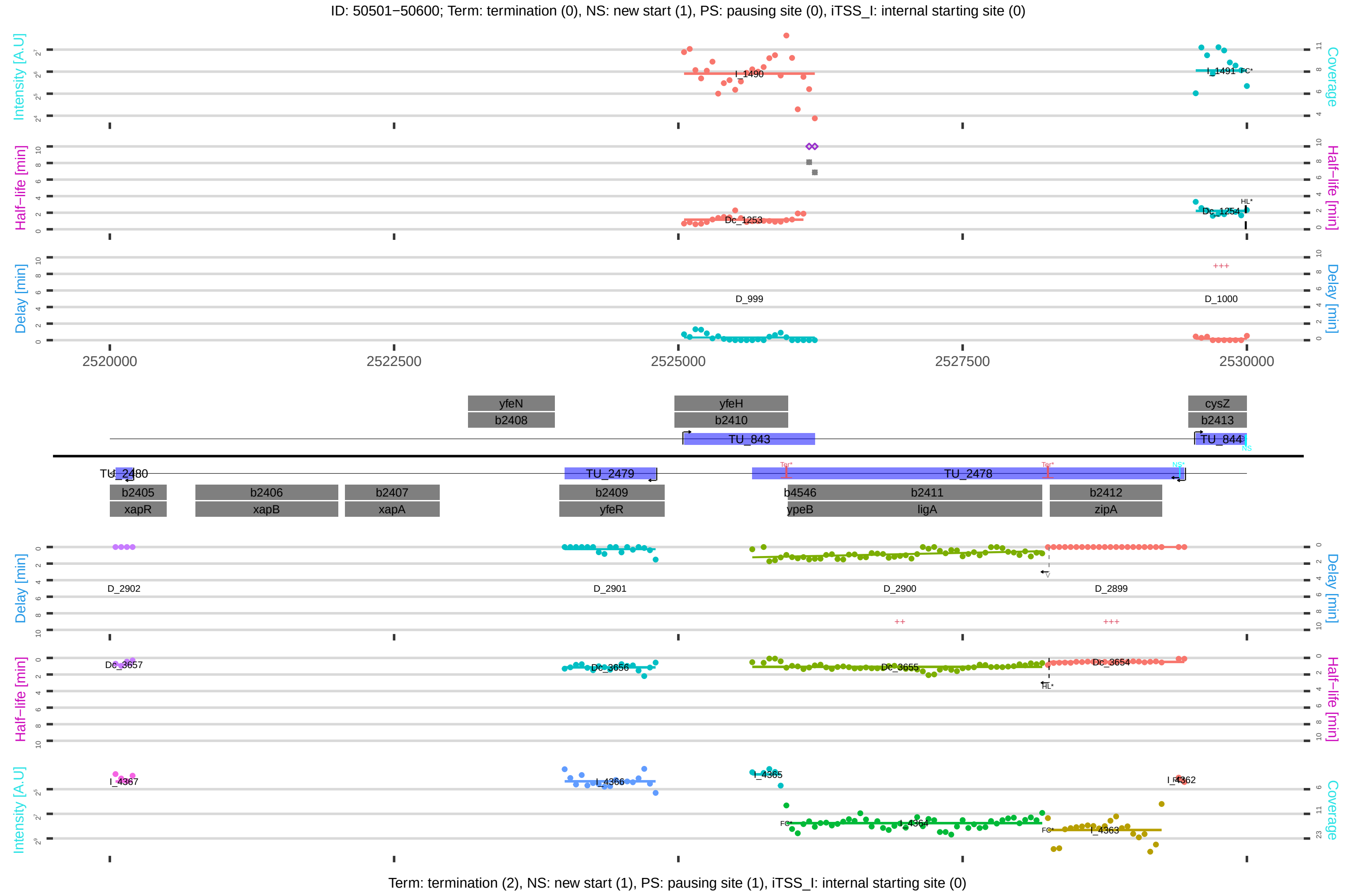
ID: 50501−50600; Term: termination (0), NS: new start (1), PS: pausing site (0), iTSS_I: internal starting site (0)
Intensity [A.U]
Coverage
Half−life [min]
Delay [min]
I_1490
I_1491
FC*
Dc_1253
Dc_1254
HL*
D_999
D_1000
+++
2520000
2522500
2525000
2527500
2530000
yfeN
b2408
yfeH
b2410
cysZ
b2413
TU_843
TU_844
NS
TU_2480
TU_2479
TU_2478
Ter*
NS*
b2405
xapR
b2406
xapB
b2407
xapA
b2409
yfeR
b4546
ypeB
b2411
ligA
b2412
zipA
D_2902
D_2901
D_2900
D_2899
++
+++
Dc_3657
Dc_3656
Dc_3655
Dc_3654
I_4367
I_4366
I_4365
I_4364
I_4363
I_4362
Term: termination (2), NS: new start (1), PS: pausing site (1), iTSS_I: internal starting site (0)

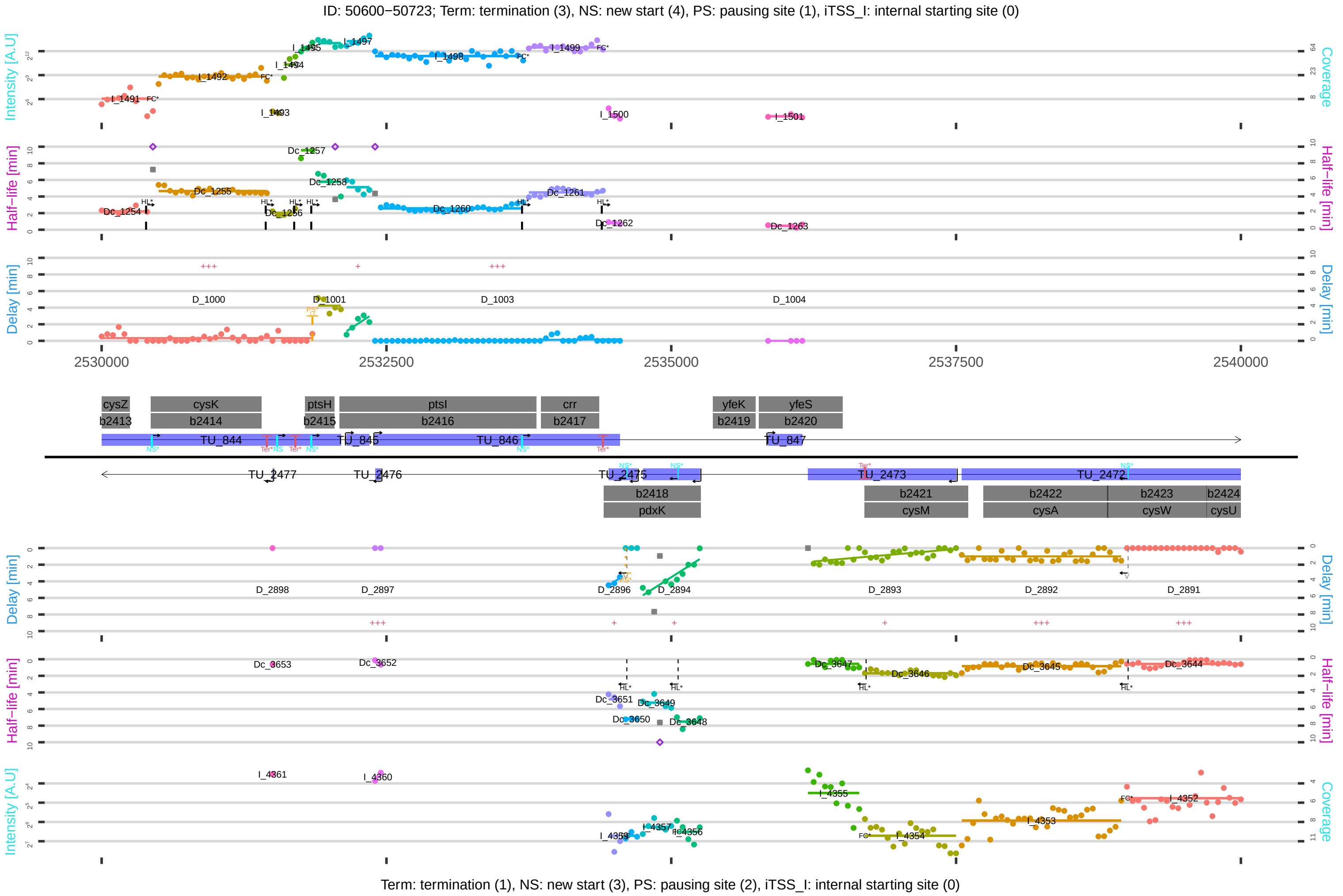

ID: 50600−50723; Term: termination (3), NS: new start (4), PS: pausing site (1), iTSS_I: internal starting site (0)
Intensity [A.U]
Coverage
Half−life [min]
Delay [min]
2530000
2532500
2535000
2537500
2540000
cysZ
b2413
cysK
b2414
ptsH
b2415
ptsI
b2416
crr
b2417
yfeK
b2419
yfeS
b2420
TU_844
TU_845
TU_846
TU_847
TU_2477
TU_2476
TU_2475
TU_2473
TU_2472
b2418
pdxK
b2421
cysM
b2422
cysA
b2423
cysW
b2424
cysU
Term: termination (1), NS: new start (3), PS: pausing site (2), iTSS_I: internal starting site (0)

ID: 50877−50952; Term: termination (1), NS: new start (3), PS: pausing site (1), iTSS_I: internal starting site (0)

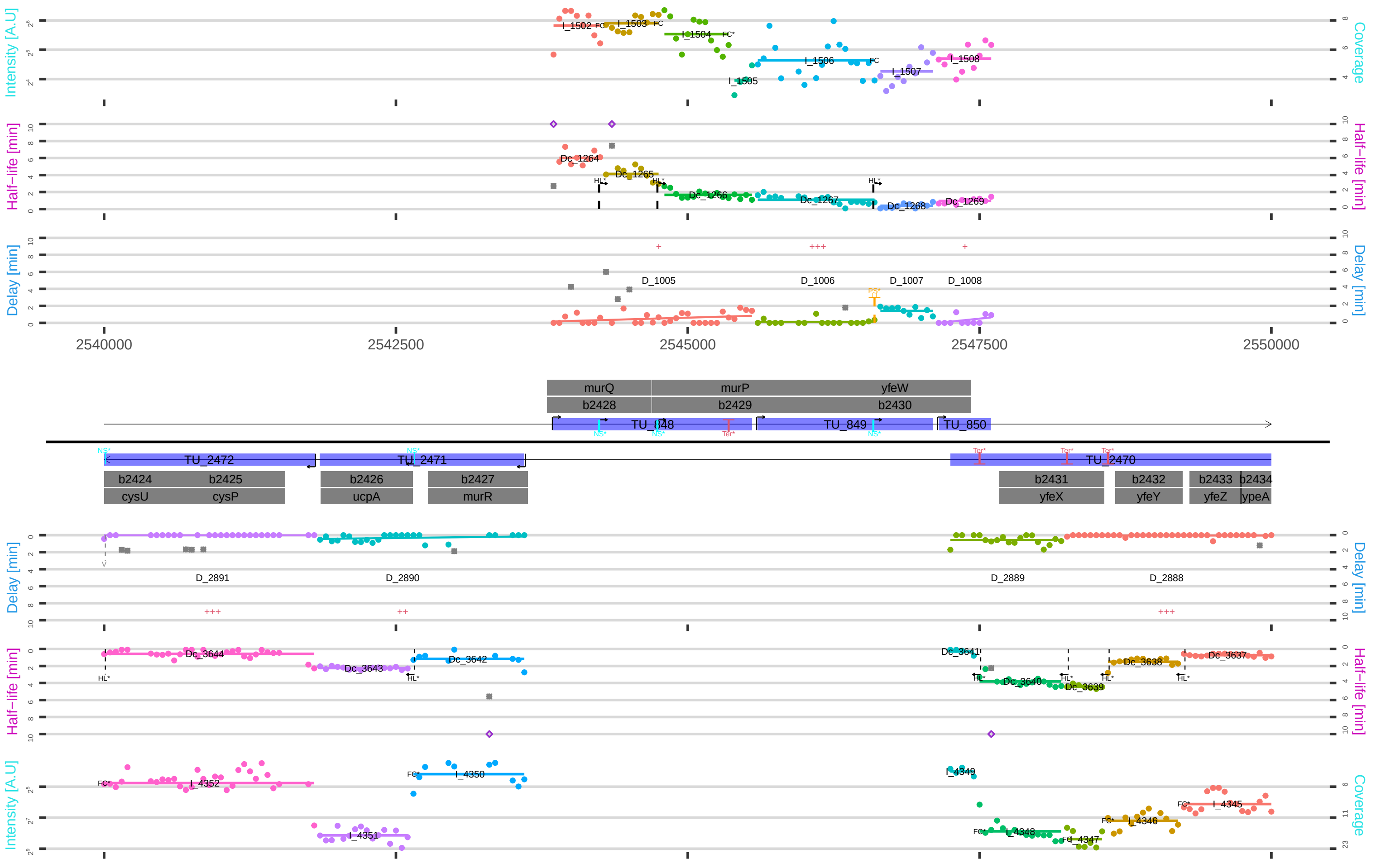

Term: termination (3), NS: new start (2), PS: pausing site (1), iTSS_I: internal starting site (0)

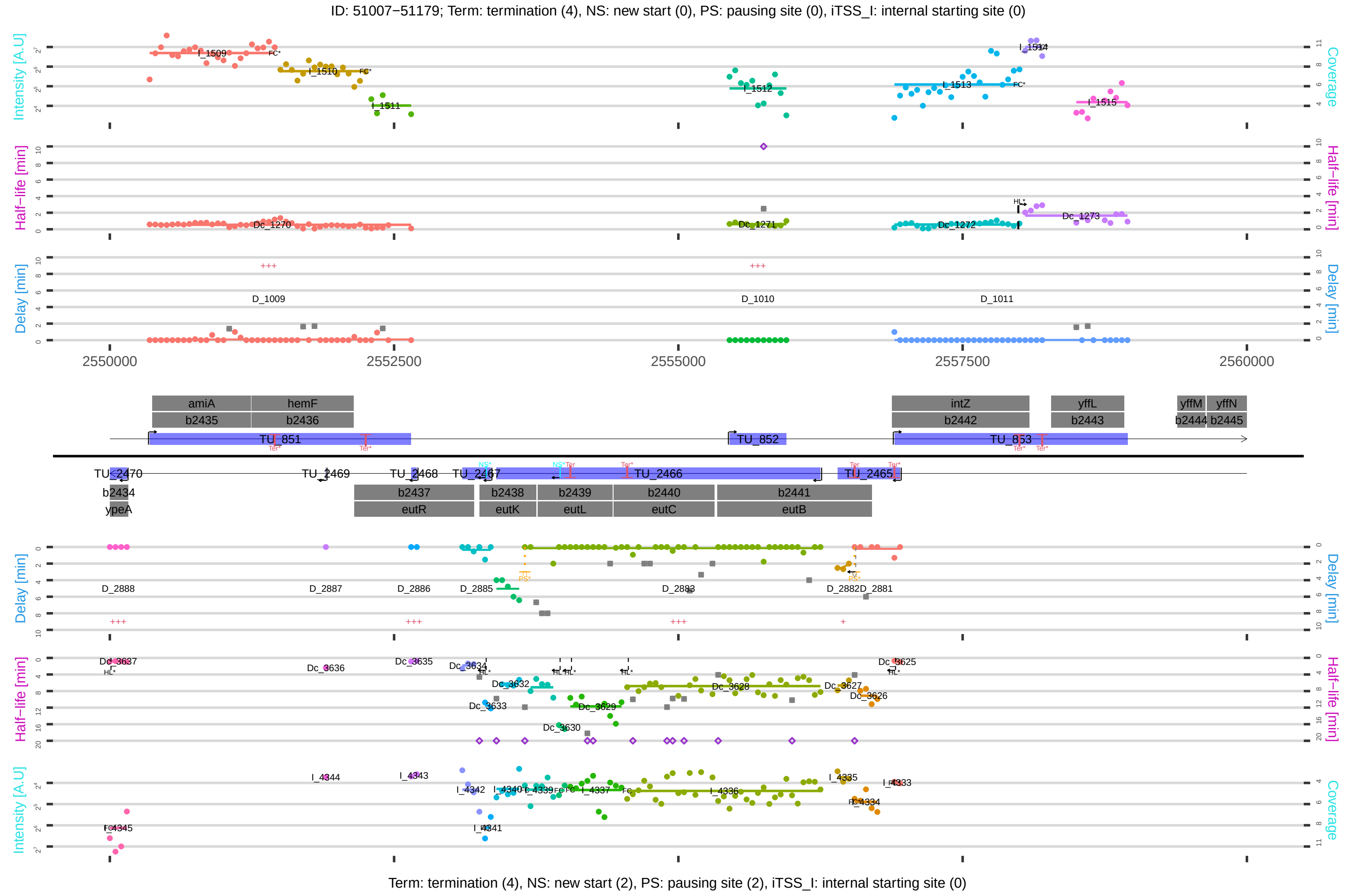

ID: 51007−51179; Term: termination (4), NS: new start (0), PS: pausing site (0), iTSS_I: internal starting site (0)
Intensity [A.U]
Coverage
Half−life [min]
Delay [min]
I_1509
I_1510
I_1511
I_1512
I_1513
I_1514
I_1515
Dc_1270
Dc_1271
Dc_1272
Dc_1273
D_1009
D_1010
D_1011
2550000
2552500
2555000
2557500
2560000
amiA
hemF
b2435
b2436
intZ
b2442
yffL
b2443
yffM
yffN
b2444
b2445
TU_851
TU_852
TU_853
TU_2470
TU_2469
TU_2468
TU_2467
TU_2466
TU_2465
b2434
ypeA
b2437
eutR
b2438
eutK
b2439
eutL
b2440
eutC
b2441
eutB
D_2888
D_2887
D_2886
D_2885
D_2883
D_2882
D_2881
Dc_3637
Dc_3636
Dc_3635
Dc_3634
Dc_3633
Dc_3632
Dc_3630
Dc_3629
Dc_3628
Dc_3627
Dc_3626
Dc_3625
I_4345
I_4344
I_4343
I_4342
I_4341
I_4340
I_4339
I_4337
I_4336
I_4335
I_4334
I_4333
Term: termination (4), NS: new start (2), PS: pausing site (2), iTSS_I: internal starting site (0)

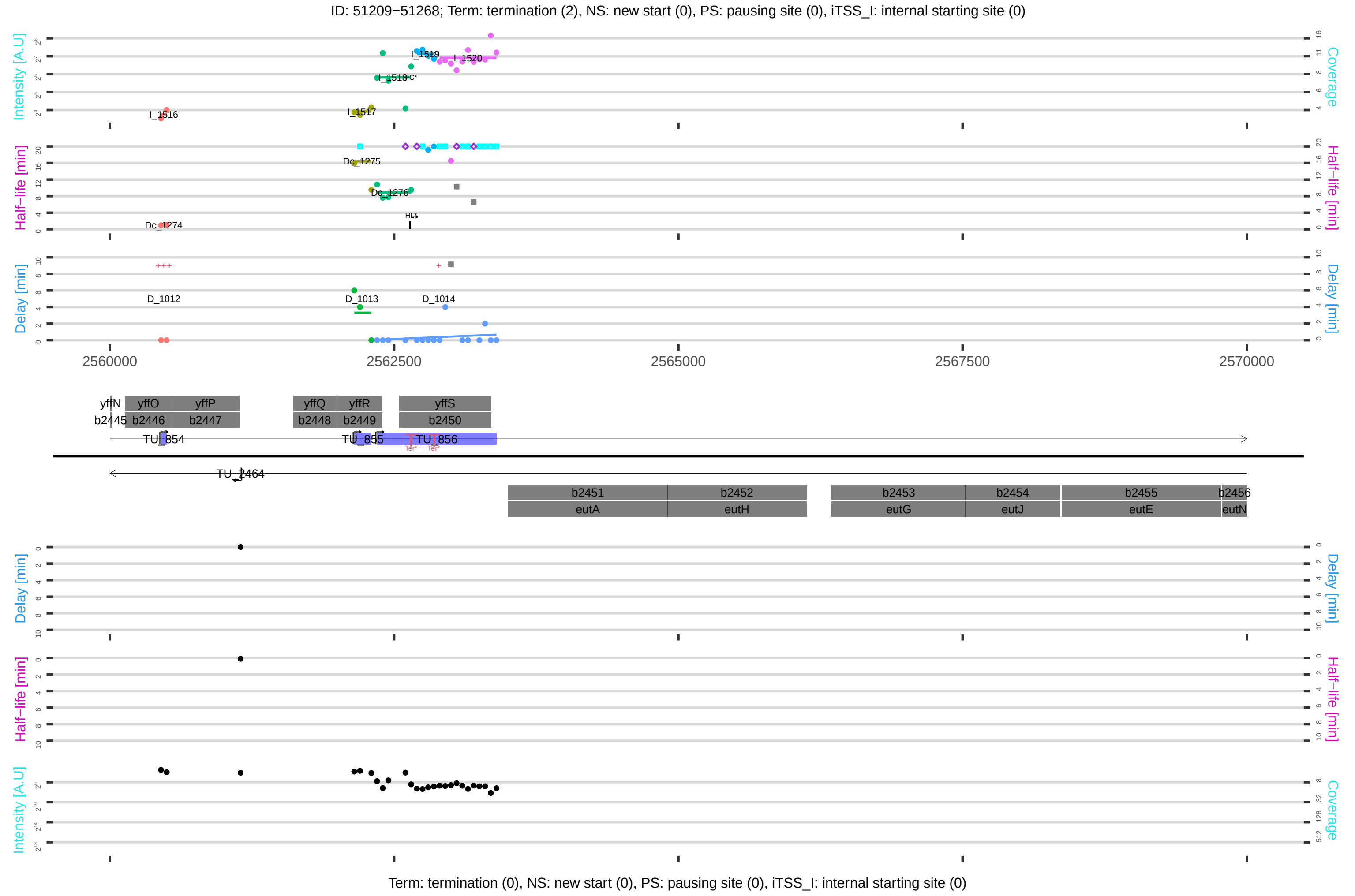

ID: 51209−51268; Term: termination (2), NS: new start (0), PS: pausing site (0), iTSS_I: internal starting site (0)
Intensity [A.U]
Coverage
Half−life [min]
Delay [min]
I_1516
I_1517
I_1518
I_1519
I_1520
Dc_1274
Dc_1275
Dc_1276
D_1012
D_1013
D_1014
2560000
2562500
2565000
2567500
2570000
yffN
yffO
yffP
yffQ
yffR
yffS
b2445
b2446
b2447
b2448
b2449
b2450
TU_854
TU_855
TU_856
TU_2464
b2451
b2452
b2453
b2454
b2455
b2456
eutA
eutH
eutG
eutJ
eutE
eutN
Term: termination (0), NS: new start (0), PS: pausing site (0), iTSS_I: internal starting site (0)

ID: 51536−51596; Term: termination (2), NS: new start (2), PS: pausing site (1), iTSS_I: internal starting site (0)

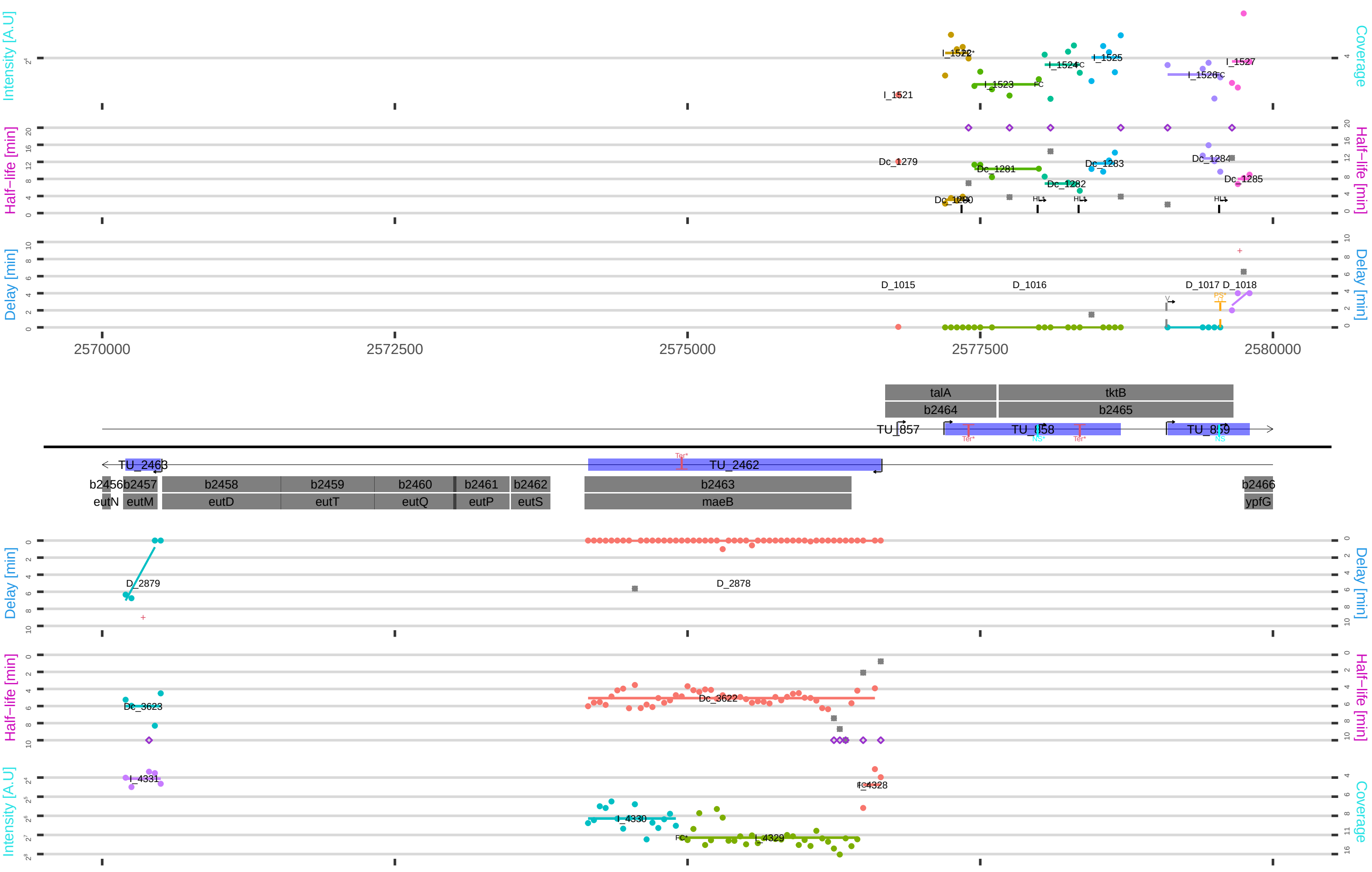

Term: termination (1), NS: new start (0), PS: pausing site (0), iTSS_I: internal starting site (0)

ID: 51675−51800; Term: termination (1), NS: new start (1), PS: pausing site (0), iTSS_I: internal starting site (0)

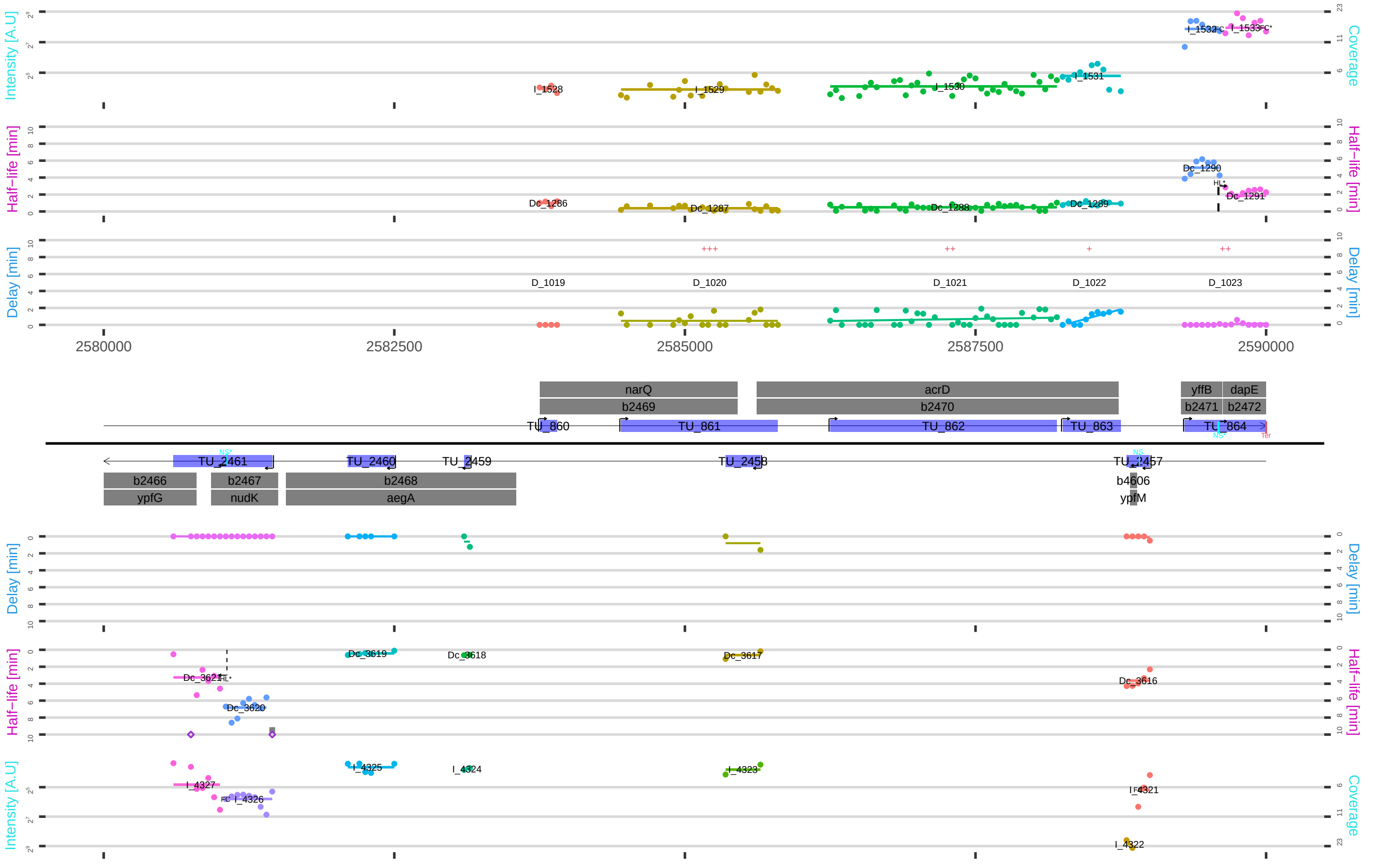

Term: termination (0), NS: new start (2), PS: pausing site (0), iTSS_I: internal starting site (0)

ID: 51800−51980; Term: termination (1), NS: new start (1), PS: pausing site (1), iTSS_I: internal starting site (0)

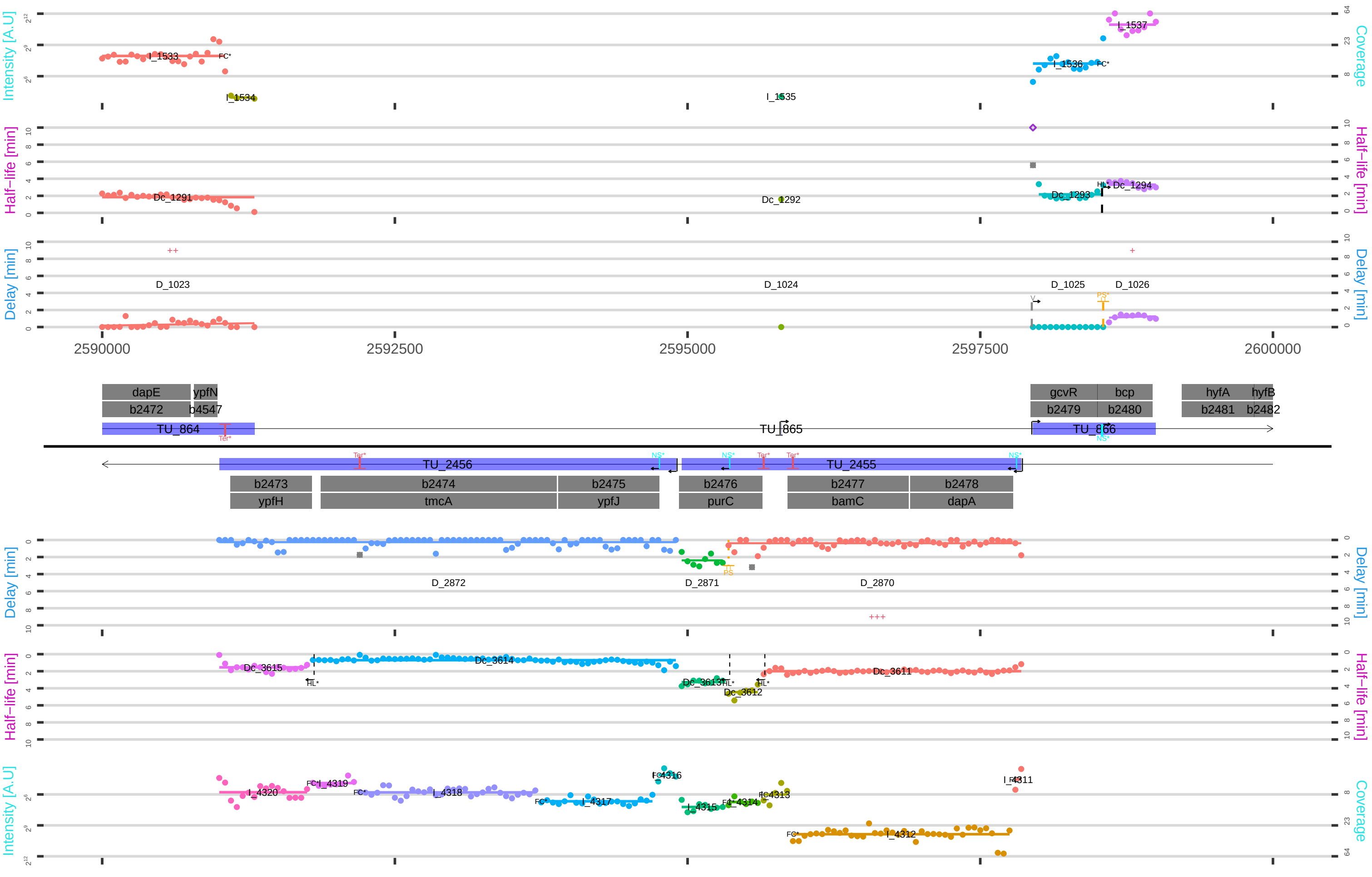

Term: termination (3), NS: new start (3), PS: pausing site (1), iTSS_I: internal starting site (0)

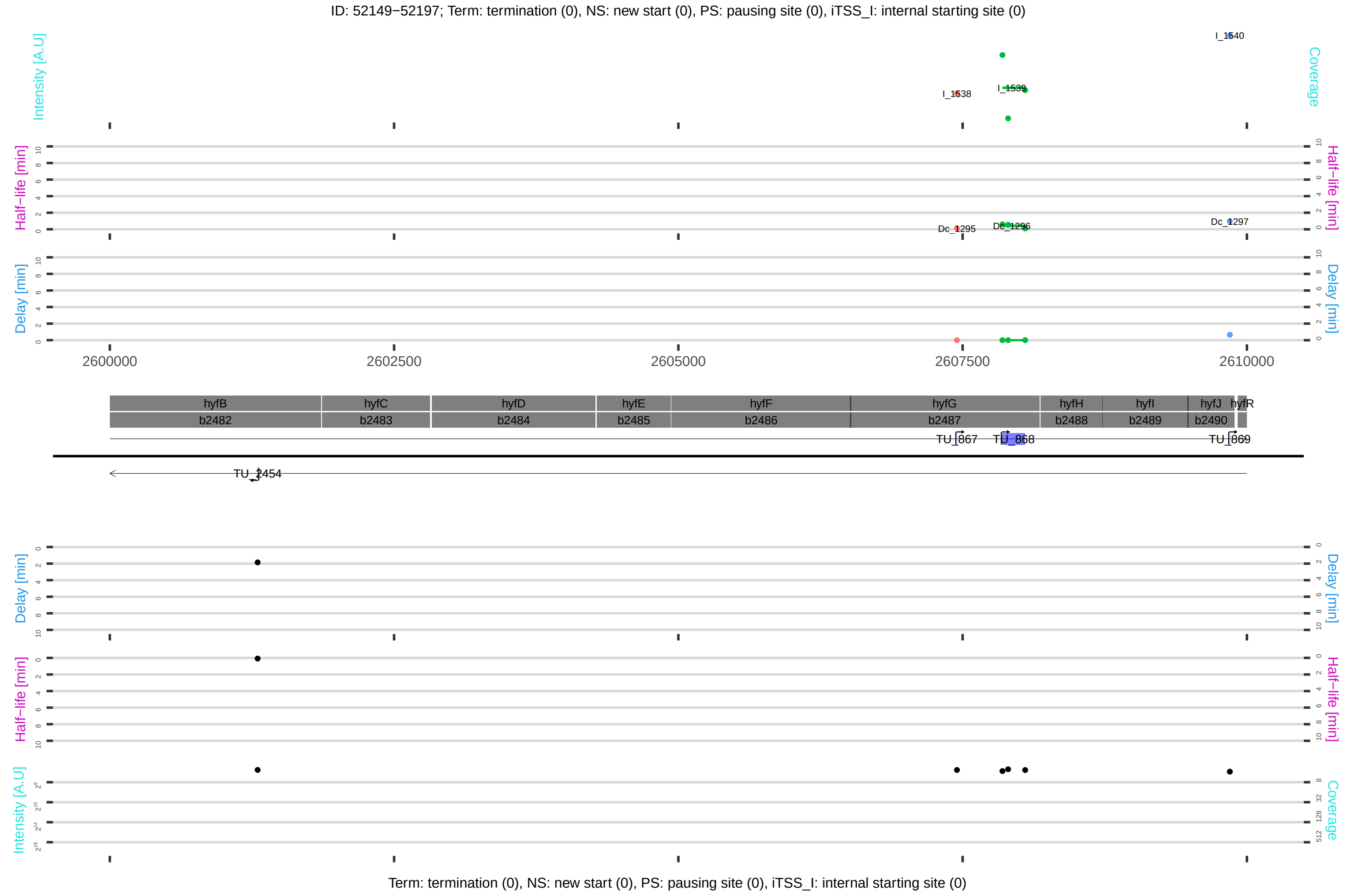
ID: 52149−52197; Term: termination (0), NS: new start (0), PS: pausing site (0), iTSS_I: internal starting site (0)
Intensity [A.U]
Coverage
I_1540
I_1538
I_1539
Half−life [min]
Dc_1295
Dc_1296
Dc_1297
Delay [min]
2600000
2602500
2605000
2607500
2610000
hyfB
hyfC
hyfD
hyfE
hyfF
hyfG
hyfH
hyfI
hyfJ
hyfR
b2482
b2483
b2484
b2485
b2486
b2487
b2488
b2489
b2490
TU_867
TU_868
TU_869
TU_2454
Term: termination (0), NS: new start (0), PS: pausing site (0), iTSS_I: internal starting site (0)

ID: 52220−52400; Term: termination (0), NS: new start (3), PS: pausing site (1), iTSS_I: internal starting site (0)

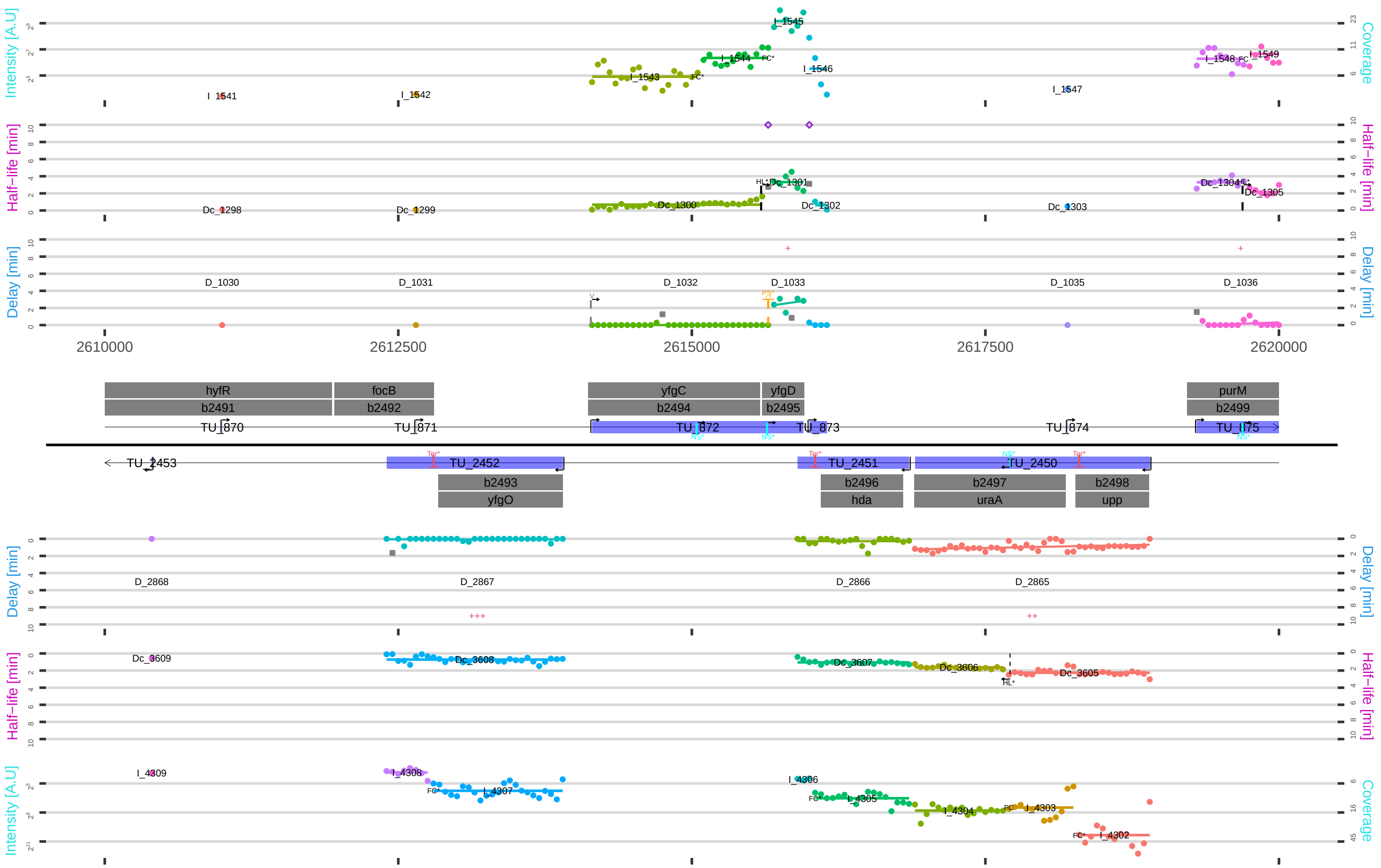

Term: termination (3), NS: new start (1), PS: pausing site (0), iTSS_I: internal starting site (0)

ID: 52400−52552; Term: termination (1), NS: new start (2), PS: pausing site (1), iTSS_I: internal starting site (0)

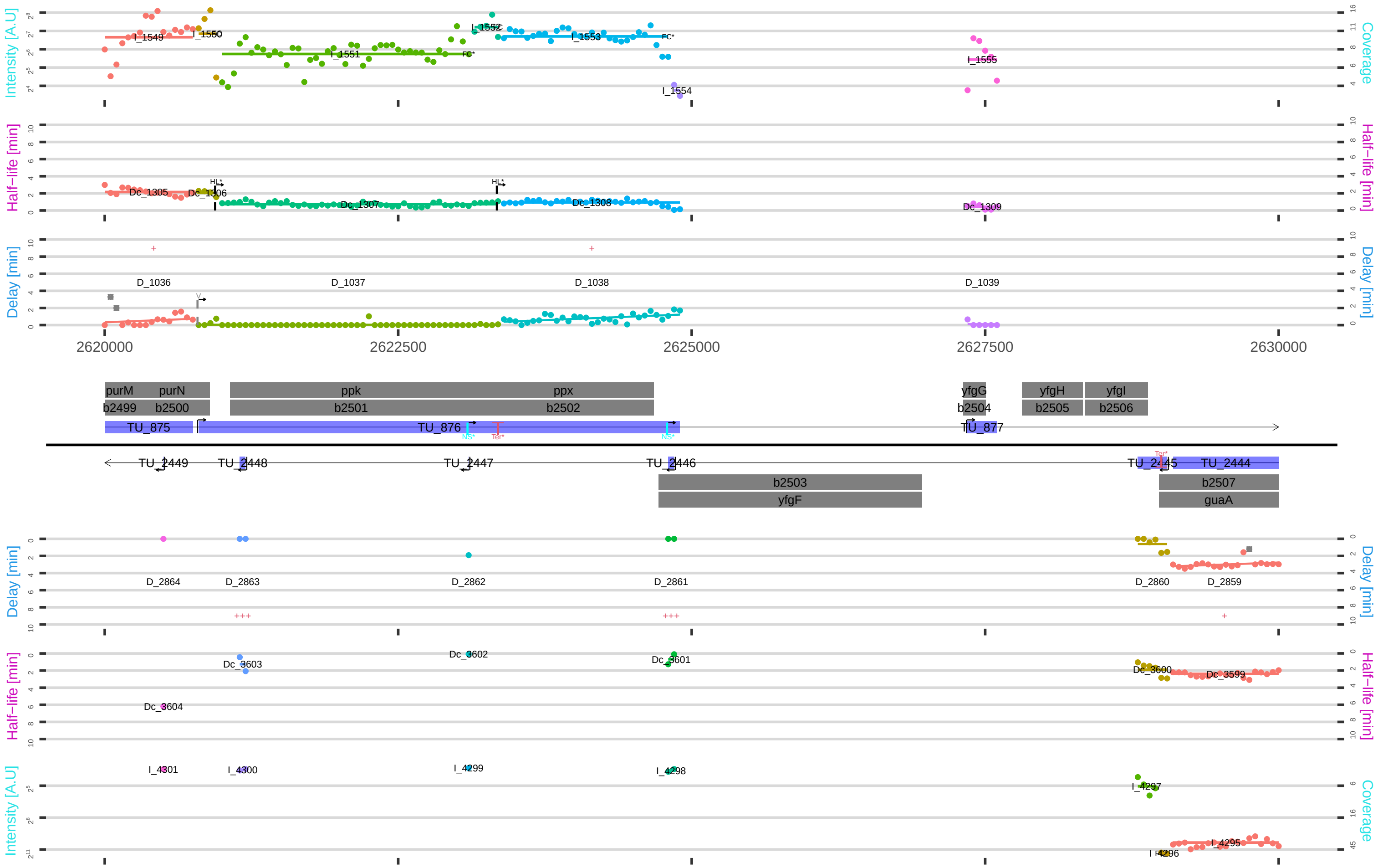

Term: termination (1), NS: new start (0), PS: pausing site (0), iTSS_I: internal starting site (0)

ID: 52646−52784; Term: termination (0), NS: new start (0), PS: pausing site (0), iTSS_I: internal starting site (0)

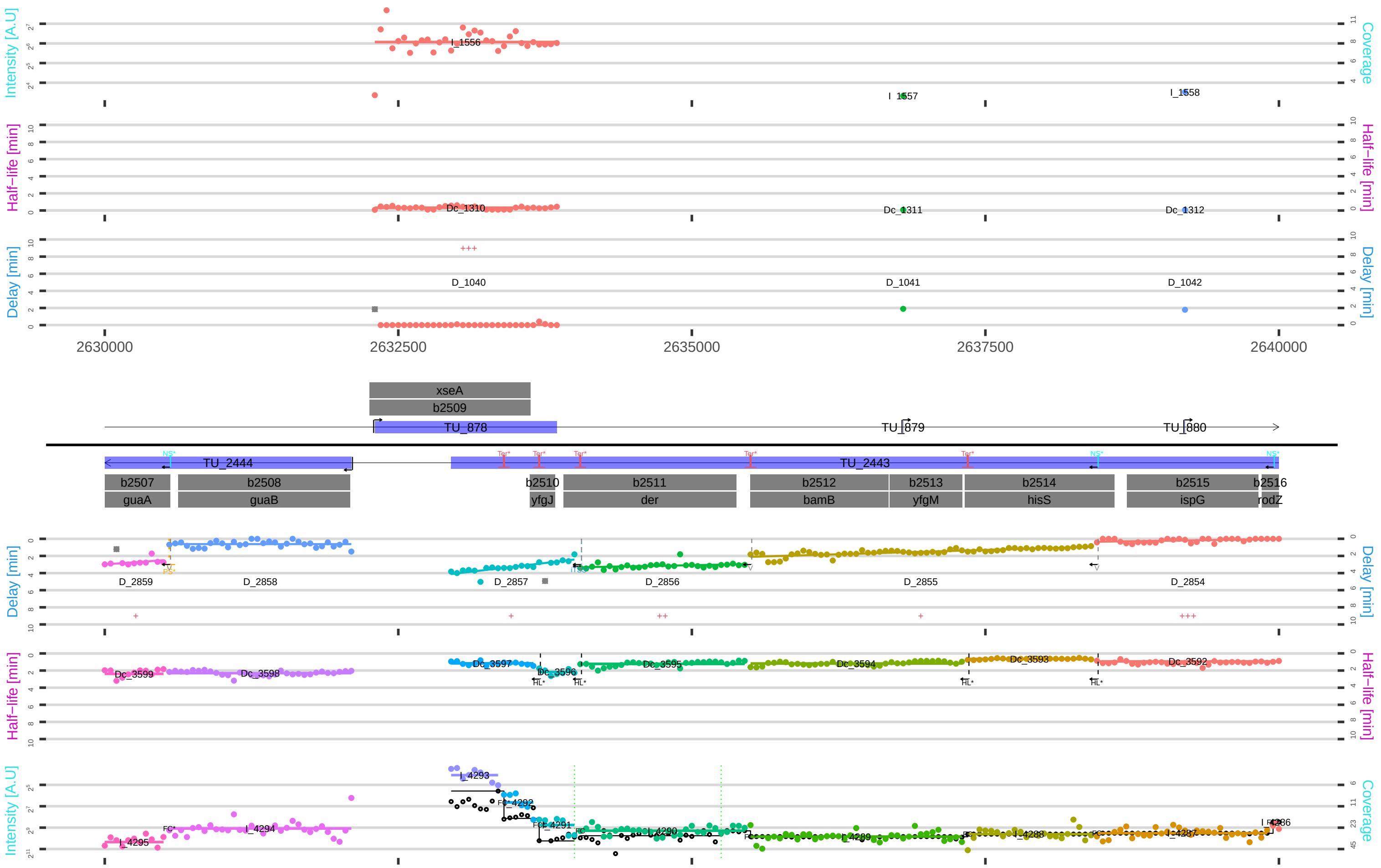

Term: termination (5), NS: new start (3), PS: pausing site (3), iTSS_I: internal starting site (1)

ID: 132789−132589; FC*: significant t−test of two consecutive segments; Term: termination, NS: new start, PS: pausing site,iTSS_I: internal starting site,TI: transcription interferance.

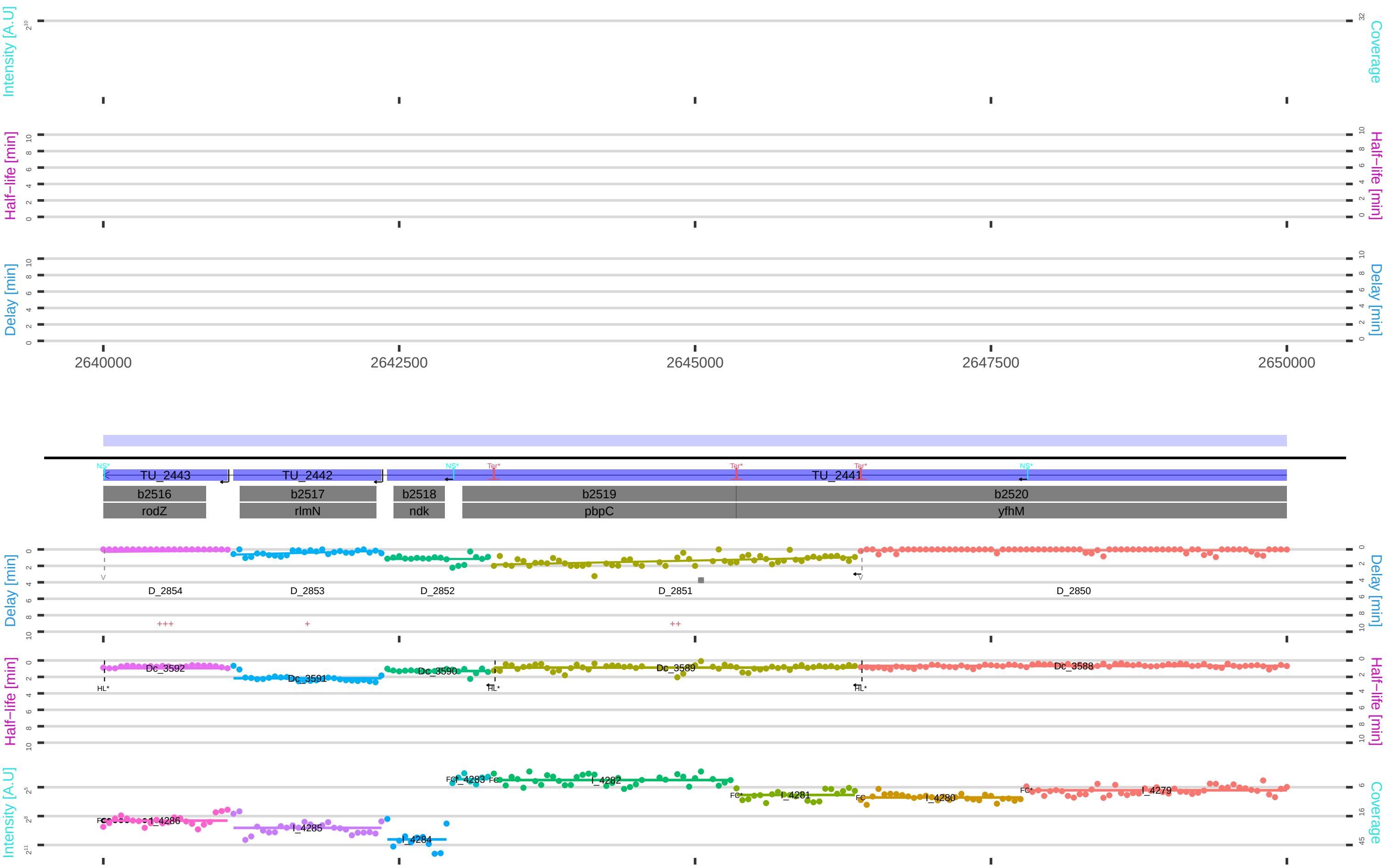

Term: termination (3), NS: new start (3), PS: pausing site (1), iTSS_I: internal starting site (1)

ID: 53011−53090; Term: termination (1), NS: new start (0), PS: pausing site (0), iTSS_I: internal starting site (0)

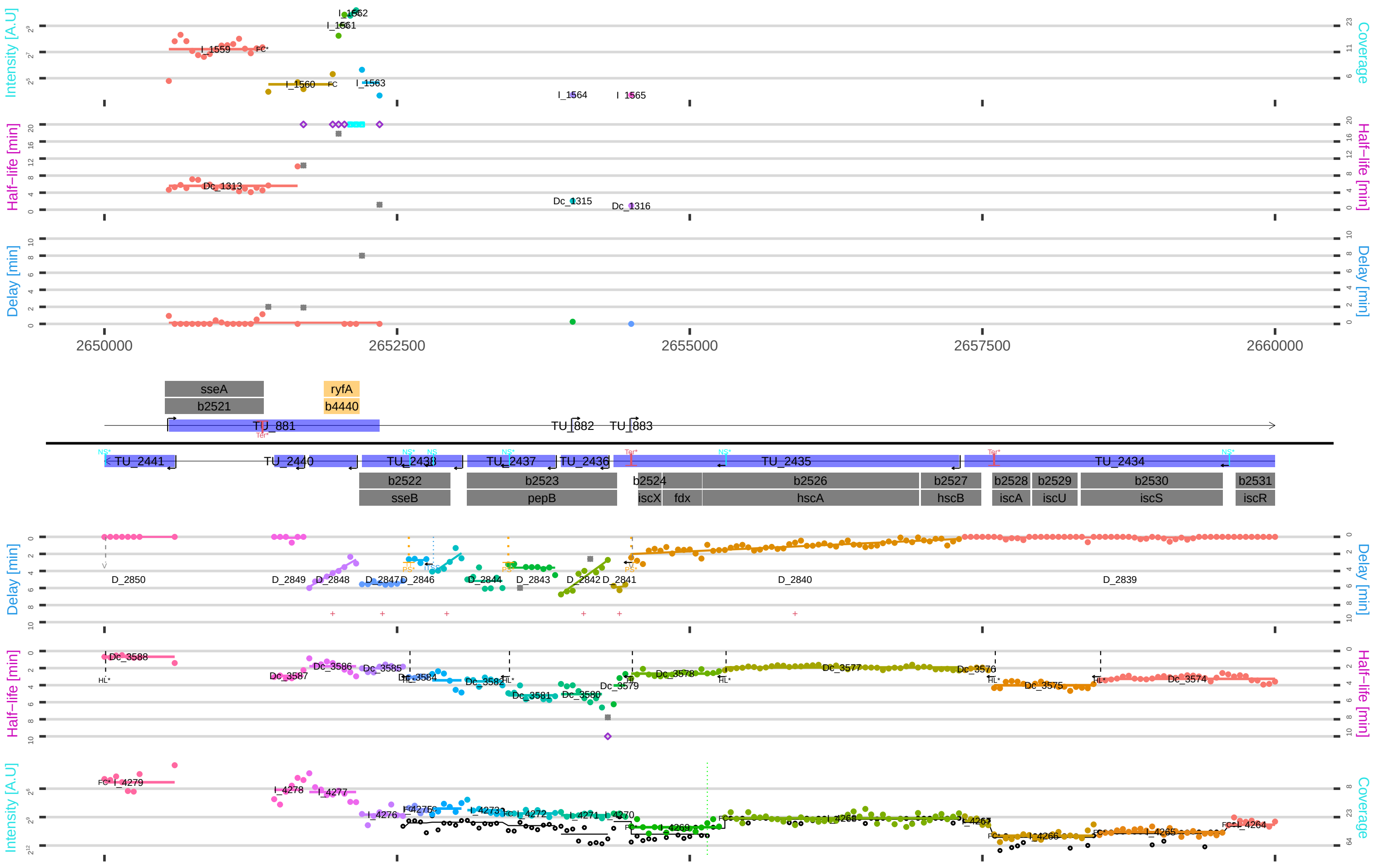

Term: termination (2), NS: new start (6), PS: pausing site (3), iTSS_I: internal starting site (1)

ID: 53203−53359; Term: termination (0), NS: new start (0), PS: pausing site (0), iTSS_I: internal starting site (0)

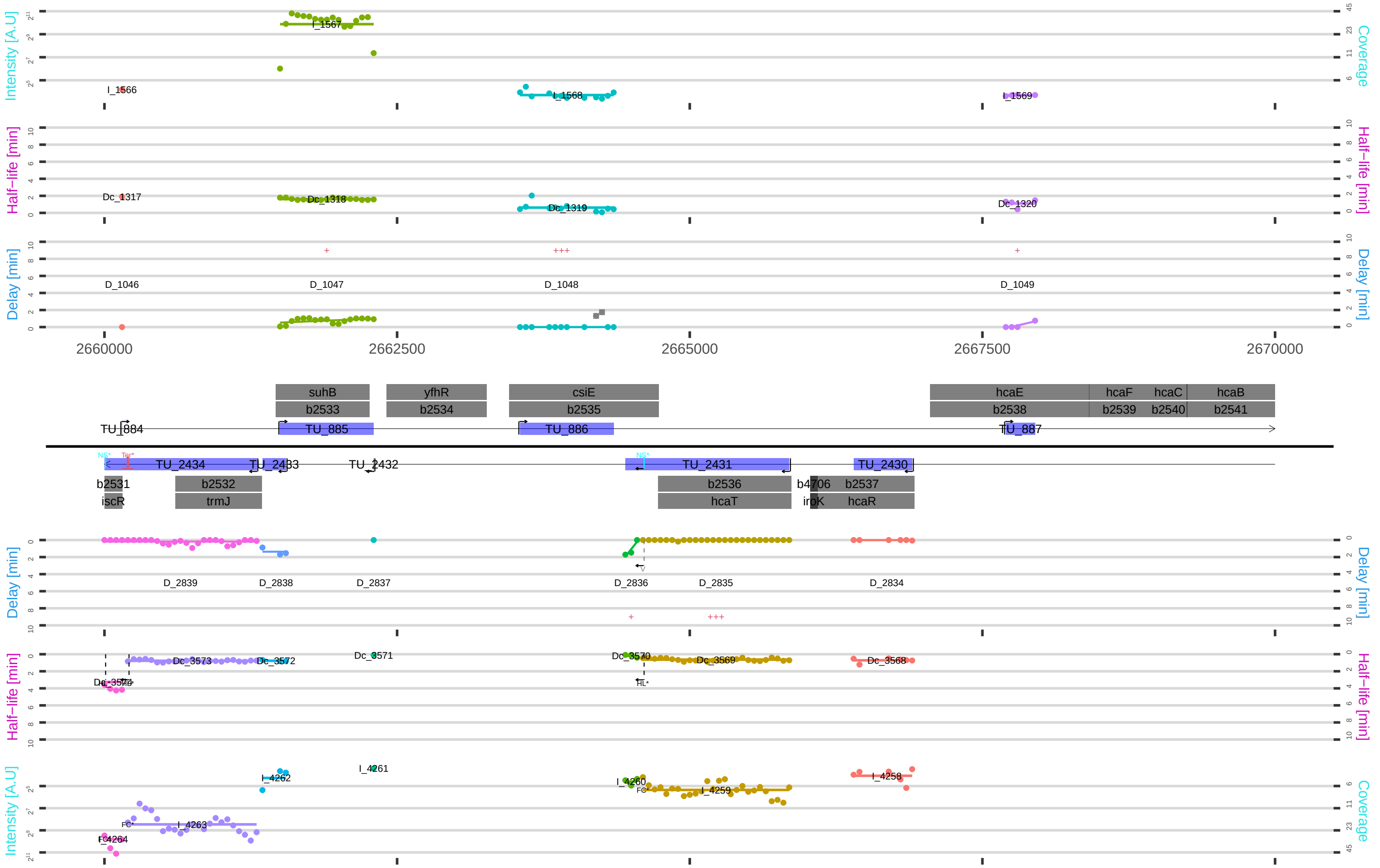

Term: termination (1), NS: new start (2), PS: pausing site (0), iTSS_I: internal starting site (1)

ID: 53406−53436; Term: termination (0), NS: new start (0), PS: pausing site (0), iTSS_I: internal starting site (0)

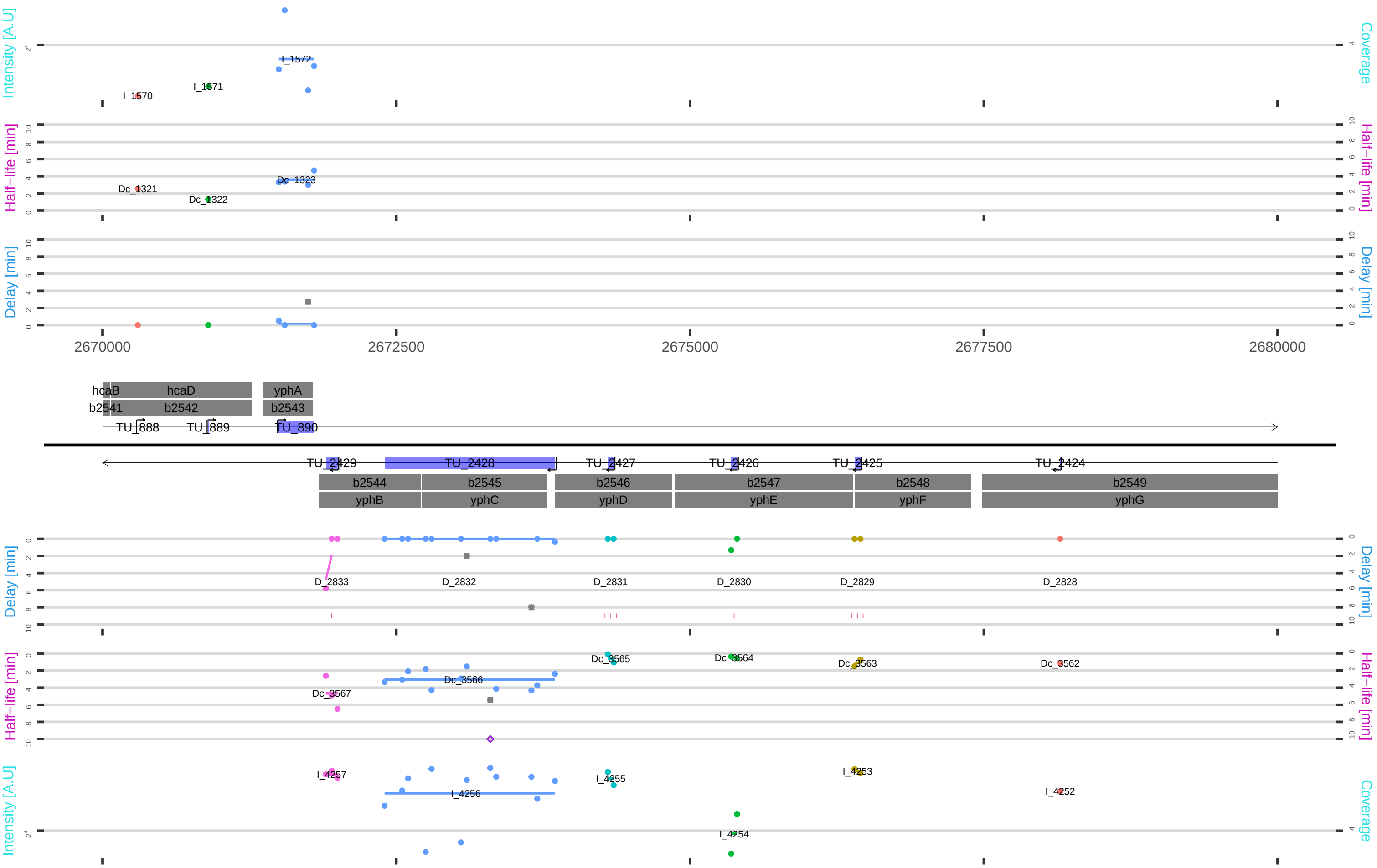

Term: termination (0), NS: new start (0), PS: pausing site (0), iTSS_I: internal starting site (0)

ID: 53621−53786; Term: termination (0), NS: new start (0), PS: pausing site (1), iTSS_I: internal starting site (0)

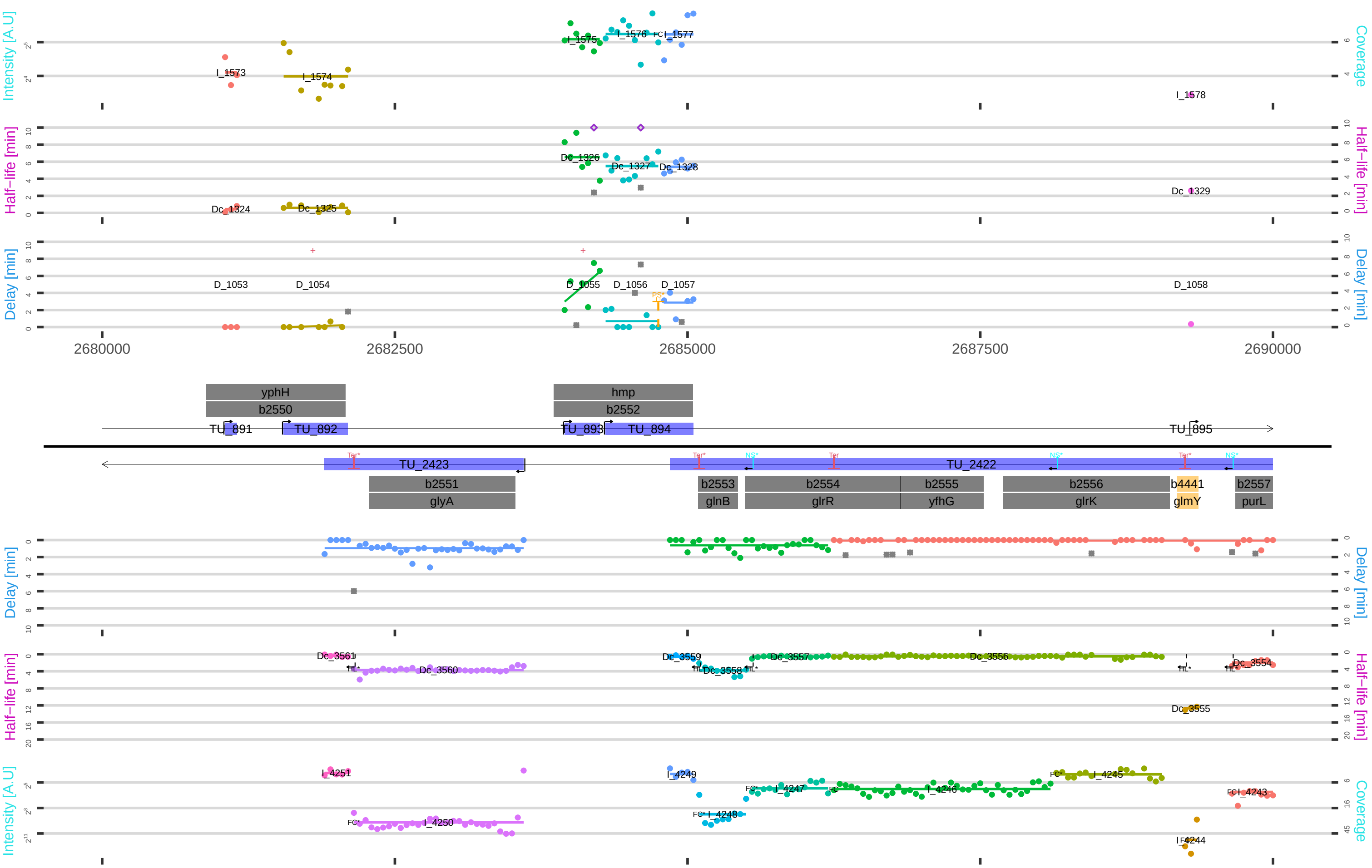

Term: termination (4), NS: new start (3), PS: pausing site (1), iTSS_I: internal starting site (0)

ID: 53878−53989; Term: termination (1), NS: new start (0), PS: pausing site (0), iTSS_I: internal starting site (0)

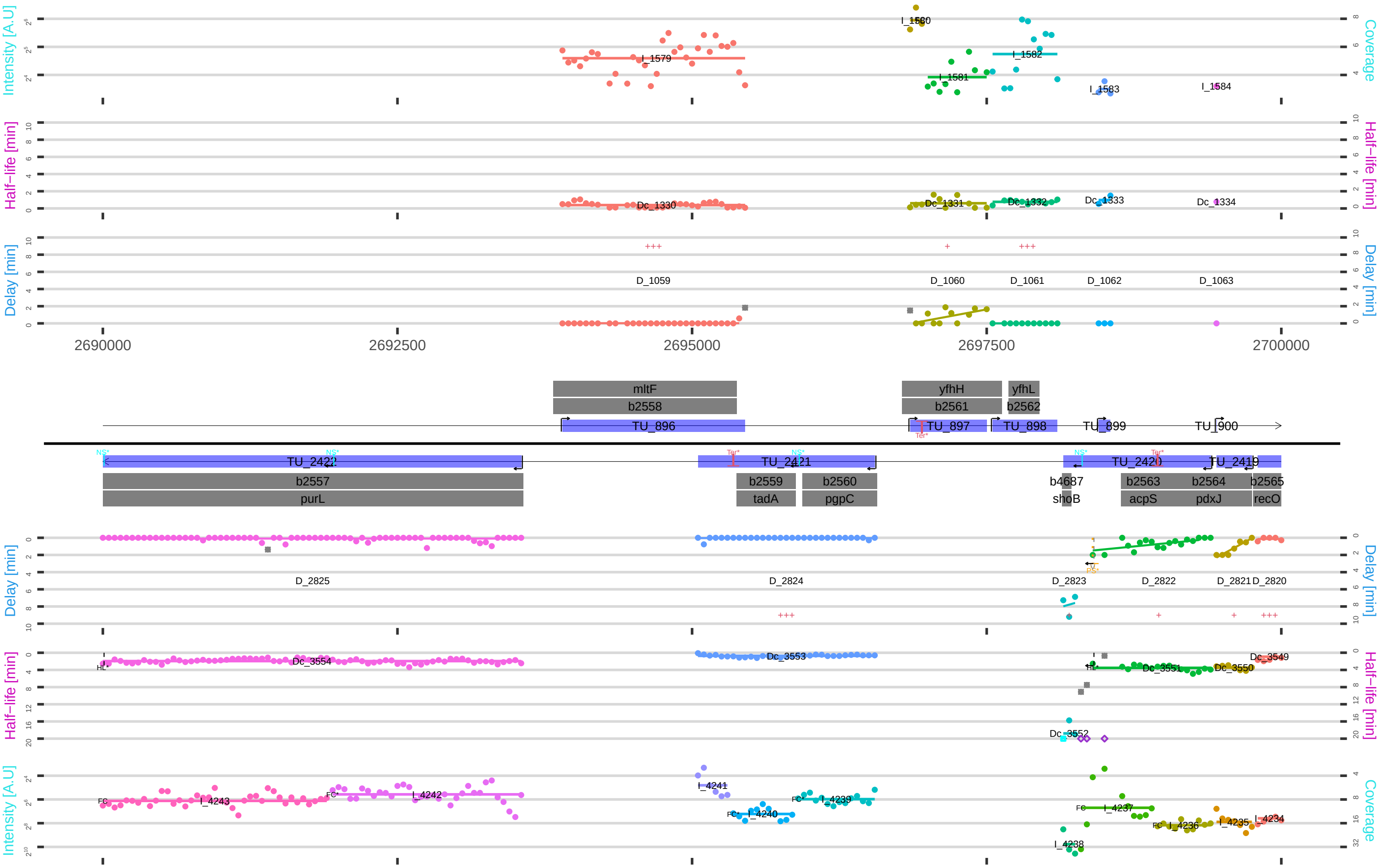

Term: termination (2), NS: new start (4), PS: pausing site (1), iTSS_I: internal starting site (0)

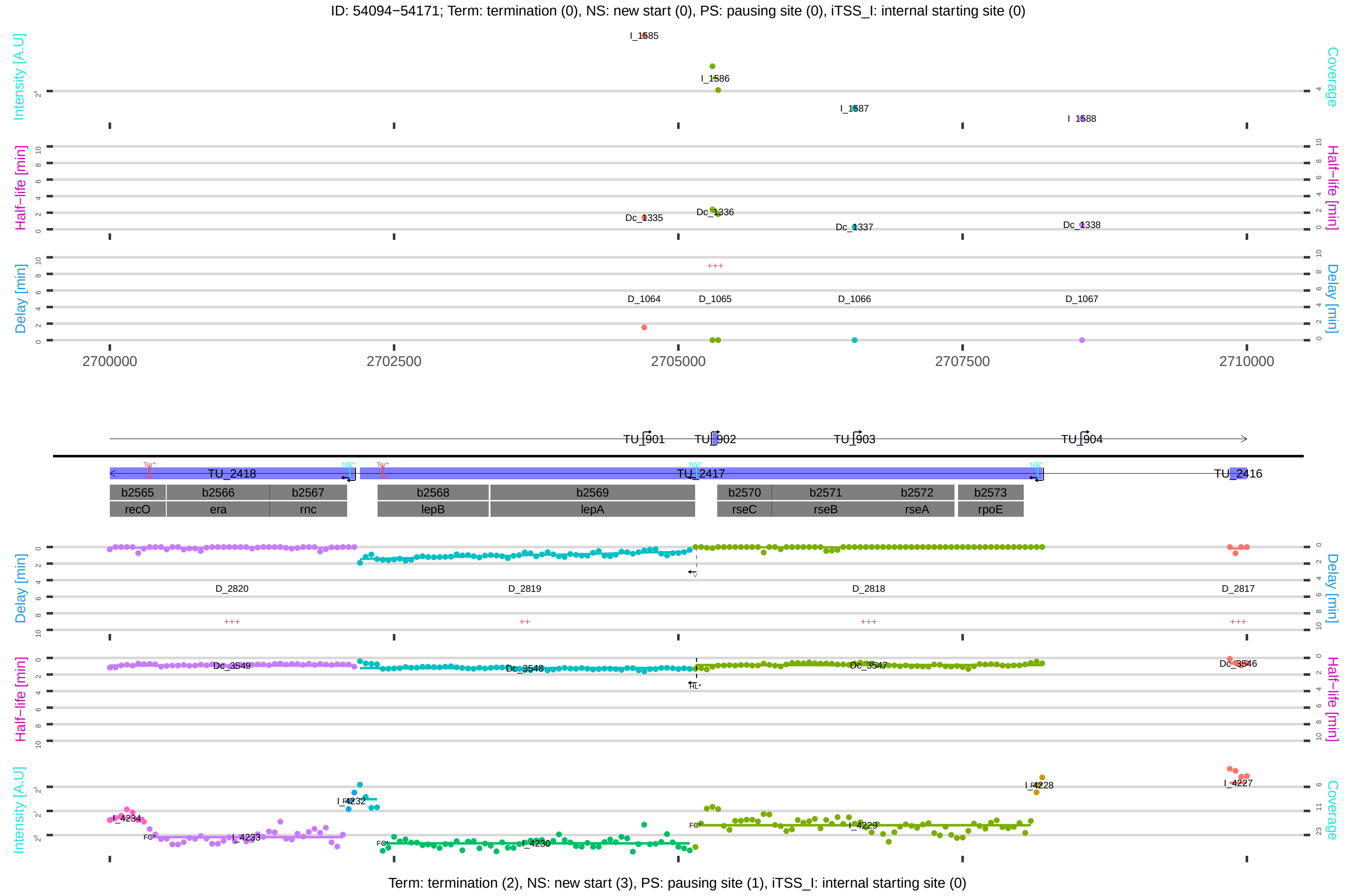
ID: 54094−54171; Term: termination (0), NS: new start (0), PS: pausing site (0), iTSS_I: internal starting site (0)
Intensity [A.U]
Coverage
Half−life [min]
Delay [min]
I_1585
I_1586
I_1587
I_1588
Dc_1335
Dc_1336
Dc_1337
Dc_1338
D_1064
D_1065
D_1066
D_1067
2700000
2702500
2705000
2707500
2710000
TU_901
TU_902
TU_903
TU_904
TU_2418
TU_2417
TU_2416
b2565
b2566
b2567
b2568
b2569
b2570
b2571
b2572
b2573
recO
era
rnc
lepB
lepA
rseC
rseB
rseA
rpoE
D_2820
D_2819
D_2818
D_2817
Dc_3549
Dc_3548
Dc_3547
Dc_3546
I_4234
I_4233
I_4232
I_4230
I_4229
I_4228
I_4227
Term: termination (2), NS: new start (3), PS: pausing site (1), iTSS_I: internal starting site (0)

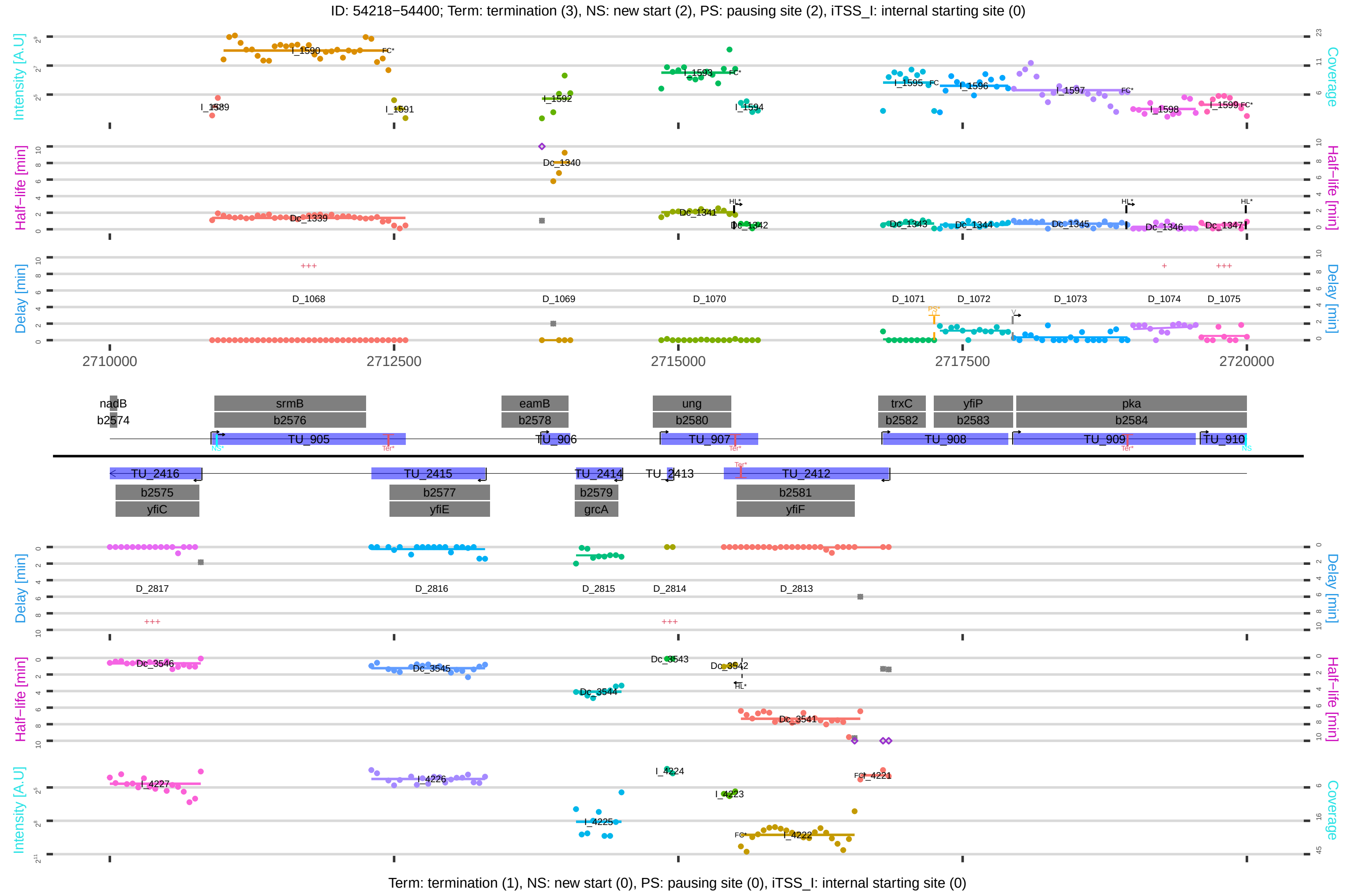
ID: 54218−54400; Term: termination (3), NS: new start (2), PS: pausing site (2), iTSS_I: internal starting site (0)
Intensity [A.U]
Coverage
Half−life [min]
Delay [min]
2710000
2712500
2715000
2717500
2720000
nadB
b2574
srmB
b2576
eamB
b2578
ung
b2580
trxC
b2582
yfiP
b2583
pka
b2584
TU_905
TU_906
TU_907
TU_908
TU_909
TU_910
TU_2416
TU_2415
TU_2414
TU_2413
TU_2412
b2575
yfiC
b2577
yfiE
b2579
grcA
b2581
yfiF
Term: termination (1), NS: new start (0), PS: pausing site (0), iTSS_I: internal starting site (0)

ID: 54400−54583; Term: termination (6), NS: new start (7), PS: pausing site (0), iTSS_I: internal starting site (0)

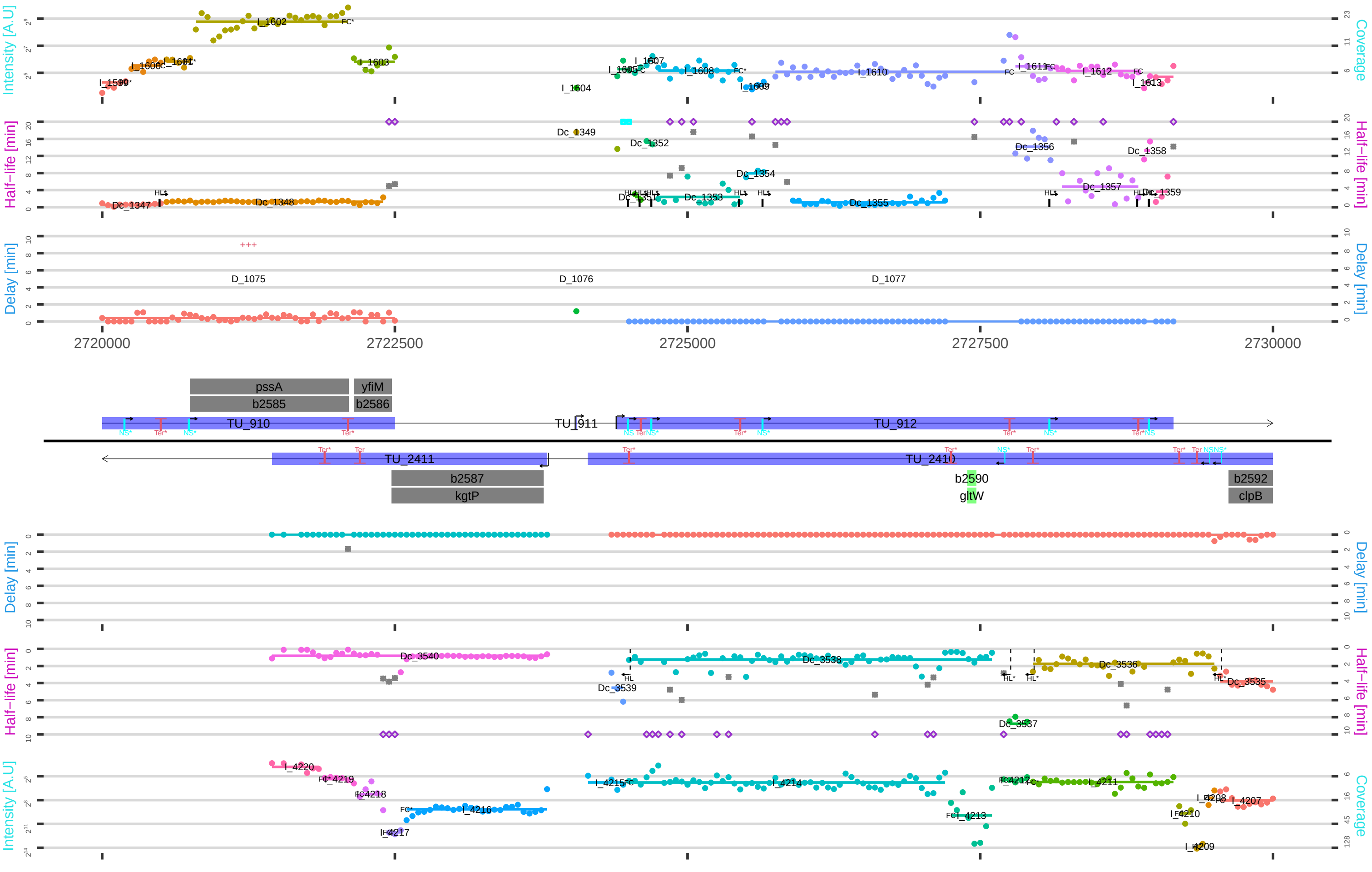

Term: termination (7), NS: new start (3), PS: pausing site (0), iTSS_I: internal starting site (0)

ID: 54682−54800; Term: termination (3), NS: new start (1), PS: pausing site (1), iTSS_I: internal starting site (0)

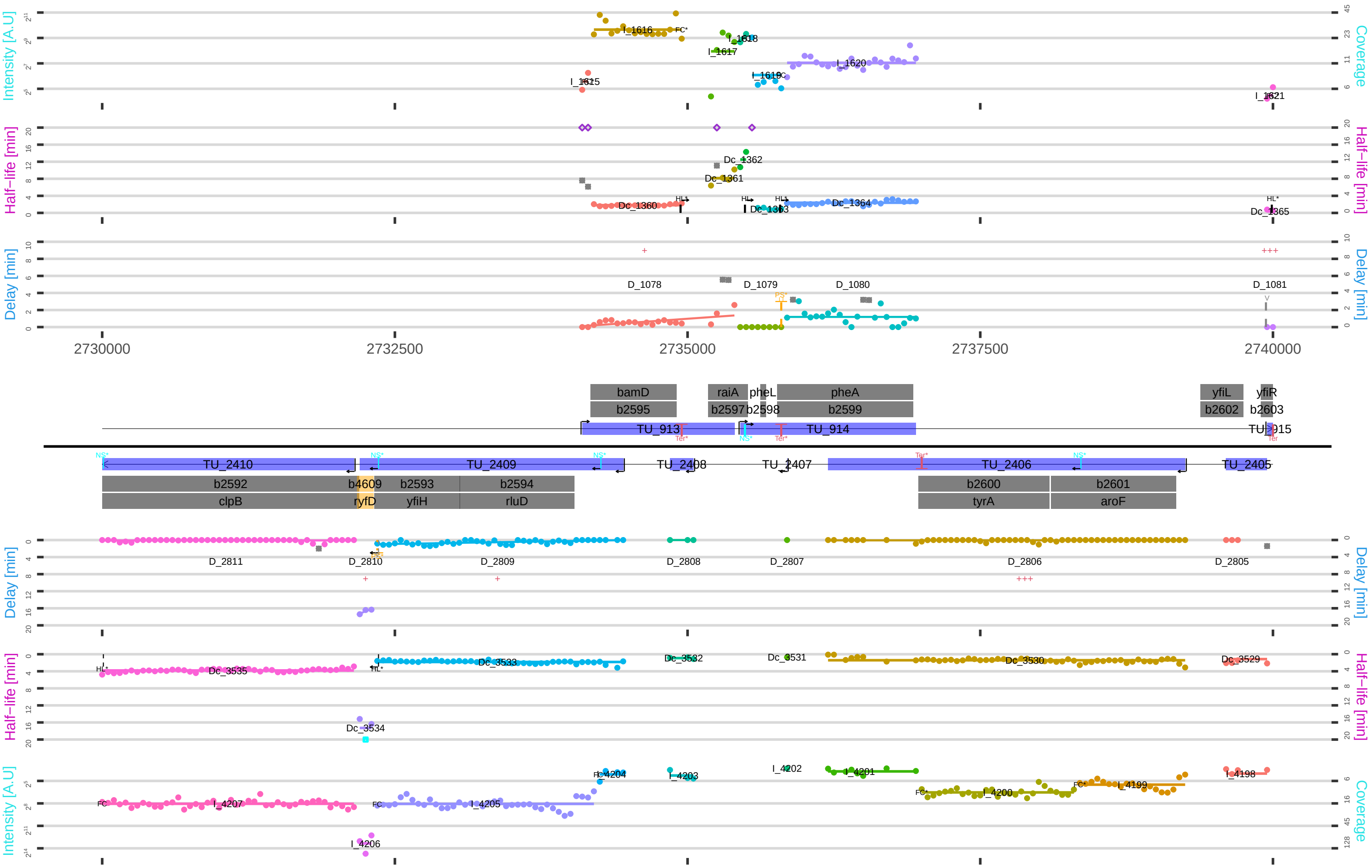

Term: termination (1), NS: new start (4), PS: pausing site (1), iTSS_I: internal starting site (0)

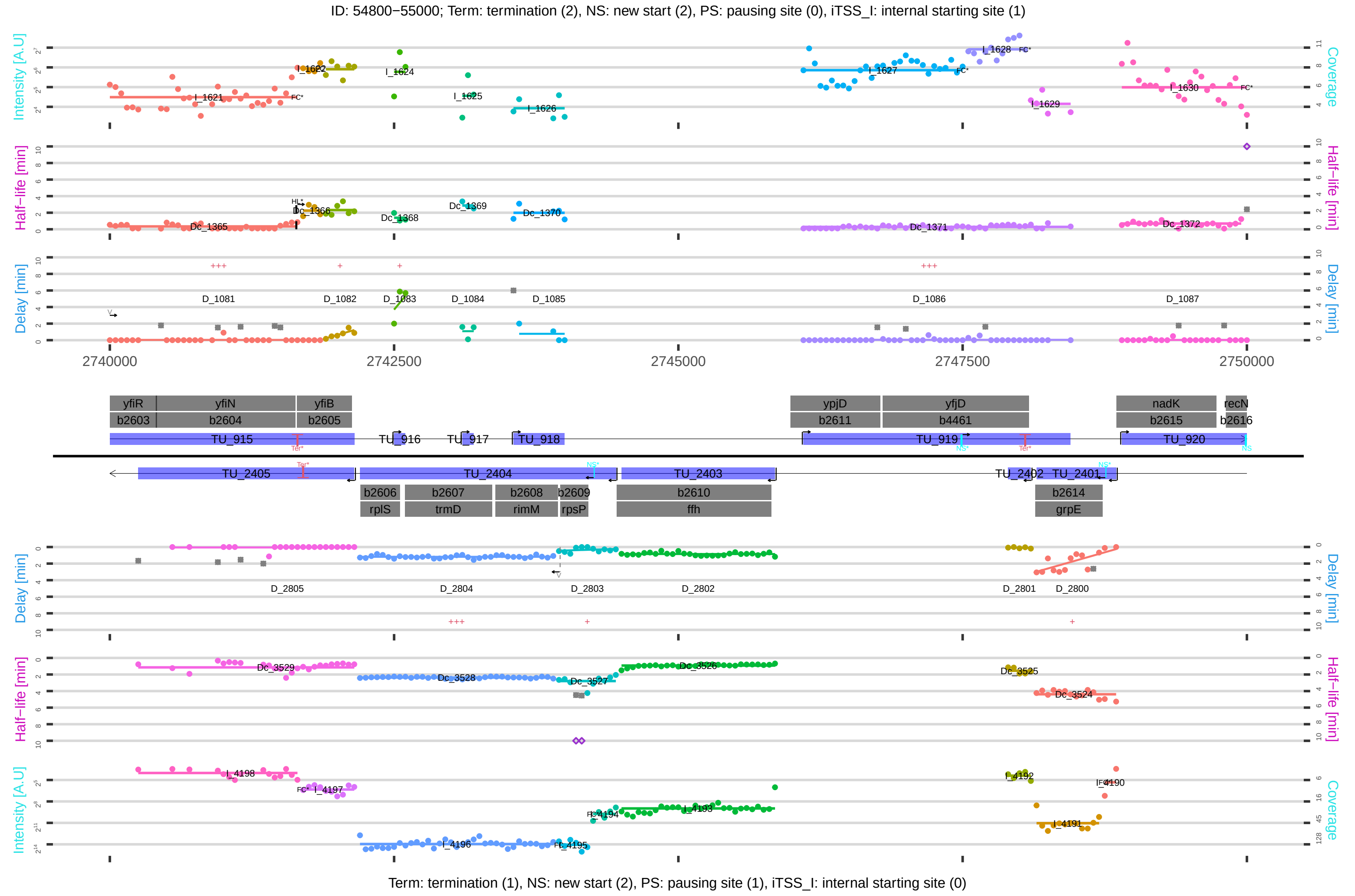
ID: 54800−55000; Term: termination (2), NS: new start (2), PS: pausing site (0), iTSS_I: internal starting site (1)
Intensity [A.U]
Coverage
Half−life [min]
Delay [min]
2740000
2742500
2745000
2747500
2750000
yfiR
yfiN
yfiB
b2603
b2604
b2605
ypjD
yfjD
b2611
b4461
nadK
recN
b2615
b2616
TU_915
TU_916
TU_917
TU_918
TU_919
TU_920
TU_2405
TU_2404
TU_2403
TU_2402
TU_2401
b2606
b2607
b2608
b2609
b2610
b2614
rplS
trmD
rimM
rpsP
ffh
grpE
Term: termination (1), NS: new start (2), PS: pausing site (1), iTSS_I: internal starting site (0)

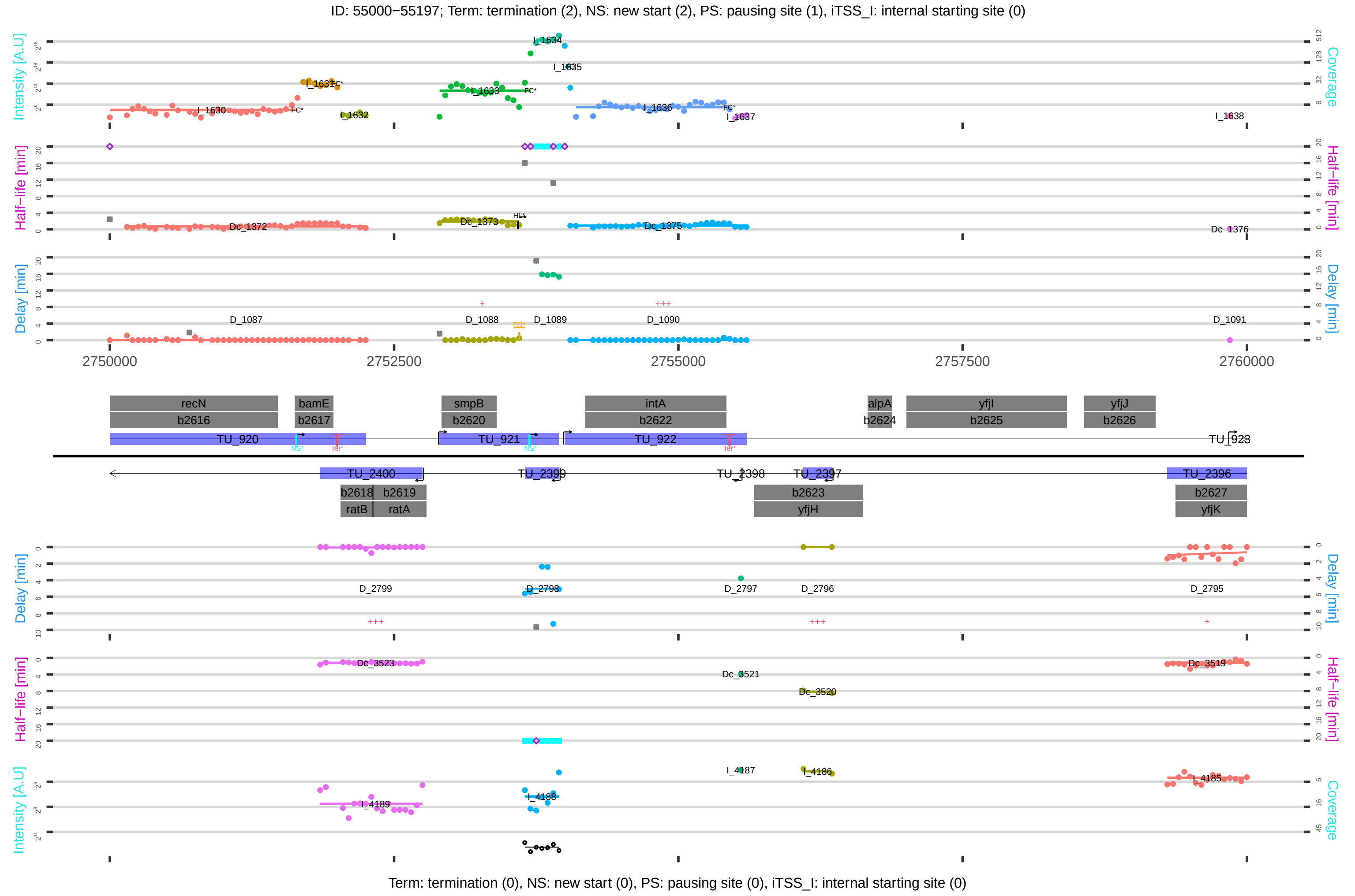
ID: 55000−55197; Term: termination (2), NS: new start (2), PS: pausing site (1), iTSS_I: internal starting site (0)
Intensity [A.U]
Coverage
Half−life [min]
Delay [min]
I_1630
I_1631
I_1632
I_1633
I_1634
I_1635
I_1636
I_1637
I_1638
Dc_1372
Dc_1373
Dc_1375
Dc_1376
D_1087
D_1088
D_1089
D_1090
D_1091
2750000
2752500
2755000
2757500
2760000
recN
b2616
bamE
b2617
smpB
b2620
intA
b2622
alpA
b2624
yfjI
b2625
yfjJ
b2626
TU_920
TU_921
TU_922
TU_923
TU_2400
TU_2399
TU_2398
TU_2397
TU_2396
b2618
b2619
ratB
ratA
b2623
yfjH
b2627
yfjK
D_2799
D_2798
D_2797
D_2796
D_2795
Dc_3523
Dc_3521
Dc_3520
Dc_3519
I_4189
I_4188
I_4187
I_4186
I_4185
Term: termination (0), NS: new start (0), PS: pausing site (0), iTSS_I: internal starting site (0)

ID: 55257−55399; Term: termination (1), NS: new start (1), PS: pausing site (0), iTSS_I: internal starting site (0)

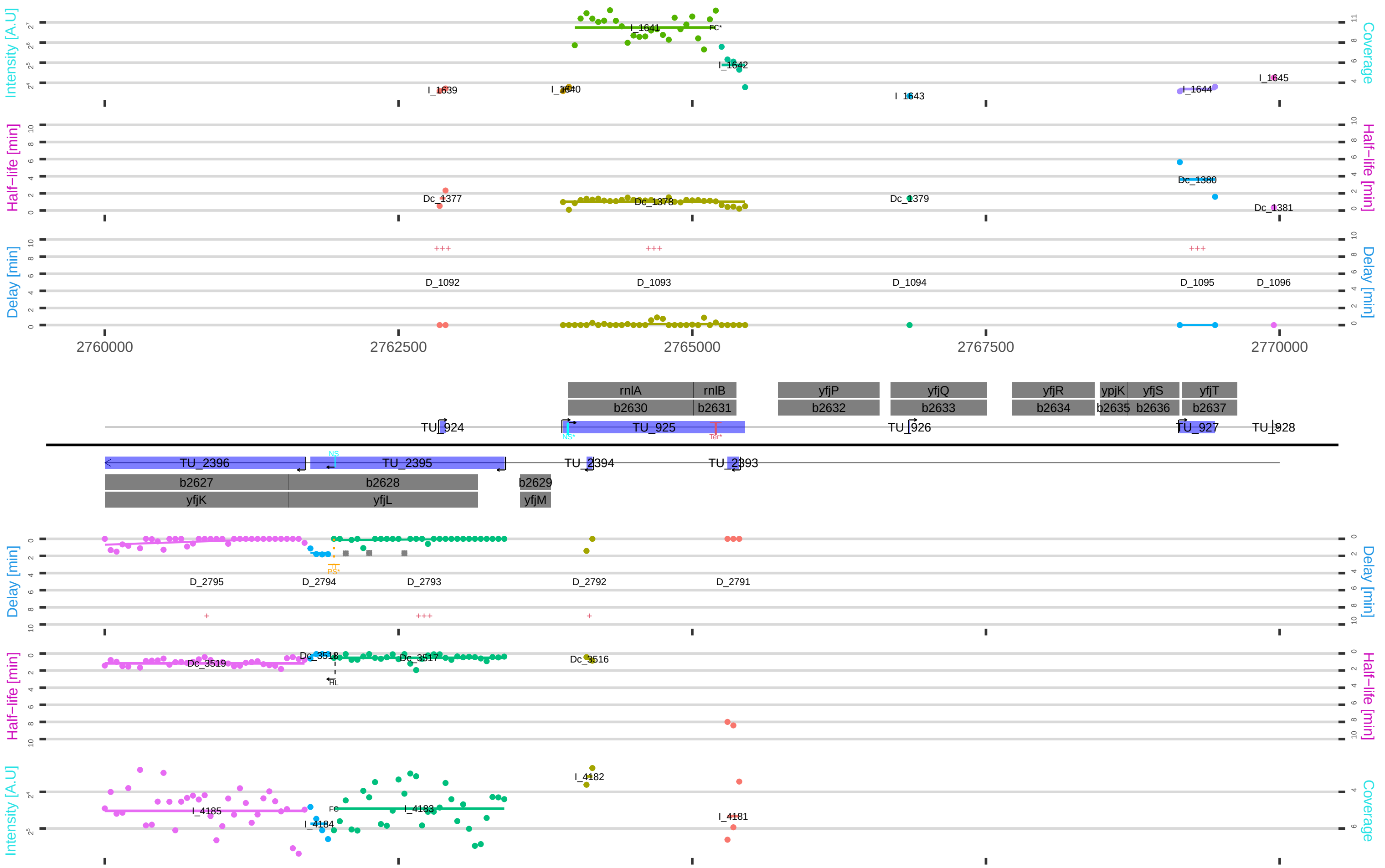

Term: termination (0), NS: new start (1), PS: pausing site (1), iTSS_I: internal starting site (0)

ID: 55456−55547; Term: termination (0), NS: new start (0), PS: pausing site (0), iTSS_I: internal starting site (0)

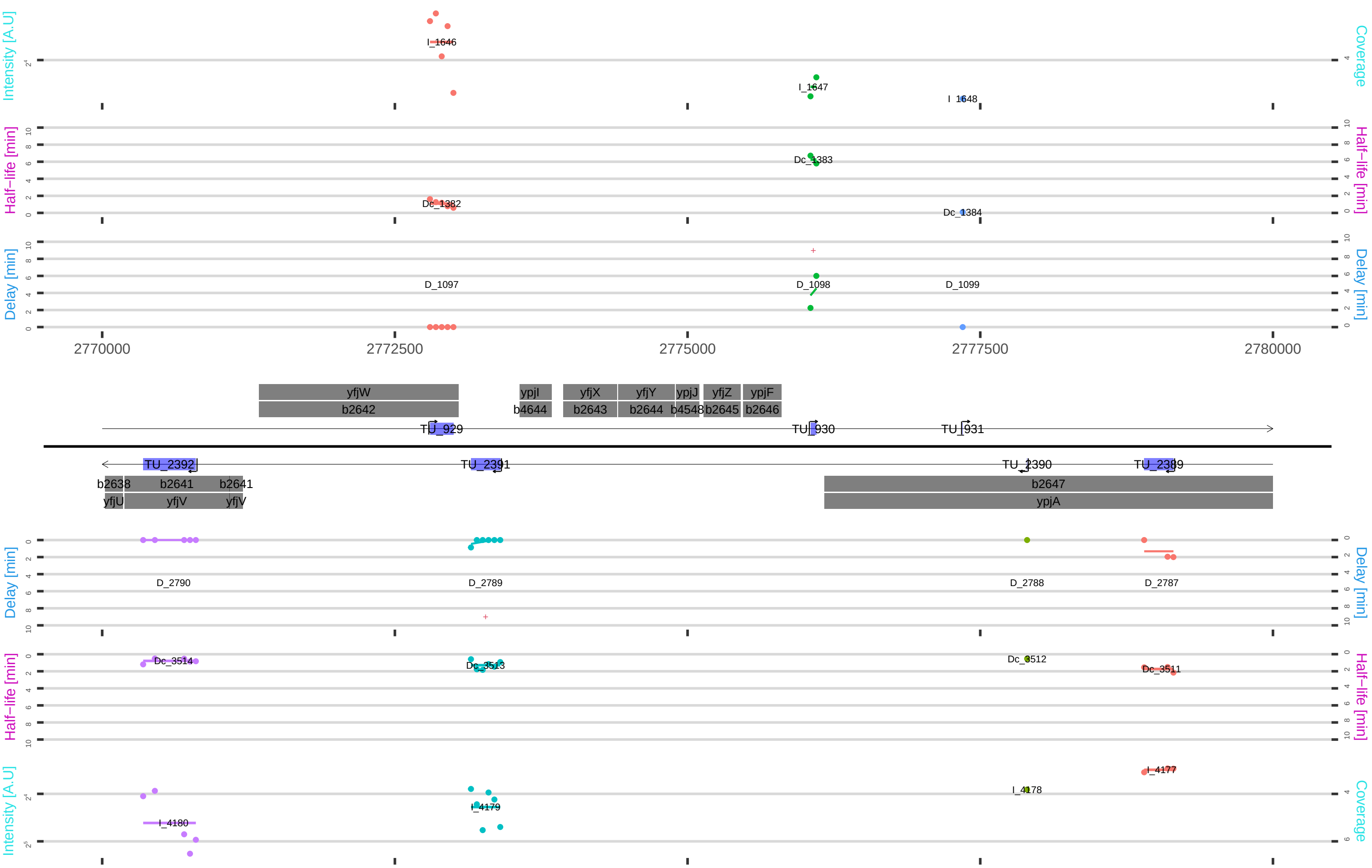

Term: termination (0), NS: new start (0), PS: pausing site (0), iTSS_I: internal starting site (0)

ID: 55625−55800; Term: termination (1), NS: new start (0), PS: pausing site (0), iTSS_I: internal starting site (0)

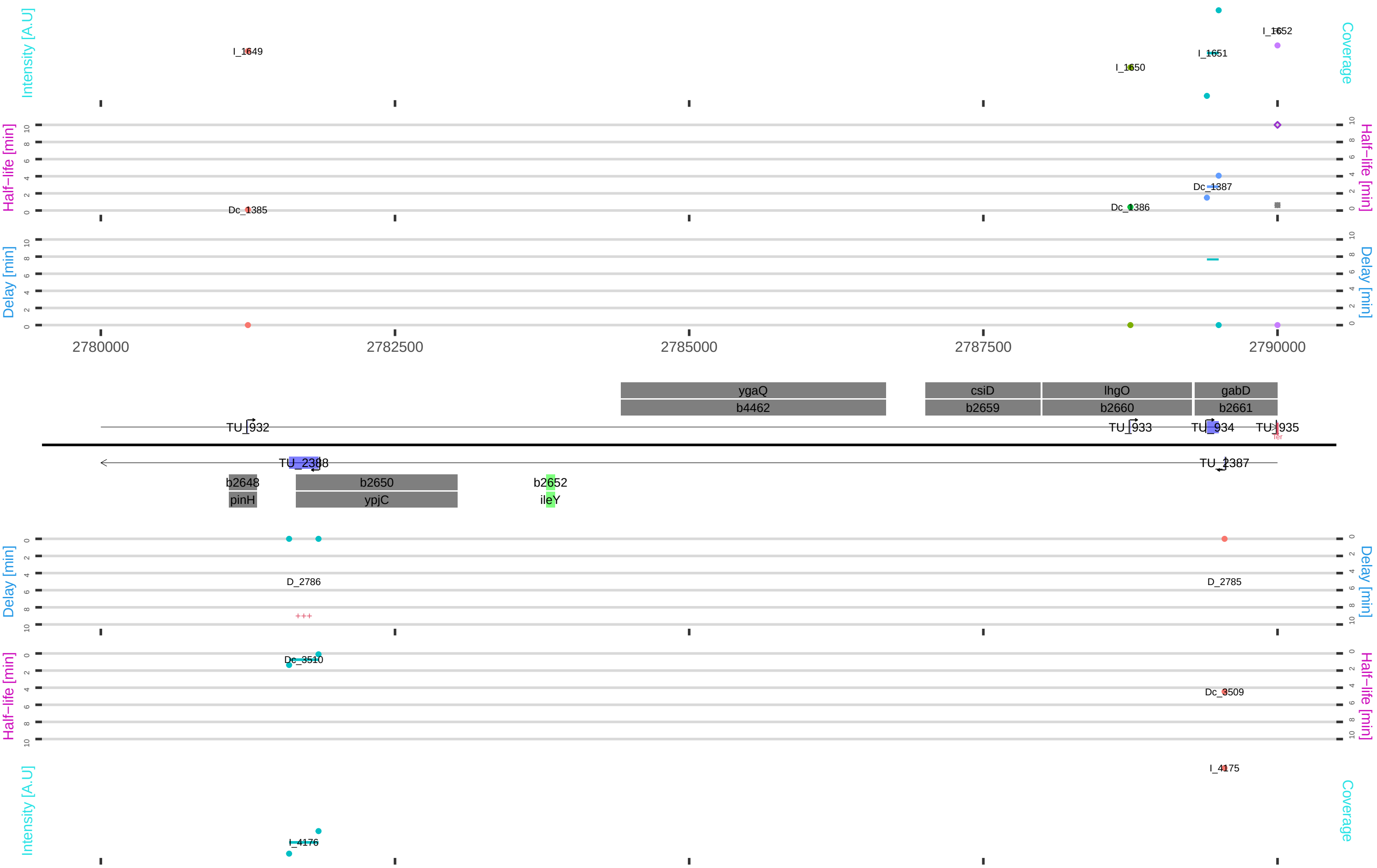

Term: termination (0), NS: new start (0), PS: pausing site (0), iTSS_I: internal starting site (0)

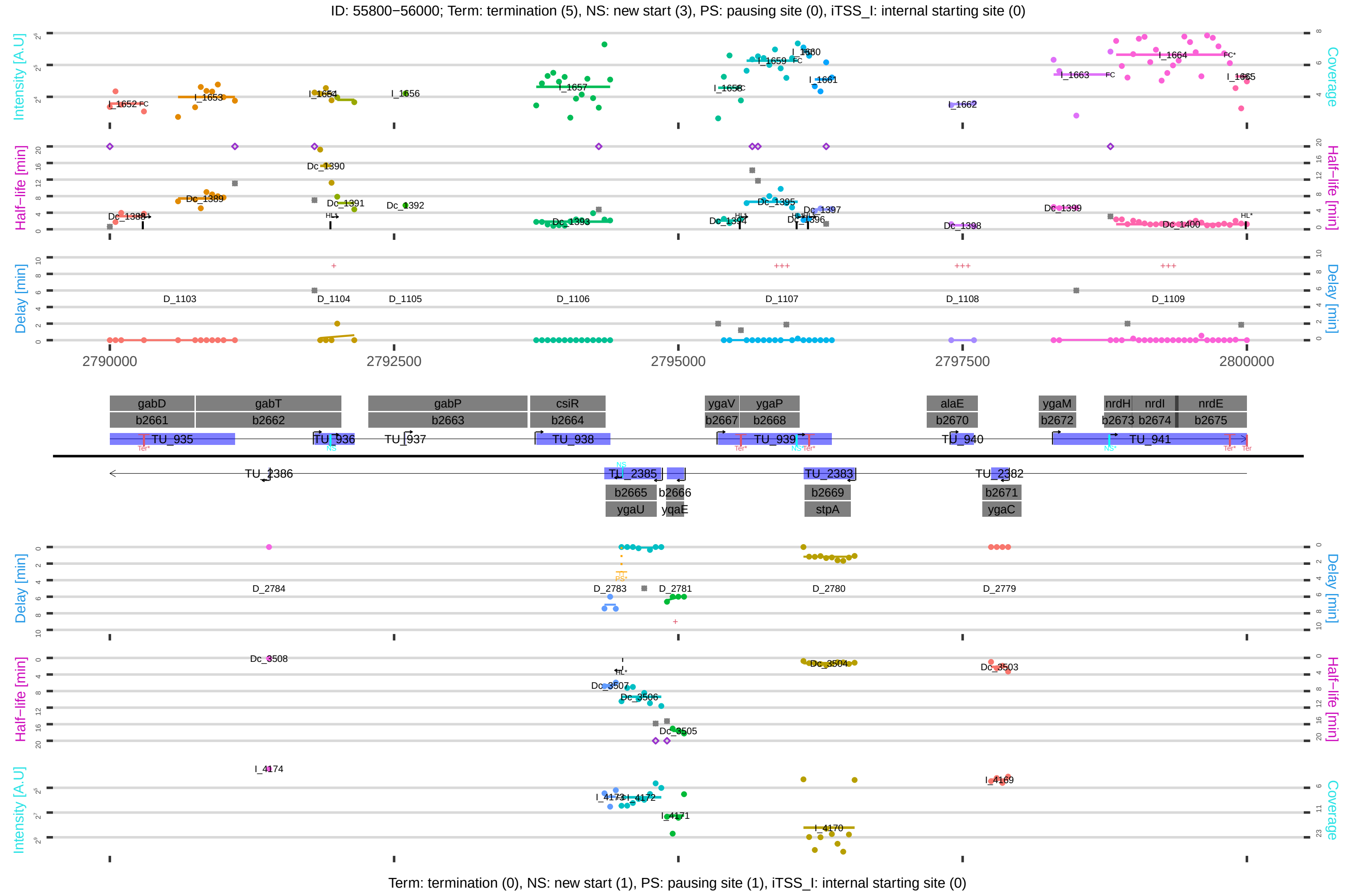
ID: 55800−56000; Term: termination (5), NS: new start (3), PS: pausing site (0), iTSS_I: internal starting site (0)
Intensity [A.U]
Coverage
Half−life [min]
Delay [min]
I_1652
I_1653
I_1654
I_1656
I_1657
I_1658
I_1659
I_1660
I_1661
I_1662
I_1663
I_1664
I_1665
Dc_1388
Dc_1389
Dc_1390
Dc_1391
Dc_1392
Dc_1393
Dc_1394
Dc_1395
Dc_1396
Dc_1397
Dc_1398
Dc_1399
Dc_1400
D_1103
D_1104
D_1105
D_1106
D_1107
D_1108
D_1109
2790000
2792500
2795000
2797500
2800000
gabD
b2661
gabT
b2662
gabP
b2663
csiR
b2664
ygaV
b2667
ygaP
b2668
alaE
b2670
ygaM
b2672
nrdH
b2673
nrdI
b2674
nrdE
b2675
TU_935
TU_936
TU_937
TU_938
TU_939
TU_940
TU_941
TU_2386
TU_2385
TU_2383
TU_2382
b2665
ygaU
b2666
ygaE
b2669
stpA
b2671
ygaC
D_2784
D_2783
D_2781
D_2780
D_2779
Dc_3508
Dc_3507
Dc_3506
Dc_3505
Dc_3504
Dc_3503
I_4174
I_4173
I_4172
I_4171
I_4170
I_4169
Term: termination (0), NS: new start (1), PS: pausing site (1), iTSS_I: internal starting site (0)

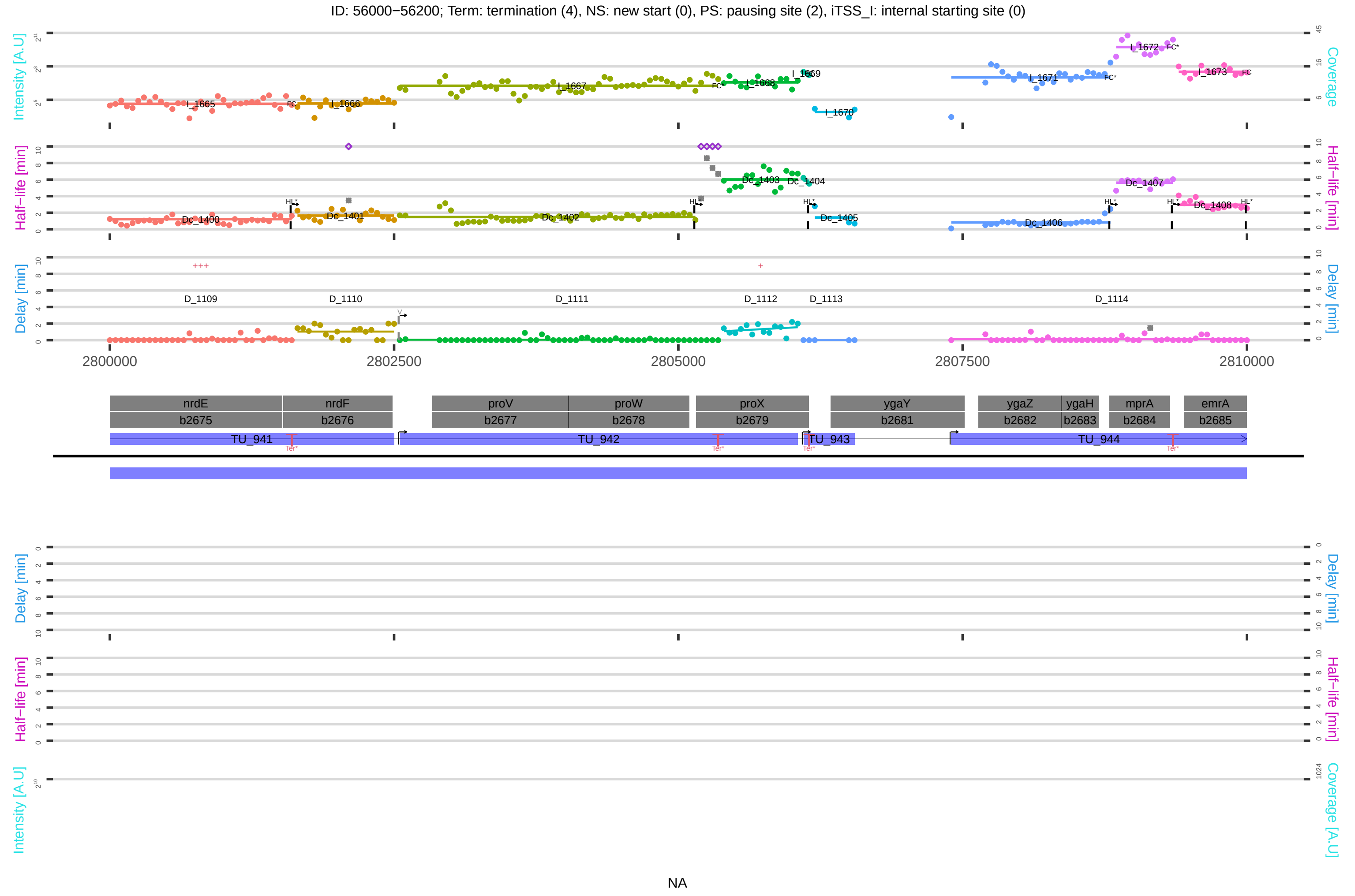

ID: 56000−56200; Term: termination (4), NS: new start (0), PS: pausing site (2), iTSS_I: internal starting site (0)
Intensity [A.U]
Coverage
Half−life [min]
Delay [min]
I_1665
I_1666
I_1667
I_1668
I_1669
I_1670
I_1671
I_1672
I_1673
Dc_1400
Dc_1401
Dc_1402
Dc_1403
Dc_1404
Dc_1405
Dc_1406
Dc_1407
Dc_1408
D_1109
D_1110
D_1111
D_1112
D_1113
D_1114
2800000
2802500
2805000
2807500
2810000
nrdE
nrdF
proV
proW
proX
ygaY
ygaZ
ygaH
mprA
emrA
b2675
b2676
b2677
b2678
b2679
b2681
b2682
b2683
b2684
b2685
TU_941
TU_942
TU_943
TU_944
Ter*
NA

ID: 56200−56344; Term: termination (1), NS: new start (0), PS: pausing site (1), iTSS_I: internal starting site (0)

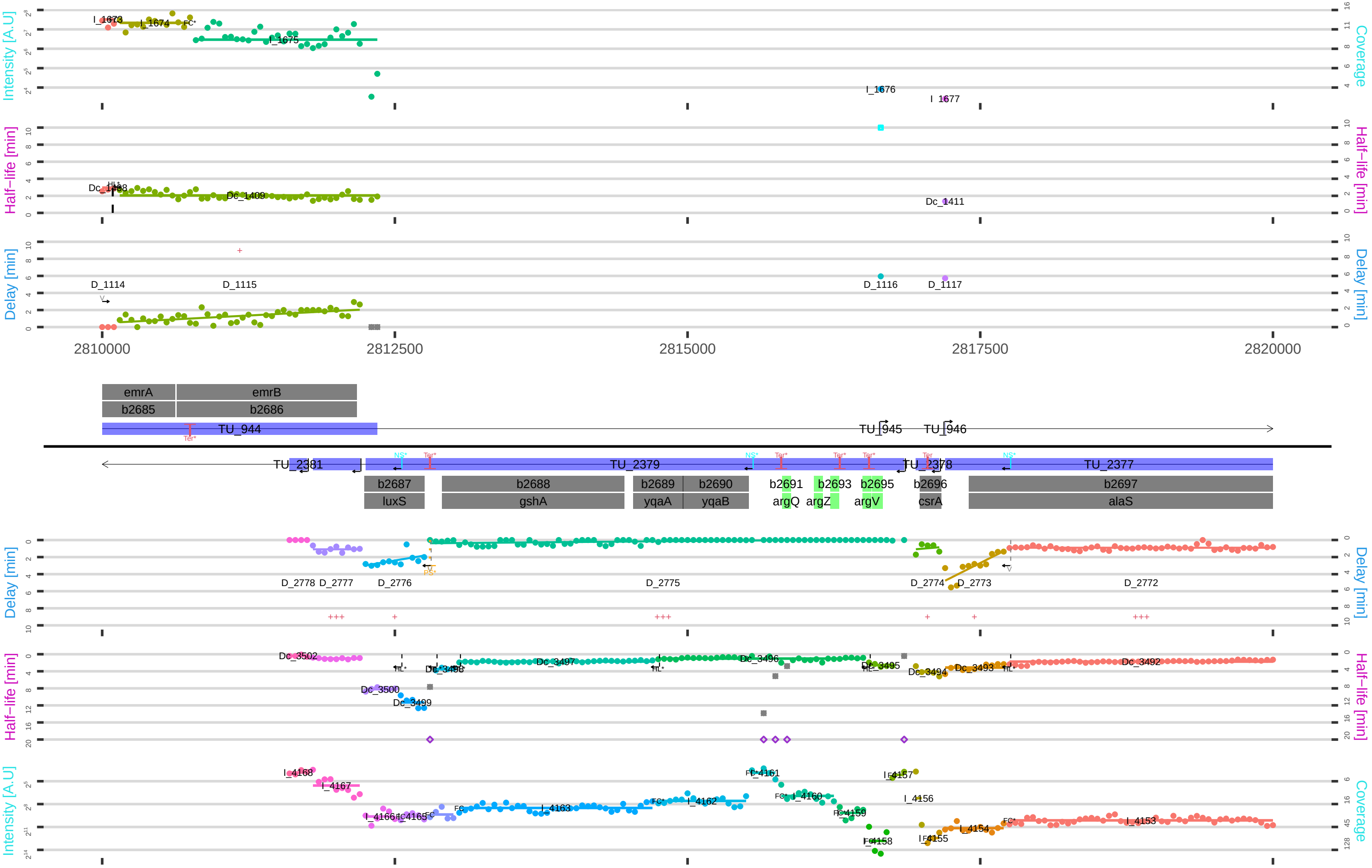

Term: termination (5), NS: new start (3), PS: pausing site (2), iTSS_I: internal starting site (0)

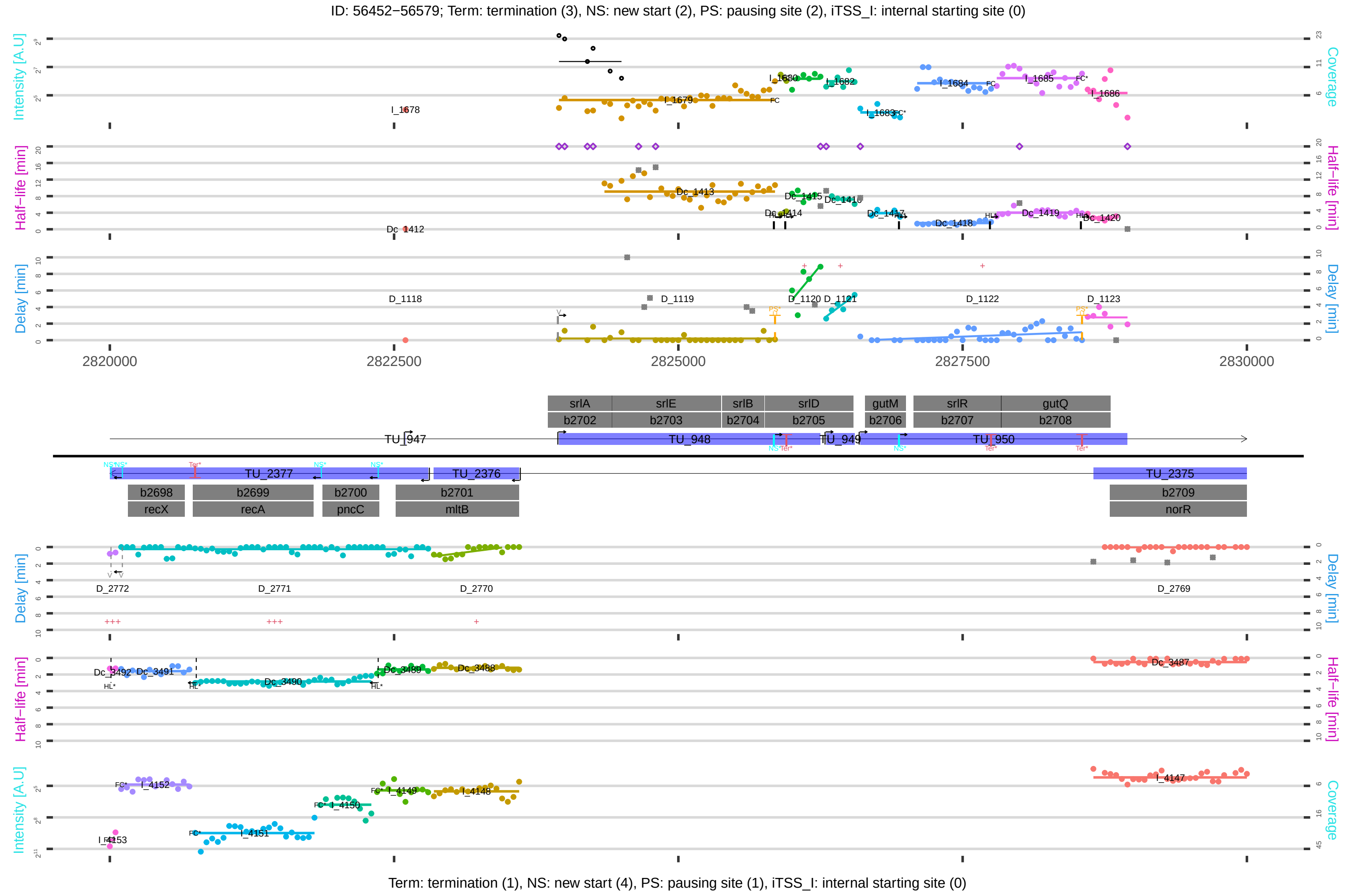

ID: 56452−56579; Term: termination (3), NS: new start (2), PS: pausing site (2), iTSS_I: internal starting site (0)
Intensity [A.U]
Coverage
Half−life [min]
Delay [min]
2820000
2822500
2825000
2827500
2830000
I_1678
I_1679
I_1680
I_1682
I_1683
I_1684
I_1685
I_1686
Dc_1412
Dc_1413
Dc_1414
Dc_1415
Dc_1416
Dc_1417
Dc_1418
Dc_1419
Dc_1420
D_1118
D_1119
D_1120
D_1121
D_1122
D_1123
srlA
srlE
srlB
srlD
gutM
srlR
gutQ
b2702
b2703
b2704
b2705
b2706
b2707
b2708
TU_947
TU_948
TU_949
TU_950
TU_2377
TU_2376
TU_2375
b2698
b2699
b2700
b2701
b2709
recX
recA
pncC
mltB
norR
D_2772
D_2771
D_2770
D_2769
Dc_3492
Dc_3491
Dc_3490
Dc_3489
Dc_3488
Dc_3487
I_4153
I_4152
I_4151
I_4150
I_4149
I_4148
I_4147
Term: termination (1), NS: new start (4), PS: pausing site (1), iTSS_I: internal starting site (0)

ID: 56709−56798; Term: termination (0), NS: new start (0), PS: pausing site (0), iTSS_I: internal starting site (0)

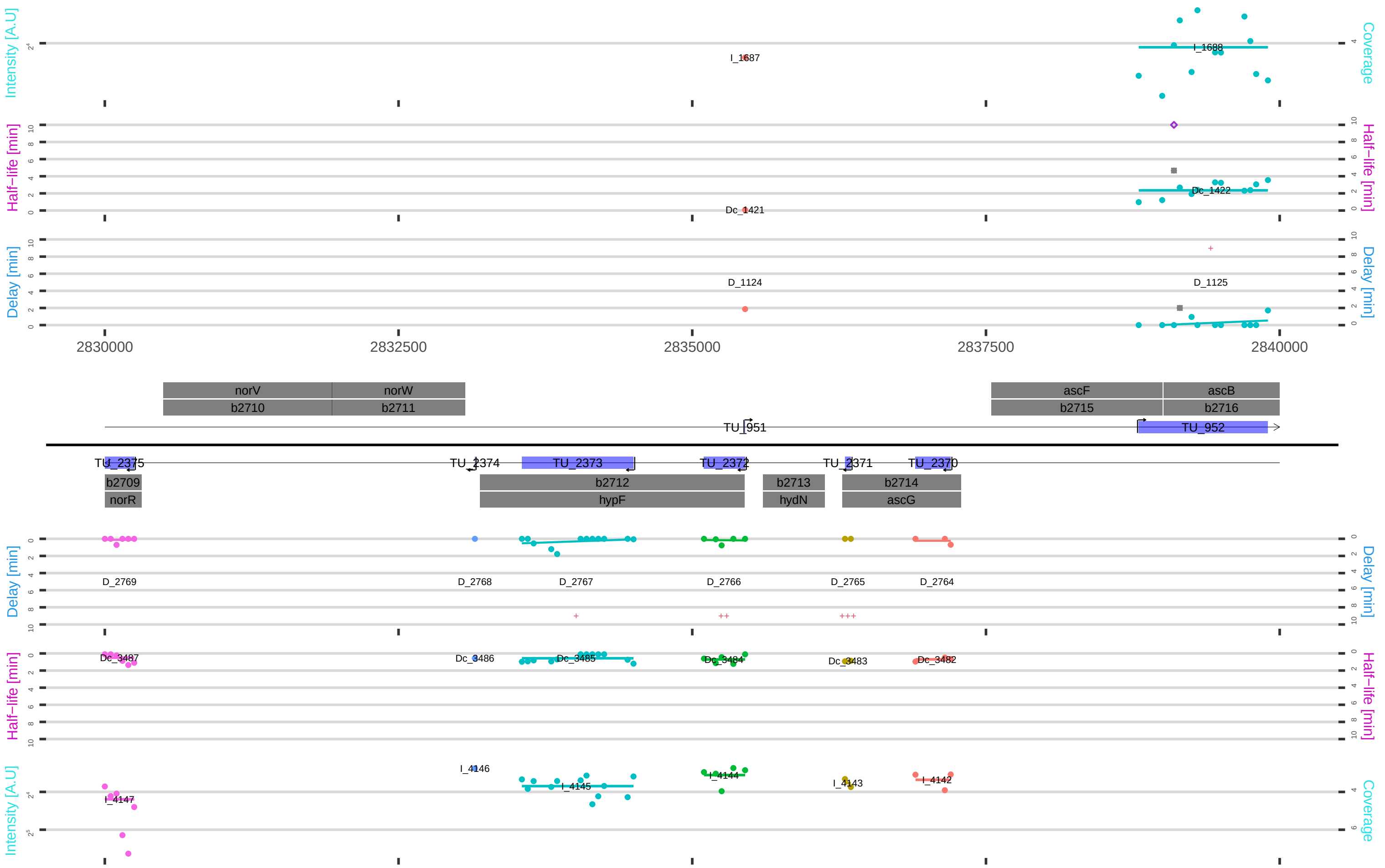

Term: termination (0), NS: new start (0), PS: pausing site (0), iTSS_I: internal starting site (0)

ID: 56804−56997; Term: termination (0), NS: new start (1), PS: pausing site (0), iTSS_I: internal starting site (0)

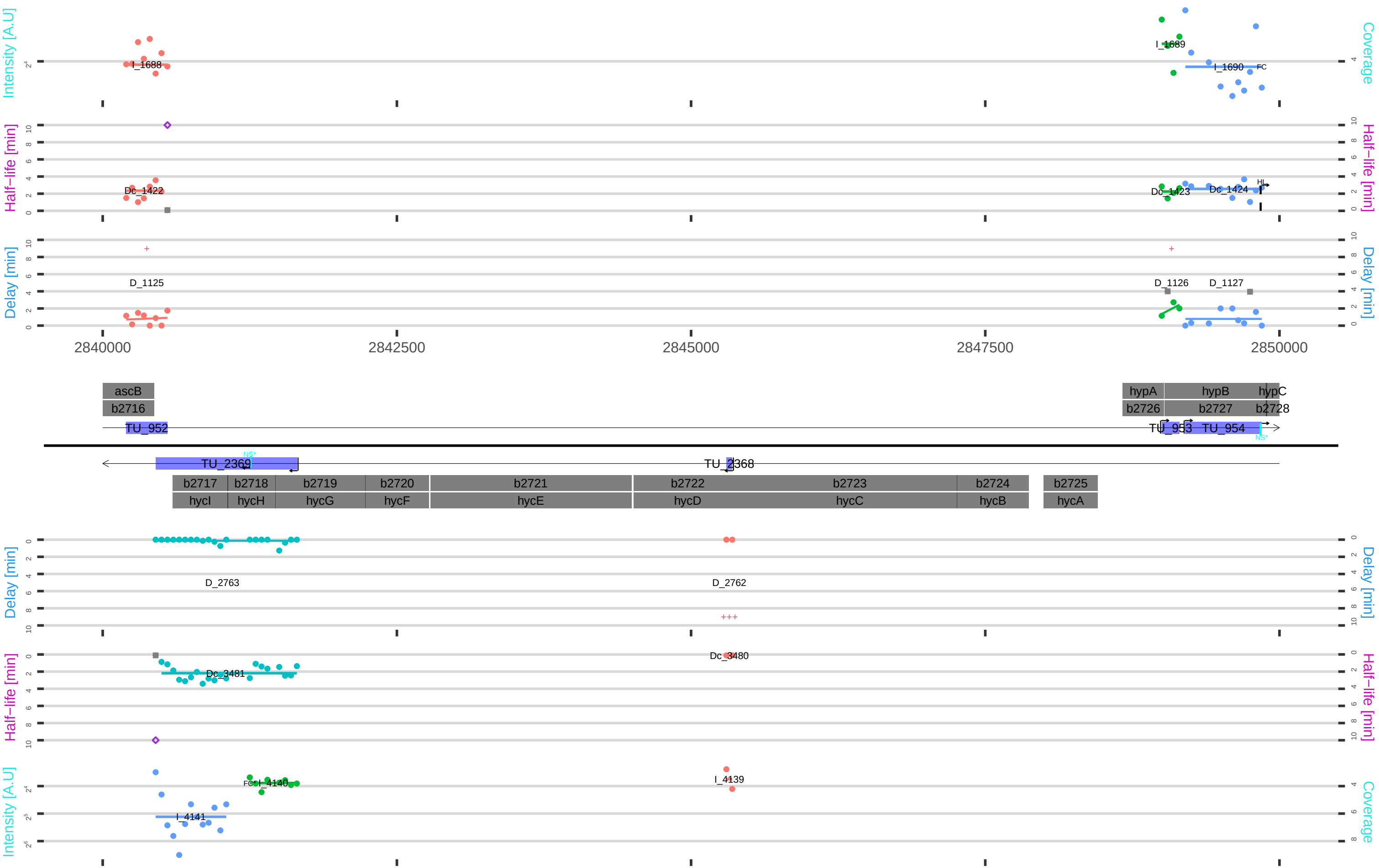

Term: termination (0), NS: new start (1), PS: pausing site (0), iTSS_I: internal starting site (0)

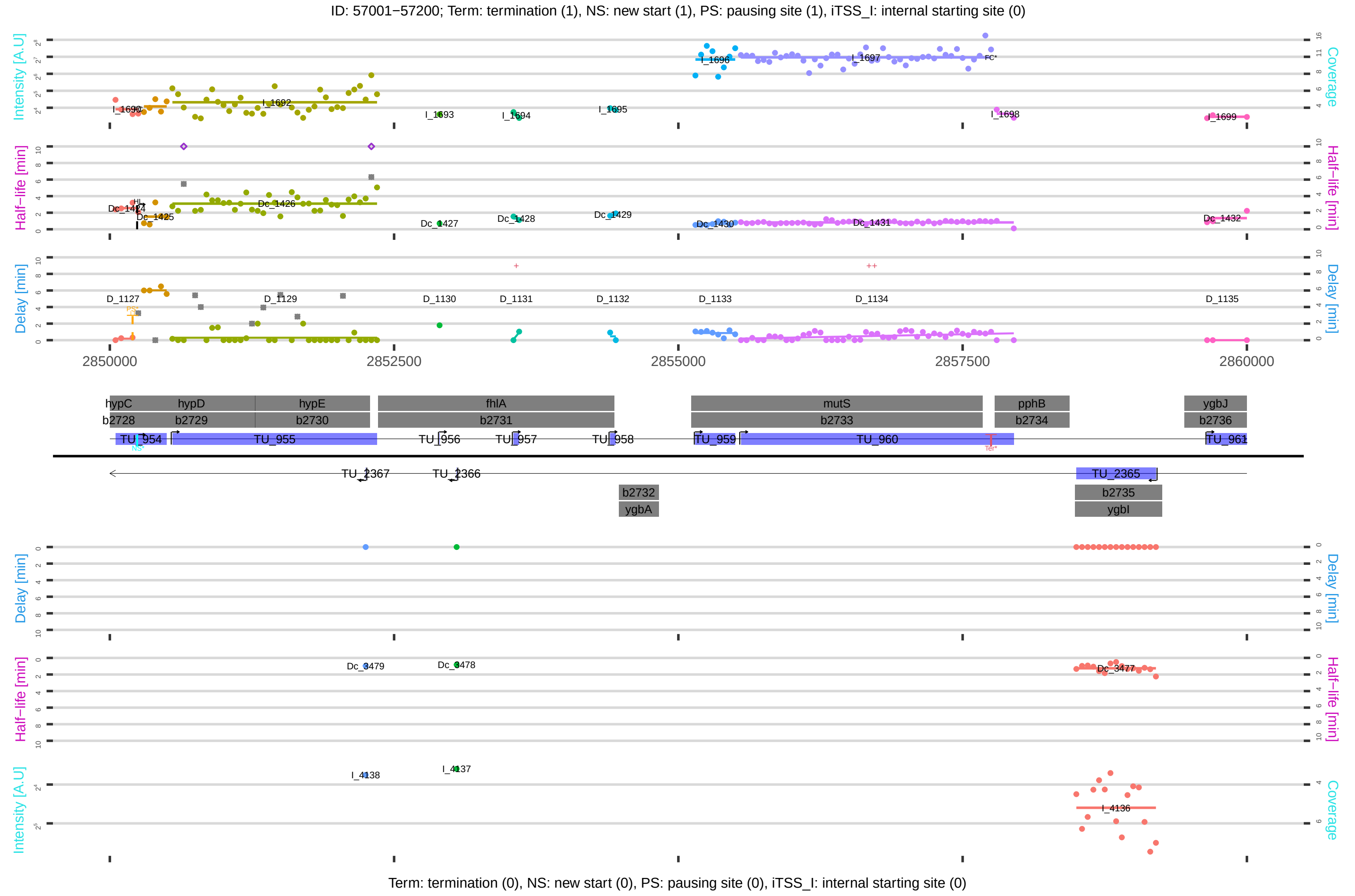

ID: 57001−57200; Term: termination (1), NS: new start (1), PS: pausing site (1), iTSS_I: internal starting site (0)
Intensity [A.U]
Coverage
Half−life [min]
Delay [min]
I_1690
I_1692
I_1693
I_1694
I_1695
I_1696
I_1697
I_1698
I_1699
FC*
Dc_1424
Dc_1425
Dc_1426
Dc_1427
Dc_1428
Dc_1429
Dc_1430
Dc_1431
Dc_1432
D_1127
D_1129
D_1130
D_1131
D_1132
D_1133
D_1134
D_1135
PS*
2850000
2852500
2855000
2857500
2860000
hypC
hypD
hypE
fhlA
mutS
pphB
ygbJ
b2728
b2729
b2730
b2731
b2733
b2734
b2736
TU_954
TU_955
TU_956
TU_957
TU_958
TU_959
TU_960
TU_961
NS*
Ter*
TU_2367
TU_2366
TU_2365
b2732
ygbA
b2735
ygbI
Dc_3479
Dc_3478
Dc_3477
I_4138
I_4137
I_4136
Term: termination (0), NS: new start (0), PS: pausing site (0), iTSS_I: internal starting site (0)

ID: 57200−57290; Term: termination (0), NS: new start (0), PS: pausing site (0), iTSS_I: internal starting site (0)

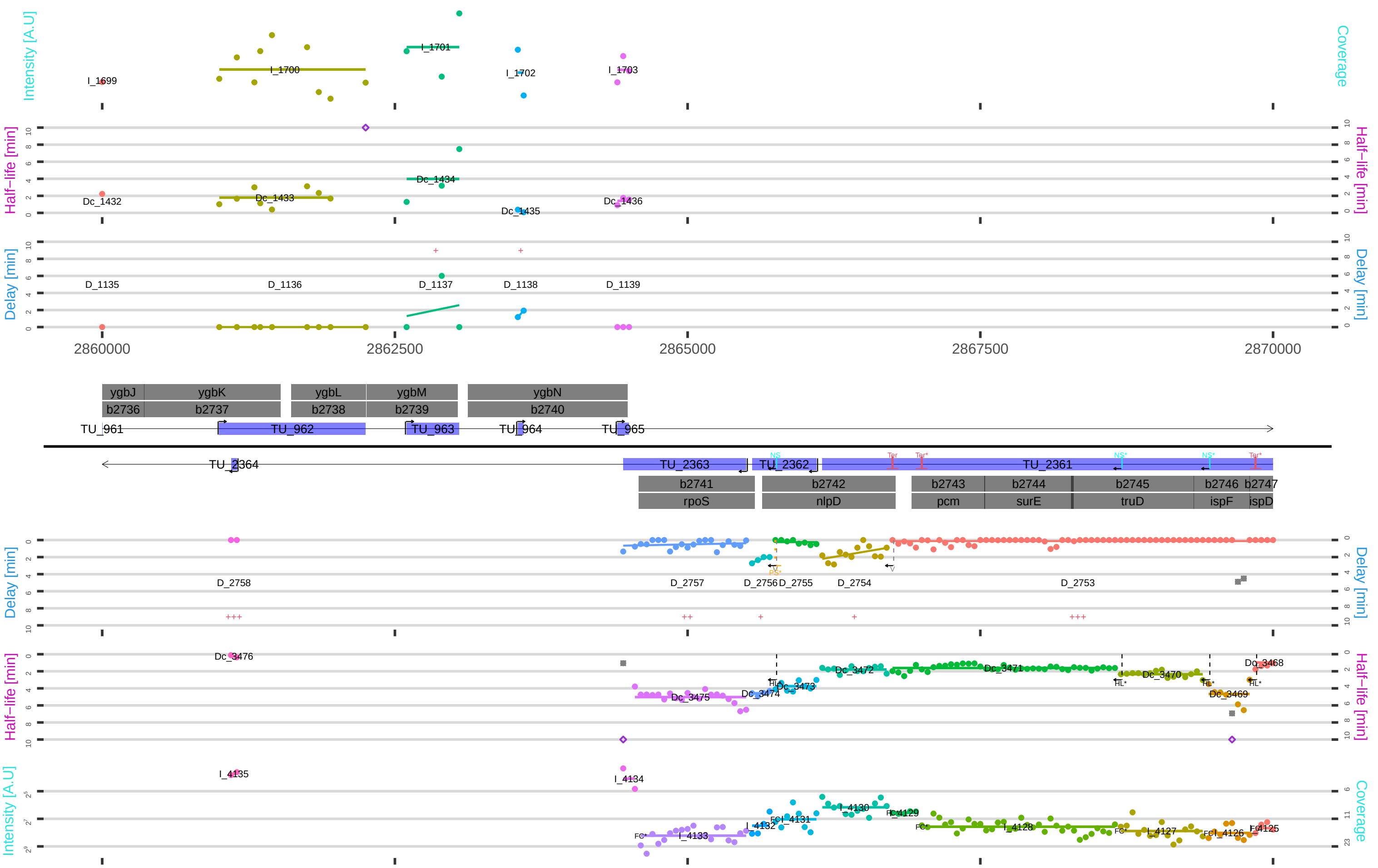

Term: termination (3), NS: new start (3), PS: pausing site (2), iTSS_I: internal starting site (0)

ID: 57493−57522; Term: termination (0), NS: new start (0), PS: pausing site (0), iTSS_I: internal starting site (0)

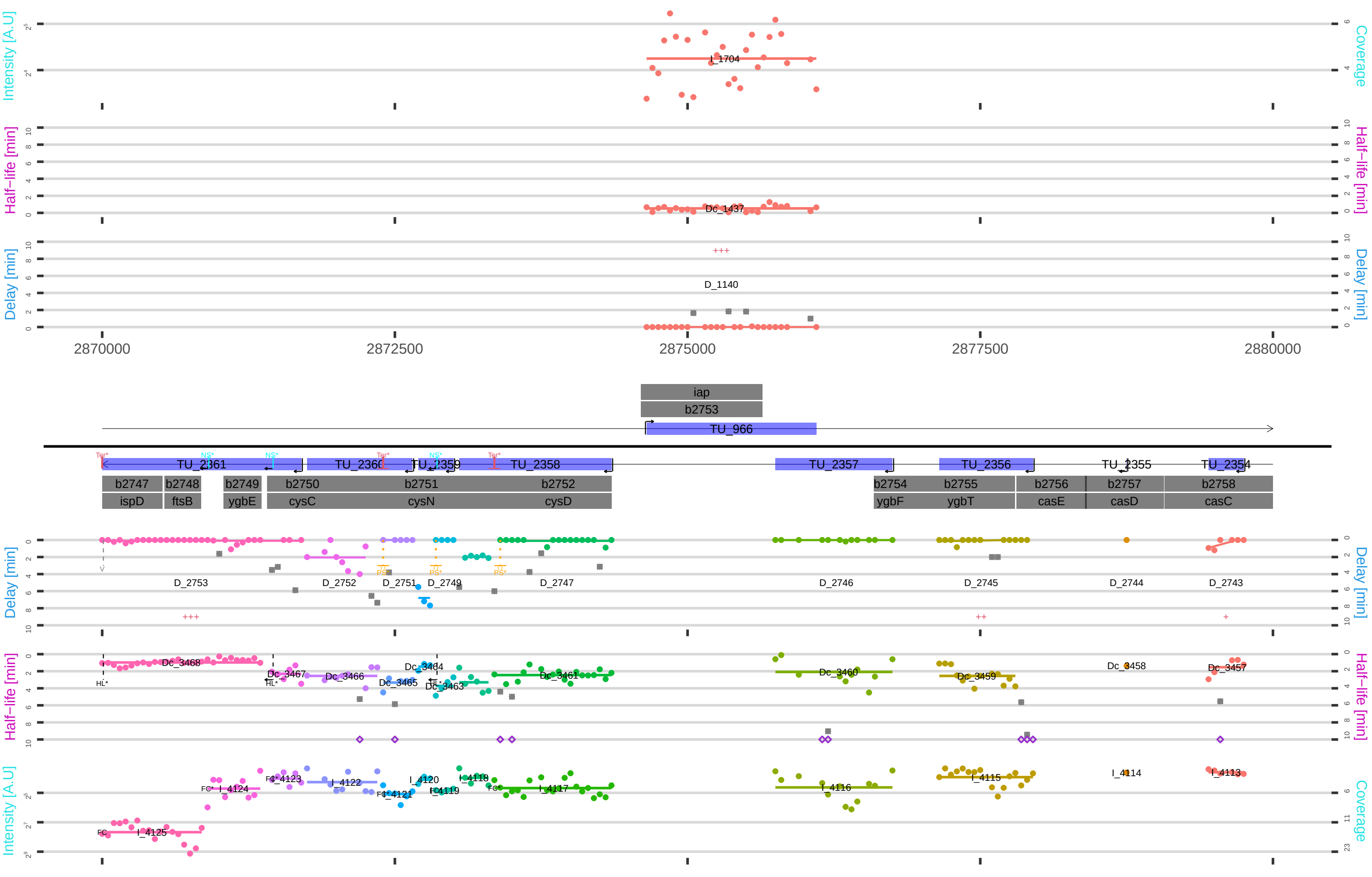

Term: termination (3), NS: new start (3), PS: pausing site (3), iTSS_I: internal starting site (0)

ID: 127987−127791; FC*: significant t−test of two consecutive segments; Term: termination, NS: new start, PS: pausing site,iTSS_I: internal starting site,TI: transcription interferance.

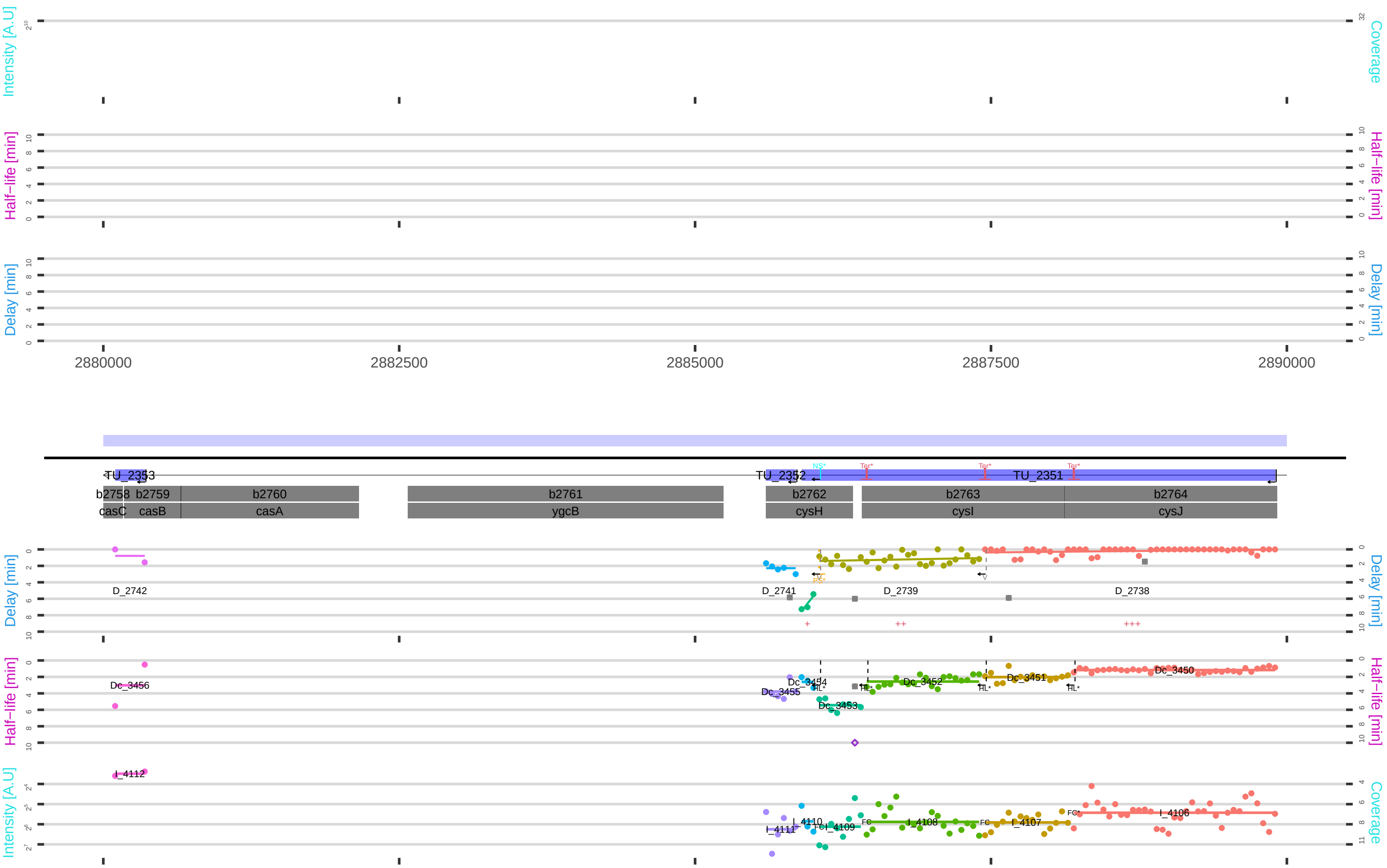

Term: termination (3), NS: new start (1), PS: pausing site (2), iTSS_I: internal starting site (0)

ID: 57806−57857; Term: termination (2), NS: new start (0), PS: pausing site (0), iTSS_I: internal starting site (0)

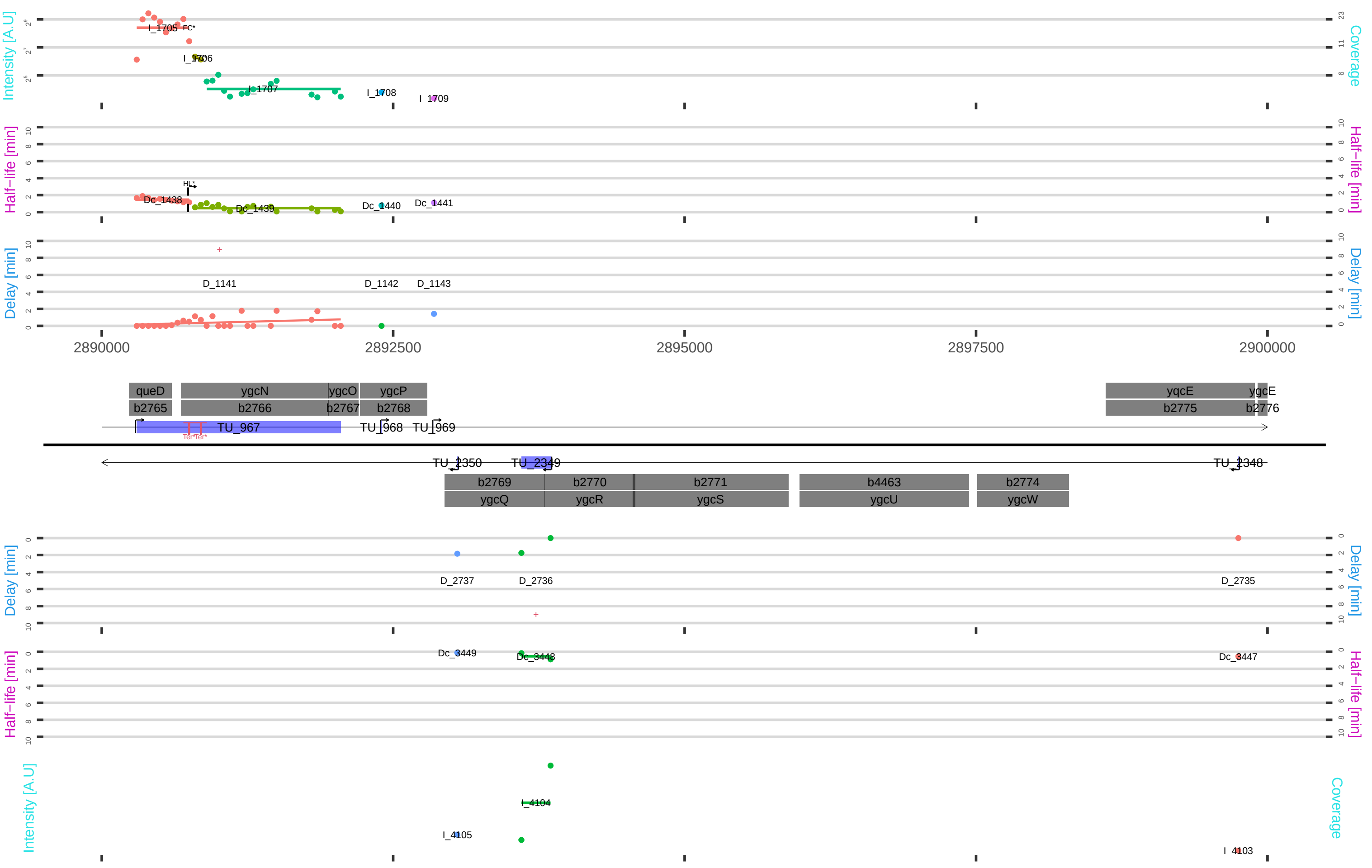

Term: termination (0), NS: new start (0), PS: pausing site (0), iTSS_I: internal starting site (0)

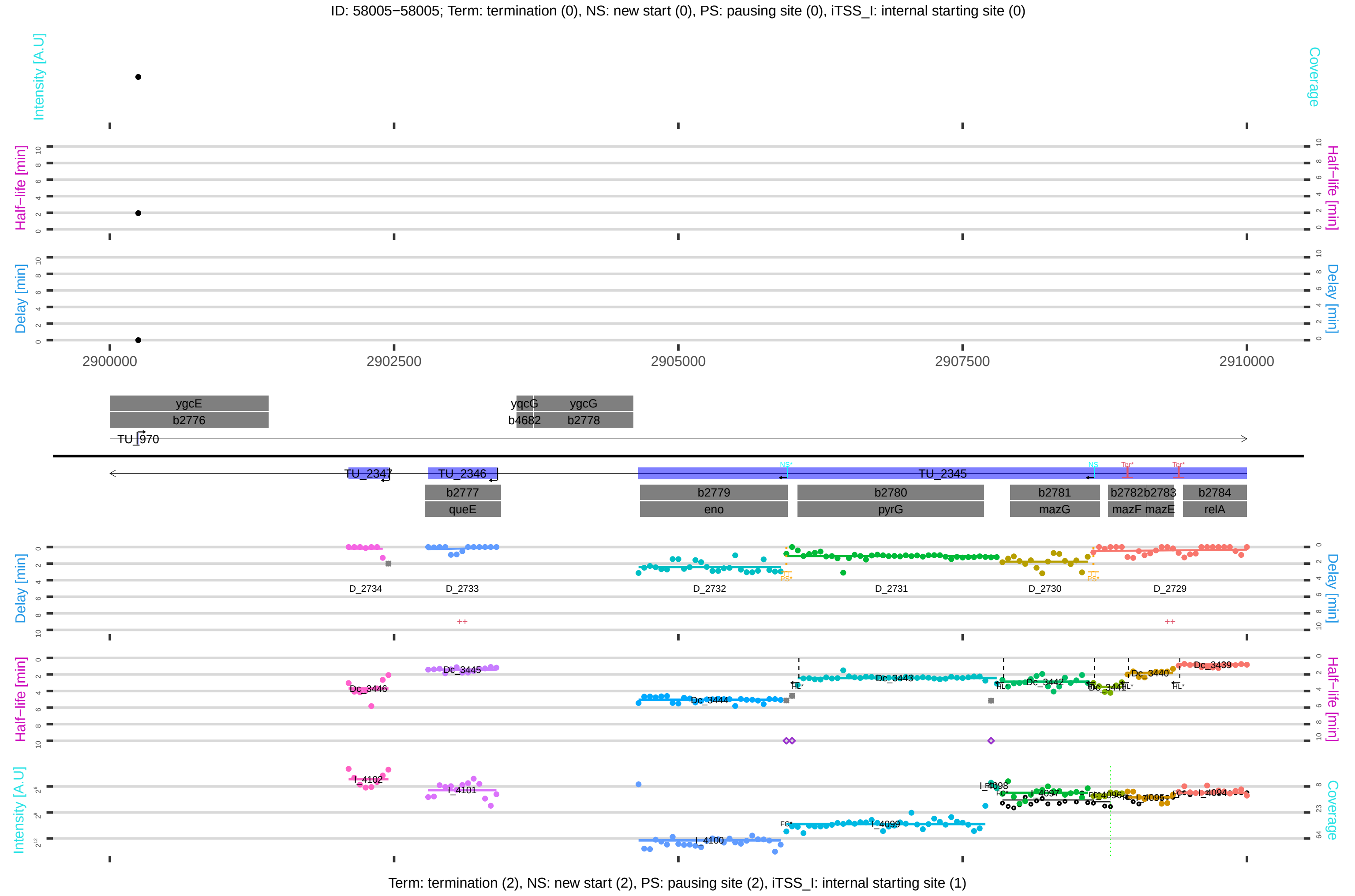
ID: 58005−58005; Term: termination (0), NS: new start (0), PS: pausing site (0), iTSS_I: internal starting site (0)
Intensity [A.U]
Coverage
Half−life [min]
Delay [min]
2900000
2902500
2905000
2907500
2910000
ygcE
b2776
ygcG
b4682
ygcG
b2778
TU_970
TU_2347
TU_2346
TU_2345
b2777
queE
b2779
eno
b2780
pyrG
b2781
mazG
b2782
mazF
b2783
mazE
b2784
relA
D_2734
D_2733
D_2732
D_2731
D_2730
D_2729
Dc_3446
Dc_3445
Dc_3444
Dc_3443
Dc_3442
Dc_3441
Dc_3440
Dc_3439
I_4102
I_4101
I_4100
I_4099
I_4098
I_4097
I_4096
I_4095
I_4094
Term: termination (2), NS: new start (2), PS: pausing site (2), iTSS_I: internal starting site (1)

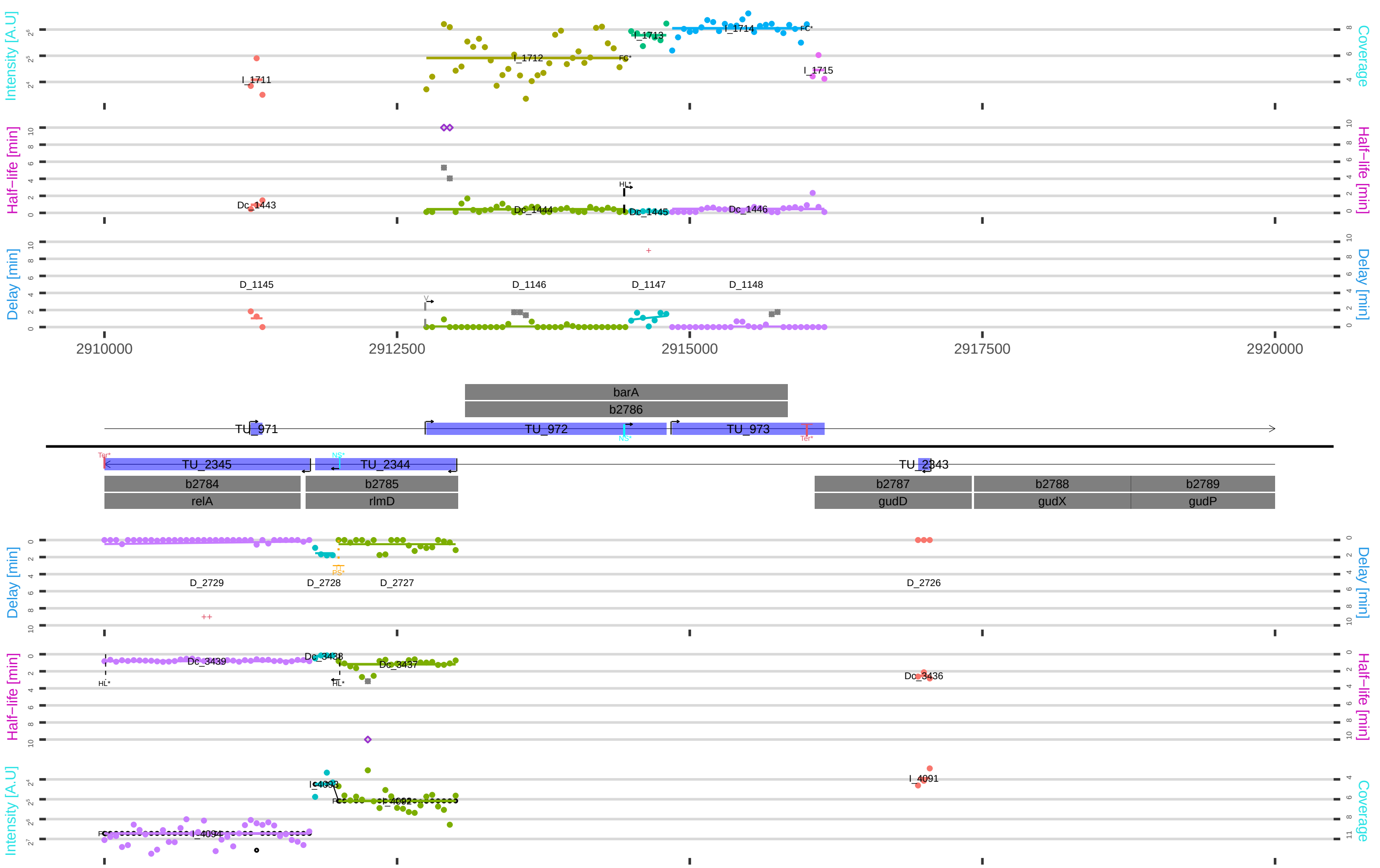

Term: termination (1), NS: new start (1), PS: pausing site (1), iTSS_I: internal starting site (0)

ID: 58447−58597; Term: termination (3), NS: new start (2), PS: pausing site (0), iTSS_I: internal starting site (0)

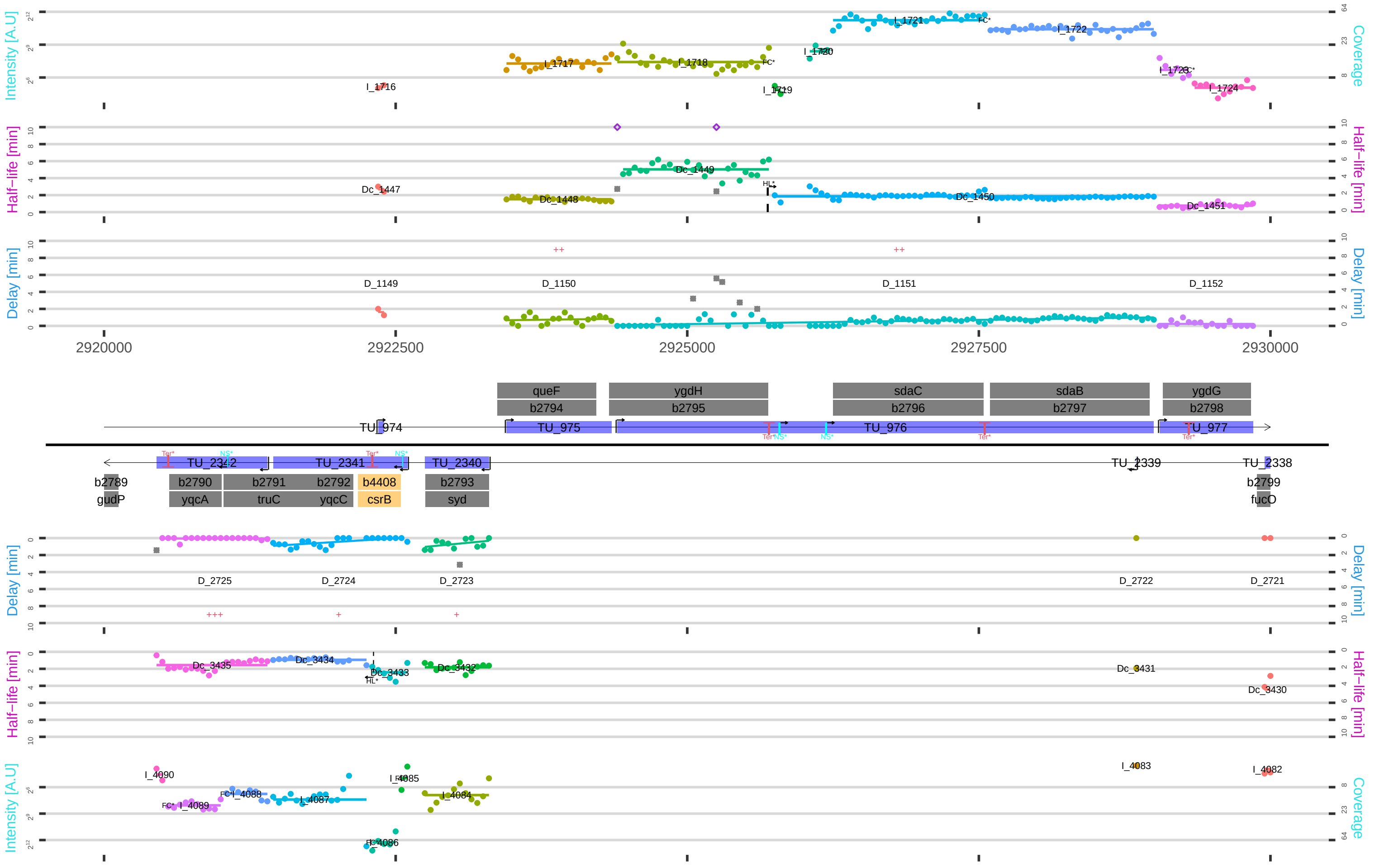

Term: termination (2), NS: new start (2), PS: pausing site (0), iTSS_I: internal starting site (0)

ID: 58674−58766; Term: termination (1), NS: new start (3), PS: pausing site (1), iTSS_I: internal starting site (0)

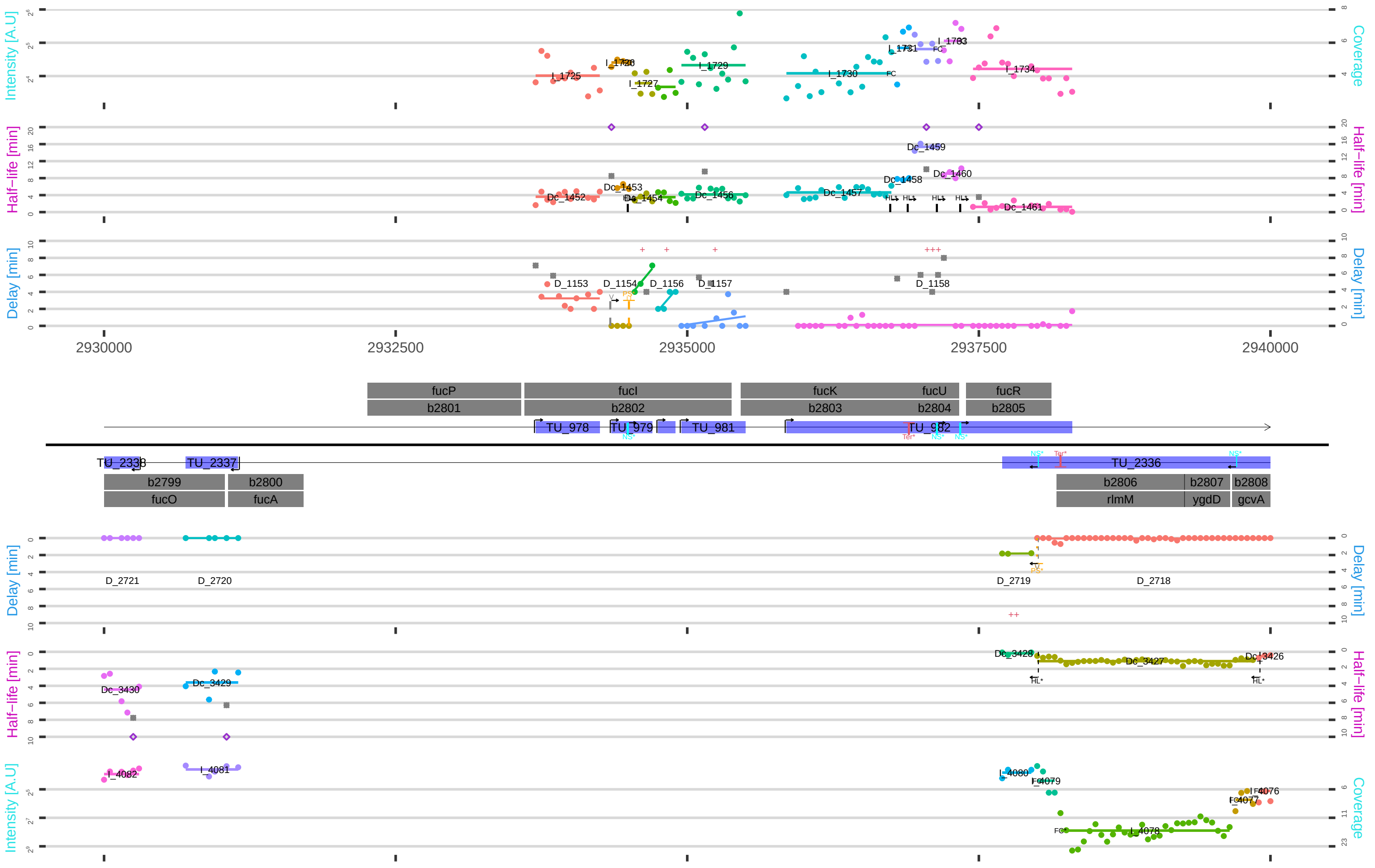

Term: termination (1), NS: new start (2), PS: pausing site (1), iTSS_I: internal starting site (0)

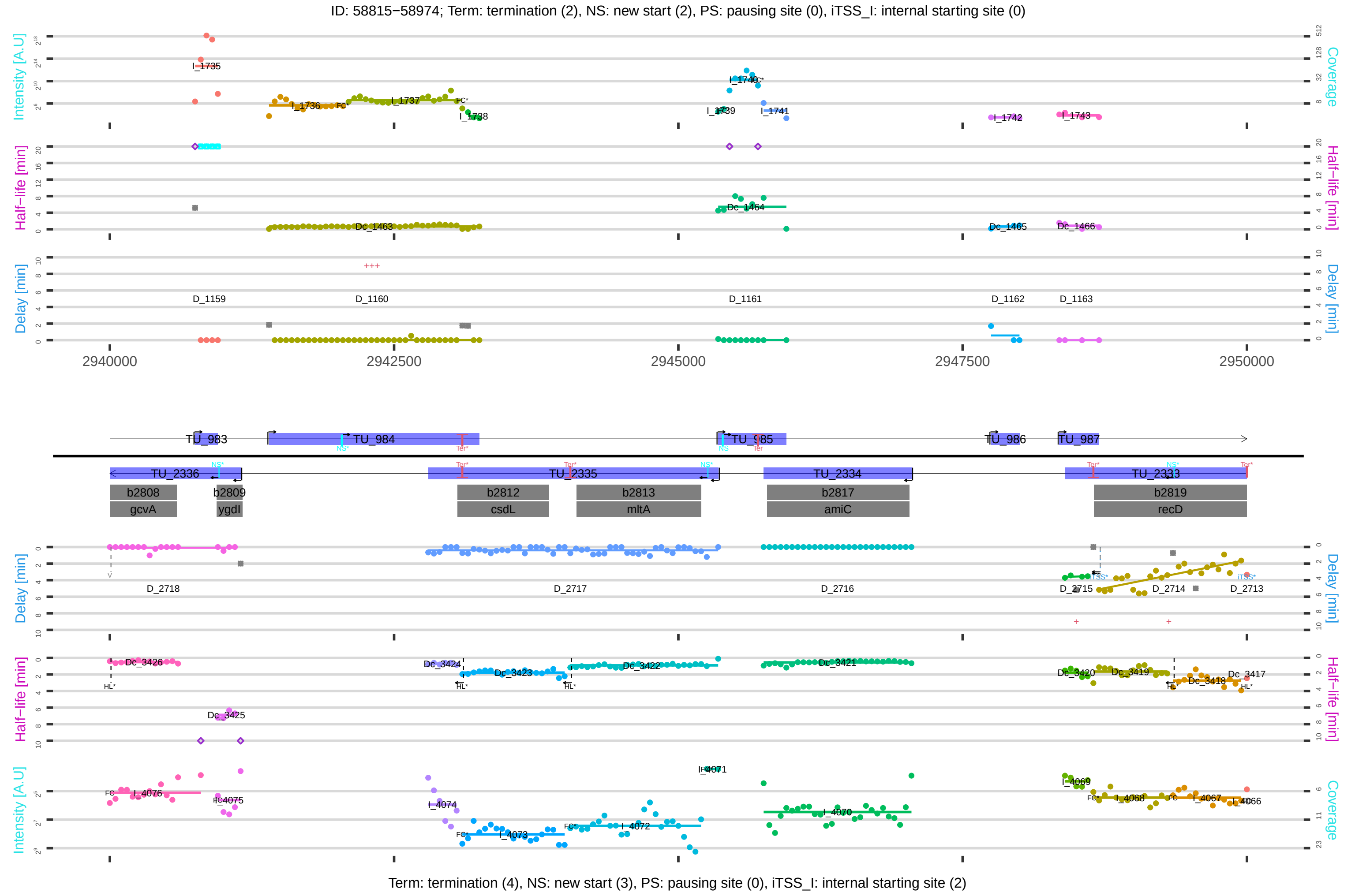

ID: 58815−58974; Term: termination (2), NS: new start (2), PS: pausing site (0), iTSS_I: internal starting site (0)
Intensity [A.U]
Coverage
Half−life [min]
Delay [min]
I_1735
I_1736
I_1737
I_1738
I_1739
I_1740
I_1741
I_1742
I_1743
Dc_1463
Dc_1464
Dc_1465
Dc_1466
D_1159
D_1160
D_1161
D_1162
D_1163
2940000
2942500
2945000
2947500
2950000
TU_983
TU_984
TU_985
TU_986
TU_987
TU_2336
TU_2335
TU_2334
TU_2333
b2808
gcvA
b2809
ygdI
b2812
csdL
b2813
mltA
b2817
amiC
b2819
recD
D_2718
D_2717
D_2716
D_2715
D_2714
D_2713
Dc_3426
Dc_3425
Dc_3424
Dc_3423
Dc_3422
Dc_3421
Dc_3420
Dc_3419
Dc_3418
Dc_3417
I_4076
I_4075
I_4074
I_4073
I_4072
I_4071
I_4070
I_4069
I_4068
I_4067
I_4066
Term: termination (4), NS: new start (3), PS: pausing site (0), iTSS_I: internal starting site (2)

ID: 59186−59186; Term: termination (0), NS: new start (0), PS: pausing site (0), iTSS_I: internal starting site (0)

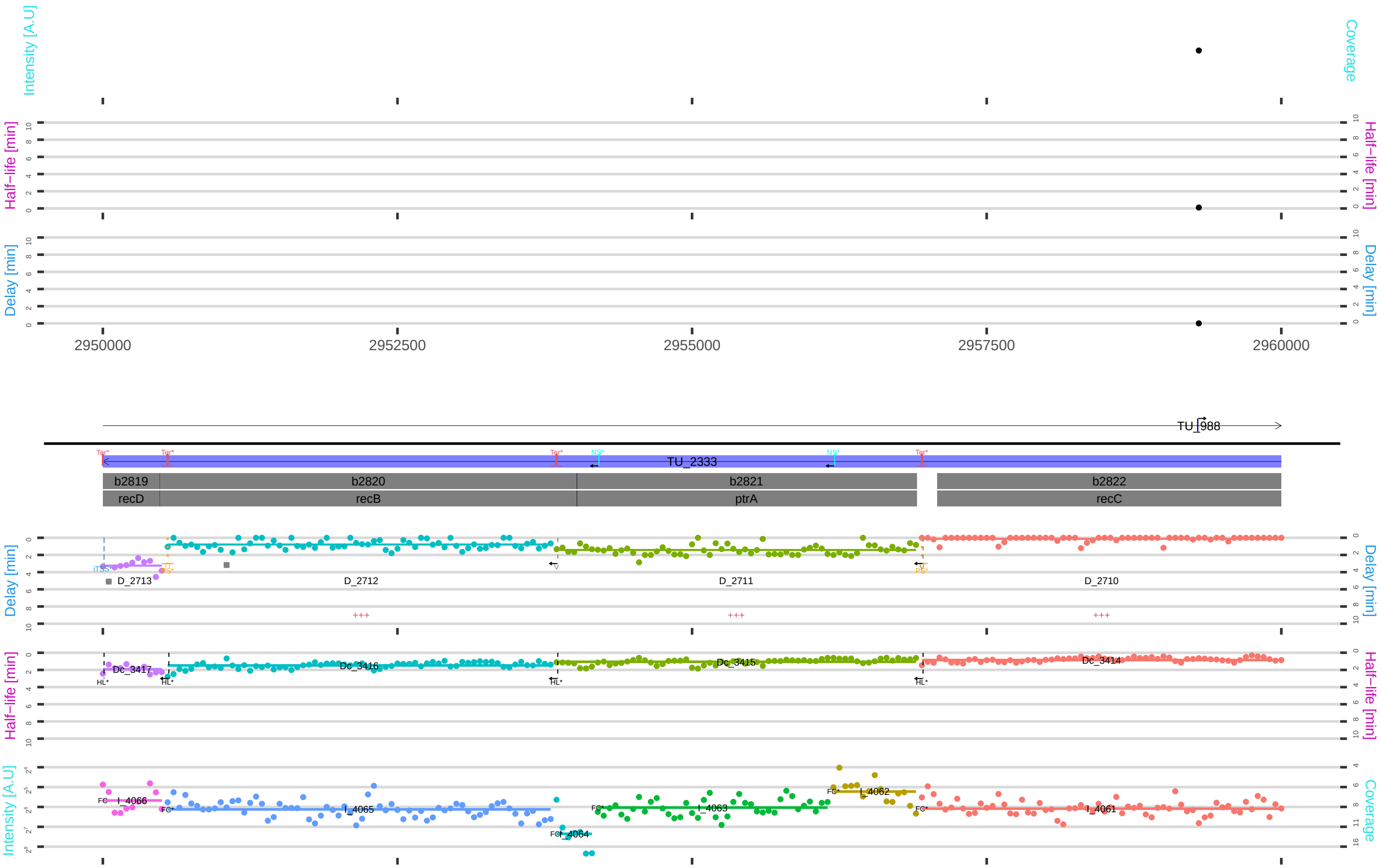

Term: termination (4), NS: new start (2), PS: pausing site (2), iTSS_I: internal starting site (2)

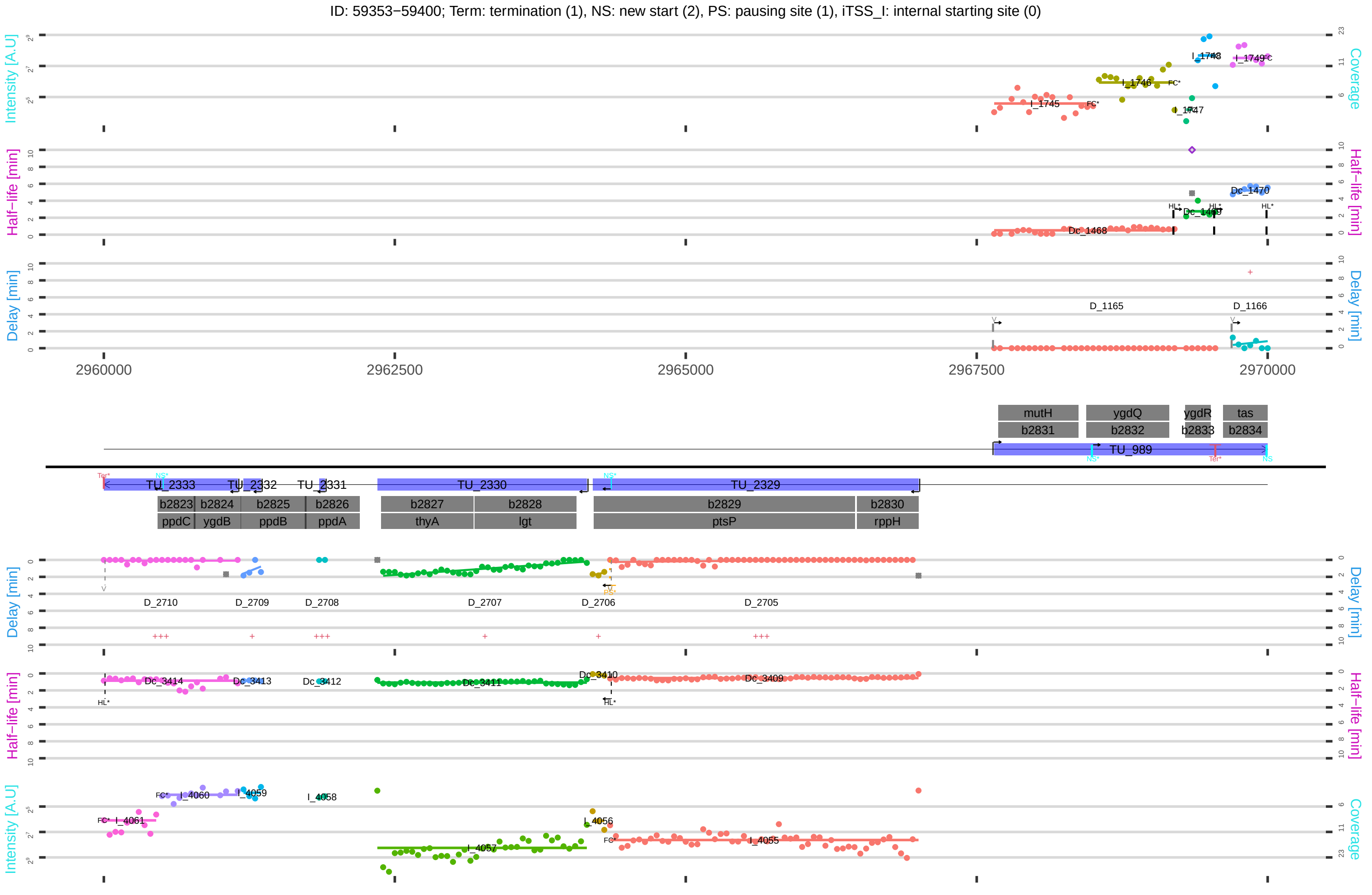

Term: termination (1), NS: new start (2), PS: pausing site (1), iTSS_I: internal starting site (0)

ID: 59400−59560; Term: termination (0), NS: new start (1), PS: pausing site (1), iTSS_I: internal starting site (0)

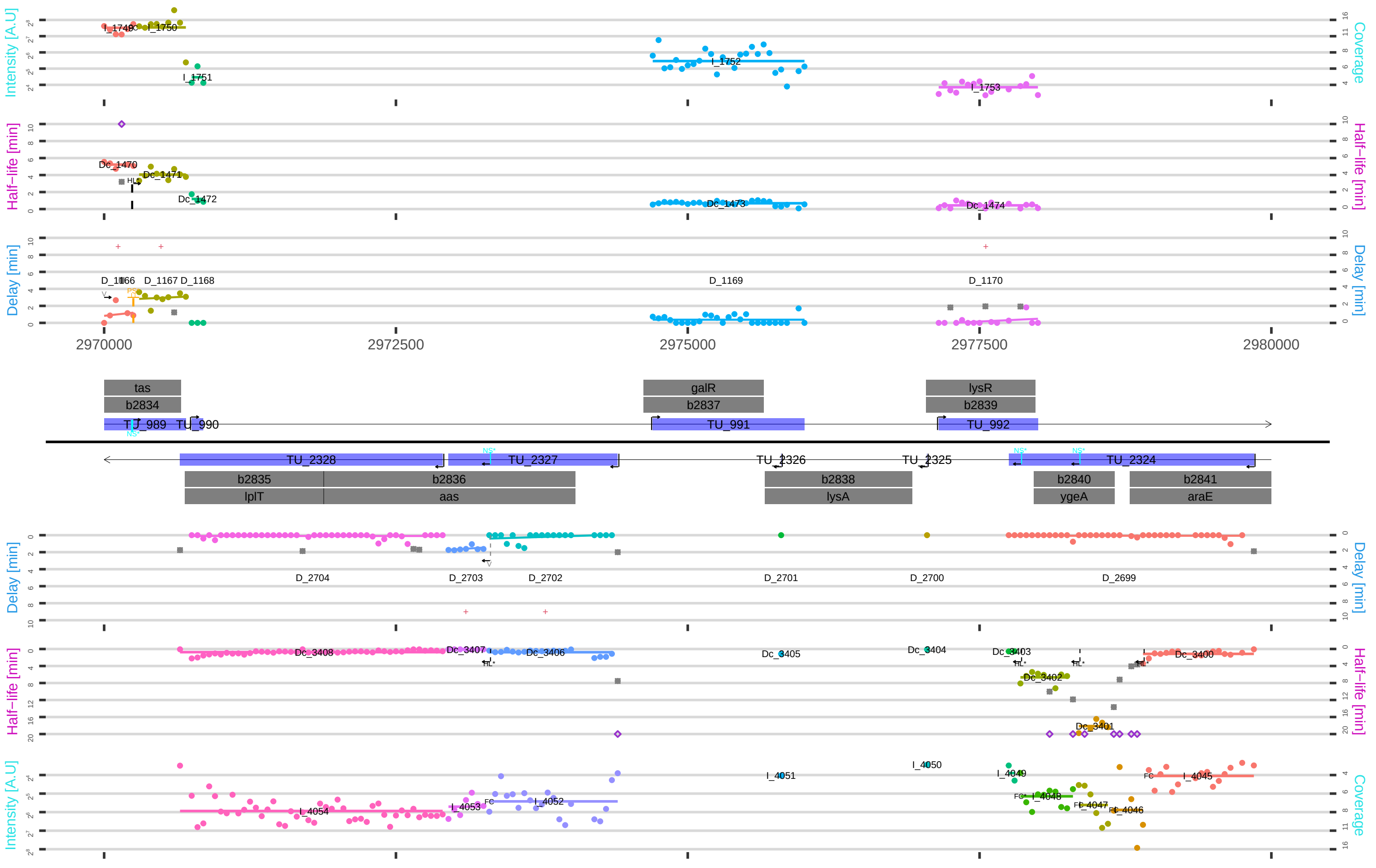

Term: termination (0), NS: new start (3), PS: pausing site (1), iTSS_I: internal starting site (0)

ID: 59679−59754; Term: termination (0), NS: new start (0), PS: pausing site (0), iTSS_I: internal starting site (0)

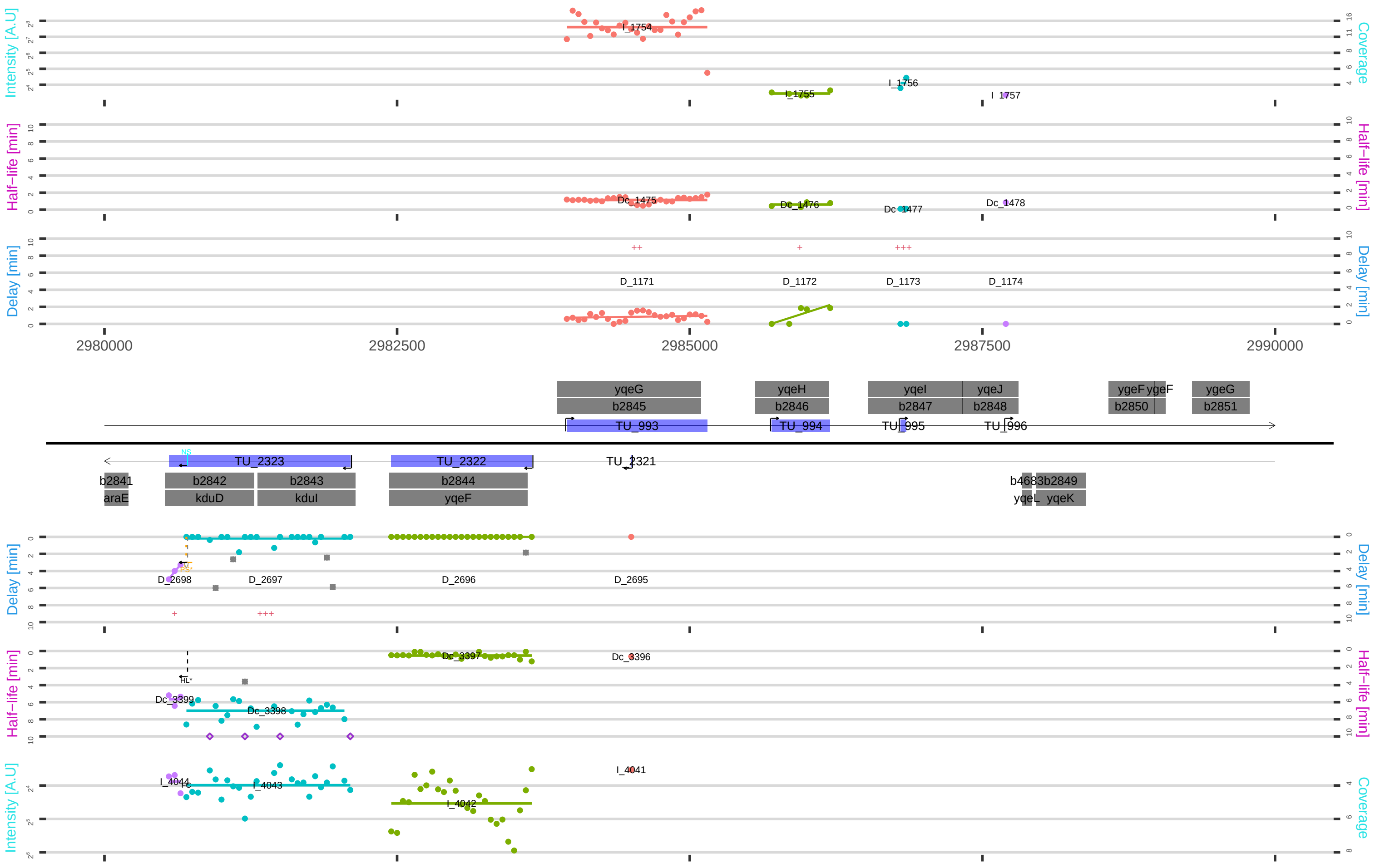

Term: termination (0), NS: new start (1), PS: pausing site (1), iTSS_I: internal starting site (0)

ID: 59837−59982; Term: termination (0), NS: new start (0), PS: pausing site (0), iTSS_I: internal starting site (0)

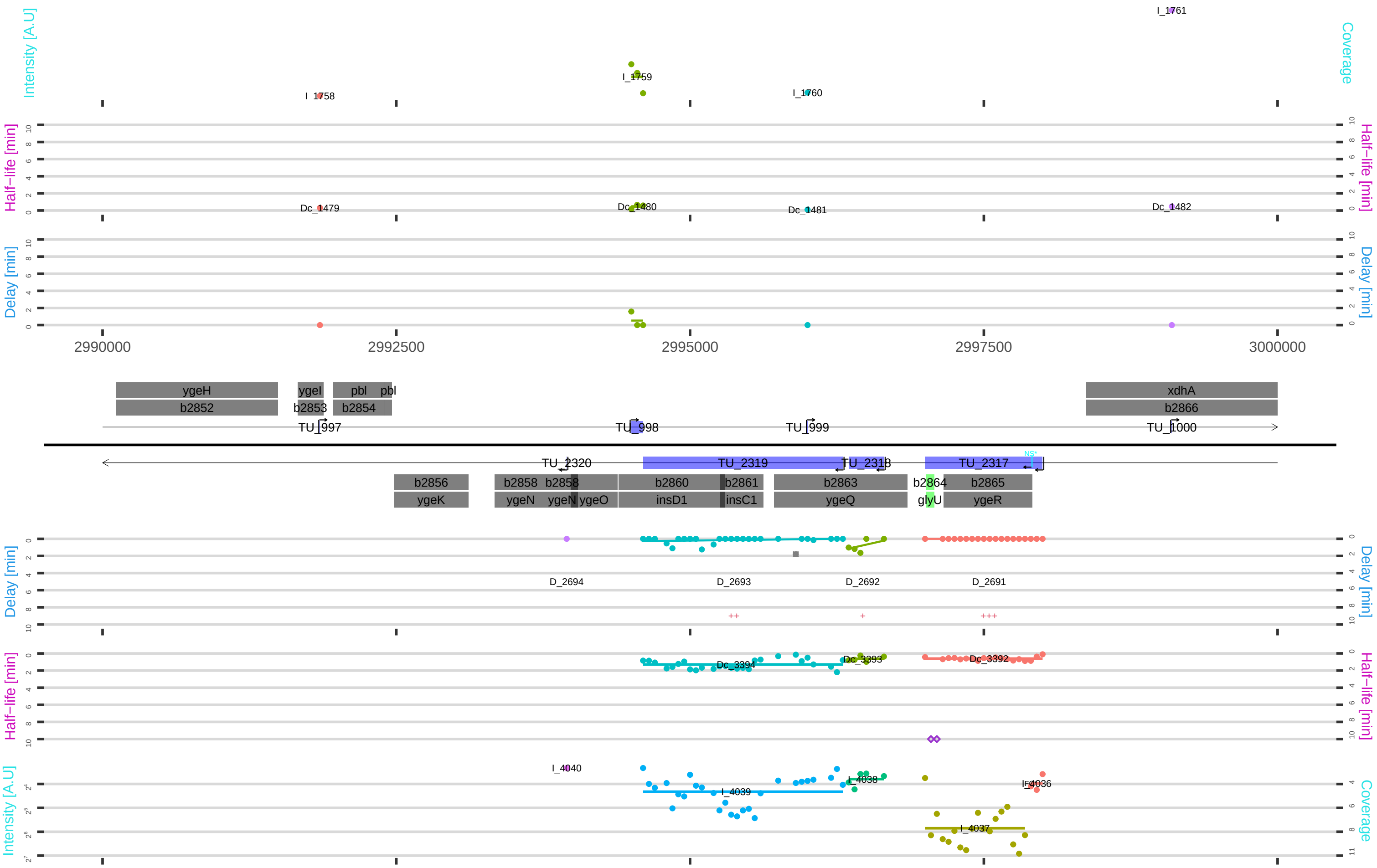

Term: termination (0), NS: new start (1), PS: pausing site (0), iTSS_I: internal starting site (0)

ID: 60008−60191; Term: termination (0), NS: new start (0), PS: pausing site (0), iTSS_I: internal starting site (0)

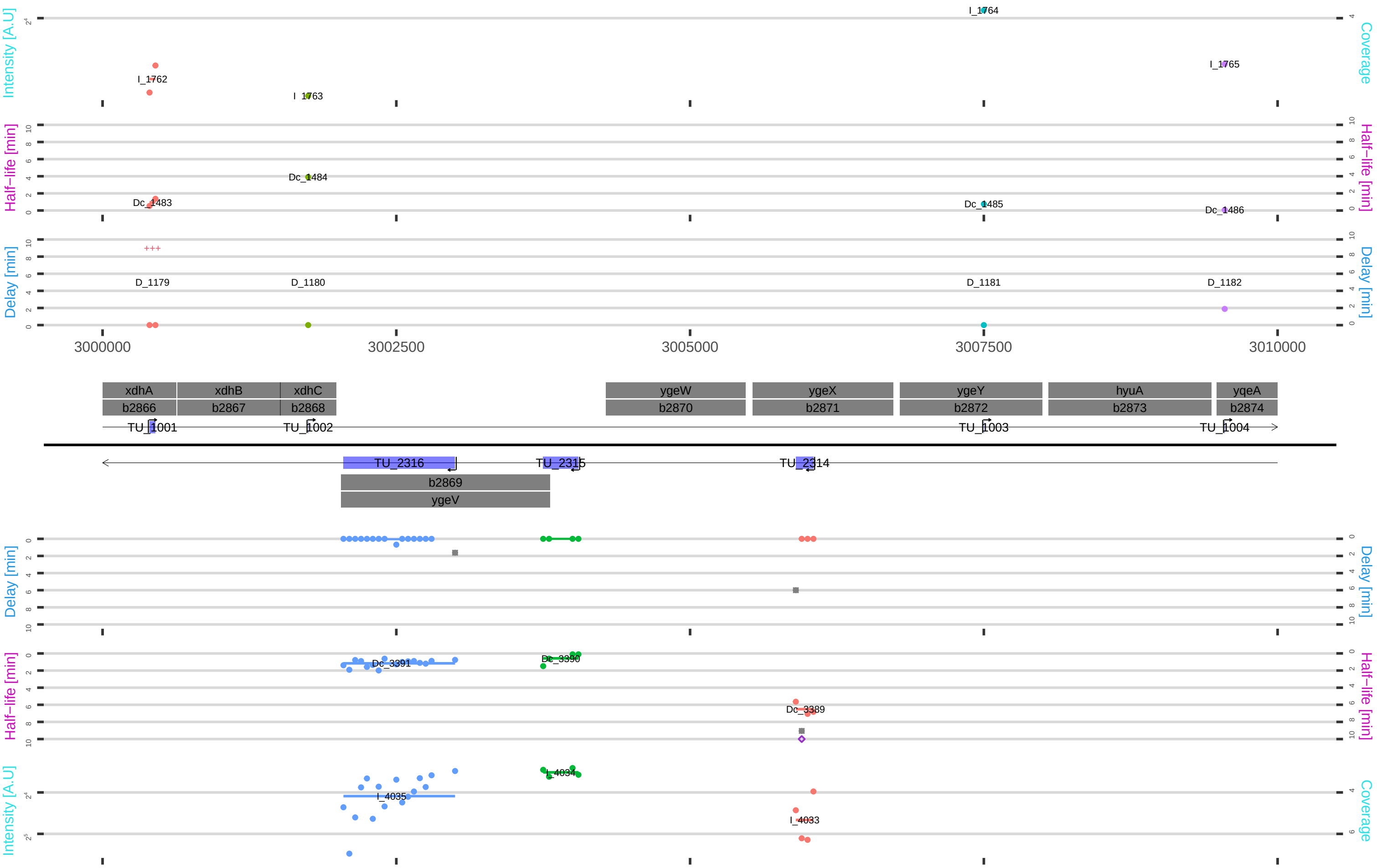

Term: termination (0), NS: new start (0), PS: pausing site (0), iTSS_I: internal starting site (0)

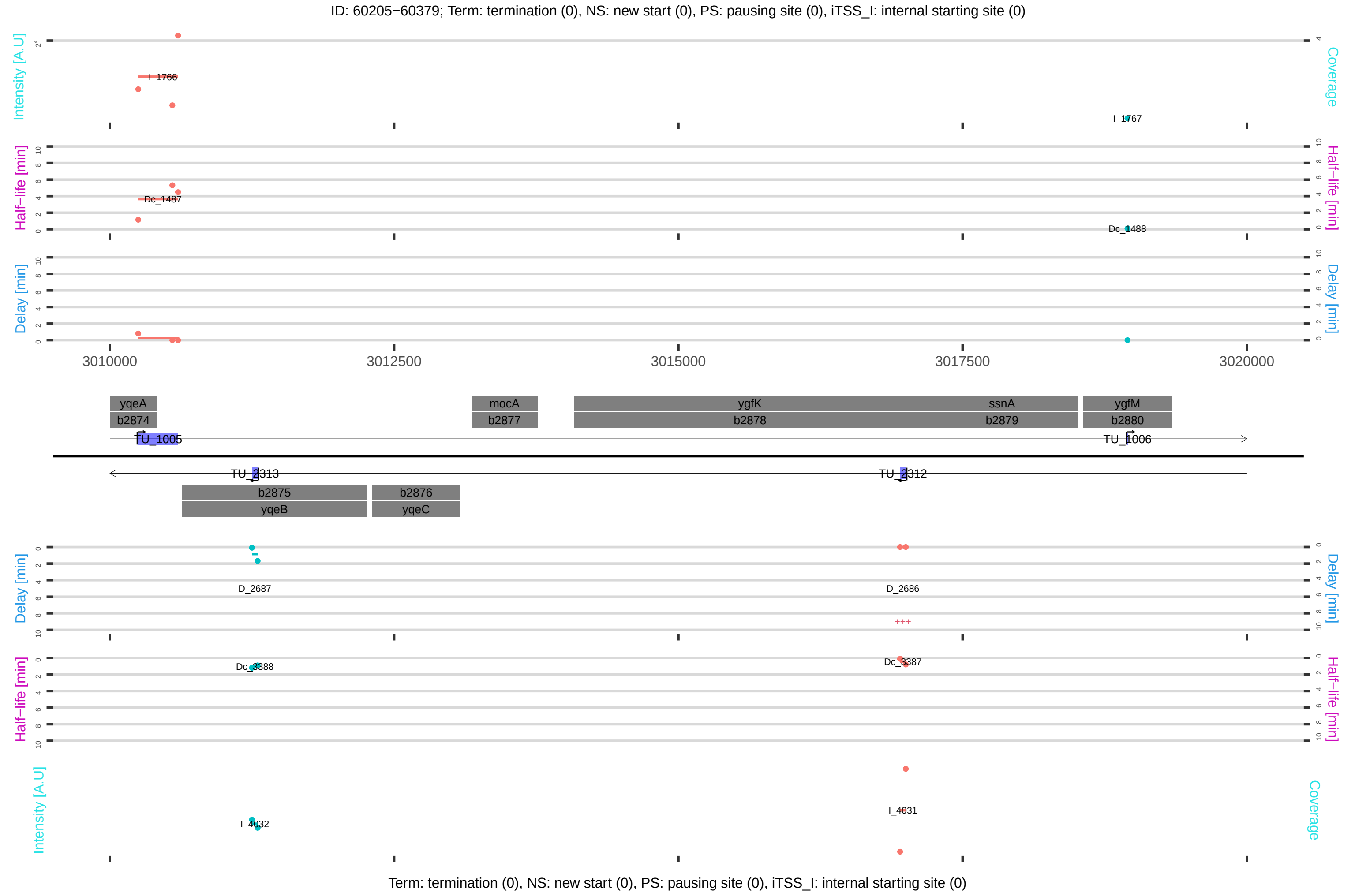

ID: 60205−60379; Term: termination (0), NS: new start (0), PS: pausing site (0), iTSS_I: internal starting site (0)
Intensity [A.U]
Coverage
I_1766
I_1767
Half−life [min]
Dc_1487
Dc_1488
Delay [min]
3010000
3012500
3015000
3017500
3020000
yqeA
b2874
mocA
b2877
ygfK
b2878
ssnA
b2879
ygfM
b2880
TU_1005
TU_1006
TU_2313
TU_2312
b2875
yqeB
b2876
yqeC
D_2687
D_2686
Dc_3388
Dc_3387
I_4032
I_4031
Term: termination (0), NS: new start (0), PS: pausing site (0), iTSS_I: internal starting site (0)

ID: 60435−60534; Term: termination (0), NS: new start (0), PS: pausing site (0), iTSS_I: internal starting site (0)

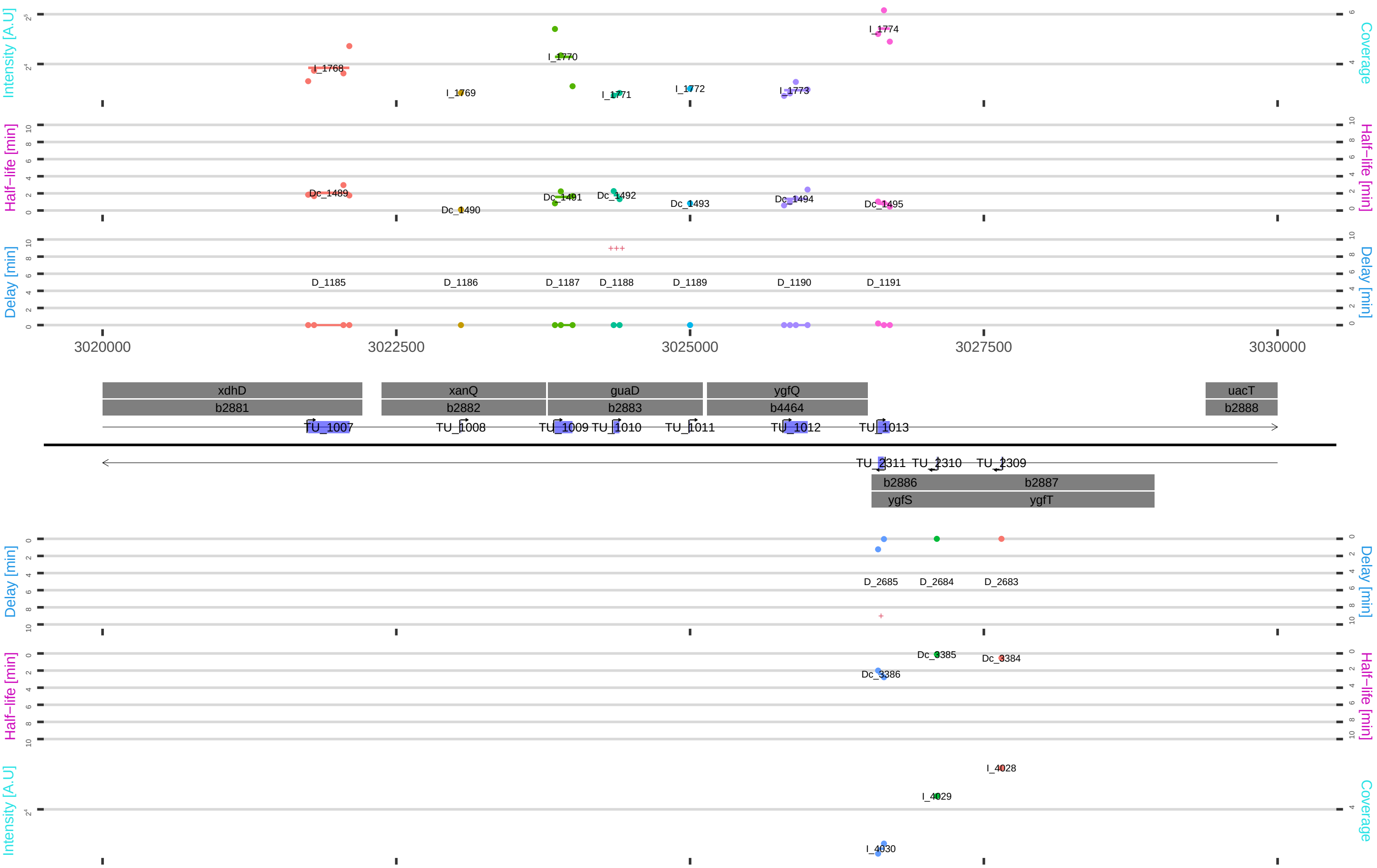

Term: termination (0), NS: new start (0), PS: pausing site (0), iTSS_I: internal starting site (0)

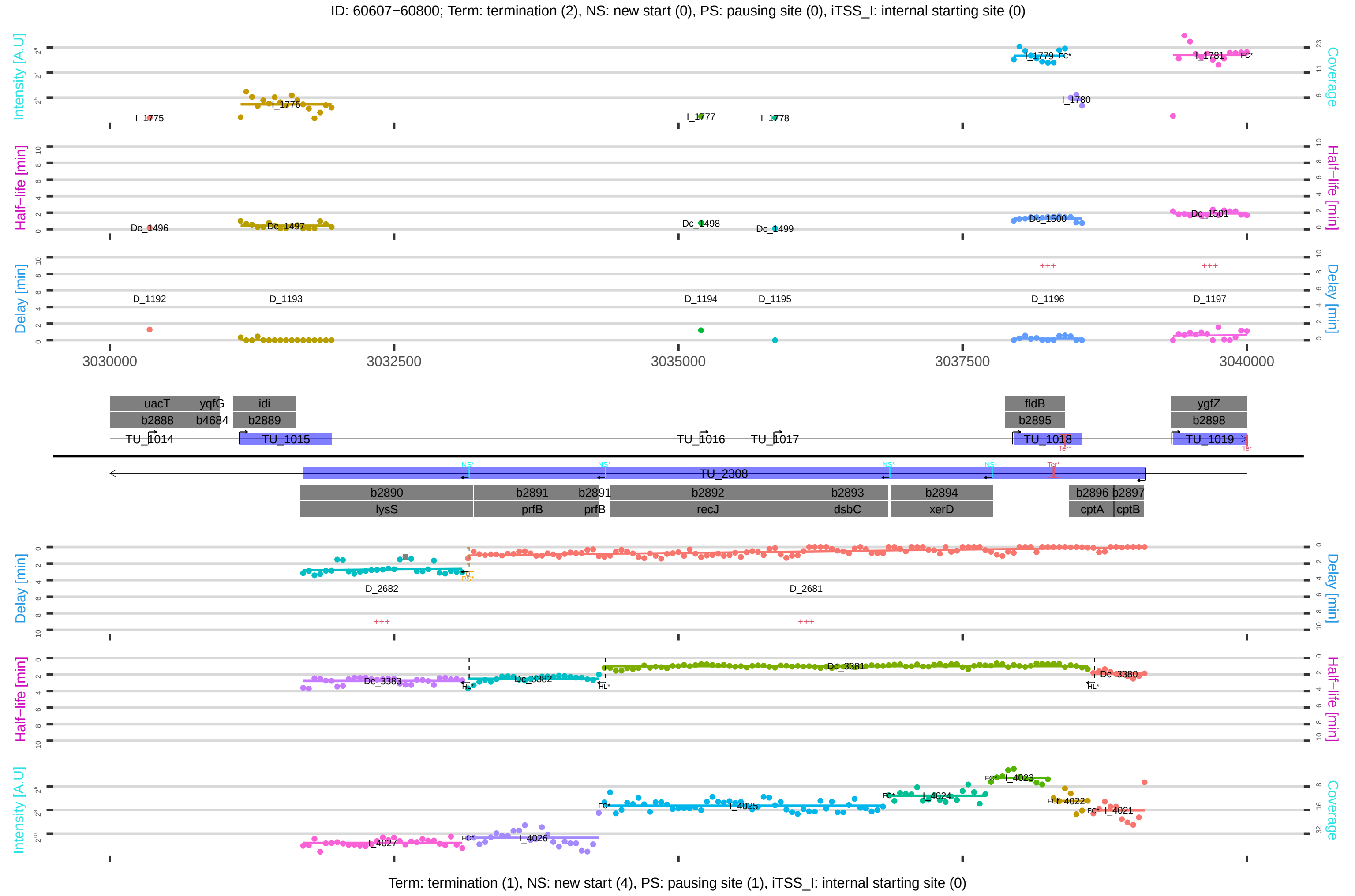
ID: 60607−60800; Term: termination (2), NS: new start (0), PS: pausing site (0), iTSS_I: internal starting site (0)
Intensity [A.U]
Coverage
Half−life [min]
Delay [min]
I_1775
I_1776
I_1777
I_1778
I_1779
I_1780
I_1781
Dc_1496
Dc_1497
Dc_1498
Dc_1499
Dc_1500
Dc_1501
D_1192
D_1193
D_1194
D_1195
D_1196
D_1197
3030000
3032500
3035000
3037500
3040000
uacT
yqfG
idi
fldB
ygfZ
b2888
b4684
b2889
b2895
b2898
TU_1014
TU_1015
TU_1016
TU_1017
TU_1018
TU_1019
TU_2308
b2890
b2891
b2892
b2893
b2894
b2896
b2897
lysS
prfB
recJ
dsbC
xerD
cptA
cptB
D_2682
D_2681
Dc_3383
Dc_3382
Dc_3381
Dc_3380
I_4027
I_4026
I_4025
I_4024
I_4023
I_4022
I_4021
Term: termination (1), NS: new start (4), PS: pausing site (1), iTSS_I: internal starting site (0)

ID: 60800−60984; Term: termination (2), NS: new start (0), PS: pausing site (1), iTSS_I: internal starting site (0)

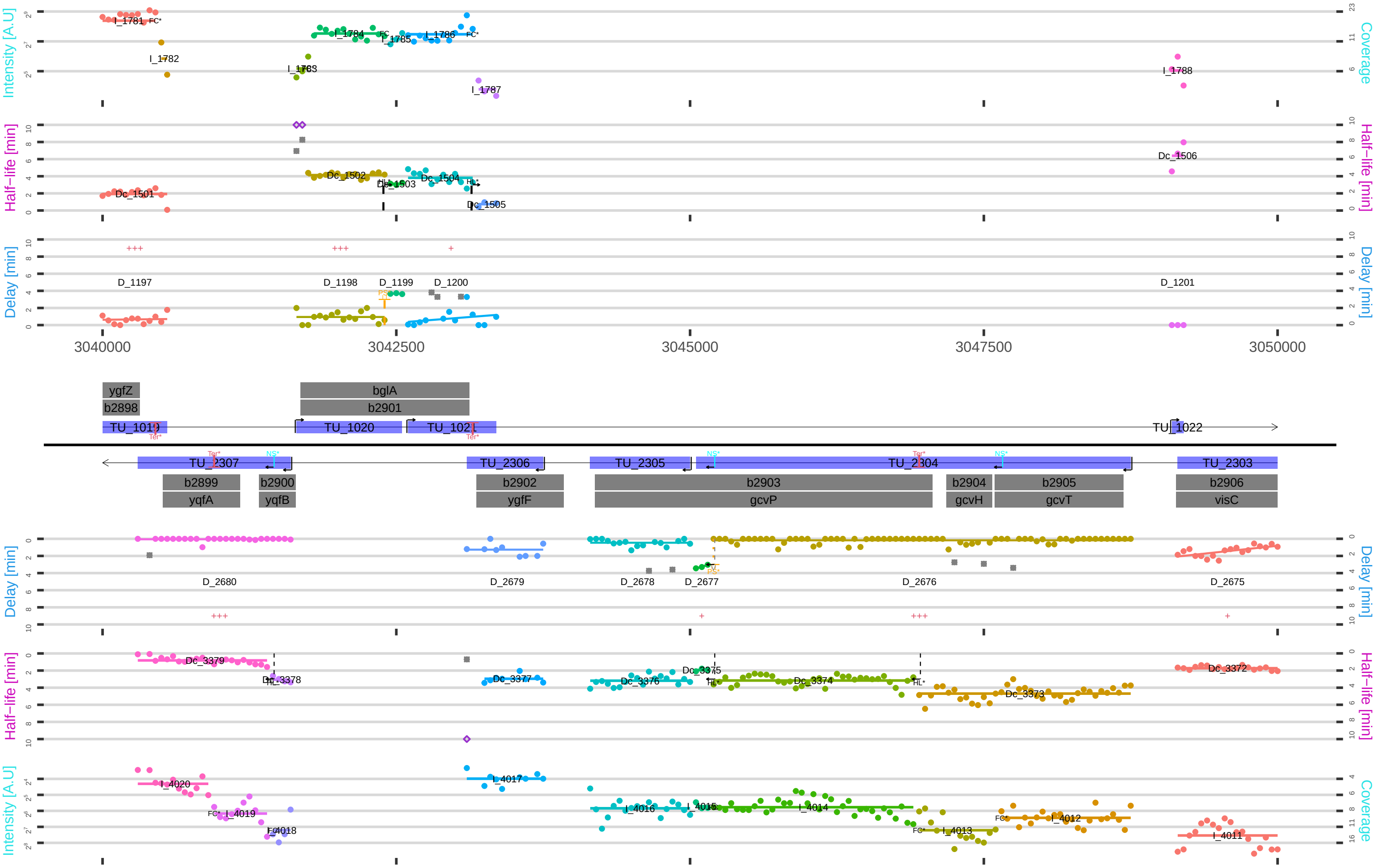

Term: termination (2), NS: new start (3), PS: pausing site (1), iTSS_I: internal starting site (0)

ID: 61072−61174; Term: termination (0), NS: new start (0), PS: pausing site (0), iTSS_I: internal starting site (0)

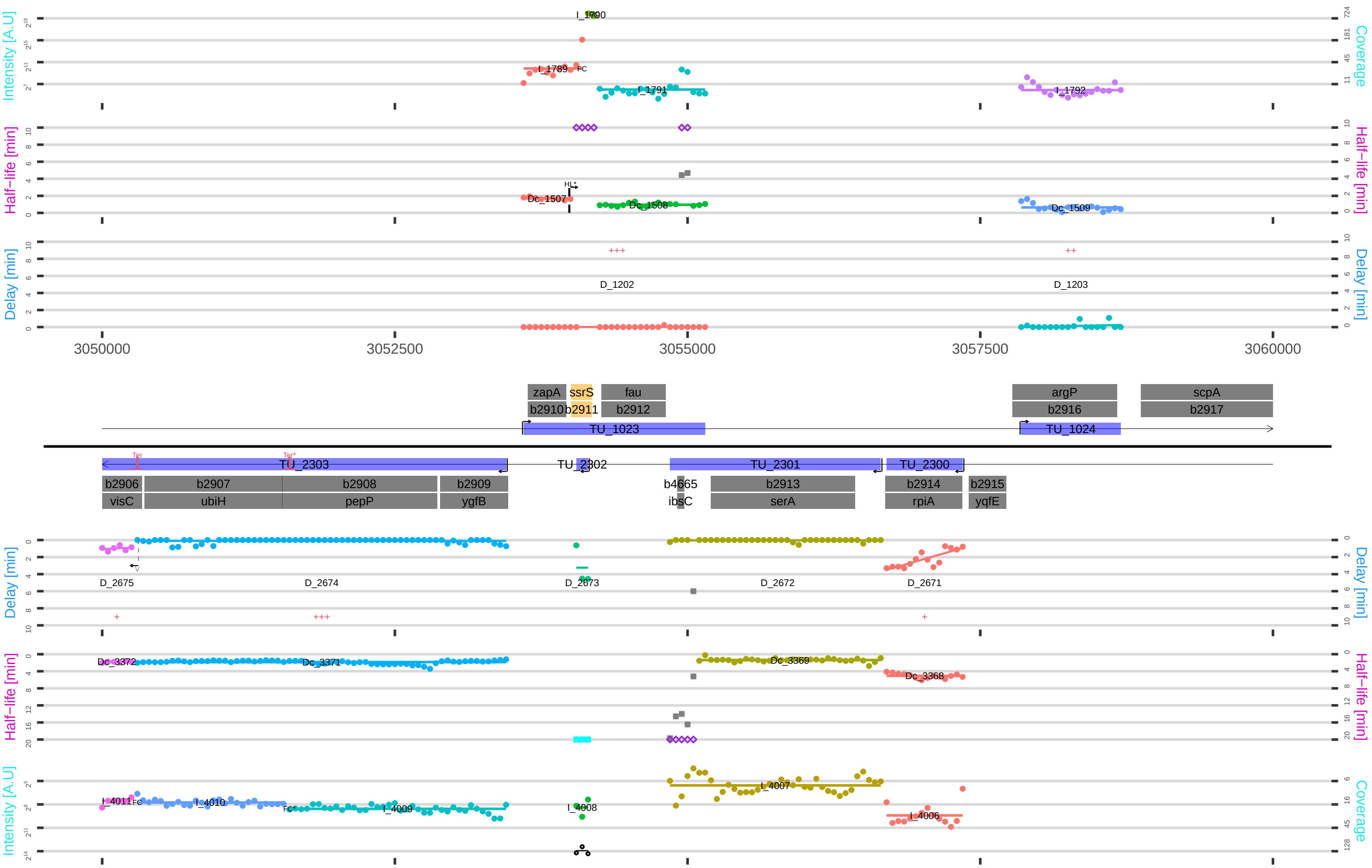

Term: termination (2), NS: new start (0), PS: pausing site (1), iTSS_I: internal starting site (0)

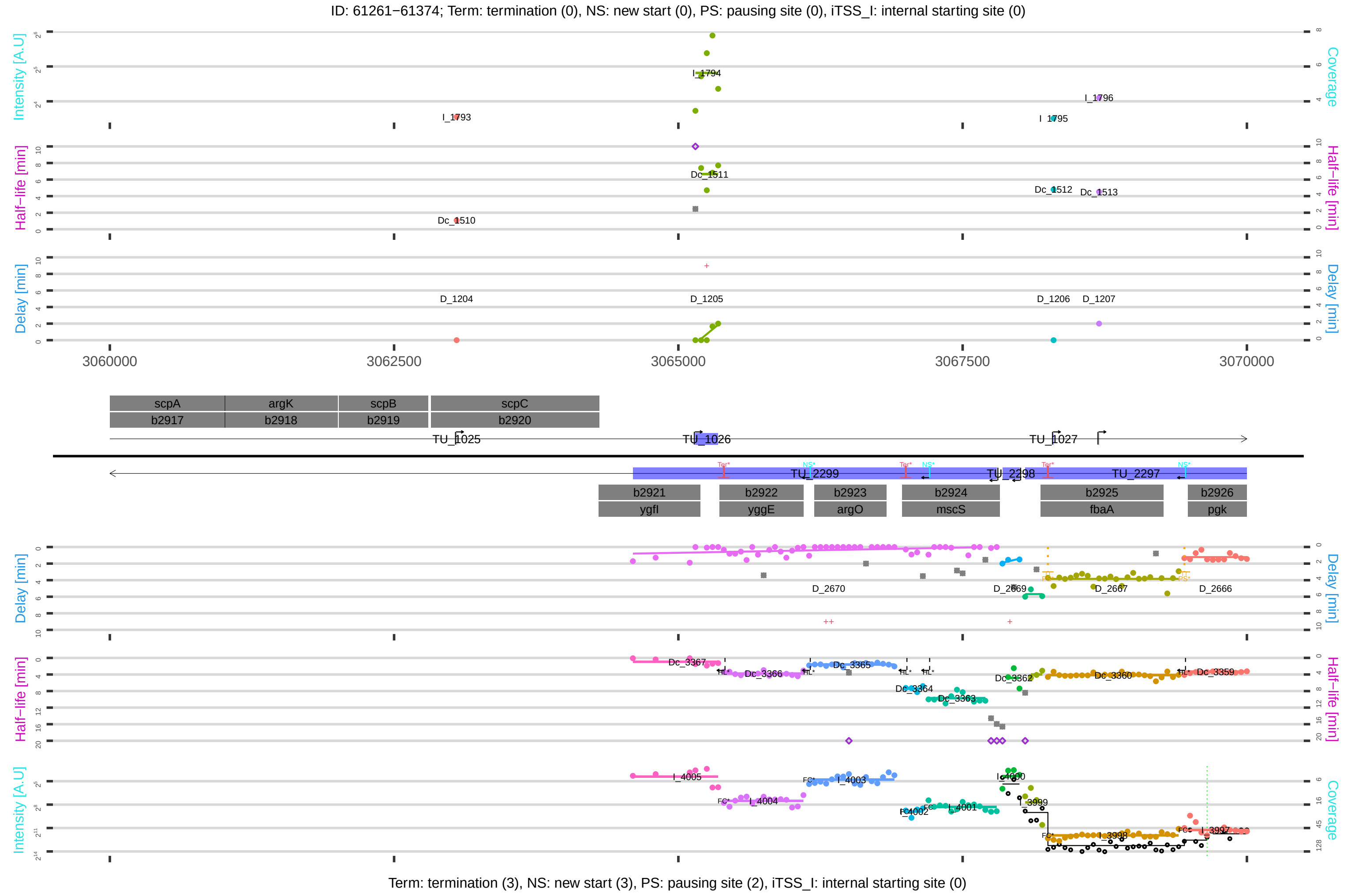

ID: 61261−61374; Term: termination (0), NS: new start (0), PS: pausing site (0), iTSS_I: internal starting site (0)
Intensity [A.U]
Coverage
Half−life [min]
Delay [min]
I_1794
I_1793
I_1796
I_1795
Dc_1511
Dc_1510
Dc_1512
Dc_1513
D_1204
D_1205
D_1206
D_1207
3060000
3062500
3065000
3067500
3070000
scpA
b2917
argK
b2918
scpB
b2919
scpC
b2920
TU_1025
TU_1026
TU_1027
TU_2299
TU_2298
TU_2297
b2921
ygfI
b2922
yggE
b2923
argO
b2924
mscS
b2925
fbaA
b2926
pgk
D_2670
D_2669
D_2667
D_2666
Dc_3367
Dc_3366
Dc_3365
Dc_3364
Dc_3363
Dc_3362
Dc_3360
Dc_3359
I_4005
I_4004
I_4003
I_4002
I_4001
I_4000
I_3999
I_3998
I_3997
Term: termination (3), NS: new start (3), PS: pausing site (2), iTSS_I: internal starting site (0)

ID: 61405−61600; Term: termination (0), NS: new start (1), PS: pausing site (0), iTSS_I: internal starting site (0)

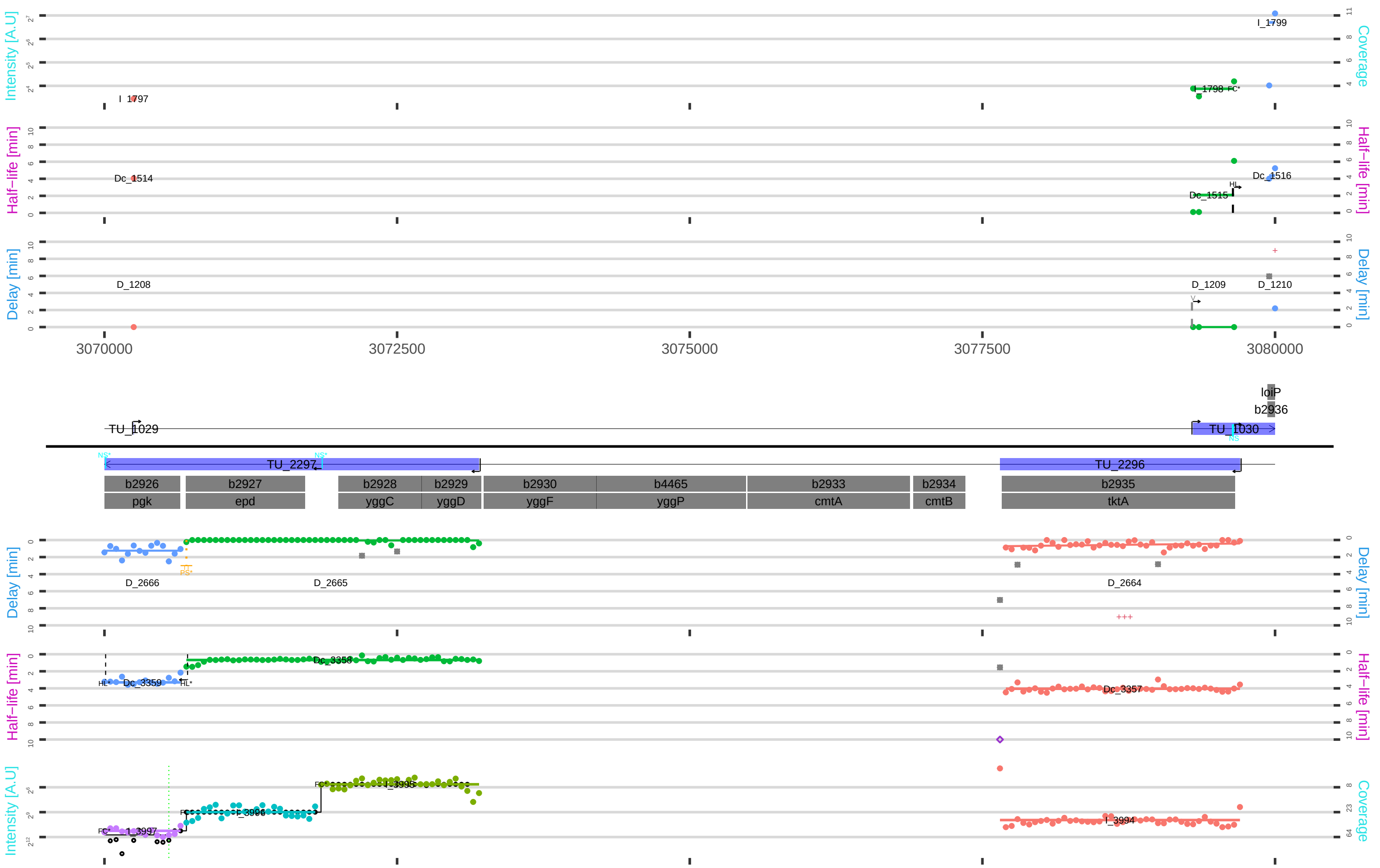

Term: termination (0), NS: new start (2), PS: pausing site (1), iTSS_I: internal starting site (0)

ID: 61600−61800; Term: termination (0), NS: new start (5), PS: pausing site (0), iTSS_I: internal starting site (0)

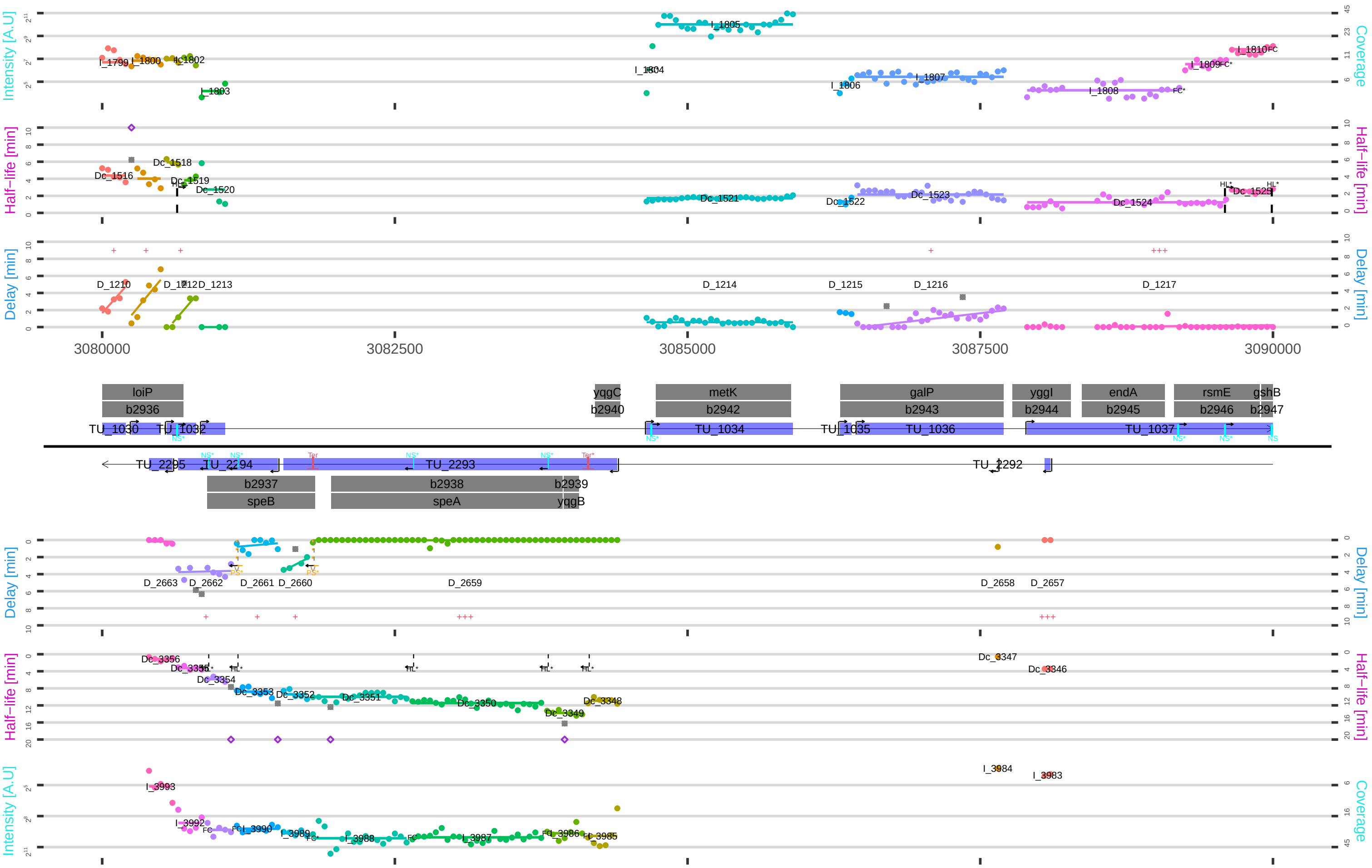

Term: termination (2), NS: new start (4), PS: pausing site (2), iTSS_I: internal starting site (0)

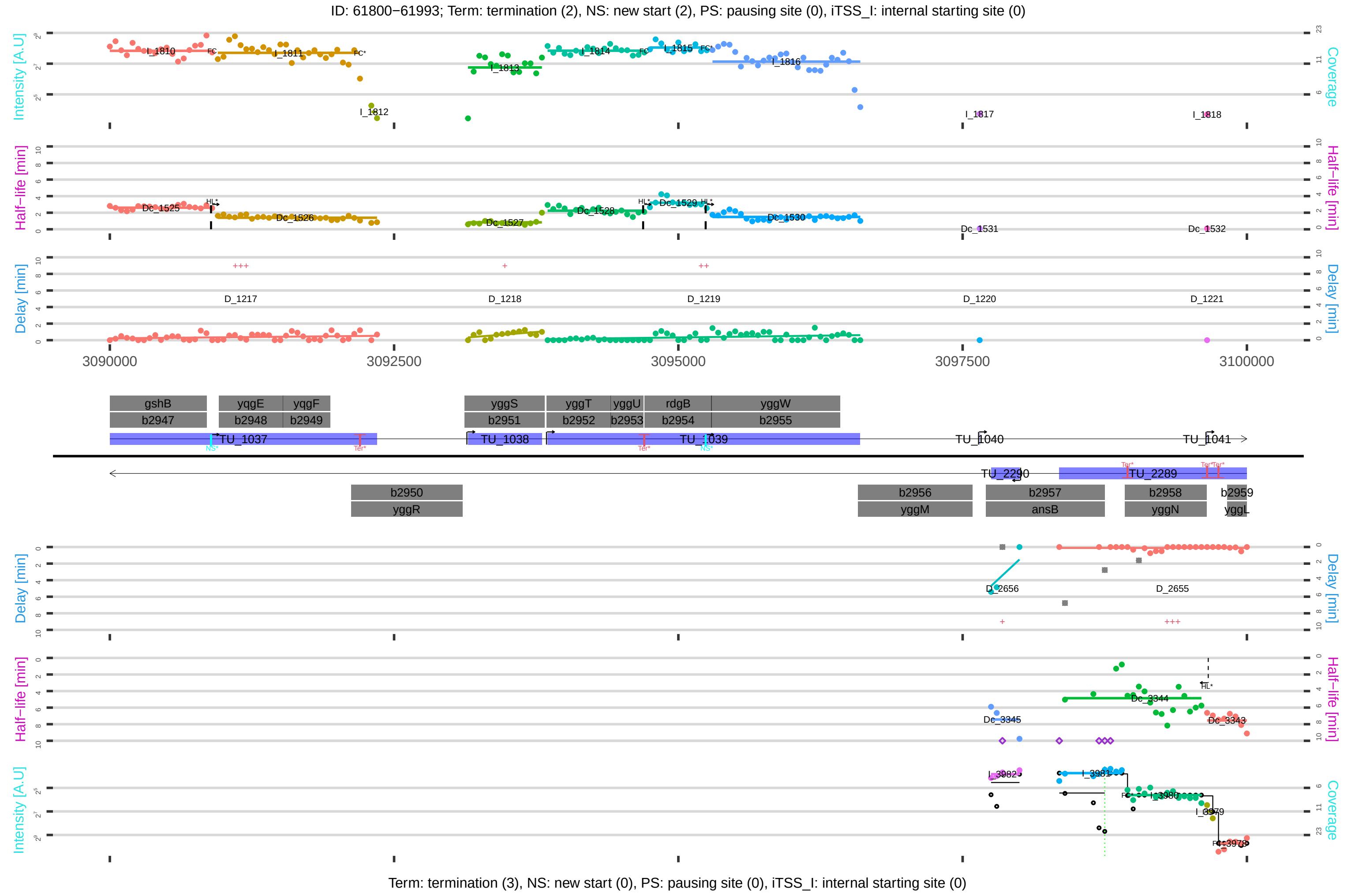
ID: 61800−61993; Term: termination (2), NS: new start (2), PS: pausing site (0), iTSS_I: internal starting site (0)
Intensity [A.U]
Coverage
Half−life [min]
Delay [min]
I_1810
I_1811
I_1812
I_1813
I_1814
I_1815
I_1816
I_1817
I_1818
Dc_1525
Dc_1526
Dc_1527
Dc_1528
Dc_1529
Dc_1530
Dc_1531
Dc_1532
D_1217
D_1218
D_1219
D_1220
D_1221
3090000
3092500
3095000
3097500
3100000
gshB
b2947
yqgE
b2948
yqgF
b2949
yggS
b2951
yggT
b2952
yggU
b2953
rdgB
b2954
yggW
b2955
TU_1037
TU_1038
TU_1039
TU_1040
TU_1041
TU_2290
TU_2289
b2950
yggR
b2956
yggM
b2957
ansB
b2958
yggN
b2959
yggL
D_2656
D_2655
Dc_3345
Dc_3344
Dc_3343
I_3982
I_3981
I_3980
I_3979
I_3978
Term: termination (3), NS: new start (0), PS: pausing site (0), iTSS_I: internal starting site (0)

ID: 62022−62200; Term: termination (3), NS: new start (1), PS: pausing site (0), iTSS_I: internal starting site (0)

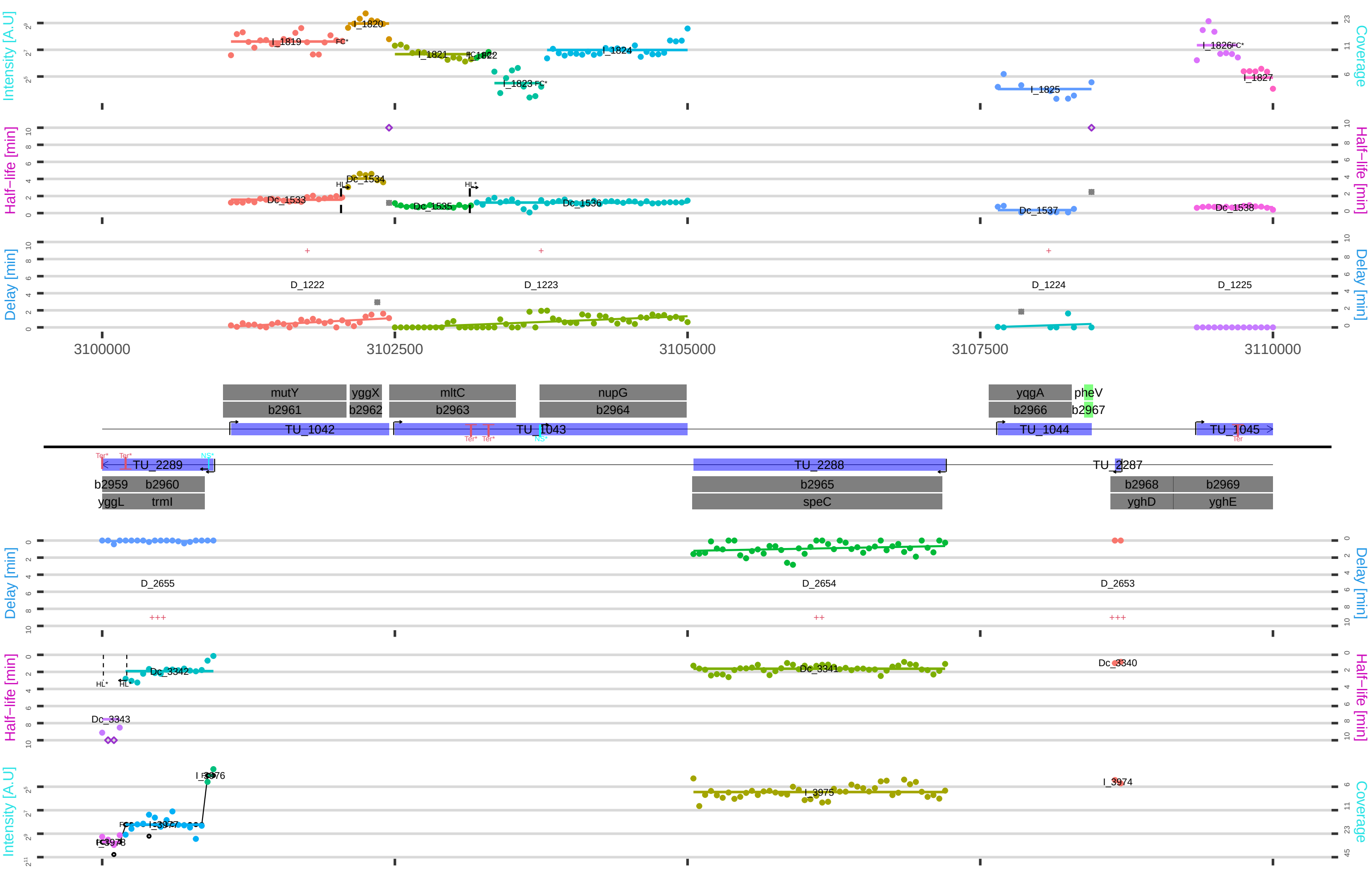

Term: termination (2), NS: new start (1), PS: pausing site (0), iTSS_I: internal starting site (0)

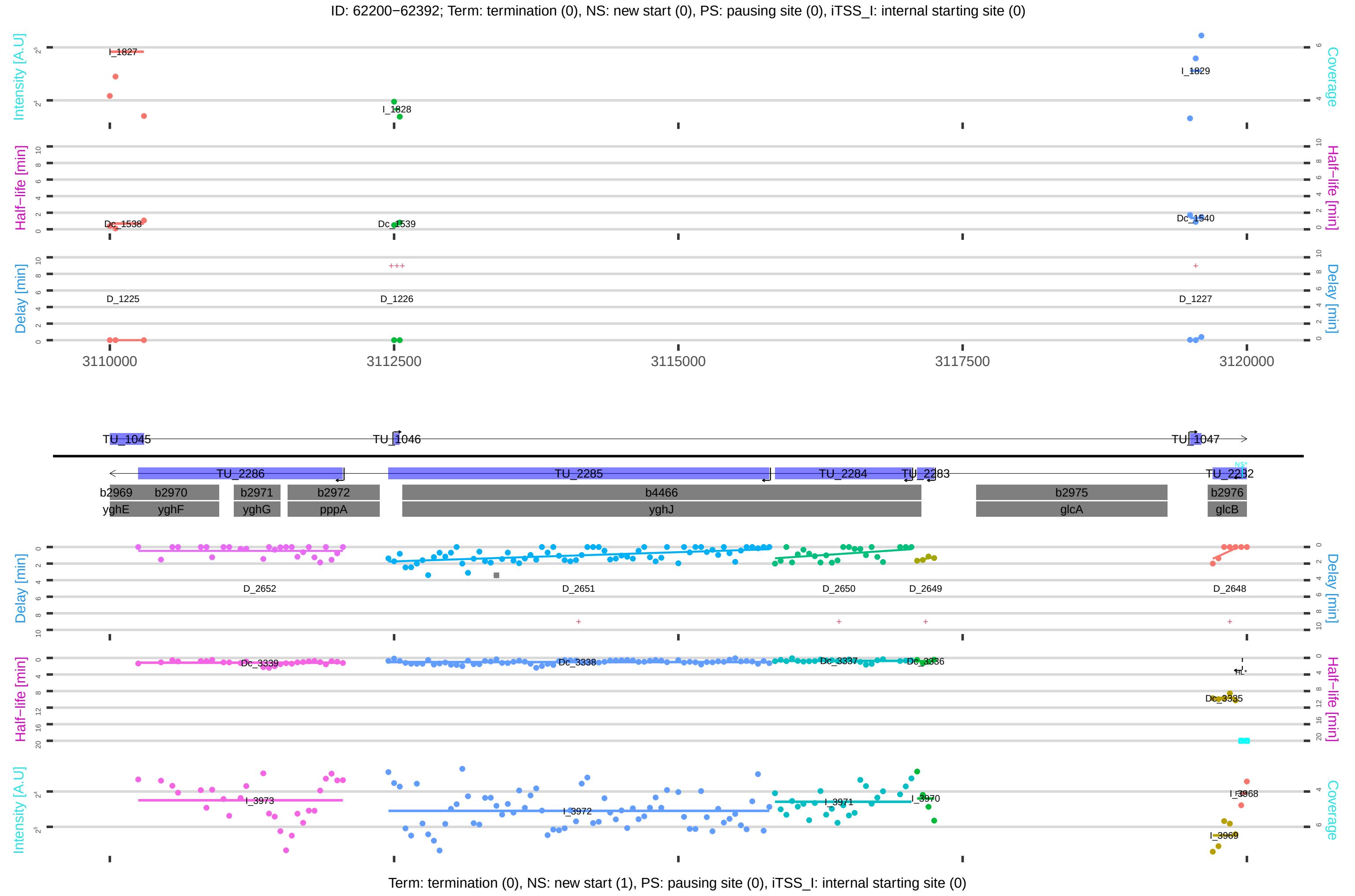

ID: 62200−62392; Term: termination (0), NS: new start (0), PS: pausing site (0), iTSS_I: internal starting site (0)
Intensity [A.U]
Coverage
I_1827
I_1828
I_1829
Half−life [min]
Dc_1538
Dc_1539
Dc_1540
Delay [min]
D_1225
D_1226
D_1227
3110000
3112500
3115000
3117500
3120000
TU_1045
TU_1046
TU_1047
TU_2286
TU_2285
TU_2284
TU_2283
TU_2232
b2969
b2970
b2971
b2972
b4466
b2975
b2976
yghE
yghF
yghG
pppA
yghJ
glcA
glcB
D_2652
D_2651
D_2650
D_2649
D_2648
Dc_3339
Dc_3338
Dc_3337
Dc_3336
Dc_3335
I_3973
I_3972
I_3971
I_3970
I_3968
I_3969
Term: termination (0), NS: new start (1), PS: pausing site (0), iTSS_I: internal starting site (0)

ID: 62458−62592; Term: termination (0), NS: new start (1), PS: pausing site (1), iTSS_I: internal starting site (0)

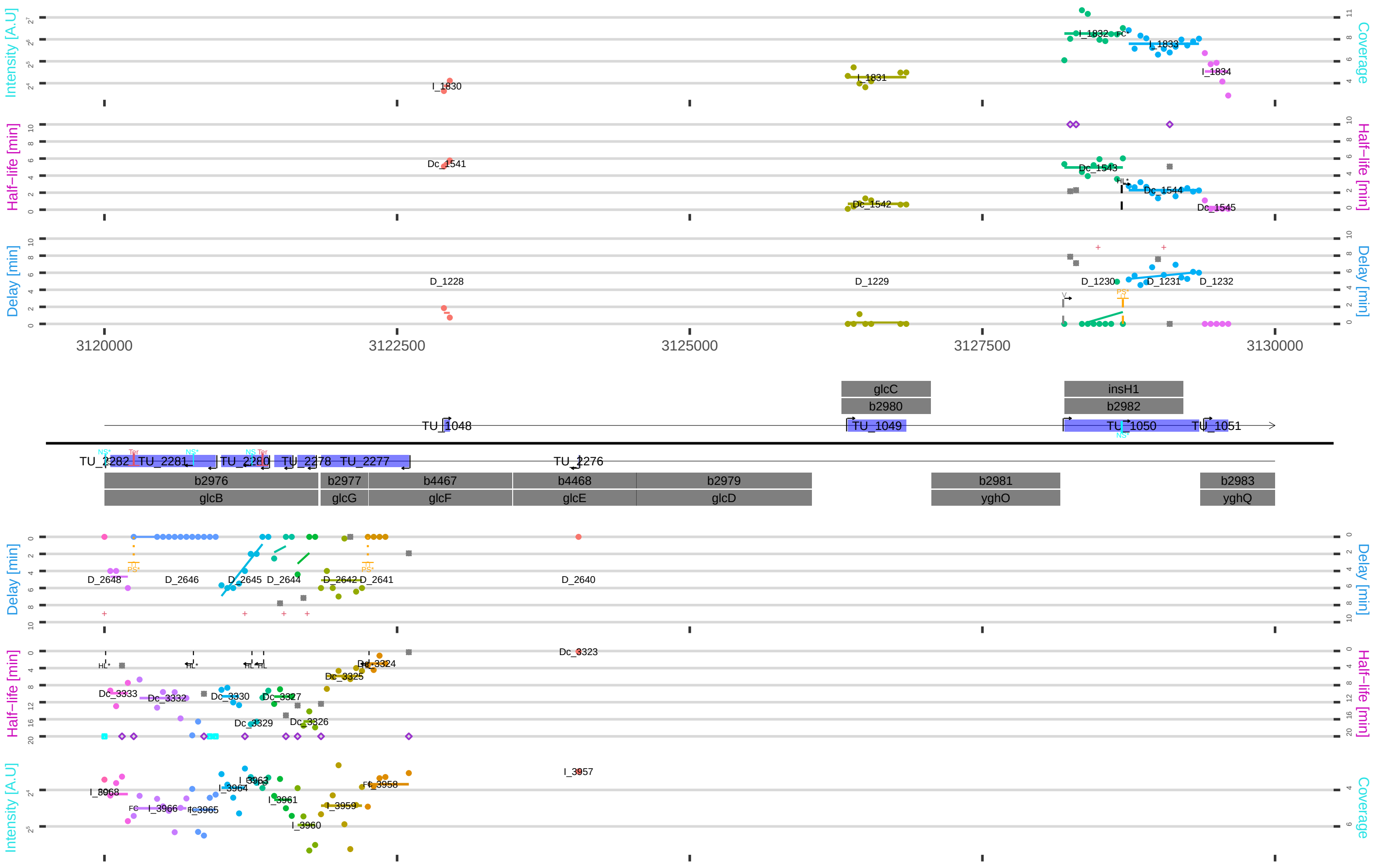

Term: termination (2), NS: new start (3), PS: pausing site (2), iTSS_I: internal starting site (0)

ID: 62736−62755; Term: termination (1), NS: new start (1), PS: pausing site (0), iTSS_I: internal starting site (0)

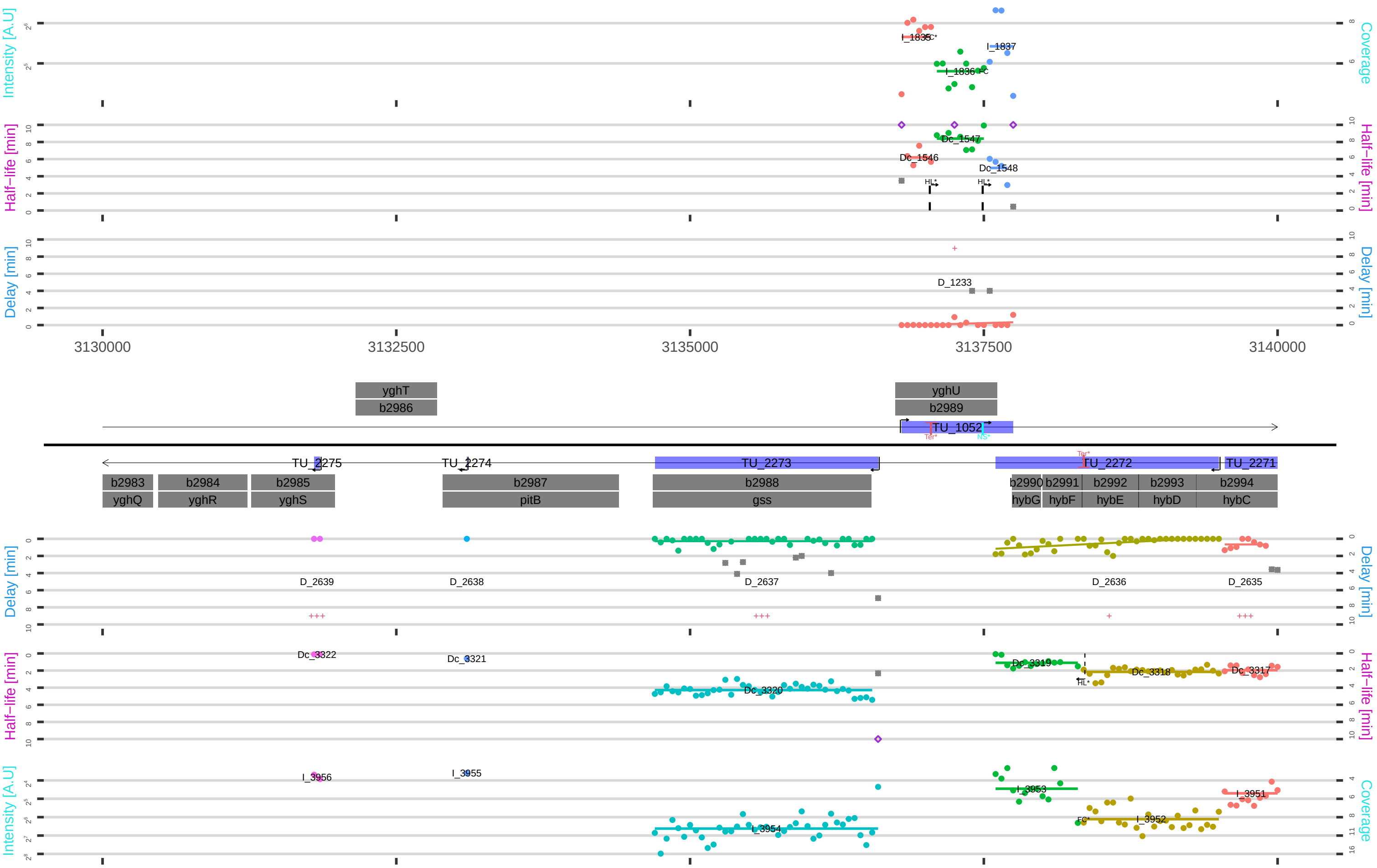

Term: termination (1), NS: new start (0), PS: pausing site (0), iTSS_I: internal starting site (0)

ID: 62891−62940; Term: termination (0), NS: new start (0), PS: pausing site (0), iTSS_I: internal starting site (0)

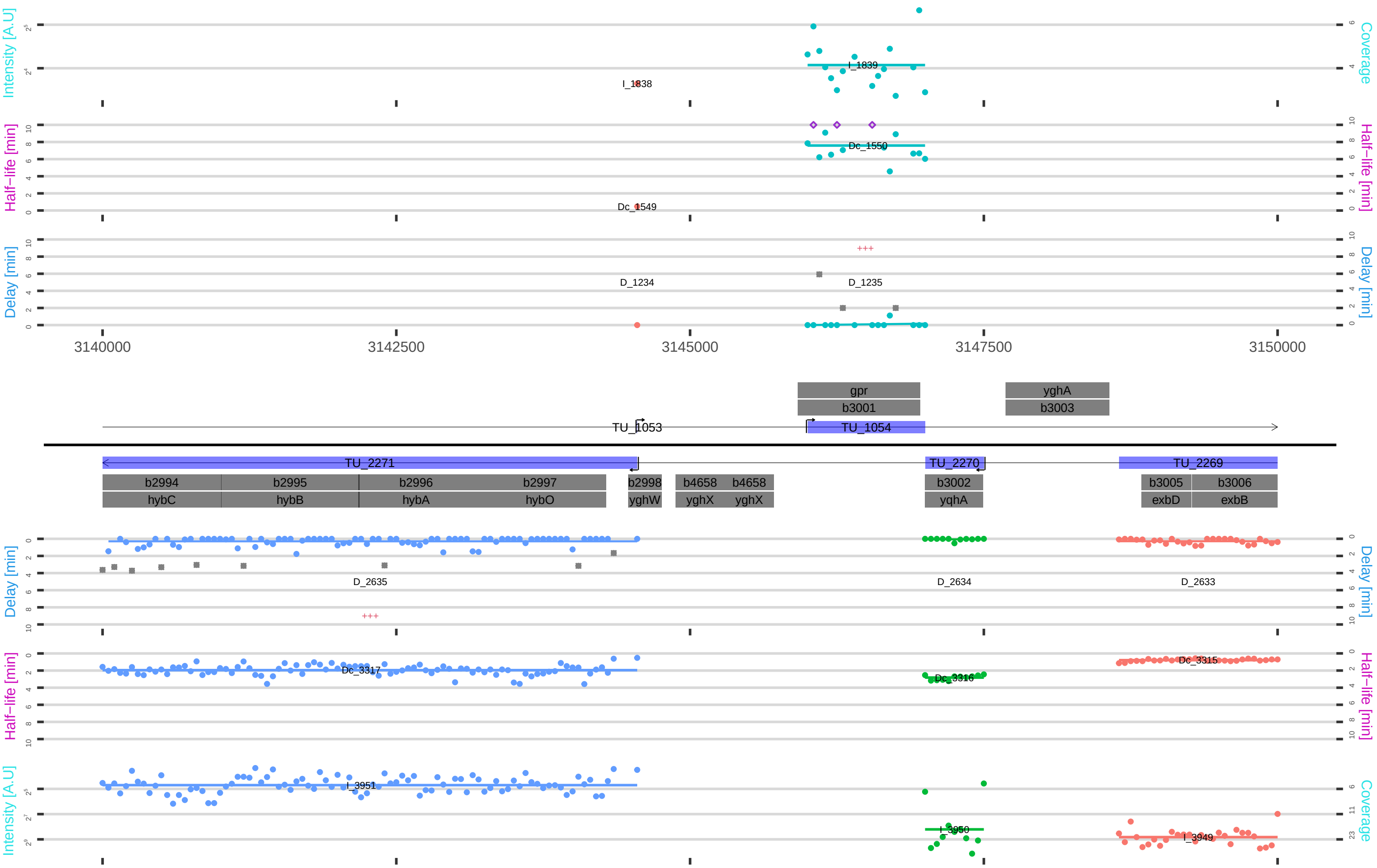

Term: termination (0), NS: new start (0), PS: pausing site (0), iTSS_I: internal starting site (0)

ID: 63008−63110; Term: termination (0), NS: new start (2), PS: pausing site (0), iTSS_I: internal starting site (0)

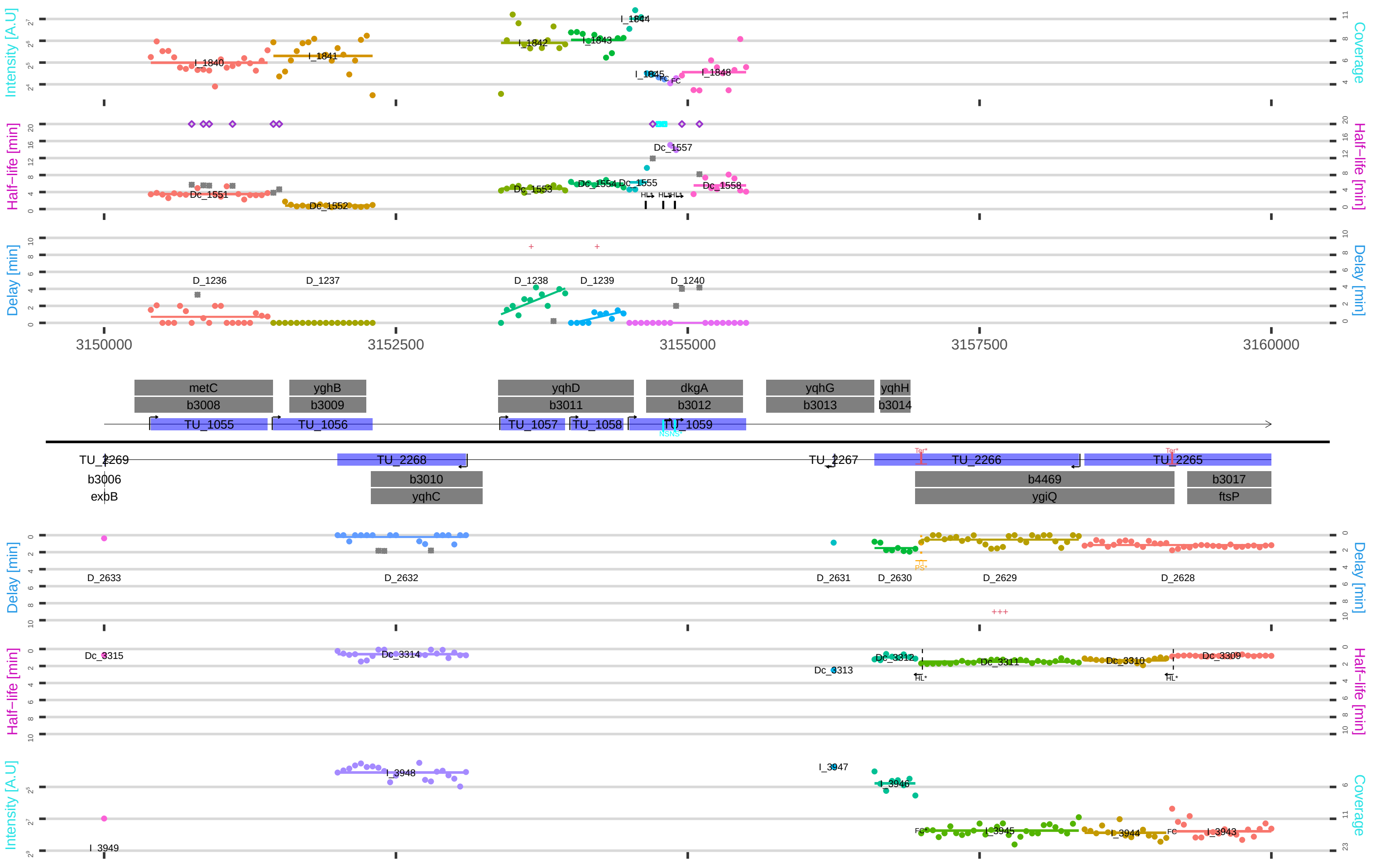

Term: termination (2), NS: new start (0), PS: pausing site (1), iTSS_I: internal starting site (0)

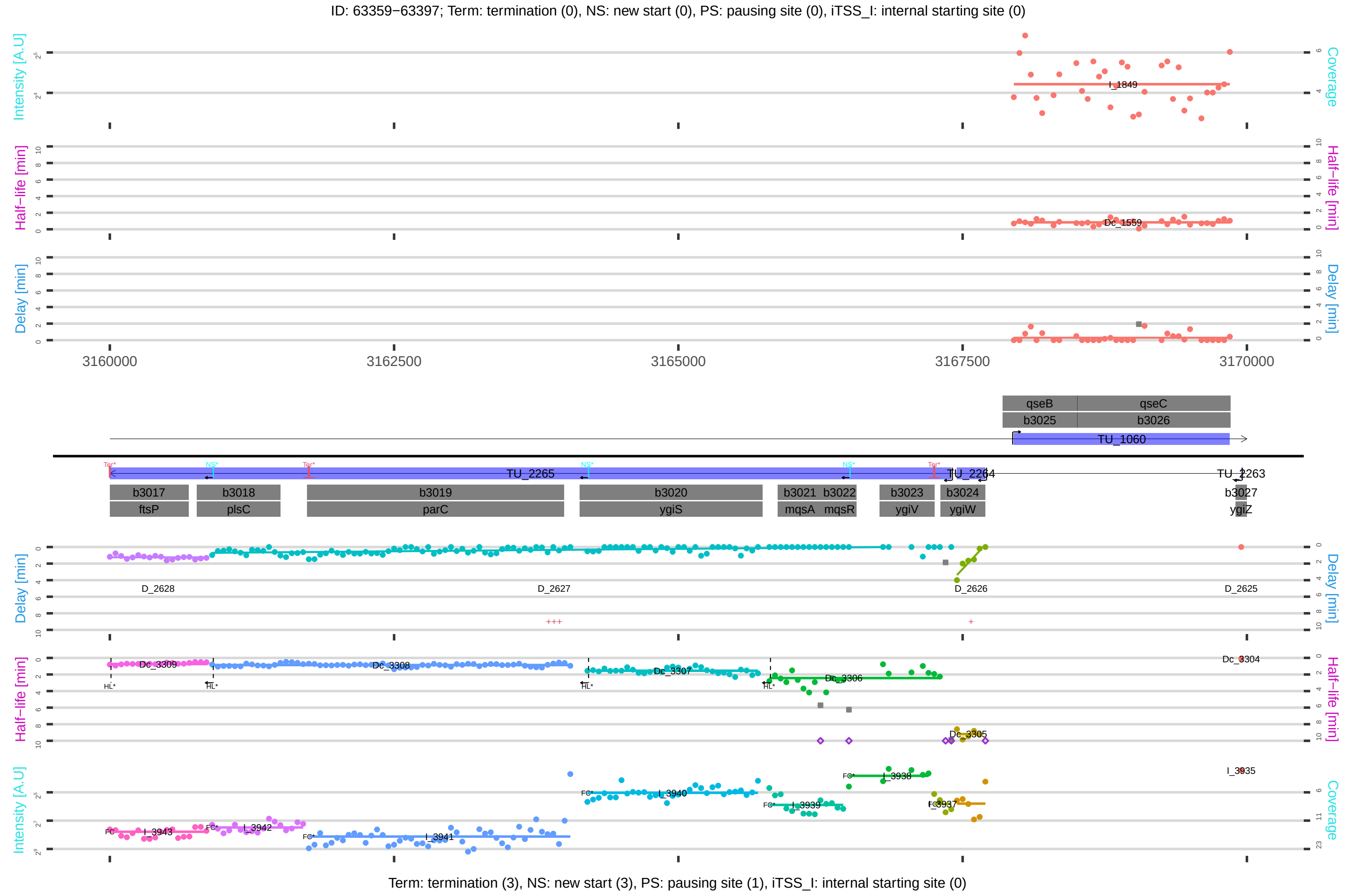
ID: 63359−63397; Term: termination (0), NS: new start (0), PS: pausing site (0), iTSS_I: internal starting site (0)
Intensity [A.U]
Coverage
Half−life [min]
Delay [min]
I_1849
Dc_1559
3160000
3162500
3165000
3167500
3170000
qseB
qseC
b3025
b3026
TU_1060
TU_2265
TU_2264
TU_2263
b3017
b3018
b3019
b3020
b3021
b3022
b3023
b3024
b3027
ftsP
plsC
parC
ygiS
mqsA
mqsR
ygiV
ygiW
ygiZ
D_2628
D_2627
D_2626
D_2625
Dc_3309
Dc_3308
Dc_3307
Dc_3306
Dc_3305
Dc_3304
I_3943
I_3942
I_3941
I_3940
I_3939
I_3938
I_3937
I_3935
Term: termination (3), NS: new start (3), PS: pausing site (1), iTSS_I: internal starting site (0)

ID: 63412−63598; Term: termination (2), NS: new start (3), PS: pausing site (0), iTSS_I: internal starting site (0)

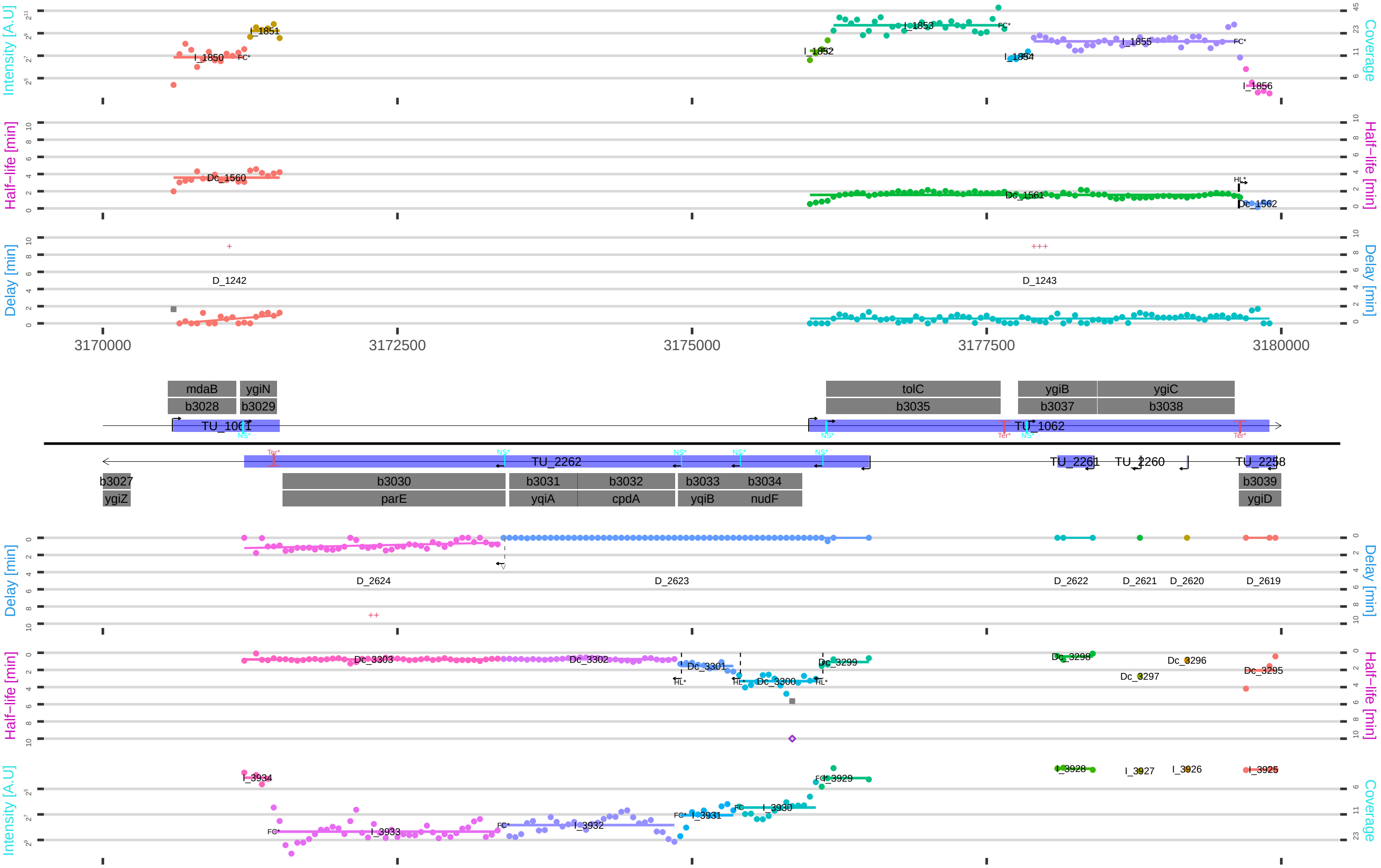

Term: termination (1), NS: new start (4), PS: pausing site (1), iTSS_I: internal starting site (0)

ID: 63611−63704; Term: termination (0), NS: new start (0), PS: pausing site (0), iTSS_I: internal starting site (0)

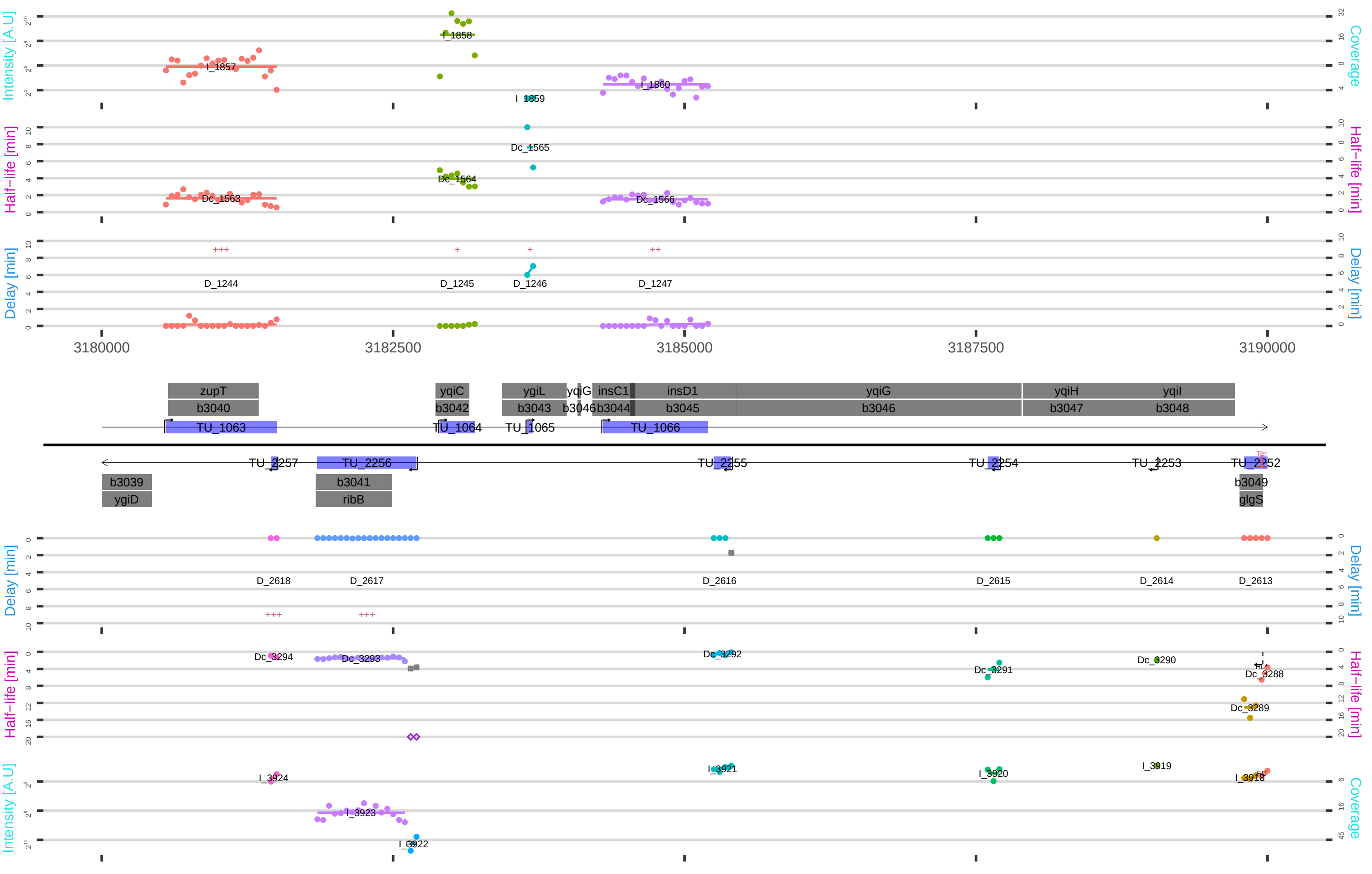

Term: termination (1), NS: new start (0), PS: pausing site (0), iTSS_I: internal starting site (0)

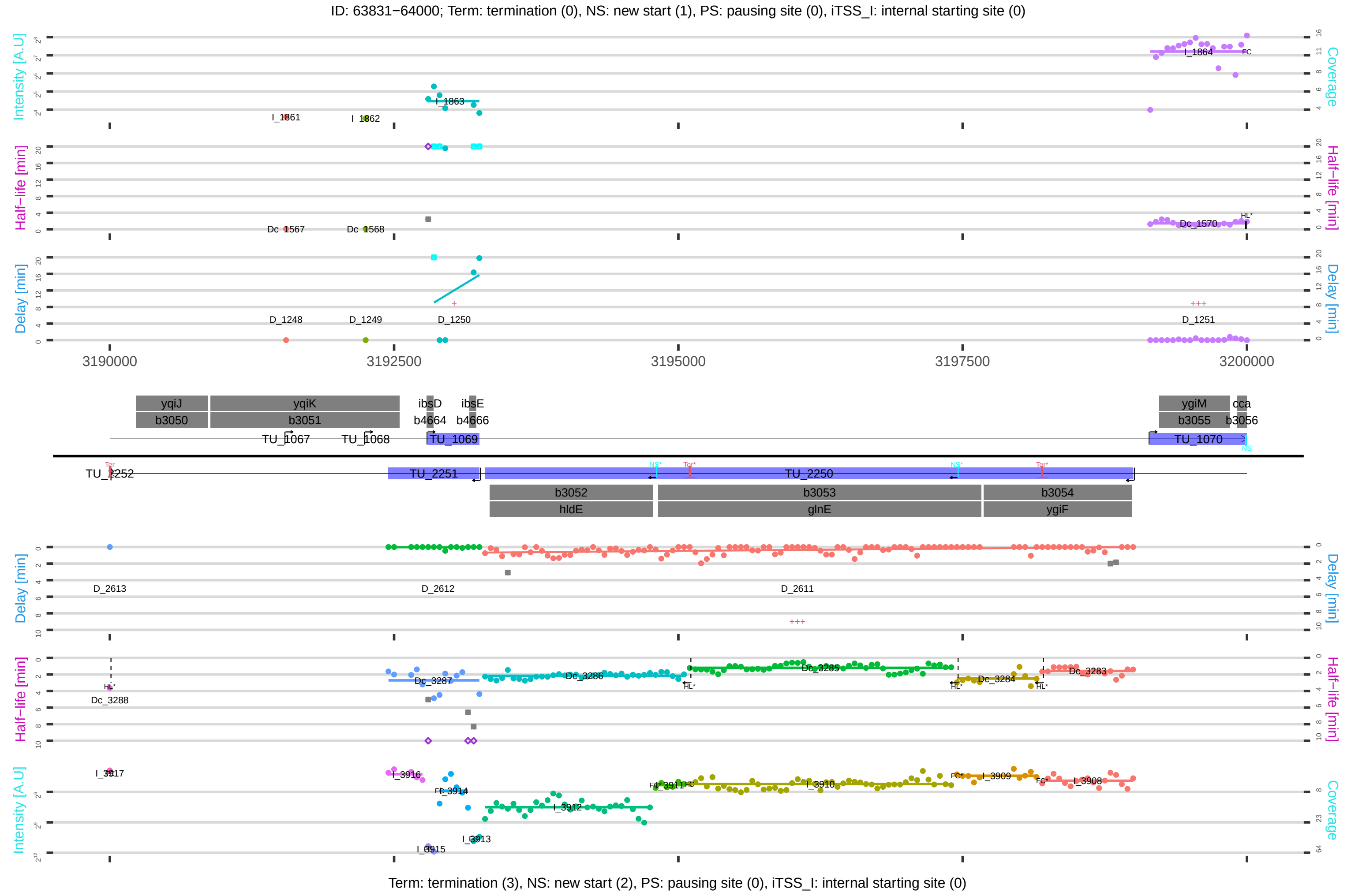
ID: 63831−64000; Term: termination (0), NS: new start (1), PS: pausing site (0), iTSS_I: internal starting site (0)
Intensity [A.U]
Coverage
Half−life [min]
Delay [min]
I_1861
I_1862
I_1863
I_1864
Dc_1567
Dc_1568
Dc_1570
D_1248
D_1249
D_1250
D_1251
3190000
3192500
3195000
3197500
3200000
yqiJ
b3050
yqiK
b3051
ibsD
b4664
ibsE
b4666
ygiM
b3055
cca
b3056
TU_1067
TU_1068
TU_1069
TU_1070
TU_2252
TU_2251
TU_2250
b3052
hldE
b3053
glnE
b3054
ygiF
D_2613
D_2612
D_2611
Dc_3288
Dc_3287
Dc_3286
Dc_3285
Dc_3284
Dc_3283
I_3917
I_3916
I_3915
I_3914
I_3913
I_3912
I_3911
I_3910
I_3909
I_3908
Term: termination (3), NS: new start (2), PS: pausing site (0), iTSS_I: internal starting site (0)

ID: 64000−64200; Term: termination (2), NS: new start (1), PS: pausing site (0), iTSS_I: internal starting site (0)

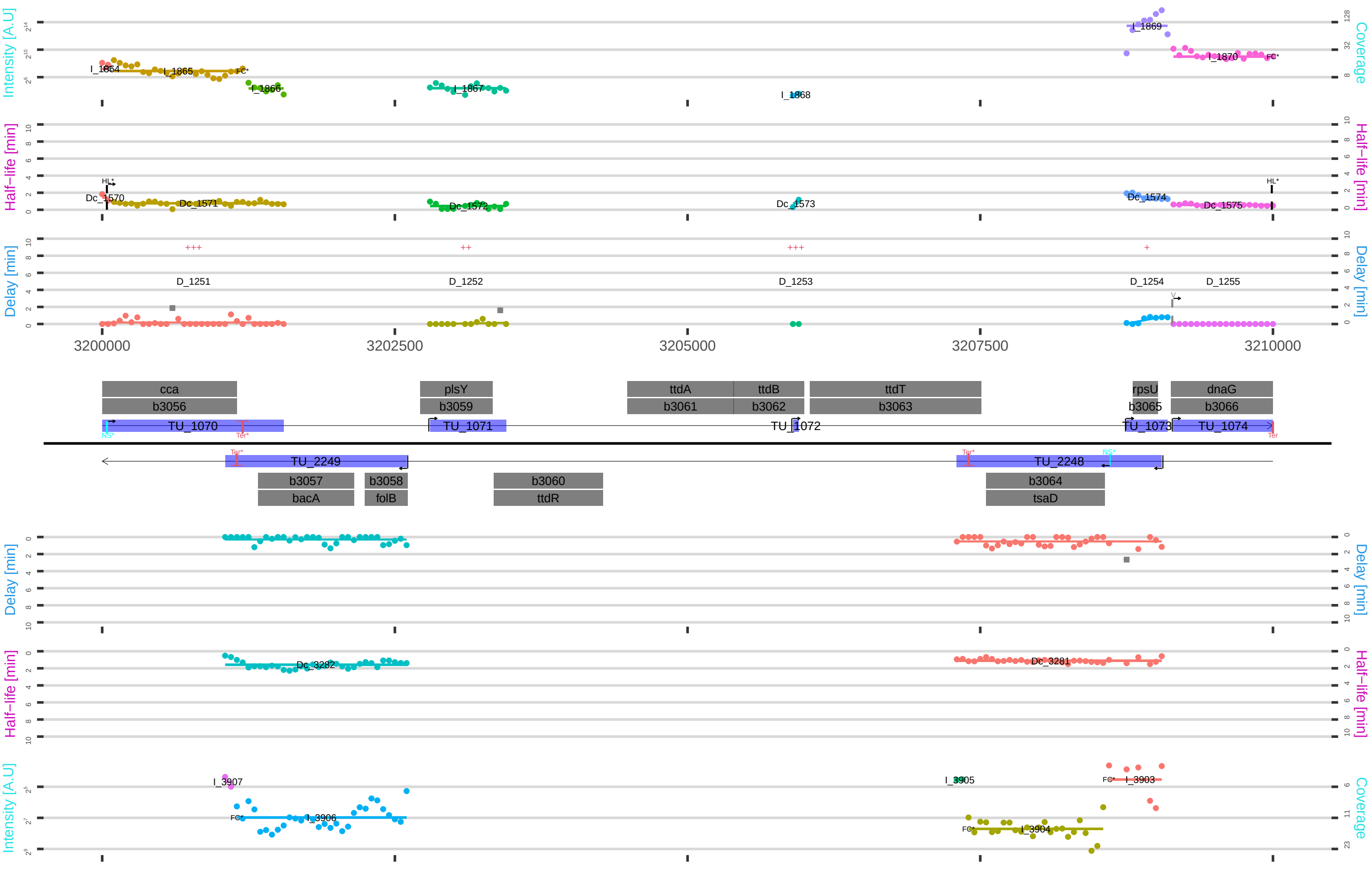

Term: termination (2), NS: new start (1), PS: pausing site (0), iTSS_I: internal starting site (0)

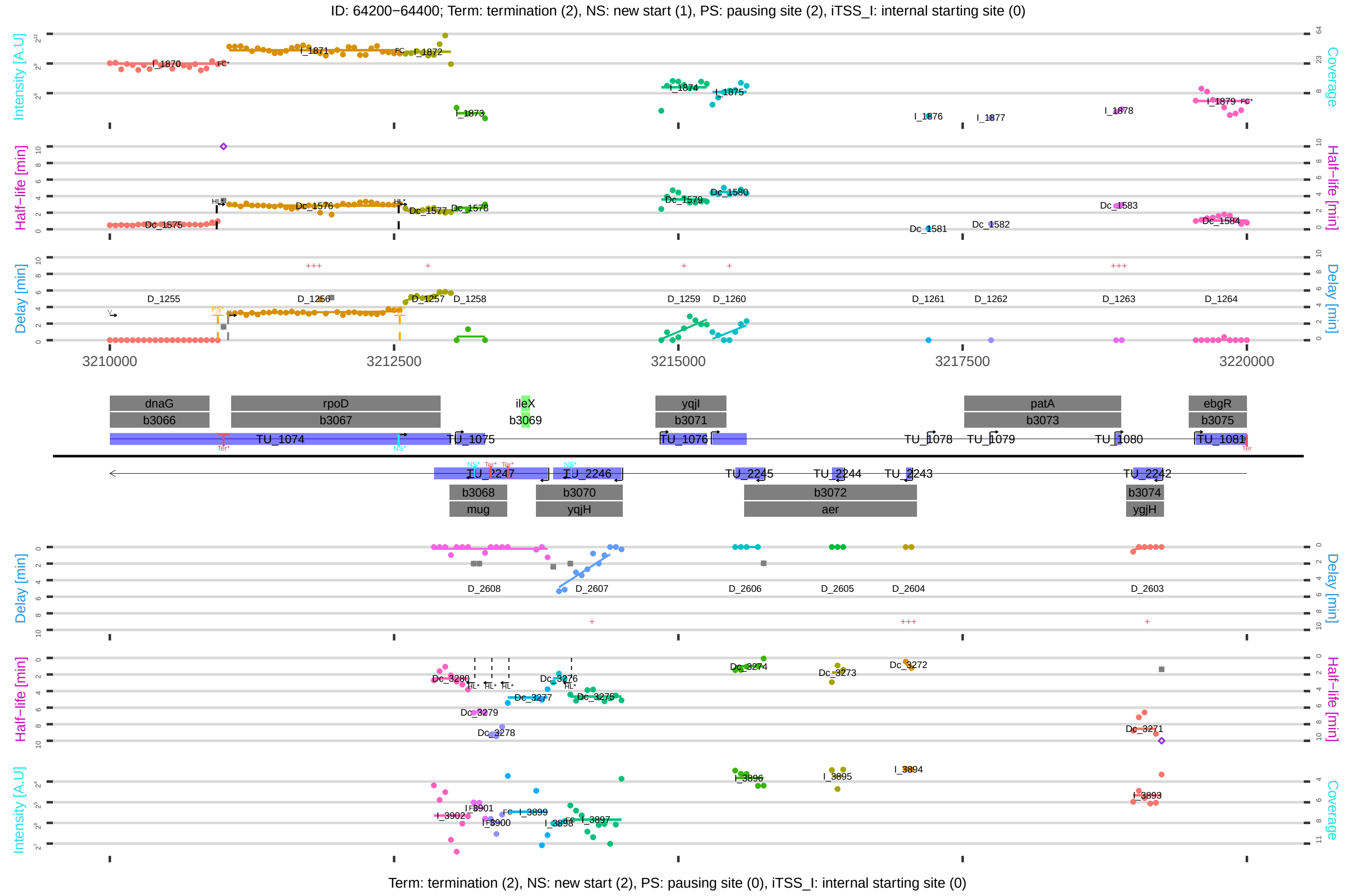

ID: 64200−64400; Term: termination (2), NS: new start (1), PS: pausing site (2), iTSS_I: internal starting site (0)
Intensity [A.U]
Coverage
Half−life [min]
Delay [min]
3210000
3212500
3215000
3217500
3220000
dnaG
b3066
rpoD
b3067
ileX
b3069
yqjI
b3071
patA
b3073
ebgR
b3075
TU_1074
TU_1075
TU_1076
TU_1078
TU_1079
TU_1080
TU_1081
TU_2247
TU_2246
TU_2245
TU_2244
TU_2243
TU_2242
b3068
mug
b3070
yqjH
b3072
aer
b3074
ygjH
Term: termination (2), NS: new start (2), PS: pausing site (0), iTSS_I: internal starting site (0)

ID: 64400−64572; Term: termination (2), NS: new start (0), PS: pausing site (1), iTSS_I: internal starting site (0)

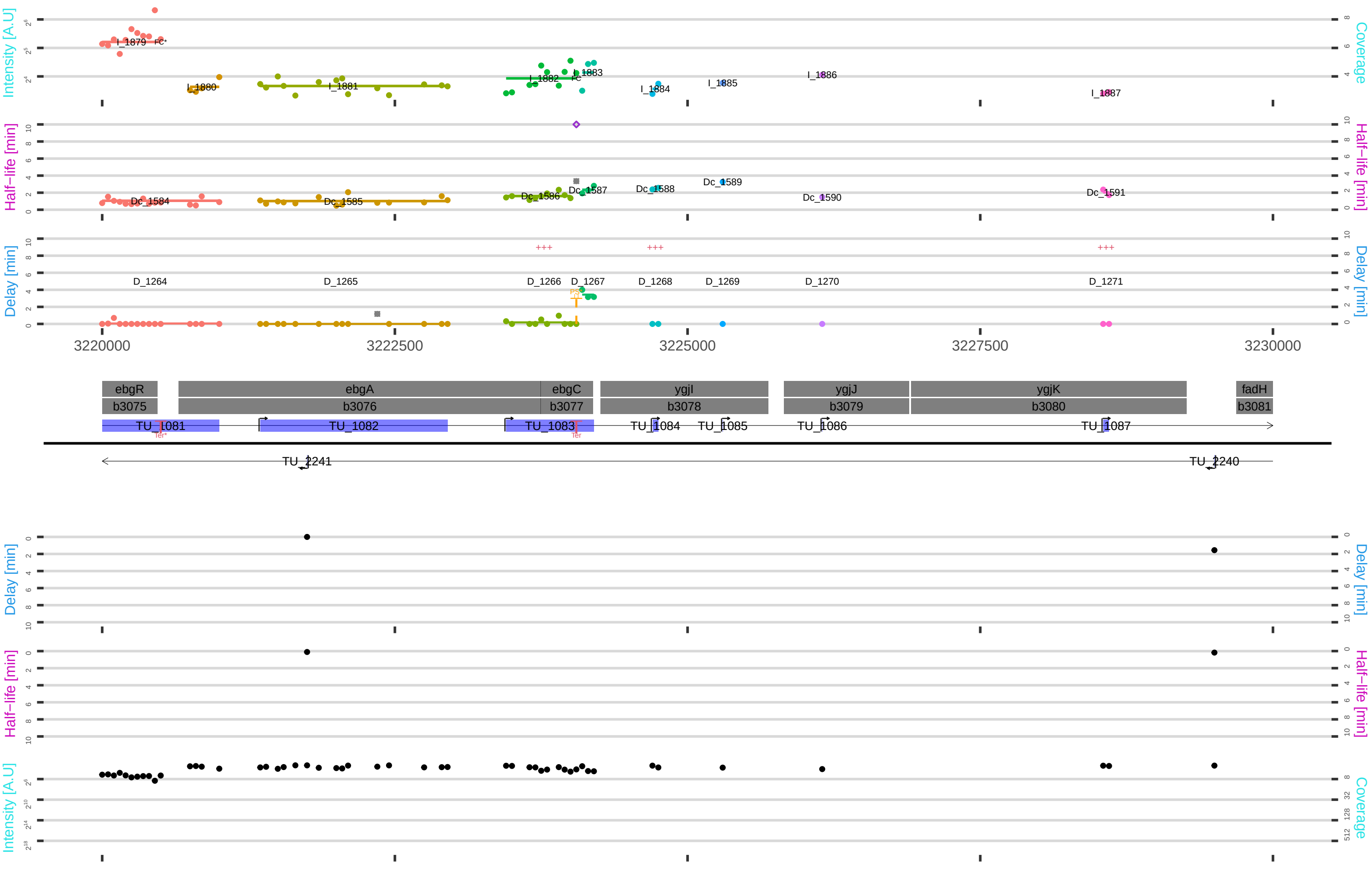

Term: termination (0), NS: new start (0), PS: pausing site (0), iTSS_I: internal starting site (0)

ID: 64631−64784; Term: termination (1), NS: new start (2), PS: pausing site (0), iTSS_I: internal starting site (0)

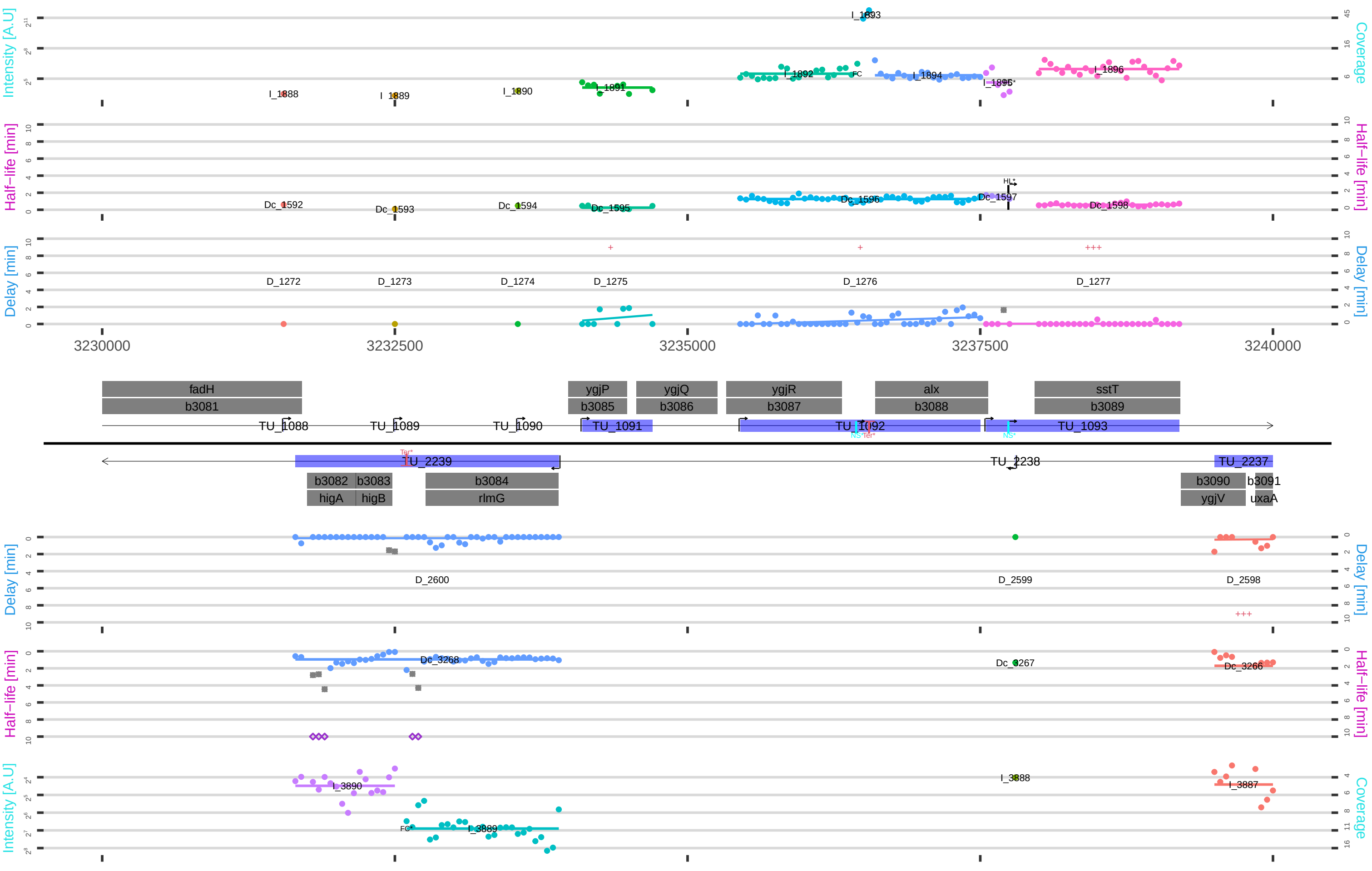

Term: termination (1), NS: new start (0), PS: pausing site (0), iTSS_I: internal starting site (0)

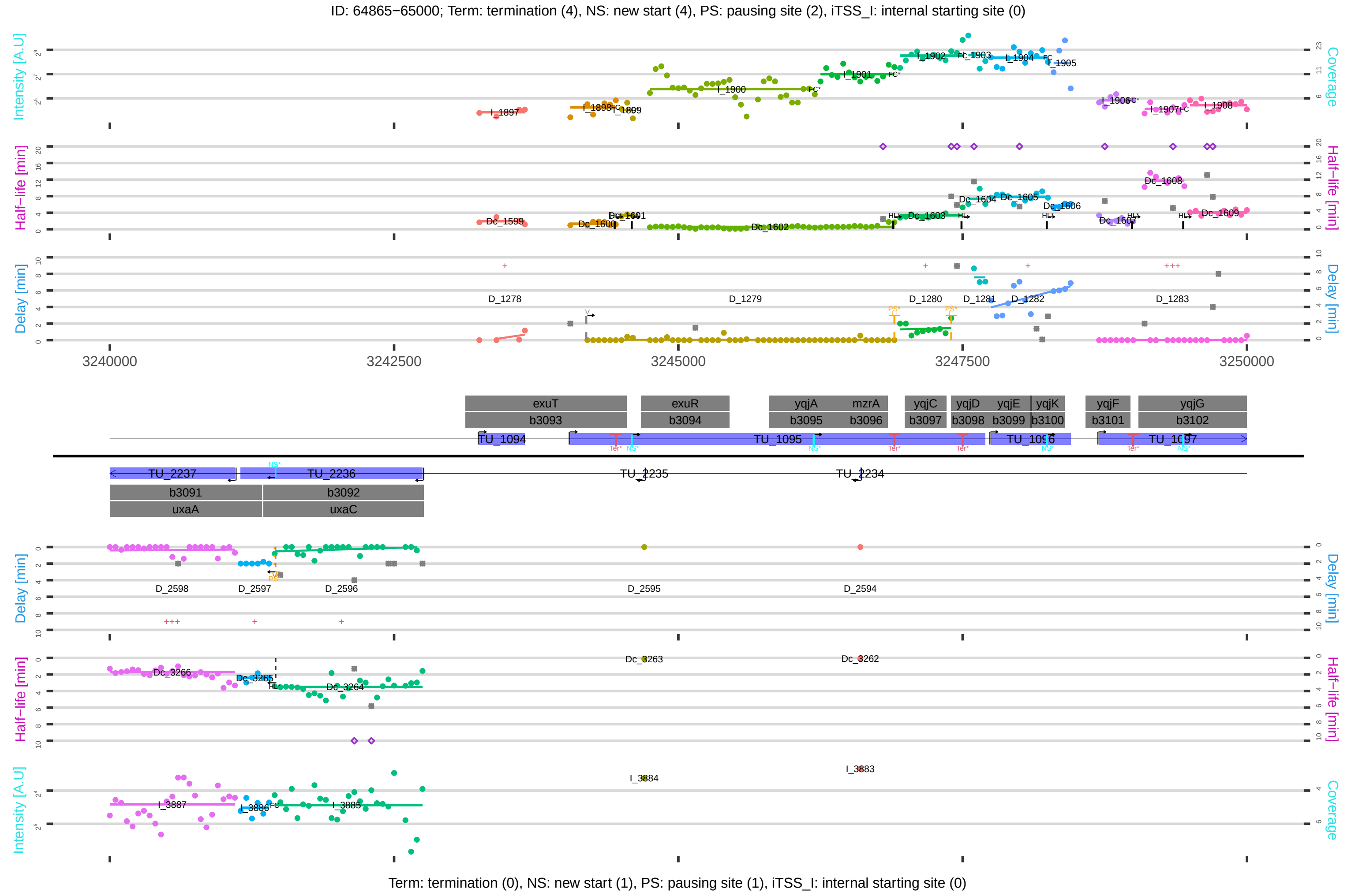

ID: 64865−65000; Term: termination (4), NS: new start (4), PS: pausing site (2), iTSS_I: internal starting site (0)
Intensity [A.U]
Coverage
Half−life [min]
Delay [min]
I_1897
I_1898
I_1899
I_1900
I_1901
I_1902
I_1903
I_1904
I_1905
I_1906
I_1907
I_1908
Dc_1599
Dc_1600
Dc_1601
Dc_1602
Dc_1603
Dc_1604
Dc_1605
Dc_1606
Dc_1607
Dc_1608
Dc_1609
D_1278
D_1279
D_1280
D_1281
D_1282
D_1283
3240000
3242500
3245000
3247500
3250000
exuT
b3093
exuR
b3094
yqjA
b3095
mzrA
b3096
yqjC
b3097
yqjD
b3098
yqjE
b3099
yqjK
b3100
yqjF
b3101
yqjG
b3102
TU_1094
TU_1095
TU_1096
TU_1097
TU_2237
TU_2236
TU_2235
TU_2234
b3091
uxaA
b3092
uxaC
D_2598
D_2597
D_2596
D_2595
D_2594
Dc_3266
Dc_3265
Dc_3264
Dc_3263
Dc_3262
I_3887
I_3886
I_3885
I_3884
I_3883
Term: termination (0), NS: new start (1), PS: pausing site (1), iTSS_I: internal starting site (0)

ID: 65000−65067; Term: termination (0), NS: new start (0), PS: pausing site (0), iTSS_I: internal starting site (0)

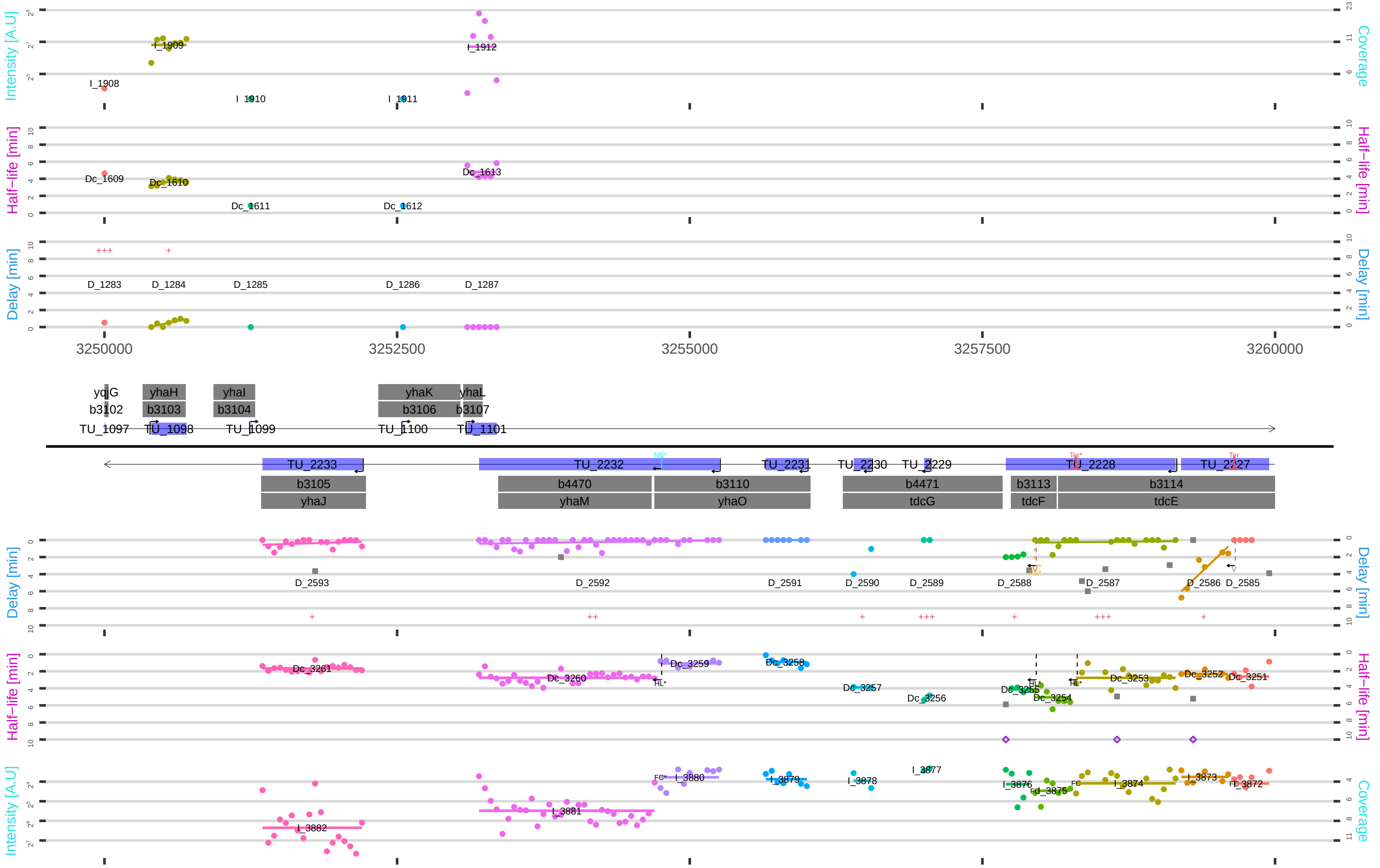

Term: termination (2), NS: new start (1), PS: pausing site (2), iTSS_I: internal starting site (0)

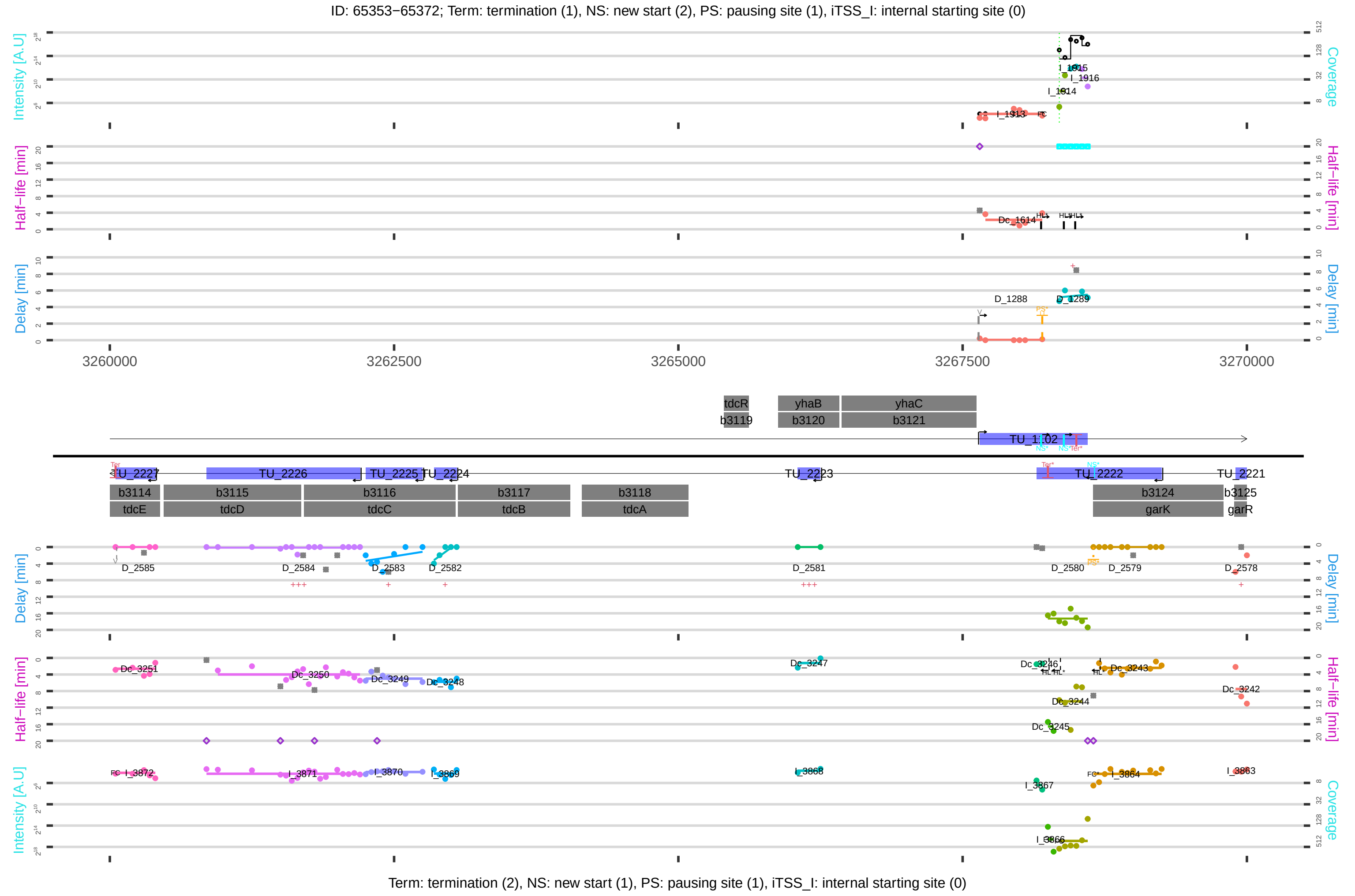

ID: 65353−65372; Term: termination (1), NS: new start (2), PS: pausing site (1), iTSS_I: internal starting site (0)
Intensity [A.U]
Coverage
Half−life [min]
Delay [min]
3260000
3262500
3265000
3267500
3270000
I_1913
I_1914
I_1915
I_1916
Dc_1614
D_1288
D_1289
tdcR
b3119
yhaB
b3120
yhaC
b3121
TU_1102
TU_2227
TU_2226
TU_2225
TU_2224
TU_2223
TU_2222
TU_2221
b3114
tdcE
b3115
tdcD
b3116
tdcC
b3117
tdcB
b3118
tdcA
b3124
garK
b3125
garR
D_2585
D_2584
D_2583
D_2582
D_2581
D_2580
D_2579
D_2578
Dc_3251
Dc_3250
Dc_3249
Dc_3248
Dc_3247
Dc_3246
Dc_3243
Dc_3244
Dc_3245
Dc_3242
I_3872
I_3871
I_3870
I_3869
I_3868
I_3867
I_3864
I_3866
I_3863
Term: termination (2), NS: new start (1), PS: pausing site (1), iTSS_I: internal starting site (0)

ID: 65473−65594; Term: termination (0), NS: new start (2), PS: pausing site (0), iTSS_I: internal starting site (0)

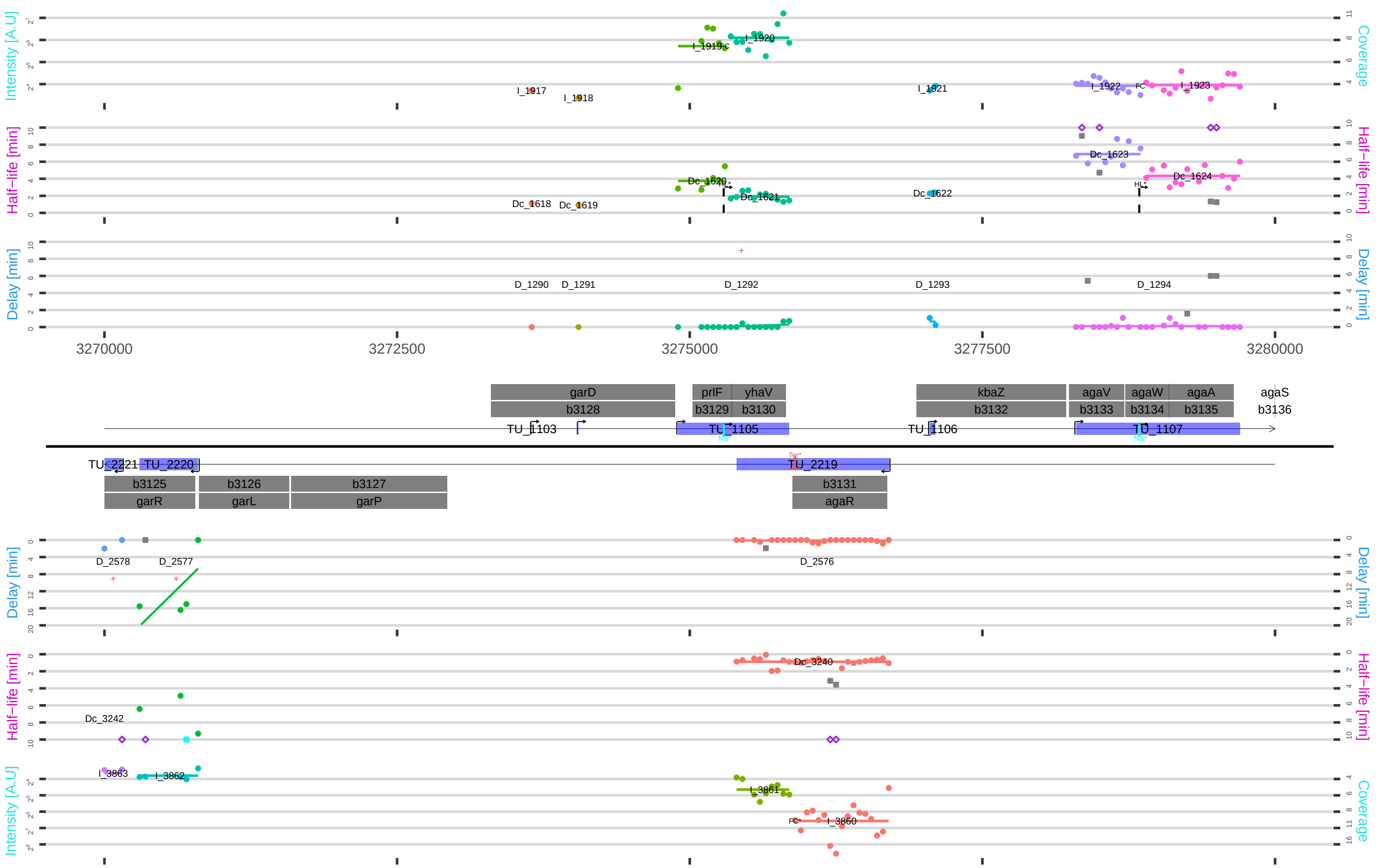

Term: termination (1), NS: new start (0), PS: pausing site (0), iTSS_I: internal starting site (0)

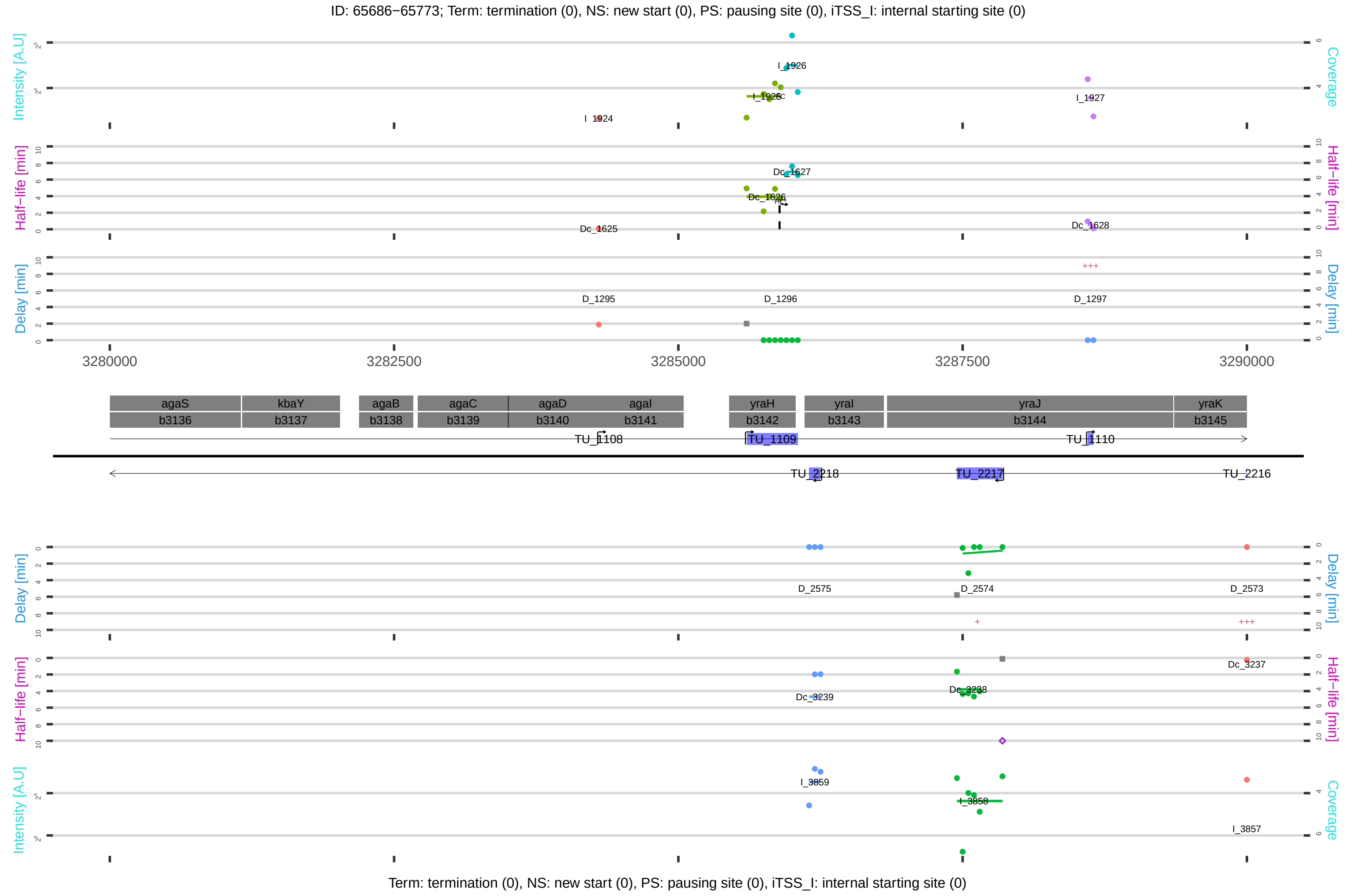

ID: 65686−65773; Term: termination (0), NS: new start (0), PS: pausing site (0), iTSS_I: internal starting site (0)
Intensity [A.U]
Coverage
Half−life [min]
Delay [min]
I_1924
I_1925c
I_1926
I_1927
Dc_1625
Dc_1626
Dc_1627
Dc_1628
D_1295
D_1296
D_1297
3280000
3282500
3285000
3287500
3290000
agaS
b3136
kbaY
b3137
agaB
b3138
agaC
b3139
agaD
b3140
agaI
b3141
yraH
b3142
yraI
b3143
yraJ
b3144
yraK
b3145
TU_1108
TU_1109
TU_1110
TU_2218
TU_2217
TU_2216
D_2575
D_2574
D_2573
Dc_3239
Dc_3238
Dc_3237
I_3859
I_3858
I_3857
Term: termination (0), NS: new start (0), PS: pausing site (0), iTSS_I: internal starting site (0)

ID: 65828−65966; Term: termination (2), NS: new start (0), PS: pausing site (0), iTSS_I: internal starting site (0)

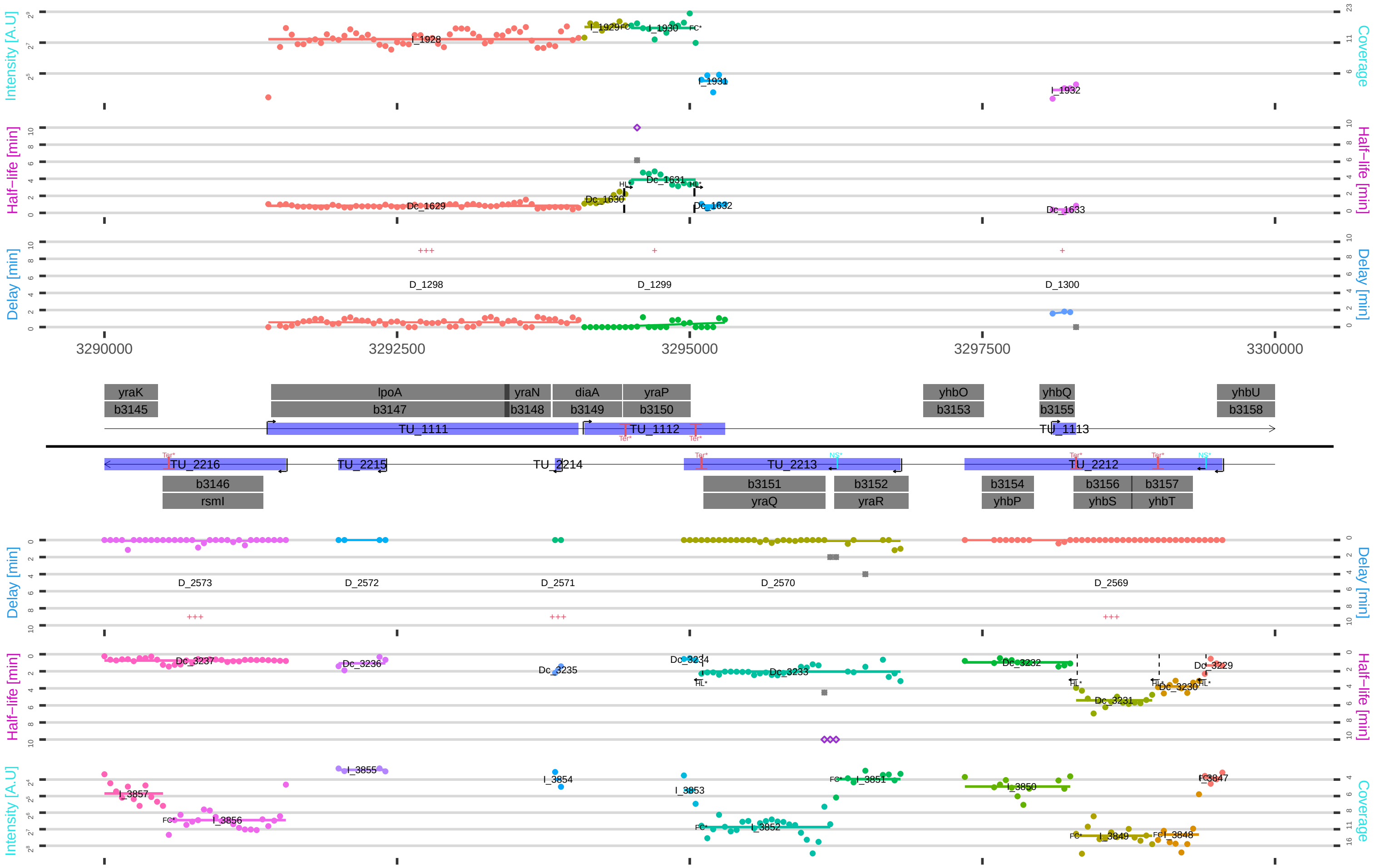

Term: termination (4), NS: new start (2), PS: pausing site (0), iTSS_I: internal starting site (0)

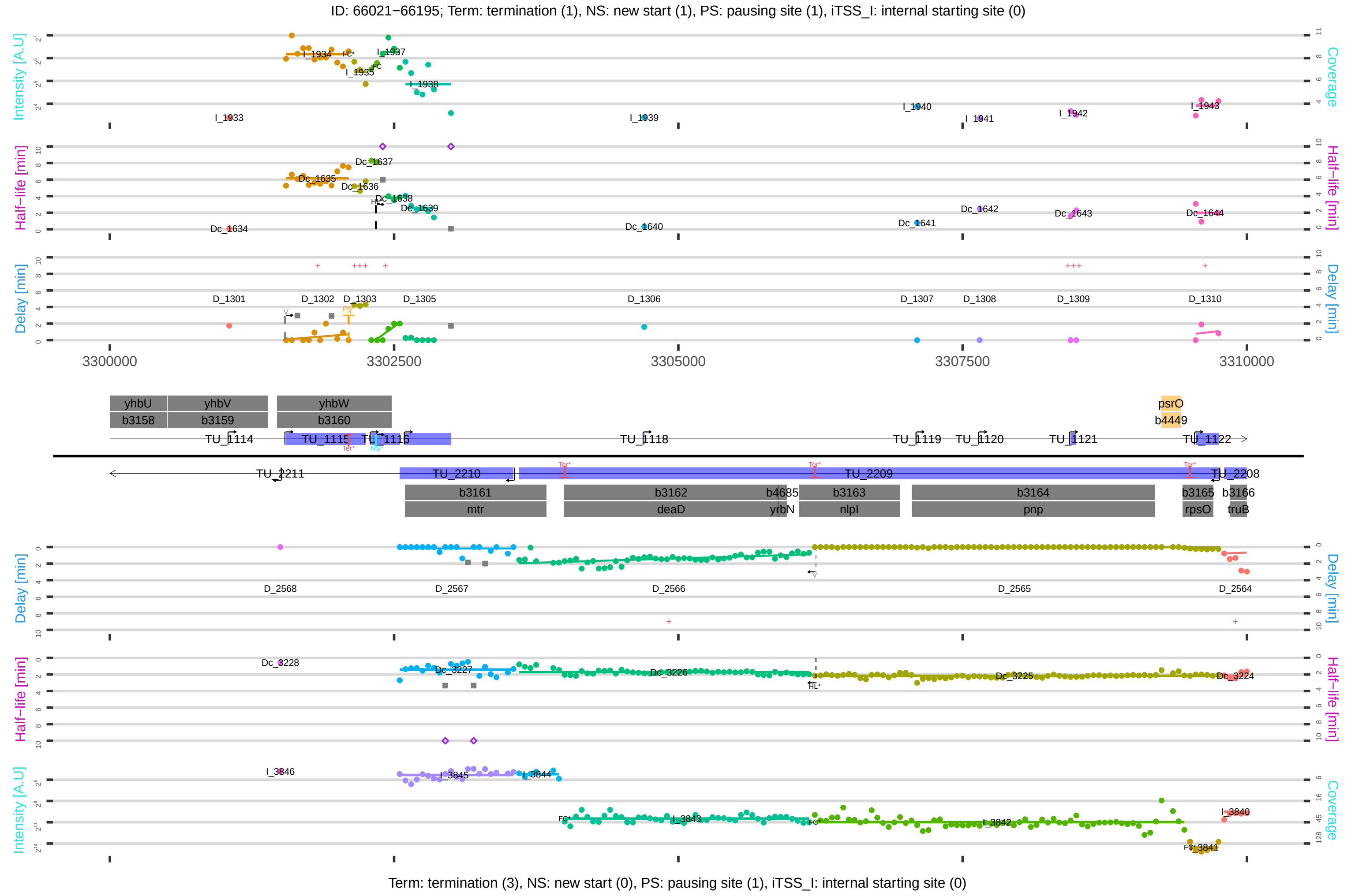
ID: 66021−66195; Term: termination (1), NS: new start (1), PS: pausing site (1), iTSS_I: internal starting site (0)
Intensity [A.U]
Coverage
Half−life [min]
Delay [min]
3300000
3302500
3305000
3307500
3310000
yhbU
b3158
yhbV
b3159
yhbW
b3160
psrO
b4449
TU_1114
TU_1115
TU_1116
TU_1118
TU_1119
TU_1120
TU_1121
TU_1122
TU_2211
TU_2210
TU_2209
TU_2208
b3161
mtr
b3162
deaD
b4685
yrbN
b3163
nlpI
b3164
pnp
b3165
rpsO
b3166
truB
Term: termination (3), NS: new start (0), PS: pausing site (1), iTSS_I: internal starting site (0)

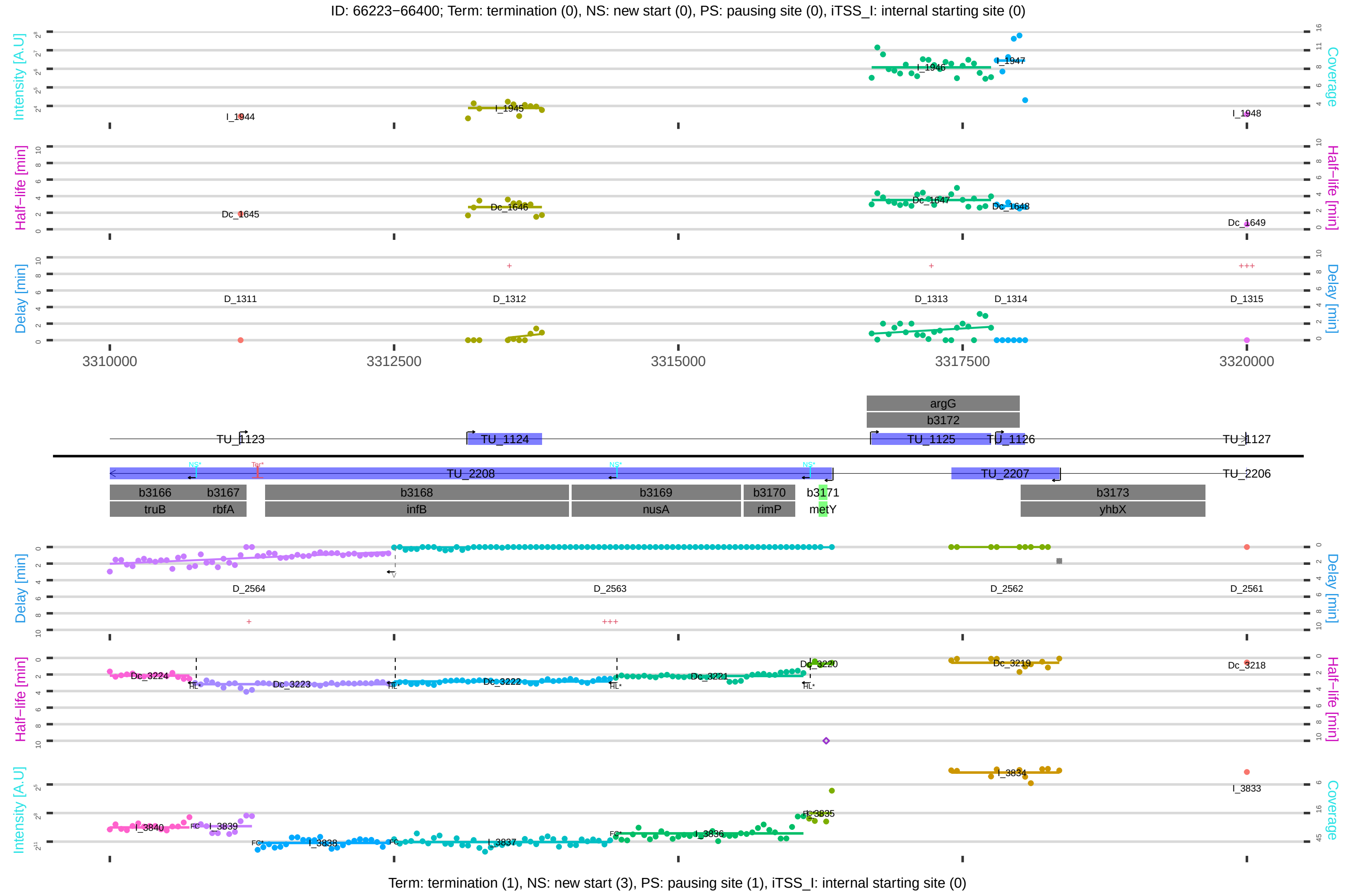

ID: 66223−66400; Term: termination (0), NS: new start (0), PS: pausing site (0), iTSS_I: internal starting site (0)
Intensity [A.U]
Coverage
Half−life [min]
Delay [min]
I_1944
I_1945
I_1946
I_1947
I_1948
Dc_1645
Dc_1646
Dc_1647
Dc_1648
Dc_1649
D_1311
D_1312
D_1313
D_1314
D_1315
3310000
3312500
3315000
3317500
3320000
argG
b3172
TU_1123
TU_1124
TU_1125
TU_1126
TU_1127
NS*
Ter*
TU_2208
TU_2207
TU_2206
b3166
b3167
b3168
b3169
b3170
b3171
b3173
truB
rbfA
infB
nusA
rimP
metY
yhbX
D_2564
D_2563
D_2562
D_2561
Dc_3224
Dc_3223
Dc_3222
Dc_3221
Dc_3220
Dc_3219
Dc_3218
HL*
I_3840
I_3839
I_3838
I_3837
I_3836
FC3835
I_3834
I_3833
FC
FC*
Term: termination (1), NS: new start (3), PS: pausing site (1), iTSS_I: internal starting site (0)

ID: 66400−66587; Term: termination (0), NS: new start (1), PS: pausing site (1), iTSS_I: internal starting site (0)

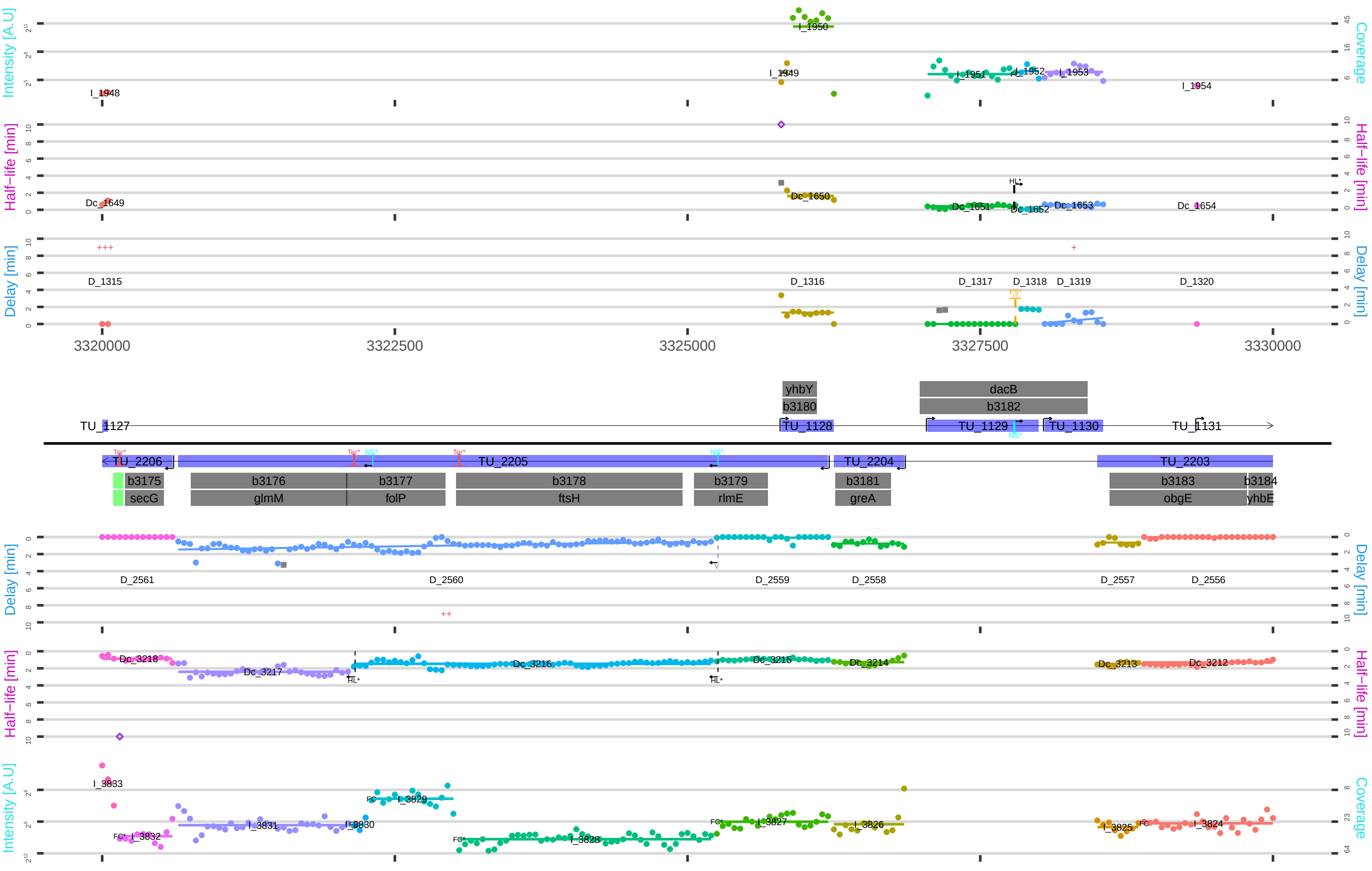

Term: termination (3), NS: new start (2), PS: pausing site (2), iTSS_I: internal starting site (0)

ID: 66620−66800; Term: termination (1), NS: new start (3), PS: pausing site (0), iTSS_I: internal starting site (0)

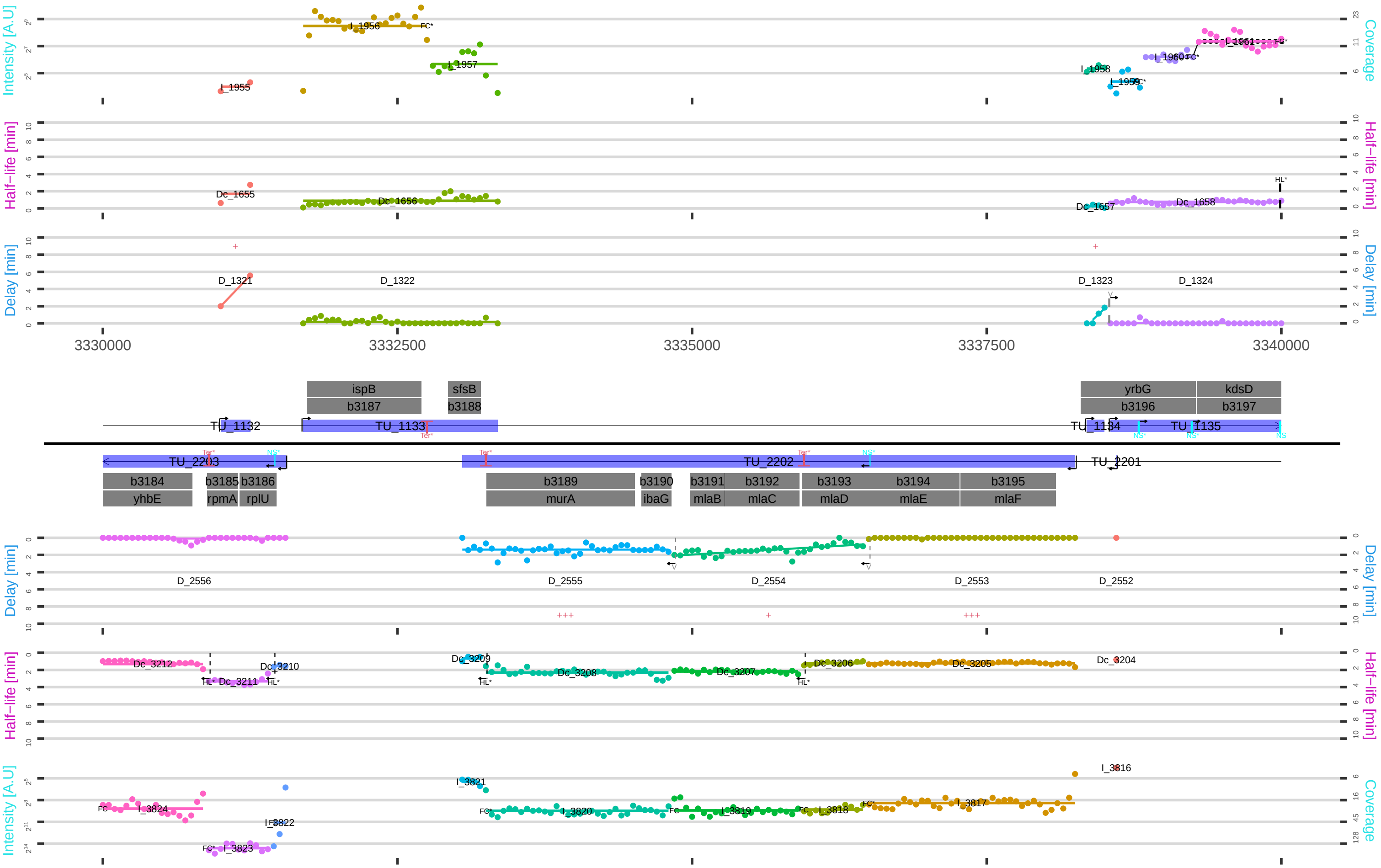

Term: termination (3), NS: new start (2), PS: pausing site (1), iTSS_I: internal starting site (1)

ID: 66800−66949; Term: termination (3), NS: new start (3), PS: pausing site (1), iTSS_I: internal starting site (0)

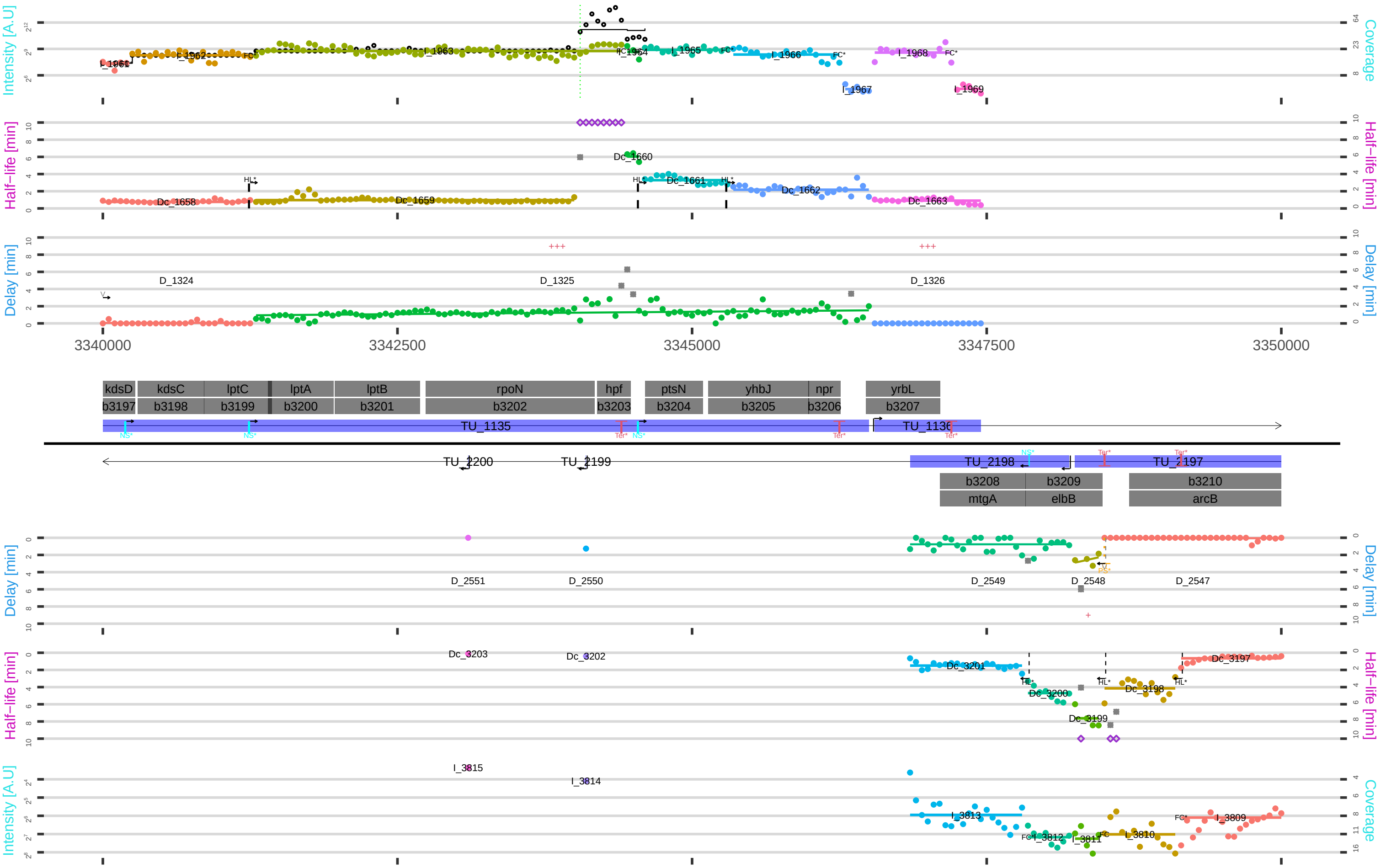

Term: termination (2), NS: new start (1), PS: pausing site (1), iTSS_I: internal starting site (0)

ID: 67053−67200; Term: termination (1), NS: new start (3), PS: pausing site (2), iTSS_I: internal starting site (0)

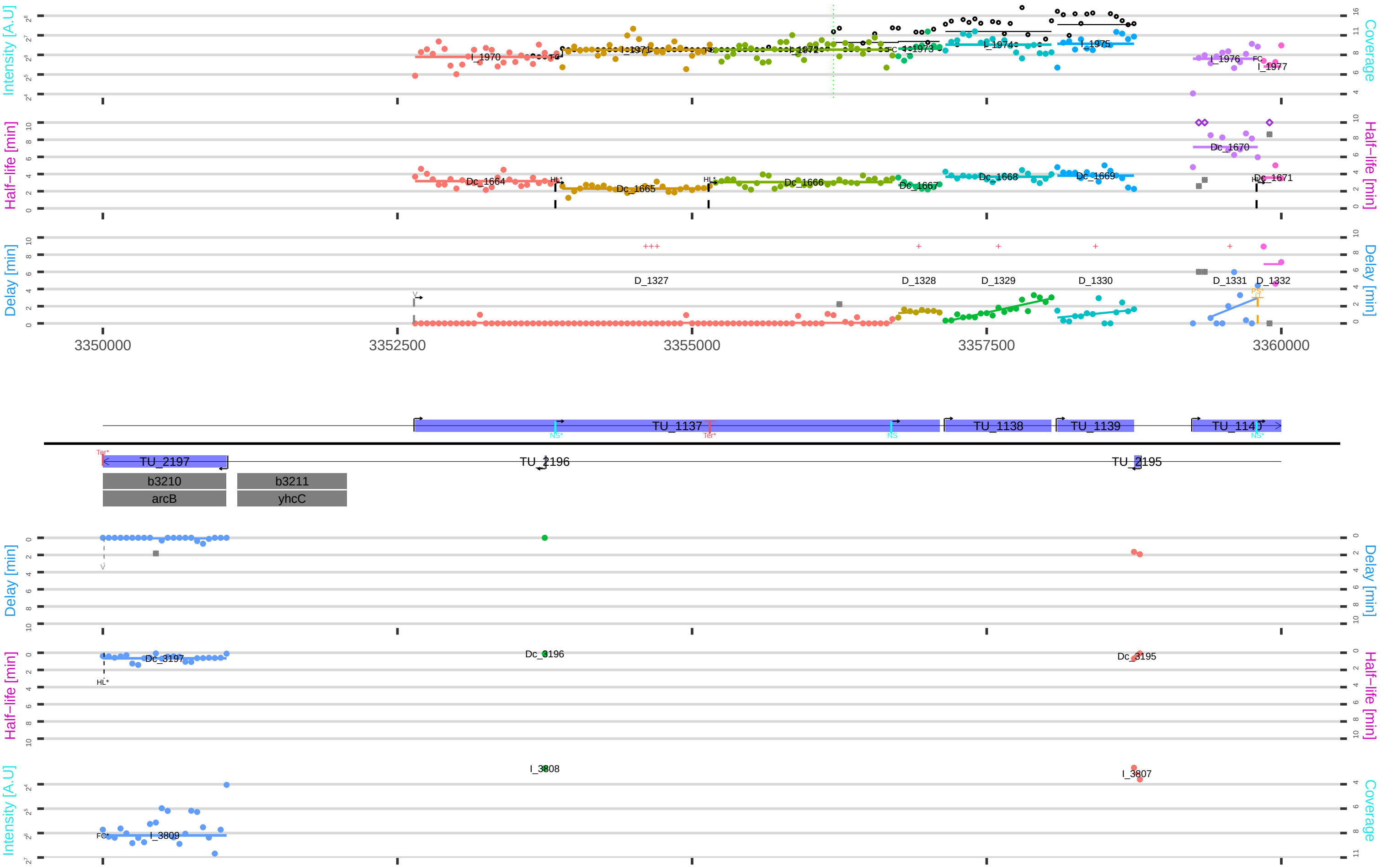

Term: termination (1), NS: new start (0), PS: pausing site (0), iTSS_I: internal starting site (0)

ID: 67200−67340; Term: termination (0), NS: new start (0), PS: pausing site (0), iTSS_I: internal starting site (0)

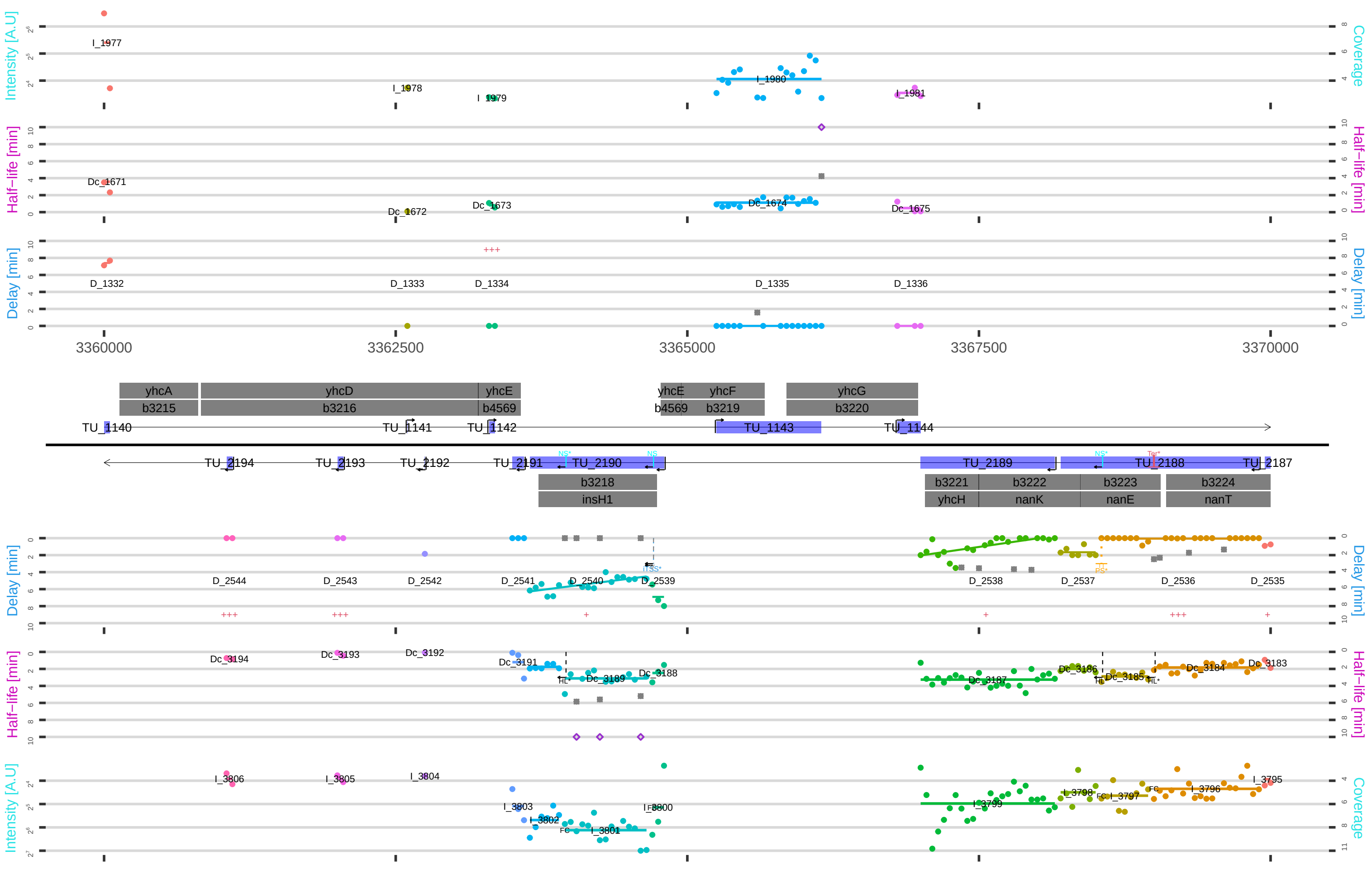

Term: termination (1), NS: new start (3), PS: pausing site (1), iTSS_I: internal starting site (1)

ID: 67515−67600; Term: termination (1), NS: new start (1), PS: pausing site (0), iTSS_I: internal starting site (0)

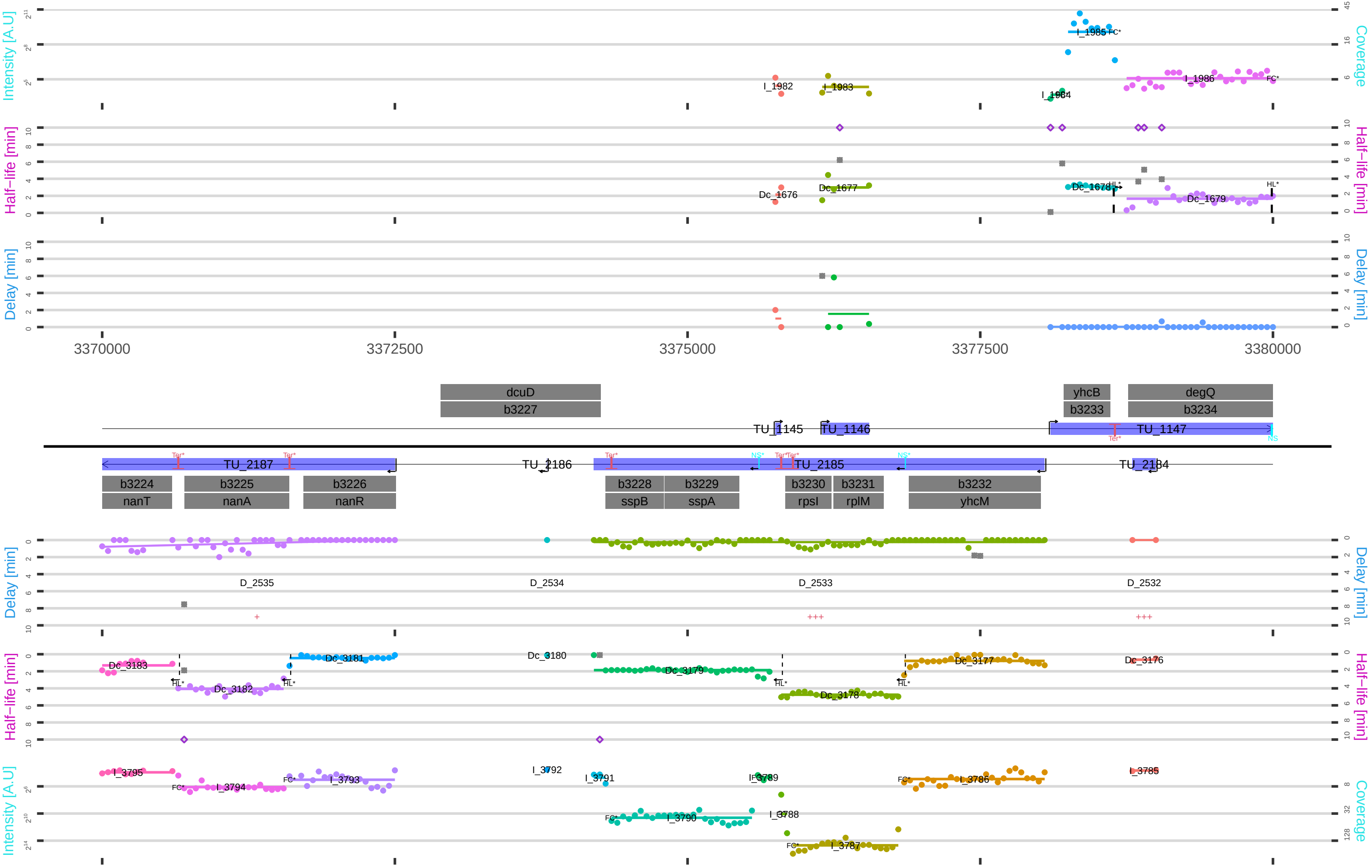

Term: termination (5), NS: new start (2), PS: pausing site (0), iTSS_I: internal starting site (0)

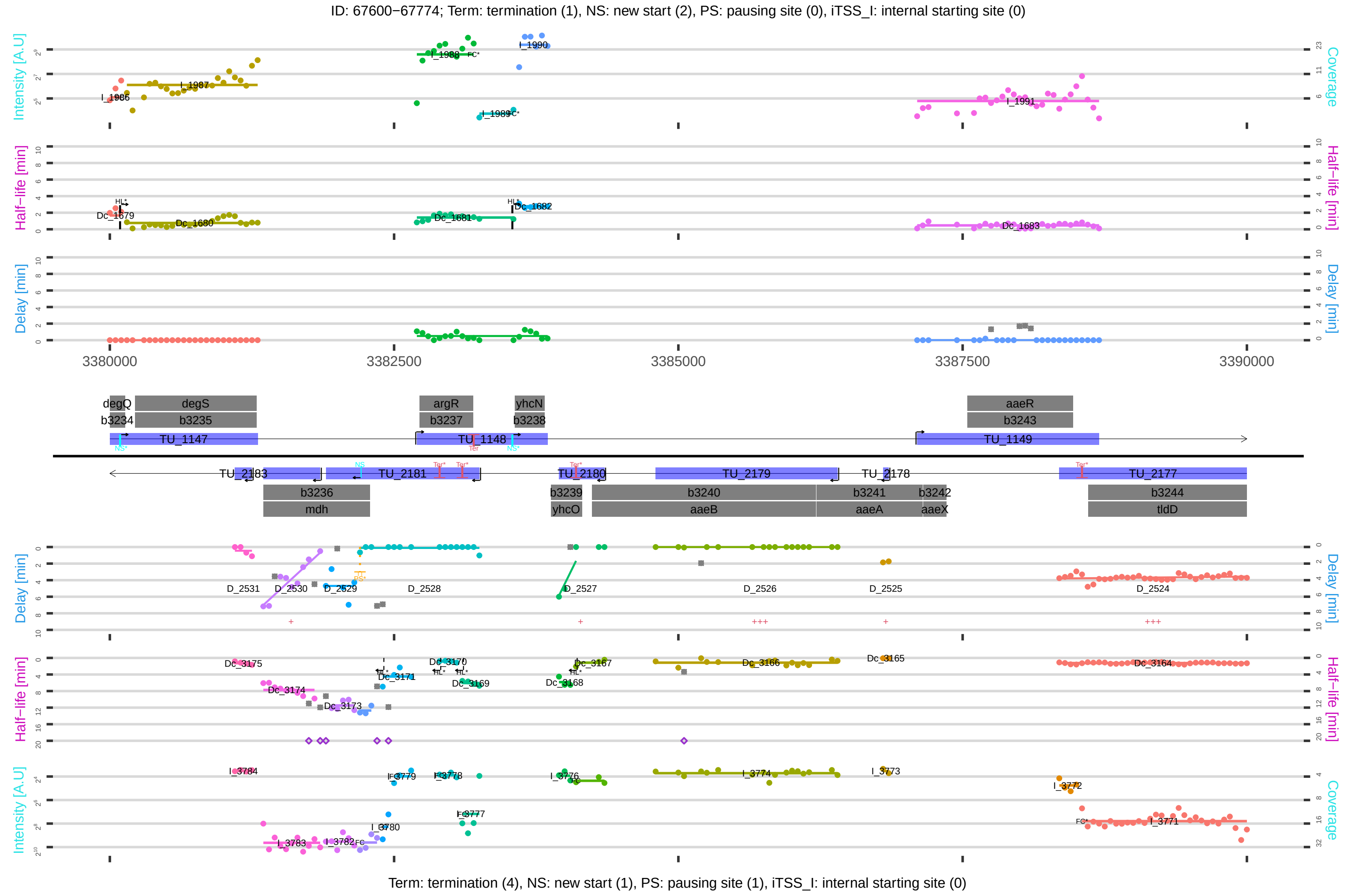
ID: 67600−67774; Term: termination (1), NS: new start (2), PS: pausing site (0), iTSS_I: internal starting site (0)
Intensity [A.U]
Coverage
Half−life [min]
Delay [min]
3380000
3382500
3385000
3387500
3390000
degQ
degS
argR
yhcN
aaeR
b3234
b3235
b3237
b3238
b3243
TU_1147
TU_1148
TU_1149
TU_2183
TU_2181
TU_2180
TU_2179
TU_2178
TU_2177
b3236
b3239
b3240
b3241
b3242
b3244
mdh
yhcO
aaeB
aaeA
aaeX
tldD
Term: termination (4), NS: new start (1), PS: pausing site (1), iTSS_I: internal starting site (0)

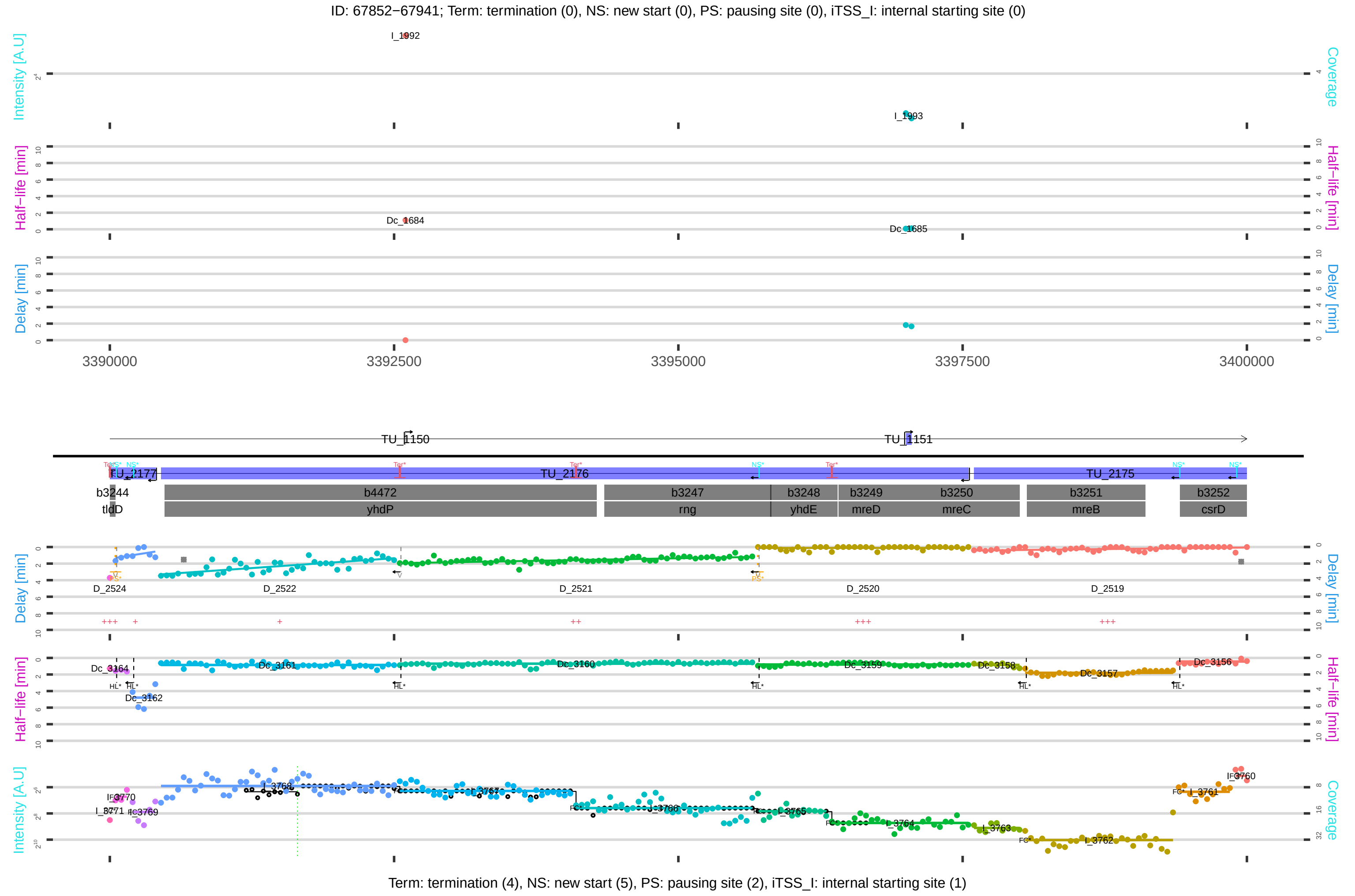

ID: 67852−67941; Term: termination (0), NS: new start (0), PS: pausing site (0), iTSS_I: internal starting site (0)
Intensity [A.U]
Coverage
Half−life [min]
Delay [min]
I_1992
I_1993
Dc_1684
Dc_1685
3390000
3392500
3395000
3397500
3400000
TU_1150
TU_1151
TU_2177
TU_2176
TU_2175
b3244
tldD
b4472
yhdP
b3247
rng
b3248
yhdE
b3249
mreD
b3250
mreC
b3251
mreB
b3252
csrD
D_2524
D_2522
D_2521
D_2520
D_2519
Dc_3164
Dc_3162
Dc_3161
Dc_3160
Dc_3159
Dc_3158
Dc_3157
Dc_3156
I_3771
I_3770
I_3769
I_3768
I_3767
I_3766
I_3765
I_3764
I_3763
I_3762
I_3761
I_3760
Term: termination (4), NS: new start (5), PS: pausing site (2), iTSS_I: internal starting site (1)

ID: 68030−68200; Term: termination (2), NS: new start (7), PS: pausing site (3), iTSS_I: internal starting site (2)

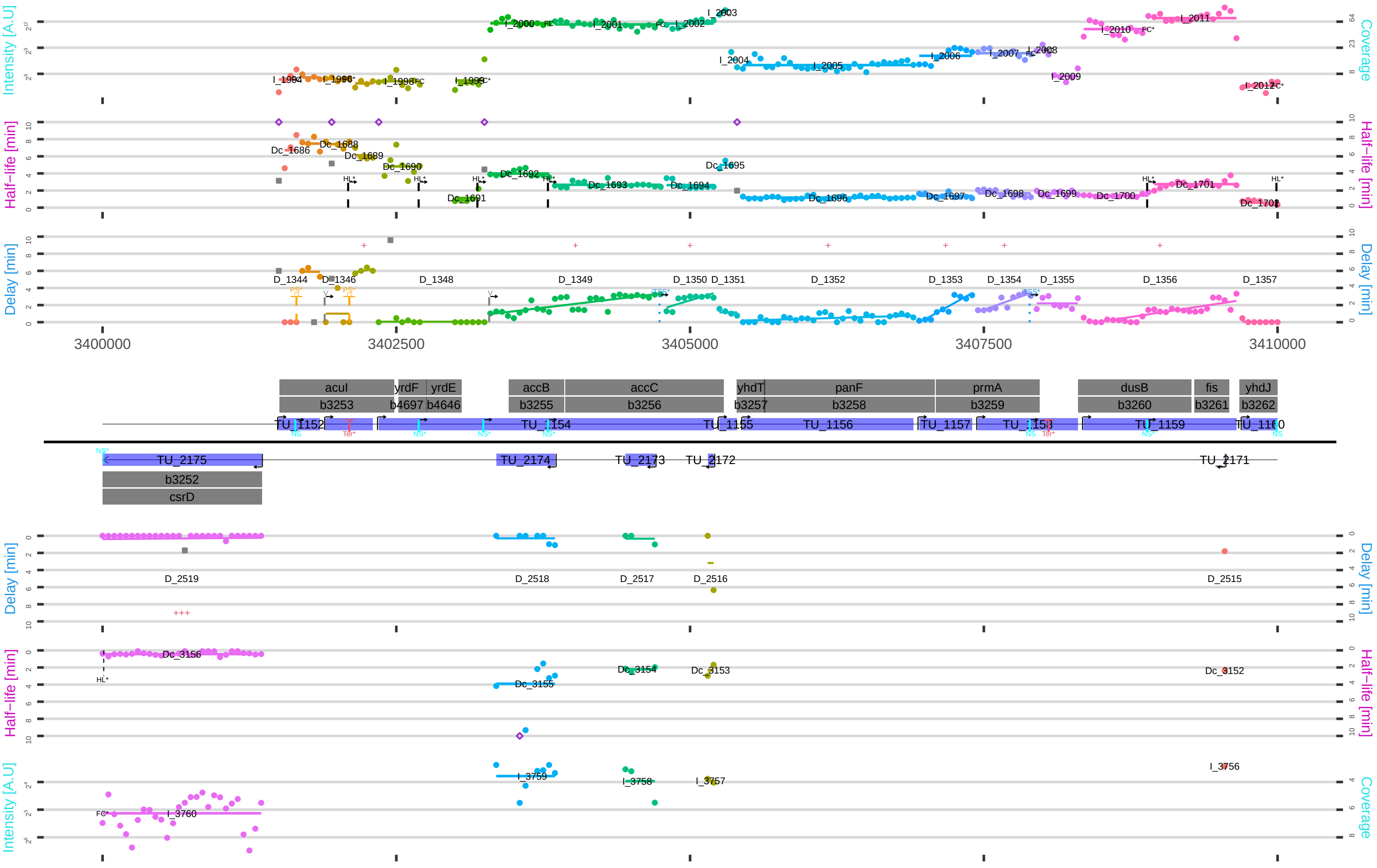

Term: termination (0), NS: new start (1), PS: pausing site (0), iTSS_I: internal starting site (0)

ID: 68200−68362; Term: termination (0), NS: new start (1), PS: pausing site (1), iTSS_I: internal starting site (0)

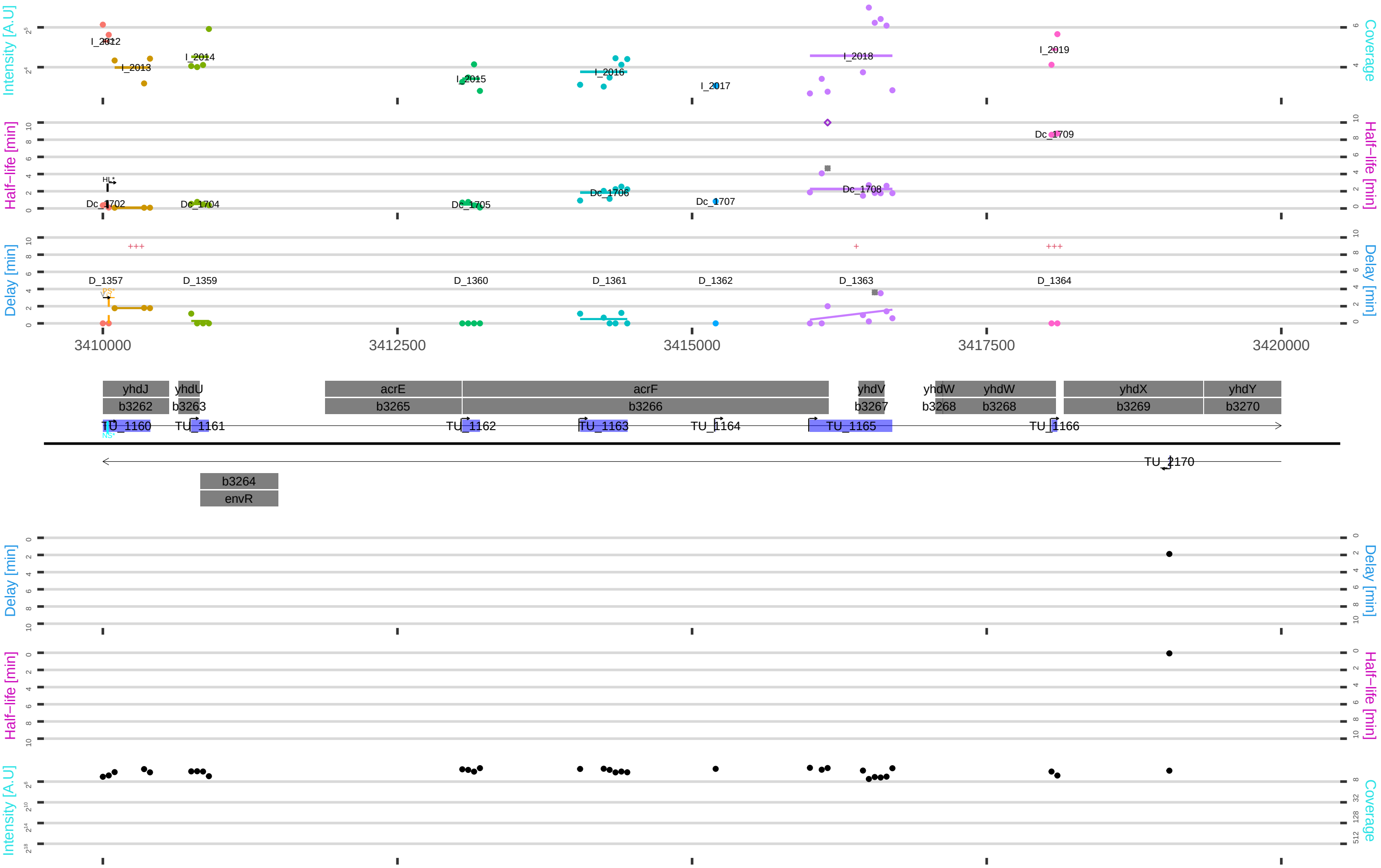

Term: termination (0), NS: new start (0), PS: pausing site (0), iTSS_I: internal starting site (0)

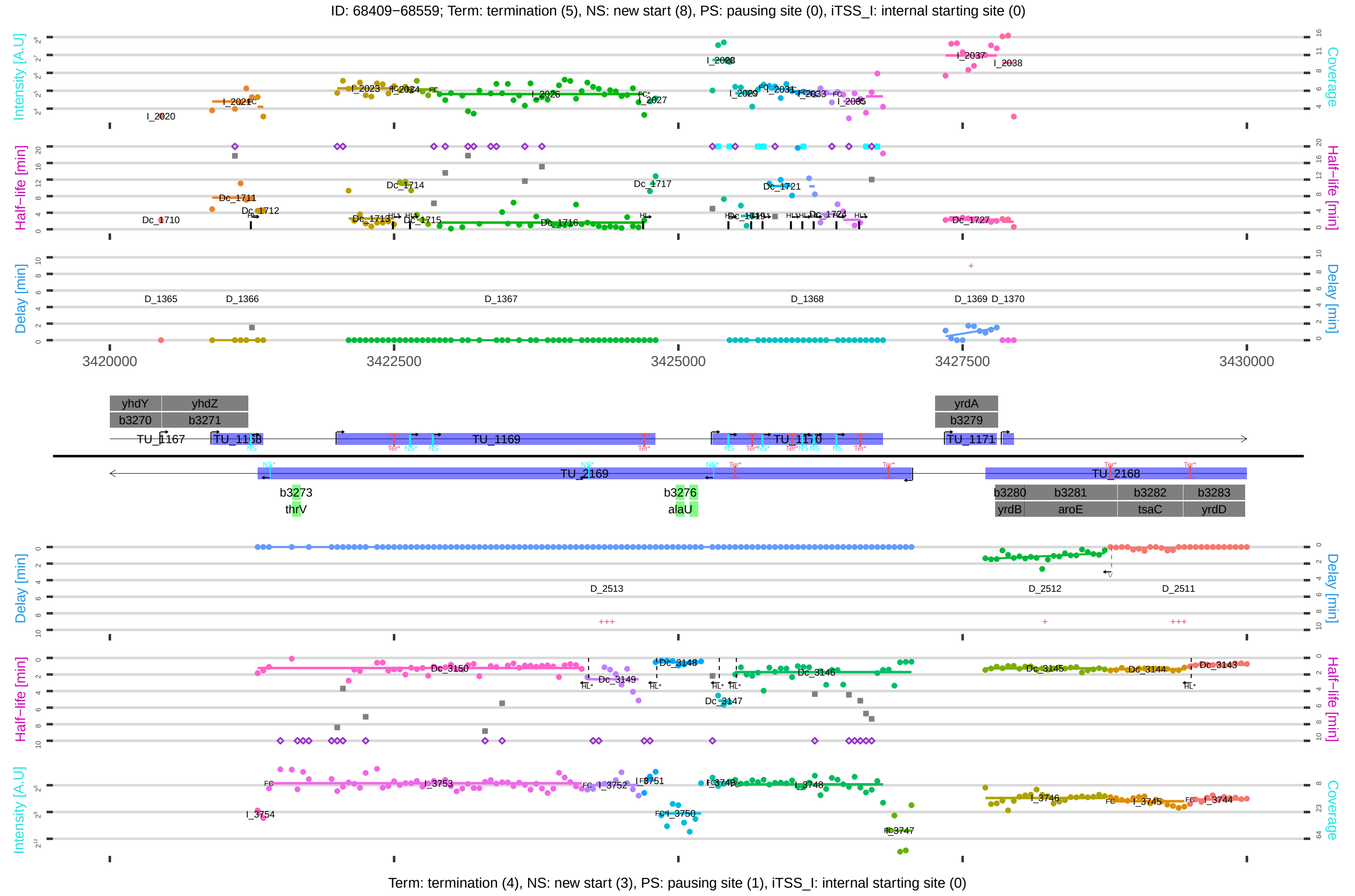
ID: 68409−68559; Term: termination (5), NS: new start (8), PS: pausing site (0), iTSS_I: internal starting site (0)
Intensity [A.U]
Coverage
Half−life [min]
Delay [min]
3420000
3422500
3425000
3427500
3430000
D_1365
D_1366
D_1367
D_1368
D_1369
D_1370
yhdY
yhdZ
b3270
b3271
yrdA
b3279
TU_1167
TU_1168
TU_1169
TU_1170
TU_1171
TU_2169
TU_2168
b3273
thrV
b3276
alaU
b3280
b3281
b3282
b3283
yrdB
aroE
tsaC
yrdD
D_2513
D_2512
D_2511
Term: termination (4), NS: new start (3), PS: pausing site (1), iTSS_I: internal starting site (0)

ID: 68604−68799; Term: termination (3), NS: new start (2), PS: pausing site (1), iTSS_I: internal starting site (1)

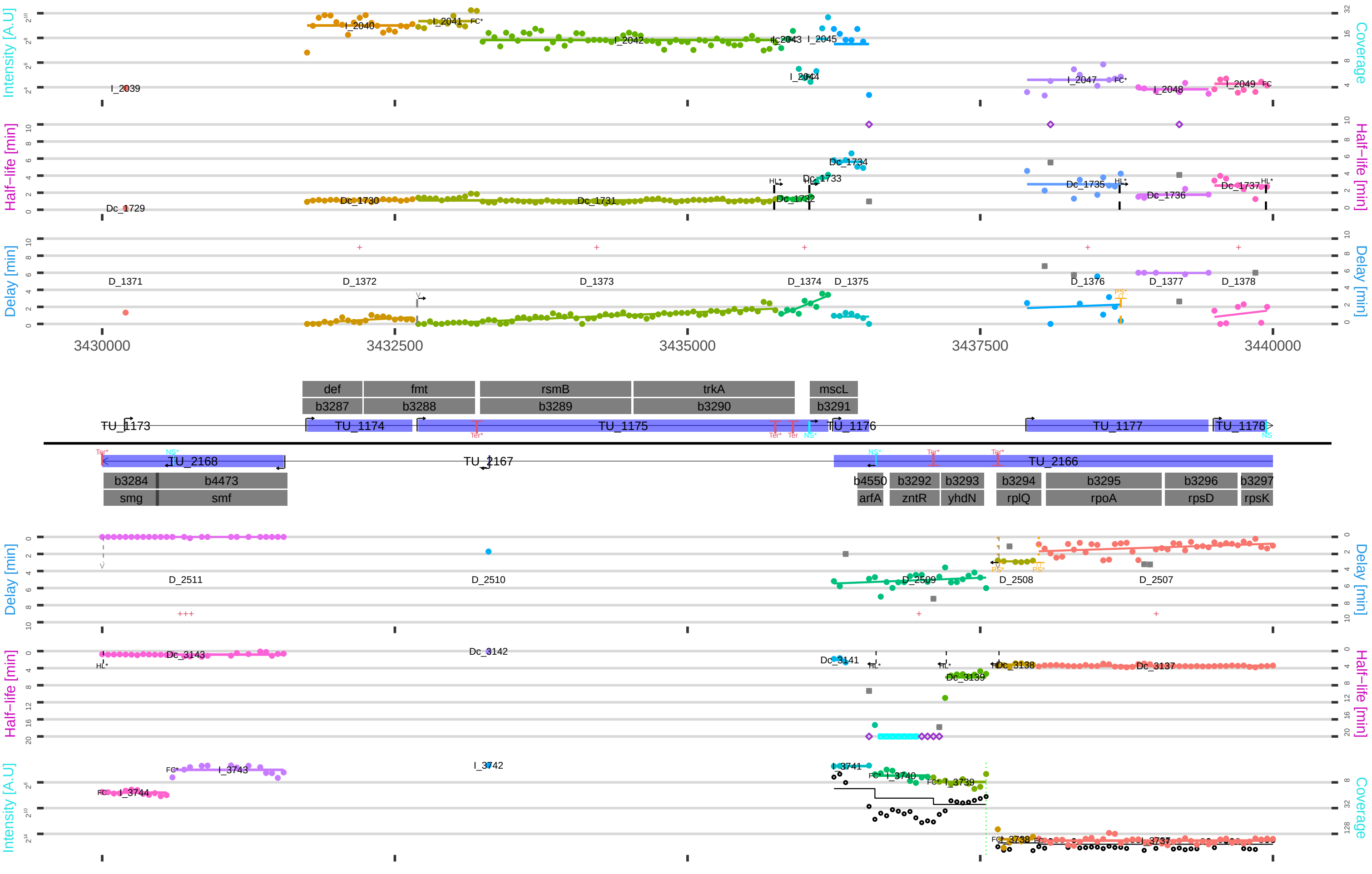

Term: termination (3), NS: new start (2), PS: pausing site (2), iTSS_I: internal starting site (0)

ID: 68803−69000; Term: termination (0), NS: new start (3), PS: pausing site (2), iTSS_I: internal starting site (1)

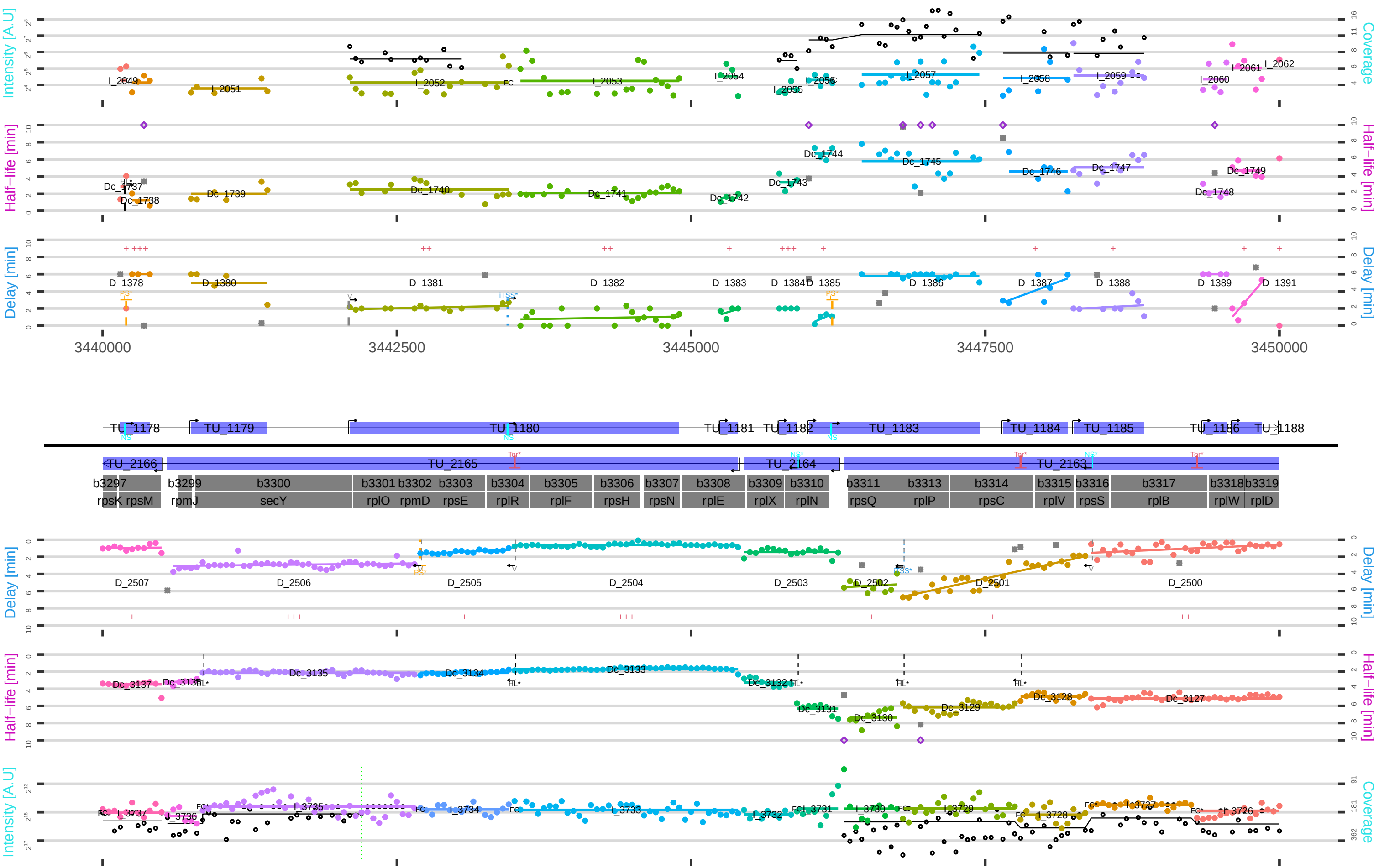

Term: termination (3), NS: new start (2), PS: pausing site (3), iTSS_I: internal starting site (1)

ID: 69000−69171; Term: termination (0), NS: new start (0), PS: pausing site (0), iTSS_I: internal starting site (0)

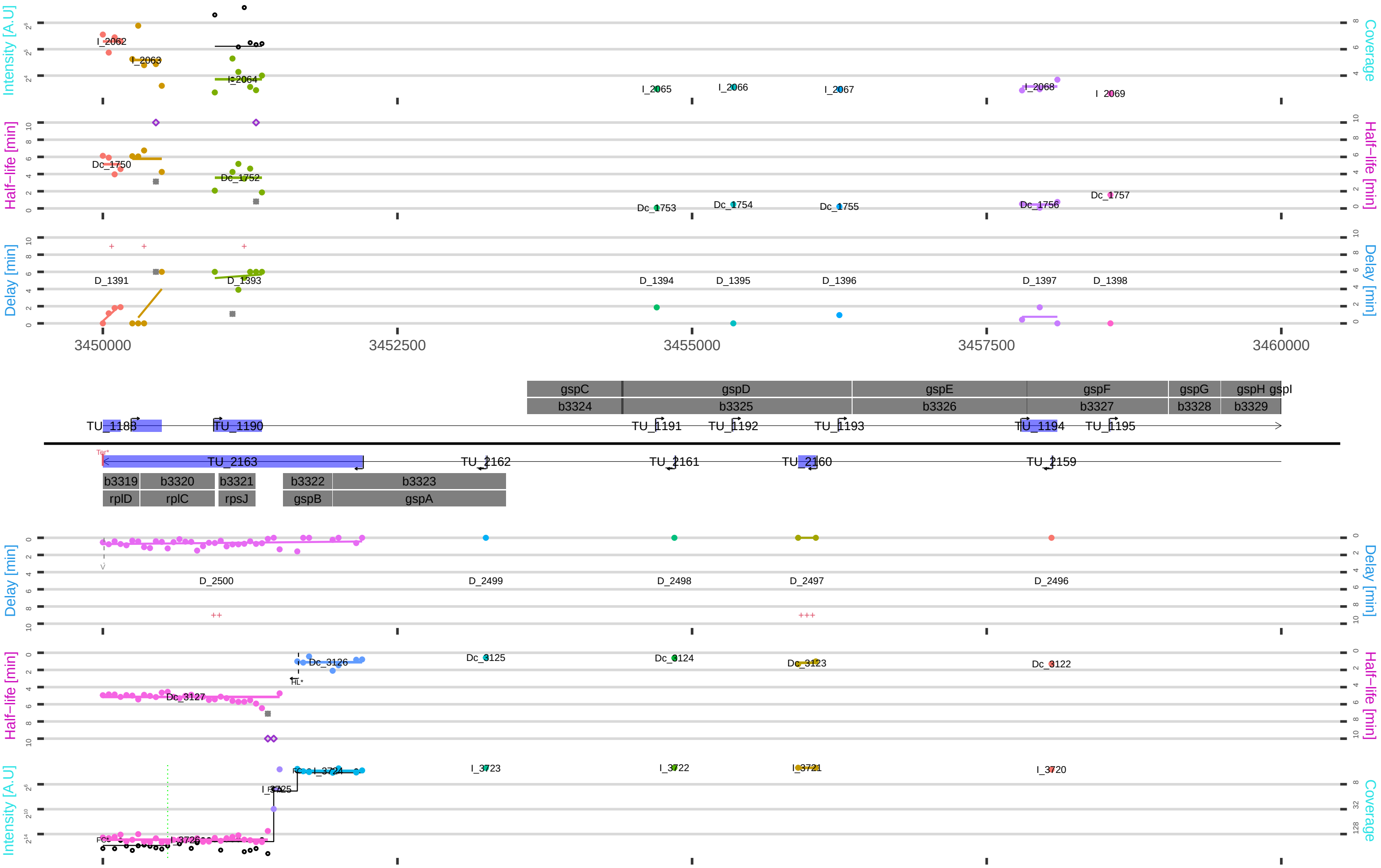

Term: termination (1), NS: new start (0), PS: pausing site (0), iTSS_I: internal starting site (0)

ID: 69282−69400; Term: termination (0), NS: new start (1), PS: pausing site (0), iTSS_I: internal starting site (1)

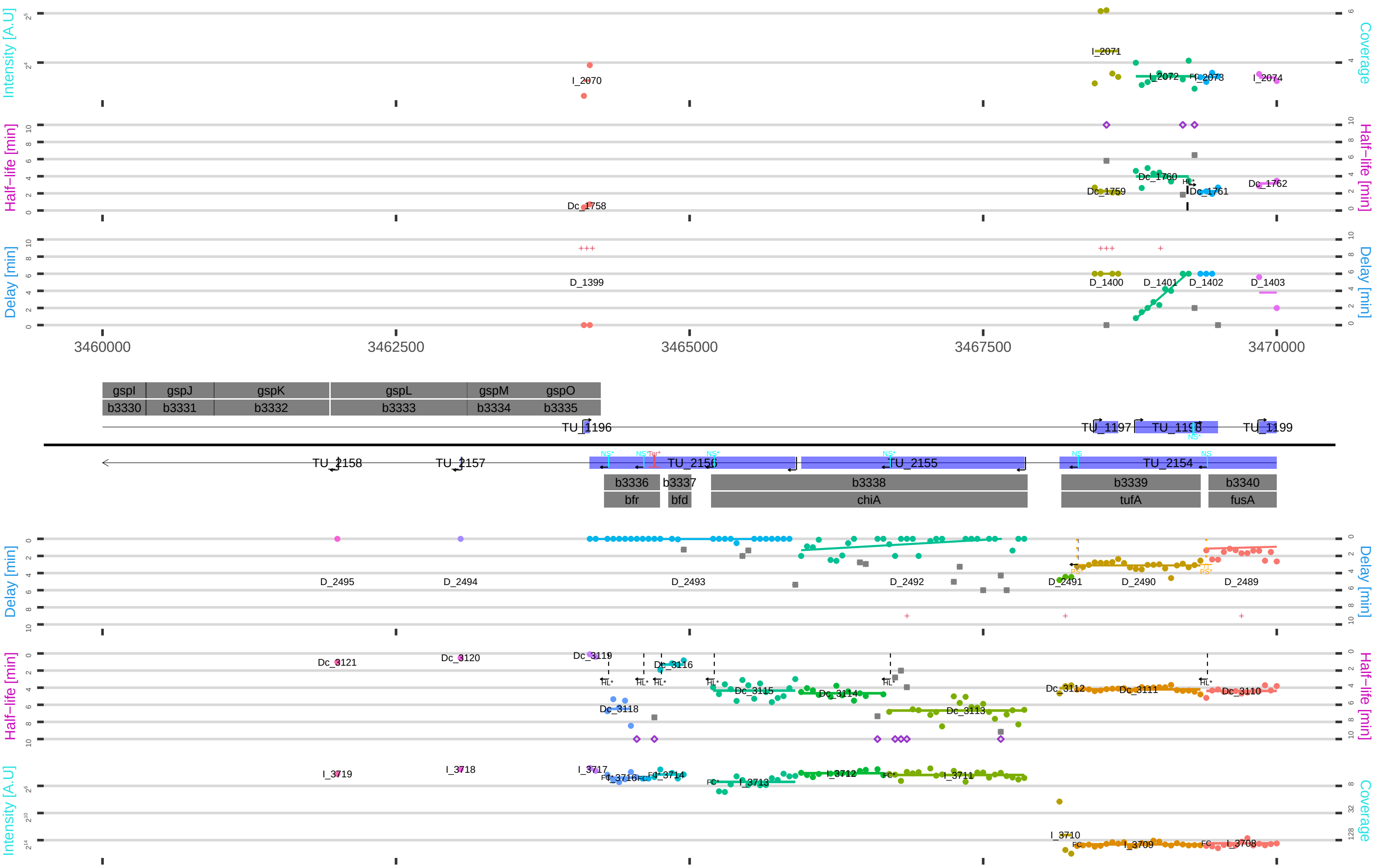

Term: termination (1), NS: new start (6), PS: pausing site (2), iTSS_I: internal starting site (0)

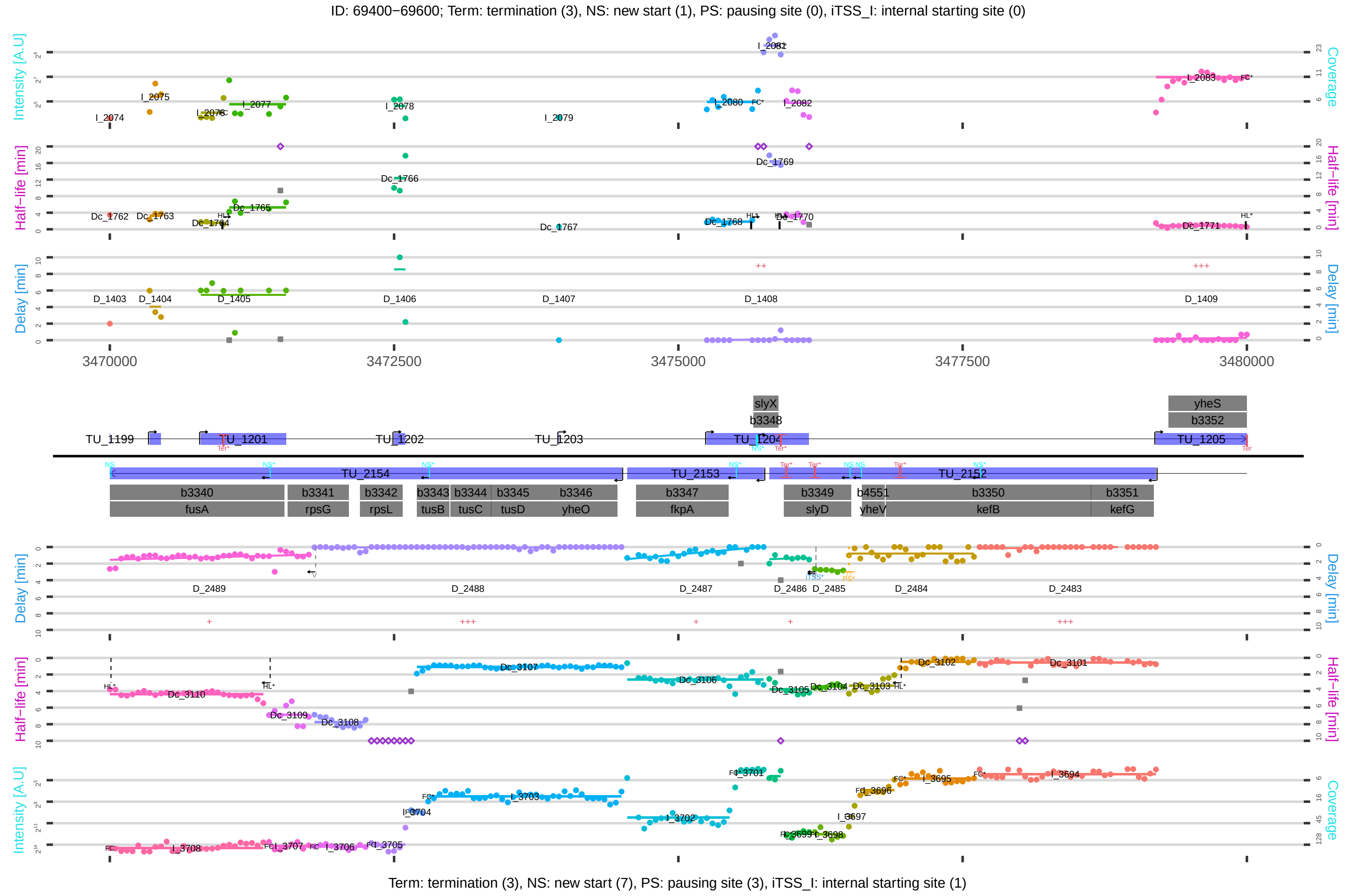
ID: 69400−69600; Term: termination (3), NS: new start (1), PS: pausing site (0), iTSS_I: internal starting site (0)
Intensity [A.U]
Coverage
Half−life [min]
Delay [min]
3470000
3472500
3475000
3477500
3480000
slyX
b3348
yheS
b3352
TU_1199
TU_1201
TU_1202
TU_1203
TU_1204
TU_1205
TU_2154
TU_2153
TU_2152
b3340
fusA
b3341
rpsG
b3342
rpsL
b3343
tusB
b3344
tusC
b3345
tusD
b3346
yheO
b3347
fkpA
b3349
slyD
b4551
yheV
b3350
kefB
b3351
kefG
D_2489
D_2488
D_2487
D_2486
D_2485
D_2484
D_2483
Term: termination (3), NS: new start (7), PS: pausing site (3), iTSS_I: internal starting site (1)

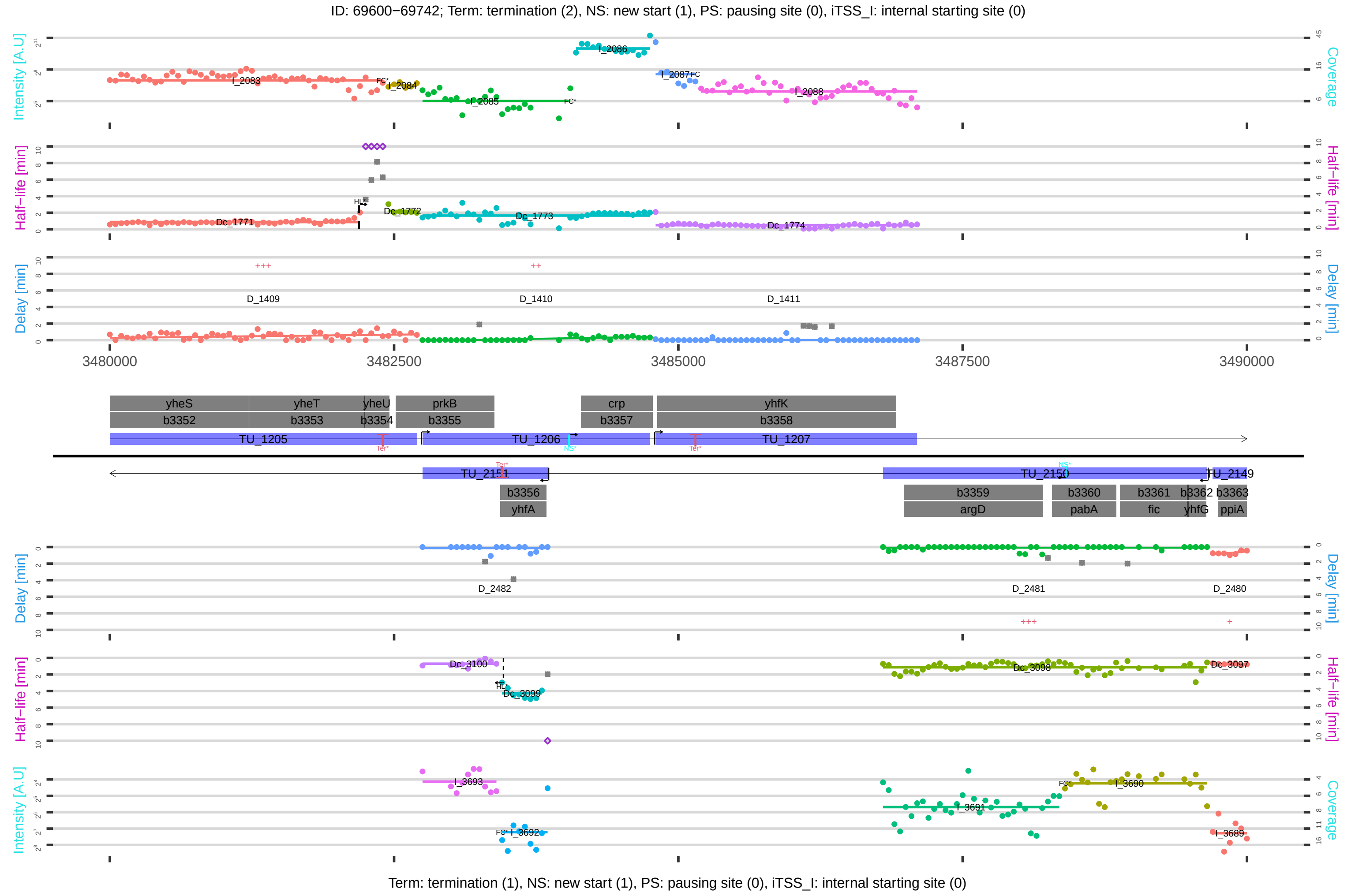
ID: 69600−69742; Term: termination (2), NS: new start (1), PS: pausing site (0), iTSS_I: internal starting site (0)
Intensity [A.U]
Coverage
Half−life [min]
Delay [min]
I_2083
I_2084
I_2085
I_2086
I_2087
I_2088
Dc_1771
Dc_1772
Dc_1773
Dc_1774
D_1409
D_1410
D_1411
3480000
3482500
3485000
3487500
3490000
yheS
yheT
yheU
prkB
crp
yhfK
b3352
b3353
b3354
b3355
b3357
b3358
TU_1205
TU_1206
TU_1207
TU_2151
TU_2150
TU_2149
b3356
yhfA
b3359
argD
b3360
pabA
b3361
fic
b3362
yhfG
b3363
ppiA
D_2482
D_2481
D_2480
Dc_3100
Dc_3099
Dc_3098
Dc_3097
I_3693
I_3692
I_3691
I_3690
I_3689
Term: termination (1), NS: new start (1), PS: pausing site (0), iTSS_I: internal starting site (0)

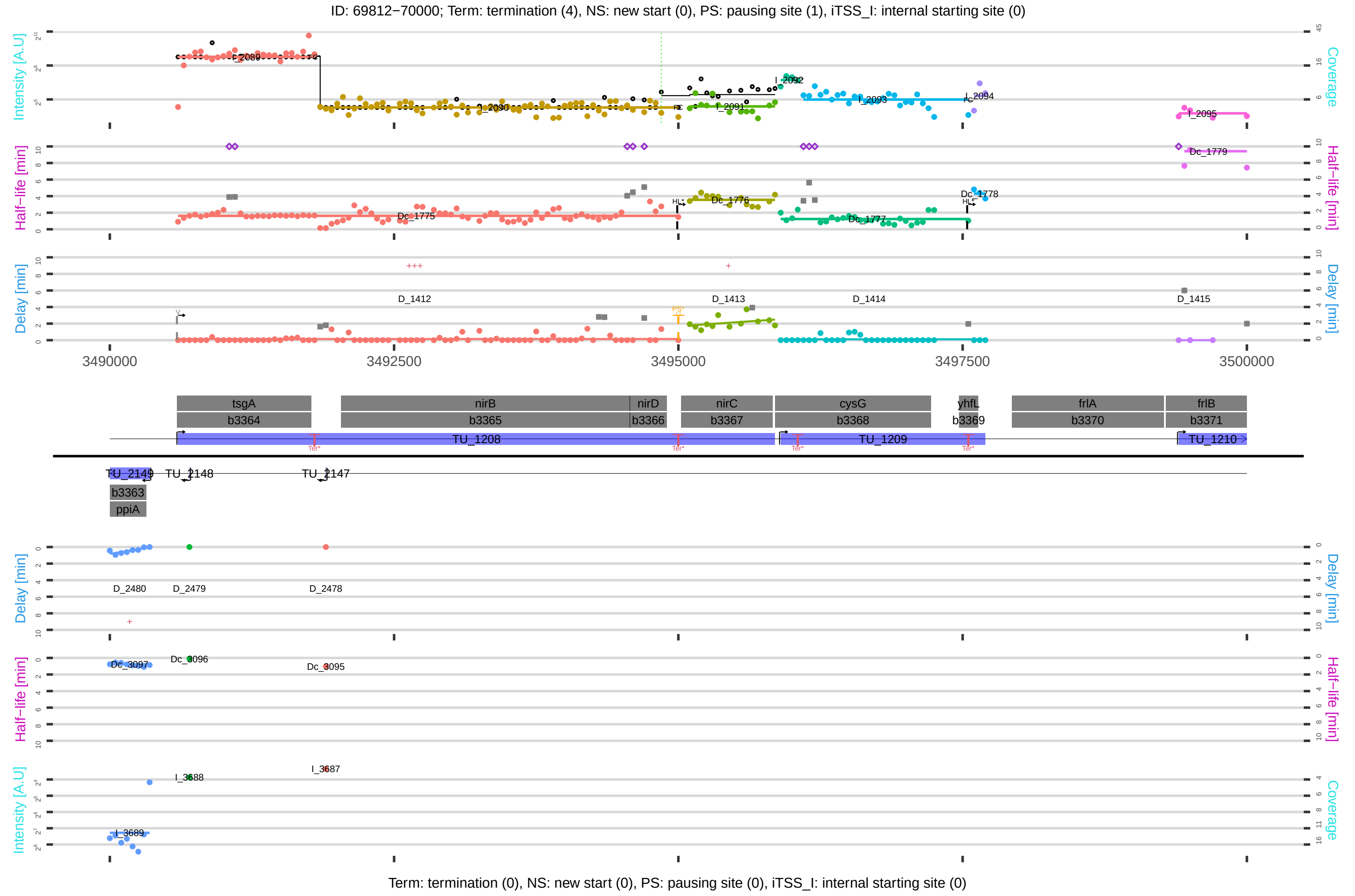

ID: 69812−70000; Term: termination (4), NS: new start (0), PS: pausing site (1), iTSS_I: internal starting site (0)
Intensity [A.U]
Coverage
Half−life [min]
Delay [min]
I_2089
I_2090
I_2091
I_2092
I_2093
I_2094
I_2095
Dc_1775
Dc_1776
Dc_1777
Dc_1778
Dc_1779
D_1412
D_1413
D_1414
D_1415
3490000
3492500
3495000
3497500
3500000
tsgA
b3364
nirB
b3365
nirD
b3366
nirC
b3367
cysG
b3368
yhfL
b3369
frlA
b3370
frlB
b3371
TU_1208
TU_1209
TU_1210
TU_2149
TU_2148
TU_2147
b3363
ppiA
D_2480
D_2479
D_2478
Dc_3097
Dc_3096
Dc_3095
I_3688
I_3687
I_3689
Term: termination (0), NS: new start (0), PS: pausing site (0), iTSS_I: internal starting site (0)

ID: 70000−70120; Term: termination (0), NS: new start (1), PS: pausing site (0), iTSS_I: internal starting site (0)

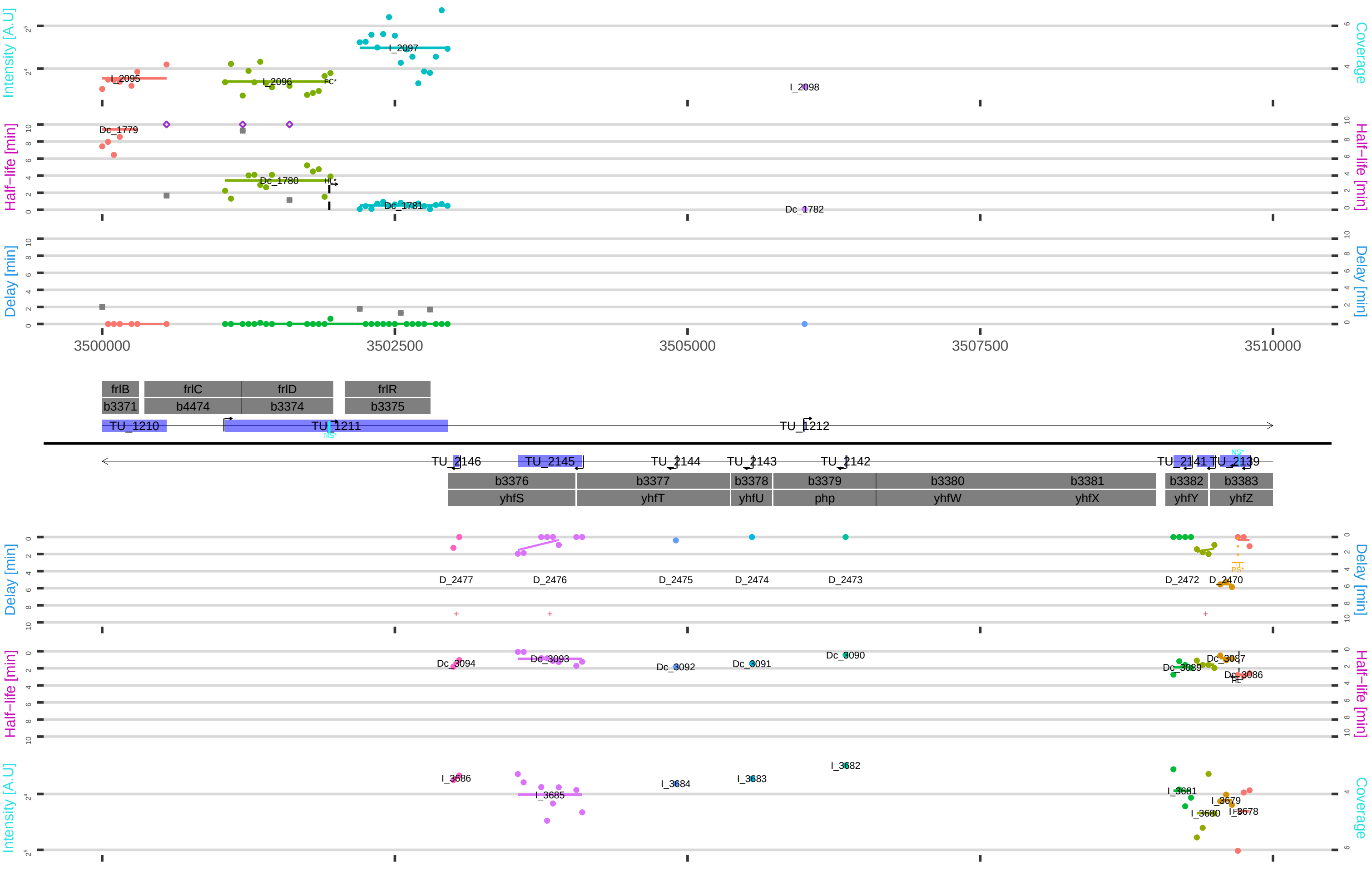

Term: termination (0), NS: new start (1), PS: pausing site (1), iTSS_I: internal starting site (0)

ID: 115386−115208; FC*: significant t−test of two consecutive segments; Term: termination, NS: new start, PS: pausing site,iTSS_I: internal starting site,TI: transcription interferance.

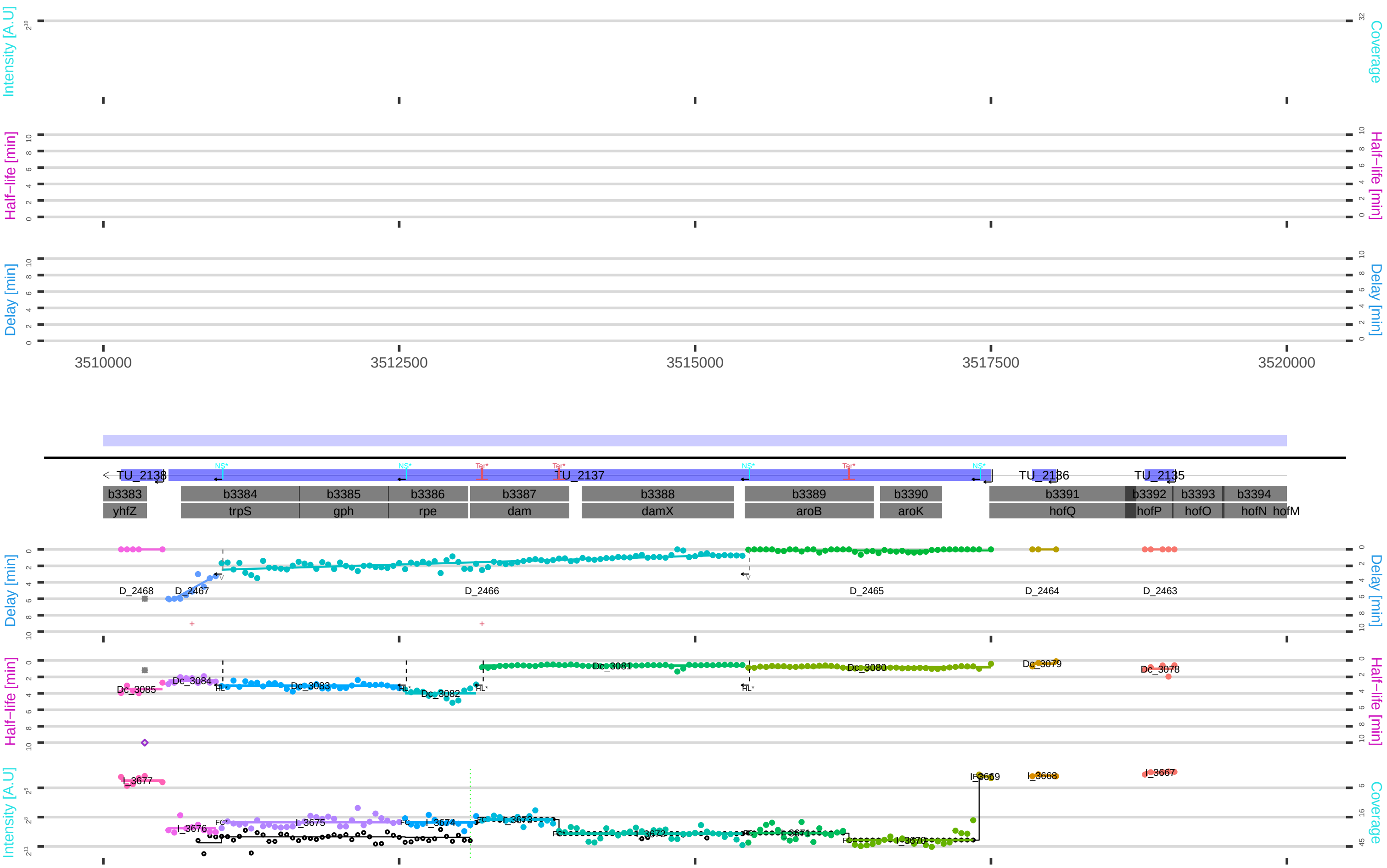

Term: termination (3), NS: new start (4), PS: pausing site (2), iTSS_I: internal starting site (0)

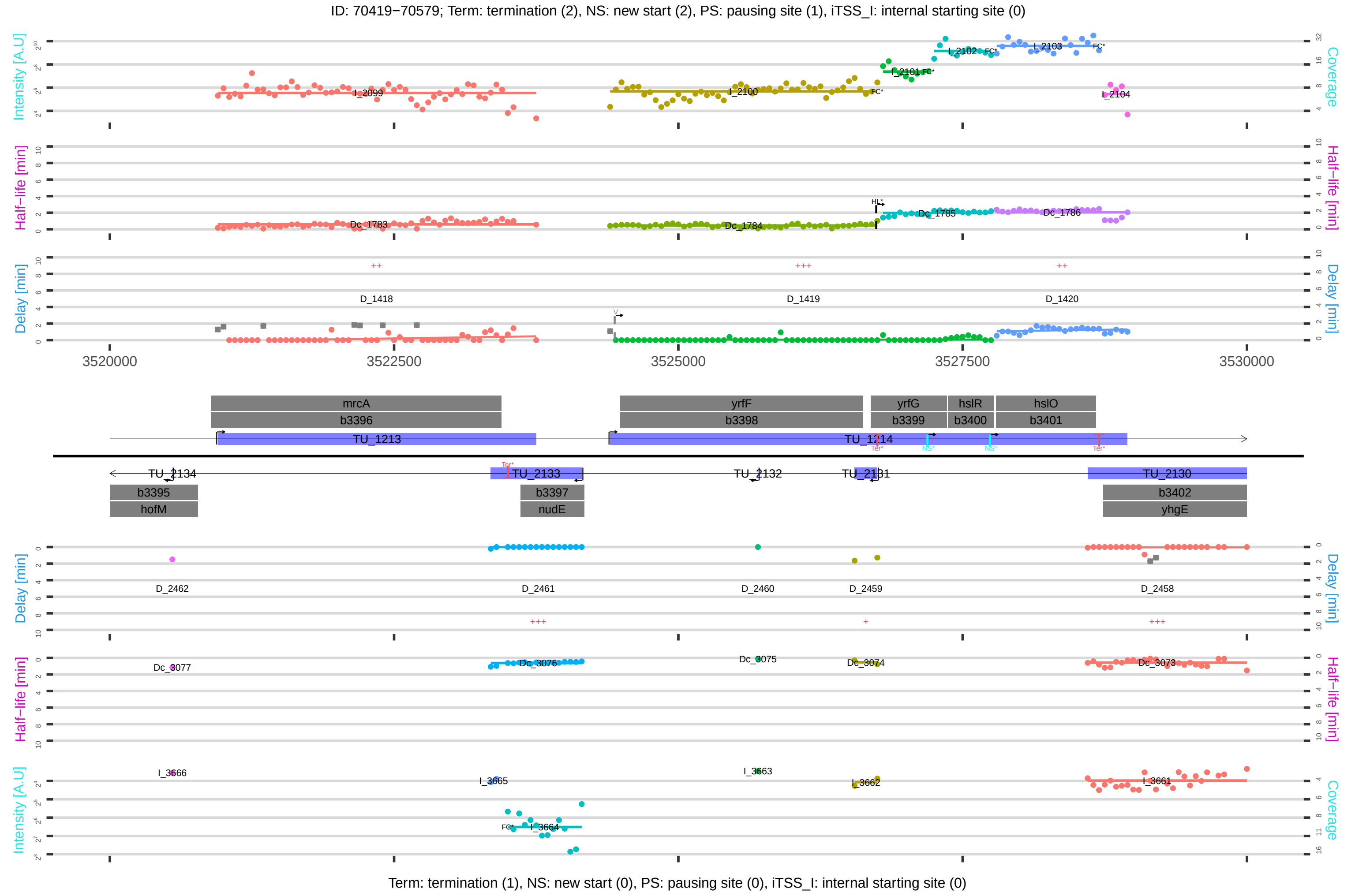

ID: 70419−70579; Term: termination (2), NS: new start (2), PS: pausing site (1), iTSS_I: internal starting site (0)
Intensity [A.U]
Coverage
Half−life [min]
Delay [min]
I_2099
I_2100
I_2101
I_2102
I_2103
I_2104
Dc_1783
Dc_1784
Dc_1785
Dc_1786
D_1418
D_1419
D_1420
3520000
3522500
3525000
3527500
3530000
mrcA
b3396
yrfF
b3398
yrfG
b3399
hslR
b3400
hslO
b3401
TU_1213
TU_1214
TU_2134
TU_2133
TU_2132
TU_2131
TU_2130
b3395
hofM
b3397
nudE
b3402
yhgE
D_2462
D_2461
D_2460
D_2459
D_2458
Dc_3077
Dc_3076
Dc_3075
Dc_3074
Dc_3073
I_3666
I_3665
I_3664
I_3663
I_3662
I_3661
Term: termination (1), NS: new start (0), PS: pausing site (0), iTSS_I: internal starting site (0)

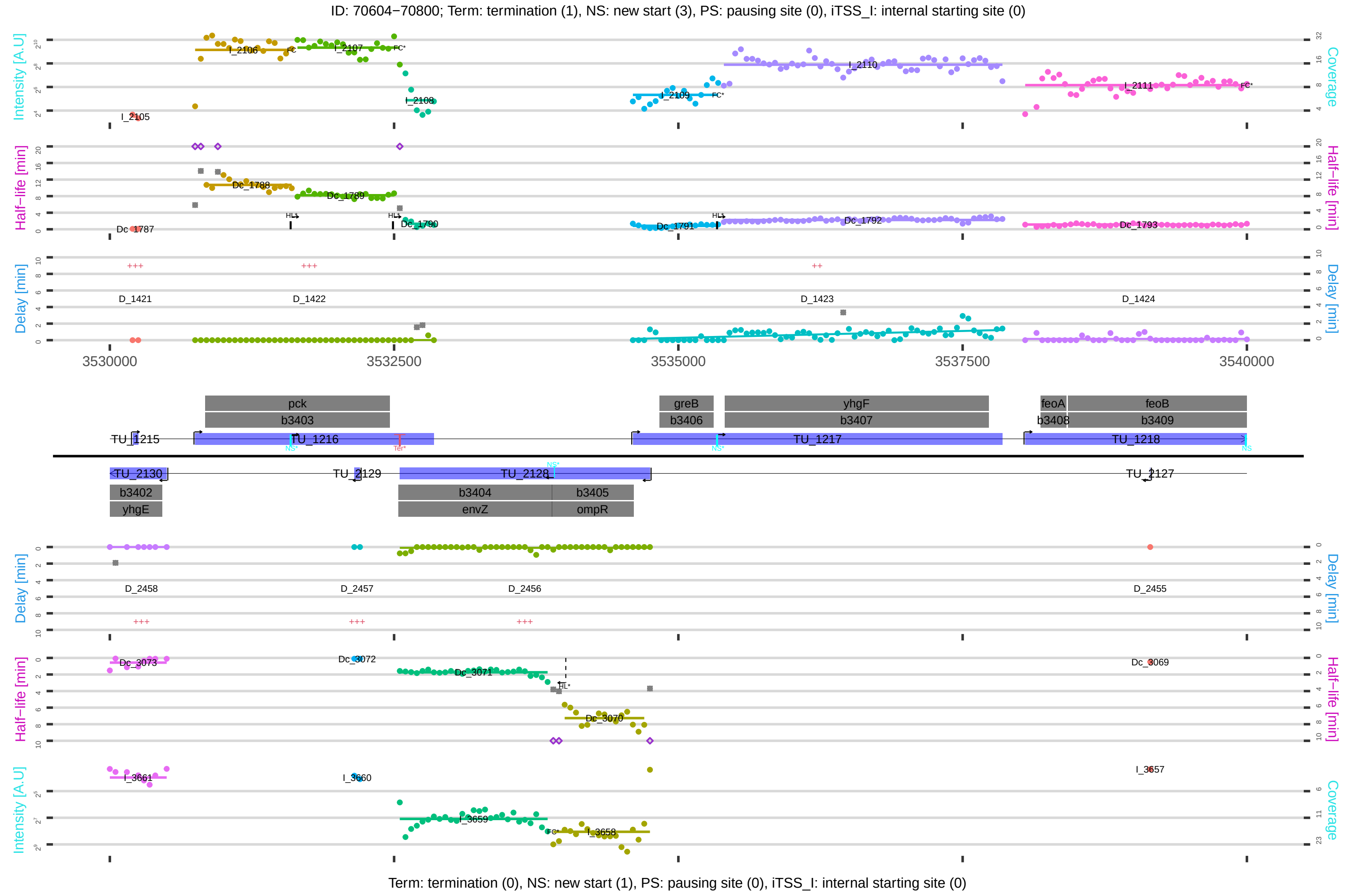
ID: 70604−70800; Term: termination (1), NS: new start (3), PS: pausing site (0), iTSS_I: internal starting site (0)
Intensity [A.U]
Coverage
Half−life [min]
Delay [min]
I_2105
I_2106
I_2107
I_2108
I_2109
I_2110
I_2111
Dc_1787
Dc_1788
Dc_1789
Dc_1790
Dc_1791
Dc_1792
Dc_1793
D_1421
D_1422
D_1423
D_1424
3530000
3532500
3535000
3537500
3540000
pck
b3403
greB
b3406
yhgF
b3407
feoA
b3408
feoB
b3409
TU_1215
TU_1216
TU_1217
TU_1218
TU_2130
TU_2129
TU_2128
TU_2127
b3402
yhgE
b3404
envZ
b3405
ompR
D_2458
D_2457
D_2456
D_2455
Dc_3073
Dc_3072
Dc_3071
Dc_3070
Dc_3069
I_3661
I_3660
I_3659
I_3658
I_3657
Term: termination (0), NS: new start (1), PS: pausing site (0), iTSS_I: internal starting site (0)

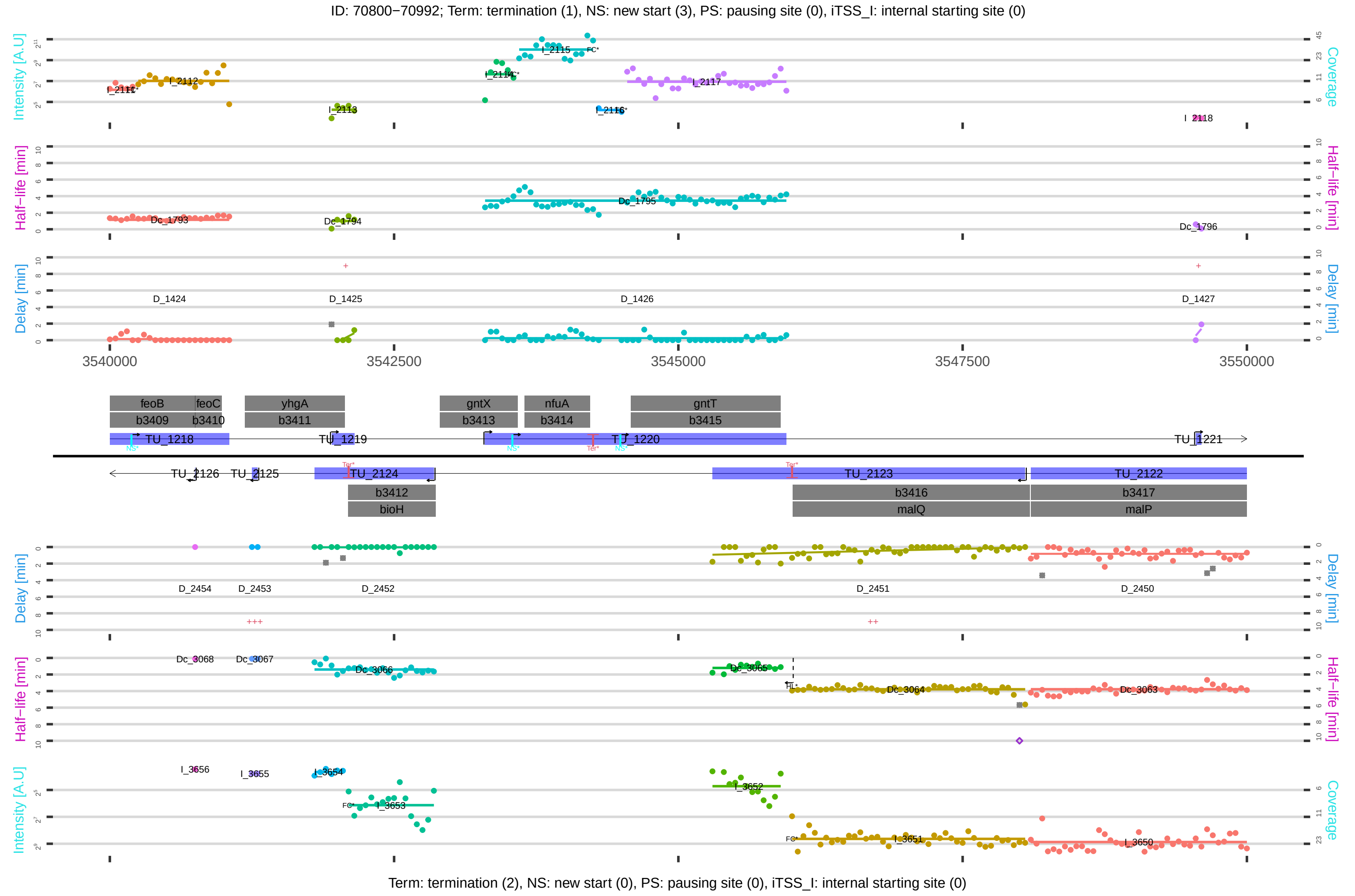

ID: 70800−70992; Term: termination (1), NS: new start (3), PS: pausing site (0), iTSS_I: internal starting site (0)
Intensity [A.U]
Coverage
Half−life [min]
Delay [min]
I_2111
I_2112
I_2113
I_2114
I_2115
I_2116
I_2117
I_2118
Dc_1793
Dc_1794
Dc_1795
Dc_1796
D_1424
D_1425
D_1426
D_1427
3540000
3542500
3545000
3547500
3550000
feoB
feoC
b3409
b3410
yhgA
b3411
gntX
b3413
nfuA
b3414
gntT
b3415
TU_1218
TU_1219
TU_1220
TU_1221
TU_2126
TU_2125
TU_2124
TU_2123
TU_2122
b3412
bioH
b3416
malQ
b3417
malP
D_2454
D_2453
D_2452
D_2451
D_2450
Dc_3068
Dc_3067
Dc_3066
Dc_3065
Dc_3064
Dc_3063
I_3656
I_3655
I_3654
I_3653
I_3652
I_3651
I_3650
Term: termination (2), NS: new start (0), PS: pausing site (0), iTSS_I: internal starting site (0)

ID: 71022−71081; Term: termination (1), NS: new start (1), PS: pausing site (1), iTSS_I: internal starting site (0)

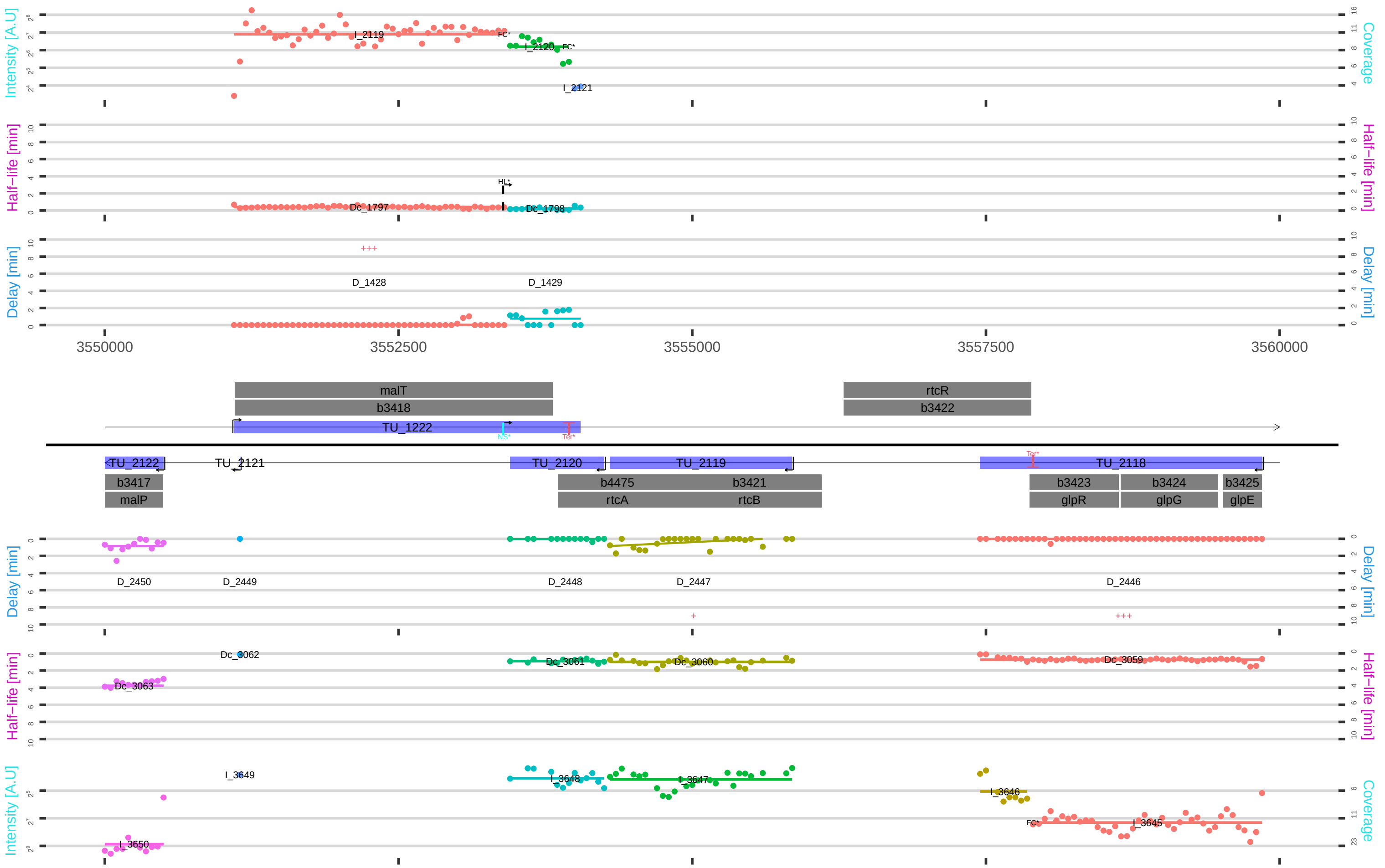

Term: termination (1), NS: new start (0), PS: pausing site (0), iTSS_I: internal starting site (0)

ID: 71202−71254; Term: termination (2), NS: new start (0), PS: pausing site (0), iTSS_I: internal starting site (0)

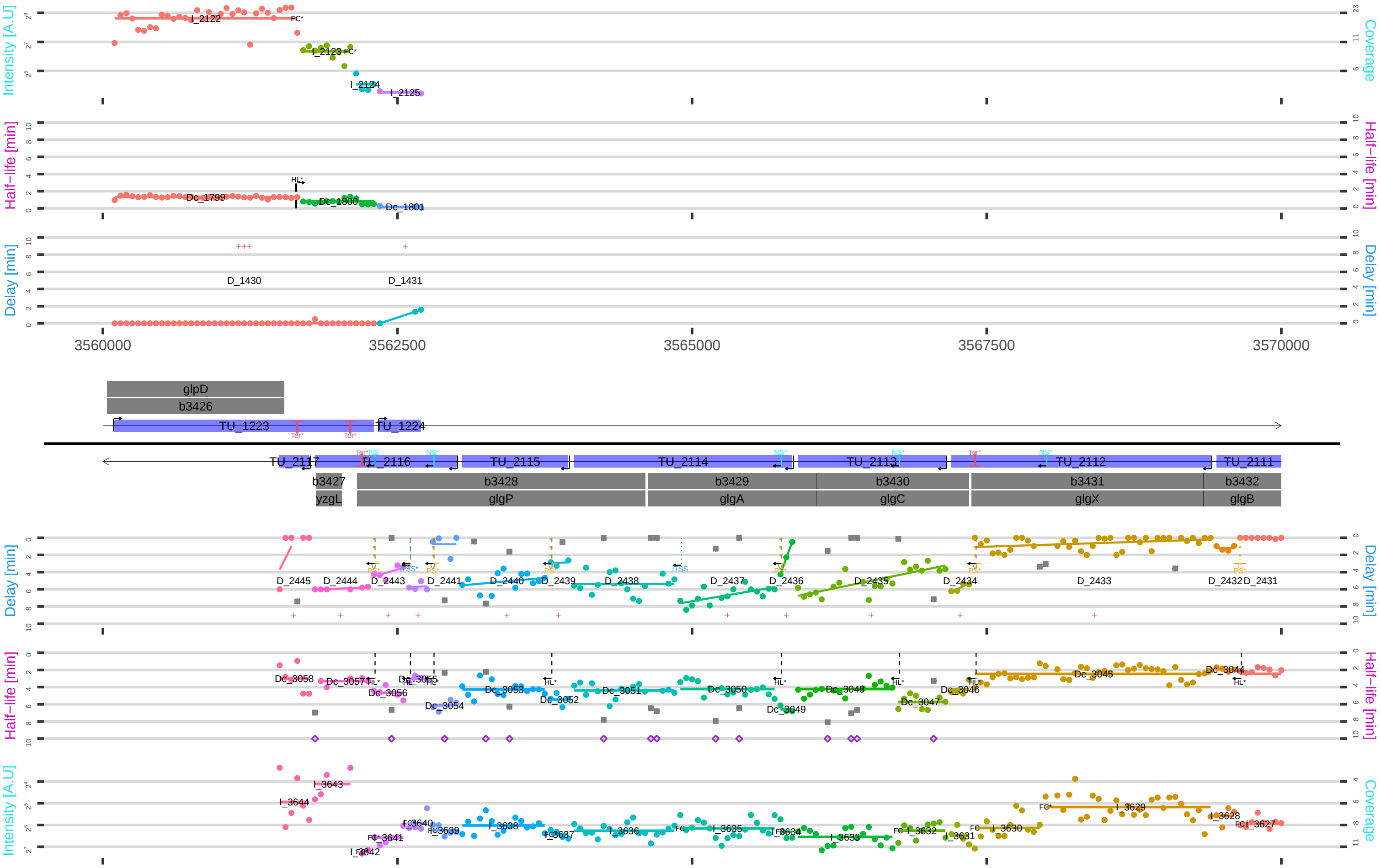

Term: termination (2), NS: new start (5), PS: pausing site (6), iTSS_I: internal starting site (2)

ID: 71455−71593; Term: termination (0), NS: new start (1), PS: pausing site (0), iTSS_I: internal starting site (0)

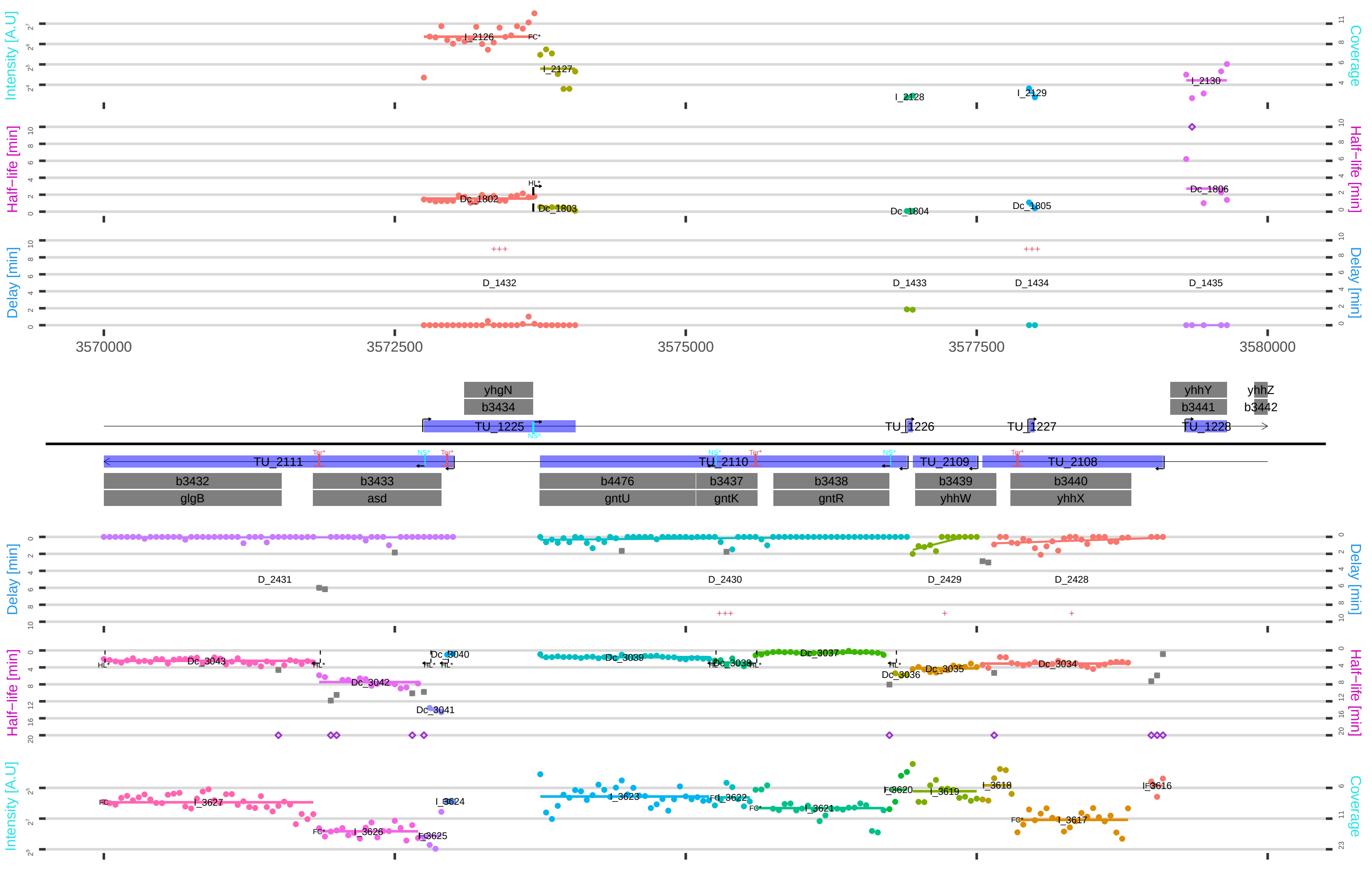

Term: termination (4), NS: new start (3), PS: pausing site (0), iTSS_I: internal starting site (0)

ID: 71631−71736; Term: termination (0), NS: new start (0), PS: pausing site (0), iTSS_I: internal starting site (0)

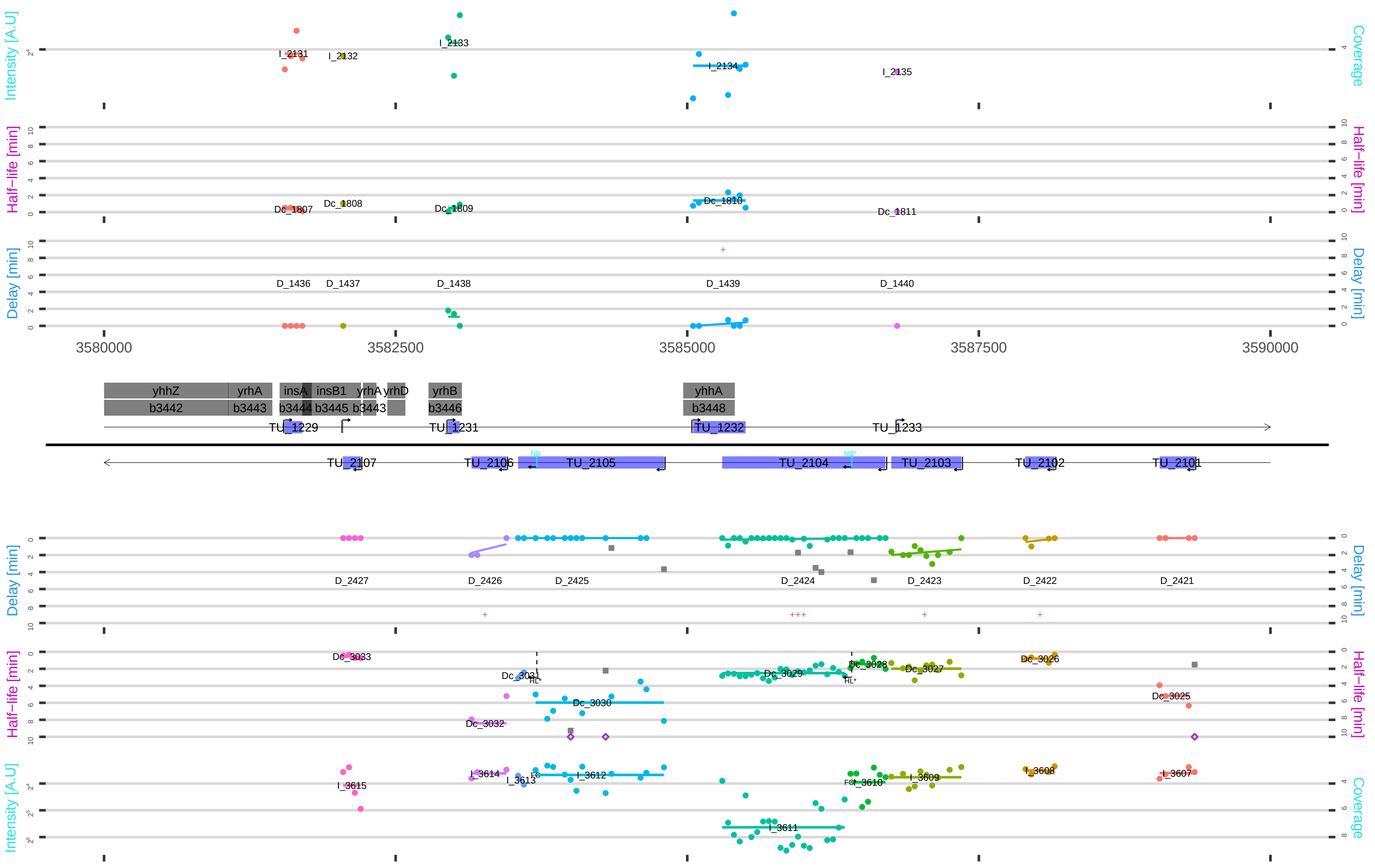

Term: termination (0), NS: new start (2), PS: pausing site (0), iTSS_I: internal starting site (0)

ID: 71801−71961; Term: termination (1), NS: new start (1), PS: pausing site (0), iTSS_I: internal starting site (0)

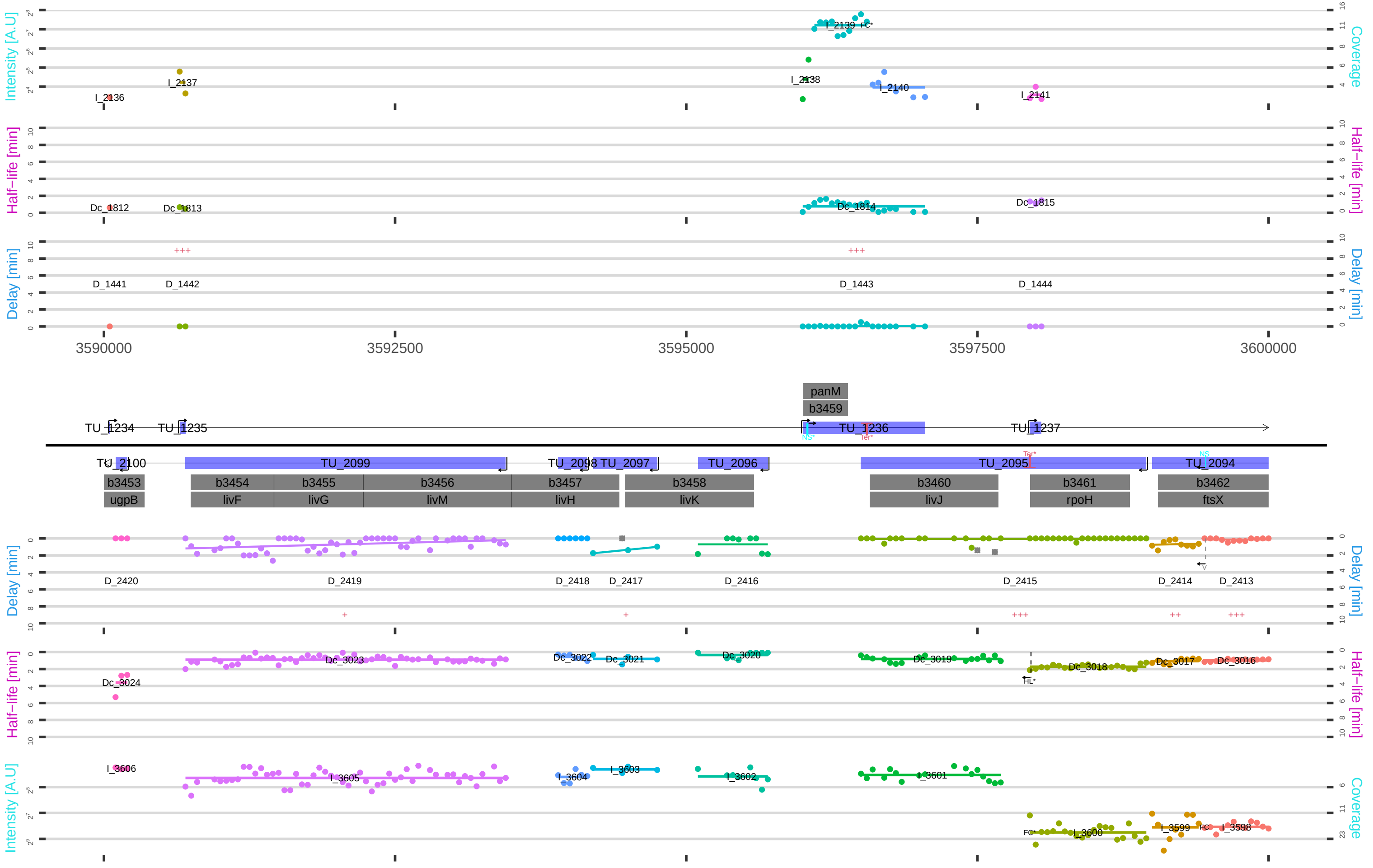

Term: termination (1), NS: new start (1), PS: pausing site (1), iTSS_I: internal starting site (0)

ID: 72048−72176; Term: termination (3), NS: new start (2), PS: pausing site (1), iTSS_I: internal starting site (0)

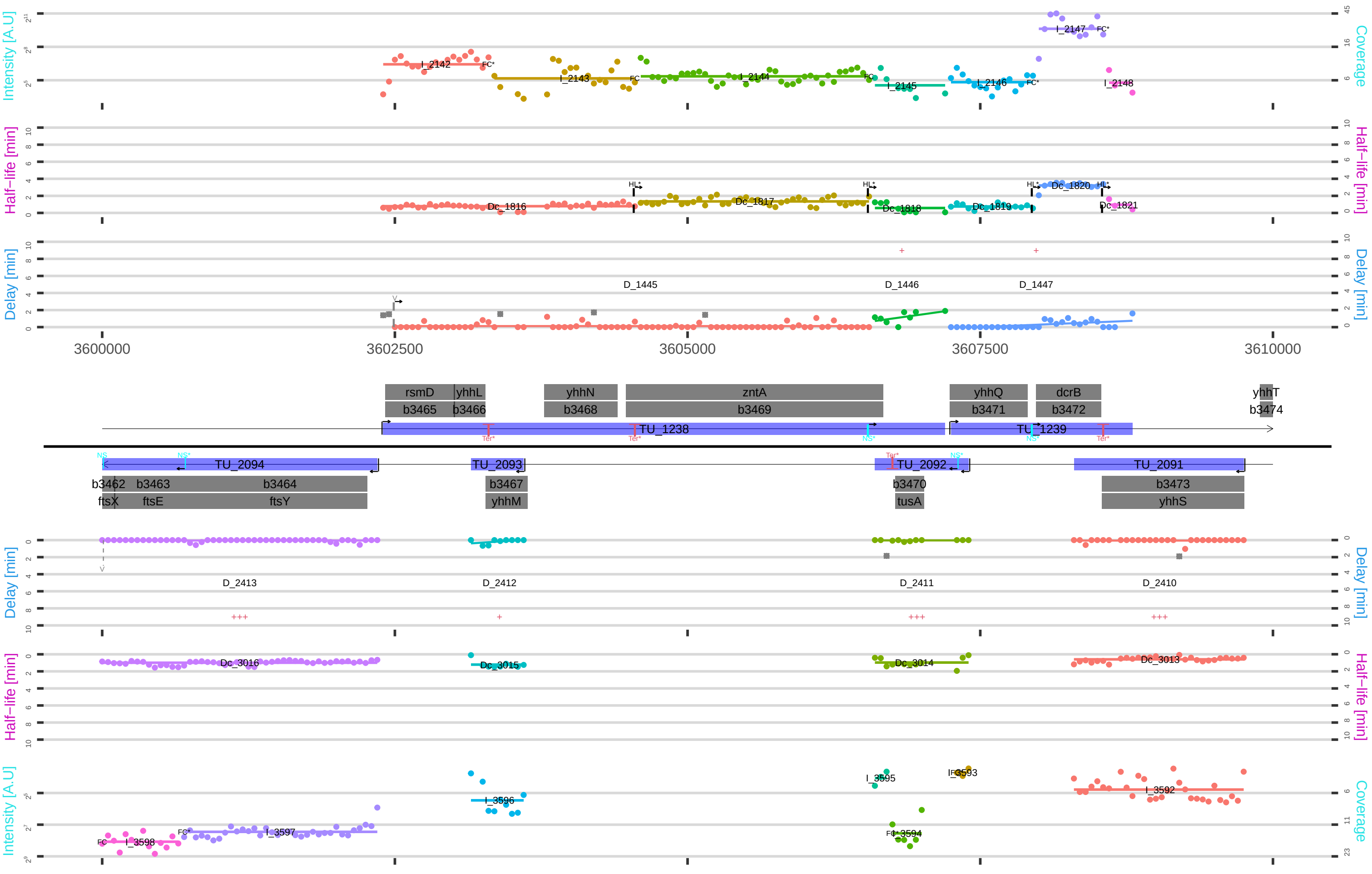

Term: termination (1), NS: new start (3), PS: pausing site (0), iTSS_I: internal starting site (0)

ID: 72214−72382; Term: termination (0), NS: new start (1), PS: pausing site (0), iTSS_I: internal starting site (0)

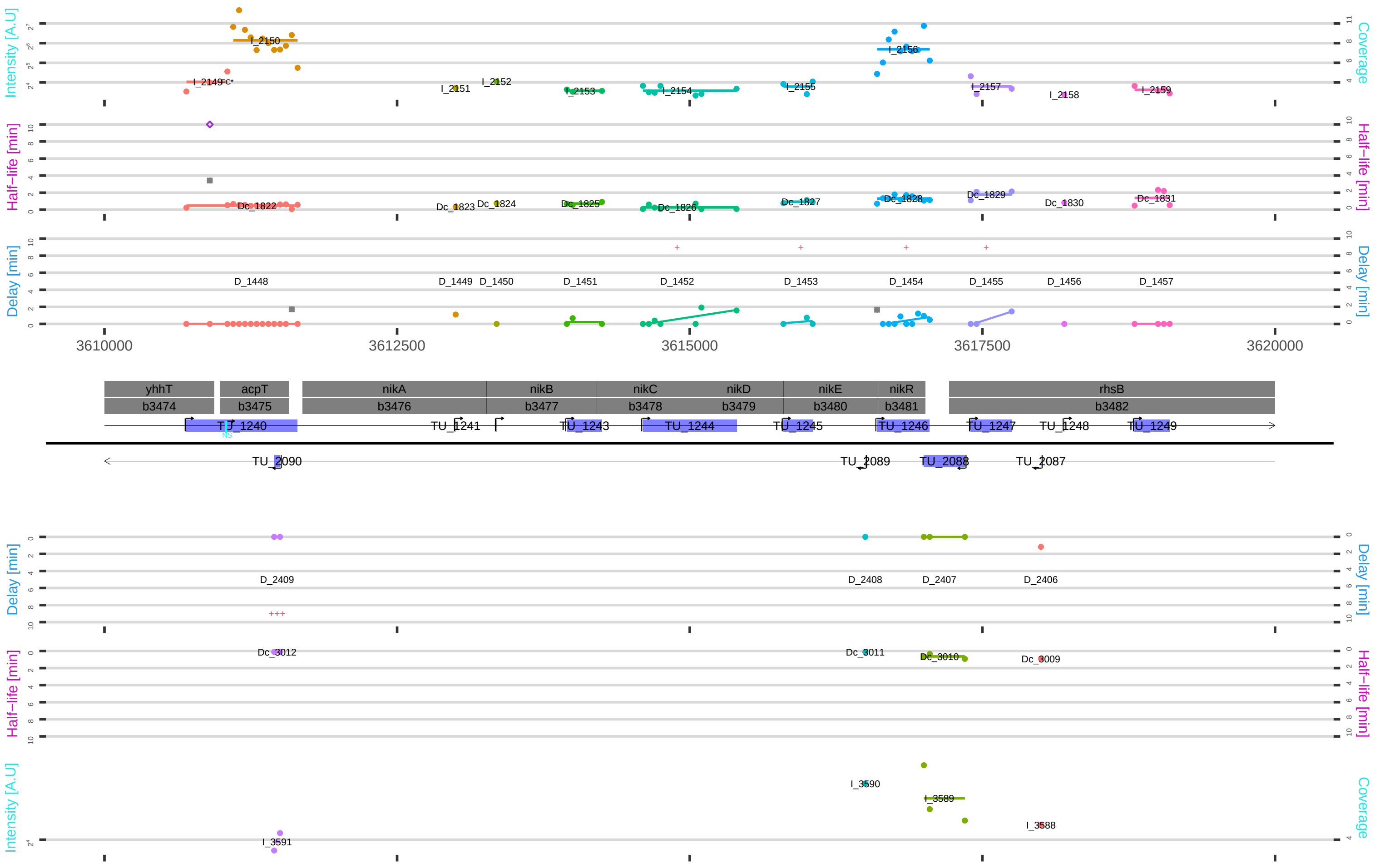

Term: termination (0), NS: new start (0), PS: pausing site (0), iTSS_I: internal starting site (0)

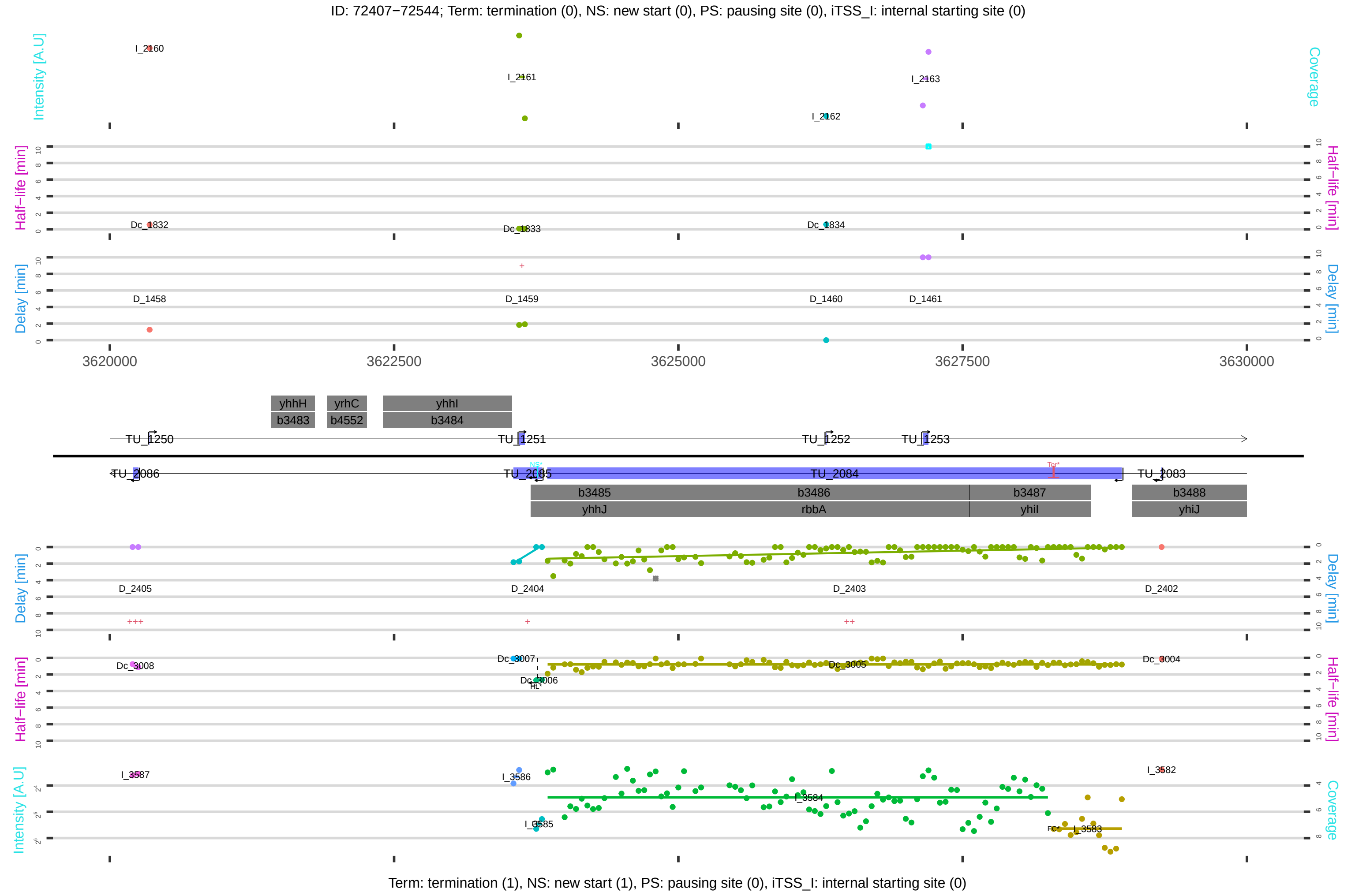
ID: 72407−72544; Term: termination (0), NS: new start (0), PS: pausing site (0), iTSS_I: internal starting site (0)
Intensity [A.U]
Coverage
Half−life [min]
Delay [min]
I_2160
I_2161
I_2162
I_2163
Dc_1832
Dc_1833
Dc_1834
D_1458
D_1459
D_1460
D_1461
3620000
3622500
3625000
3627500
3630000
yhhH
b3483
yrhC
b4552
yhhI
b3484
TU_1250
TU_1251
TU_1252
TU_1253
TU_2086
TU_2085
TU_2084
TU_2083
b3485
yhhJ
b3486
rbbA
b3487
yhiI
b3488
yhiJ
D_2405
D_2404
D_2403
D_2402
Dc_3008
Dc_3007
Dc_3006
Dc_3005
Dc_3004
I_3587
I_3586
I_3585
I_3584
I_3583
I_3582
Term: termination (1), NS: new start (1), PS: pausing site (0), iTSS_I: internal starting site (0)

ID: 72662−72800; Term: termination (2), NS: new start (2), PS: pausing site (1), iTSS_I: internal starting site (0)

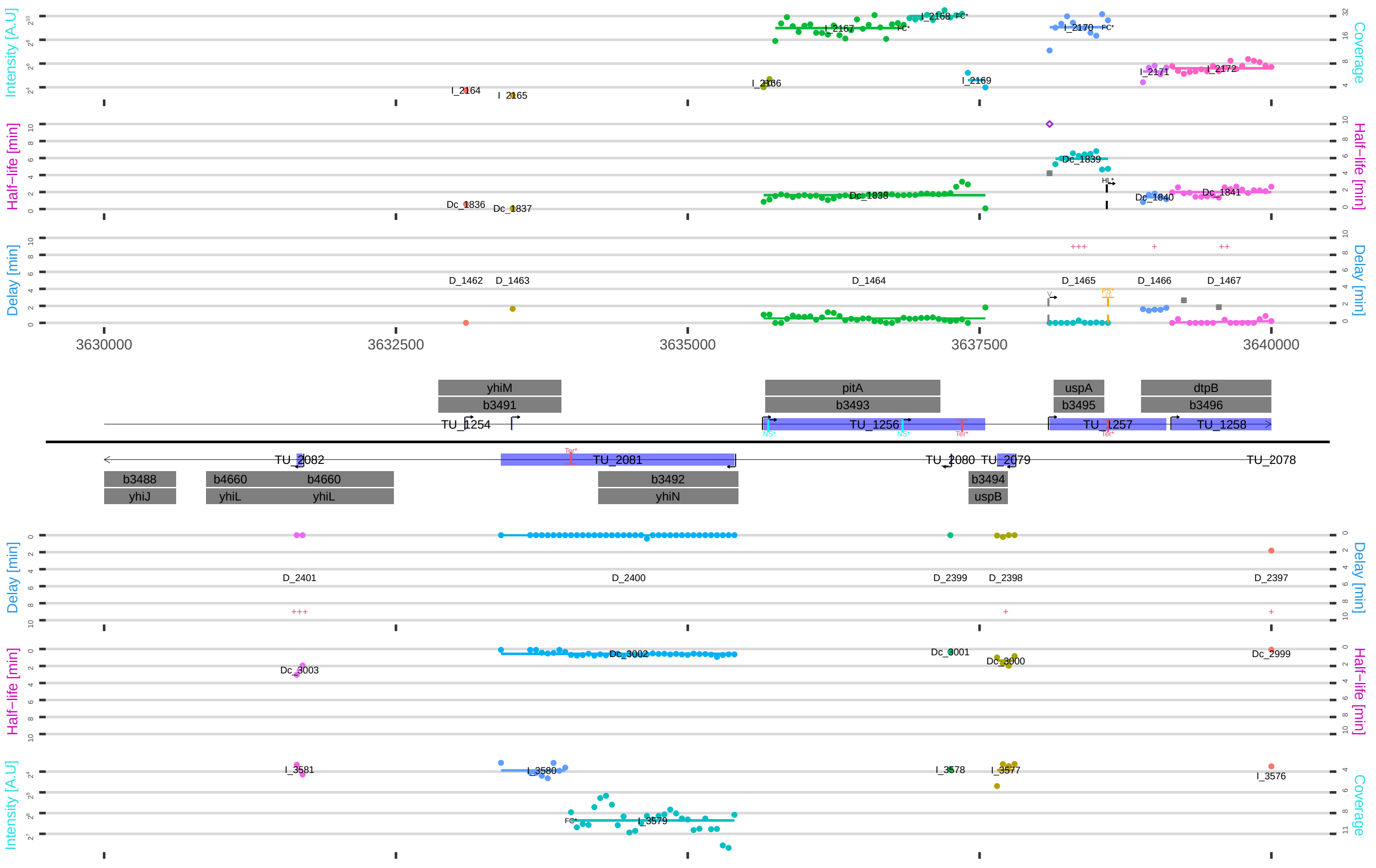

Term: termination (1), NS: new start (0), PS: pausing site (0), iTSS_I: internal starting site (0)

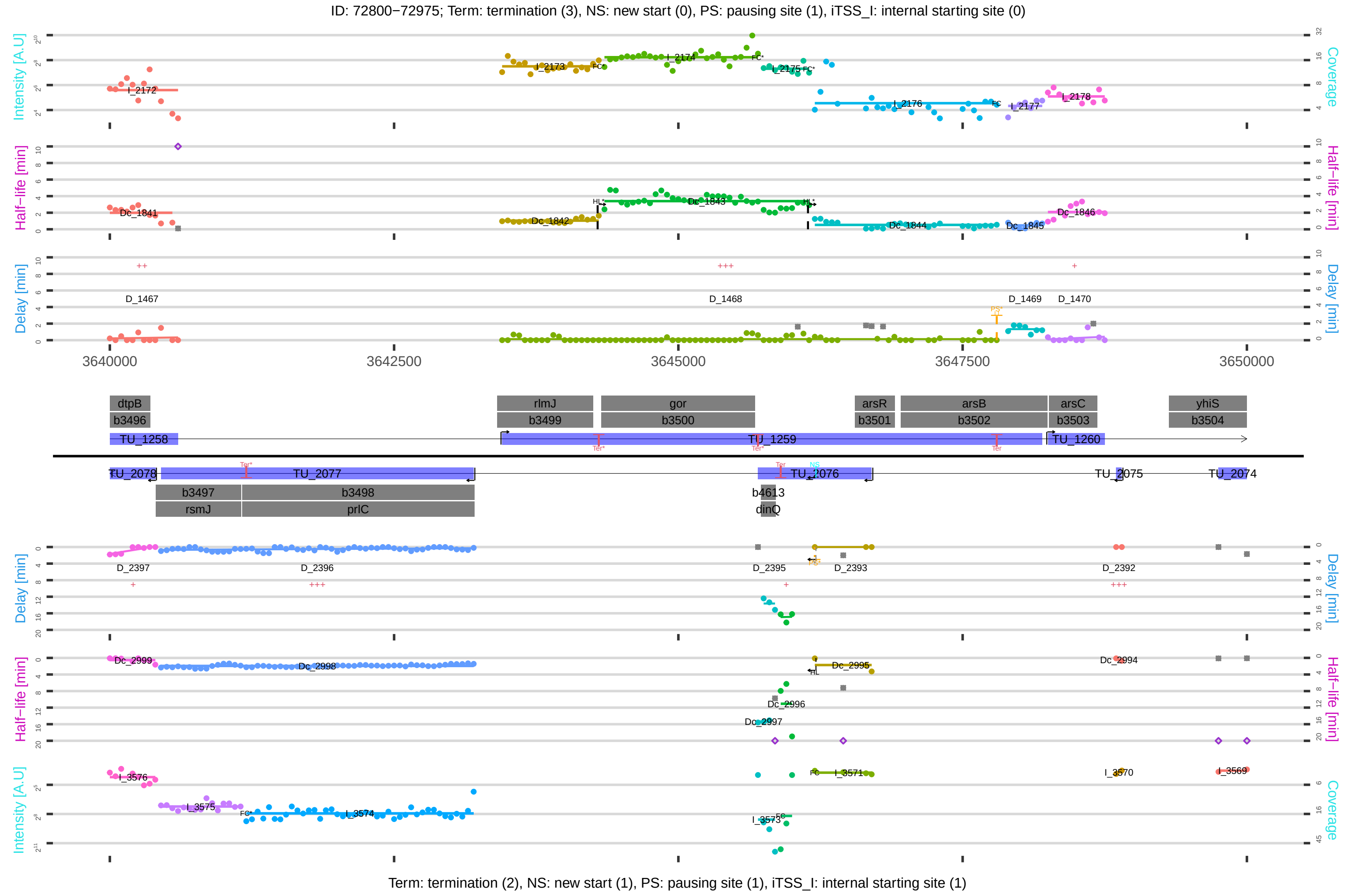

ID: 72800−72975; Term: termination (3), NS: new start (0), PS: pausing site (1), iTSS_I: internal starting site (0)
Intensity [A.U]
Coverage
Half−life [min]
Delay [min]
I_2172
I_2173
I_2174
I_2175
I_2176
I_2177
I_2178
Dc_1841
Dc_1842
Dc_1843
Dc_1844
Dc_1845
Dc_1846
D_1467
D_1468
D_1469
D_1470
3640000
3642500
3645000
3647500
3650000
dtpB
b3496
rlmJ
b3499
gor
b3500
arsR
b3501
arsB
b3502
arsC
b3503
yhiS
b3504
TU_1258
TU_1259
TU_1260
TU_2078
TU_2077
TU_2076
TU_2075
TU_2074
b3497
rsmJ
b3498
prlC
b4613
dinQ
D_2397
D_2396
D_2395
D_2393
D_2392
Dc_2999
Dc_2998
Dc_2995
Dc_2996
Dc_2997
Dc_2994
I_3576
I_3575
I_3574
I_3573
I_3571
I_3570
I_3569
Term: termination (2), NS: new start (1), PS: pausing site (1), iTSS_I: internal starting site (1)

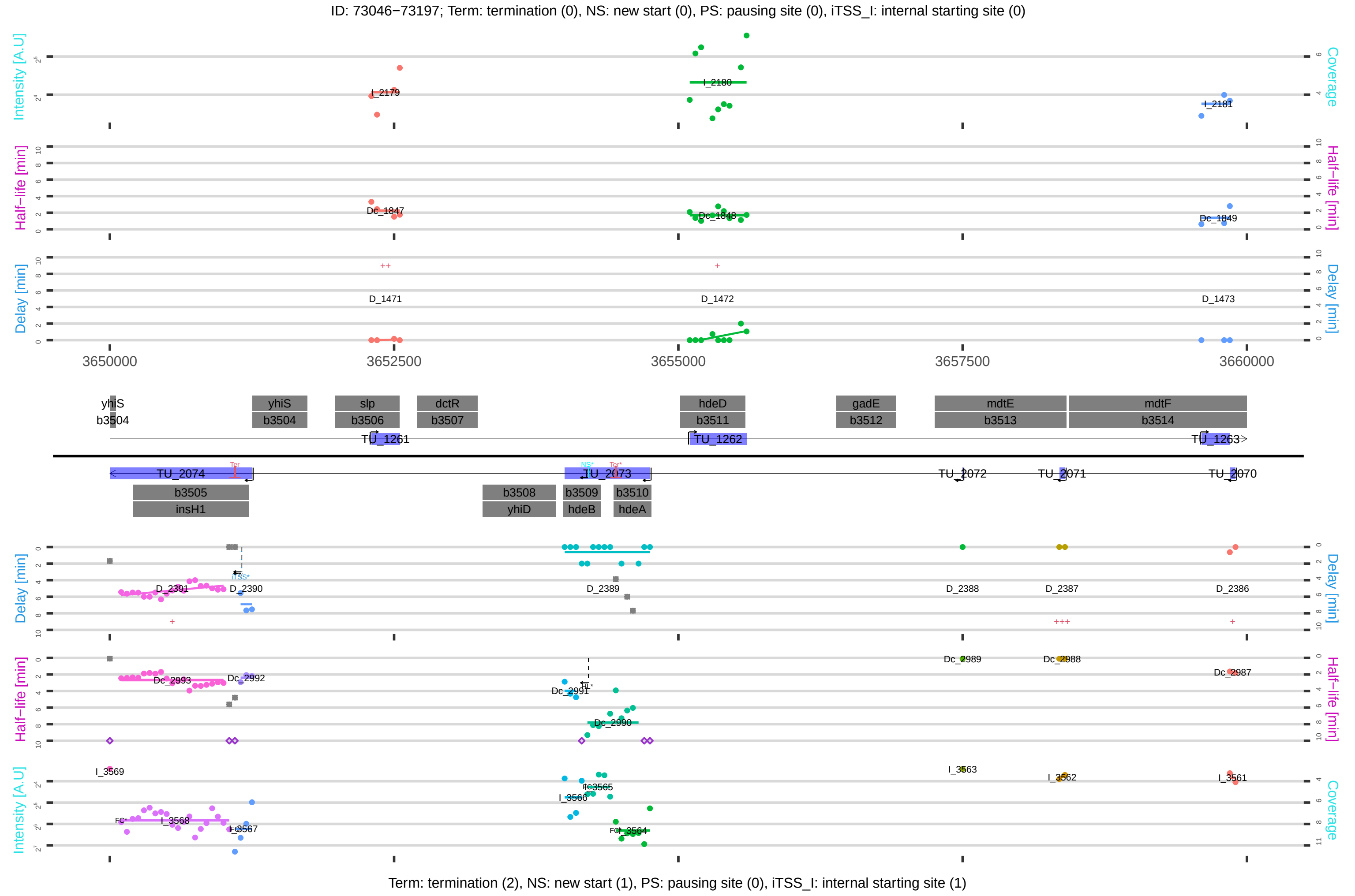

ID: 73046−73197; Term: termination (0), NS: new start (0), PS: pausing site (0), iTSS_I: internal starting site (0)
Intensity [A.U]
Coverage
Half−life [min]
Delay [min]
I_2179
I_2180
I_2181
Dc_1847
Dc_1848
Dc_1849
D_1471
D_1472
D_1473
3650000
3652500
3655000
3657500
3660000
yhiS
b3504
slp
b3506
dctR
b3507
hdeD
b3511
gadE
b3512
mdtE
b3513
mdtF
b3514
TU_1261
TU_1262
TU_1263
TU_2074
TU_2073
TU_2072
TU_2071
TU_2070
b3505
insH1
b3508
yhiD
b3509
hdeB
b3510
hdeA
D_2391
D_2390
D_2389
D_2388
D_2387
D_2386
Dc_2993
Dc_2992
Dc_2991
Dc_2990
Dc_2989
Dc_2988
Dc_2987
I_3569
I_3568
I_3567
I_3566
I_3565
I_3564
I_3563
I_3562
I_3561
Term: termination (2), NS: new start (1), PS: pausing site (0), iTSS_I: internal starting site (1)

ID: 73221−73384; Term: termination (0), NS: new start (0), PS: pausing site (0), iTSS_I: internal starting site (0)

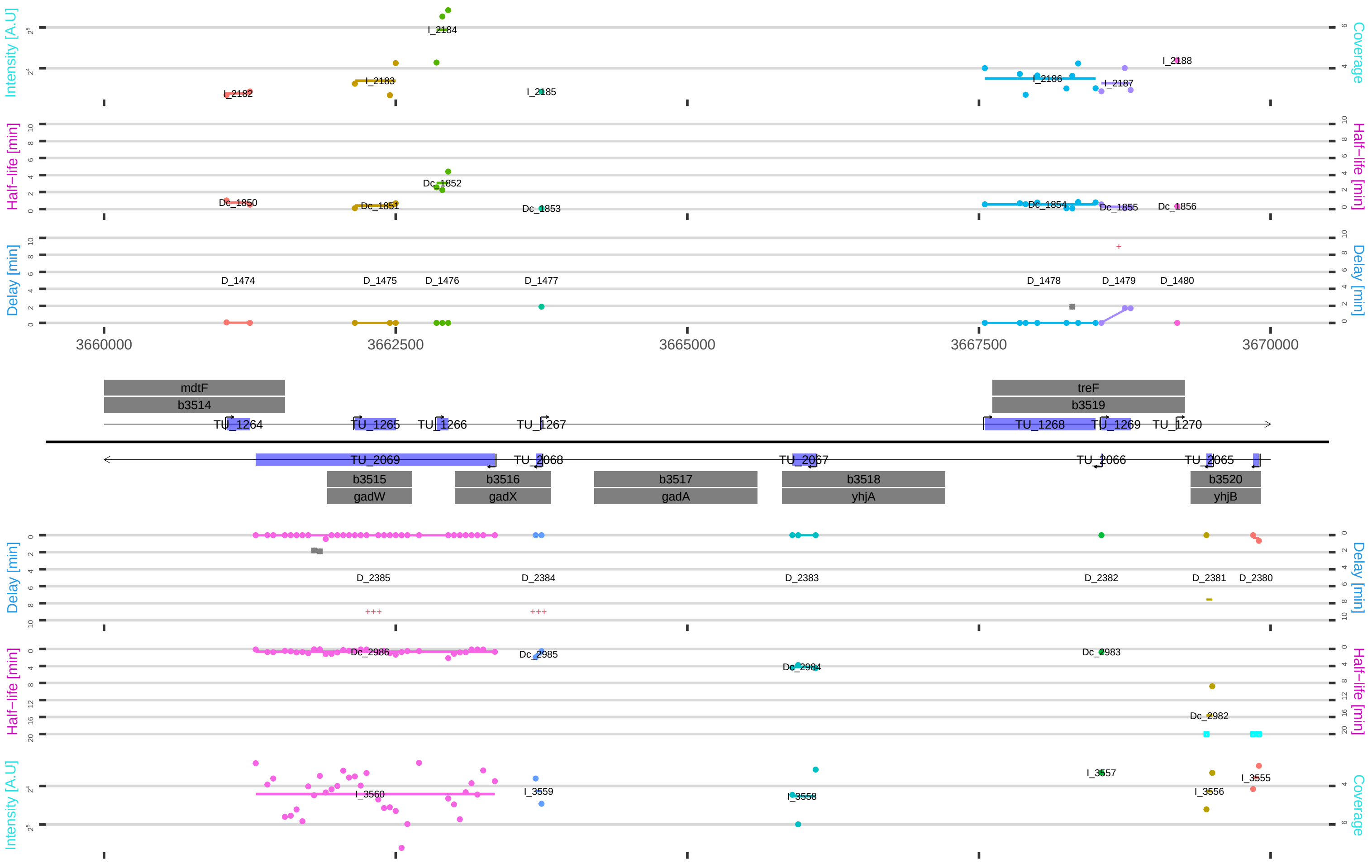

Term: termination (0), NS: new start (0), PS: pausing site (0), iTSS_I: internal starting site (0)

ID: 73410−73573; Term: termination (2), NS: new start (0), PS: pausing site (0), iTSS_I: internal starting site (0)

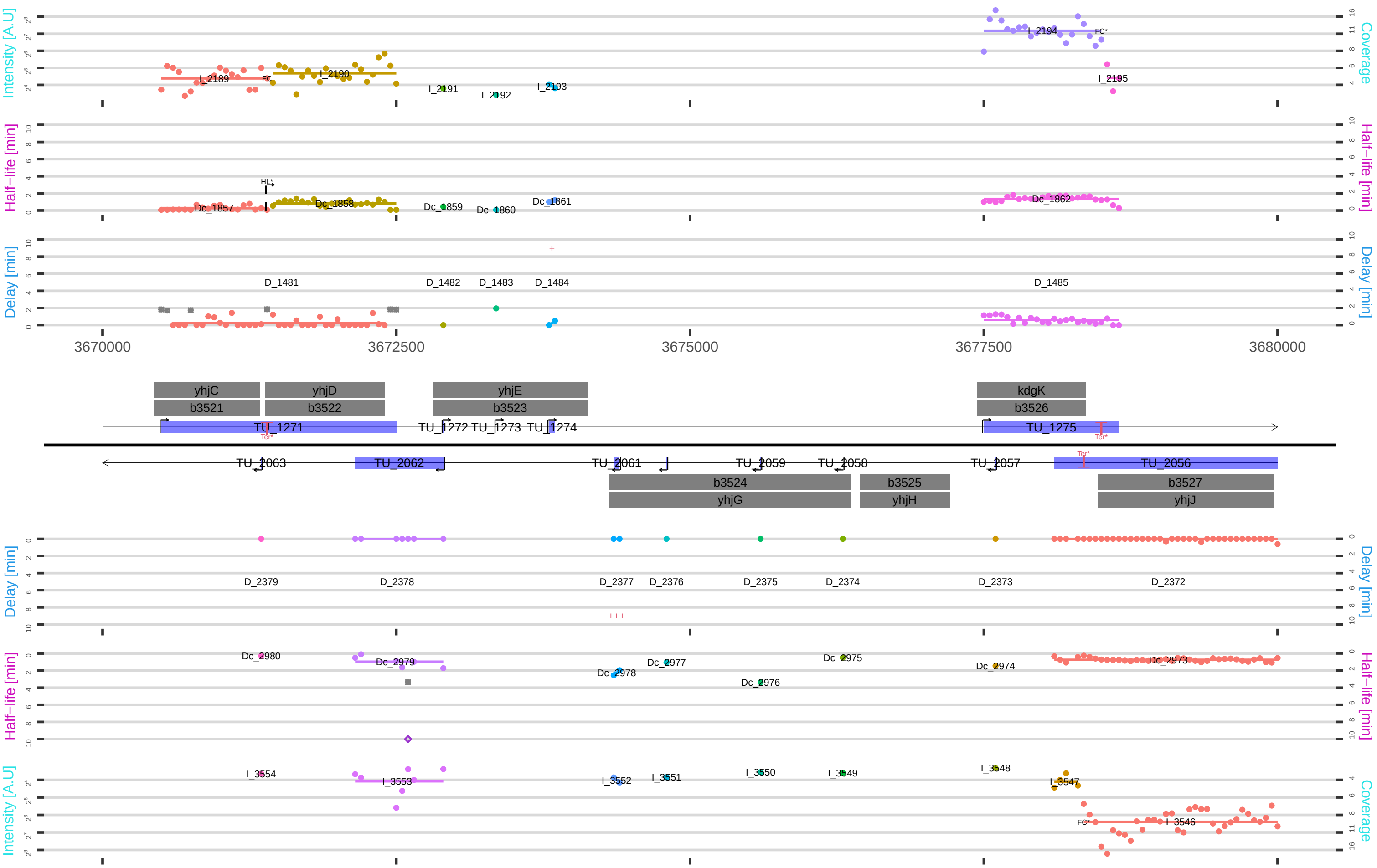

Term: termination (1), NS: new start (0), PS: pausing site (0), iTSS_I: internal starting site (0)

ID: 73666−73666; Term: termination (0), NS: new start (0), PS: pausing site (0), iTSS_I: internal starting site (0)

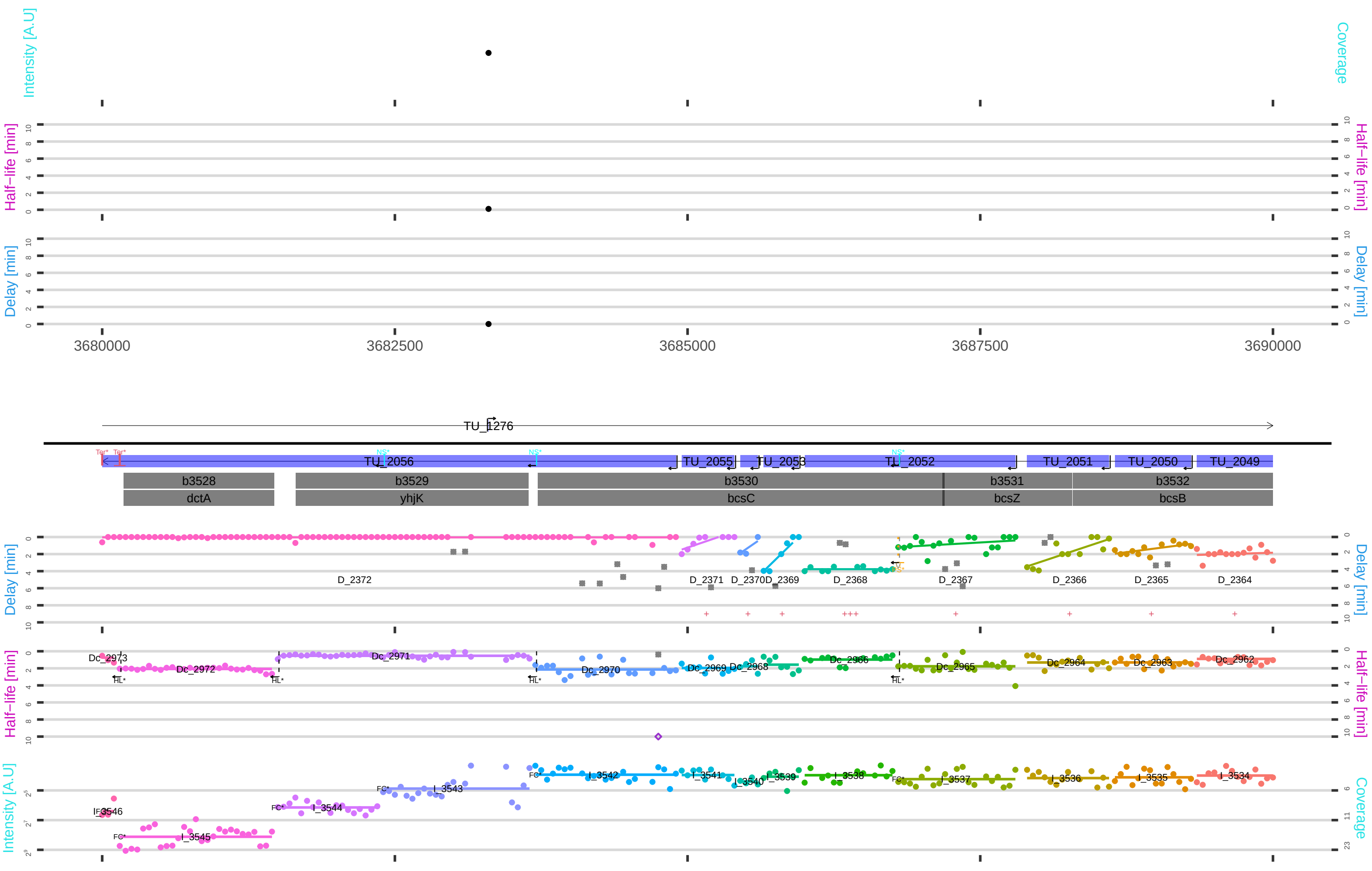

Term: termination (2), NS: new start (3), PS: pausing site (1), iTSS_I: internal starting site (0)

ID: 73891−74000; Term: termination (0), NS: new start (1), PS: pausing site (0), iTSS_I: internal starting site (0)

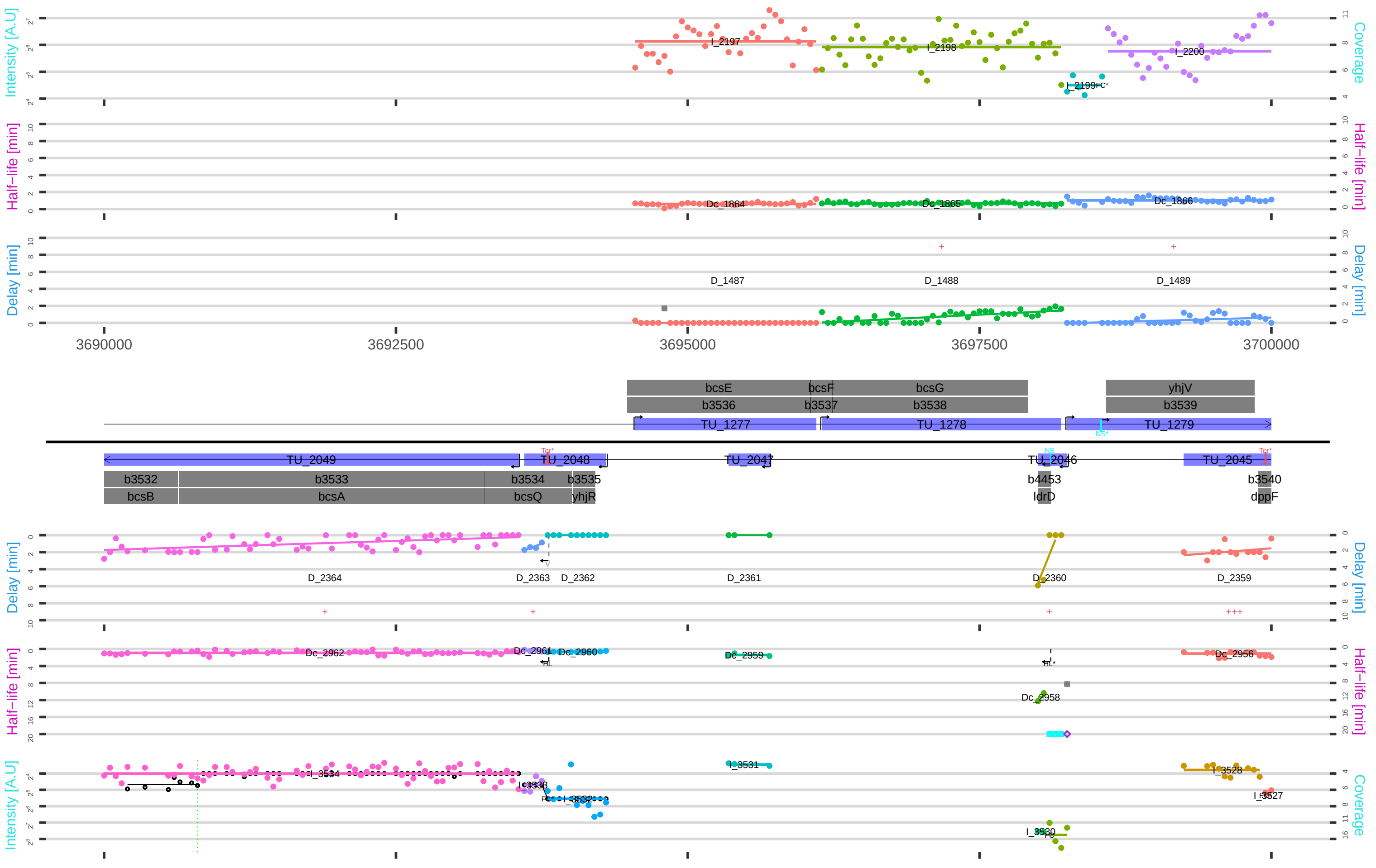

Term: termination (2), NS: new start (1), PS: pausing site (1), iTSS_I: internal starting site (0)

ID: 74000−74175; Term: termination (0), NS: new start (0), PS: pausing site (0), iTSS_I: internal starting site (0)

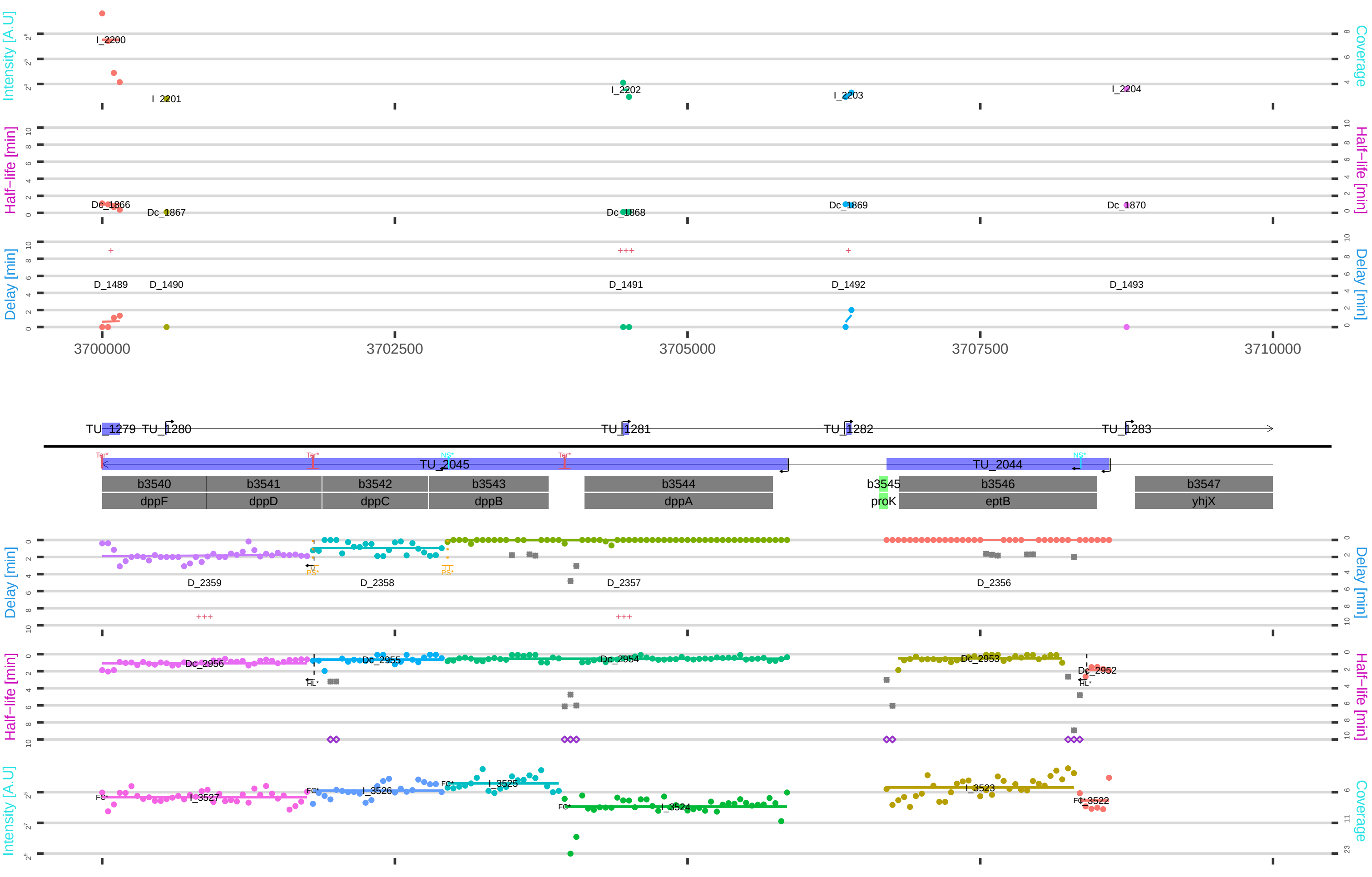

Term: termination (3), NS: new start (2), PS: pausing site (2), iTSS_I: internal starting site (0)

ID: 74209−74400; Term: termination (2), NS: new start (2), PS: pausing site (0), iTSS_I: internal starting site (0)

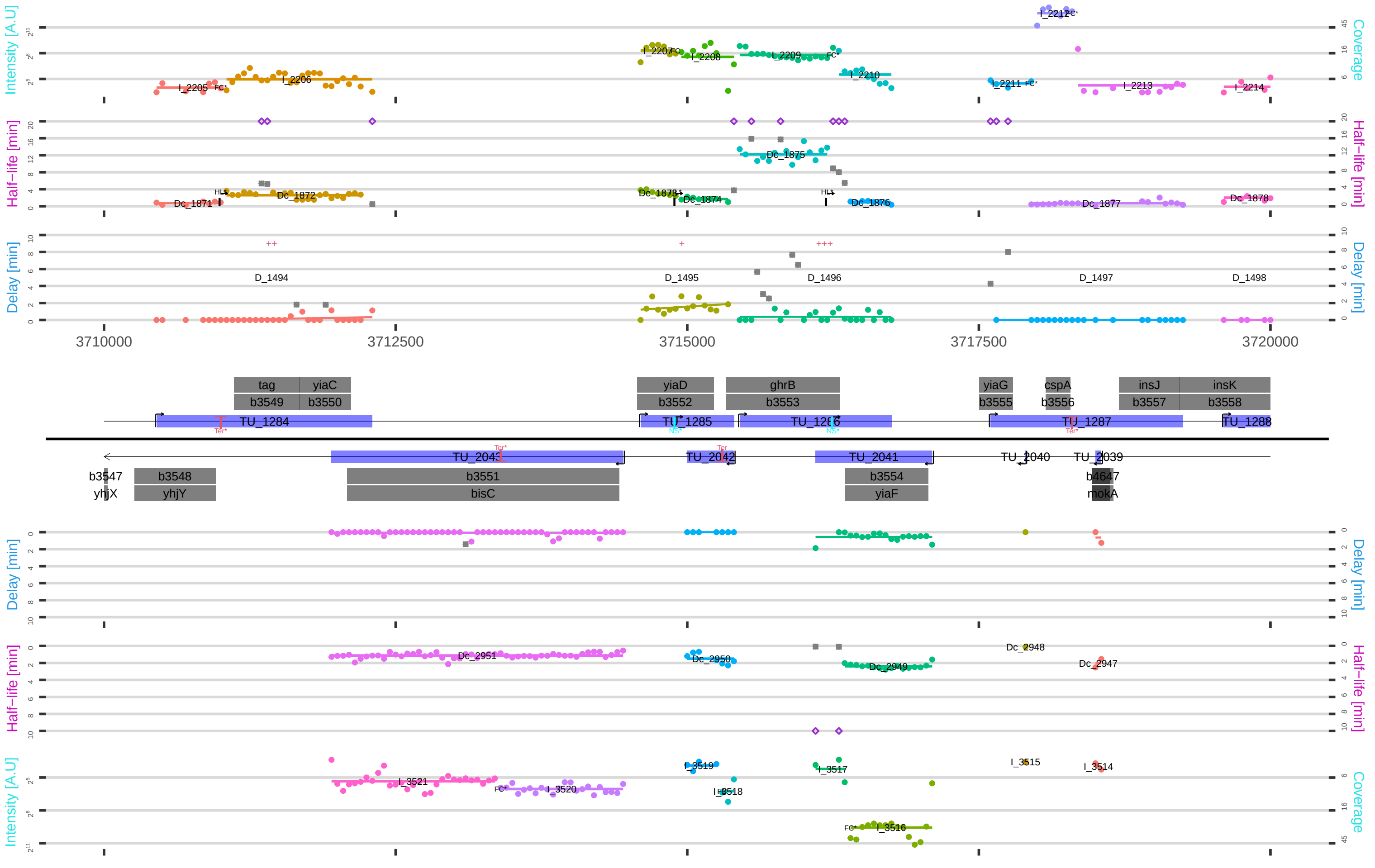

Term: termination (2), NS: new start (0), PS: pausing site (0), iTSS_I: internal starting site (0)

ID: 74400−74545; Term: termination (0), NS: new start (0), PS: pausing site (0), iTSS_I: internal starting site (0)

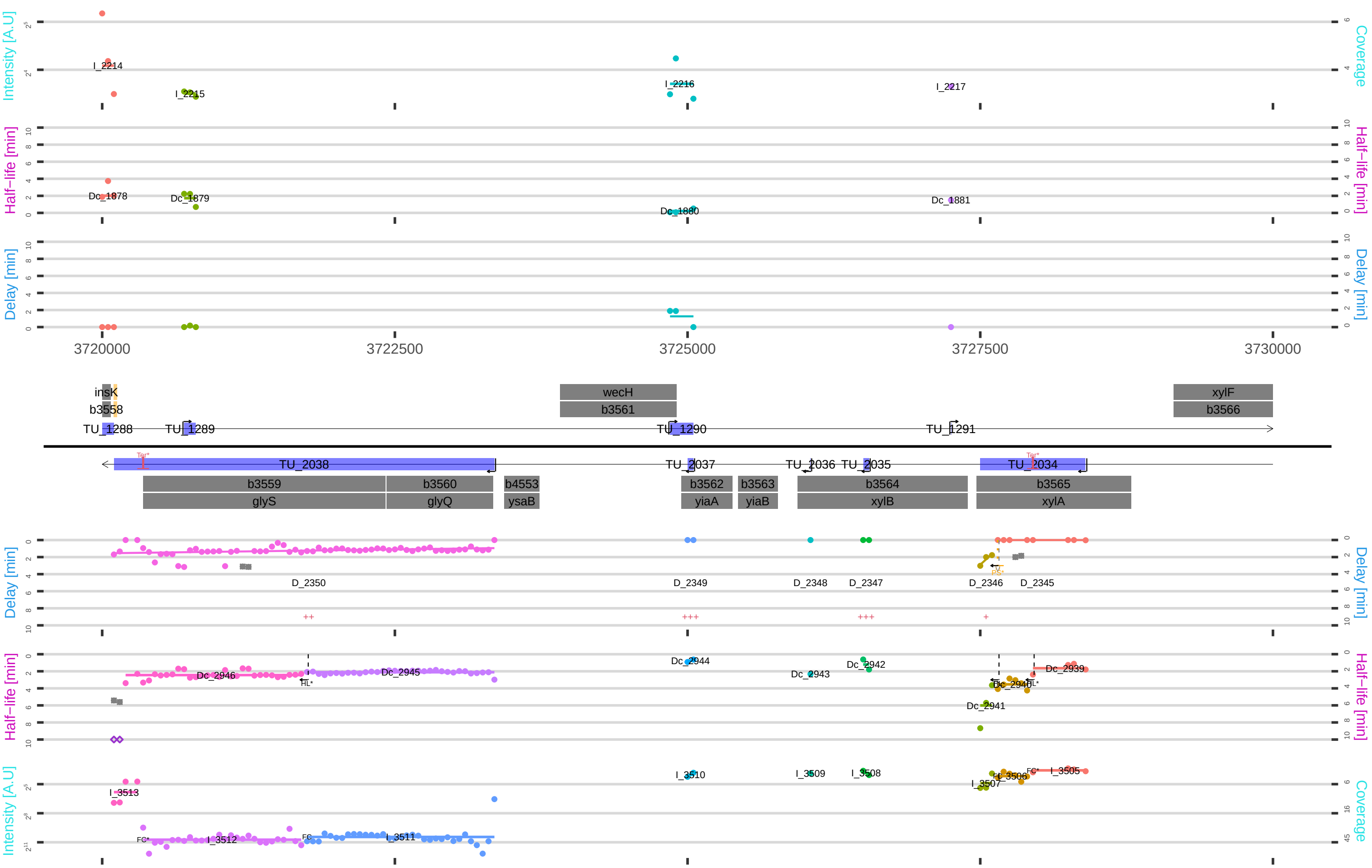

Term: termination (2), NS: new start (0), PS: pausing site (1), iTSS_I: internal starting site (0)

ID: 74610−74787; Term: termination (0), NS: new start (2), PS: pausing site (1), iTSS_I: internal starting site (0)

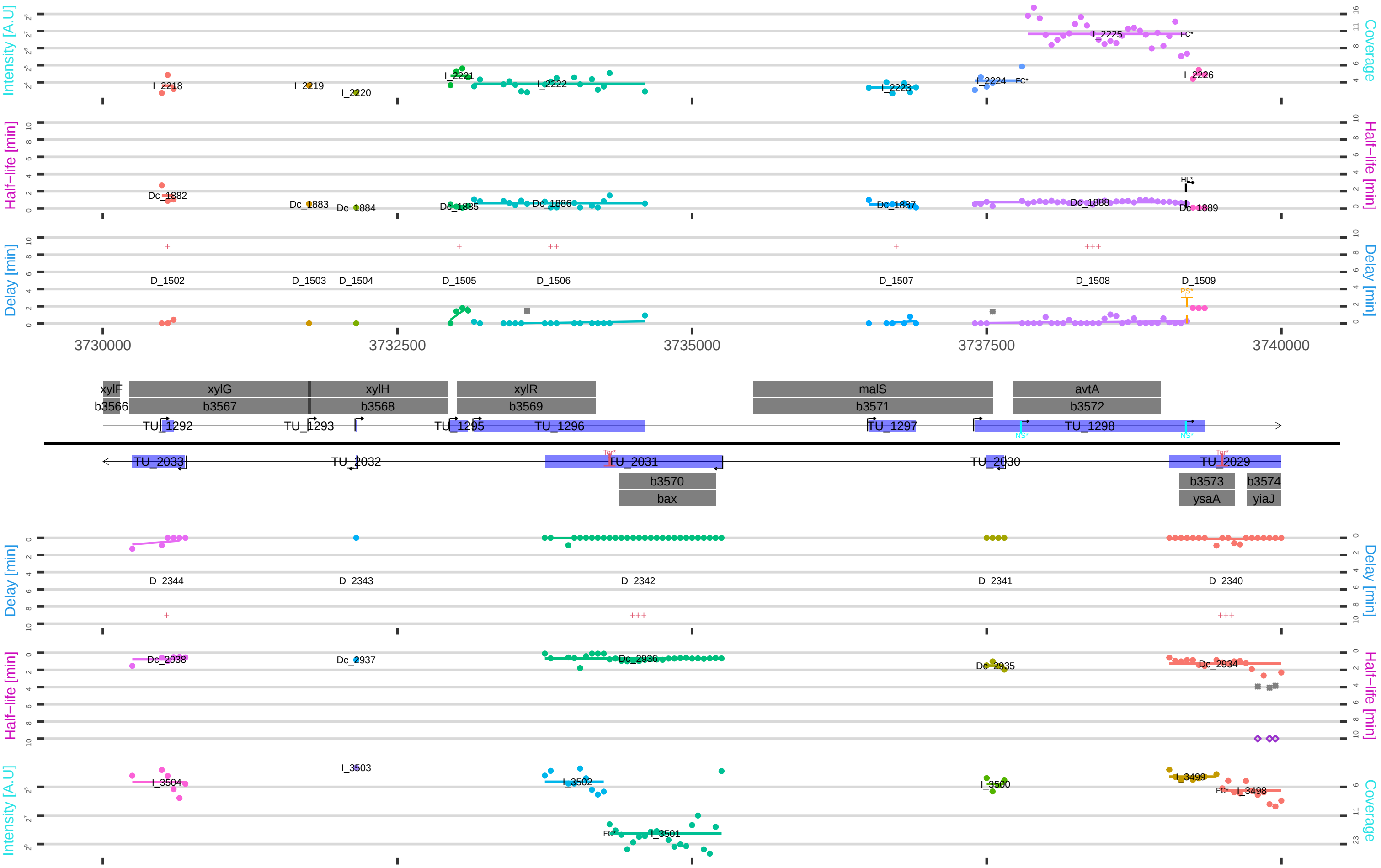

Term: termination (2), NS: new start (0), PS: pausing site (0), iTSS_I: internal starting site (0)

ID: 74867−74976; Term: termination (0), NS: new start (0), PS: pausing site (0), iTSS_I: internal starting site (0)

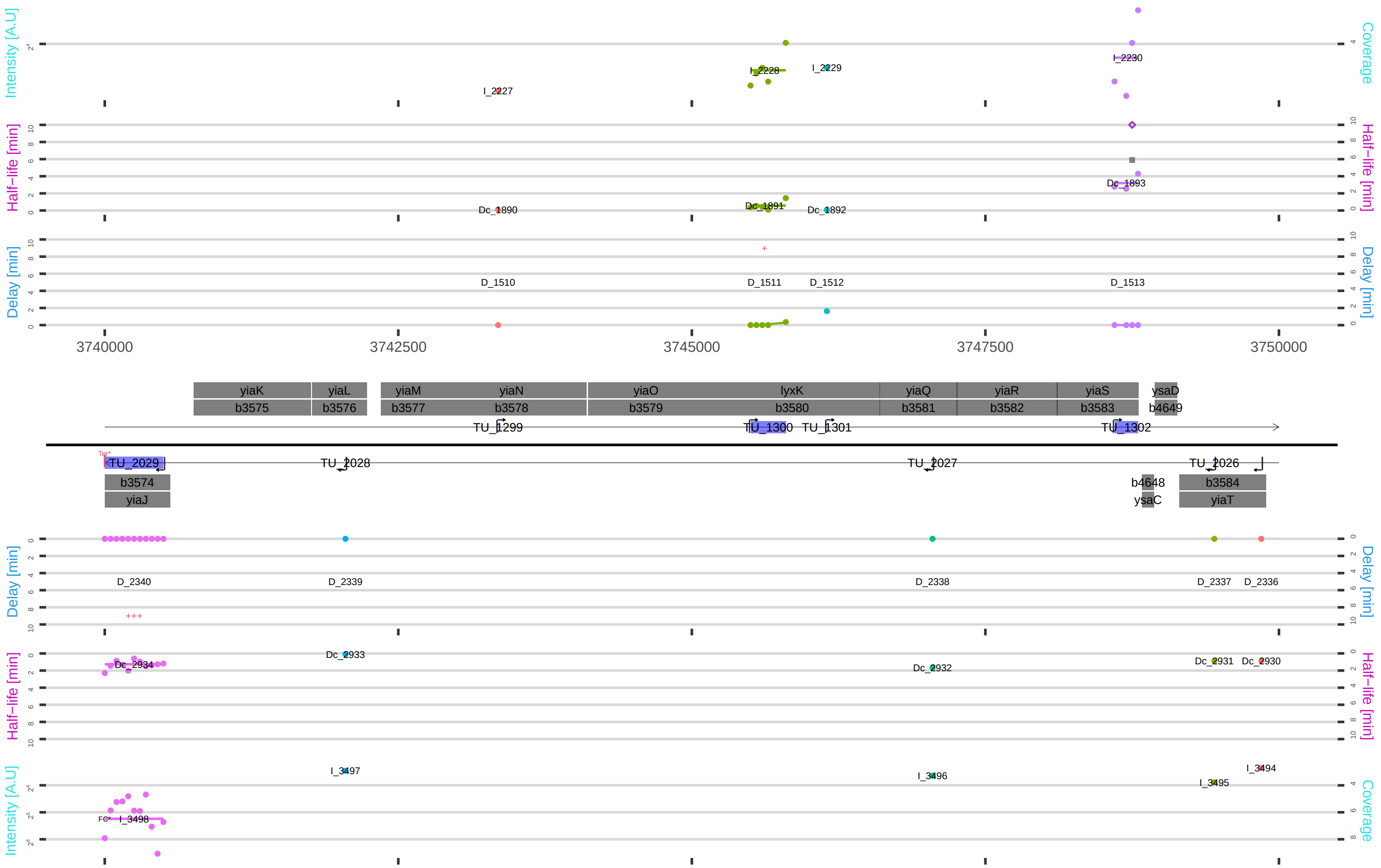

Term: termination (1), NS: new start (0), PS: pausing site (0), iTSS_I: internal starting site (0)

ID: 75004−75118; Term: termination (1), NS: new start (0), PS: pausing site (0), iTSS_I: internal starting site (1)

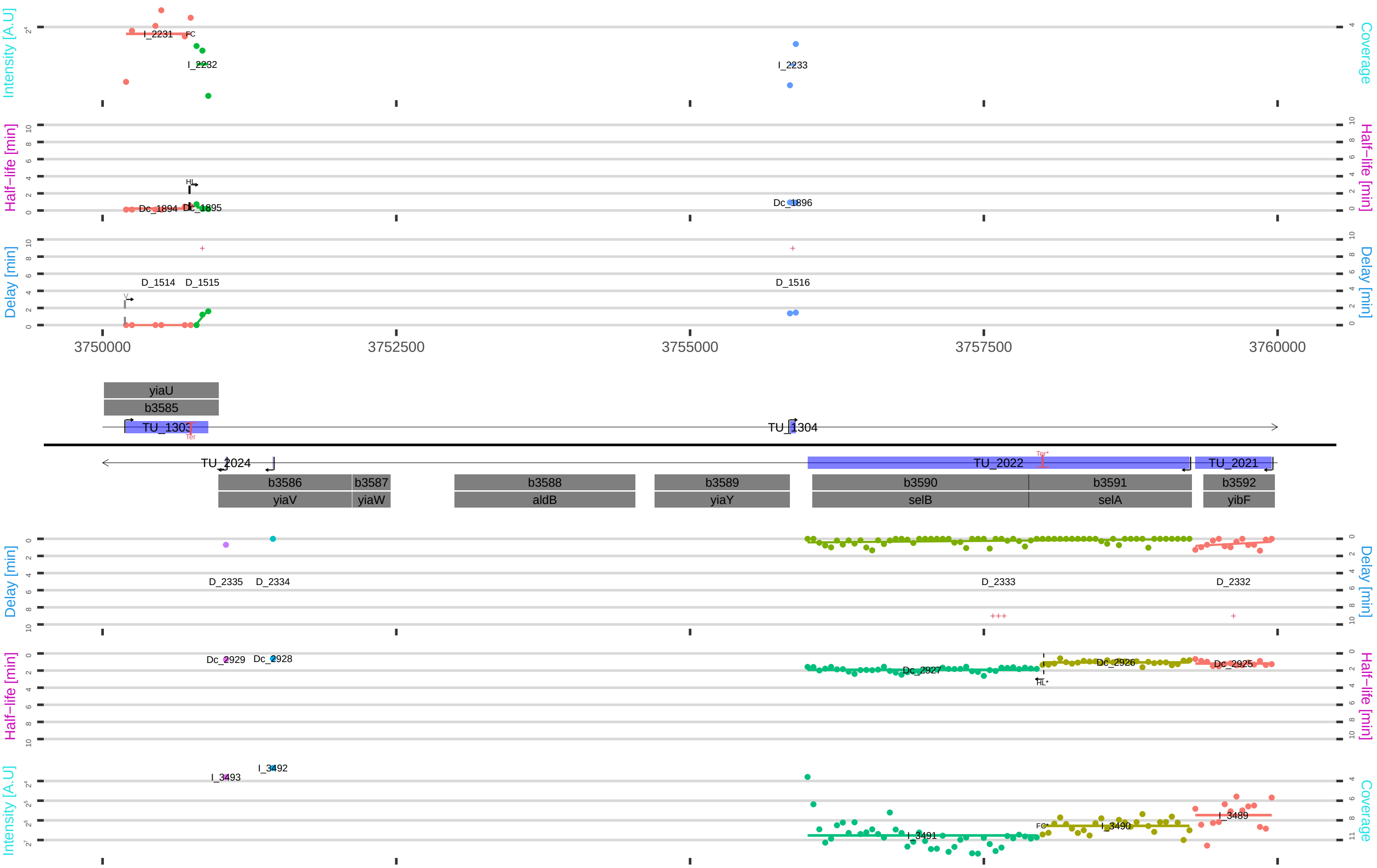

Term: termination (1), NS: new start (0), PS: pausing site (0), iTSS_I: internal starting site (0)

ID: 75203−75343; Term: termination (0), NS: new start (0), PS: pausing site (0), iTSS_I: internal starting site (0)

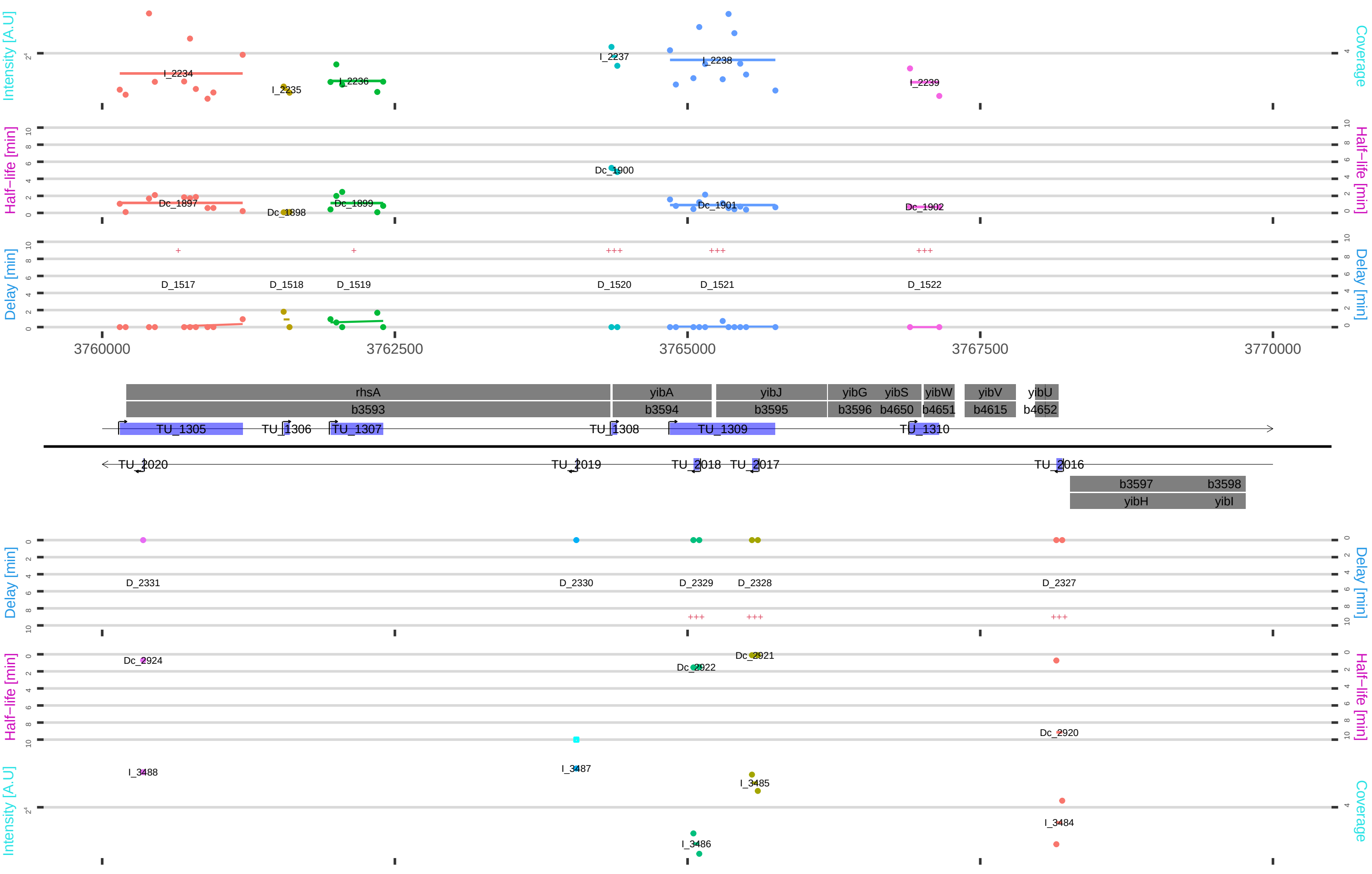

Term: termination (0), NS: new start (0), PS: pausing site (0), iTSS_I: internal starting site (0)

ID: 75406−75598; Term: termination (4), NS: new start (2), PS: pausing site (0), iTSS_I: internal starting site (0)

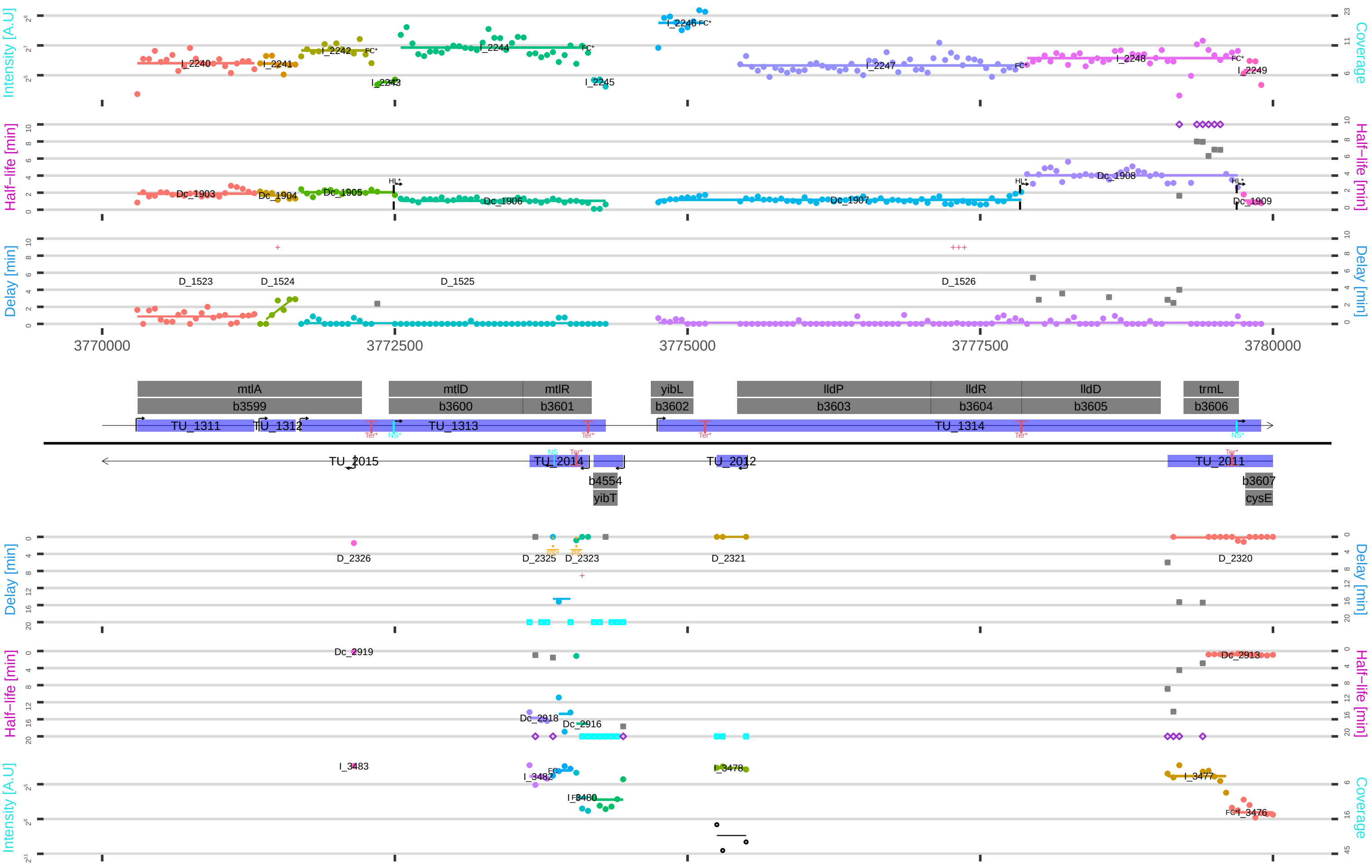

Term: termination (2), NS: new start (1), PS: pausing site (2), iTSS_I: internal starting site (0)

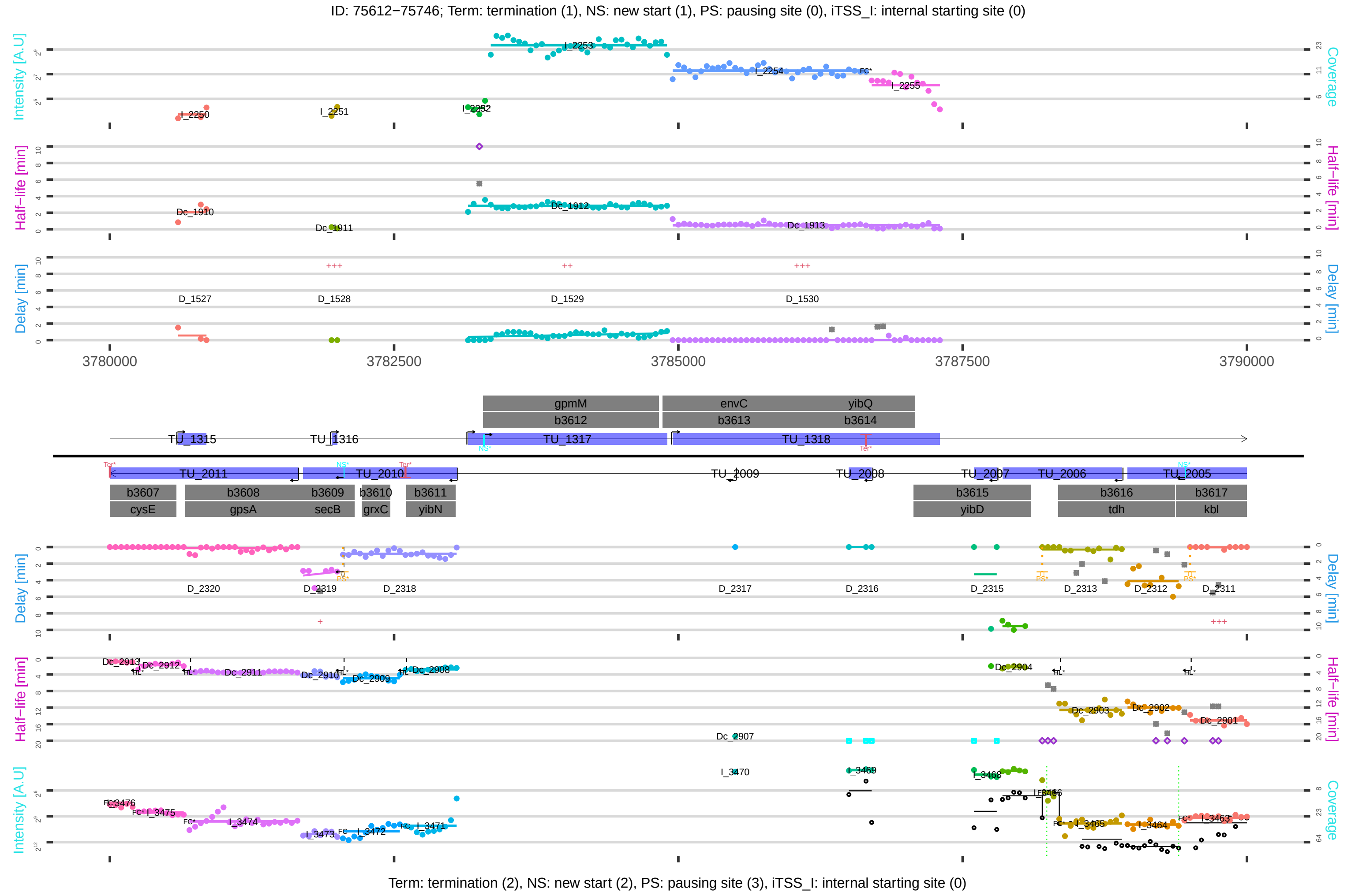
ID: 75612−75746; Term: termination (1), NS: new start (1), PS: pausing site (0), iTSS_I: internal starting site (0)
Intensity [A.U]
Coverage
Half−life [min]
Delay [min]
I_2250
I_2251
I_2252
I_2253
I_2254
I_2255
Dc_1910
Dc_1911
Dc_1912
Dc_1913
D_1527
D_1528
D_1529
D_1530
3780000
3782500
3785000
3787500
3790000
gpmM
b3612
envC
b3613
yibQ
b3614
TU_1315
TU_1316
TU_1317
TU_1318
TU_2011
TU_2010
TU_2009
TU_2008
TU_2007
TU_2006
TU_2005
b3607
cysE
b3608
gpsA
b3609
secB
b3610
grxC
b3611
yibN
b3615
yibD
b3616
tdh
b3617
kbl
D_2320
D_2319
D_2318
D_2317
D_2316
D_2315
D_2313
D_2312
D_2311
Dc_2913
Dc_2912
Dc_2911
Dc_2910
Dc_2909
Dc_2908
Dc_2907
Dc_2904
Dc_2903
Dc_2902
Dc_2901
I_3476
I_3475
I_3474
I_3473
I_3472
I_3471
I_3470
I_3469
I_3468
I_3466
I_3465
I_3464
I_3463
Term: termination (2), NS: new start (2), PS: pausing site (3), iTSS_I: internal starting site (0)

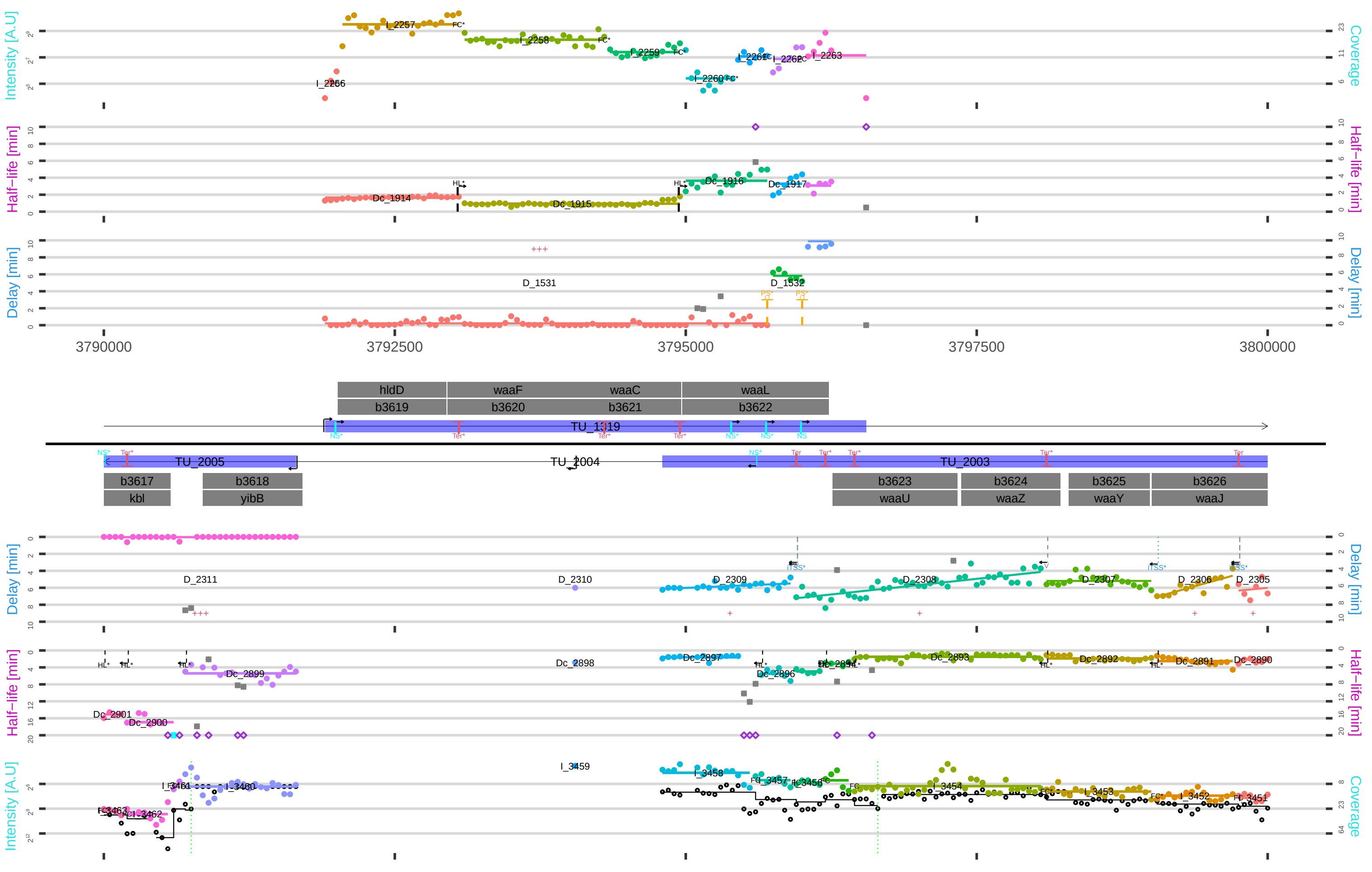

Term: termination (6), NS: new start (2), PS: pausing site (0), iTSS_I: internal starting site (4)

ID: 76129−76189; Term: termination (1), NS: new start (2), PS: pausing site (0), iTSS_I: internal starting site (0)

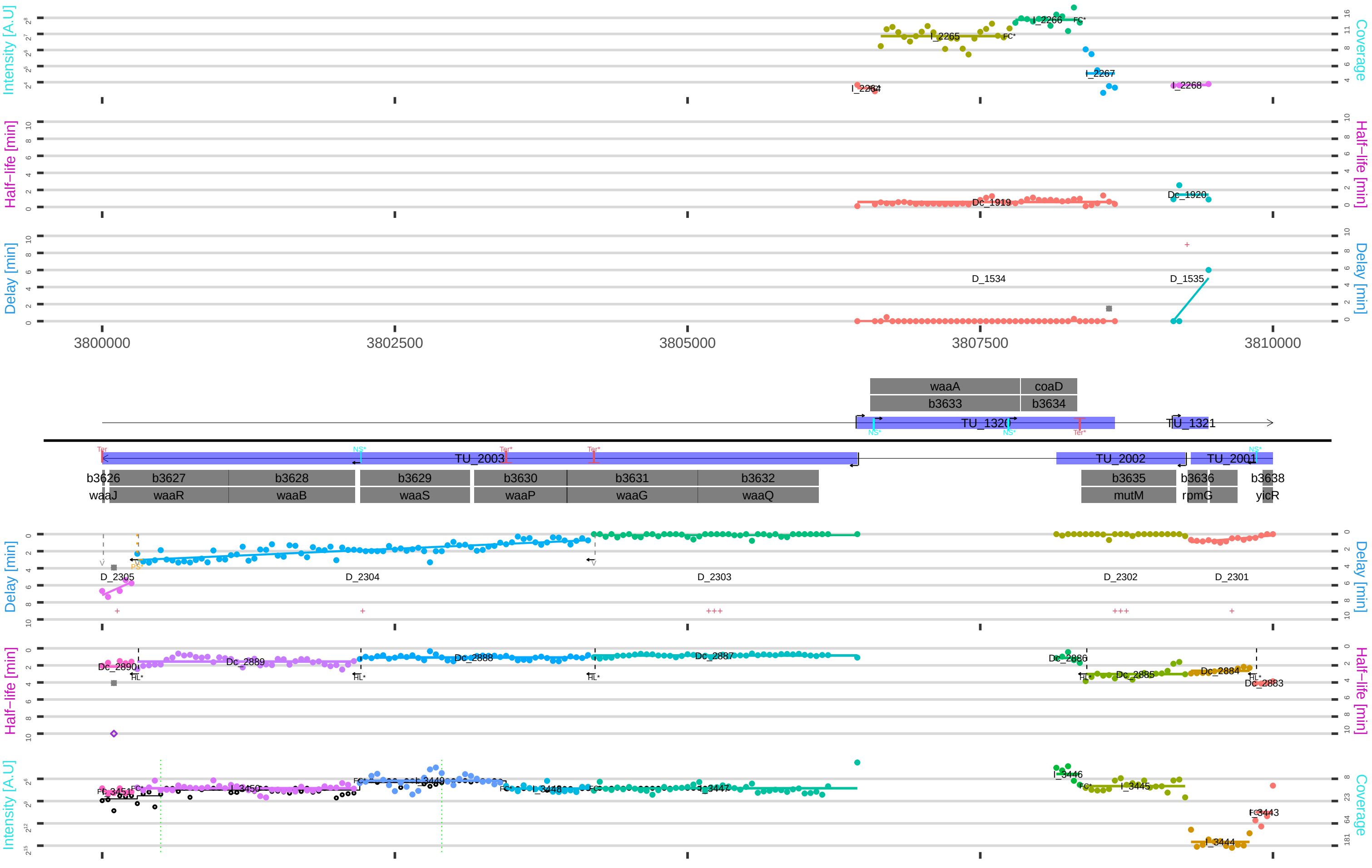

Term: termination (3), NS: new start (2), PS: pausing site (2), iTSS_I: internal starting site (0)

ID: 76216−76400; Term: termination (2), NS: new start (2), PS: pausing site (1), iTSS_I: internal starting site (0)

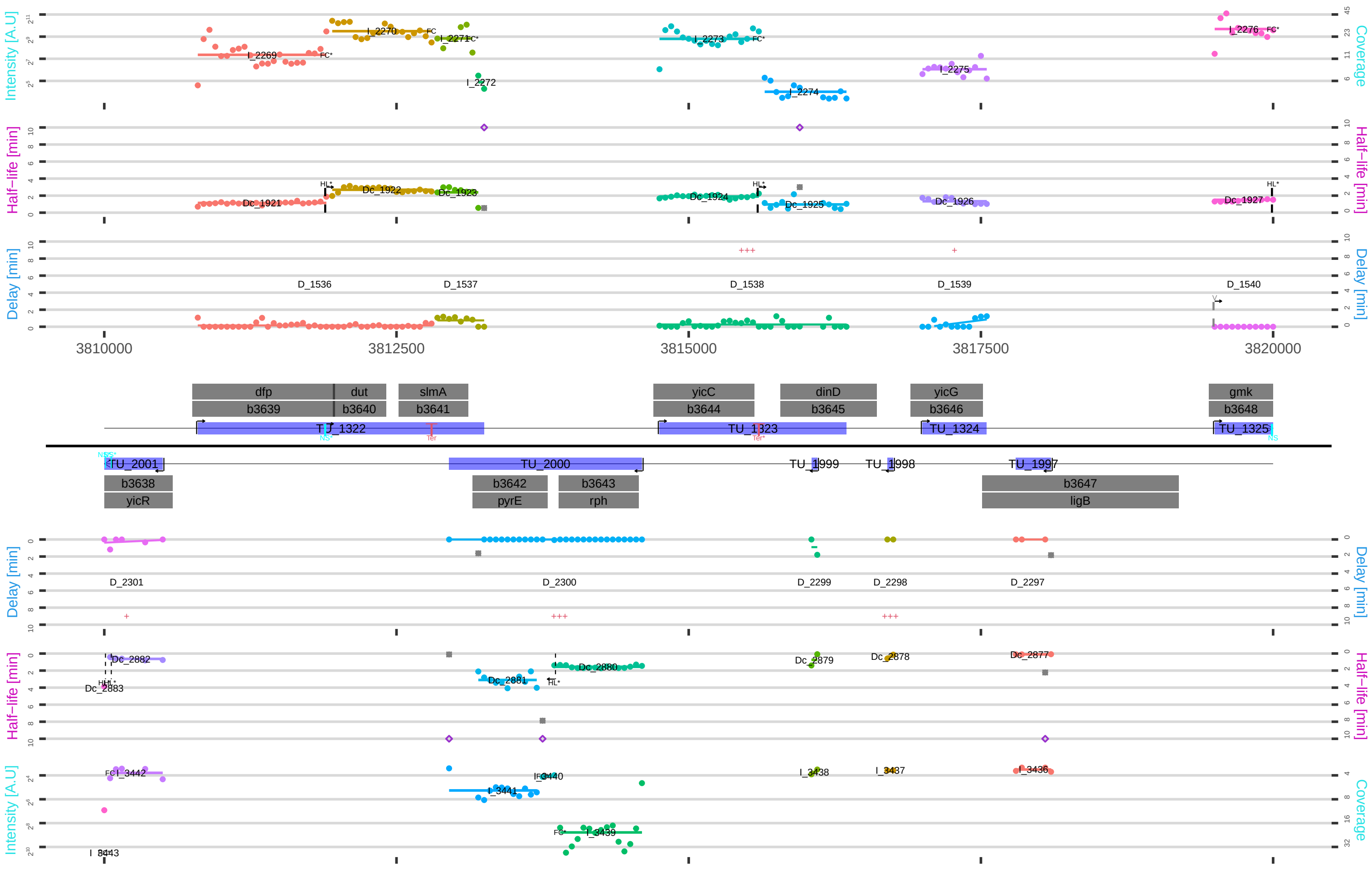

Term: termination (0), NS: new start (2), PS: pausing site (0), iTSS_I: internal starting site (0)

ID: 76400−76600; Term: termination (4), NS: new start (3), PS: pausing site (1), iTSS_I: internal starting site (0)

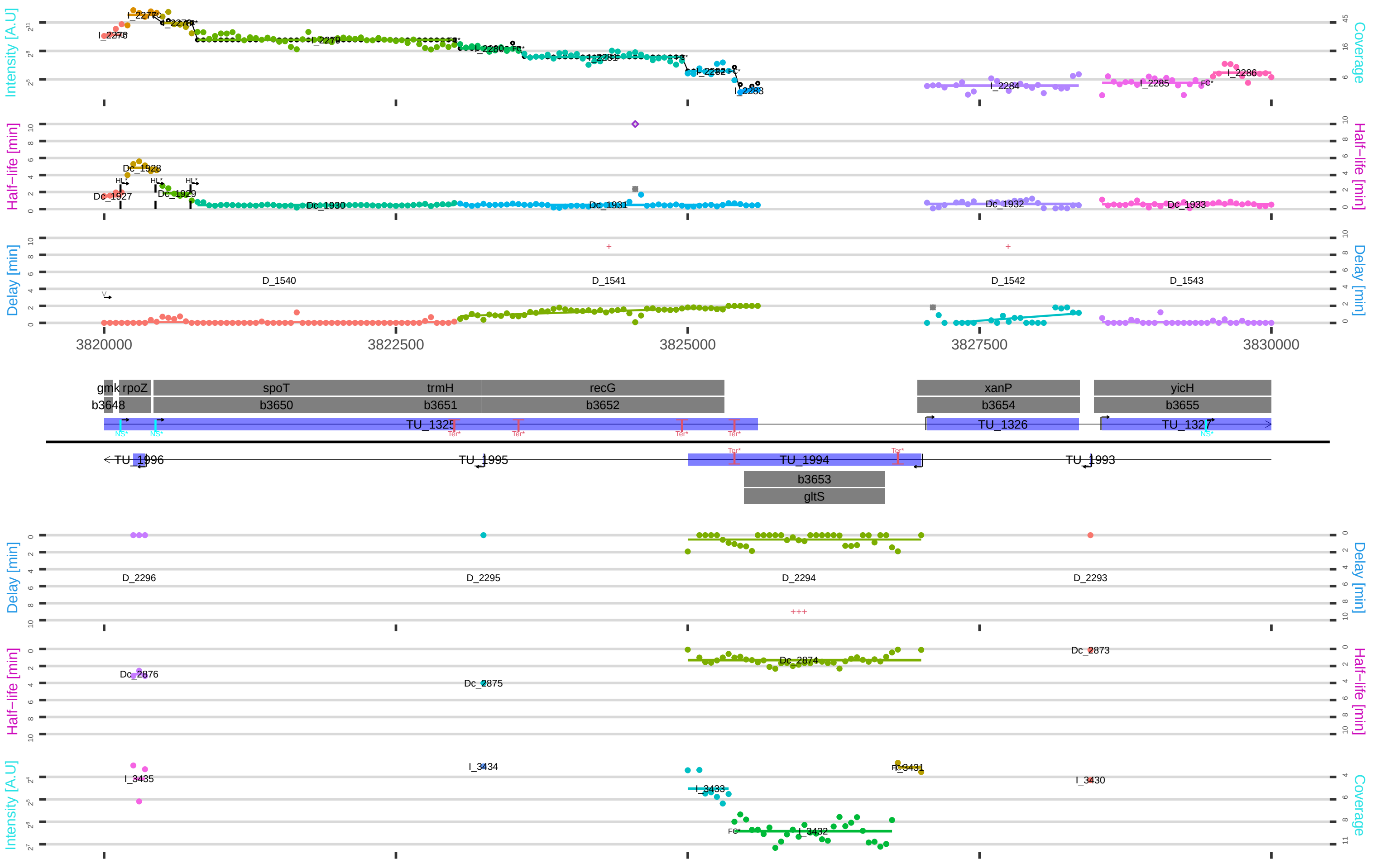

Term: termination (2), NS: new start (0), PS: pausing site (0), iTSS_I: internal starting site (0)

ID: 76600−76746; Term: termination (1), NS: new start (0), PS: pausing site (0), iTSS_I: internal starting site (0)

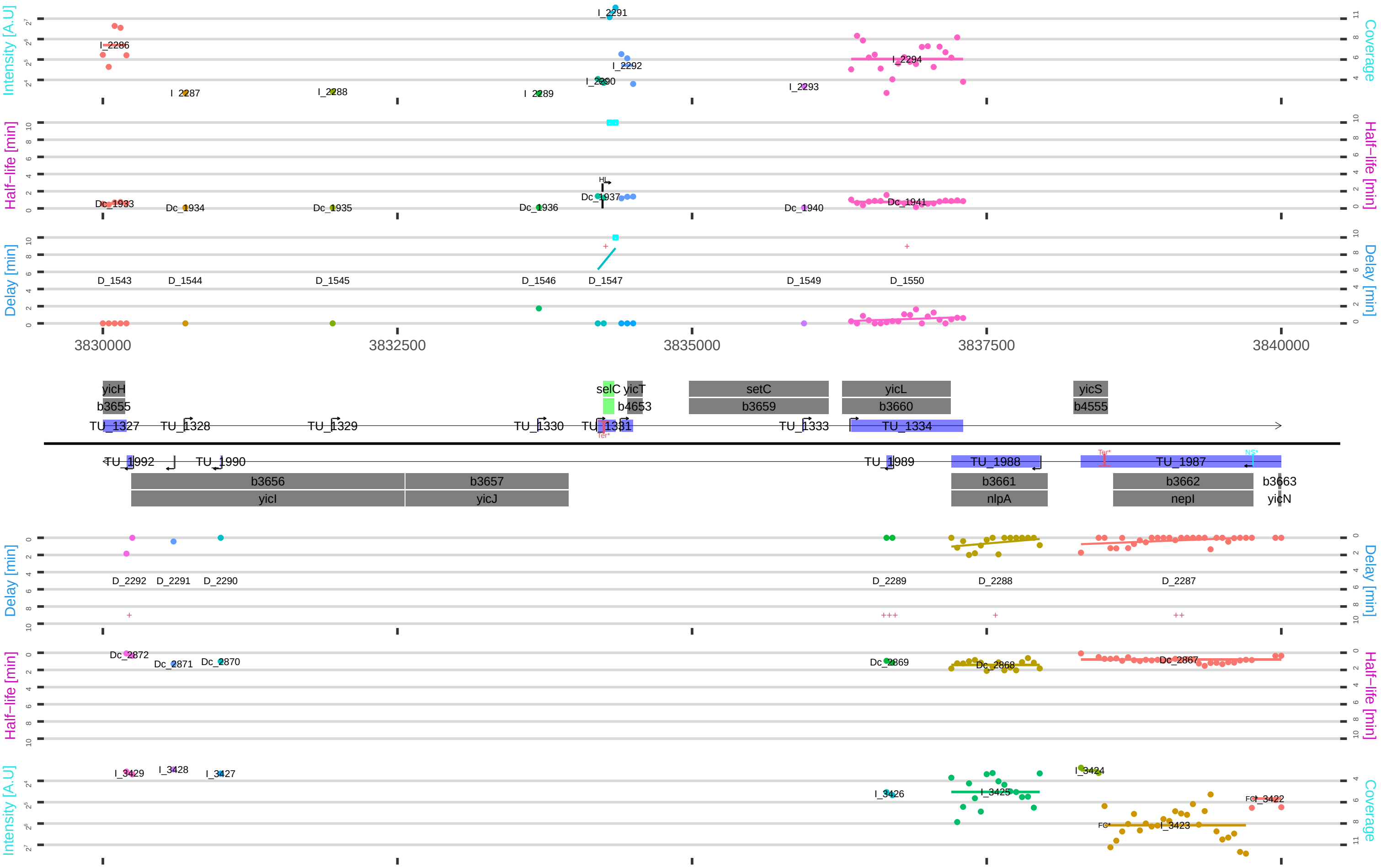

Term: termination (1), NS: new start (1), PS: pausing site (0), iTSS_I: internal starting site (0)

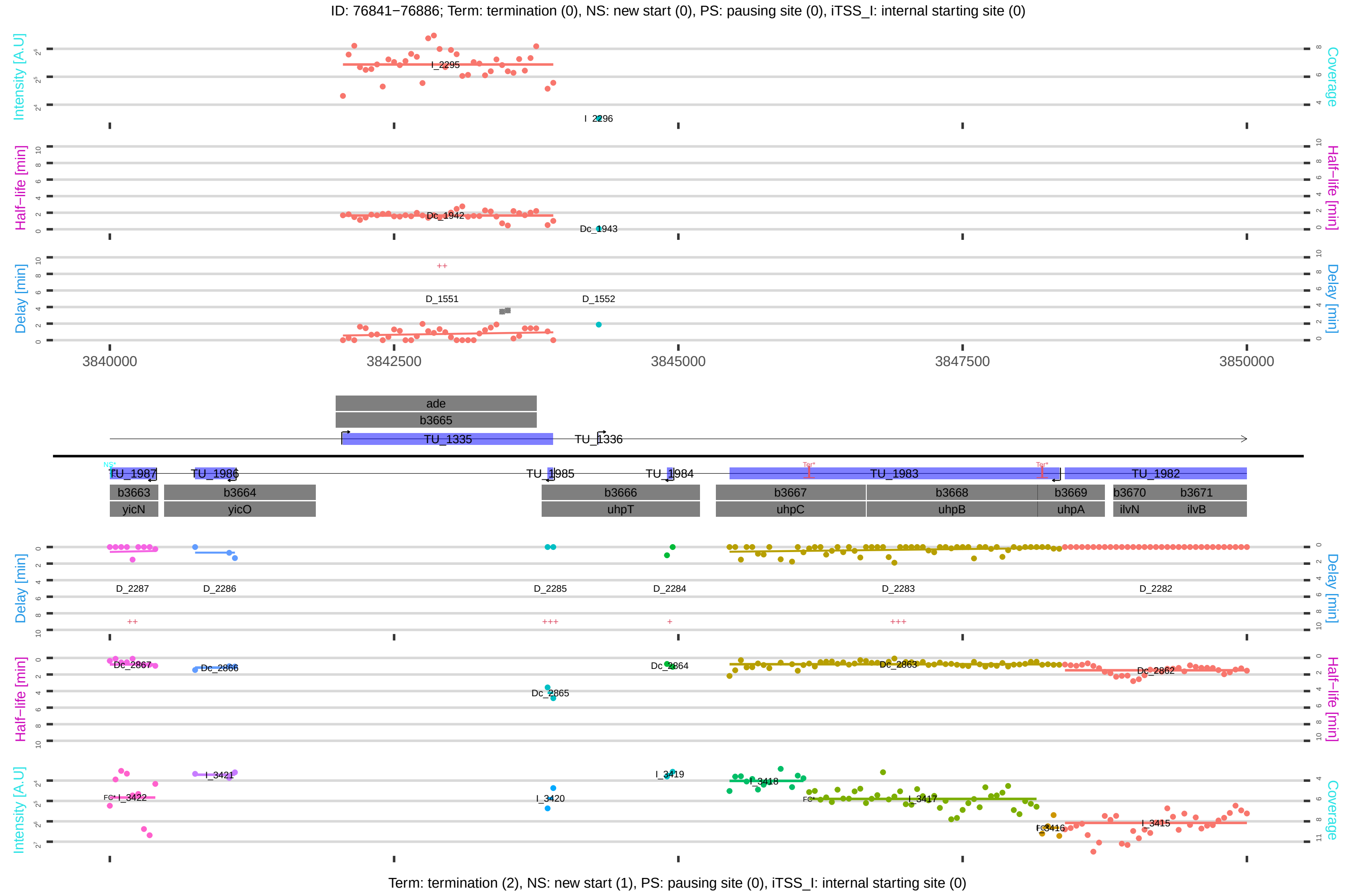

ID: 76841−76886; Term: termination (0), NS: new start (0), PS: pausing site (0), iTSS_I: internal starting site (0)
Intensity [A.U]
Coverage
I_2295
I_2296
Half−life [min]
Dc_1942
Dc_1943
Delay [min]
D_1551
D_1552
3840000
3842500
3845000
3847500
3850000
ade
b3665
TU_1335
TU_1336
TU_1987
TU_1986
TU_1985
TU_1984
TU_1983
TU_1982
b3663
yicN
b3664
yicO
b3666
uhpT
b3667
uhpC
b3668
uhpB
b3669
uhpA
b3670
ilvN
b3671
ilvB
D_2287
D_2286
D_2285
D_2284
D_2283
D_2282
Dc_2867
Dc_2866
Dc_2865
Dc_2864
Dc_2863
Dc_2862
I_3422
I_3421
I_3420
I_3419
I_3418
I_3417
I_3416
I_3415
Term: termination (2), NS: new start (1), PS: pausing site (0), iTSS_I: internal starting site (0)

ID: 77029−77124; Term: termination (1), NS: new start (0), PS: pausing site (0), iTSS_I: internal starting site (0)

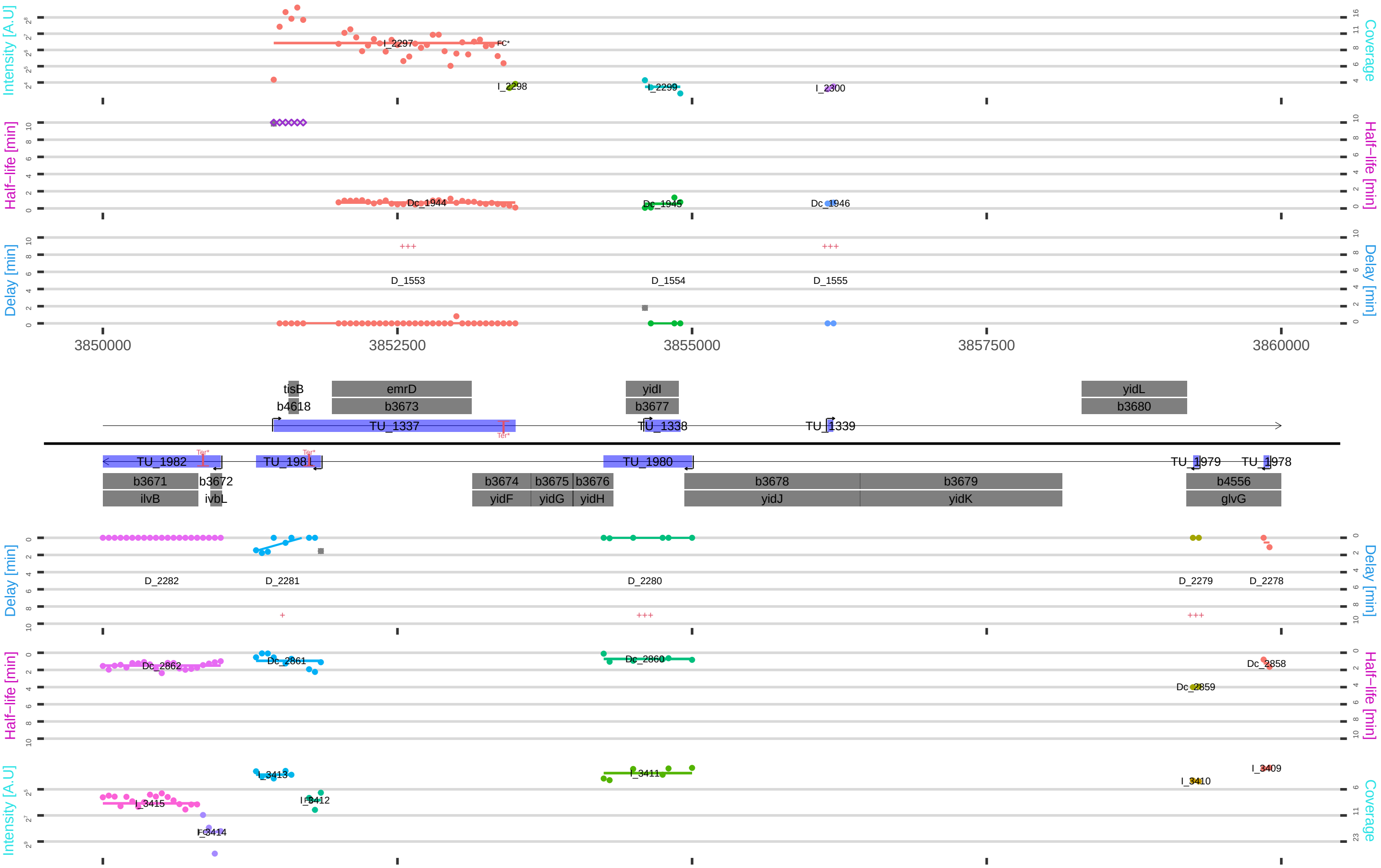

Term: termination (2), NS: new start (0), PS: pausing site (0), iTSS_I: internal starting site (0)

ID: 77311−77331; Term: termination (0), NS: new start (0), PS: pausing site (0), iTSS_I: internal starting site (0)

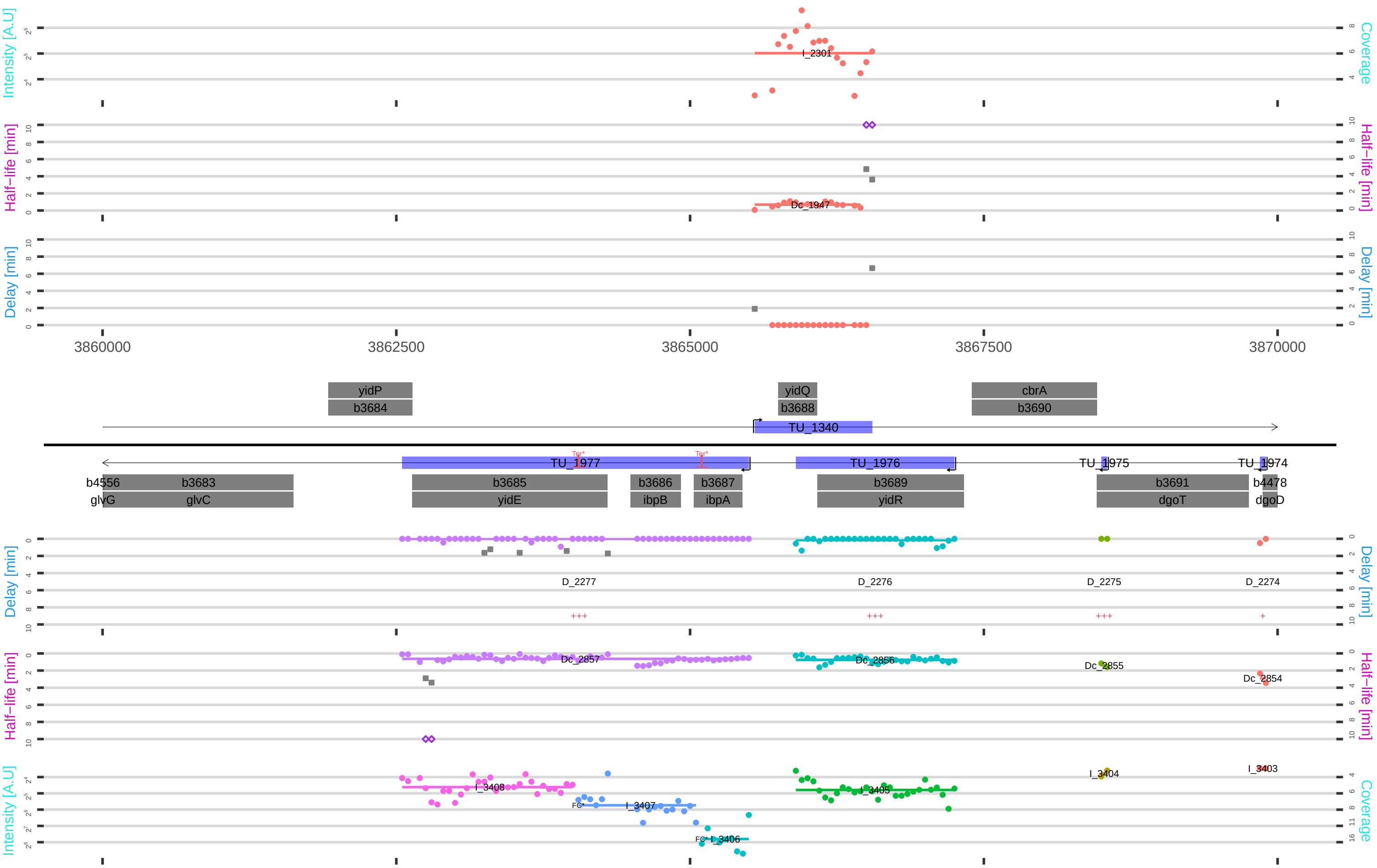

Term: termination (2), NS: new start (0), PS: pausing site (0), iTSS_I: internal starting site (0)

ID: 77423−77600; Term: termination (0), NS: new start (0), PS: pausing site (0), iTSS_I: internal starting site (0)

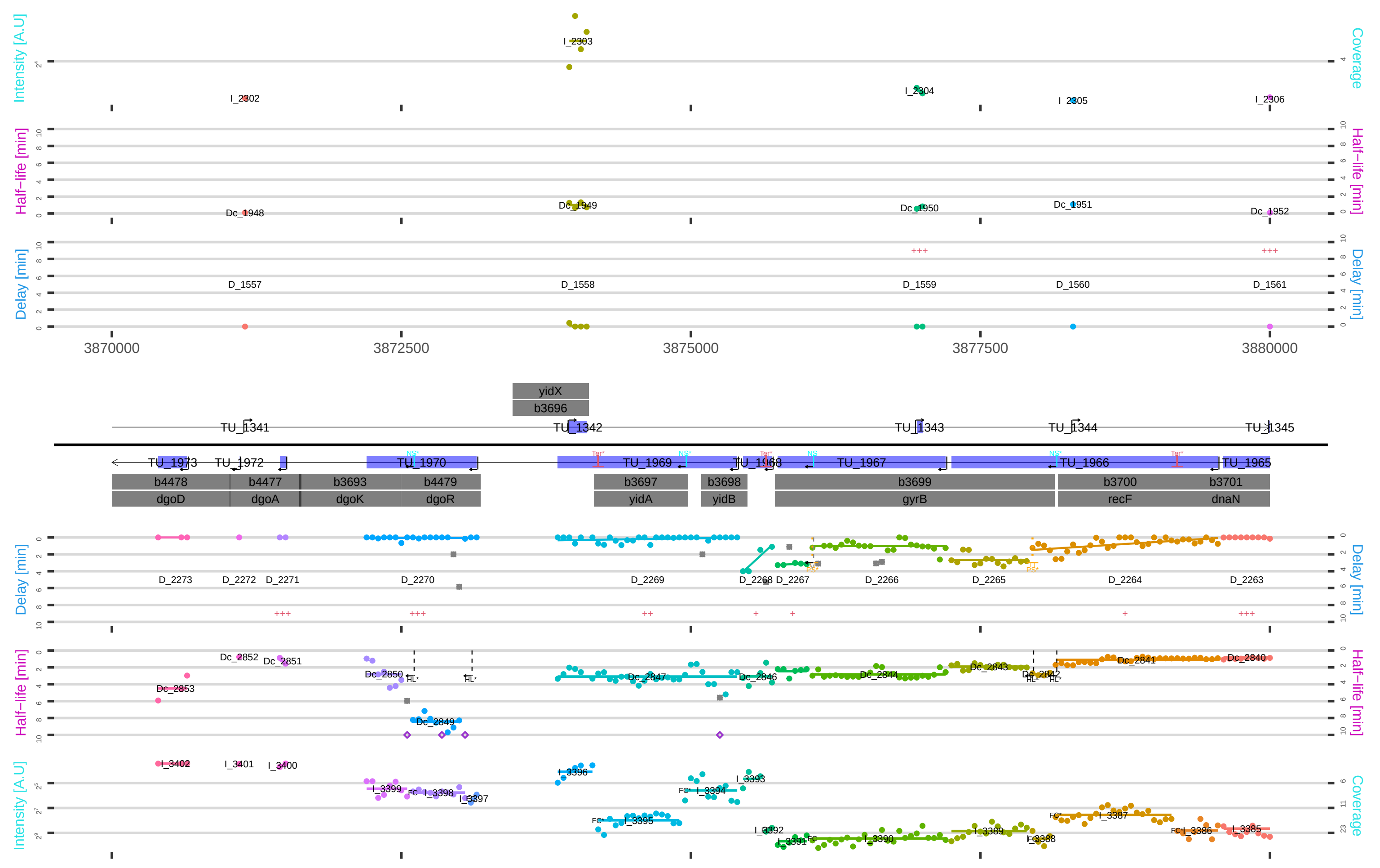

Term: termination (3), NS: new start (4), PS: pausing site (2), iTSS_I: internal starting site (0)

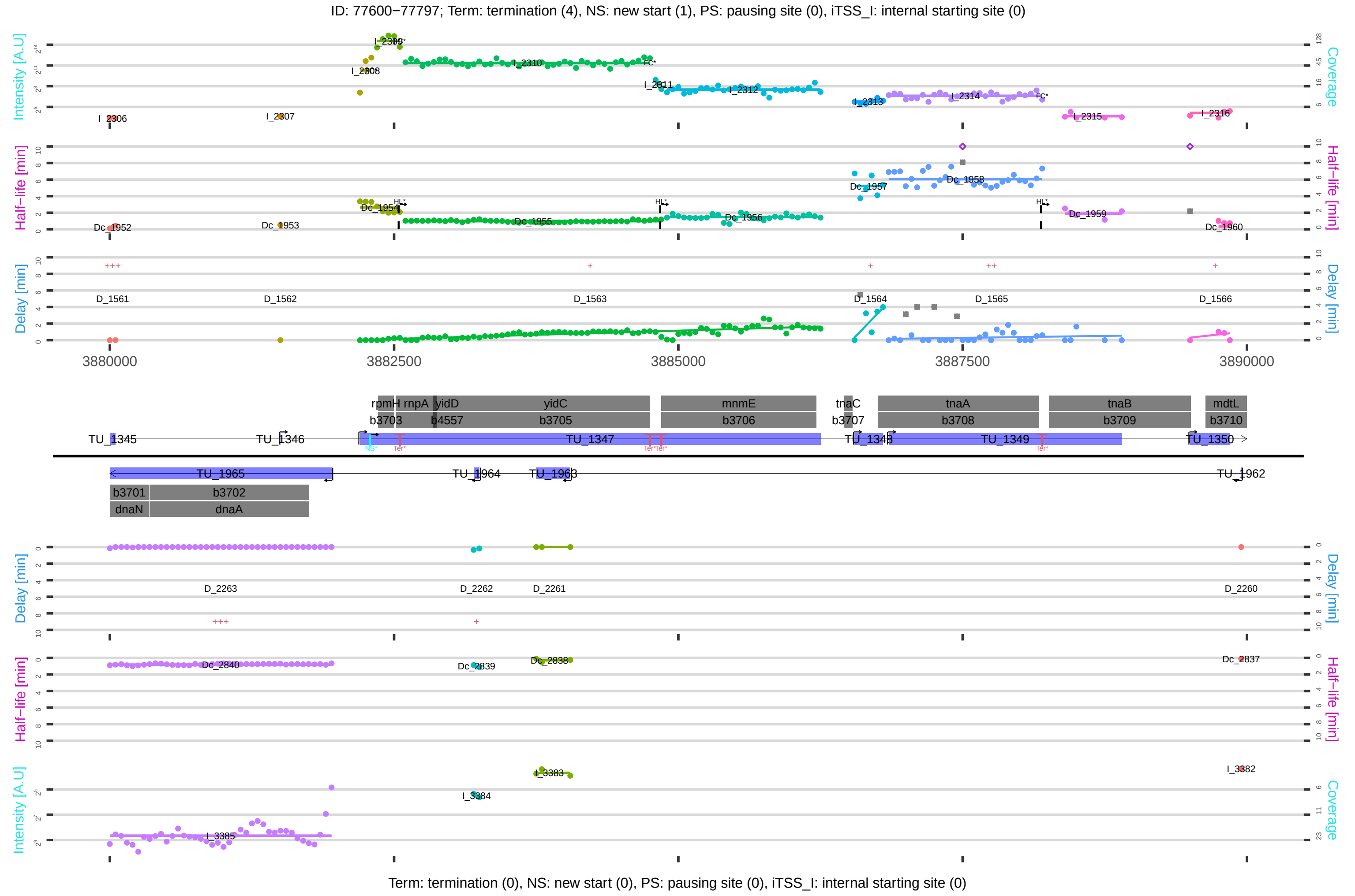

ID: 77600−77797; Term: termination (4), NS: new start (1), PS: pausing site (0), iTSS_I: internal starting site (0)
Intensity [A.U]
Coverage
Half−life [min]
Delay [min]
3880000
3882500
3885000
3887500
3890000
rpmH rnpA yidD yidC mnmE tnaC tnaA tnaB mdtL
b3703 b4557 b3705 b3706 b3707 b3708 b3709 b3710
TU_1345 TU_1346 TU_1347 TU_1348 TU_1349 TU_1350
TU_1965 TU_1964 TU_1963 TU_1962
b3701 b3702
dnaN dnaA
D_2263 D_2262 D_2261 D_2260
Dc_2840 Dc_2839 Dc_2838 Dc_2837
I_3385 I_3384 I_3383 I_3382
Term: termination (0), NS: new start (0), PS: pausing site (0), iTSS_I: internal starting site (0)

ID: 77802−77938; Term: termination (2), NS: new start (2), PS: pausing site (2), iTSS_I: internal starting site (0)

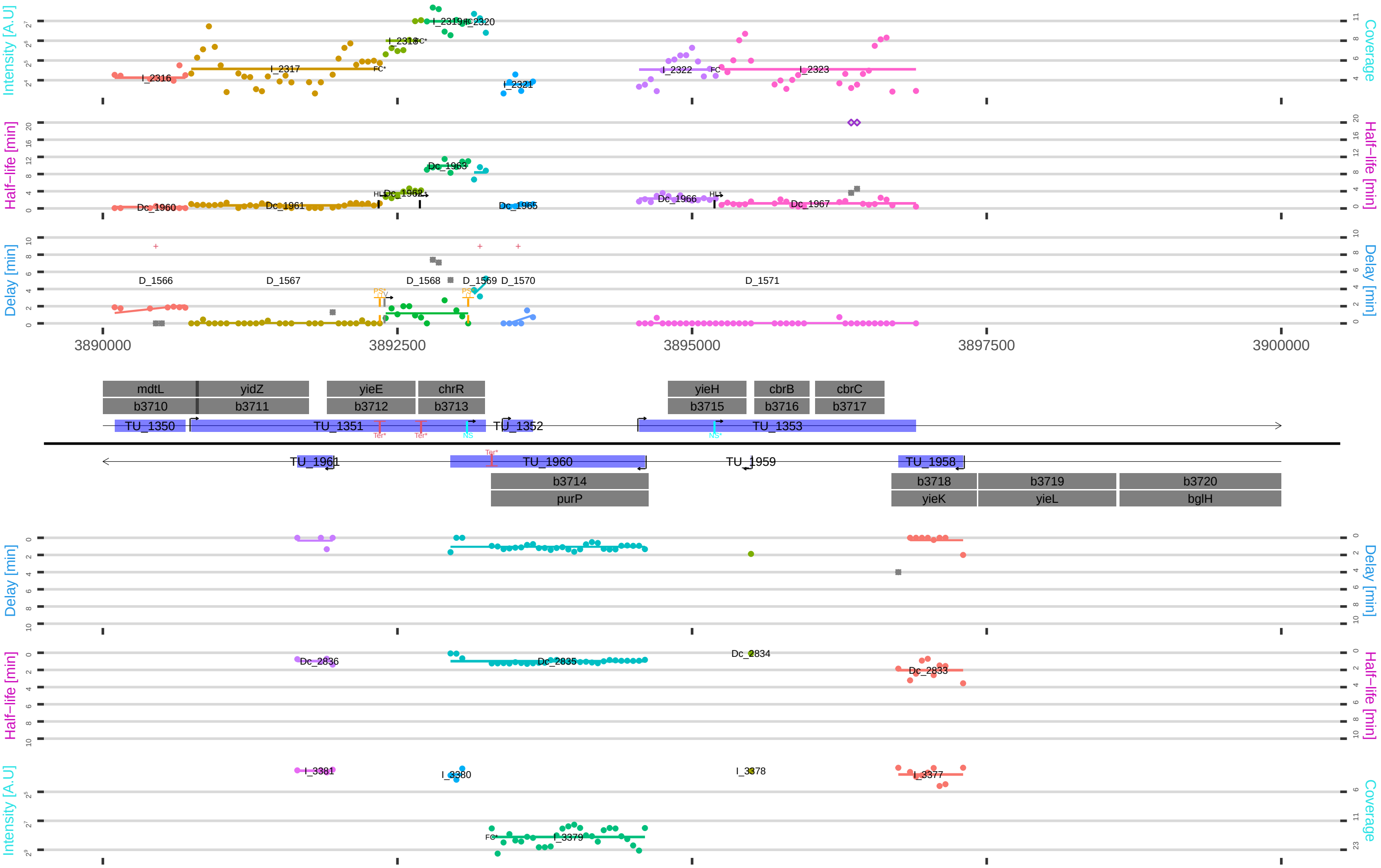

Term: termination (1), NS: new start (0), PS: pausing site (0), iTSS_I: internal starting site (0)

ID: 78051−78197; Term: termination (0), NS: new start (0), PS: pausing site (0), iTSS_I: internal starting site (0)

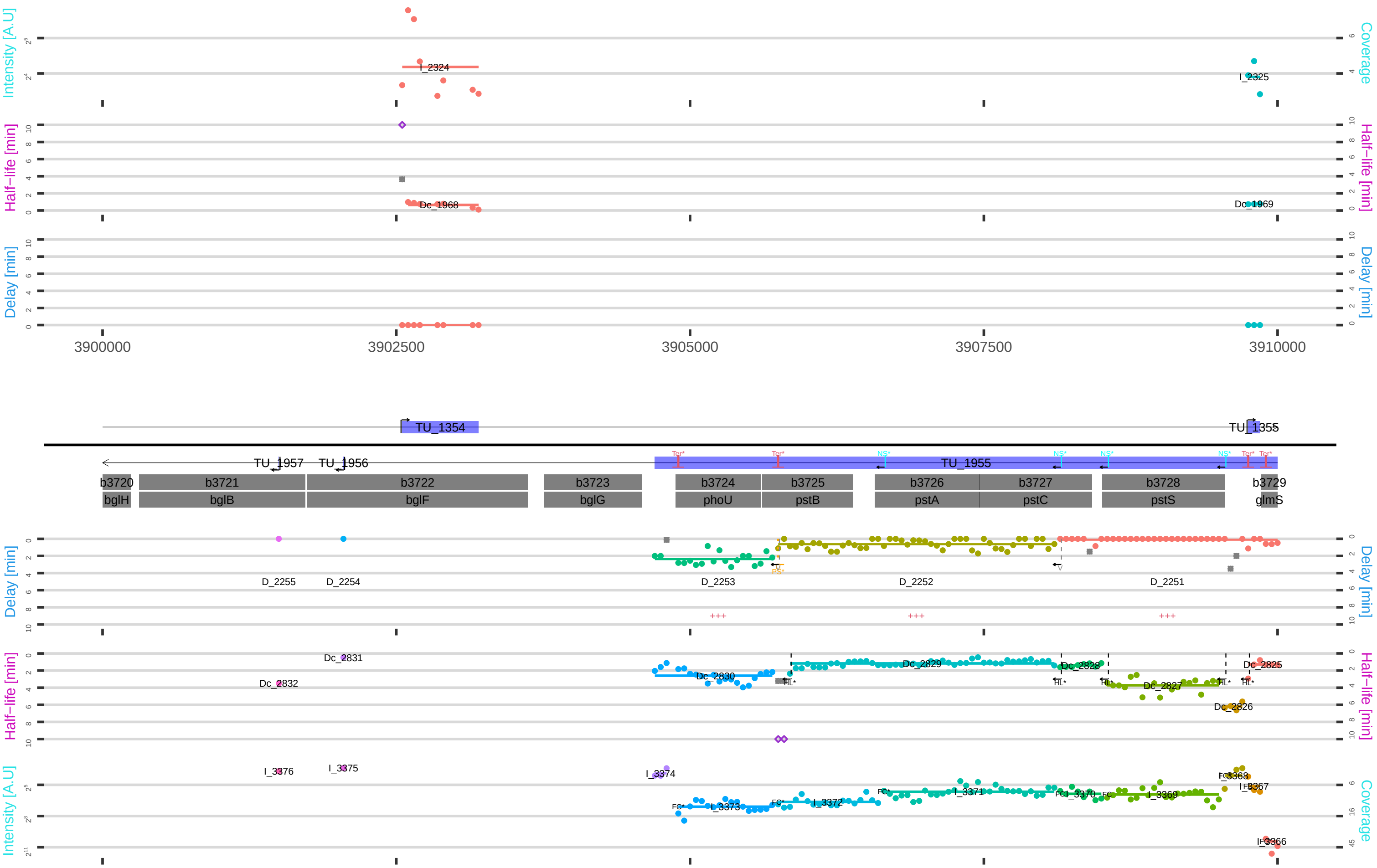

Term: termination (4), NS: new start (4), PS: pausing site (2), iTSS_I: internal starting site (0)

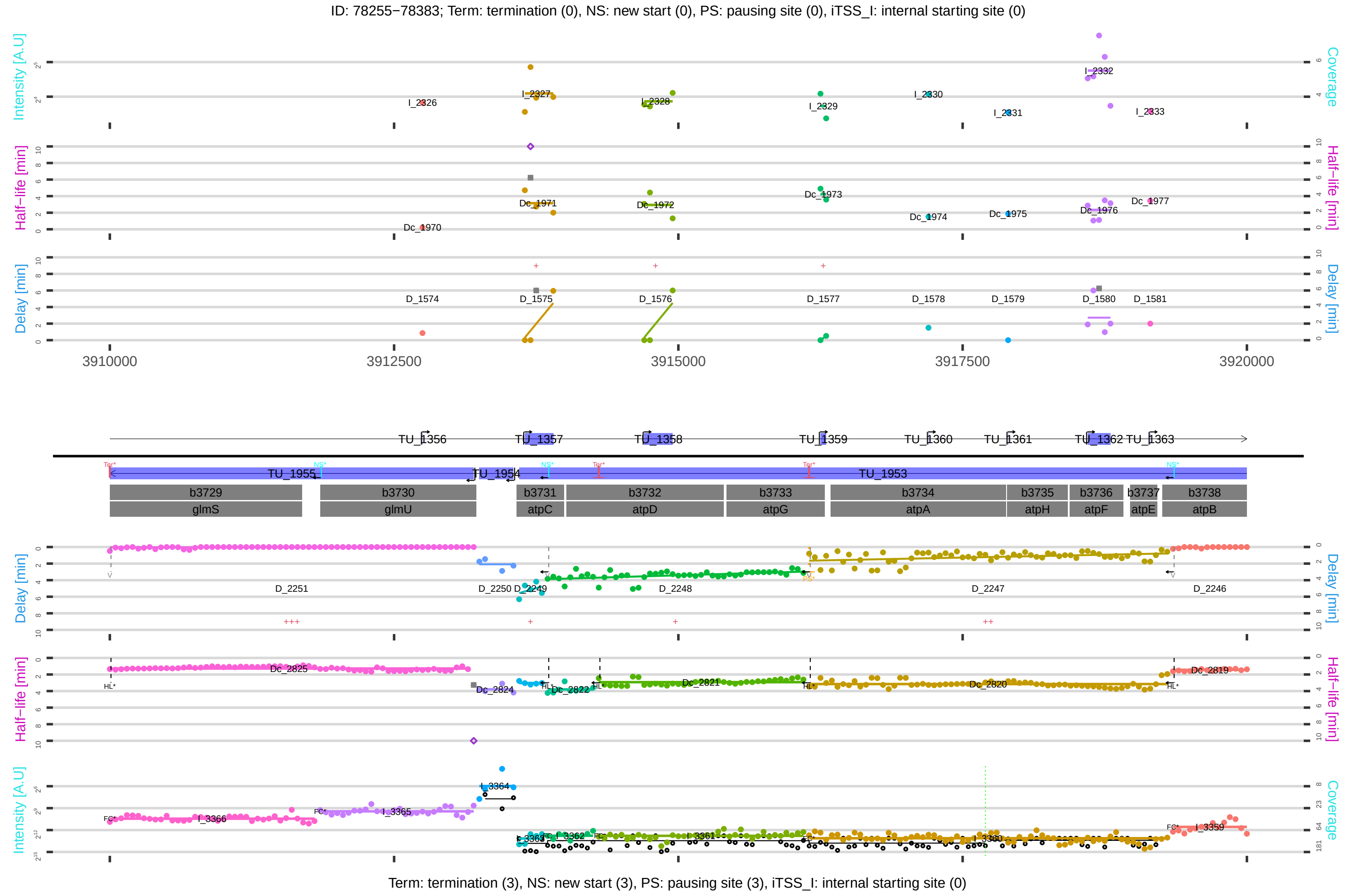
ID: 78255−78383; Term: termination (0), NS: new start (0), PS: pausing site (0), iTSS_I: internal starting site (0)
Intensity [A.U]
Coverage
Half−life [min]
Delay [min]
I_2326
I_2327
I_2328
I_2329
I_2330
I_2331
I_2332
I_2333
Dc_1970
Dc_1971
Dc_1972
Dc_1973
Dc_1974
Dc_1975
Dc_1976
Dc_1977
D_1574
D_1575
D_1576
D_1577
D_1578
D_1579
D_1580
D_1581
3910000
3912500
3915000
3917500
3920000
TU_1356
TU_1357
TU_1358
TU_1359
TU_1360
TU_1361
TU_1362
TU_1363
TU_1955
TU_1954
TU_1953
b3729
glmS
b3730
glmU
b3731
atpC
b3732
atpD
b3733
atpG
b3734
atpA
b3735
atpH
b3736
atpF
b3737
atpE
b3738
atpB
D_2251
D_2250
D_2249
D_2248
D_2247
D_2246
Dc_2825
Dc_2824
Dc_2822
Dc_2821
Dc_2820
Dc_2819
I_3366
I_3365
I_3364
I_3363
I_3362
I_3361
I_3360
I_3359
Term: termination (3), NS: new start (3), PS: pausing site (3), iTSS_I: internal starting site (0)

ID: 78424−78600; Term: termination (3), NS: new start (0), PS: pausing site (0), iTSS_I: internal starting site (0)

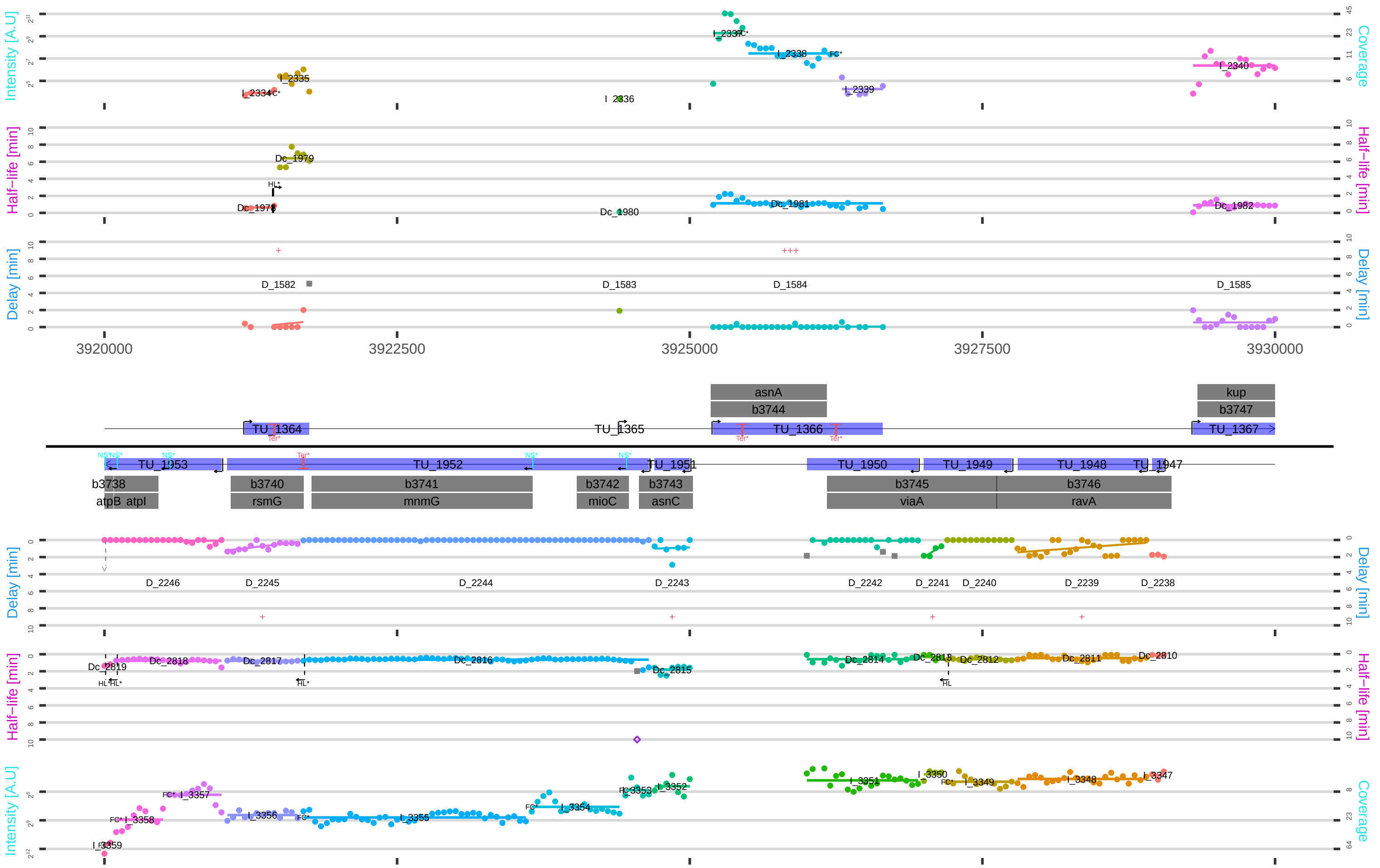

Term: termination (1), NS: new start (5), PS: pausing site (2), iTSS_I: internal starting site (0)

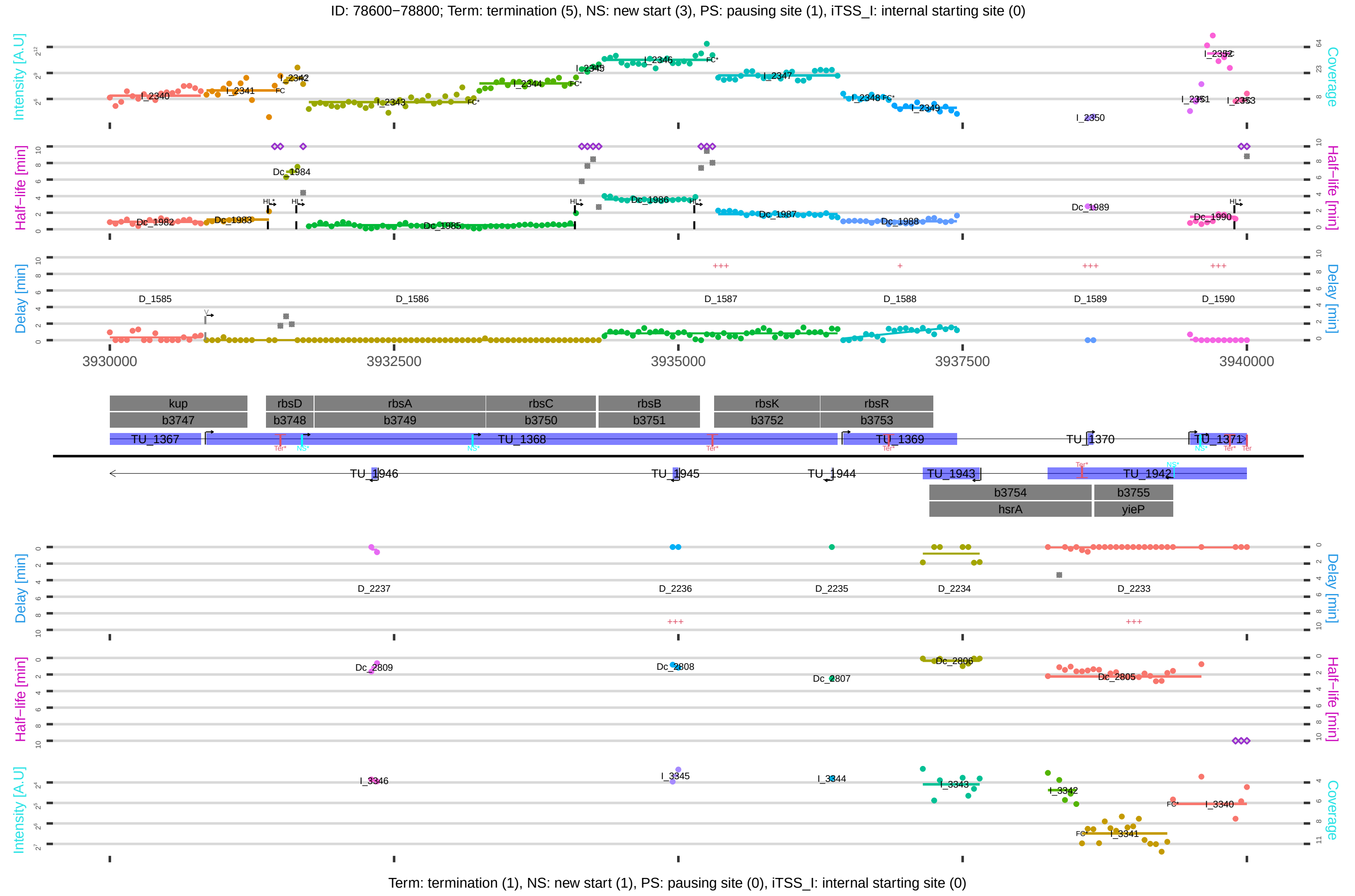

ID: 78600−78800; Term: termination (5), NS: new start (3), PS: pausing site (1), iTSS_I: internal starting site (0)
Intensity [A.U]
Coverage
Half−life [min]
Delay [min]
I_2340
I_2341
I_2342
I_2343
I_2344
I_2345
I_2346
I_2347
I_2348
I_2349
I_2350
I_2351
I_2352
I_2353
Dc_1982
Dc_1983
Dc_1984
Dc_1985
Dc_1986
Dc_1987
Dc_1988
Dc_1989
Dc_1990
D_1585
D_1586
D_1587
D_1588
D_1589
D_1590
3930000
3932500
3935000
3937500
3940000
kup
b3747
rbsD
b3748
rbsA
b3749
rbsC
b3750
rbsB
b3751
rbsK
b3752
rbsR
b3753
TU_1367
TU_1368
TU_1369
TU_1370
TU_1371
TU_1946
TU_1945
TU_1944
TU_1943
TU_1942
b3754
hsrA
b3755
yieP
D_2237
D_2236
D_2235
D_2234
D_2233
Dc_2809
Dc_2808
Dc_2807
Dc_2806
Dc_2805
I_3346
I_3345
I_3344
I_3343
I_3342
I_3341
I_3340
Term: termination (1), NS: new start (1), PS: pausing site (0), iTSS_I: internal starting site (0)

ID: 78800−79000; Term: termination (10), NS: new start (3), PS: pausing site (0), iTSS_I: internal starting site (0)

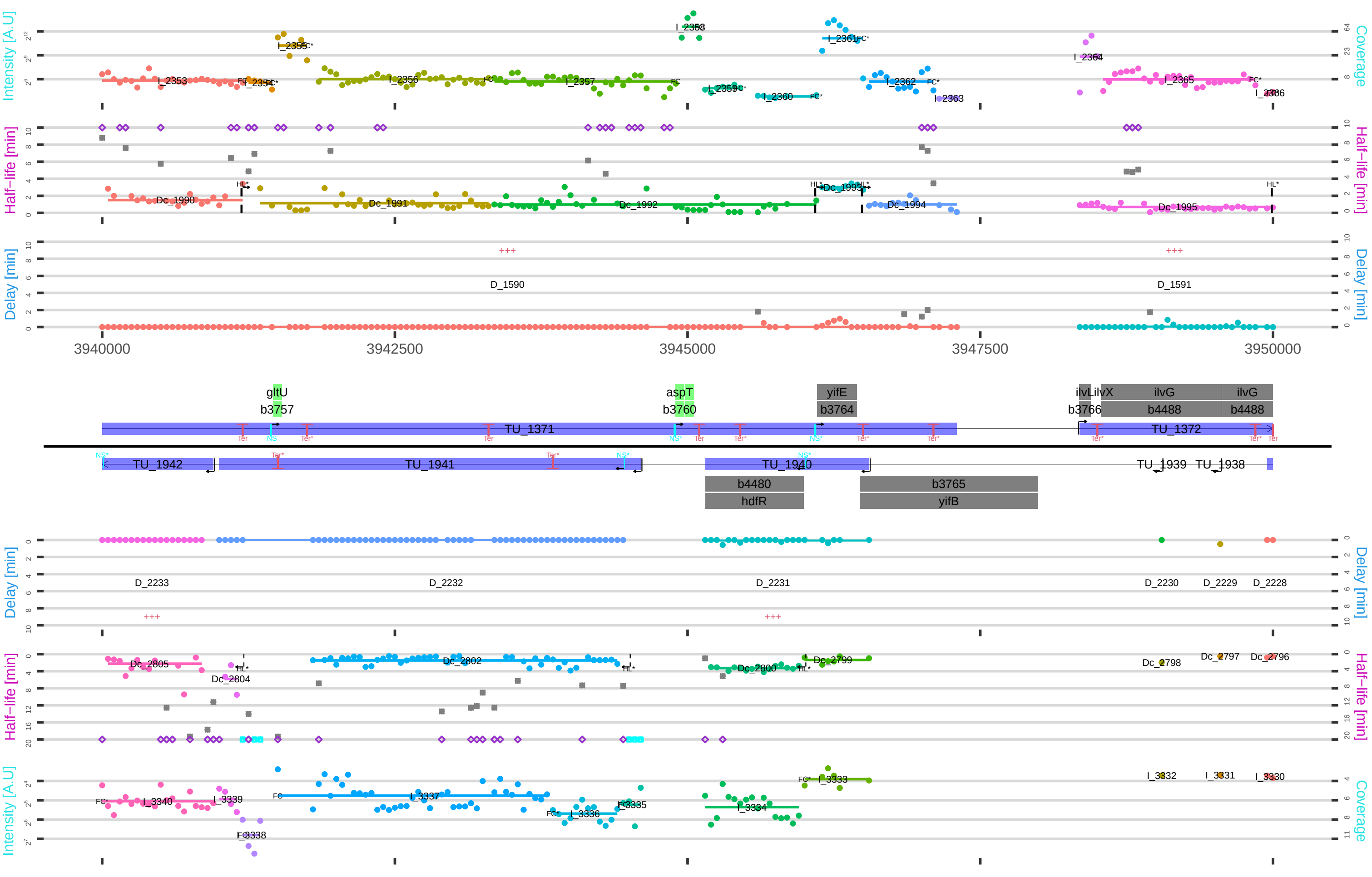

Term: termination (2), NS: new start (3), PS: pausing site (0), iTSS_I: internal starting site (0)

ID: 79000−79200; Term: termination (2), NS: new start (0), PS: pausing site (1), iTSS_I: internal starting site (0)

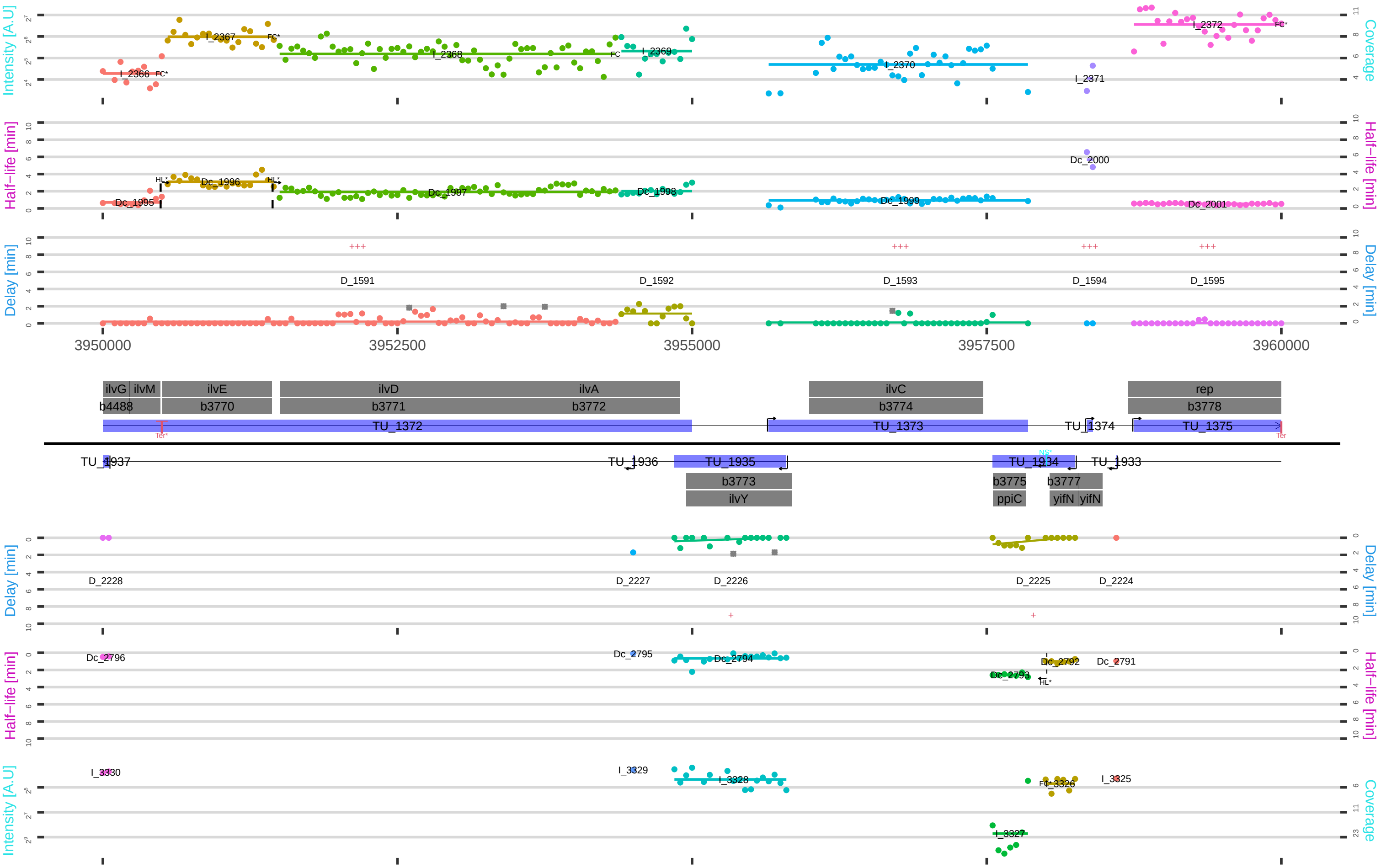

Term: termination (0), NS: new start (1), PS: pausing site (0), iTSS_I: internal starting site (0)

ID: 79200−79400; Term: termination (5), NS: new start (1), PS: pausing site (1), iTSS_I: internal starting site (0)

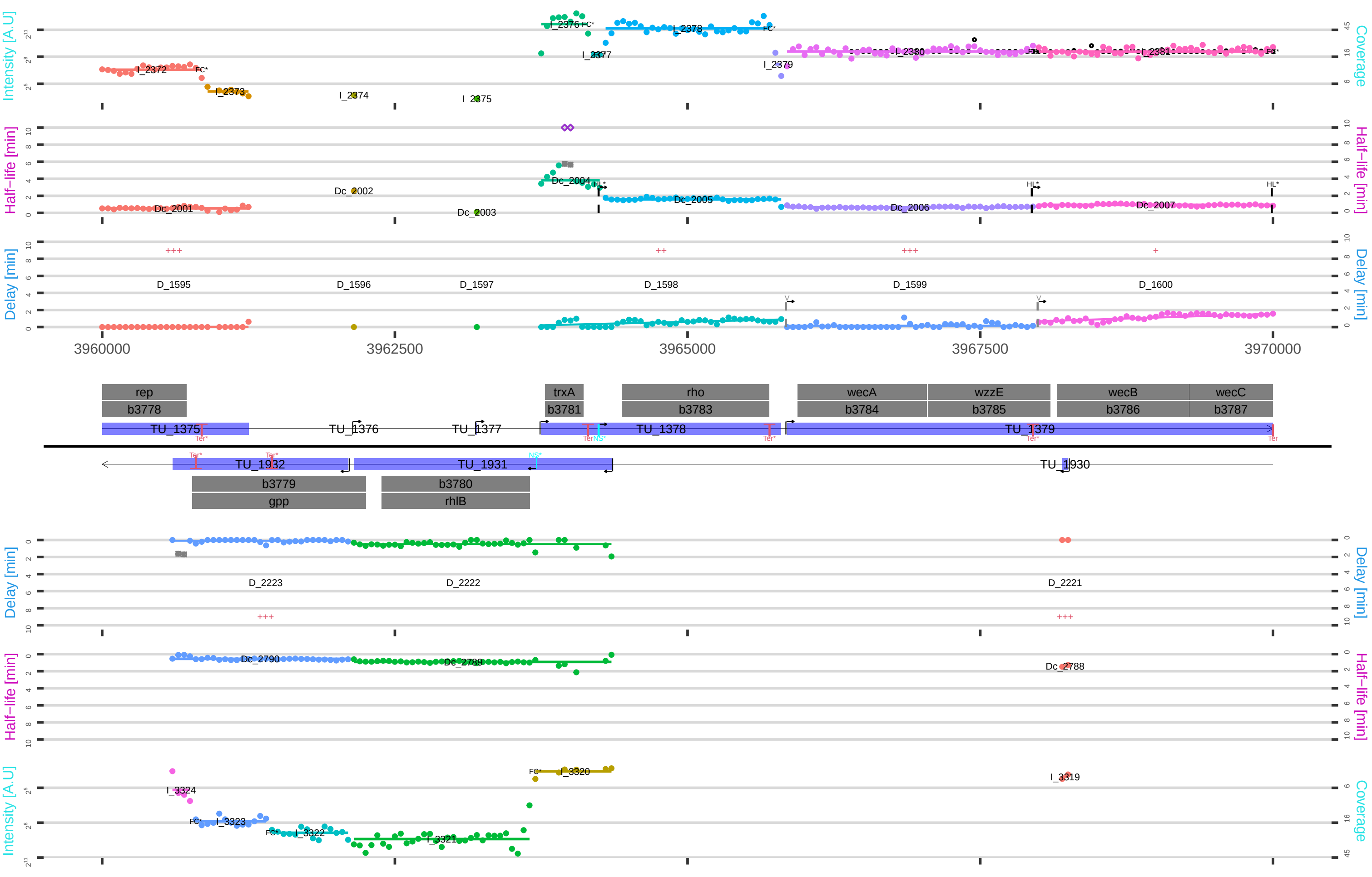

Term: termination (2), NS: new start (1), PS: pausing site (0), iTSS_I: internal starting site (0)

ID: 79400−79600; Term: termination (4), NS: new start (2), PS: pausing site (3), iTSS_I: internal starting site (1)

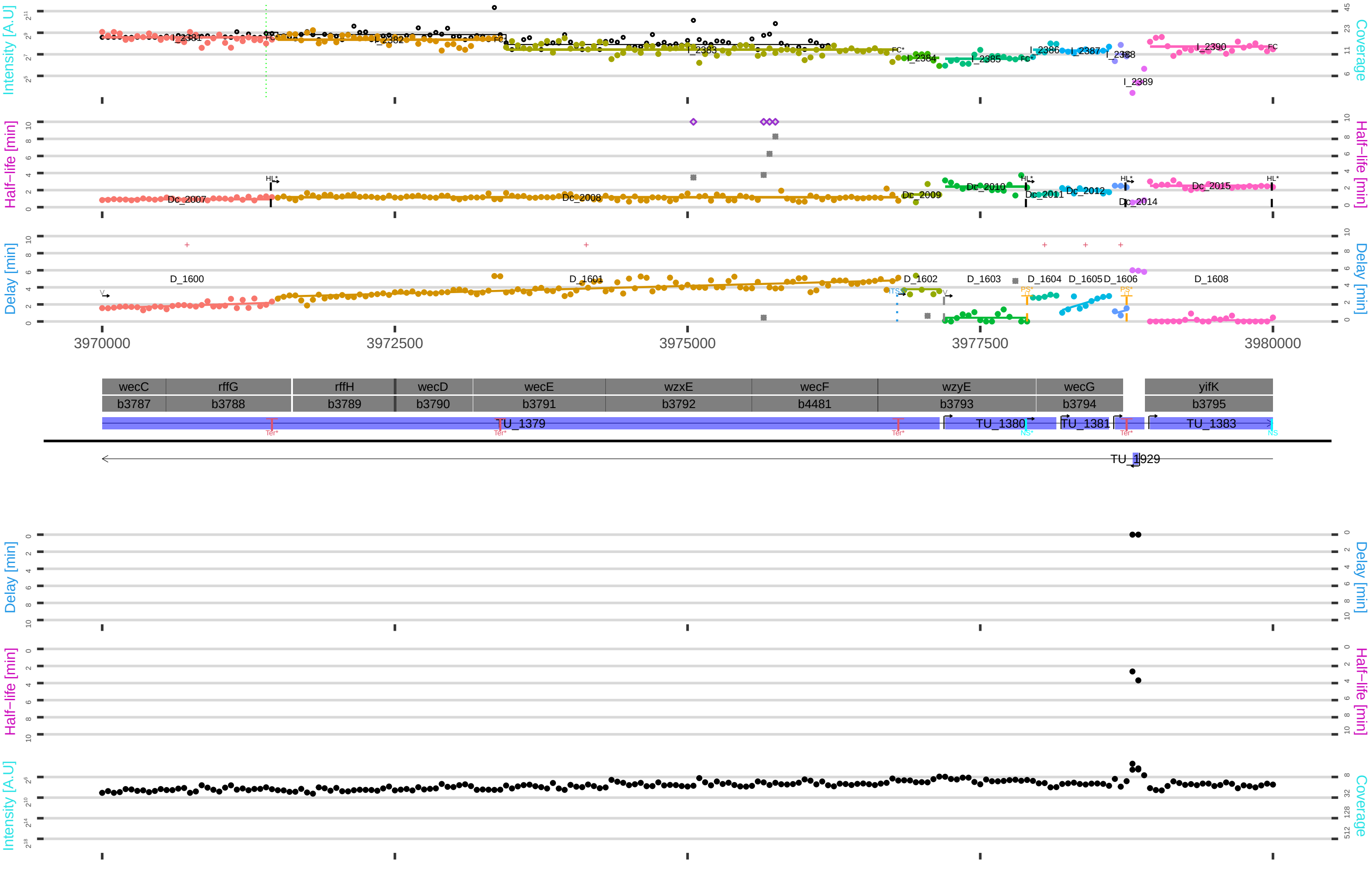

Term: termination (0), NS: new start (0), PS: pausing site (0), iTSS_I: internal starting site (0)

ID: 79600−79800; Term: termination (2), NS: new start (3), PS: pausing site (0), iTSS_I: internal starting site (0)

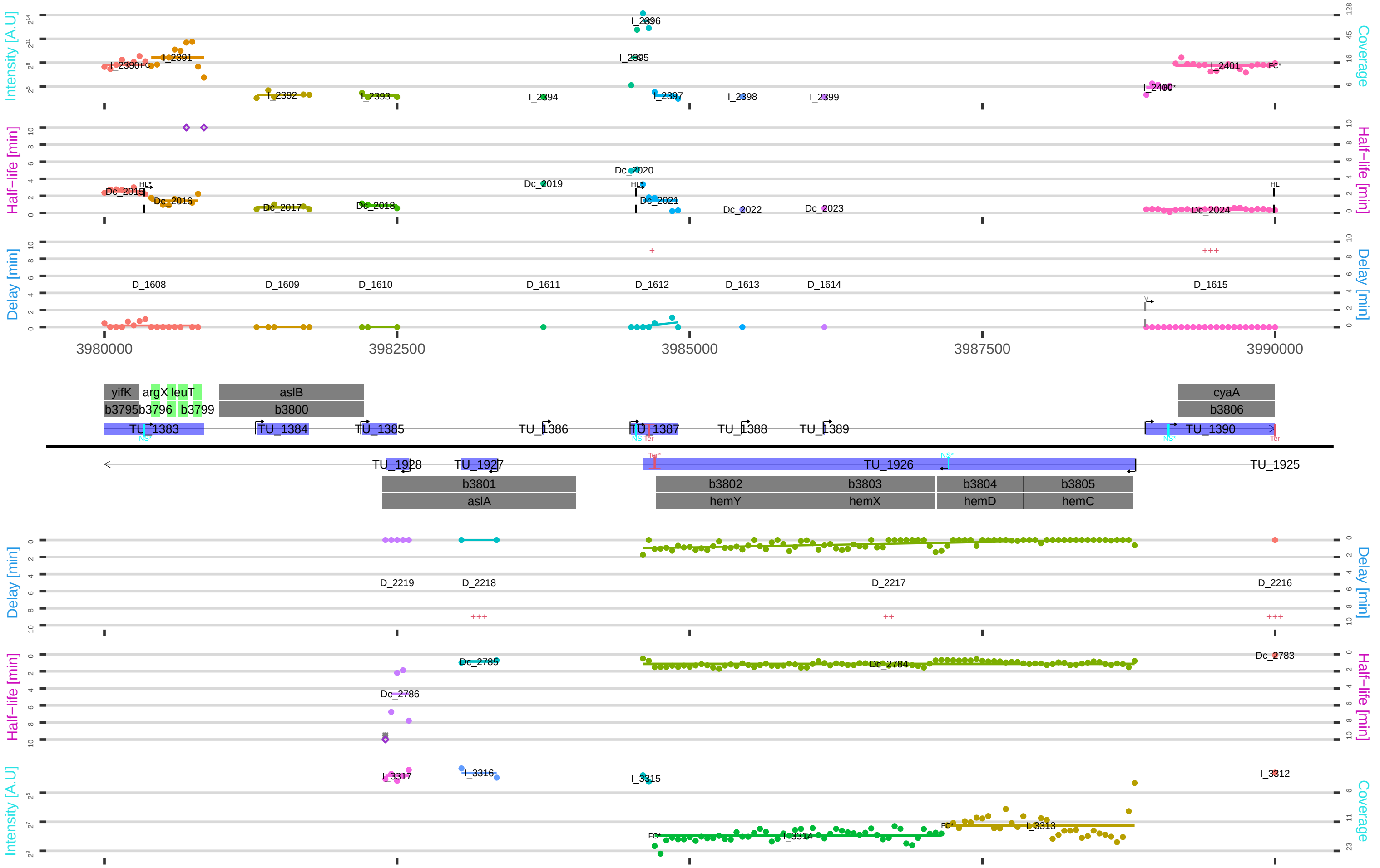

Term: termination (1), NS: new start (1), PS: pausing site (0), iTSS_I: internal starting site (0)

ID: 79800−80000; Term: termination (5), NS: new start (2), PS: pausing site (1), iTSS_I: internal starting site (2)

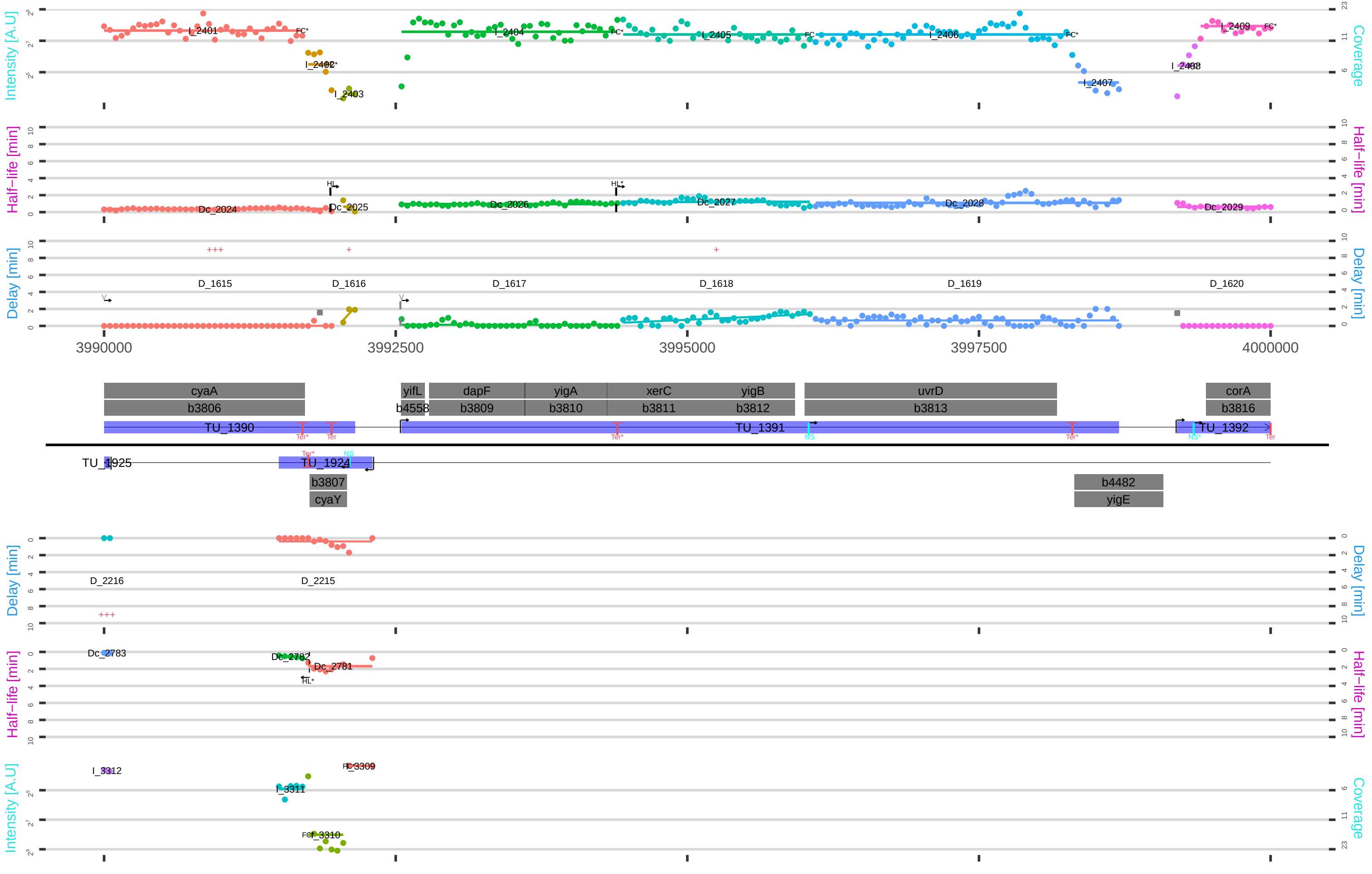

Term: termination (1), NS: new start (1), PS: pausing site (0), iTSS_I: internal starting site (0)

ID: 80000−80200; Term: termination (5), NS: new start (0), PS: pausing site (0), iTSS_I: internal starting site (0)

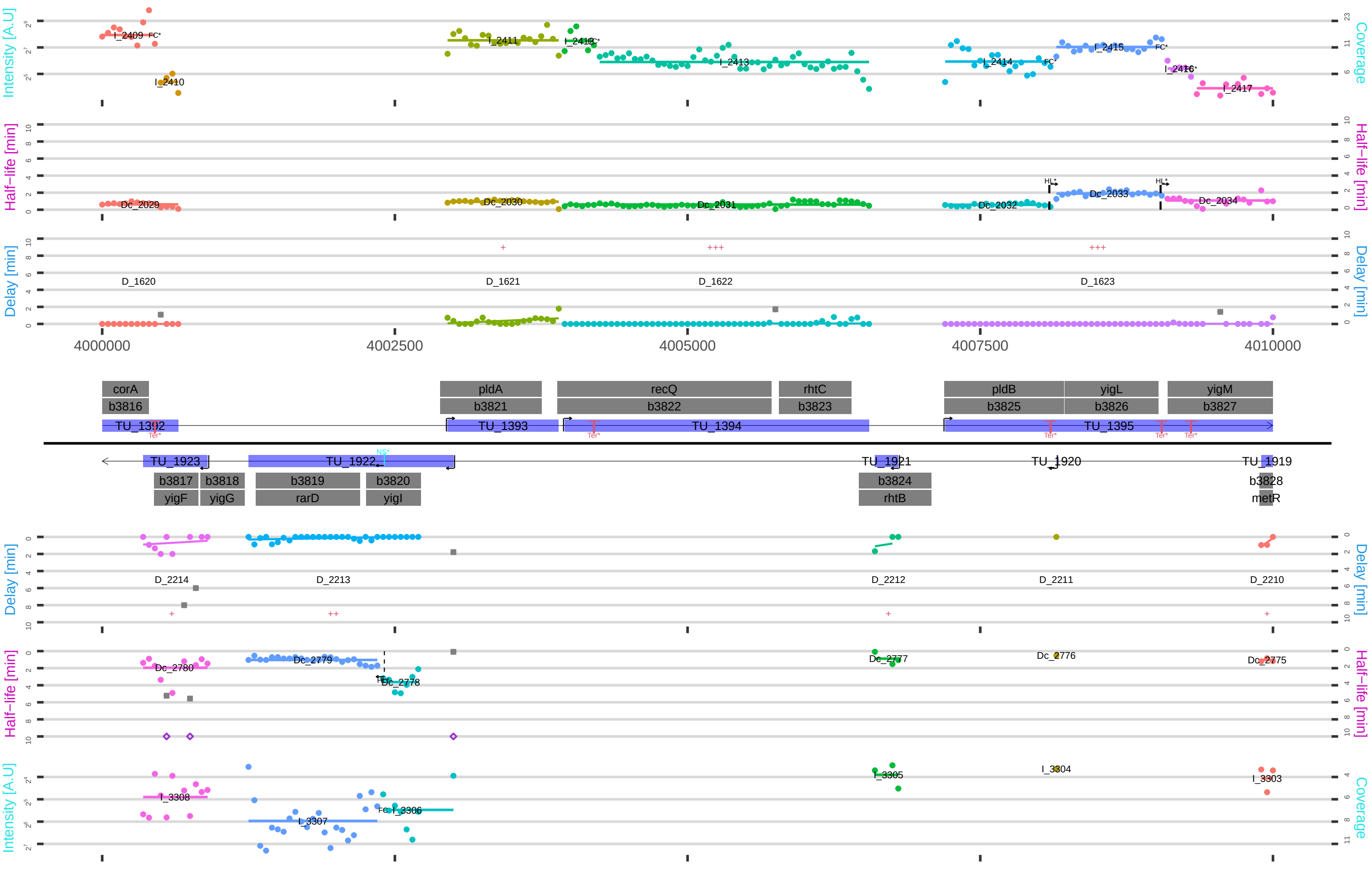

Term: termination (0), NS: new start (1), PS: pausing site (0), iTSS_I: internal starting site (0)

ID: 80200−80400; Term: termination (3), NS: new start (6), PS: pausing site (2), iTSS_I: internal starting site (0)

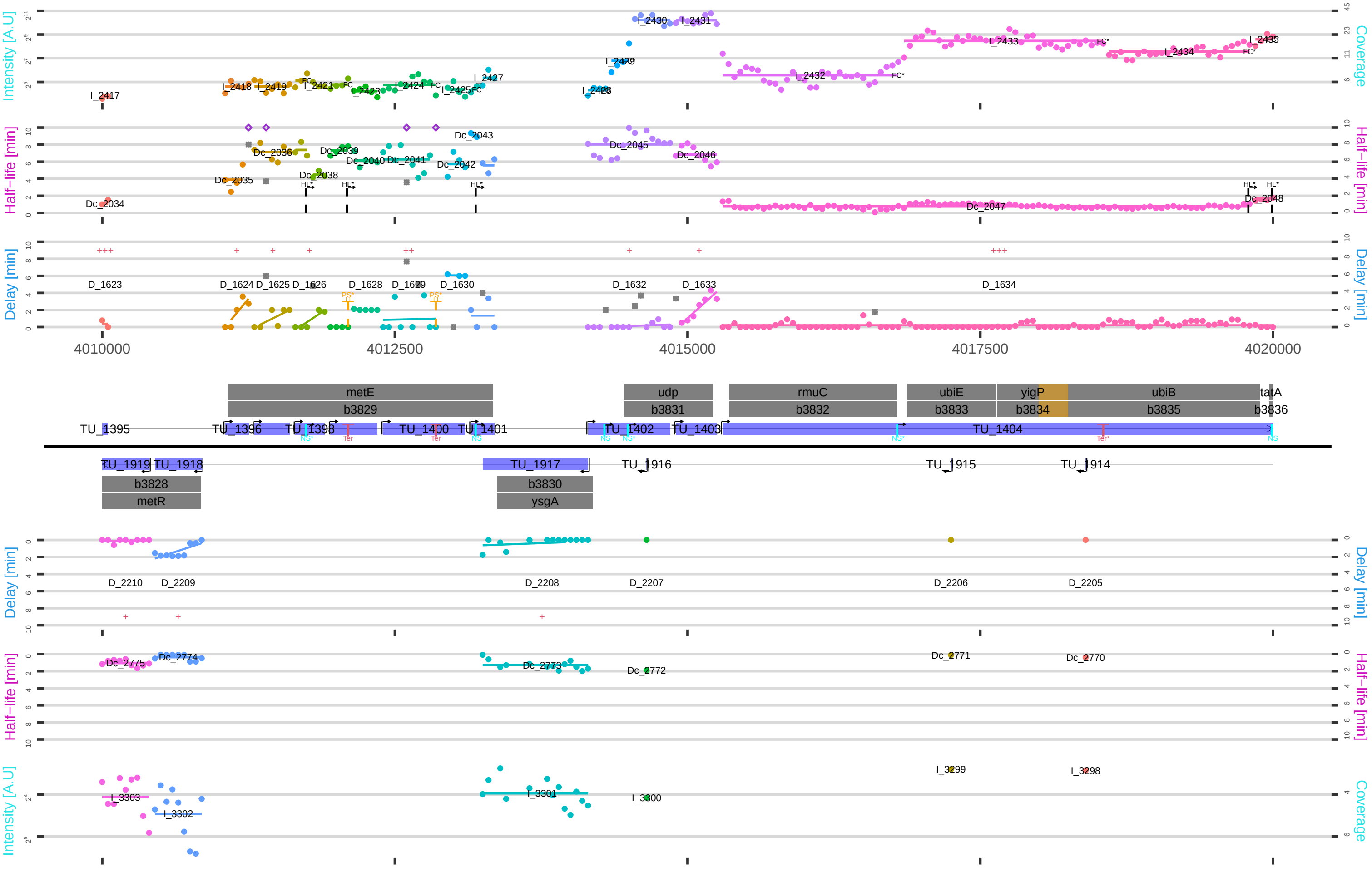

Term: termination (0), NS: new start (0), PS: pausing site (0), iTSS_I: internal starting site (0)

ID: 80400−80600; Term: termination (6), NS: new start (1), PS: pausing site (2), iTSS_I: internal starting site (0)

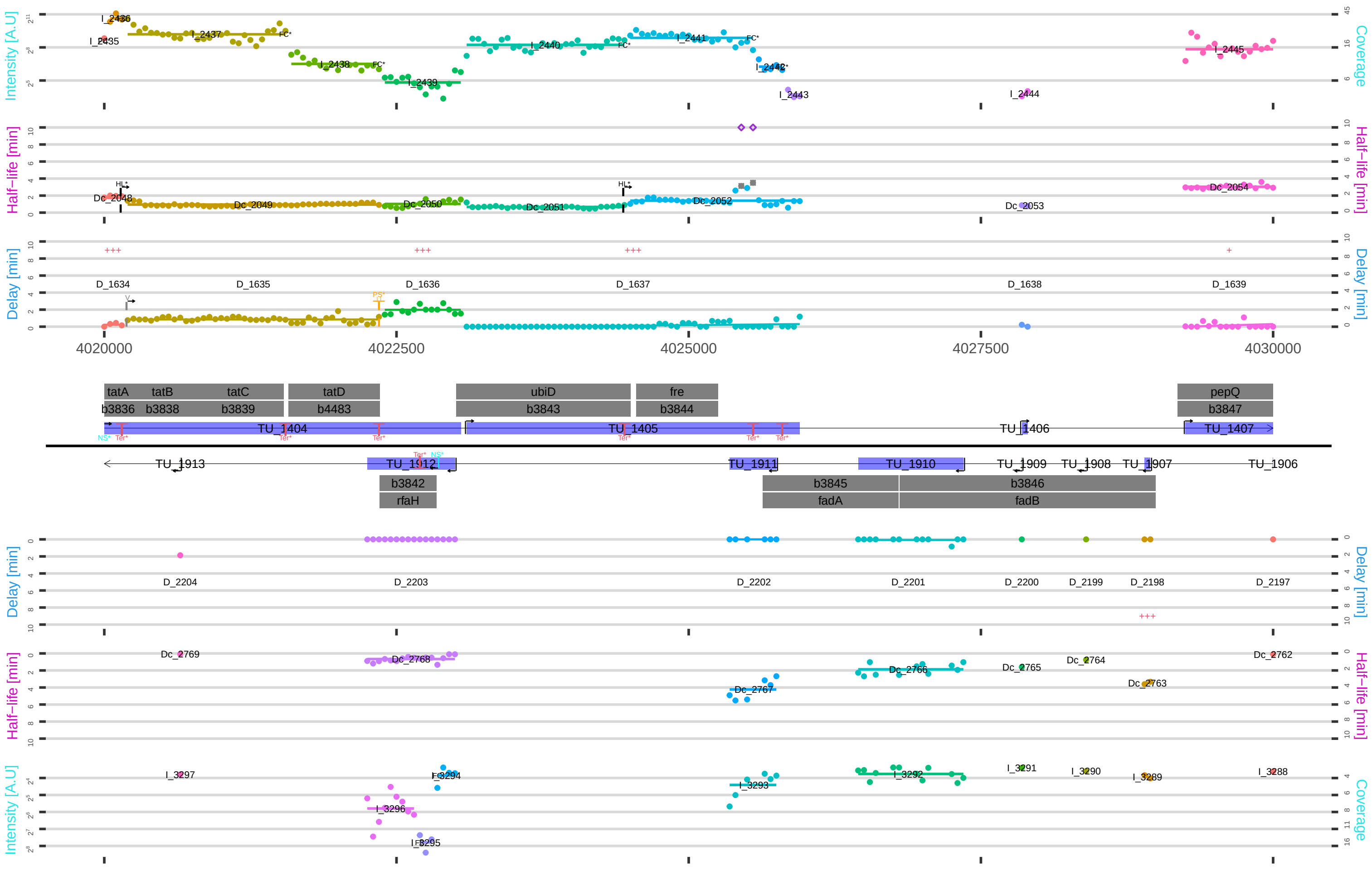

Term: termination (1), NS: new start (1), PS: pausing site (0), iTSS_I: internal starting site (0)

ID: 80600−80778; Term: termination (2), NS: new start (1), PS: pausing site (0), iTSS_I: internal starting site (0)

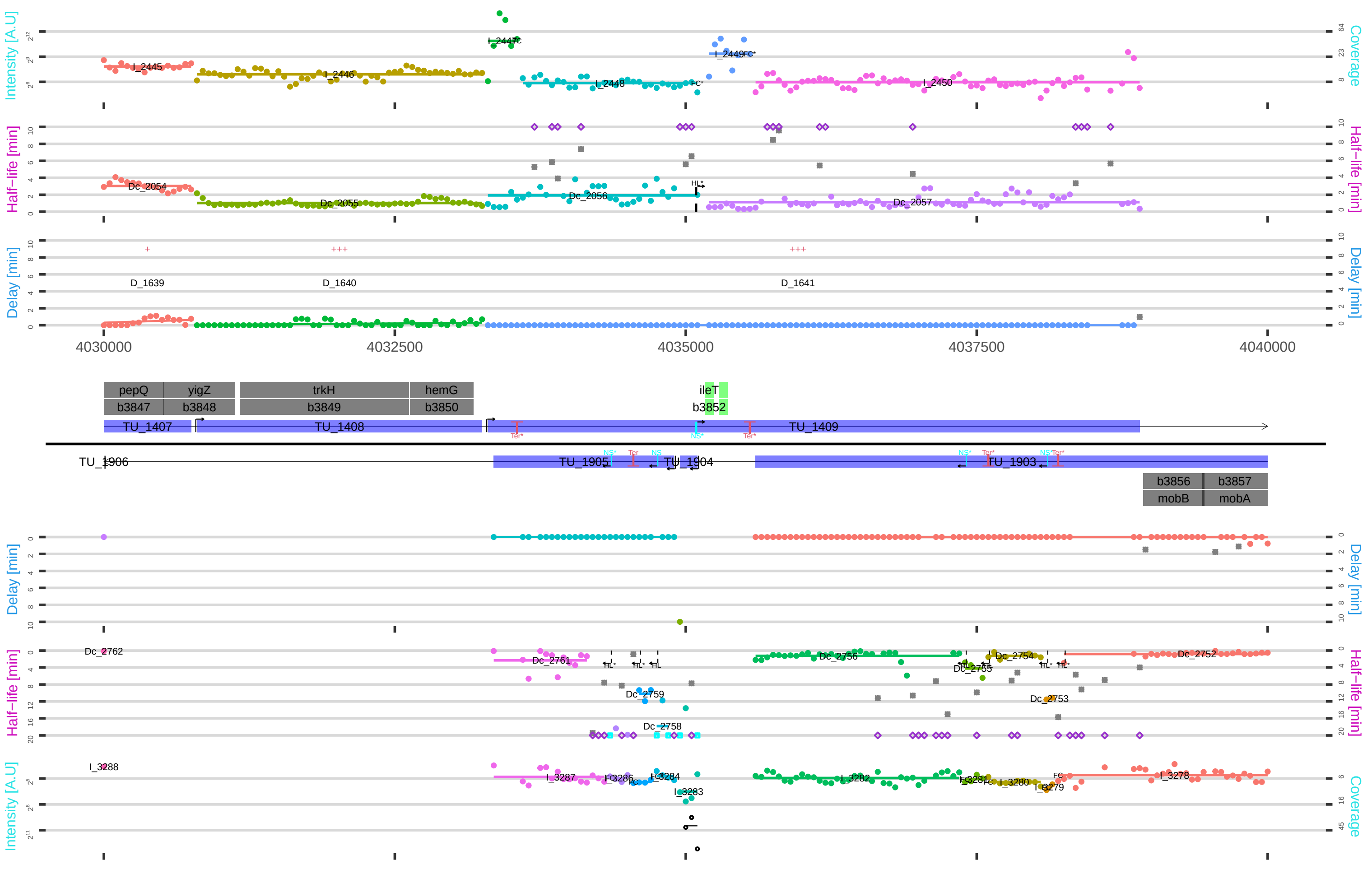

Term: termination (3), NS: new start (4), PS: pausing site (0), iTSS_I: internal starting site (0)

ID: 80803−81000; Term: termination (4), NS: new start (2), PS: pausing site (1), iTSS_I: internal starting site (0)

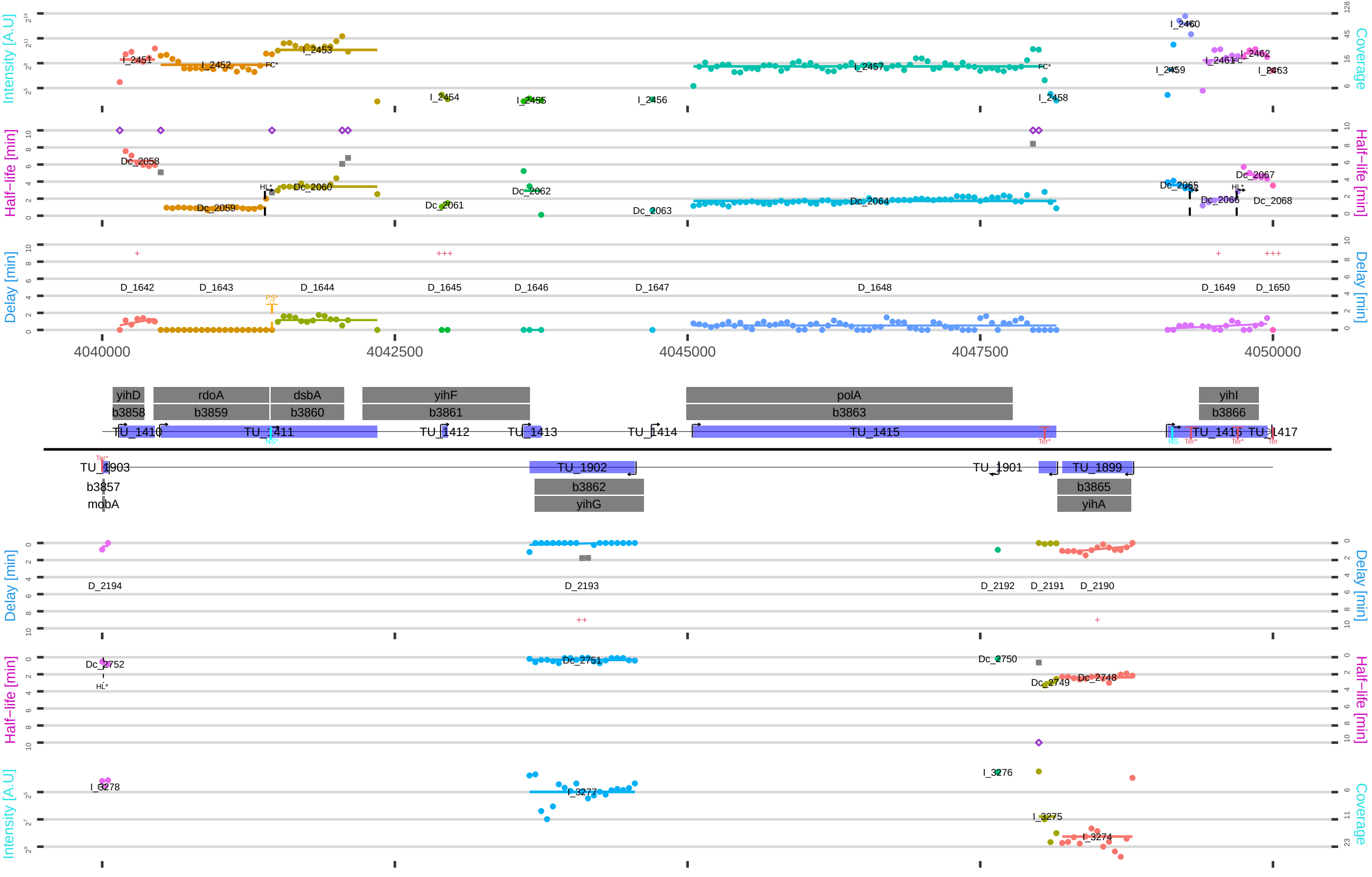

Term: termination (1), NS: new start (0), PS: pausing site (0), iTSS_I: internal starting site (0)

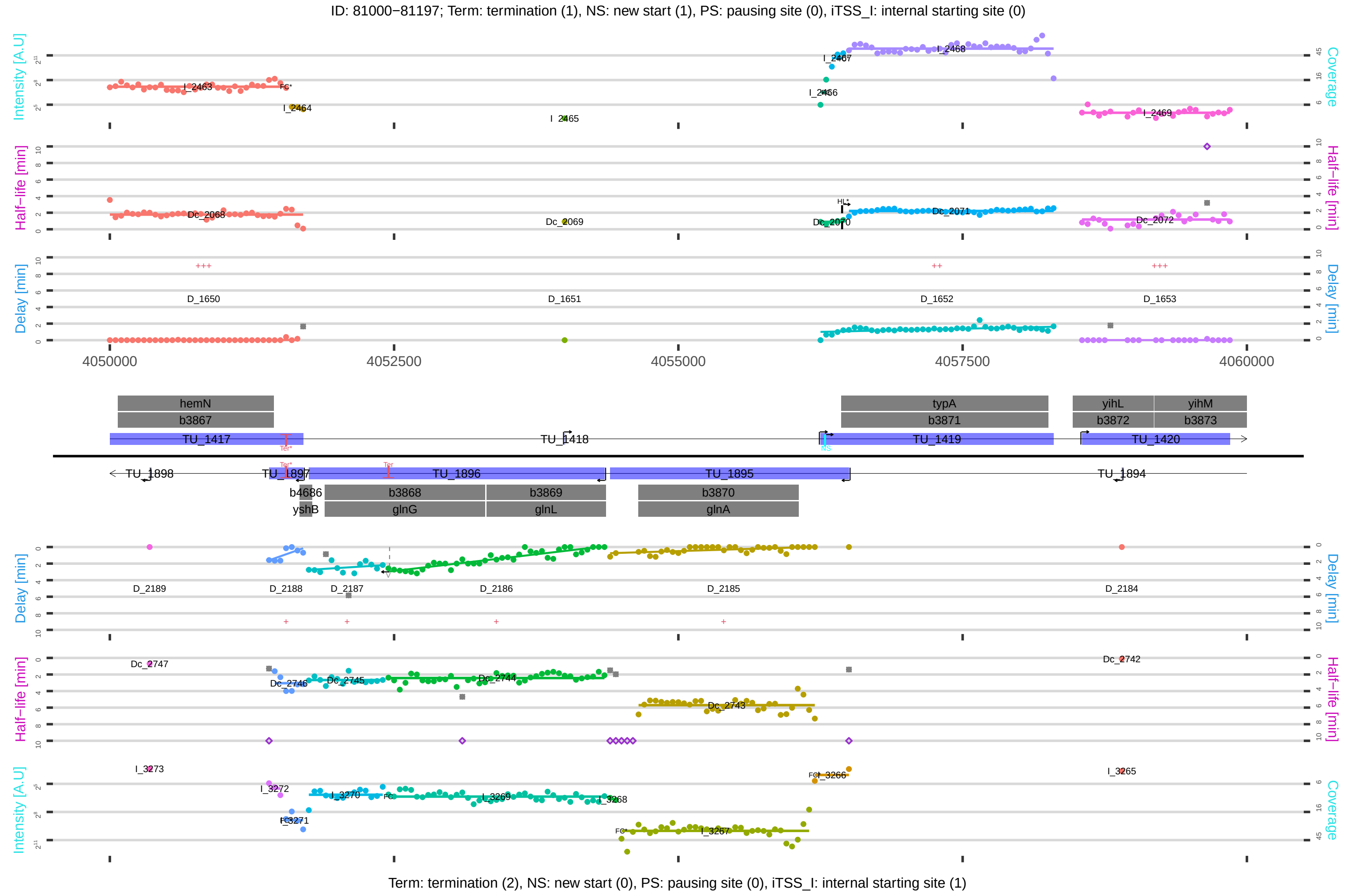
ID: 81000−81197; Term: termination (1), NS: new start (1), PS: pausing site (0), iTSS_I: internal starting site (0)
Intensity [A.U]
Coverage
Half−life [min]
Delay [min]
4050000
4052500
4055000
4057500
4060000
hemN
b3867
typA
b3871
yihL
b3872
yihM
b3873
TU_1417
TU_1418
TU_1419
TU_1420
TU_1898
TU_1897
TU_1896
TU_1895
TU_1894
b4686
yshB
b3868
glnG
b3869
glnL
b3870
glnA
Term: termination (2), NS: new start (0), PS: pausing site (0), iTSS_I: internal starting site (1)

ID: 81201−81374; Term: termination (0), NS: new start (0), PS: pausing site (0), iTSS_I: internal starting site (0)

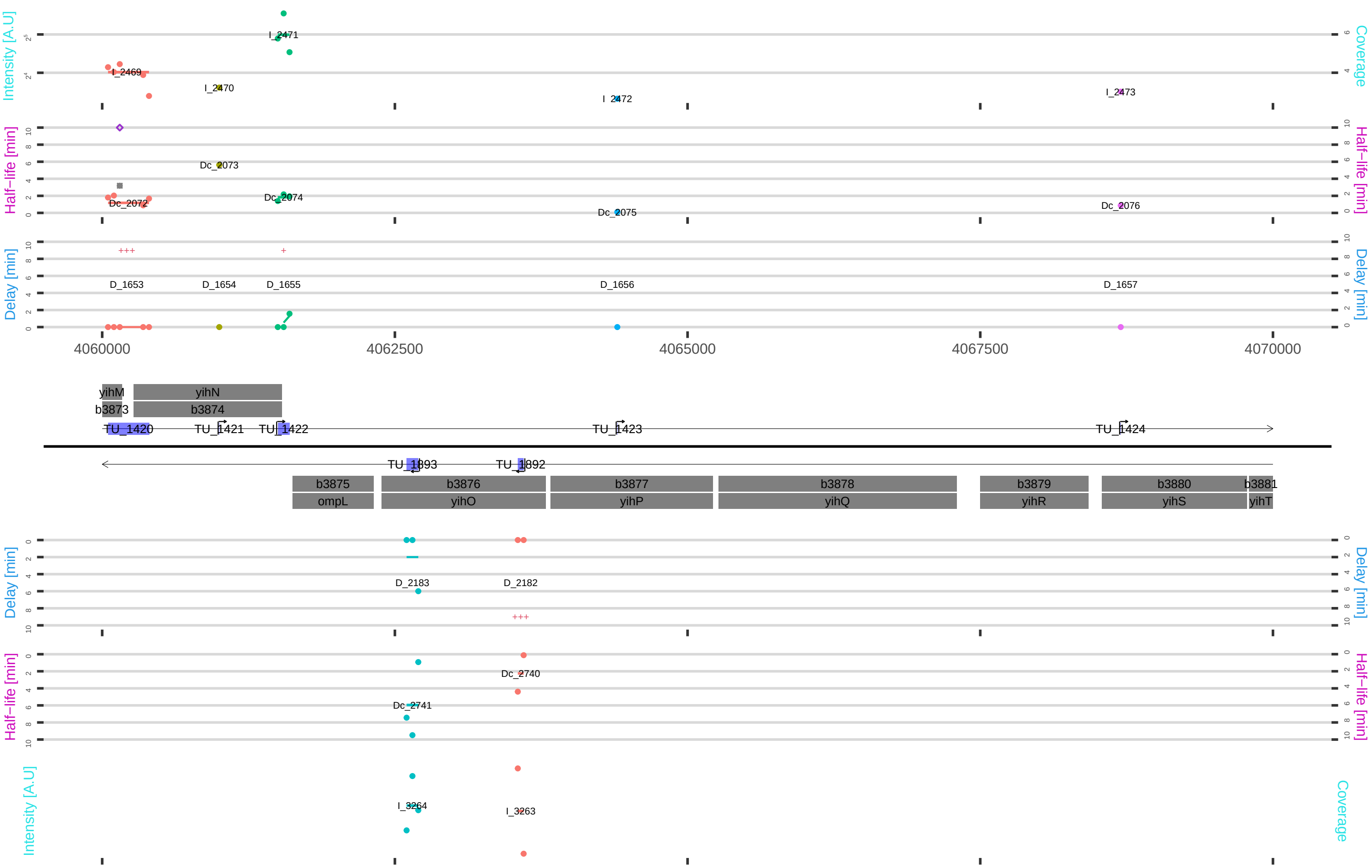

Term: termination (0), NS: new start (0), PS: pausing site (0), iTSS_I: internal starting site (0)

ID: 81445−81568; Term: termination (1), NS: new start (2), PS: pausing site (0), iTSS_I: internal starting site (0)

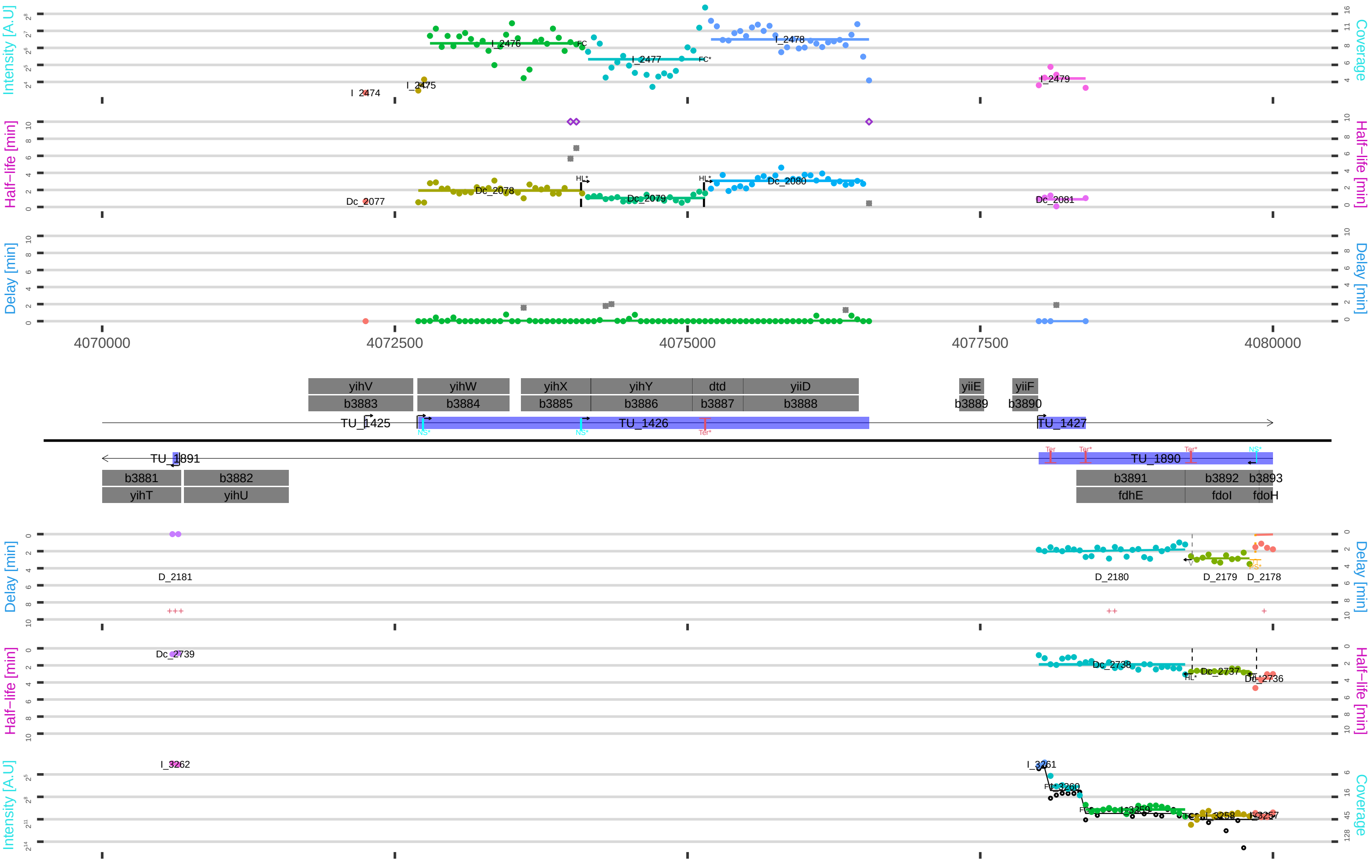

Term: termination (3), NS: new start (1), PS: pausing site (1), iTSS_I: internal starting site (1)

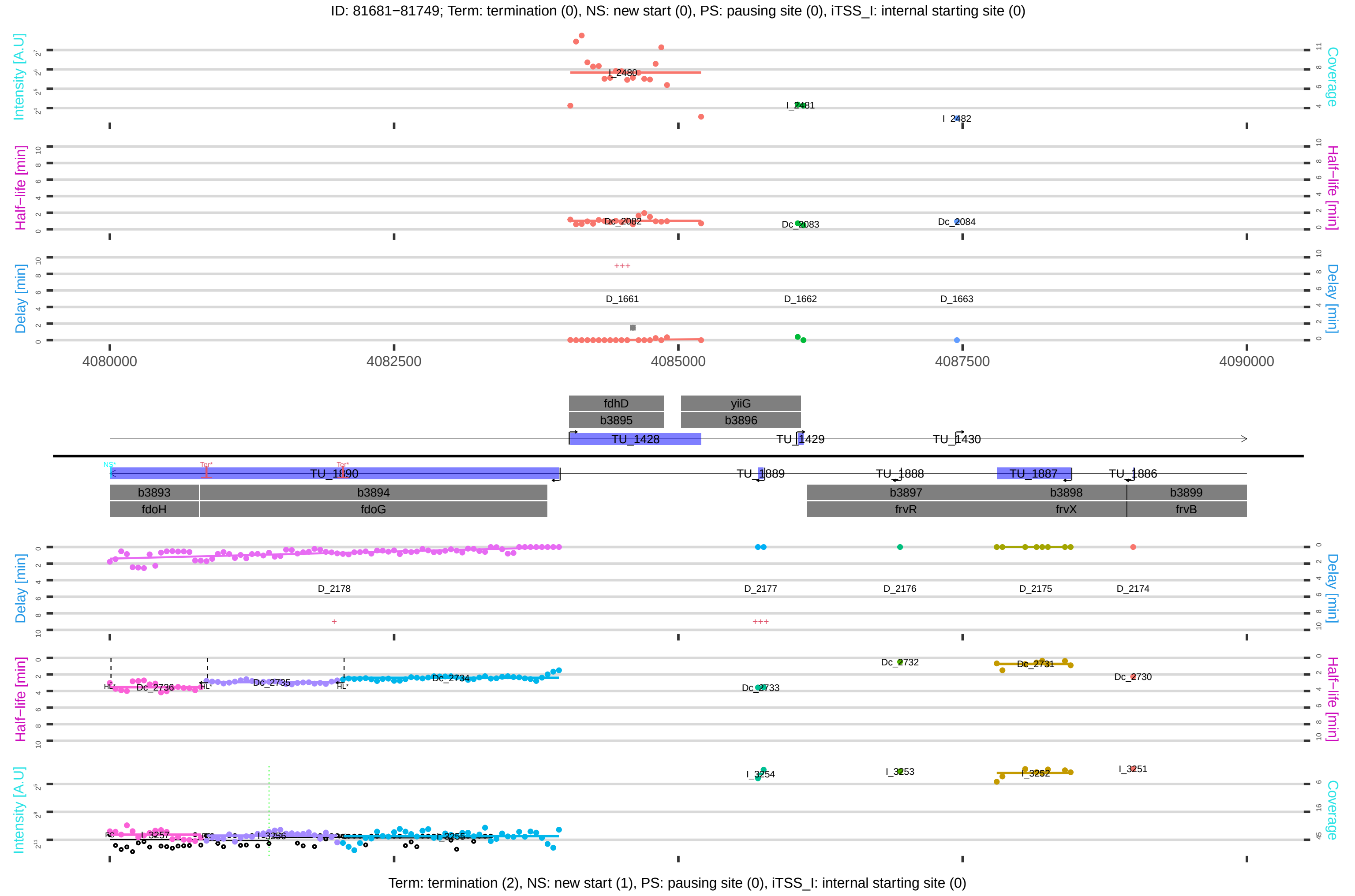

ID: 81681−81749; Term: termination (0), NS: new start (0), PS: pausing site (0), iTSS_I: internal starting site (0)
Intensity [A.U]
Coverage
I_2480
I_2481
I_2482
Half−life [min]
Dc_2082
Dc_2083
Dc_2084
Delay [min]
D_1661
D_1662
D_1663
4080000
4082500
4085000
4087500
4090000
fdhD
b3895
yiiG
b3896
TU_1428
TU_1429
TU_1430
TU_1890
TU_1889
TU_1888
TU_1887
TU_1886
b3893
fdoH
b3894
fdoG
b3897
frvR
b3898
frvX
b3899
frvB
D_2178
D_2177
D_2176
D_2175
D_2174
Dc_2736
Dc_2735
Dc_2734
Dc_2733
Dc_2732
Dc_2731
Dc_2730
I_3257
I_3256
I_3255
I_3254
I_3253
I_3252
I_3251
Term: termination (2), NS: new start (1), PS: pausing site (0), iTSS_I: internal starting site (0)

ID: 81840−82000; Term: termination (1), NS: new start (1), PS: pausing site (0), iTSS_I: internal starting site (1)

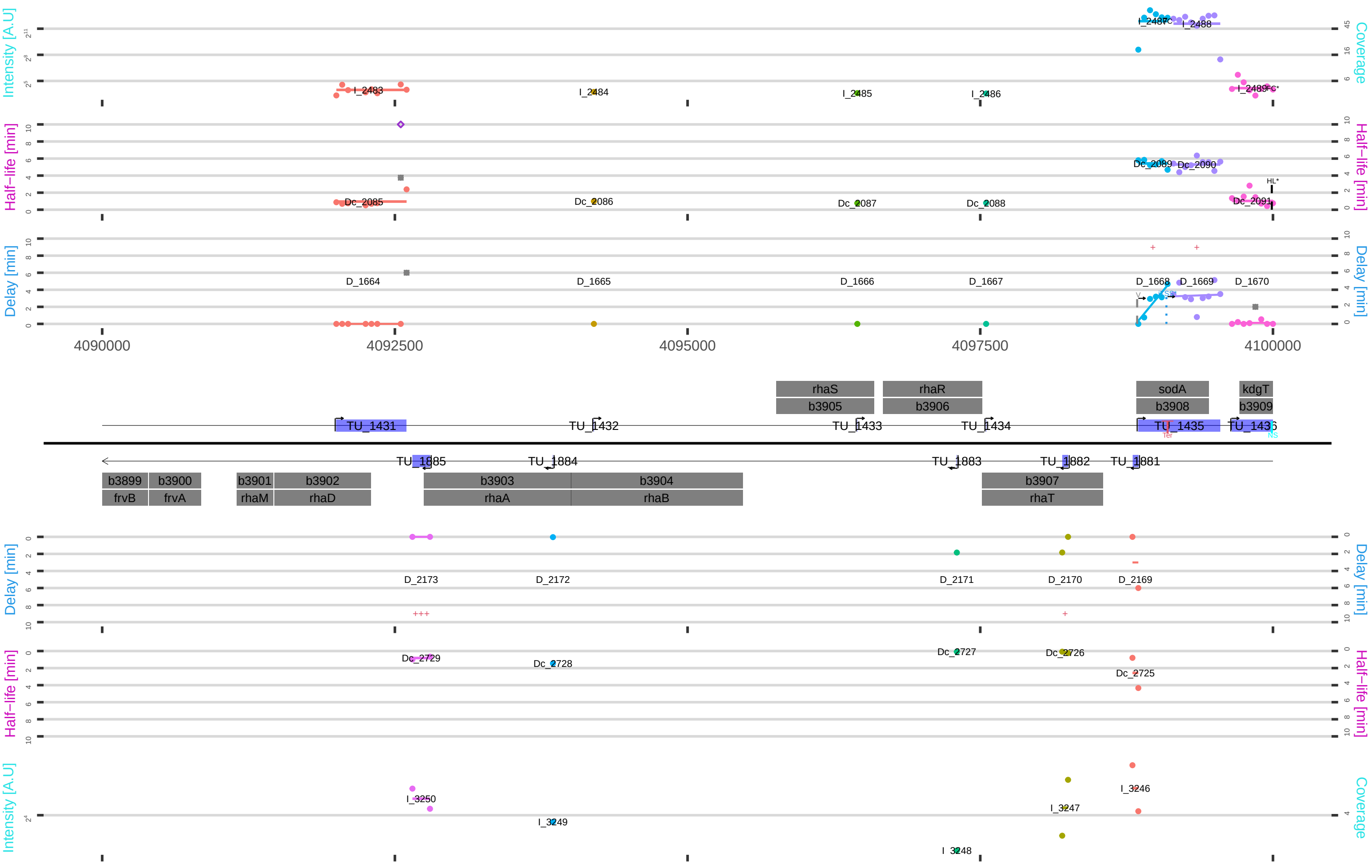

Term: termination (0), NS: new start (0), PS: pausing site (0), iTSS_I: internal starting site (0)

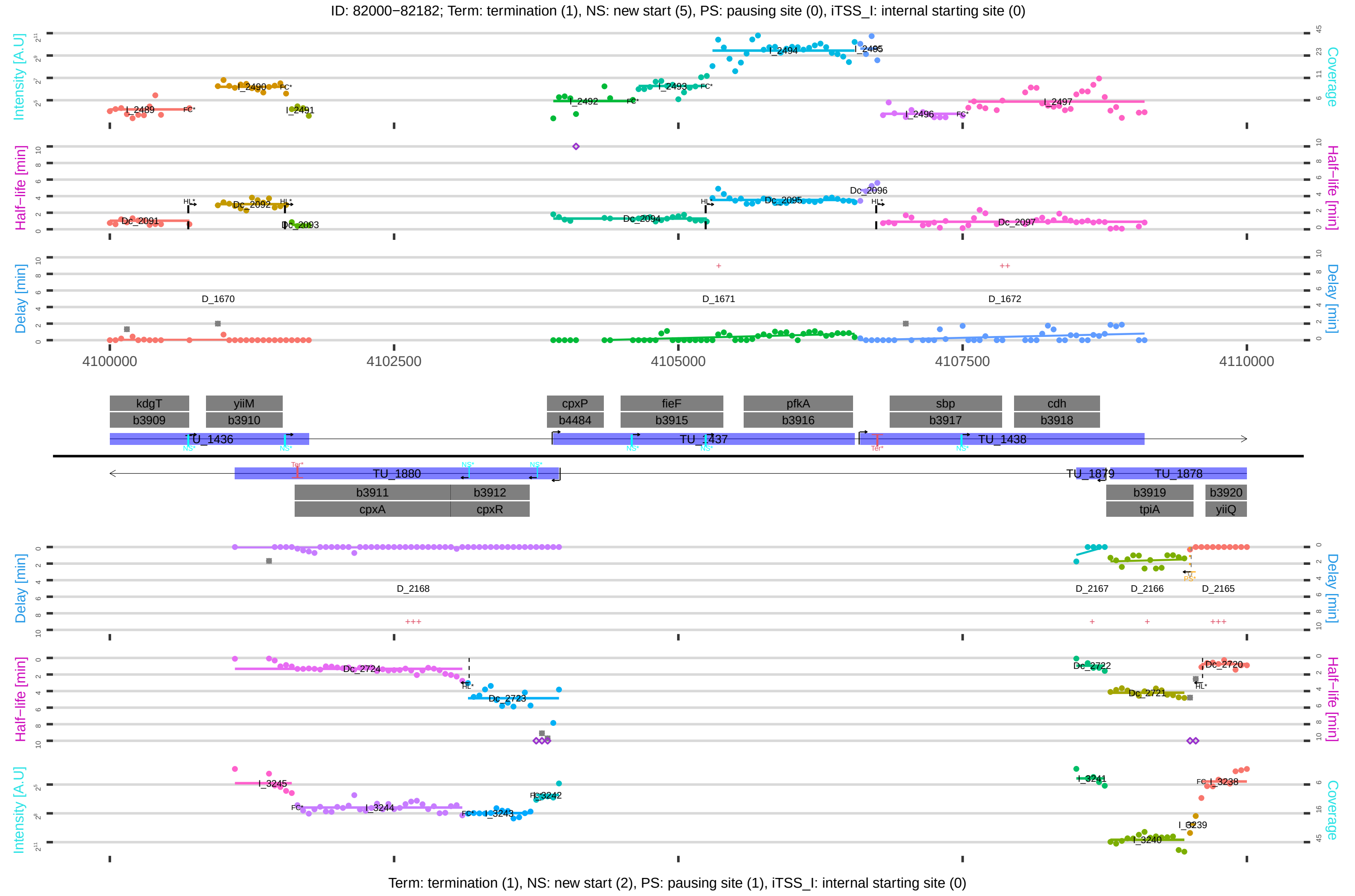
ID: 82000−82182; Term: termination (1), NS: new start (5), PS: pausing site (0), iTSS_I: internal starting site (0)
Intensity [A.U]
Coverage
Half−life [min]
Delay [min]
I_2489
I_2490
I_2491
I_2492
I_2493
I_2494
I_2495
I_2496
I_2497
Dc_2091
Dc_2092
Dc_2093
Dc_2094
Dc_2095
Dc_2096
Dc_2097
D_1670
D_1671
D_1672
4100000
4102500
4105000
4107500
4110000
kdgT
b3909
yiiM
b3910
cpxP
b4484
fieF
b3915
pfkA
b3916
sbp
b3917
cdh
b3918
TU_1436
TU_1437
TU_1438
TU_1880
TU_1879
TU_1878
b3911
cpxA
b3912
cpxR
b3919
tpiA
b3920
yiiQ
D_2168
D_2167
D_2166
D_2165
Dc_2724
Dc_2723
Dc_2722
Dc_2721
Dc_2720
I_3245
I_3244
I_3243
I_3242
I_3241
I_3240
I_3239
I_3238
Term: termination (1), NS: new start (2), PS: pausing site (1), iTSS_I: internal starting site (0)

ID: 82204−82342; Term: termination (2), NS: new start (0), PS: pausing site (0), iTSS_I: internal starting site (0)

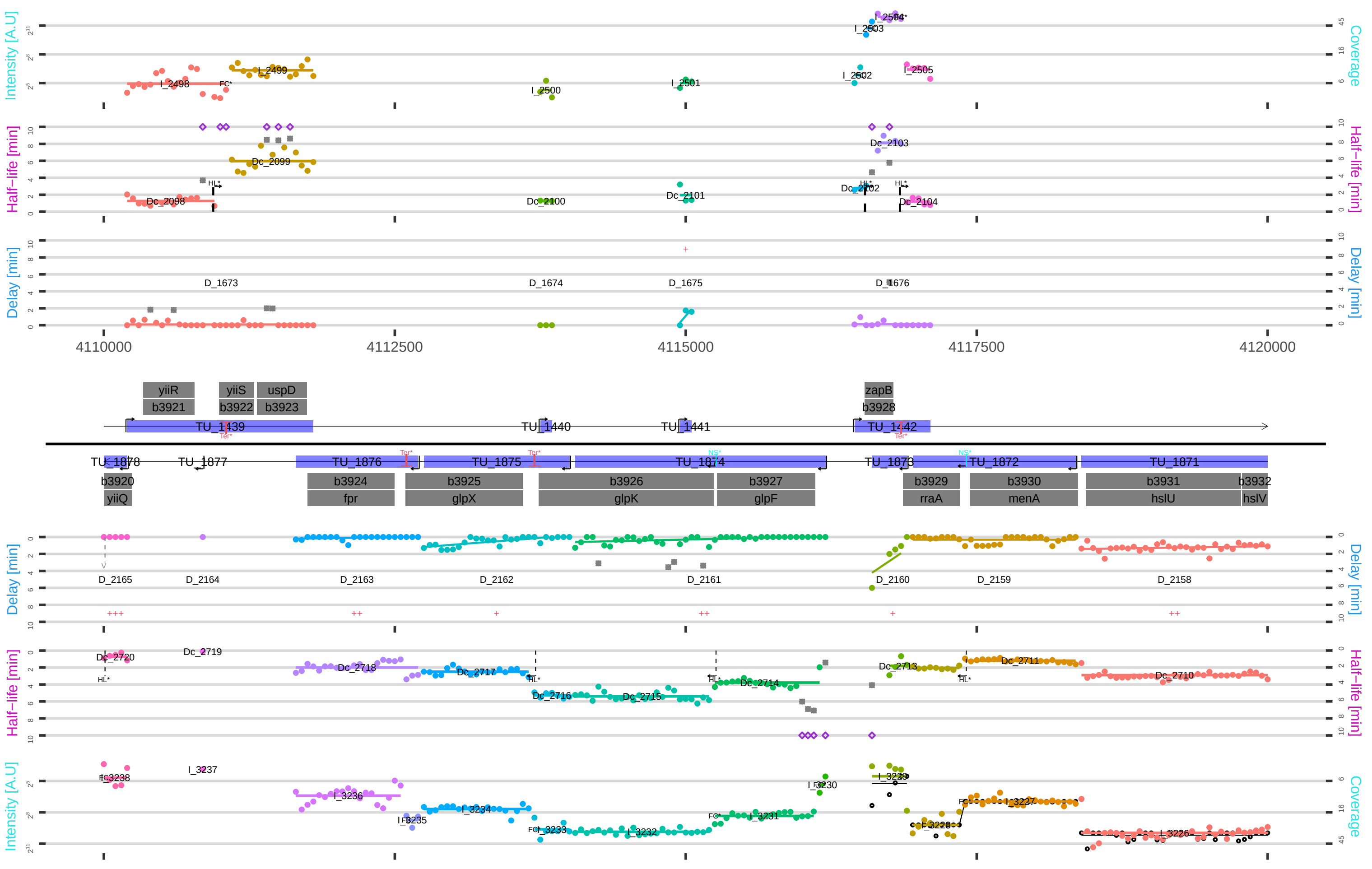

Term: termination (2), NS: new start (2), PS: pausing site (0), iTSS_I: internal starting site (0)

ID: 82413−82600; Term: termination (2), NS: new start (2), PS: pausing site (1), iTSS_I: internal starting site (0)

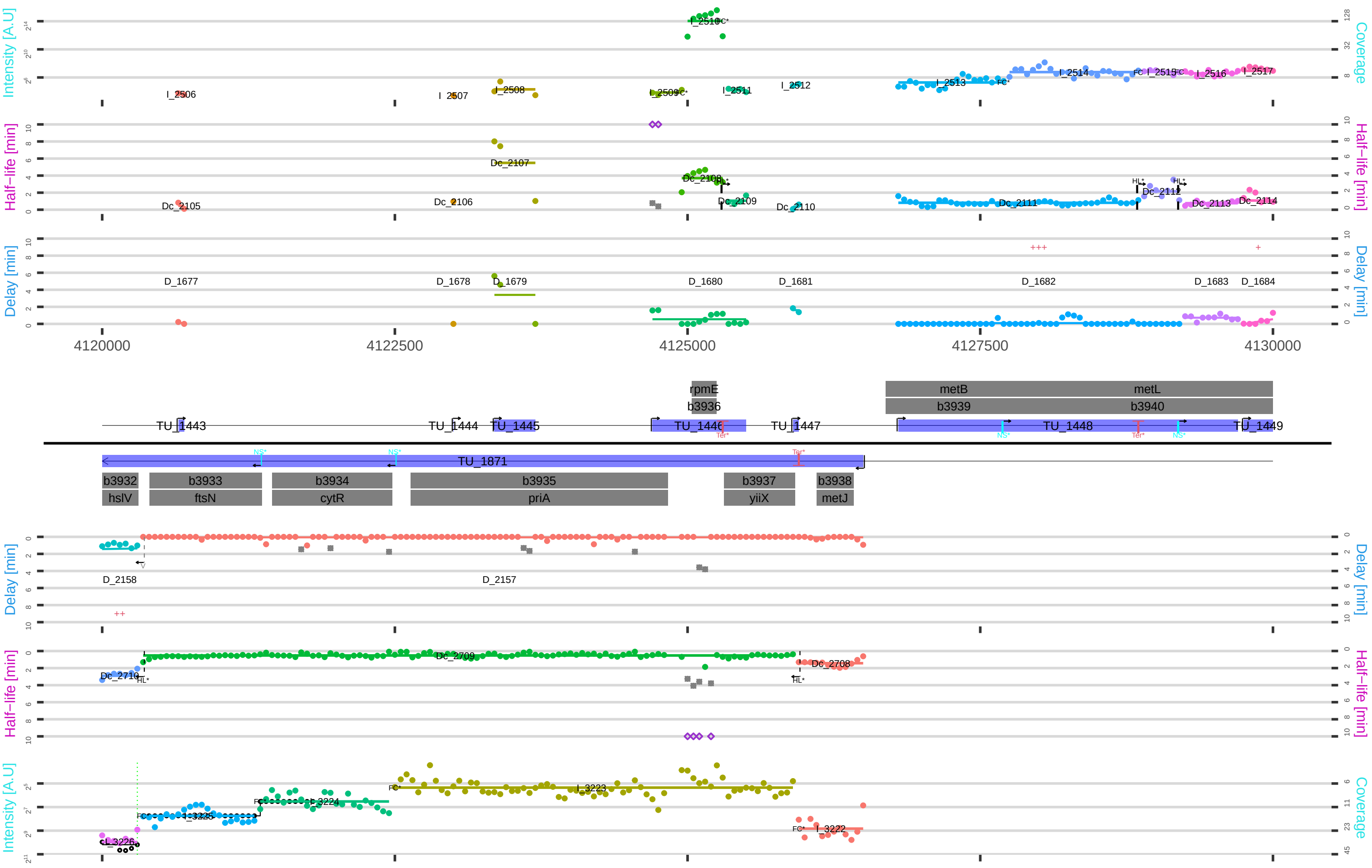

Term: termination (1), NS: new start (2), PS: pausing site (1), iTSS_I: internal starting site (0)

ID: 82600−82717; Term: termination (2), NS: new start (1), PS: pausing site (1), iTSS_I: internal starting site (0)

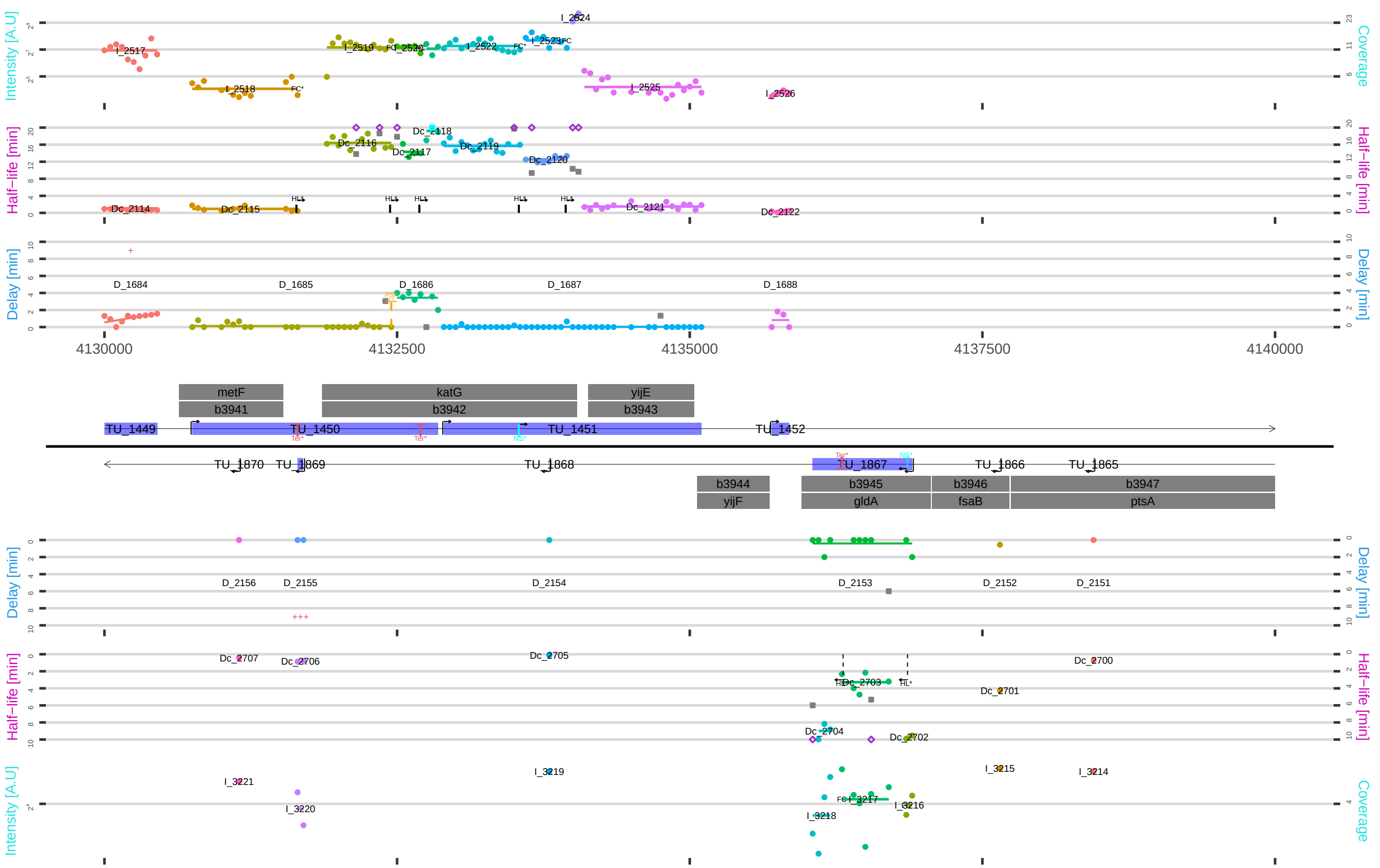

Term: termination (1), NS: new start (1), PS: pausing site (0), iTSS_I: internal starting site (0)

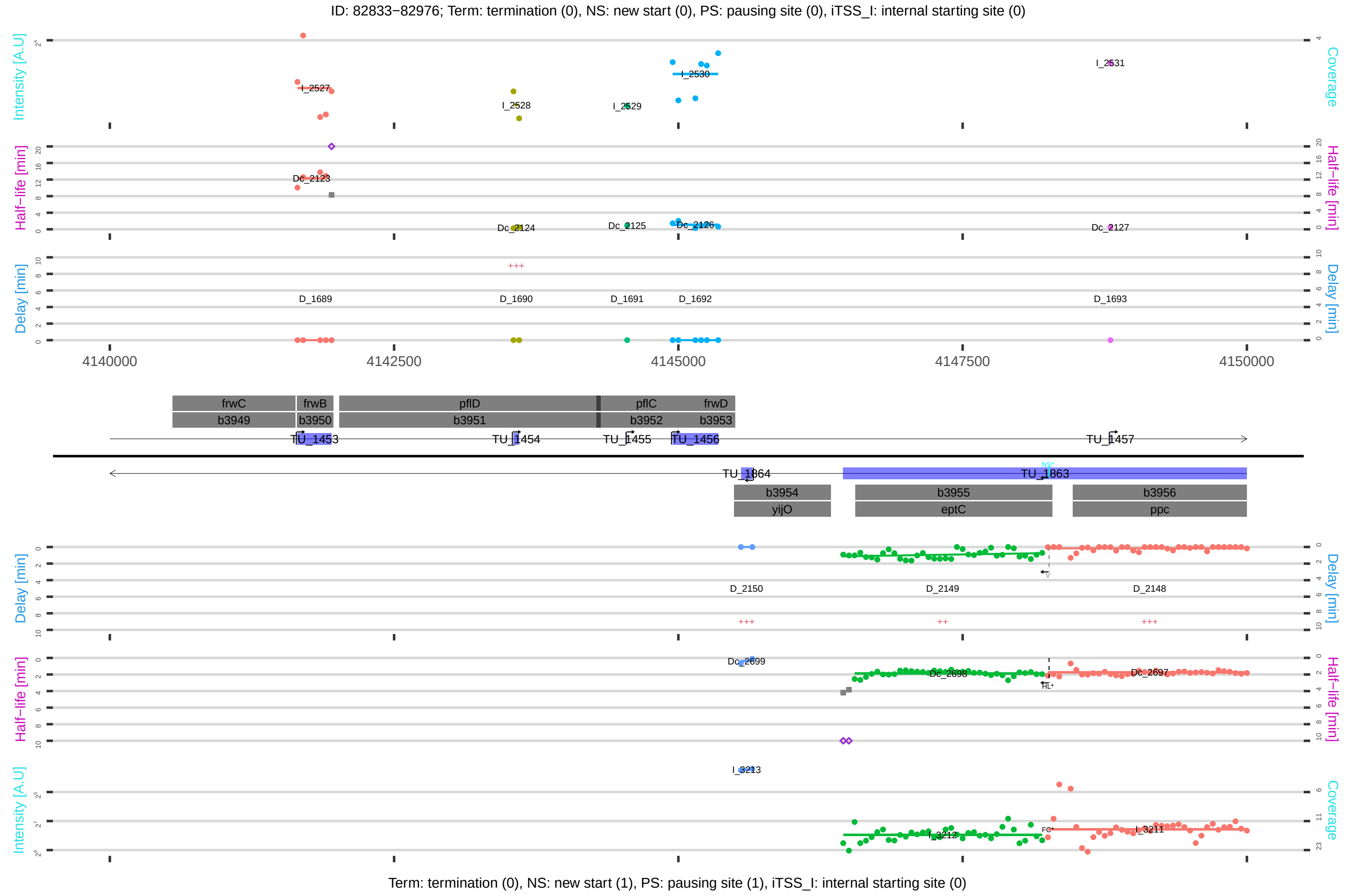
ID: 82833−82976; Term: termination (0), NS: new start (0), PS: pausing site (0), iTSS_I: internal starting site (0)
Intensity [A.U]
Coverage
Half−life [min]
Delay [min]
I_2527
I_2528
I_2529
I_2530
I_2531
Dc_2123
Dc_2124
Dc_2125
Dc_2126
Dc_2127
D_1689
D_1690
D_1691
D_1692
D_1693
4140000
4142500
4145000
4147500
4150000
frwC
b3949
frwB
b3950
pflD
b3951
pflC
b3952
frwD
b3953
TU_1453
TU_1454
TU_1455
TU_1456
TU_1457
TU_1864
TU_1863
b3954
yijO
b3955
eptC
b3956
ppc
D_2150
D_2149
D_2148
Dc_2699
Dc_2698
Dc_2697
I_3213
I_3212
I_3211
Term: termination (0), NS: new start (1), PS: pausing site (1), iTSS_I: internal starting site (0)

ID: 83027−83200; Term: termination (2), NS: new start (5), PS: pausing site (0), iTSS_I: internal starting site (0)

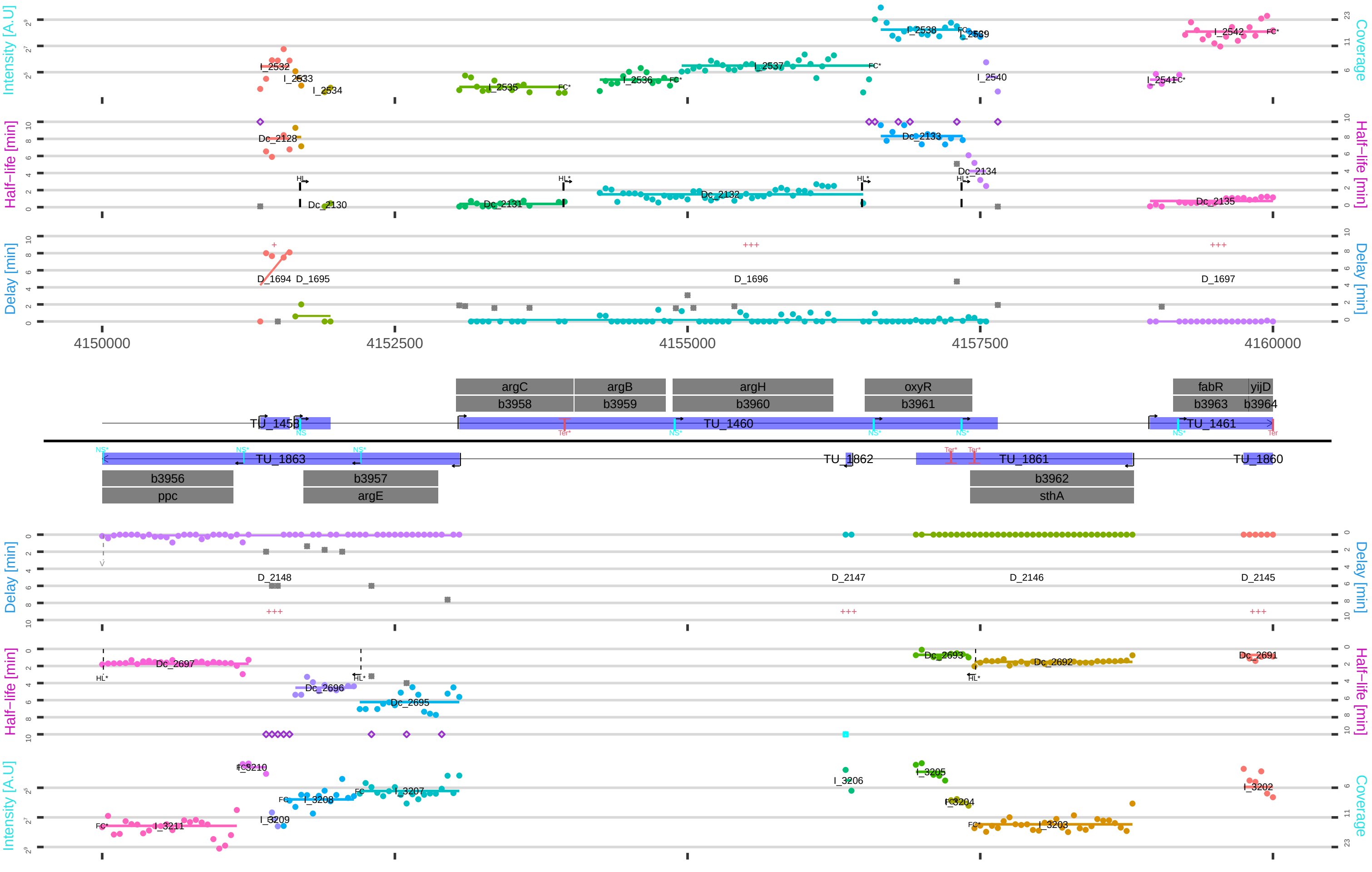

Term: termination (2), NS: new start (3), PS: pausing site (0), iTSS_I: internal starting site (0)

ID: 83200−83400; Term: termination (5), NS: new start (5), PS: pausing site (1), iTSS_I: internal starting site (0)

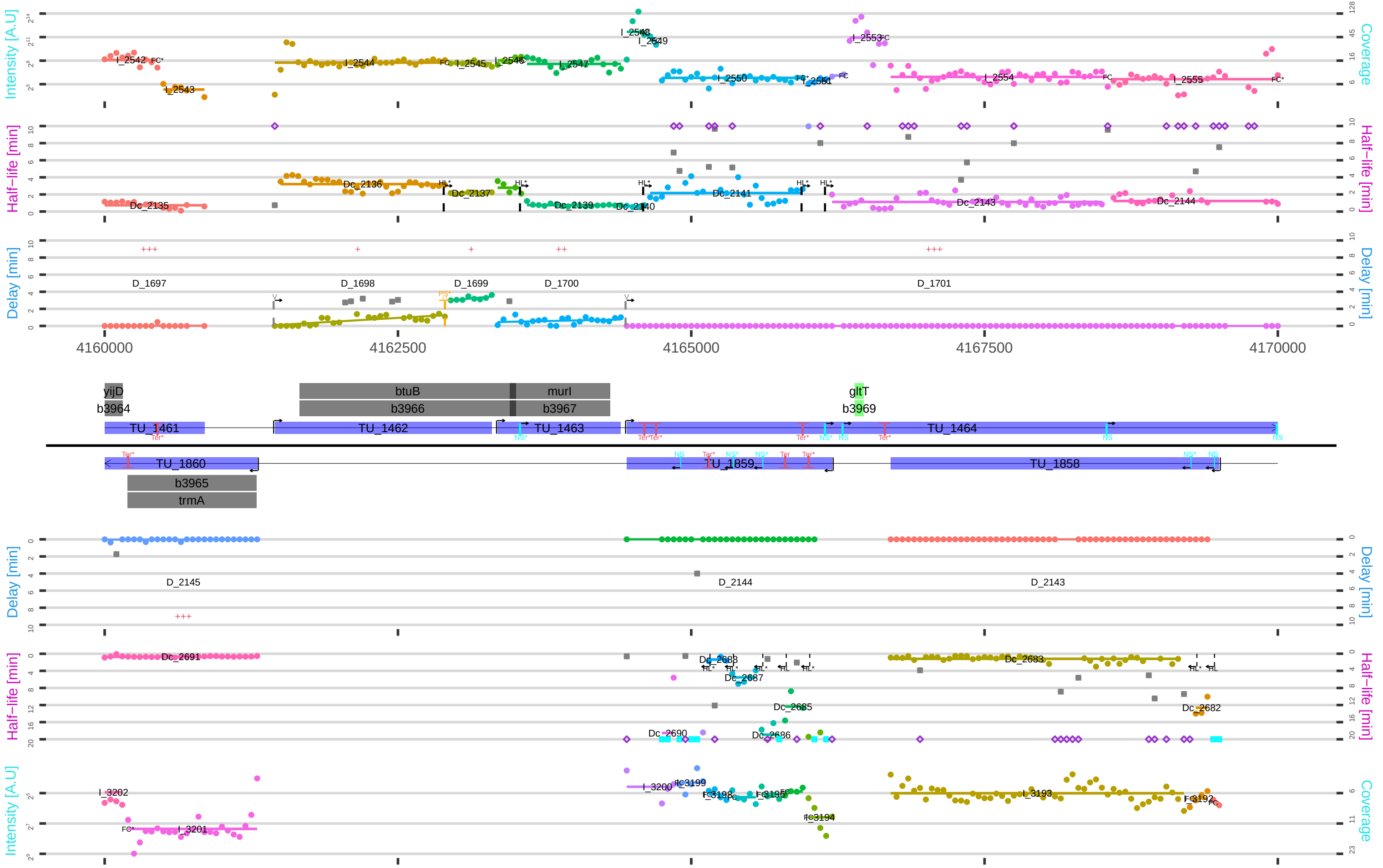

Term: termination (4), NS: new start (5), PS: pausing site (0), iTSS_I: internal starting site (0)

ID: 83400−83600; Term: termination (4), NS: new start (4), PS: pausing site (2), iTSS_I: internal starting site (0)

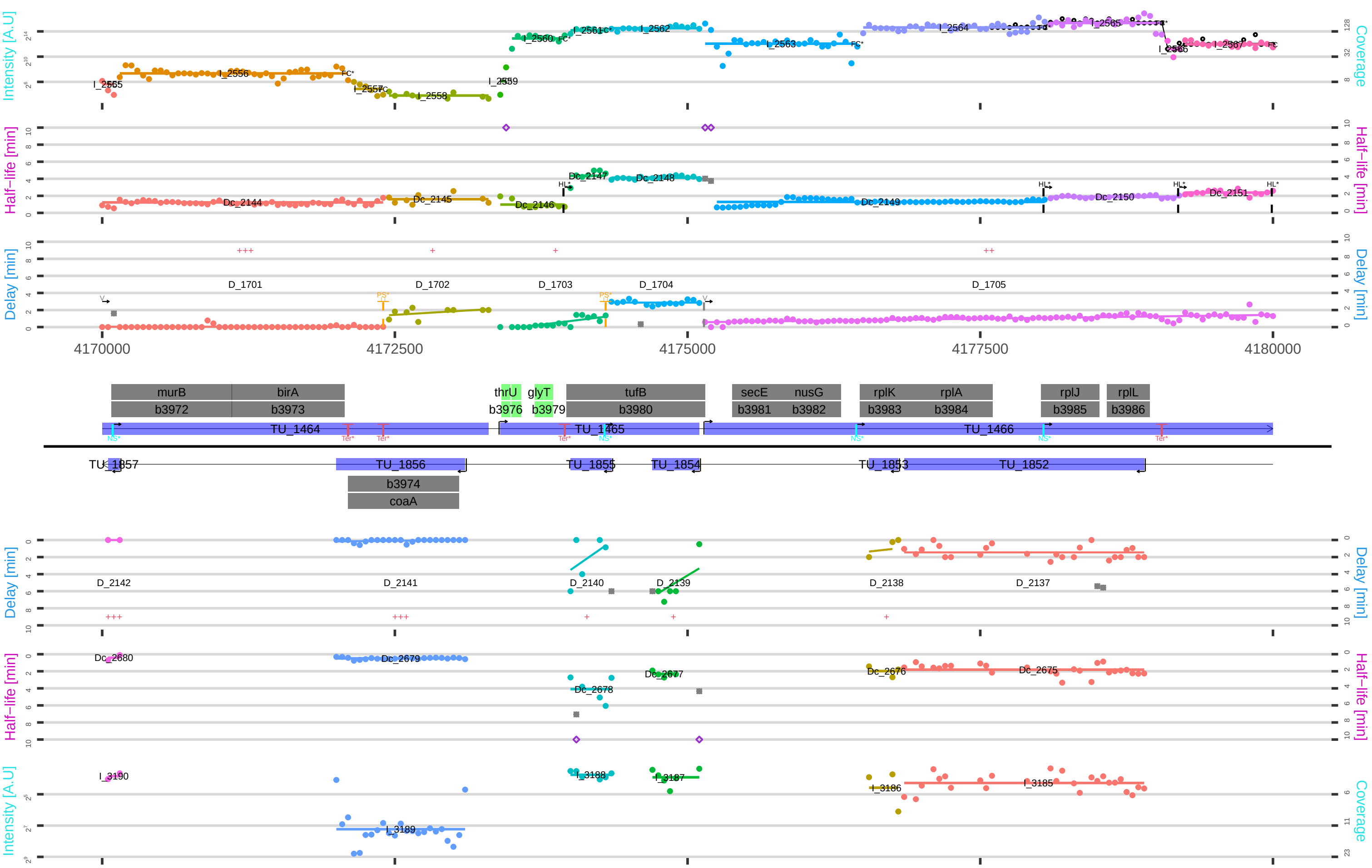

Term: termination (0), NS: new start (0), PS: pausing site (0), iTSS_I: internal starting site (0)

ID: 83600−83769; Term: termination (3), NS: new start (1), PS: pausing site (2), iTSS_I: internal starting site (1)

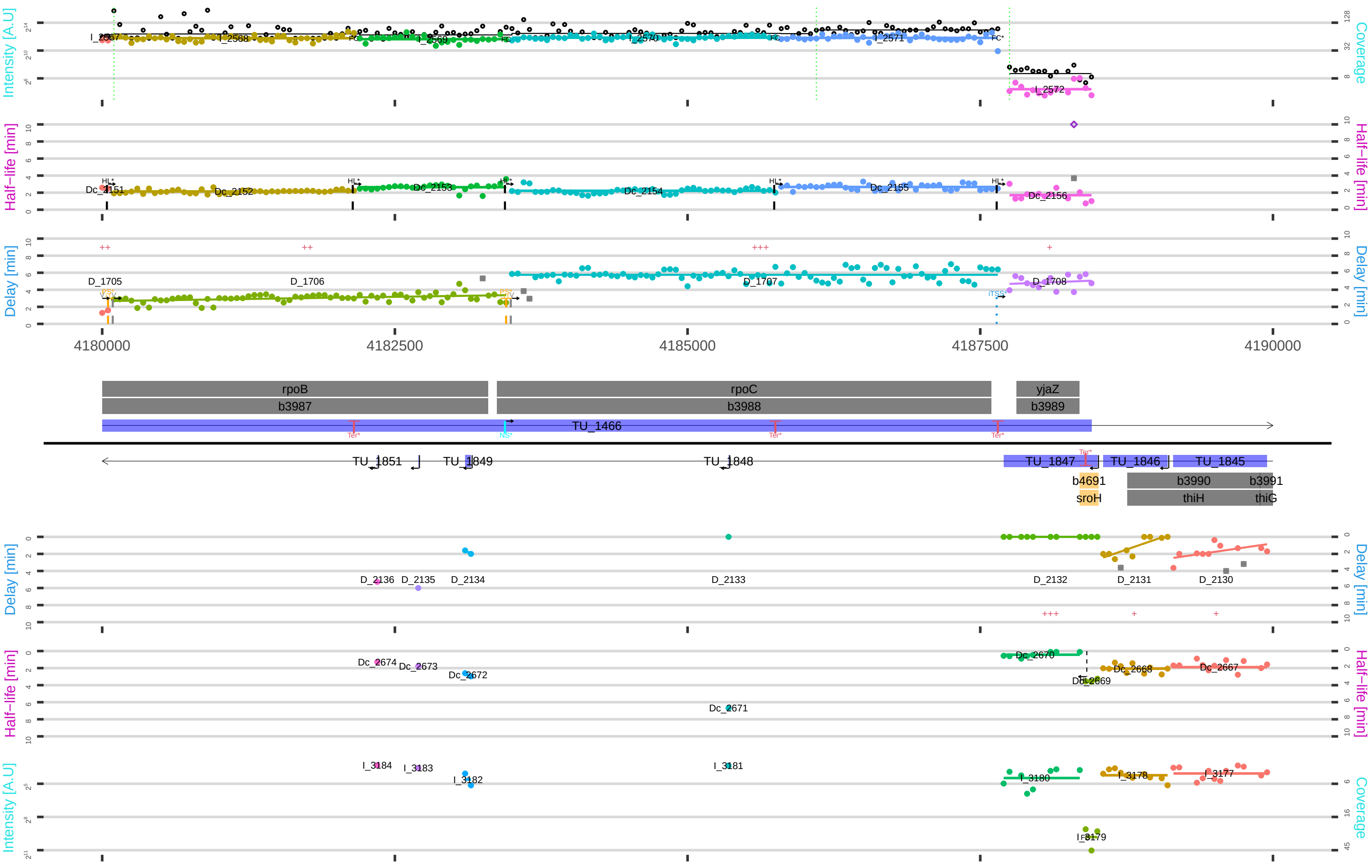

Term: termination (1), NS: new start (0), PS: pausing site (0), iTSS_I: internal starting site (0)

ID: 83896−83995; Term: termination (4), NS: new start (4), PS: pausing site (1), iTSS_I: internal starting site (0)

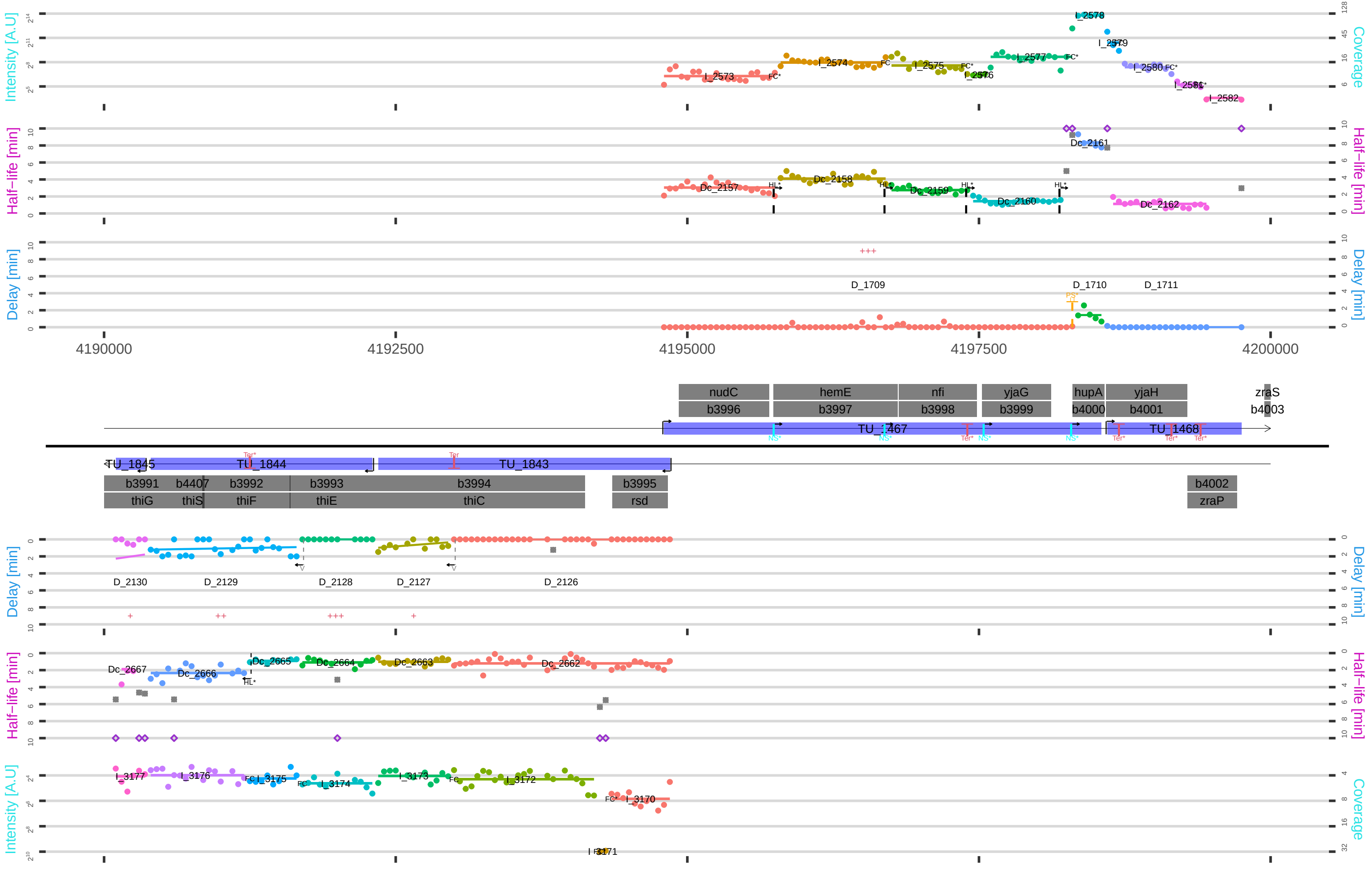

Term: termination (2), NS: new start (0), PS: pausing site (2), iTSS_I: internal starting site (0)

ID: 84001−84200; Term: termination (2), NS: new start (1), PS: pausing site (0), iTSS_I: internal starting site (0)

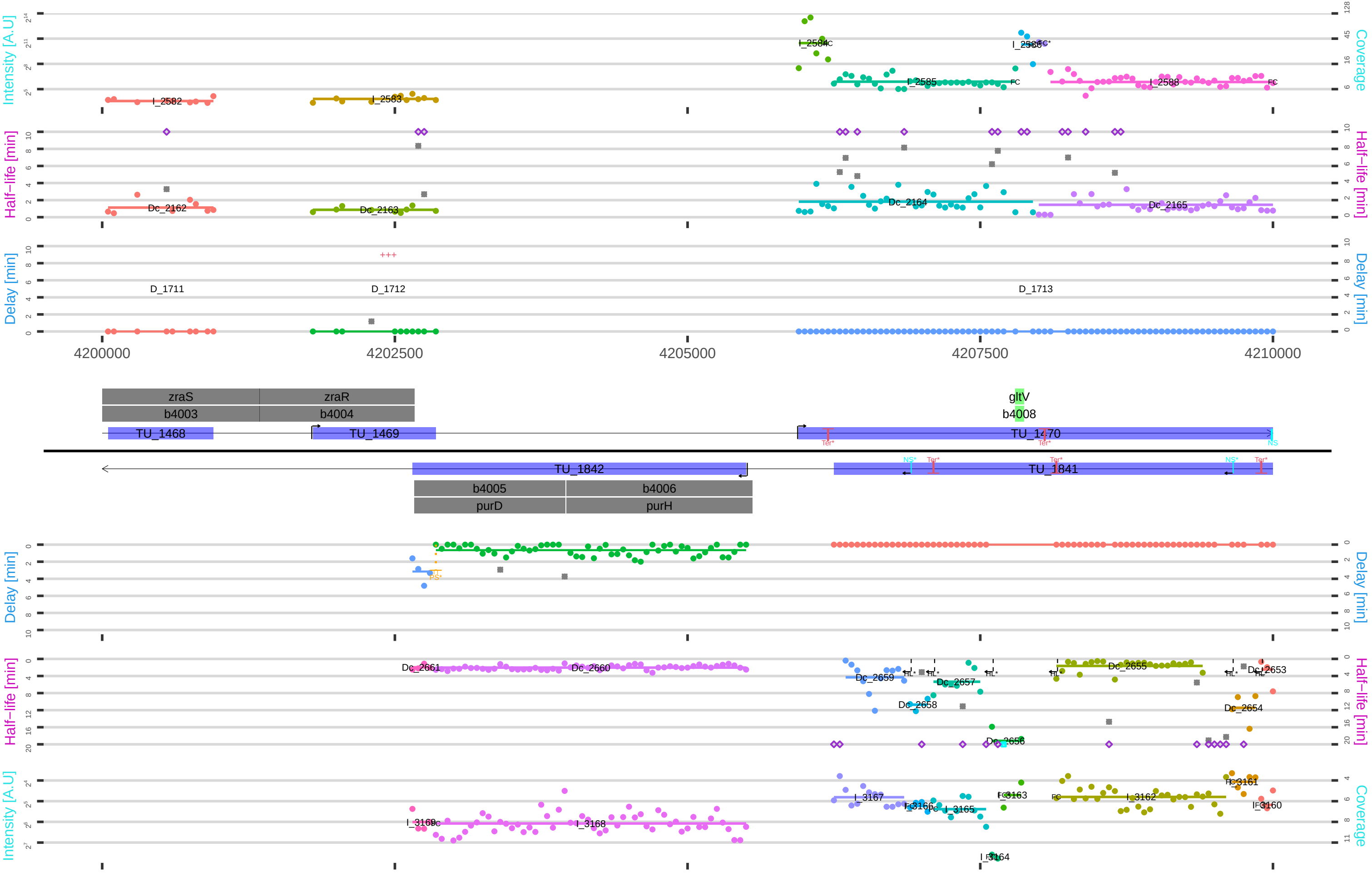

Term: termination (3), NS: new start (2), PS: pausing site (1), iTSS_I: internal starting site (0)

ID: 84200−84365; Term: termination (1), NS: new start (4), PS: pausing site (0), iTSS_I: internal starting site (0)

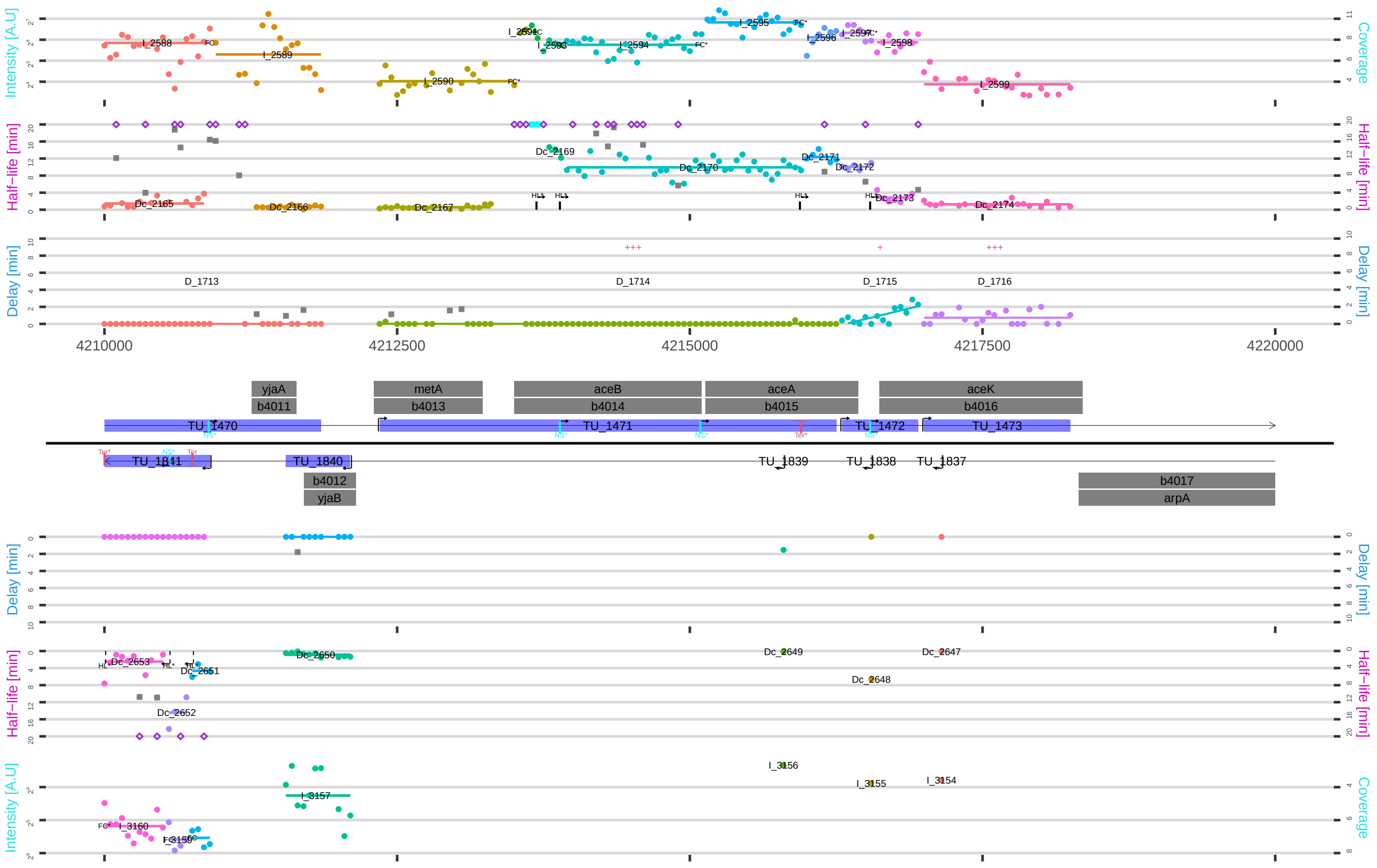

Term: termination (2), NS: new start (1), PS: pausing site (0), iTSS_I: internal starting site (0)

ID: 84438−84596; Term: termination (1), NS: new start (1), PS: pausing site (1), iTSS_I: internal starting site (0)

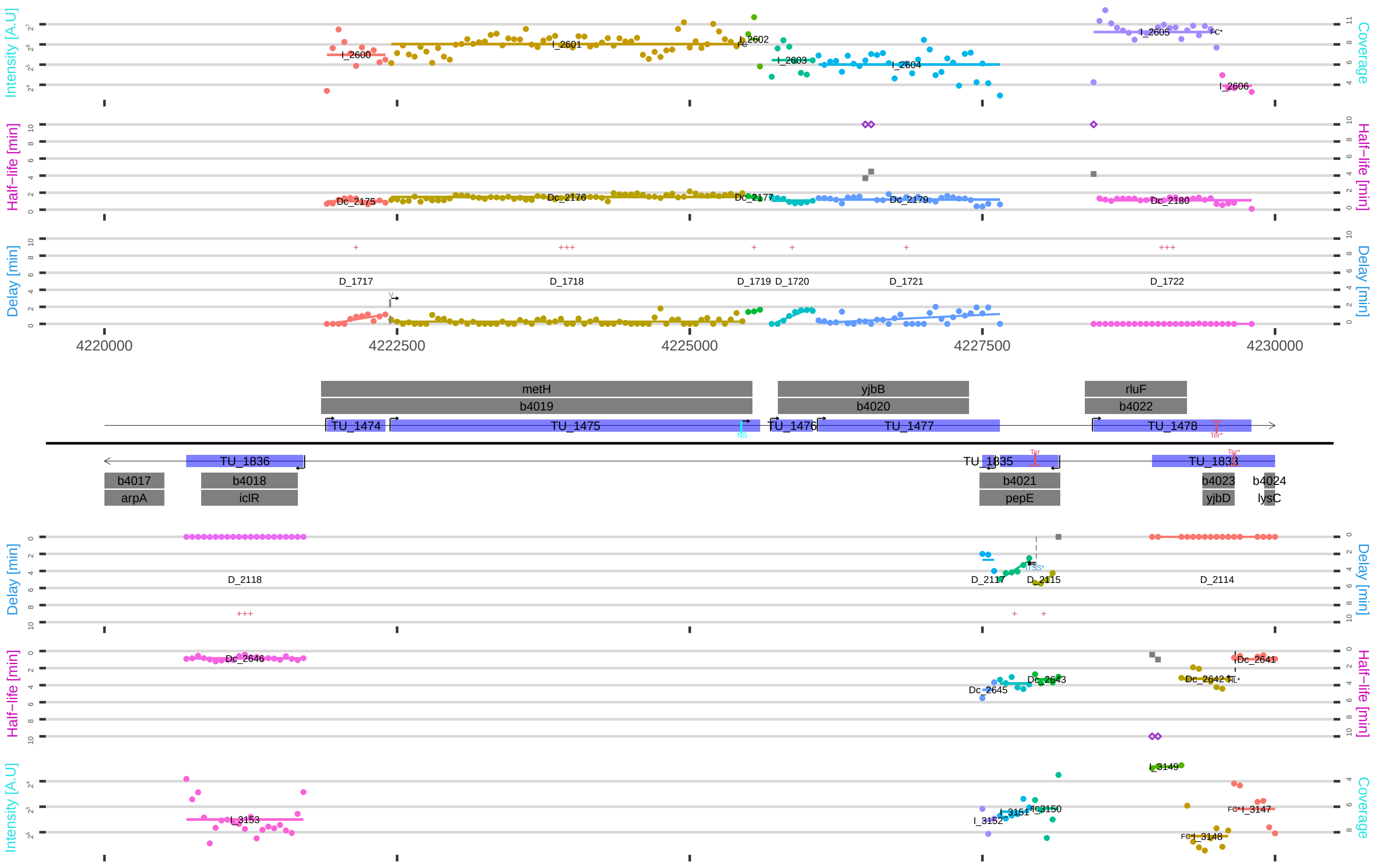

Term: termination (2), NS: new start (0), PS: pausing site (0), iTSS_I: internal starting site (1)

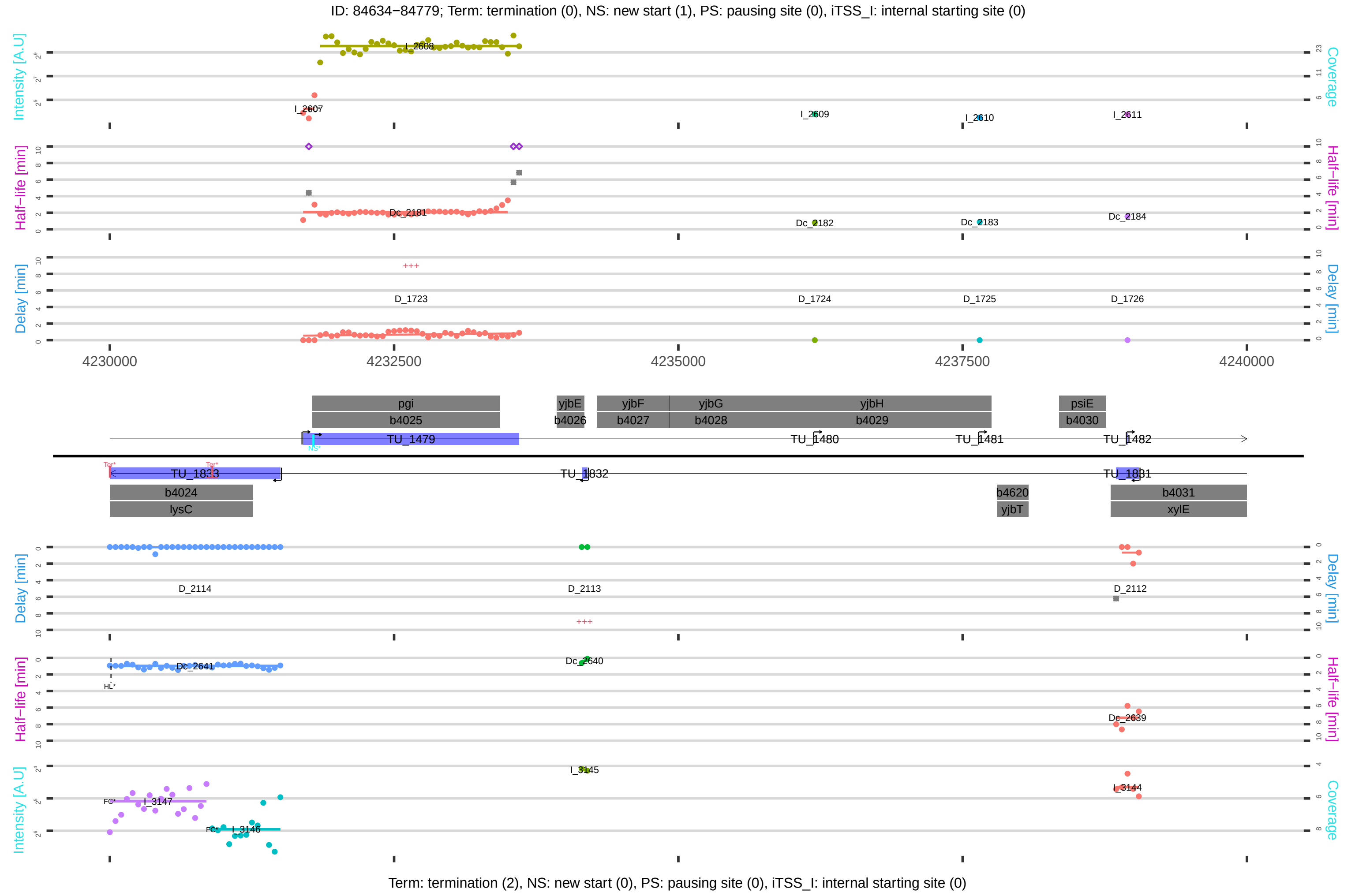
ID: 84634−84779; Term: termination (0), NS: new start (1), PS: pausing site (0), iTSS_I: internal starting site (0)
Intensity [A.U]
Coverage
Half−life [min]
Delay [min]
I_2607
I_2608
I_2609
I_2610
I_2611
Dc_2181
Dc_2182
Dc_2183
Dc_2184
D_1723
D_1724
D_1725
D_1726
4230000
4232500
4235000
4237500
4240000
pgi
b4025
yjbE
b4026
yjbF
b4027
yjbG
b4028
yjbH
b4029
psiE
b4030
TU_1479
TU_1480
TU_1481
TU_1482
NS*
Ter*
TU_1833
TU_1832
TU_1831
b4024
lysC
b4620
yjbT
b4031
xylE
D_2114
D_2113
D_2112
Dc_2641
Dc_2640
Dc_2639
HL*
I_3145
I_3144
I_3147
I_3146
FC*
Term: termination (2), NS: new start (0), PS: pausing site (0), iTSS_I: internal starting site (0)

ID: 84897−84974; Term: termination (1), NS: new start (2), PS: pausing site (2), iTSS_I: internal starting site (0)

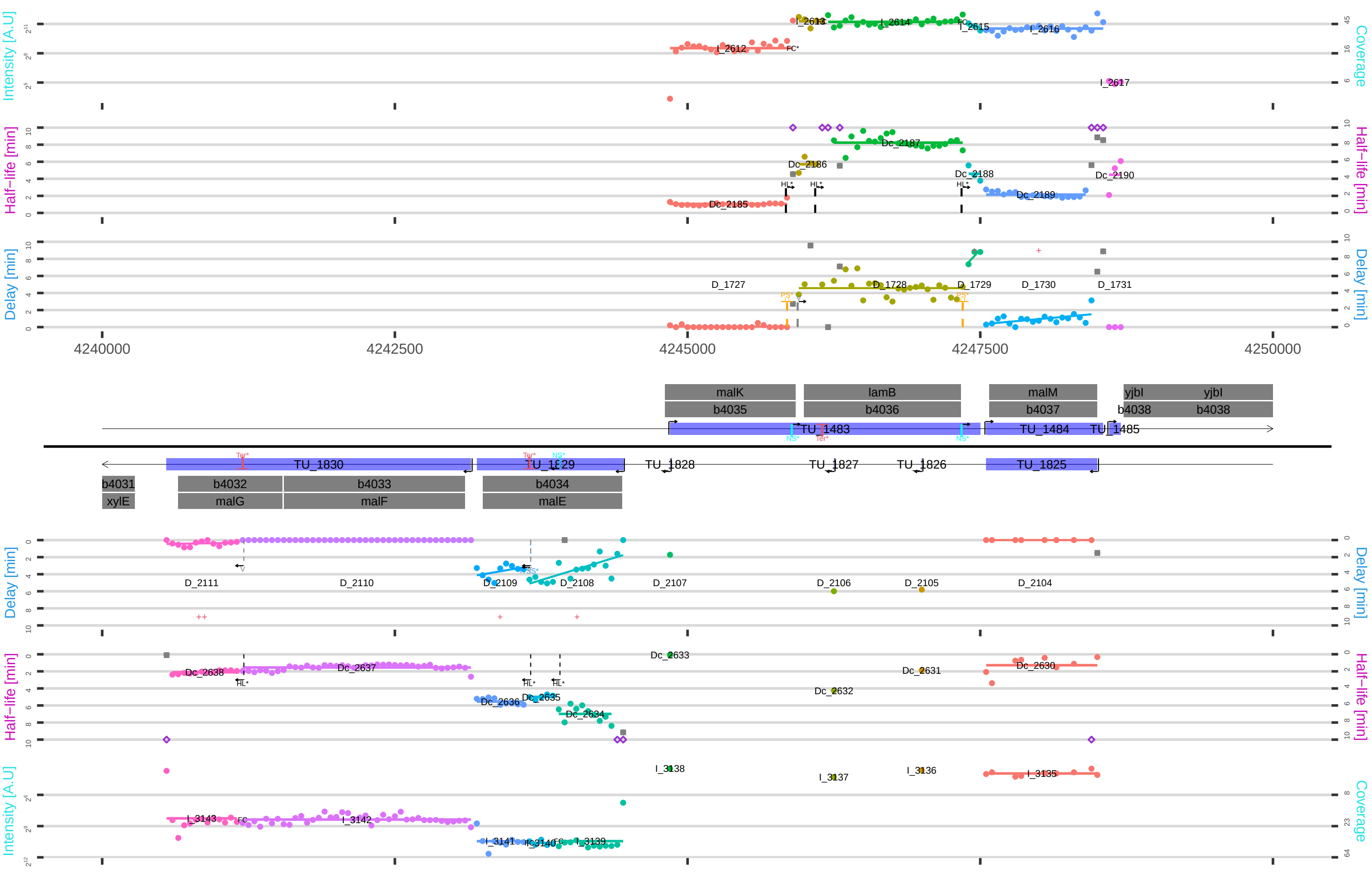

Term: termination (2), NS: new start (1), PS: pausing site (1), iTSS_I: internal starting site (1)

ID: 85005−85200; Term: termination (3), NS: new start (2), PS: pausing site (0), iTSS_I: internal starting site (0)

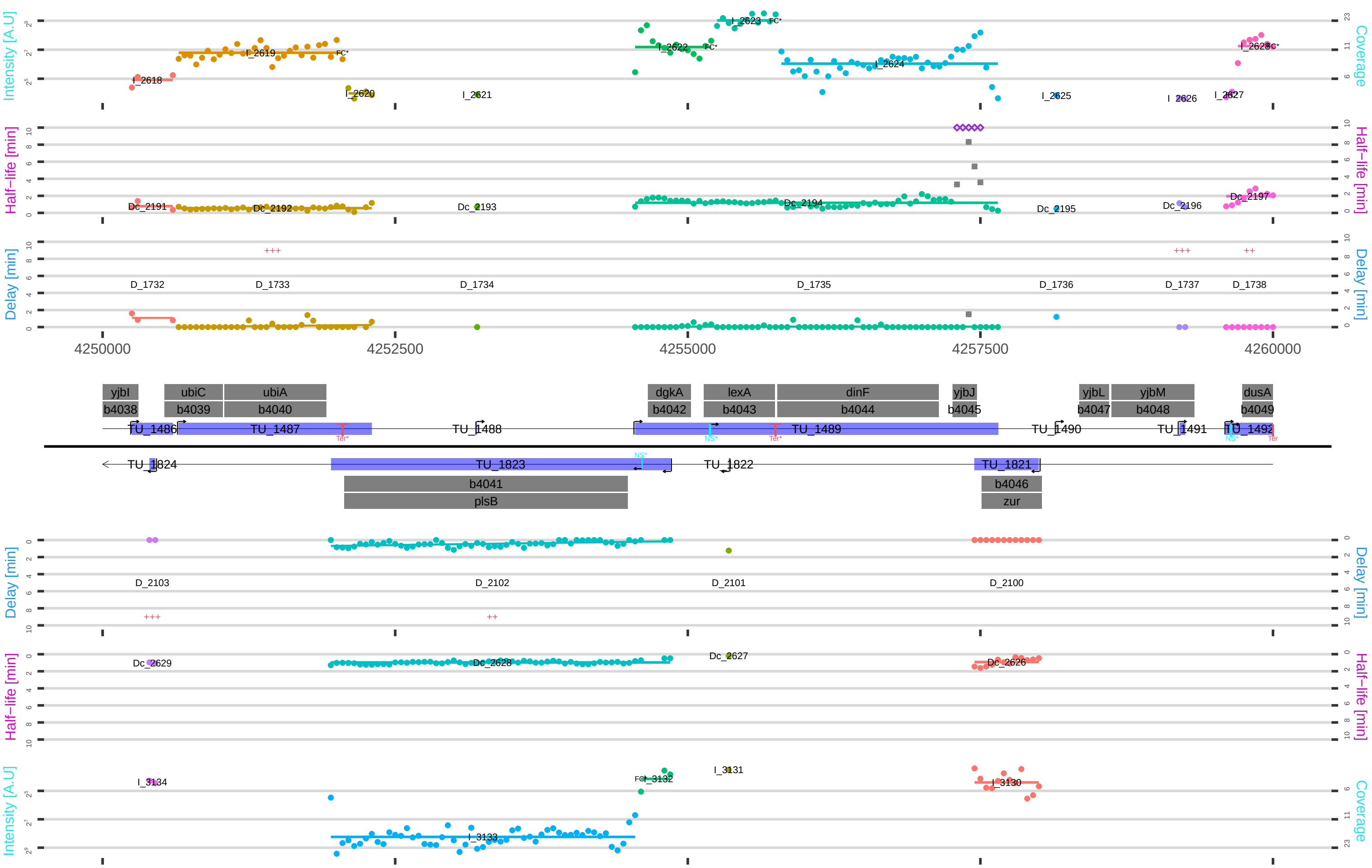

Term: termination (0), NS: new start (1), PS: pausing site (0), iTSS_I: internal starting site (0)

ID: 85200−85385; Term: termination (3), NS: new start (1), PS: pausing site (0), iTSS_I: internal starting site (0)

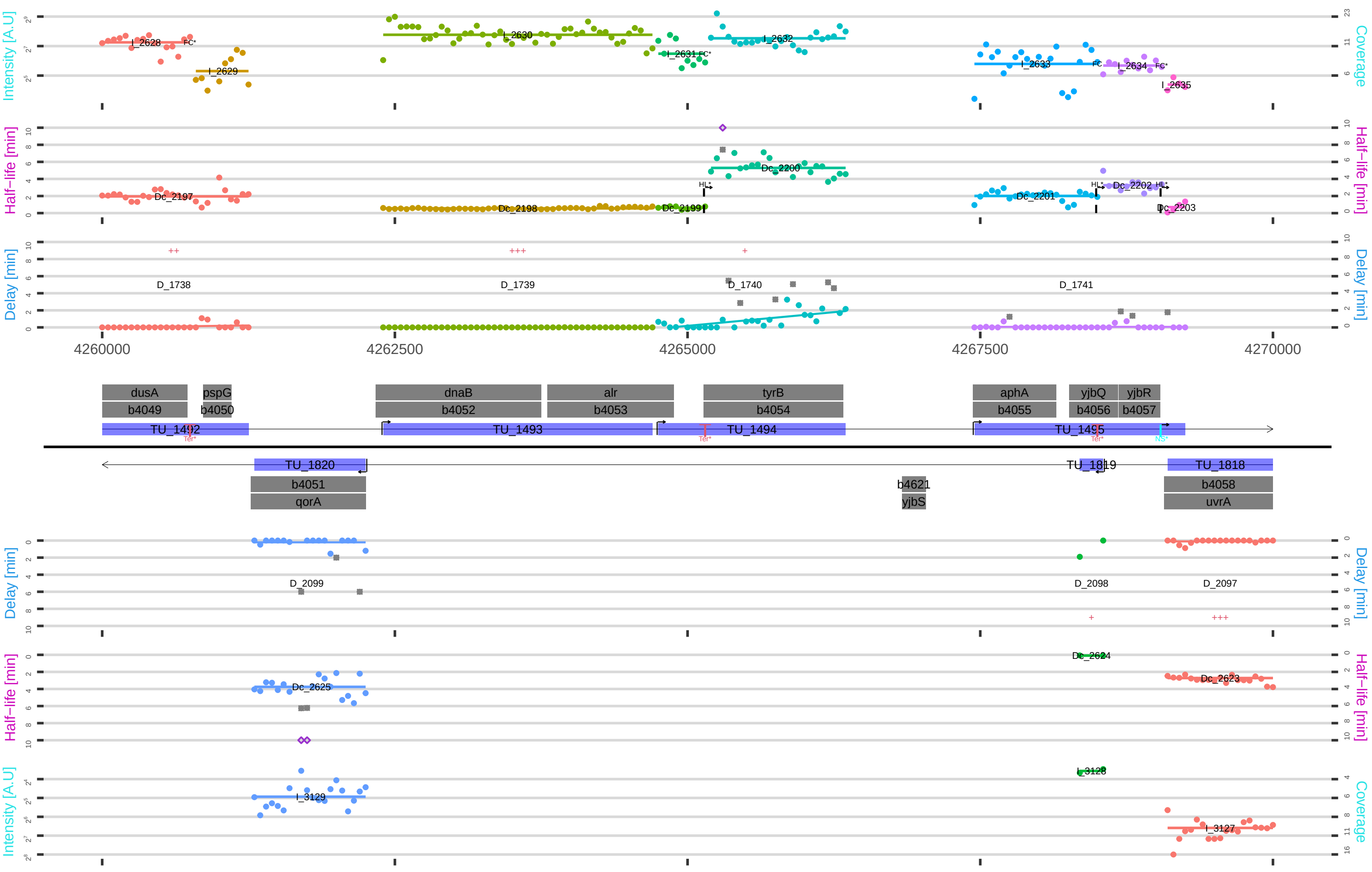

Term: termination (0), NS: new start (0), PS: pausing site (0), iTSS_I: internal starting site (0)

ID: 85428−85594; Term: termination (1), NS: new start (1), PS: pausing site (0), iTSS_I: internal starting site (1)

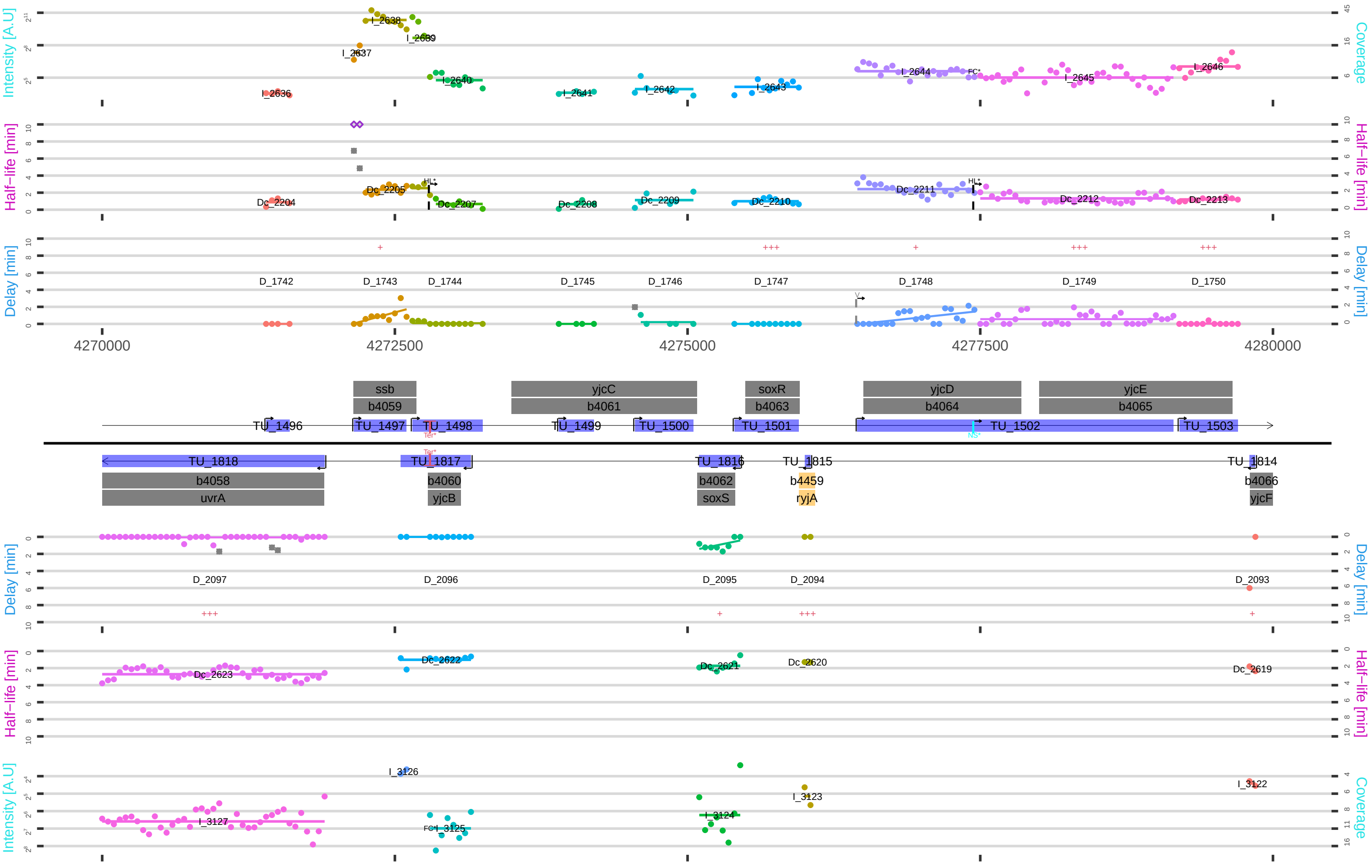

Term: termination (1), NS: new start (0), PS: pausing site (0), iTSS_I: internal starting site (0)

ID: 85711−85789; Term: termination (0), NS: new start (0), PS: pausing site (0), iTSS_I: internal starting site (0)

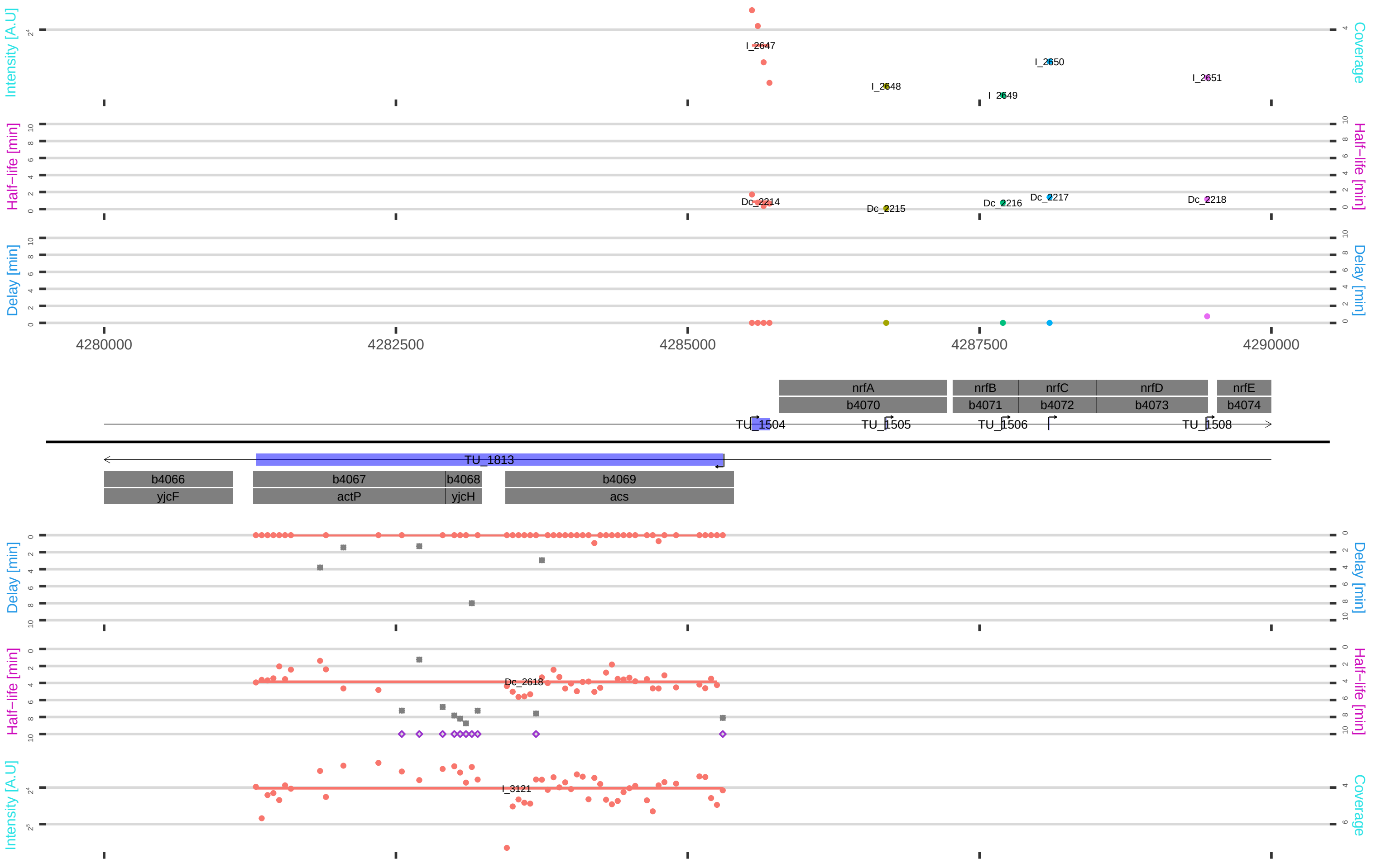

Term: termination (0), NS: new start (0), PS: pausing site (0), iTSS_I: internal starting site (0)

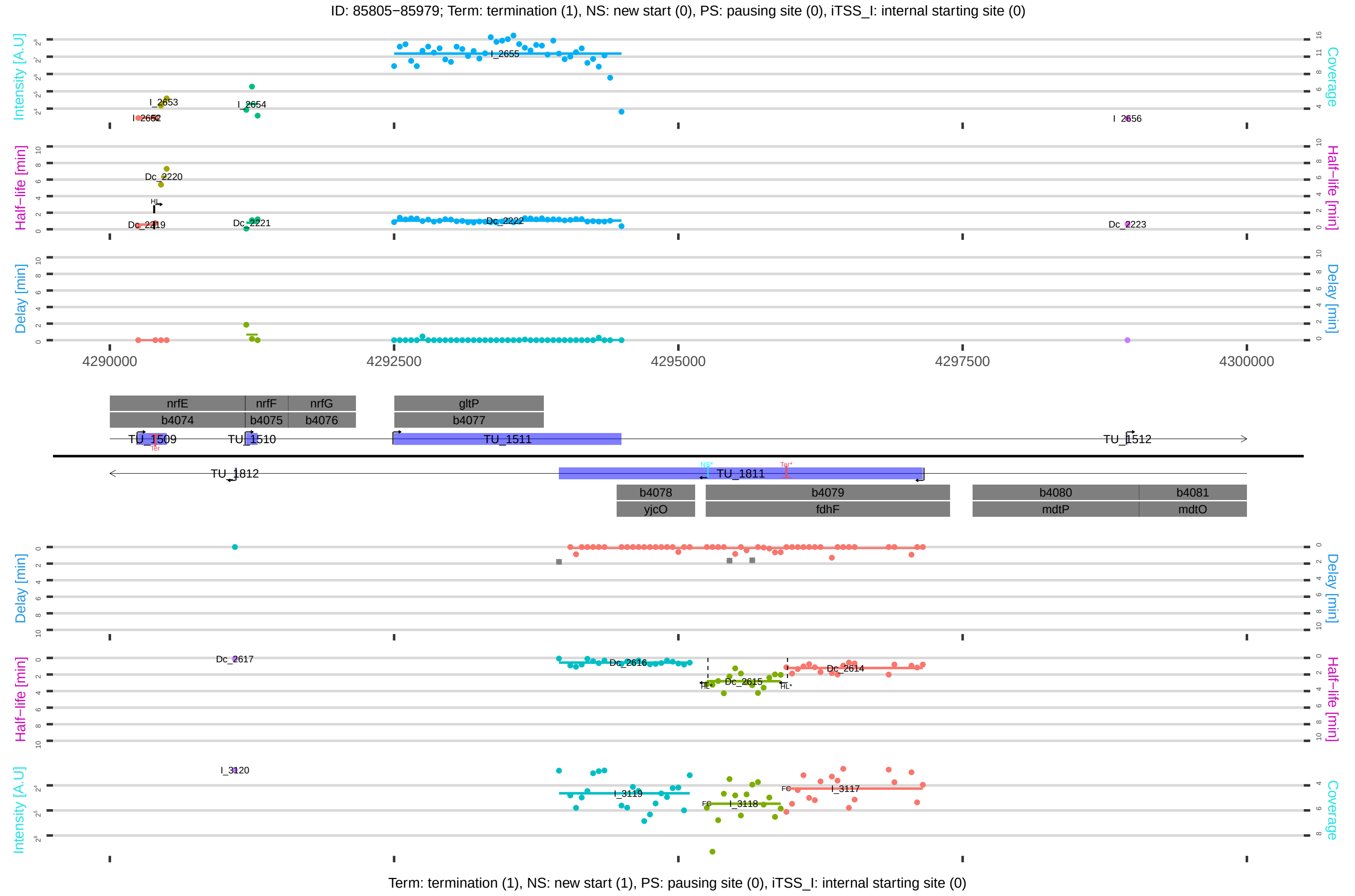

ID: 85805−85979; Term: termination (1), NS: new start (0), PS: pausing site (0), iTSS_I: internal starting site (0)
Intensity [A.U]
Coverage
I_2655
I_2653
I_2654
I_2652
I_2656
Half−life [min]
Dc_2220
Dc_2219
Dc_2221
Dc_2222
Dc_2223
Delay [min]
4290000
4292500
4295000
4297500
4300000
nrfE
nrfF
nrfG
gltP
b4074
b4075
b4076
b4077
TU_1509
TU_1510
TU_1511
TU_1512
Ter
TU_1812
TU_1811
NS*
Ter*
b4078
b4079
b4080
b4081
yjcO
fdhF
mdtP
mdtO
Dc_2617
Dc_2616
Dc_2615
Dc_2614
HL*
I_3120
I_3119
I_3118
I_3117
FC
Term: termination (1), NS: new start (1), PS: pausing site (0), iTSS_I: internal starting site (0)

ID: 86009−86106; Term: termination (0), NS: new start (0), PS: pausing site (0), iTSS_I: internal starting site (0)

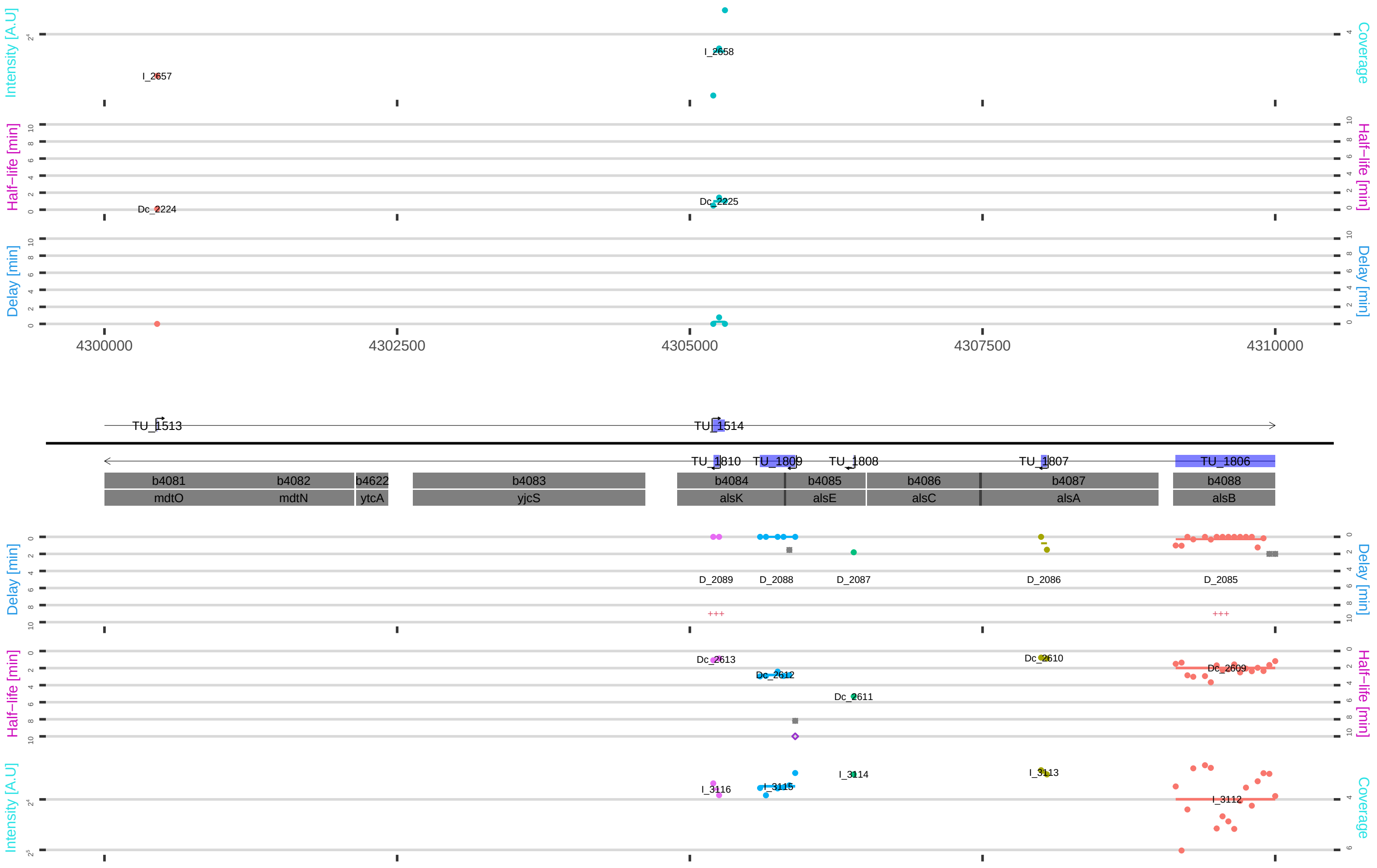

Term: termination (0), NS: new start (0), PS: pausing site (0), iTSS_I: internal starting site (0)

ID: 86229−86400; Term: termination (0), NS: new start (0), PS: pausing site (0), iTSS_I: internal starting site (0)

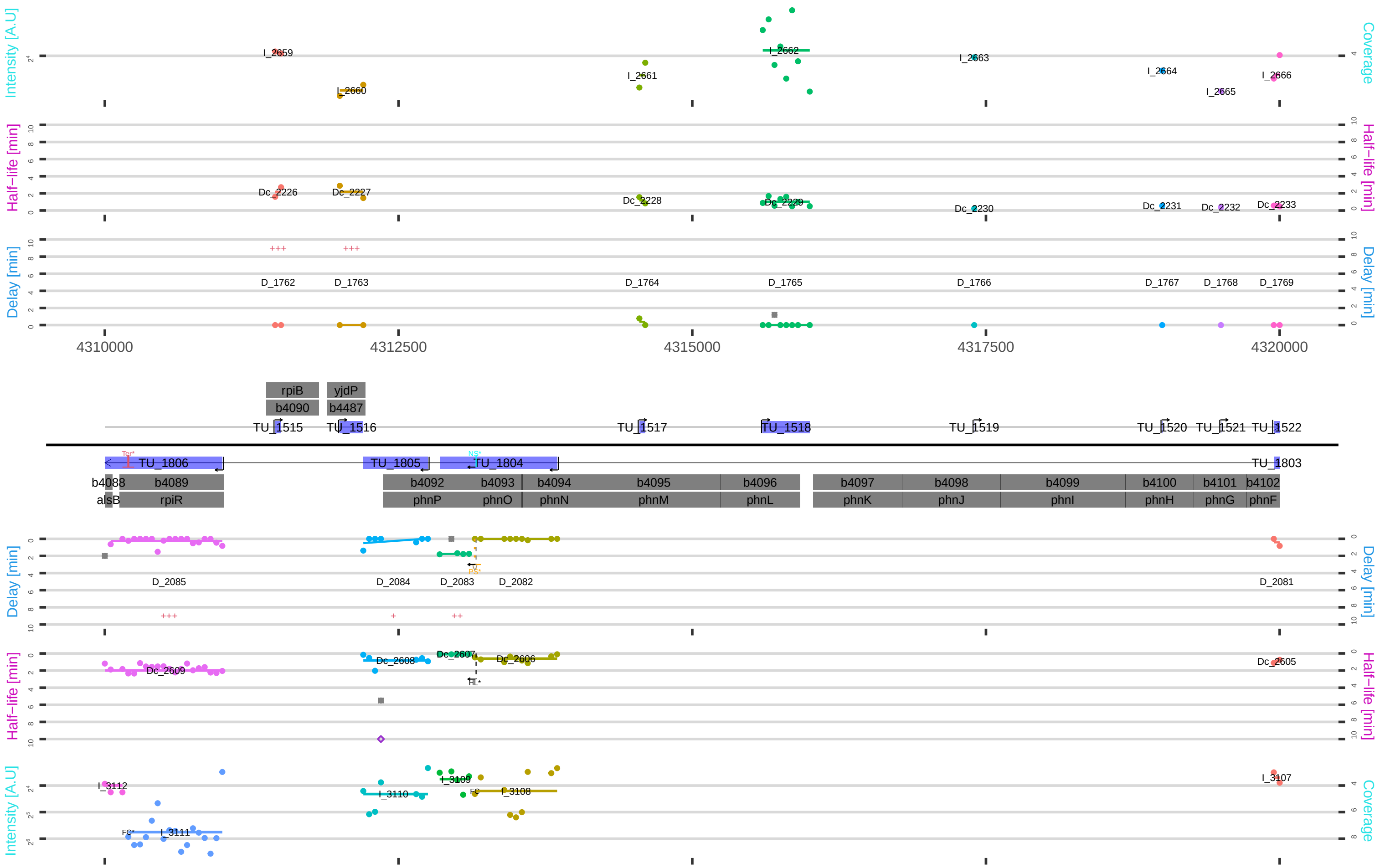

Term: termination (1), NS: new start (1), PS: pausing site (1), iTSS_I: internal starting site (0)

ID: 86400−86600; Term: termination (1), NS: new start (0), PS: pausing site (0), iTSS_I: internal starting site (0)

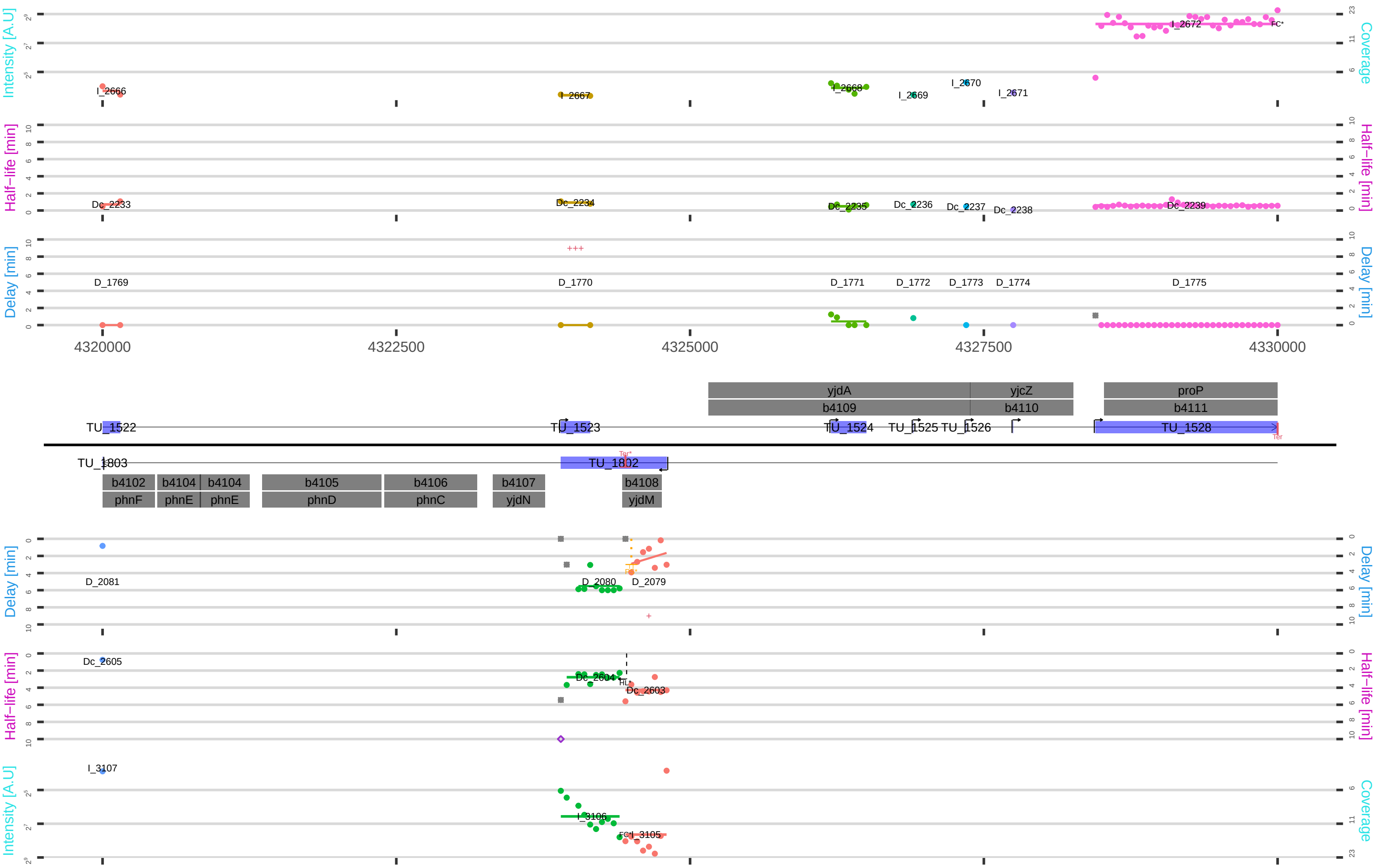

Term: termination (1), NS: new start (0), PS: pausing site (1), iTSS_I: internal starting site (0)

ID: 86600−86742; Term: termination (2), NS: new start (0), PS: pausing site (0), iTSS_I: internal starting site (0)

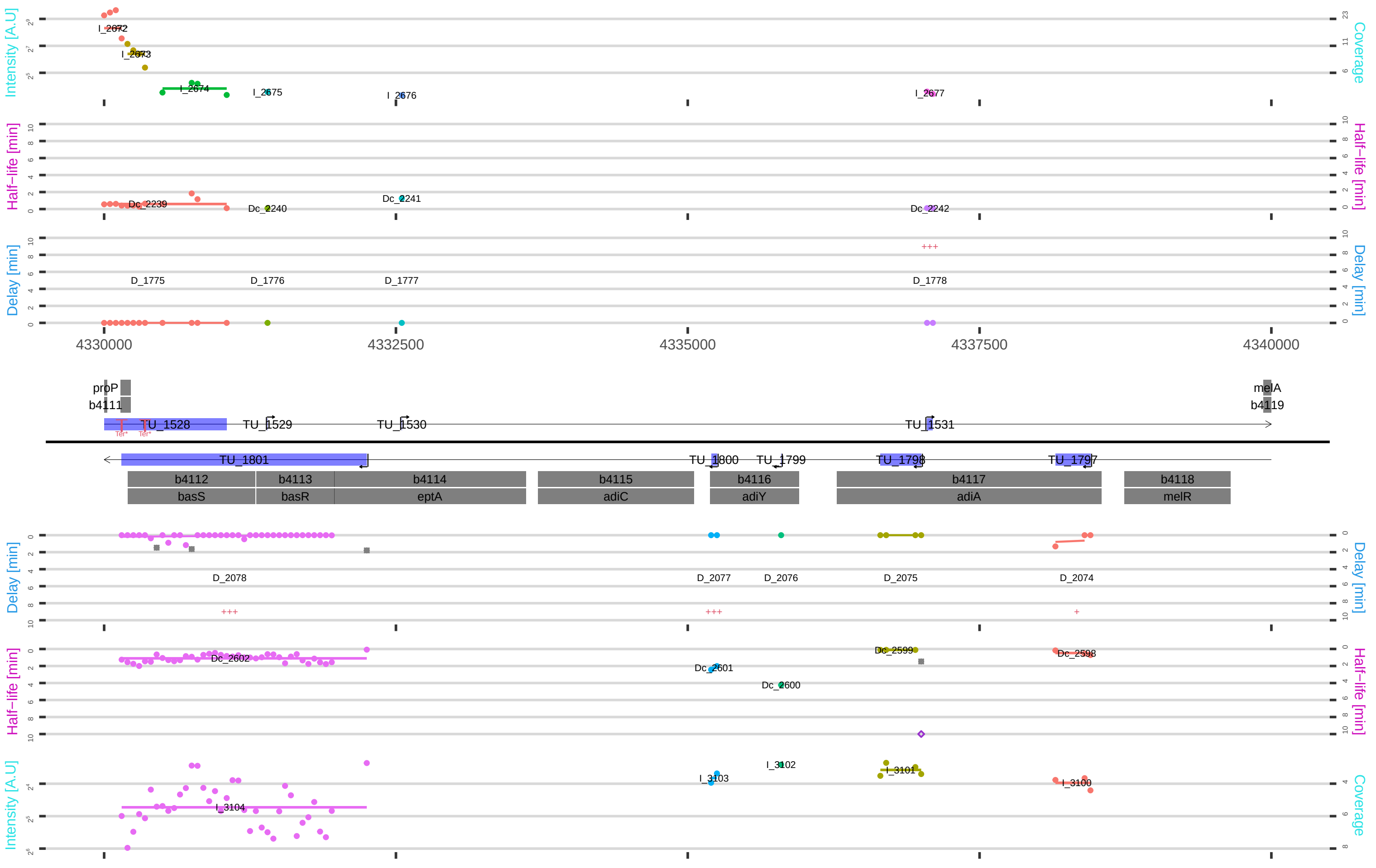

Term: termination (0), NS: new start (0), PS: pausing site (0), iTSS_I: internal starting site (0)

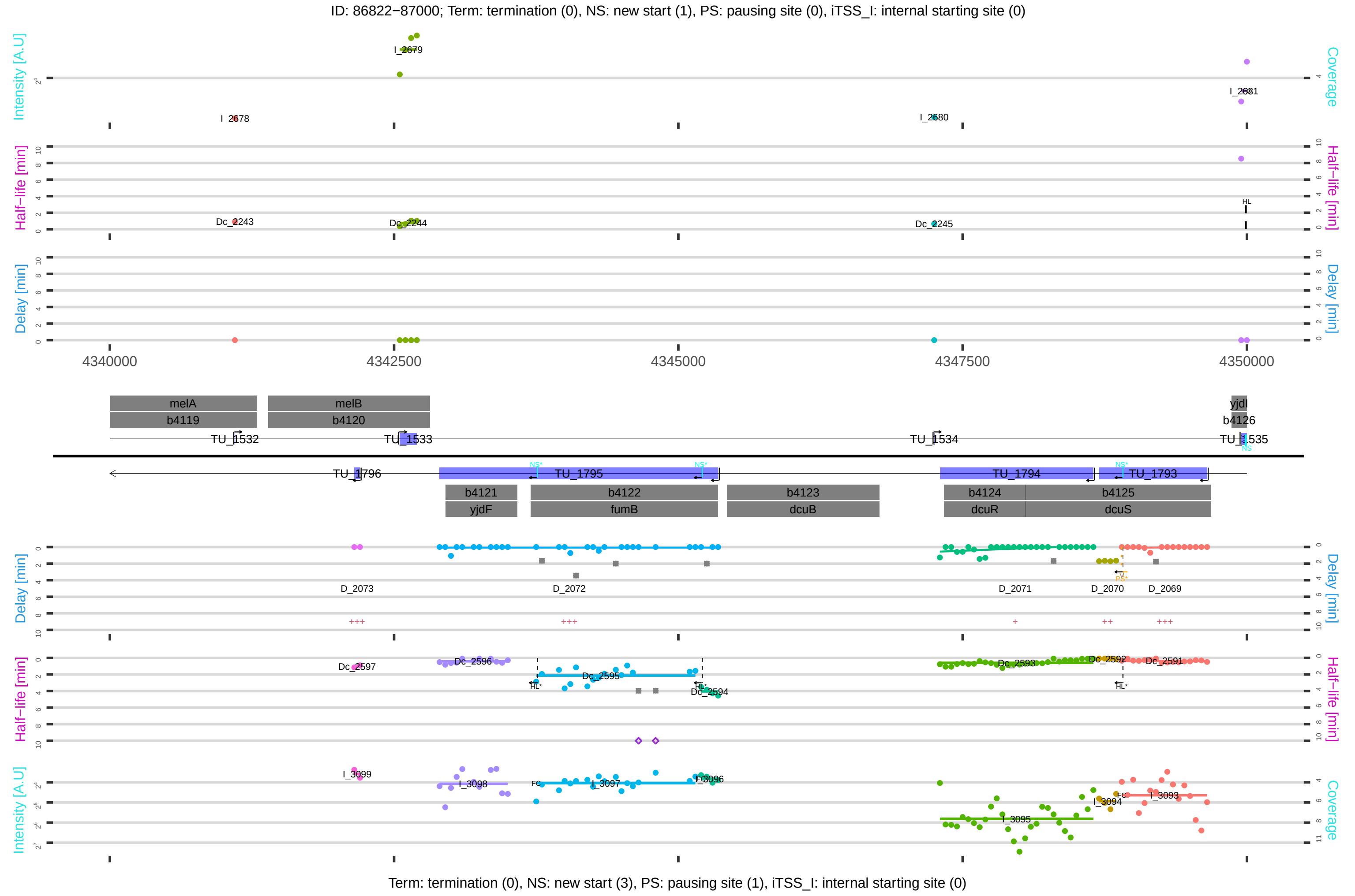

ID: 86822−87000; Term: termination (0), NS: new start (1), PS: pausing site (0), iTSS_I: internal starting site (0)
Intensity [A.U]
Coverage
I_2679
I_2678
I_2680
I_2681
Half−life [min]
Dc_2243
Dc_2244
Dc_2245
HL
Delay [min]
4340000
4342500
4345000
4347500
4350000
melA
b4119
melB
b4120
yjdI
b4126
TU_1532
TU_1533
TU_1534
TU_1535
NS
TU_1796
TU_1795
TU_1794
TU_1793
NS*
b4121
yjdF
b4122
fumB
b4123
dcuB
b4124
dcuR
b4125
dcuS
D_2073
D_2072
D_2071
D_2070
D_2069
PS*
+++
+
++
Dc_2597
Dc_2596
Dc_2595
Dc_2594
Dc_2593
Dc_2592
Dc_2591
HL*
I_3099
I_3098
I_3097
I_3096
I_3095
I_3094
I_3093
FC
Term: termination (0), NS: new start (3), PS: pausing site (1), iTSS_I: internal starting site (0)

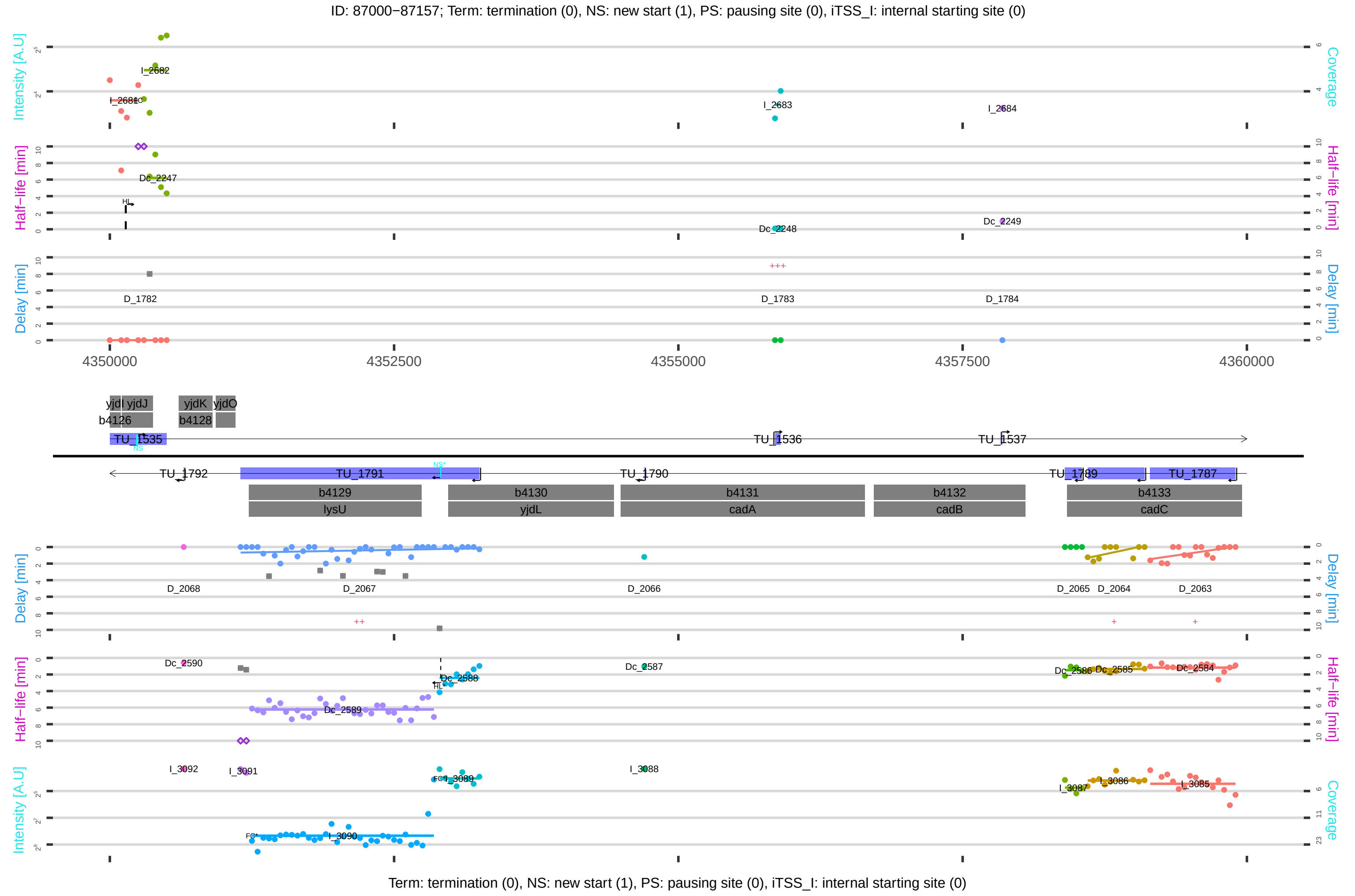

ID: 87000−87157; Term: termination (0), NS: new start (1), PS: pausing site (0), iTSS_I: internal starting site (0)
Intensity [A.U]
Coverage
I_2682
I_2681
I_2683
I_2684
Half−life [min]
Dc_2247
HL
Dc_2248
Dc_2249
Delay [min]
D_1782
D_1783
D_1784
4350000
4352500
4355000
4357500
4360000
yjdI
yjdJ
yjdK
yjdO
b4126
b4128
TU_1535
NS
TU_1536
TU_1537
TU_1792
TU_1791
NS*
TU_1790
TU_1789
TU_1787
b4129
lysU
b4130
yjdL
b4131
cadA
b4132
cadB
b4133
cadC
D_2068
D_2067
D_2066
D_2065
D_2064
D_2063
Dc_2590
Dc_2588
HL
Dc_2587
Dc_2586
Dc_2585
Dc_2584
Dc_2589
I_3092
I_3091
I_3089
I_3088
I_3087
I_3086
I_3085
I_3090
Term: termination (0), NS: new start (1), PS: pausing site (0), iTSS_I: internal starting site (0)

ID: 87302−87400; Term: termination (1), NS: new start (0), PS: pausing site (1), iTSS_I: internal starting site (0)

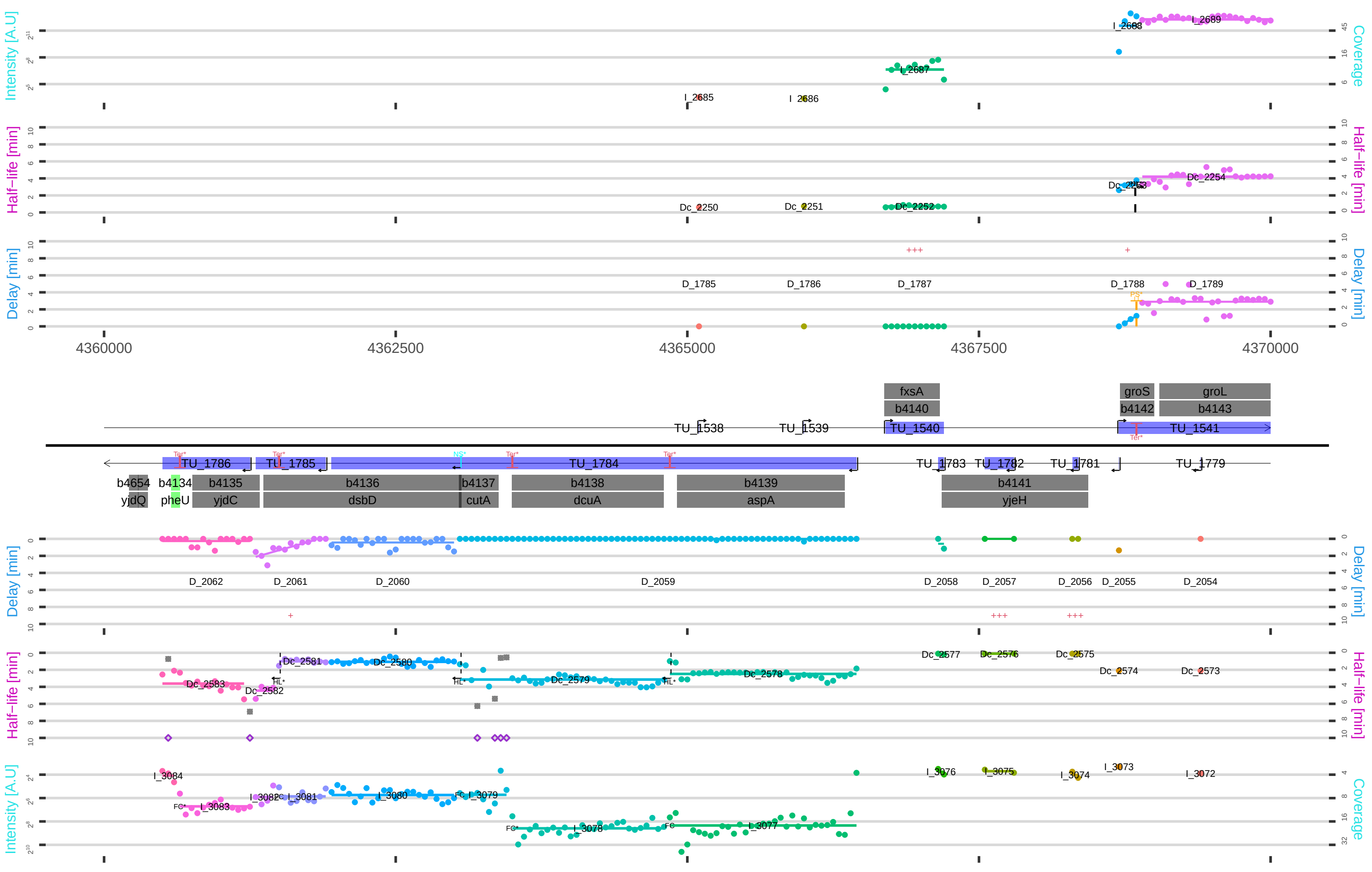

Term: termination (4), NS: new start (1), PS: pausing site (1), iTSS_I: internal starting site (0)

ID: 87400−87539; Term: termination (3), NS: new start (0), PS: pausing site (0), iTSS_I: internal starting site (0)

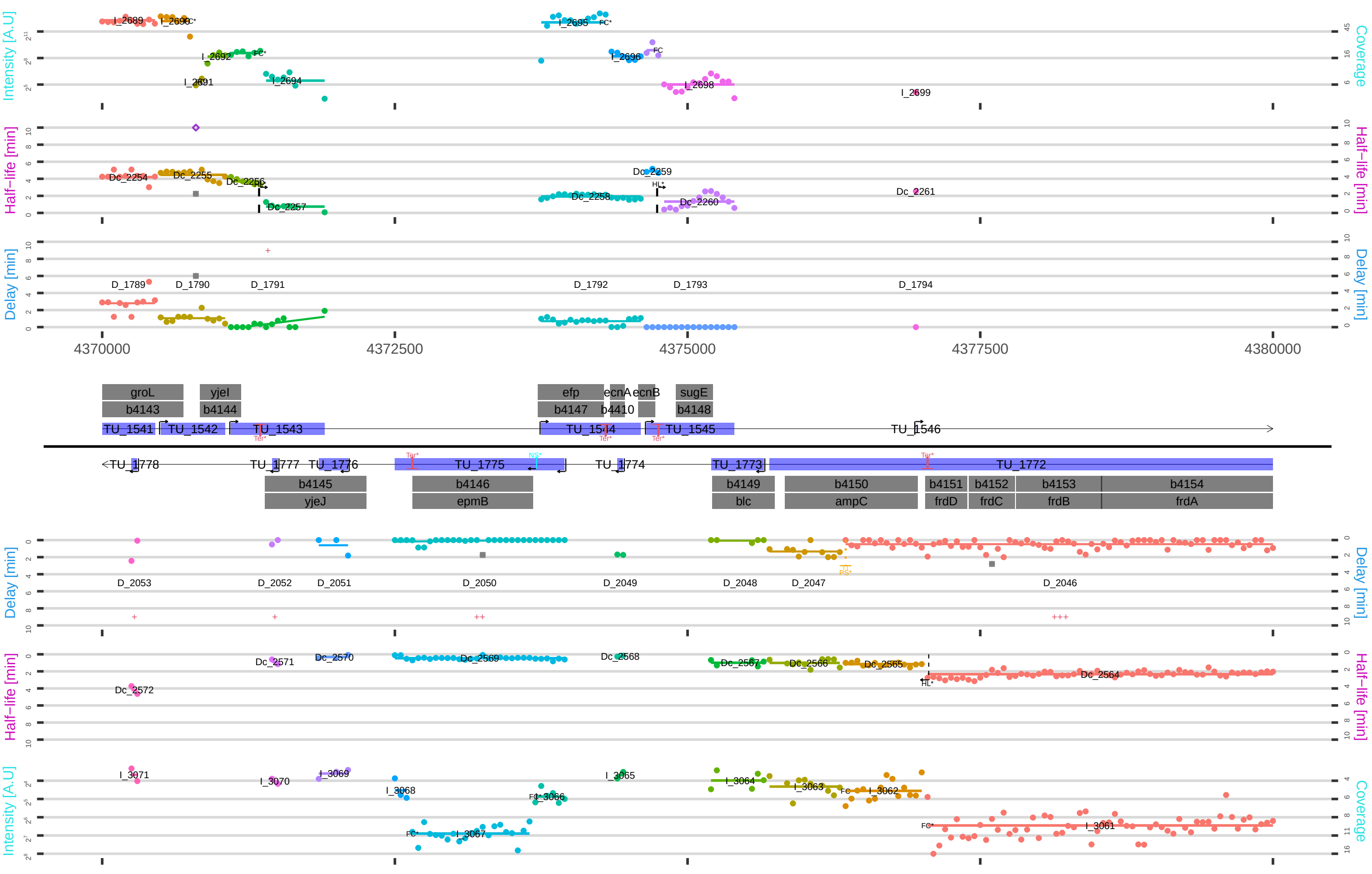

Term: termination (2), NS: new start (1), PS: pausing site (1), iTSS_I: internal starting site (0)

ID: 87615−87800; Term: termination (1), NS: new start (0), PS: pausing site (0), iTSS_I: internal starting site (0)

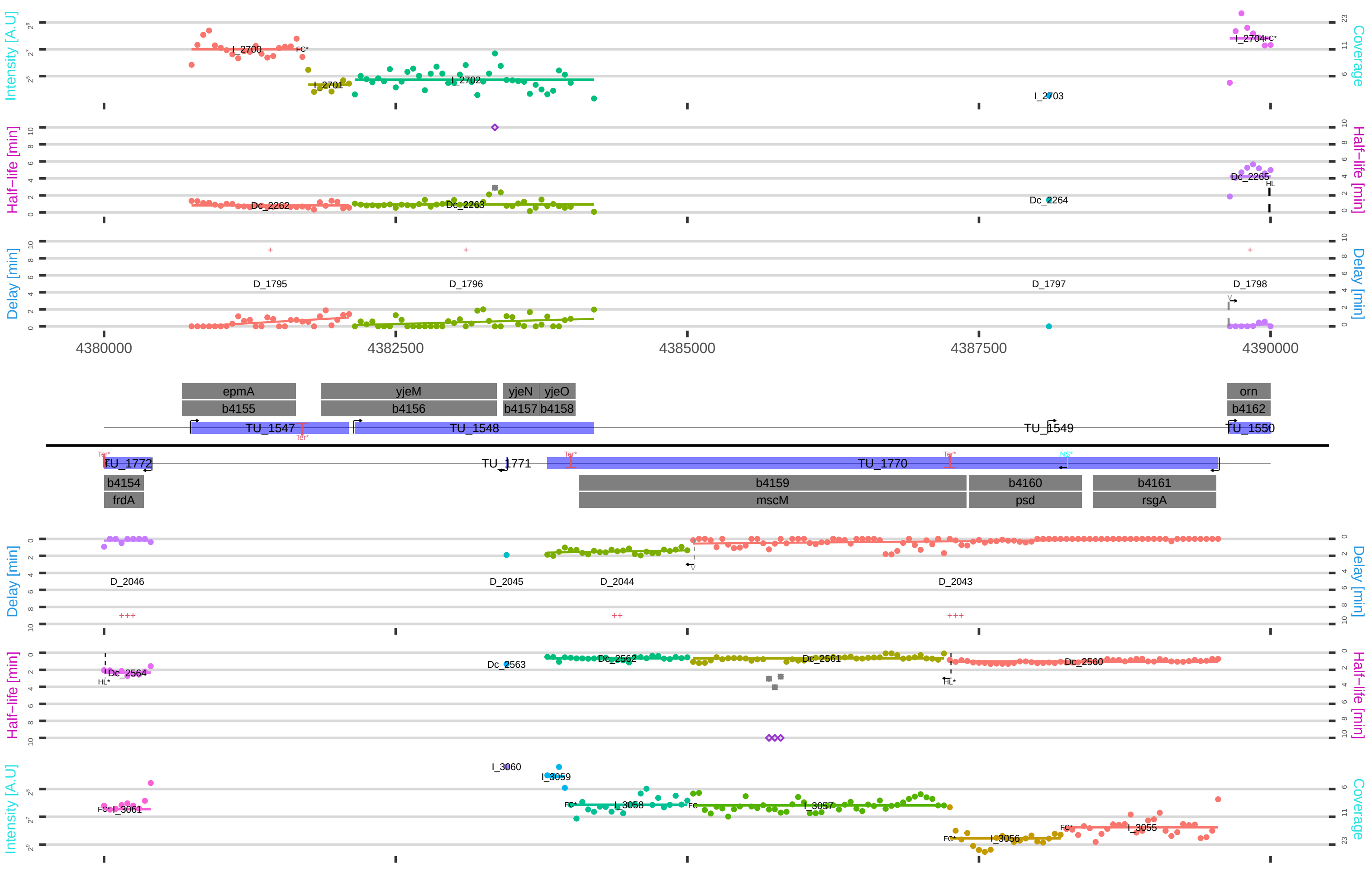

Term: termination (3), NS: new start (1), PS: pausing site (1), iTSS_I: internal starting site (0)

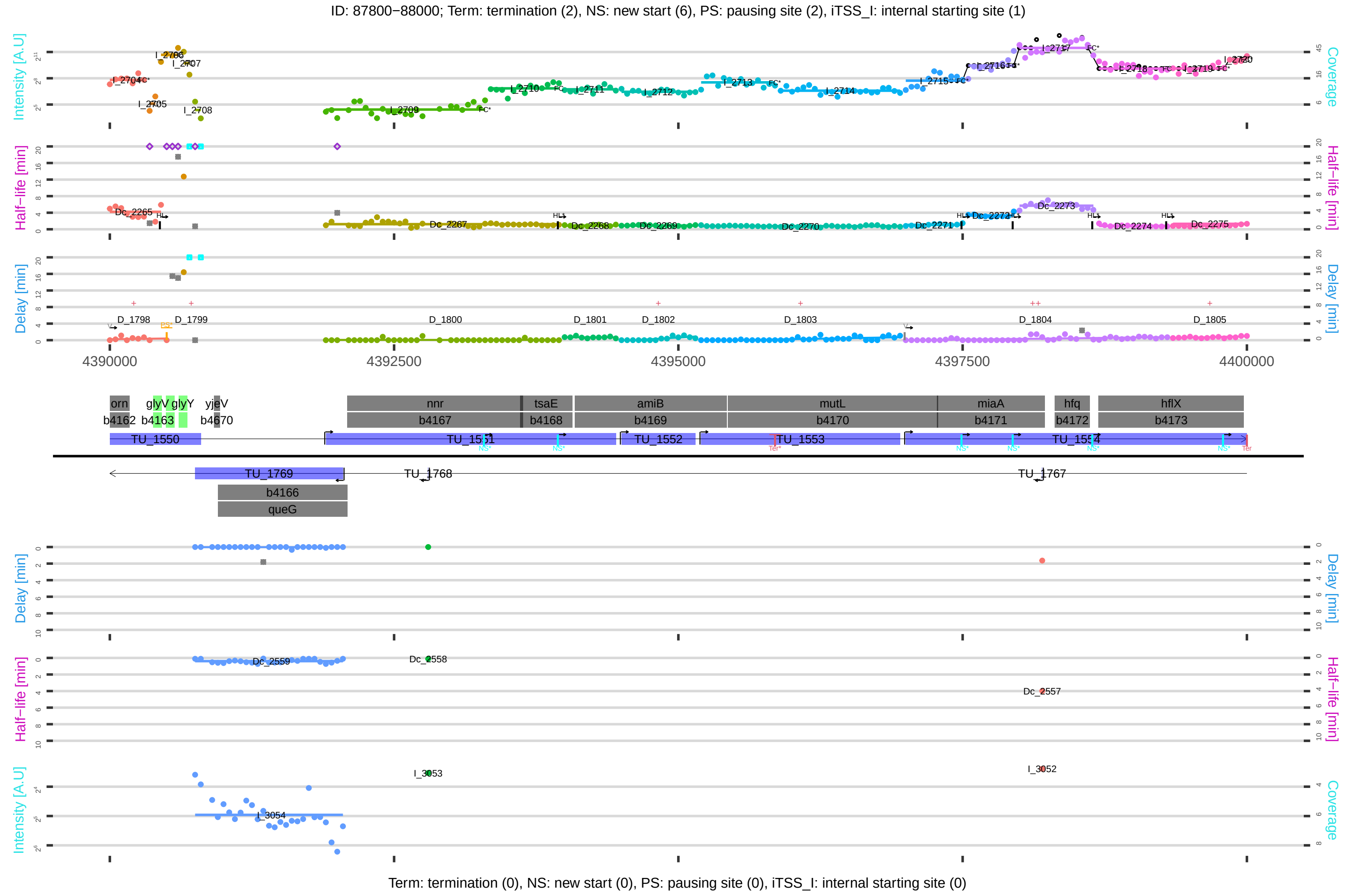

ID: 87800−88000; Term: termination (2), NS: new start (6), PS: pausing site (2), iTSS_I: internal starting site (1)
Intensity [A.U]
Coverage
Half−life [min]
Delay [min]
4390000
4392500
4395000
4397500
4400000
orn
glyV
glyY
yjeV
b4162
b4163
b4670
nnr
b4167
tsaE
b4168
amiB
b4169
mutL
b4170
miaA
b4171
hfq
b4172
hflX
b4173
TU_1550
TU_1551
TU_1552
TU_1553
TU_1554
TU_1769
TU_1768
TU_1767
b4166
queG
Term: termination (0), NS: new start (0), PS: pausing site (0), iTSS_I: internal starting site (0)

ID: 88000−88192; Term: termination (1), NS: new start (3), PS: pausing site (2), iTSS_I: internal starting site (0)

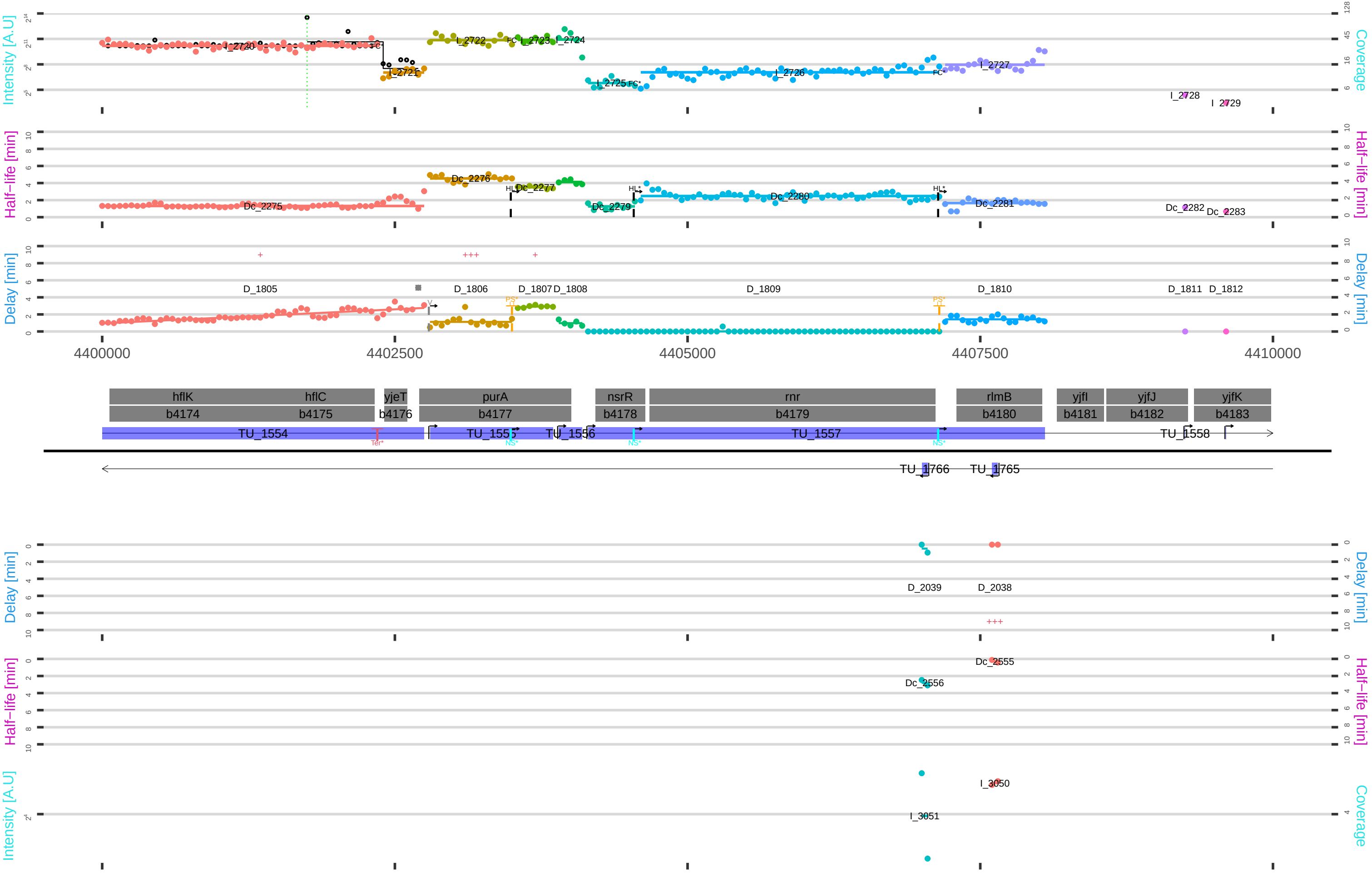

Term: termination (0), NS: new start (0), PS: pausing site (0), iTSS_I: internal starting site (0)

ID: 88230−88400; Term: termination (1), NS: new start (0), PS: pausing site (0), iTSS_I: internal starting site (0)

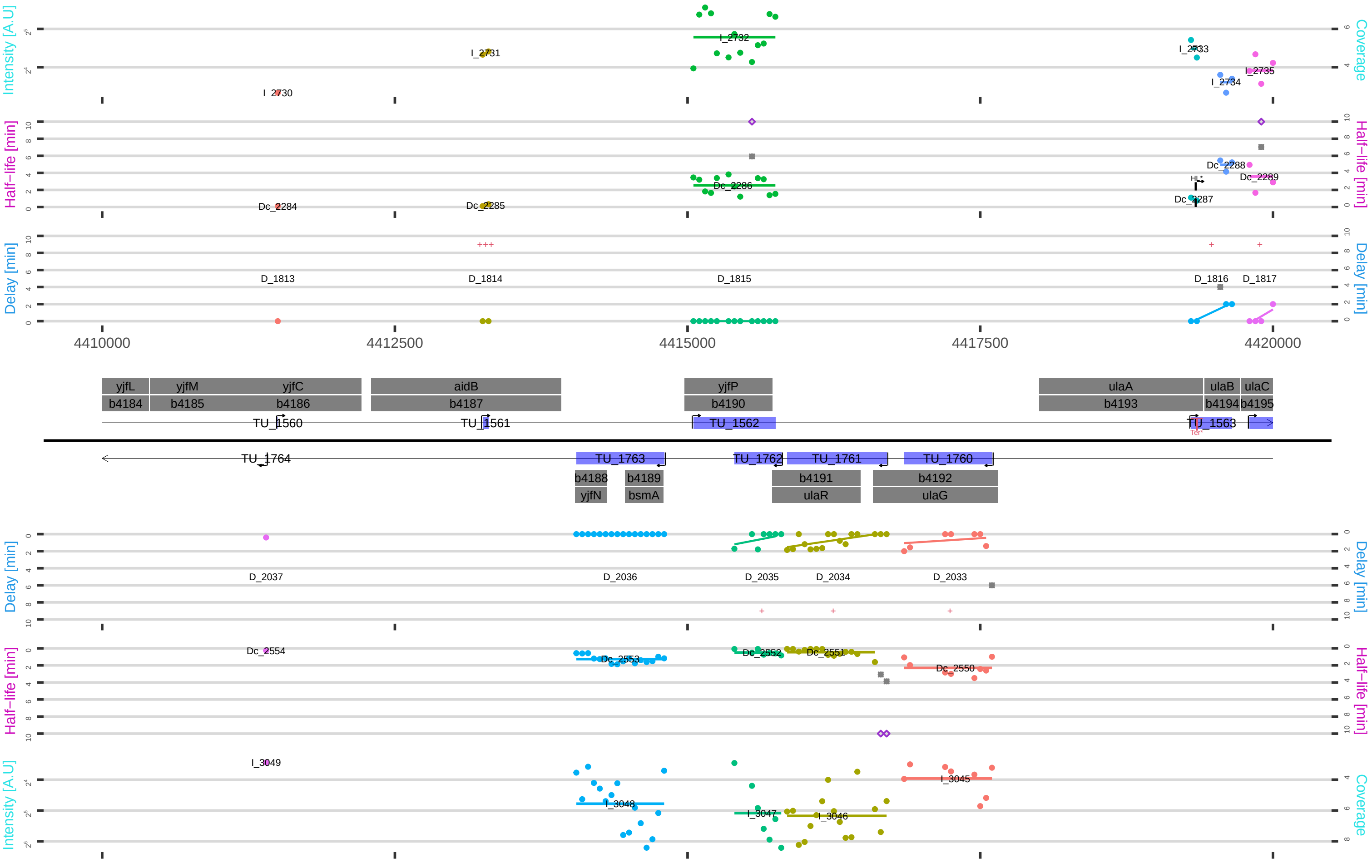

Term: termination (0), NS: new start (0), PS: pausing site (0), iTSS_I: internal starting site (0)

ID: 88400−88592; Term: termination (2), NS: new start (2), PS: pausing site (1), iTSS_I: internal starting site (0)

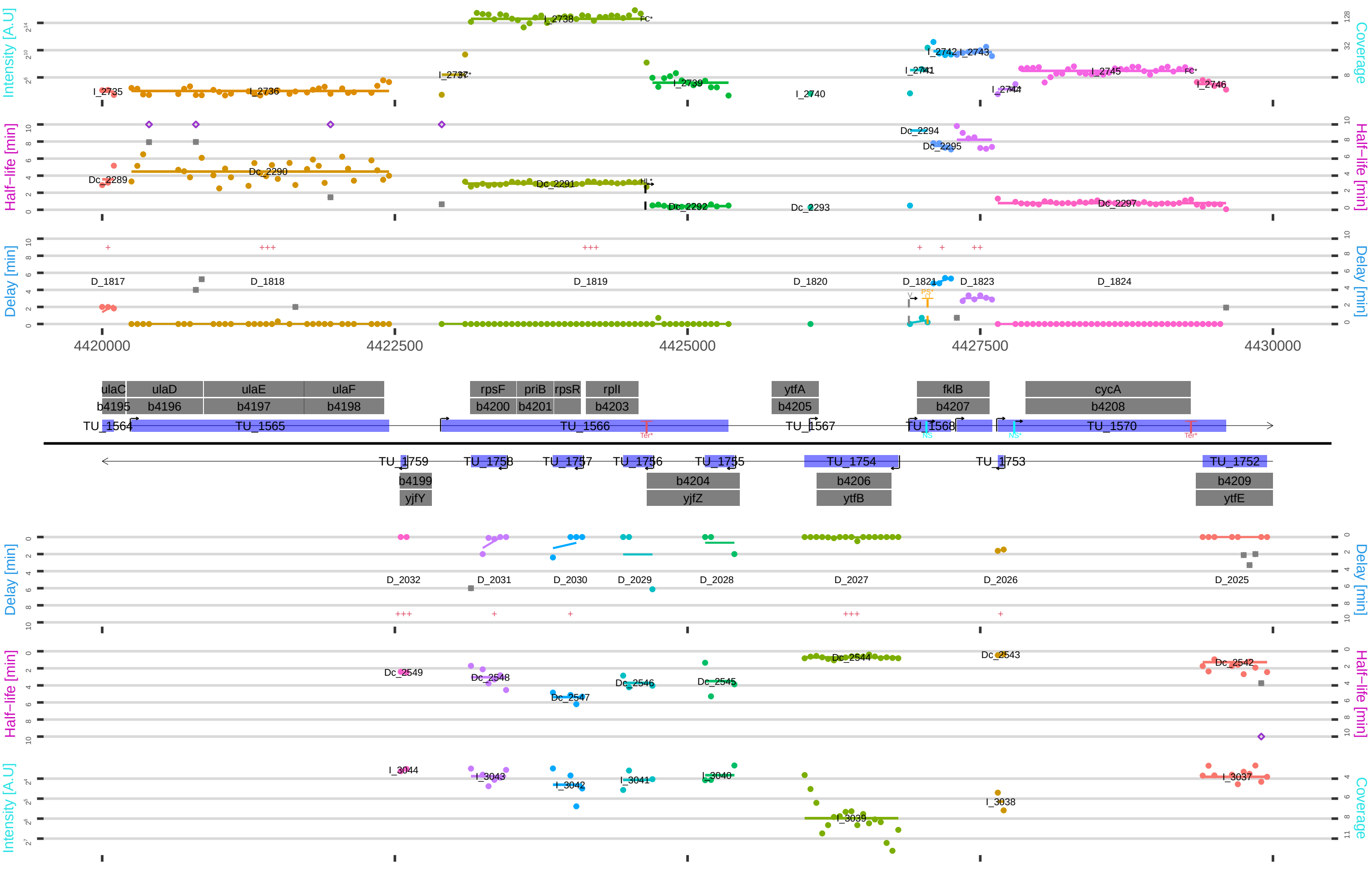

Term: termination (0), NS: new start (0), PS: pausing site (0), iTSS_I: internal starting site (0)

ID: 88612−88788; Term: termination (0), NS: new start (0), PS: pausing site (0), iTSS_I: internal starting site (0)

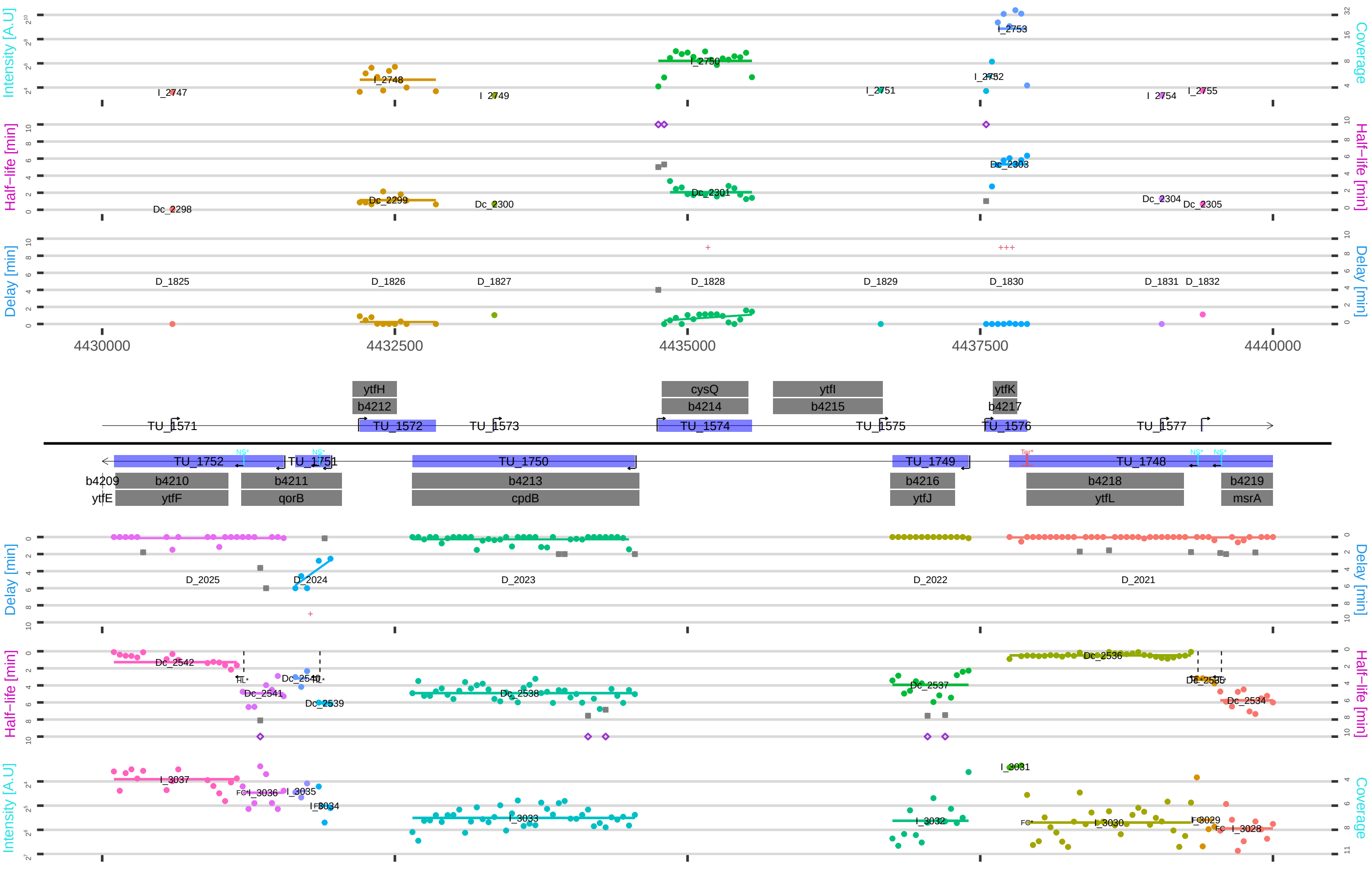

Term: termination (1), NS: new start (4), PS: pausing site (0), iTSS_I: internal starting site (0)

ID: 88807−88987; Term: termination (2), NS: new start (3), PS: pausing site (1), iTSS_I: internal starting site (0)

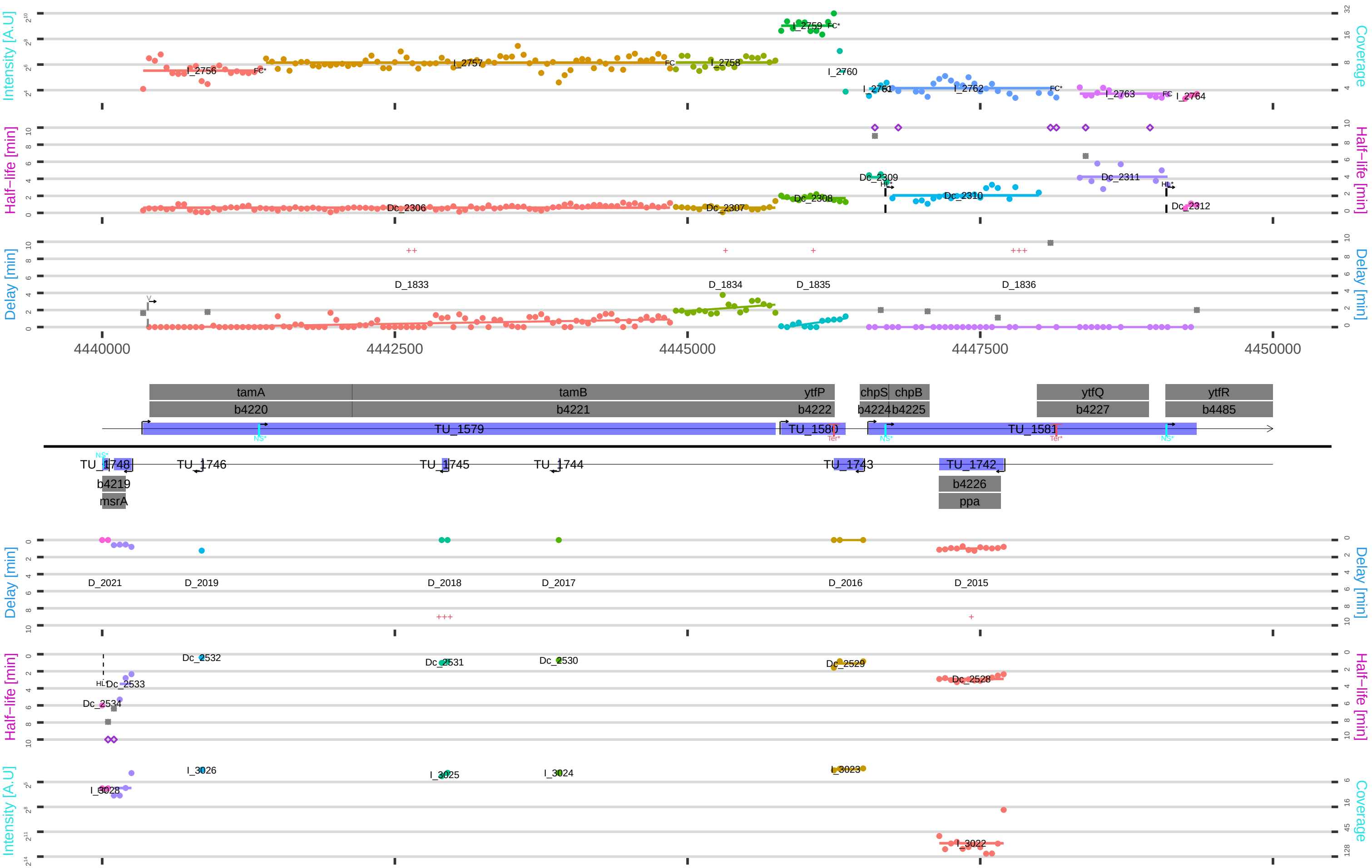

Term: termination (0), NS: new start (1), PS: pausing site (0), iTSS_I: internal starting site (0)

ID: 89002−89159; Term: termination (1), NS: new start (1), PS: pausing site (0), iTSS_I: internal starting site (0)

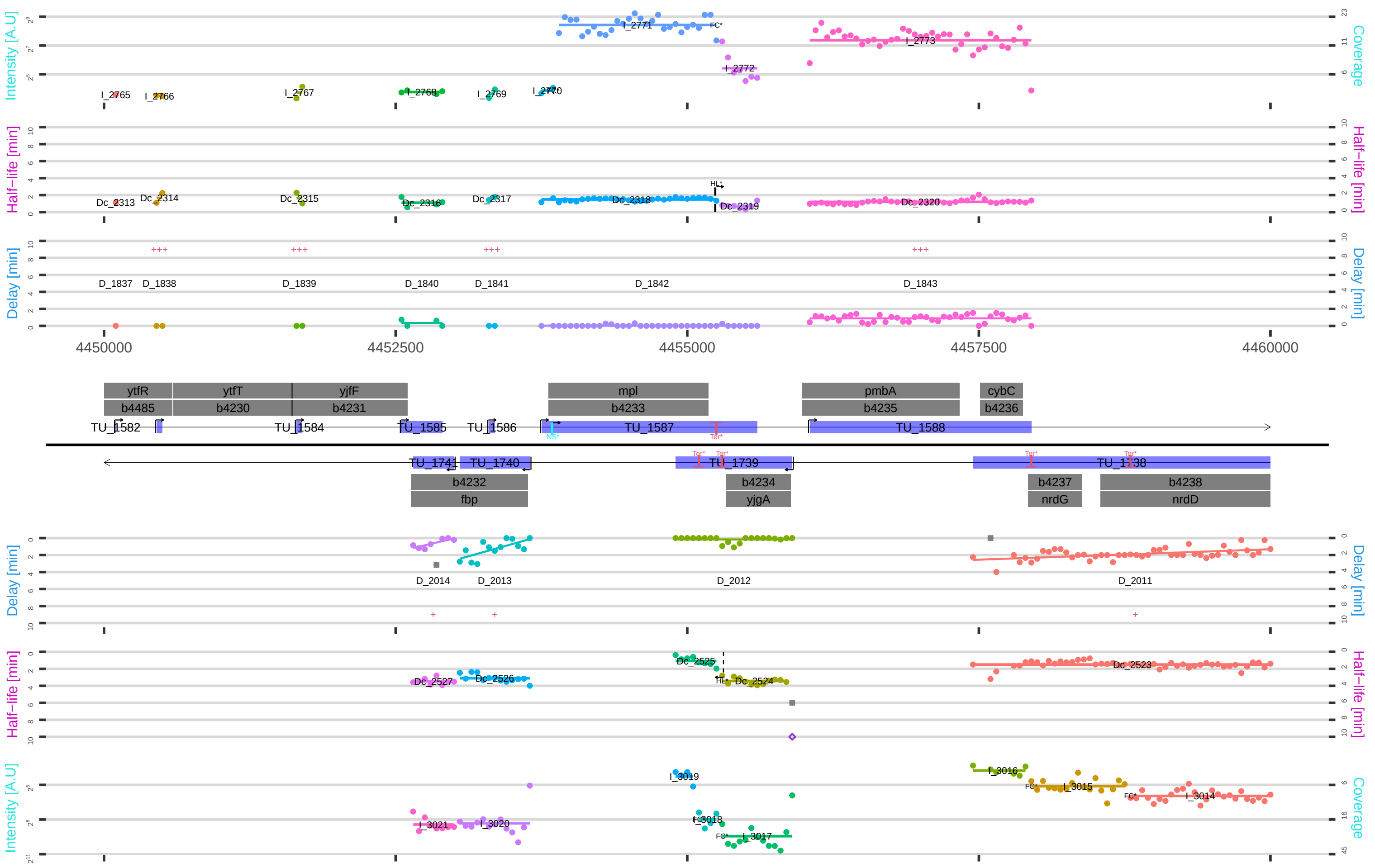

Term: termination (4), NS: new start (0), PS: pausing site (0), iTSS_I: internal starting site (0)

ID: 89216−89392; Term: termination (3), NS: new start (2), PS: pausing site (0), iTSS_I: internal starting site (0)

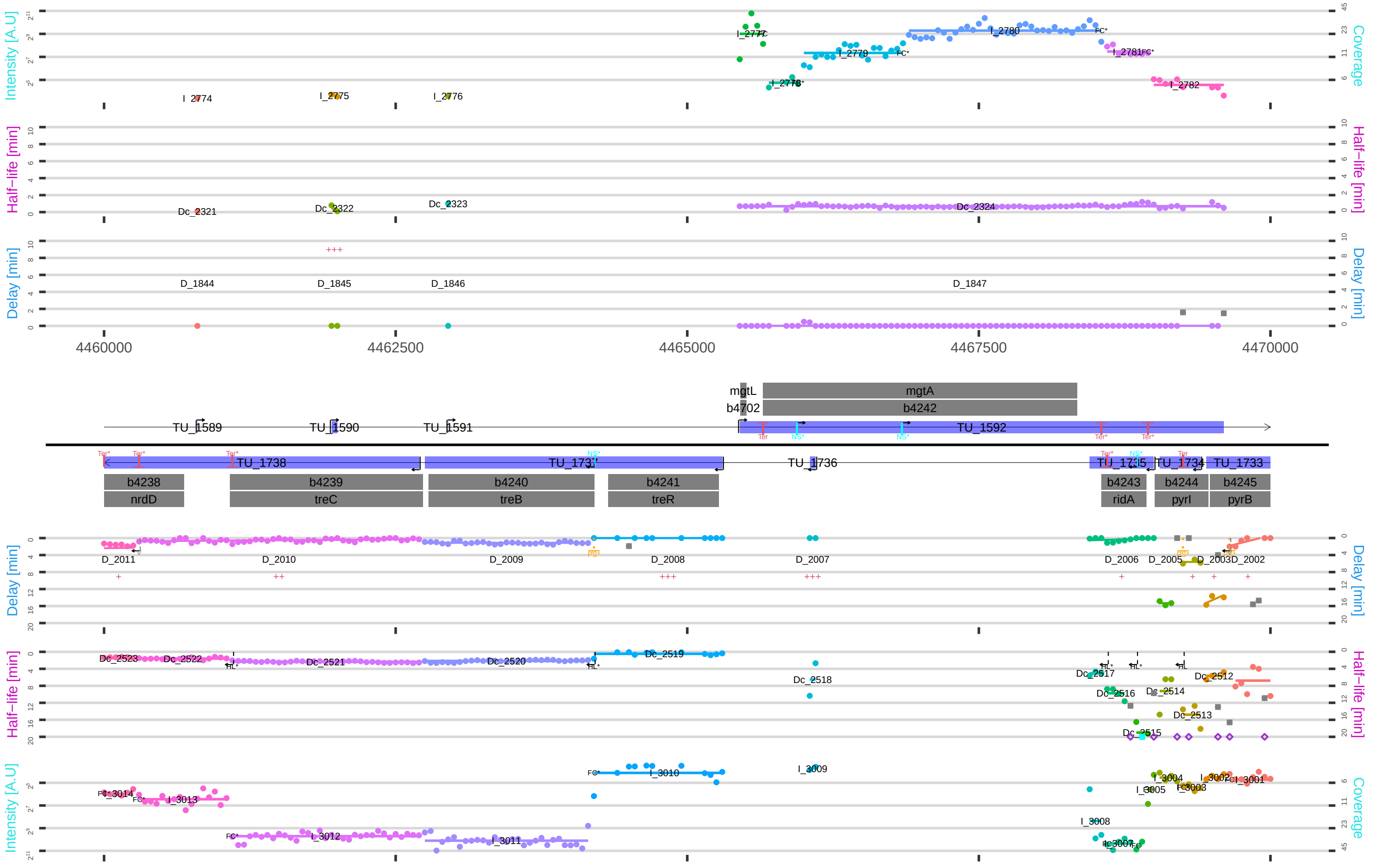

Term: termination (5), NS: new start (2), PS: pausing site (4), iTSS_I: internal starting site (0)

ID: 89425−89589; Term: termination (0), NS: new start (0), PS: pausing site (0), iTSS_I: internal starting site (0)

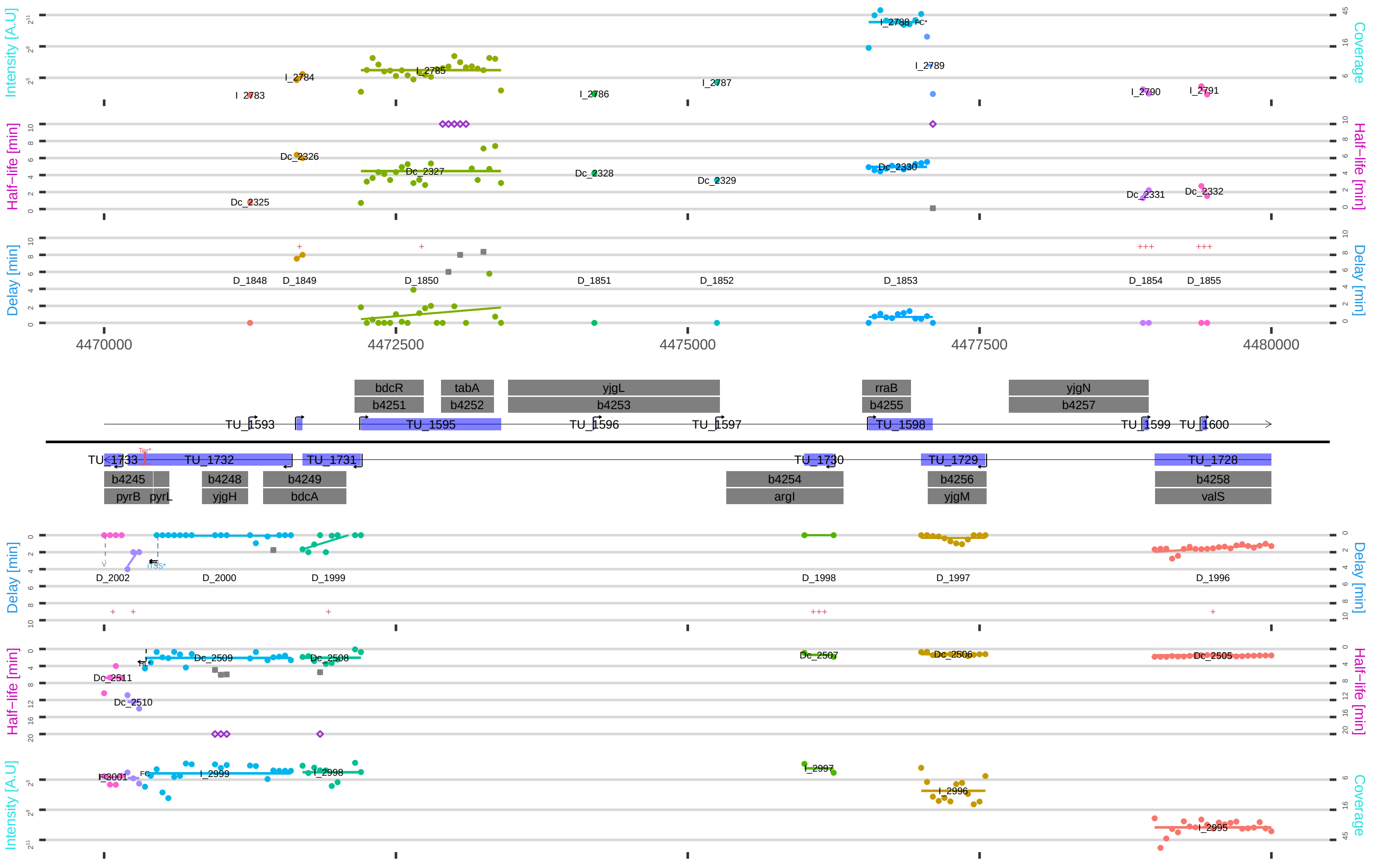

Term: termination (1), NS: new start (0), PS: pausing site (0), iTSS_I: internal starting site (1)

ID: 89639−89779; Term: termination (1), NS: new start (2), PS: pausing site (0), iTSS_I: internal starting site (0)

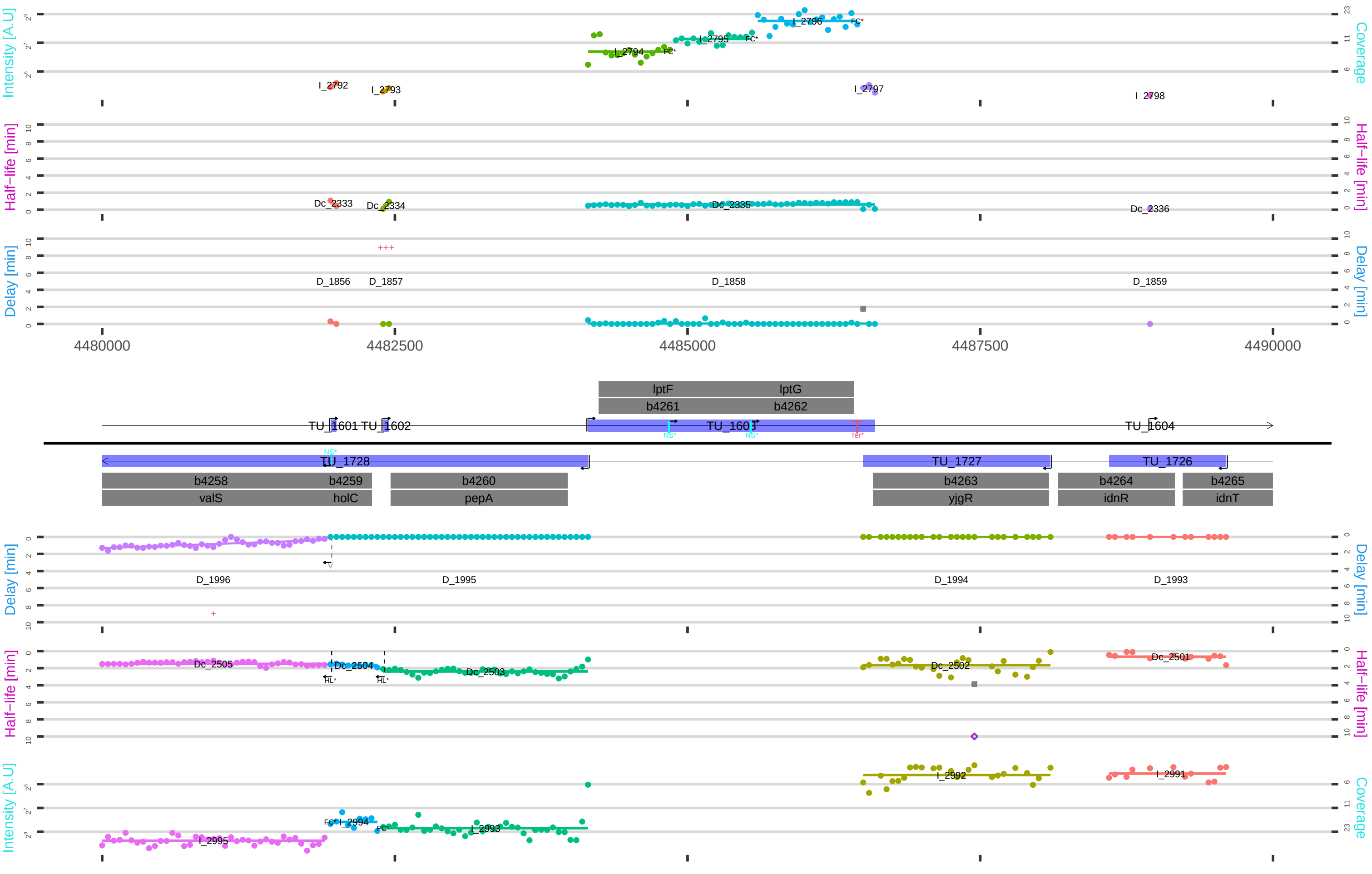

Term: termination (0), NS: new start (1), PS: pausing site (1), iTSS_I: internal starting site (0)

ID: 89855−90000; Term: termination (1), NS: new start (2), PS: pausing site (0), iTSS_I: internal starting site (0)

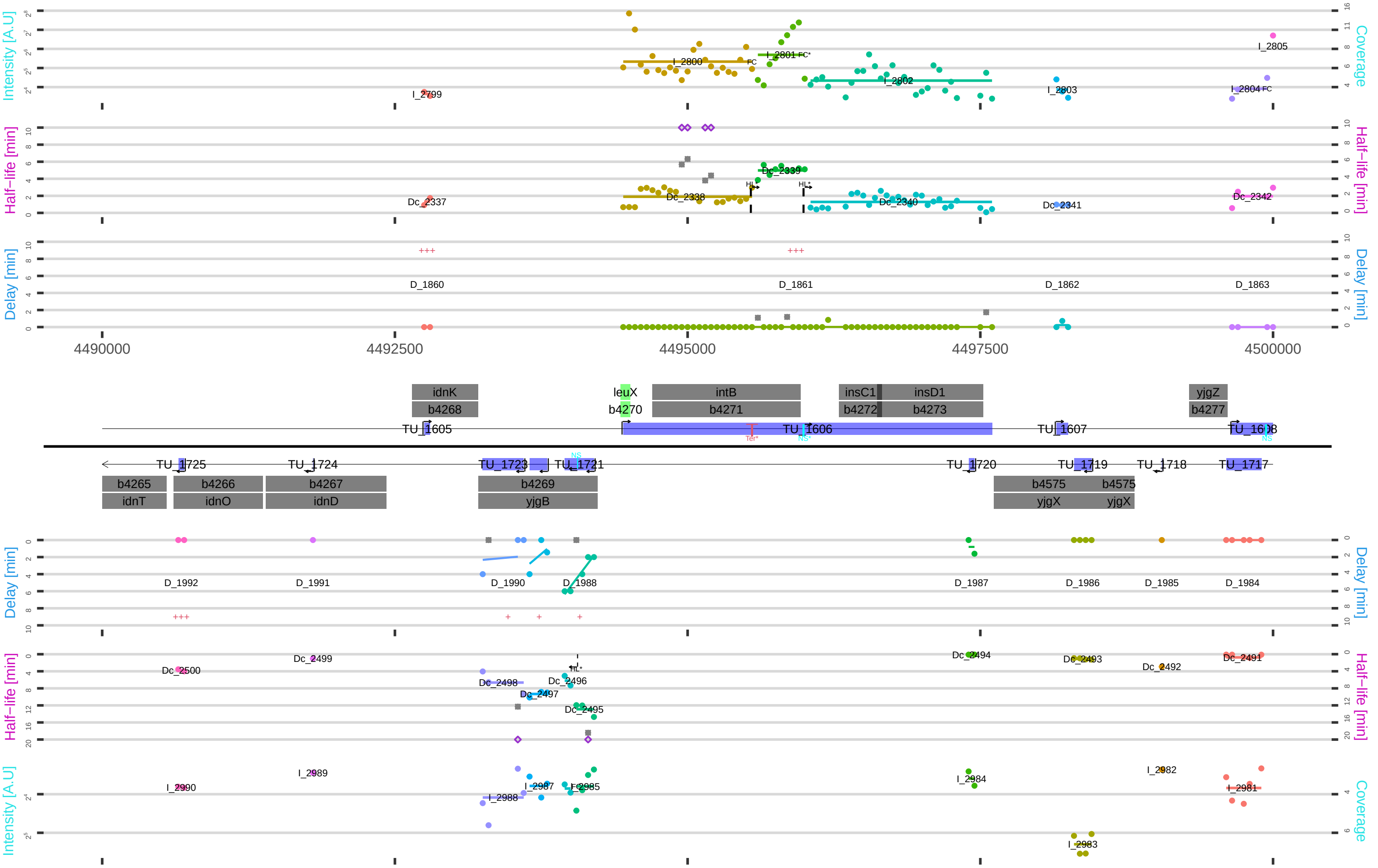

Term: termination (0), NS: new start (1), PS: pausing site (0), iTSS_I: internal starting site (0)

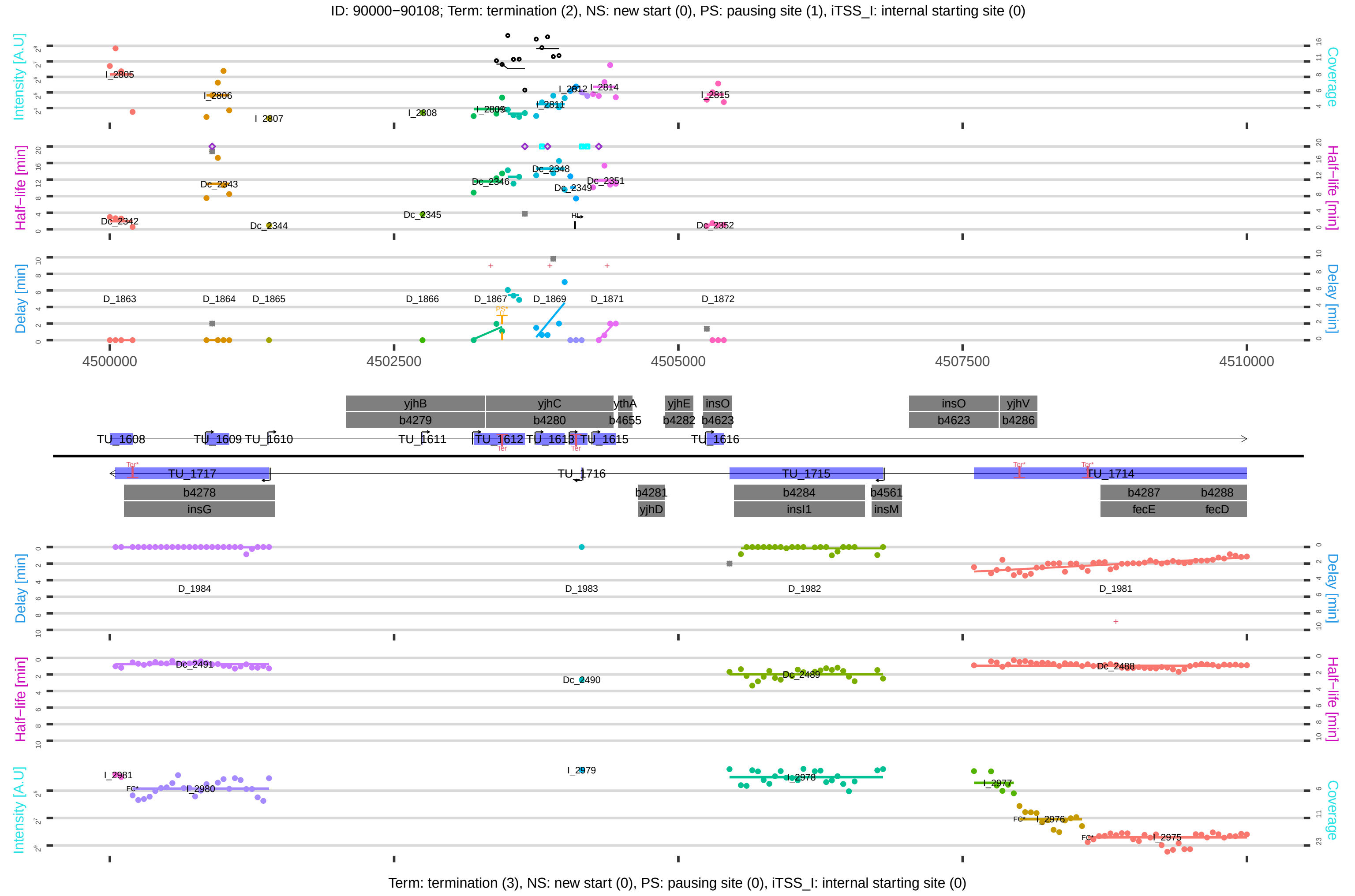
ID: 90000−90108; Term: termination (2), NS: new start (0), PS: pausing site (1), iTSS_I: internal starting site (0)
Intensity [A.U]
Coverage
Half−life [min]
Delay [min]
I_2805
I_2806
I_2807
I_2808
I_2809
I_2811
I_2812
I_2814
I_2815
Dc_2342
Dc_2343
Dc_2344
Dc_2345
Dc_2346
Dc_2348
Dc_2349
Dc_2351
Dc_2352
D_1863
D_1864
D_1865
D_1866
D_1867
D_1869
D_1871
D_1872
4500000
4502500
4505000
4507500
4510000
yjhB
b4279
yjhC
b4280
ythA
b4655
yjhE
b4282
insO
b4623
insO
b4623
yjhV
b4286
TU_1608
TU_1609
TU_1610
TU_1611
TU_1612
TU_1613
TU_1615
TU_1616
TU_1717
TU_1716
TU_1715
TU_1714
b4278
insG
b4281
yjhD
b4284
insI1
b4561
insM
b4287
fecE
b4288
fecD
D_1984
D_1983
D_1982
D_1981
Dc_2491
Dc_2490
Dc_2489
Dc_2488
I_2981
I_2980
I_2979
I_2978
I_2977
I_2976
I_2975
Term: termination (3), NS: new start (0), PS: pausing site (0), iTSS_I: internal starting site (0)

ID: 90225−90346; Term: termination (1), NS: new start (0), PS: pausing site (0), iTSS_I: internal starting site (0)

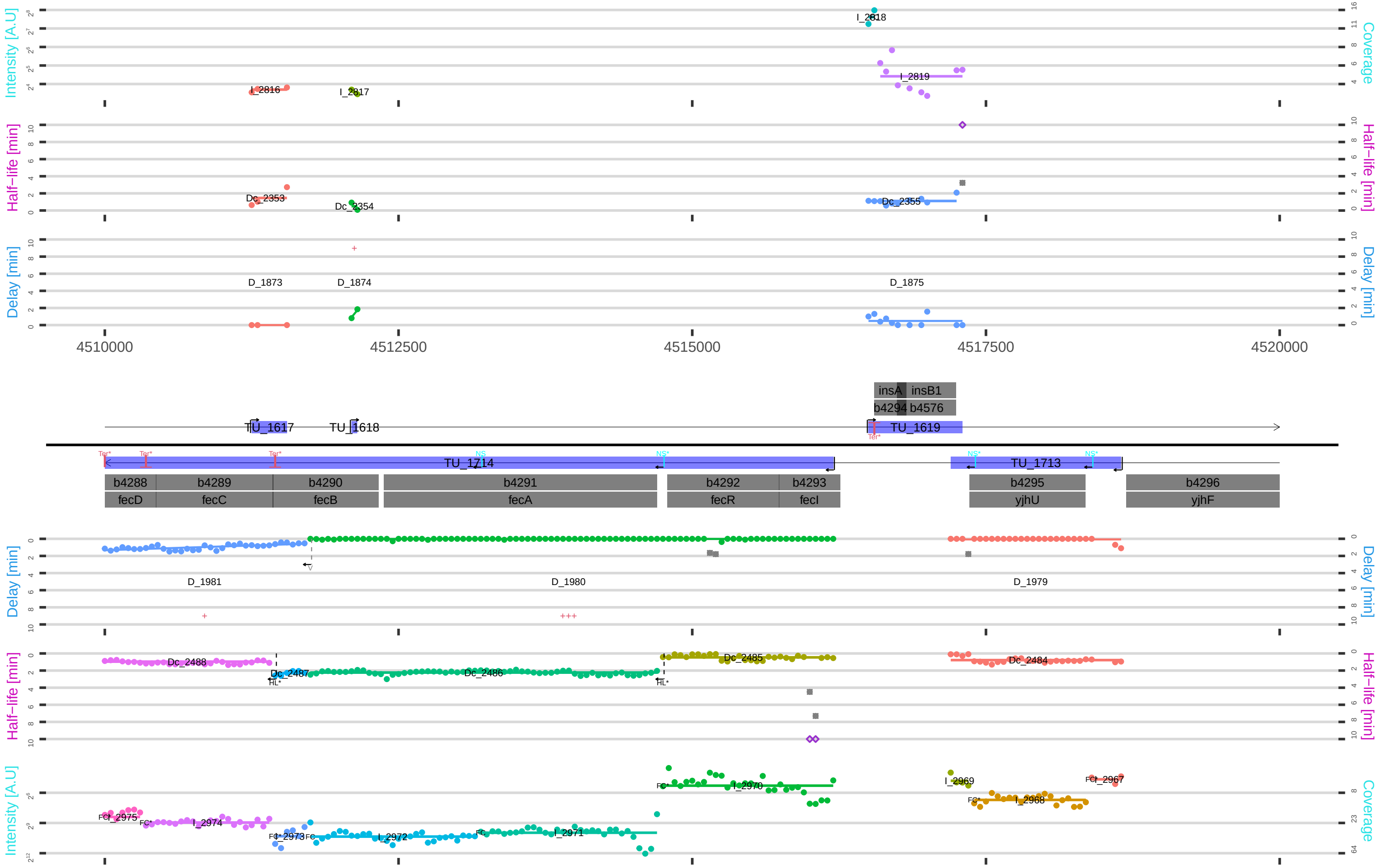

Term: termination (3), NS: new start (4), PS: pausing site (1), iTSS_I: internal starting site (0)

ID: 90423−90531; Term: termination (0), NS: new start (0), PS: pausing site (0), iTSS_I: internal starting site (0)

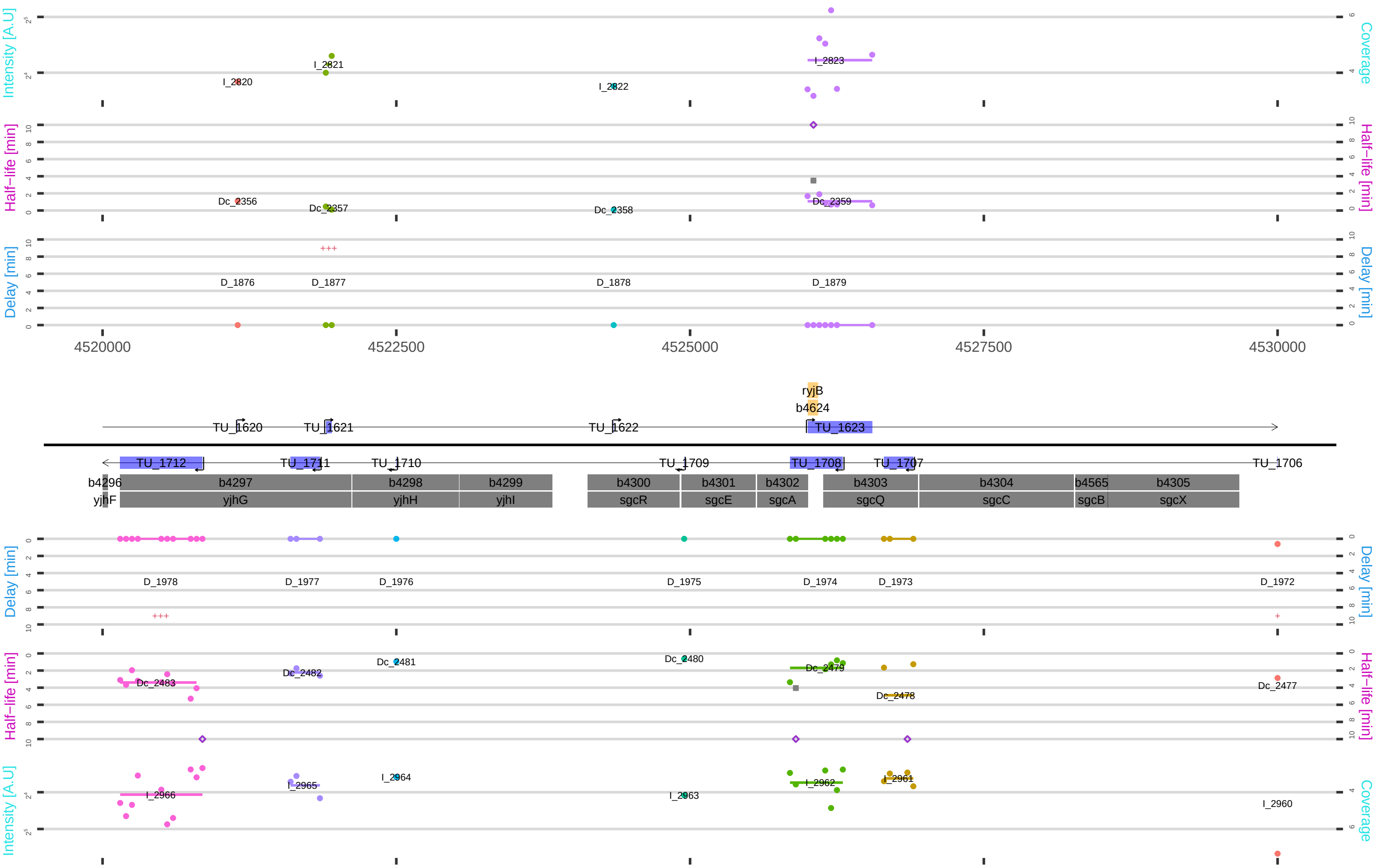

Term: termination (0), NS: new start (0), PS: pausing site (0), iTSS_I: internal starting site (0)

ID: 90639−90792; Term: termination (0), NS: new start (1), PS: pausing site (0), iTSS_I: internal starting site (0)

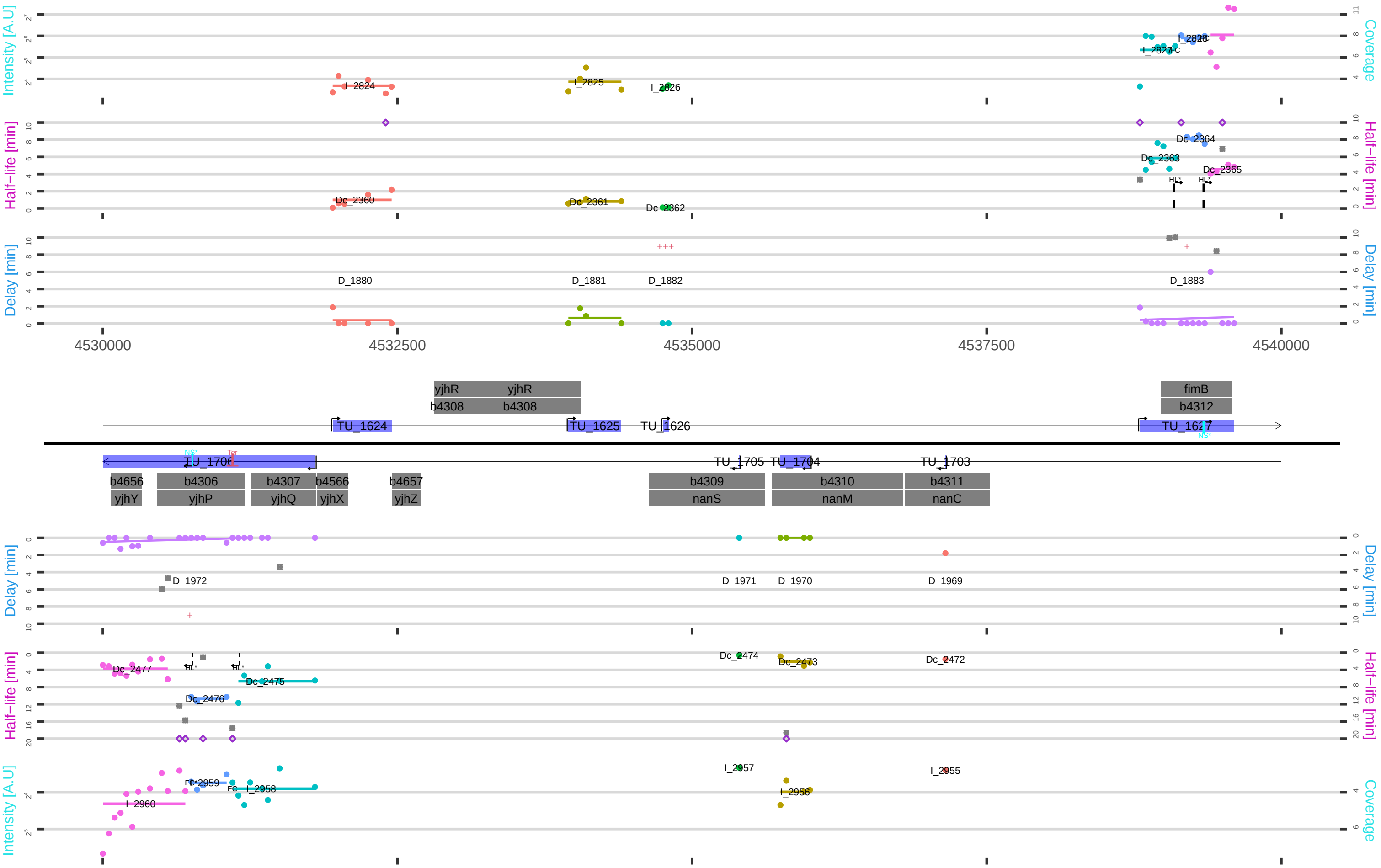

Term: termination (1), NS: new start (1), PS: pausing site (0), iTSS_I: internal starting site (0)

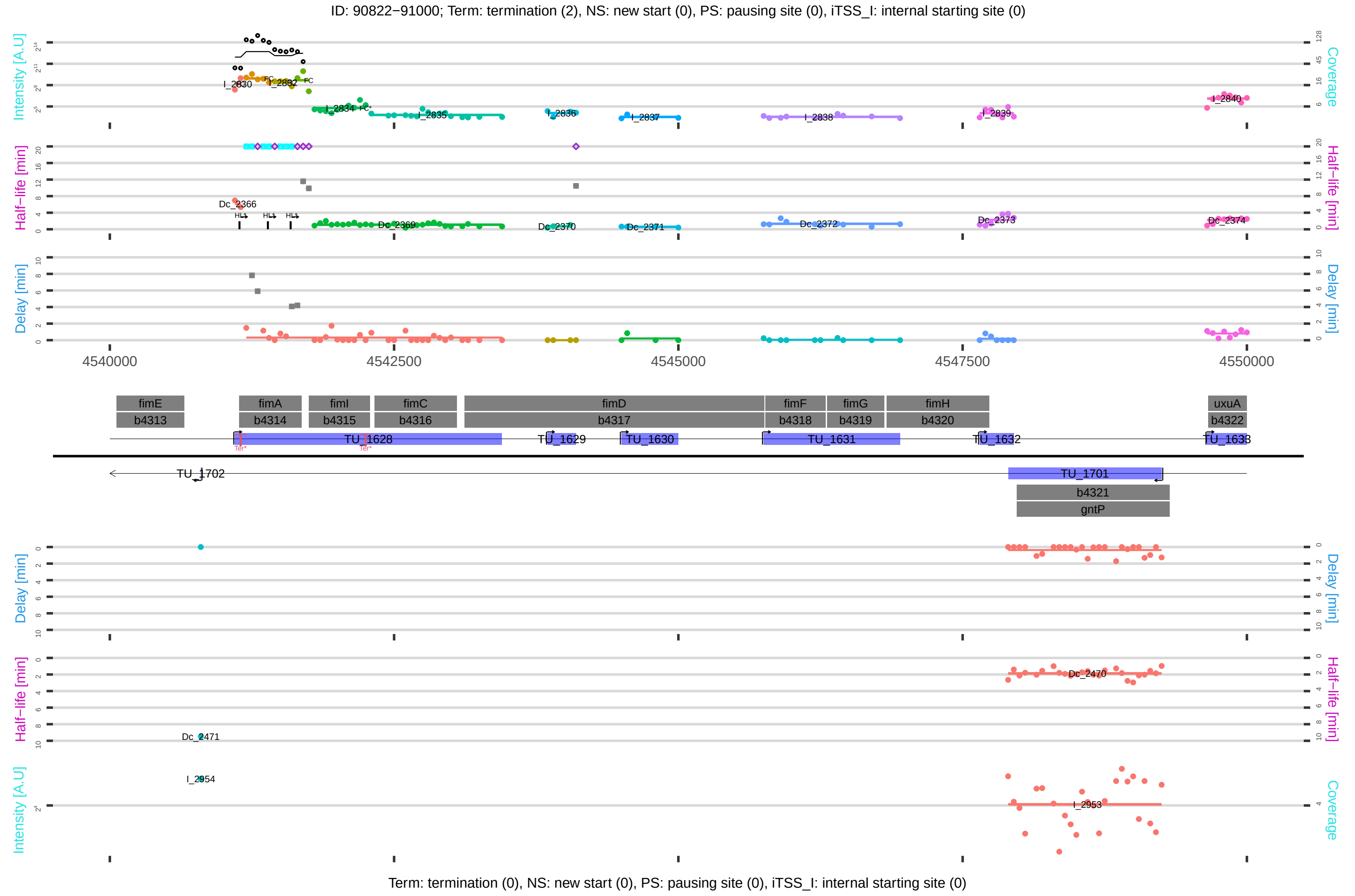

ID: 90822−91000; Term: termination (2), NS: new start (0), PS: pausing site (0), iTSS_I: internal starting site (0)
Intensity [A.U]
Coverage
Half−life [min]
Delay [min]
I_2830
I_2832
I_2834
I_2835
I_2836
I_2837
I_2838
I_2839
I_2840
Dc_2366
Dc_2369
Dc_2370
Dc_2371
Dc_2372
Dc_2373
Dc_2374
4540000
4542500
4545000
4547500
4550000
fimE
b4313
fimA
b4314
fimI
b4315
fimC
b4316
fimD
b4317
fimF
b4318
fimG
b4319
fimH
b4320
uxuA
b4322
TU_1628
TU_1629
TU_1630
TU_1631
TU_1632
TU_1633
TU_1702
TU_1701
b4321
gntP
Dc_2470
Dc_2471
I_2954
I_2953
Term: termination (0), NS: new start (0), PS: pausing site (0), iTSS_I: internal starting site (0)

ID: 91000−91188; Term: termination (0), NS: new start (0), PS: pausing site (1), iTSS_I: internal starting site (0)

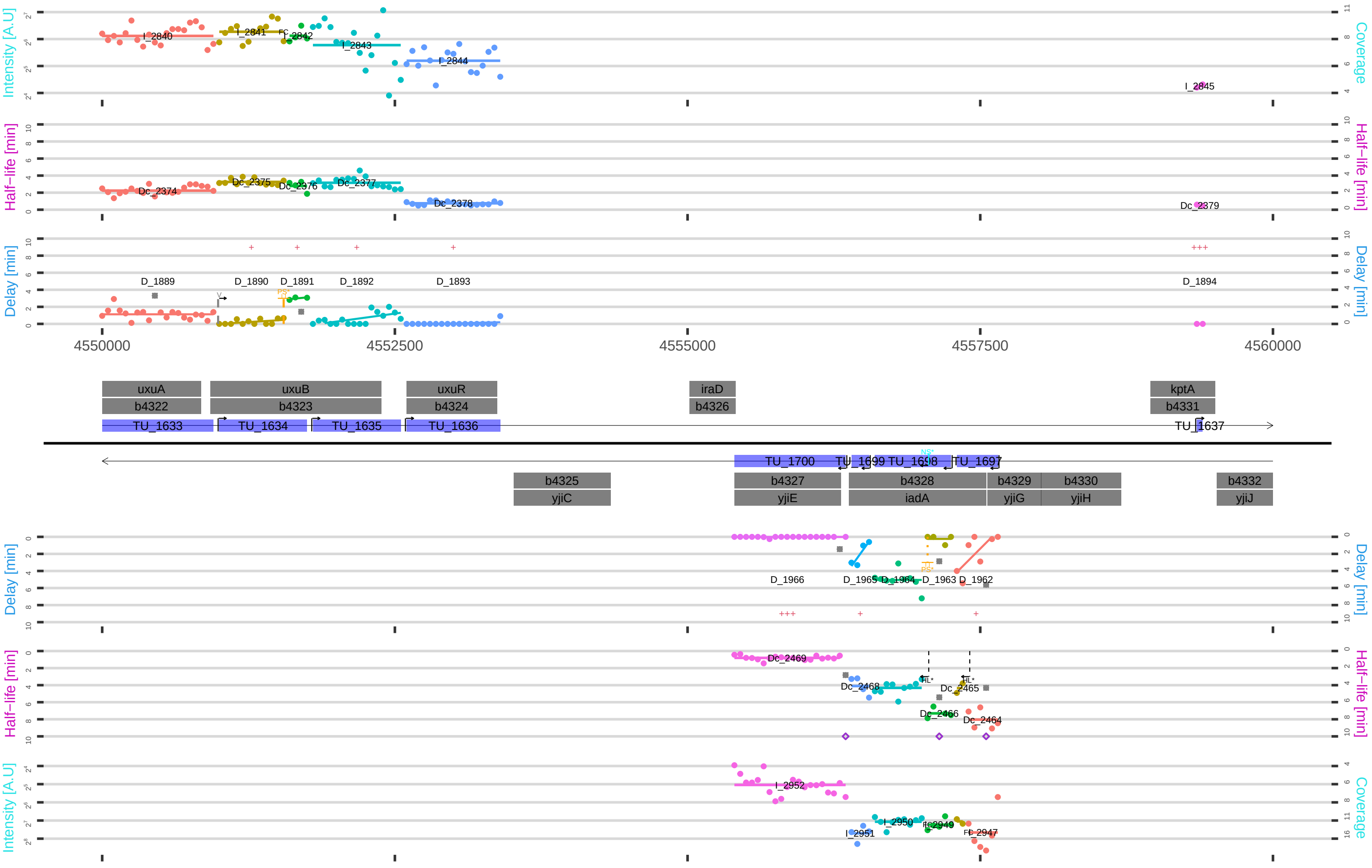

Term: termination (0), NS: new start (1), PS: pausing site (1), iTSS_I: internal starting site (0)

ID: 91340−91366; Term: termination (0), NS: new start (0), PS: pausing site (0), iTSS_I: internal starting site (0)

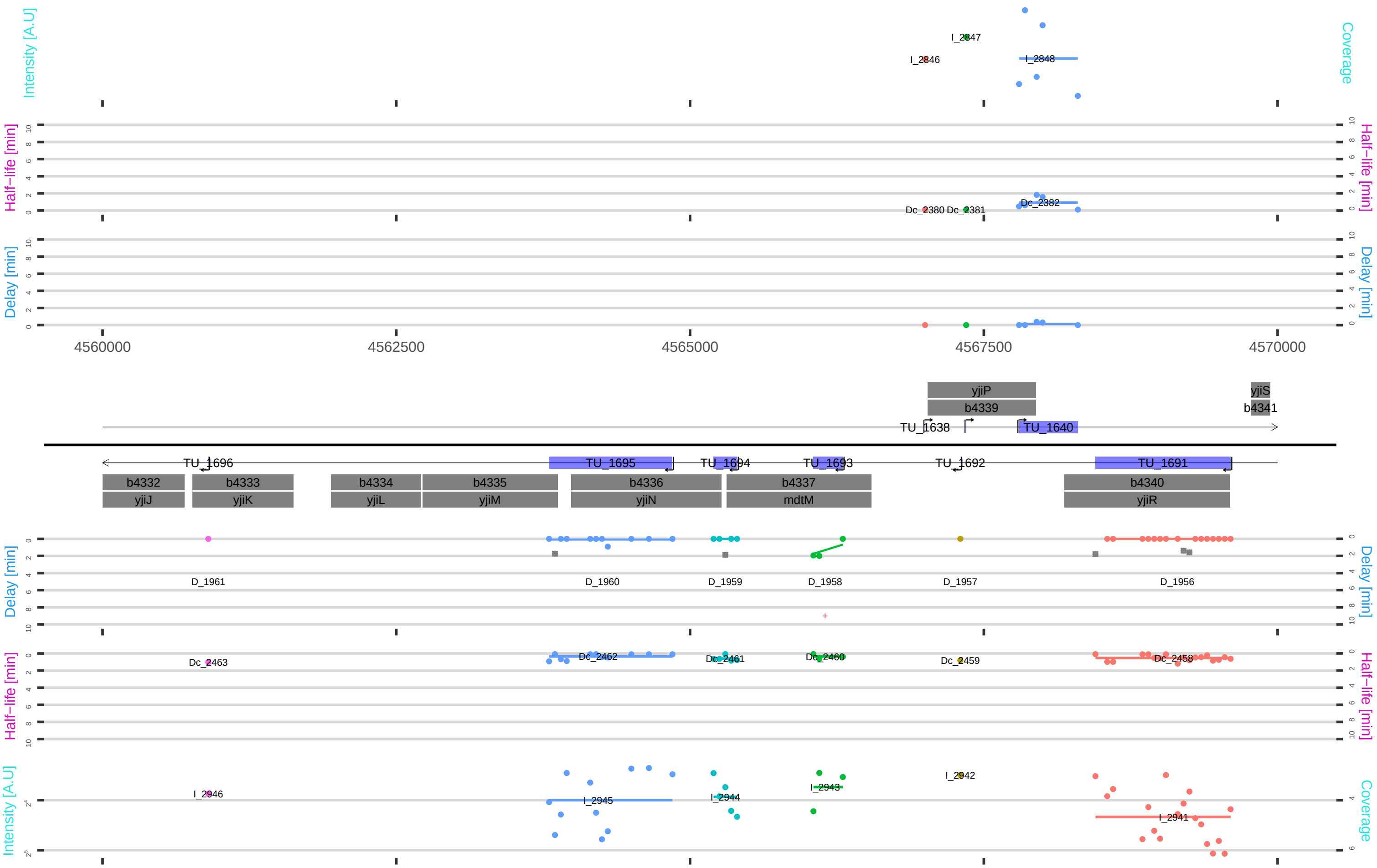

Term: termination (0), NS: new start (0), PS: pausing site (0), iTSS_I: internal starting site (0)

ID: 91408−91572; Term: termination (2), NS: new start (2), PS: pausing site (0), iTSS_I: internal starting site (1)

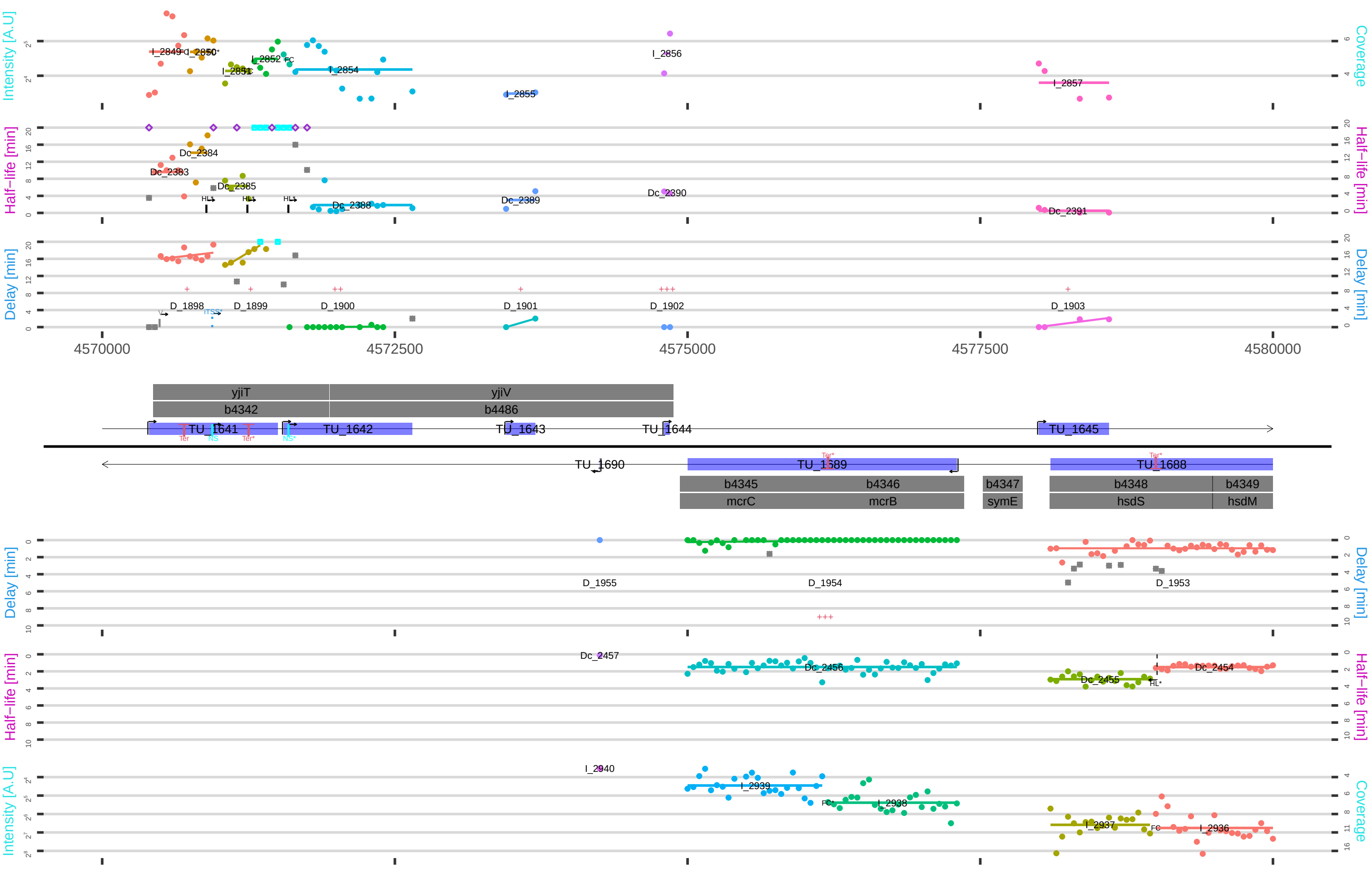

Term: termination (2), NS: new start (0), PS: pausing site (0), iTSS_I: internal starting site (0)

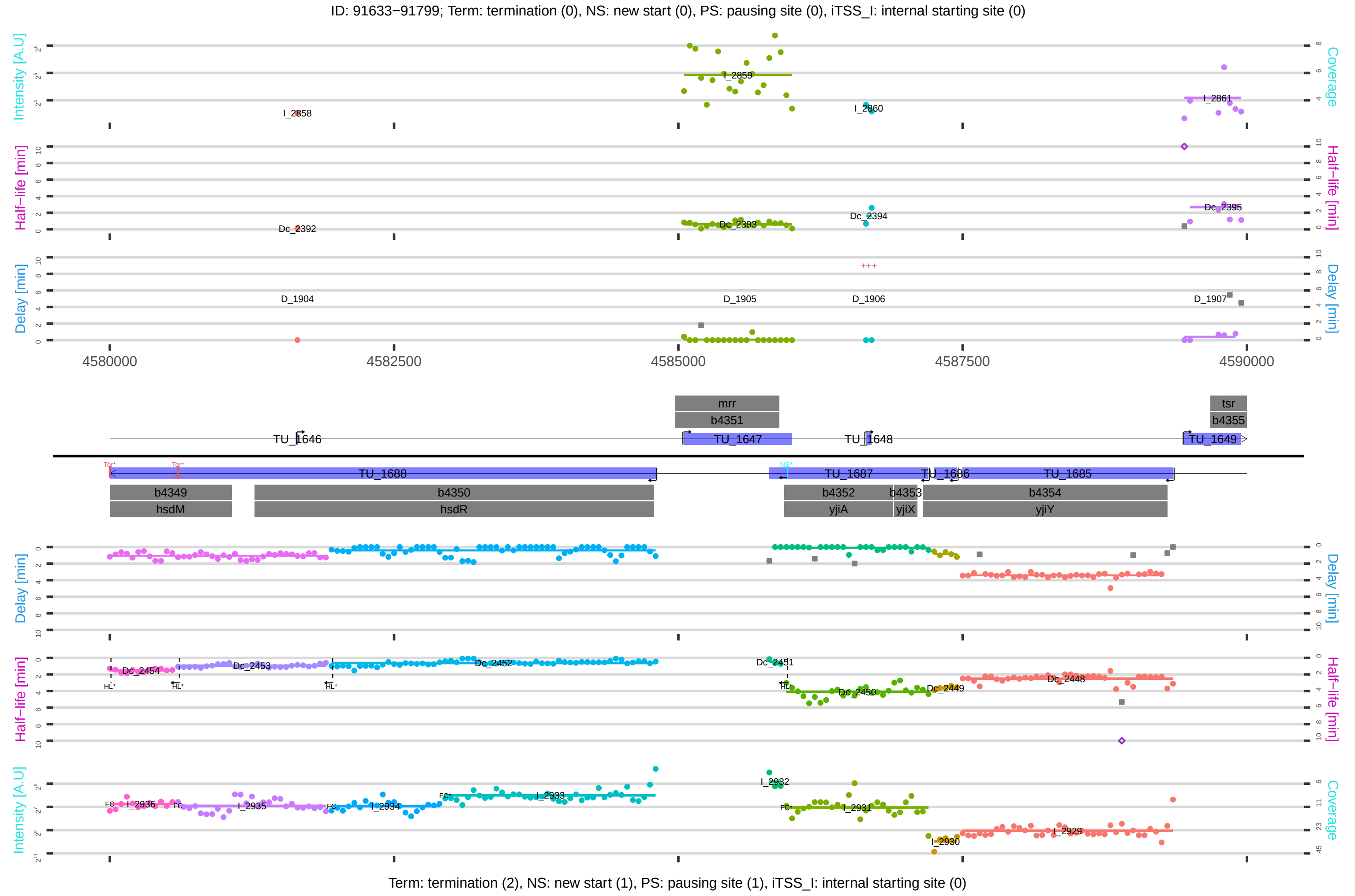
ID: 91633−91799; Term: termination (0), NS: new start (0), PS: pausing site (0), iTSS_I: internal starting site (0)
Intensity [A.U]
Coverage
Half−life [min]
Delay [min]
I_2858
I_2859
I_2860
I_2861
Dc_2392
Dc_2393
Dc_2394
Dc_2395
D_1904
D_1905
D_1906
D_1907
4580000
4582500
4585000
4587500
4590000
mrr
b4351
tsr
b4355
TU_1646
TU_1647
TU_1648
TU_1649
TU_1688
TU_1687
TU_1686
TU_1685
b4349
hsdM
b4350
hsdR
b4352
yjiA
b4353
yjiX
b4354
yjiY
Dc_2454
Dc_2453
Dc_2452
Dc_2451
Dc_2450
Dc_2449
Dc_2448
I_2936
I_2935
I_2934
I_2933
I_2932
I_2931
I_2930
I_2929
Term: termination (2), NS: new start (1), PS: pausing site (1), iTSS_I: internal starting site (0)

ID: 91801−91902; Term: termination (0), NS: new start (0), PS: pausing site (0), iTSS_I: internal starting site (0)

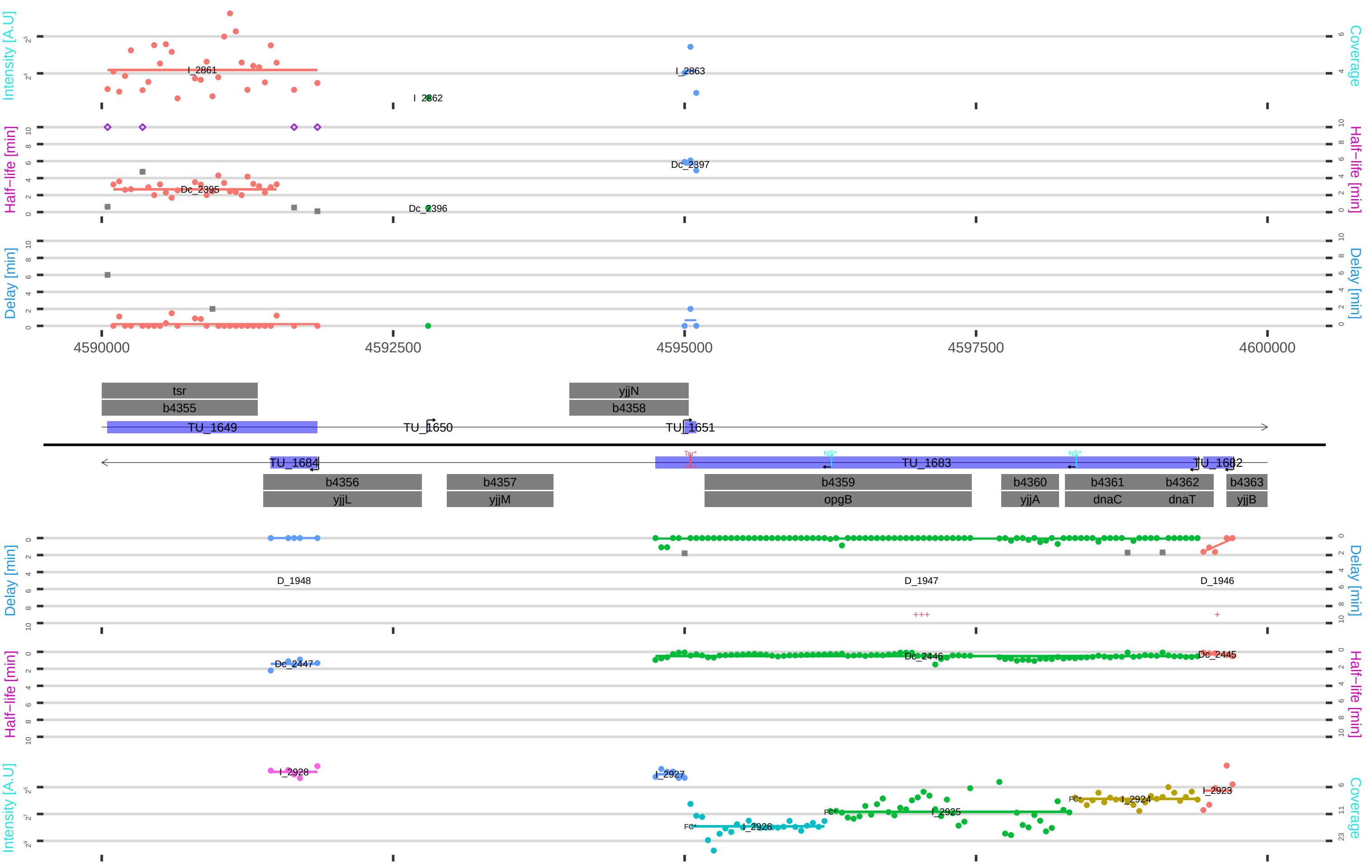

Term: termination (1), NS: new start (2), PS: pausing site (0), iTSS_I: internal starting site (0)

ID: 92055−92200; Term: termination (4), NS: new start (1), PS: pausing site (2), iTSS_I: internal starting site (0)

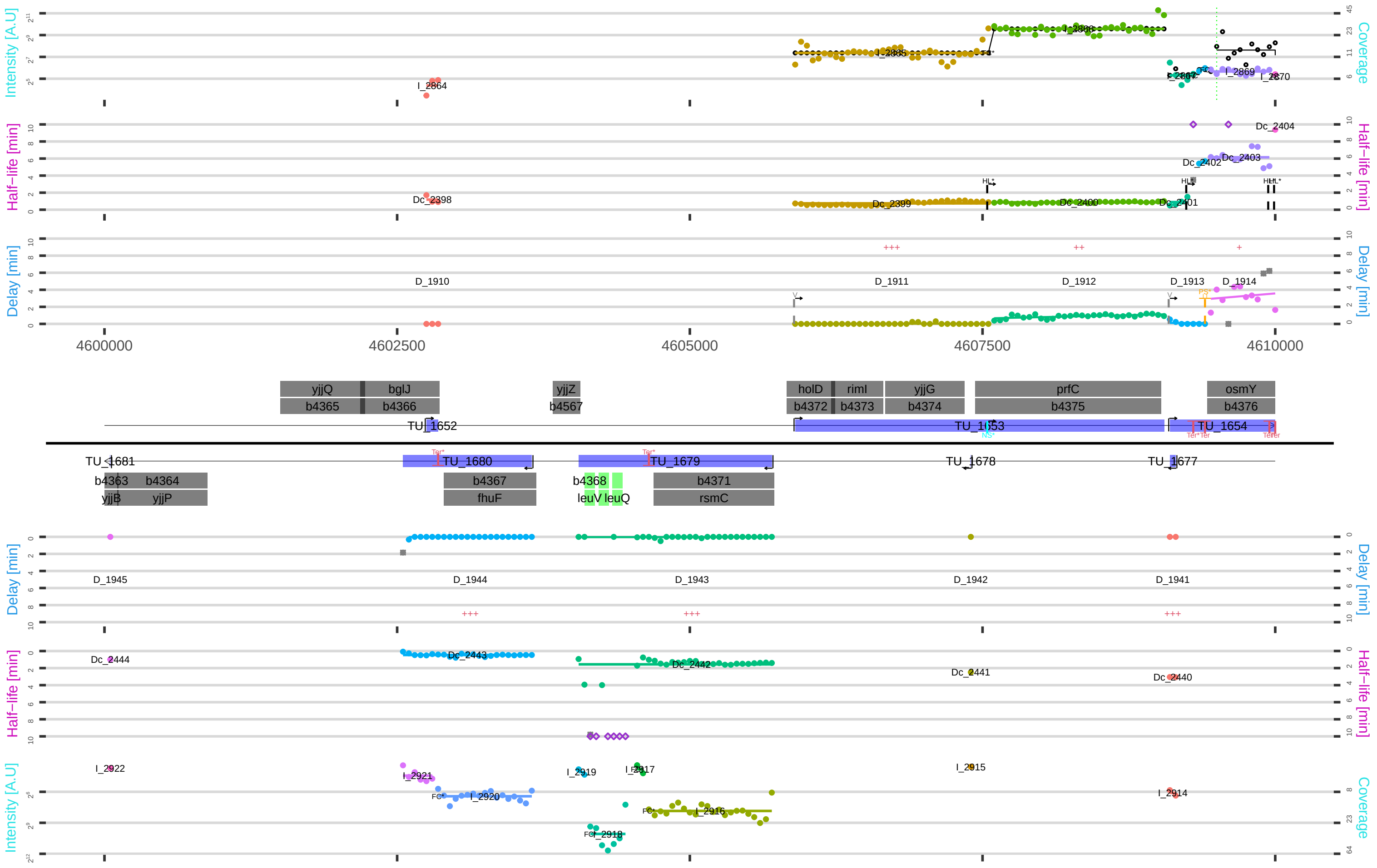

Term: termination (2), NS: new start (0), PS: pausing site (0), iTSS_I: internal starting site (0)

ID: 92200−92400; Term: termination (3), NS: new start (5), PS: pausing site (1), iTSS_I: internal starting site (2)

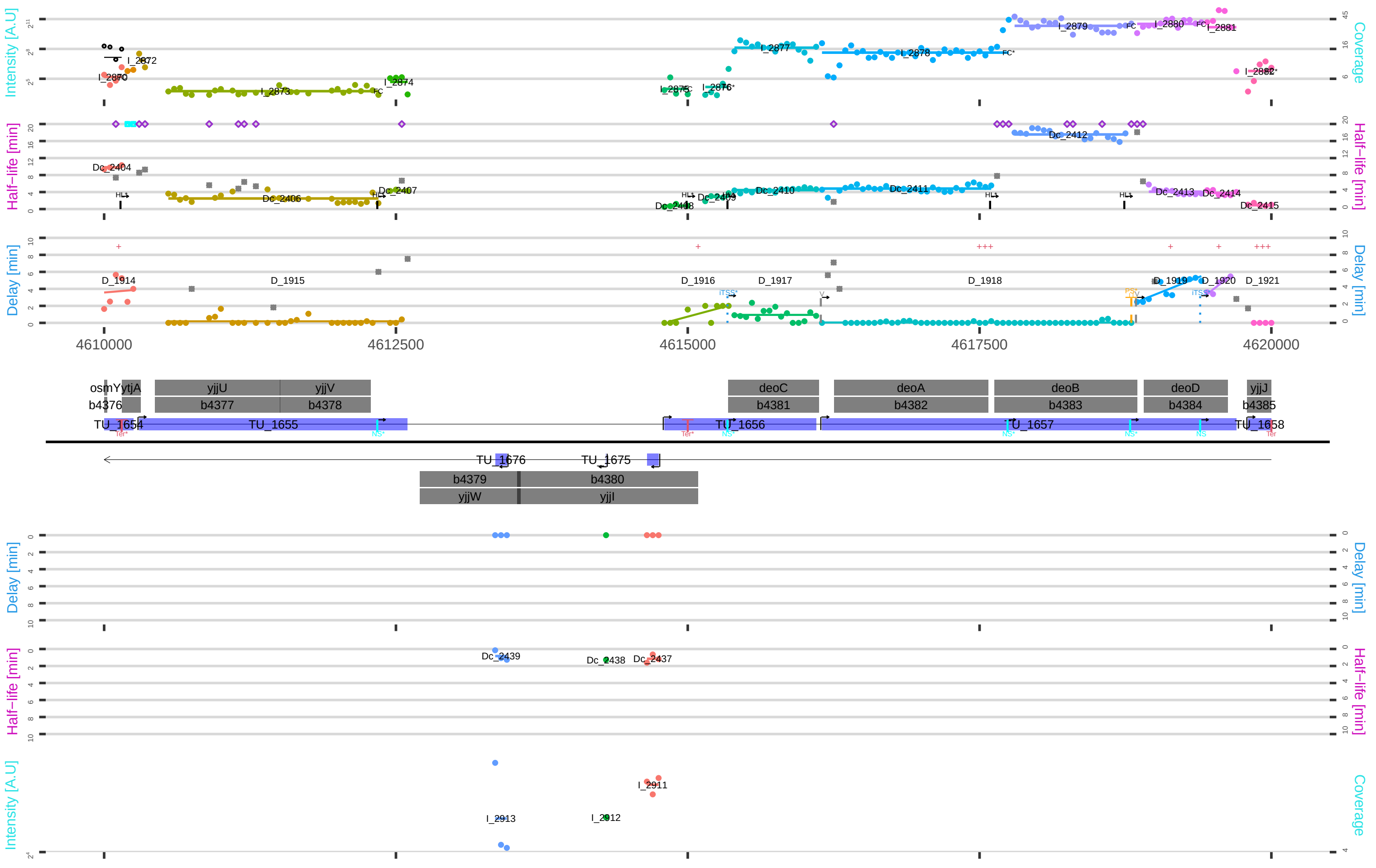

Term: termination (0), NS: new start (0), PS: pausing site (0), iTSS_I: internal starting site (0)

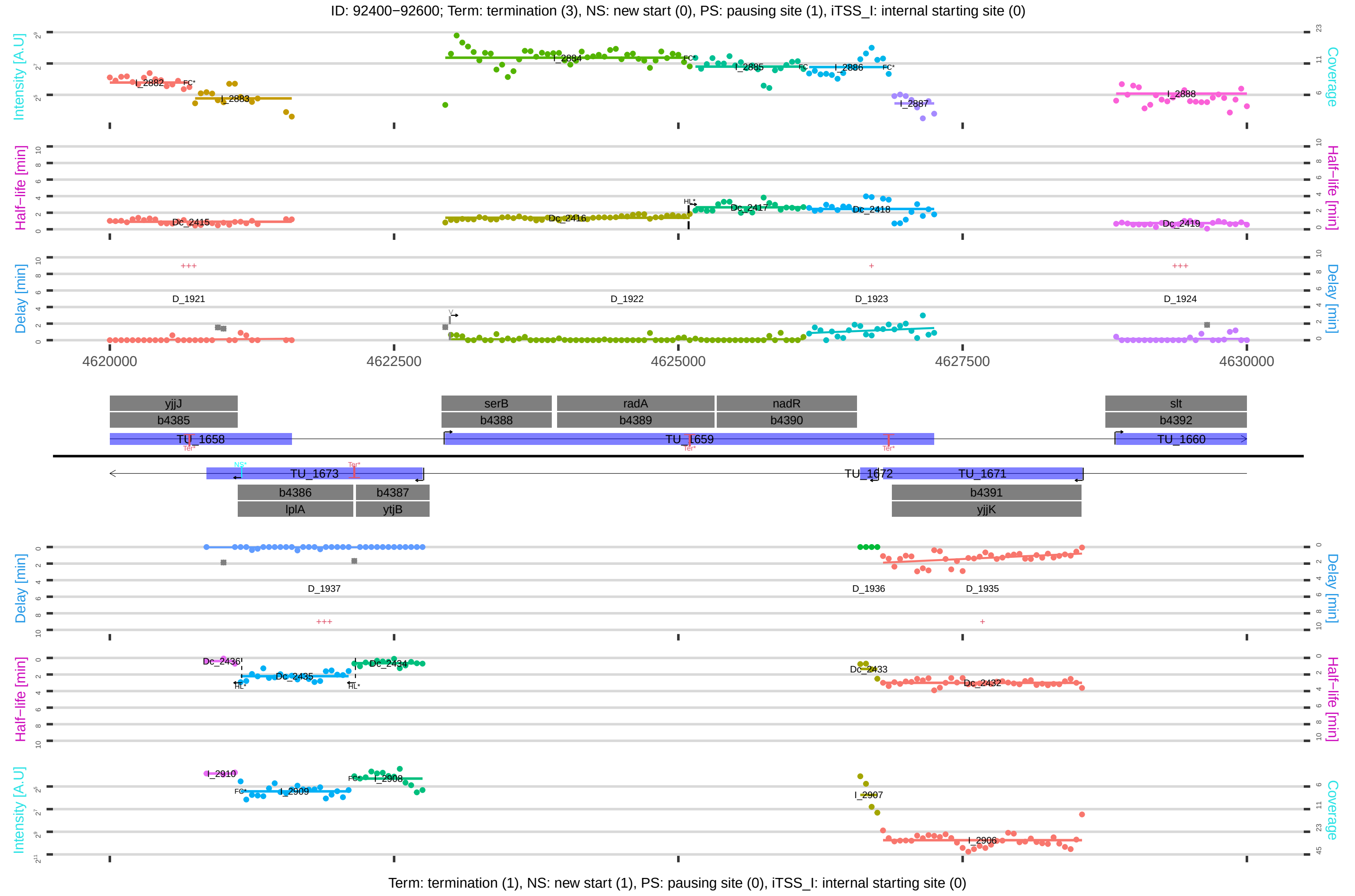

ID: 92400−92600; Term: termination (3), NS: new start (0), PS: pausing site (1), iTSS_I: internal starting site (0)
Intensity [A.U]
Coverage
Half−life [min]
Delay [min]
I_2882
I_2883
I_2884
I_2885
I_2886
I_2887
I_2888
Dc_2415
Dc_2416
Dc_2417
Dc_2418
Dc_2419
D_1921
D_1922
D_1923
D_1924
4620000
4622500
4625000
4627500
4630000
yjjJ
b4385
serB
b4388
radA
b4389
nadR
b4390
slt
b4392
TU_1658
TU_1659
TU_1660
TU_1673
TU_1672
TU_1671
b4386
lplA
b4387
ytjB
b4391
yjjK
D_1937
D_1936
D_1935
Dc_2436
Dc_2435
Dc_2434
Dc_2433
Dc_2432
I_2910
I_2909
I_2908
I_2907
I_2906
Term: termination (1), NS: new start (1), PS: pausing site (0), iTSS_I: internal starting site (0)

ID: 92600−92793; Term: termination (2), NS: new start (1), PS: pausing site (0), iTSS_I: internal starting site (0)

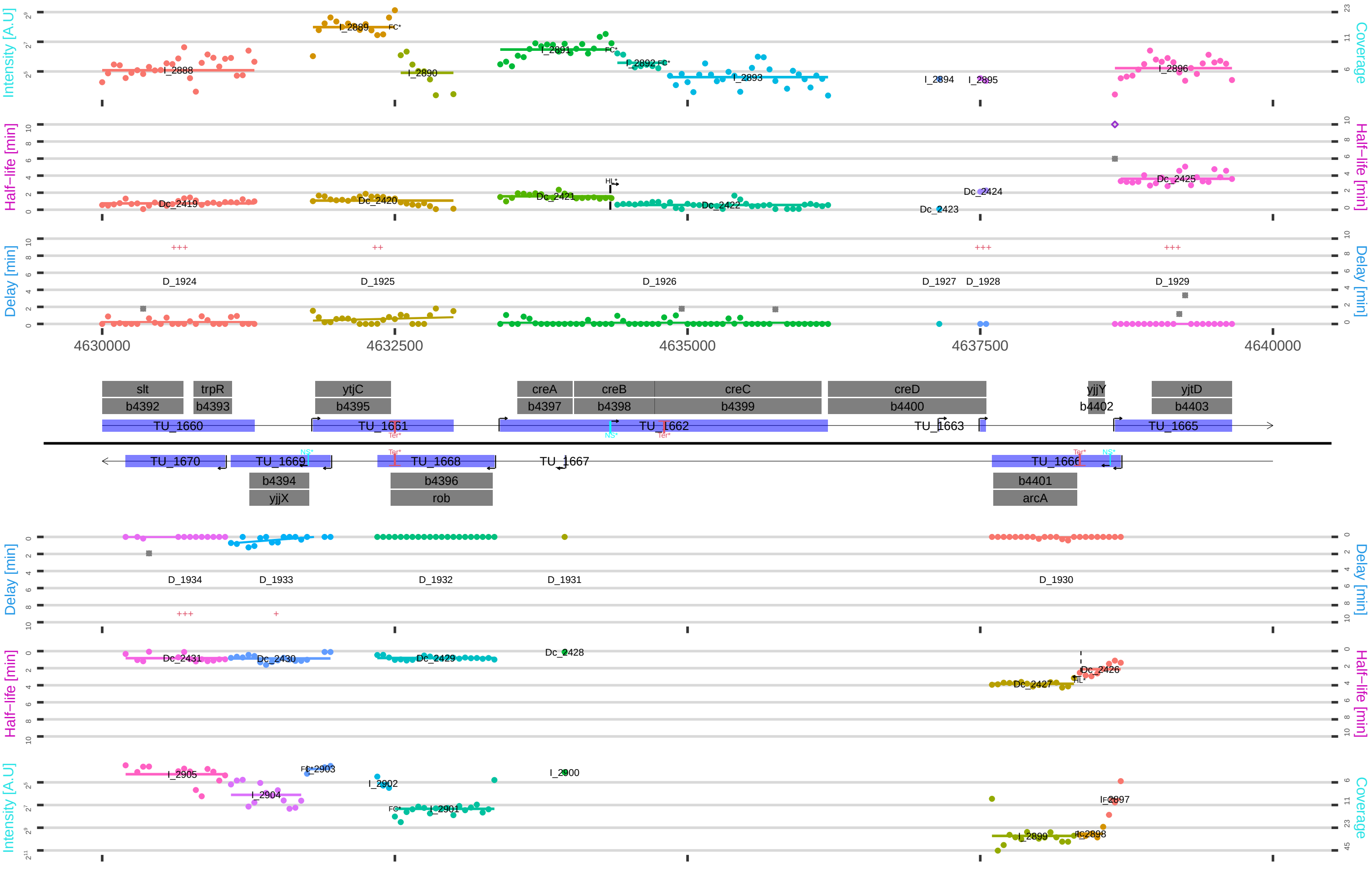

Term: termination (2), NS: new start (2), PS: pausing site (0), iTSS_I: internal starting site (0)
