# Supplementary material for: Improved RNA stability estimation indicates that transcriptional interference is frequent in diverse bacteria
Source: Commun Biol. 2023 Jul 15;6:732. doi: 10.1038/s42003-023-05097-2 (PMC10349824; doi:10.1038/s42003-023-05097-2)
Supplement: Supplementary file 6 — Supplementary Data 3 [file 42003_2023_5097_MOESM6_ESM.zip › Klebsiella_aerogenes_KCTC2190.pdf]

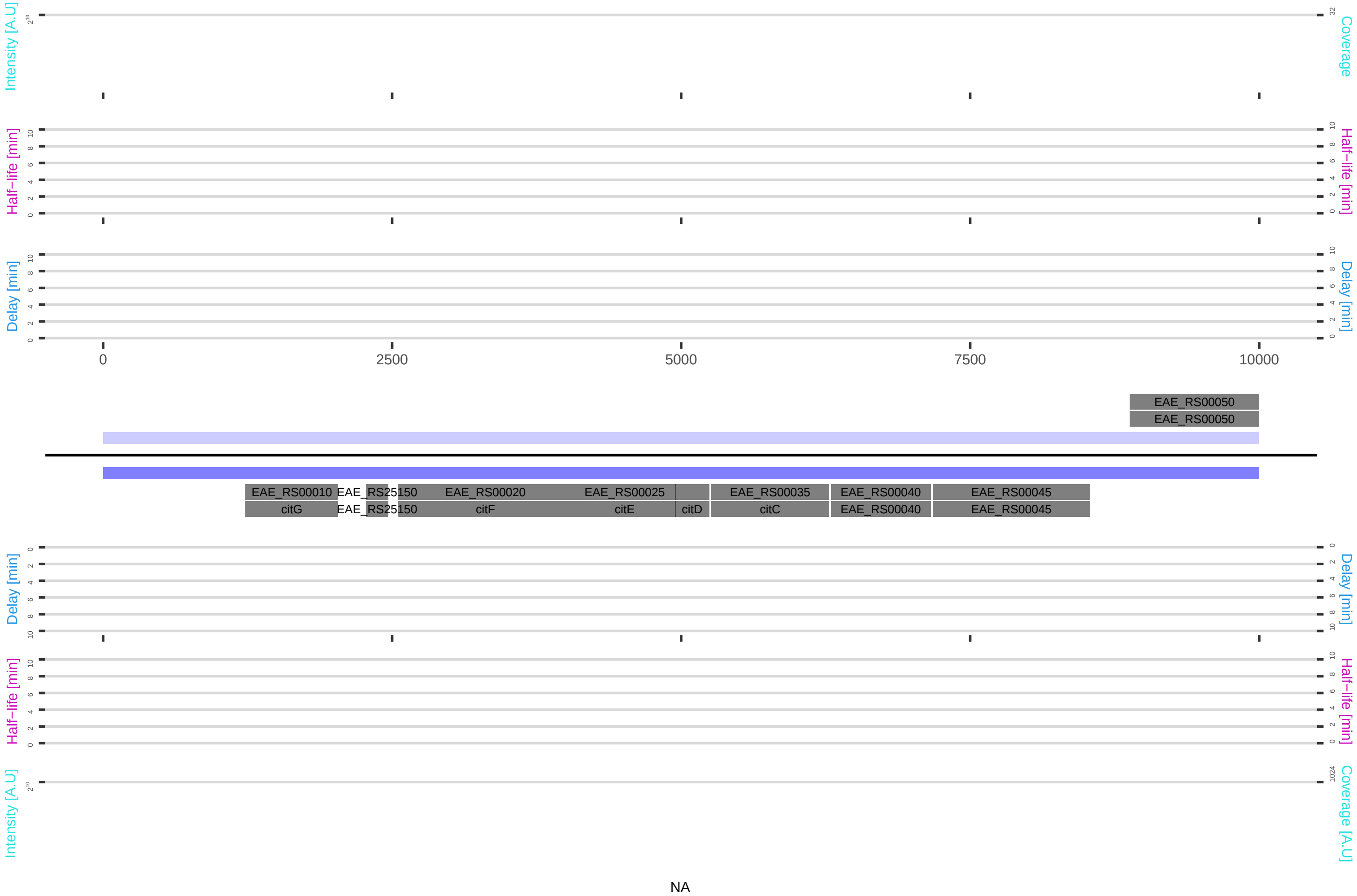

Intensity [A.U]
Coverage
Half−life [min]
Delay [min]
0
2500
5000
7500
10000
EAE_RS00050
EAE_RS00010
citG
EAE_RS25150
EAE_RS00020
citF
EAE_RS00025
citE
citD
EAE_RS00035
citC
EAE_RS00040
EAE_RS00045
Coverage [A.U]
NA

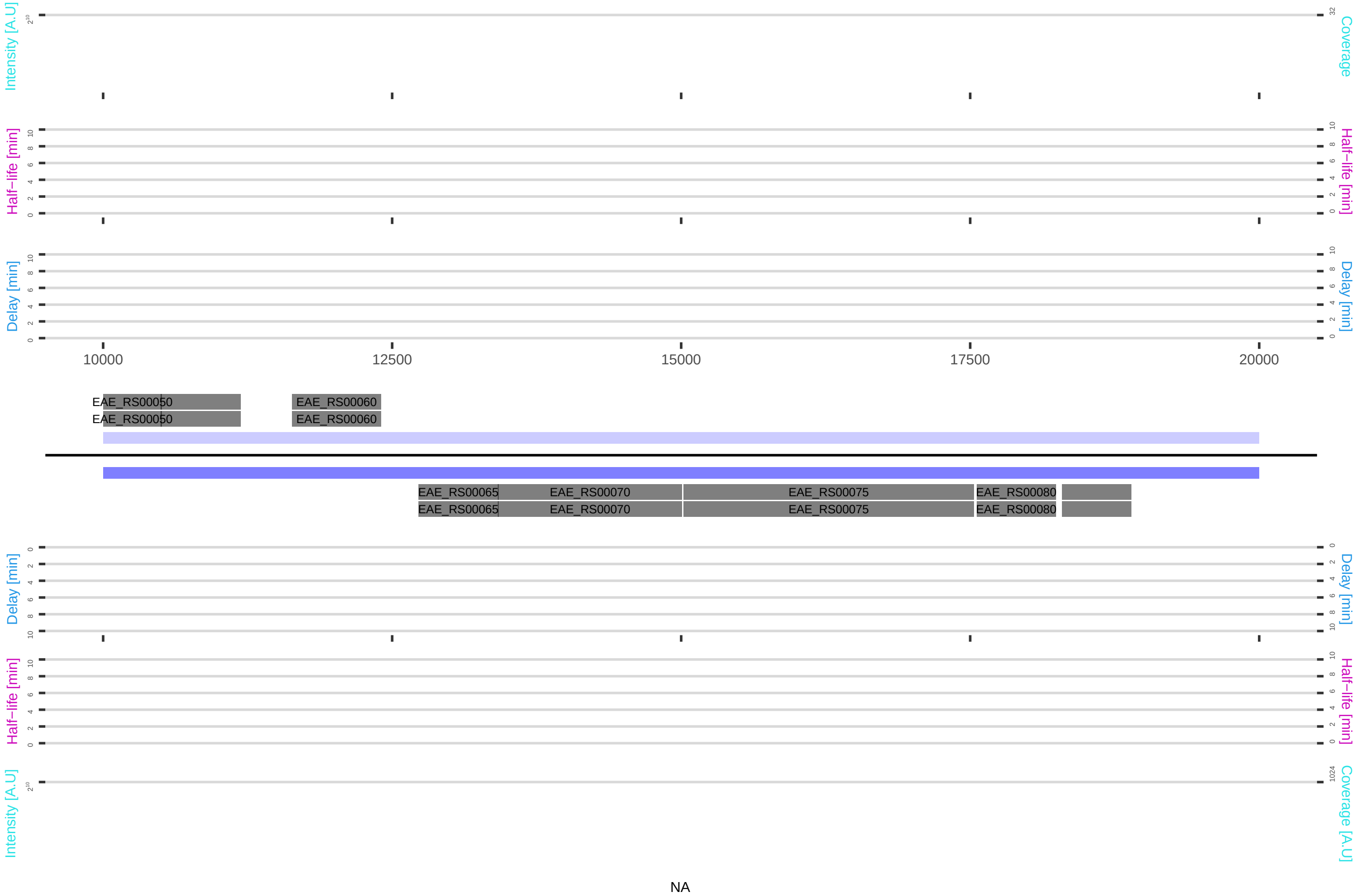

Intensity [A.U]
Coverage
Half−life [min]
Delay [min]
10000
12500
15000
17500
20000
EAE_RS00050
EAE_RS00060
EAE_RS00065
EAE_RS00070
EAE_RS00075
EAE_RS00080
Coverage [A.U]
NA

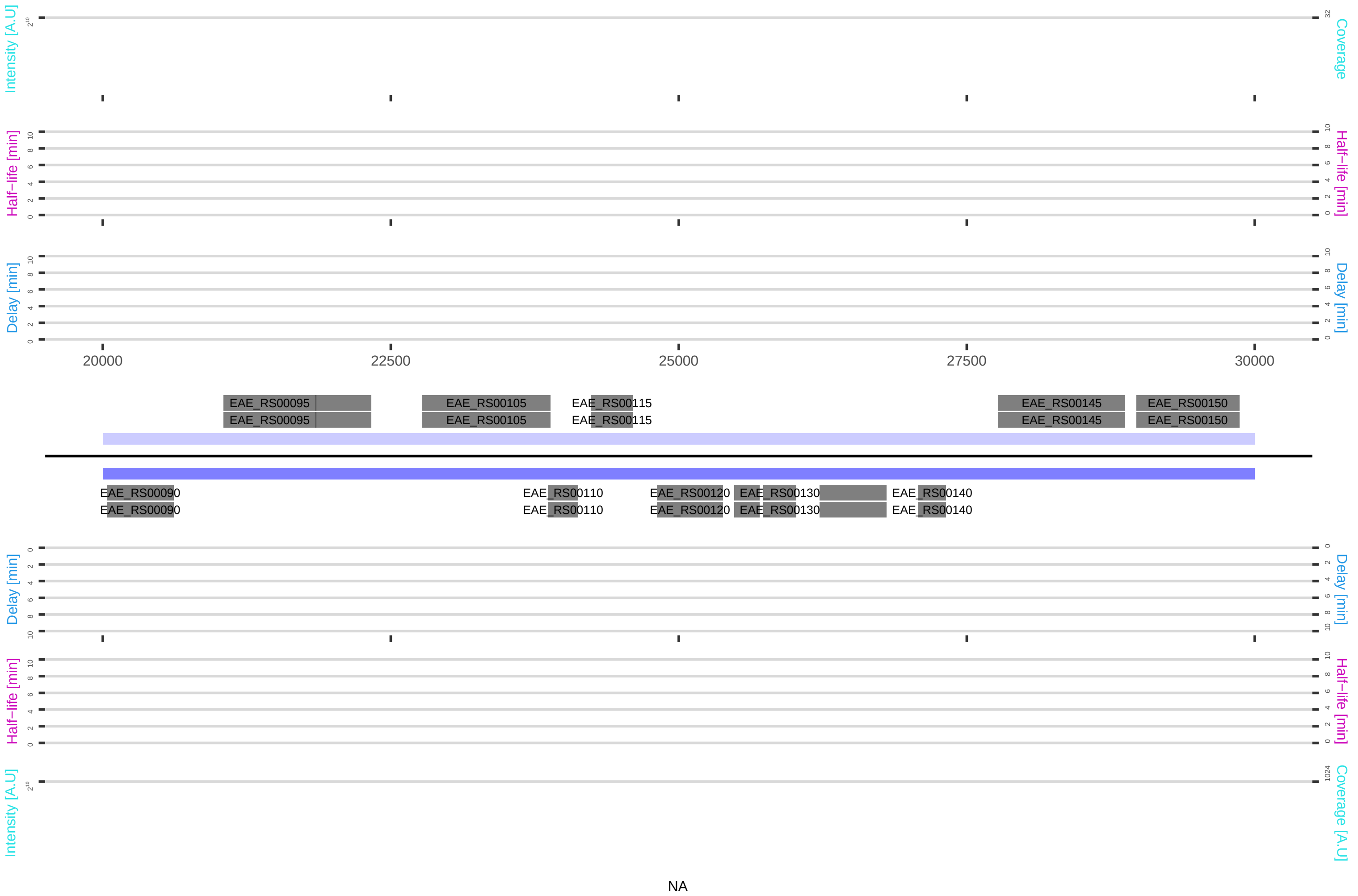

Intensity [A.U]
Coverage
Half−life [min]
Delay [min]
20000
22500
25000
27500
30000
EAE_RS00095
EAE_RS00105
EAE_RS00115
EAE_RS00145
EAE_RS00150
EAE_RS00090
EAE_RS00110
EAE_RS00120
EAE_RS00130
EAE_RS00140
Coverage [A.U]
NA

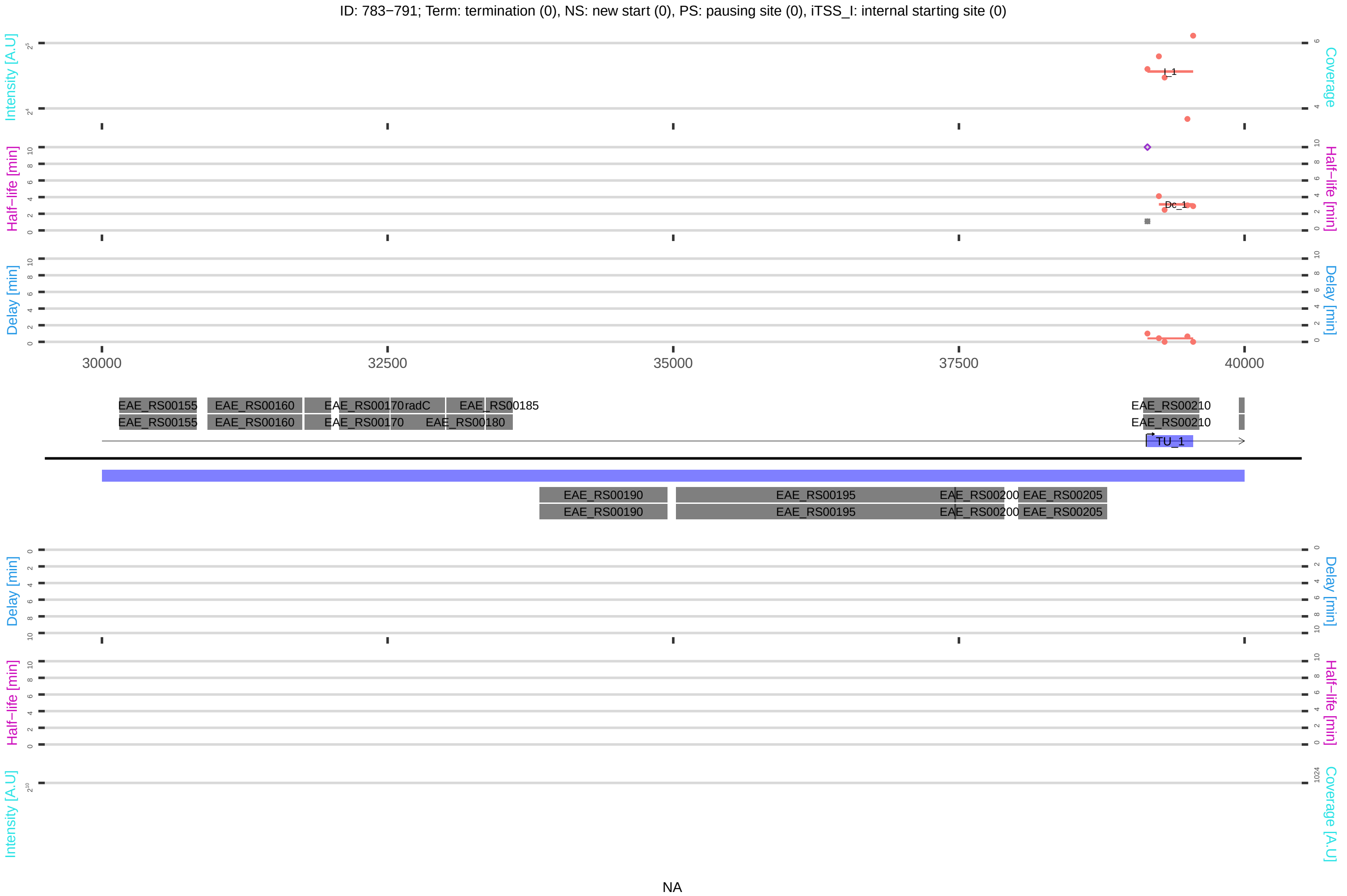

ID: 783−791; Term: termination (0), NS: new start (0), PS: pausing site (0), iTSS_I: internal starting site (0)
Intensity [A.U]
Coverage
I_1
Half−life [min]
Dc_1
Delay [min]
30000
32500
35000
37500
40000
EAE_RS00155
EAE_RS00160
EAE_RS00170
radC
EAE_RS00185
EAE_RS00180
EAE_RS00210
TU_1
EAE_RS00190
EAE_RS00195
EAE_RS00200
EAE_RS00205
Coverage [A.U]
NA

ID: 880−1000; Term: termination (0), NS: new start (0), PS: pausing site (0), iTSS_I: internal starting site (0)

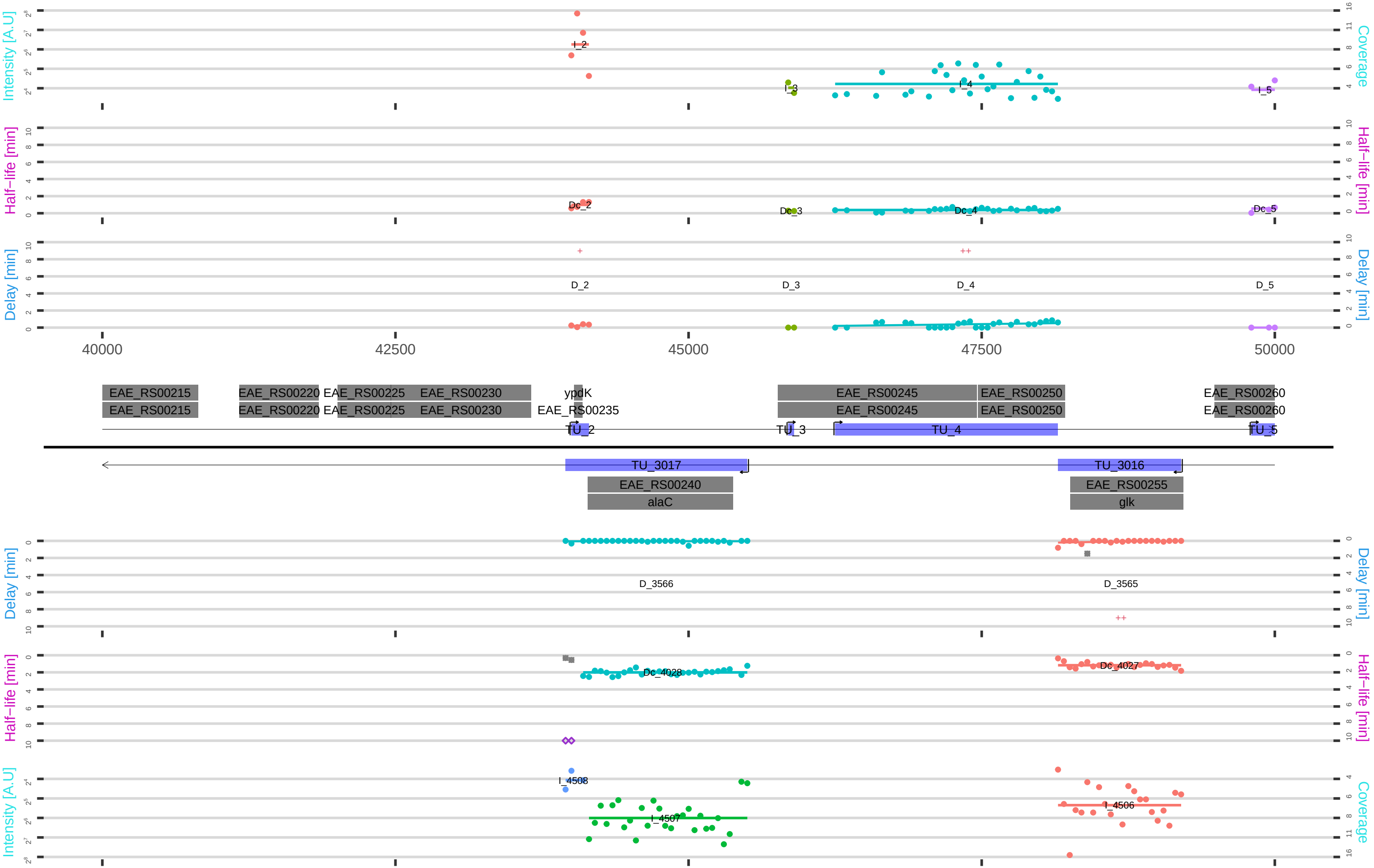

Term: termination (0), NS: new start (0), PS: pausing site (0), iTSS_I: internal starting site (0)

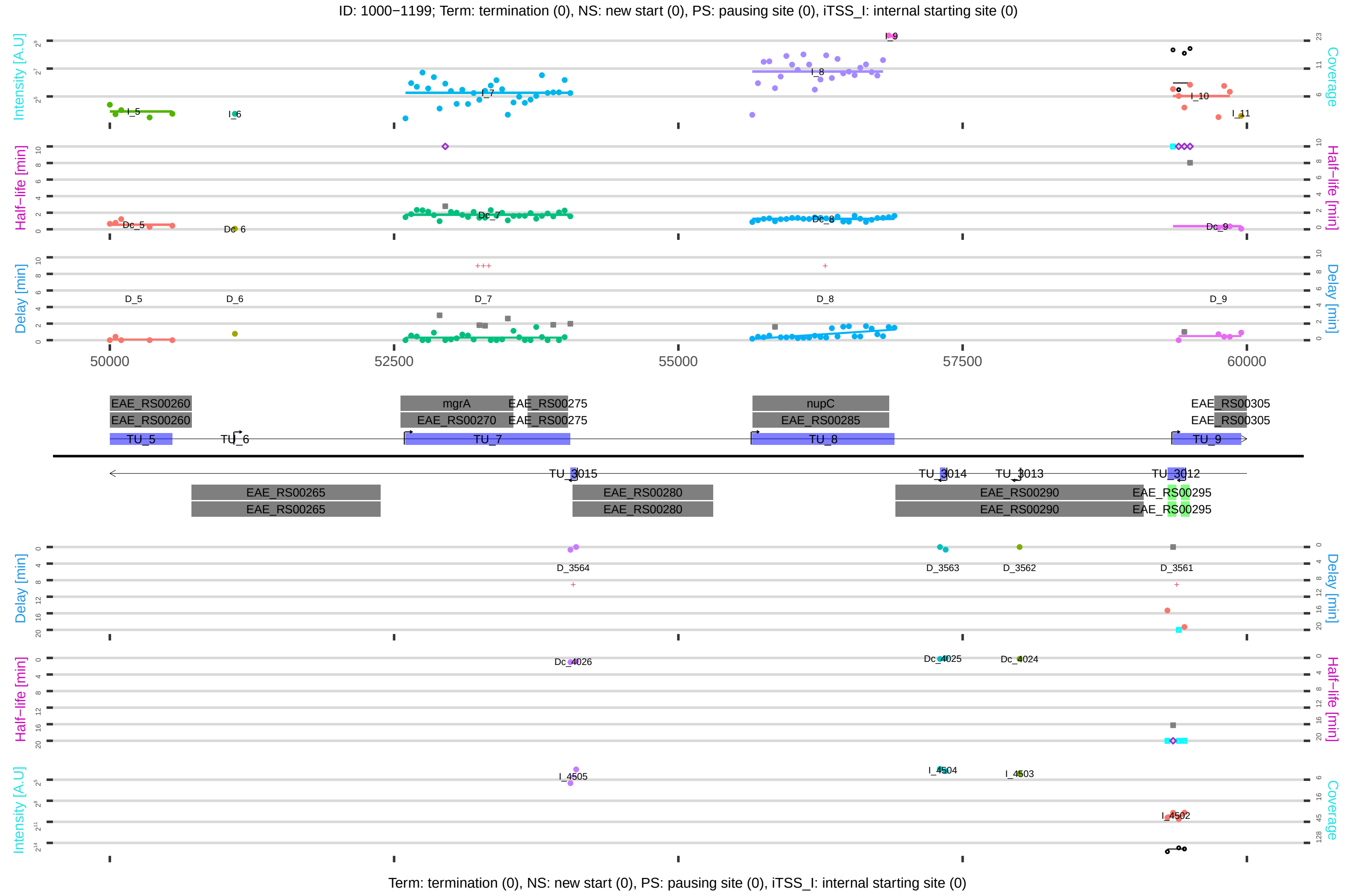
ID: 1000−1199; Term: termination (0), NS: new start (0), PS: pausing site (0), iTSS_I: internal starting site (0)
Intensity [A.U]
Coverage
Half−life [min]
Delay [min]
50000
52500
55000
57500
60000
EAE_RS00260
mgrA
EAE_RS00270
EAE_RS00275
nupC
EAE_RS00285
EAE_RS00305
TU_5
TU_6
TU_7
TU_8
TU_9
TU_3015
TU_3014
TU_3013
TU_3012
EAE_RS00265
EAE_RS00280
EAE_RS00290
EAE_RS00295
Term: termination (0), NS: new start (0), PS: pausing site (0), iTSS_I: internal starting site (0)

ID: 1201−1344; Term: termination (1), NS: new start (0), PS: pausing site (0), iTSS_I: internal starting site (0)

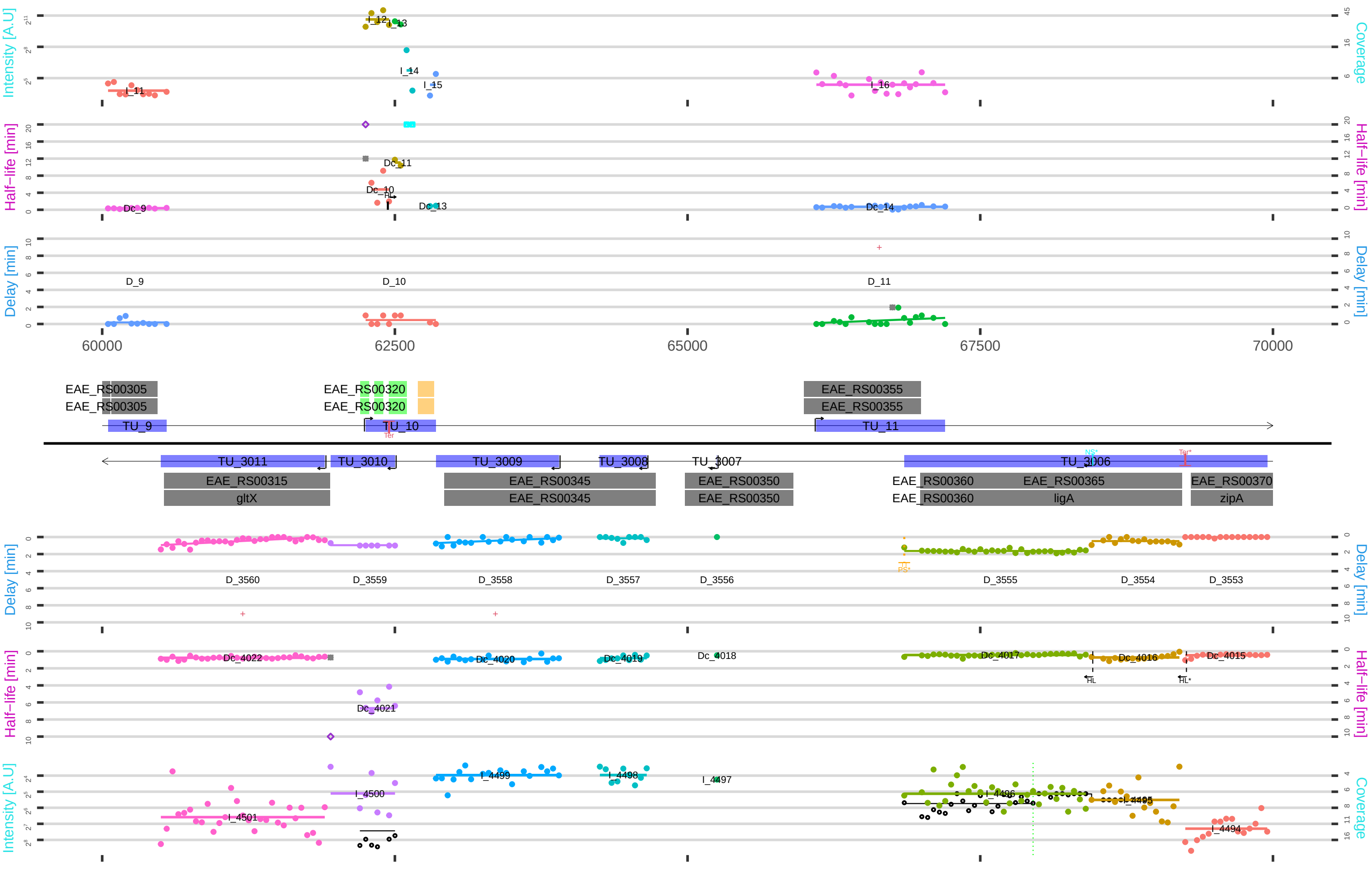

Term: termination (1), NS: new start (1), PS: pausing site (2), iTSS_I: internal starting site (0)

ID: 1413−1574; Term: termination (1), NS: new start (2), PS: pausing site (1), iTSS_I: internal starting site (0)

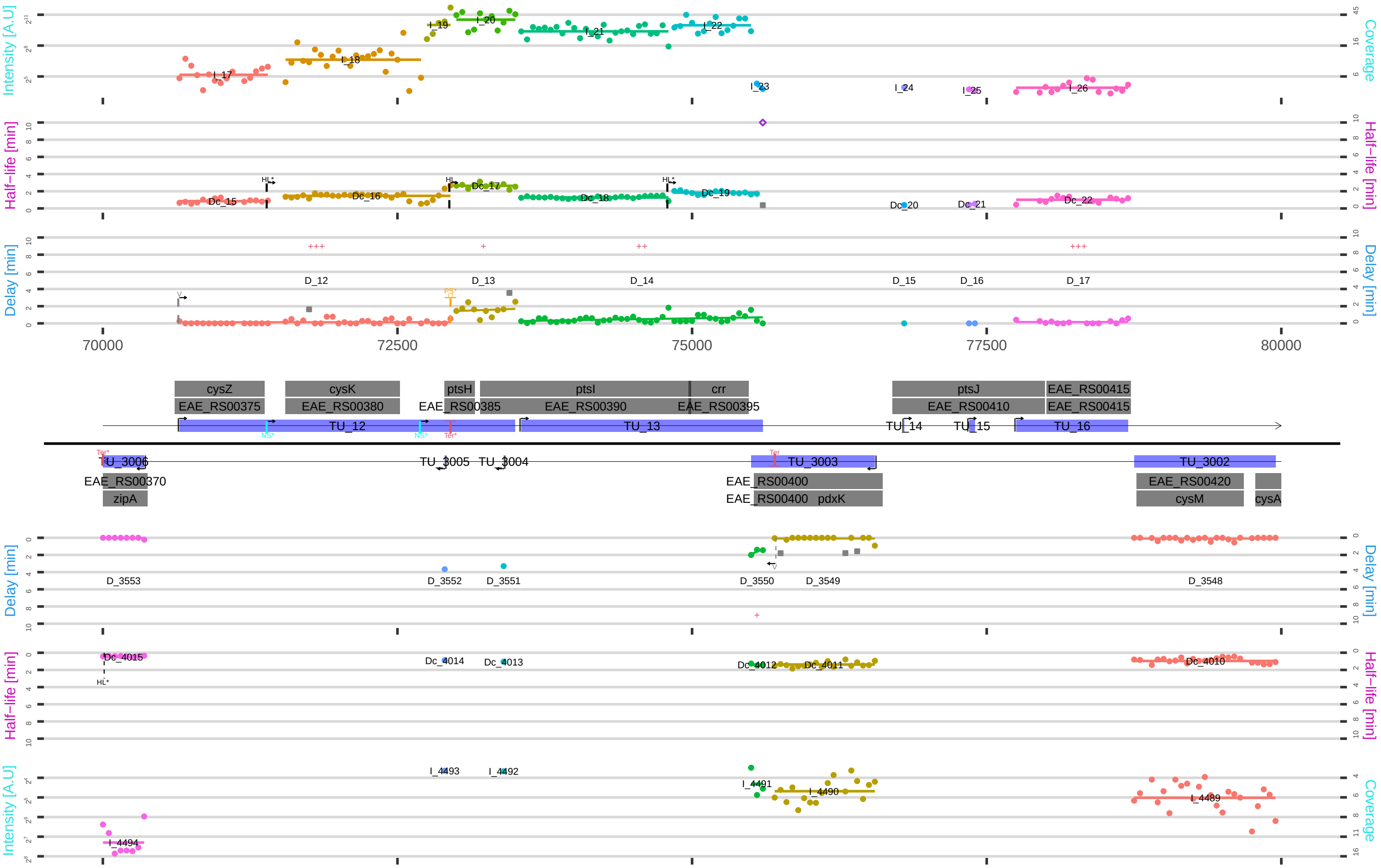

Term: termination (2), NS: new start (0), PS: pausing site (1), iTSS_I: internal starting site (0)

ID: 1730−1766; Term: termination (0), NS: new start (0), PS: pausing site (0), iTSS_I: internal starting site (0)

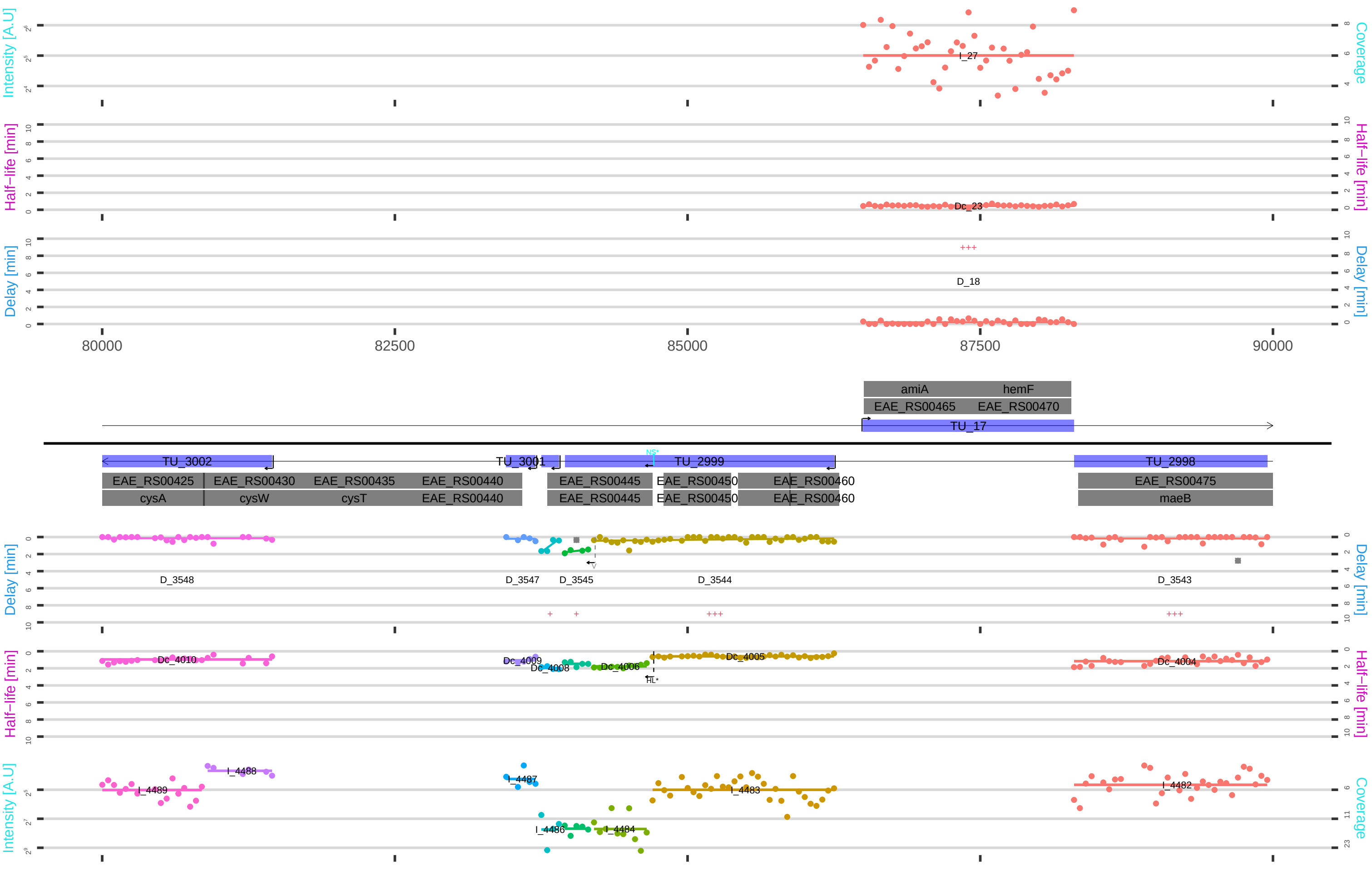

Term: termination (0), NS: new start (1), PS: pausing site (1), iTSS_I: internal starting site (0)

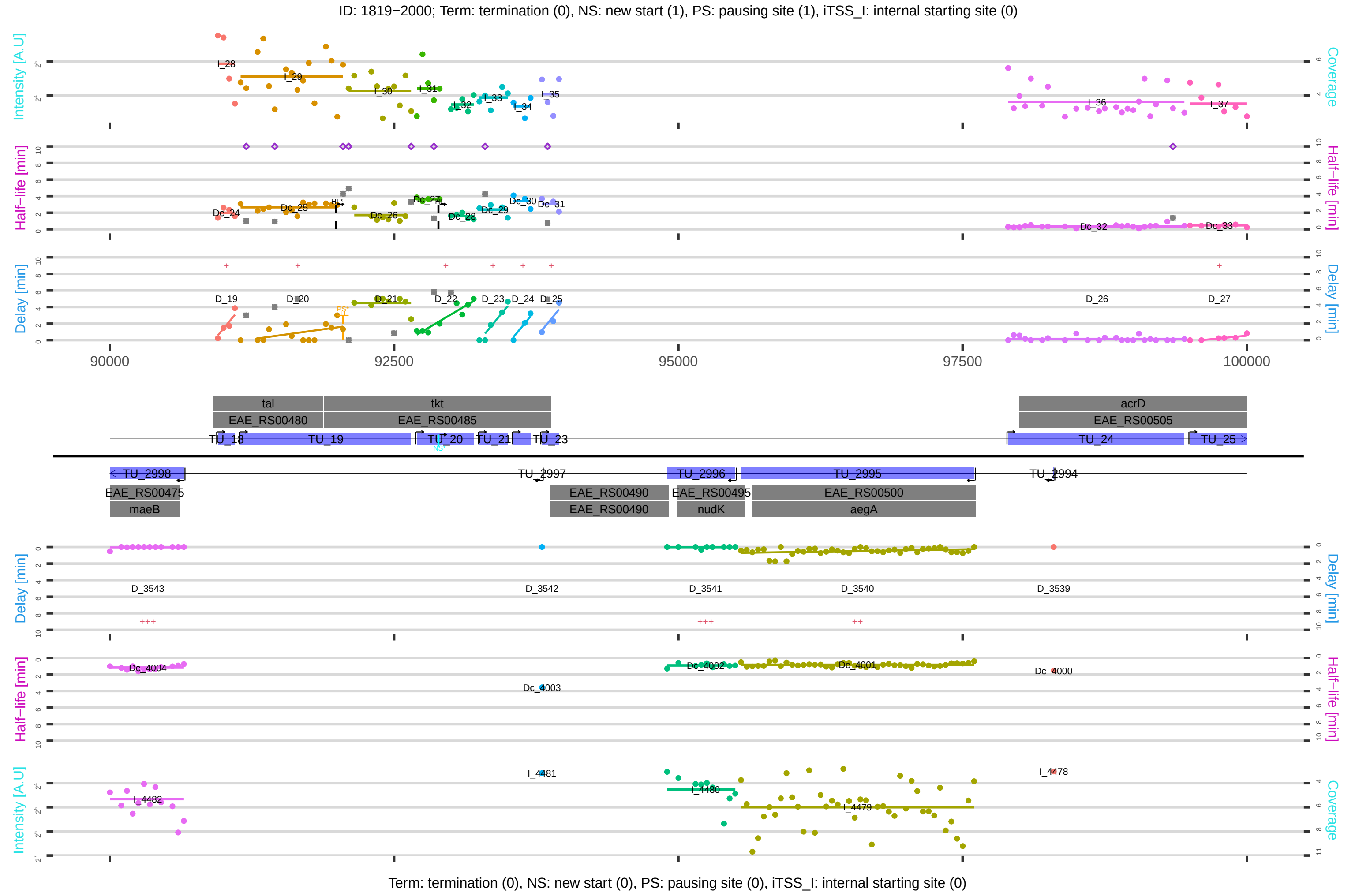
ID: 1819−2000; Term: termination (0), NS: new start (1), PS: pausing site (1), iTSS_I: internal starting site (0)
Intensity [A.U]
Coverage
Half−life [min]
Delay [min]
90000
92500
95000
97500
100000
tal
tkt
acrD
EAE_RS00480
EAE_RS00485
EAE_RS00505
TU_18
TU_19
TU_20
TU_21
TU_23
TU_24
TU_25
TU_2998
TU_2997
TU_2996
TU_2995
TU_2994
EAE_RS00475
EAE_RS00490
EAE_RS00495
EAE_RS00500
maeB
nudK
aegA
Term: termination (0), NS: new start (0), PS: pausing site (0), iTSS_I: internal starting site (0)

ID: 2000−2070; Term: termination (0), NS: new start (0), PS: pausing site (0), iTSS_I: internal starting site (0)

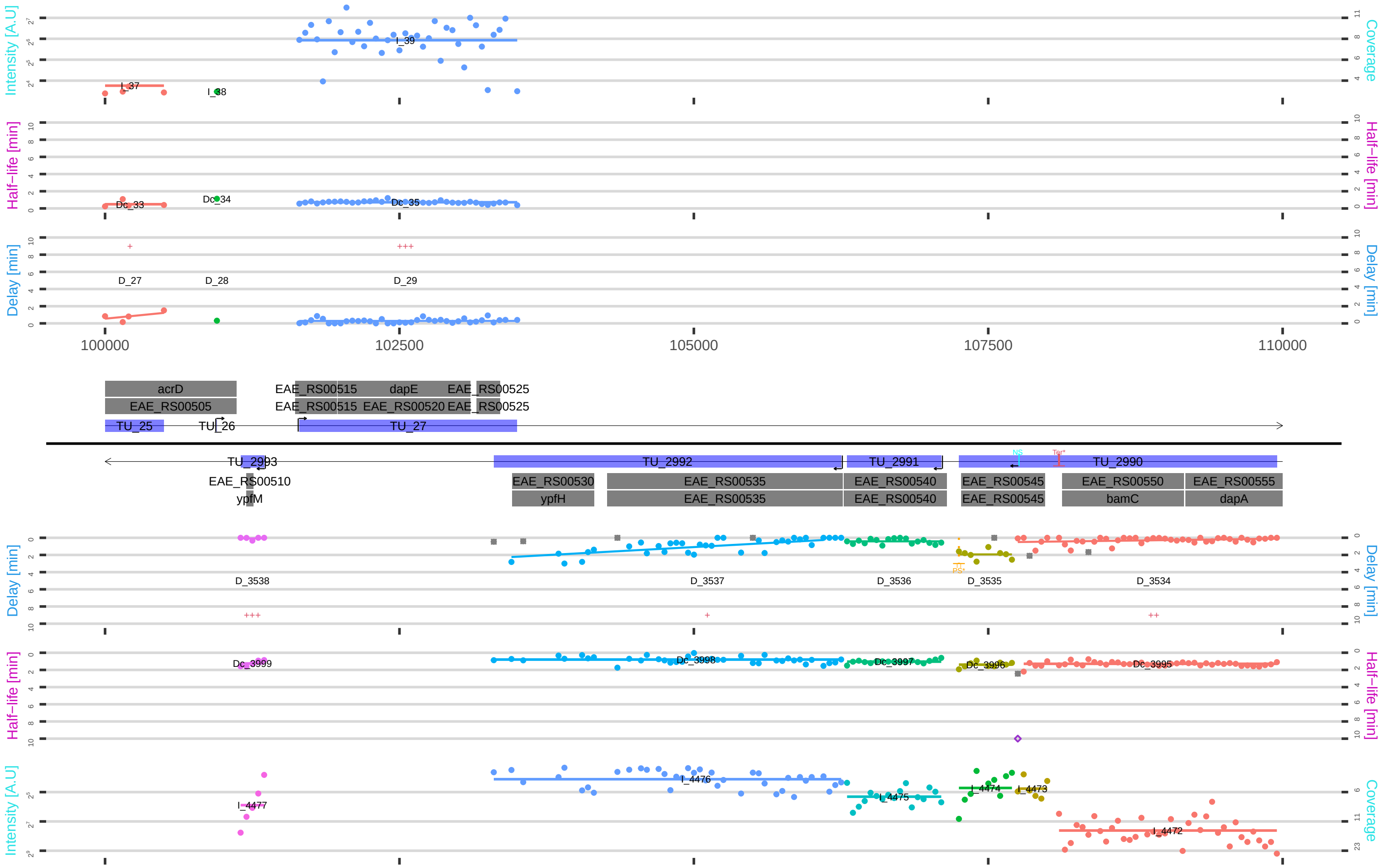

Term: termination (1), NS: new start (1), PS: pausing site (1), iTSS_I: internal starting site (0)

ID: 2205−2366; Term: termination (0), NS: new start (1), PS: pausing site (1), iTSS_I: internal starting site (0)

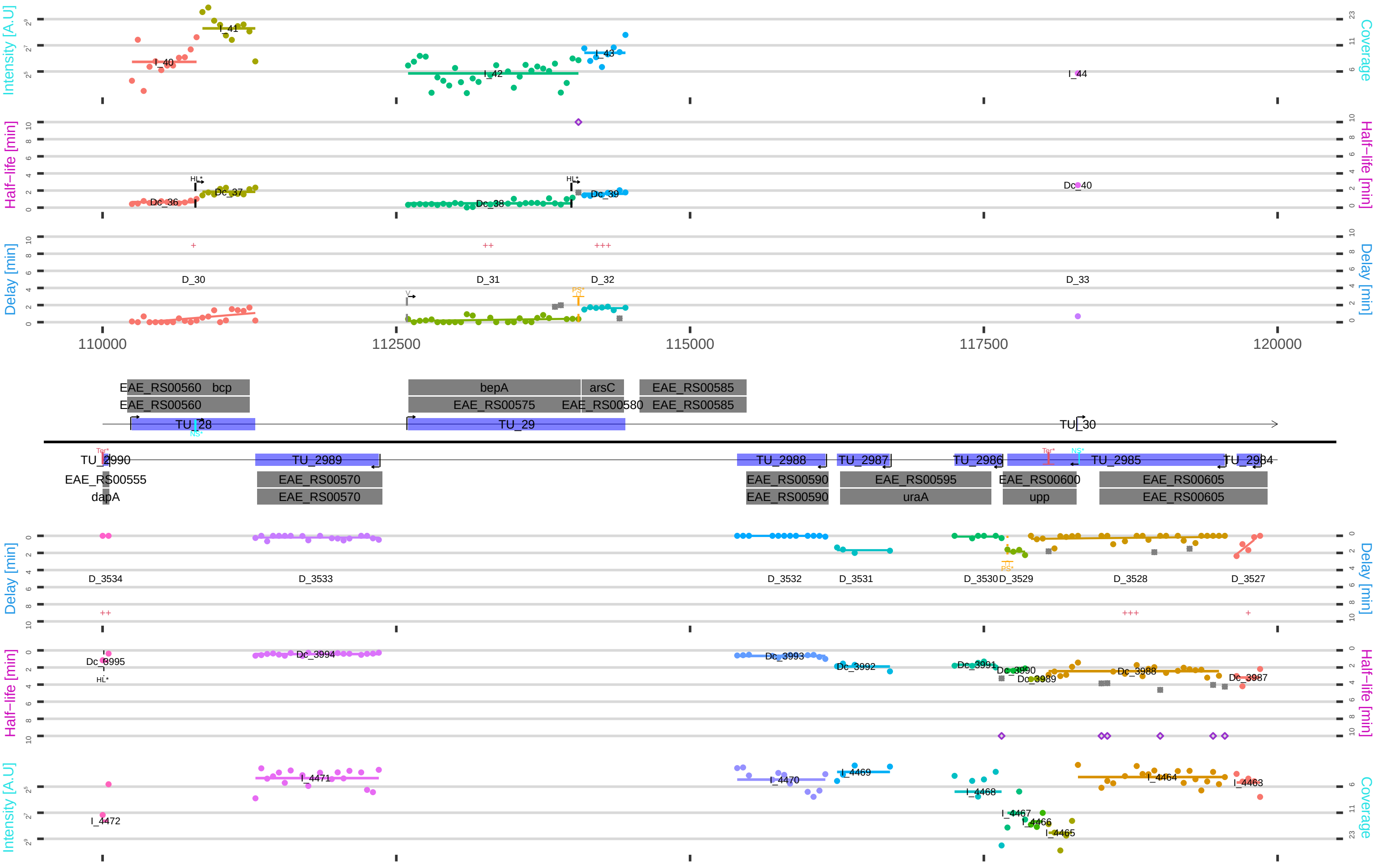

Term: termination (2), NS: new start (1), PS: pausing site (1), iTSS_I: internal starting site (0)

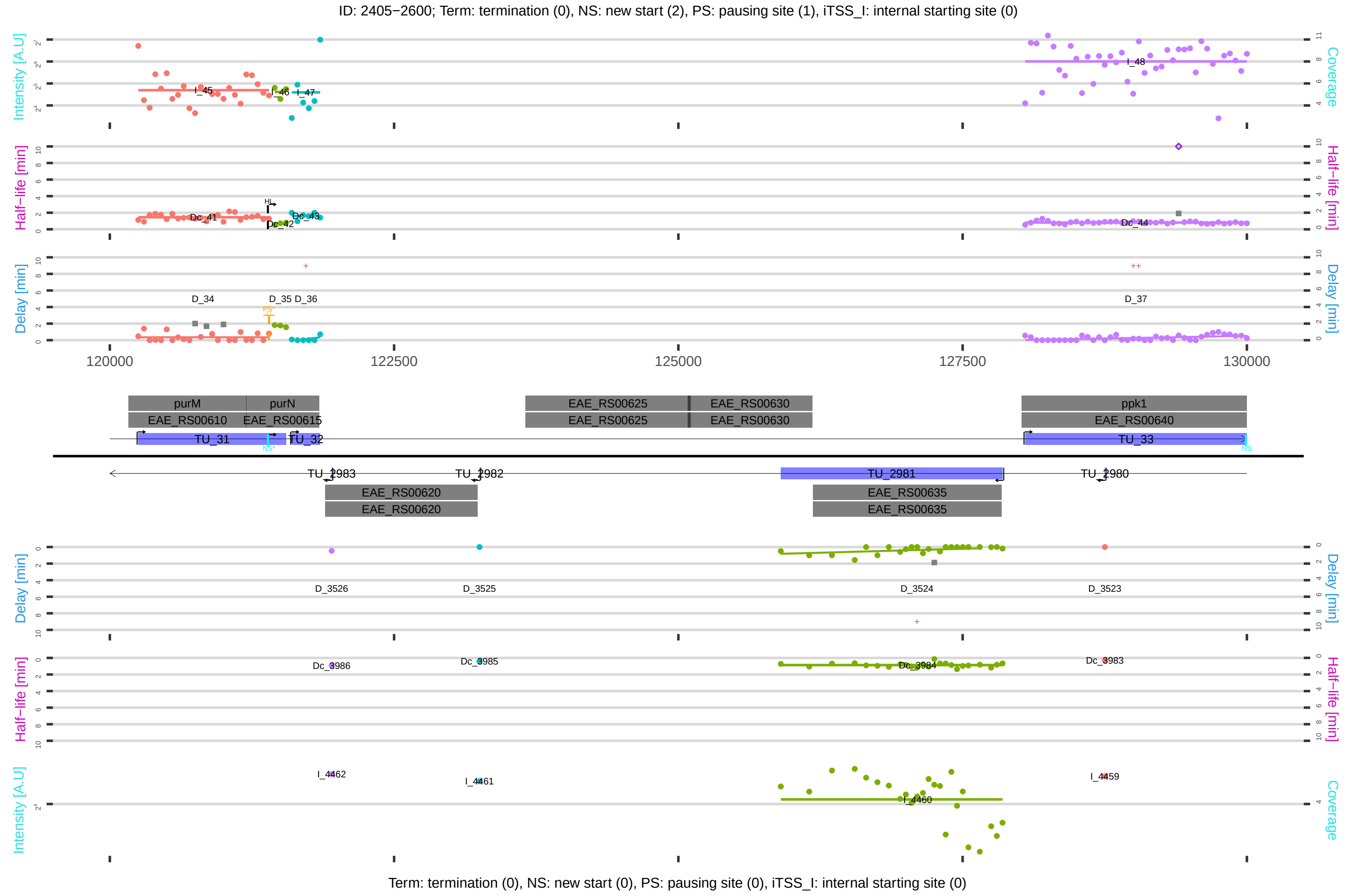

ID: 2405−2600; Term: termination (0), NS: new start (2), PS: pausing site (1), iTSS_I: internal starting site (0)
Intensity [A.U]
Coverage
Half−life [min]
Delay [min]
I_45
I_46
I_47
I_48
Dc_41
Dc_42
Dc_43
Dc_44
D_34
D_35
D_36
D_37
120000
122500
125000
127500
130000
purM
purN
EAE_RS00610
EAE_RS00615
EAE_RS00625
EAE_RS00630
ppk1
EAE_RS00640
TU_31
TU_32
TU_33
TU_2983
TU_2982
TU_2981
TU_2980
EAE_RS00620
EAE_RS00635
D_3526
D_3525
D_3524
D_3523
Dc_3986
Dc_3985
Dc_3984
Dc_3983
I_4462
I_4461
I_4460
I_4459
Term: termination (0), NS: new start (0), PS: pausing site (0), iTSS_I: internal starting site (0)

ID: 2600−2691; Term: termination (0), NS: new start (1), PS: pausing site (1), iTSS_I: internal starting site (0)

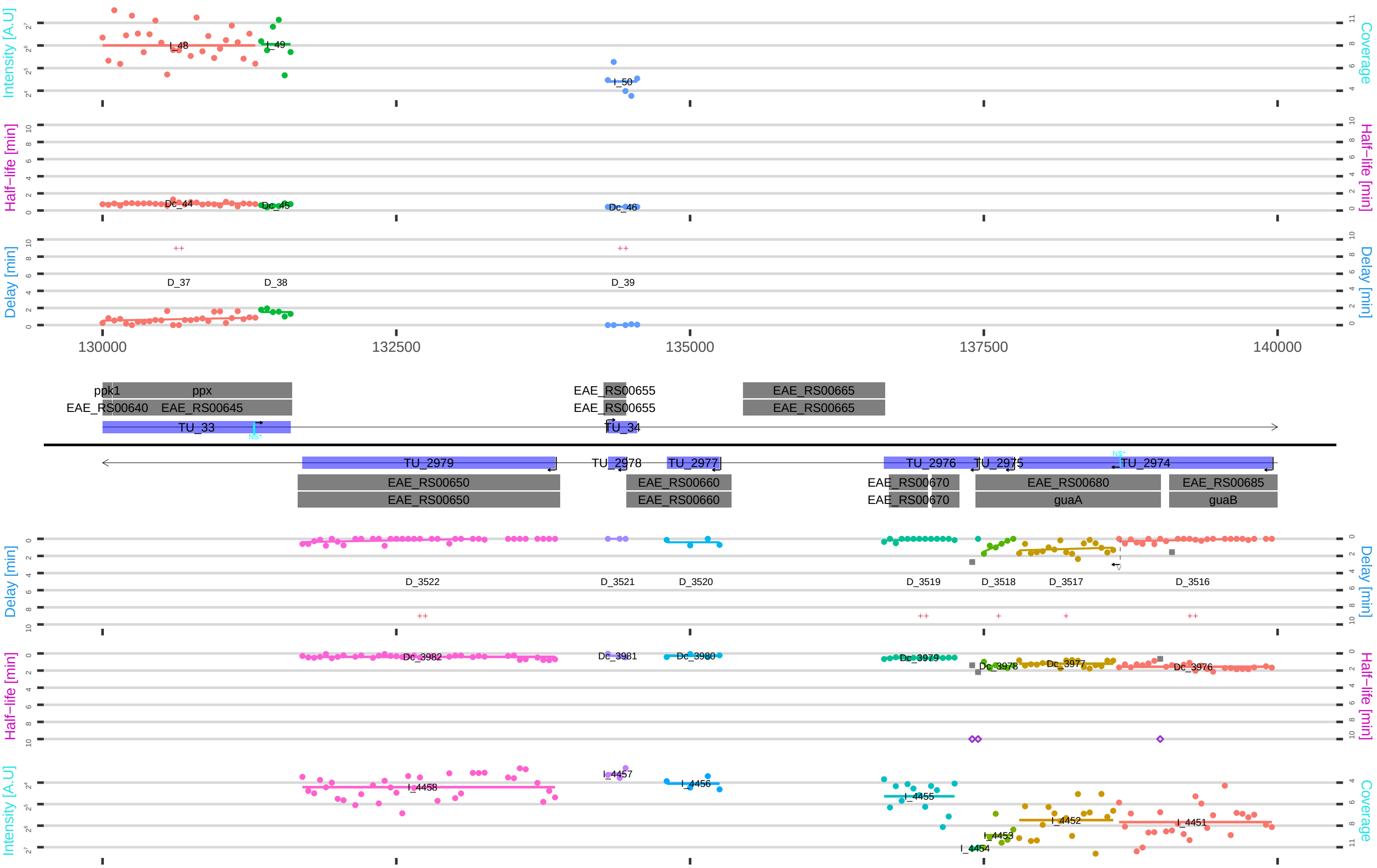

Term: termination (0), NS: new start (1), PS: pausing site (1), iTSS_I: internal starting site (0)

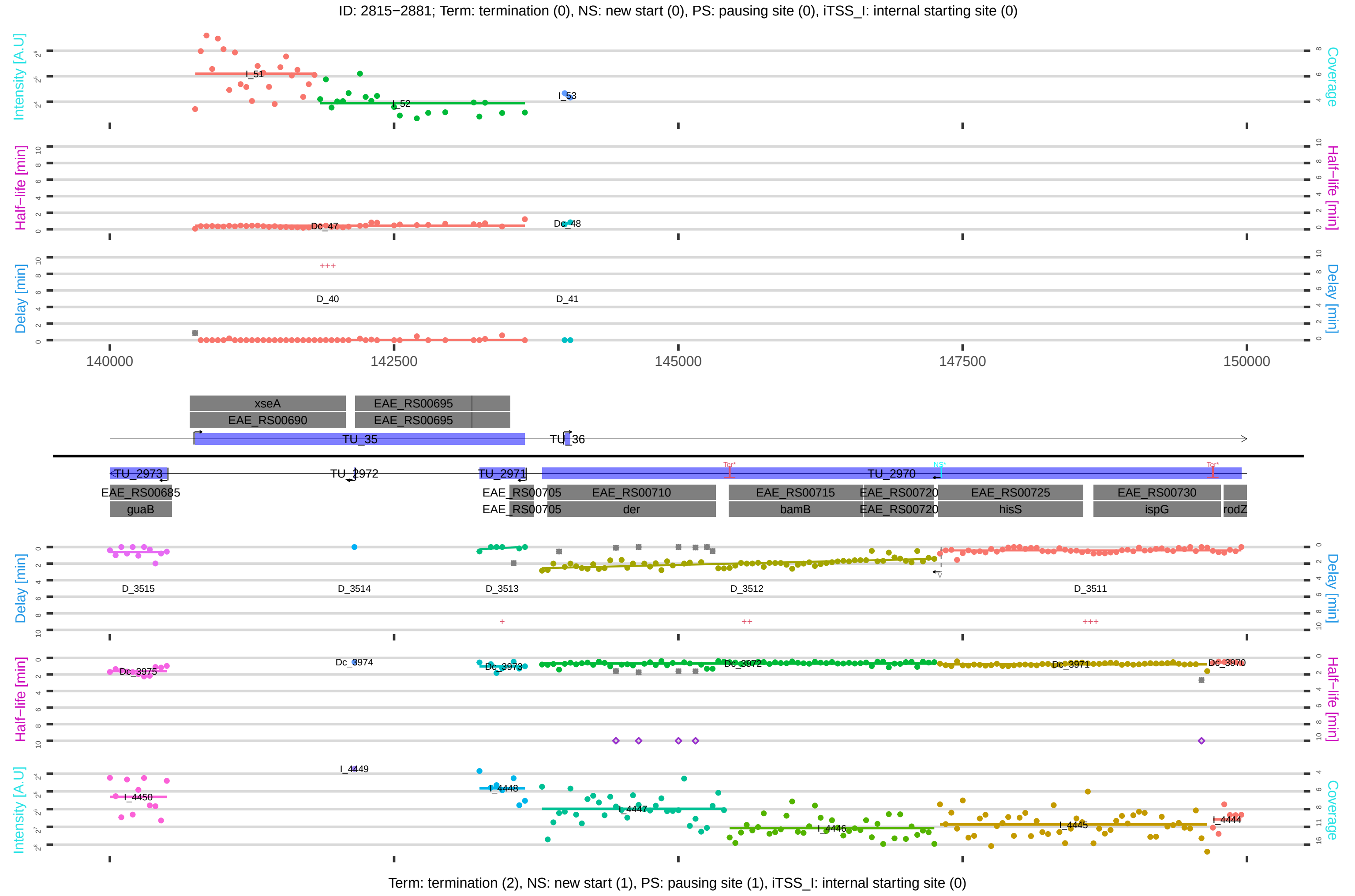

ID: 2815−2881; Term: termination (0), NS: new start (0), PS: pausing site (0), iTSS_I: internal starting site (0)
Intensity [A.U]
Coverage
Half−life [min]
Delay [min]
I_51
I_52
I_53
Dc_47
Dc_48
D_40
D_41
140000
142500
145000
147500
150000
xseA
EAE_RS00690
EAE_RS00695
TU_35
TU_36
TU_2973
TU_2972
TU_2971
TU_2970
EAE_RS00685
guaB
EAE_RS00705
EAE_RS00710
der
EAE_RS00715
bamB
EAE_RS00720
EAE_RS00725
hisS
EAE_RS00730
ispG
rodZ
D_3515
D_3514
D_3513
D_3512
D_3511
Dc_3975
Dc_3974
Dc_3973
Dc_3972
Dc_3971
Dc_3970
I_4450
I_4449
I_4448
I_4447
I_4446
I_4445
I_4444
Term: termination (2), NS: new start (1), PS: pausing site (1), iTSS_I: internal starting site (0)

ID: 3050−3050; Term: termination (0), NS: new start (0), PS: pausing site (0), iTSS_I: internal starting site (0)

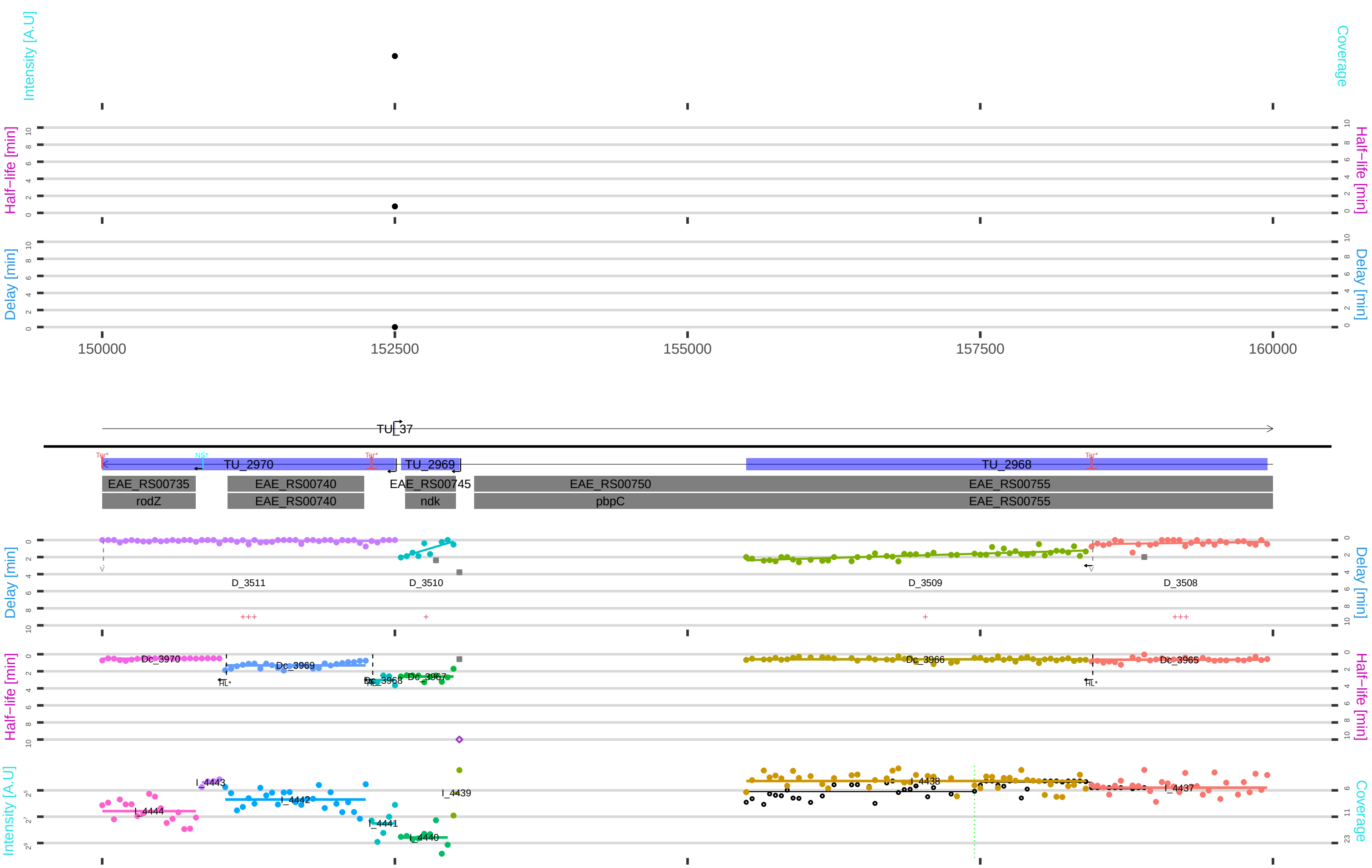

Term: termination (3), NS: new start (1), PS: pausing site (1), iTSS_I: internal starting site (0)

ID: 3214−3391; Term: termination (0), NS: new start (0), PS: pausing site (1), iTSS_I: internal starting site (0)

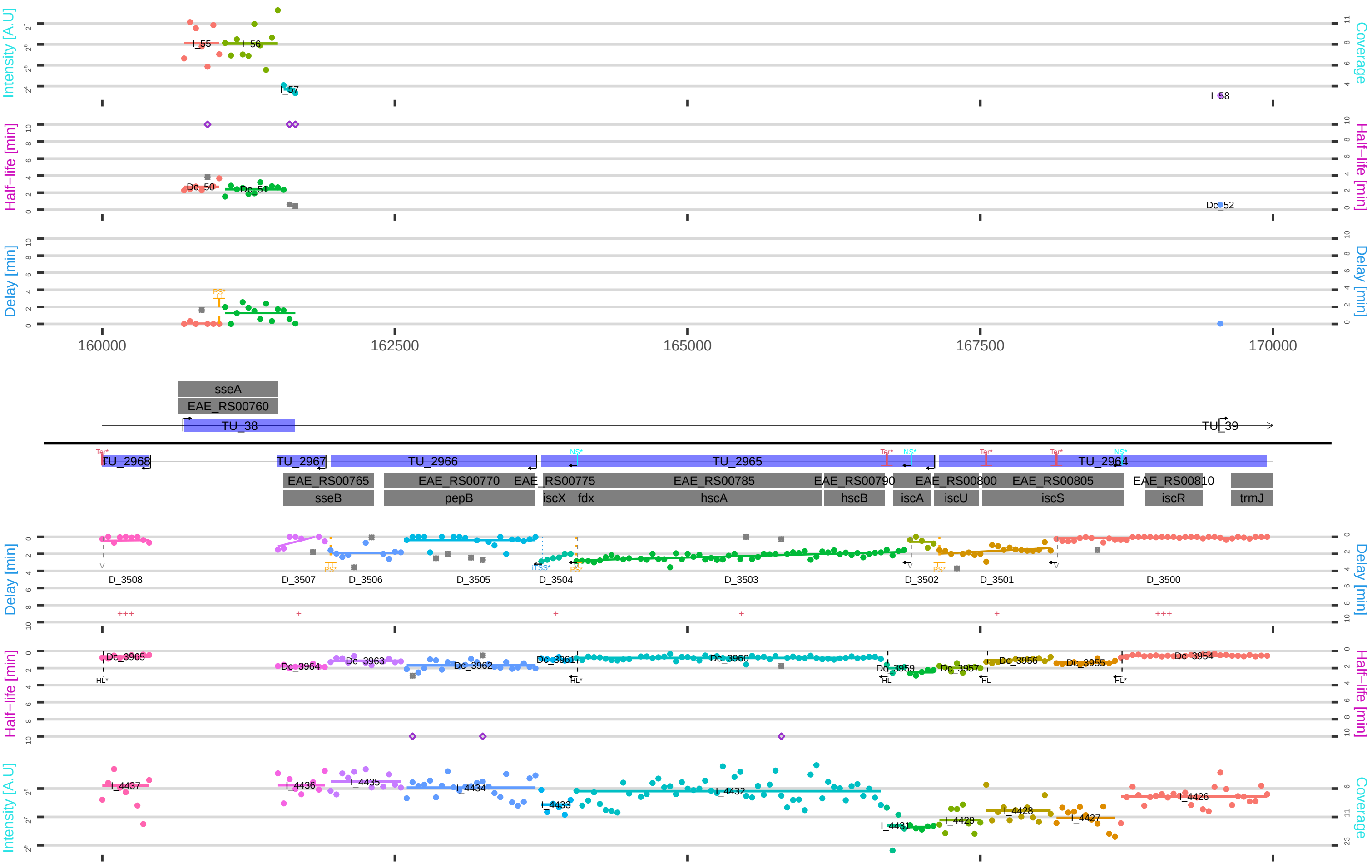

Term: termination (4), NS: new start (3), PS: pausing site (3), iTSS_I: internal starting site (1)

ID: 3411−3583; Term: termination (0), NS: new start (0), PS: pausing site (0), iTSS_I: internal starting site (0)

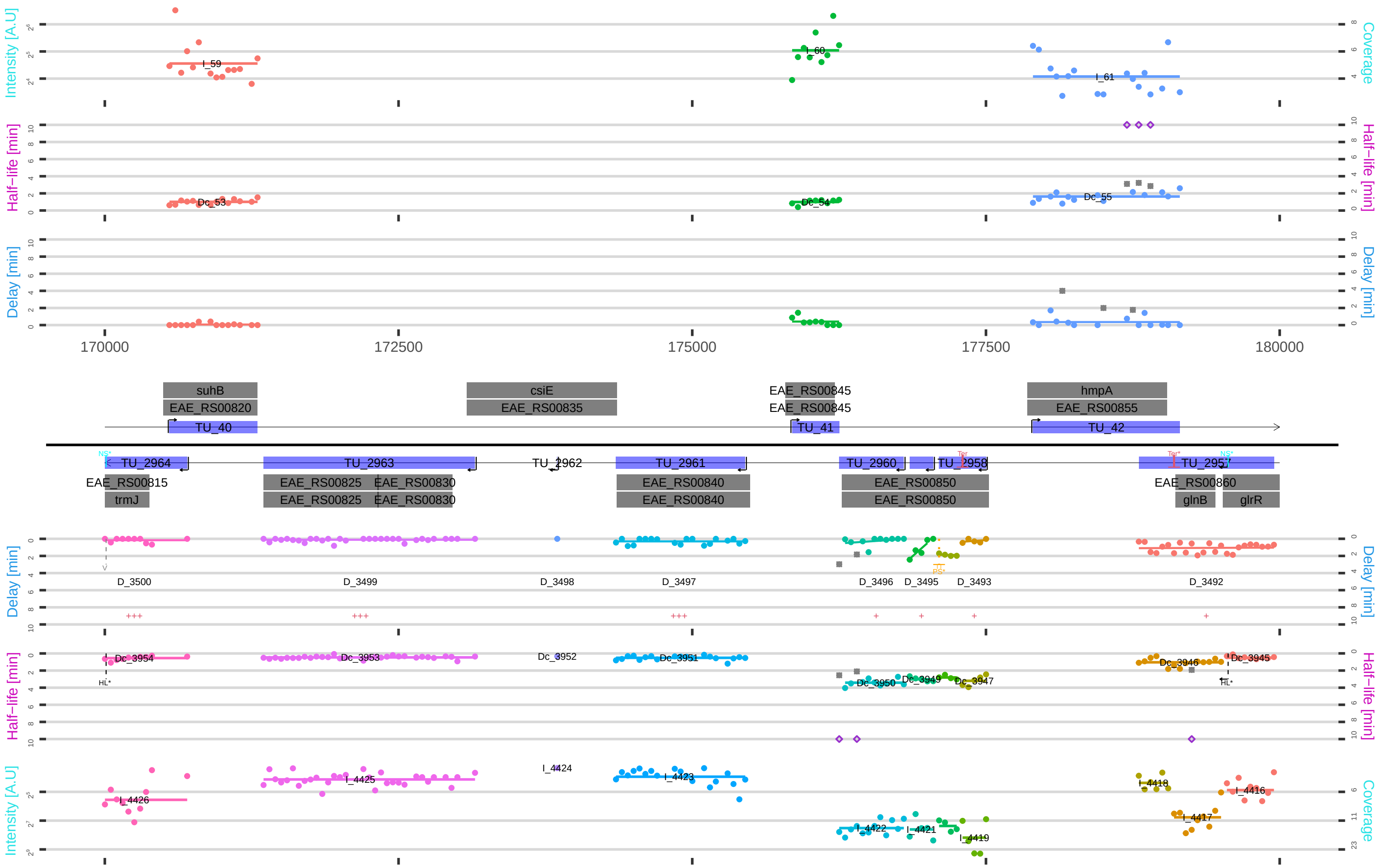

Term: termination (2), NS: new start (2), PS: pausing site (1), iTSS_I: internal starting site (0)

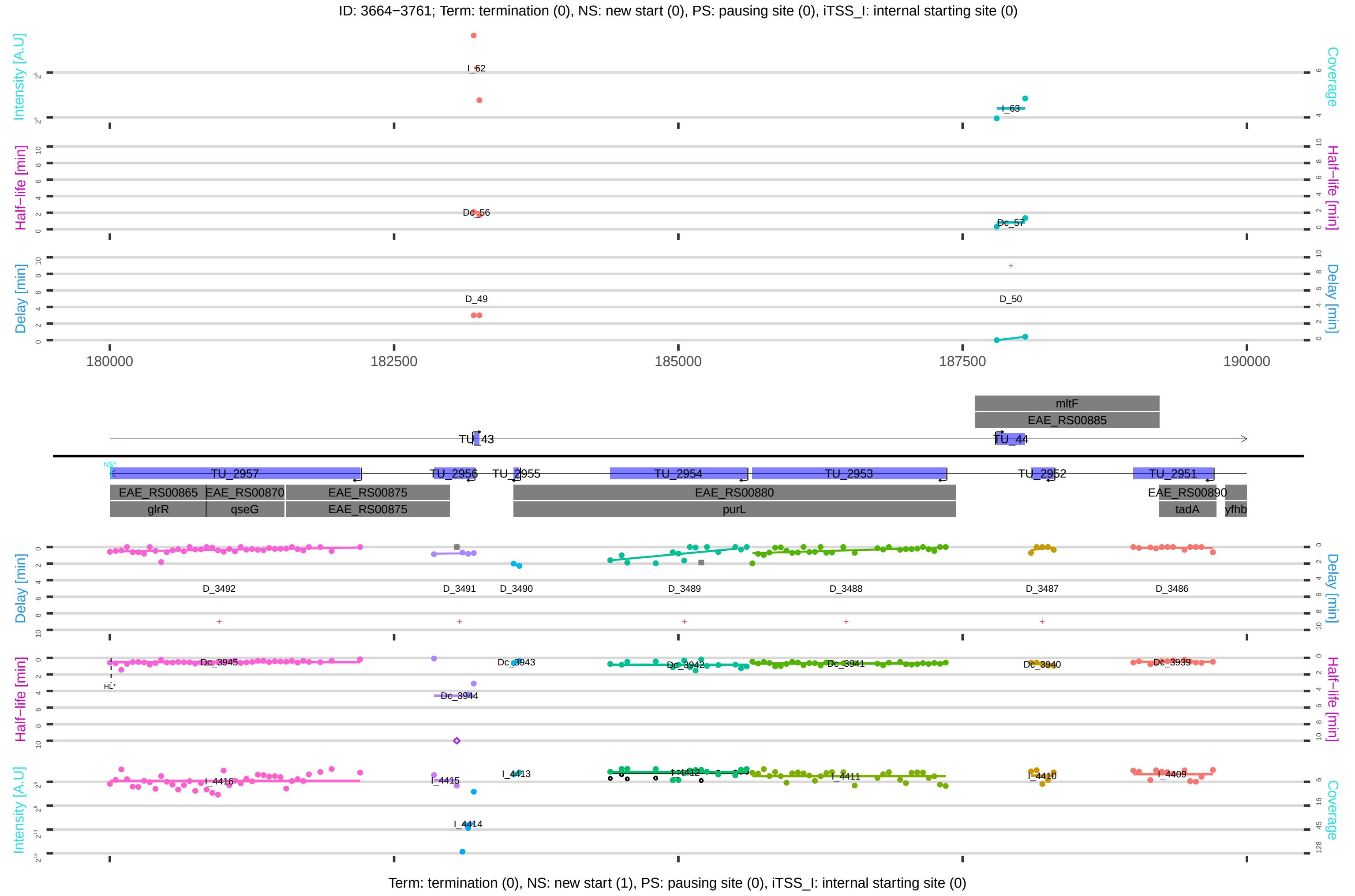

ID: 3664−3761; Term: termination (0), NS: new start (0), PS: pausing site (0), iTSS_I: internal starting site (0)
Intensity [A.U]
Coverage
Half−life [min]
Delay [min]
I_62
I_63
Dc_56
Dc_57
D_49
D_50
180000
182500
185000
187500
190000
mltF
EAE_RS00885
TU_43
TU_44
TU_2957
TU_2956
TU_2955
TU_2954
TU_2953
TU_2952
TU_2951
EAE_RS00865
EAE_RS00870
EAE_RS00875
EAE_RS00880
EAE_RS00890
glrR
qseG
purL
tadA
yfhb
D_3492
D_3491
D_3490
D_3489
D_3488
D_3487
D_3486
Dc_3945
Dc_3944
Dc_3943
Dc_3942
Dc_3941
Dc_3940
Dc_3939
I_4416
I_4415
I_4414
I_4413
I_4412
I_4411
I_4410
I_4409
Term: termination (0), NS: new start (1), PS: pausing site (0), iTSS_I: internal starting site (0)

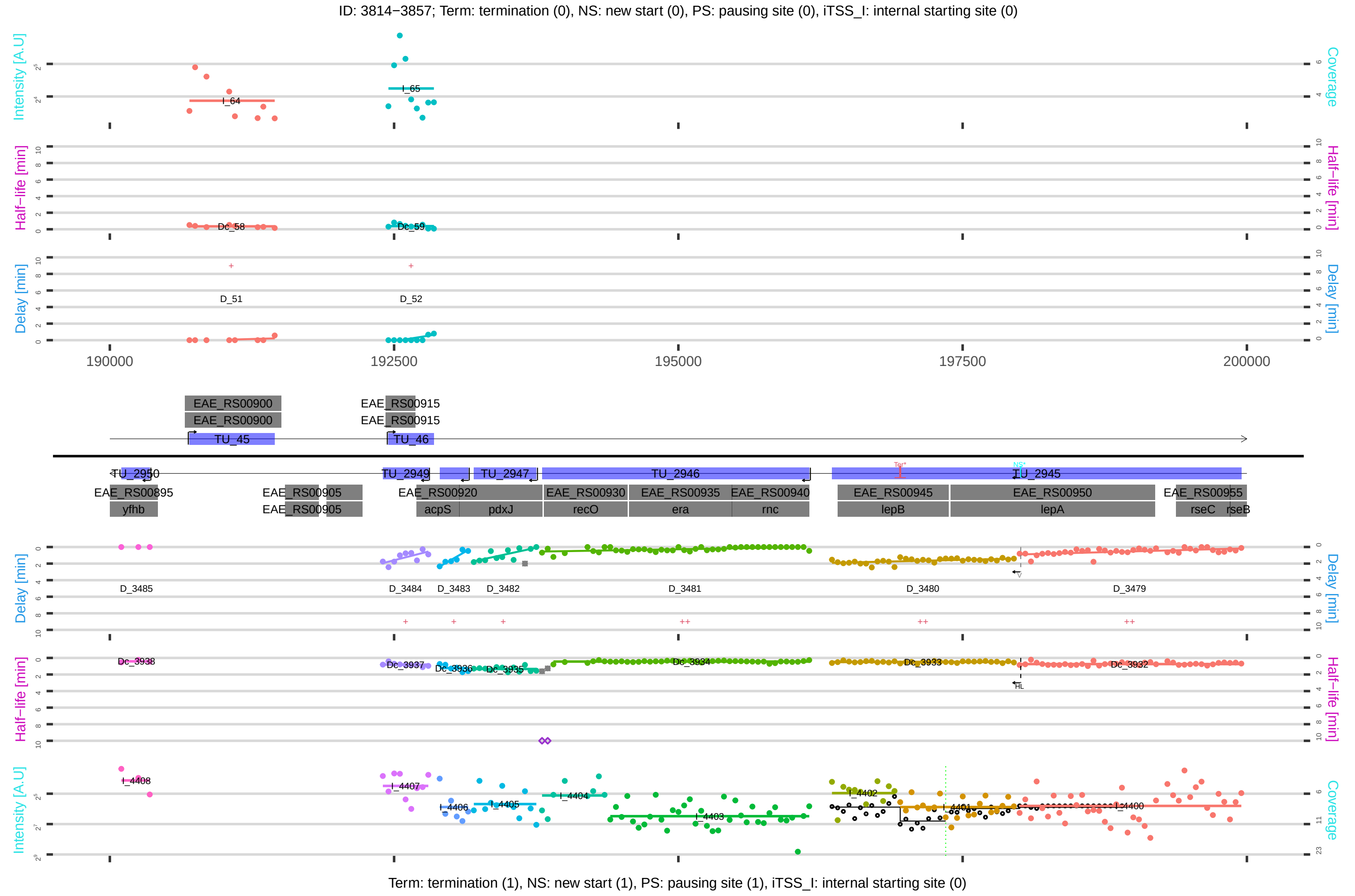

ID: 3814−3857; Term: termination (0), NS: new start (0), PS: pausing site (0), iTSS_I: internal starting site (0)
Intensity [A.U]
Coverage
I_64
I_65
Half−life [min]
Dc_58
Dc_59
Delay [min]
D_51
D_52
190000
192500
195000
197500
200000
EAE_RS00900
EAE_RS00915
TU_45
TU_46
TU_2950
TU_2949
TU_2947
TU_2946
TU_2945
EAE_RS00895
EAE_RS00905
EAE_RS00920
EAE_RS00930
EAE_RS00935
EAE_RS00940
EAE_RS00945
EAE_RS00950
EAE_RS00955
yfhb
acpS
pdxJ
recO
era
rnc
lepB
lepA
rseC
rseB
D_3485
D_3484
D_3483
D_3482
D_3481
D_3480
D_3479
Dc_3938
Dc_3937
Dc_3936
Dc_3935
Dc_3934
Dc_3933
Dc_3932
I_4408
I_4407
I_4406
I_4405
I_4404
I_4403
I_4402
I_4401
I_4400
Term: termination (1), NS: new start (1), PS: pausing site (1), iTSS_I: internal starting site (0)

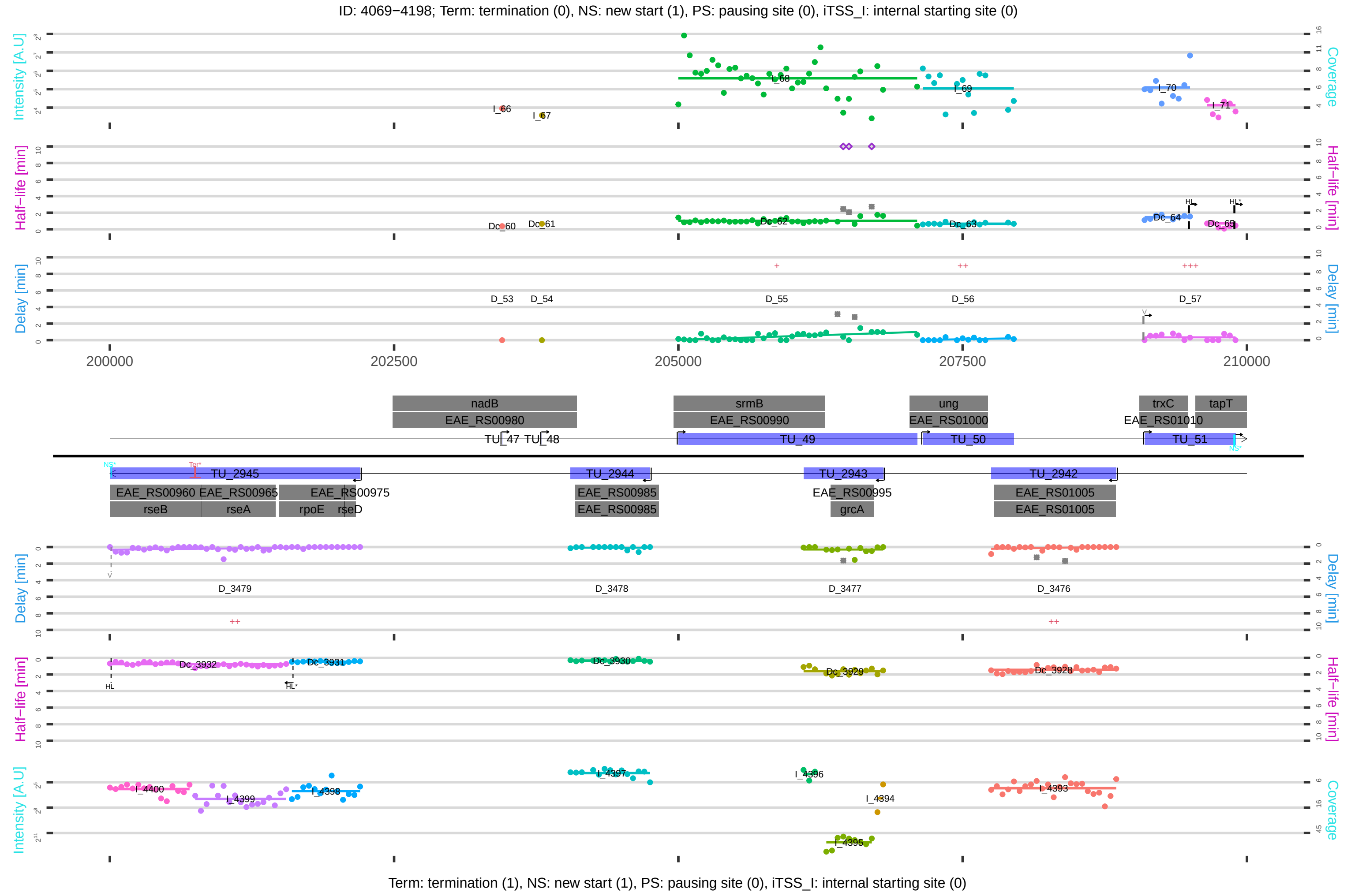

ID: 4069−4198; Term: termination (0), NS: new start (1), PS: pausing site (0), iTSS_I: internal starting site (0)
Intensity [A.U]
Coverage
Half−life [min]
Delay [min]
200000
202500
205000
207500
210000
nadB
EAE_RS00980
srmB
EAE_RS00990
ung
EAE_RS01000
trxC
tapT
EAE_RS01010
TU_47
TU_48
TU_49
TU_50
TU_51
TU_2945
TU_2944
TU_2943
TU_2942
EAE_RS00960
EAE_RS00965
EAE_RS00975
EAE_RS00985
EAE_RS00995
EAE_RS01005
rseB
rseA
rpoE
rseD
grcA
D_3479
D_3478
D_3477
D_3476
Term: termination (1), NS: new start (1), PS: pausing site (0), iTSS_I: internal starting site (0)

ID: 4202−4400; Term: termination (1), NS: new start (3), PS: pausing site (1), iTSS_I: internal starting site (1)

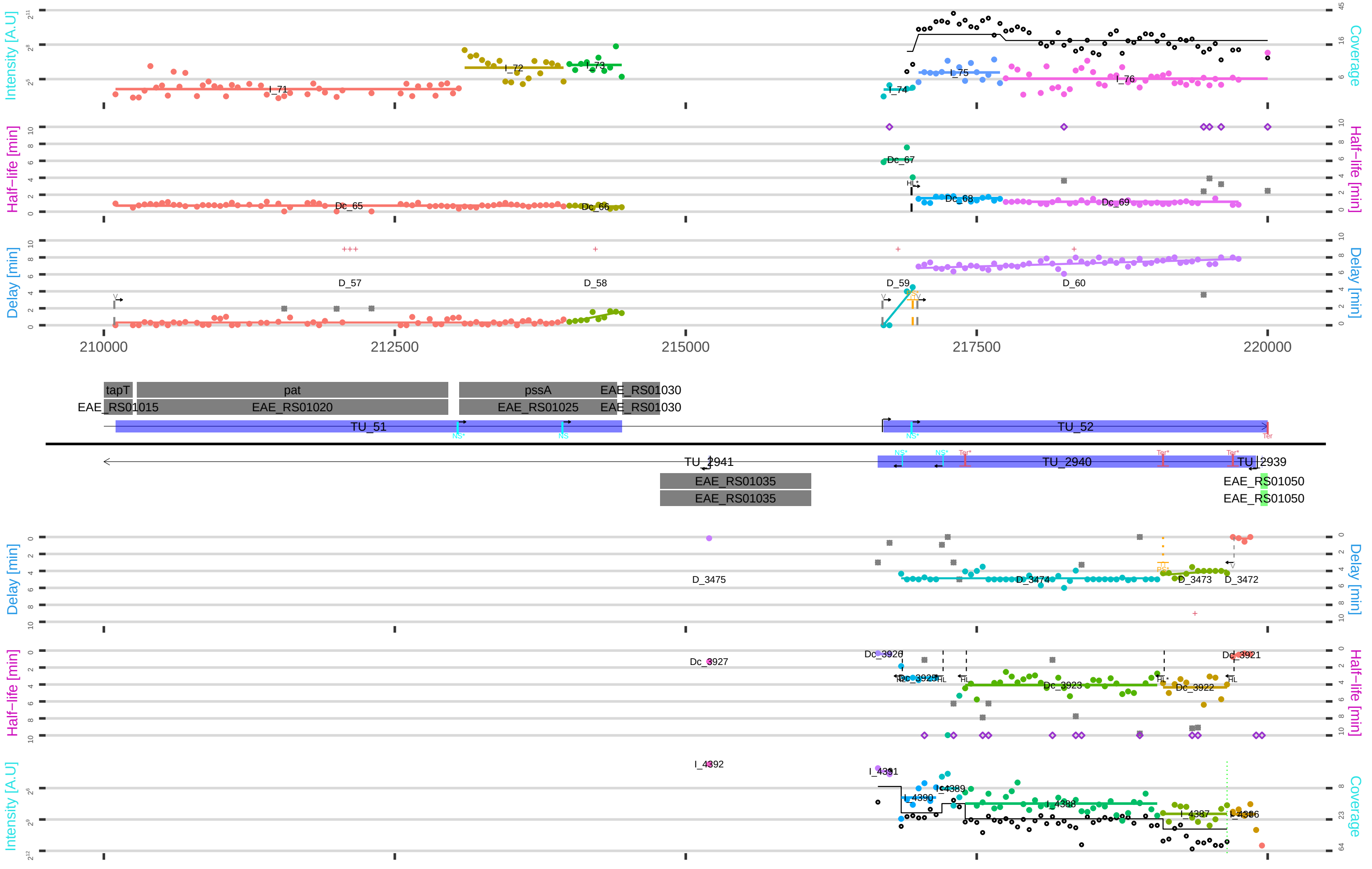

Term: termination (3), NS: new start (2), PS: pausing site (2), iTSS_I: internal starting site (0)

ID: 4400−4589; Term: termination (1), NS: new start (1), PS: pausing site (1), iTSS_I: internal starting site (0)

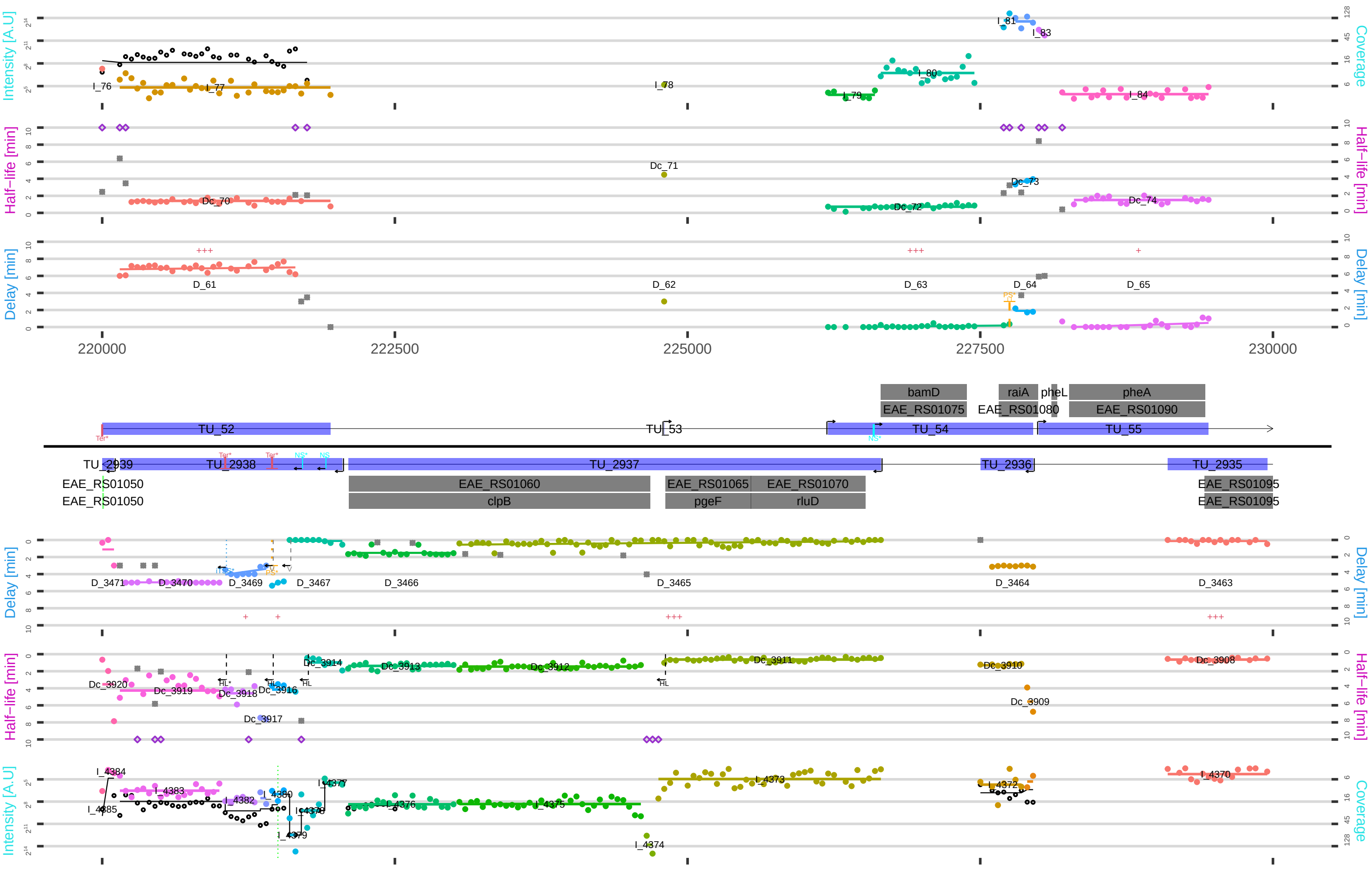

Term: termination (2), NS: new start (2), PS: pausing site (3), iTSS_I: internal starting site (1)

ID: 4659−4751; Term: termination (1), NS: new start (1), PS: pausing site (0), iTSS_I: internal starting site (0)

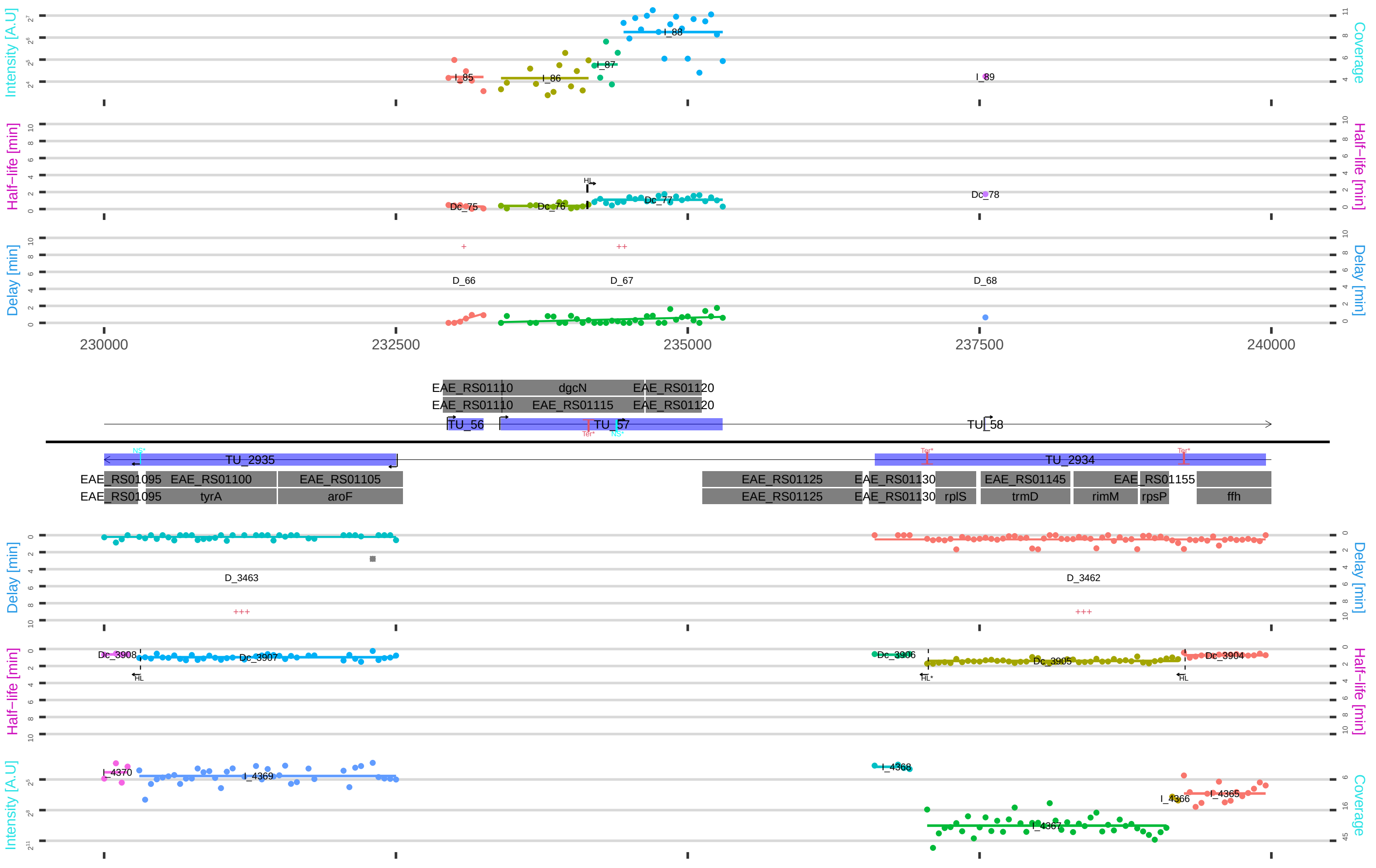

Term: termination (2), NS: new start (1), PS: pausing site (0), iTSS_I: internal starting site (0)

ID: 4819−4989; Term: termination (1), NS: new start (1), PS: pausing site (0), iTSS_I: internal starting site (0)

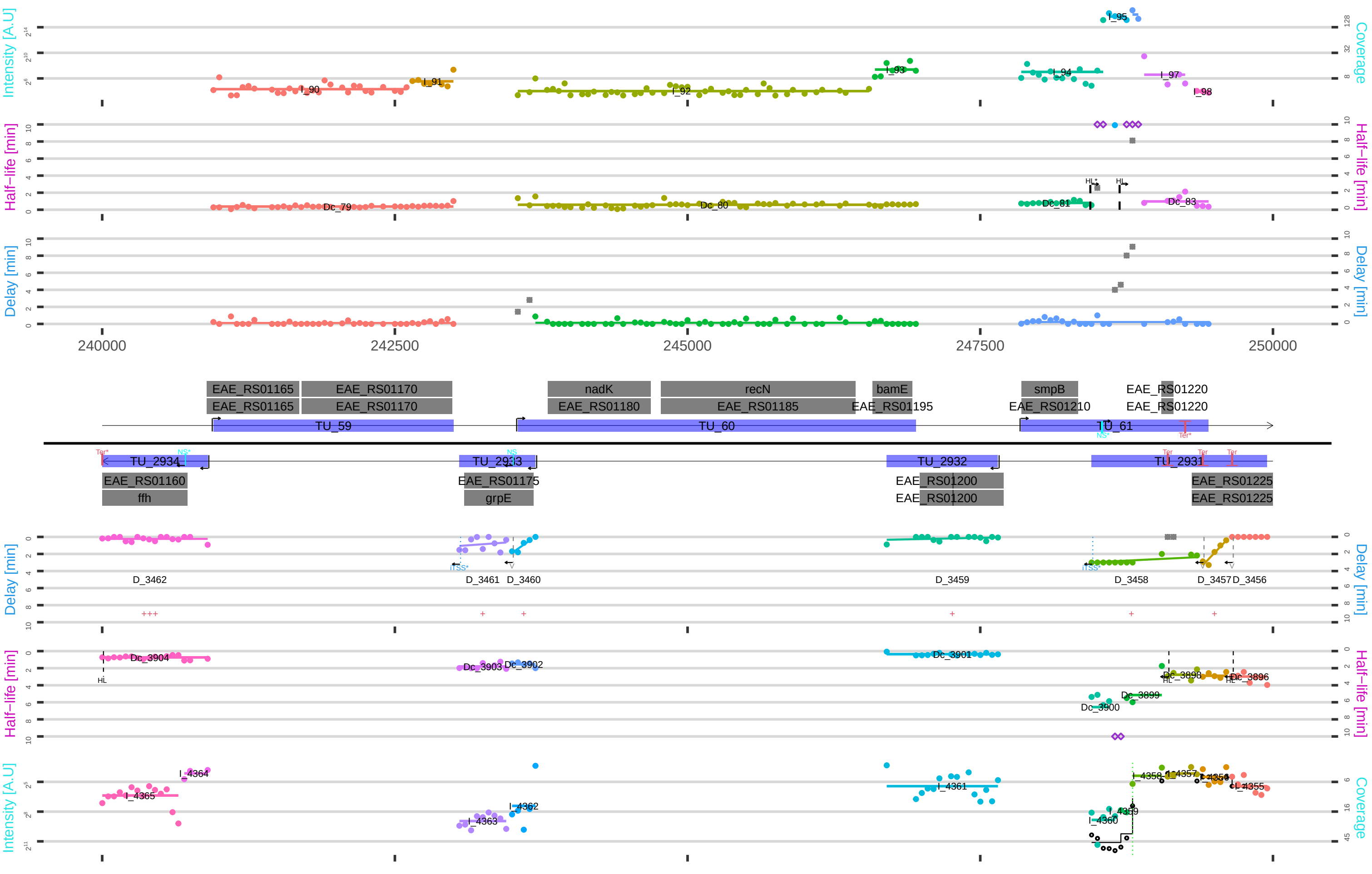

Term: termination (4), NS: new start (2), PS: pausing site (0), iTSS_I: internal starting site (3)

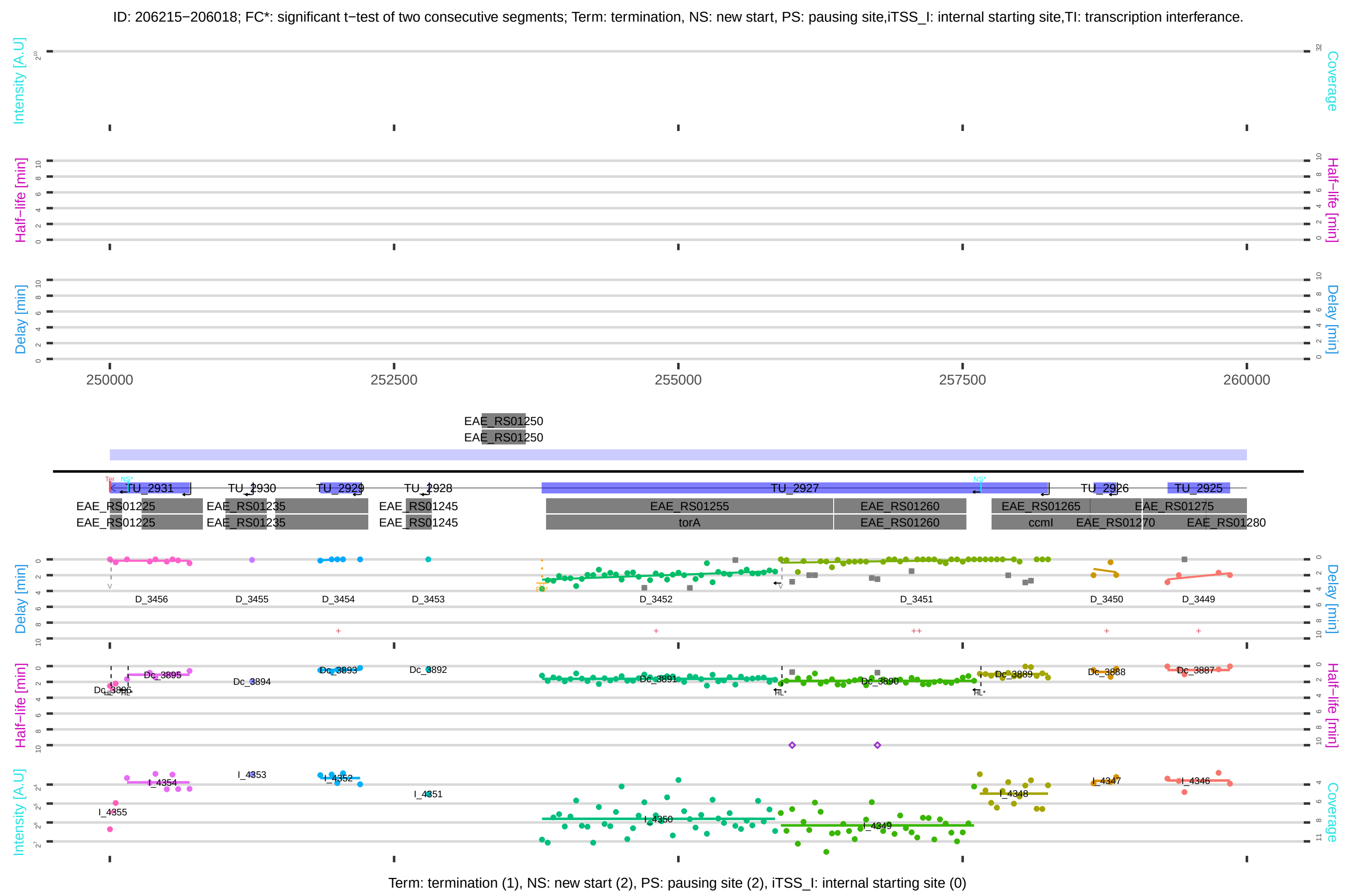

ID: 206215−206018; FC*: significant t−test of two consecutive segments; Term: termination, NS: new start, PS: pausing site,iTSS_I: internal starting site,TI: transcription interferance.
Intensity [A.U]
Coverage
Half−life [min]
Delay [min]
250000
252500
255000
257500
260000
EAE_RS01250
TU_2931
TU_2930
TU_2929
TU_2928
TU_2927
TU_2926
TU_2925
EAE_RS01225
EAE_RS01235
EAE_RS01245
EAE_RS01255
torA
EAE_RS01260
EAE_RS01265
ccmI
EAE_RS01270
EAE_RS01275
EAE_RS01280
D_3456
D_3455
D_3454
D_3453
D_3452
D_3451
D_3450
D_3449
Dc_3896
Dc_3895
Dc_3894
Dc_3893
Dc_3892
Dc_3891
Dc_3890
Dc_3889
Dc_3888
Dc_3887
I_4355
I_4354
I_4353
I_4352
I_4351
I_4350
I_4349
I_4348
I_4347
I_4346
Term: termination (1), NS: new start (2), PS: pausing site (2), iTSS_I: internal starting site (0)

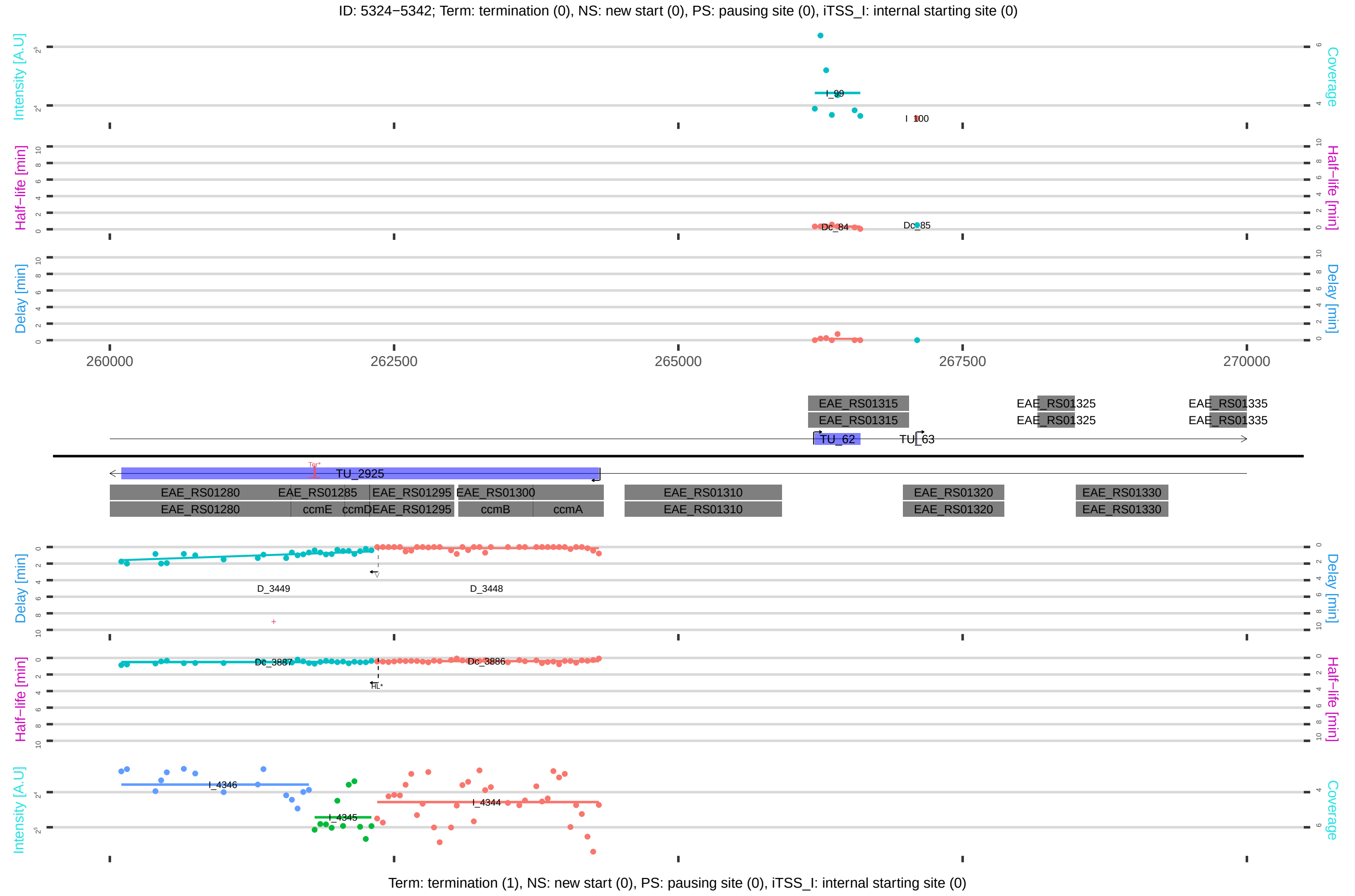

ID: 5324−5342; Term: termination (0), NS: new start (0), PS: pausing site (0), iTSS_I: internal starting site (0)
Intensity [A.U]
Coverage
I_99
I_100
Half−life [min]
Dc_84
Dc_85
Delay [min]
260000
262500
265000
267500
270000
EAE_RS01315
EAE_RS01325
EAE_RS01335
TU_62
TU_63
TU_2925
Ter*
EAE_RS01280
EAE_RS01285
EAE_RS01295
EAE_RS01300
EAE_RS01310
EAE_RS01320
EAE_RS01330
ccmE
ccmD
ccmB
ccmA
D_3449
D_3448
Dc_3887
Dc_3886
HL*
I_4346
I_4345
I_4344
Term: termination (1), NS: new start (0), PS: pausing site (0), iTSS_I: internal starting site (0)

ID: 205794−205761; FC*: significant t−test of two consecutive segments; Term: termination, NS: new start, PS: pausing site,iTSS_I: internal starting site,TI: transcription interferance.

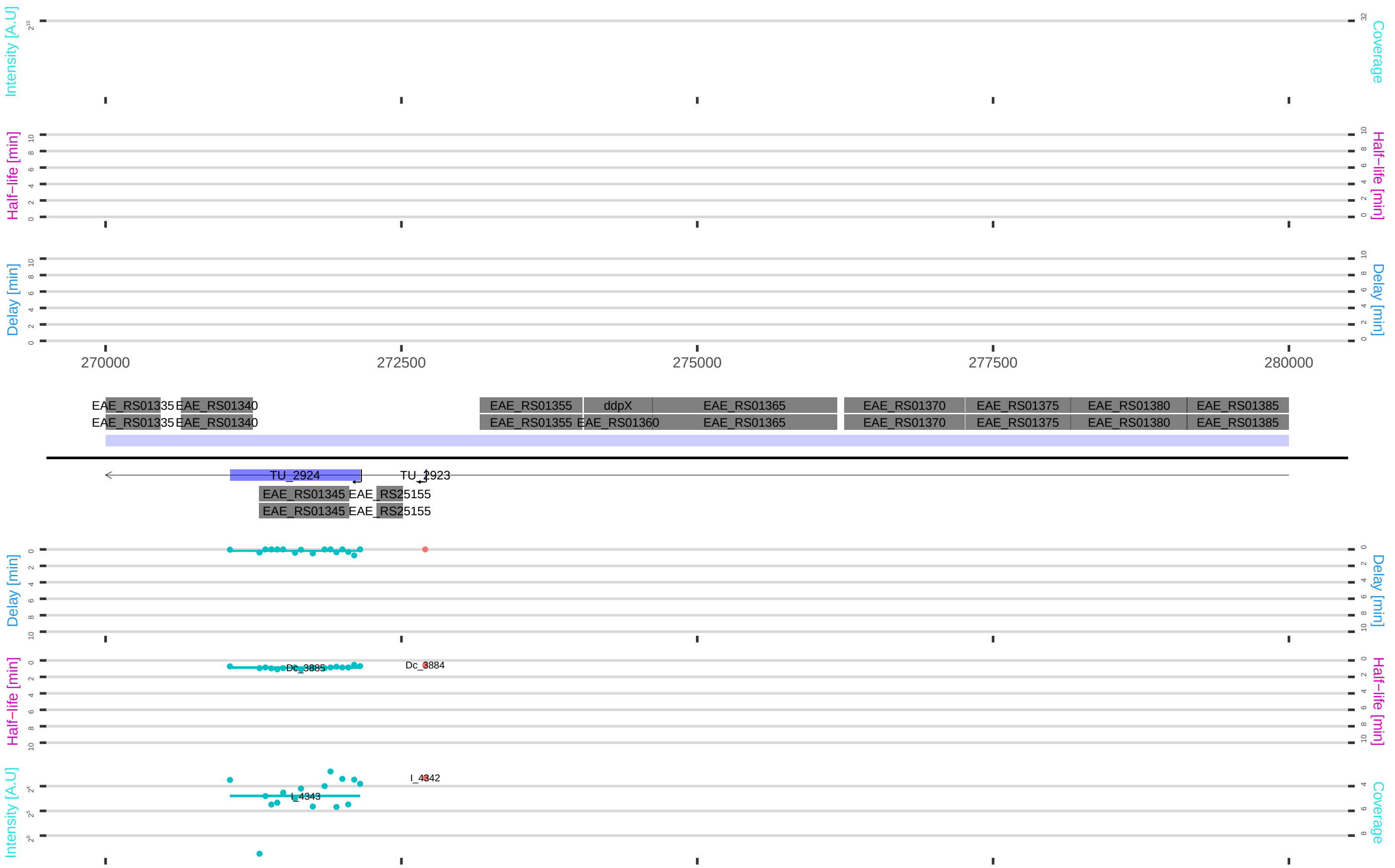

Term: termination (0), NS: new start (0), PS: pausing site (0), iTSS_I: internal starting site (0)

ID: 5607−5800; Term: termination (0), NS: new start (0), PS: pausing site (0), iTSS_I: internal starting site (0)

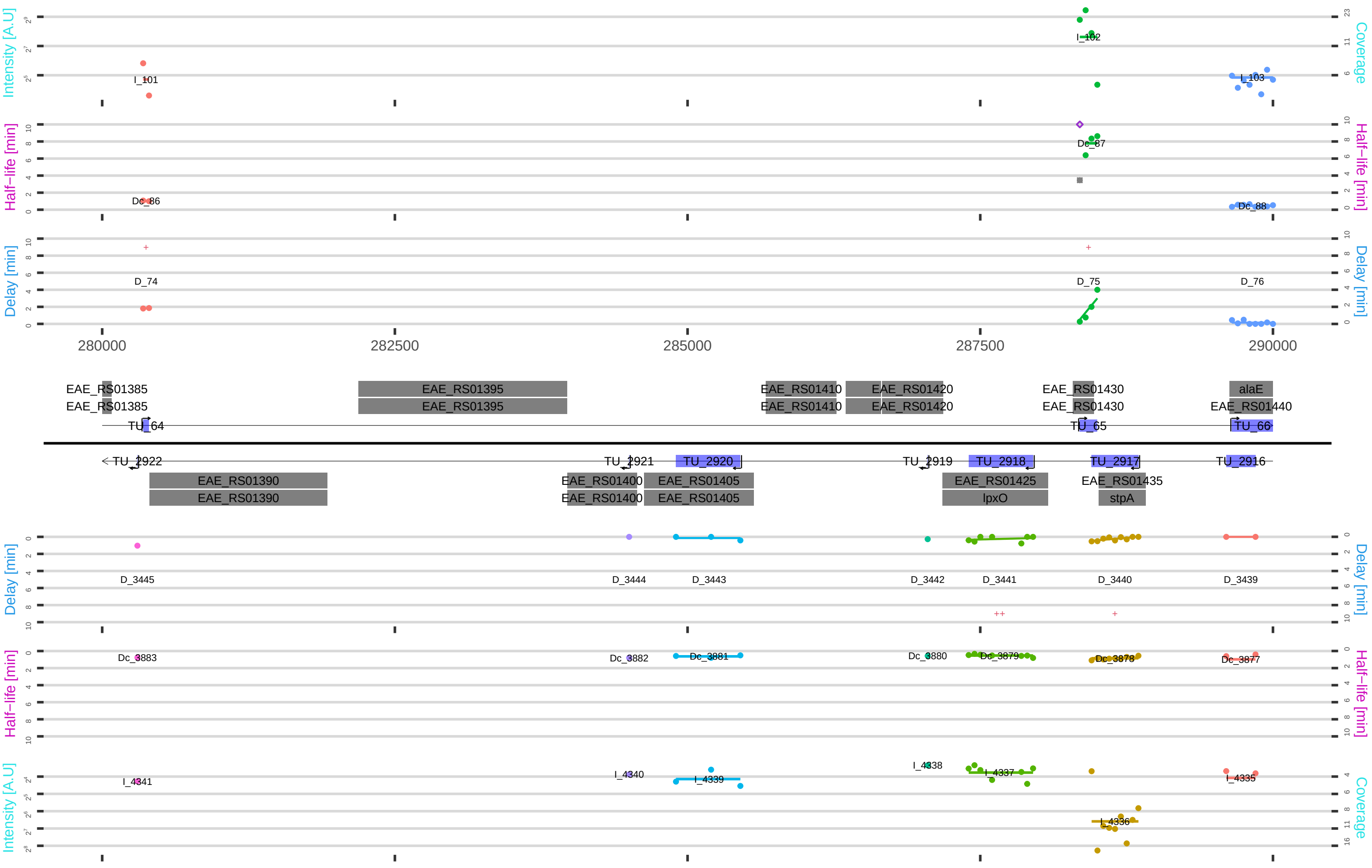

Term: termination (0), NS: new start (0), PS: pausing site (0), iTSS_I: internal starting site (0)

ID: 5800−6000; Term: termination (1), NS: new start (1), PS: pausing site (2), iTSS_I: internal starting site (0)

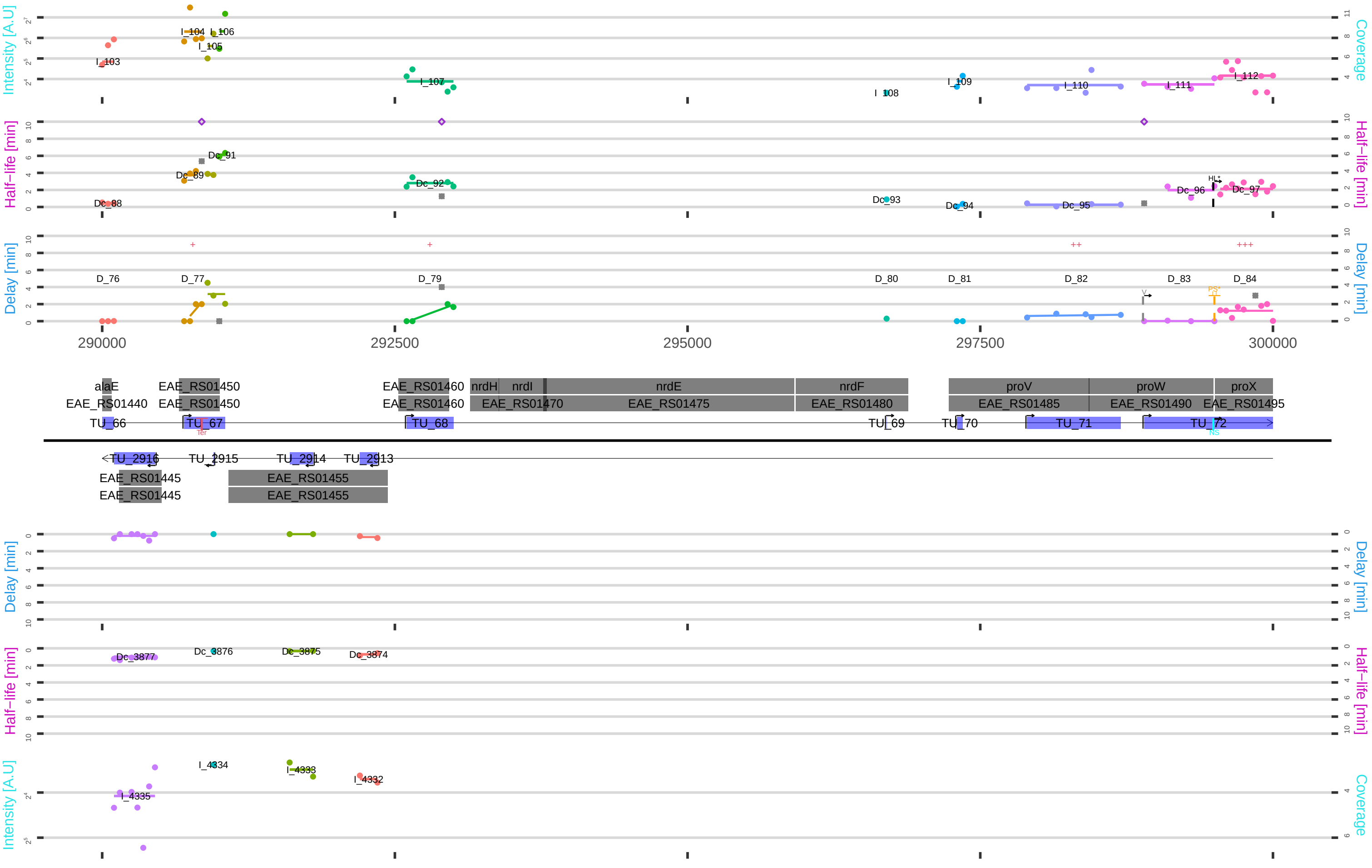

Term: termination (0), NS: new start (0), PS: pausing site (0), iTSS_I: internal starting site (0)

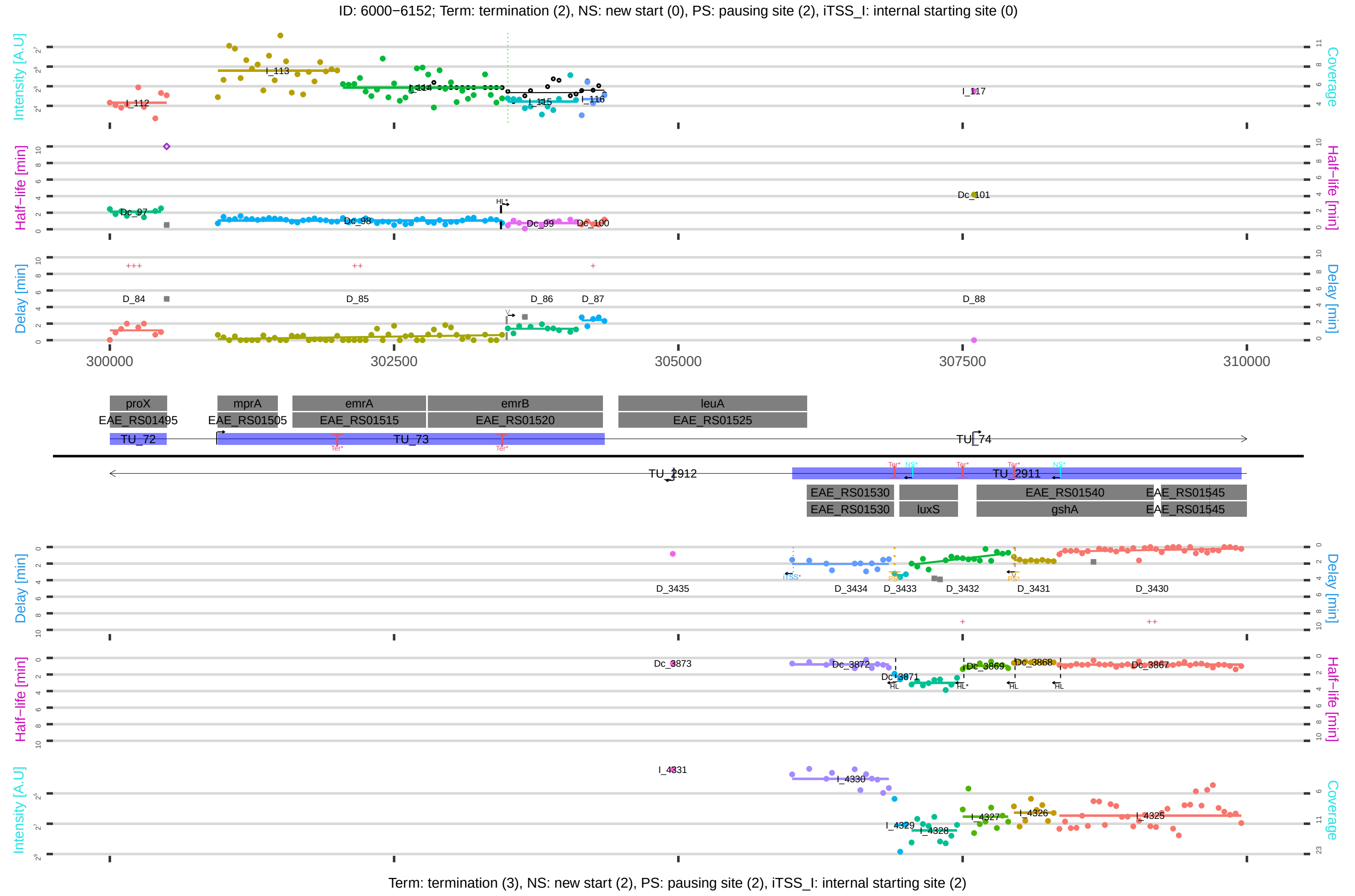

ID: 6000−6152; Term: termination (2), NS: new start (0), PS: pausing site (2), iTSS_I: internal starting site (0)
Intensity [A.U]
Coverage
Half−life [min]
Delay [min]
I_112
I_113
I_114
I_115
I_116
I_117
Dc_97
Dc_98
Dc_99
Dc_100
Dc_101
D_84
D_85
D_86
D_87
D_88
300000
302500
305000
307500
310000
proX
EAE_RS01495
mprA
EAE_RS01505
emrA
EAE_RS01515
emrB
EAE_RS01520
leuA
EAE_RS01525
TU_72
TU_73
TU_74
TU_2912
TU_2911
EAE_RS01530
luxS
EAE_RS01540
gshA
EAE_RS01545
D_3435
D_3434
D_3433
D_3432
D_3431
D_3430
Dc_3873
Dc_3872
Dc_3871
Dc_3869
Dc_3868
Dc_3867
I_4331
I_4330
I_4329
I_4328
I_4327
I_4326
I_4325
Term: termination (3), NS: new start (2), PS: pausing site (2), iTSS_I: internal starting site (2)

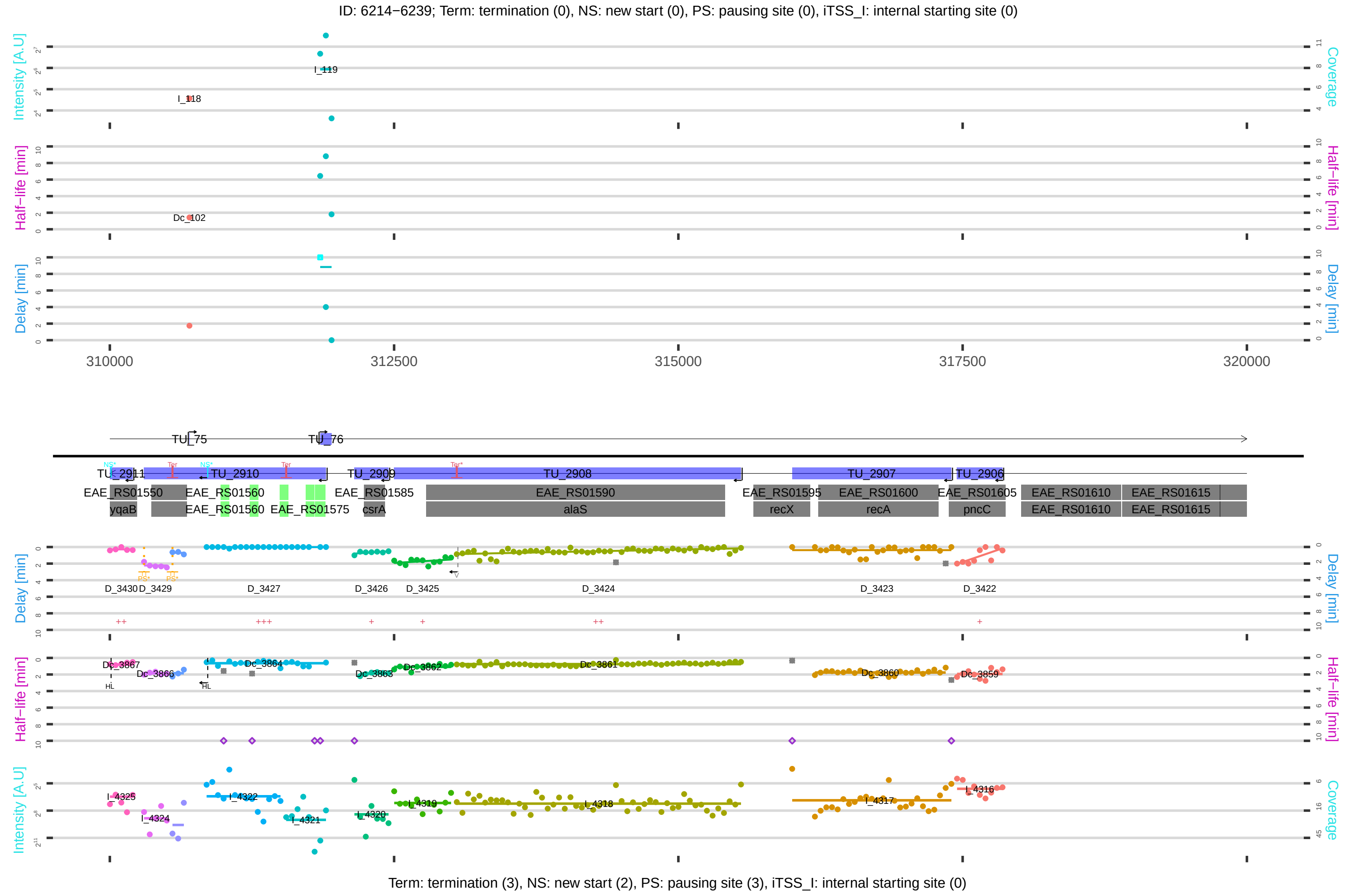
ID: 6214−6239; Term: termination (0), NS: new start (0), PS: pausing site (0), iTSS_I: internal starting site (0)
Intensity [A.U]
Coverage
Half−life [min]
Delay [min]
I_119
I_118
Dc_102
310000
312500
315000
317500
320000
TU_75
TU_76
TU_2911
TU_2910
TU_2909
TU_2908
TU_2907
TU_2906
EAE_RS01550
EAE_RS01560
EAE_RS01575
EAE_RS01585
EAE_RS01590
EAE_RS01595
EAE_RS01600
EAE_RS01605
EAE_RS01610
EAE_RS01615
yqaB
csrA
alaS
recX
recA
pncC
D_3430
D_3429
D_3427
D_3426
D_3425
D_3424
D_3423
D_3422
Dc_3867
Dc_3866
Dc_3864
Dc_3863
Dc_3862
Dc_3861
Dc_3860
Dc_3859
I_4325
I_4324
I_4322
I_4321
I_4320
I_4319
I_4318
I_4317
I_4316
Term: termination (3), NS: new start (2), PS: pausing site (3), iTSS_I: internal starting site (0)

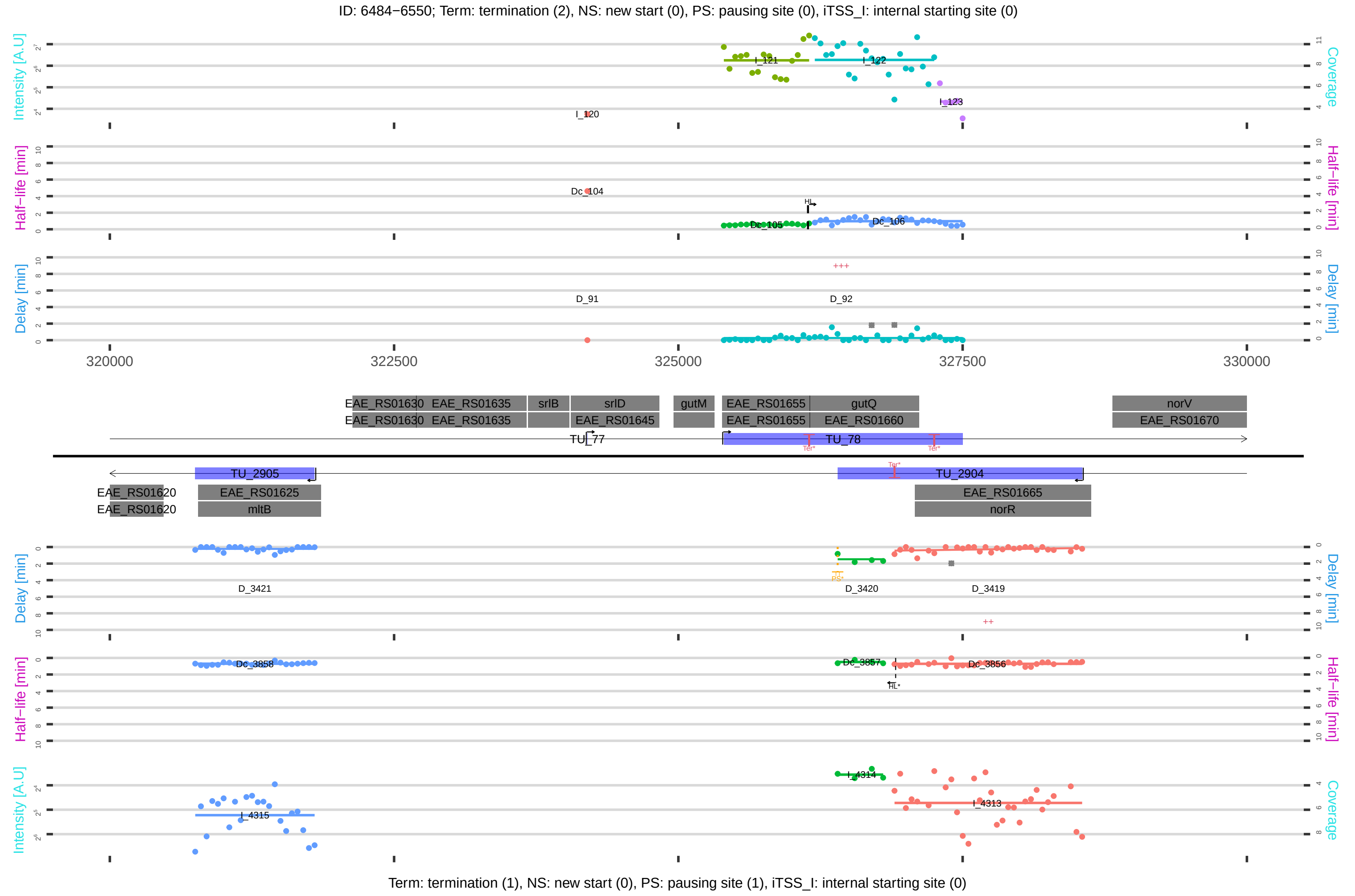

ID: 6484−6550; Term: termination (2), NS: new start (0), PS: pausing site (0), iTSS_I: internal starting site (0)
Intensity [A.U]
Coverage
I_120
I_121
I_122
I_123
Half−life [min]
Dc_104
Dc_105
Dc_106
HL*
Delay [min]
D_91
D_92
+++
320000
322500
325000
327500
330000
EAE_RS01630
EAE_RS01635
srlB
srlD
EAE_RS01645
gutM
EAE_RS01655
gutQ
EAE_RS01660
norV
EAE_RS01670
TU_77
TU_78
Ter*
TU_2905
TU_2904
EAE_RS01620
EAE_RS01625
mltB
EAE_RS01665
norR
PS*
D_3421
D_3420
D_3419
++
Dc_3858
Dc_3857
Dc_3856
I_4314
I_4313
I_4315
Term: termination (1), NS: new start (0), PS: pausing site (1), iTSS_I: internal starting site (0)

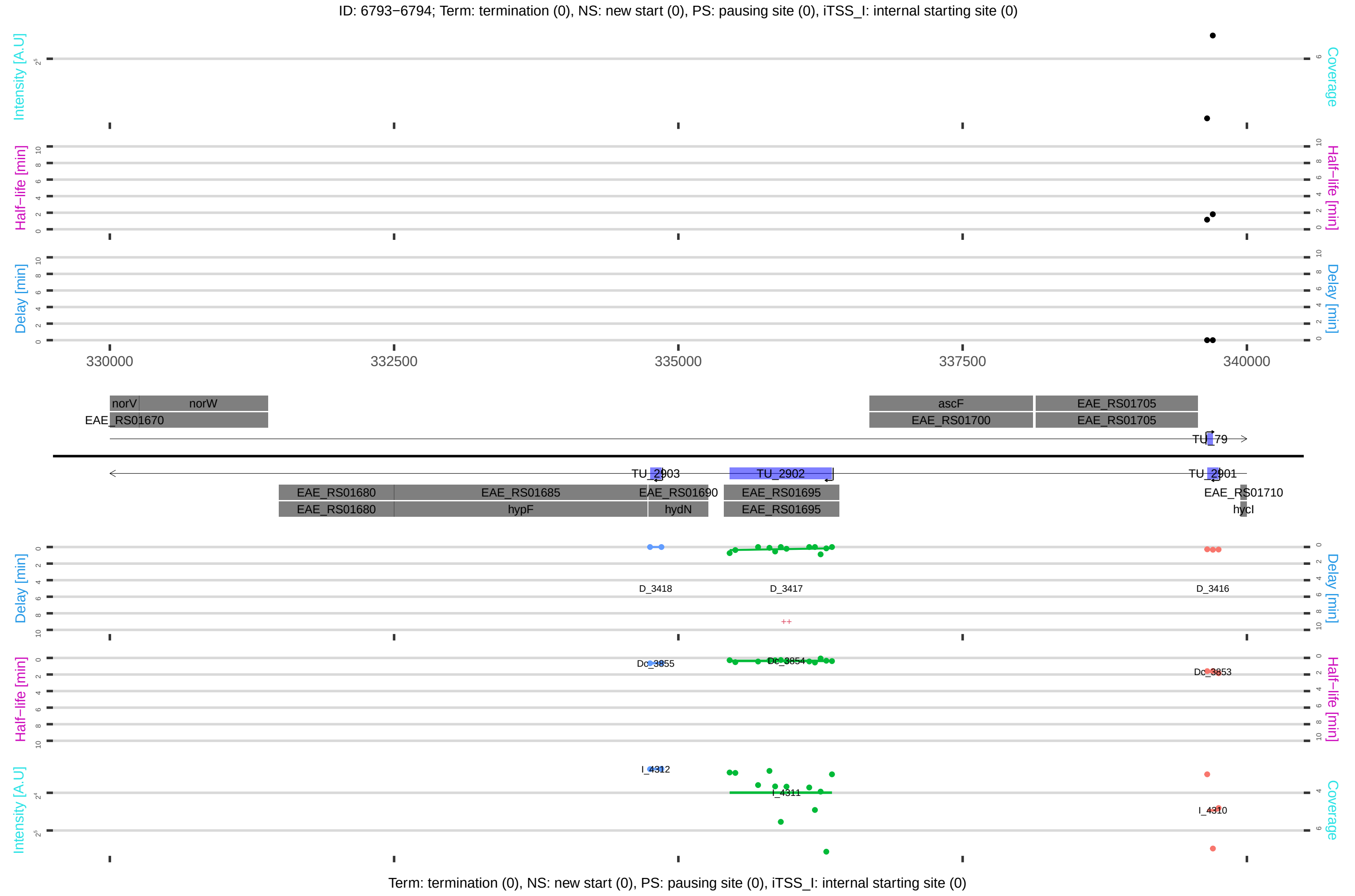
ID: 6793−6794; Term: termination (0), NS: new start (0), PS: pausing site (0), iTSS_I: internal starting site (0)
Intensity [A.U]
Coverage
Half−life [min]
Delay [min]
330000
332500
335000
337500
340000
norV
norW
EAE_RS01670
ascF
EAE_RS01700
EAE_RS01705
TU_79
TU_2903
TU_2902
TU_2901
EAE_RS01680
EAE_RS01685
hypF
EAE_RS01690
hydN
EAE_RS01695
EAE_RS01710
hycI
D_3418
D_3417
D_3416
Dc_3855
Dc_3854
Dc_3853
I_4312
I_4311
I_4310
Term: termination (0), NS: new start (0), PS: pausing site (0), iTSS_I: internal starting site (0)

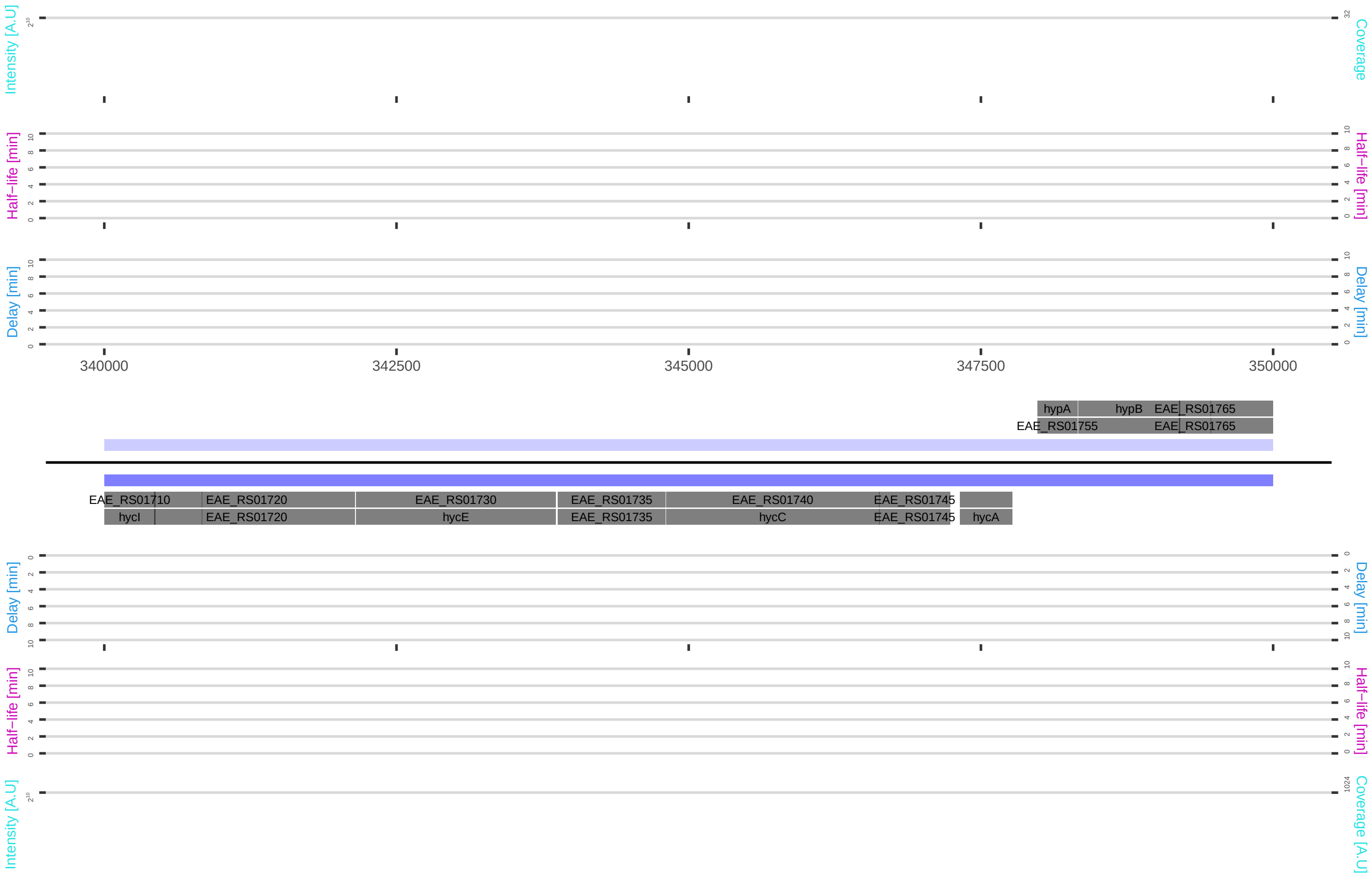

Intensity [A.U]
Coverage
Half−life [min]
Delay [min]
340000
342500
345000
347500
350000
hypA
hypB
EAE_RS01765
EAE_RS01755
EAE_RS01710
EAE_RS01720
EAE_RS01730
EAE_RS01735
EAE_RS01740
EAE_RS01745
hycI
hycE
hycC
hycA
Coverage [A.U]
NA

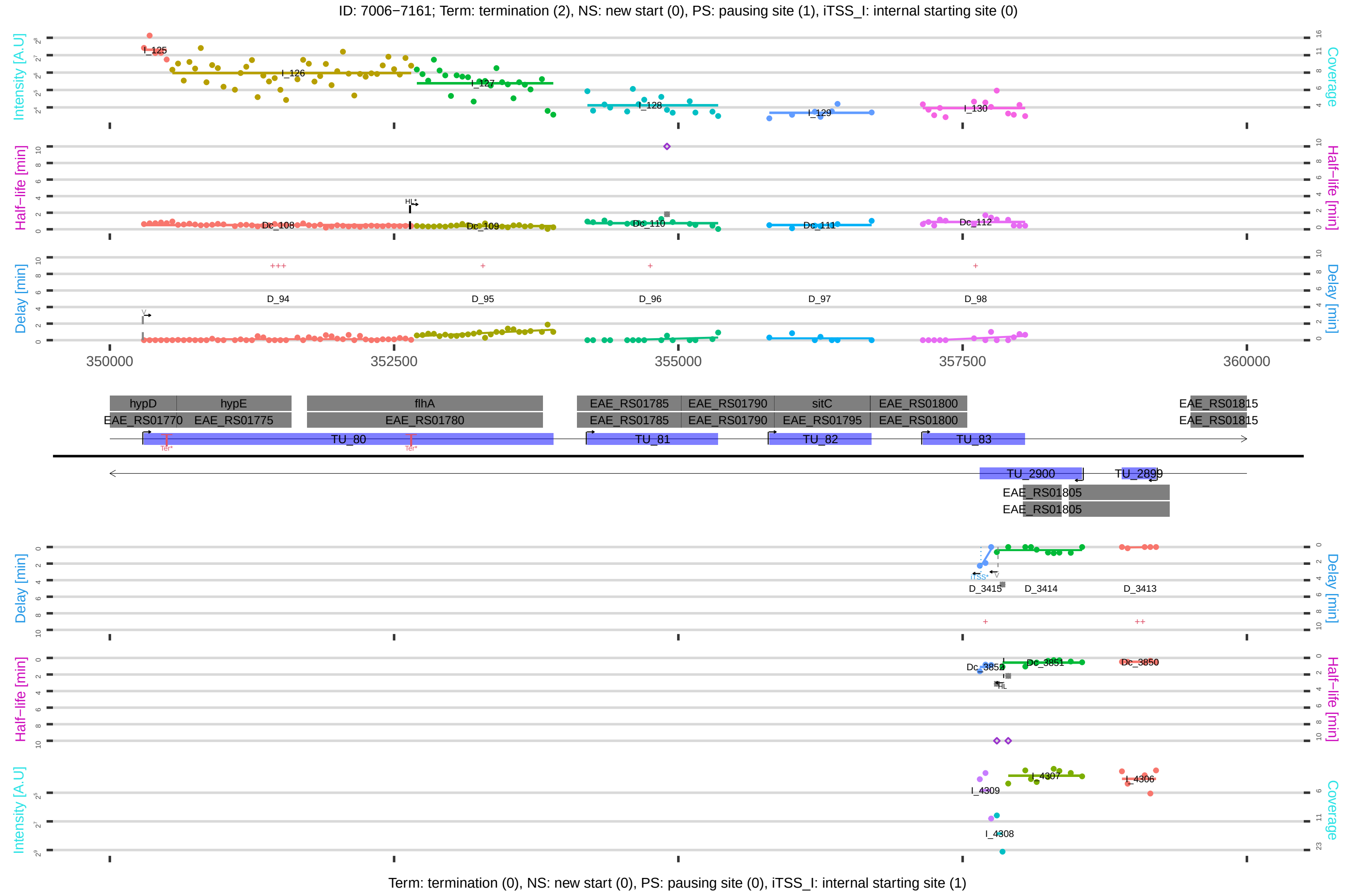
ID: 7006−7161; Term: termination (2), NS: new start (0), PS: pausing site (1), iTSS_I: internal starting site (0)
Intensity [A.U]
Coverage
Half−life [min]
Delay [min]
I_125
I_126
I_127
I_128
I_129
I_130
Dc_108
Dc_109
Dc_110
Dc_111
Dc_112
D_94
D_95
D_96
D_97
D_98
350000
352500
355000
357500
360000
hypD
hypE
flhA
EAE_RS01770
EAE_RS01775
EAE_RS01780
EAE_RS01785
EAE_RS01790
sitC
EAE_RS01795
EAE_RS01800
EAE_RS01815
TU_80
TU_81
TU_82
TU_83
TU_2900
TU_2899
EAE_RS01805
D_3415
D_3414
D_3413
Dc_3852
Dc_3851
Dc_3850
I_4309
I_4307
I_4306
I_4308
Term: termination (0), NS: new start (0), PS: pausing site (0), iTSS_I: internal starting site (1)

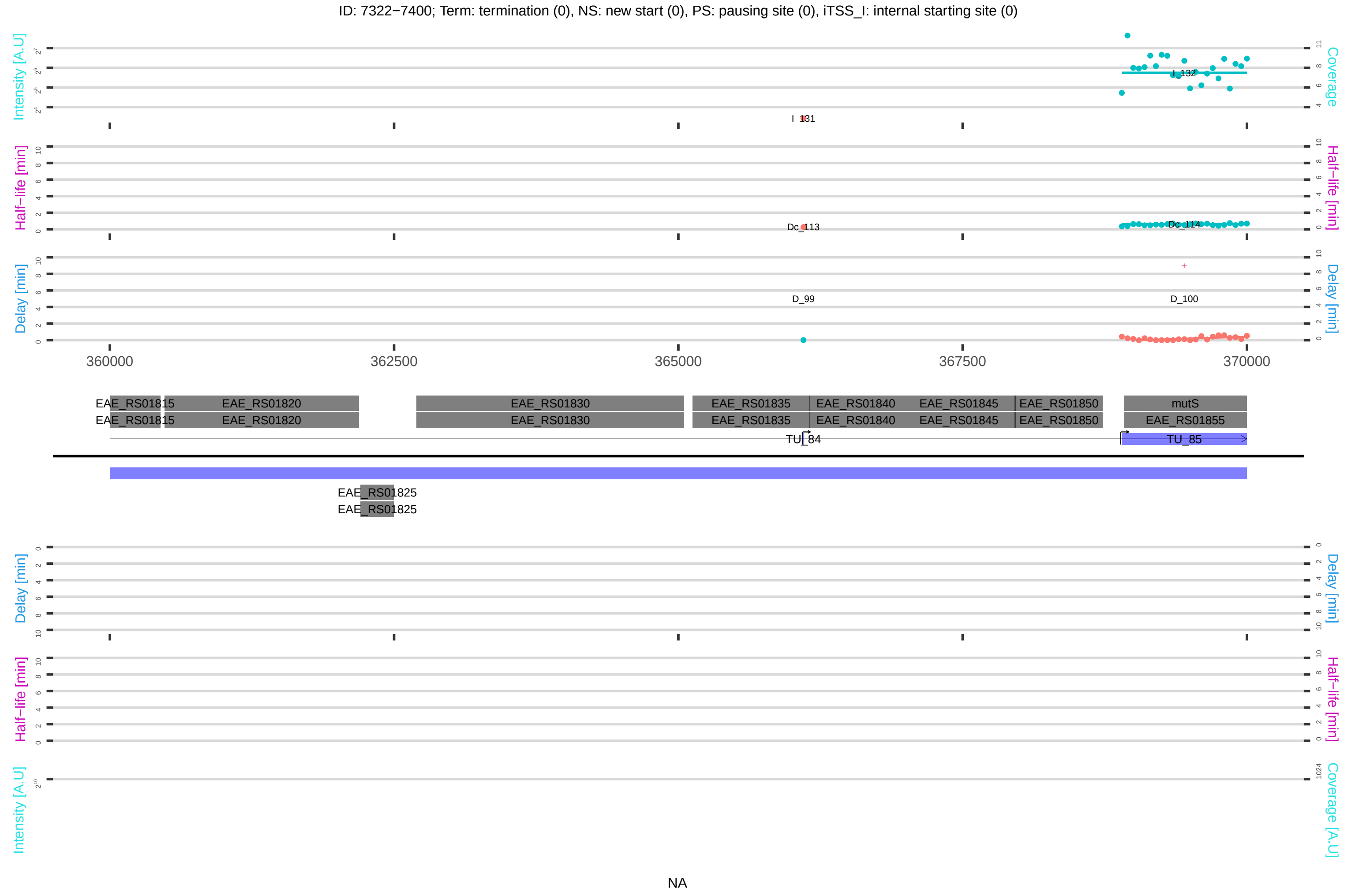

ID: 7322−7400; Term: termination (0), NS: new start (0), PS: pausing site (0), iTSS_I: internal starting site (0)
Intensity [A.U]
Coverage
I_132
I_131
Half−life [min]
Dc_113
Dc_114
Delay [min]
D_99
D_100
360000
362500
365000
367500
370000
EAE_RS01815
EAE_RS01820
EAE_RS01830
EAE_RS01835
EAE_RS01840
EAE_RS01845
EAE_RS01850
mutS
EAE_RS01855
TU_84
TU_85
EAE_RS01825
Coverage [A.U]
NA

ID: 7400−7562; Term: termination (0), NS: new start (0), PS: pausing site (0), iTSS_I: internal starting site (0)

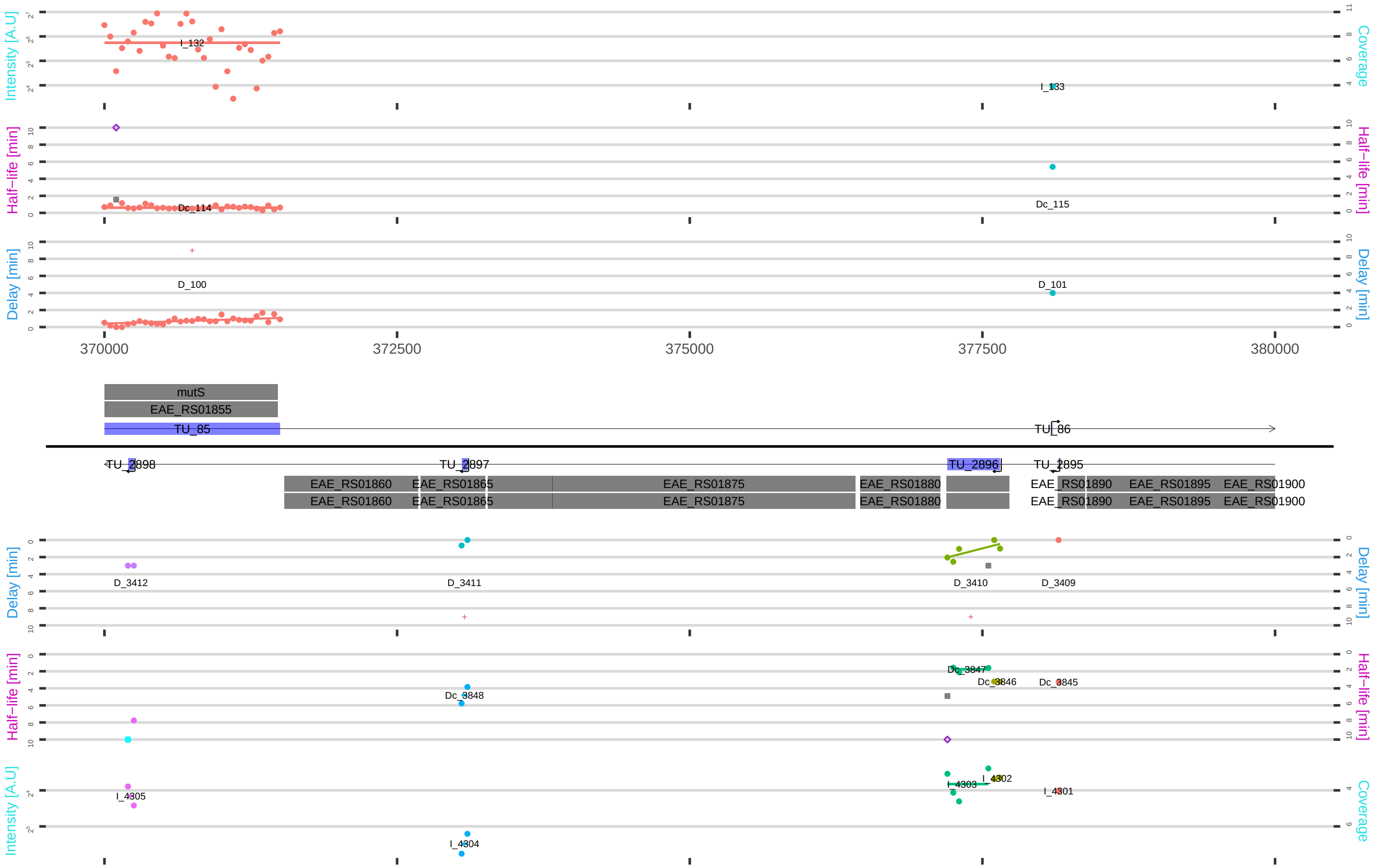

Term: termination (0), NS: new start (0), PS: pausing site (0), iTSS_I: internal starting site (0)

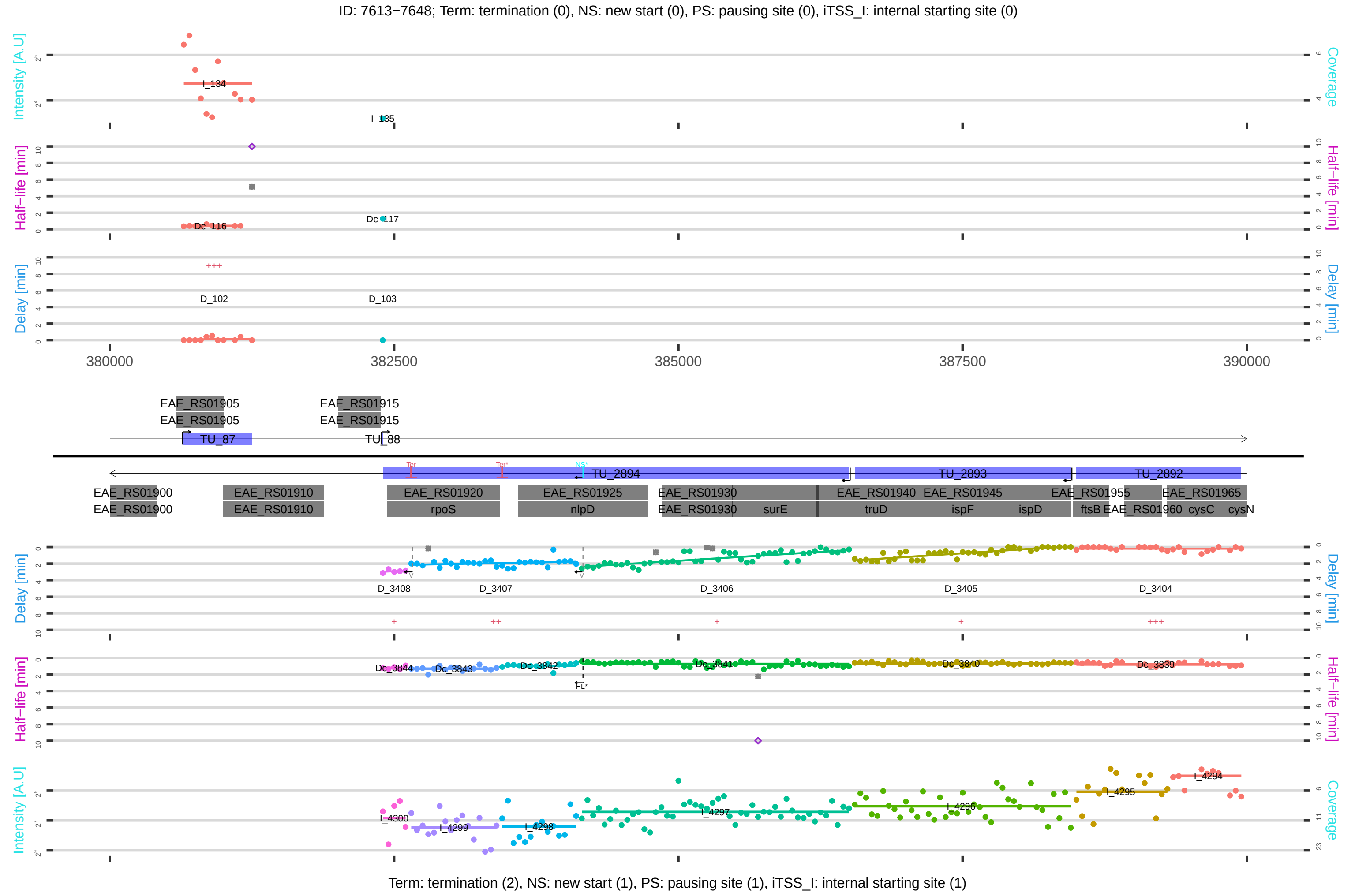

ID: 7613−7648; Term: termination (0), NS: new start (0), PS: pausing site (0), iTSS_I: internal starting site (0)
Intensity [A.U]
Coverage
Half−life [min]
Delay [min]
I_134
I_135
Dc_116
Dc_117
D_102
D_103
380000
382500
385000
387500
390000
EAE_RS01905
EAE_RS01915
TU_87
TU_88
TU_2894
TU_2893
TU_2892
EAE_RS01900
EAE_RS01910
EAE_RS01920
rpoS
EAE_RS01925
nlpD
EAE_RS01930
surE
EAE_RS01940
truD
EAE_RS01945
ispF
ispD
EAE_RS01955
ftsB
EAE_RS01960
cysC
EAE_RS01965
cysN
D_3408
D_3407
D_3406
D_3405
D_3404
Dc_3844
Dc_3843
Dc_3842
Dc_3841
Dc_3840
Dc_3839
I_4300
I_4299
I_4298
I_4297
I_4296
I_4295
I_4294
Term: termination (2), NS: new start (1), PS: pausing site (1), iTSS_I: internal starting site (1)

ID: 7882−8000; Term: termination (0), NS: new start (0), PS: pausing site (0), iTSS_I: internal starting site (0)

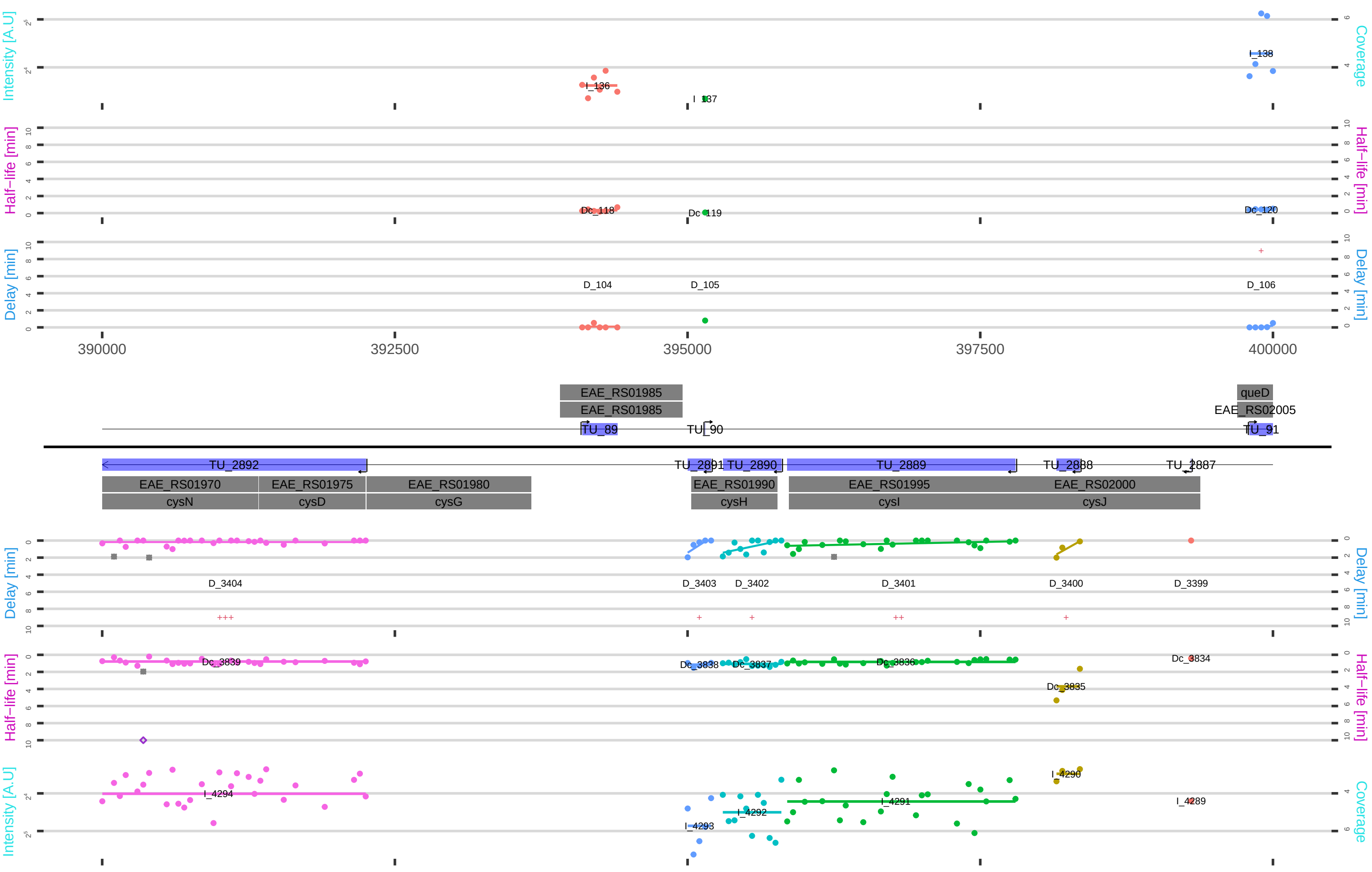

Term: termination (0), NS: new start (0), PS: pausing site (0), iTSS_I: internal starting site (0)

ID: 8000−8096; Term: termination (0), NS: new start (0), PS: pausing site (0), iTSS_I: internal starting site (0)

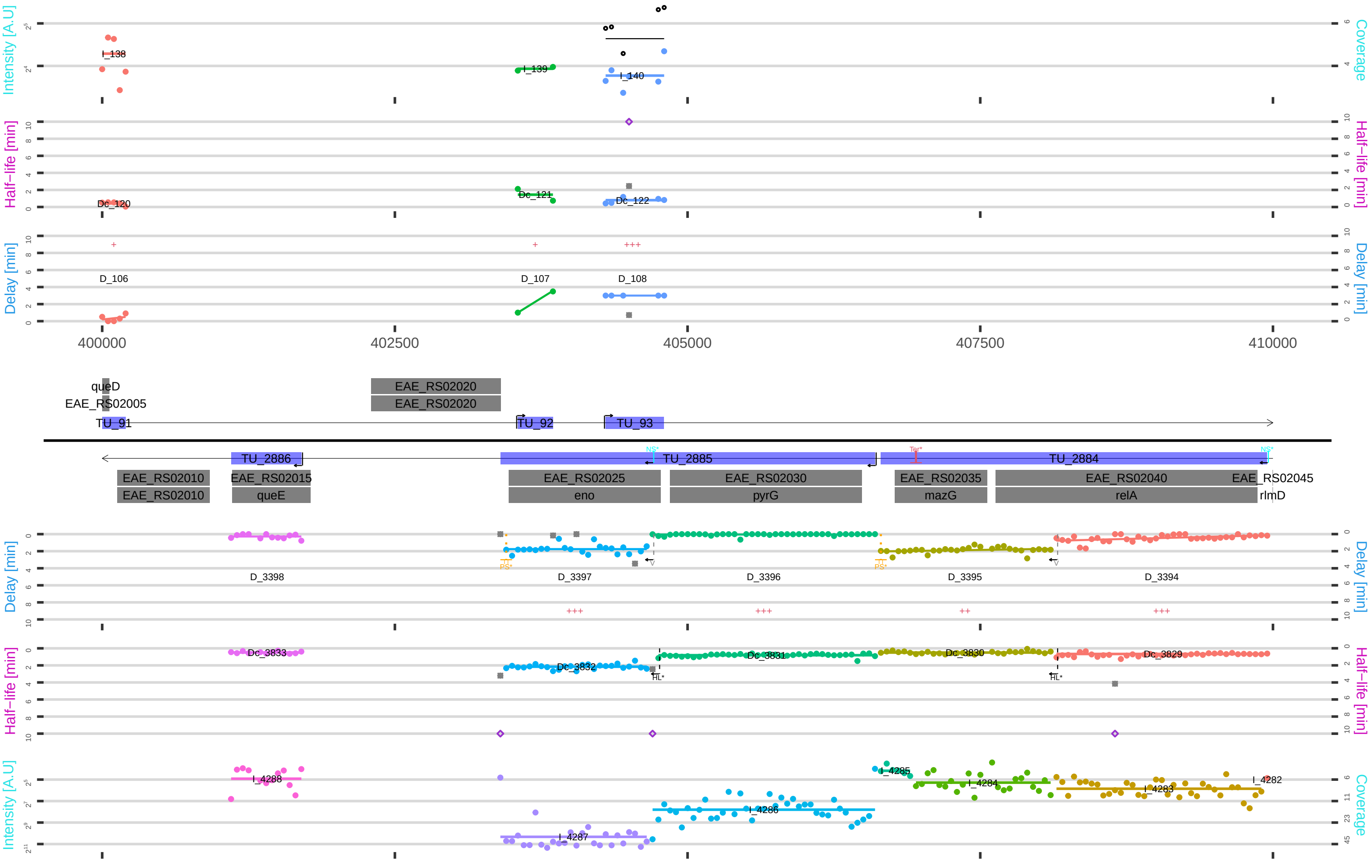

Term: termination (1), NS: new start (2), PS: pausing site (2), iTSS_I: internal starting site (0)

ID: 8224−8284; Term: termination (0), NS: new start (0), PS: pausing site (0), iTSS_I: internal starting site (0)

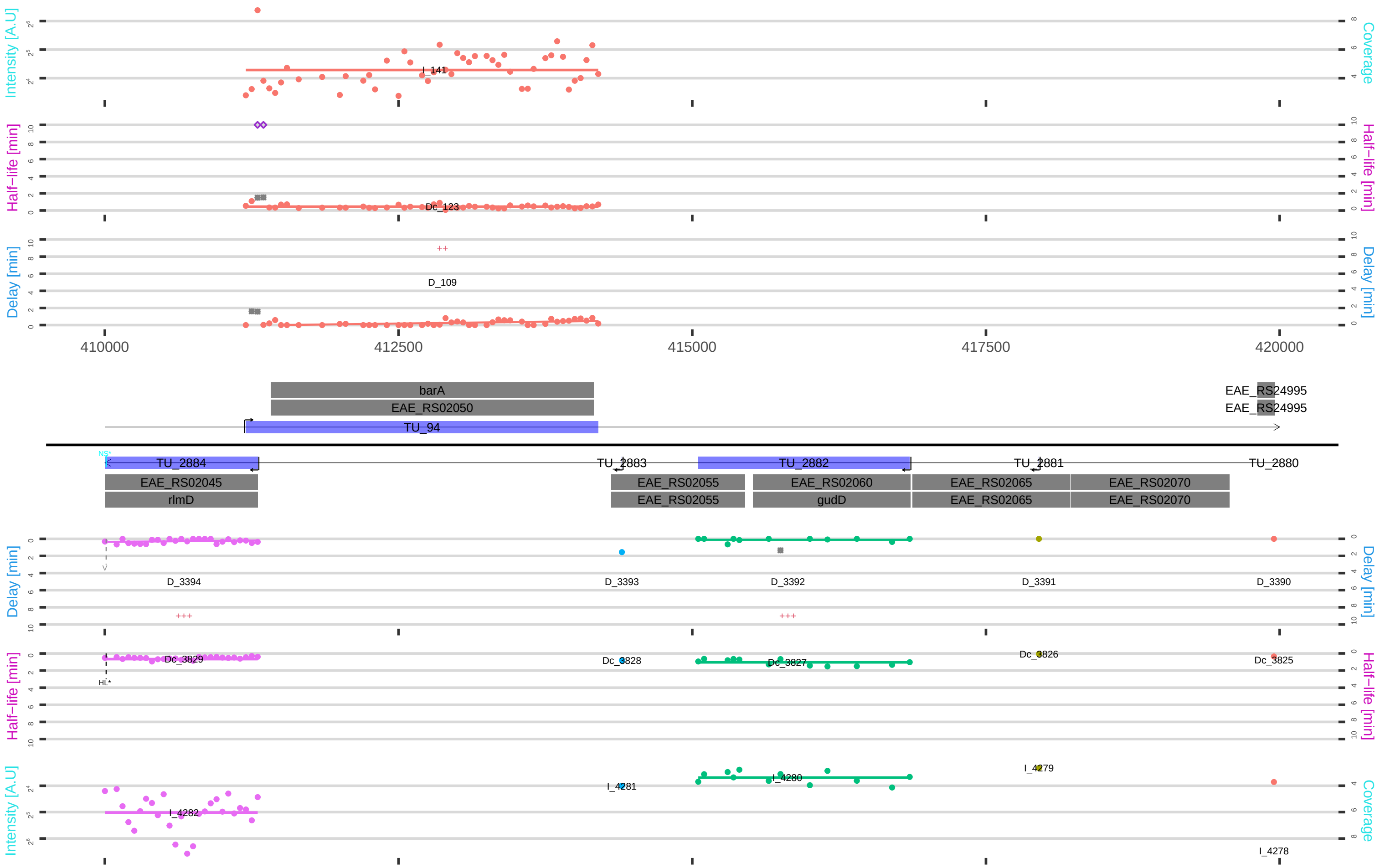

Term: termination (0), NS: new start (1), PS: pausing site (0), iTSS_I: internal starting site (0)

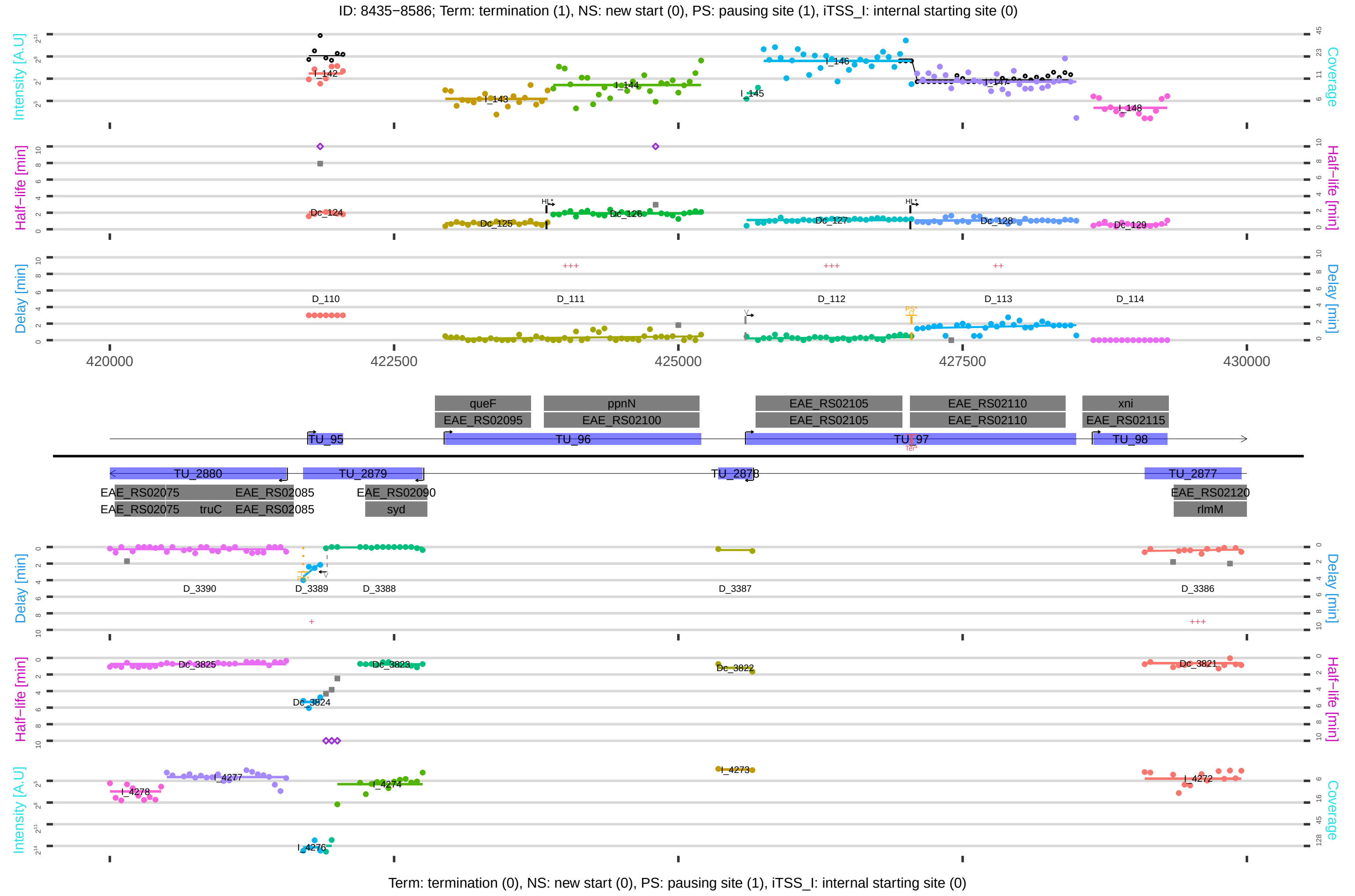

ID: 8435−8586; Term: termination (1), NS: new start (0), PS: pausing site (1), iTSS_I: internal starting site (0)
Intensity [A.U]
Coverage
Half−life [min]
Delay [min]
420000
422500
425000
427500
430000
queF
EAE_RS02095
ppnN
EAE_RS02100
EAE_RS02105
EAE_RS02110
xni
EAE_RS02115
TU_95
TU_96
TU_97
TU_98
TU_2880
TU_2879
TU_2878
TU_2877
EAE_RS02075
EAE_RS02085
EAE_RS02090
truC
syd
EAE_RS02120
rlmM
Term: termination (0), NS: new start (0), PS: pausing site (1), iTSS_I: internal starting site (0)

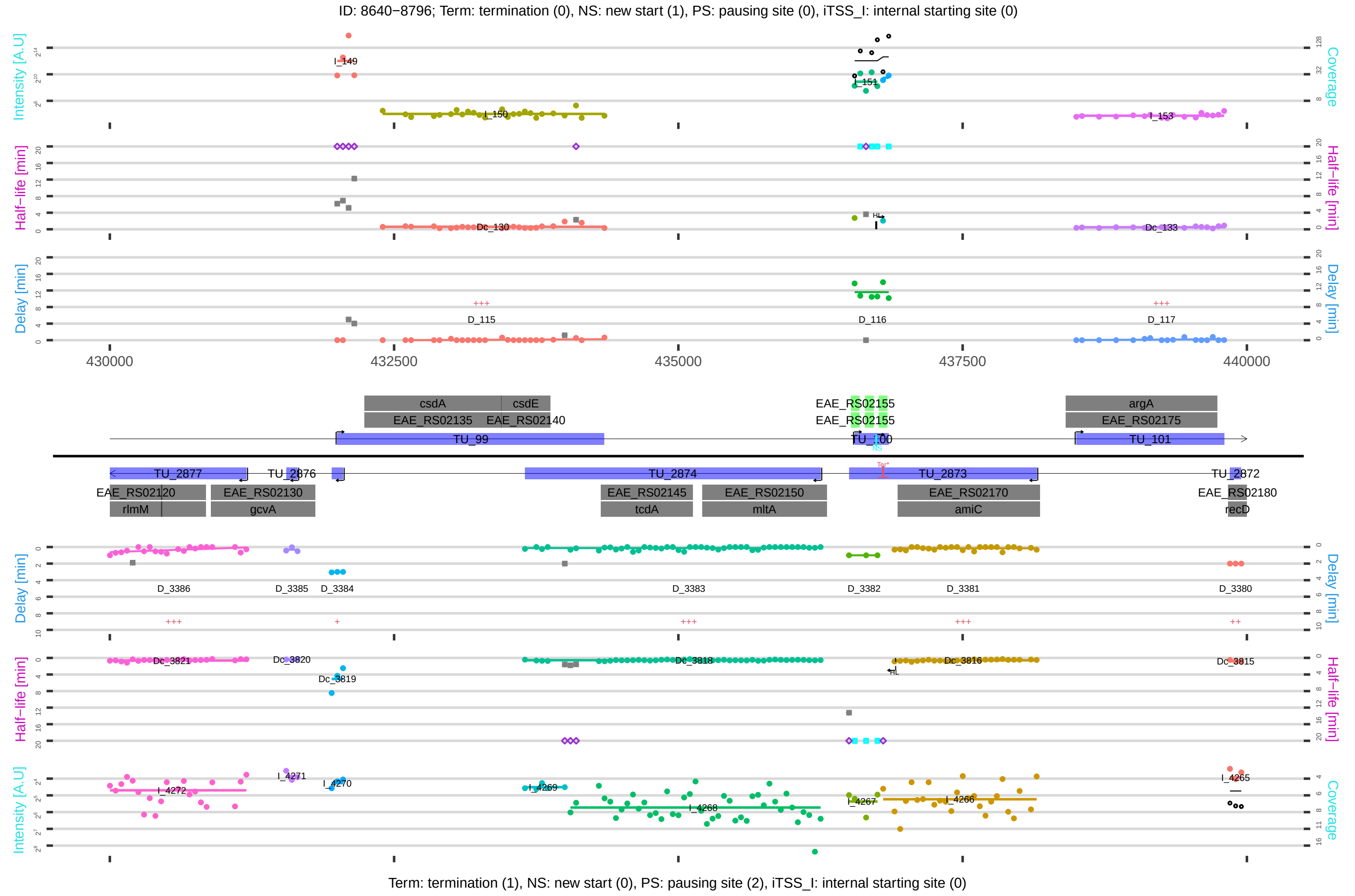
ID: 8640−8796; Term: termination (0), NS: new start (1), PS: pausing site (0), iTSS_I: internal starting site (0)
Intensity [A.U]
Coverage
Half−life [min]
Delay [min]
I_149
I_150
I_151
I_153
Dc_130
Dc_133
D_115
D_116
D_117
430000
432500
435000
437500
440000
csdA
csdE
EAE_RS02135
EAE_RS02140
EAE_RS02155
argA
EAE_RS02175
TU_99
TU_100
TU_101
NS
Ter*
TU_2877
TU_2876
TU_2874
TU_2873
TU_2872
EAE_RS02120
EAE_RS02130
EAE_RS02145
EAE_RS02150
EAE_RS02170
EAE_RS02180
rlmM
gcvA
tcdA
mltA
amiC
recD
D_3386
D_3385
D_3384
D_3383
D_3382
D_3381
D_3380
Dc_3821
Dc_3820
Dc_3819
Dc_3818
Dc_3816
Dc_3815
I_4272
I_4271
I_4270
I_4269
I_4268
I_4267
I_4266
I_4265
Term: termination (1), NS: new start (0), PS: pausing site (2), iTSS_I: internal starting site (0)

ID: 202414−202216; FC*: significant t−test of two consecutive segments; Term: termination, NS: new start, PS: pausing site,iTSS_I: internal starting site,TI: transcription interferance.

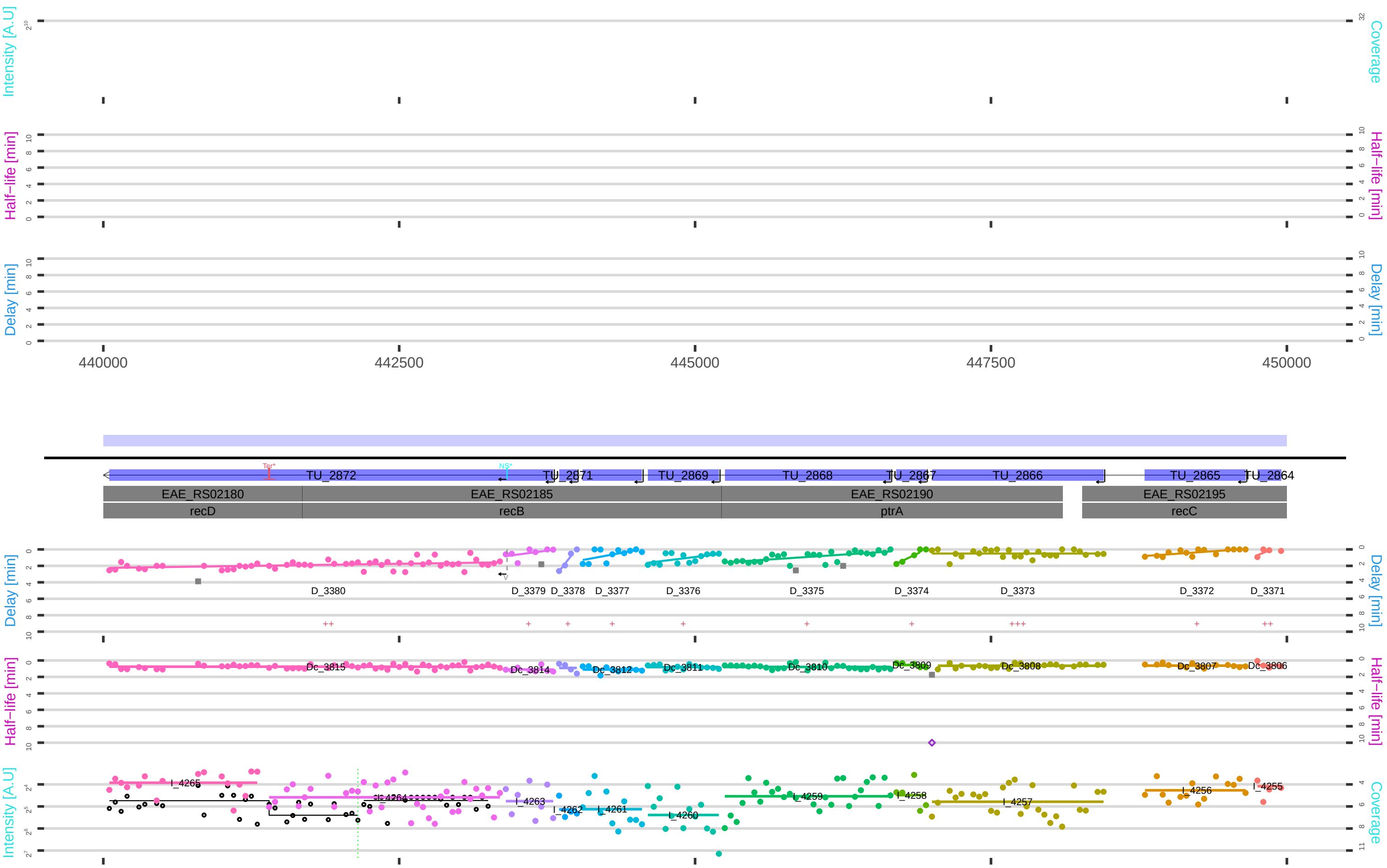

Term: termination (1), NS: new start (1), PS: pausing site (0), iTSS_I: internal starting site (0)

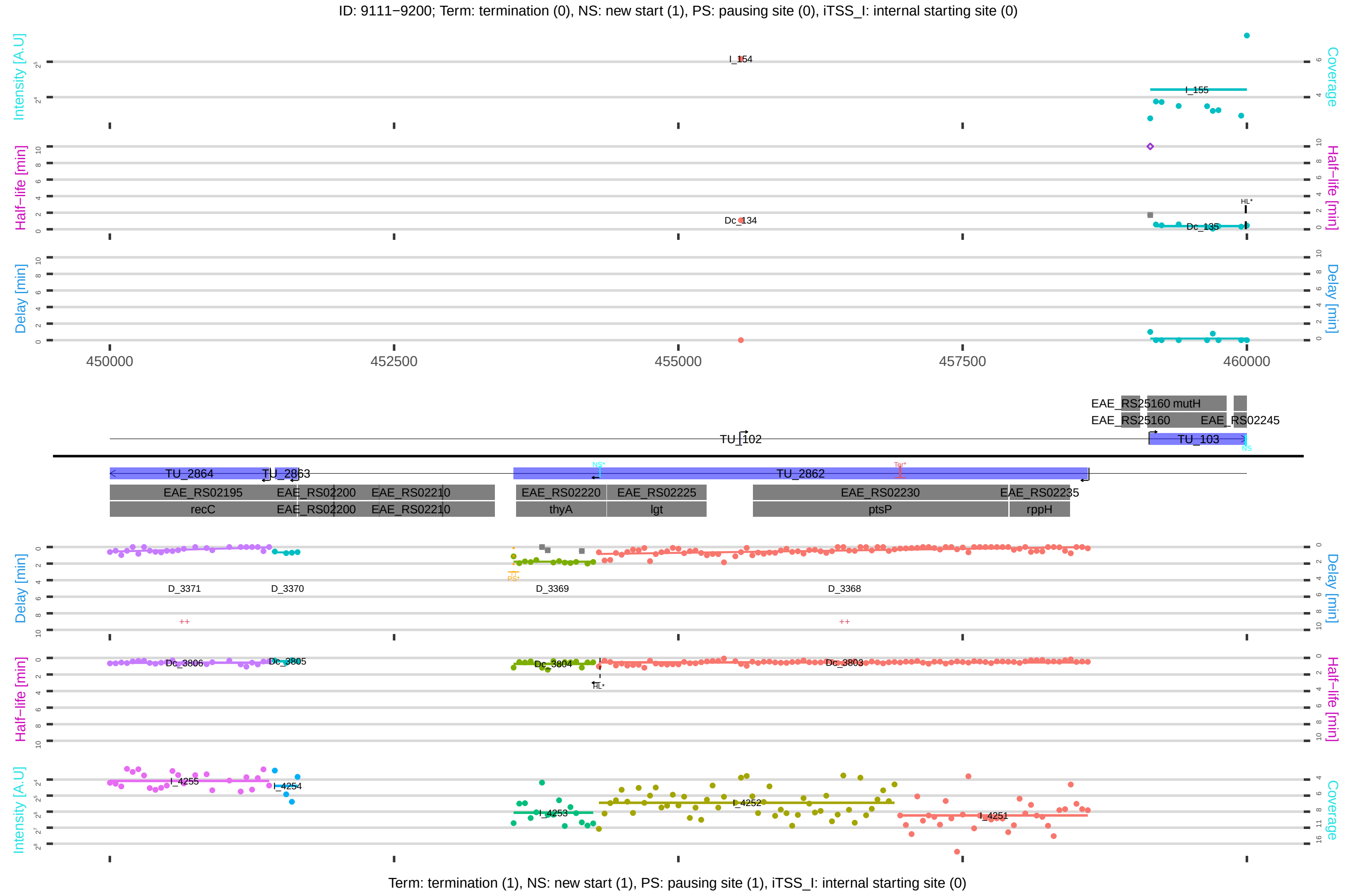
ID: 9111−9200; Term: termination (0), NS: new start (1), PS: pausing site (0), iTSS_I: internal starting site (0)
Intensity [A.U]
Coverage
Half−life [min]
Delay [min]
I_154
I_155
Dc_134
Dc_135
HL*
450000
452500
455000
457500
460000
EAE_RS25160 mutH
EAE_RS25160
EAE_RS02245
TU_102
TU_103
TU_2864
TU_2863
TU_2862
EAE_RS02195
recC
EAE_RS02200
EAE_RS02210
EAE_RS02220
thyA
EAE_RS02225
lgt
EAE_RS02230
ptsP
EAE_RS02235
rppH
D_3371
D_3370
D_3369
D_3368
Dc_3806
Dc_3805
Dc_3804
Dc_3803
I_4255
I_4254
I_4253
I_4252
I_4251
Term: termination (1), NS: new start (1), PS: pausing site (1), iTSS_I: internal starting site (0)

ID: 9200−9382; Term: termination (1), NS: new start (1), PS: pausing site (0), iTSS_I: internal starting site (0)

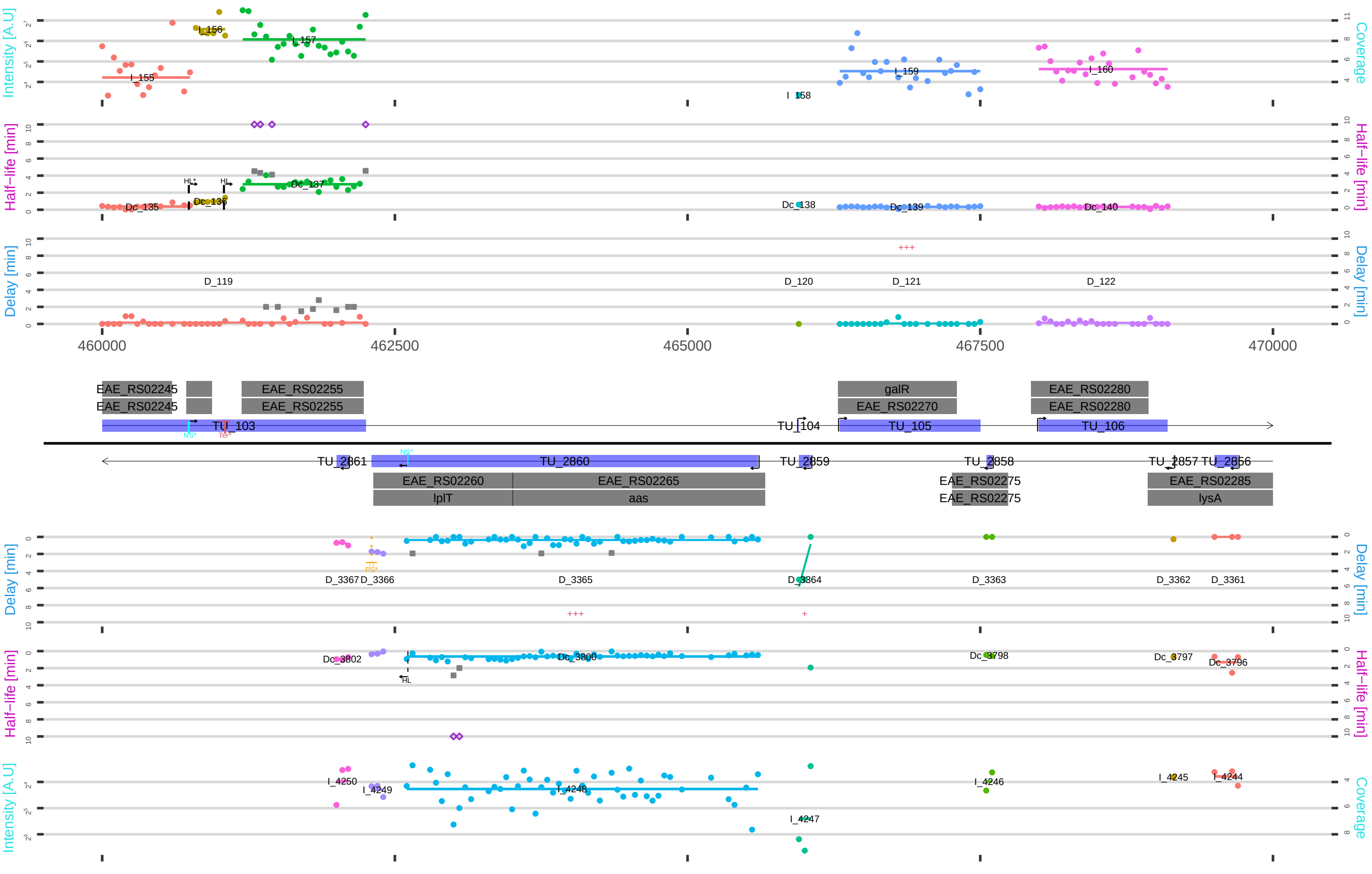

Term: termination (0), NS: new start (1), PS: pausing site (1), iTSS_I: internal starting site (0)

ID: 9408−9553; Term: termination (0), NS: new start (0), PS: pausing site (0), iTSS_I: internal starting site (0)

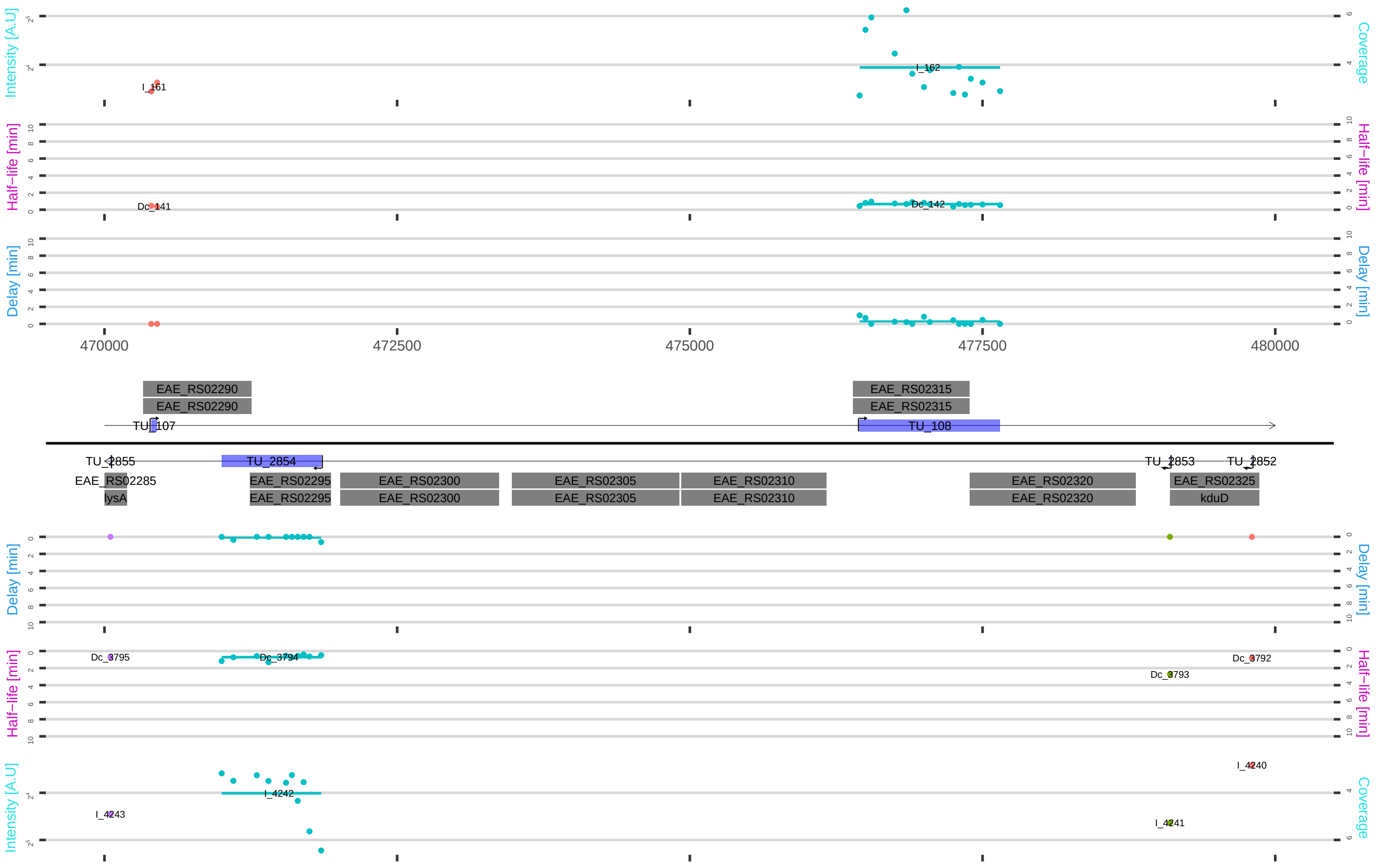

Term: termination (0), NS: new start (0), PS: pausing site (0), iTSS_I: internal starting site (0)

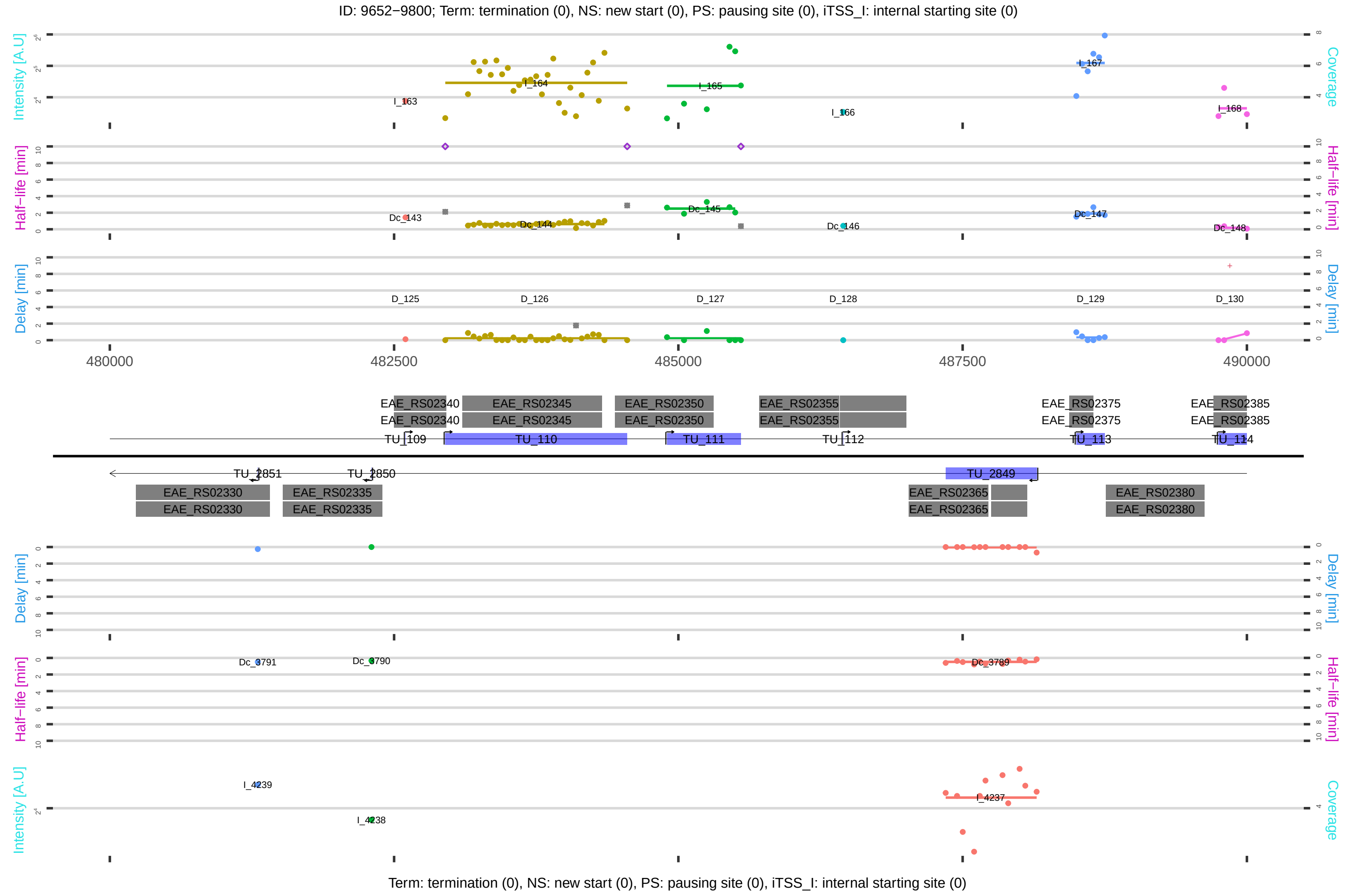

ID: 9652−9800; Term: termination (0), NS: new start (0), PS: pausing site (0), iTSS_I: internal starting site (0)
Intensity [A.U]
Coverage
Half−life [min]
Delay [min]
I_163
I_164
I_165
I_166
I_167
I_168
Dc_143
Dc_144
Dc_145
Dc_146
Dc_147
Dc_148
D_125
D_126
D_127
D_128
D_129
D_130
480000
482500
485000
487500
490000
EAE_RS02340
EAE_RS02345
EAE_RS02350
EAE_RS02355
EAE_RS02375
EAE_RS02385
TU_109
TU_110
TU_111
TU_112
TU_113
TU_114
TU_2851
TU_2850
TU_2849
EAE_RS02330
EAE_RS02335
EAE_RS02365
EAE_RS02380
Dc_3791
Dc_3790
Dc_3789
I_4239
I_4238
I_4237
Term: termination (0), NS: new start (0), PS: pausing site (0), iTSS_I: internal starting site (0)

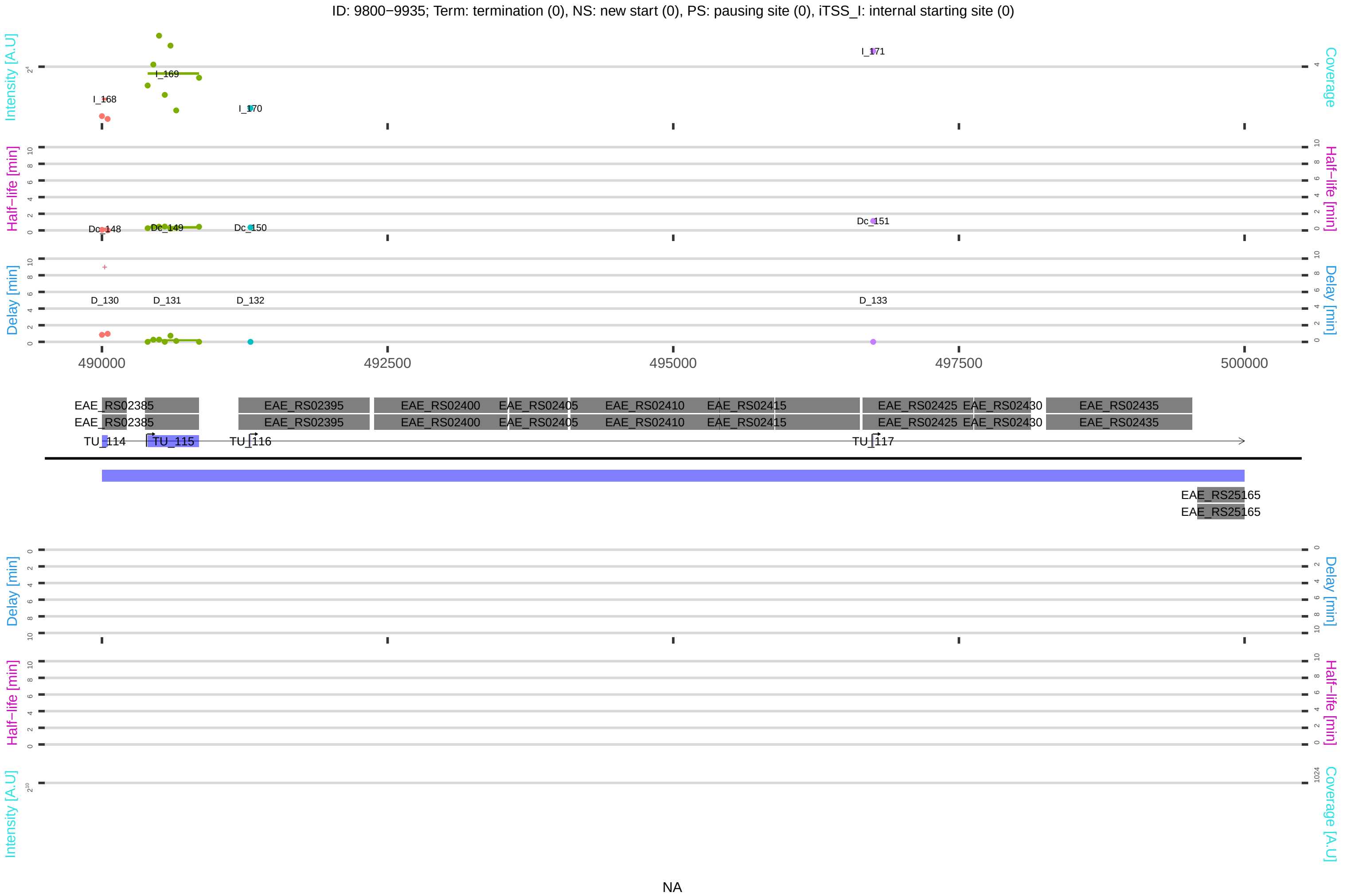
ID: 9800−9935; Term: termination (0), NS: new start (0), PS: pausing site (0), iTSS_I: internal starting site (0)
Intensity [A.U]
Coverage
Half−life [min]
Delay [min]
I_168
I_169
I_170
I_171
Dc_148
Dc_149
Dc_150
Dc_151
D_130
D_131
D_132
D_133
490000
492500
495000
497500
500000
EAE_RS02385
EAE_RS02395
EAE_RS02400
EAE_RS02405
EAE_RS02410
EAE_RS02415
EAE_RS02425
EAE_RS02430
EAE_RS02435
TU_114
TU_115
TU_116
TU_117
EAE_RS25165
Coverage [A.U]
NA

ID: 10114−10199; Term: termination (0), NS: new start (1), PS: pausing site (1), iTSS_I: internal starting site (0)

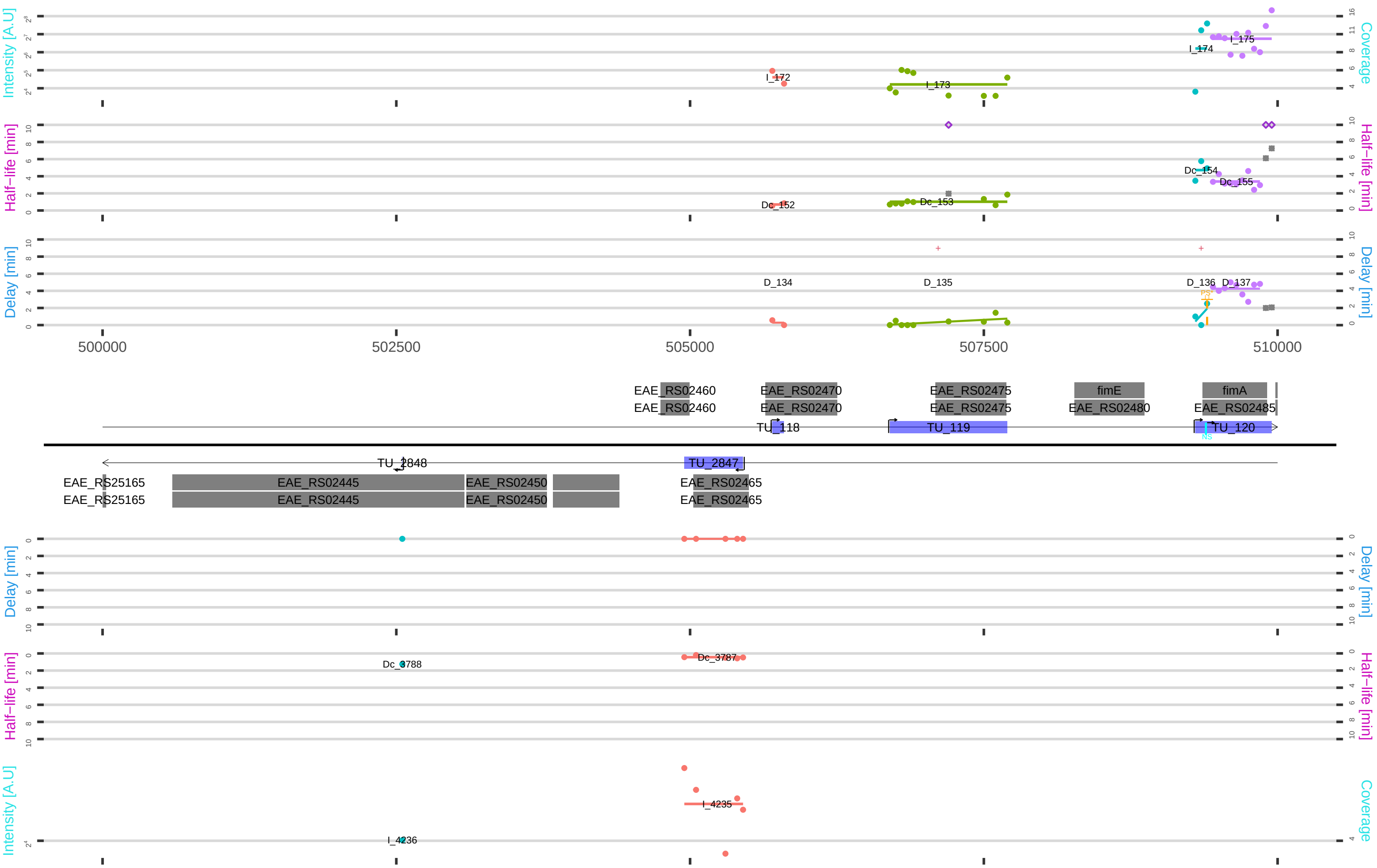

Term: termination (0), NS: new start (0), PS: pausing site (0), iTSS_I: internal starting site (0)

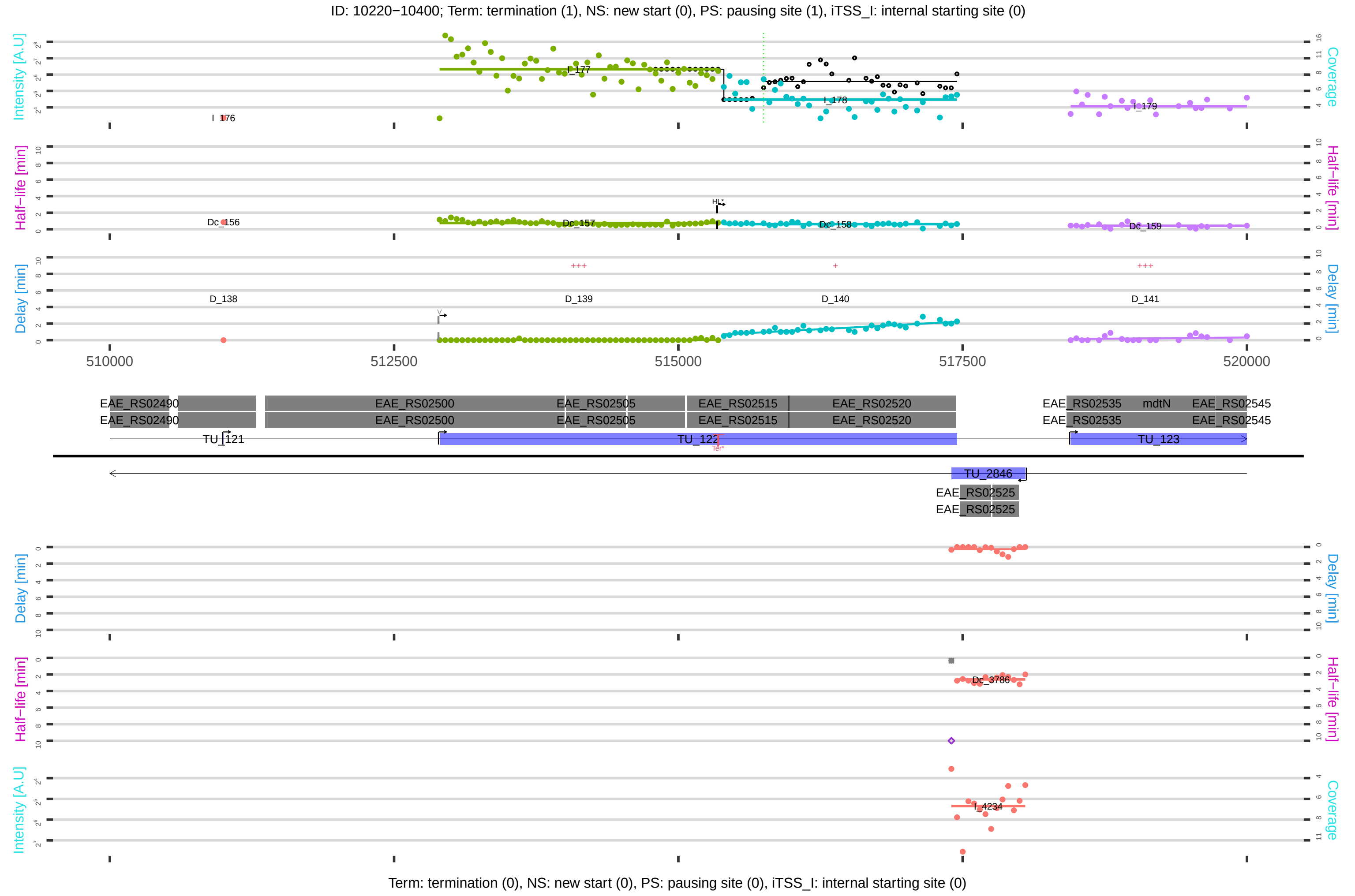

ID: 10220−10400; Term: termination (1), NS: new start (0), PS: pausing site (1), iTSS_I: internal starting site (0)
Intensity [A.U]
Coverage
Half−life [min]
Delay [min]
I_176
I_177
I_178
I_179
Dc_156
Dc_157
Dc_158
Dc_159
D_138
D_139
D_140
D_141
510000
512500
515000
517500
520000
EAE_RS02490
EAE_RS02500
EAE_RS02505
EAE_RS02515
EAE_RS02520
EAE_RS02535
mdtN
EAE_RS02545
TU_121
TU_122
TU_123
TU_2846
EAE_RS02525
Dc_3786
I_4234
Term: termination (0), NS: new start (0), PS: pausing site (0), iTSS_I: internal starting site (0)

ID: 10400−10598; Term: termination (1), NS: new start (1), PS: pausing site (1), iTSS_I: internal starting site (0)

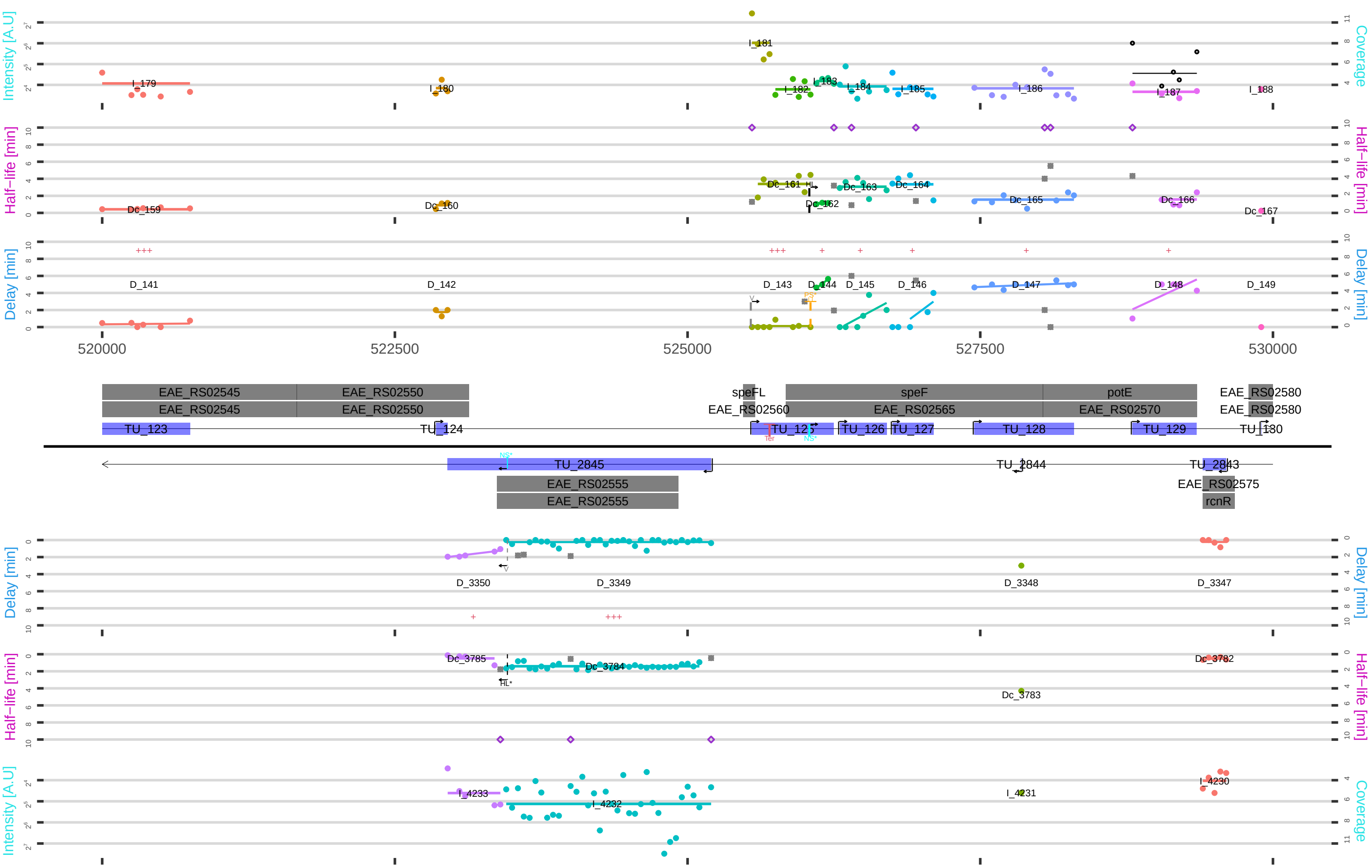

Term: termination (0), NS: new start (1), PS: pausing site (1), iTSS_I: internal starting site (0)

ID: 10622−10634; Term: termination (0), NS: new start (0), PS: pausing site (0), iTSS_I: internal starting site (0)

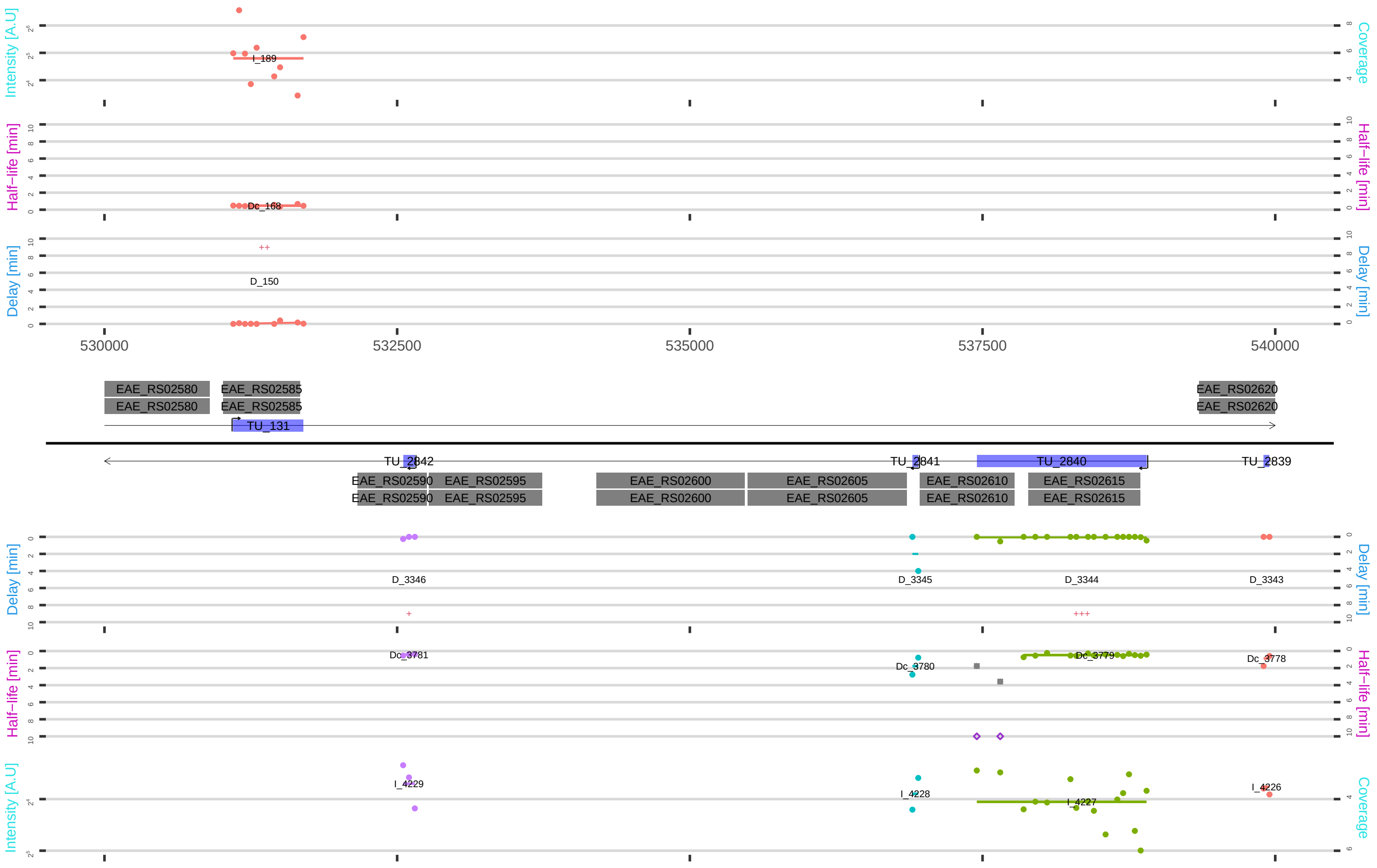

Term: termination (0), NS: new start (0), PS: pausing site (0), iTSS_I: internal starting site (0)

ID: 10802−10864; Term: termination (0), NS: new start (0), PS: pausing site (0), iTSS_I: internal starting site (0)

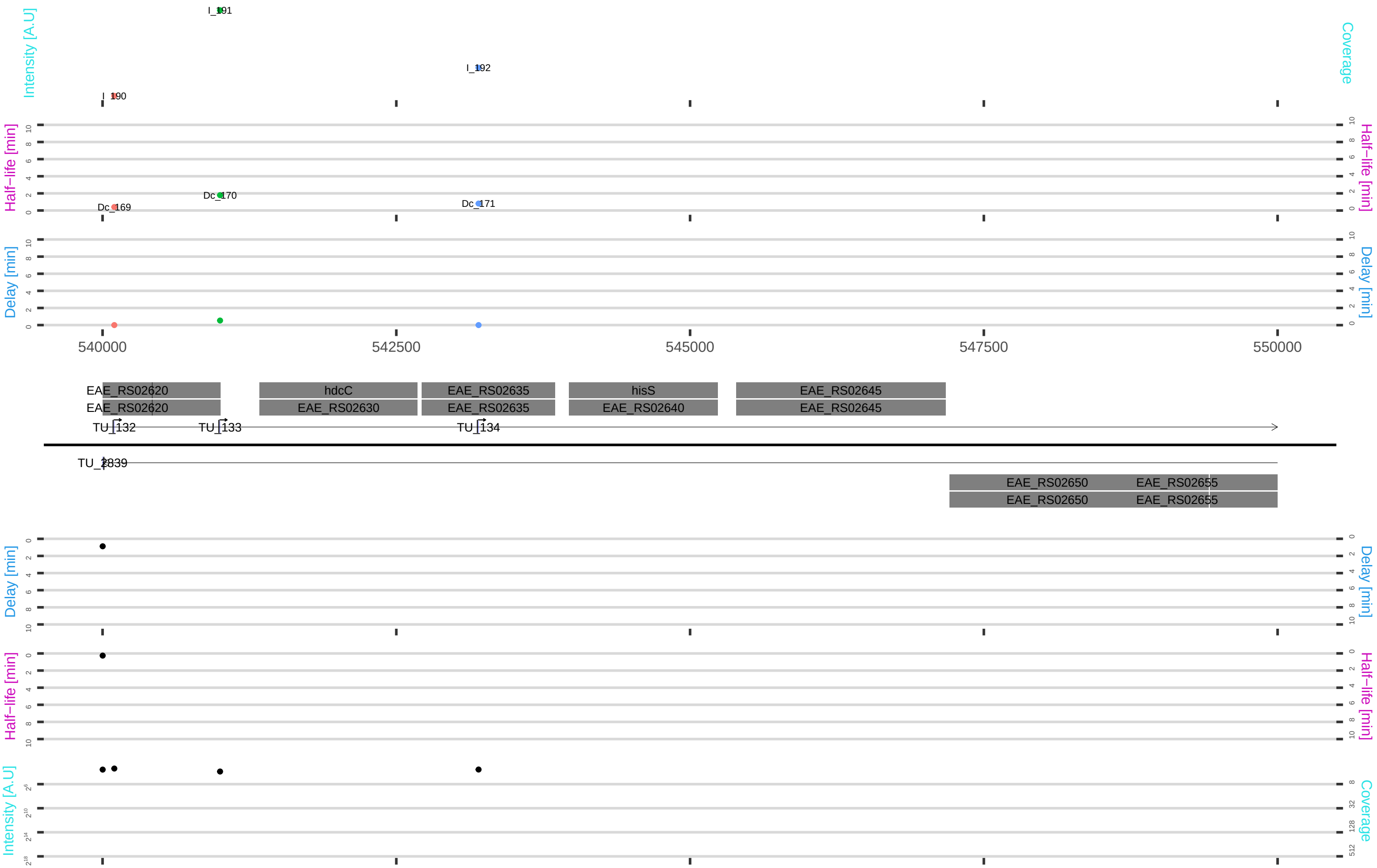

Term: termination (0), NS: new start (0), PS: pausing site (0), iTSS_I: internal starting site (0)

ID: 11126−11151; Term: termination (0), NS: new start (0), PS: pausing site (0), iTSS_I: internal starting site (0)

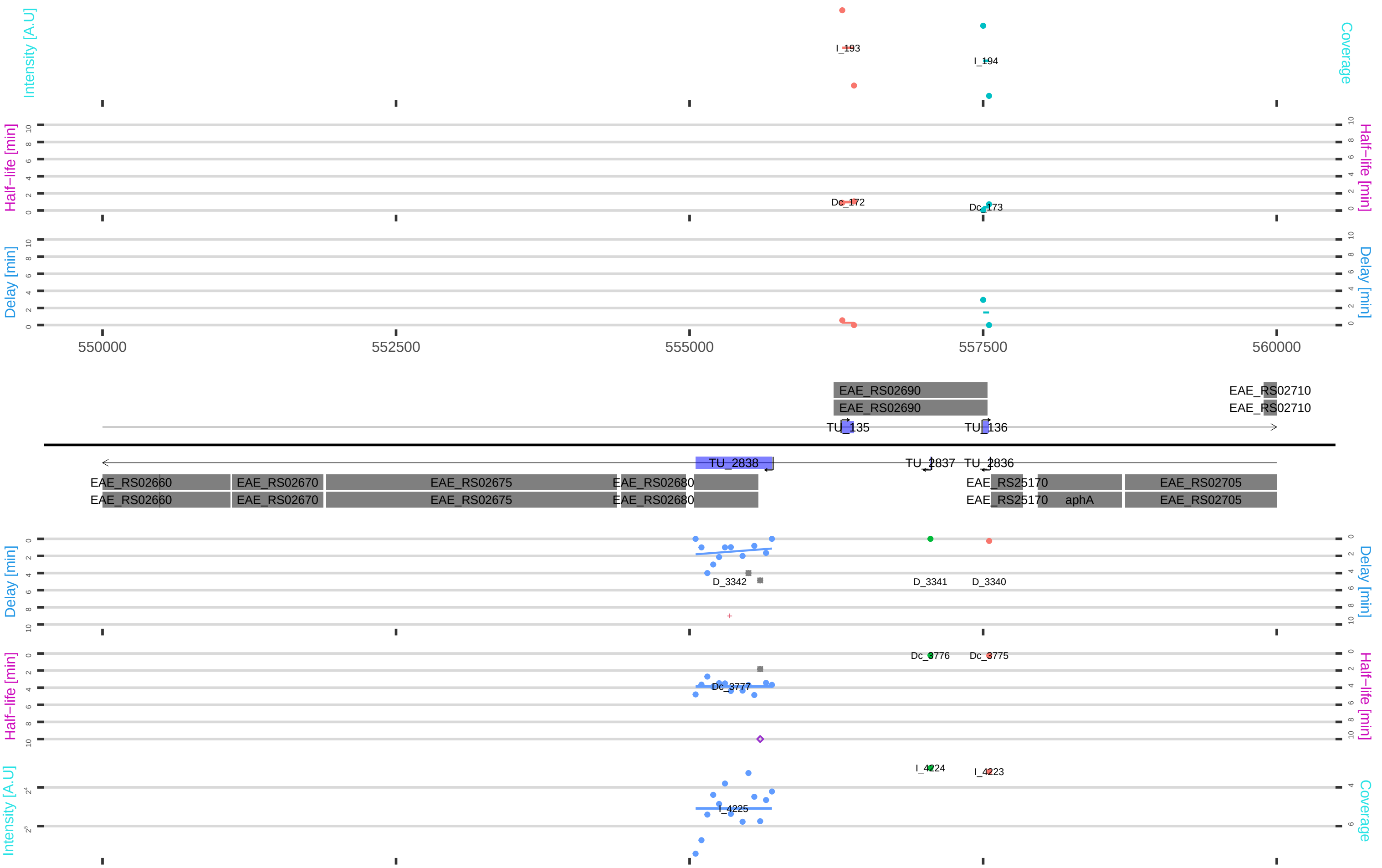

Term: termination (0), NS: new start (0), PS: pausing site (0), iTSS_I: internal starting site (0)

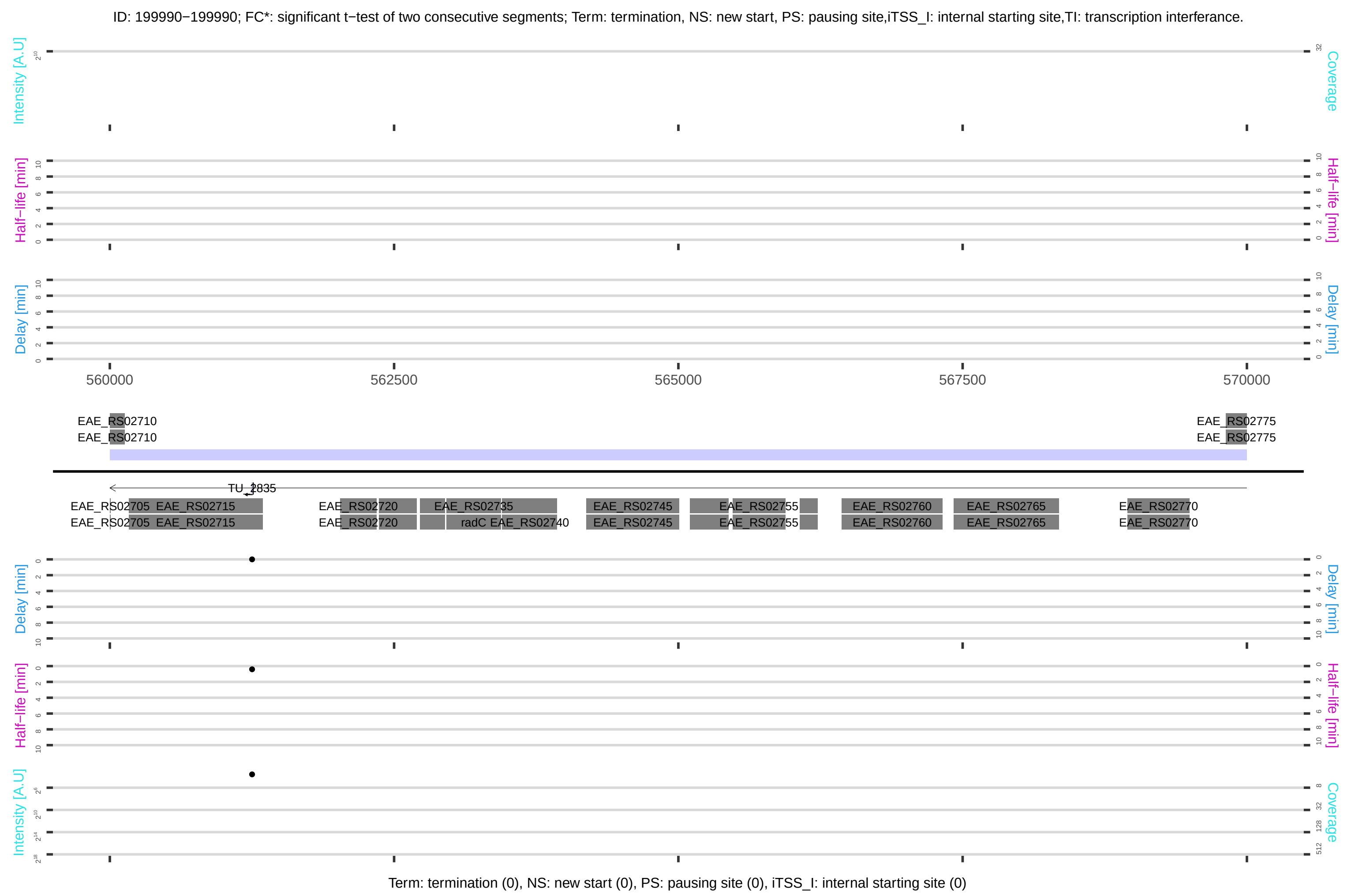
ID: 199990−199990; FC*: significant t−test of two consecutive segments; Term: termination, NS: new start, PS: pausing site,iTSS_I: internal starting site,TI: transcription interferance.
Intensity [A.U]
Coverage
Half−life [min]
Delay [min]
560000
562500
565000
567500
570000
EAE_RS02710
EAE_RS02775
TU_2835
EAE_RS02705
EAE_RS02715
EAE_RS02720
EAE_RS02735
radC
EAE_RS02740
EAE_RS02745
EAE_RS02755
EAE_RS02760
EAE_RS02765
EAE_RS02770
Term: termination (0), NS: new start (0), PS: pausing site (0), iTSS_I: internal starting site (0)

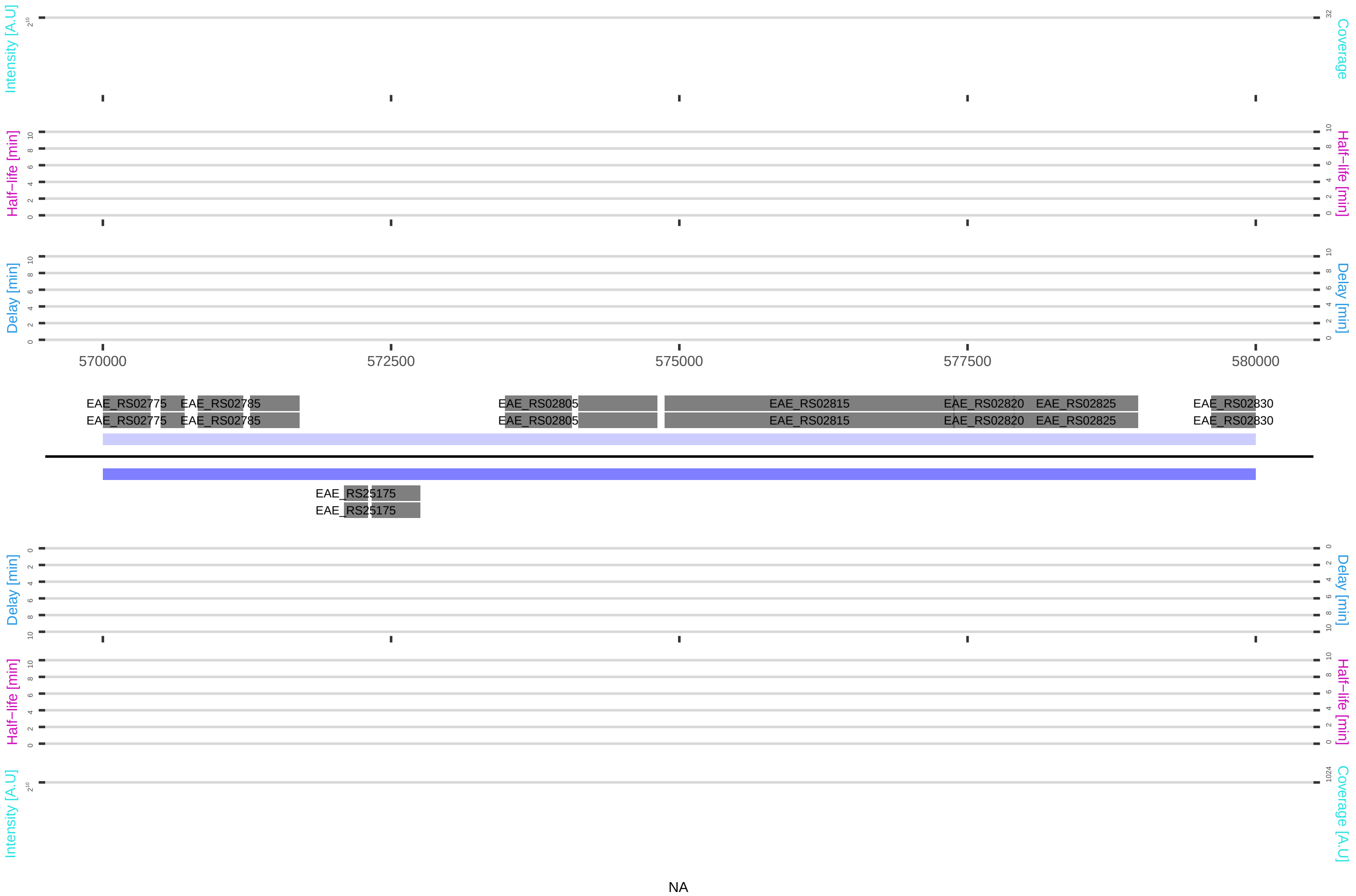
Intensity [A.U]
Coverage
Half−life [min]
Delay [min]
570000
572500
575000
577500
580000
EAE_RS02775
EAE_RS02785
EAE_RS02805
EAE_RS02815
EAE_RS02820
EAE_RS02825
EAE_RS02830
EAE_RS25175
Coverage [A.U]
NA

ID: 11696−11730; Term: termination (0), NS: new start (0), PS: pausing site (0), iTSS_I: internal starting site (0)

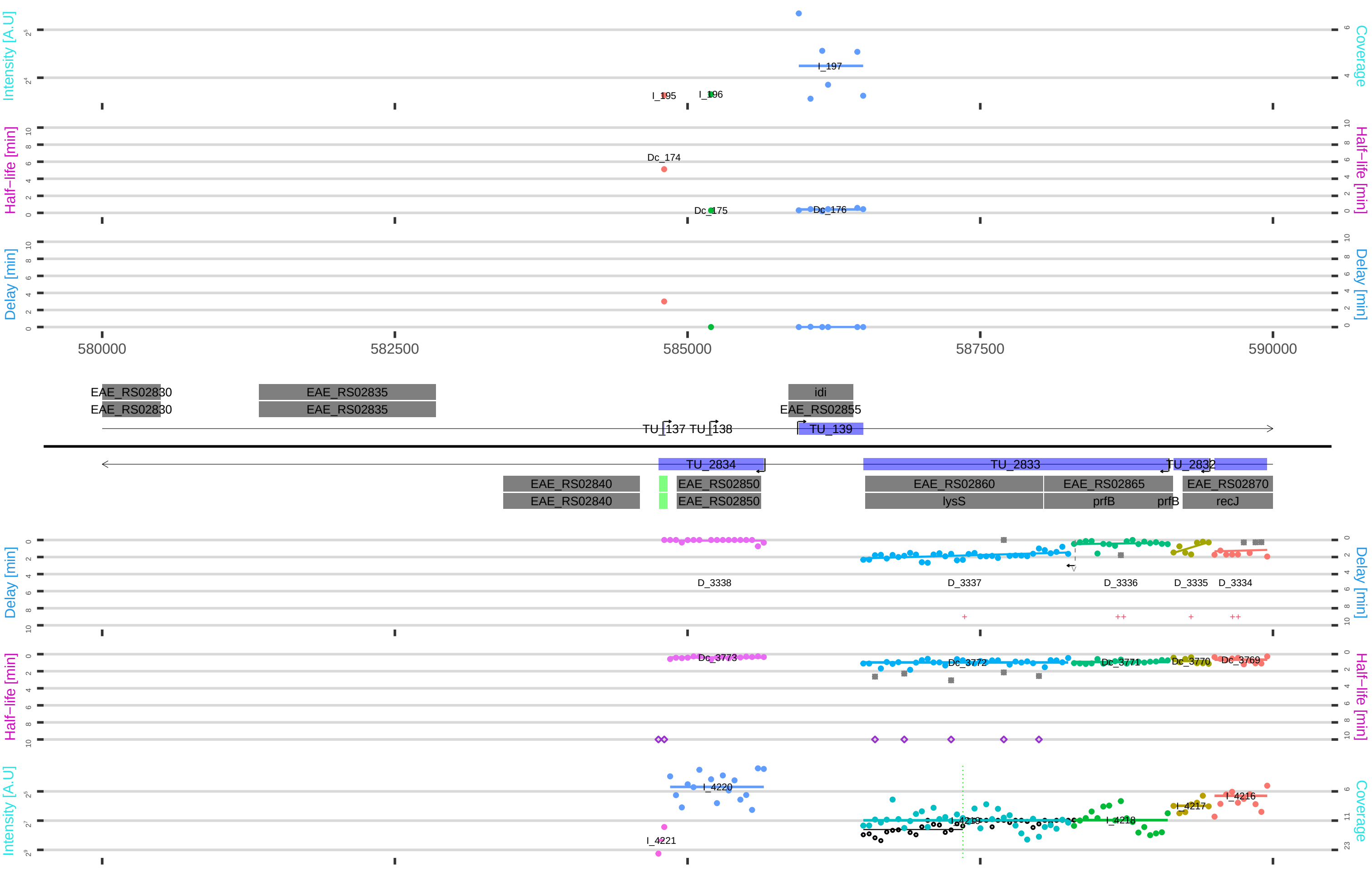

Term: termination (0), NS: new start (0), PS: pausing site (2), iTSS_I: internal starting site (0)

ID: 11855−11982; Term: termination (1), NS: new start (0), PS: pausing site (0), iTSS_I: internal starting site (0)

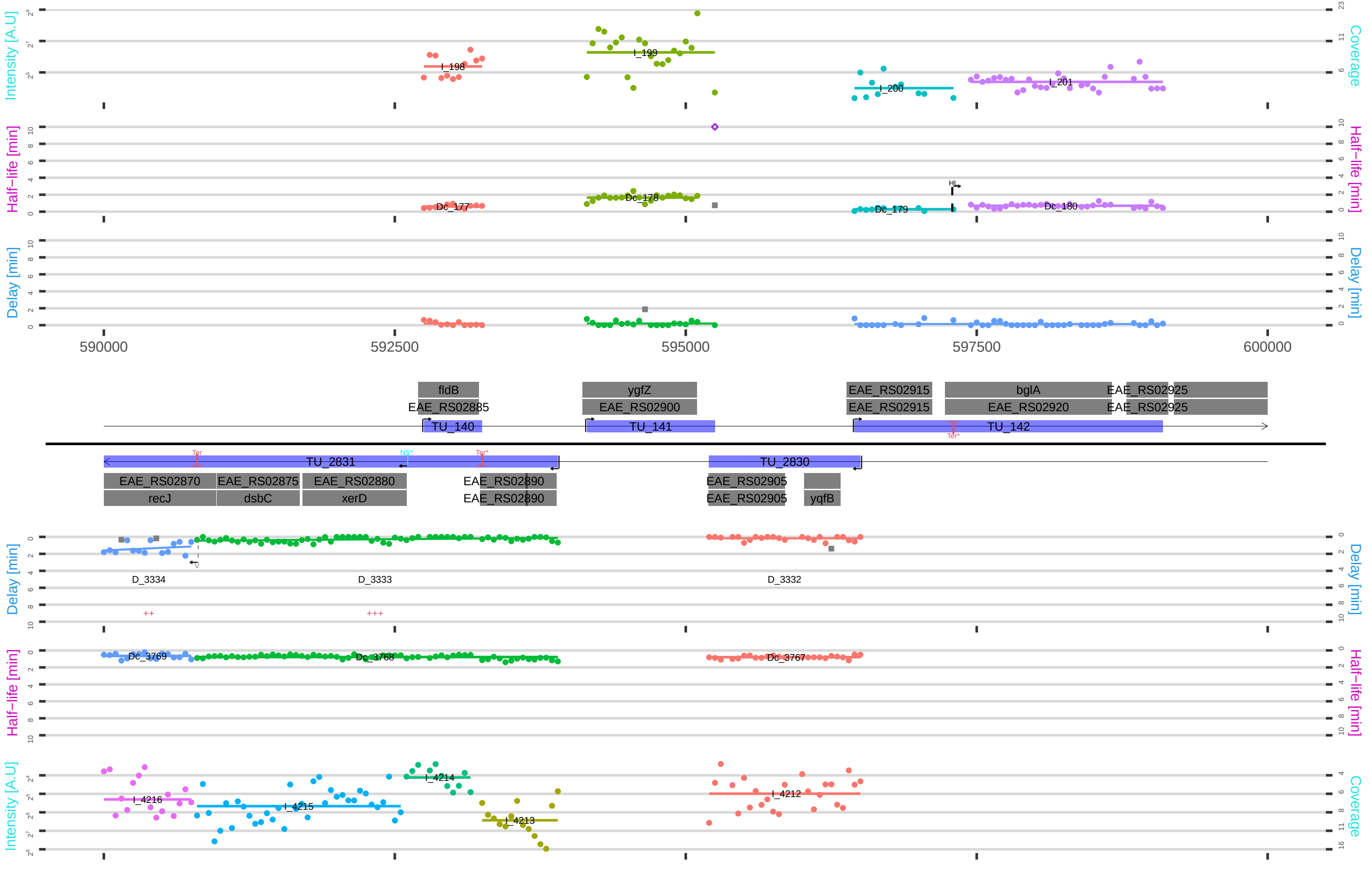

Term: termination (2), NS: new start (1), PS: pausing site (0), iTSS_I: internal starting site (0)

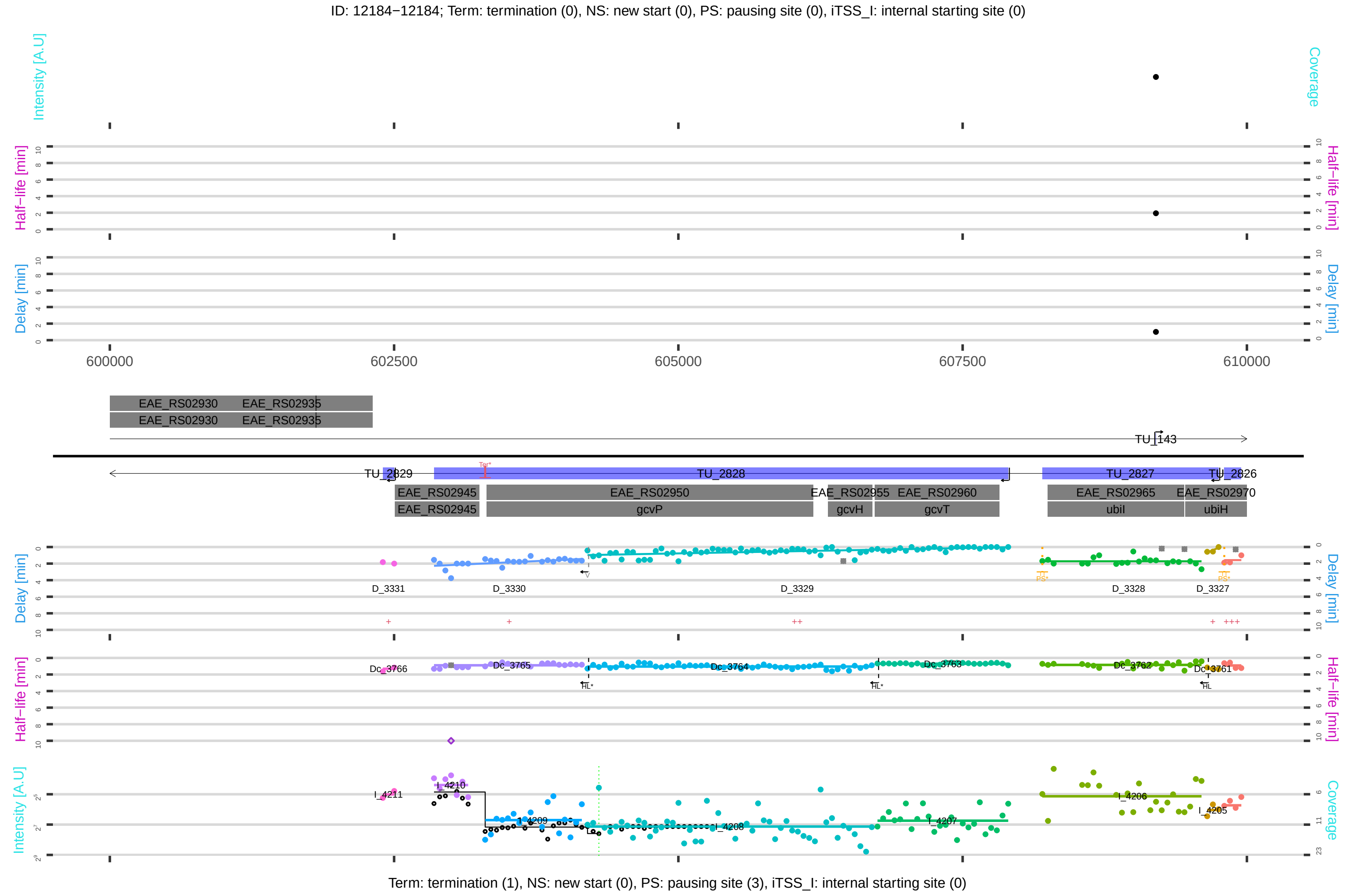

ID: 12184−12184; Term: termination (0), NS: new start (0), PS: pausing site (0), iTSS_I: internal starting site (0)
Intensity [A.U]
Coverage
Half−life [min]
Delay [min]
600000
602500
605000
607500
610000
EAE_RS02930
EAE_RS02935
TU_143
TU_2829
TU_2828
TU_2827
TU_2826
EAE_RS02945
EAE_RS02950
EAE_RS02955
EAE_RS02960
EAE_RS02965
EAE_RS02970
gcvP
gcvH
gcvT
ubiI
ubiH
D_3331
D_3330
D_3329
D_3328
D_3327
Dc_3766
Dc_3765
Dc_3764
Dc_3763
Dc_3762
Dc_3761
I_4211
I_4210
I_4209
I_4208
I_4207
I_4206
I_4205
Term: termination (1), NS: new start (0), PS: pausing site (3), iTSS_I: internal starting site (0)

ID: 12253−12346; Term: termination (0), NS: new start (0), PS: pausing site (0), iTSS_I: internal starting site (0)

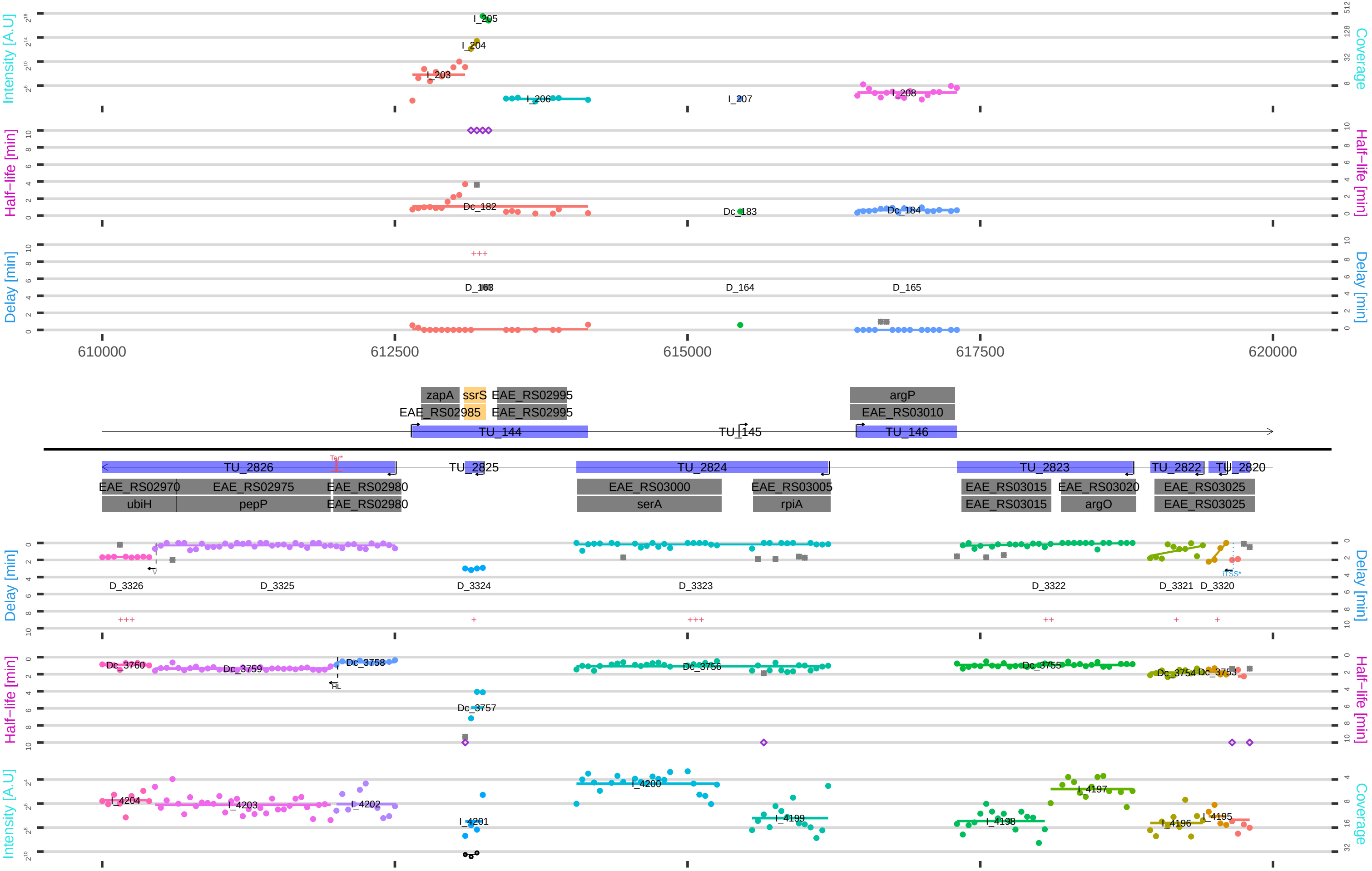

Term: termination (1), NS: new start (0), PS: pausing site (0), iTSS_I: internal starting site (1)

ID: 12404−12571; Term: termination (0), NS: new start (0), PS: pausing site (0), iTSS_I: internal starting site (0)

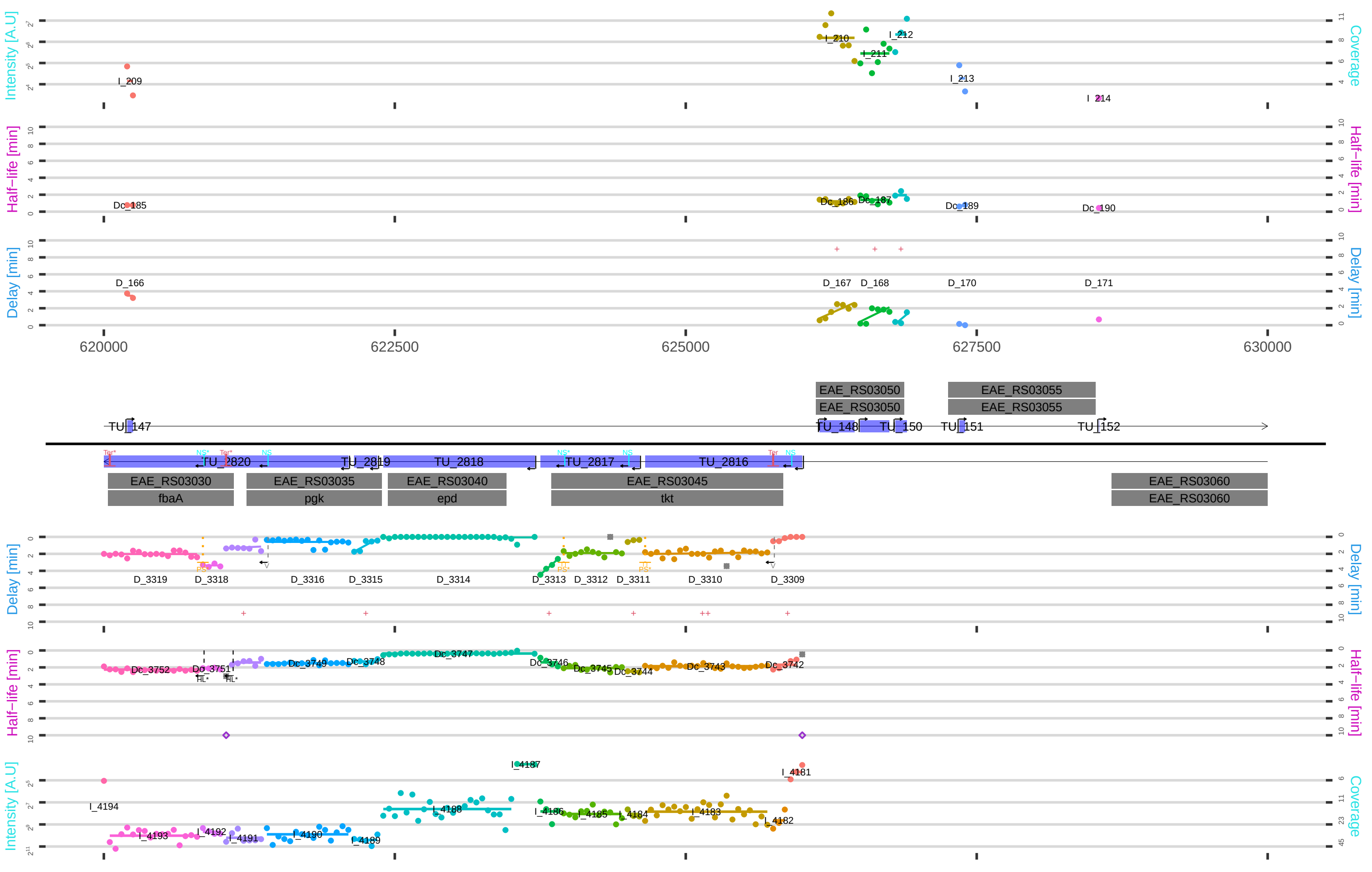

Term: termination (3), NS: new start (5), PS: pausing site (5), iTSS_I: internal starting site (0)

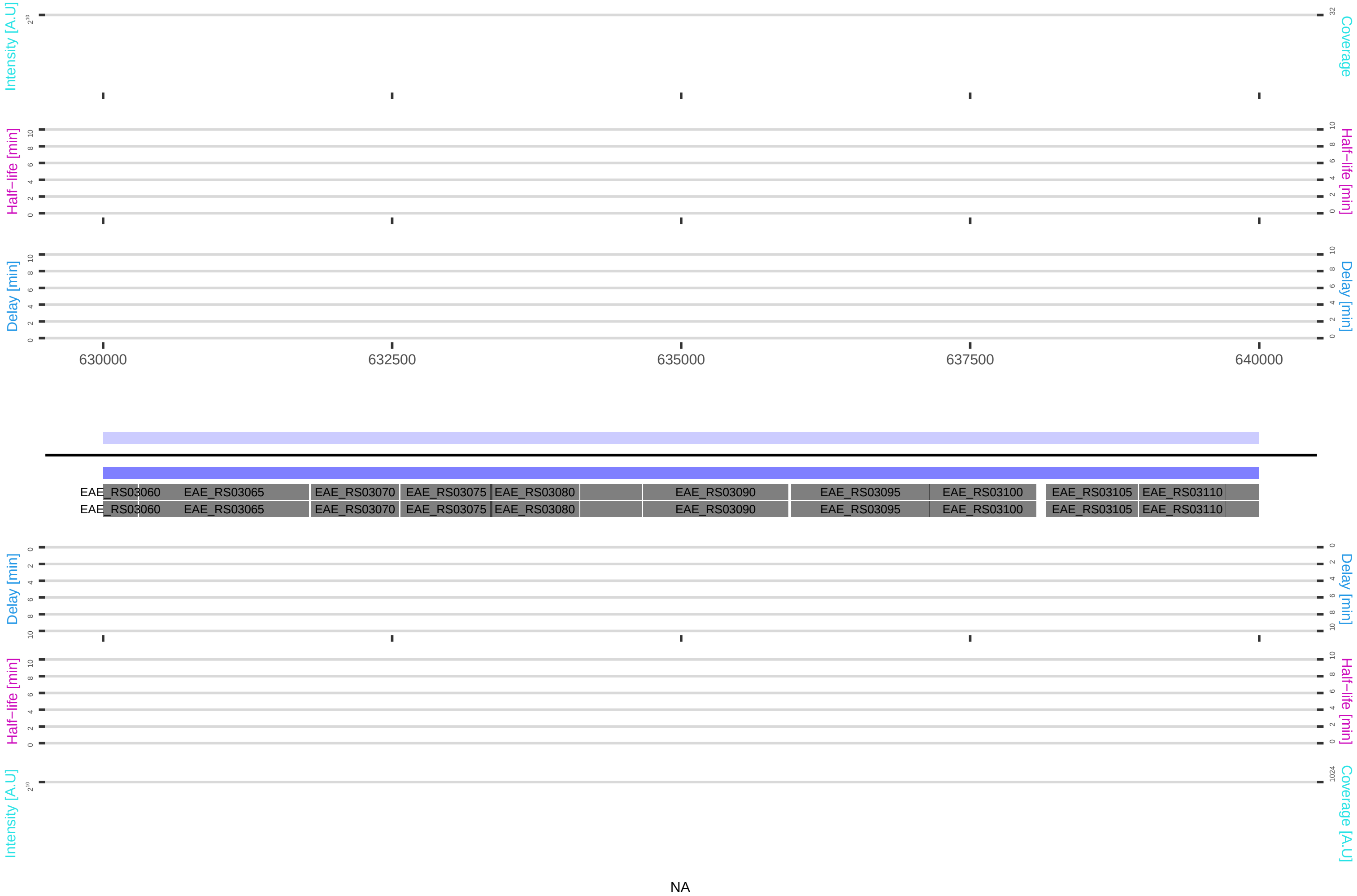
Intensity [A.U]
Coverage
Half−life [min]
Delay [min]
630000
632500
635000
637500
640000
EAE_RS03060
EAE_RS03065
EAE_RS03070
EAE_RS03075
EAE_RS03080
EAE_RS03090
EAE_RS03095
EAE_RS03100
EAE_RS03105
EAE_RS03110
Coverage [A.U]
NA

ID: 12862−13000; Term: termination (0), NS: new start (0), PS: pausing site (0), iTSS_I: internal starting site (0)

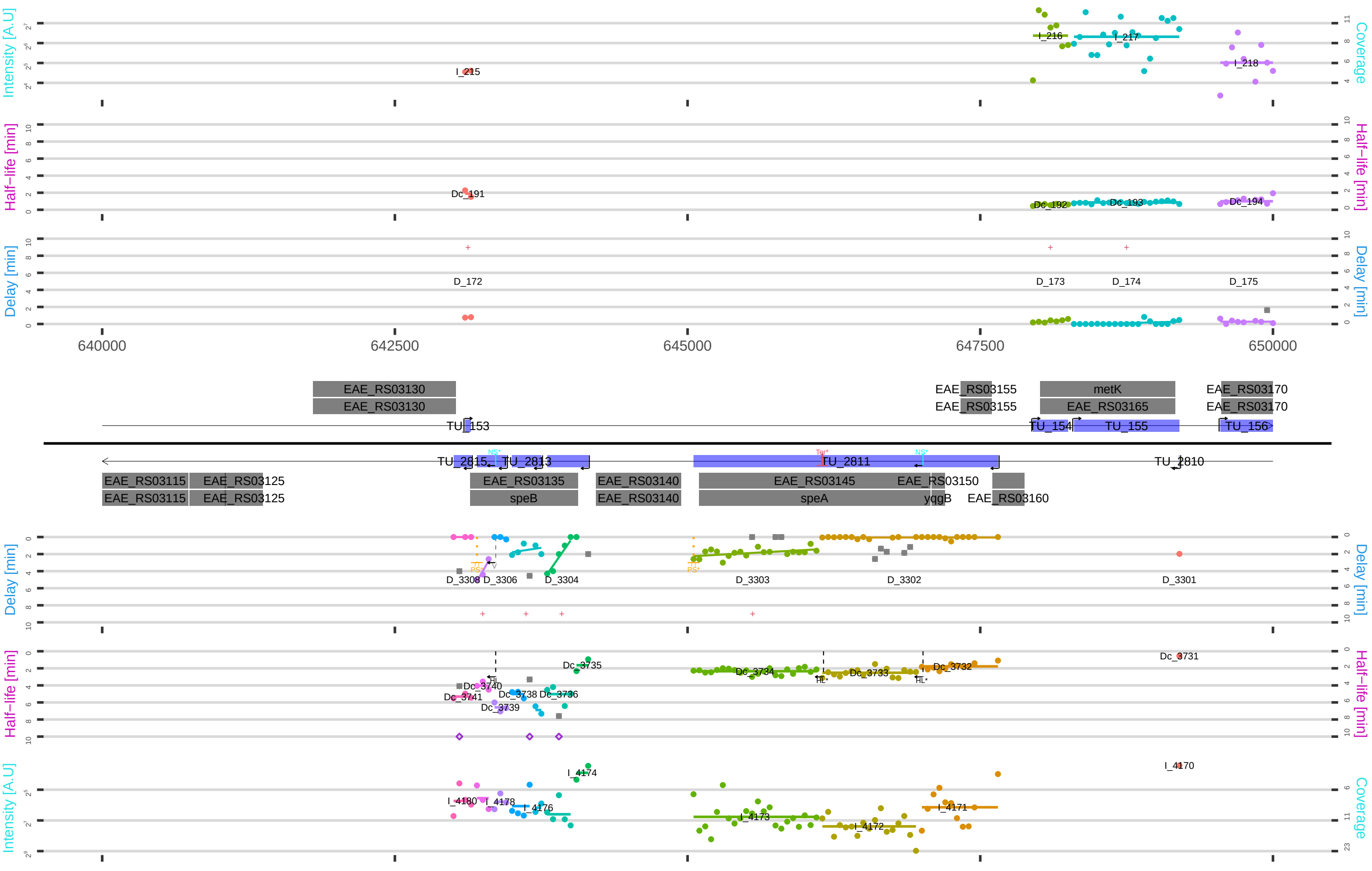

Term: termination (1), NS: new start (2), PS: pausing site (2), iTSS_I: internal starting site (0)

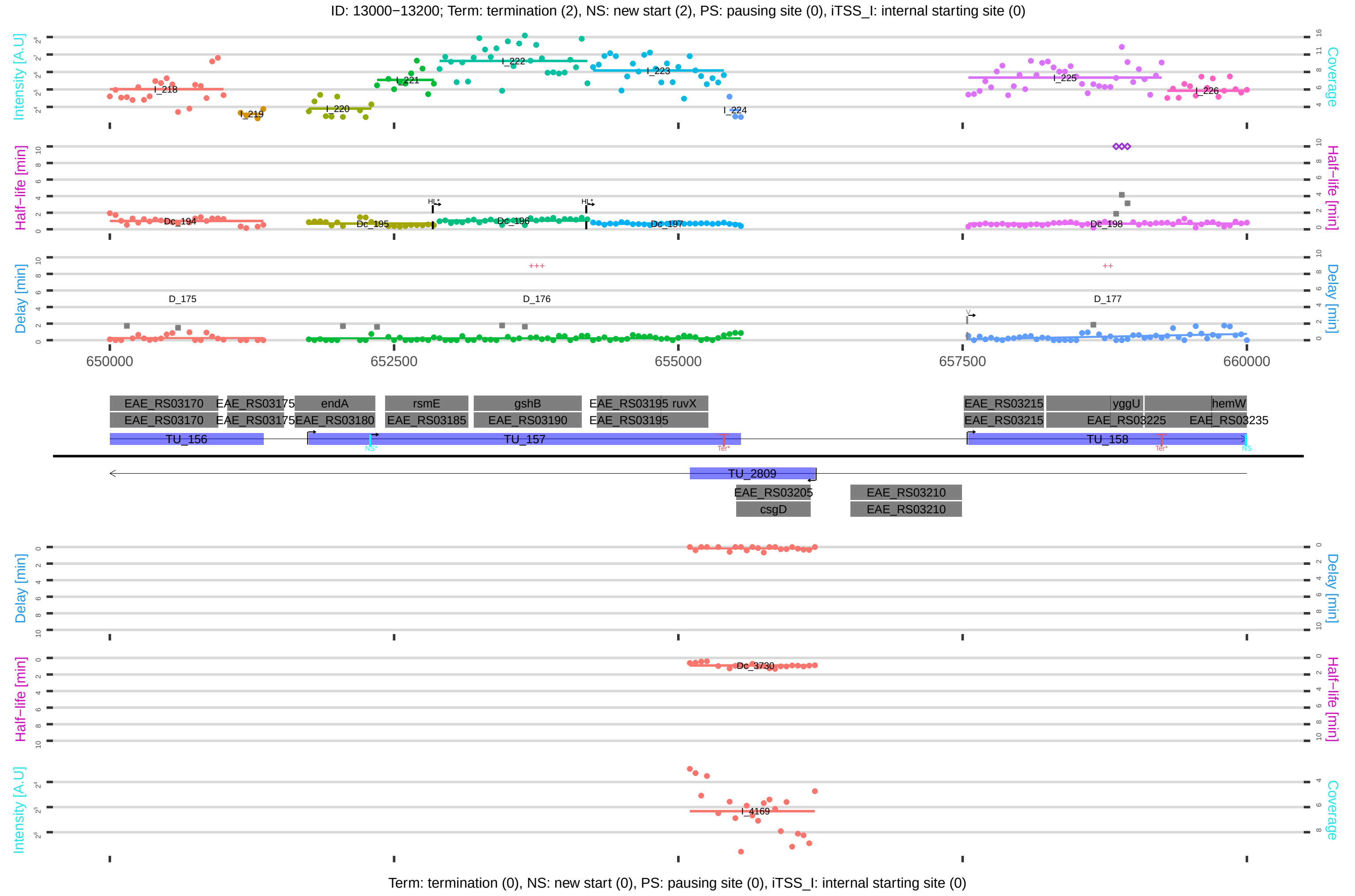

ID: 13000−13200; Term: termination (2), NS: new start (2), PS: pausing site (0), iTSS_I: internal starting site (0)
Intensity [A.U]
Coverage
Half−life [min]
Delay [min]
I_218
I_219
I_220
I_221
I_222
I_223
I_224
I_225
I_226
Dc_194
Dc_195
Dc_196
Dc_197
Dc_198
D_175
D_176
D_177
650000
652500
655000
657500
660000
EAE_RS03170
EAE_RS03175
endA
EAE_RS03180
rsmE
EAE_RS03185
gshB
EAE_RS03190
EAE_RS03195
ruvX
EAE_RS03215
yggU
EAE_RS03225
hemW
EAE_RS03235
TU_156
TU_157
TU_158
TU_2809
EAE_RS03205
csgD
EAE_RS03210
Dc_3730
I_4169
Term: termination (0), NS: new start (0), PS: pausing site (0), iTSS_I: internal starting site (0)

ID: 13200−13400; Term: termination (0), NS: new start (1), PS: pausing site (1), iTSS_I: internal starting site (0)

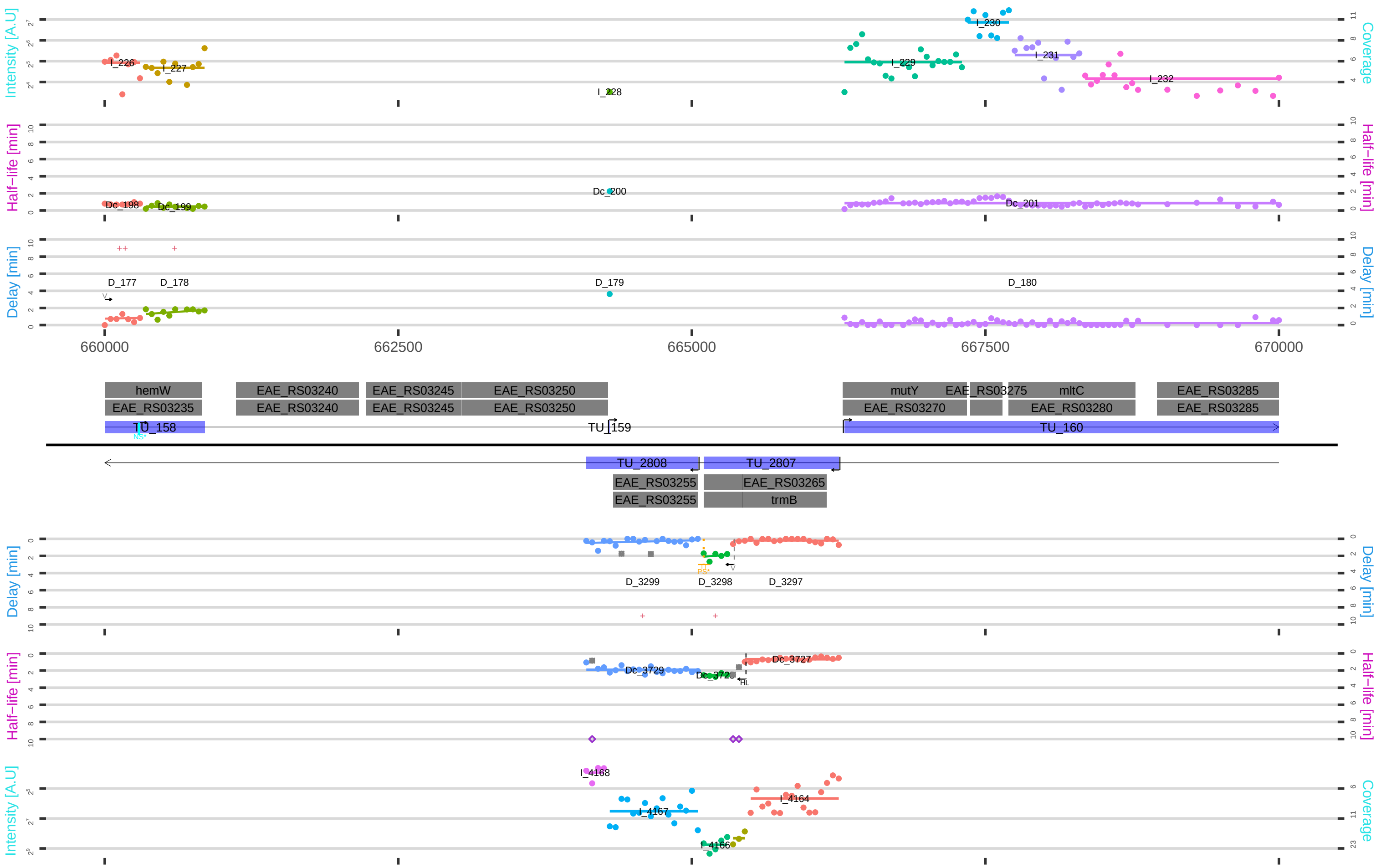

Term: termination (0), NS: new start (0), PS: pausing site (1), iTSS_I: internal starting site (0)

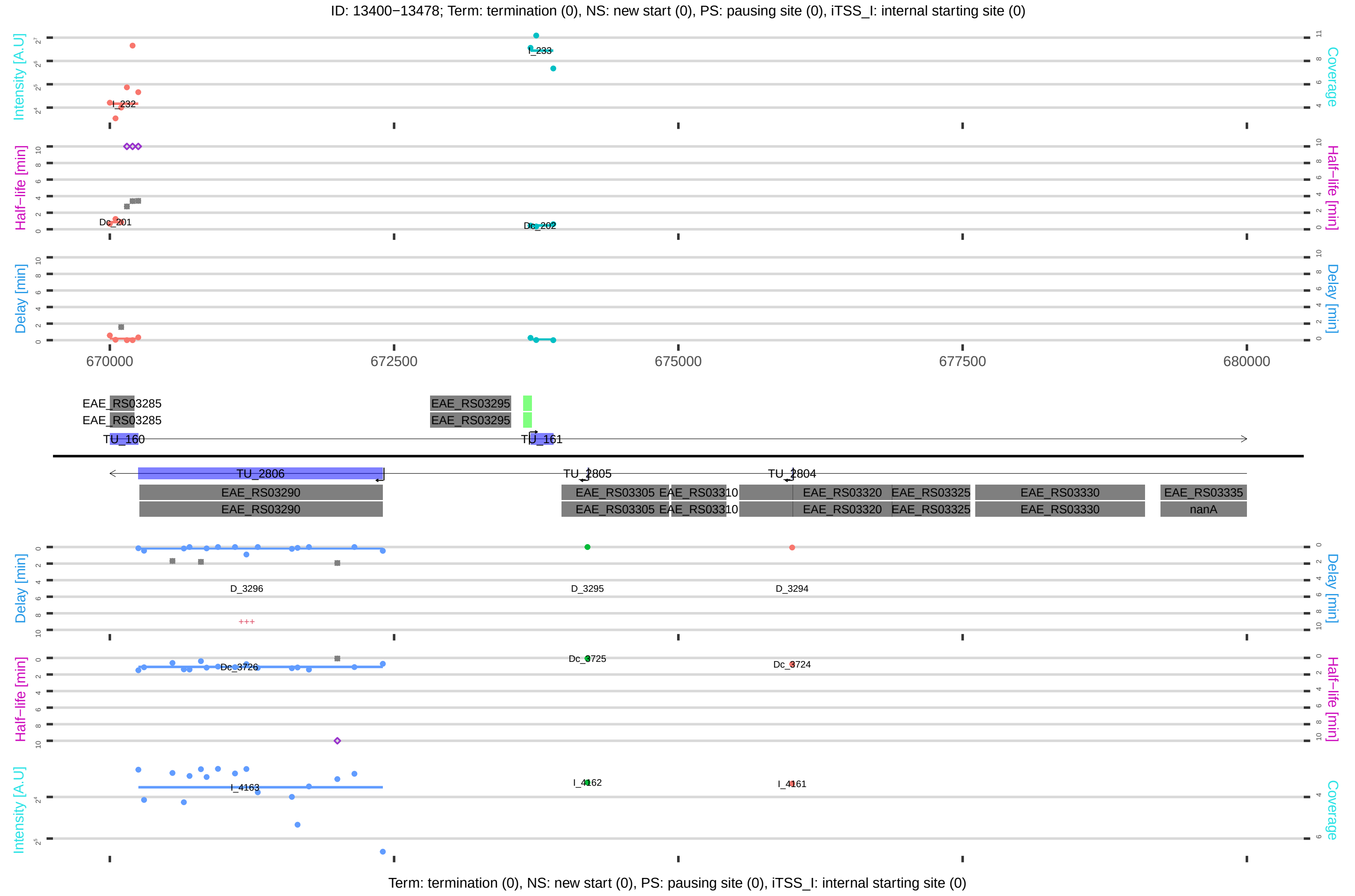

ID: 13400−13478; Term: termination (0), NS: new start (0), PS: pausing site (0), iTSS_I: internal starting site (0)
Intensity [A.U]
Coverage
I_233
I_232
Half−life [min]
Dc_201
Dc_202
Delay [min]
670000
672500
675000
677500
680000
EAE_RS03285
EAE_RS03295
TU_160
TU_161
TU_2806
TU_2805
TU_2804
EAE_RS03290
EAE_RS03305
EAE_RS03310
EAE_RS03320
EAE_RS03325
EAE_RS03330
EAE_RS03335
nanA
D_3296
D_3295
D_3294
Dc_3726
Dc_3725
Dc_3724
I_4163
I_4162
I_4161
Term: termination (0), NS: new start (0), PS: pausing site (0), iTSS_I: internal starting site (0)

ID: 13630−13780; Term: termination (0), NS: new start (0), PS: pausing site (0), iTSS_I: internal starting site (0)

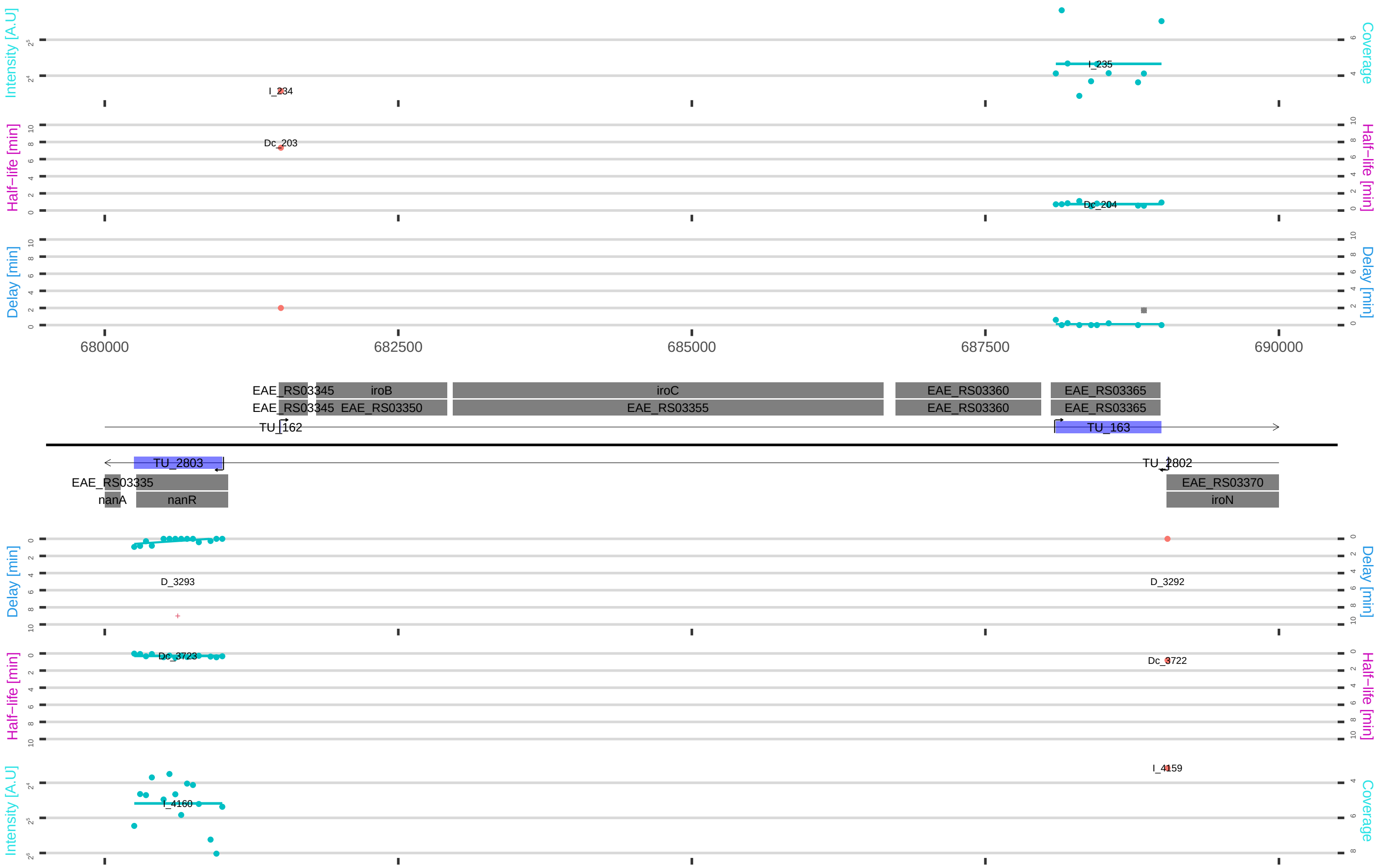

Term: termination (0), NS: new start (0), PS: pausing site (0), iTSS_I: internal starting site (0)

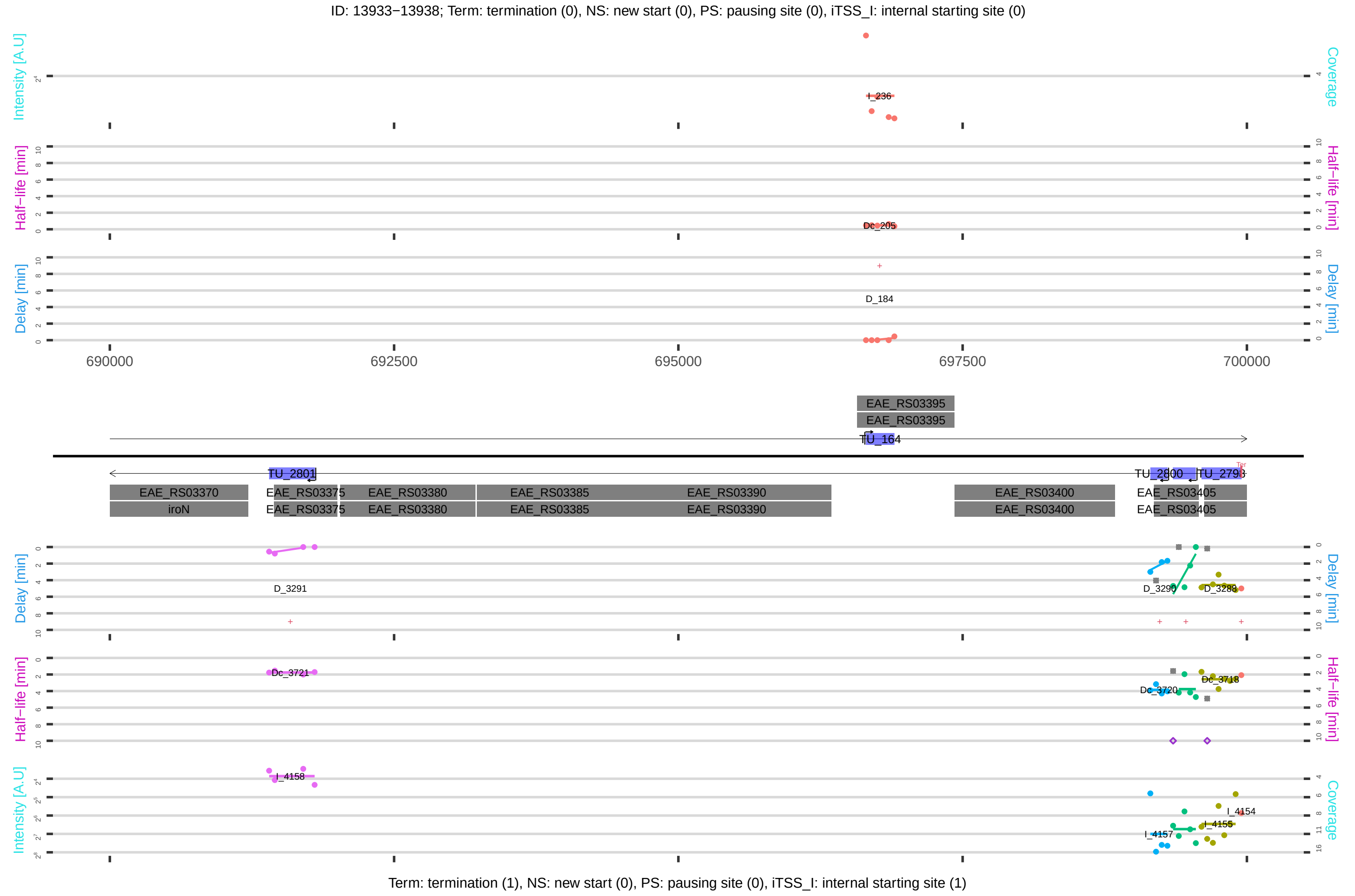

ID: 13933−13938; Term: termination (0), NS: new start (0), PS: pausing site (0), iTSS_I: internal starting site (0)
Intensity [A.U]
Coverage
I_236
Half−life [min]
Dc_205
Delay [min]
D_184
690000
692500
695000
697500
700000
EAE_RS03395
EAE_RS03395
TU_164
TU_2801
TU_2800
TU_2798
EAE_RS03370
iroN
EAE_RS03375
EAE_RS03375
EAE_RS03380
EAE_RS03380
EAE_RS03385
EAE_RS03385
EAE_RS03390
EAE_RS03390
EAE_RS03400
EAE_RS03400
EAE_RS03405
EAE_RS03405
D_3291
D_3290
D_3288
Dc_3721
Dc_3720
Dc_3718
I_4158
I_4154
I_4155
I_4157
Term: termination (1), NS: new start (0), PS: pausing site (0), iTSS_I: internal starting site (1)

ID: 197215−197047; FC*: significant t−test of two consecutive segments; Term: termination, NS: new start, PS: pausing site,iTSS_I: internal starting site,TI: transcription interferance.

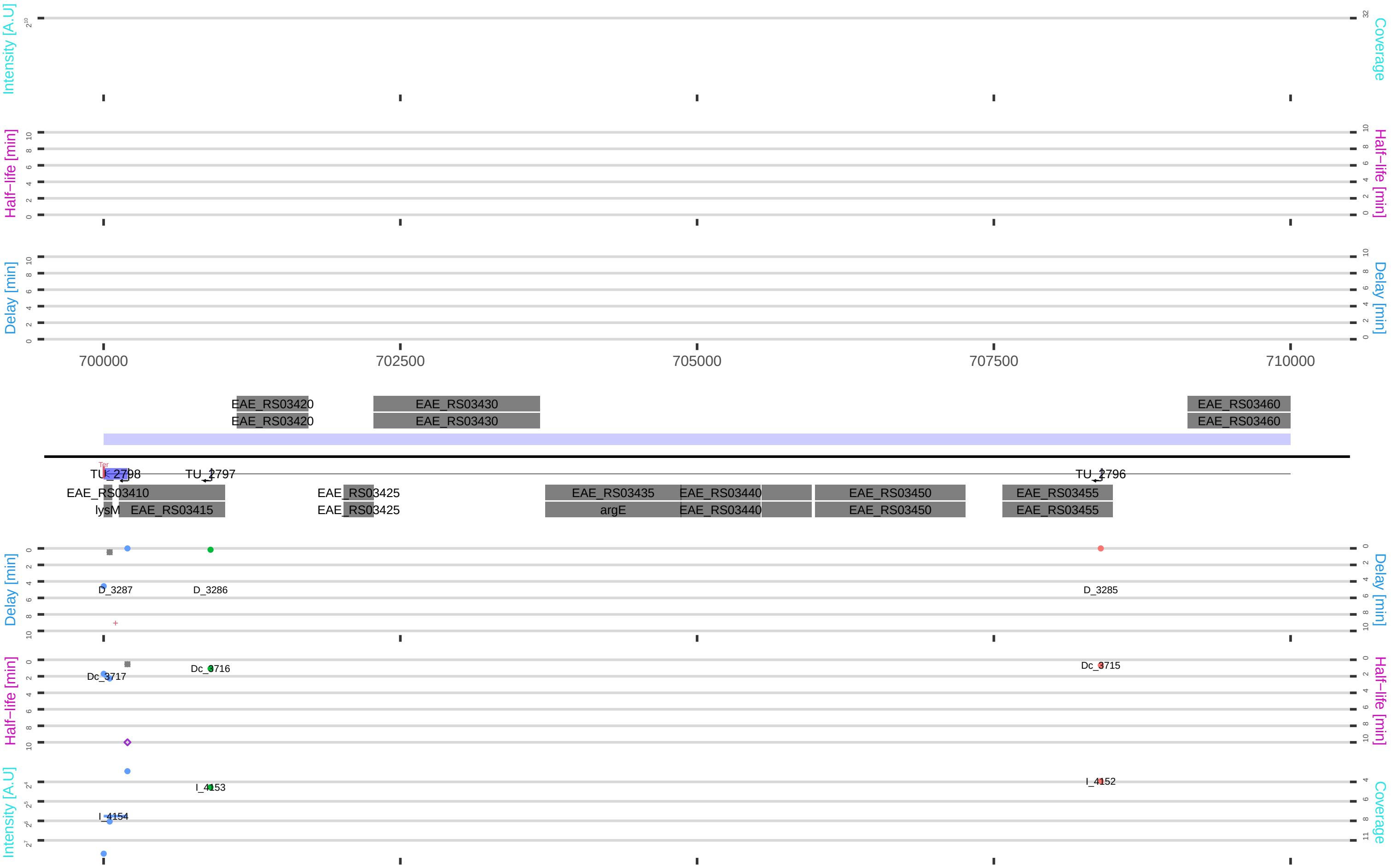

Term: termination (1), NS: new start (0), PS: pausing site (0), iTSS_I: internal starting site (0)

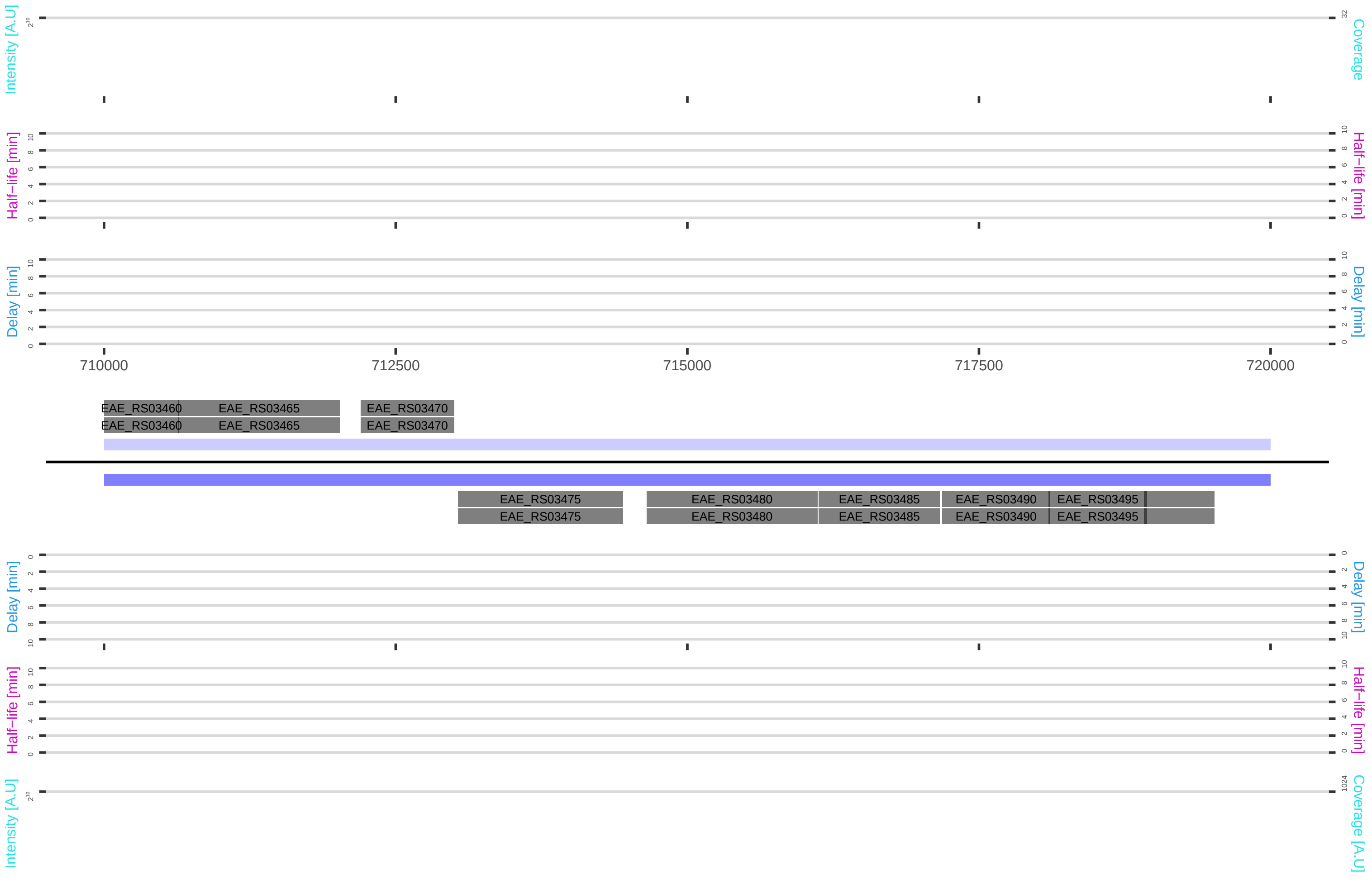
Intensity [A.U]
Coverage
Half−life [min]
Delay [min]
710000
712500
715000
717500
720000
EAE_RS03460
EAE_RS03465
EAE_RS03470
EAE_RS03475
EAE_RS03480
EAE_RS03485
EAE_RS03490
EAE_RS03495
Coverage [A.U]
NA

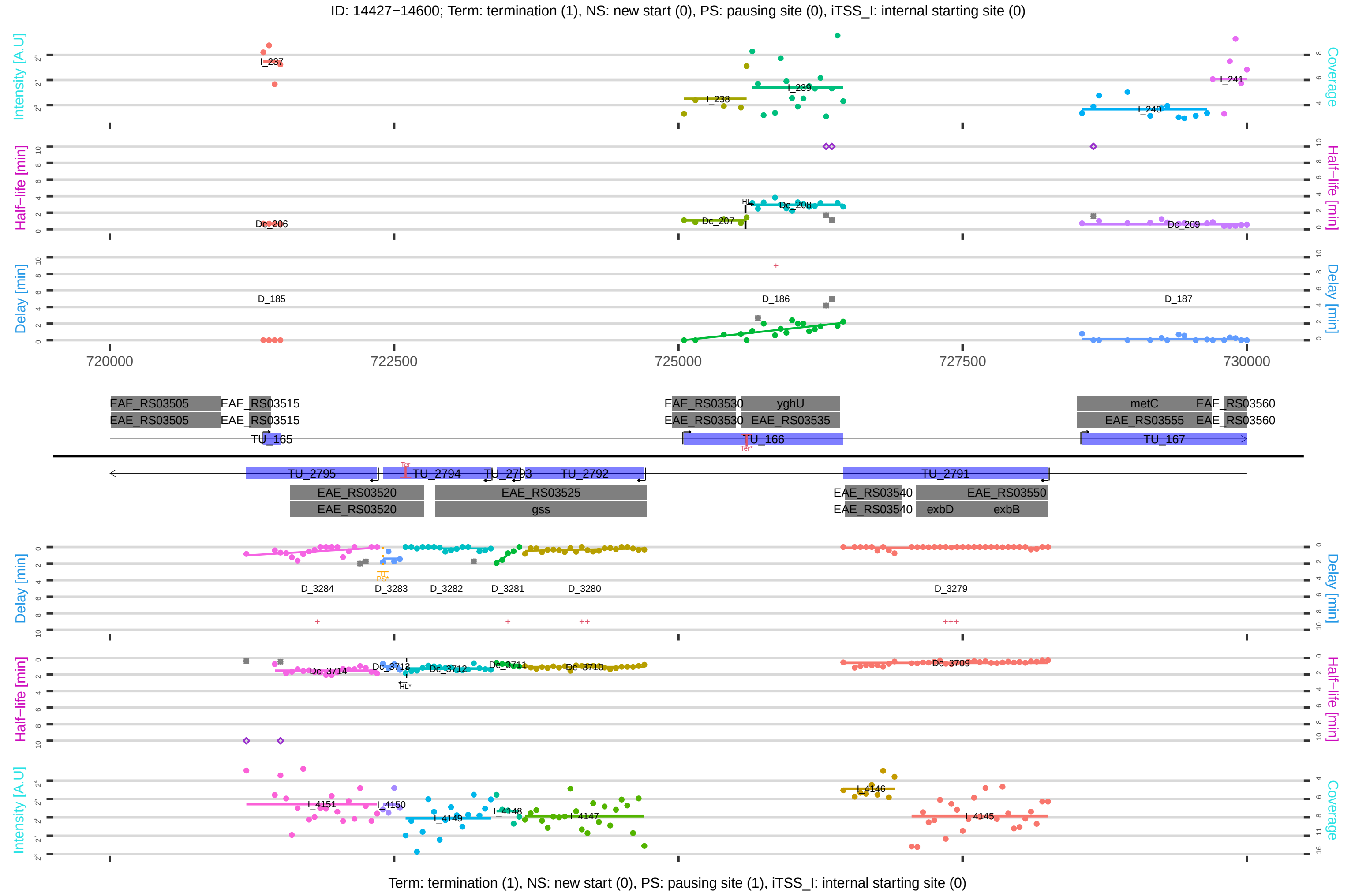

ID: 14427−14600; Term: termination (1), NS: new start (0), PS: pausing site (0), iTSS_I: internal starting site (0)
Intensity [A.U]
Coverage
Half−life [min]
Delay [min]
720000
722500
725000
727500
730000
EAE_RS03505
EAE_RS03515
EAE_RS03530
yghU
EAE_RS03535
metC
EAE_RS03555
EAE_RS03560
TU_165
TU_166
TU_167
TU_2795
TU_2794
TU_2793
TU_2792
TU_2791
EAE_RS03520
EAE_RS03525
gss
EAE_RS03540
exbD
EAE_RS03550
exbB
Term: termination (1), NS: new start (0), PS: pausing site (1), iTSS_I: internal starting site (0)

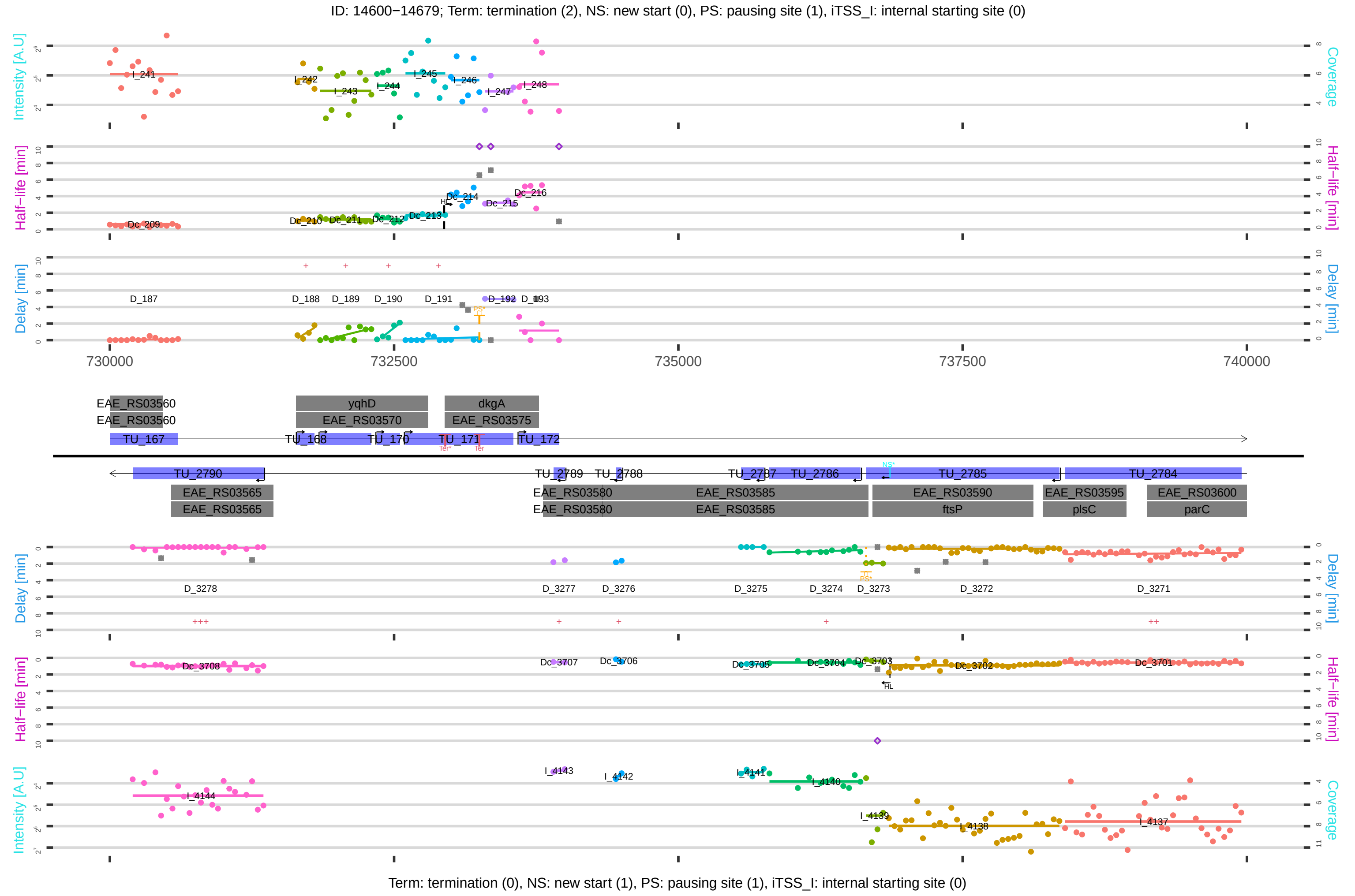
ID: 14600−14679; Term: termination (2), NS: new start (0), PS: pausing site (1), iTSS_I: internal starting site (0)
Intensity [A.U]
Coverage
Half−life [min]
Delay [min]
730000
732500
735000
737500
740000
EAE_RS03560
yqhD
EAE_RS03570
dkgA
EAE_RS03575
TU_167
TU_168
TU_170
TU_171
TU_172
TU_2790
TU_2789
TU_2788
TU_2787
TU_2786
TU_2785
TU_2784
EAE_RS03565
EAE_RS03580
EAE_RS03585
EAE_RS03590
ftsP
EAE_RS03595
plsC
EAE_RS03600
parC
Term: termination (0), NS: new start (1), PS: pausing site (1), iTSS_I: internal starting site (0)

ID: 14861−14922; Term: termination (0), NS: new start (1), PS: pausing site (1), iTSS_I: internal starting site (0)

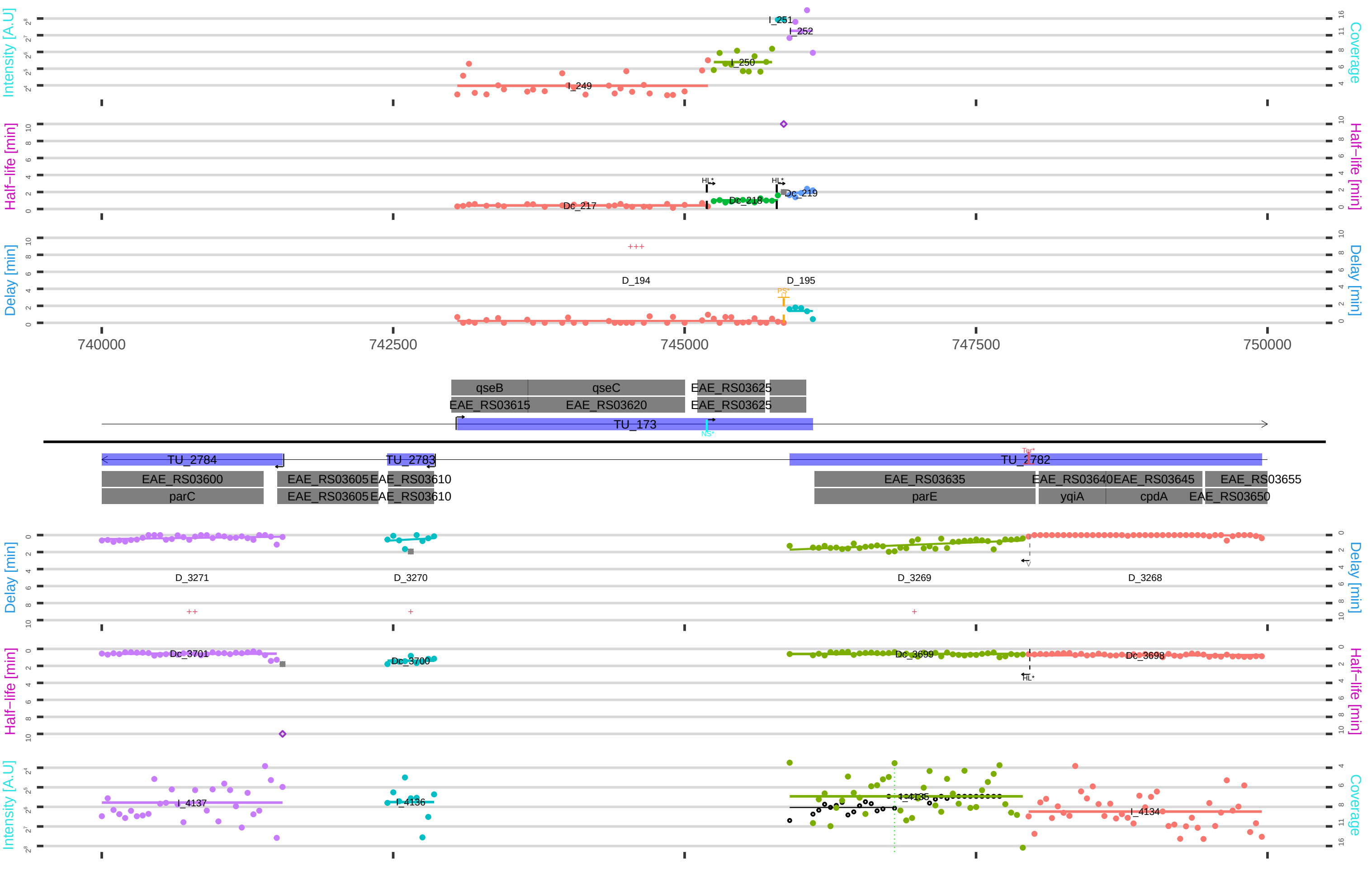

Term: termination (1), NS: new start (0), PS: pausing site (1), iTSS_I: internal starting site (0)

ID: 15012−15134; Term: termination (0), NS: new start (0), PS: pausing site (0), iTSS_I: internal starting site (0)

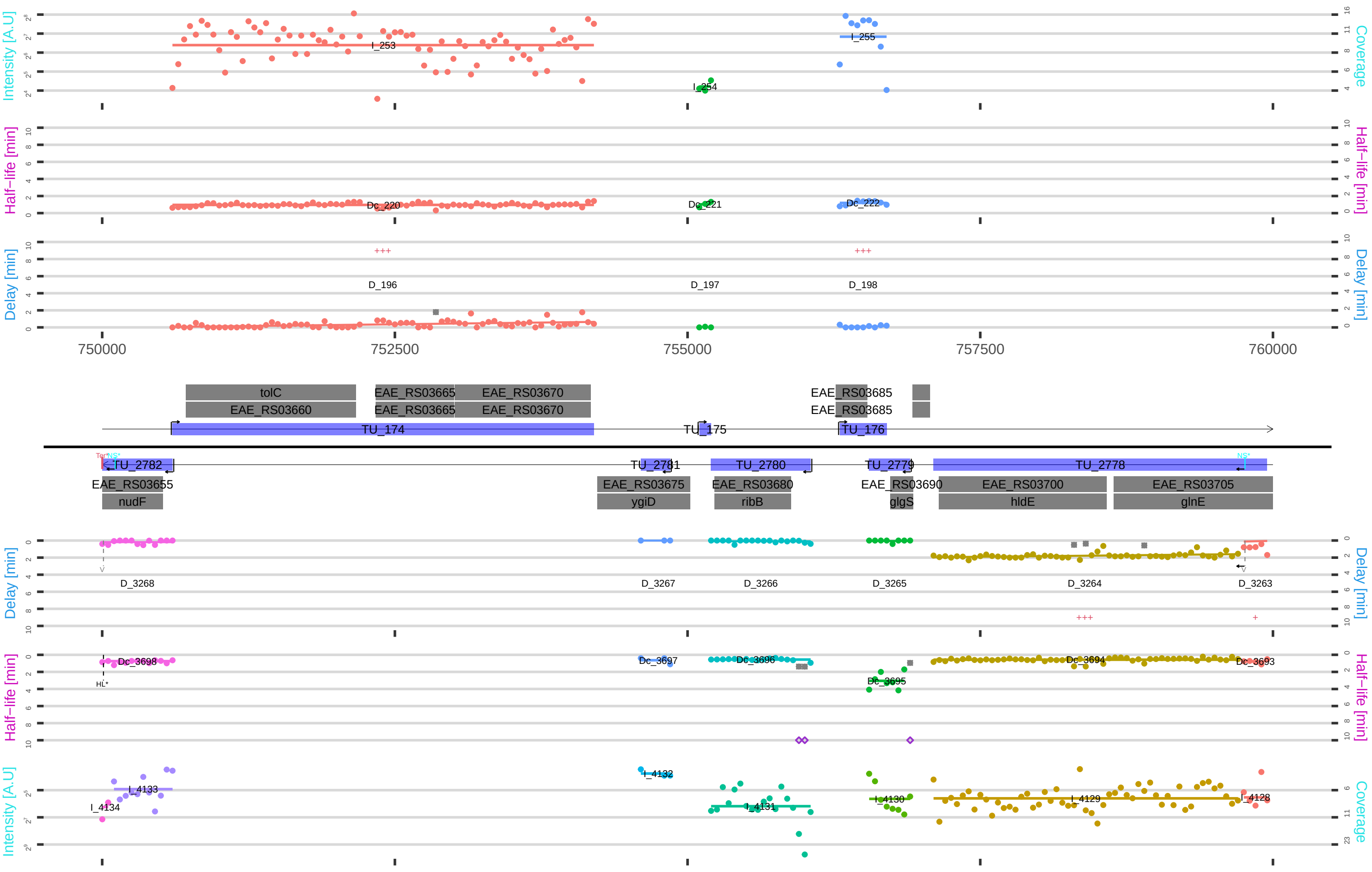

Term: termination (1), NS: new start (2), PS: pausing site (1), iTSS_I: internal starting site (0)

ID: 15260−15342; Term: termination (0), NS: new start (0), PS: pausing site (0), iTSS_I: internal starting site (0)

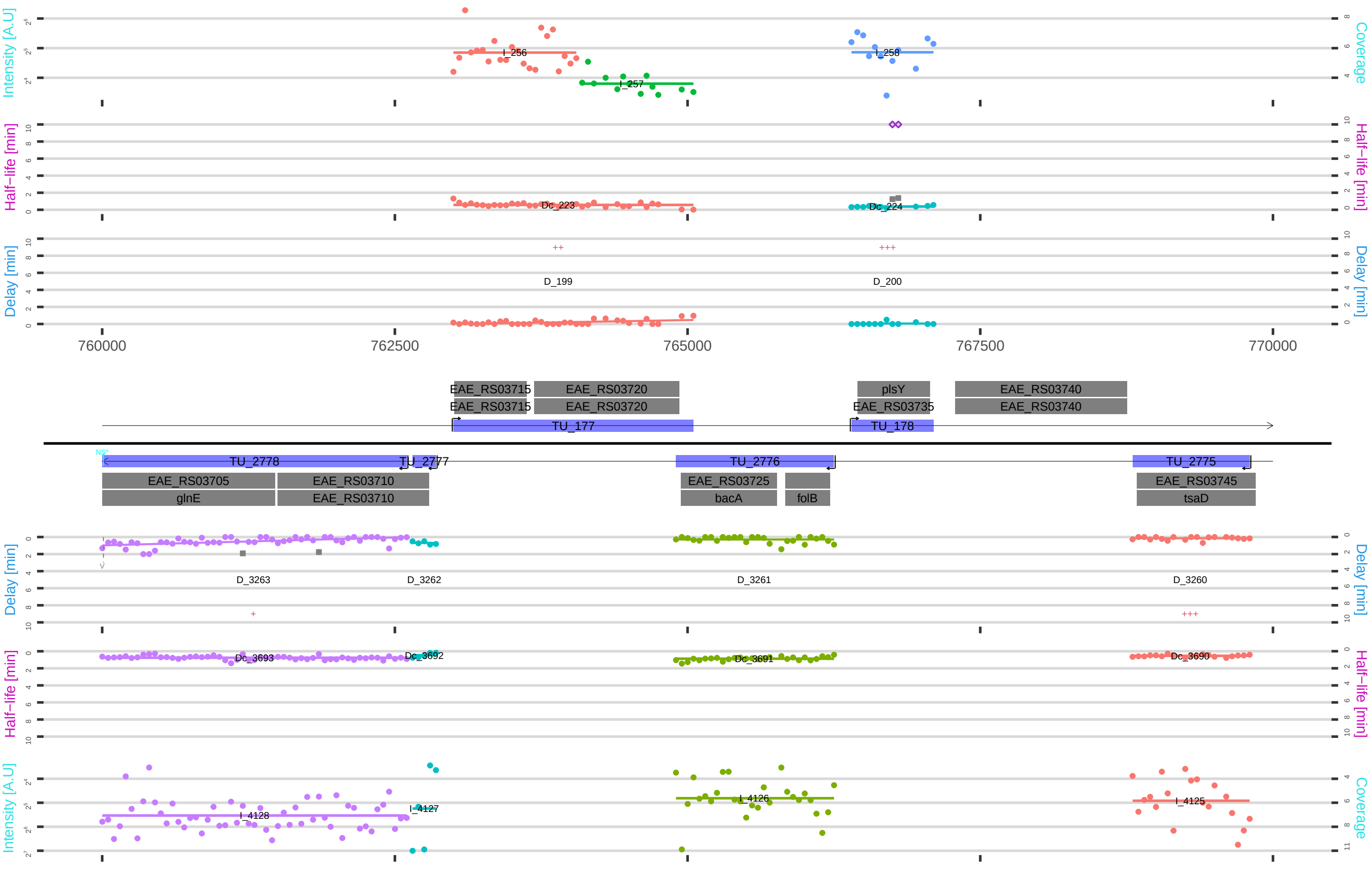

Term: termination (0), NS: new start (1), PS: pausing site (0), iTSS_I: internal starting site (0)

ID: 15401−15500; Term: termination (1), NS: new start (1), PS: pausing site (1), iTSS_I: internal starting site (0)

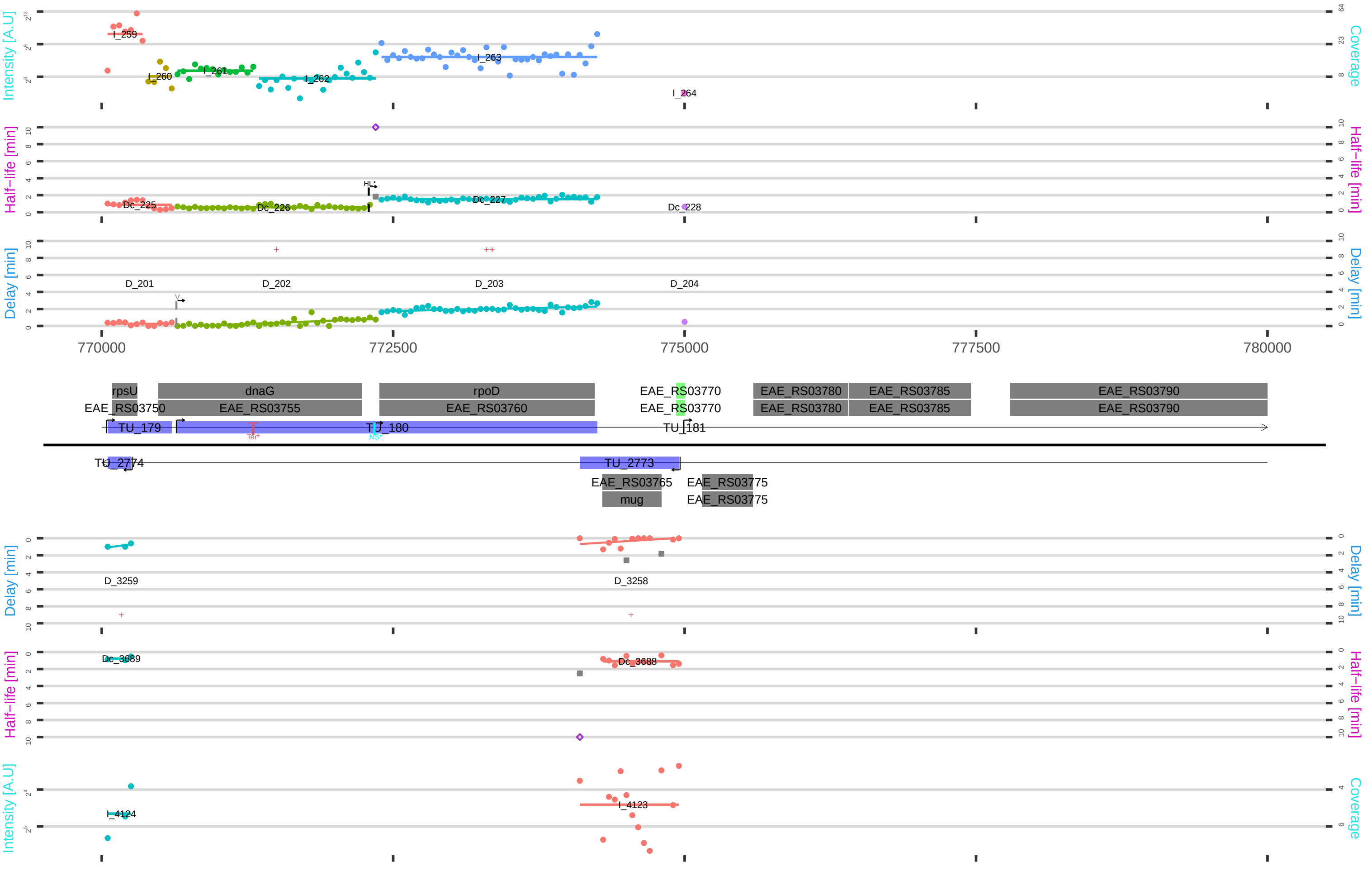

Term: termination (0), NS: new start (0), PS: pausing site (0), iTSS_I: internal starting site (0)

ID: 195547−195498; FC*: significant t−test of two consecutive segments; Term: termination, NS: new start, PS: pausing site,iTSS_I: internal starting site,TI: transcription interferance.

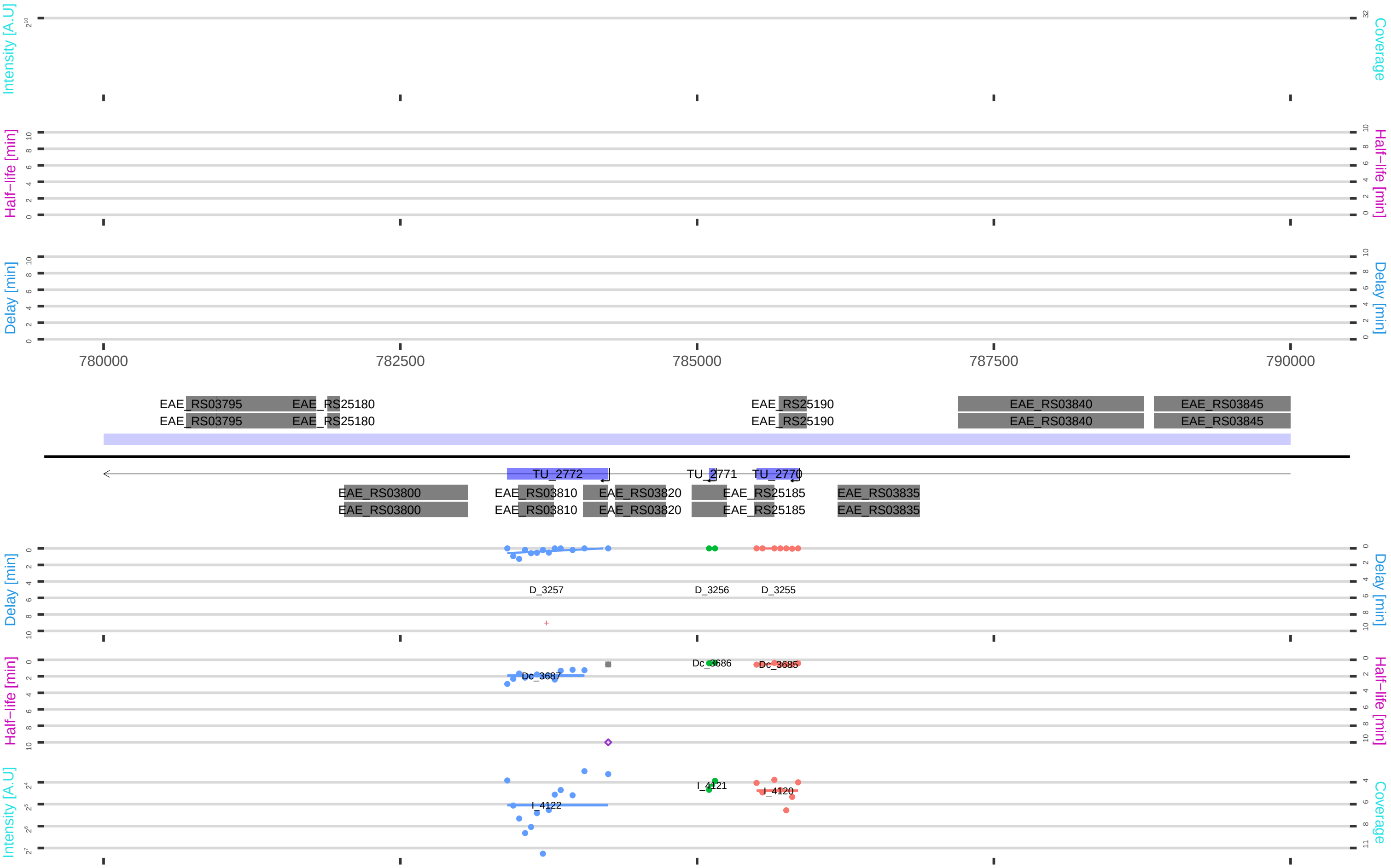

Term: termination (0), NS: new start (0), PS: pausing site (0), iTSS_I: internal starting site (0)

ID: 15897−15917; Term: termination (0), NS: new start (0), PS: pausing site (0), iTSS_I: internal starting site (0)

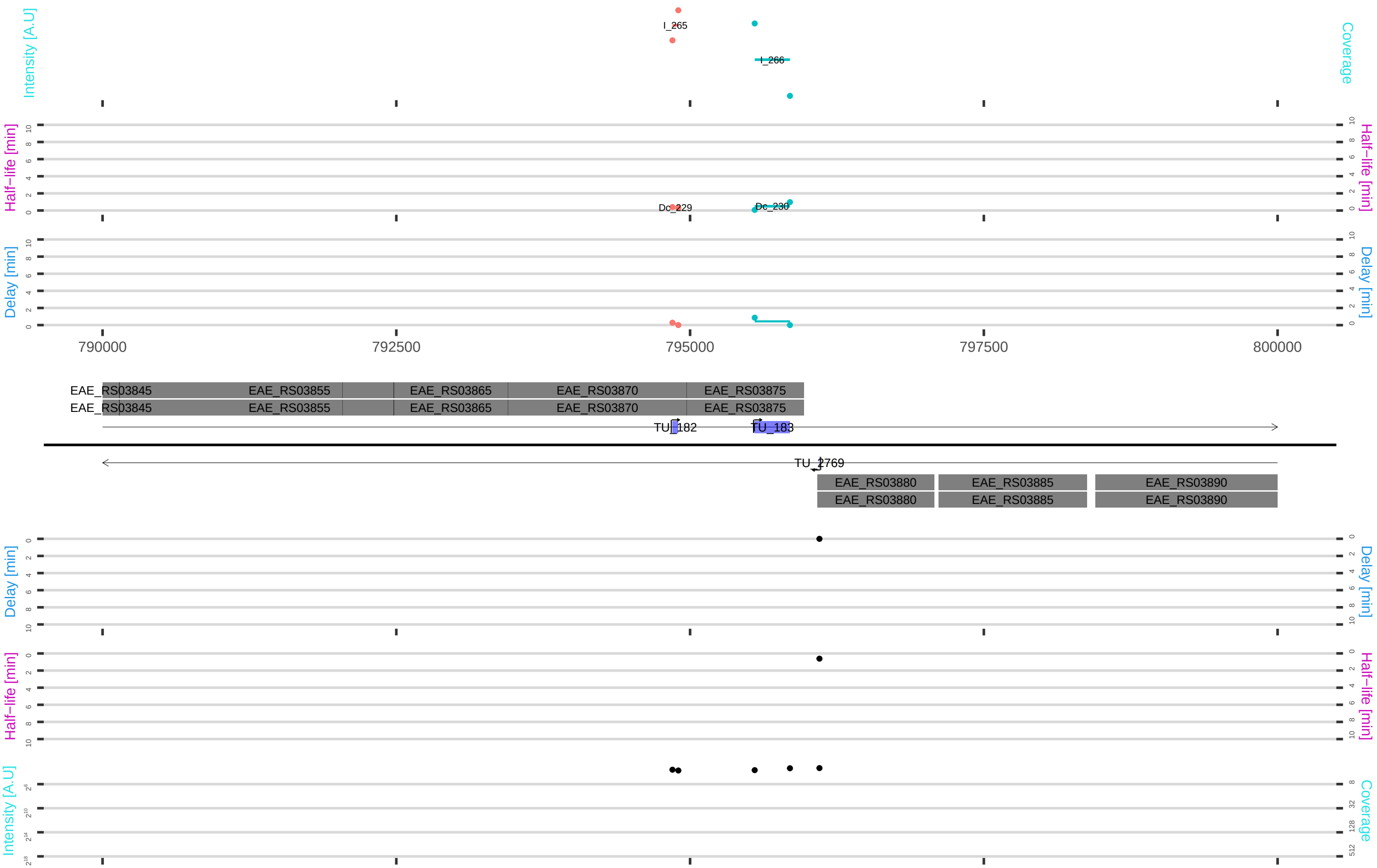

Term: termination (0), NS: new start (0), PS: pausing site (0), iTSS_I: internal starting site (0)

ID: 16102−16200; Term: termination (1), NS: new start (0), PS: pausing site (2), iTSS_I: internal starting site (0)

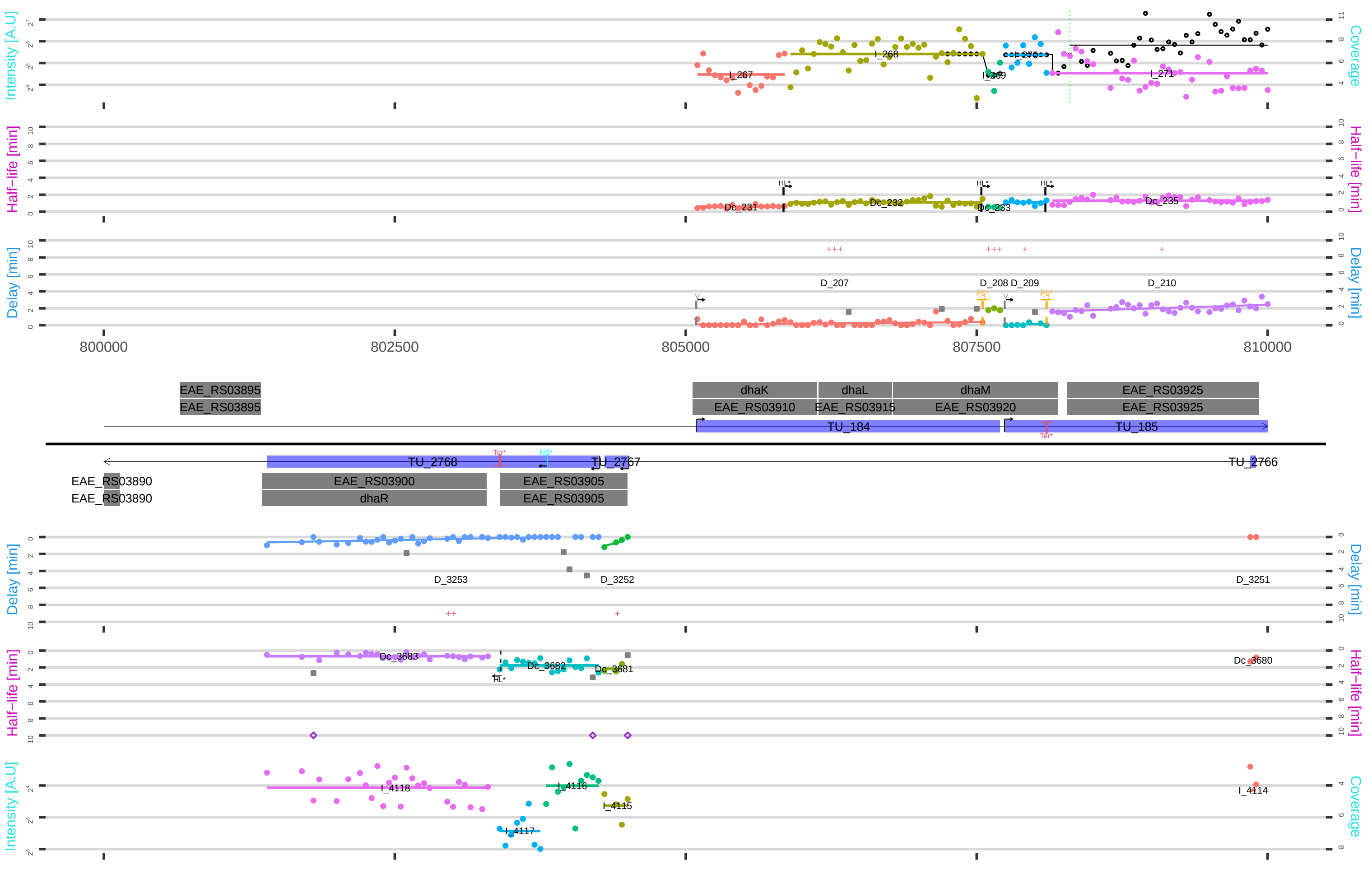

Term: termination (1), NS: new start (1), PS: pausing site (0), iTSS_I: internal starting site (0)

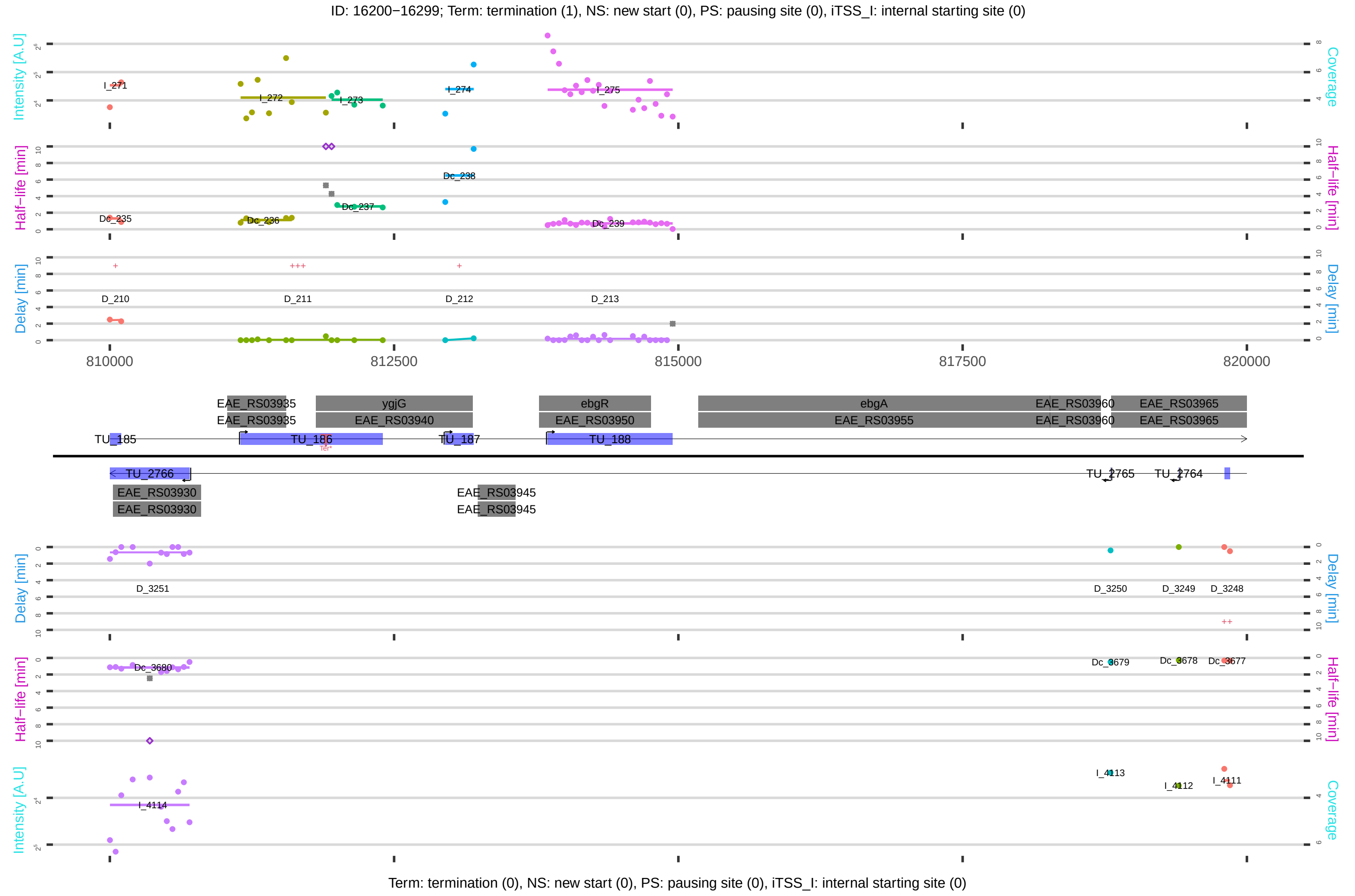

ID: 16200−16299; Term: termination (1), NS: new start (0), PS: pausing site (0), iTSS_I: internal starting site (0)
Intensity [A.U]
Coverage
Half−life [min]
Delay [min]
I_271
I_272
I_273
I_274
I_275
Dc_235
Dc_236
Dc_237
Dc_238
Dc_239
D_210
D_211
D_212
D_213
810000
812500
815000
817500
820000
EAE_RS03935
ygjG
EAE_RS03940
ebgR
EAE_RS03950
ebgA
EAE_RS03955
EAE_RS03960
EAE_RS03965
TU_185
TU_186
TU_187
TU_188
TU_2766
TU_2765
TU_2764
EAE_RS03930
EAE_RS03945
D_3251
D_3250
D_3249
D_3248
Dc_3680
Dc_3679
Dc_3678
Dc_3677
I_4114
I_4113
I_4112
I_4111
Term: termination (0), NS: new start (0), PS: pausing site (0), iTSS_I: internal starting site (0)

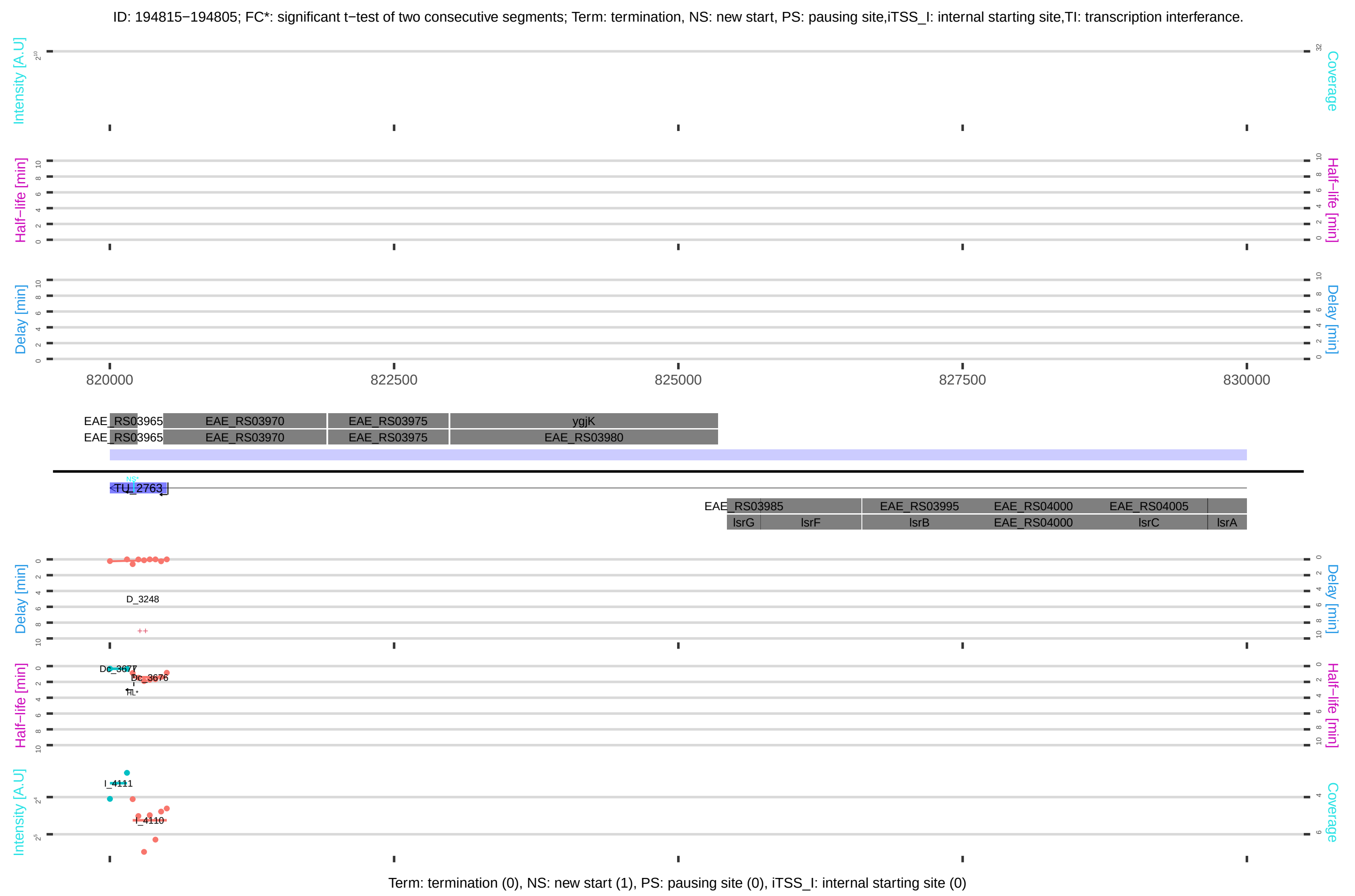

ID: 194815−194805; FC*: significant t−test of two consecutive segments; Term: termination, NS: new start, PS: pausing site,iTSS_I: internal starting site,TI: transcription interferance.
Intensity [A.U]
Coverage
Half−life [min]
Delay [min]
820000
822500
825000
827500
830000
EAE_RS03965
EAE_RS03970
EAE_RS03975
ygjK
EAE_RS03980
NS*
TU_2763
EAE_RS03985
EAE_RS03995
EAE_RS04000
EAE_RS04005
lsrG
lsrF
lsrB
lsrC
lsrA
D_3248
Dc_3677
Dc_3676
HL*
I_4111
I_4110
Term: termination (0), NS: new start (1), PS: pausing site (0), iTSS_I: internal starting site (0)

ID: 16626−16789; Term: termination (0), NS: new start (0), PS: pausing site (0), iTSS_I: internal starting site (0)

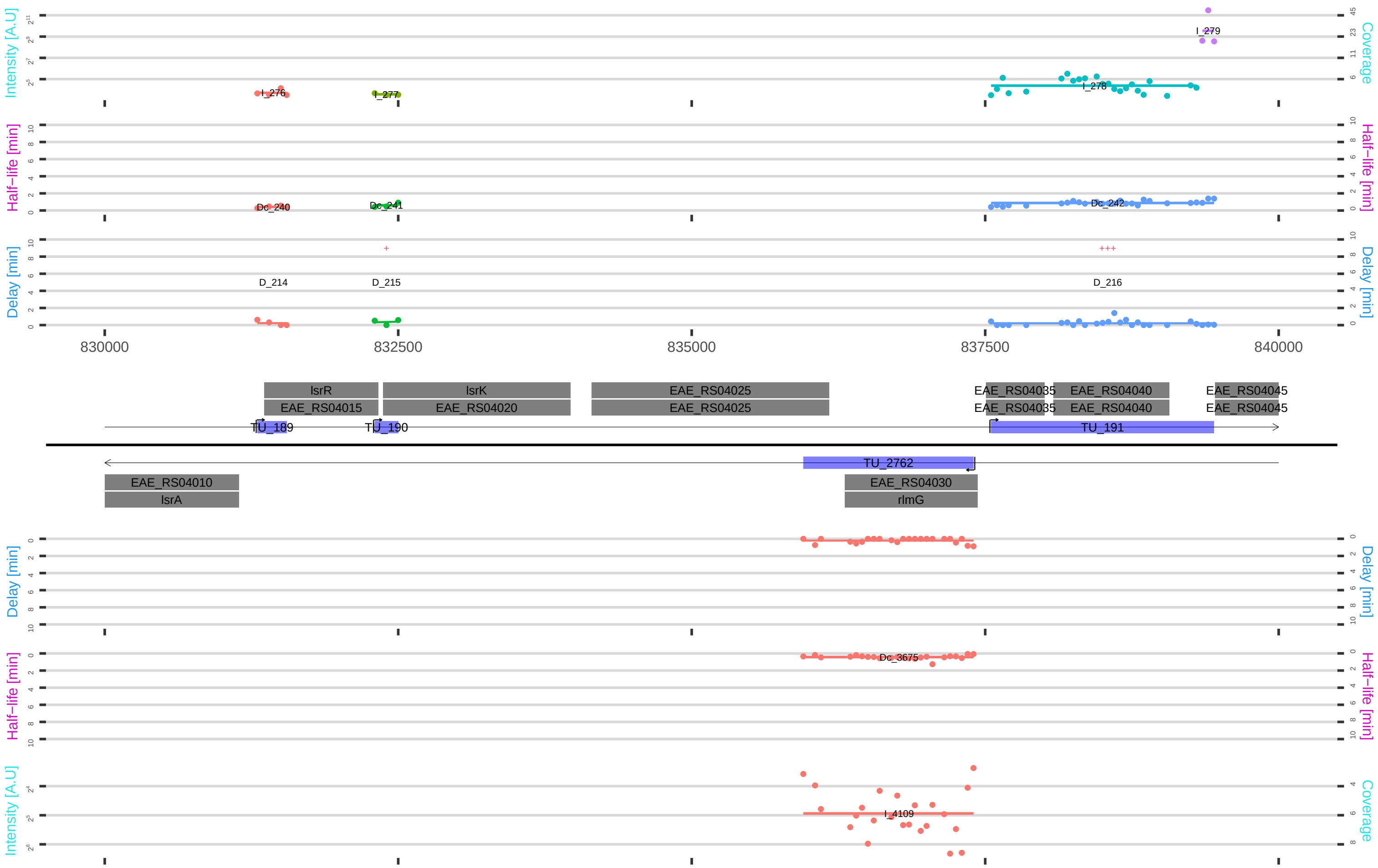

Term: termination (0), NS: new start (0), PS: pausing site (0), iTSS_I: internal starting site (0)

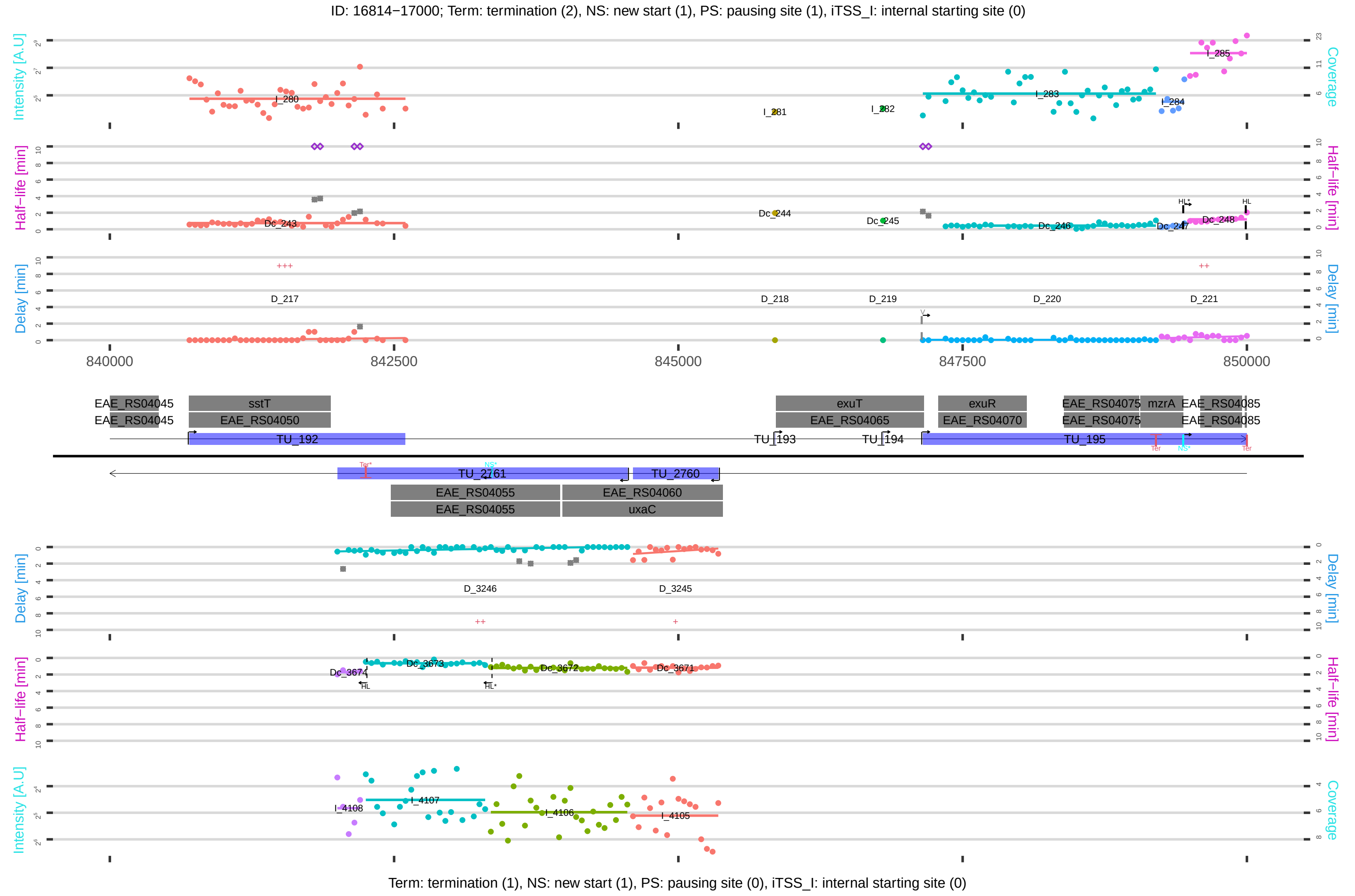

ID: 16814−17000; Term: termination (2), NS: new start (1), PS: pausing site (1), iTSS_I: internal starting site (0)
Intensity [A.U]
Coverage
Half−life [min]
Delay [min]
I_280
I_281
I_282
I_283
I_284
I_285
Dc_243
Dc_244
Dc_245
Dc_246
Dc_247
Dc_248
D_217
D_218
D_219
D_220
D_221
840000
842500
845000
847500
850000
EAE_RS04045
sstT
EAE_RS04050
exuT
EAE_RS04065
exuR
EAE_RS04070
EAE_RS04075
mzrA
EAE_RS04085
TU_192
TU_193
TU_194
TU_195
Ter
NS*
TU_2761
TU_2760
Ter*
EAE_RS04055
EAE_RS04060
uxaC
D_3246
D_3245
Dc_3674
Dc_3673
Dc_3672
Dc_3671
HL
HL*
I_4108
I_4107
I_4106
I_4105
Term: termination (1), NS: new start (1), PS: pausing site (0), iTSS_I: internal starting site (0)

ID: 17000−17168; Term: termination (2), NS: new start (0), PS: pausing site (1), iTSS_I: internal starting site (0)

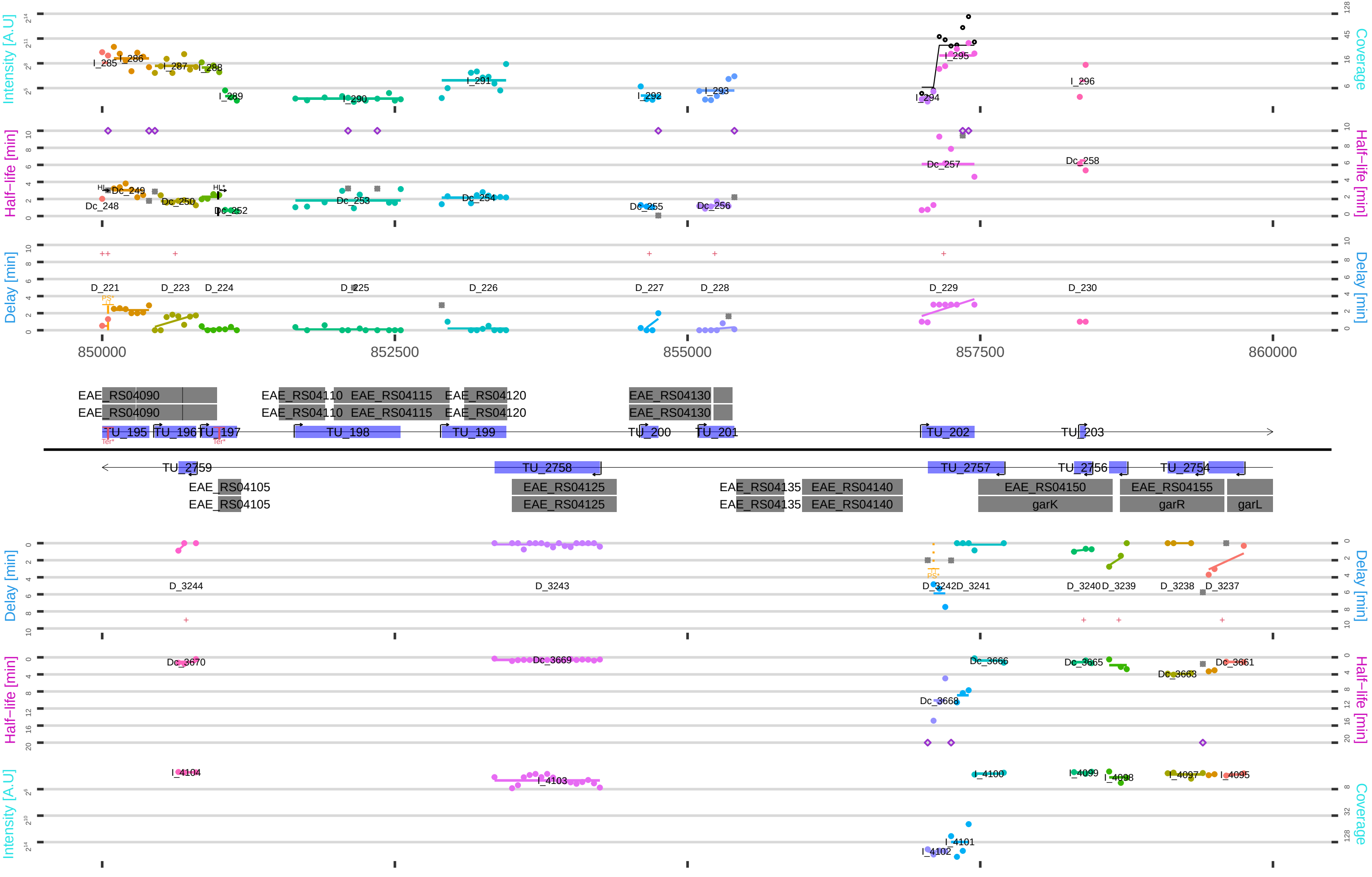

Term: termination (0), NS: new start (0), PS: pausing site (1), iTSS_I: internal starting site (0)

ID: 17218−17372; Term: termination (0), NS: new start (0), PS: pausing site (0), iTSS_I: internal starting site (0)

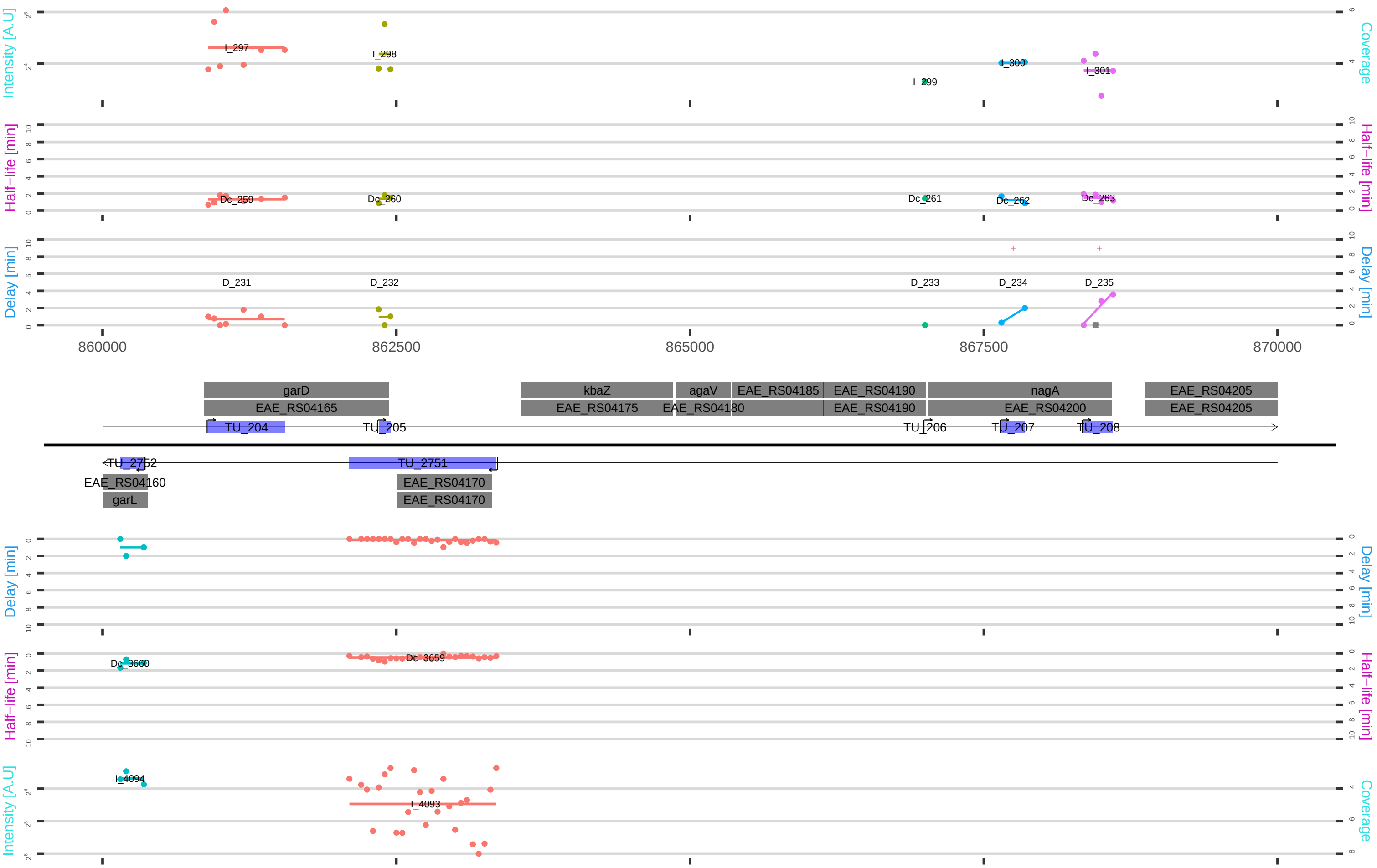

Term: termination (0), NS: new start (0), PS: pausing site (0), iTSS_I: internal starting site (0)

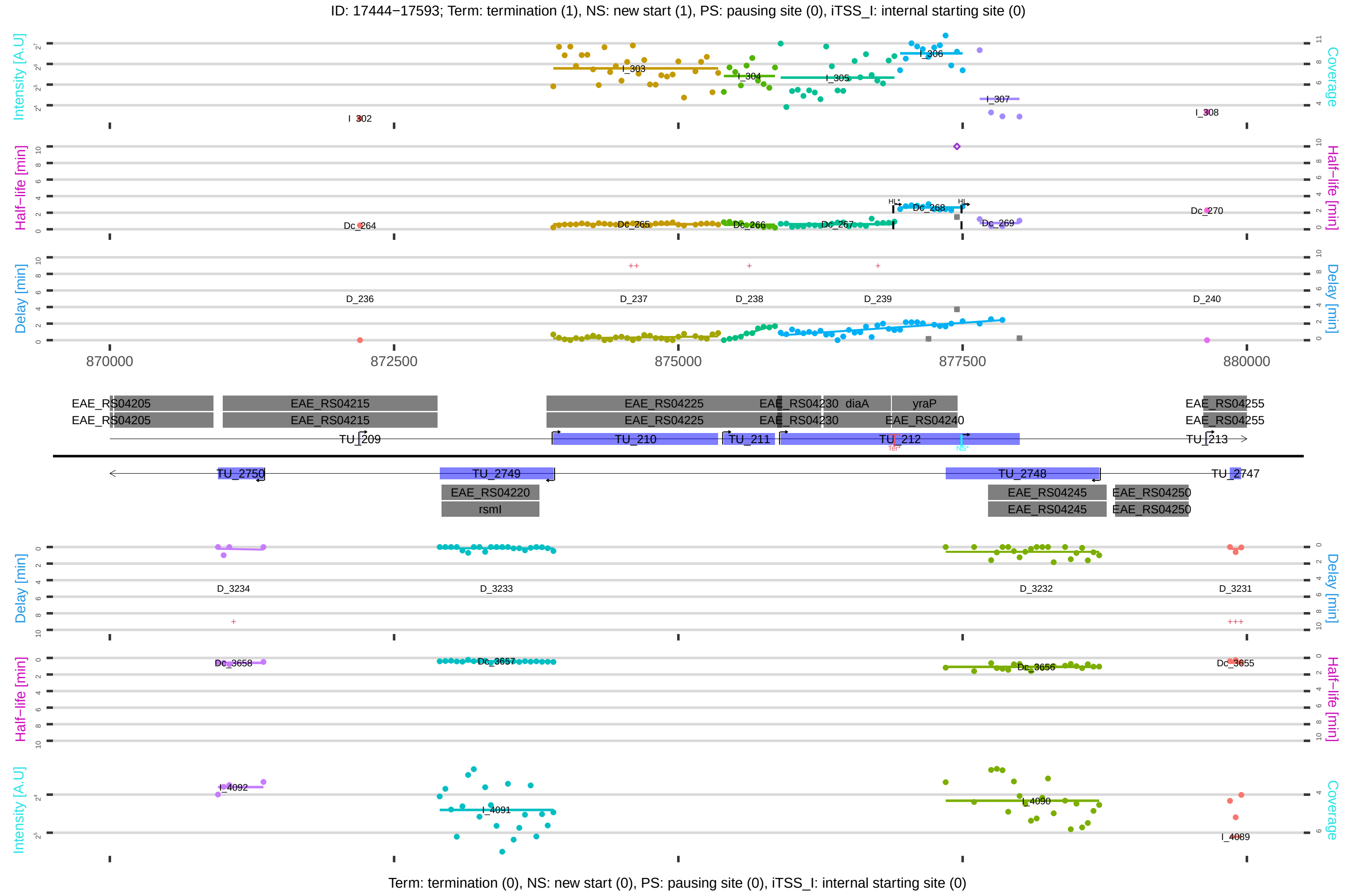
ID: 17444−17593; Term: termination (1), NS: new start (1), PS: pausing site (0), iTSS_I: internal starting site (0)
Intensity [A.U]
Coverage
Half−life [min]
Delay [min]
870000
872500
875000
877500
880000
EAE_RS04205
EAE_RS04215
EAE_RS04225
EAE_RS04230
diaA
yraP
EAE_RS04240
EAE_RS04255
TU_209
TU_210
TU_211
TU_212
TU_213
TU_2750
TU_2749
TU_2748
TU_2747
EAE_RS04220
rsmI
EAE_RS04245
EAE_RS04250
Term: termination (0), NS: new start (0), PS: pausing site (0), iTSS_I: internal starting site (0)

ID: 17606−17702; Term: termination (0), NS: new start (0), PS: pausing site (0), iTSS_I: internal starting site (0)

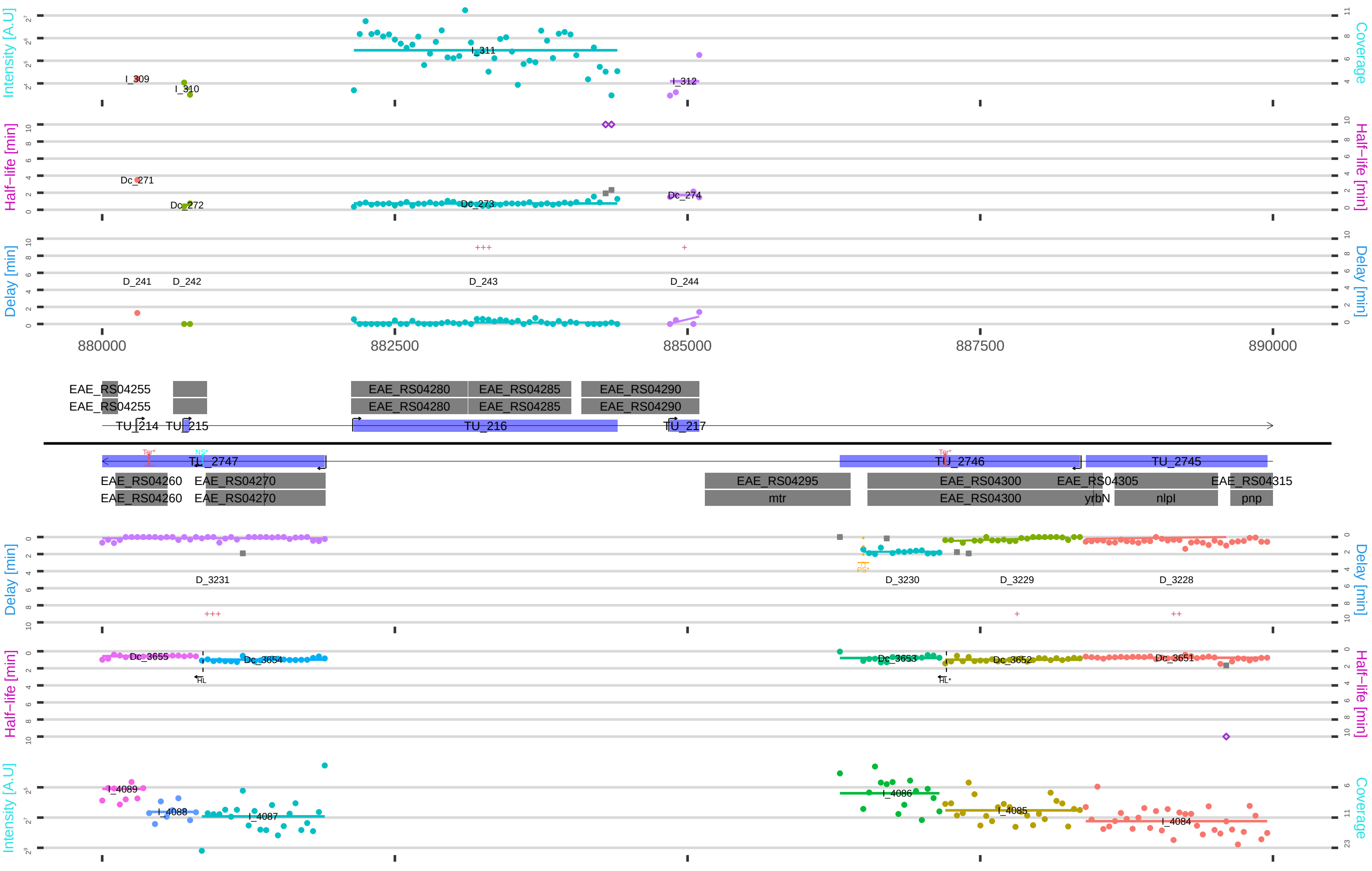

Term: termination (2), NS: new start (1), PS: pausing site (1), iTSS_I: internal starting site (0)

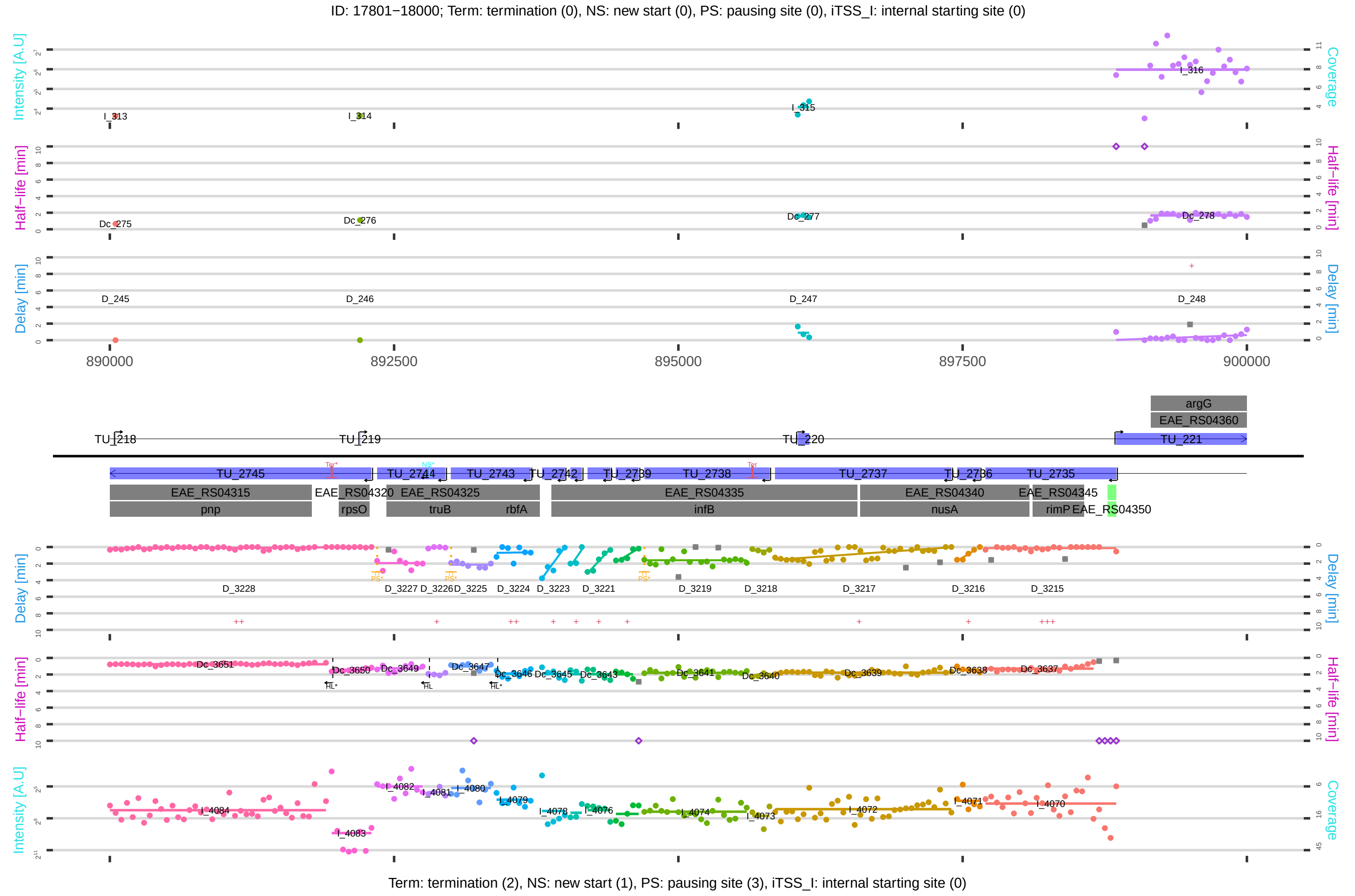

ID: 17801−18000; Term: termination (0), NS: new start (0), PS: pausing site (0), iTSS_I: internal starting site (0)
Intensity [A.U]
Coverage
Half−life [min]
Delay [min]
I_313
I_314
I_315
I_316
Dc_275
Dc_276
Dc_277
Dc_278
D_245
D_246
D_247
D_248
890000
892500
895000
897500
900000
argG
EAE_RS04360
TU_218
TU_219
TU_220
TU_221
TU_2745
TU_2744
TU_2743
TU_2742
TU_2739
TU_2738
TU_2737
TU_2736
TU_2735
EAE_RS04315
EAE_RS04320
EAE_RS04325
EAE_RS04335
EAE_RS04340
EAE_RS04345
pnp
rpsO
truB
rbfA
infB
nusA
rimP
EAE_RS04350
D_3228
D_3227
D_3226
D_3225
D_3224
D_3223
D_3221
D_3219
D_3218
D_3217
D_3216
D_3215
Dc_3651
Dc_3650
Dc_3649
Dc_3647
Dc_3646
Dc_3645
Dc_3643
Dc_3641
Dc_3640
Dc_3639
Dc_3638
Dc_3637
I_4084
I_4083
I_4082
I_4081
I_4080
I_4079
I_4078
I_4076
I_4074
I_4073
I_4072
I_4071
I_4070
Term: termination (2), NS: new start (1), PS: pausing site (3), iTSS_I: internal starting site (0)

ID: 18000−18200; Term: termination (1), NS: new start (0), PS: pausing site (0), iTSS_I: internal starting site (0)

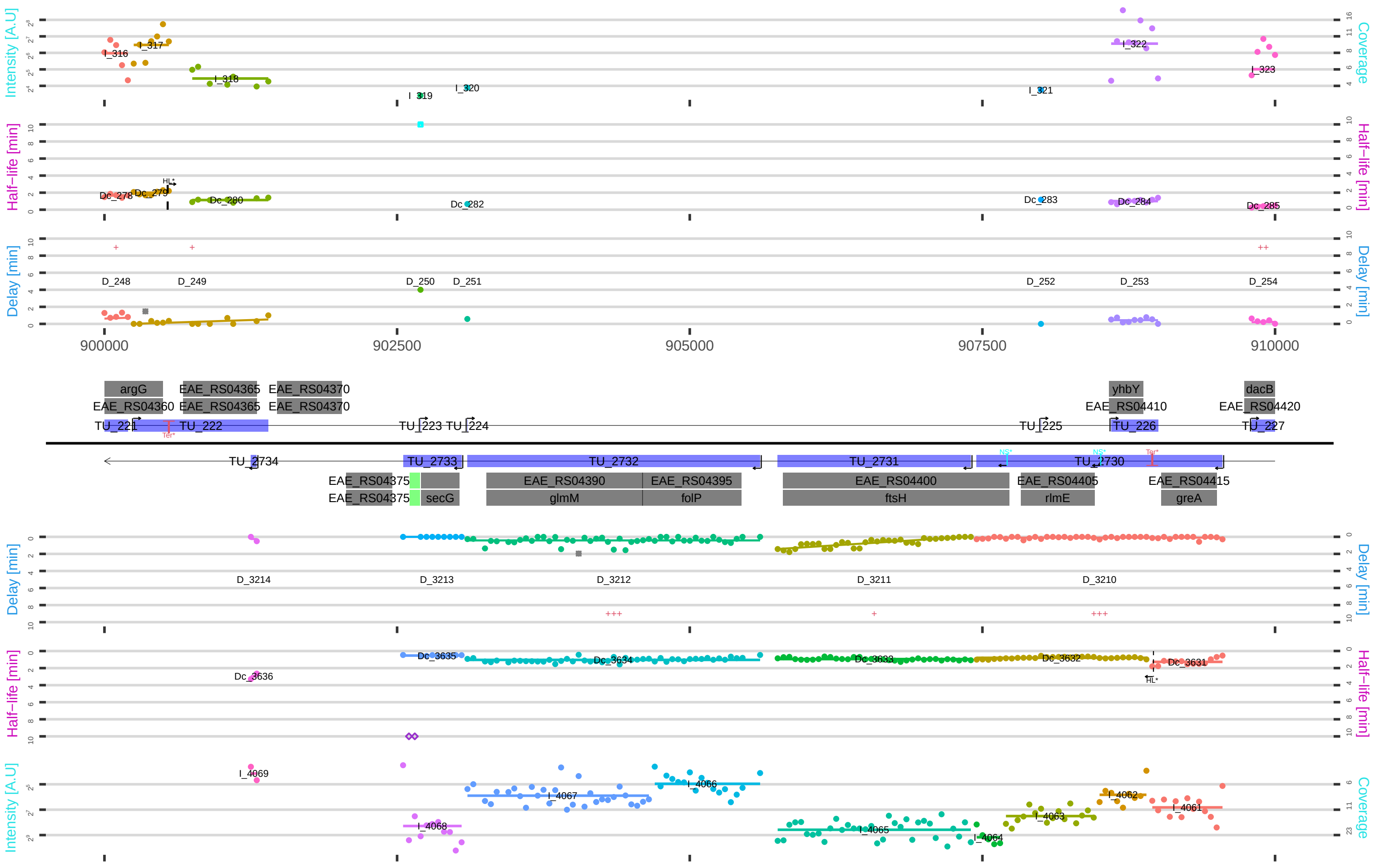

Term: termination (1), NS: new start (2), PS: pausing site (0), iTSS_I: internal starting site (0)

ID: 18200−18316; Term: termination (2), NS: new start (1), PS: pausing site (0), iTSS_I: internal starting site (0)

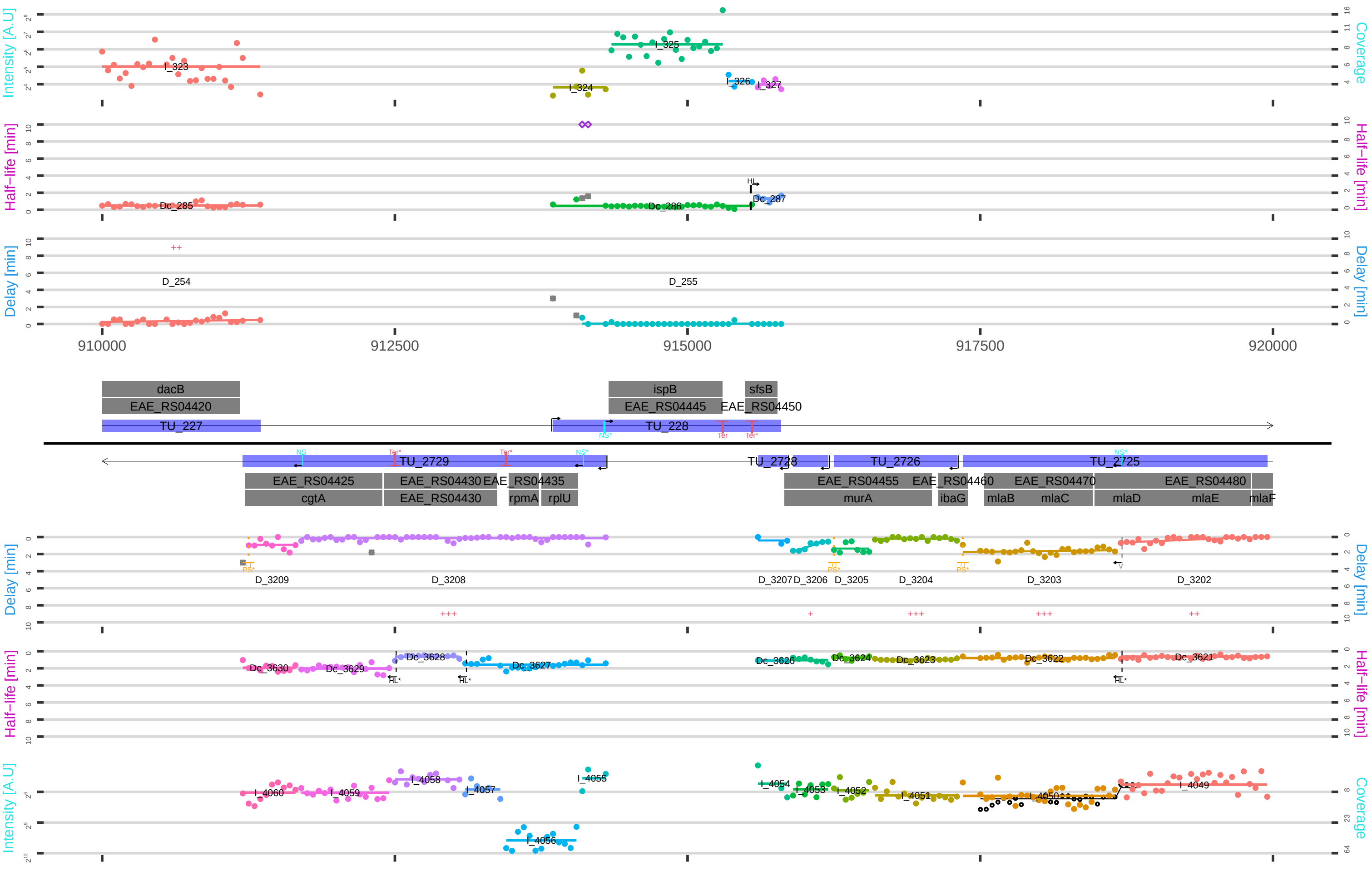

Term: termination (2), NS: new start (3), PS: pausing site (3), iTSS_I: internal starting site (0)

ID: 18418−18582; Term: termination (1), NS: new start (3), PS: pausing site (1), iTSS_I: internal starting site (1)

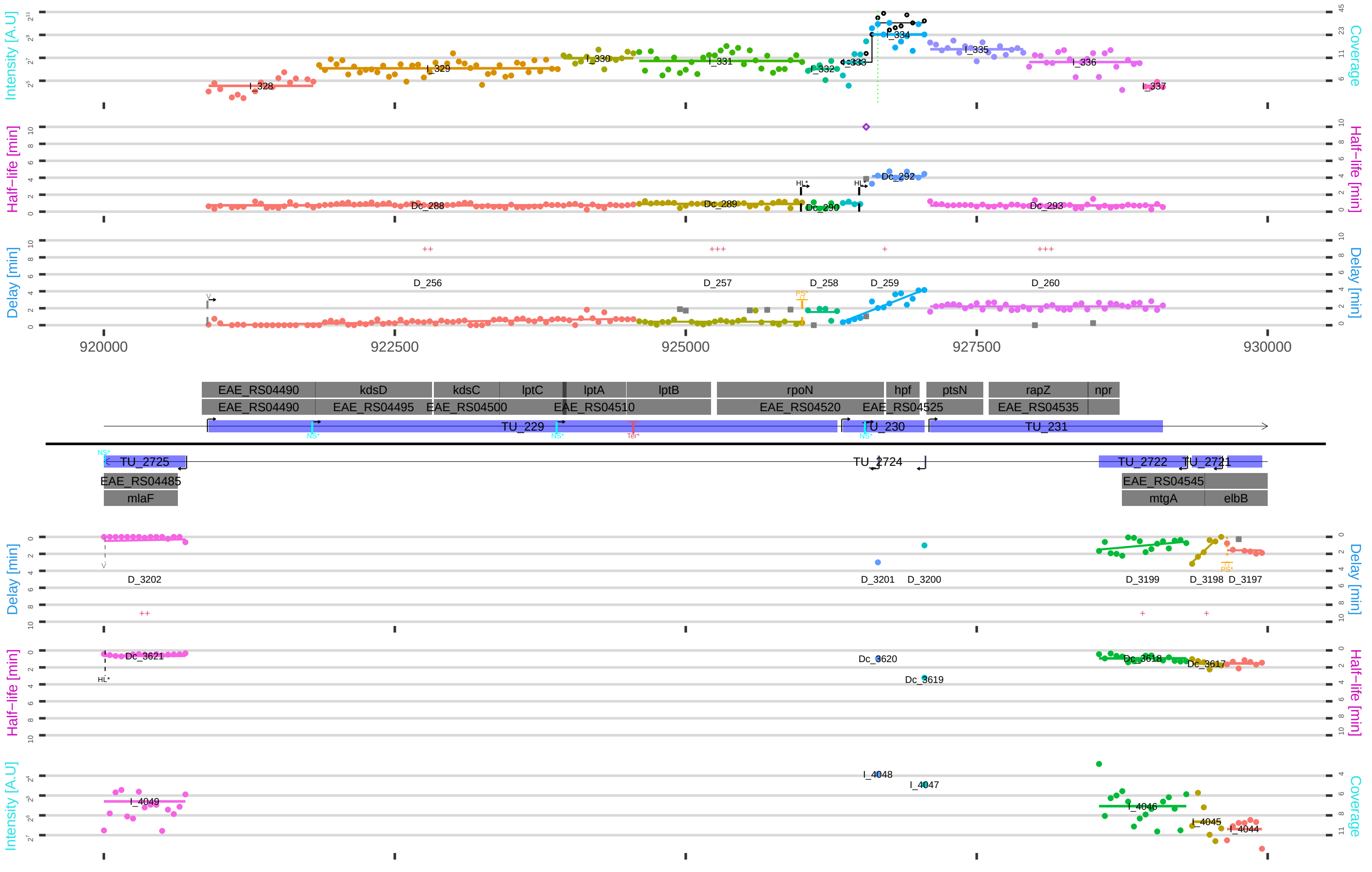

Term: termination (0), NS: new start (1), PS: pausing site (1), iTSS_I: internal starting site (0)

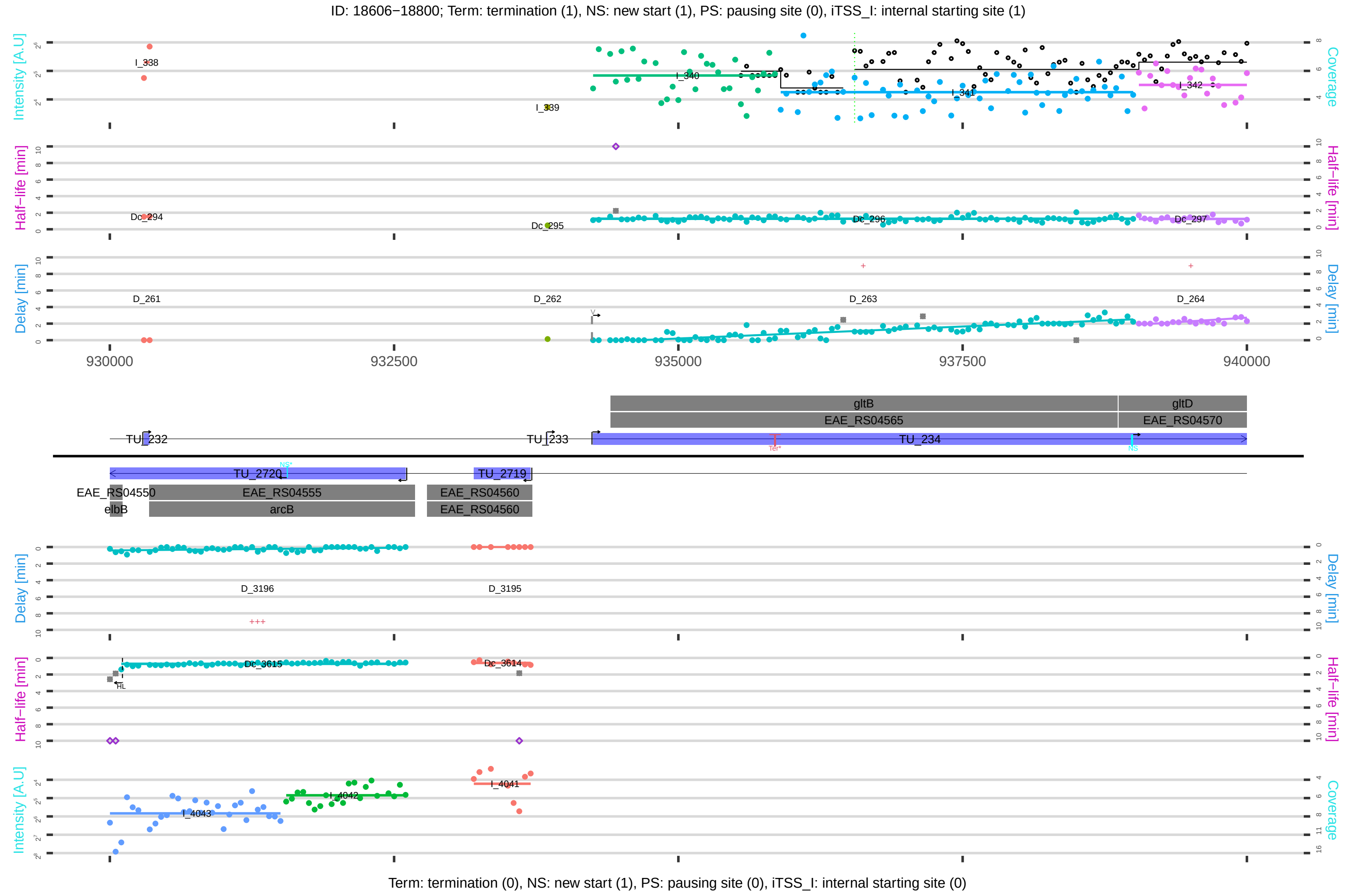
ID: 18606−18800; Term: termination (1), NS: new start (1), PS: pausing site (0), iTSS_I: internal starting site (1)
Intensity [A.U]
Coverage
Half−life [min]
Delay [min]
I_338
I_339
I_340
I_341
I_342
Dc_294
Dc_295
Dc_296
Dc_297
D_261
D_262
D_263
D_264
930000
932500
935000
937500
940000
gltB
gltD
EAE_RS04565
EAE_RS04570
TU_232
TU_233
TU_234
Ter*
NS
TU_2720
NS*
TU_2719
EAE_RS04550
EAE_RS04555
EAE_RS04560
elbB
arcB
D_3196
D_3195
Dc_3615
Dc_3614
HL
I_4043
I_4042
I_4041
Term: termination (0), NS: new start (1), PS: pausing site (0), iTSS_I: internal starting site (0)

ID: 18800−18951; Term: termination (2), NS: new start (0), PS: pausing site (0), iTSS_I: internal starting site (0)

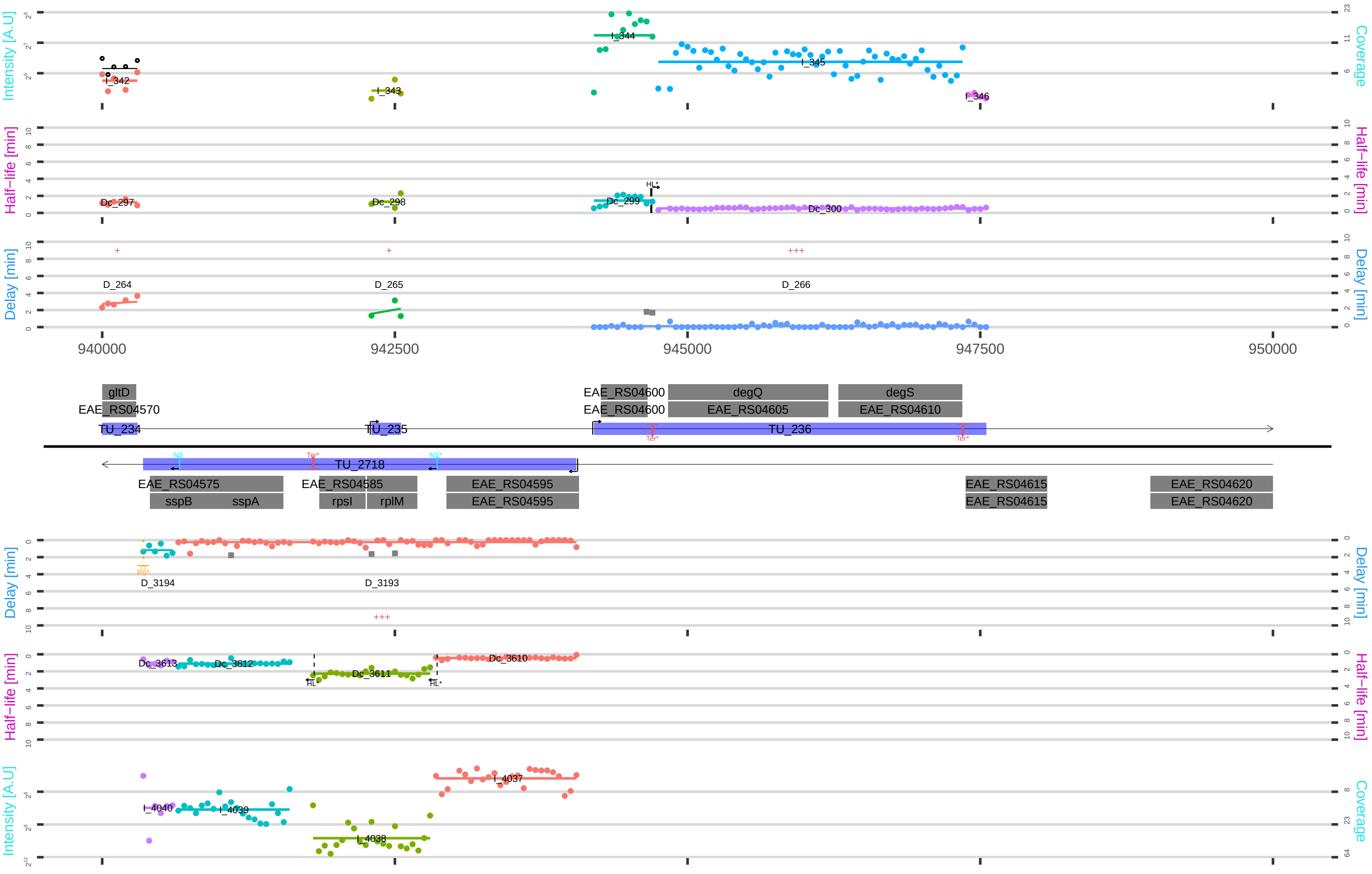

Term: termination (1), NS: new start (2), PS: pausing site (1), iTSS_I: internal starting site (0)

ID: 19165−19195; Term: termination (0), NS: new start (0), PS: pausing site (0), iTSS_I: internal starting site (0)

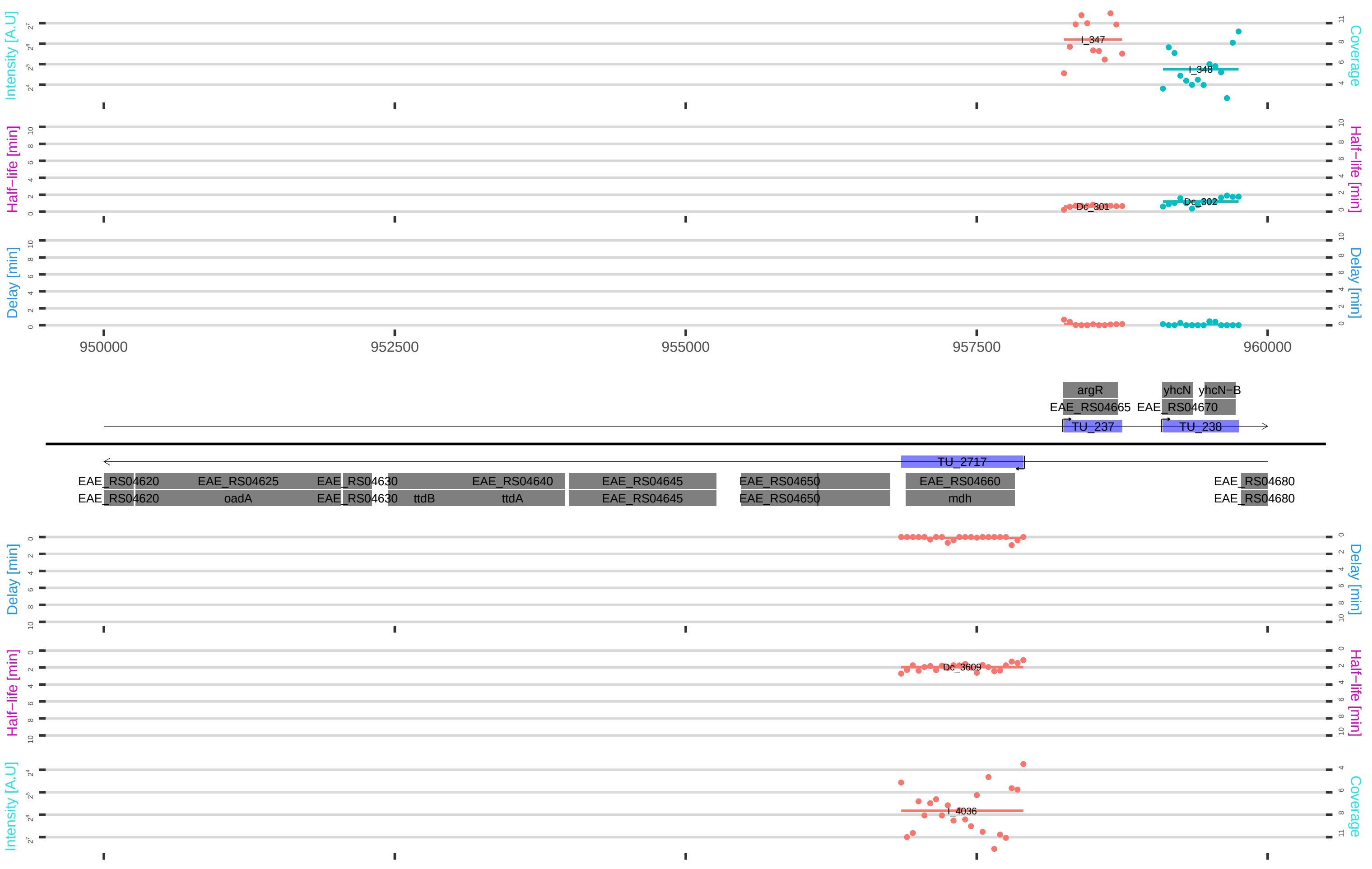

Term: termination (0), NS: new start (0), PS: pausing site (0), iTSS_I: internal starting site (0)

ID: 19260−19286; Term: termination (0), NS: new start (0), PS: pausing site (0), iTSS_I: internal starting site (0)

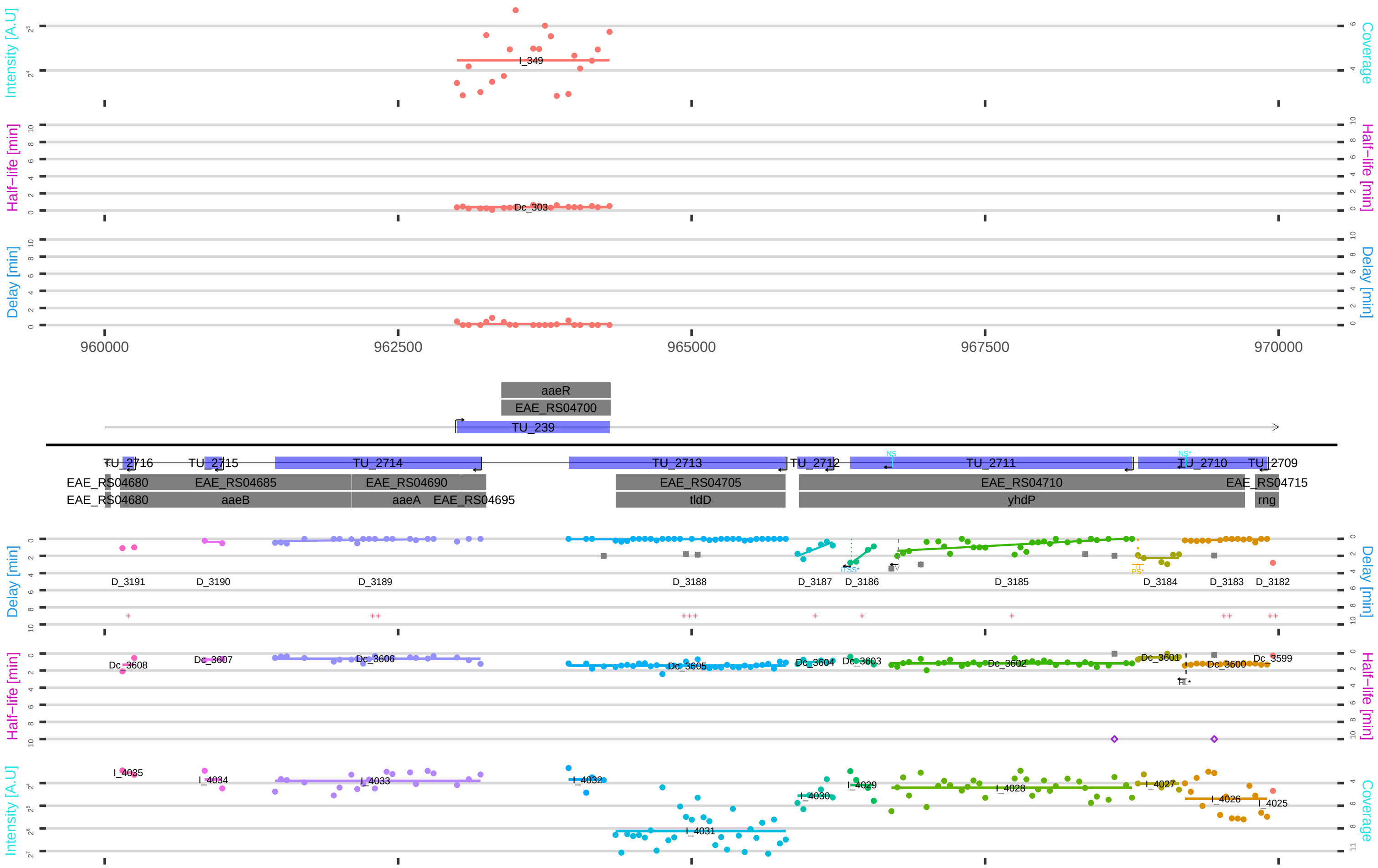

Term: termination (0), NS: new start (2), PS: pausing site (2), iTSS_I: internal starting site (1)

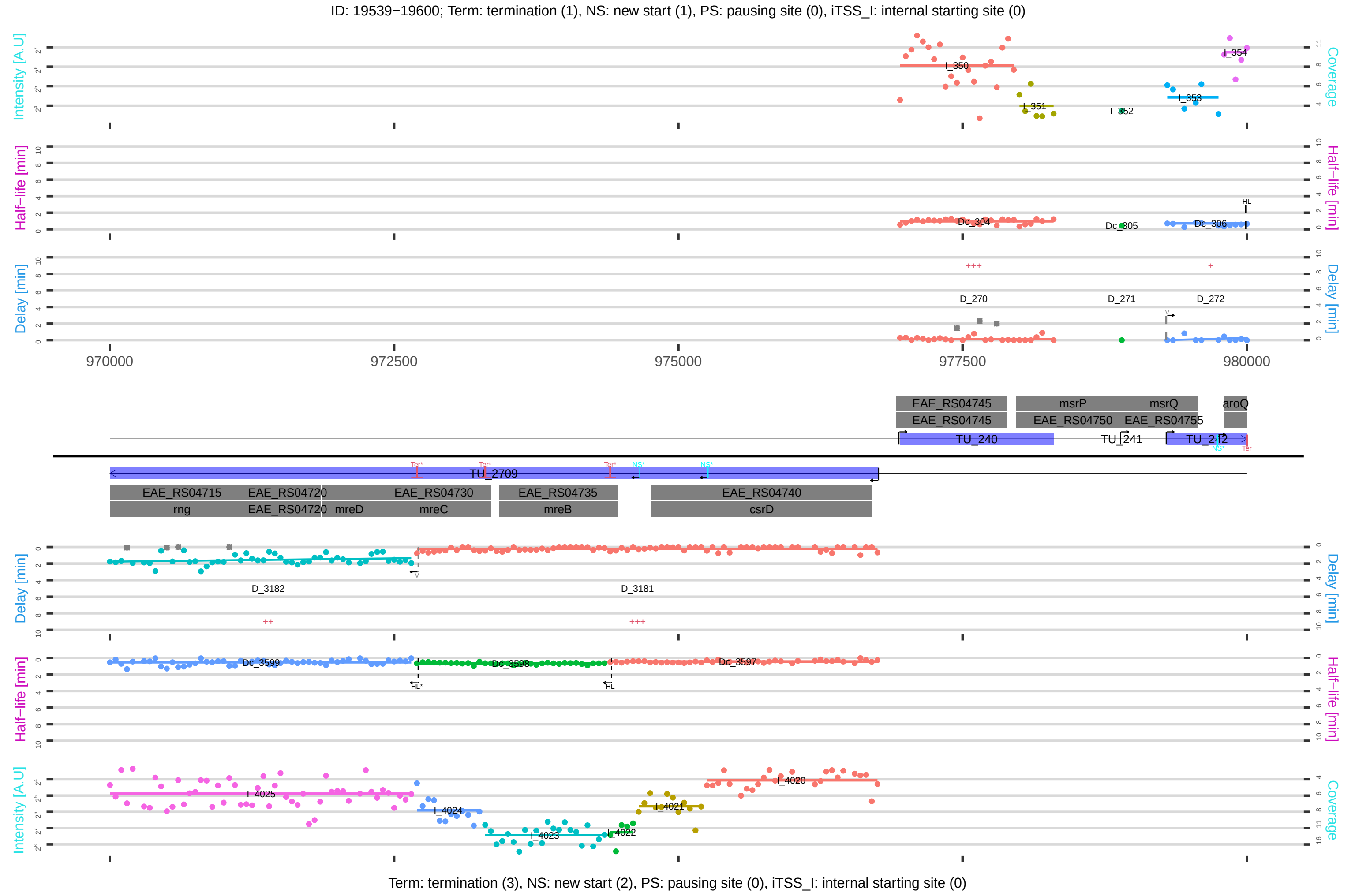
ID: 19539−19600; Term: termination (1), NS: new start (1), PS: pausing site (0), iTSS_I: internal starting site (0)
Intensity [A.U]
Coverage
Half−life [min]
Delay [min]
970000
972500
975000
977500
980000
I_350
I_351
I_352
I_353
I_354
Dc_304
Dc_305
Dc_306
D_270
D_271
D_272
EAE_RS04745
msrP
msrQ
EAE_RS04750
EAE_RS04755
aroQ
TU_240
TU_241
TU_242
TU_2709
EAE_RS04715
EAE_RS04720
EAE_RS04730
EAE_RS04735
EAE_RS04740
rng
mreD
mreC
mreB
csrD
D_3182
D_3181
Dc_3599
Dc_3598
Dc_3597
I_4025
I_4024
I_4023
I_4022
I_4021
I_4020
Term: termination (3), NS: new start (2), PS: pausing site (0), iTSS_I: internal starting site (0)

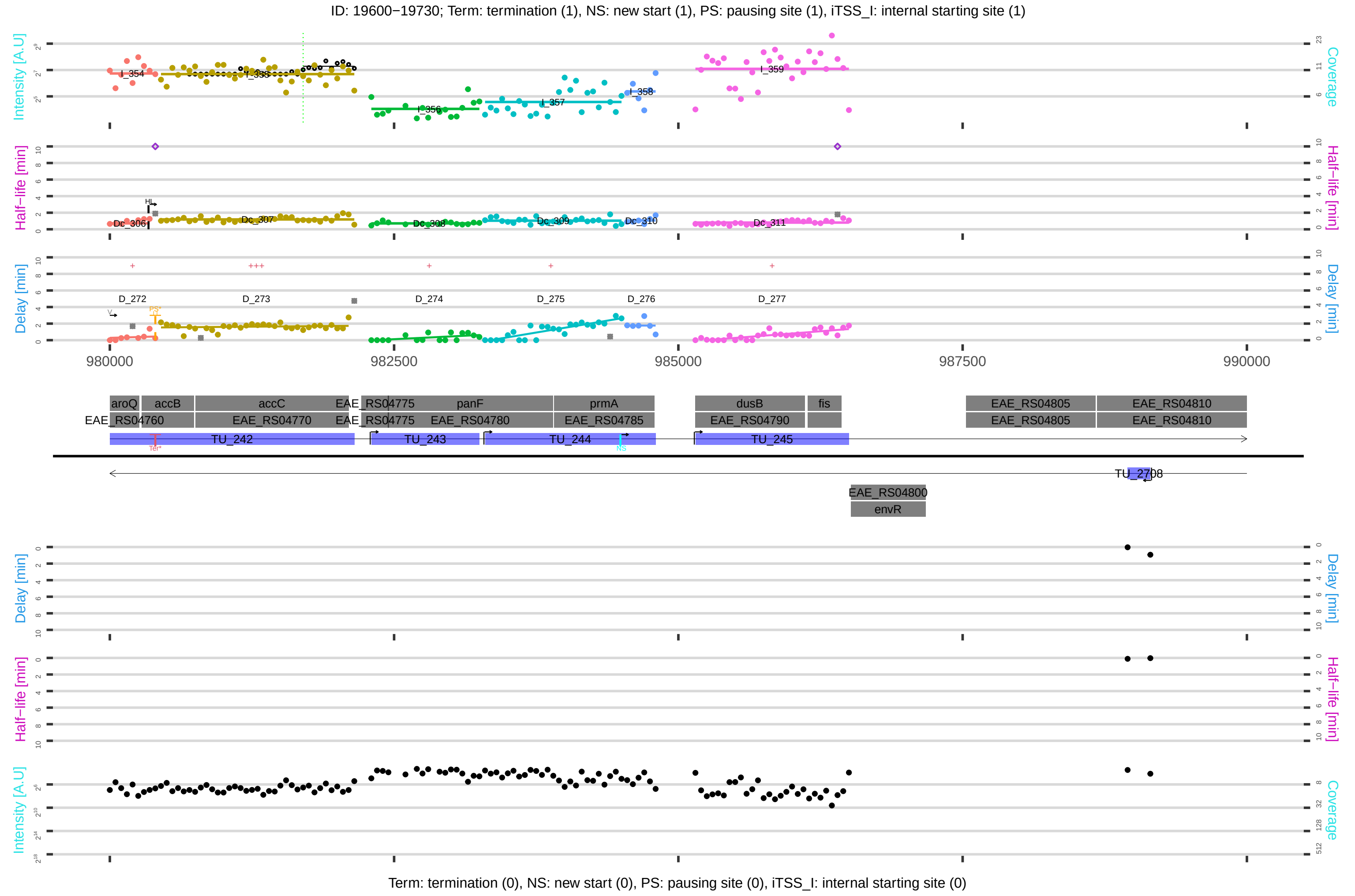

ID: 19600−19730; Term: termination (1), NS: new start (1), PS: pausing site (1), iTSS_I: internal starting site (1)
Intensity [A.U]
Coverage
Half−life [min]
Delay [min]
I_354
I_355
I_356
I_357
I_358
I_359
Dc_306
Dc_307
Dc_308
Dc_309
Dc_310
Dc_311
D_272
D_273
D_274
D_275
D_276
D_277
980000
982500
985000
987500
990000
aroQ
accB
accC
EAE_RS04775
panF
prmA
dusB
fis
EAE_RS04805
EAE_RS04810
EAE_RS04760
EAE_RS04770
EAE_RS04780
EAE_RS04785
EAE_RS04790
TU_242
TU_243
TU_244
TU_245
TU_2708
EAE_RS04800
envR
Term: termination (0), NS: new start (0), PS: pausing site (0), iTSS_I: internal starting site (0)

ID: 19843−19975; Term: termination (3), NS: new start (2), PS: pausing site (1), iTSS_I: internal starting site (1)

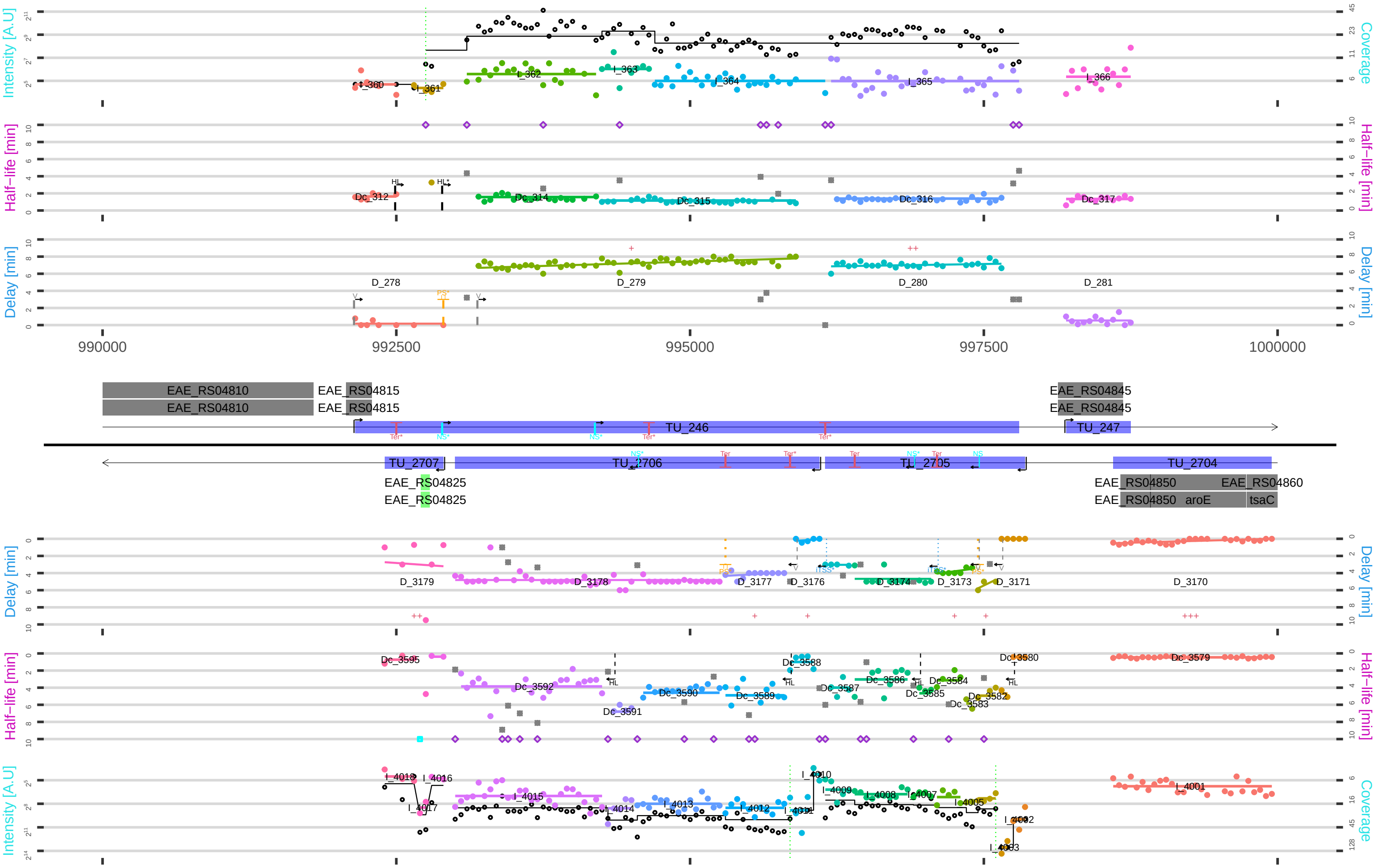

Term: termination (4), NS: new start (3), PS: pausing site (4), iTSS_I: internal starting site (2)

ID: 20053−20200; Term: termination (1), NS: new start (1), PS: pausing site (1), iTSS_I: internal starting site (0)

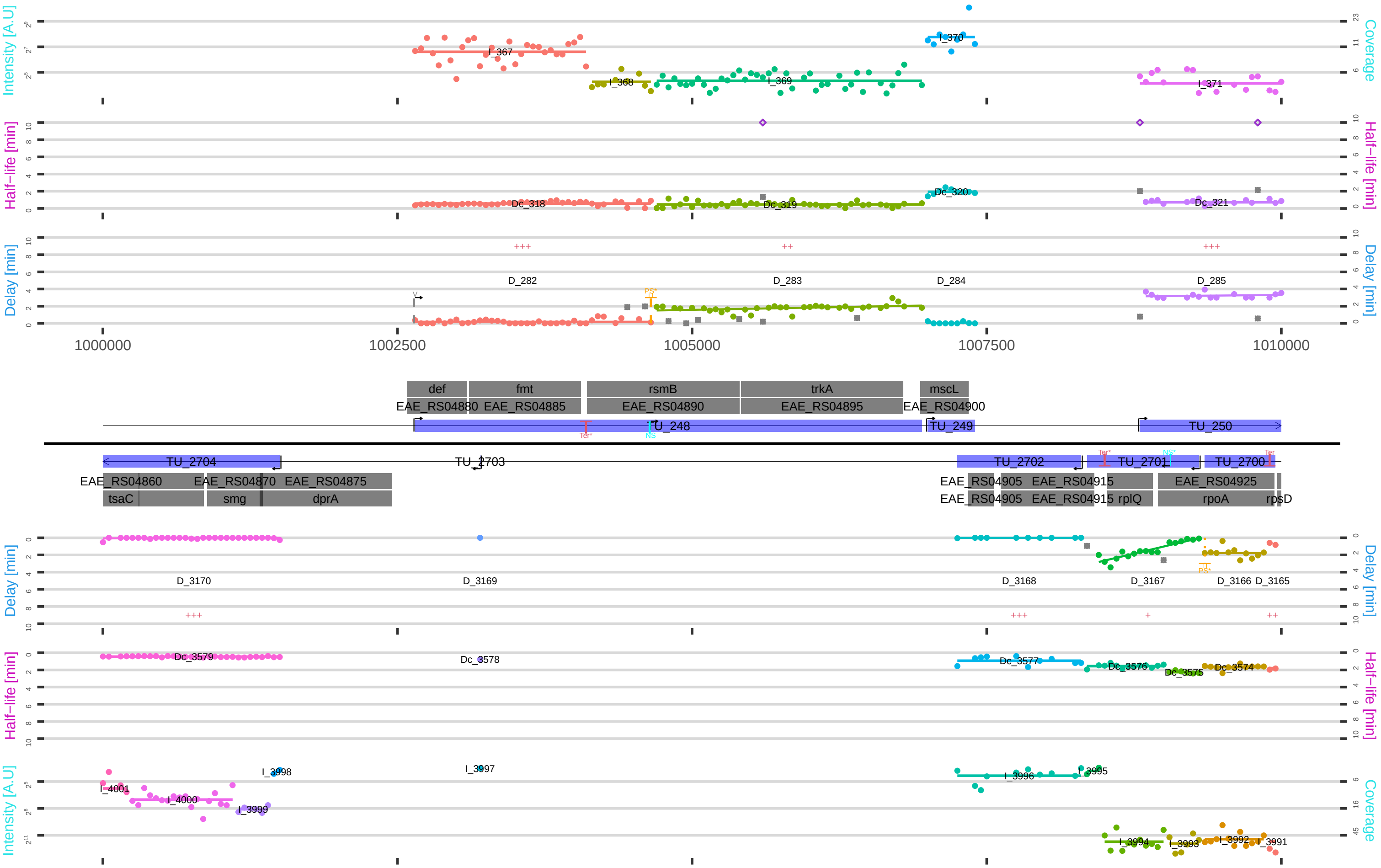

Term: termination (2), NS: new start (1), PS: pausing site (1), iTSS_I: internal starting site (0)

ID: 20200−20399; Term: termination (0), NS: new start (2), PS: pausing site (2), iTSS_I: internal starting site (0)

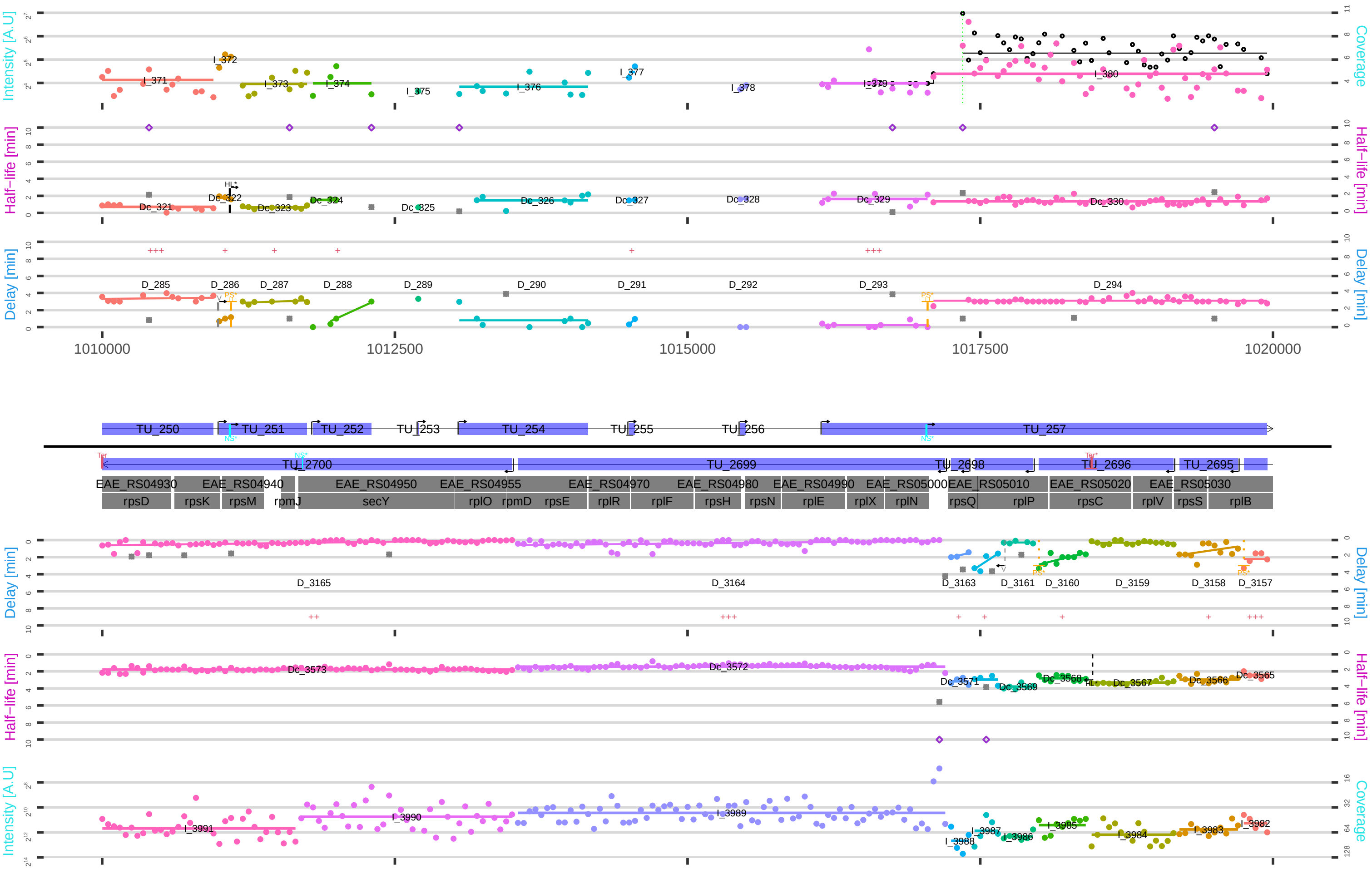

Term: termination (2), NS: new start (1), PS: pausing site (3), iTSS_I: internal starting site (0)

ID: 20401−20567; Term: termination (0), NS: new start (0), PS: pausing site (0), iTSS_I: internal starting site (0)

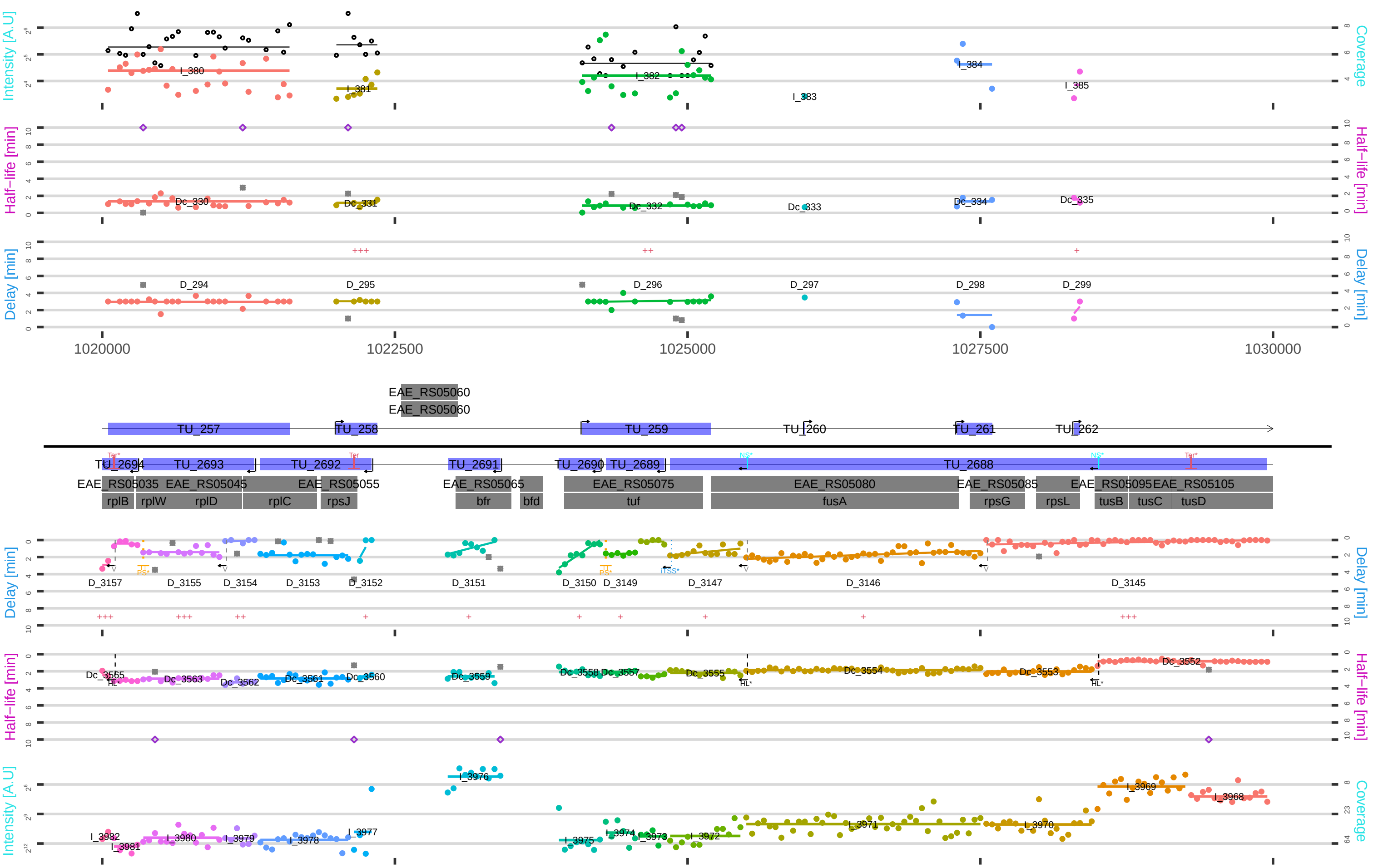

Term: termination (3), NS: new start (2), PS: pausing site (4), iTSS_I: internal starting site (1)

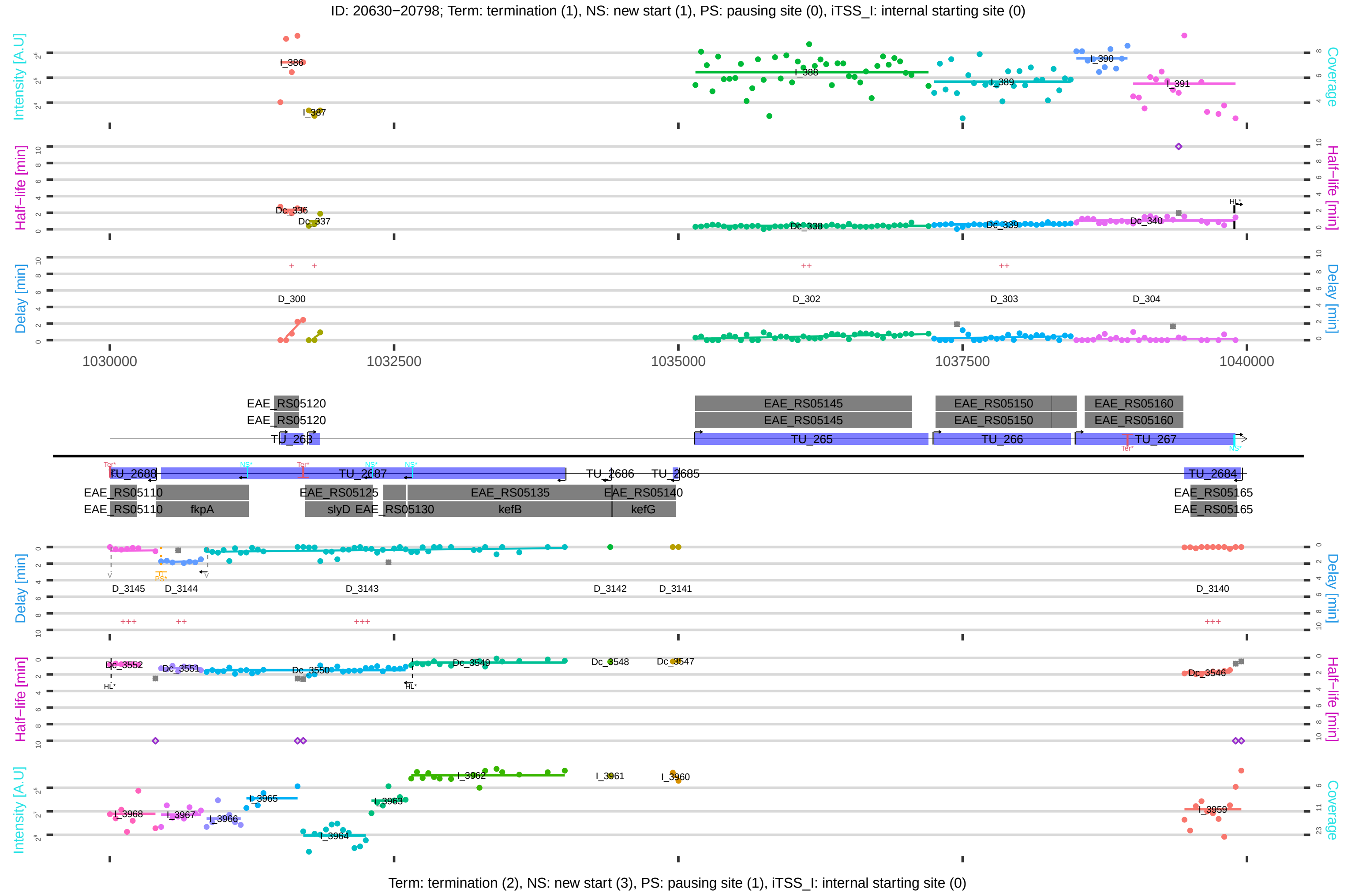
ID: 20630−20798; Term: termination (1), NS: new start (1), PS: pausing site (0), iTSS_I: internal starting site (0)
Intensity [A.U]
Coverage
Half−life [min]
Delay [min]
1030000
1032500
1035000
1037500
1040000
EAE_RS05120
EAE_RS05145
EAE_RS05150
EAE_RS05160
TU_263
TU_265
TU_266
TU_267
TU_2688
TU_2687
TU_2686
TU_2685
TU_2684
EAE_RS05110
fkpA
EAE_RS05125
slyD
EAE_RS05130
EAE_RS05135
kefB
EAE_RS05140
kefG
EAE_RS05165
Term: termination (2), NS: new start (3), PS: pausing site (1), iTSS_I: internal starting site (0)

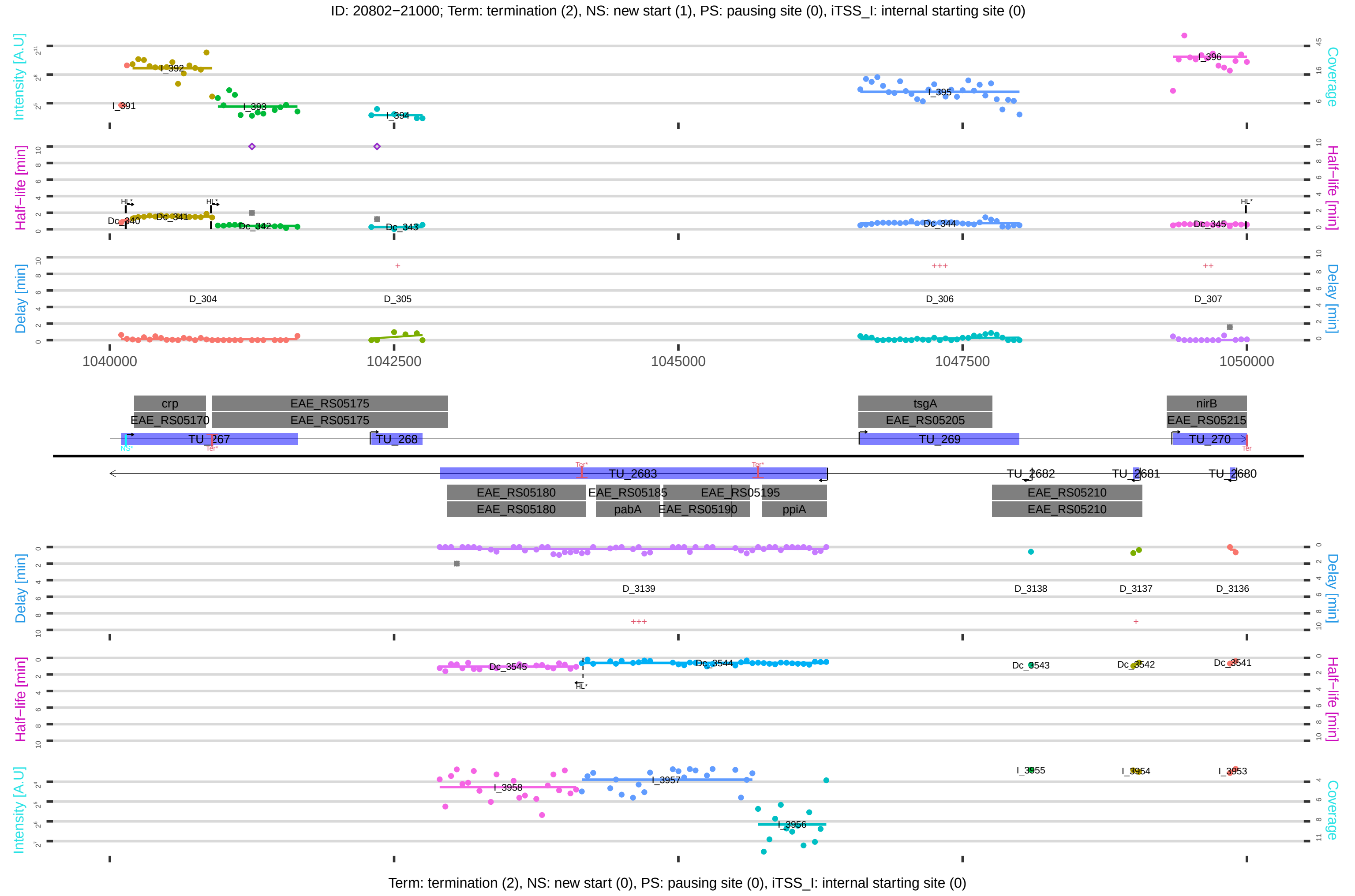

ID: 20802−21000; Term: termination (2), NS: new start (1), PS: pausing site (0), iTSS_I: internal starting site (0)
Intensity [A.U]
Coverage
Half−life [min]
Delay [min]
I_391
I_392
I_393
I_394
I_395
I_396
Dc_340
Dc_341
Dc_342
Dc_343
Dc_344
Dc_345
D_304
D_305
D_306
D_307
1040000
1042500
1045000
1047500
1050000
crp
EAE_RS05170
EAE_RS05175
tsgA
EAE_RS05205
nirB
EAE_RS05215
TU_267
TU_268
TU_269
TU_270
TU_2683
TU_2682
TU_2681
TU_2680
EAE_RS05180
EAE_RS05185
EAE_RS05195
pabA
EAE_RS05190
ppiA
EAE_RS05210
D_3139
D_3138
D_3137
D_3136
Dc_3545
Dc_3544
Dc_3543
Dc_3542
Dc_3541
I_3958
I_3957
I_3956
I_3955
I_3954
I_3953
Term: termination (2), NS: new start (0), PS: pausing site (0), iTSS_I: internal starting site (0)

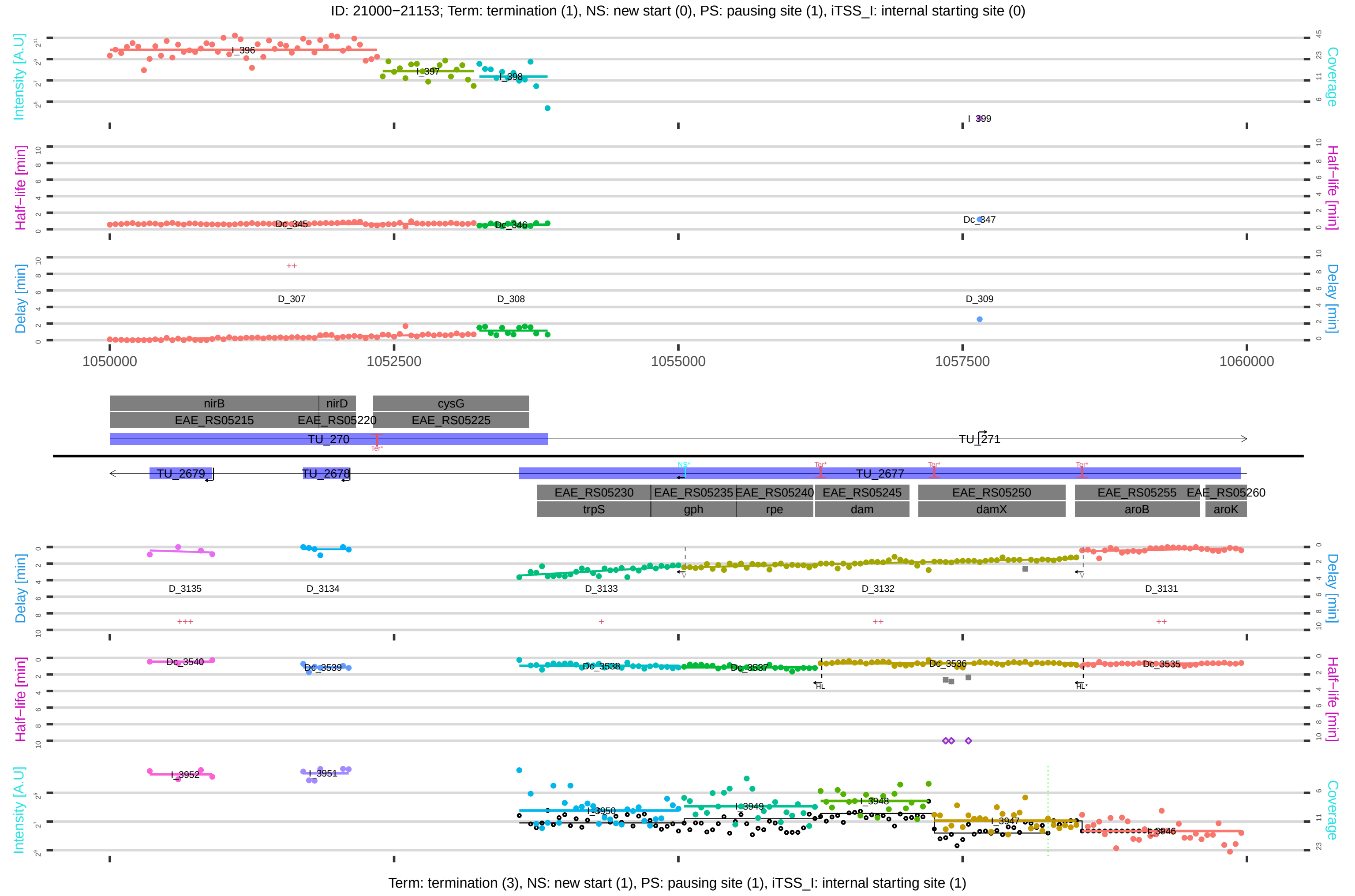
ID: 21000−21153; Term: termination (1), NS: new start (0), PS: pausing site (1), iTSS_I: internal starting site (0)
Intensity [A.U]
Coverage
I_396
I_397
I_398
I_399
Half−life [min]
Dc_345
Dc_346
Dc_347
Delay [min]
D_307
D_308
D_309
1050000
1052500
1055000
1057500
1060000
nirB
nirD
cysG
EAE_RS05215
EAE_RS05220
EAE_RS05225
TU_270
TU_271
TU_2679
TU_2678
TU_2677
EAE_RS05230
EAE_RS05235
EAE_RS05240
EAE_RS05245
EAE_RS05250
EAE_RS05255
EAE_RS05260
trpS
gph
rpe
dam
damX
aroB
aroK
D_3135
D_3134
D_3133
D_3132
D_3131
Dc_3540
Dc_3539
Dc_3538
Dc_3537
Dc_3536
Dc_3535
I_3952
I_3951
I_3950
I_3949
I_3948
I_3947
I_3946
Term: termination (3), NS: new start (1), PS: pausing site (1), iTSS_I: internal starting site (1)

ID: 21280−21400; Term: termination (0), NS: new start (1), PS: pausing site (1), iTSS_I: internal starting site (0)

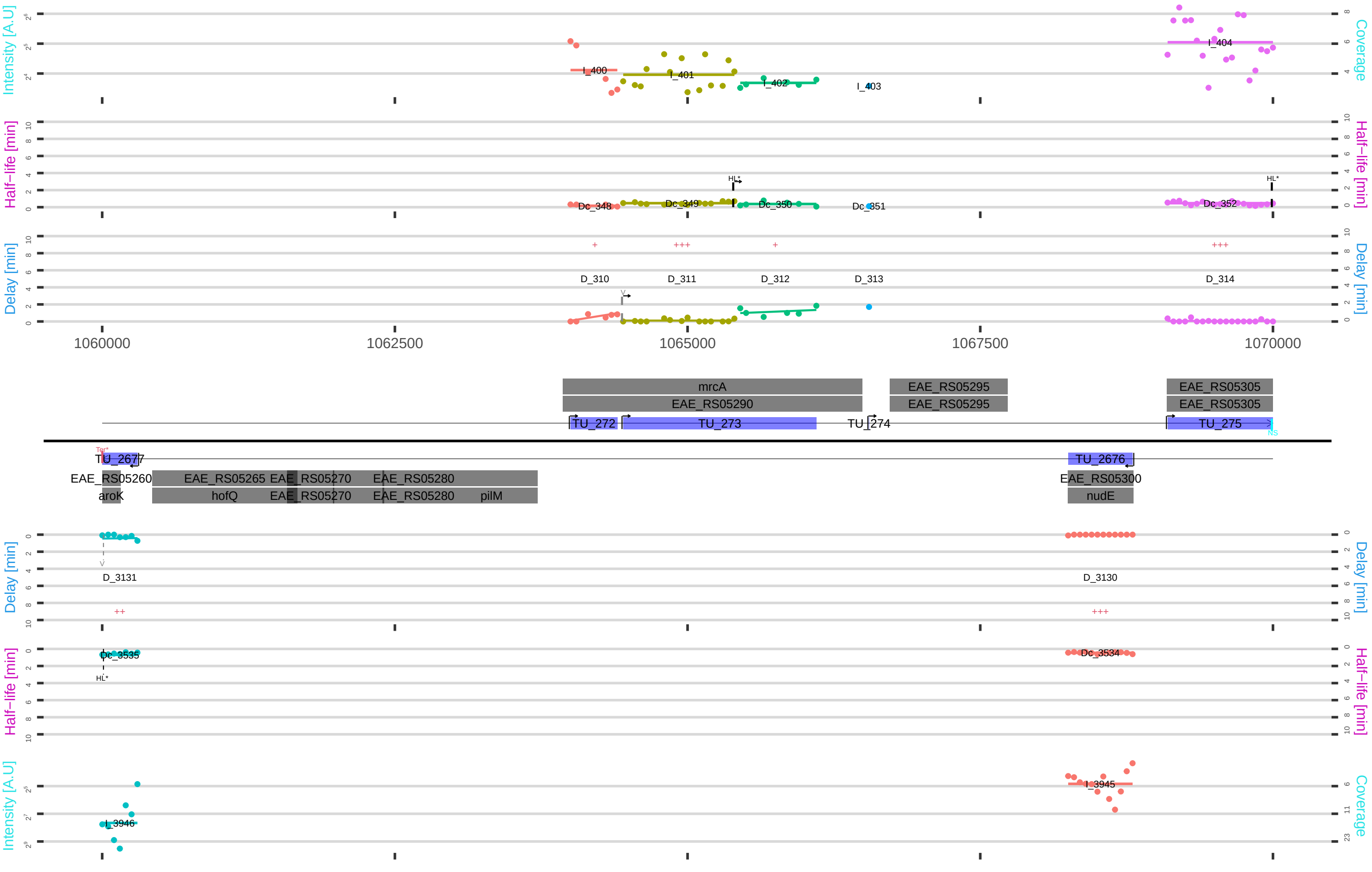

Term: termination (1), NS: new start (0), PS: pausing site (0), iTSS_I: internal starting site (0)

ID: 21400−21554; Term: termination (2), NS: new start (3), PS: pausing site (2), iTSS_I: internal starting site (0)

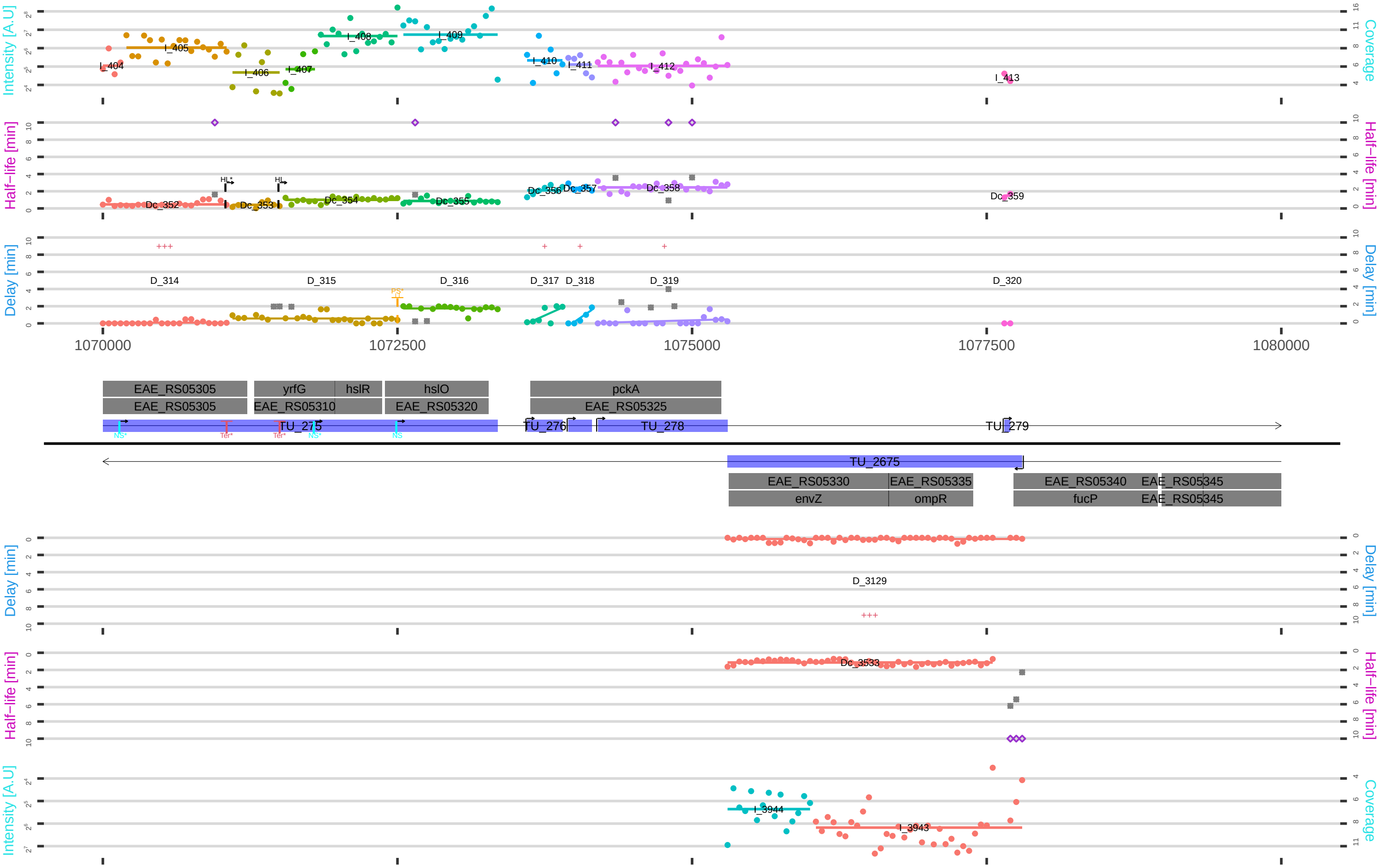

Term: termination (0), NS: new start (0), PS: pausing site (0), iTSS_I: internal starting site (0)

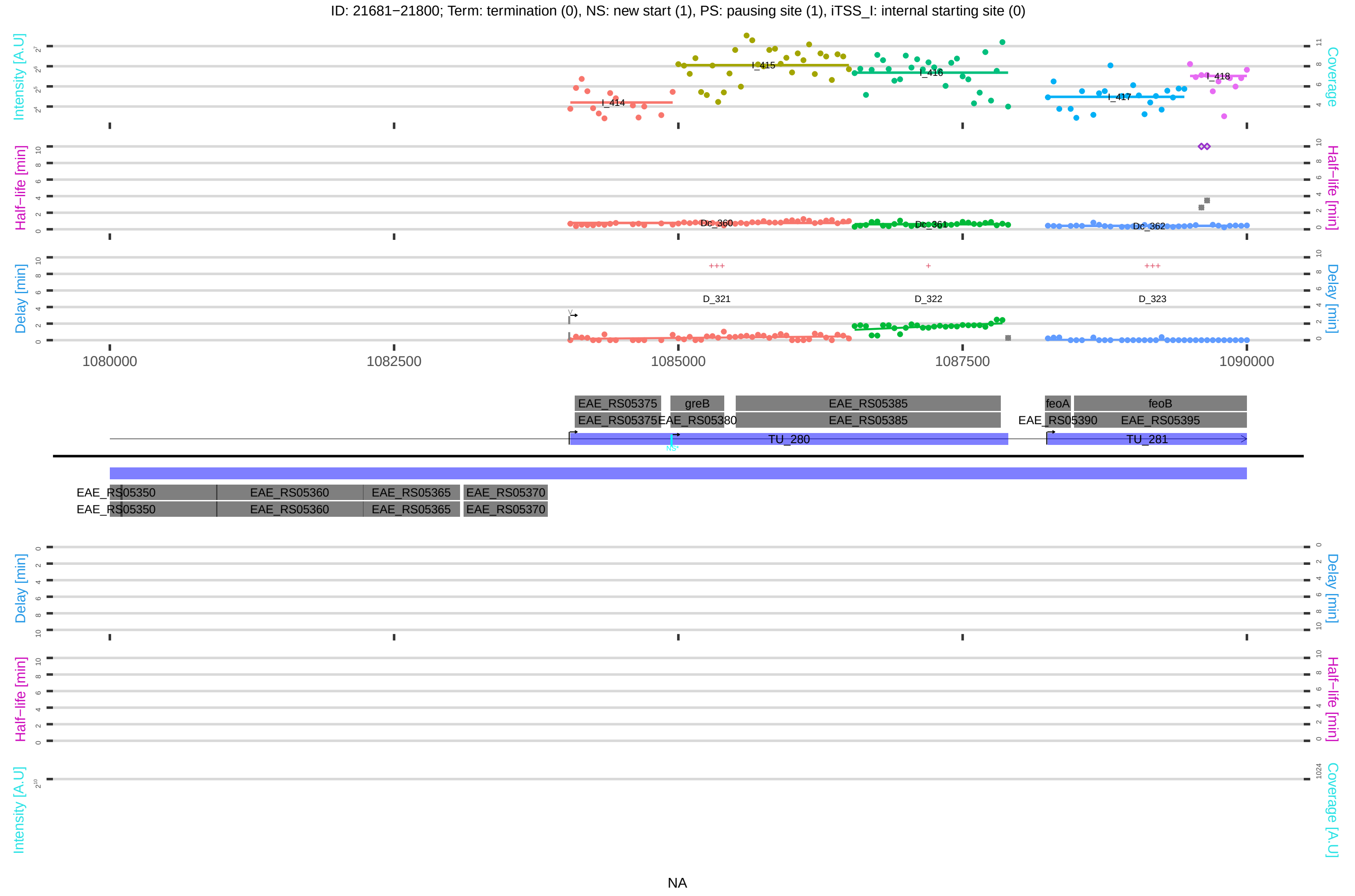

ID: 21681−21800; Term: termination (0), NS: new start (1), PS: pausing site (1), iTSS_I: internal starting site (0)
Intensity [A.U]
Coverage
Half−life [min]
Delay [min]
I_414
I_415
I_416
I_417
I_418
Dc_360
Dc_361
Dc_362
D_321
D_322
D_323
1080000
1082500
1085000
1087500
1090000
EAE_RS05375
greB
EAE_RS05385
feoA
feoB
EAE_RS05380
EAE_RS05390
EAE_RS05395
TU_280
TU_281
NS
EAE_RS05350
EAE_RS05360
EAE_RS05365
EAE_RS05370
Coverage [A.U]
NA

ID: 21800−21911; Term: termination (2), NS: new start (0), PS: pausing site (1), iTSS_I: internal starting site (0)

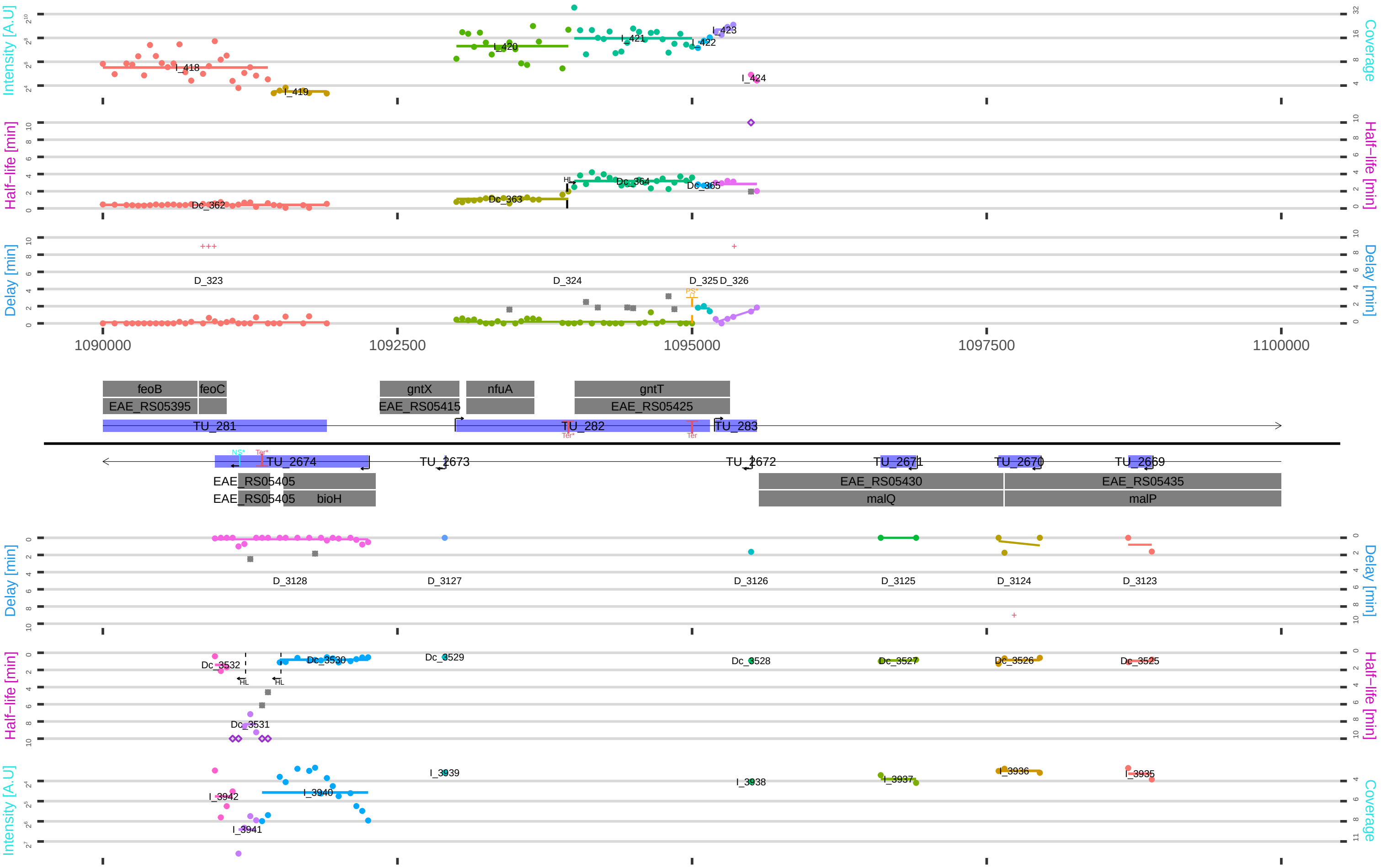

Term: termination (1), NS: new start (1), PS: pausing site (0), iTSS_I: internal starting site (0)

ID: 22014−22147; Term: termination (0), NS: new start (0), PS: pausing site (0), iTSS_I: internal starting site (0)

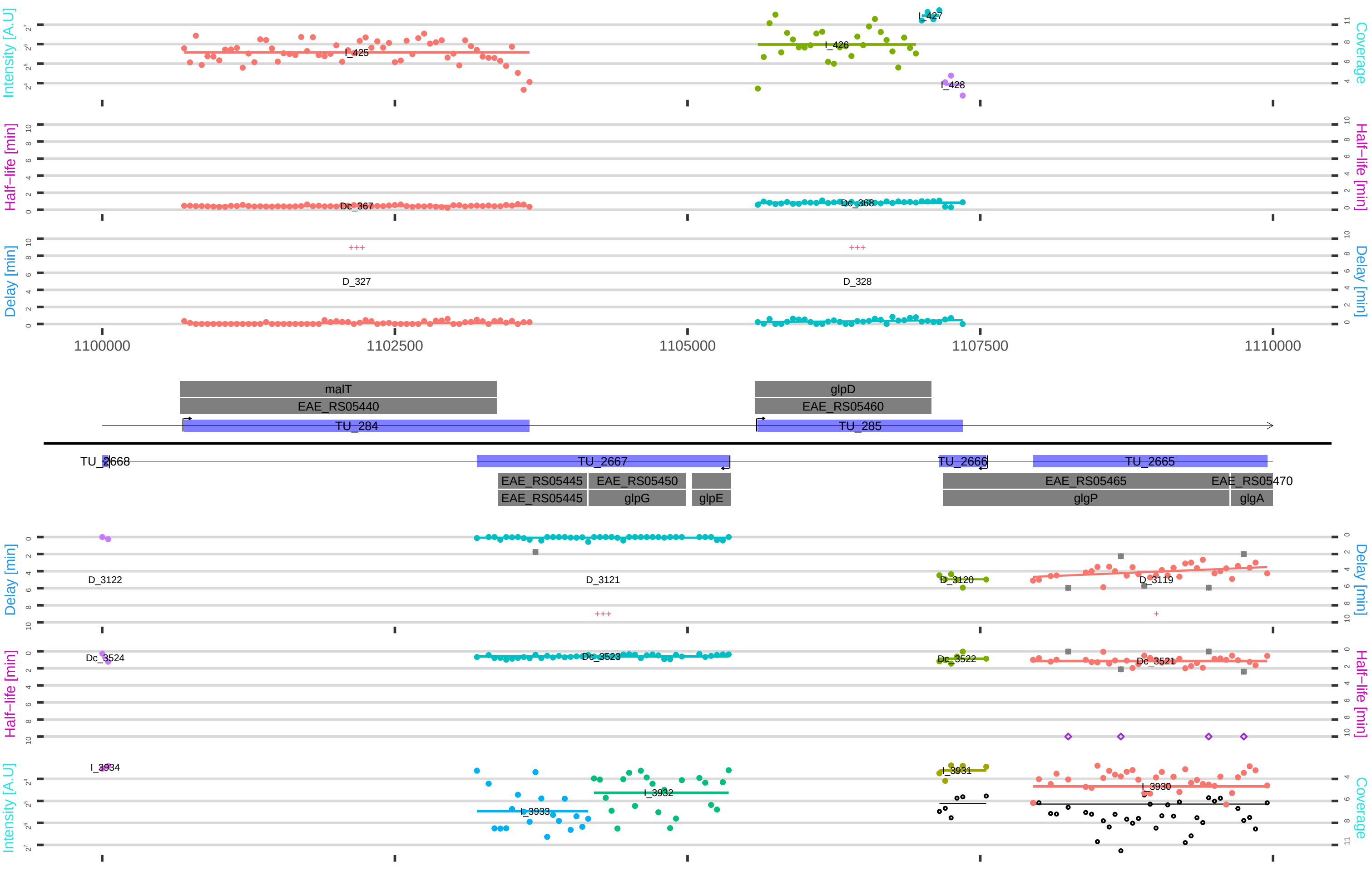

Term: termination (0), NS: new start (0), PS: pausing site (0), iTSS_I: internal starting site (1)

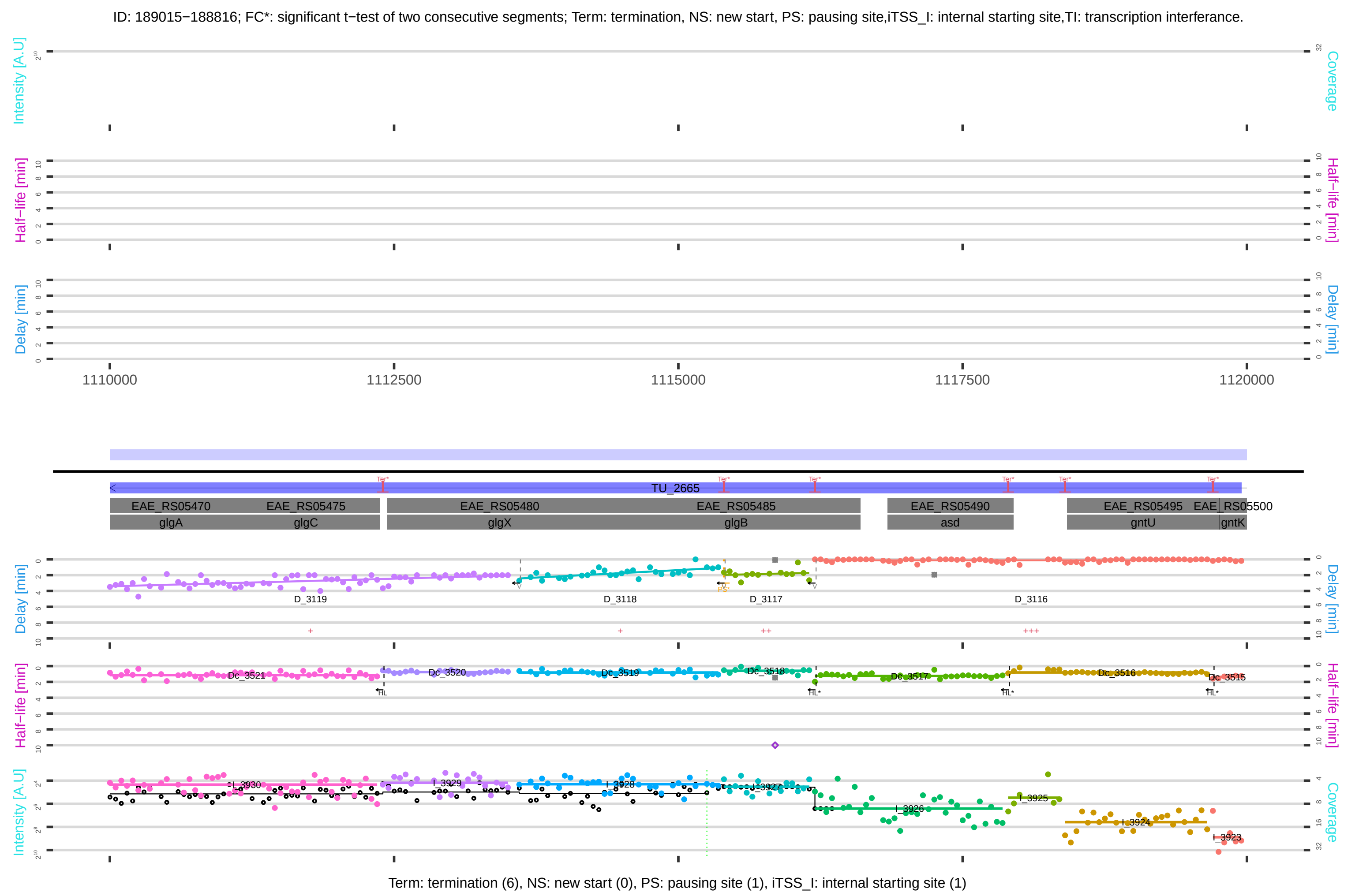

ID: 189015−188816; FC*: significant t−test of two consecutive segments; Term: termination, NS: new start, PS: pausing site,iTSS_I: internal starting site,TI: transcription interferance.
Intensity [A.U]
Coverage
Half−life [min]
Delay [min]
1110000
1112500
1115000
1117500
1120000
TU_2665
EAE_RS05470
EAE_RS05475
EAE_RS05480
EAE_RS05485
EAE_RS05490
EAE_RS05495
EAE_RS05500
glgA
glgC
glgX
glgB
asd
gntU
gntK
D_3119
D_3118
D_3117
D_3116
Dc_3521
Dc_3520
Dc_3519
Dc_3518
Dc_3517
Dc_3516
Dc_3515
I_3930
I_3929
I_3928
I_3927
I_3926
I_3925
I_3924
I_3923
Term: termination (6), NS: new start (0), PS: pausing site (1), iTSS_I: internal starting site (1)

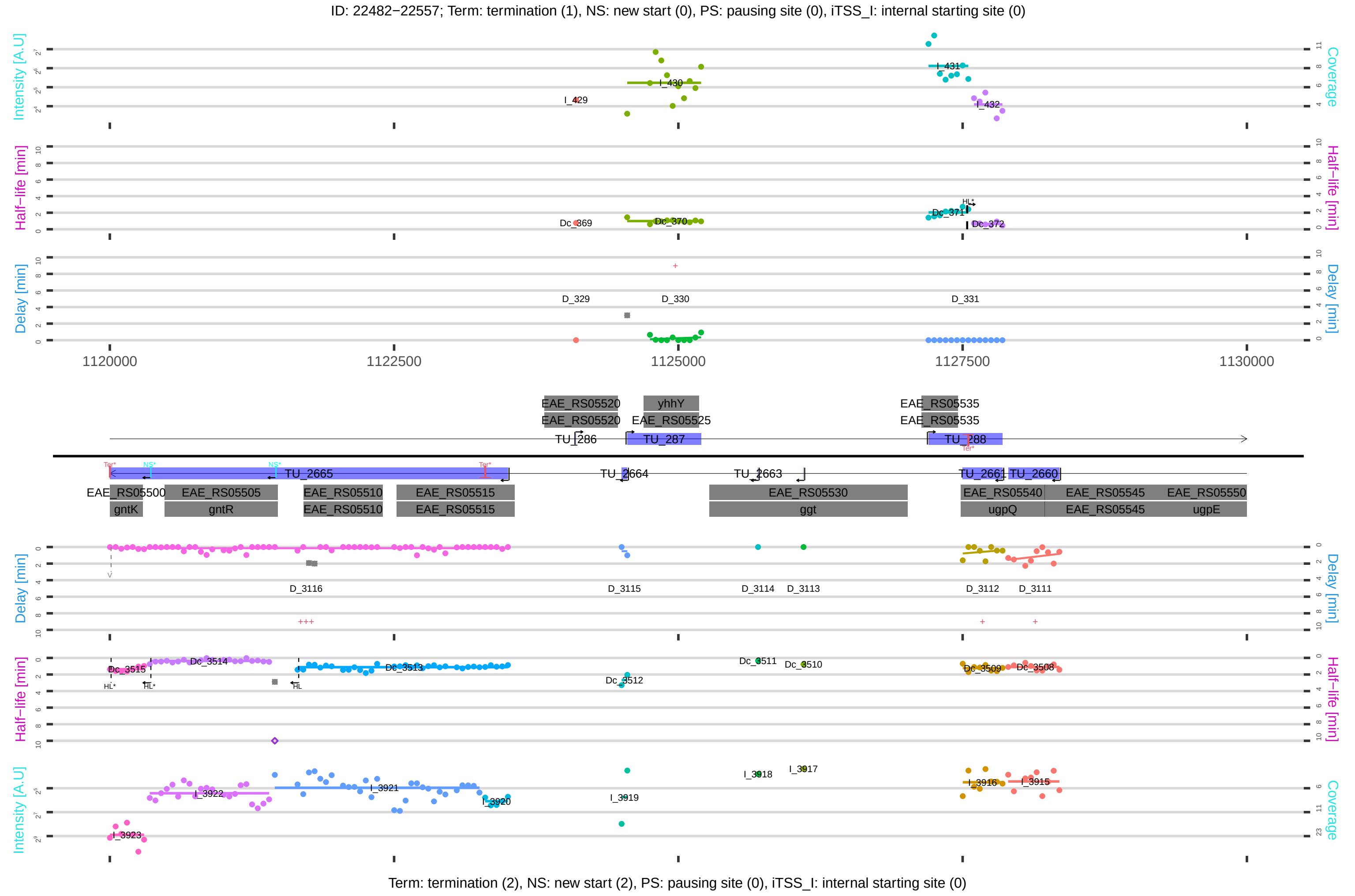

ID: 22482−22557; Term: termination (1), NS: new start (0), PS: pausing site (0), iTSS_I: internal starting site (0)
Intensity [A.U]
Coverage
Half−life [min]
Delay [min]
1120000
1122500
1125000
1127500
1130000
EAE_RS05520
yhhY
EAE_RS05525
EAE_RS05535
TU_286
TU_287
TU_288
TU_2665
TU_2664
TU_2663
TU_2661
TU_2660
EAE_RS05500
gntK
EAE_RS05505
gntR
EAE_RS05510
EAE_RS05515
EAE_RS05530
ggt
EAE_RS05540
ugpQ
EAE_RS05545
EAE_RS05550
ugpE
Term: termination (2), NS: new start (2), PS: pausing site (0), iTSS_I: internal starting site (0)

ID: 22762−22775; Term: termination (0), NS: new start (0), PS: pausing site (0), iTSS_I: internal starting site (0)

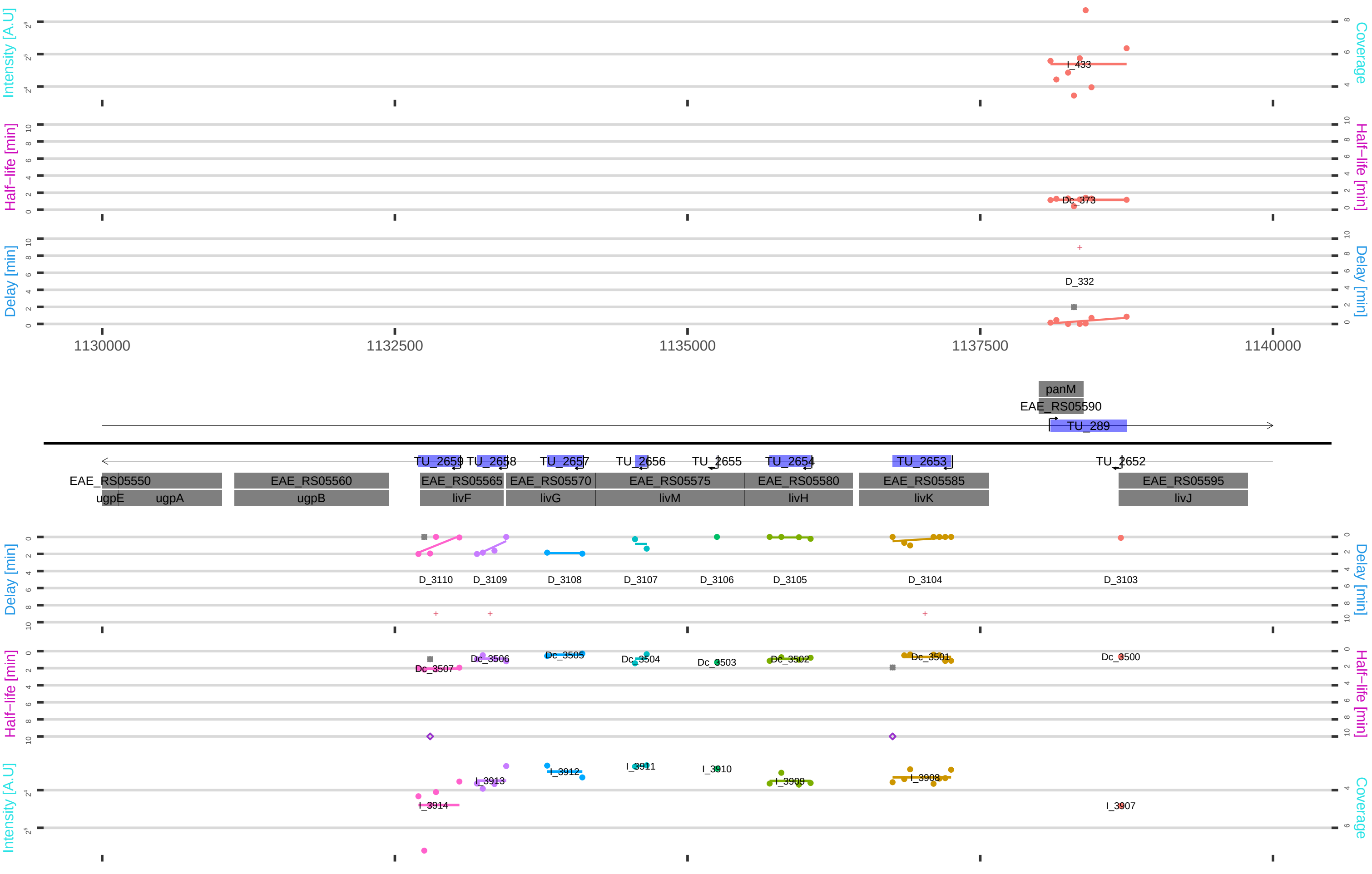

Term: termination (0), NS: new start (0), PS: pausing site (0), iTSS_I: internal starting site (0)

ID: 22894−23000; Term: termination (2), NS: new start (2), PS: pausing site (0), iTSS_I: internal starting site (1)

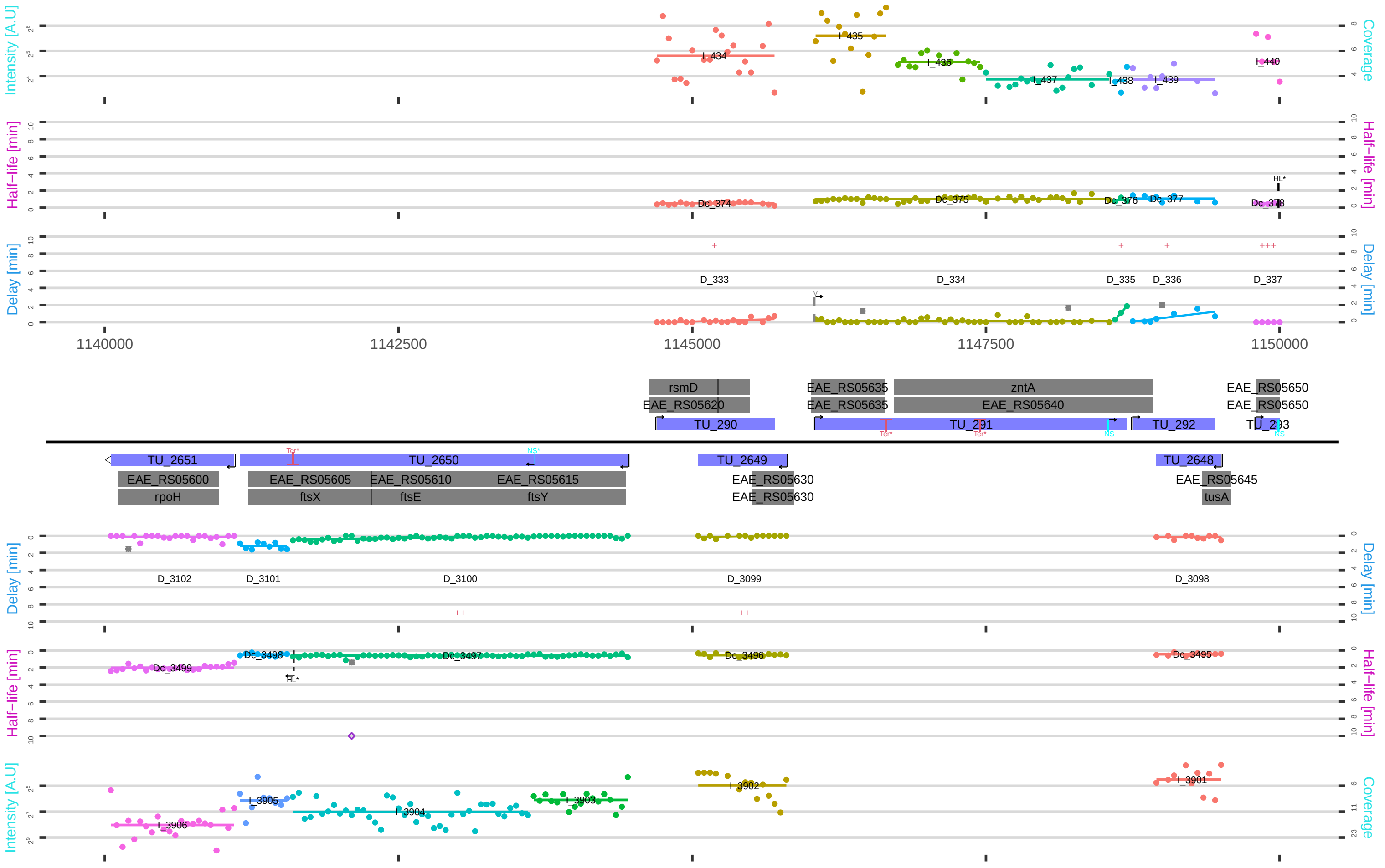

Term: termination (1), NS: new start (1), PS: pausing site (1), iTSS_I: internal starting site (0)

ID: 23000−23187; Term: termination (2), NS: new start (2), PS: pausing site (1), iTSS_I: internal starting site (0)

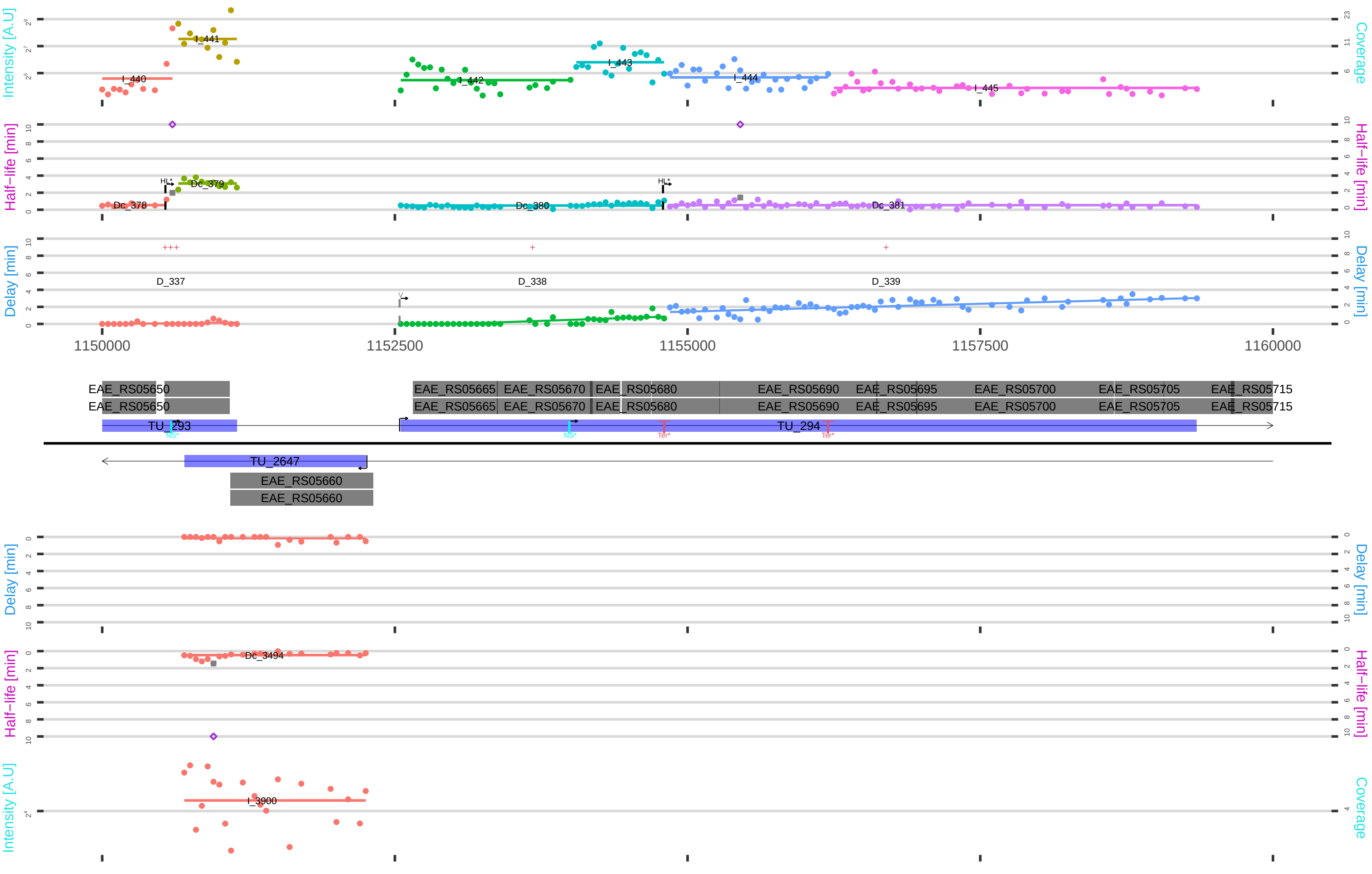

Term: termination (0), NS: new start (0), PS: pausing site (0), iTSS_I: internal starting site (0)

ID: 23236−23323; Term: termination (0), NS: new start (0), PS: pausing site (0), iTSS_I: internal starting site (0)

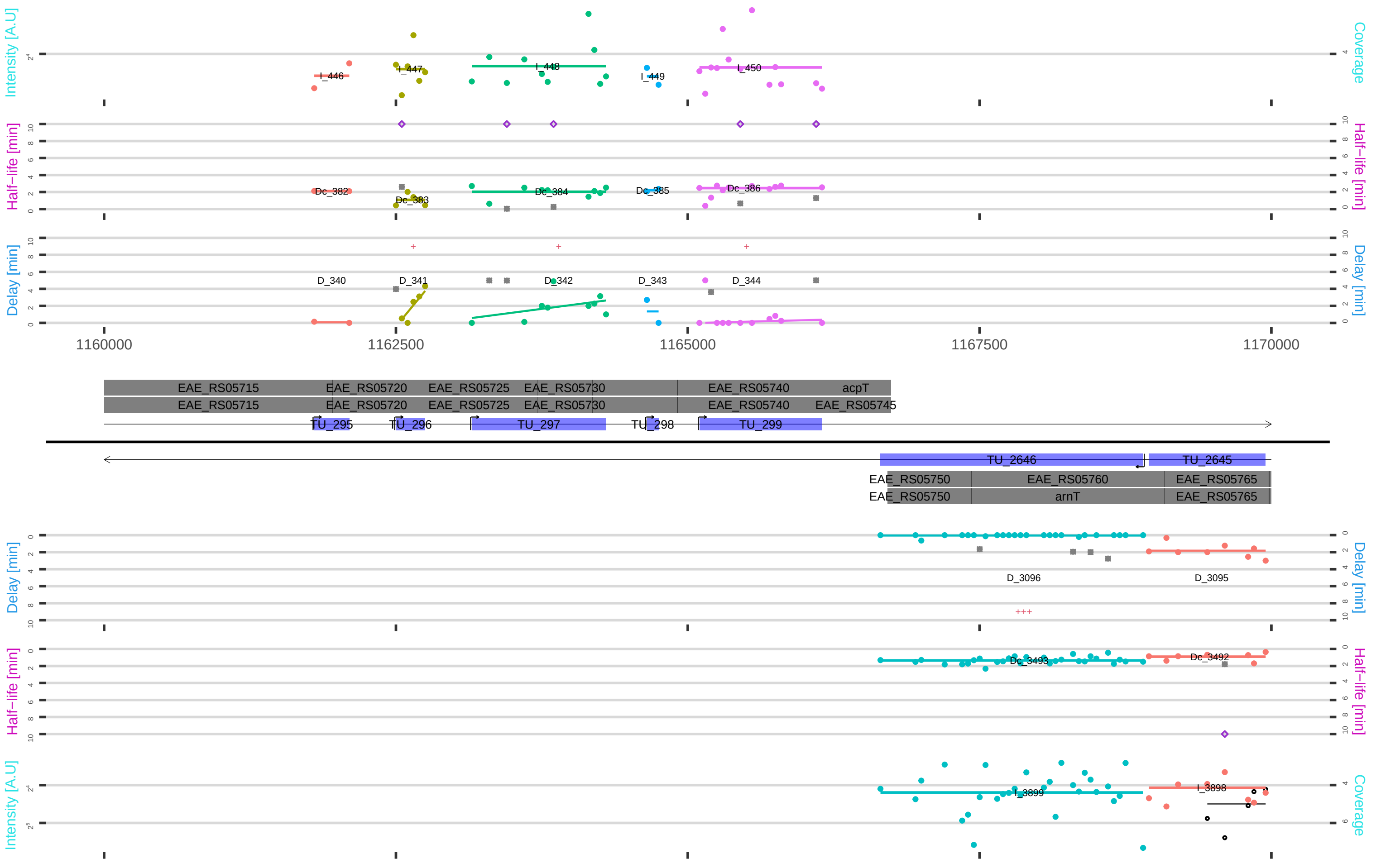

Term: termination (0), NS: new start (0), PS: pausing site (0), iTSS_I: internal starting site (0)

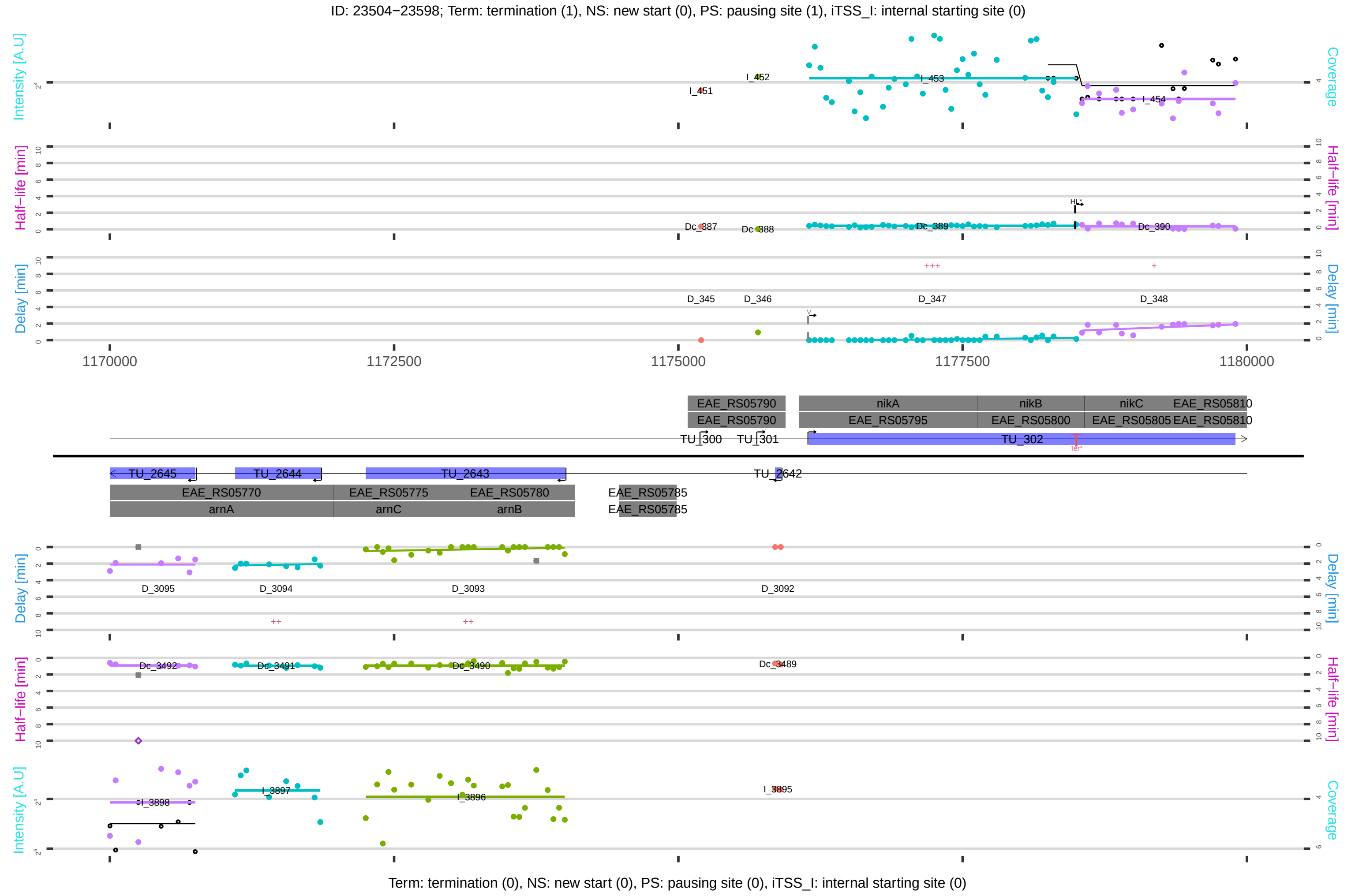
ID: 23504−23598; Term: termination (1), NS: new start (0), PS: pausing site (1), iTSS_I: internal starting site (0)
Intensity [A.U]
Coverage
Half−life [min]
Delay [min]
I_451
I_452
I_453
I_454
Dc_387
Dc_388
Dc_389
Dc_390
D_345
D_346
D_347
D_348
1170000
1172500
1175000
1177500
1180000
EAE_RS05790
nikA
nikB
nikC
EAE_RS05810
EAE_RS05795
EAE_RS05800
EAE_RS05805
TU_300
TU_301
TU_302
TU_2645
TU_2644
TU_2643
TU_2642
EAE_RS05770
EAE_RS05775
EAE_RS05780
EAE_RS05785
arnA
arnC
arnB
D_3095
D_3094
D_3093
D_3092
Dc_3492
Dc_3491
Dc_3490
Dc_3489
I_3898
I_3897
I_3896
I_3895
Term: termination (0), NS: new start (0), PS: pausing site (0), iTSS_I: internal starting site (0)

ID: 23604−23792; Term: termination (2), NS: new start (0), PS: pausing site (0), iTSS_I: internal starting site (0)

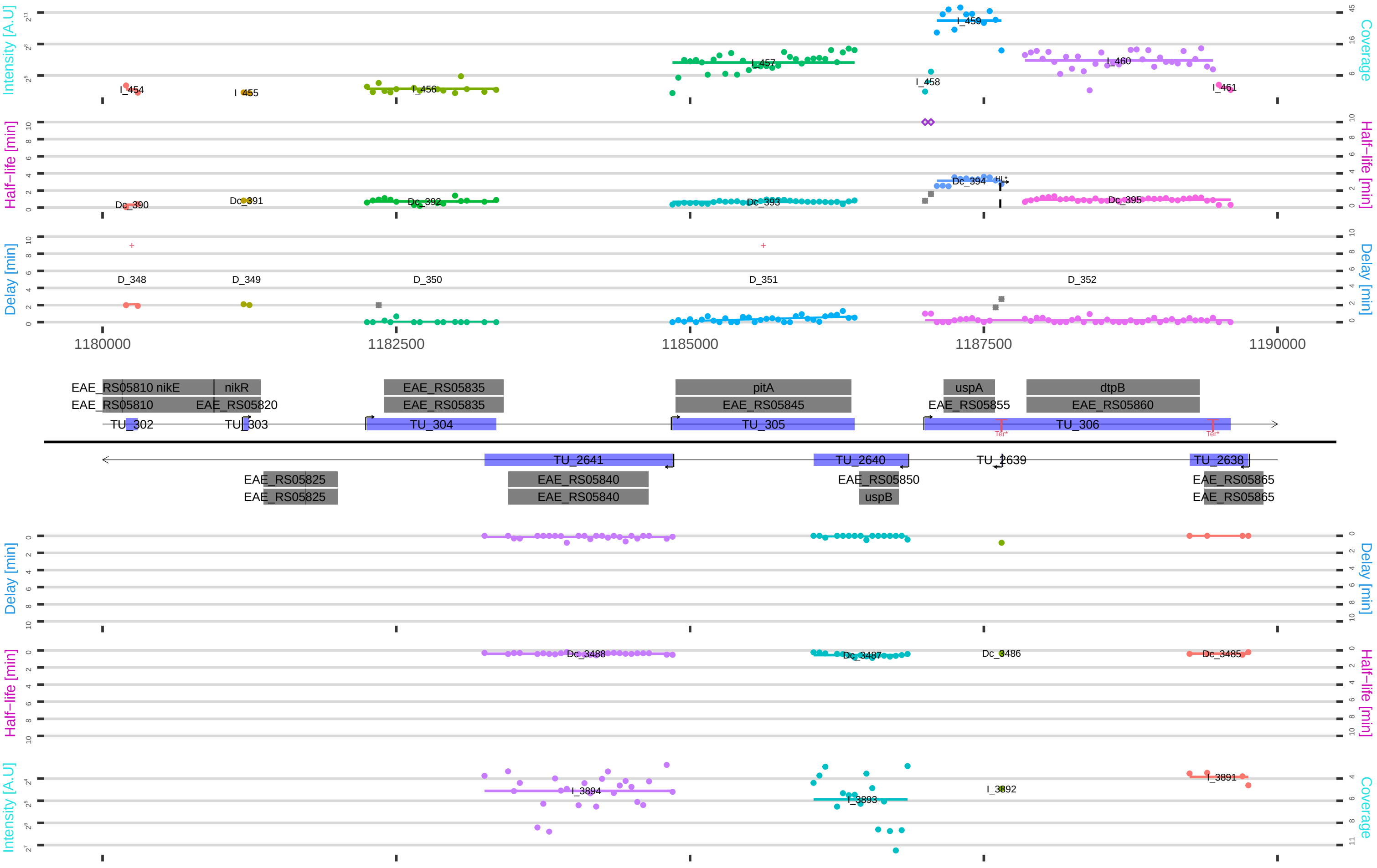

Term: termination (0), NS: new start (0), PS: pausing site (0), iTSS_I: internal starting site (0)

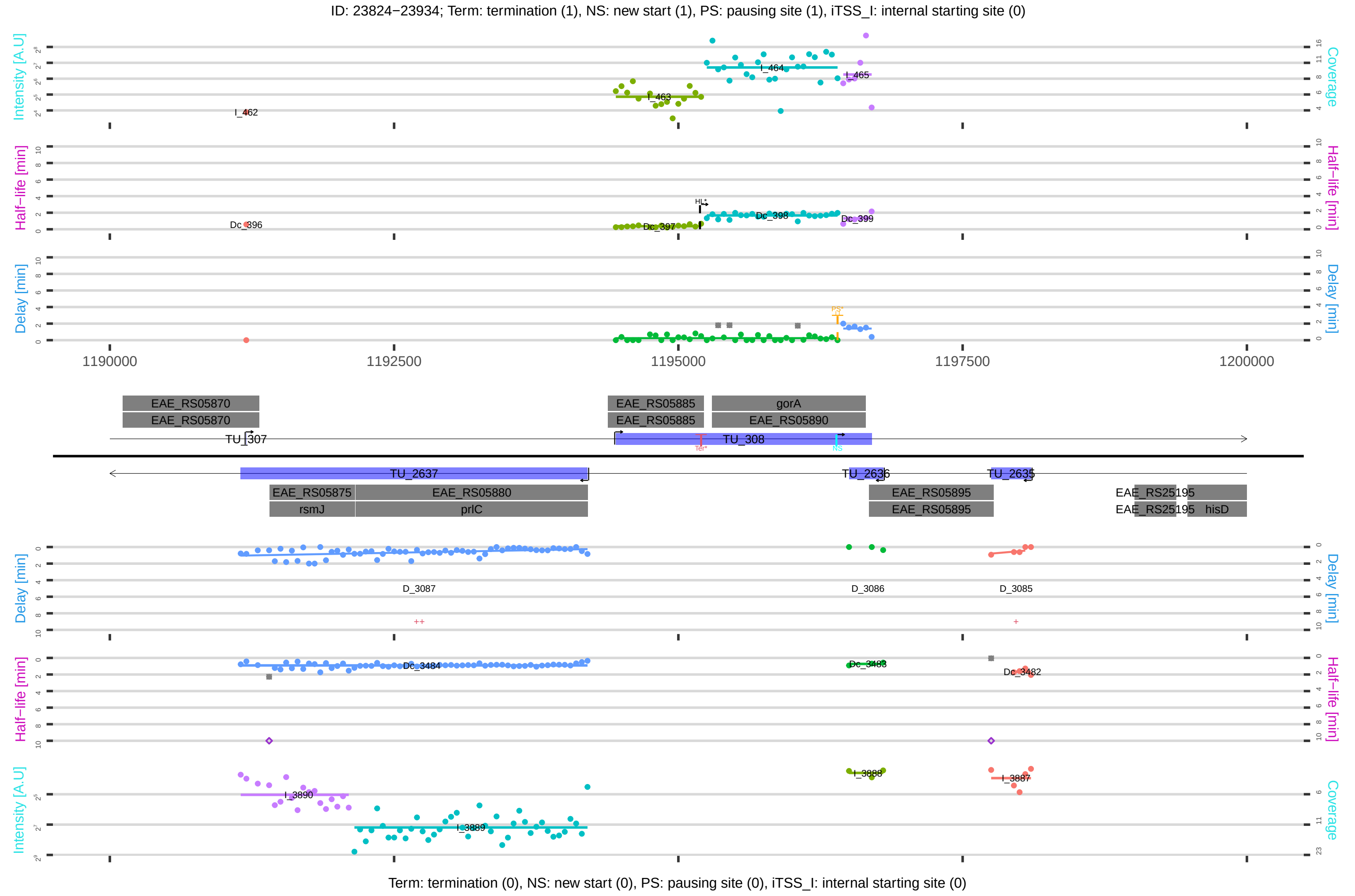

ID: 23824−23934; Term: termination (1), NS: new start (1), PS: pausing site (1), iTSS_I: internal starting site (0)
Intensity [A.U]
Coverage
Half−life [min]
Delay [min]
I_462
I_463
I_464
I_465
Dc_396
Dc_397
Dc_398
Dc_399
1190000
1192500
1195000
1197500
1200000
EAE_RS05870
EAE_RS05885
gorA
EAE_RS05890
TU_307
TU_308
TU_2637
TU_2636
TU_2635
EAE_RS05875
EAE_RS05880
rsmJ
prlC
EAE_RS05895
EAE_RS25195
hisD
D_3087
D_3086
D_3085
Dc_3484
Dc_3483
Dc_3482
I_3890
I_3889
I_3888
I_3887
Term: termination (0), NS: new start (0), PS: pausing site (0), iTSS_I: internal starting site (0)

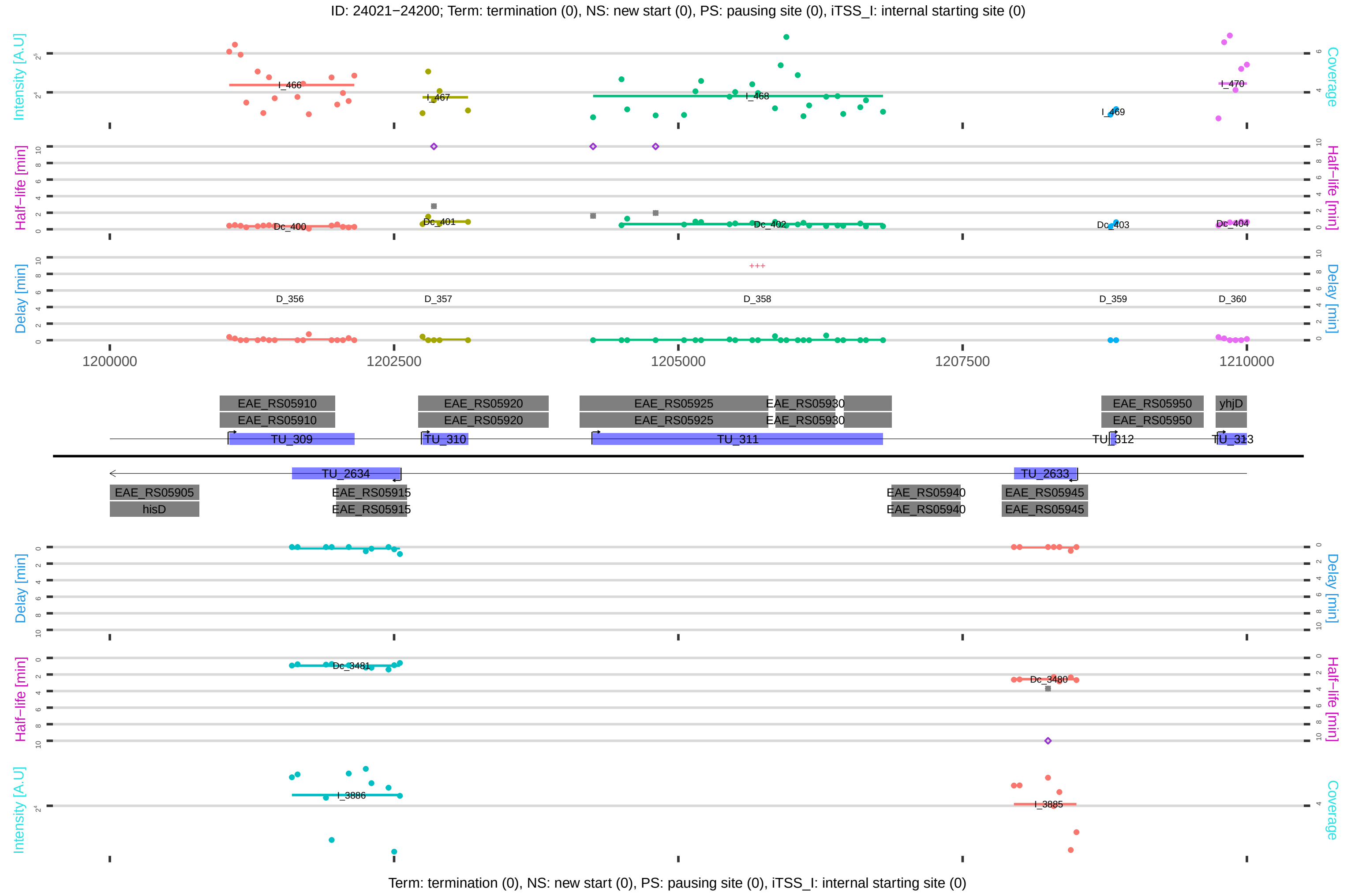

ID: 24021−24200; Term: termination (0), NS: new start (0), PS: pausing site (0), iTSS_I: internal starting site (0)
Intensity [A.U]
Coverage
Half−life [min]
Delay [min]
I_466
I_467
I_468
I_469
I_470
Dc_400
Dc_401
Dc_402
Dc_403
Dc_404
D_356
D_357
D_358
D_359
D_360
1200000
1202500
1205000
1207500
1210000
EAE_RS05910
EAE_RS05920
EAE_RS05925
EAE_RS05930
EAE_RS05950
yhjD
TU_309
TU_310
TU_311
TU_312
TU_313
TU_2634
TU_2633
EAE_RS05905
hisD
EAE_RS05915
EAE_RS05940
EAE_RS05945
Dc_3481
Dc_3480
I_3886
I_3885
Term: termination (0), NS: new start (0), PS: pausing site (0), iTSS_I: internal starting site (0)

ID: 24200−24317; Term: termination (0), NS: new start (0), PS: pausing site (0), iTSS_I: internal starting site (0)

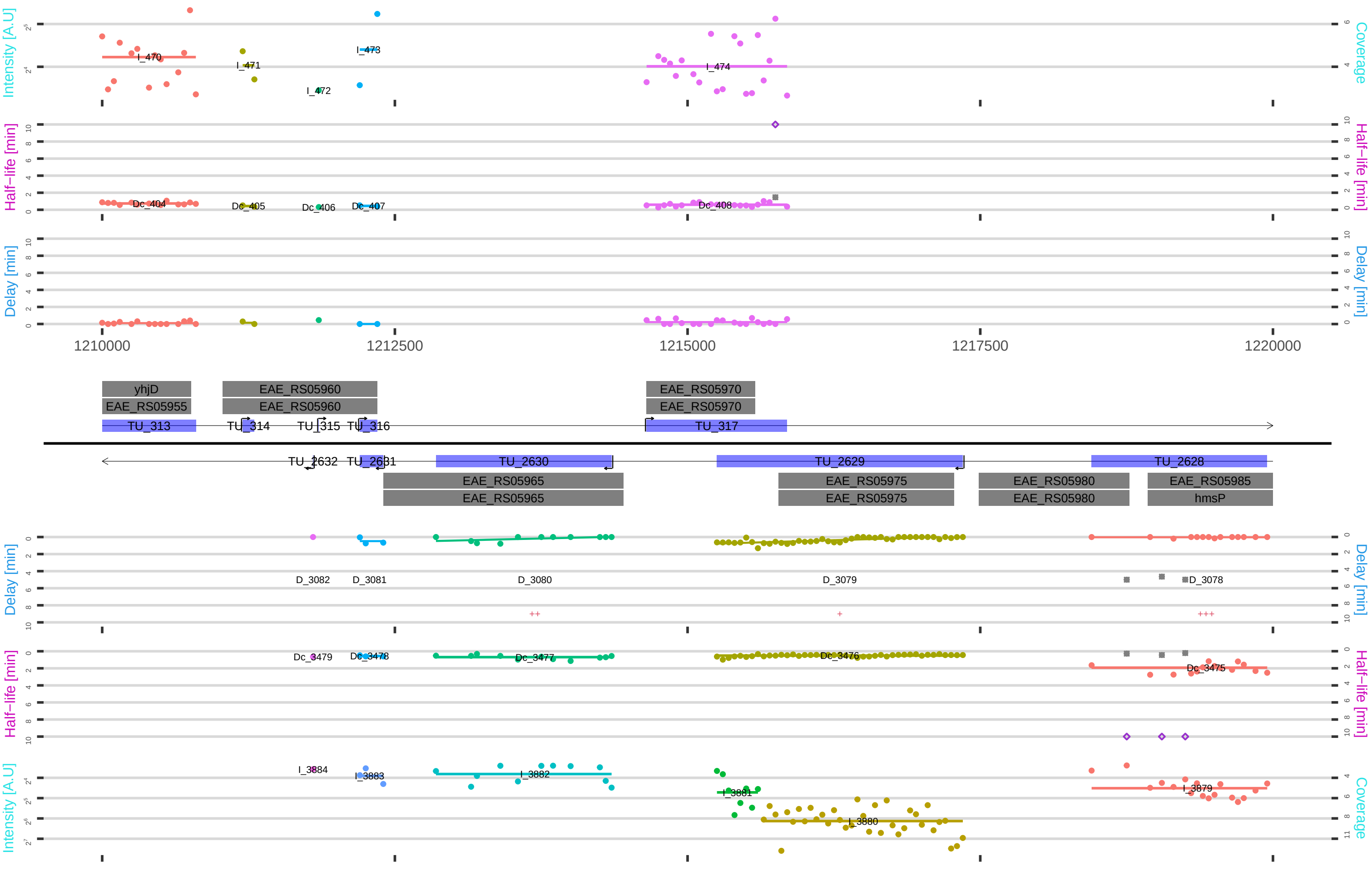

Term: termination (0), NS: new start (0), PS: pausing site (0), iTSS_I: internal starting site (0)

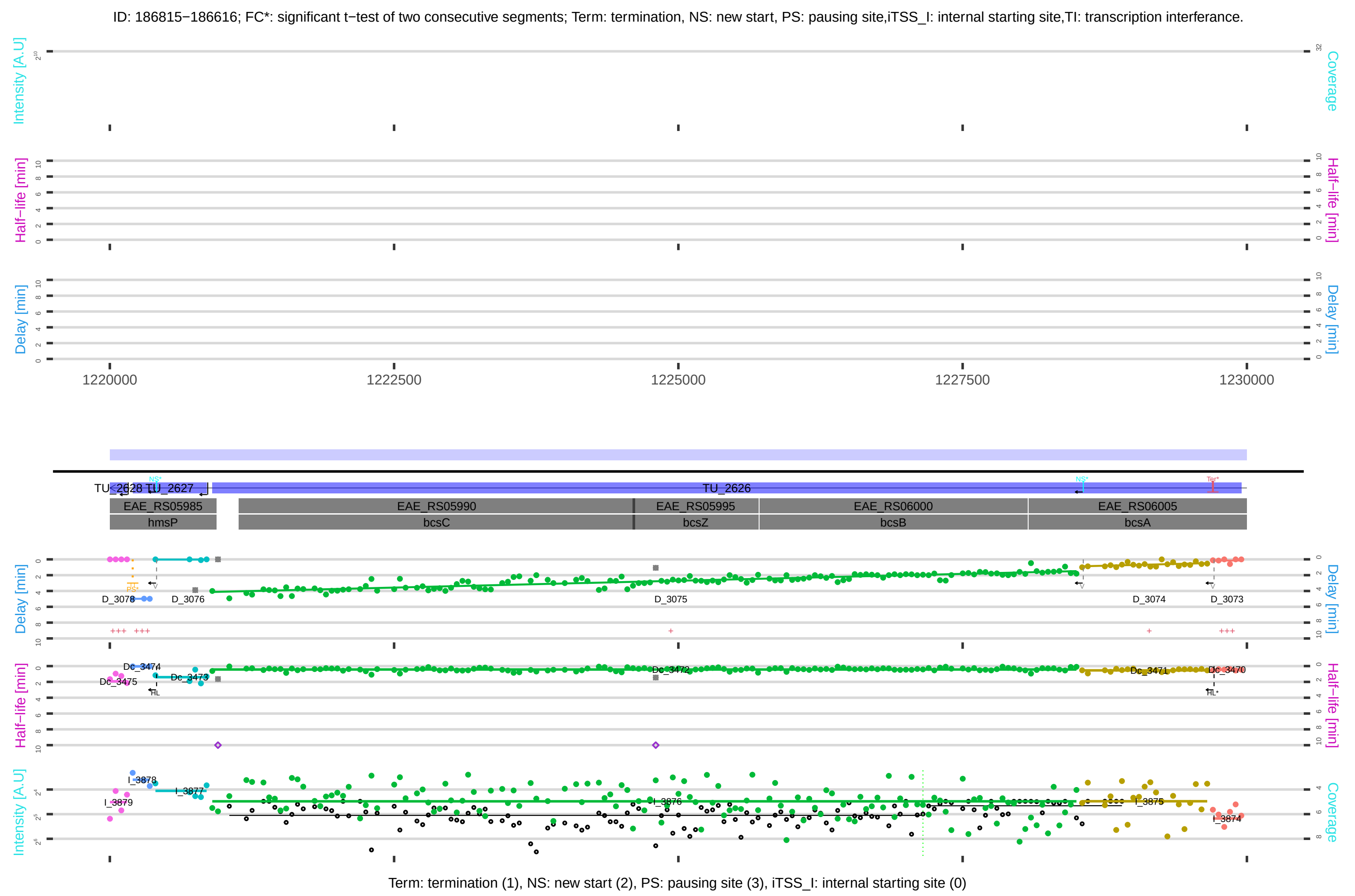

ID: 186815−186616; FC*: significant t−test of two consecutive segments; Term: termination, NS: new start, PS: pausing site,iTSS_I: internal starting site,TI: transcription interferance.
Intensity [A.U]
Coverage
Half−life [min]
Delay [min]
1220000
1222500
1225000
1227500
1230000
TU_2628
TU_2627
TU_2626
EAE_RS05985
hmsP
EAE_RS05990
bcsC
EAE_RS05995
bcsZ
EAE_RS06000
bcsB
EAE_RS06005
bcsA
D_3078
D_3076
D_3075
D_3074
D_3073
Dc_3474
Dc_3475
Dc_3473
Dc_3472
Dc_3471
Dc_3470
I_3878
I_3879
I_3877
I_3876
I_3875
I_3874
Term: termination (1), NS: new start (2), PS: pausing site (3), iTSS_I: internal starting site (0)

ID: 24637−24722; Term: termination (1), NS: new start (0), PS: pausing site (1), iTSS_I: internal starting site (0)

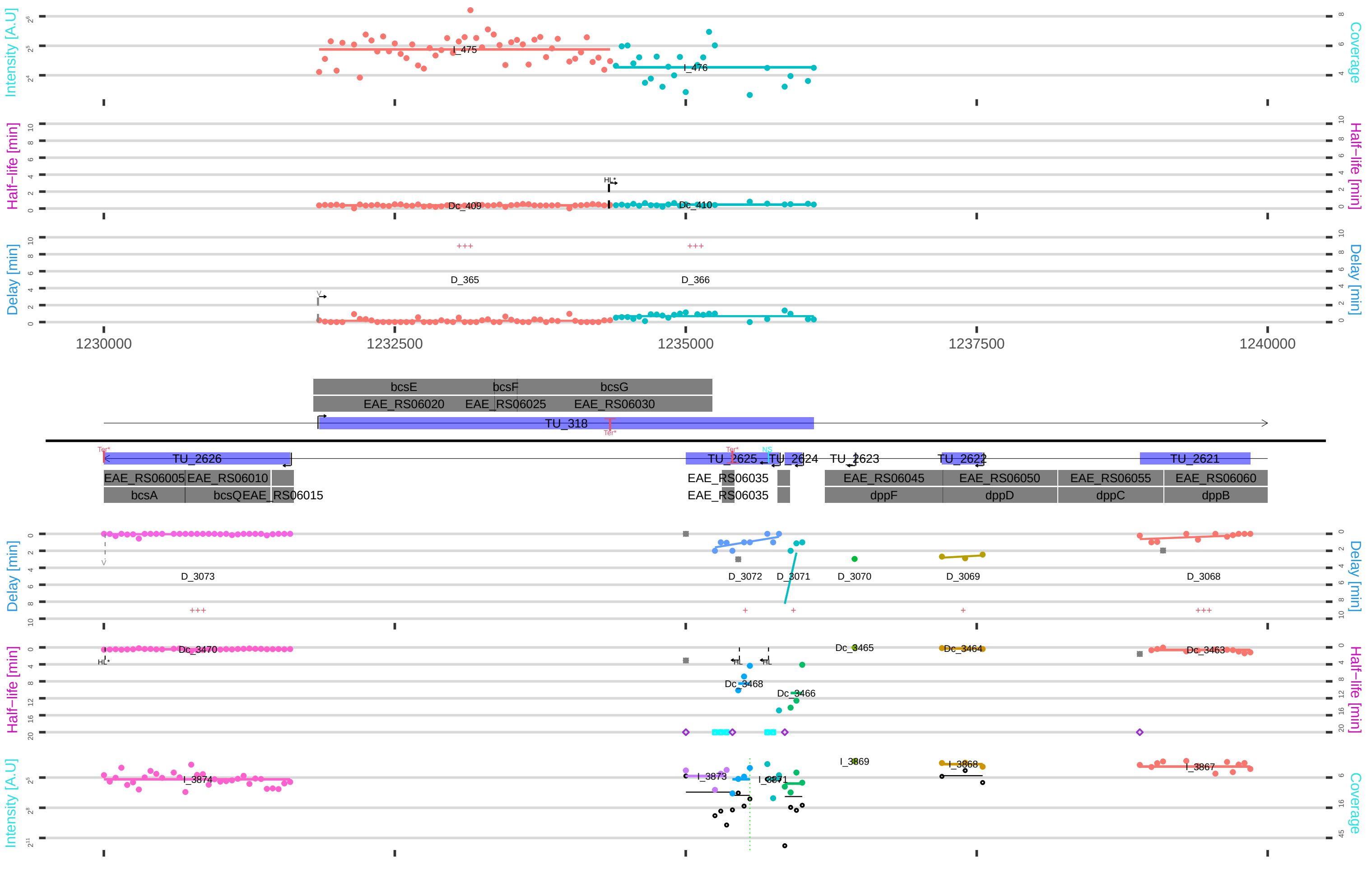

Term: termination (2), NS: new start (1), PS: pausing site (0), iTSS_I: internal starting site (0)

ID: 24944−24965; Term: termination (0), NS: new start (0), PS: pausing site (0), iTSS_I: internal starting site (0)

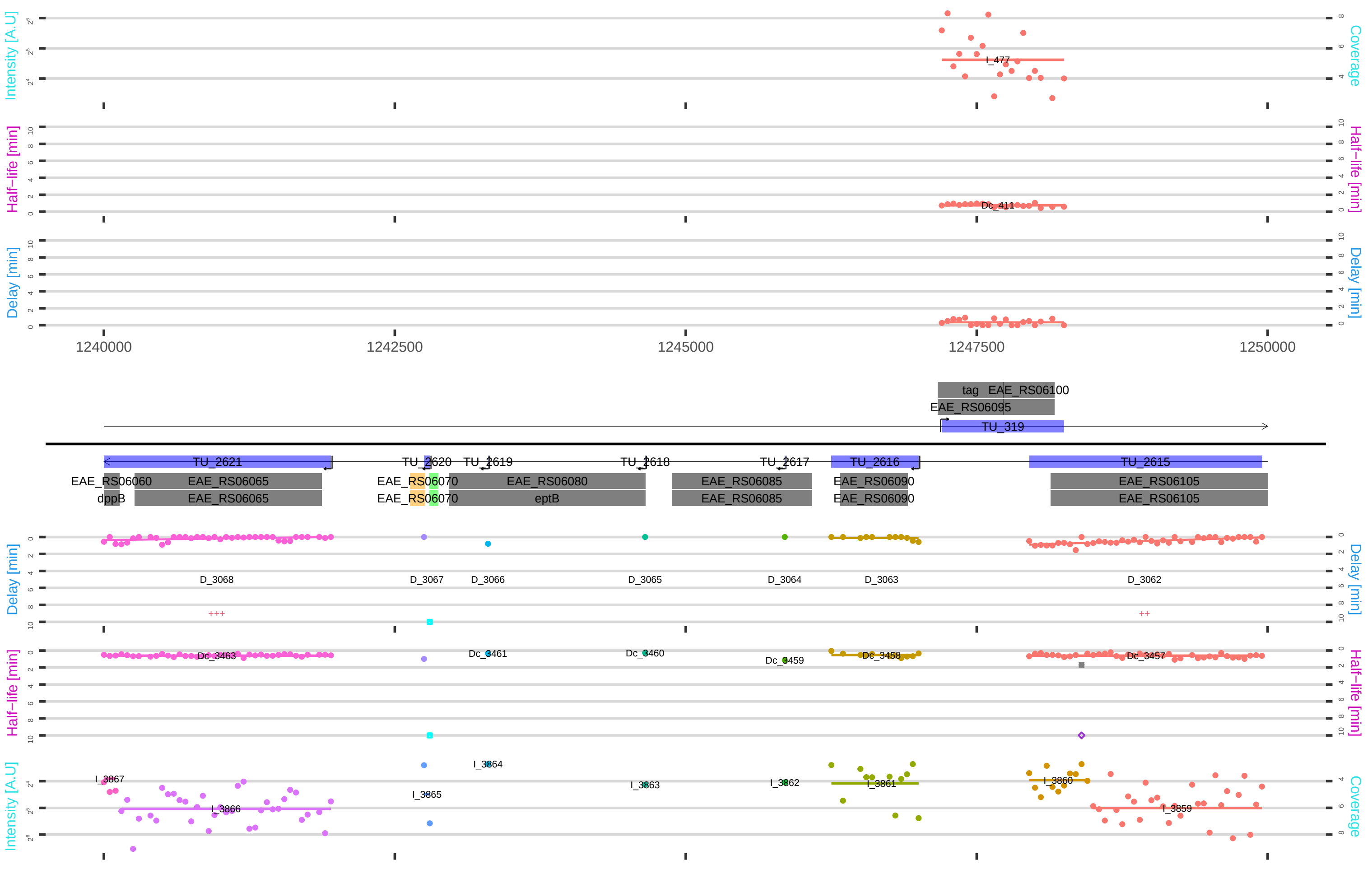

Term: termination (0), NS: new start (0), PS: pausing site (0), iTSS_I: internal starting site (0)

ID: 25013−25200; Term: termination (1), NS: new start (2), PS: pausing site (1), iTSS_I: internal starting site (0)

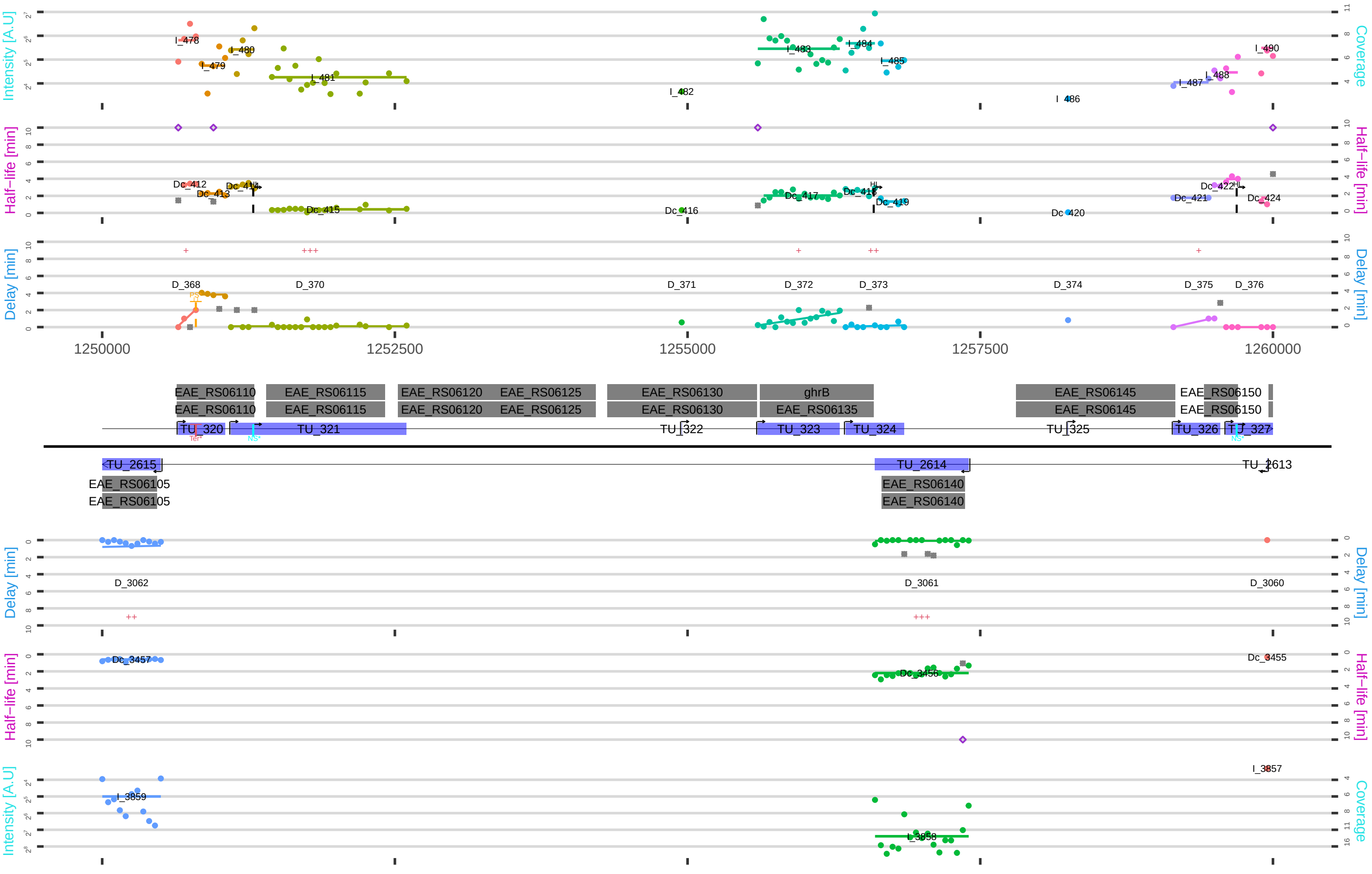

Term: termination (0), NS: new start (0), PS: pausing site (0), iTSS_I: internal starting site (0)

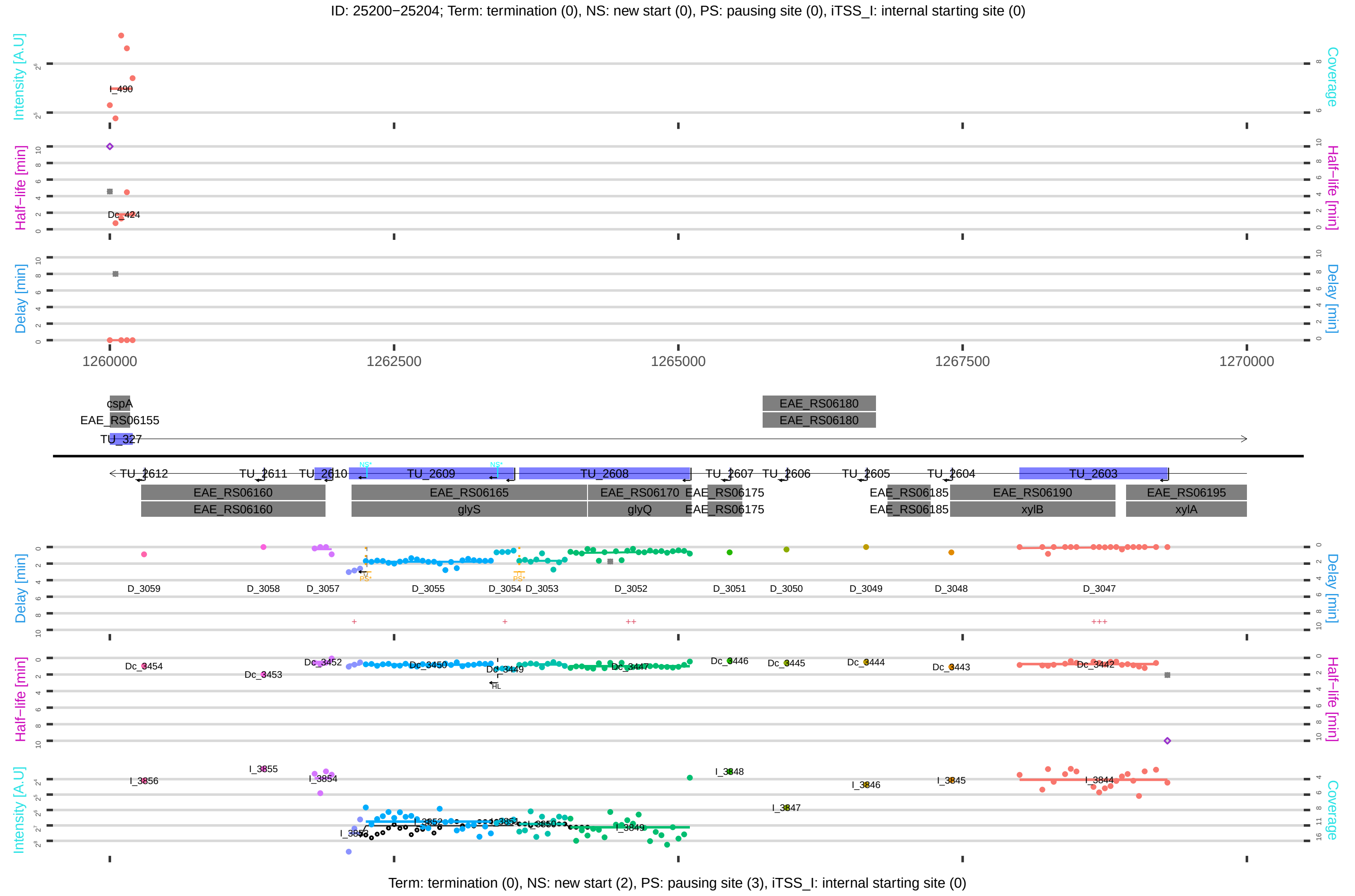
ID: 25200−25204; Term: termination (0), NS: new start (0), PS: pausing site (0), iTSS_I: internal starting site (0)
Intensity [A.U]
Coverage
Half−life [min]
Delay [min]
I_490
Dc_424
1260000
1262500
1265000
1267500
1270000
cspA
EAE_RS06155
TU_327
EAE_RS06180
TU_2612
TU_2611
TU_2610
TU_2609
TU_2608
TU_2607
TU_2606
TU_2605
TU_2604
TU_2603
EAE_RS06160
EAE_RS06165
glyS
EAE_RS06170
glyQ
EAE_RS06175
EAE_RS06185
EAE_RS06190
xylB
EAE_RS06195
xylA
D_3059
D_3058
D_3057
D_3055
D_3054
D_3053
D_3052
D_3051
D_3050
D_3049
D_3048
D_3047
Dc_3454
Dc_3453
Dc_3452
Dc_3450
Dc_3449
Dc_3447
Dc_3446
Dc_3445
Dc_3444
Dc_3443
Dc_3442
I_3856
I_3855
I_3854
I_3853
I_3852
I_3851
I_3850
I_3849
I_3848
I_3847
I_3846
I_3845
I_3844
Term: termination (0), NS: new start (2), PS: pausing site (3), iTSS_I: internal starting site (0)

ID: 25492−25599; Term: termination (0), NS: new start (0), PS: pausing site (0), iTSS_I: internal starting site (0)

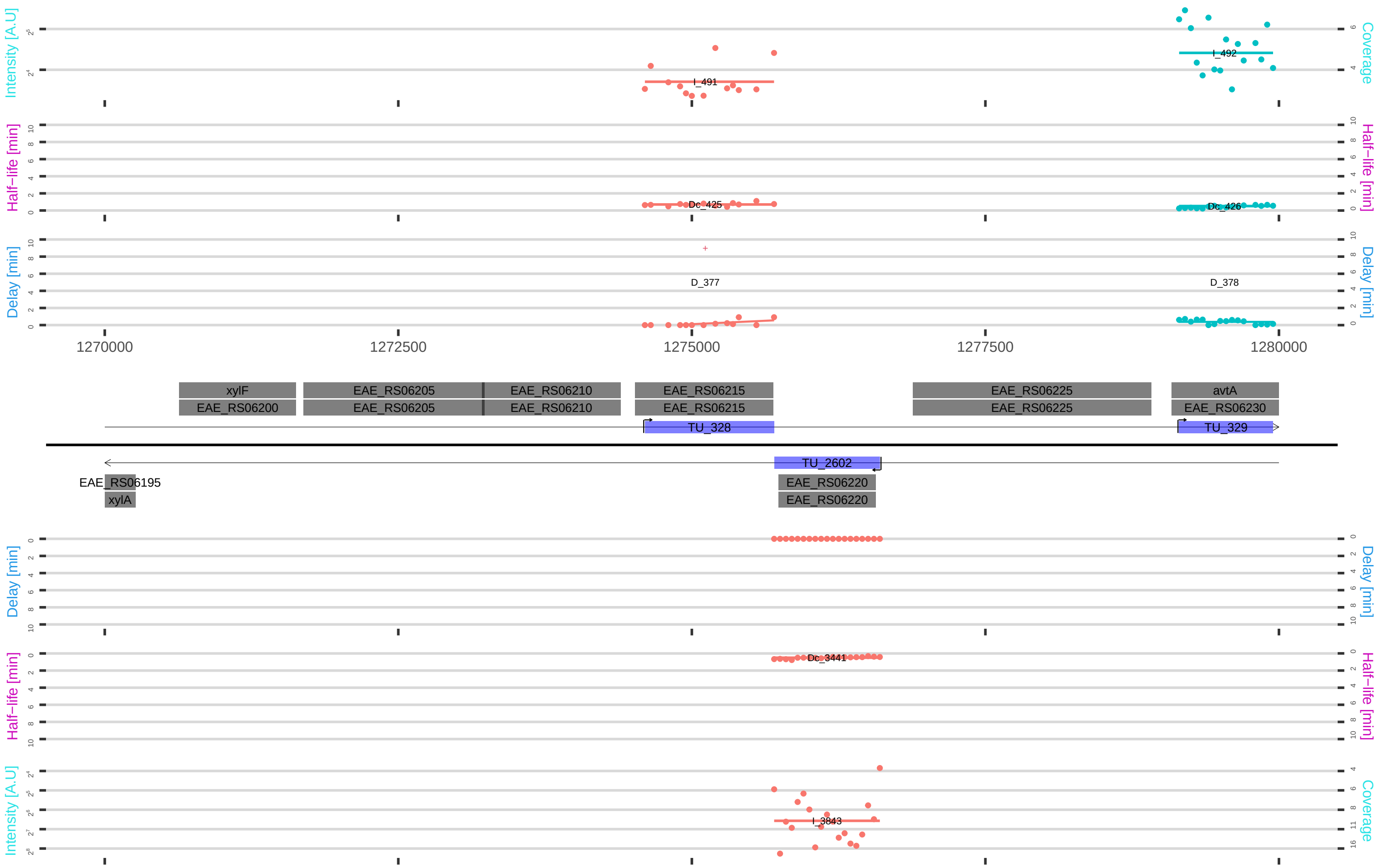

Term: termination (0), NS: new start (0), PS: pausing site (0), iTSS_I: internal starting site (0)

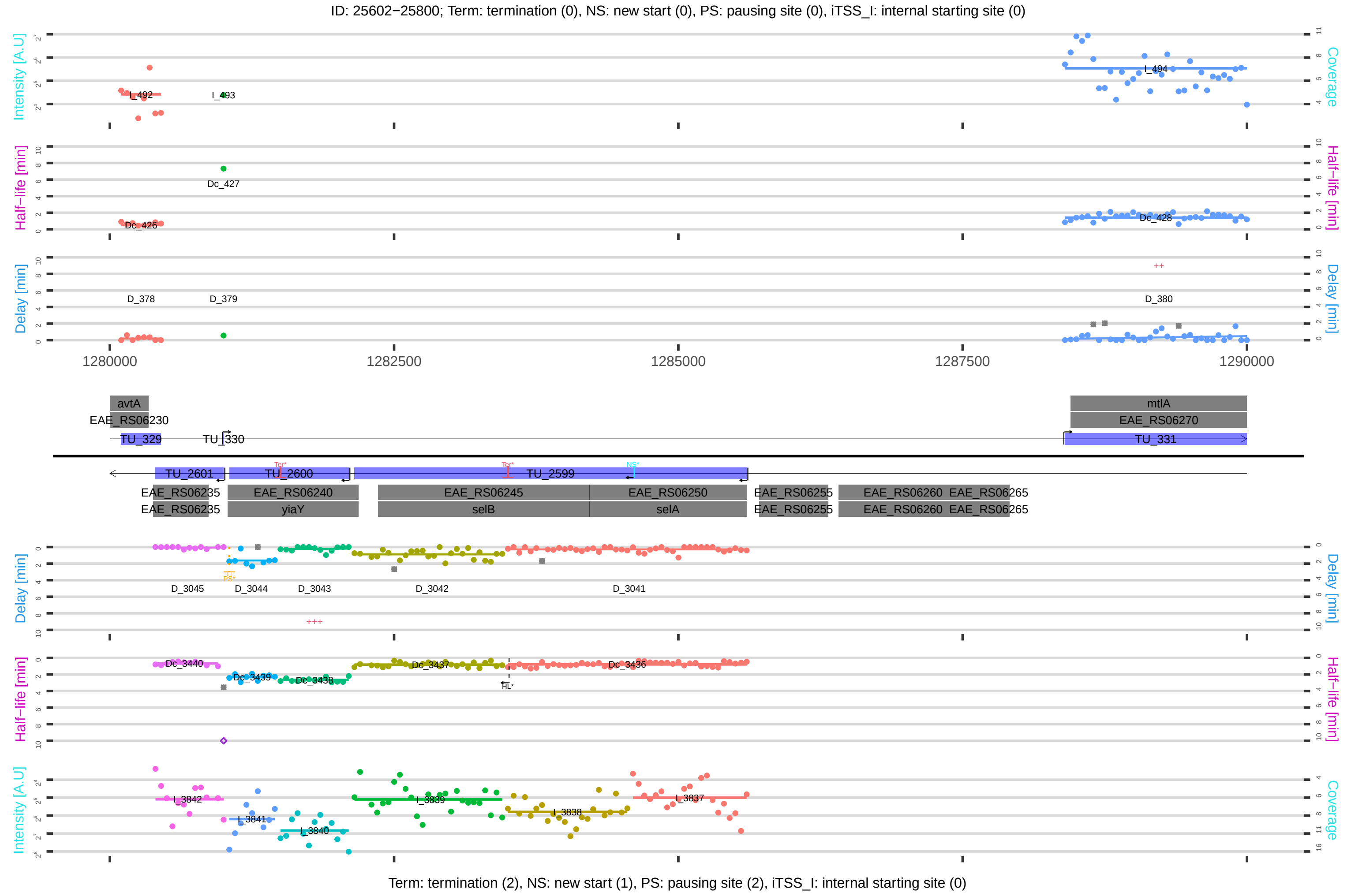

ID: 25602−25800; Term: termination (0), NS: new start (0), PS: pausing site (0), iTSS_I: internal starting site (0)
Intensity [A.U]
Coverage
I_492
I_493
I_494
Half−life [min]
Dc_427
Dc_426
Dc_428
Delay [min]
D_378
D_379
D_380
1280000
1282500
1285000
1287500
1290000
avtA
EAE_RS06230
TU_329
TU_330
mtlA
EAE_RS06270
TU_331
TU_2601
TU_2600
TU_2599
Ter*
NS*
EAE_RS06235
EAE_RS06240
yiaY
EAE_RS06245
selB
EAE_RS06250
selA
EAE_RS06255
EAE_RS06260
EAE_RS06265
PS*
D_3045
D_3044
D_3043
D_3042
D_3041
Dc_3440
Dc_3439
Dc_3438
Dc_3437
Dc_3436
HL*
I_3842
I_3841
I_3840
I_3839
I_3838
I_3837
Term: termination (2), NS: new start (1), PS: pausing site (2), iTSS_I: internal starting site (0)

ID: 25800−25970; Term: termination (1), NS: new start (1), PS: pausing site (0), iTSS_I: internal starting site (1)

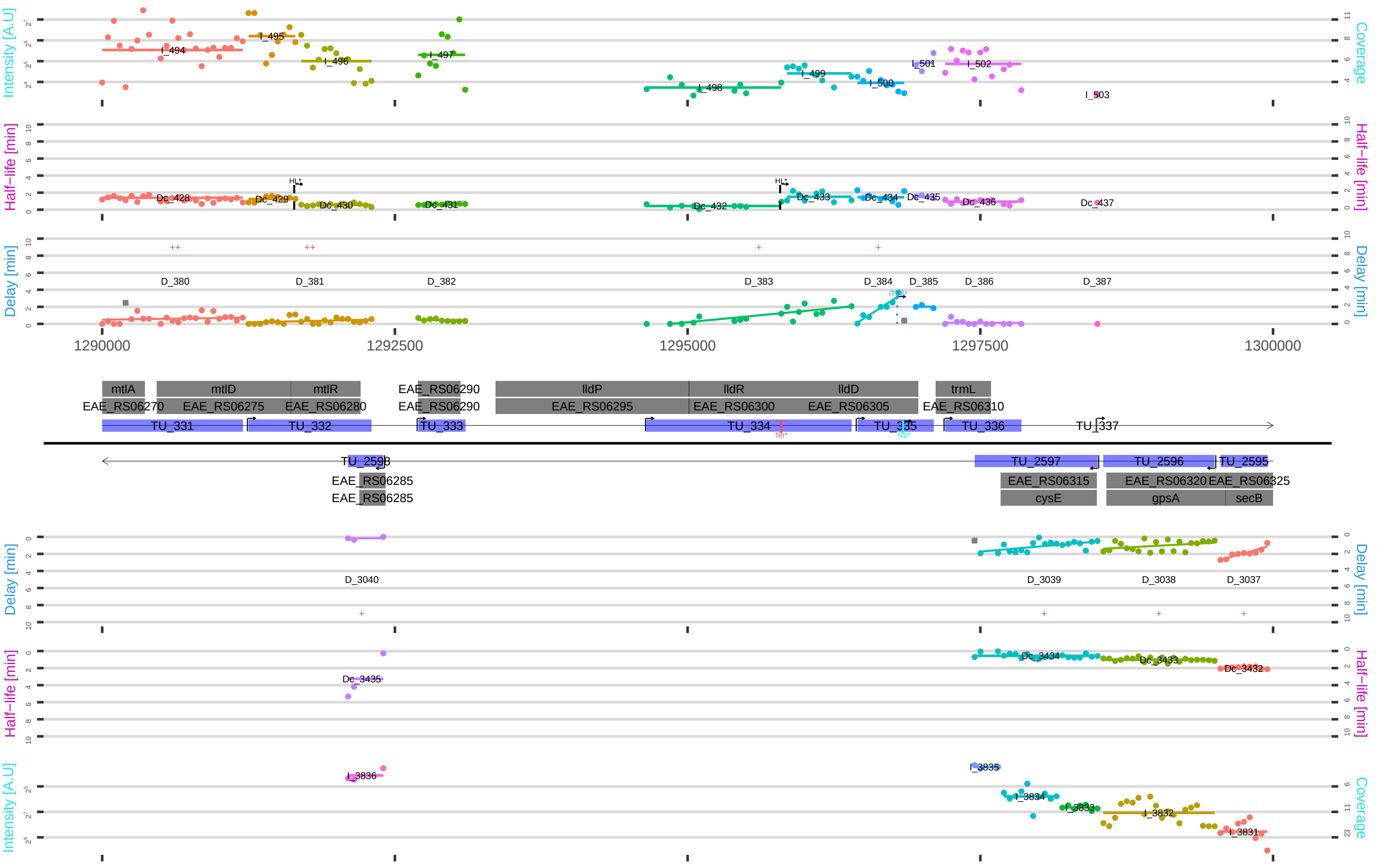

Term: termination (0), NS: new start (0), PS: pausing site (0), iTSS_I: internal starting site (0)

ID: 26024−26200; Term: termination (2), NS: new start (0), PS: pausing site (0), iTSS_I: internal starting site (0)

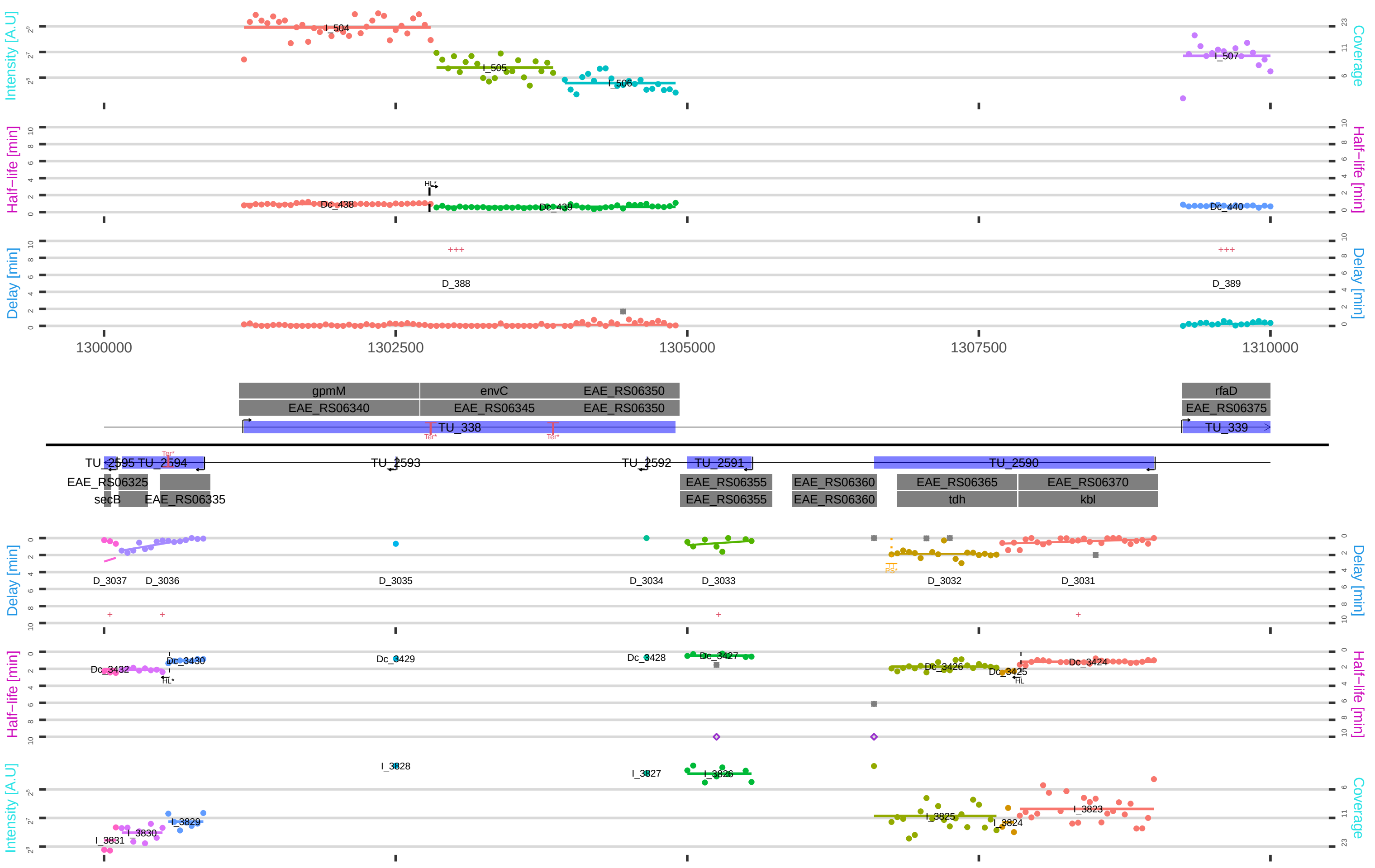

Term: termination (1), NS: new start (0), PS: pausing site (1), iTSS_I: internal starting site (0)

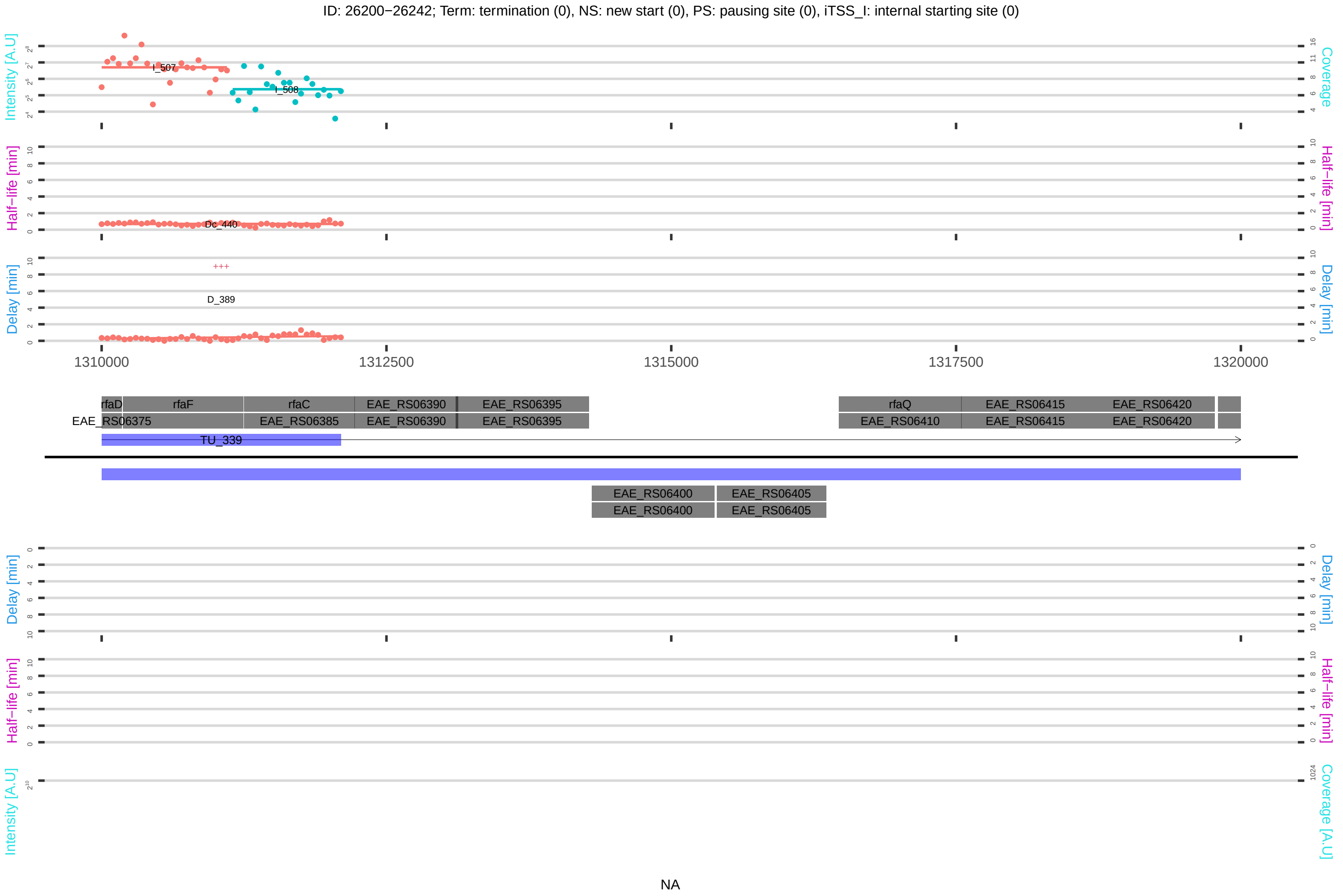

ID: 26200−26242; Term: termination (0), NS: new start (0), PS: pausing site (0), iTSS_I: internal starting site (0)
Intensity [A.U]
Coverage
I_507
I_508
Half−life [min]
Dc_440
Delay [min]
D_389
1310000
1312500
1315000
1317500
1320000
rfaD
rfaF
rfaC
EAE_RS06390
EAE_RS06395
rfaQ
EAE_RS06415
EAE_RS06420
EAE_RS06375
EAE_RS06385
EAE_RS06410
TU_339
EAE_RS06400
EAE_RS06405
Coverage [A.U]
NA

ID: 26418−26571; Term: termination (0), NS: new start (1), PS: pausing site (1), iTSS_I: internal starting site (0)

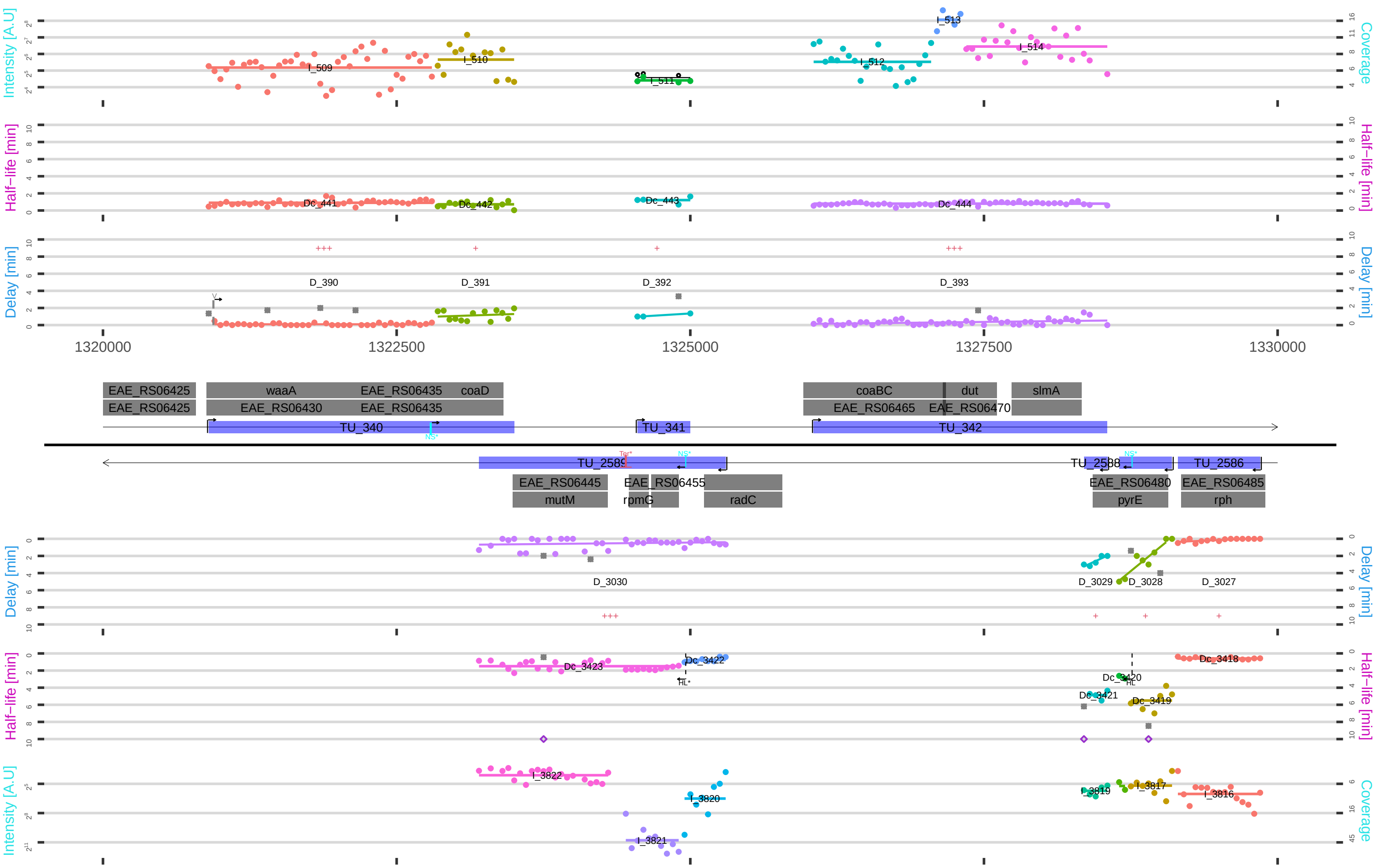

Term: termination (1), NS: new start (2), PS: pausing site (0), iTSS_I: internal starting site (0)

ID: 26602−26800; Term: termination (3), NS: new start (2), PS: pausing site (1), iTSS_I: internal starting site (0)

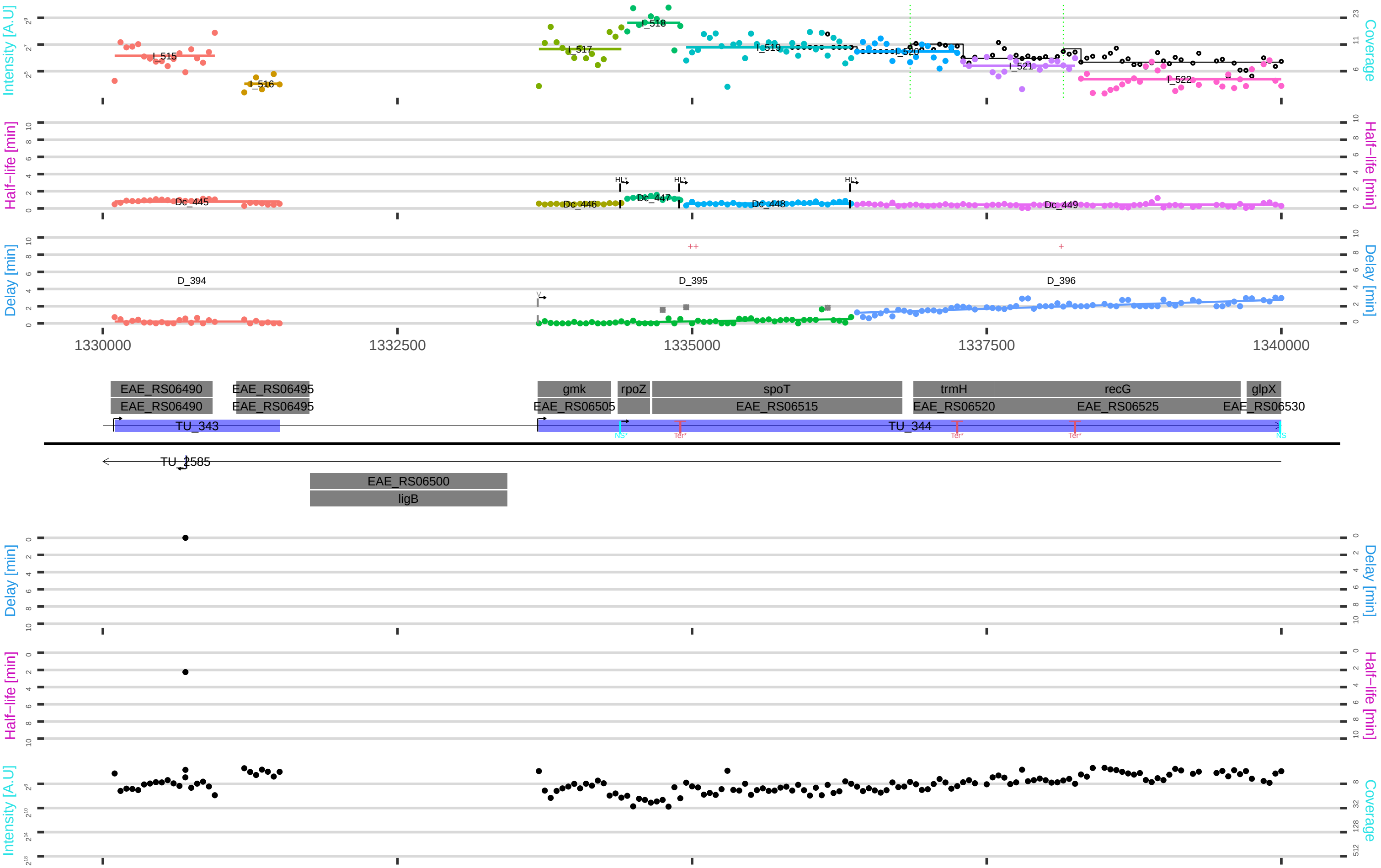

Term: termination (0), NS: new start (0), PS: pausing site (0), iTSS_I: internal starting site (0)

ID: 26800−27000; Term: termination (1), NS: new start (2), PS: pausing site (2), iTSS_I: internal starting site (0)

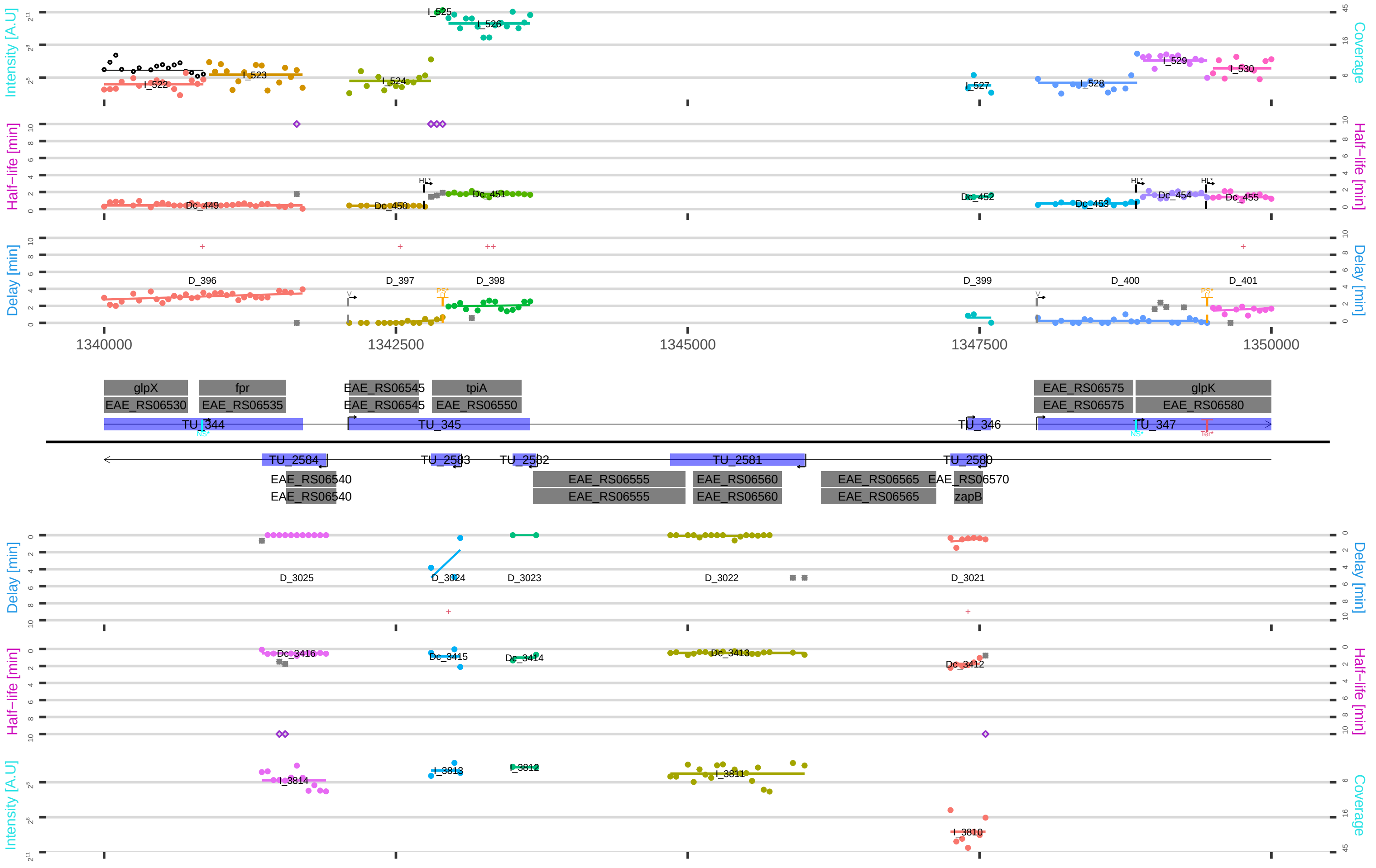

Term: termination (0), NS: new start (0), PS: pausing site (0), iTSS_I: internal starting site (0)

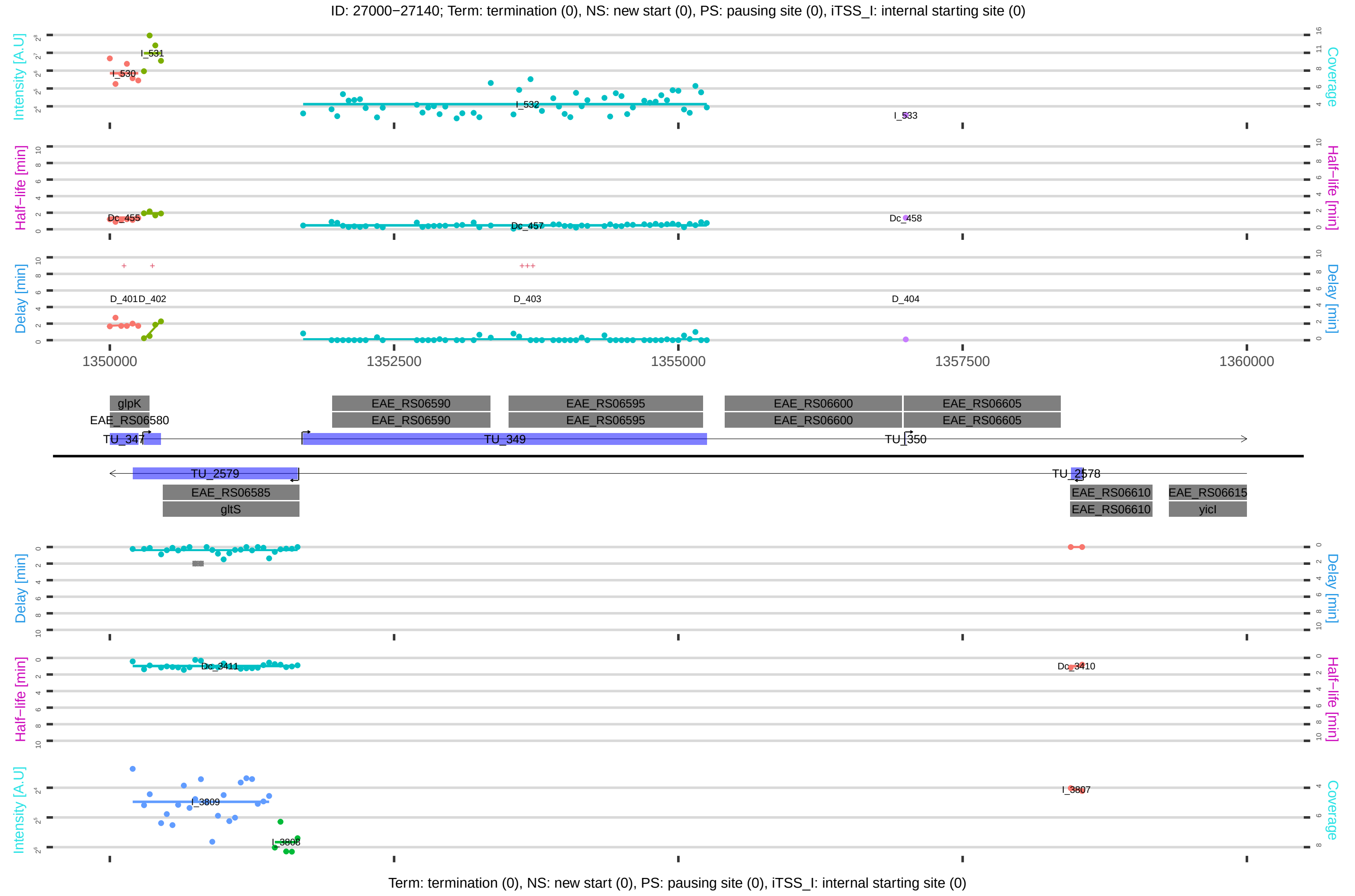

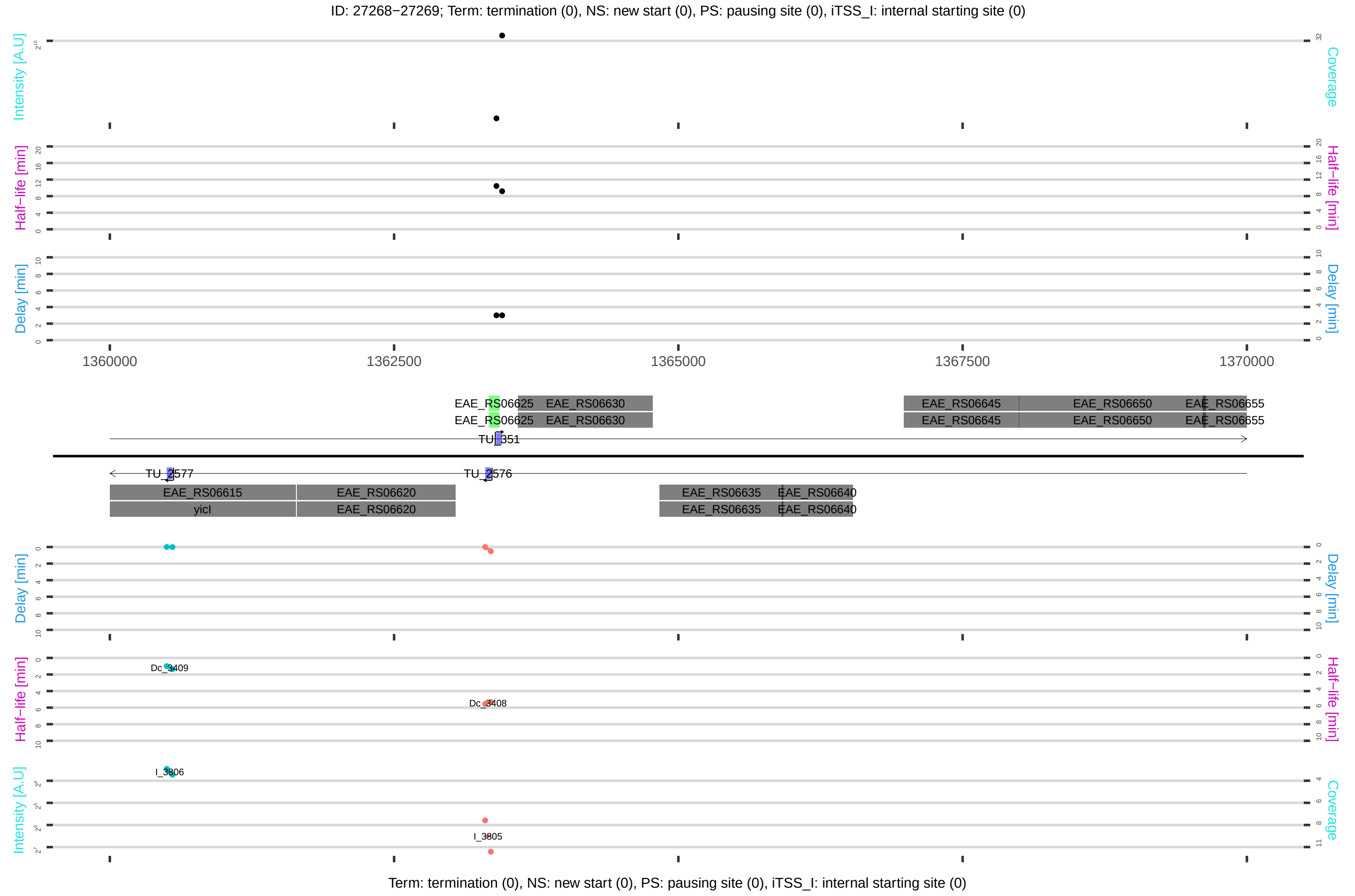

ID: 27268−27269; Term: termination (0), NS: new start (0), PS: pausing site (0), iTSS_I: internal starting site (0)
Intensity [A.U]
Coverage
Half−life [min]
Delay [min]
1360000
1362500
1365000
1367500
1370000
EAE_RS06625
EAE_RS06630
EAE_RS06645
EAE_RS06650
EAE_RS06655
TU_1351
TU_2577
TU_2576
EAE_RS06615
EAE_RS06620
yicI
EAE_RS06635
EAE_RS06640
Dc_3409
Dc_3408
I_3806
I_3805
Term: termination (0), NS: new start (0), PS: pausing site (0), iTSS_I: internal starting site (0)

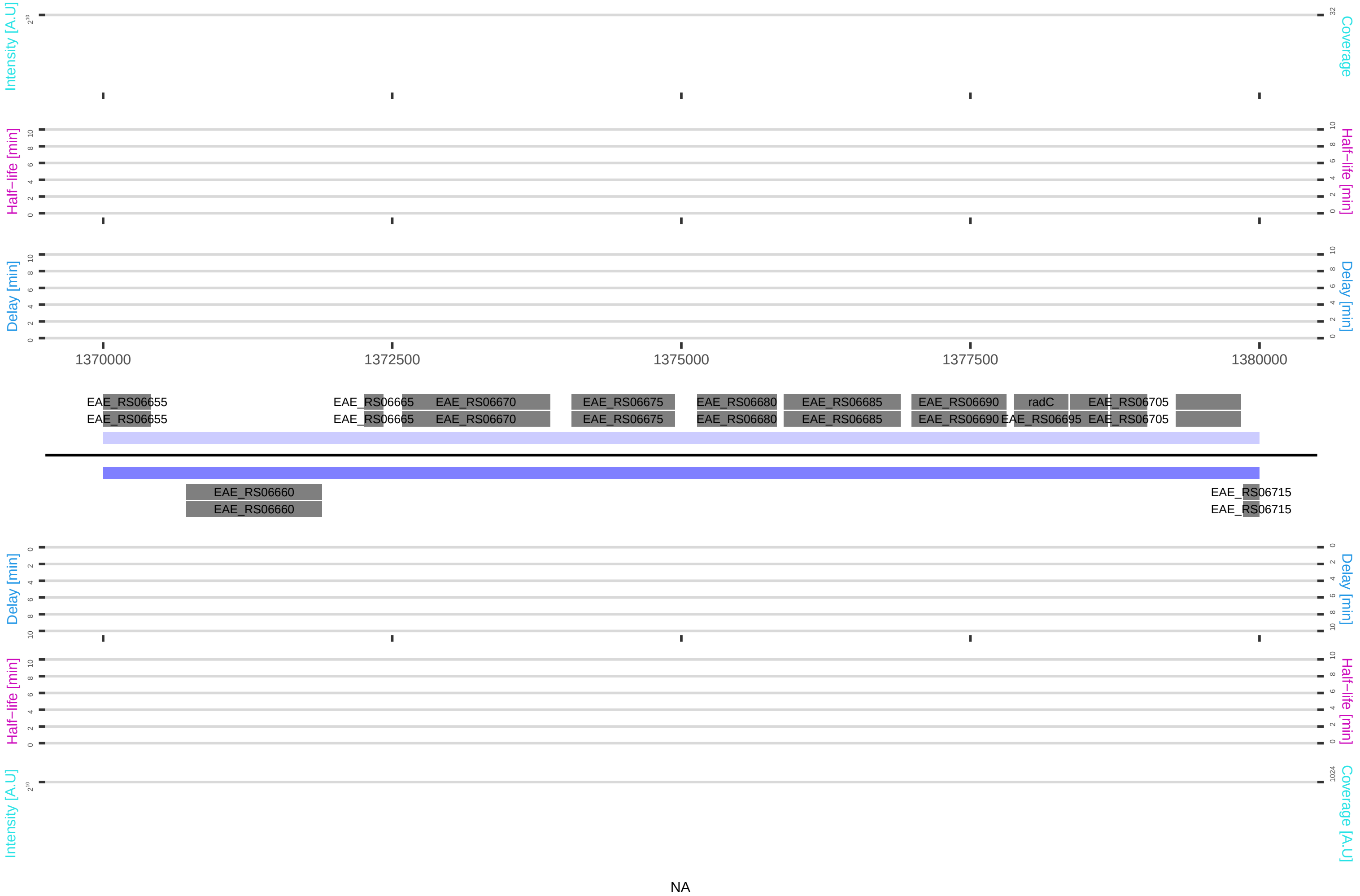
Intensity [A.U]
Coverage
Half−life [min]
Delay [min]
1370000
1372500
1375000
1377500
1380000
EAE_RS06655
EAE_RS06665
EAE_RS06670
EAE_RS06675
EAE_RS06680
EAE_RS06685
EAE_RS06690
radC
EAE_RS06695
EAE_RS06705
EAE_RS06660
EAE_RS06715
Coverage [A.U]
NA

ID: 27623−27784; Term: termination (0), NS: new start (0), PS: pausing site (0), iTSS_I: internal starting site (0)

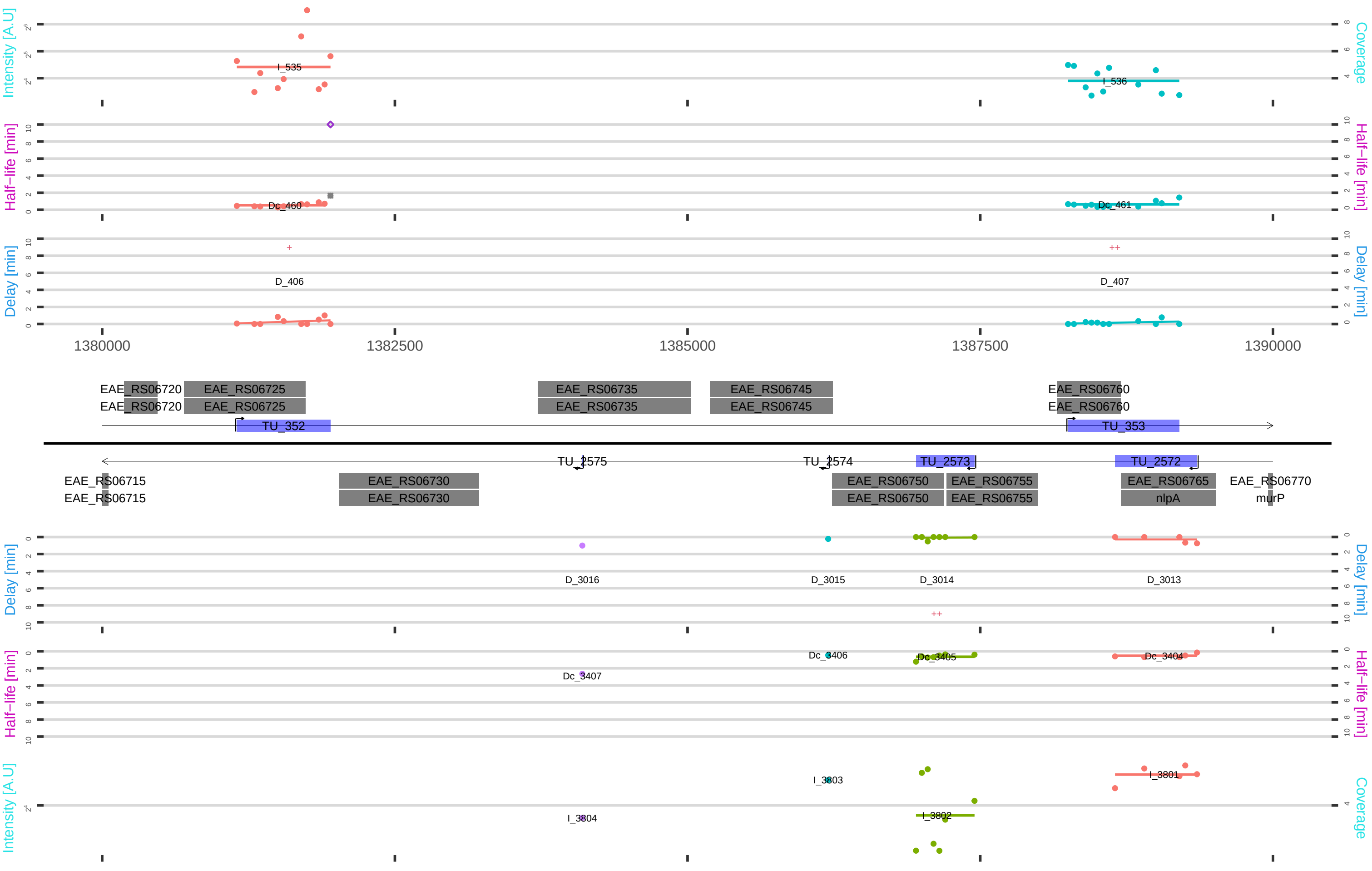

Term: termination (0), NS: new start (0), PS: pausing site (0), iTSS_I: internal starting site (0)

ID: 27970−27983; Term: termination (0), NS: new start (0), PS: pausing site (0), iTSS_I: internal starting site (0)

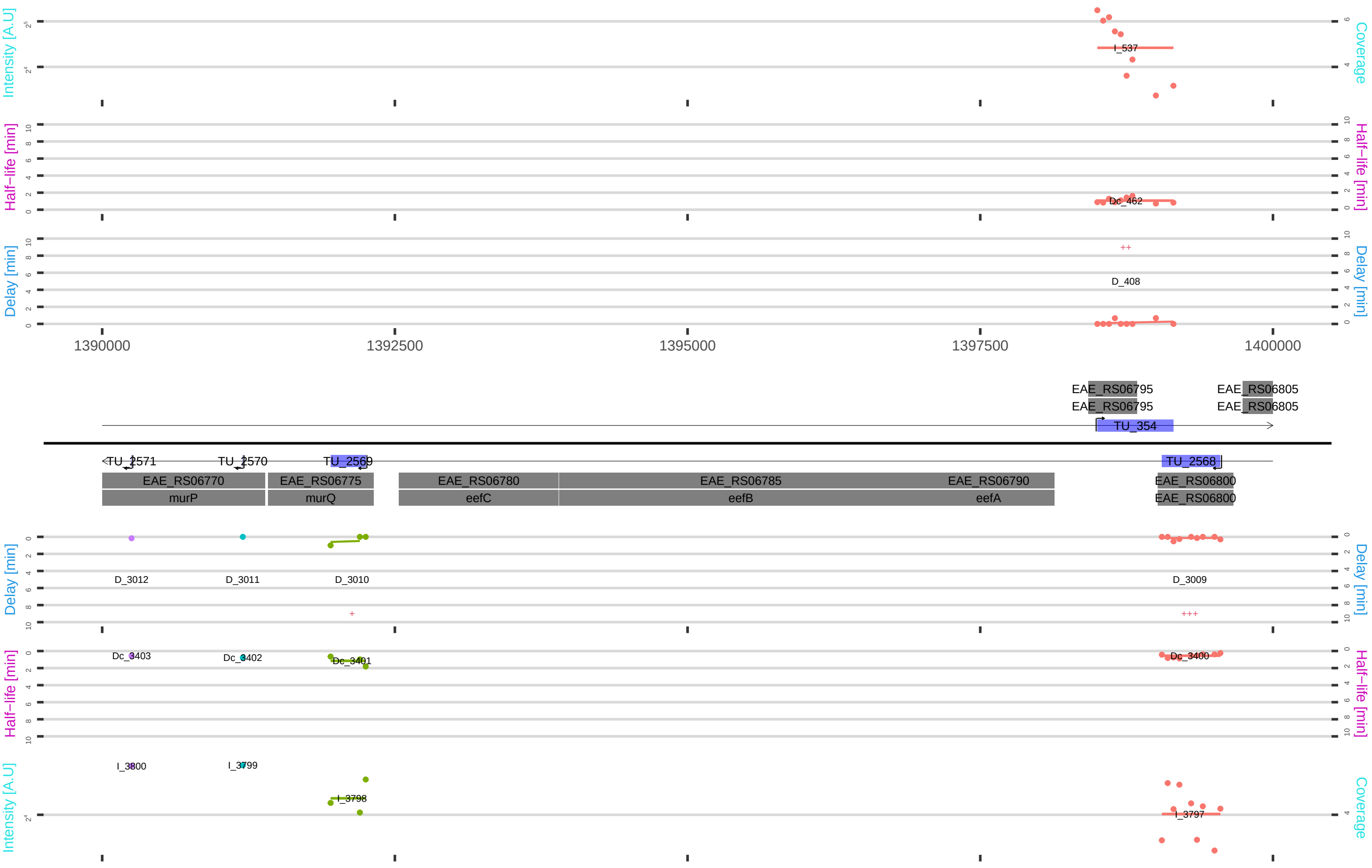

Term: termination (0), NS: new start (0), PS: pausing site (0), iTSS_I: internal starting site (0)

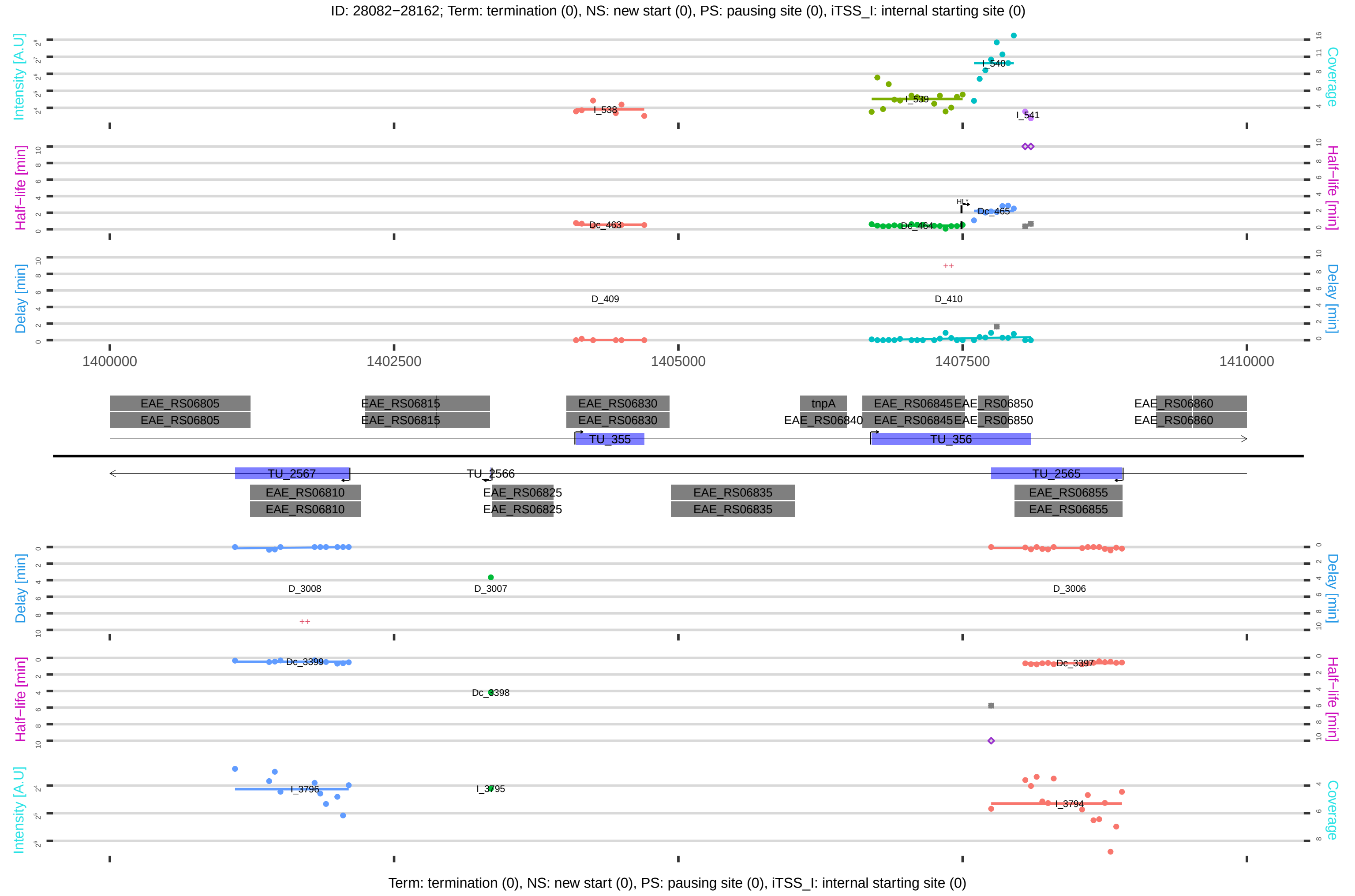

ID: 28082−28162; Term: termination (0), NS: new start (0), PS: pausing site (0), iTSS_I: internal starting site (0)
Intensity [A.U]
Coverage
Half−life [min]
Delay [min]
I_538
I_539
I_540
I_541
Dc_463
Dc_464
Dc_465
D_409
D_410
1400000
1402500
1405000
1407500
1410000
EAE_RS06805
EAE_RS06815
EAE_RS06830
tnpA
EAE_RS06840
EAE_RS06845
EAE_RS06850
EAE_RS06860
TU_355
TU_356
TU_2567
TU_2566
TU_2565
EAE_RS06810
EAE_RS06825
EAE_RS06835
EAE_RS06855
D_3008
D_3007
D_3006
Dc_3399
Dc_3398
Dc_3397
I_3796
I_3795
I_3794
Term: termination (0), NS: new start (0), PS: pausing site (0), iTSS_I: internal starting site (0)

ID: 182962−182817; FC*: significant t−test of two consecutive segments; Term: termination, NS: new start, PS: pausing site,iTSS_I: internal starting site,TI: transcription interferance.

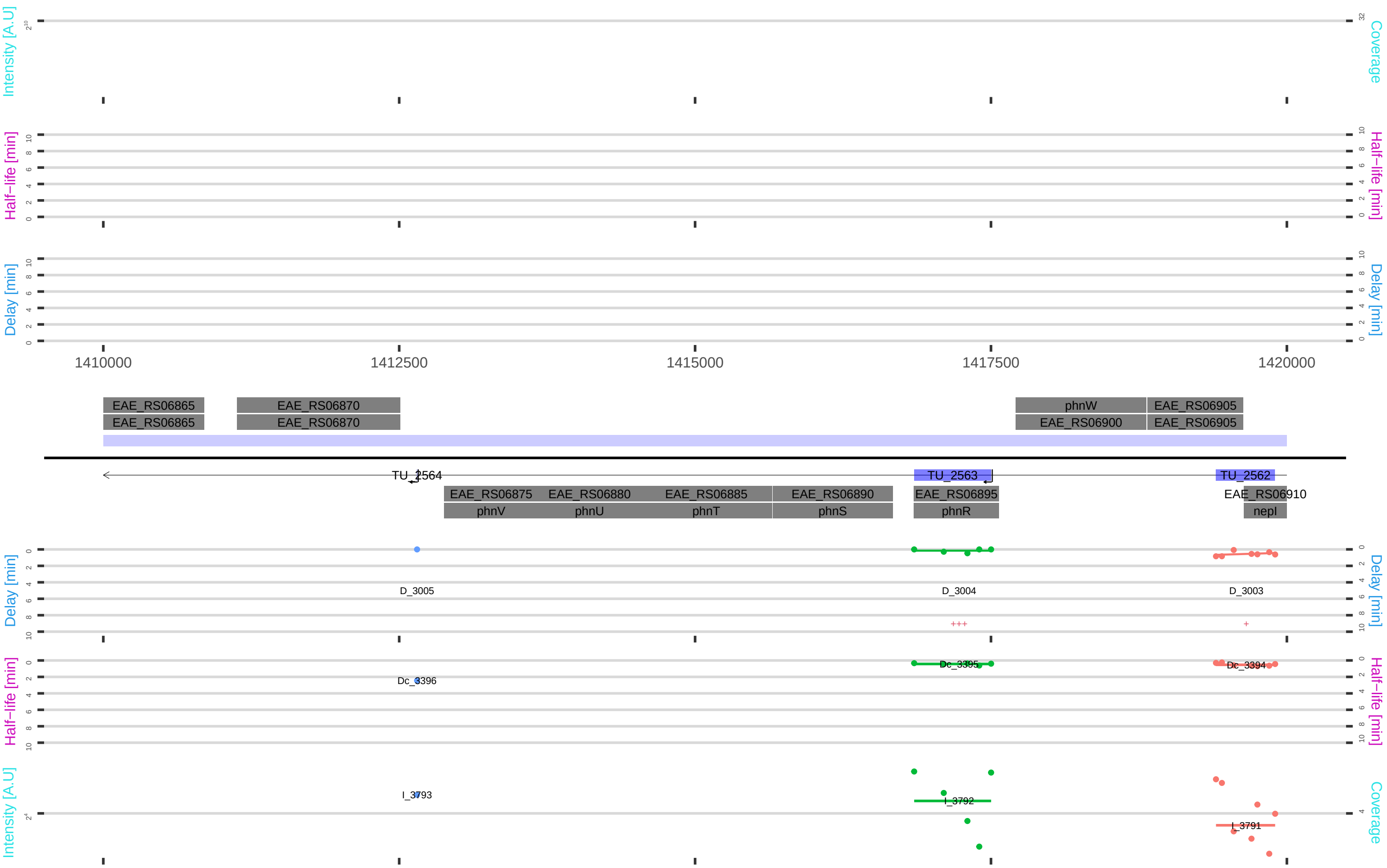

Term: termination (0), NS: new start (0), PS: pausing site (0), iTSS_I: internal starting site (0)

ID: 182815−182624; FC*: significant t−test of two consecutive segments; Term: termination, NS: new start, PS: pausing site,iTSS_I: internal starting site,TI: transcription interferance.

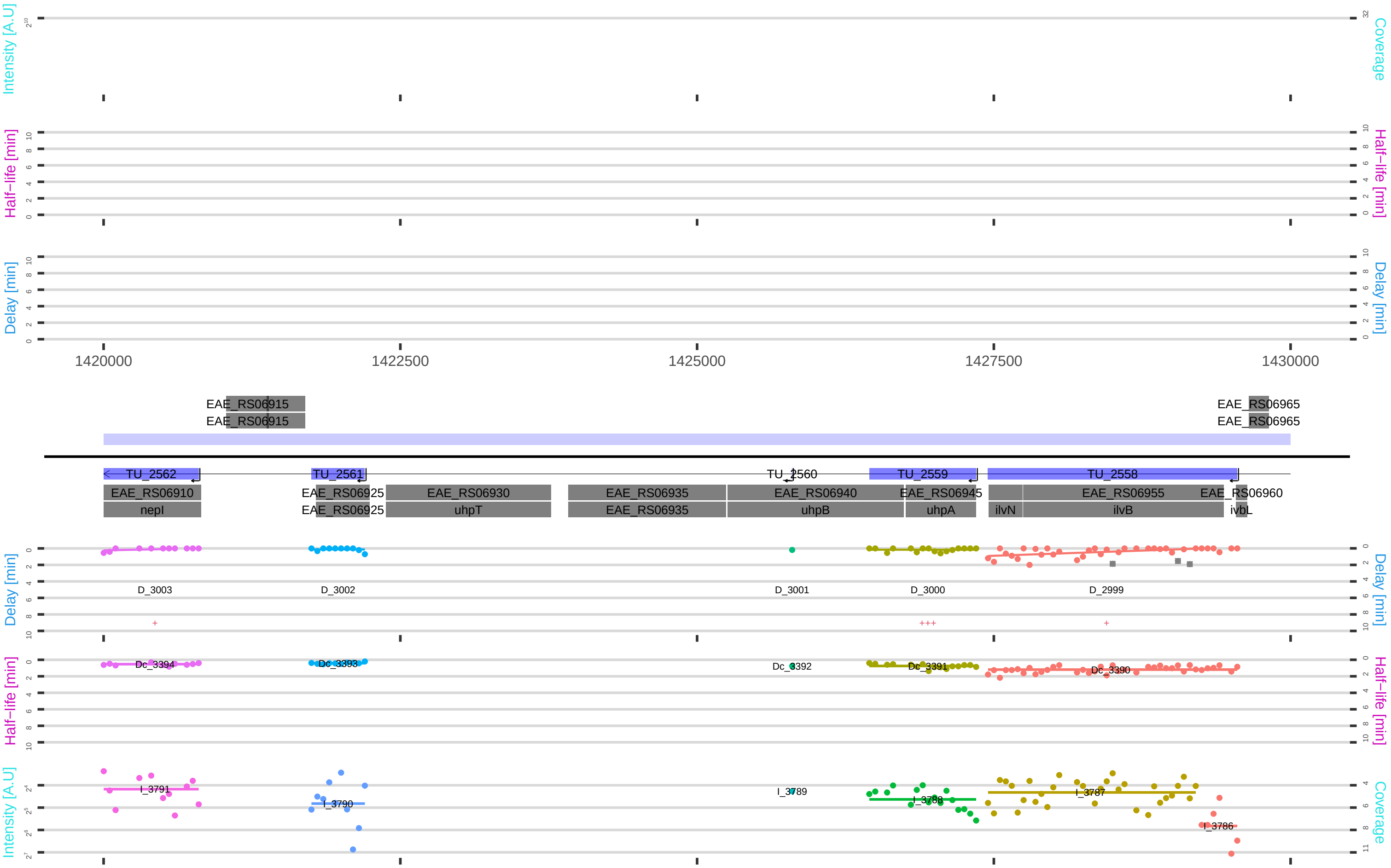

Term: termination (0), NS: new start (0), PS: pausing site (0), iTSS_I: internal starting site (0)

ID: 28601−28743; Term: termination (0), NS: new start (0), PS: pausing site (0), iTSS_I: internal starting site (0)

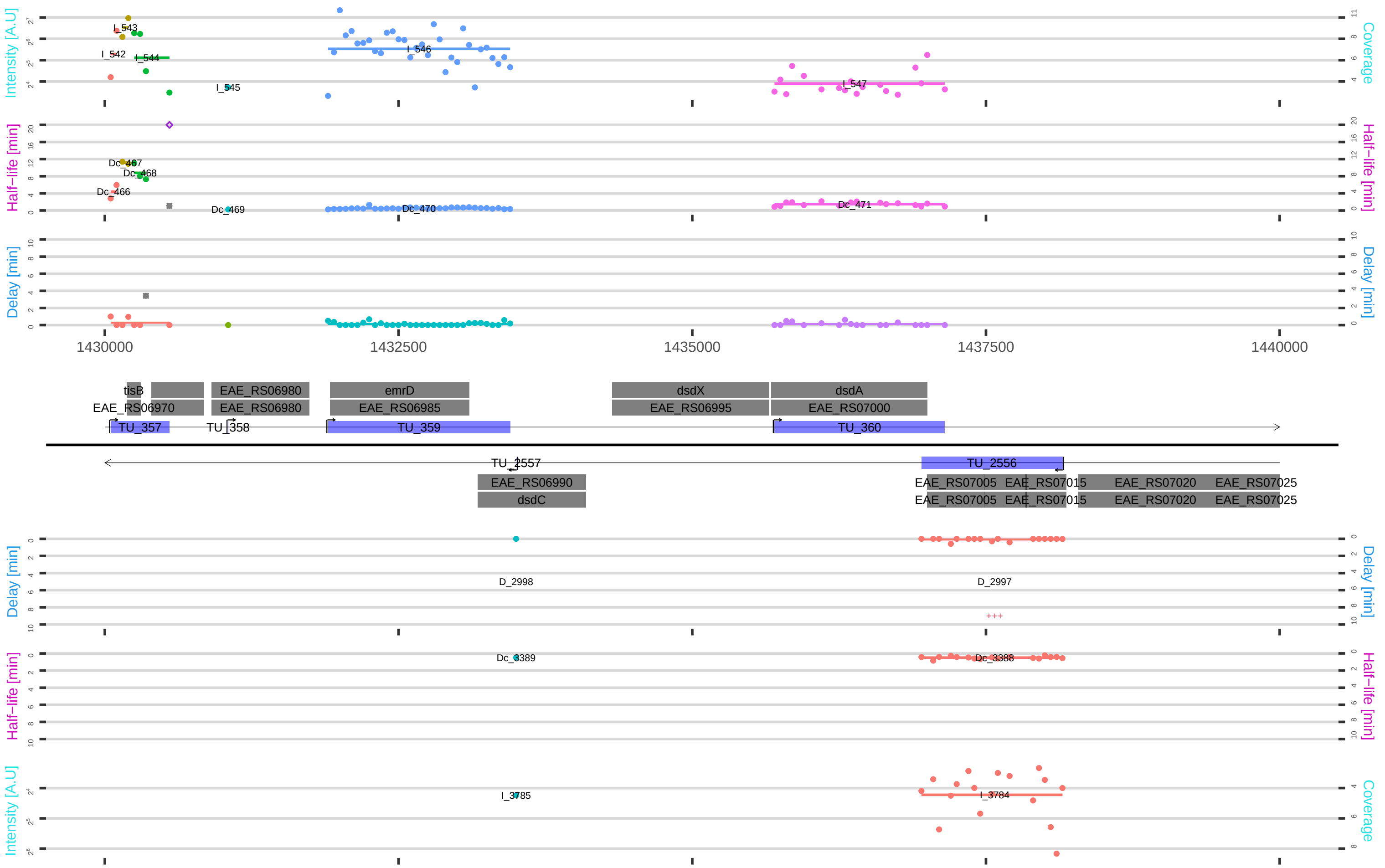

Term: termination (0), NS: new start (0), PS: pausing site (0), iTSS_I: internal starting site (0)

ID: 28831−28914; Term: termination (0), NS: new start (0), PS: pausing site (0), iTSS_I: internal starting site (0)

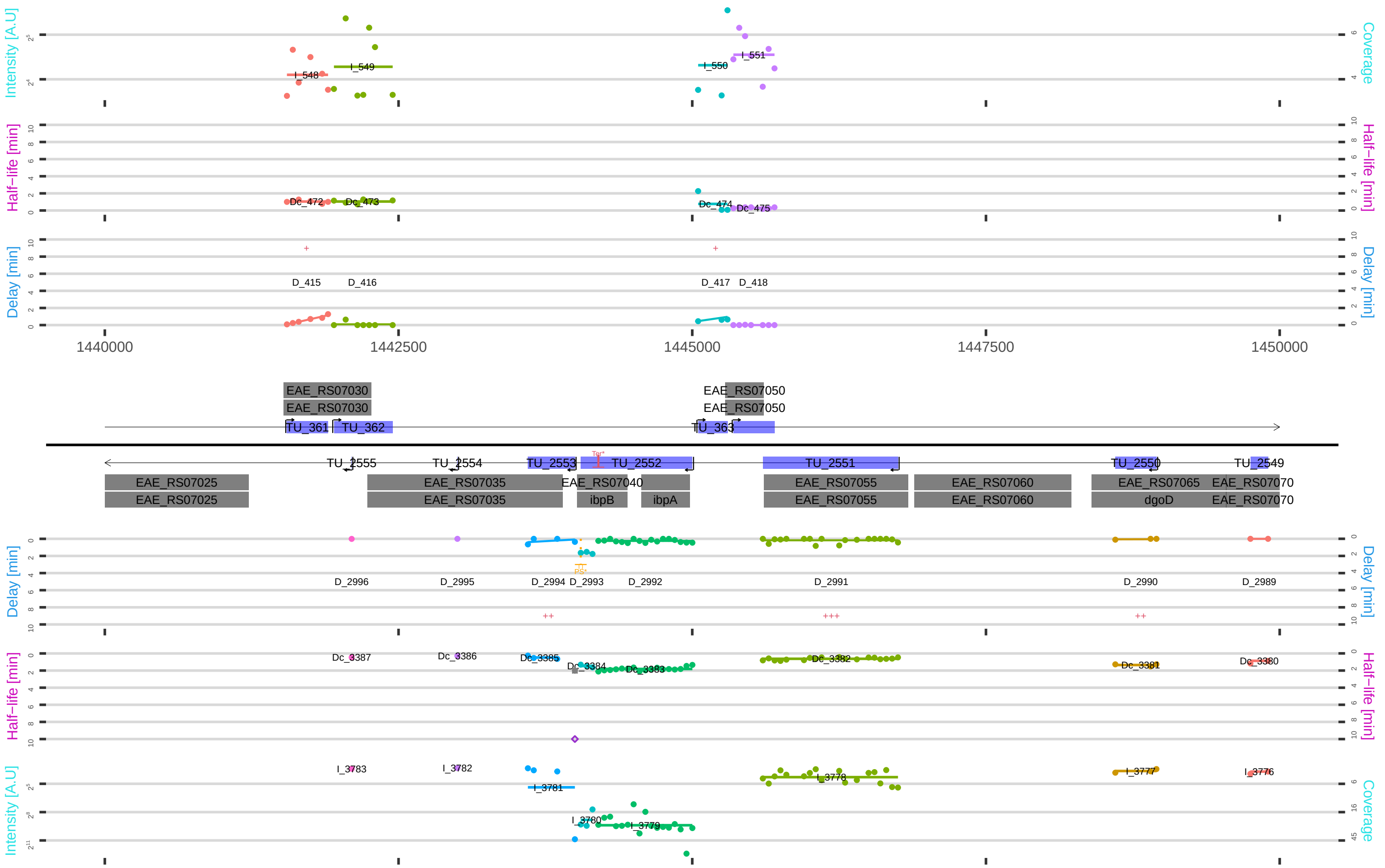

Term: termination (1), NS: new start (0), PS: pausing site (1), iTSS_I: internal starting site (0)

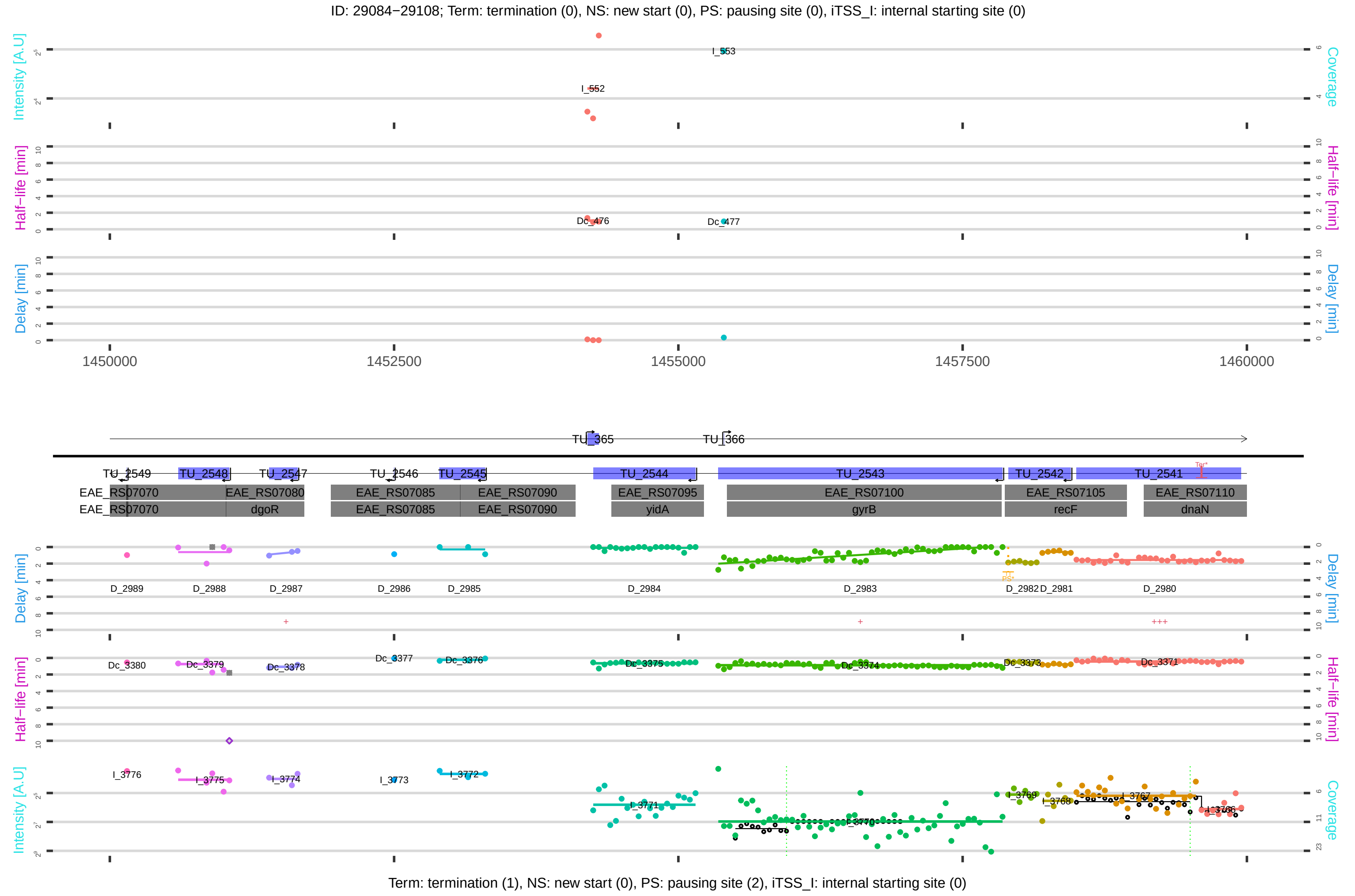

ID: 29084−29108; Term: termination (0), NS: new start (0), PS: pausing site (0), iTSS_I: internal starting site (0)
Intensity [A.U]
Coverage
Half−life [min]
Delay [min]
I_553
I_552
Dc_476
Dc_477
1450000
1452500
1455000
1457500
1460000
TU_365
TU_366
TU_2549
TU_2548
TU_2547
TU_2546
TU_2545
TU_2544
TU_2543
TU_2542
TU_2541
EAE_RS07070
EAE_RS07080
EAE_RS07085
EAE_RS07090
EAE_RS07095
EAE_RS07100
EAE_RS07105
EAE_RS07110
dgoR
yidA
gyrB
recF
dnaN
D_2989
D_2988
D_2987
D_2986
D_2985
D_2984
D_2983
D_2982
D_2981
D_2980
Dc_3380
Dc_3379
Dc_3378
Dc_3377
Dc_3376
Dc_3375
Dc_3374
Dc_3373
Dc_3371
I_3776
I_3775
I_3774
I_3773
I_3772
I_3771
I_3770
I_3769
I_3768
I_3767
I_3766
Term: termination (1), NS: new start (0), PS: pausing site (2), iTSS_I: internal starting site (0)

ID: 29241−29400; Term: termination (2), NS: new start (0), PS: pausing site (2), iTSS_I: internal starting site (0)

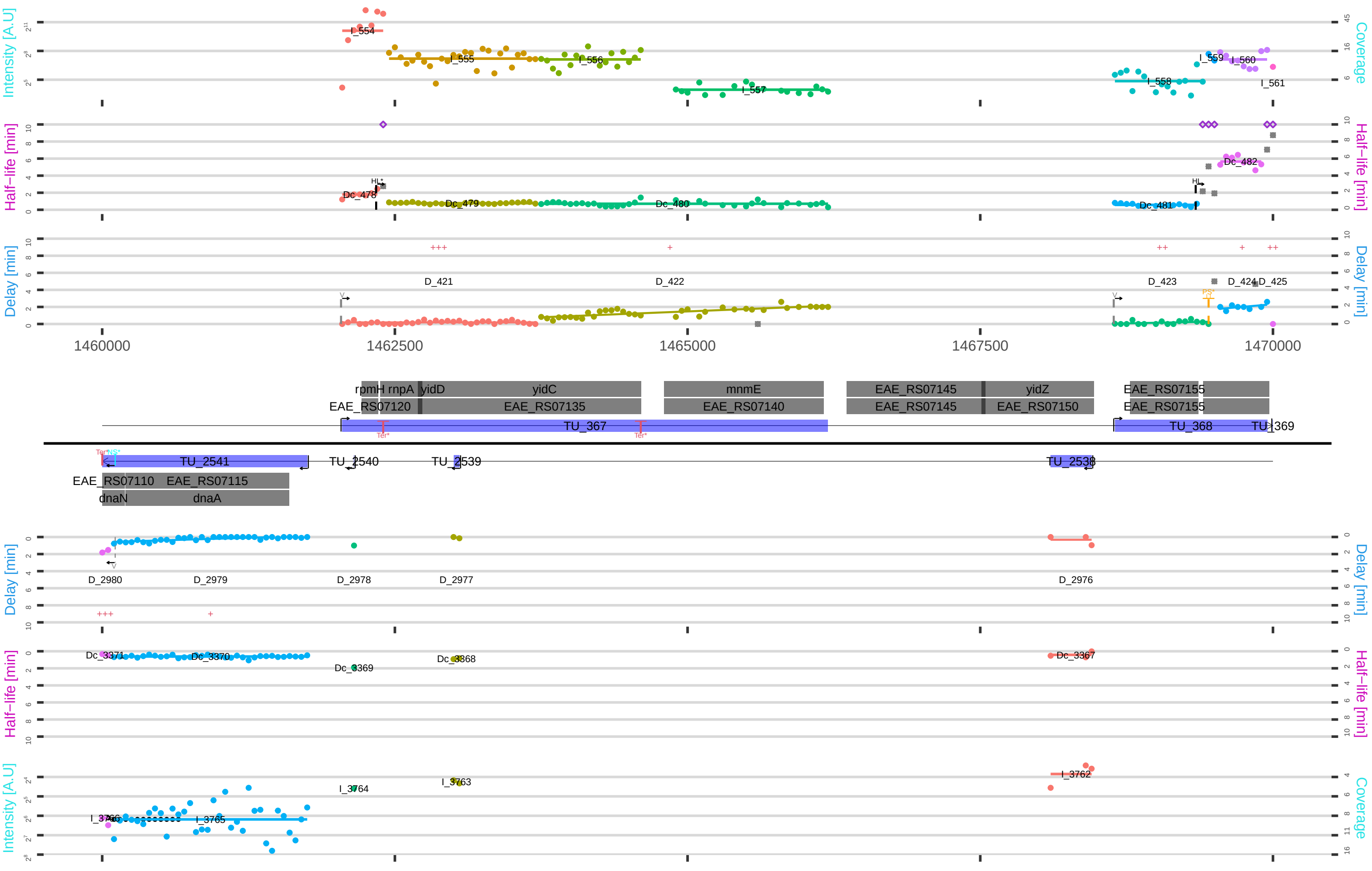

Term: termination (1), NS: new start (1), PS: pausing site (0), iTSS_I: internal starting site (0)

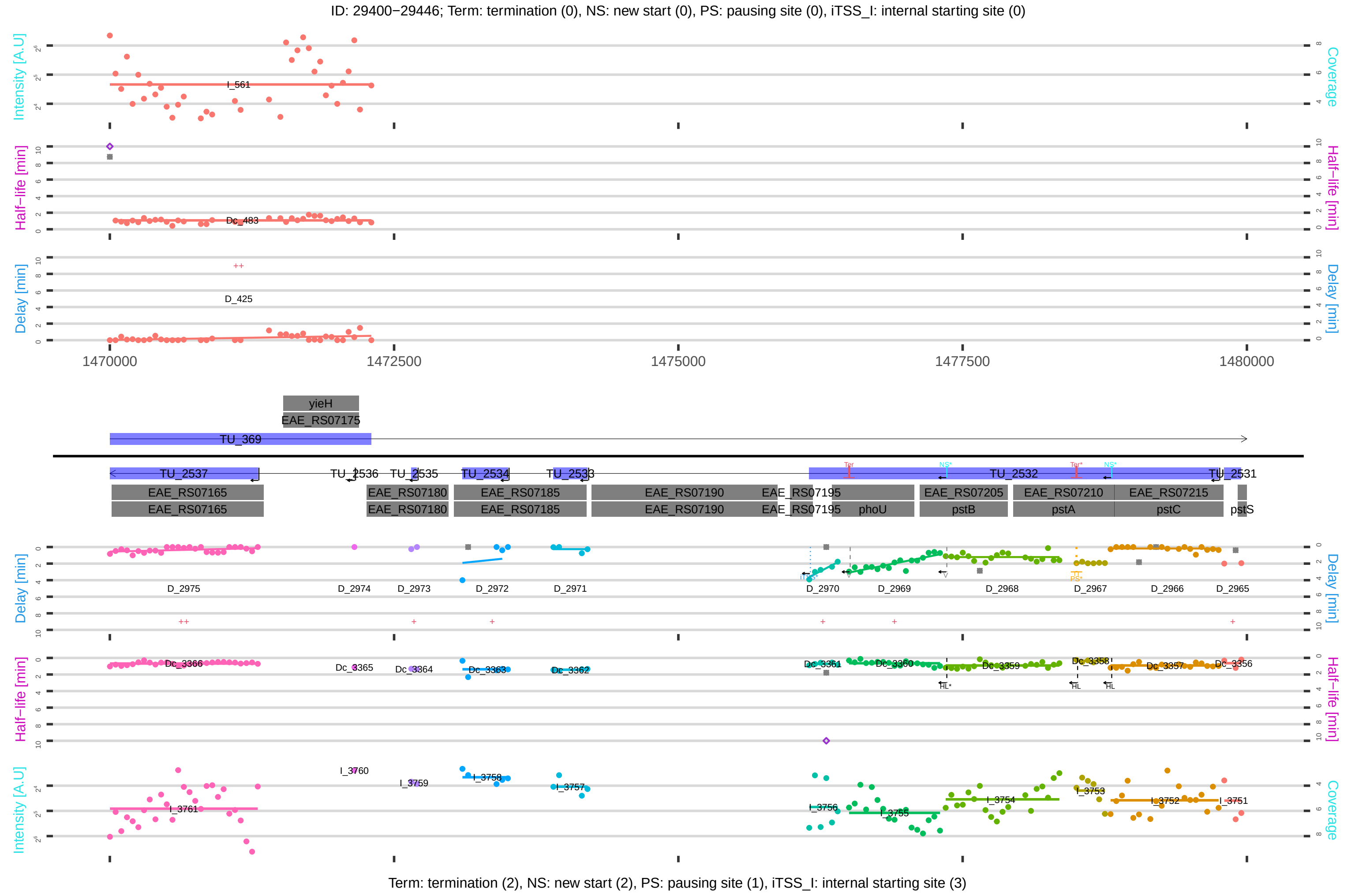
ID: 29400−29446; Term: termination (0), NS: new start (0), PS: pausing site (0), iTSS_I: internal starting site (0)
Intensity [A.U]
Coverage
Half−life [min]
Delay [min]
I_561
Dc_483
D_425
1470000
1472500
1475000
1477500
1480000
yieH
EAE_RS07175
TU_369
TU_2537
TU_2536
TU_2535
TU_2534
TU_2533
TU_2532
TU_2531
EAE_RS07165
EAE_RS07180
EAE_RS07185
EAE_RS07190
EAE_RS07195
EAE_RS07205
EAE_RS07210
EAE_RS07215
phoU
pstB
pstA
pstC
pstS
D_2975
D_2974
D_2973
D_2972
D_2971
D_2970
D_2969
D_2968
D_2967
D_2966
D_2965
Dc_3366
Dc_3365
Dc_3364
Dc_3363
Dc_3362
Dc_3361
Dc_3360
Dc_3359
Dc_3358
Dc_3357
Dc_3356
I_3761
I_3760
I_3759
I_3758
I_3757
I_3756
I_3755
I_3754
I_3753
I_3752
I_3751
Term: termination (2), NS: new start (2), PS: pausing site (1), iTSS_I: internal starting site (3)

ID: 29637−29782; Term: termination (0), NS: new start (0), PS: pausing site (0), iTSS_I: internal starting site (0)

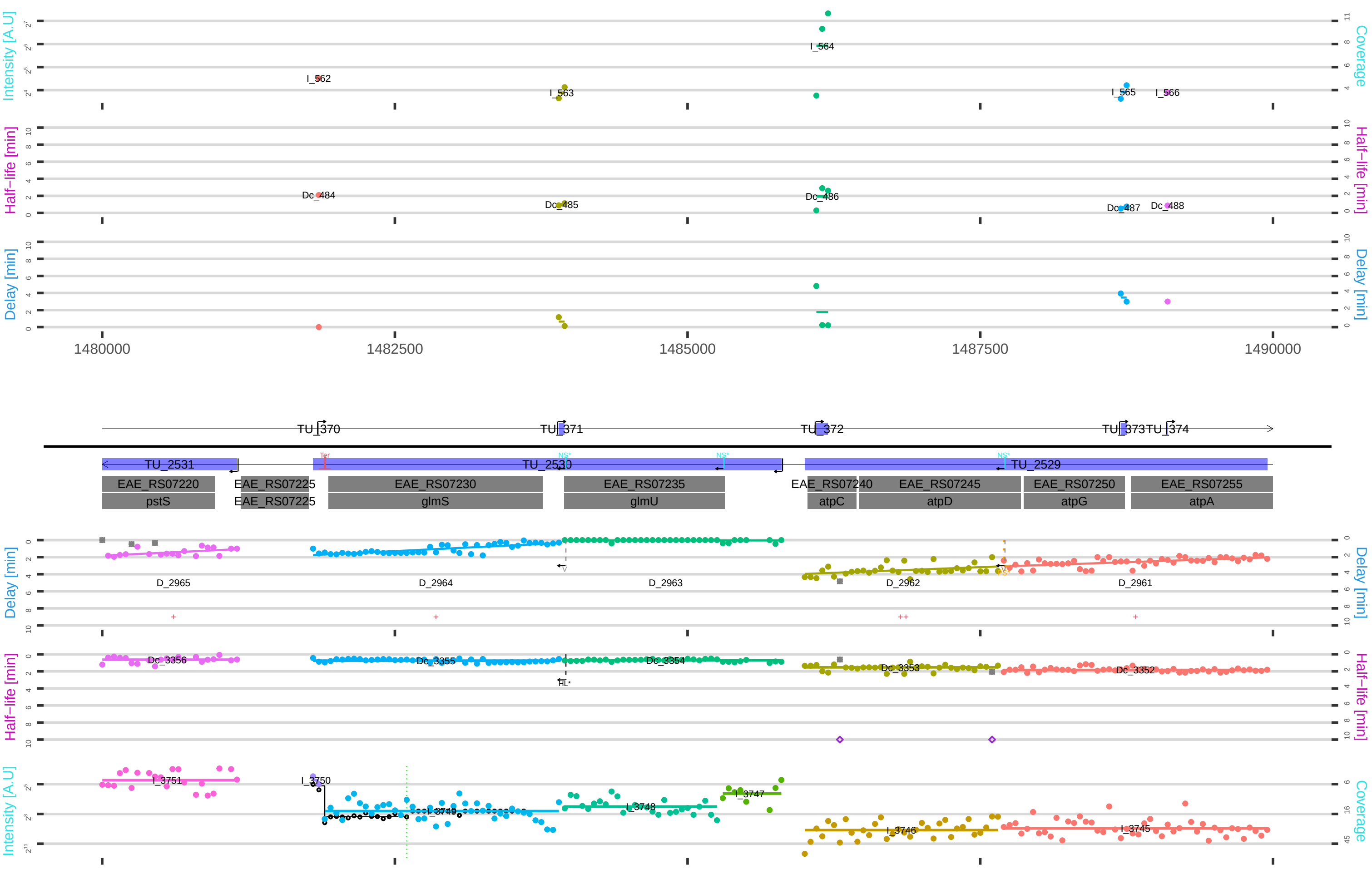

Term: termination (1), NS: new start (3), PS: pausing site (3), iTSS_I: internal starting site (0)

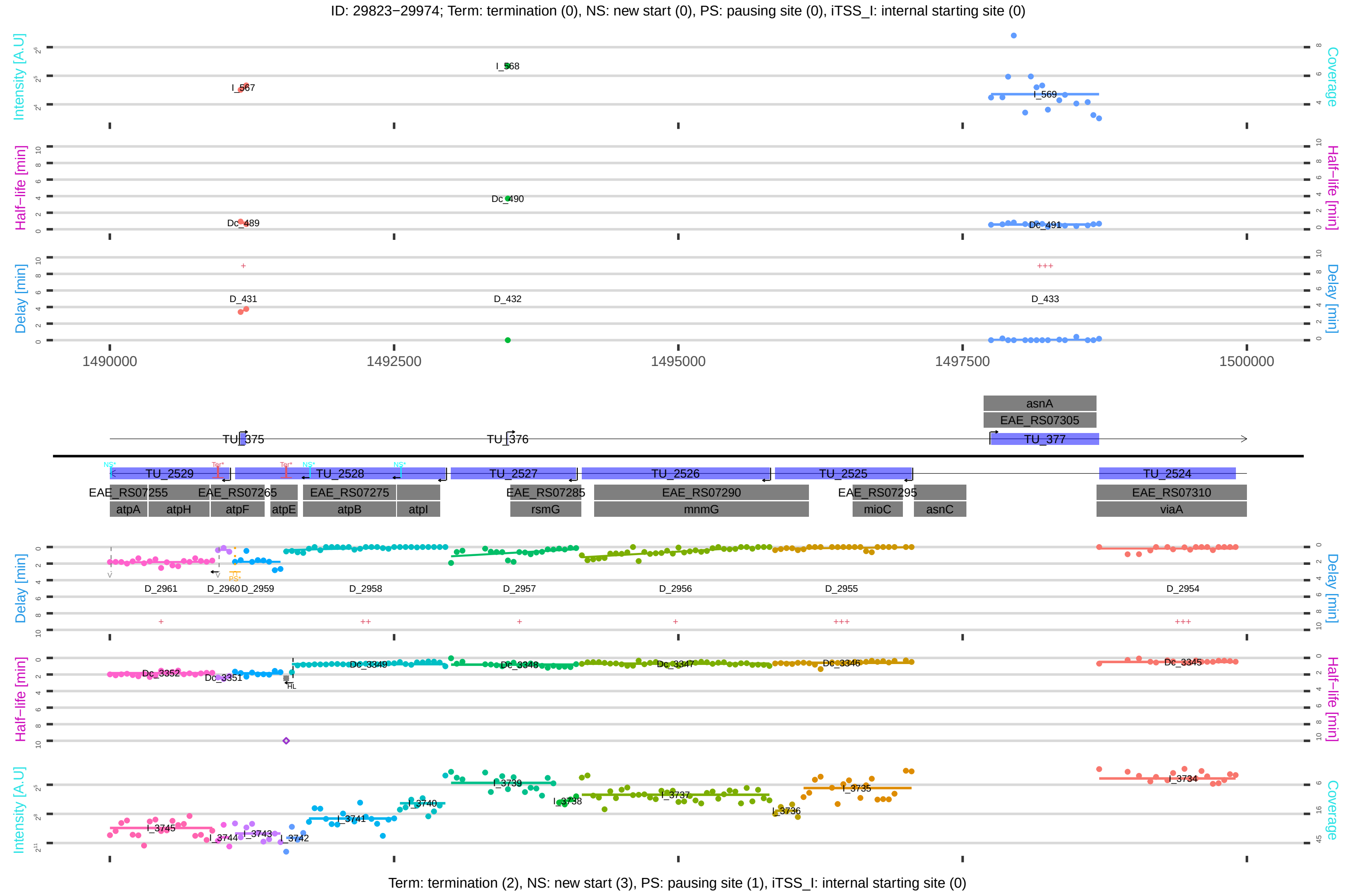

ID: 29823−29974; Term: termination (0), NS: new start (0), PS: pausing site (0), iTSS_I: internal starting site (0)
Intensity [A.U]
Coverage
Half−life [min]
Delay [min]
I_567
I_568
I_569
Dc_489
Dc_490
Dc_491
D_431
D_432
D_433
1490000
1492500
1495000
1497500
1500000
asnA
EAE_RS07305
TU_375
TU_376
TU_377
TU_2529
TU_2528
TU_2527
TU_2526
TU_2525
TU_2524
EAE_RS07255
EAE_RS07265
EAE_RS07275
EAE_RS07285
EAE_RS07290
EAE_RS07295
EAE_RS07310
atpA
atpH
atpF
atpE
atpB
atpI
rsmG
mnmG
mioC
asnC
viaA
D_2961
D_2960
D_2959
D_2958
D_2957
D_2956
D_2955
D_2954
Dc_3352
Dc_3351
Dc_3349
Dc_3348
Dc_3347
Dc_3346
Dc_3345
I_3745
I_3744
I_3743
I_3742
I_3741
I_3740
I_3739
I_3738
I_3737
I_3736
I_3735
I_3734
Term: termination (2), NS: new start (3), PS: pausing site (1), iTSS_I: internal starting site (0)

ID: 30035−30199; Term: termination (2), NS: new start (3), PS: pausing site (1), iTSS_I: internal starting site (0)

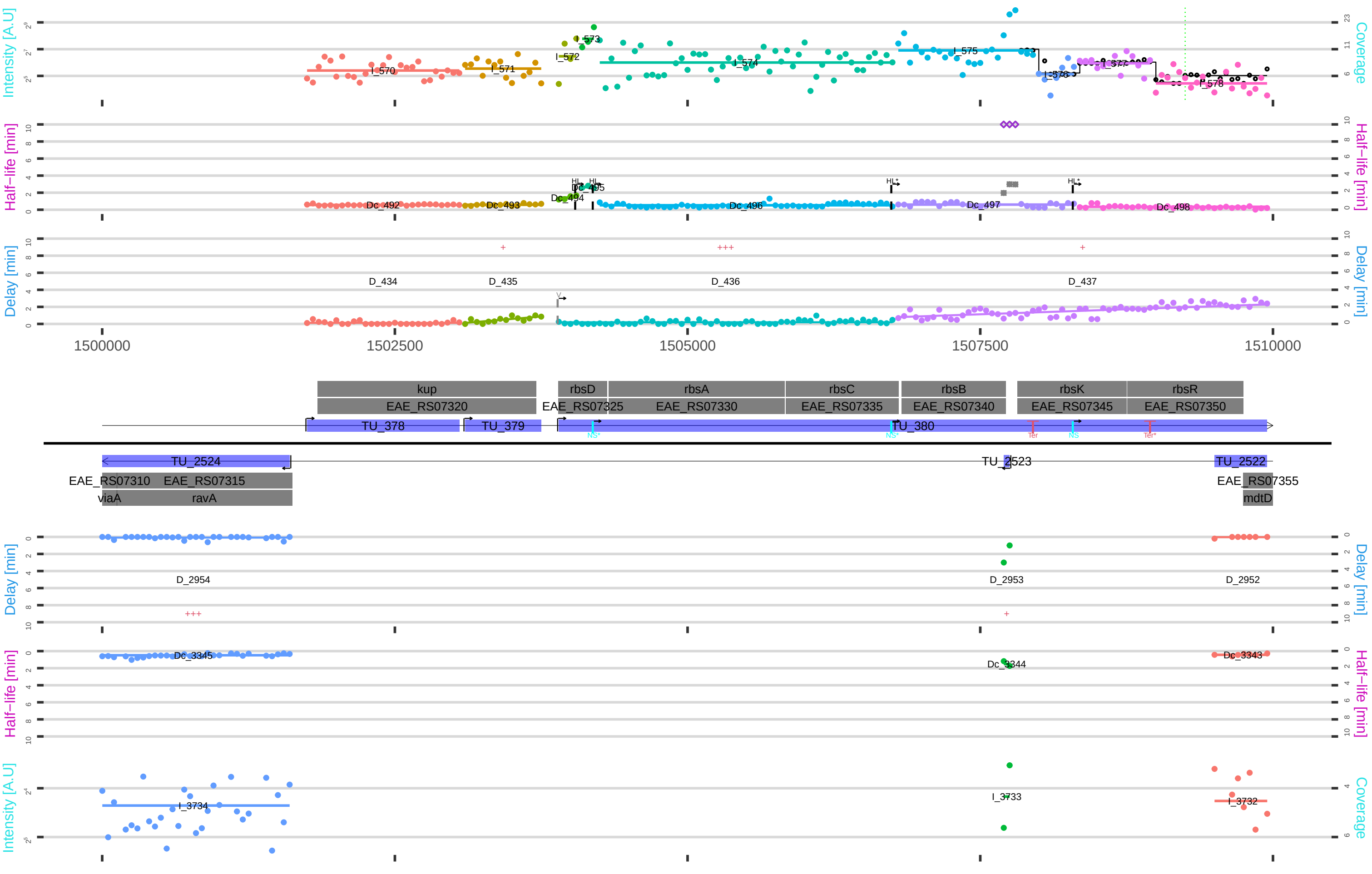

Term: termination (0), NS: new start (0), PS: pausing site (0), iTSS_I: internal starting site (0)

ID: 30201−30400; Term: termination (6), NS: new start (3), PS: pausing site (5), iTSS_I: internal starting site (1)

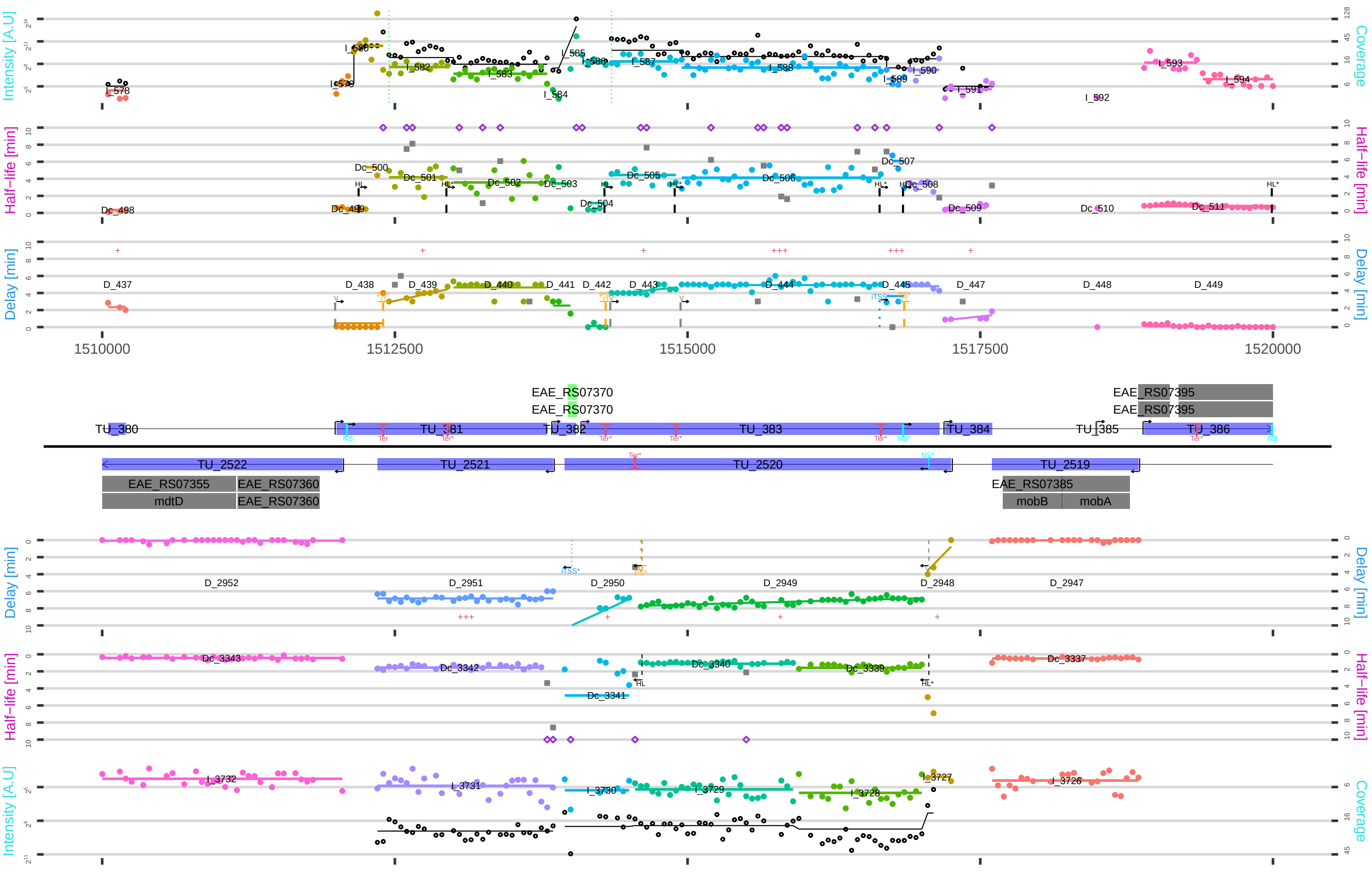

Term: termination (1), NS: new start (1), PS: pausing site (1), iTSS_I: internal starting site (1)

ID: 30400−30574; Term: termination (1), NS: new start (2), PS: pausing site (3), iTSS_I: internal starting site (0)

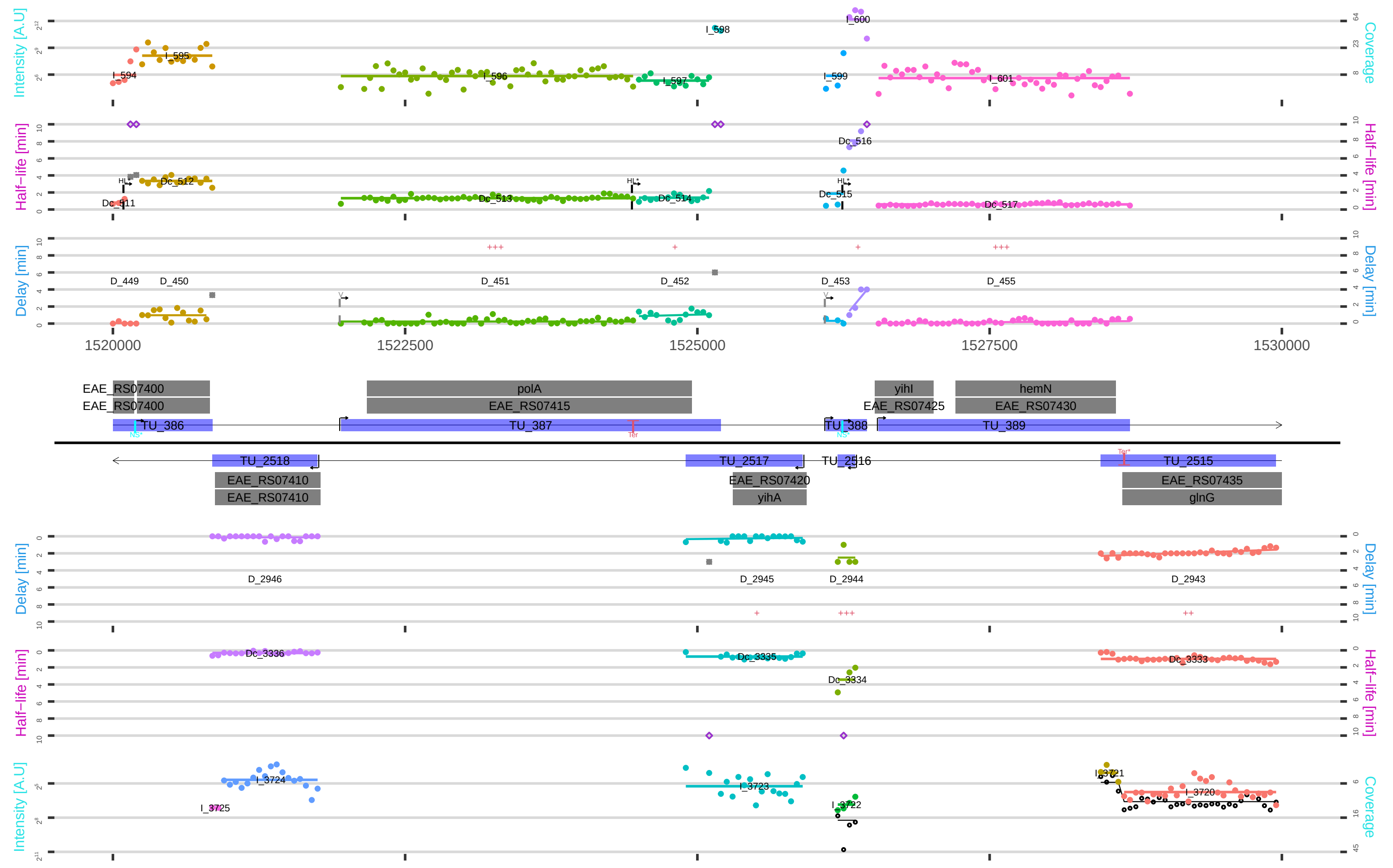

Term: termination (1), NS: new start (0), PS: pausing site (1), iTSS_I: internal starting site (0)

ID: 30642−30800; Term: termination (0), NS: new start (1), PS: pausing site (0), iTSS_I: internal starting site (0)

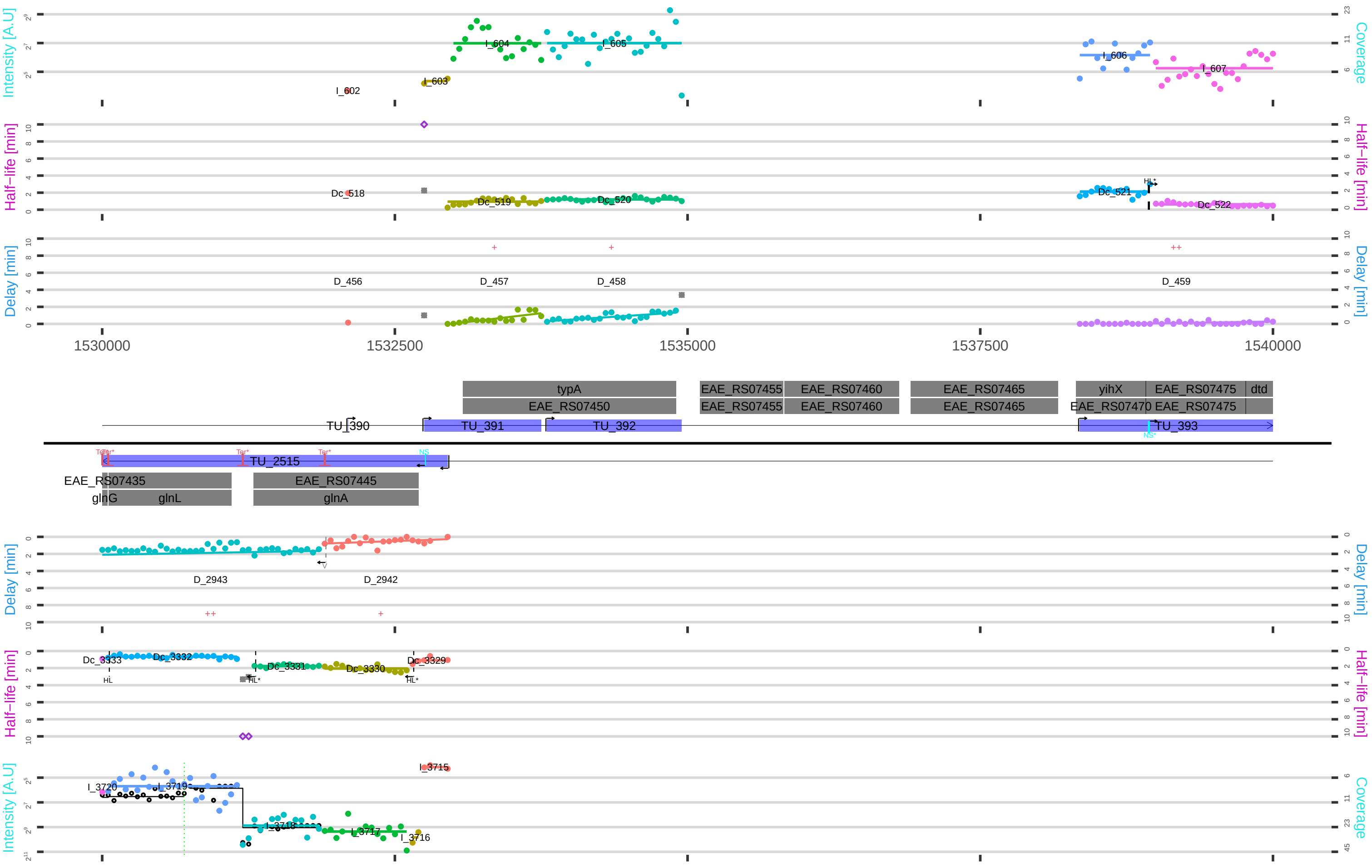

Term: termination (4), NS: new start (1), PS: pausing site (0), iTSS_I: internal starting site (0)

ID: 30800−30985; Term: termination (0), NS: new start (0), PS: pausing site (0), iTSS_I: internal starting site (0)

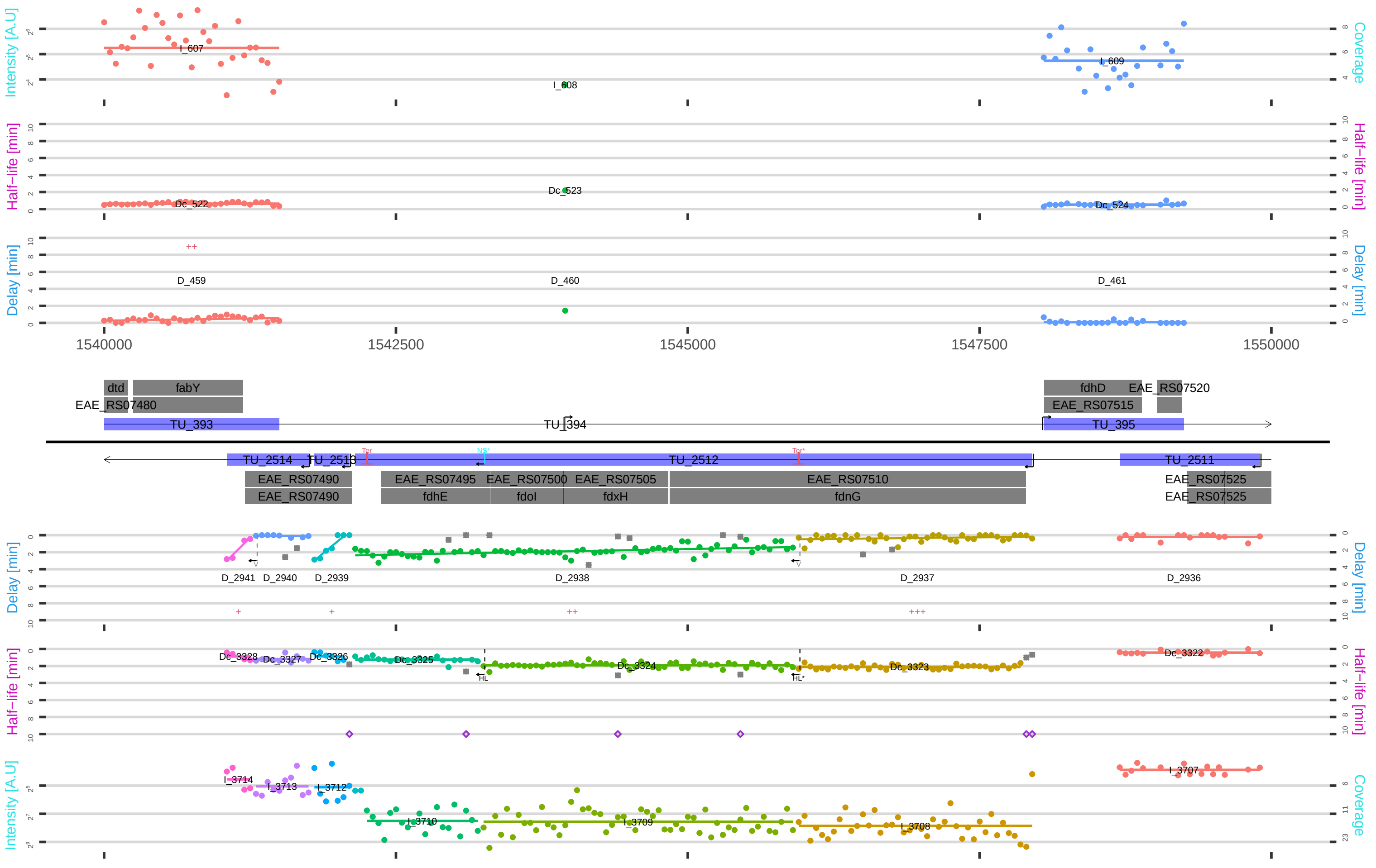

Term: termination (2), NS: new start (1), PS: pausing site (1), iTSS_I: internal starting site (1)

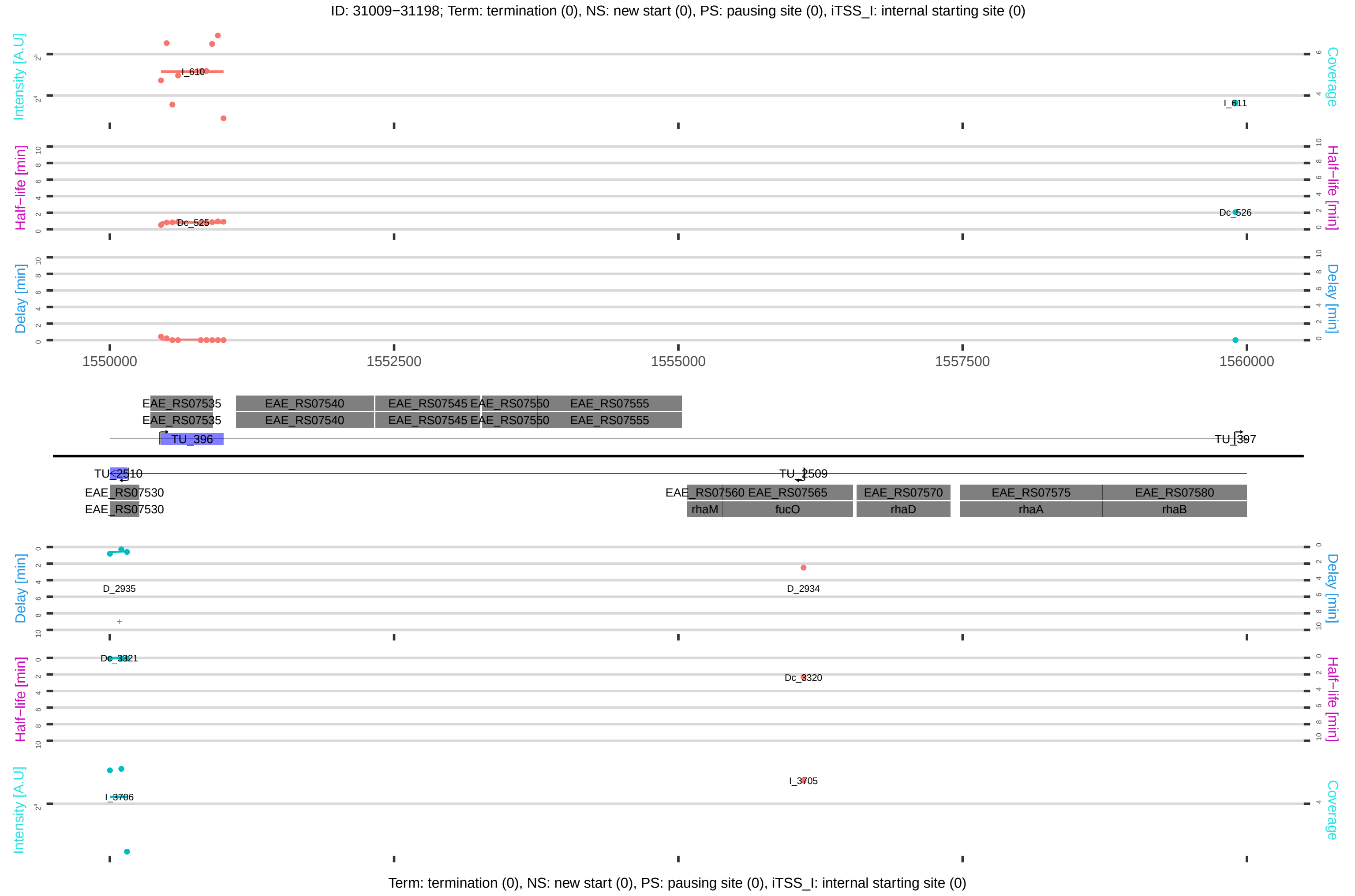

ID: 31009−31198; Term: termination (0), NS: new start (0), PS: pausing site (0), iTSS_I: internal starting site (0)
Intensity [A.U]
Coverage
I_610
I_611
Half−life [min]
Dc_525
Dc_526
Delay [min]
1550000
1552500
1555000
1557500
1560000
EAE_RS07535
EAE_RS07540
EAE_RS07545
EAE_RS07550
EAE_RS07555
TU_396
TU_397
TU_2510
TU_2509
EAE_RS07530
EAE_RS07560
EAE_RS07565
EAE_RS07570
EAE_RS07575
EAE_RS07580
rhaM
fucO
rhaD
rhaA
rhaB
D_2935
D_2934
Dc_3321
Dc_3320
I_3706
I_3705
Term: termination (0), NS: new start (0), PS: pausing site (0), iTSS_I: internal starting site (0)

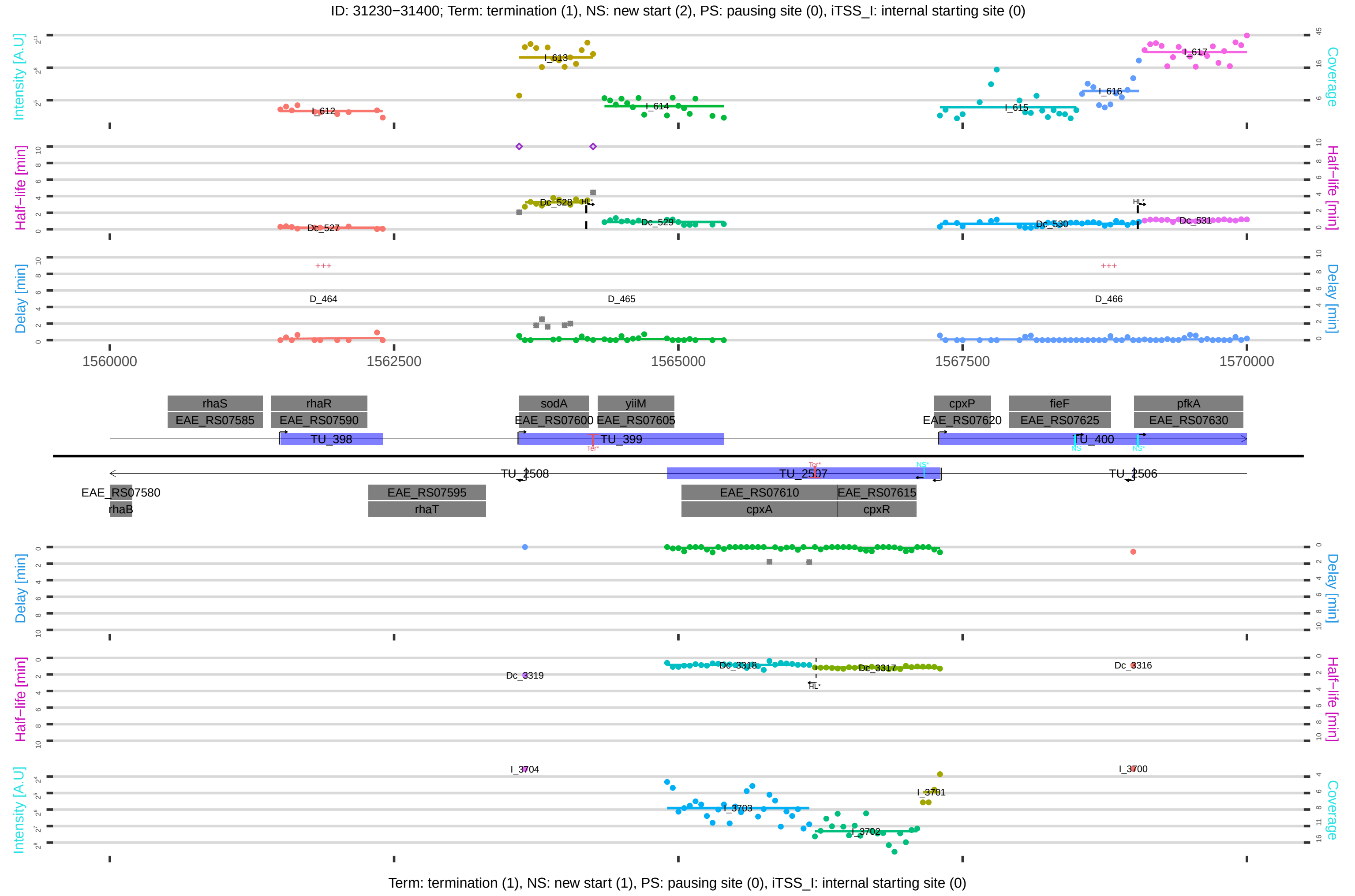

ID: 31230−31400; Term: termination (1), NS: new start (2), PS: pausing site (0), iTSS_I: internal starting site (0)
Intensity [A.U]
Coverage
Half−life [min]
Delay [min]
I_612
I_613
I_614
I_615
I_616
I_617
Dc_527
Dc_528
Dc_529
Dc_530
Dc_531
D_464
D_465
D_466
1560000
1562500
1565000
1567500
1570000
rhaS
EAE_RS07585
rhaR
EAE_RS07590
sodA
EAE_RS07600
yiiM
EAE_RS07605
cpxP
EAE_RS07620
fieF
EAE_RS07625
pfkA
EAE_RS07630
TU_398
TU_399
TU_400
TU_2508
TU_2507
TU_2506
EAE_RS07580
rhaB
EAE_RS07595
rhaT
EAE_RS07610
cpxA
EAE_RS07615
cpxR
Dc_3319
Dc_3318
Dc_3317
Dc_3316
I_3704
I_3703
I_3702
I_3701
I_3700
Term: termination (1), NS: new start (1), PS: pausing site (0), iTSS_I: internal starting site (0)

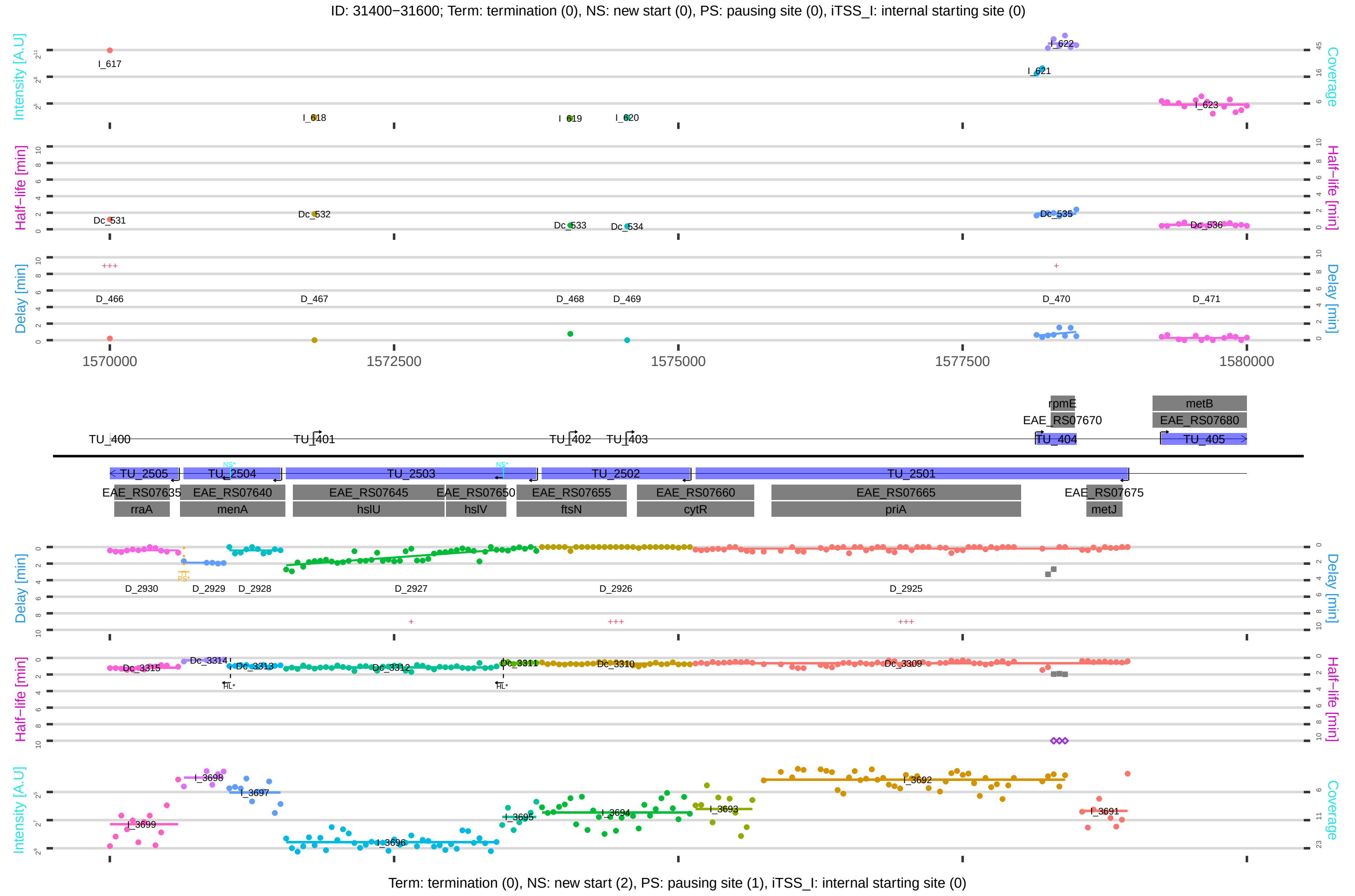

ID: 31400−31600; Term: termination (0), NS: new start (0), PS: pausing site (0), iTSS_I: internal starting site (0)
Intensity [A.U]
Coverage
Half−life [min]
Delay [min]
I_617
I_618
I_619
I_620
I_621
I_622
I_623
Dc_531
Dc_532
Dc_533
Dc_534
Dc_535
Dc_536
D_466
D_467
D_468
D_469
D_470
D_471
1570000
1572500
1575000
1577500
1580000
rpmE
EAE_RS07670
metB
EAE_RS07680
TU_400
TU_401
TU_402
TU_403
TU_404
TU_405
TU_2505
TU_2504
TU_2503
TU_2502
TU_2501
EAE_RS07635
EAE_RS07640
EAE_RS07645
EAE_RS07650
EAE_RS07655
EAE_RS07660
EAE_RS07665
EAE_RS07675
rraA
menA
hslU
hslV
ftsN
cytR
priA
metJ
D_2930
D_2929
D_2928
D_2927
D_2926
D_2925
Dc_3315
Dc_3314
Dc_3313
Dc_3312
Dc_3311
Dc_3310
Dc_3309
I_3699
I_3698
I_3697
I_3696
I_3695
I_3694
I_3693
I_3692
I_3691
Term: termination (0), NS: new start (2), PS: pausing site (1), iTSS_I: internal starting site (0)

ID: 31600−31775; Term: termination (0), NS: new start (0), PS: pausing site (0), iTSS_I: internal starting site (0)

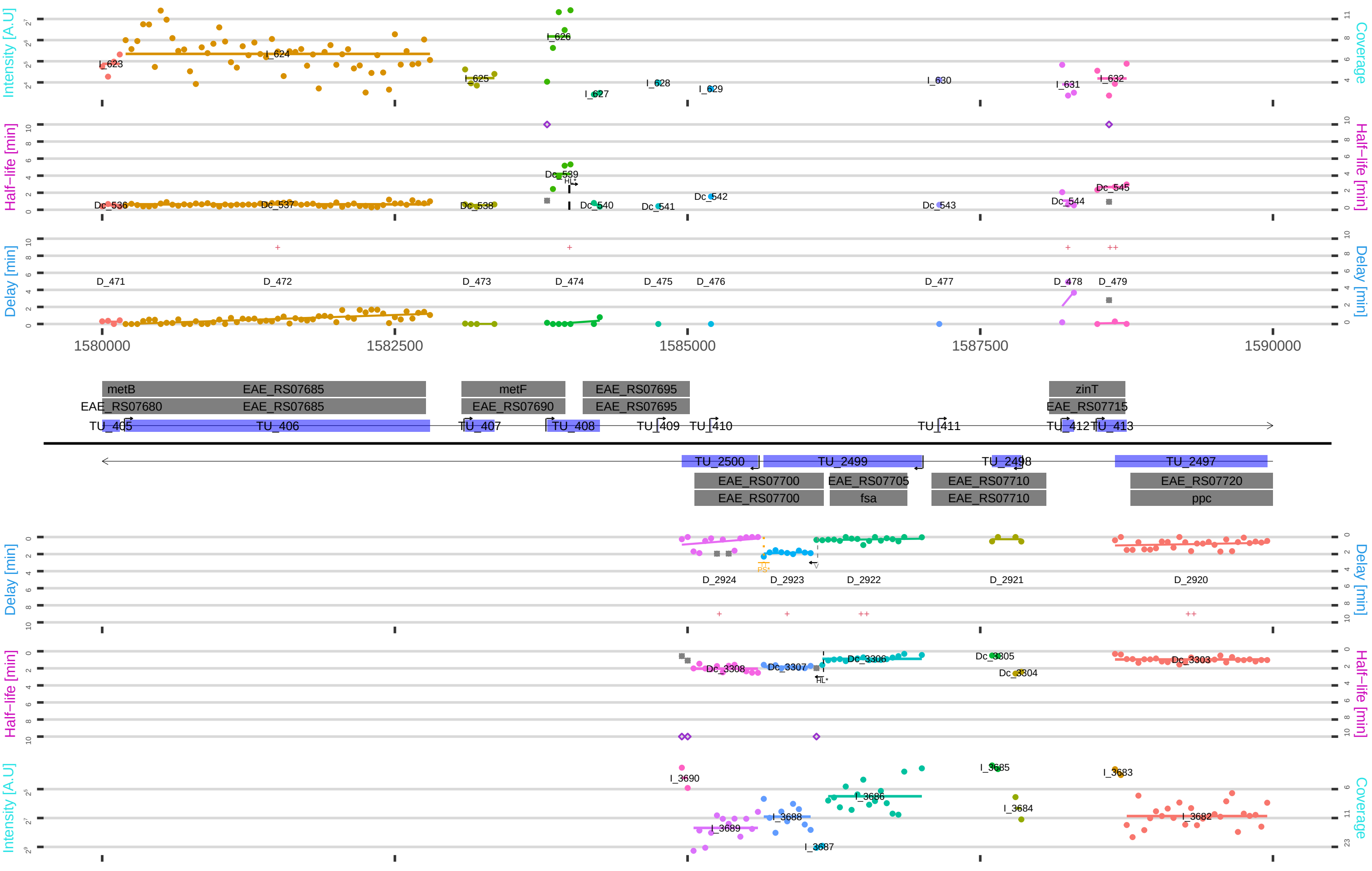

Term: termination (0), NS: new start (0), PS: pausing site (1), iTSS_I: internal starting site (0)

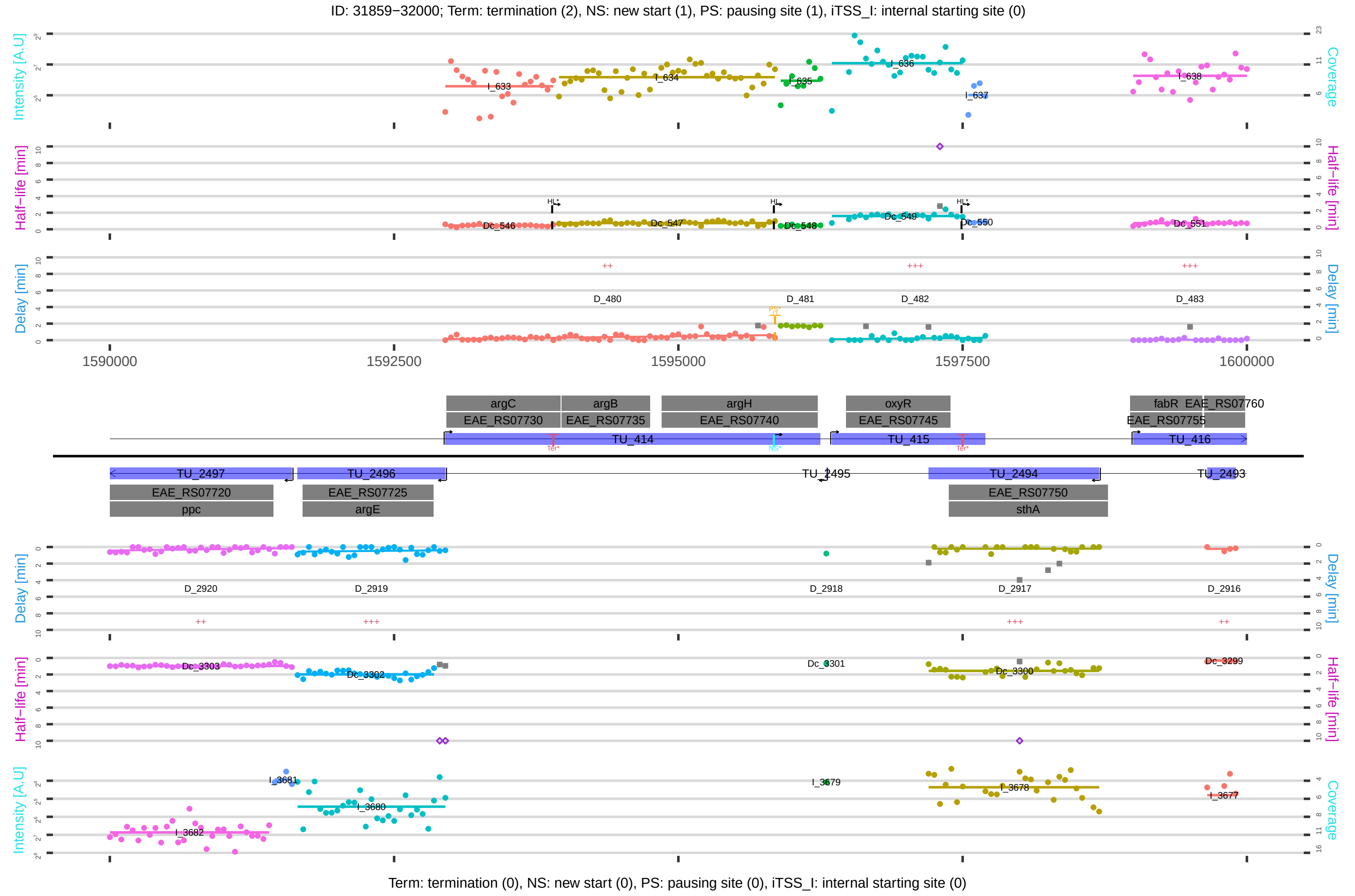

ID: 31859−32000; Term: termination (2), NS: new start (1), PS: pausing site (1), iTSS_I: internal starting site (0)
Intensity [A.U]
Coverage
Half−life [min]
Delay [min]
I_633
I_634
I_635
I_636
I_637
I_638
Dc_546
Dc_547
Dc_548
Dc_549
Dc_550
Dc_551
D_480
D_481
D_482
D_483
1590000
1592500
1595000
1597500
1600000
argC
argB
argH
oxyR
fabR
EAE_RS07760
EAE_RS07730
EAE_RS07735
EAE_RS07740
EAE_RS07745
EAE_RS07755
TU_414
TU_415
TU_416
TU_2497
TU_2496
TU_2495
TU_2494
TU_2493
EAE_RS07720
EAE_RS07725
EAE_RS07750
ppc
argE
sthA
D_2920
D_2919
D_2918
D_2917
D_2916
Dc_3303
Dc_3302
Dc_3301
Dc_3300
Dc_3299
I_3681
I_3680
I_3679
I_3678
I_3677
I_3682
Term: termination (0), NS: new start (0), PS: pausing site (0), iTSS_I: internal starting site (0)

ID: 32000−32200; Term: termination (7), NS: new start (3), PS: pausing site (4), iTSS_I: internal starting site (1)

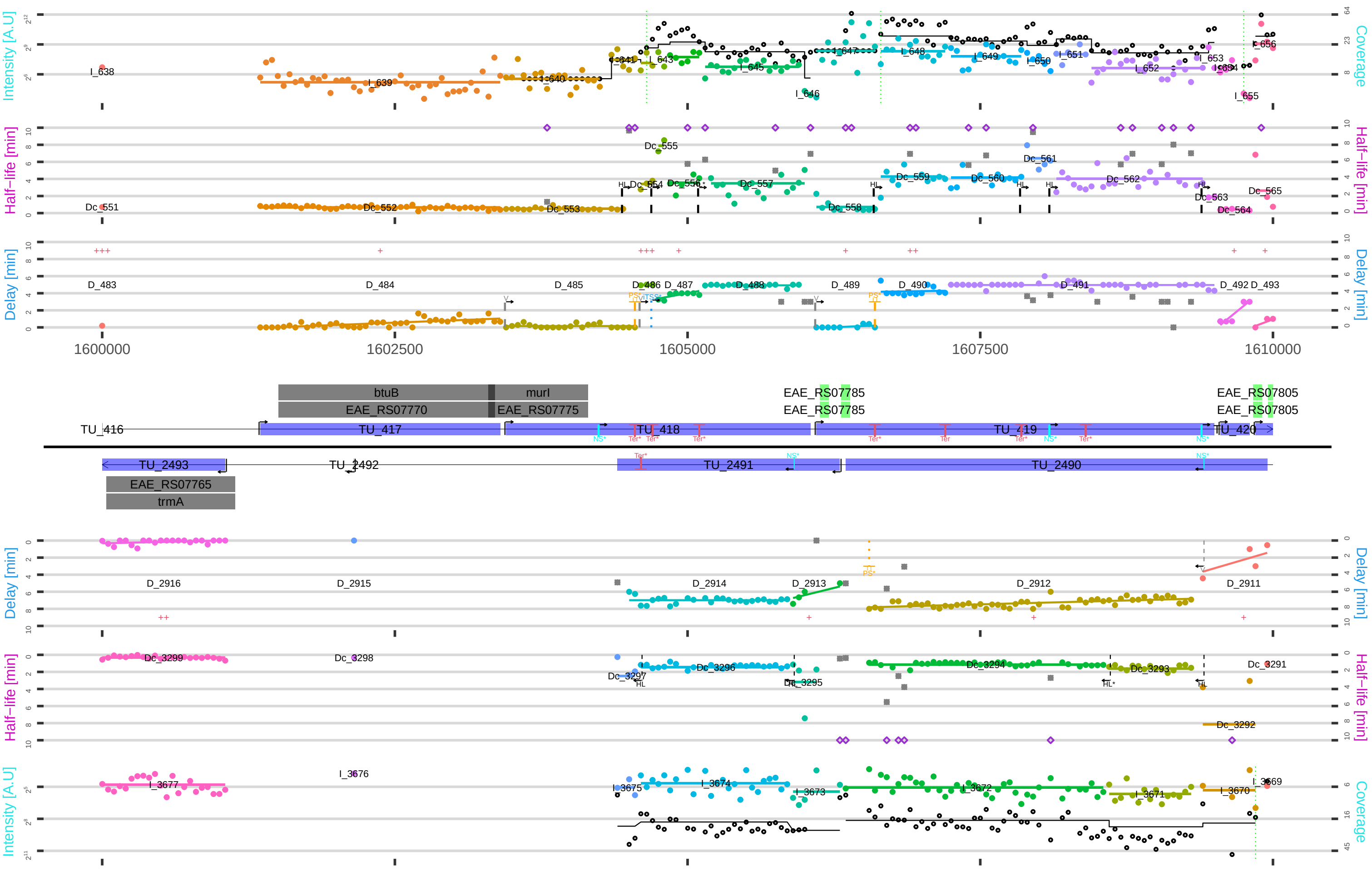

Term: termination (1), NS: new start (2), PS: pausing site (2), iTSS_I: internal starting site (1)

ID: 32200−32398; Term: termination (2), NS: new start (0), PS: pausing site (2), iTSS_I: internal starting site (0)

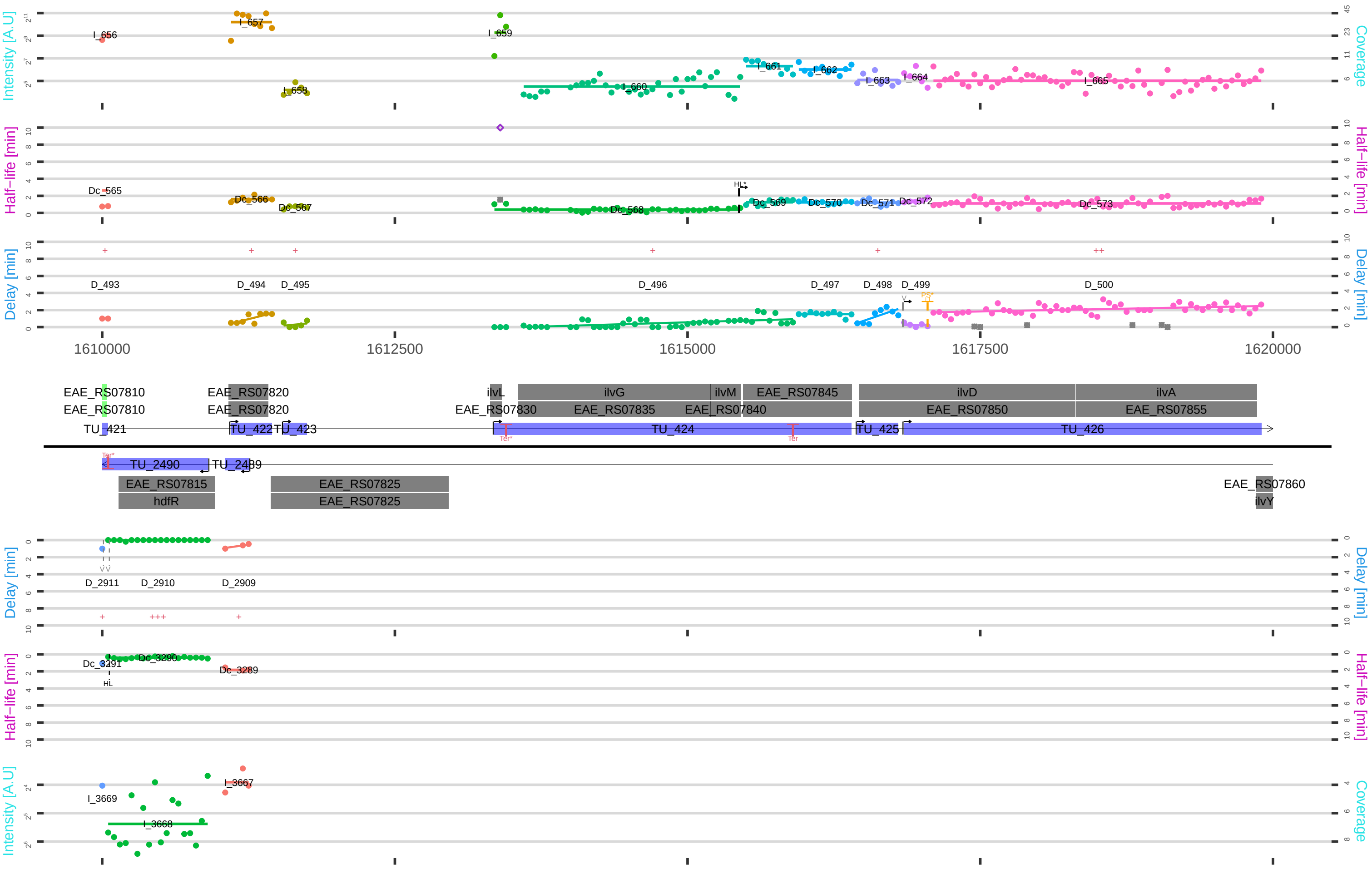

Term: termination (1), NS: new start (0), PS: pausing site (0), iTSS_I: internal starting site (0)

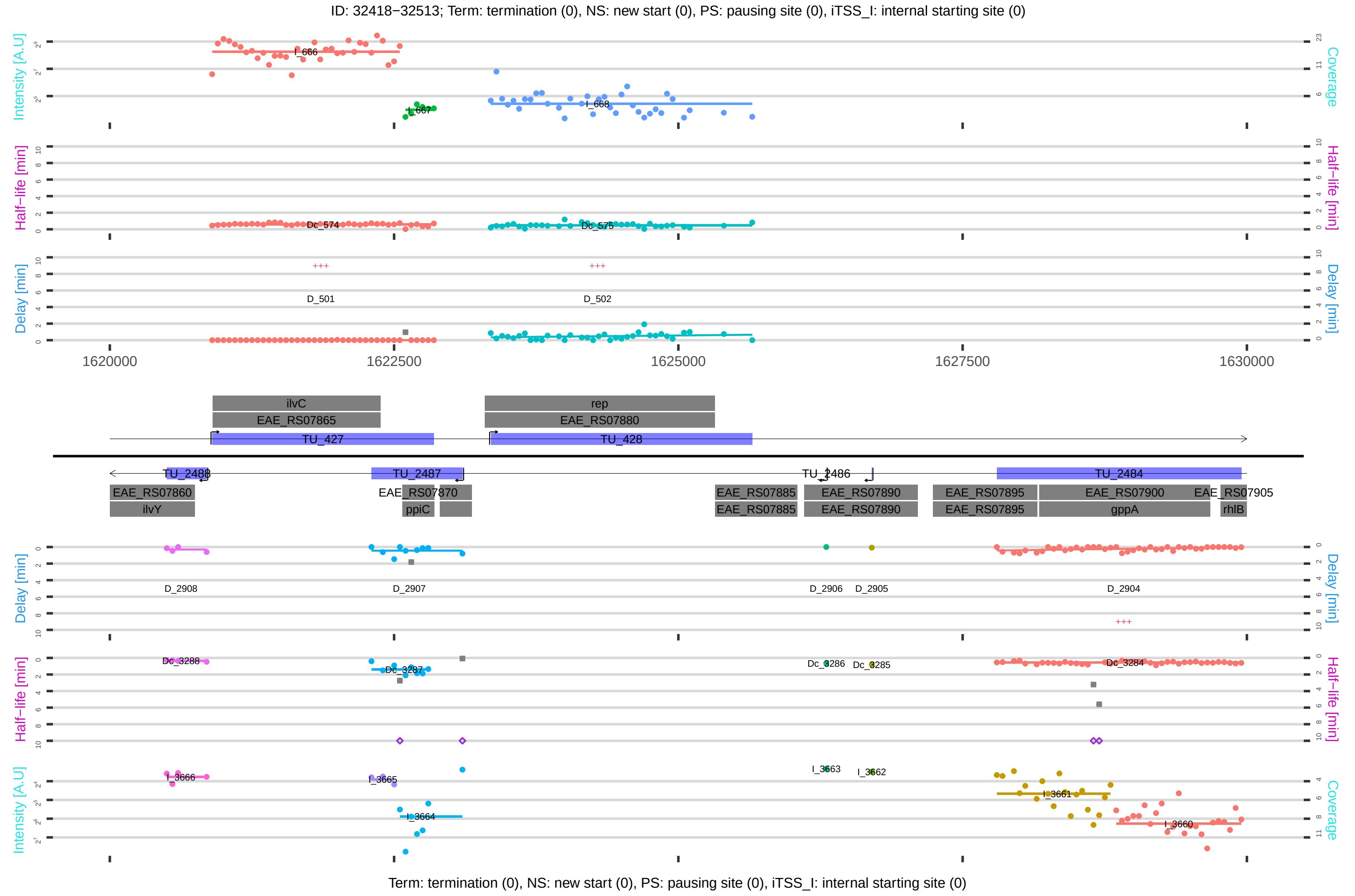

ID: 32418−32513; Term: termination (0), NS: new start (0), PS: pausing site (0), iTSS_I: internal starting site (0)
Intensity [A.U]
Coverage
I_666
I_667
I_668
Half−life [min]
Dc_574
Dc_575
Delay [min]
D_501
D_502
1620000
1622500
1625000
1627500
1630000
ilvC
EAE_RS07865
rep
EAE_RS07880
TU_427
TU_428
TU_2488
TU_2487
TU_2486
TU_2484
EAE_RS07860
ilvY
EAE_RS07870
ppiC
EAE_RS07885
EAE_RS07890
EAE_RS07895
EAE_RS07900
gppA
EAE_RS07905
rhlB
D_2908
D_2907
D_2906
D_2905
D_2904
Dc_3288
Dc_3287
Dc_3286
Dc_3285
Dc_3284
I_3666
I_3665
I_3664
I_3663
I_3662
I_3661
I_3660
Term: termination (0), NS: new start (0), PS: pausing site (0), iTSS_I: internal starting site (0)

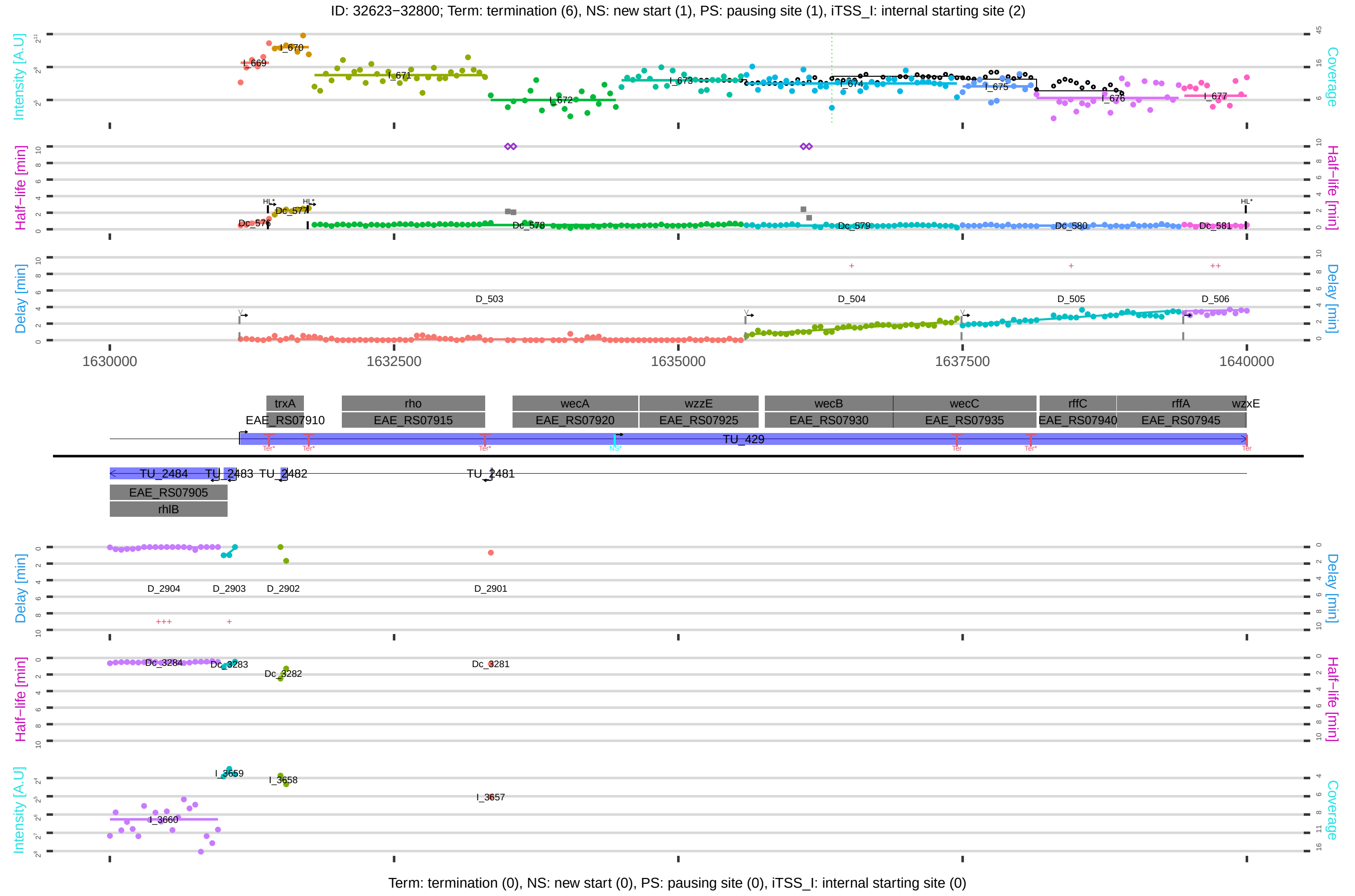
ID: 32623−32800; Term: termination (6), NS: new start (1), PS: pausing site (1), iTSS_I: internal starting site (2)
Intensity [A.U]
Coverage
Half−life [min]
Delay [min]
I_669
I_670
I_671
I_672
I_673
I_674
I_675
I_676
I_677
Dc_576
Dc_577
Dc_578
Dc_579
Dc_580
Dc_581
D_503
D_504
D_505
D_506
1630000
1632500
1635000
1637500
1640000
trxA
rho
wecA
wzzE
wecB
wecC
rffC
rffA
wzxE
EAE_RS07910
EAE_RS07915
EAE_RS07920
EAE_RS07925
EAE_RS07930
EAE_RS07935
EAE_RS07940
EAE_RS07945
TU_429
TU_2484
TU_2483
TU_2482
TU_2481
EAE_RS07905
rhlB
D_2904
D_2903
D_2902
D_2901
Dc_3284
Dc_3283
Dc_3282
Dc_3281
I_3659
I_3658
I_3657
I_3660
Term: termination (0), NS: new start (0), PS: pausing site (0), iTSS_I: internal starting site (0)

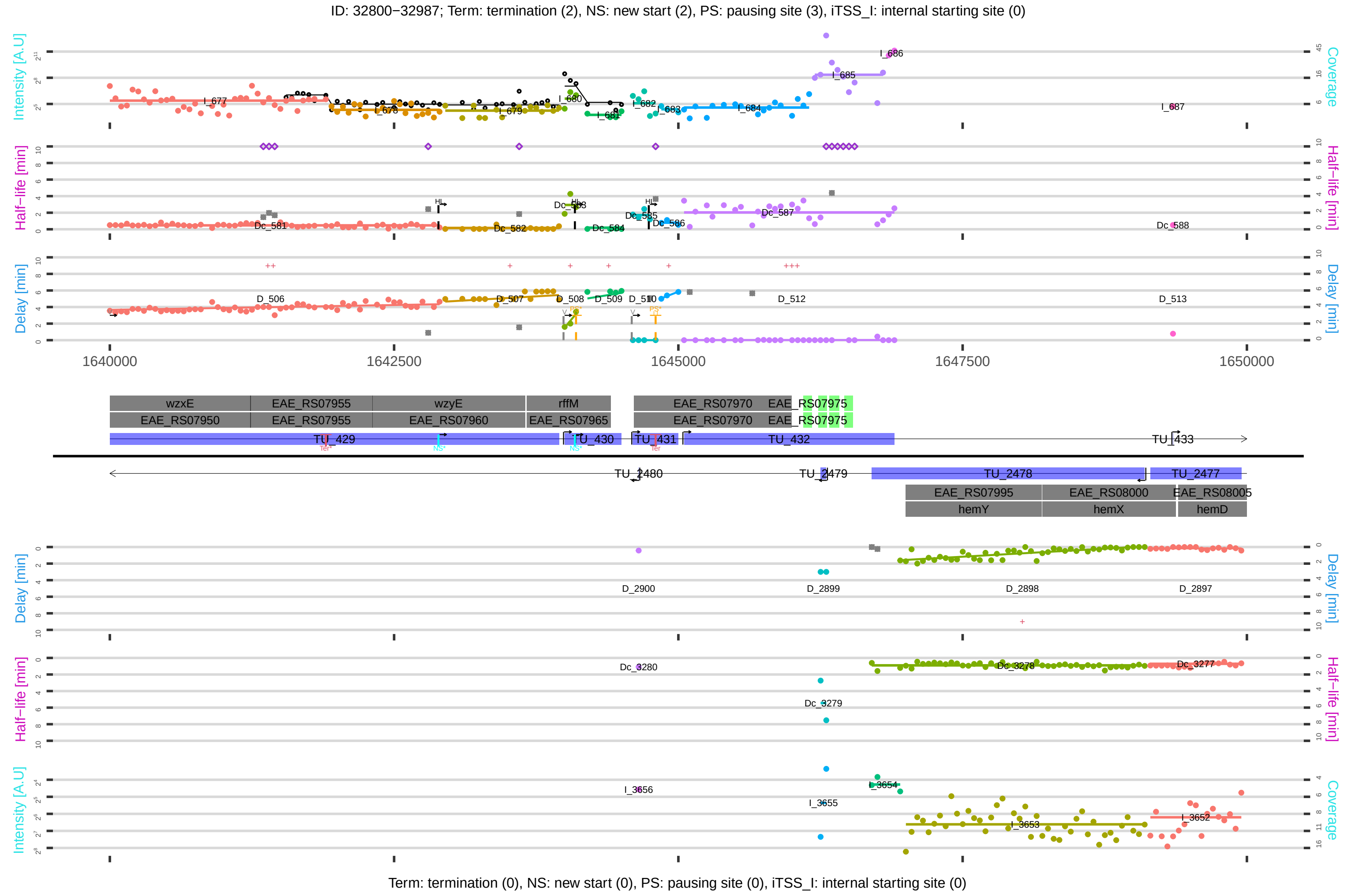
ID: 32800−32987; Term: termination (2), NS: new start (2), PS: pausing site (3), iTSS_I: internal starting site (0)
Intensity [A.U]
Coverage
Half−life [min]
Delay [min]
1640000
1642500
1645000
1647500
1650000
wzxE
EAE_RS07955
wzyE
rffM
EAE_RS07970
EAE_RS07975
EAE_RS07950
EAE_RS07960
EAE_RS07965
TU_429
TU_430
TU_431
TU_432
TU_433
TU_2480
TU_2479
TU_2478
TU_2477
EAE_RS07995
EAE_RS08000
EAE_RS08005
hemY
hemX
hemD
Term: termination (0), NS: new start (0), PS: pausing site (0), iTSS_I: internal starting site (0)

ID: 33023−33200; Term: termination (1), NS: new start (0), PS: pausing site (1), iTSS_I: internal starting site (0)

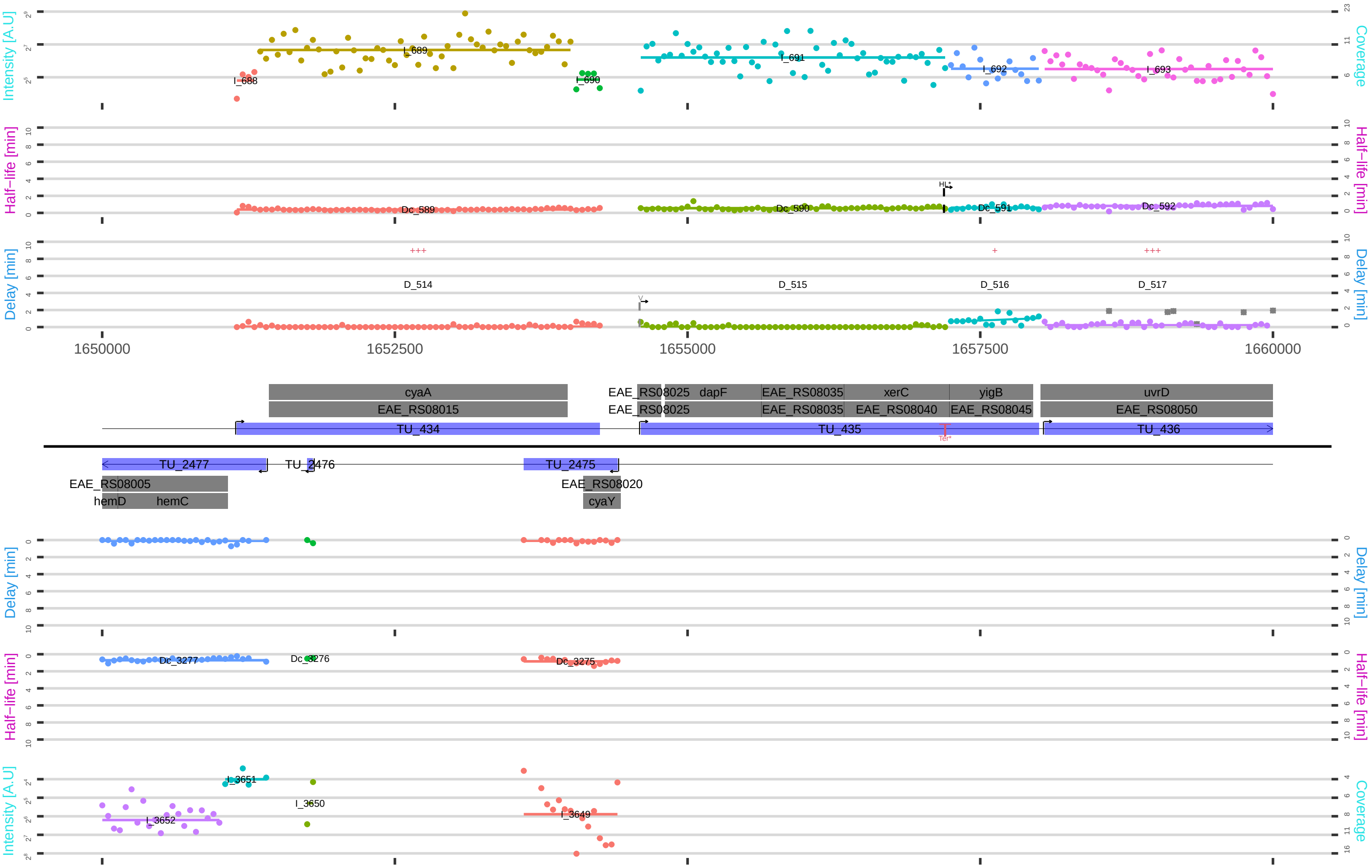

Term: termination (0), NS: new start (0), PS: pausing site (0), iTSS_I: internal starting site (0)

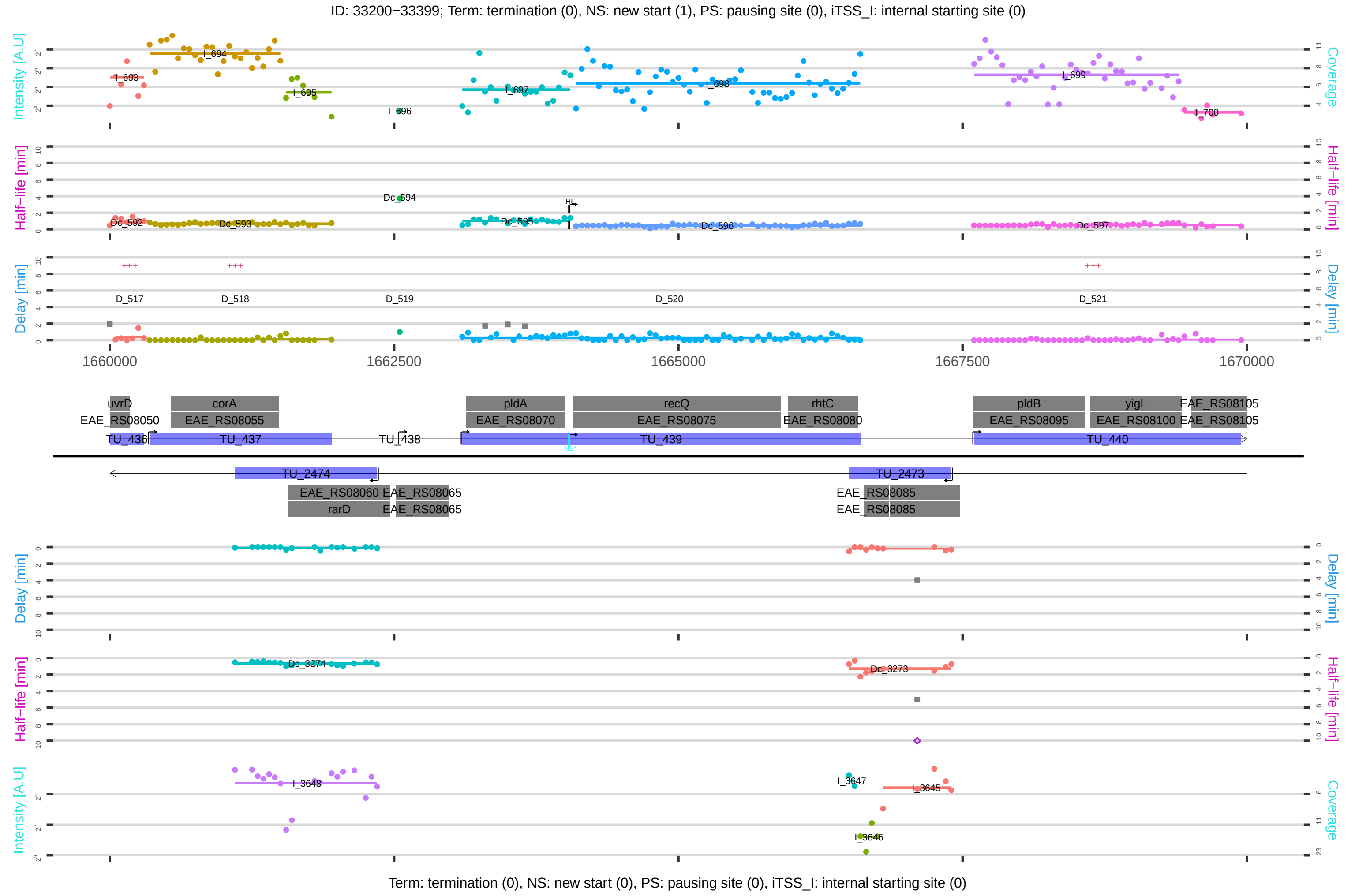

ID: 33200−33399; Term: termination (0), NS: new start (1), PS: pausing site (0), iTSS_I: internal starting site (0)
Intensity [A.U]
Coverage
Half−life [min]
Delay [min]
I_693
I_694
I_695
I_696
I_697
I_698
I_699
I_700
Dc_592
Dc_593
Dc_594
Dc_595
Dc_596
Dc_597
HL
D_517
D_518
D_519
D_520
D_521
1660000
1662500
1665000
1667500
1670000
uvrD
EAE_RS08050
corA
EAE_RS08055
pldA
EAE_RS08070
recQ
EAE_RS08075
rhtC
EAE_RS08080
pldB
EAE_RS08095
yigL
EAE_RS08100
EAE_RS08105
TU_436
TU_437
TU_438
TU_439
TU_440
NS
TU_2474
TU_2473
EAE_RS08060
EAE_RS08065
rarD
EAE_RS08085
Dc_3274
Dc_3273
I_3648
I_3647
I_3645
I_3646
Term: termination (0), NS: new start (0), PS: pausing site (0), iTSS_I: internal starting site (0)

ID: 33429−33600; Term: termination (2), NS: new start (2), PS: pausing site (2), iTSS_I: internal starting site (0)

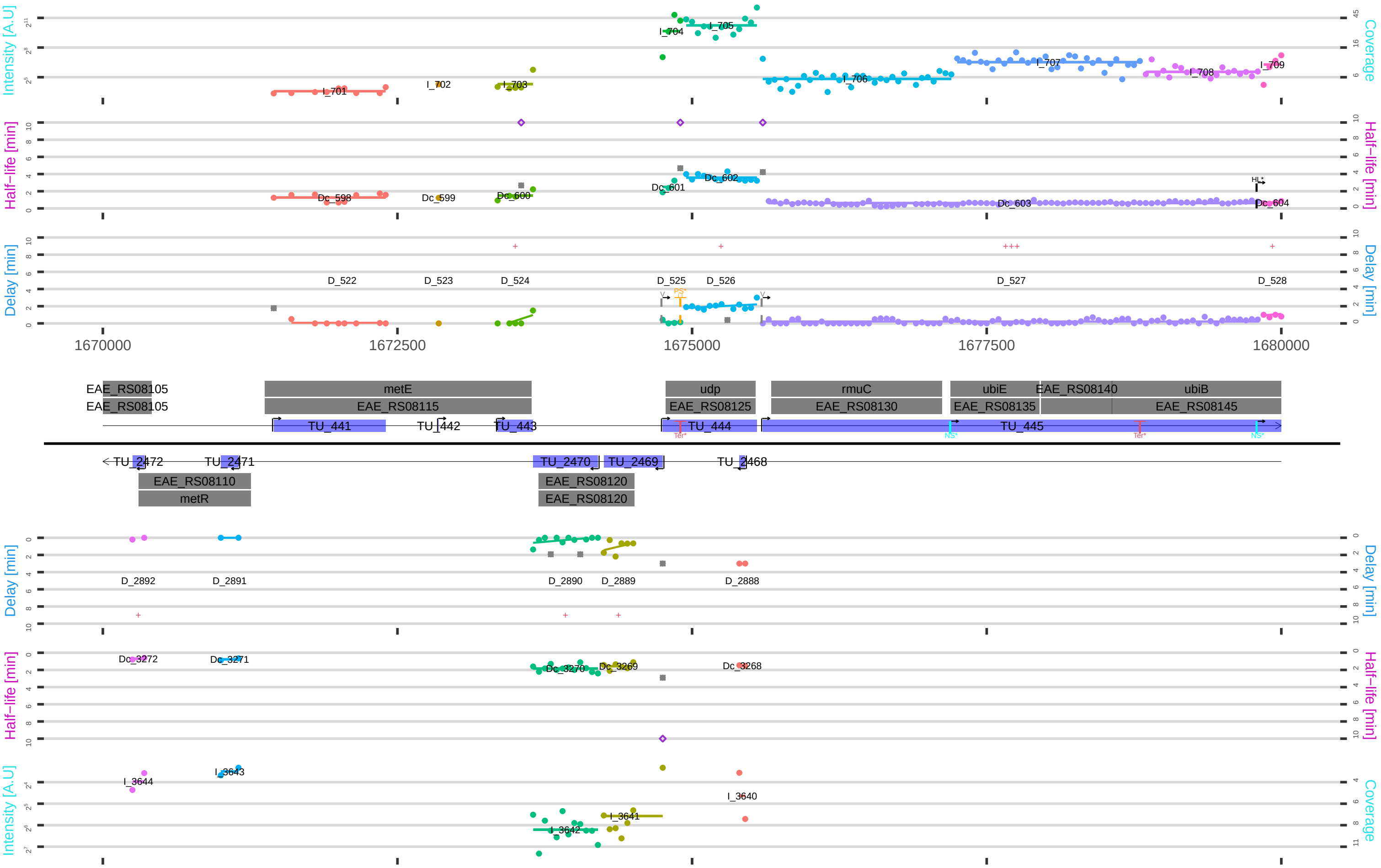

Term: termination (0), NS: new start (0), PS: pausing site (0), iTSS_I: internal starting site (0)

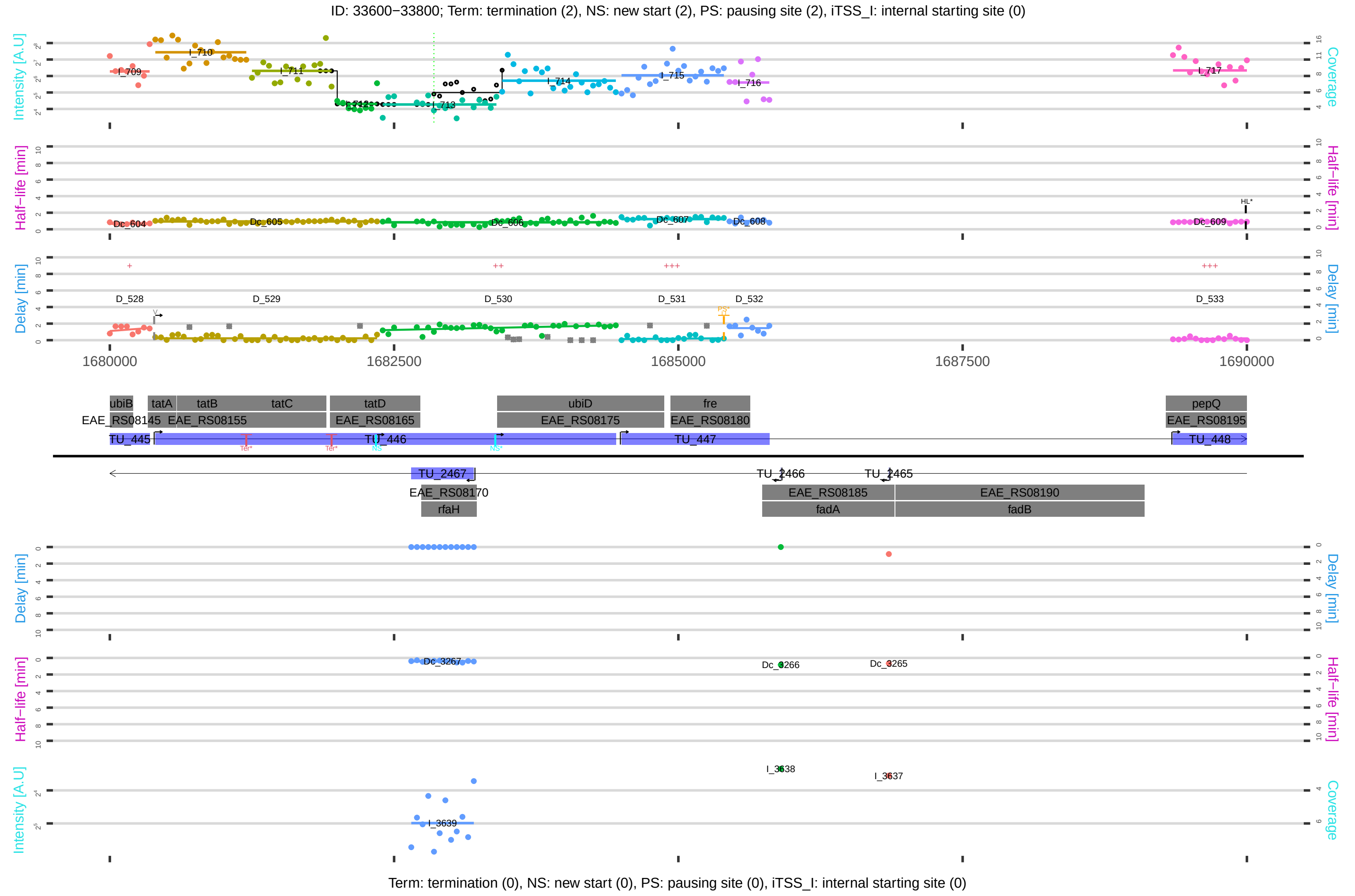

ID: 33600−33800; Term: termination (2), NS: new start (2), PS: pausing site (2), iTSS_I: internal starting site (0)
Intensity [A.U]
Coverage
Half−life [min]
Delay [min]
1680000
1682500
1685000
1687500
1690000
ubiB
tatA
tatB
tatC
tatD
ubiD
fre
pepQ
EAE_RS08145
EAE_RS08155
EAE_RS08165
EAE_RS08175
EAE_RS08180
EAE_RS08195
TU_445
TU_446
TU_447
TU_448
TU_2467
TU_2466
TU_2465
EAE_RS08170
rfaH
EAE_RS08185
EAE_RS08190
fadA
fadB
Term: termination (0), NS: new start (0), PS: pausing site (0), iTSS_I: internal starting site (0)

ID: 33800−34000; Term: termination (6), NS: new start (3), PS: pausing site (4), iTSS_I: internal starting site (3)

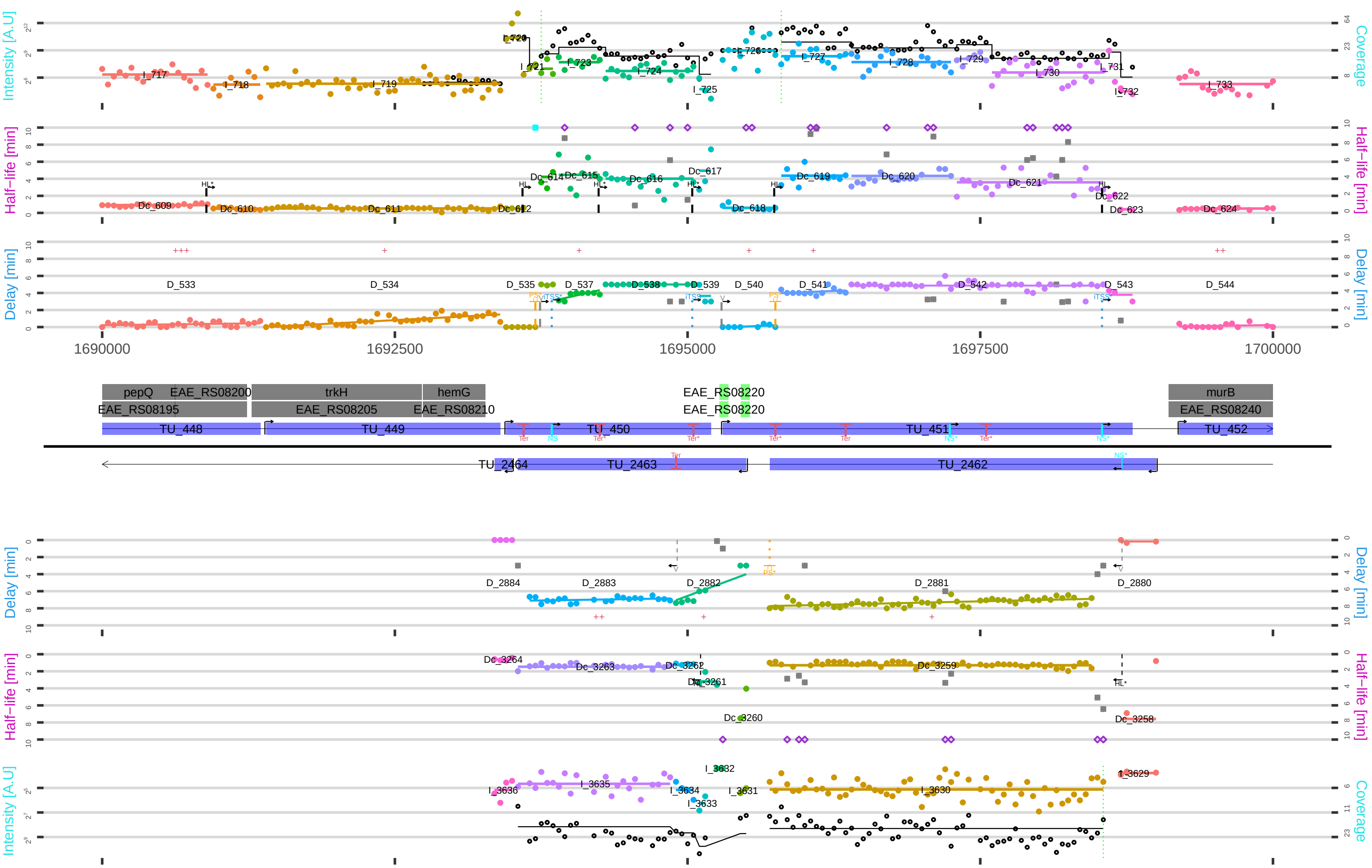

Term: termination (1), NS: new start (1), PS: pausing site (1), iTSS_I: internal starting site (1)

ID: 34000−34200; Term: termination (3), NS: new start (3), PS: pausing site (1), iTSS_I: internal starting site (0)

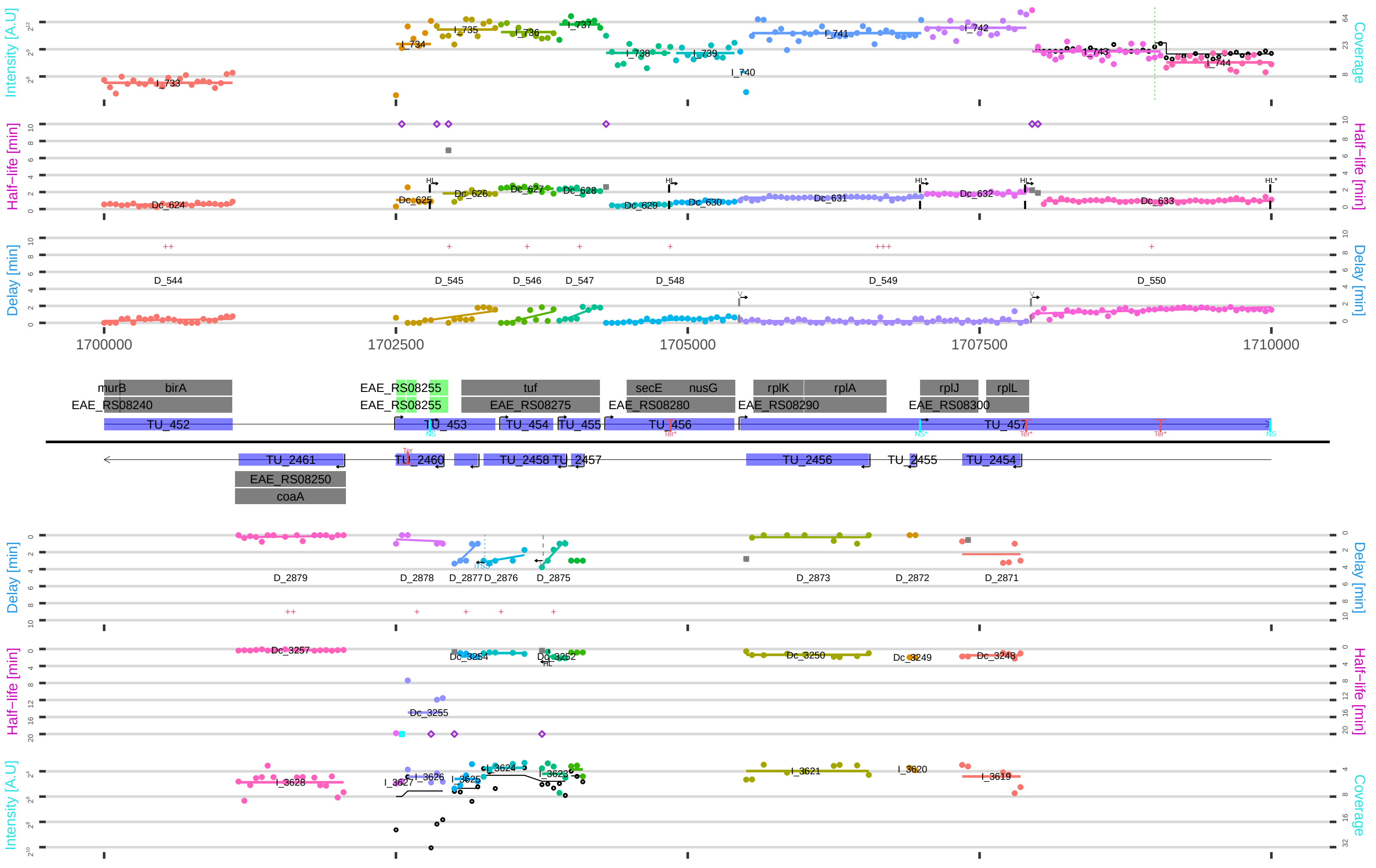

Term: termination (1), NS: new start (0), PS: pausing site (0), iTSS_I: internal starting site (1)

ID: 34200−34364; Term: termination (0), NS: new start (2), PS: pausing site (2), iTSS_I: internal starting site (0)

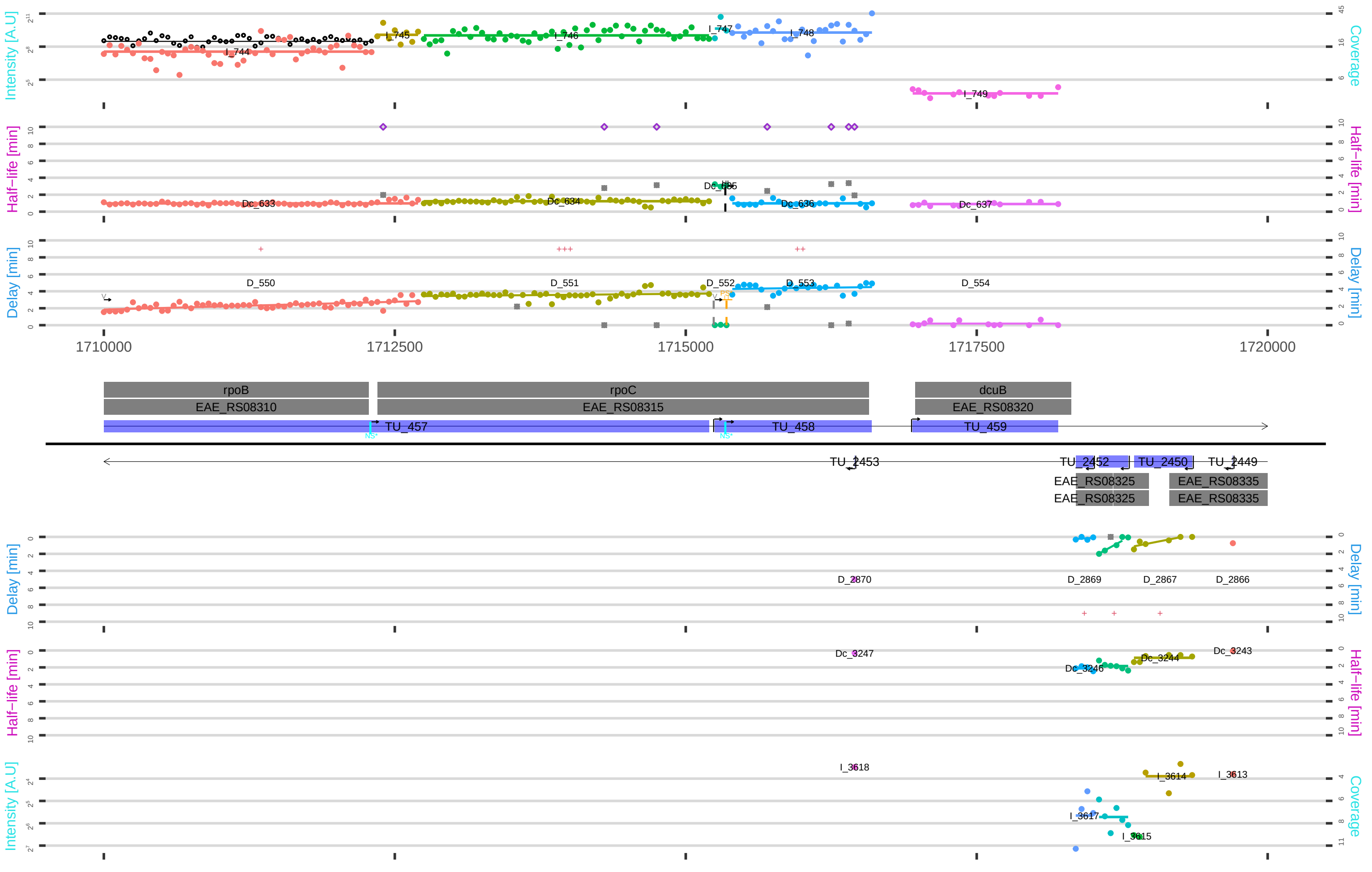

Term: termination (0), NS: new start (0), PS: pausing site (0), iTSS_I: internal starting site (0)

ID: 34415−34600; Term: termination (0), NS: new start (2), PS: pausing site (0), iTSS_I: internal starting site (0)

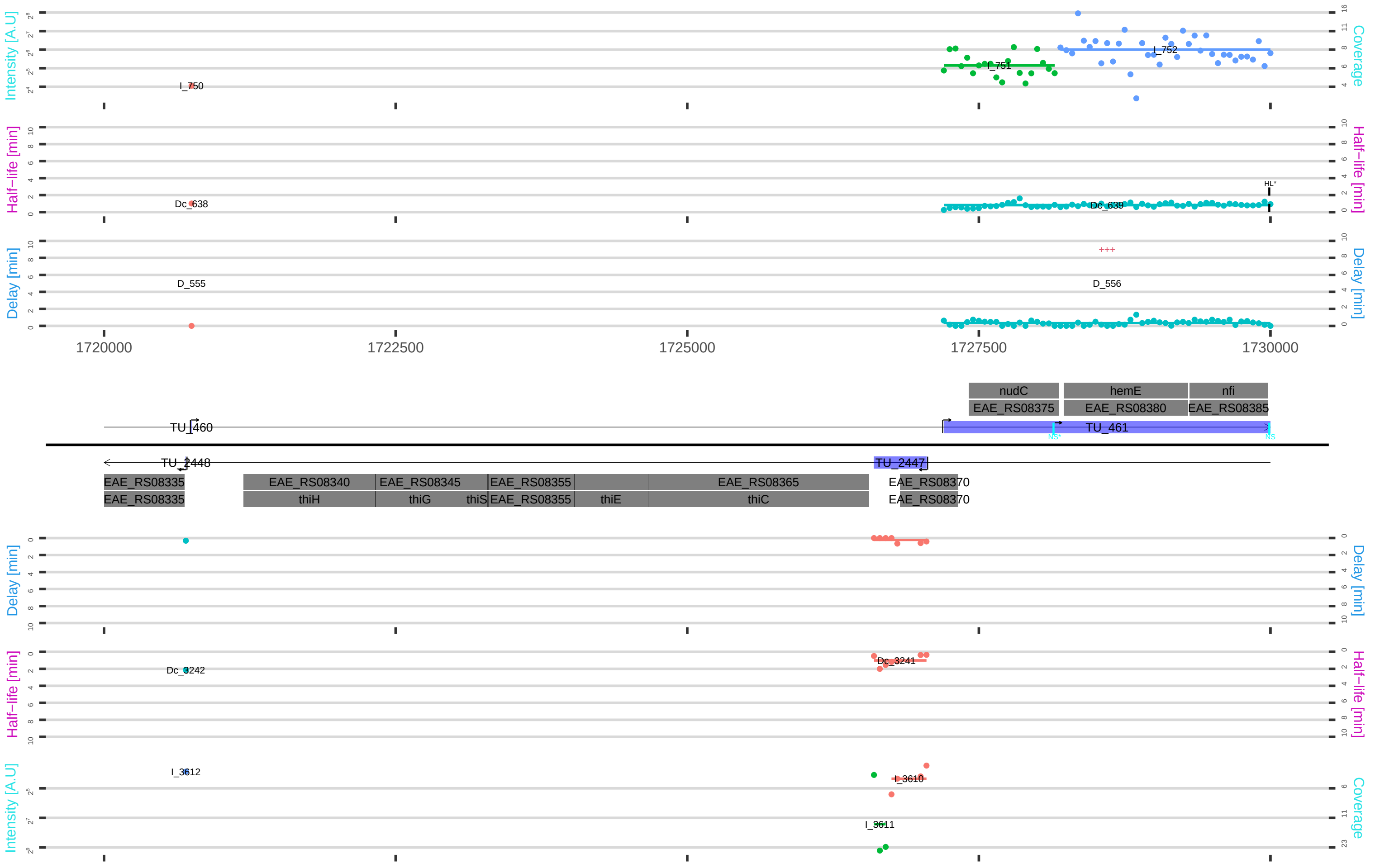

Term: termination (0), NS: new start (0), PS: pausing site (0), iTSS_I: internal starting site (0)

ID: 34600−34800; Term: termination (3), NS: new start (2), PS: pausing site (2), iTSS_I: internal starting site (2)

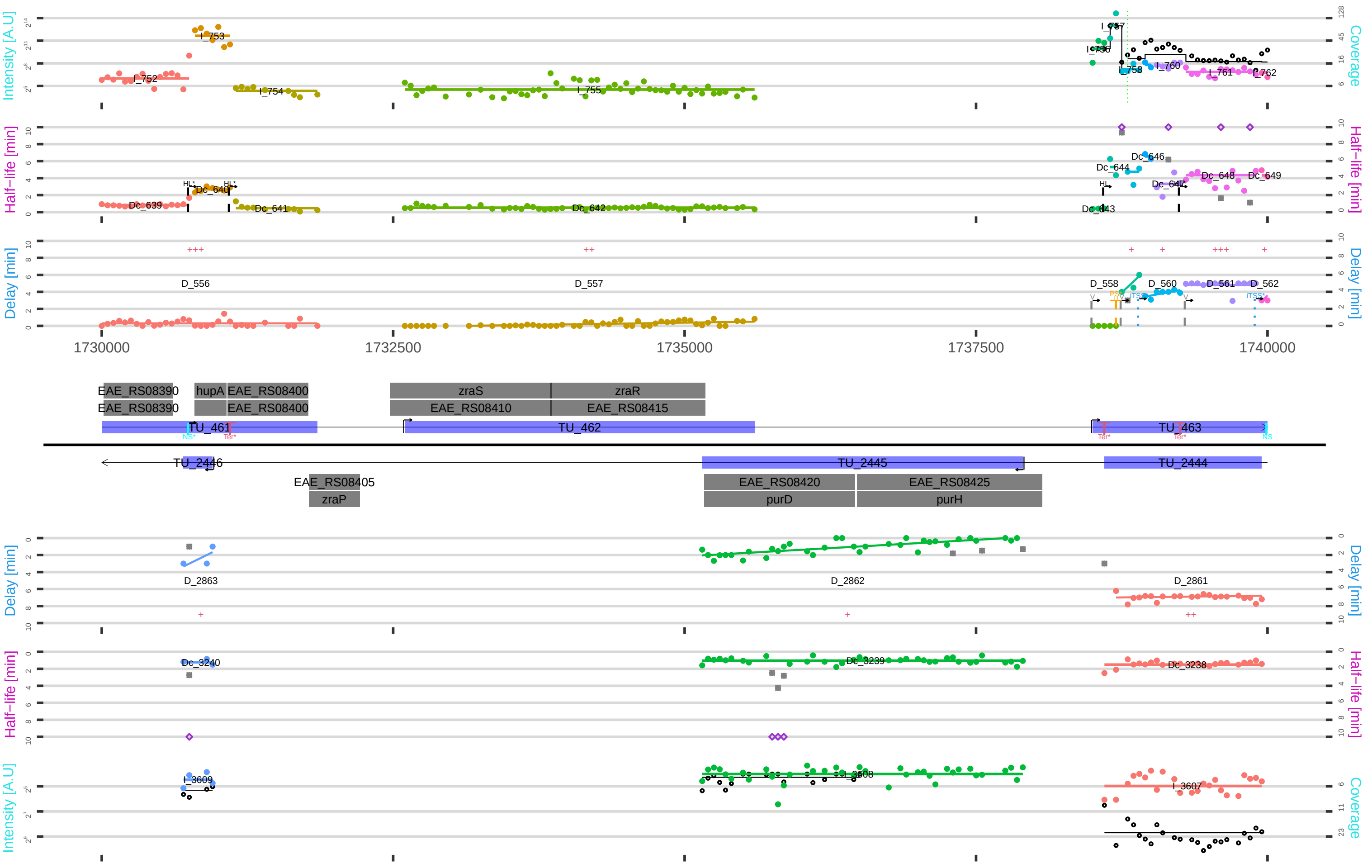

Term: termination (0), NS: new start (0), PS: pausing site (0), iTSS_I: internal starting site (0)

ID: 34800−34928; Term: termination (5), NS: new start (2), PS: pausing site (2), iTSS_I: internal starting site (1)

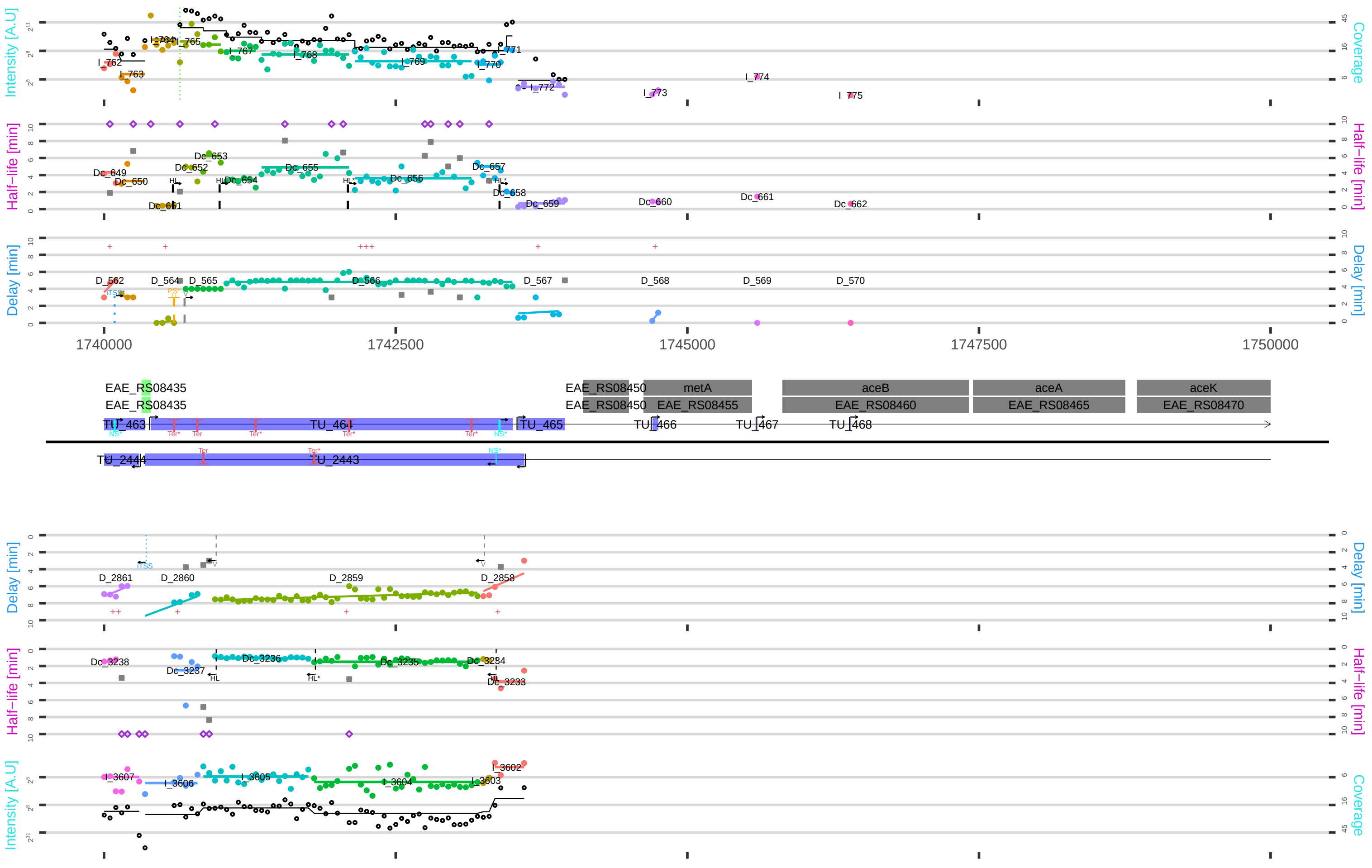

Term: termination (2), NS: new start (1), PS: pausing site (0), iTSS_I: internal starting site (2)

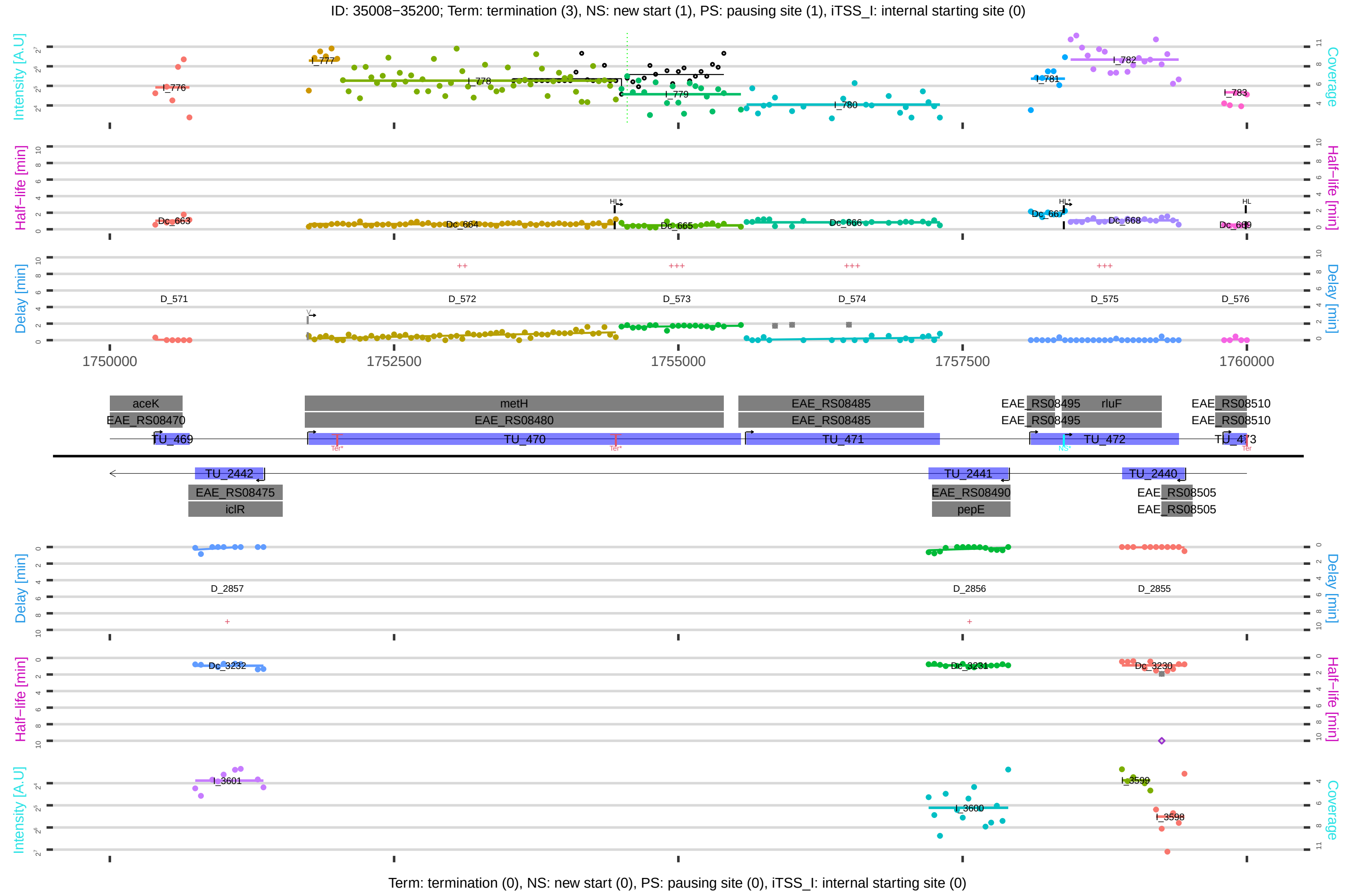

ID: 35008−35200; Term: termination (3), NS: new start (1), PS: pausing site (1), iTSS_I: internal starting site (0)
Intensity [A.U]
Coverage
Half−life [min]
Delay [min]
I_776
I_777
I_778
I_779
I_780
I_781
I_782
I_783
Dc_663
Dc_664
Dc_665
Dc_666
Dc_667
Dc_668
Dc_669
D_571
D_572
D_573
D_574
D_575
D_576
1750000
1752500
1755000
1757500
1760000
aceK
EAE_RS08470
metH
EAE_RS08480
EAE_RS08485
EAE_RS08485
EAE_RS08495
rluF
EAE_RS08495
EAE_RS08510
EAE_RS08510
TU_469
TU_470
TU_471
TU_472
TU_473
TU_2442
EAE_RS08475
iclR
TU_2441
EAE_RS08490
pepE
TU_2440
EAE_RS08505
EAE_RS08505
D_2857
D_2856
D_2855
Dc_3232
Dc_3231
Dc_3230
I_3601
I_3600
I_3599
I_3598
Term: termination (0), NS: new start (0), PS: pausing site (0), iTSS_I: internal starting site (0)

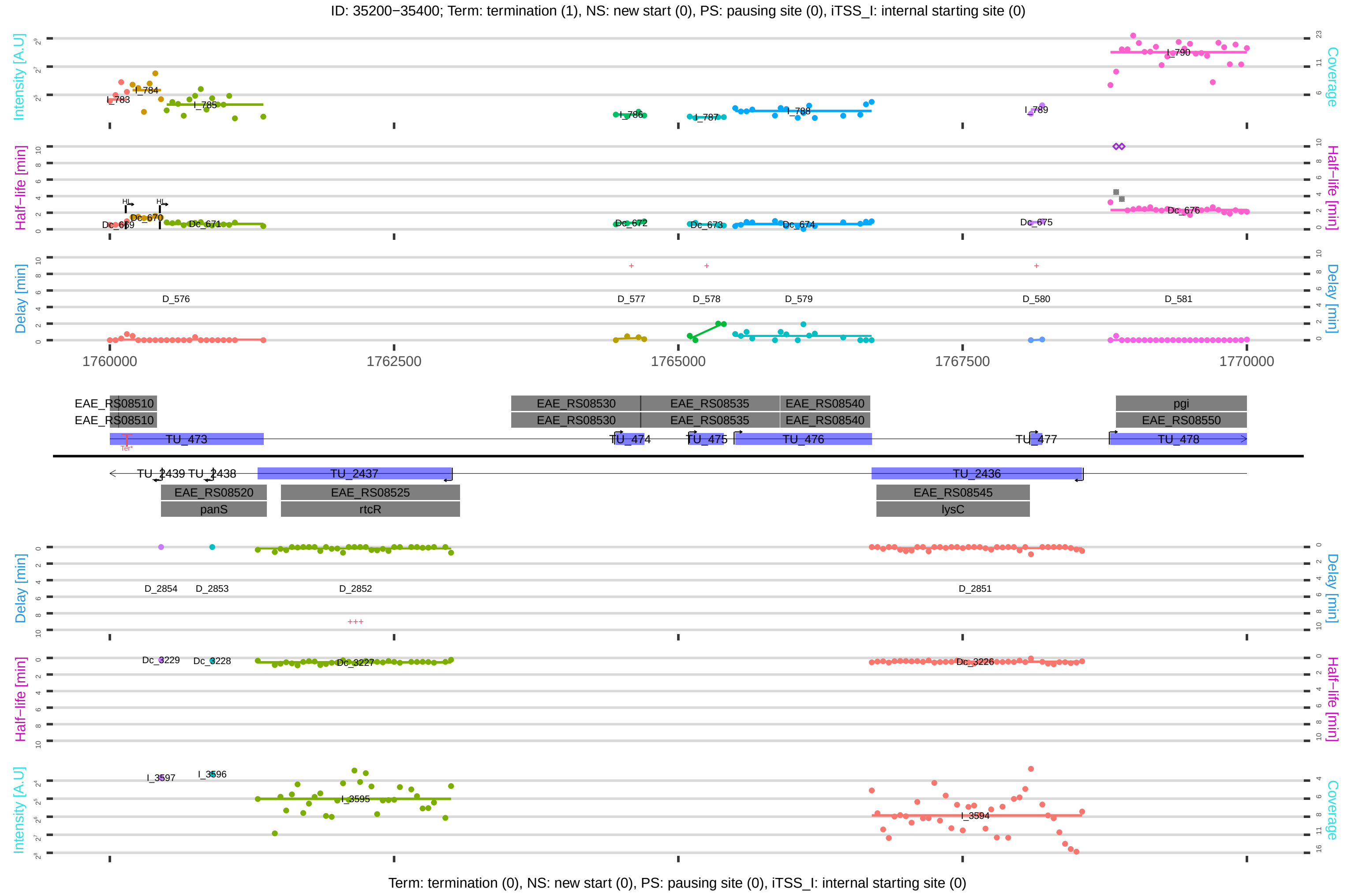

ID: 35200−35400; Term: termination (1), NS: new start (0), PS: pausing site (0), iTSS_I: internal starting site (0)
Intensity [A.U]
Coverage
Half−life [min]
Delay [min]
I_783
I_784
I_785
I_786
I_787
I_788
I_789
I_790
Dc_669
Dc_670
Dc_671
Dc_672
Dc_673
Dc_674
Dc_675
Dc_676
D_576
D_577
D_578
D_579
D_580
D_581
1760000
1762500
1765000
1767500
1770000
EAE_RS08510
EAE_RS08530
EAE_RS08535
EAE_RS08540
pgi
EAE_RS08550
TU_473
TU_474
TU_475
TU_476
TU_477
TU_478
TU_2439
TU_2438
TU_2437
TU_2436
EAE_RS08520
panS
EAE_RS08525
rtcR
EAE_RS08545
lysC
D_2854
D_2853
D_2852
D_2851
Dc_3229
Dc_3228
Dc_3227
Dc_3226
I_3597
I_3596
I_3595
I_3594
Term: termination (0), NS: new start (0), PS: pausing site (0), iTSS_I: internal starting site (0)

ID: 35400−35548; Term: termination (0), NS: new start (0), PS: pausing site (0), iTSS_I: internal starting site (0)

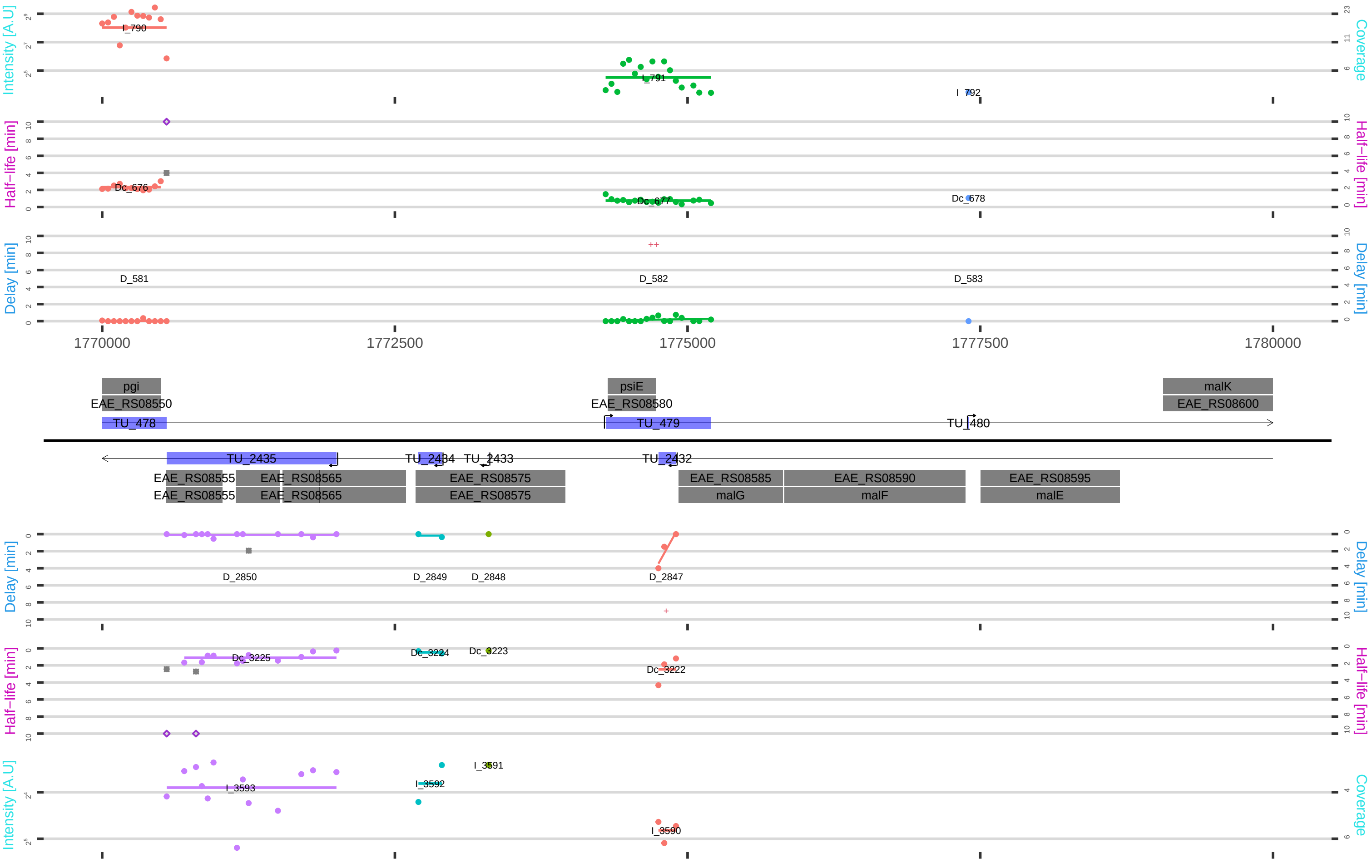

Term: termination (0), NS: new start (0), PS: pausing site (0), iTSS_I: internal starting site (0)

ID: 35653−35796; Term: termination (2), NS: new start (1), PS: pausing site (1), iTSS_I: internal starting site (0)

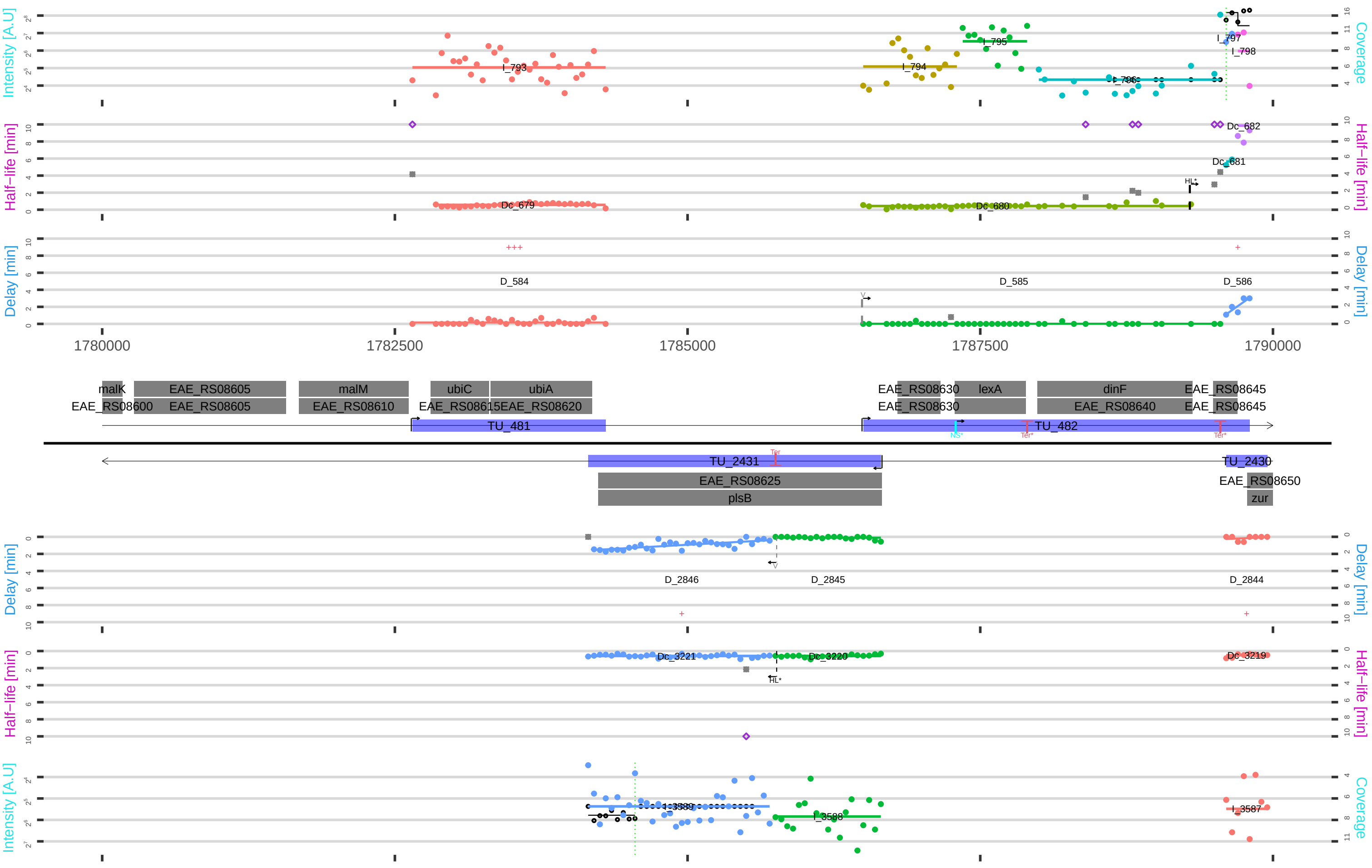

Term: termination (1), NS: new start (0), PS: pausing site (1), iTSS_I: internal starting site (0)

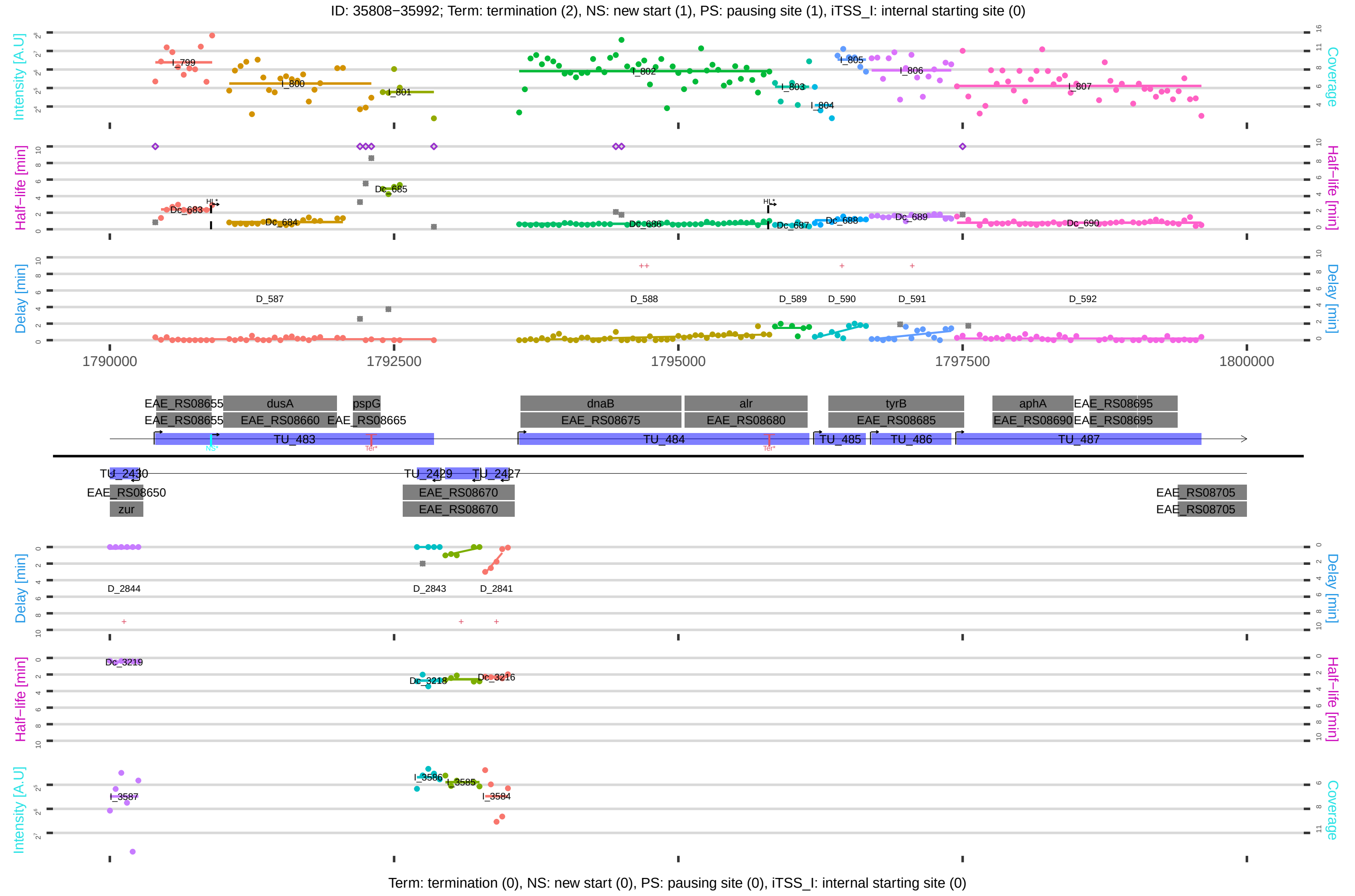
ID: 35808−35992; Term: termination (2), NS: new start (1), PS: pausing site (1), iTSS_I: internal starting site (0)
Intensity [A.U]
Coverage
Half−life [min]
Delay [min]
I_799
I_800
I_801
I_802
I_803
I_804
I_805
I_806
I_807
Dc_683
Dc_684
Dc_685
Dc_686
Dc_687
Dc_688
Dc_689
Dc_690
D_587
D_588
D_589
D_590
D_591
D_592
1790000
1792500
1795000
1797500
1800000
EAE_RS08655
dusA
pspG
dnaB
alr
tyrB
aphA
EAE_RS08695
EAE_RS08660
EAE_RS08665
EAE_RS08675
EAE_RS08680
EAE_RS08685
EAE_RS08690
TU_483
TU_484
TU_485
TU_486
TU_487
NS*
Ter*
TU_2430
TU_2429
TU_2427
EAE_RS08650
zur
EAE_RS08670
EAE_RS08705
D_2844
D_2843
D_2841
Dc_3219
Dc_3218
Dc_3216
I_3587
I_3586
I_3585
I_3584
Term: termination (0), NS: new start (0), PS: pausing site (0), iTSS_I: internal starting site (0)

ID: 36067−36200; Term: termination (1), NS: new start (1), PS: pausing site (1), iTSS_I: internal starting site (0)

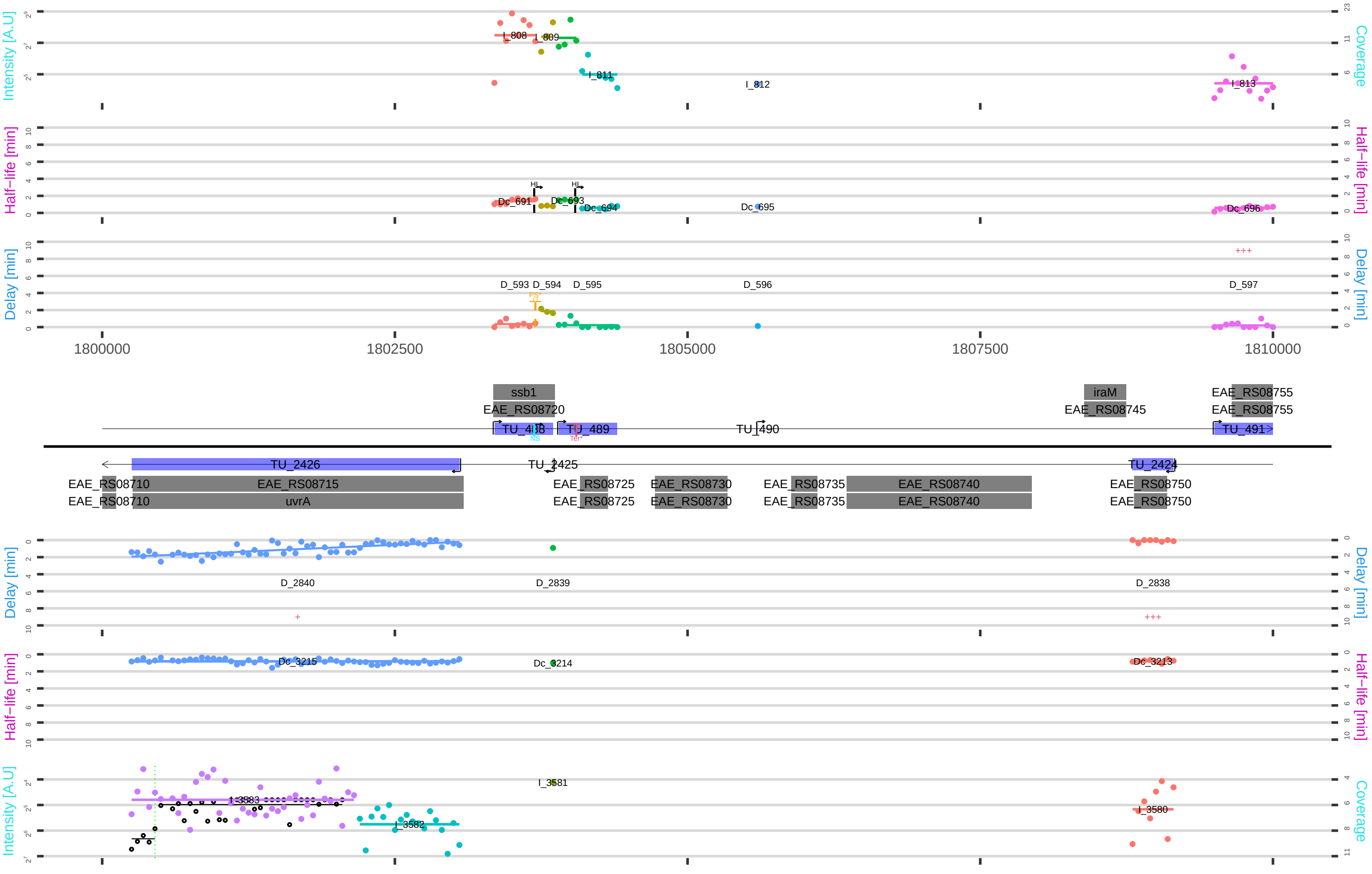

Term: termination (0), NS: new start (0), PS: pausing site (0), iTSS_I: internal starting site (0)

ID: 36200−36400; Term: termination (0), NS: new start (1), PS: pausing site (0), iTSS_I: internal starting site (1)

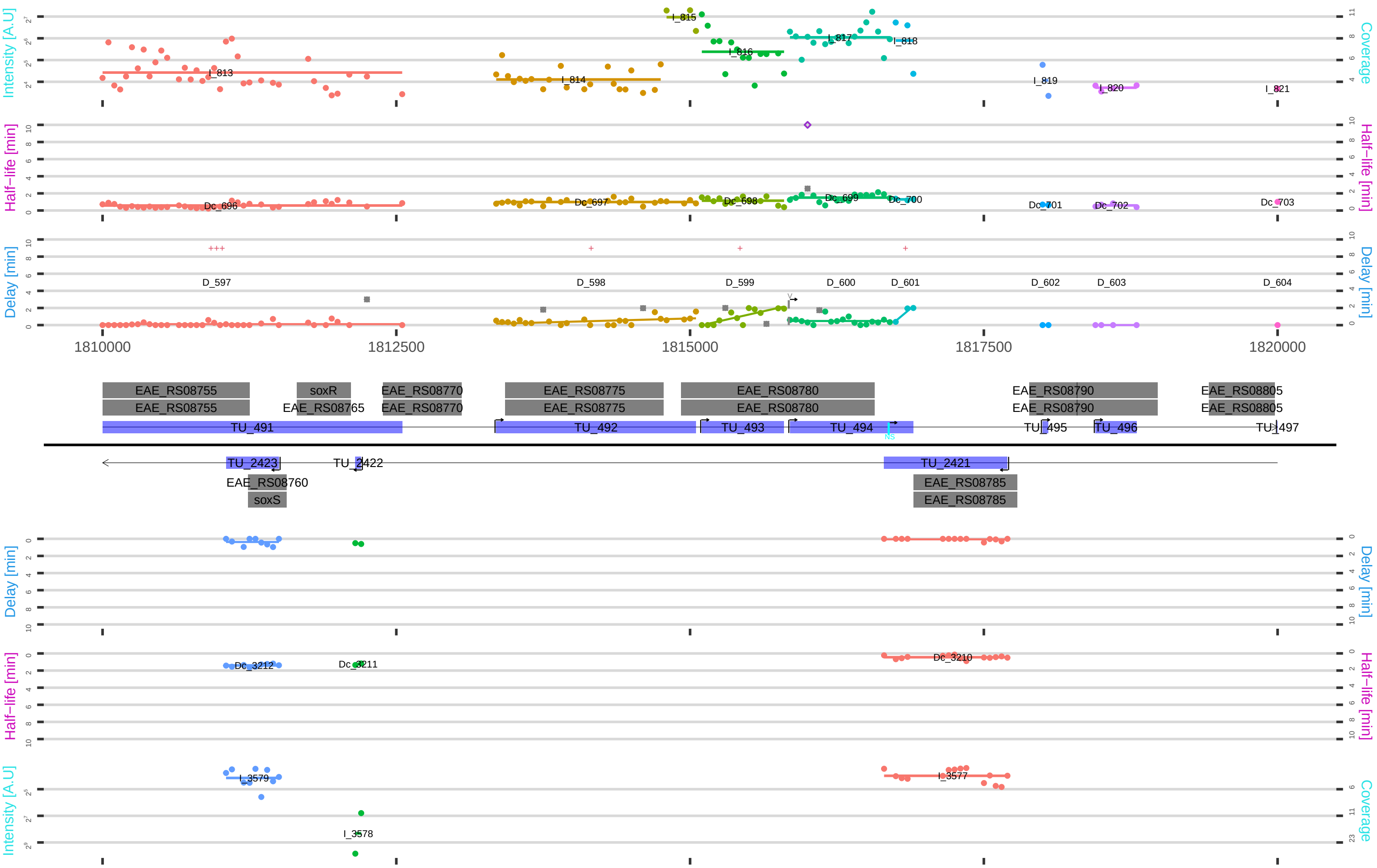

Term: termination (0), NS: new start (0), PS: pausing site (0), iTSS_I: internal starting site (0)

ID: 36400−36600; Term: termination (0), NS: new start (0), PS: pausing site (0), iTSS_I: internal starting site (0)

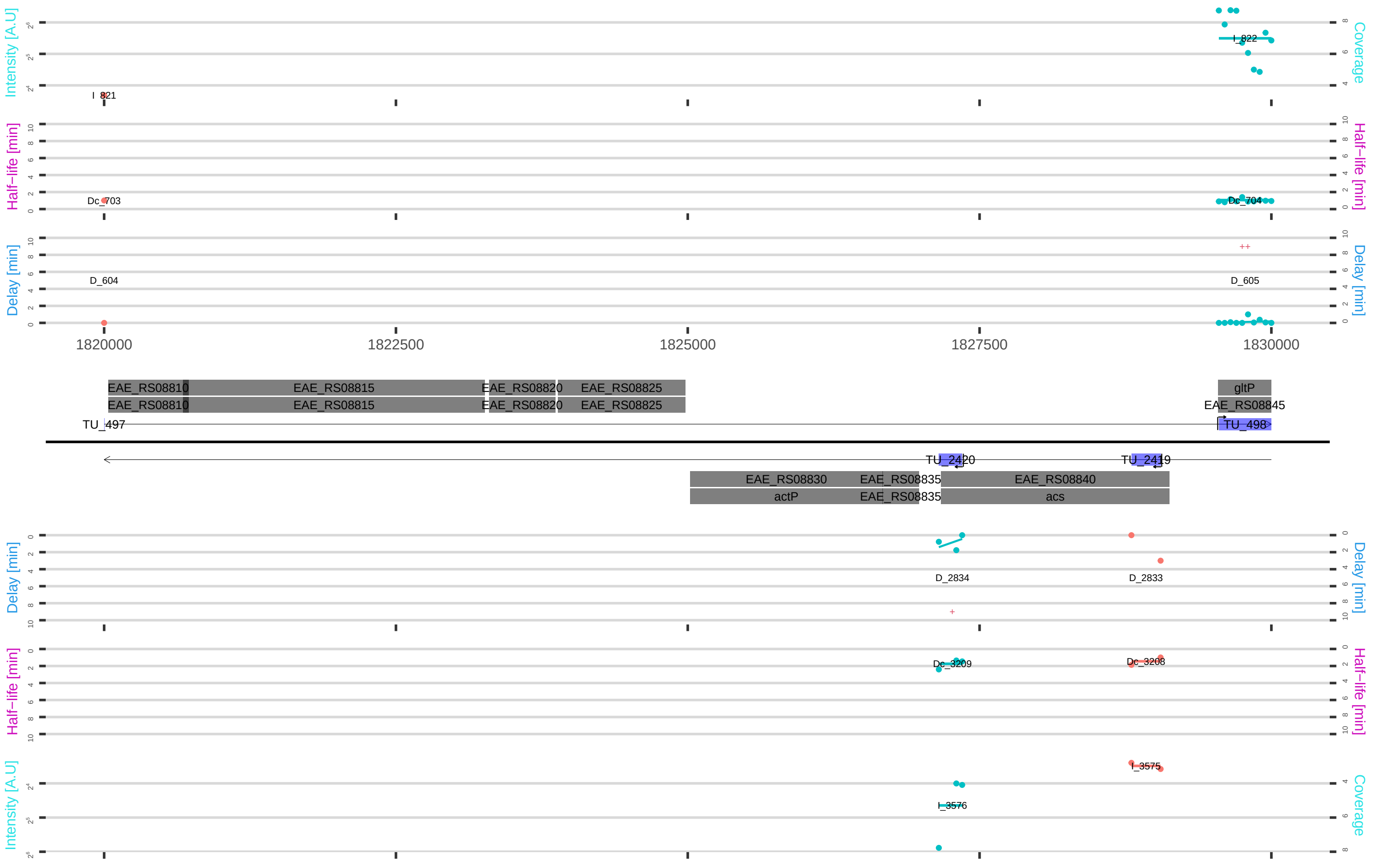

Term: termination (0), NS: new start (0), PS: pausing site (0), iTSS_I: internal starting site (0)

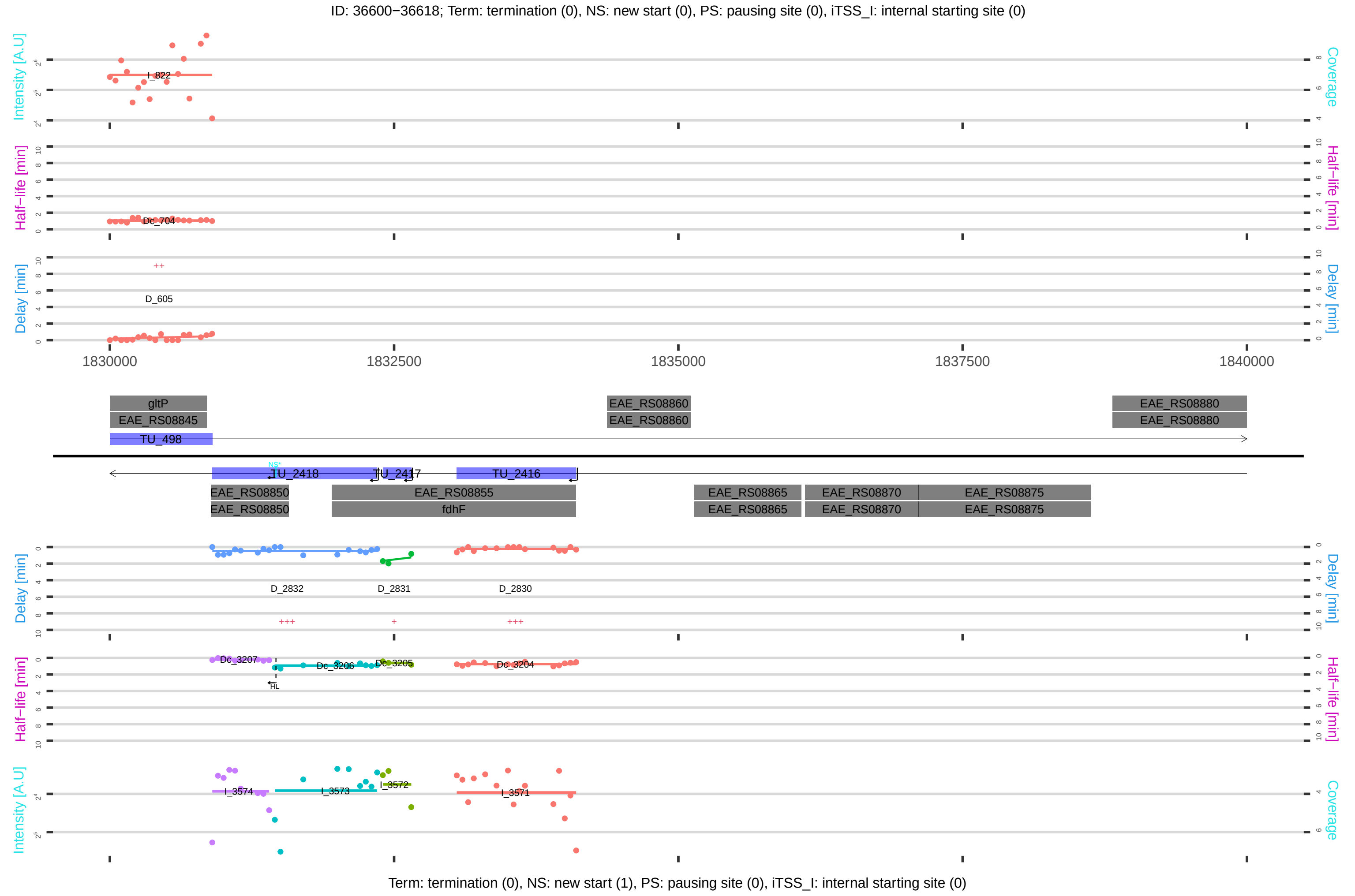

ID: 36600−36618; Term: termination (0), NS: new start (0), PS: pausing site (0), iTSS_I: internal starting site (0)
Intensity [A.U]
Coverage
I_822
Half−life [min]
Dc_704
Delay [min]
D_605
1830000
1832500
1835000
1837500
1840000
gltP
EAE_RS08845
TU_498
EAE_RS08860
EAE_RS08860
EAE_RS08880
EAE_RS08880
TU_2418
TU_2417
TU_2416
EAE_RS08850
EAE_RS08850
EAE_RS08855
fdhF
EAE_RS08865
EAE_RS08865
EAE_RS08870
EAE_RS08870
EAE_RS08875
EAE_RS08875
D_2832
D_2831
D_2830
Dc_3207
Dc_3206
Dc_3205
Dc_3204
HL
I_3574
I_3573
I_3572
I_3571
Term: termination (0), NS: new start (1), PS: pausing site (0), iTSS_I: internal starting site (0)

ID: 174217−174217; FC*: significant t−test of two consecutive segments; Term: termination, NS: new start, PS: pausing site,iTSS_I: internal starting site,TI: transcription interferance.

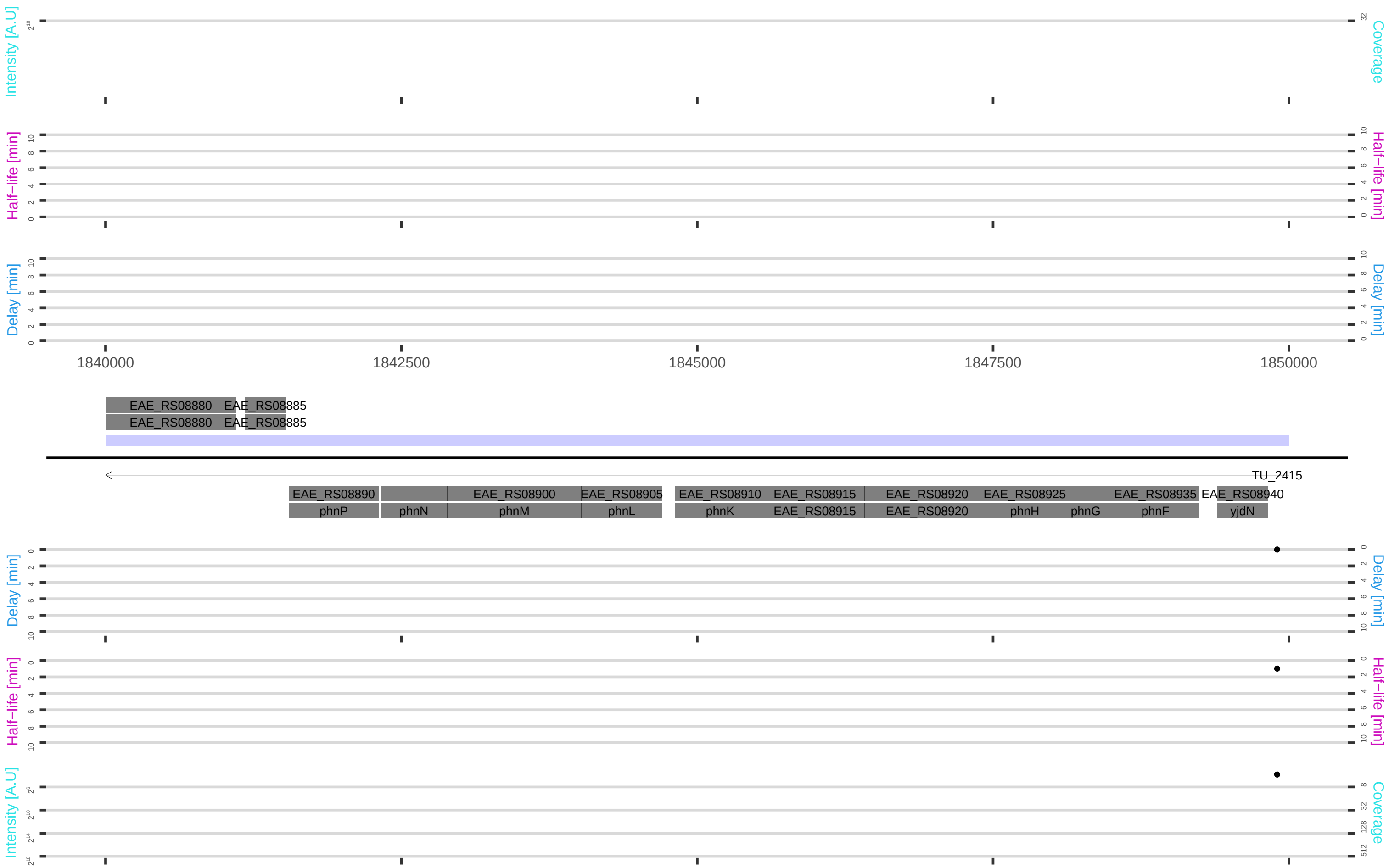

Term: termination (0), NS: new start (0), PS: pausing site (0), iTSS_I: internal starting site (0)

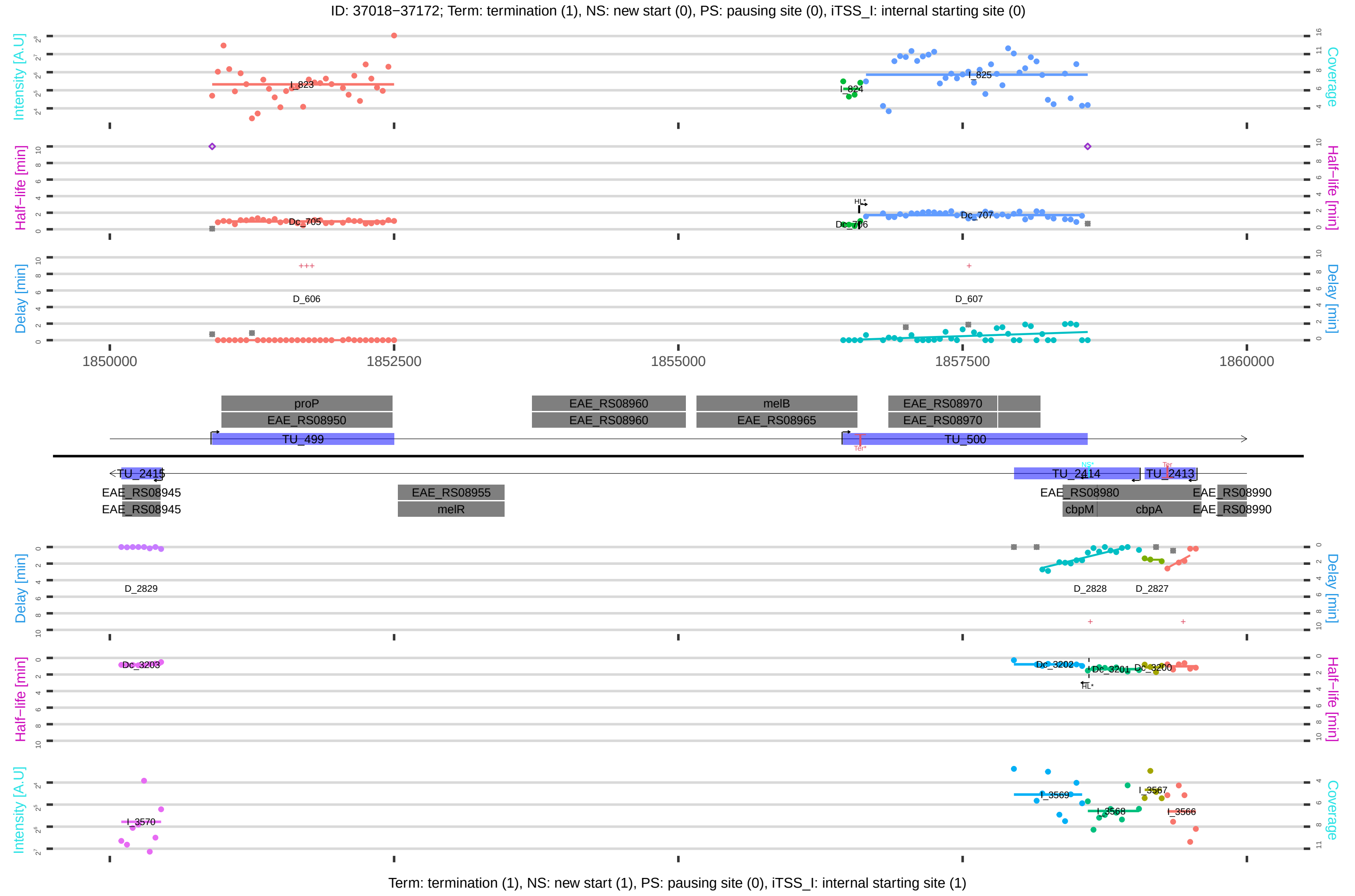

ID: 37018−37172; Term: termination (1), NS: new start (0), PS: pausing site (0), iTSS_I: internal starting site (0)
Intensity [A.U]
Coverage
Half−life [min]
Delay [min]
I_823
I_824
I_825
Dc_705
Dc_706
Dc_707
D_606
D_607
1850000
1852500
1855000
1857500
1860000
proP
EAE_RS08950
EAE_RS08960
melB
EAE_RS08965
EAE_RS08970
TU_499
TU_500
TU_2415
TU_2414
TU_2413
EAE_RS08945
EAE_RS08955
melR
EAE_RS08980
cbpM
cbpA
EAE_RS08990
D_2829
D_2828
D_2827
Dc_3203
Dc_3202
Dc_3201
Dc_3200
I_3570
I_3569
I_3568
I_3567
I_3566
Term: termination (1), NS: new start (1), PS: pausing site (0), iTSS_I: internal starting site (1)

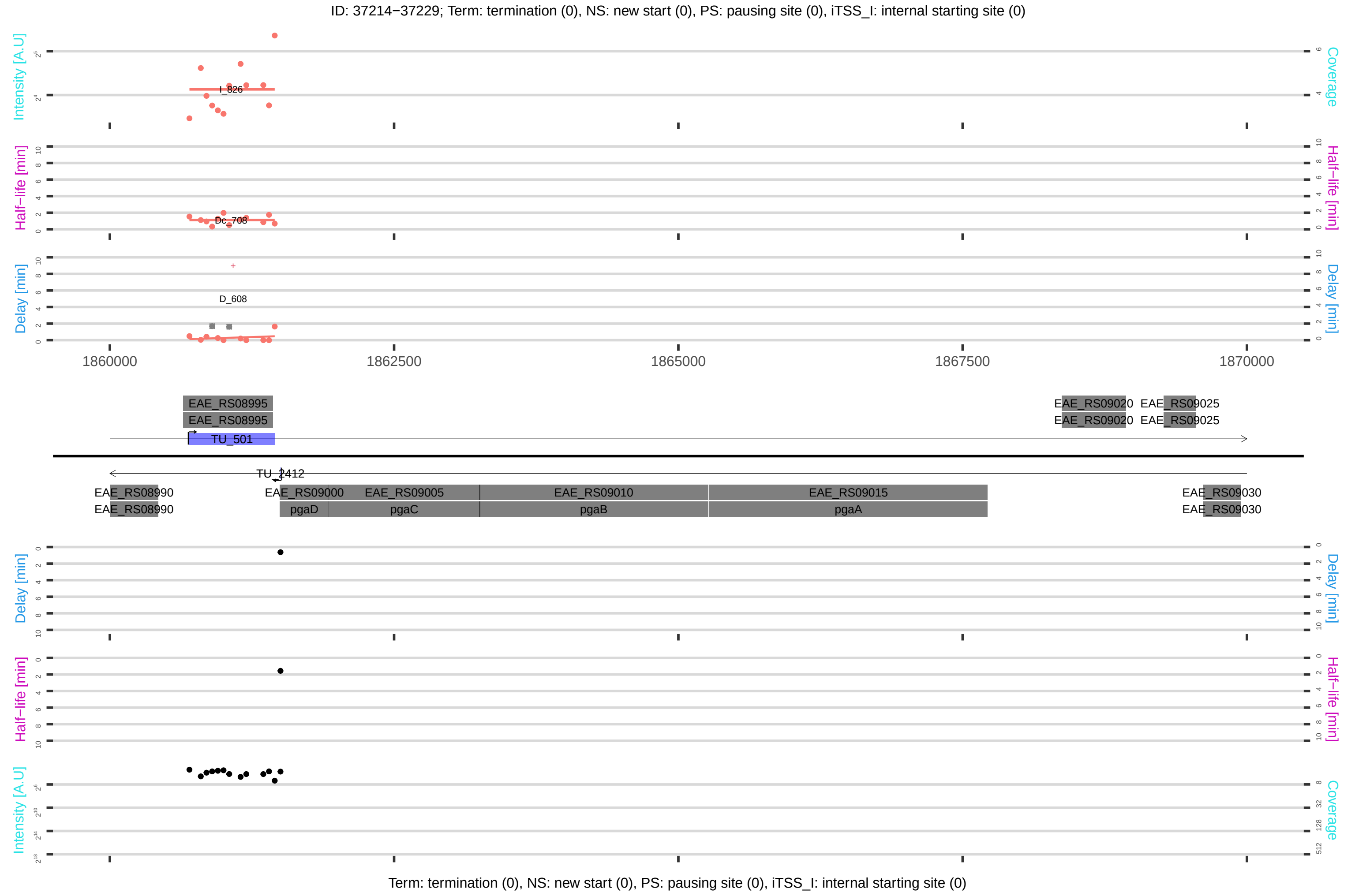

ID: 37214−37229; Term: termination (0), NS: new start (0), PS: pausing site (0), iTSS_I: internal starting site (0)
Intensity [A.U]
Coverage
I_826
Half−life [min]
Dc_708
Delay [min]
D_608
1860000
1862500
1865000
1867500
1870000
EAE_RS08995
TU_501
EAE_RS09020
EAE_RS09025
TU_2412
EAE_RS08990
EAE_RS09000
EAE_RS09005
EAE_RS09010
EAE_RS09015
EAE_RS09030
pgaD
pgaC
pgaB
pgaA
Term: termination (0), NS: new start (0), PS: pausing site (0), iTSS_I: internal starting site (0)

ID: 173811−173811; FC*: significant t−test of two consecutive segments; Term: termination, NS: new start, PS: pausing site,iTSS_I: internal starting site,TI: transcription interferance.

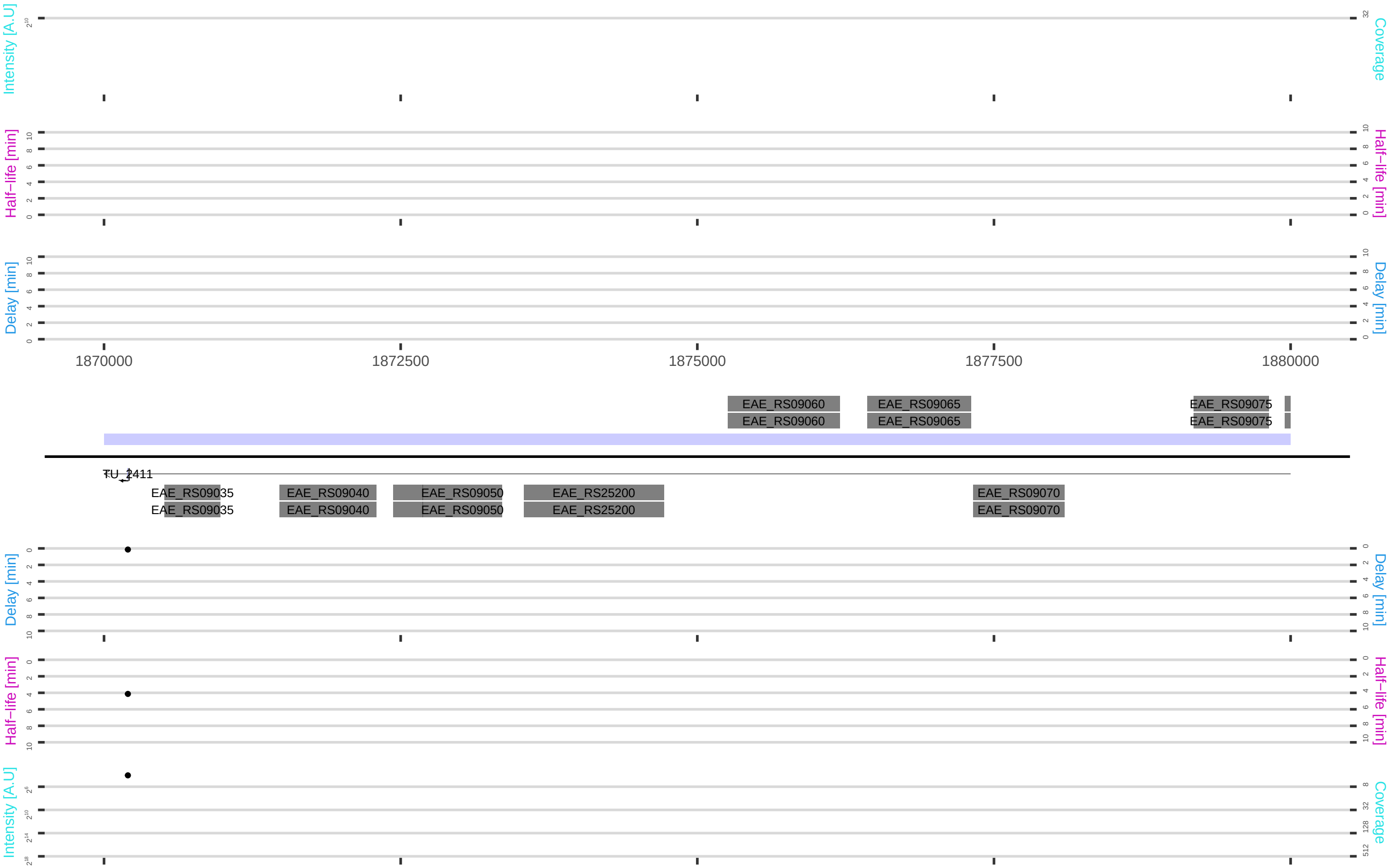

Term: termination (0), NS: new start (0), PS: pausing site (0), iTSS_I: internal starting site (0)

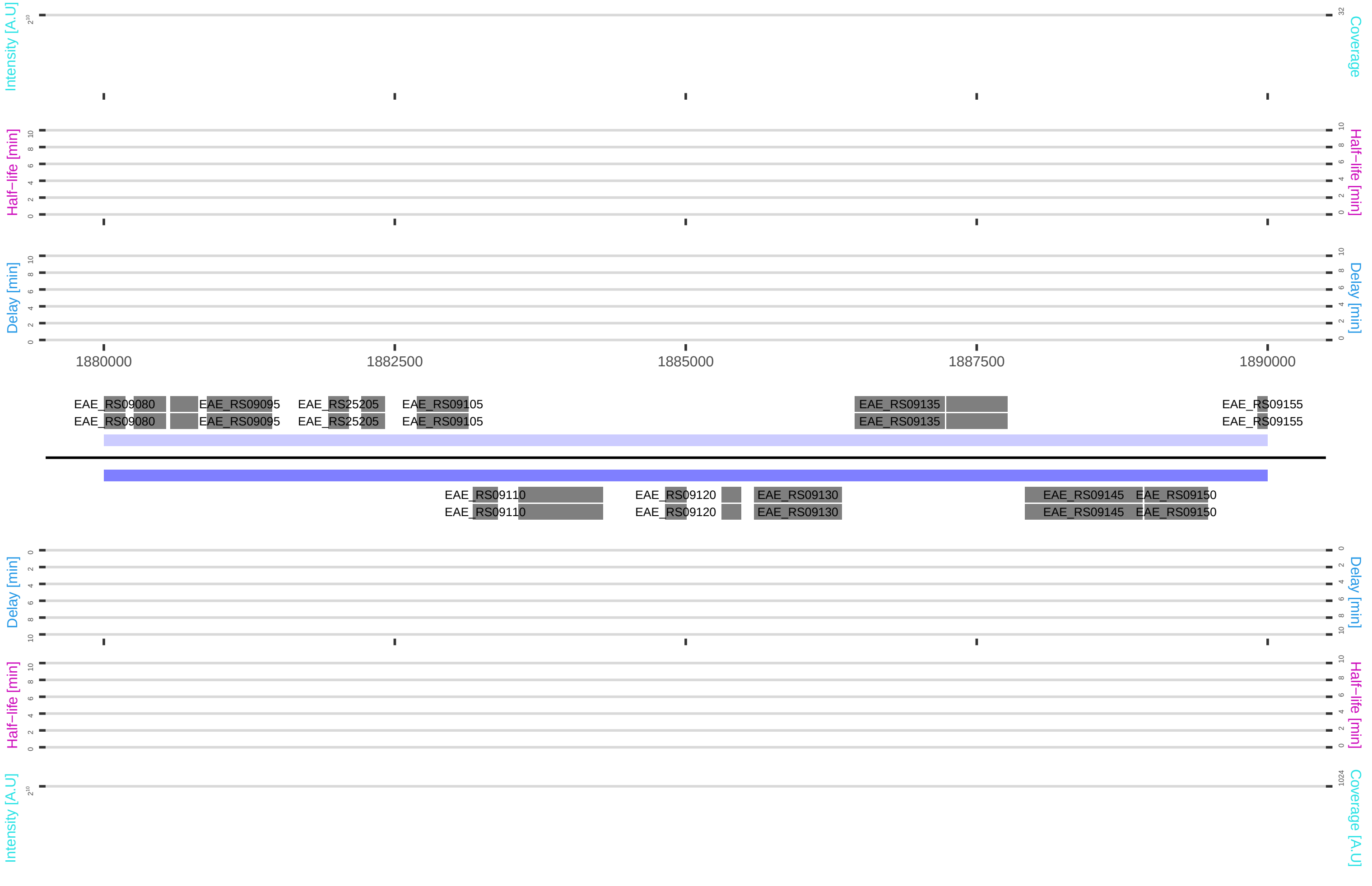
Intensity [A.U]
Coverage
Half−life [min]
Delay [min]
1880000
1882500
1885000
1887500
1890000
EAE_RS09080
EAE_RS09095
EAE_RS25205
EAE_RS09105
EAE_RS09135
EAE_RS09155
EAE_RS09110
EAE_RS09120
EAE_RS09130
EAE_RS09145
EAE_RS09150
Delay [min]
Half−life [min]
Intensity [A.U]
Coverage [A.U]
NA

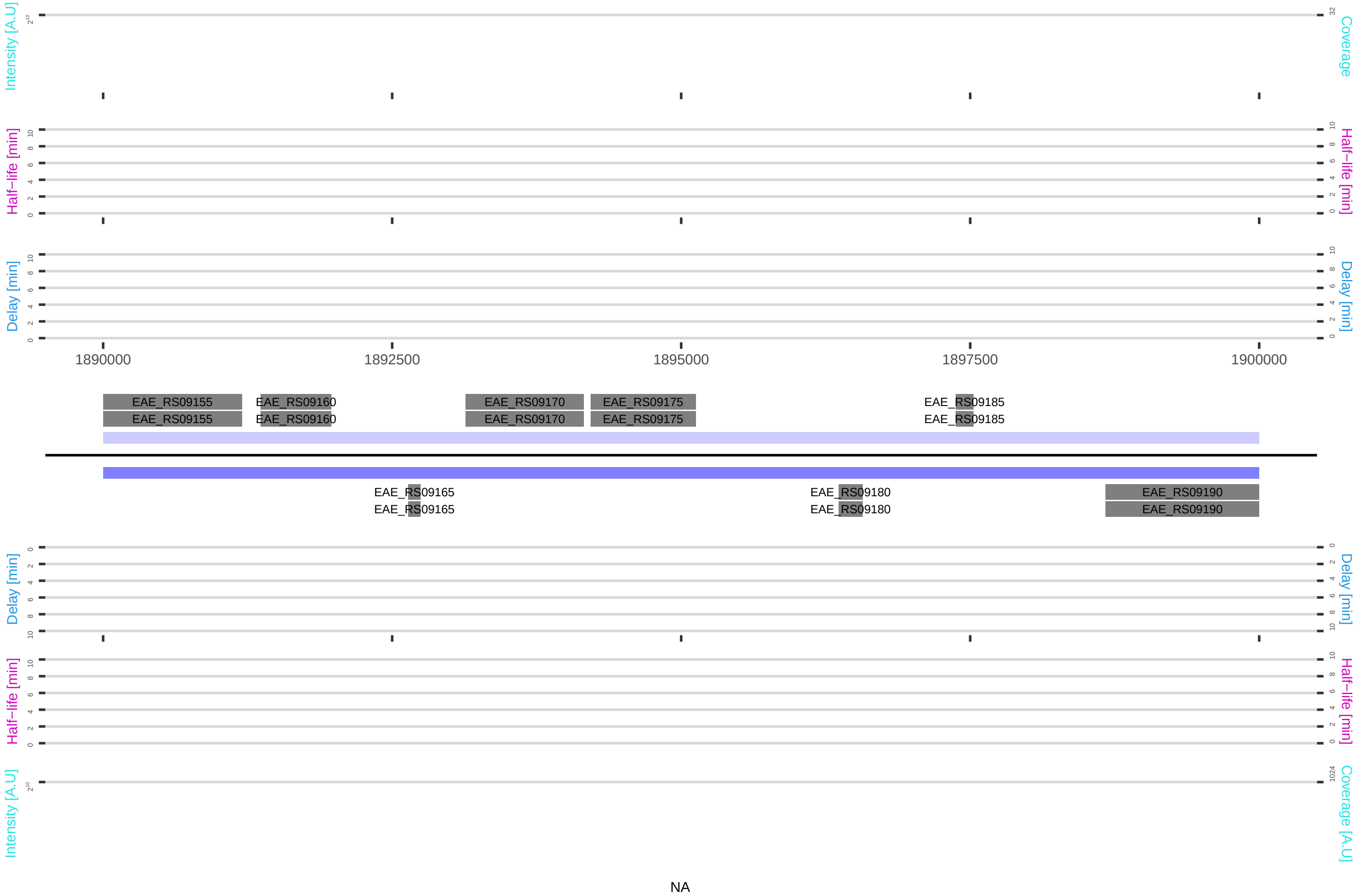
Intensity [A.U]
Coverage
Half−life [min]
Delay [min]
1890000
1892500
1895000
1897500
1900000
EAE_RS09155
EAE_RS09160
EAE_RS09170
EAE_RS09175
EAE_RS09185
EAE_RS09165
EAE_RS09180
EAE_RS09190
Coverage [A.U]
NA

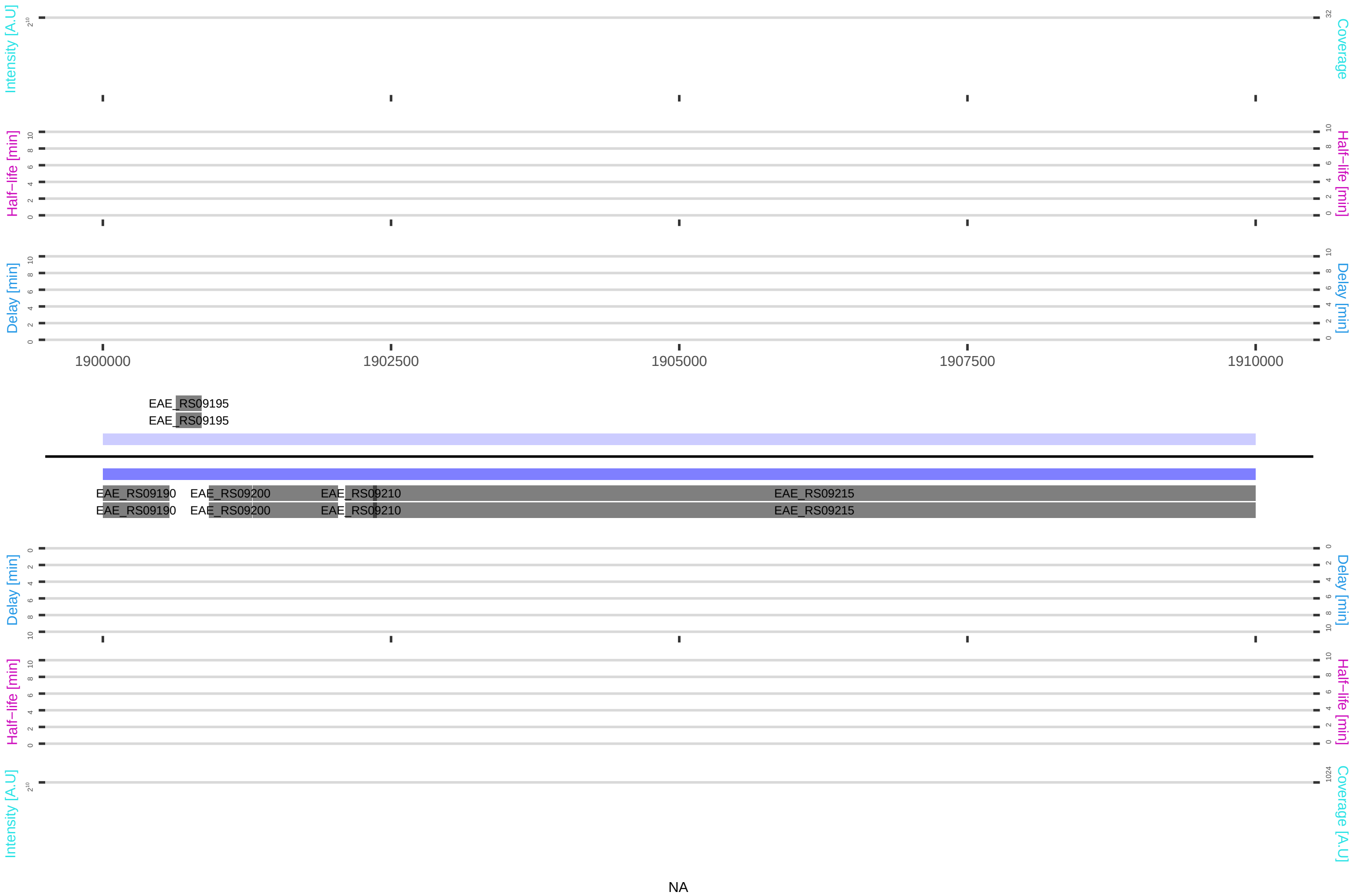

Intensity [A.U]
Coverage
Half−life [min]
Delay [min]
1900000
1902500
1905000
1907500
1910000
EAE_RS09195
EAE_RS09195
EAE_RS09190
EAE_RS09200
EAE_RS09210
EAE_RS09215
EAE_RS09190
EAE_RS09200
EAE_RS09210
EAE_RS09215
Delay [min]
Half−life [min]
Intensity [A.U]
Coverage [A.U]
NA

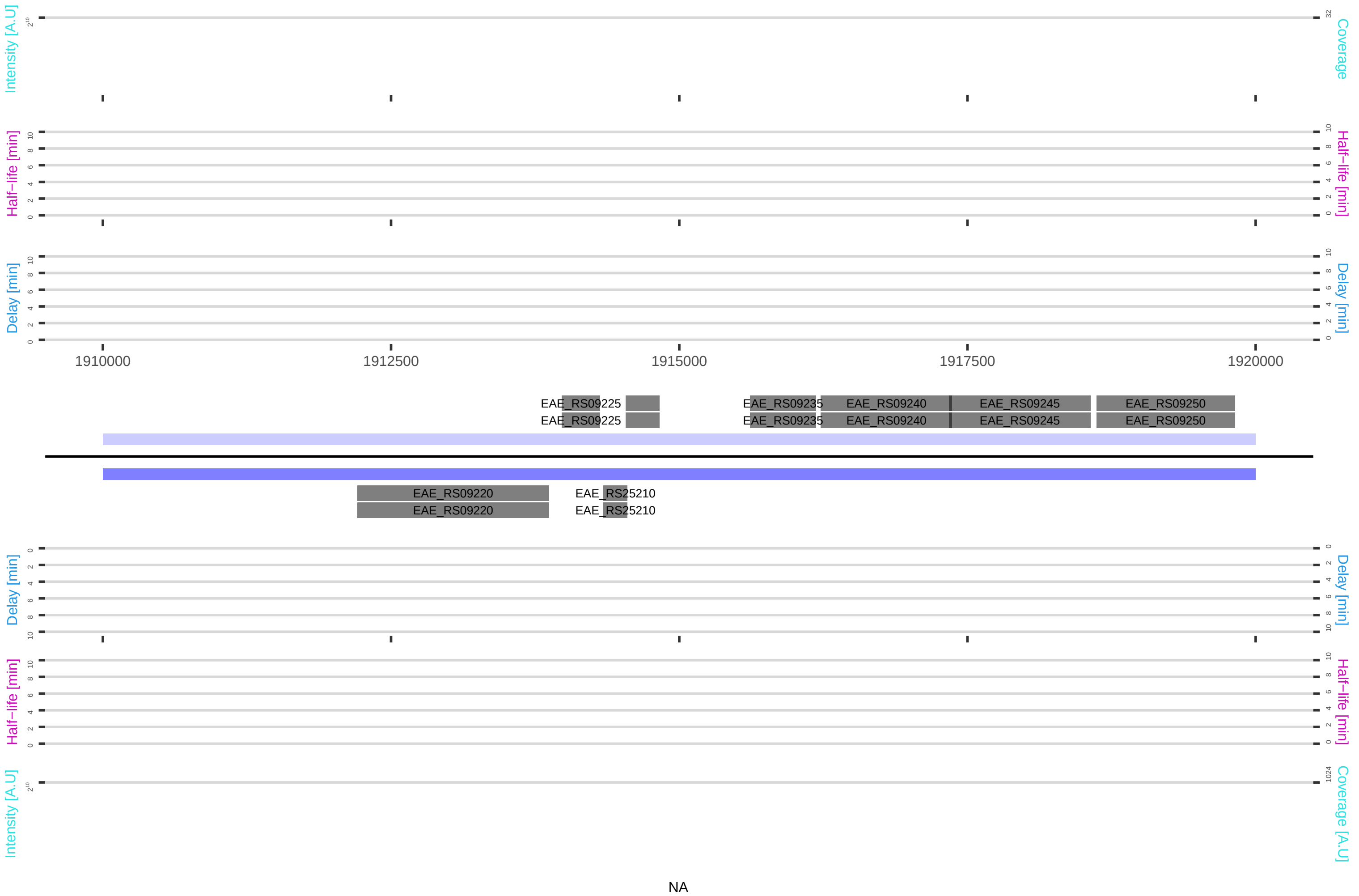

Intensity [A.U]
Coverage
Half−life [min]
Delay [min]
1910000
1912500
1915000
1917500
1920000
EAE_RS09225
EAE_RS09235
EAE_RS09240
EAE_RS09245
EAE_RS09250
EAE_RS09220
EAE_RS25210
Coverage [A.U]
NA

ID: 172730−172616; FC*: significant t−test of two consecutive segments; Term: termination, NS: new start, PS: pausing site,iTSS_I: internal starting site,TI: transcription interferance.

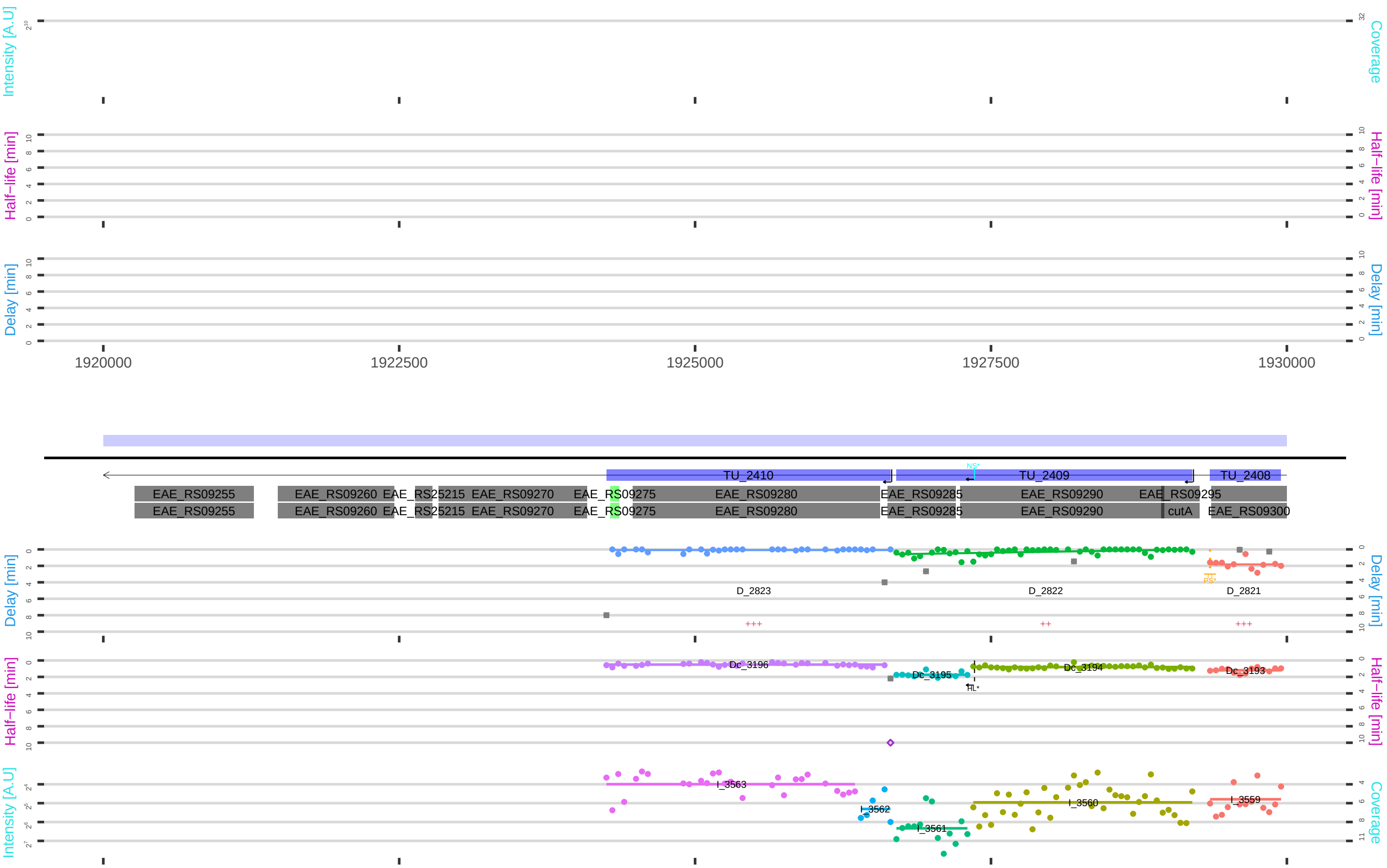

Term: termination (0), NS: new start (1), PS: pausing site (1), iTSS_I: internal starting site (0)

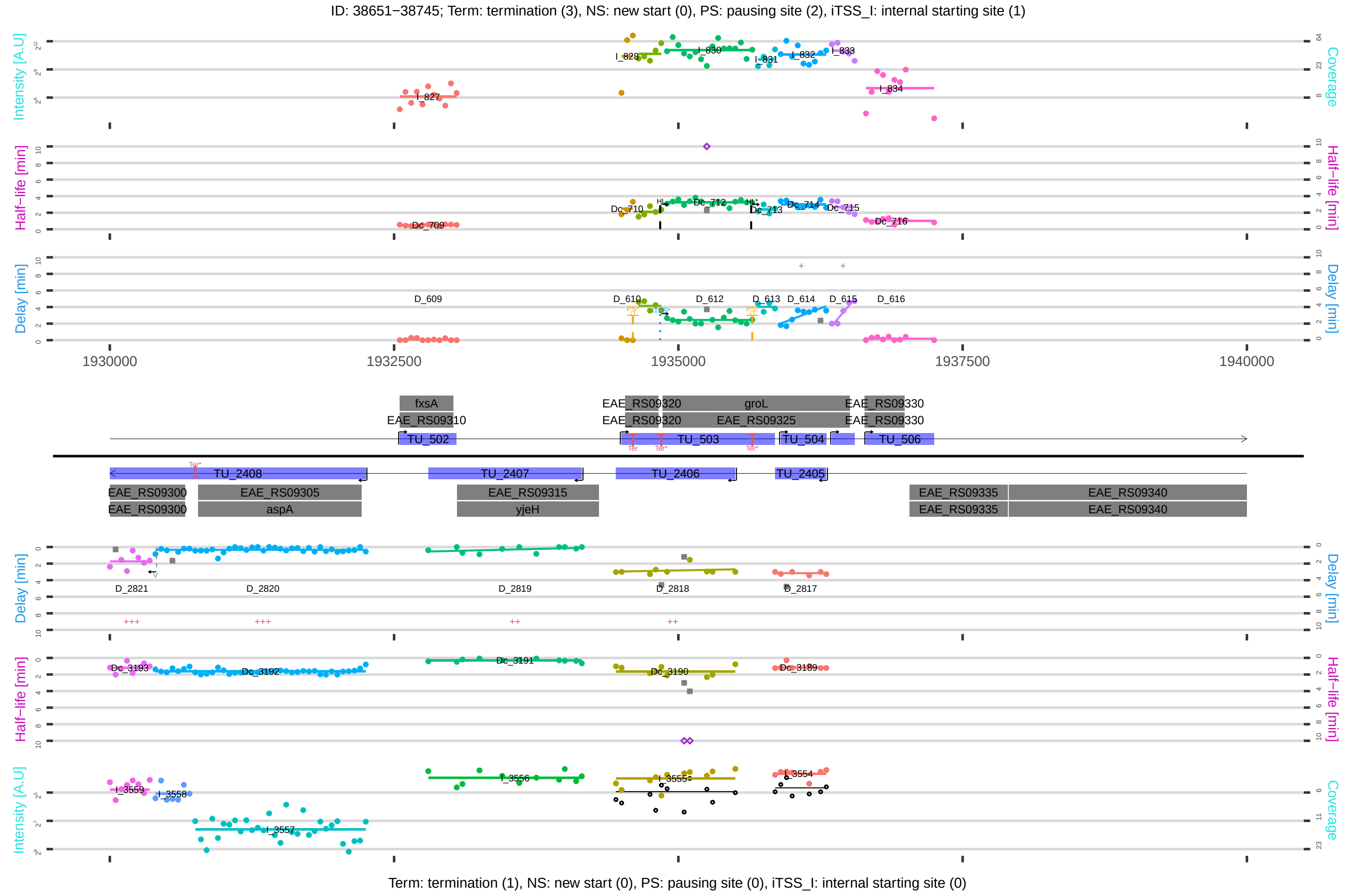
ID: 38651−38745; Term: termination (3), NS: new start (0), PS: pausing site (2), iTSS_I: internal starting site (1)
Intensity [A.U]
Coverage
Half−life [min]
Delay [min]
I_827
I_828
I_830
I_831
I_832
I_833
I_834
Dc_709
Dc_710
Dc_712
Dc_713
Dc_714
Dc_715
Dc_716
D_609
D_610
D_612
D_613
D_614
D_615
D_616
1930000
1932500
1935000
1937500
1940000
fxsA
EAE_RS09310
EAE_RS09320
groL
EAE_RS09325
EAE_RS09330
TU_502
TU_503
TU_504
TU_506
TU_2408
TU_2407
TU_2406
TU_2405
EAE_RS09300
EAE_RS09305
aspA
EAE_RS09315
yjeH
EAE_RS09335
EAE_RS09340
D_2821
D_2820
D_2819
D_2818
D_2817
Dc_3193
Dc_3192
Dc_3191
Dc_3190
Dc_3189
I_3559
I_3558
I_3557
I_3556
I_3555
I_3554
Term: termination (1), NS: new start (0), PS: pausing site (0), iTSS_I: internal starting site (0)

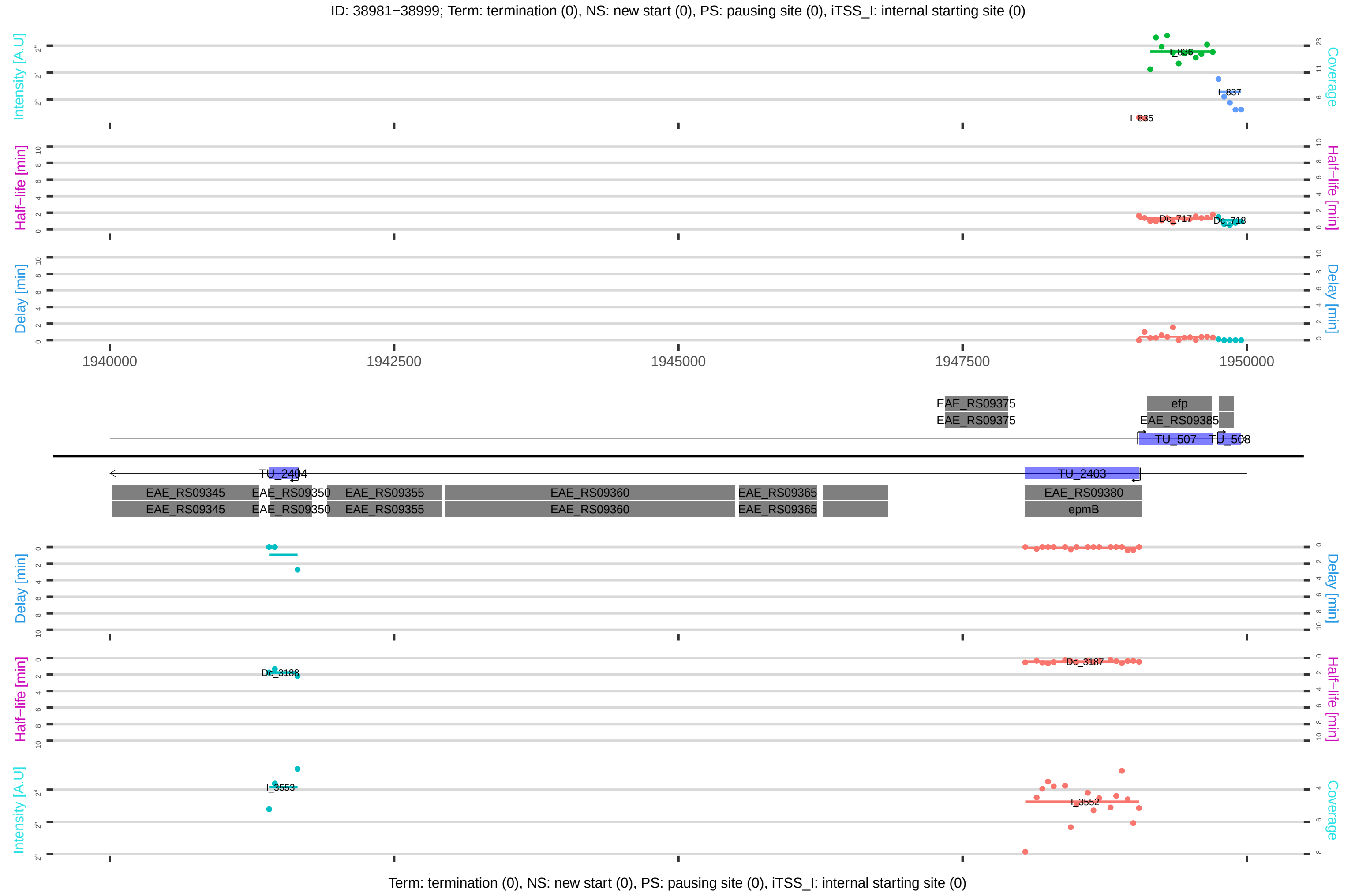

ID: 38981−38999; Term: termination (0), NS: new start (0), PS: pausing site (0), iTSS_I: internal starting site (0)
Intensity [A.U]
Coverage
Half−life [min]
Delay [min]
I_836
I_837
I_835
Dc_717
Dc_718
1940000
1942500
1945000
1947500
1950000
EAE_RS09375
efp
EAE_RS09385
TU_507
TU_508
TU_2404
TU_2403
EAE_RS09345
EAE_RS09350
EAE_RS09355
EAE_RS09360
EAE_RS09365
EAE_RS09380
epmB
Dc_3188
Dc_3187
I_3553
I_3552
Term: termination (0), NS: new start (0), PS: pausing site (0), iTSS_I: internal starting site (0)

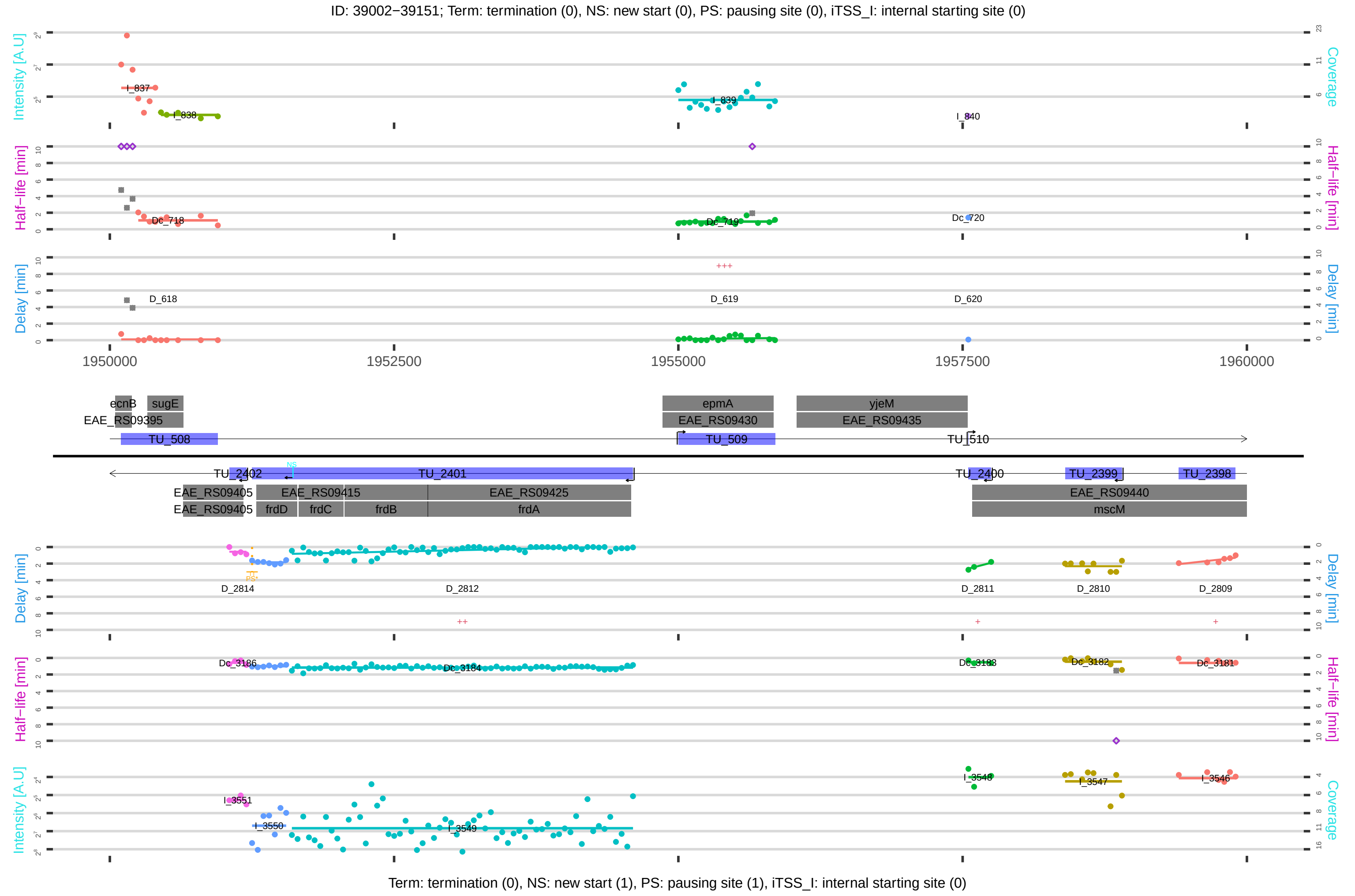
ID: 39002−39151; Term: termination (0), NS: new start (0), PS: pausing site (0), iTSS_I: internal starting site (0)
Intensity [A.U]
Coverage
Half−life [min]
Delay [min]
I_837
I_838
I_839
I_840
Dc_718
Dc_719
Dc_720
D_618
D_619
D_620
1950000
1952500
1955000
1957500
1960000
ecnB
sugE
EAE_RS09395
epmA
EAE_RS09430
yjeM
EAE_RS09435
TU_508
TU_509
TU_510
TU_2402
TU_2401
TU_2400
TU_2399
TU_2398
NS
EAE_RS09405
EAE_RS09415
EAE_RS09425
EAE_RS09440
frdD
frdC
frdB
frdA
mscM
PS
D_2814
D_2812
D_2811
D_2810
D_2809
Dc_3186
Dc_3184
Dc_3183
Dc_3182
Dc_3181
I_3551
I_3550
I_3549
I_3548
I_3547
I_3546
Term: termination (0), NS: new start (1), PS: pausing site (1), iTSS_I: internal starting site (0)

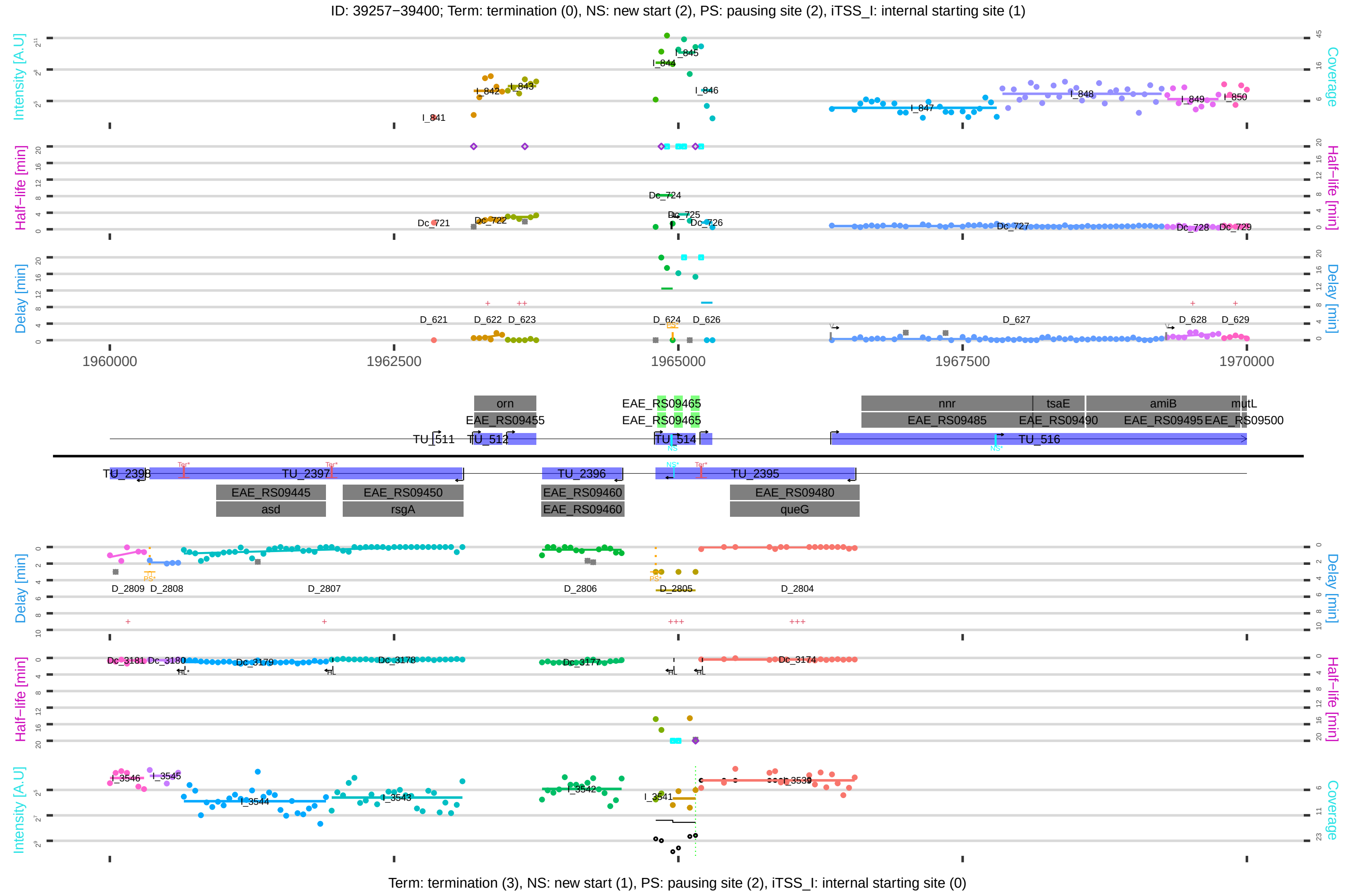

ID: 39257−39400; Term: termination (0), NS: new start (2), PS: pausing site (2), iTSS_I: internal starting site (1)
Intensity [A.U]
Coverage
Half−life [min]
Delay [min]
I_841
I_842
I_843
I_844
I_845
I_846
I_847
I_848
I_849
I_850
Dc_721
Dc_722
Dc_724
Dc_725
Dc_726
Dc_727
Dc_728
Dc_729
D_621
D_622
D_623
D_624
D_626
D_627
D_628
D_629
1960000
1962500
1965000
1967500
1970000
orn
EAE_RS09455
EAE_RS09465
nnr
tsaE
amiB
mutL
EAE_RS09485
EAE_RS09490
EAE_RS09495
EAE_RS09500
TU_511
TU_512
TU_514
TU_516
TU_2398
TU_2397
TU_2396
TU_2395
EAE_RS09445
EAE_RS09450
EAE_RS09460
EAE_RS09480
asd
rsgA
queG
D_2809
D_2808
D_2807
D_2806
D_2805
D_2804
Dc_3181
Dc_3180
Dc_3179
Dc_3178
Dc_3177
Dc_3174
I_3546
I_3545
I_3544
I_3543
I_3542
I_3541
I_3539
Term: termination (3), NS: new start (1), PS: pausing site (2), iTSS_I: internal starting site (0)

ID: 39400−39600; Term: termination (3), NS: new start (3), PS: pausing site (2), iTSS_I: internal starting site (0)

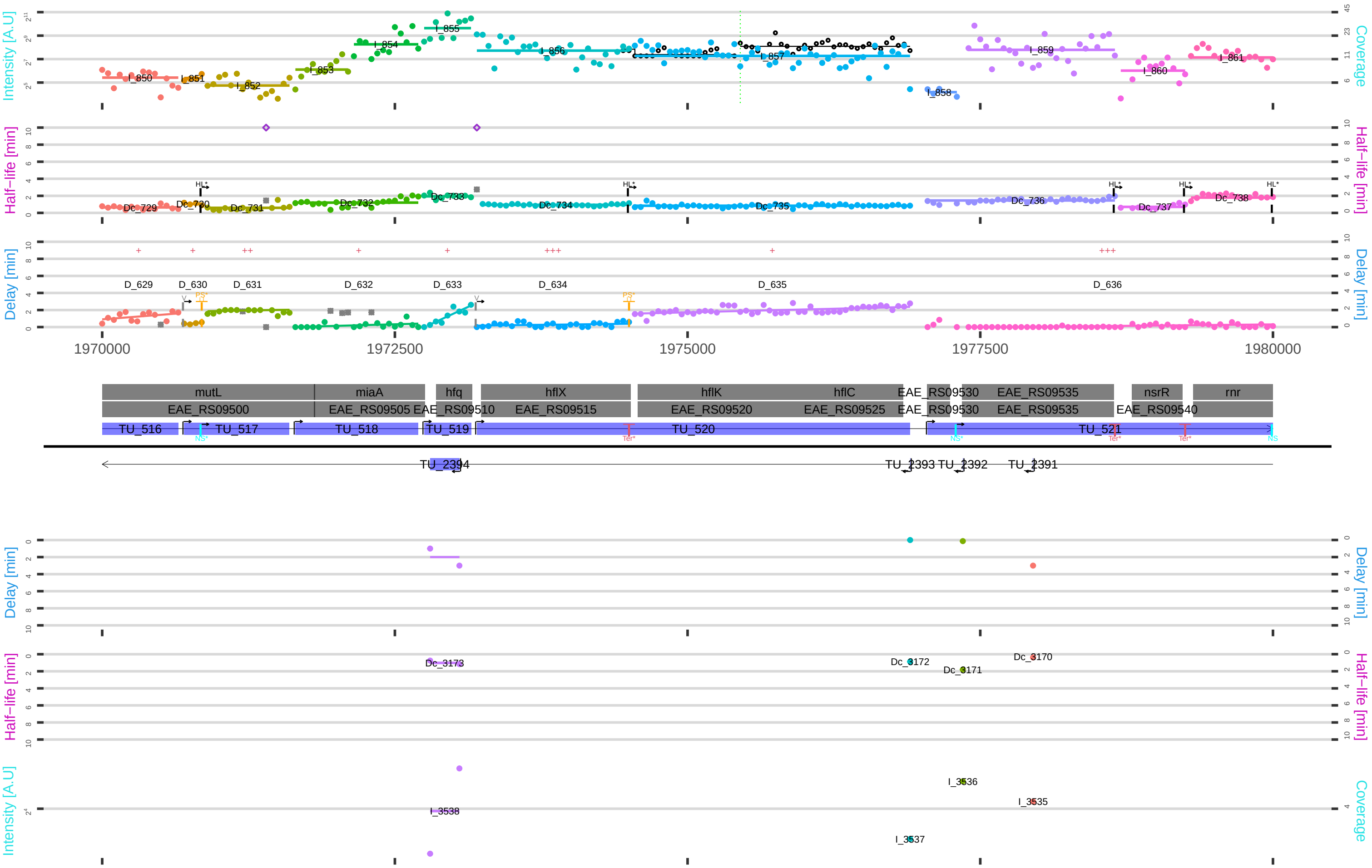

Term: termination (0), NS: new start (0), PS: pausing site (0), iTSS_I: internal starting site (0)

ID: 39600−39753; Term: termination (0), NS: new start (1), PS: pausing site (1), iTSS_I: internal starting site (0)

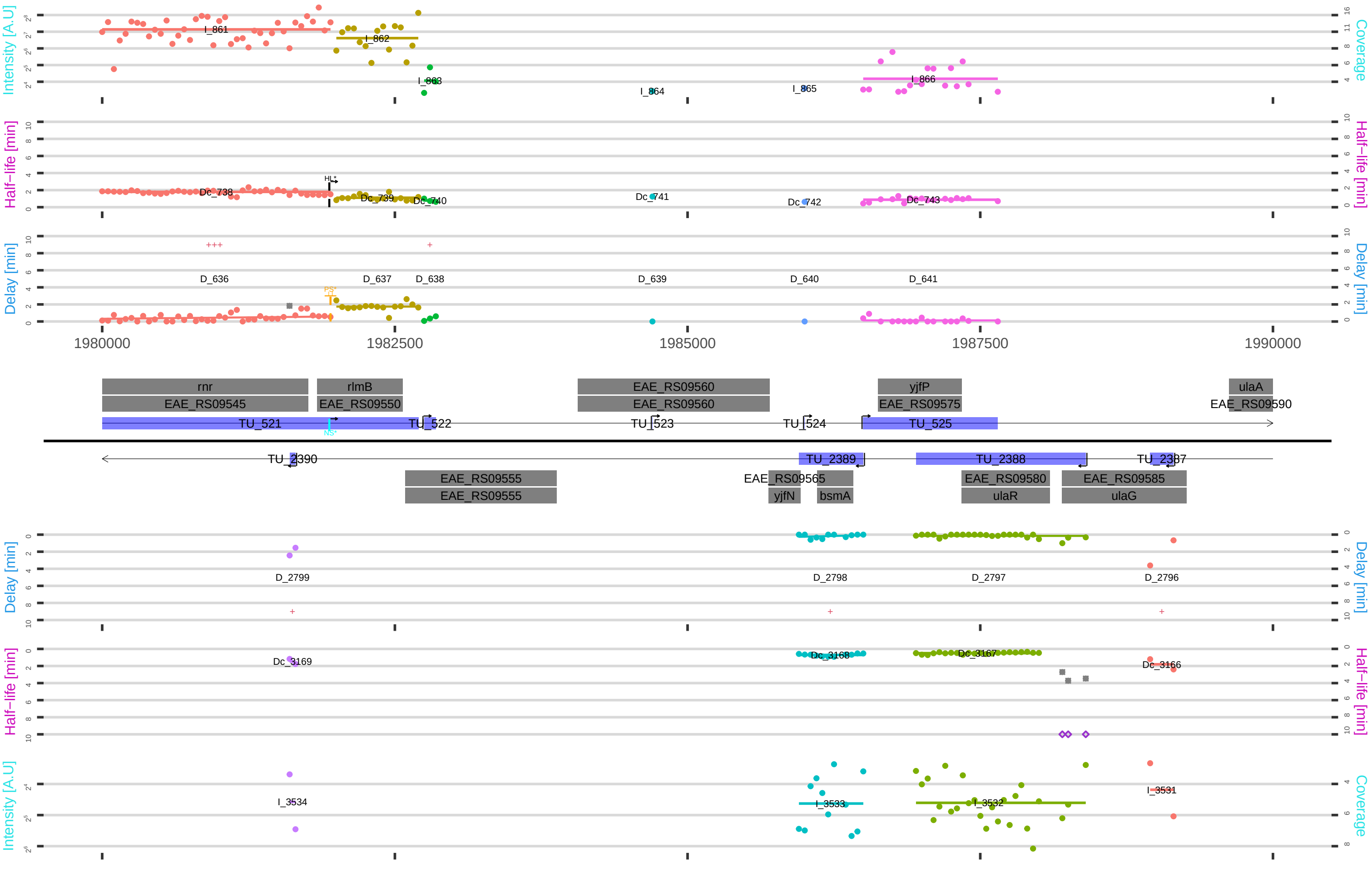

Term: termination (0), NS: new start (0), PS: pausing site (0), iTSS_I: internal starting site (0)

ID: 39835−40000; Term: termination (1), NS: new start (0), PS: pausing site (0), iTSS_I: internal starting site (0)

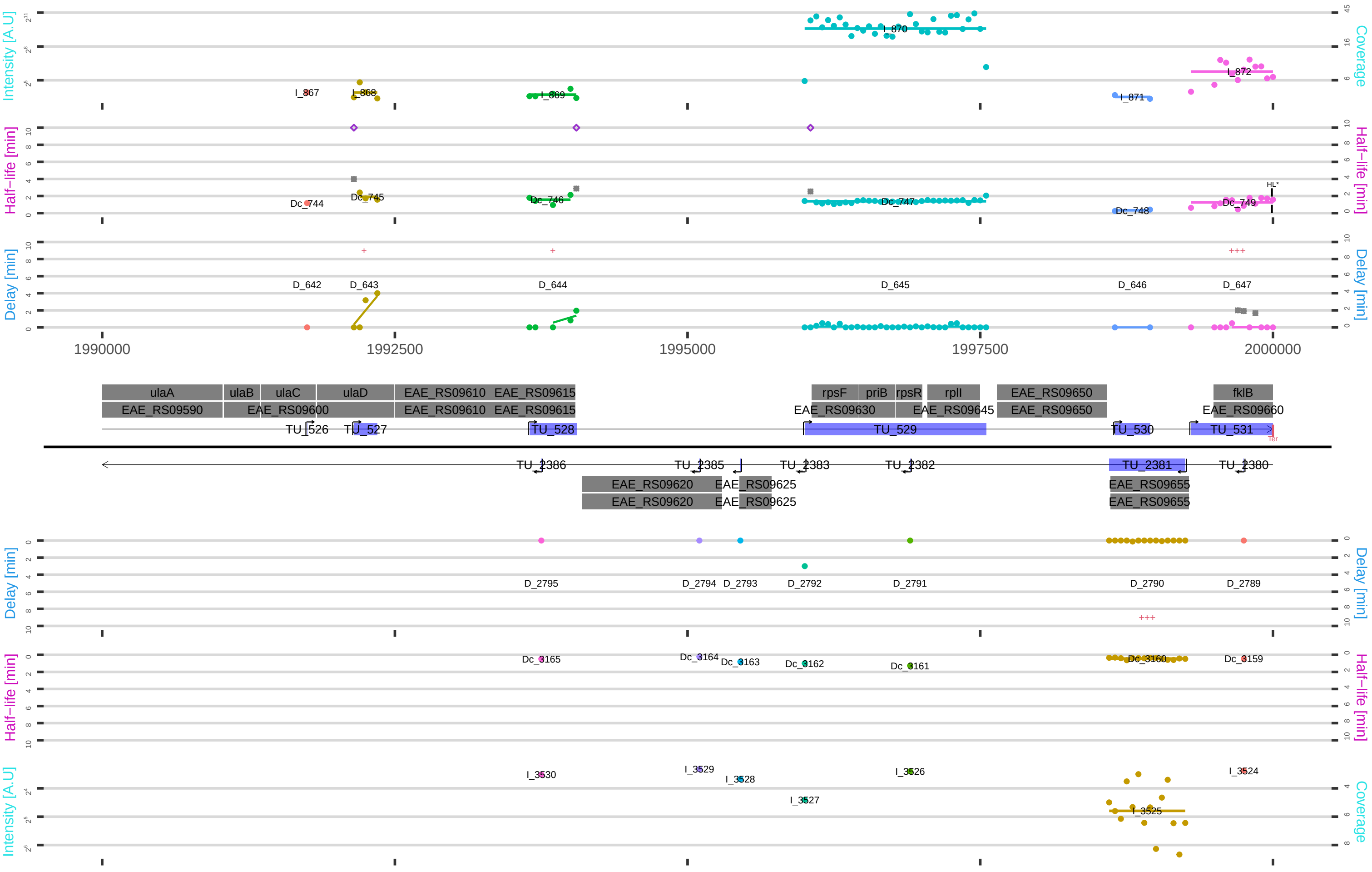

Term: termination (0), NS: new start (0), PS: pausing site (0), iTSS_I: internal starting site (0)

ID: 40000−40200; Term: termination (1), NS: new start (0), PS: pausing site (0), iTSS_I: internal starting site (0)

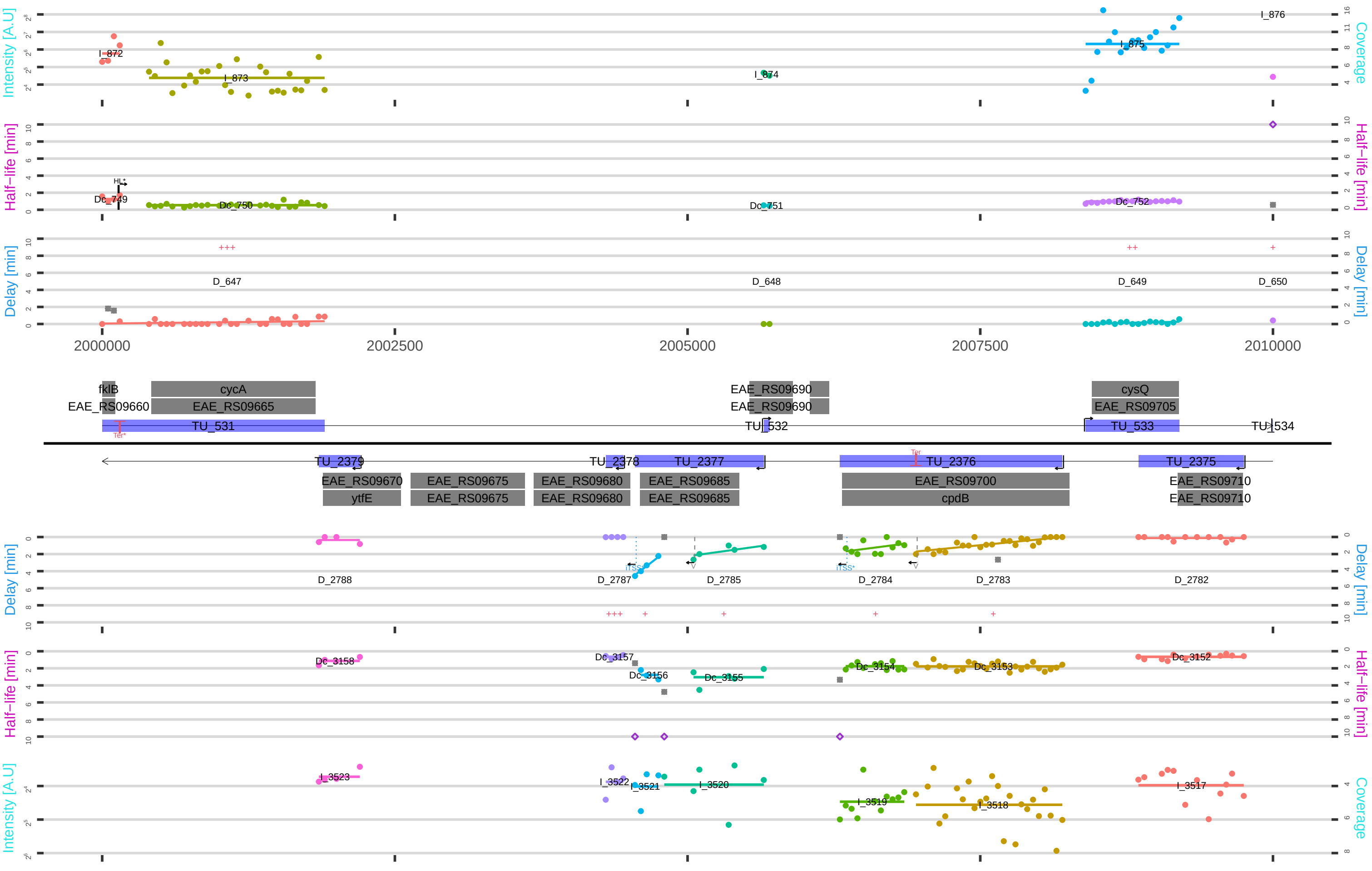

Term: termination (1), NS: new start (0), PS: pausing site (0), iTSS_I: internal starting site (2)

ID: 40200−40384; Term: termination (1), NS: new start (0), PS: pausing site (1), iTSS_I: internal starting site (0)

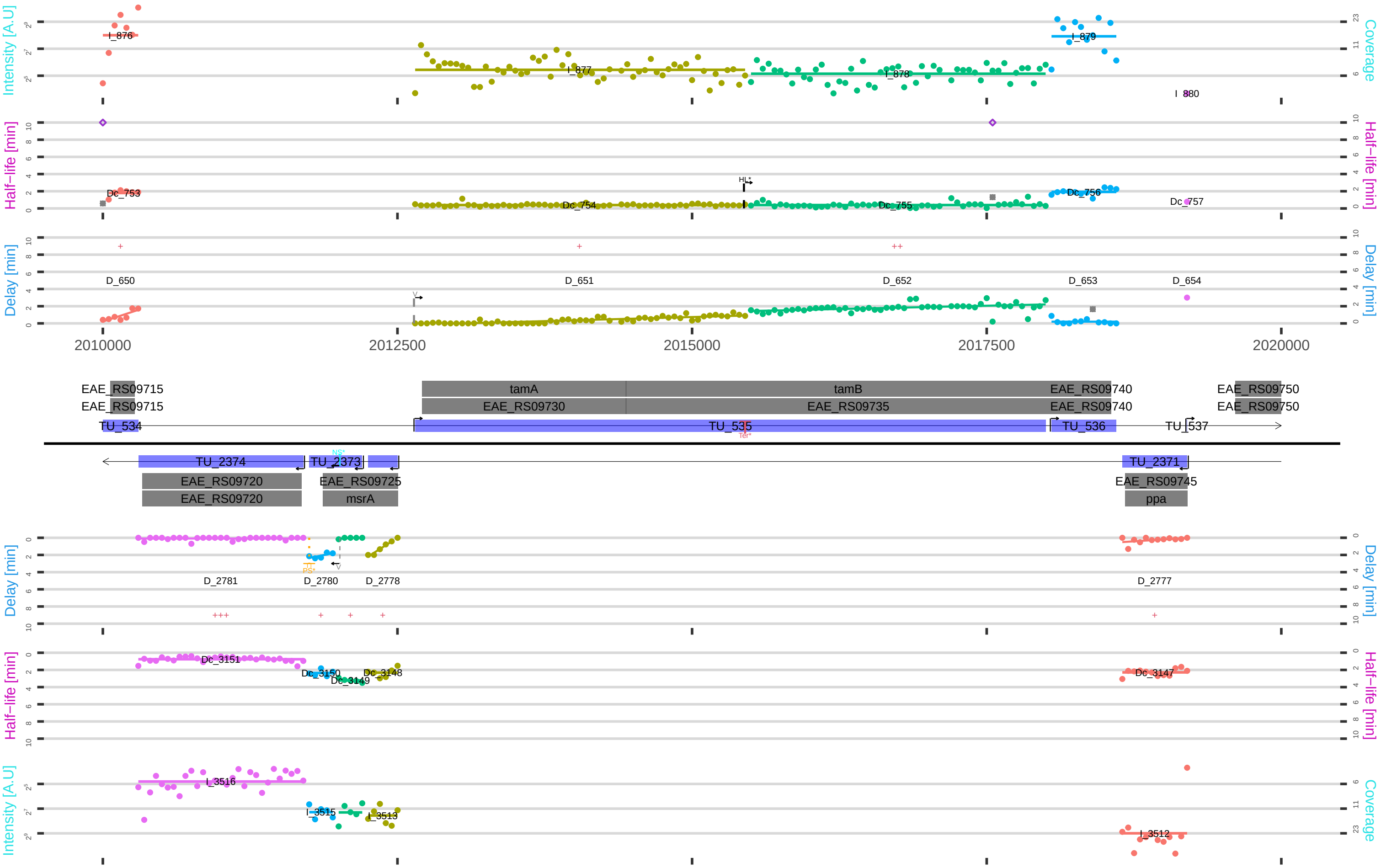

Term: termination (0), NS: new start (1), PS: pausing site (1), iTSS_I: internal starting site (0)

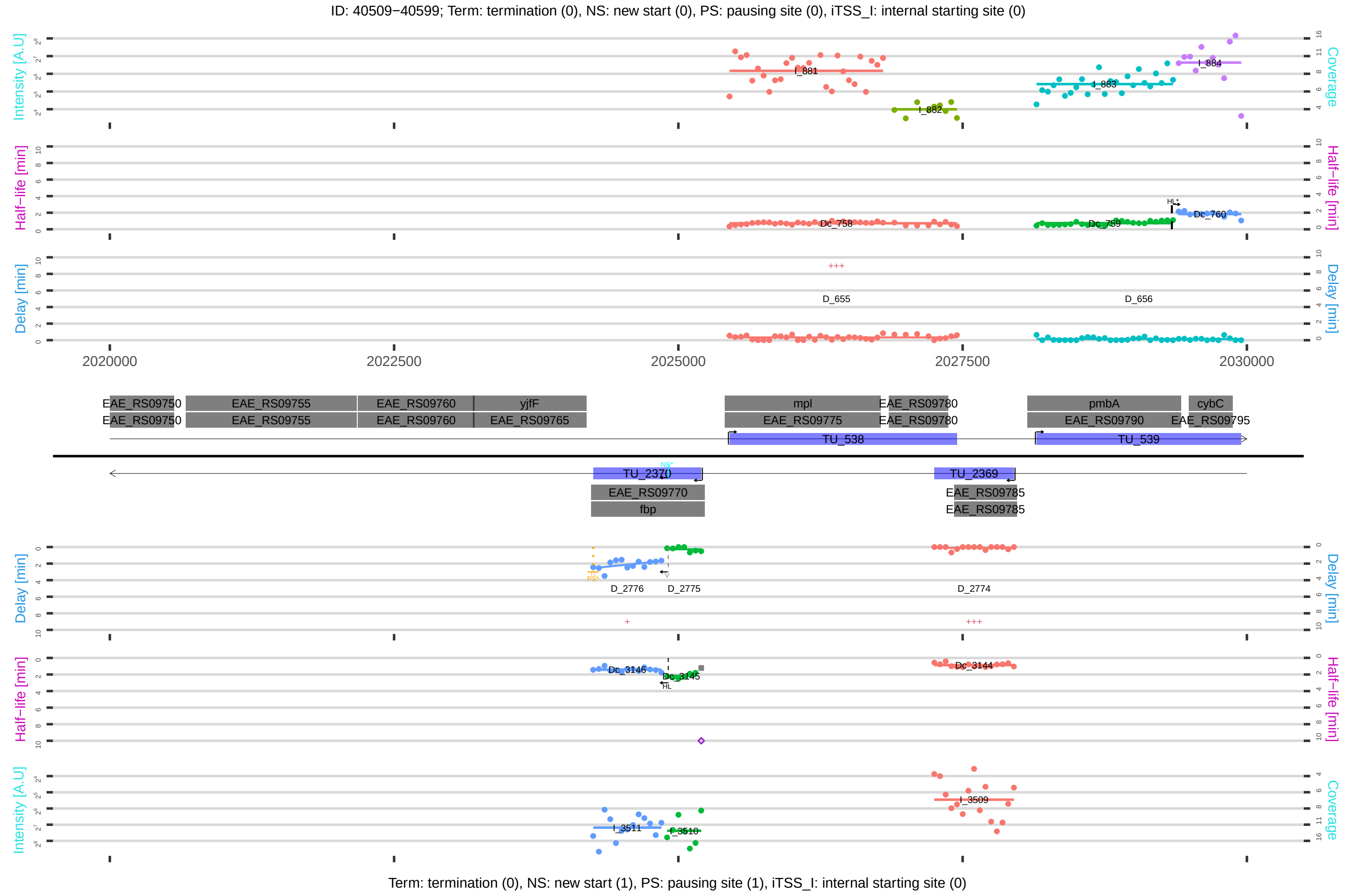

ID: 40509−40599; Term: termination (0), NS: new start (0), PS: pausing site (0), iTSS_I: internal starting site (0)
Intensity [A.U]
Coverage
I_881
I_882
I_883
I_884
Half−life [min]
Dc_758
Dc_759
Dc_760
HL*
Delay [min]
D_655
D_656
2020000
2022500
2025000
2027500
2030000
EAE_RS09750
EAE_RS09755
EAE_RS09760
yjfF
EAE_RS09765
mpl
EAE_RS09775
EAE_RS09780
pmbA
EAE_RS09790
cybC
EAE_RS09795
TU_538
TU_539
TU_2370
NS*
TU_2369
EAE_RS09770
fbp
EAE_RS09785
D_2776
D_2775
D_2774
PS*
Dc_3146
Dc_3145
Dc_3144
HL
I_3511
I_3510
I_3509
Term: termination (0), NS: new start (1), PS: pausing site (1), iTSS_I: internal starting site (0)

ID: 40610−40771; Term: termination (1), NS: new start (2), PS: pausing site (1), iTSS_I: internal starting site (0)

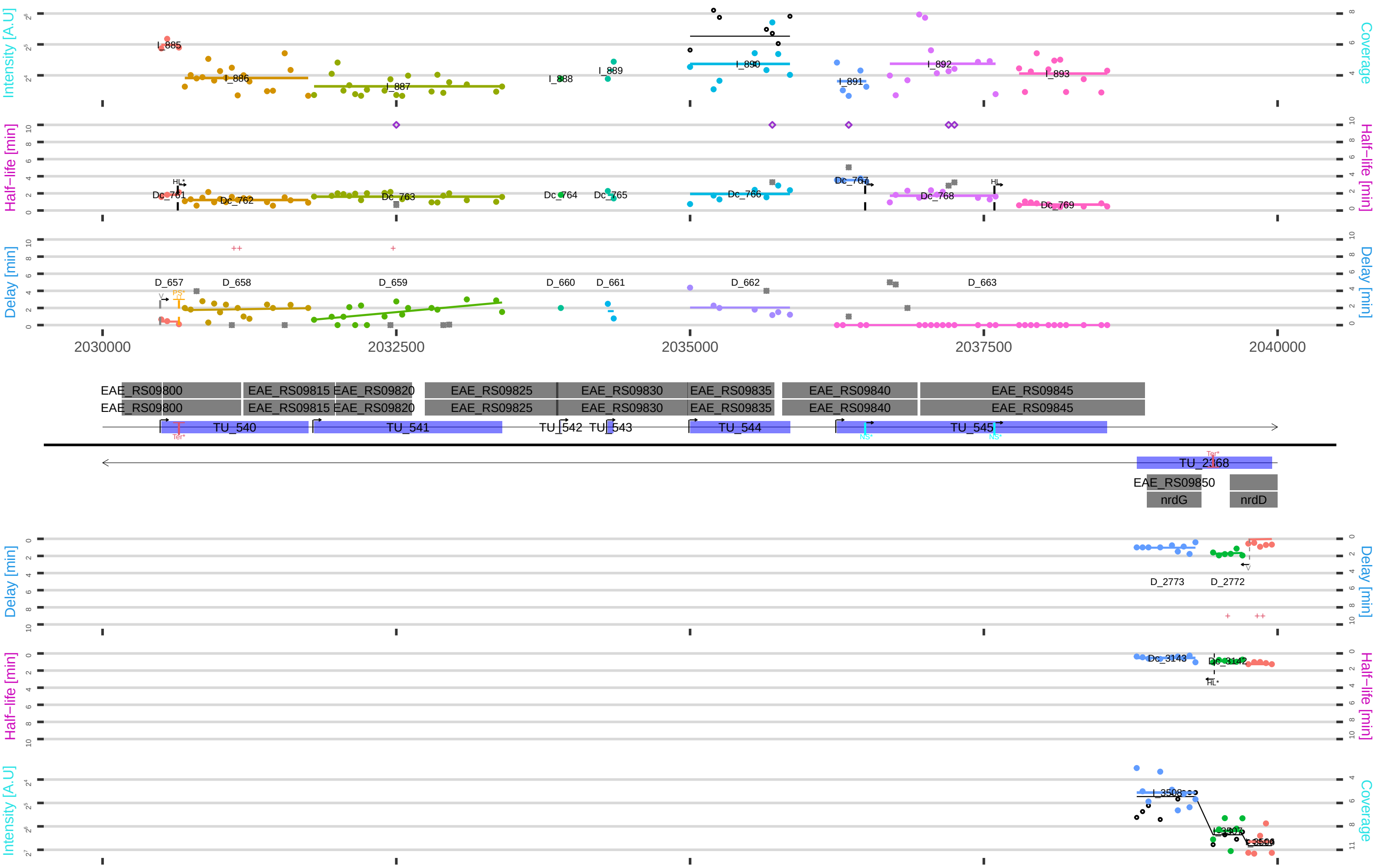

Term: termination (1), NS: new start (0), PS: pausing site (1), iTSS_I: internal starting site (1)

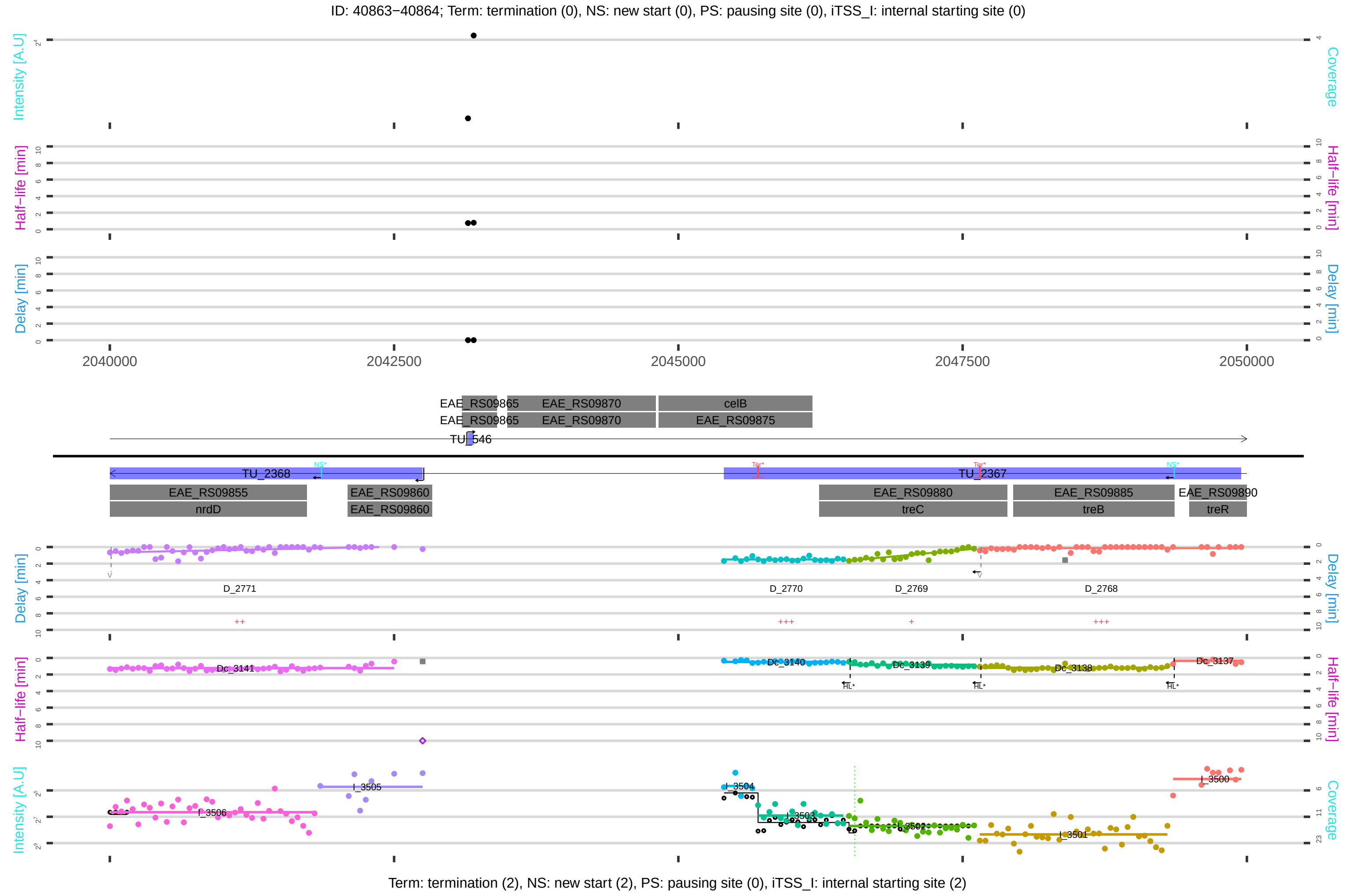

ID: 40863−40864; Term: termination (0), NS: new start (0), PS: pausing site (0), iTSS_I: internal starting site (0)
Intensity [A.U]
Coverage
Half−life [min]
Delay [min]
2040000
2042500
2045000
2047500
2050000
EAE_RS09865
EAE_RS09870
celB
EAE_RS09875
TU_546
TU_2368
TU_2367
EAE_RS09855
nrdD
EAE_RS09860
EAE_RS09880
treC
EAE_RS09885
treB
EAE_RS09890
treR
D_2771
D_2770
D_2769
D_2768
Dc_3141
Dc_3140
Dc_3139
Dc_3138
Dc_3137
I_3506
I_3505
I_3504
I_3503
I_3502
I_3501
I_3500
Term: termination (2), NS: new start (2), PS: pausing site (0), iTSS_I: internal starting site (2)

ID: 41013−41183; Term: termination (0), NS: new start (0), PS: pausing site (0), iTSS_I: internal starting site (0)

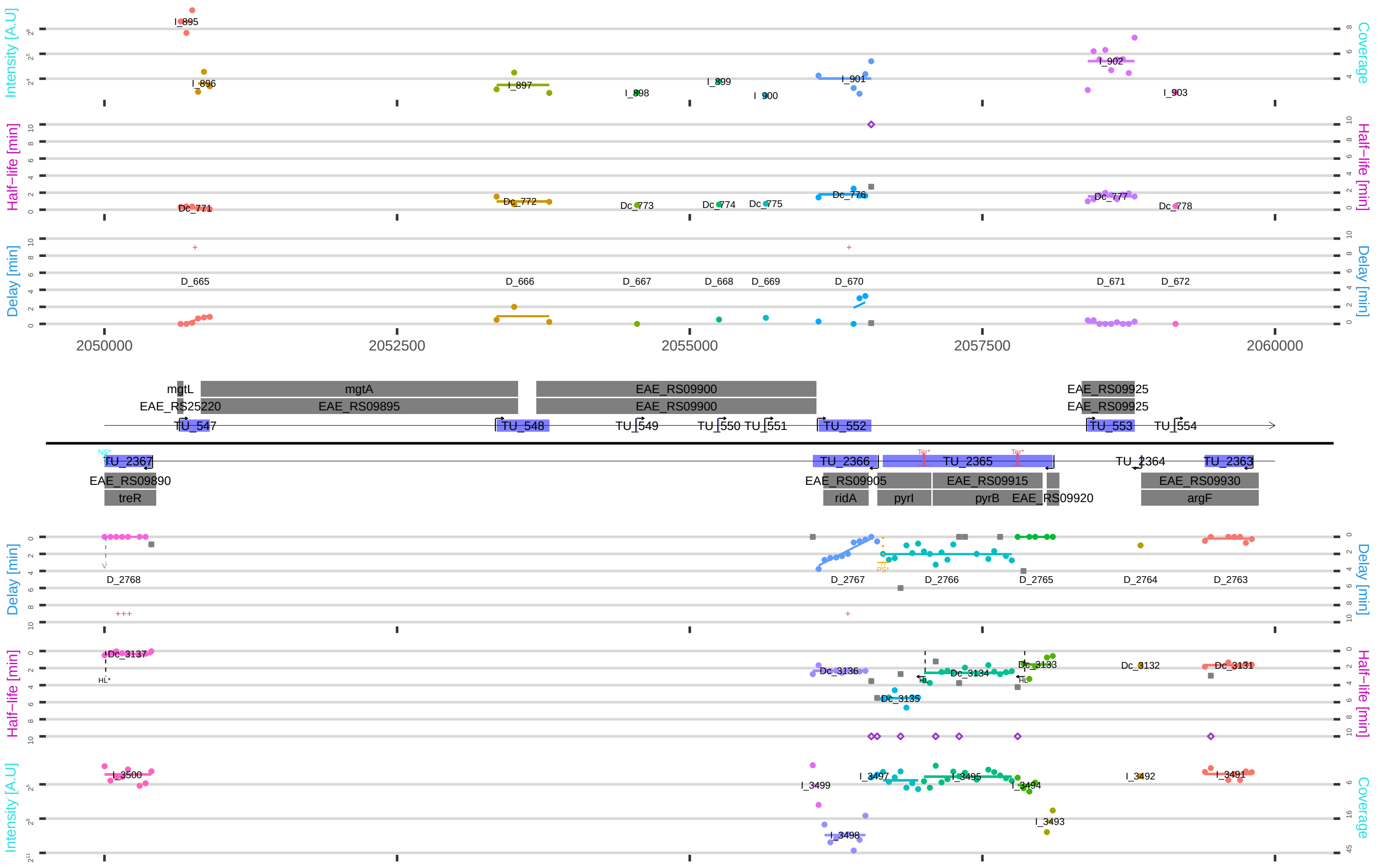

Term: termination (2), NS: new start (1), PS: pausing site (1), iTSS_I: internal starting site (0)

ID: 41201−41398; Term: termination (2), NS: new start (0), PS: pausing site (1), iTSS_I: internal starting site (0)

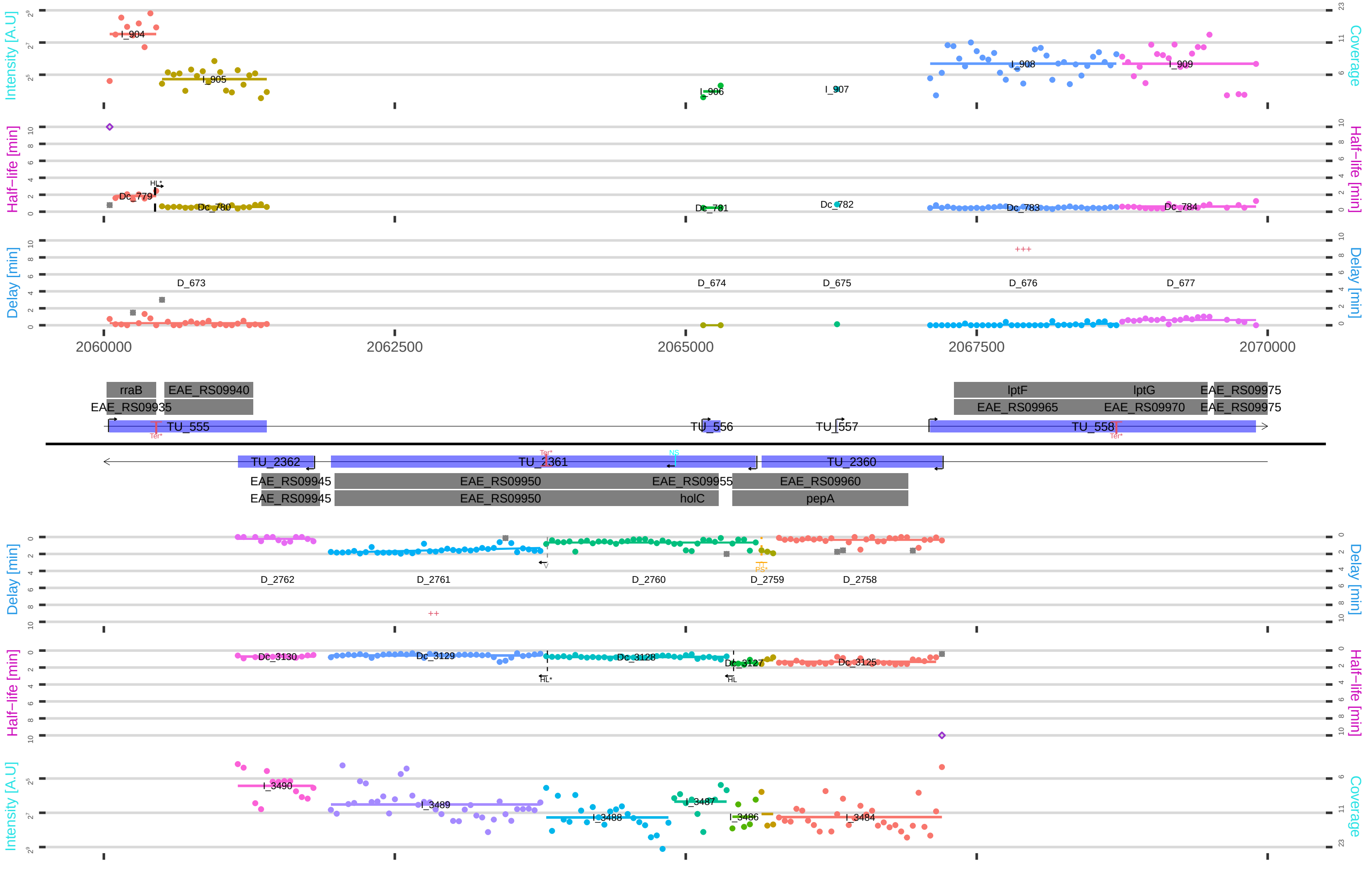

Term: termination (1), NS: new start (1), PS: pausing site (2), iTSS_I: internal starting site (0)

ID: 41401−41575; Term: termination (0), NS: new start (0), PS: pausing site (0), iTSS_I: internal starting site (0)

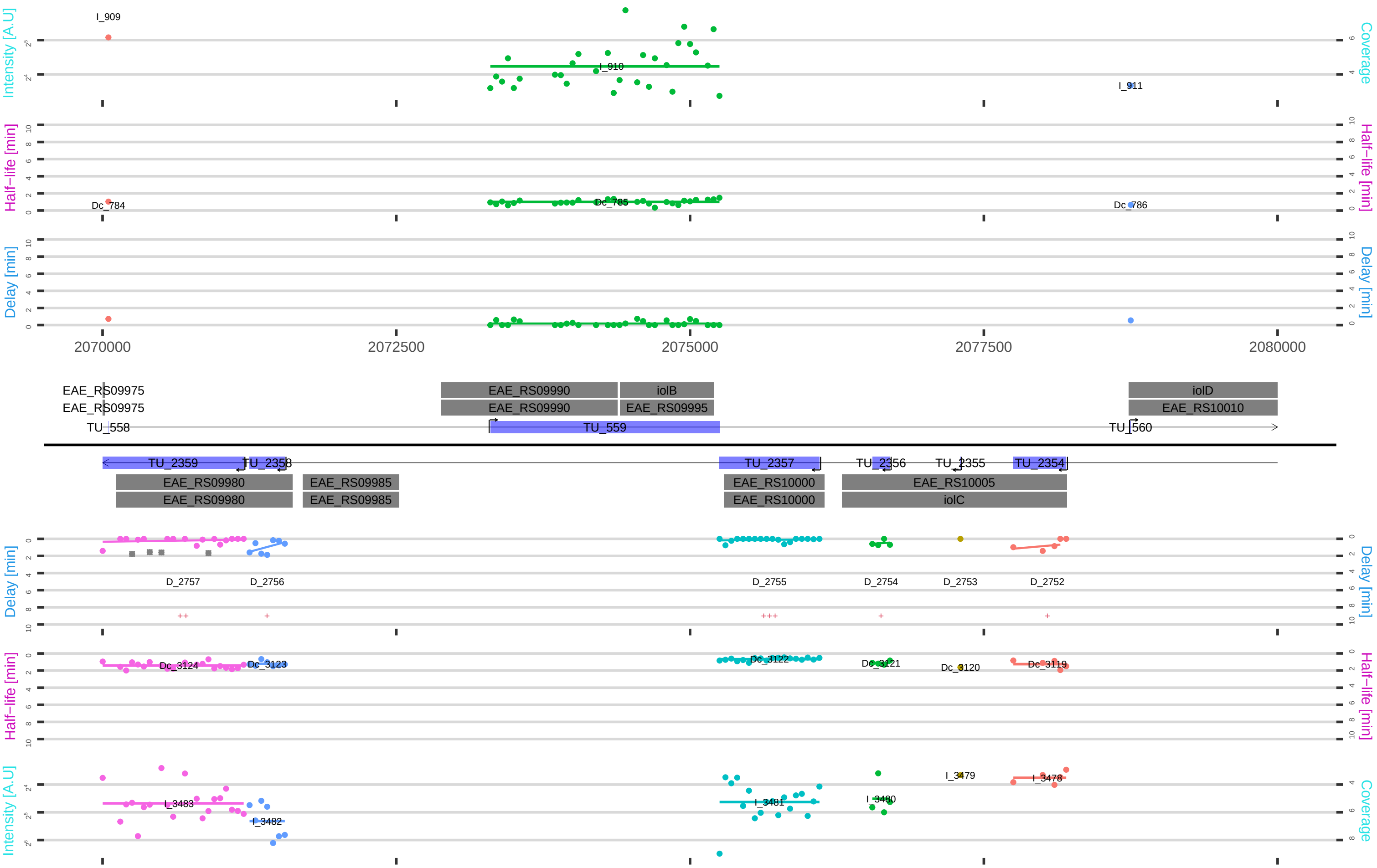

Term: termination (0), NS: new start (0), PS: pausing site (0), iTSS_I: internal starting site (0)

ID: 169542−169542; FC*: significant t−test of two consecutive segments; Term: termination, NS: new start, PS: pausing site,iTSS_I: internal starting site,TI: transcription interferance.

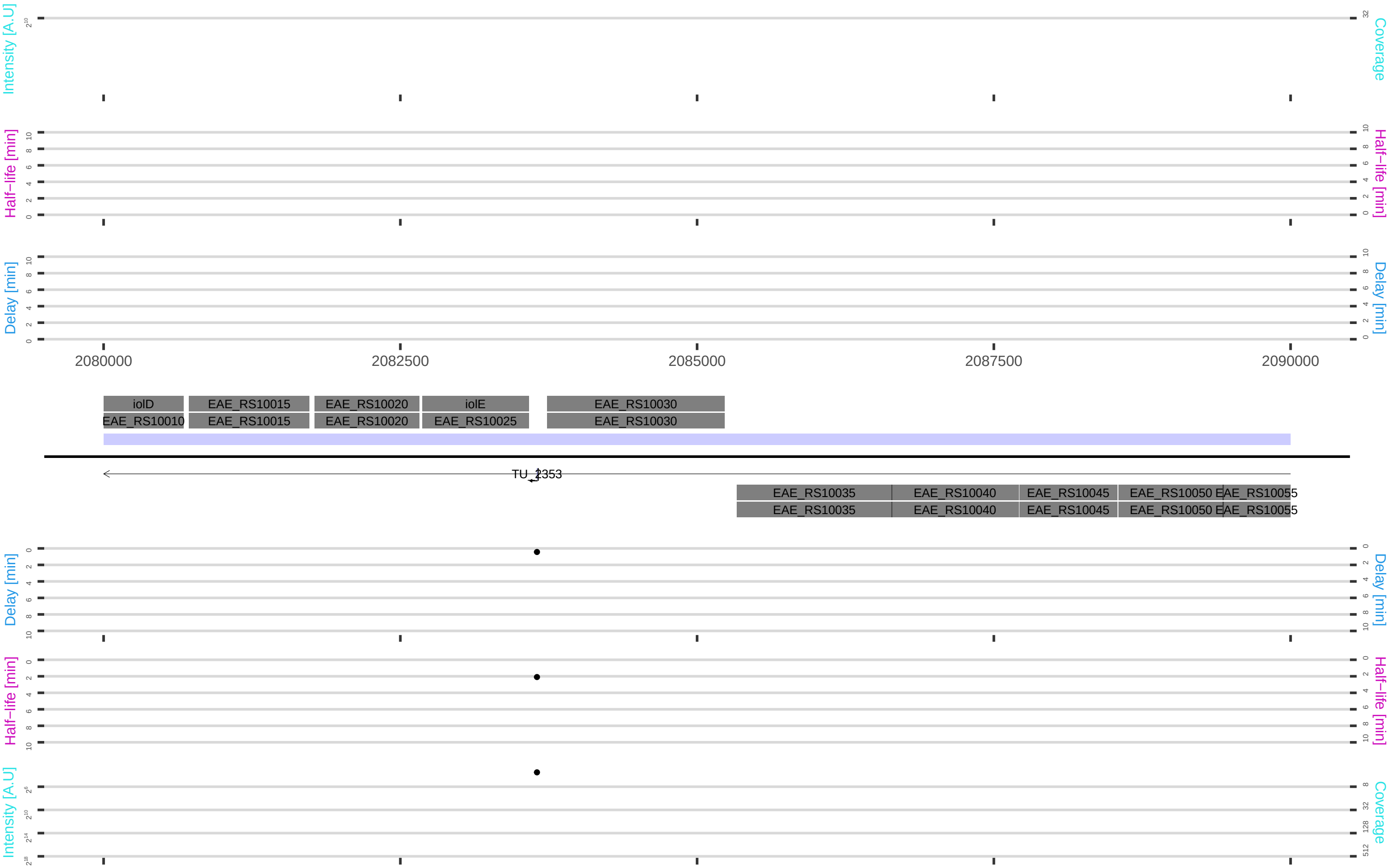

Term: termination (0), NS: new start (0), PS: pausing site (0), iTSS_I: internal starting site (0)

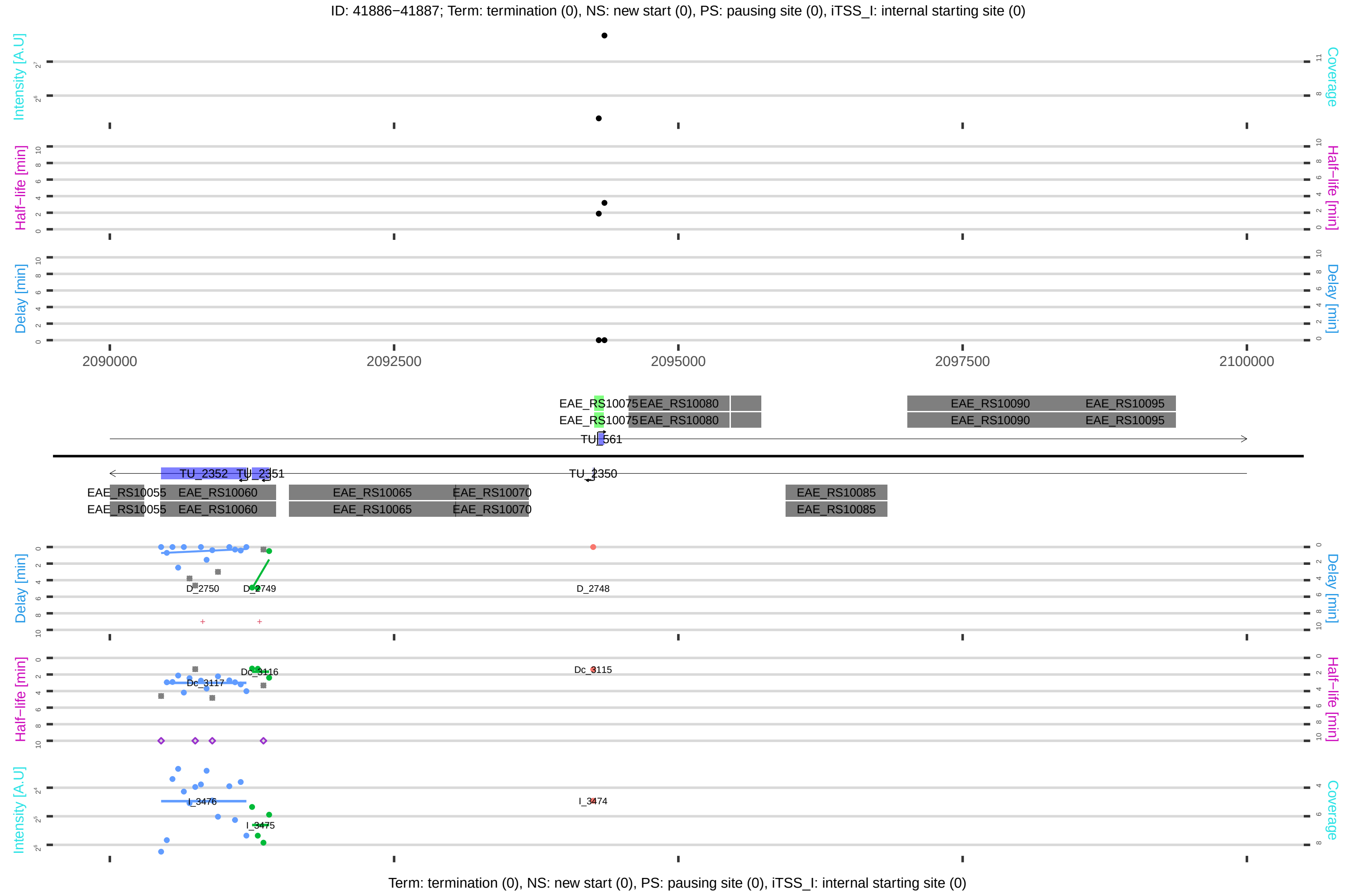

ID: 41886−41887; Term: termination (0), NS: new start (0), PS: pausing site (0), iTSS_I: internal starting site (0)
Intensity [A.U]
Coverage
Half−life [min]
Delay [min]
2090000
2092500
2095000
2097500
2100000
EAE_RS10075
EAE_RS10080
EAE_RS10090
EAE_RS10095
TU_561
TU_2352
TU_2351
TU_2350
EAE_RS10055
EAE_RS10060
EAE_RS10065
EAE_RS10070
EAE_RS10085
D_2750
D_2749
D_2748
Dc_3117
Dc_3116
Dc_3115
I_3476
I_3475
I_3474
Term: termination (0), NS: new start (0), PS: pausing site (0), iTSS_I: internal starting site (0)

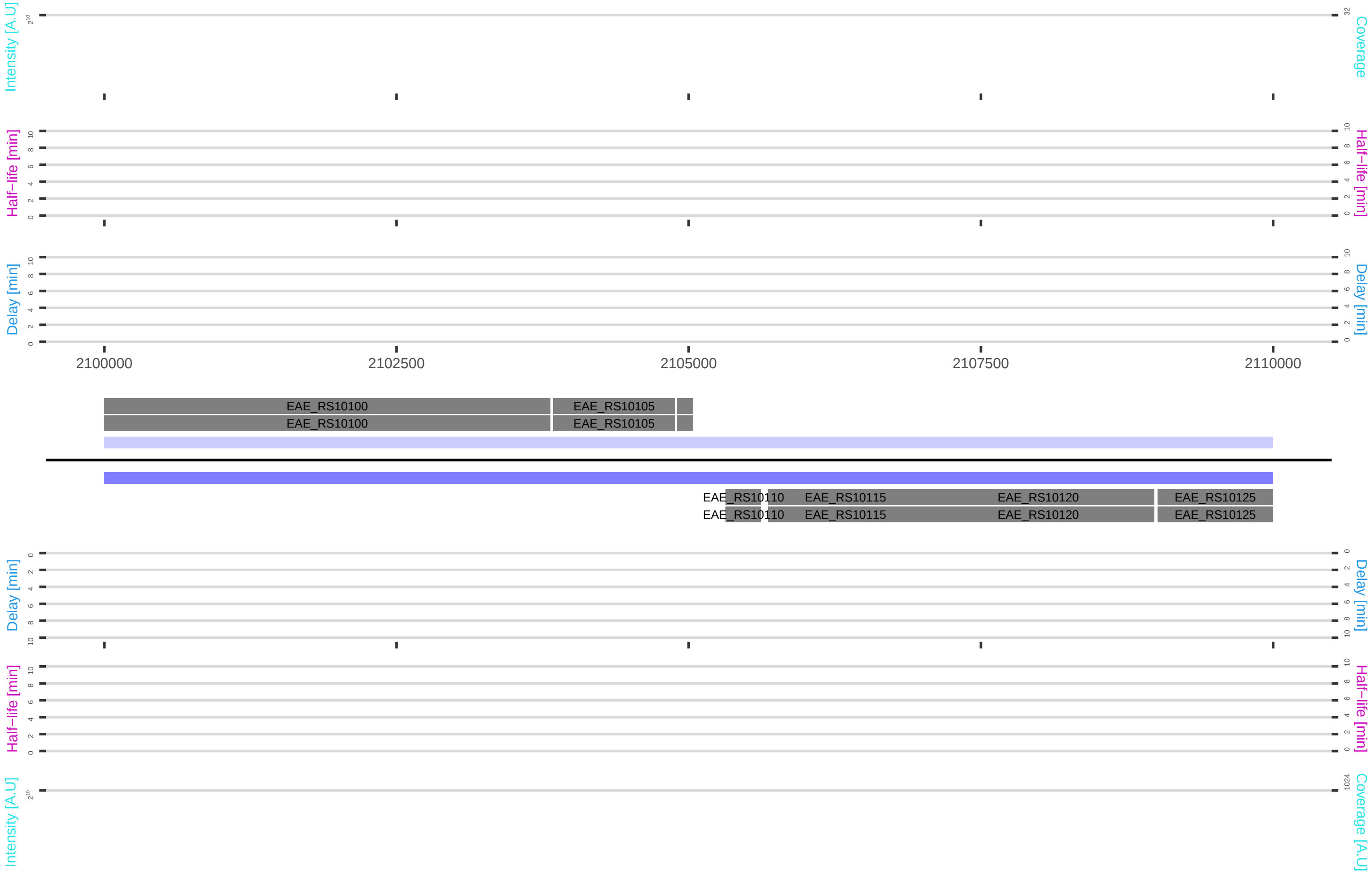

Intensity [A.U]
Coverage
Half−life [min]
Delay [min]
2100000
2102500
2105000
2107500
2110000
EAE_RS10100
EAE_RS10105
EAE_RS10110
EAE_RS10115
EAE_RS10120
EAE_RS10125
Coverage [A.U]

NA

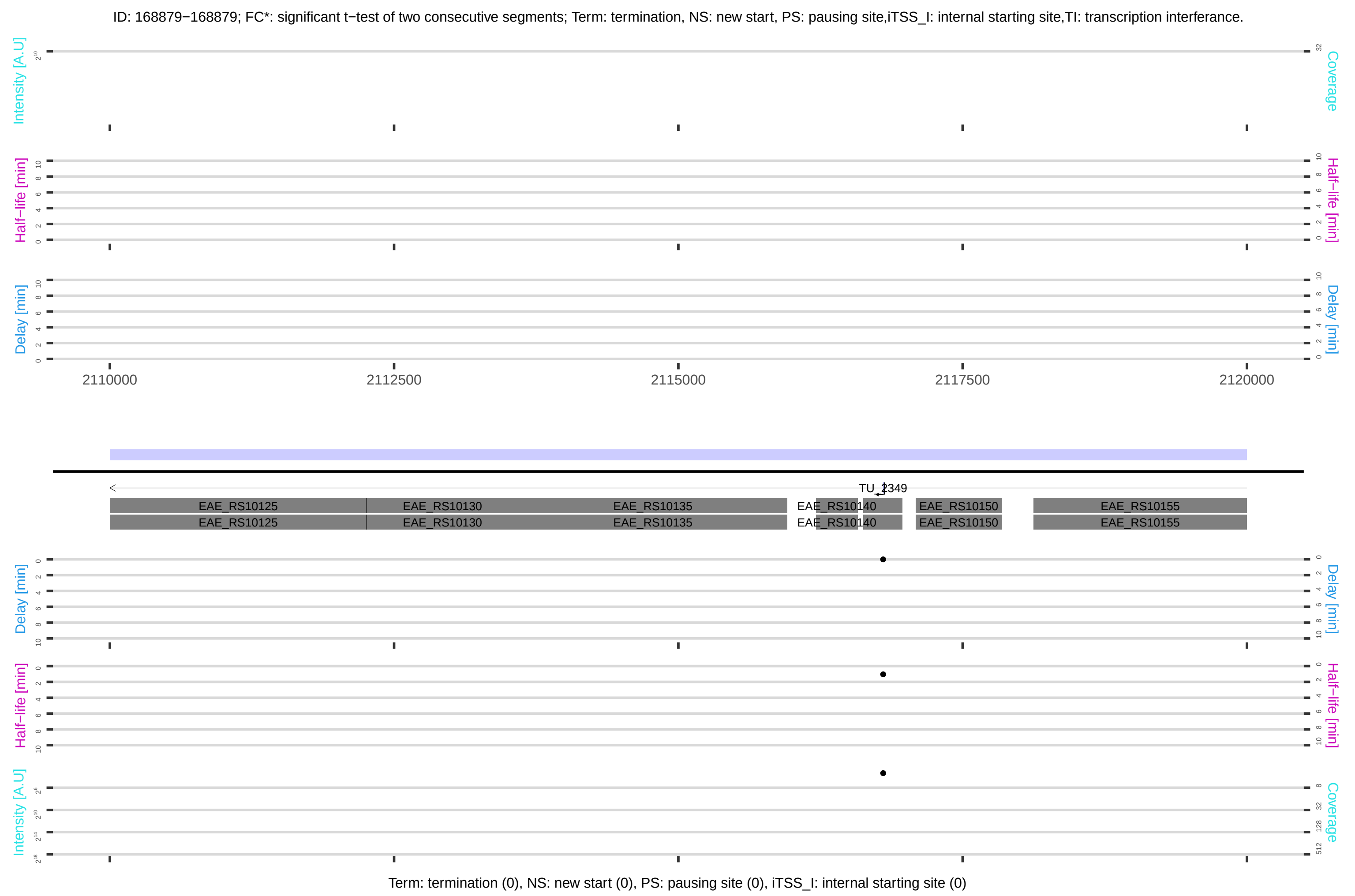
ID: 168879−168879; FC*: significant t−test of two consecutive segments; Term: termination, NS: new start, PS: pausing site,iTSS_I: internal starting site,TI: transcription interferance.
Intensity [A.U]
Coverage
Half−life [min]
Delay [min]
2110000
2112500
2115000
2117500
2120000
TU_2349
EAE_RS10125
EAE_RS10130
EAE_RS10135
EAE_RS10140
EAE_RS10150
EAE_RS10155
Term: termination (0), NS: new start (0), PS: pausing site (0), iTSS_I: internal starting site (0)

ID: 42491−42599; Term: termination (2), NS: new start (2), PS: pausing site (2), iTSS_I: internal starting site (1)

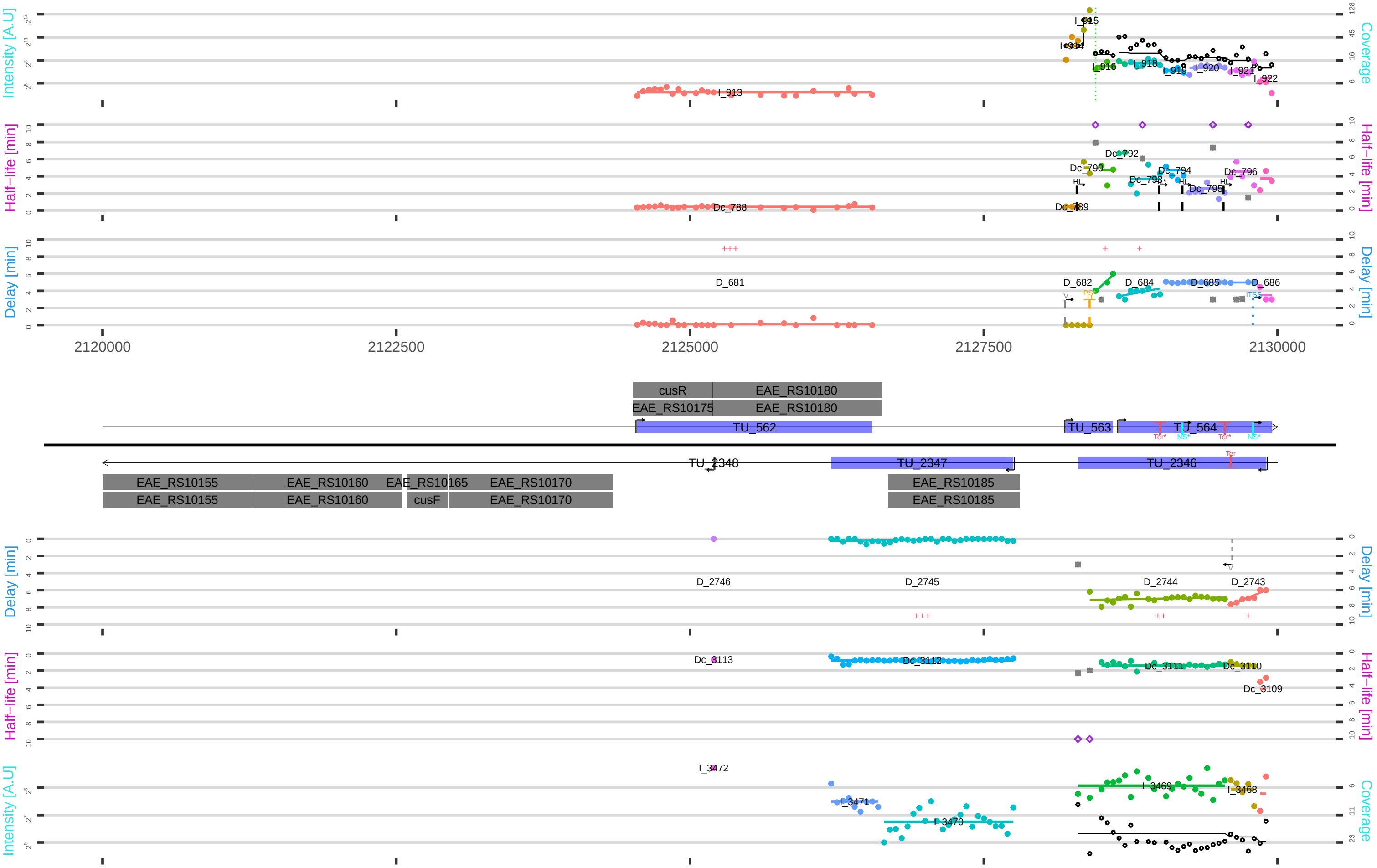

Term: termination (1), NS: new start (0), PS: pausing site (0), iTSS_I: internal starting site (1)

ID: 42601−42687; Term: termination (3), NS: new start (0), PS: pausing site (2), iTSS_I: internal starting site (0)

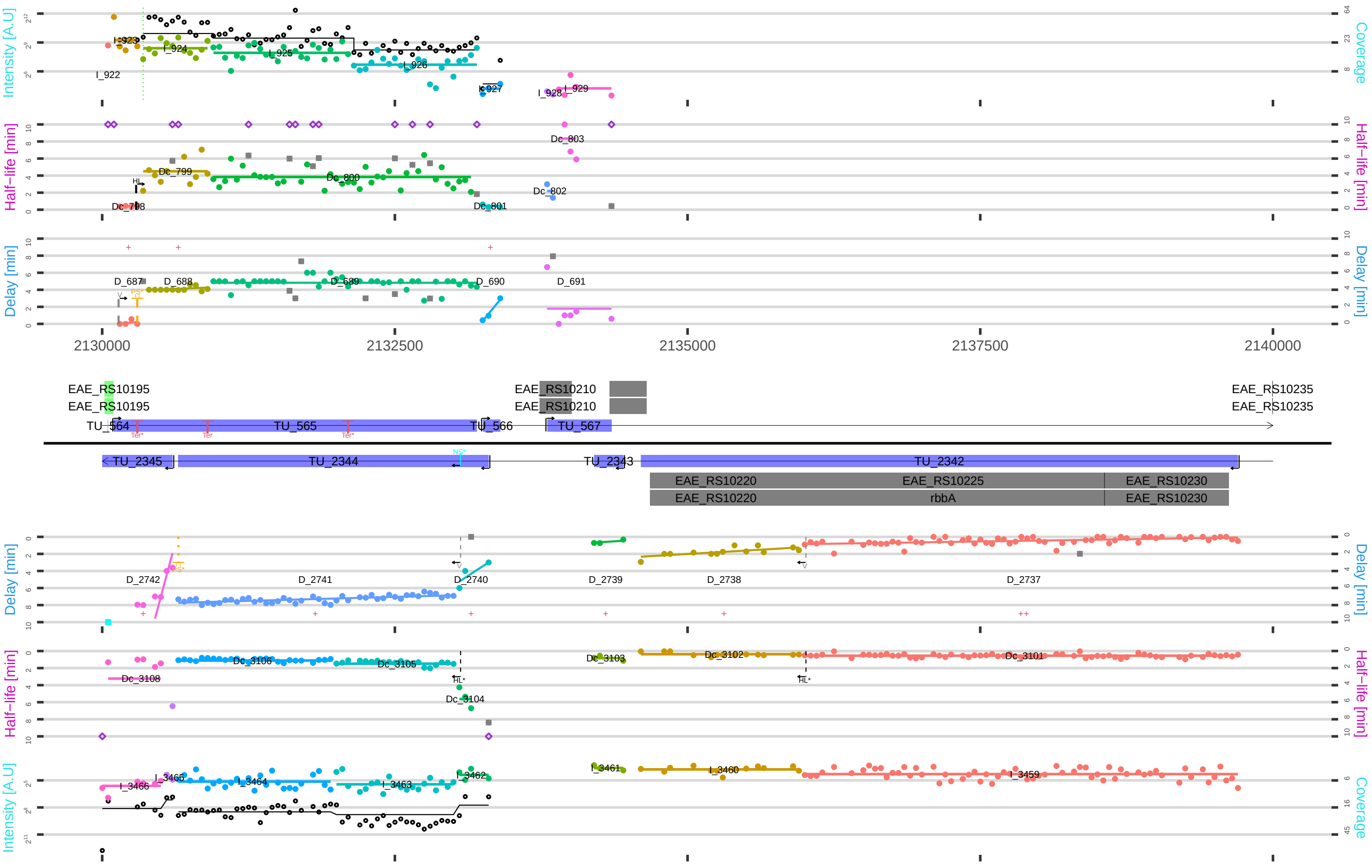

Term: termination (0), NS: new start (1), PS: pausing site (2), iTSS_I: internal starting site (0)

ID: 42802−42911; Term: termination (0), NS: new start (0), PS: pausing site (0), iTSS_I: internal starting site (0)

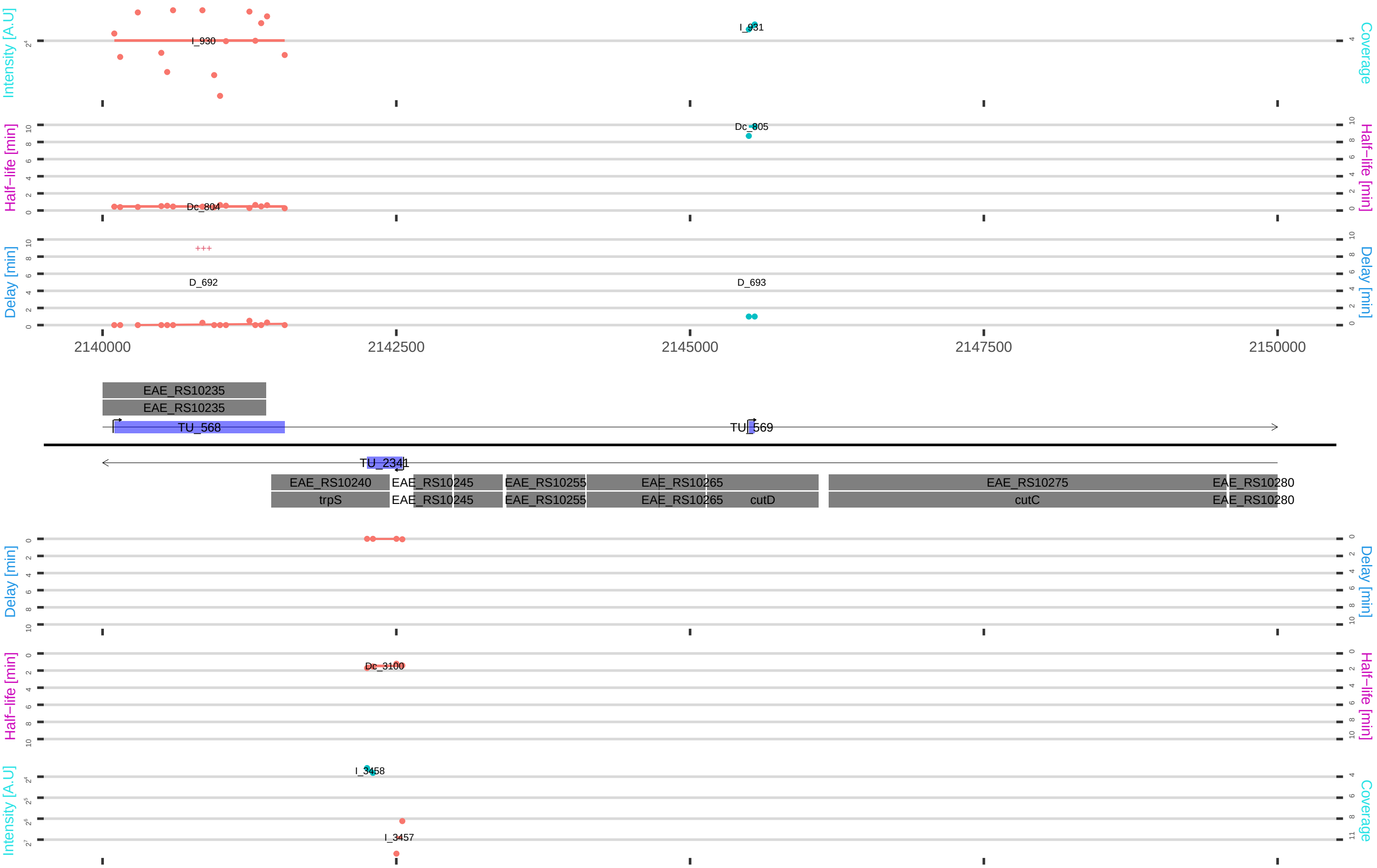

Term: termination (0), NS: new start (0), PS: pausing site (0), iTSS_I: internal starting site (0)

ID: 168135−168099; FC*: significant t−test of two consecutive segments; Term: termination, NS: new start, PS: pausing site,iTSS_I: internal starting site,TI: transcription interferance.

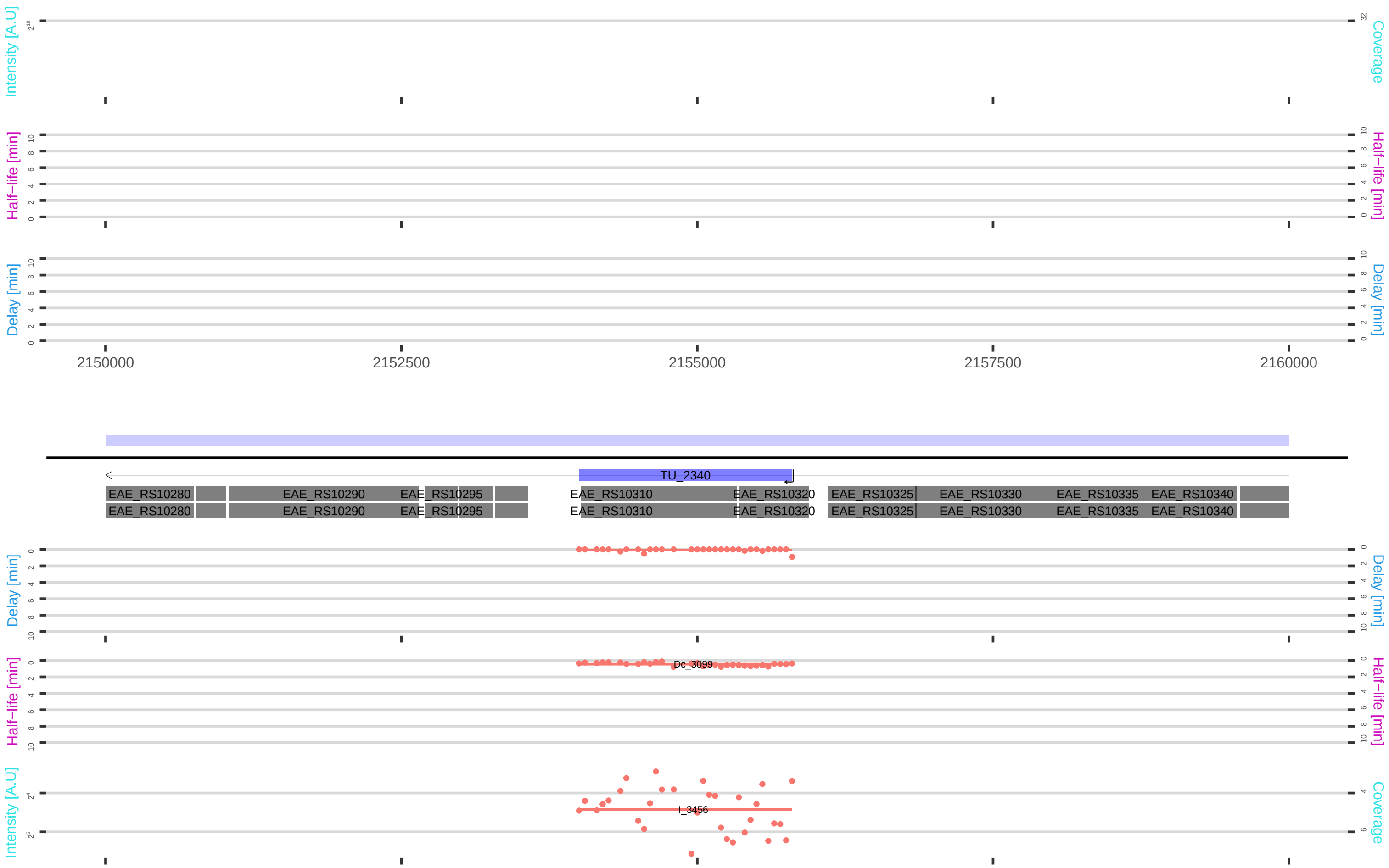

Term: termination (0), NS: new start (0), PS: pausing site (0), iTSS_I: internal starting site (0)

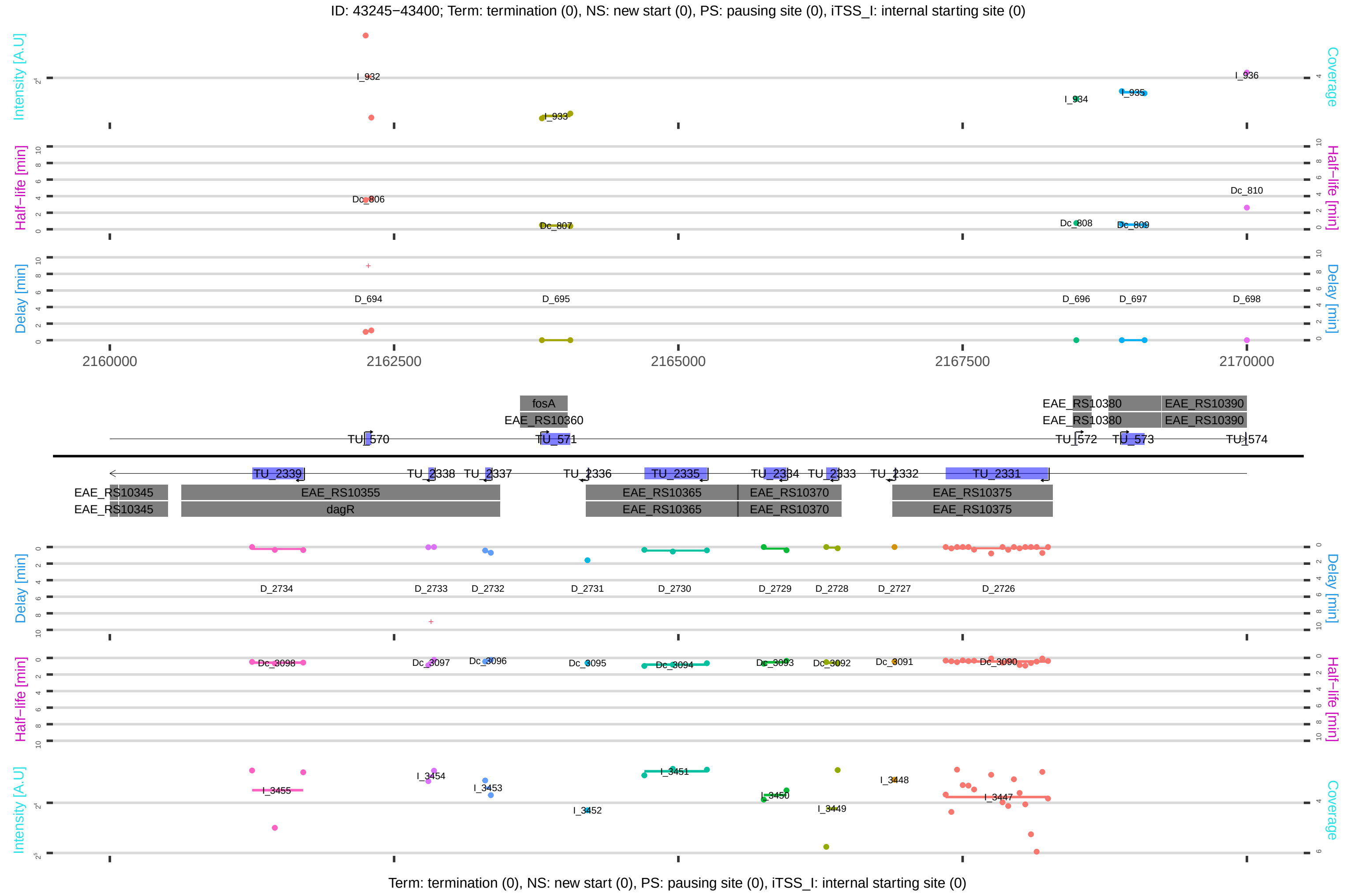

ID: 43245−43400; Term: termination (0), NS: new start (0), PS: pausing site (0), iTSS_I: internal starting site (0)
Intensity [A.U]
Coverage
Half−life [min]
Delay [min]
I_932
I_933
I_934
I_935
I_936
Dc_806
Dc_807
Dc_808
Dc_809
Dc_810
D_694
D_695
D_696
D_697
D_698
2160000
2162500
2165000
2167500
2170000
fosA
EAE_RS10360
EAE_RS10380
EAE_RS10390
TU_570
TU_571
TU_572
TU_573
TU_574
TU_2339
TU_2338
TU_2337
TU_2336
TU_2335
TU_2334
TU_2333
TU_2332
TU_2331
EAE_RS10345
EAE_RS10355
dagR
EAE_RS10365
EAE_RS10370
EAE_RS10375
D_2734
D_2733
D_2732
D_2731
D_2730
D_2729
D_2728
D_2727
D_2726
Dc_3098
Dc_3097
Dc_3096
Dc_3095
Dc_3094
Dc_3093
Dc_3092
Dc_3091
Dc_3090
I_3455
I_3454
I_3453
I_3452
I_3451
I_3450
I_3449
I_3448
I_3447
Term: termination (0), NS: new start (0), PS: pausing site (0), iTSS_I: internal starting site (0)

ID: 43400−43600; Term: termination (0), NS: new start (0), PS: pausing site (0), iTSS_I: internal starting site (0)

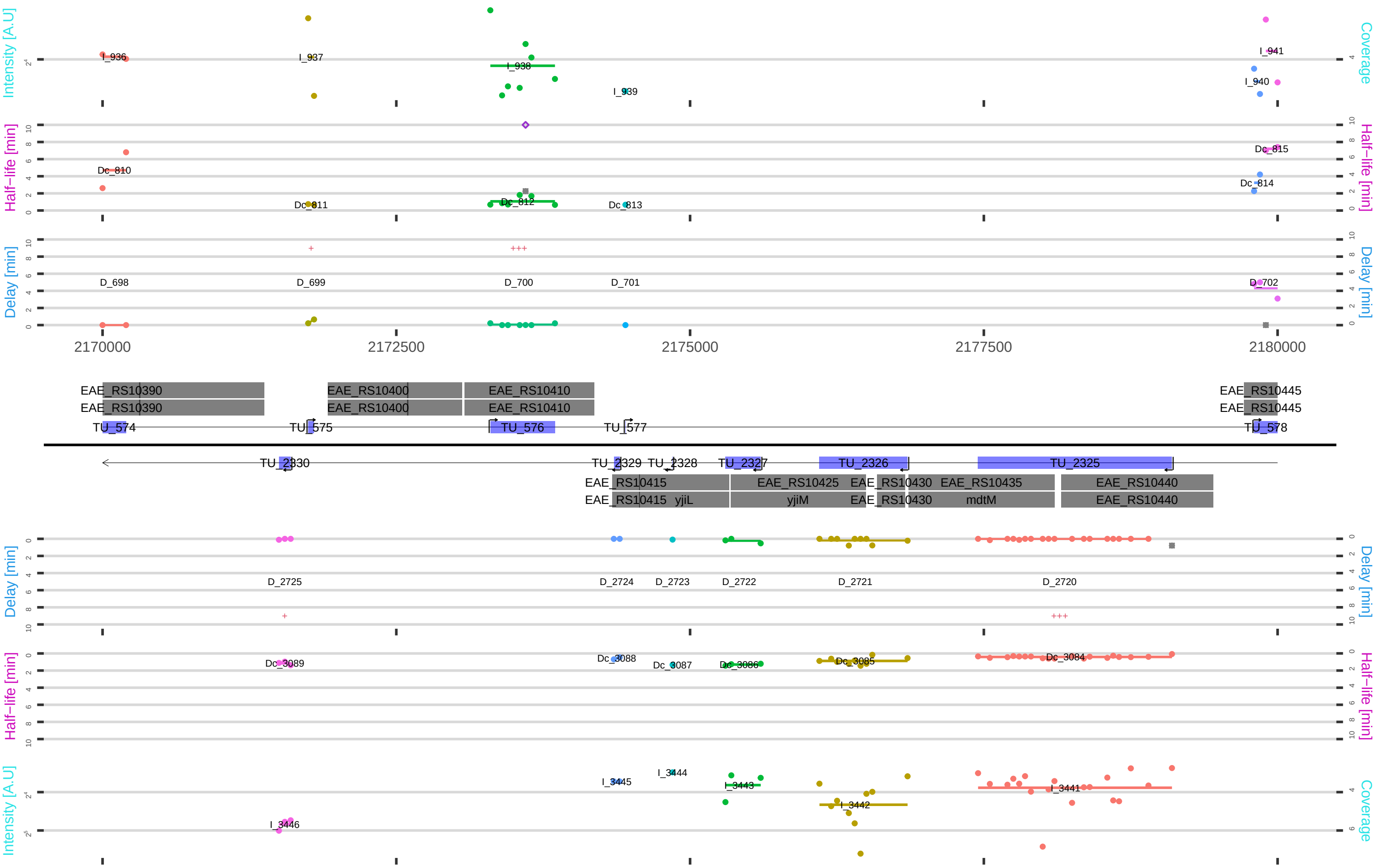

Term: termination (0), NS: new start (0), PS: pausing site (0), iTSS_I: internal starting site (0)

ID: 43600−43622; Term: termination (0), NS: new start (0), PS: pausing site (0), iTSS_I: internal starting site (0)

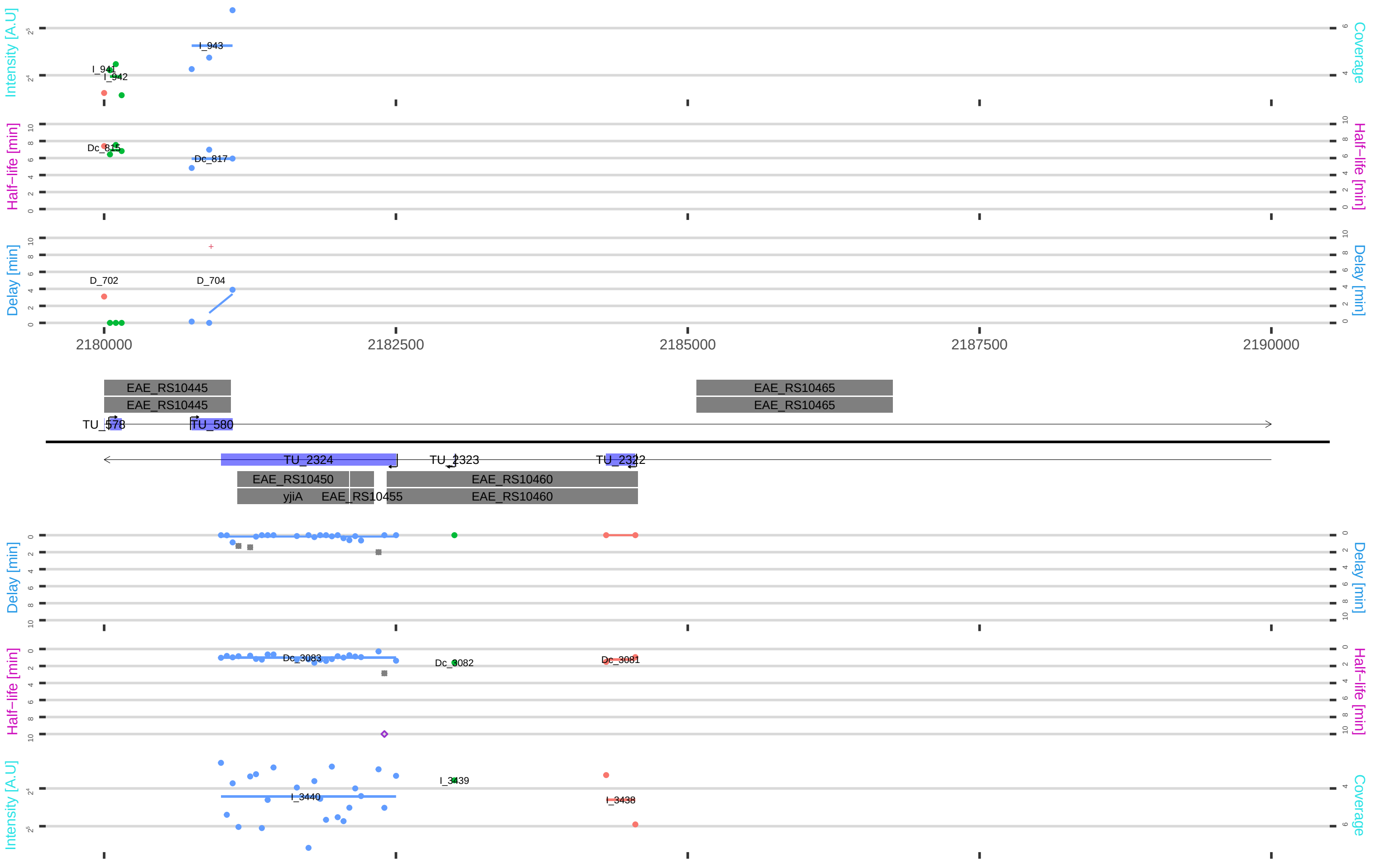

Term: termination (0), NS: new start (0), PS: pausing site (0), iTSS_I: internal starting site (0)

ID: 167225−167225; FC*: significant t−test of two consecutive segments; Term: termination, NS: new start, PS: pausing site,iTSS_I: internal starting site,TI: transcription interferance.

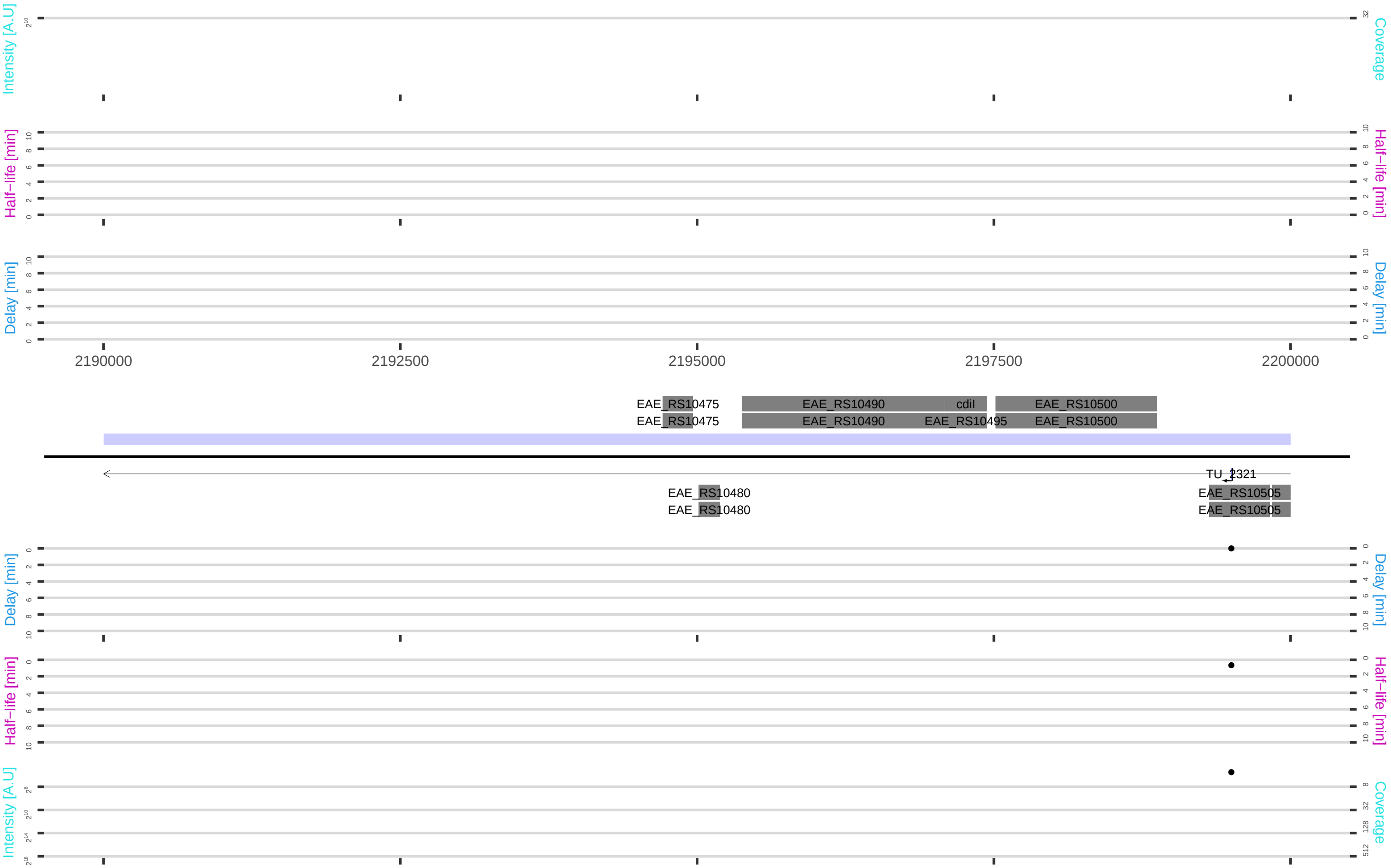

Term: termination (0), NS: new start (0), PS: pausing site (0), iTSS_I: internal starting site (0)

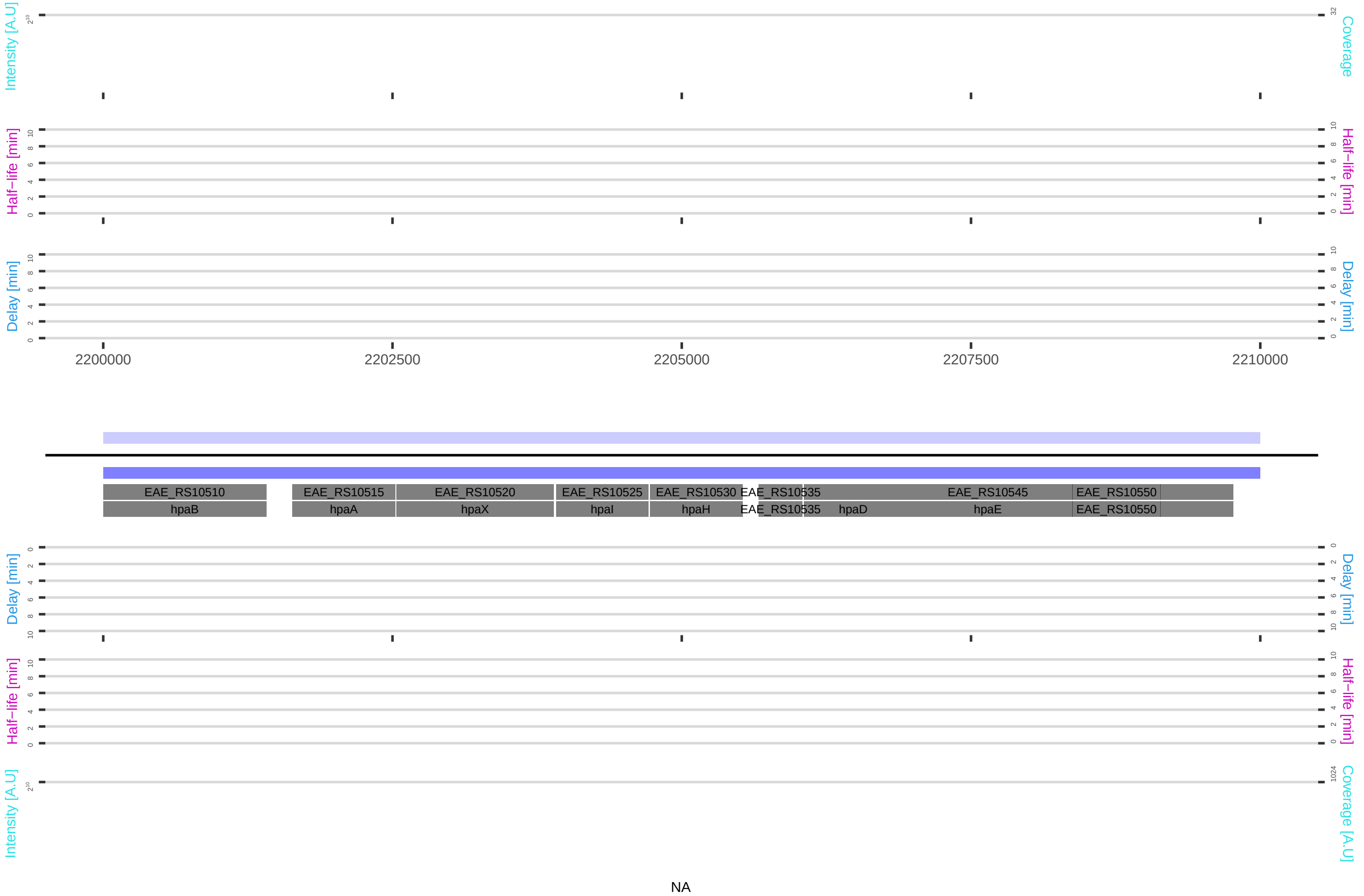

Intensity [A.U]
Coverage
Half−life [min]
Delay [min]
2200000
2202500
2205000
2207500
2210000
EAE_RS10510
EAE_RS10515
EAE_RS10520
EAE_RS10525
EAE_RS10530
EAE_RS10535
EAE_RS10545
EAE_RS10550
hpaB
hpaA
hpaX
hpaI
hpaH
EAE_RS10535
hpaD
hpaE
EAE_RS10550
Coverage [A.U]
NA

ID: 44203−44400; Term: termination (0), NS: new start (0), PS: pausing site (0), iTSS_I: internal starting site (0)

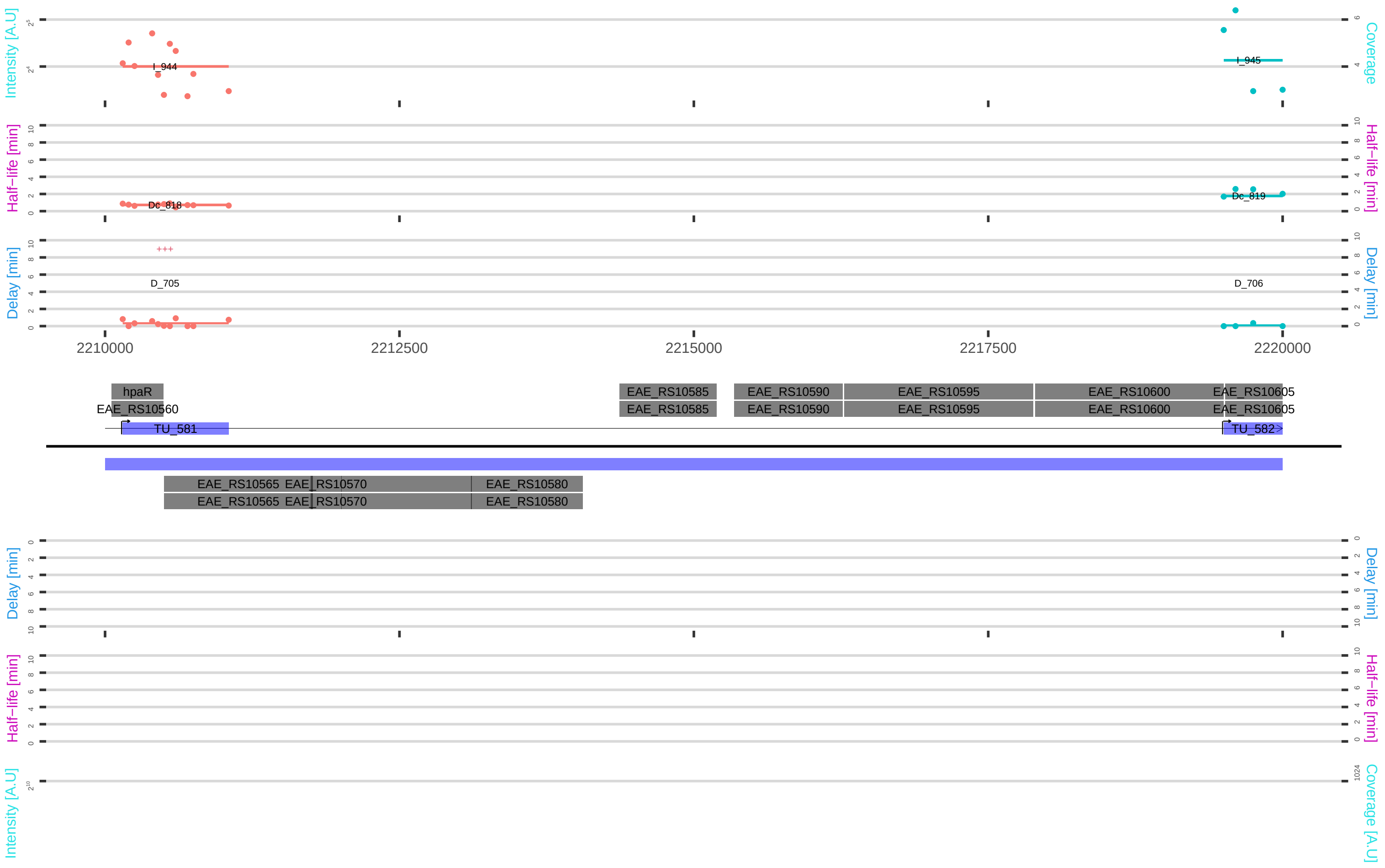

NA

ID: 44400−44584; Term: termination (0), NS: new start (1), PS: pausing site (1), iTSS_I: internal starting site (0)

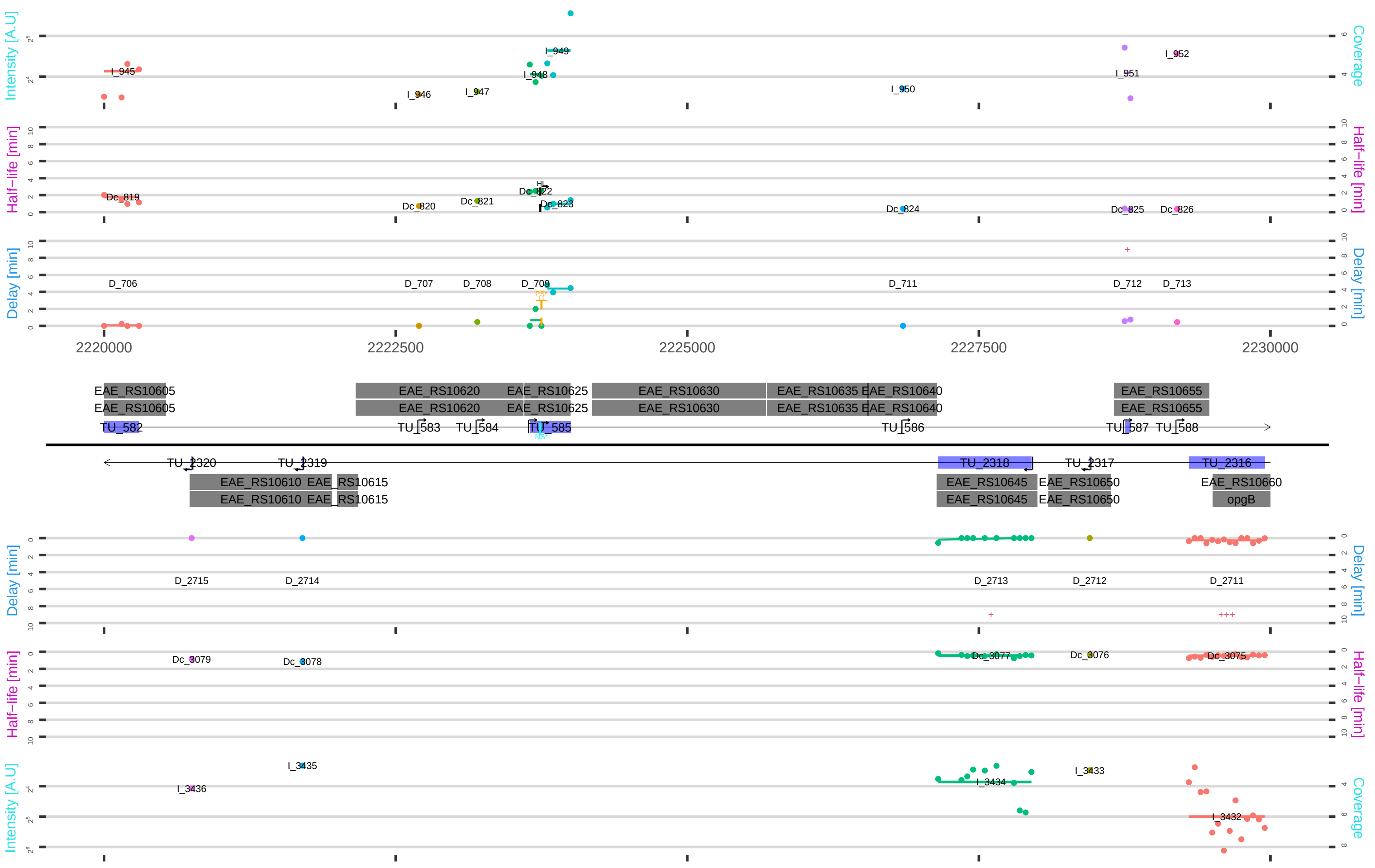

Term: termination (0), NS: new start (0), PS: pausing site (0), iTSS_I: internal starting site (0)

ID: 44799−44800; Term: termination (0), NS: new start (0), PS: pausing site (0), iTSS_I: internal starting site (0)

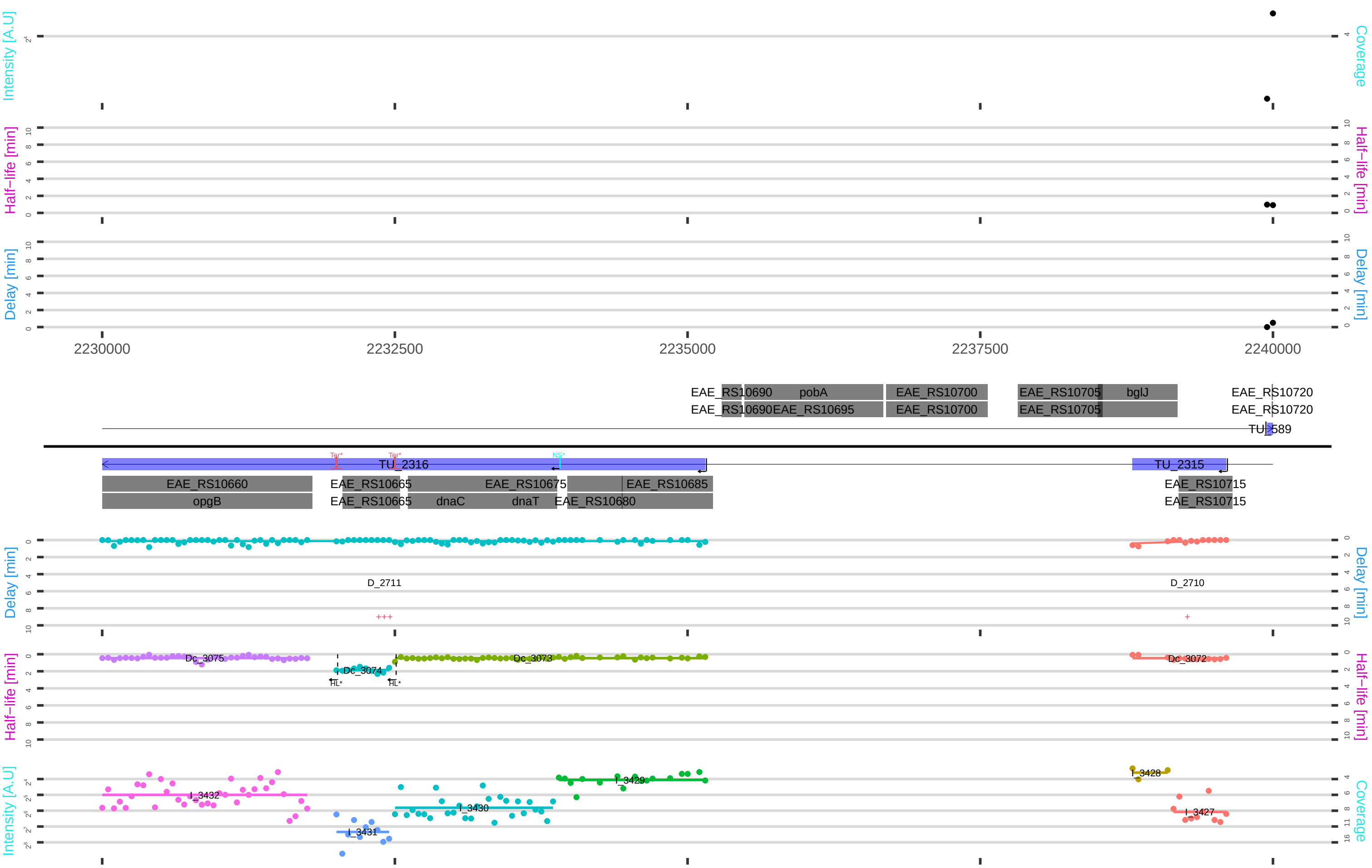

Term: termination (2), NS: new start (1), PS: pausing site (0), iTSS_I: internal starting site (0)

ID: 44800−45000; Term: termination (0), NS: new start (0), PS: pausing site (1), iTSS_I: internal starting site (0)

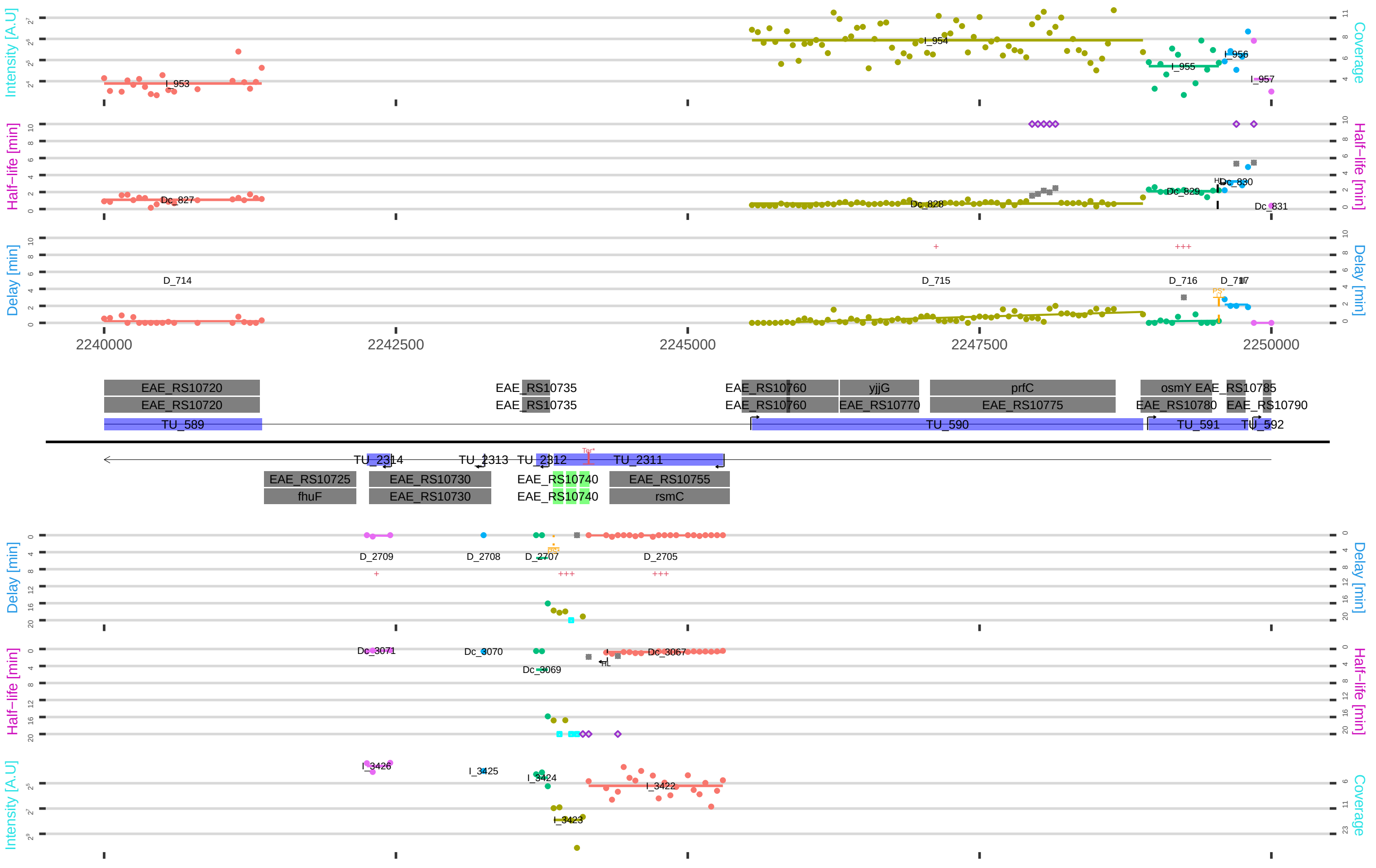

Term: termination (1), NS: new start (0), PS: pausing site (1), iTSS_I: internal starting site (0)

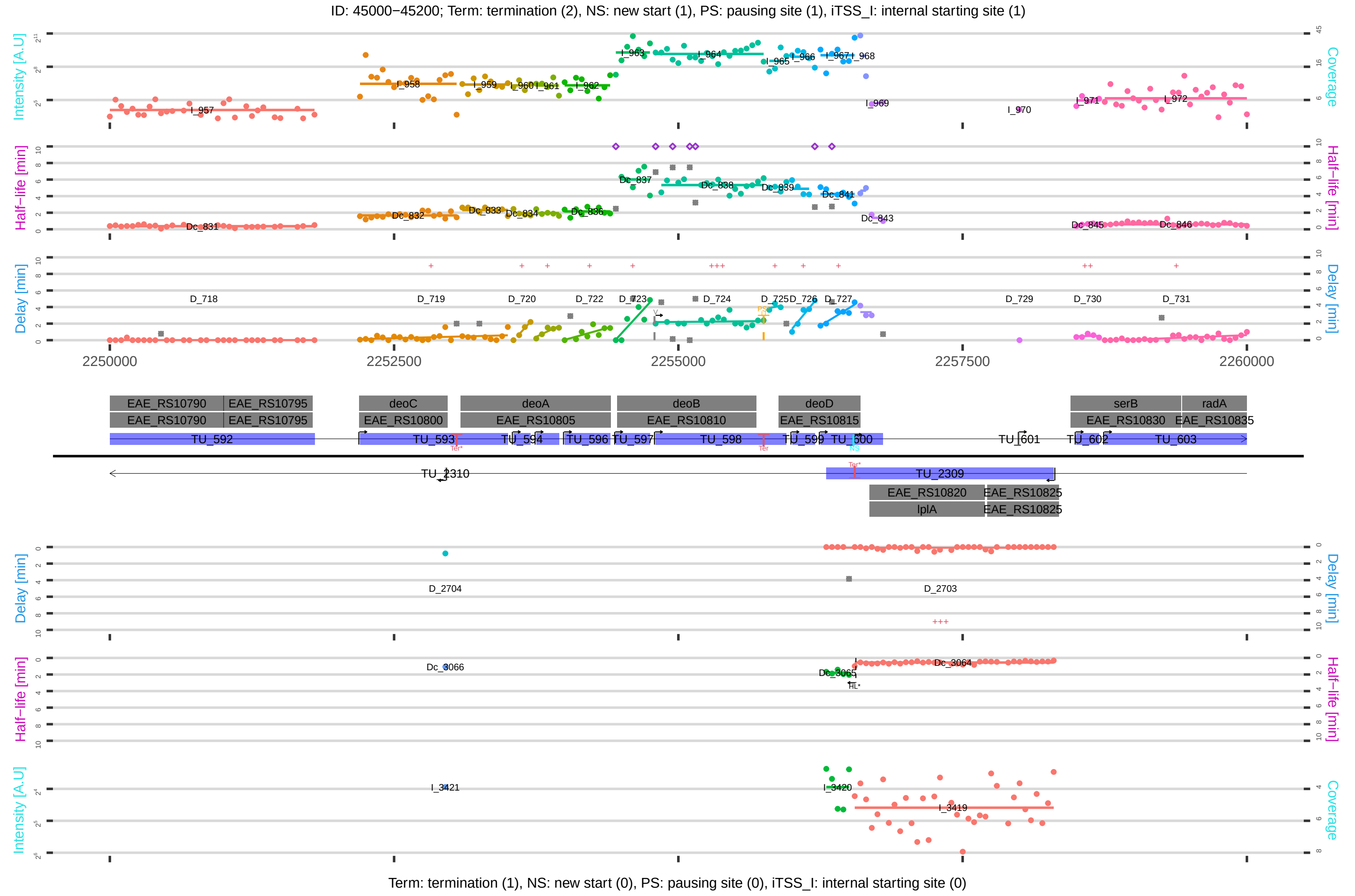
ID: 45000−45200; Term: termination (2), NS: new start (1), PS: pausing site (1), iTSS_I: internal starting site (1)
Intensity [A.U]
Coverage
Half−life [min]
Delay [min]
2250000
2252500
2255000
2257500
2260000
EAE_RS10790
EAE_RS10795
deoC
EAE_RS10800
deoA
EAE_RS10805
deoB
EAE_RS10810
deoD
EAE_RS10815
serB
EAE_RS10830
radA
EAE_RS10835
TU_592
TU_593
TU_594
TU_596
TU_597
TU_598
TU_599
TU_600
TU_601
TU_602
TU_603
TU_2310
TU_2309
EAE_RS10820
lplA
EAE_RS10825
Term: termination (1), NS: new start (0), PS: pausing site (0), iTSS_I: internal starting site (0)

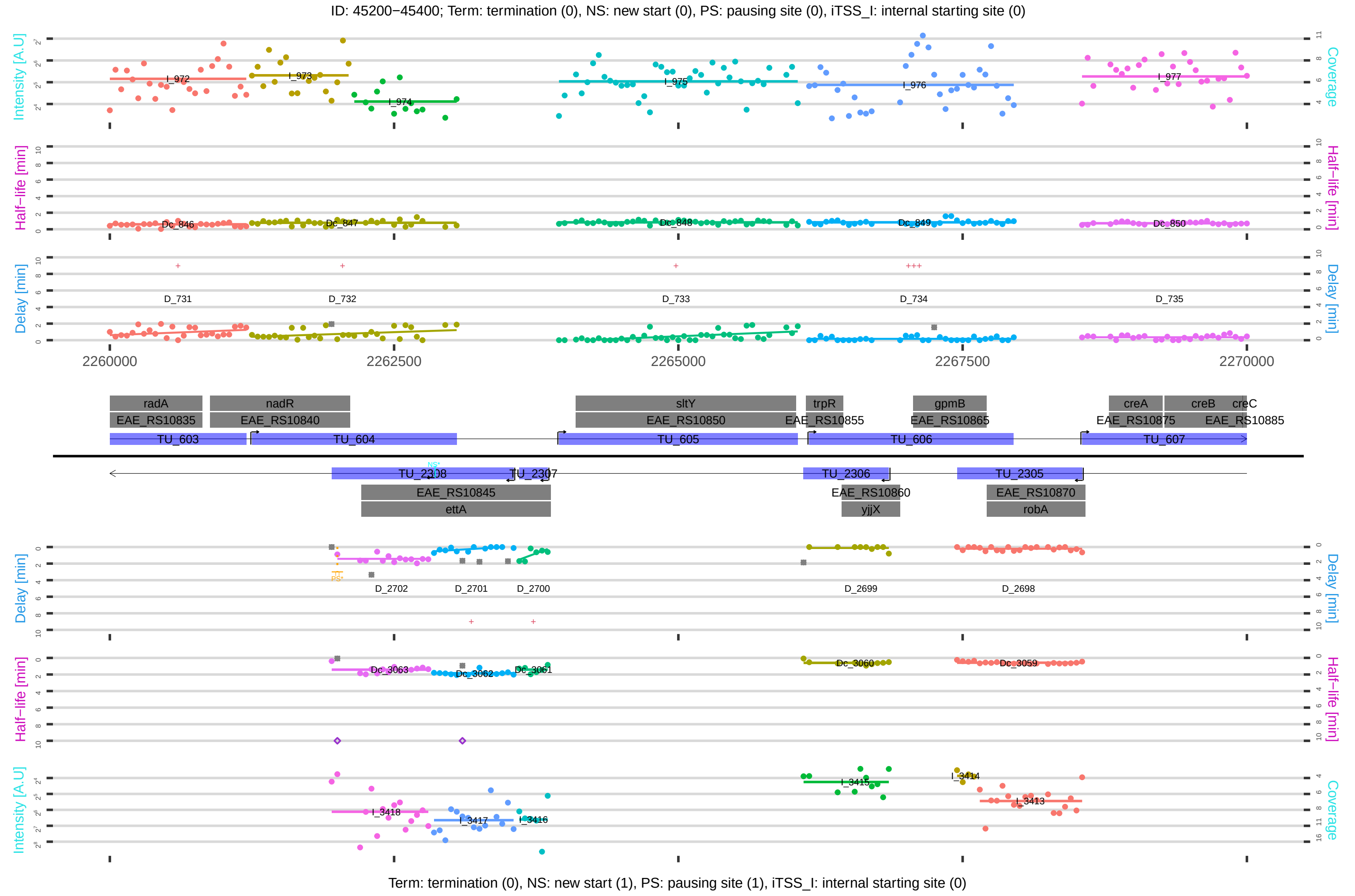
ID: 45200−45400; Term: termination (0), NS: new start (0), PS: pausing site (0), iTSS_I: internal starting site (0)
Intensity [A.U]
Coverage
Half−life [min]
Delay [min]
2260000
2262500
2265000
2267500
2270000
radA
EAE_RS10835
nadR
EAE_RS10840
sltY
EAE_RS10850
trpR
EAE_RS10855
gpmB
EAE_RS10865
creA
EAE_RS10875
creB
creC
EAE_RS10885
TU_603
TU_604
TU_605
TU_606
TU_607
TU_2308
TU_2307
TU_2306
TU_2305
EAE_RS10845
ettA
EAE_RS10860
yjjX
EAE_RS10870
robA
Term: termination (0), NS: new start (1), PS: pausing site (1), iTSS_I: internal starting site (0)

ID: 45400−45599; Term: termination (0), NS: new start (1), PS: pausing site (0), iTSS_I: internal starting site (0)

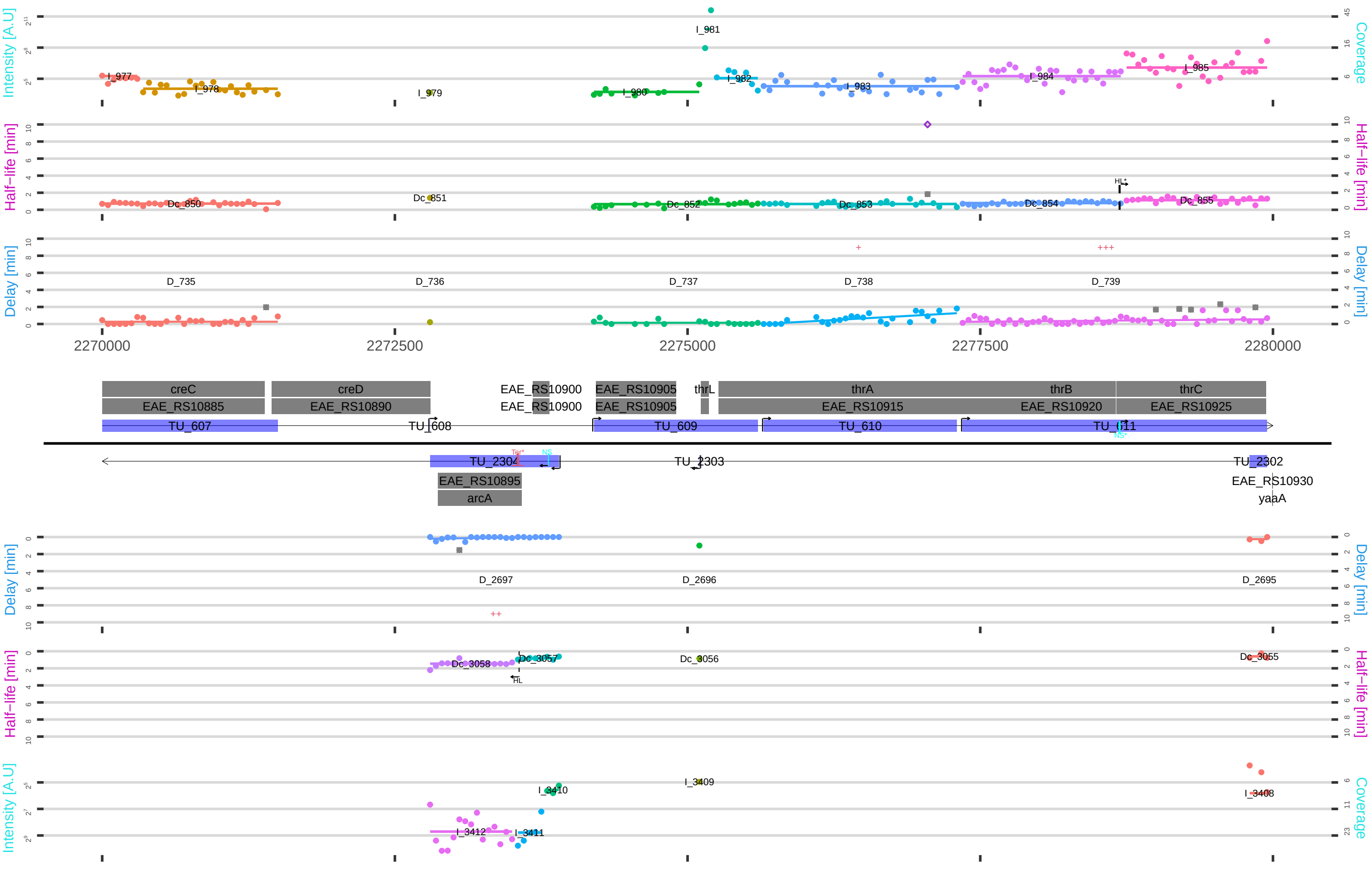

Term: termination (1), NS: new start (1), PS: pausing site (0), iTSS_I: internal starting site (0)

ID: 45601−45800; Term: termination (2), NS: new start (0), PS: pausing site (1), iTSS_I: internal starting site (0)

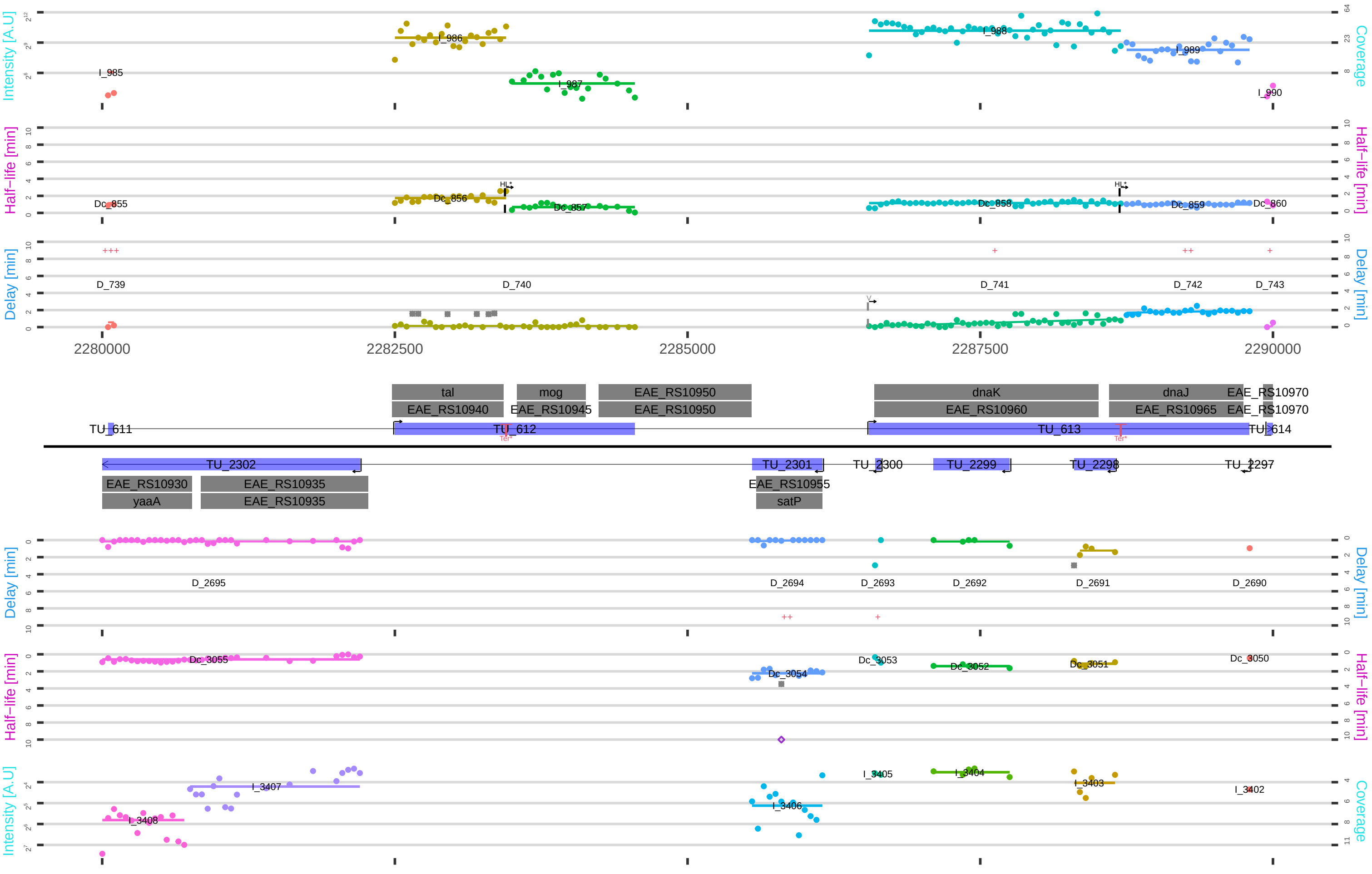

Term: termination (0), NS: new start (0), PS: pausing site (0), iTSS_I: internal starting site (0)

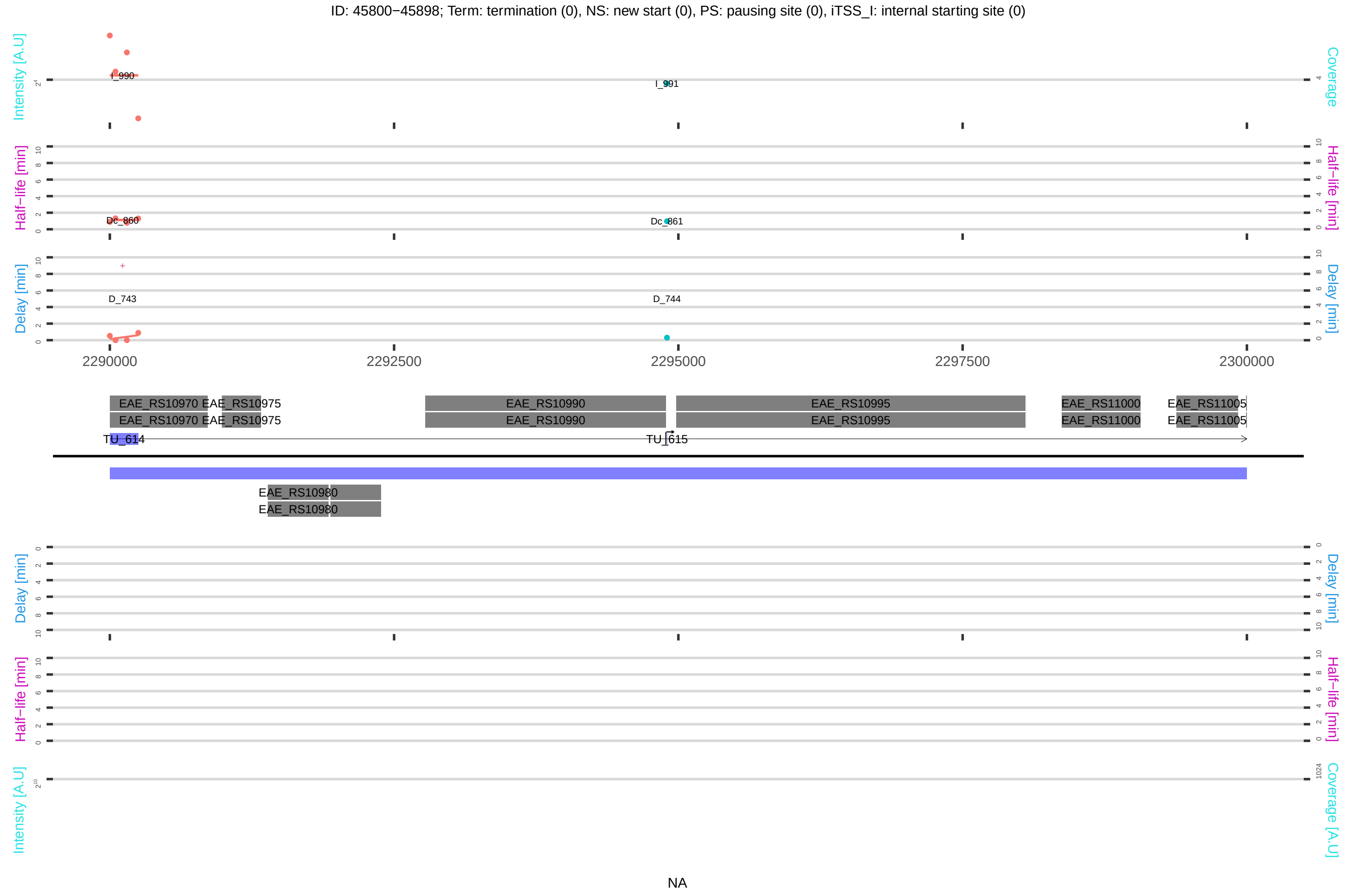

ID: 45800−45898; Term: termination (0), NS: new start (0), PS: pausing site (0), iTSS_I: internal starting site (0)
Intensity [A.U]
Coverage
I_990
I_991
Half−life [min]
Dc_860
Dc_861
Delay [min]
D_743
D_744
2290000
2292500
2295000
2297500
2300000
EAE_RS10970
EAE_RS10975
EAE_RS10990
EAE_RS10995
EAE_RS11000
EAE_RS11005
TU_614
TU_615
EAE_RS10980
Coverage [A.U]
NA

ID: 46150−46199; Term: termination (1), NS: new start (2), PS: pausing site (1), iTSS_I: internal starting site (1)

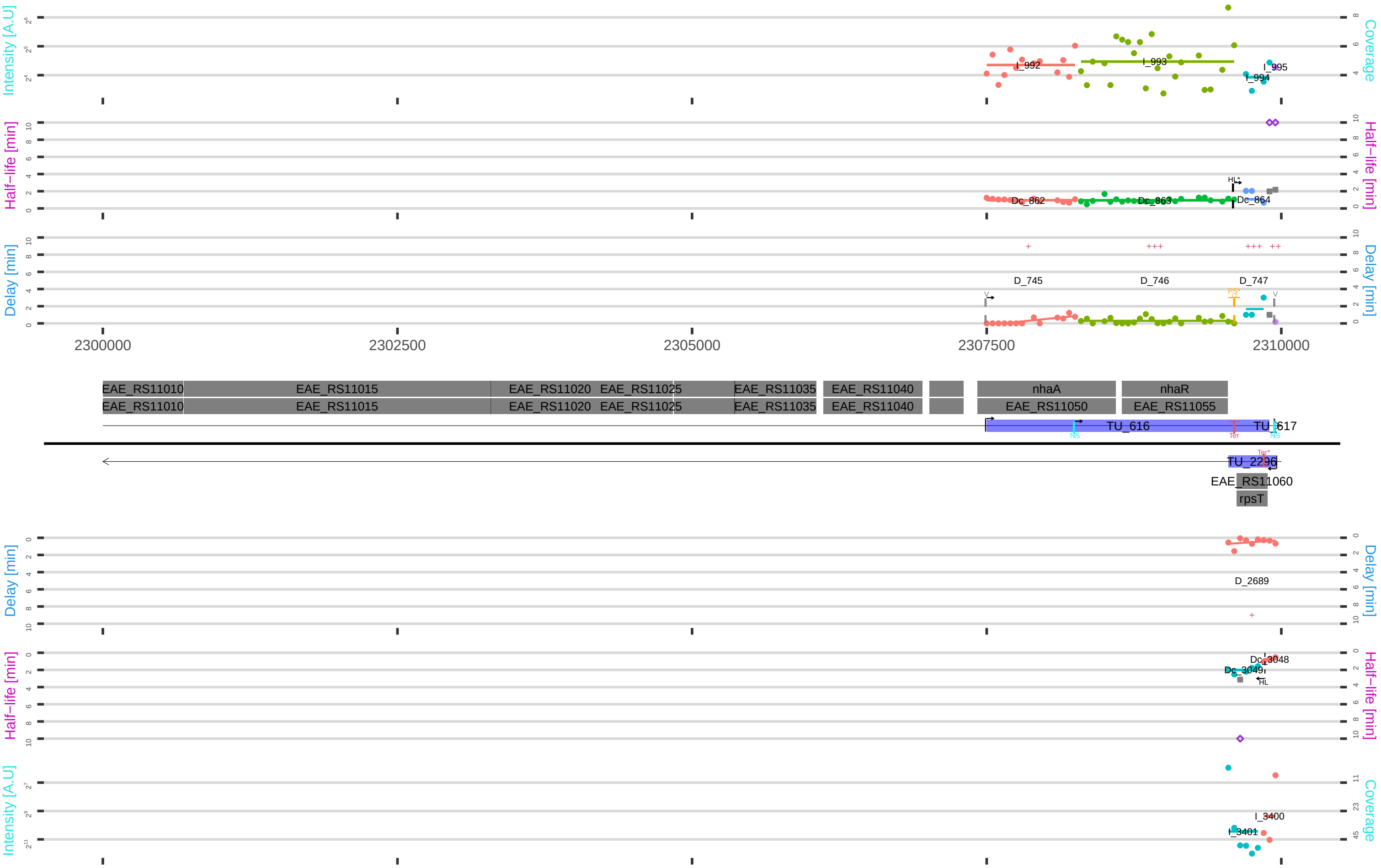

Term: termination (1), NS: new start (0), PS: pausing site (0), iTSS_I: internal starting site (0)

ID: 46201−46345; Term: termination (2), NS: new start (2), PS: pausing site (1), iTSS_I: internal starting site (0)

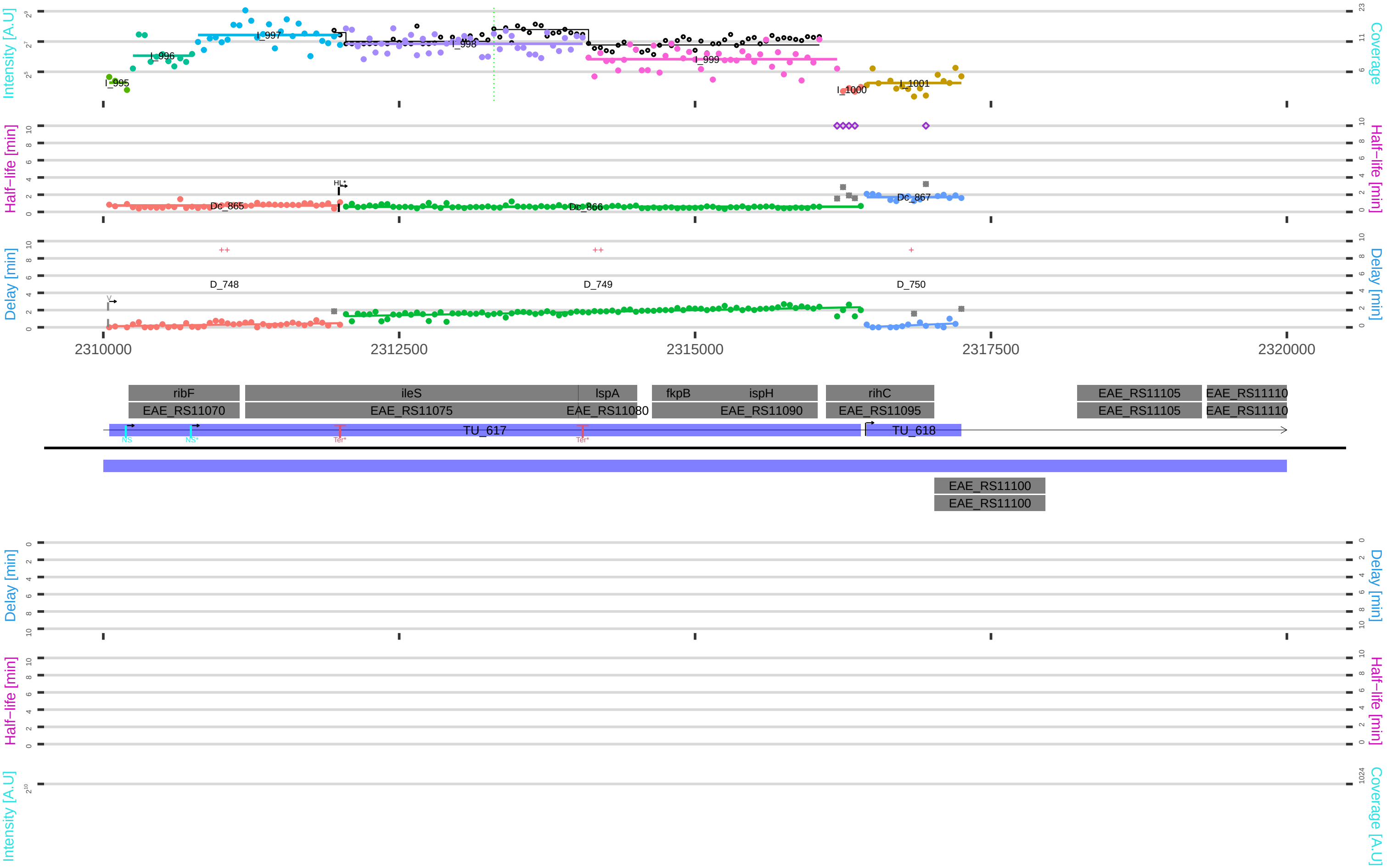

NA

ID: 46513−46540; Term: termination (0), NS: new start (0), PS: pausing site (0), iTSS_I: internal starting site (0)

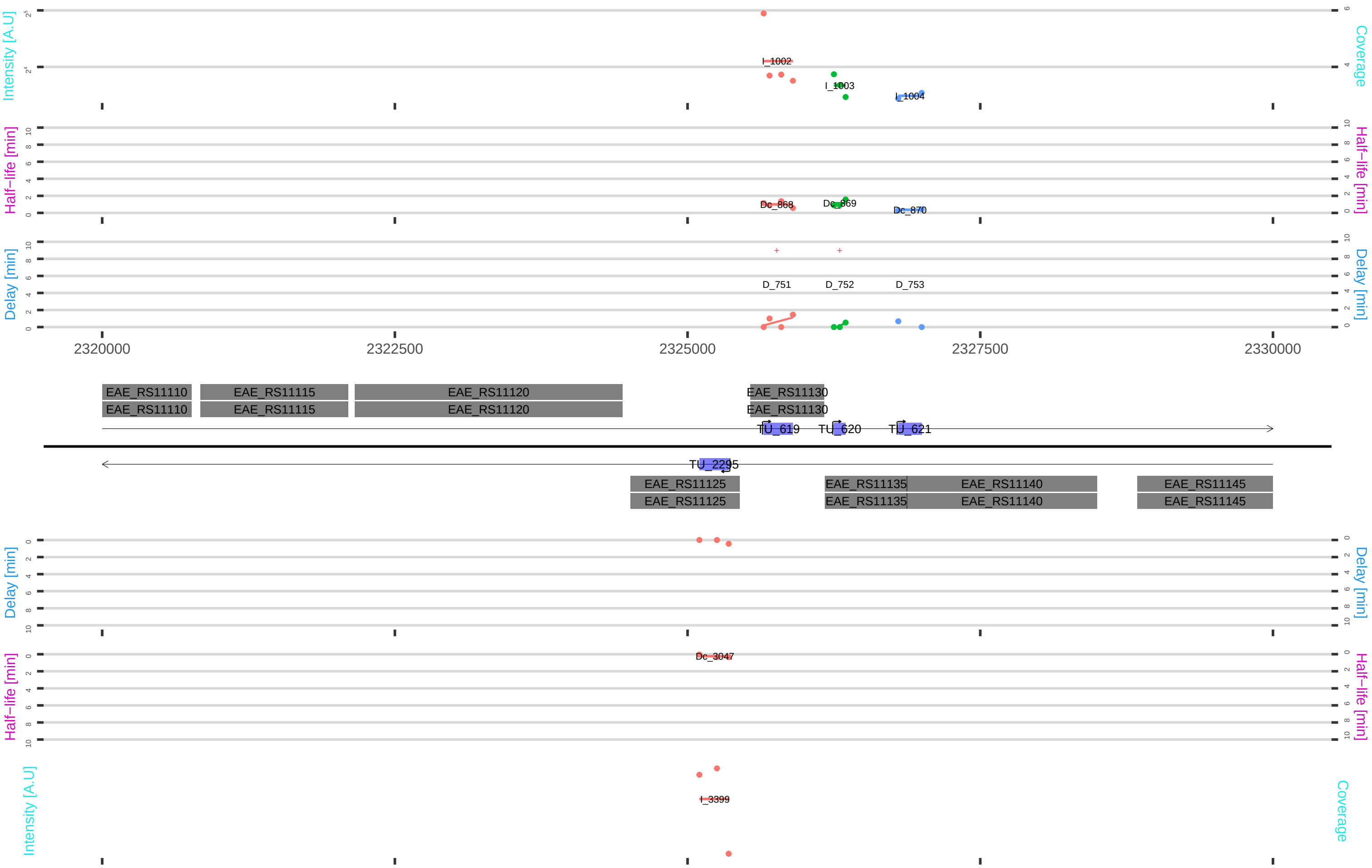

Term: termination (0), NS: new start (0), PS: pausing site (0), iTSS_I: internal starting site (0)

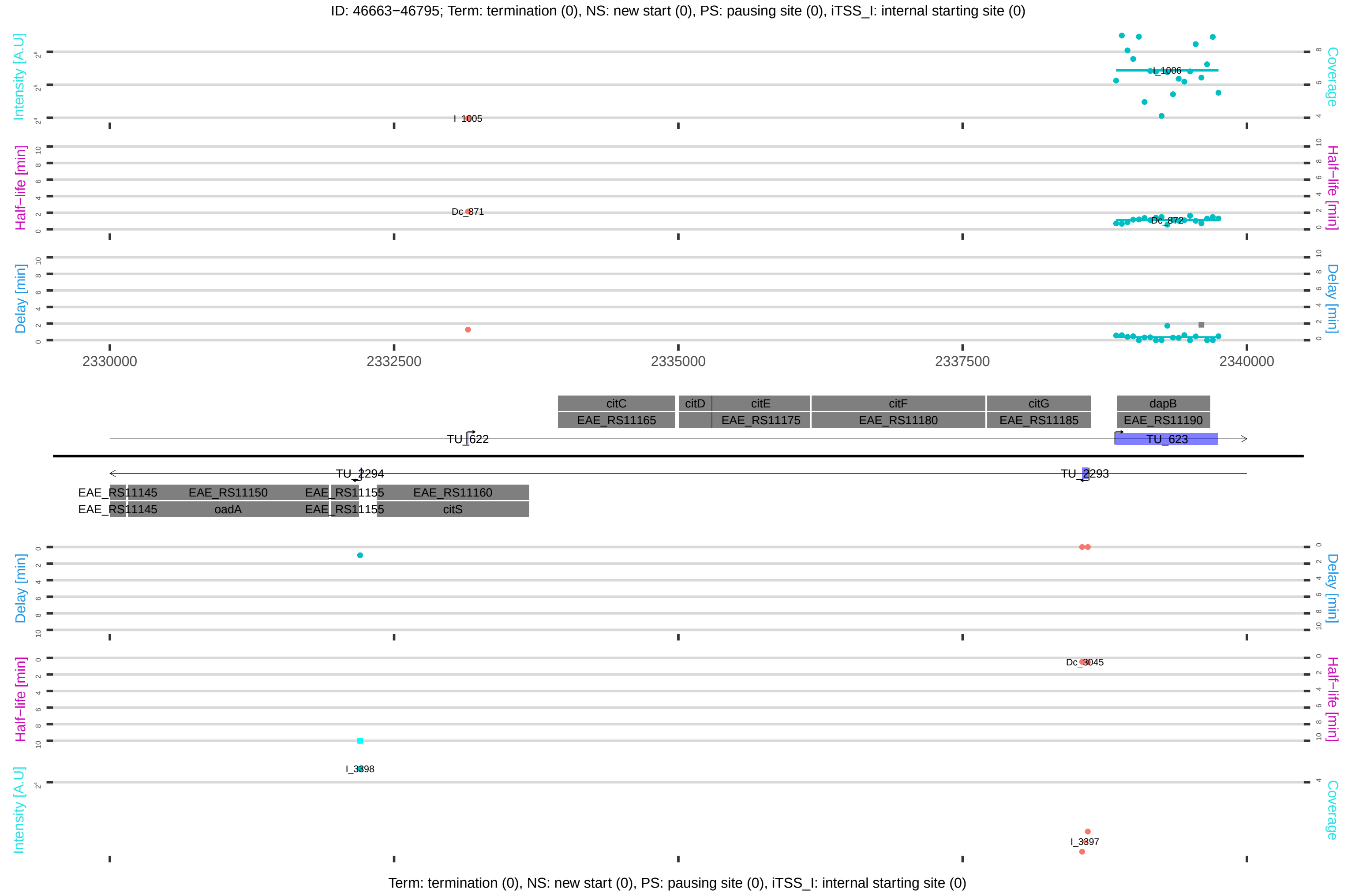
ID: 46663−46795; Term: termination (0), NS: new start (0), PS: pausing site (0), iTSS_I: internal starting site (0)
Intensity [A.U]
Coverage
I_1006
I_1005
Half−life [min]
Dc_871
Dc_872
Delay [min]
2330000
2332500
2335000
2337500
2340000
citC
citD
citE
citF
citG
dapB
EAE_RS11165
EAE_RS11175
EAE_RS11180
EAE_RS11185
EAE_RS11190
TU_622
TU_623
TU_2294
TU_2293
EAE_RS11145
EAE_RS11150
EAE_RS11155
EAE_RS11160
oadA
citS
Dc_3045
I_3398
I_3397
Term: termination (0), NS: new start (0), PS: pausing site (0), iTSS_I: internal starting site (0)

ID: 46803−47000; Term: termination (1), NS: new start (1), PS: pausing site (1), iTSS_I: internal starting site (0)

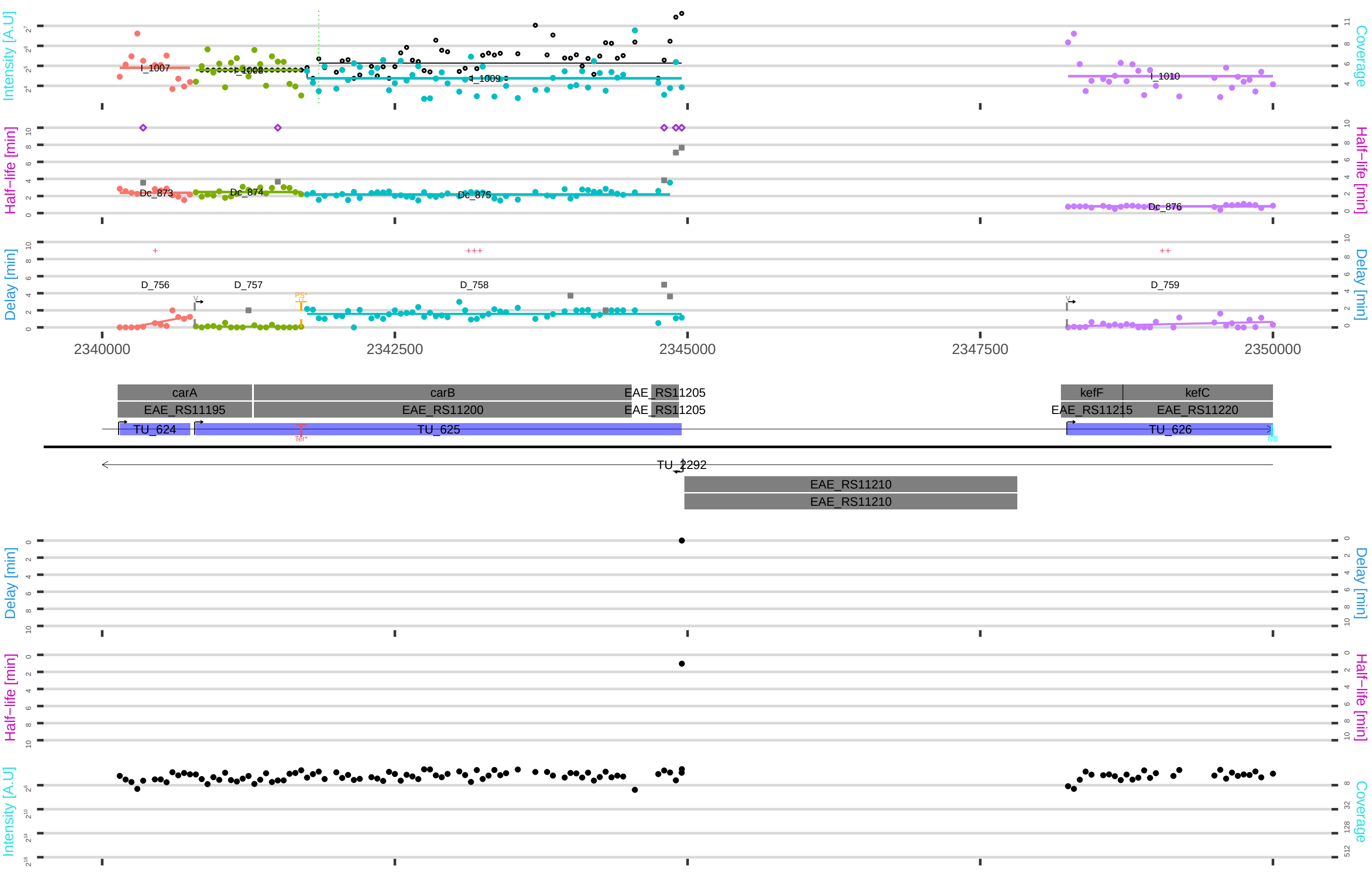

Term: termination (0), NS: new start (0), PS: pausing site (0), iTSS_I: internal starting site (0)

ID: 47000−47196; Term: termination (1), NS: new start (1), PS: pausing site (0), iTSS_I: internal starting site (1)

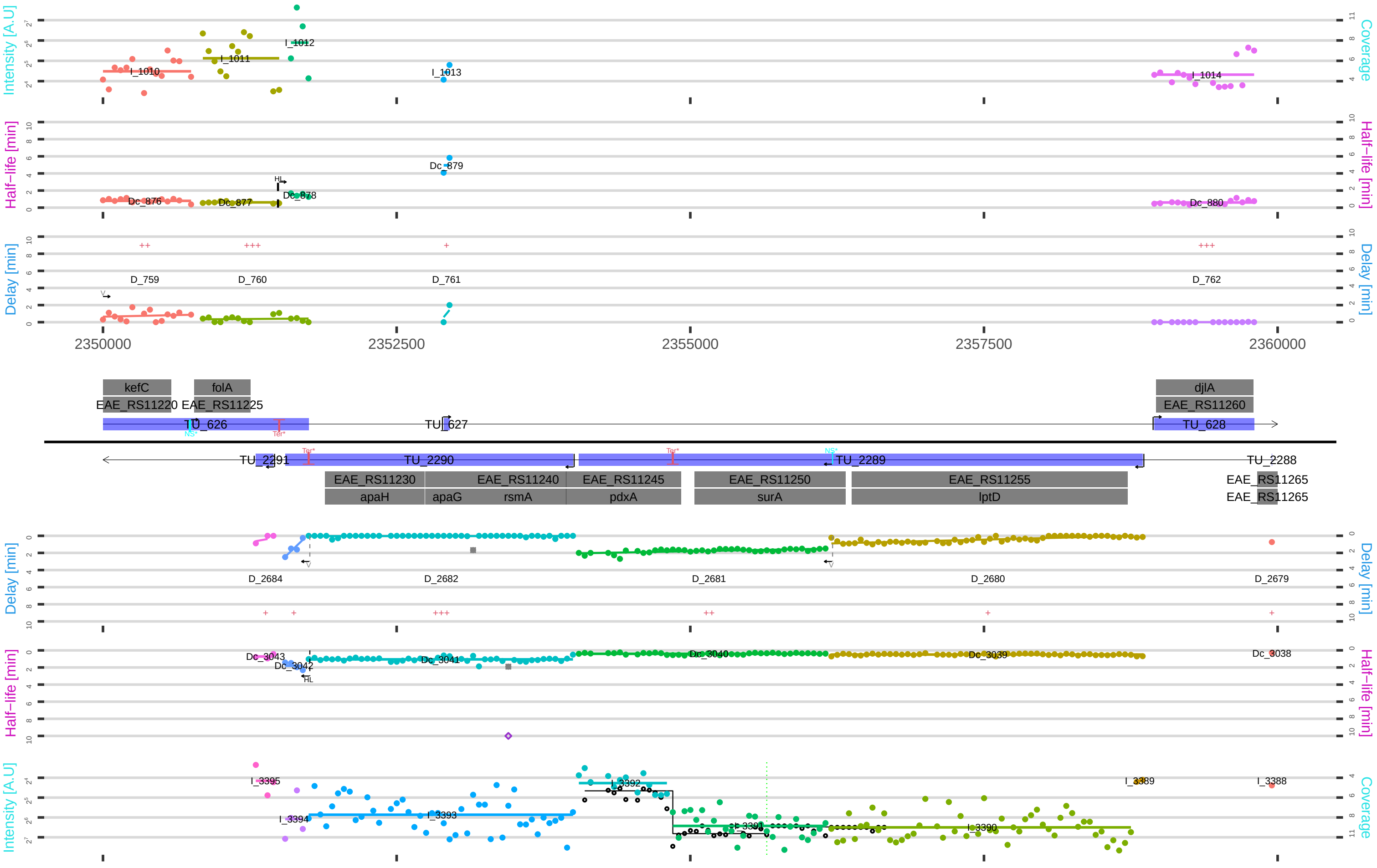

Term: termination (2), NS: new start (1), PS: pausing site (1), iTSS_I: internal starting site (1)

ID: 47279−47298; Term: termination (0), NS: new start (0), PS: pausing site (0), iTSS_I: internal starting site (0)

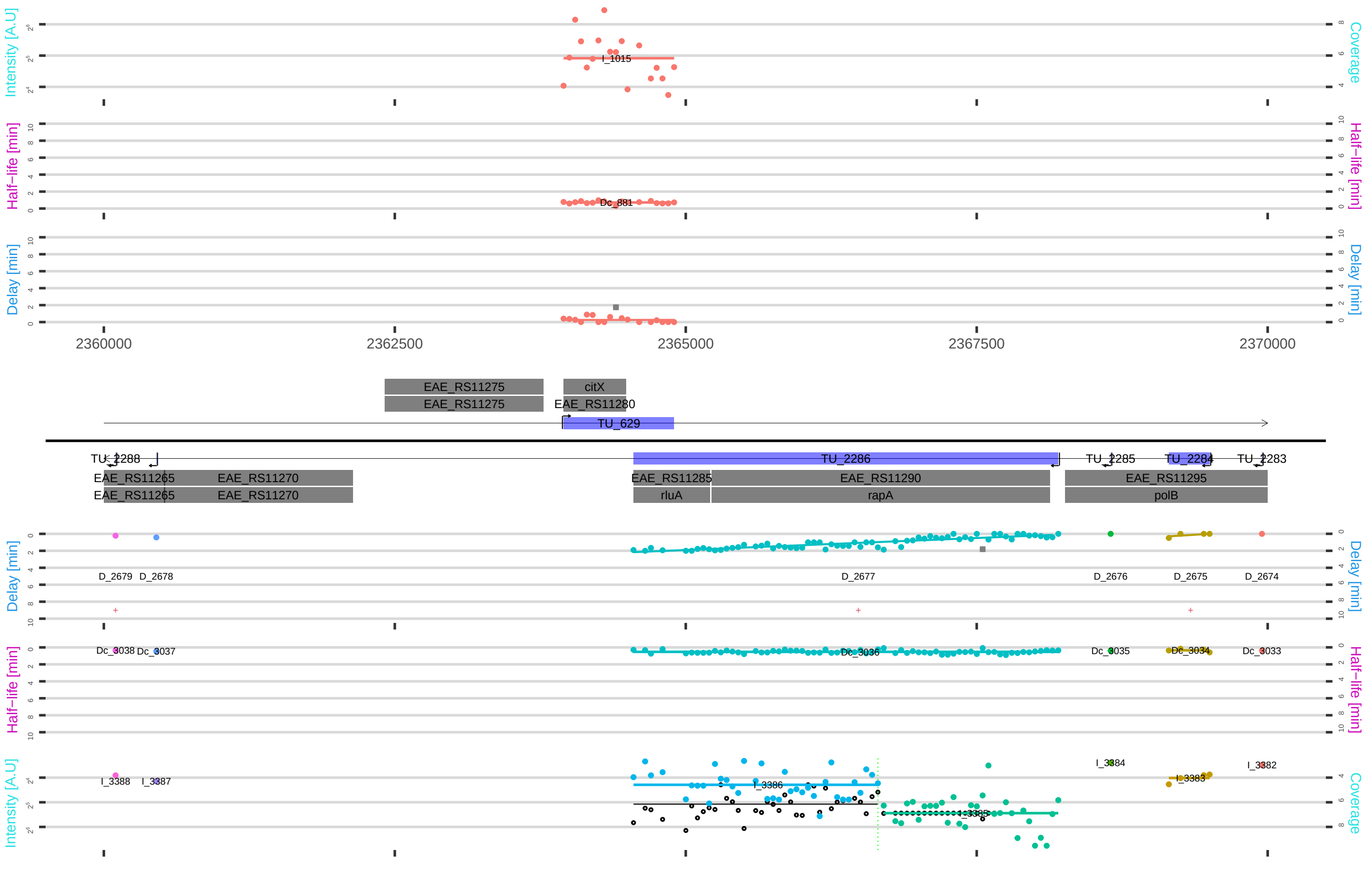

Term: termination (0), NS: new start (0), PS: pausing site (0), iTSS_I: internal starting site (0)

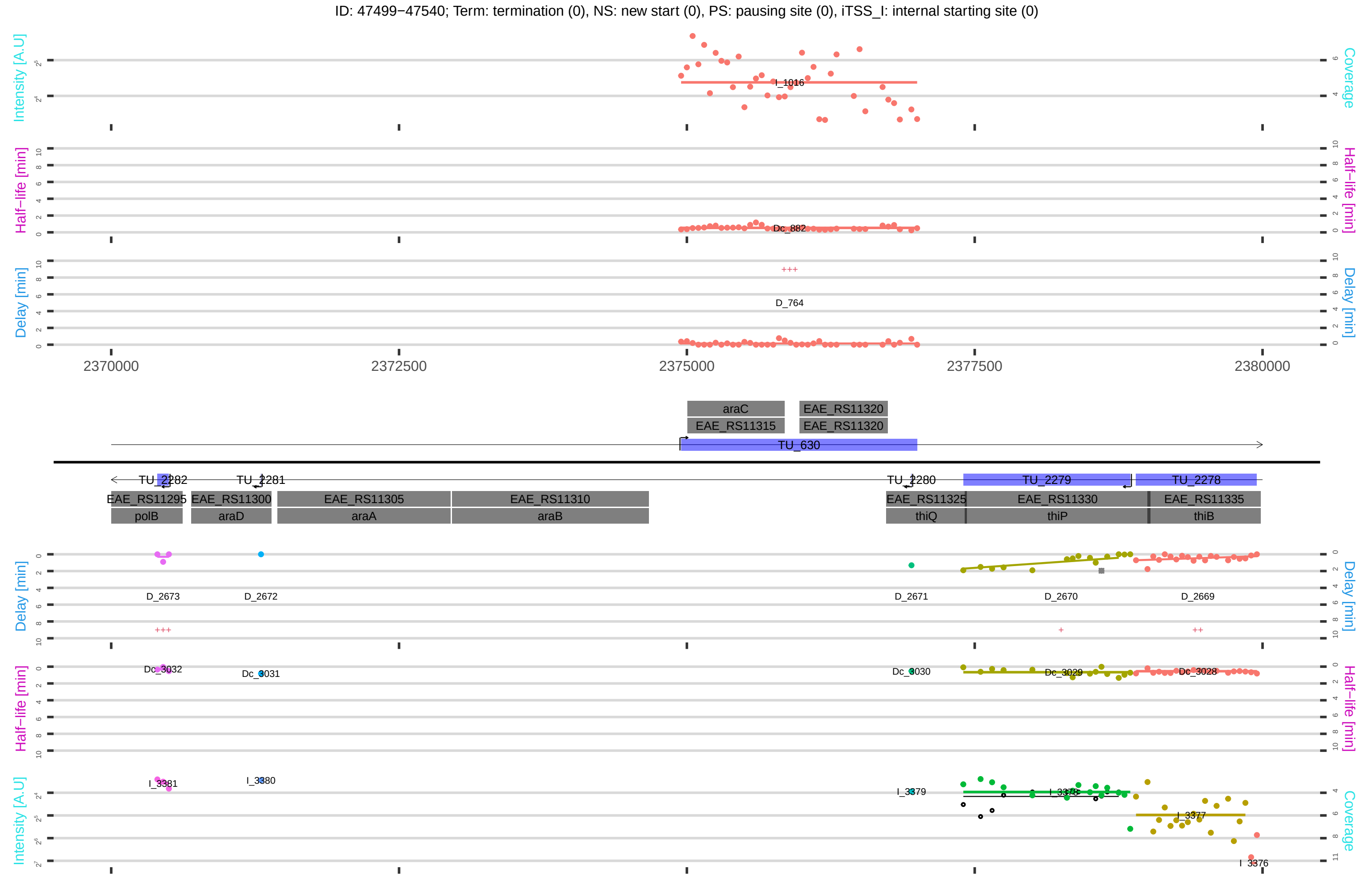

Term: termination (0), NS: new start (0), PS: pausing site (0), iTSS_I: internal starting site (0)

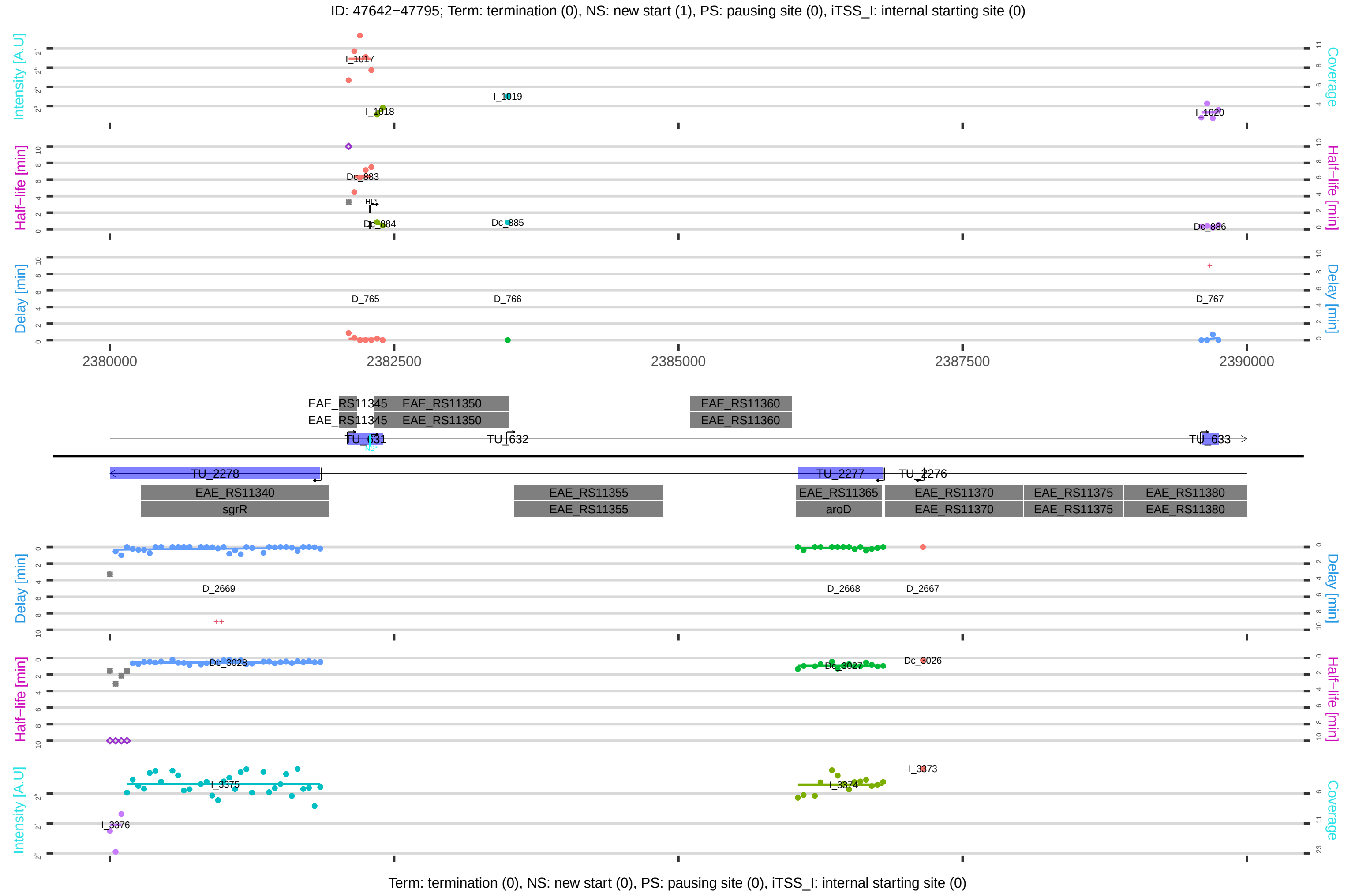
ID: 47642−47795; Term: termination (0), NS: new start (1), PS: pausing site (0), iTSS_I: internal starting site (0)
Intensity [A.U]
Coverage
I_1017
I_1018
I_1019
I_1020
Half−life [min]
Dc_883
Dc_884
Dc_885
Dc_886
HL*
Delay [min]
D_765
D_766
D_767
2380000
2382500
2385000
2387500
2390000
EAE_RS11345
EAE_RS11350
EAE_RS11360
TU_631
TU_632
TU_633
NS*
TU_2278
TU_2277
TU_2276
EAE_RS11340
sgrR
EAE_RS11355
EAE_RS11365
aroD
EAE_RS11370
EAE_RS11375
EAE_RS11380
D_2669
D_2668
D_2667
Dc_3028
Dc_3027
Dc_3026
I_3375
I_3376
I_3374
I_3373
Term: termination (0), NS: new start (0), PS: pausing site (0), iTSS_I: internal starting site (0)

ID: 47816−48000; Term: termination (0), NS: new start (0), PS: pausing site (0), iTSS_I: internal starting site (0)

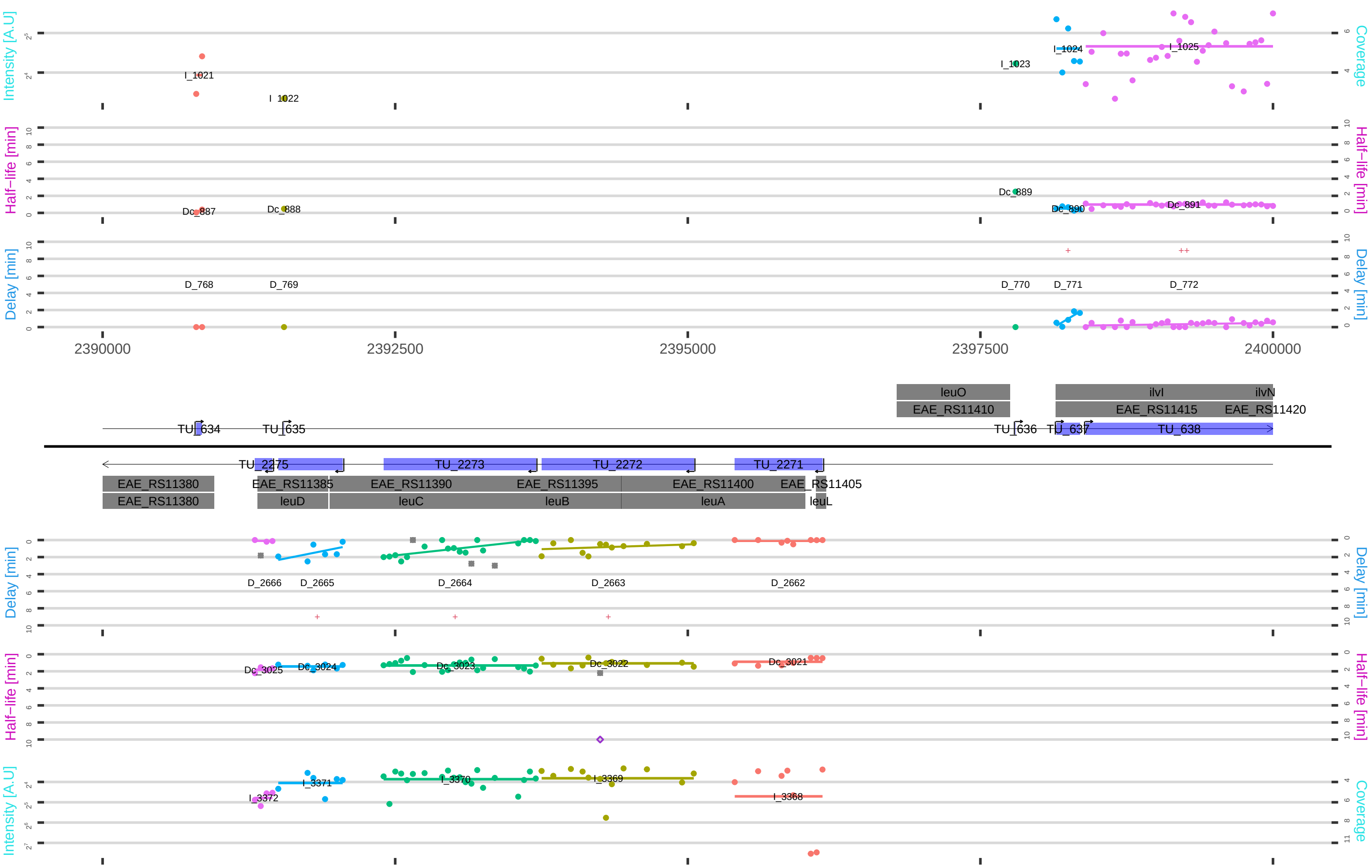

Term: termination (0), NS: new start (0), PS: pausing site (0), iTSS_I: internal starting site (0)

ID: 48000−48200; Term: termination (4), NS: new start (1), PS: pausing site (2), iTSS_I: internal starting site (1)

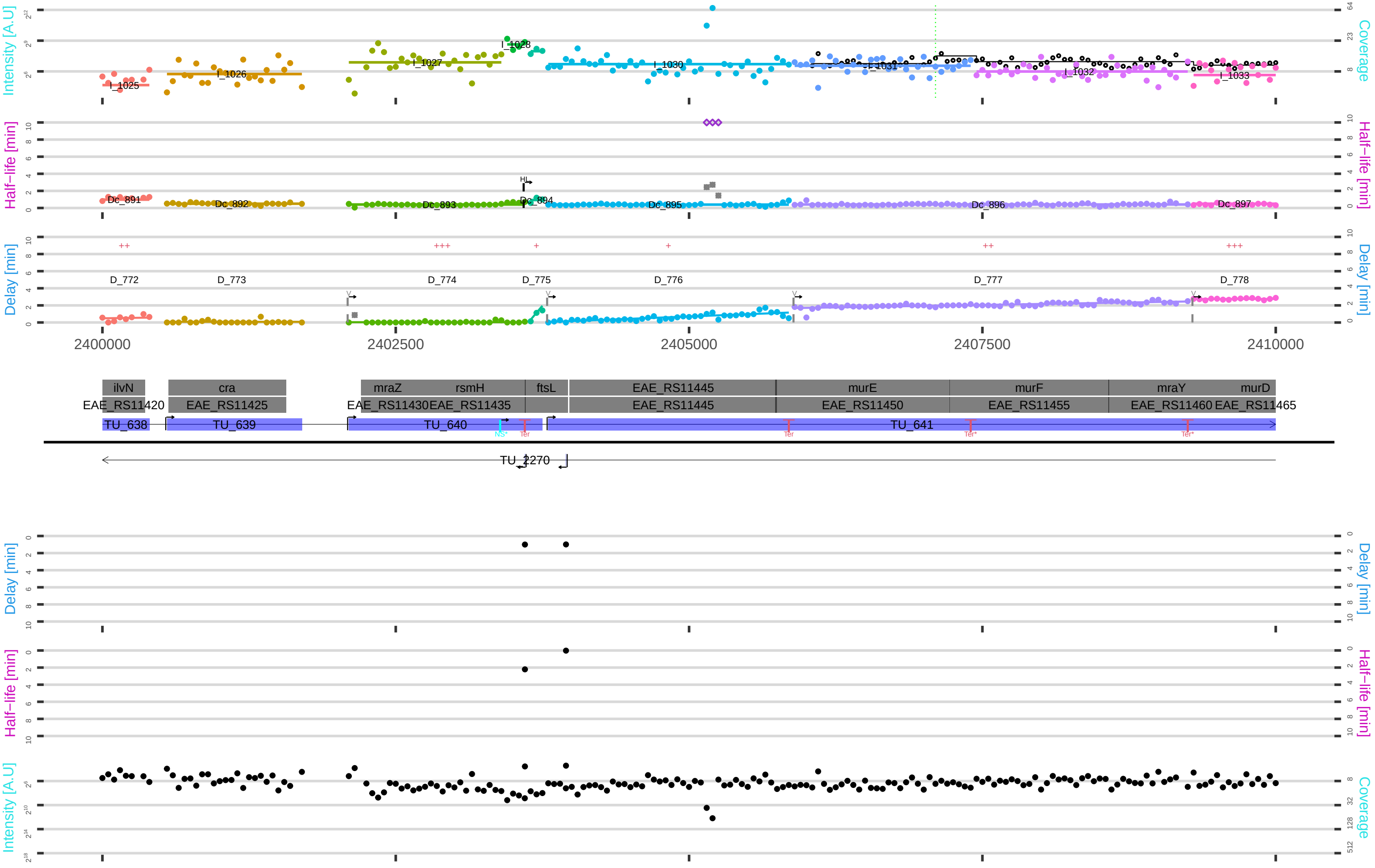

Term: termination (0), NS: new start (0), PS: pausing site (0), iTSS_I: internal starting site (0)

ID: 48200−48400; Term: termination (3), NS: new start (3), PS: pausing site (2), iTSS_I: internal starting site (2)

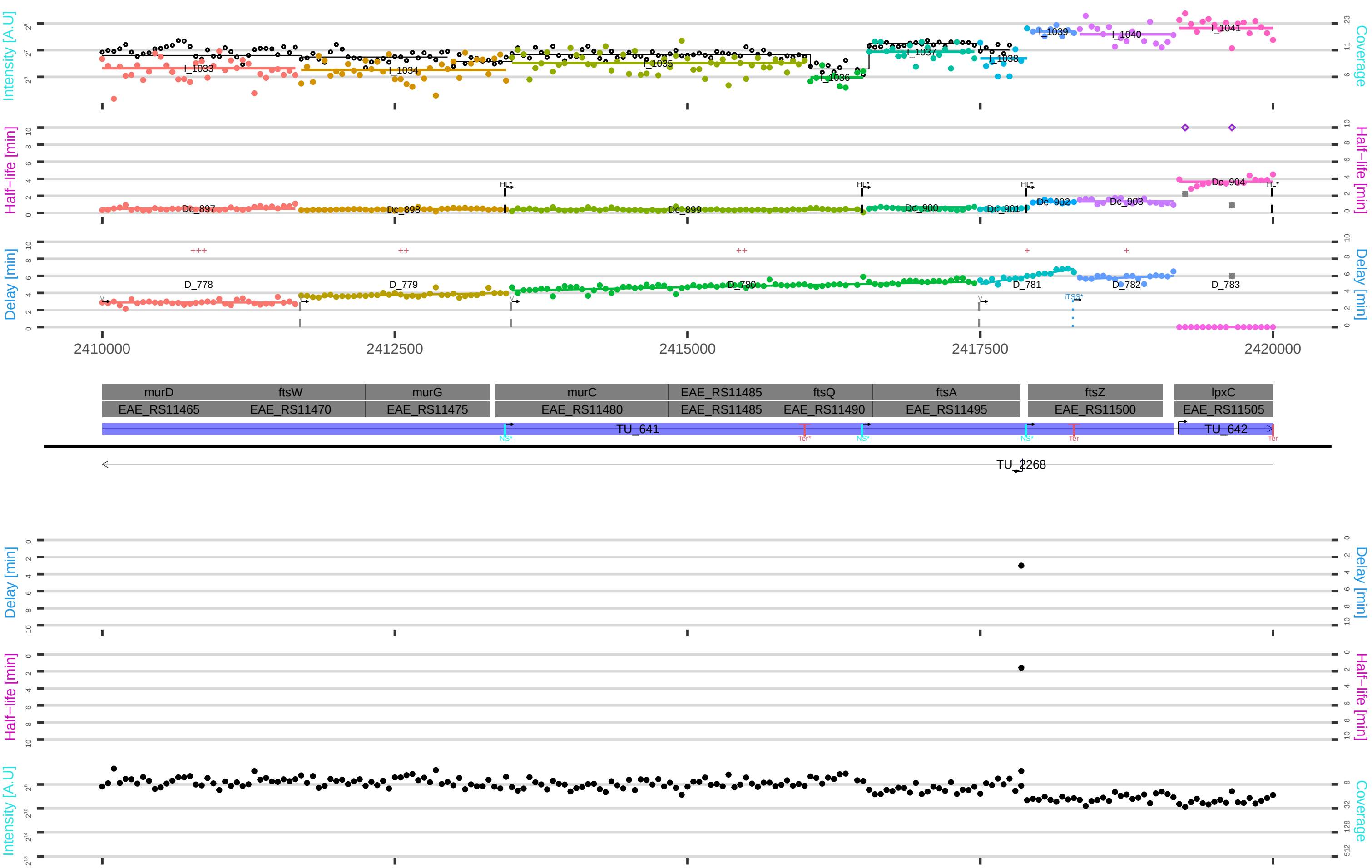

Term: termination (0), NS: new start (0), PS: pausing site (0), iTSS_I: internal starting site (0)

ID: 48400−48546; Term: termination (1), NS: new start (1), PS: pausing site (1), iTSS_I: internal starting site (0)

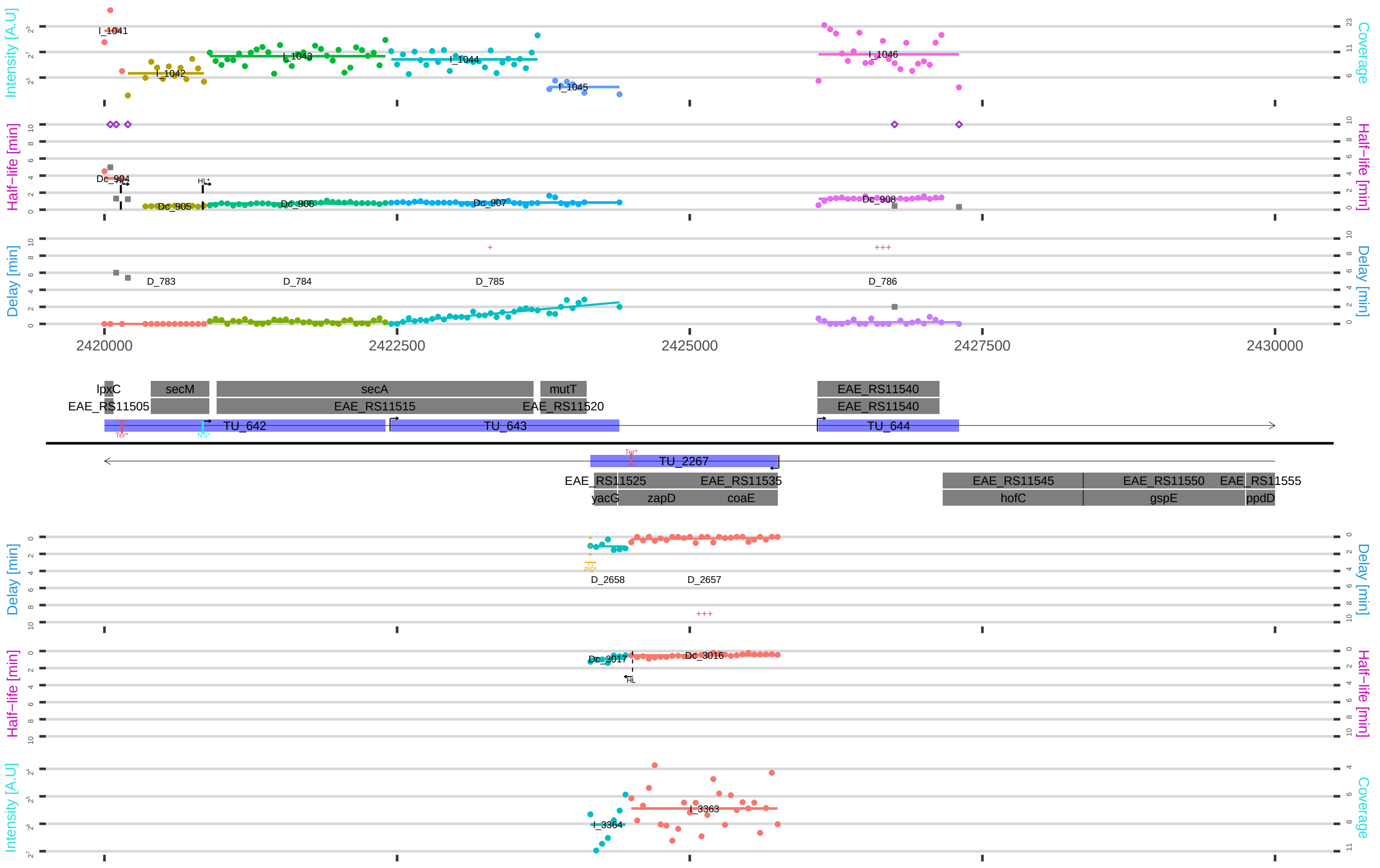

Term: termination (1), NS: new start (0), PS: pausing site (1), iTSS_I: internal starting site (0)

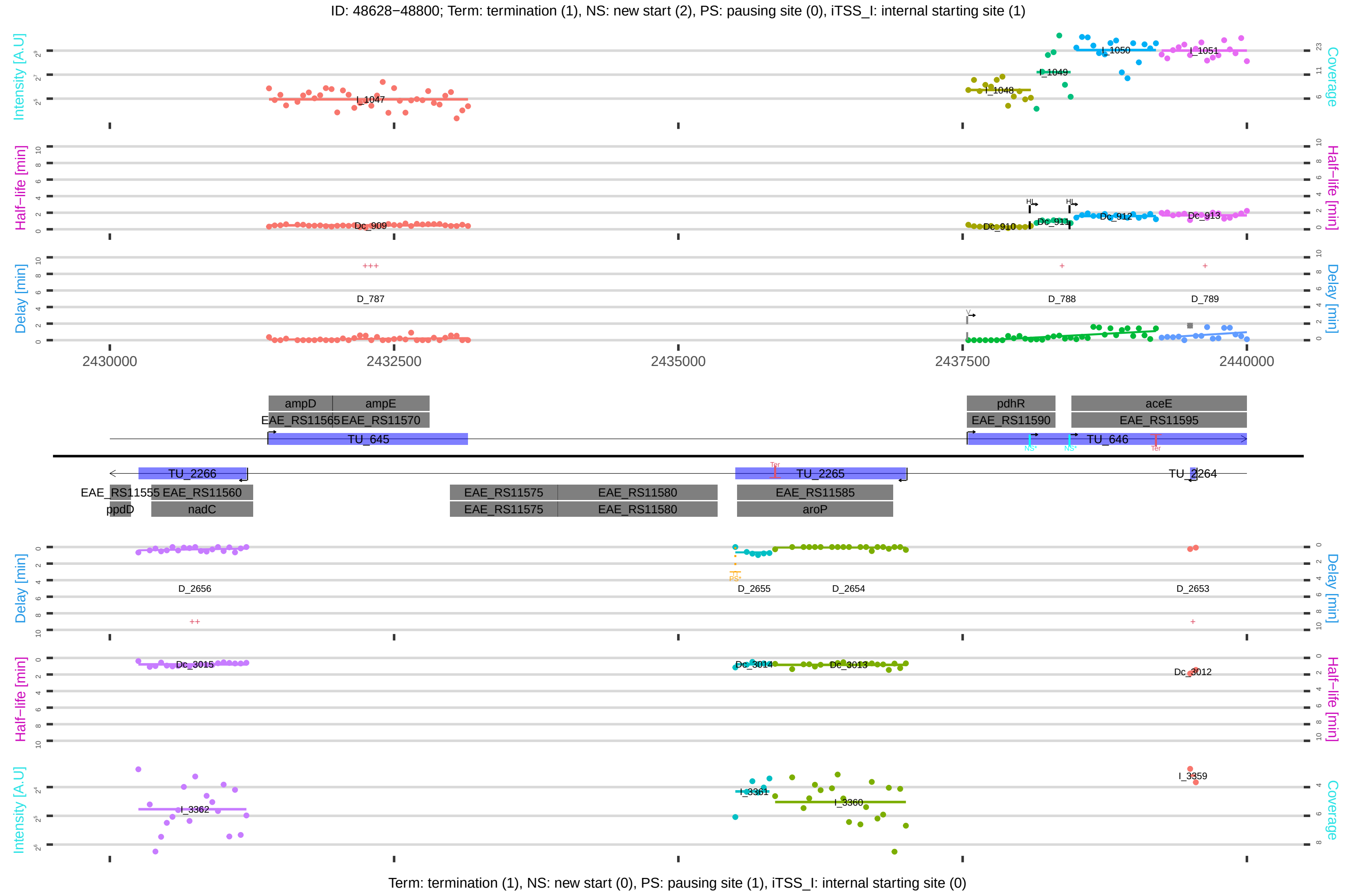

ID: 48628−48800; Term: termination (1), NS: new start (2), PS: pausing site (0), iTSS_I: internal starting site (1)
Intensity [A.U]
Coverage
Half−life [min]
Delay [min]
I_1047
I_1048
I_1049
I_1050
I_1051
Dc_909
Dc_910
Dc_911
Dc_912
Dc_913
D_787
D_788
D_789
2430000
2432500
2435000
2437500
2440000
ampD
ampE
EAE_RS11565
EAE_RS11570
TU_645
pdhR
EAE_RS11590
aceE
EAE_RS11595
TU_646
TU_2266
TU_2265
TU_2264
EAE_RS11555
EAE_RS11560
ppdD
nadC
EAE_RS11575
EAE_RS11580
EAE_RS11585
aroP
D_2656
D_2655
D_2654
D_2653
Dc_3015
Dc_3014
Dc_3013
Dc_3012
I_3362
I_3361
I_3360
I_3359
Term: termination (1), NS: new start (0), PS: pausing site (1), iTSS_I: internal starting site (0)

ID: 48800−49000; Term: termination (3), NS: new start (1), PS: pausing site (3), iTSS_I: internal starting site (0)

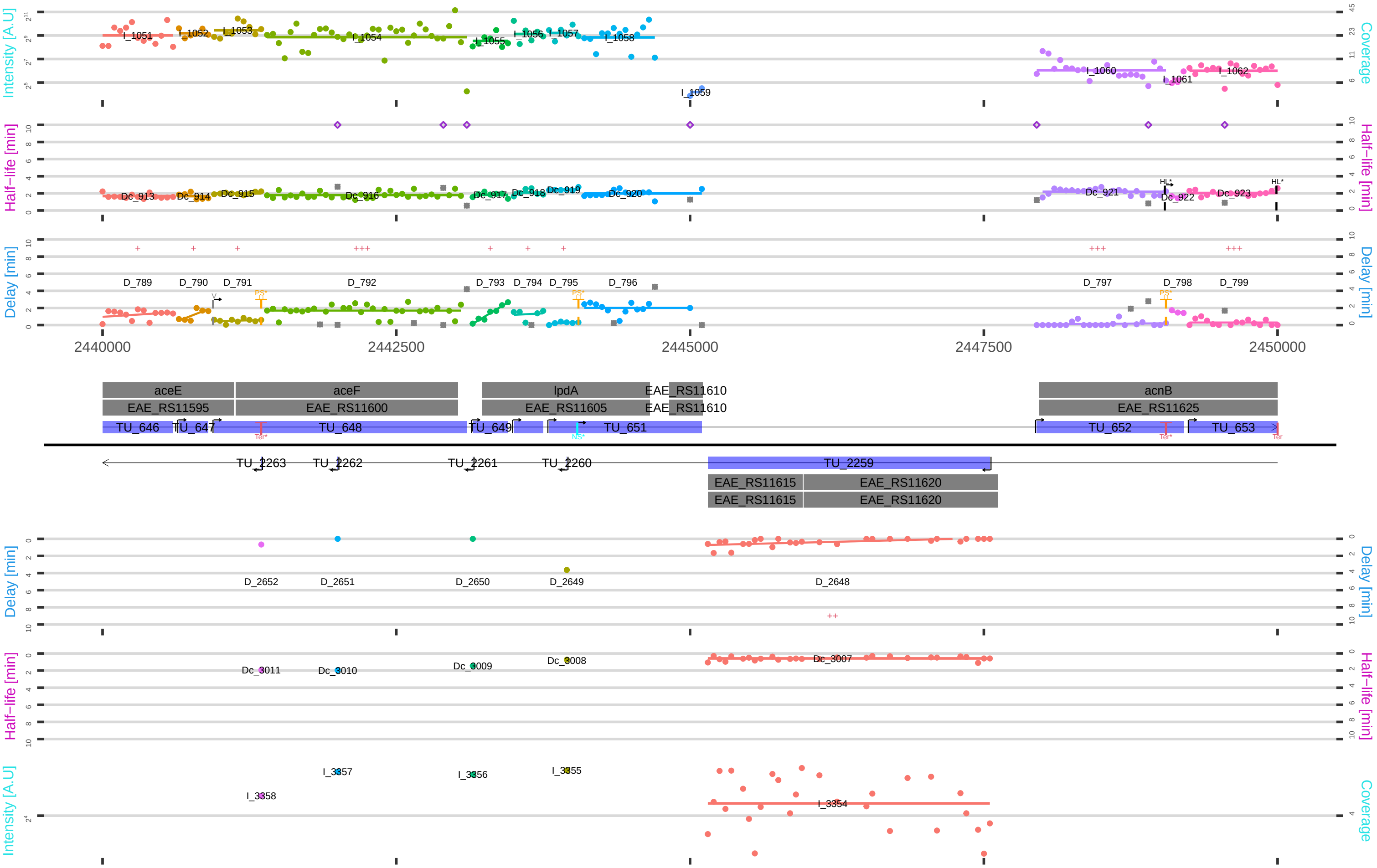

Term: termination (0), NS: new start (0), PS: pausing site (0), iTSS_I: internal starting site (0)

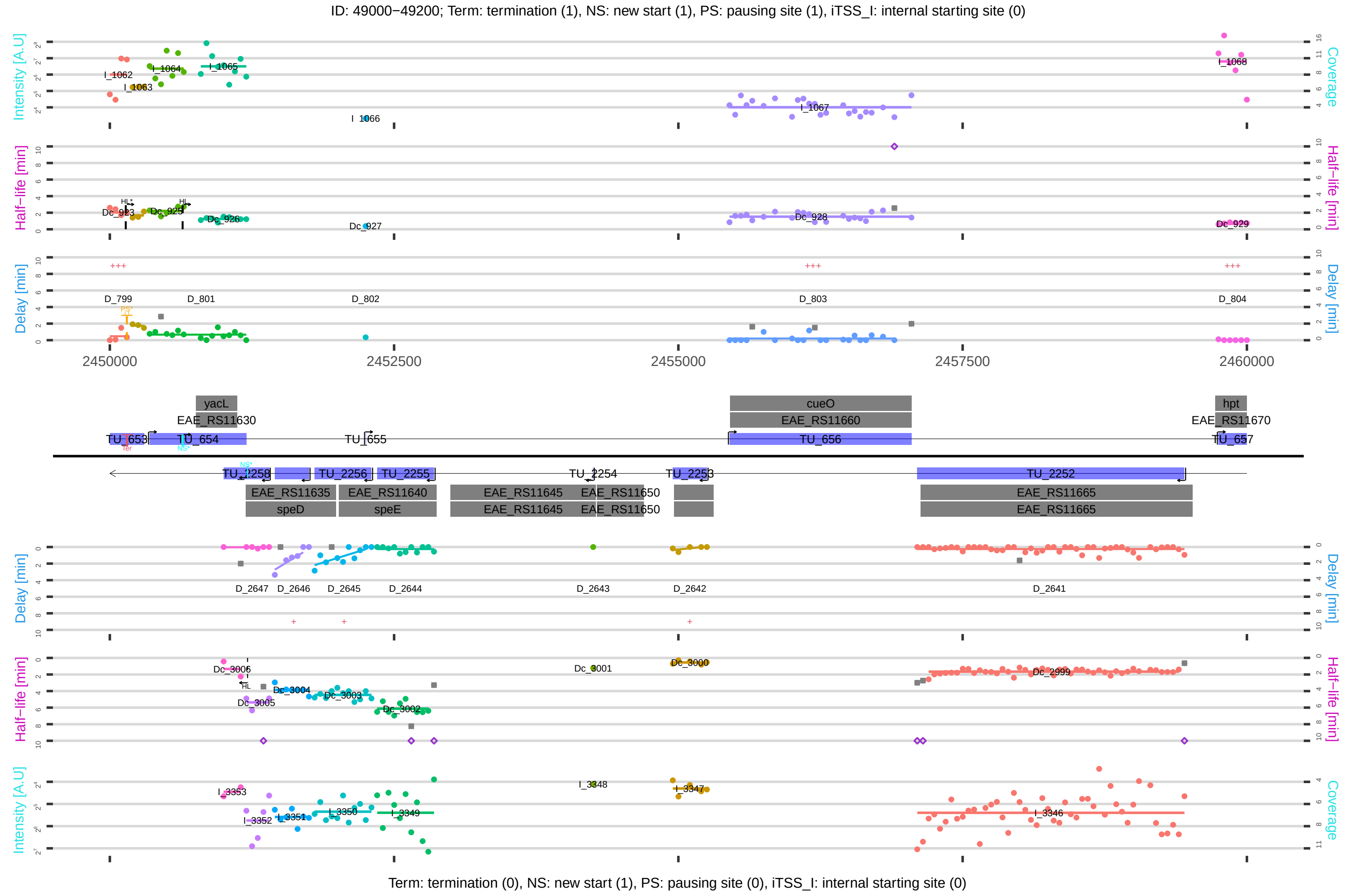

ID: 49000−49200; Term: termination (1), NS: new start (1), PS: pausing site (1), iTSS_I: internal starting site (0)
Intensity [A.U]
Coverage
Half−life [min]
Delay [min]
I_1062
I_1063
I_1064
I_1065
I_1066
I_1067
I_1068
Dc_923
Dc_925
Dc_926
Dc_927
Dc_928
Dc_929
D_799
D_801
D_802
D_803
D_804
2450000
2452500
2455000
2457500
2460000
yacL
EAE_RS11630
cueO
EAE_RS11660
hpt
EAE_RS11670
TU_653
TU_654
TU_655
TU_656
TU_657
TU_2258
TU_2256
TU_2255
TU_2254
TU_2253
TU_2252
EAE_RS11635
EAE_RS11640
EAE_RS11645
EAE_RS11650
EAE_RS11665
speD
speE
D_2647
D_2646
D_2645
D_2644
D_2643
D_2642
D_2641
Dc_3006
Dc_3005
Dc_3004
Dc_3003
Dc_3002
Dc_3001
Dc_3000
Dc_2999
I_3353
I_3352
I_3351
I_3350
I_3349
I_3348
I_3347
I_3346
Term: termination (0), NS: new start (1), PS: pausing site (0), iTSS_I: internal starting site (0)

ID: 49200−49302; Term: termination (0), NS: new start (0), PS: pausing site (0), iTSS_I: internal starting site (0)

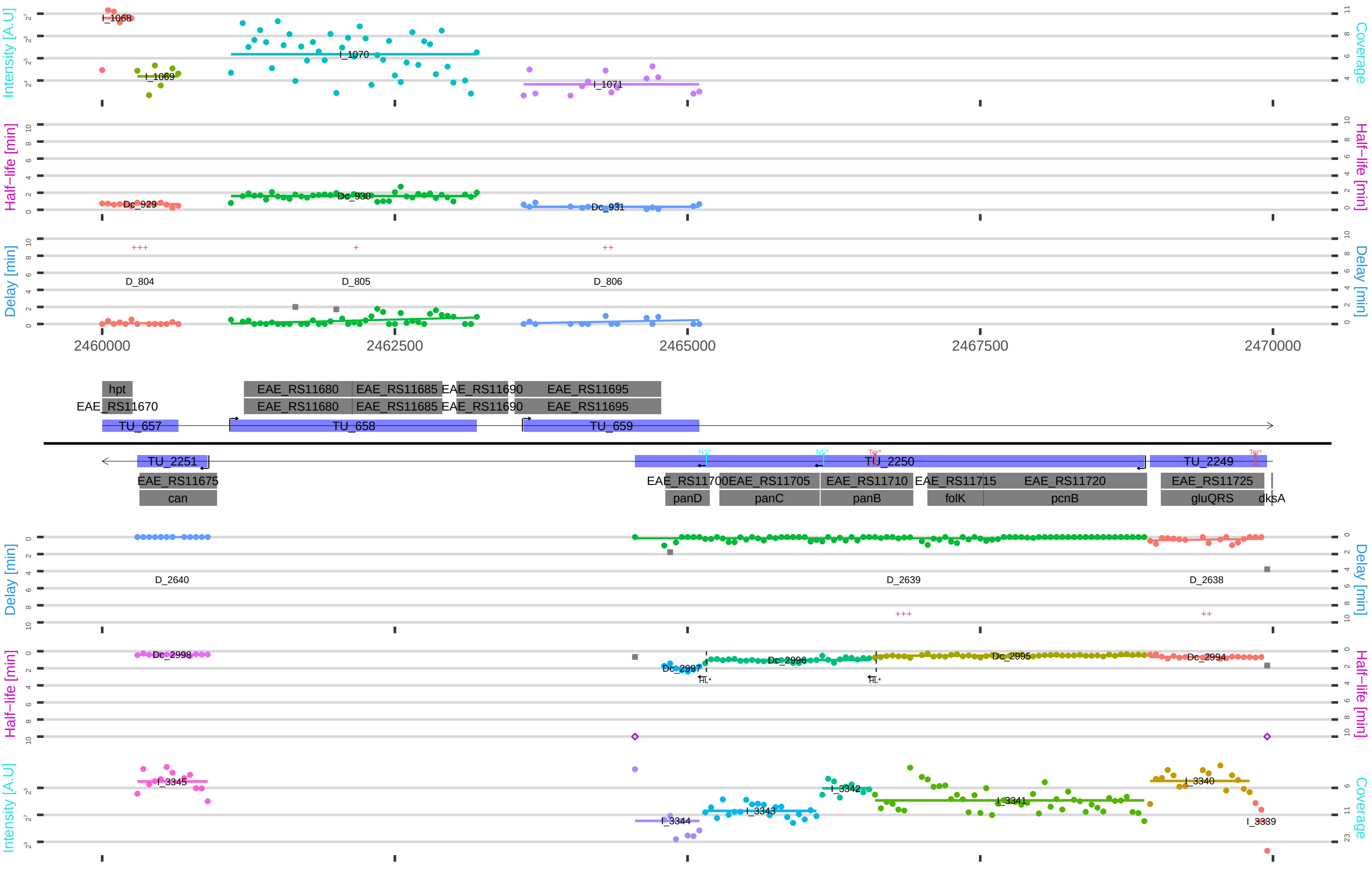

Term: termination (2), NS: new start (2), PS: pausing site (0), iTSS_I: internal starting site (0)

ID: 49409−49600; Term: termination (0), NS: new start (0), PS: pausing site (0), iTSS_I: internal starting site (0)

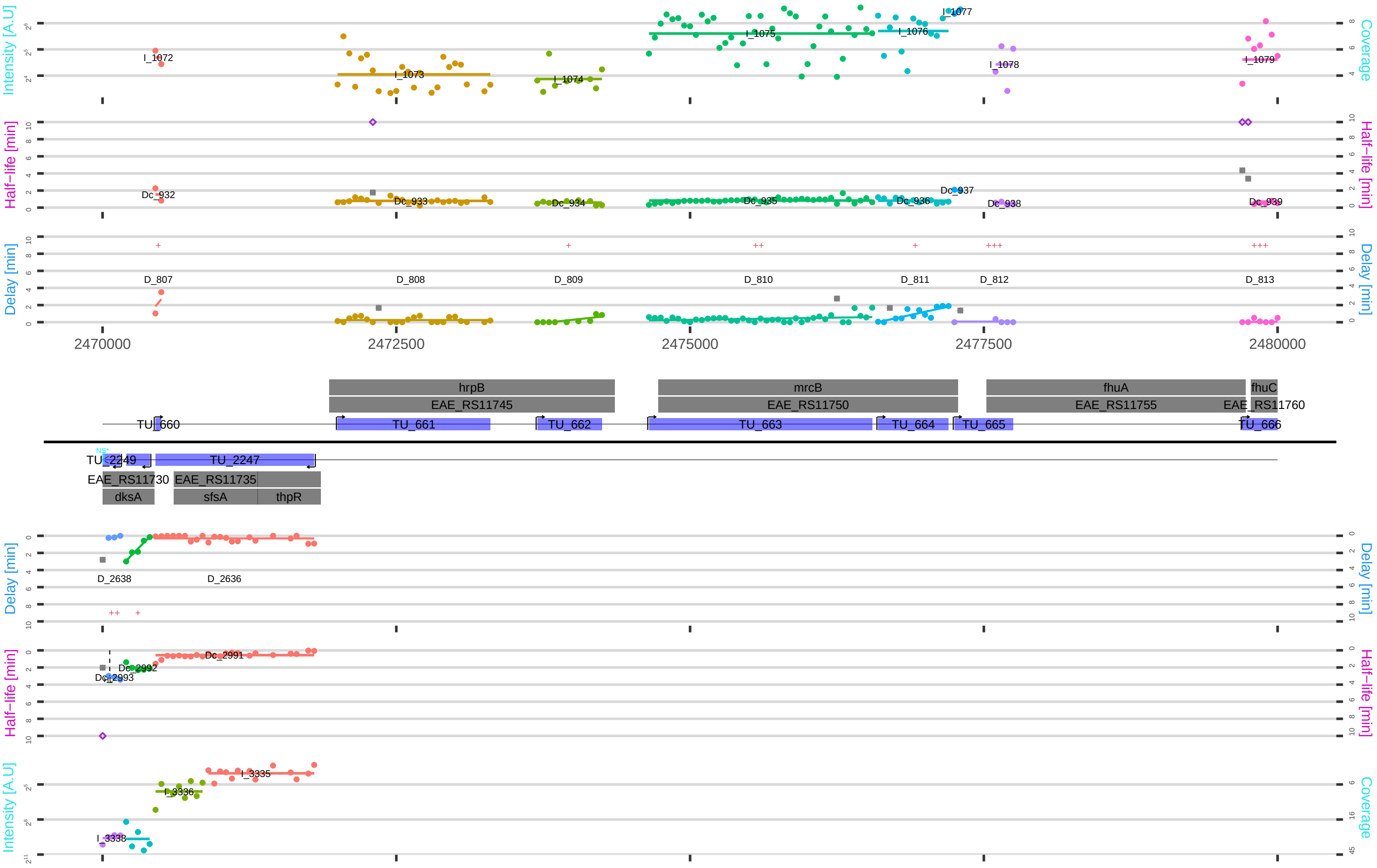

Term: termination (0), NS: new start (1), PS: pausing site (0), iTSS_I: internal starting site (0)

ID: 49600−49796; Term: termination (1), NS: new start (1), PS: pausing site (0), iTSS_I: internal starting site (0)

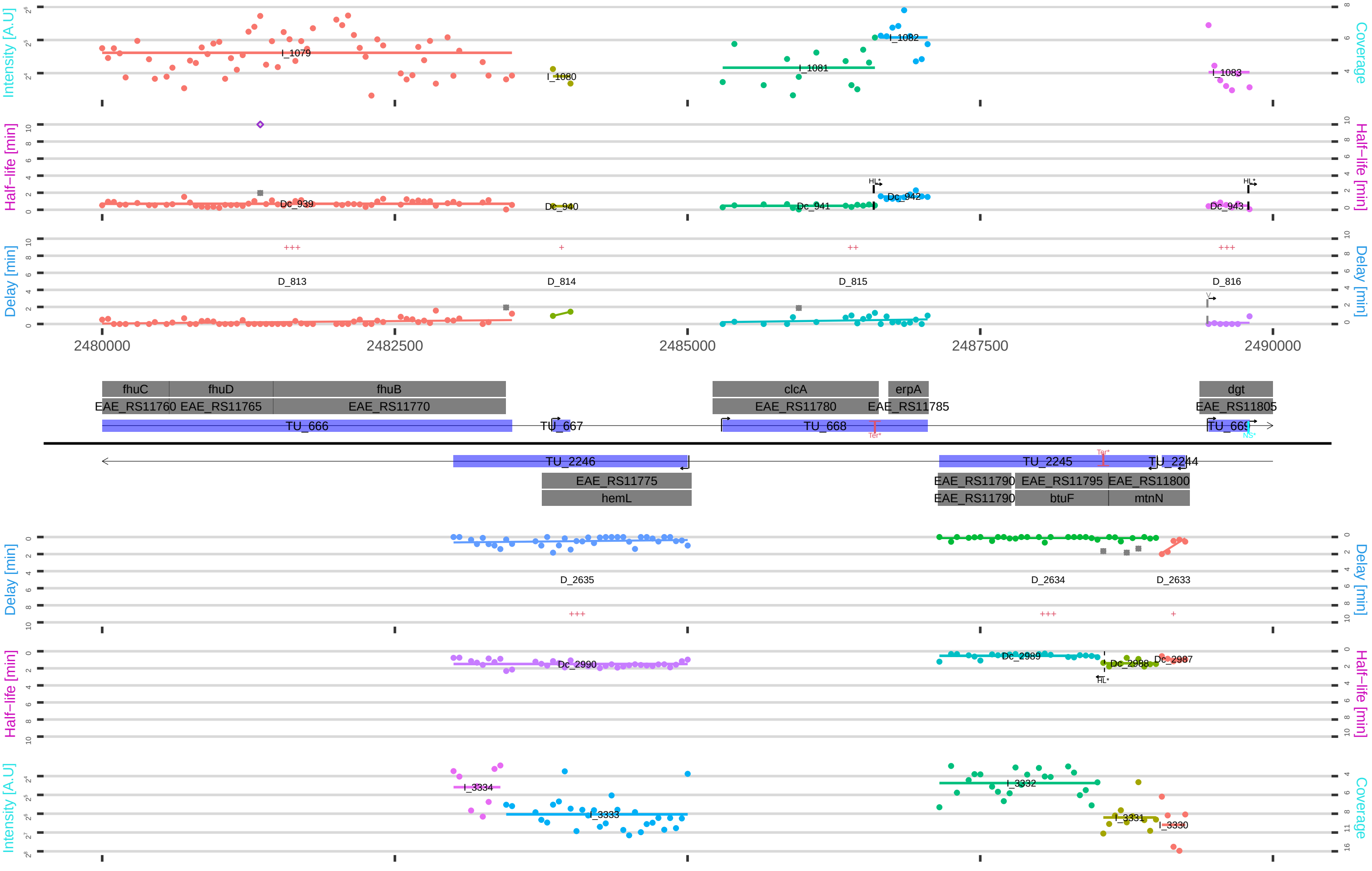

Term: termination (1), NS: new start (0), PS: pausing site (0), iTSS_I: internal starting site (0)

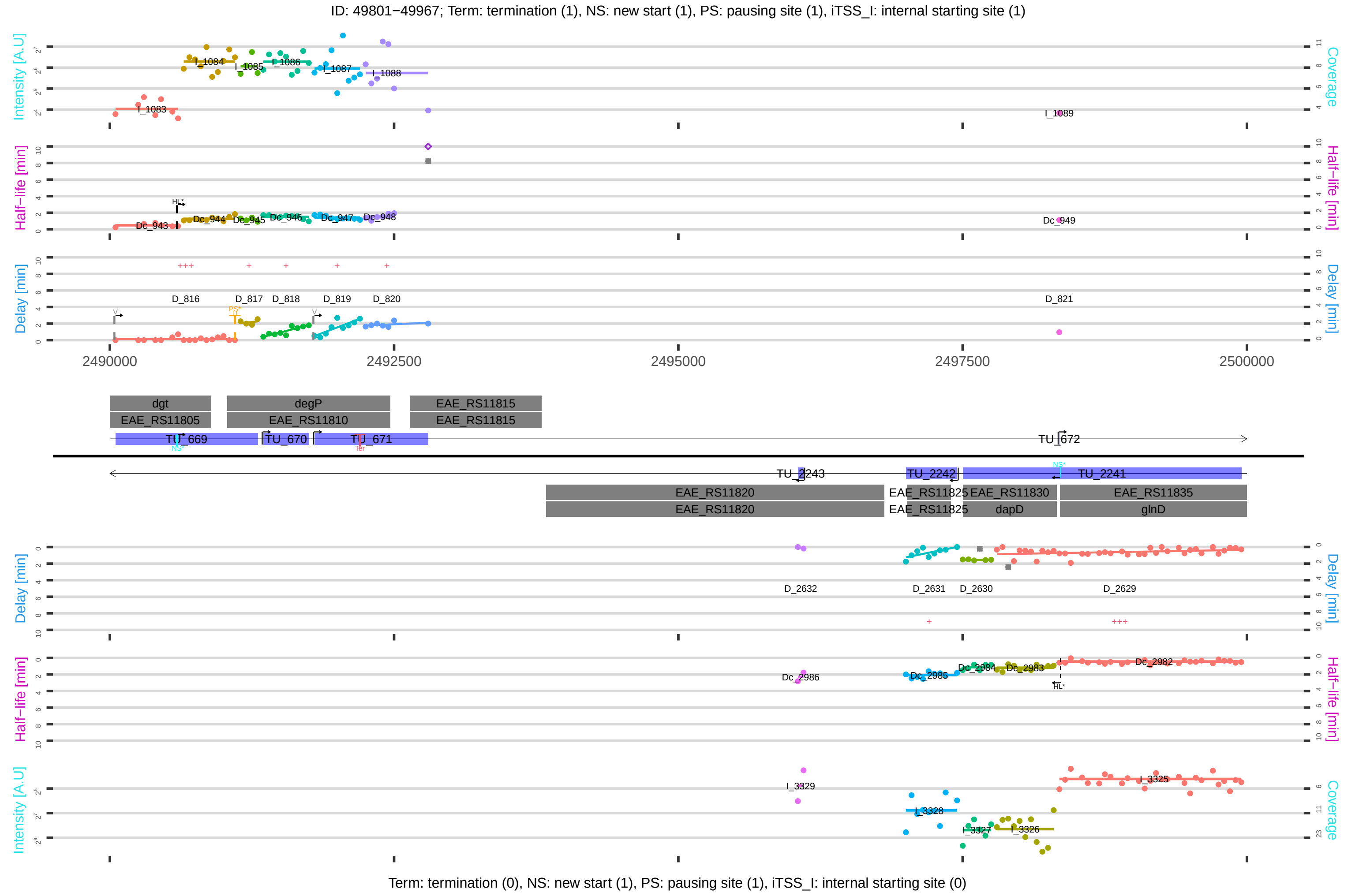
ID: 49801−49967; Term: termination (1), NS: new start (1), PS: pausing site (1), iTSS_I: internal starting site (1)
Intensity [A.U]
Coverage
I_1083
I_1084
I_1085
I_1086
I_1087
I_1088
I_1089
Half−life [min]
Dc_943
Dc_944
Dc_945
Dc_946
Dc_947
Dc_948
Dc_949
Delay [min]
D_816
D_817
D_818
D_819
D_820
D_821
2490000
2492500
2495000
2497500
2500000
dgt
EAE_RS11805
degP
EAE_RS11810
EAE_RS11815
EAE_RS11815
TU_669
TU_670
TU_671
TU_672
TU_2243
TU_2242
TU_2241
EAE_RS11820
EAE_RS11820
EAE_RS11825
EAE_RS11825
EAE_RS11830
dapD
EAE_RS11835
glnD
D_2632
D_2631
D_2630
D_2629
Dc_2986
Dc_2985
Dc_2984
Dc_2983
Dc_2982
I_3329
I_3328
I_3327
I_3326
I_3325
Term: termination (0), NS: new start (1), PS: pausing site (1), iTSS_I: internal starting site (0)

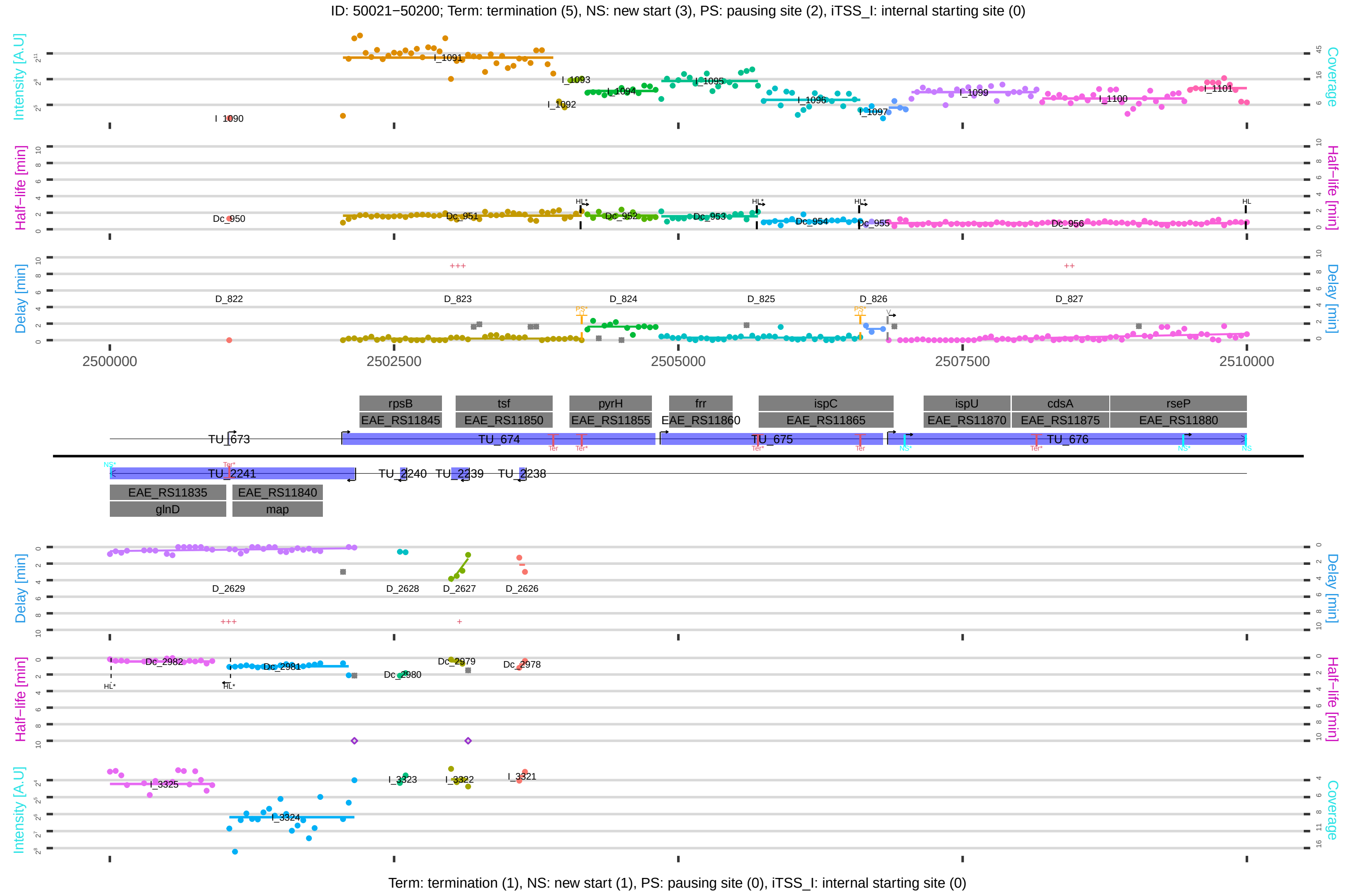

ID: 50021−50200; Term: termination (5), NS: new start (3), PS: pausing site (2), iTSS_I: internal starting site (0)
Intensity [A.U]
Coverage
Half−life [min]
Delay [min]
I_1090
I_1091
I_1092
I_1093
I_1094
I_1095
I_1096
I_1097
I_1099
I_1100
I_1101
Dc_950
Dc_951
Dc_952
Dc_953
Dc_954
Dc_955
Dc_956
D_822
D_823
D_824
D_825
D_826
D_827
2500000
2502500
2505000
2507500
2510000
rpsB
EAE_RS11845
tsf
EAE_RS11850
pyrH
EAE_RS11855
frr
EAE_RS11860
ispC
EAE_RS11865
ispU
EAE_RS11870
cdsA
EAE_RS11875
rseP
EAE_RS11880
TU_673
TU_674
TU_675
TU_676
TU_2241
TU_2240
TU_2239
TU_2238
EAE_RS11835
glnD
EAE_RS11840
map
D_2629
D_2628
D_2627
D_2626
Dc_2982
Dc_2981
Dc_2980
Dc_2979
Dc_2978
I_3325
I_3324
I_3323
I_3322
I_3321
Term: termination (1), NS: new start (1), PS: pausing site (0), iTSS_I: internal starting site (0)

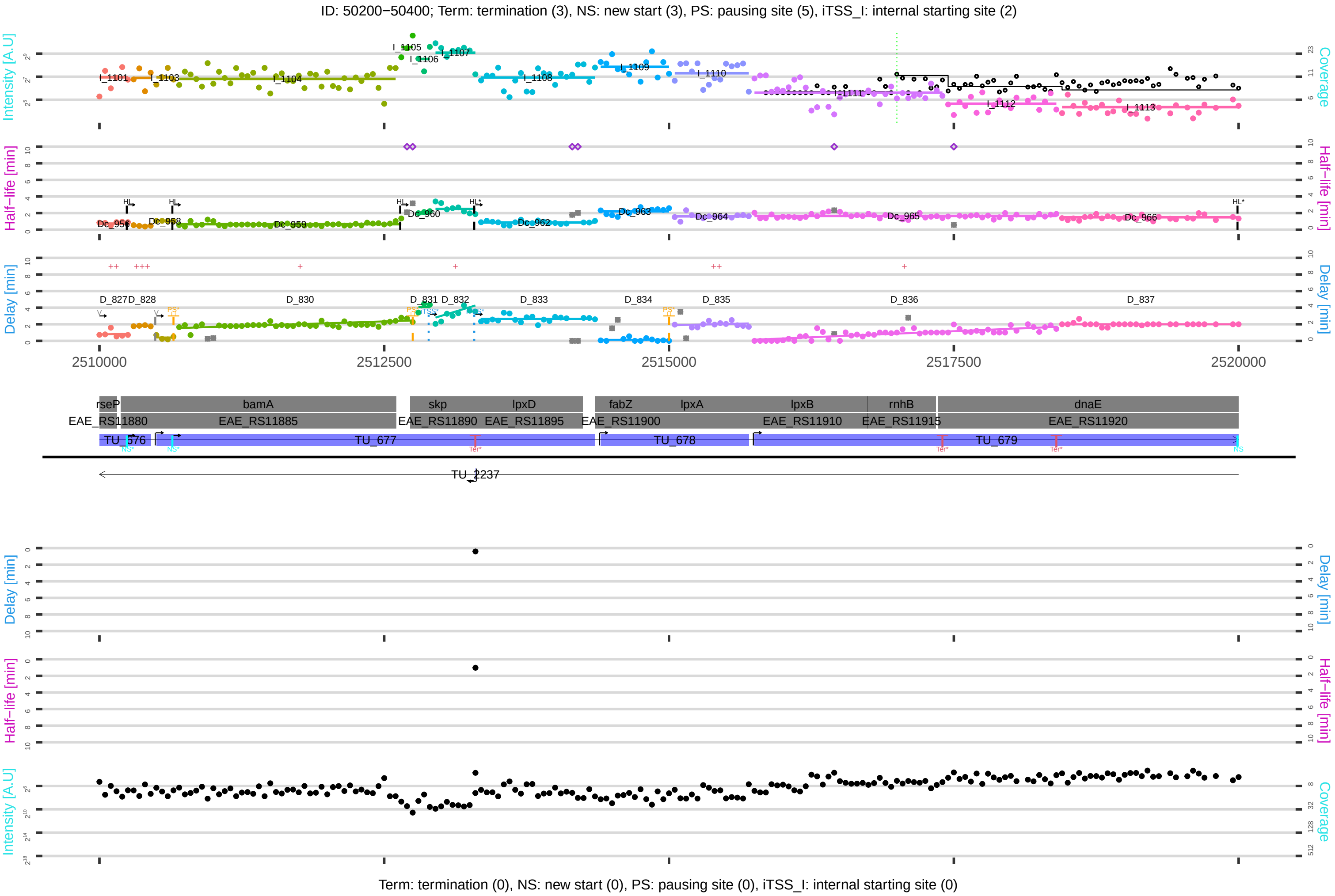

ID: 50200−50400; Term: termination (3), NS: new start (3), PS: pausing site (5), iTSS_I: internal starting site (2)
Intensity [A.U]
Coverage
Half−life [min]
Delay [min]
2510000
2512500
2515000
2517500
2520000
rseP
bamA
skp
lpxD
fabZ
lpxA
lpxB
rnhB
dnaE
EAE_RS11880
EAE_RS11885
EAE_RS11890
EAE_RS11895
EAE_RS11900
EAE_RS11910
EAE_RS11915
EAE_RS11920
TU_676
TU_677
TU_678
TU_679
TU_2237
Term: termination (0), NS: new start (0), PS: pausing site (0), iTSS_I: internal starting site (0)

ID: 50400−50600; Term: termination (1), NS: new start (2), PS: pausing site (1), iTSS_I: internal starting site (0)

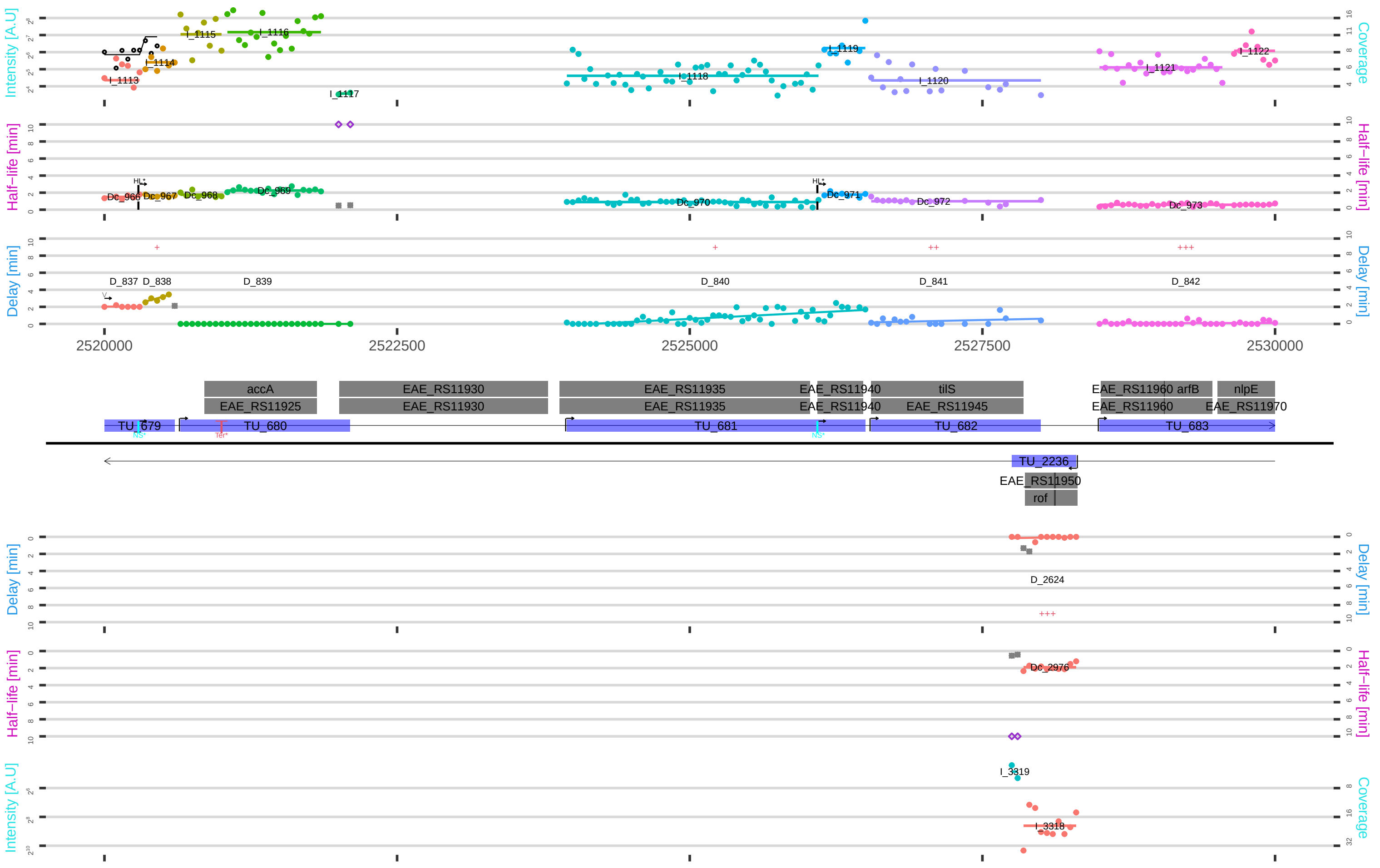

Term: termination (0), NS: new start (0), PS: pausing site (0), iTSS_I: internal starting site (0)

ID: 50600−50800; Term: termination (7), NS: new start (3), PS: pausing site (4), iTSS_I: internal starting site (1)

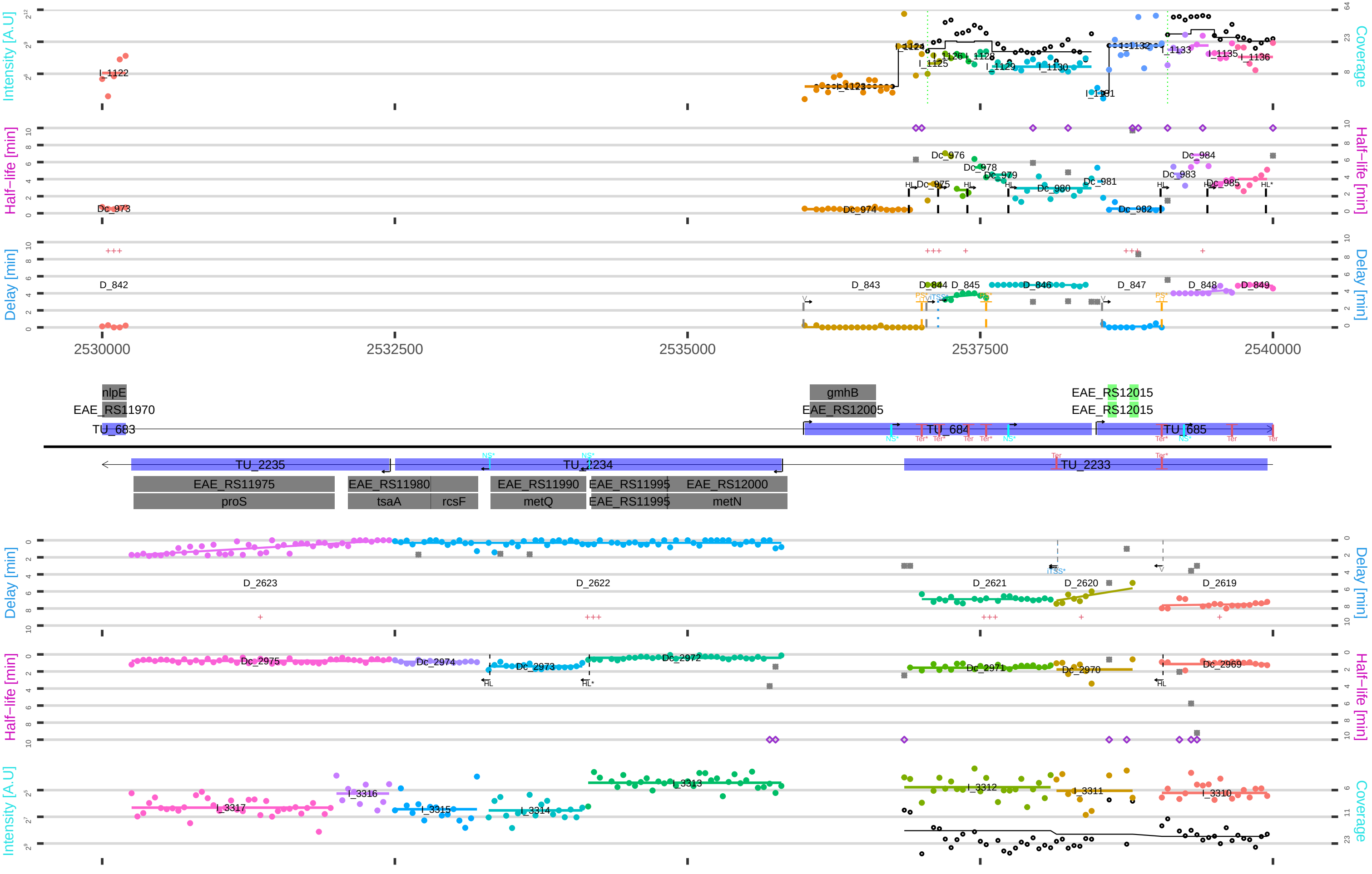

Term: termination (2), NS: new start (2), PS: pausing site (0), iTSS_I: internal starting site (3)

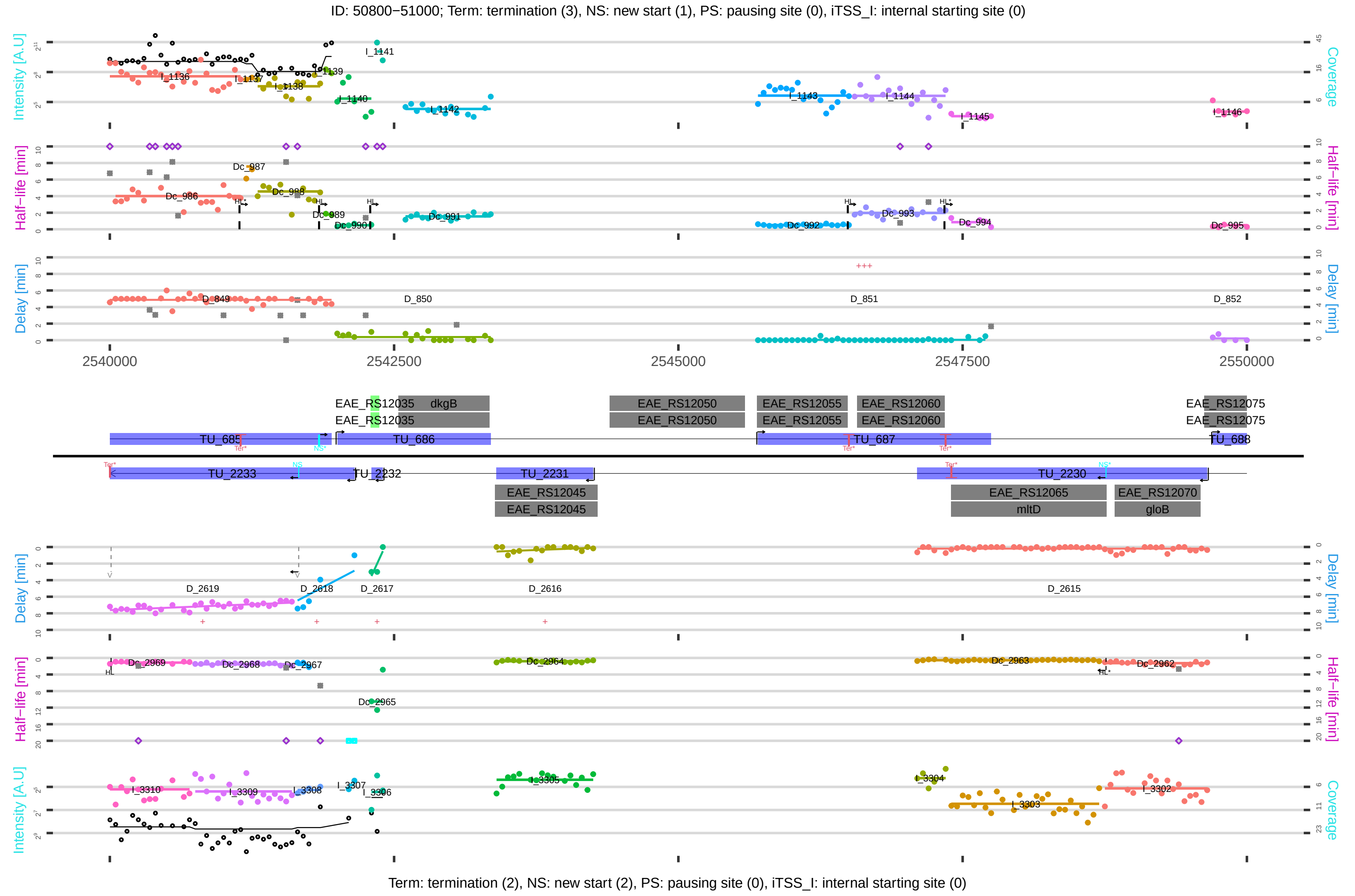

ID: 50800−51000; Term: termination (3), NS: new start (1), PS: pausing site (0), iTSS_I: internal starting site (0)
Intensity [A.U]
Coverage
Half−life [min]
Delay [min]
2540000
2542500
2545000
2547500
2550000
TU_685
TU_686
TU_687
TU_688
TU_2233
TU_2232
TU_2231
TU_2230
EAE_RS12035
dkgB
EAE_RS12050
EAE_RS12055
EAE_RS12060
EAE_RS12075
EAE_RS12045
EAE_RS12065
mltD
EAE_RS12070
globB
Term: termination (2), NS: new start (2), PS: pausing site (0), iTSS_I: internal starting site (0)

ID: 51000−51200; Term: termination (3), NS: new start (2), PS: pausing site (0), iTSS_I: internal starting site (0)

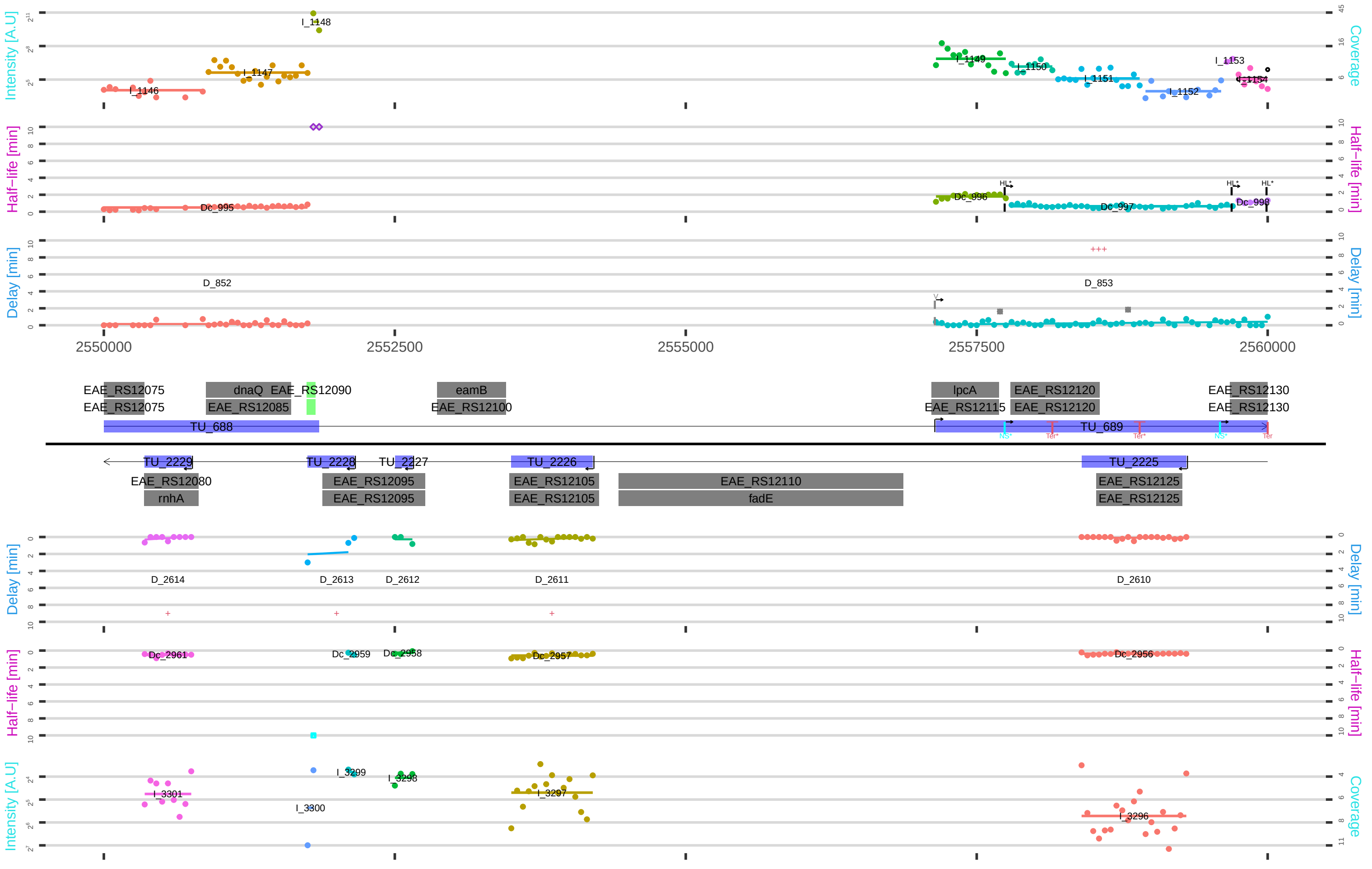

Term: termination (0), NS: new start (0), PS: pausing site (0), iTSS_I: internal starting site (0)

ID: 51200−51378; Term: termination (2), NS: new start (0), PS: pausing site (2), iTSS_I: internal starting site (0)

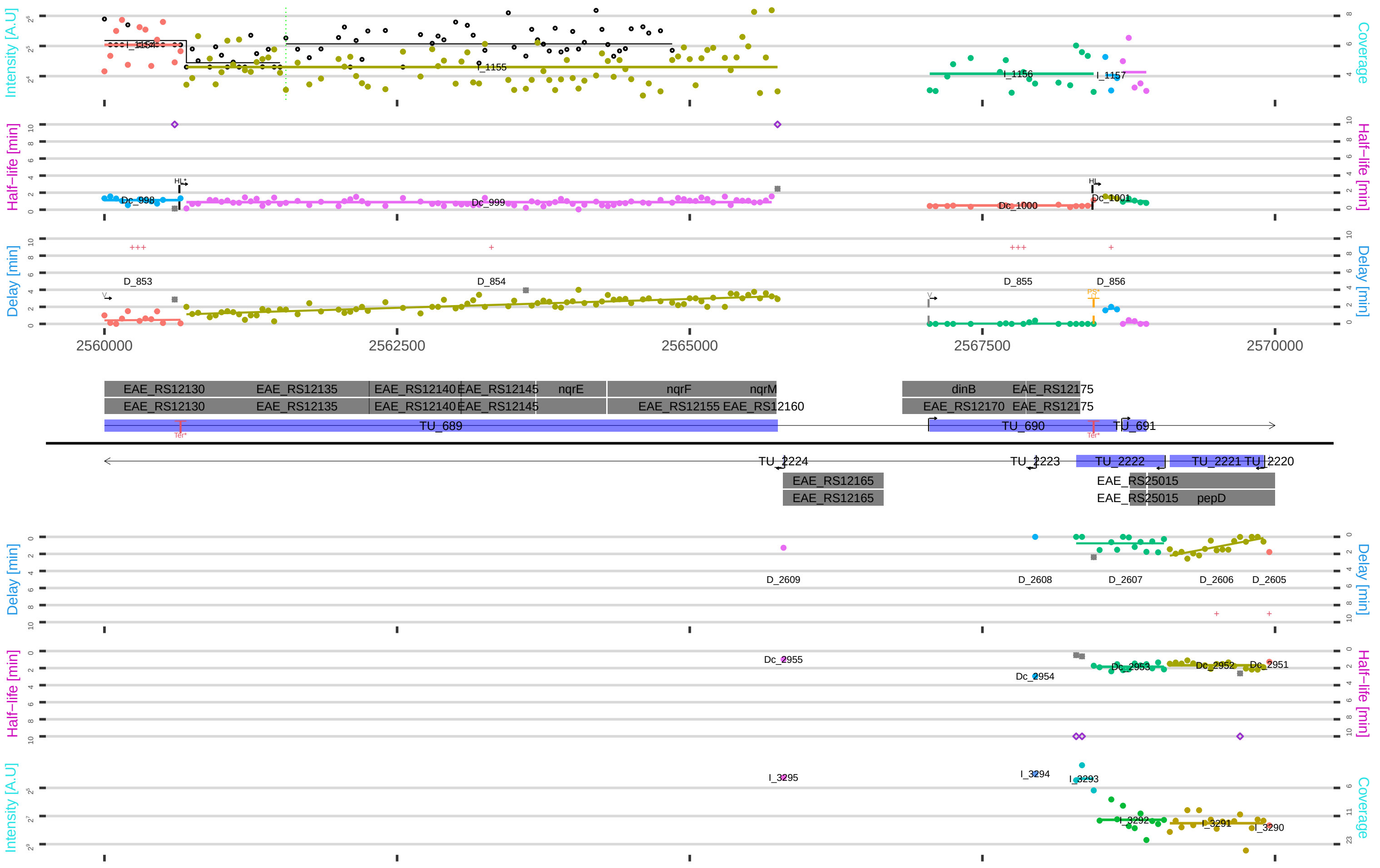

Term: termination (0), NS: new start (0), PS: pausing site (0), iTSS_I: internal starting site (0)

ID: 51413−51482; Term: termination (2), NS: new start (1), PS: pausing site (0), iTSS_I: internal starting site (0)

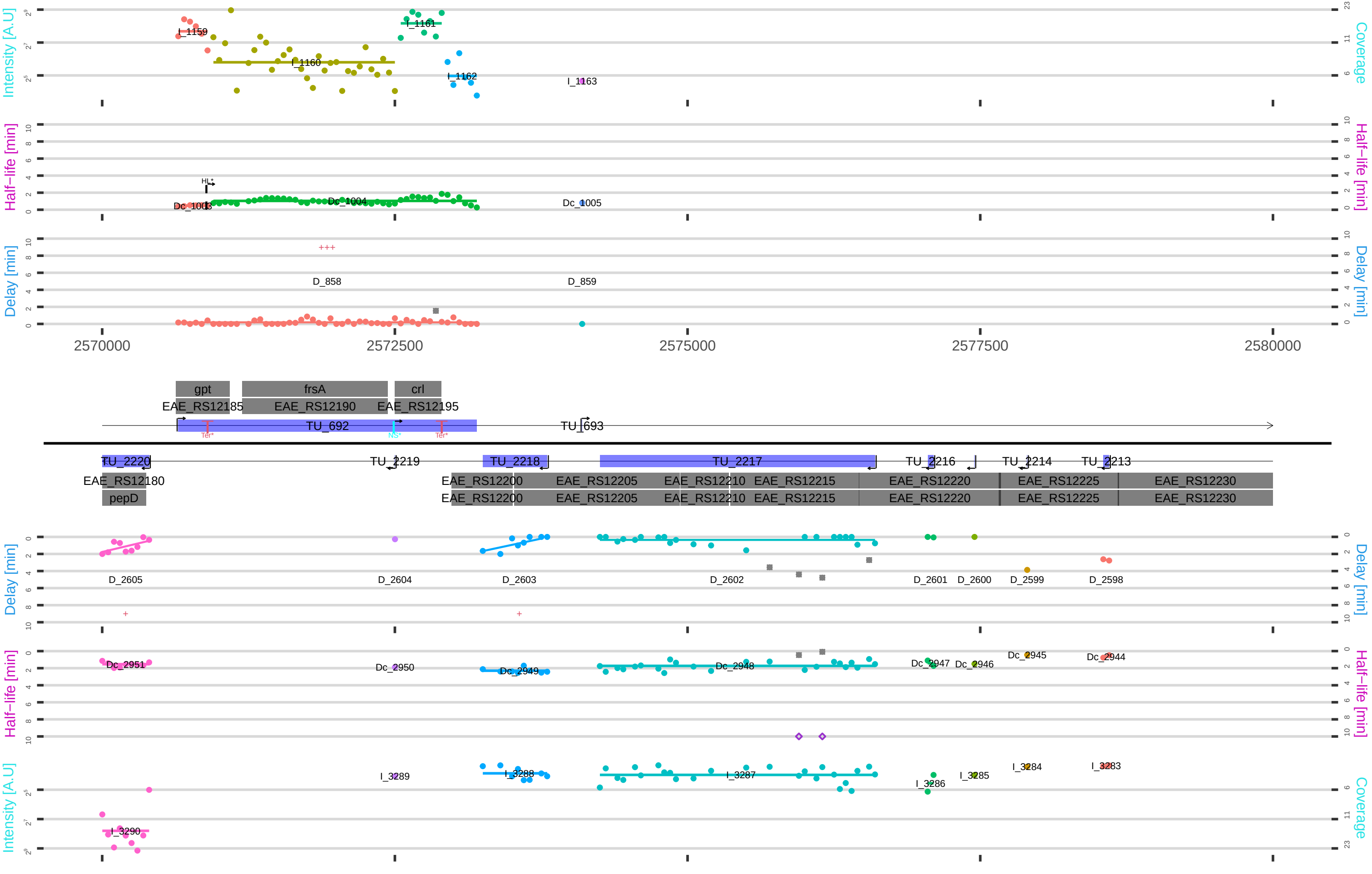

Term: termination (0), NS: new start (0), PS: pausing site (0), iTSS_I: internal starting site (0)

ID: 51798−51800; Term: termination (0), NS: new start (0), PS: pausing site (0), iTSS_I: internal starting site (0)

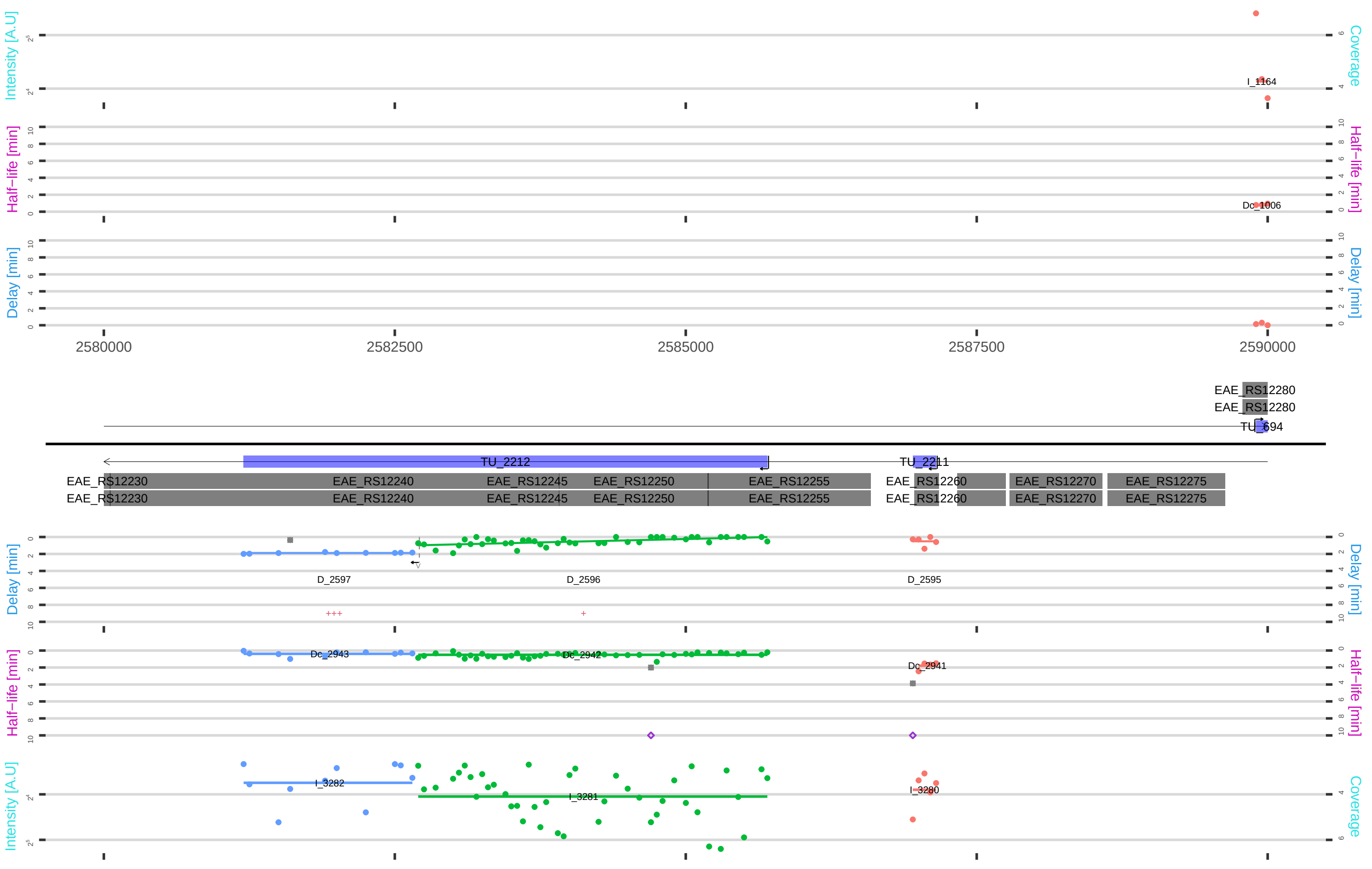

Term: termination (0), NS: new start (0), PS: pausing site (1), iTSS_I: internal starting site (0)

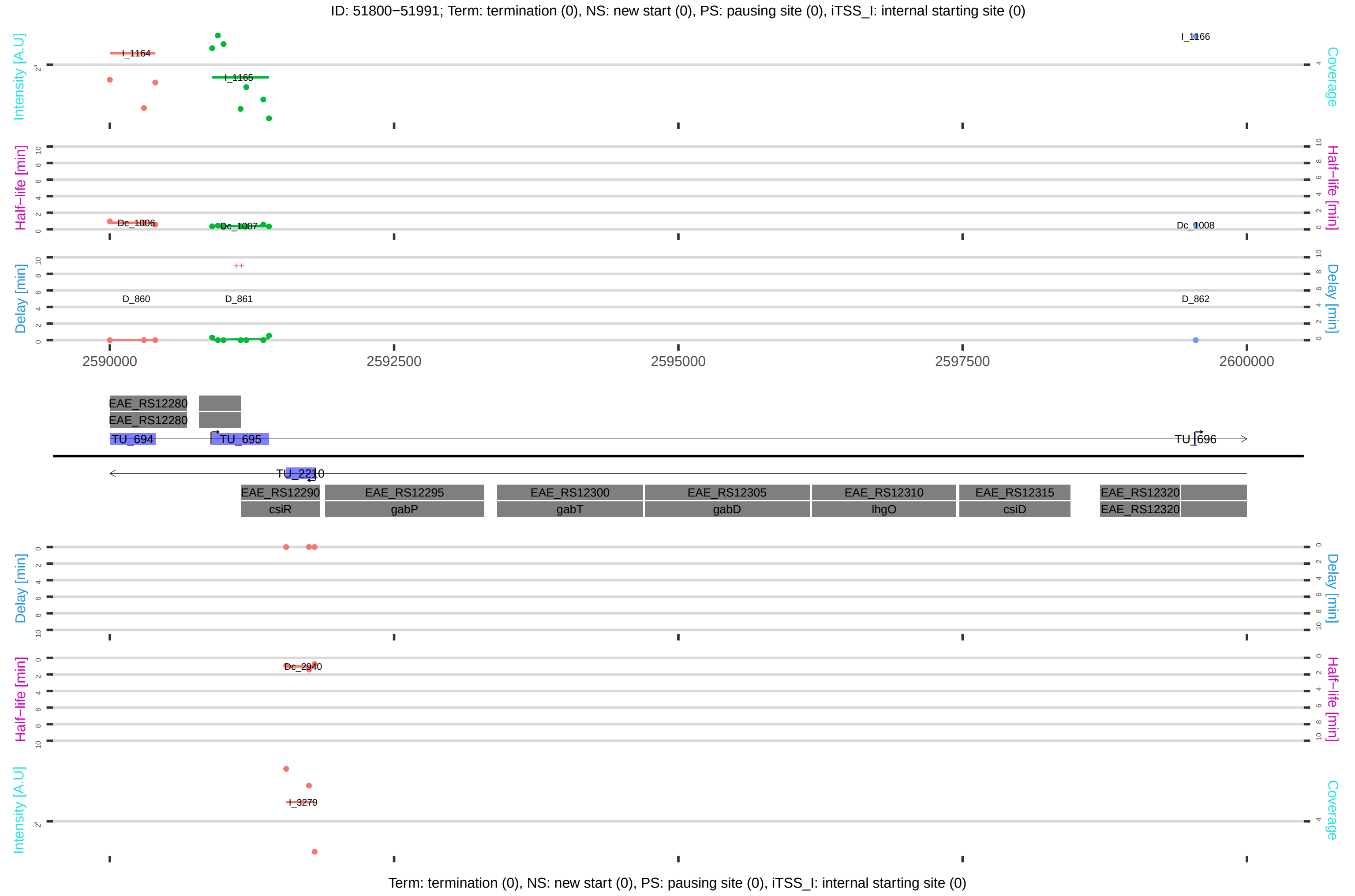

ID: 51800−51991; Term: termination (0), NS: new start (0), PS: pausing site (0), iTSS_I: internal starting site (0)
Intensity [A.U]
Coverage
I_1164
I_1165
I_1166
Half−life [min]
Dc_1006
Dc_1007
Dc_1008
Delay [min]
D_860
D_861
D_862
2590000
2592500
2595000
2597500
2600000
EAE_RS12280
EAE_RS12280
TU_694
TU_695
TU_696
TU_2210
EAE_RS12290
EAE_RS12295
EAE_RS12300
EAE_RS12305
EAE_RS12310
EAE_RS12315
EAE_RS12320
csiR
gabP
gabT
gabD
lhgO
csiD
EAE_RS12320
Dc_2940
I_3279
Term: termination (0), NS: new start (0), PS: pausing site (0), iTSS_I: internal starting site (0)

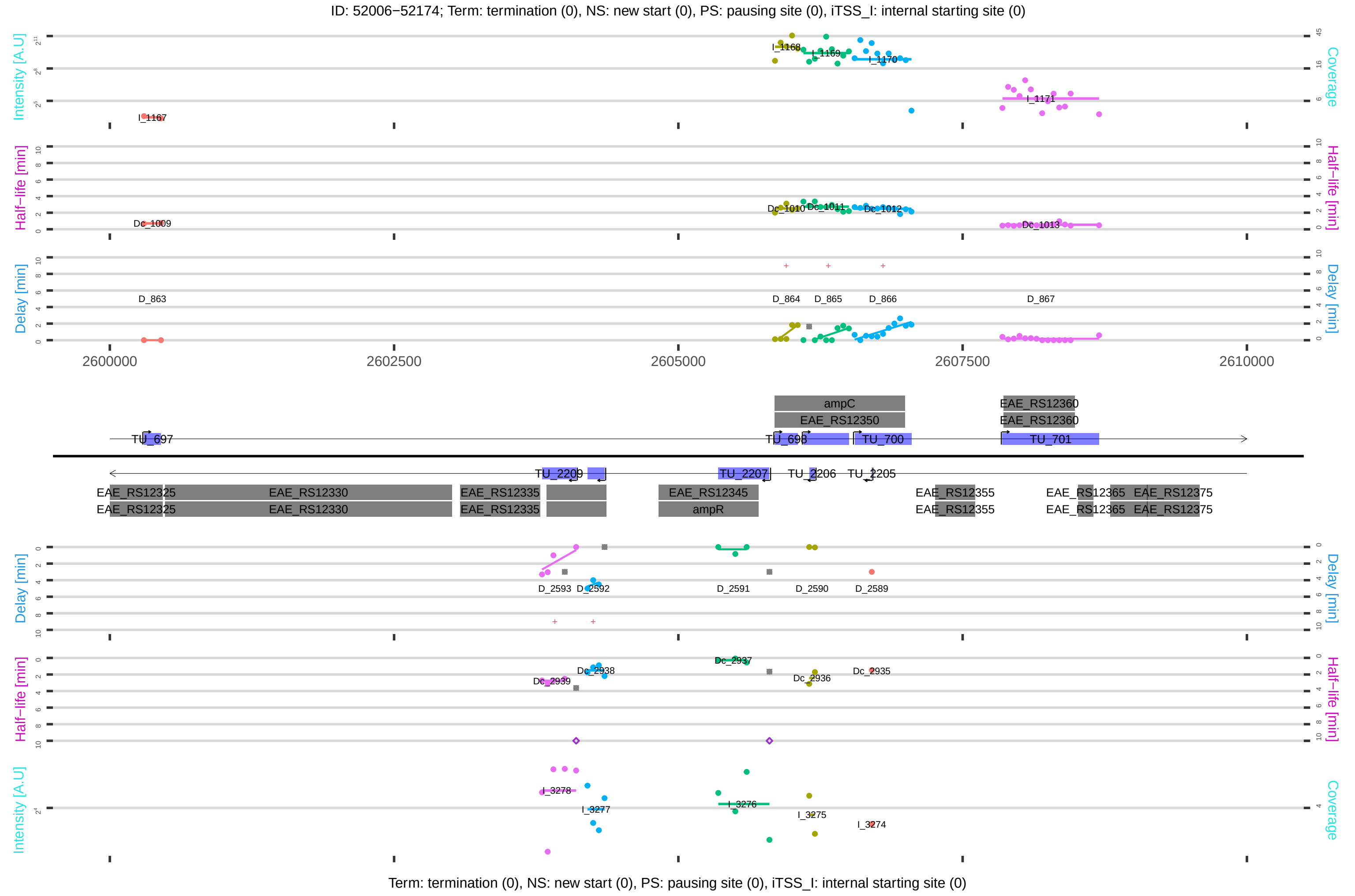

ID: 52006−52174; Term: termination (0), NS: new start (0), PS: pausing site (0), iTSS_I: internal starting site (0)
Intensity [A.U]
Coverage
Half−life [min]
Delay [min]
I_1167
I_1168
I_1169
I_1170
I_1171
Dc_1009
Dc_1010
Dc_1011
Dc_1012
Dc_1013
D_863
D_864
D_865
D_866
D_867
2600000
2602500
2605000
2607500
2610000
ampC
EAE_RS12350
EAE_RS12360
TU_697
TU_698
TU_700
TU_701
TU_2209
TU_2207
TU_2206
TU_2205
EAE_RS12325
EAE_RS12330
EAE_RS12335
EAE_RS12345
ampR
EAE_RS12355
EAE_RS12365
EAE_RS12375
D_2593
D_2592
D_2591
D_2590
D_2589
Dc_2939
Dc_2938
Dc_2937
Dc_2936
Dc_2935
I_3278
I_3277
I_3276
I_3275
I_3274
Term: termination (0), NS: new start (0), PS: pausing site (0), iTSS_I: internal starting site (0)

ID: 52208−52351; Term: termination (0), NS: new start (0), PS: pausing site (0), iTSS_I: internal starting site (0)

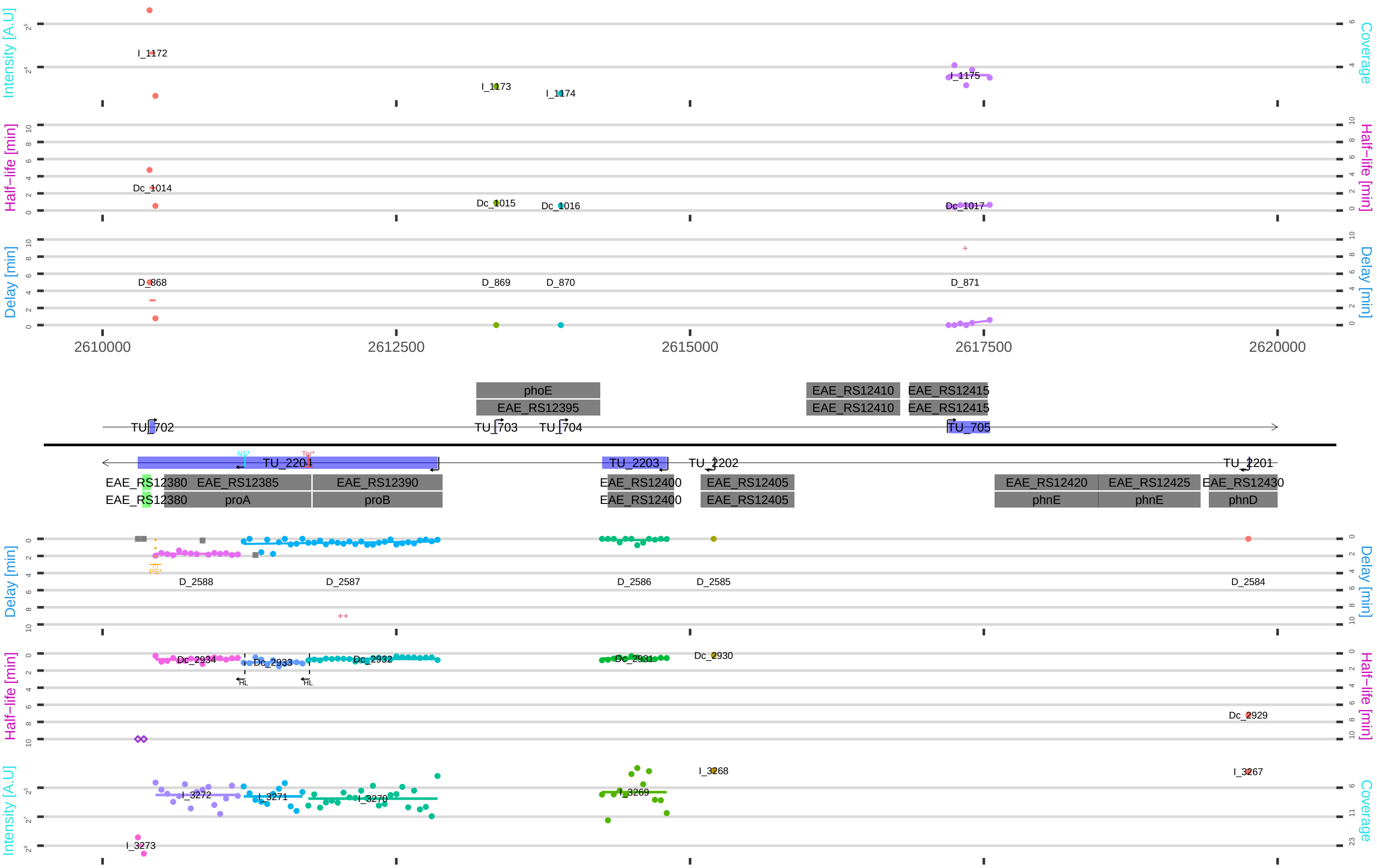

Term: termination (1), NS: new start (1), PS: pausing site (1), iTSS_I: internal starting site (0)

ID: 52454−52574; Term: termination (0), NS: new start (0), PS: pausing site (0), iTSS_I: internal starting site (0)

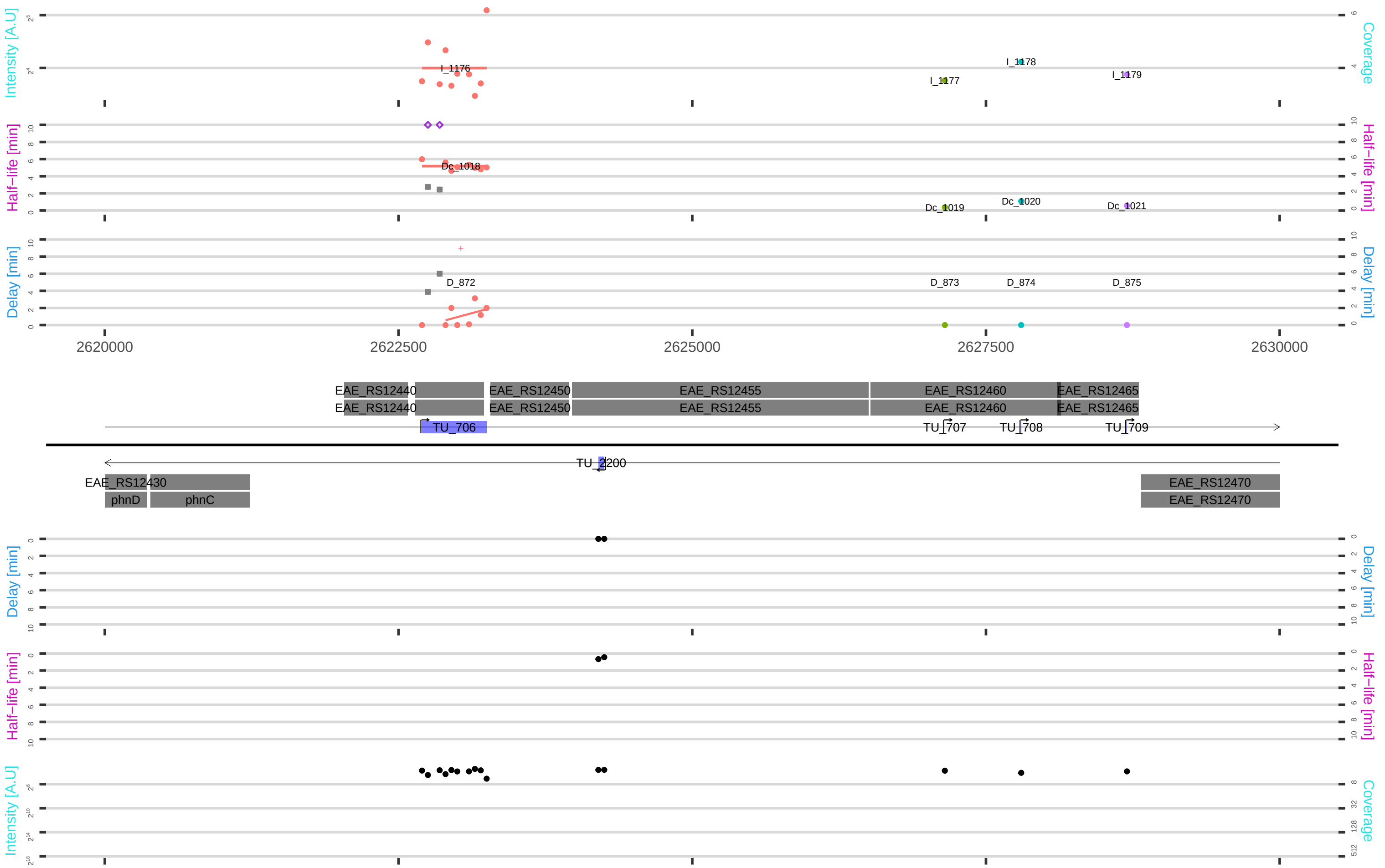

Term: termination (0), NS: new start (0), PS: pausing site (0), iTSS_I: internal starting site (0)

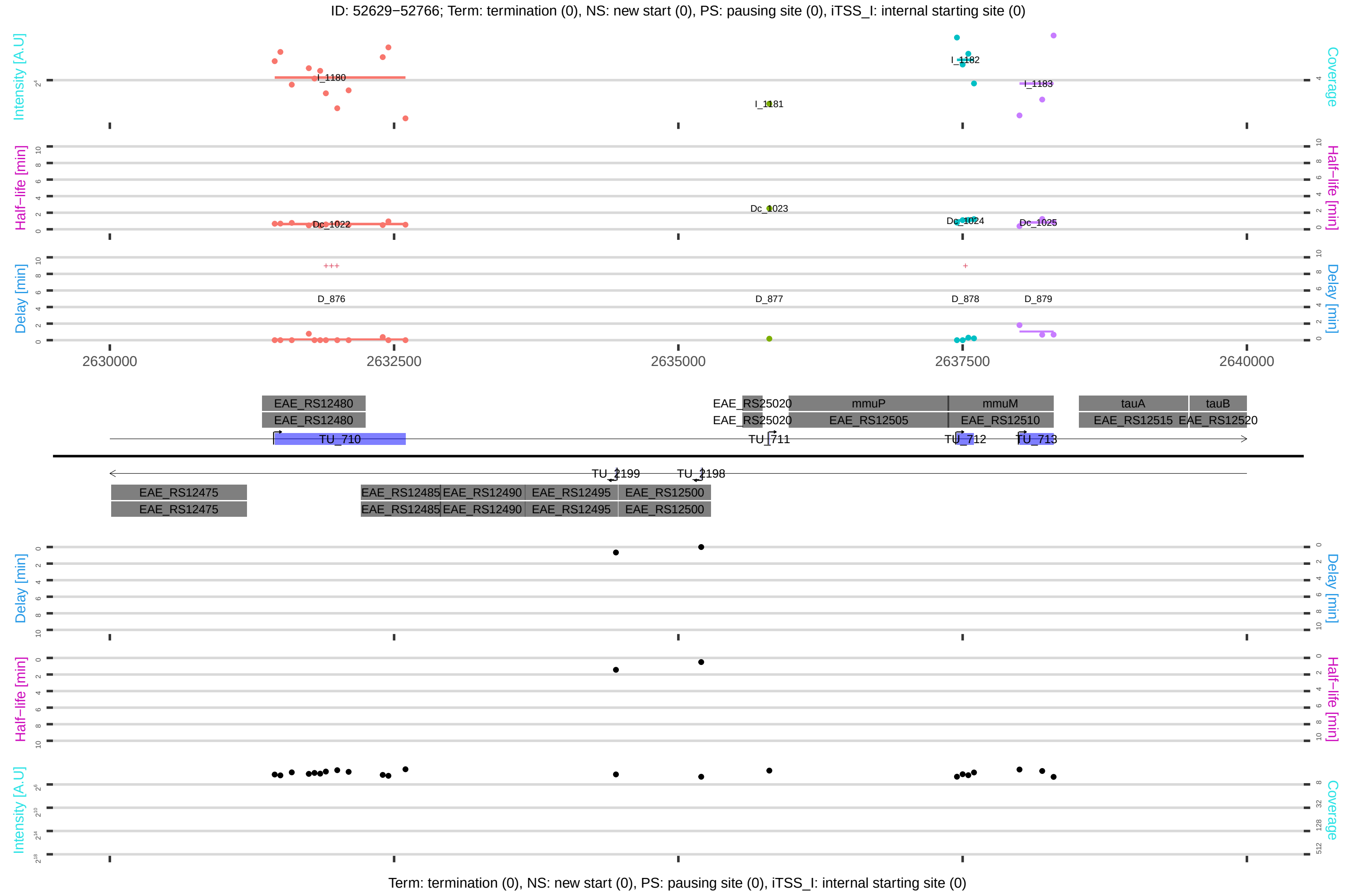

ID: 52629−52766; Term: termination (0), NS: new start (0), PS: pausing site (0), iTSS_I: internal starting site (0)
Intensity [A.U]
Coverage
I_1180
I_1181
I_1182
I_1183
Half−life [min]
Dc_1022
Dc_1023
Dc_1024
Dc_1025
Delay [min]
D_876
D_877
D_878
D_879
2630000
2632500
2635000
2637500
2640000
EAE_RS12480
EAE_RS25020
mmuP
mmuM
tauA
tauB
EAE_RS12505
EAE_RS12510
EAE_RS12515
EAE_RS12520
TU_710
TU_711
TU_712
TU_713
TU_2199
TU_2198
EAE_RS12475
EAE_RS12485
EAE_RS12490
EAE_RS12495
EAE_RS12500
Term: termination (0), NS: new start (0), PS: pausing site (0), iTSS_I: internal starting site (0)

ID: 52948−53000; Term: termination (0), NS: new start (0), PS: pausing site (0), iTSS_I: internal starting site (0)

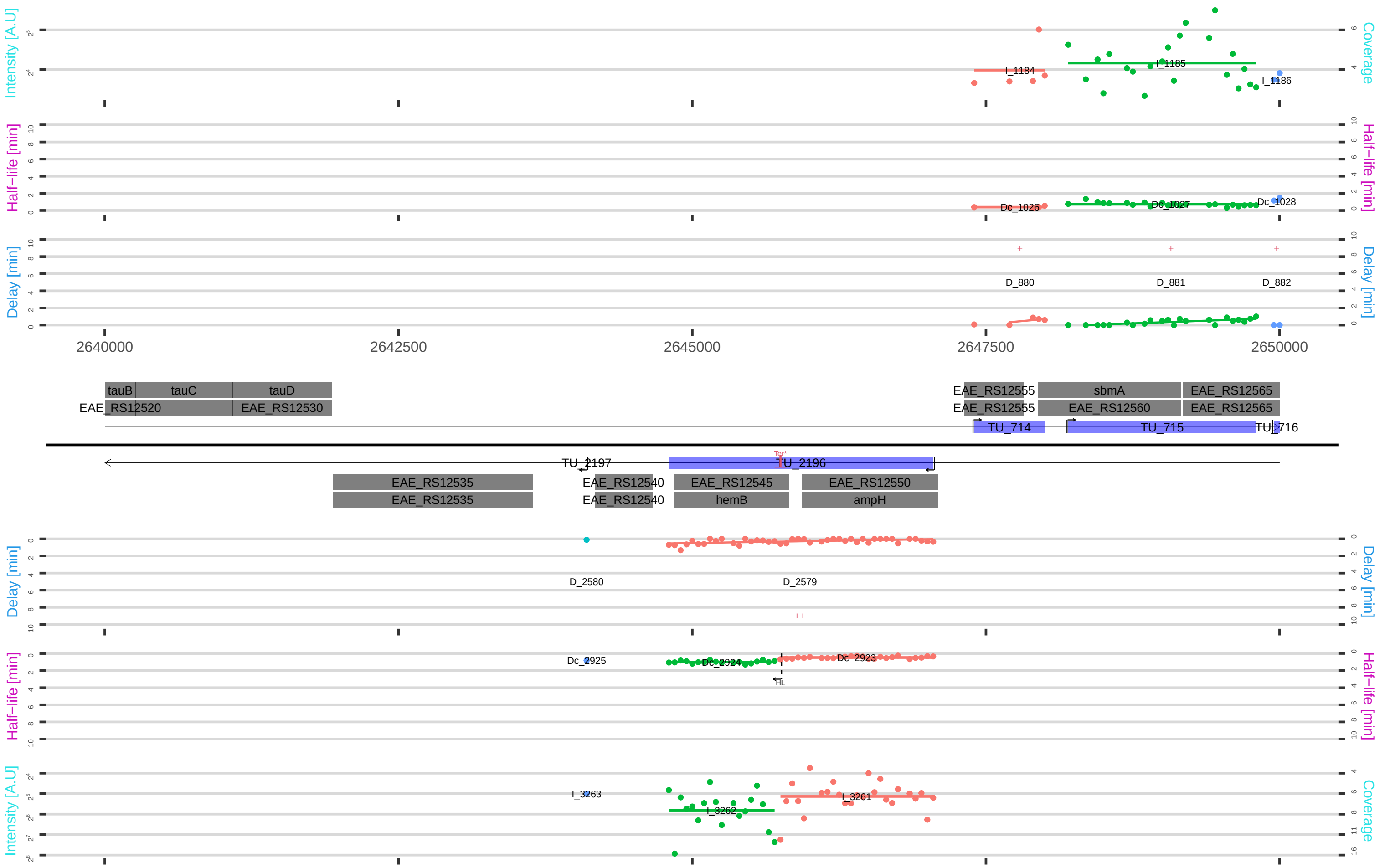

Term: termination (1), NS: new start (0), PS: pausing site (0), iTSS_I: internal starting site (0)

ID: 53000−53200; Term: termination (3), NS: new start (1), PS: pausing site (1), iTSS_I: internal starting site (0)

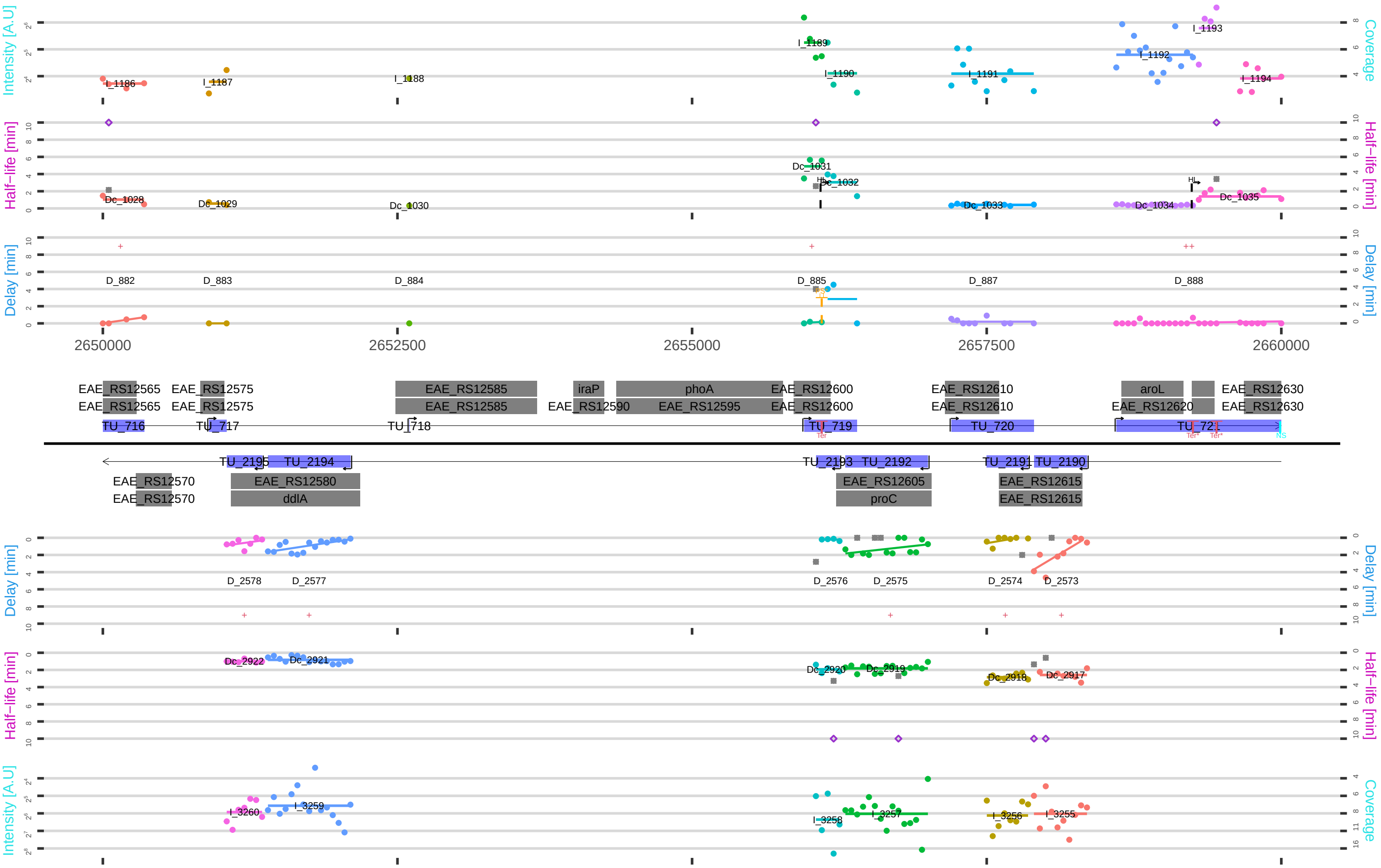

Term: termination (0), NS: new start (0), PS: pausing site (0), iTSS_I: internal starting site (0)

ID: 53200−53400; Term: termination (0), NS: new start (1), PS: pausing site (0), iTSS_I: internal starting site (0)

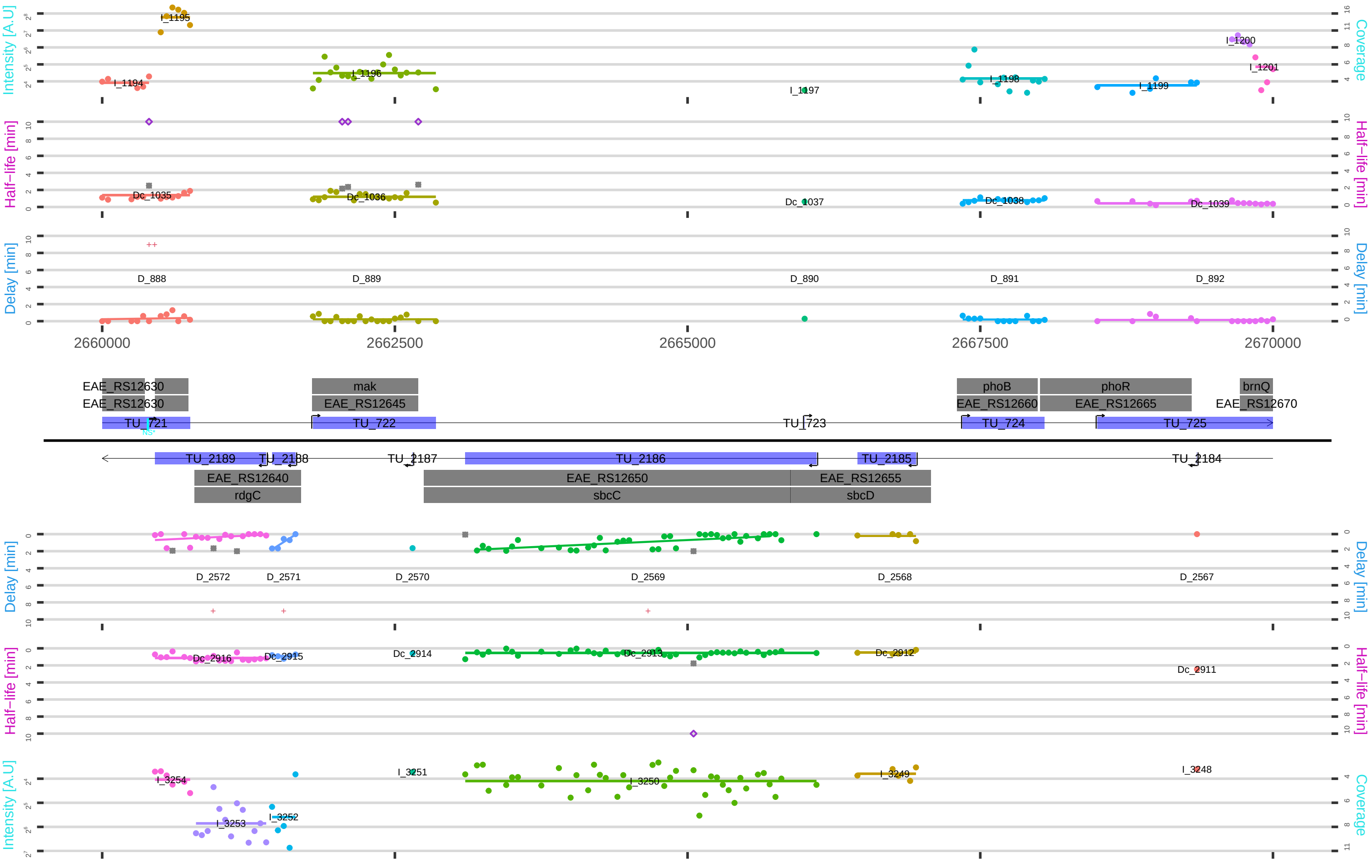

Term: termination (0), NS: new start (0), PS: pausing site (0), iTSS_I: internal starting site (0)

ID: 53400−53451; Term: termination (0), NS: new start (0), PS: pausing site (0), iTSS_I: internal starting site (0)

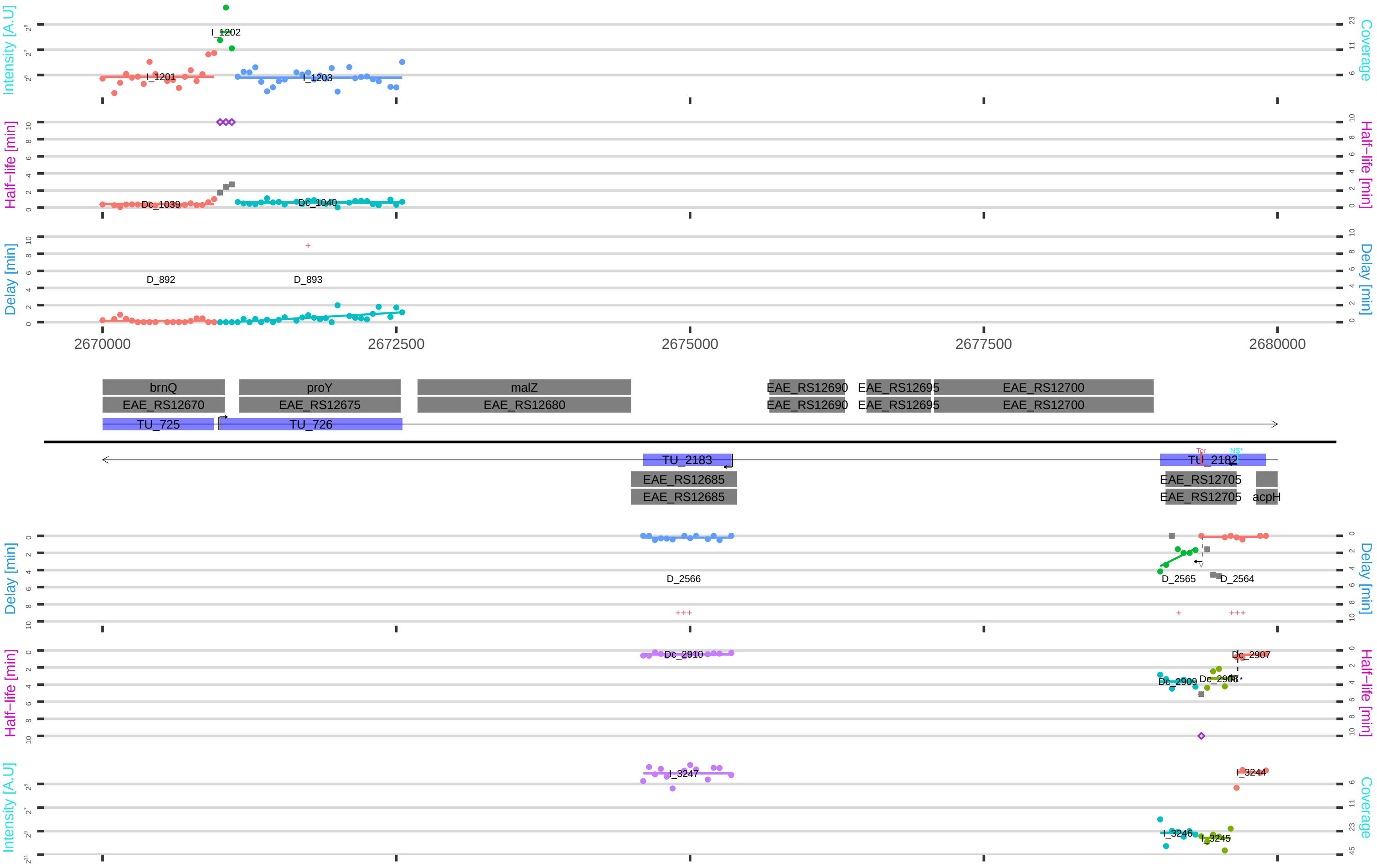

Term: termination (1), NS: new start (1), PS: pausing site (1), iTSS_I: internal starting site (0)

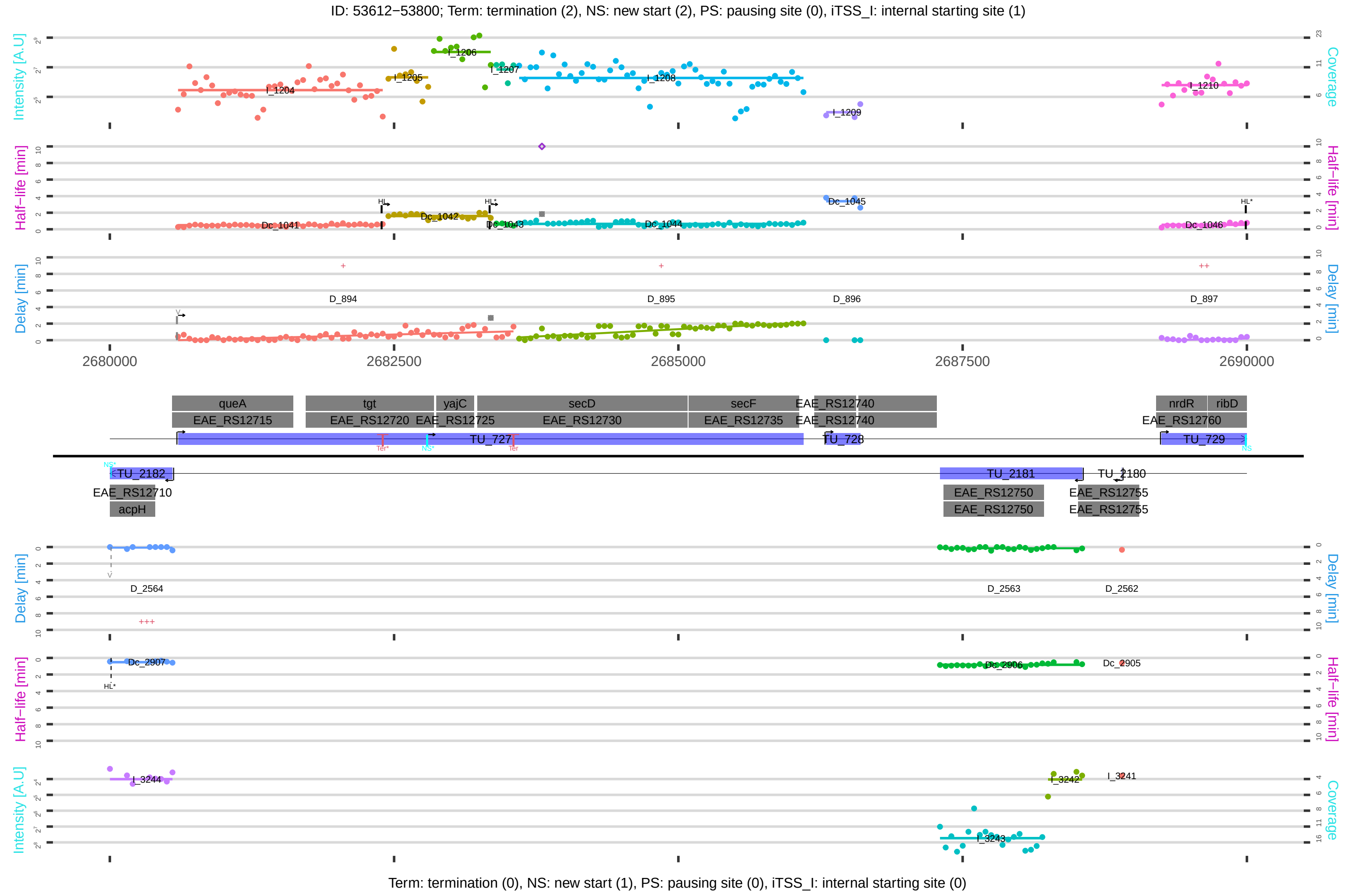

ID: 53612−53800; Term: termination (2), NS: new start (2), PS: pausing site (0), iTSS_I: internal starting site (1)
Intensity [A.U]
Coverage
Half−life [min]
Delay [min]
I_1204
I_1205
I_1206
I_1207
I_1208
I_1209
I_1210
Dc_1041
Dc_1042
Dc_1043
Dc_1044
Dc_1045
Dc_1046
D_894
D_895
D_896
D_897
2680000
2682500
2685000
2687500
2690000
queA
EAE_RS12715
tgt
EAE_RS12720
yajC
EAE_RS12725
secD
EAE_RS12730
secF
EAE_RS12735
EAE_RS12740
nrdR
ribD
EAE_RS12760
TU_727
TU_728
TU_729
TU_2182
TU_2181
TU_2180
EAE_RS12710
acpH
EAE_RS12750
EAE_RS12755
D_2564
D_2563
D_2562
Dc_2907
Dc_2906
Dc_2905
I_3244
I_3243
I_3242
I_3241
Term: termination (0), NS: new start (1), PS: pausing site (0), iTSS_I: internal starting site (0)

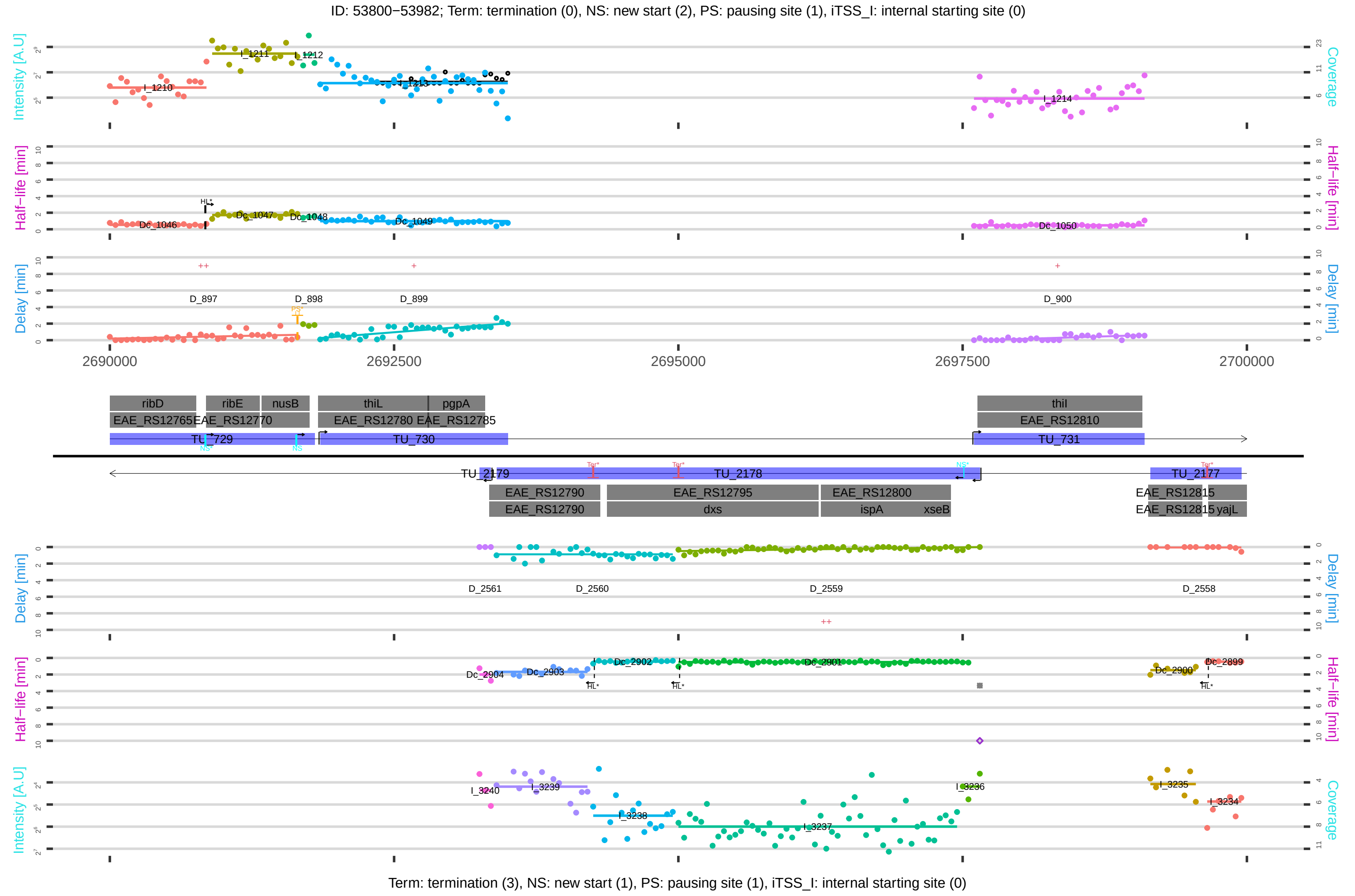

ID: 53800−53982; Term: termination (0), NS: new start (2), PS: pausing site (1), iTSS_I: internal starting site (0)
Intensity [A.U]
Coverage
Half−life [min]
Delay [min]
I_1210
I_1211
I_1212
I_1213
I_1214
Dc_1046
Dc_1047
Dc_1048
Dc_1049
Dc_1050
D_897
D_898
D_899
D_900
2690000
2692500
2695000
2697500
2700000
ribD
ribE
nusB
thiL
pgpA
thiI
EAE_RS12765
EAE_RS12770
EAE_RS12780
EAE_RS12785
EAE_RS12810
TU_729
TU_730
TU_731
TU_2179
TU_2178
TU_2177
EAE_RS12790
EAE_RS12795
EAE_RS12800
EAE_RS12815
dxs
ispA
xseB
yajL
D_2561
D_2560
D_2559
D_2558
Dc_2904
Dc_2903
Dc_2902
Dc_2901
Dc_2900
Dc_2899
I_3240
I_3239
I_3238
I_3237
I_3236
I_3235
I_3234
Term: termination (3), NS: new start (1), PS: pausing site (1), iTSS_I: internal starting site (0)

ID: 54025−54036; Term: termination (1), NS: new start (0), PS: pausing site (0), iTSS_I: internal starting site (1)

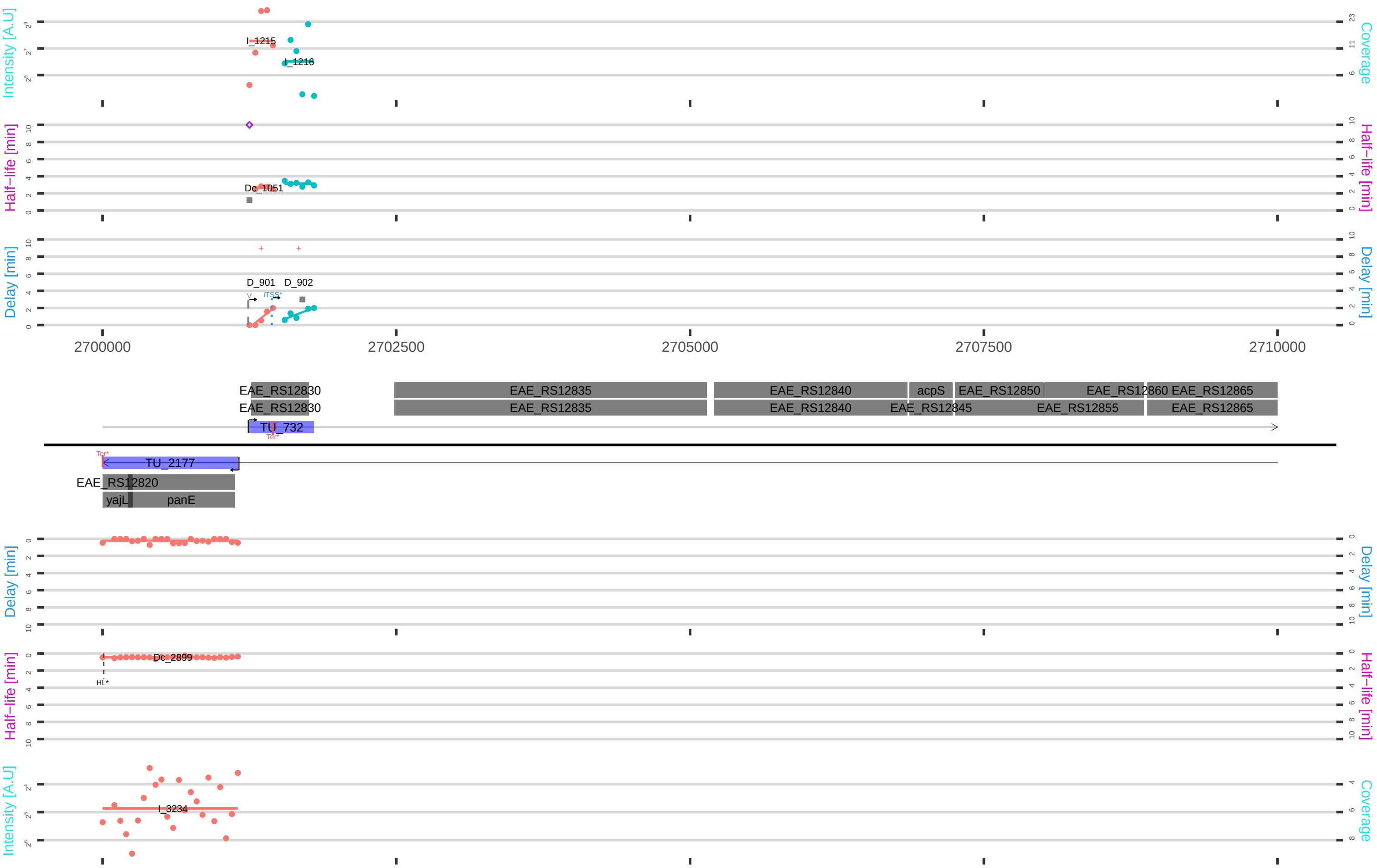

Term: termination (1), NS: new start (0), PS: pausing site (0), iTSS_I: internal starting site (0)

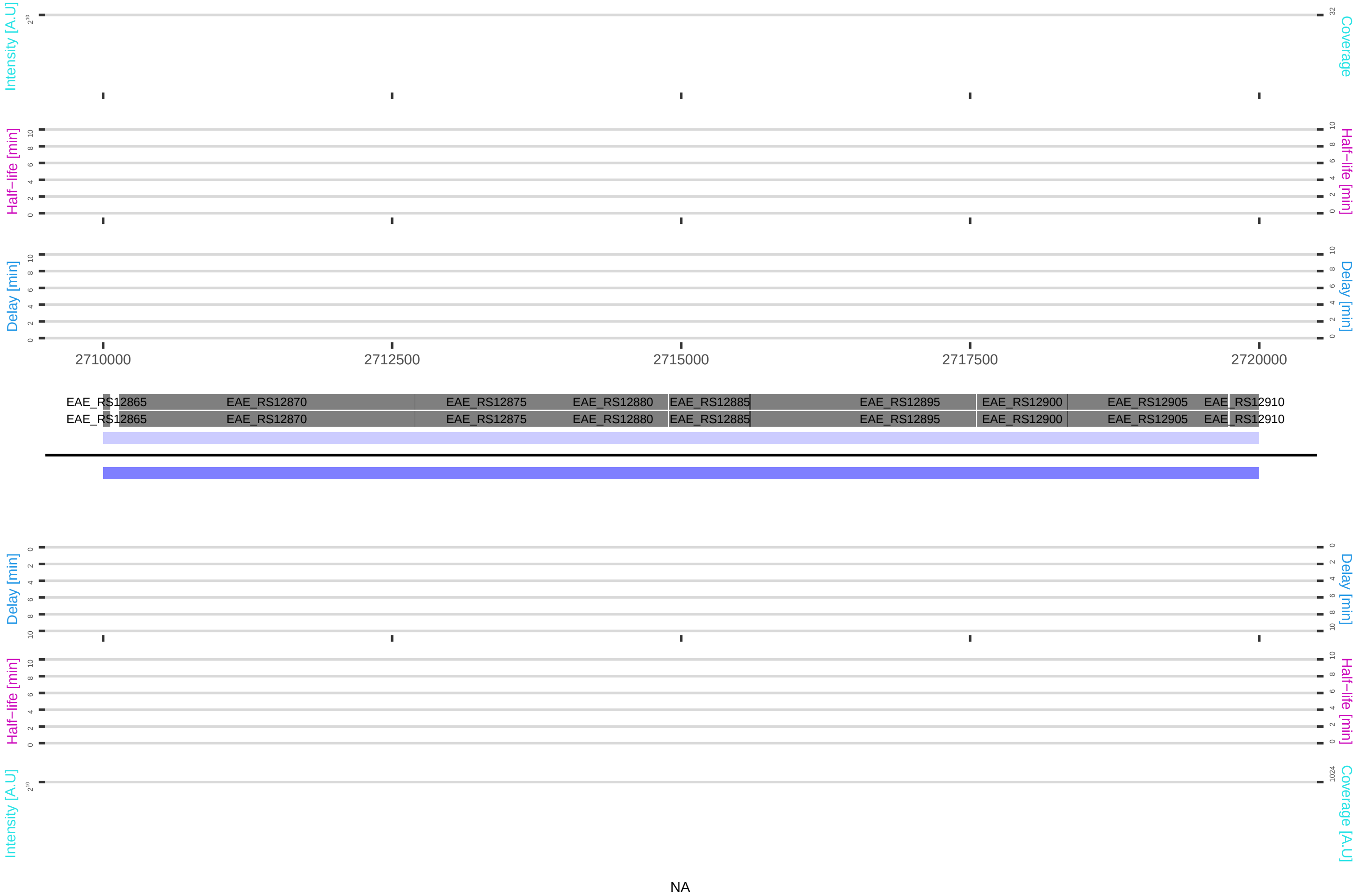
Intensity [A.U]
Coverage
Half−life [min]
Delay [min]
2710000
2712500
2715000
2717500
2720000
EAE_RS12865
EAE_RS12870
EAE_RS12875
EAE_RS12880
EAE_RS12885
EAE_RS12895
EAE_RS12900
EAE_RS12905
EAE_RS12910
Coverage [A.U]
NA

ID: 54476−54476; Term: termination (0), NS: new start (0), PS: pausing site (0), iTSS_I: internal starting site (0)

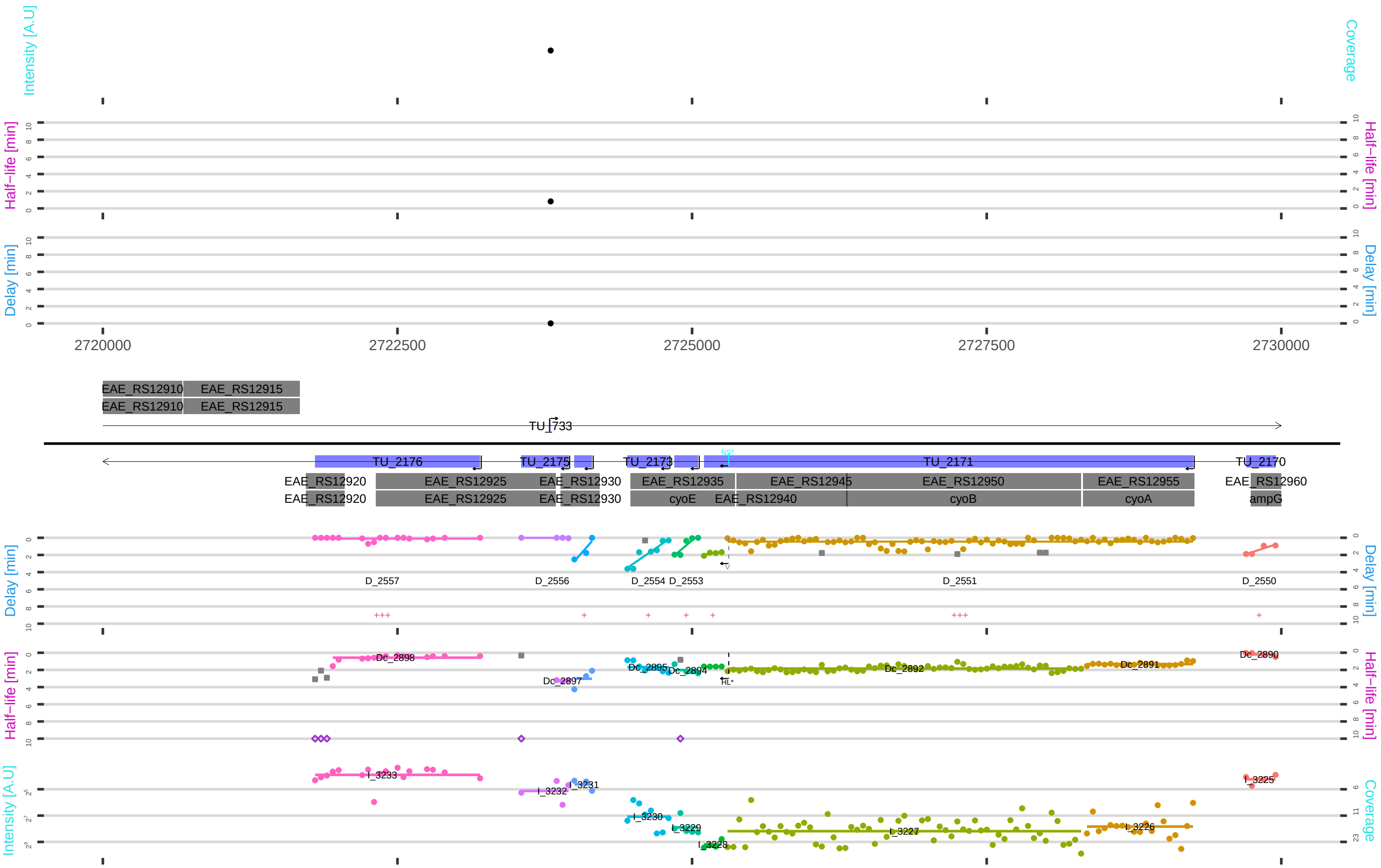

Term: termination (0), NS: new start (1), PS: pausing site (2), iTSS_I: internal starting site (0)

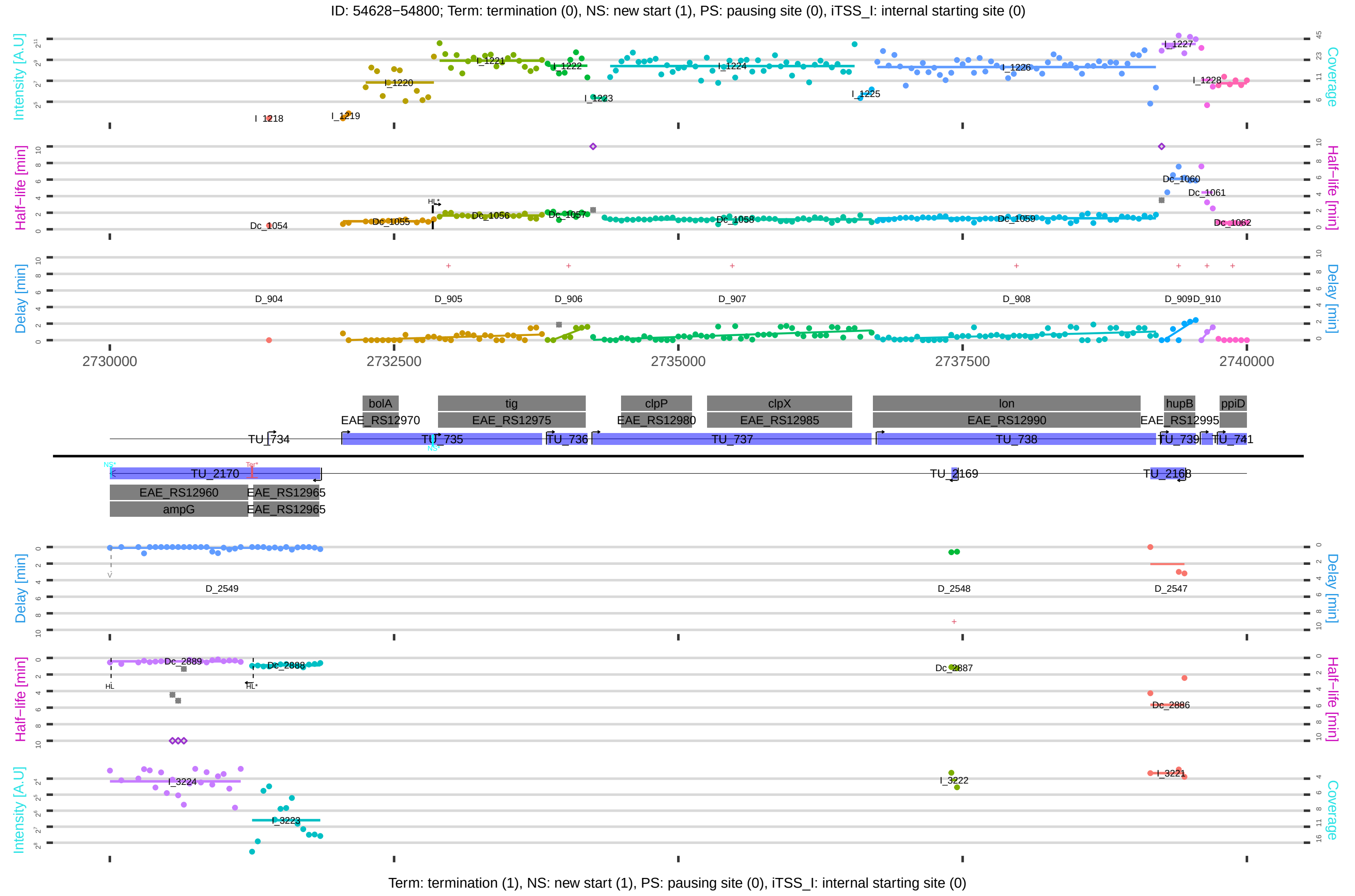

ID: 54628−54800; Term: termination (0), NS: new start (1), PS: pausing site (0), iTSS_I: internal starting site (0)
Intensity [A.U]
Coverage
Half−life [min]
Delay [min]
2730000
2732500
2735000
2737500
2740000
bolA
tig
clpP
clpX
lon
hupB
ppiD
EAE_RS12970
EAE_RS12975
EAE_RS12980
EAE_RS12985
EAE_RS12990
EAE_RS12995
TU_734
TU_735
TU_736
TU_737
TU_738
TU_739
TU_741
TU_2170
TU_2169
TU_2168
EAE_RS12960
EAE_RS12965
ampG
Term: termination (1), NS: new start (1), PS: pausing site (0), iTSS_I: internal starting site (0)

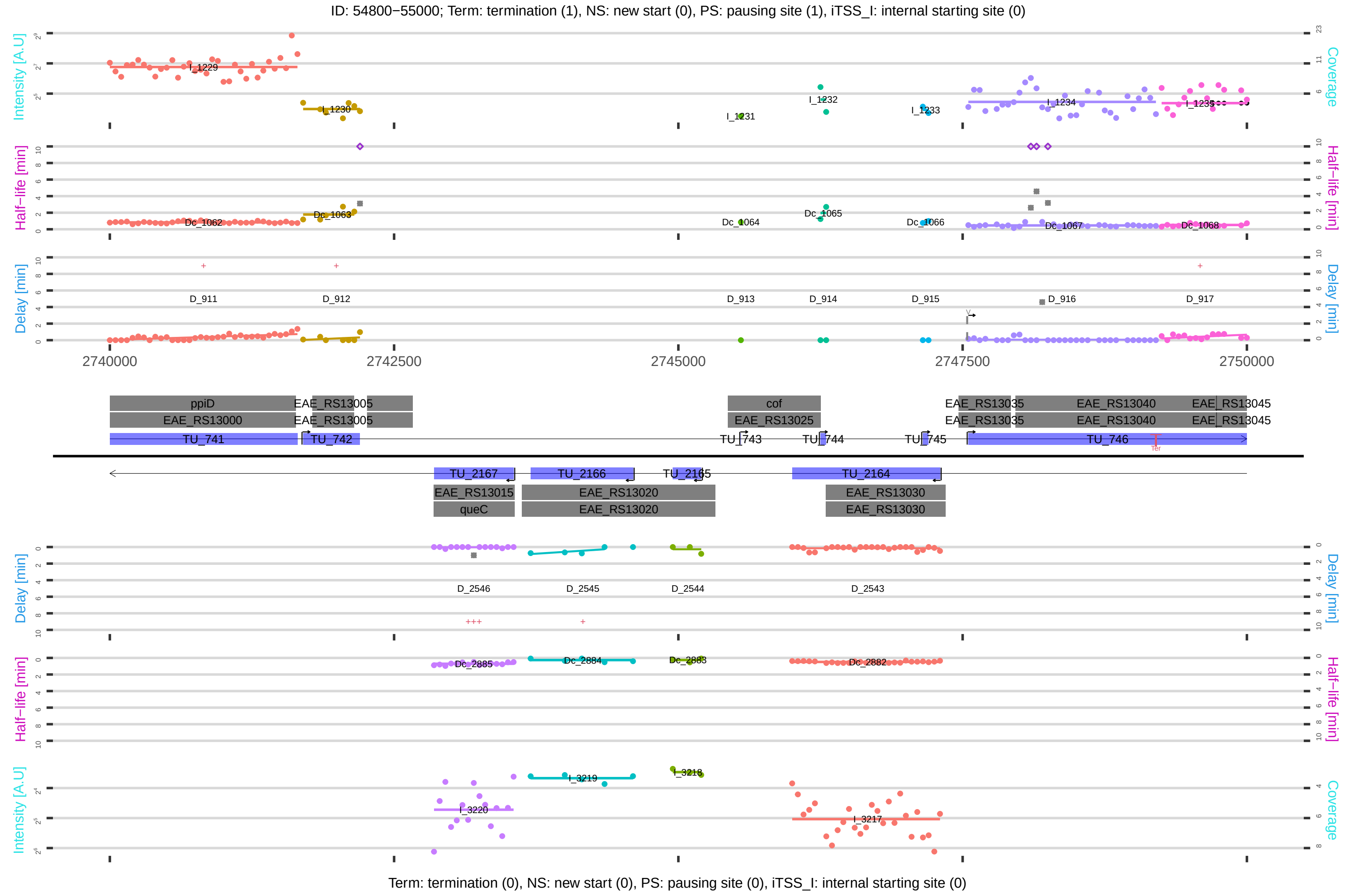
ID: 54800−55000; Term: termination (1), NS: new start (0), PS: pausing site (1), iTSS_I: internal starting site (0)
Intensity [A.U]
Coverage
Half−life [min]
Delay [min]
I_1229
I_1230
I_1231
I_1232
I_1233
I_1234
Dc_1062
Dc_1063
Dc_1064
Dc_1065
Dc_1066
Dc_1067
Dc_1068
D_911
D_912
D_913
D_914
D_915
D_916
D_917
2740000
2742500
2745000
2747500
2750000
ppiD
EAE_RS13000
EAE_RS13005
cof
EAE_RS13025
EAE_RS13035
EAE_RS13040
EAE_RS13045
TU_741
TU_742
TU_743
TU_744
TU_745
TU_746
Ter
TU_2167
TU_2166
TU_2165
TU_2164
EAE_RS13015
queC
EAE_RS13020
EAE_RS13030
D_2546
D_2545
D_2544
D_2543
Dc_2885
Dc_2884
Dc_2883
Dc_2882
I_3220
I_3219
I_3218
I_3217
Term: termination (0), NS: new start (0), PS: pausing site (0), iTSS_I: internal starting site (0)

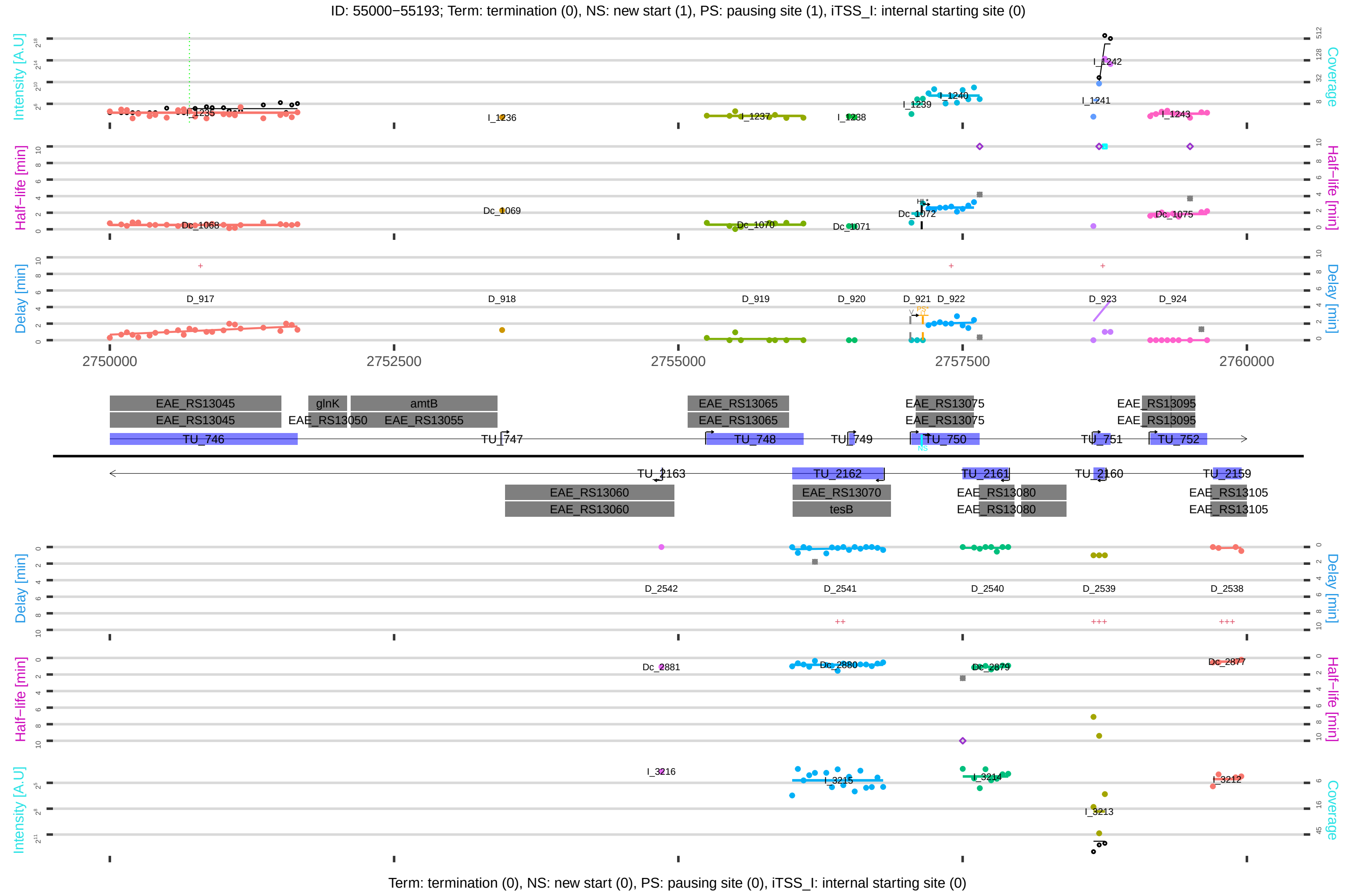
ID: 55000−55193; Term: termination (0), NS: new start (1), PS: pausing site (1), iTSS_I: internal starting site (0)
Intensity [A.U]
Coverage
Half−life [min]
Delay [min]
2750000
2752500
2755000
2757500
2760000
EAE_RS13045
glnK
amtB
EAE_RS13050
EAE_RS13055
EAE_RS13065
EAE_RS13075
EAE_RS13095
TU_746
TU_747
TU_748
TU_749
TU_750
TU_751
TU_752
TU_2163
TU_2162
TU_2161
TU_2160
TU_2159
EAE_RS13060
EAE_RS13070
tesB
EAE_RS13080
EAE_RS13105
Term: termination (0), NS: new start (0), PS: pausing site (0), iTSS_I: internal starting site (0)

ID: 55226−55349; Term: termination (0), NS: new start (0), PS: pausing site (0), iTSS_I: internal starting site (0)

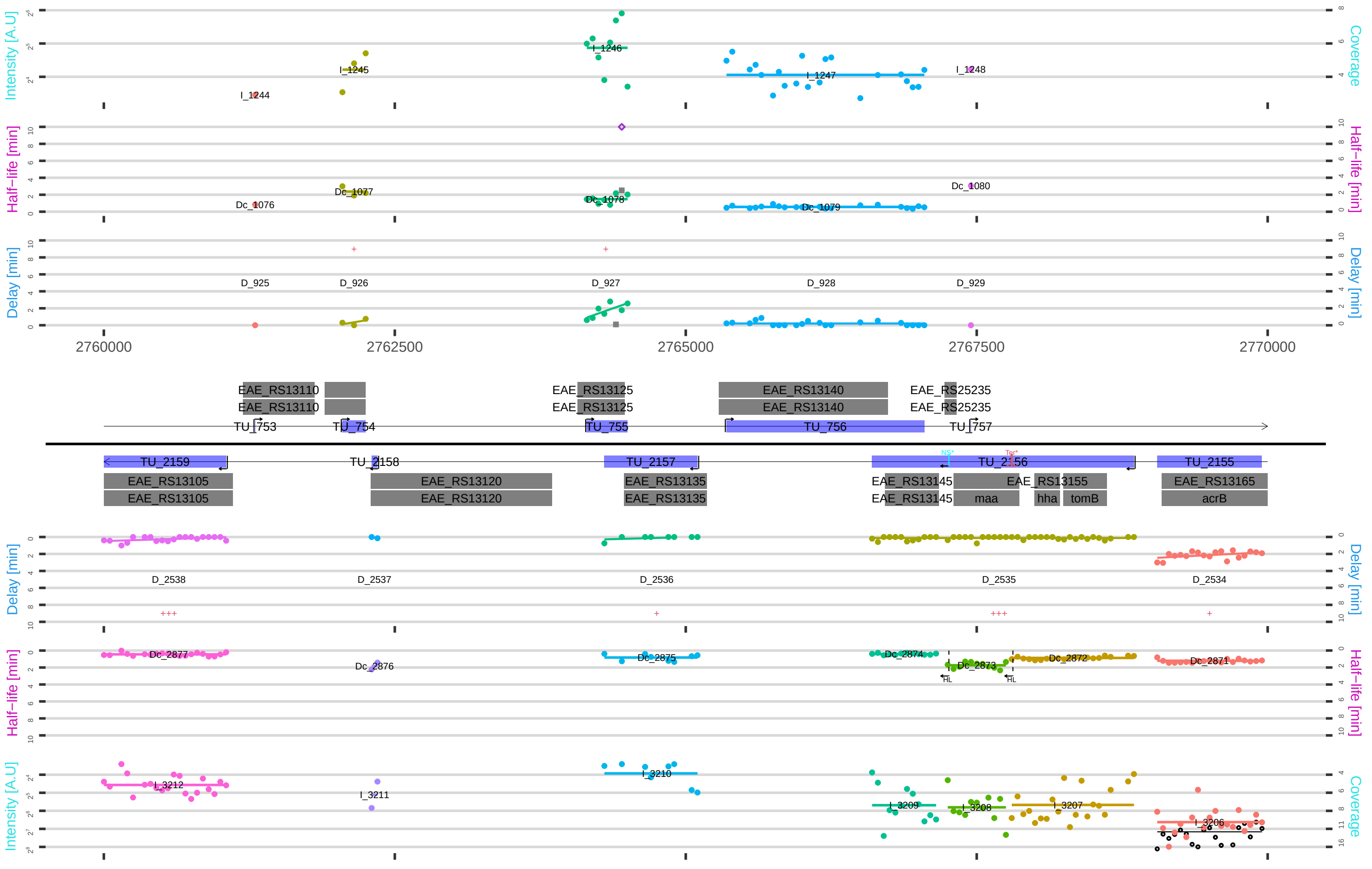

Term: termination (1), NS: new start (1), PS: pausing site (1), iTSS_I: internal starting site (0)

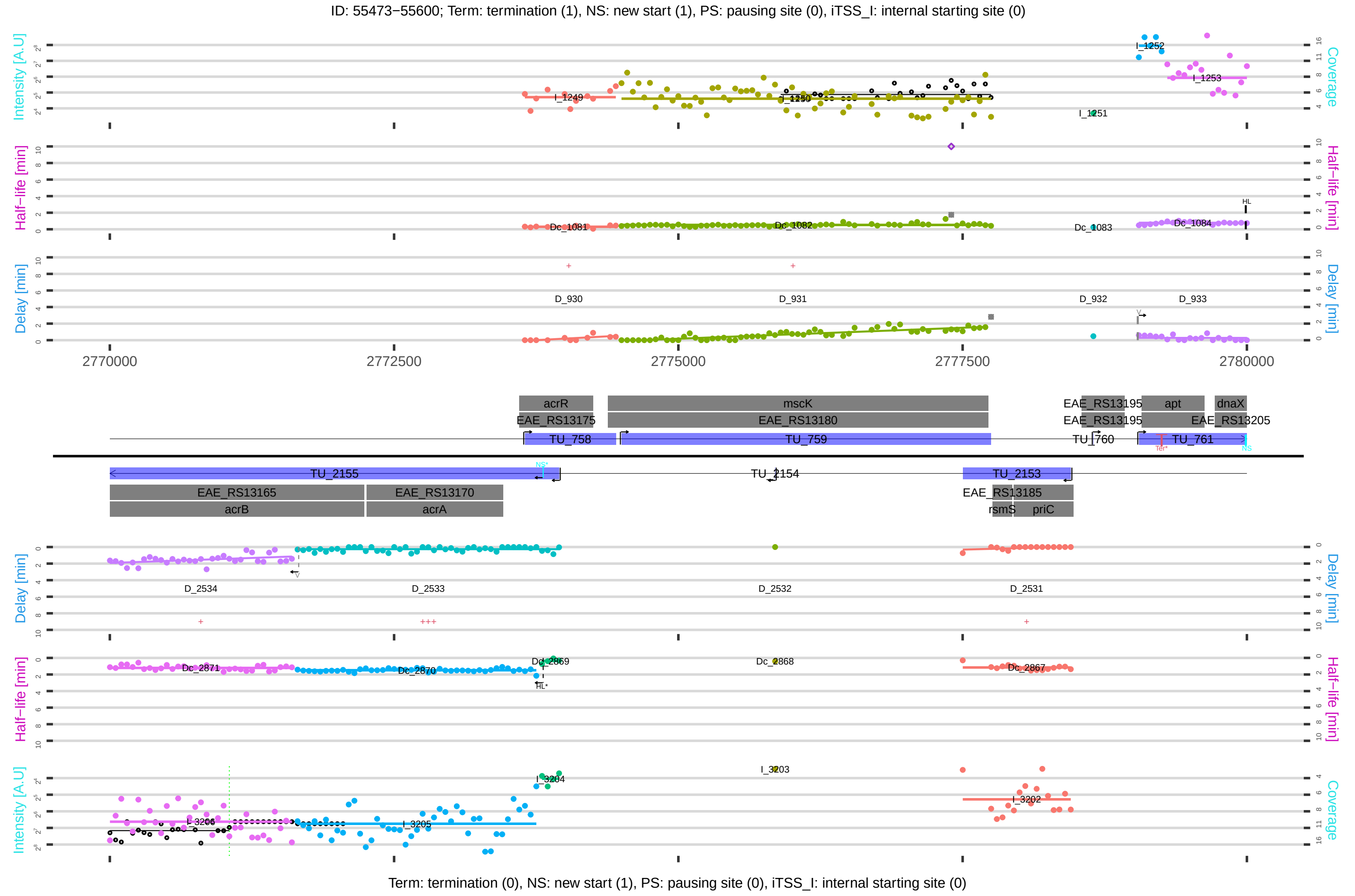
ID: 55473−55600; Term: termination (1), NS: new start (1), PS: pausing site (0), iTSS_I: internal starting site (0)
Intensity [A.U]
Coverage
Half−life [min]
Delay [min]
I_1249
I_1250
I_1251
I_1252
I_1253
Dc_1081
Dc_1082
Dc_1083
Dc_1084
D_930
D_931
D_932
D_933
2770000
2772500
2775000
2777500
2780000
acrR
EAE_RS13175
mscK
EAE_RS13180
EAE_RS13195
apt
dnaX
EAE_RS13205
TU_758
TU_759
TU_760
TU_761
TU_2155
TU_2154
TU_2153
EAE_RS13165
EAE_RS13170
acrB
acrA
EAE_RS13185
rsmS
priC
D_2534
D_2533
D_2532
D_2531
Dc_2871
Dc_2870
Dc_2869
Dc_2868
Dc_2867
I_3206
I_3205
I_3204
I_3203
I_3202
Term: termination (0), NS: new start (1), PS: pausing site (0), iTSS_I: internal starting site (0)

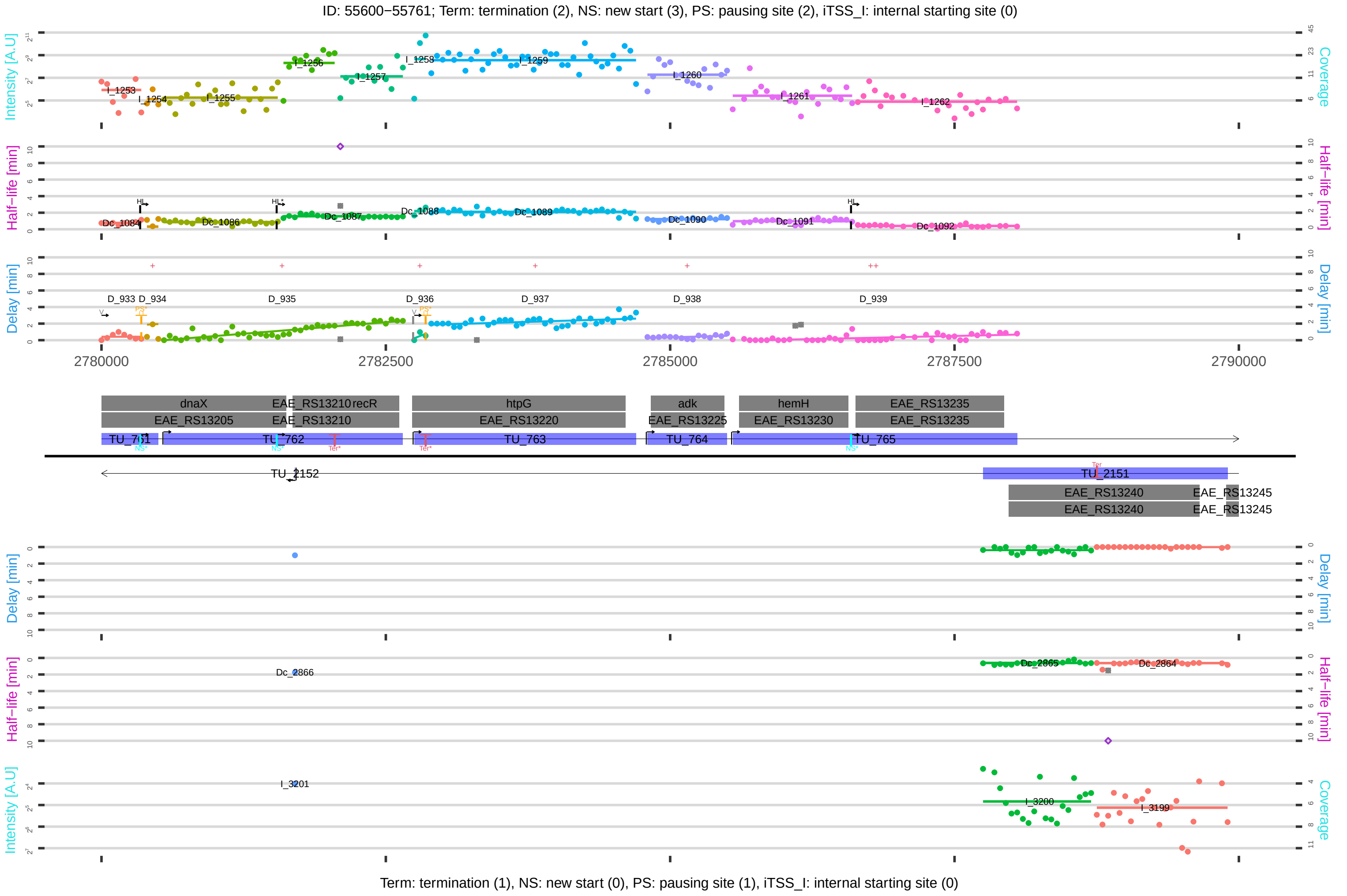

Term: termination (1), NS: new start (0), PS: pausing site (1), iTSS_I: internal starting site (0)

ID: 55826−56000; Term: termination (0), NS: new start (0), PS: pausing site (0), iTSS_I: internal starting site (0)

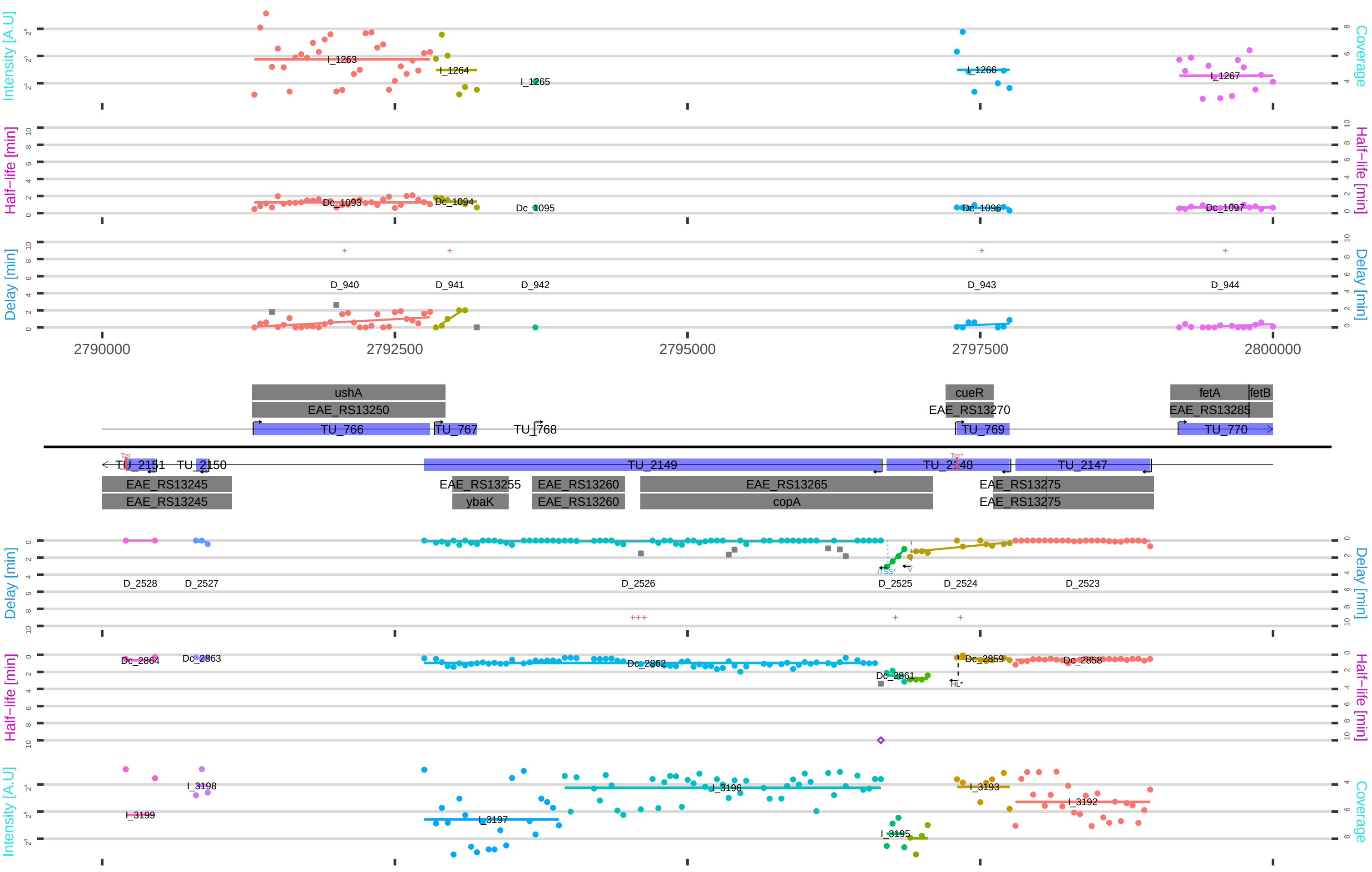

Term: termination (2), NS: new start (0), PS: pausing site (0), iTSS_I: internal starting site (1)

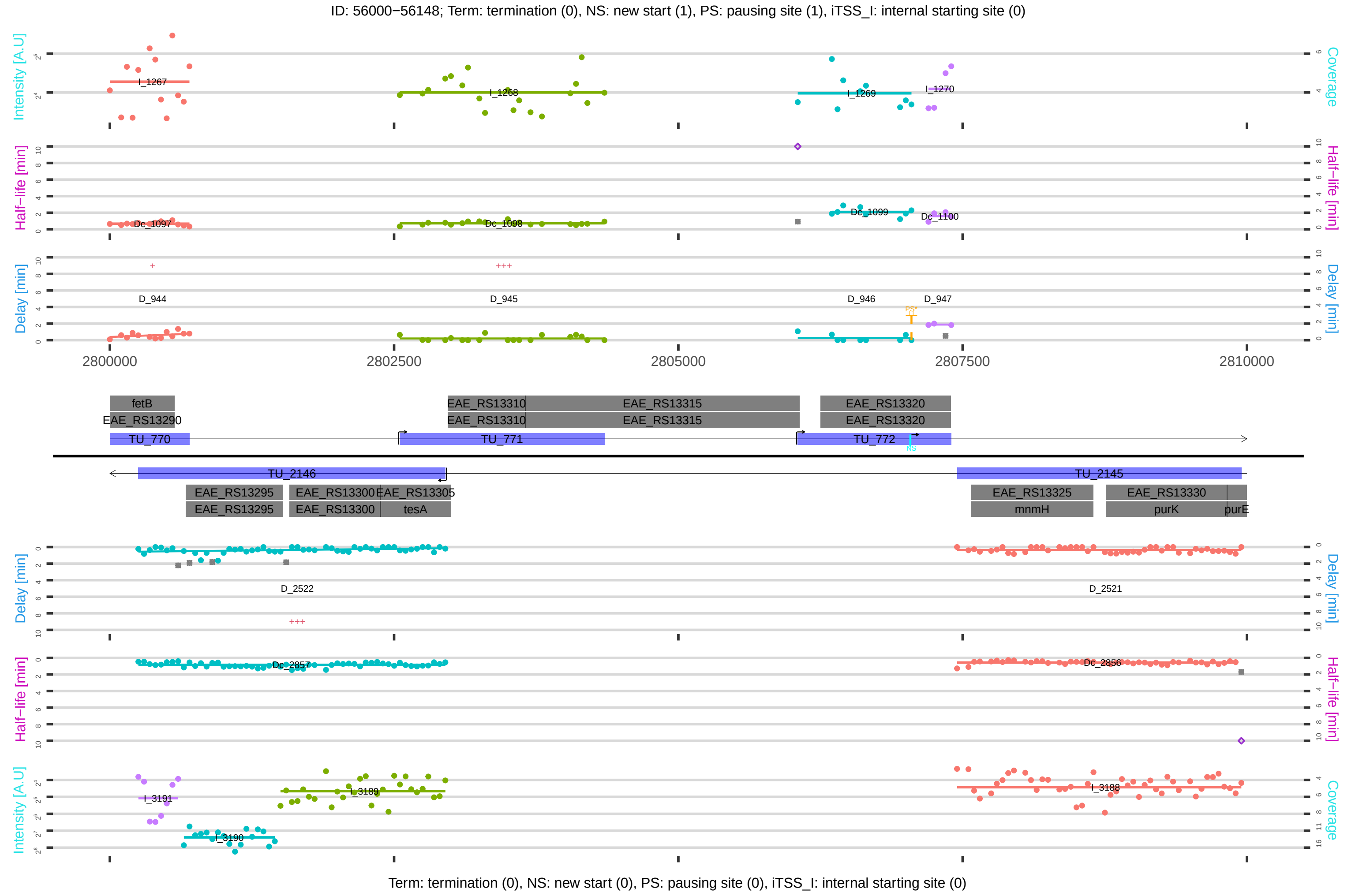

ID: 56000−56148; Term: termination (0), NS: new start (1), PS: pausing site (1), iTSS_I: internal starting site (0)
Intensity [A.U]
Coverage
I_1267
I_1268
I_1269
I_1270
Half−life [min]
Dc_1097
Dc_1098
Dc_1099
Dc_1100
Delay [min]
D_944
D_945
D_946
D_947
PS
2800000
2802500
2805000
2807500
2810000
fetB
EAE_RS13290
EAE_RS13310
EAE_RS13315
EAE_RS13320
TU_770
TU_771
TU_772
NS
TU_2146
TU_2145
EAE_RS13295
EAE_RS13300
EAE_RS13305
tesA
EAE_RS13325
mnmH
EAE_RS13330
purK
purE
D_2522
D_2521
Dc_2857
Dc_2856
I_3191
I_3190
I_3189
I_3188
Term: termination (0), NS: new start (0), PS: pausing site (0), iTSS_I: internal starting site (0)

ID: 56226−56295; Term: termination (0), NS: new start (0), PS: pausing site (0), iTSS_I: internal starting site (0)

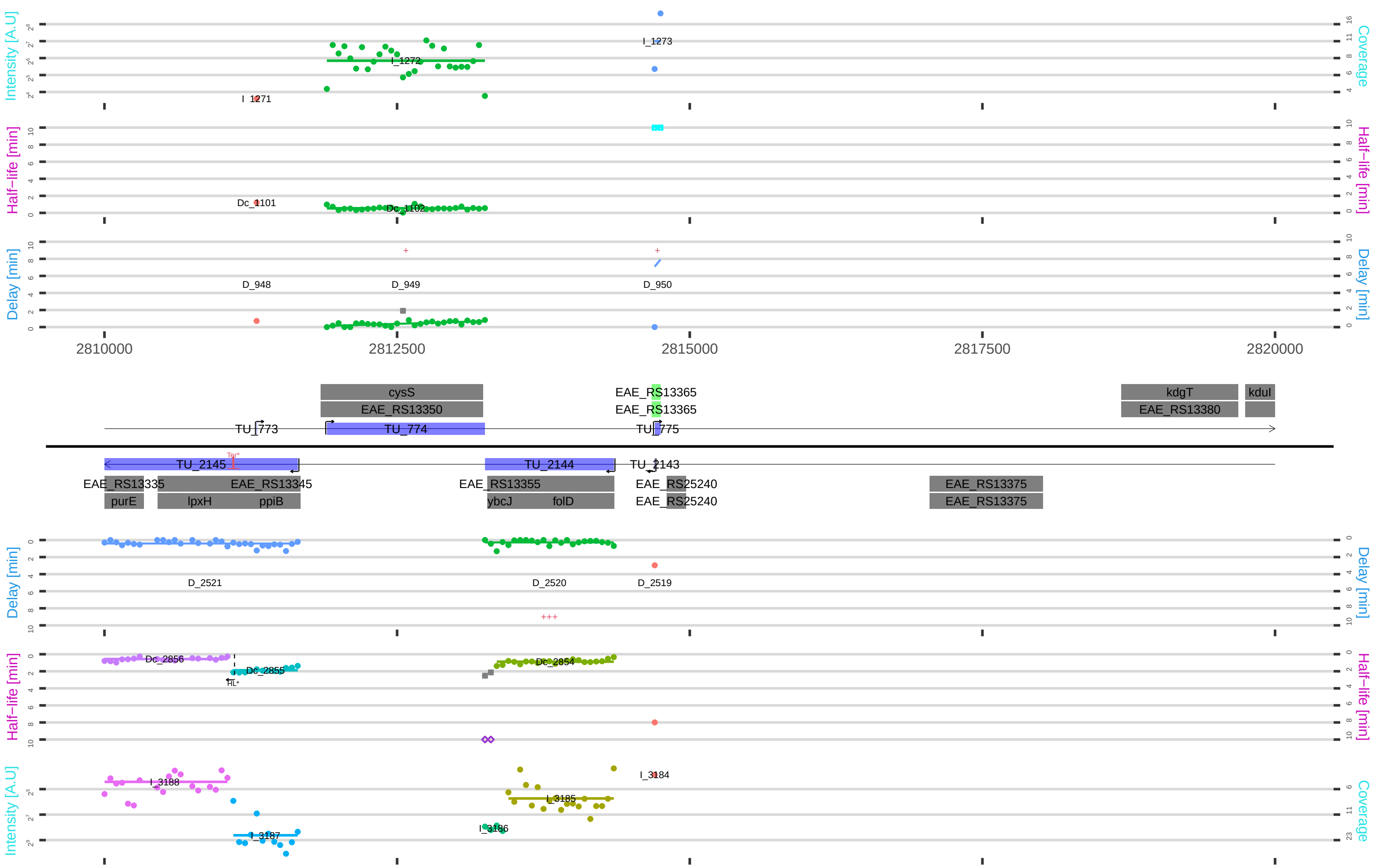

Term: termination (1), NS: new start (0), PS: pausing site (0), iTSS_I: internal starting site (0)

ID: 56509−56526; Term: termination (0), NS: new start (0), PS: pausing site (0), iTSS_I: internal starting site (0)

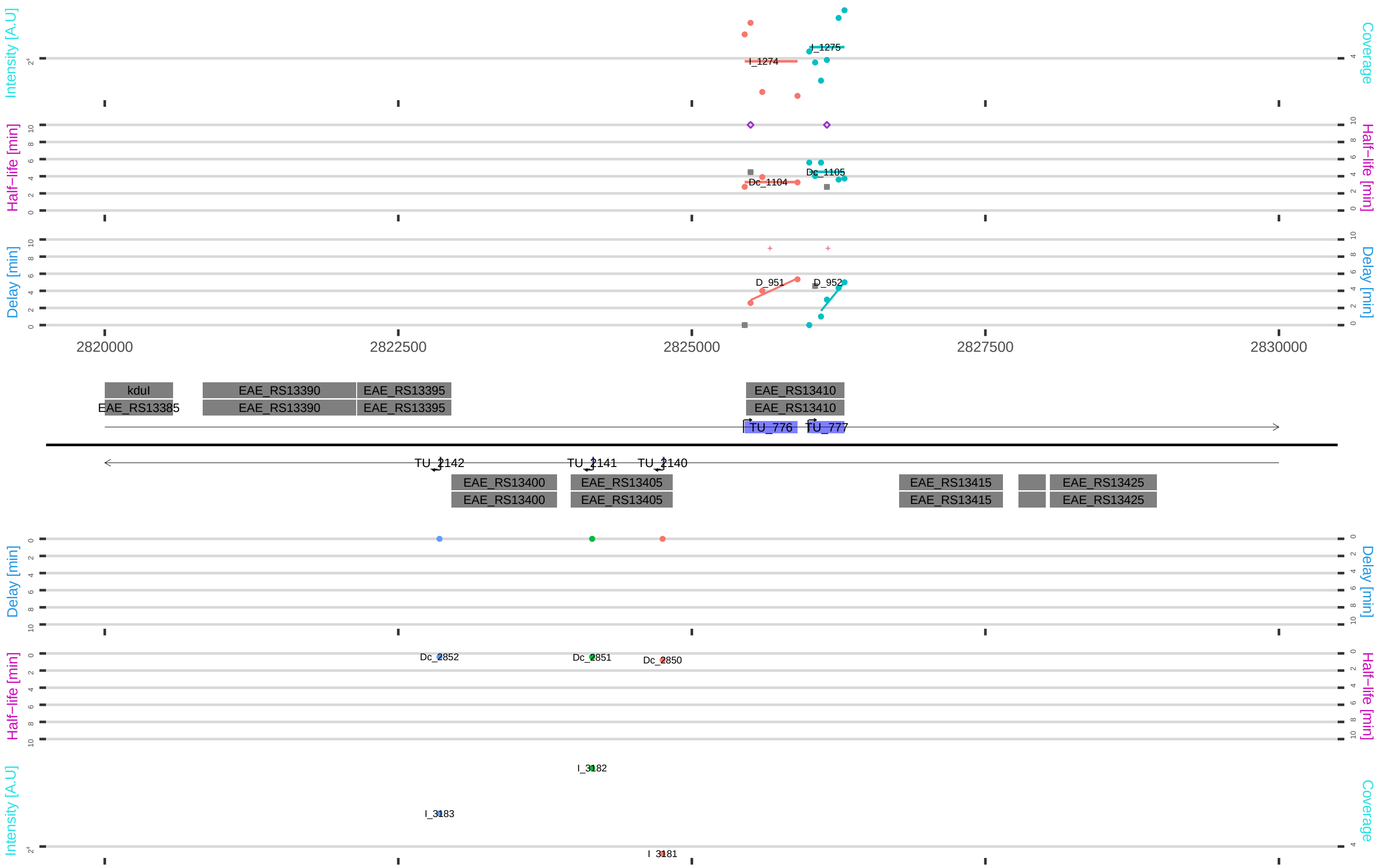

Term: termination (0), NS: new start (0), PS: pausing site (0), iTSS_I: internal starting site (0)

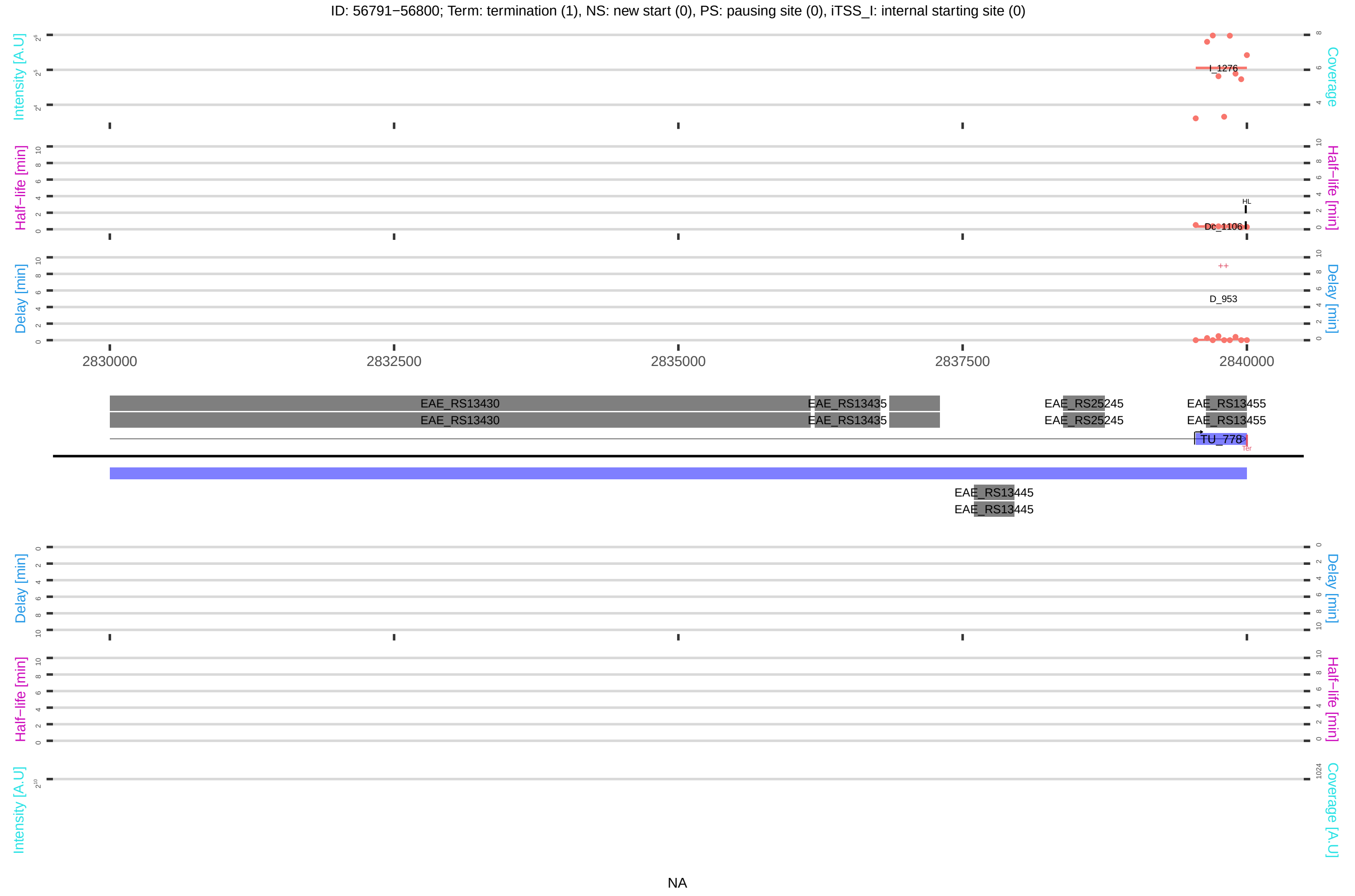

ID: 56791−56800; Term: termination (1), NS: new start (0), PS: pausing site (0), iTSS_I: internal starting site (0)
Intensity [A.U]
Coverage
Half−life [min]
Delay [min]
I_1276
HL
Dc_1106
D_953
2830000
2832500
2835000
2837500
2840000
EAE_RS13430
EAE_RS13435
EAE_RS25245
EAE_RS13455
TU_778
Ter
EAE_RS13445
Coverage [A.U]
NA

ID: 56800−57000; Term: termination (4), NS: new start (2), PS: pausing site (2), iTSS_I: internal starting site (3)

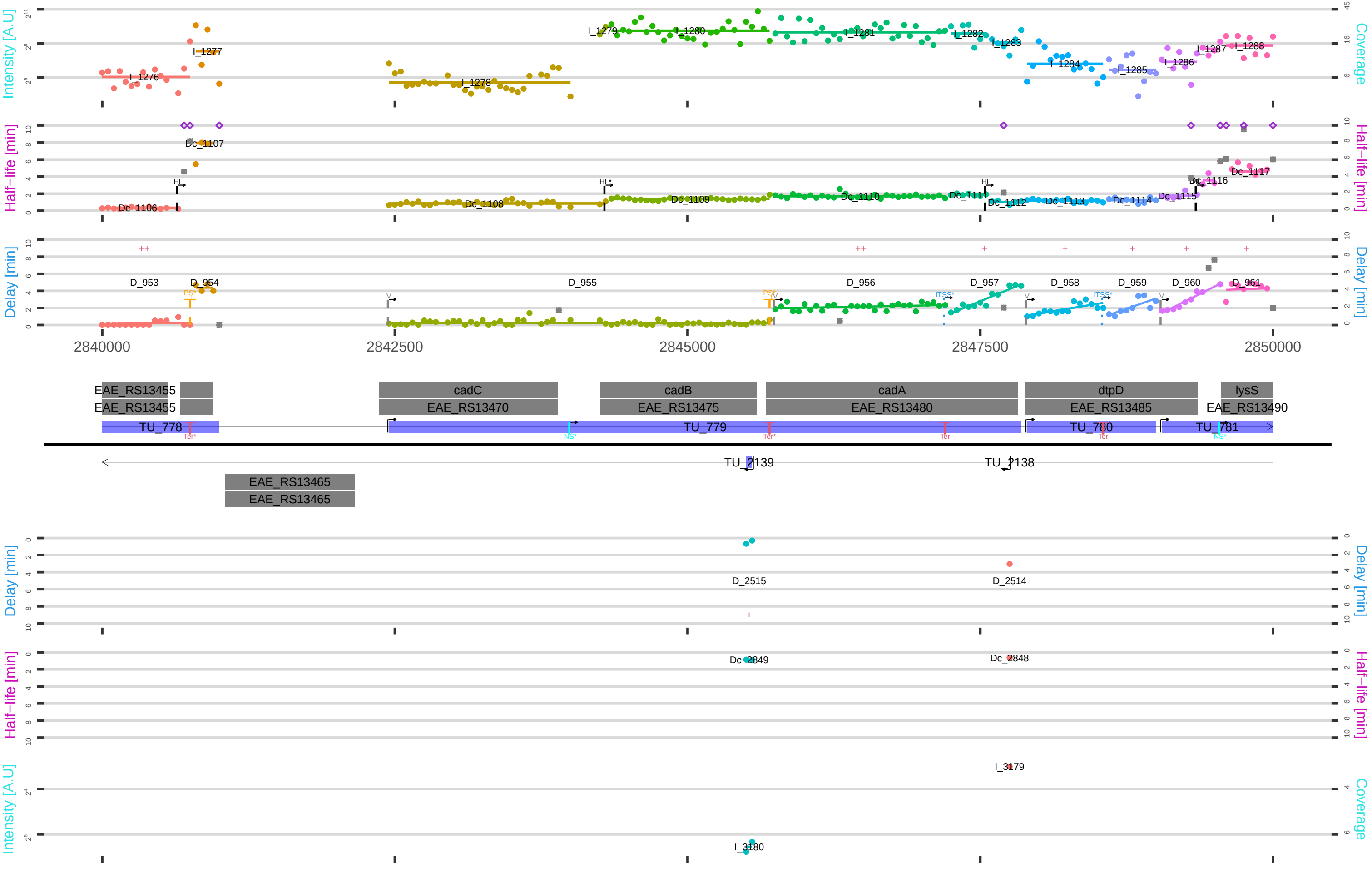

Term: termination (0), NS: new start (0), PS: pausing site (0), iTSS_I: internal starting site (0)

ID: 57000−57200; Term: termination (1), NS: new start (0), PS: pausing site (0), iTSS_I: internal starting site (0)

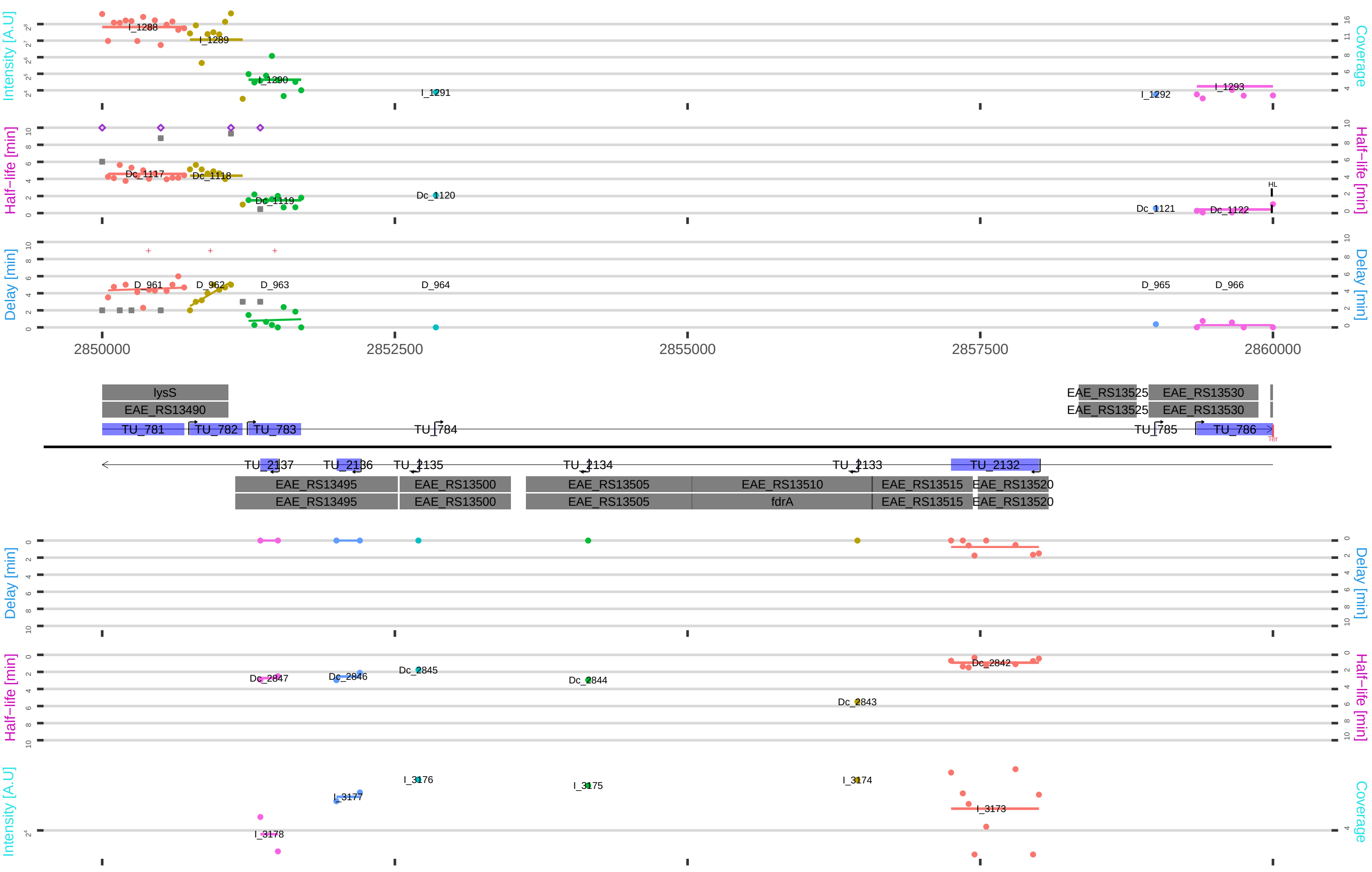

Term: termination (0), NS: new start (0), PS: pausing site (0), iTSS_I: internal starting site (0)

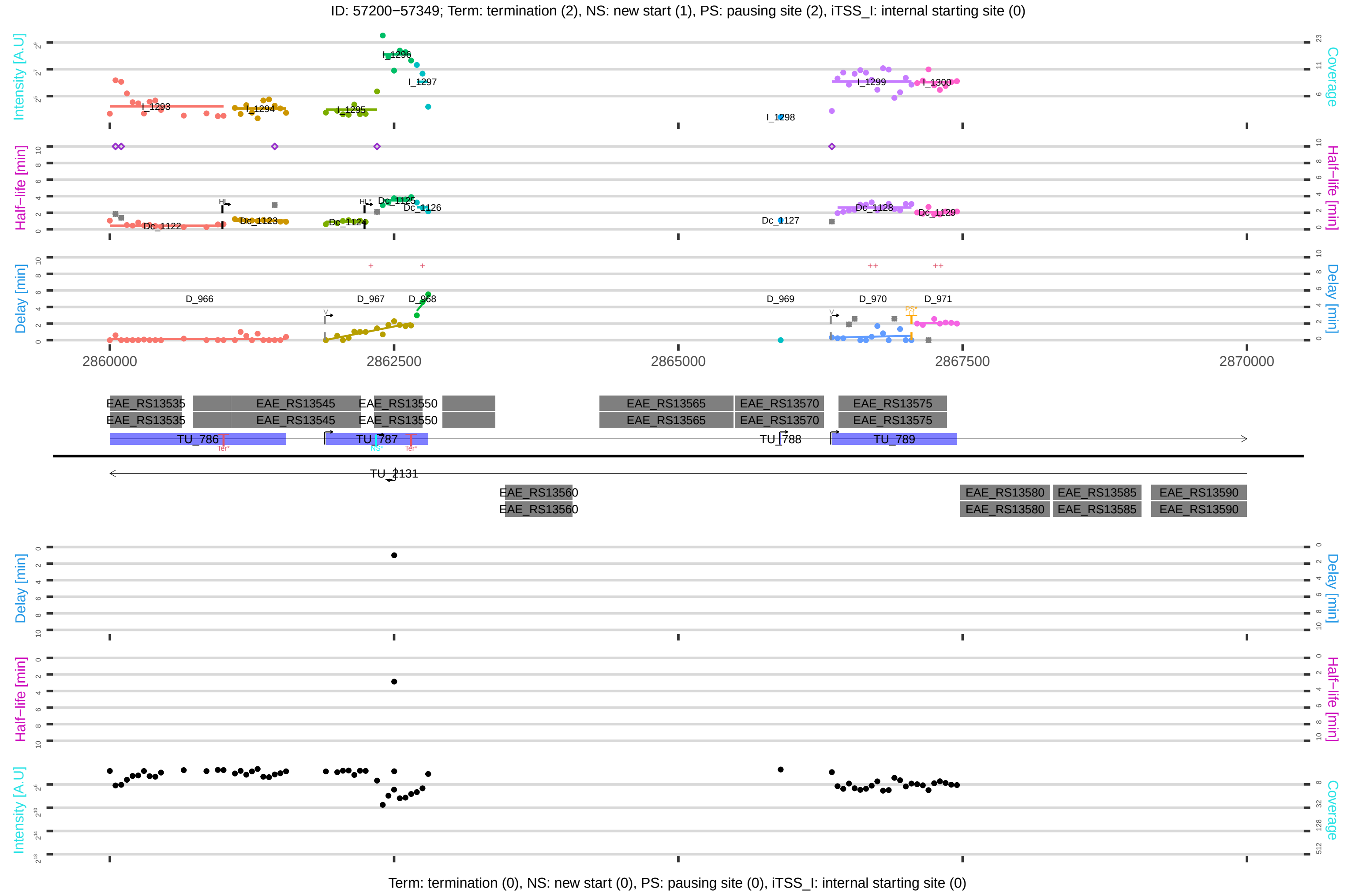

ID: 57200−57349; Term: termination (2), NS: new start (1), PS: pausing site (2), iTSS_I: internal starting site (0)
Intensity [A.U]
Coverage
Half−life [min]
Delay [min]
I_1293
I_1294
I_1295
I_1296
I_1297
I_1298
I_1299
I_1300
Dc_1122
Dc_1123
Dc_1124
Dc_1125
Dc_1126
Dc_1127
Dc_1128
Dc_1129
D_966
D_967
D_968
D_969
D_970
D_971
2860000
2862500
2865000
2867500
2870000
EAE_RS13535
EAE_RS13545
EAE_RS13550
EAE_RS13565
EAE_RS13570
EAE_RS13575
TU_786
TU_787
TU_788
TU_789
TU_2131
EAE_RS13560
EAE_RS13580
EAE_RS13585
EAE_RS13590
Term: termination (0), NS: new start (0), PS: pausing site (0), iTSS_I: internal starting site (0)

ID: 153631−153631; FC*: significant t−test of two consecutive segments; Term: termination, NS: new start, PS: pausing site,iTSS_I: internal starting site,TI: transcription interferance.

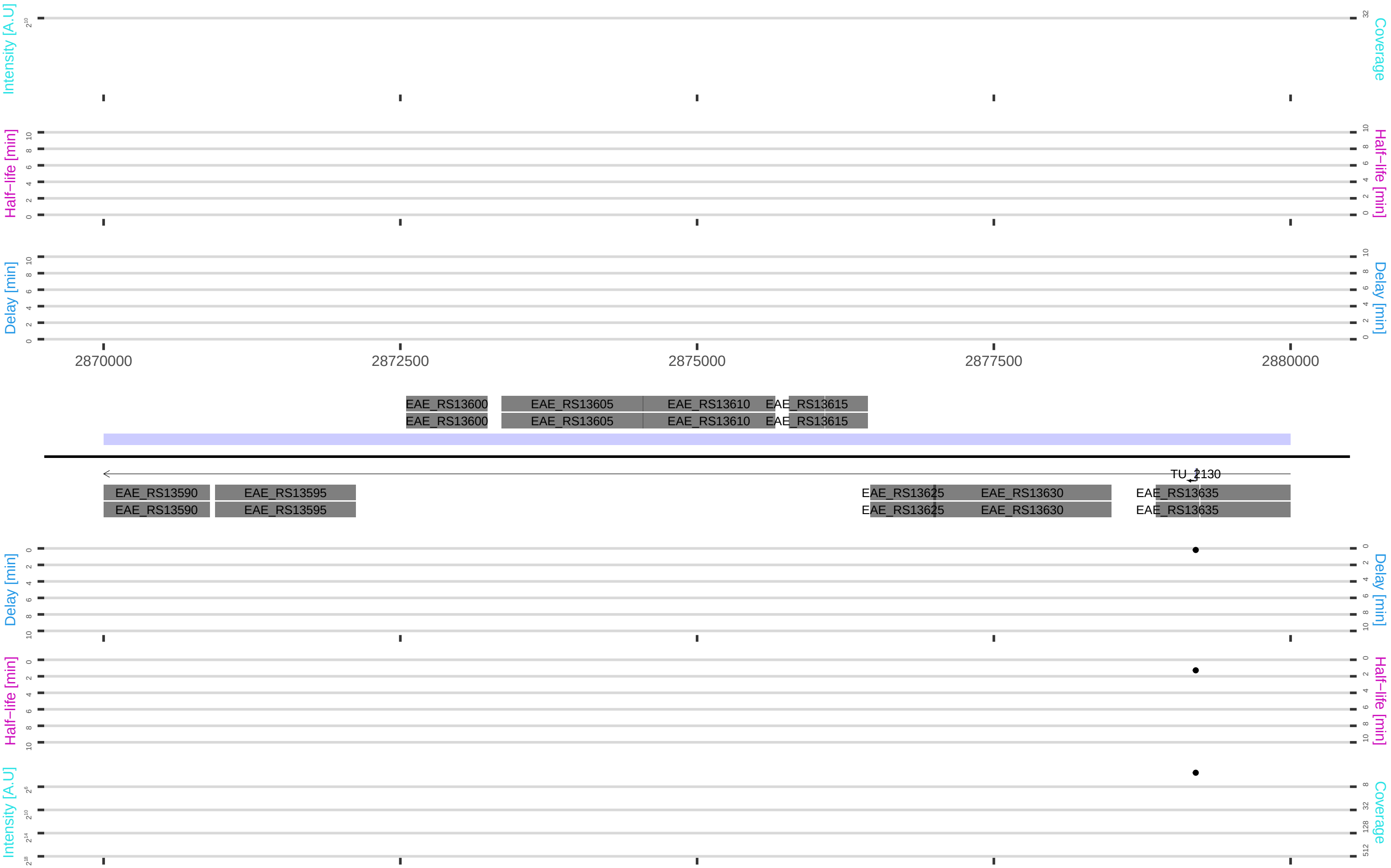

Term: termination (0), NS: new start (0), PS: pausing site (0), iTSS_I: internal starting site (0)

ID: 57612−57780; Term: termination (0), NS: new start (0), PS: pausing site (0), iTSS_I: internal starting site (0)

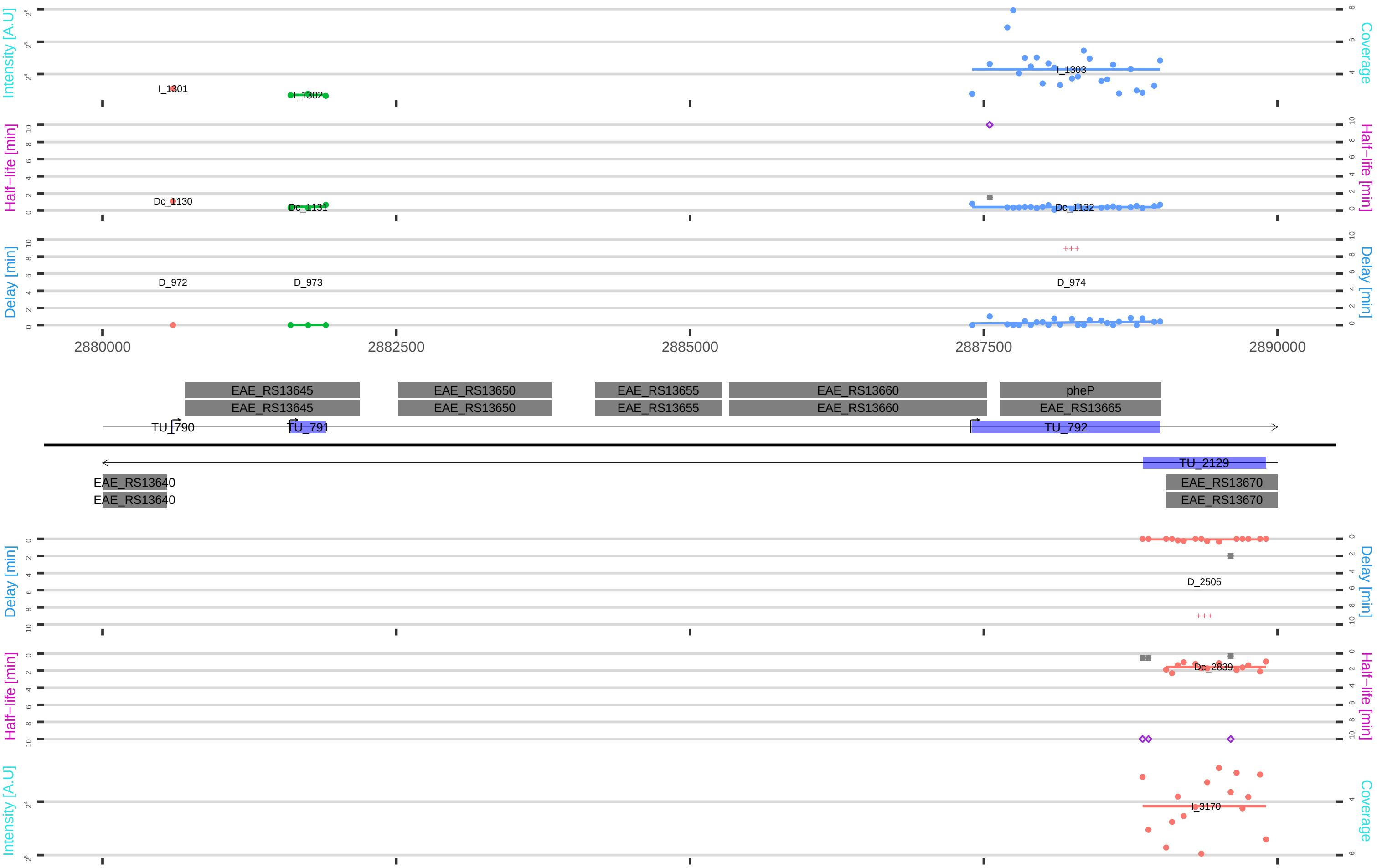

Term: termination (0), NS: new start (0), PS: pausing site (0), iTSS_I: internal starting site (0)

ID: 57839−57998; Term: termination (1), NS: new start (1), PS: pausing site (1), iTSS_I: internal starting site (0)

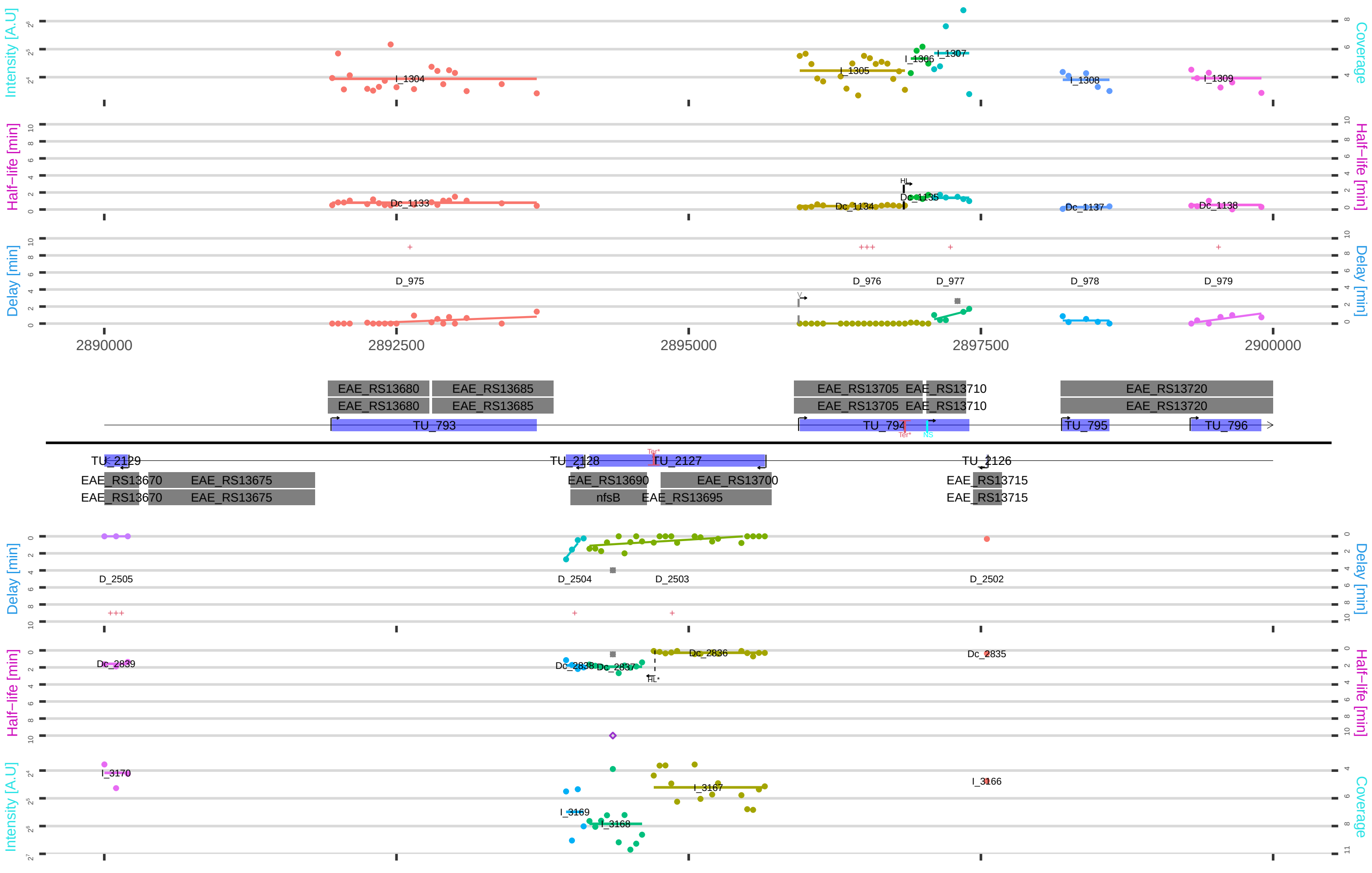

Term: termination (1), NS: new start (0), PS: pausing site (0), iTSS_I: internal starting site (0)

ID: 58004−58184; Term: termination (0), NS: new start (0), PS: pausing site (0), iTSS_I: internal starting site (0)

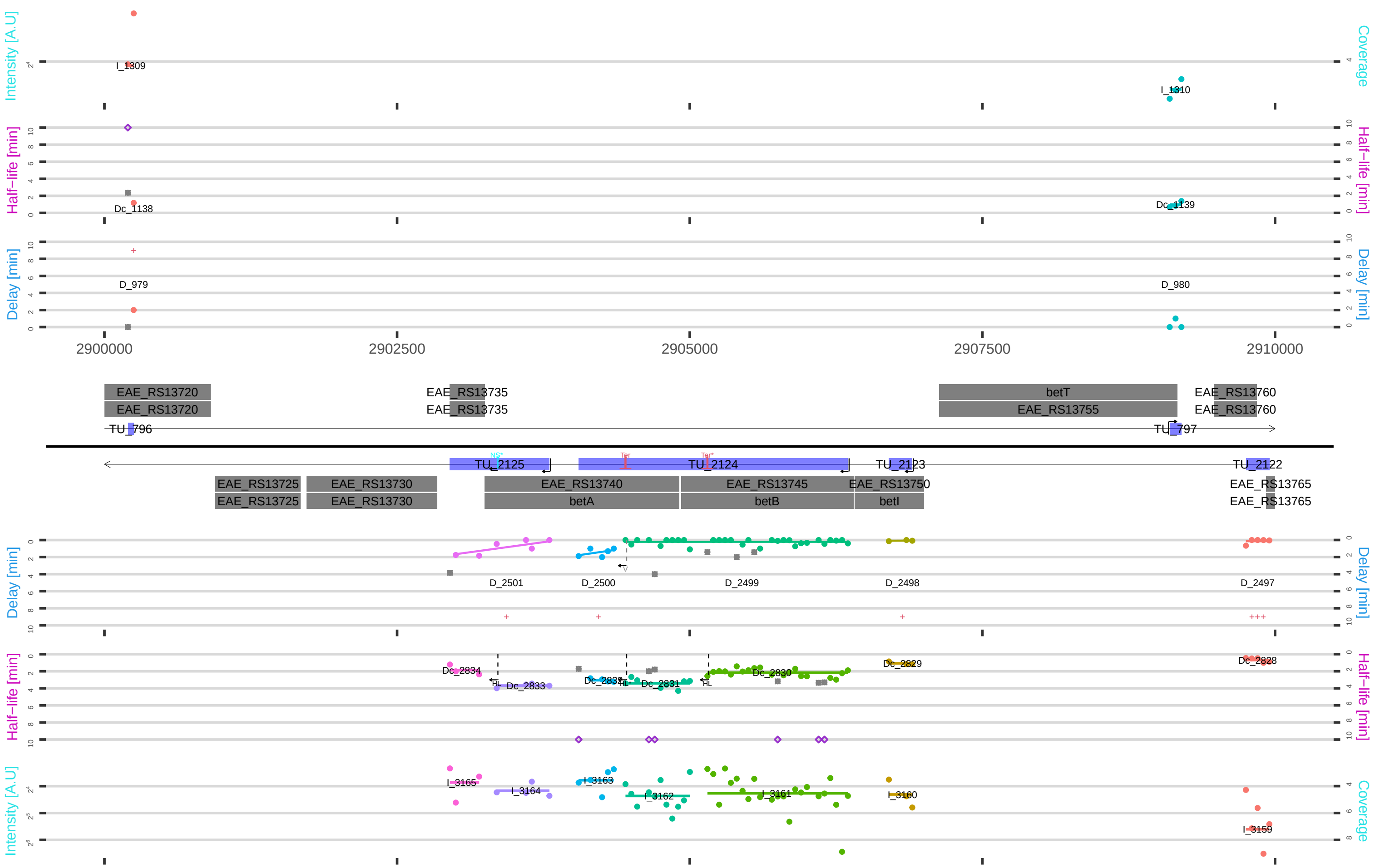

Term: termination (2), NS: new start (1), PS: pausing site (1), iTSS_I: internal starting site (0)

ID: 153015−152866; FC*: significant t−test of two consecutive segments; Term: termination, NS: new start, PS: pausing site,iTSS_I: internal starting site,TI: transcription interferance.

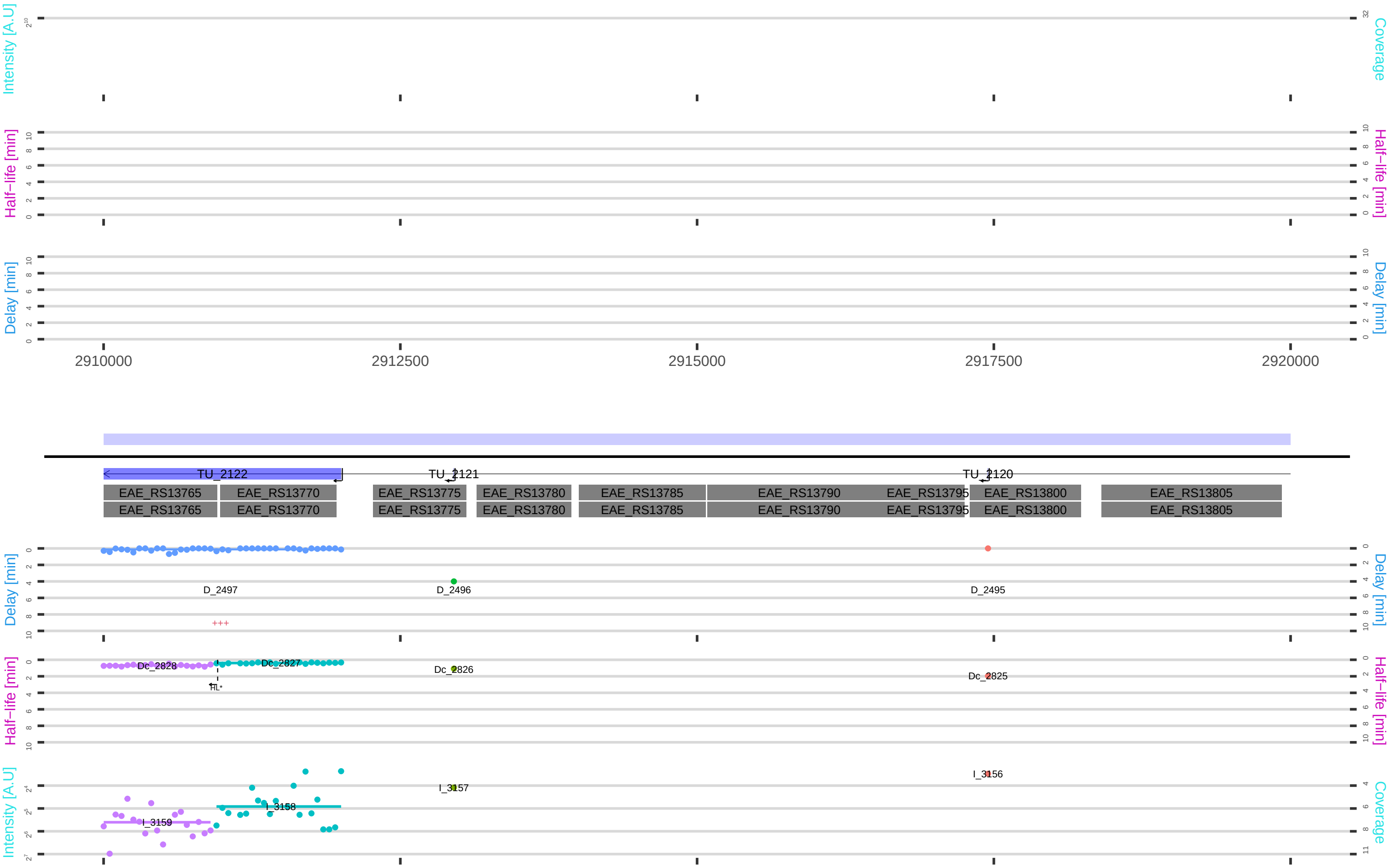

Term: termination (0), NS: new start (0), PS: pausing site (0), iTSS_I: internal starting site (0)

ID: 58543−58600; Term: termination (0), NS: new start (0), PS: pausing site (0), iTSS_I: internal starting site (0)

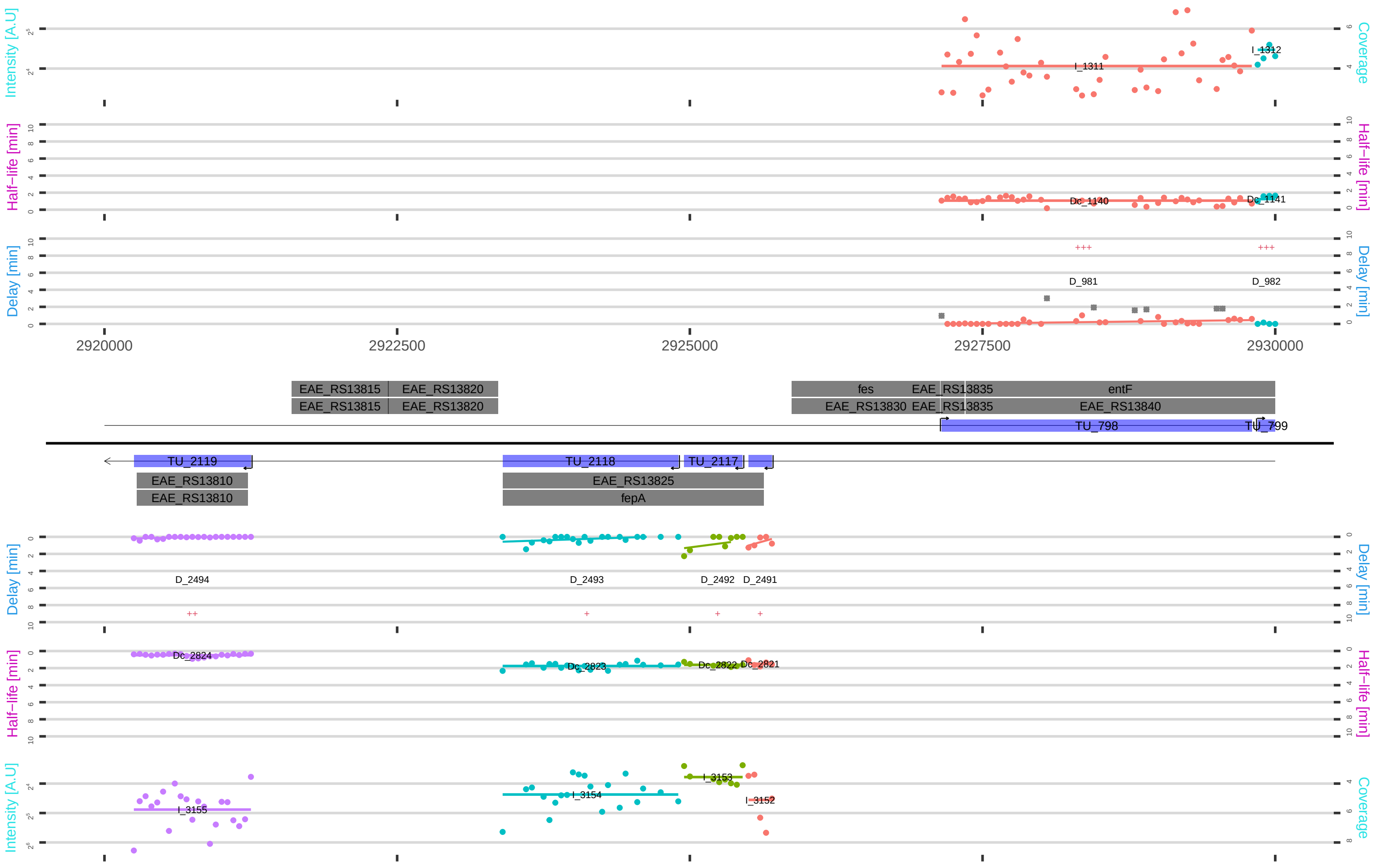

Term: termination (0), NS: new start (0), PS: pausing site (0), iTSS_I: internal starting site (0)

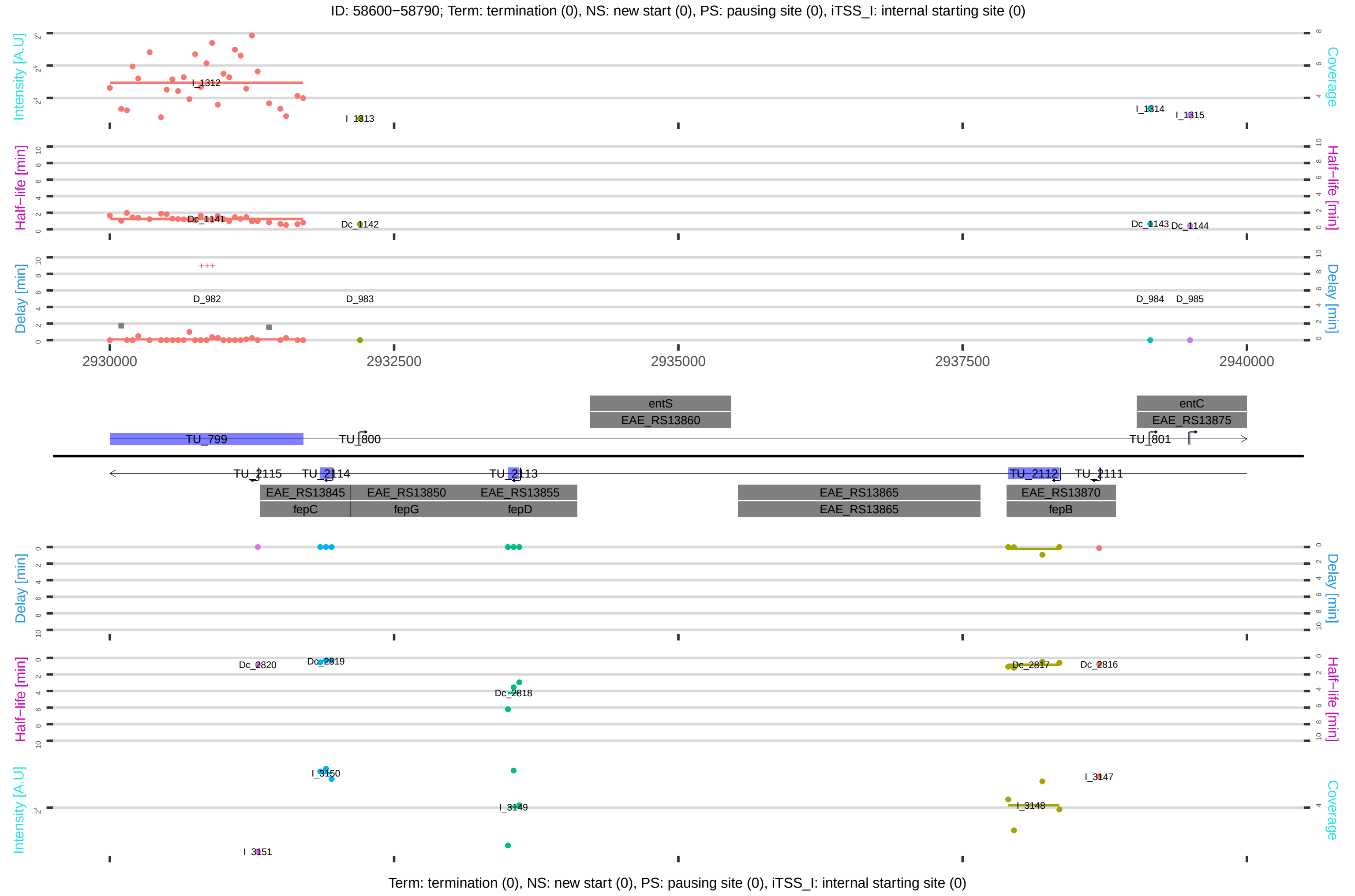
ID: 58600−58790; Term: termination (0), NS: new start (0), PS: pausing site (0), iTSS_I: internal starting site (0)
Intensity [A.U]
Coverage
Half−life [min]
Delay [min]
I_1312
I_1313
I_1314
I_1315
Dc_1141
Dc_1142
Dc_1143
Dc_1144
D_982
D_983
D_984
D_985
+++
2930000
2932500
2935000
2937500
2940000
entS
EAE_RS13860
entC
EAE_RS13875
TU_799
TU_800
TU_801
TU_2115
TU_2114
TU_2113
TU_2112
TU_2111
EAE_RS13845
EAE_RS13850
EAE_RS13855
EAE_RS13865
EAE_RS13870
fepC
fepG
fepD
fepB
Dc_2820
Dc_2819
Dc_2818
Dc_2817
Dc_2816
I_3150
I_3149
I_3148
I_3147
I_3151
Term: termination (0), NS: new start (0), PS: pausing site (0), iTSS_I: internal starting site (0)

ID: 58805−58925; Term: termination (0), NS: new start (0), PS: pausing site (0), iTSS_I: internal starting site (0)

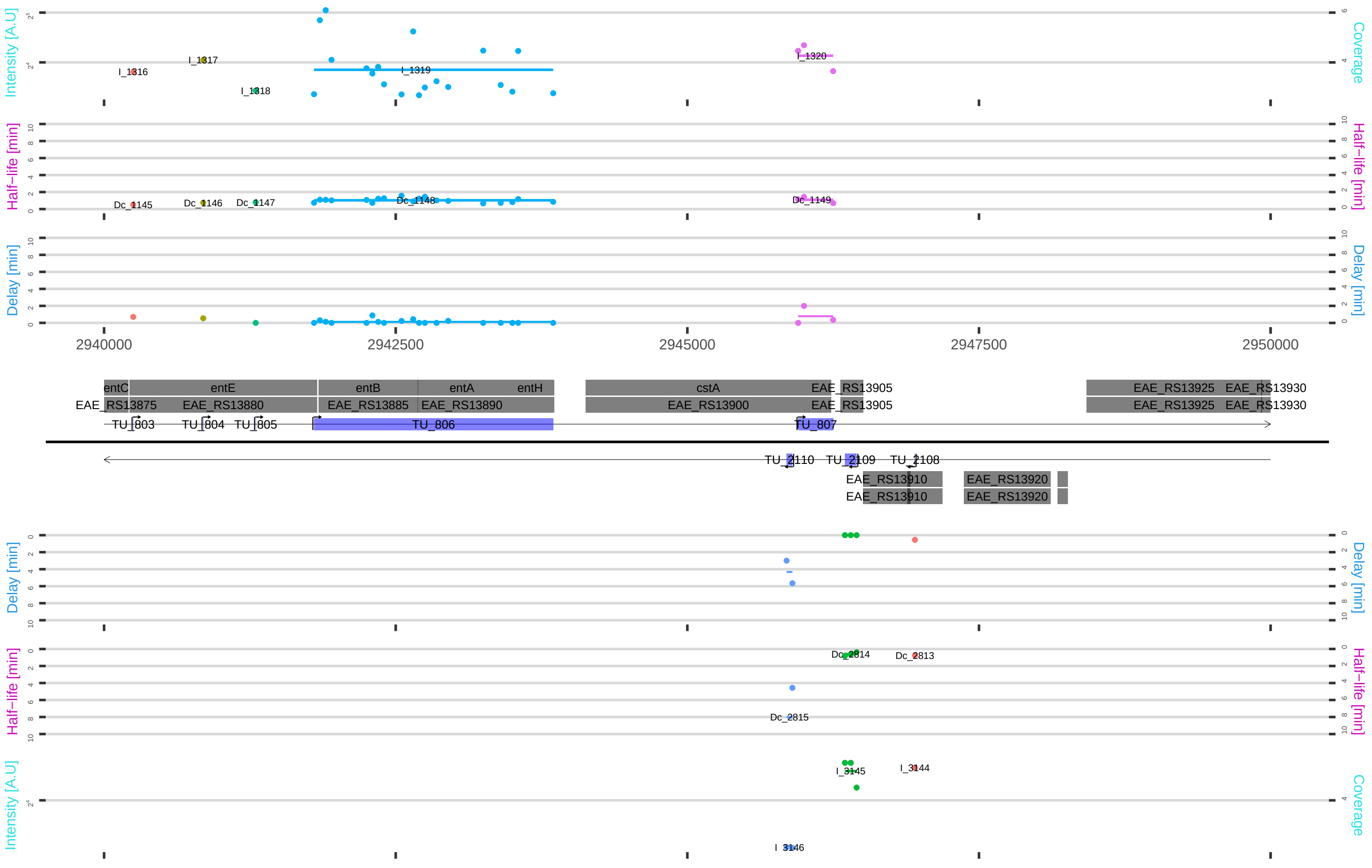

Term: termination (0), NS: new start (0), PS: pausing site (0), iTSS_I: internal starting site (0)

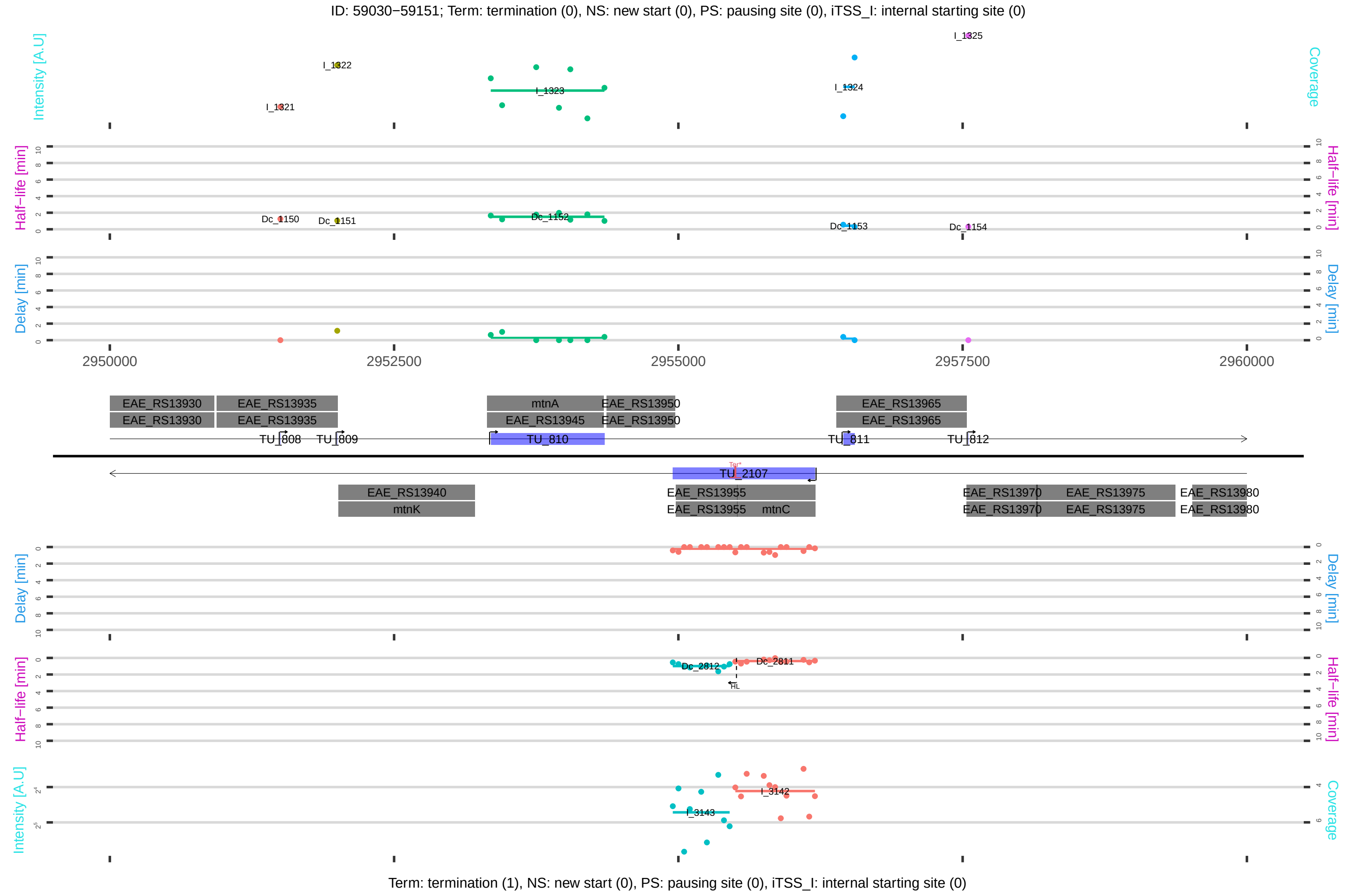

ID: 59030−59151; Term: termination (0), NS: new start (0), PS: pausing site (0), iTSS_I: internal starting site (0)
Intensity [A.U]
Coverage
I_1321
I_1322
I_1323
I_1324
I_1325
Half−life [min]
Dc_1150
Dc_1151
Dc_1152
Dc_1153
Dc_1154
Delay [min]
2950000
2952500
2955000
2957500
2960000
EAE_RS13930
EAE_RS13935
mtnA
EAE_RS13945
EAE_RS13950
EAE_RS13965
TU_808
TU_809
TU_810
TU_811
TU_812
TU_2107
EAE_RS13940
mtnK
EAE_RS13955
mtnC
EAE_RS13970
EAE_RS13975
EAE_RS13980
Dc_2811
Dc_2812
HL
I_3142
I_3143
Term: termination (1), NS: new start (0), PS: pausing site (0), iTSS_I: internal starting site (0)

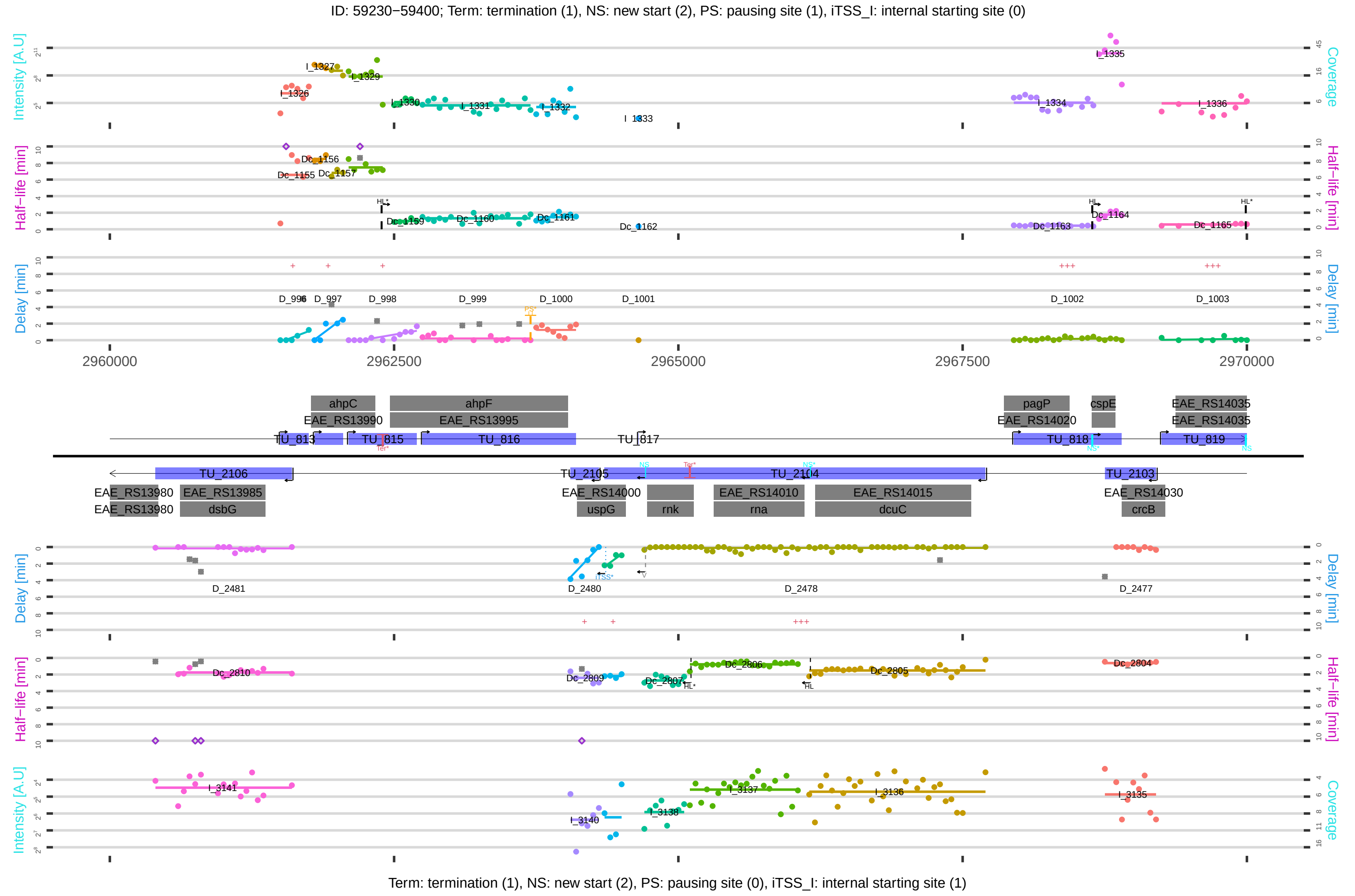
ID: 59230−59400; Term: termination (1), NS: new start (2), PS: pausing site (1), iTSS_I: internal starting site (0)
Intensity [A.U]
Coverage
Half−life [min]
Delay [min]
2960000
2962500
2965000
2967500
2970000
ahpC
ahpF
EAE_RS13990
EAE_RS13995
pagP
cspE
EAE_RS14035
EAE_RS14020
TU_813
TU_815
TU_816
TU_817
TU_818
TU_819
TU_2106
TU_2105
TU_2104
TU_2103
EAE_RS13980
EAE_RS13985
EAE_RS14000
EAE_RS14010
EAE_RS14015
EAE_RS14030
dsbG
uspG
rnk
rna
dcuC
crcB
D_2481
D_2480
D_2478
D_2477
Dc_2810
Dc_2809
Dc_2807
Dc_2806
Dc_2805
Dc_2804
I_3141
I_3140
I_3138
I_3137
I_3136
I_3135
Term: termination (1), NS: new start (2), PS: pausing site (0), iTSS_I: internal starting site (1)

ID: 59400−59474; Term: termination (1), NS: new start (1), PS: pausing site (0), iTSS_I: internal starting site (0)

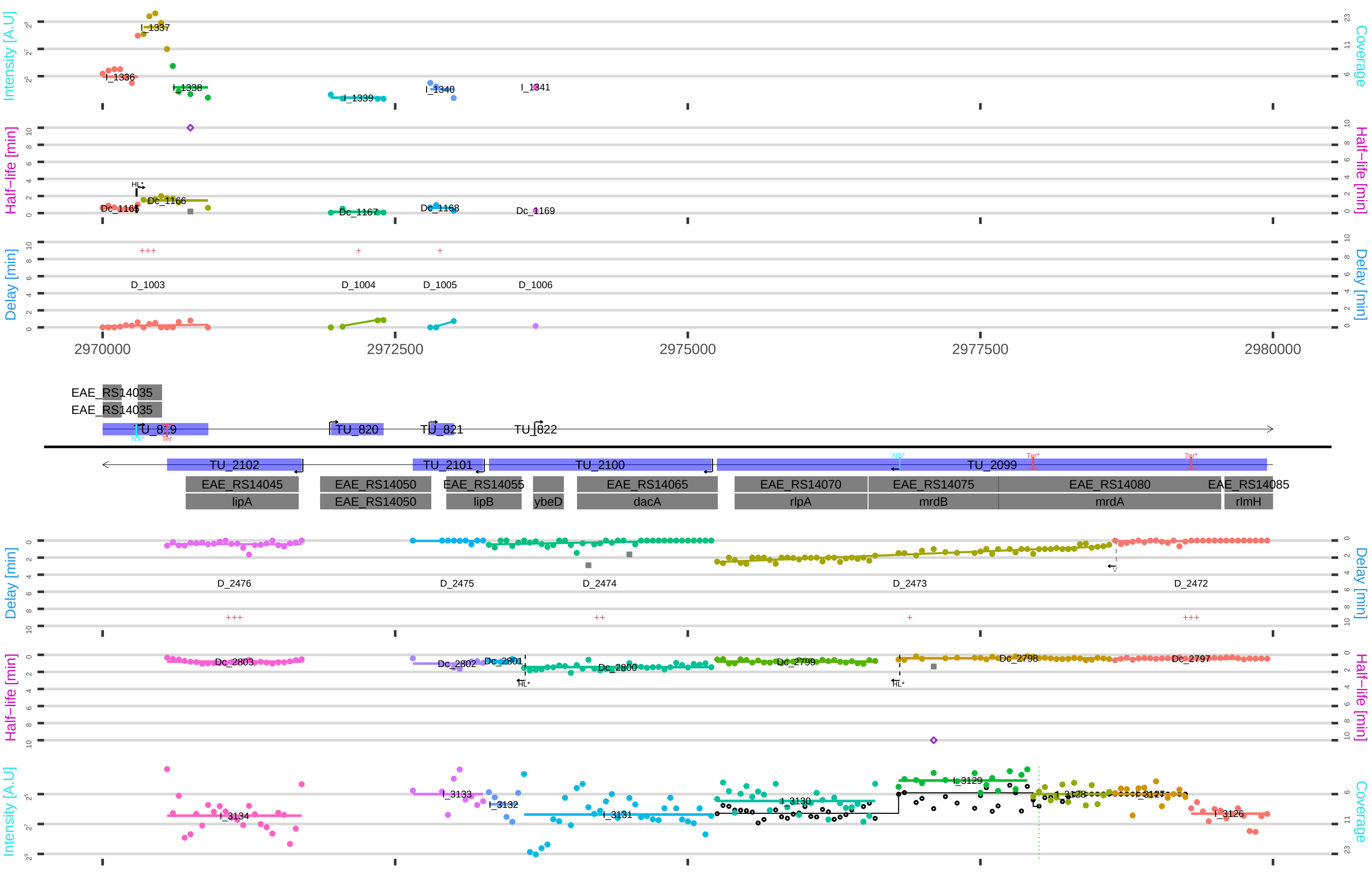

Term: termination (2), NS: new start (1), PS: pausing site (1), iTSS_I: internal starting site (0)

ID: 59730−59736; Term: termination (0), NS: new start (0), PS: pausing site (0), iTSS_I: internal starting site (0)

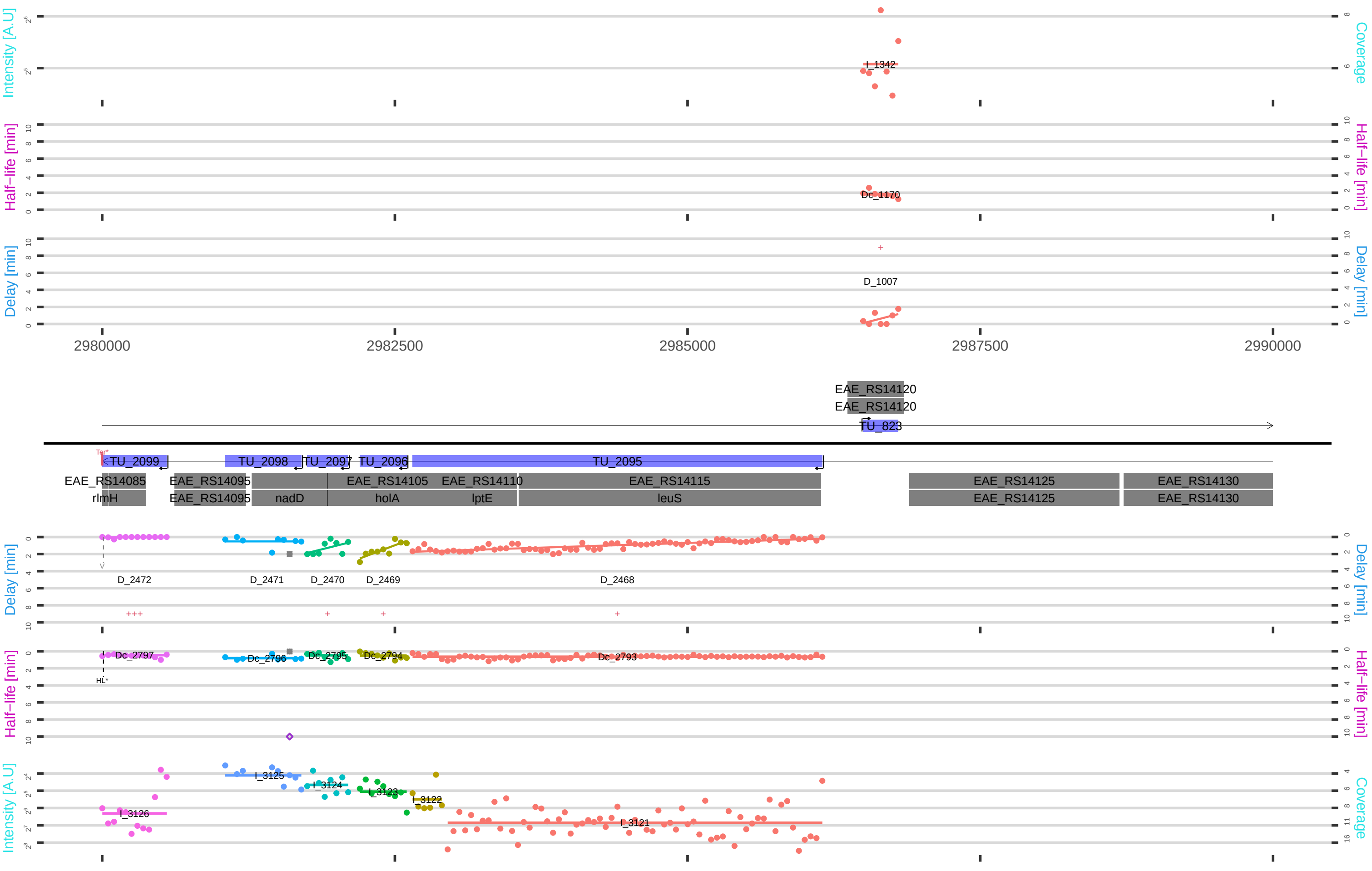

Term: termination (1), NS: new start (0), PS: pausing site (0), iTSS_I: internal starting site (0)

ID: 59951−59951; Term: termination (0), NS: new start (0), PS: pausing site (0), iTSS_I: internal starting site (0)

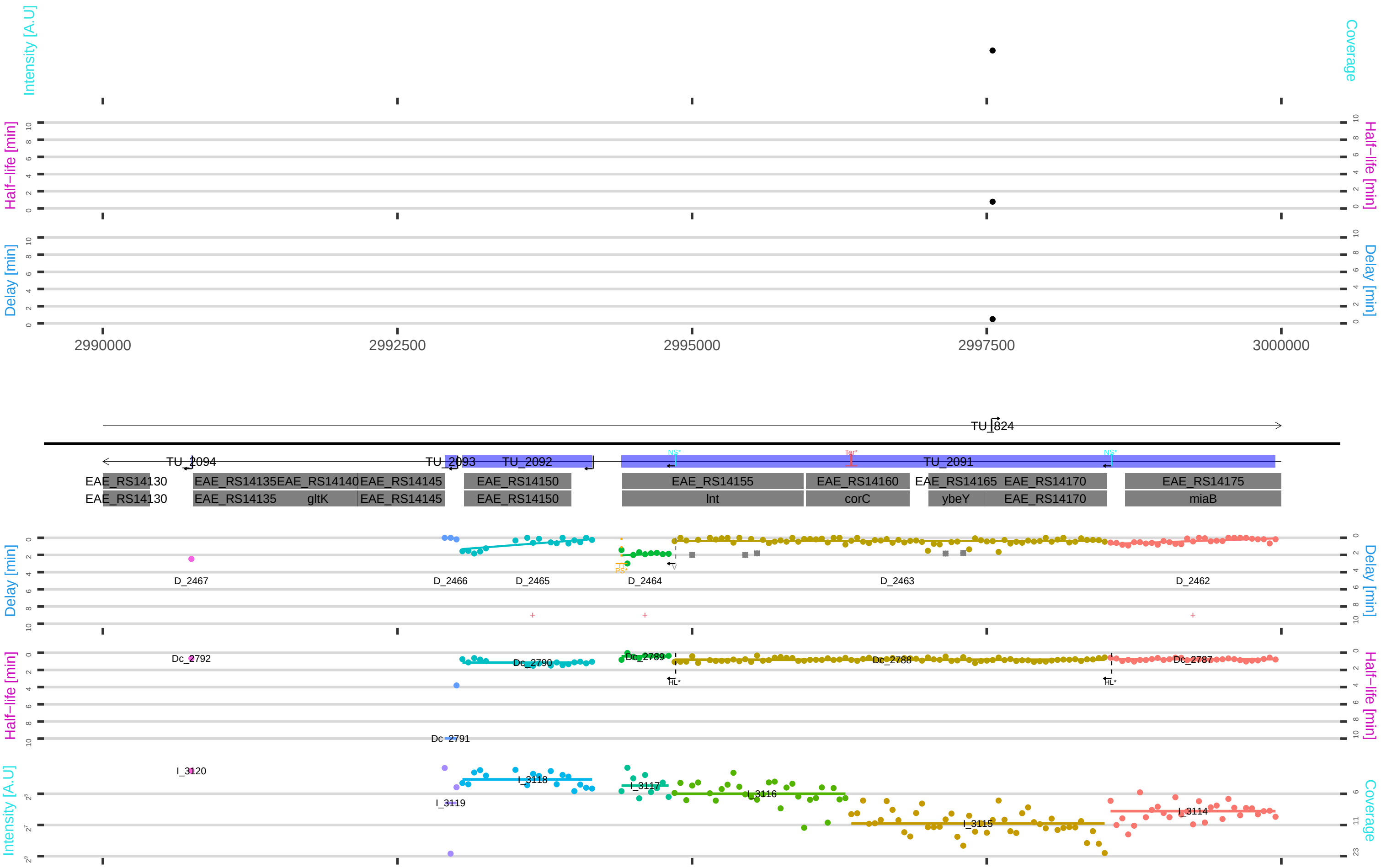

Term: termination (1), NS: new start (2), PS: pausing site (1), iTSS_I: internal starting site (1)

ID: 60005−60200; Term: termination (2), NS: new start (4), PS: pausing site (1), iTSS_I: internal starting site (0)

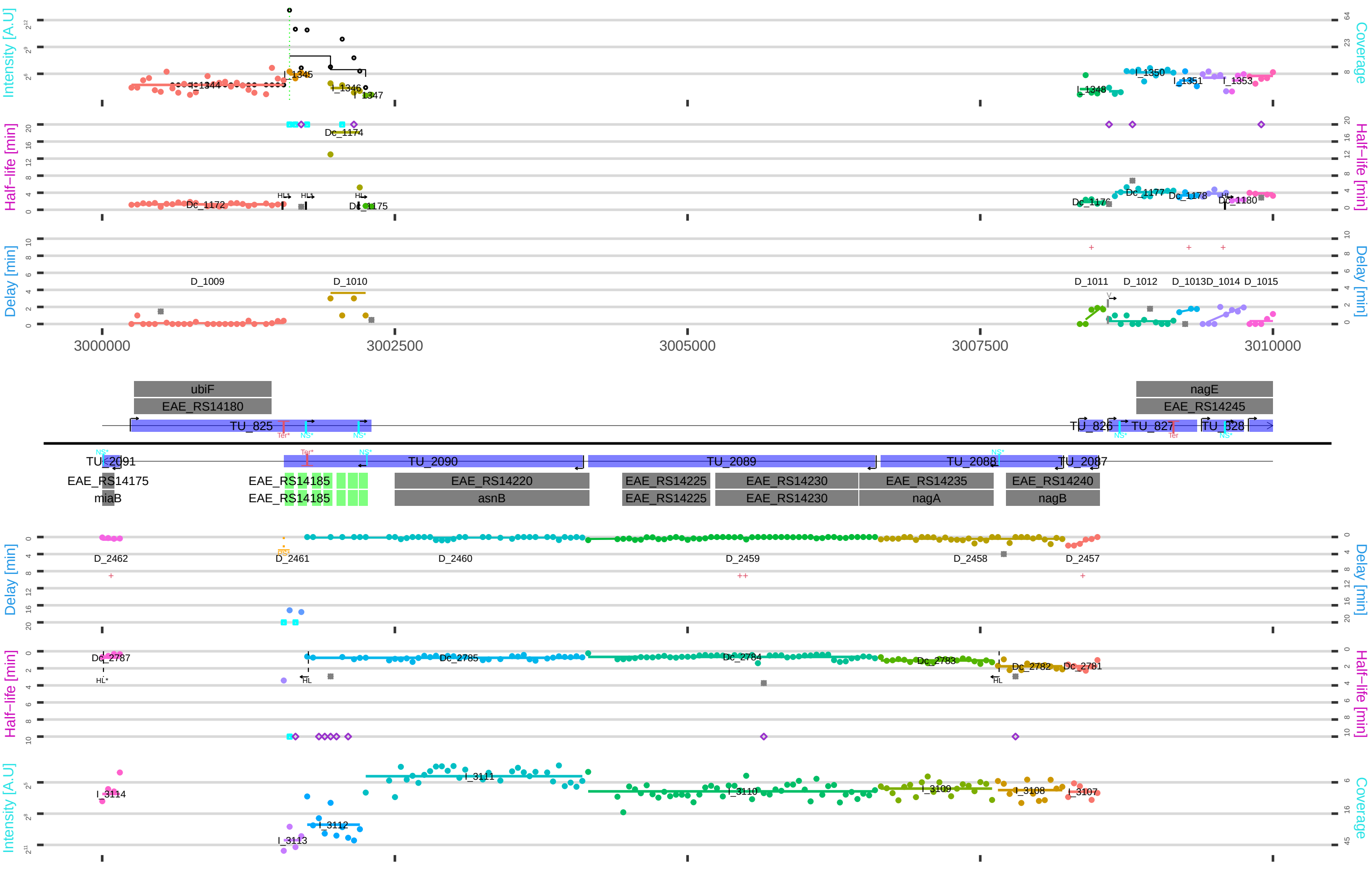

Term: termination (1), NS: new start (3), PS: pausing site (1), iTSS_I: internal starting site (0)

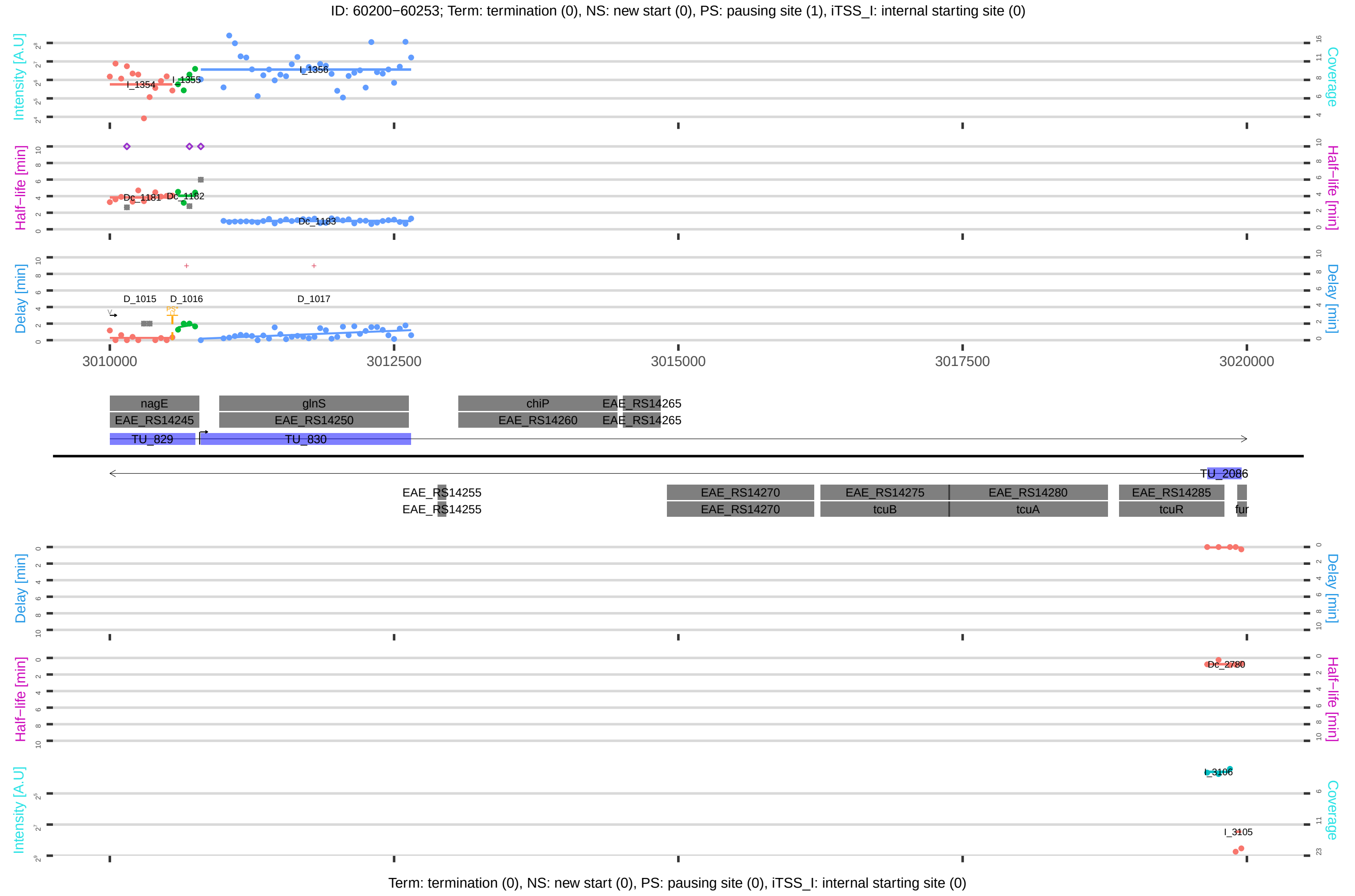
ID: 60200−60253; Term: termination (0), NS: new start (0), PS: pausing site (1), iTSS_I: internal starting site (0)
Intensity [A.U]
Coverage
I_1354
I_1355
I_1356
Half−life [min]
Dc_1181
Dc_1182
Dc_1183
Delay [min]
D_1015
D_1016
D_1017
3010000
3012500
3015000
3017500
3020000
nagE
EAE_RS14245
glnS
EAE_RS14250
chiP
EAE_RS14260
EAE_RS14265
TU_829
TU_830
TU_2086
EAE_RS14255
EAE_RS14270
EAE_RS14275
tcuB
EAE_RS14280
tcuA
EAE_RS14285
tcuR
fur
Dc_2780
I_3106
I_3105
Term: termination (0), NS: new start (0), PS: pausing site (0), iTSS_I: internal starting site (0)

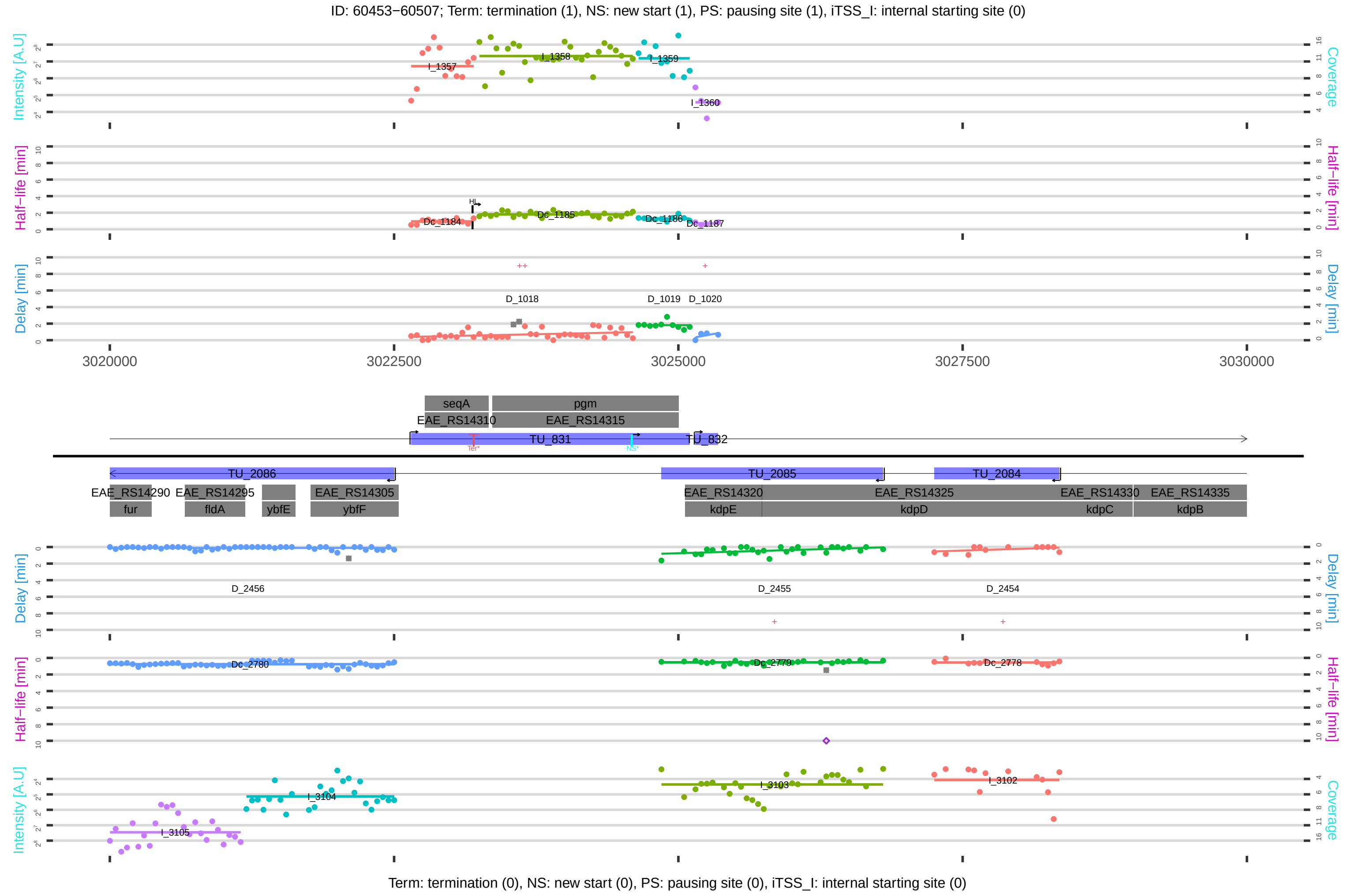

ID: 60453−60507; Term: termination (1), NS: new start (1), PS: pausing site (1), iTSS_I: internal starting site (0)
Intensity [A.U]
Coverage
I_1357
I_1358
I_1359
I_1360
Half−life [min]
Dc_1184
Dc_1185
Dc_1186
Dc_1187
HL
Delay [min]
D_1018
D_1019
D_1020
3020000
3022500
3025000
3027500
3030000
seqA
pgm
EAE_RS14310
EAE_RS14315
TU_831
TU_832
Ter
NS
TU_2086
TU_2085
TU_2084
EAE_RS14290
EAE_RS14295
EAE_RS14305
EAE_RS14320
EAE_RS14325
EAE_RS14330
EAE_RS14335
fur
fldA
ybfE
ybfF
kdpE
kdpD
kdpC
kdpB
D_2456
D_2455
D_2454
Dc_2780
Dc_2779
Dc_2778
I_3105
I_3104
I_3103
I_3102
Term: termination (0), NS: new start (0), PS: pausing site (0), iTSS_I: internal starting site (0)

ID: 60663−60800; Term: termination (2), NS: new start (2), PS: pausing site (2), iTSS_I: internal starting site (1)

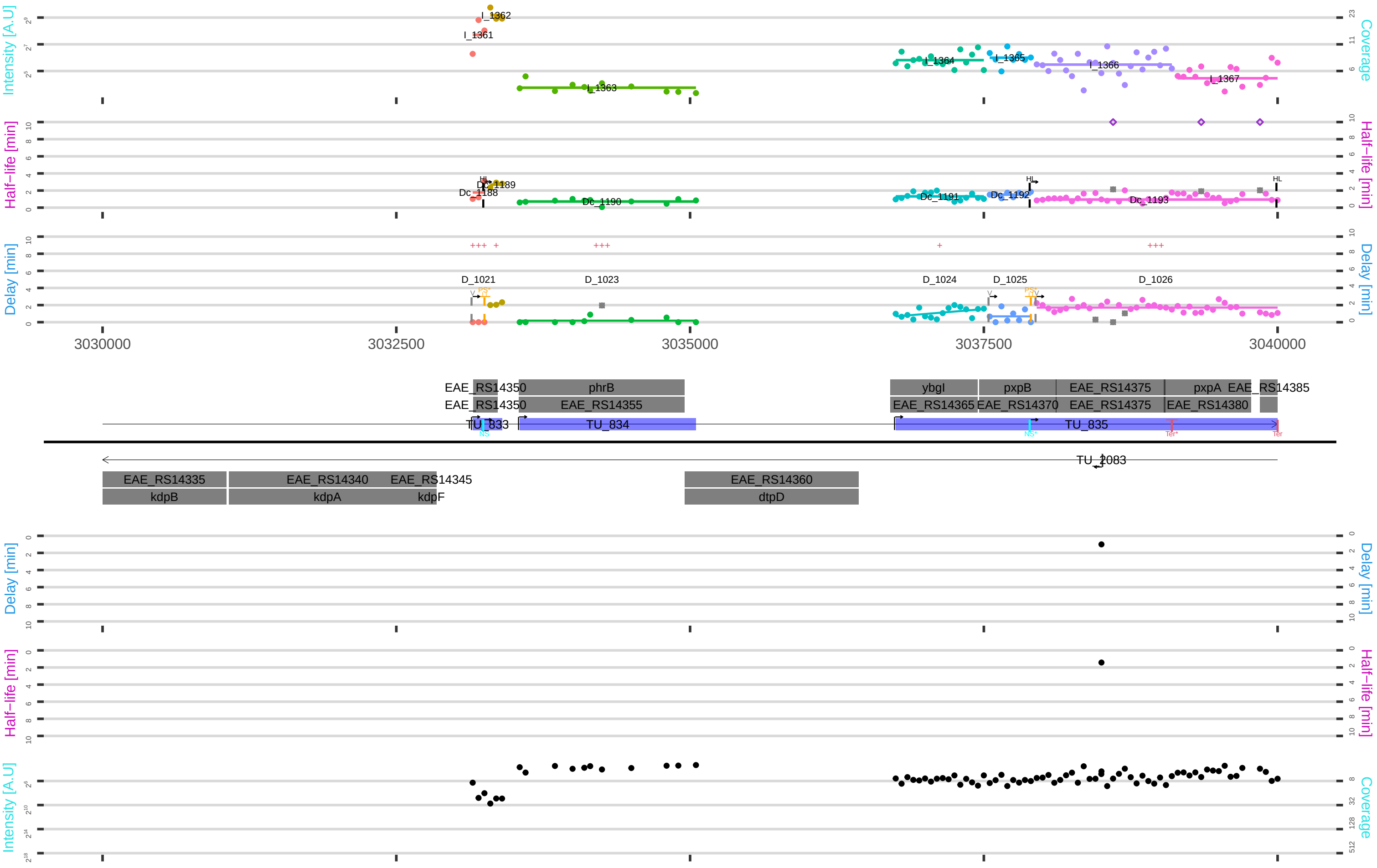

Term: termination (0), NS: new start (0), PS: pausing site (0), iTSS_I: internal starting site (0)

ID: 60800−61000; Term: termination (2), NS: new start (4), PS: pausing site (2), iTSS_I: internal starting site (3)

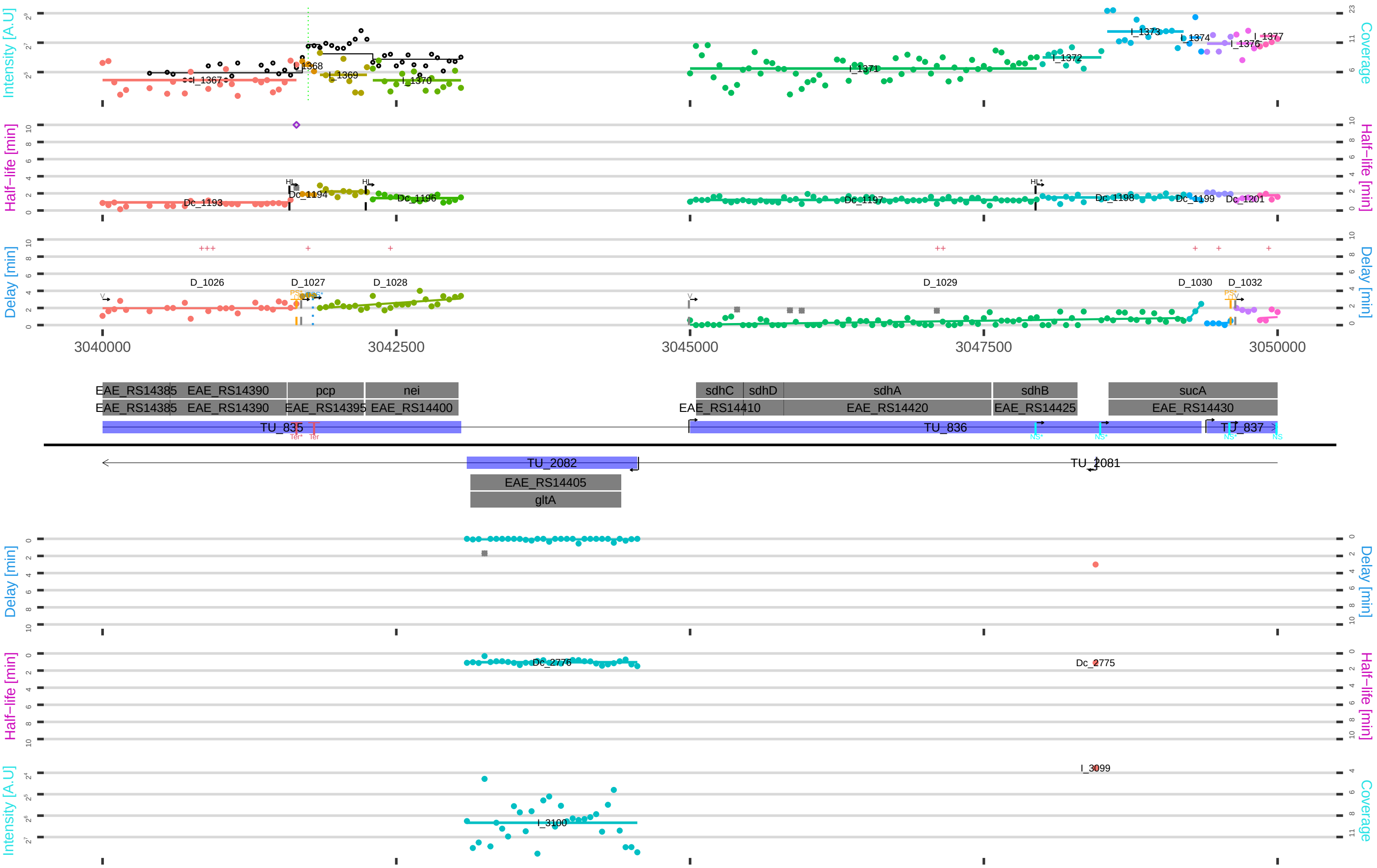

Term: termination (0), NS: new start (0), PS: pausing site (0), iTSS_I: internal starting site (0)

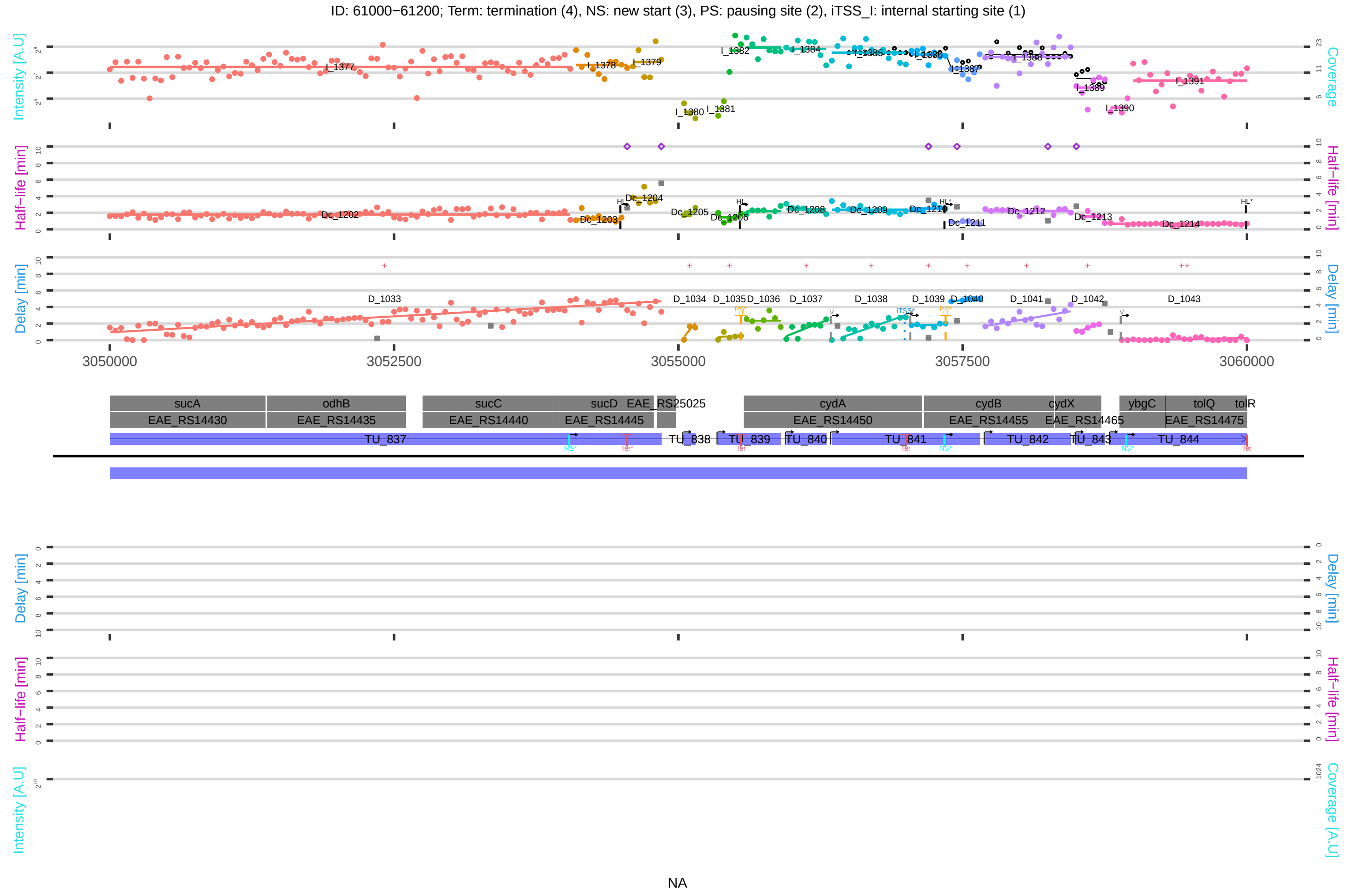

ID: 61000−61200; Term: termination (4), NS: new start (3), PS: pausing site (2), iTSS_I: internal starting site (1)
Intensity [A.U]
Coverage
Half−life [min]
Delay [min]
3050000
3052500
3055000
3057500
3060000
sucA
odhB
sucC
sucD
EAE_RS25025
cydA
cydB
cydX
ybgC
tolQ
tolR
EAE_RS14430
EAE_RS14435
EAE_RS14440
EAE_RS14445
EAE_RS14450
EAE_RS14455
EAE_RS14465
EAE_RS14475
TU_837
TU_838
TU_839
TU_840
TU_841
TU_842
TU_843
TU_844
NA

ID: 61200−61400; Term: termination (4), NS: new start (1), PS: pausing site (2), iTSS_I: internal starting site (1)

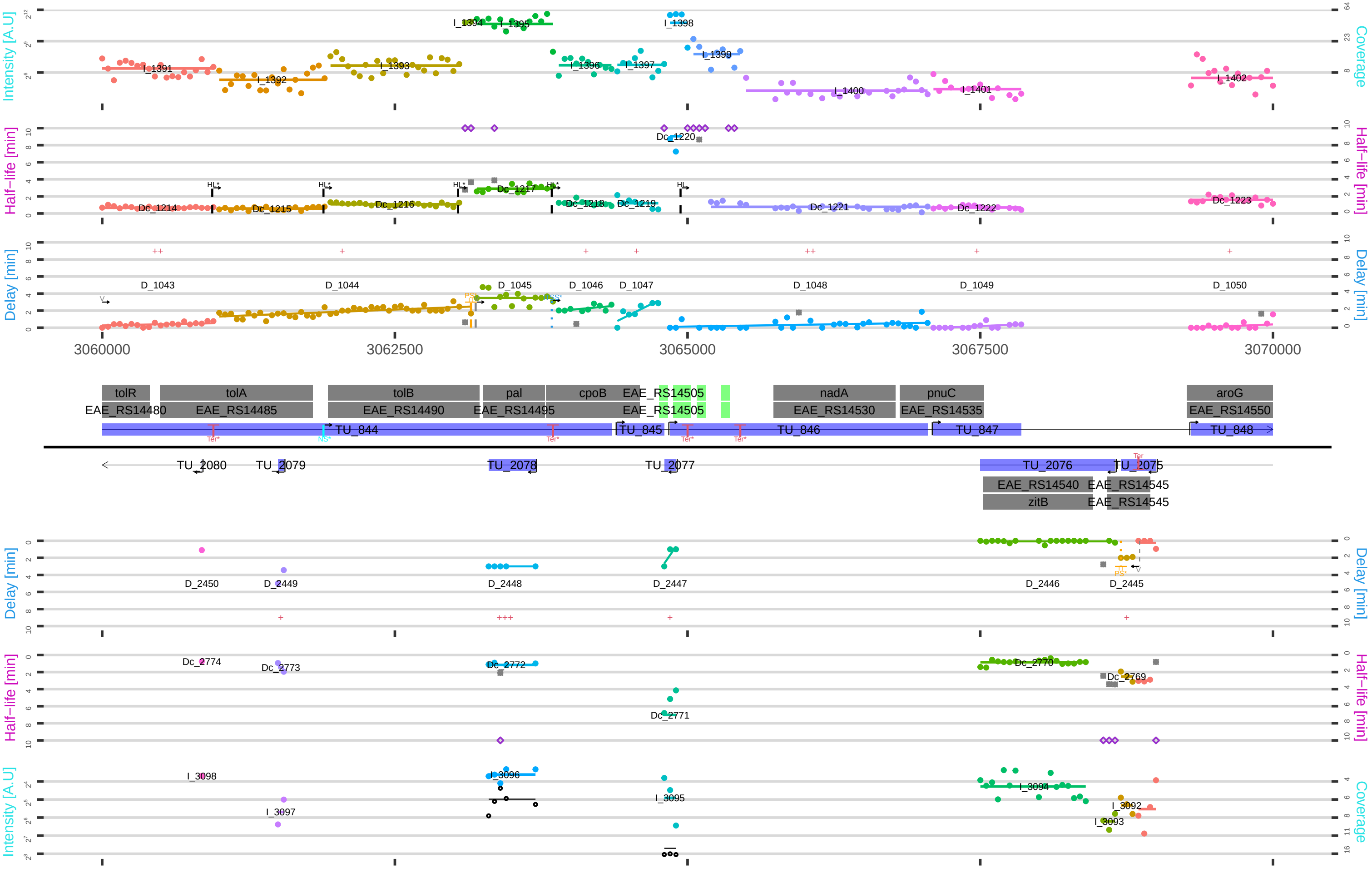

Term: termination (1), NS: new start (0), PS: pausing site (1), iTSS_I: internal starting site (0)

ID: 61400−61600; Term: termination (0), NS: new start (0), PS: pausing site (0), iTSS_I: internal starting site (0)

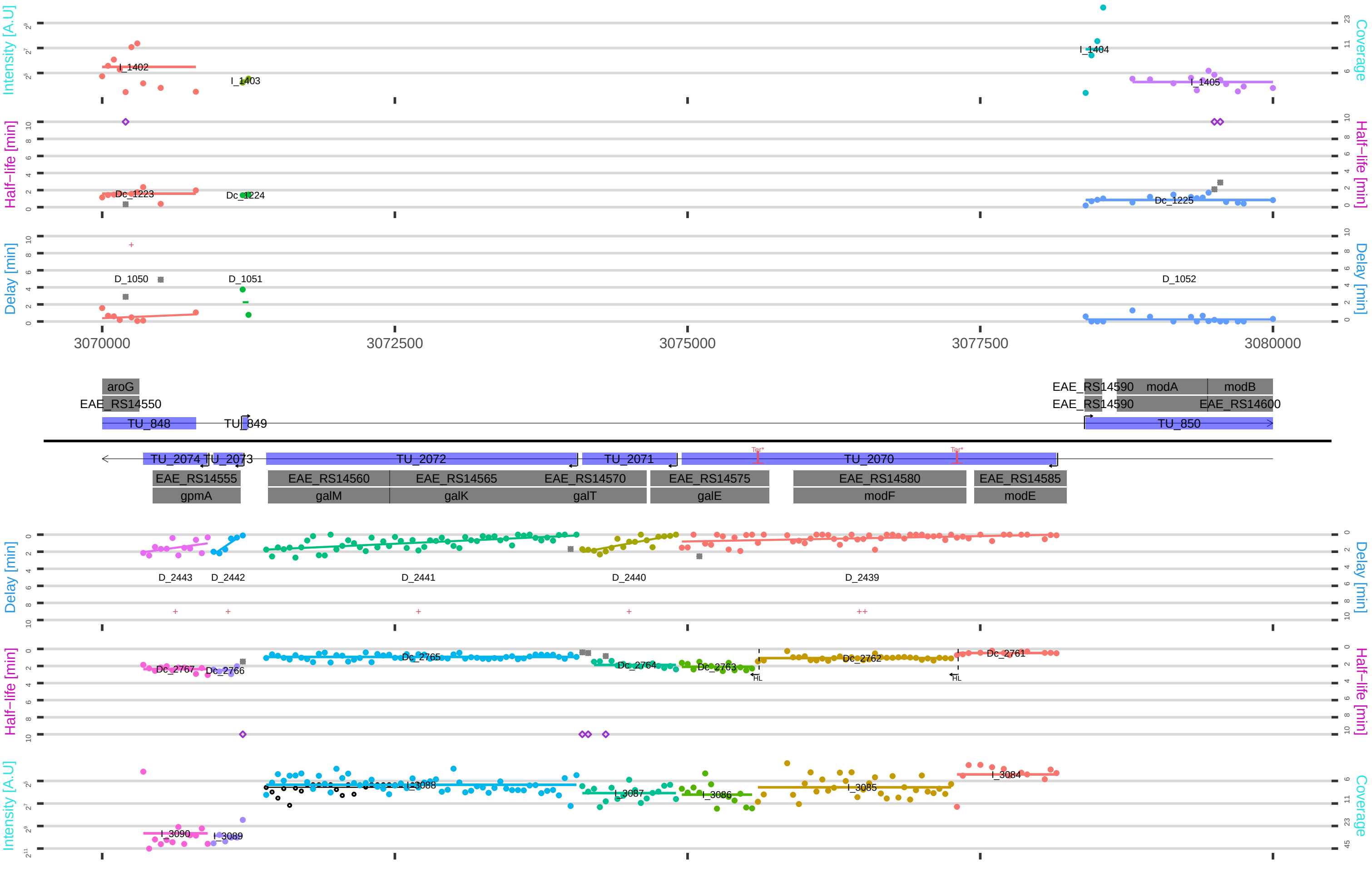

Term: termination (2), NS: new start (0), PS: pausing site (0), iTSS_I: internal starting site (0)

ID: 61600−61784; Term: termination (1), NS: new start (1), PS: pausing site (0), iTSS_I: internal starting site (0)

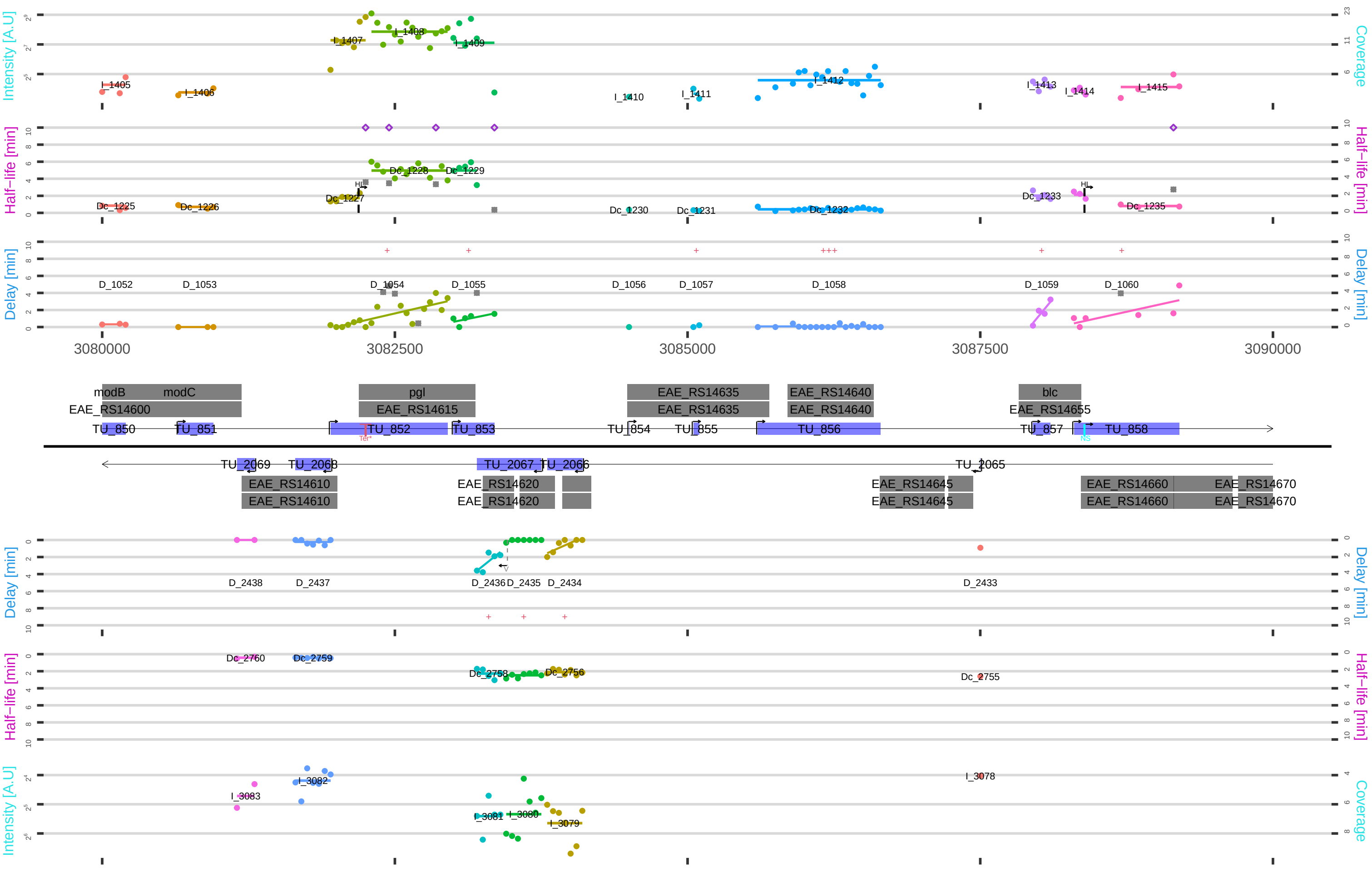

Term: termination (0), NS: new start (0), PS: pausing site (1), iTSS_I: internal starting site (0)

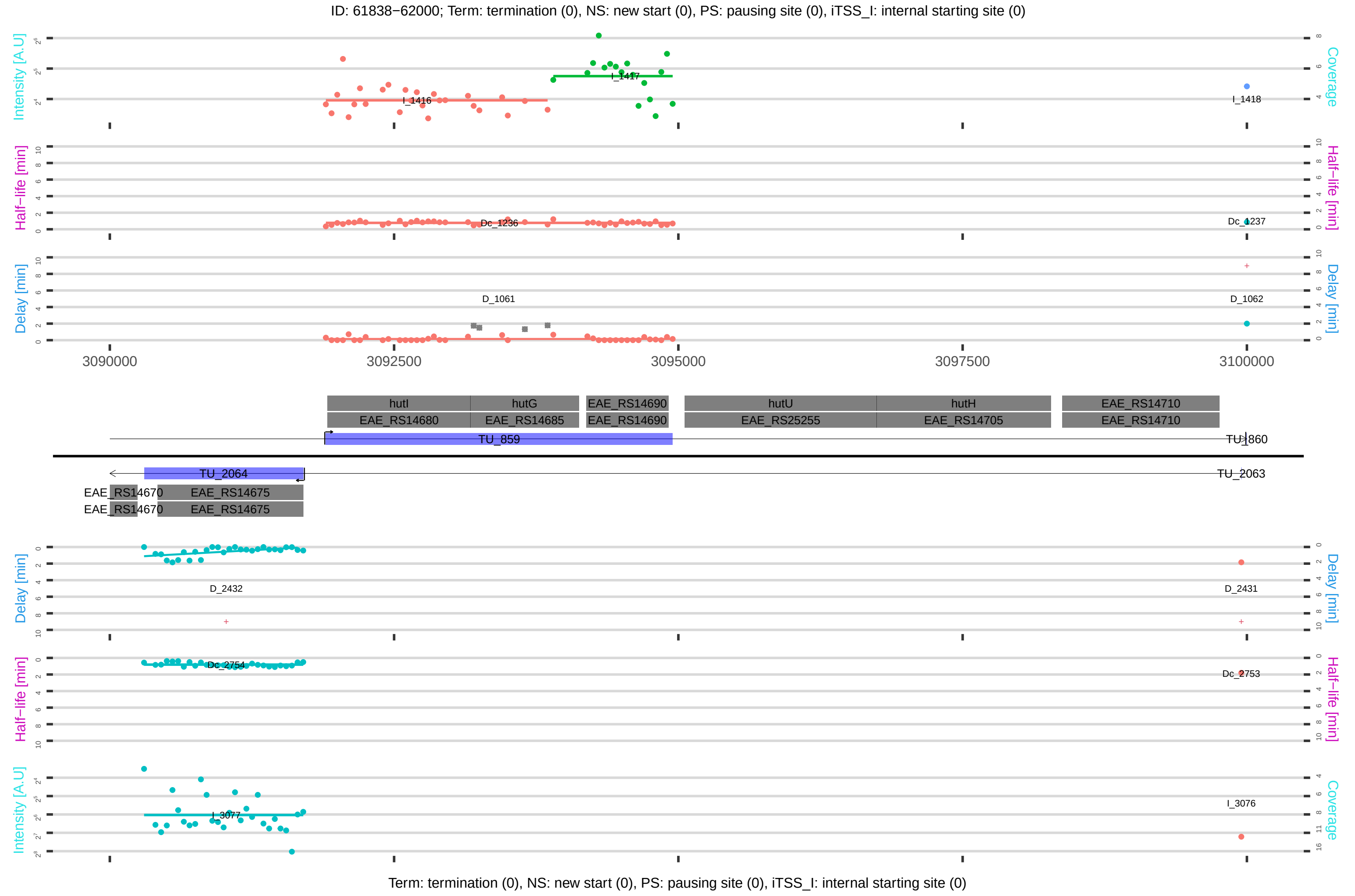
ID: 61838−62000; Term: termination (0), NS: new start (0), PS: pausing site (0), iTSS_I: internal starting site (0)
Intensity [A.U]
Coverage
I_1416
I_1417
I_1418
Half−life [min]
Dc_1236
Dc_1237
Delay [min]
D_1061
D_1062
3090000
3092500
3095000
3097500
3100000
hutI
hutG
EAE_RS14690
hutU
hutH
EAE_RS14710
EAE_RS14680
EAE_RS14685
EAE_RS25255
EAE_RS14705
TU_859
TU_860
TU_2064
TU_2063
EAE_RS14670
EAE_RS14675
D_2432
D_2431
Dc_2754
Dc_2753
I_3077
I_3076
Term: termination (0), NS: new start (0), PS: pausing site (0), iTSS_I: internal starting site (0)

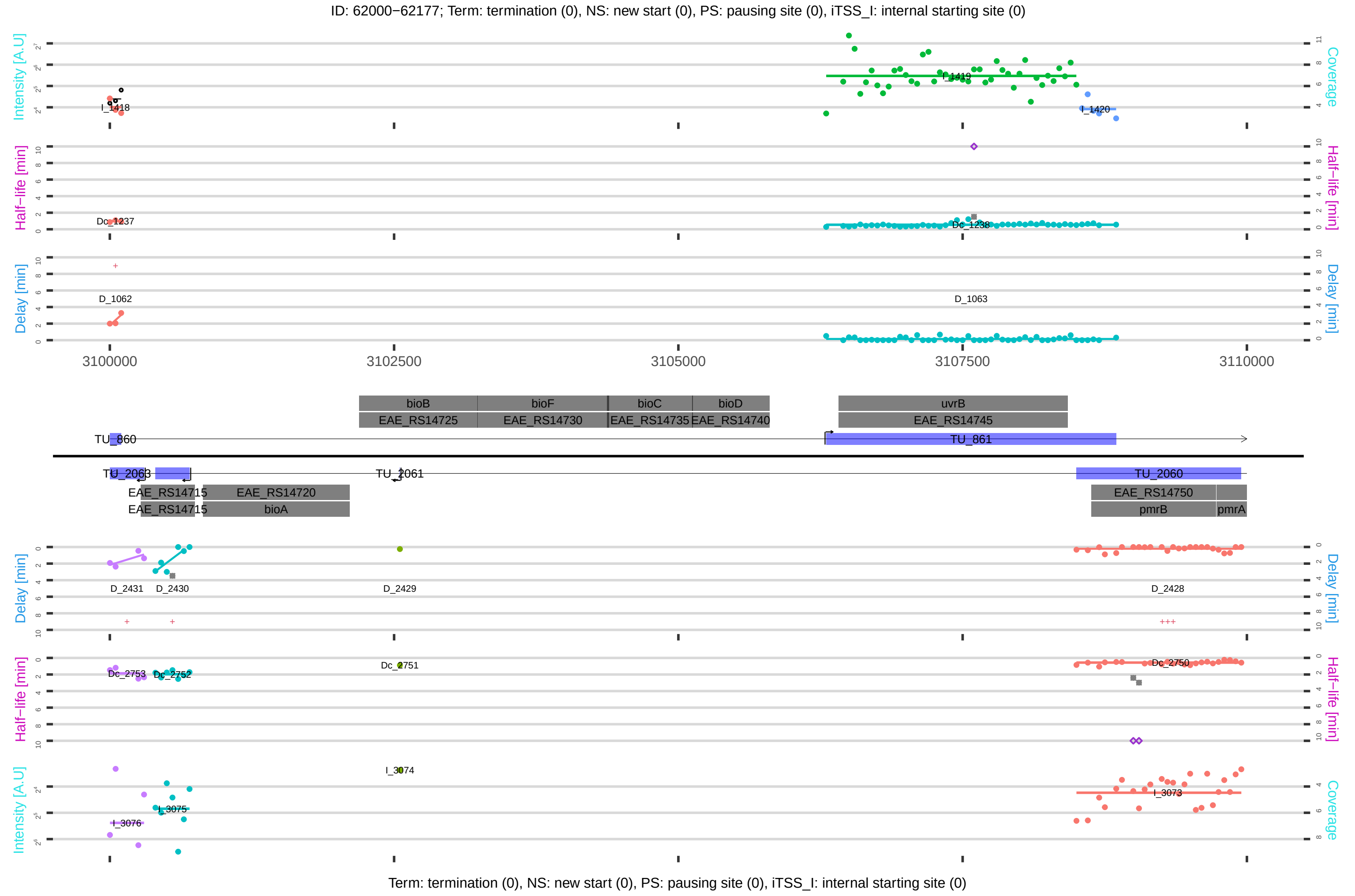
ID: 62000−62177; Term: termination (0), NS: new start (0), PS: pausing site (0), iTSS_I: internal starting site (0)
Intensity [A.U]
Coverage
Half−life [min]
Delay [min]
I_1418
I_1419
I_1420
Dc_1237
Dc_1238
D_1062
D_1063
3100000
3102500
3105000
3107500
3110000
bioB
bioF
bioC
bioD
uvrB
EAE_RS14725
EAE_RS14730
EAE_RS14735
EAE_RS14740
EAE_RS14745
TU_860
TU_861
TU_2063
TU_2061
TU_2060
EAE_RS14715
EAE_RS14720
EAE_RS14750
bioA
pmrB
pmrA
D_2431
D_2430
D_2429
D_2428
Dc_2753
Dc_2752
Dc_2751
Dc_2750
I_3074
I_3075
I_3076
I_3073
Term: termination (0), NS: new start (0), PS: pausing site (0), iTSS_I: internal starting site (0)

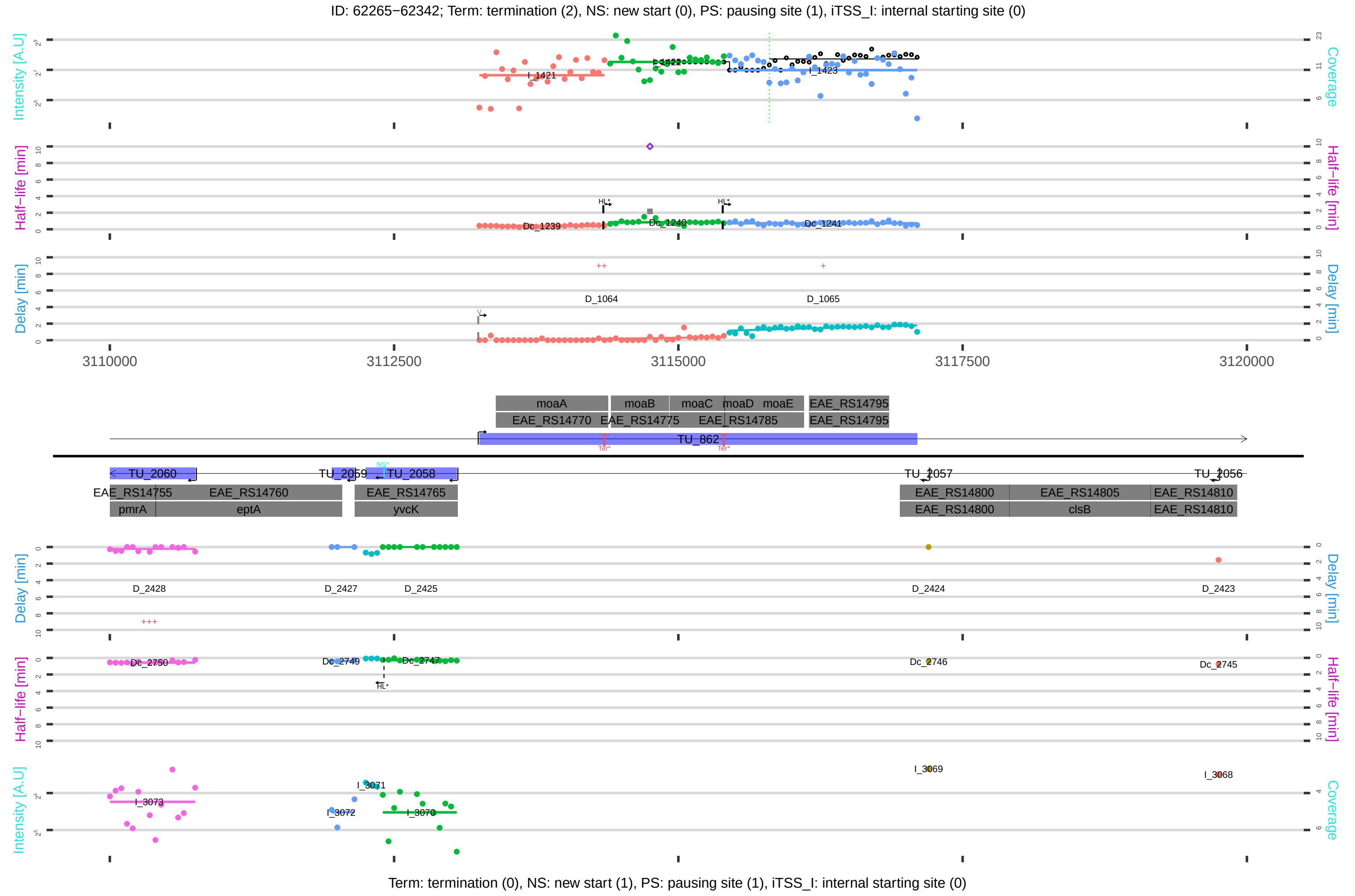
ID: 62265−62342; Term: termination (2), NS: new start (0), PS: pausing site (1), iTSS_I: internal starting site (0)
Intensity [A.U]
Coverage
Half−life [min]
Delay [min]
I_1421
I_1422
I_1423
Dc_1239
Dc_1240
Dc_1241
D_1064
D_1065
3110000
3112500
3115000
3117500
3120000
moaA
moaB
moaC
moaD
moaE
EAE_RS14795
EAE_RS14770
EAE_RS14775
EAE_RS14785
TU_862
TU_2060
TU_2059
TU_2058
TU_2057
TU_2056
EAE_RS14755
EAE_RS14760
EAE_RS14765
EAE_RS14800
EAE_RS14805
EAE_RS14810
pmrA
eptA
yvcK
clsB
D_2428
D_2427
D_2425
D_2424
D_2423
Dc_2750
Dc_2749
Dc_2747
Dc_2746
Dc_2745
I_3073
I_3072
I_3071
I_3070
I_3069
I_3068
Term: termination (0), NS: new start (1), PS: pausing site (1), iTSS_I: internal starting site (0)

ID: 62402−62598; Term: termination (0), NS: new start (0), PS: pausing site (0), iTSS_I: internal starting site (0)

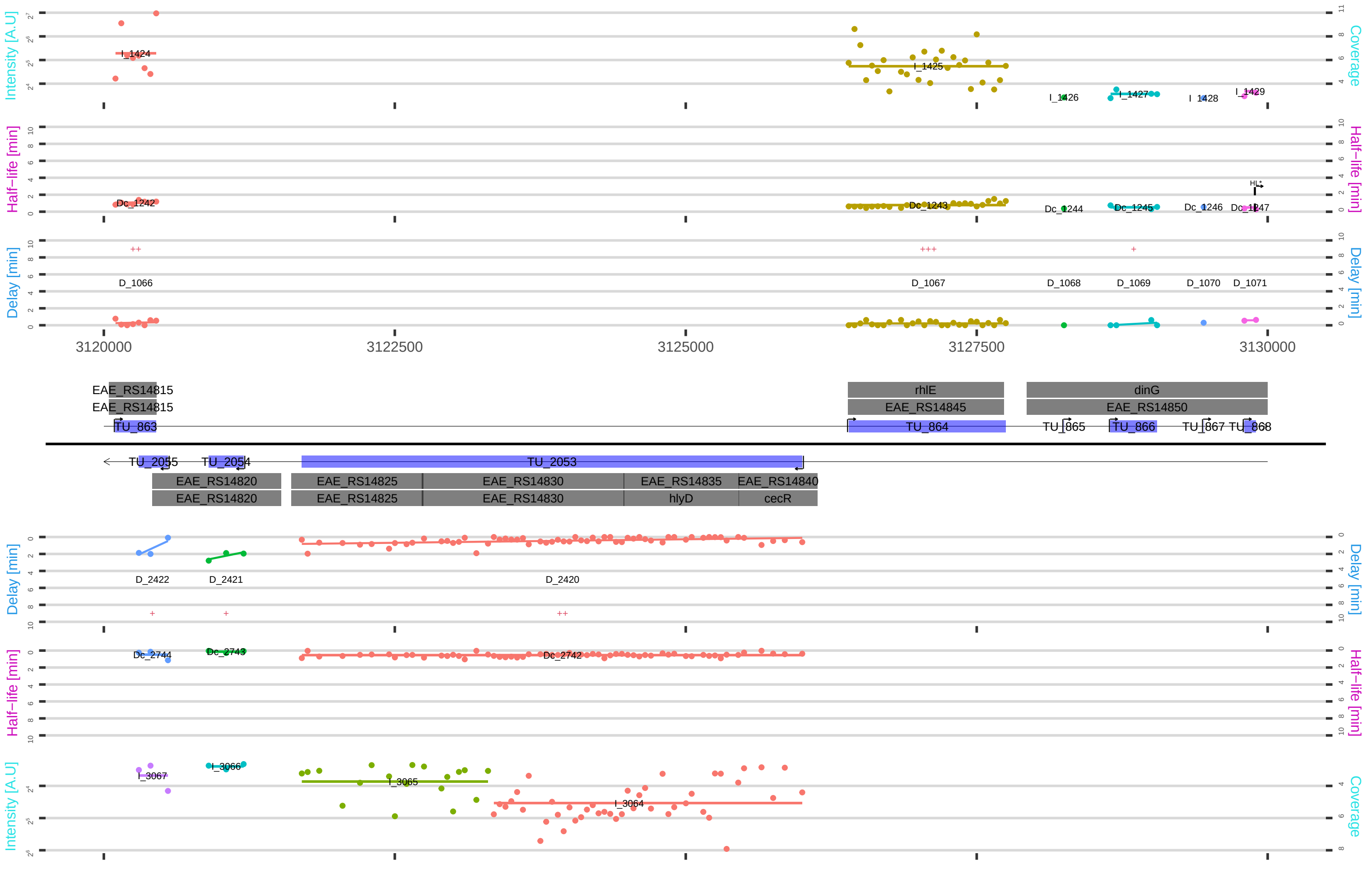

Term: termination (0), NS: new start (0), PS: pausing site (0), iTSS_I: internal starting site (0)

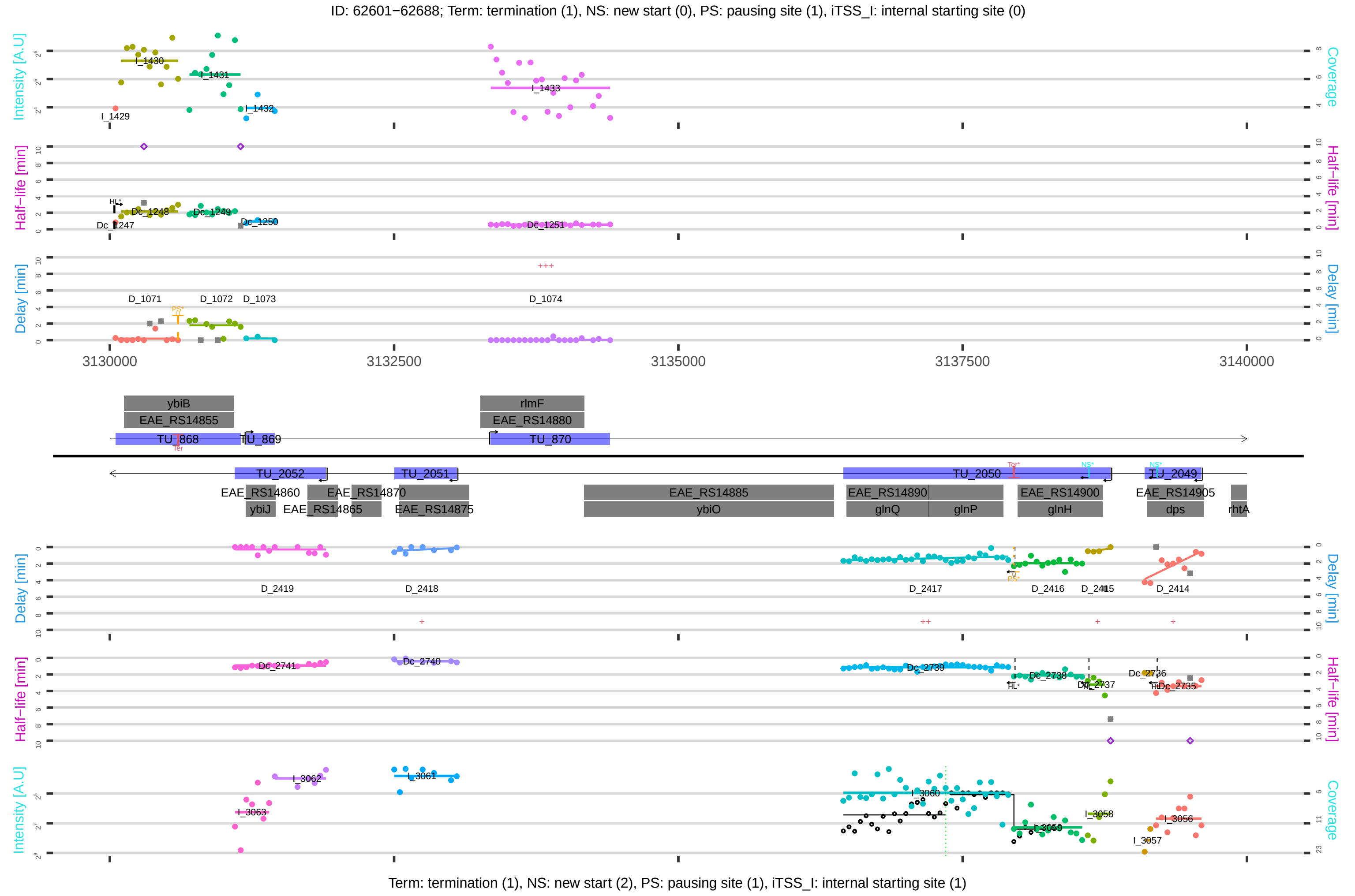

ID: 62601−62688; Term: termination (1), NS: new start (0), PS: pausing site (1), iTSS_I: internal starting site (0)
Intensity [A.U]
Coverage
Half−life [min]
Delay [min]
I_1429
I_1430
I_1431
I_1432
I_1433
Dc_1247
Dc_1248
Dc_1249
Dc_1250
Dc_1251
D_1071
D_1072
D_1073
D_1074
3130000
3132500
3135000
3137500
3140000
ybiB
EAE_RS14855
TU_868
TU_869
rlmF
EAE_RS14880
TU_870
TU_2052
TU_2051
TU_2050
TU_2049
EAE_RS14860
EAE_RS14870
EAE_RS14885
EAE_RS14890
EAE_RS14900
EAE_RS14905
ybiJ
EAE_RS14865
EAE_RS14875
ybiO
glnQ
glnP
glnH
dps
rhtA
D_2419
D_2418
D_2417
D_2416
D_2415
D_2414
Dc_2741
Dc_2740
Dc_2739
Dc_2738
Dc_2737
Dc_2736
Dc_2735
I_3063
I_3062
I_3061
I_3060
I_3059
I_3058
I_3057
I_3056
Term: termination (1), NS: new start (2), PS: pausing site (1), iTSS_I: internal starting site (1)

ID: 62814−62978; Term: termination (0), NS: new start (2), PS: pausing site (1), iTSS_I: internal starting site (0)

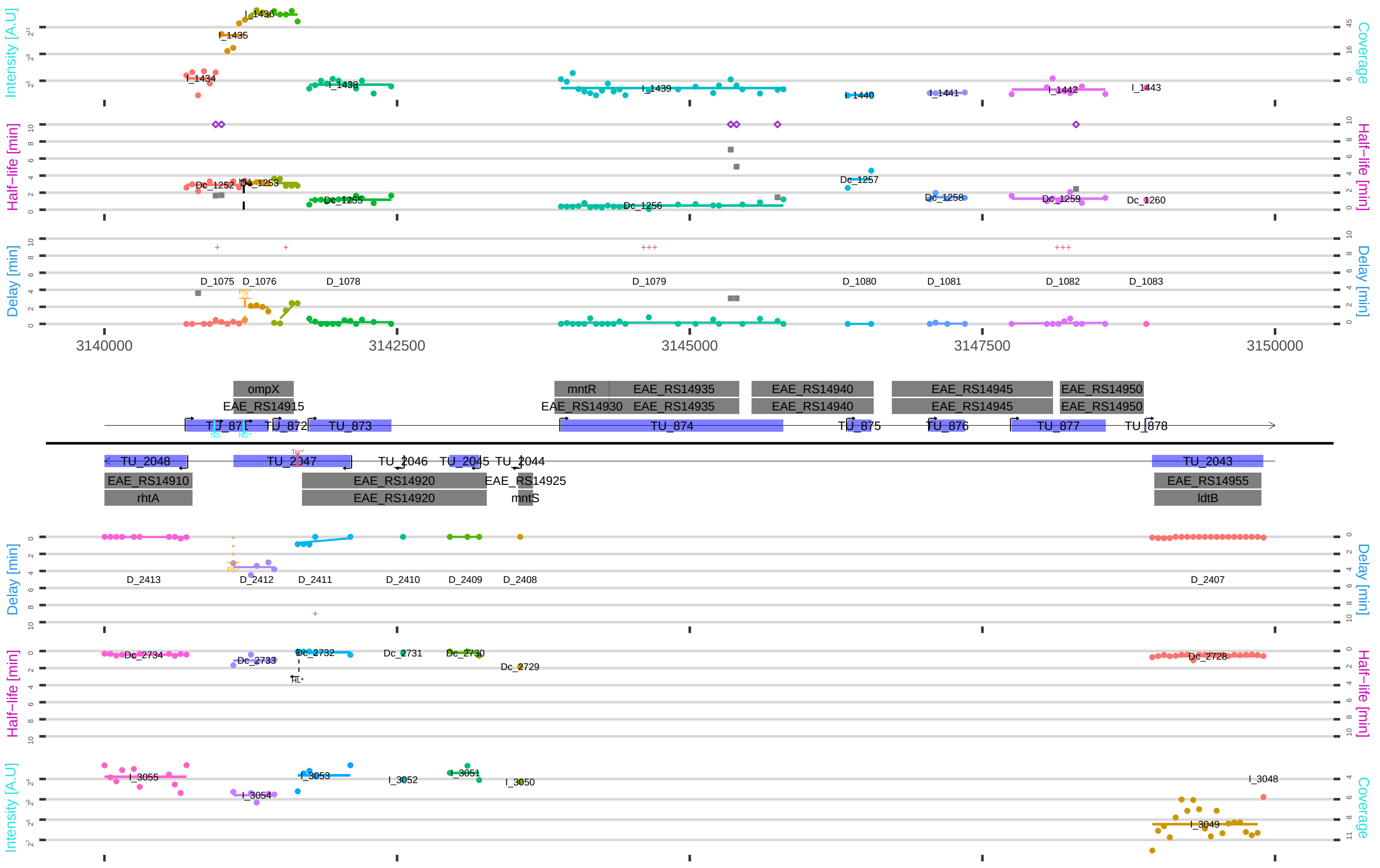

Term: termination (1), NS: new start (0), PS: pausing site (1), iTSS_I: internal starting site (0)

ID: 63022−63168; Term: termination (0), NS: new start (0), PS: pausing site (0), iTSS_I: internal starting site (0)

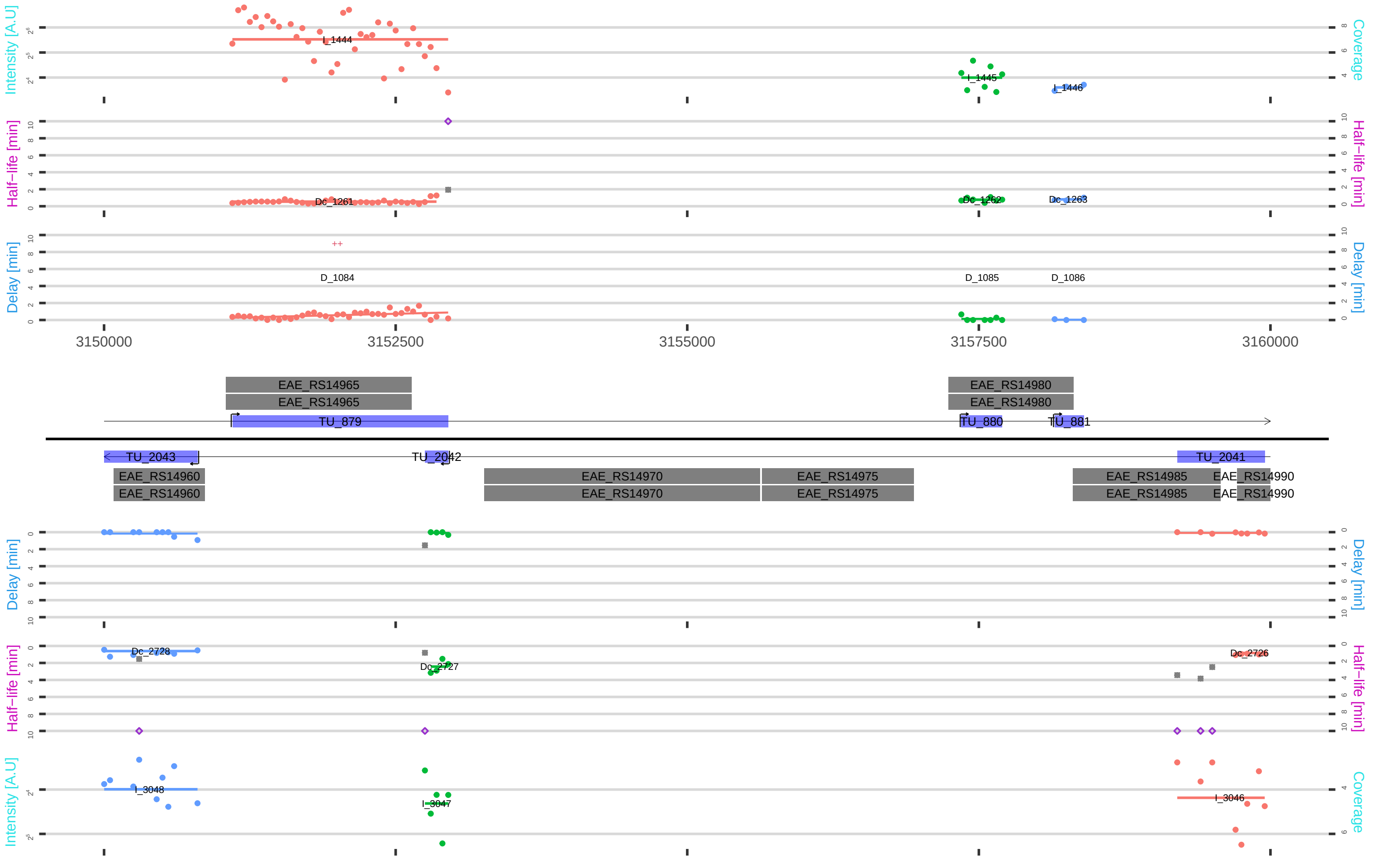

Term: termination (0), NS: new start (0), PS: pausing site (0), iTSS_I: internal starting site (0)

ID: 63327−63400; Term: termination (2), NS: new start (0), PS: pausing site (1), iTSS_I: internal starting site (0)

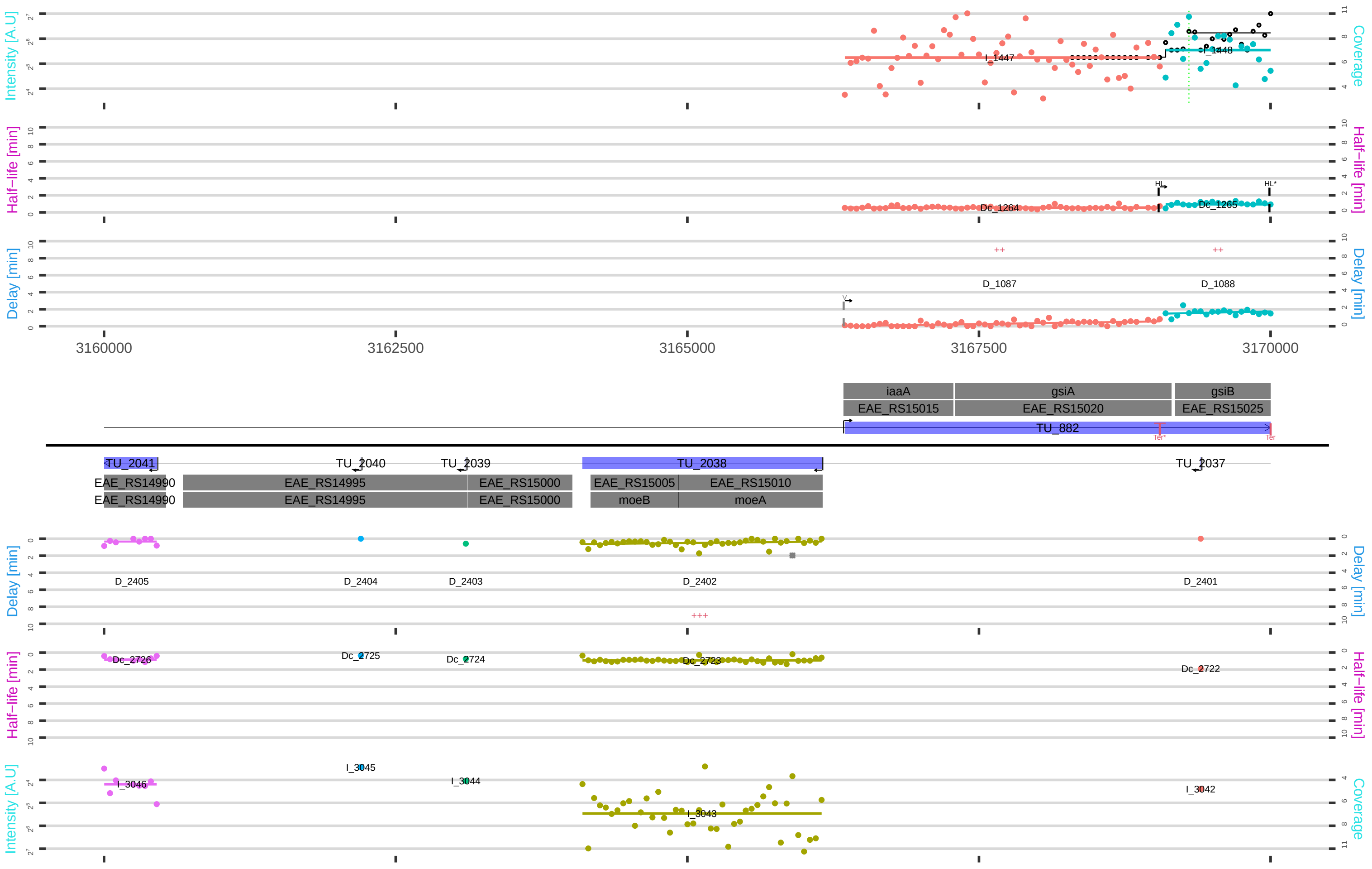

Term: termination (0), NS: new start (0), PS: pausing site (0), iTSS_I: internal starting site (0)

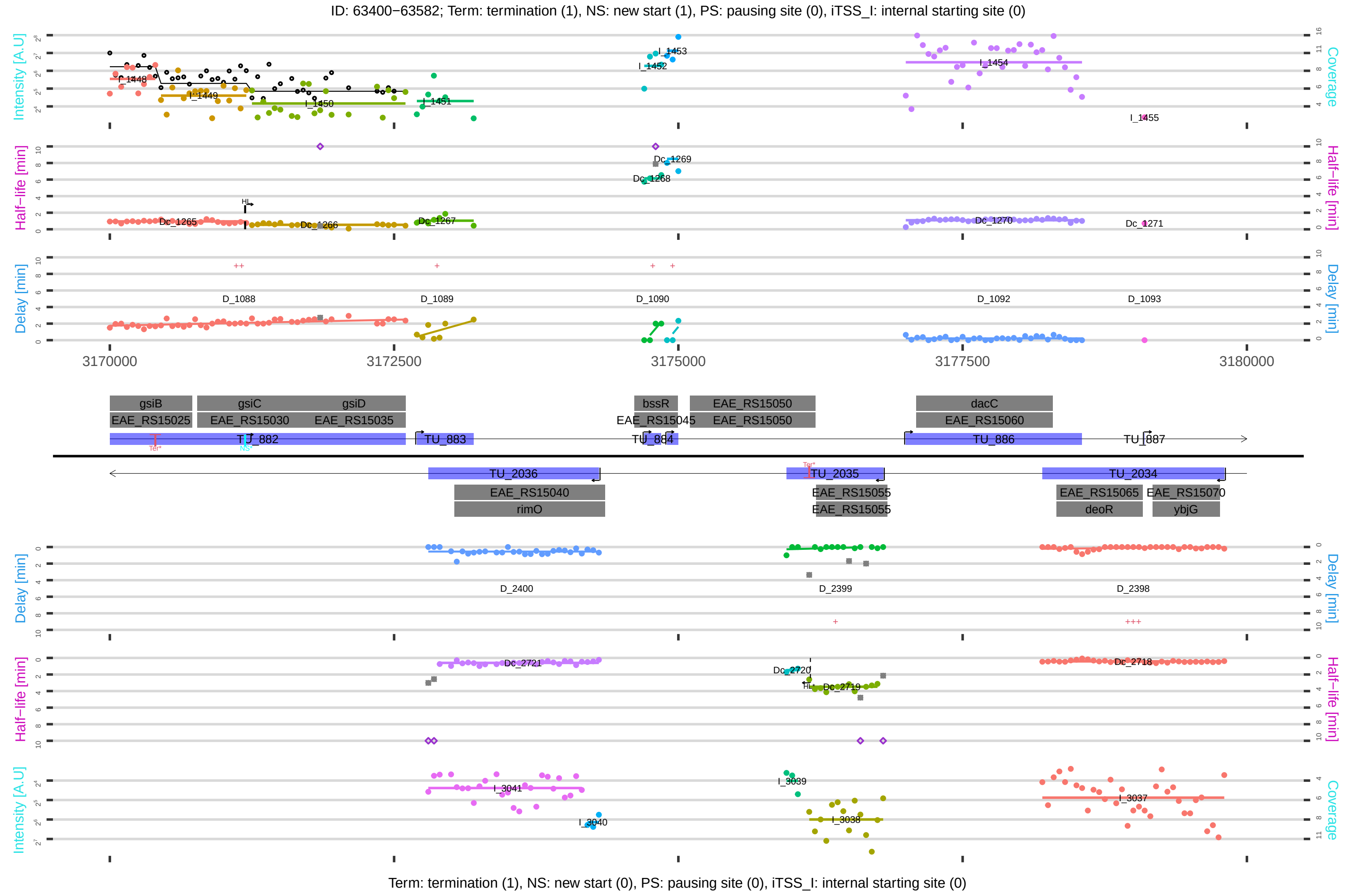

ID: 63400−63582; Term: termination (1), NS: new start (1), PS: pausing site (0), iTSS_I: internal starting site (0)
Intensity [A.U]
Coverage
Half−life [min]
Delay [min]
3170000
3172500
3175000
3177500
3180000
gsiB
gsiC
gsiD
EAE_RS15025
EAE_RS15030
EAE_RS15035
bssR
EAE_RS15045
EAE_RS15050
dacC
EAE_RS15060
TU_882
TU_883
TU_884
TU_886
TU_887
TU_2036
TU_2035
TU_2034
EAE_RS15040
rimO
EAE_RS15055
EAE_RS15065
EAE_RS15070
deoR
ybjG
D_1088
D_1089
D_1090
D_1092
D_1093
D_2400
D_2399
D_2398
Dc_1265
Dc_1266
Dc_1267
Dc_1268
Dc_1269
Dc_1270
Dc_1271
Dc_2721
Dc_2720
Dc_2719
Dc_2718
I_1448
I_1449
I_1450
I_1451
I_1452
I_1453
I_1454
I_1455
I_3041
I_3040
I_3039
I_3038
I_3037
Term: termination (1), NS: new start (0), PS: pausing site (0), iTSS_I: internal starting site (0)

ID: 63603−63800; Term: termination (1), NS: new start (0), PS: pausing site (0), iTSS_I: internal starting site (0)

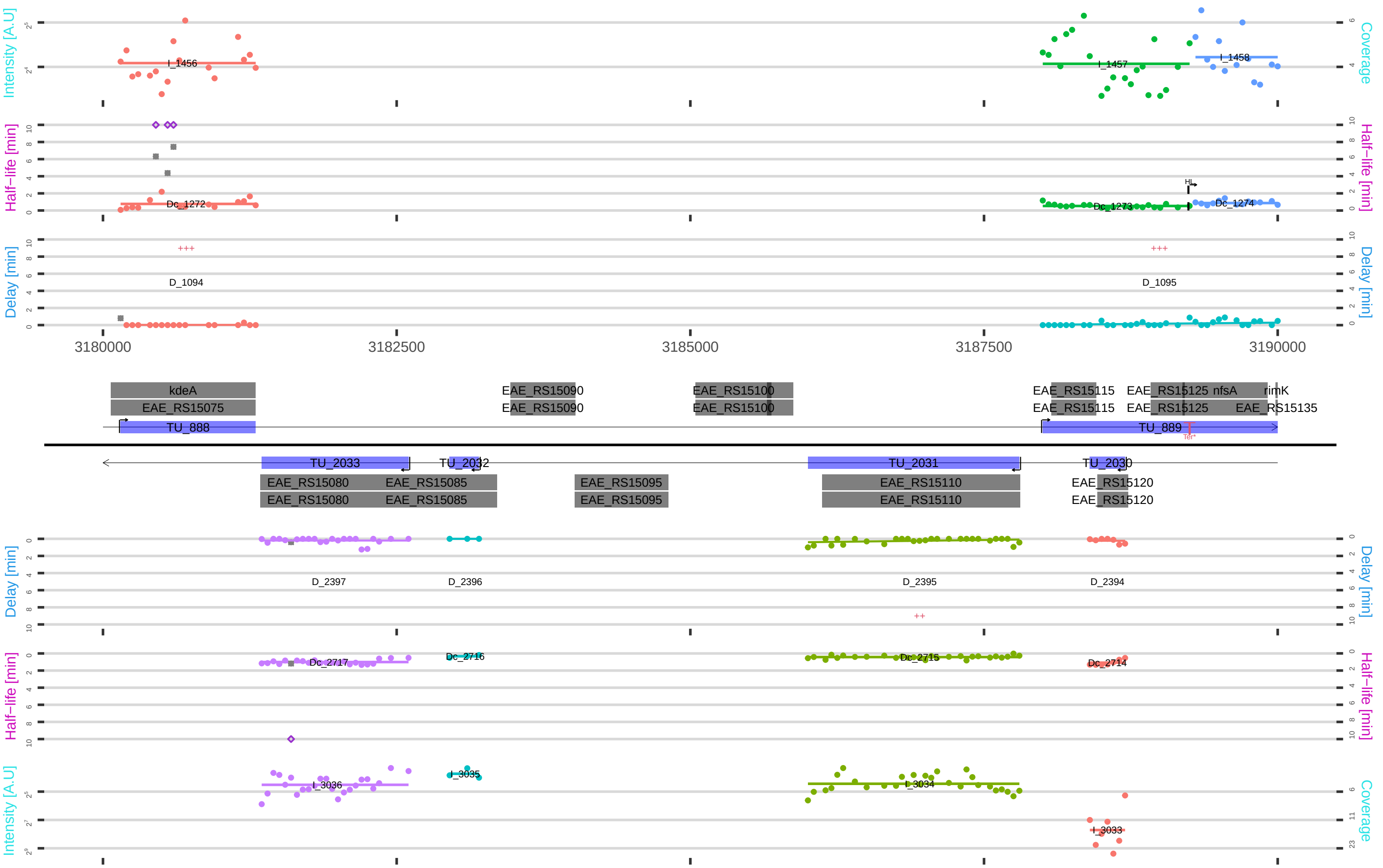

Term: termination (0), NS: new start (0), PS: pausing site (0), iTSS_I: internal starting site (0)

ID: 63800−64000; Term: termination (1), NS: new start (0), PS: pausing site (0), iTSS_I: internal starting site (0)

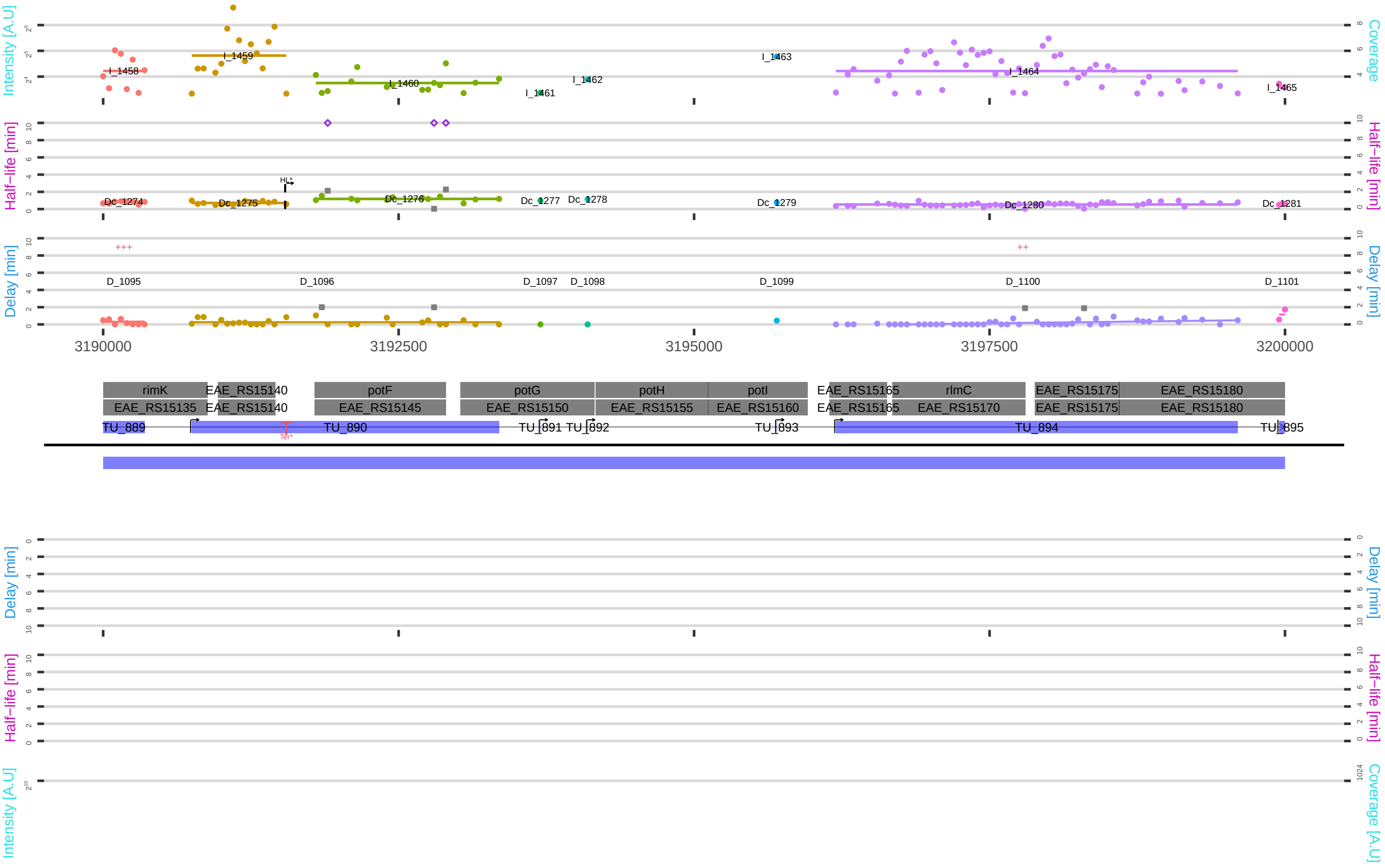

NA

ID: 64000−64181; Term: termination (0), NS: new start (0), PS: pausing site (0), iTSS_I: internal starting site (0)

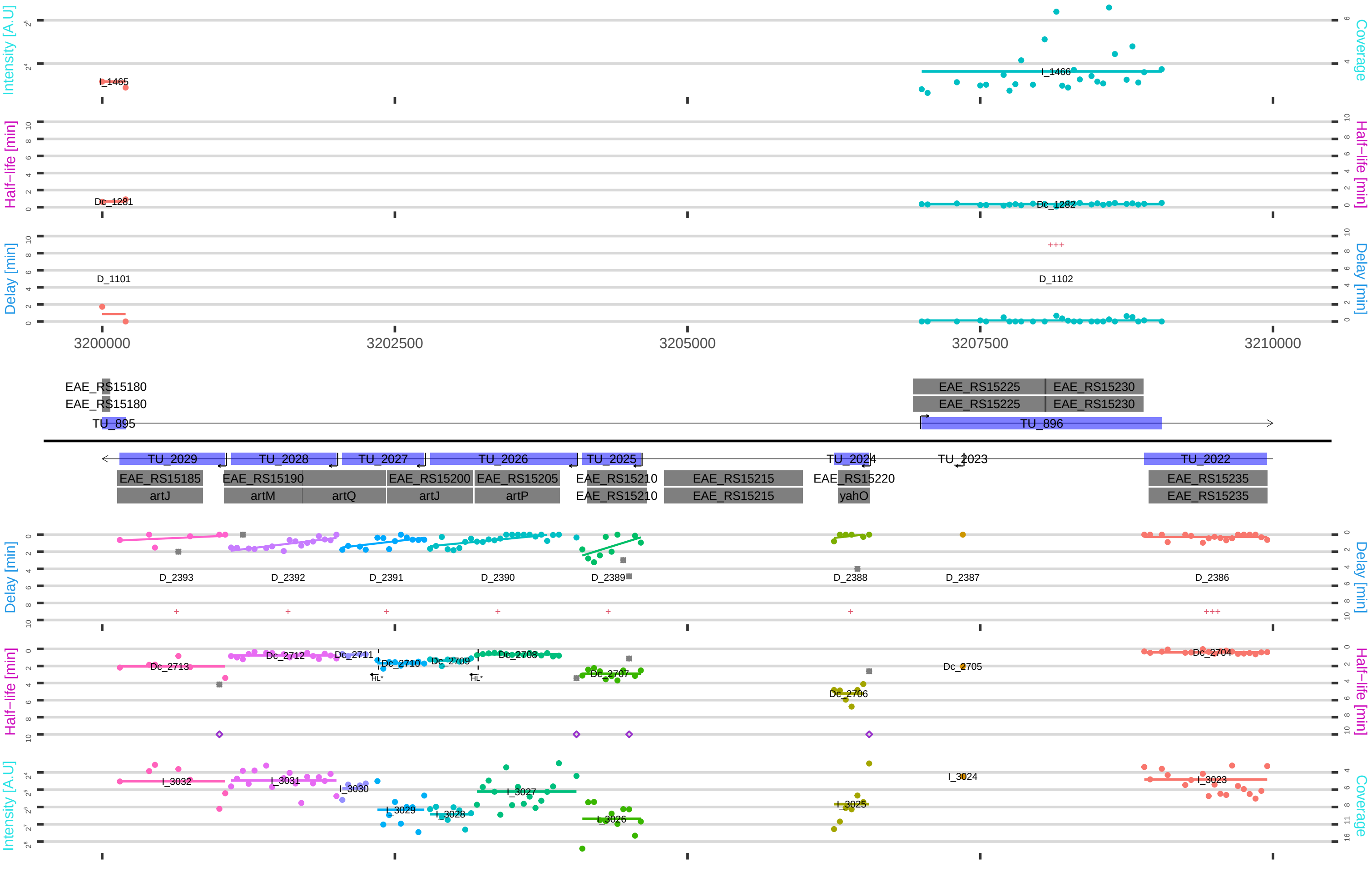

Term: termination (0), NS: new start (0), PS: pausing site (0), iTSS_I: internal starting site (0)

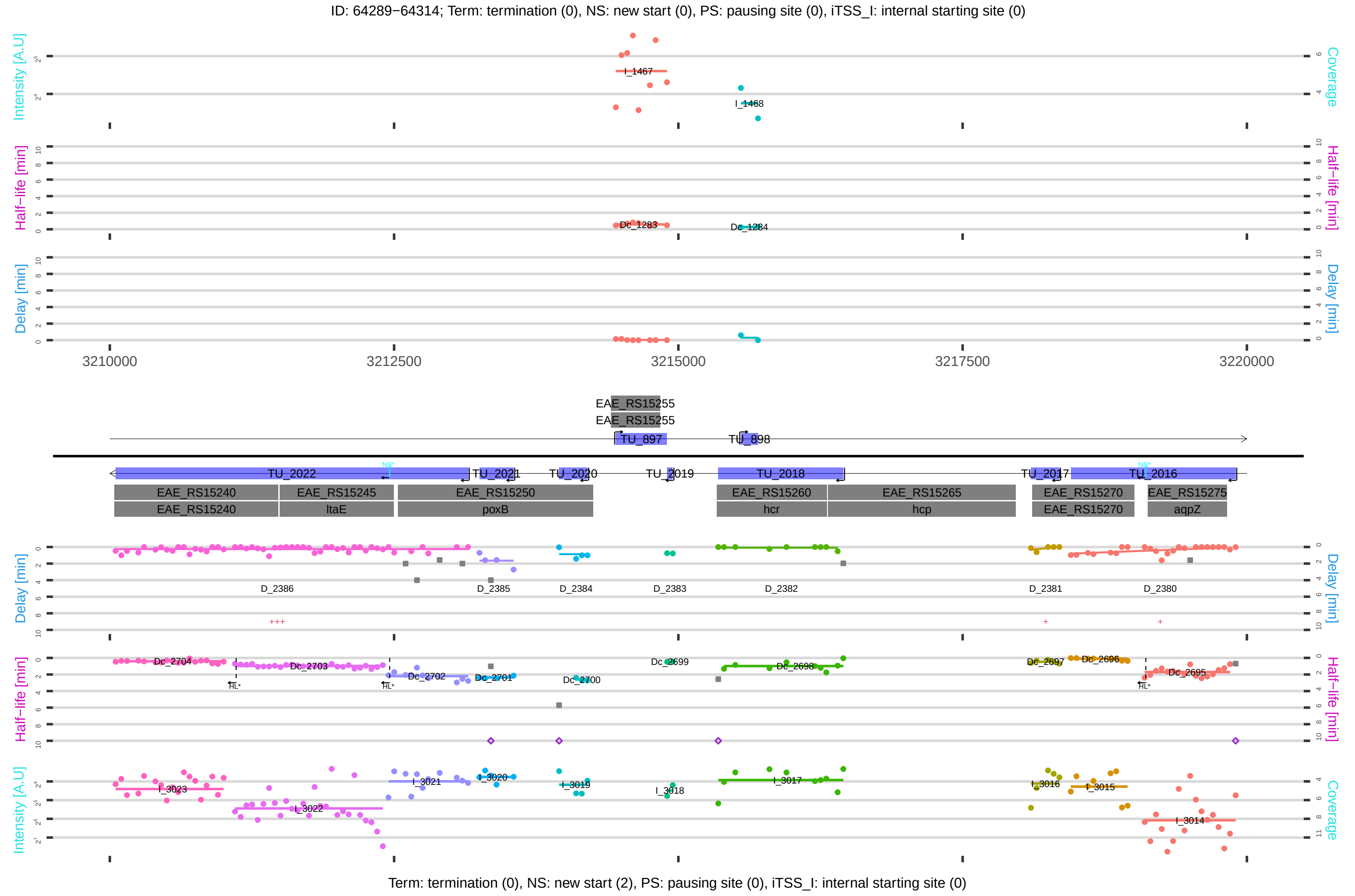

ID: 64289−64314; Term: termination (0), NS: new start (0), PS: pausing site (0), iTSS_I: internal starting site (0)
Intensity [A.U]
Coverage
Half−life [min]
Delay [min]
I_1467
I_1468
Dc_1283
Dc_1284
3210000
3212500
3215000
3217500
3220000
EAE_RS15255
TU_897
TU_898
TU_2022
TU_2021
TU_2020
TU_2019
TU_2018
TU_2017
TU_2016
EAE_RS15240
EAE_RS15245
EAE_RS15250
EAE_RS15260
EAE_RS15265
EAE_RS15270
EAE_RS15275
ltaE
poxB
hcr
hcp
aqpZ
D_2386
D_2385
D_2384
D_2383
D_2382
D_2381
D_2380
Dc_2704
Dc_2703
Dc_2702
Dc_2701
Dc_2700
Dc_2699
Dc_2698
Dc_2697
Dc_2696
Dc_2695
I_3023
I_3022
I_3021
I_3020
I_3019
I_3018
I_3017
I_3016
I_3015
I_3014
Term: termination (0), NS: new start (2), PS: pausing site (0), iTSS_I: internal starting site (0)

ID: 64406−64567; Term: termination (0), NS: new start (2), PS: pausing site (1), iTSS_I: internal starting site (0)

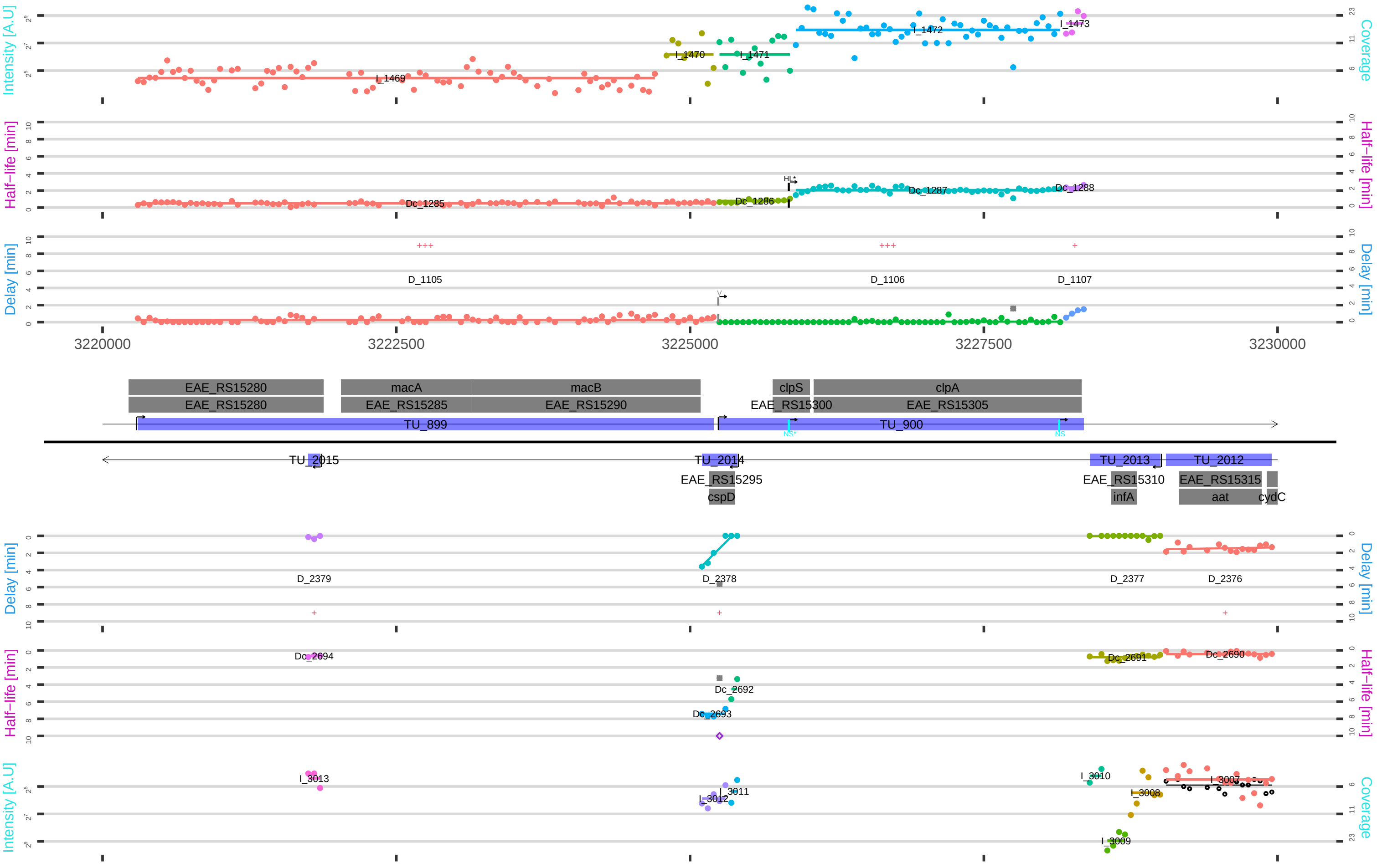

Term: termination (0), NS: new start (0), PS: pausing site (1), iTSS_I: internal starting site (0)

ID: 64696−64800; Term: termination (3), NS: new start (1), PS: pausing site (1), iTSS_I: internal starting site (1)

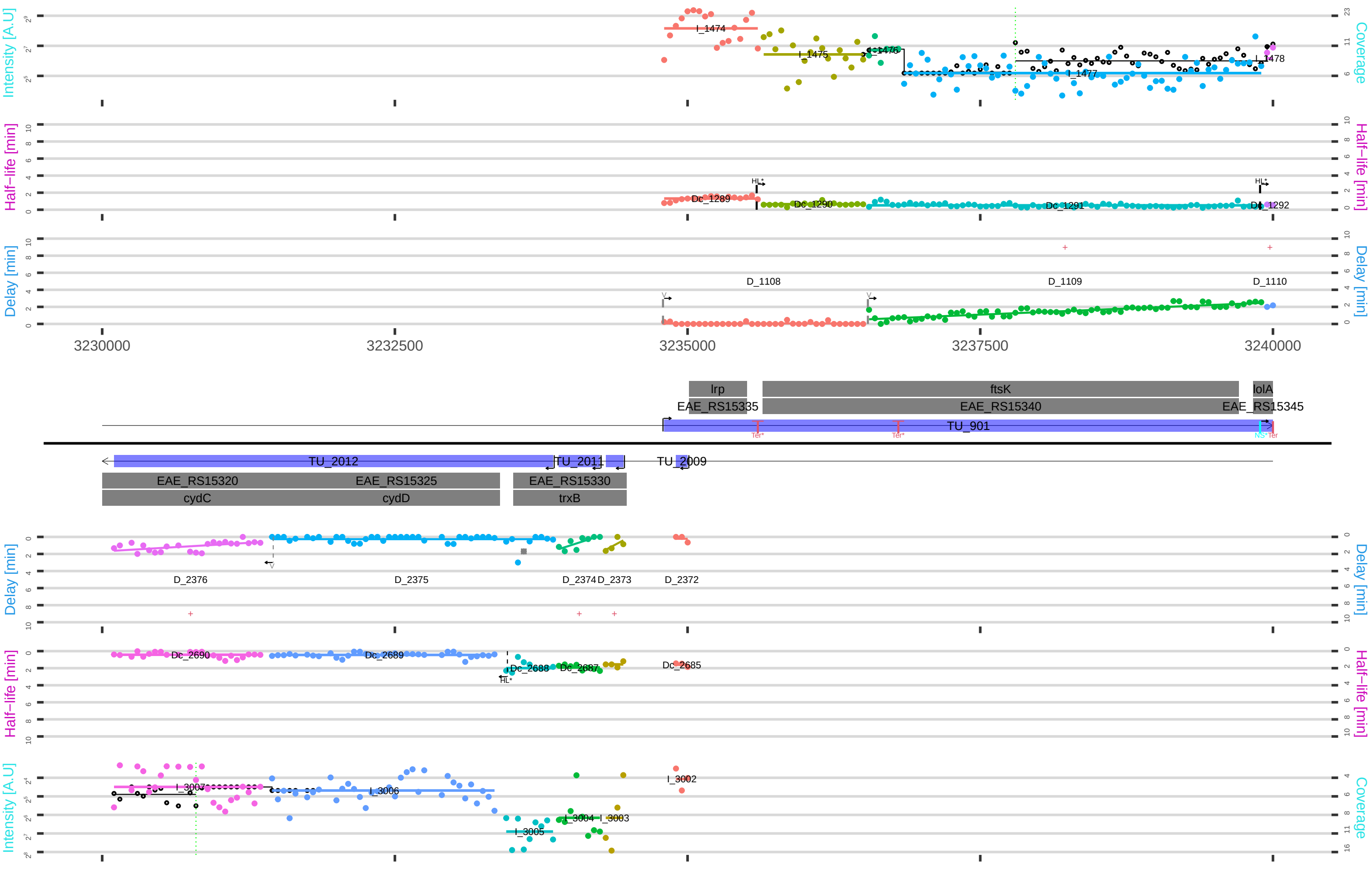

Term: termination (0), NS: new start (0), PS: pausing site (0), iTSS_I: internal starting site (0)

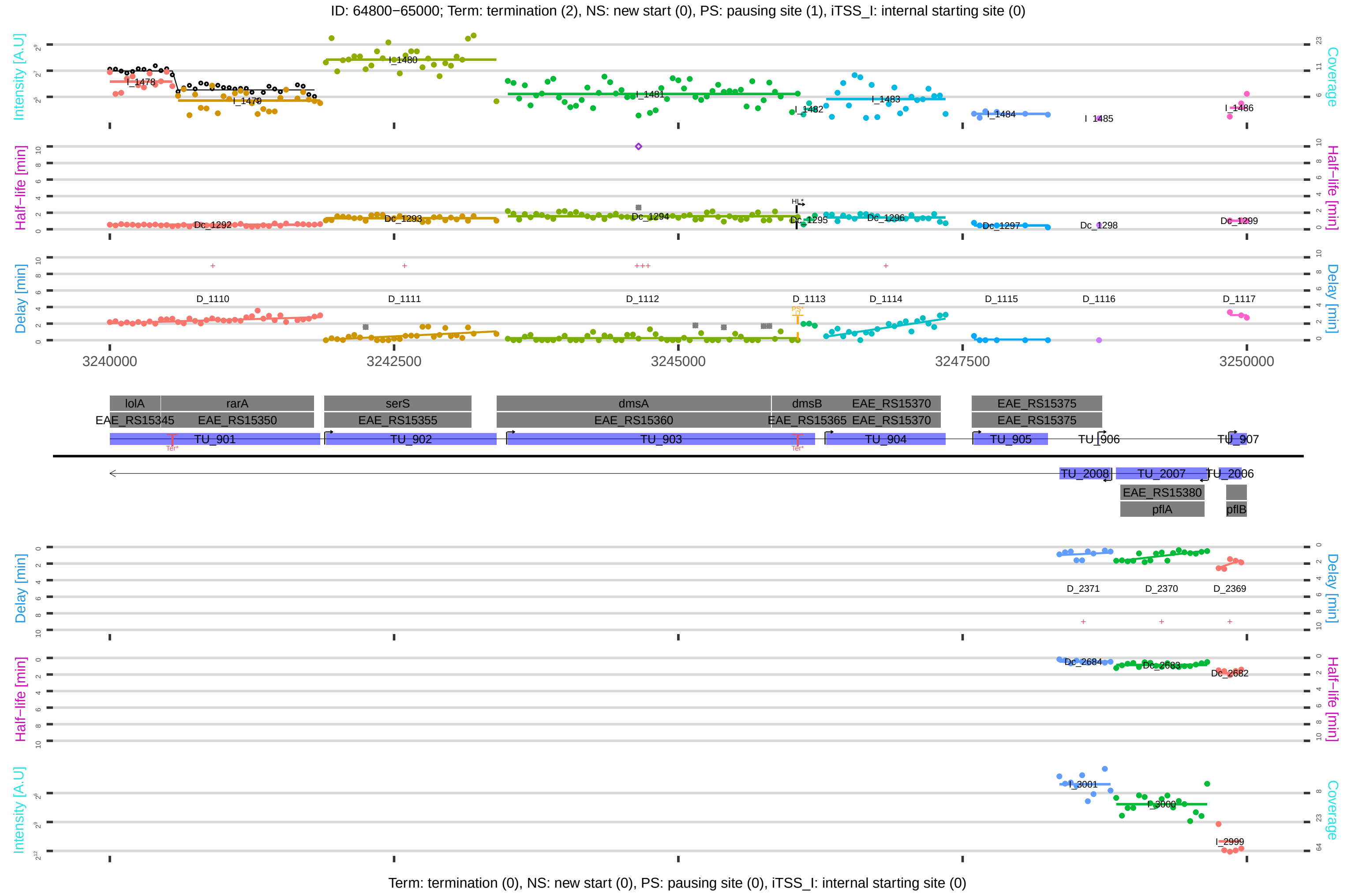
ID: 64800−65000; Term: termination (2), NS: new start (0), PS: pausing site (1), iTSS_I: internal starting site (0)
Intensity [A.U]
Coverage
Half−life [min]
Delay [min]
3240000
3242500
3245000
3247500
3250000
lolA
rarA
serS
dmsA
dmsB
EAE_RS15370
EAE_RS15375
EAE_RS15345
EAE_RS15350
EAE_RS15355
EAE_RS15360
EAE_RS15365
TU_901
TU_902
TU_903
TU_904
TU_905
TU_906
TU_907
TU_2008
TU_2007
TU_2006
EAE_RS15380
pflA
pflB
Term: termination (0), NS: new start (0), PS: pausing site (0), iTSS_I: internal starting site (0)

ID: 65000−65200; Term: termination (0), NS: new start (2), PS: pausing site (2), iTSS_I: internal starting site (0)

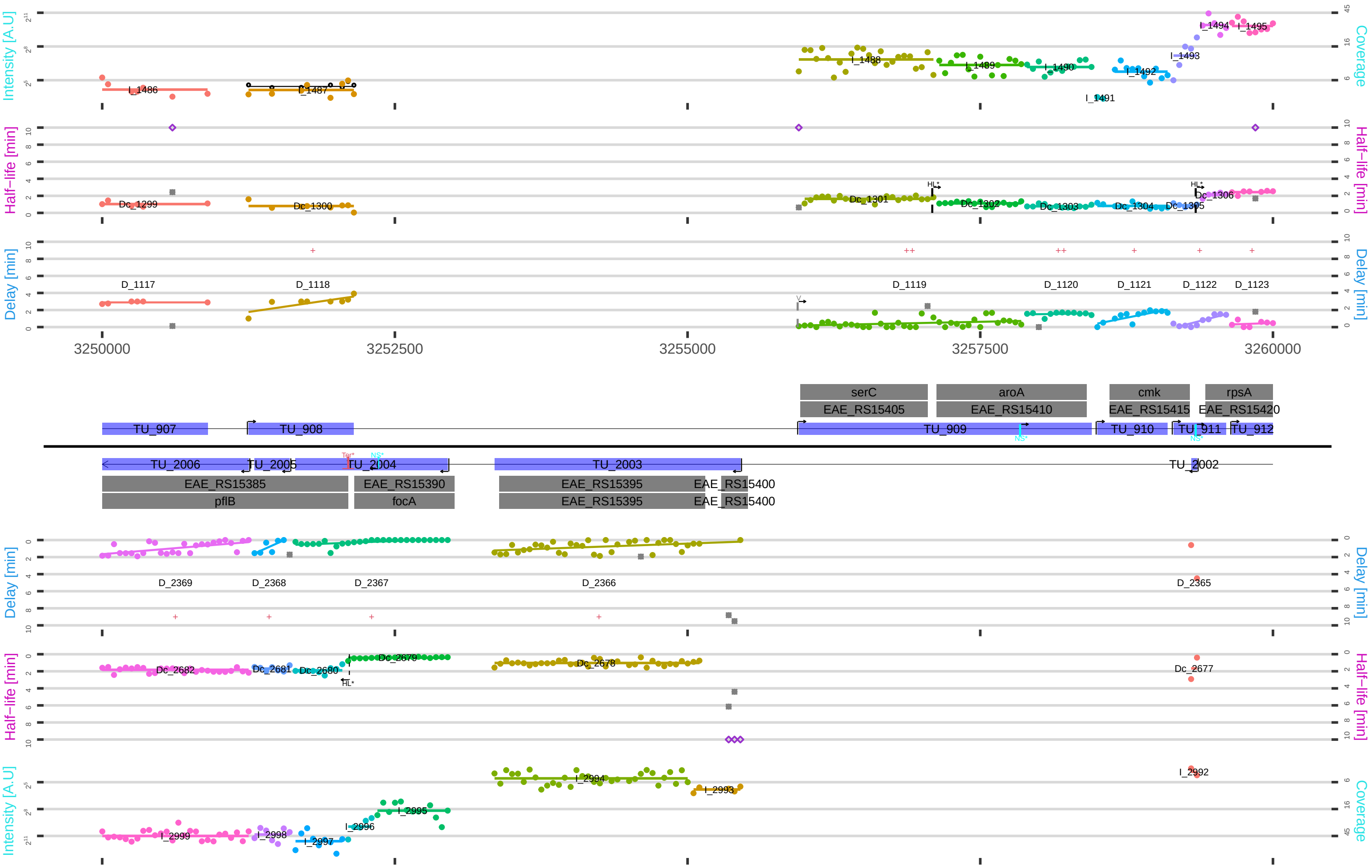

Term: termination (1), NS: new start (1), PS: pausing site (0), iTSS_I: internal starting site (0)

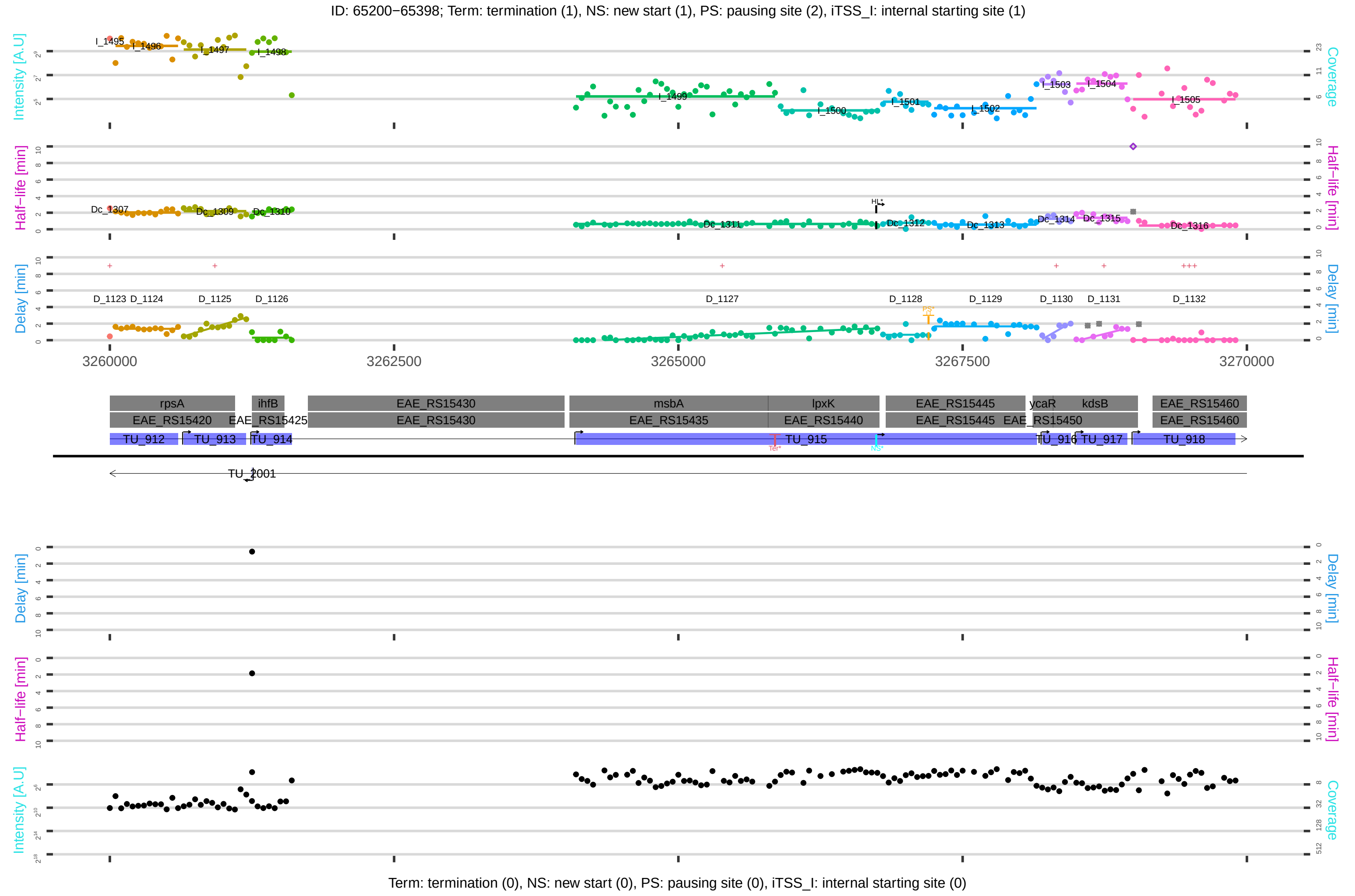

ID: 65200−65398; Term: termination (1), NS: new start (1), PS: pausing site (2), iTSS_I: internal starting site (1)
Intensity [A.U]
Coverage
Half−life [min]
Delay [min]
I_1495 I_1496 I_1497 I_1498 I_1499 I_1500 I_1501 I_1502 I_1503 I_1504 I_1505
Dc_1307 Dc_1309 Dc_1310 Dc_1311 Dc_1312 Dc_1313 Dc_1314 Dc_1315 Dc_1316
D_1123 D_1124 D_1125 D_1126 D_1127 D_1128 D_1129 D_1130 D_1131 D_1132
3260000 3262500 3265000 3267500 3270000
rpsA ihfB EAE_RS15430 msbA lpxK EAE_RS15445 ycaR kdsB EAE_RS15460
EAE_RS15420 EAE_RS15425 EAE_RS15430 EAE_RS15435 EAE_RS15440 EAE_RS15445 EAE_RS15450 EAE_RS15460
TU_912 TU_913 TU_914 TU_915 TU_916 TU_917 TU_918
TU_2001
Term: termination (0), NS: new start (0), PS: pausing site (0), iTSS_I: internal starting site (0)

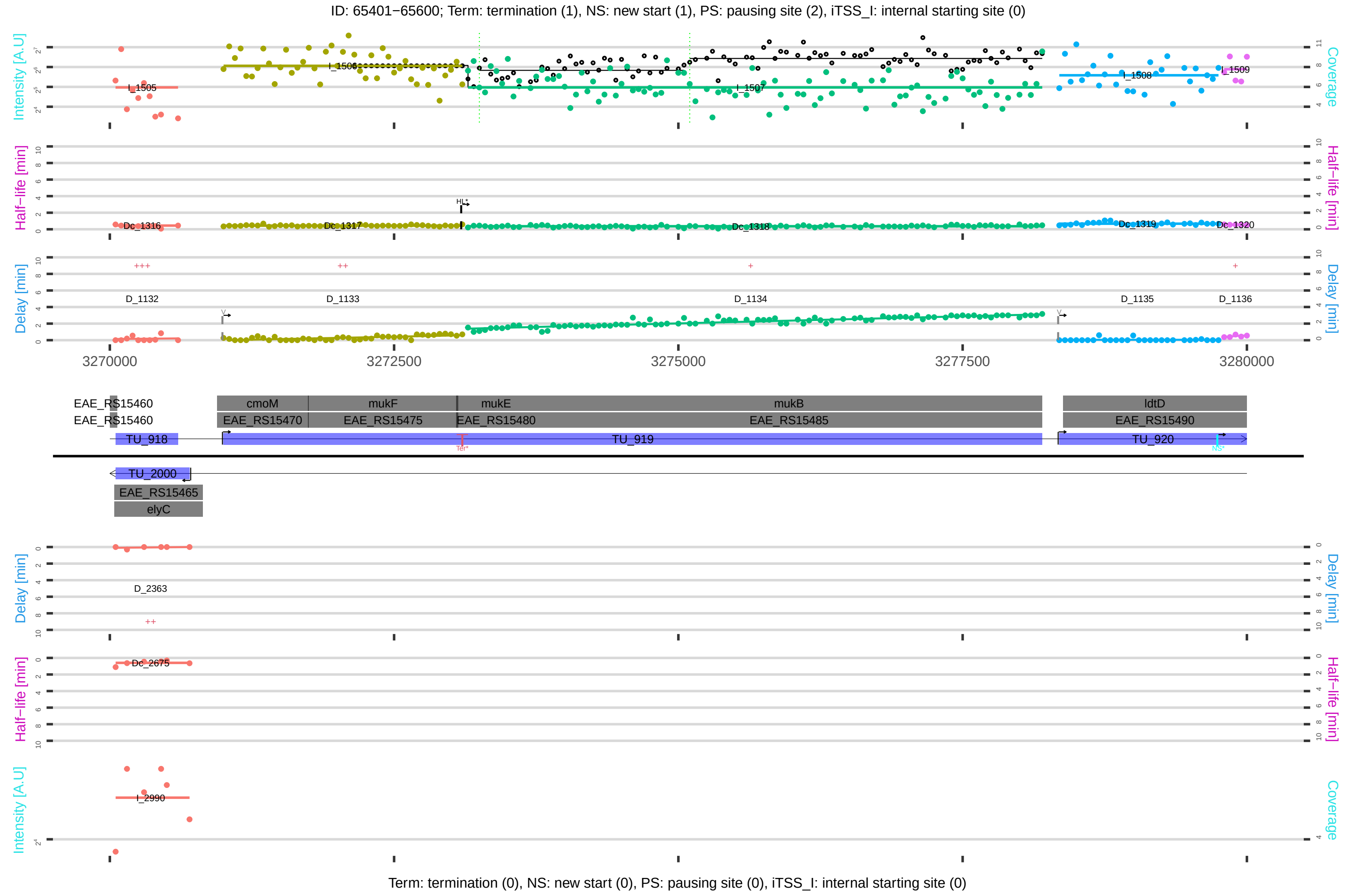
ID: 65401−65600; Term: termination (1), NS: new start (1), PS: pausing site (2), iTSS_I: internal starting site (0)
Intensity [A.U]
Coverage
Half−life [min]
Delay [min]
I_1505
I_1506
I_1507
I_1508
I_1509
Dc_1316
Dc_1317
Dc_1318
Dc_1319
Dc_1320
D_1132
D_1133
D_1134
D_1135
D_1136
3270000
3272500
3275000
3277500
3280000
EAE_RS15460
cmoM
mukF
mukE
mukB
ldtD
EAE_RS15470
EAE_RS15475
EAE_RS15480
EAE_RS15485
EAE_RS15490
TU_918
TU_919
TU_920
TU_2000
EAE_RS15465
elyC
D_2363
Dc_2675
I_2990
Term: termination (0), NS: new start (0), PS: pausing site (0), iTSS_I: internal starting site (0)

ID: 65600−65800; Term: termination (0), NS: new start (1), PS: pausing site (1), iTSS_I: internal starting site (0)

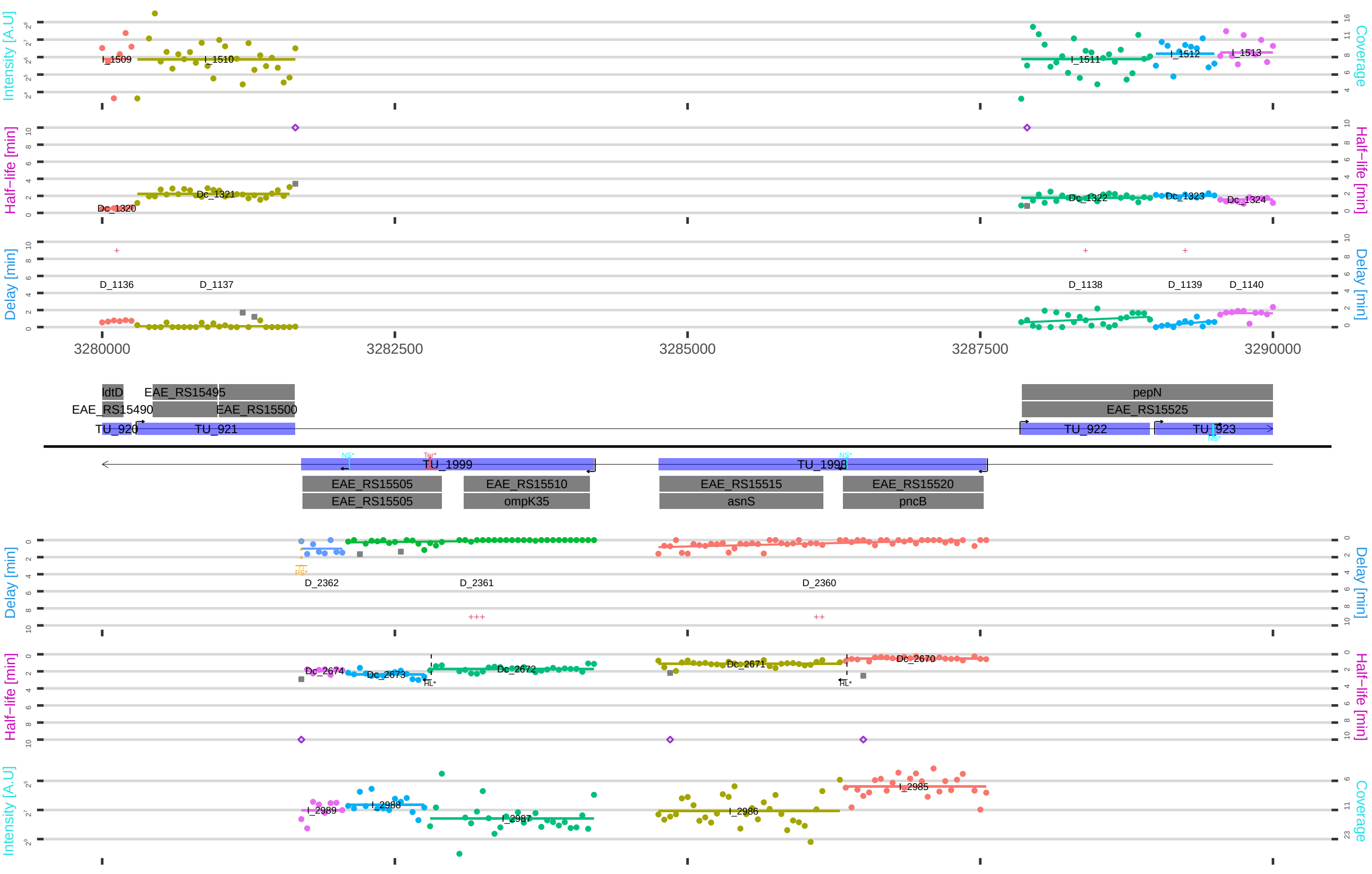

Term: termination (1), NS: new start (2), PS: pausing site (1), iTSS_I: internal starting site (0)

ID: 65800−66000; Term: termination (0), NS: new start (1), PS: pausing site (1), iTSS_I: internal starting site (0)

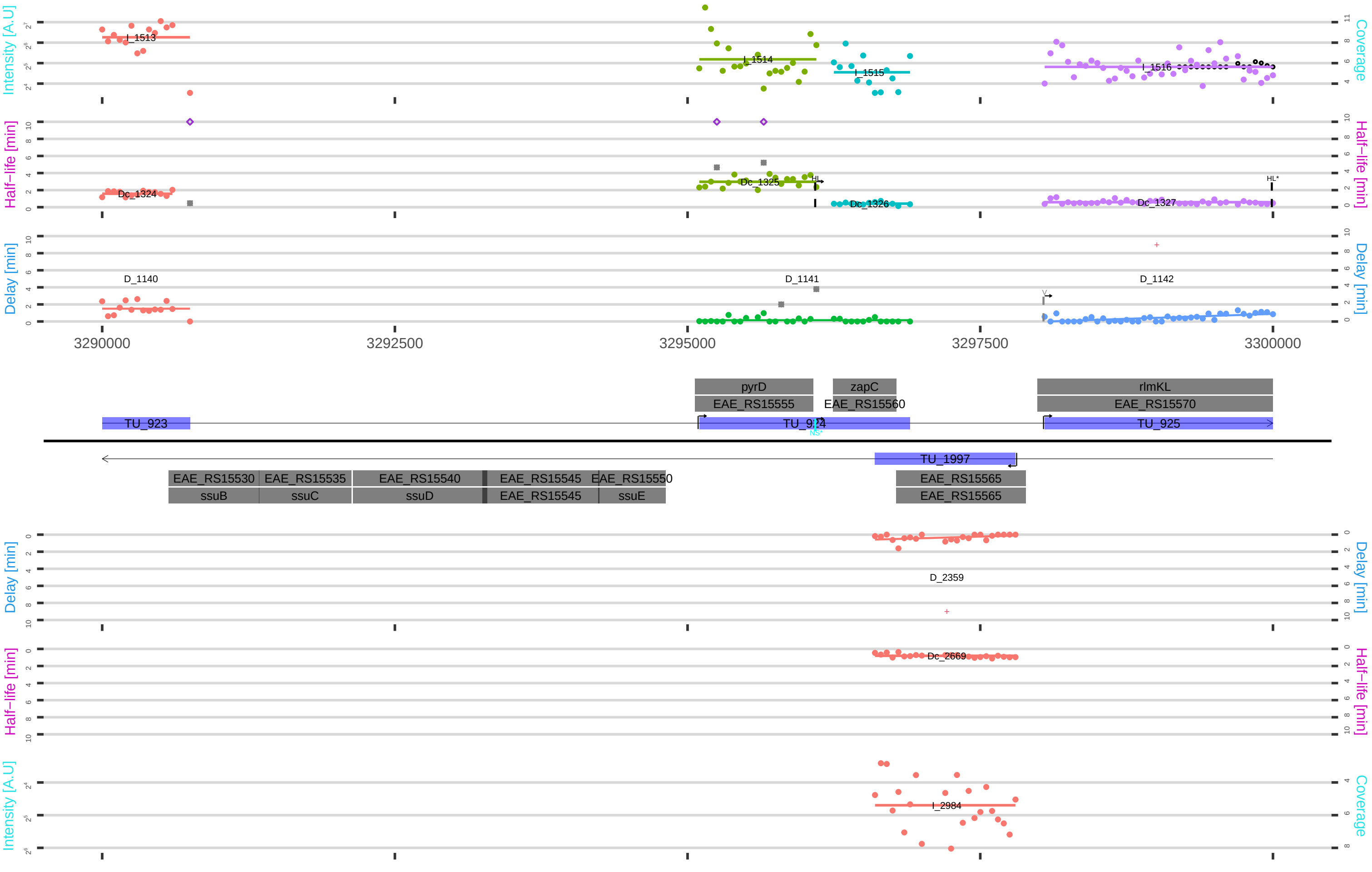

Term: termination (0), NS: new start (0), PS: pausing site (0), iTSS_I: internal starting site (0)

ID: 66000−66200; Term: termination (3), NS: new start (2), PS: pausing site (3), iTSS_I: internal starting site (1)

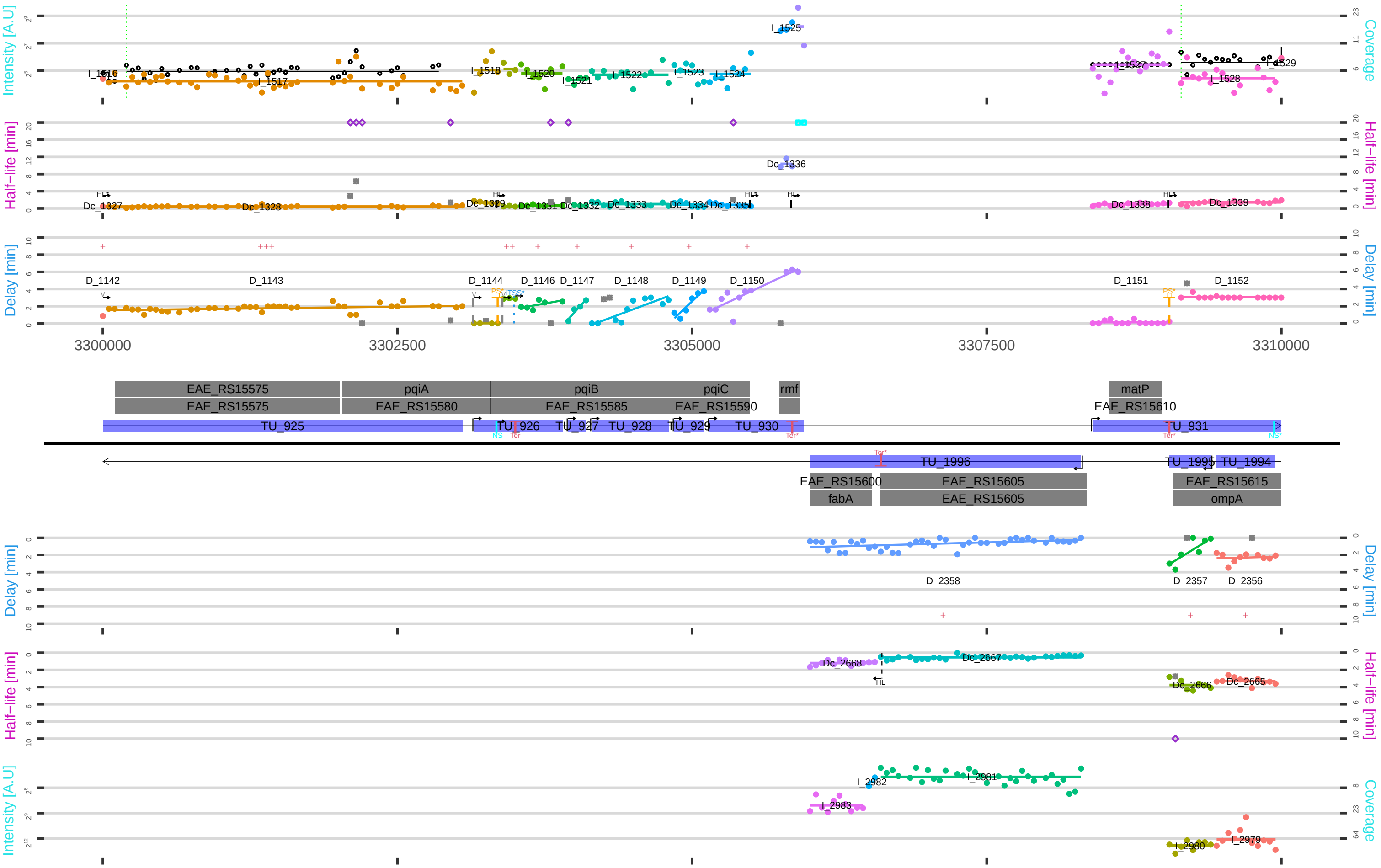

Term: termination (1), NS: new start (0), PS: pausing site (0), iTSS_I: internal starting site (0)

ID: 66200−66358; Term: termination (0), NS: new start (0), PS: pausing site (0), iTSS_I: internal starting site (0)

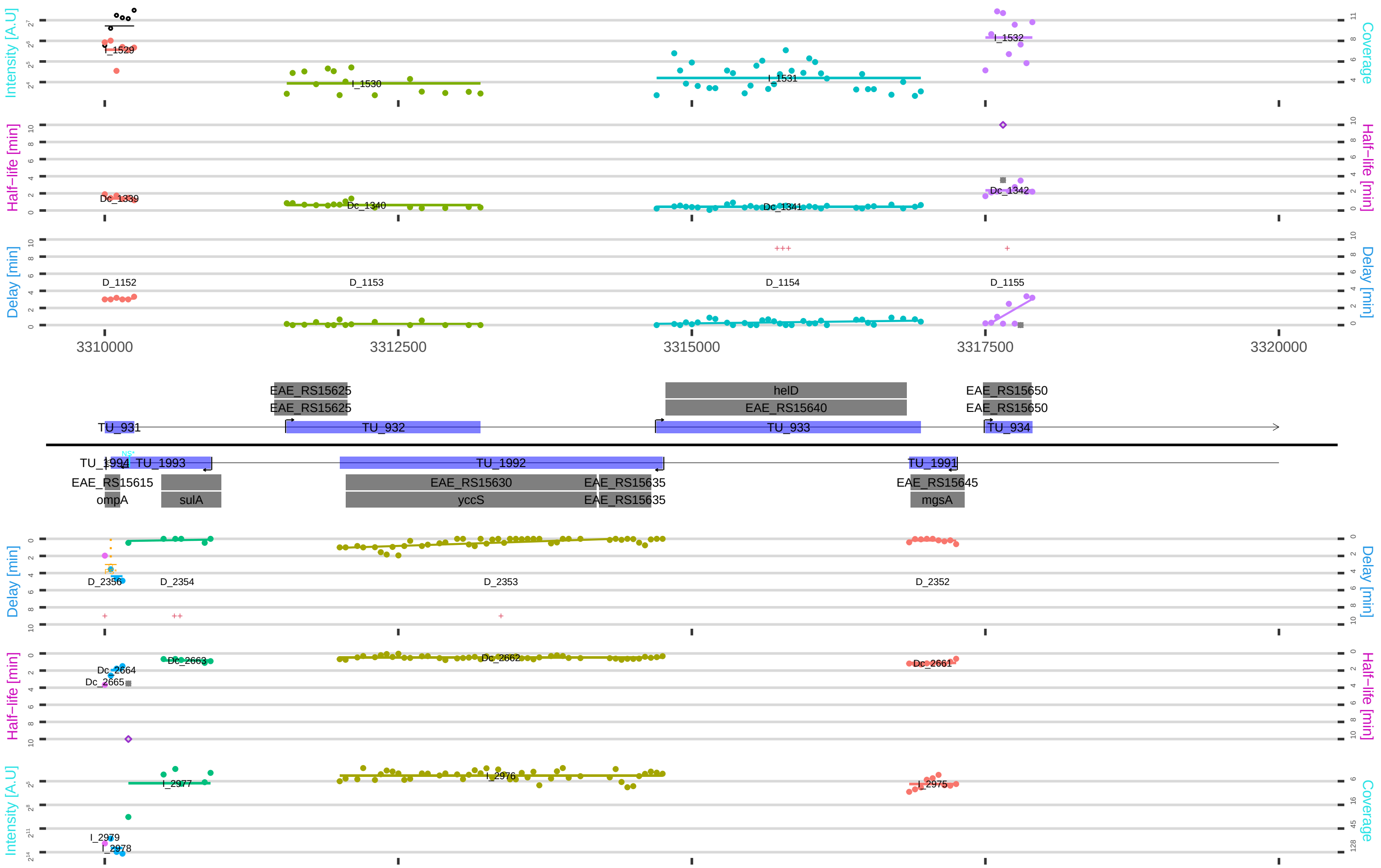

Term: termination (0), NS: new start (1), PS: pausing site (1), iTSS_I: internal starting site (0)

ID: 66450−66572; Term: termination (0), NS: new start (0), PS: pausing site (0), iTSS_I: internal starting site (0)

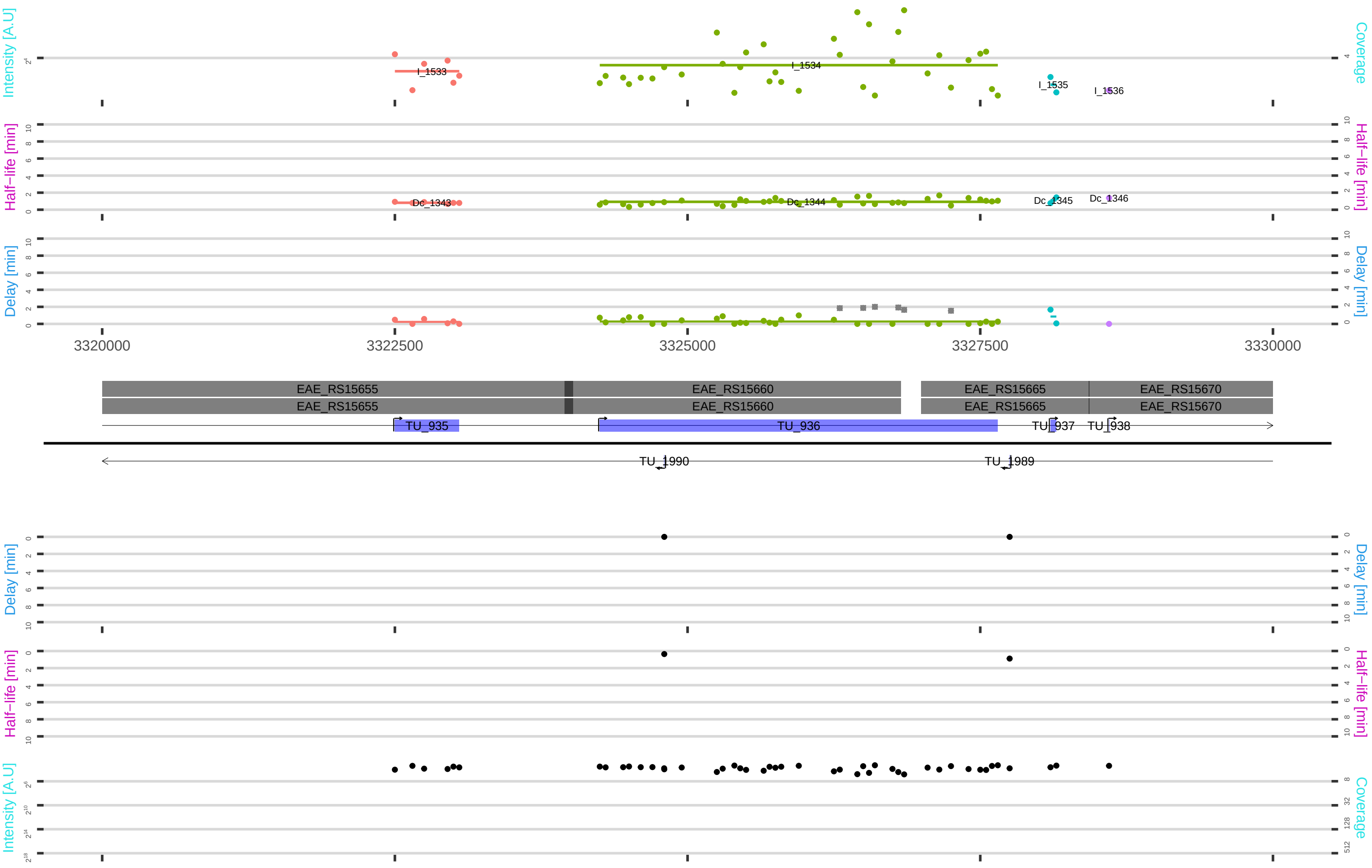

Term: termination (0), NS: new start (0), PS: pausing site (0), iTSS_I: internal starting site (0)

ID: 66636−66795; Term: termination (0), NS: new start (0), PS: pausing site (0), iTSS_I: internal starting site (0)

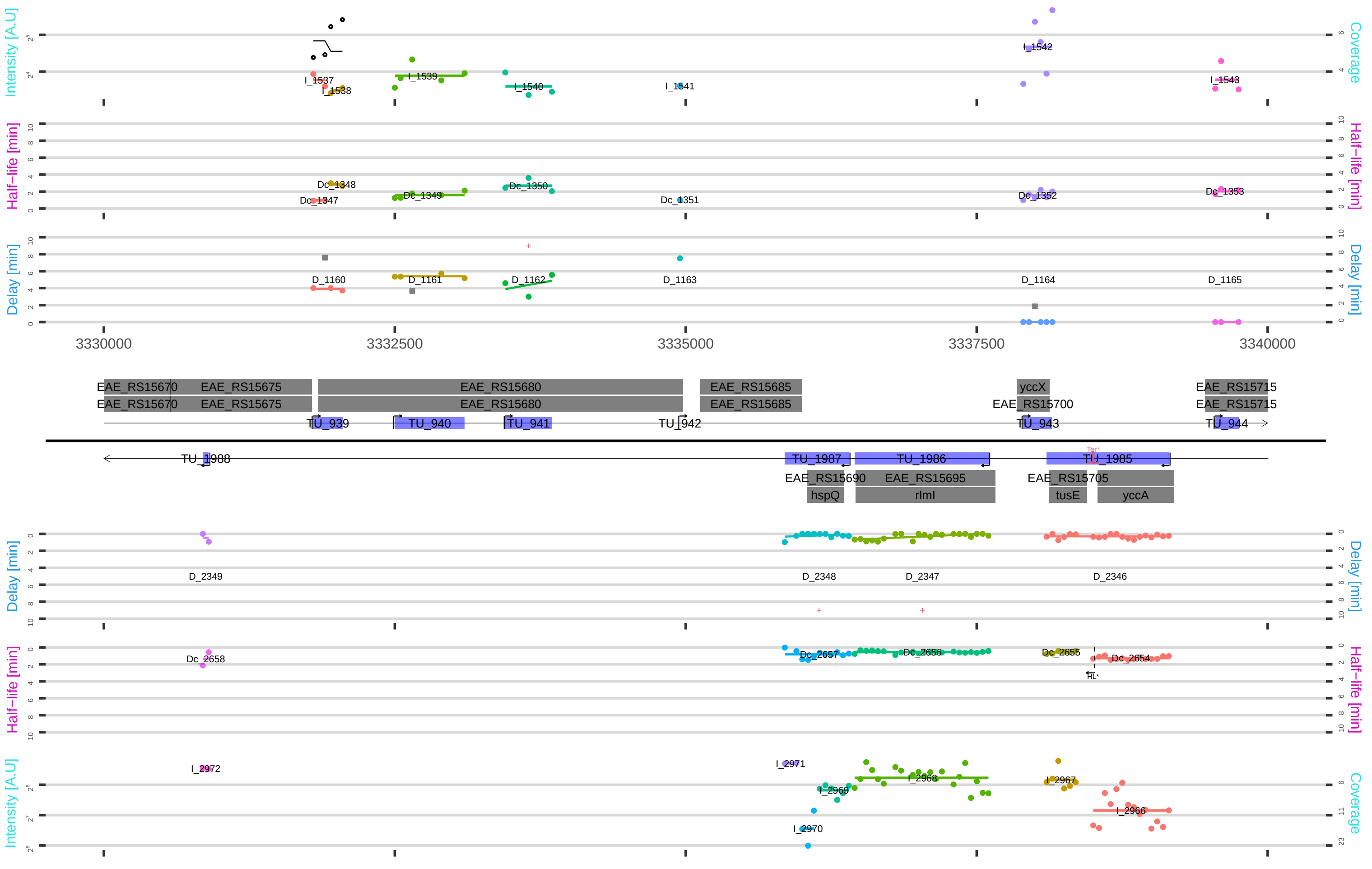

Term: termination (1), NS: new start (0), PS: pausing site (0), iTSS_I: internal starting site (0)

ID: 66802−66933; Term: termination (0), NS: new start (1), PS: pausing site (0), iTSS_I: internal starting site (0)

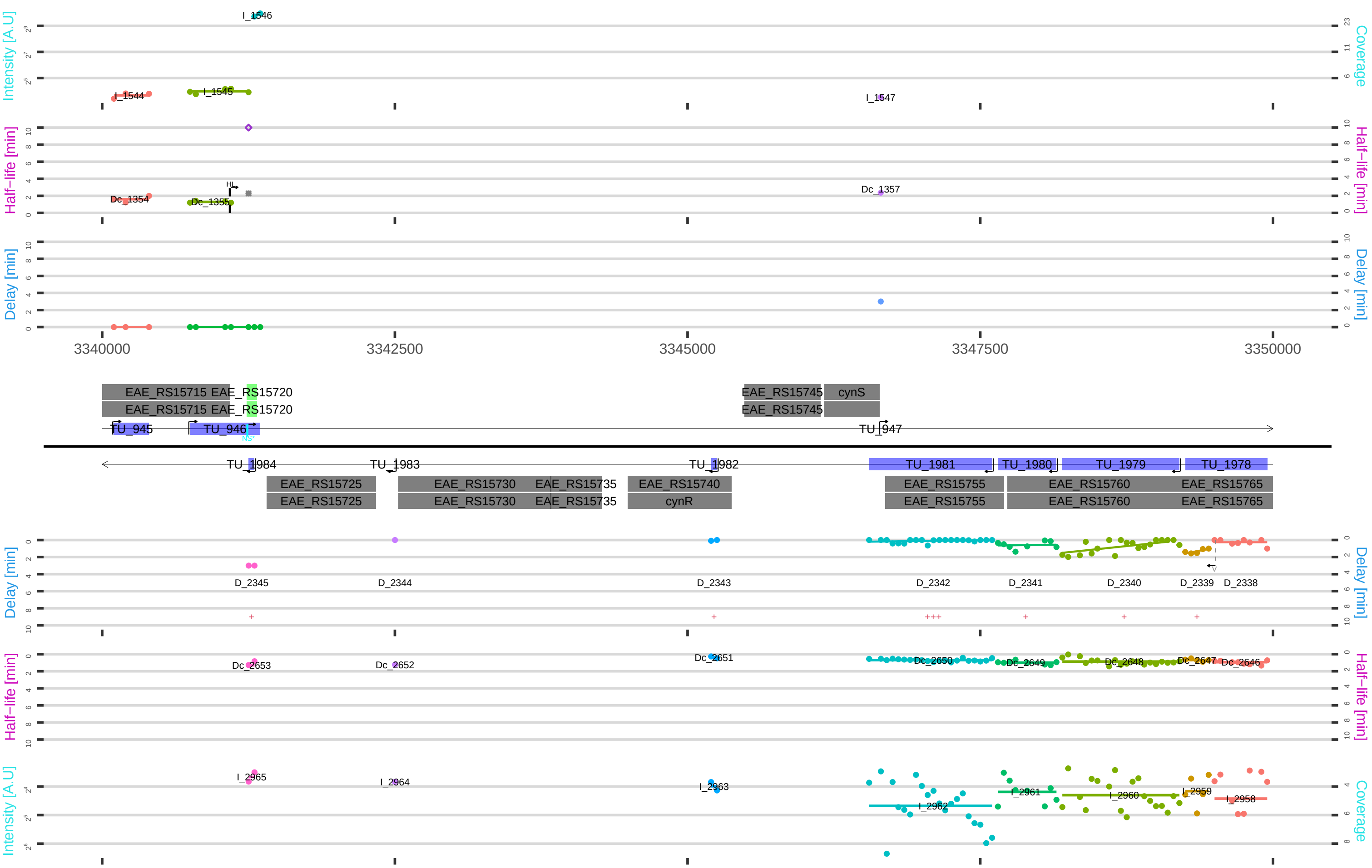

Term: termination (0), NS: new start (0), PS: pausing site (1), iTSS_I: internal starting site (0)

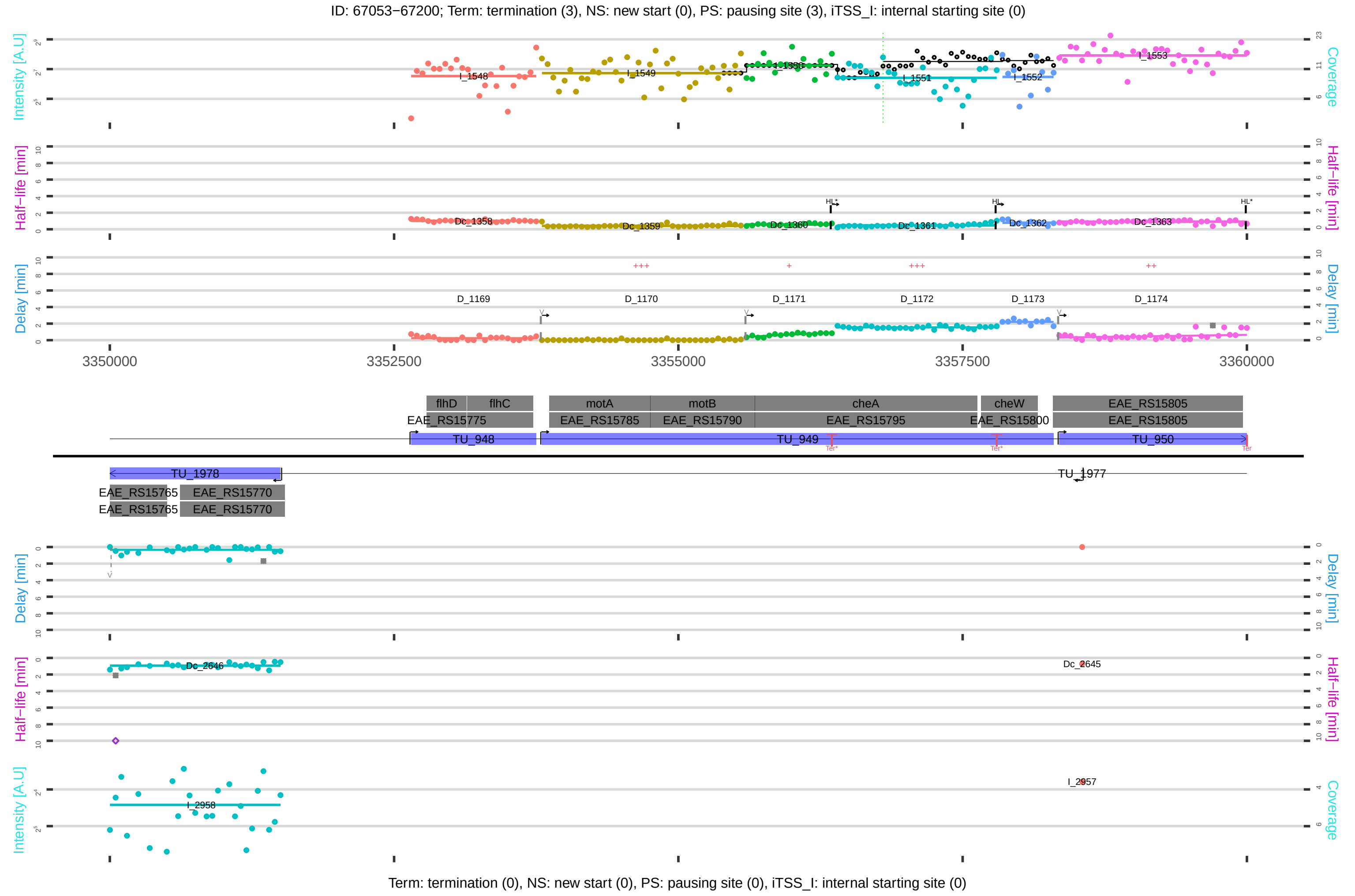
ID: 67053−67200; Term: termination (3), NS: new start (0), PS: pausing site (3), iTSS_I: internal starting site (0)
Intensity [A.U]
Coverage
Half−life [min]
Delay [min]
I_1548
I_1549
I_1550
I_1551
I_1552
I_1553
Dc_1358
Dc_1359
Dc_1360
Dc_1361
Dc_1362
Dc_1363
D_1169
D_1170
D_1171
D_1172
D_1173
D_1174
3350000
3352500
3355000
3357500
3360000
flhD
flhC
motA
motB
cheA
cheW
EAE_RS15775
EAE_RS15785
EAE_RS15790
EAE_RS15795
EAE_RS15800
EAE_RS15805
TU_948
TU_949
TU_950
TU_1978
TU_1977
EAE_RS15765
EAE_RS15770
Dc_2646
Dc_2645
I_2958
I_2957
Term: termination (0), NS: new start (0), PS: pausing site (0), iTSS_I: internal starting site (0)

ID: 67200−67380; Term: termination (4), NS: new start (1), PS: pausing site (1), iTSS_I: internal starting site (0)

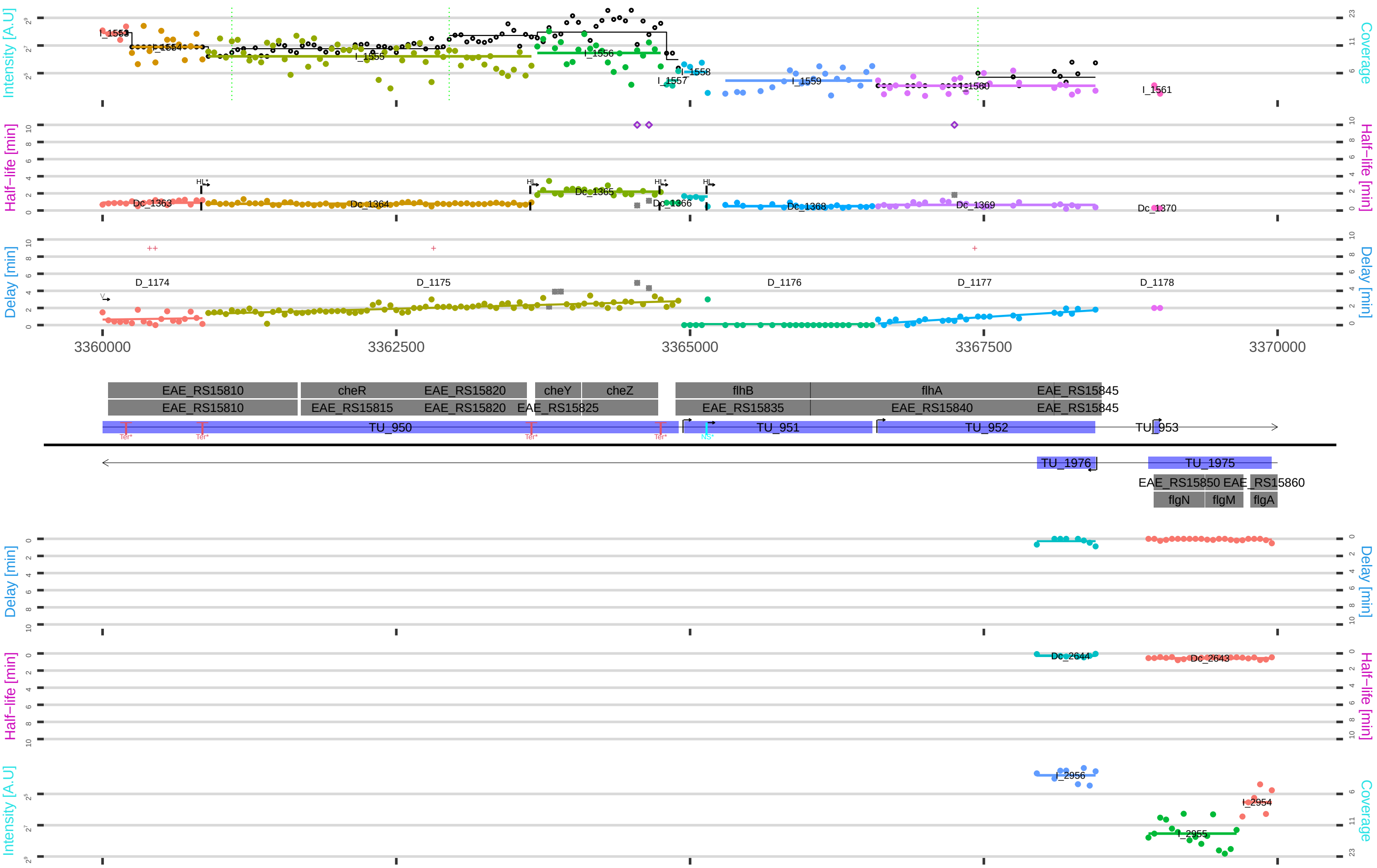

Term: termination (0), NS: new start (0), PS: pausing site (0), iTSS_I: internal starting site (0)

ID: 67413−67600; Term: termination (4), NS: new start (2), PS: pausing site (2), iTSS_I: internal starting site (0)

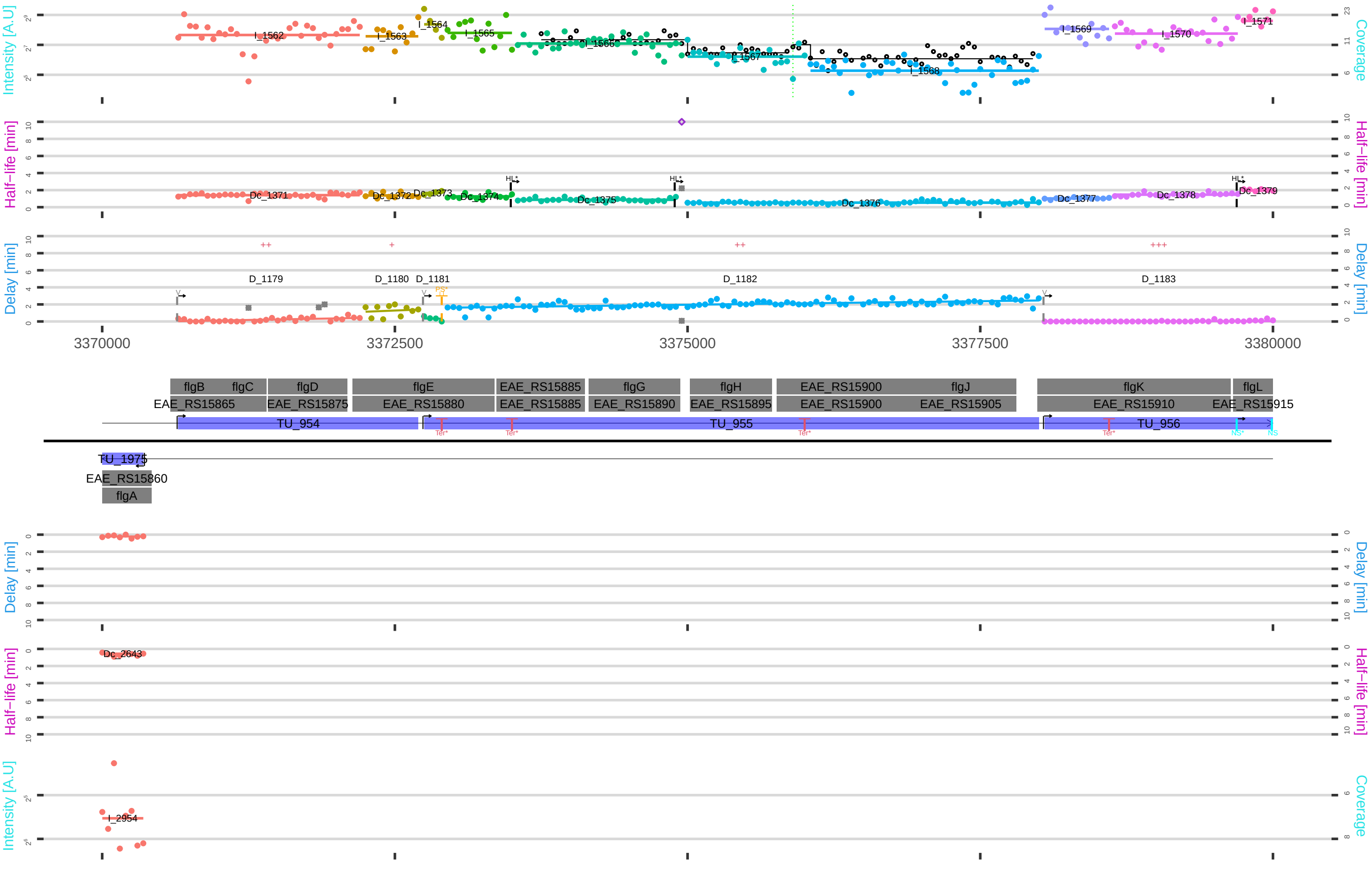

Term: termination (0), NS: new start (0), PS: pausing site (0), iTSS_I: internal starting site (0)

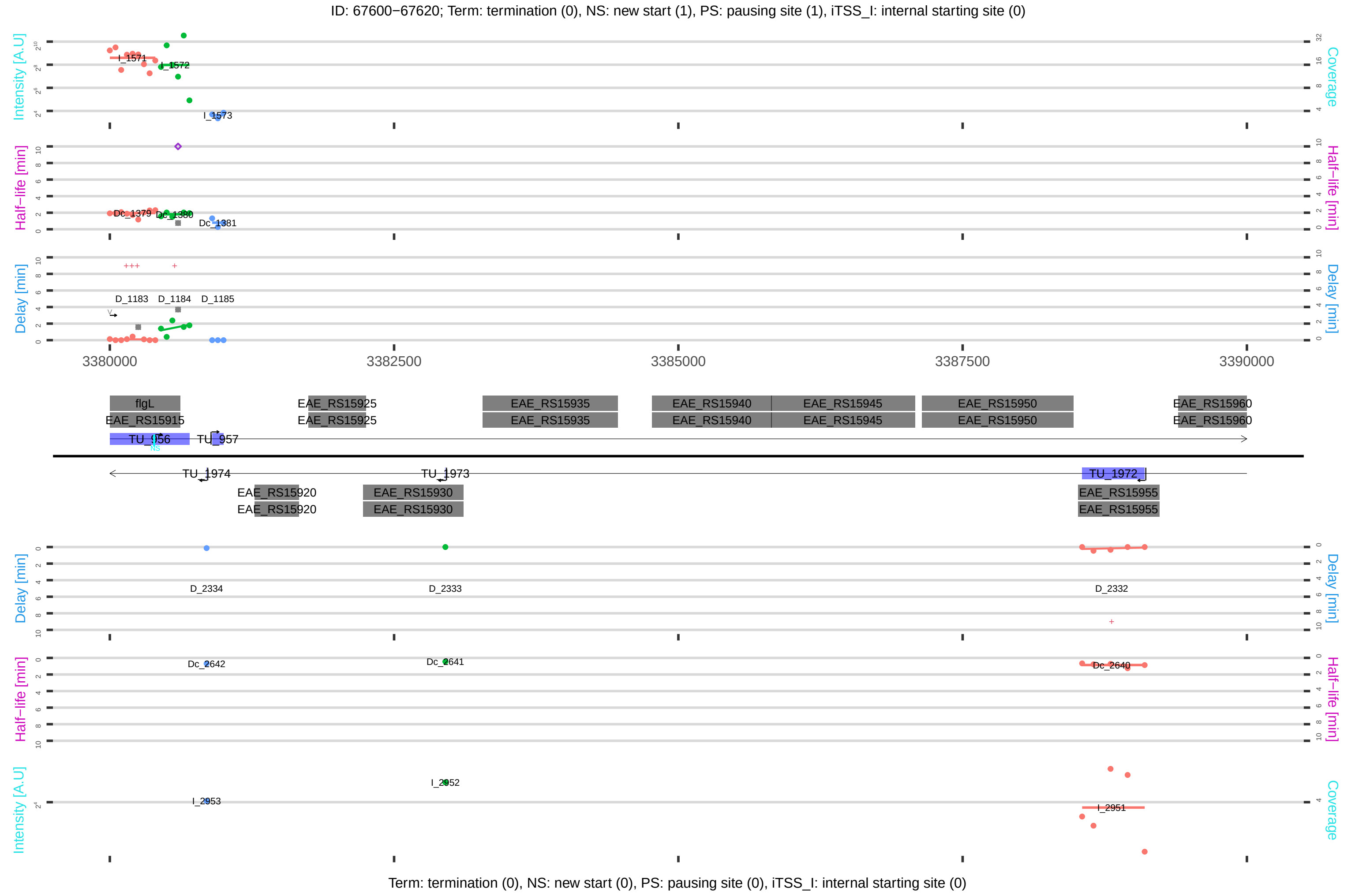

ID: 67600−67620; Term: termination (0), NS: new start (1), PS: pausing site (1), iTSS_I: internal starting site (0)
Intensity [A.U]
Coverage
I_1571
I_1572
I_1573
Half−life [min]
Dc_1379
Dc_1380
Dc_1381
Delay [min]
D_1183
D_1184
D_1185
3380000
3382500
3385000
3387500
3390000
flgL
EAE_RS15915
EAE_RS15925
EAE_RS15935
EAE_RS15940
EAE_RS15945
EAE_RS15950
EAE_RS15960
TU_956
TU_957
NS
TU_1974
TU_1973
TU_1972
EAE_RS15920
EAE_RS15930
EAE_RS15955
D_2334
D_2333
D_2332
Dc_2642
Dc_2641
Dc_2640
I_2953
I_2952
I_2951
Term: termination (0), NS: new start (0), PS: pausing site (0), iTSS_I: internal starting site (0)

ID: 67835−67880; Term: termination (0), NS: new start (0), PS: pausing site (0), iTSS_I: internal starting site (0)

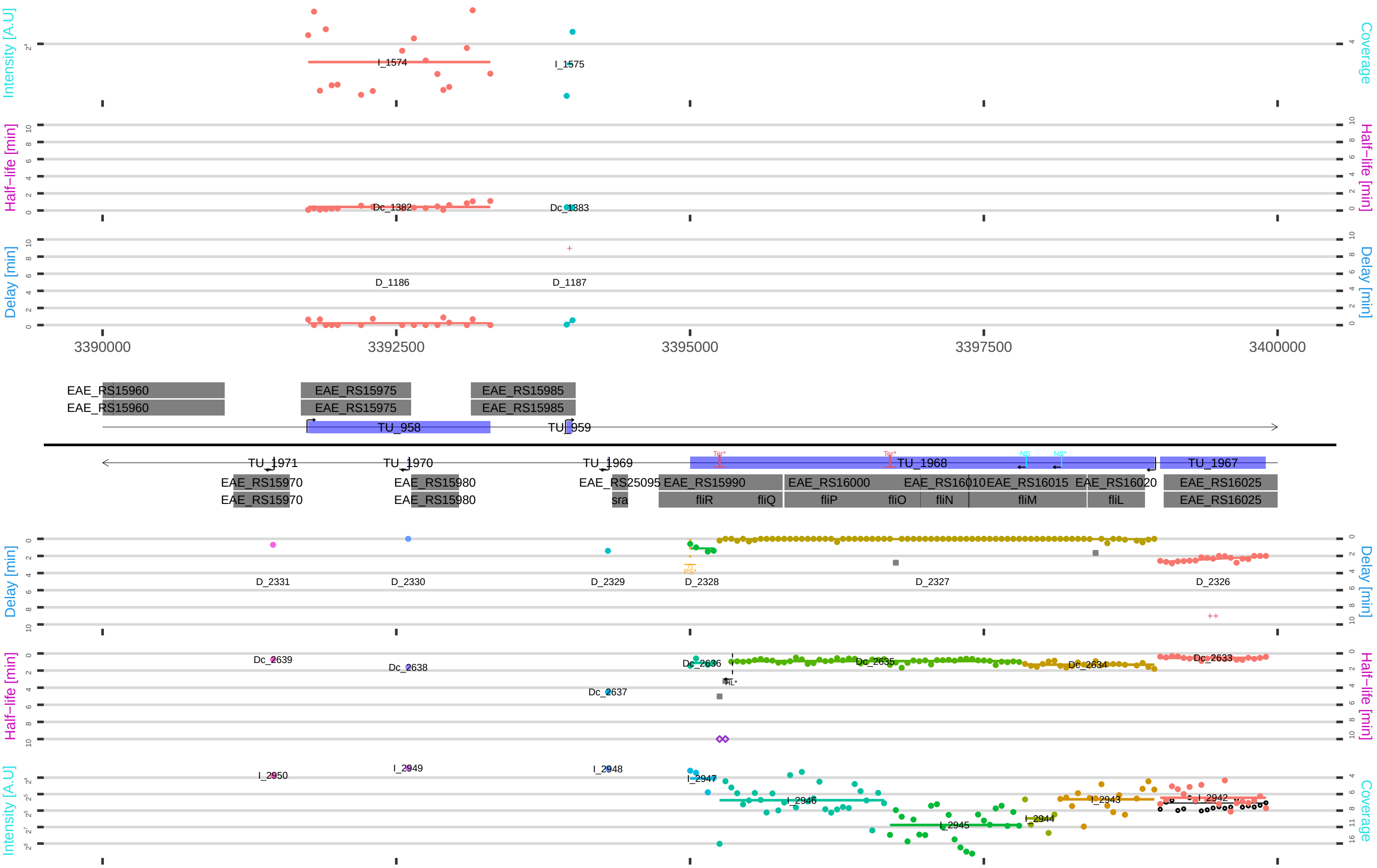

Term: termination (2), NS: new start (2), PS: pausing site (2), iTSS_I: internal starting site (0)

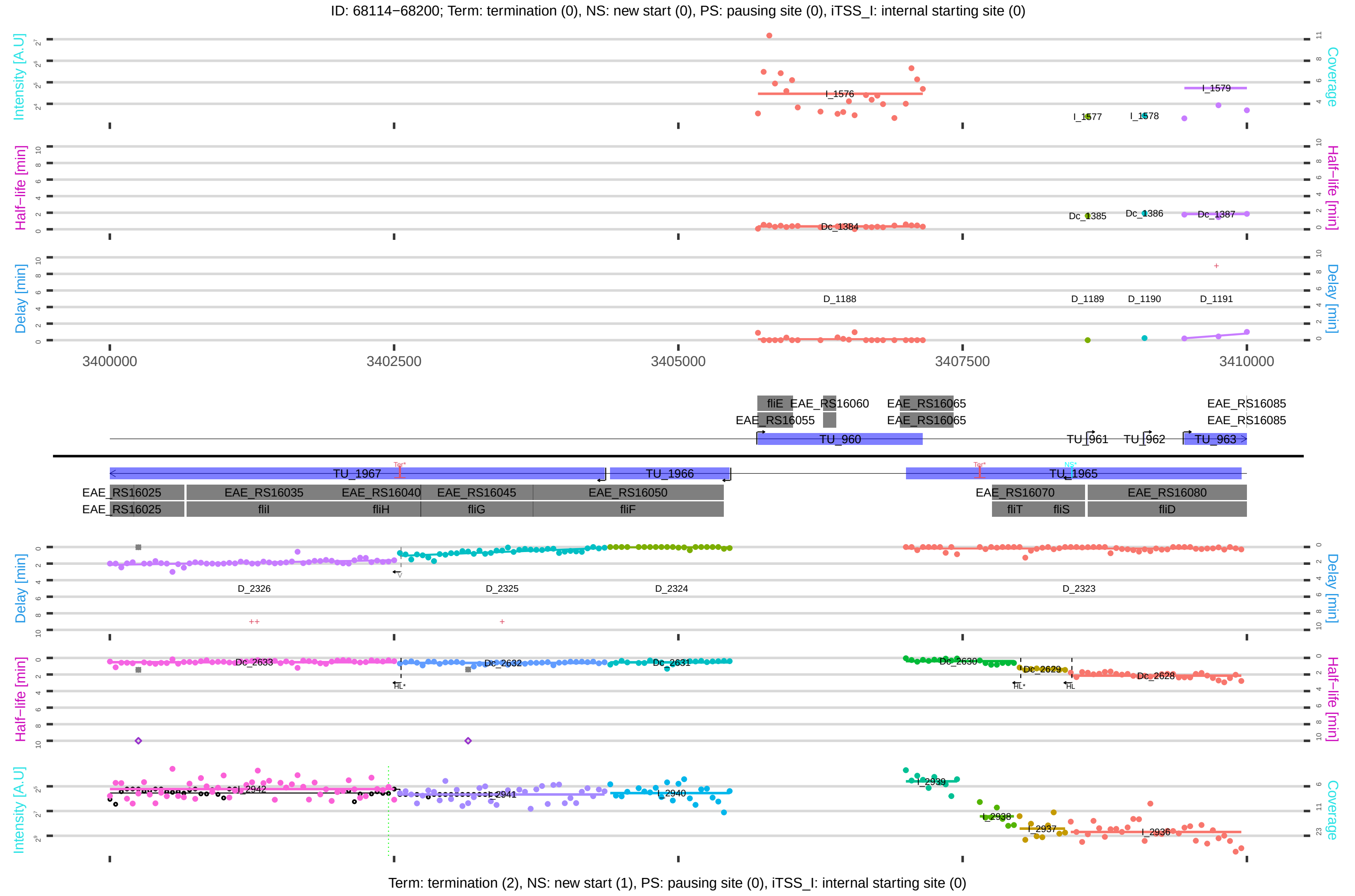
ID: 68114−68200; Term: termination (0), NS: new start (0), PS: pausing site (0), iTSS_I: internal starting site (0)
Intensity [A.U]
Coverage
Half−life [min]
Delay [min]
I_1576
I_1577
I_1578
I_1579
Dc_1384
Dc_1385
Dc_1386
Dc_1387
D_1188
D_1189
D_1190
D_1191
3400000
3402500
3405000
3407500
3410000
fliE
EAE_RS16060
EAE_RS16065
EAE_RS16055
EAE_RS16085
TU_960
TU_961
TU_962
TU_963
TU_1967
TU_1966
TU_1965
EAE_RS16025
EAE_RS16035
EAE_RS16040
EAE_RS16045
EAE_RS16050
EAE_RS16070
EAE_RS16080
fliI
fliH
fliG
fliF
fliT
fliS
fliD
D_2326
D_2325
D_2324
D_2323
Dc_2633
Dc_2632
Dc_2631
Dc_2630
Dc_2629
Dc_2628
I_2942
I_2941
I_2940
I_2939
I_2938
I_2937
I_2936
Term: termination (2), NS: new start (1), PS: pausing site (0), iTSS_I: internal starting site (0)

ID: 68200−68384; Term: termination (0), NS: new start (0), PS: pausing site (0), iTSS_I: internal starting site (0)

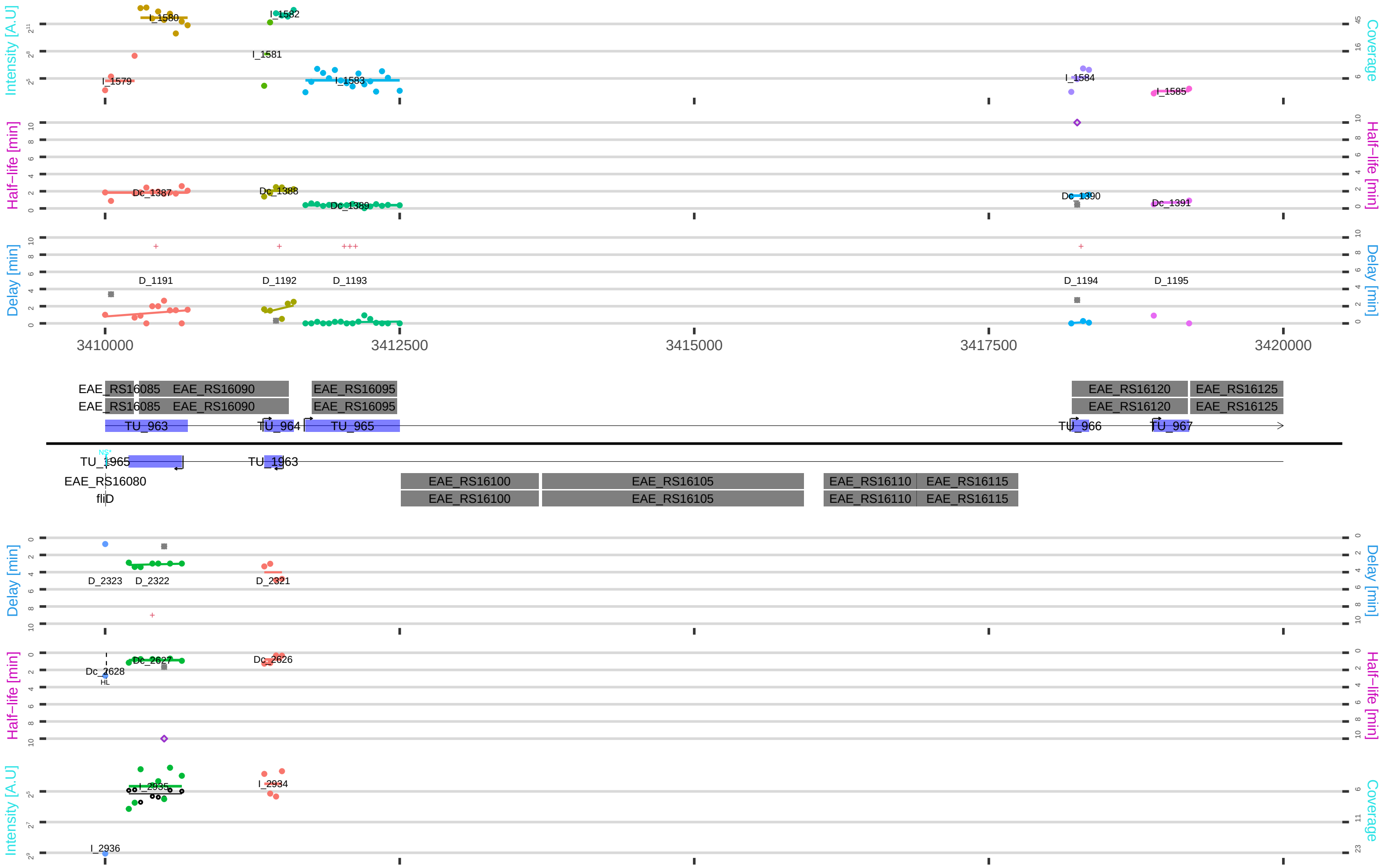

Term: termination (0), NS: new start (1), PS: pausing site (0), iTSS_I: internal starting site (0)

ID: 68440−68558; Term: termination (0), NS: new start (0), PS: pausing site (0), iTSS_I: internal starting site (0)

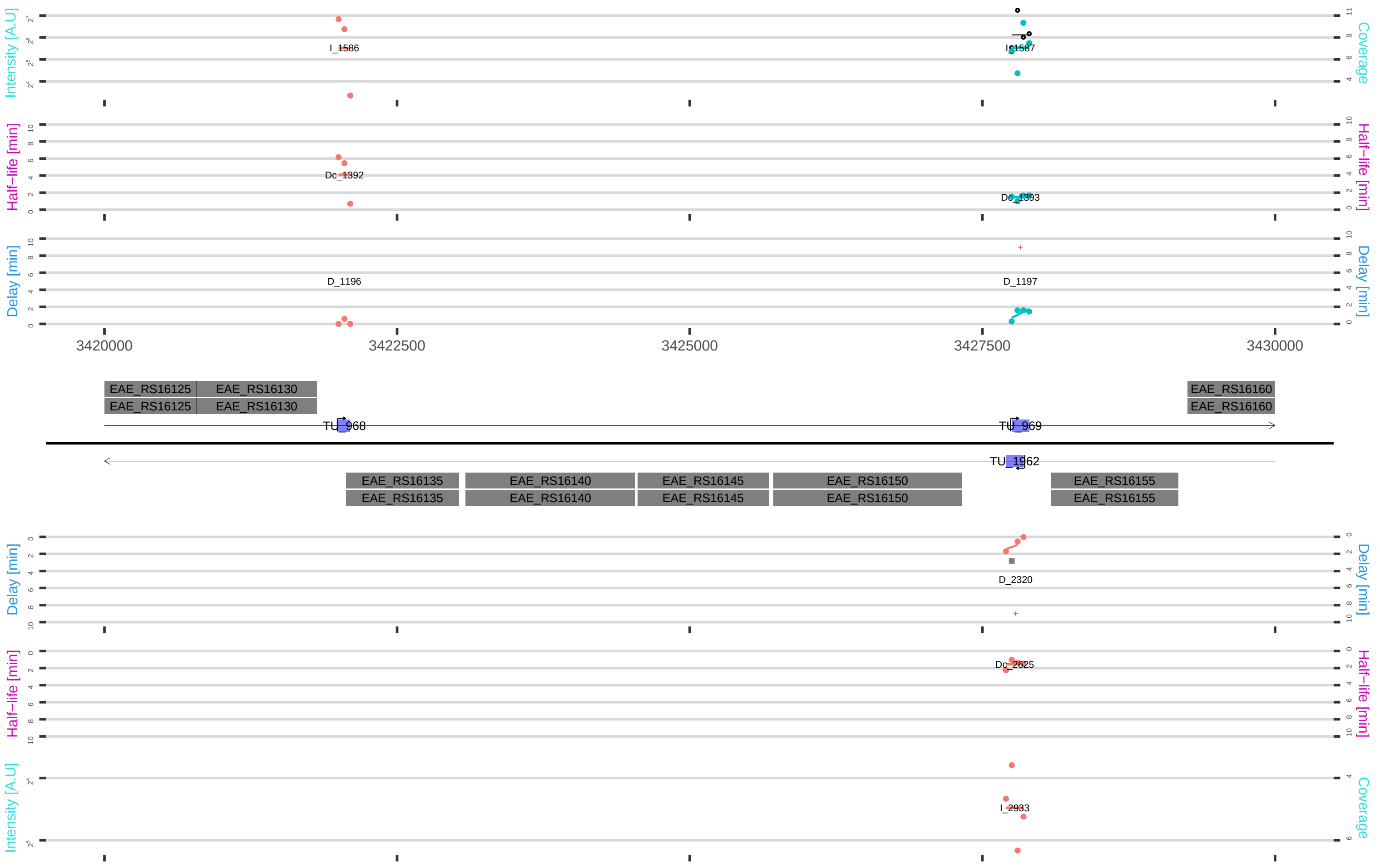

Term: termination (0), NS: new start (0), PS: pausing site (0), iTSS_I: internal starting site (0)

ID: 68785−68786; Term: termination (0), NS: new start (0), PS: pausing site (0), iTSS_I: internal starting site (0)

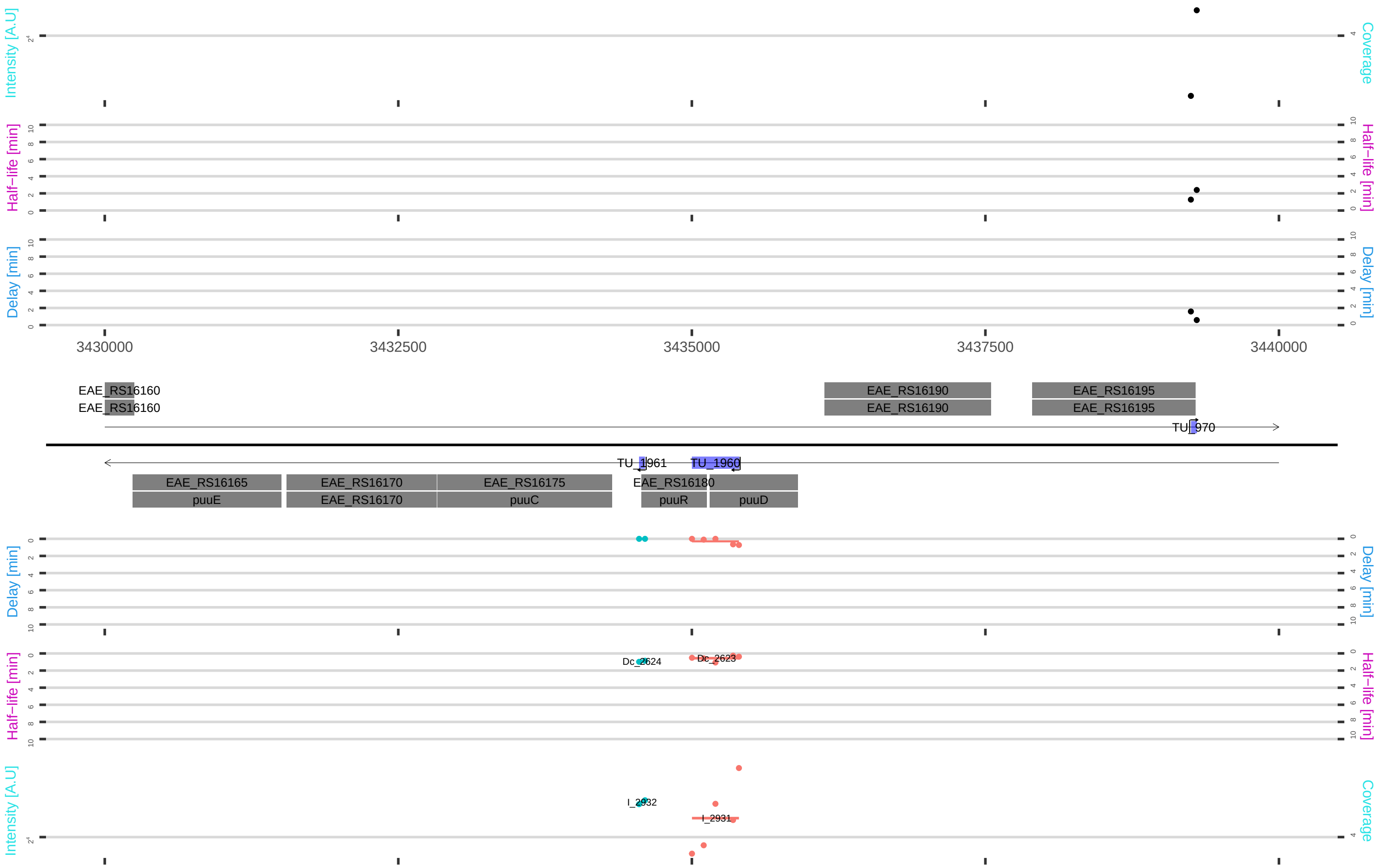

Term: termination (0), NS: new start (0), PS: pausing site (0), iTSS_I: internal starting site (0)

ID: 68910−68993; Term: termination (0), NS: new start (0), PS: pausing site (0), iTSS_I: internal starting site (0)

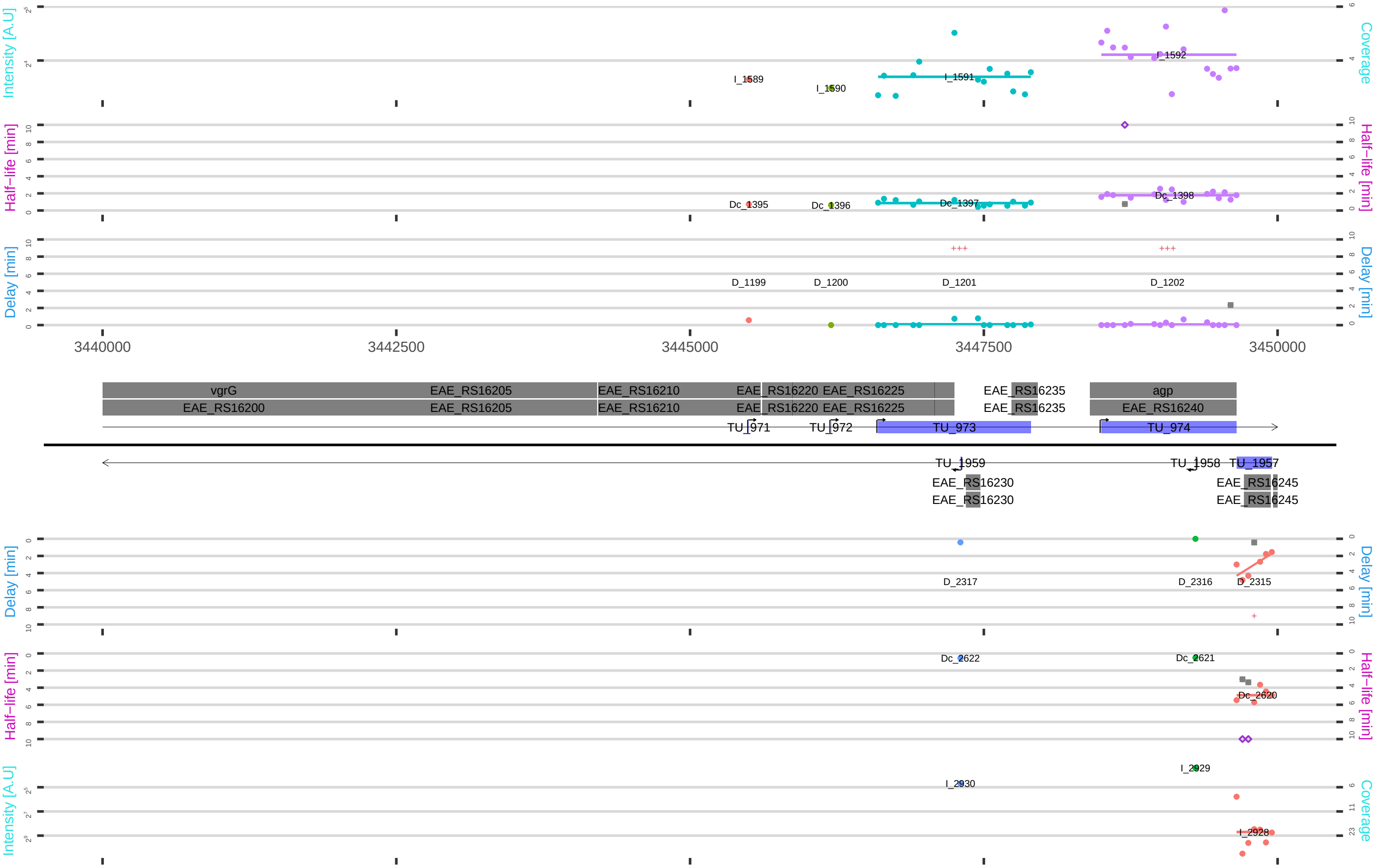

Term: termination (0), NS: new start (0), PS: pausing site (0), iTSS_I: internal starting site (0)

ID: 69020−69180; Term: termination (0), NS: new start (1), PS: pausing site (0), iTSS_I: internal starting site (0)

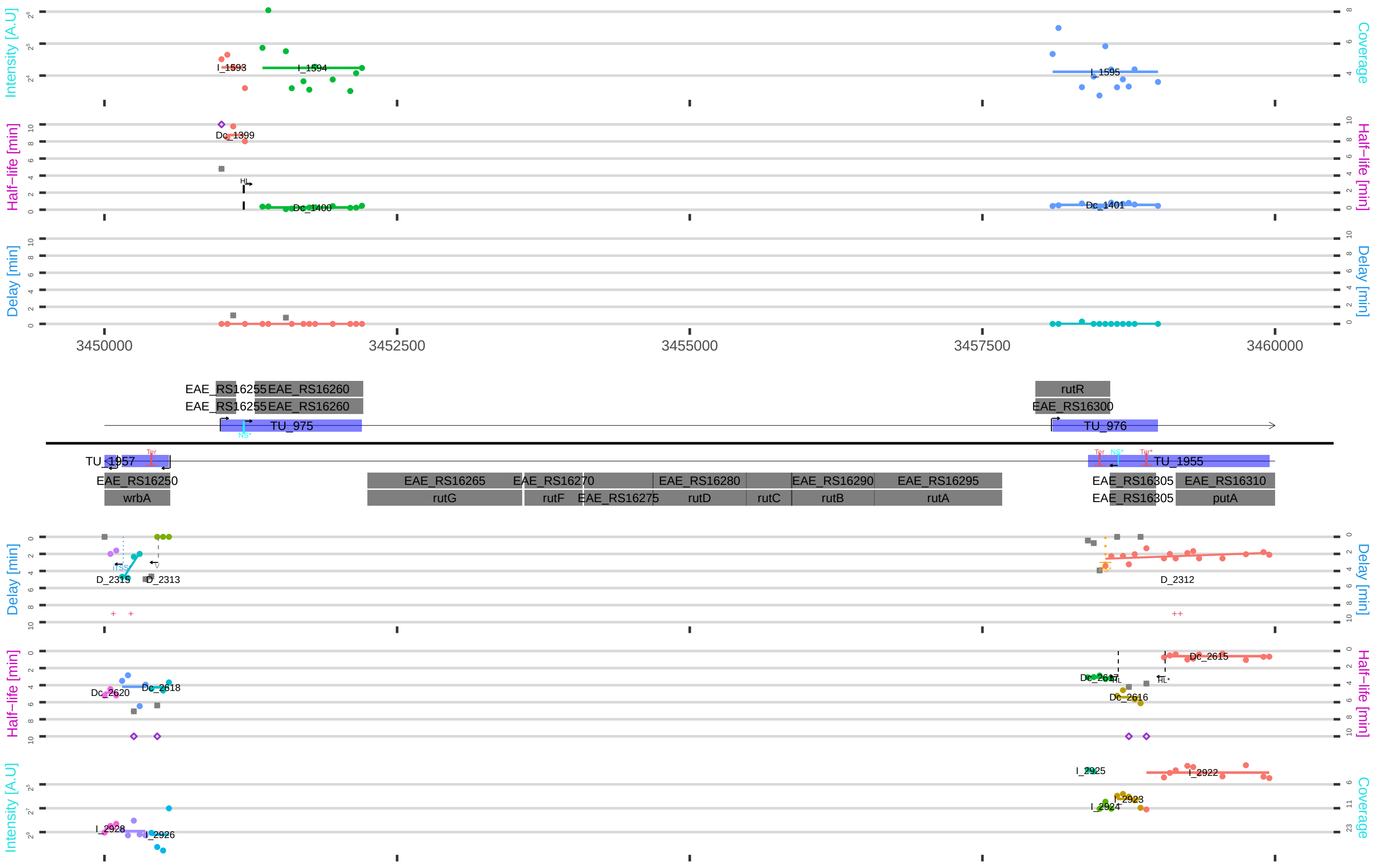

Term: termination (3), NS: new start (1), PS: pausing site (1), iTSS_I: internal starting site (1)

ID: 69271−69400; Term: termination (1), NS: new start (0), PS: pausing site (0), iTSS_I: internal starting site (0)

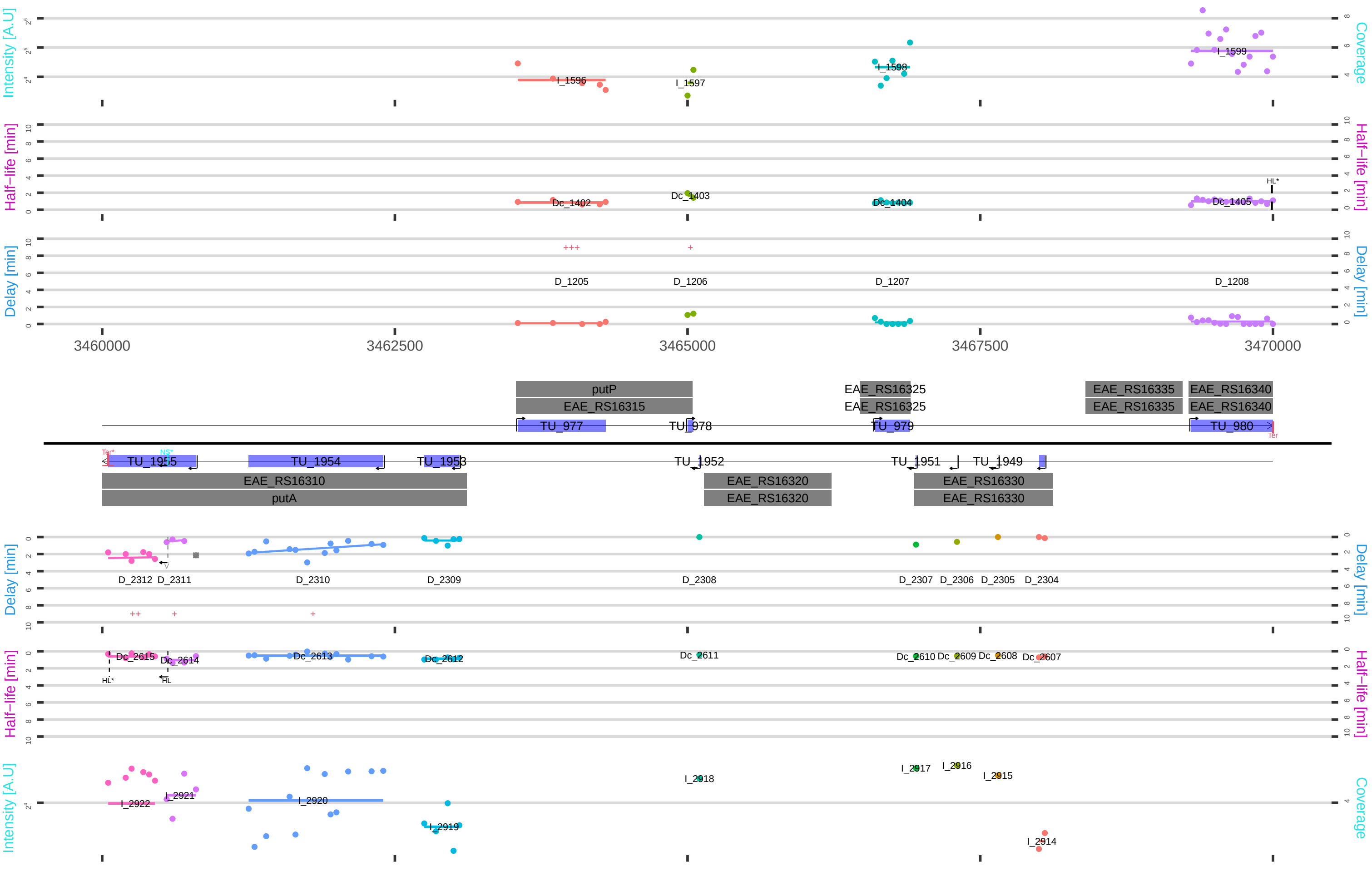

Term: termination (1), NS: new start (1), PS: pausing site (0), iTSS_I: internal starting site (0)

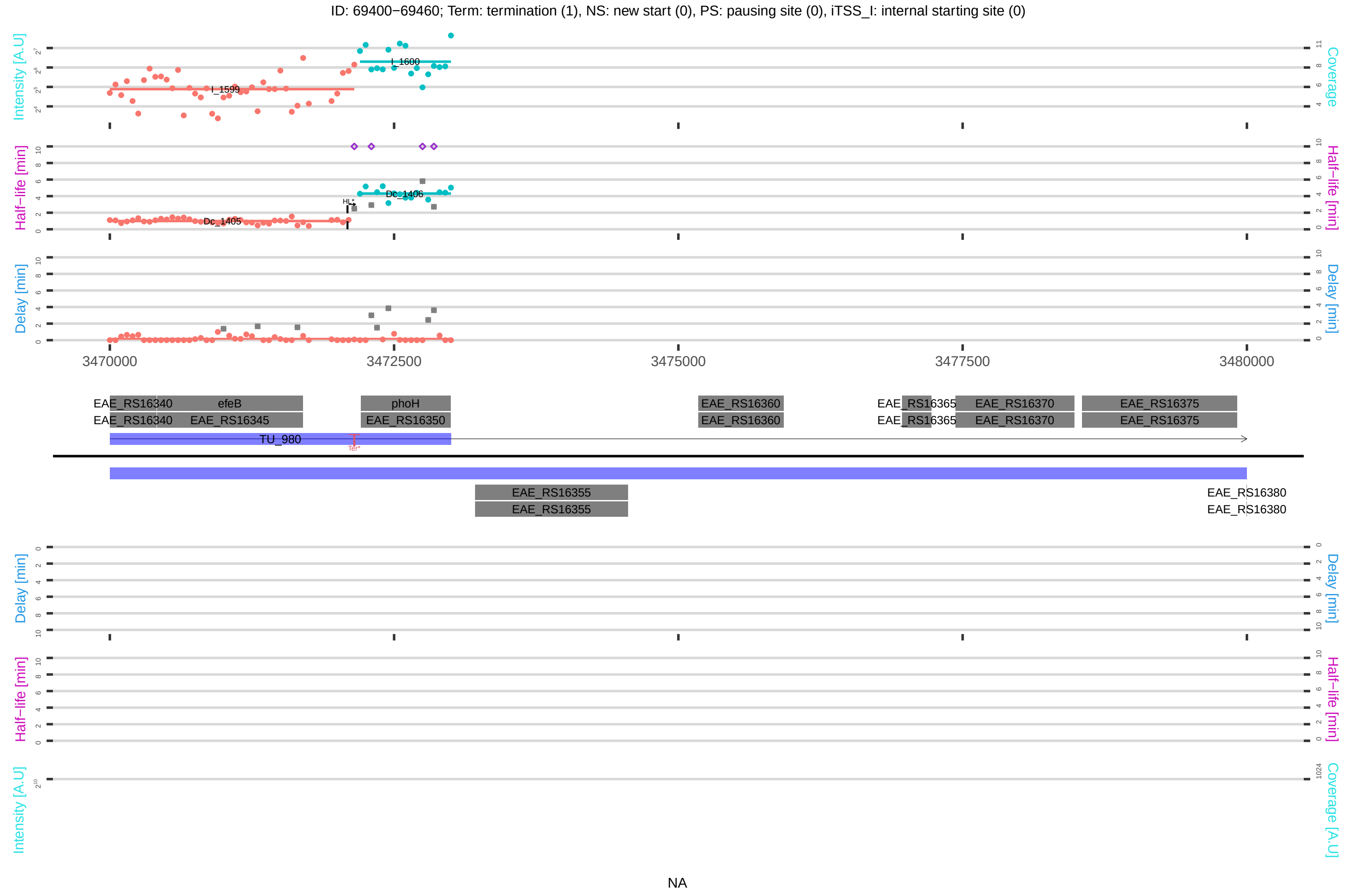

ID: 69400−69460; Term: termination (1), NS: new start (0), PS: pausing site (0), iTSS_I: internal starting site (0)
Intensity [A.U]
Coverage
I_1599
I_1600
Half−life [min]
Dc_1405
Dc_1406
HL*
Delay [min]
3470000
3472500
3475000
3477500
3480000
EAE_RS16340
efeB
EAE_RS16345
phoH
EAE_RS16350
EAE_RS16360
EAE_RS16365
EAE_RS16370
EAE_RS16375
TU_980
Ter*
EAE_RS16355
EAE_RS16380
NA

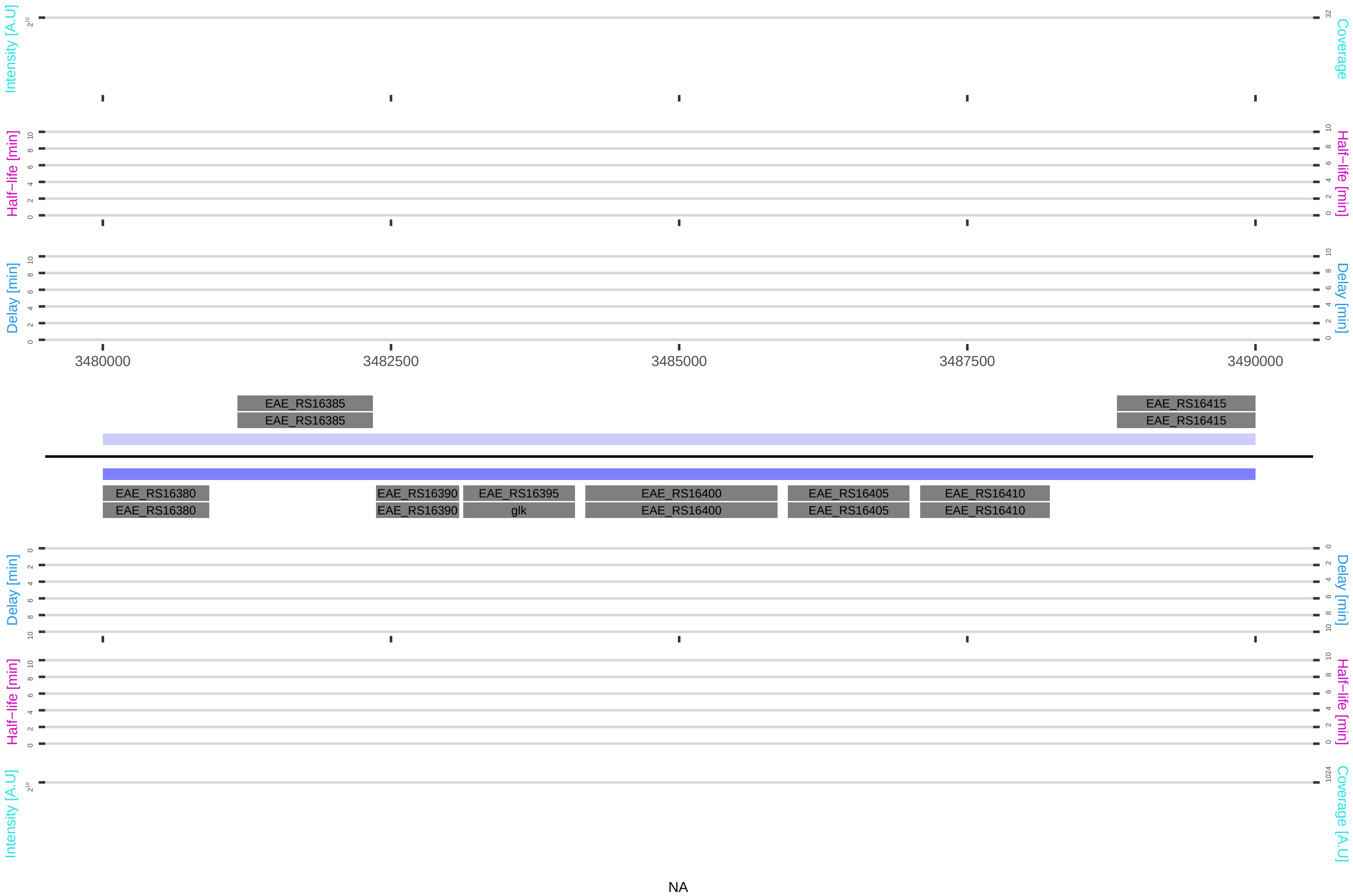
Intensity [A.U]
Coverage
Half−life [min]
Delay [min]
3480000
3482500
3485000
3487500
3490000
EAE_RS16385
EAE_RS16415
EAE_RS16380
EAE_RS16390
EAE_RS16395
glk
EAE_RS16400
EAE_RS16405
EAE_RS16410
Coverage [A.U]
NA

ID: 69915−70000; Term: termination (0), NS: new start (0), PS: pausing site (0), iTSS_I: internal starting site (0)

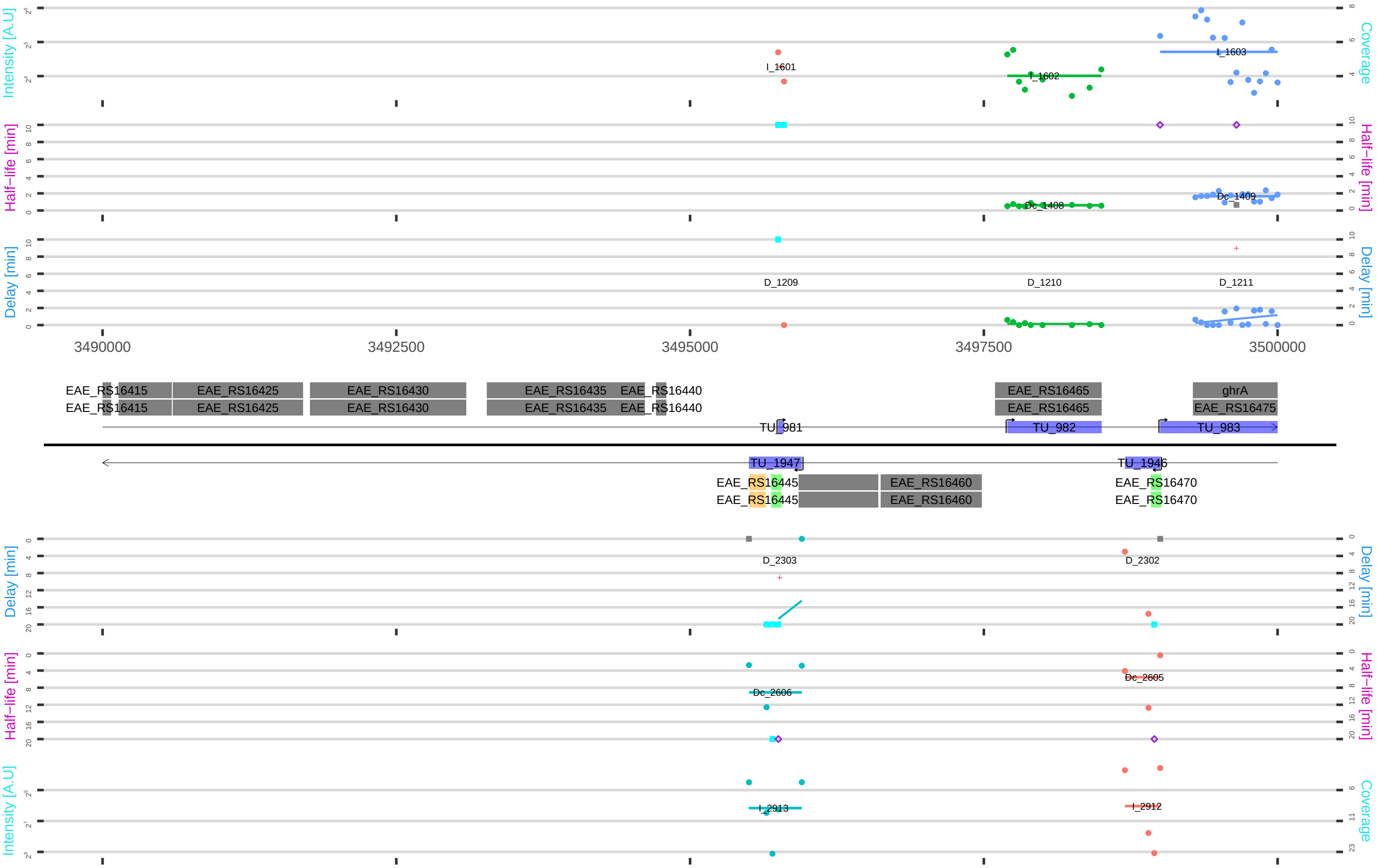

Term: termination (0), NS: new start (0), PS: pausing site (0), iTSS_I: internal starting site (0)

ID: 70000−70190; Term: termination (2), NS: new start (0), PS: pausing site (1), iTSS_I: internal starting site (0)

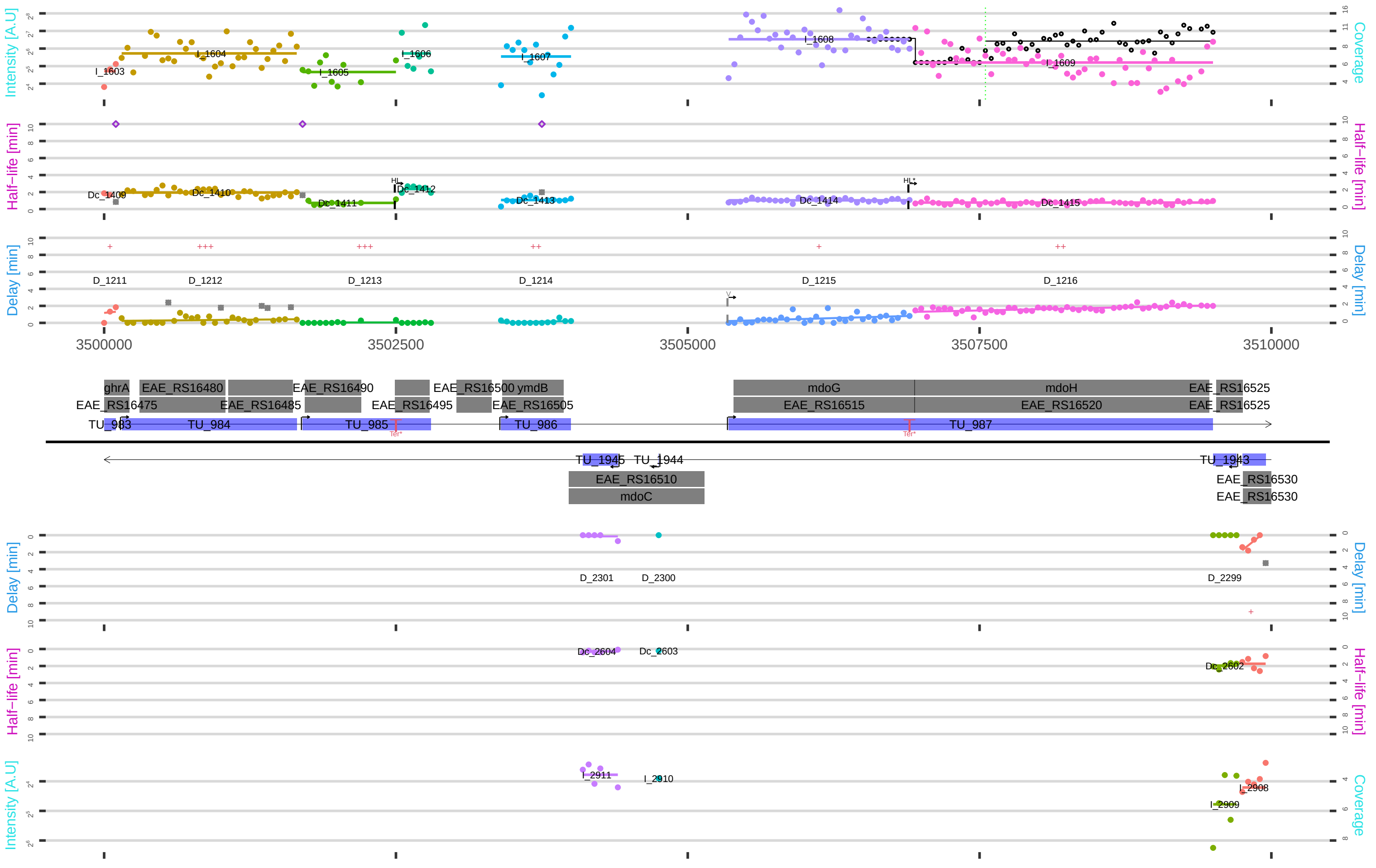

Term: termination (0), NS: new start (0), PS: pausing site (0), iTSS_I: internal starting site (0)

ID: 70257−70400; Term: termination (0), NS: new start (0), PS: pausing site (0), iTSS_I: internal starting site (0)

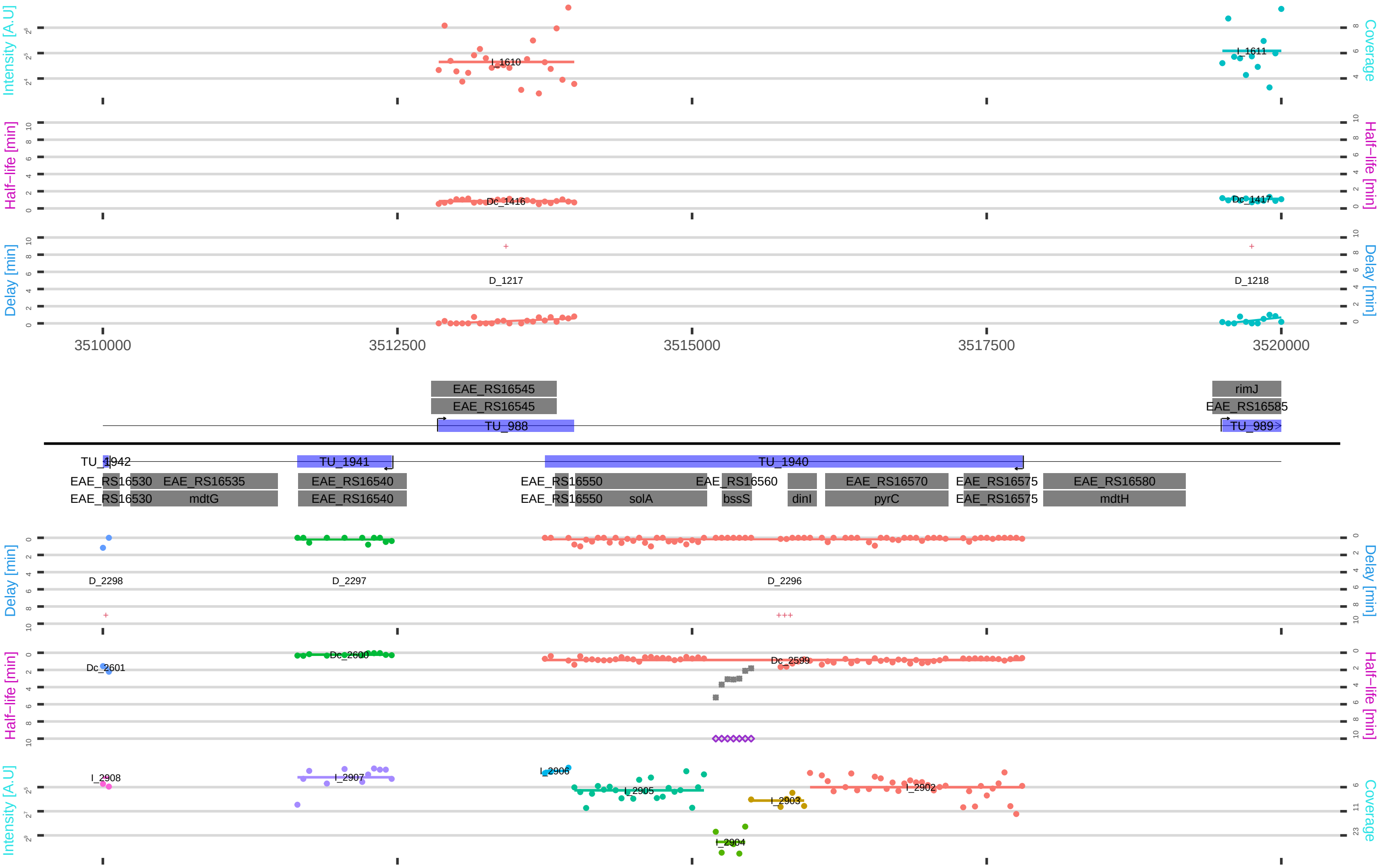

Term: termination (0), NS: new start (0), PS: pausing site (0), iTSS_I: internal starting site (0)

ID: 70400−70600; Term: termination (0), NS: new start (1), PS: pausing site (0), iTSS_I: internal starting site (0)

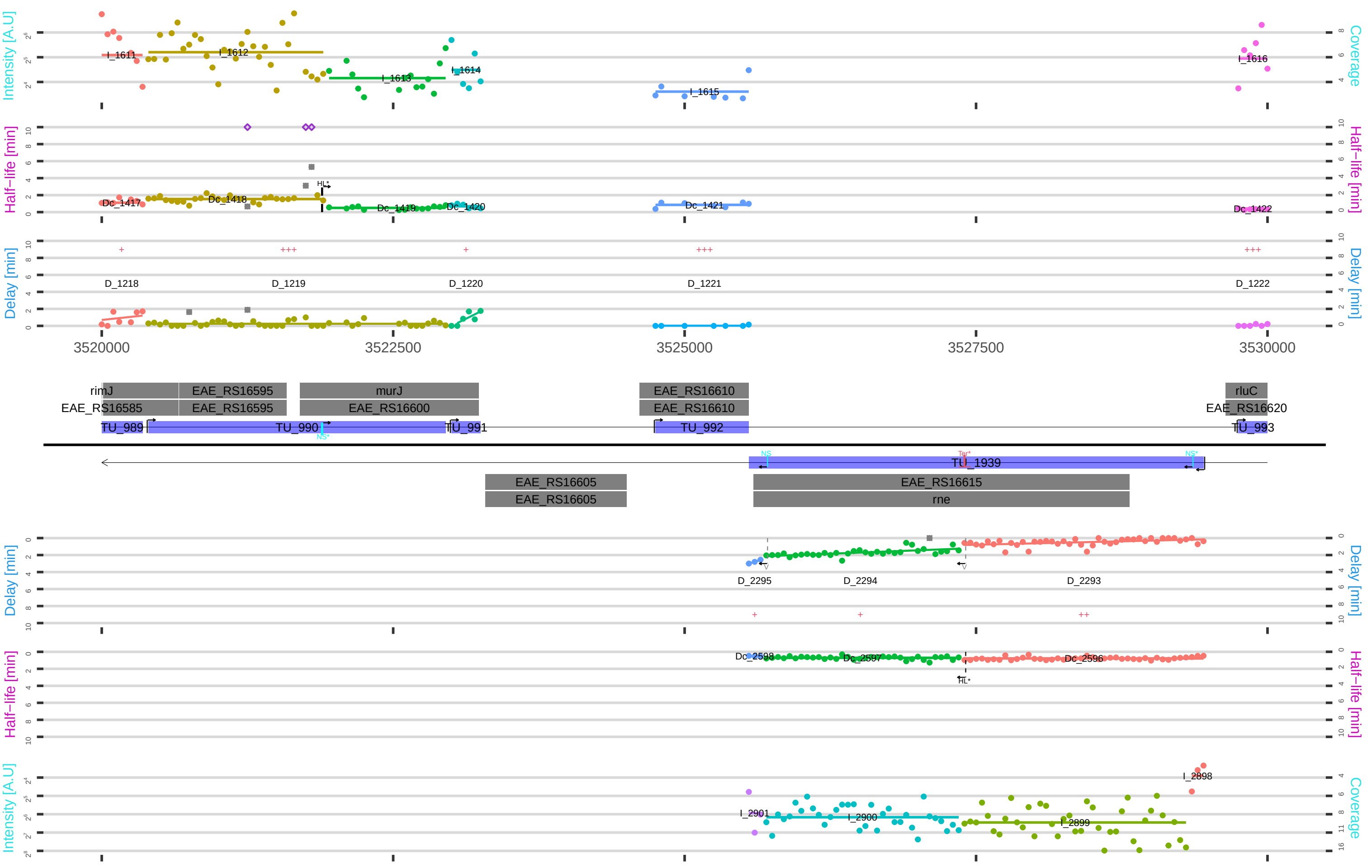

Term: termination (1), NS: new start (2), PS: pausing site (2), iTSS_I: internal starting site (0)

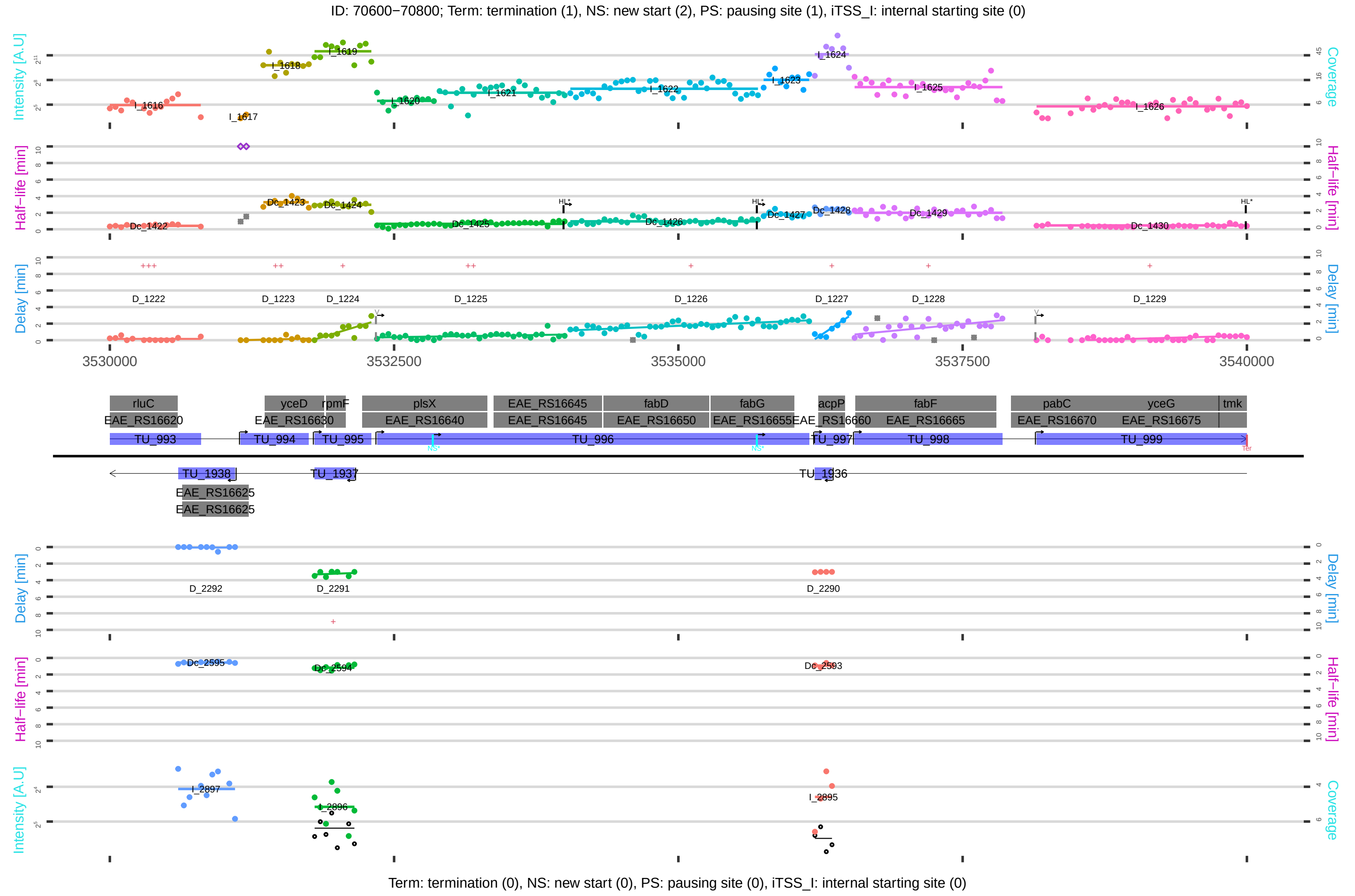

ID: 70600−70800; Term: termination (1), NS: new start (2), PS: pausing site (1), iTSS_I: internal starting site (0)
Intensity [A.U]
Coverage
Half−life [min]
Delay [min]
3530000
3532500
3535000
3537500
3540000
rluC
EAE_RS16620
yceD
EAE_RS16630
rpmF
plsX
EAE_RS16640
EAE_RS16645
fabD
EAE_RS16650
fabG
EAE_RS16655
acpP
EAE_RS16660
fabF
EAE_RS16665
pabC
EAE_RS16670
yceG
EAE_RS16675
tmk
TU_993
TU_994
TU_995
TU_996
TU_997
TU_998
TU_999
TU_1938
TU_1937
TU_1936
EAE_RS16625
Term: termination (0), NS: new start (0), PS: pausing site (0), iTSS_I: internal starting site (0)

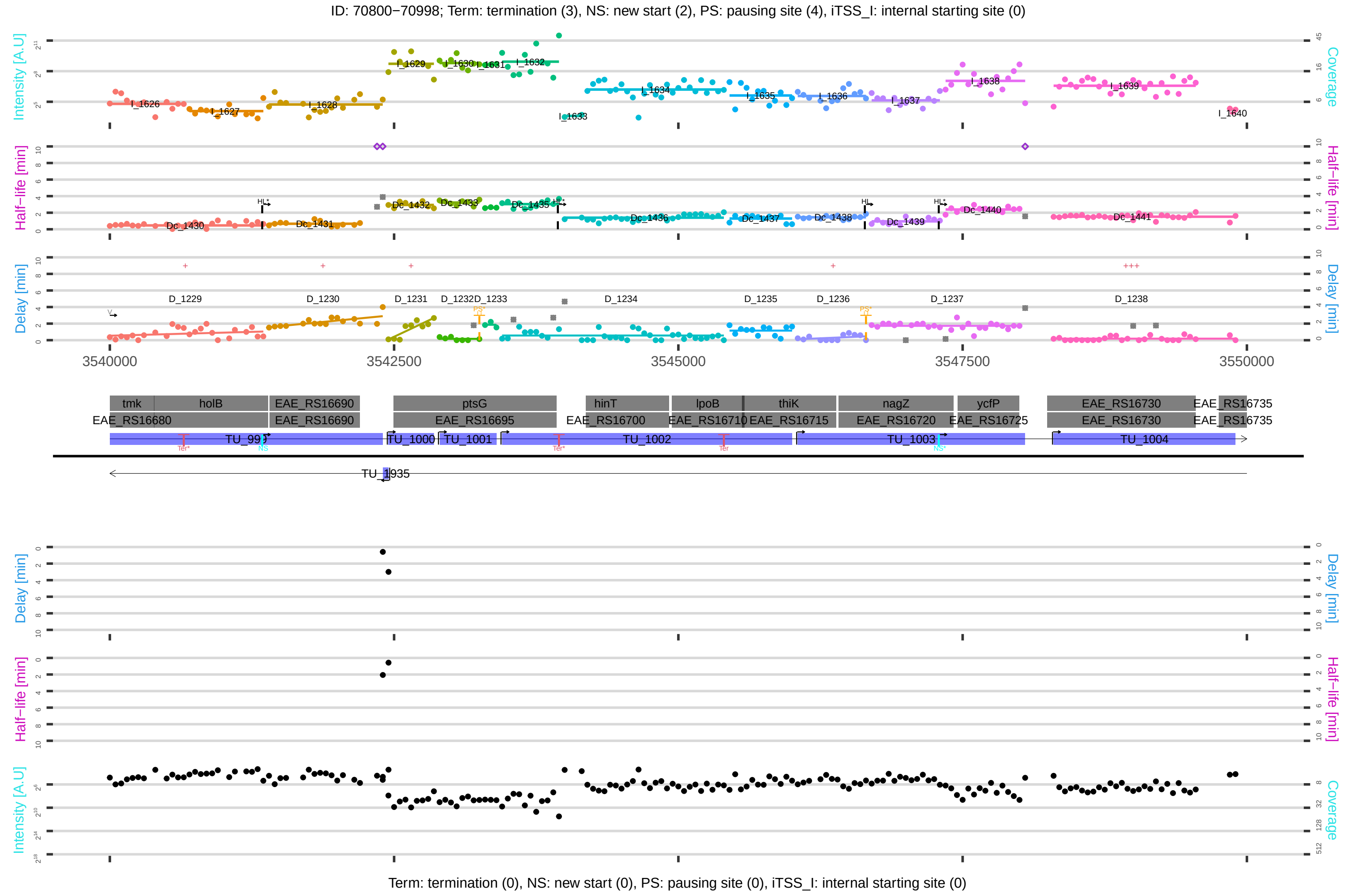
ID: 70800−70998; Term: termination (3), NS: new start (2), PS: pausing site (4), iTSS_I: internal starting site (0)
Intensity [A.U]
Coverage
Half−life [min]
Delay [min]
I_1626
I_1627
I_1628
I_1629
I_1630
I_1631
I_1632
I_1633
I_1634
I_1635
I_1636
I_1637
I_1638
I_1639
I_1640
Dc_1430
Dc_1431
Dc_1432
Dc_1433
Dc_1435
Dc_1436
Dc_1437
Dc_1438
Dc_1439
Dc_1440
Dc_1441
D_1229
D_1230
D_1231
D_1232
D_1233
D_1234
D_1235
D_1236
D_1237
D_1238
3540000
3542500
3545000
3547500
3550000
tmk
holB
EAE_RS16690
ptsG
hinT
lpoB
thiK
nagZ
ycfP
EAE_RS16730
EAE_RS16735
EAE_RS16680
EAE_RS16695
EAE_RS16700
EAE_RS16710
EAE_RS16715
EAE_RS16720
EAE_RS16725
TU_999
TU_1000
TU_1001
TU_1002
TU_1003
TU_1004
TU_1935
Term: termination (0), NS: new start (0), PS: pausing site (0), iTSS_I: internal starting site (0)

ID: 71004−71200; Term: termination (0), NS: new start (1), PS: pausing site (0), iTSS_I: internal starting site (0)

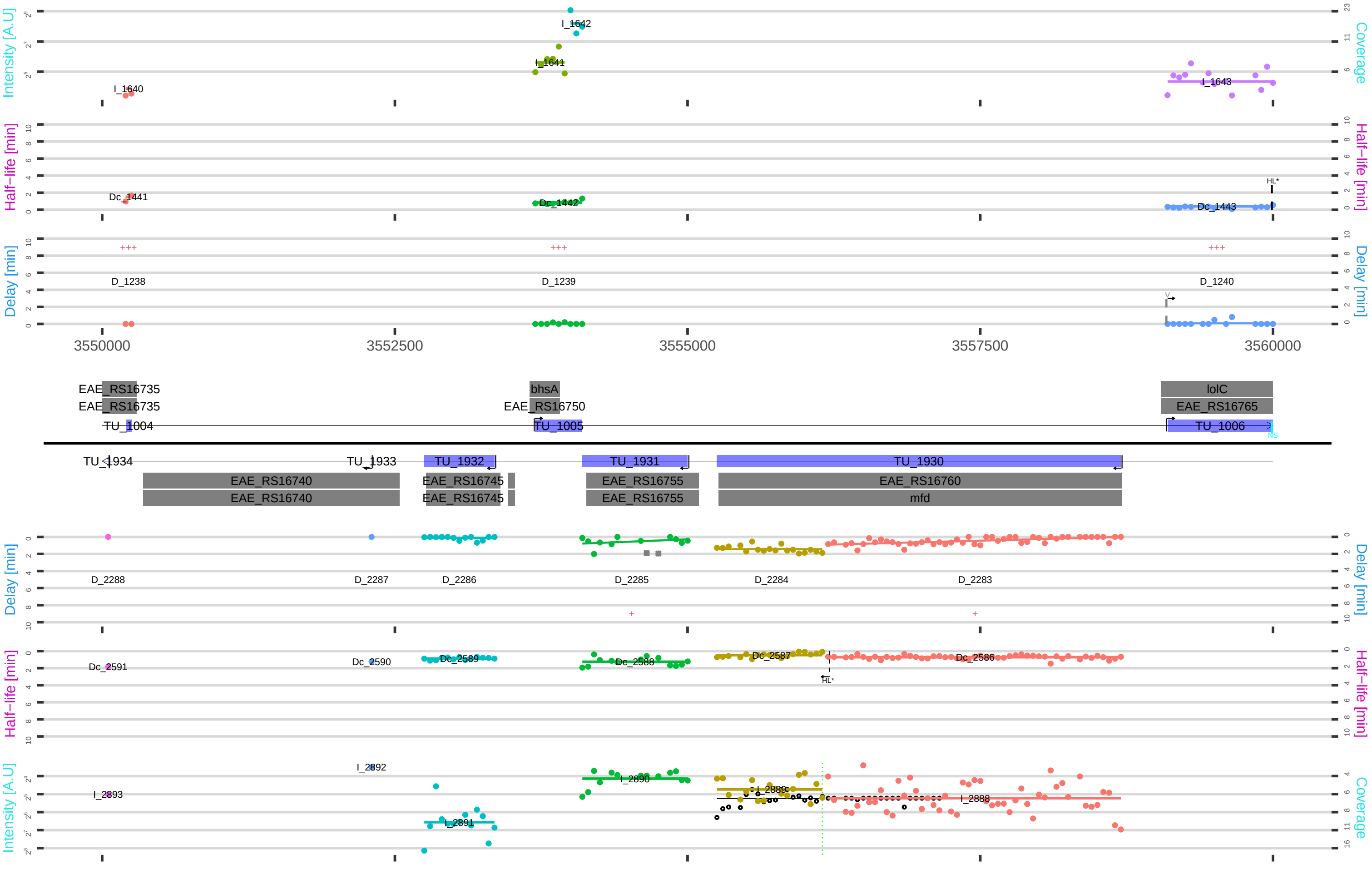

Term: termination (0), NS: new start (0), PS: pausing site (1), iTSS_I: internal starting site (0)

ID: 71200−71281; Term: termination (1), NS: new start (2), PS: pausing site (1), iTSS_I: internal starting site (0)

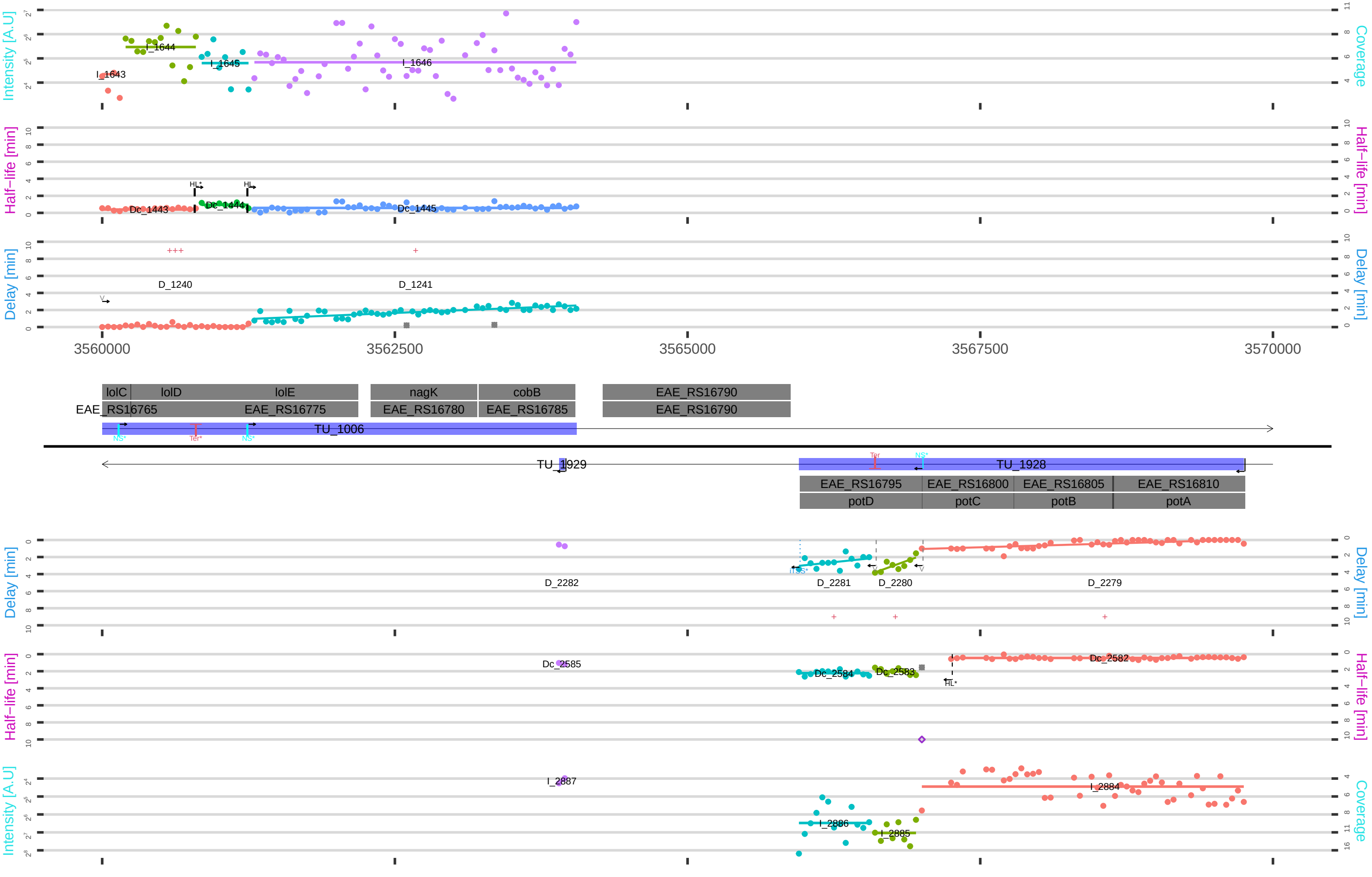

Term: termination (1), NS: new start (1), PS: pausing site (1), iTSS_I: internal starting site (1)

ID: 71401−71460; Term: termination (1), NS: new start (1), PS: pausing site (0), iTSS_I: internal starting site (0)

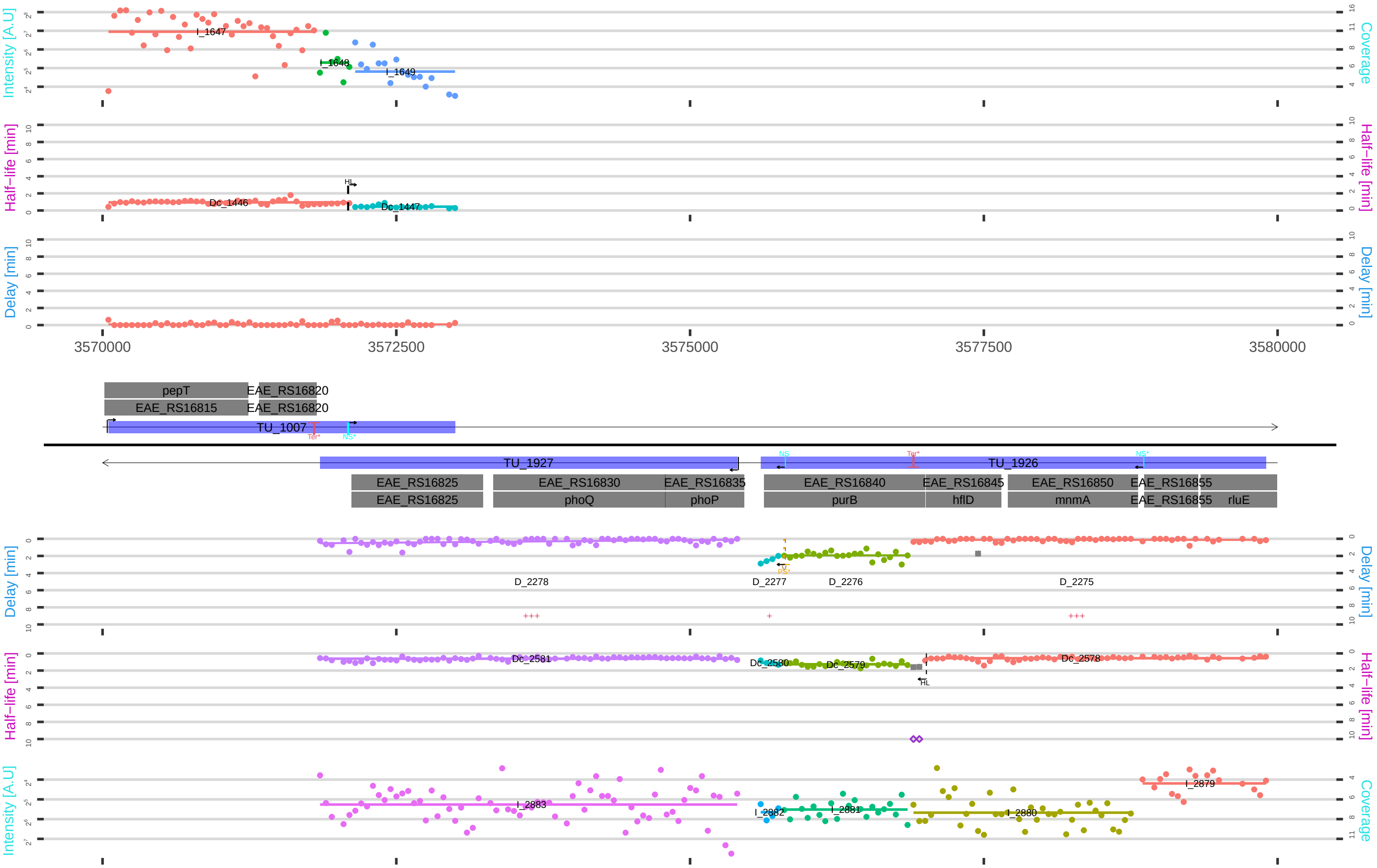

Term: termination (1), NS: new start (2), PS: pausing site (1), iTSS_I: internal starting site (1)

ID: 71601−71799; Term: termination (1), NS: new start (2), PS: pausing site (1), iTSS_I: internal starting site (2)

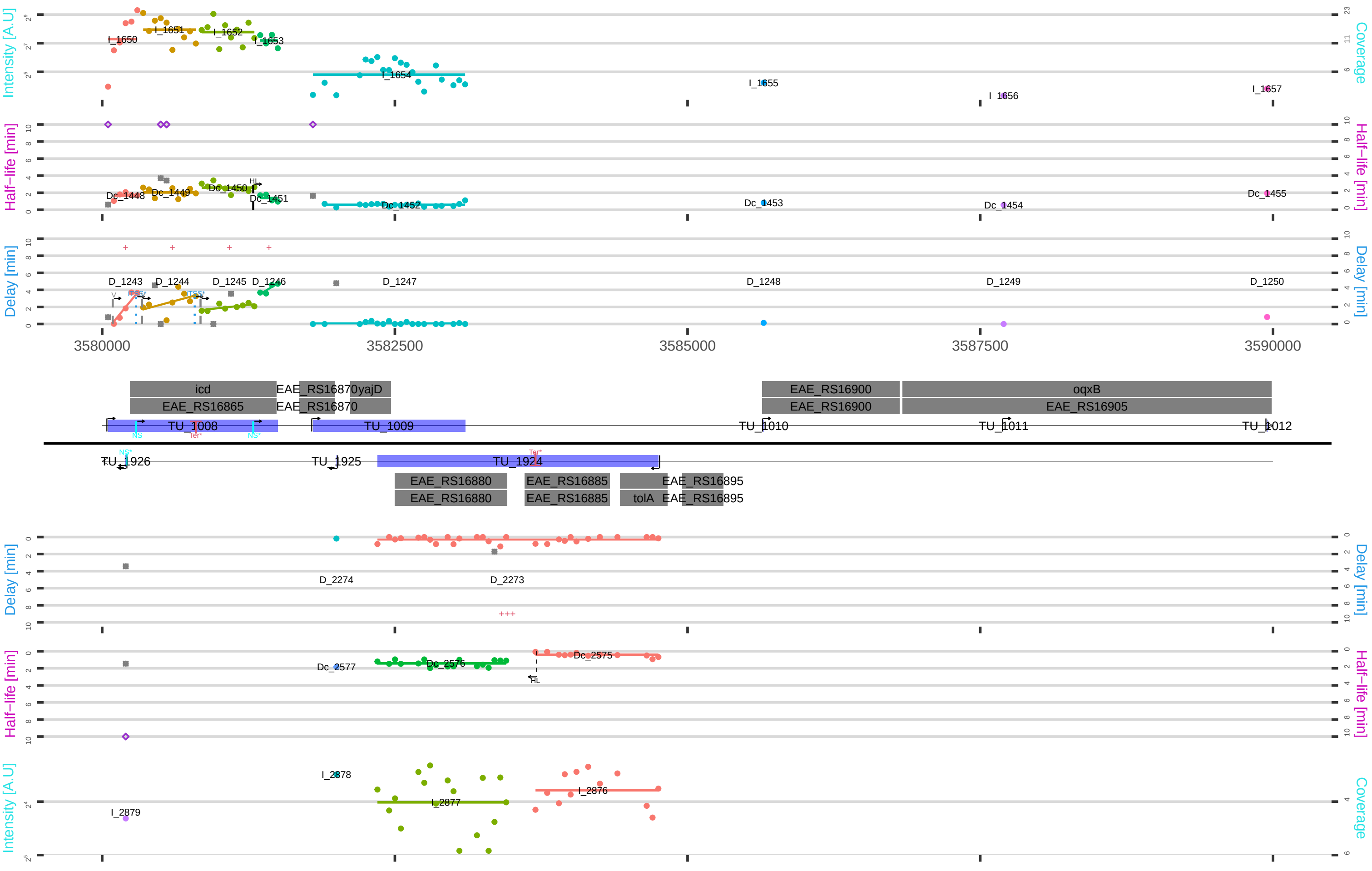

Term: termination (1), NS: new start (1), PS: pausing site (0), iTSS_I: internal starting site (0)

ID: 71835−71889; Term: termination (1), NS: new start (1), PS: pausing site (1), iTSS_I: internal starting site (0)

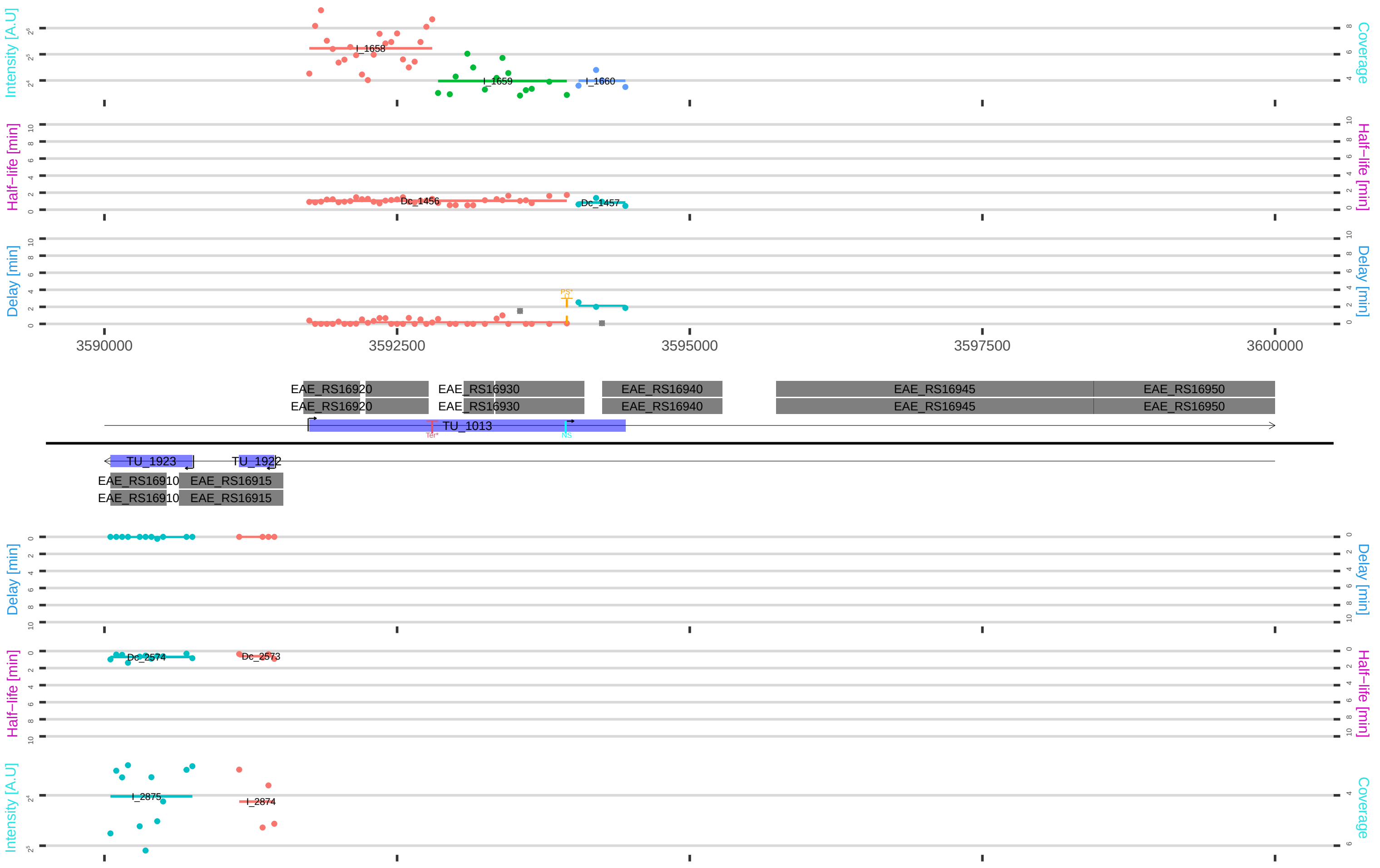

Term: termination (0), NS: new start (0), PS: pausing site (0), iTSS_I: internal starting site (0)

ID: 72041−72200; Term: termination (0), NS: new start (1), PS: pausing site (0), iTSS_I: internal starting site (0)

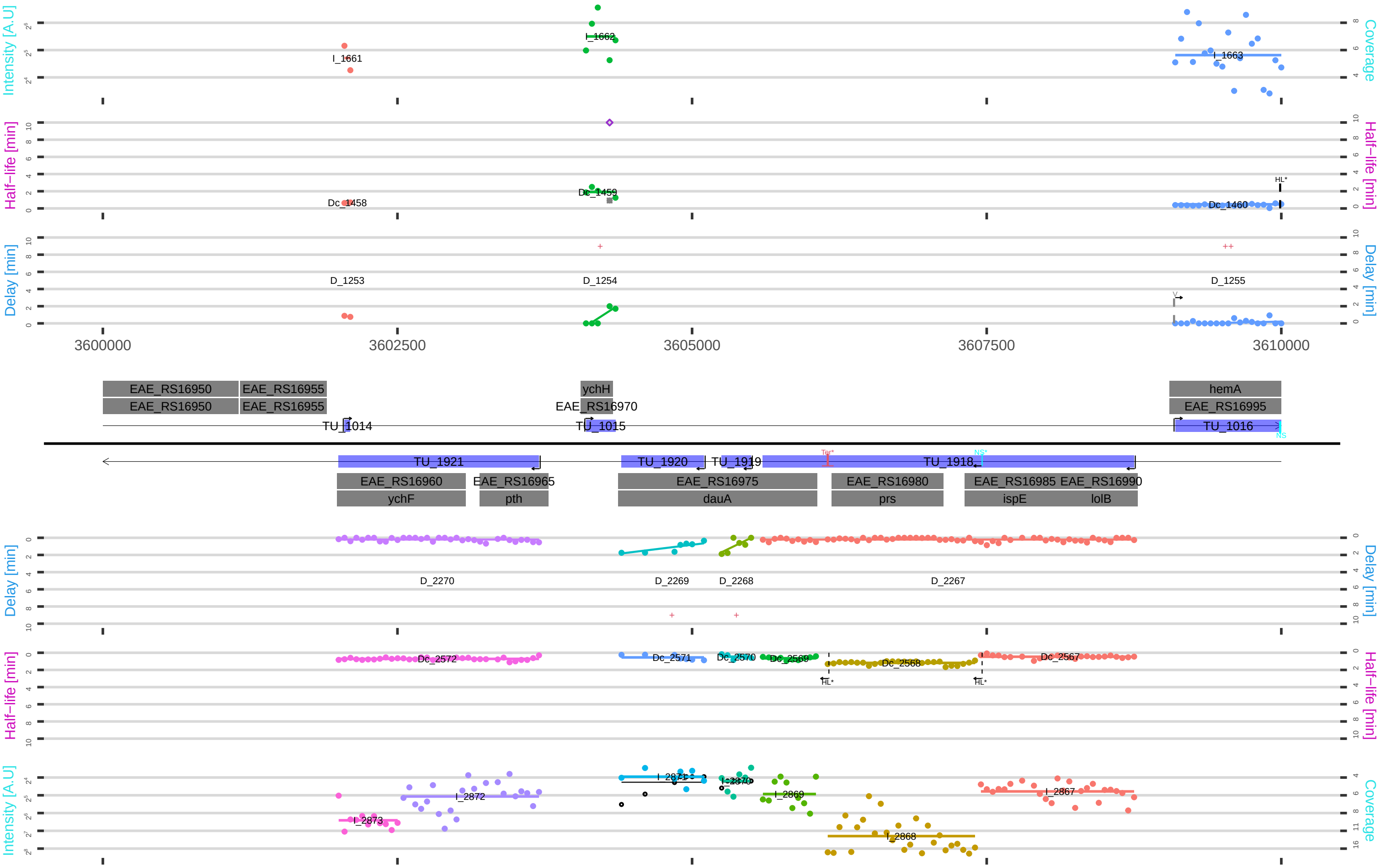

Term: termination (1), NS: new start (1), PS: pausing site (0), iTSS_I: internal starting site (0)

ID: 72200−72343; Term: termination (2), NS: new start (2), PS: pausing site (2), iTSS_I: internal starting site (1)

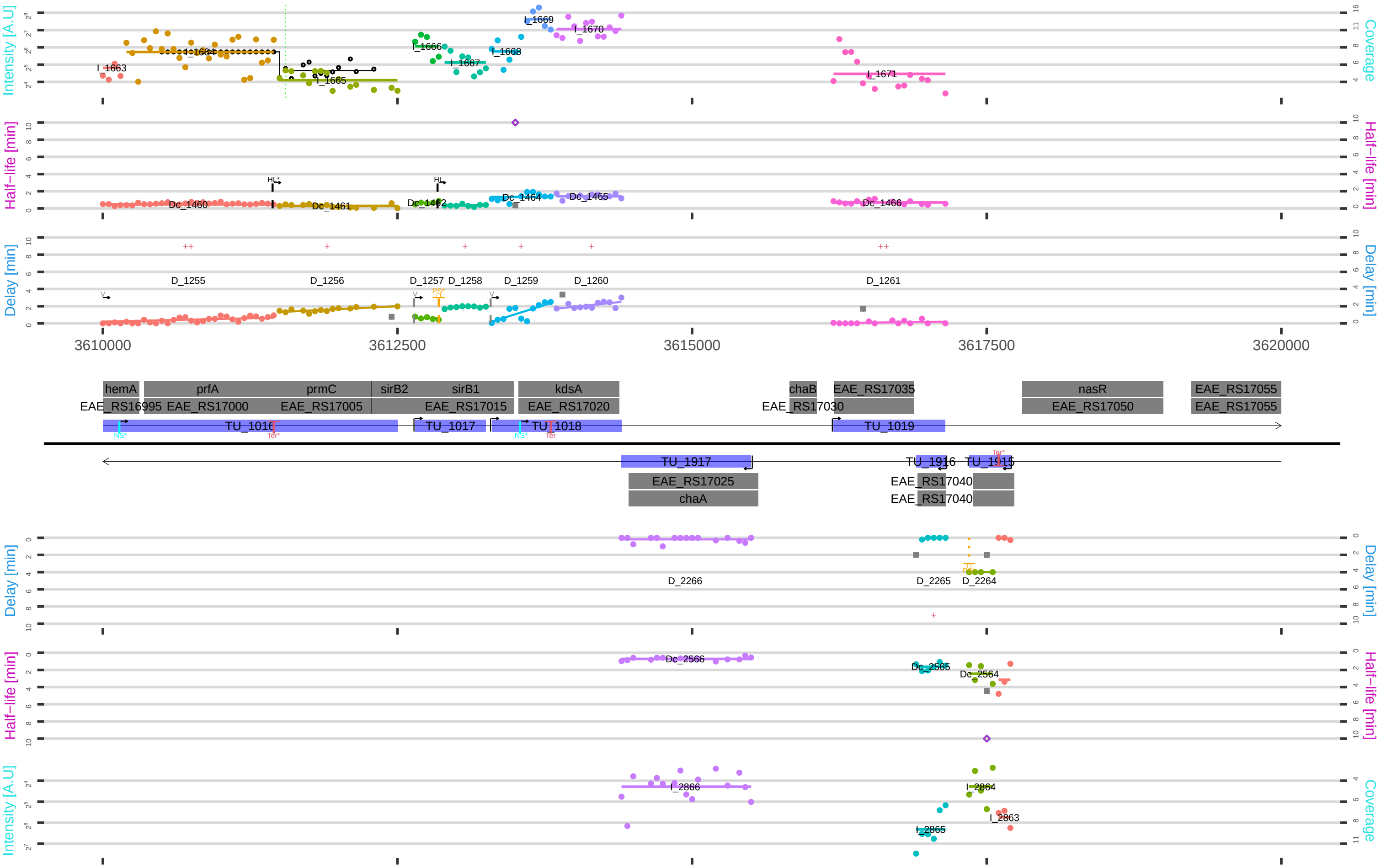

Term: termination (1), NS: new start (0), PS: pausing site (1), iTSS_I: internal starting site (0)

ID: 72564−72600; Term: termination (0), NS: new start (0), PS: pausing site (0), iTSS_I: internal starting site (0)

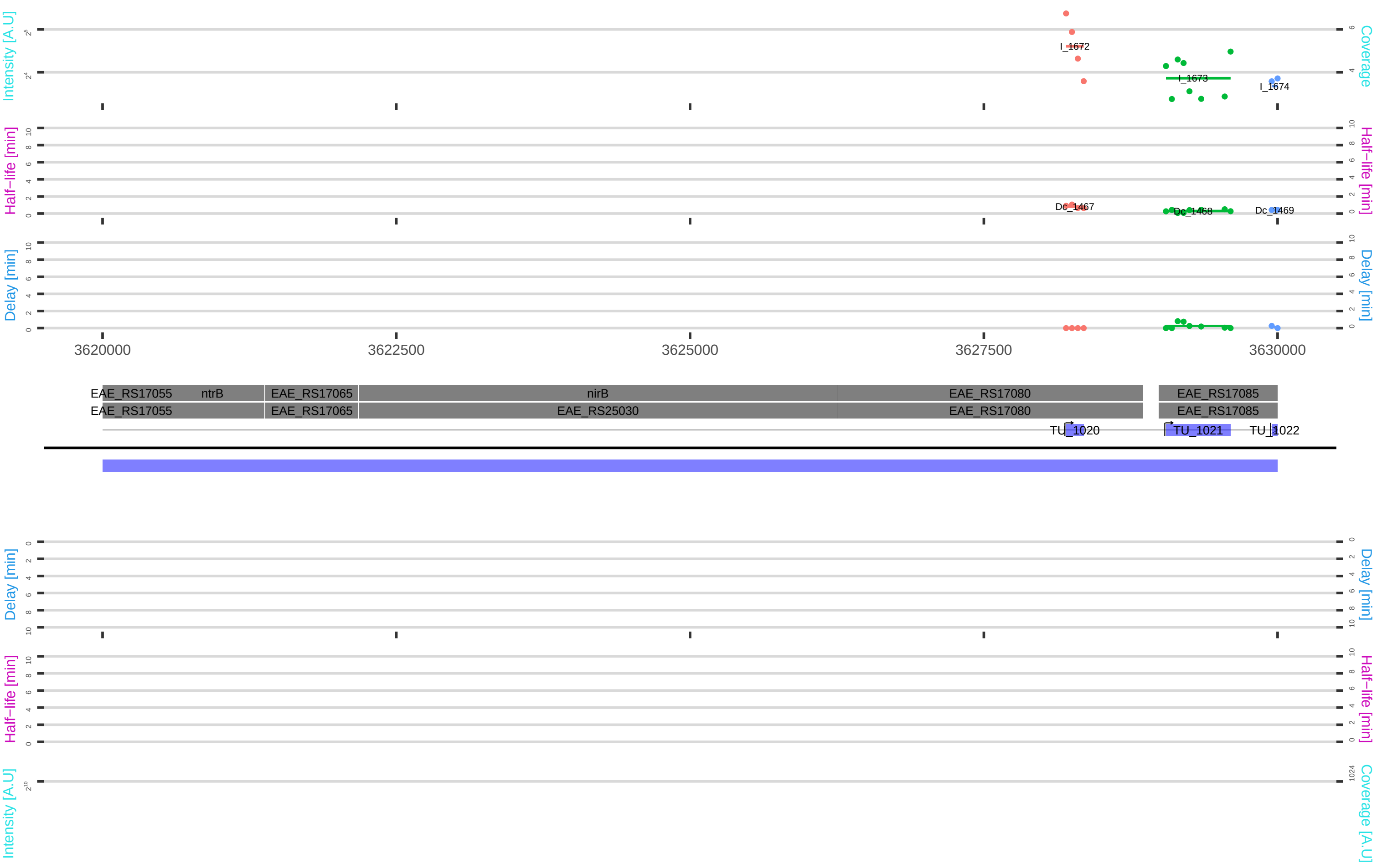

NA

ID: 72600−72800; Term: termination (2), NS: new start (0), PS: pausing site (0), iTSS_I: internal starting site (3)

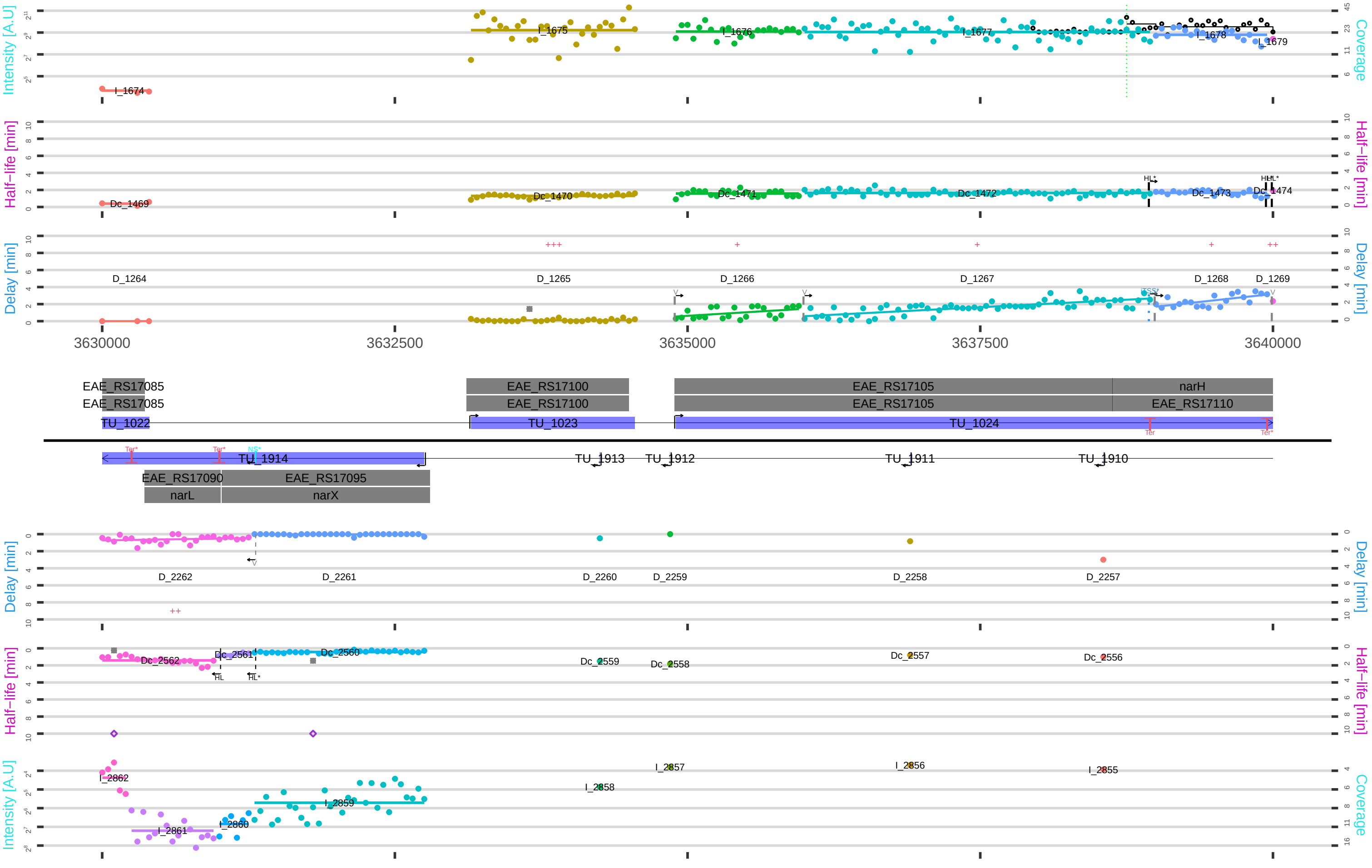

Term: termination (2), NS: new start (1), PS: pausing site (1), iTSS_I: internal starting site (0)

ID: 72800−73000; Term: termination (1), NS: new start (2), PS: pausing site (2), iTSS_I: internal starting site (1)

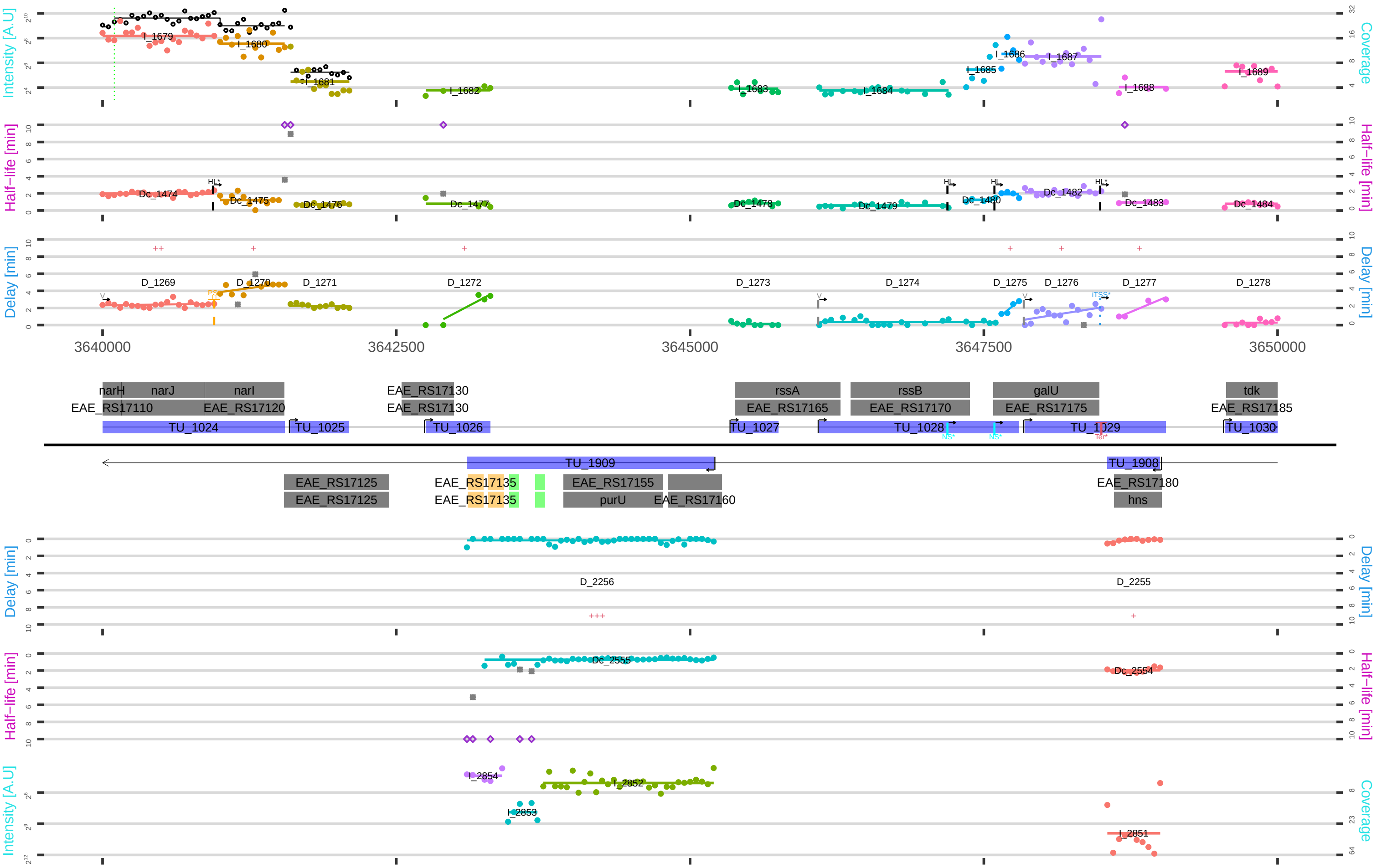

Term: termination (0), NS: new start (0), PS: pausing site (0), iTSS_I: internal starting site (0)

ID: 73000−73200; Term: termination (2), NS: new start (2), PS: pausing site (1), iTSS_I: internal starting site (0)

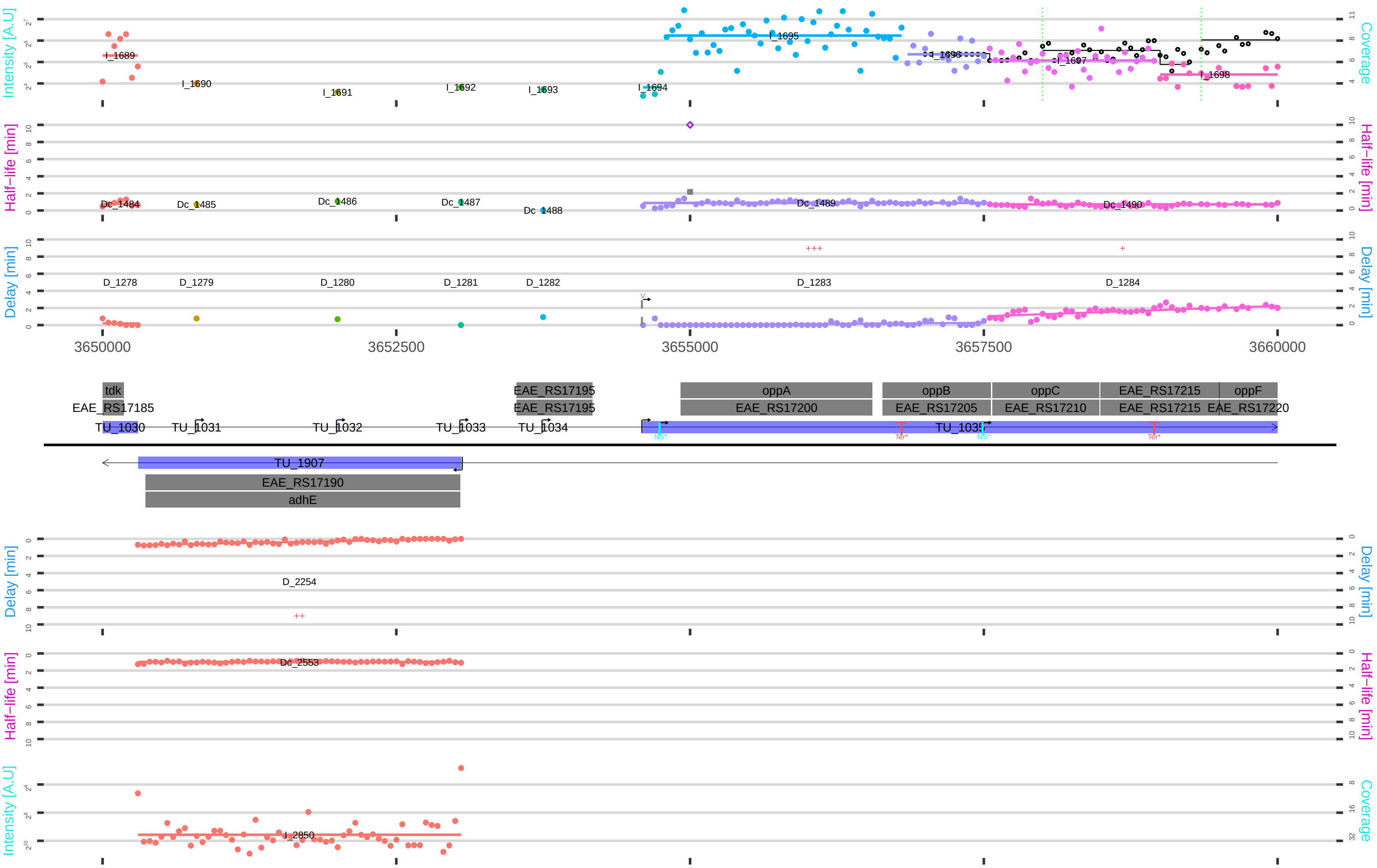

Term: termination (0), NS: new start (0), PS: pausing site (0), iTSS_I: internal starting site (0)

ID: 73200−73400; Term: termination (1), NS: new start (0), PS: pausing site (1), iTSS_I: internal starting site (0)

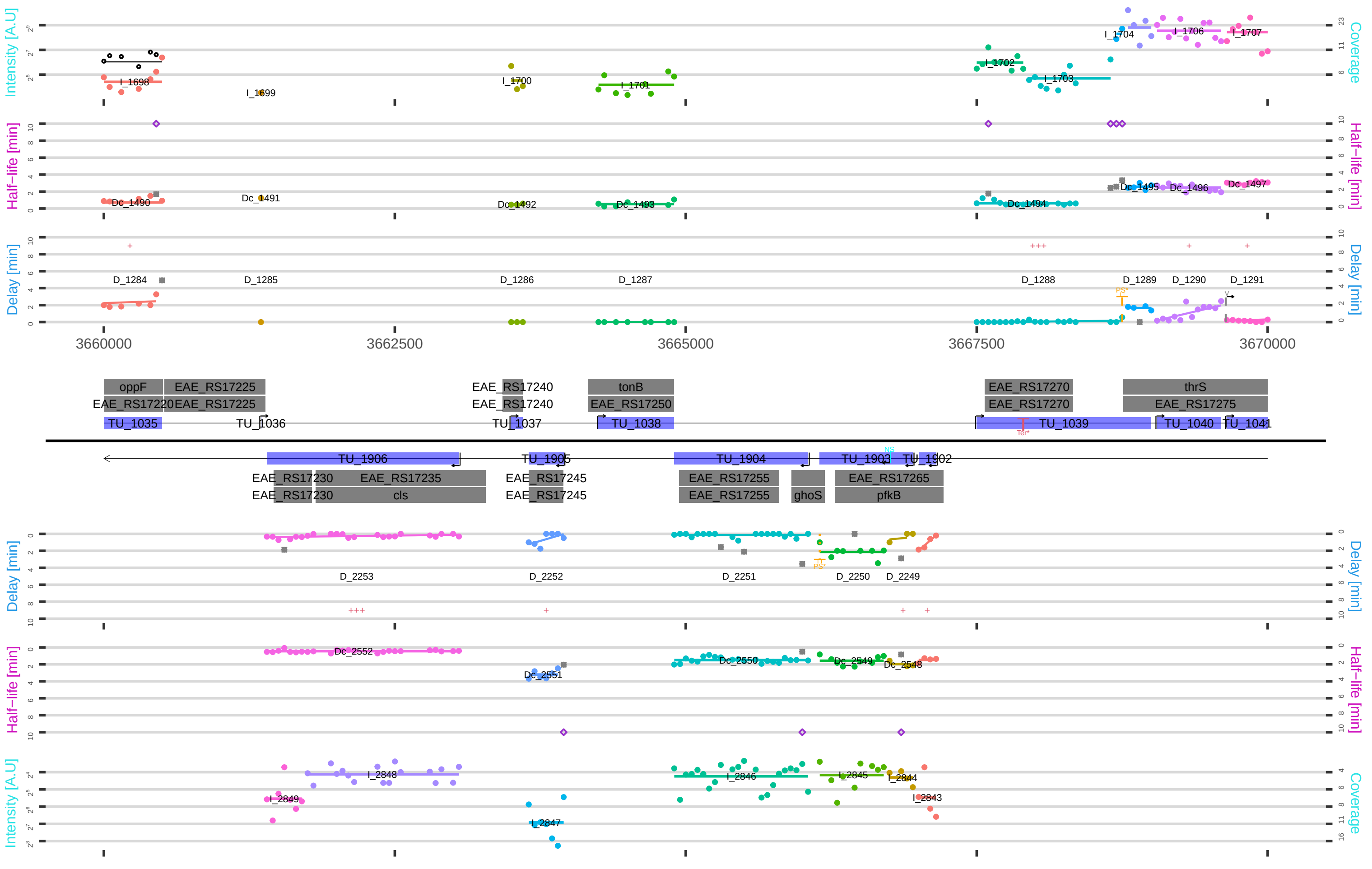

Term: termination (0), NS: new start (1), PS: pausing site (1), iTSS_I: internal starting site (0)

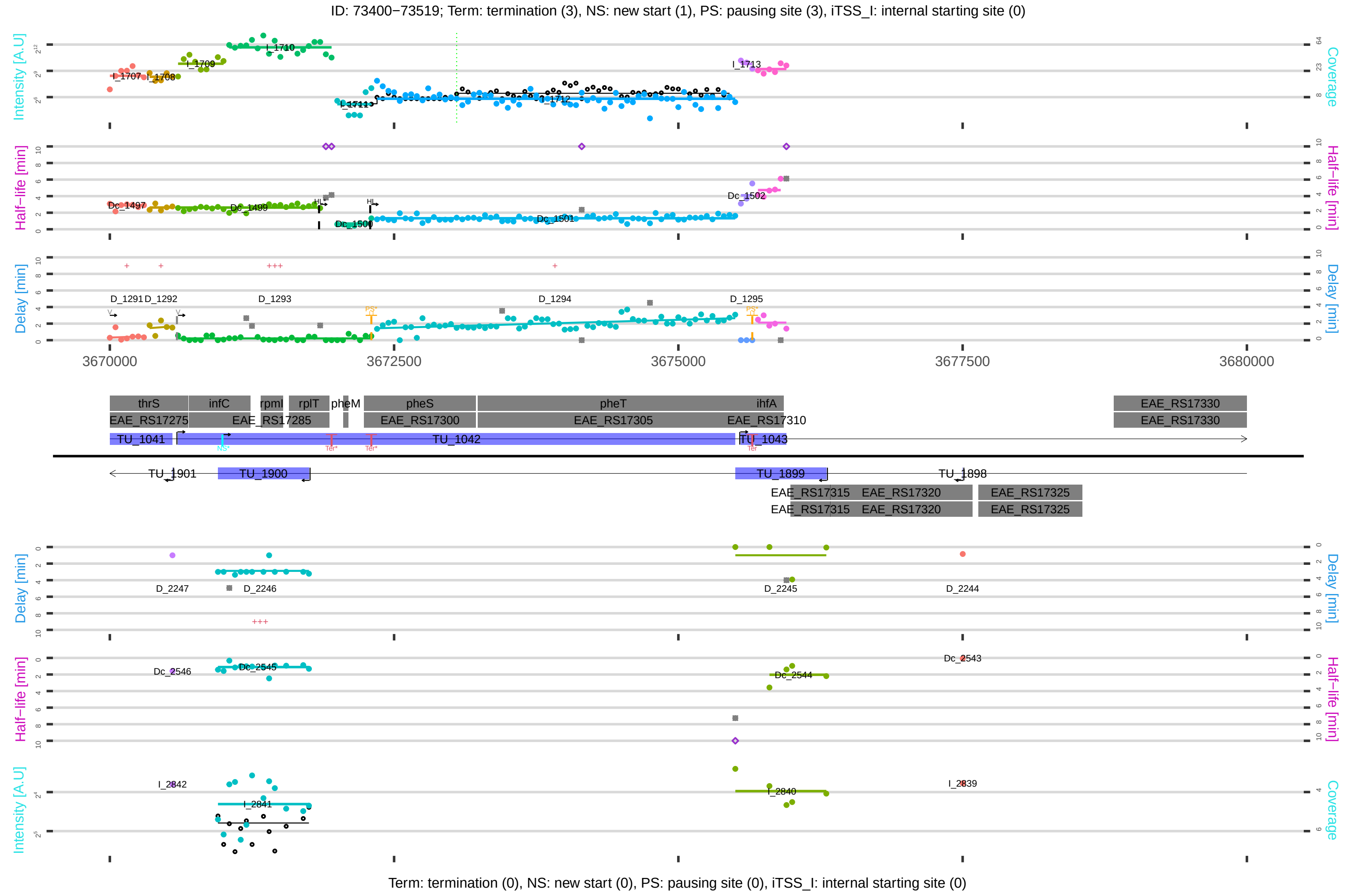

ID: 73400−73519; Term: termination (3), NS: new start (1), PS: pausing site (3), iTSS_I: internal starting site (0)
Intensity [A.U]
Coverage
Half−life [min]
Delay [min]
I_1707
I_1708
I_1709
I_1710
I_1711
I_1712
I_1713
Dc_1497
Dc_1499
Dc_1500
Dc_1501
Dc_1502
D_1291
D_1292
D_1293
D_1294
D_1295
3670000
3672500
3675000
3677500
3680000
thrS
infC
rpmI
rplT
pheM
pheS
pheT
ihfA
EAE_RS17275
EAE_RS17285
EAE_RS17300
EAE_RS17305
EAE_RS17310
EAE_RS17330
TU_1041
TU_1042
TU_1043
TU_1901
TU_1900
TU_1899
TU_1898
EAE_RS17315
EAE_RS17320
EAE_RS17325
D_2247
D_2246
D_2245
D_2244
Dc_2546
Dc_2545
Dc_2544
Dc_2543
I_2842
I_2841
I_2840
I_2839
Term: termination (0), NS: new start (0), PS: pausing site (0), iTSS_I: internal starting site (0)

ID: 73610−73799; Term: termination (1), NS: new start (2), PS: pausing site (0), iTSS_I: internal starting site (0)

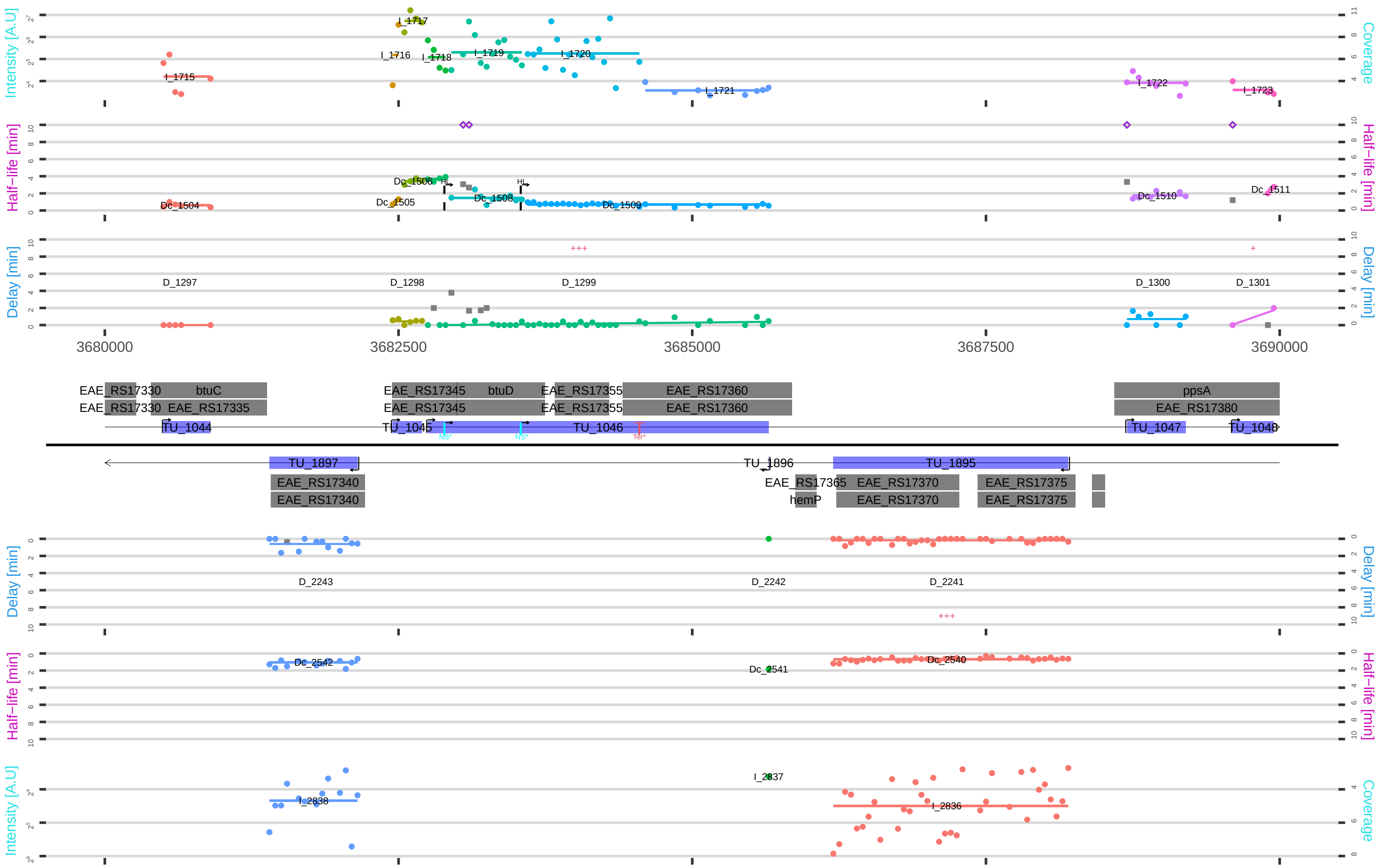

Term: termination (0), NS: new start (0), PS: pausing site (0), iTSS_I: internal starting site (0)

ID: 73801−74000; Term: termination (0), NS: new start (0), PS: pausing site (1), iTSS_I: internal starting site (0)

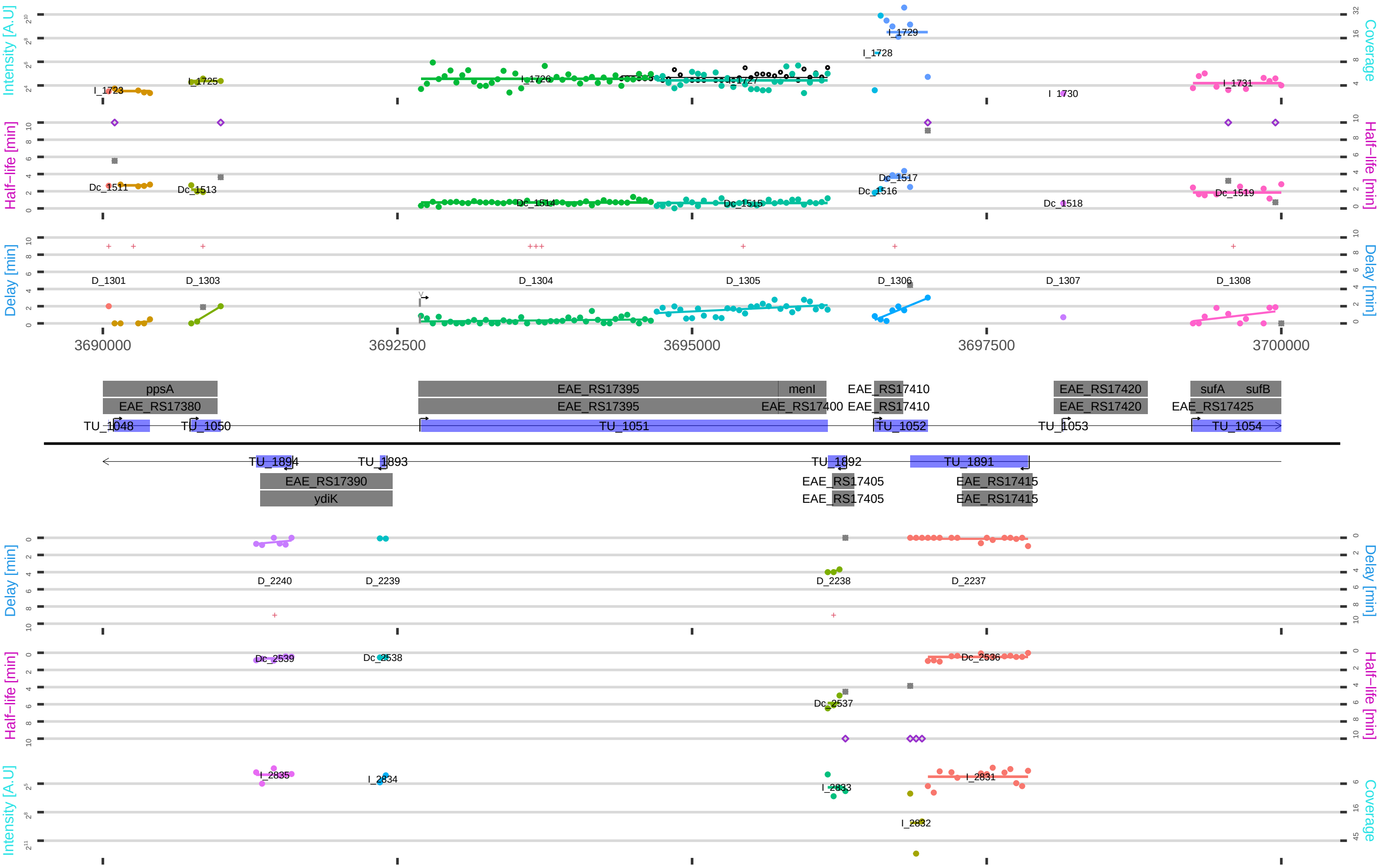

Term: termination (0), NS: new start (0), PS: pausing site (0), iTSS_I: internal starting site (0)

ID: 74000−74176; Term: termination (0), NS: new start (2), PS: pausing site (1), iTSS_I: internal starting site (0)

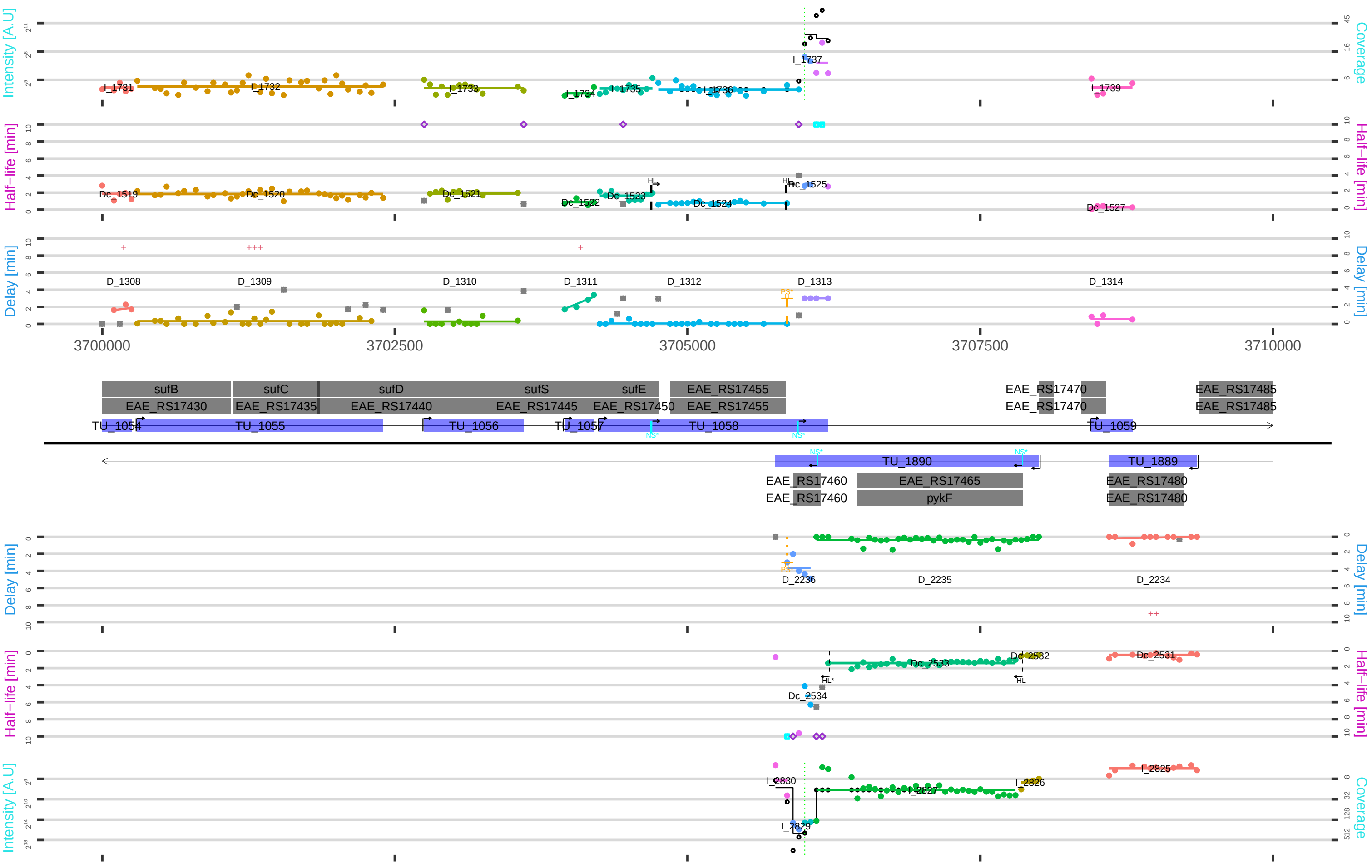

Term: termination (0), NS: new start (2), PS: pausing site (1), iTSS_I: internal starting site (0)

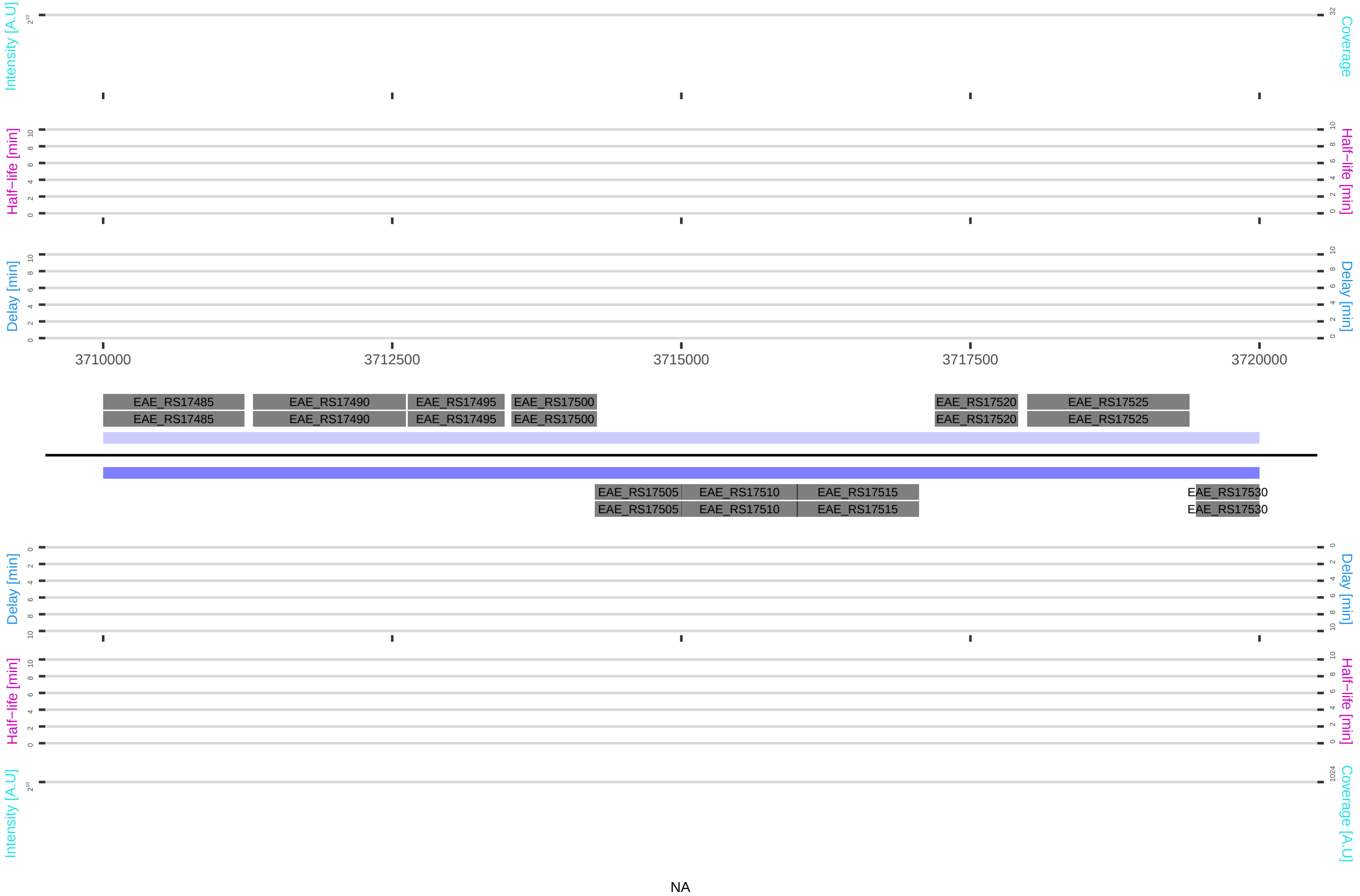

Intensity [A.U]
Coverage
Half−life [min]
Delay [min]
3710000
3712500
3715000
3717500
3720000
EAE_RS17485
EAE_RS17490
EAE_RS17495
EAE_RS17500
EAE_RS17520
EAE_RS17525
EAE_RS17505
EAE_RS17510
EAE_RS17515
EAE_RS17530
Coverage [A.U]
NA

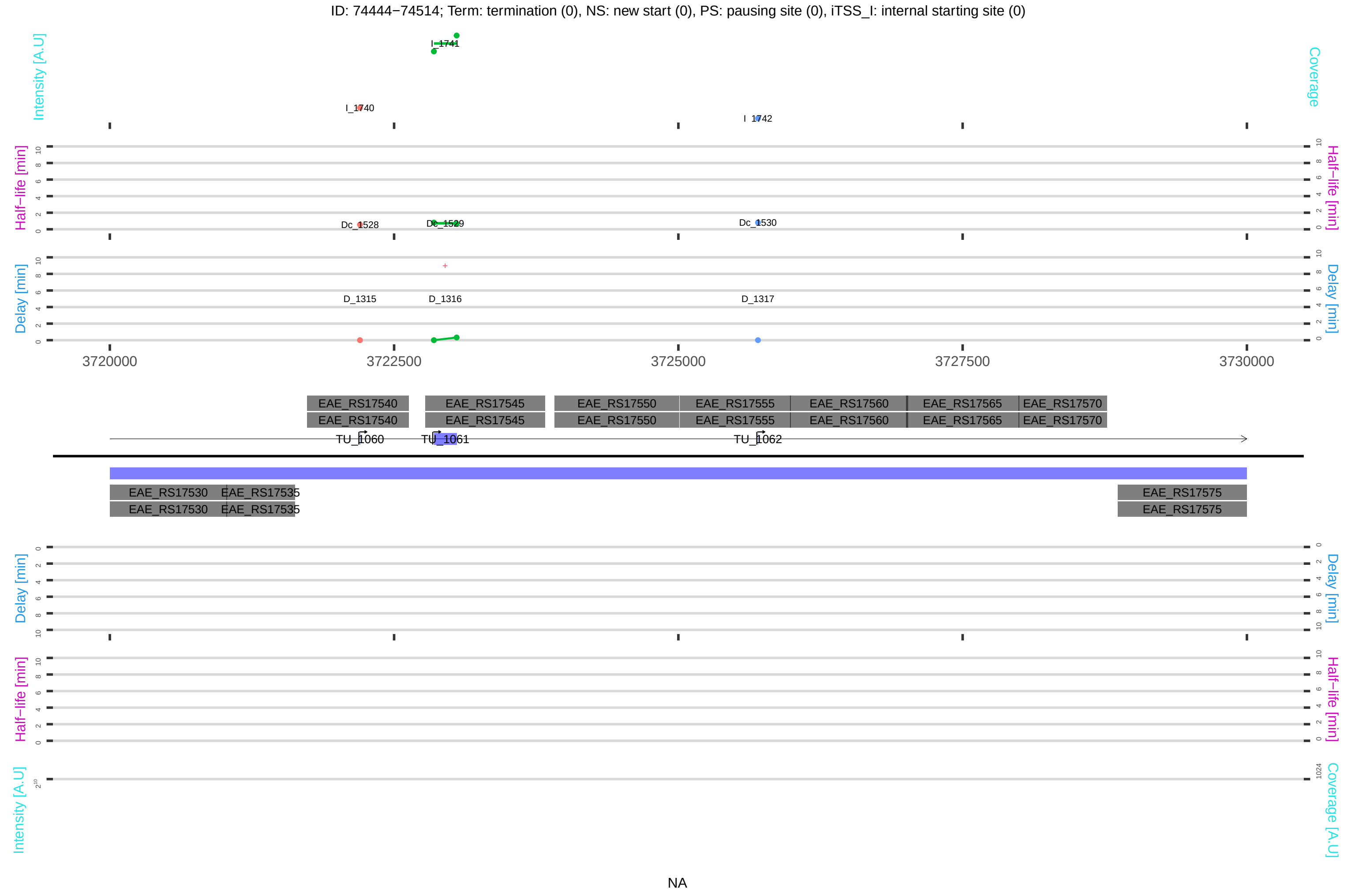
ID: 74444−74514; Term: termination (0), NS: new start (0), PS: pausing site (0), iTSS_I: internal starting site (0)
Intensity [A.U]
Coverage
I_1741
I_1740
I_1742
Half−life [min]
Dc_1528
Dc_1529
Dc_1530
Delay [min]
D_1315
D_1316
D_1317
3720000
3722500
3725000
3727500
3730000
EAE_RS17540
EAE_RS17545
EAE_RS17550
EAE_RS17555
EAE_RS17560
EAE_RS17565
EAE_RS17570
TU_1060
TU_1061
TU_1062
EAE_RS17530
EAE_RS17535
EAE_RS17575
Delay [min]
Half−life [min]
Intensity [A.U]
Coverage [A.U]
NA

ID: 74728−74741; Term: termination (0), NS: new start (0), PS: pausing site (0), iTSS_I: internal starting site (0)

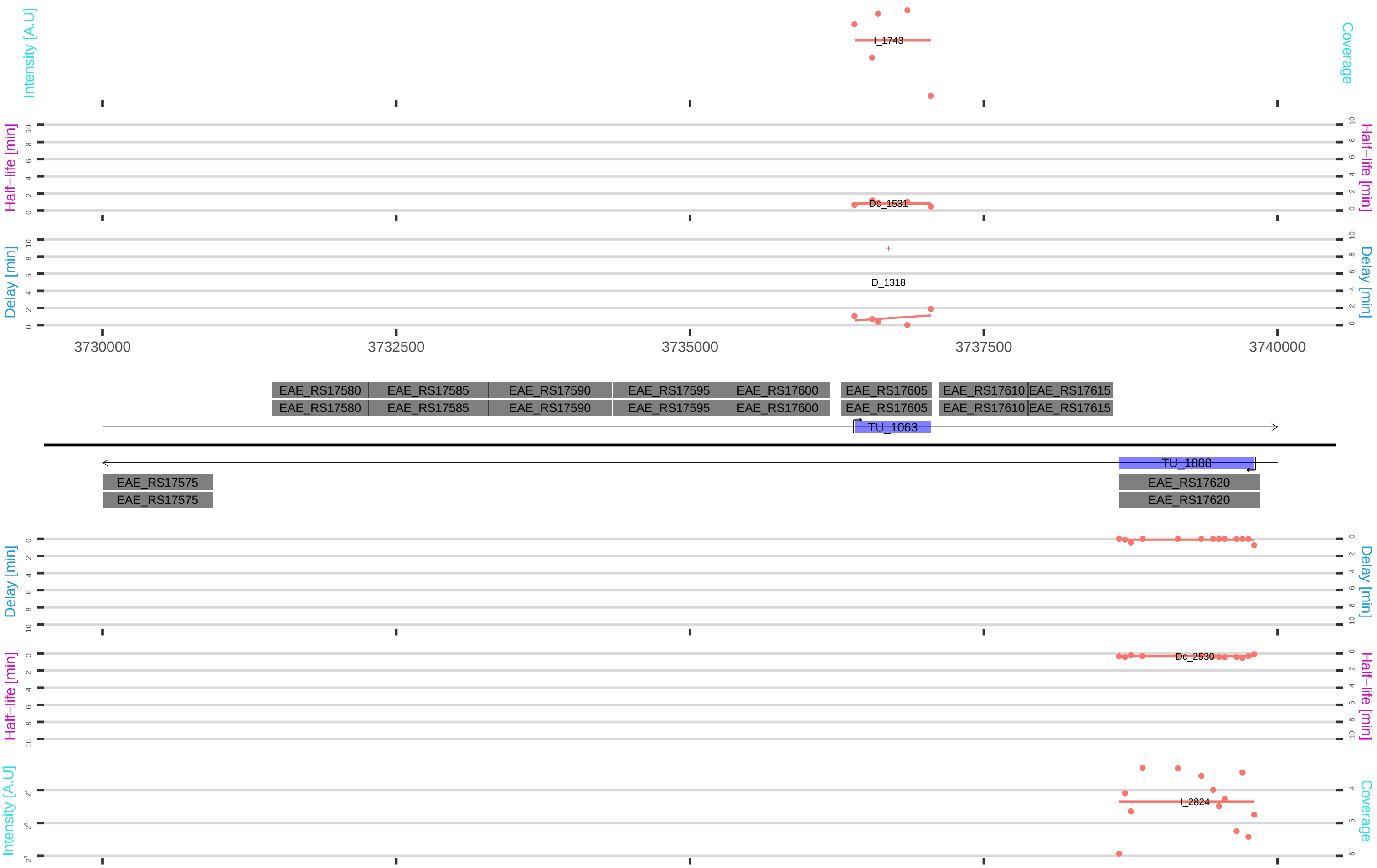

Term: termination (0), NS: new start (0), PS: pausing site (0), iTSS_I: internal starting site (0)

ID: 74802−74992; Term: termination (0), NS: new start (0), PS: pausing site (0), iTSS_I: internal starting site (0)

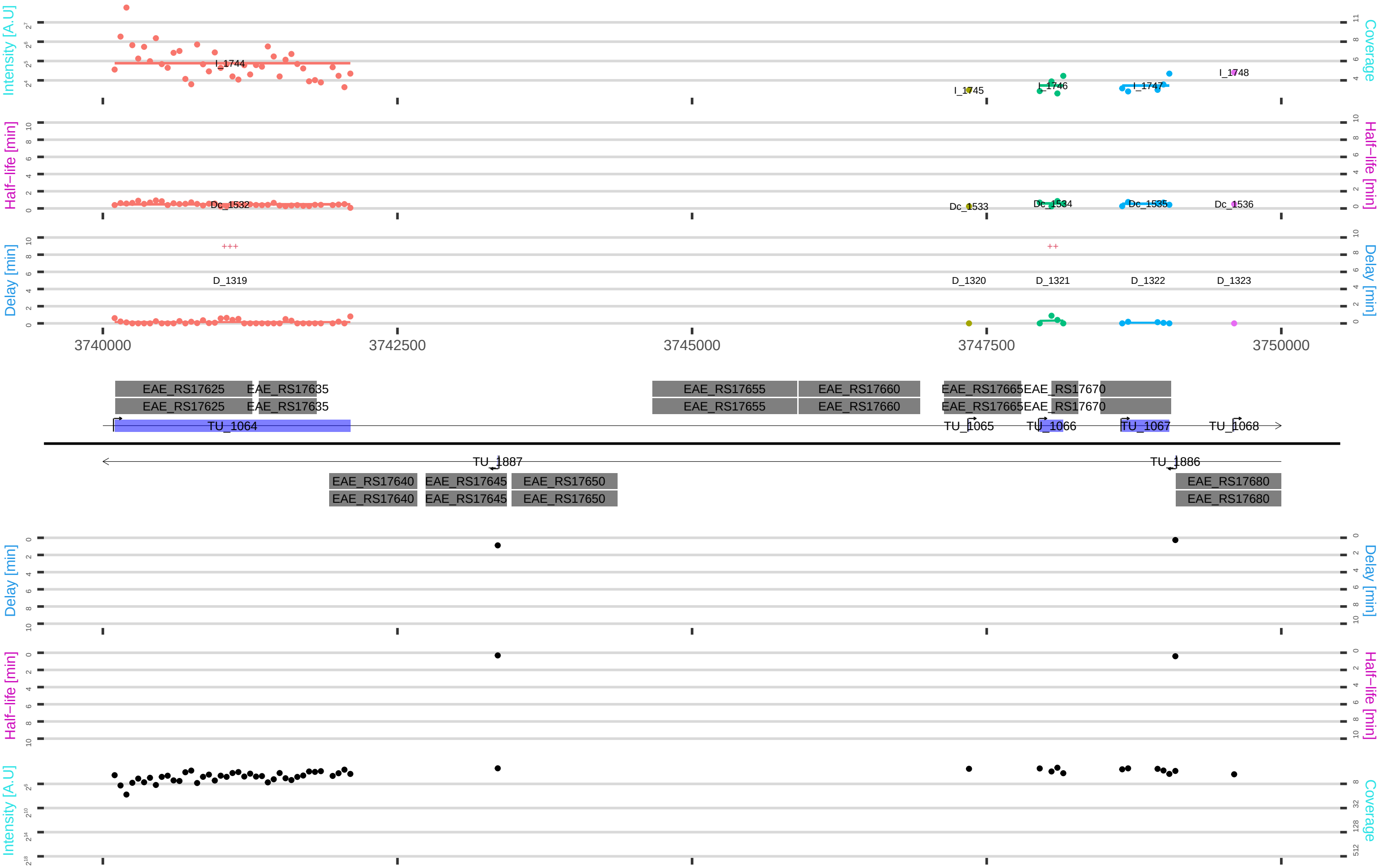

Term: termination (0), NS: new start (0), PS: pausing site (0), iTSS_I: internal starting site (0)

ID: 75050−75155; Term: termination (0), NS: new start (0), PS: pausing site (0), iTSS_I: internal starting site (0)

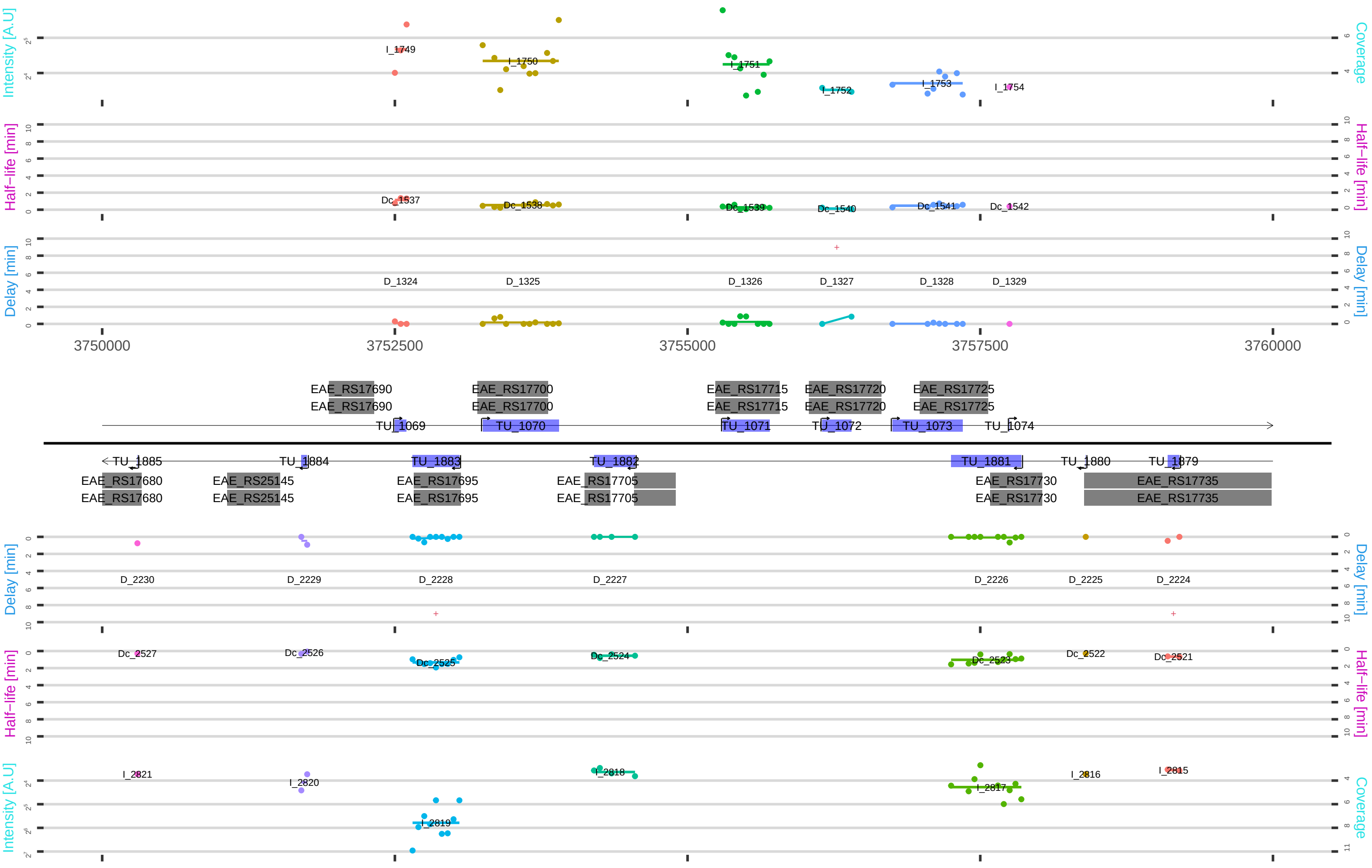

Term: termination (0), NS: new start (0), PS: pausing site (0), iTSS_I: internal starting site (0)

ID: 135973−135817; FC*: significant t−test of two consecutive segments; Term: termination, NS: new start, PS: pausing site,iTSS_I: internal starting site,TI: transcription interferance.

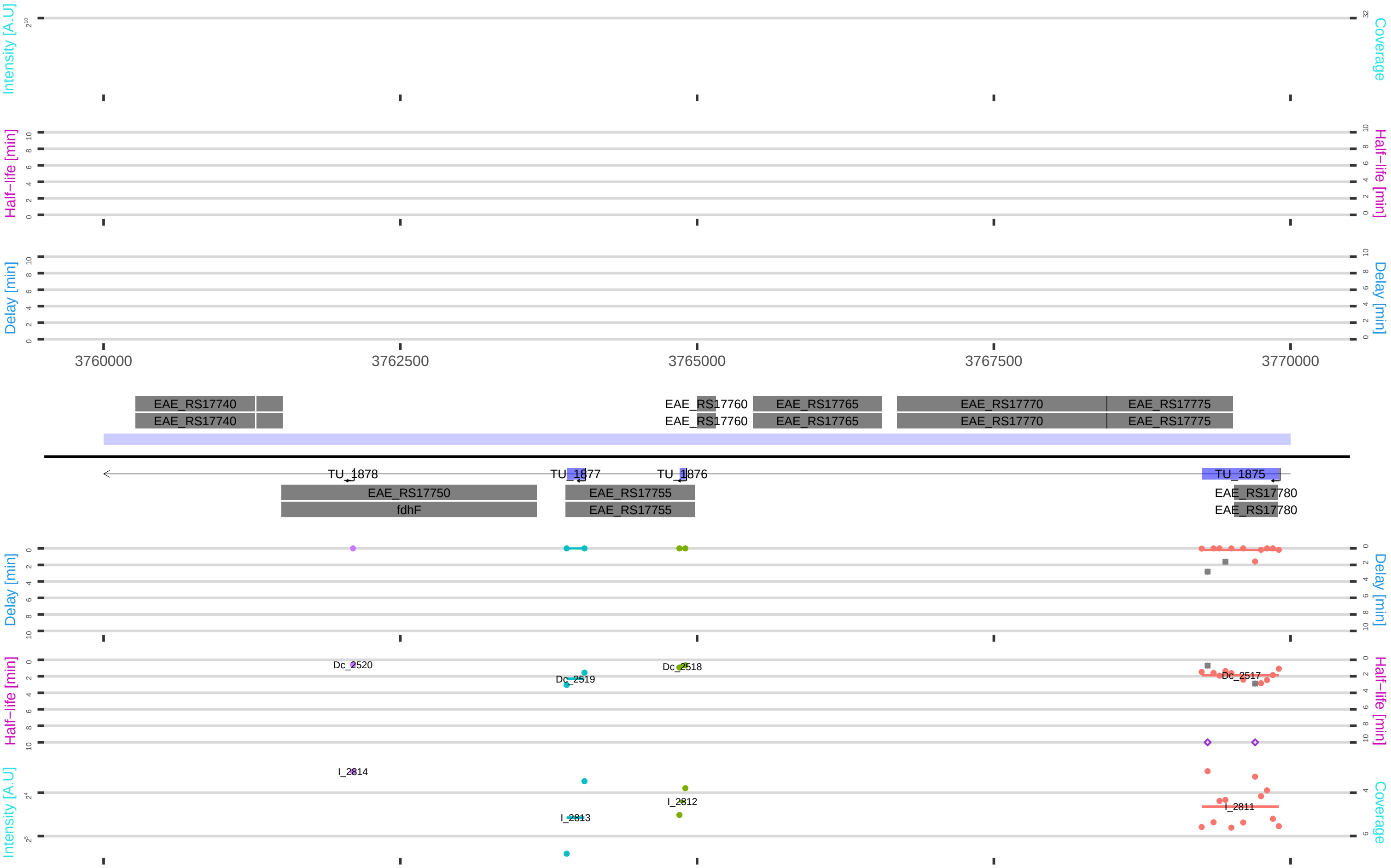

Term: termination (0), NS: new start (0), PS: pausing site (0), iTSS_I: internal starting site (0)

ID: 75404−75584; Term: termination (0), NS: new start (0), PS: pausing site (0), iTSS_I: internal starting site (0)

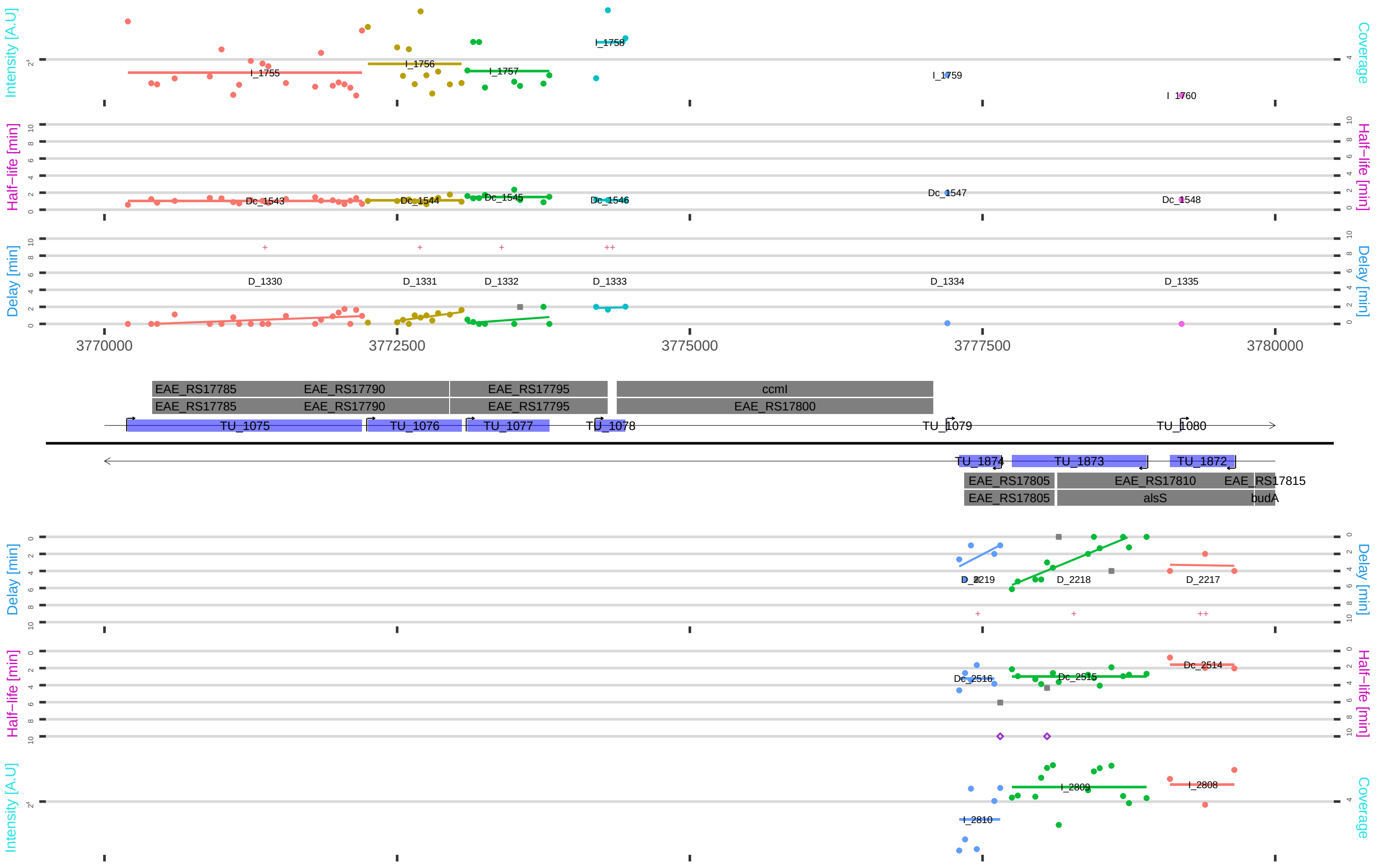

Term: termination (0), NS: new start (0), PS: pausing site (0), iTSS_I: internal starting site (0)

ID: 75616−75630; Term: termination (0), NS: new start (0), PS: pausing site (0), iTSS_I: internal starting site (0)

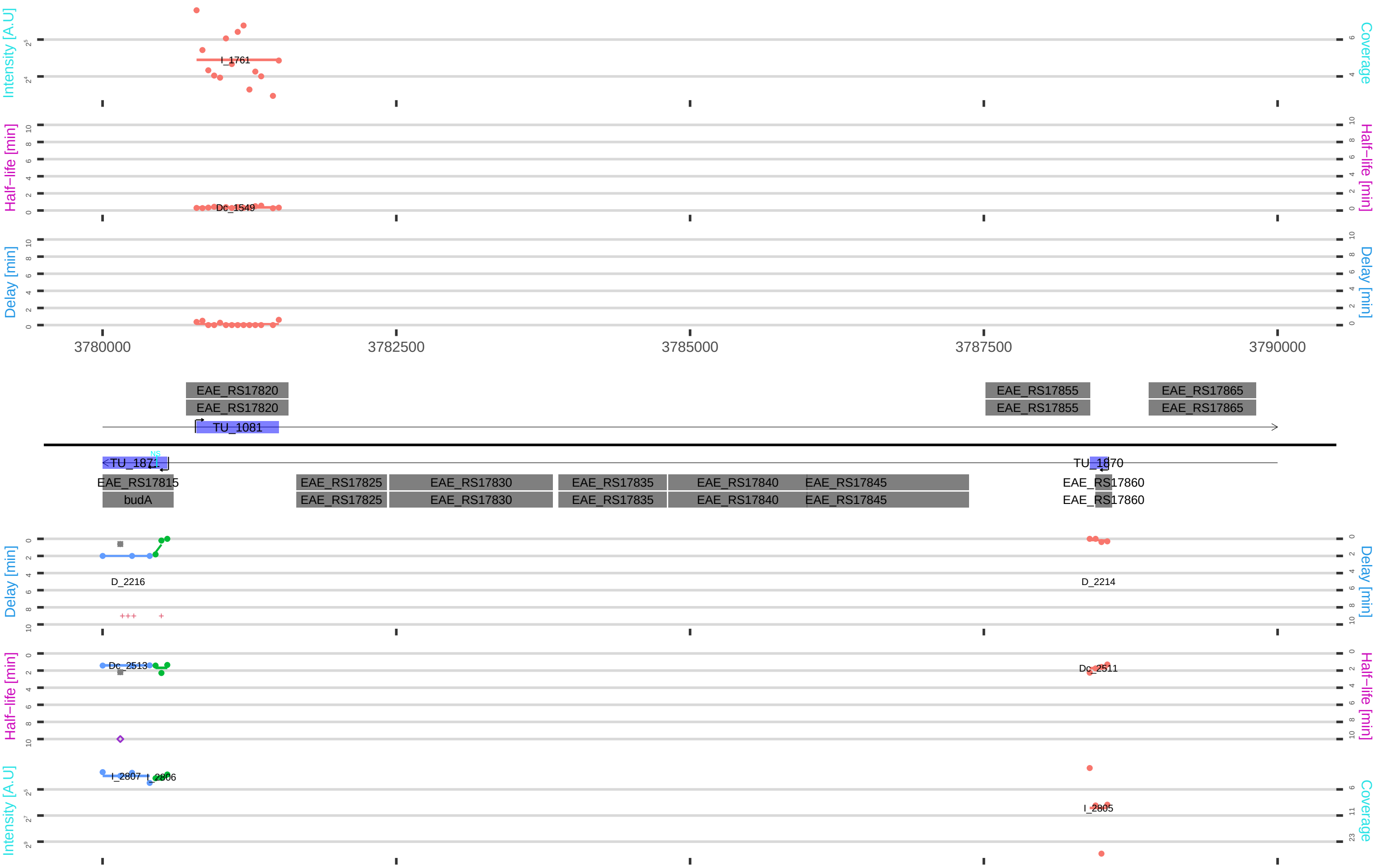

Term: termination (0), NS: new start (1), PS: pausing site (1), iTSS_I: internal starting site (0)

ID: 135363−135217; FC*: significant t−test of two consecutive segments; Term: termination, NS: new start, PS: pausing site,iTSS_I: internal starting site,TI: transcription interferance.

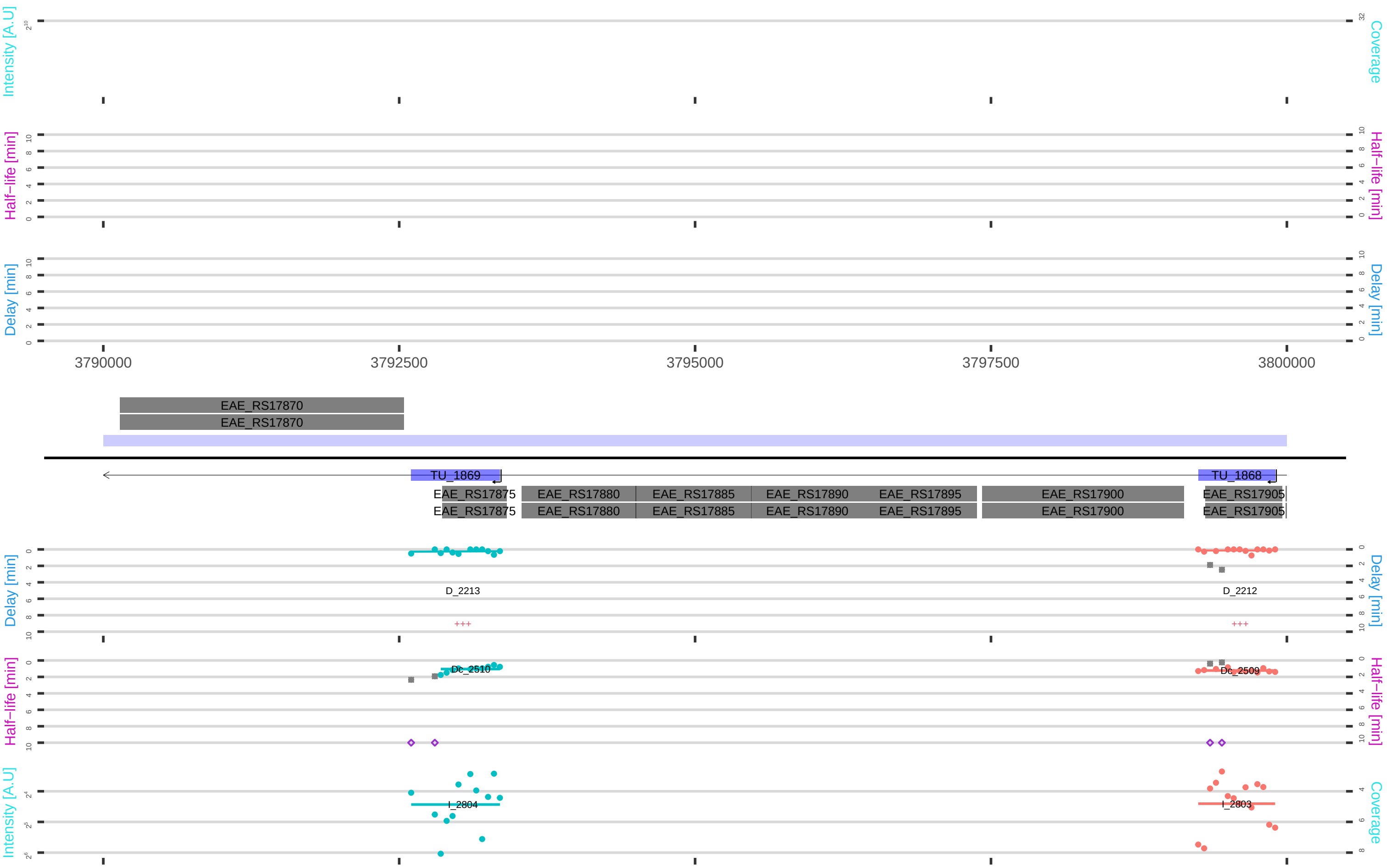

Term: termination (0), NS: new start (0), PS: pausing site (0), iTSS_I: internal starting site (0)

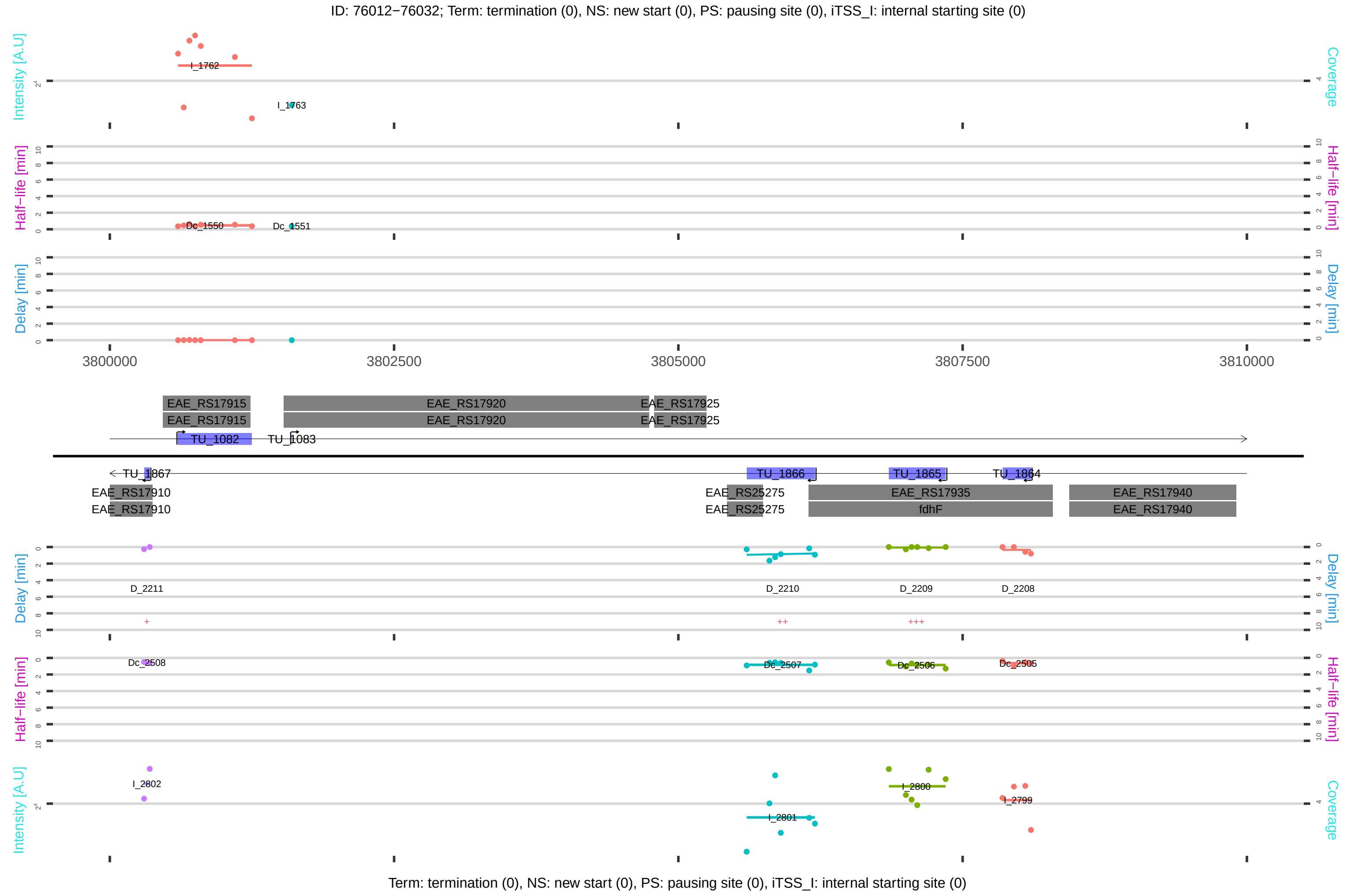
ID: 76012−76032; Term: termination (0), NS: new start (0), PS: pausing site (0), iTSS_I: internal starting site (0)
Intensity [A.U]
Coverage
Half−life [min]
Delay [min]
I_1762
I_1763
Dc_1550
Dc_1551
3800000
3802500
3805000
3807500
3810000
EAE_RS17915
EAE_RS17920
EAE_RS17925
TU_1082
TU_1083
TU_1867
TU_1866
TU_1865
TU_1864
EAE_RS17910
EAE_RS25275
EAE_RS17935
fdhF
EAE_RS17940
D_2211
D_2210
D_2209
D_2208
Dc_2508
Dc_2507
Dc_2506
Dc_2505
I_2802
I_2801
I_2800
I_2799
Term: termination (0), NS: new start (0), PS: pausing site (0), iTSS_I: internal starting site (0)

ID: 76366−76397; Term: termination (0), NS: new start (0), PS: pausing site (0), iTSS_I: internal starting site (0)

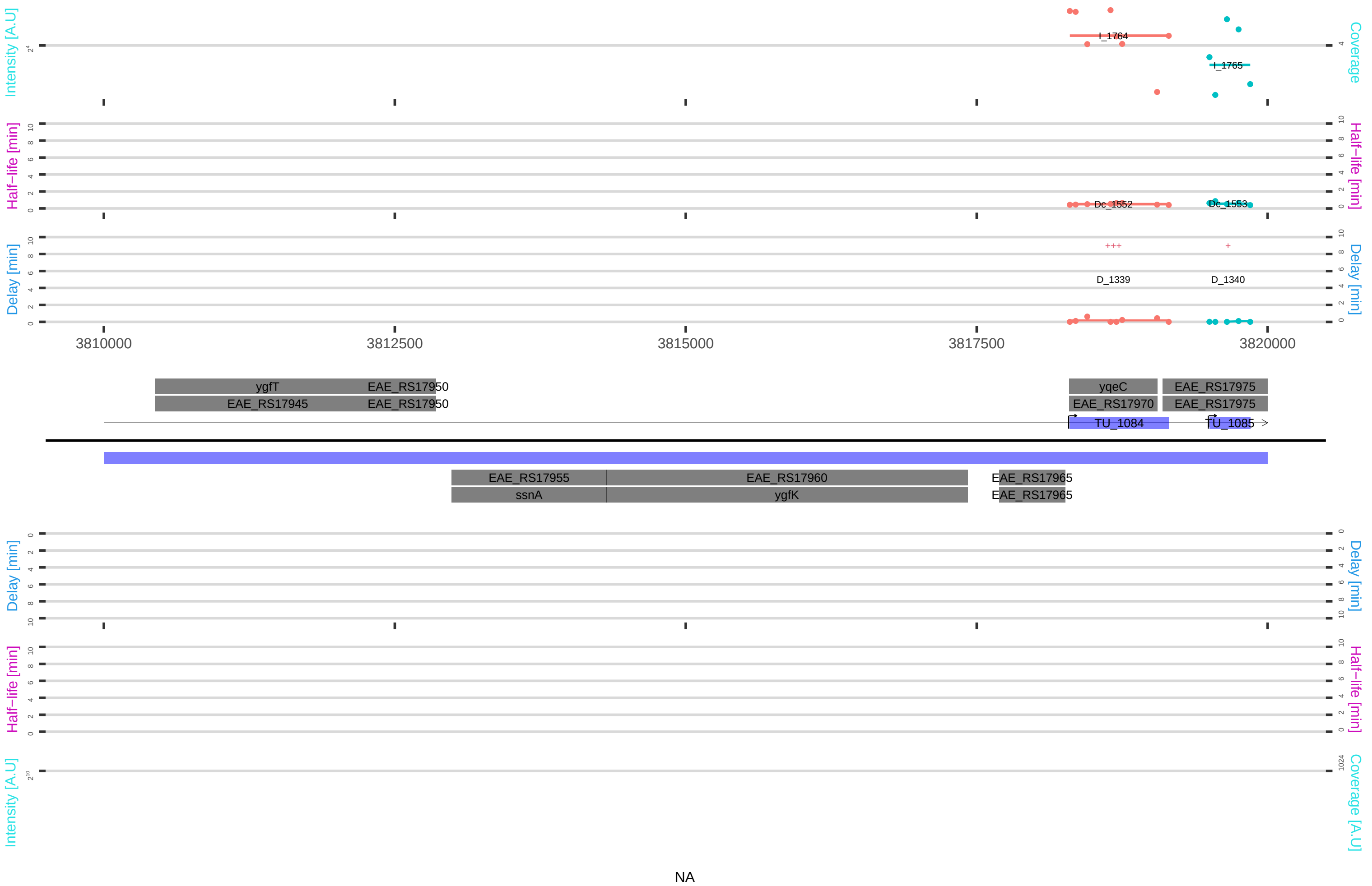

ID: 76401−76585; Term: termination (0), NS: new start (0), PS: pausing site (0), iTSS_I: internal starting site (0)

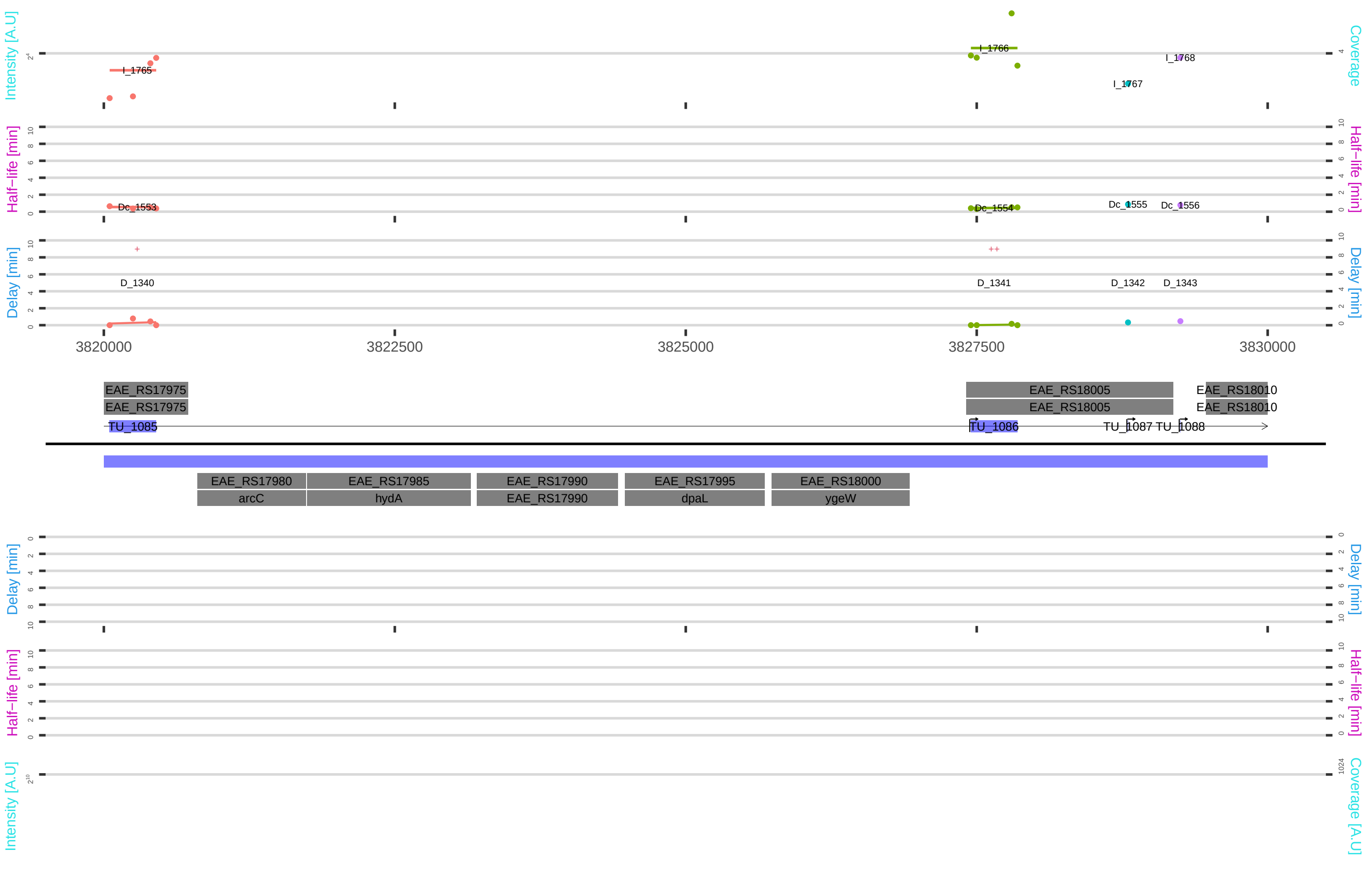

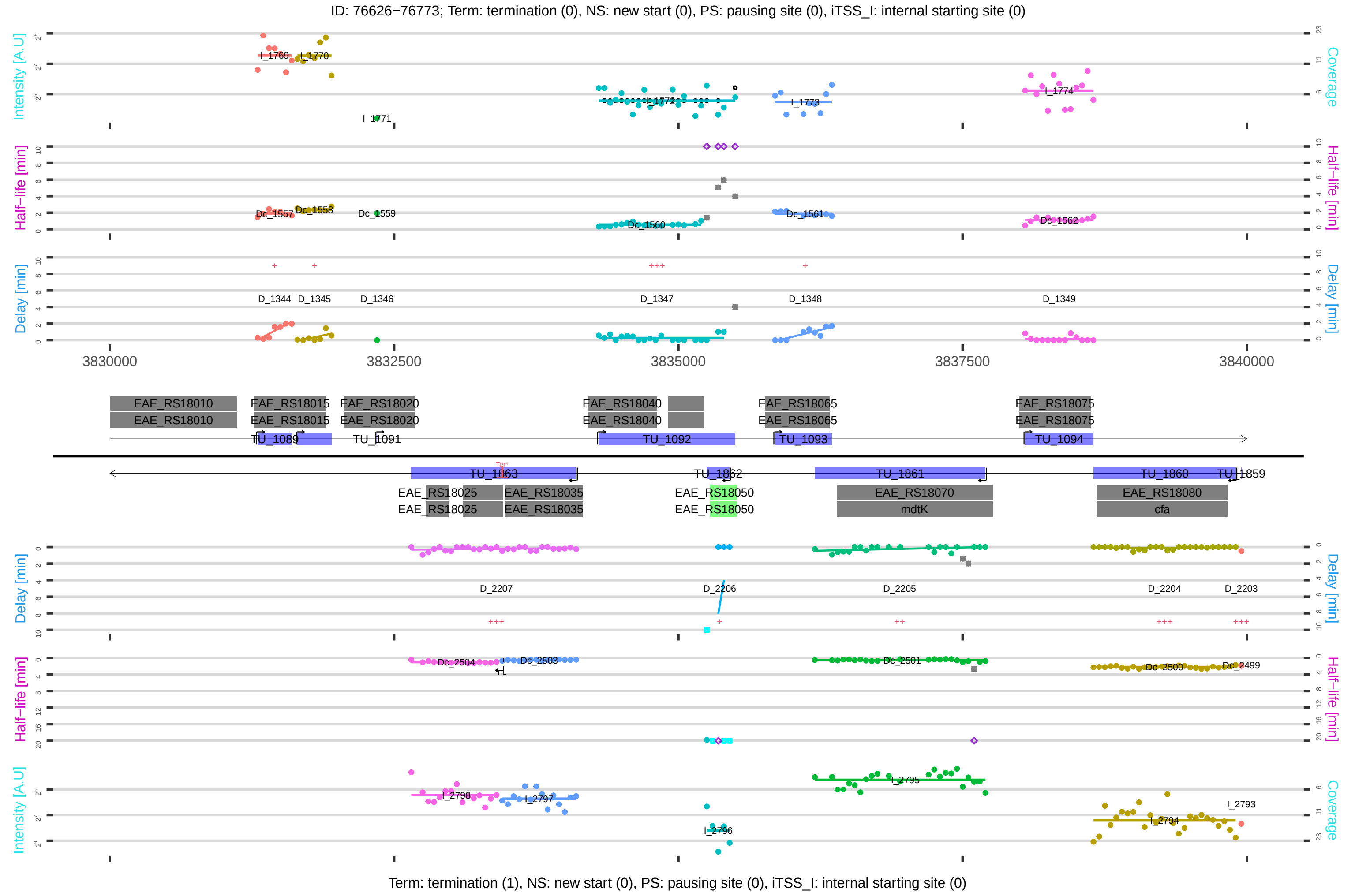
ID: 76626−76773; Term: termination (0), NS: new start (0), PS: pausing site (0), iTSS_I: internal starting site (0)
Intensity [A.U]
Coverage
Half−life [min]
Delay [min]
3830000
3832500
3835000
3837500
3840000
EAE_RS18010
EAE_RS18015
EAE_RS18020
EAE_RS18040
EAE_RS18065
EAE_RS18075
TU_1089
TU_1091
TU_1092
TU_1093
TU_1094
TU_1863
TU_1862
TU_1861
TU_1860
TU_1859
EAE_RS18025
EAE_RS18035
EAE_RS18050
EAE_RS18070
mdtK
EAE_RS18080
cfa
Term: termination (1), NS: new start (0), PS: pausing site (0), iTSS_I: internal starting site (0)

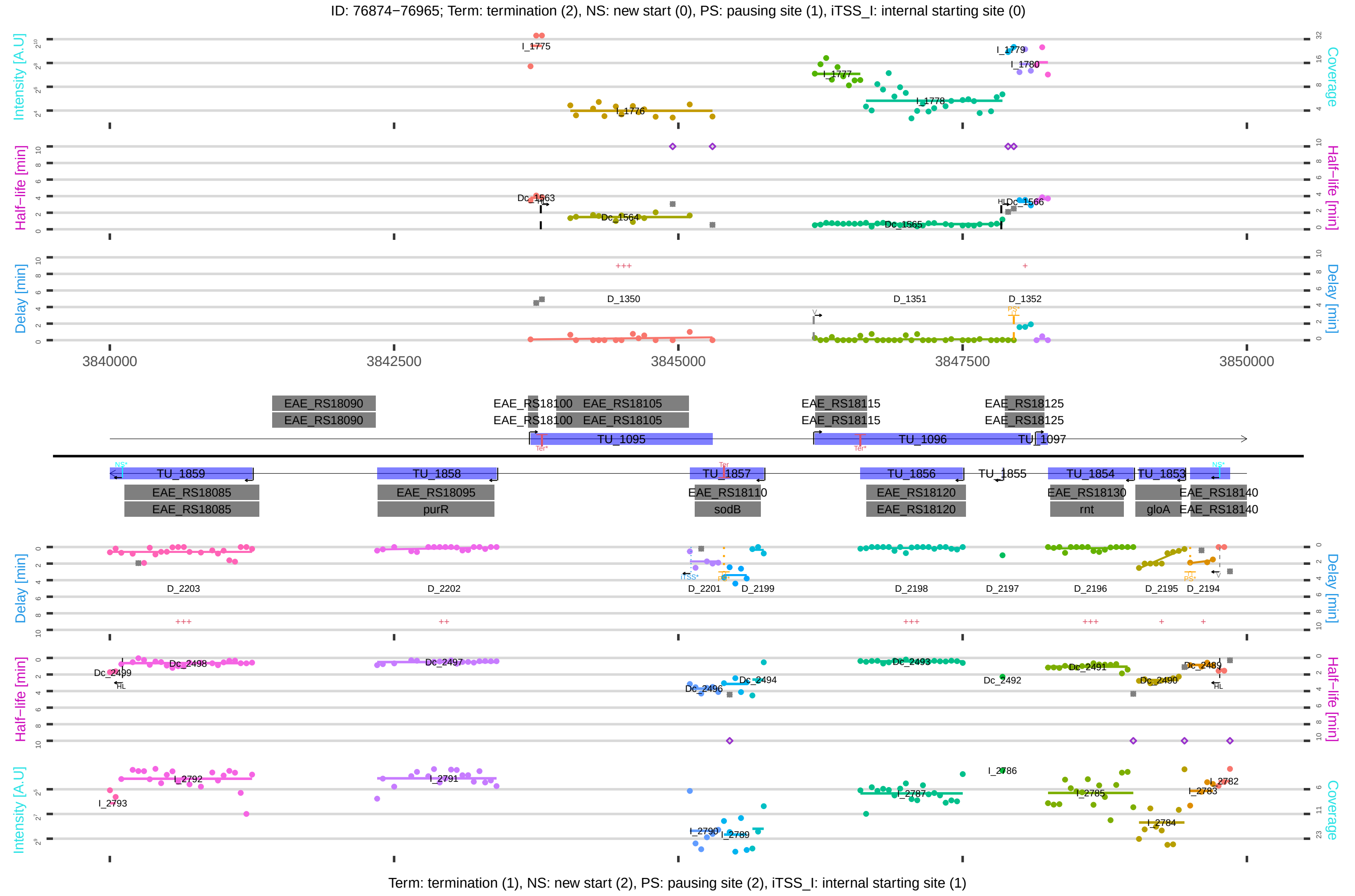

ID: 76874−76965; Term: termination (2), NS: new start (0), PS: pausing site (1), iTSS_I: internal starting site (0)
Intensity [A.U]
Coverage
Half−life [min]
Delay [min]
I_1775
I_1776
I_1777
I_1778
I_1779
I_1780
Dc_1563
Dc_1564
Dc_1565
Dc_1566
D_1350
D_1351
D_1352
3840000
3842500
3845000
3847500
3850000
EAE_RS18090
EAE_RS18100
EAE_RS18105
EAE_RS18115
EAE_RS18125
TU_1095
TU_1096
TU_1097
TU_1859
TU_1858
TU_1857
TU_1856
TU_1855
TU_1854
TU_1853
EAE_RS18085
EAE_RS18095
purR
EAE_RS18110
sodB
EAE_RS18120
EAE_RS18130
rnt
gloA
EAE_RS18140
D_2203
D_2202
D_2201
D_2199
D_2198
D_2197
D_2196
D_2195
D_2194
Dc_2499
Dc_2498
Dc_2497
Dc_2496
Dc_2494
Dc_2493
Dc_2492
Dc_2491
Dc_2490
Dc_2489
I_2793
I_2792
I_2791
I_2790
I_2789
I_2787
I_2786
I_2785
I_2784
I_2783
I_2782
Term: termination (1), NS: new start (2), PS: pausing site (2), iTSS_I: internal starting site (1)

ID: 77030−77200; Term: termination (2), NS: new start (1), PS: pausing site (0), iTSS_I: internal starting site (0)

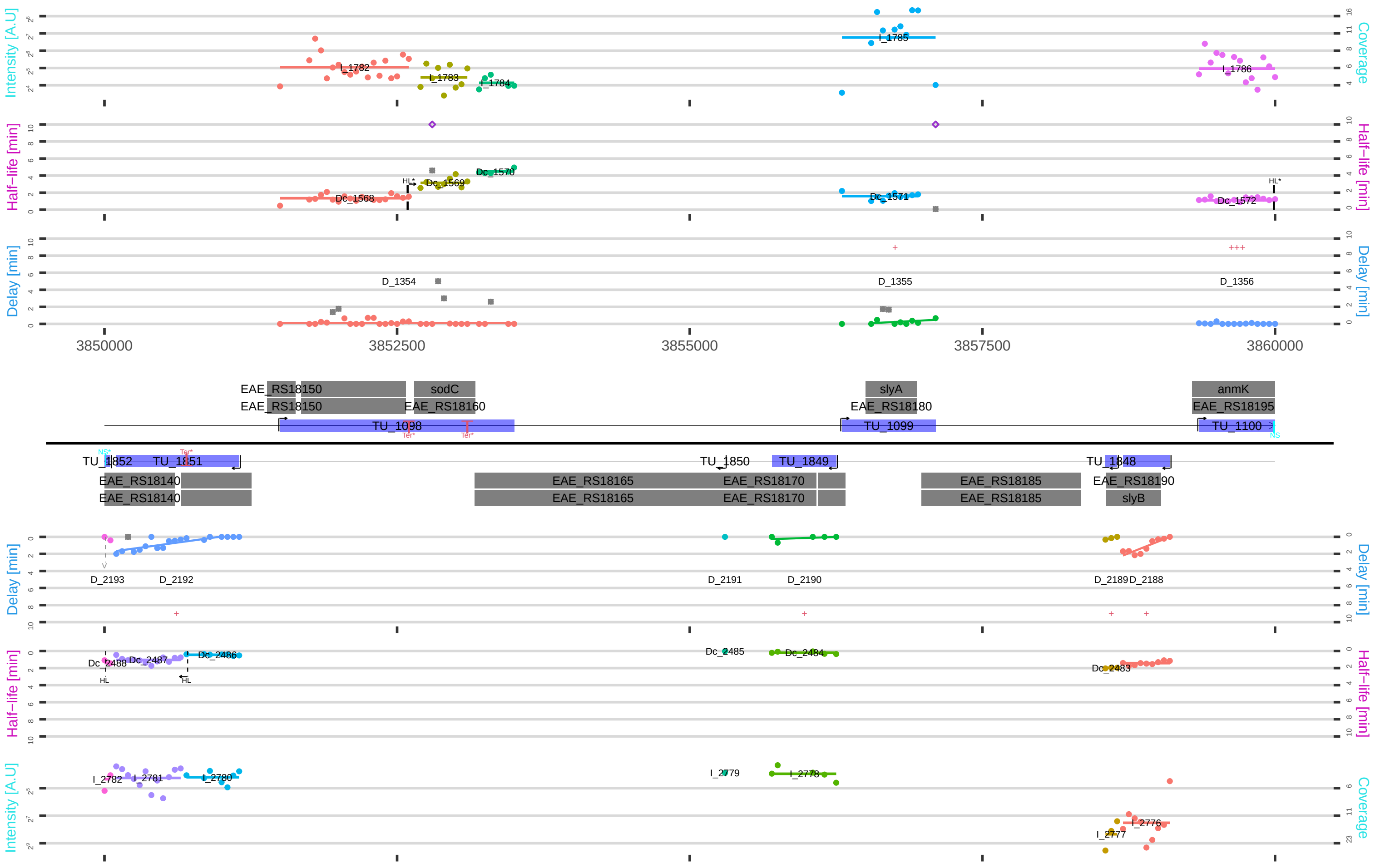

Term: termination (1), NS: new start (1), PS: pausing site (0), iTSS_I: internal starting site (0)

ID: 77200−77277; Term: termination (2), NS: new start (1), PS: pausing site (1), iTSS_I: internal starting site (0)

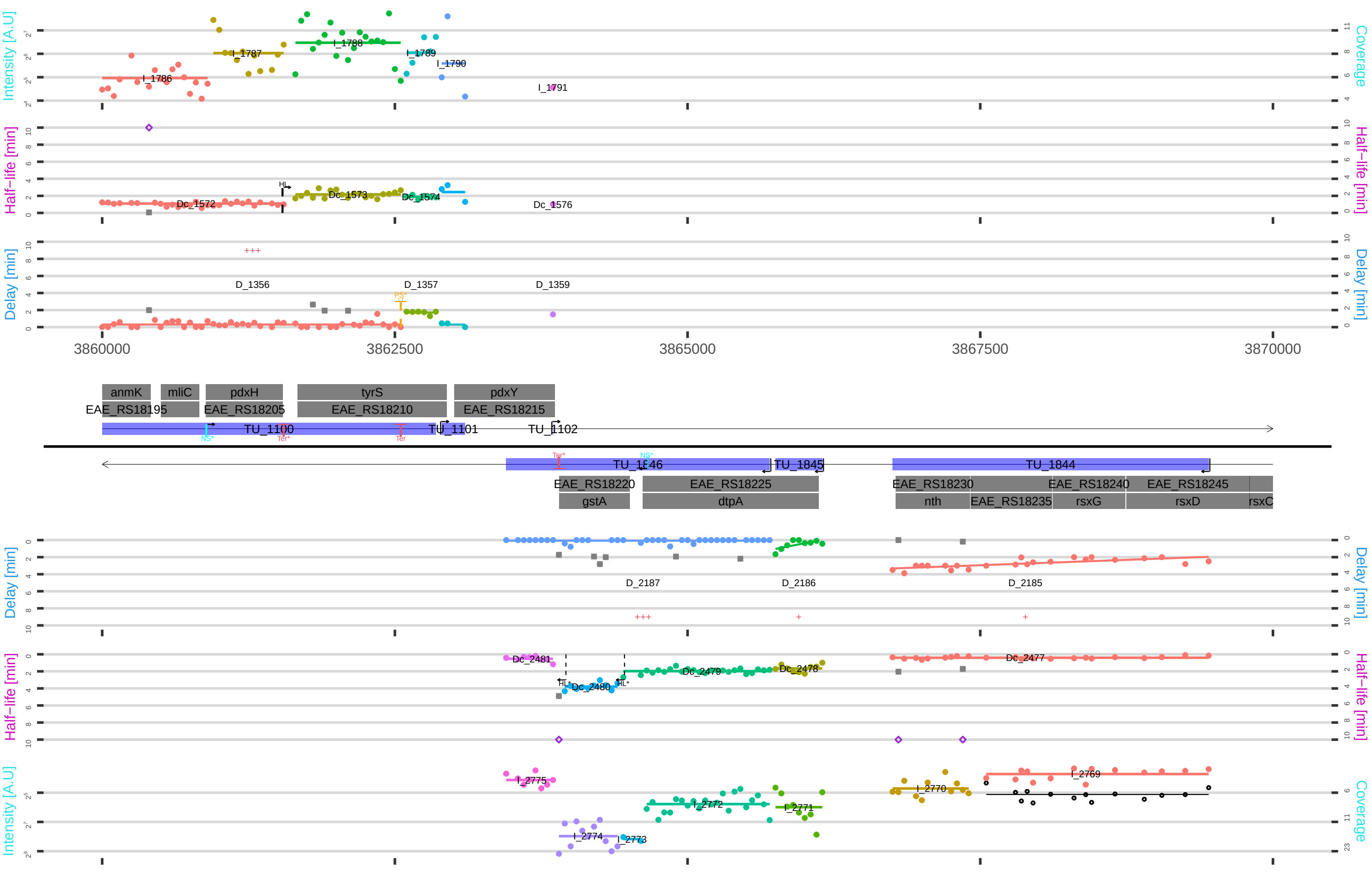

Term: termination (1), NS: new start (1), PS: pausing site (0), iTSS_I: internal starting site (0)

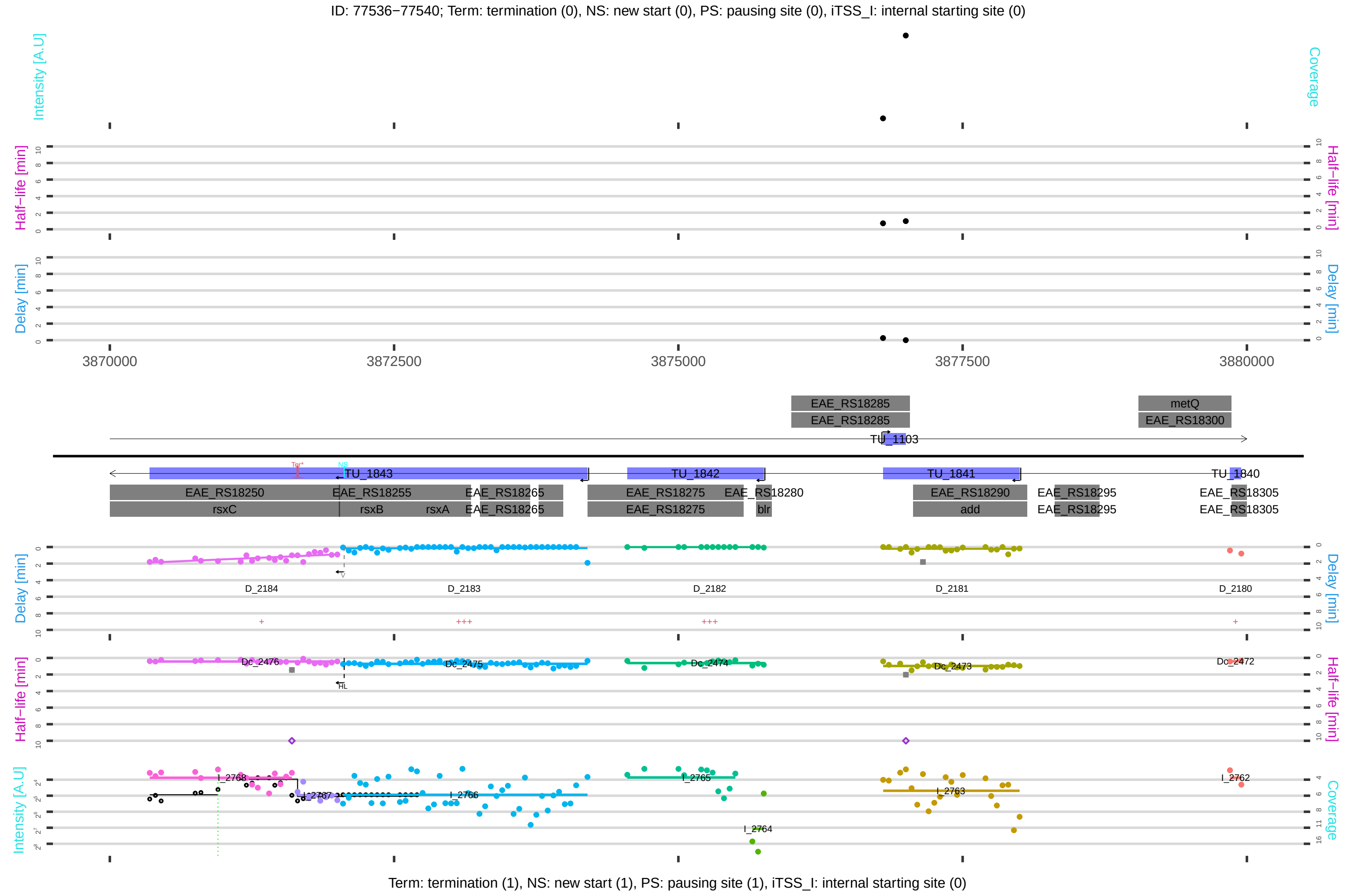
ID: 77536−77540; Term: termination (0), NS: new start (0), PS: pausing site (0), iTSS_I: internal starting site (0)
Intensity [A.U]
Coverage
Half−life [min]
Delay [min]
3870000
3872500
3875000
3877500
3880000
EAE_RS18285
metQ
EAE_RS18300
TU_1103
TU_1843
TU_1842
TU_1841
TU_1840
EAE_RS18250
rsxC
EAE_RS18255
rsxB
rsxA
EAE_RS18265
EAE_RS18275
EAE_RS18280
blr
EAE_RS18290
add
EAE_RS18295
EAE_RS18305
D_2184
D_2183
D_2182
D_2181
D_2180
Dc_2476
Dc_2475
Dc_2474
Dc_2473
Dc_2472
I_2768
I_2767
I_2766
I_2765
I_2764
I_2763
I_2762
Term: termination (1), NS: new start (1), PS: pausing site (1), iTSS_I: internal starting site (0)

ID: 77670−77754; Term: termination (0), NS: new start (0), PS: pausing site (0), iTSS_I: internal starting site (0)

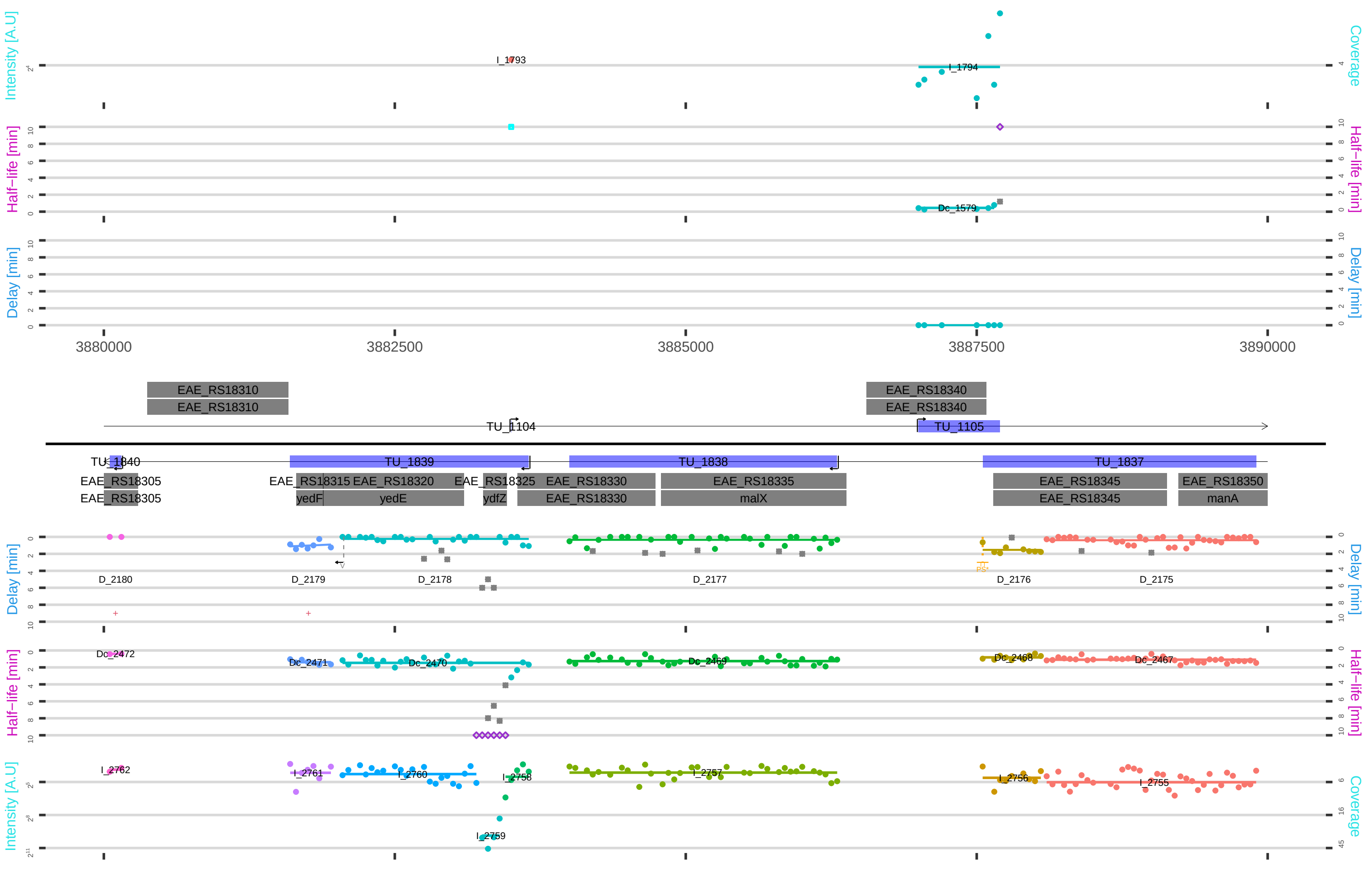

Term: termination (0), NS: new start (0), PS: pausing site (2), iTSS_I: internal starting site (0)

ID: 77812−77902; Term: termination (0), NS: new start (0), PS: pausing site (0), iTSS_I: internal starting site (0)

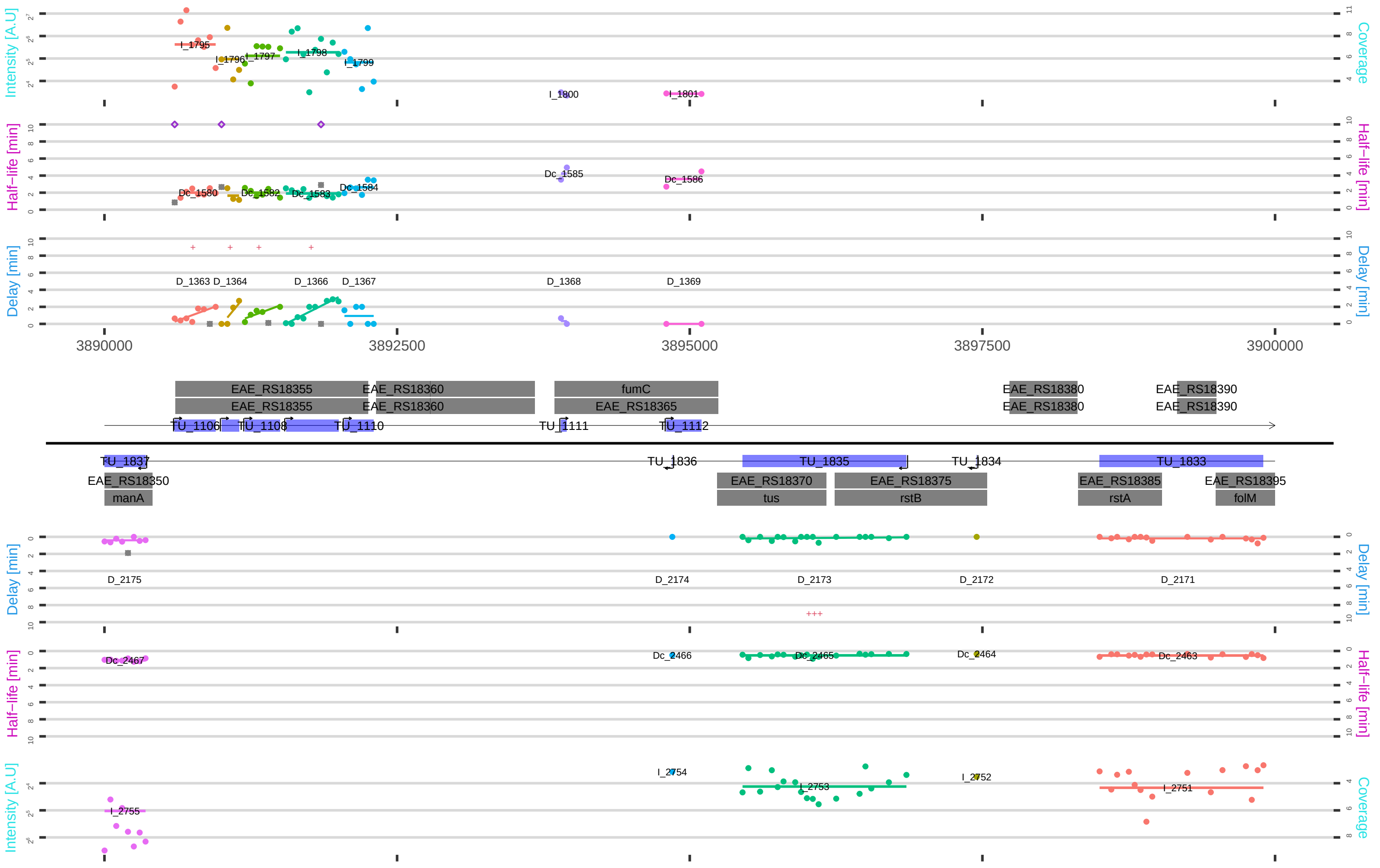

Term: termination (0), NS: new start (0), PS: pausing site (0), iTSS_I: internal starting site (0)

ID: 78065−78193; Term: termination (1), NS: new start (1), PS: pausing site (1), iTSS_I: internal starting site (2)

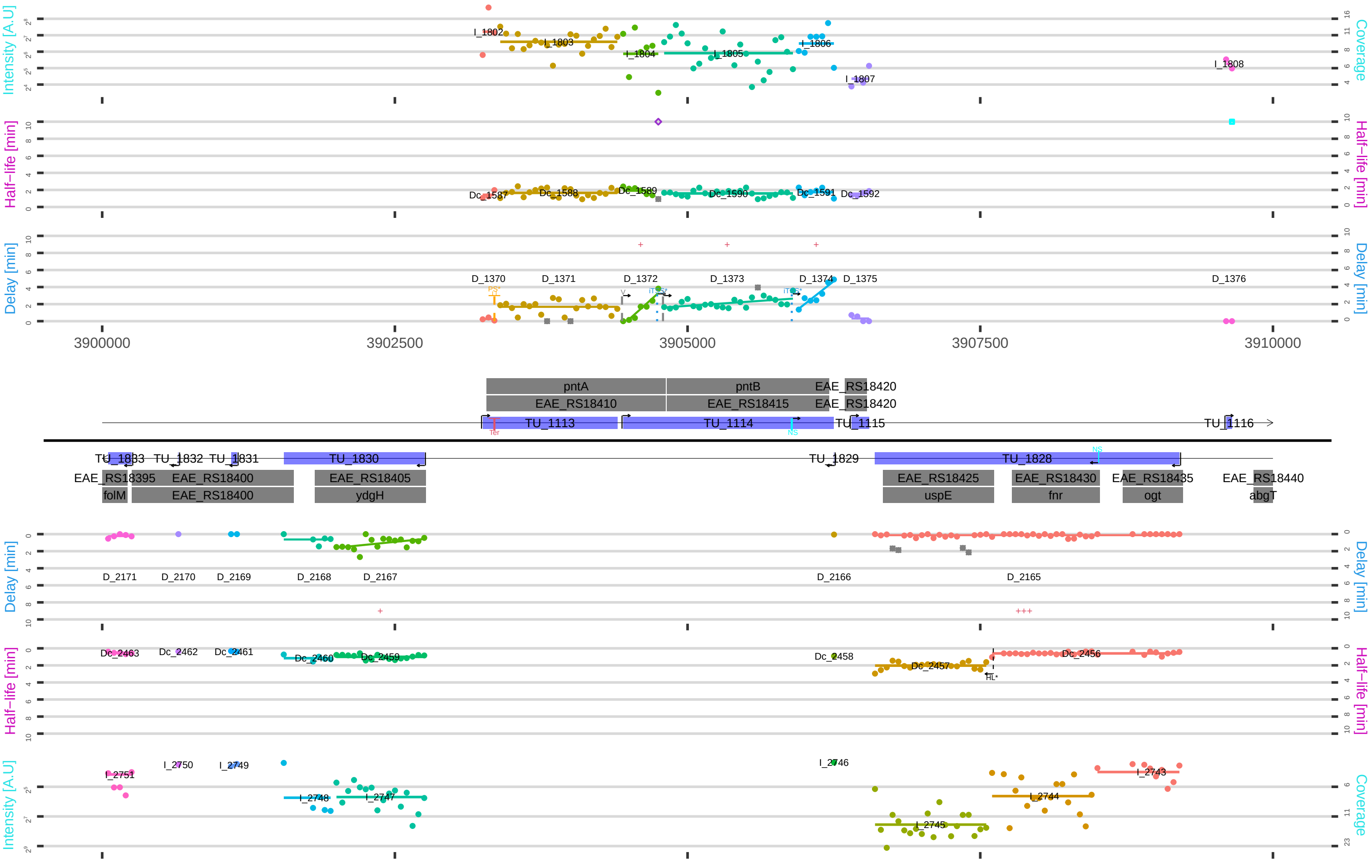

Term: termination (0), NS: new start (1), PS: pausing site (0), iTSS_I: internal starting site (1)

ID: 78291−78398; Term: termination (0), NS: new start (0), PS: pausing site (0), iTSS_I: internal starting site (0)

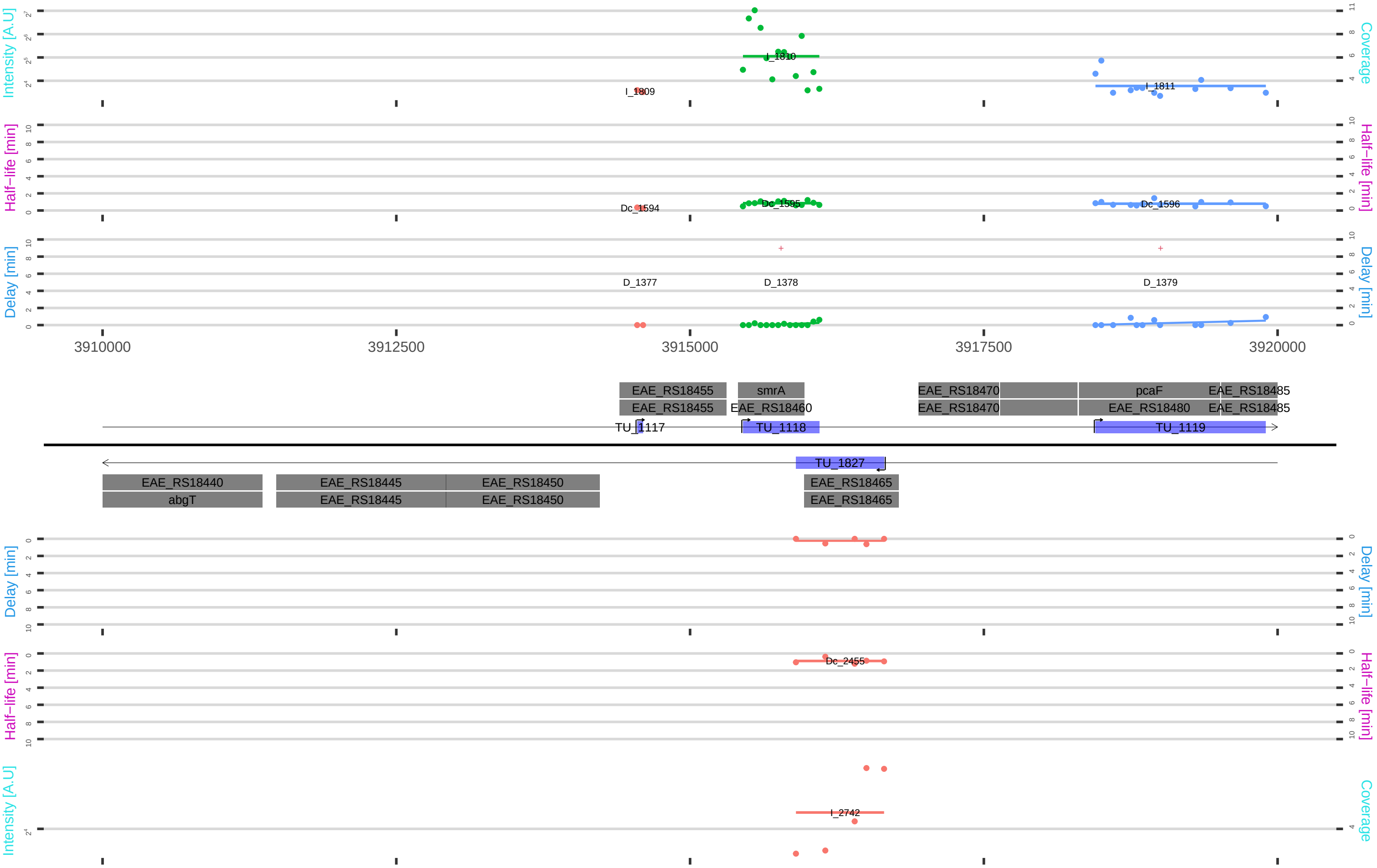

Term: termination (0), NS: new start (0), PS: pausing site (0), iTSS_I: internal starting site (0)

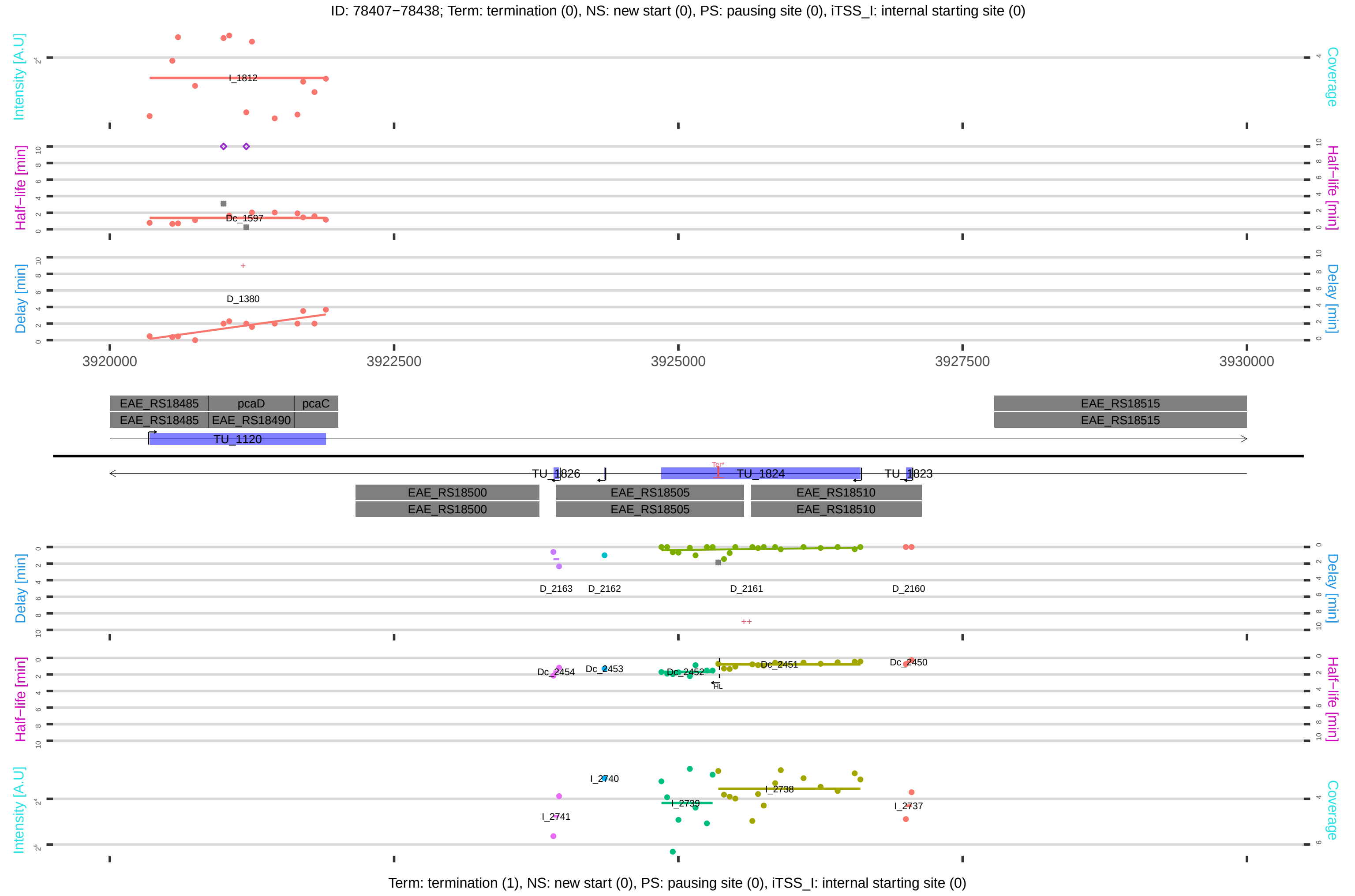

ID: 78407−78438; Term: termination (0), NS: new start (0), PS: pausing site (0), iTSS_I: internal starting site (0)
Intensity [A.U]
Coverage
I_1812
Half−life [min]
Dc_1597
Delay [min]
D_1380
3920000
3922500
3925000
3927500
3930000
EAE_RS18485
pcaD
pcaC
EAE_RS18490
EAE_RS18515
TU_1120
TU_1826
TU_1824
TU_1823
EAE_RS18500
EAE_RS18505
EAE_RS18510
D_2163
D_2162
D_2161
D_2160
Dc_2454
Dc_2453
Dc_2452
Dc_2451
Dc_2450
HL
I_2740
I_2741
I_2739
I_2738
I_2737
Term: termination (1), NS: new start (0), PS: pausing site (0), iTSS_I: internal starting site (0)

ID: 78606−78760; Term: termination (0), NS: new start (0), PS: pausing site (0), iTSS_I: internal starting site (0)

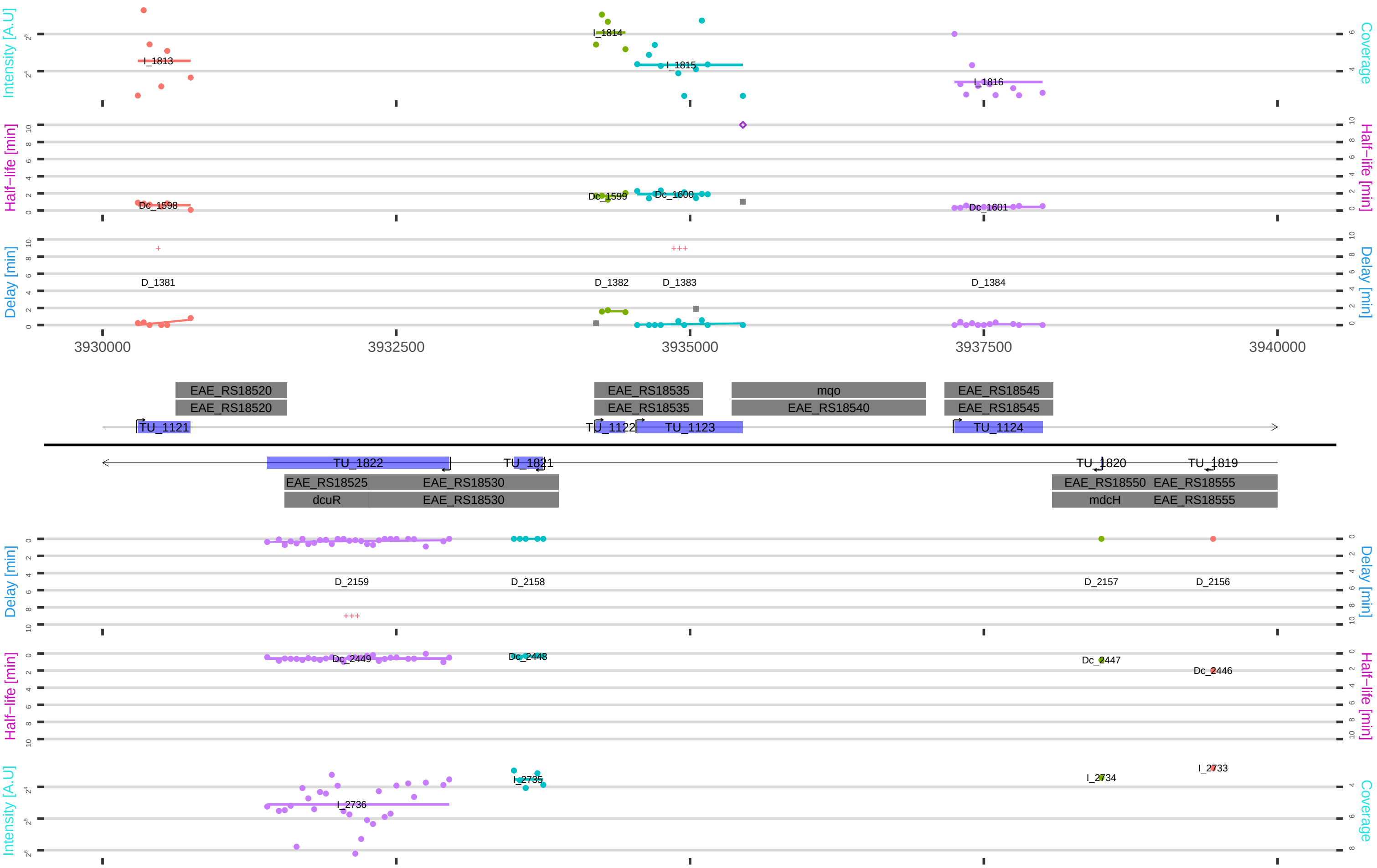

Term: termination (0), NS: new start (0), PS: pausing site (0), iTSS_I: internal starting site (0)

ID: 78933−78984; Term: termination (0), NS: new start (0), PS: pausing site (0), iTSS_I: internal starting site (0)

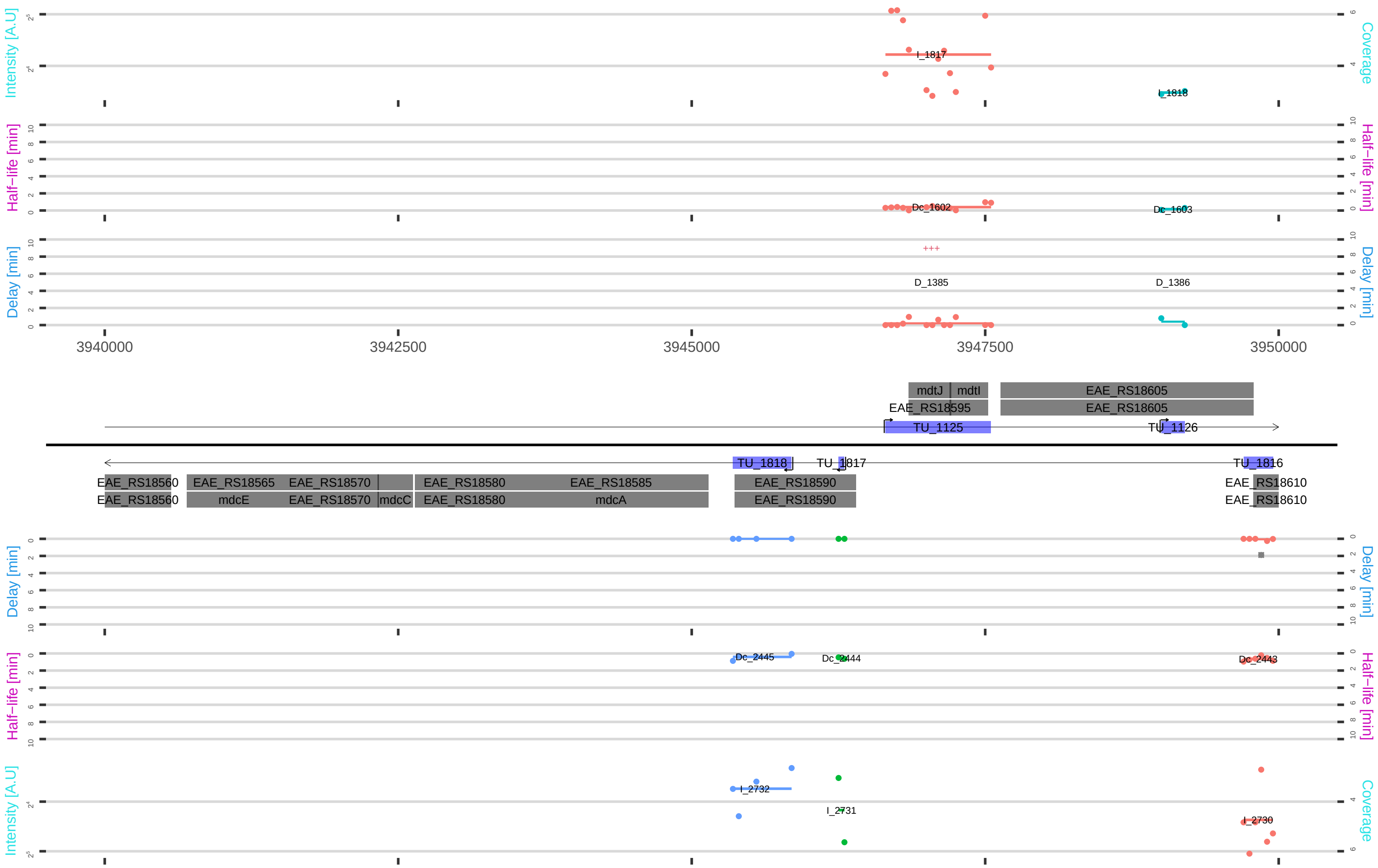

Term: termination (0), NS: new start (0), PS: pausing site (0), iTSS_I: internal starting site (0)

ID: 79023−79174; Term: termination (0), NS: new start (0), PS: pausing site (0), iTSS_I: internal starting site (0)

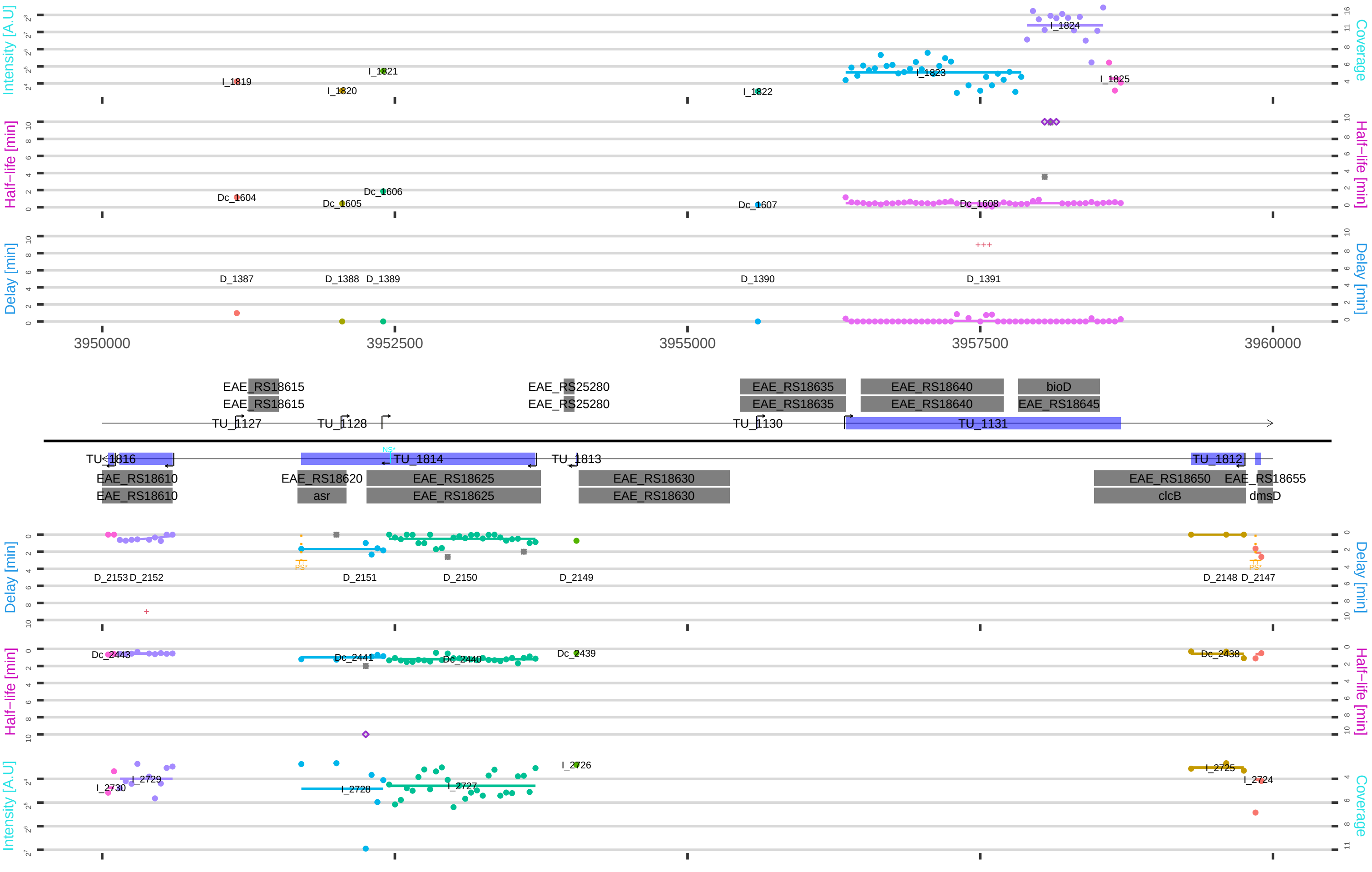

Term: termination (0), NS: new start (1), PS: pausing site (2), iTSS_I: internal starting site (0)

ID: 79301−79339; Term: termination (0), NS: new start (0), PS: pausing site (0), iTSS_I: internal starting site (0)

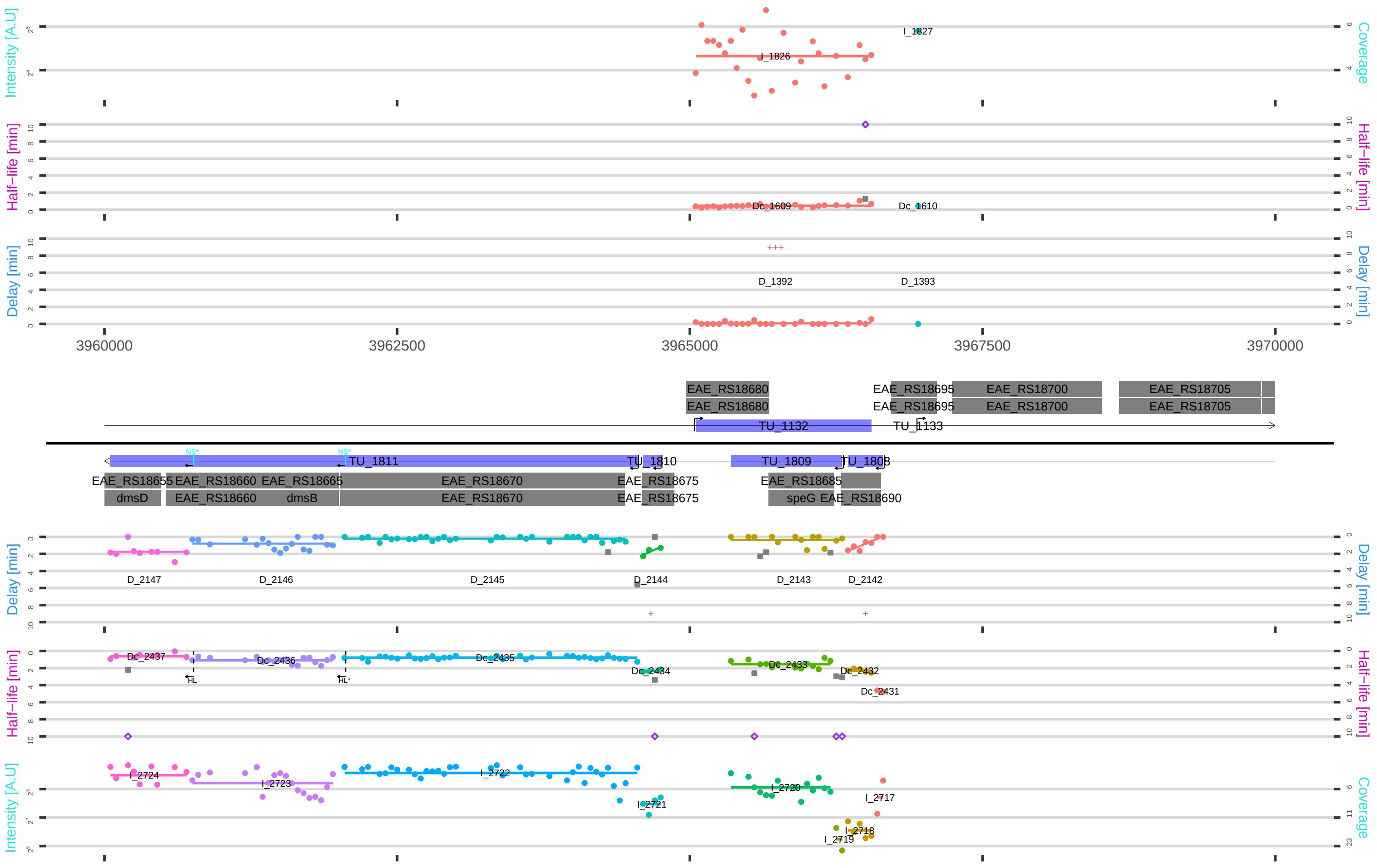

Term: termination (0), NS: new start (2), PS: pausing site (1), iTSS_I: internal starting site (0)

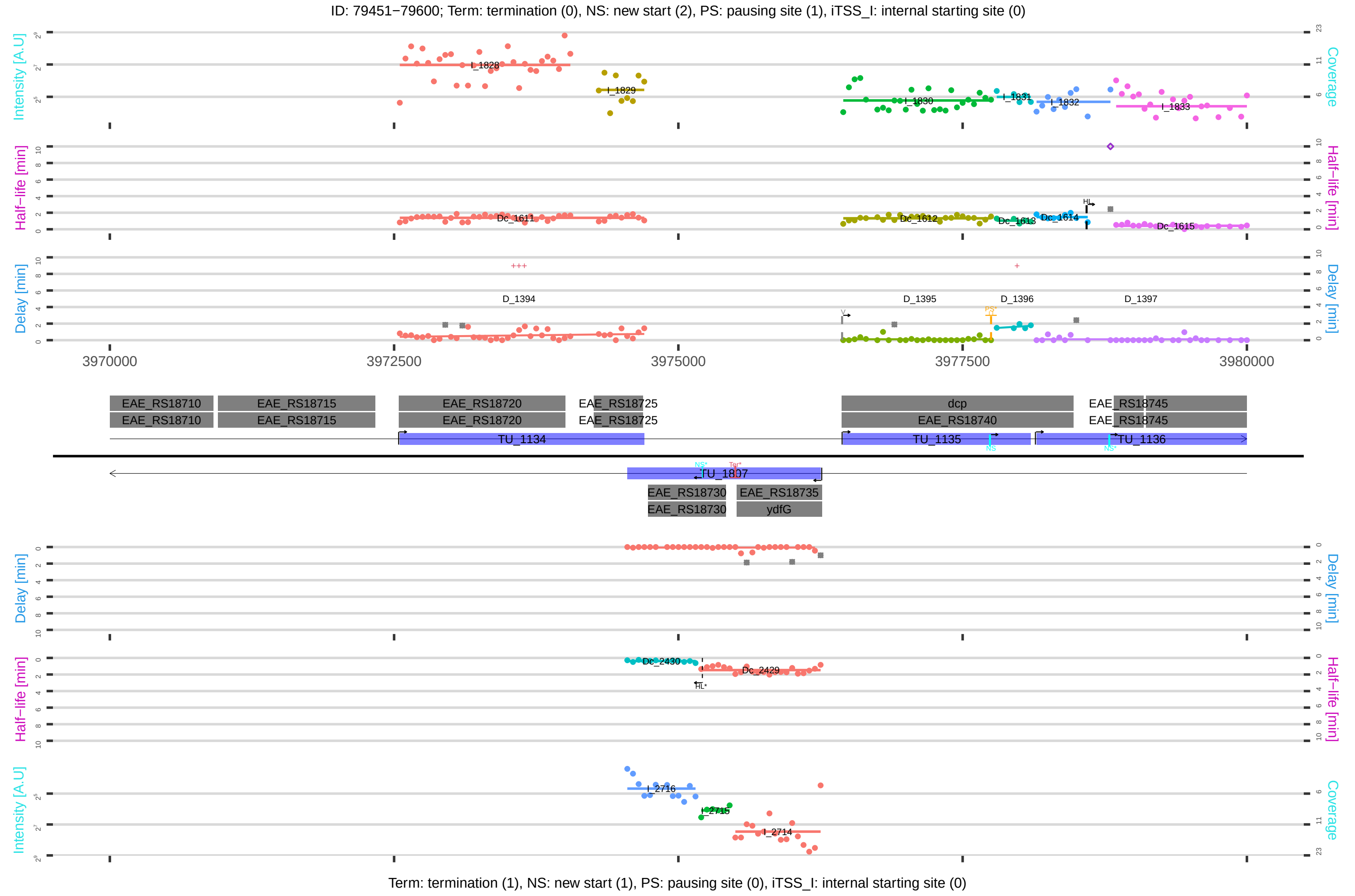

ID: 79451−79600; Term: termination (0), NS: new start (2), PS: pausing site (1), iTSS_I: internal starting site (0)
Intensity [A.U]
Coverage
Half−life [min]
Delay [min]
I_1828
I_1829
I_1830
I_1831
I_1832
I_1833
Dc_1611
Dc_1612
Dc_1613
Dc_1614
Dc_1615
D_1394
D_1395
D_1396
D_1397
3970000
3972500
3975000
3977500
3980000
EAE_RS18710
EAE_RS18715
EAE_RS18720
EAE_RS18725
dcp
EAE_RS18740
EAE_RS18745
TU_1134
TU_1135
TU_1136
TU_1807
EAE_RS18730
EAE_RS18735
ydfG
Dc_2430
Dc_2429
I_2716
I_2715
I_2714
Term: termination (1), NS: new start (1), PS: pausing site (0), iTSS_I: internal starting site (0)

ID: 79600−79752; Term: termination (1), NS: new start (0), PS: pausing site (0), iTSS_I: internal starting site (0)

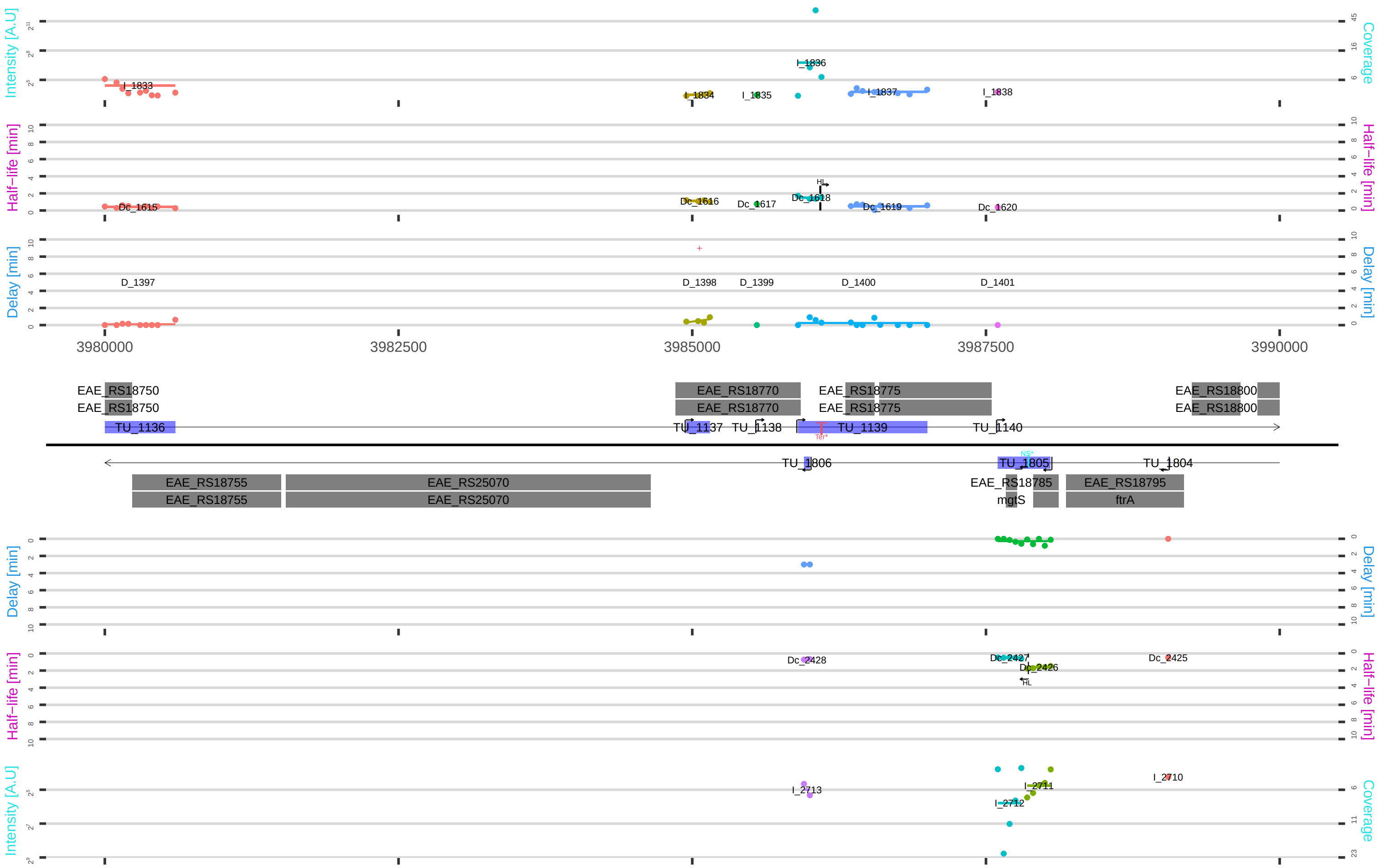

Term: termination (0), NS: new start (1), PS: pausing site (0), iTSS_I: internal starting site (0)

ID: 79860−79966; Term: termination (0), NS: new start (0), PS: pausing site (0), iTSS_I: internal starting site (0)

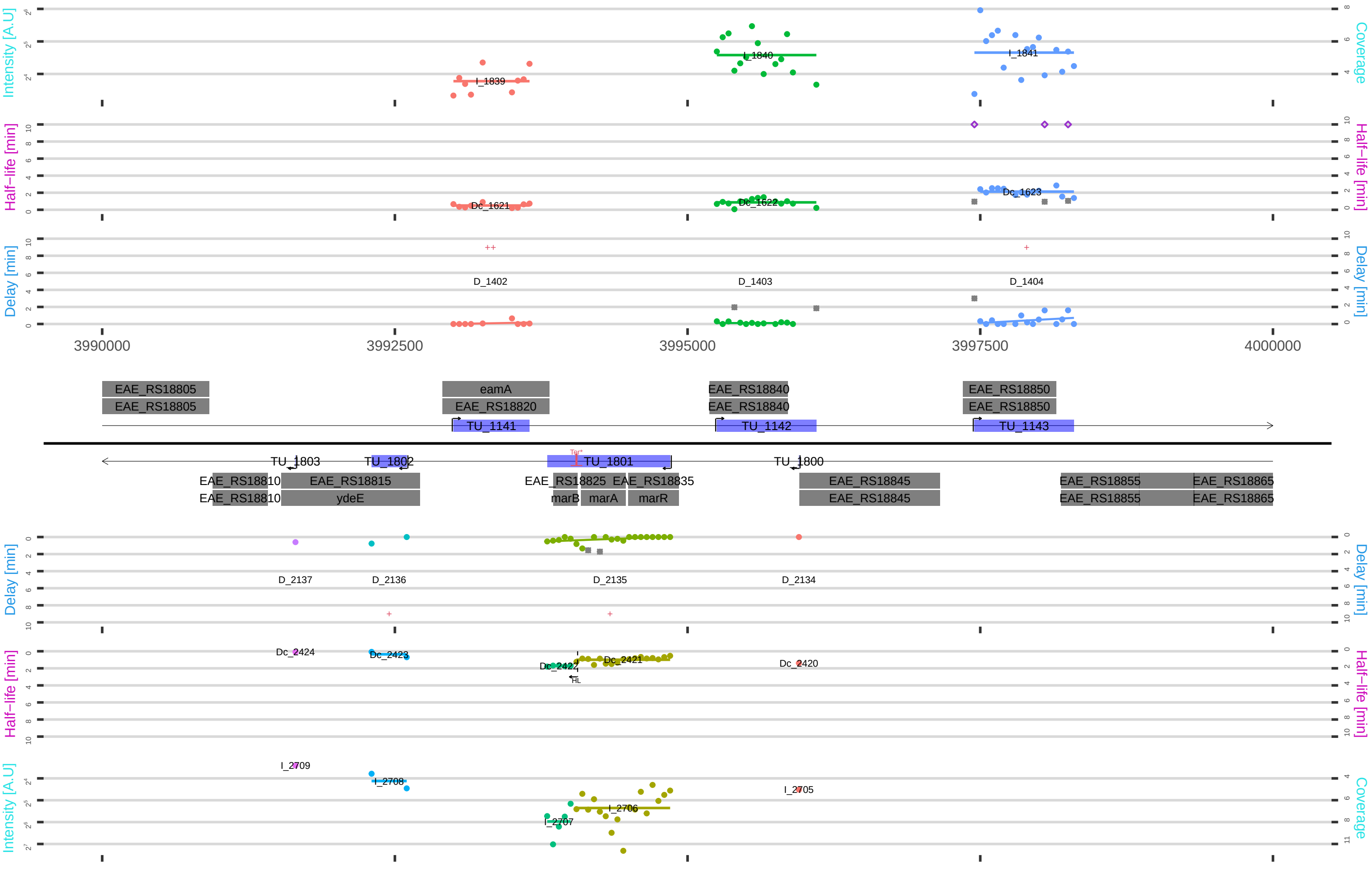

Term: termination (1), NS: new start (0), PS: pausing site (0), iTSS_I: internal starting site (0)

ID: 80130−80200; Term: termination (0), NS: new start (0), PS: pausing site (0), iTSS_I: internal starting site (0)

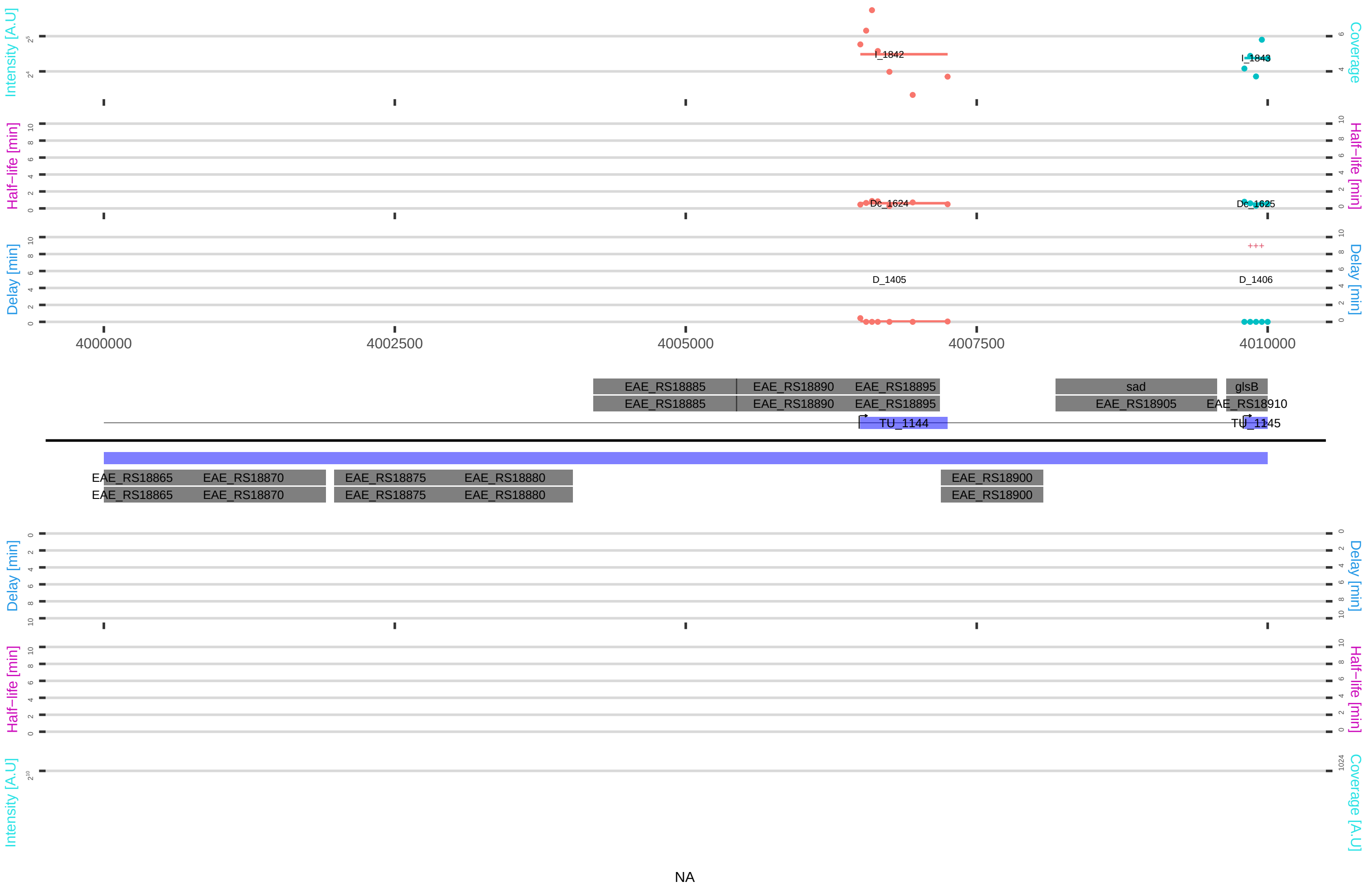

ID: 80200−80316; Term: termination (0), NS: new start (1), PS: pausing site (0), iTSS_I: internal starting site (0)

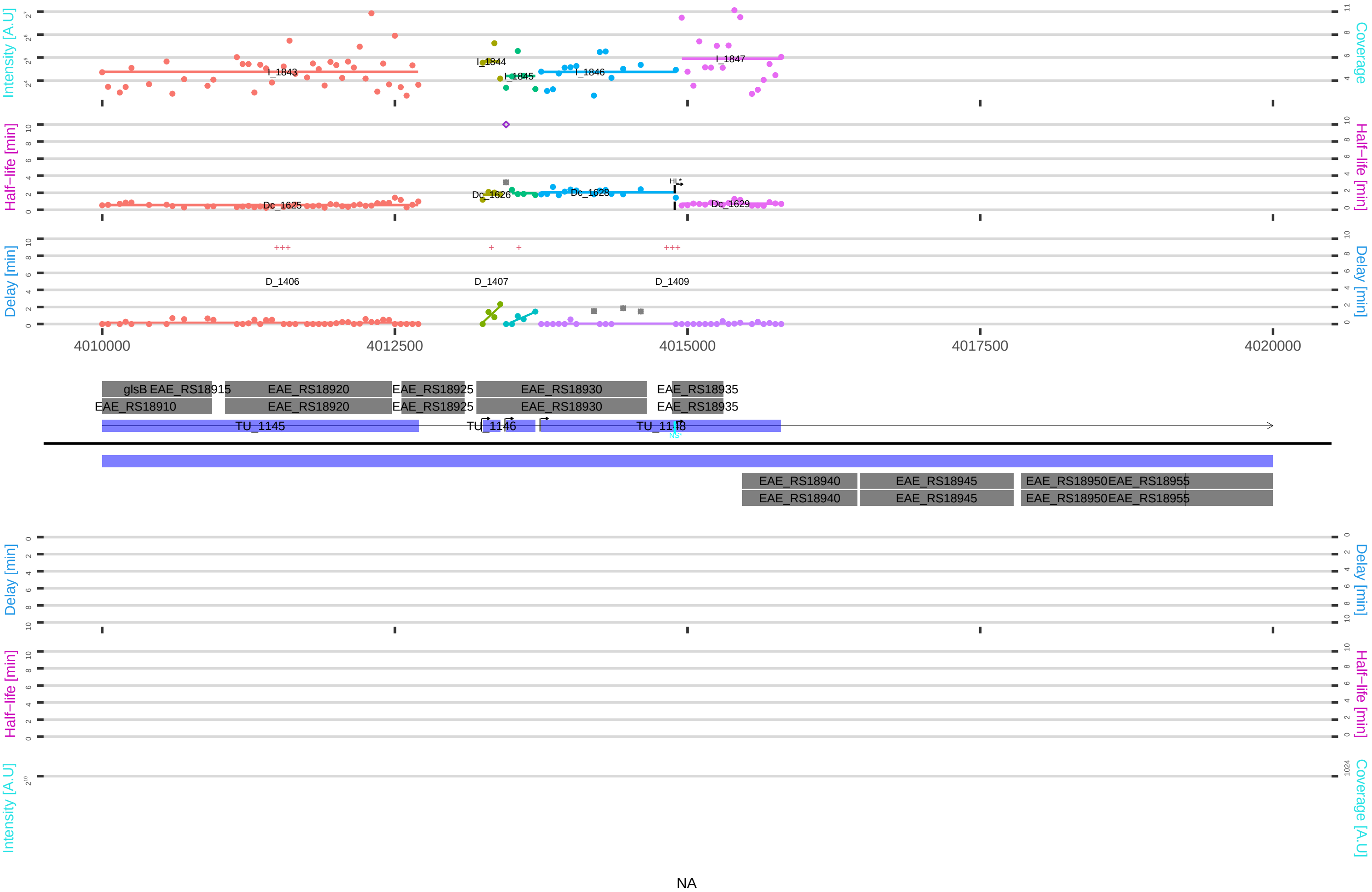

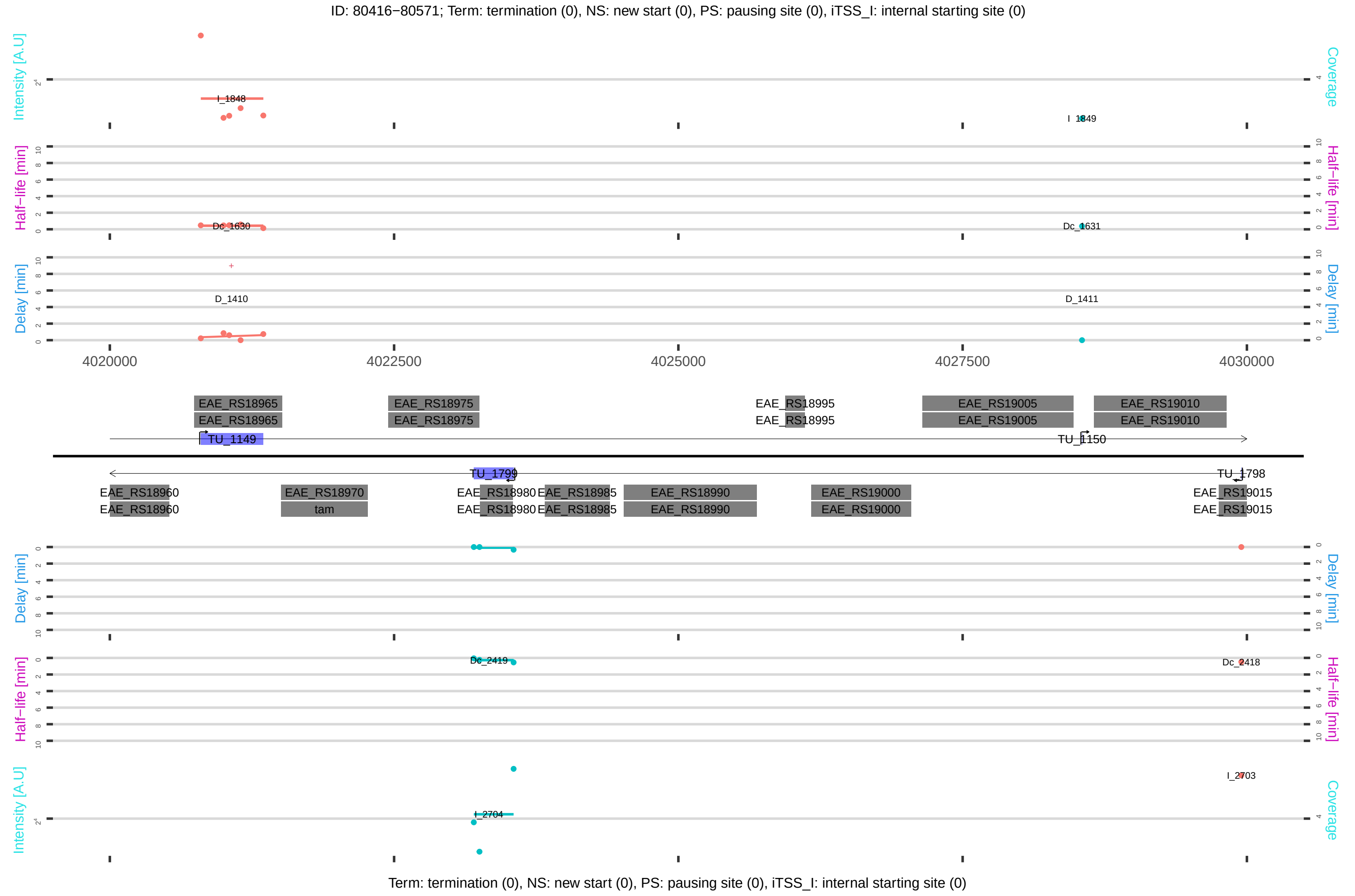

ID: 80416−80571; Term: termination (0), NS: new start (0), PS: pausing site (0), iTSS_I: internal starting site (0)
Intensity [A.U]
Coverage
Half−life [min]
Delay [min]
I_1848
I_1849
Dc_1630
Dc_1631
D_1410
D_1411
4020000
4022500
4025000
4027500
4030000
EAE_RS18965
EAE_RS18975
EAE_RS18995
EAE_RS19005
EAE_RS19010
TU_1149
TU_1150
TU_1799
TU_1798
EAE_RS18960
EAE_RS18970
tam
EAE_RS18980
EAE_RS18985
EAE_RS18990
EAE_RS19000
EAE_RS19015
Dc_2419
Dc_2418
I_2703
I_2704
Term: termination (0), NS: new start (0), PS: pausing site (0), iTSS_I: internal starting site (0)

ID: 80618−80758; Term: termination (0), NS: new start (0), PS: pausing site (0), iTSS_I: internal starting site (0)

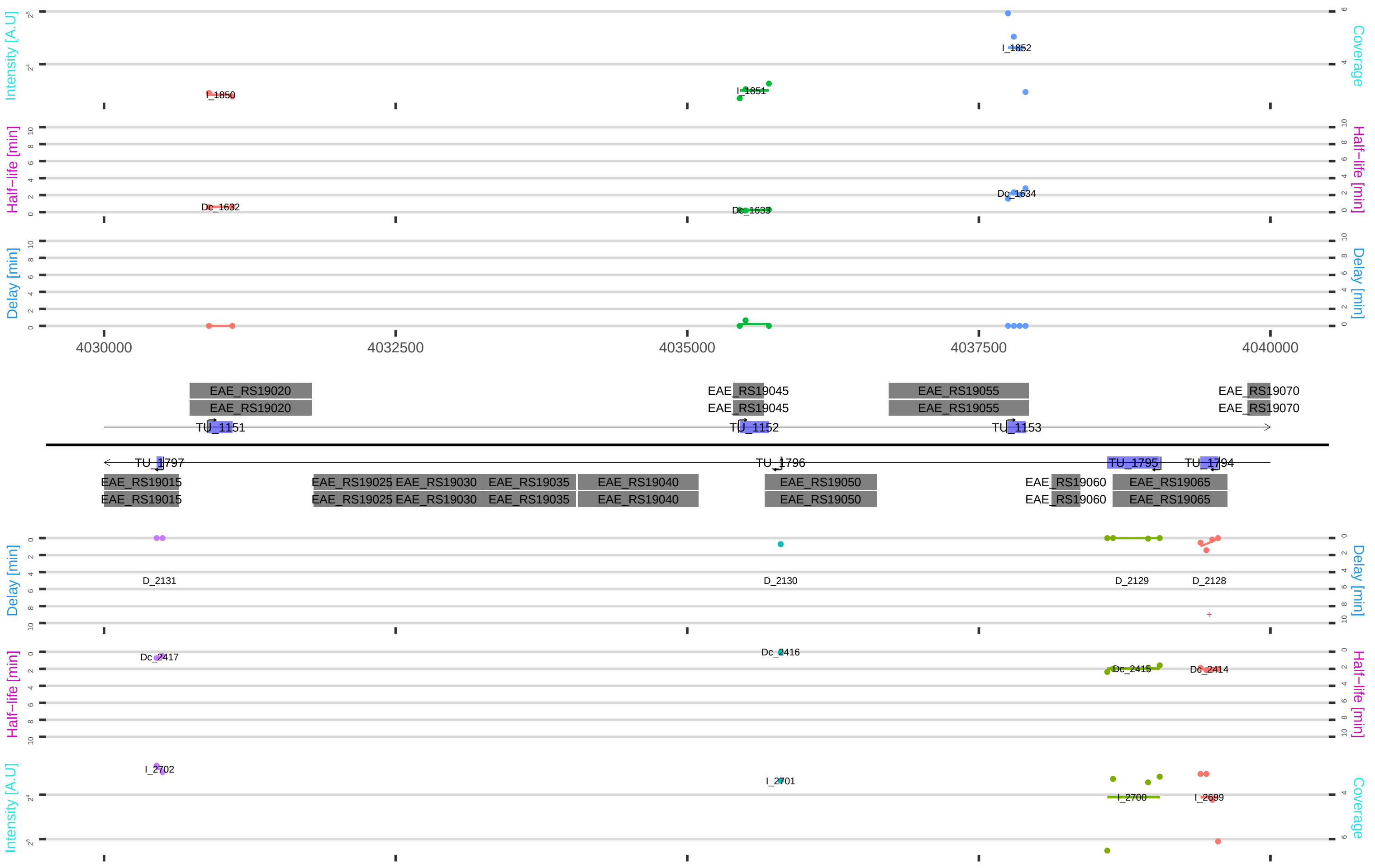

Term: termination (0), NS: new start (0), PS: pausing site (0), iTSS_I: internal starting site (0)

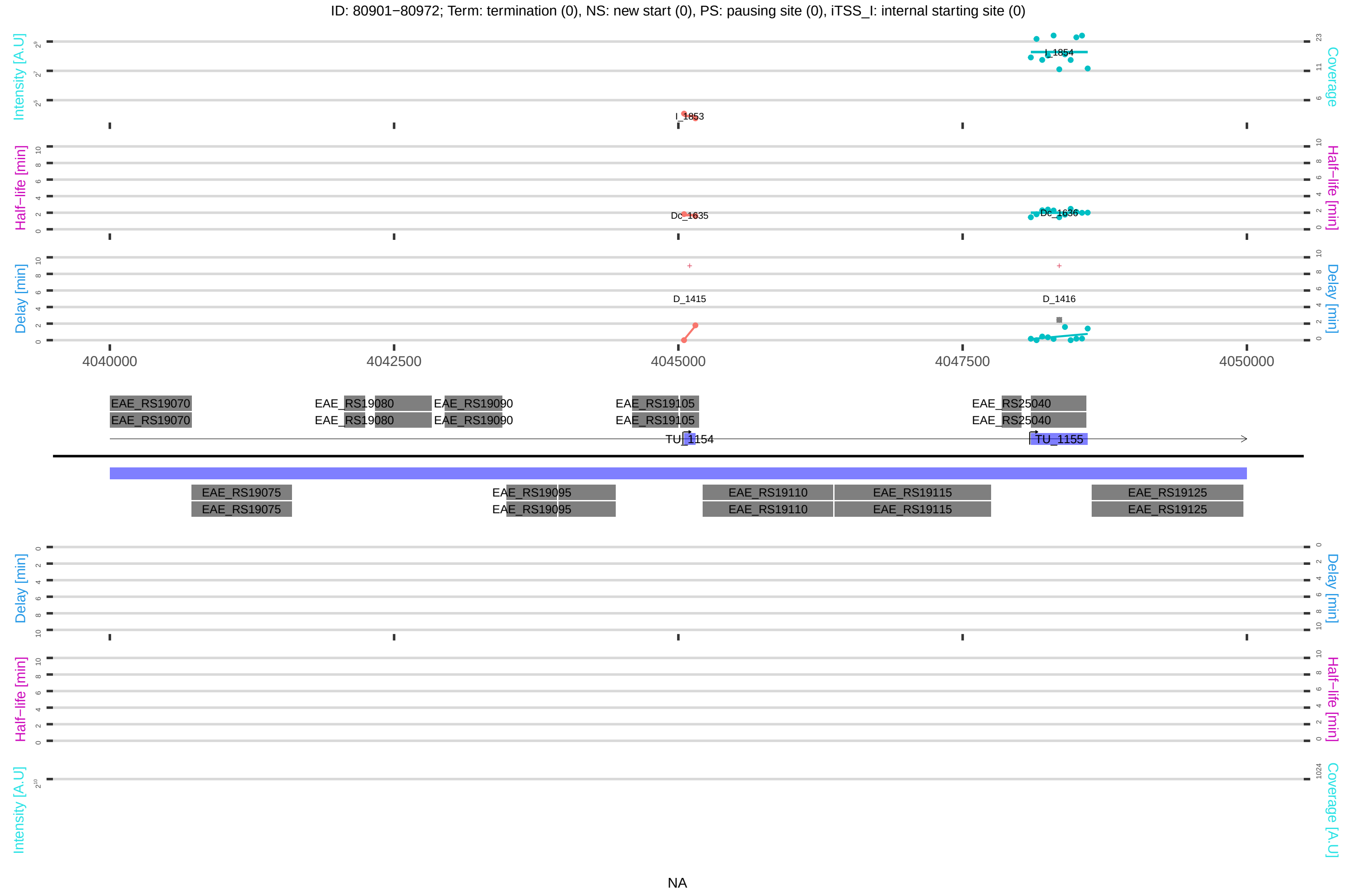

ID: 80901−80972; Term: termination (0), NS: new start (0), PS: pausing site (0), iTSS_I: internal starting site (0)
Intensity [A.U]
Coverage
I_1854
I_1853
Half−life [min]
Dc_1635
Dc_1636
Delay [min]
D_1415
D_1416
4040000
4042500
4045000
4047500
4050000
EAE_RS19070
EAE_RS19080
EAE_RS19090
EAE_RS19105
EAE_RS25040
TU_1154
TU_1155
EAE_RS19075
EAE_RS19095
EAE_RS19110
EAE_RS19115
EAE_RS19125
Coverage [A.U]
NA

ID: 81029−81031; Term: termination (0), NS: new start (0), PS: pausing site (0), iTSS_I: internal starting site (0)

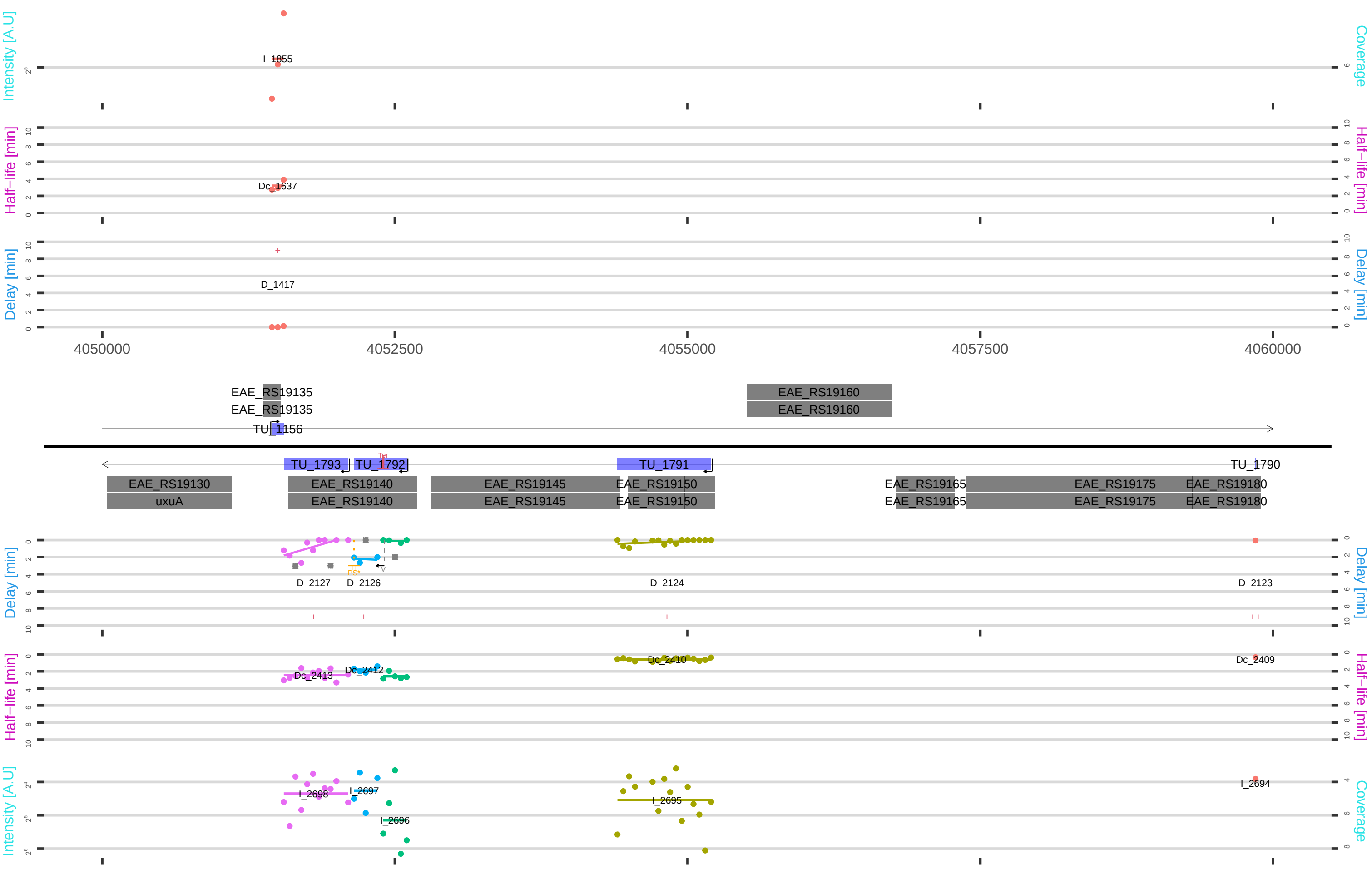

Term: termination (1), NS: new start (0), PS: pausing site (1), iTSS_I: internal starting site (0)

ID: 81268−81268; Term: termination (0), NS: new start (0), PS: pausing site (0), iTSS_I: internal starting site (0)

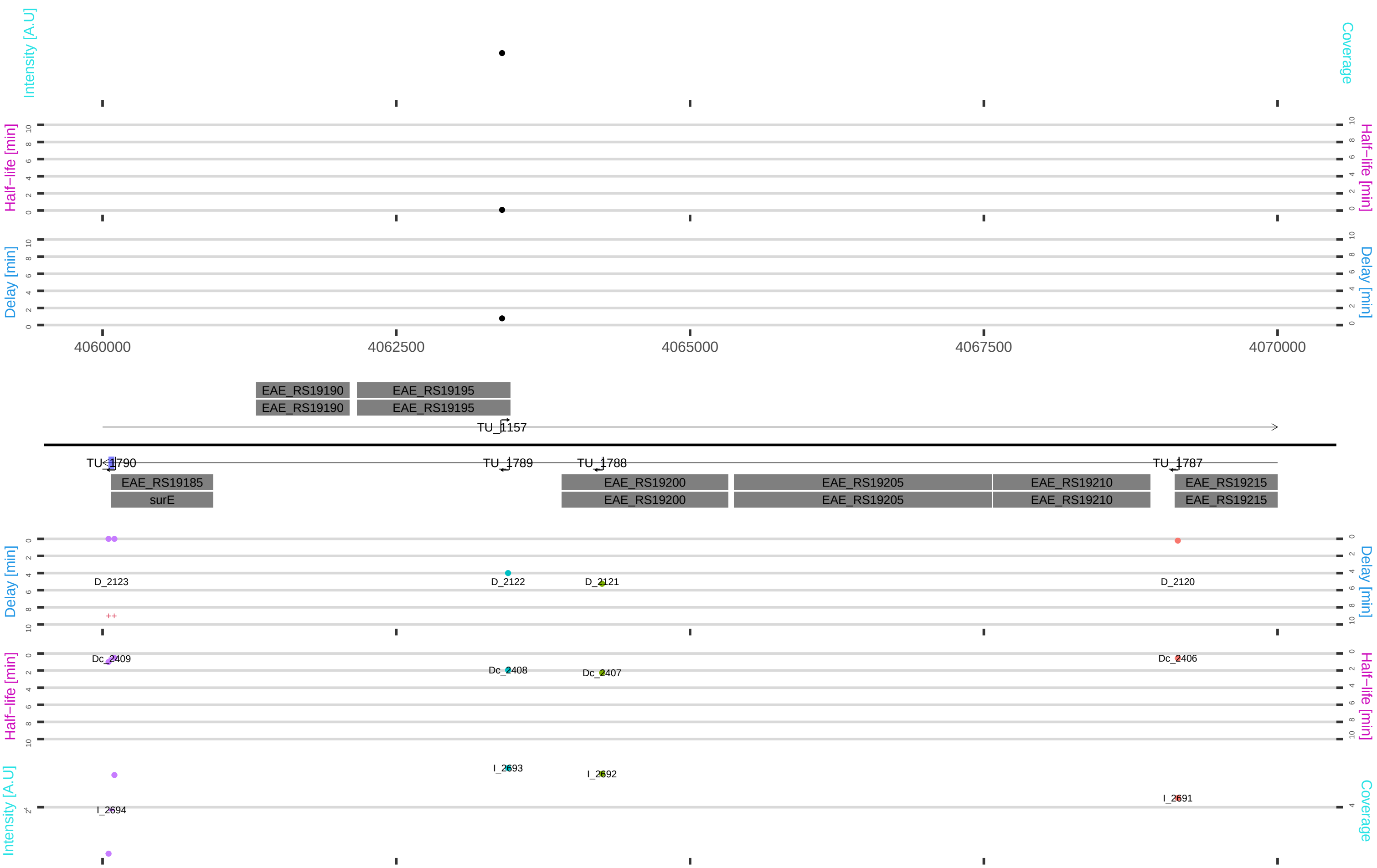

Term: termination (0), NS: new start (0), PS: pausing site (0), iTSS_I: internal starting site (0)

ID: 81465−81576; Term: termination (0), NS: new start (0), PS: pausing site (0), iTSS_I: internal starting site (0)

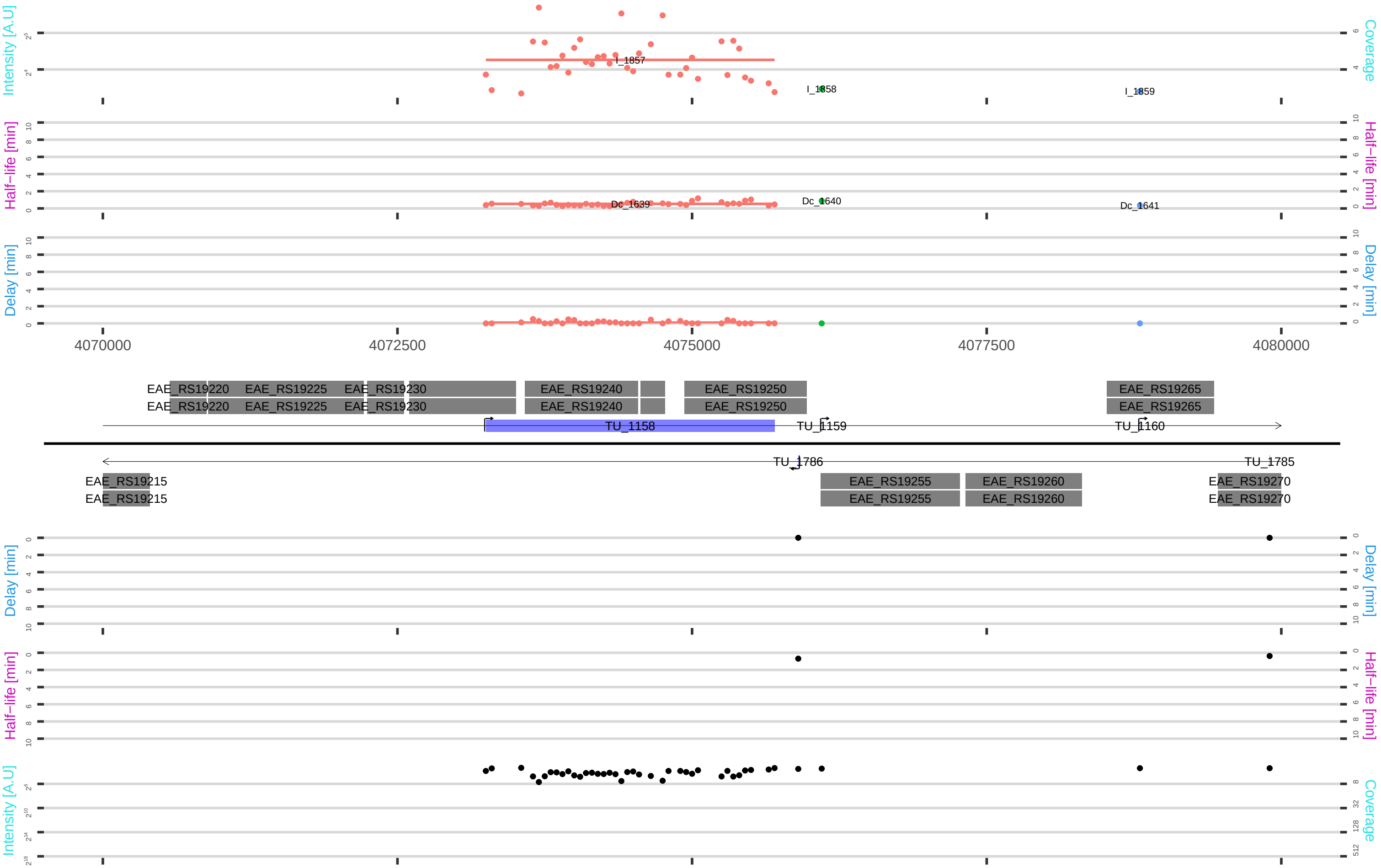

Term: termination (0), NS: new start (0), PS: pausing site (0), iTSS_I: internal starting site (0)

ID: 81737−81779; Term: termination (0), NS: new start (0), PS: pausing site (0), iTSS_I: internal starting site (0)

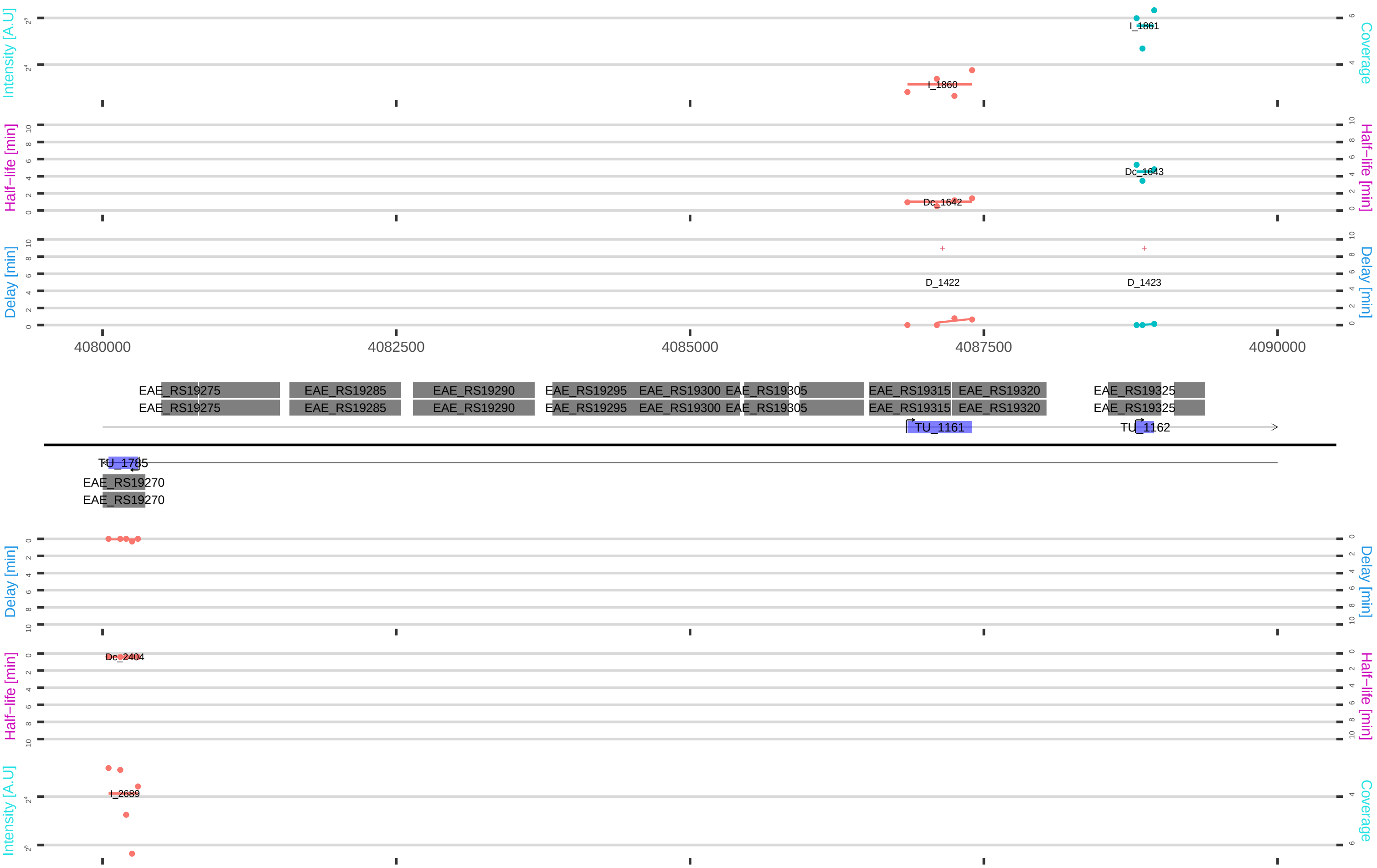

Term: termination (0), NS: new start (0), PS: pausing site (0), iTSS_I: internal starting site (0)

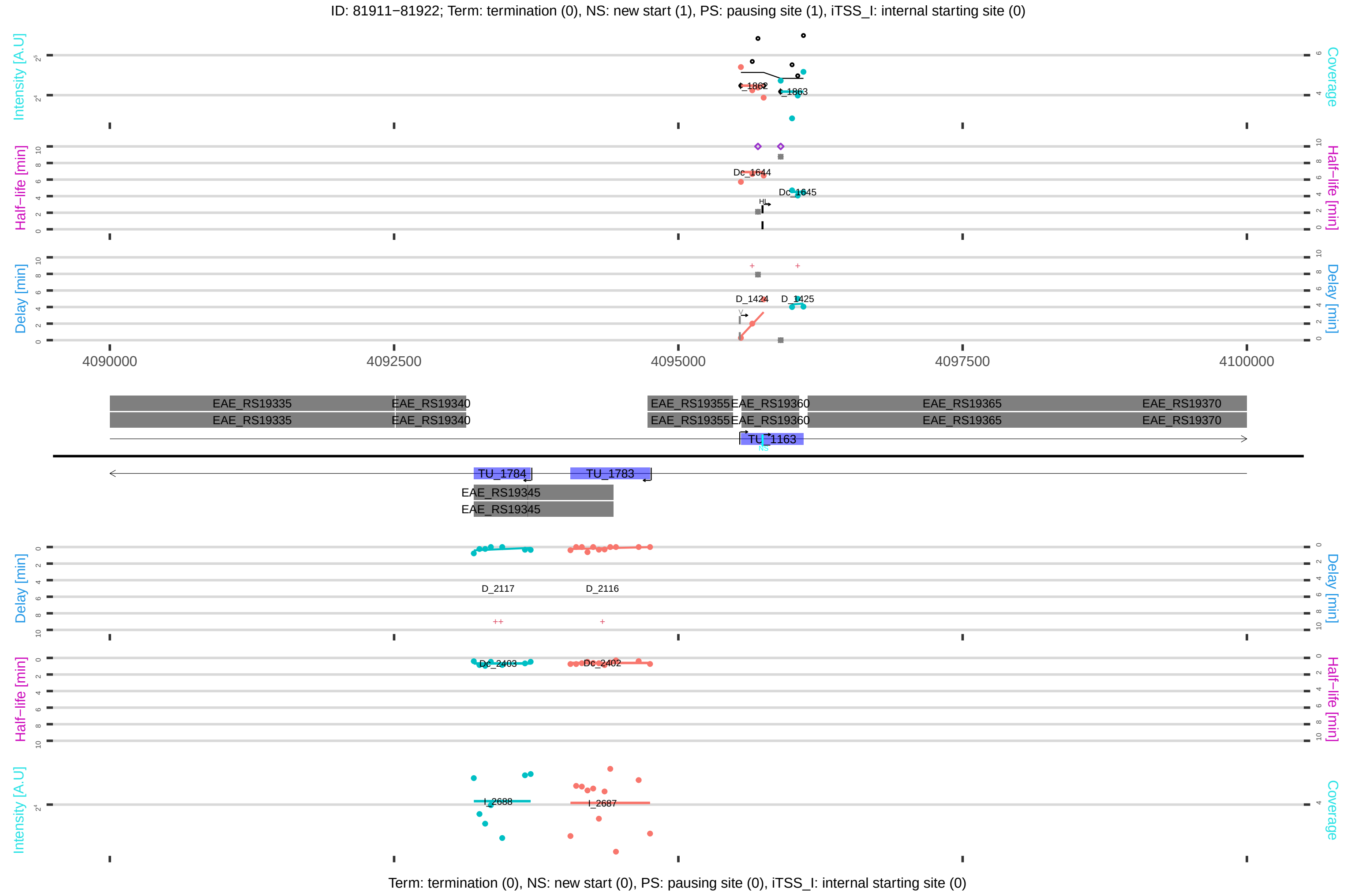

ID: 81911−81922; Term: termination (0), NS: new start (1), PS: pausing site (1), iTSS_I: internal starting site (0)
Intensity [A.U]
Coverage
I_1862
I_1863
Half−life [min]
Dc_1644
Dc_1645
HL
Delay [min]
D_1424
D_1425
4090000
4092500
4095000
4097500
4100000
EAE_RS19335
EAE_RS19340
EAE_RS19355
EAE_RS19360
EAE_RS19365
EAE_RS19370
TU_1163
NS
TU_1784
TU_1783
EAE_RS19345
D_2117
D_2116
Dc_2403
Dc_2402
I_2688
I_2687
Term: termination (0), NS: new start (0), PS: pausing site (0), iTSS_I: internal starting site (0)

ID: 129187−129029; FC*: significant t−test of two consecutive segments; Term: termination, NS: new start, PS: pausing site,iTSS_I: internal starting site,TI: transcription interferance.

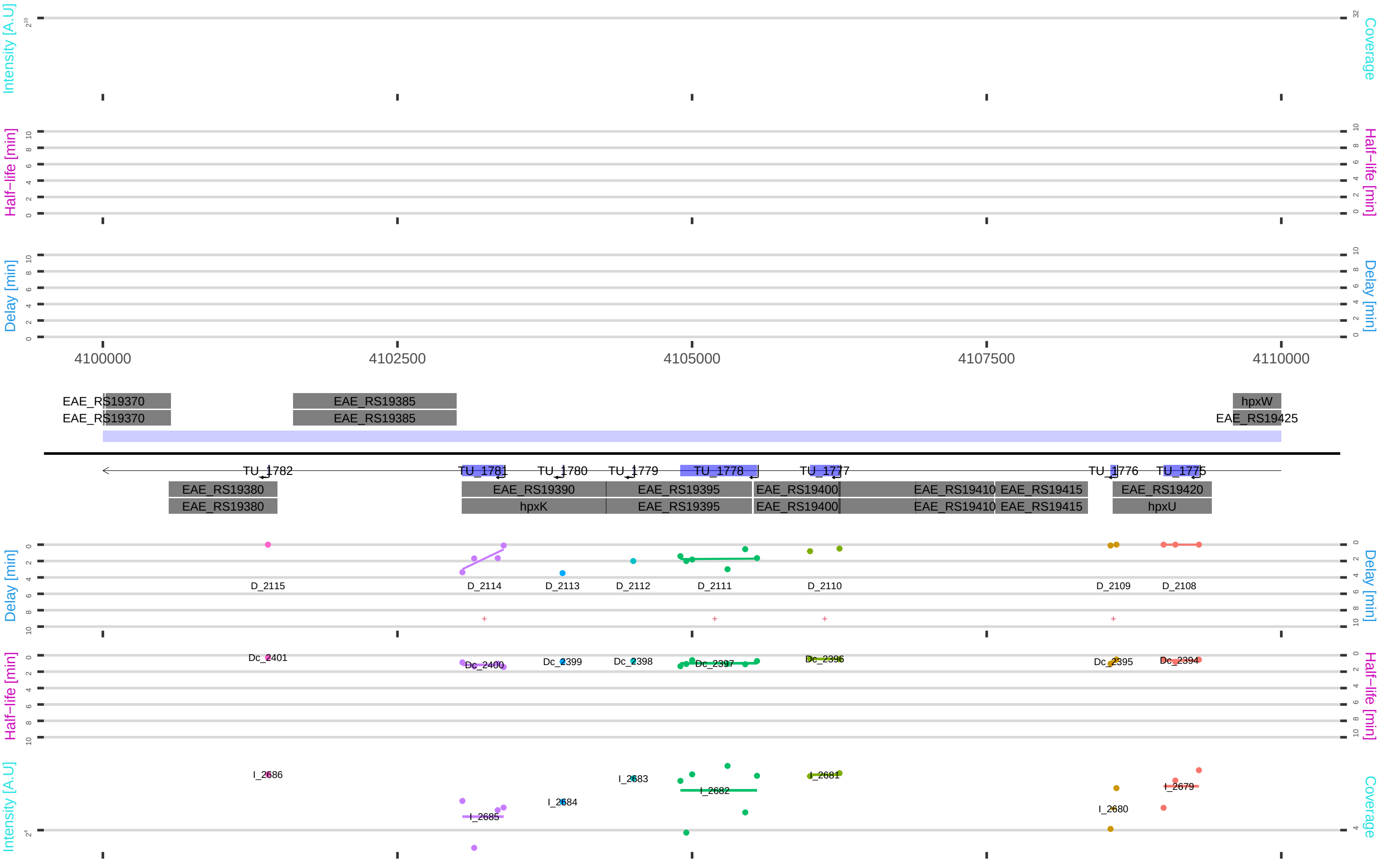

Term: termination (0), NS: new start (0), PS: pausing site (0), iTSS_I: internal starting site (0)

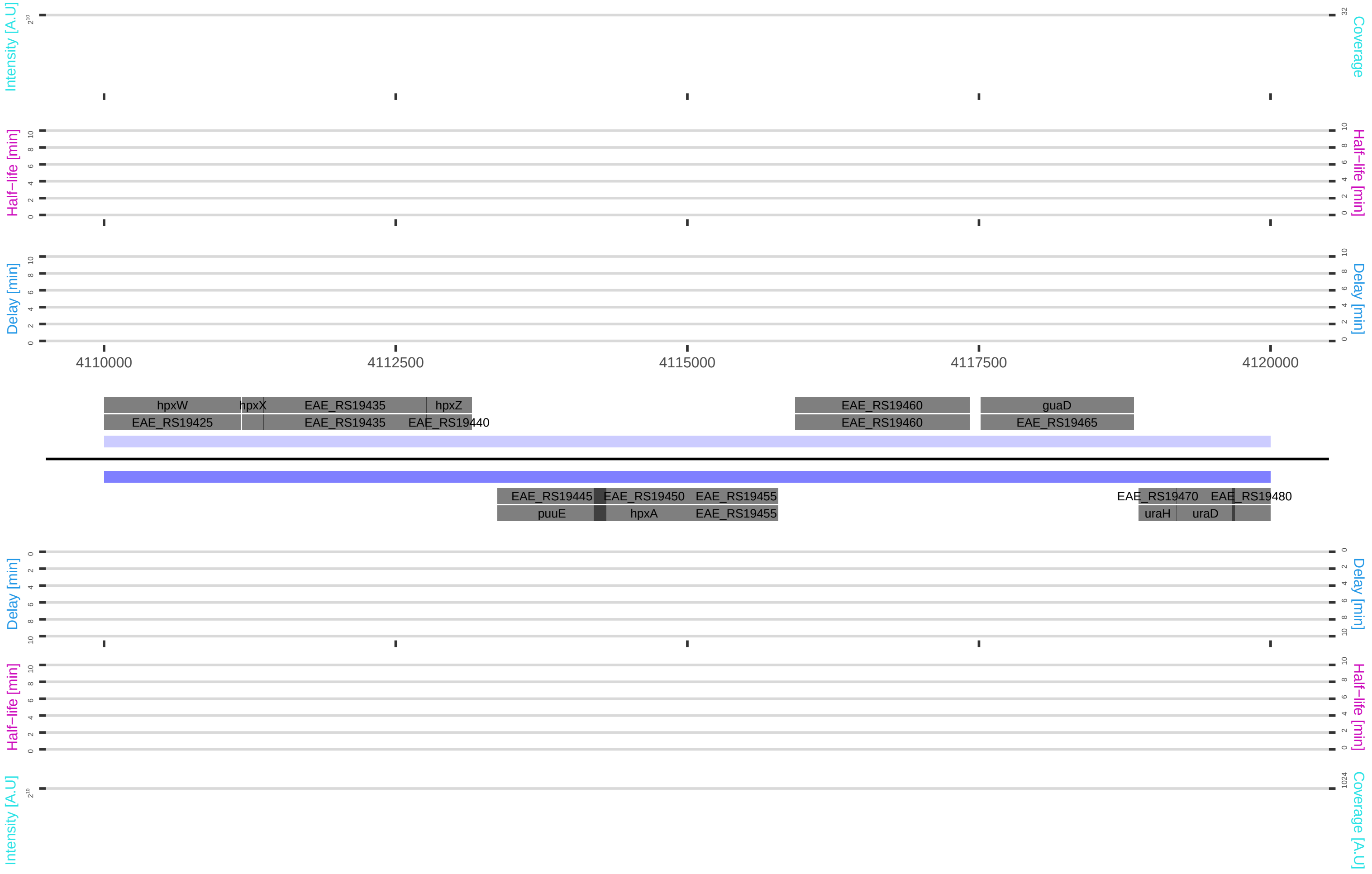
Intensity [A.U]
Coverage
Half−life [min]
Delay [min]
4110000
4112500
4115000
4117500
4120000
hpxW
hpxX
EAE_RS19435
hpxZ
EAE_RS19425
EAE_RS19440
EAE_RS19460
guaD
EAE_RS19465
EAE_RS19445
EAE_RS19450
EAE_RS19455
puuE
hpxA
EAE_RS19470
EAE_RS19480
uraH
uraD
Coverage [A.U]

NA

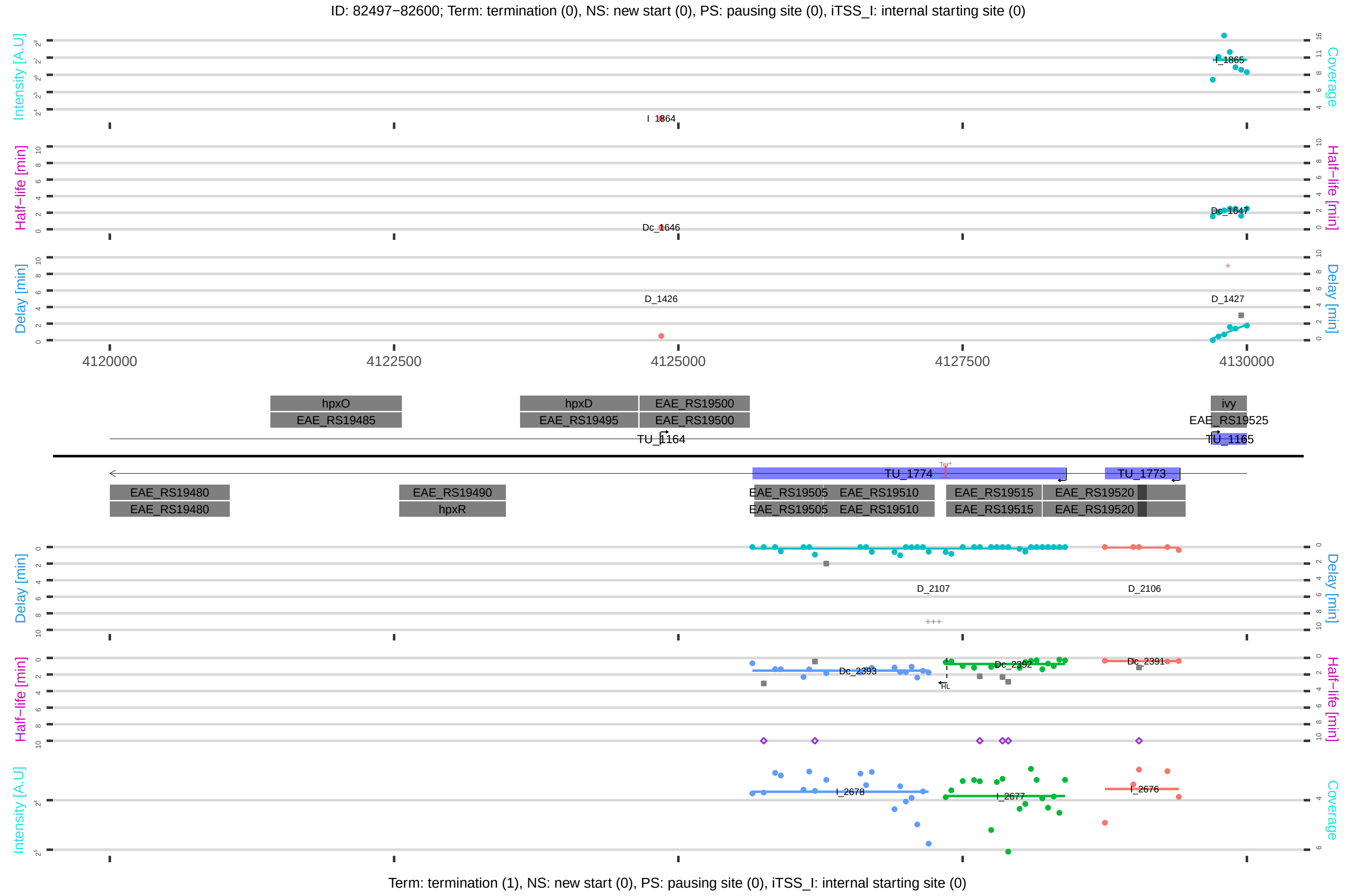

ID: 82497−82600; Term: termination (0), NS: new start (0), PS: pausing site (0), iTSS_I: internal starting site (0)
Intensity [A.U]
Coverage
Half−life [min]
Delay [min]
I_1865
I_1864
Dc_1647
Dc_1646
D_1426
D_1427
4120000
4122500
4125000
4127500
4130000
hpxO
EAE_RS19485
hpxD
EAE_RS19495
EAE_RS19500
ivy
EAE_RS19525
TU_1164
TU_1165
TU_1774
TU_1773
EAE_RS19480
EAE_RS19490
hpxR
EAE_RS19505
EAE_RS19510
EAE_RS19515
EAE_RS19520
D_2107
D_2106
Dc_2393
Dc_2392
Dc_2391
HL
I_2678
I_2677
I_2676
Term: termination (1), NS: new start (0), PS: pausing site (0), iTSS_I: internal starting site (0)

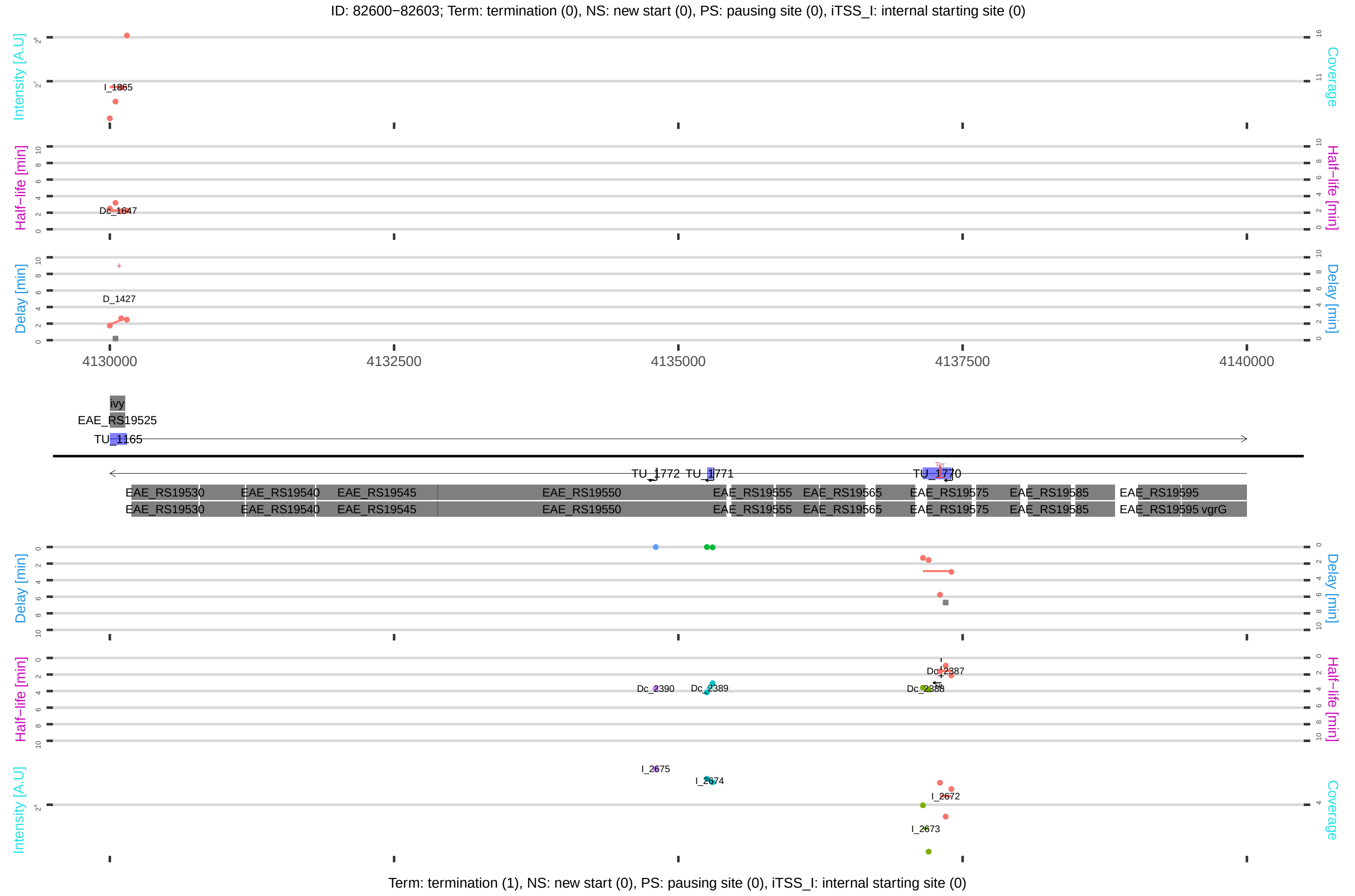
ID: 82600−82603; Term: termination (0), NS: new start (0), PS: pausing site (0), iTSS_I: internal starting site (0)
Intensity [A.U]
Coverage
I_1865
Half−life [min]
Dc_1647
Delay [min]
D_1427
4130000
4132500
4135000
4137500
4140000
ivy
EAE_RS19525
TU_1165
TU_1772
TU_1771
TU_1770
EAE_RS19530
EAE_RS19540
EAE_RS19545
EAE_RS19550
EAE_RS19555
EAE_RS19565
EAE_RS19575
EAE_RS19585
EAE_RS19595
EAE_RS19595 vgrG
Dc_2387
Dc_2390
Dc_2389
Dc_2388
I_2675
I_2674
I_2672
I_2673
Term: termination (1), NS: new start (0), PS: pausing site (0), iTSS_I: internal starting site (0)

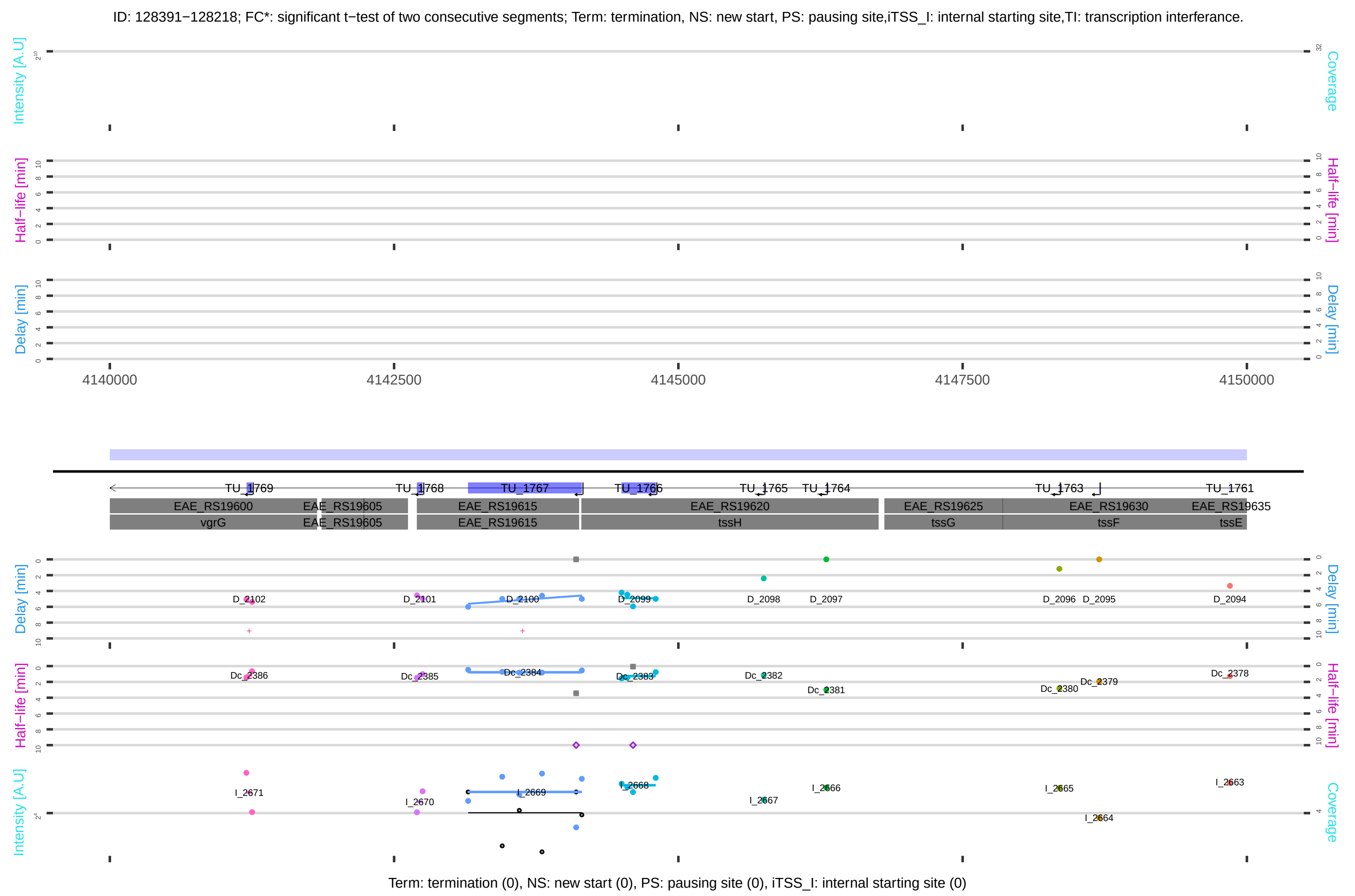
ID: 128391−128218; FC*: significant t−test of two consecutive segments; Term: termination, NS: new start, PS: pausing site,iTSS_I: internal starting site,TI: transcription interferance.
Intensity [A.U]
Coverage
Half−life [min]
Delay [min]
4140000
4142500
4145000
4147500
4150000
TU_1769
TU_1768
TU_1767
TU_1766
TU_1765
TU_1764
TU_1763
TU_1761
EAE_RS19600
EAE_RS19605
EAE_RS19615
EAE_RS19620
EAE_RS19625
EAE_RS19630
EAE_RS19635
vgrG
tssH
tssG
tssF
tssE
D_2102
D_2101
D_2100
D_2099
D_2098
D_2097
D_2096
D_2095
D_2094
Dc_2386
Dc_2385
Dc_2384
Dc_2383
Dc_2382
Dc_2381
Dc_2380
Dc_2379
Dc_2378
I_2671
I_2670
I_2669
I_2668
I_2667
I_2666
I_2665
I_2664
I_2663
Term: termination (0), NS: new start (0), PS: pausing site (0), iTSS_I: internal starting site (0)

ID: 128214−128026; FC*: significant t−test of two consecutive segments; Term: termination, NS: new start, PS: pausing site,iTSS_I: internal starting site,TI: transcription interferance.

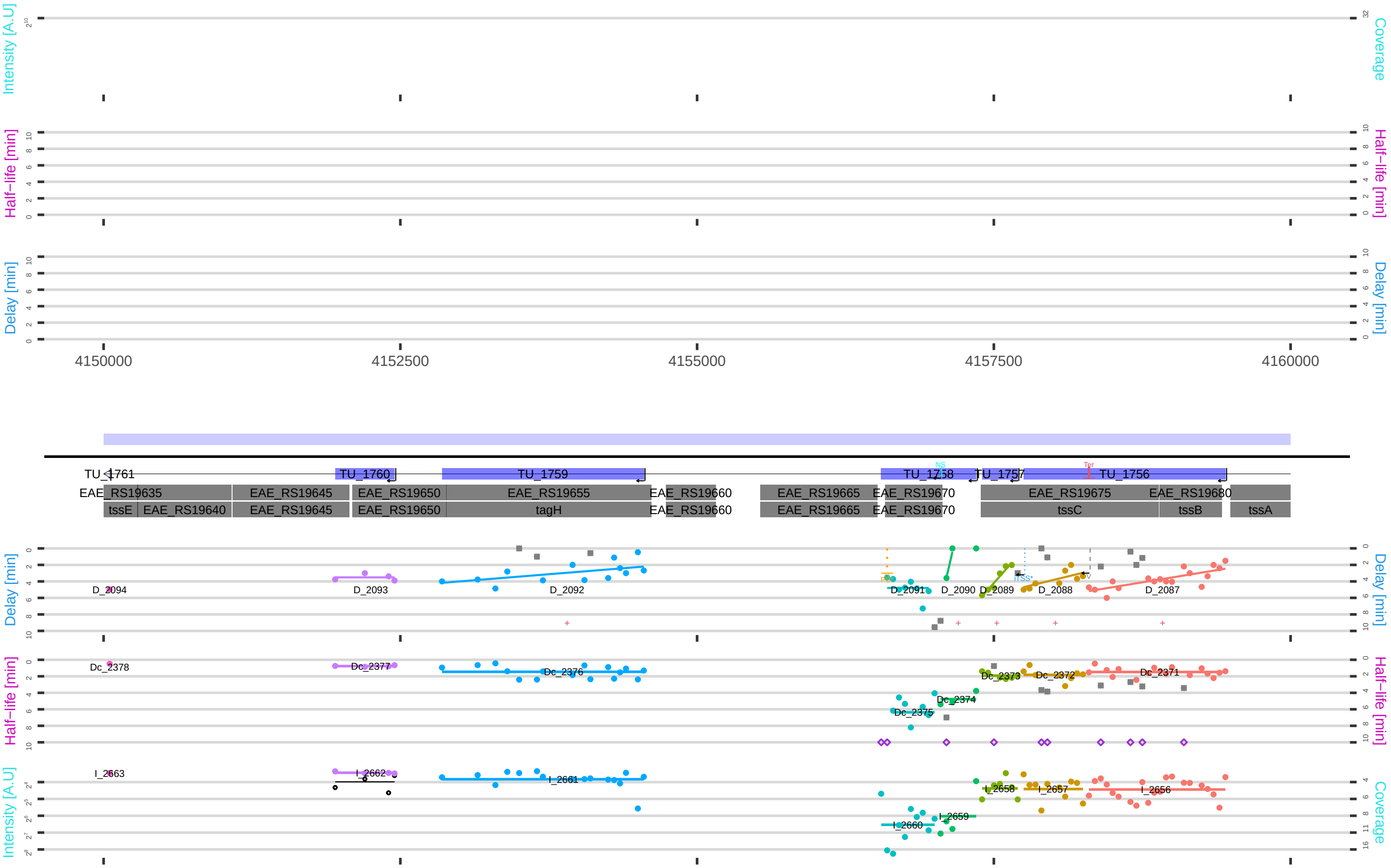

Term: termination (1), NS: new start (1), PS: pausing site (1), iTSS_I: internal starting site (1)

ID: 83396−83400; Term: termination (0), NS: new start (0), PS: pausing site (0), iTSS_I: internal starting site (0)

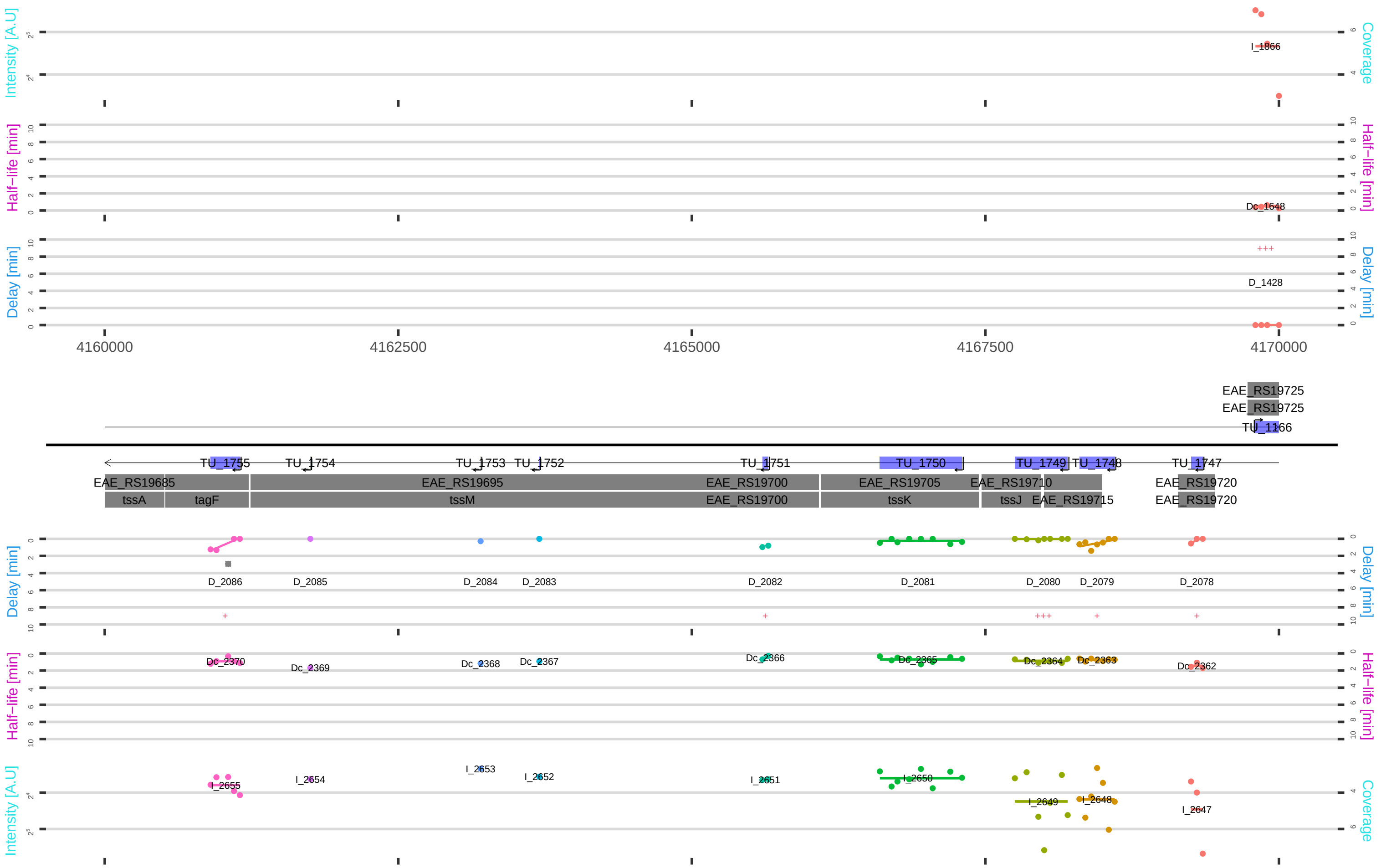

Term: termination (0), NS: new start (0), PS: pausing site (0), iTSS_I: internal starting site (0)

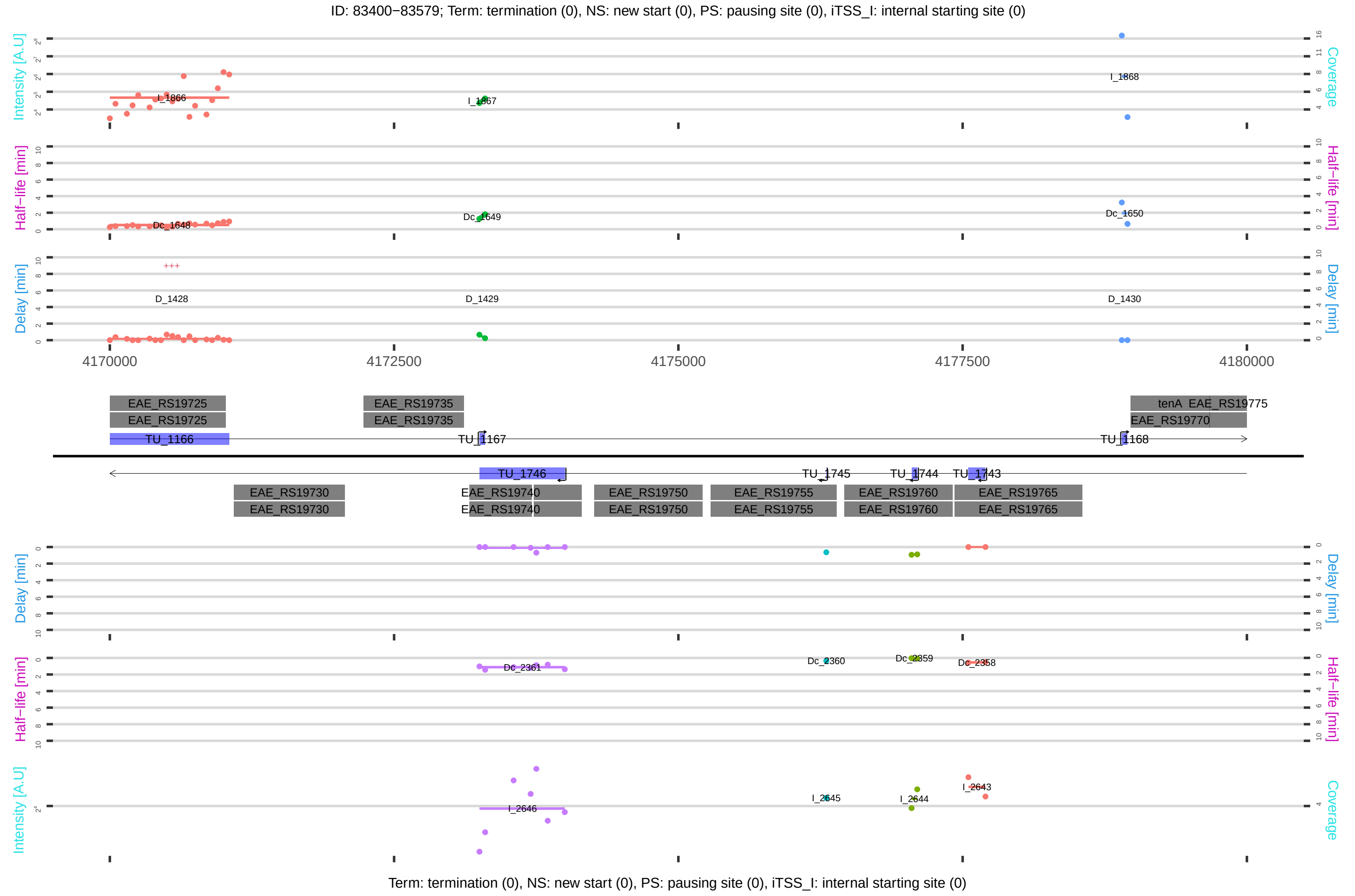

ID: 83400−83579; Term: termination (0), NS: new start (0), PS: pausing site (0), iTSS_I: internal starting site (0)
Intensity [A.U]
Coverage
Half−life [min]
Delay [min]
I_1866
I_1867
I_1868
Dc_1648
Dc_1649
Dc_1650
D_1428
D_1429
D_1430
4170000
4172500
4175000
4177500
4180000
EAE_RS19725
EAE_RS19735
tenA EAE_RS19775
EAE_RS19770
TU_1166
TU_1167
TU_1168
TU_1746
TU_1745
TU_1744
TU_1743
EAE_RS19730
EAE_RS19740
EAE_RS19750
EAE_RS19755
EAE_RS19760
EAE_RS19765
Dc_2361
Dc_2360
Dc_2359
Dc_2358
I_2646
I_2645
I_2644
I_2643
Term: termination (0), NS: new start (0), PS: pausing site (0), iTSS_I: internal starting site (0)

ID: 83649−83706; Term: termination (0), NS: new start (0), PS: pausing site (0), iTSS_I: internal starting site (0)

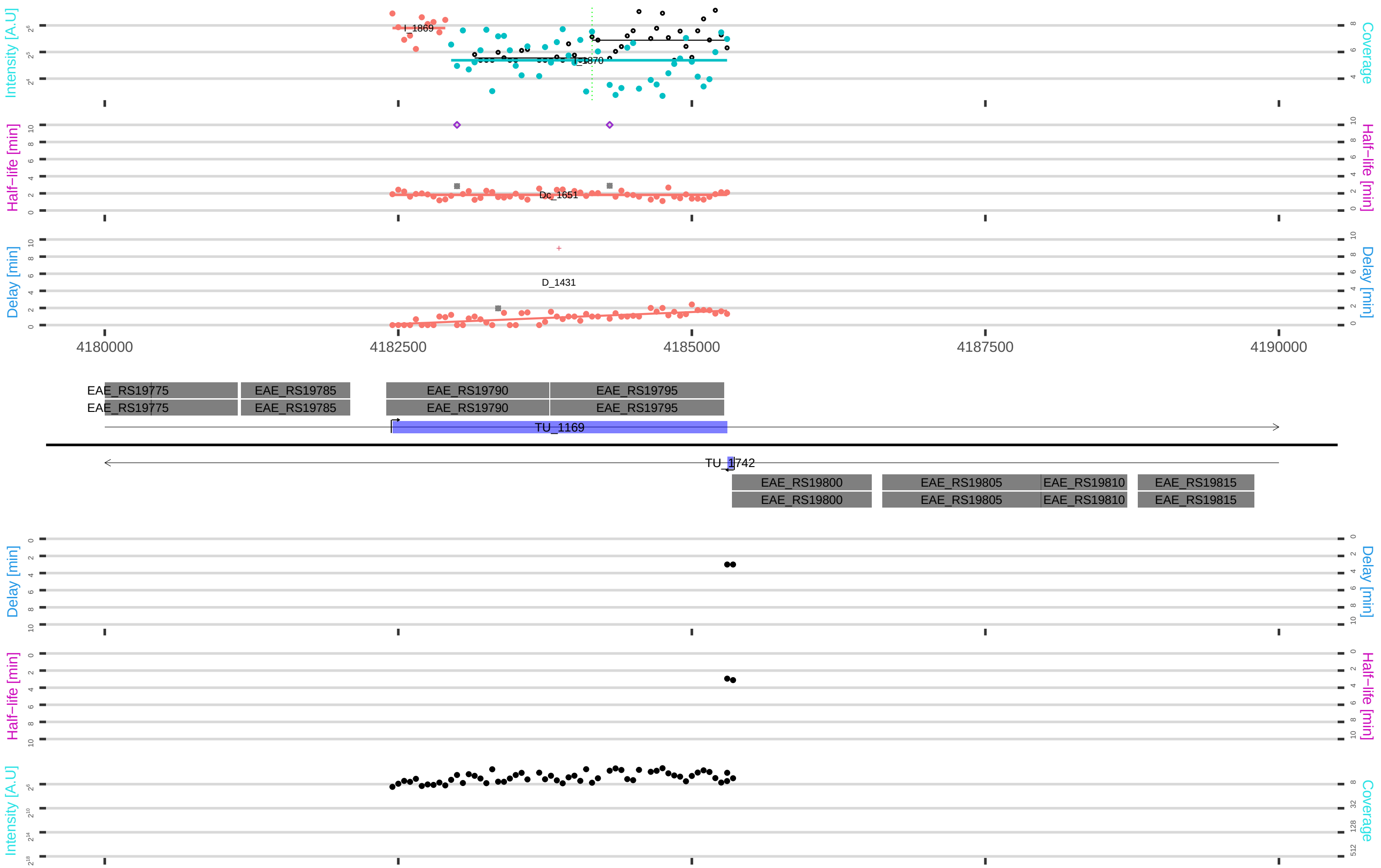

Term: termination (0), NS: new start (0), PS: pausing site (0), iTSS_I: internal starting site (0)

ID: 83823−83880; Term: termination (0), NS: new start (0), PS: pausing site (0), iTSS_I: internal starting site (0)

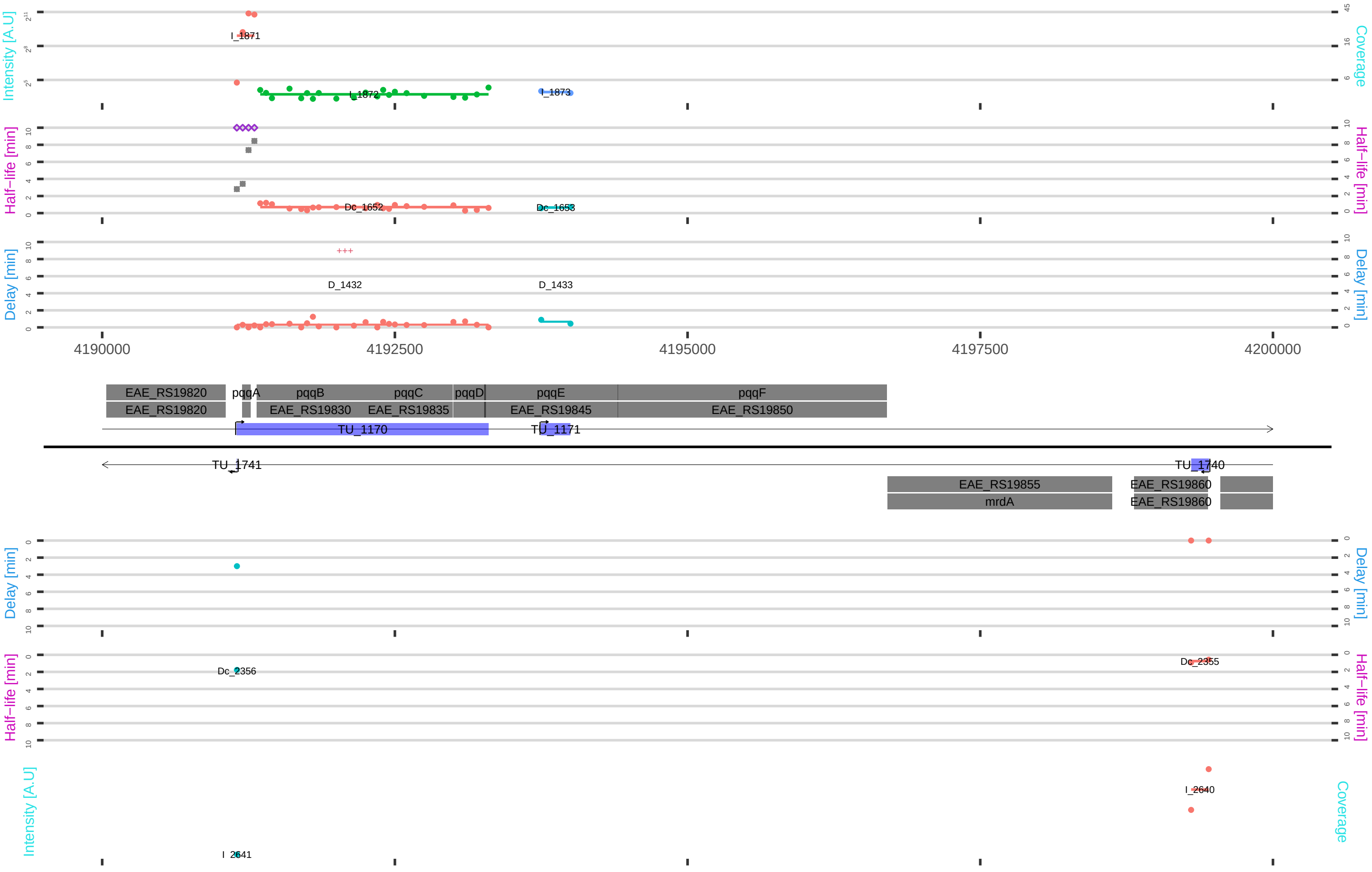

Term: termination (0), NS: new start (0), PS: pausing site (0), iTSS_I: internal starting site (0)

ID: 84035−84135; Term: termination (3), NS: new start (3), PS: pausing site (4), iTSS_I: internal starting site (0)

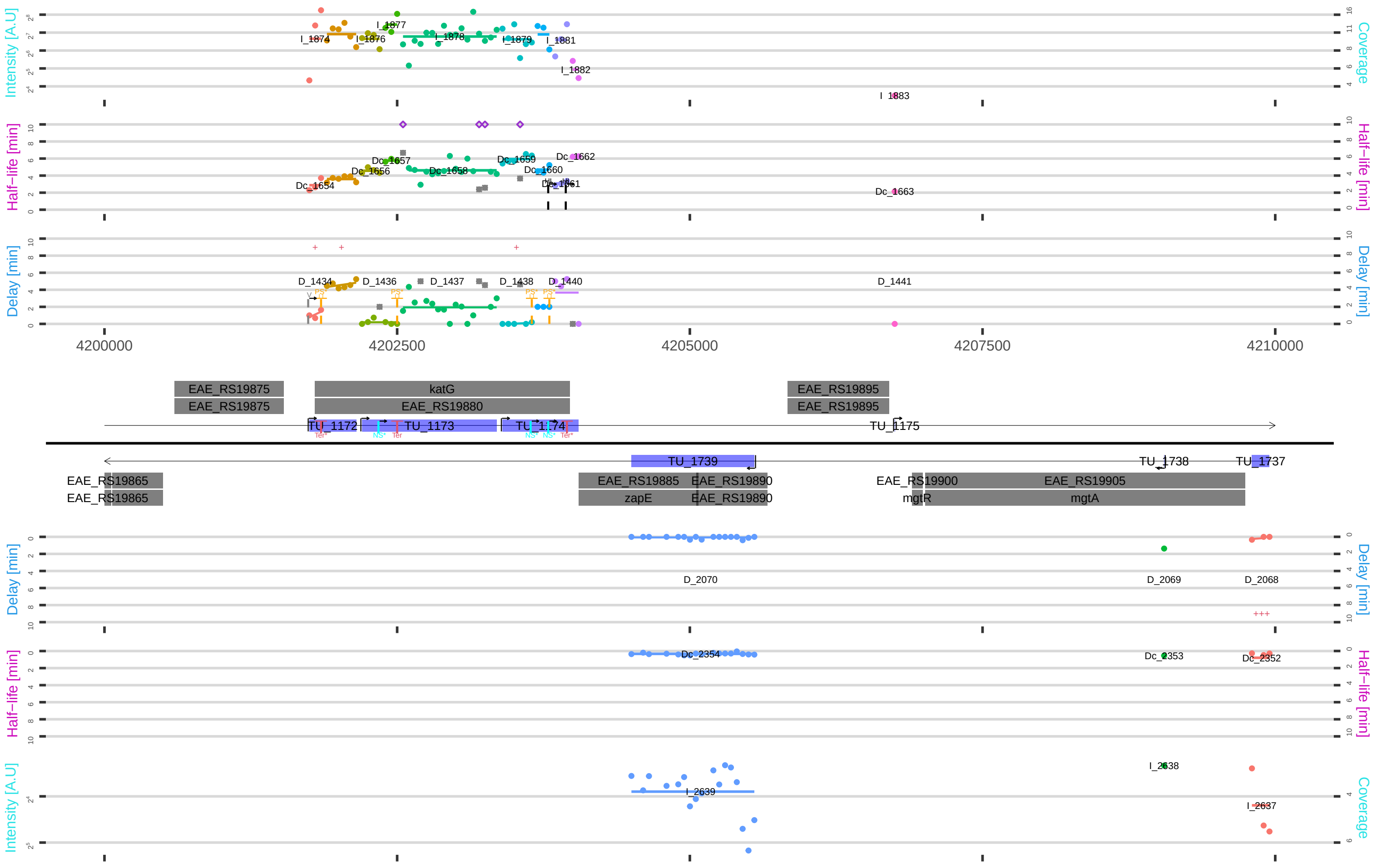

Term: termination (0), NS: new start (0), PS: pausing site (0), iTSS_I: internal starting site (0)

ID: 84368−84400; Term: termination (0), NS: new start (0), PS: pausing site (0), iTSS_I: internal starting site (0)

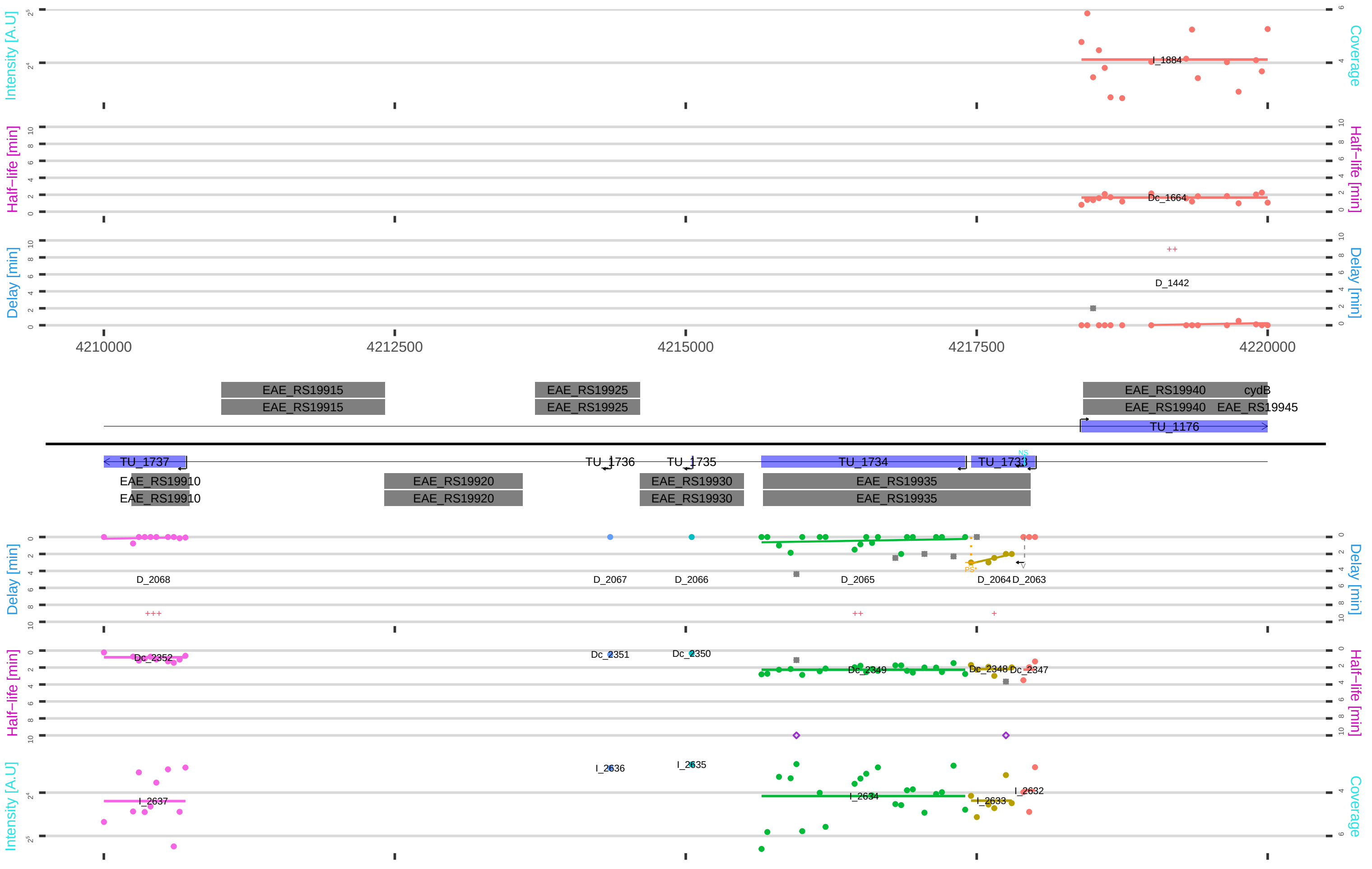

Term: termination (0), NS: new start (1), PS: pausing site (1), iTSS_I: internal starting site (0)

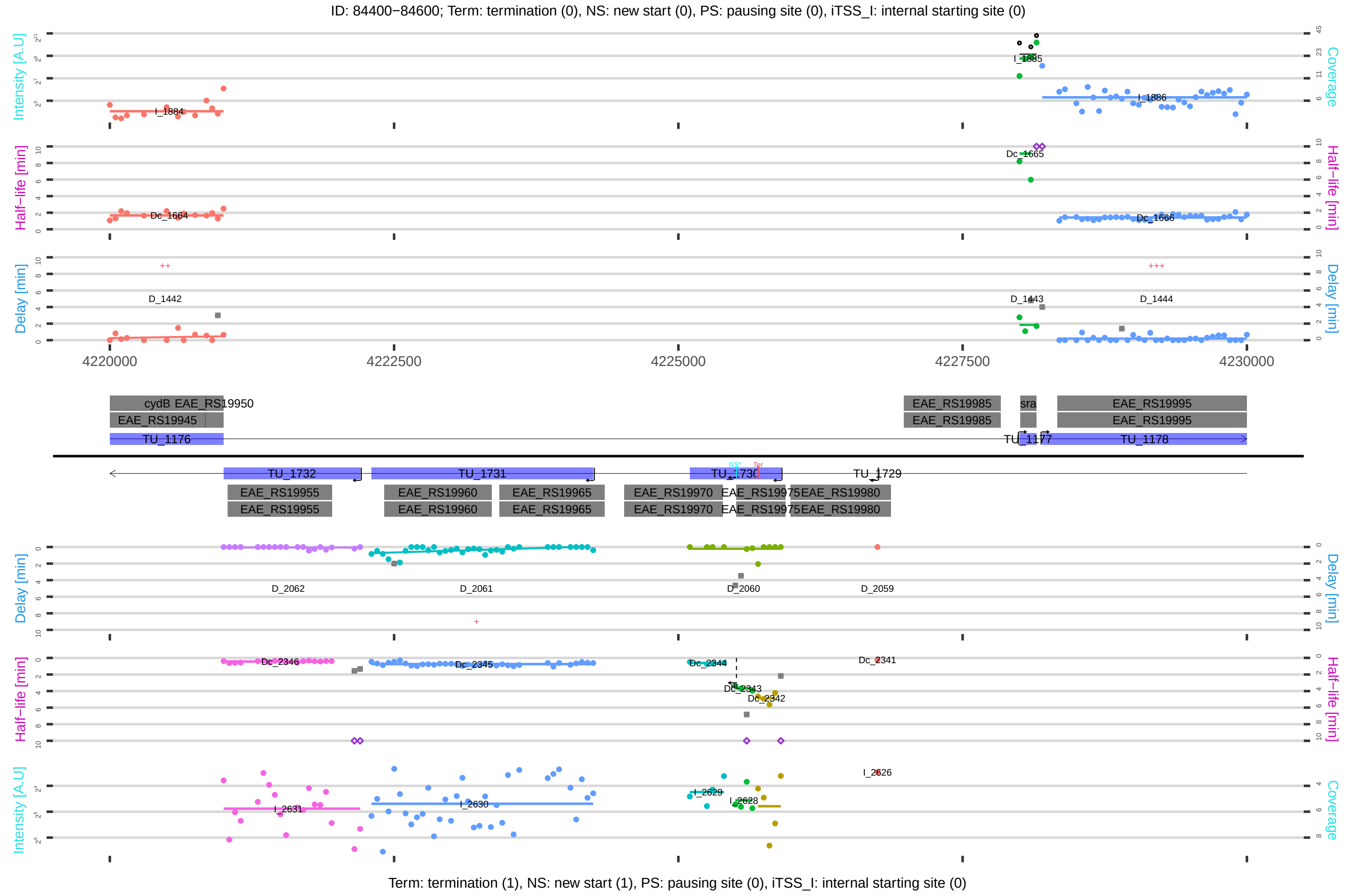

ID: 84400−84600; Term: termination (0), NS: new start (0), PS: pausing site (0), iTSS_I: internal starting site (0)
Intensity [A.U]
Coverage
Half−life [min]
Delay [min]
4220000
4222500
4225000
4227500
4230000
cydB EAE_RS19950
EAE_RS19945
EAE_RS19985
sra
EAE_RS19995
TU_1176
TU_1177
TU_1178
TU_1732
TU_1731
TU_1730
TU_1729
EAE_RS19955
EAE_RS19960
EAE_RS19965
EAE_RS19970
EAE_RS19975
EAE_RS19980
Term: termination (1), NS: new start (1), PS: pausing site (0), iTSS_I: internal starting site (0)

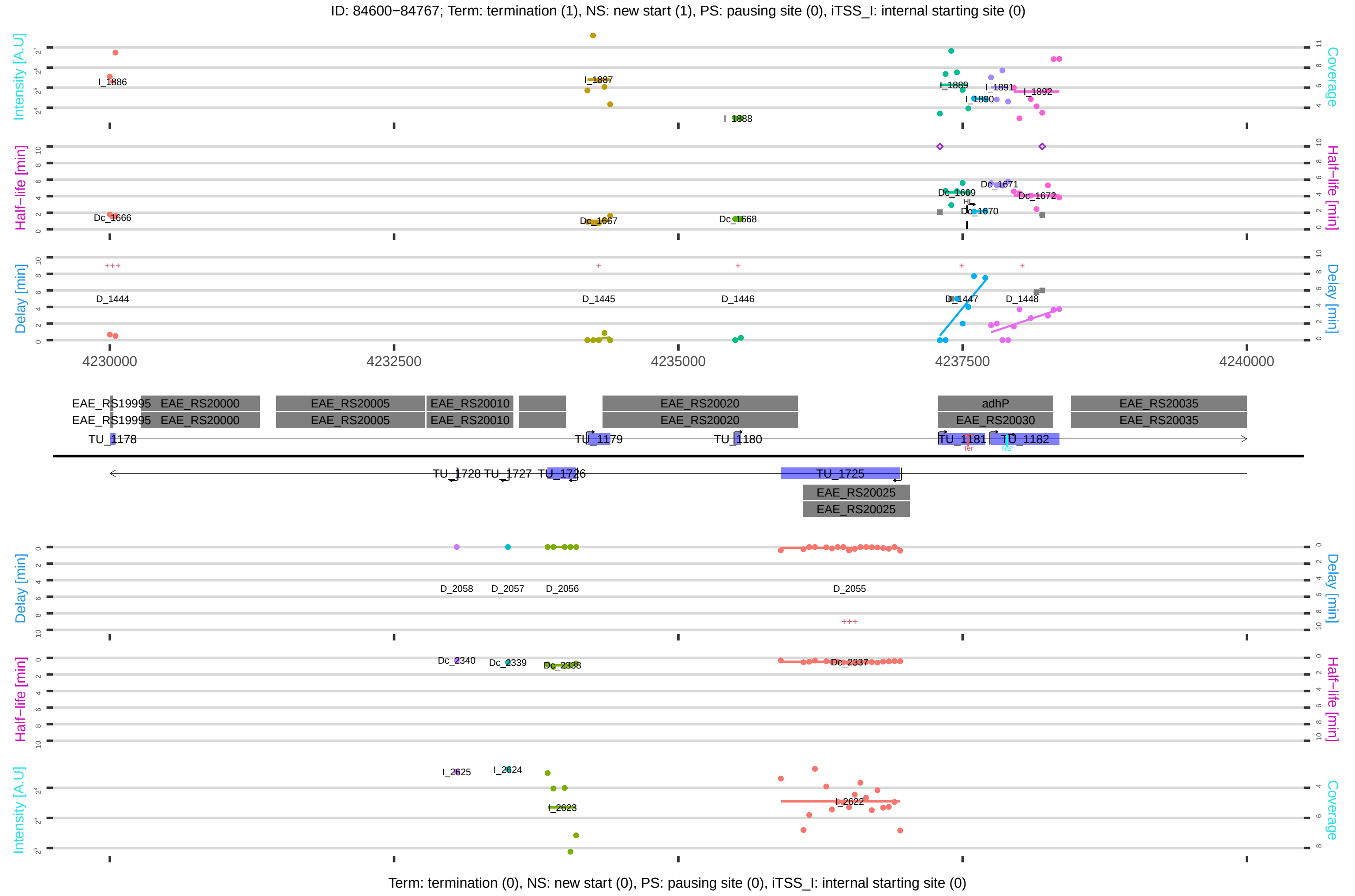

ID: 84600−84767; Term: termination (1), NS: new start (1), PS: pausing site (0), iTSS_I: internal starting site (0)
Intensity [A.U]
Coverage
Half−life [min]
Delay [min]
I_1886
I_1887
I_1888
I_1889
I_1890
I_1891
I_1892
Dc_1666
Dc_1667
Dc_1668
Dc_1669
Dc_1670
Dc_1671
Dc_1672
D_1444
D_1445
D_1446
D_1447
D_1448
4230000
4232500
4235000
4237500
4240000
EAE_RS19995
EAE_RS20000
EAE_RS20005
EAE_RS20010
EAE_RS20020
adhP
EAE_RS20030
EAE_RS20035
TU_1178
TU_1179
TU_1180
TU_1181
TU_1182
Ter
NS
TU_1728
TU_1727
TU_1726
TU_1725
EAE_RS20025
D_2058
D_2057
D_2056
D_2055
Dc_2340
Dc_2339
Dc_2338
Dc_2337
I_2625
I_2624
I_2623
I_2622
Term: termination (0), NS: new start (0), PS: pausing site (0), iTSS_I: internal starting site (0)

ID: 126299−126281; FC*: significant t−test of two consecutive segments; Term: termination, NS: new start, PS: pausing site,iTSS_I: internal starting site,TI: transcription interferance.

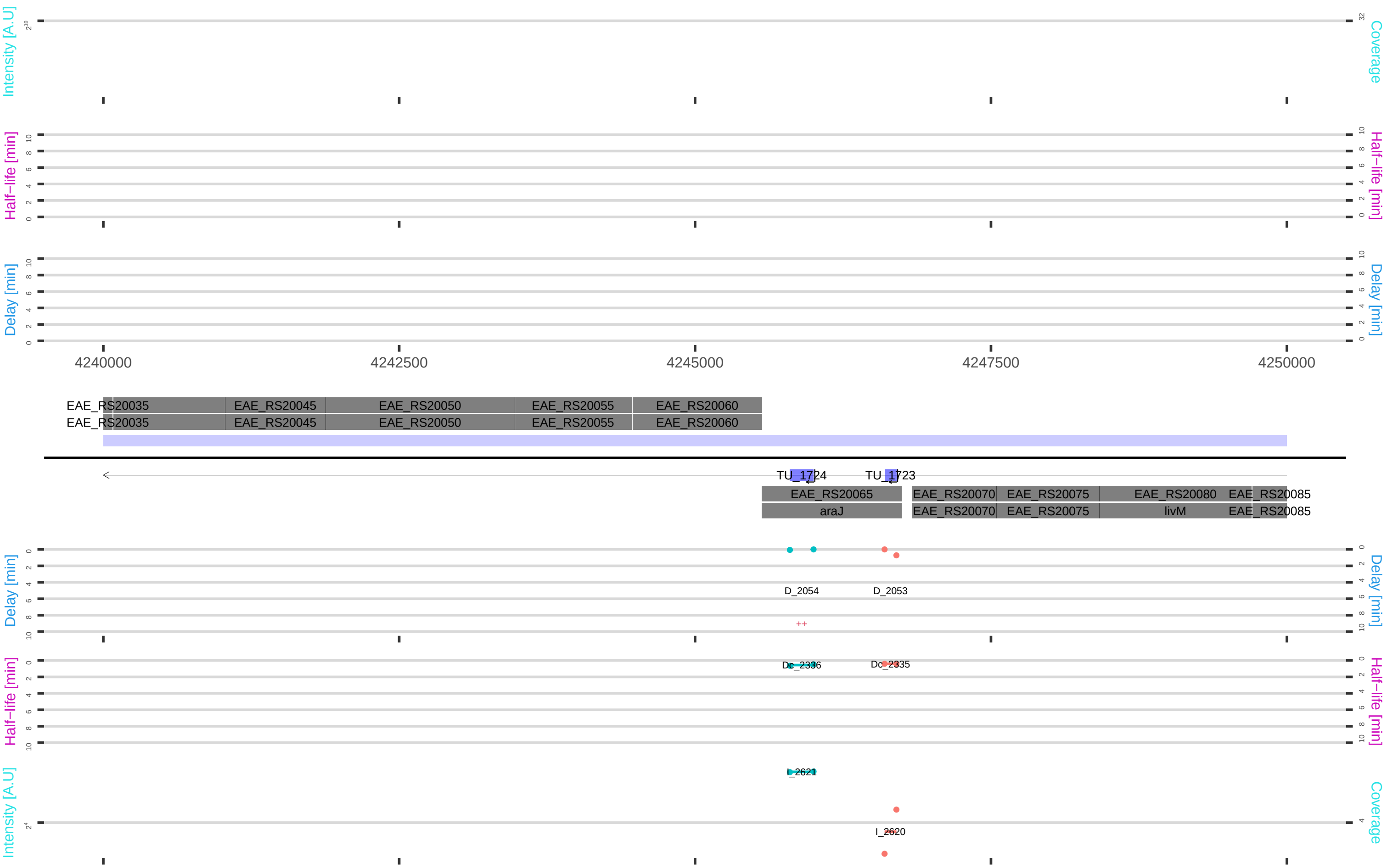

Term: termination (0), NS: new start (0), PS: pausing site (0), iTSS_I: internal starting site (0)

ID: 85049−85200; Term: termination (0), NS: new start (0), PS: pausing site (0), iTSS_I: internal starting site (0)

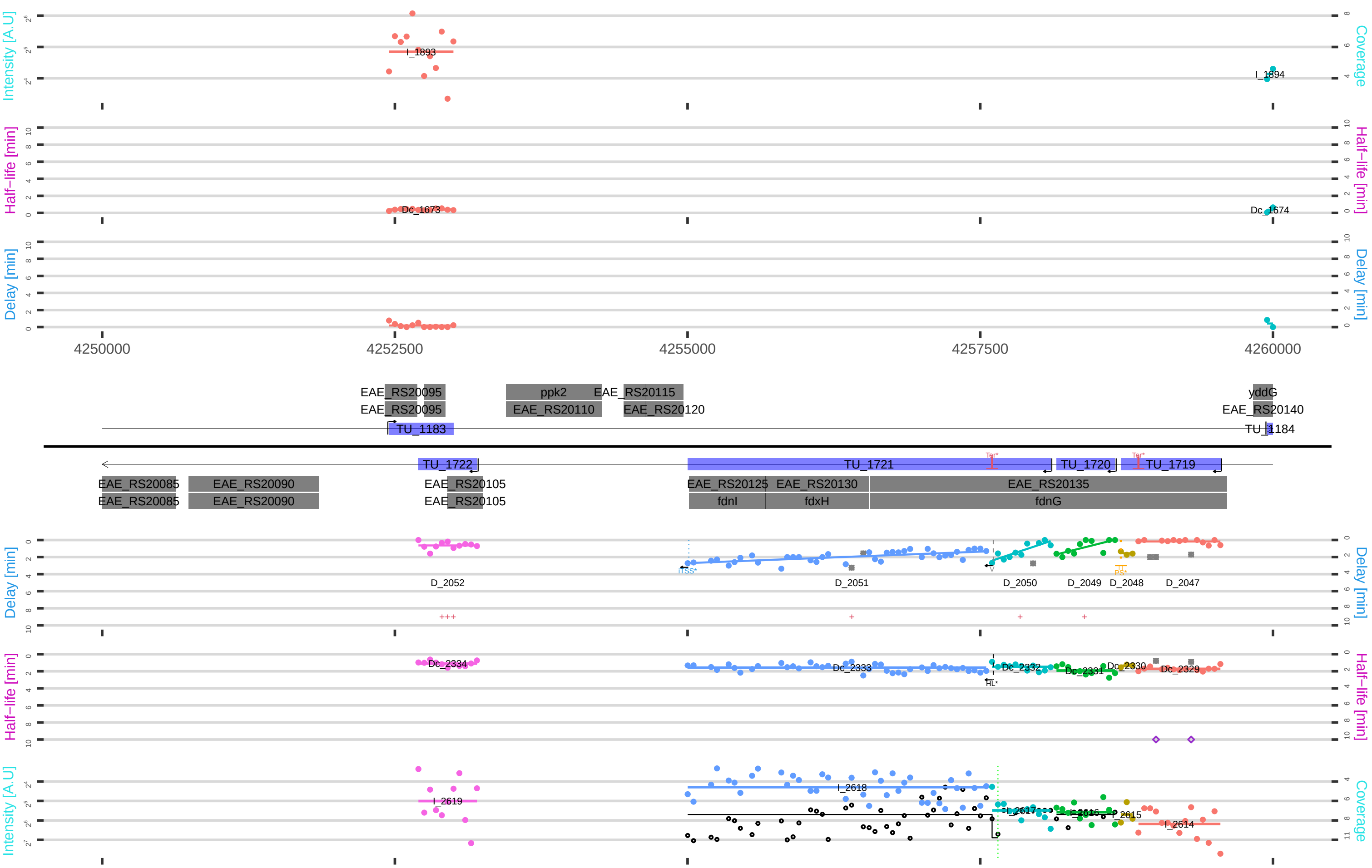

Term: termination (2), NS: new start (0), PS: pausing site (1), iTSS_I: internal starting site (1)

ID: 85200−85217; Term: termination (0), NS: new start (0), PS: pausing site (0), iTSS_I: internal starting site (0)

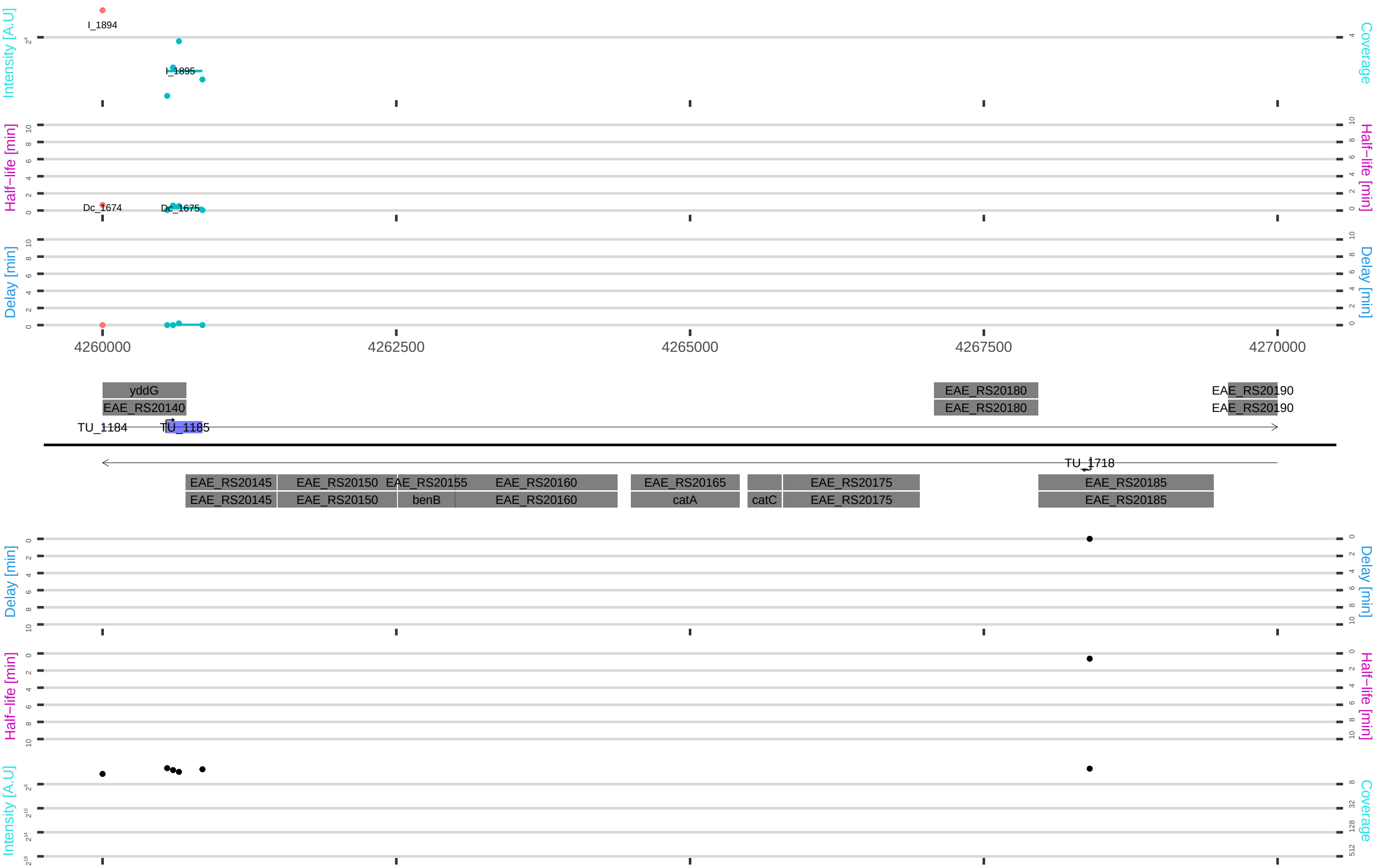

Term: termination (0), NS: new start (0), PS: pausing site (0), iTSS_I: internal starting site (0)

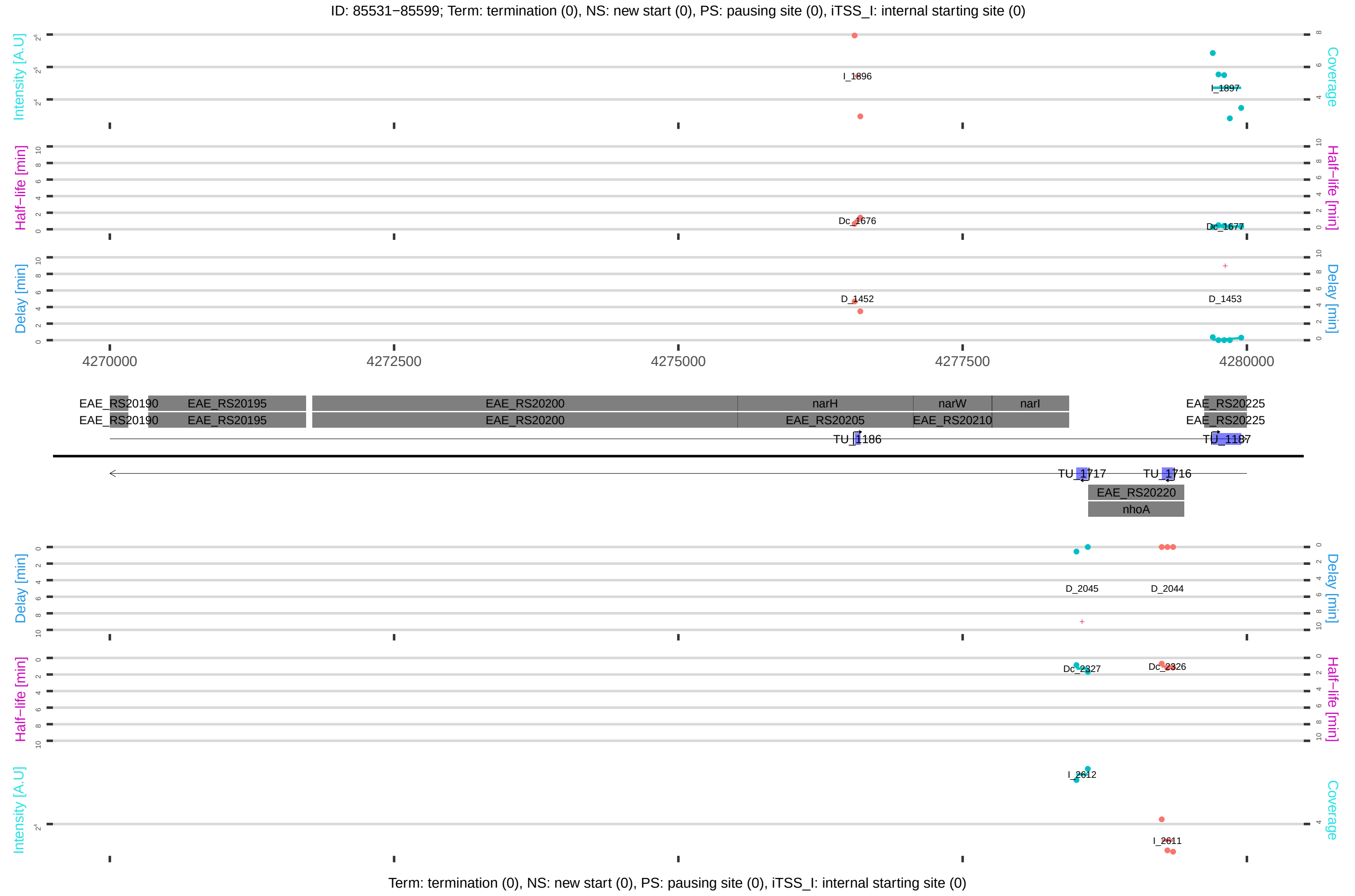

ID: 85531−85599; Term: termination (0), NS: new start (0), PS: pausing site (0), iTSS_I: internal starting site (0)
Intensity [A.U]
Coverage
I_1896
I_1897
Half−life [min]
Dc_1676
Dc_1677
Delay [min]
D_1452
D_1453
4270000
4272500
4275000
4277500
4280000
EAE_RS20190
EAE_RS20195
EAE_RS20200
narH
narW
narI
EAE_RS20205
EAE_RS20210
EAE_RS20225
TU_1186
TU_1187
TU_1717
TU_1716
EAE_RS20220
nhoA
D_2045
D_2044
Dc_2327
Dc_2326
I_2612
I_2611
Term: termination (0), NS: new start (0), PS: pausing site (0), iTSS_I: internal starting site (0)

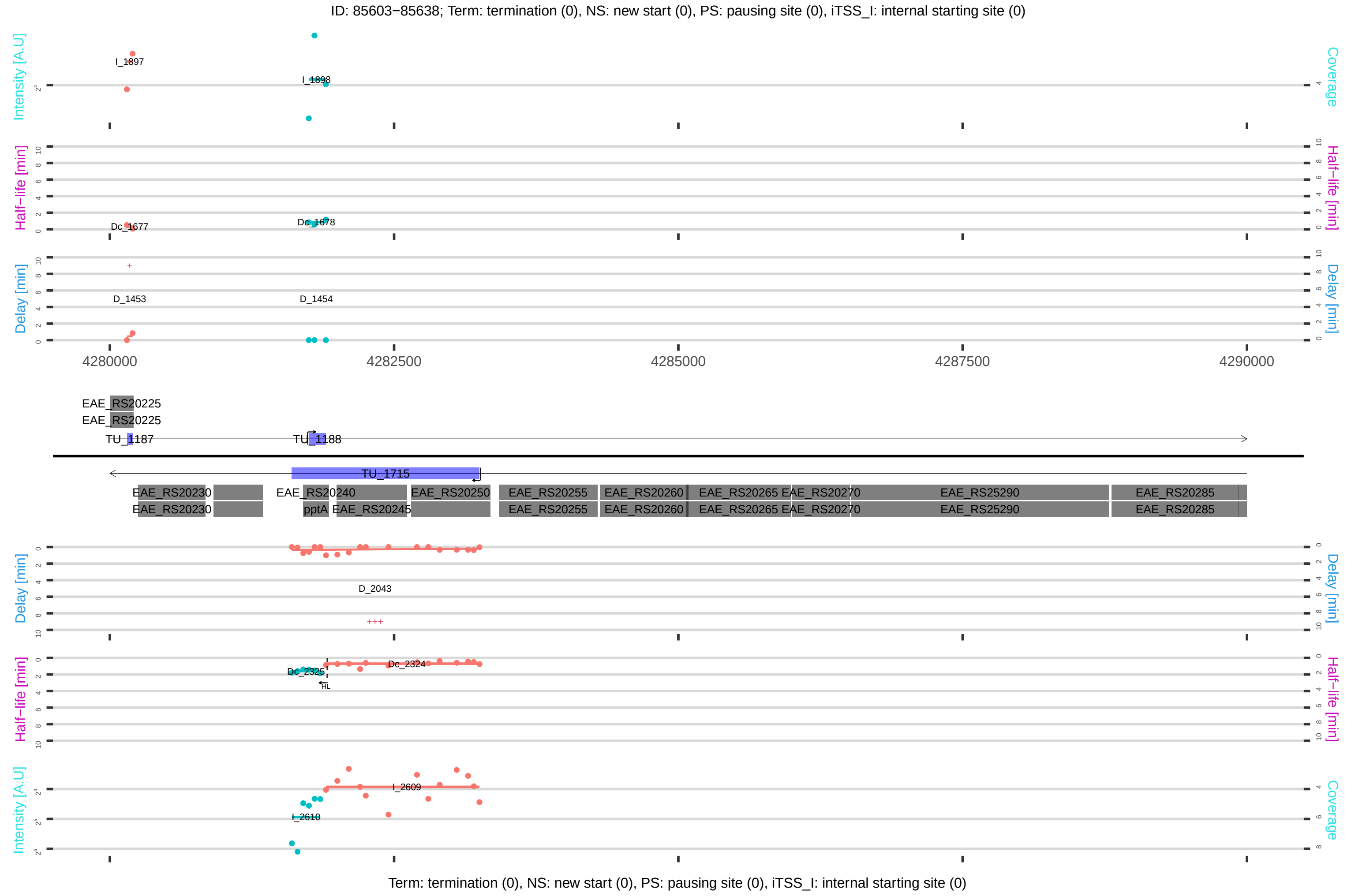
ID: 85603−85638; Term: termination (0), NS: new start (0), PS: pausing site (0), iTSS_I: internal starting site (0)
Intensity [A.U]
Coverage
I_1897
I_1898
Half−life [min]
Dc_1677
Dc_1678
Delay [min]
D_1453
D_1454
4280000
4282500
4285000
4287500
4290000
EAE_RS20225
EAE_RS20225
TU_1187
TU_1188
TU_1715
EAE_RS20230
EAE_RS20240
EAE_RS20250
EAE_RS20255
EAE_RS20260
EAE_RS20265
EAE_RS20270
EAE_RS25290
EAE_RS20285
pptA
EAE_RS20245
D_2043
Dc_2324
Dc_2325
HL
I_2609
I_2610
Term: termination (0), NS: new start (0), PS: pausing site (0), iTSS_I: internal starting site (0)

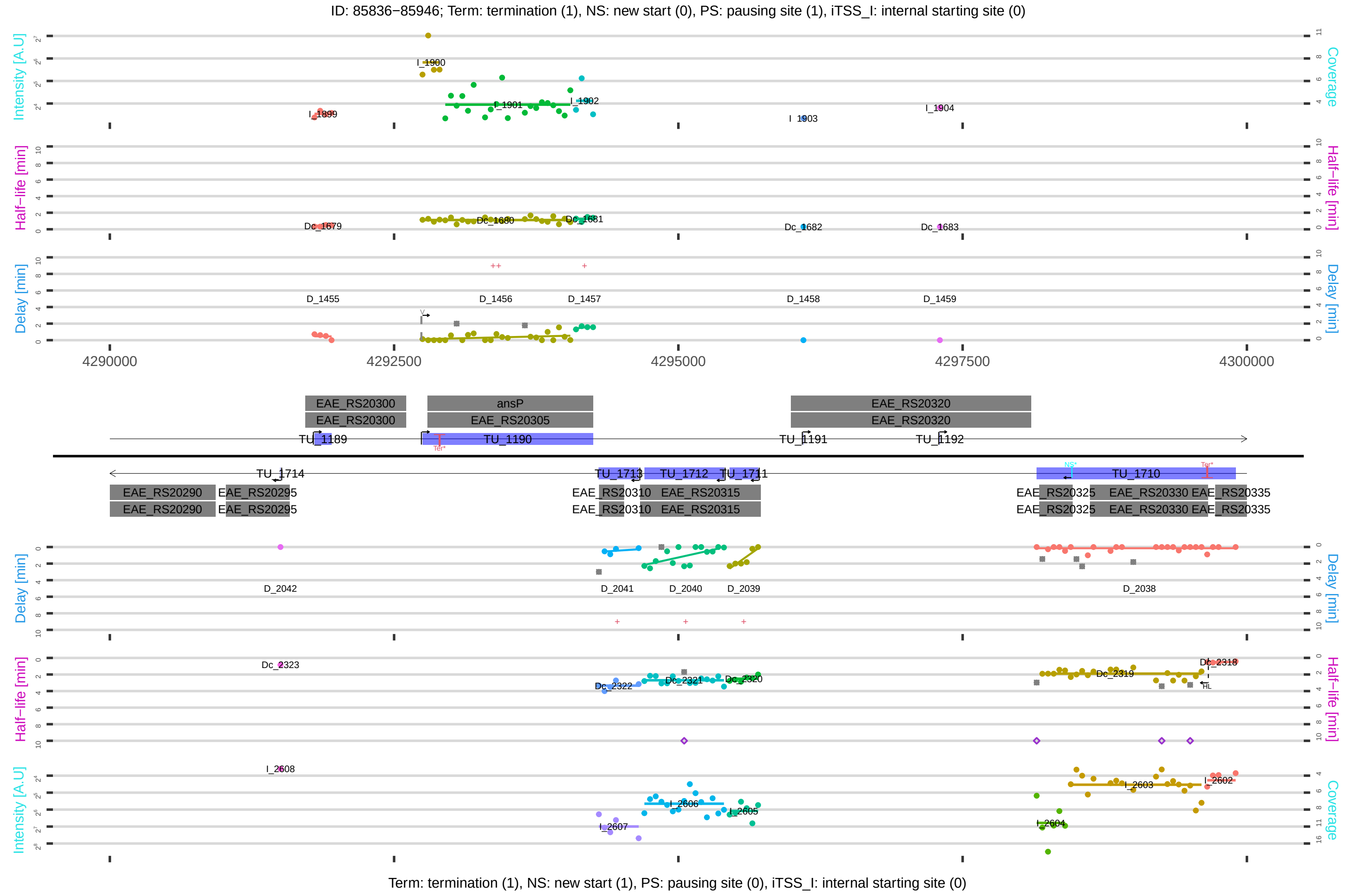

ID: 85836−85946; Term: termination (1), NS: new start (0), PS: pausing site (1), iTSS_I: internal starting site (0)
Intensity [A.U]
Coverage
Half−life [min]
Delay [min]
I_1899
I_1900
I_1901
I_1902
I_1903
I_1904
Dc_1679
Dc_1680
Dc_1681
Dc_1682
Dc_1683
D_1455
D_1456
D_1457
D_1458
D_1459
4290000
4292500
4295000
4297500
4300000
EAE_RS20300
ansP
EAE_RS20305
EAE_RS20320
TU_1189
TU_1190
TU_1191
TU_1192
Ter*
TU_1714
TU_1713
TU_1712
TU_1711
TU_1710
NS*
EAE_RS20290
EAE_RS20295
EAE_RS20310
EAE_RS20315
EAE_RS20325
EAE_RS20330
EAE_RS20335
D_2042
D_2041
D_2040
D_2039
D_2038
Dc_2323
Dc_2322
Dc_2321
Dc_2320
Dc_2319
Dc_2318
HL
I_2608
I_2607
I_2606
I_2605
I_2604
I_2603
I_2602
Term: termination (1), NS: new start (1), PS: pausing site (0), iTSS_I: internal starting site (0)

ID: 86011−86155; Term: termination (0), NS: new start (0), PS: pausing site (0), iTSS_I: internal starting site (0)

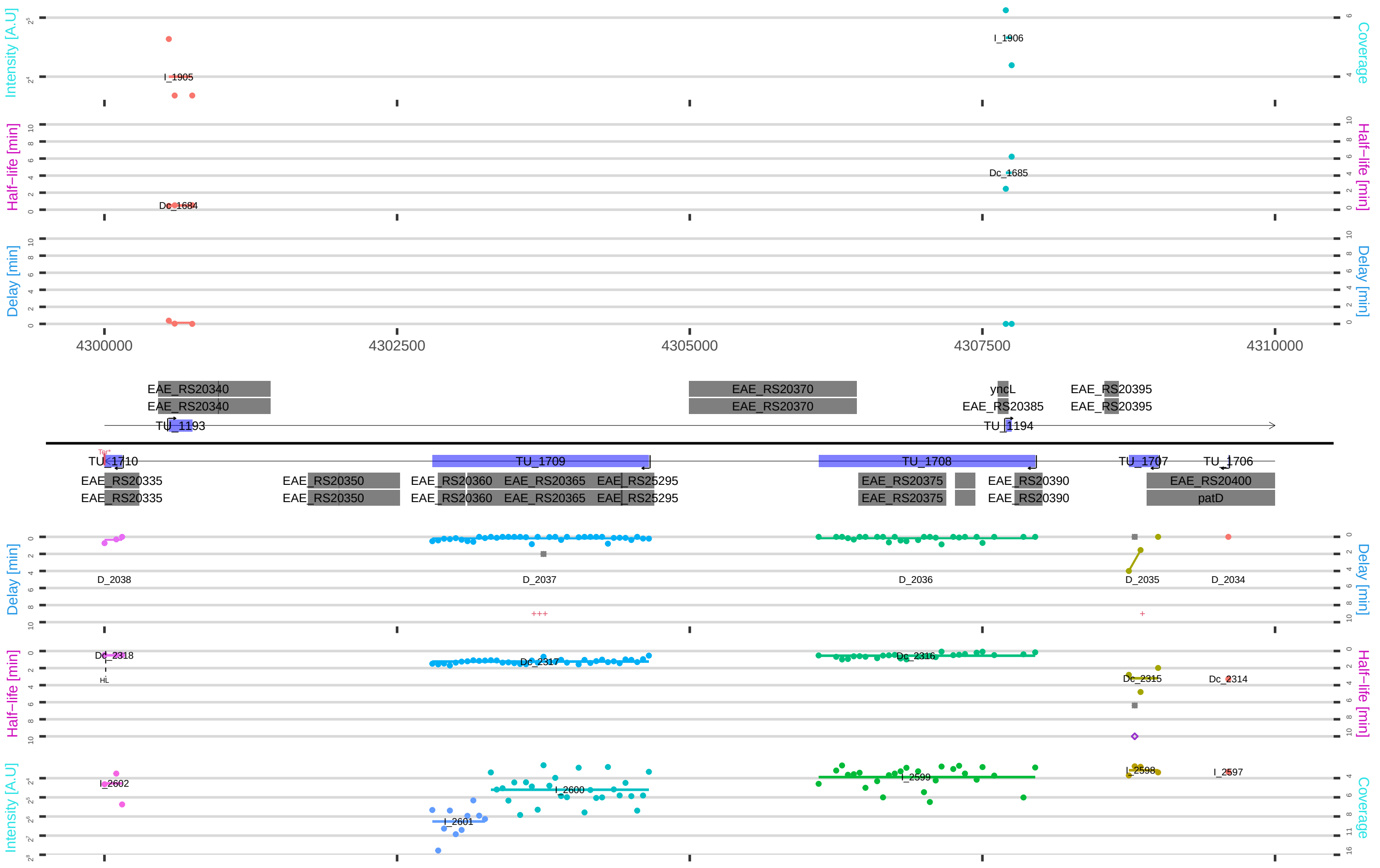

Term: termination (1), NS: new start (0), PS: pausing site (0), iTSS_I: internal starting site (0)

ID: 86389−86390; Term: termination (0), NS: new start (0), PS: pausing site (0), iTSS_I: internal starting site (0)

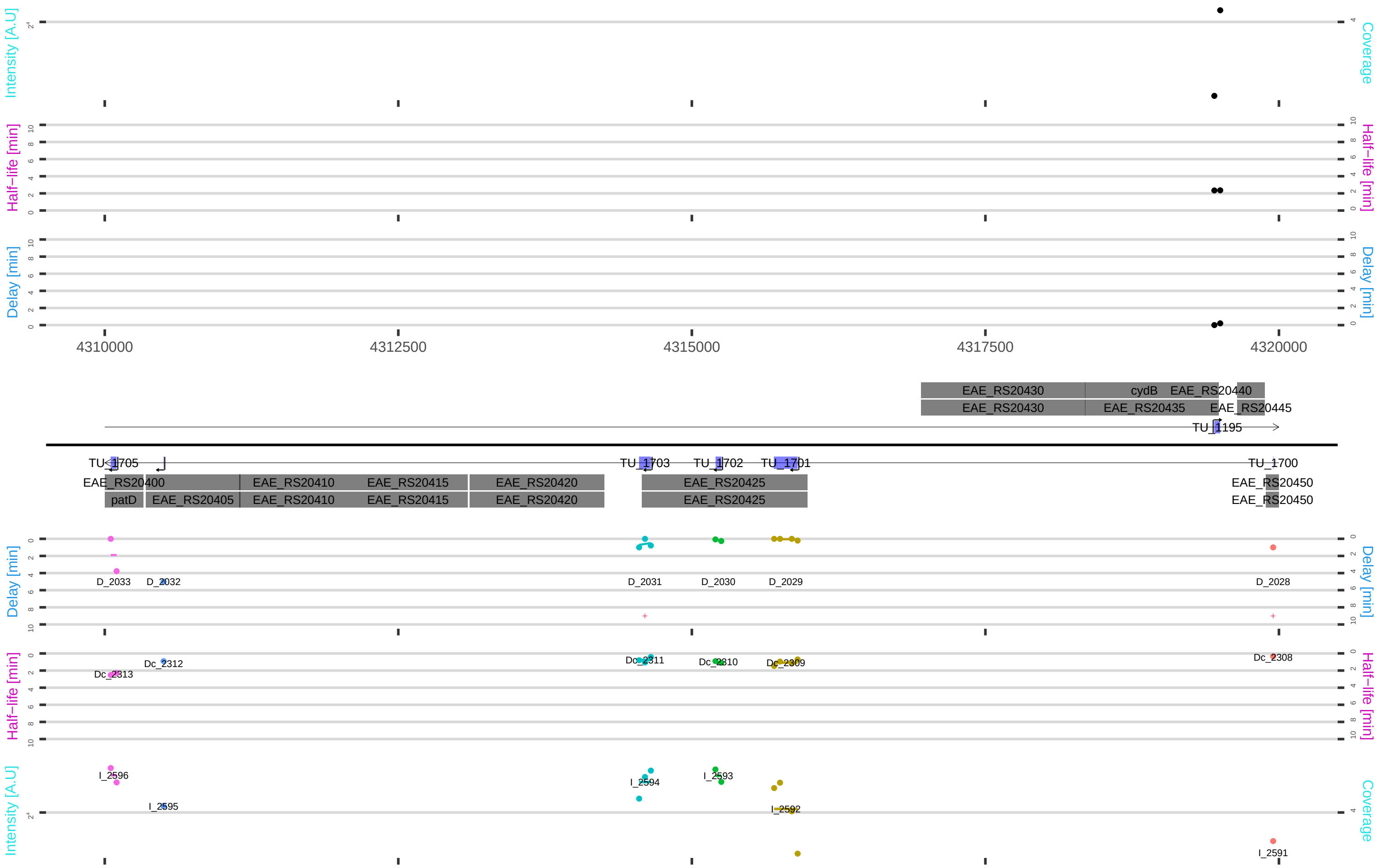

Term: termination (0), NS: new start (0), PS: pausing site (0), iTSS_I: internal starting site (0)

ID: 86459−86599; Term: termination (0), NS: new start (0), PS: pausing site (0), iTSS_I: internal starting site (0)

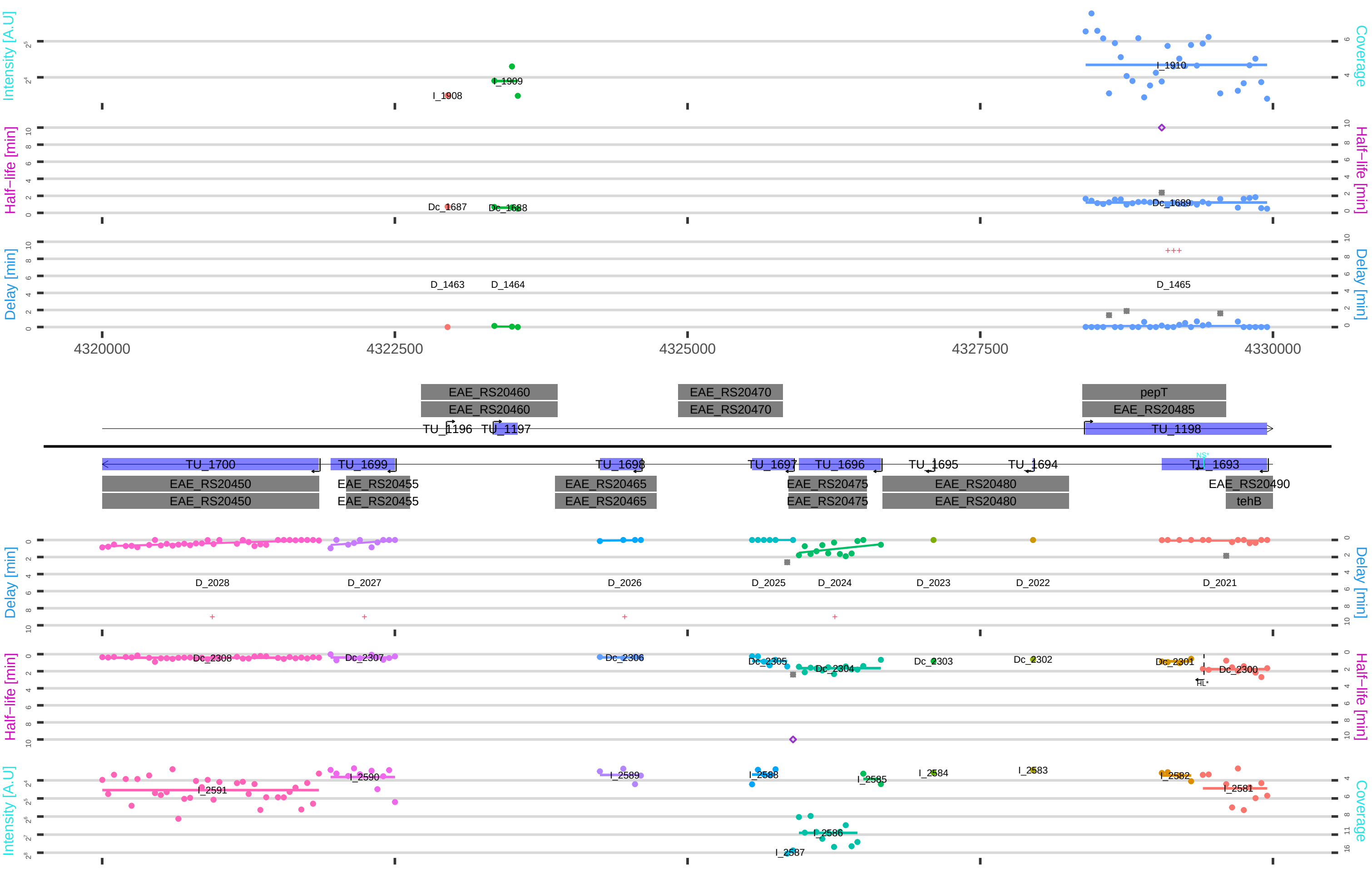

Term: termination (0), NS: new start (1), PS: pausing site (0), iTSS_I: internal starting site (0)

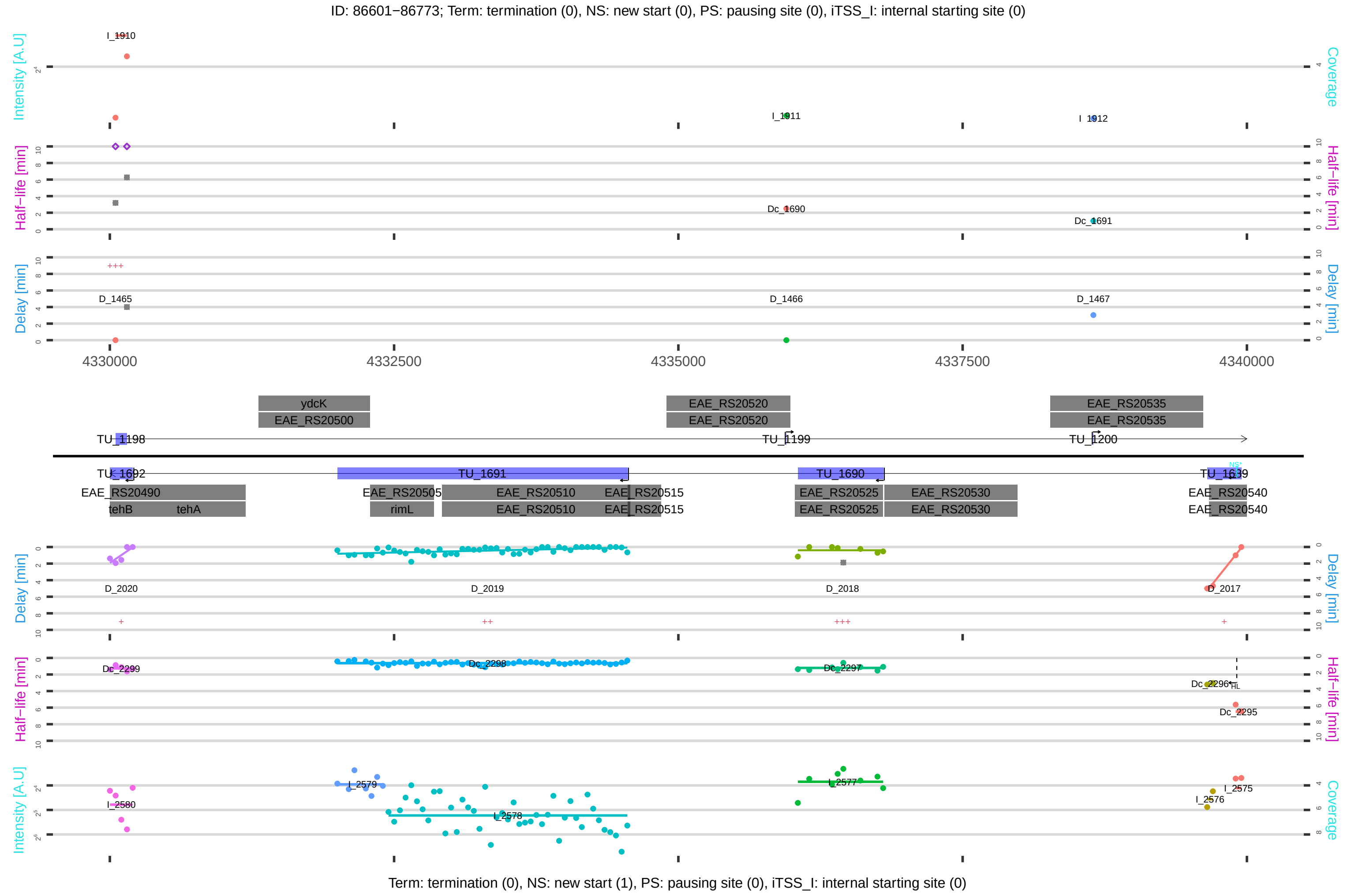

ID: 86601−86773; Term: termination (0), NS: new start (0), PS: pausing site (0), iTSS_I: internal starting site (0)
Intensity [A.U]
Coverage
Half−life [min]
Delay [min]
I_1910
I_1911
I_1912
Dc_1690
Dc_1691
D_1465
D_1466
D_1467
4330000
4332500
4335000
4337500
4340000
ydcK
EAE_RS20500
EAE_RS20520
EAE_RS20535
TU_1198
TU_1199
TU_1200
TU_1692
TU_1691
TU_1690
TU_1689
EAE_RS20490
tehB
tehA
EAE_RS20505
rimL
EAE_RS20510
EAE_RS20515
EAE_RS20525
EAE_RS20530
EAE_RS20540
D_2020
D_2019
D_2018
D_2017
Dc_2299
Dc_2298
Dc_2297
Dc_2296
Dc_2295
I_2580
I_2579
I_2578
I_2577
I_2576
I_2575
Term: termination (0), NS: new start (1), PS: pausing site (0), iTSS_I: internal starting site (0)

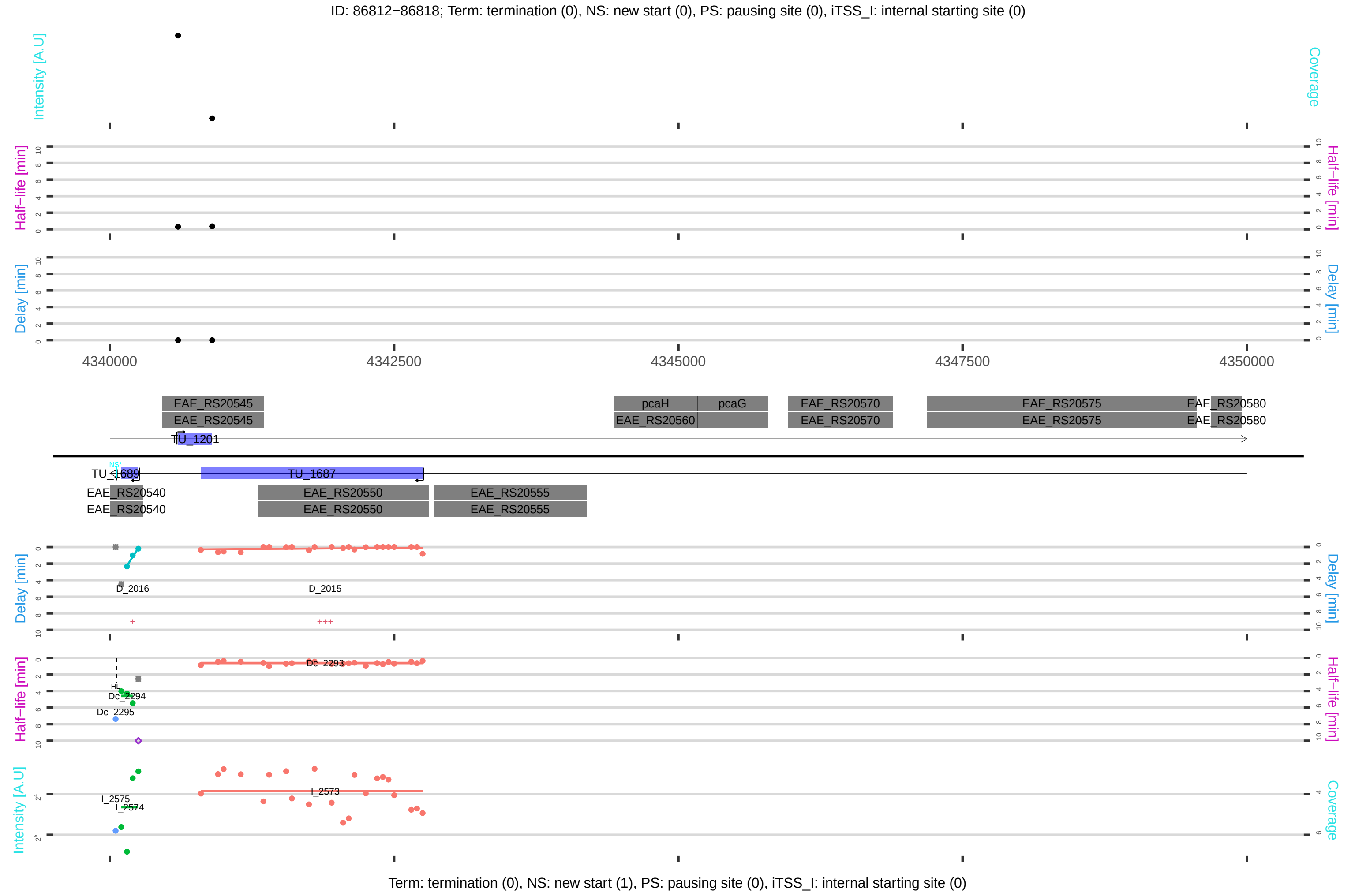
ID: 86812−86818; Term: termination (0), NS: new start (0), PS: pausing site (0), iTSS_I: internal starting site (0)
Intensity [A.U]
Coverage
Half−life [min]
Delay [min]
4340000
4342500
4345000
4347500
4350000
EAE_RS20545
pcaH
pcaG
EAE_RS20560
EAE_RS20570
EAE_RS20575
EAE_RS20580
TU_1201
TU_1689
TU_1687
EAE_RS20540
EAE_RS20550
EAE_RS20555
D_2016
D_2015
Dc_2293
Dc_2294
Dc_2295
I_2575
I_2574
I_2573
Term: termination (0), NS: new start (1), PS: pausing site (0), iTSS_I: internal starting site (0)

ID: 87005−87075; Term: termination (0), NS: new start (0), PS: pausing site (0), iTSS_I: internal starting site (0)

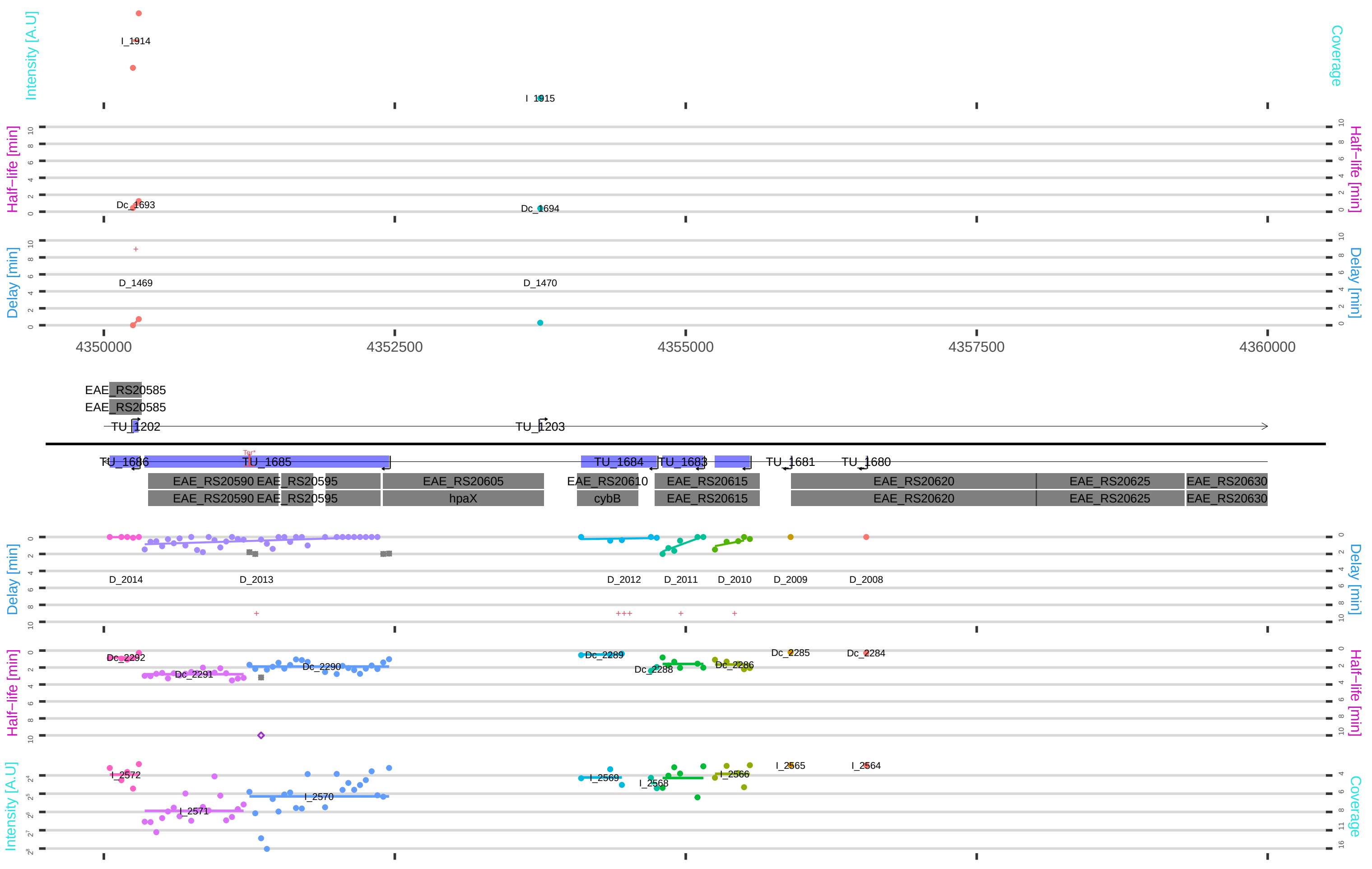

Term: termination (1), NS: new start (0), PS: pausing site (0), iTSS_I: internal starting site (0)

ID: 87224−87246; Term: termination (1), NS: new start (0), PS: pausing site (0), iTSS_I: internal starting site (1)

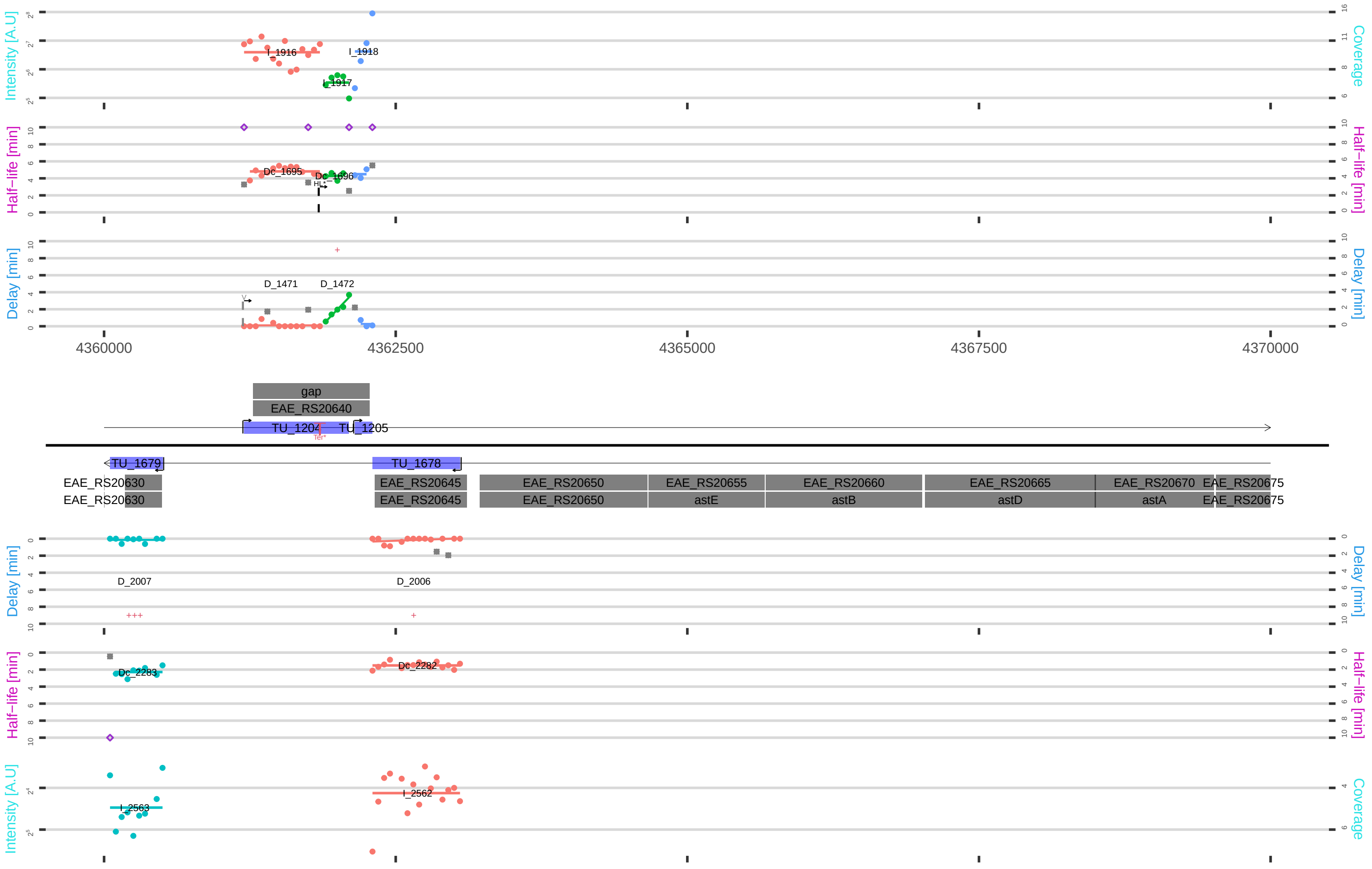

Term: termination (0), NS: new start (0), PS: pausing site (0), iTSS_I: internal starting site (0)

ID: 87426−87555; Term: termination (0), NS: new start (0), PS: pausing site (0), iTSS_I: internal starting site (0)

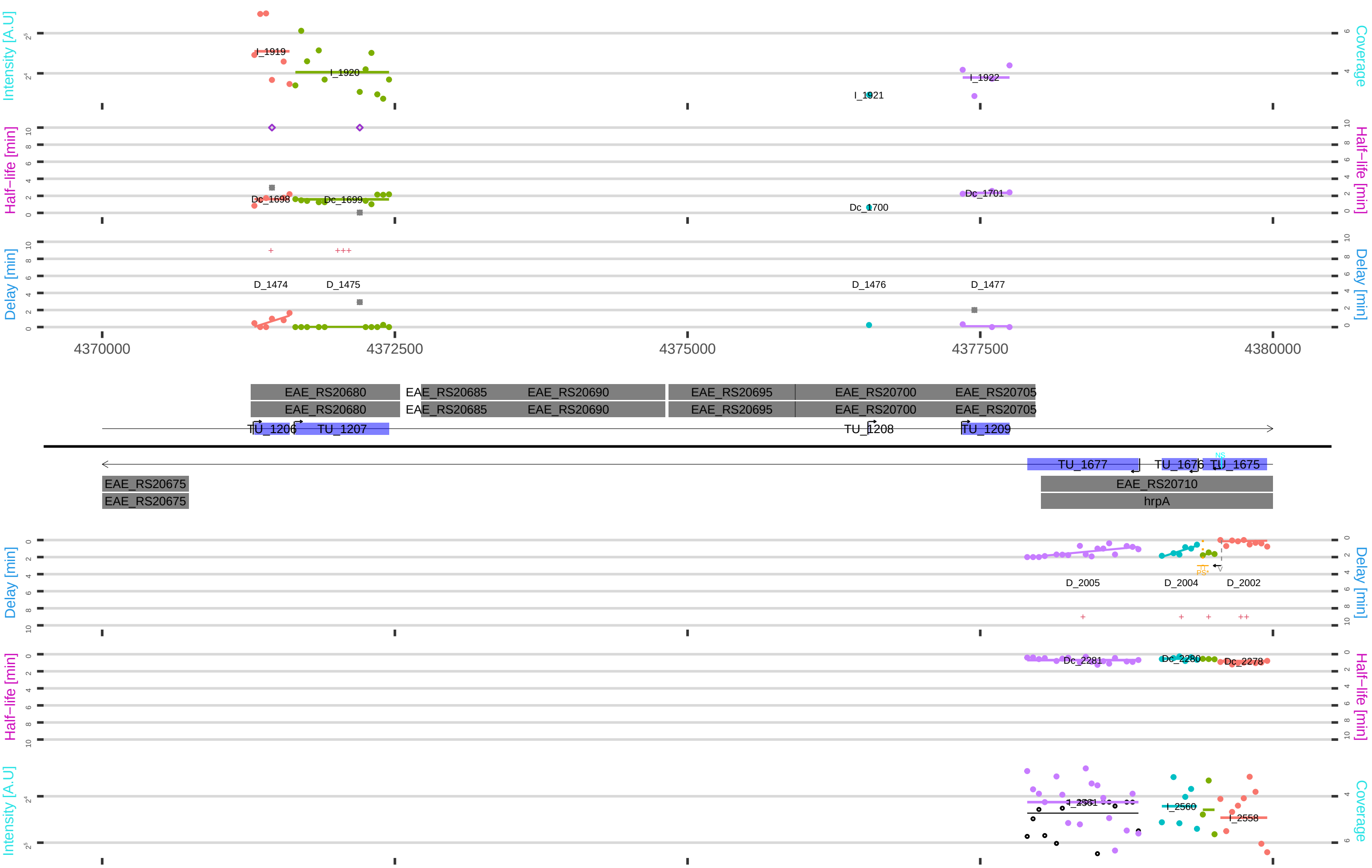

Term: termination (0), NS: new start (1), PS: pausing site (1), iTSS_I: internal starting site (0)

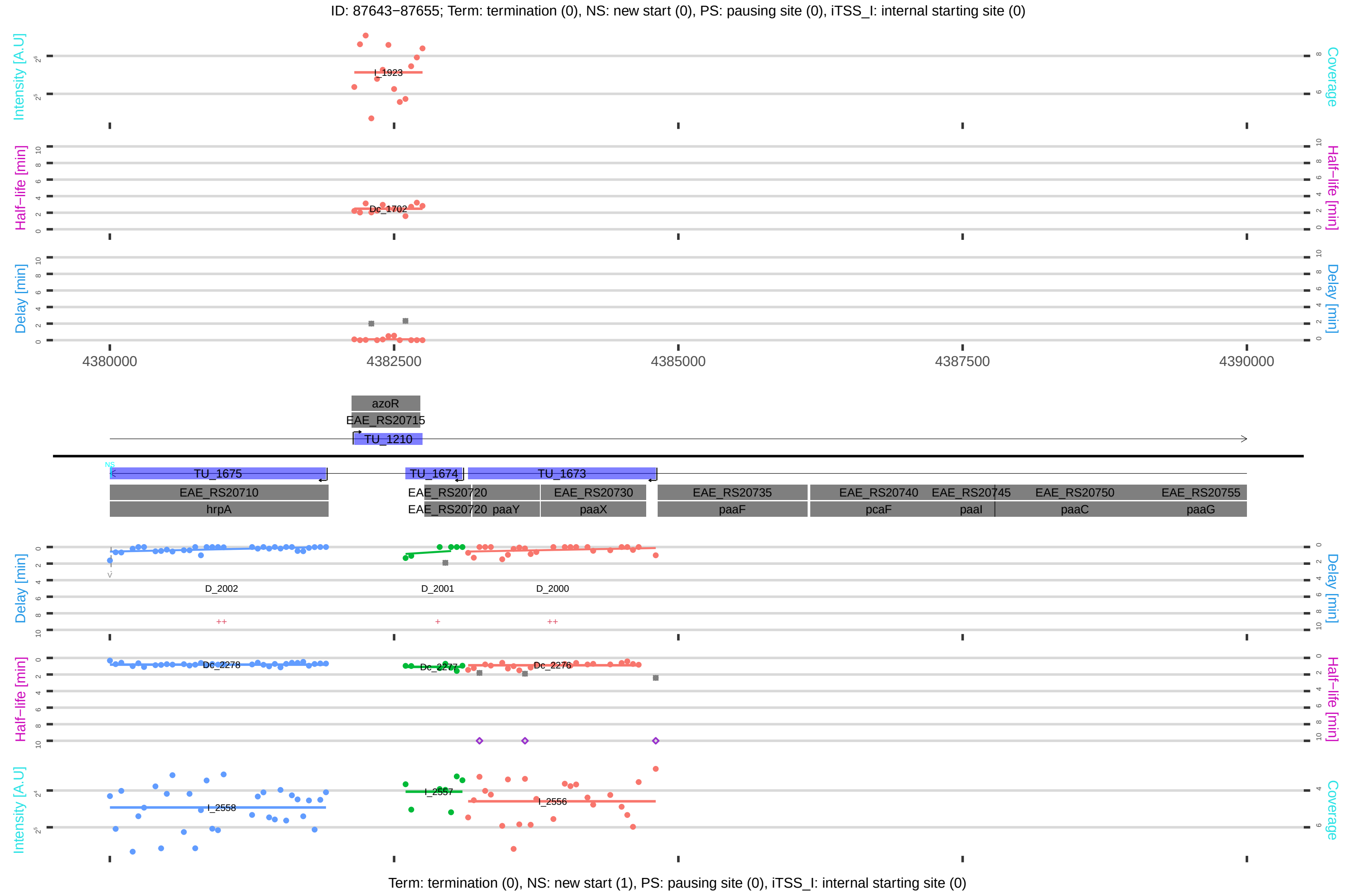
ID: 87643−87655; Term: termination (0), NS: new start (0), PS: pausing site (0), iTSS_I: internal starting site (0)
Intensity [A.U]
Coverage
I_1923
Half−life [min]
Dc_1702
Delay [min]
4380000
4382500
4385000
4387500
4390000
azoR
EAE_RS20715
TU_1210
TU_1675
TU_1674
TU_1673
EAE_RS20710
hrpA
EAE_RS20720
paaY
EAE_RS20730
paaX
EAE_RS20735
paaF
EAE_RS20740
pcaF
EAE_RS20745
paaI
EAE_RS20750
paaC
EAE_RS20755
paaG
D_2002
D_2001
D_2000
Dc_2278
Dc_2277
Dc_2276
I_2558
I_2557
I_2556
Term: termination (0), NS: new start (1), PS: pausing site (0), iTSS_I: internal starting site (0)

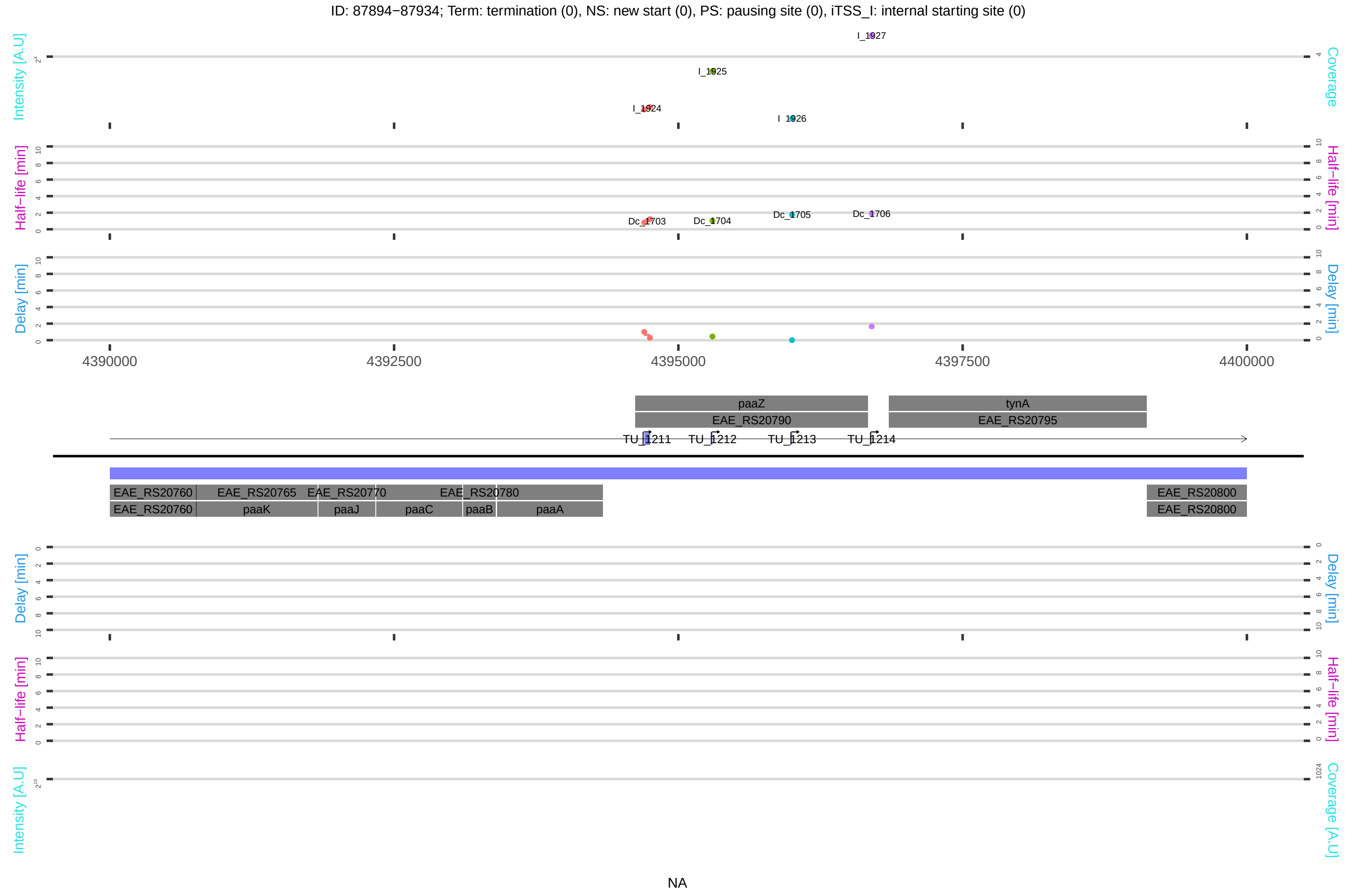
ID: 87894−87934; Term: termination (0), NS: new start (0), PS: pausing site (0), iTSS_I: internal starting site (0)
Intensity [A.U]
Coverage
I_1927
I_1925
I_1924
I_1926
Half−life [min]
Dc_1703
Dc_1704
Dc_1705
Dc_1706
Delay [min]
4390000
4392500
4395000
4397500
4400000
paaZ
EAE_RS20790
tynA
EAE_RS20795
TU_1211
TU_1212
TU_1213
TU_1214
EAE_RS20760
EAE_RS20765
EAE_RS20770
EAE_RS20780
EAE_RS20800
paaK
paaJ
paaC
paaB
paaA
Coverage [A.U]
NA

ID: 88005−88200; Term: termination (1), NS: new start (0), PS: pausing site (0), iTSS_I: internal starting site (0)

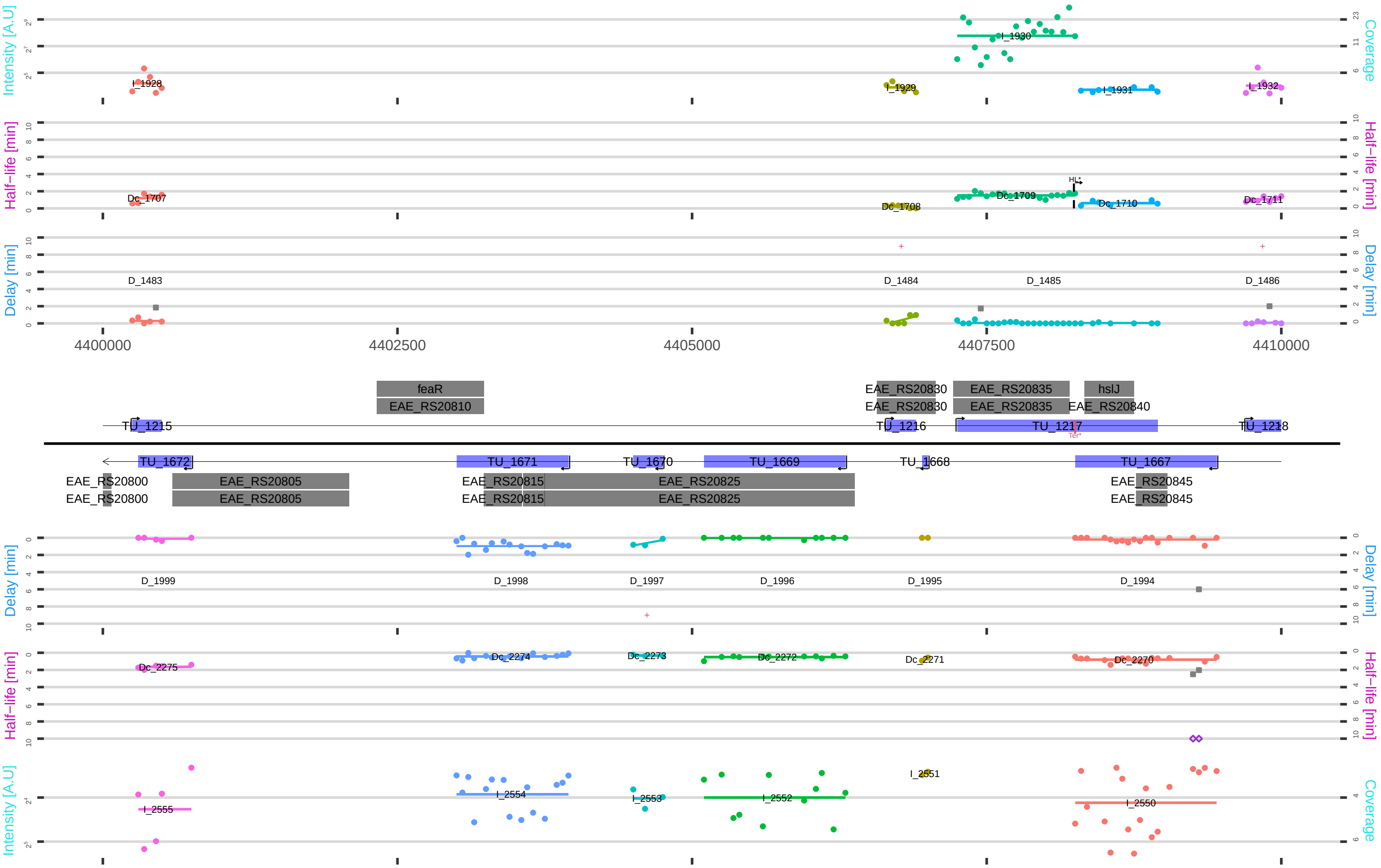

Term: termination (0), NS: new start (0), PS: pausing site (0), iTSS_I: internal starting site (0)

ID: 88200−88365; Term: termination (0), NS: new start (1), PS: pausing site (0), iTSS_I: internal starting site (0)

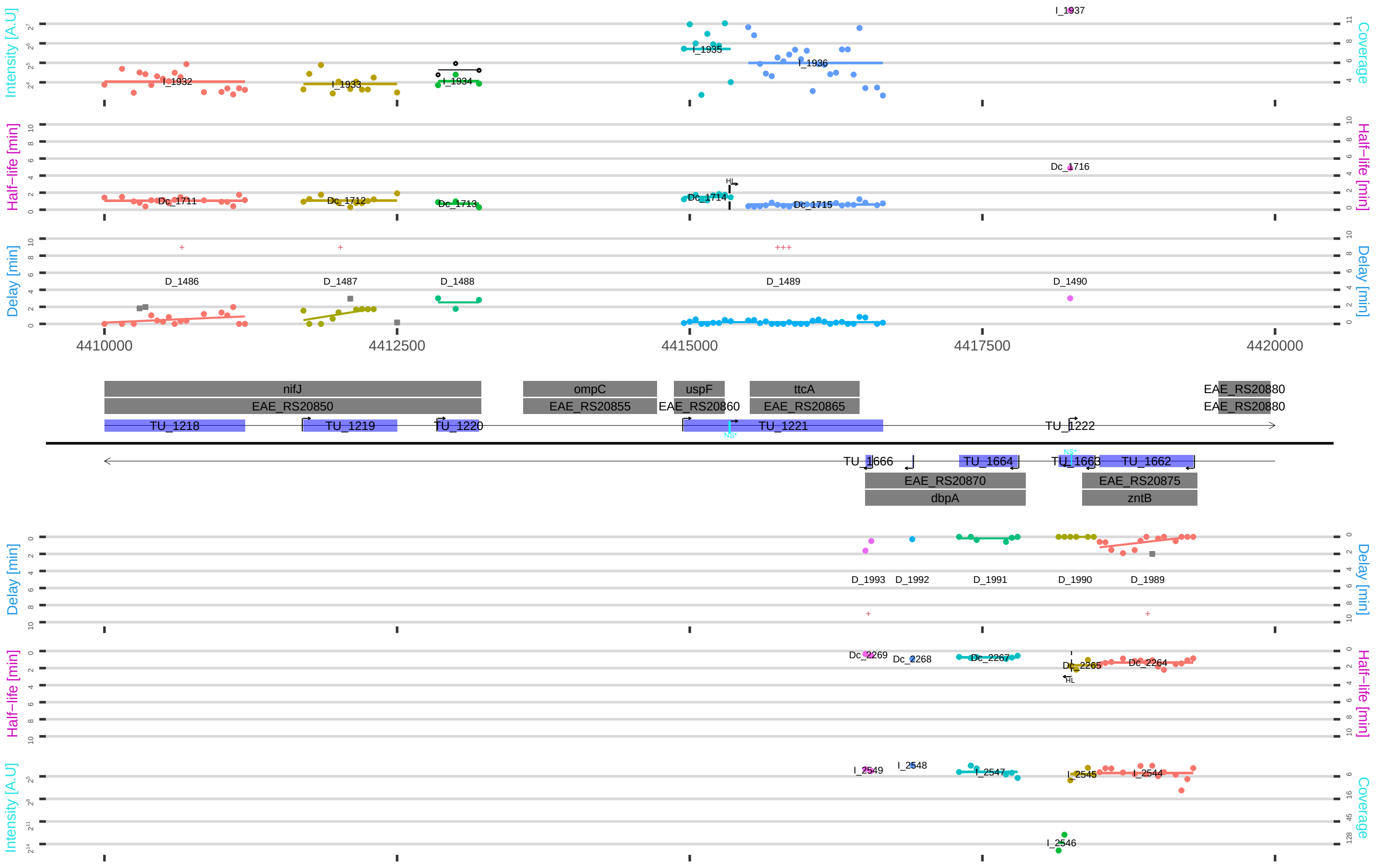

Term: termination (0), NS: new start (1), PS: pausing site (0), iTSS_I: internal starting site (0)

ID: 88524−88600; Term: termination (1), NS: new start (0), PS: pausing site (0), iTSS_I: internal starting site (0)

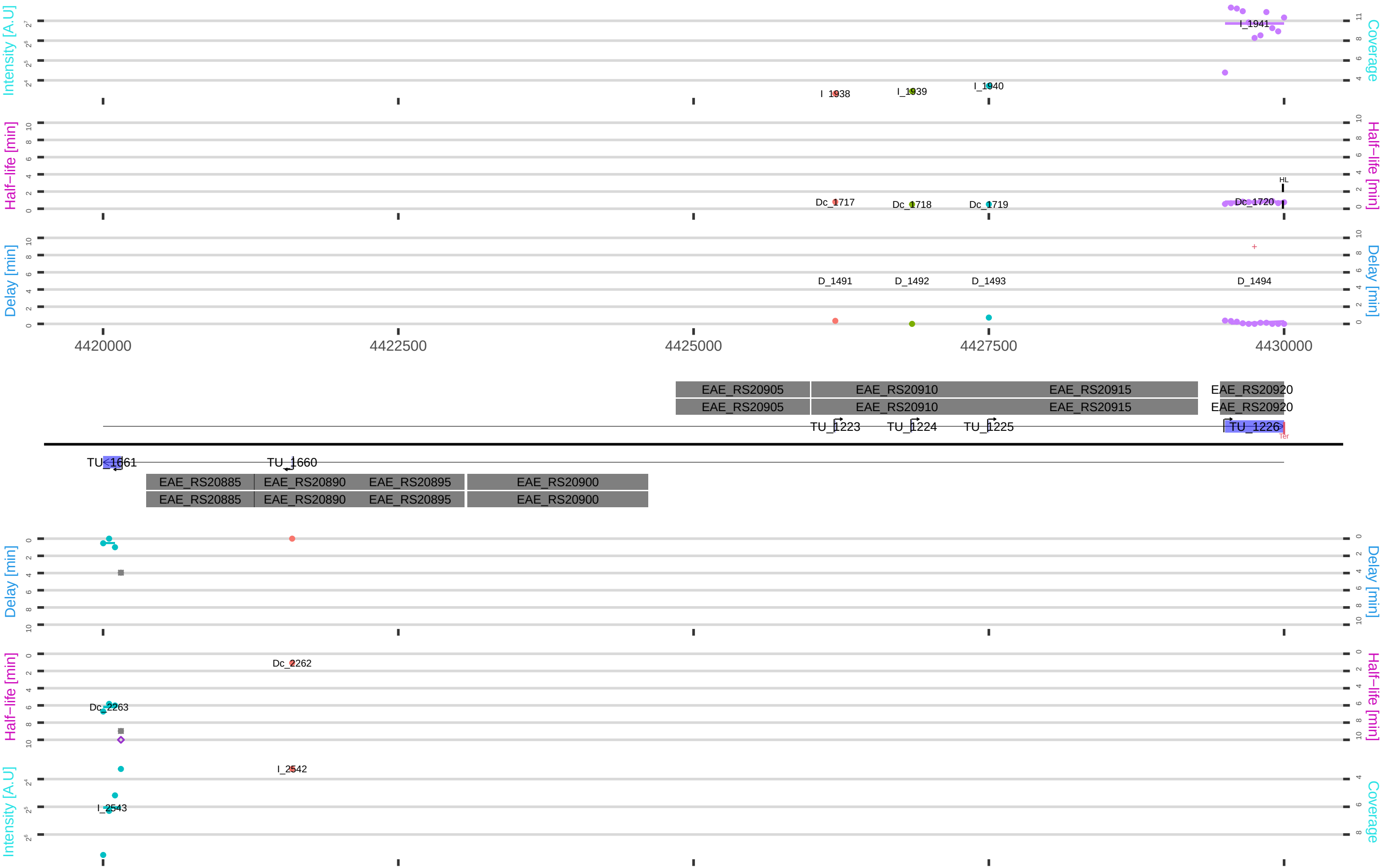

Term: termination (0), NS: new start (0), PS: pausing site (0), iTSS_I: internal starting site (0)

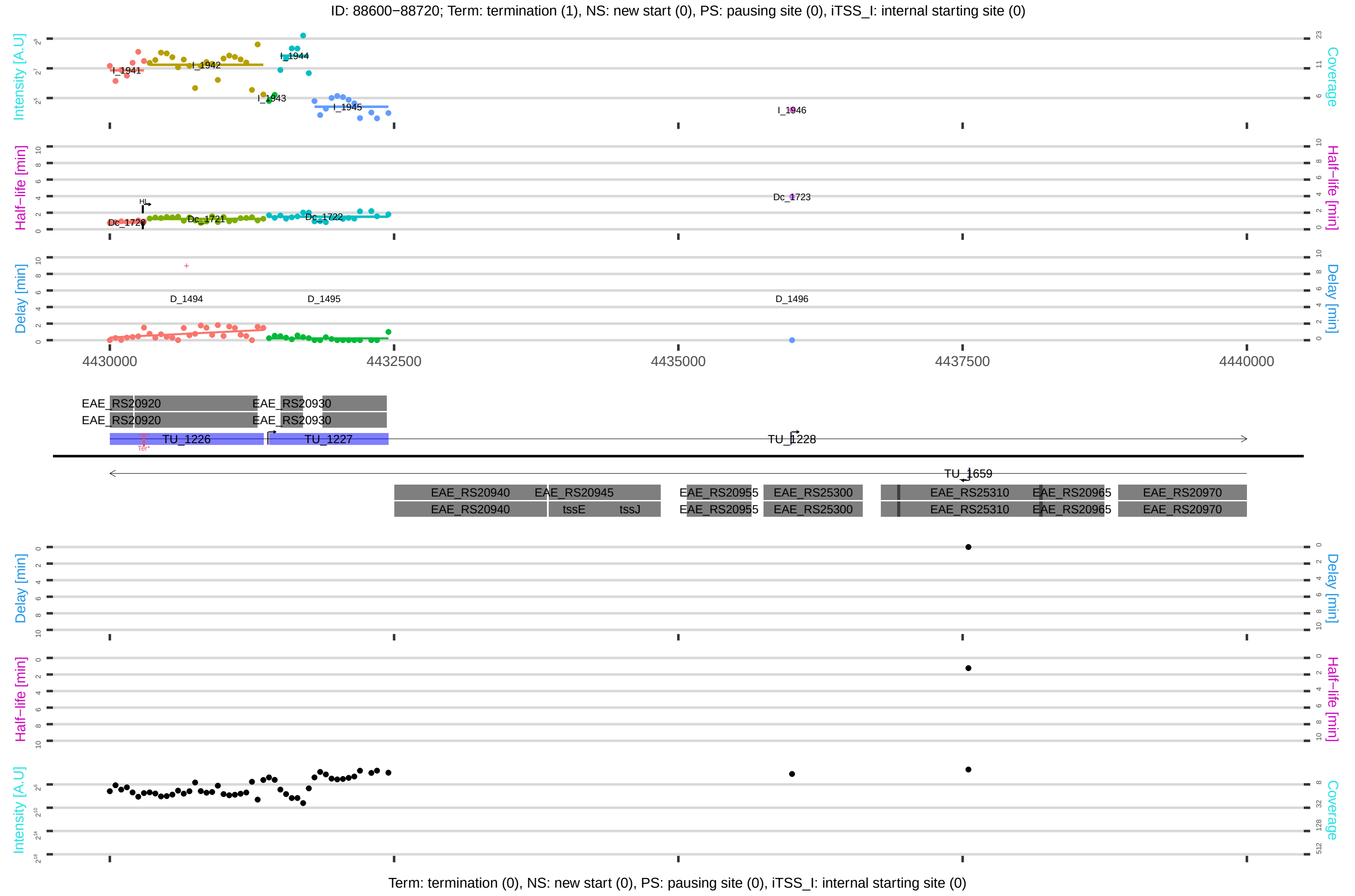

ID: 88600−88720; Term: termination (1), NS: new start (0), PS: pausing site (0), iTSS_I: internal starting site (0)
Intensity [A.U]
Coverage
I_1941
I_1942
I_1943
I_1944
I_1945
I_1946
Half−life [min]
Dc_1720
Dc_1721
Dc_1722
Dc_1723
HL
Delay [min]
D_1494
D_1495
D_1496
4430000
4432500
4435000
4437500
4440000
EAE_RS20920
EAE_RS20930
TU_1226
TU_1227
TU_1228
Ter
TU_1659
EAE_RS20940
EAE_RS20945
tssE
tssJ
EAE_RS20955
EAE_RS25300
EAE_RS25310
EAE_RS20965
EAE_RS20970
Term: termination (0), NS: new start (0), PS: pausing site (0), iTSS_I: internal starting site (0)

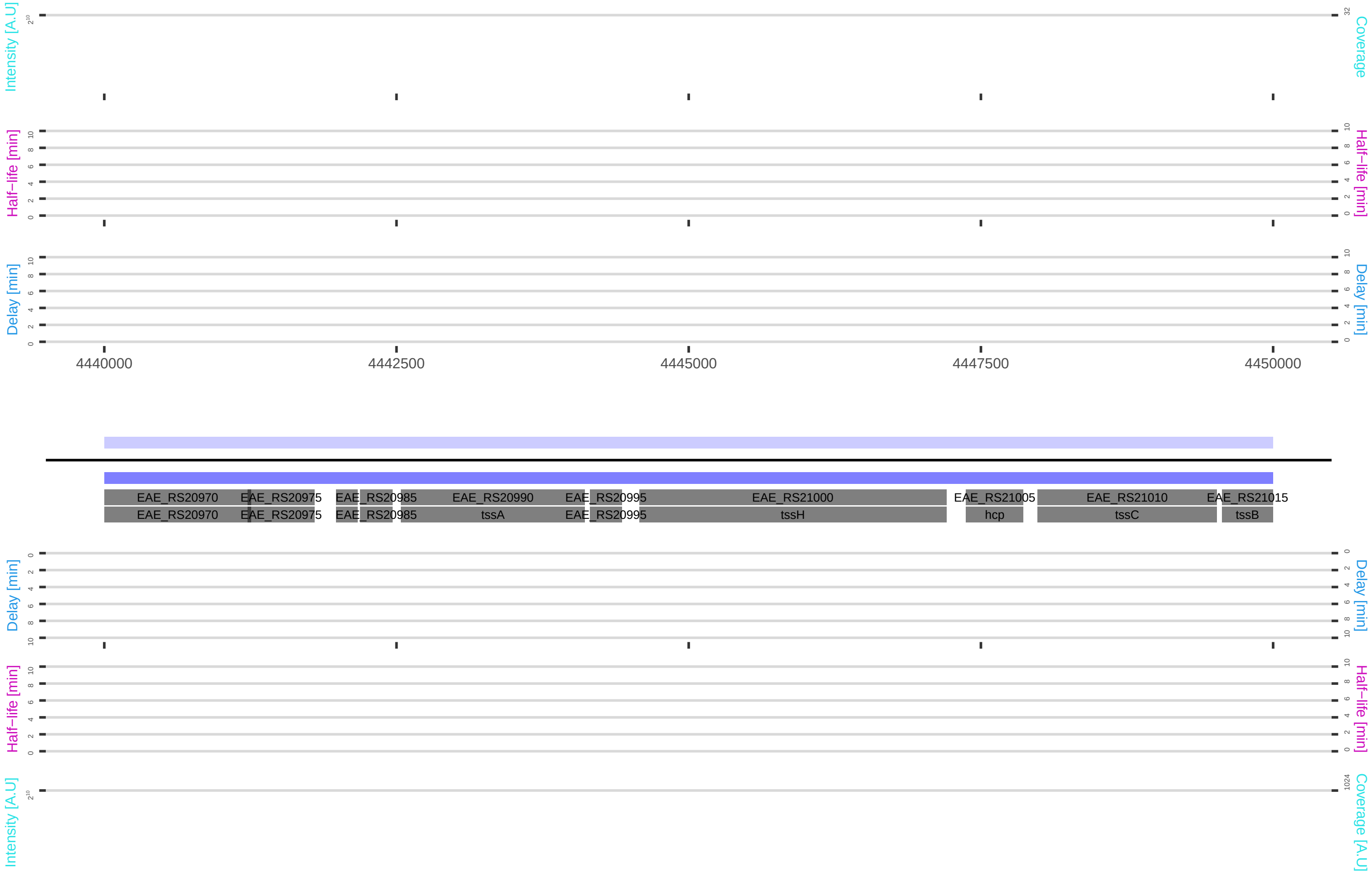
Intensity [A.U]
Coverage
Half−life [min]
Delay [min]
4440000
4442500
4445000
4447500
4450000
EAE_RS20970
EAE_RS20975
EAE_RS20985
EAE_RS20990
EAE_RS20995
EAE_RS21000
EAE_RS21005
EAE_RS21010
EAE_RS21015
tssA
tssH
hcp
tssC
tssB
Coverage [A.U]
NA

ID: 122085−122019; FC*: significant t−test of two consecutive segments; Term: termination, NS: new start, PS: pausing site,iTSS_I: internal starting site,TI: transcription interferance.

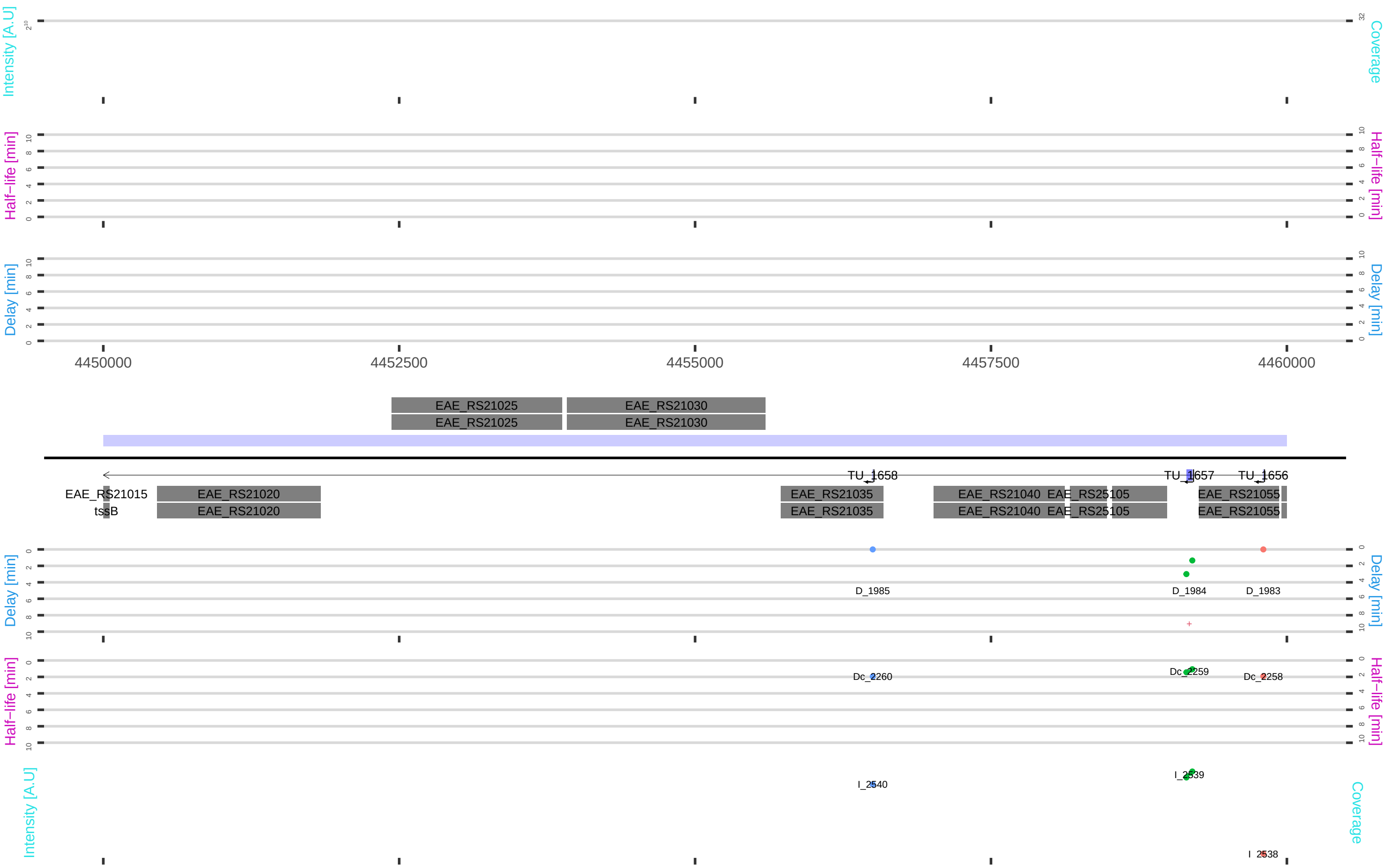

Term: termination (0), NS: new start (0), PS: pausing site (0), iTSS_I: internal starting site (0)

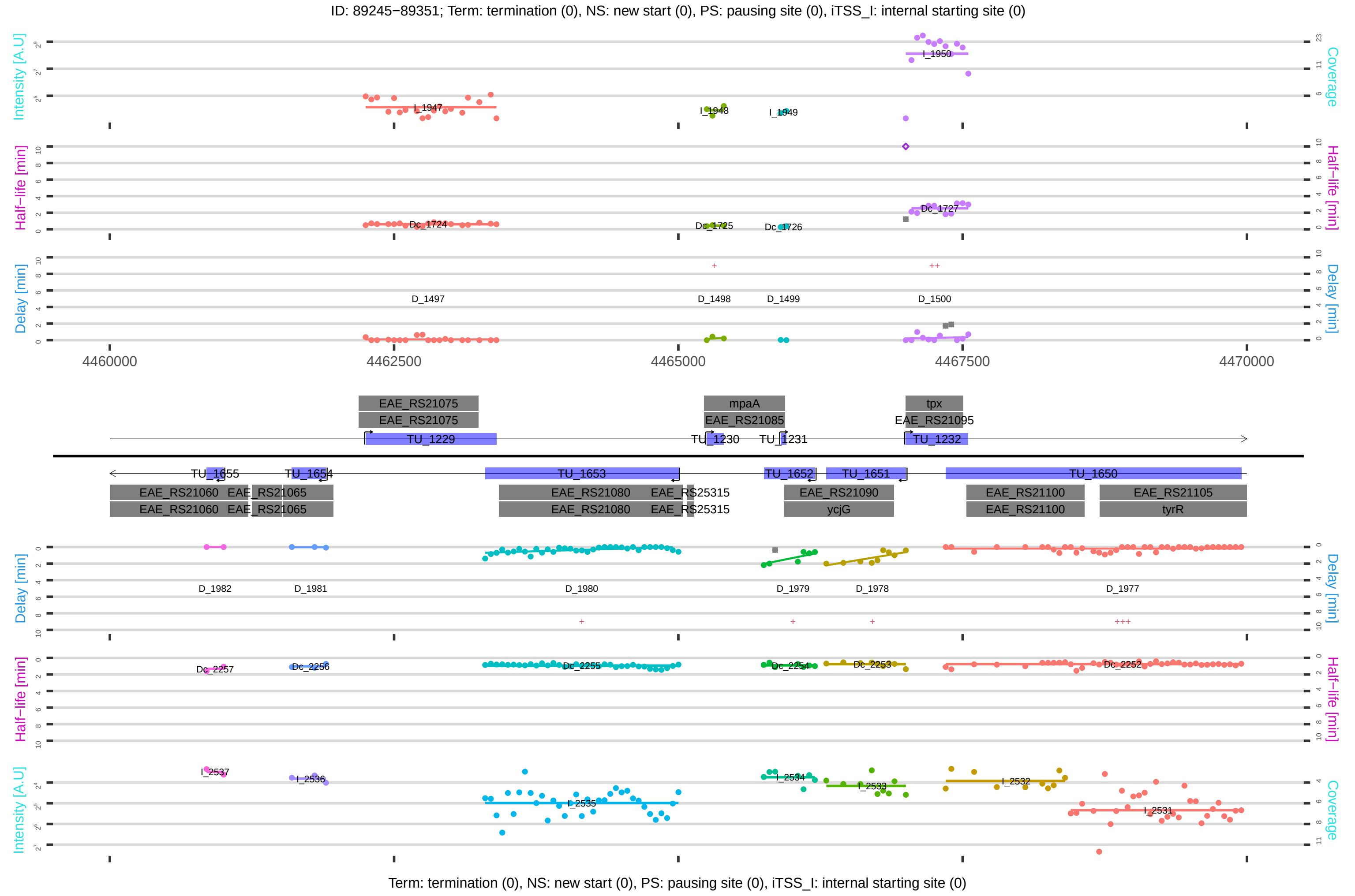

ID: 89245−89351; Term: termination (0), NS: new start (0), PS: pausing site (0), iTSS_I: internal starting site (0)
Intensity [A.U]
Coverage
I_1947
I_1948
I_1949
I_1950
Half−life [min]
Dc_1724
Dc_1725
Dc_1726
Dc_1727
Delay [min]
D_1497
D_1498
D_1499
D_1500
4460000
4462500
4465000
4467500
4470000
EAE_RS21075
mpaA
EAE_RS21085
tpx
EAE_RS21095
TU_1229
TU_1230
TU_1231
TU_1232
TU_1655
TU_1654
TU_1653
TU_1652
TU_1651
TU_1650
EAE_RS21060
EAE_RS21065
EAE_RS21080
EAE_RS25315
EAE_RS21090
ycjG
EAE_RS21100
EAE_RS21105
tyrR
D_1982
D_1981
D_1980
D_1979
D_1978
D_1977
Dc_2257
Dc_2256
Dc_2255
Dc_2254
Dc_2253
Dc_2252
I_2537
I_2536
I_2535
I_2534
I_2533
I_2532
I_2531
Term: termination (0), NS: new start (0), PS: pausing site (0), iTSS_I: internal starting site (0)

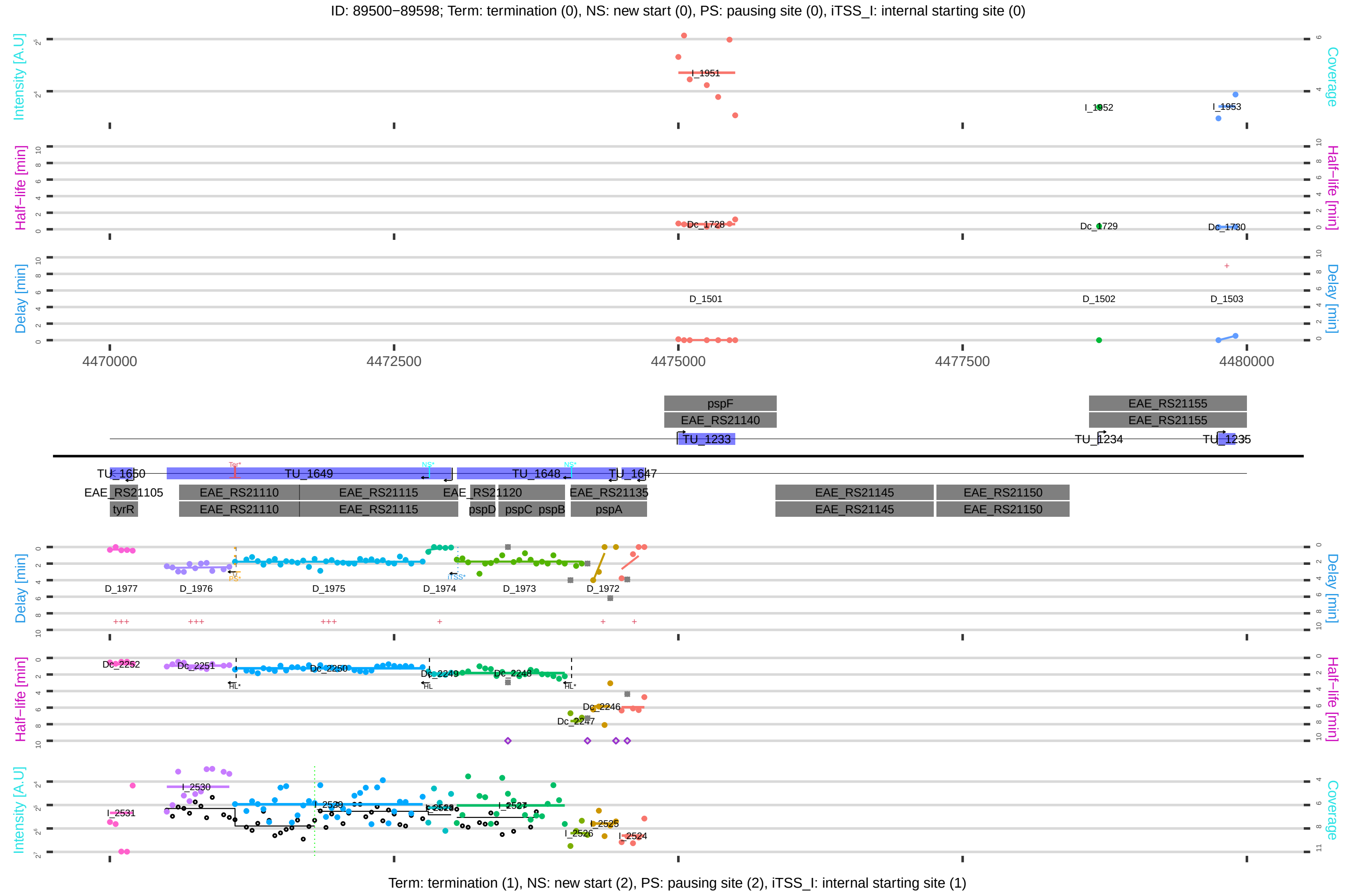

ID: 89500−89598; Term: termination (0), NS: new start (0), PS: pausing site (0), iTSS_I: internal starting site (0)
Intensity [A.U]
Coverage
Half−life [min]
Delay [min]
I_1951
I_1952
I_1953
Dc_1728
Dc_1729
Dc_1730
D_1501
D_1502
D_1503
4470000
4472500
4475000
4477500
4480000
pspF
EAE_RS21140
EAE_RS21155
EAE_RS21155
TU_1233
TU_1234
TU_1235
TU_1650
TU_1649
TU_1648
TU_1647
EAE_RS21105
tyrR
EAE_RS21110
EAE_RS21110
EAE_RS21115
EAE_RS21115
EAE_RS21120
pspD
pspC
pspB
EAE_RS21135
pspA
EAE_RS21145
EAE_RS21145
EAE_RS21150
EAE_RS21150
D_1977
D_1976
D_1975
D_1974
D_1973
D_1972
Dc_2252
Dc_2251
Dc_2250
Dc_2249
Dc_2248
Dc_2247
Dc_2246
I_2531
I_2530
I_2529
I_2528
I_2527
I_2526
I_2525
I_2524
Term: termination (1), NS: new start (2), PS: pausing site (2), iTSS_I: internal starting site (1)

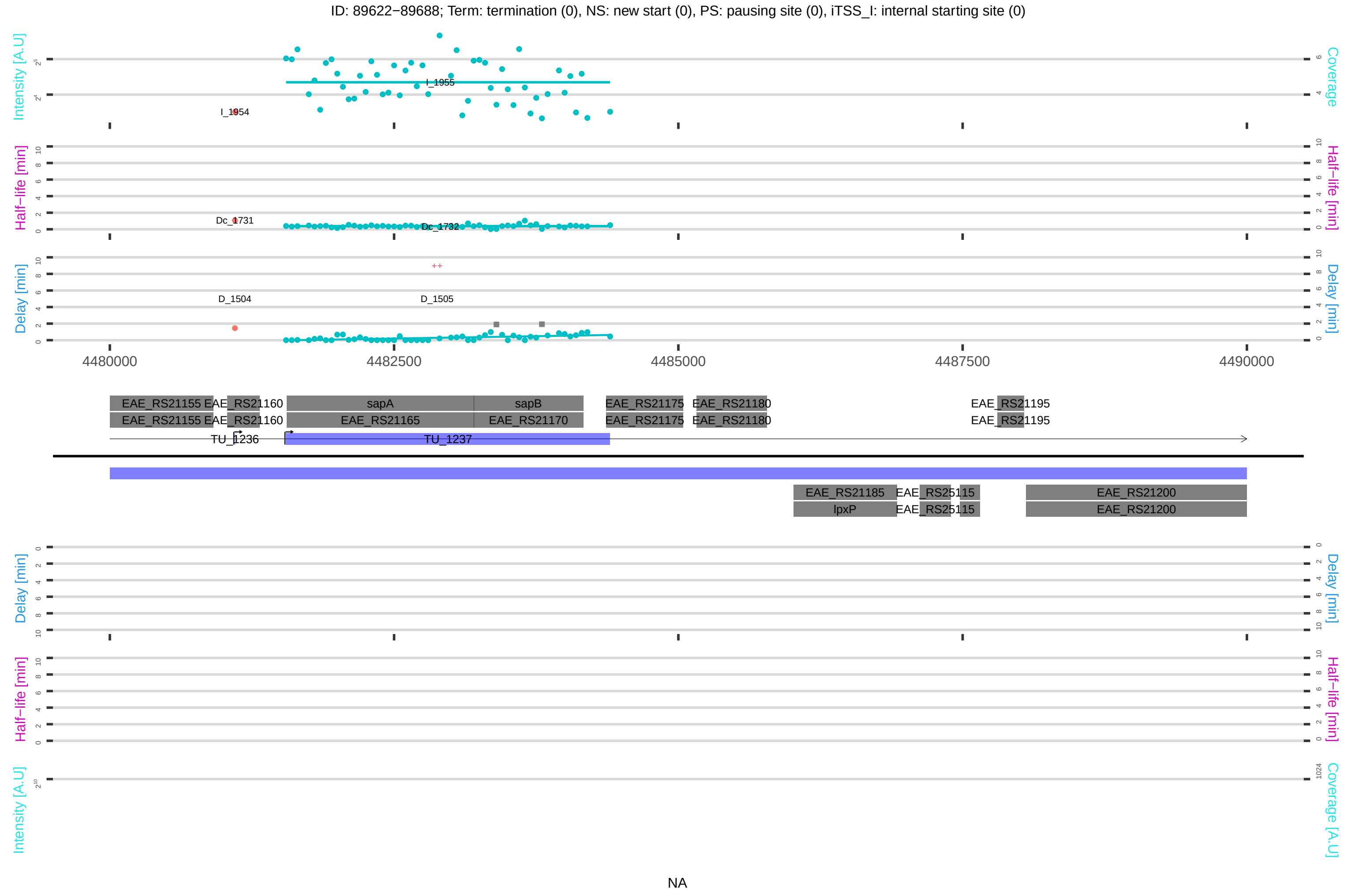

ID: 89622−89688; Term: termination (0), NS: new start (0), PS: pausing site (0), iTSS_I: internal starting site (0)
Intensity [A.U]
Coverage
I_1954
I_1955
Half−life [min]
Dc_1731
Dc_1732
Delay [min]
D_1504
D_1505
4480000
4482500
4485000
4487500
4490000
EAE_RS21155
EAE_RS21160
sapA
EAE_RS21165
sapB
EAE_RS21170
EAE_RS21175
EAE_RS21180
EAE_RS21195
TU_1236
TU_1237
EAE_RS21185
lpxP
EAE_RS25115
EAE_RS21200
NA

ID: 121224−121224; FC*: significant t−test of two consecutive segments; Term: termination, NS: new start, PS: pausing site,iTSS_I: internal starting site,TI: transcription interferance.

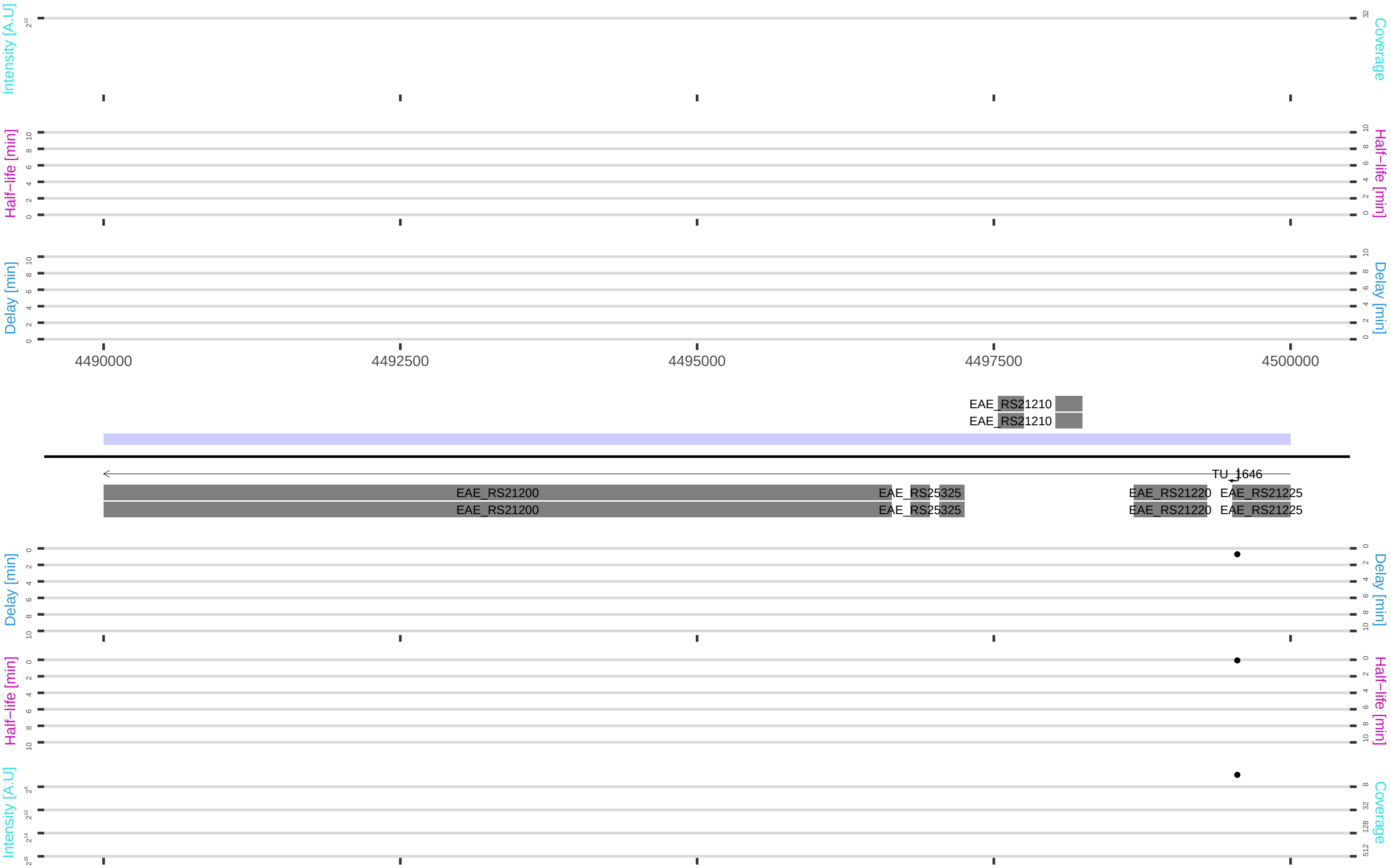

Term: termination (0), NS: new start (0), PS: pausing site (0), iTSS_I: internal starting site (0)

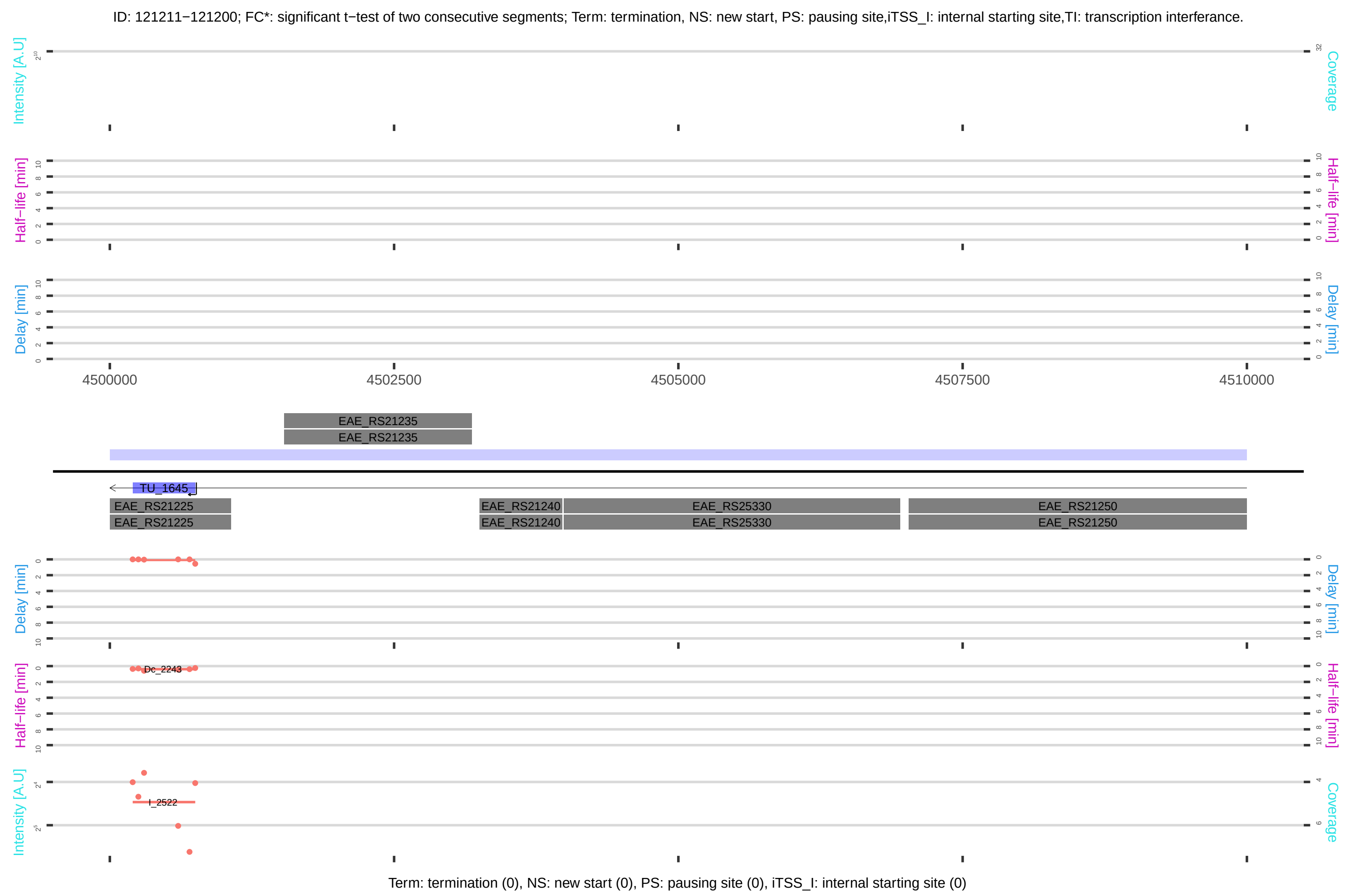
ID: 121211−121200; FC*: significant t−test of two consecutive segments; Term: termination, NS: new start, PS: pausing site,iTSS_I: internal starting site,TI: transcription interferance.
Intensity [A.U]
Coverage
Half−life [min]
Delay [min]
4500000
4502500
4505000
4507500
4510000
EAE_RS21235
TU_1645
EAE_RS21225
EAE_RS21240
EAE_RS25330
EAE_RS21250
Dc_2243
I_2522
Term: termination (0), NS: new start (0), PS: pausing site (0), iTSS_I: internal starting site (0)

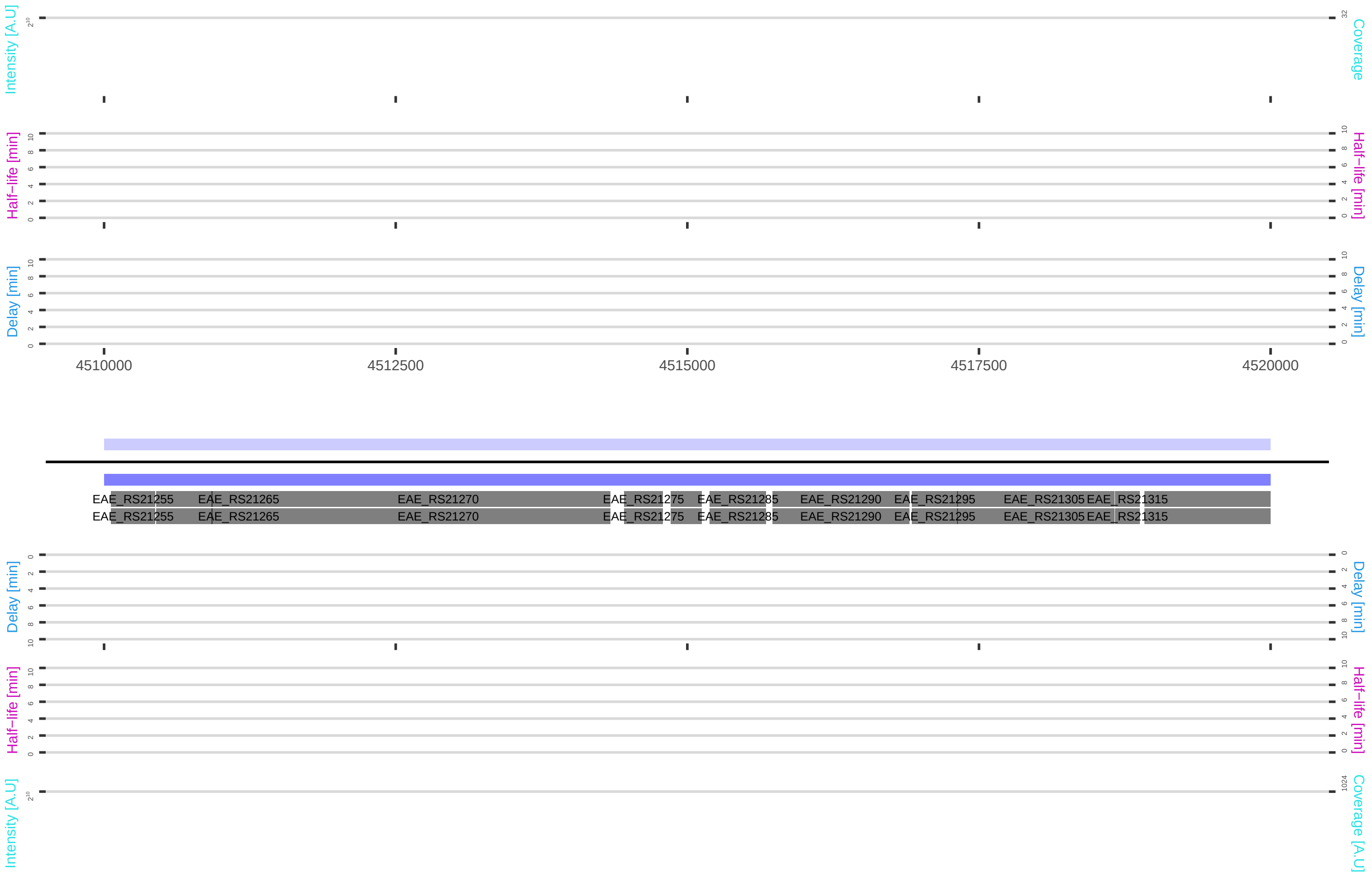

Intensity [A.U]
Coverage
Half−life [min]
Delay [min]
4510000
4512500
4515000
4517500
4520000
EAE_RS21255
EAE_RS21265
EAE_RS21270
EAE_RS21275
EAE_RS21285
EAE_RS21290
EAE_RS21295
EAE_RS21305
EAE_RS21315
Coverage [A.U]
NA

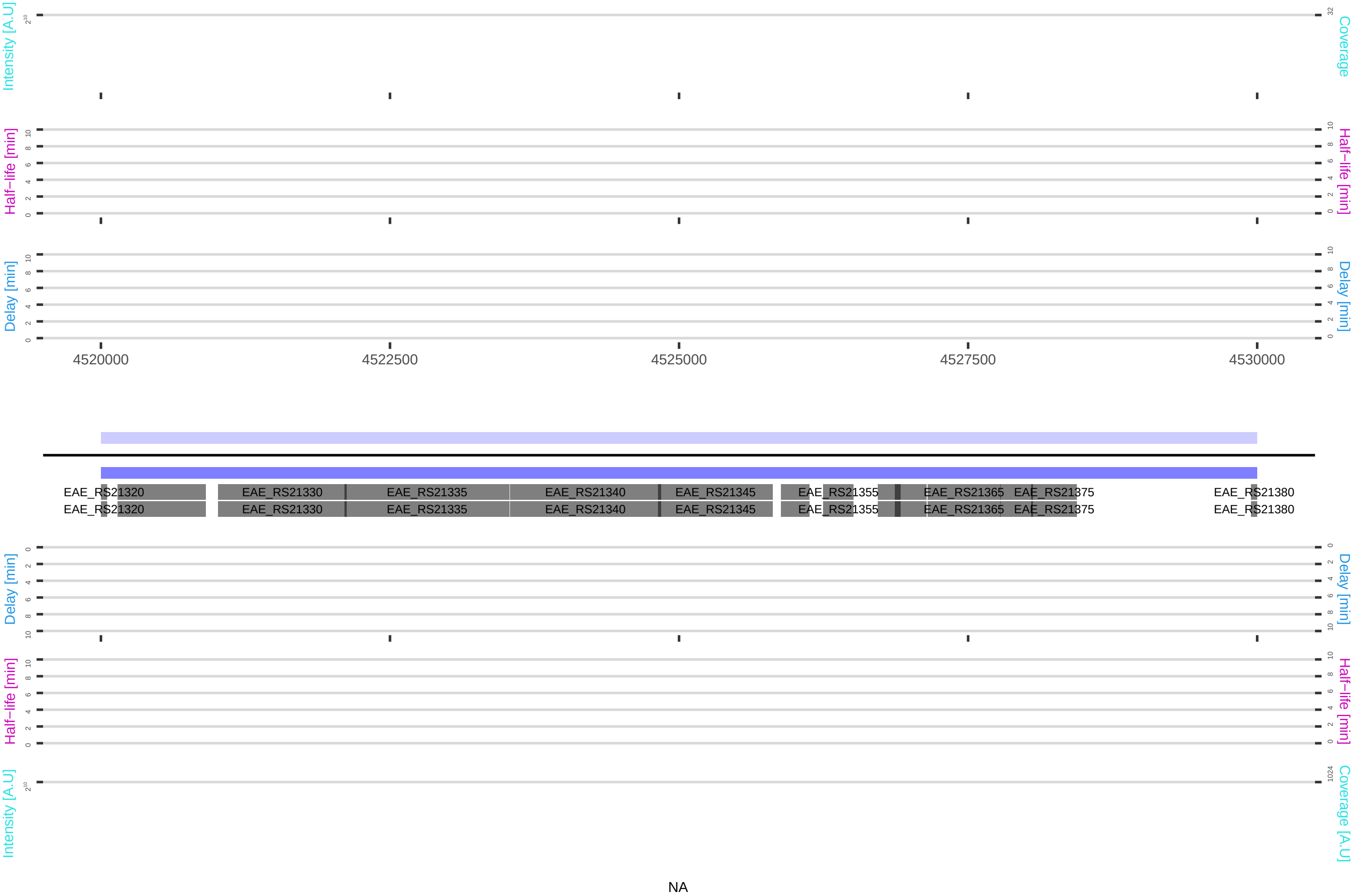

Intensity [A.U]
Coverage
Half−life [min]
Delay [min]
4520000
4522500
4525000
4527500
4530000
EAE_RS21320
EAE_RS21330
EAE_RS21335
EAE_RS21340
EAE_RS21345
EAE_RS21355
EAE_RS21365
EAE_RS21375
EAE_RS21380
Coverage [A.U]
NA

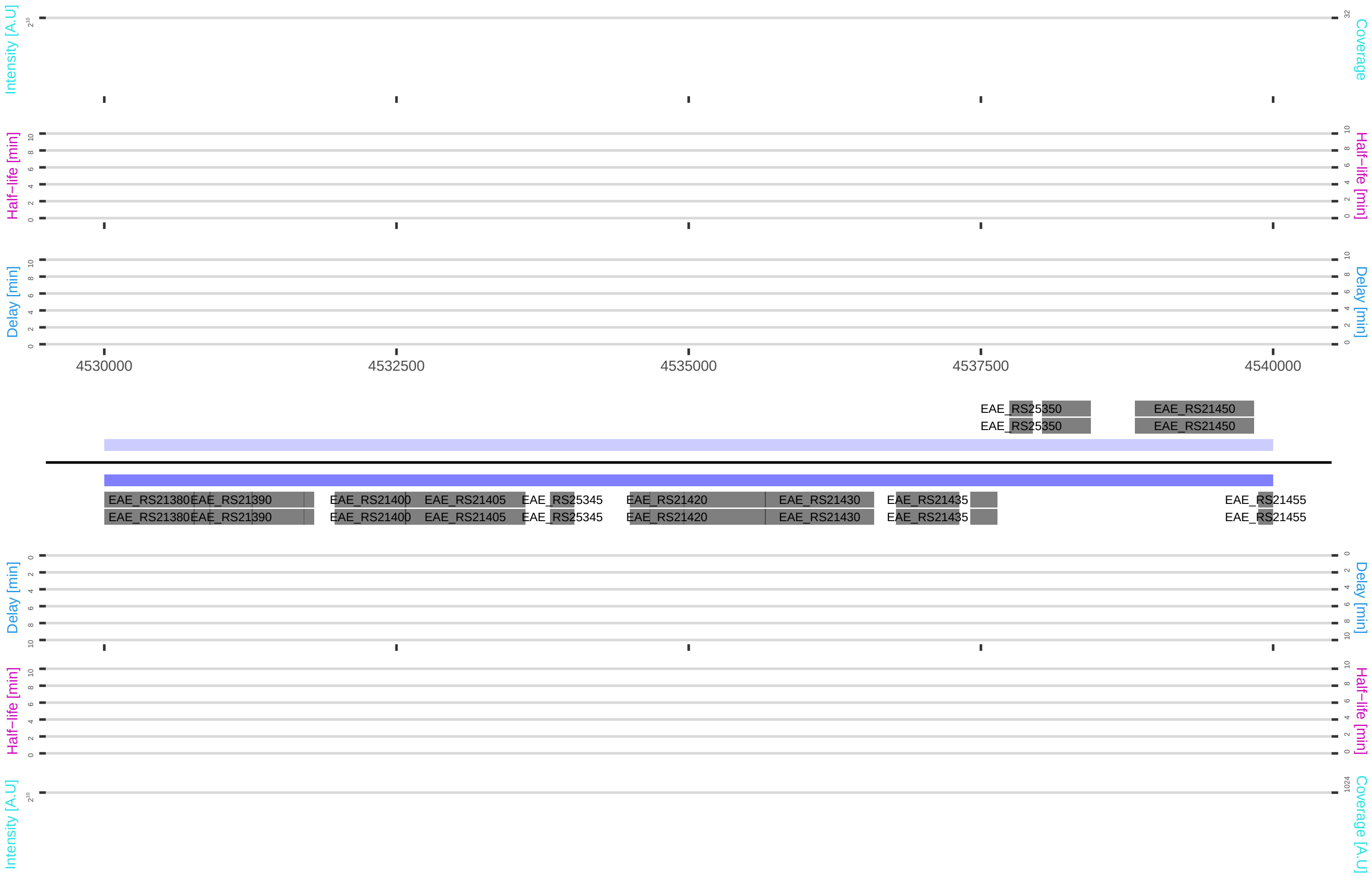
Intensity [A.U]
Coverage
Half−life [min]
Delay [min]
4530000
4532500
4535000
4537500
4540000
EAE_RS25350
EAE_RS21450
EAE_RS21380
EAE_RS21390
EAE_RS21400
EAE_RS21405
EAE_RS25345
EAE_RS21420
EAE_RS21430
EAE_RS21435
EAE_RS21455
Coverage [A.U]
NA

ID: 90940−90998; Term: termination (0), NS: new start (0), PS: pausing site (0), iTSS_I: internal starting site (0)

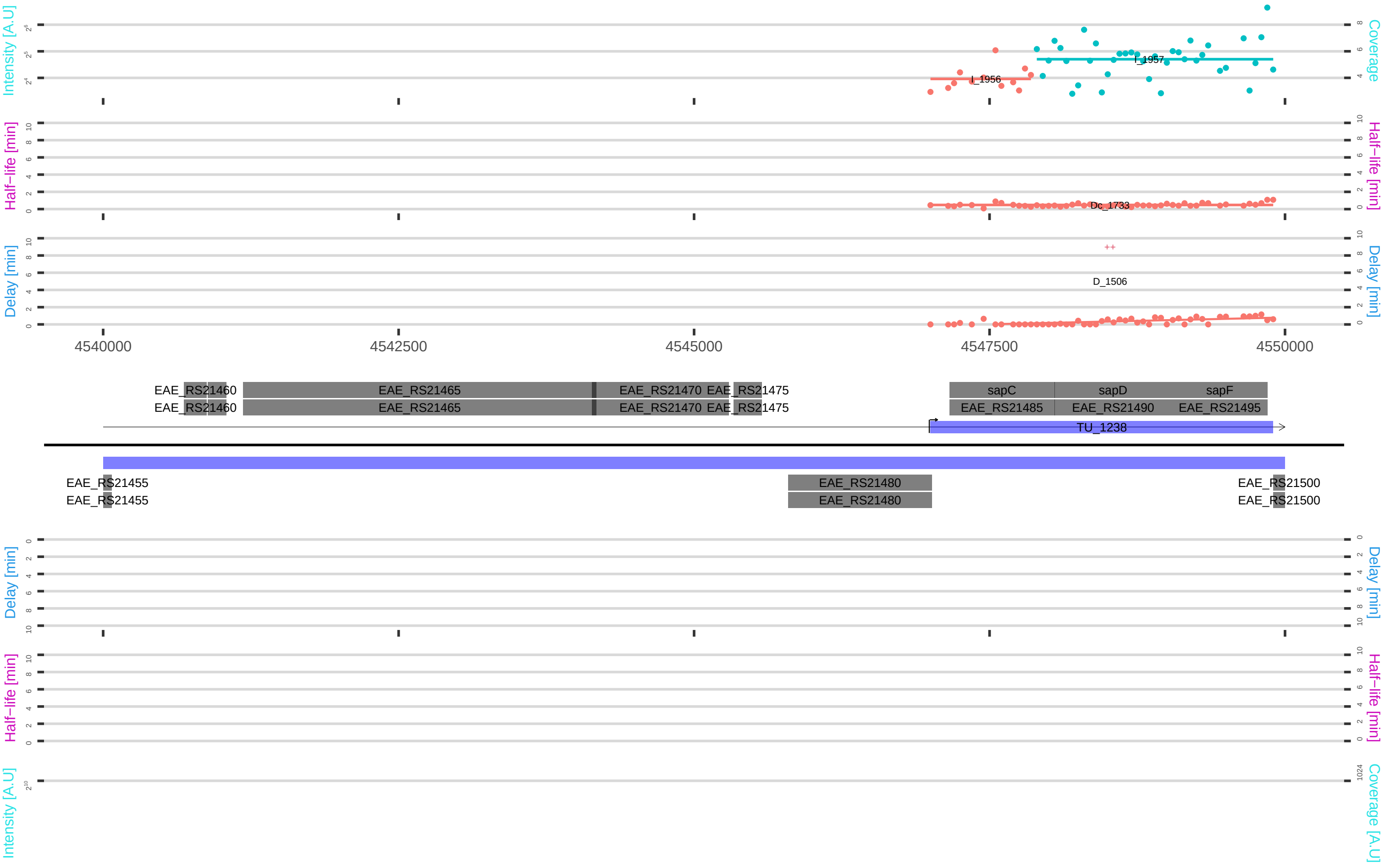

NA

ID: 91044−91164; Term: termination (1), NS: new start (0), PS: pausing site (1), iTSS_I: internal starting site (0)

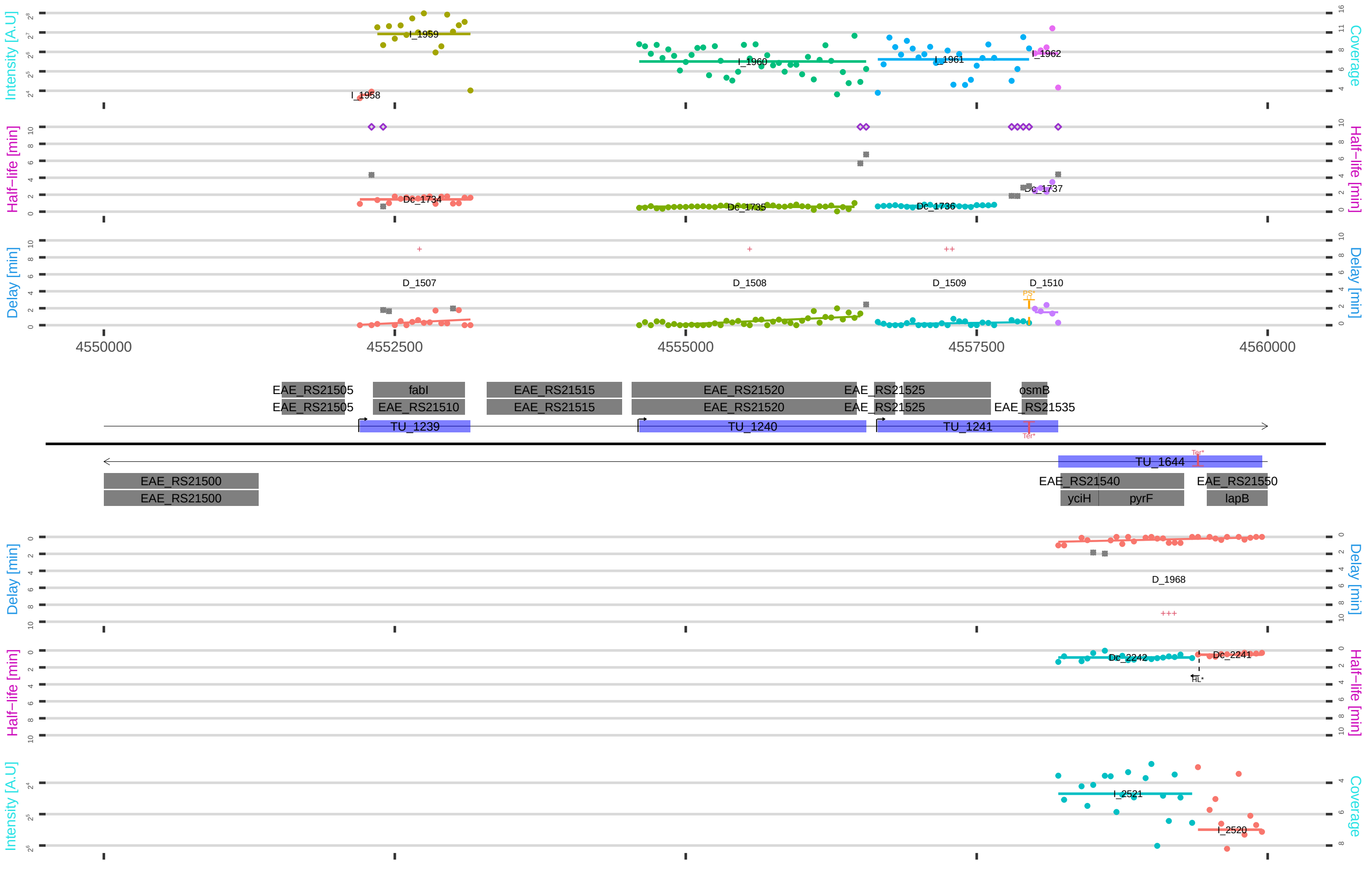

Term: termination (1), NS: new start (0), PS: pausing site (0), iTSS_I: internal starting site (0)

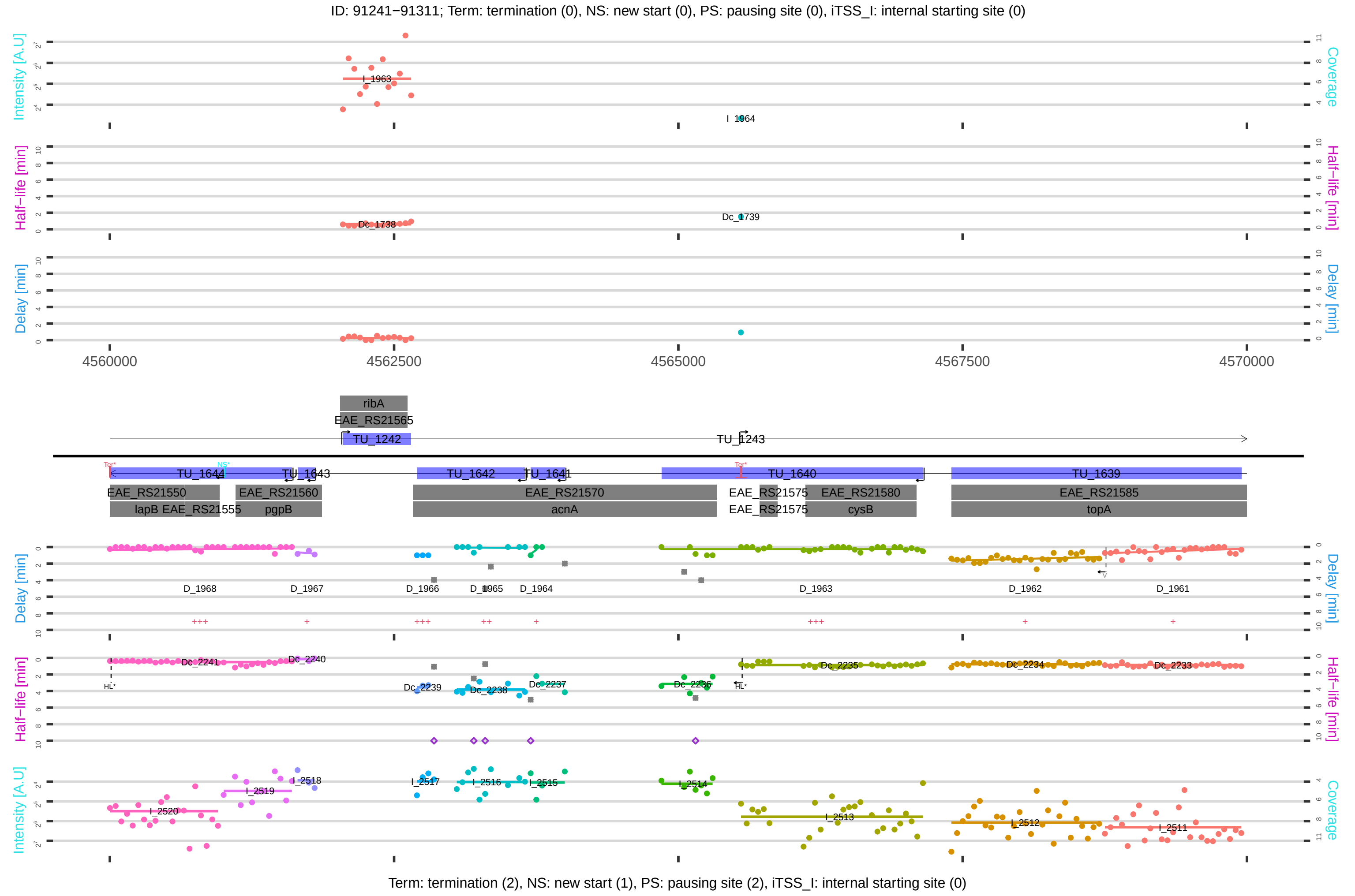

ID: 91241−91311; Term: termination (0), NS: new start (0), PS: pausing site (0), iTSS_I: internal starting site (0)
Intensity [A.U]
Coverage
Half−life [min]
Delay [min]
I_1963
I_1964
Dc_1738
Dc_1739
4560000
4562500
4565000
4567500
4570000
ribA
EAE_RS21565
TU_1242
TU_1243
TU_1644
TU_1643
TU_1642
TU_1641
TU_1640
TU_1639
EAE_RS21550
EAE_RS21560
EAE_RS21570
EAE_RS21575
EAE_RS21580
EAE_RS21585
lapB
EAE_RS21555
pgpB
acnA
cysB
topA
D_1968
D_1967
D_1966
D_1965
D_1964
D_1963
D_1962
D_1961
Dc_2241
Dc_2240
Dc_2239
Dc_2238
Dc_2237
Dc_2236
Dc_2235
Dc_2234
Dc_2233
I_2520
I_2519
I_2518
I_2517
I_2516
I_2515
I_2514
I_2513
I_2512
I_2511
Term: termination (2), NS: new start (1), PS: pausing site (2), iTSS_I: internal starting site (0)

ID: 91408−91476; Term: termination (0), NS: new start (1), PS: pausing site (0), iTSS_I: internal starting site (0)

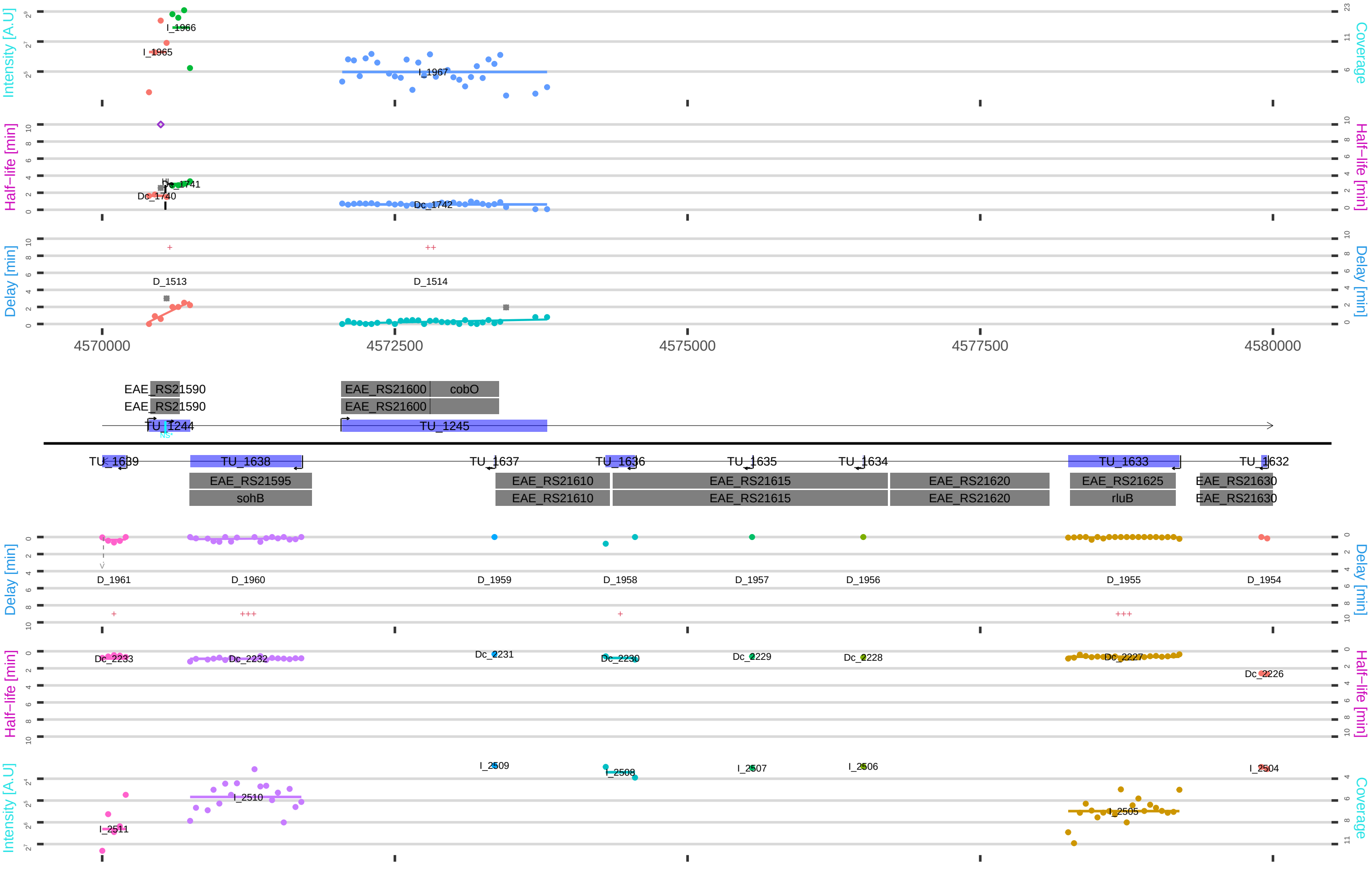

Term: termination (0), NS: new start (0), PS: pausing site (0), iTSS_I: internal starting site (0)

ID: 91729−91800; Term: termination (0), NS: new start (0), PS: pausing site (0), iTSS_I: internal starting site (0)

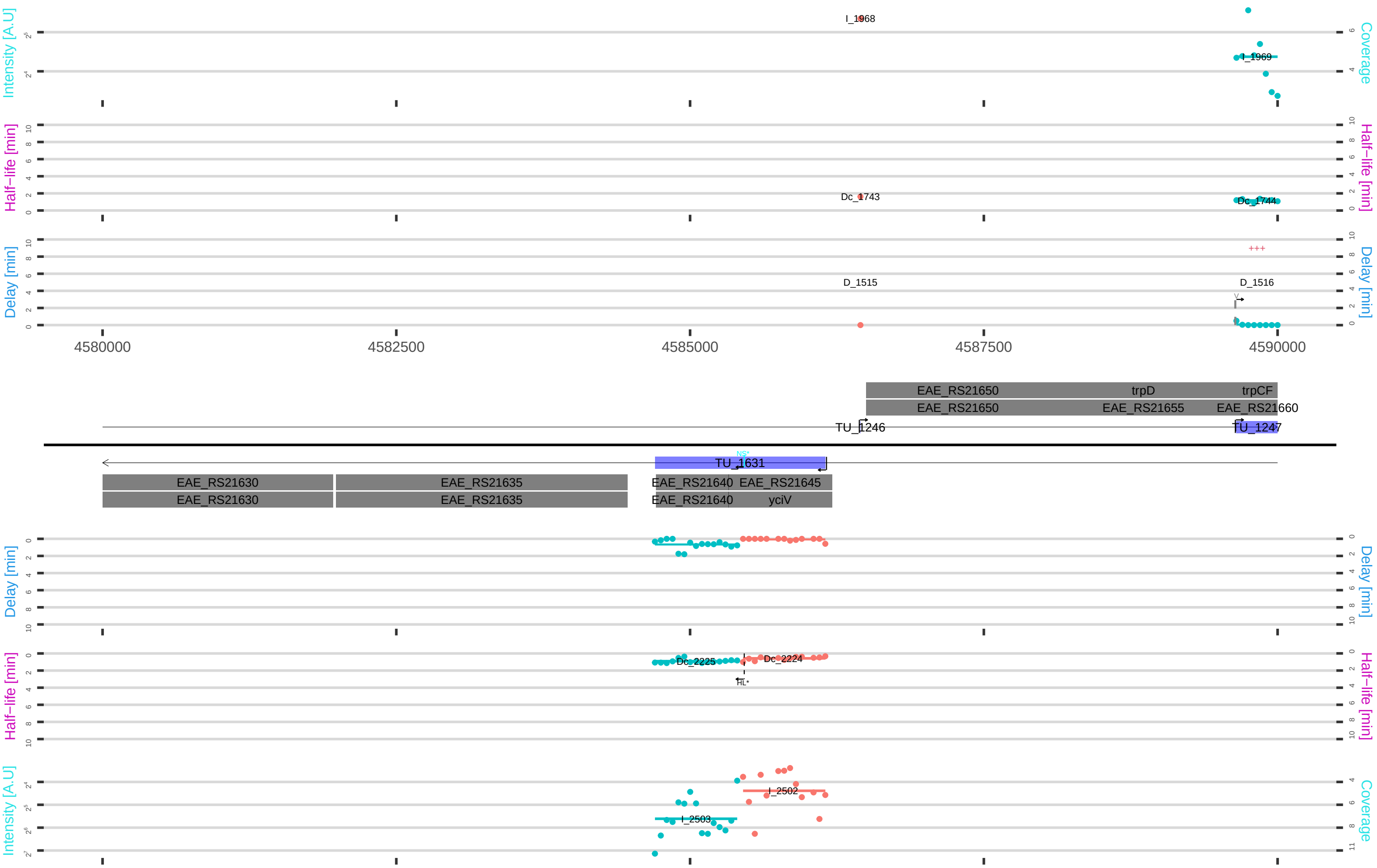

Term: termination (0), NS: new start (1), PS: pausing site (1), iTSS_I: internal starting site (0)

ID: 91800−92000; Term: termination (0), NS: new start (0), PS: pausing site (1), iTSS_I: internal starting site (0)

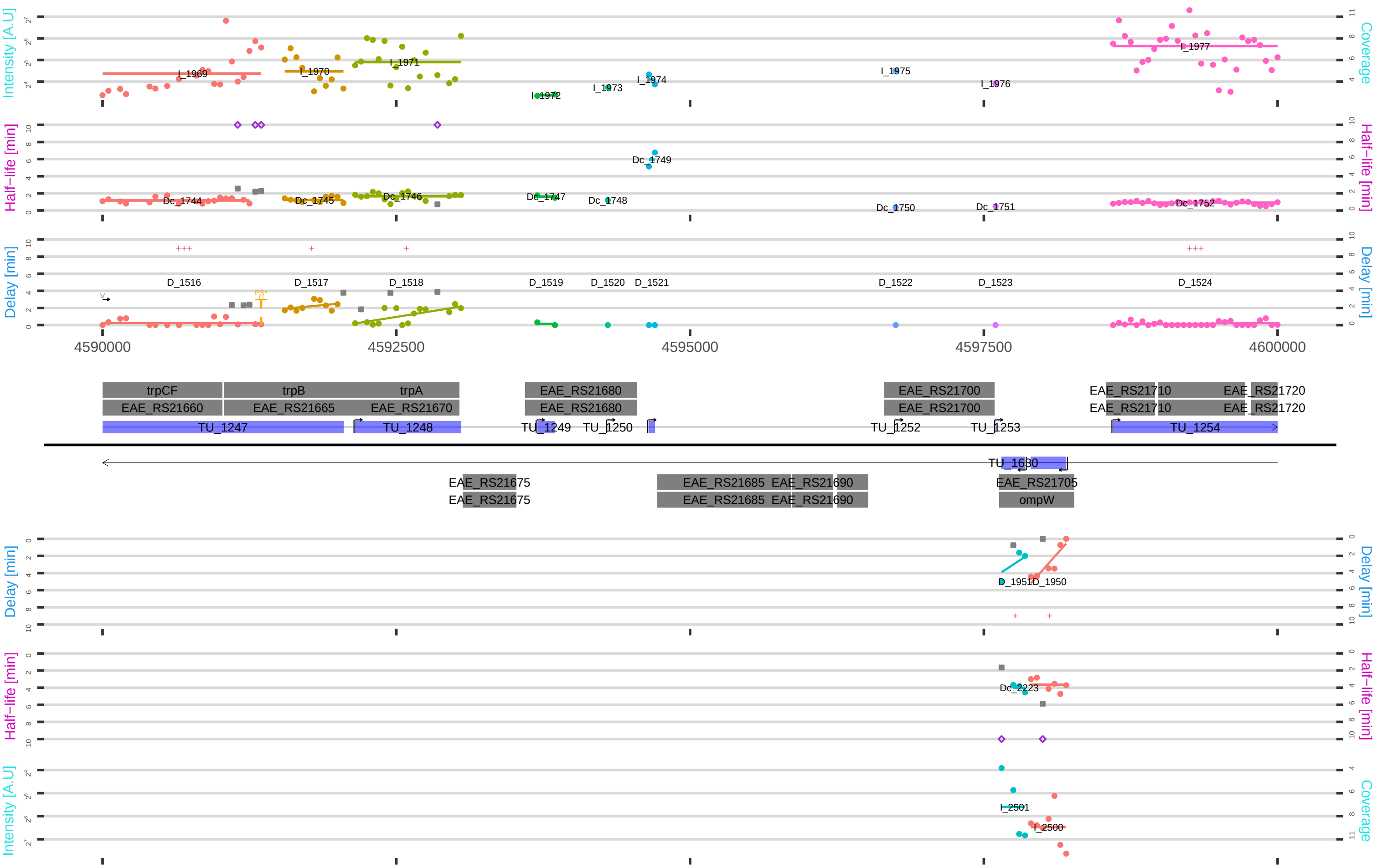

Term: termination (0), NS: new start (0), PS: pausing site (0), iTSS_I: internal starting site (0)

ID: 92000−92194; Term: termination (0), NS: new start (0), PS: pausing site (0), iTSS_I: internal starting site (0)

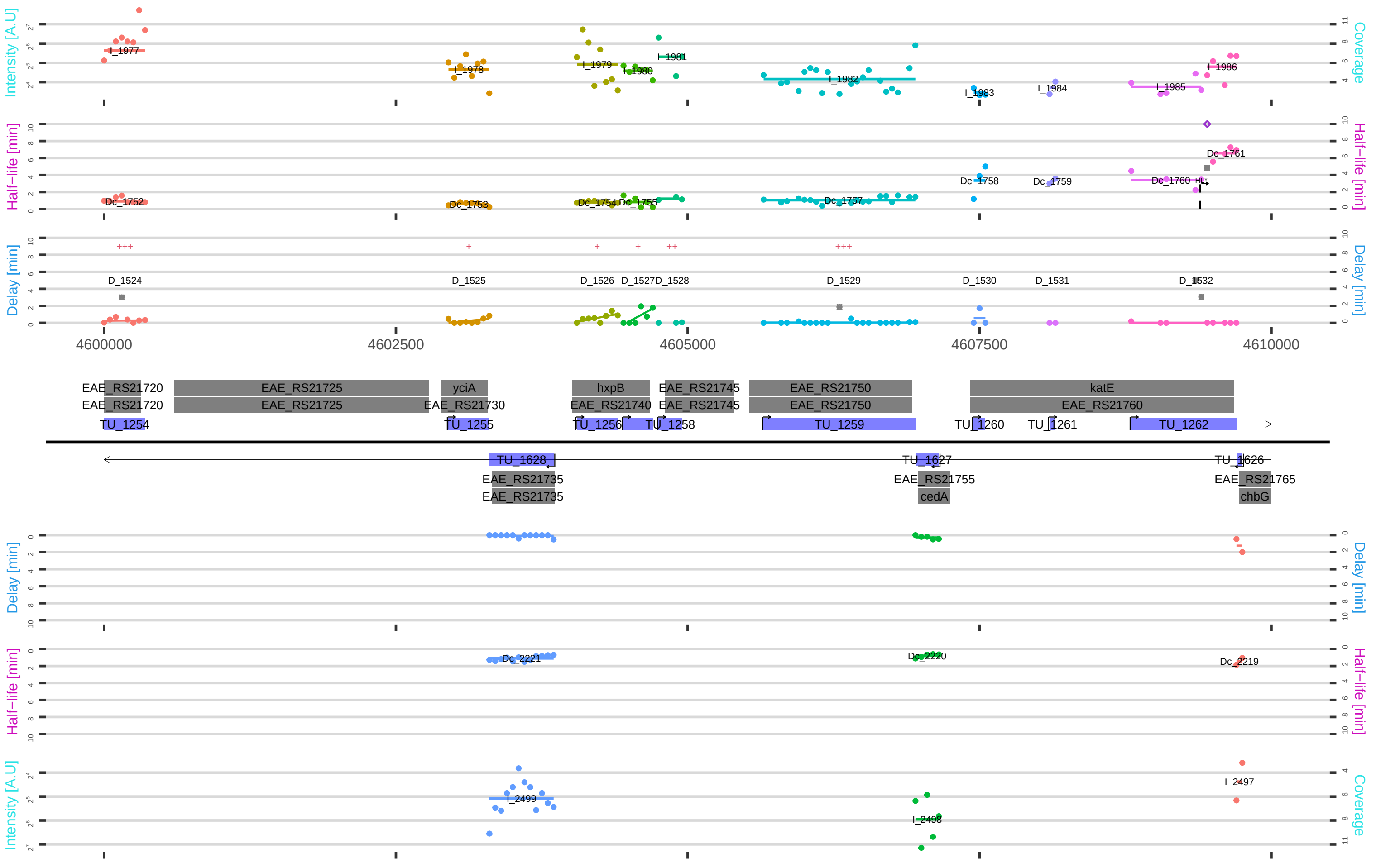

Term: termination (0), NS: new start (0), PS: pausing site (0), iTSS_I: internal starting site (0)

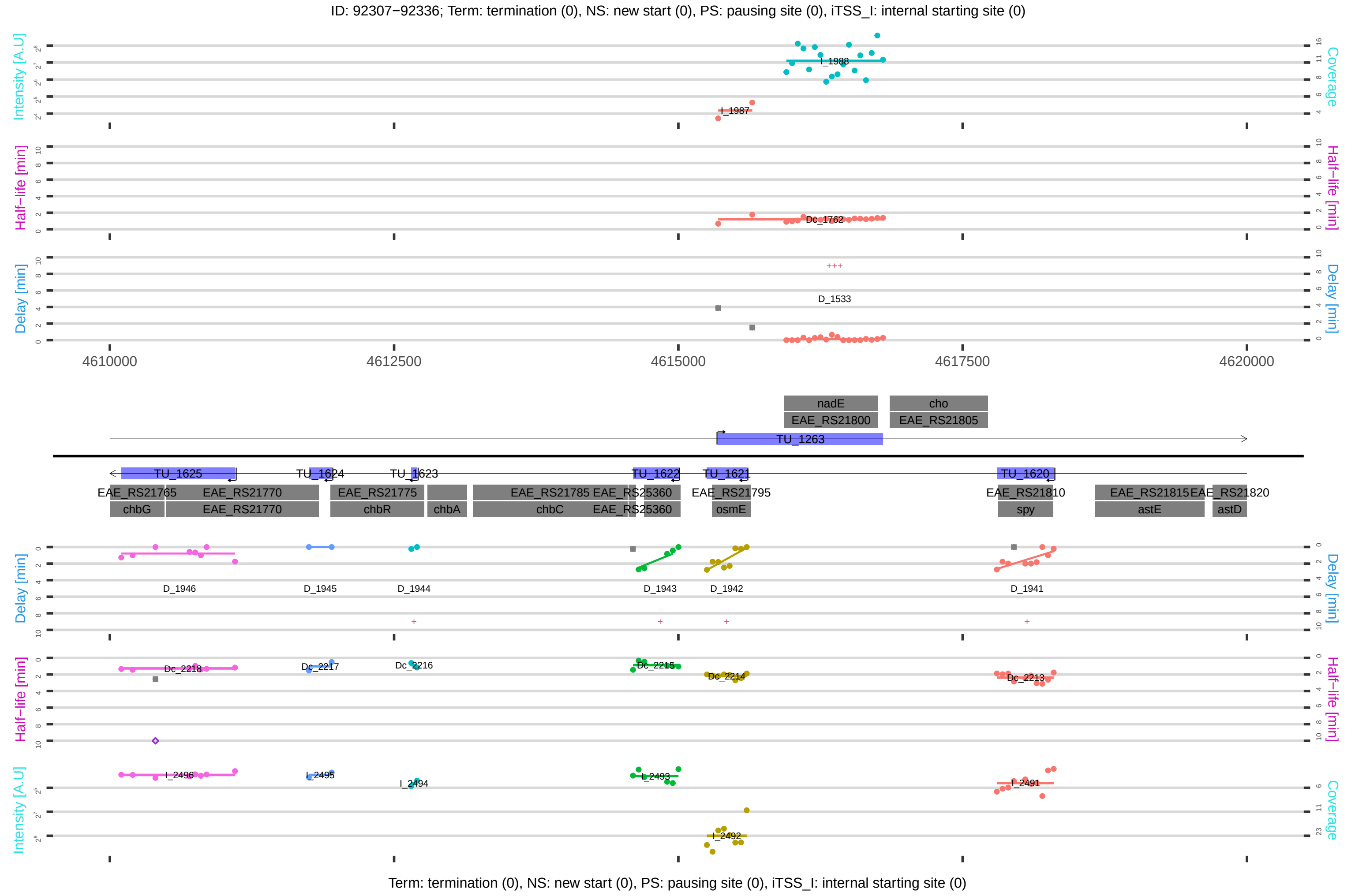
ID: 92307−92336; Term: termination (0), NS: new start (0), PS: pausing site (0), iTSS_I: internal starting site (0)
Intensity [A.U]
Coverage
Half−life [min]
Delay [min]
I_1988
I_1987
Dc_1762
D_1533
4610000
4612500
4615000
4617500
4620000
nadE
EAE_RS21800
cho
EAE_RS21805
TU_1263
TU_1625
TU_1624
TU_1623
TU_1622
TU_1621
TU_1620
EAE_RS21765
chbG
EAE_RS21770
EAE_RS21775
chbR
chbA
EAE_RS21785
chbC
EAE_RS25360
EAE_RS21795
osmE
EAE_RS21810
spy
EAE_RS21815
astE
EAE_RS21820
astD
D_1946
D_1945
D_1944
D_1943
D_1942
D_1941
Dc_2218
Dc_2217
Dc_2216
Dc_2215
Dc_2214
Dc_2213
I_2496
I_2495
I_2494
I_2493
I_2492
I_2491
Term: termination (0), NS: new start (0), PS: pausing site (0), iTSS_I: internal starting site (0)

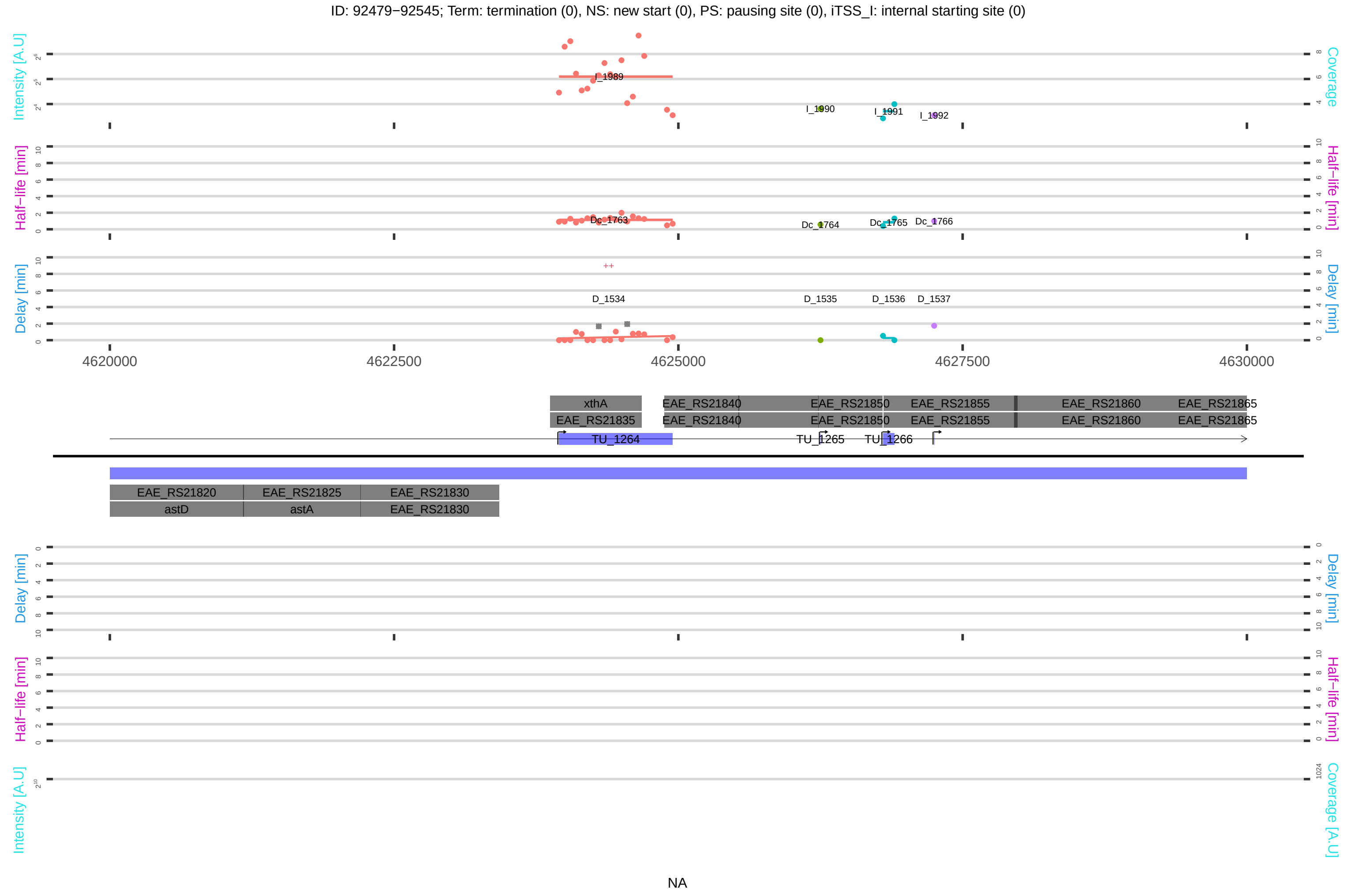
ID: 92479−92545; Term: termination (0), NS: new start (0), PS: pausing site (0), iTSS_I: internal starting site (0)
Intensity [A.U]
Coverage
I_1989
I_1990
I_1991
I_1992
Half−life [min]
Dc_1763
Dc_1764
Dc_1765
Dc_1766
Delay [min]
D_1534
D_1535
D_1536
D_1537
4620000
4622500
4625000
4627500
4630000
xthA
EAE_RS21835
EAE_RS21840
EAE_RS21850
EAE_RS21855
EAE_RS21860
EAE_RS21865
TU_1264
TU_1265
TU_1266
EAE_RS21820
EAE_RS21825
EAE_RS21830
astD
astA
Coverage [A.U]
NA

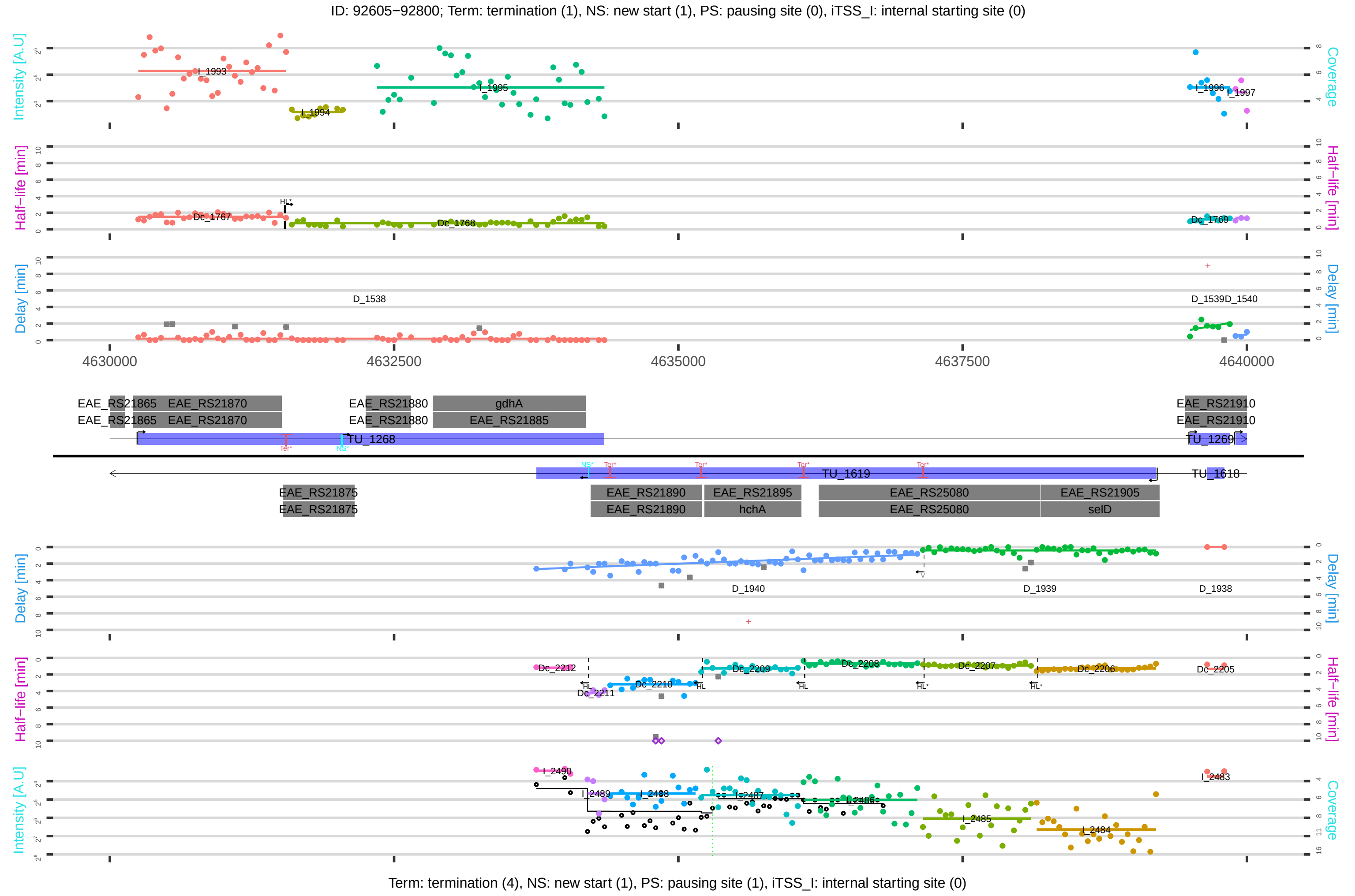

ID: 92605−92800; Term: termination (1), NS: new start (1), PS: pausing site (0), iTSS_I: internal starting site (0)
Intensity [A.U]
Coverage
Half−life [min]
Delay [min]
I_1993
I_1994
I_1995
I_1996
I_1997
Dc_1767
Dc_1768
Dc_1769
D_1538
D_1539D_1540
4630000
4632500
4635000
4637500
4640000
EAE_RS21865
EAE_RS21870
EAE_RS21880
gdhA
EAE_RS21885
EAE_RS21910
TU_1268
TU_1269
TU_1619
TU_1618
EAE_RS21875
EAE_RS21890
EAE_RS21895
hchA
EAE_RS25080
EAE_RS21905
selD
D_1940
D_1939
D_1938
Dc_2212
Dc_2211
Dc_2210
Dc_2209
Dc_2208
Dc_2207
Dc_2206
Dc_2205
I_2490
I_2489
I_2488
I_2487
I_2486
I_2485
I_2484
I_2483
Term: termination (4), NS: new start (1), PS: pausing site (1), iTSS_I: internal starting site (0)

ID: 92800−92994; Term: termination (0), NS: new start (2), PS: pausing site (2), iTSS_I: internal starting site (0)

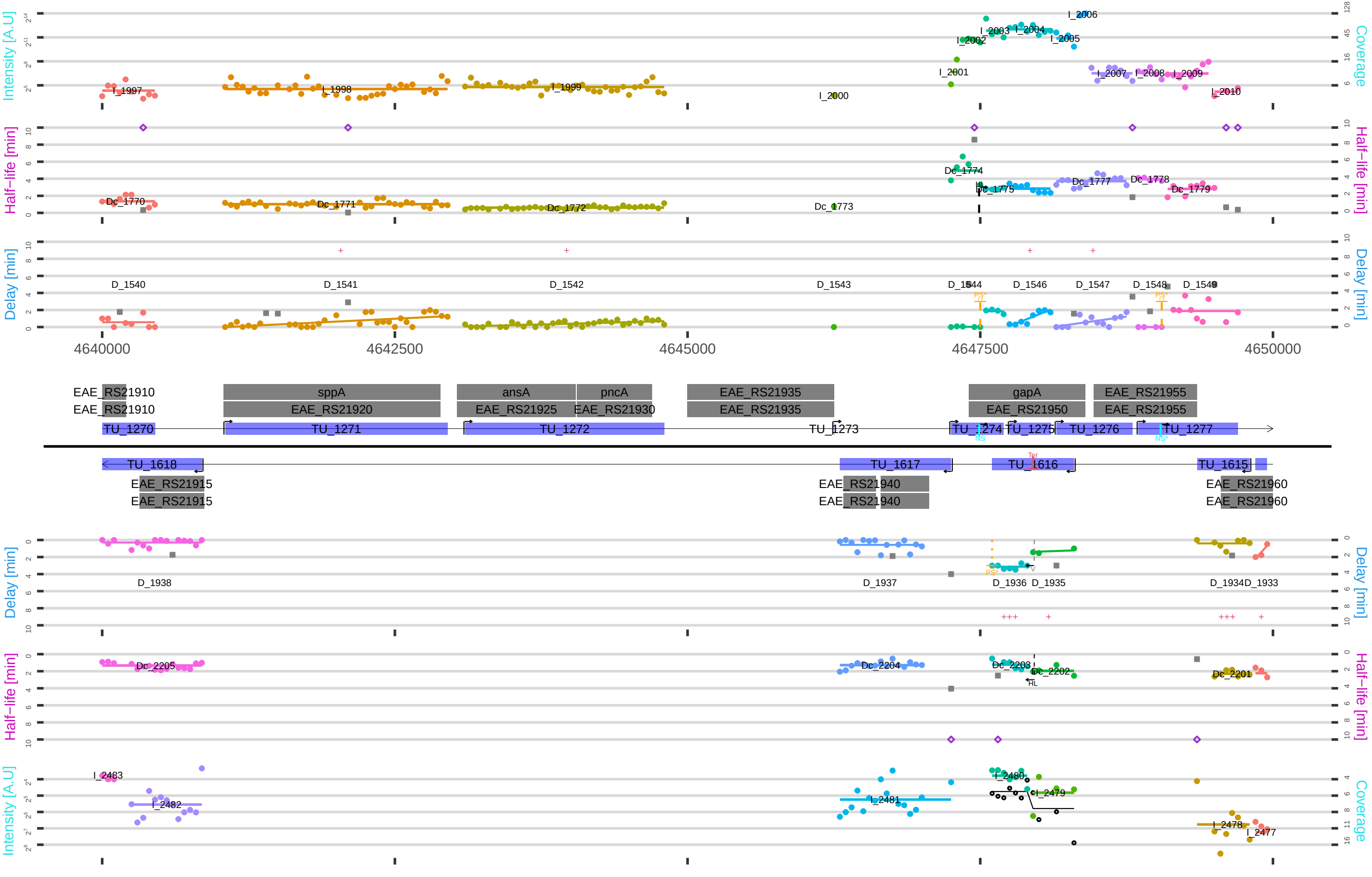

Term: termination (1), NS: new start (0), PS: pausing site (1), iTSS_I: internal starting site (0)

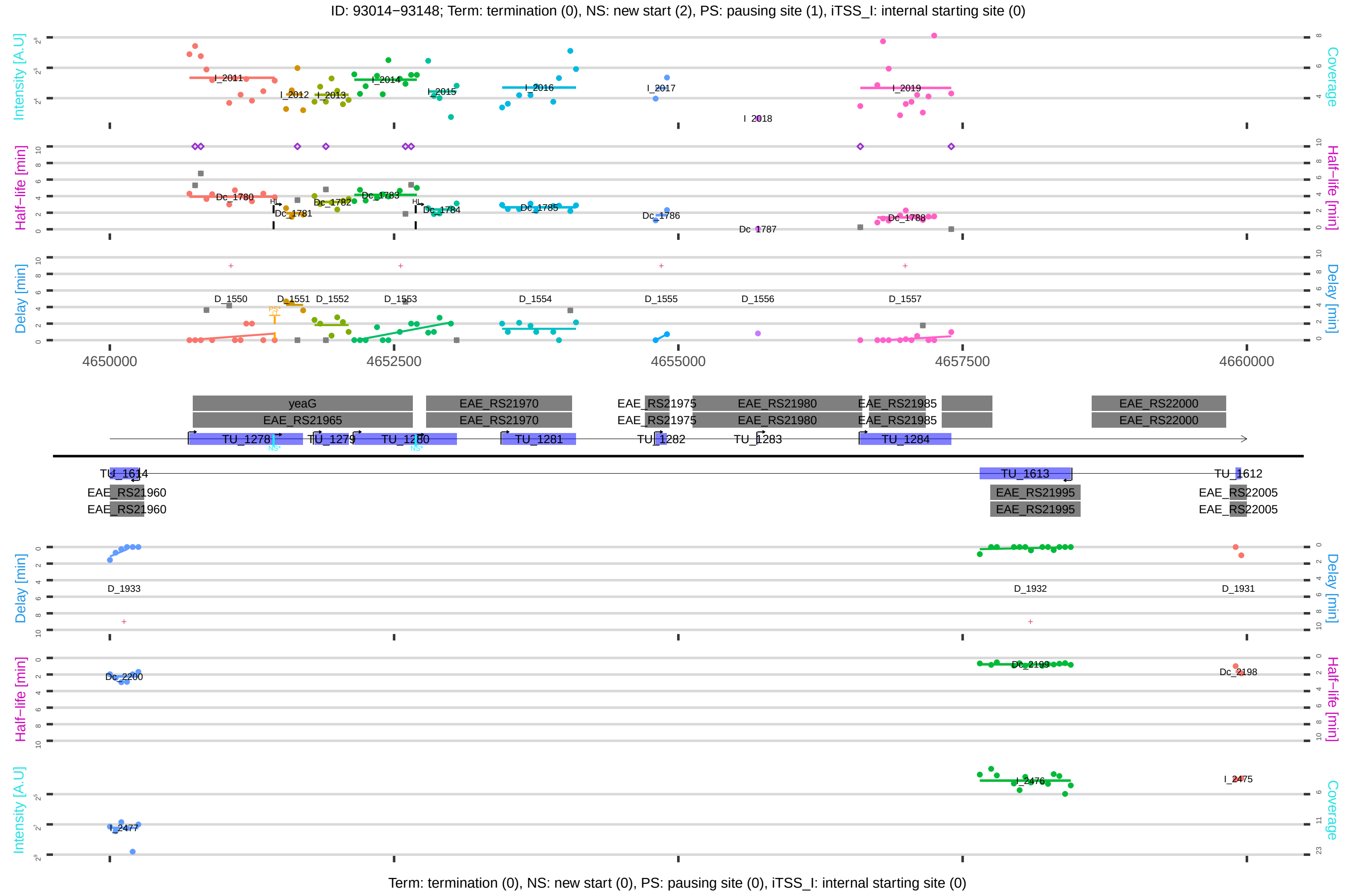

ID: 93014−93148; Term: termination (0), NS: new start (2), PS: pausing site (1), iTSS_I: internal starting site (0)
Intensity [A.U]
Coverage
Half−life [min]
Delay [min]
I_2011
I_2012
I_2013
I_2014
I_2015
I_2016
I_2017
I_2018
I_2019
Dc_1780
Dc_1781
Dc_1782
Dc_1783
Dc_1784
Dc_1785
Dc_1786
Dc_1787
Dc_1788
D_1550
D_1551
D_1552
D_1553
D_1554
D_1555
D_1556
D_1557
4650000
4652500
4655000
4657500
4660000
yeaG
EAE_RS21965
EAE_RS21970
EAE_RS21975
EAE_RS21980
EAE_RS21985
EAE_RS22000
TU_1278
TU_1279
TU_1280
TU_1281
TU_1282
TU_1283
TU_1284
TU_1614
TU_1613
TU_1612
EAE_RS21960
EAE_RS21995
EAE_RS22005
D_1933
D_1932
D_1931
Dc_2200
Dc_2199
Dc_2198
I_2477
I_2476
I_2475
Term: termination (0), NS: new start (0), PS: pausing site (0), iTSS_I: internal starting site (0)

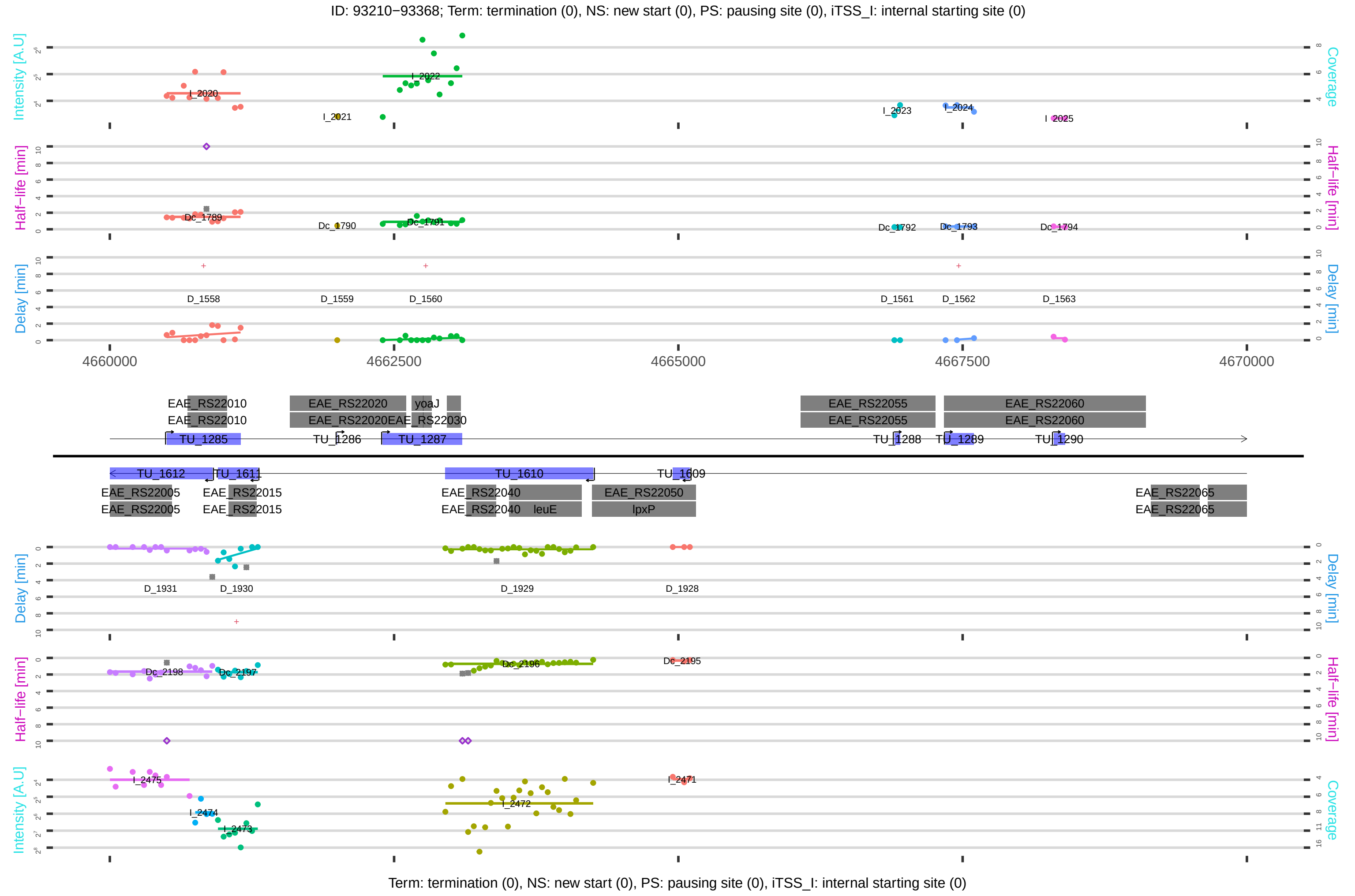

ID: 93210−93368; Term: termination (0), NS: new start (0), PS: pausing site (0), iTSS_I: internal starting site (0)
Intensity [A.U]
Coverage
Half−life [min]
Delay [min]
I_2020
I_2021
I_2022
I_2023
I_2024
I_2025
Dc_1789
Dc_1790
Dc_1791
Dc_1792
Dc_1793
Dc_1794
D_1558
D_1559
D_1560
D_1561
D_1562
D_1563
4660000
4662500
4665000
4667500
4670000
EAE_RS22010
EAE_RS22020
yoaJ
EAE_RS22030
EAE_RS22055
EAE_RS22060
TU_1285
TU_1286
TU_1287
TU_1288
TU_1289
TU_1290
TU_1612
TU_1611
TU_1610
TU_1609
EAE_RS22005
EAE_RS22015
EAE_RS22040
leuE
EAE_RS22050
lpxP
EAE_RS22065
D_1931
D_1930
D_1929
D_1928
Dc_2198
Dc_2197
Dc_2196
Dc_2195
I_2475
I_2474
I_2473
I_2472
I_2471
Term: termination (0), NS: new start (0), PS: pausing site (0), iTSS_I: internal starting site (0)

ID: 93439−93449; Term: termination (0), NS: new start (0), PS: pausing site (0), iTSS_I: internal starting site (0)

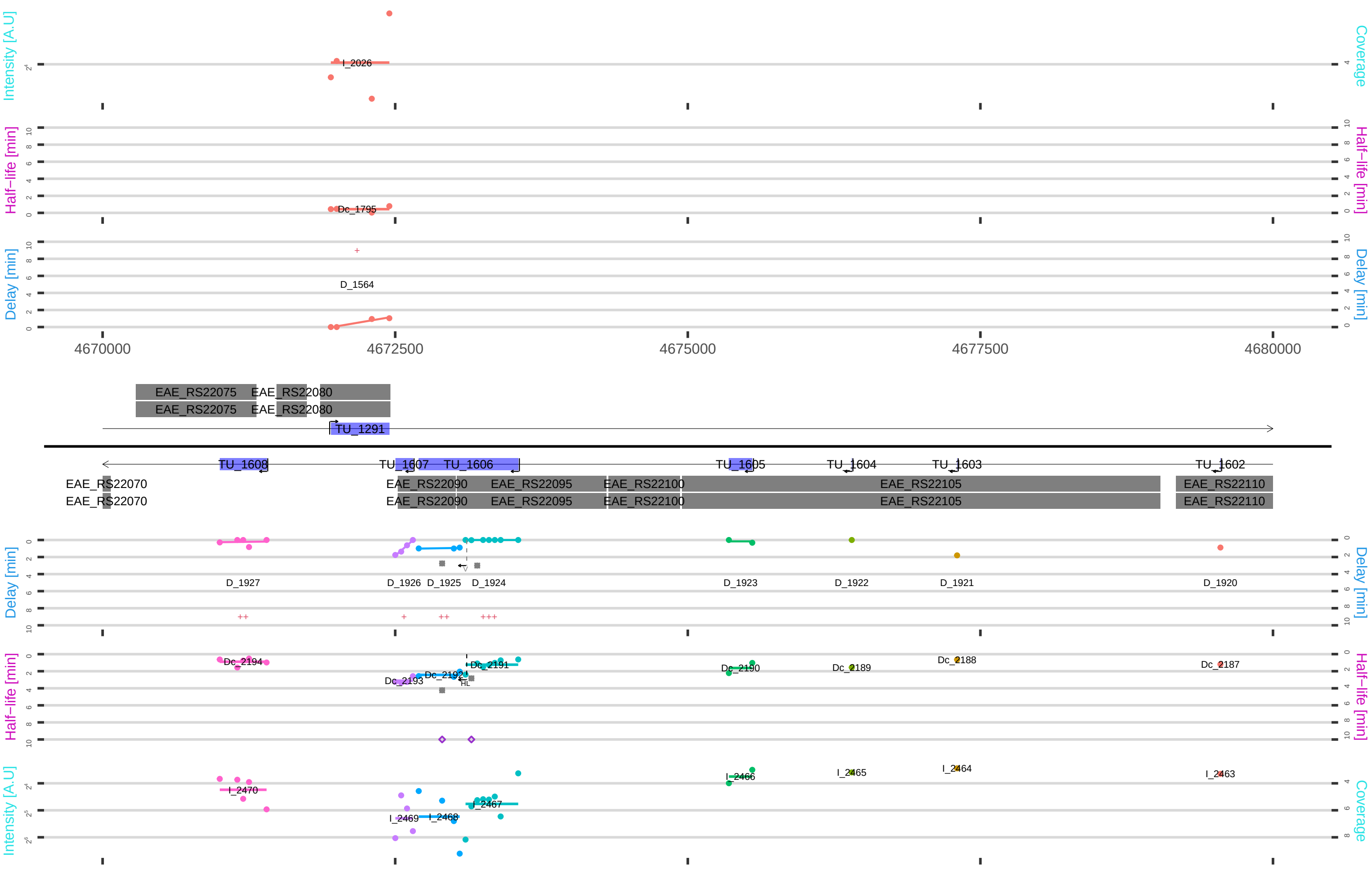

Term: termination (0), NS: new start (0), PS: pausing site (1), iTSS_I: internal starting site (0)

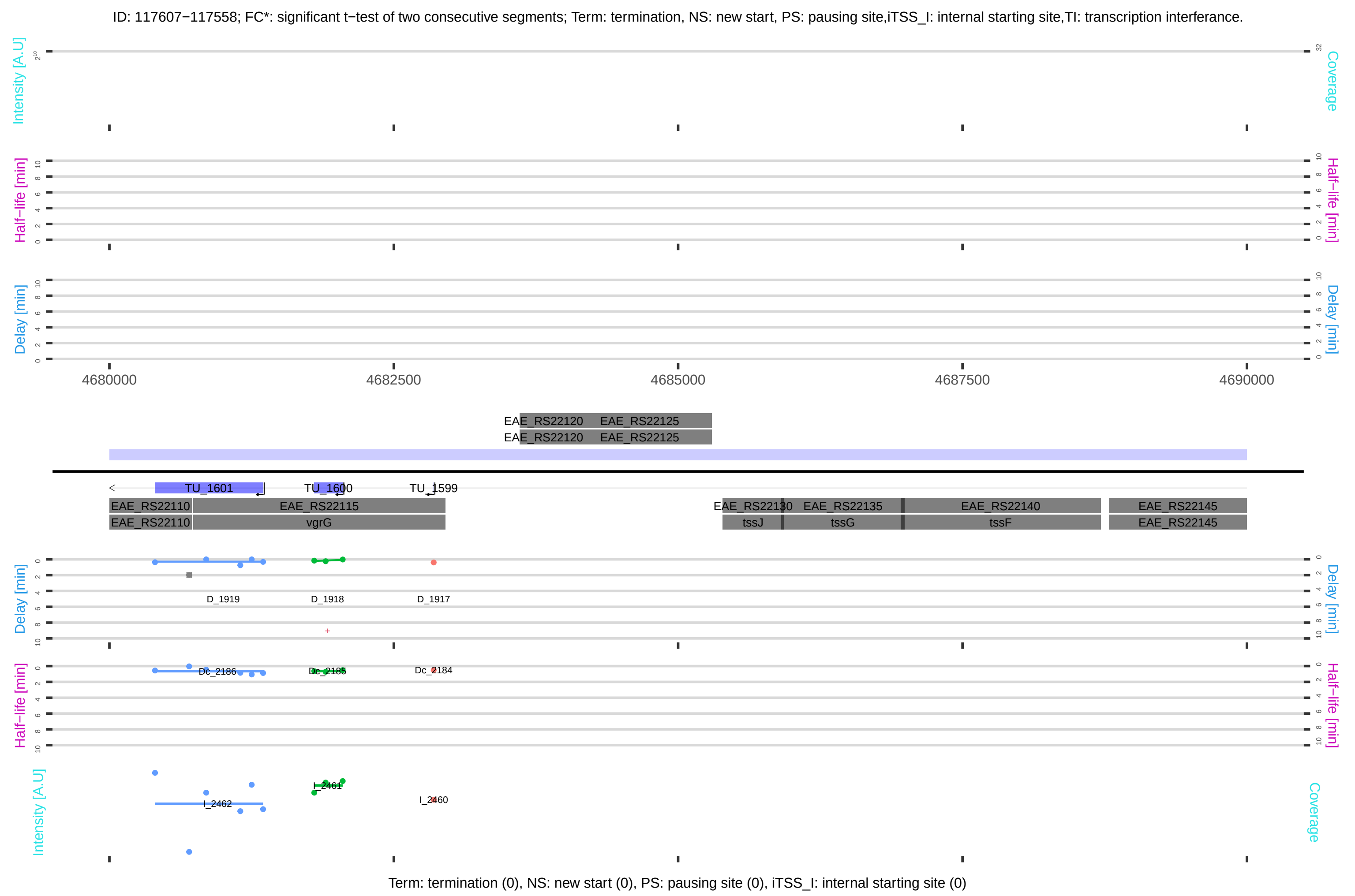
ID: 117607−117558; FC*: significant t−test of two consecutive segments; Term: termination, NS: new start, PS: pausing site,iTSS_I: internal starting site,TI: transcription interferance.
Intensity [A.U]
Coverage
Half−life [min]
Delay [min]
4680000
4682500
4685000
4687500
4690000
EAE_RS22120
EAE_RS22125
TU_1601
TU_1600
TU_1599
EAE_RS22110
EAE_RS22115
vgrG
EAE_RS22130
tssJ
EAE_RS22135
tssG
EAE_RS22140
tssF
EAE_RS22145
D_1919
D_1918
D_1917
Dc_2186
Dc_2185
Dc_2184
I_2462
I_2461
I_2460
Term: termination (0), NS: new start (0), PS: pausing site (0), iTSS_I: internal starting site (0)

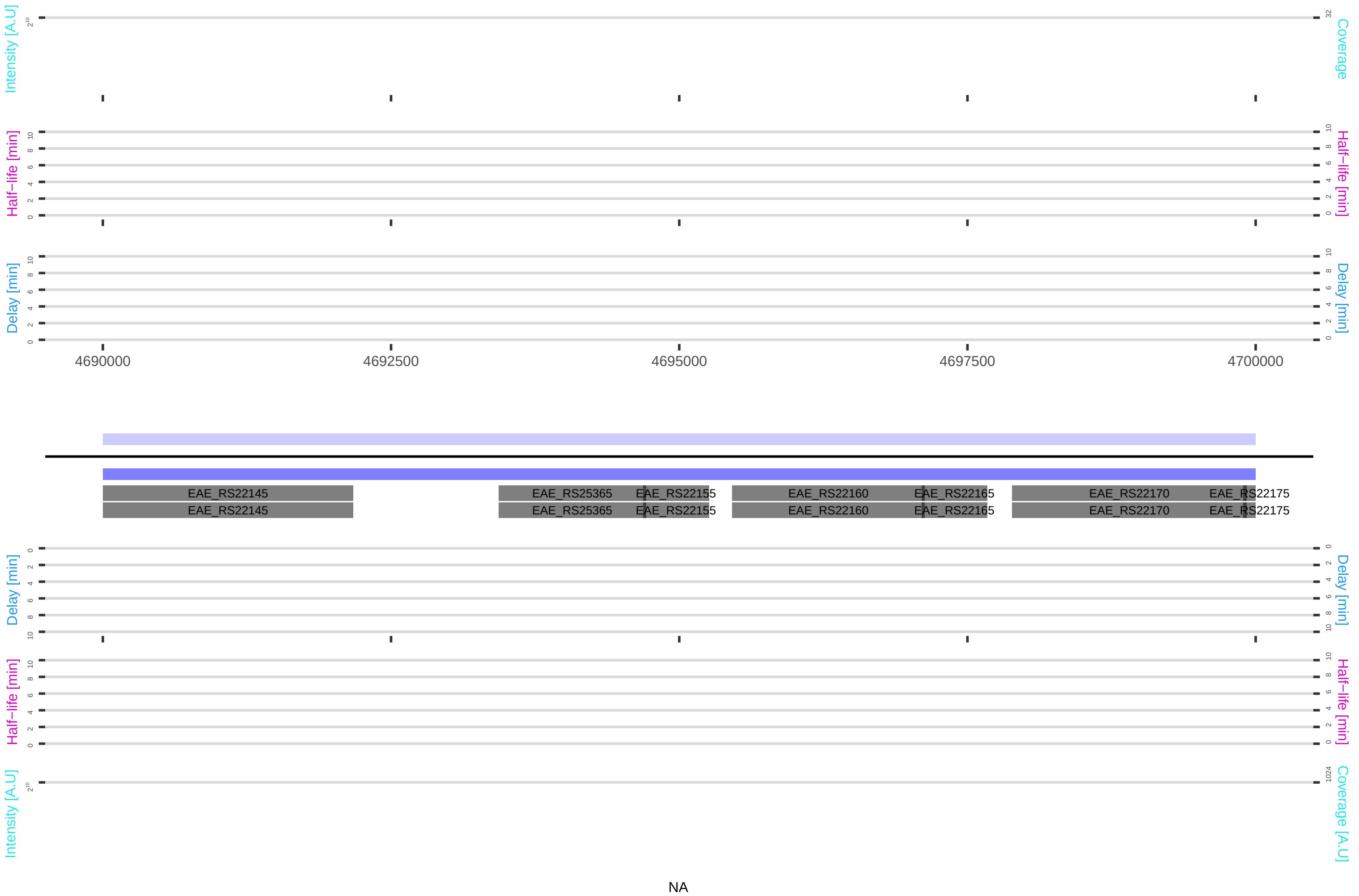

Intensity [A.U]
Coverage
Half−life [min]
Delay [min]
4690000
4692500
4695000
4697500
4700000
EAE_RS22145
EAE_RS25365
EAE_RS22155
EAE_RS22160
EAE_RS22165
EAE_RS22170
EAE_RS22175
Coverage [A.U]
NA

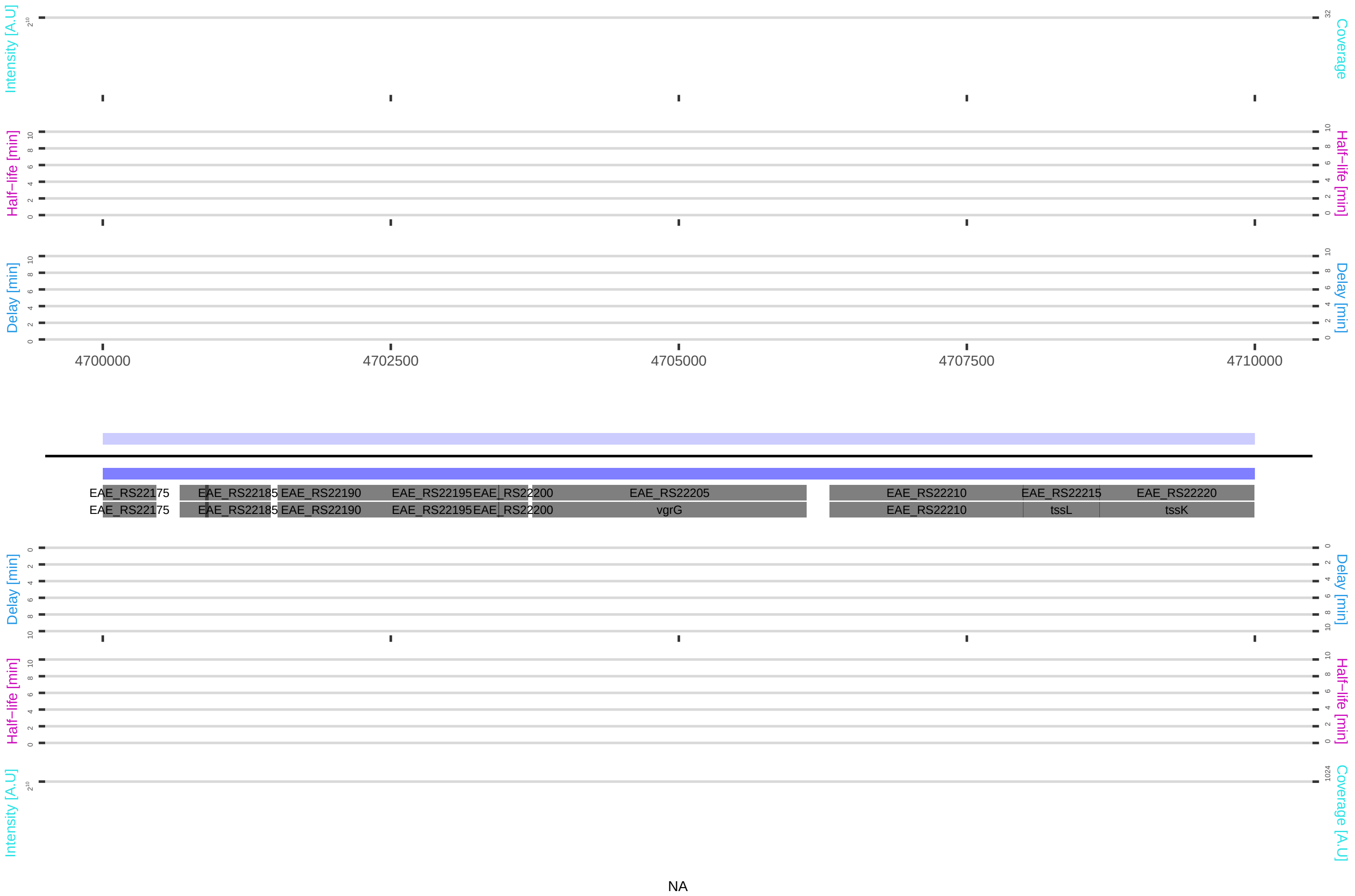
Intensity [A.U]
Coverage
Half−life [min]
Delay [min]
4700000
4702500
4705000
4707500
4710000
EAE_RS22175
EAE_RS22185
EAE_RS22190
EAE_RS22195
EAE_RS22200
EAE_RS22205
vgrG
EAE_RS22210
EAE_RS22215
tssL
EAE_RS22220
tssK
Coverage [A.U]
NA

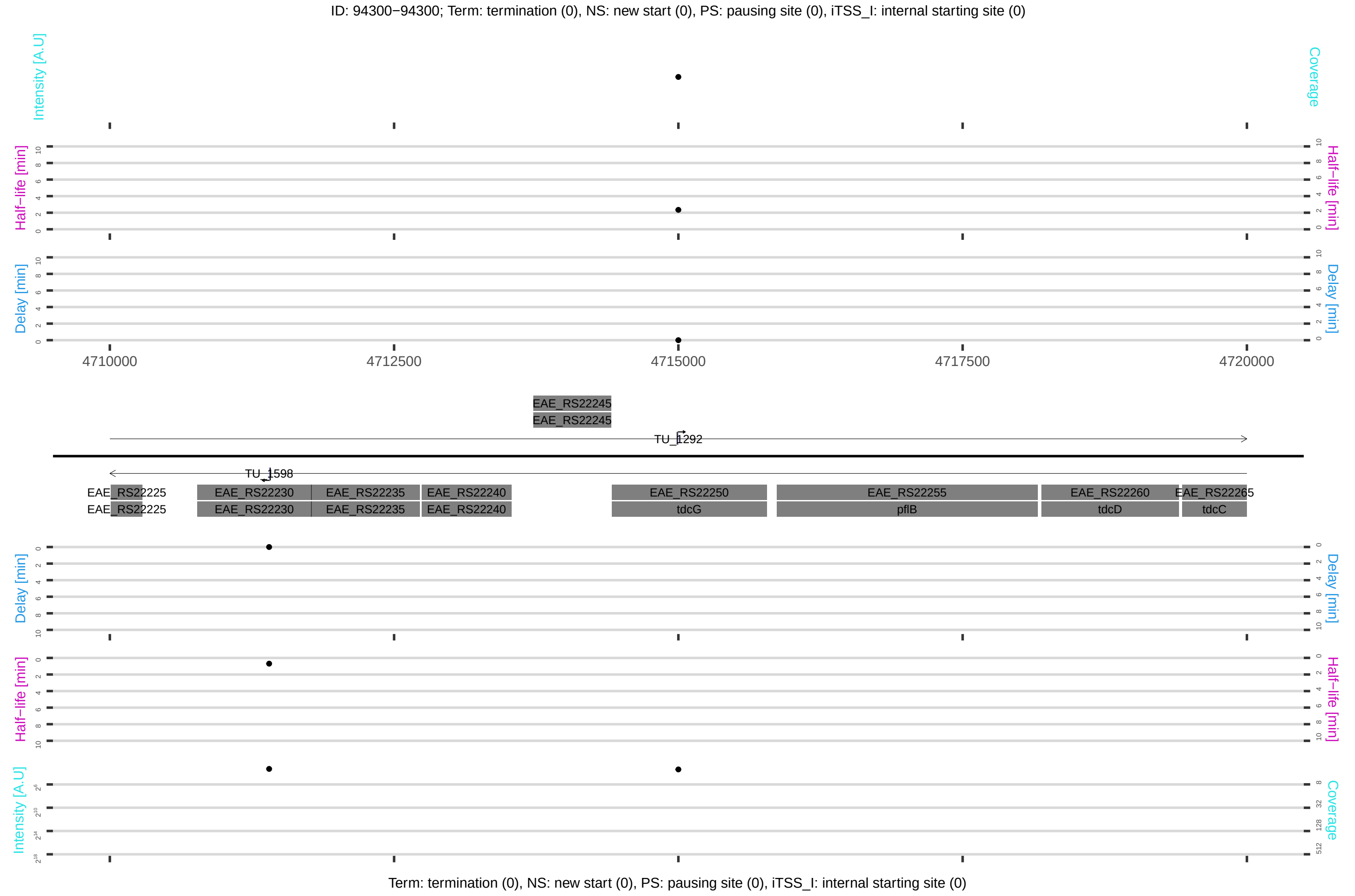
ID: 94300−94300; Term: termination (0), NS: new start (0), PS: pausing site (0), iTSS_I: internal starting site (0)
Intensity [A.U]
Coverage
Half−life [min]
Delay [min]
4710000
4712500
4715000
4717500
4720000
EAE_RS22245
EAE_RS22245
TU_1292
TU_1598
EAE_RS22225
EAE_RS22230
EAE_RS22235
EAE_RS22240
EAE_RS22250
tdcG
EAE_RS22255
pflB
EAE_RS22260
tdcD
EAE_RS22265
tdcC
Term: termination (0), NS: new start (0), PS: pausing site (0), iTSS_I: internal starting site (0)

ID: 94511−94545; Term: termination (0), NS: new start (0), PS: pausing site (0), iTSS_I: internal starting site (0)

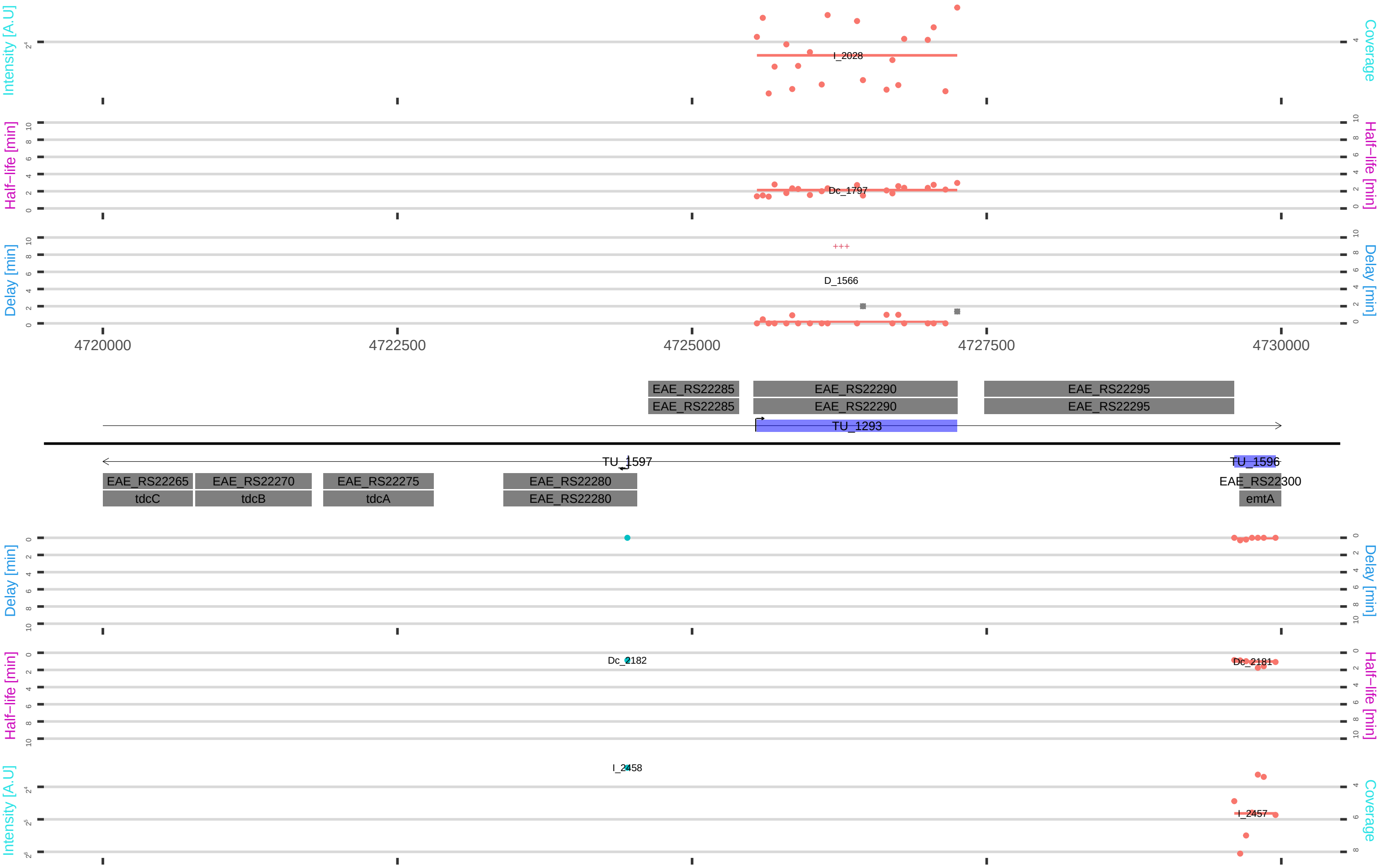

Term: termination (0), NS: new start (0), PS: pausing site (0), iTSS_I: internal starting site (0)

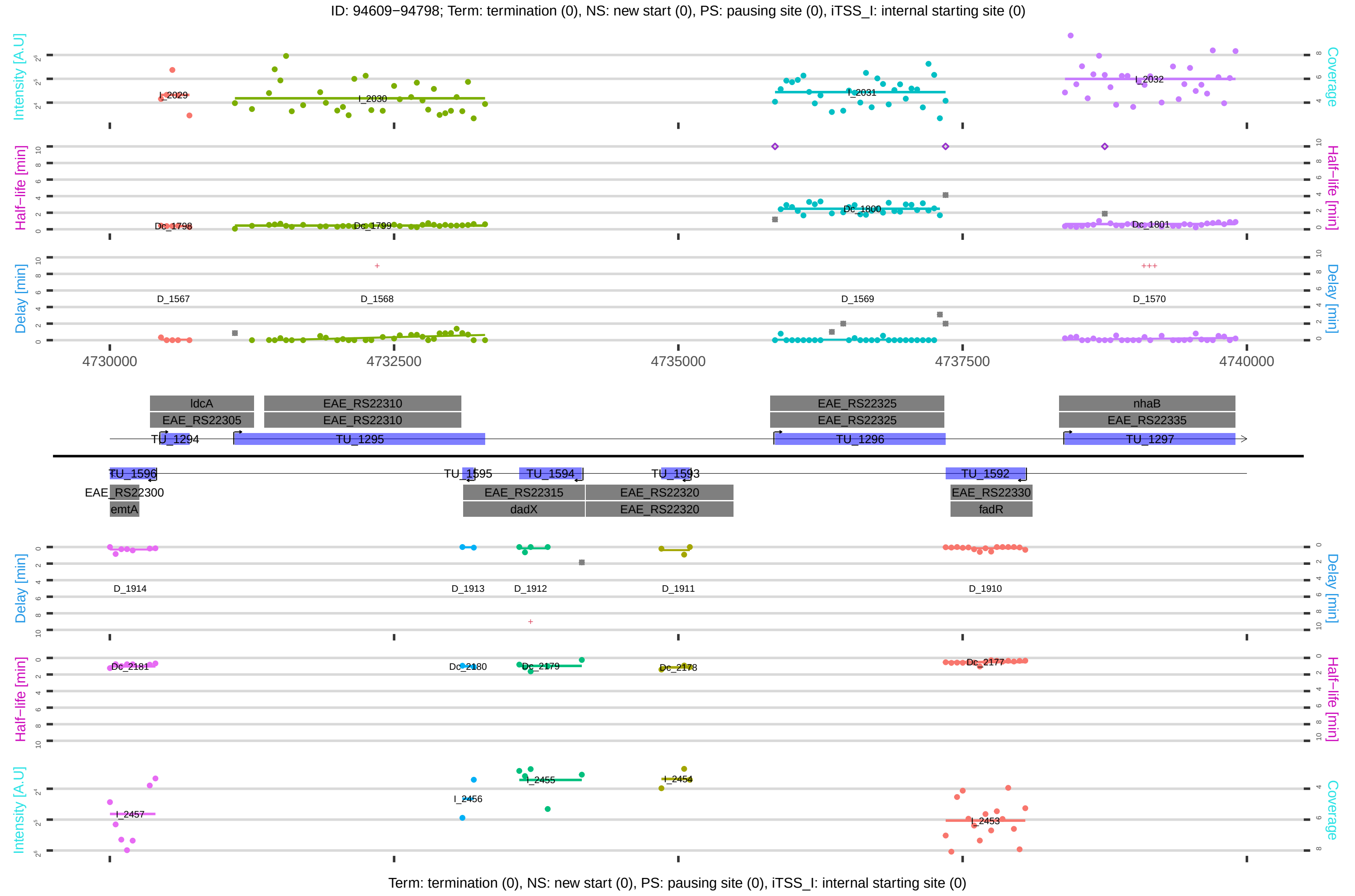
ID: 94609−94798; Term: termination (0), NS: new start (0), PS: pausing site (0), iTSS_I: internal starting site (0)
Intensity [A.U]
Coverage
Half−life [min]
Delay [min]
I_2029
I_2030
I_2031
I_2032
Dc_1798
Dc_1799
Dc_1800
Dc_1801
D_1567
D_1568
D_1569
D_1570
4730000
4732500
4735000
4737500
4740000
ldcA
EAE_RS22305
EAE_RS22310
EAE_RS22325
nhaB
EAE_RS22335
TU_1294
TU_1295
TU_1296
TU_1297
TU_1596
TU_1595
TU_1594
TU_1593
TU_1592
EAE_RS22300
emtA
EAE_RS22315
dadX
EAE_RS22320
EAE_RS22330
fadR
D_1914
D_1913
D_1912
D_1911
D_1910
Dc_2181
Dc_2180
Dc_2179
Dc_2178
Dc_2177
I_2457
I_2456
I_2455
I_2454
I_2453
Term: termination (0), NS: new start (0), PS: pausing site (0), iTSS_I: internal starting site (0)

ID: 94801−94831; Term: termination (1), NS: new start (0), PS: pausing site (0), iTSS_I: internal starting site (0)

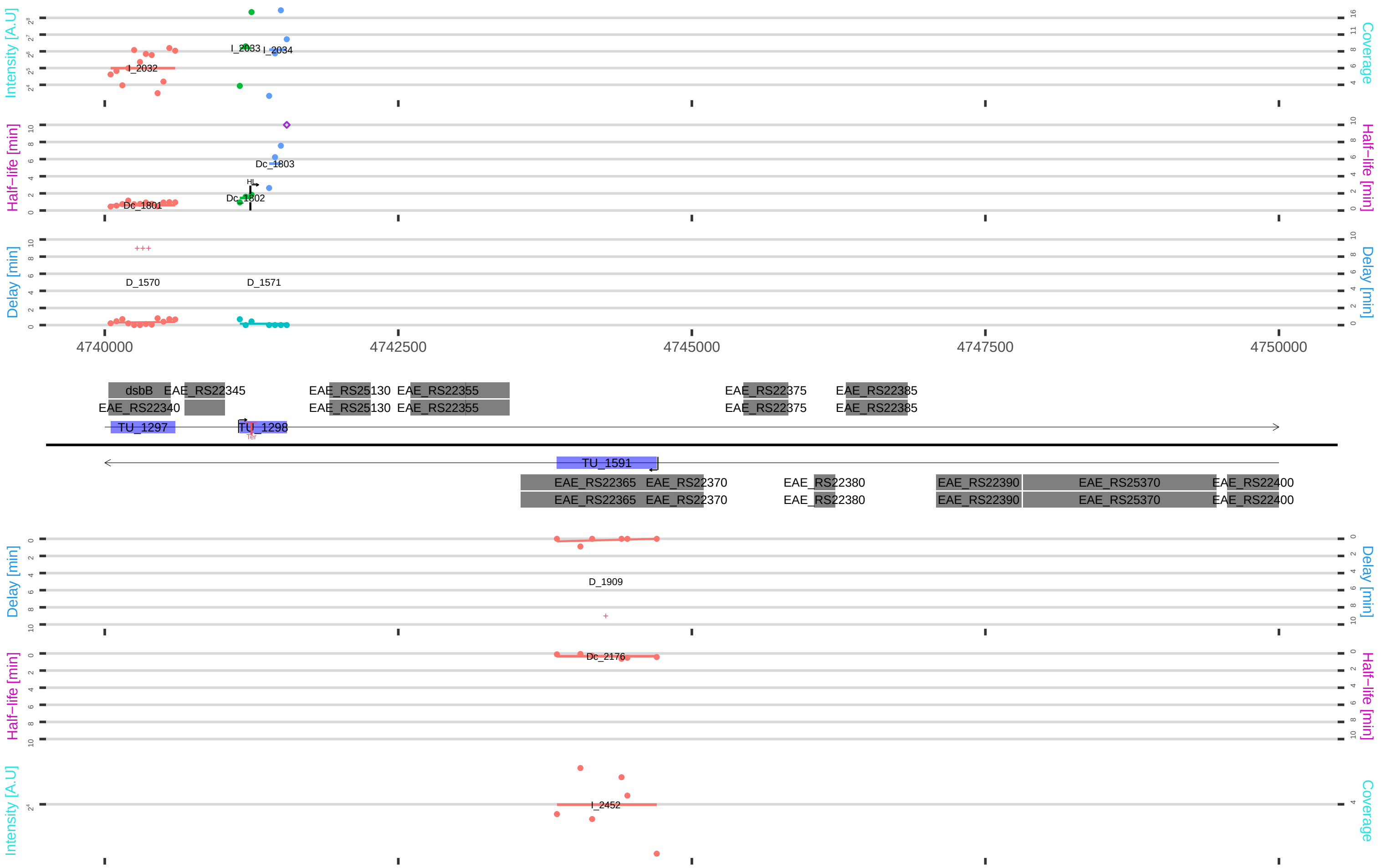

Term: termination (0), NS: new start (0), PS: pausing site (0), iTSS_I: internal starting site (0)

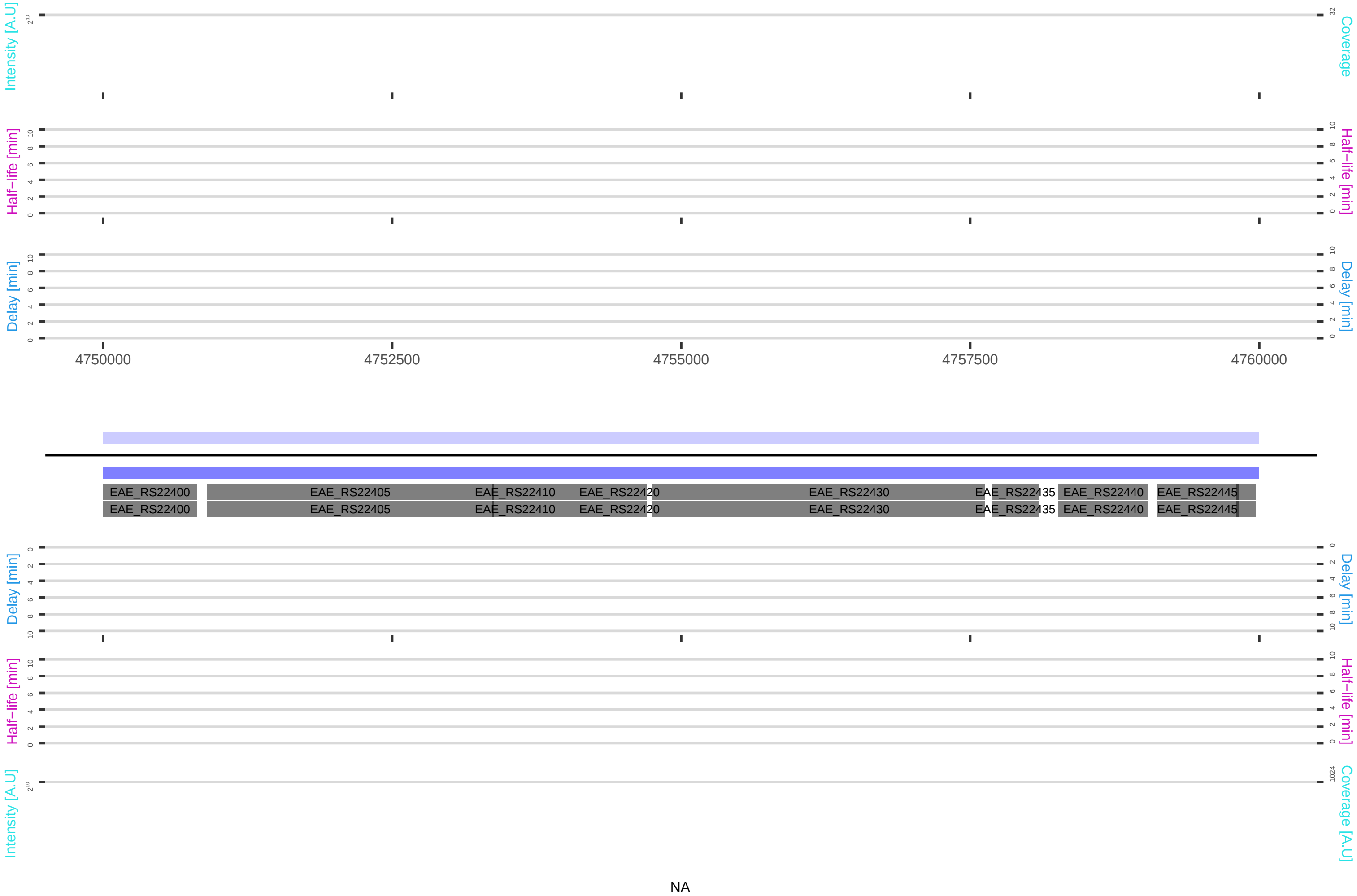
Intensity [A.U]
Coverage
Half−life [min]
Delay [min]
4750000
4752500
4755000
4757500
4760000
EAE_RS22400
EAE_RS22405
EAE_RS22410
EAE_RS22420
EAE_RS22430
EAE_RS22435
EAE_RS22440
EAE_RS22445
Coverage [A.U]
NA

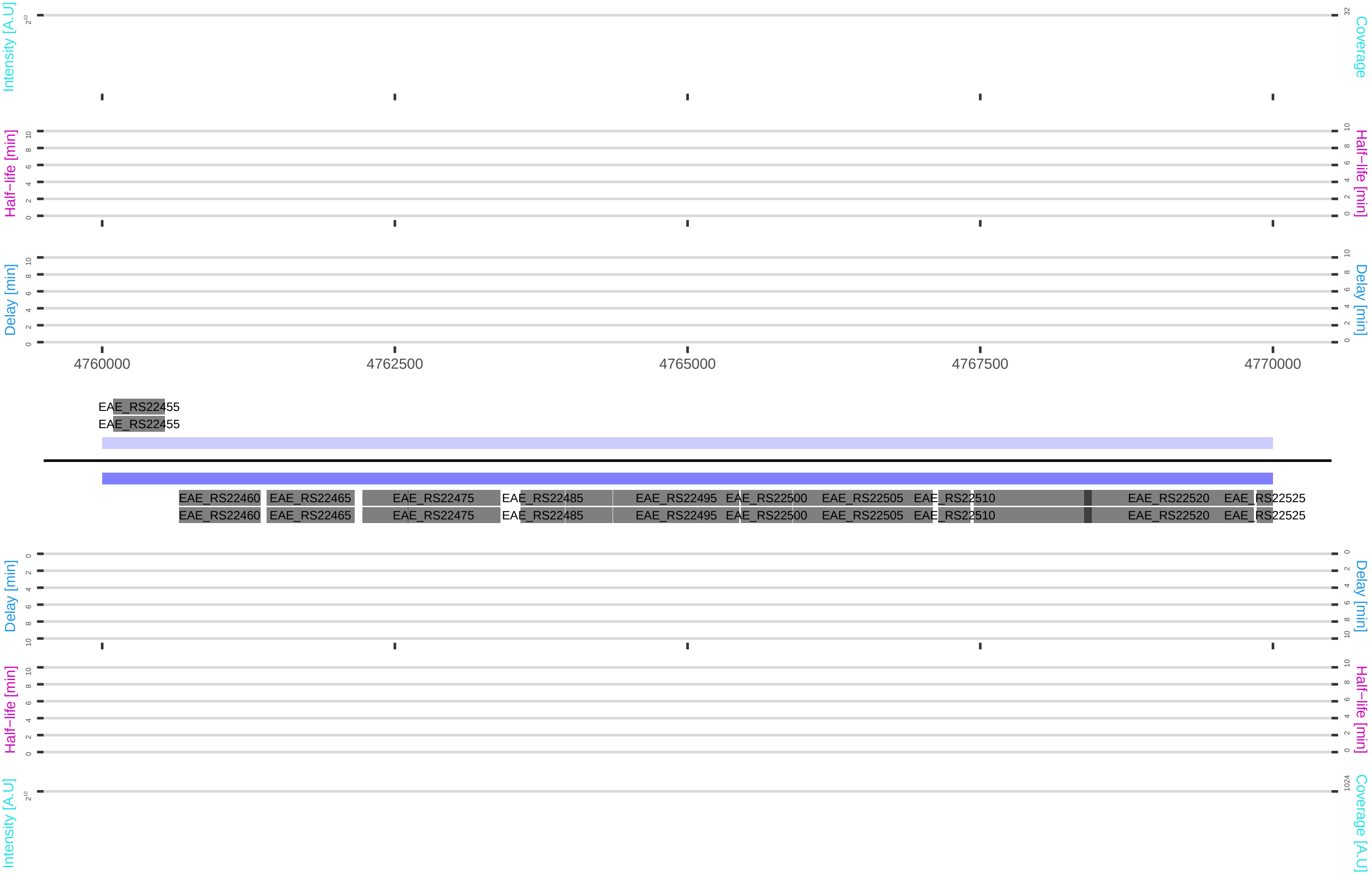

Intensity [A.U]
Coverage
Half−life [min]
Delay [min]
4760000
4762500
4765000
4767500
4770000
EAE_RS22455
EAE_RS22460
EAE_RS22465
EAE_RS22475
EAE_RS22485
EAE_RS22495
EAE_RS22500
EAE_RS22505
EAE_RS22510
EAE_RS22520
EAE_RS22525
Coverage [A.U]

NA

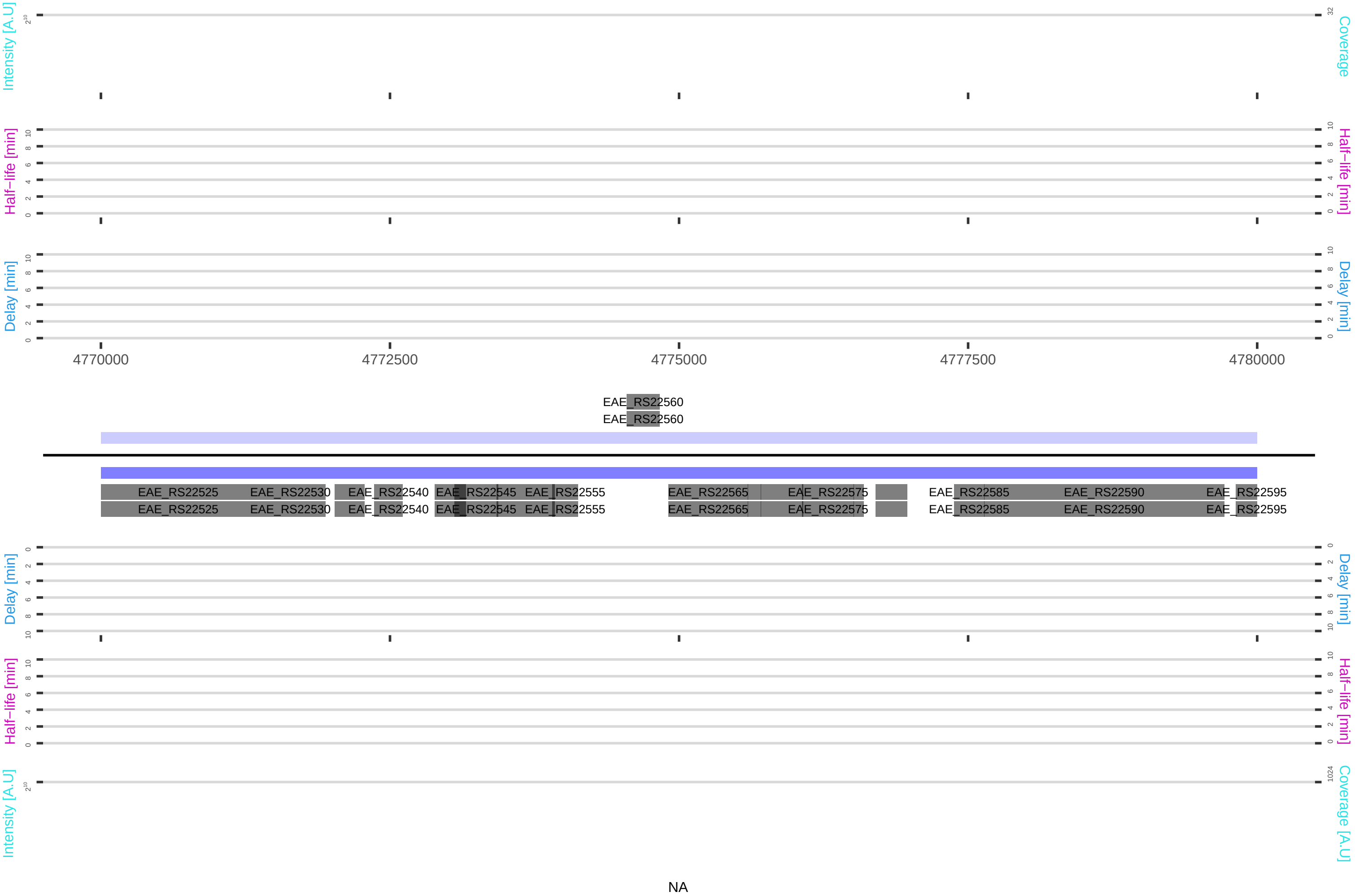
Intensity [A.U]
Coverage
Half−life [min]
Delay [min]
4770000
4772500
4775000
4777500
4780000
EAE_RS22560
EAE_RS22525
EAE_RS22530
EAE_RS22540
EAE_RS22545
EAE_RS22555
EAE_RS22565
EAE_RS22575
EAE_RS22585
EAE_RS22590
EAE_RS22595
Coverage [A.U]
NA

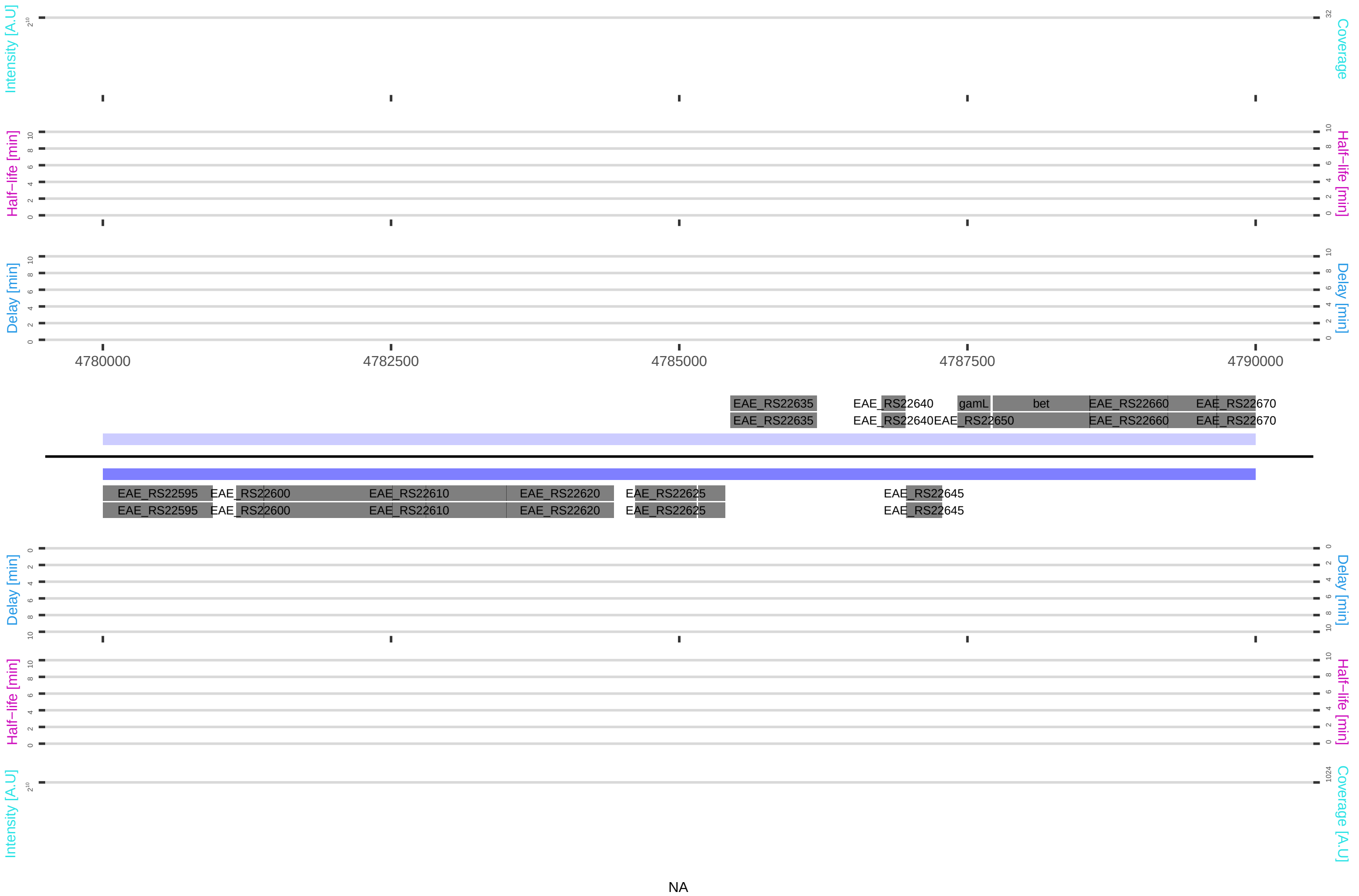
Intensity [A.U]
Coverage
Half−life [min]
Delay [min]
4780000
4782500
4785000
4787500
4790000
EAE_RS22635
EAE_RS22640
gamL
bet
EAE_RS22650
EAE_RS22660
EAE_RS22670
EAE_RS22595
EAE_RS22600
EAE_RS22610
EAE_RS22620
EAE_RS22625
EAE_RS22645
Coverage [A.U]
NA

ID: 95895−95932; Term: termination (1), NS: new start (0), PS: pausing site (1), iTSS_I: internal starting site (0)

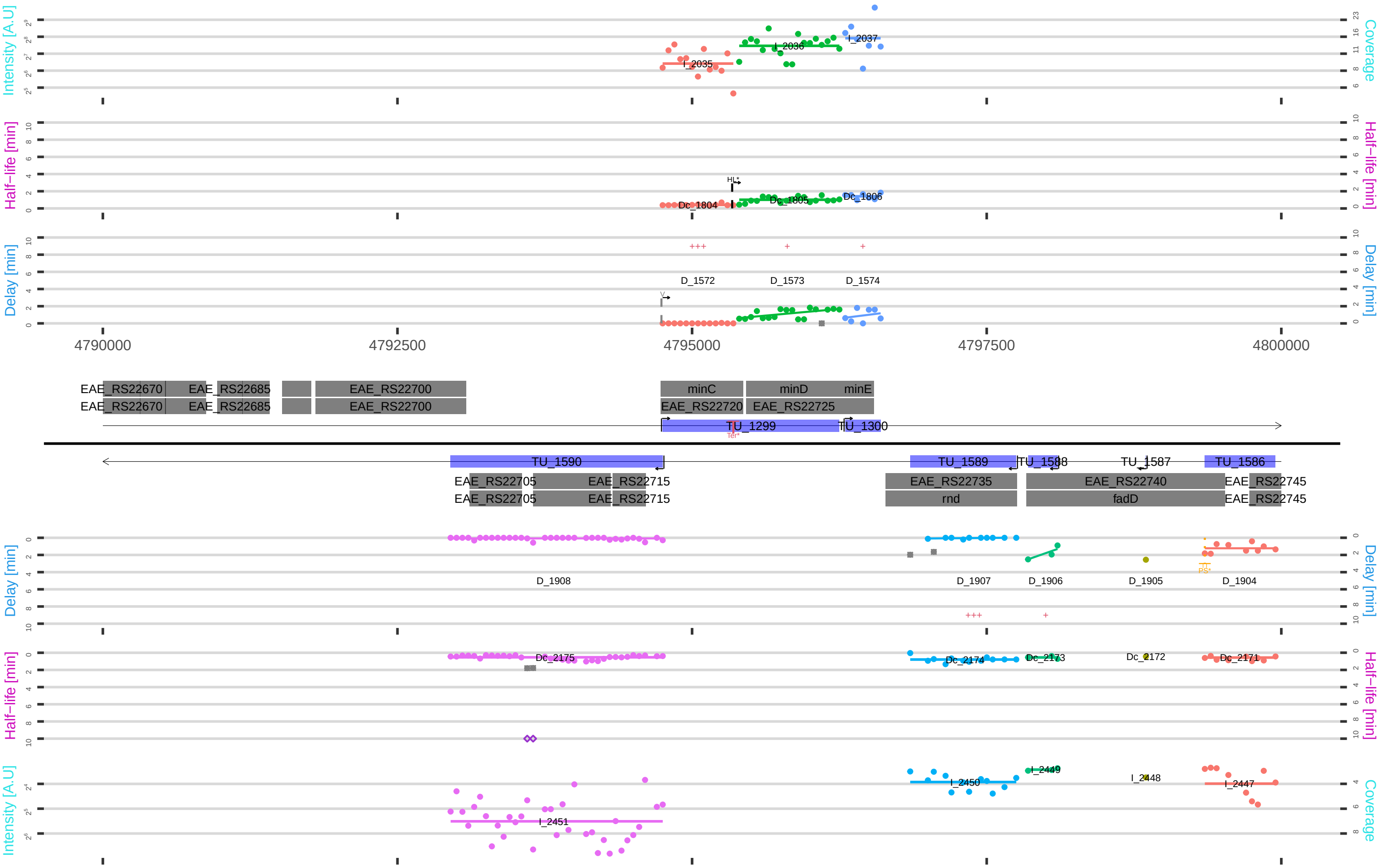

Term: termination (0), NS: new start (0), PS: pausing site (1), iTSS_I: internal starting site (0)

ID: 96065−96181; Term: termination (0), NS: new start (2), PS: pausing site (1), iTSS_I: internal starting site (1)

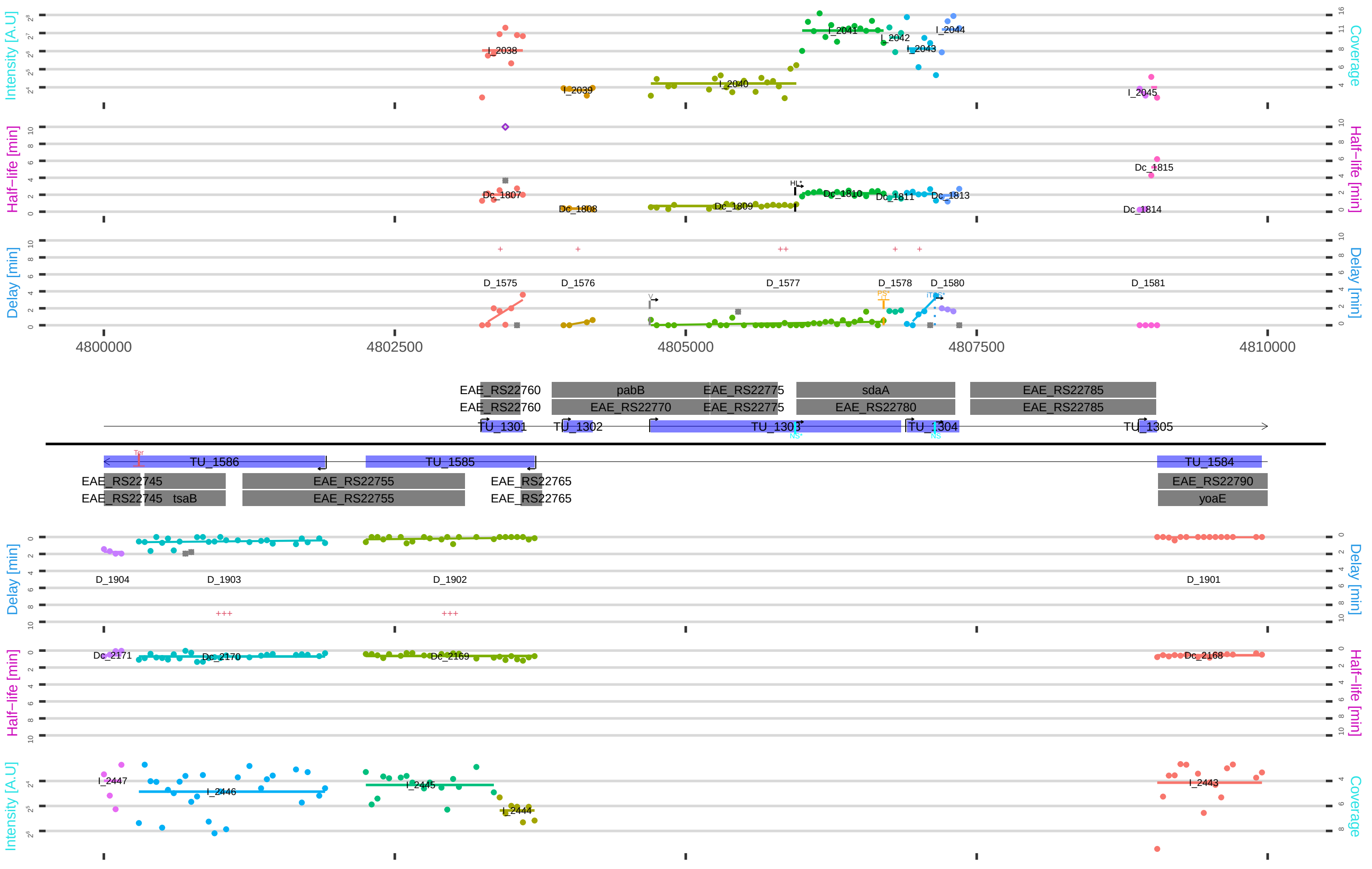

Term: termination (1), NS: new start (0), PS: pausing site (0), iTSS_I: internal starting site (0)

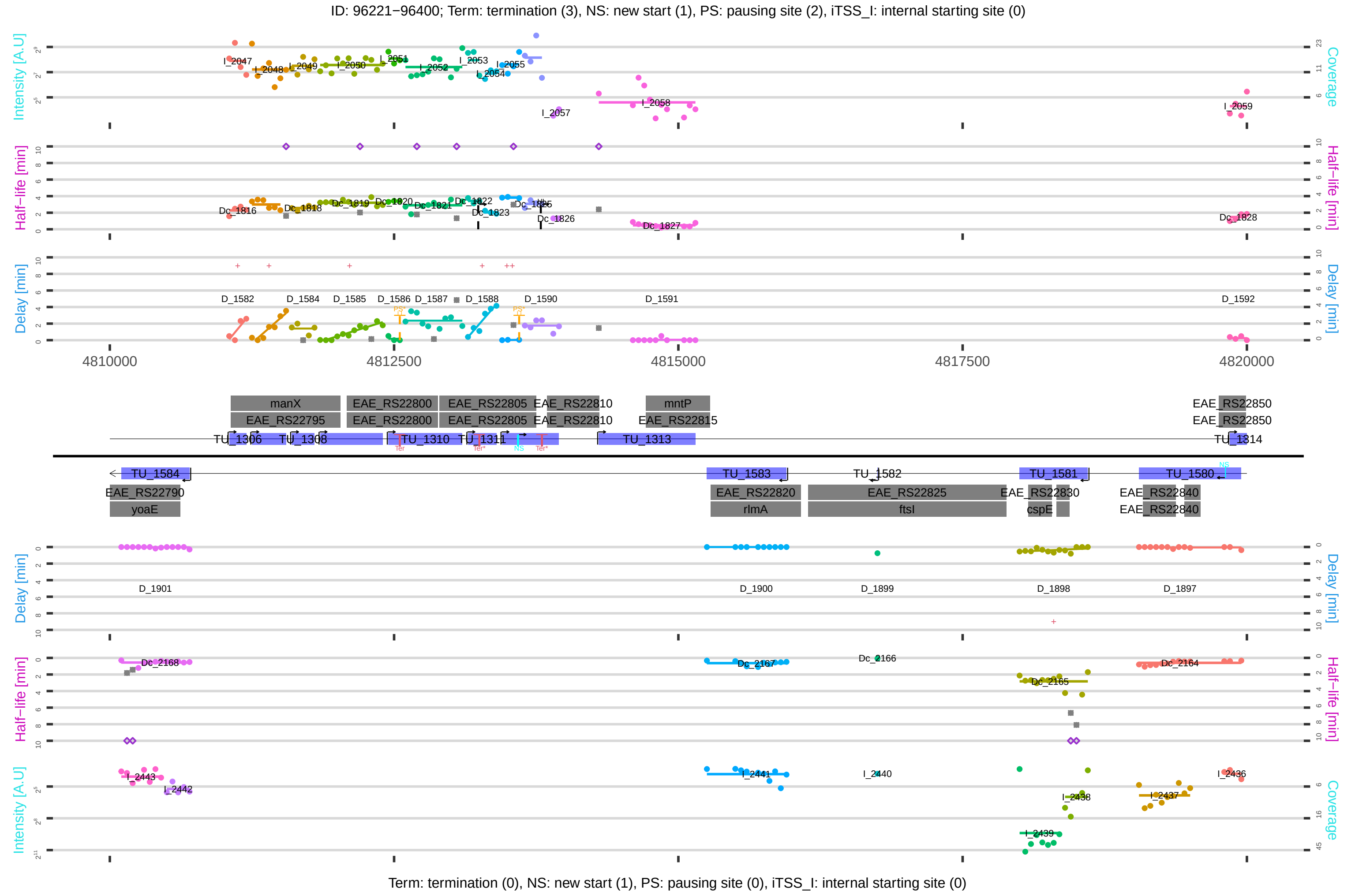
ID: 96221−96400; Term: termination (3), NS: new start (1), PS: pausing site (2), iTSS_I: internal starting site (0)
Intensity [A.U]
Coverage
Half−life [min]
Delay [min]
4810000
4812500
4815000
4817500
4820000
manX
EAE_RS22795
EAE_RS22800
EAE_RS22805
EAE_RS22810
mntP
EAE_RS22815
EAE_RS22850
TU_1306
TU_1308
TU_1310
TU_1311
TU_1313
TU_1314
TU_1584
TU_1583
TU_1582
TU_1581
TU_1580
EAE_RS22790
yoaE
EAE_RS22820
rlmA
EAE_RS22825
ftsI
EAE_RS22830
cspE
EAE_RS22840
Term: termination (0), NS: new start (1), PS: pausing site (0), iTSS_I: internal starting site (0)

ID: 96400−96600; Term: termination (0), NS: new start (0), PS: pausing site (0), iTSS_I: internal starting site (0)

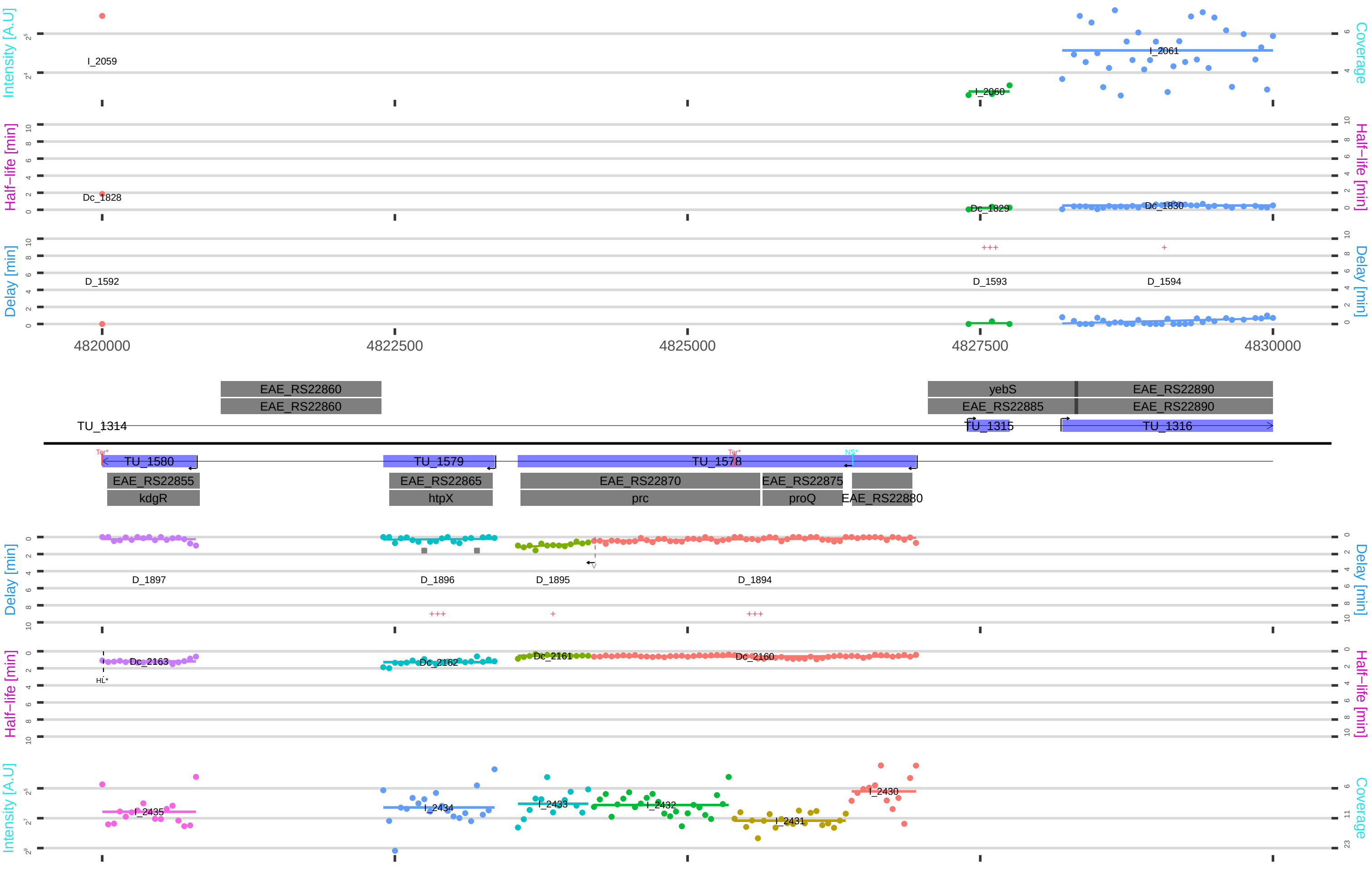

Term: termination (2), NS: new start (1), PS: pausing site (1), iTSS_I: internal starting site (0)

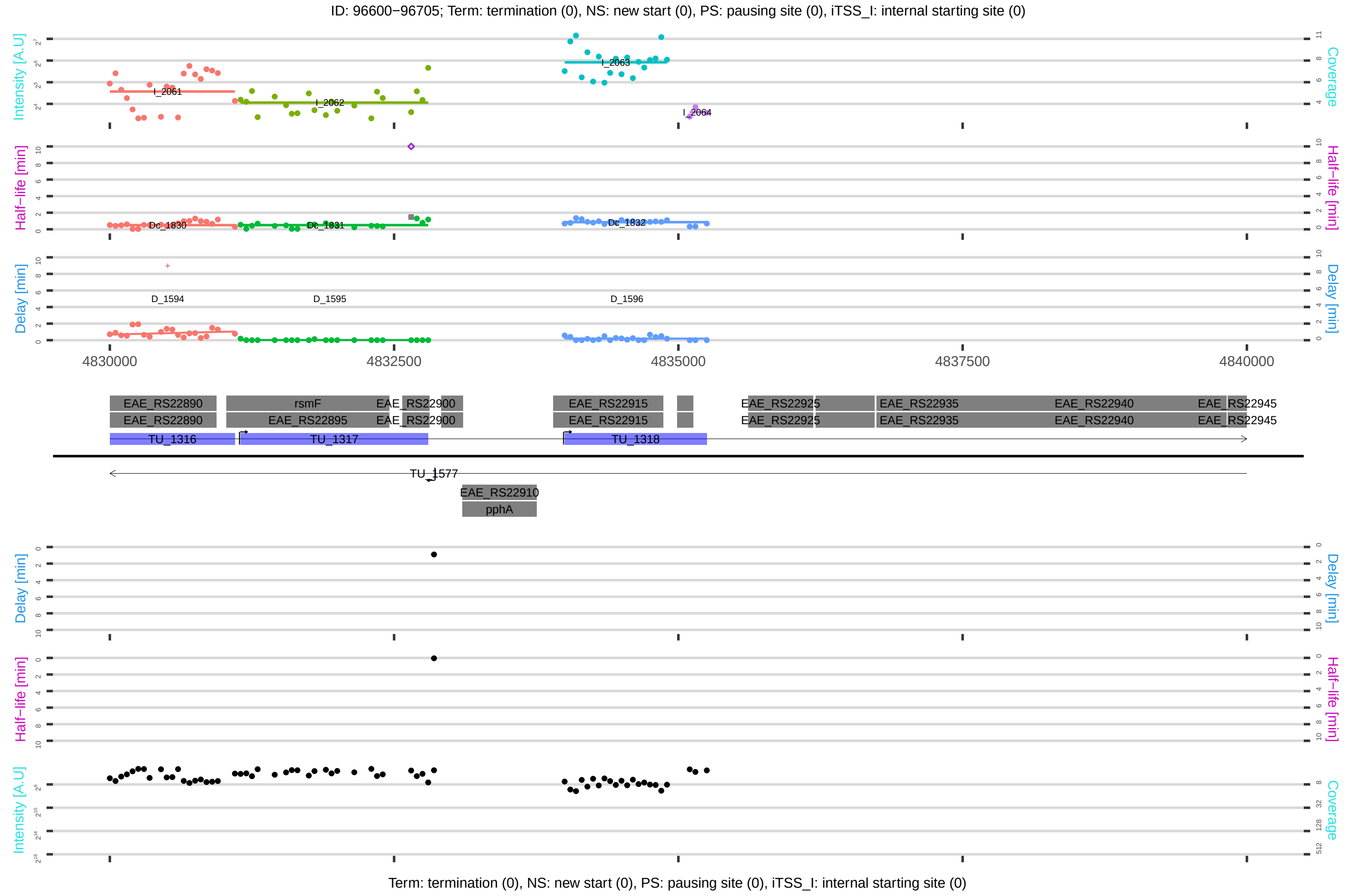
ID: 96600−96705; Term: termination (0), NS: new start (0), PS: pausing site (0), iTSS_I: internal starting site (0)
Intensity [A.U]
Coverage
I_2061
I_2062
I_2063
I_2064
Half−life [min]
Dc_1830
Dc_1831
Dc_1832
Delay [min]
D_1594
D_1595
D_1596
4830000
4832500
4835000
4837500
4840000
EAE_RS22890
rsmF
EAE_RS22895
EAE_RS22900
EAE_RS22915
EAE_RS22925
EAE_RS22935
EAE_RS22940
EAE_RS22945
TU_1316
TU_1317
TU_1318
TU_1577
EAE_RS22910
pphA
Term: termination (0), NS: new start (0), PS: pausing site (0), iTSS_I: internal starting site (0)

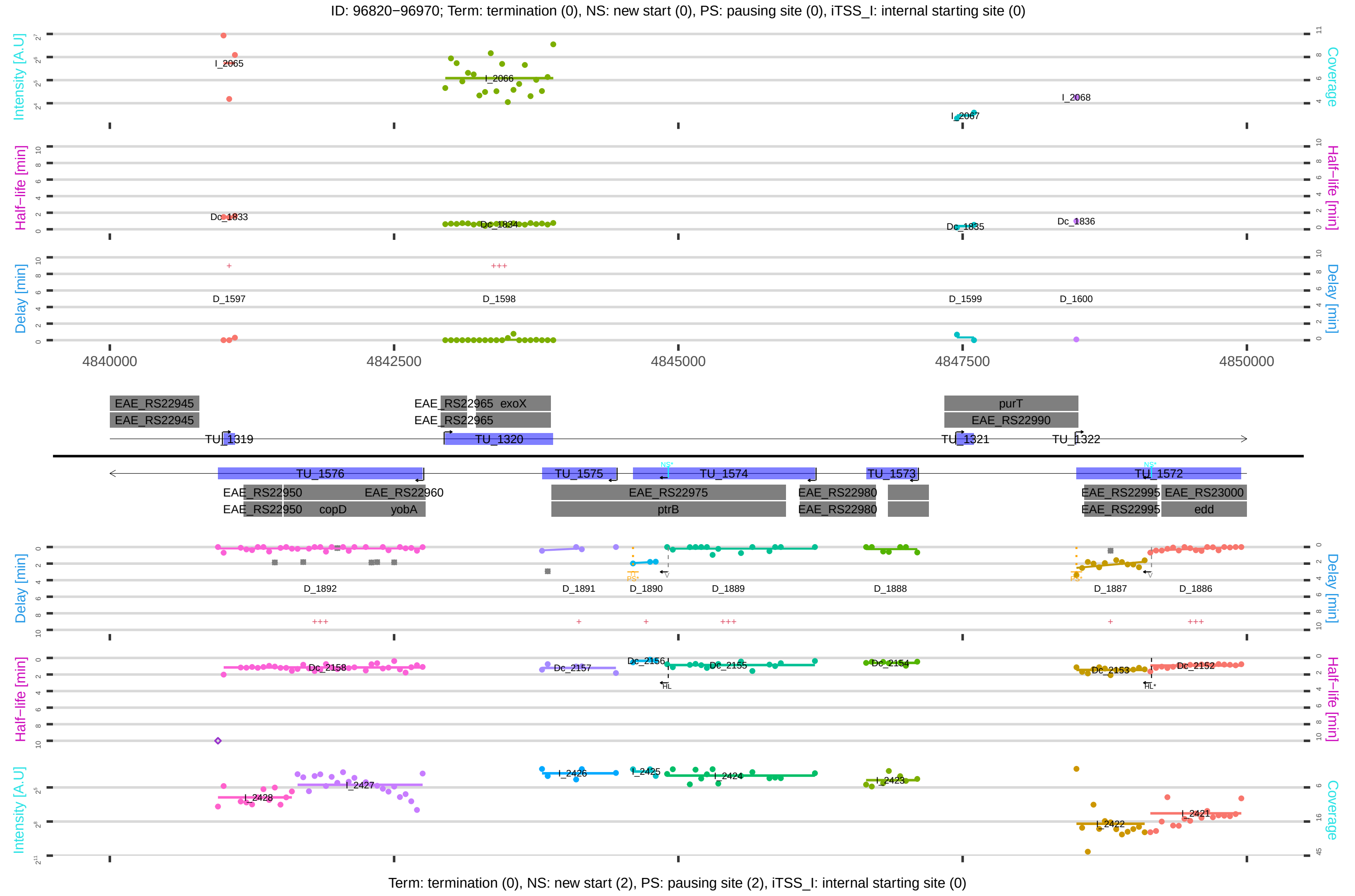
ID: 96820−96970; Term: termination (0), NS: new start (0), PS: pausing site (0), iTSS_I: internal starting site (0)
Intensity [A.U]
Coverage
Half−life [min]
Delay [min]
I_2065
I_2066
I_2067
I_2068
Dc_1833
Dc_1834
Dc_1835
Dc_1836
D_1597
D_1598
D_1599
D_1600
4840000
4842500
4845000
4847500
4850000
EAE_RS22945
EAE_RS22965
exoX
purT
EAE_RS22990
TU_1319
TU_1320
TU_1321
TU_1322
TU_1576
TU_1575
TU_1574
TU_1573
TU_1572
EAE_RS22950
EAE_RS22960
copD
yobA
EAE_RS22975
ptrB
EAE_RS22980
EAE_RS22995
EAE_RS23000
edd
D_1892
D_1891
D_1890
D_1889
D_1888
D_1887
D_1886
Dc_2158
Dc_2157
Dc_2156
Dc_2155
Dc_2154
Dc_2153
Dc_2152
I_2428
I_2427
I_2426
I_2425
I_2424
I_2423
I_2422
I_2421
Term: termination (0), NS: new start (2), PS: pausing site (2), iTSS_I: internal starting site (0)

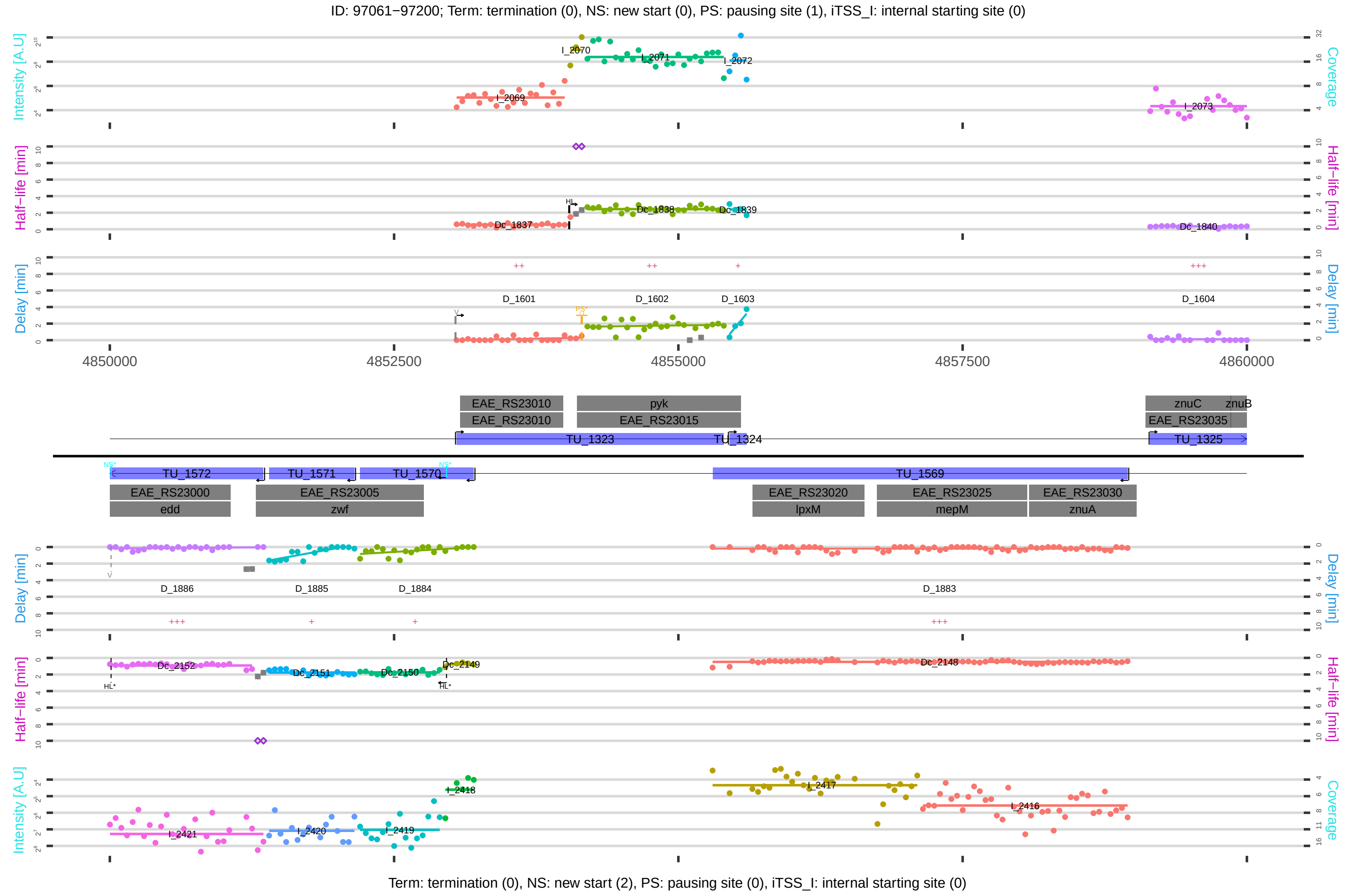

ID: 97061−97200; Term: termination (0), NS: new start (0), PS: pausing site (1), iTSS_I: internal starting site (0)
Intensity [A.U]
Coverage
Half−life [min]
Delay [min]
I_2069
I_2070
I_2071
I_2072
I_2073
Dc_1837
Dc_1838
Dc_1839
Dc_1840
D_1601
D_1602
D_1603
D_1604
4850000
4852500
4855000
4857500
4860000
EAE_RS23010
pyk
EAE_RS23015
znuC
znuB
EAE_RS23035
TU_1323
TU_1324
TU_1325
TU_1572
TU_1571
TU_1570
TU_1569
EAE_RS23000
edd
EAE_RS23005
zwf
EAE_RS23020
lpxM
EAE_RS23025
mepM
EAE_RS23030
znuA
D_1886
D_1885
D_1884
D_1883
Dc_2152
Dc_2151
Dc_2150
Dc_2149
Dc_2148
I_2421
I_2420
I_2419
I_2418
I_2417
I_2416
Term: termination (0), NS: new start (2), PS: pausing site (0), iTSS_I: internal starting site (0)

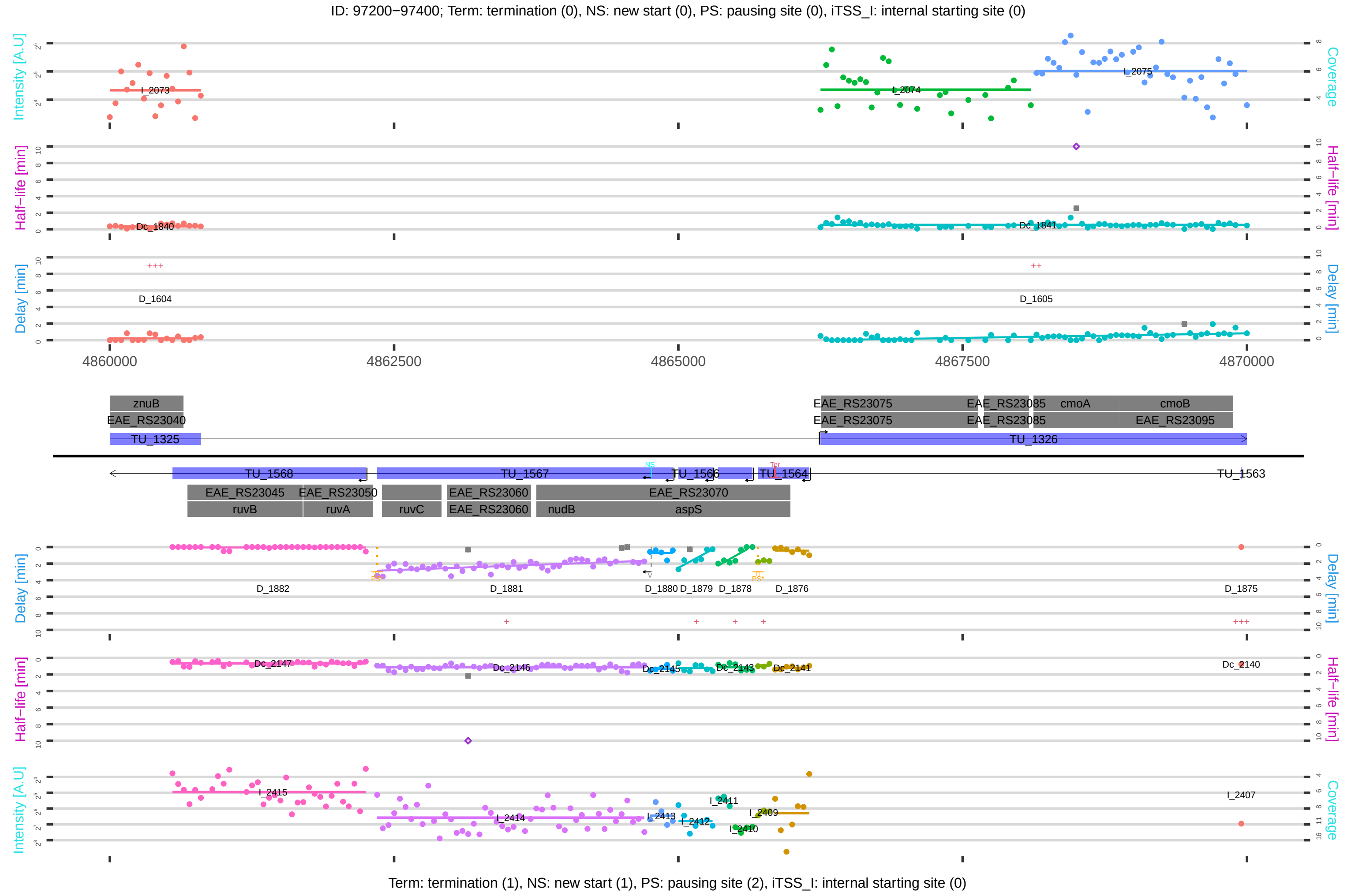

ID: 97200−97400; Term: termination (0), NS: new start (0), PS: pausing site (0), iTSS_I: internal starting site (0)
Intensity [A.U]
Coverage
Half−life [min]
Delay [min]
I_2073
I_2074
I_2075
Dc_1840
Dc_1841
D_1604
D_1605
4860000
4862500
4865000
4867500
4870000
znuB
EAE_RS23040
TU_1325
EAE_RS23075
EAE_RS23085
cmoA
cmoB
EAE_RS23095
TU_1326
TU_1568
TU_1567
TU_1566
TU_1564
TU_1563
EAE_RS23045
EAE_RS23050
EAE_RS23060
EAE_RS23070
ruvB
ruvA
ruvC
nudB
aspS
D_1882
D_1881
D_1880
D_1879
D_1878
D_1876
D_1875
Dc_2147
Dc_2146
Dc_2145
Dc_2143
Dc_2141
Dc_2140
I_2415
I_2414
I_2413
I_2412
I_2411
I_2410
I_2409
I_2407
Term: termination (1), NS: new start (1), PS: pausing site (2), iTSS_I: internal starting site (0)

ID: 97400−97550; Term: termination (0), NS: new start (0), PS: pausing site (0), iTSS_I: internal starting site (0)

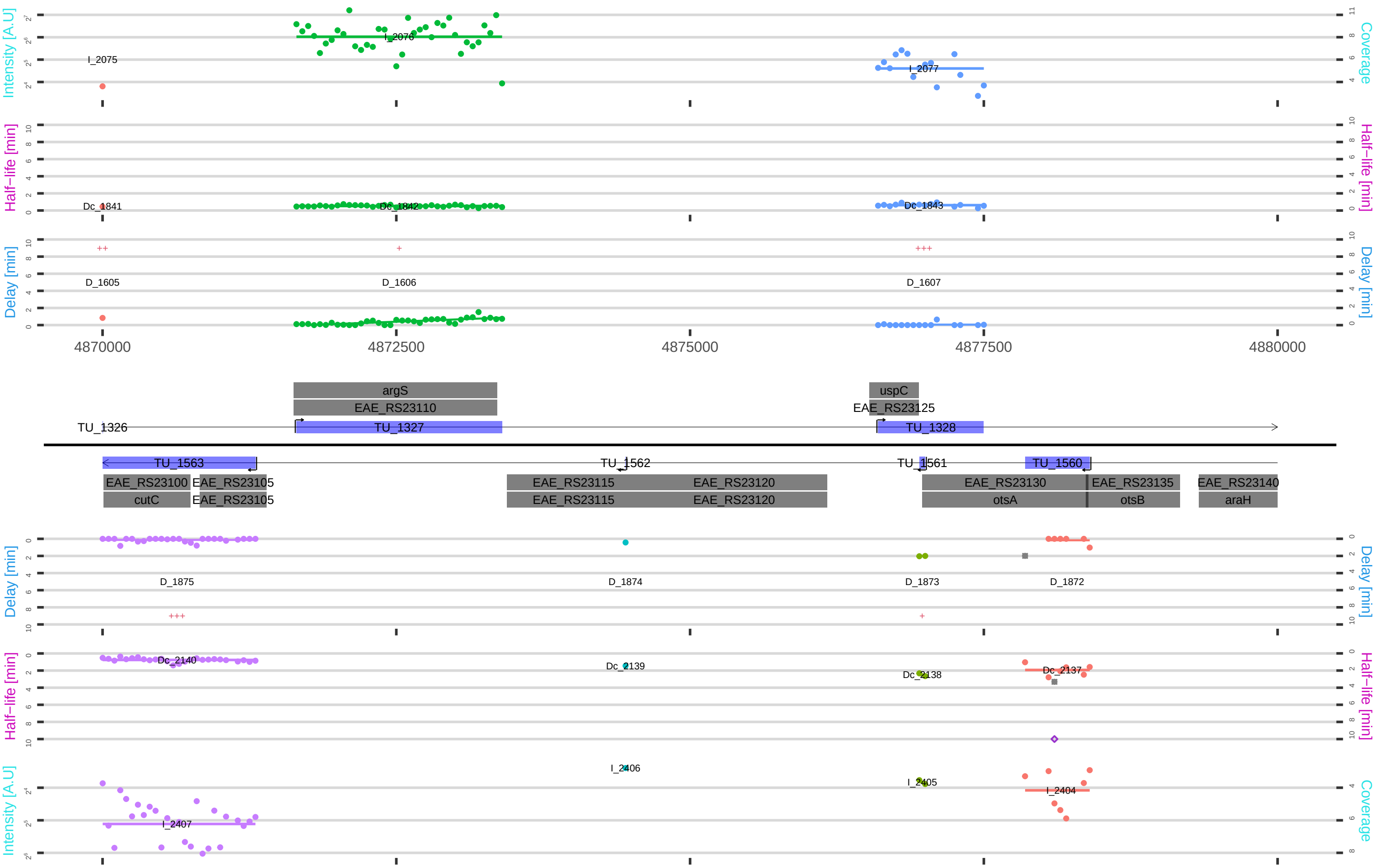

Term: termination (0), NS: new start (0), PS: pausing site (0), iTSS_I: internal starting site (0)

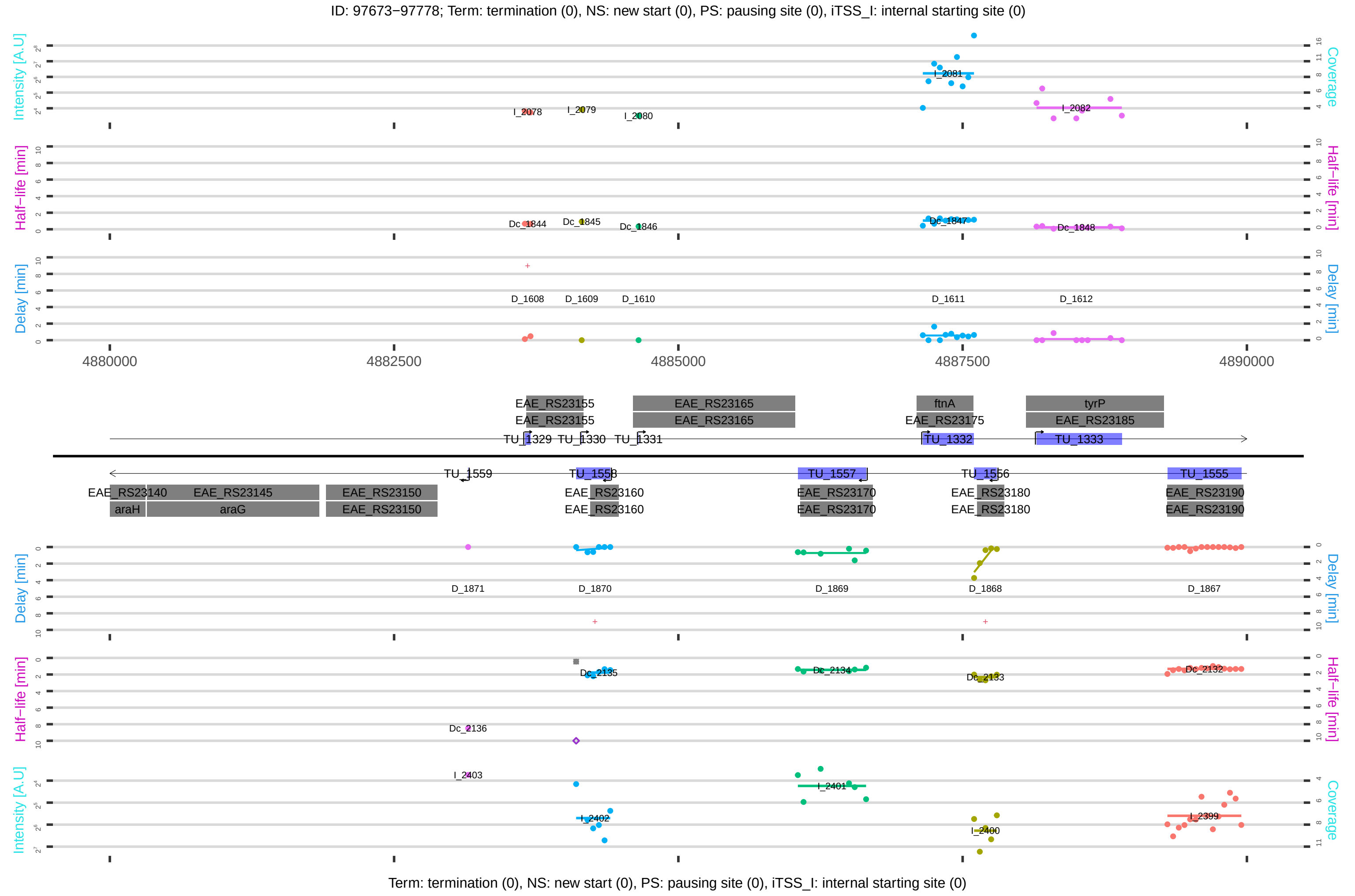

ID: 97673−97778; Term: termination (0), NS: new start (0), PS: pausing site (0), iTSS_I: internal starting site (0)
Intensity [A.U]
Coverage
Half−life [min]
Delay [min]
I_2078
I_2079
I_2080
I_2081
I_2082
Dc_1844
Dc_1845
Dc_1846
Dc_1847
Dc_1848
D_1608
D_1609
D_1610
D_1611
D_1612
4880000
4882500
4885000
4887500
4890000
EAE_RS23155
EAE_RS23165
ftnA
EAE_RS23175
tyrP
EAE_RS23185
TU_1329
TU_1330
TU_1331
TU_1332
TU_1333
TU_1559
TU_1558
TU_1557
TU_1556
TU_1555
EAE_RS23140
EAE_RS23145
EAE_RS23150
EAE_RS23160
EAE_RS23170
EAE_RS23180
EAE_RS23190
araH
araG
D_1871
D_1870
D_1869
D_1868
D_1867
Dc_2135
Dc_2134
Dc_2133
Dc_2132
Dc_2136
I_2403
I_2402
I_2401
I_2400
I_2399
Term: termination (0), NS: new start (0), PS: pausing site (0), iTSS_I: internal starting site (0)

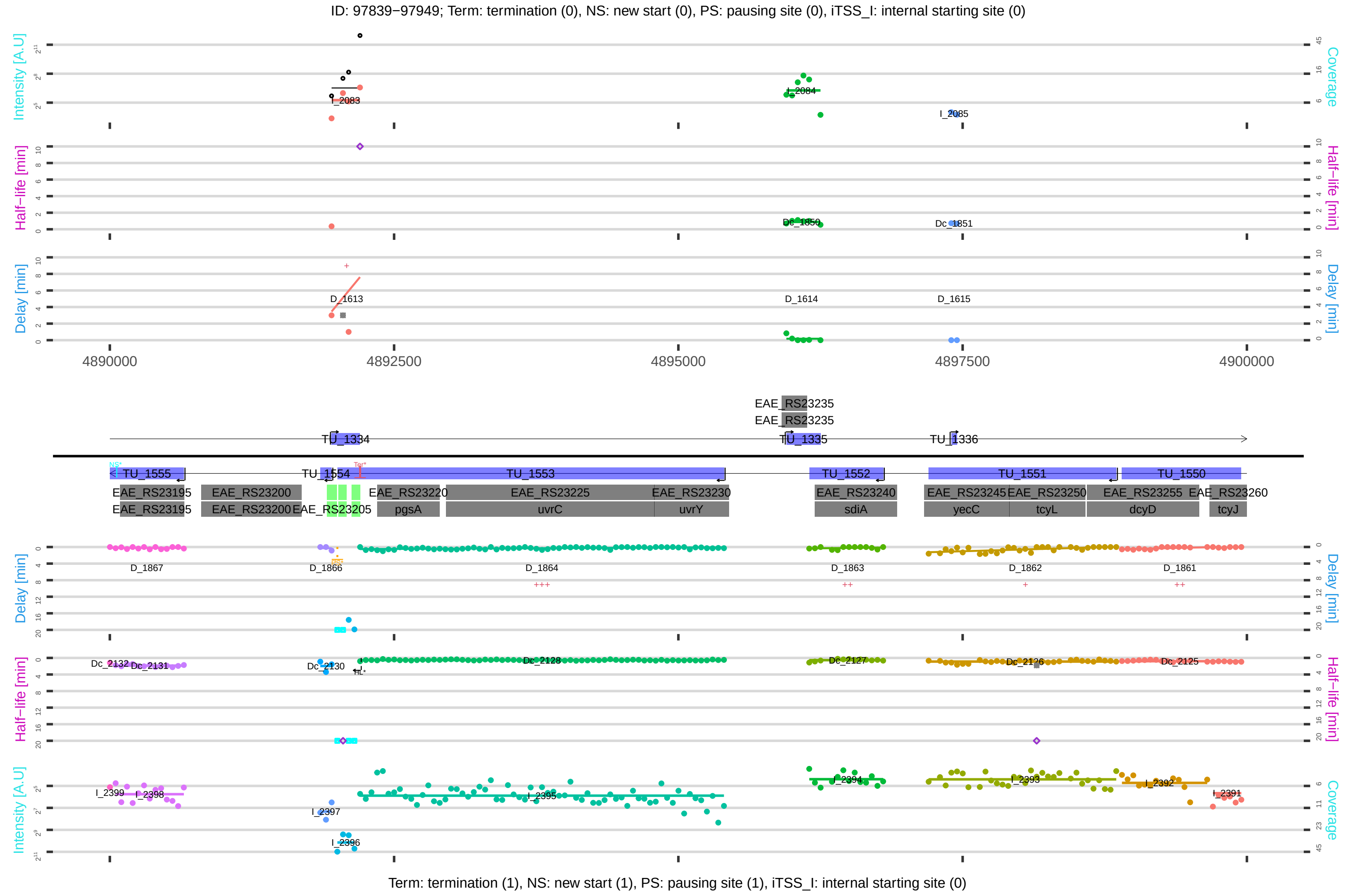

ID: 97839−97949; Term: termination (0), NS: new start (0), PS: pausing site (0), iTSS_I: internal starting site (0)
Intensity [A.U]
Coverage
Half−life [min]
Delay [min]
I_2083
I_2084
I_2085
Dc_1850
Dc_1851
D_1613
D_1614
D_1615
4890000
4892500
4895000
4897500
4900000
EAE_RS23235
EAE_RS23235
TU_1334
TU_1335
TU_1336
TU_1555
TU_1554
TU_1553
TU_1552
TU_1551
TU_1550
EAE_RS23195
EAE_RS23195
EAE_RS23200
EAE_RS23200
EAE_RS23205
EAE_RS23220
pgsA
EAE_RS23225
uvrC
EAE_RS23230
uvrY
EAE_RS23240
sdiA
EAE_RS23245
yecC
EAE_RS23250
tcyL
EAE_RS23255
dcyD
EAE_RS23260
tcyJ
D_1867
D_1866
D_1864
D_1863
D_1862
D_1861
Dc_2132
Dc_2131
Dc_2130
Dc_2128
Dc_2127
Dc_2126
Dc_2125
I_2399
I_2398
I_2397
I_2396
I_2395
I_2394
I_2393
I_2392
I_2391
Term: termination (1), NS: new start (1), PS: pausing site (1), iTSS_I: internal starting site (0)

ID: 98015−98074; Term: termination (0), NS: new start (0), PS: pausing site (0), iTSS_I: internal starting site (0)

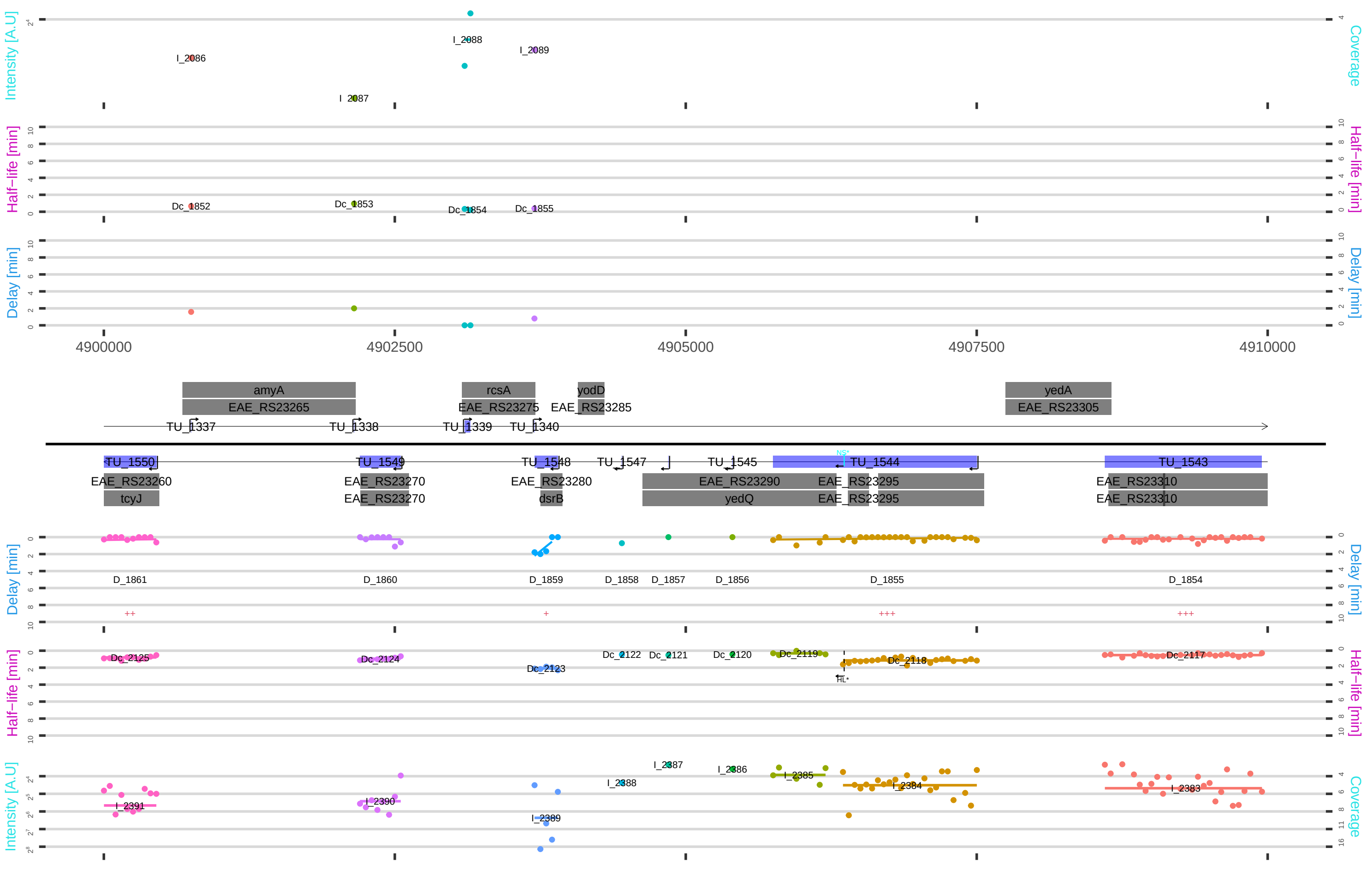

Term: termination (0), NS: new start (1), PS: pausing site (0), iTSS_I: internal starting site (0)

ID: 98244−98266; Term: termination (0), NS: new start (0), PS: pausing site (0), iTSS_I: internal starting site (0)

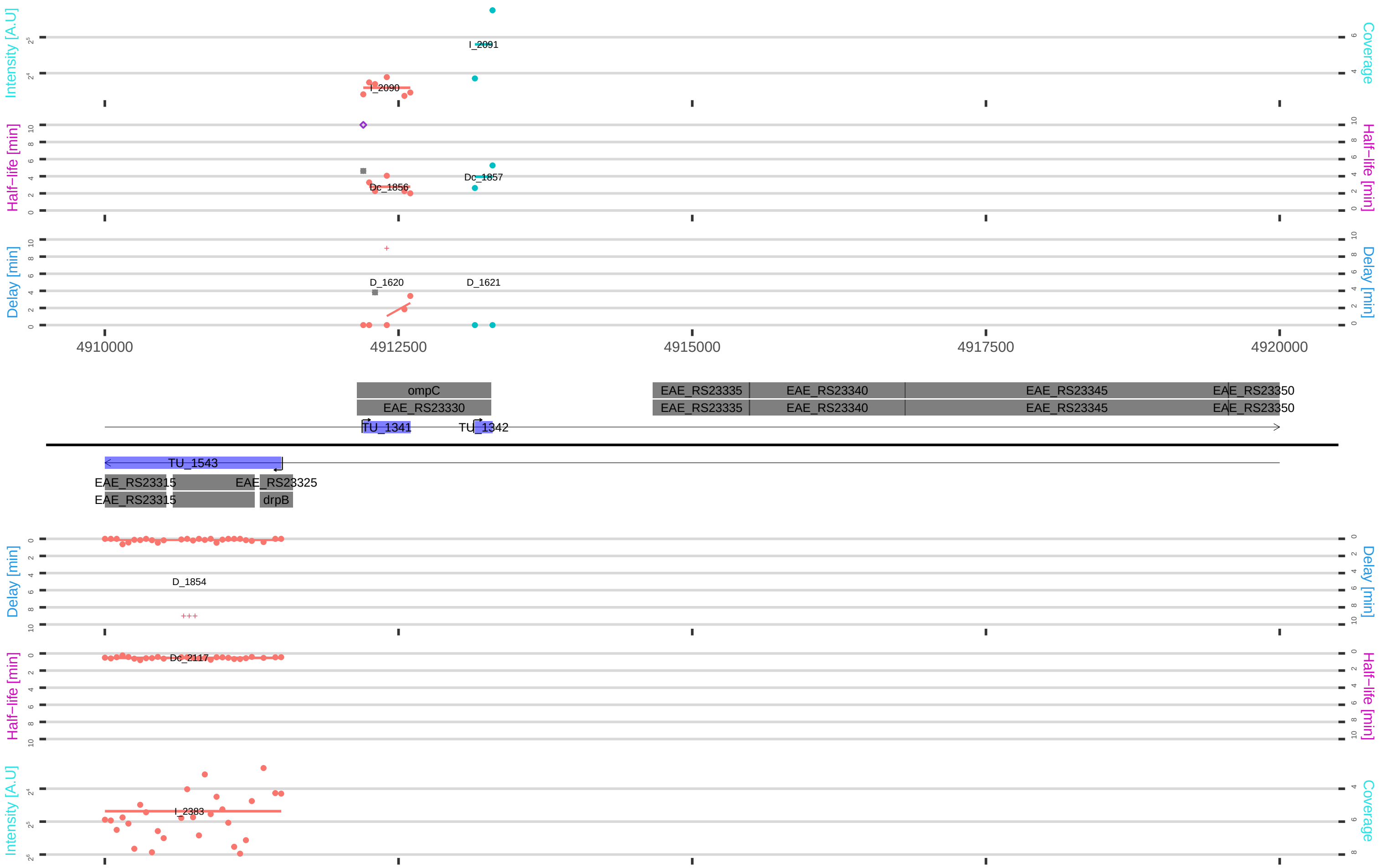

Term: termination (0), NS: new start (0), PS: pausing site (0), iTSS_I: internal starting site (0)

ID: 98448−98539; Term: termination (0), NS: new start (0), PS: pausing site (0), iTSS_I: internal starting site (0)

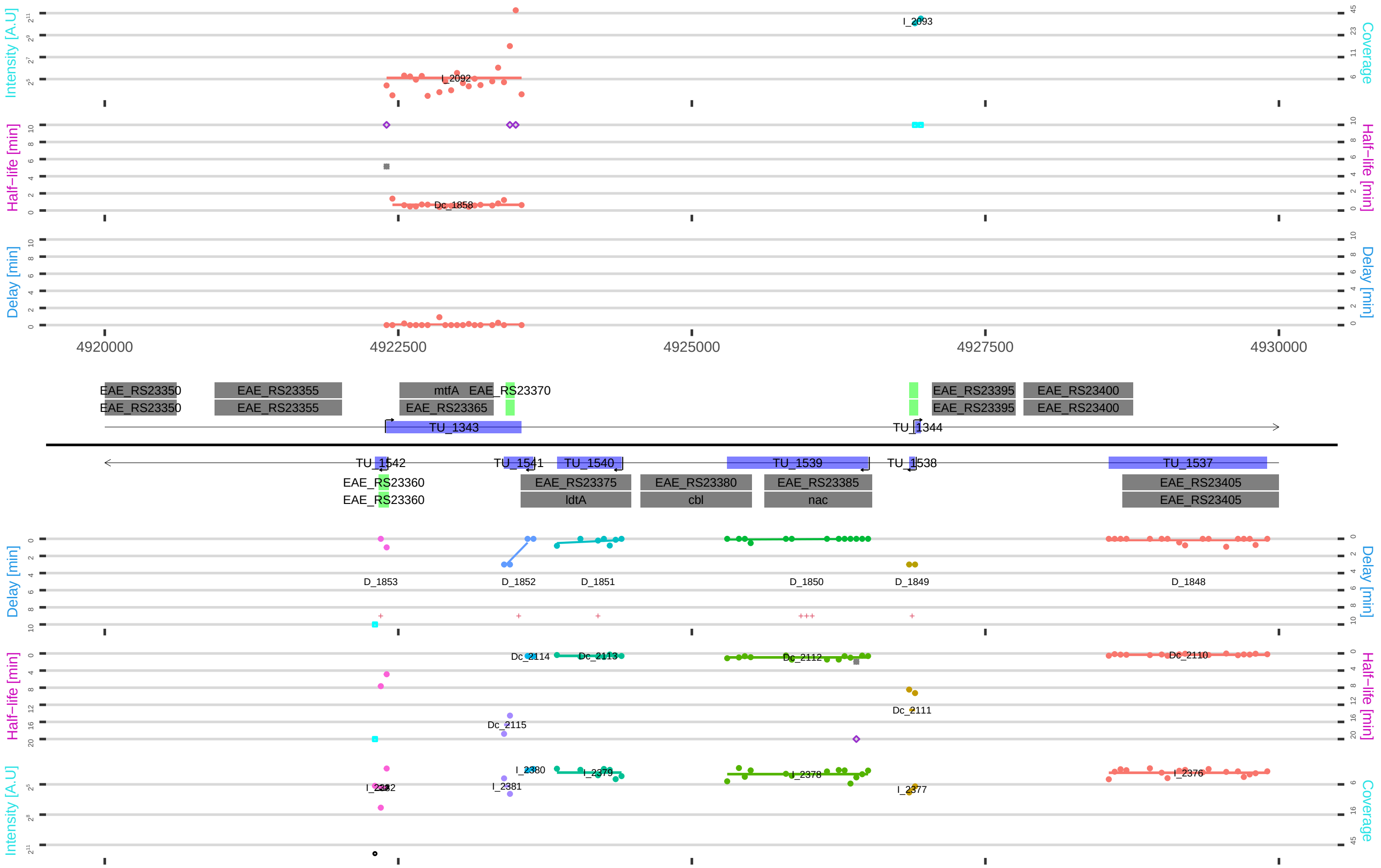

Term: termination (0), NS: new start (0), PS: pausing site (0), iTSS_I: internal starting site (0)

ID: 98616−98789; Term: termination (0), NS: new start (0), PS: pausing site (0), iTSS_I: internal starting site (0)

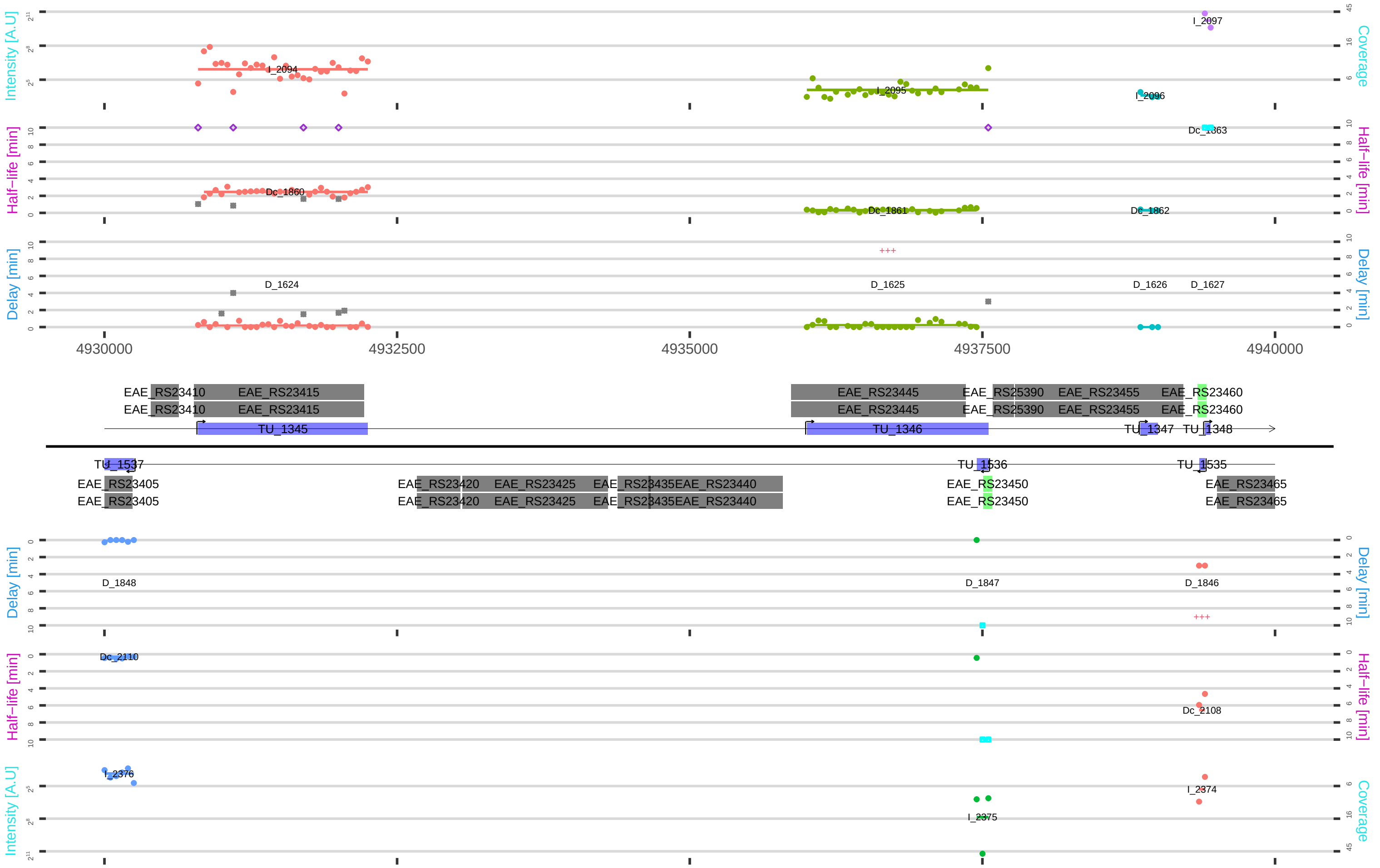

Term: termination (0), NS: new start (0), PS: pausing site (0), iTSS_I: internal starting site (0)

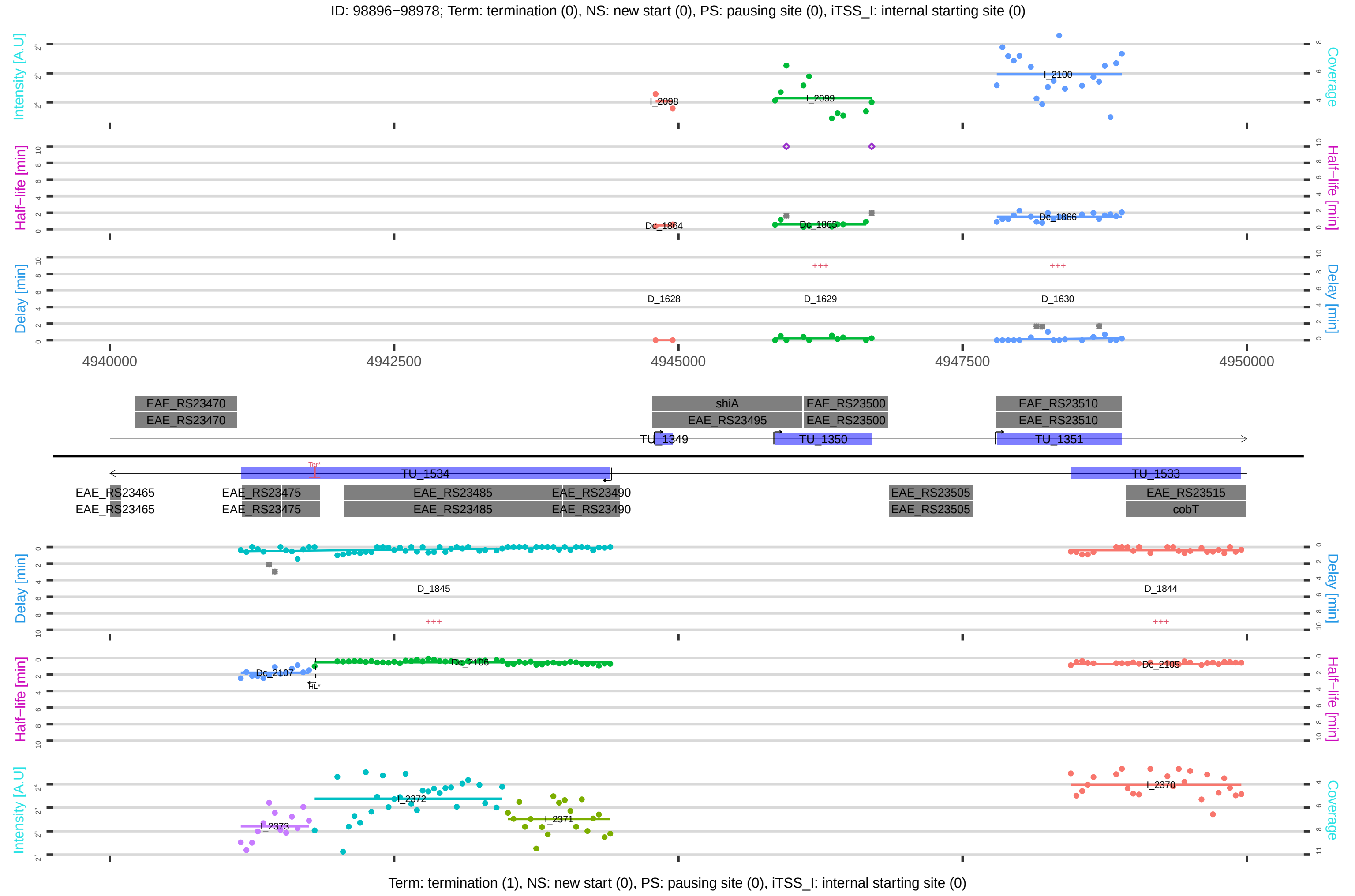

ID: 98896−98978; Term: termination (0), NS: new start (0), PS: pausing site (0), iTSS_I: internal starting site (0)
Intensity [A.U]
Coverage
Half−life [min]
Delay [min]
I_2098
I_2099
I_2100
Dc_1864
Dc_1865
Dc_1866
D_1628
D_1629
D_1630
4940000
4942500
4945000
4947500
4950000
EAE_RS23470
shiA
EAE_RS23495
EAE_RS23500
EAE_RS23510
TU_1349
TU_1350
TU_1351
TU_1534
TU_1533
EAE_RS23465
EAE_RS23475
EAE_RS23485
EAE_RS23490
EAE_RS23505
EAE_RS23515
cobT
D_1845
D_1844
Dc_2107
Dc_2106
Dc_2105
HL*
Ter*
I_2373
I_2372
I_2371
I_2370
Term: termination (1), NS: new start (0), PS: pausing site (0), iTSS_I: internal starting site (0)

ID: 99182−99200; Term: termination (0), NS: new start (0), PS: pausing site (0), iTSS_I: internal starting site (0)

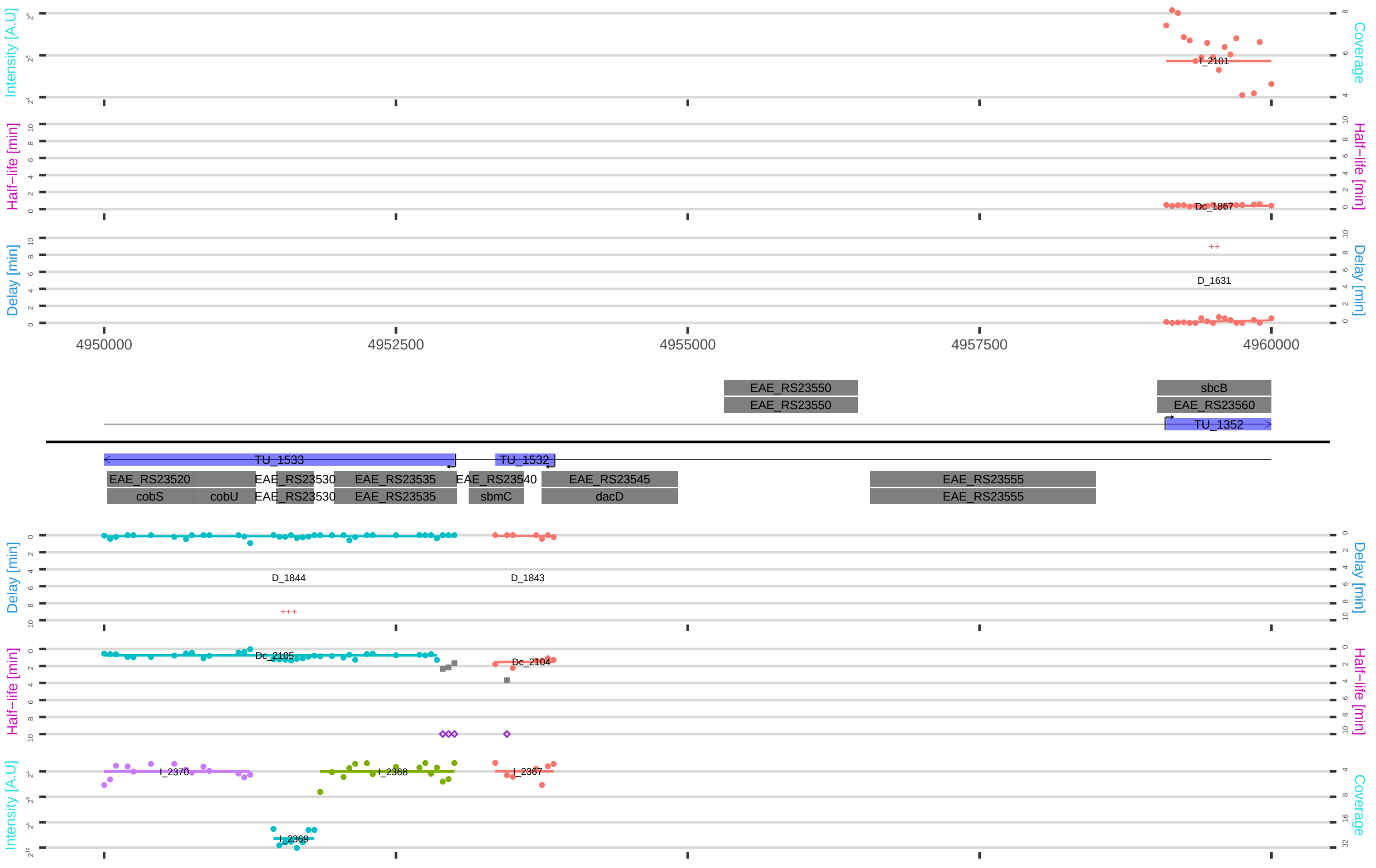

Term: termination (0), NS: new start (0), PS: pausing site (0), iTSS_I: internal starting site (0)

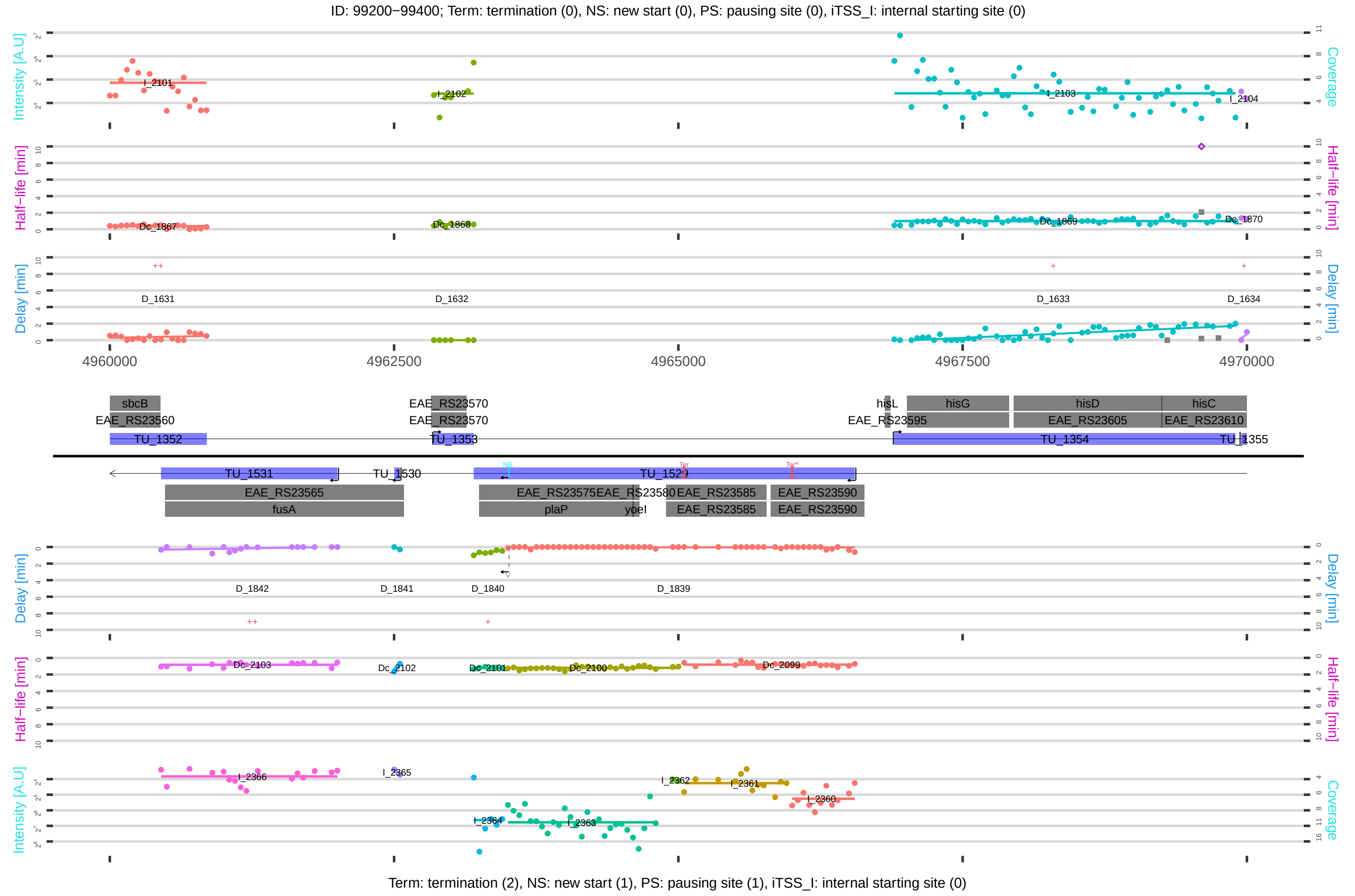

ID: 99200−99400; Term: termination (0), NS: new start (0), PS: pausing site (0), iTSS_I: internal starting site (0)
Intensity [A.U]
Coverage
Half−life [min]
Delay [min]
I_2101
I_2102
I_2103
I_2104
Dc_1867
Dc_1868
Dc_1869
Dc_1870
D_1631
D_1632
D_1633
D_1634
4960000
4962500
4965000
4967500
4970000
sbcB
EAE_RS23560
EAE_RS23570
hisL
hisG
hisD
hisC
EAE_RS23595
EAE_RS23605
EAE_RS23610
TU_1352
TU_1353
TU_1354
TU_1355
TU_1531
TU_1530
TU_1529
EAE_RS23565
fusA
EAE_RS23575
EAE_RS23580
EAE_RS23585
EAE_RS23590
plaP
yoeI
D_1842
D_1841
D_1840
D_1839
Dc_2103
Dc_2102
Dc_2101
Dc_2100
Dc_2099
I_2366
I_2365
I_2364
I_2363
I_2362
I_2361
I_2360
Term: termination (2), NS: new start (1), PS: pausing site (1), iTSS_I: internal starting site (0)

ID: 99400−99481; Term: termination (1), NS: new start (0), PS: pausing site (0), iTSS_I: internal starting site (1)

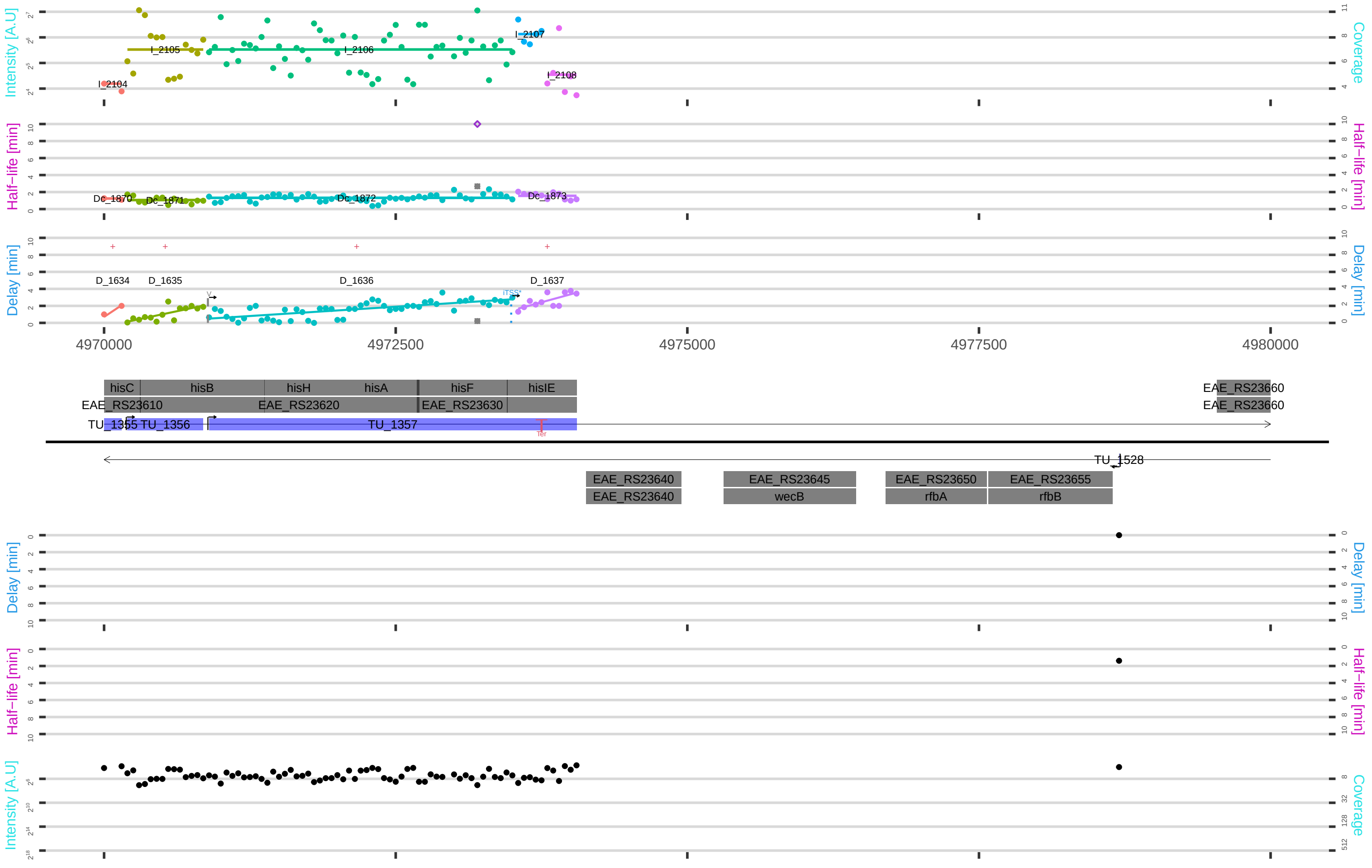

Term: termination (0), NS: new start (0), PS: pausing site (0), iTSS_I: internal starting site (0)

ID: 99604−99605; Term: termination (0), NS: new start (0), PS: pausing site (0), iTSS_I: internal starting site (0)

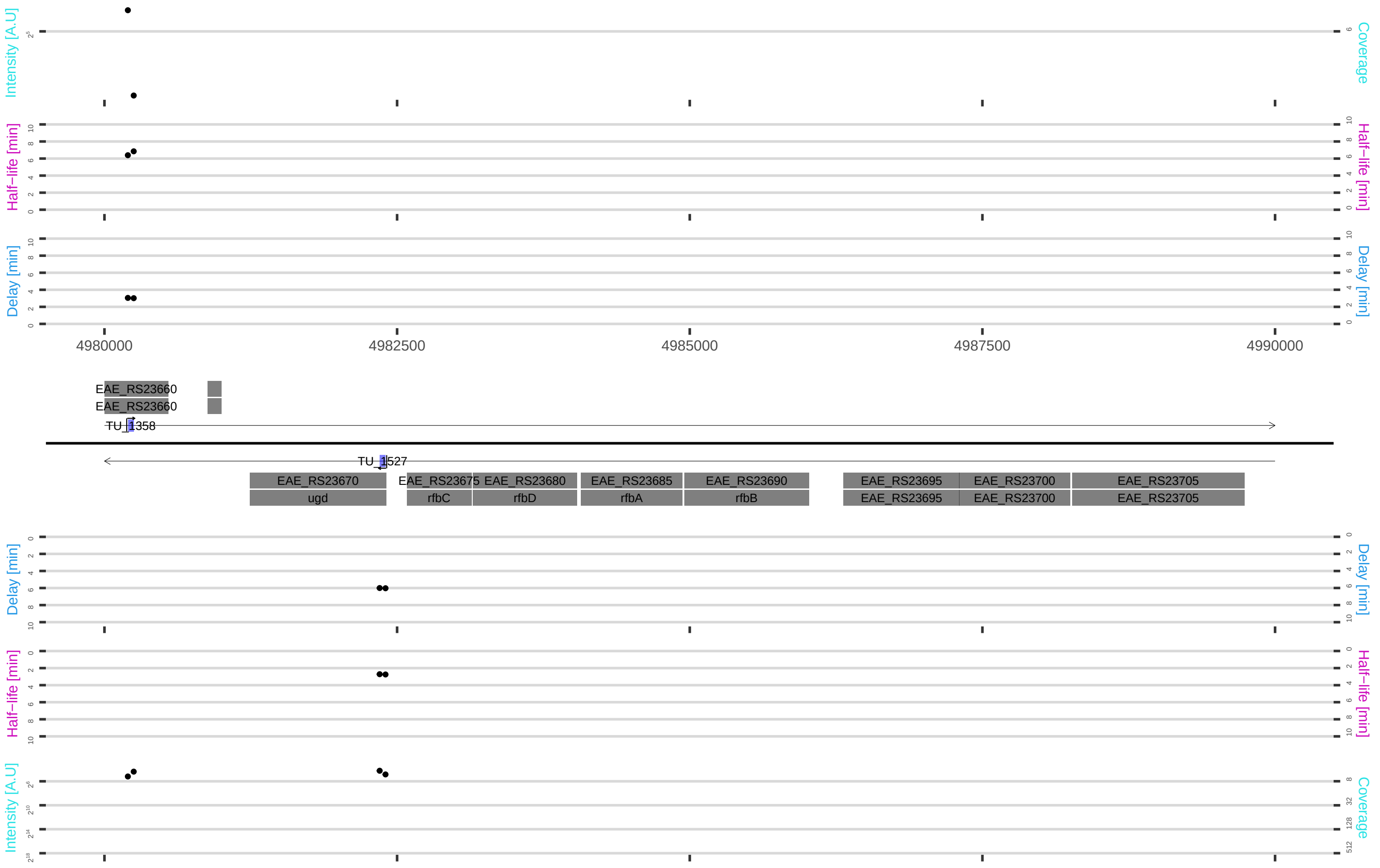

Term: termination (0), NS: new start (0), PS: pausing site (0), iTSS_I: internal starting site (0)

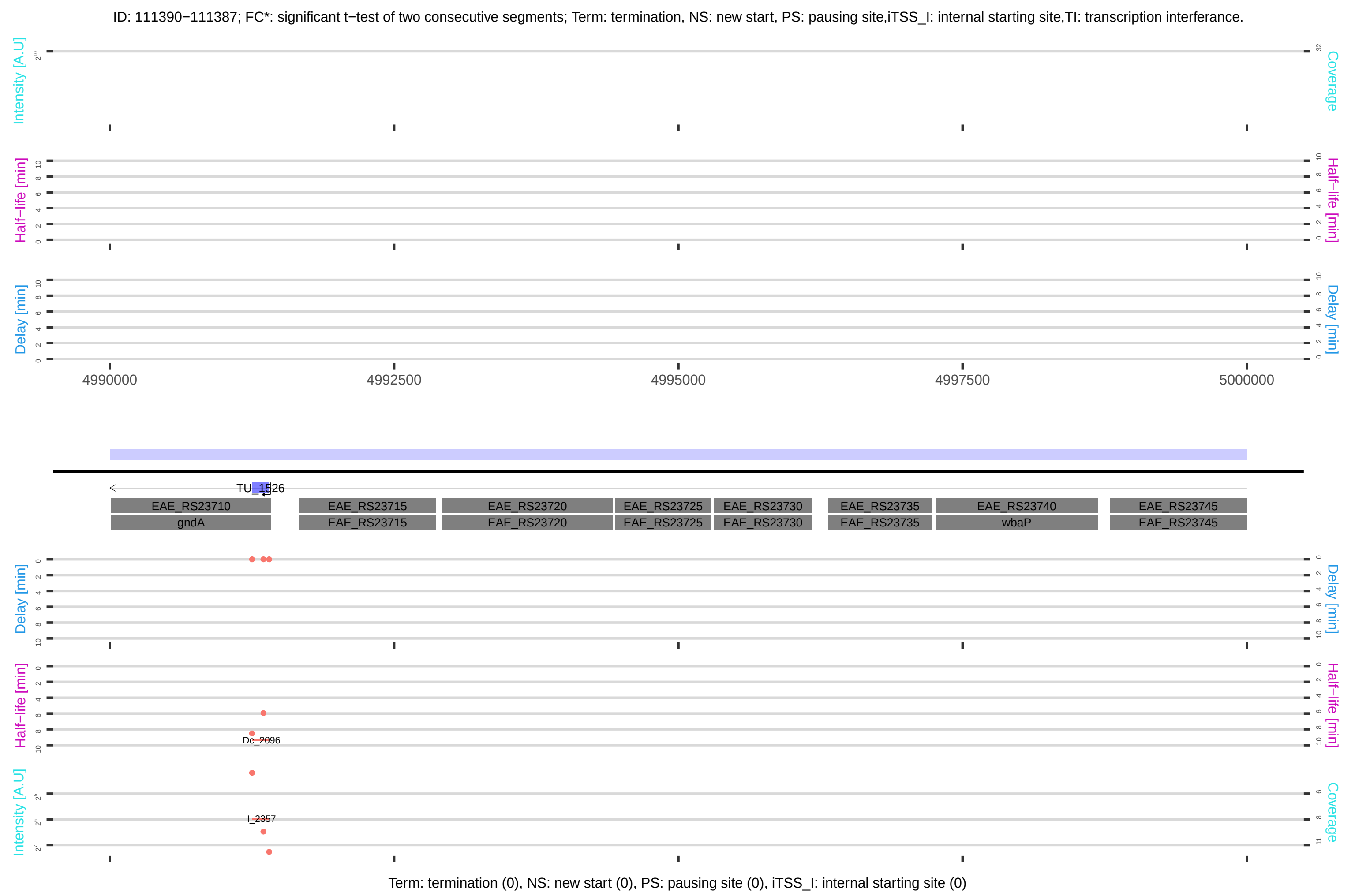
ID: 111390−111387; FC*: significant t−test of two consecutive segments; Term: termination, NS: new start, PS: pausing site,iTSS_I: internal starting site,TI: transcription interferance.
Intensity [A.U]
Coverage
Half−life [min]
Delay [min]
4990000
4992500
4995000
4997500
5000000
TU_1526
EAE_RS23710
gndA
EAE_RS23715
EAE_RS23720
EAE_RS23725
EAE_RS23730
EAE_RS23735
EAE_RS23740
wbaP
EAE_RS23745
Dc_2096
I_2357
Term: termination (0), NS: new start (0), PS: pausing site (0), iTSS_I: internal starting site (0)

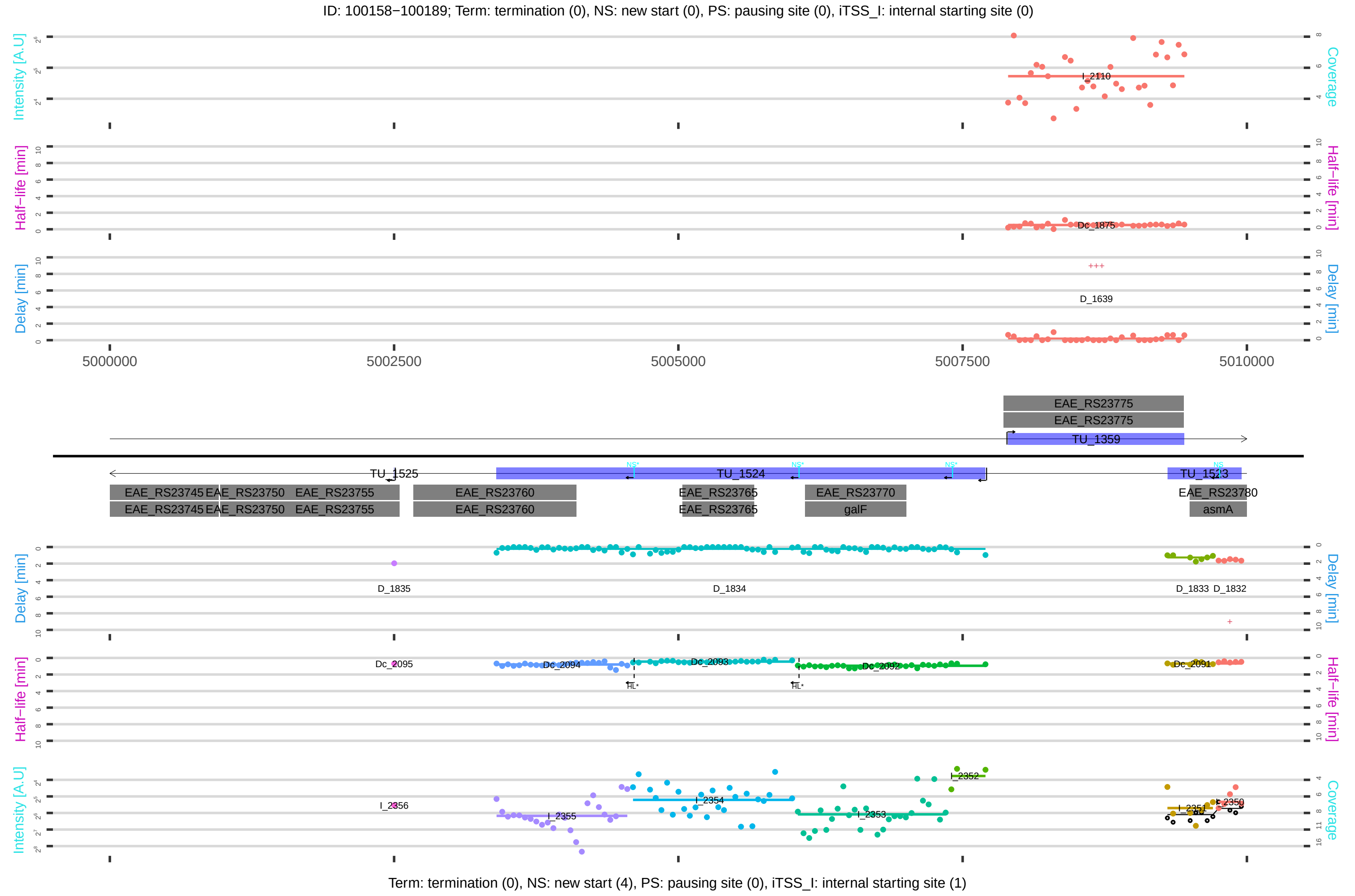

ID: 100158−100189; Term: termination (0), NS: new start (0), PS: pausing site (0), iTSS_I: internal starting site (0)
Intensity [A.U]
Coverage
Half−life [min]
Delay [min]
I_2110
Dc_1875
D_1639
5000000
5002500
5005000
5007500
5010000
EAE_RS23775
TU_1359
TU_1525
TU_1524
TU_1523
EAE_RS23745
EAE_RS23750
EAE_RS23755
EAE_RS23760
EAE_RS23765
EAE_RS23770
galF
EAE_RS23780
asmA
D_1835
D_1834
D_1833
D_1832
Dc_2095
Dc_2094
Dc_2093
Dc_2092
Dc_2091
I_2356
I_2355
I_2354
I_2353
I_2352
I_2351
I_2350
Term: termination (0), NS: new start (4), PS: pausing site (0), iTSS_I: internal starting site (1)

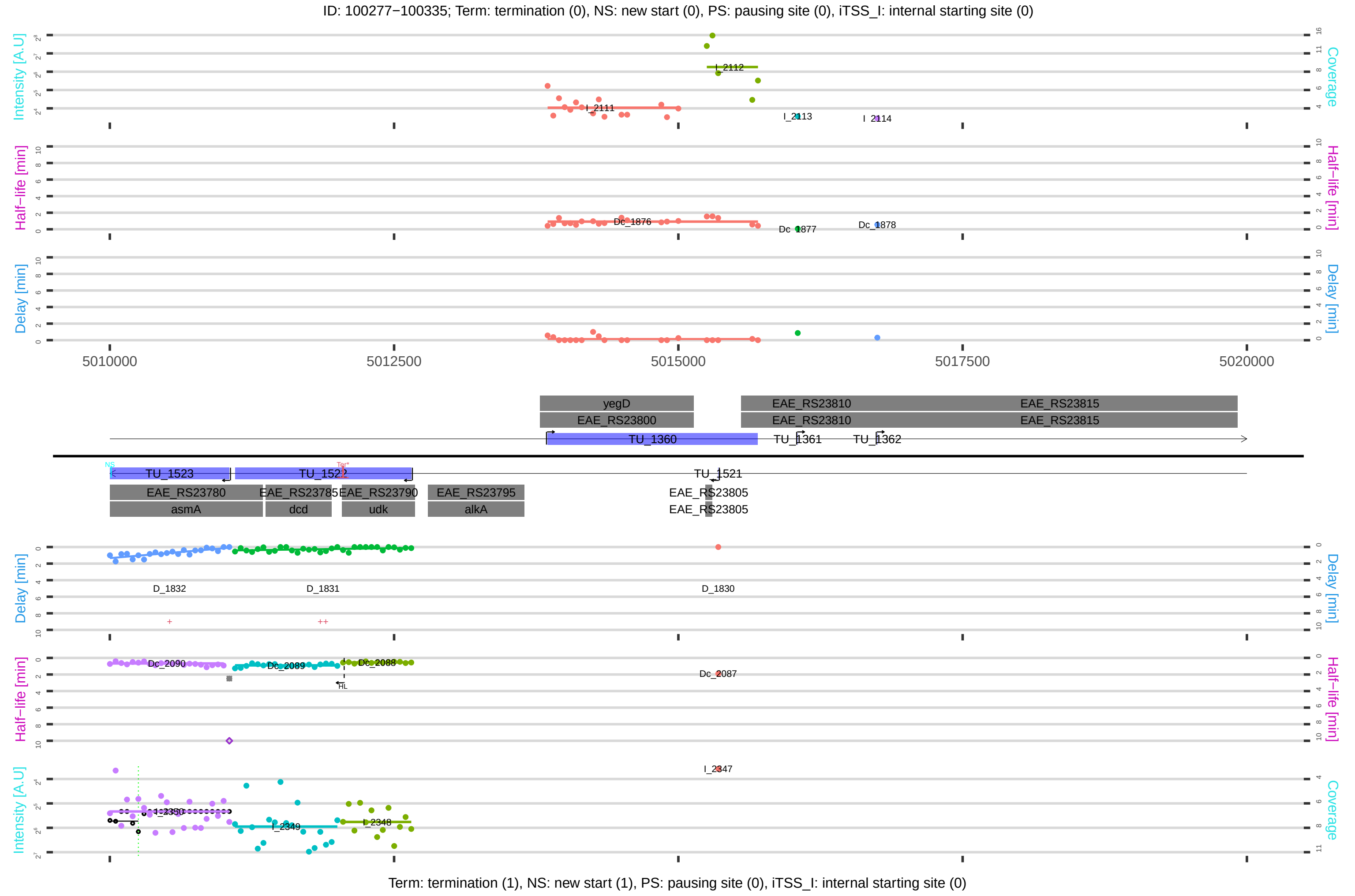

ID: 100277−100335; Term: termination (0), NS: new start (0), PS: pausing site (0), iTSS_I: internal starting site (0)
Intensity [A.U]
Coverage
Half−life [min]
Delay [min]
I_2111
I_2112
I_2113
I_2114
Dc_1876
Dc_1877
Dc_1878
5010000
5012500
5015000
5017500
5020000
yegD
EAE_RS23800
EAE_RS23810
EAE_RS23815
TU_1360
TU_1361
TU_1362
TU_1523
TU_1522
TU_1521
EAE_RS23780
EAE_RS23785
EAE_RS23790
EAE_RS23795
EAE_RS23805
asmA
dcd
udk
alkA
D_1832
D_1831
D_1830
Dc_2090
Dc_2089
Dc_2088
Dc_2087
I_2350
I_2349
I_2348
I_2347
Term: termination (1), NS: new start (1), PS: pausing site (0), iTSS_I: internal starting site (0)

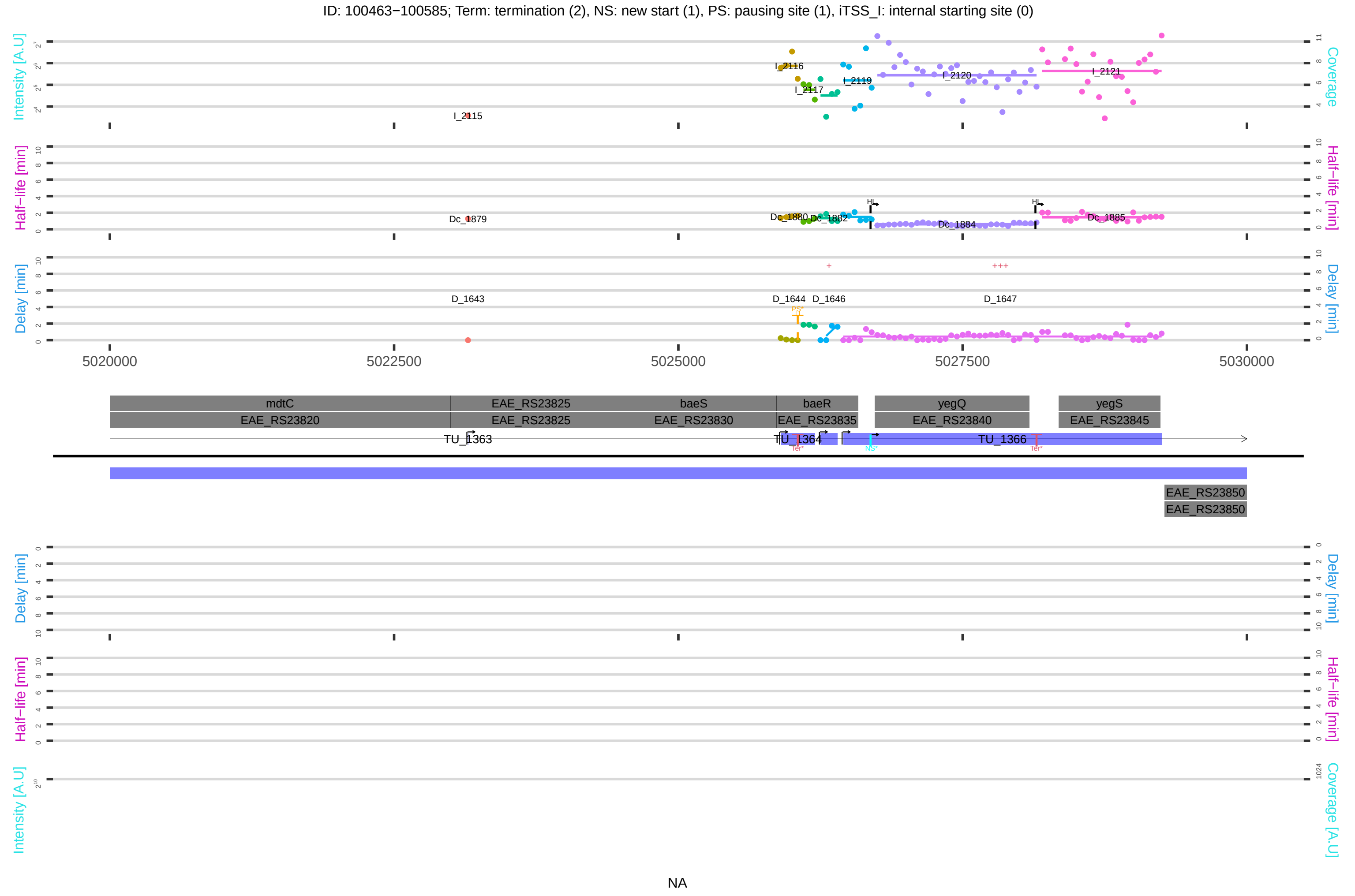

ID: 100463−100585; Term: termination (2), NS: new start (1), PS: pausing site (1), iTSS_I: internal starting site (0)
Intensity [A.U]
Coverage
Half−life [min]
Delay [min]
I_2115
I_2116
I_2117
I_2119
I_2120
I_2121
Dc_1879
Dc_1880
Dc_1882
Dc_1884
Dc_1885
D_1643
D_1644
D_1646
D_1647
5020000
5022500
5025000
5027500
5030000
mdtC
EAE_RS23820
EAE_RS23825
baeS
EAE_RS23830
baeR
EAE_RS23835
yegQ
EAE_RS23840
yegS
EAE_RS23845
TU_1363
TU_1364
TU_1366
EAE_RS23850
NA

ID: 100675−100766; Term: termination (0), NS: new start (0), PS: pausing site (0), iTSS_I: internal starting site (0)

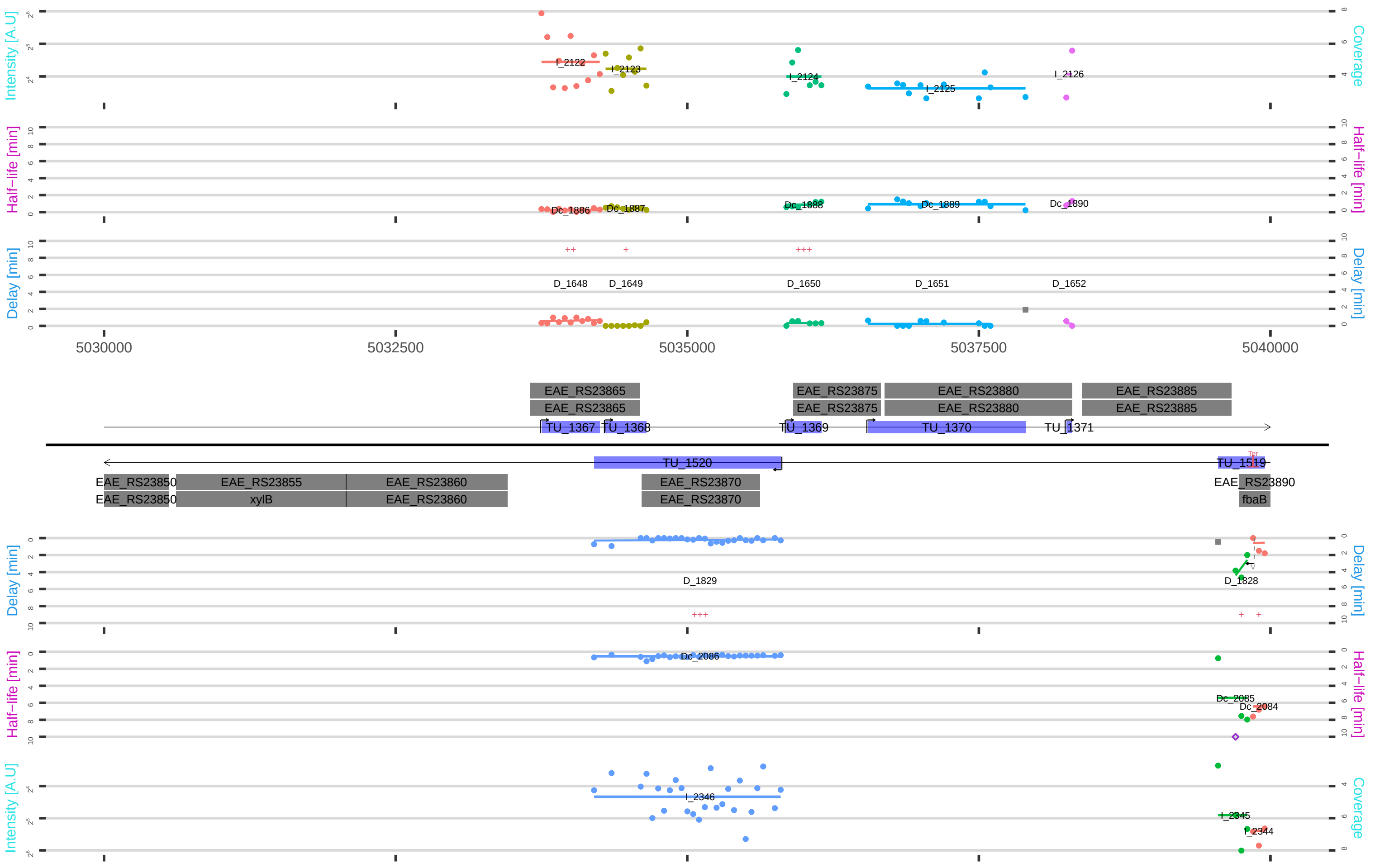

Term: termination (1), NS: new start (0), PS: pausing site (1), iTSS_I: internal starting site (0)

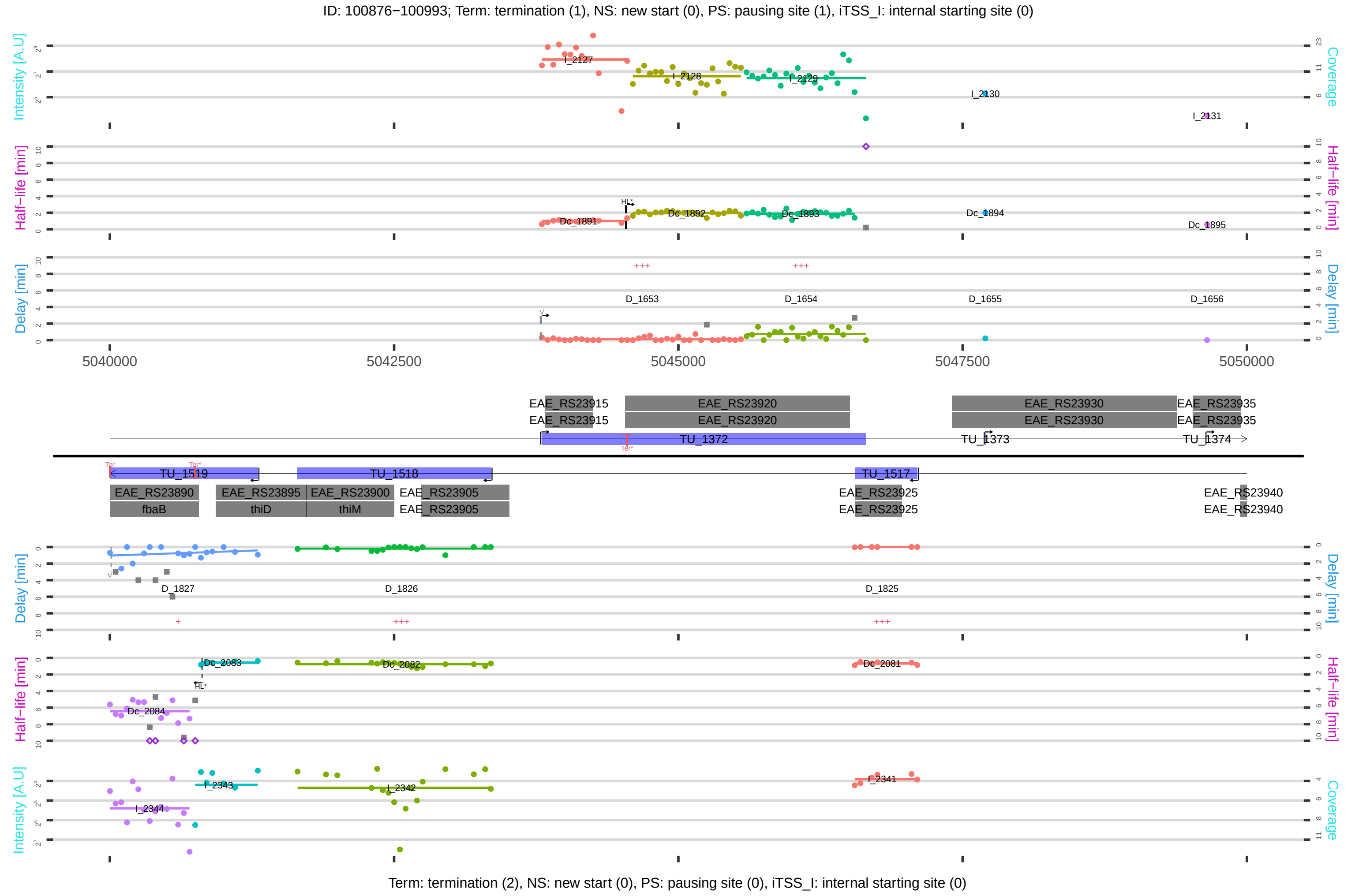
ID: 100876−100993; Term: termination (1), NS: new start (0), PS: pausing site (1), iTSS_I: internal starting site (0)
Intensity [A.U]
Coverage
Half−life [min]
Delay [min]
I_2127
I_2128
I_2129
I_2130
I_2131
Dc_1891
Dc_1892
Dc_1893
Dc_1894
Dc_1895
D_1653
D_1654
D_1655
D_1656
5040000
5042500
5045000
5047500
5050000
EAE_RS23915
EAE_RS23920
EAE_RS23930
EAE_RS23935
TU_1372
TU_1373
TU_1374
TU_1519
TU_1518
TU_1517
EAE_RS23890
fbaB
EAE_RS23895
thiD
EAE_RS23900
thiM
EAE_RS23905
EAE_RS23925
EAE_RS23940
D_1827
D_1826
D_1825
Dc_2083
Dc_2082
Dc_2081
Dc_2084
I_2343
I_2342
I_2341
I_2344
Term: termination (2), NS: new start (0), PS: pausing site (0), iTSS_I: internal starting site (0)

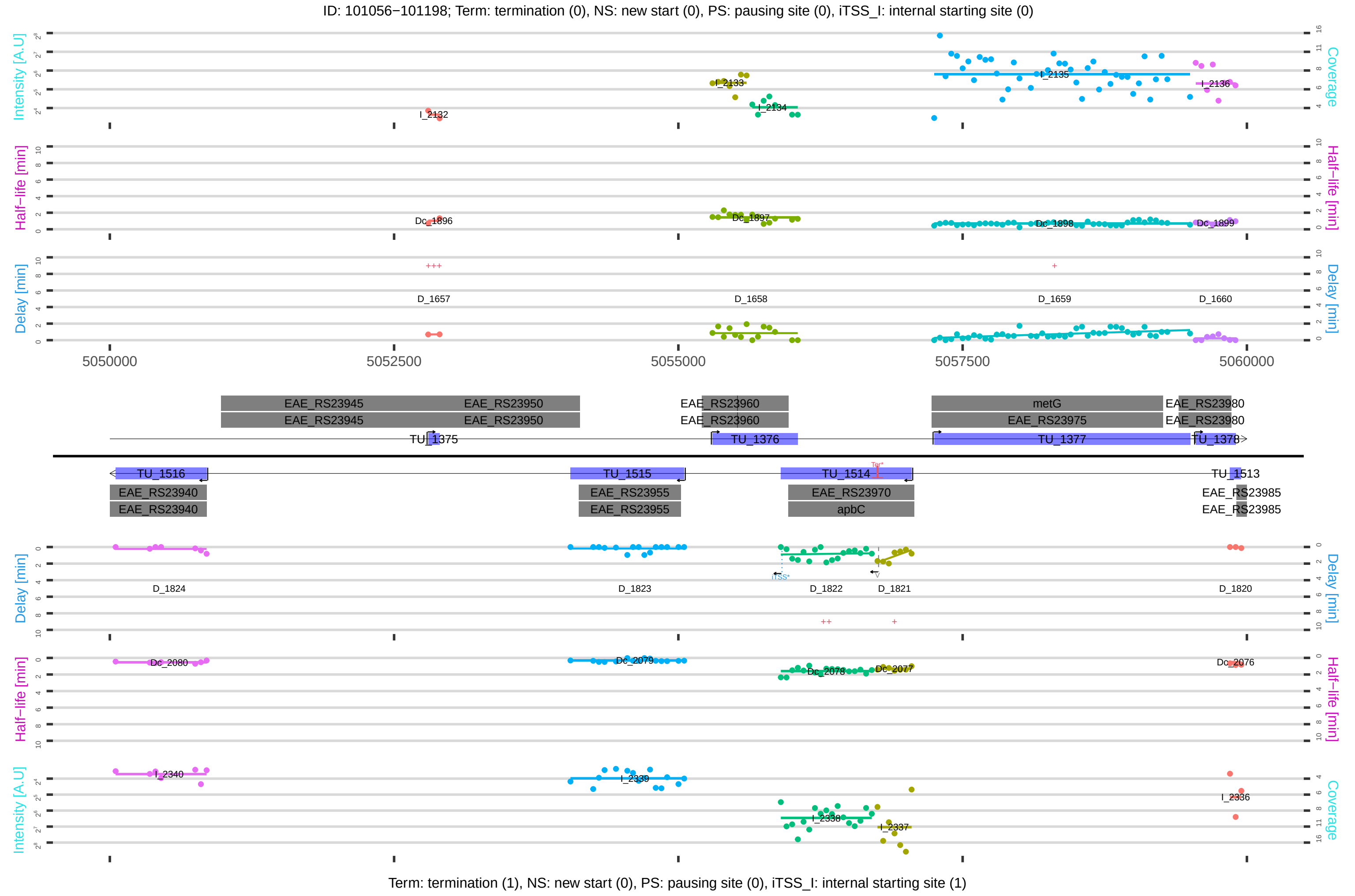
ID: 101056−101198; Term: termination (0), NS: new start (0), PS: pausing site (0), iTSS_I: internal starting site (0)
Intensity [A.U]
Coverage
Half−life [min]
Delay [min]
I_2132
I_2133
I_2134
I_2135
I_2136
Dc_1896
Dc_1897
Dc_1898
Dc_1899
D_1657
D_1658
D_1659
D_1660
5050000
5052500
5055000
5057500
5060000
EAE_RS23945
EAE_RS23950
EAE_RS23960
metG
EAE_RS23975
EAE_RS23980
TU_1375
TU_1376
TU_1377
TU_1378
TU_1516
TU_1515
TU_1514
TU_1513
EAE_RS23940
EAE_RS23955
EAE_RS23970
apbC
EAE_RS23985
D_1824
D_1823
D_1822
D_1821
D_1820
Dc_2080
Dc_2079
Dc_2078
Dc_2077
Dc_2076
I_2340
I_2339
I_2338
I_2337
I_2336
Term: termination (1), NS: new start (0), PS: pausing site (0), iTSS_I: internal starting site (1)

ID: 101201−101282; Term: termination (0), NS: new start (0), PS: pausing site (0), iTSS_I: internal starting site (0)

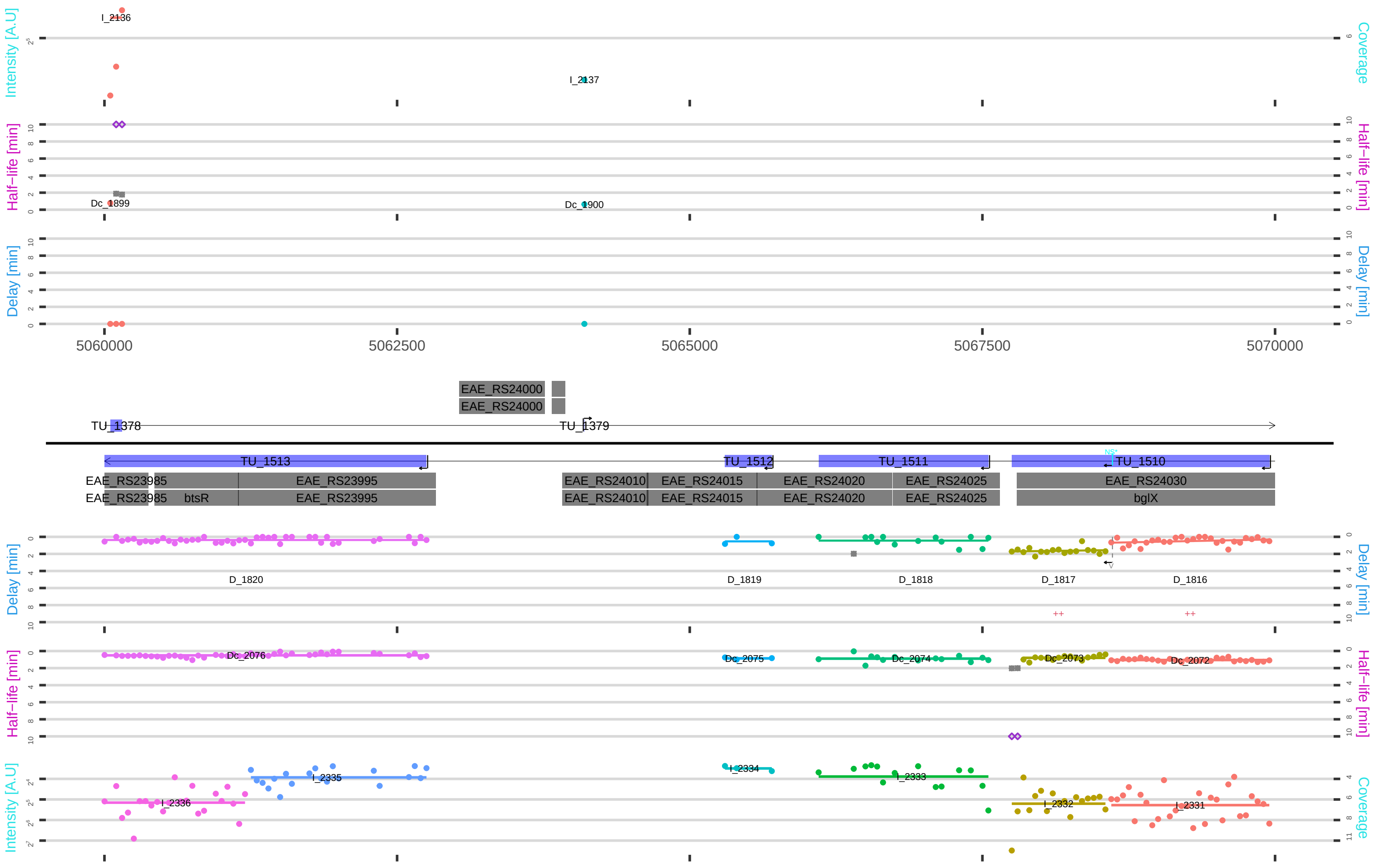

Term: termination (0), NS: new start (1), PS: pausing site (1), iTSS_I: internal starting site (0)

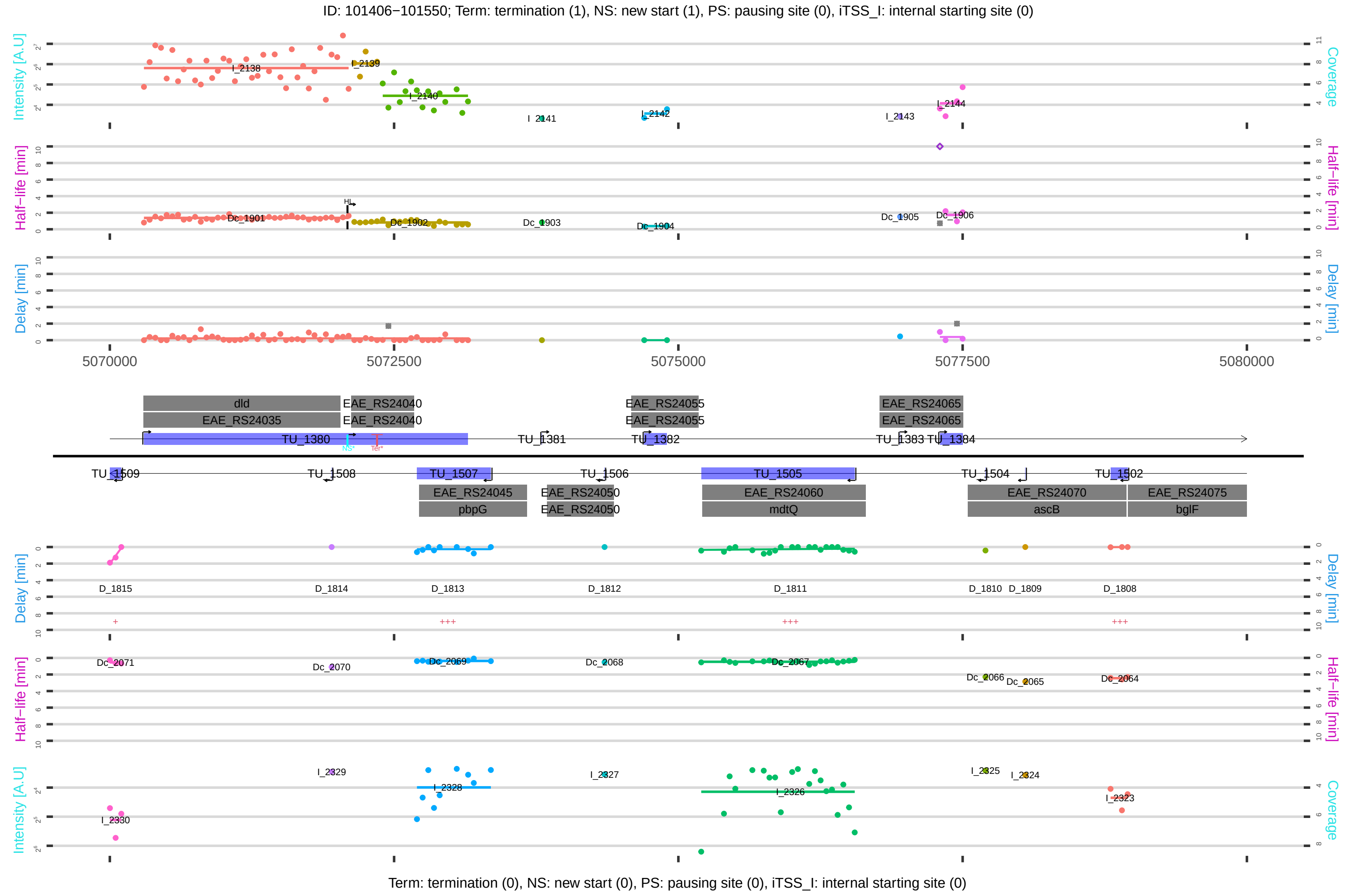

ID: 101406−101550; Term: termination (1), NS: new start (1), PS: pausing site (0), iTSS_I: internal starting site (0)
Intensity [A.U]
Coverage
Half−life [min]
Delay [min]
5070000
5072500
5075000
5077500
5080000
dld
EAE_RS24035
EAE_RS24040
EAE_RS24055
EAE_RS24065
TU_1380
TU_1381
TU_1382
TU_1383
TU_1384
TU_1509
TU_1508
TU_1507
TU_1506
TU_1505
TU_1504
TU_1502
EAE_RS24045
pbpG
EAE_RS24050
EAE_RS24060
mdtQ
EAE_RS24070
ascB
EAE_RS24075
bglF
Term: termination (0), NS: new start (0), PS: pausing site (0), iTSS_I: internal starting site (0)

ID: 101781−101800; Term: termination (0), NS: new start (2), PS: pausing site (0), iTSS_I: internal starting site (0)

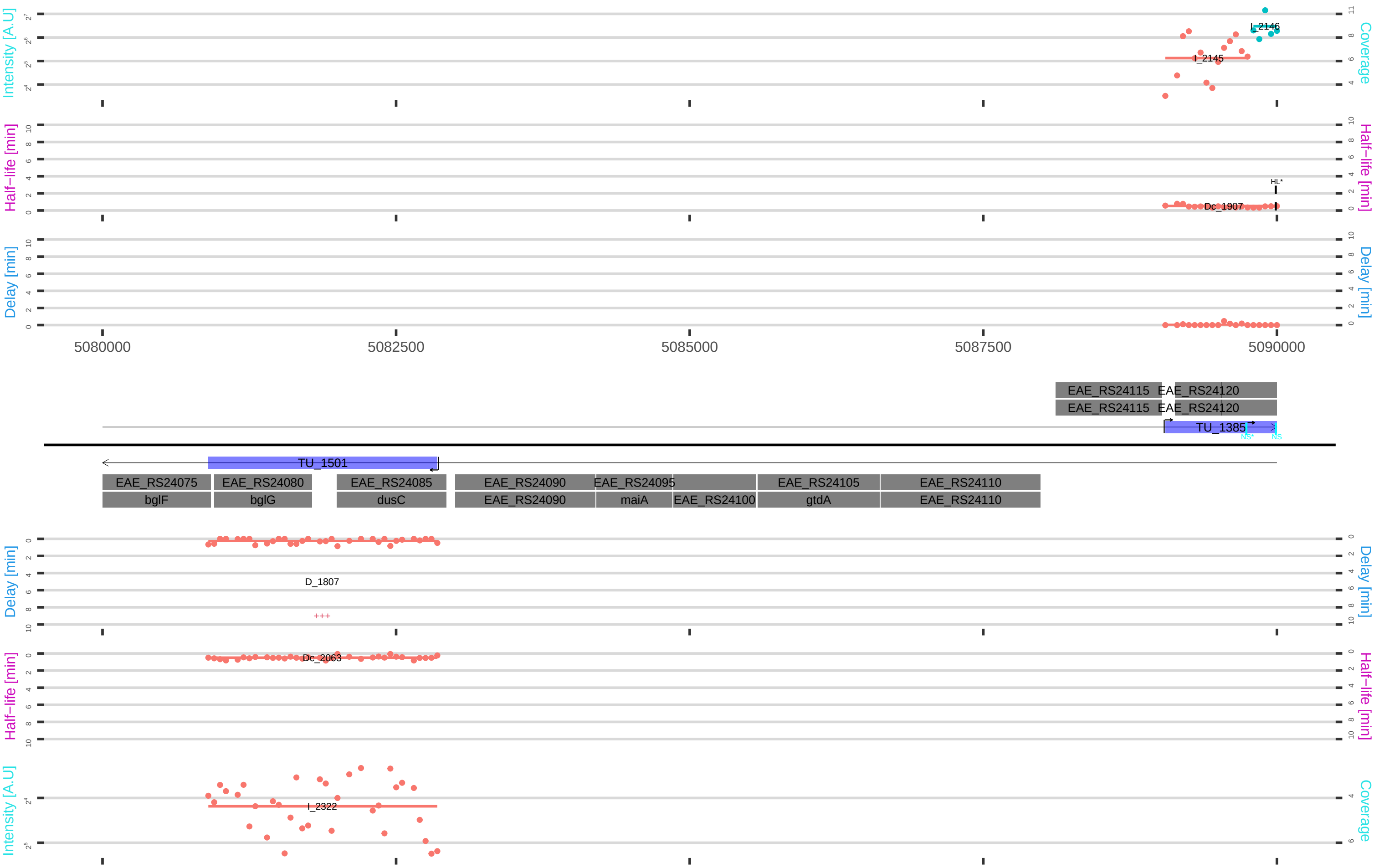

Term: termination (0), NS: new start (0), PS: pausing site (0), iTSS_I: internal starting site (0)

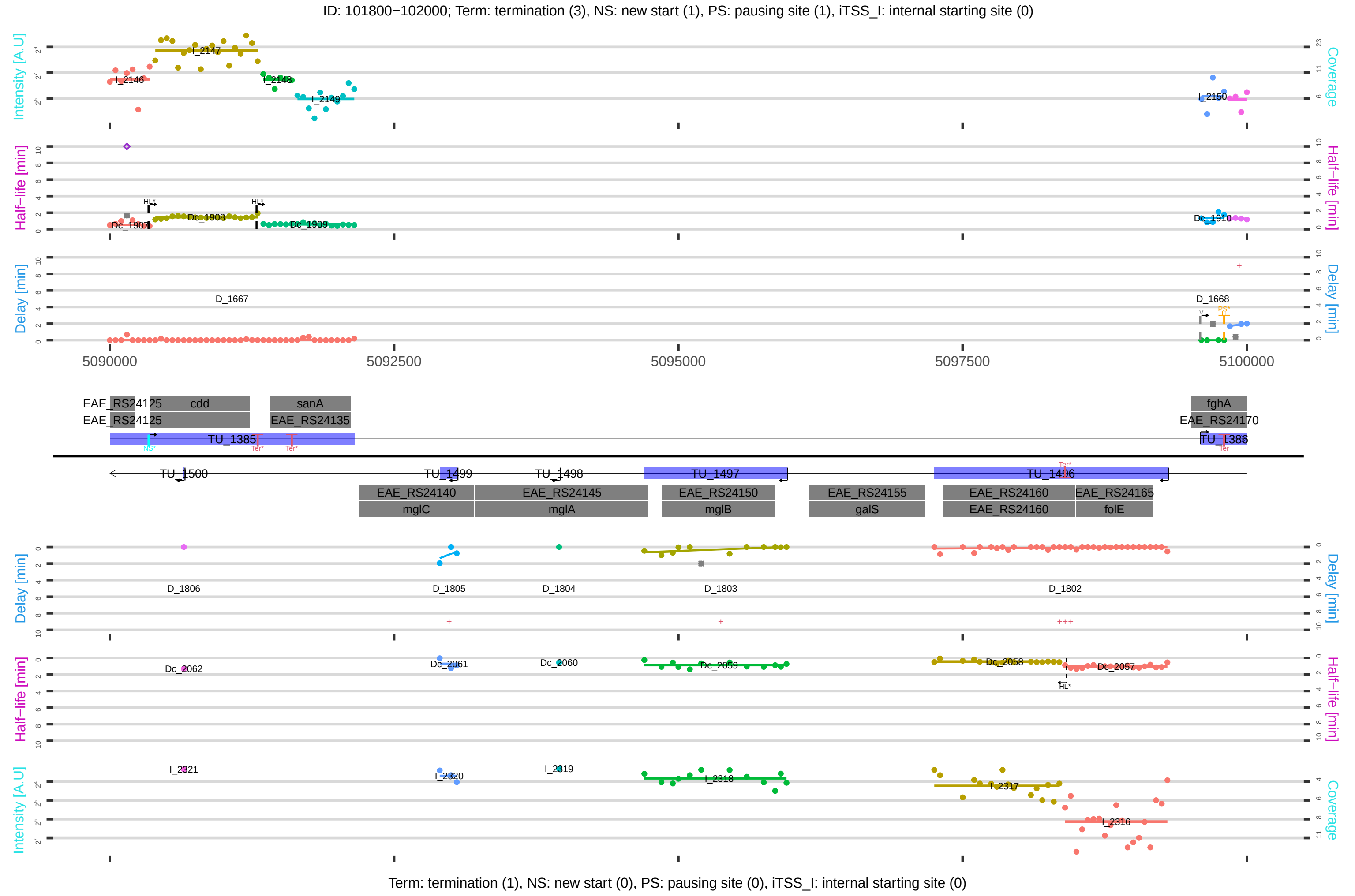
ID: 101800−102000; Term: termination (3), NS: new start (1), PS: pausing site (1), iTSS_I: internal starting site (0)
Intensity [A.U]
Coverage
Half−life [min]
Delay [min]
I_2146
I_2147
I_2148
I_2149
I_2150
Dc_1907
Dc_1908
Dc_1909
Dc_1910
D_1667
D_1668
5090000
5092500
5095000
5097500
5100000
EAE_RS24125
cdd
sanA
EAE_RS24135
fghA
EAE_RS24170
TU_1385
TU_1386
TU_1500
TU_1499
TU_1498
TU_1497
TU_1496
EAE_RS24140
mglC
EAE_RS24145
mglA
EAE_RS24150
mglB
EAE_RS24155
galS
EAE_RS24160
EAE_RS24165
folE
D_1806
D_1805
D_1804
D_1803
D_1802
Dc_2062
Dc_2061
Dc_2060
Dc_2059
Dc_2058
Dc_2057
I_2321
I_2320
I_2319
I_2318
I_2317
I_2316
Term: termination (1), NS: new start (0), PS: pausing site (0), iTSS_I: internal starting site (0)

ID: 102000−102148; Term: termination (0), NS: new start (0), PS: pausing site (0), iTSS_I: internal starting site (0)

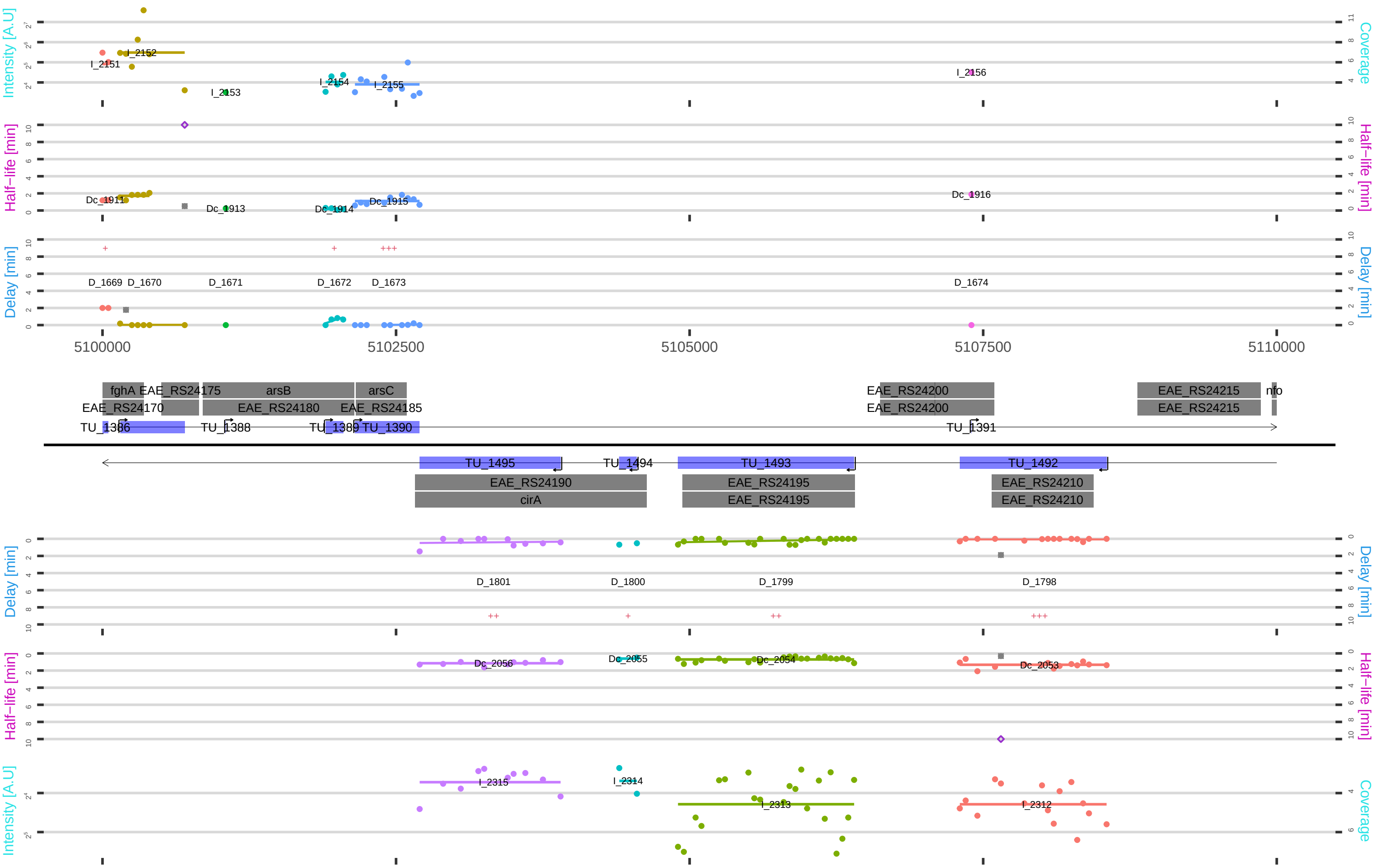

Term: termination (0), NS: new start (0), PS: pausing site (0), iTSS_I: internal starting site (0)

ID: 102201−102346; Term: termination (0), NS: new start (0), PS: pausing site (0), iTSS_I: internal starting site (0)

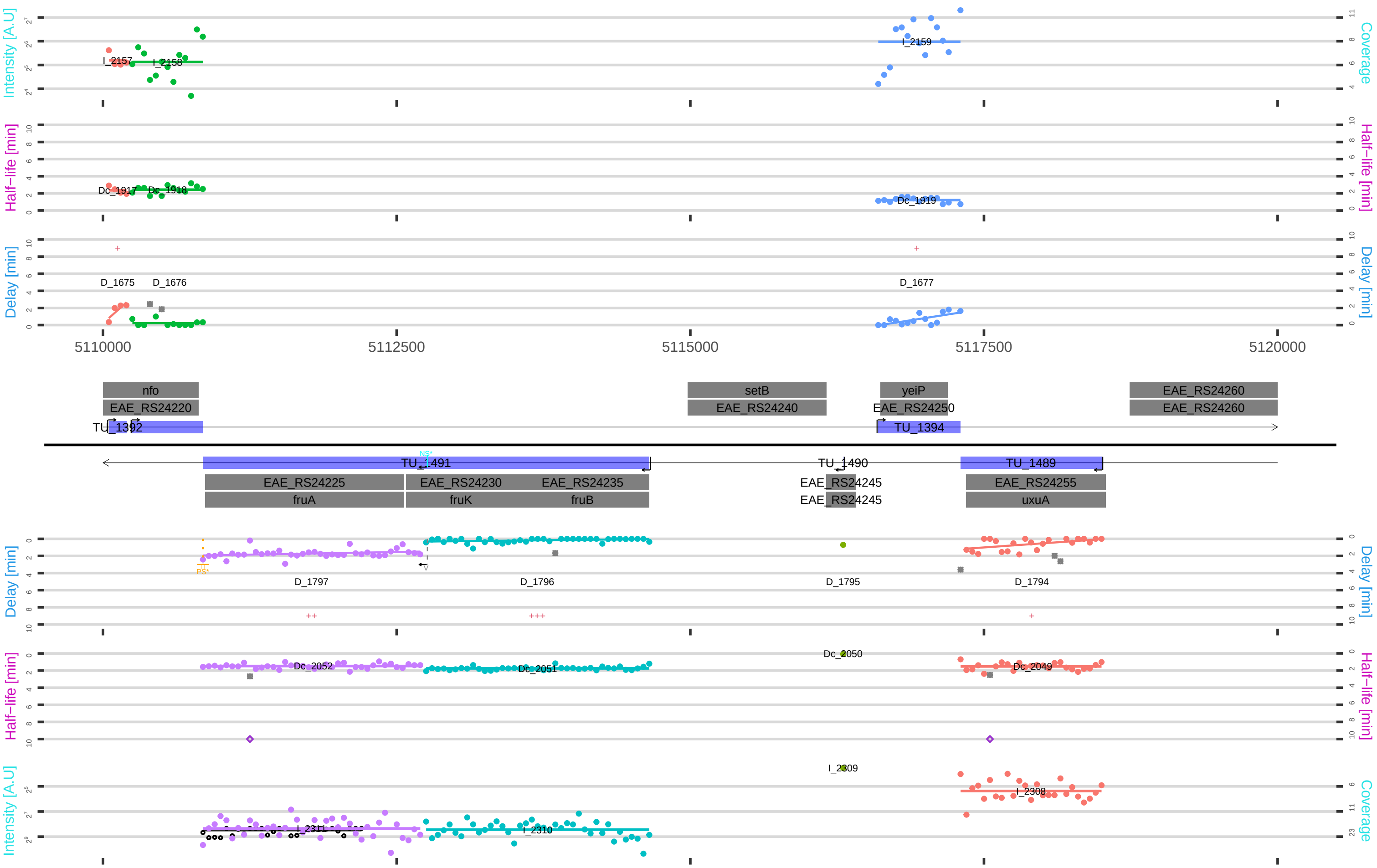

Term: termination (0), NS: new start (1), PS: pausing site (1), iTSS_I: internal starting site (0)

ID: 102408−102588; Term: termination (1), NS: new start (2), PS: pausing site (0), iTSS_I: internal starting site (0)

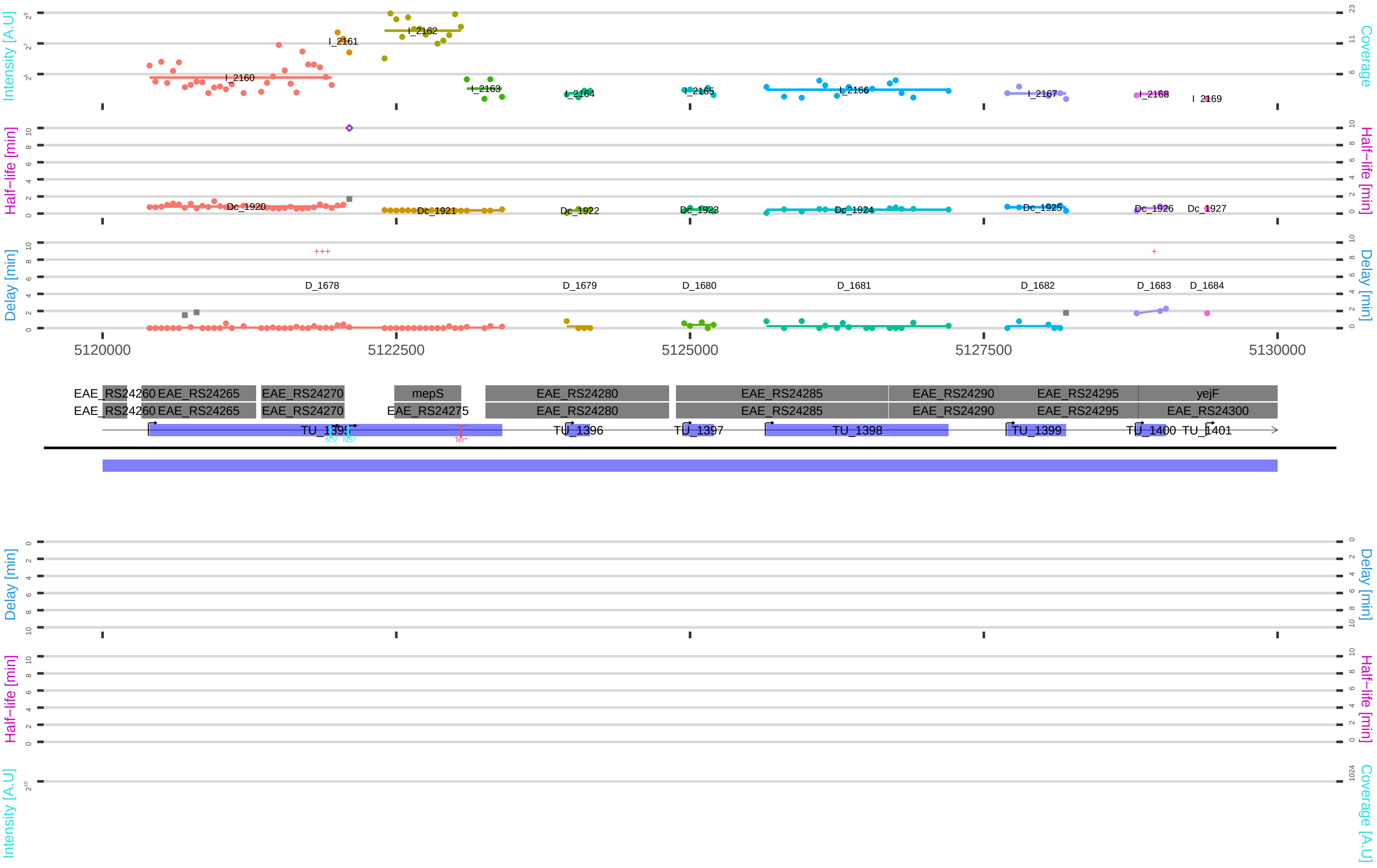

NA

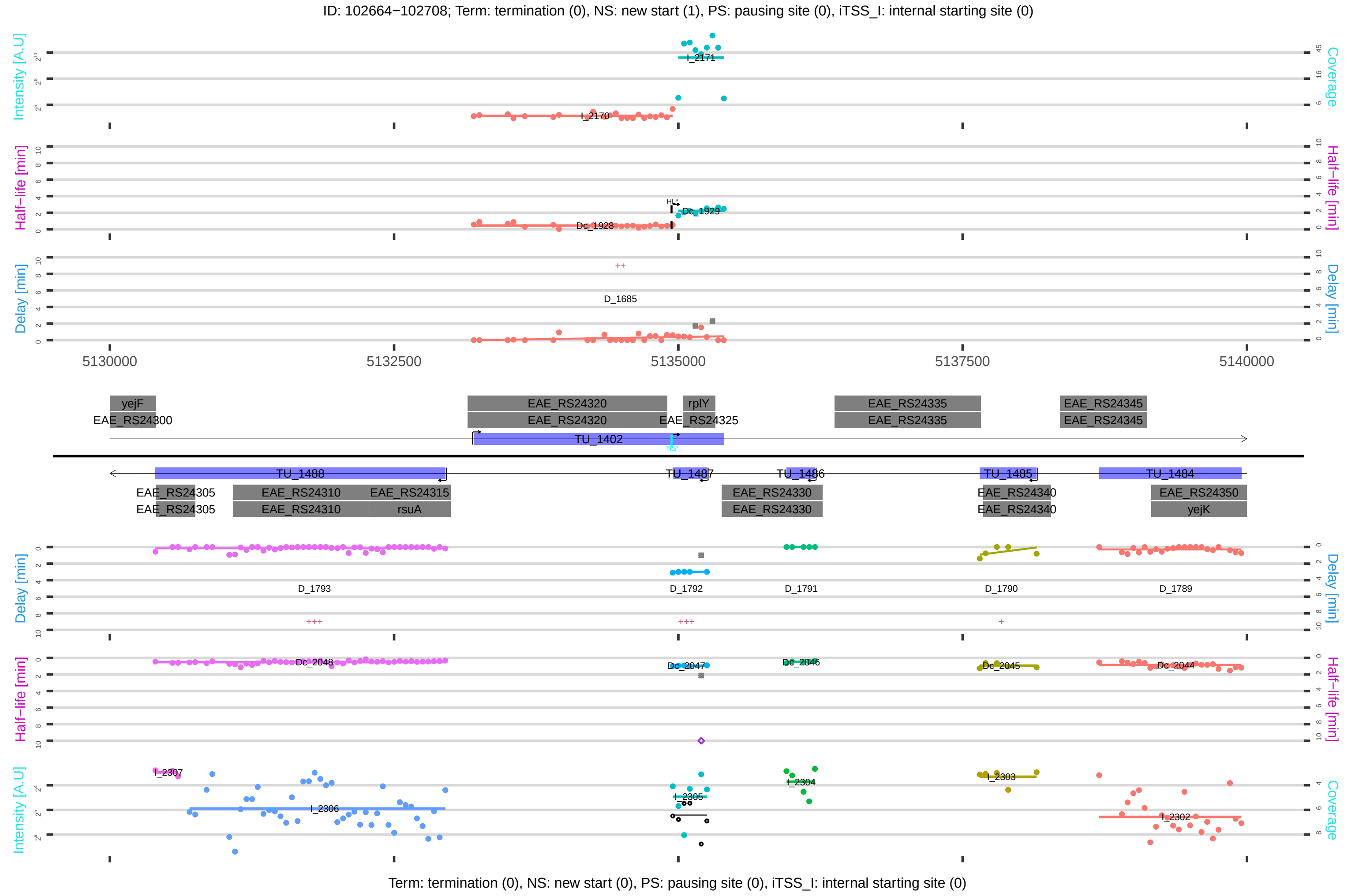

ID: 102664−102708; Term: termination (0), NS: new start (1), PS: pausing site (0), iTSS_I: internal starting site (0)
Intensity [A.U]
Coverage
I_2171
I_2170
Half−life [min]
Dc_1929
Dc_1928
Delay [min]
D_1685
5130000
5132500
5135000
5137500
5140000
yejF
EAE_RS24300
EAE_RS24320
rplY
EAE_RS24325
EAE_RS24335
EAE_RS24345
TU_1402
TU_1488
TU_1487
TU_1486
TU_1485
TU_1484
EAE_RS24305
EAE_RS24310
EAE_RS24315
rsuA
EAE_RS24330
EAE_RS24340
EAE_RS24350
yejK
D_1793
D_1792
D_1791
D_1790
D_1789
Dc_2048
Dc_2047
Dc_2046
Dc_2045
Dc_2044
I_2307
I_2306
I_2305
I_2304
I_2303
I_2302
Term: termination (0), NS: new start (0), PS: pausing site (0), iTSS_I: internal starting site (0)

ID: 102807−102952; Term: termination (3), NS: new start (2), PS: pausing site (1), iTSS_I: internal starting site (0)

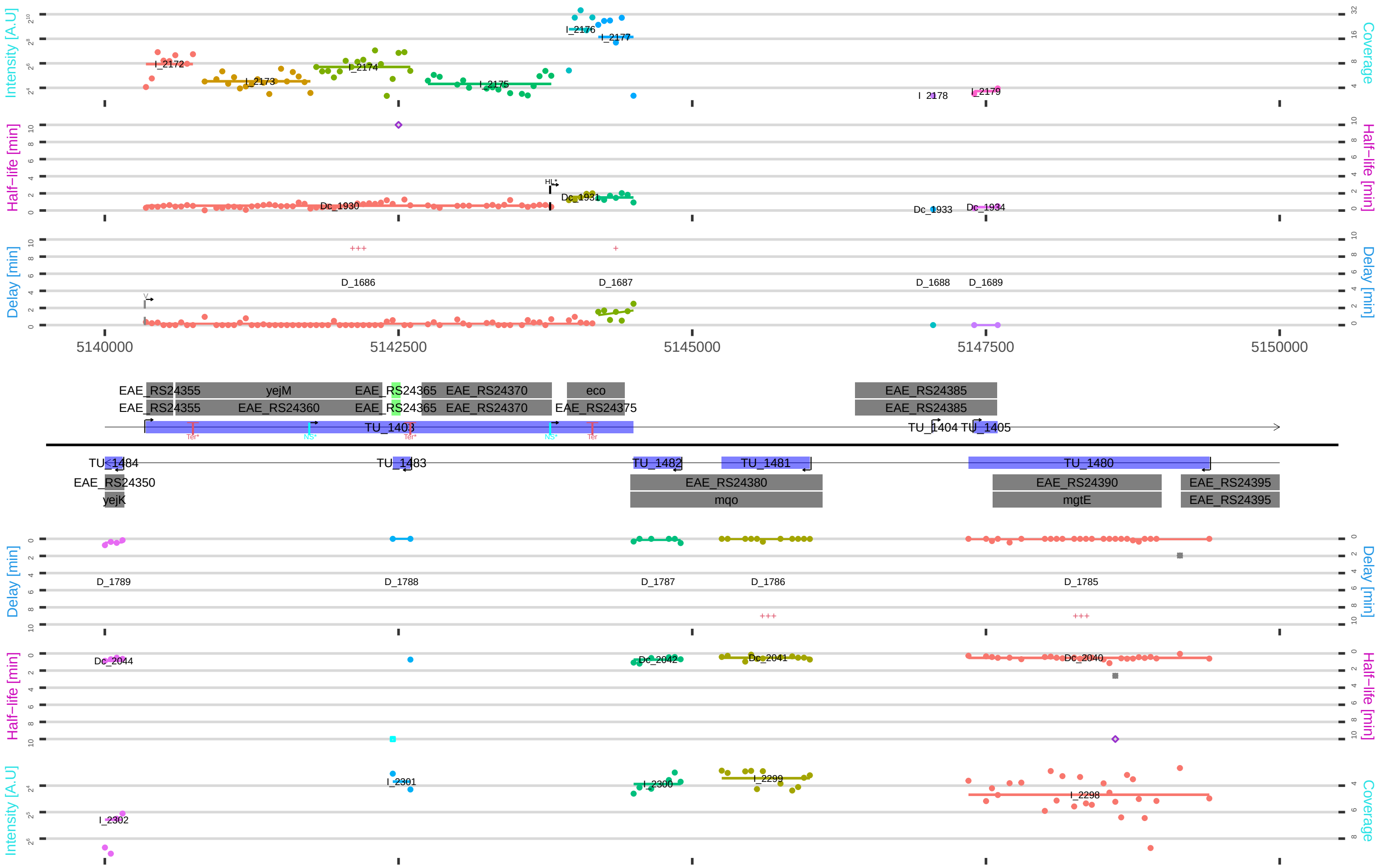

Term: termination (0), NS: new start (0), PS: pausing site (0), iTSS_I: internal starting site (0)

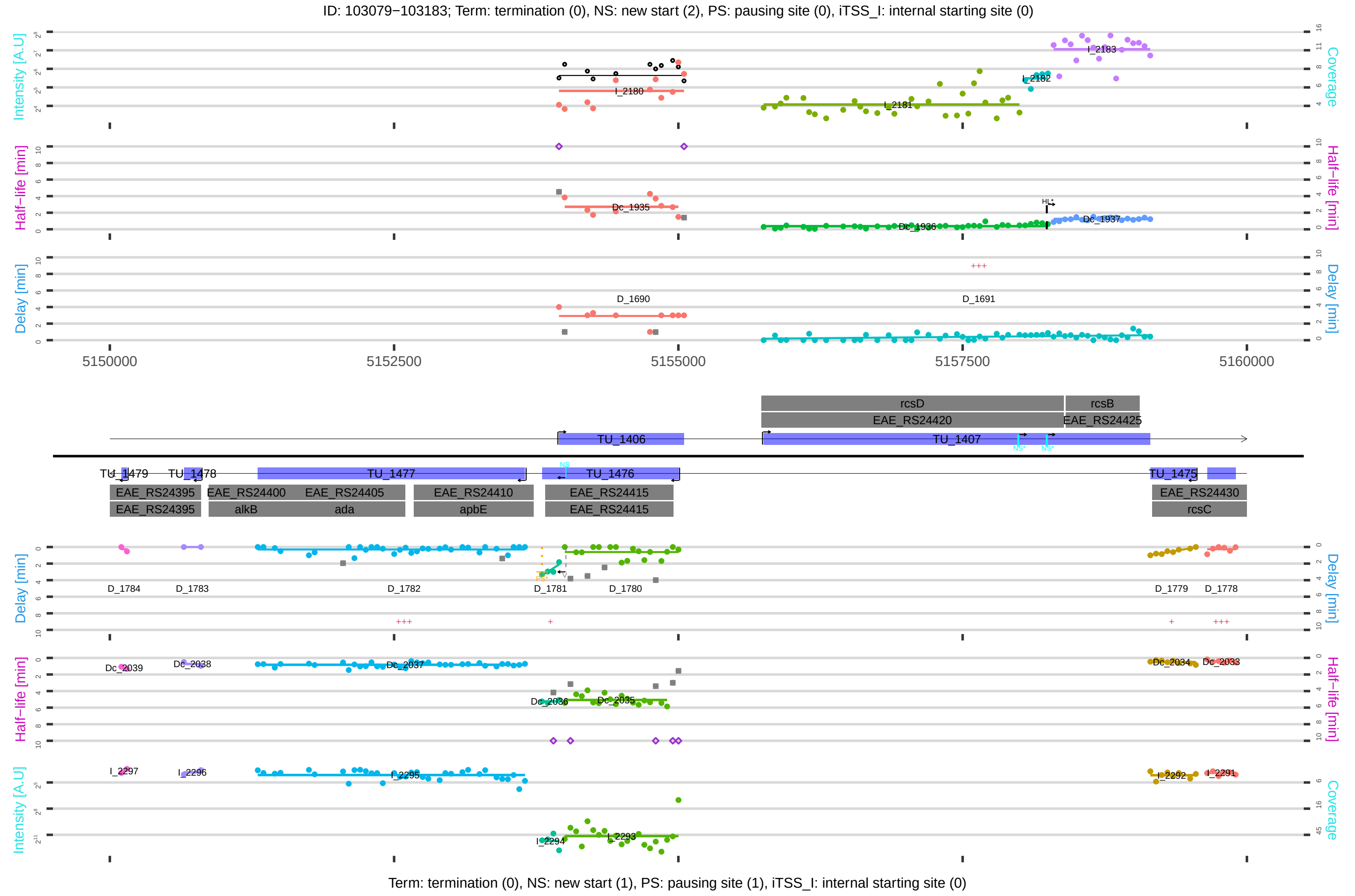

ID: 103079−103183; Term: termination (0), NS: new start (2), PS: pausing site (0), iTSS_I: internal starting site (0)
Intensity [A.U]
Coverage
Half−life [min]
Delay [min]
I_2180
I_2181
I_2182
I_2183
Dc_1935
Dc_1936
Dc_1937
D_1690
D_1691
5150000
5152500
5155000
5157500
5160000
rcsD
rcsB
EAE_RS24420
EAE_RS24425
TU_1406
TU_1407
TU_1479
TU_1478
TU_1477
TU_1476
TU_1475
EAE_RS24395
EAE_RS24400
EAE_RS24405
EAE_RS24410
EAE_RS24415
EAE_RS24430
alkB
ada
apbE
rcsC
D_1784
D_1783
D_1782
D_1781
D_1780
D_1779
D_1778
Dc_2039
Dc_2038
Dc_2037
Dc_2036
Dc_2035
Dc_2034
Dc_2033
I_2297
I_2296
I_2295
I_2294
I_2293
I_2292
I_2291
Term: termination (0), NS: new start (1), PS: pausing site (1), iTSS_I: internal starting site (0)

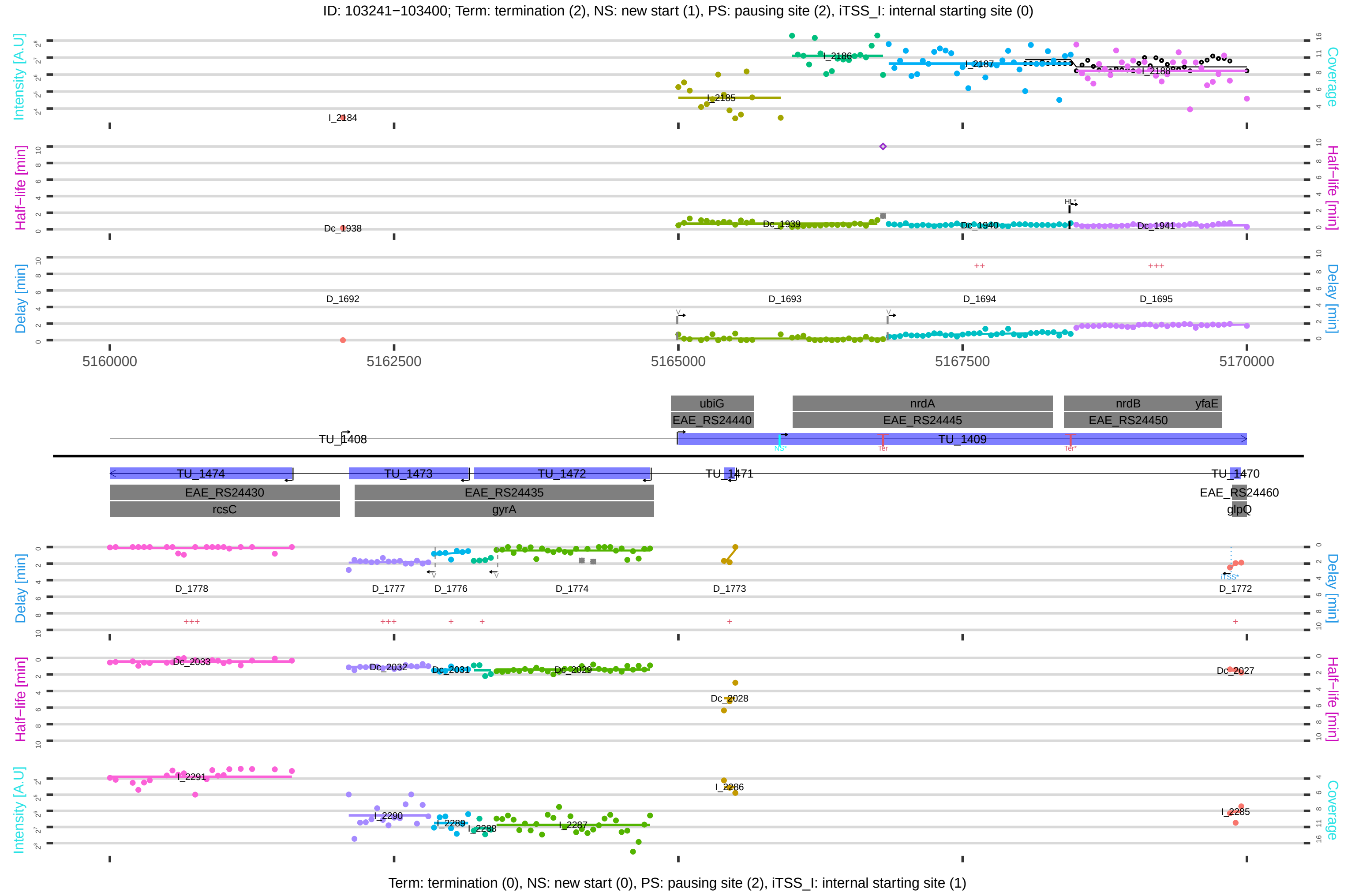
ID: 103241−103400; Term: termination (2), NS: new start (1), PS: pausing site (2), iTSS_I: internal starting site (0)
Intensity [A.U]
Coverage
Half−life [min]
Delay [min]
I_2184
I_2185
I_2186
I_2187
I_2188
Dc_1938
Dc_1939
Dc_1940
Dc_1941
D_1692
D_1693
D_1694
D_1695
5160000
5162500
5165000
5167500
5170000
ubiG
EAE_RS24440
nrdA
EAE_RS24445
nrdB
yfaE
EAE_RS24450
TU_1408
TU_1409
TU_1474
TU_1473
TU_1472
TU_1471
TU_1470
EAE_RS24430
rcsC
EAE_RS24435
gyrA
EAE_RS24460
glpQ
D_1778
D_1777
D_1776
D_1774
D_1773
D_1772
Dc_2033
Dc_2032
Dc_2031
Dc_2029
Dc_2028
Dc_2027
I_2291
I_2290
I_2289
I_2288
I_2287
I_2286
I_2285
Term: termination (0), NS: new start (0), PS: pausing site (2), iTSS_I: internal starting site (1)

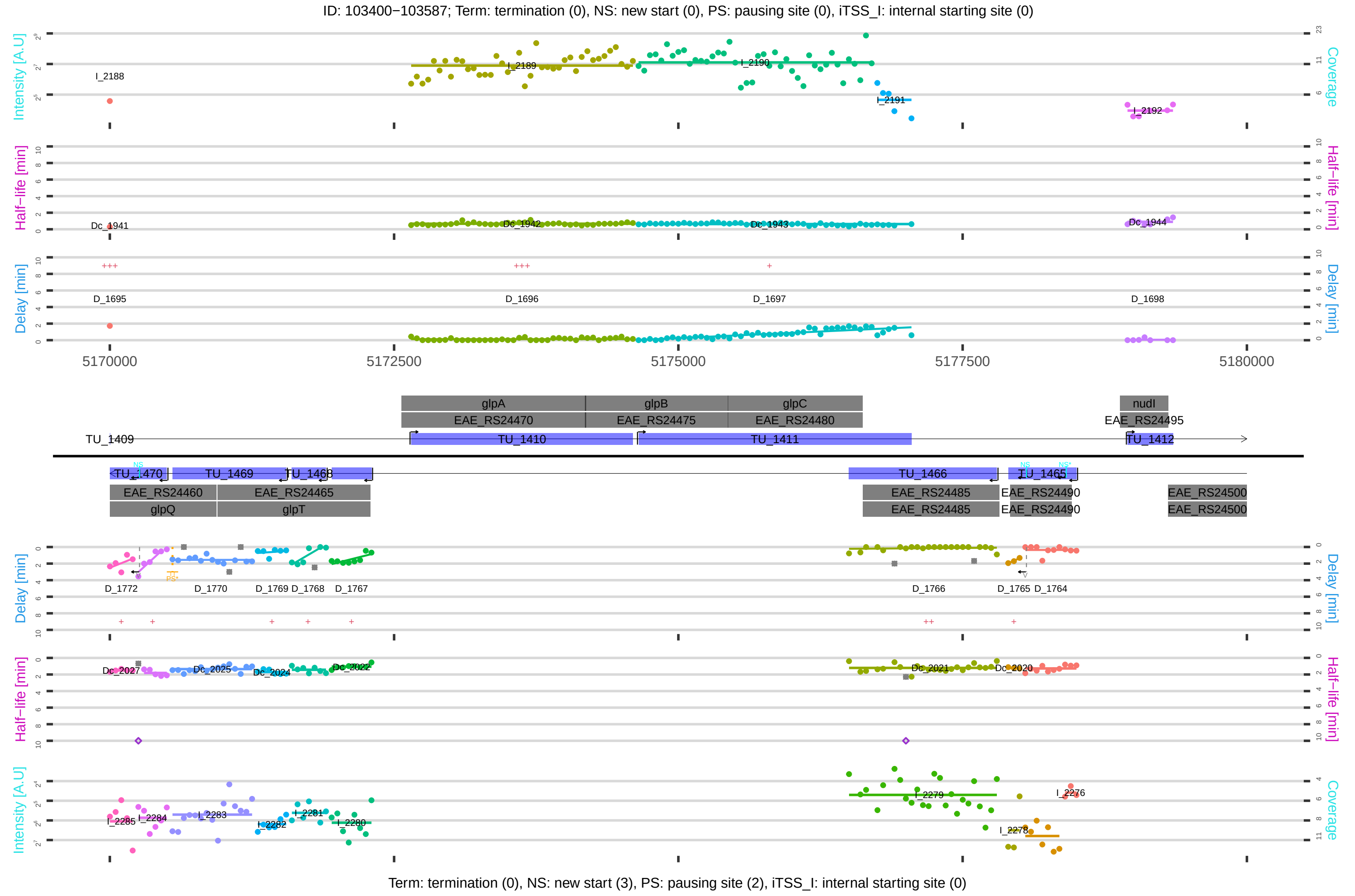

ID: 103400−103587; Term: termination (0), NS: new start (0), PS: pausing site (0), iTSS_I: internal starting site (0)
Intensity [A.U]
Coverage
Half−life [min]
Delay [min]
I_2188
I_2189
I_2190
I_2191
I_2192
Dc_1941
Dc_1942
Dc_1943
Dc_1944
D_1695
D_1696
D_1697
D_1698
5170000
5172500
5175000
5177500
5180000
glpA
glpB
glpC
nudI
EAE_RS24470
EAE_RS24475
EAE_RS24480
EAE_RS24495
TU_1409
TU_1410
TU_1411
TU_1412
TU_1470
TU_1469
TU_1468
TU_1466
TU_1465
EAE_RS24460
EAE_RS24465
EAE_RS24485
EAE_RS24490
EAE_RS24500
glpQ
glpT
D_1772
D_1770
D_1769
D_1768
D_1767
D_1766
D_1765
D_1764
Dc_2027
Dc_2025
Dc_2024
Dc_2022
Dc_2021
Dc_2020
I_2285
I_2284
I_2283
I_2282
I_2281
I_2280
I_2279
I_2278
I_2276
Term: termination (0), NS: new start (3), PS: pausing site (2), iTSS_I: internal starting site (0)

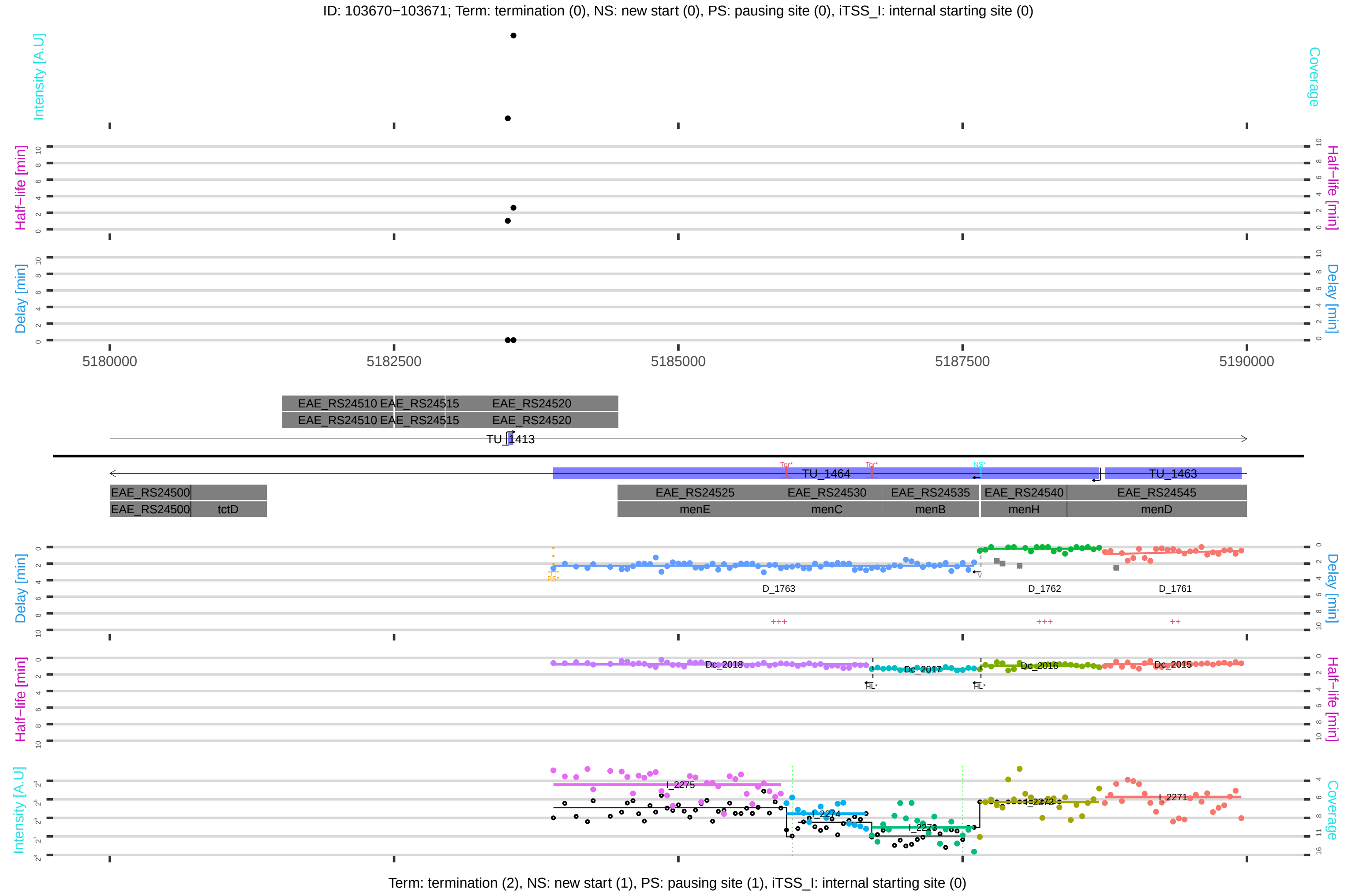
ID: 103670−103671; Term: termination (0), NS: new start (0), PS: pausing site (0), iTSS_I: internal starting site (0)
Intensity [A.U]
Coverage
Half−life [min]
Delay [min]
5180000
5182500
5185000
5187500
5190000
EAE_RS24510 EAE_RS24515 EAE_RS24520
TU_1413
TU_1464
TU_1463
EAE_RS24500
tctD
EAE_RS24525
menE
EAE_RS24530
menC
EAE_RS24535
menB
EAE_RS24540
menH
EAE_RS24545
menD
D_1763
D_1762
D_1761
Dc_2018
Dc_2017
Dc_2016
Dc_2015
I_2275
I_2274
I_2273
I_2272
I_2271
Term: termination (2), NS: new start (1), PS: pausing site (1), iTSS_I: internal starting site (0)

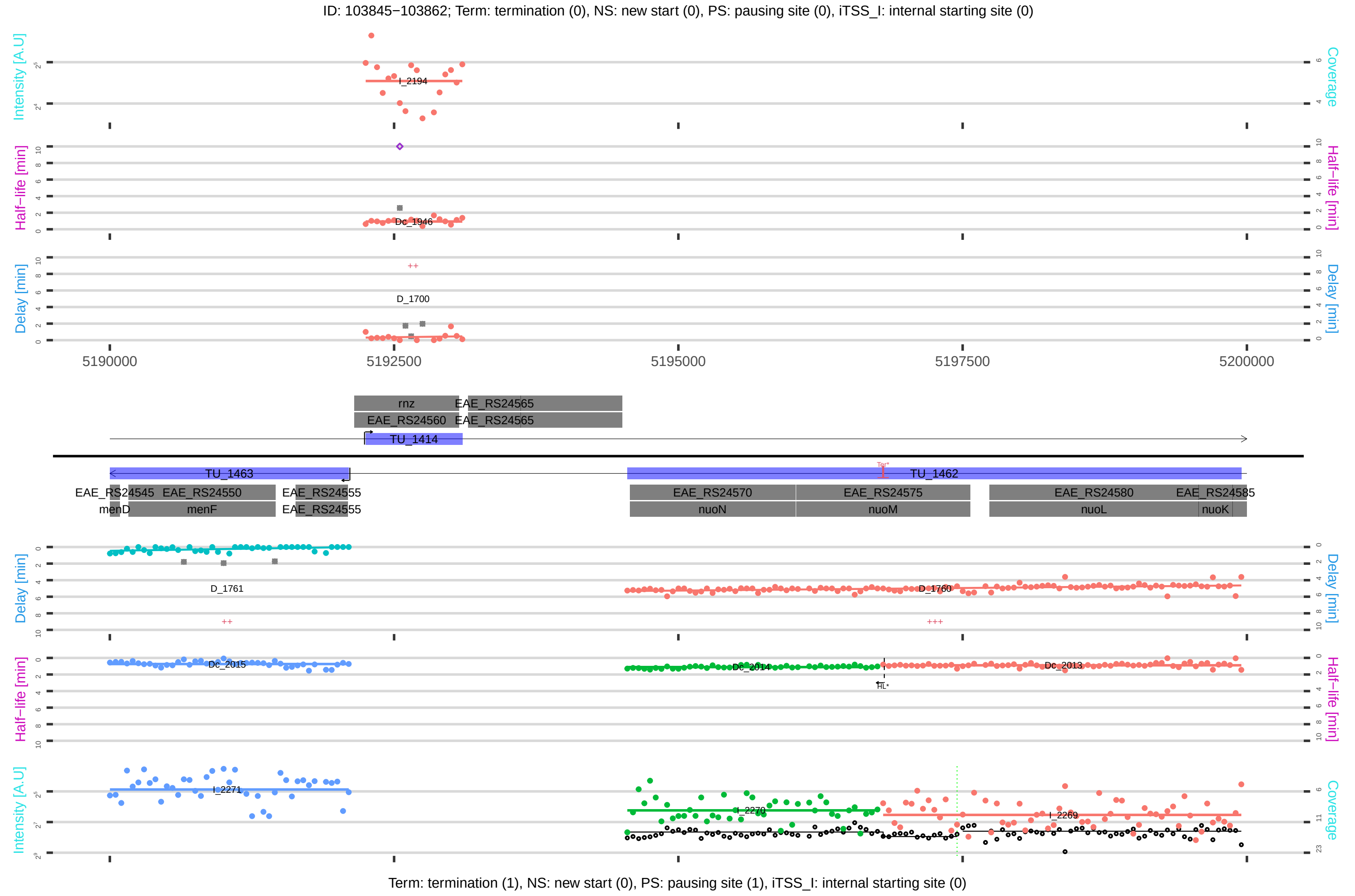

ID: 103845−103862; Term: termination (0), NS: new start (0), PS: pausing site (0), iTSS_I: internal starting site (0)
Intensity [A.U]
Coverage
I_2194
Half−life [min]
Dc_1946
Delay [min]
D_1700
5190000
5192500
5195000
5197500
5200000
rnz
EAE_RS24565
EAE_RS24560
TU_1414
TU_1463
TU_1462
EAE_RS24545
EAE_RS24550
EAE_RS24555
EAE_RS24570
EAE_RS24575
EAE_RS24580
EAE_RS24585
menD
menF
nuoN
nuoM
nuoL
nuoK
D_1761
D_1760
Dc_2015
Dc_2014
Dc_2013
I_2271
I_2270
I_2269
Term: termination (1), NS: new start (0), PS: pausing site (1), iTSS_I: internal starting site (0)

ID: 107215−107018; FC*: significant t−test of two consecutive segments; Term: termination, NS: new start, PS: pausing site,iTSS_I: internal starting site,TI: transcription interferance.

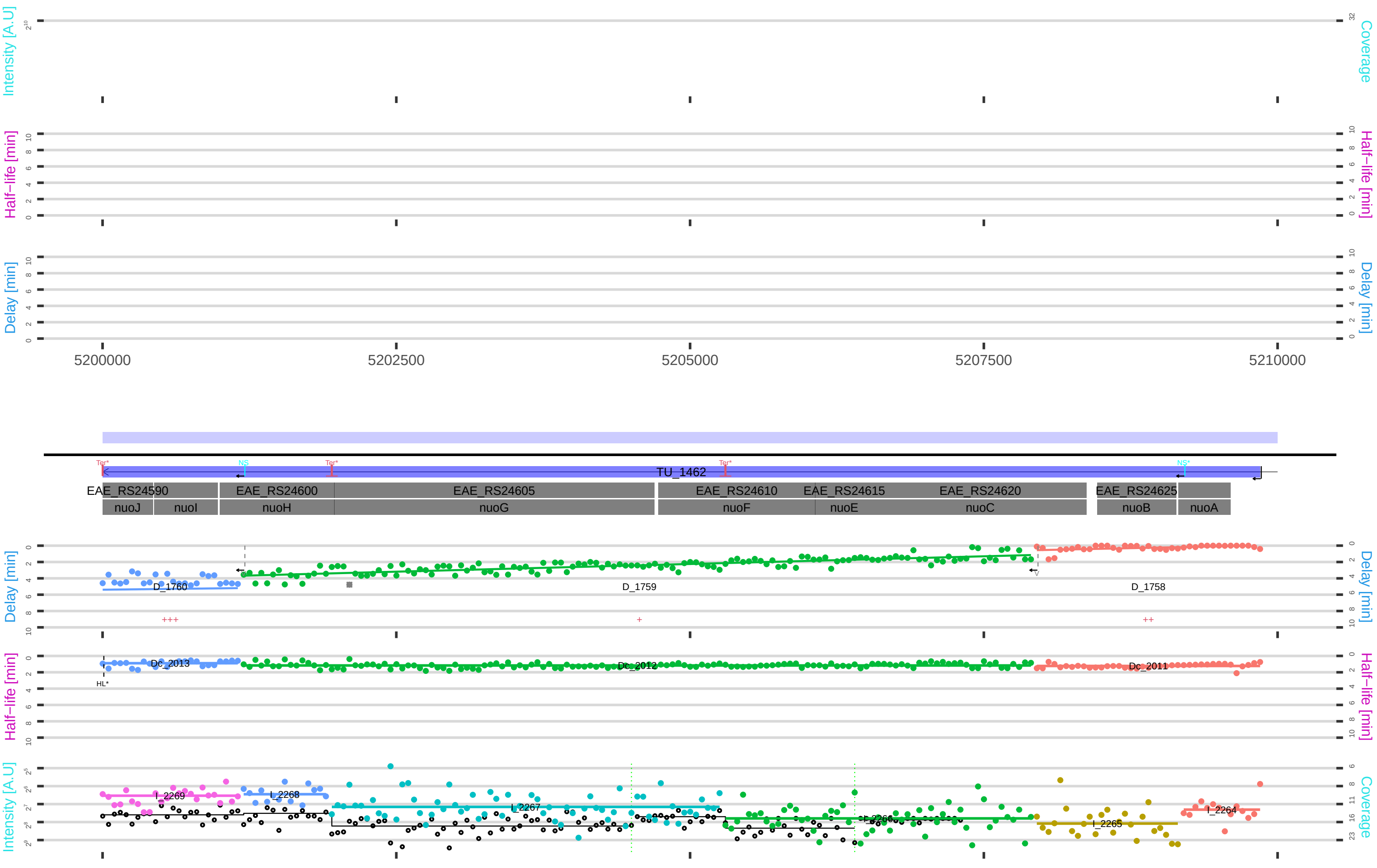

Term: termination (3), NS: new start (2), PS: pausing site (1), iTSS_I: internal starting site (0)

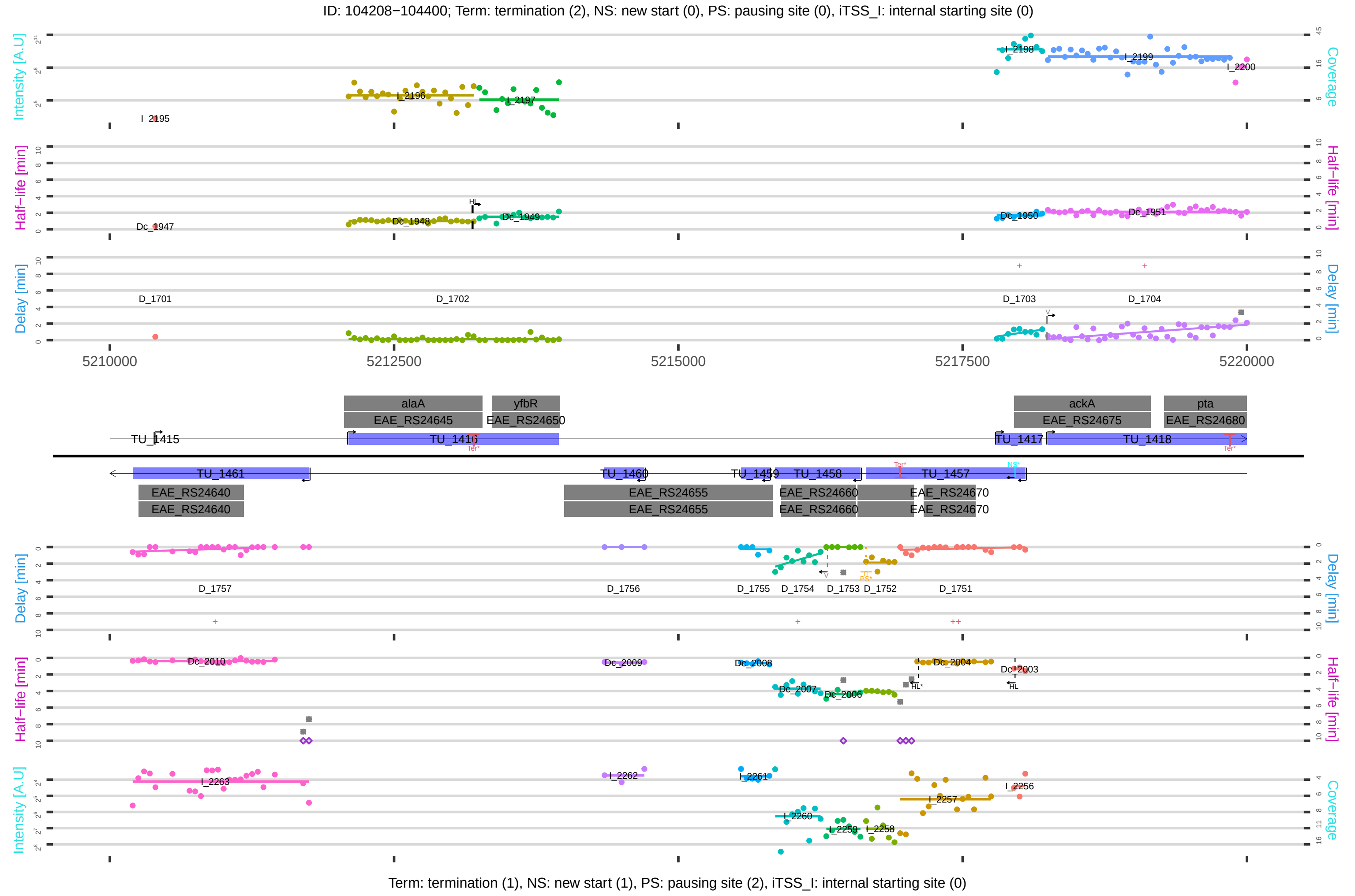

ID: 104208−104400; Term: termination (2), NS: new start (0), PS: pausing site (0), iTSS_I: internal starting site (0)
Intensity [A.U]
Coverage
Half−life [min]
Delay [min]
5210000
5212500
5215000
5217500
5220000
alaA
yfbR
ackA
pta
EAE_RS24645
EAE_RS24650
EAE_RS24675
EAE_RS24680
TU_1415
TU_1416
TU_1417
TU_1418
TU_1461
TU_1460
TU_1459
TU_1458
TU_1457
EAE_RS24640
EAE_RS24655
EAE_RS24660
EAE_RS24670
Term: termination (1), NS: new start (1), PS: pausing site (2), iTSS_I: internal starting site (0)

ID: 104400−104544; Term: termination (1), NS: new start (2), PS: pausing site (0), iTSS_I: internal starting site (2)

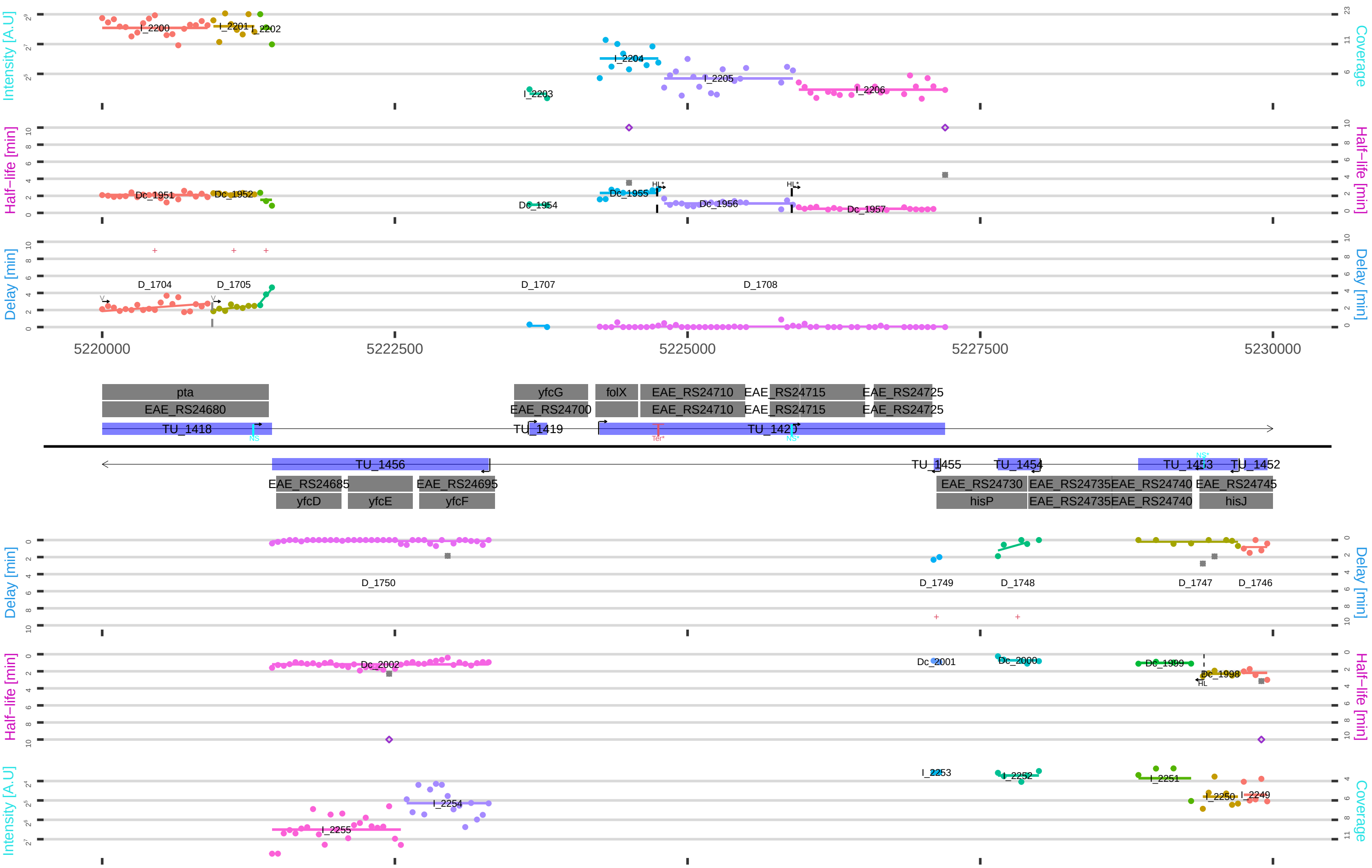

Term: termination (0), NS: new start (1), PS: pausing site (0), iTSS_I: internal starting site (0)

ID: 106614−106416; FC*: significant t−test of two consecutive segments; Term: termination, NS: new start, PS: pausing site,iTSS_I: internal starting site,TI: transcription interferance.

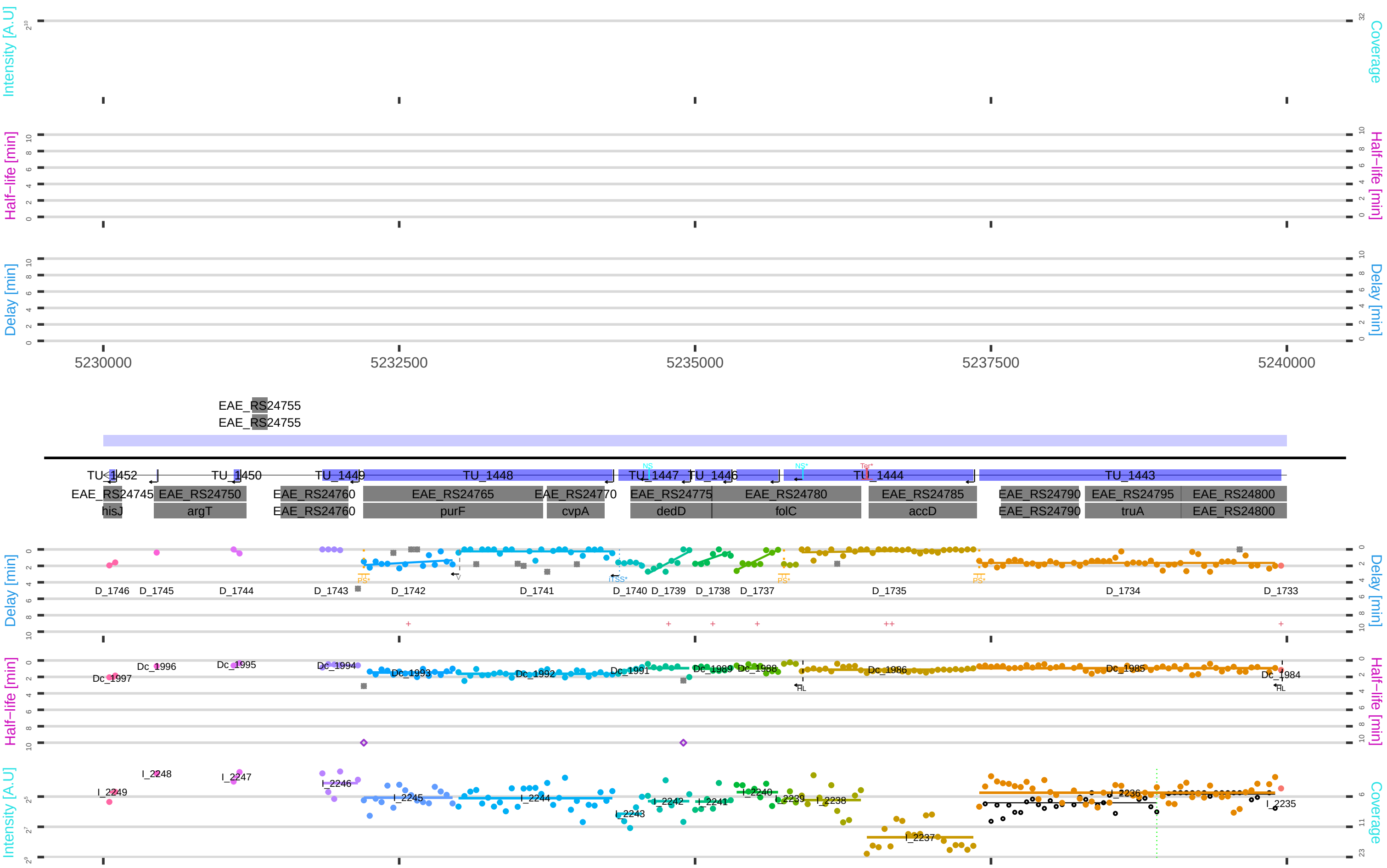

Term: termination (1), NS: new start (2), PS: pausing site (3), iTSS_I: internal starting site (1)

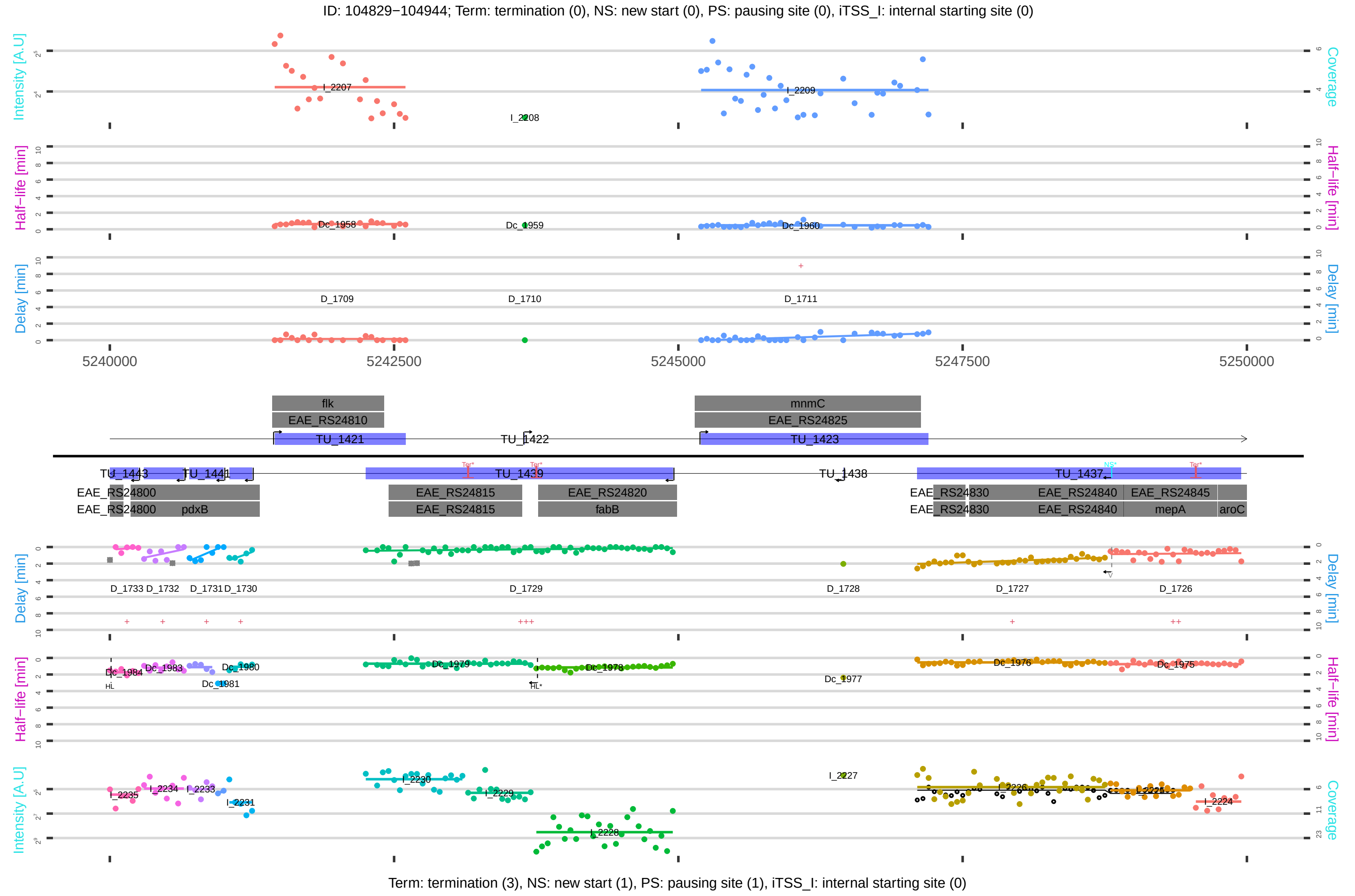

ID: 104829−104944; Term: termination (0), NS: new start (0), PS: pausing site (0), iTSS_I: internal starting site (0)
Intensity [A.U]
Coverage
Half−life [min]
Delay [min]
I_2207
I_2208
I_2209
Dc_1958
Dc_1959
Dc_1960
D_1709
D_1710
D_1711
5240000
5242500
5245000
5247500
5250000
flk
EAE_RS24810
TU_1421
TU_1422
mnmC
EAE_RS24825
TU_1423
TU_1443
TU_1441
TU_1439
TU_1438
TU_1437
EAE_RS24800
pdxB
EAE_RS24815
EAE_RS24820
fabB
EAE_RS24830
EAE_RS24840
EAE_RS24845
mepA
aroC
D_1733
D_1732
D_1731
D_1730
D_1729
D_1728
D_1727
D_1726
Dc_1984
Dc_1983
Dc_1980
Dc_1981
Dc_1979
Dc_1978
Dc_1977
Dc_1976
Dc_1975
I_2235
I_2234
I_2233
I_2231
I_2230
I_2229
I_2228
I_2227
I_2226
I_2225
I_2224
Term: termination (3), NS: new start (1), PS: pausing site (1), iTSS_I: internal starting site (0)

ID: 105041−105177; Term: termination (0), NS: new start (1), PS: pausing site (1), iTSS_I: internal starting site (0)

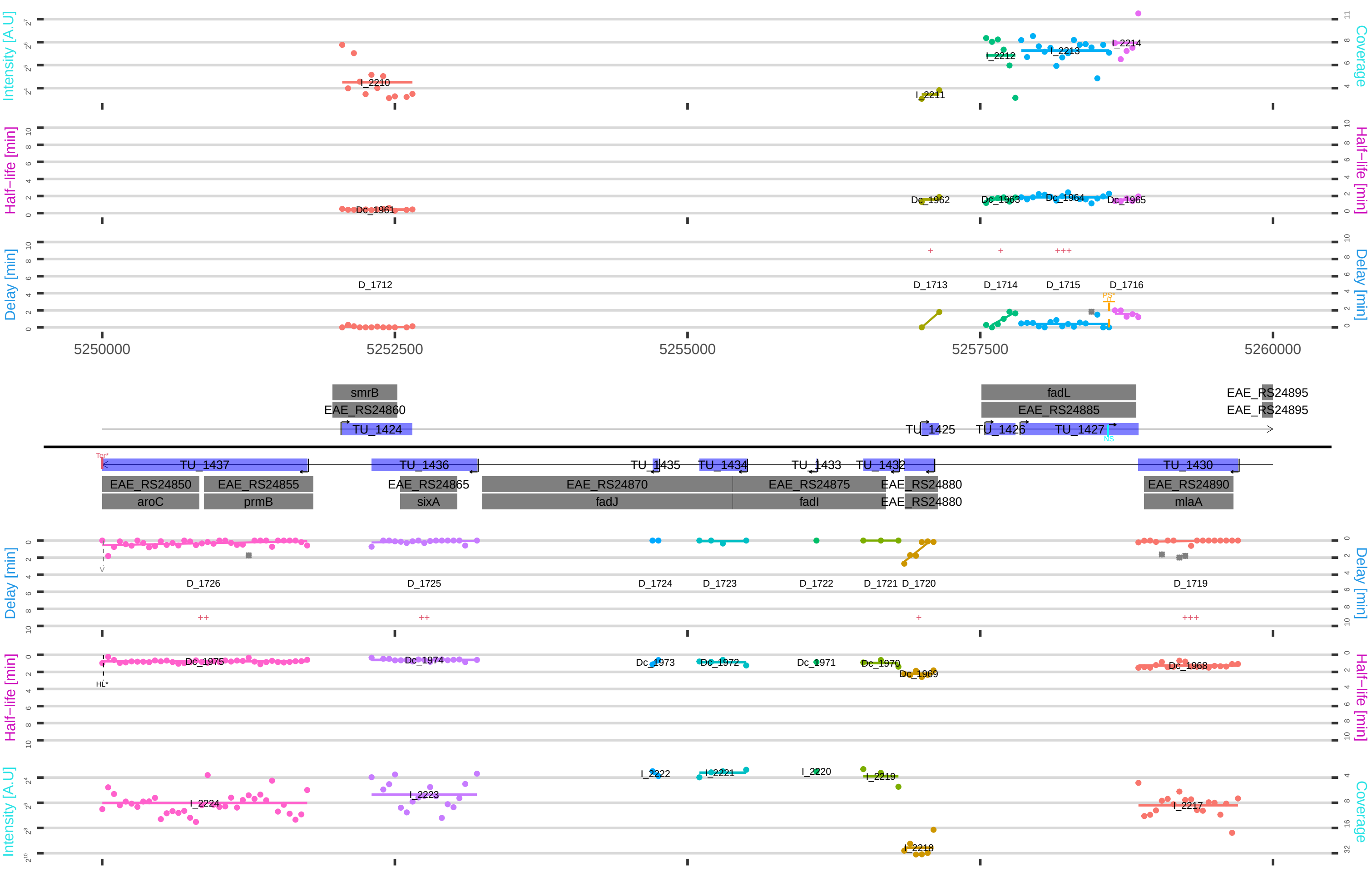

Term: termination (1), NS: new start (0), PS: pausing site (0), iTSS_I: internal starting site (0)

ID: 105219−105220; Term: termination (0), NS: new start (0), PS: pausing site (0), iTSS_I: internal starting site (0)

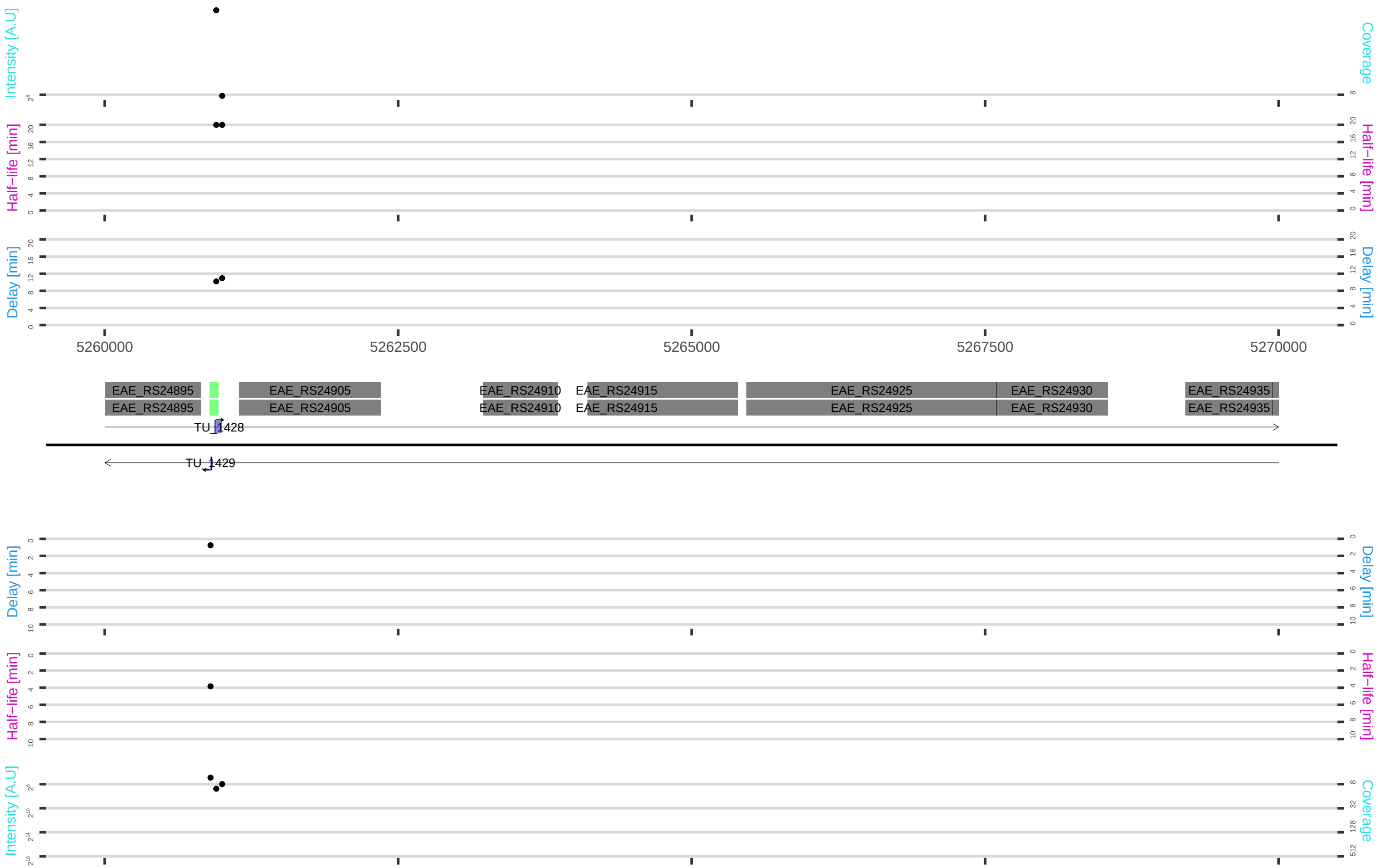

Term: termination (0), NS: new start (0), PS: pausing site (0), iTSS_I: internal starting site (0)

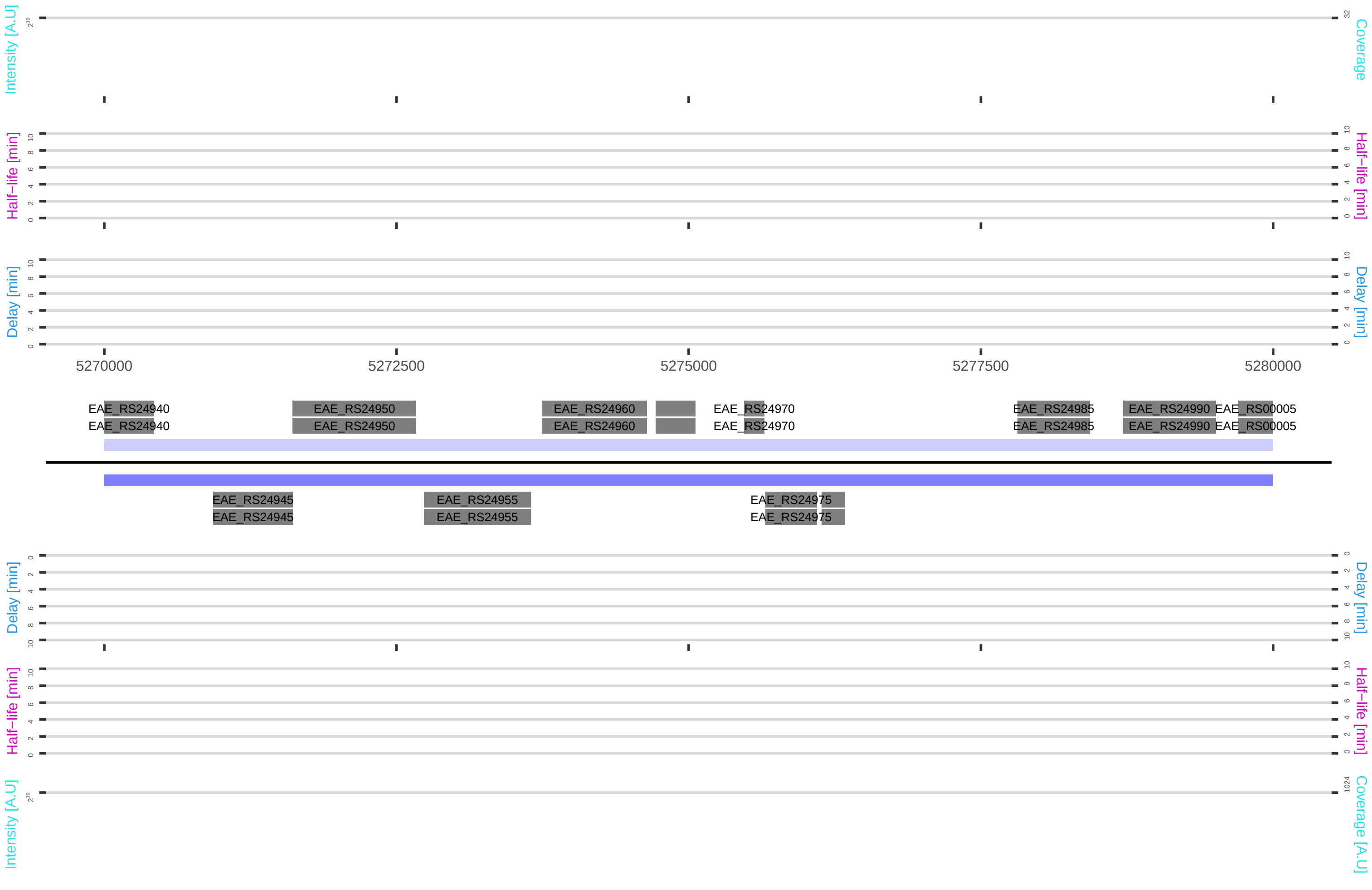
Intensity [A.U]
Coverage
Half−life [min]
Delay [min]
5270000
5272500
5275000
5277500
5280000
EAE_RS24940
EAE_RS24950
EAE_RS24960
EAE_RS24970
EAE_RS24985
EAE_RS24990
EAE_RS00005
EAE_RS24945
EAE_RS24955
EAE_RS24975
Delay [min]
Half−life [min]
Intensity [A.U]
Coverage [A.U]
NA
