# Supplementary material for: Improved RNA stability estimation indicates that transcriptional interference is frequent in diverse bacteria
Source: Commun Biol. 2023 Jul 15;6:732. doi: 10.1038/s42003-023-05097-2 (PMC10349824; doi:10.1038/s42003-023-05097-2)
Supplement: Supplementary file 6 — Supplementary Data 3 [file 42003_2023_5097_MOESM6_ESM.zip › SynechococcusPCC7002.pdf]

ID: 1−200; FC*: significant t−test of two consecutive segments;
Term: termination (4), NS: new start (4), PS: pausing site (2), iTSS_I: internal starting site (0), (*): p_value below 0.05; TI: transcription interferance.

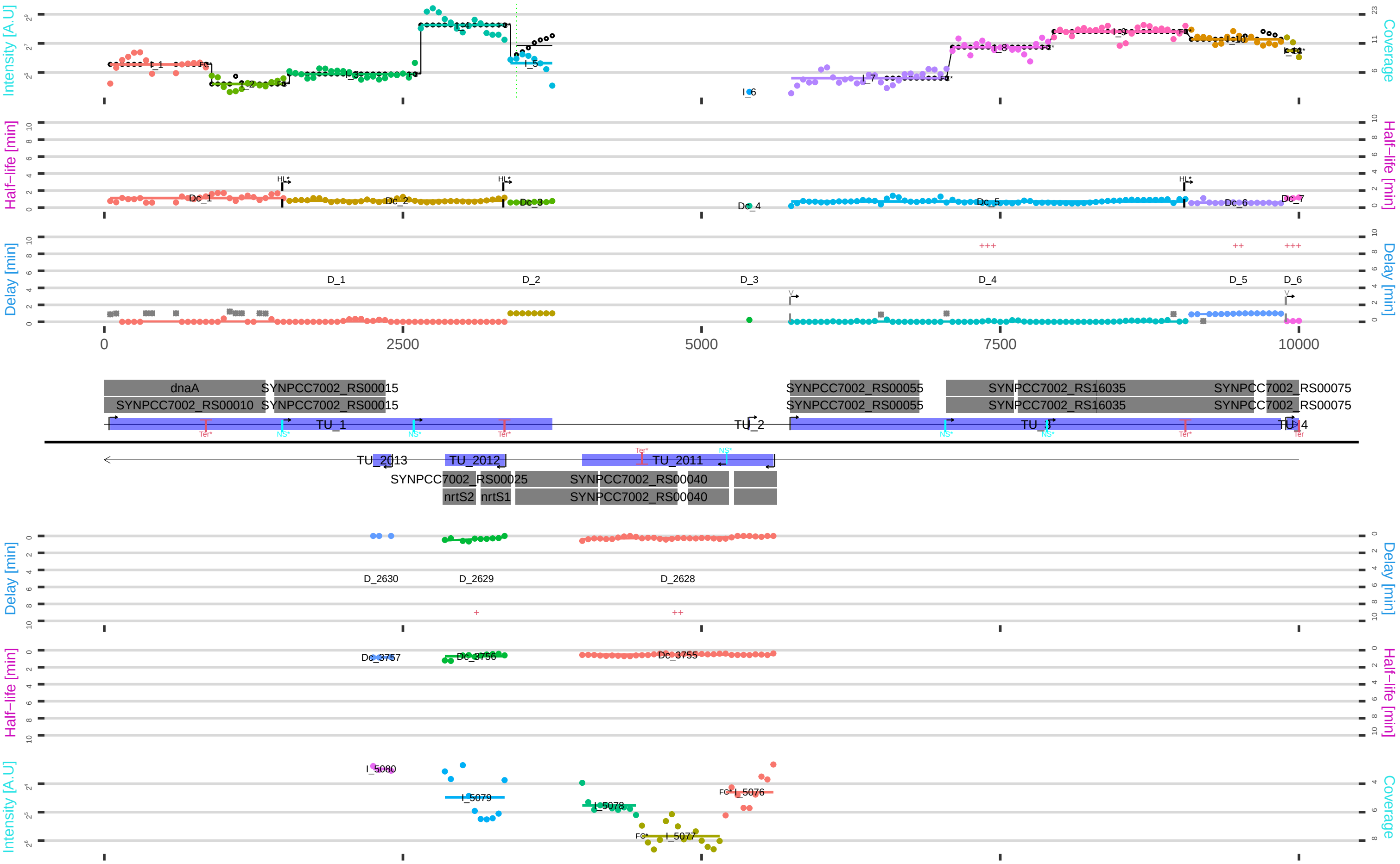

Term: termination (1), NS: new start (1), PS: pausing site (0), iTSS_I: internal starting site (0)

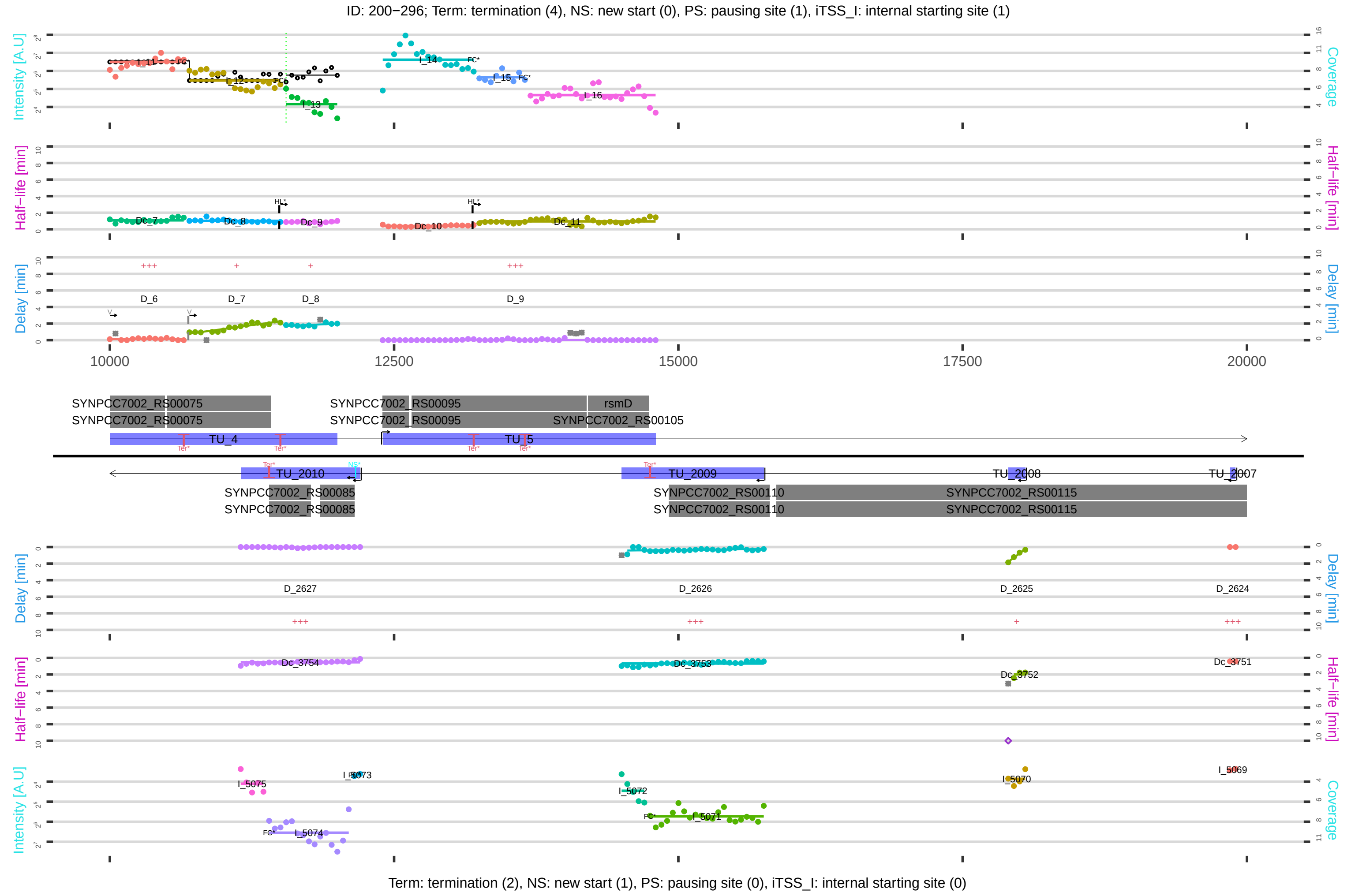

ID: 200−296; Term: termination (4), NS: new start (0), PS: pausing site (1), iTSS_I: internal starting site (1)
Intensity [A.U]
Coverage
Half−life [min]
Delay [min]
10000
12500
15000
17500
20000
I_11
I_12
I_13
I_14
I_15
I_16
Dc_7
Dc_8
Dc_9
Dc_10
Dc_11
D_6
D_7
D_8
D_9
SYNPCC7002_RS00075
SYNPCC7002_RS00095
rsmD
SYNPCC7002_RS00105
TU_4
TU_5
TU_2010
TU_2009
TU_2008
TU_2007
SYNPCC7002_RS00085
SYNPCC7002_RS00110
SYNPCC7002_RS00115
D_2627
D_2626
D_2625
D_2624
Dc_3754
Dc_3753
Dc_3752
Dc_3751
I_5075
I_5073
I_5074
I_5072
I_5071
I_5070
I_5069
Term: termination (2), NS: new start (1), PS: pausing site (0), iTSS_I: internal starting site (0)

ID: 404−600; Term: termination (7), NS: new start (1), PS: pausing site (1), iTSS_I: internal starting site (0)

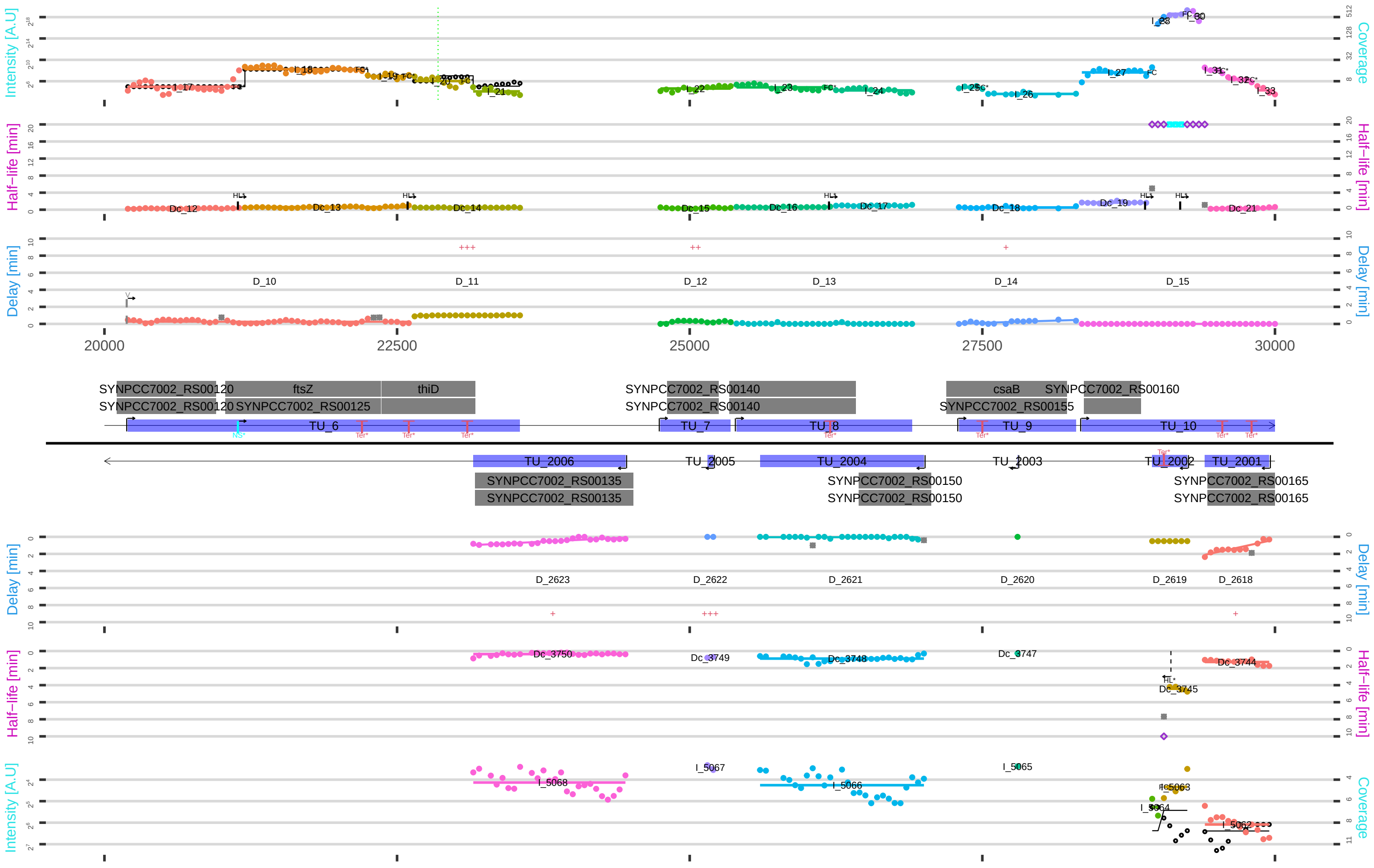

Term: termination (1), NS: new start (0), PS: pausing site (0), iTSS_I: internal starting site (0)

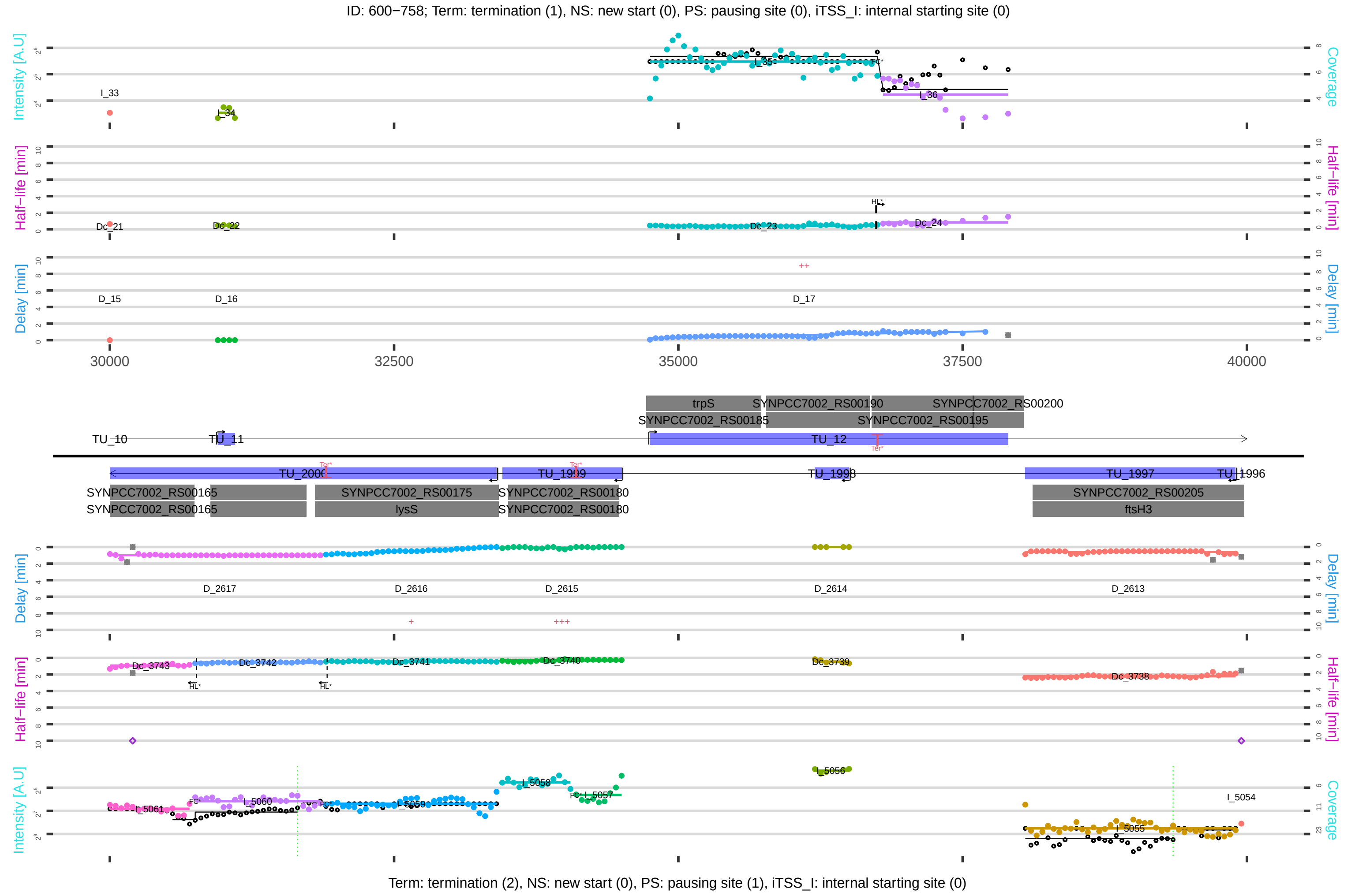
ID: 600−758; Term: termination (1), NS: new start (0), PS: pausing site (0), iTSS_I: internal starting site (0)
Intensity [A.U]
Coverage
Half−life [min]
Delay [min]
I_33
I_34
I_35
I_36
Dc_21
Dc_22
Dc_23
Dc_24
D_15
D_16
D_17
30000
32500
35000
37500
40000
trpS
SYNPCC7002_RS00190
SYNPCC7002_RS00200
SYNPCC7002_RS00185
SYNPCC7002_RS00195
TU_10
TU_11
TU_12
TU_2000
TU_1999
TU_1998
TU_1997
TU_1996
SYNPCC7002_RS00165
SYNPCC7002_RS00175
SYNPCC7002_RS00180
SYNPCC7002_RS00205
lysS
ftsH3
D_2617
D_2616
D_2615
D_2614
D_2613
Dc_3743
Dc_3742
Dc_3741
Dc_3740
Dc_3739
Dc_3738
I_5061
I_5060
I_5059
I_5058
I_5057
I_5056
I_5055
I_5054
Term: termination (2), NS: new start (0), PS: pausing site (1), iTSS_I: internal starting site (0)

ID: 803−1000; Term: termination (2), NS: new start (1), PS: pausing site (0), iTSS_I: internal starting site (0)

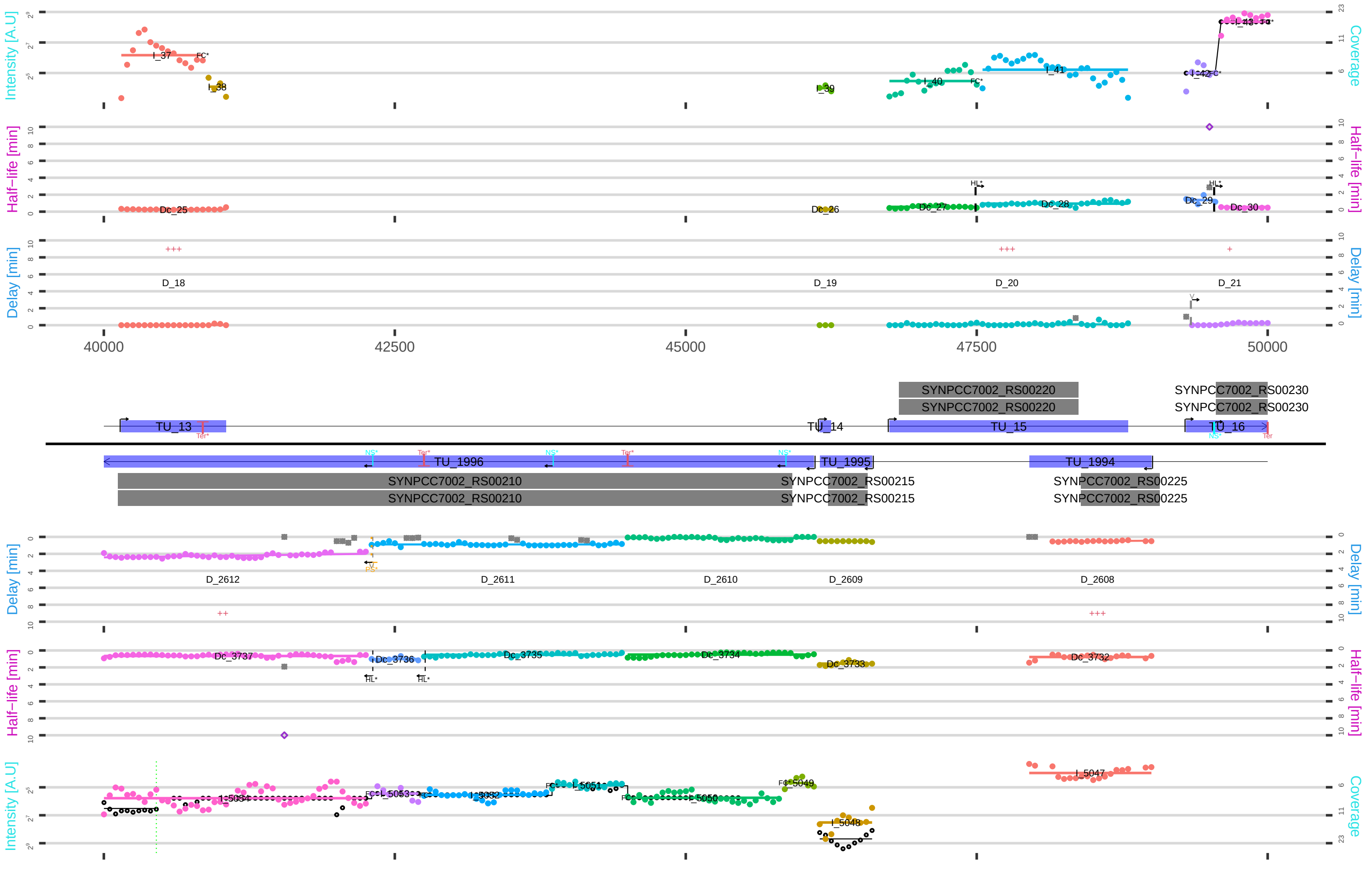

Term: termination (2), NS: new start (3), PS: pausing site (2), iTSS_I: internal starting site (0)

ID: 1000−1197; Term: termination (7), NS: new start (3), PS: pausing site (4), iTSS_I: internal starting site (1)

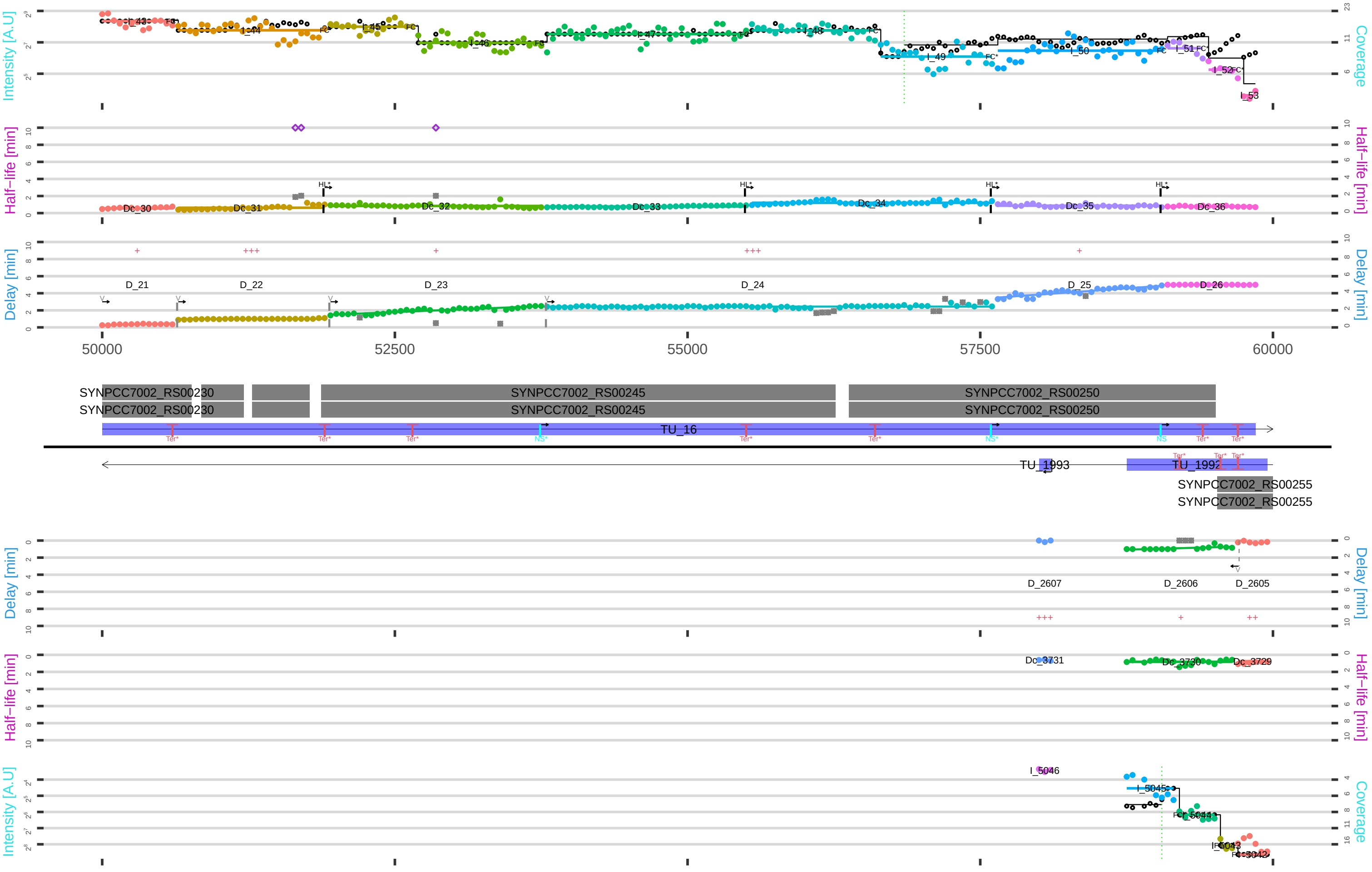

Term: termination (3), NS: new start (0), PS: pausing site (1), iTSS_I: internal starting site (0)

ID: 1255−1400; Term: termination (2), NS: new start (0), PS: pausing site (1), iTSS_I: internal starting site (0)

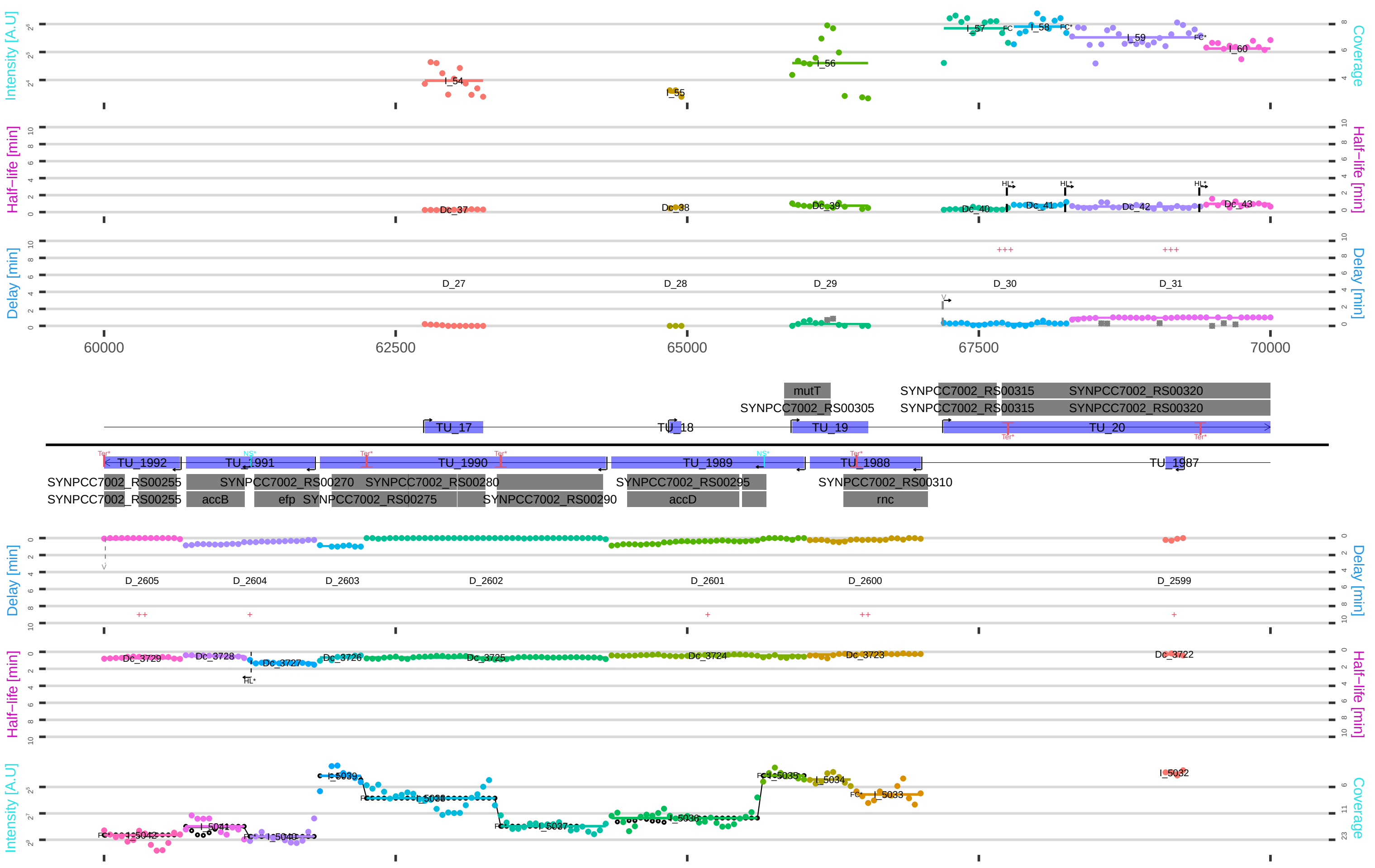

Term: termination (4), NS: new start (2), PS: pausing site (1), iTSS_I: internal starting site (0)

ID: 1400−1600; Term: termination (3), NS: new start (0), PS: pausing site (1), iTSS_I: internal starting site (0)

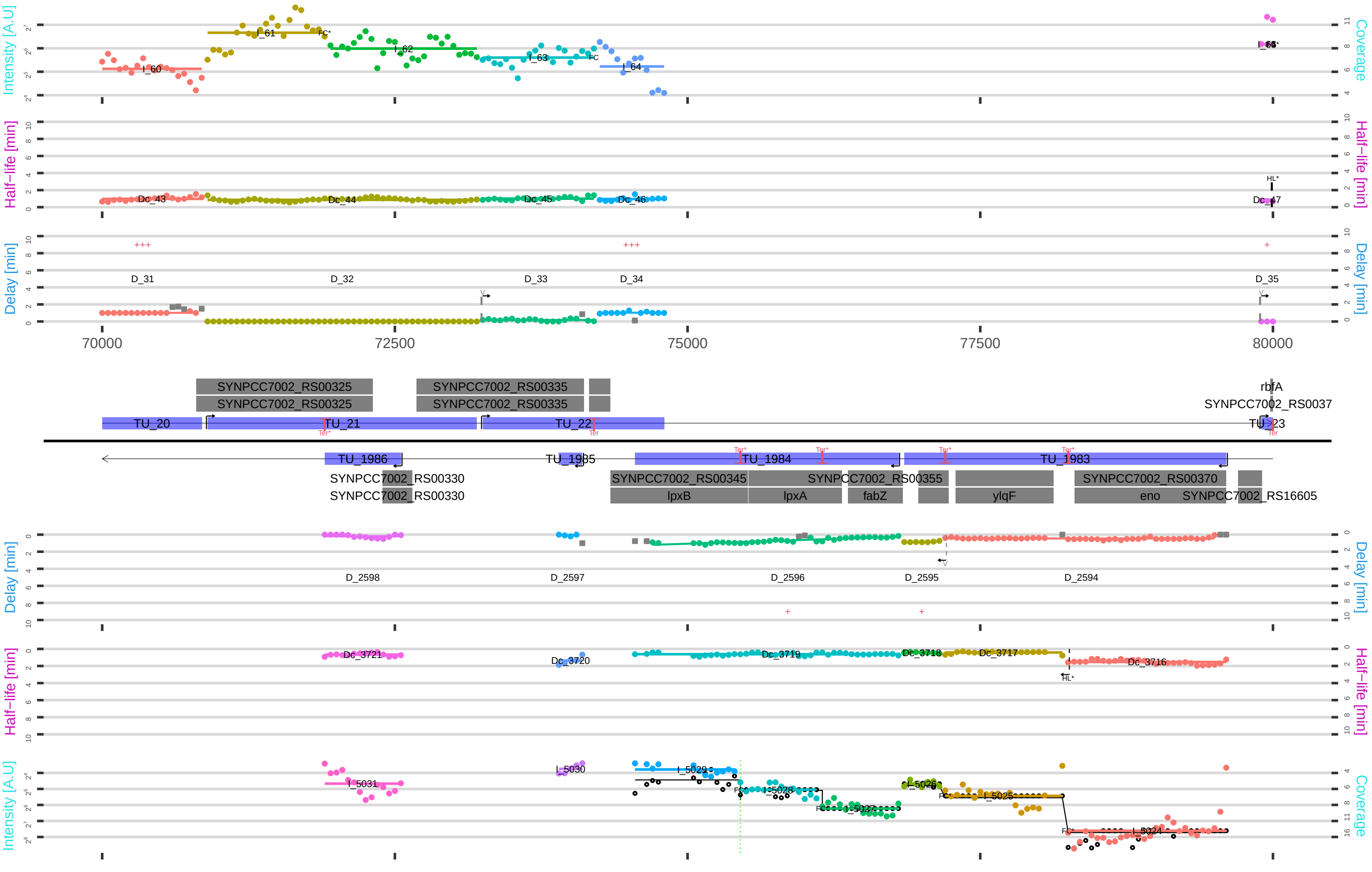

Term: termination (4), NS: new start (0), PS: pausing site (1), iTSS_I: internal starting site (0)

ID: 1600−1800; Term: termination (2), NS: new start (1), PS: pausing site (1), iTSS_I: internal starting site (0)

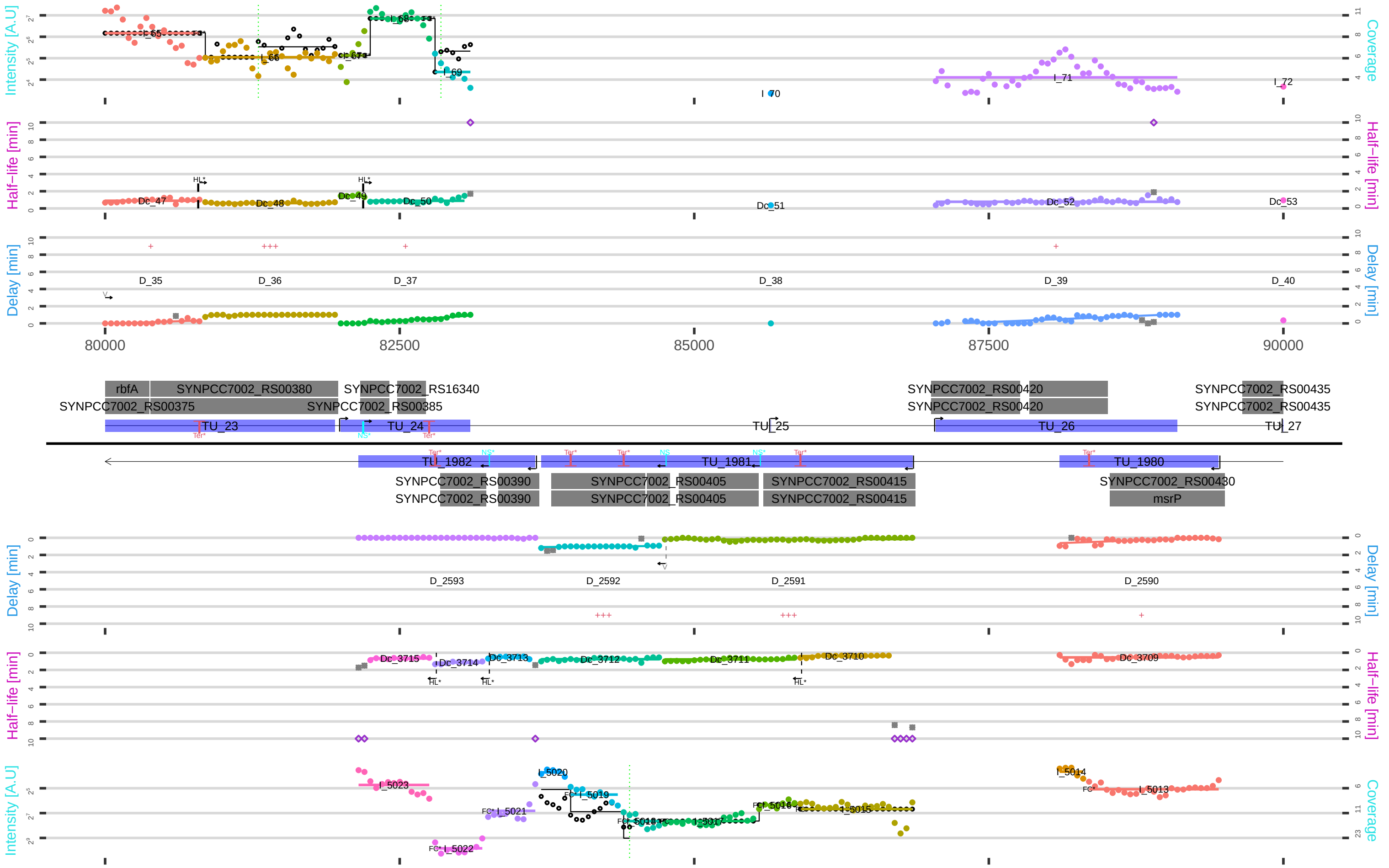

Term: termination (5), NS: new start (3), PS: pausing site (1), iTSS_I: internal starting site (0)

ID: 1800−2000; Term: termination (4), NS: new start (4), PS: pausing site (1), iTSS_I: internal starting site (0)

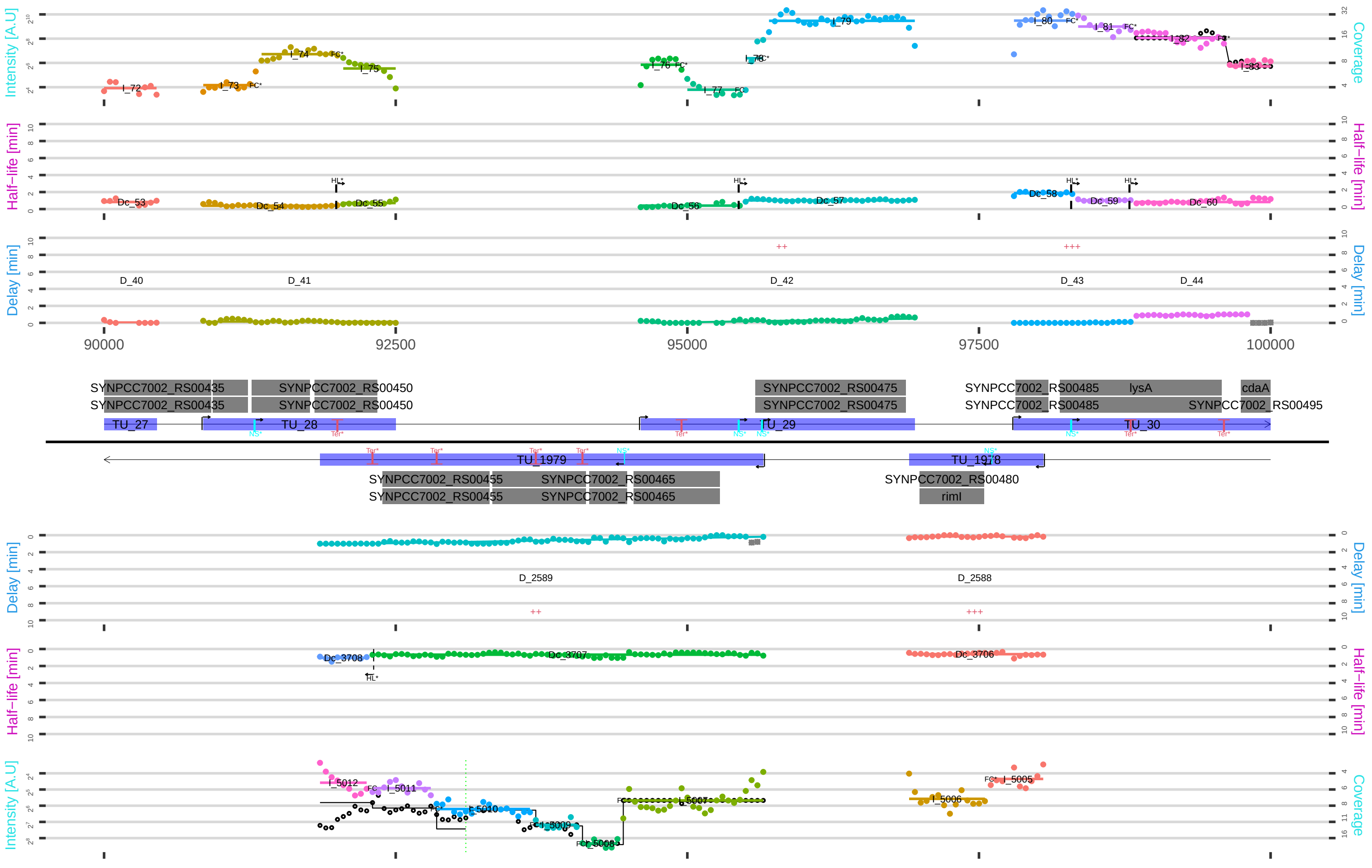

Term: termination (4), NS: new start (2), PS: pausing site (0), iTSS_I: internal starting site (0)

ID: 2000−2200; Term: termination (3), NS: new start (4), PS: pausing site (1), iTSS_I: internal starting site (0)

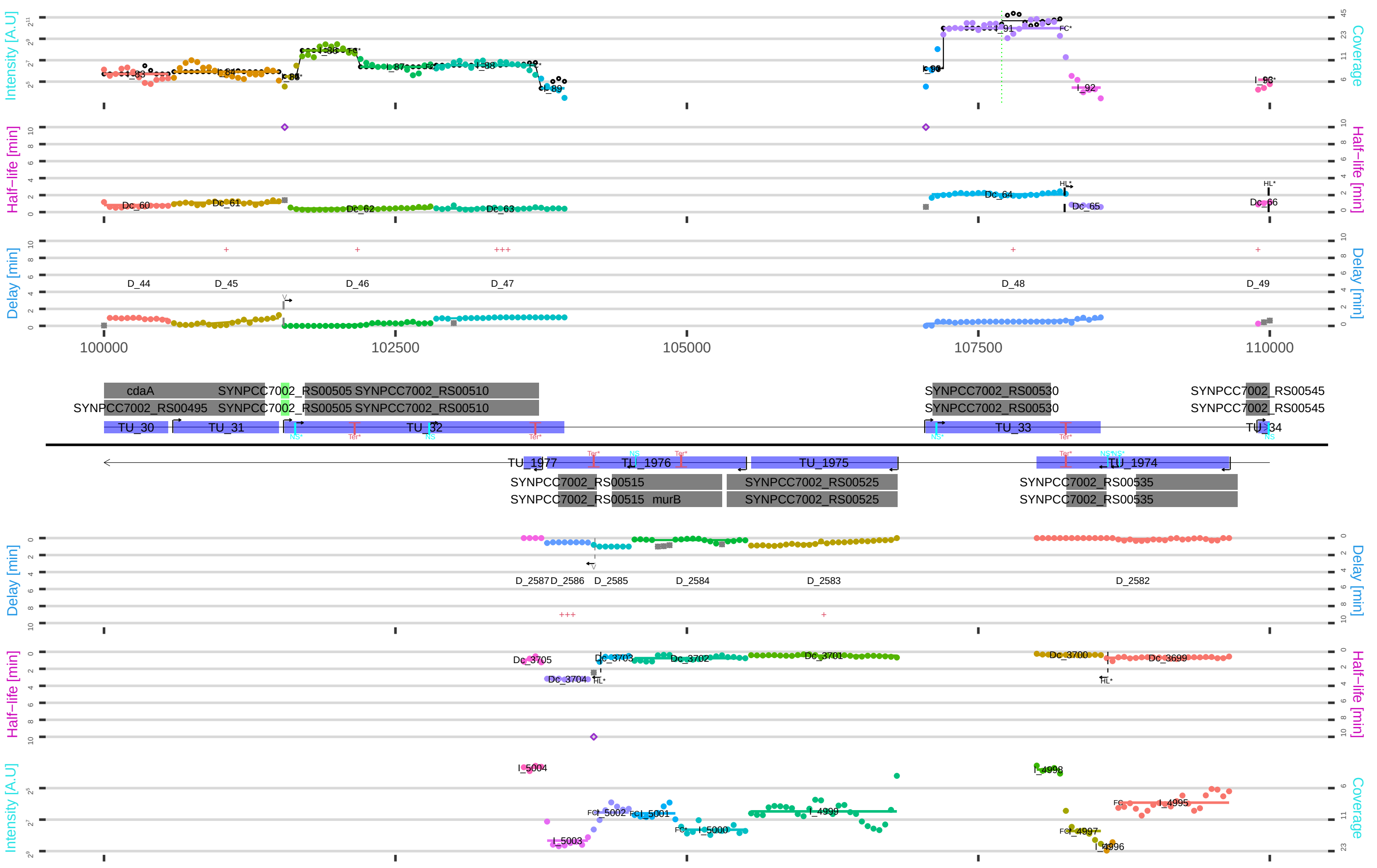

Term: termination (3), NS: new start (3), PS: pausing site (1), iTSS_I: internal starting site (1)

ID: 2200−2400; Term: termination (4), NS: new start (2), PS: pausing site (1), iTSS_I: internal starting site (0)

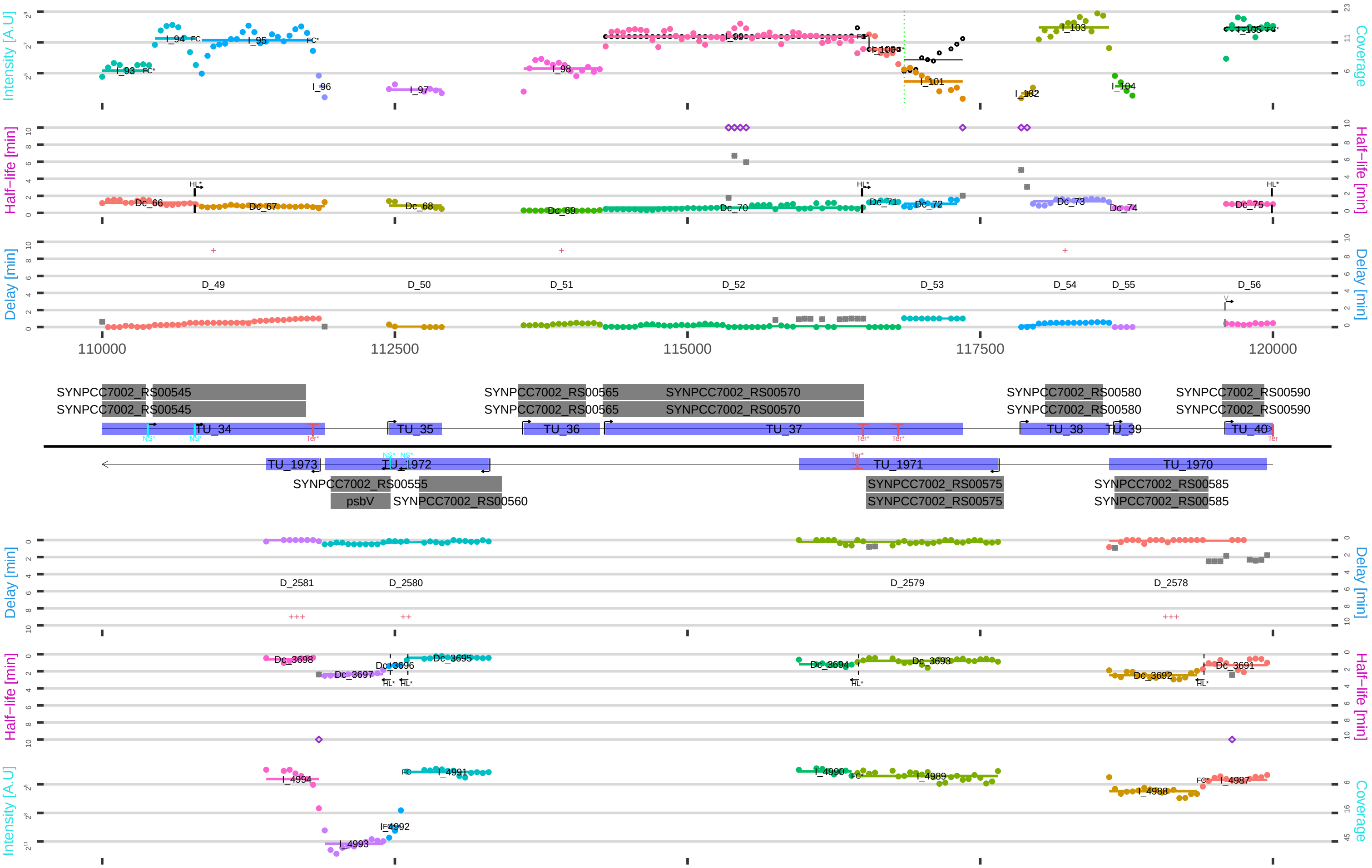

Term: termination (1), NS: new start (2), PS: pausing site (0), iTSS_I: internal starting site (0)

ID: 2400−2592; Term: termination (4), NS: new start (1), PS: pausing site (1), iTSS_I: internal starting site (0)

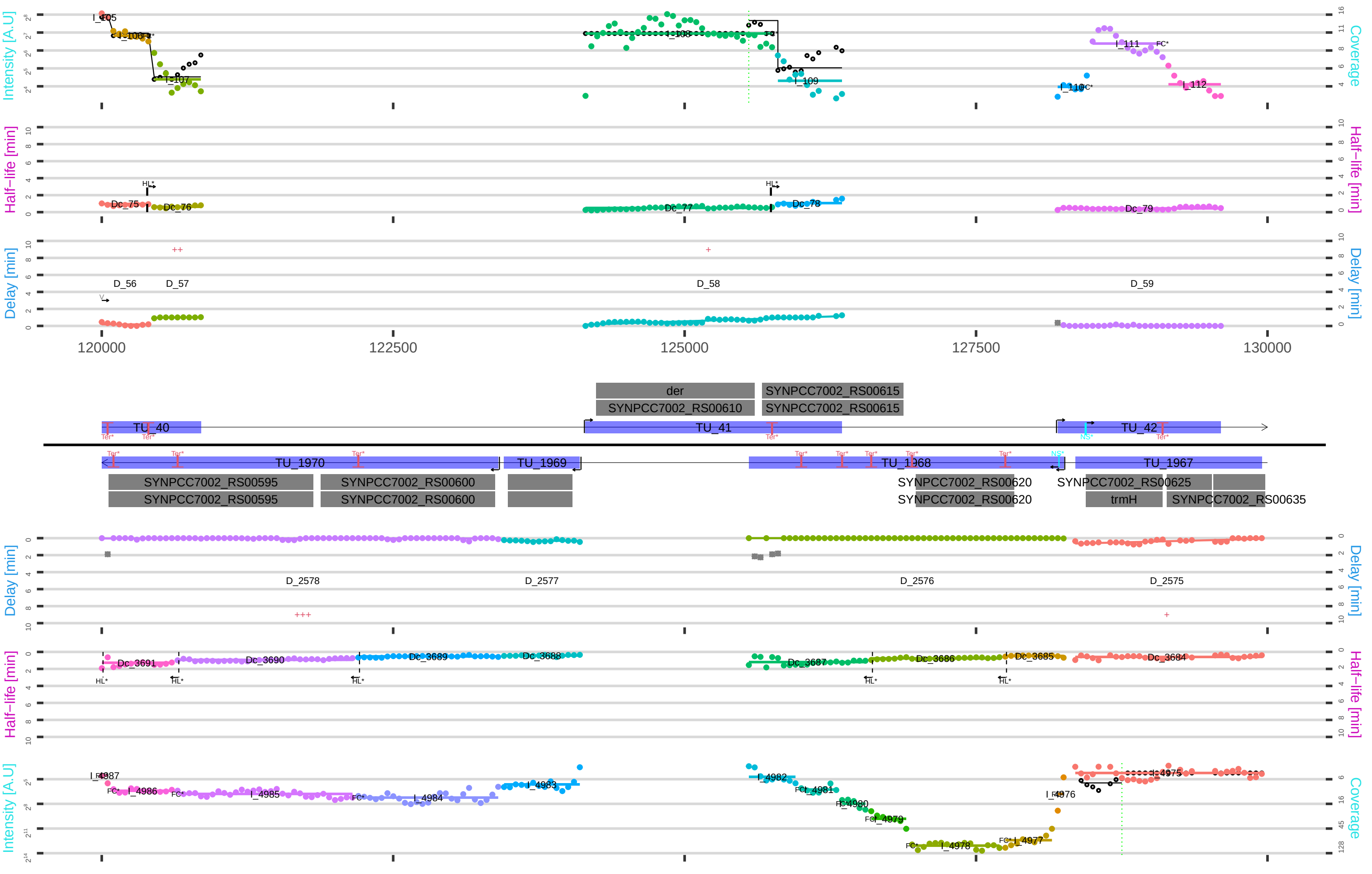

Term: termination (8), NS: new start (1), PS: pausing site (0), iTSS_I: internal starting site (0)

ID: 2603−2797; Term: termination (4), NS: new start (2), PS: pausing site (1), iTSS_I: internal starting site (0)

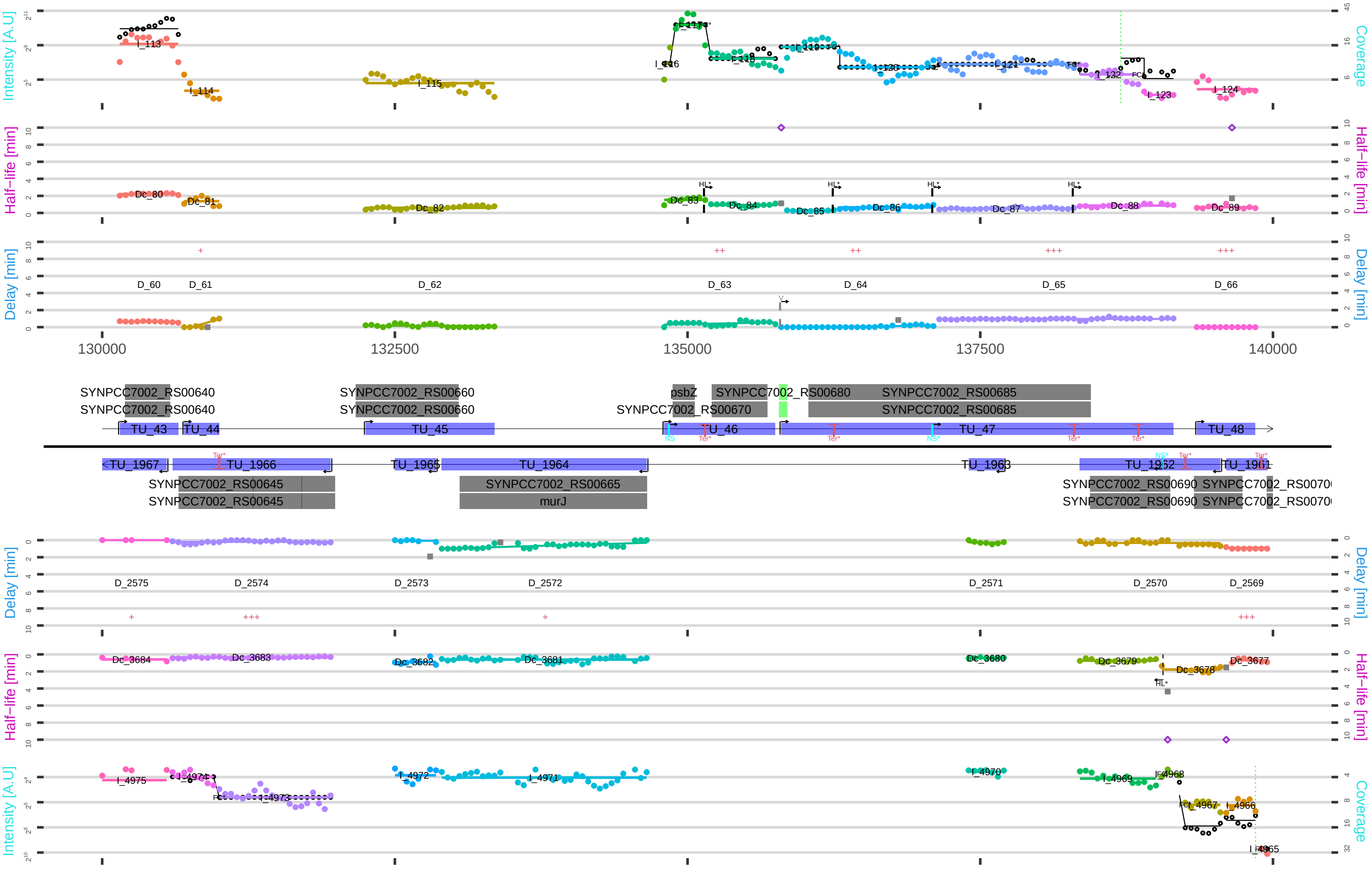

Term: termination (3), NS: new start (1), PS: pausing site (0), iTSS_I: internal starting site (0)

ID: 2868−2995; Term: termination (2), NS: new start (1), PS: pausing site (1), iTSS_I: internal starting site (0)

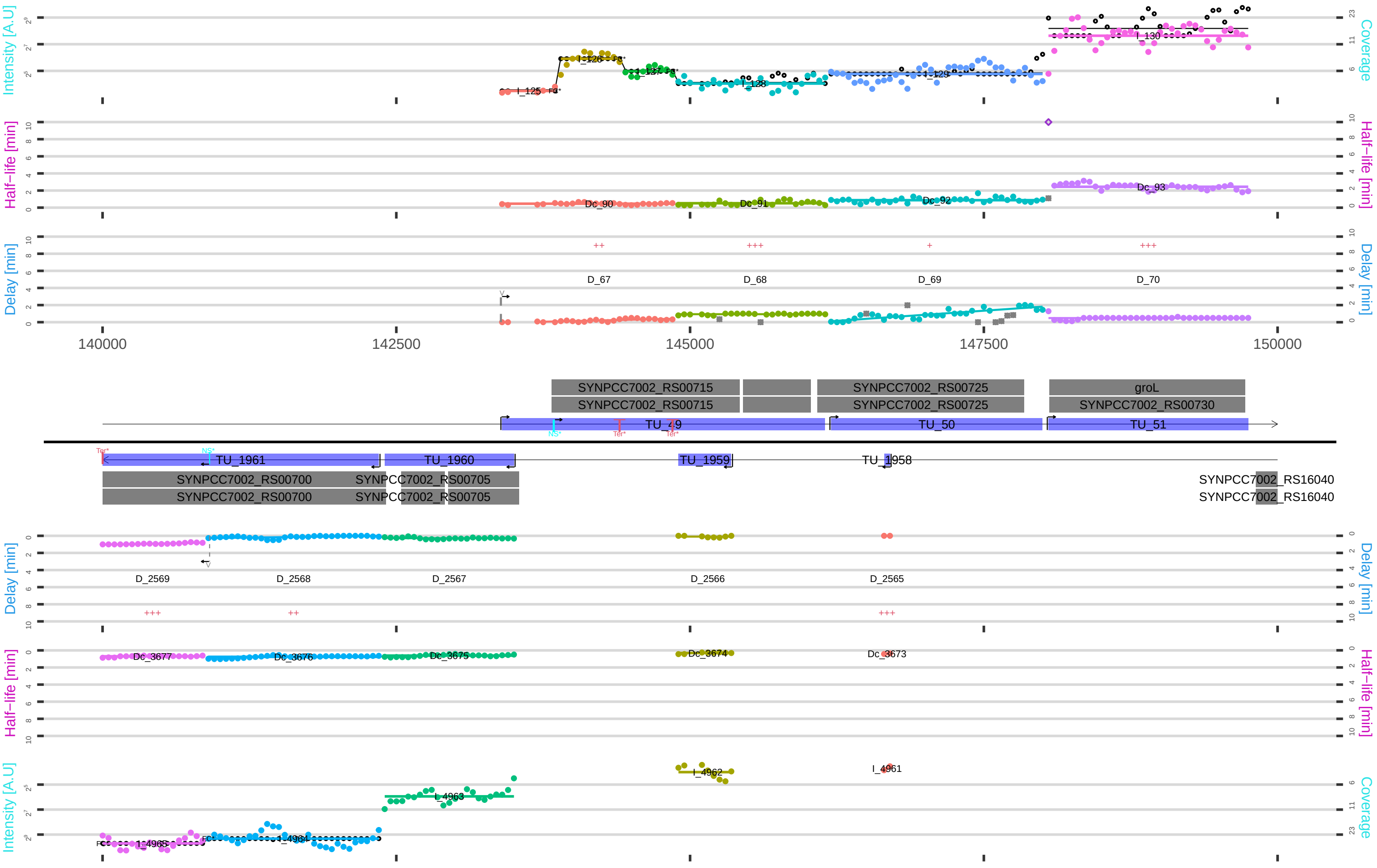

Term: termination (1), NS: new start (1), PS: pausing site (1), iTSS_I: internal starting site (0)

ID: 3021−3200; Term: termination (1), NS: new start (1), PS: pausing site (0), iTSS_I: internal starting site (0)

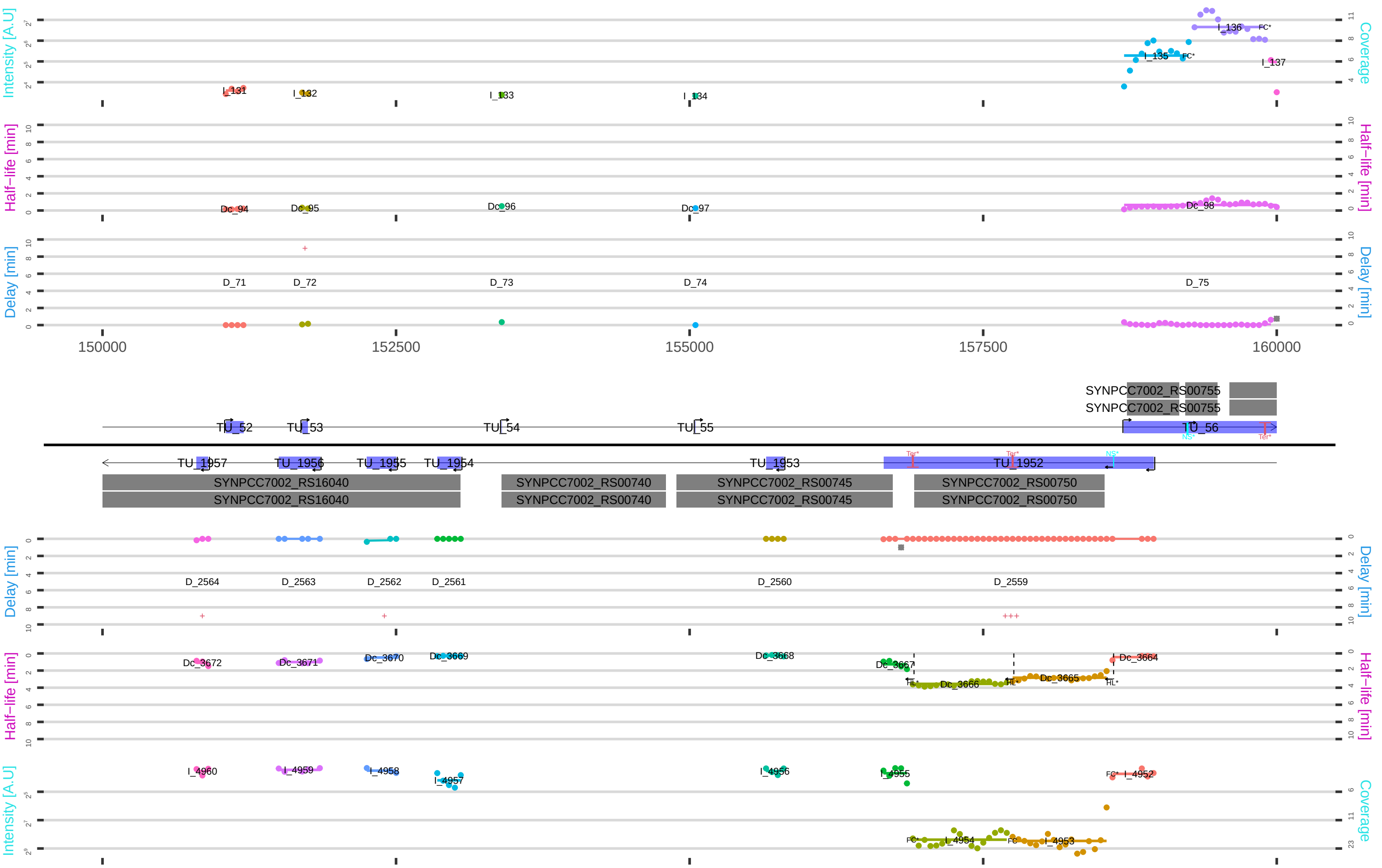

Term: termination (2), NS: new start (1), PS: pausing site (0), iTSS_I: internal starting site (0)

ID: 3200−3364; Term: termination (2), NS: new start (0), PS: pausing site (0), iTSS_I: internal starting site (0)

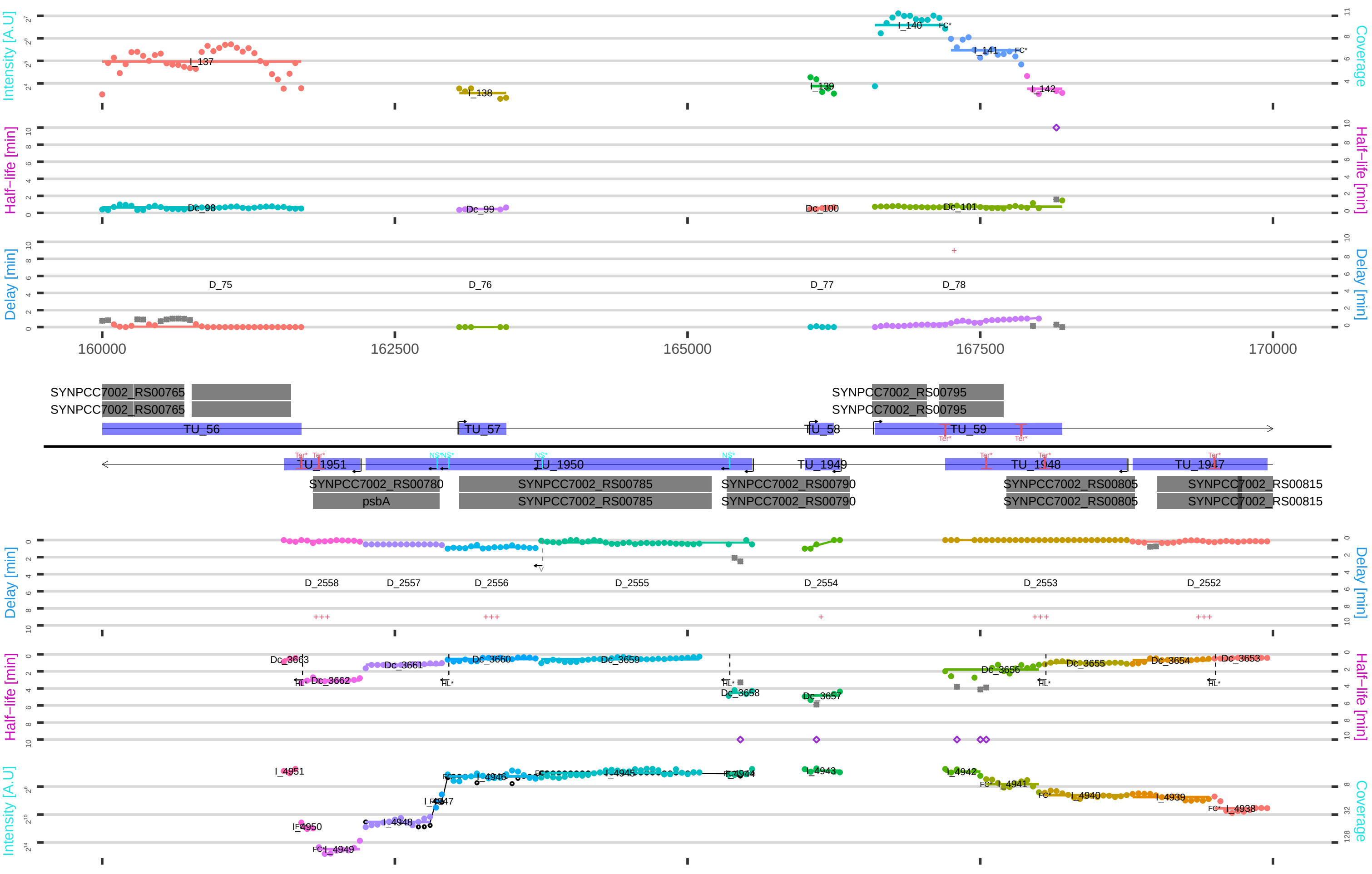

Term: termination (5), NS: new start (4), PS: pausing site (1), iTSS_I: internal starting site (1)

ID: 3411−3598; Term: termination (3), NS: new start (0), PS: pausing site (0), iTSS_I: internal starting site (0)

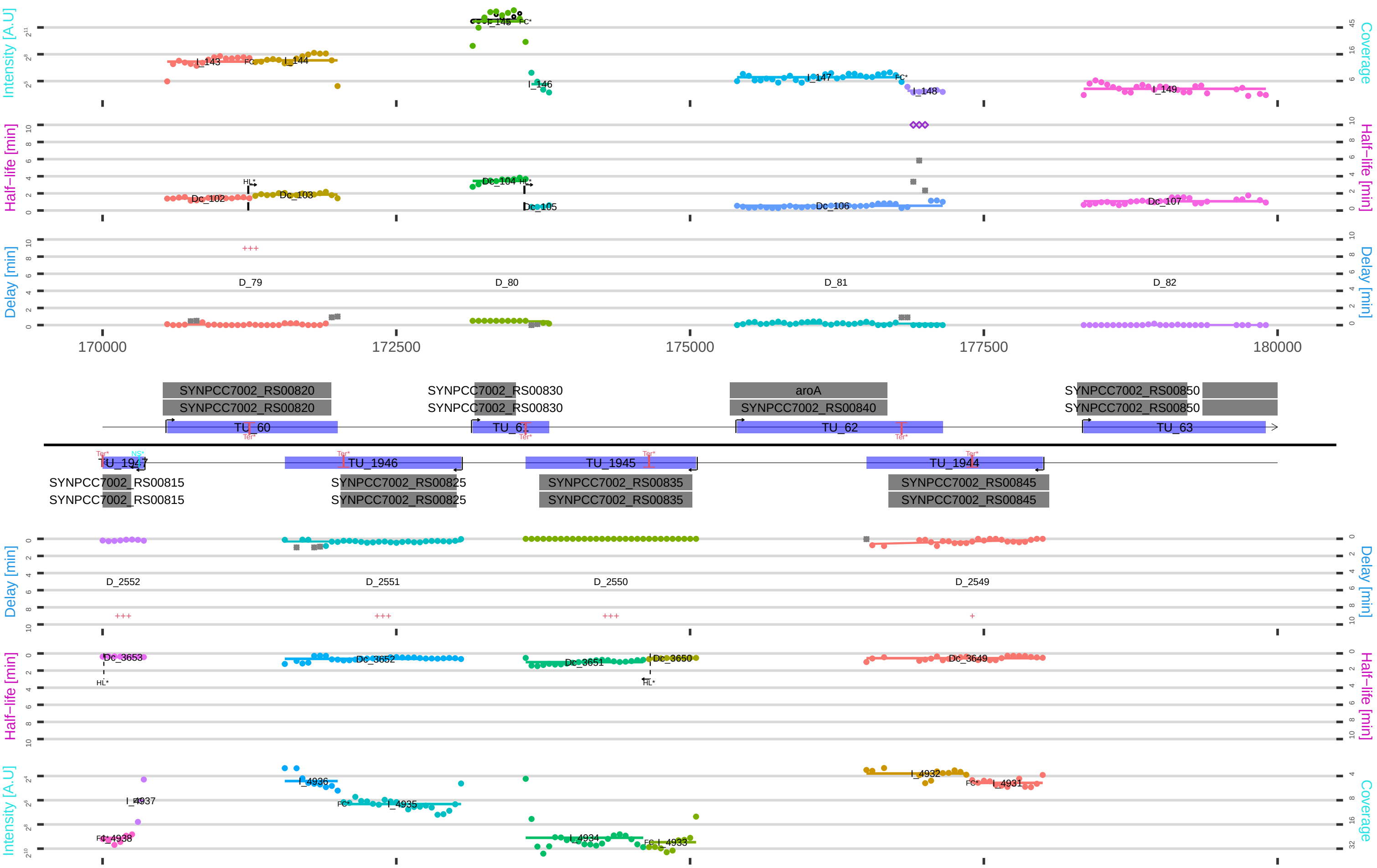

Term: termination (4), NS: new start (1), PS: pausing site (0), iTSS_I: internal starting site (0)

ID: 3607−3800; Term: termination (1), NS: new start (1), PS: pausing site (0), iTSS_I: internal starting site (0)

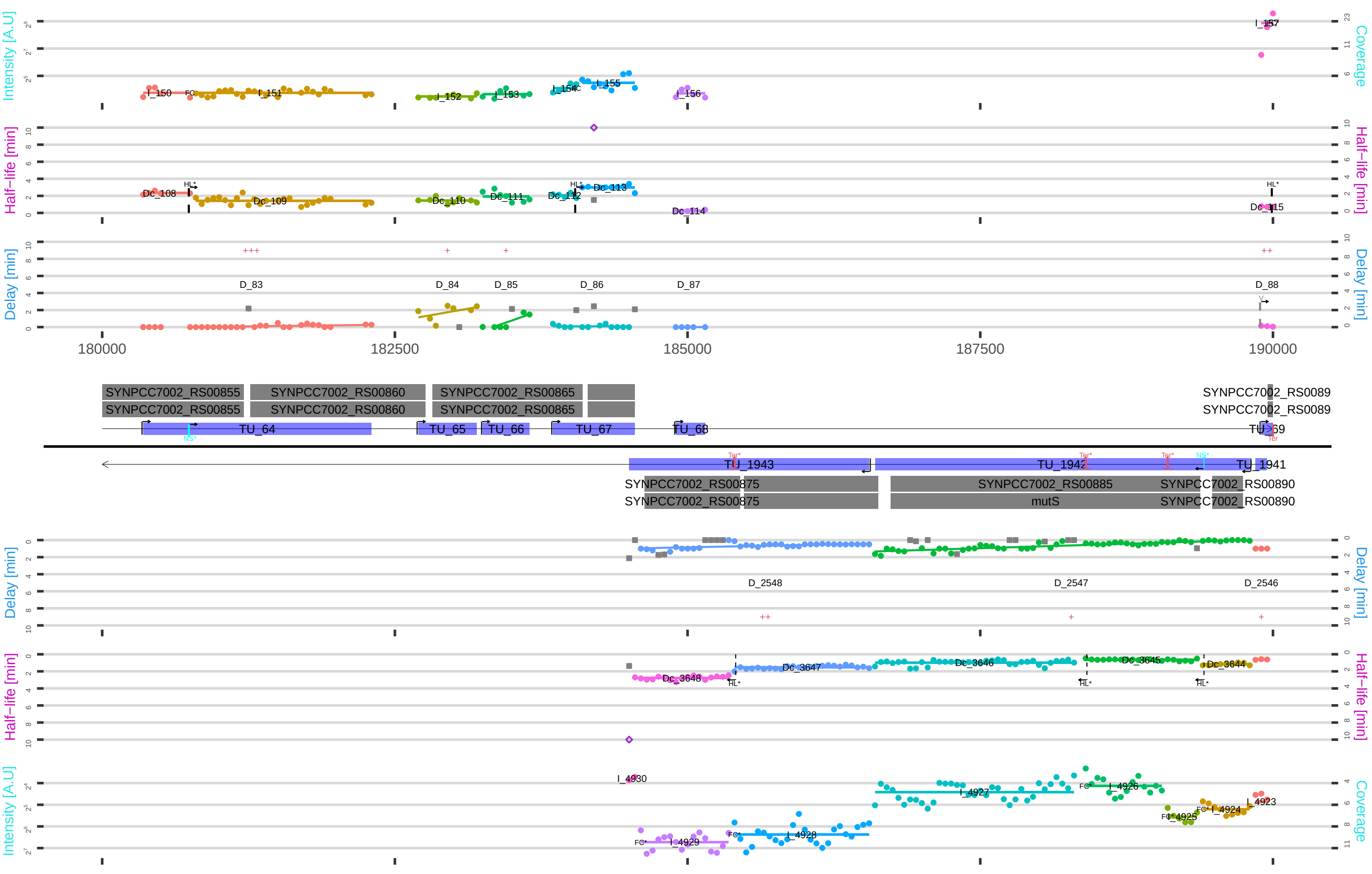

Term: termination (3), NS: new start (1), PS: pausing site (0), iTSS_I: internal starting site (0)

ID: 3800−4000; Term: termination (4), NS: new start (2), PS: pausing site (2), iTSS_I: internal starting site (0)

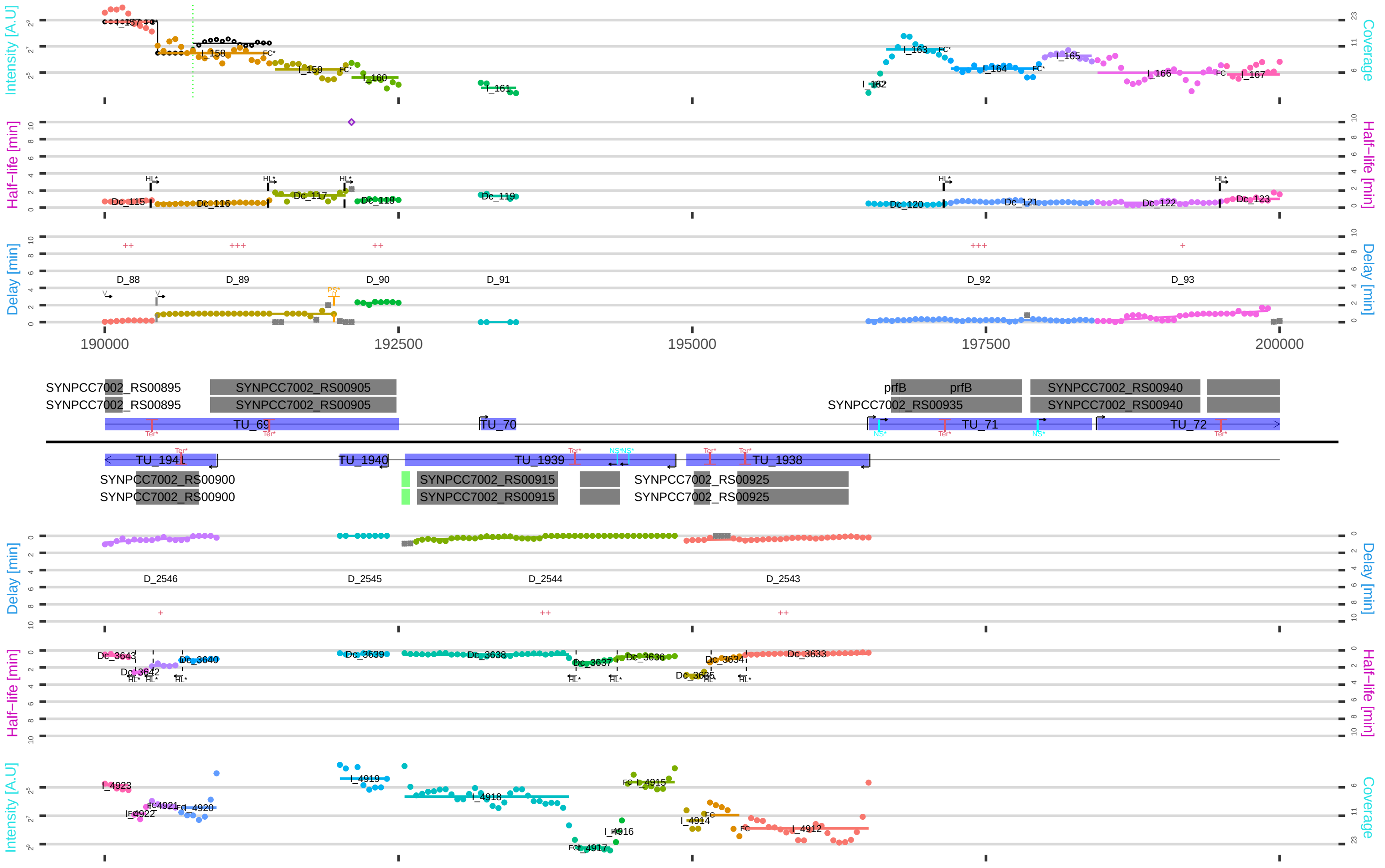

Term: termination (4), NS: new start (2), PS: pausing site (0), iTSS_I: internal starting site (0)

ID: 4000−4200; Term: termination (1), NS: new start (4), PS: pausing site (1), iTSS_I: internal starting site (0)

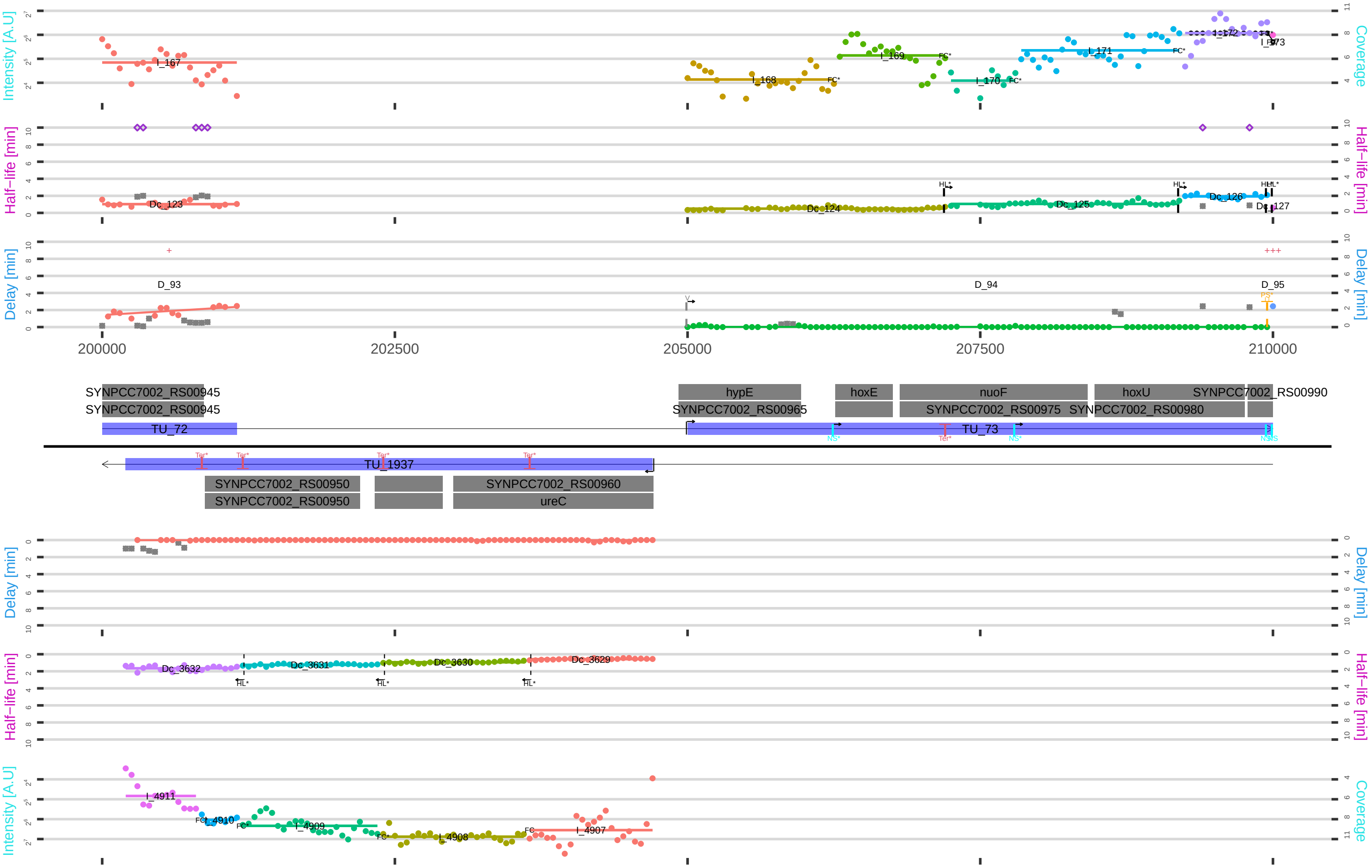

Term: termination (4), NS: new start (0), PS: pausing site (0), iTSS_I: internal starting site (0)

ID: 4200−4400; Term: termination (6), NS: new start (2), PS: pausing site (2), iTSS_I: internal starting site (1)

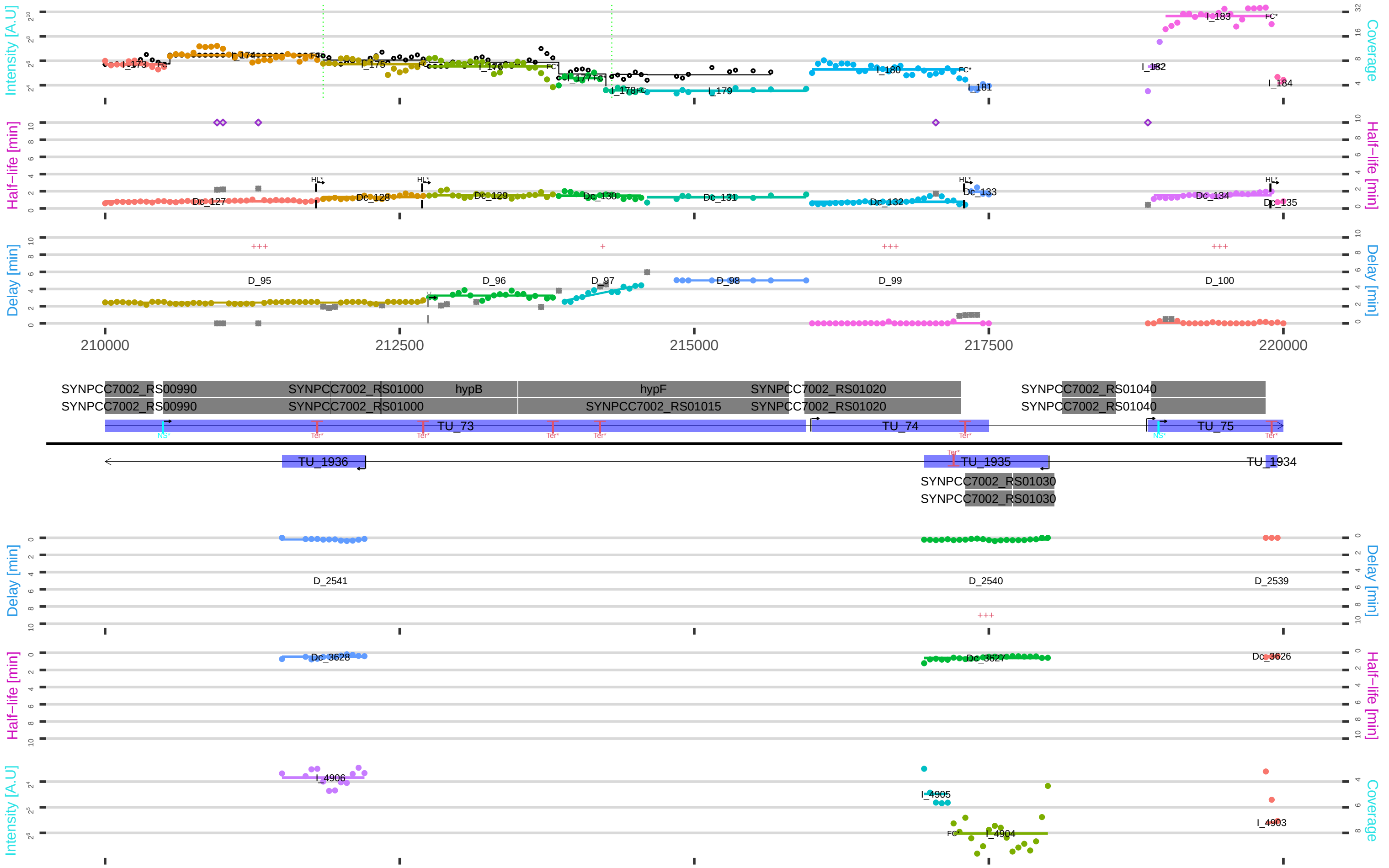

Term: termination (1), NS: new start (0), PS: pausing site (0), iTSS_I: internal starting site (0)

ID: 4400−4600; Term: termination (5), NS: new start (1), PS: pausing site (1), iTSS_I: internal starting site (0)

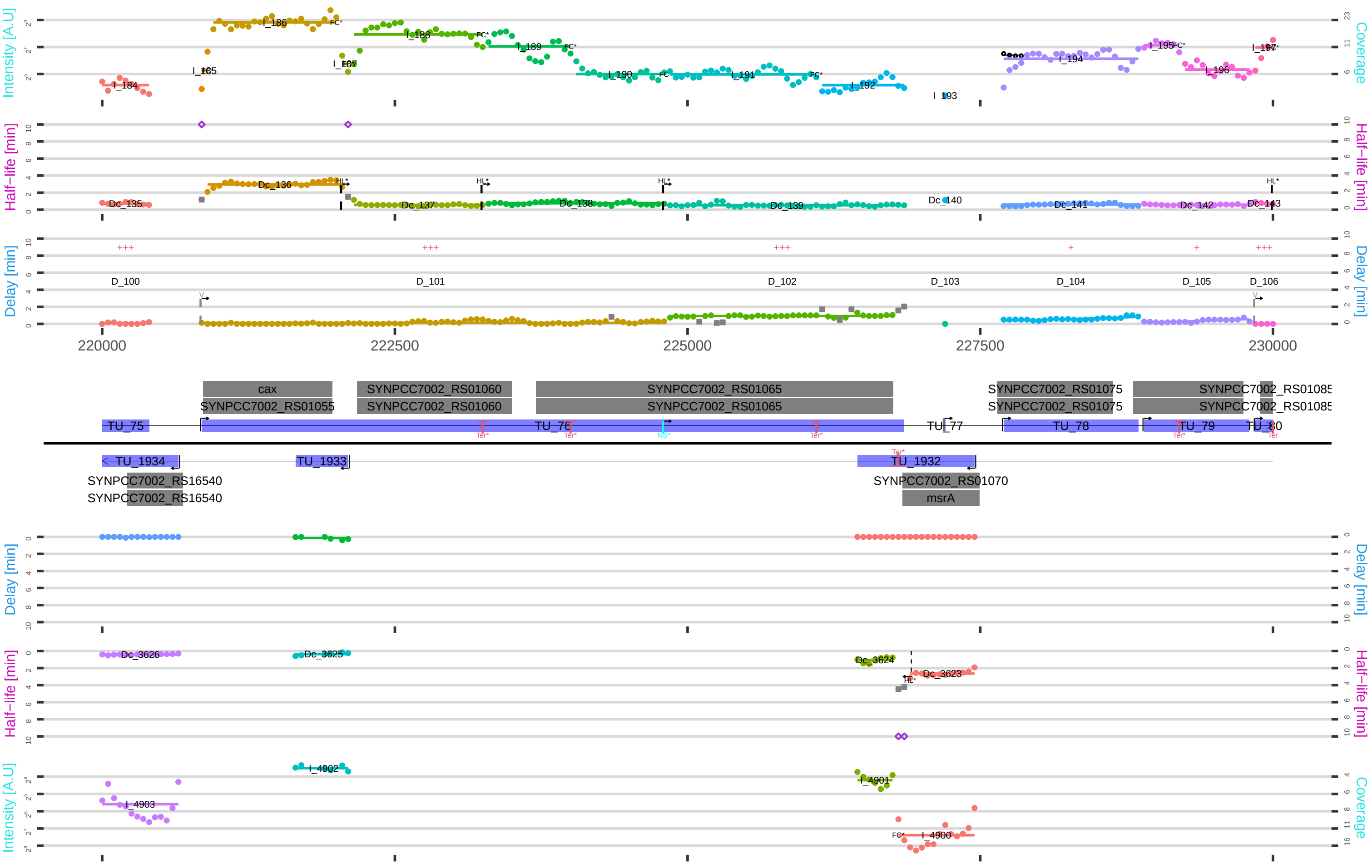

Term: termination (1), NS: new start (0), PS: pausing site (0), iTSS_I: internal starting site (0)

ID: 4600−4800; Term: termination (4), NS: new start (3), PS: pausing site (1), iTSS_I: internal starting site (0)

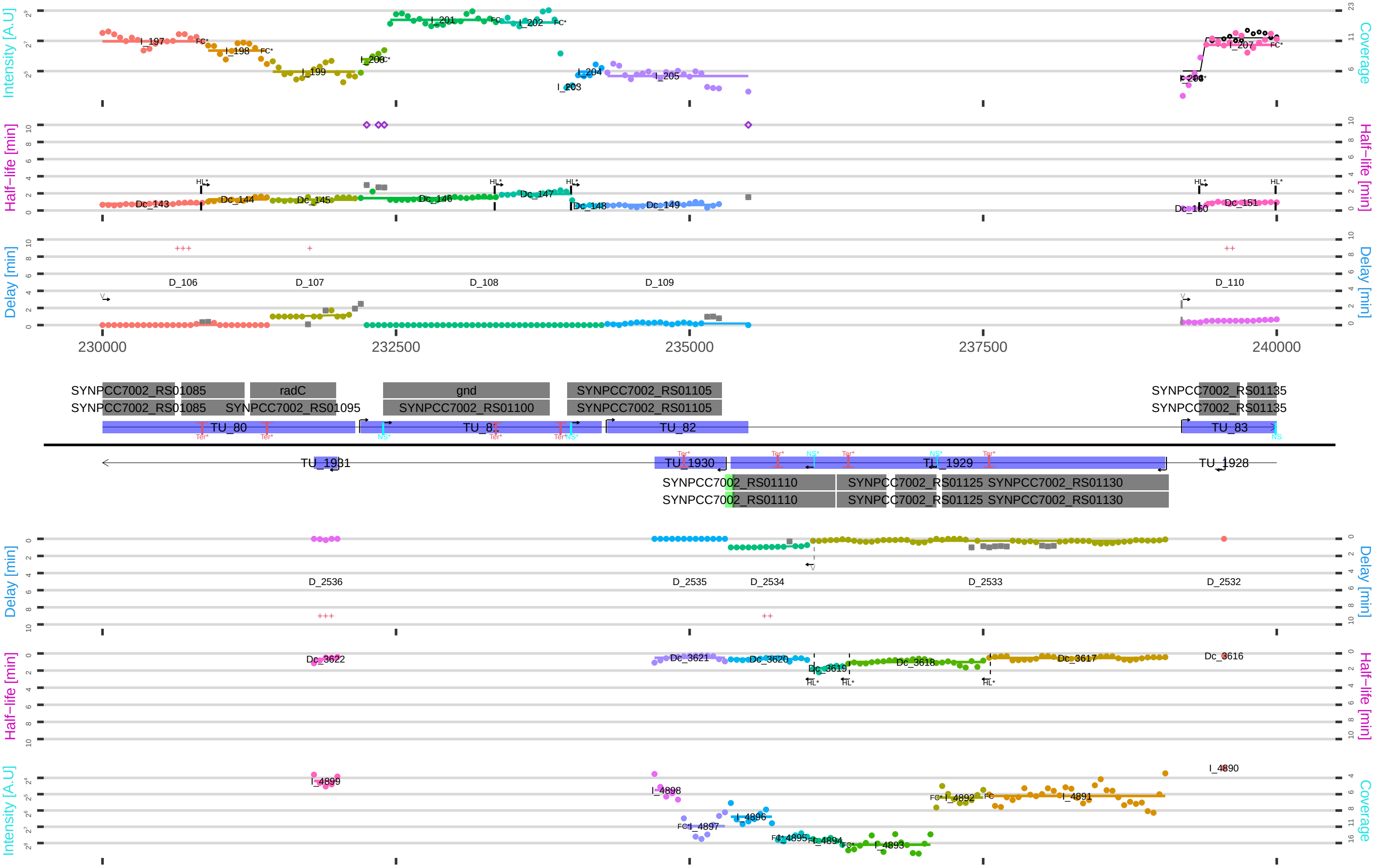

Term: termination (4), NS: new start (2), PS: pausing site (1), iTSS_I: internal starting site (0)

ID: 4800−5000; Term: termination (5), NS: new start (2), PS: pausing site (1), iTSS_I: internal starting site (1)

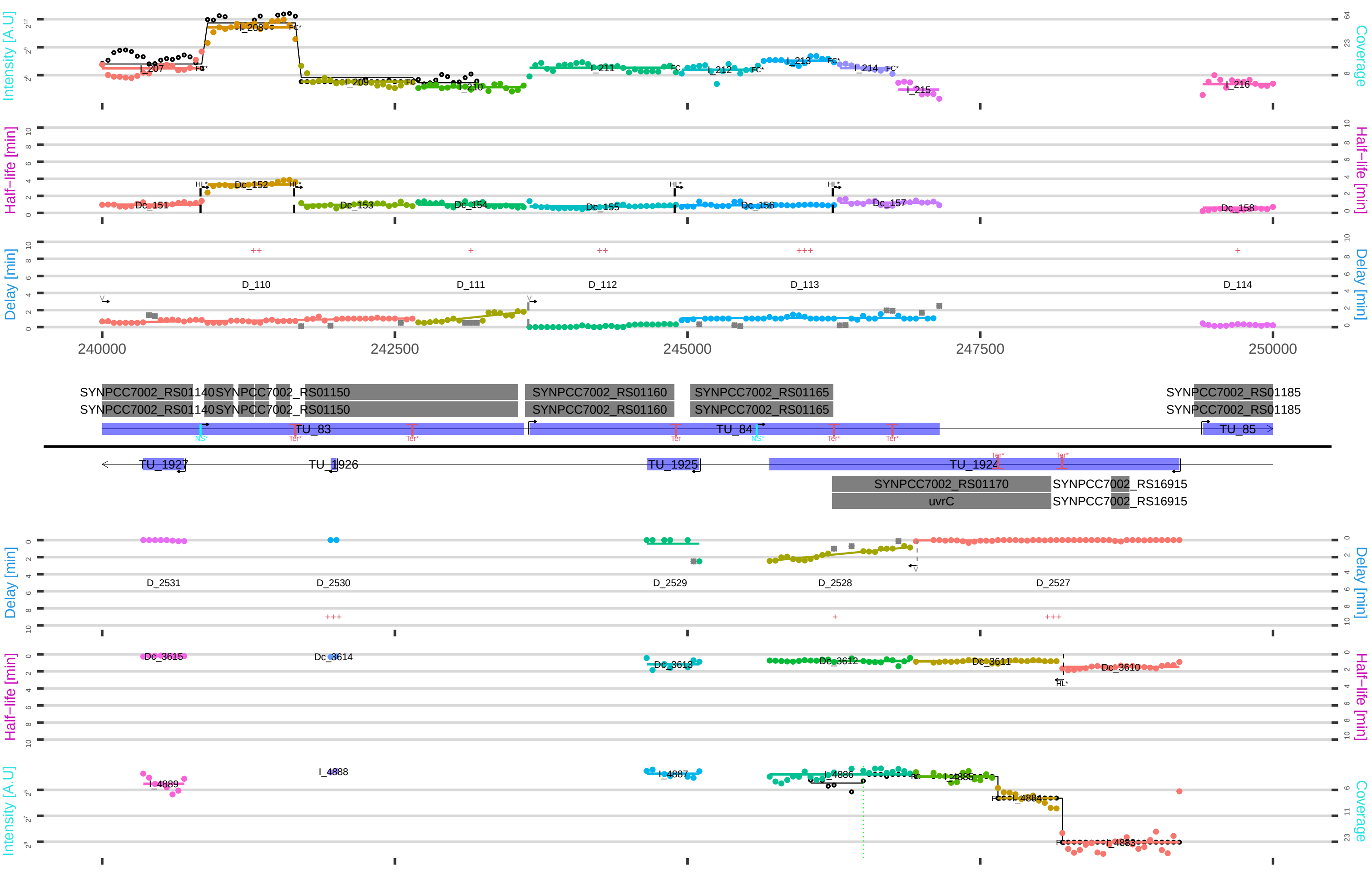

Term: termination (2), NS: new start (0), PS: pausing site (1), iTSS_I: internal starting site (0)

ID: 5000−5200; Term: termination (3), NS: new start (3), PS: pausing site (1), iTSS_I: internal starting site (0)

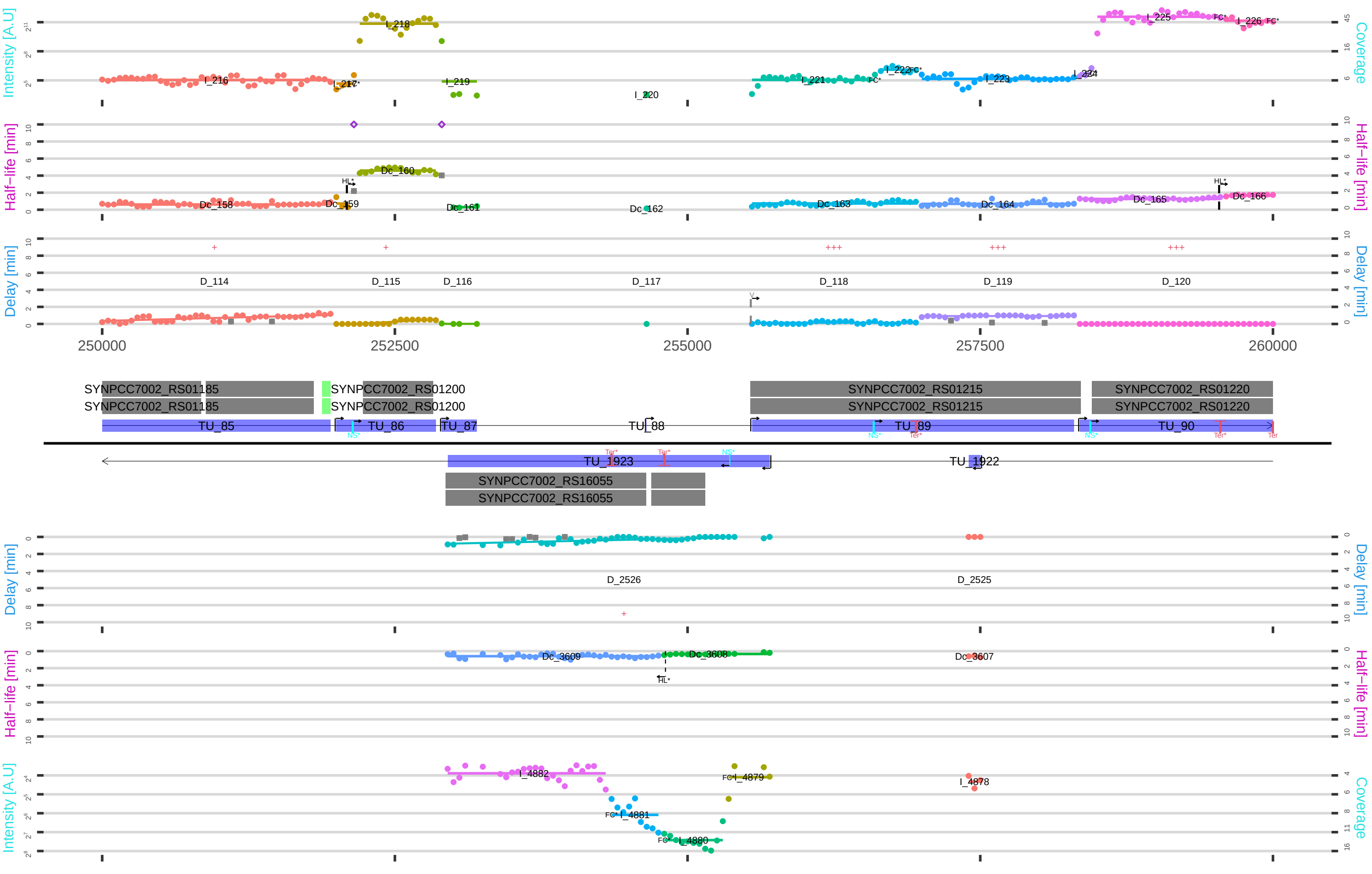

Term: termination (2), NS: new start (1), PS: pausing site (0), iTSS_I: internal starting site (0)

ID: 5200−5400; Term: termination (2), NS: new start (0), PS: pausing site (0), iTSS_I: internal starting site (0)

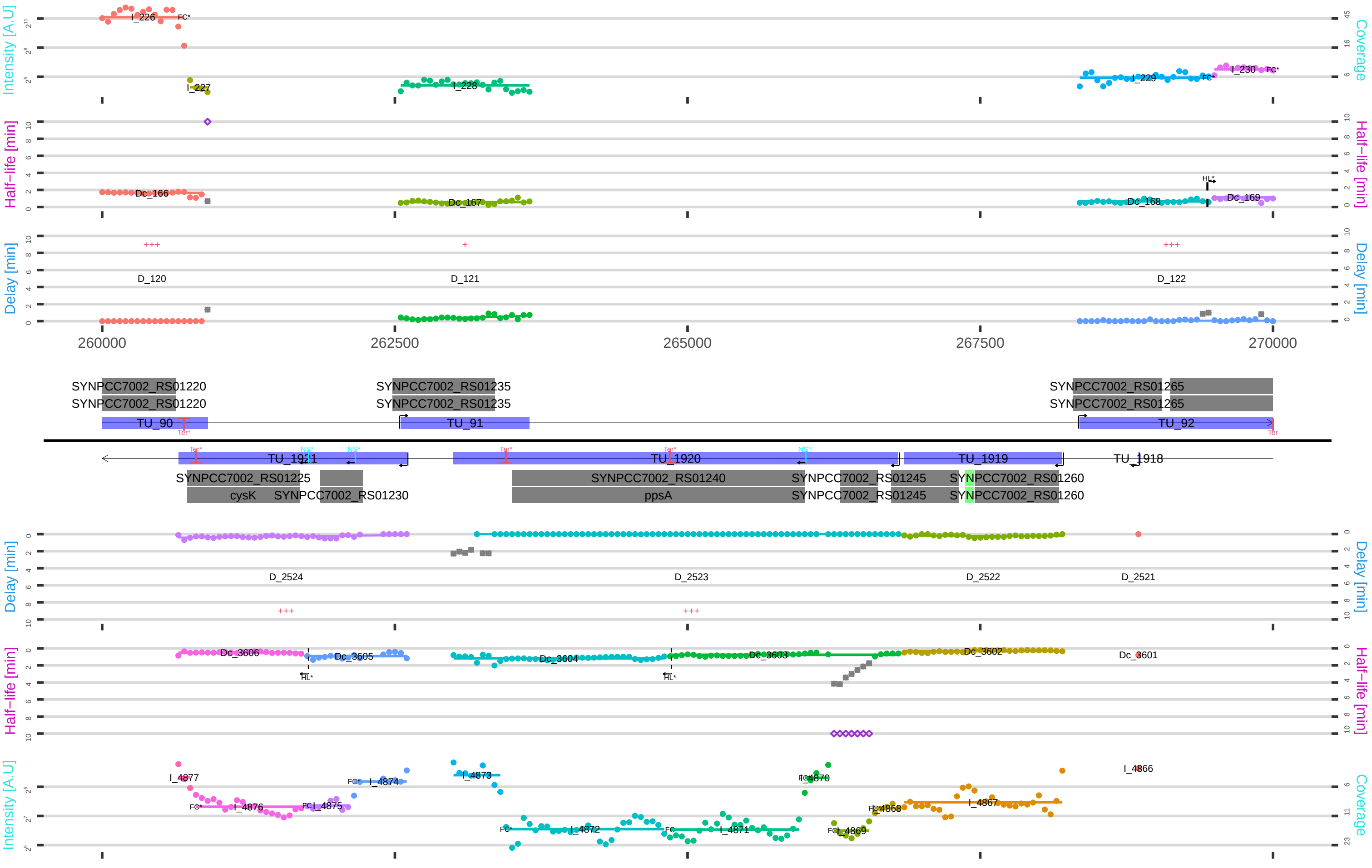

Term: termination (3), NS: new start (3), PS: pausing site (0), iTSS_I: internal starting site (0)

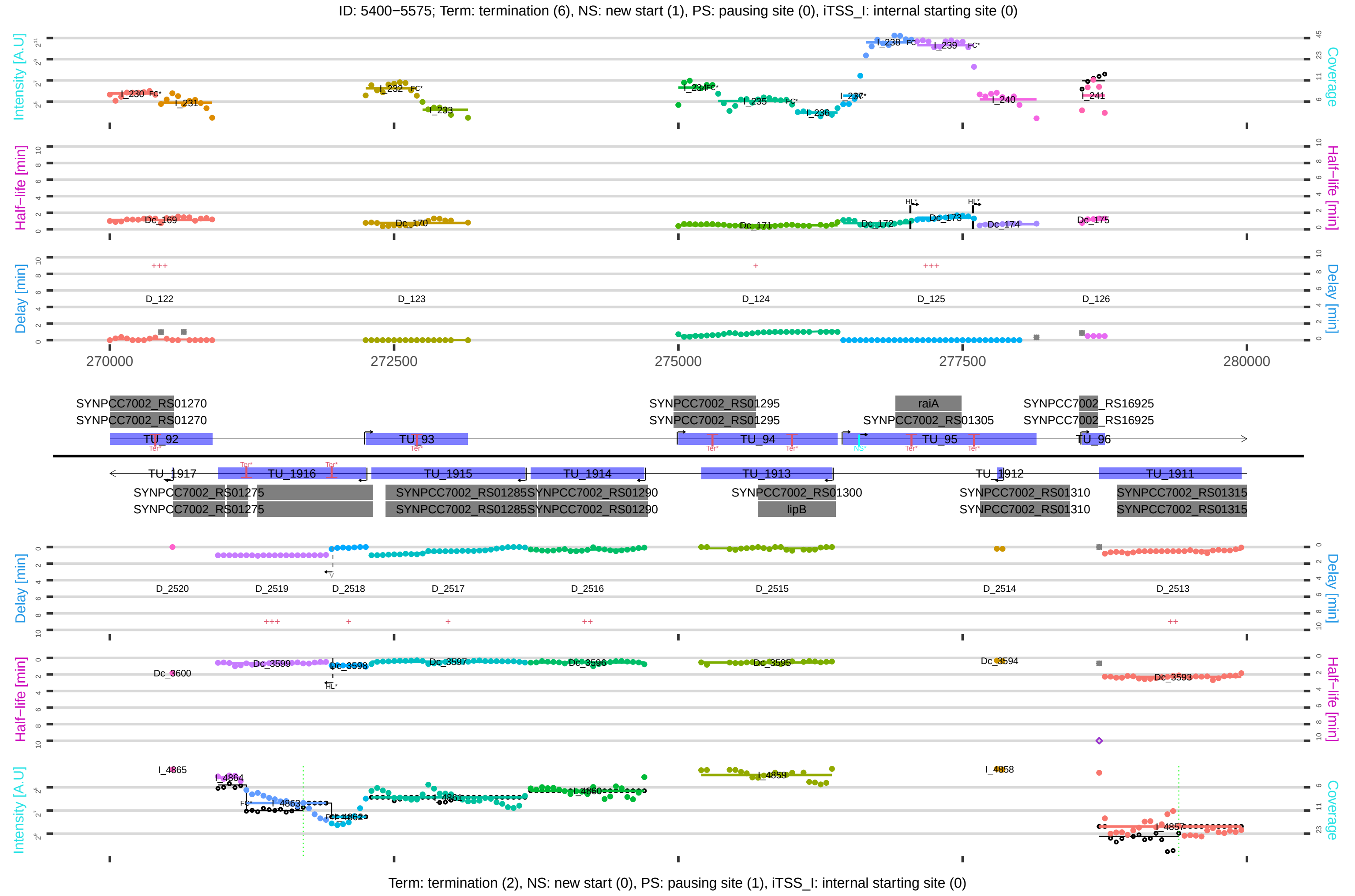

ID: 5400−5575; Term: termination (6), NS: new start (1), PS: pausing site (0), iTSS_I: internal starting site (0)
Intensity [A.U]
Coverage
Half−life [min]
Delay [min]
270000
272500
275000
277500
280000
SYNPCC7002_RS01270
TU_92
TU_93
SYNPCC7002_RS01295
TU_94
raiA
SYNPCC7002_RS01305
TU_95
SYNPCC7002_RS16925
TU_96
TU_1917
TU_1916
TU_1915
TU_1914
TU_1913
TU_1912
TU_1911
SYNPCC7002_RS01275
SYNPCC7002_RS01285
SYNPCC7002_RS01290
SYNPCC7002_RS01300
lipB
SYNPCC7002_RS01310
SYNPCC7002_RS01315
Term: termination (2), NS: new start (0), PS: pausing site (1), iTSS_I: internal starting site (0)

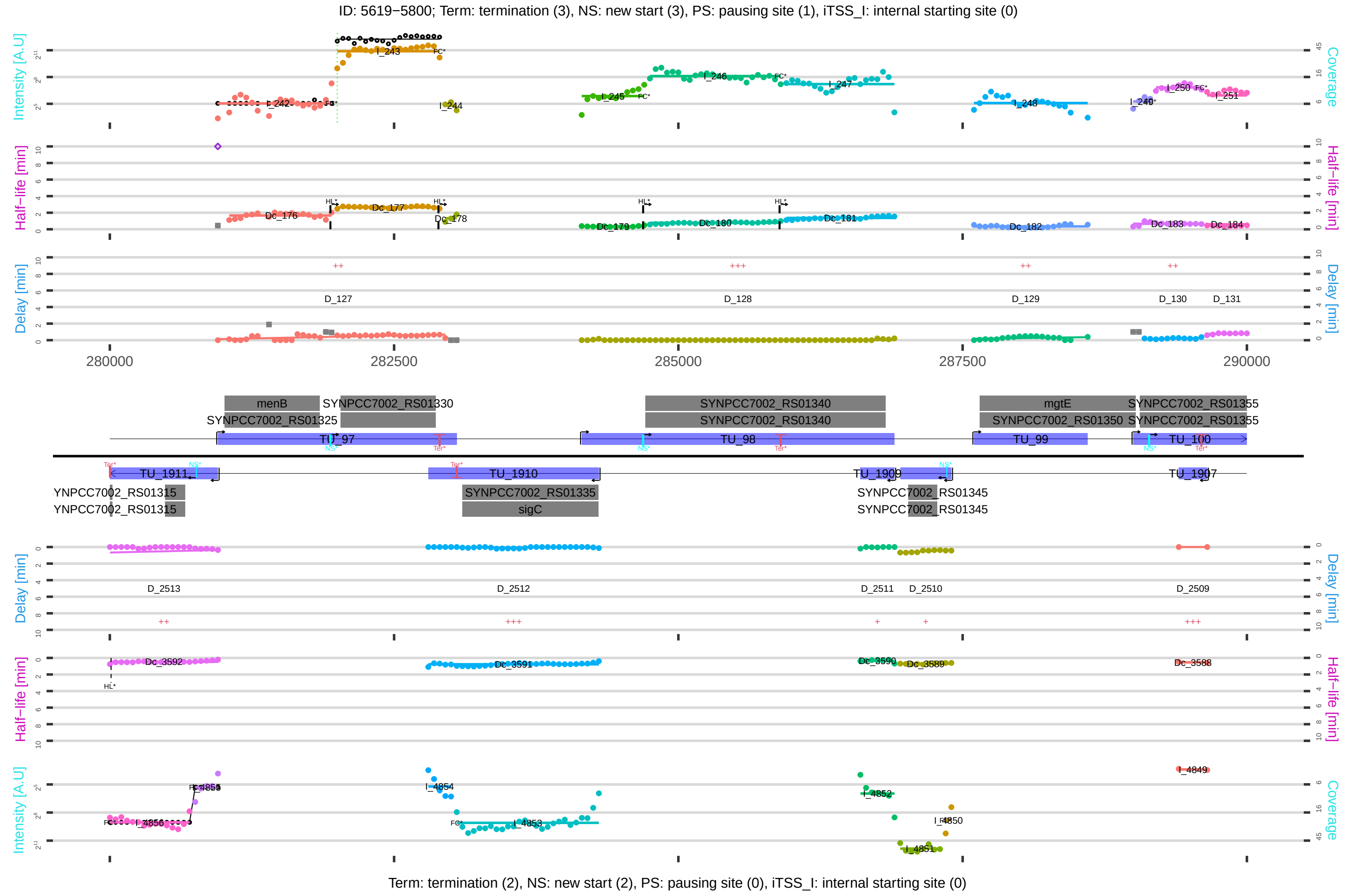
ID: 5619−5800; Term: termination (3), NS: new start (3), PS: pausing site (1), iTSS_I: internal starting site (0)
Intensity [A.U]
Coverage
Half−life [min]
Delay [min]
280000
282500
285000
287500
290000
menB
SYNPCC7002_RS01325
SYNPCC7002_RS01330
SYNPCC7002_RS01340
mgtE
SYNPCC7002_RS01350
SYNPCC7002_RS01355
TU_97
TU_98
TU_99
TU_100
TU_1911
TU_1910
TU_1909
TU_1907
SYNPCC7002_RS01315
SYNPCC7002_RS01335
sigC
SYNPCC7002_RS01345
D_127
D_128
D_129
D_130
D_131
D_2513
D_2512
D_2511
D_2510
D_2509
Term: termination (2), NS: new start (2), PS: pausing site (0), iTSS_I: internal starting site (0)

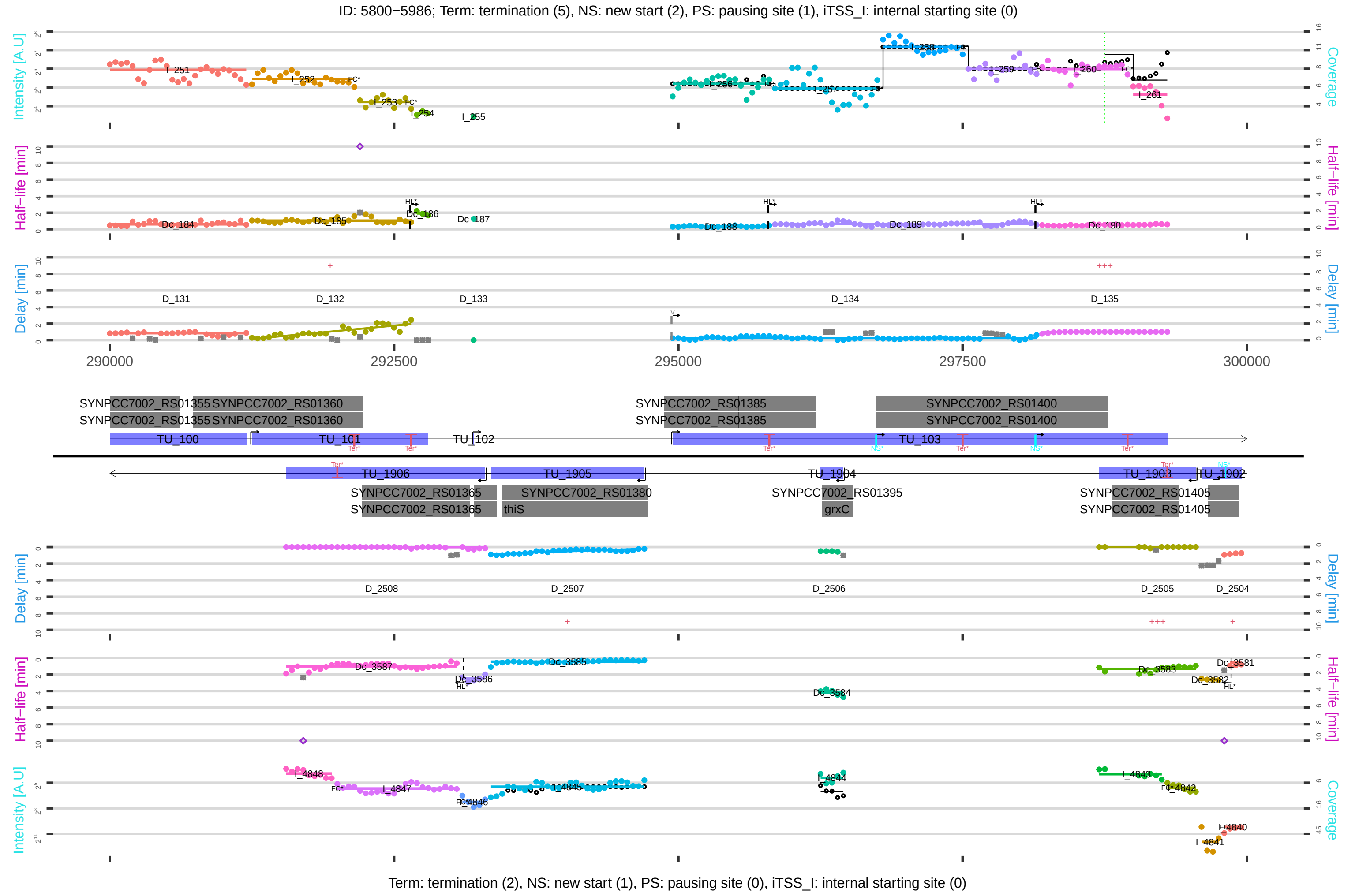

ID: 5800−5986; Term: termination (5), NS: new start (2), PS: pausing site (1), iTSS_I: internal starting site (0)
Intensity [A.U]
Coverage
Half−life [min]
Delay [min]
290000
292500
295000
297500
300000
SYNPCC7002_RS01355
SYNPCC7002_RS01360
SYNPCC7002_RS01385
SYNPCC7002_RS01400
TU_100
TU_101
TU_102
TU_103
TU_1906
TU_1905
TU_1904
TU_1903
TU_1902
SYNPCC7002_RS01365
SYNPCC7002_RS01380
thiS
SYNPCC7002_RS01395
grxC
SYNPCC7002_RS01405
Term: termination (2), NS: new start (1), PS: pausing site (0), iTSS_I: internal starting site (0)

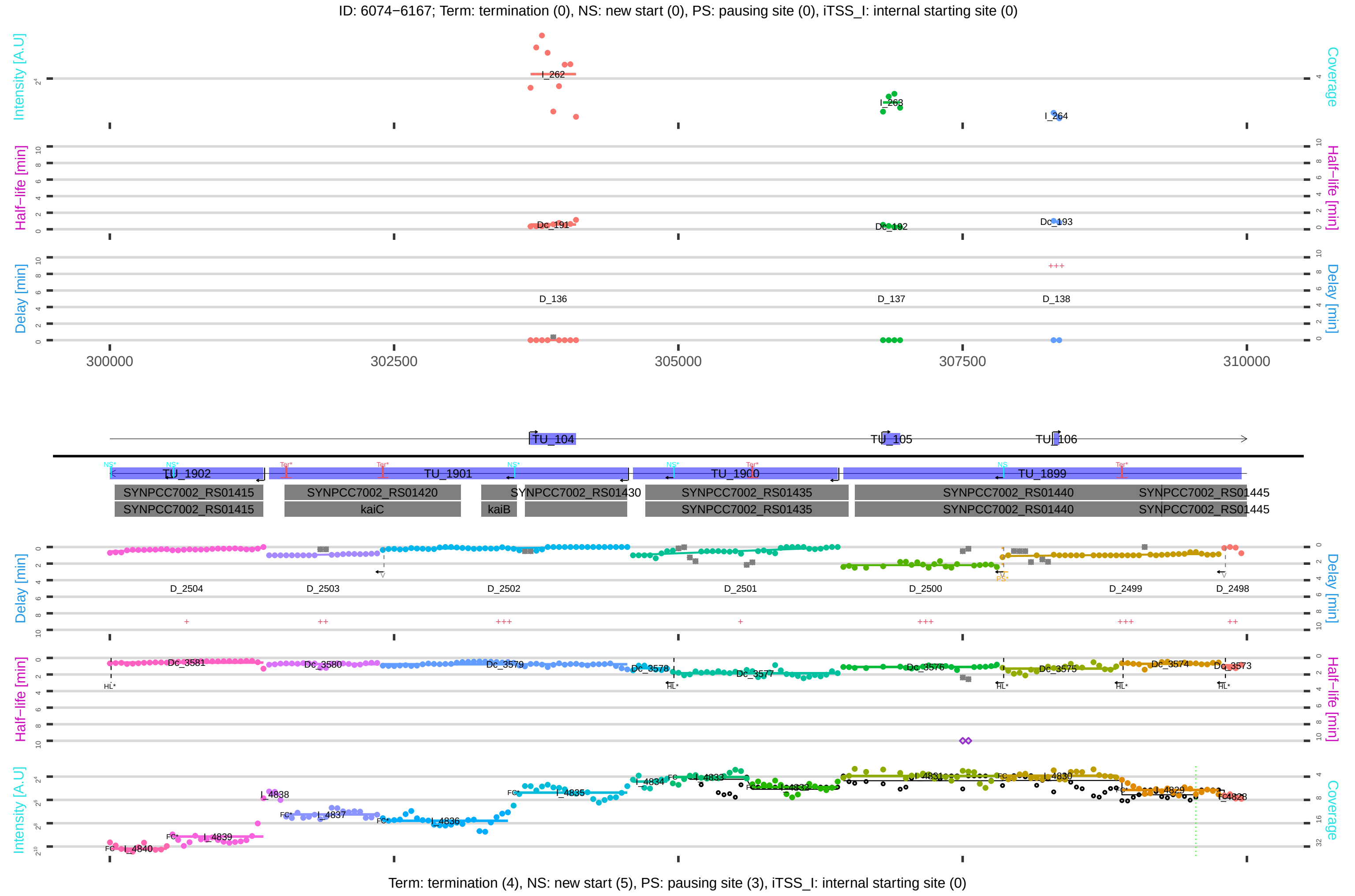

ID: 6074−6167; Term: termination (0), NS: new start (0), PS: pausing site (0), iTSS_I: internal starting site (0)
Intensity [A.U]
Coverage
Half−life [min]
Delay [min]
I_262
I_263
I_264
Dc_191
Dc_192
Dc_193
D_136
D_137
D_138
300000
302500
305000
307500
310000
TU_104
TU_105
TU_106
TU_1902
TU_1901
TU_1900
TU_1899
SYNPCC7002_RS01415
SYNPCC7002_RS01420
SYNPCC7002_RS01430
SYNPCC7002_RS01435
SYNPCC7002_RS01440
SYNPCC7002_RS01445
kaiC
kaiB
D_2504
D_2503
D_2502
D_2501
D_2500
D_2499
D_2498
Dc_3581
Dc_3580
Dc_3579
Dc_3578
Dc_3577
Dc_3576
Dc_3575
Dc_3574
Dc_3573
I_4840
I_4839
I_4838
I_4837
I_4836
I_4835
I_4834
I_4833
I_4832
I_4831
I_4830
I_4829
I_4828
Term: termination (4), NS: new start (5), PS: pausing site (3), iTSS_I: internal starting site (0)

ID: 6238−6358; Term: termination (4), NS: new start (1), PS: pausing site (0), iTSS_I: internal starting site (0)

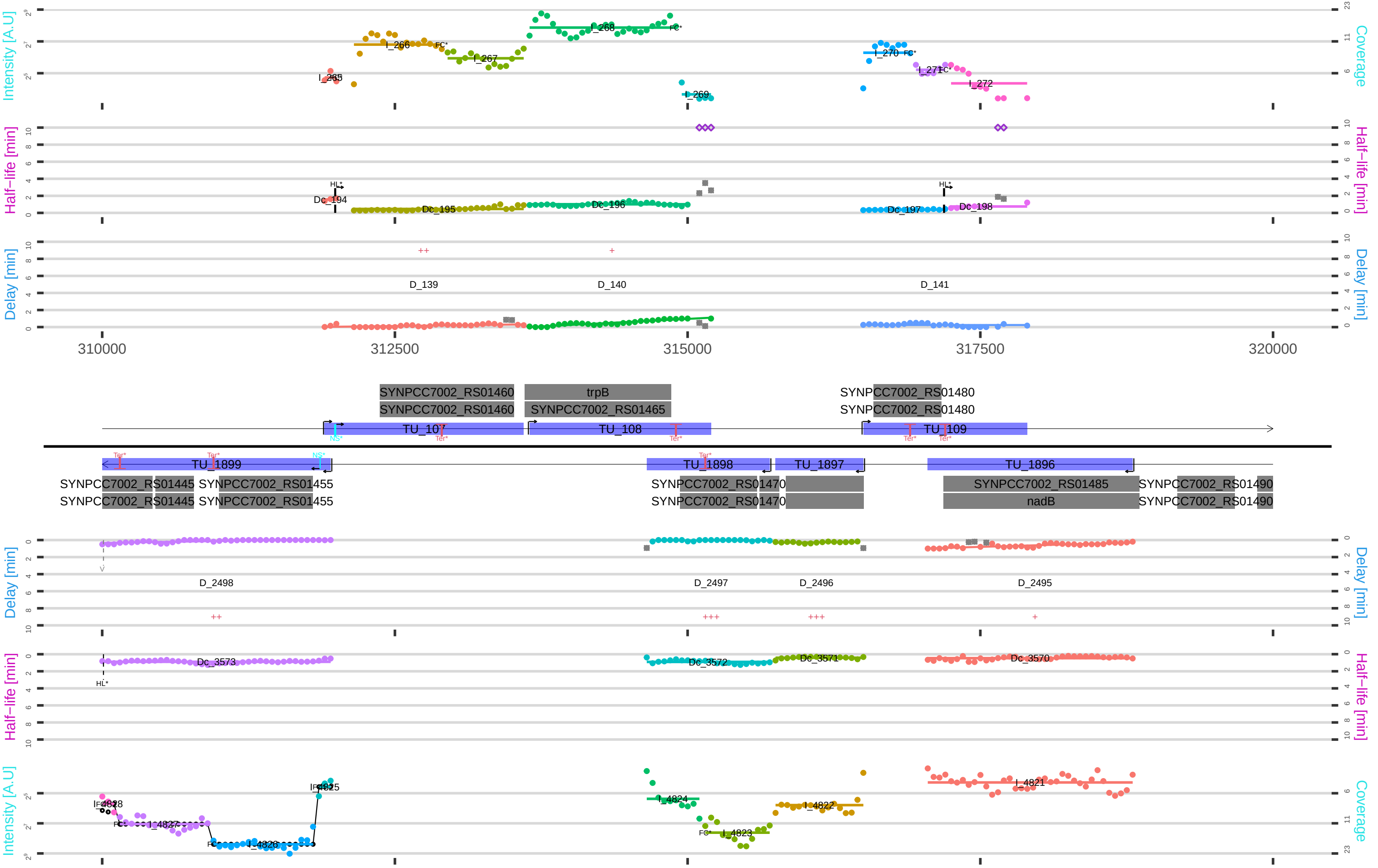

Term: termination (3), NS: new start (1), PS: pausing site (0), iTSS_I: internal starting site (0)

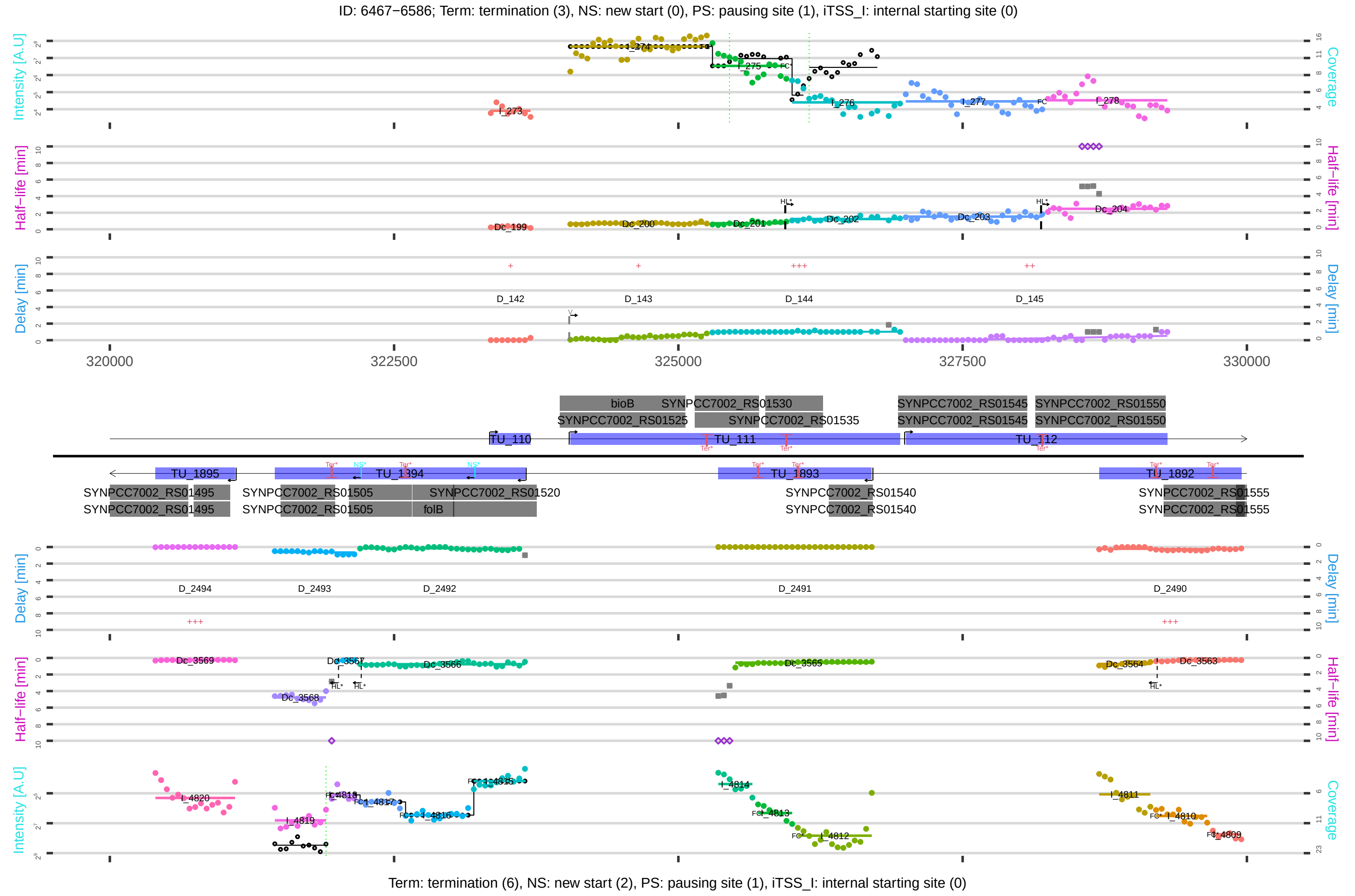

ID: 6467−6586; Term: termination (3), NS: new start (0), PS: pausing site (1), iTSS_I: internal starting site (0)
Intensity [A.U]
Coverage
Half−life [min]
Delay [min]
I_273
I_274
I_275
I_276
I_277
I_278
Dc_199
Dc_200
Dc_201
Dc_202
Dc_203
Dc_204
D_142
D_143
D_144
D_145
320000
322500
325000
327500
330000
bioB
SYNPCC7002_RS01530
SYNPCC7002_RS01525
SYNPCC7002_RS01535
SYNPCC7002_RS01545
SYNPCC7002_RS01550
TU_110
TU_111
TU_112
TU_1895
TU_1894
TU_1893
TU_1892
SYNPCC7002_RS01495
SYNPCC7002_RS01505
SYNPCC7002_RS01520
folB
SYNPCC7002_RS01540
SYNPCC7002_RS01555
D_2494
D_2493
D_2492
D_2491
D_2490
Dc_3569
Dc_3568
Dc_3567
Dc_3566
Dc_3565
Dc_3564
Dc_3563
I_4820
I_4819
I_4818
I_4817
I_4816
I_4815
I_4814
I_4813
I_4812
I_4811
I_4810
I_4809
Term: termination (6), NS: new start (2), PS: pausing site (1), iTSS_I: internal starting site (0)

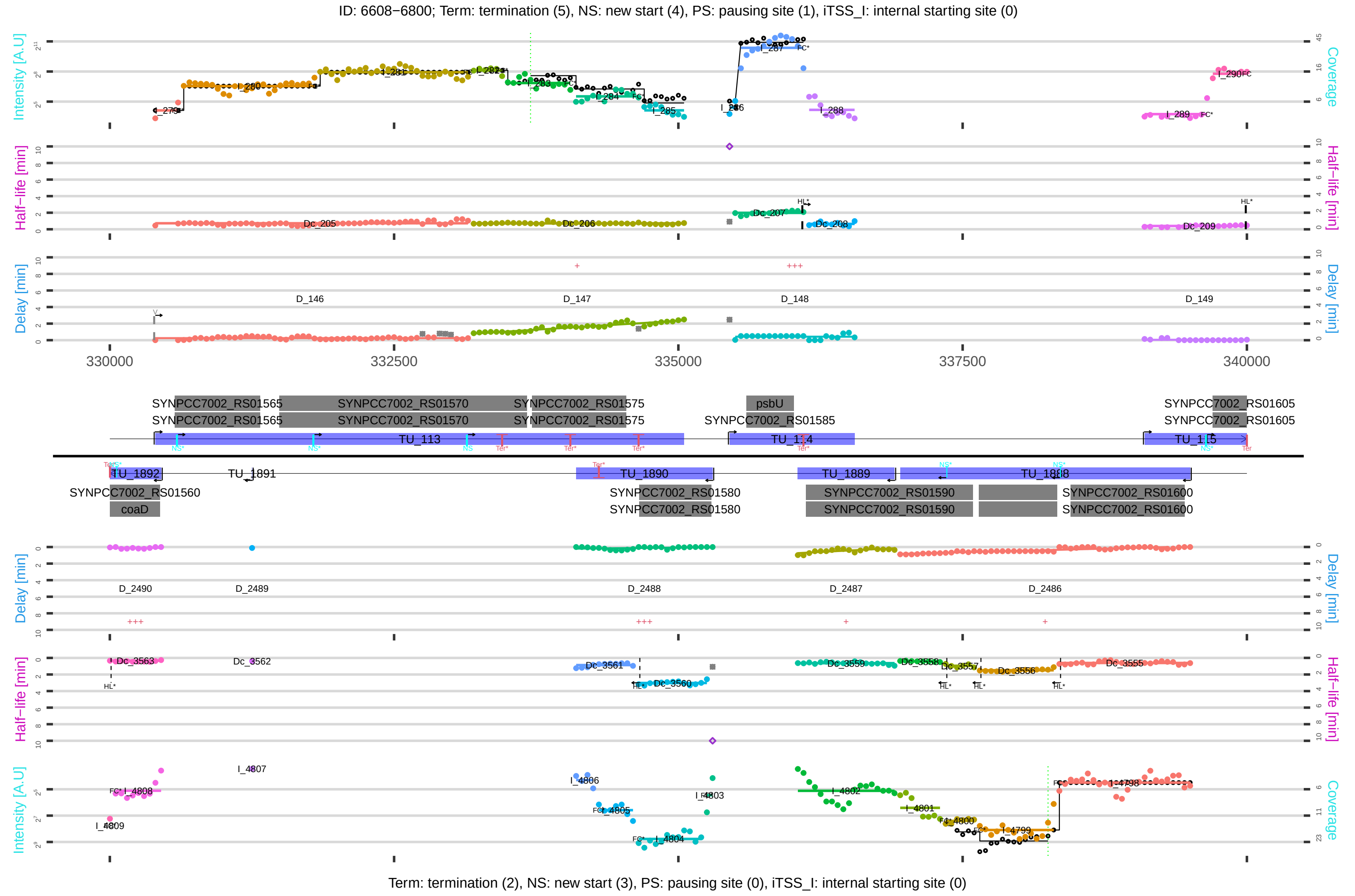
ID: 6608−6800; Term: termination (5), NS: new start (4), PS: pausing site (1), iTSS_I: internal starting site (0)
Intensity [A.U]
Coverage
Half−life [min]
Delay [min]
330000
332500
335000
337500
340000
SYNPCC7002_RS01565
SYNPCC7002_RS01570
SYNPCC7002_RS01575
psbU
SYNPCC7002_RS01585
SYNPCC7002_RS01605
TU_113
TU_114
TU_115
TU_1892
TU_1891
TU_1890
TU_1889
TU_1888
SYNPCC7002_RS01560
coaD
SYNPCC7002_RS01580
SYNPCC7002_RS01590
SYNPCC7002_RS01600
D_146
D_147
D_148
D_149
D_2490
D_2489
D_2488
D_2487
D_2486
Term: termination (2), NS: new start (3), PS: pausing site (0), iTSS_I: internal starting site (0)

ID: 6800−6987; Term: termination (4), NS: new start (0), PS: pausing site (1), iTSS_I: internal starting site (1)

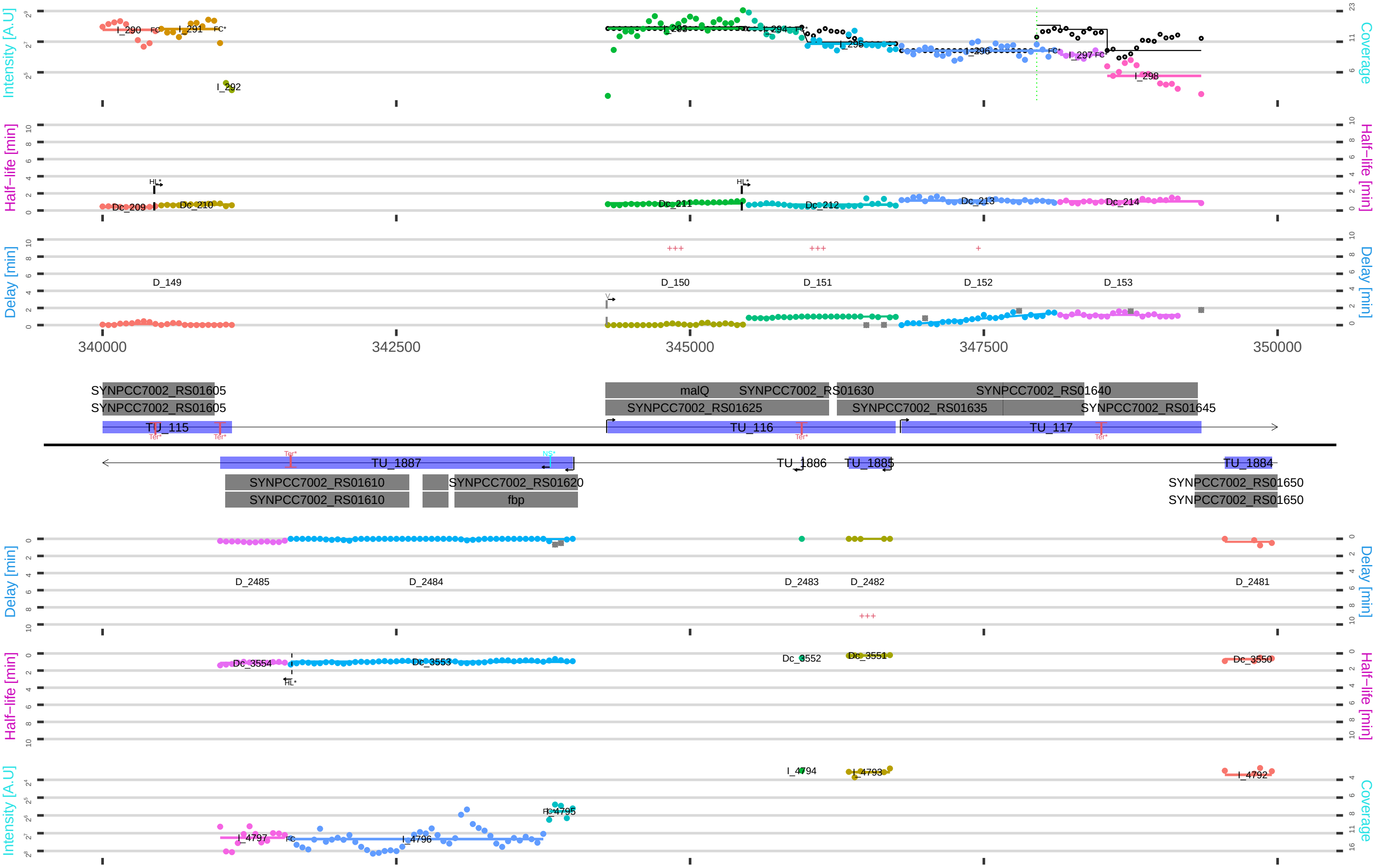

Term: termination (1), NS: new start (1), PS: pausing site (1), iTSS_I: internal starting site (0)

ID: 7021−7200; Term: termination (3), NS: new start (0), PS: pausing site (0), iTSS_I: internal starting site (0)

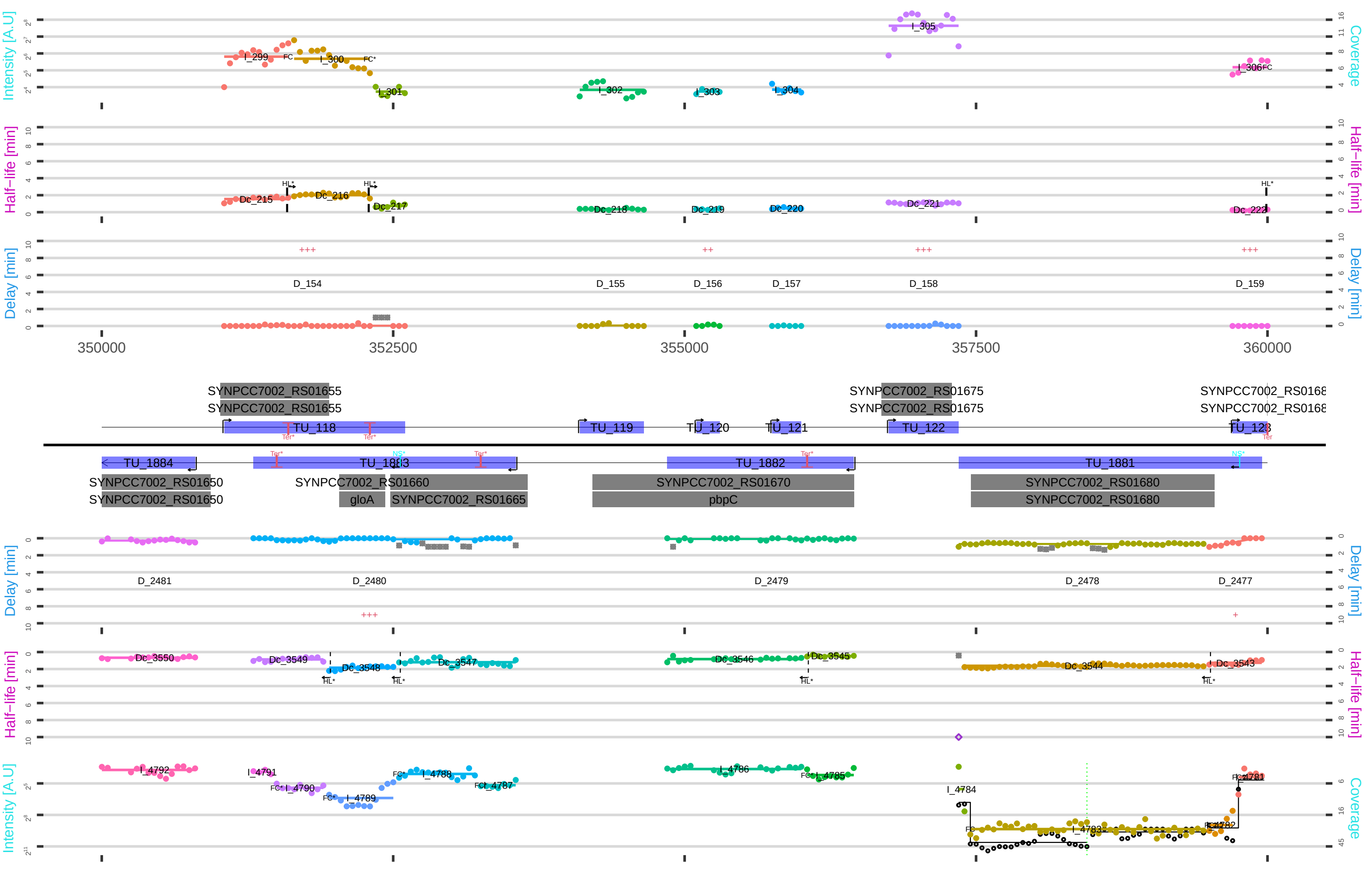

Term: termination (3), NS: new start (2), PS: pausing site (0), iTSS_I: internal starting site (1)

ID: 7200−7400; Term: termination (4), NS: new start (1), PS: pausing site (0), iTSS_I: internal starting site (0)

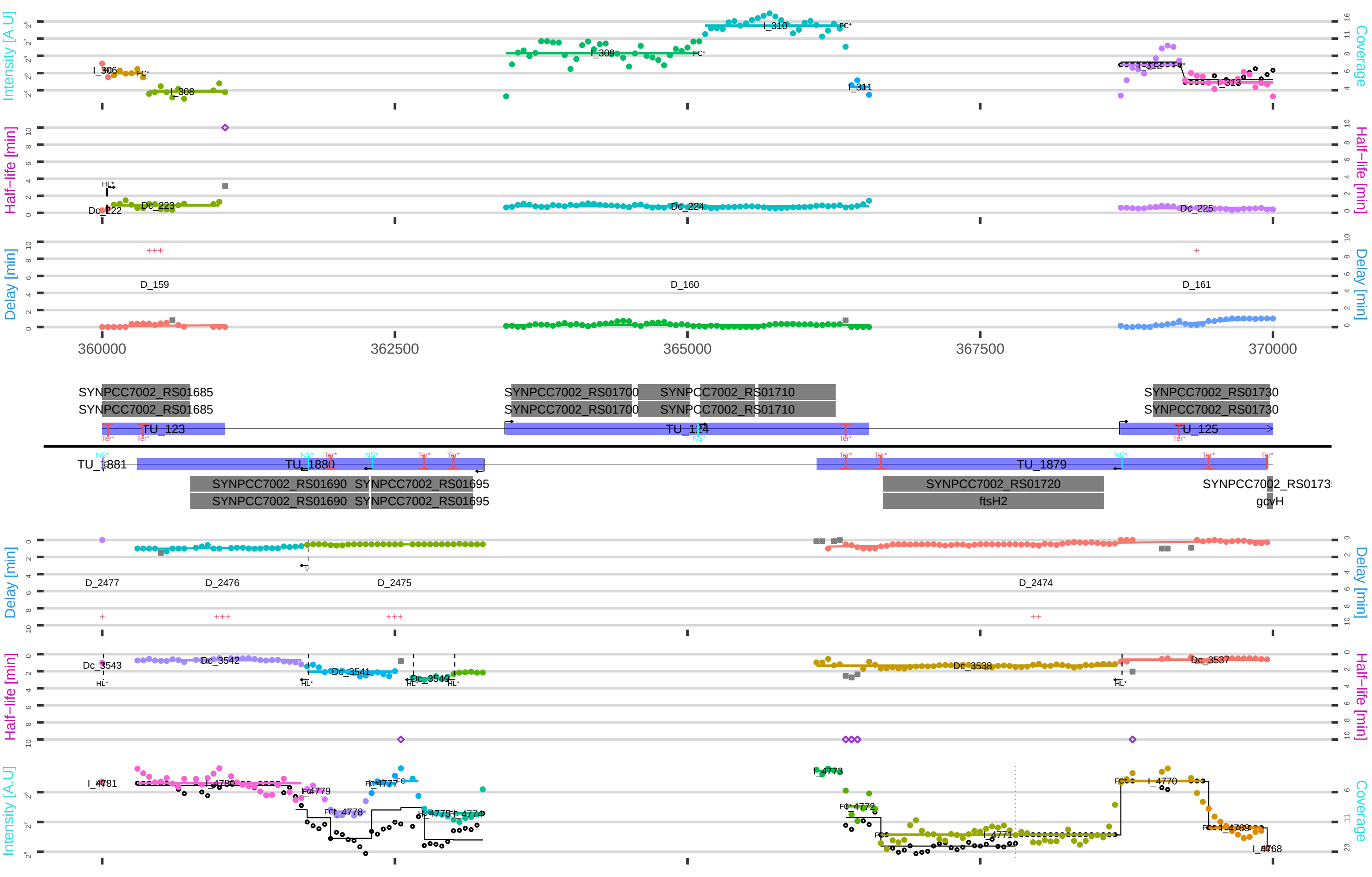

Term: termination (7), NS: new start (4), PS: pausing site (1), iTSS_I: internal starting site (0)

ID: 7400−7600; Term: termination (5), NS: new start (1), PS: pausing site (1), iTSS_I: internal starting site (0)

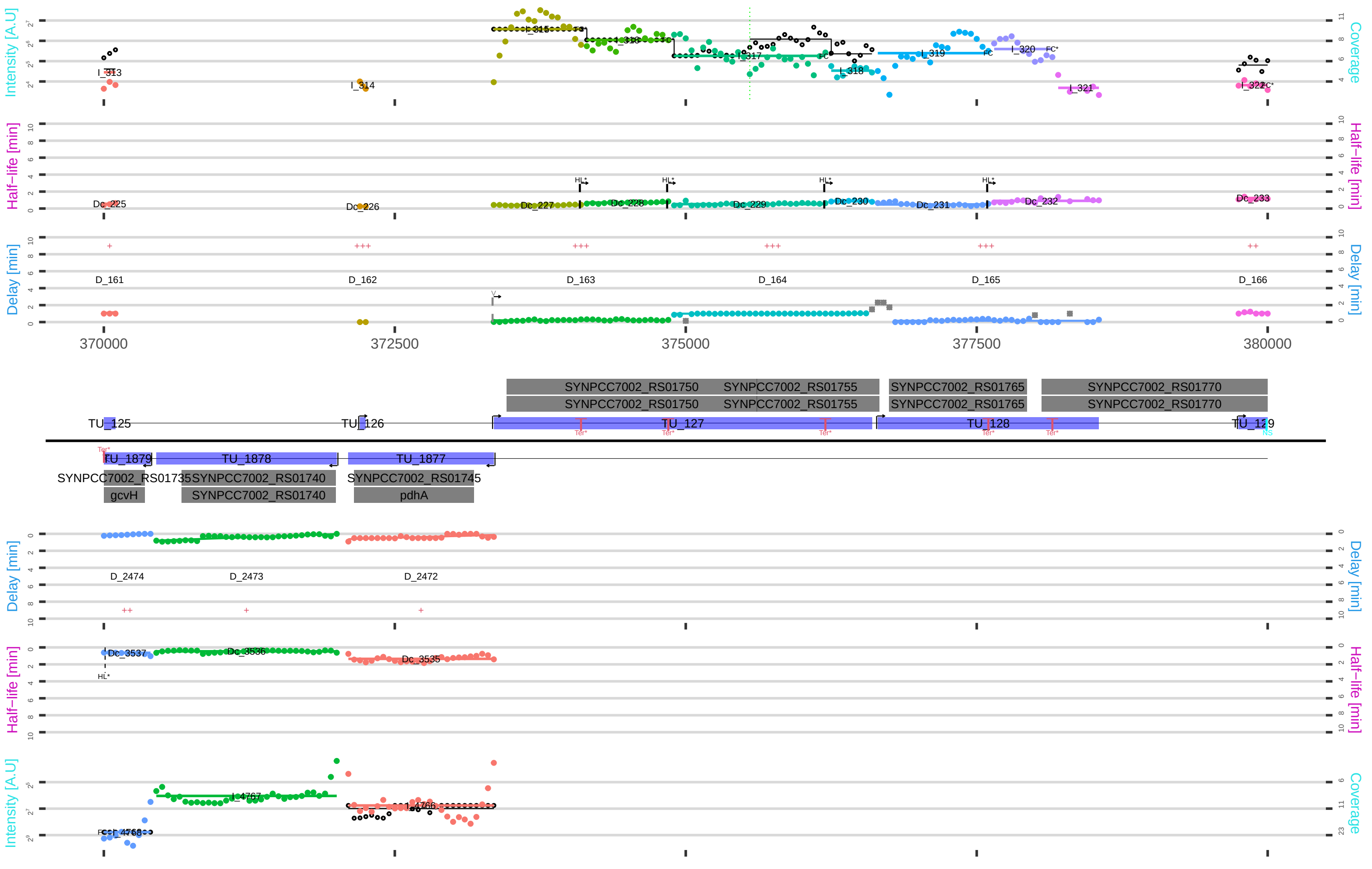

Term: termination (1), NS: new start (0), PS: pausing site (0), iTSS_I: internal starting site (0)

ID: 7600−7770; Term: termination (0), NS: new start (2), PS: pausing site (0), iTSS_I: internal starting site (0)

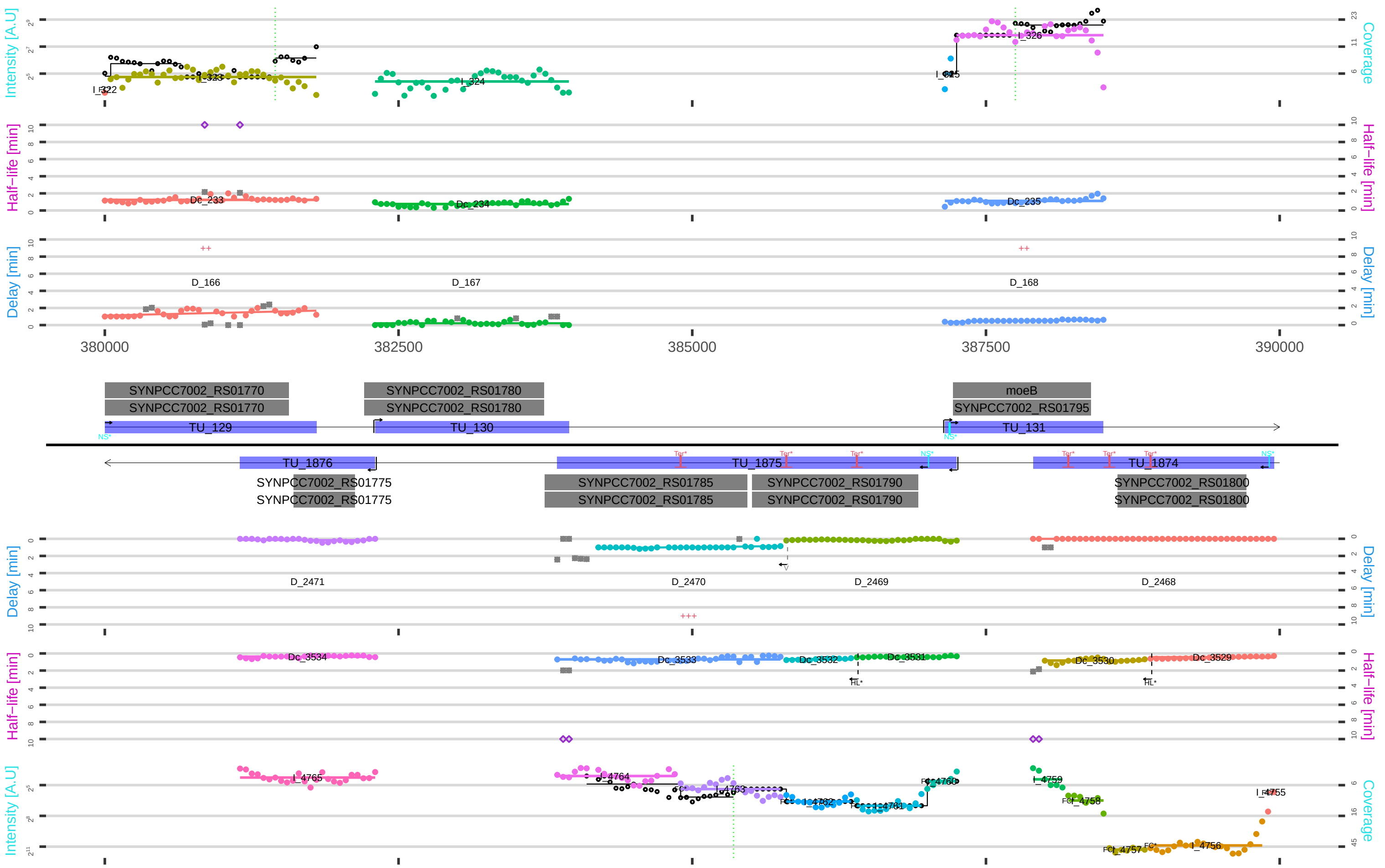

Term: termination (6), NS: new start (2), PS: pausing site (1), iTSS_I: internal starting site (0)

ID: 7803−7999; Term: termination (1), NS: new start (0), PS: pausing site (0), iTSS_I: internal starting site (0)

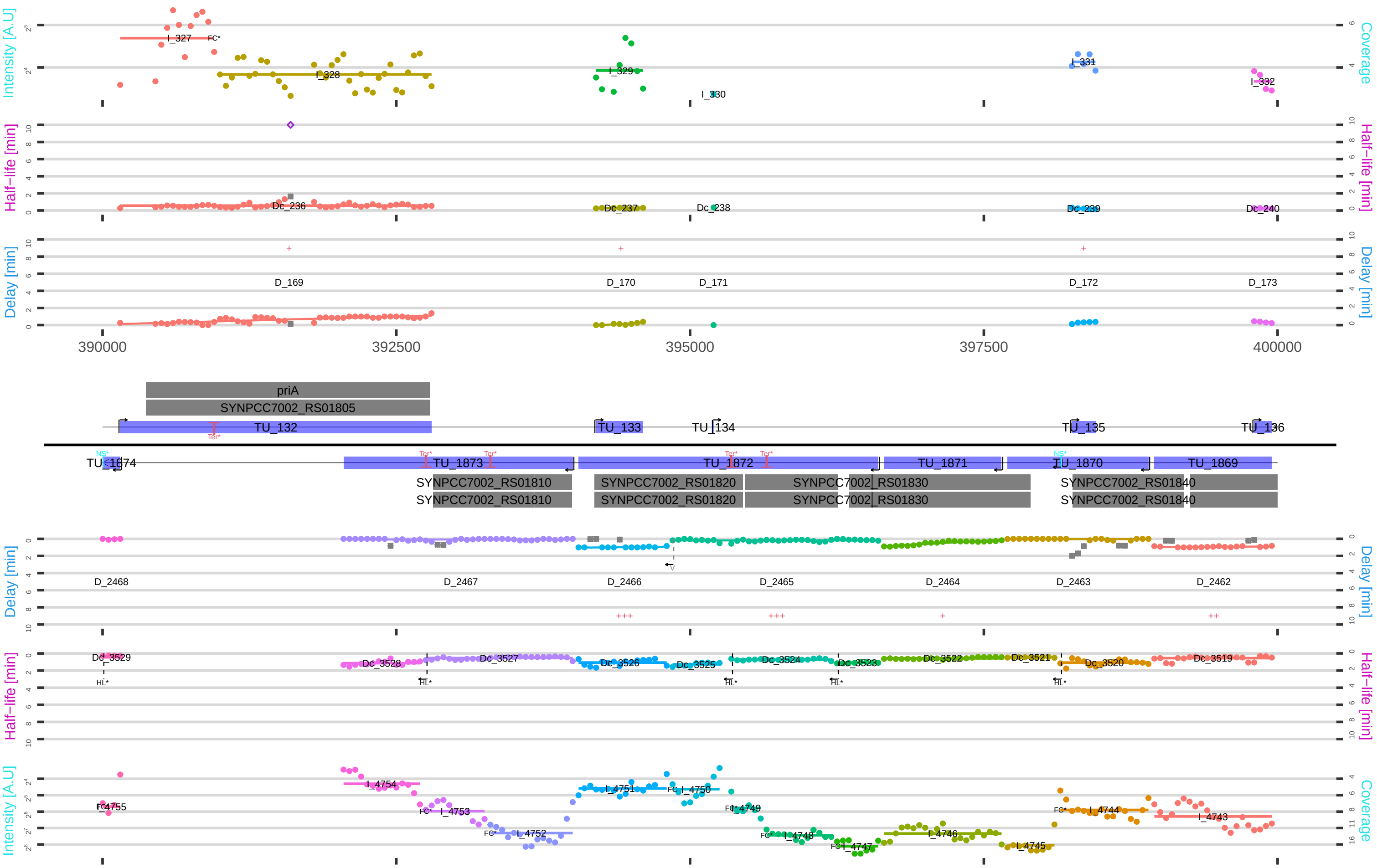

Term: termination (4), NS: new start (2), PS: pausing site (1), iTSS_I: internal starting site (0)

ID: 8035−8200; Term: termination (3), NS: new start (4), PS: pausing site (0), iTSS_I: internal starting site (0)

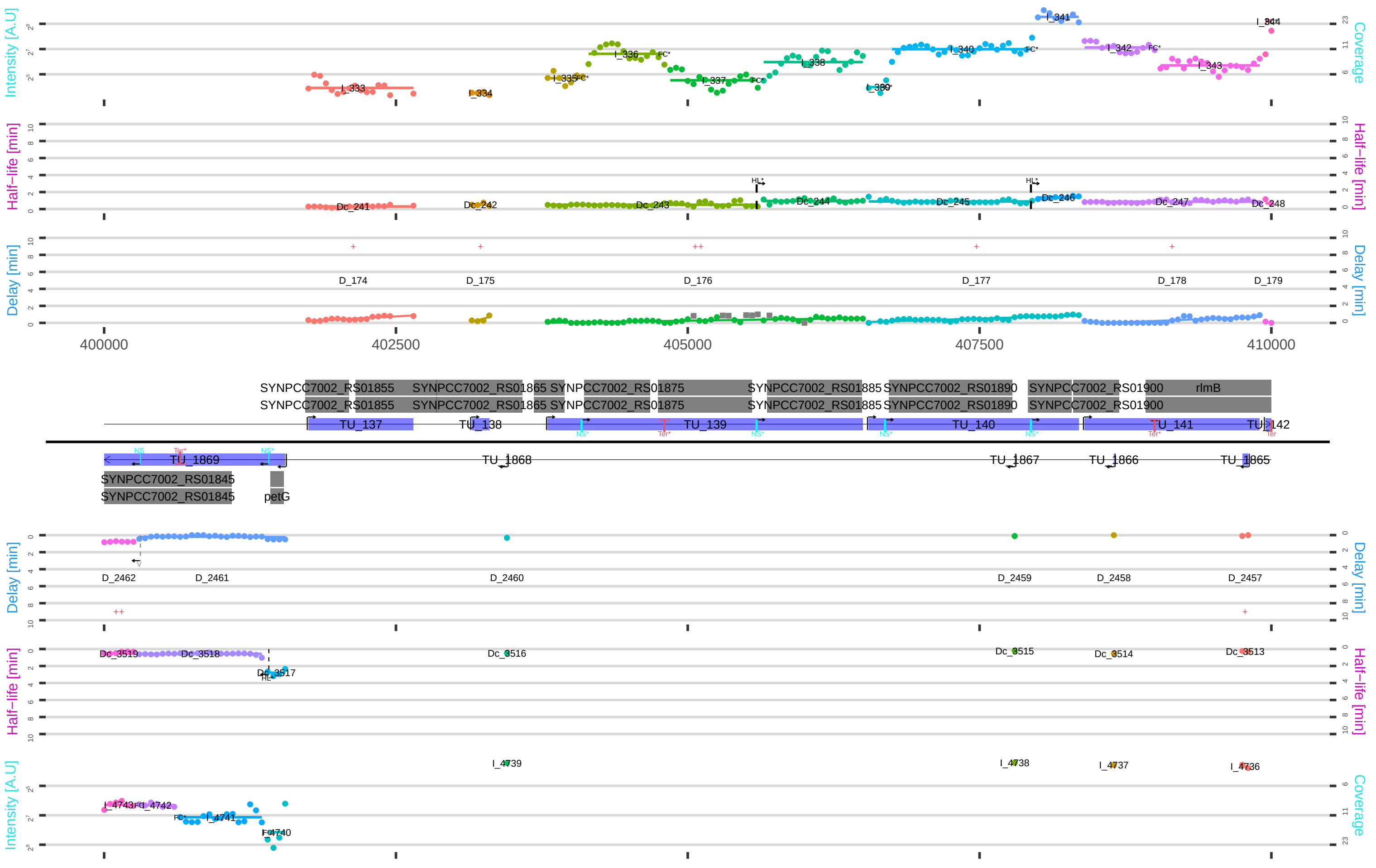

Term: termination (1), NS: new start (2), PS: pausing site (1), iTSS_I: internal starting site (0)

ID: 8200−8308; Term: termination (3), NS: new start (0), PS: pausing site (0), iTSS_I: internal starting site (0)

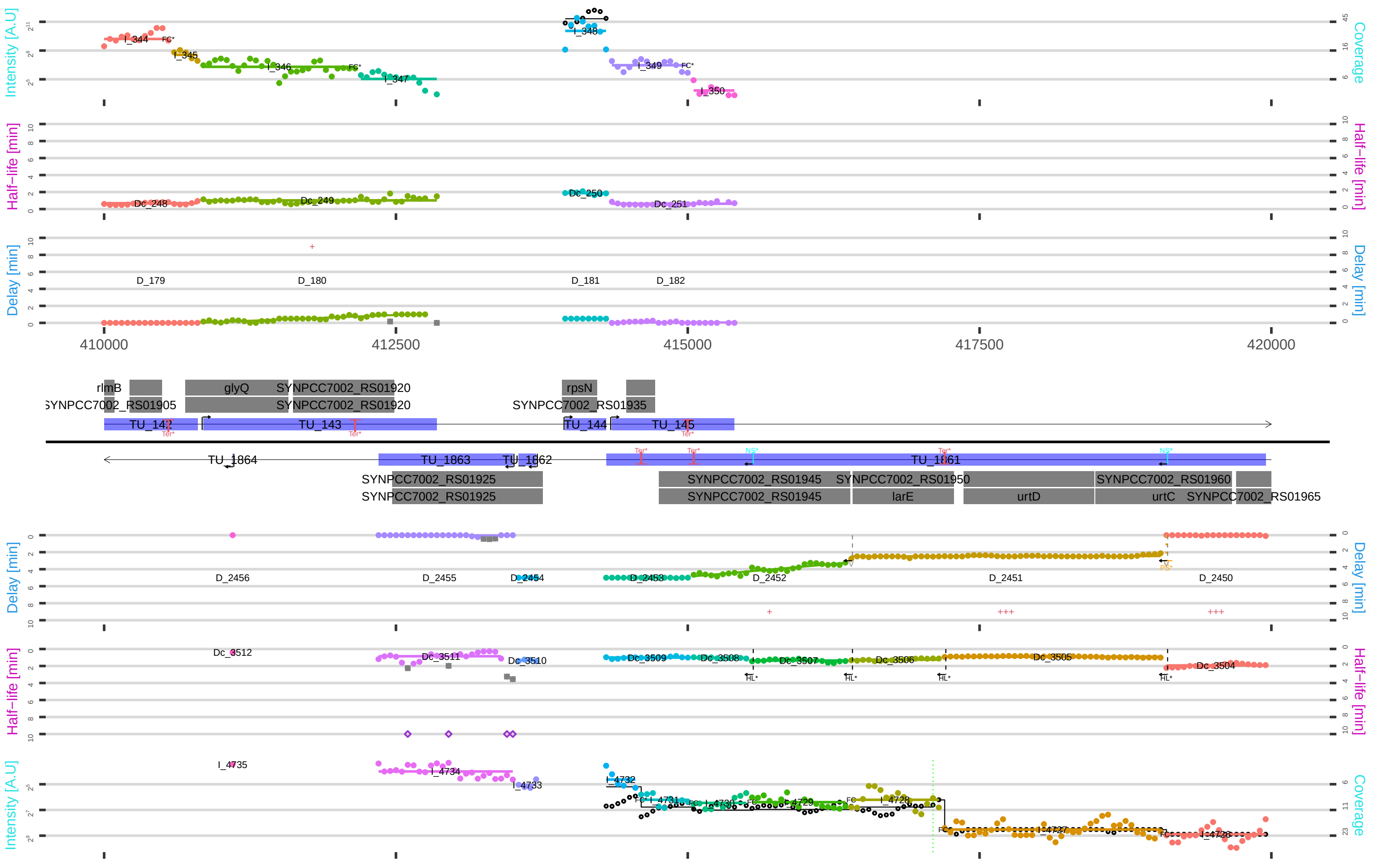

Term: termination (3), NS: new start (2), PS: pausing site (3), iTSS_I: internal starting site (0)

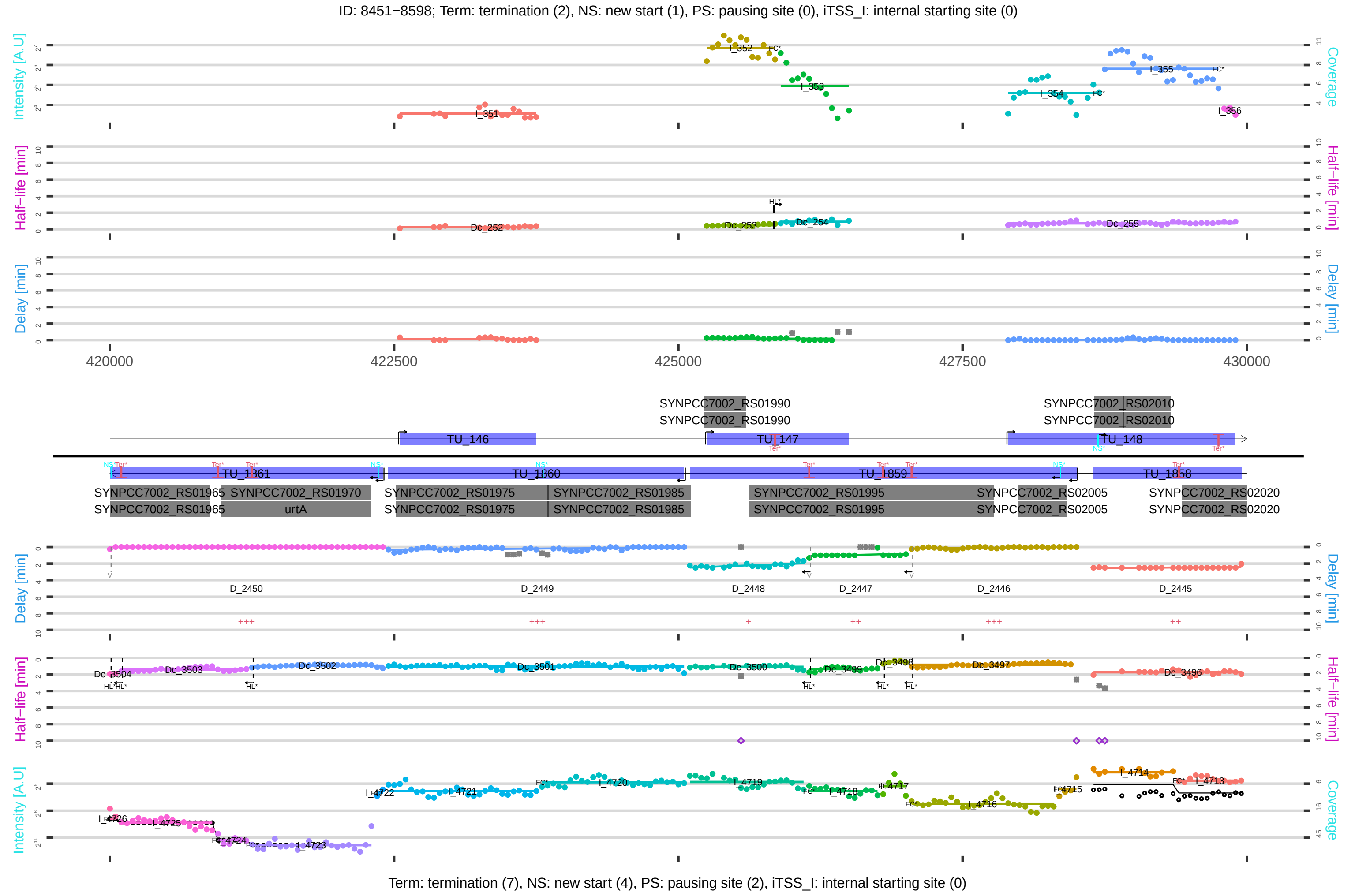

ID: 8451−8598; Term: termination (2), NS: new start (1), PS: pausing site (0), iTSS_I: internal starting site (0)
Intensity [A.U]
Coverage
Half−life [min]
Delay [min]
420000
422500
425000
427500
430000
TU_146
TU_147
TU_148
TU_1361
TU_1360
TU_1859
TU_1858
SYNPCC7002_RS01990
SYNPCC7002_RS02010
SYNPCC7002_RS01965
SYNPCC7002_RS01970
urtA
SYNPCC7002_RS01975
SYNPCC7002_RS01985
SYNPCC7002_RS01995
SYNPCC7002_RS02005
SYNPCC7002_RS02020
D_2450
D_2449
D_2448
D_2447
D_2446
D_2445
Term: termination (7), NS: new start (4), PS: pausing site (2), iTSS_I: internal starting site (0)

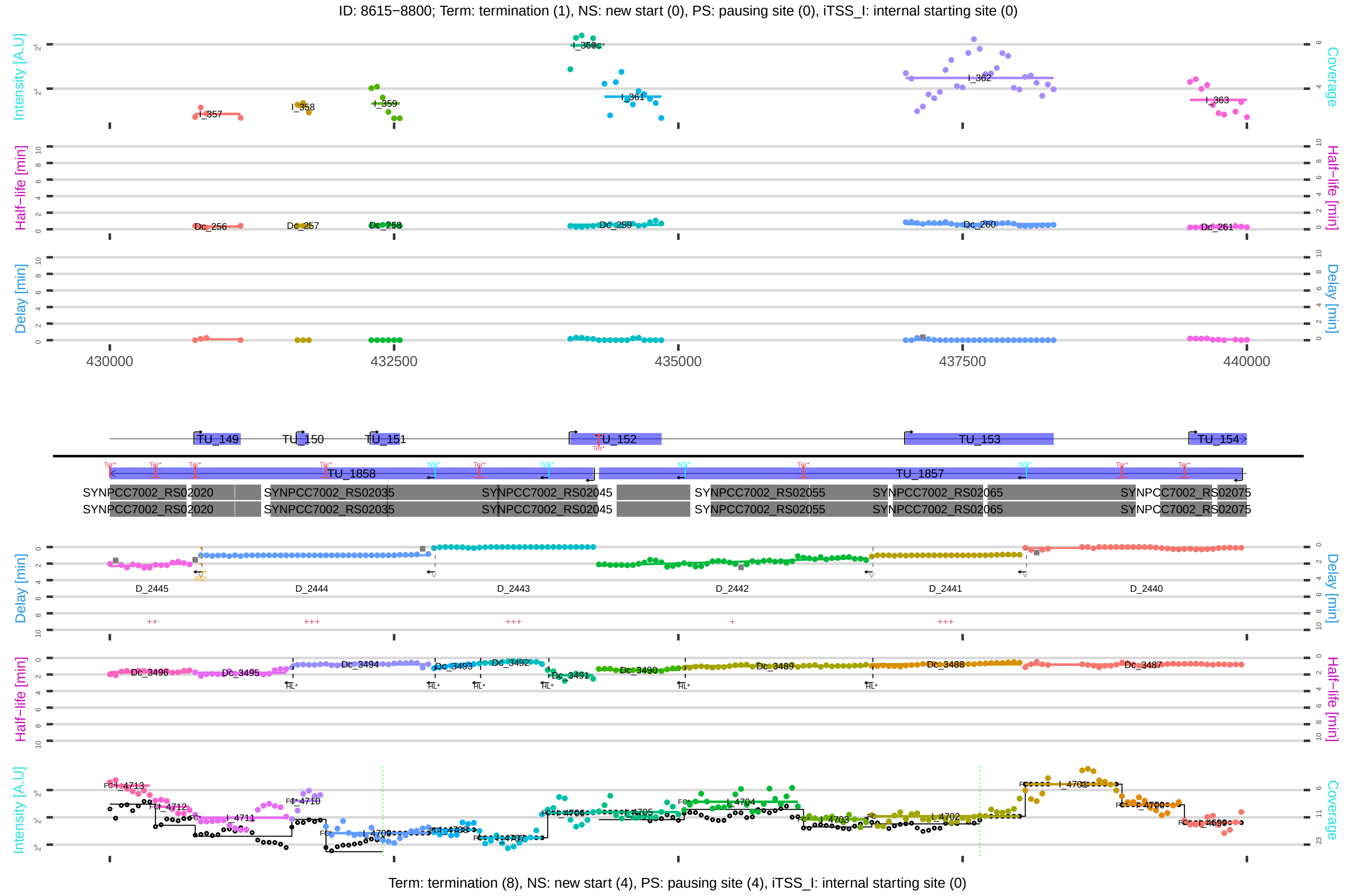

ID: 8615−8800; Term: termination (1), NS: new start (0), PS: pausing site (0), iTSS_I: internal starting site (0)
Intensity [A.U]
Coverage
Half−life [min]
Delay [min]
430000
432500
435000
437500
440000
TU_149
TU_150
TU_151
TU_152
TU_153
TU_154
TU_1858
TU_1857
SYNPCC7002_RS02020
SYNPCC7002_RS02035
SYNPCC7002_RS02045
SYNPCC7002_RS02055
SYNPCC7002_RS02065
SYNPCC7002_RS02075
D_2445
D_2444
D_2443
D_2442
D_2441
D_2440
Term: termination (8), NS: new start (4), PS: pausing site (4), iTSS_I: internal starting site (0)

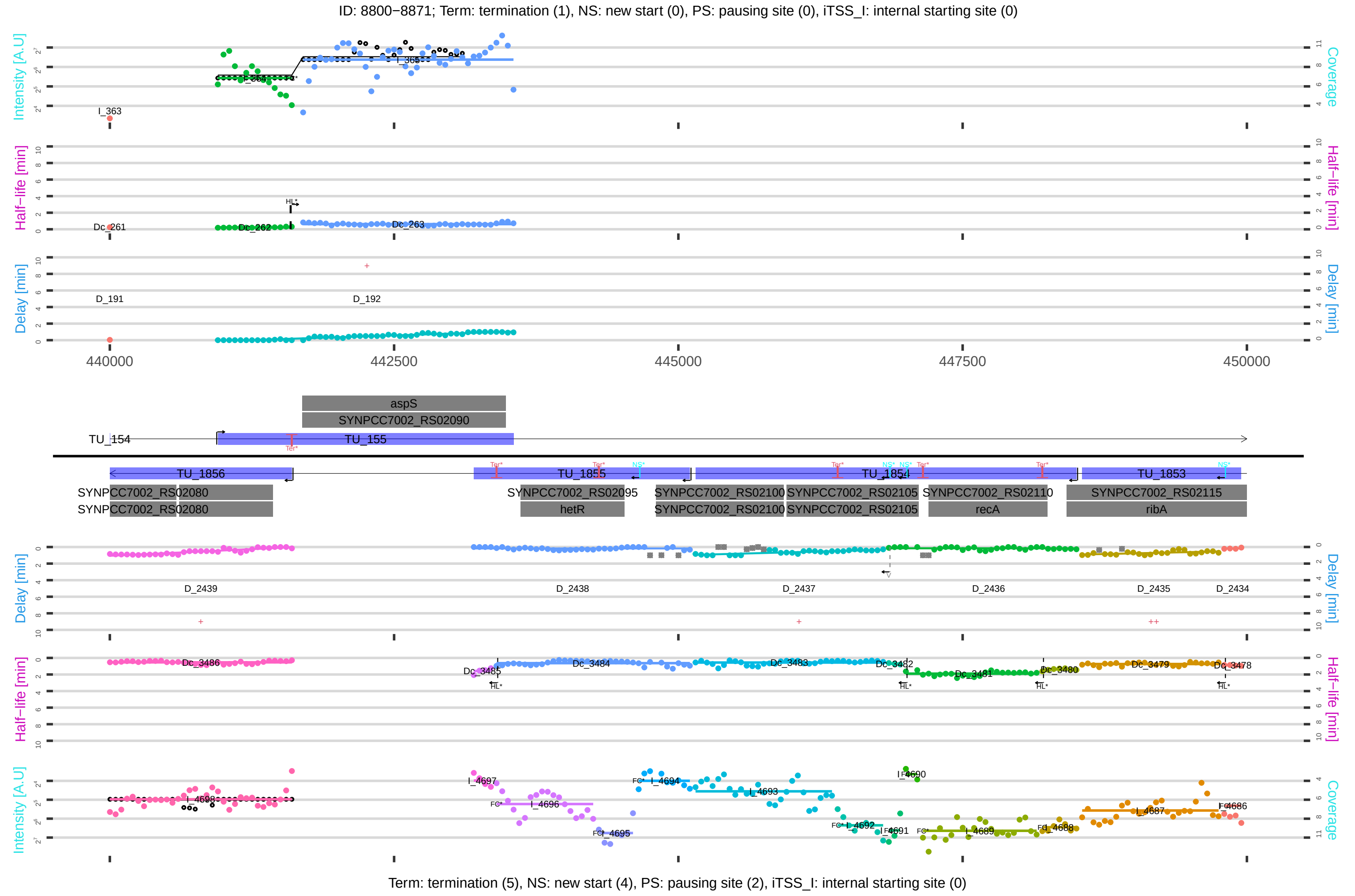
ID: 8800−8871; Term: termination (1), NS: new start (0), PS: pausing site (0), iTSS_I: internal starting site (0)
Intensity [A.U]
Coverage
Half−life [min]
Delay [min]
440000
442500
445000
447500
450000
aspS
SYNPCC7002_RS02090
TU_154
TU_155
TU_1856
TU_1855
TU_1854
TU_1853
SYNPCC7002_RS02080
SYNPCC7002_RS02095
hetR
SYNPCC7002_RS02100
SYNPCC7002_RS02105
SYNPCC7002_RS02110
recA
SYNPCC7002_RS02115
ribA
D_2439
D_2438
D_2437
D_2436
D_2435
D_2434
Term: termination (5), NS: new start (4), PS: pausing site (2), iTSS_I: internal starting site (0)

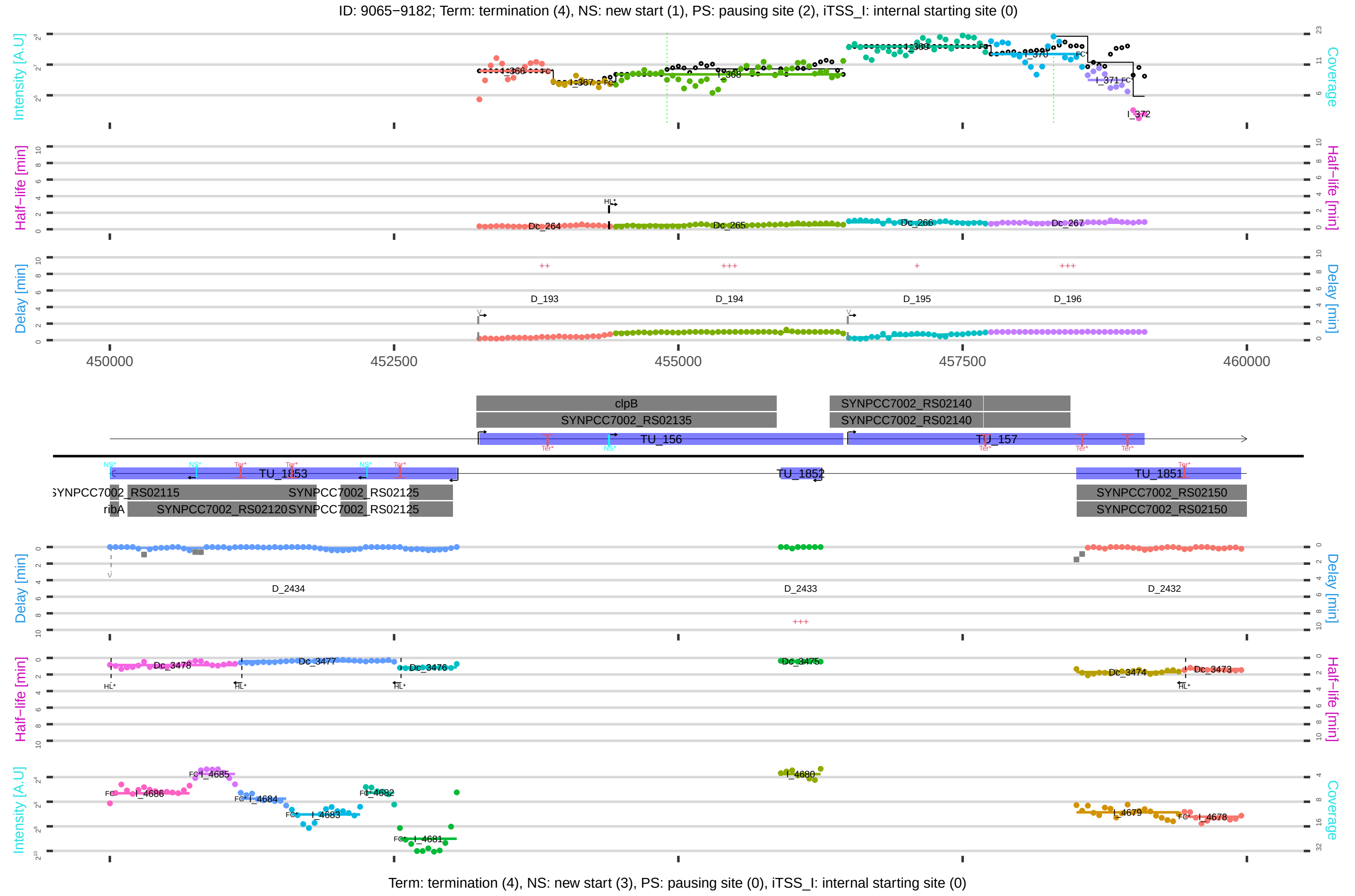
ID: 9065−9182; Term: termination (4), NS: new start (1), PS: pausing site (2), iTSS_I: internal starting site (0)
Intensity [A.U]
Coverage
Half−life [min]
Delay [min]
450000
452500
455000
457500
460000
clpB
SYNPCC7002_RS02135
SYNPCC7002_RS02140
TU_156
TU_157
TU_1853
TU_1852
TU_1851
SYNPCC7002_RS02115
SYNPCC7002_RS02120
SYNPCC7002_RS02125
SYNPCC7002_RS02150
ribA
D_193
D_194
D_195
D_196
D_2434
D_2433
D_2432
Term: termination (4), NS: new start (3), PS: pausing site (0), iTSS_I: internal starting site (0)

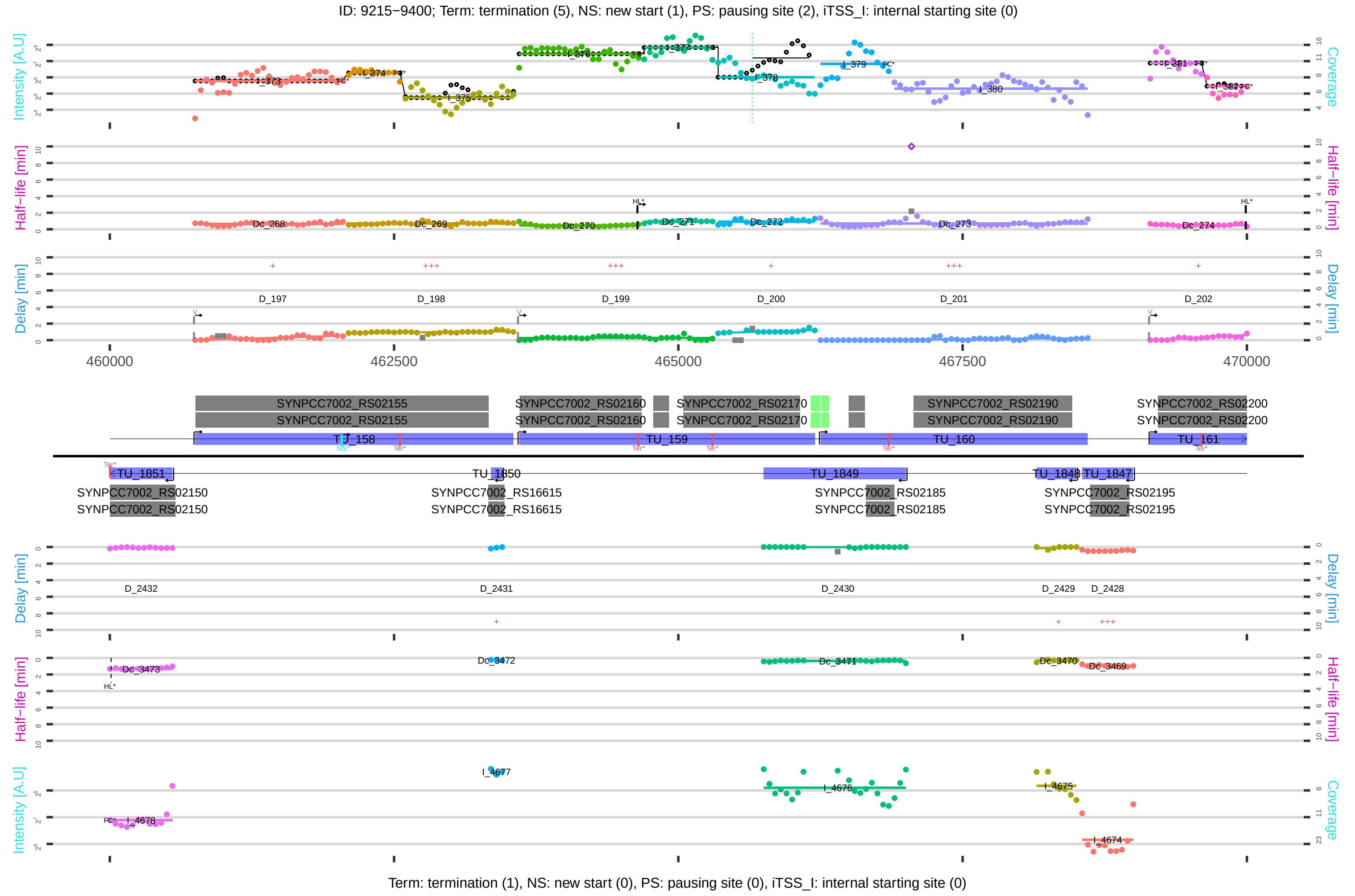

ID: 9215−9400; Term: termination (5), NS: new start (1), PS: pausing site (2), iTSS_I: internal starting site (0)
Intensity [A.U]
Coverage
Half−life [min]
Delay [min]
I_373
I_374
I_375
I_376
I_377
I_378
I_379
I_380
I_381
I_382
Dc_268
Dc_269
Dc_270
Dc_271
Dc_272
Dc_273
Dc_274
D_197
D_198
D_199
D_200
D_201
D_202
460000
462500
465000
467500
470000
SYNPCC7002_RS02155
SYNPCC7002_RS02160
SYNPCC7002_RS02170
SYNPCC7002_RS02190
SYNPCC7002_RS02200
TU_158
TU_159
TU_160
TU_161
TU_1851
TU_1850
TU_1849
TU_1848
TU_1847
SYNPCC7002_RS02150
SYNPCC7002_RS16615
SYNPCC7002_RS02185
SYNPCC7002_RS02195
D_2432
D_2431
D_2430
D_2429
D_2428
Dc_3473
Dc_3472
Dc_3471
Dc_3470
Dc_3469
I_4678
I_4677
I_4676
I_4675
I_4674
Term: termination (1), NS: new start (0), PS: pausing site (0), iTSS_I: internal starting site (0)

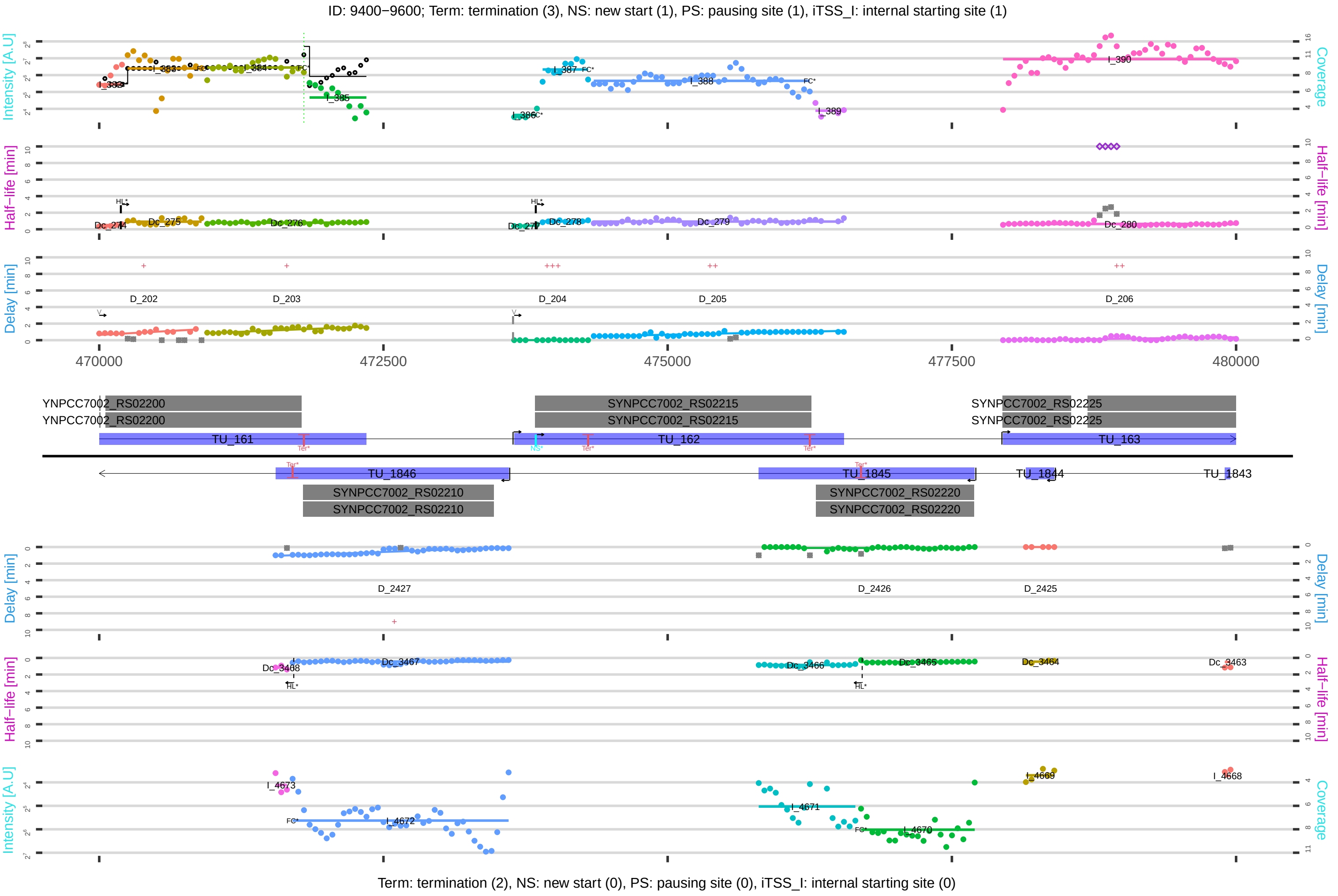
ID: 9400−9600; Term: termination (3), NS: new start (1), PS: pausing site (1), iTSS_I: internal starting site (1)
Intensity [A.U]
Coverage
Half−life [min]
Delay [min]
470000
472500
475000
477500
480000
TU_161
TU_162
TU_163
TU_1846
TU_1845
TU_1844
TU_1843
SYNPCC7002_RS02215
SYNPCC7002_RS02225
SYNPCC7002_RS02210
SYNPCC7002_RS02220
D_202
D_203
D_204
D_205
D_206
D_2427
D_2426
D_2425
Term: termination (2), NS: new start (0), PS: pausing site (0), iTSS_I: internal starting site (0)

ID: 9600−9763; Term: termination (3), NS: new start (1), PS: pausing site (0), iTSS_I: internal starting site (0)

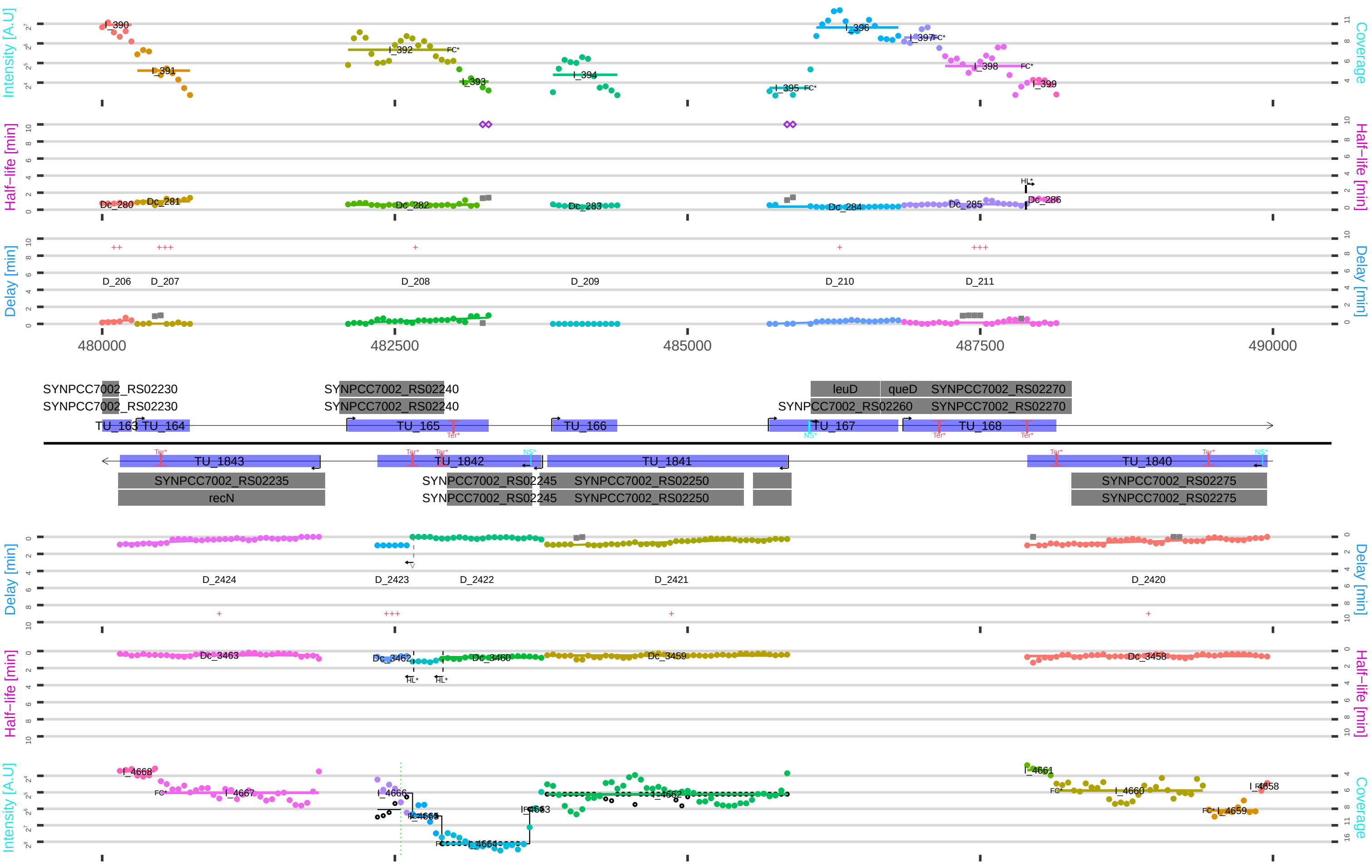

Term: termination (5), NS: new start (2), PS: pausing site (1), iTSS_I: internal starting site (0)

ID: 9803−9982; Term: termination (3), NS: new start (0), PS: pausing site (1), iTSS_I: internal starting site (0)

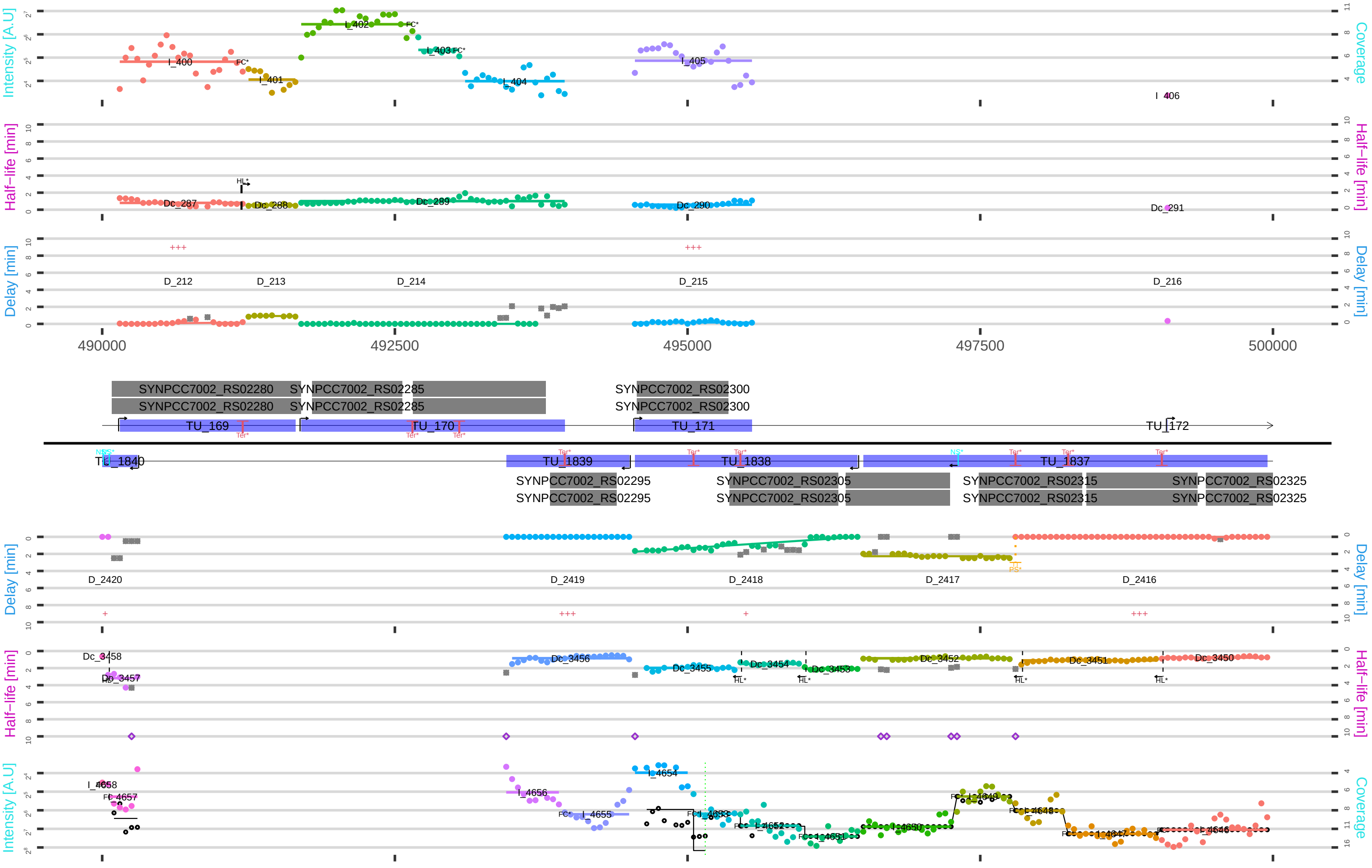

Term: termination (6), NS: new start (3), PS: pausing site (1), iTSS_I: internal starting site (0)

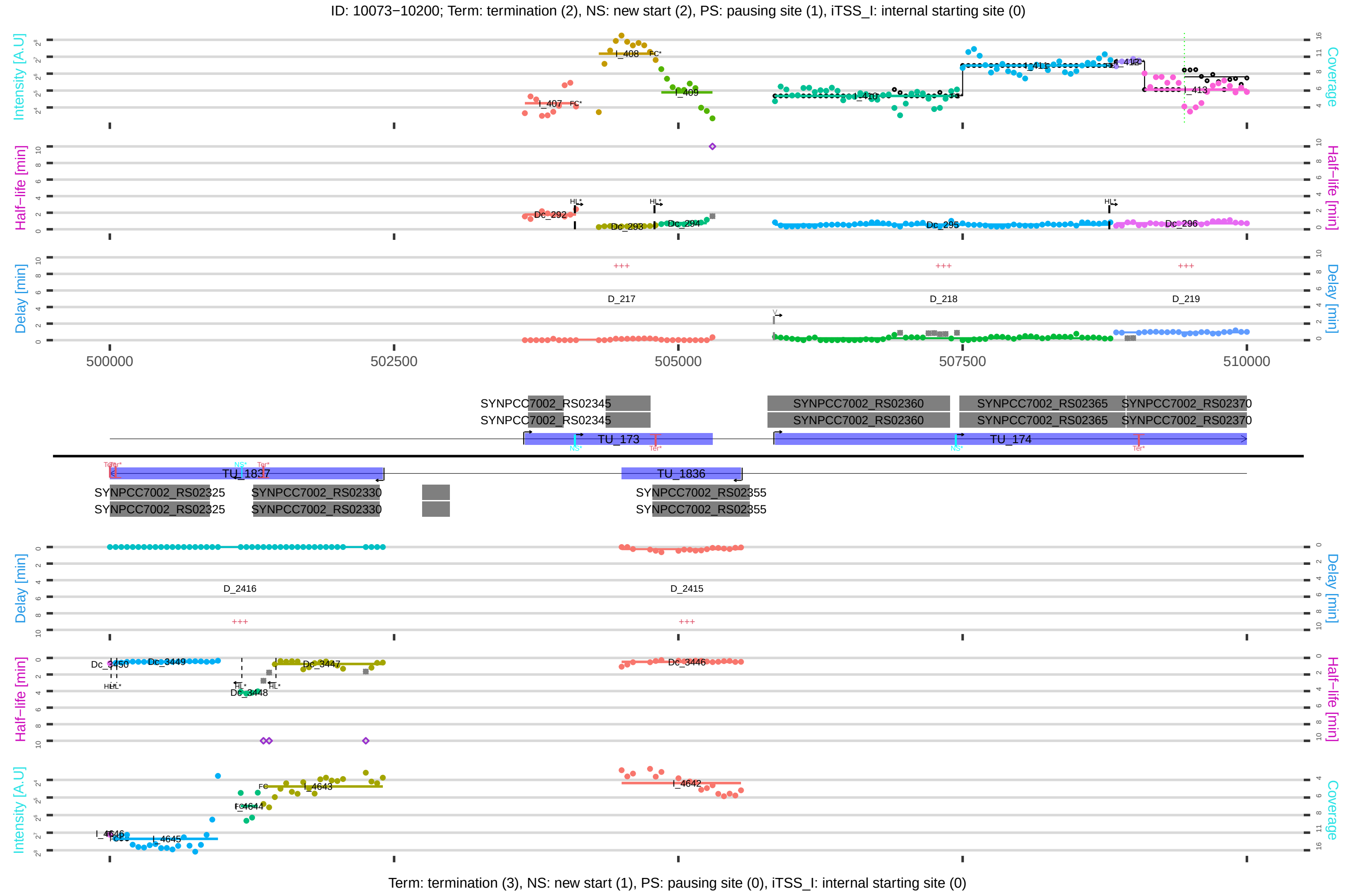
ID: 10073−10200; Term: termination (2), NS: new start (2), PS: pausing site (1), iTSS_I: internal starting site (0)
Intensity [A.U]
Coverage
Half−life [min]
Delay [min]
500000
502500
505000
507500
510000
SYNPCC7002_RS02345
SYNPCC7002_RS02360
SYNPCC7002_RS02365
SYNPCC7002_RS02370
TU_173
TU_174
TU_1837
TU_1836
SYNPCC7002_RS02325
SYNPCC7002_RS02330
SYNPCC7002_RS02355
D_217
D_218
D_219
D_2416
D_2415
Dc_292
Dc_293
Dc_294
Dc_295
Dc_296
Dc_3449
Dc_3447
Dc_3448
Dc_3446
I_407
I_408
I_409
I_410
I_411
I_413
I_4642
I_4643
I_4644
I_4645
Term: termination (3), NS: new start (1), PS: pausing site (0), iTSS_I: internal starting site (0)

ID: 10200−10351; Term: termination (3), NS: new start (0), PS: pausing site (0), iTSS_I: internal starting site (0)

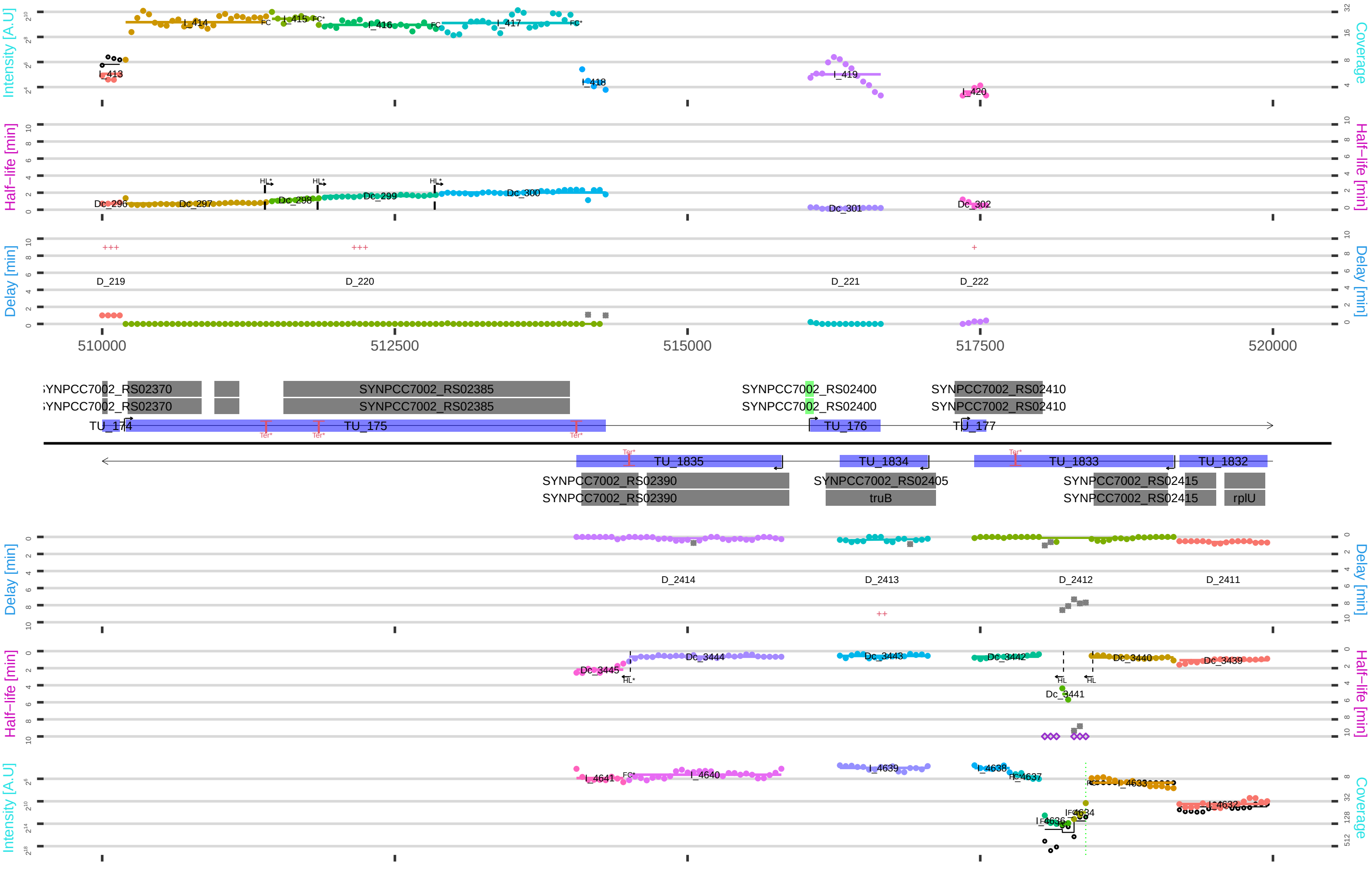

Term: termination (2), NS: new start (0), PS: pausing site (0), iTSS_I: internal starting site (0)

ID: 10403−10600; Term: termination (4), NS: new start (3), PS: pausing site (1), iTSS_I: internal starting site (0)

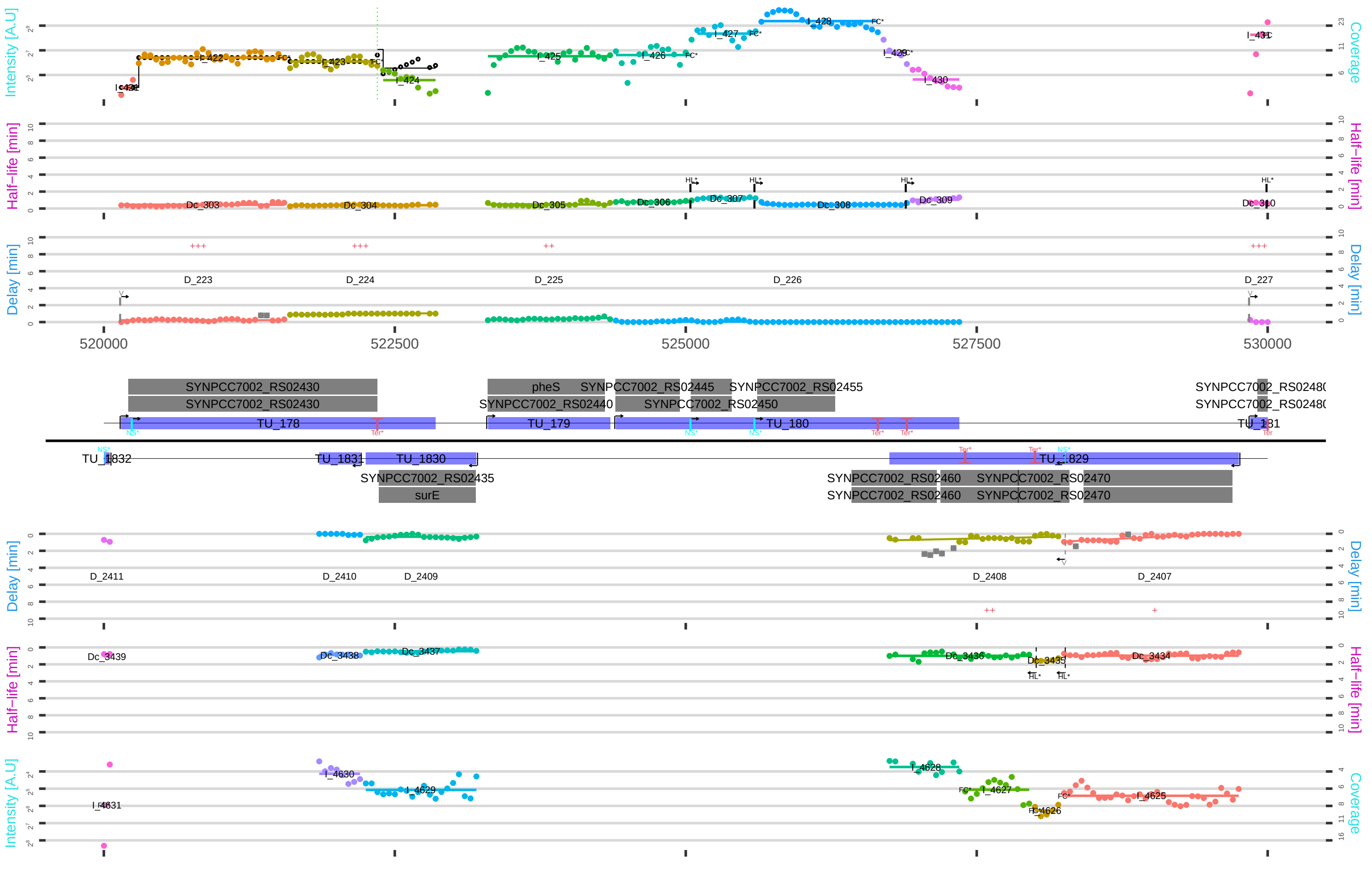

Term: termination (2), NS: new start (2), PS: pausing site (0), iTSS_I: internal starting site (1)

ID: 10600−10800; Term: termination (5), NS: new start (3), PS: pausing site (2), iTSS_I: internal starting site (0)

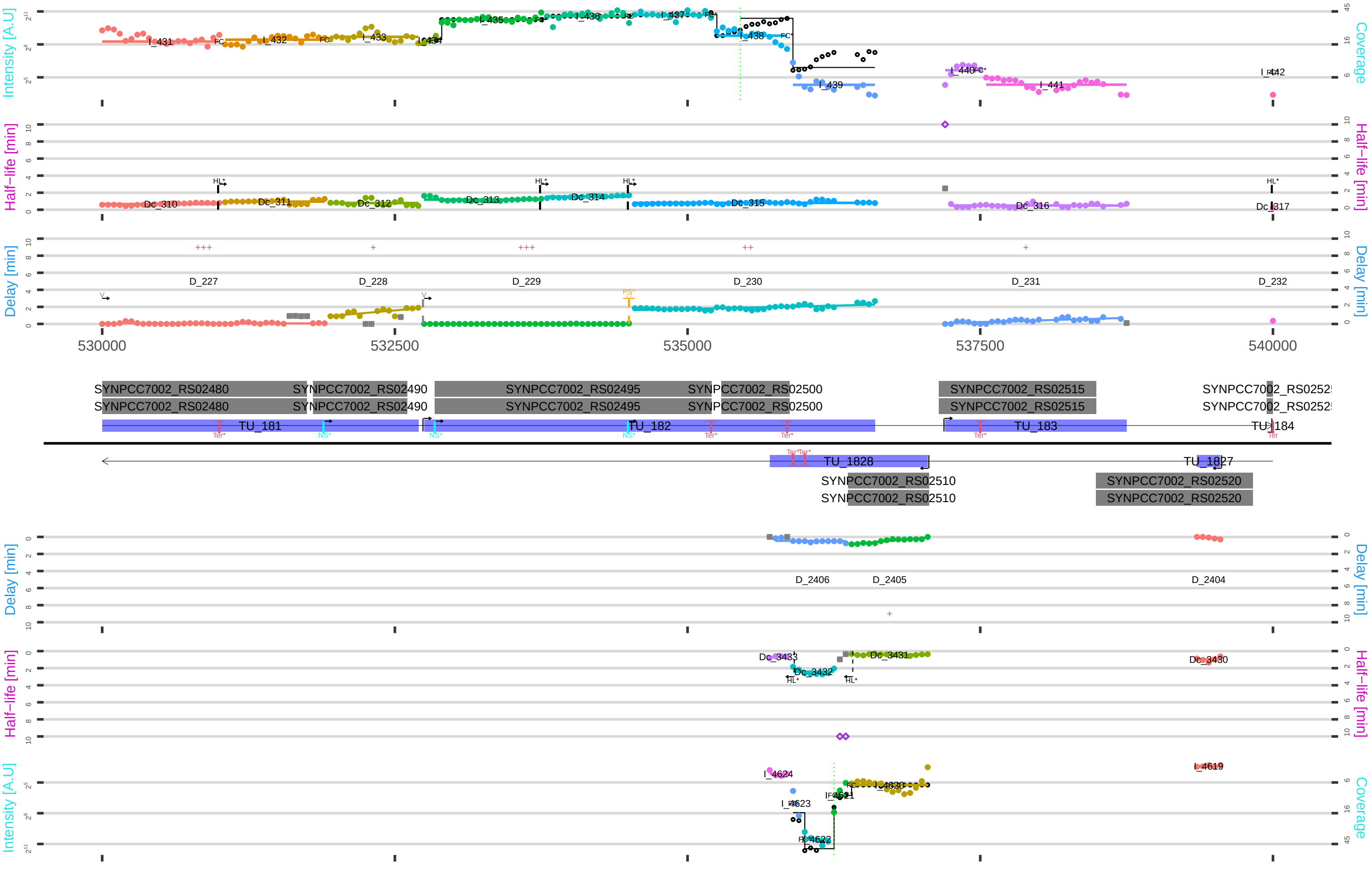

Term: termination (2), NS: new start (0), PS: pausing site (0), iTSS_I: internal starting site (1)

ID: 10800−11000; Term: termination (6), NS: new start (1), PS: pausing site (2), iTSS_I: internal starting site (0)

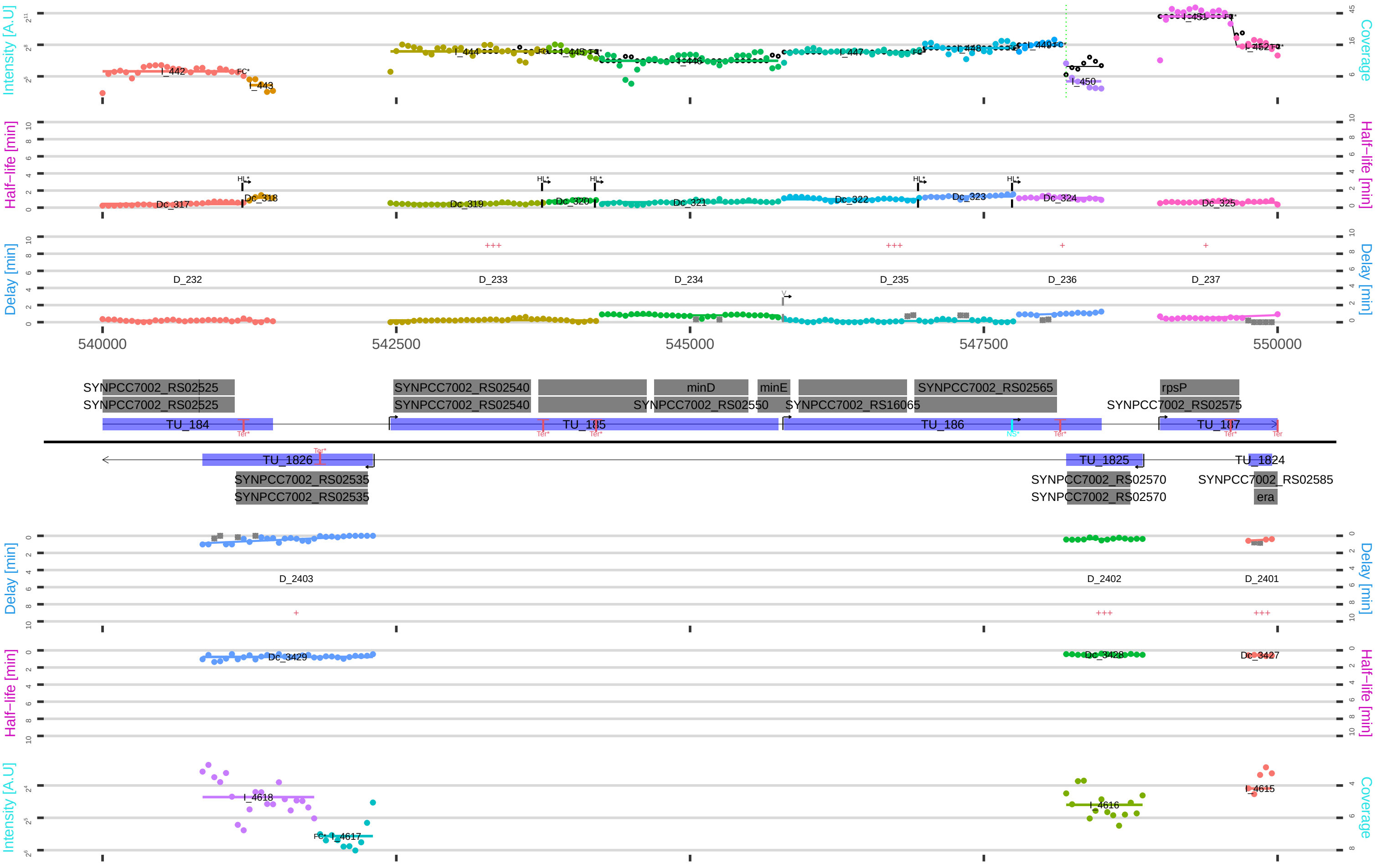

Term: termination (1), NS: new start (0), PS: pausing site (0), iTSS_I: internal starting site (0)

ID: 11000−11182; Term: termination (4), NS: new start (1), PS: pausing site (0), iTSS_I: internal starting site (0)

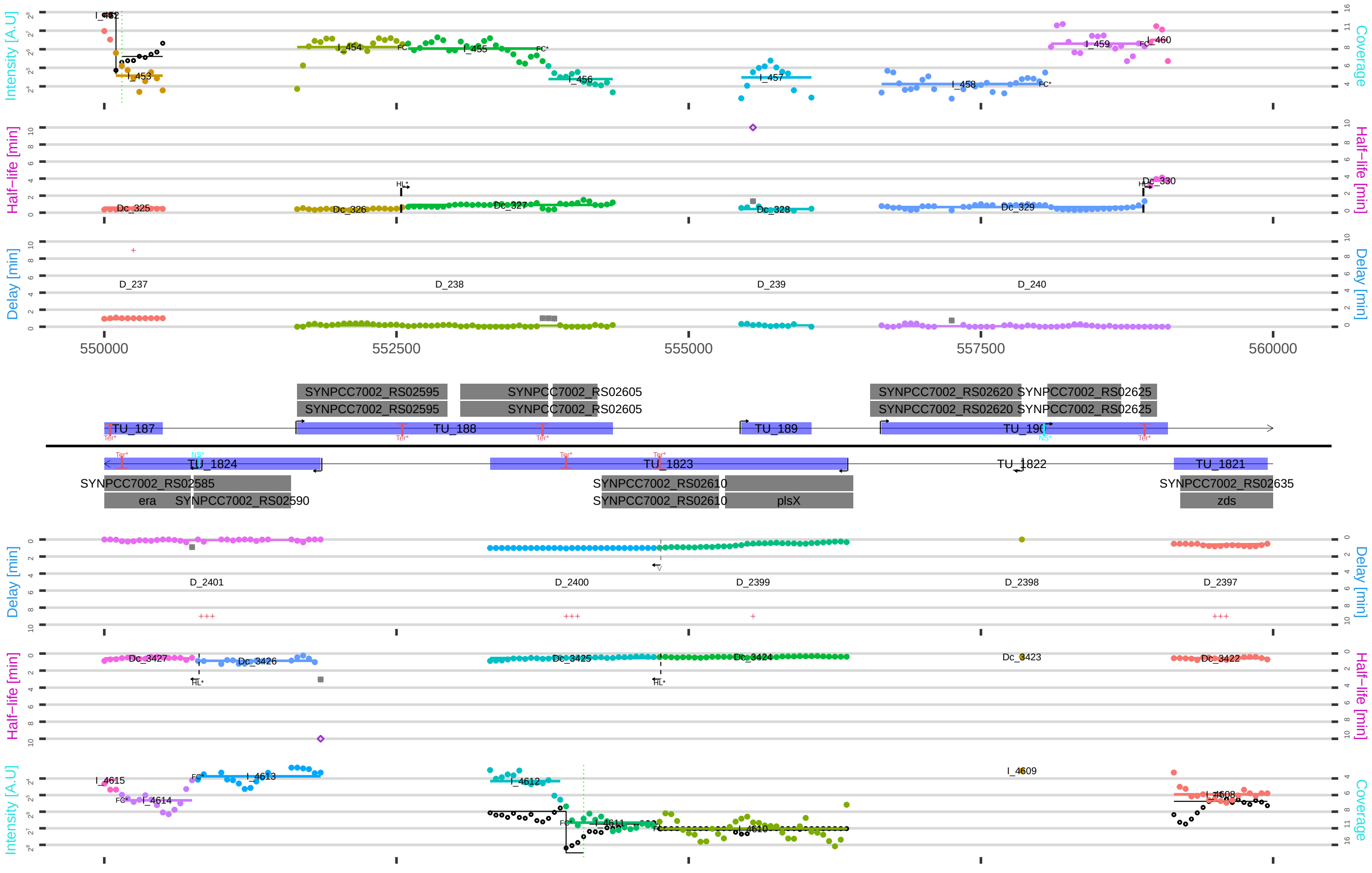

Term: termination (3), NS: new start (1), PS: pausing site (1), iTSS_I: internal starting site (0)

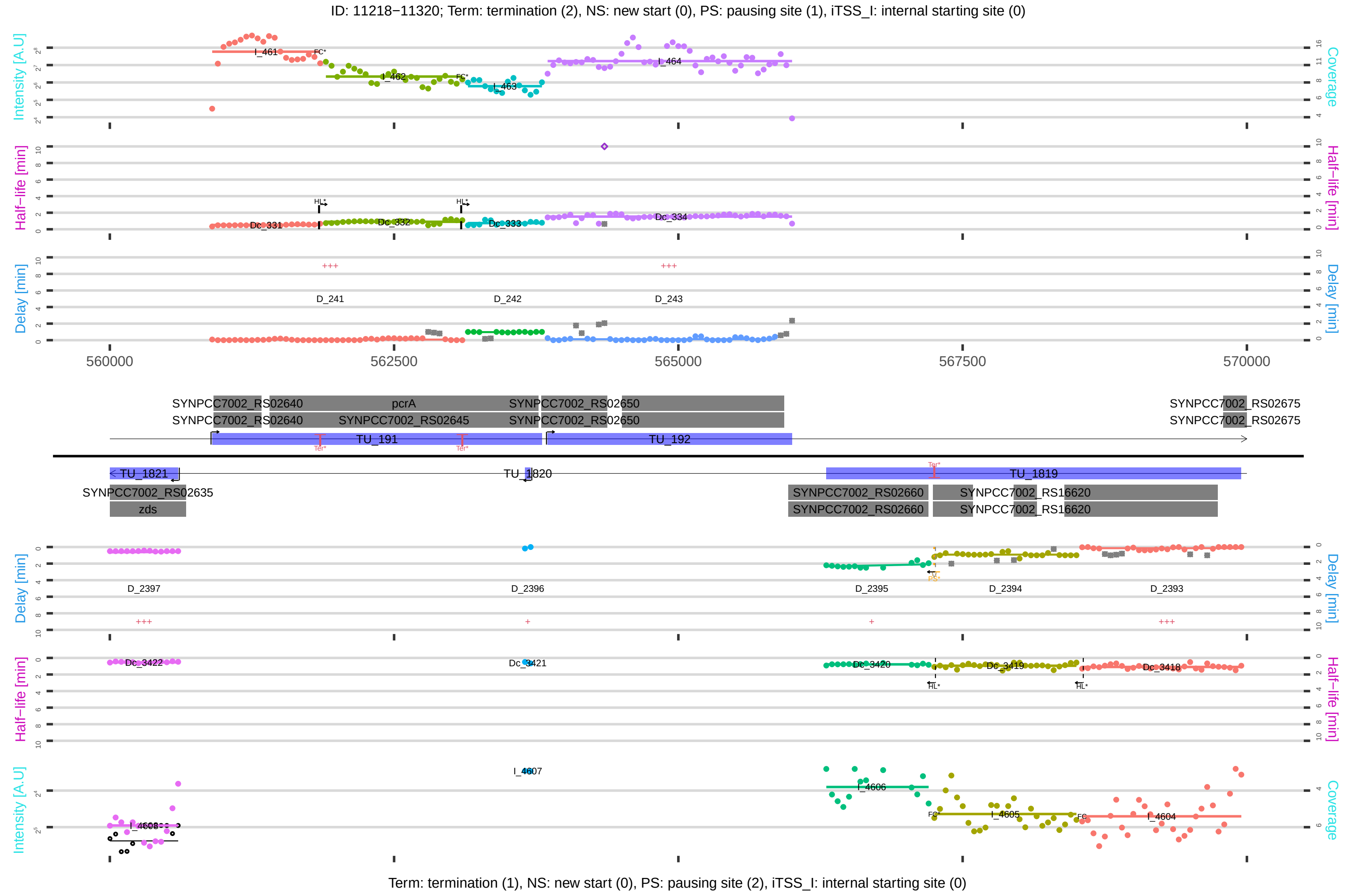
ID: 11218−11320; Term: termination (2), NS: new start (0), PS: pausing site (1), iTSS_I: internal starting site (0)
Intensity [A.U]
Coverage
Half−life [min]
Delay [min]
I_461
I_462
I_463
I_464
Dc_331
Dc_332
Dc_333
Dc_334
D_241
D_242
D_243
560000
562500
565000
567500
570000
SYNPCC7002_RS02640
pcrA
SYNPCC7002_RS02645
SYNPCC7002_RS02650
SYNPCC7002_RS02675
TU_191
TU_192
TU_1821
TU_1820
TU_1819
SYNPCC7002_RS02635
zds
SYNPCC7002_RS02660
SYNPCC7002_RS16620
D_2397
D_2396
D_2395
D_2394
D_2393
Dc_3422
Dc_3421
Dc_3420
Dc_3419
Dc_3418
I_4607
I_4606
I_4605
I_4604
I_4608
Term: termination (1), NS: new start (0), PS: pausing site (2), iTSS_I: internal starting site (0)

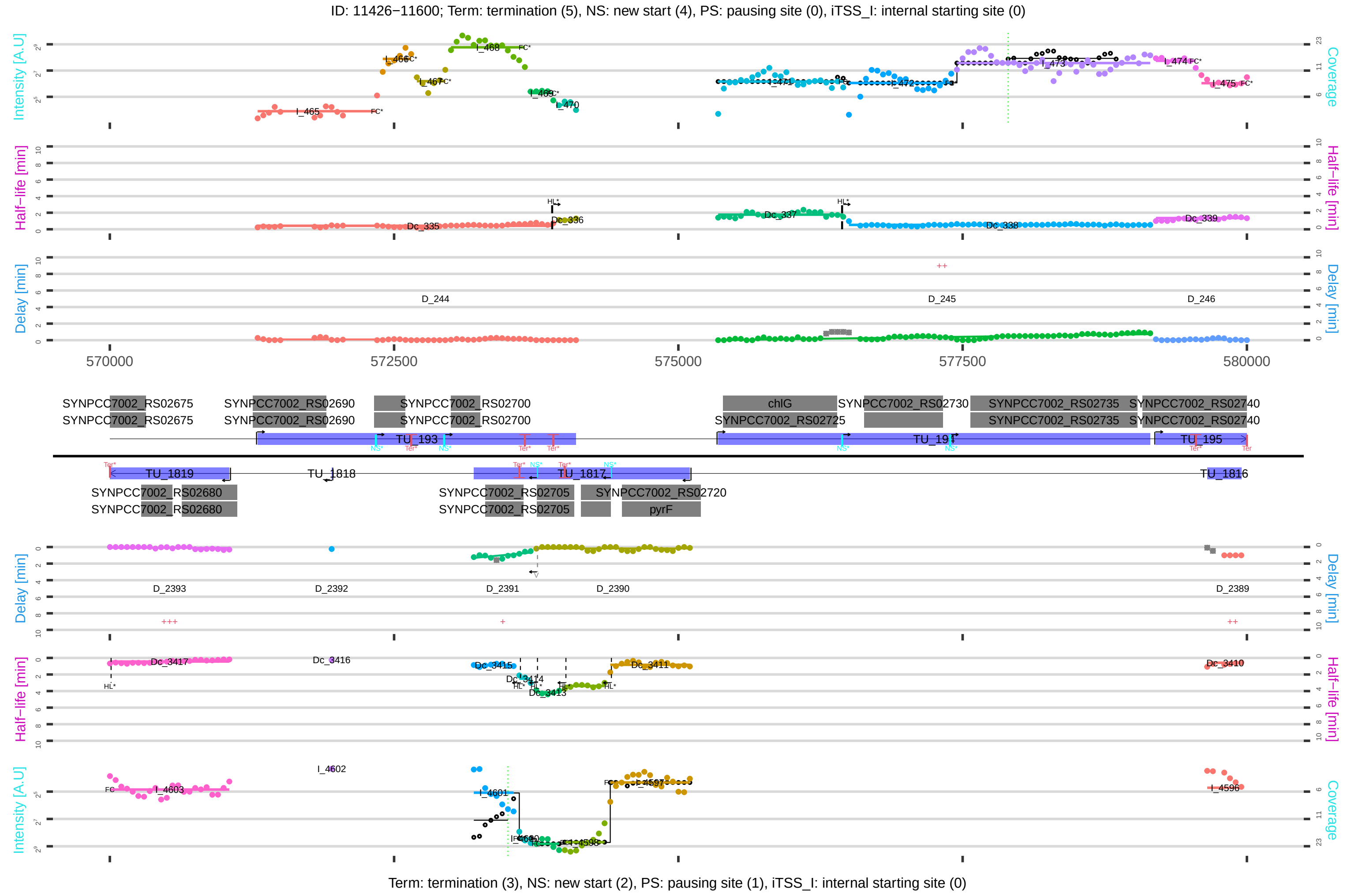

ID: 11426−11600; Term: termination (5), NS: new start (4), PS: pausing site (0), iTSS_I: internal starting site (0)
Intensity [A.U]
Coverage
Half−life [min]
Delay [min]
I_465
I_466
I_467
I_468
I_469
I_470
I_471
I_472
I_473
I_474
I_475
Dc_335
Dc_336
Dc_337
Dc_338
Dc_339
D_244
D_245
D_246
570000
572500
575000
577500
580000
SYNPCC7002_RS02675
SYNPCC7002_RS02690
SYNPCC7002_RS02700
chlG
SYNPCC7002_RS02730
SYNPCC7002_RS02735
SYNPCC7002_RS02740
SYNPCC7002_RS02725
TU_193
TU_194
TU_195
TU_1819
TU_1818
TU_1817
TU_1816
SYNPCC7002_RS02680
SYNPCC7002_RS02705
SYNPCC7002_RS02720
pyrF
D_2393
D_2392
D_2391
D_2390
D_2389
Dc_3417
Dc_3416
Dc_3415
Dc_3414
Dc_3413
Dc_3411
Dc_3410
I_4602
I_4603
I_4601
I_4600
I_4598
I_4597
I_4596
Term: termination (3), NS: new start (2), PS: pausing site (1), iTSS_I: internal starting site (0)

ID: 11600−11799; Term: termination (5), NS: new start (1), PS: pausing site (0), iTSS_I: internal starting site (0)

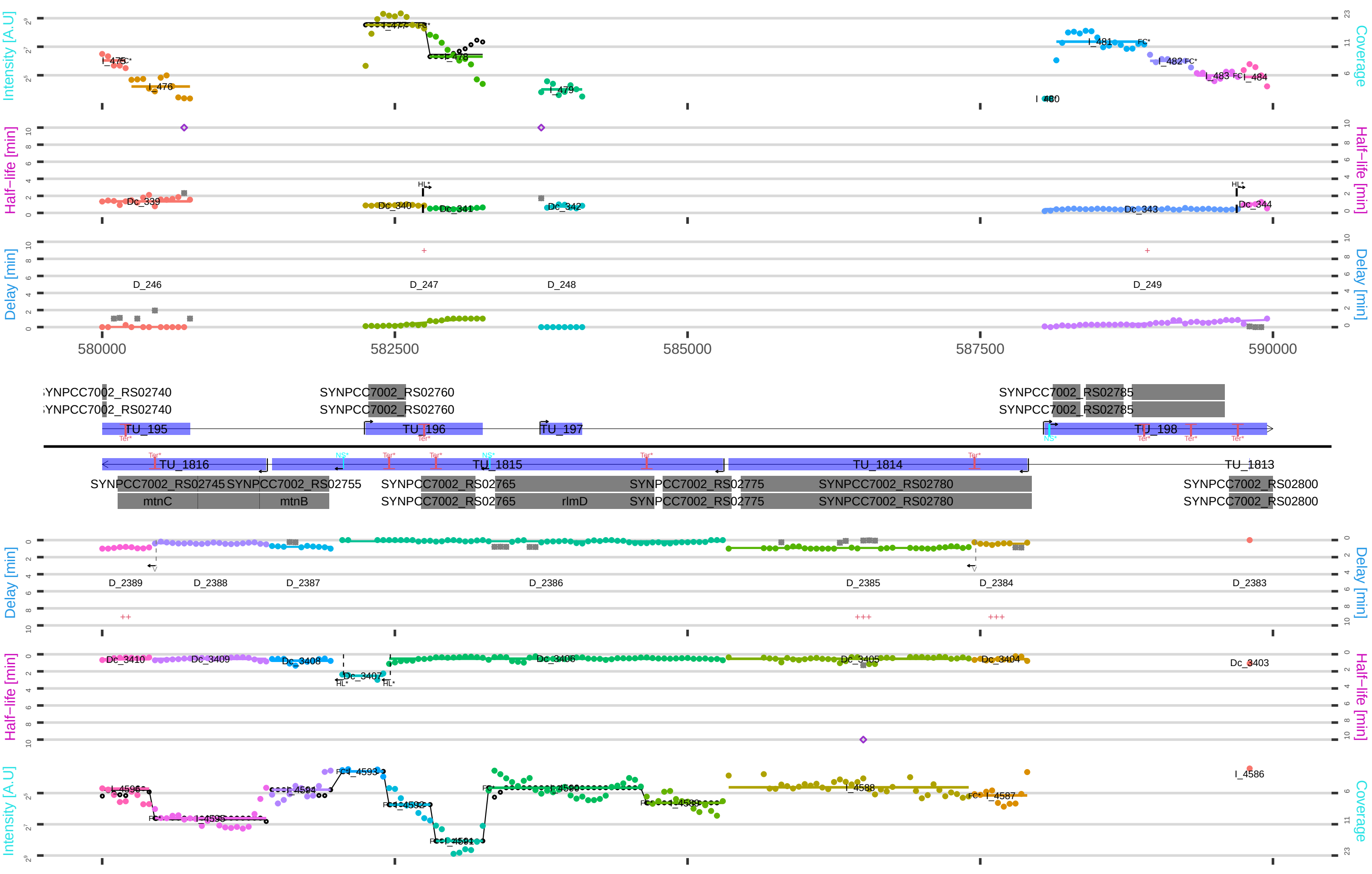

Term: termination (5), NS: new start (2), PS: pausing site (3), iTSS_I: internal starting site (0)

ID: 11804−12000; Term: termination (5), NS: new start (4), PS: pausing site (1), iTSS_I: internal starting site (0)

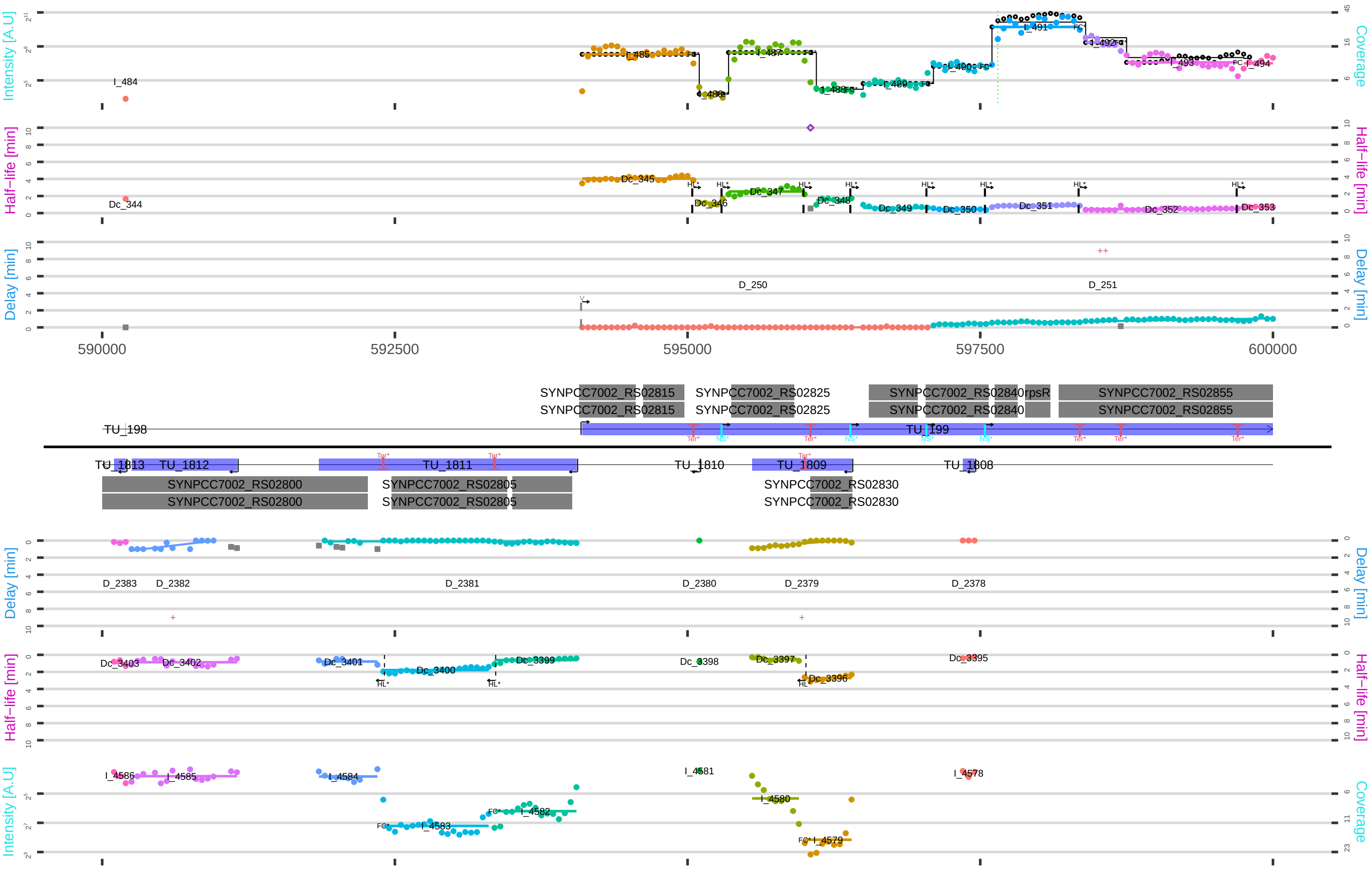

Term: termination (3), NS: new start (0), PS: pausing site (0), iTSS_I: internal starting site (0)

ID: 12000−12200; Term: termination (8), NS: new start (1), PS: pausing site (2), iTSS_I: internal starting site (0)

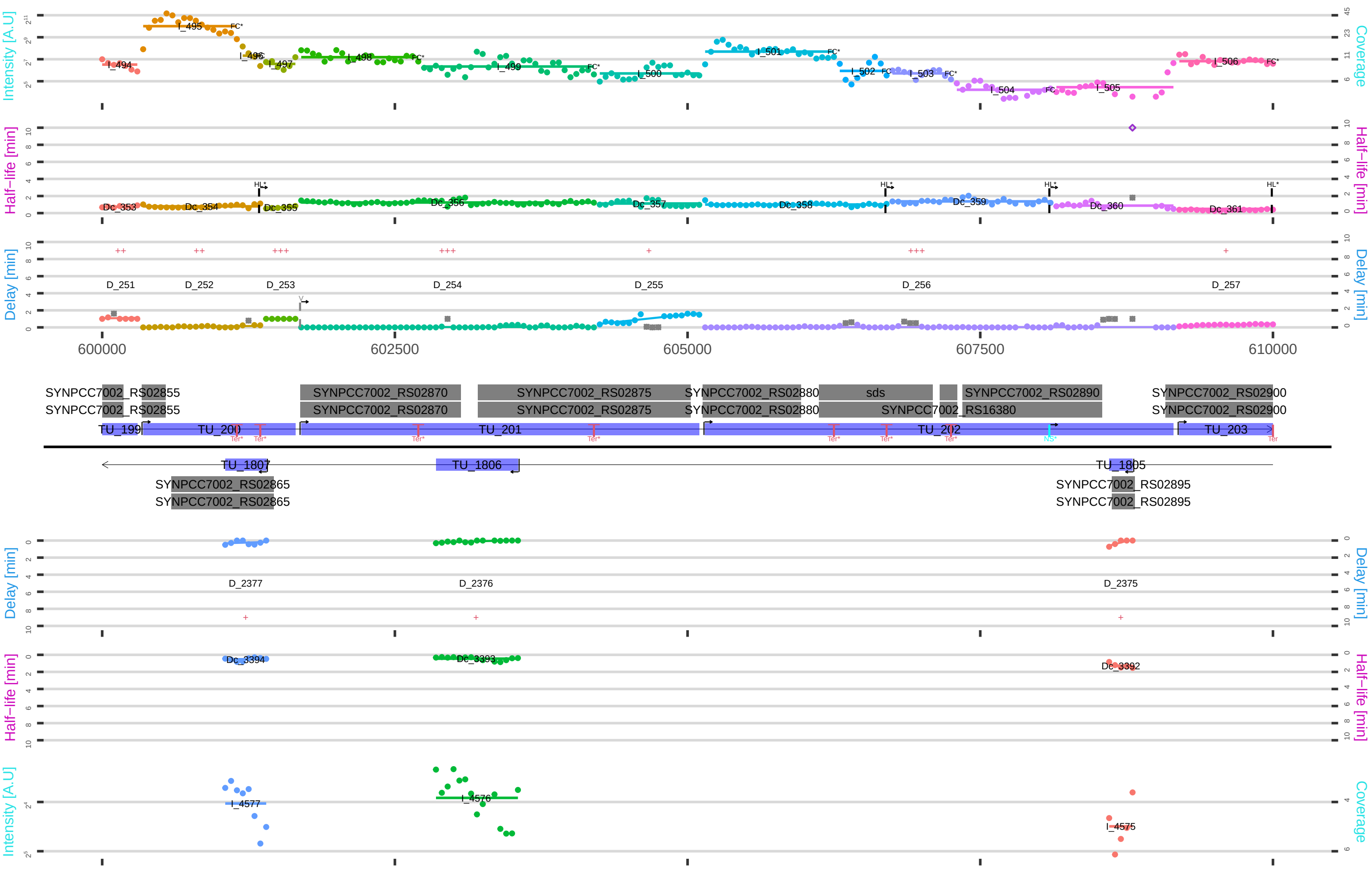

Term: termination (0), NS: new start (0), PS: pausing site (0), iTSS_I: internal starting site (0)

ID: 12200−12350; Term: termination (3), NS: new start (1), PS: pausing site (0), iTSS_I: internal starting site (0)

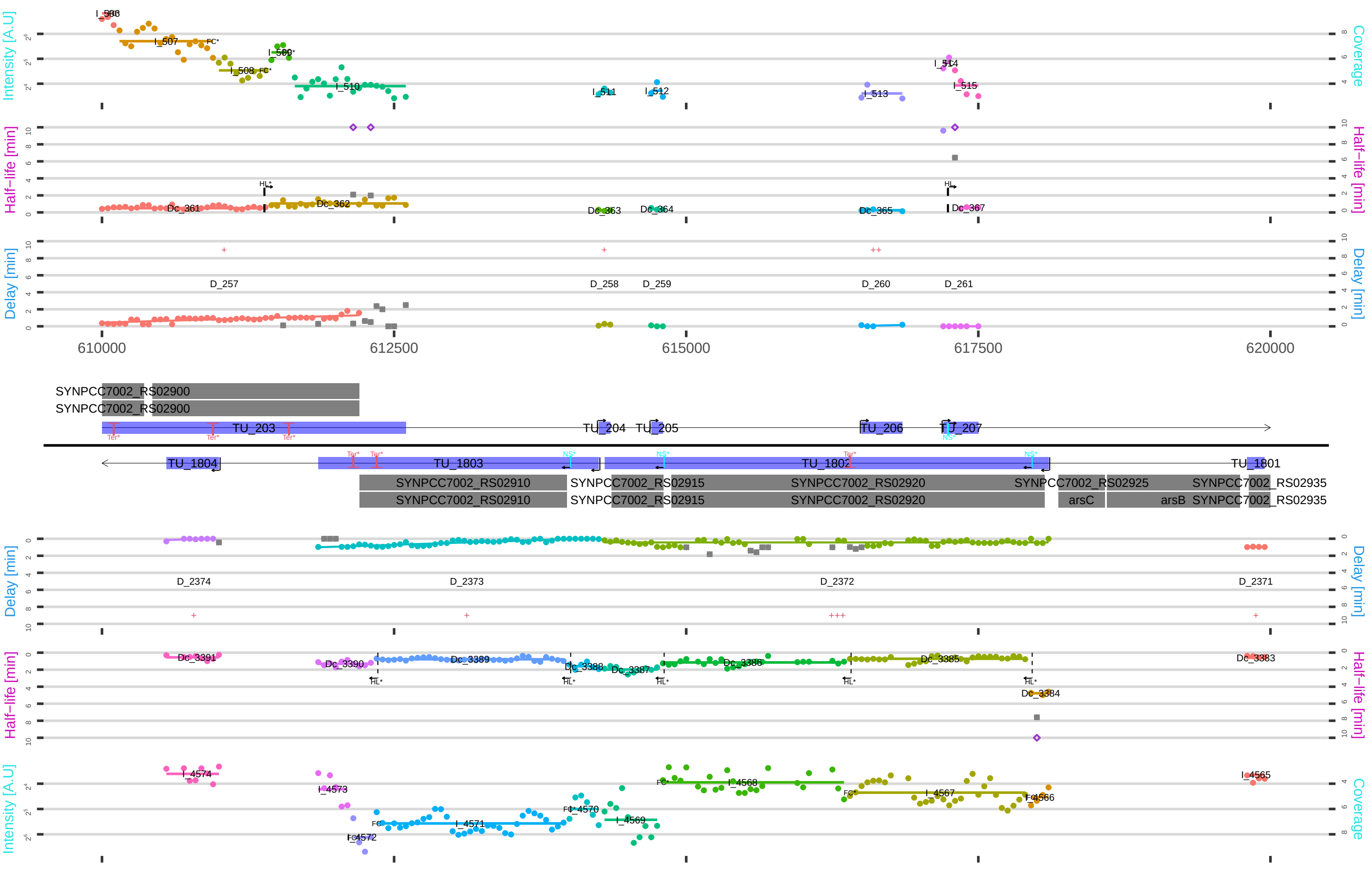

Term: termination (3), NS: new start (3), PS: pausing site (0), iTSS_I: internal starting site (0)

ID: 12433−12600; Term: termination (4), NS: new start (0), PS: pausing site (2), iTSS_I: internal starting site (0)

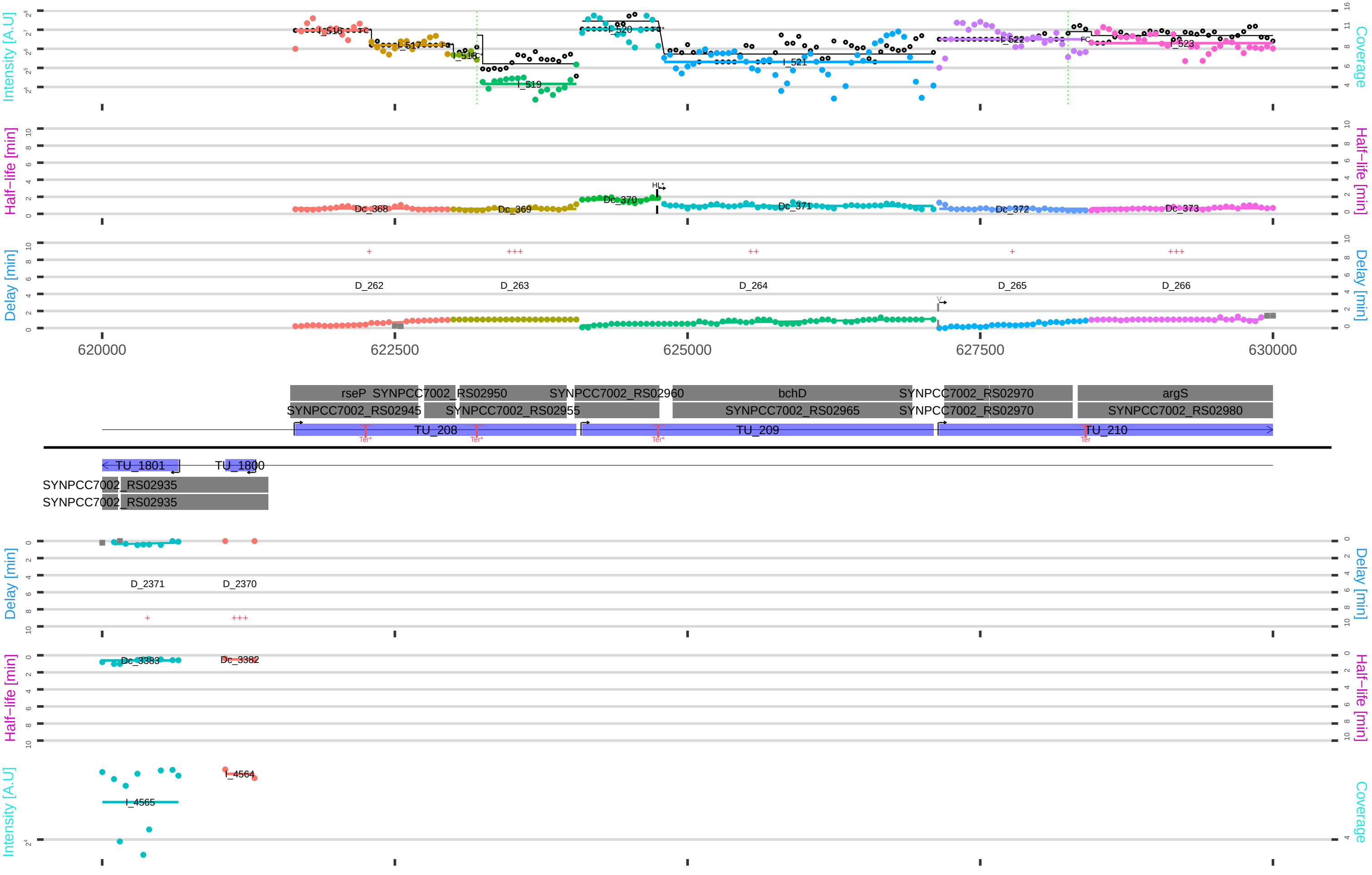

Term: termination (0), NS: new start (0), PS: pausing site (0), iTSS_I: internal starting site (0)

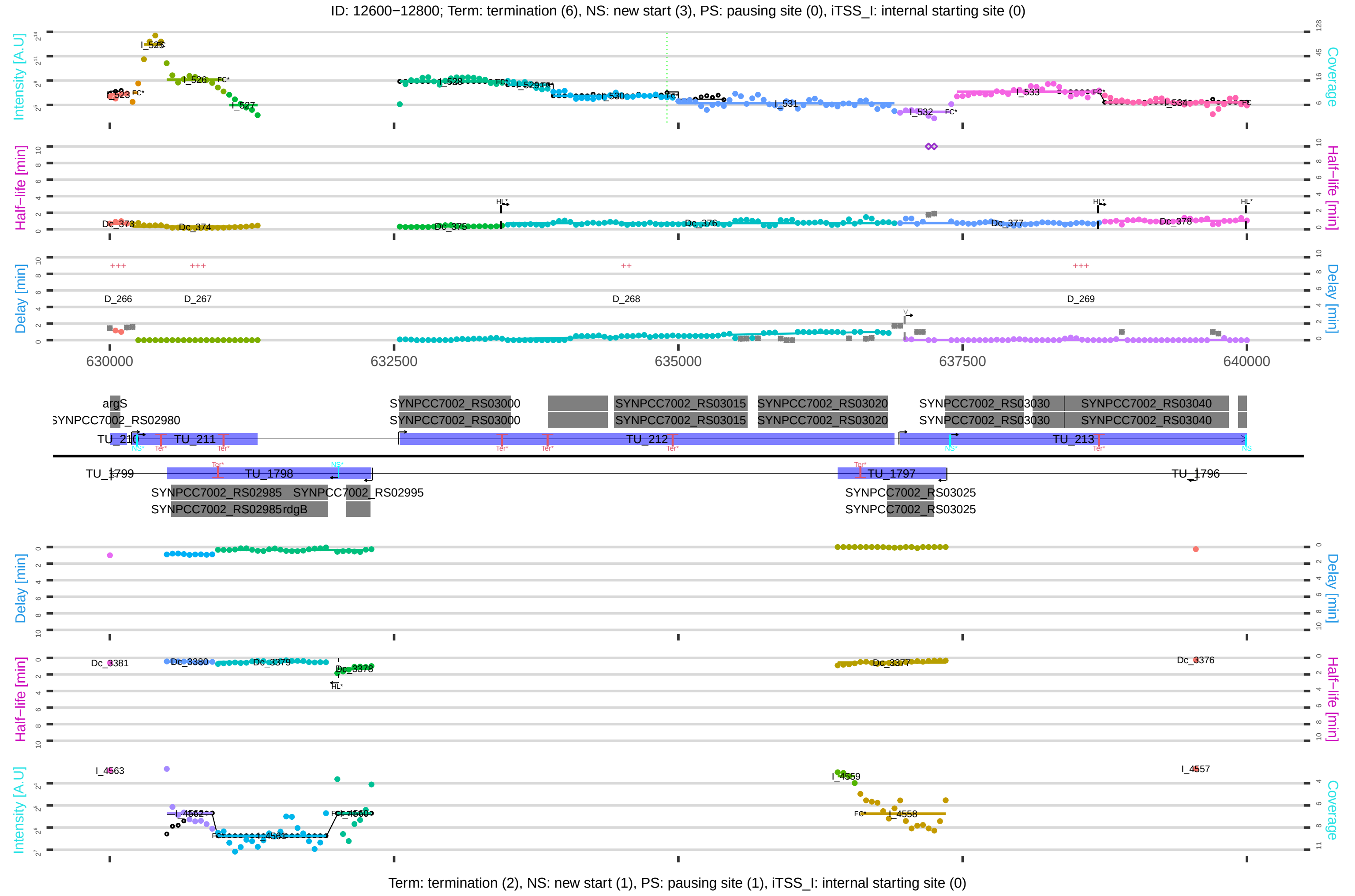

ID: 12600−12800; Term: termination (6), NS: new start (3), PS: pausing site (0), iTSS_I: internal starting site (0)
Intensity [A.U]
Coverage
Half−life [min]
Delay [min]
630000
632500
635000
637500
640000
argS
SYNPCC7002_RS02980
SYNPCC7002_RS03000
SYNPCC7002_RS03015
SYNPCC7002_RS03020
SYNPCC7002_RS03030
SYNPCC7002_RS03040
TU_211
TU_212
TU_213
TU_1799
TU_1798
TU_1797
TU_1796
SYNPCC7002_RS02985
SYNPCC7002_RS02995
SYNPCC7002_RS02985rdgB
SYNPCC7002_RS03025
D_266
D_267
D_268
D_269
Dc_373
Dc_374
Dc_375
Dc_376
Dc_377
Dc_378
Dc_3381
Dc_3380
Dc_3379
Dc_3378
Dc_3377
Dc_3376
I_4563
I_4562
I_4561
I_4560
I_4559
I_4558
I_4557
Term: termination (2), NS: new start (1), PS: pausing site (1), iTSS_I: internal starting site (0)

ID: 12800−12988; Term: termination (3), NS: new start (3), PS: pausing site (1), iTSS_I: internal starting site (0)

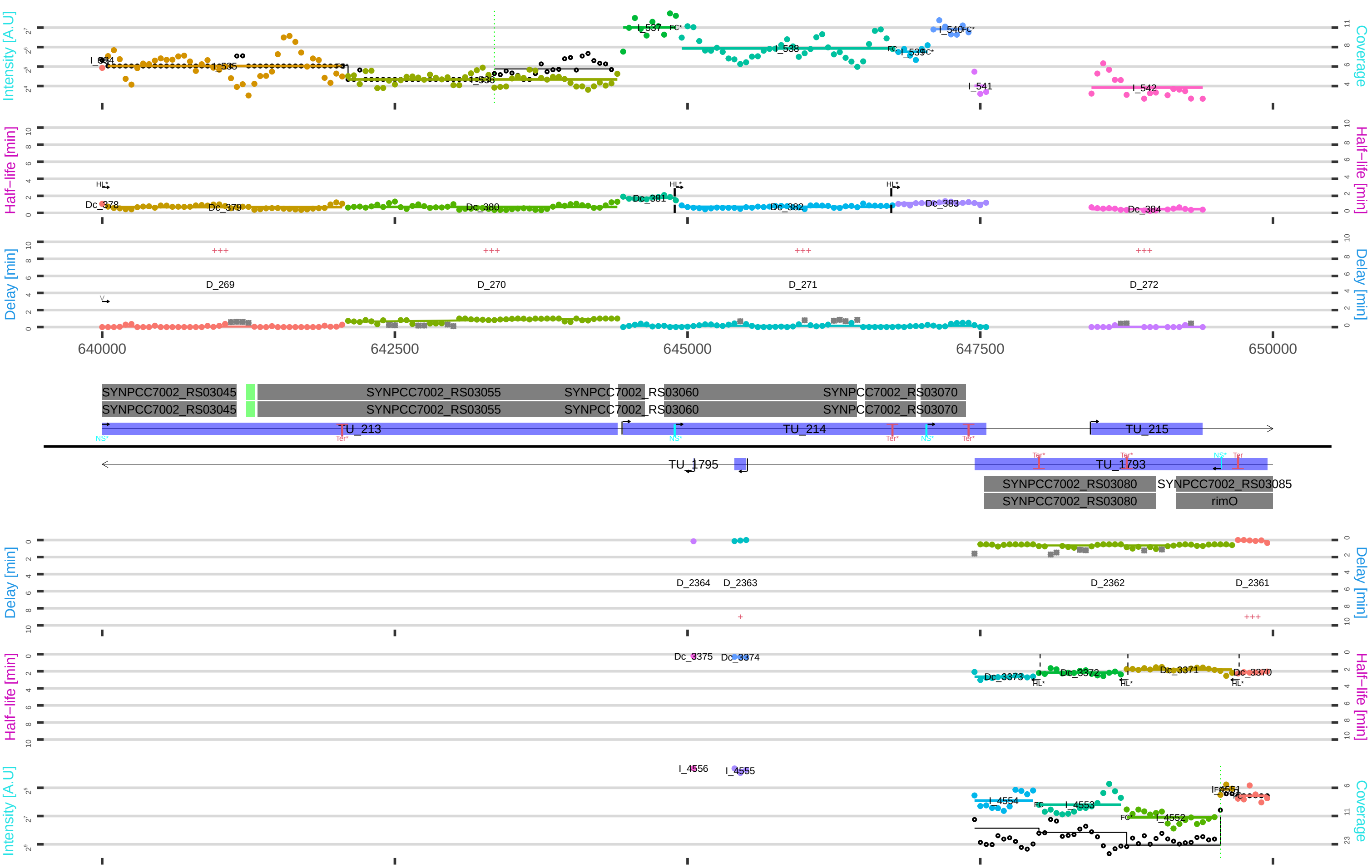

Term: termination (3), NS: new start (1), PS: pausing site (1), iTSS_I: internal starting site (0)

ID: 13097−13113; Term: termination (0), NS: new start (0), PS: pausing site (0), iTSS_I: internal starting site (0)

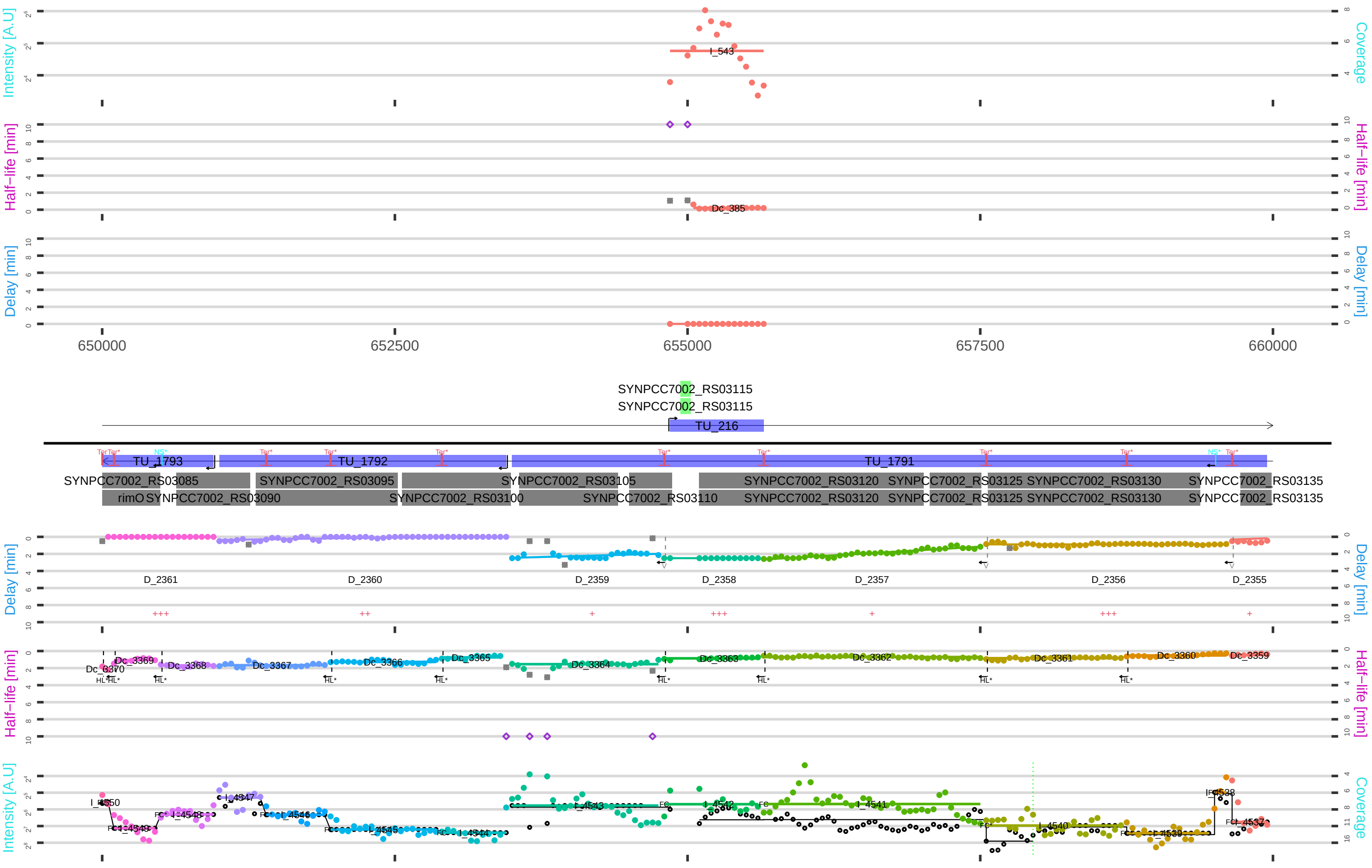

Term: termination (10), NS: new start (2), PS: pausing site (2), iTSS_I: internal starting site (2)

ID: 13202−13390; Term: termination (0), NS: new start (0), PS: pausing site (0), iTSS_I: internal starting site (0)

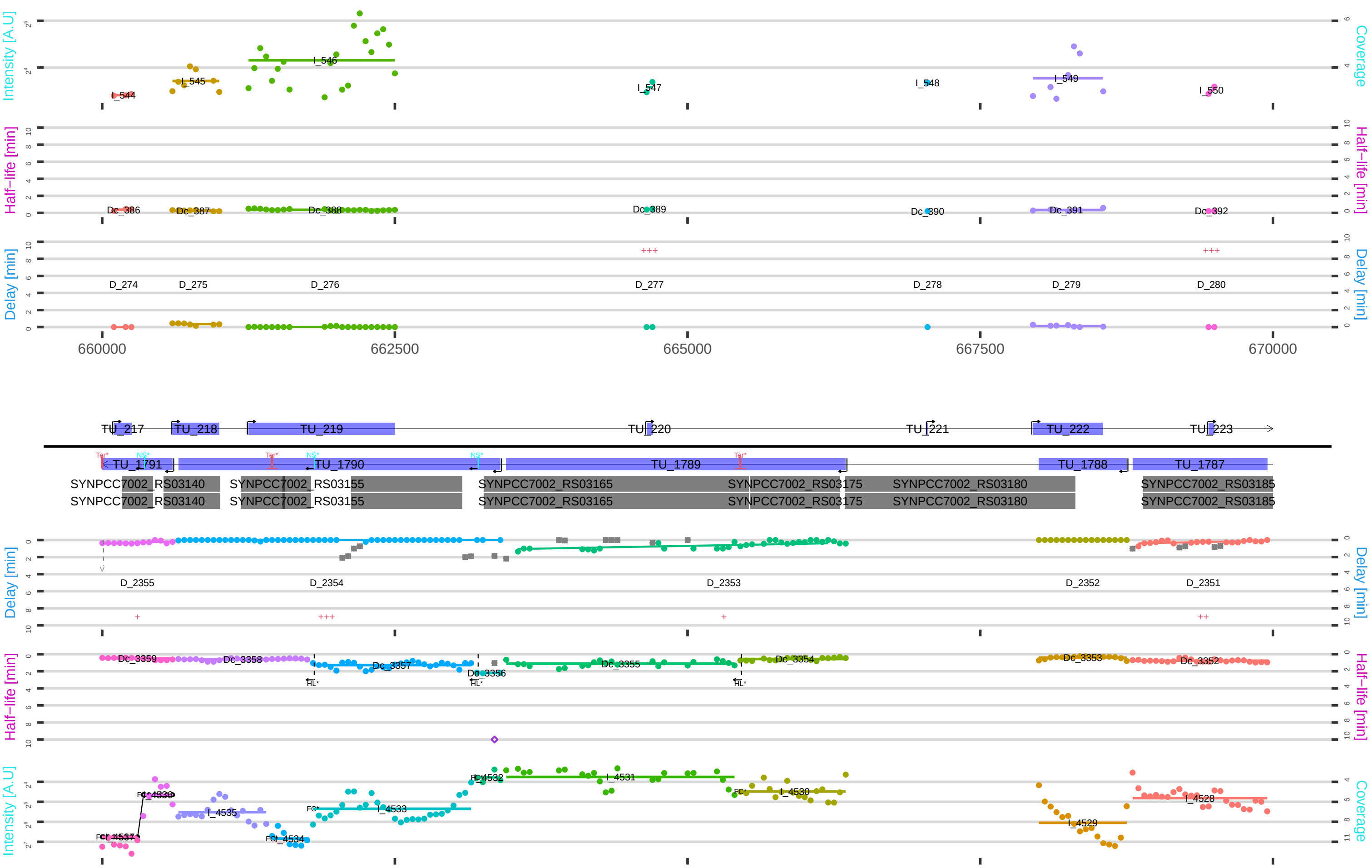

Term: termination (3), NS: new start (3), PS: pausing site (0), iTSS_I: internal starting site (0)

ID: 13404−13564; Term: termination (4), NS: new start (2), PS: pausing site (1), iTSS_I: internal starting site (0)

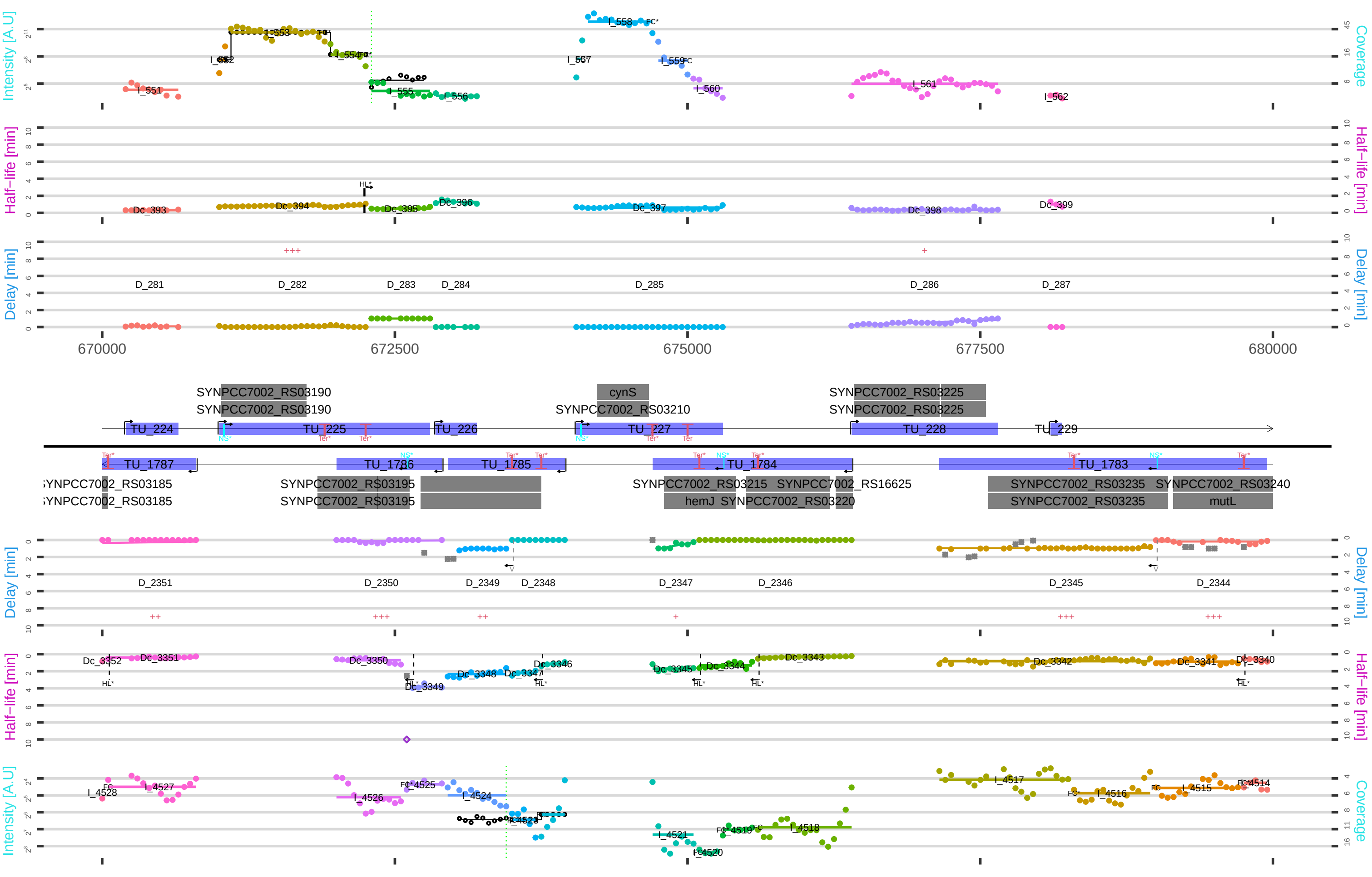

Term: termination (7), NS: new start (3), PS: pausing site (3), iTSS_I: internal starting site (0)

ID: 13624−13800; Term: termination (1), NS: new start (1), PS: pausing site (1), iTSS_I: internal starting site (0)

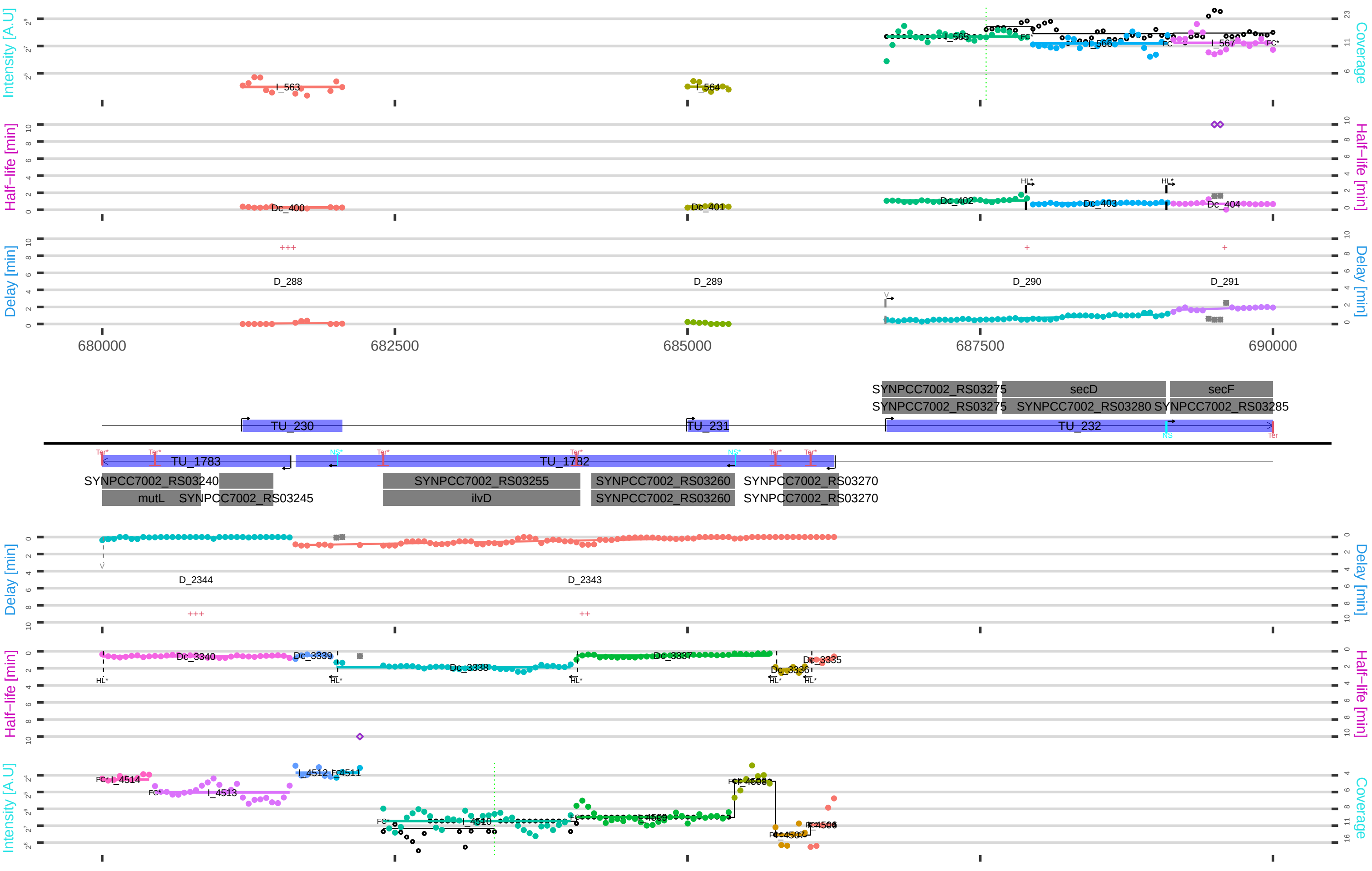

Term: termination (6), NS: new start (2), PS: pausing site (0), iTSS_I: internal starting site (0)

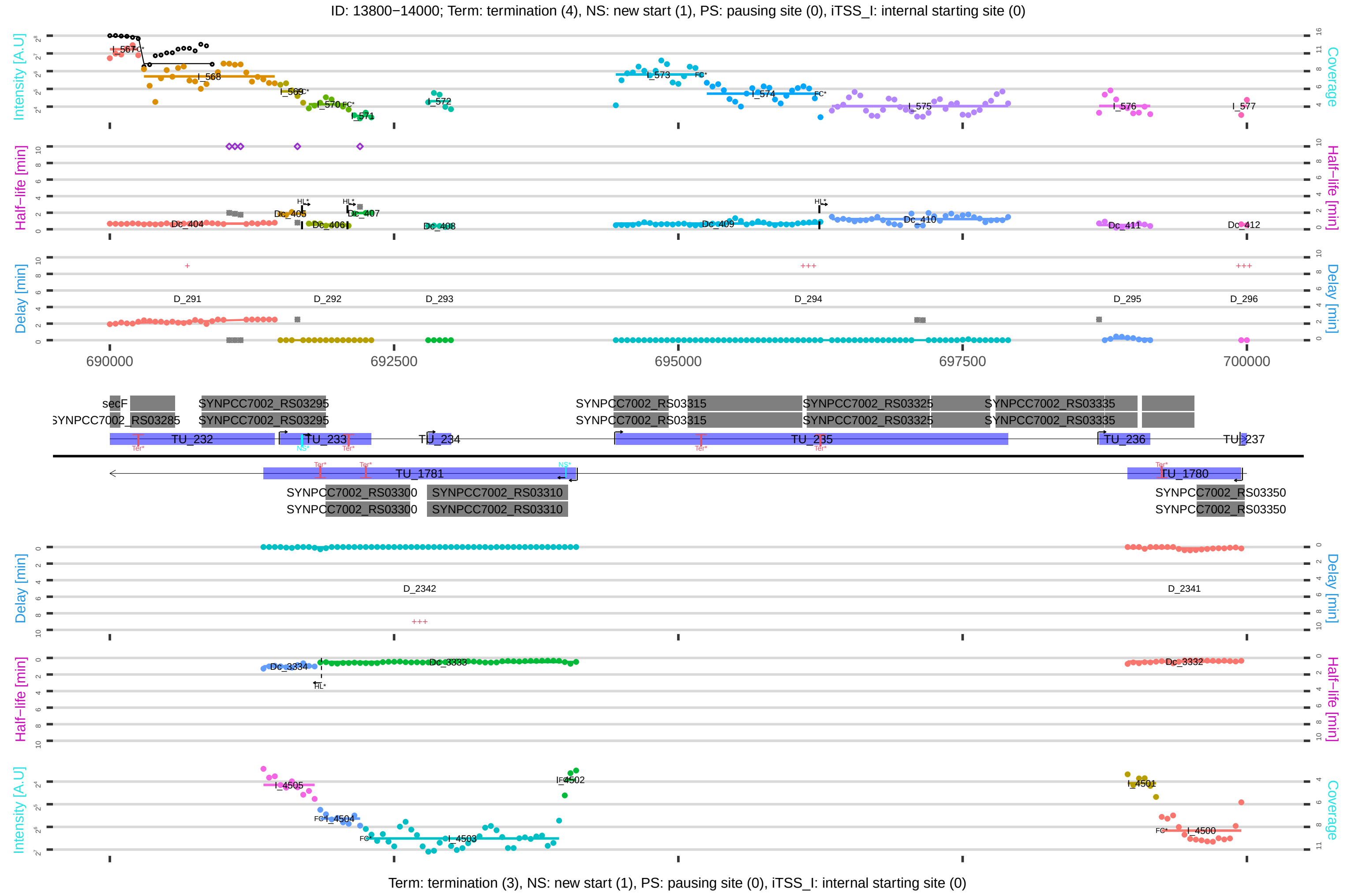

ID: 13800−14000; Term: termination (4), NS: new start (1), PS: pausing site (0), iTSS_I: internal starting site (0)
Intensity [A.U]
Coverage
Half−life [min]
Delay [min]
690000
692500
695000
697500
700000
secF
SYNPCC7002_RS03285
SYNPCC7002_RS03295
SYNPCC7002_RS03315
SYNPCC7002_RS03325
SYNPCC7002_RS03335
TU_232
TU_233
TU_234
TU_235
TU_236
TU_237
TU_1781
TU_1780
SYNPCC7002_RS03300
SYNPCC7002_RS03310
SYNPCC7002_RS03350
D_291
D_292
D_293
D_294
D_295
D_296
D_2342
D_2341
Term: termination (3), NS: new start (1), PS: pausing site (0), iTSS_I: internal starting site (0)

ID: 14000−14199; Term: termination (5), NS: new start (5), PS: pausing site (1), iTSS_I: internal starting site (0)

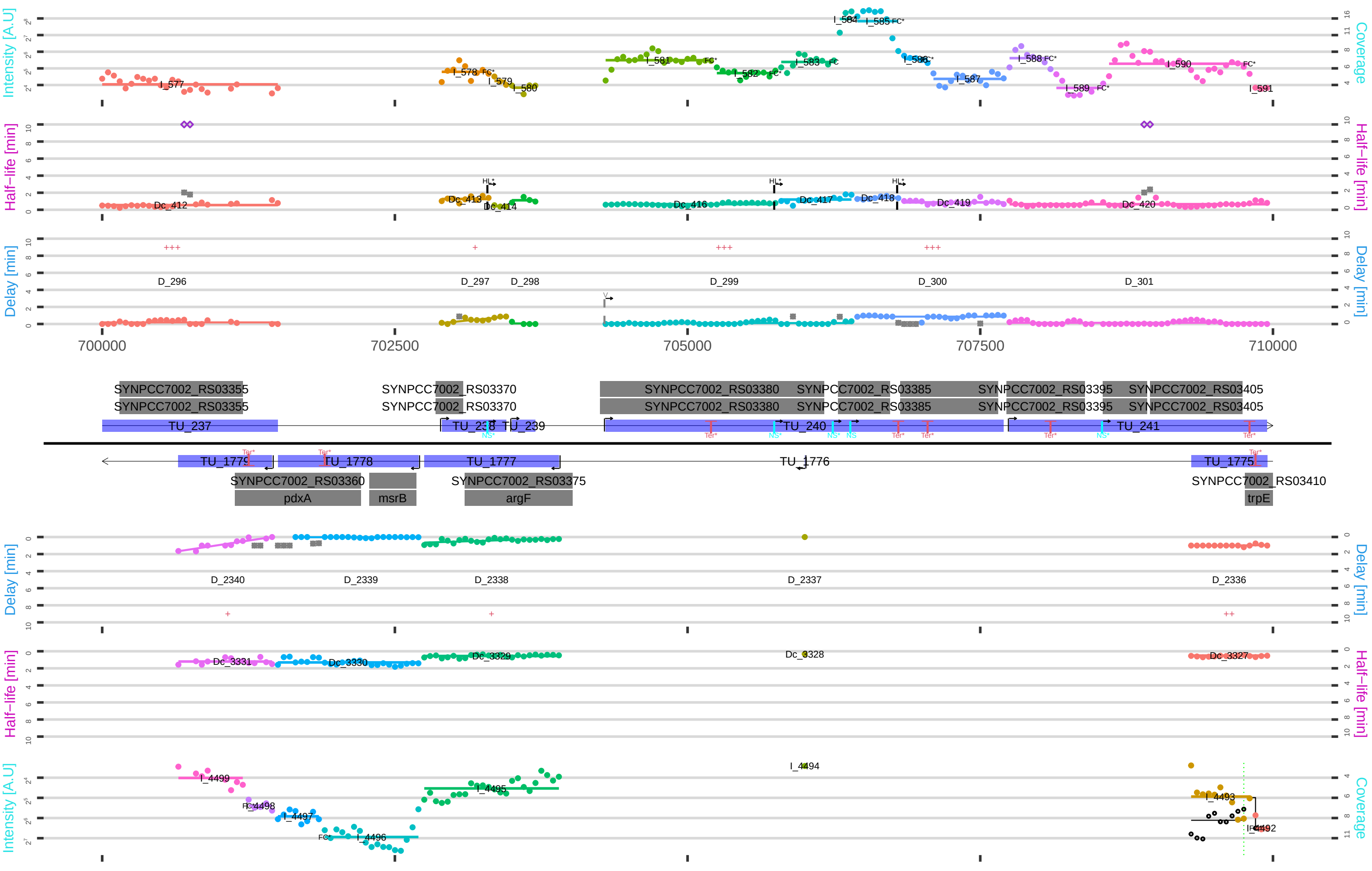

Term: termination (3), NS: new start (0), PS: pausing site (0), iTSS_I: internal starting site (0)

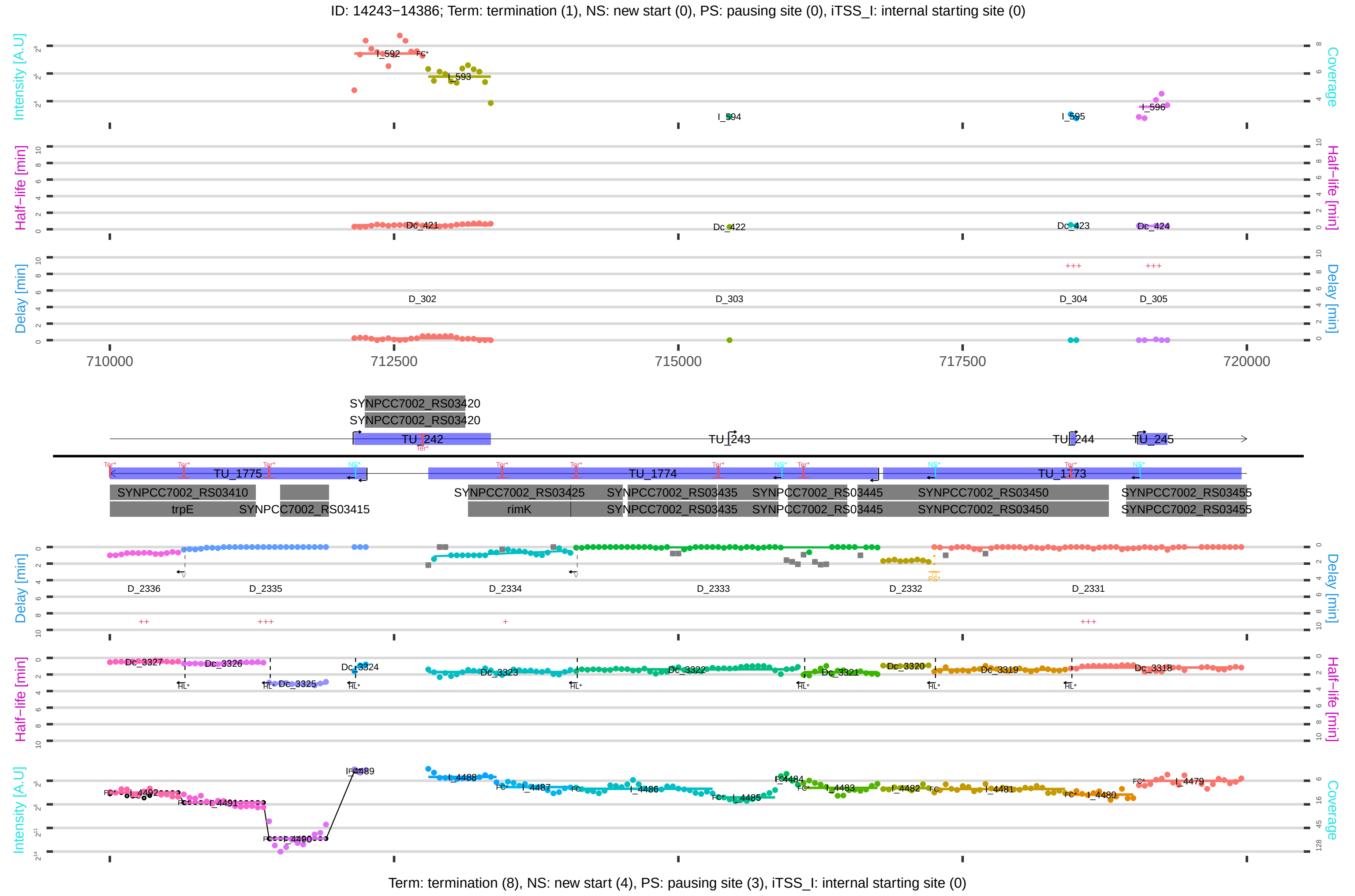
ID: 14243−14386; Term: termination (1), NS: new start (0), PS: pausing site (0), iTSS_I: internal starting site (0)
Intensity [A.U]
Coverage
Half−life [min]
Delay [min]
I_592
I_593
I_594
I_595
I_596
Dc_421
Dc_422
Dc_423
Dc_424
D_302
D_303
D_304
D_305
710000
712500
715000
717500
720000
SYNPCC7002_RS03420
TU_242
TU_243
TU_244
TU_245
TU_1775
TU_1774
TU_1773
SYNPCC7002_RS03410
trpE
SYNPCC7002_RS03415
SYNPCC7002_RS03425
rimK
SYNPCC7002_RS03435
SYNPCC7002_RS03445
SYNPCC7002_RS03450
SYNPCC7002_RS03455
D_2336
D_2335
D_2334
D_2333
D_2332
D_2331
Dc_3327
Dc_3326
Dc_3325
Dc_3324
Dc_3323
Dc_3322
Dc_3321
Dc_3320
Dc_3319
Dc_3318
I_4492
I_4491
I_4490
I_4489
I_4488
I_4487
I_4486
I_4485
I_4484
I_4483
I_4482
I_4481
I_4480
I_4479
Term: termination (8), NS: new start (4), PS: pausing site (3), iTSS_I: internal starting site (0)

ID: 14519−14600; Term: termination (2), NS: new start (3), PS: pausing site (1), iTSS_I: internal starting site (0)

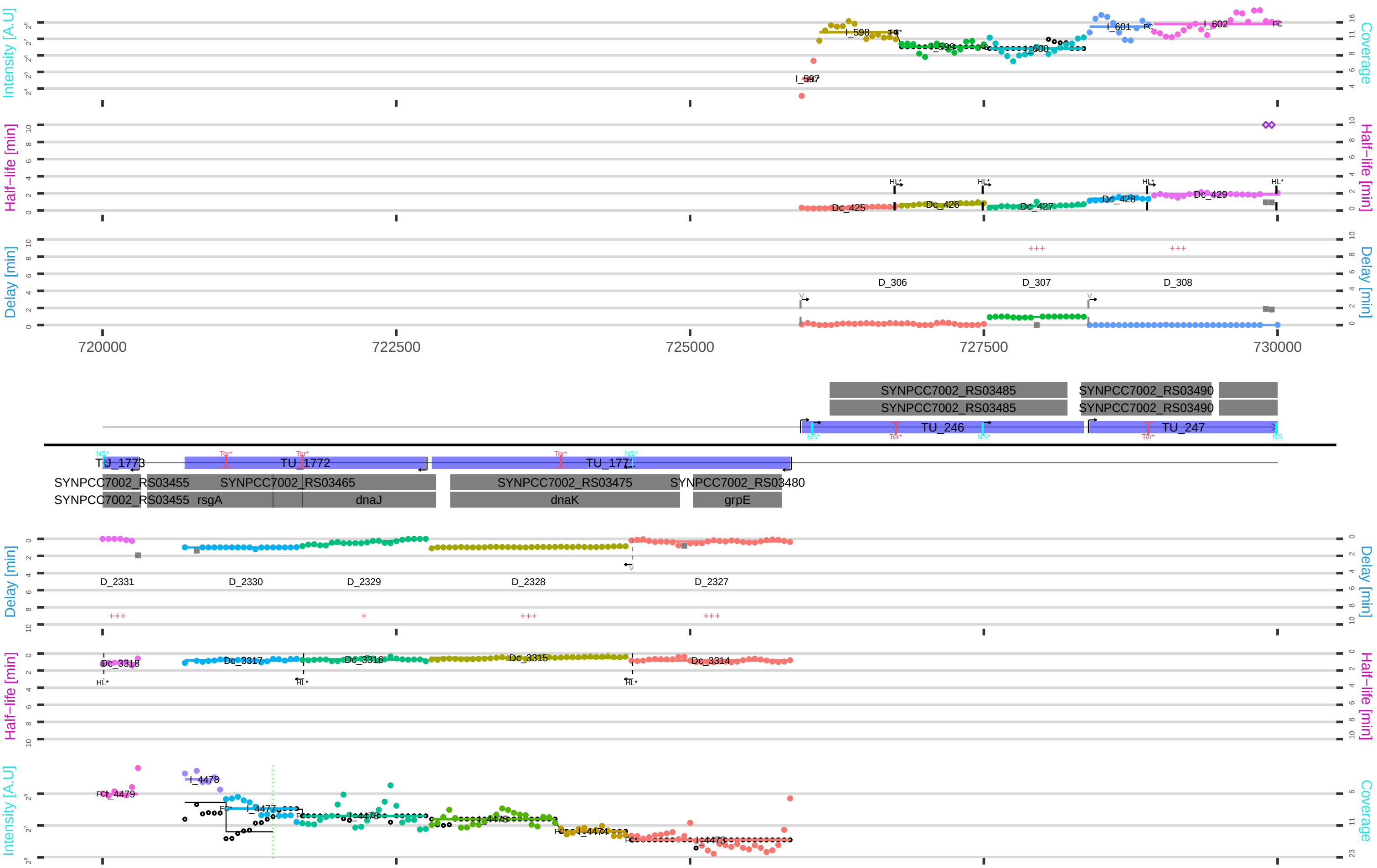

Term: termination (3), NS: new start (2), PS: pausing site (2), iTSS_I: internal starting site (0)

ID: 14600−14781; Term: termination (4), NS: new start (1), PS: pausing site (1), iTSS_I: internal starting site (0)

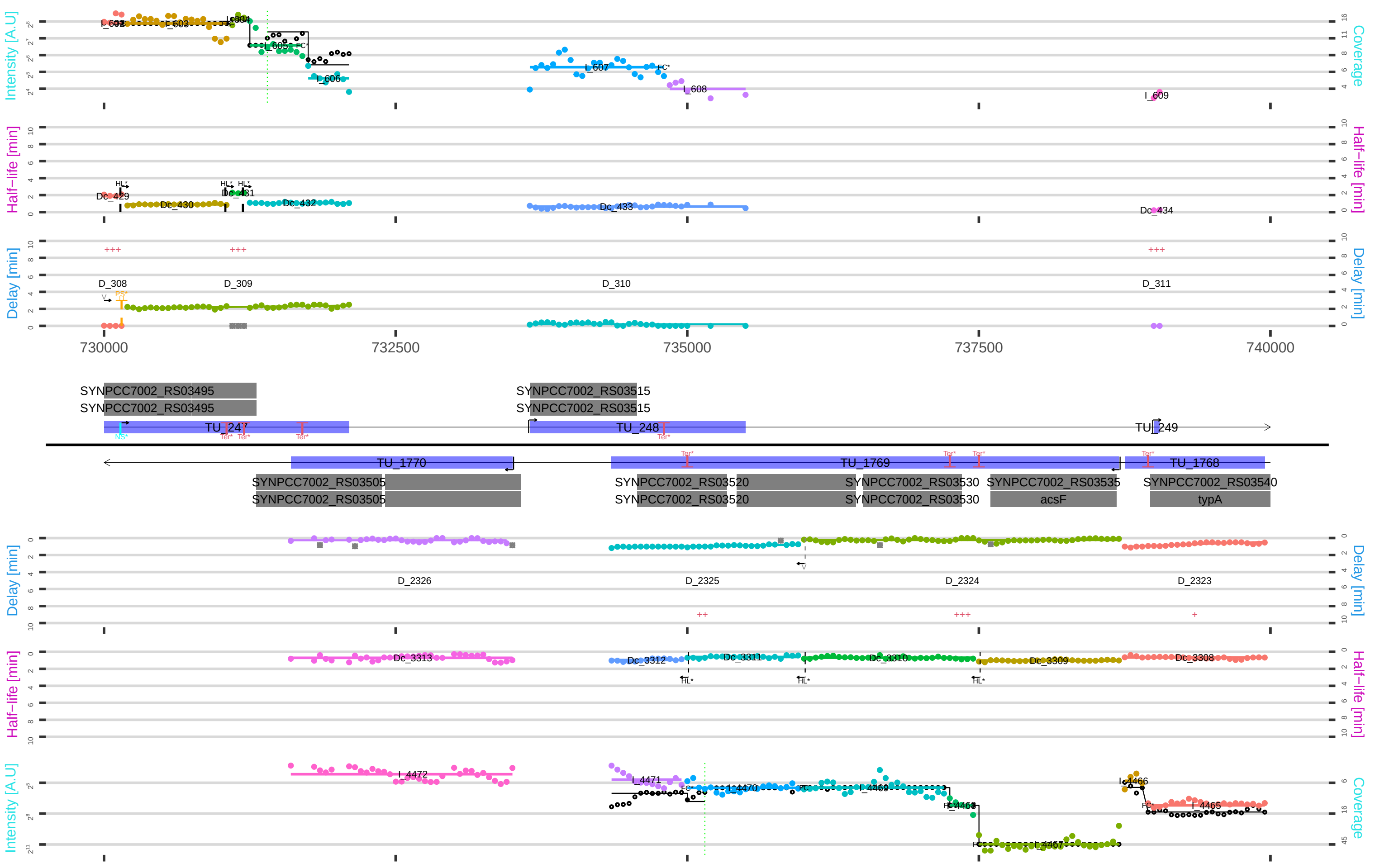

Term: termination (4), NS: new start (0), PS: pausing site (1), iTSS_I: internal starting site (0)

ID: 14822−14999; Term: termination (3), NS: new start (0), PS: pausing site (0), iTSS_I: internal starting site (0)

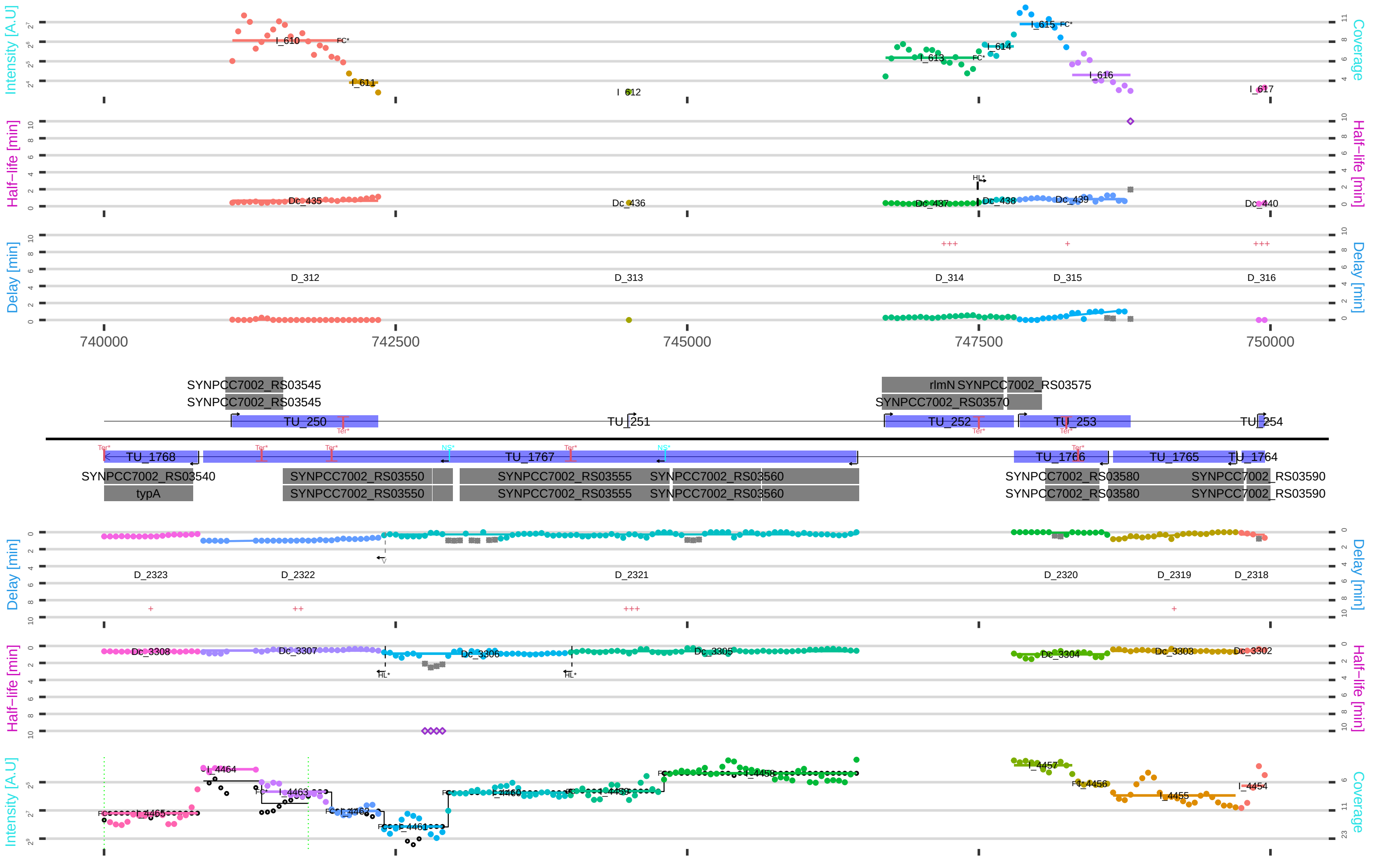

Term: termination (5), NS: new start (2), PS: pausing site (1), iTSS_I: internal starting site (0)

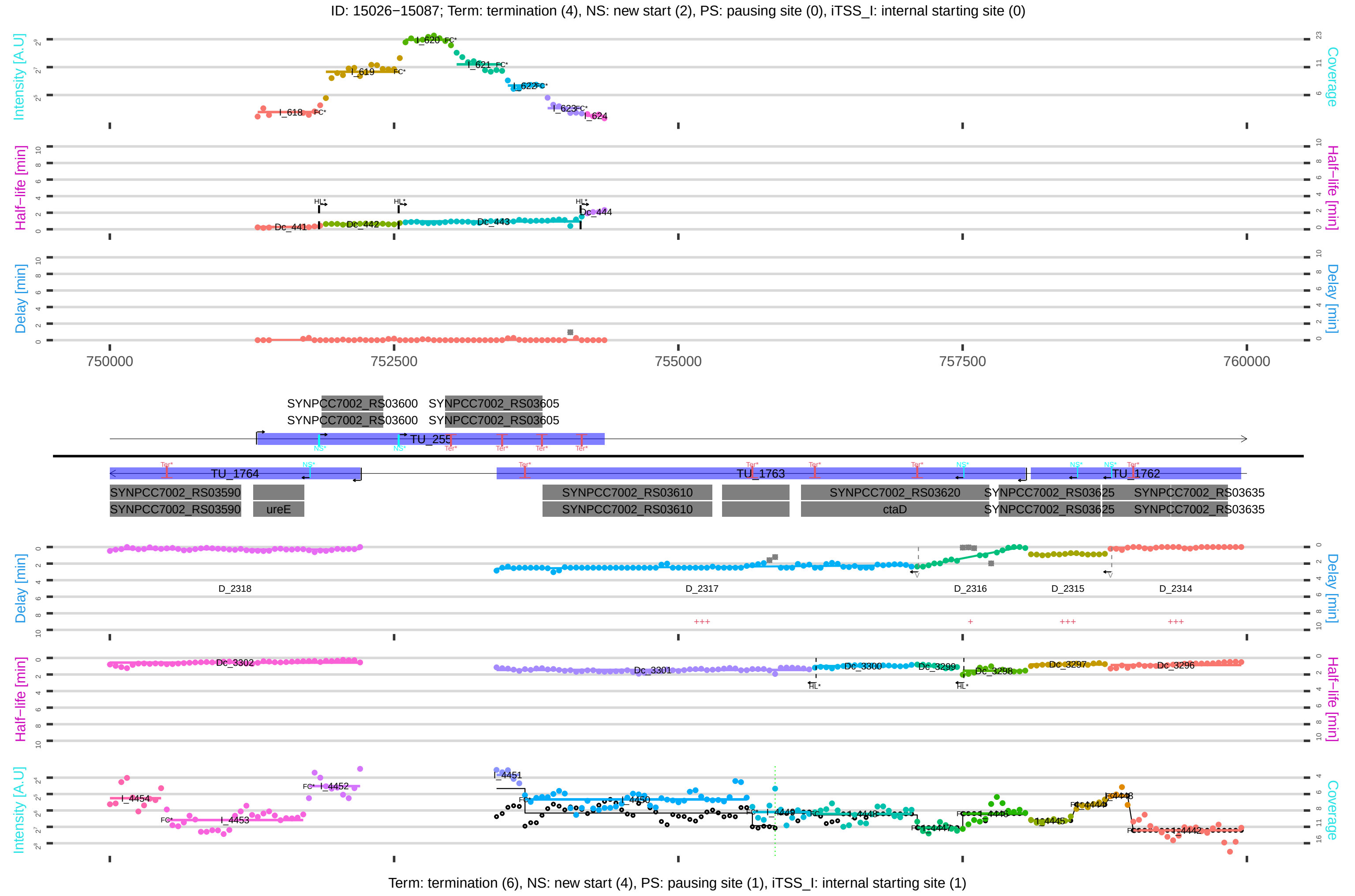
ID: 15026−15087; Term: termination (4), NS: new start (2), PS: pausing site (0), iTSS_I: internal starting site (0)
Intensity [A.U]
Coverage
Half−life [min]
Delay [min]
750000
752500
755000
757500
760000
SYNPCC7002_RS03600
SYNPCC7002_RS03605
TU_255
TU_1764
TU_1763
TU_1762
SYNPCC7002_RS03590
ureE
SYNPCC7002_RS03610
SYNPCC7002_RS03620
ctaD
SYNPCC7002_RS03625
SYNPCC7002_RS03635
D_2318
D_2317
D_2316
D_2315
D_2314
Term: termination (6), NS: new start (4), PS: pausing site (1), iTSS_I: internal starting site (1)

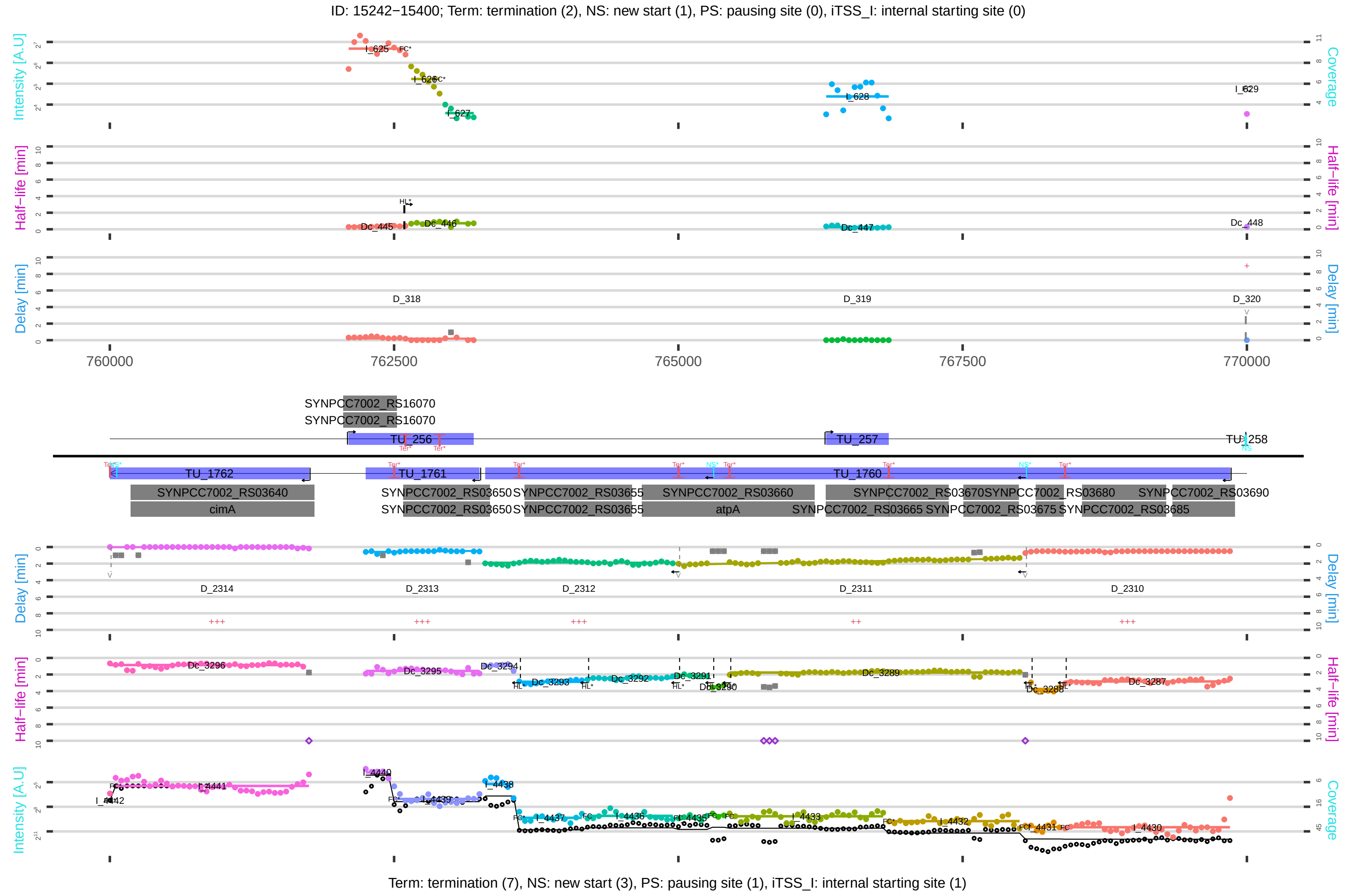

ID: 15242−15400; Term: termination (2), NS: new start (1), PS: pausing site (0), iTSS_I: internal starting site (0)
Intensity [A.U]
Coverage
Half−life [min]
Delay [min]
I_625
I_626
I_627
I_628
I_629
Dc_445
Dc_446
Dc_447
Dc_448
D_318
D_319
D_320
760000
762500
765000
767500
770000
SYNPCC7002_RS16070
TU_256
TU_257
TU_258
TU_1762
TU_1761
TU_1760
SYNPCC7002_RS03640
cimA
SYNPCC7002_RS03650
SYNPCC7002_RS03655
SYNPCC7002_RS03660
atpA
SYNPCC7002_RS03665
SYNPCC7002_RS03670
SYNPCC7002_RS03675
SYNPCC7002_RS03680
SYNPCC7002_RS03685
SYNPCC7002_RS03690
D_2314
D_2313
D_2312
D_2311
D_2310
Dc_3296
Dc_3295
Dc_3294
Dc_3293
Dc_3292
Dc_3291
Dc_3290
Dc_3289
Dc_3288
Dc_3287
I_4442
I_4441
I_4440
I_4439
I_4438
I_4437
I_4436
I_4435
I_4433
I_4432
I_4431
I_4430
Term: termination (7), NS: new start (3), PS: pausing site (1), iTSS_I: internal starting site (1)

ID: 15400−15600; Term: termination (4), NS: new start (3), PS: pausing site (4), iTSS_I: internal starting site (0)

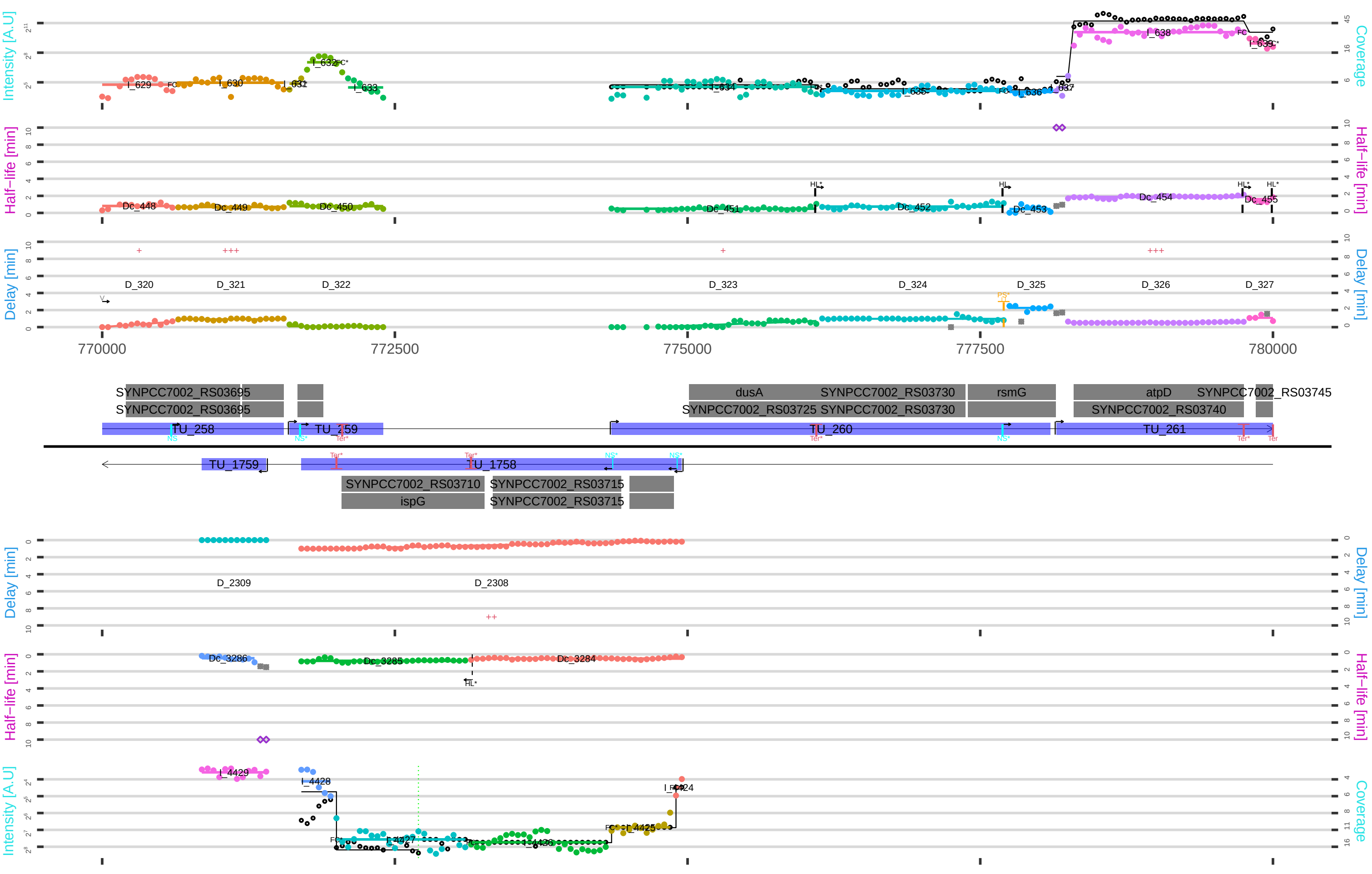

Term: termination (2), NS: new start (2), PS: pausing site (0), iTSS_I: internal starting site (0)

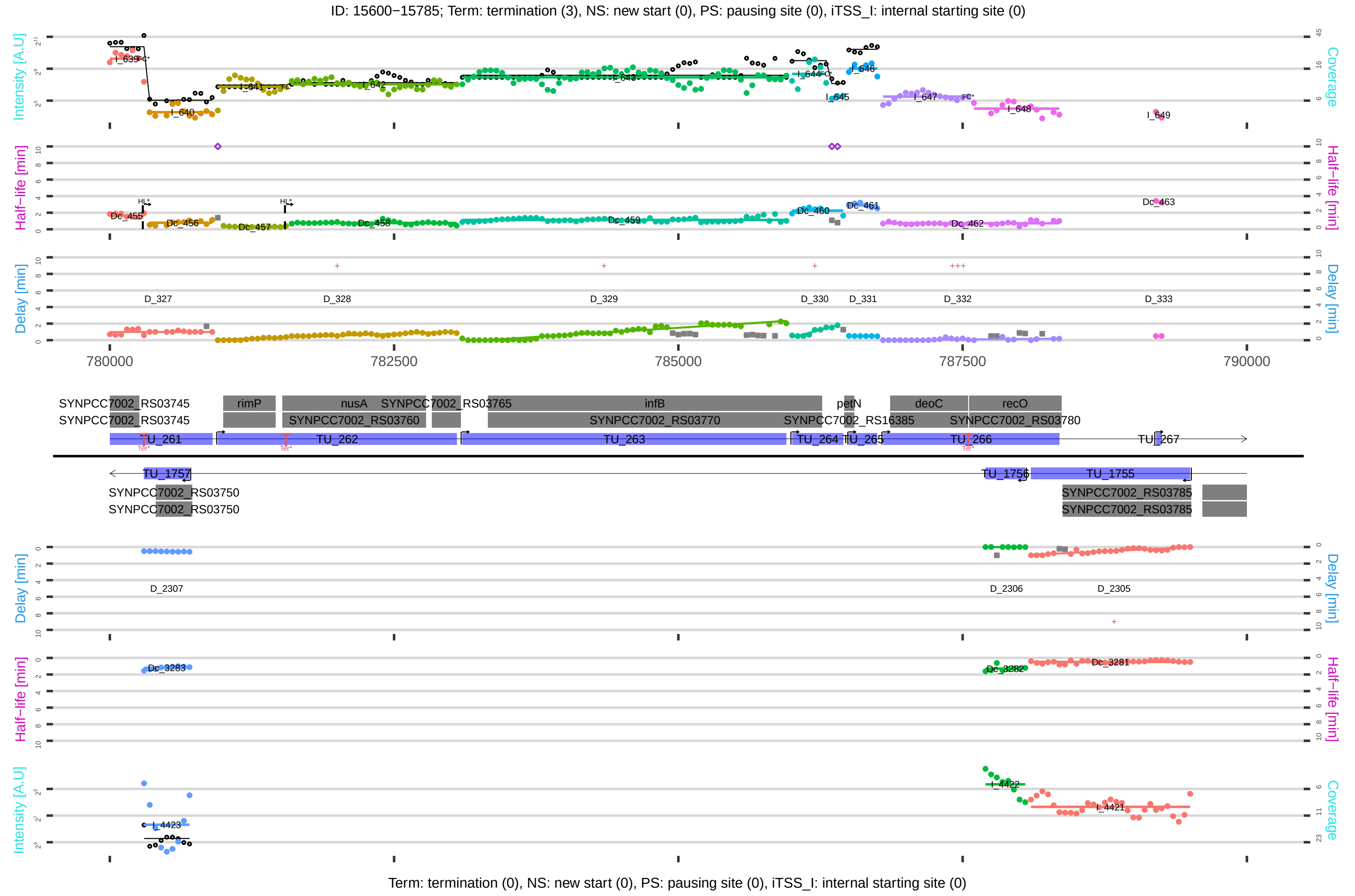
ID: 15600−15785; Term: termination (3), NS: new start (0), PS: pausing site (0), iTSS_I: internal starting site (0)
Intensity [A.U]
Coverage
Half−life [min]
Delay [min]
780000
782500
785000
787500
790000
SYNPCC7002_RS03745
rimP
nusA
SYNPCC7002_RS03765
infB
petN
deoC
recO
SYNPCC7002_RS03760
SYNPCC7002_RS03770
SYNPCC7002_RS16385
SYNPCC7002_RS03780
TU_261
TU_262
TU_263
TU_264
TU_265
TU_266
TU_267
TU_1757
TU_1756
TU_1755
SYNPCC7002_RS03750
SYNPCC7002_RS03785
D_327
D_328
D_329
D_330
D_331
D_332
D_333
D_2307
D_2306
D_2305
Dc_455
Dc_456
Dc_457
Dc_458
Dc_459
Dc_460
Dc_461
Dc_462
Dc_463
Dc_3283
Dc_3282
Dc_3281
I_639
I_640
I_641
I_642
I_643
I_644
I_645
I_646
I_647
I_648
I_649
I_4423
I_4422
I_4421
Term: termination (0), NS: new start (0), PS: pausing site (0), iTSS_I: internal starting site (0)

ID: 15827−15950; Term: termination (3), NS: new start (0), PS: pausing site (0), iTSS_I: internal starting site (0)

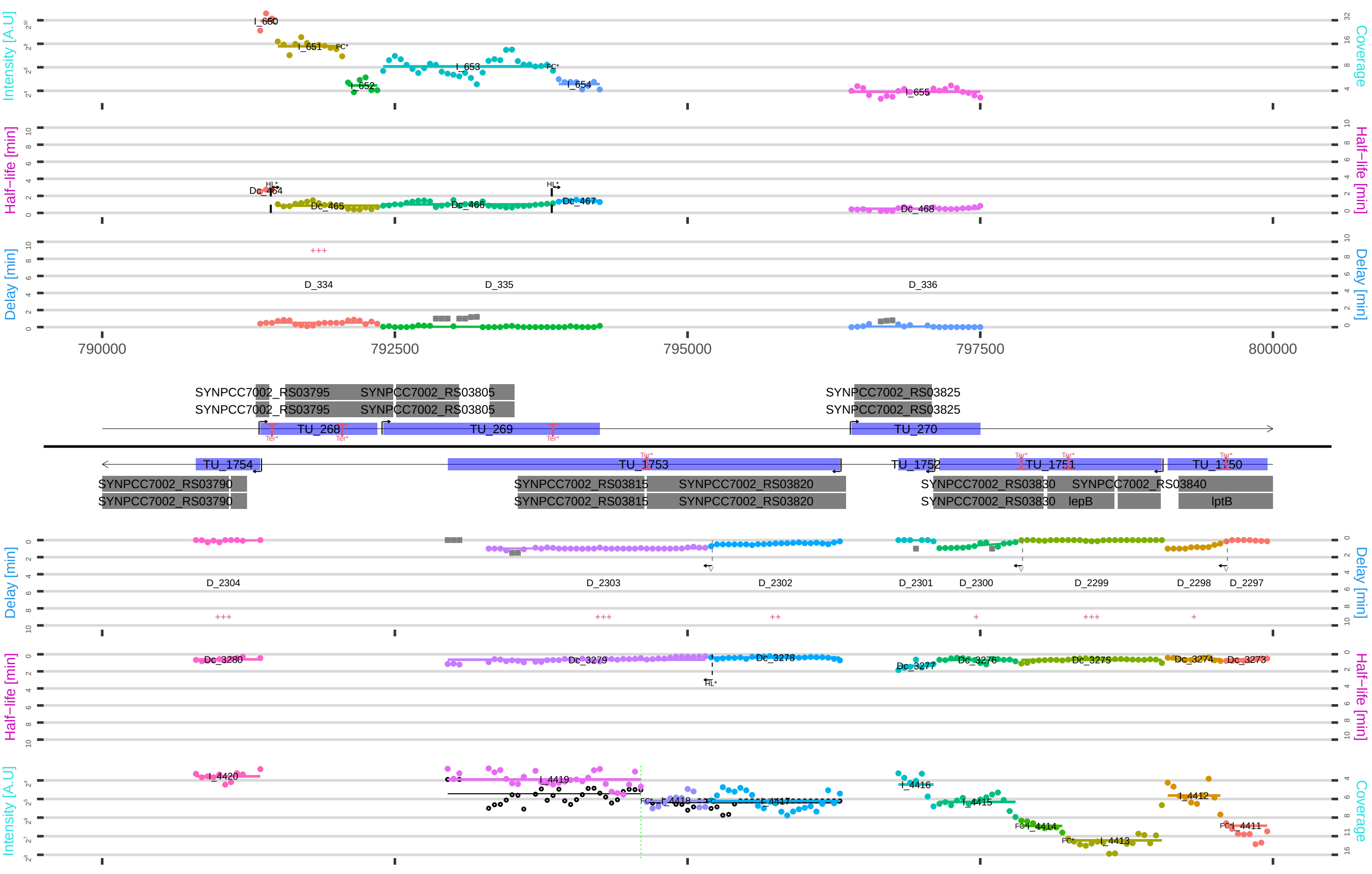

Term: termination (4), NS: new start (0), PS: pausing site (3), iTSS_I: internal starting site (0)

ID: 16053−16188; Term: termination (4), NS: new start (1), PS: pausing site (1), iTSS_I: internal starting site (0)

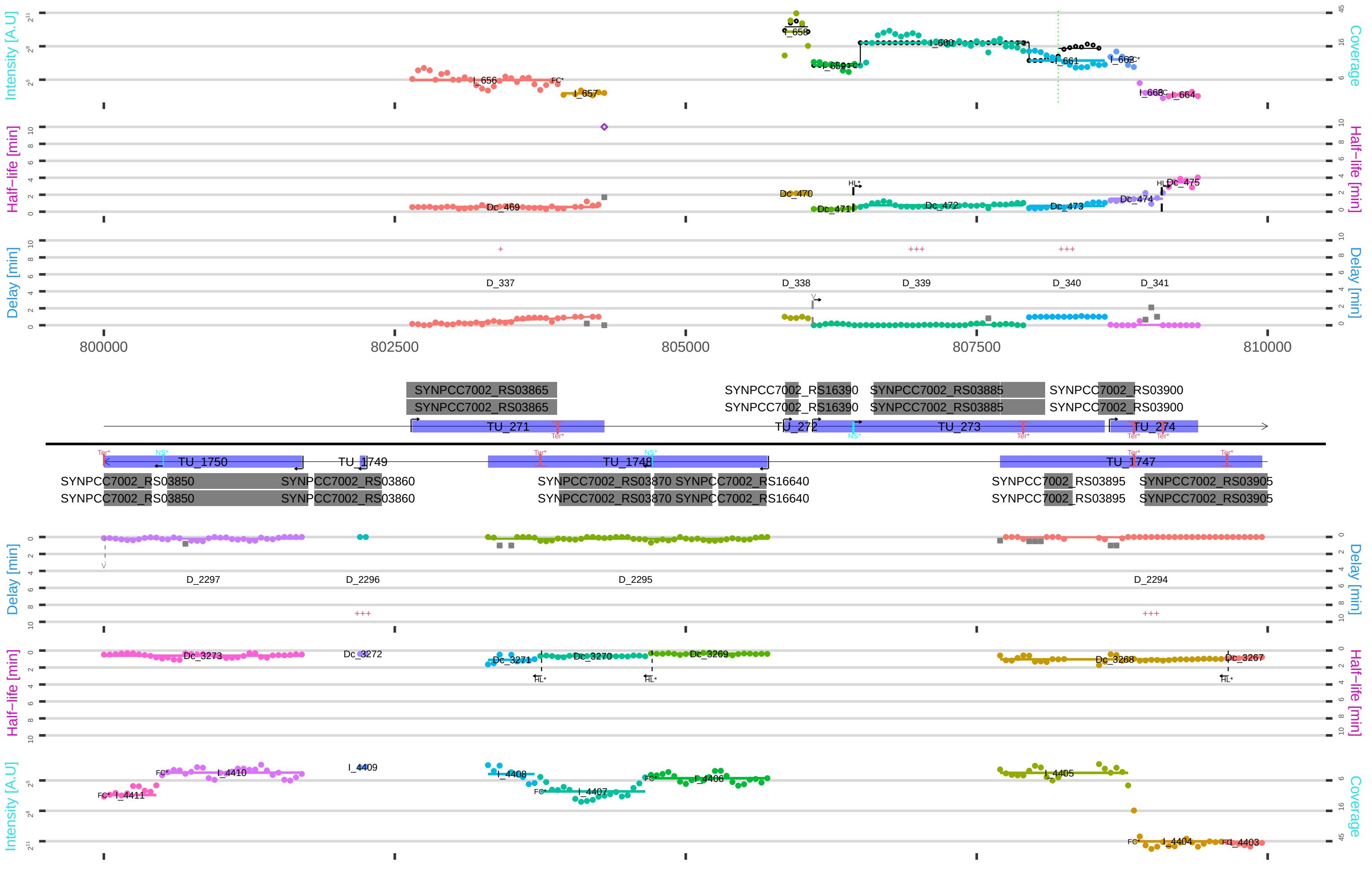

Term: termination (4), NS: new start (2), PS: pausing site (0), iTSS_I: internal starting site (0)

ID: 16216−16395; Term: termination (6), NS: new start (3), PS: pausing site (2), iTSS_I: internal starting site (0)

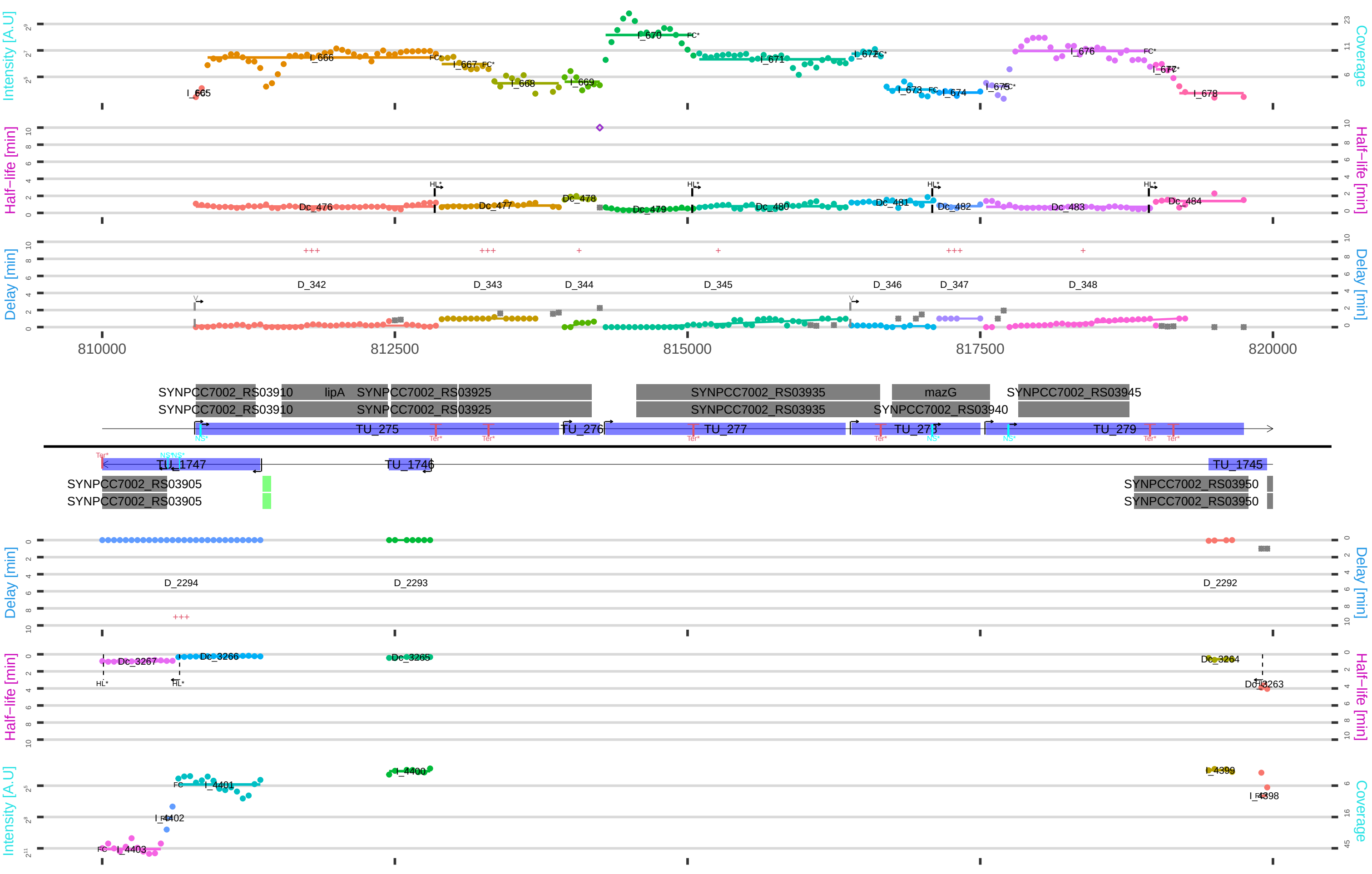

Term: termination (1), NS: new start (2), PS: pausing site (0), iTSS_I: internal starting site (0)

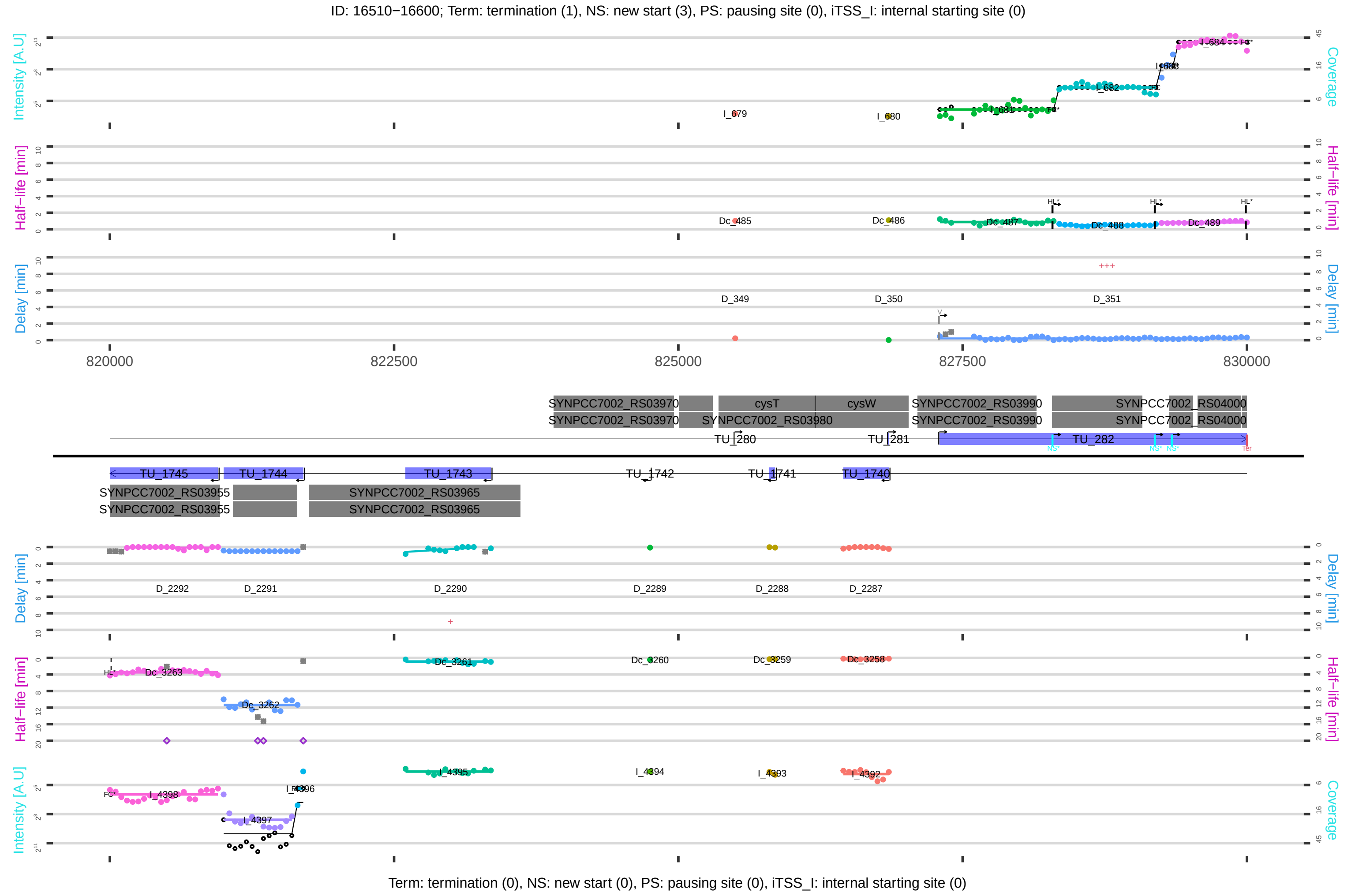

ID: 16510−16600; Term: termination (1), NS: new start (3), PS: pausing site (0), iTSS_I: internal starting site (0)
Intensity [A.U]
Coverage
Half−life [min]
Delay [min]
I_679
I_680
I_681
I_682
I_683
I_684
Dc_485
Dc_486
Dc_487
Dc_488
Dc_489
D_349
D_350
D_351
820000
822500
825000
827500
830000
SYNPCC7002_RS03970
cysT
cysW
SYNPCC7002_RS03980
SYNPCC7002_RS03990
SYNPCC7002_RS04000
TU_280
TU_281
TU_282
TU_1745
TU_1744
TU_1743
TU_1742
TU_1741
TU_1740
SYNPCC7002_RS03955
SYNPCC7002_RS03965
D_2292
D_2291
D_2290
D_2289
D_2288
D_2287
Dc_3263
Dc_3262
Dc_3261
Dc_3260
Dc_3259
Dc_3258
I_4398
I_4397
I_4396
I_4395
I_4394
I_4393
I_4392
Term: termination (0), NS: new start (0), PS: pausing site (0), iTSS_I: internal starting site (0)

ID: 16600−16800; Term: termination (8), NS: new start (0), PS: pausing site (1), iTSS_I: internal starting site (0)

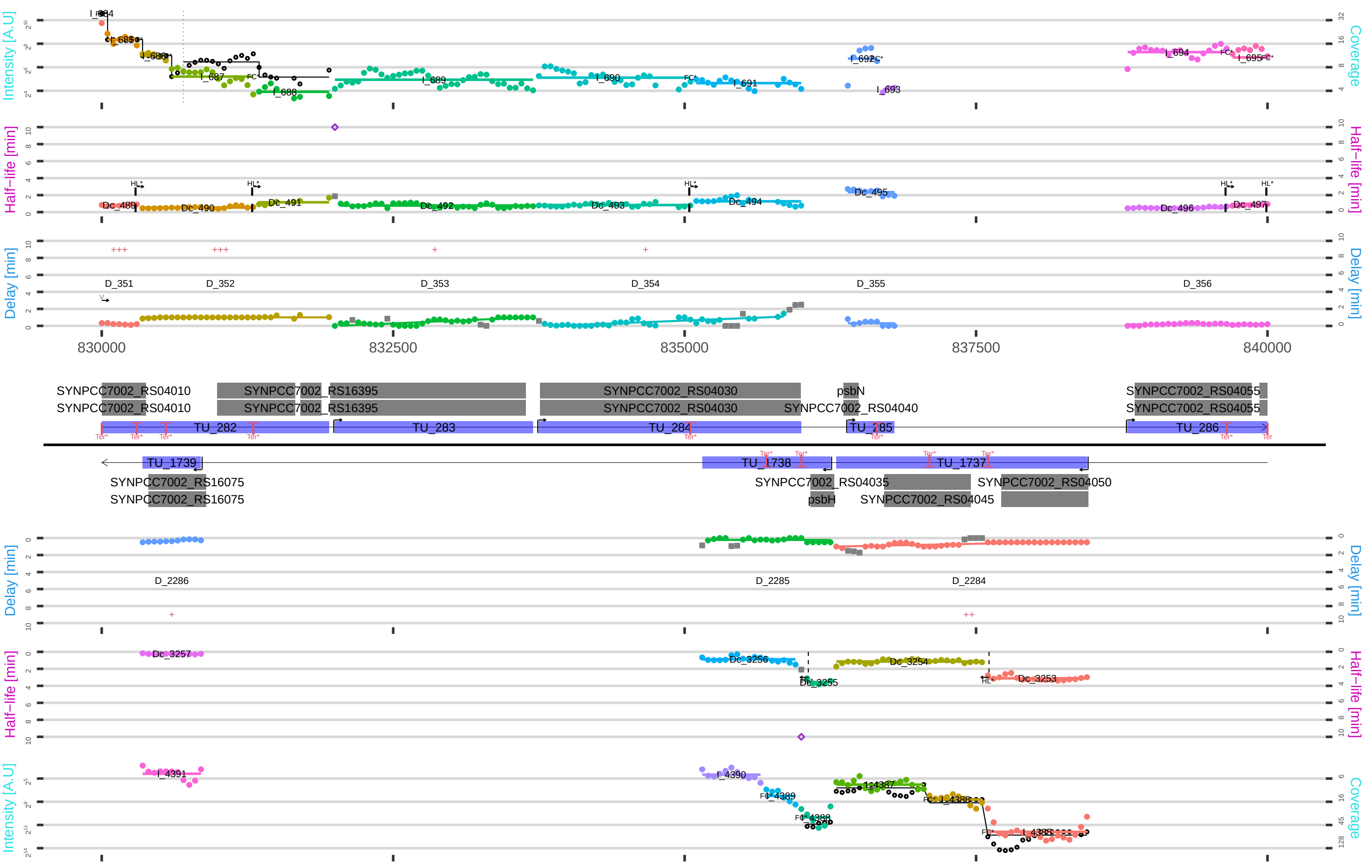

Term: termination (4), NS: new start (0), PS: pausing site (0), iTSS_I: internal starting site (0)

ID: 16800−17000; Term: termination (5), NS: new start (2), PS: pausing site (1), iTSS_I: internal starting site (1)

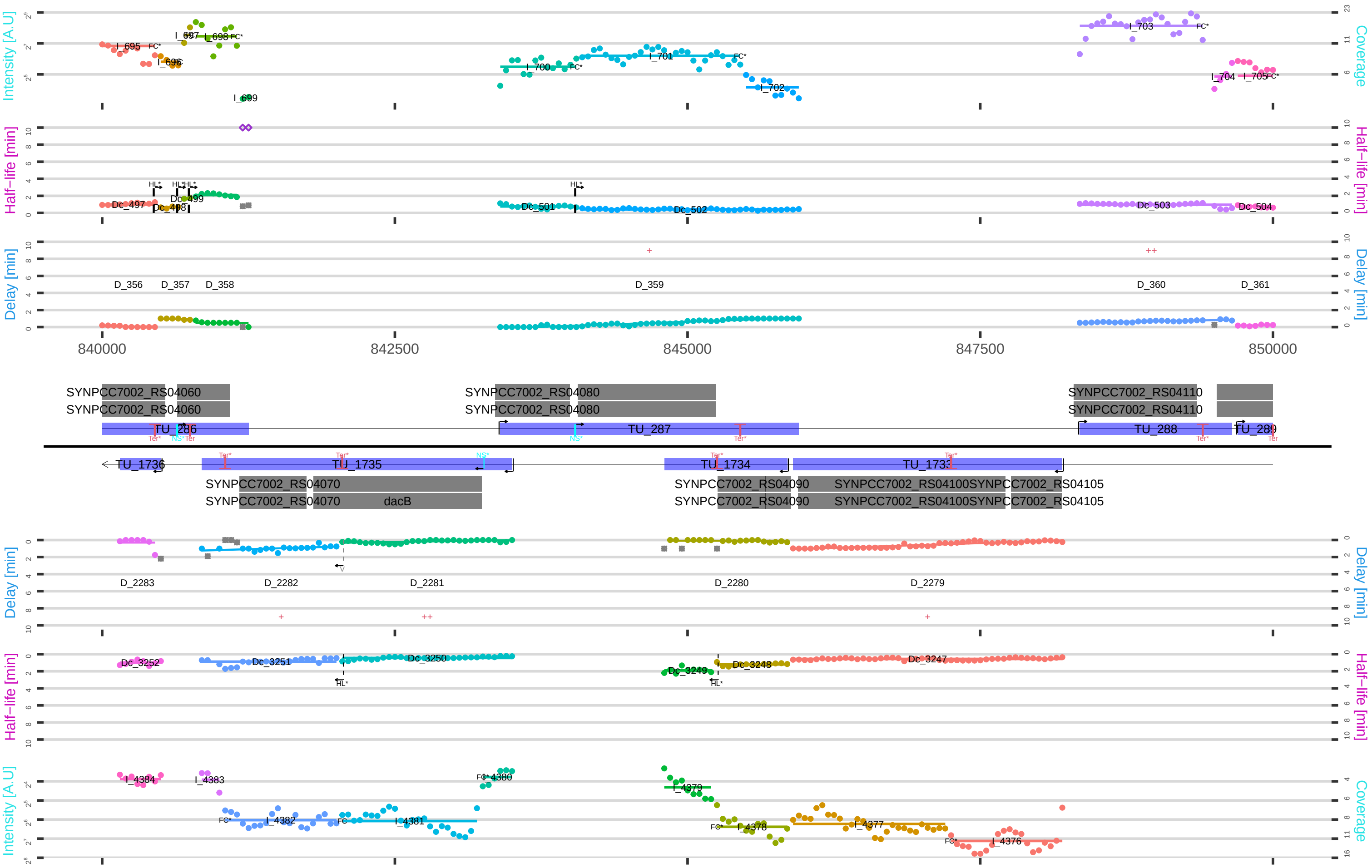

Term: termination (4), NS: new start (1), PS: pausing site (1), iTSS_I: internal starting site (0)

ID: 17000−17161; Term: termination (1), NS: new start (0), PS: pausing site (1), iTSS_I: internal starting site (0)

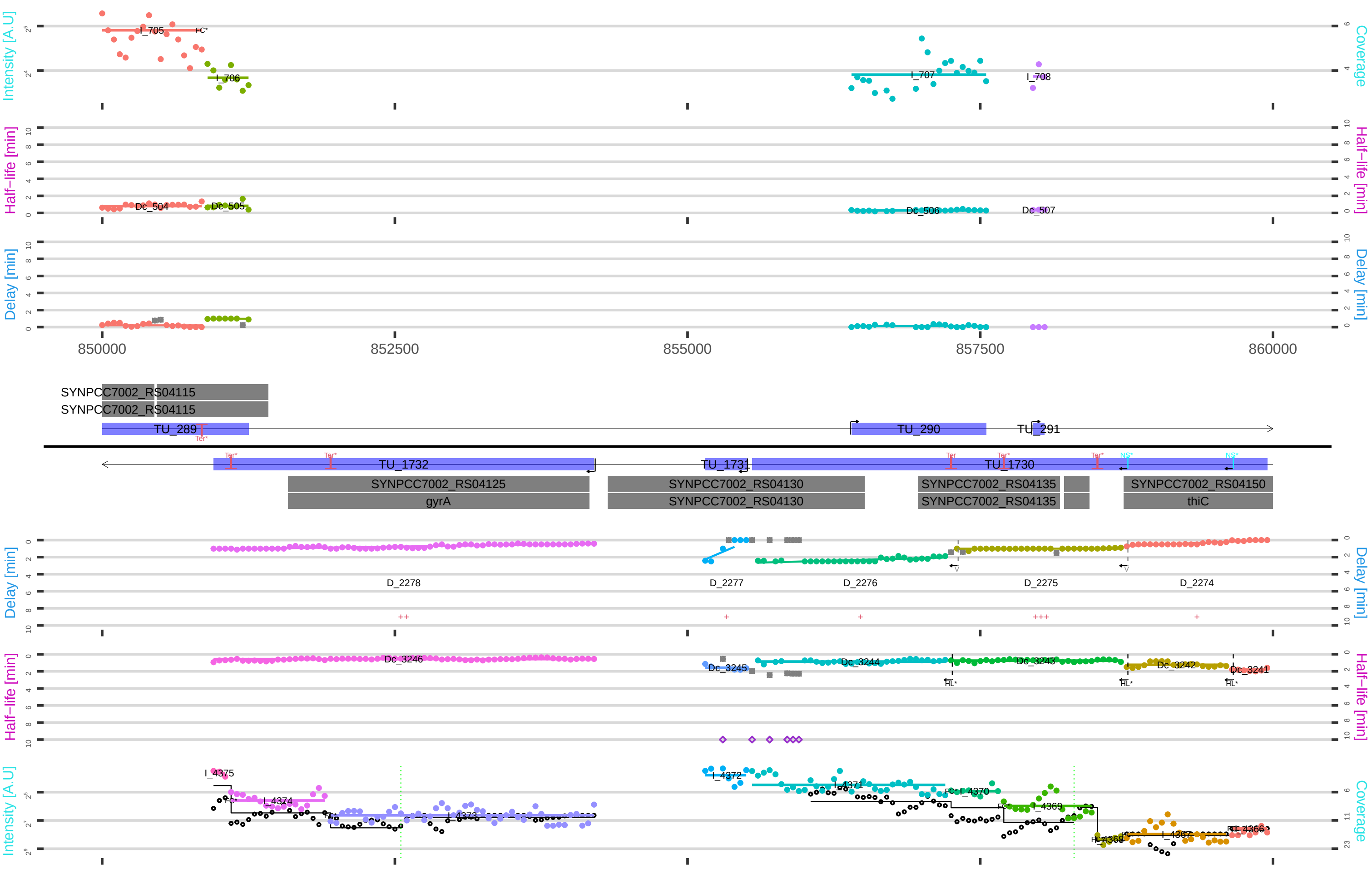

Term: termination (5), NS: new start (2), PS: pausing site (2), iTSS_I: internal starting site (0)

ID: 17210−17400; Term: termination (4), NS: new start (2), PS: pausing site (0), iTSS_I: internal starting site (0)

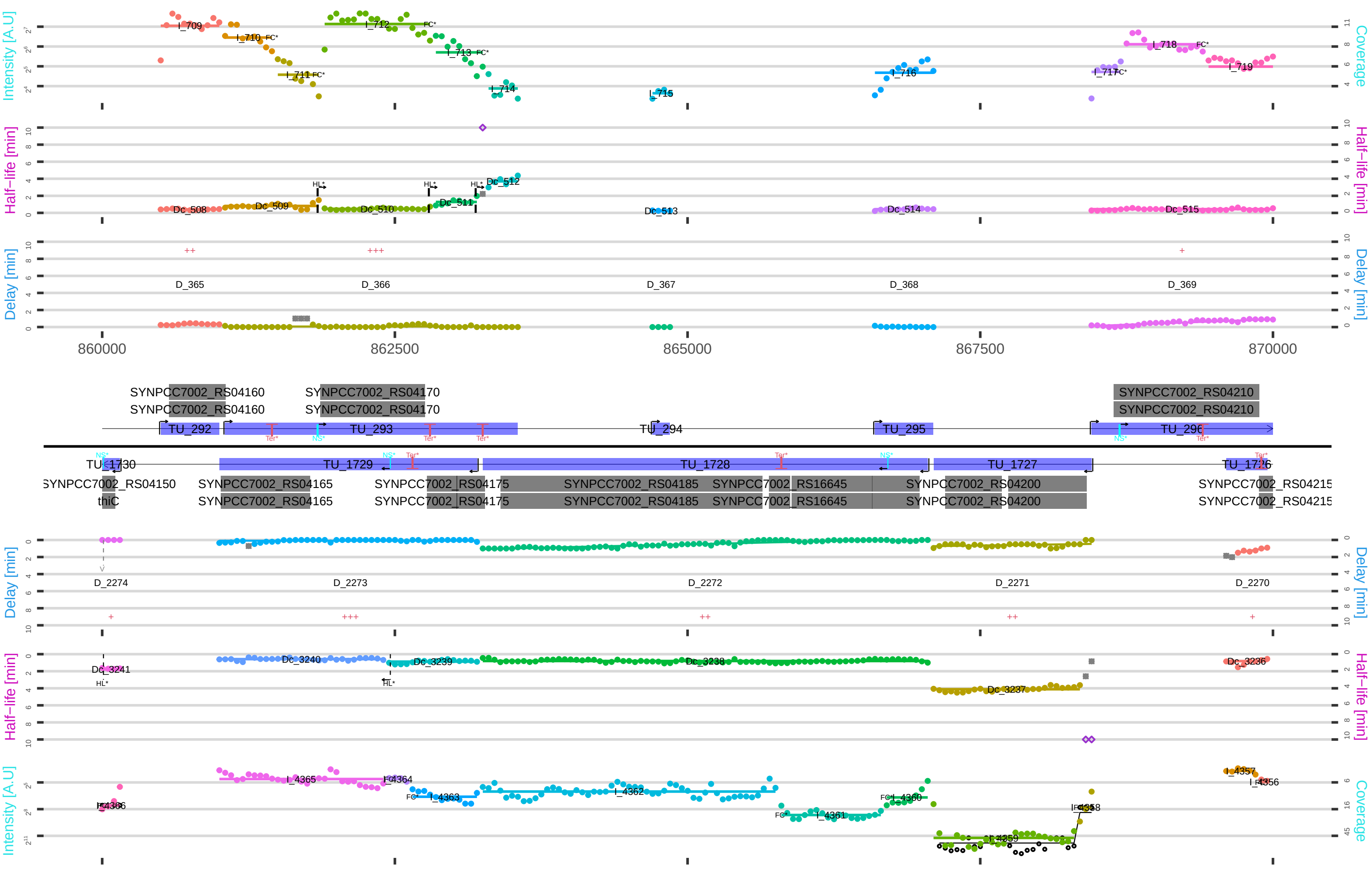

Term: termination (3), NS: new start (3), PS: pausing site (0), iTSS_I: internal starting site (0)

ID: 17400−17516; Term: termination (1), NS: new start (0), PS: pausing site (0), iTSS_I: internal starting site (0)

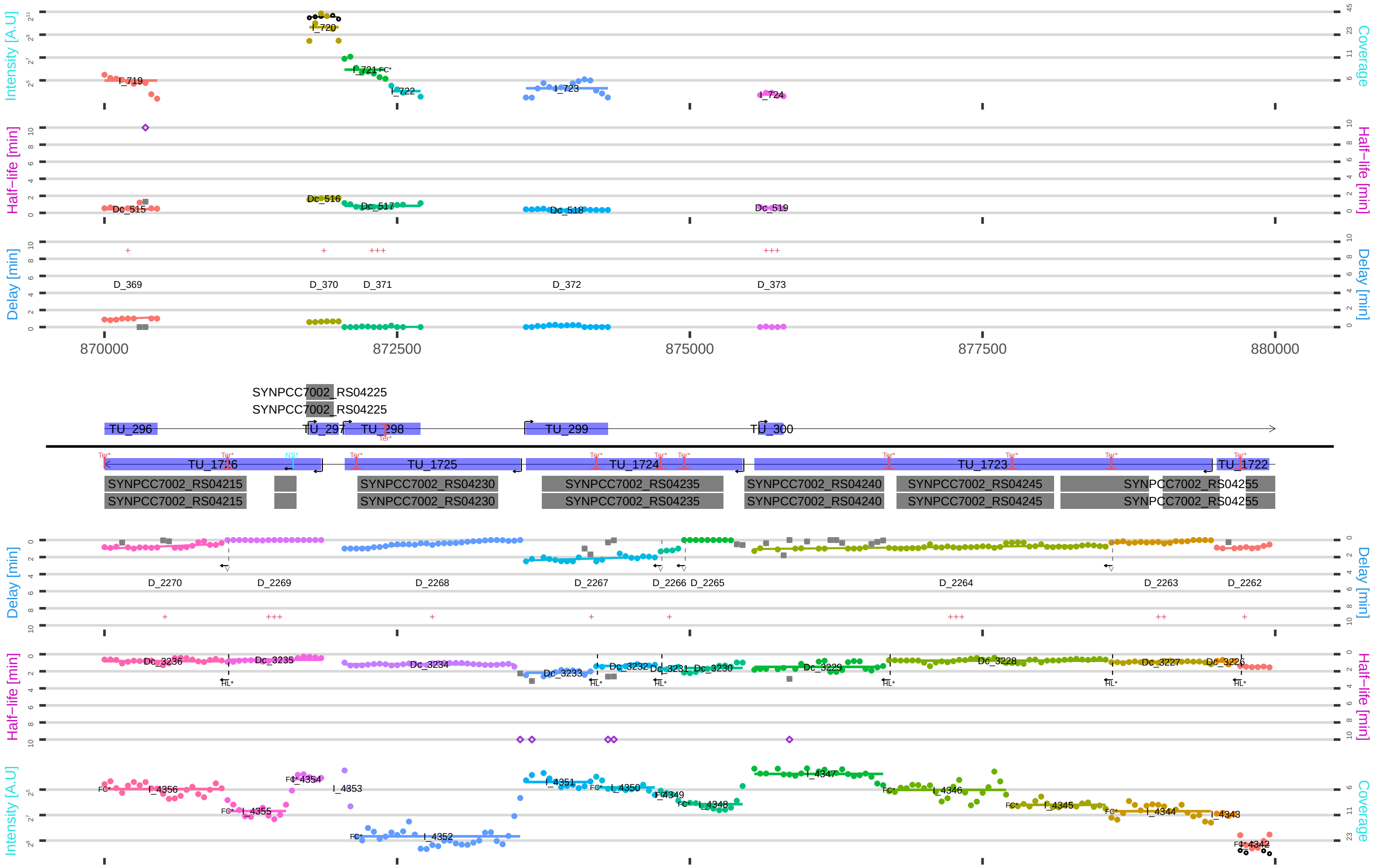

Term: termination (10), NS: new start (1), PS: pausing site (4), iTSS_I: internal starting site (0)

ID: 17623−17800; Term: termination (3), NS: new start (3), PS: pausing site (2), iTSS_I: internal starting site (0)

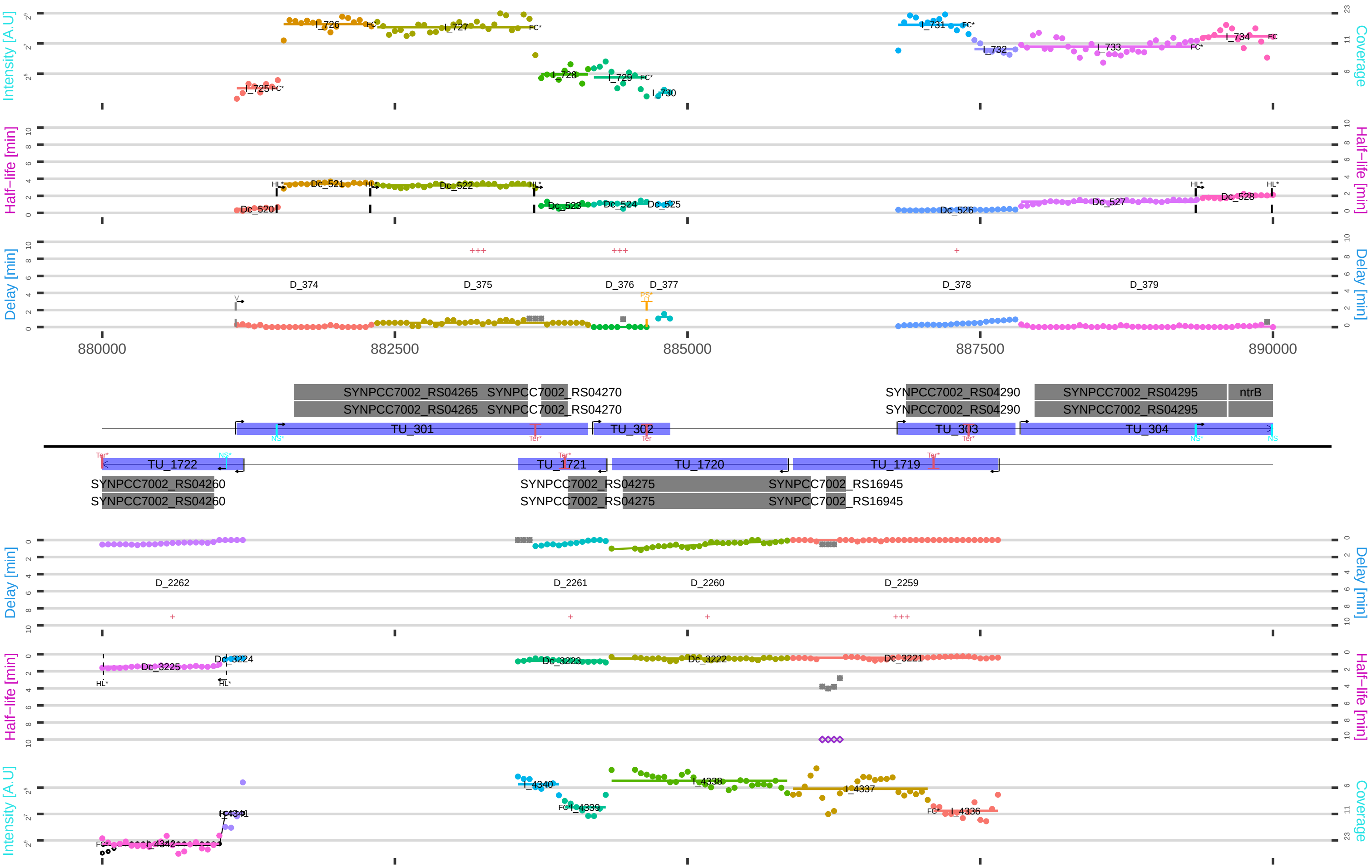

Term: termination (3), NS: new start (1), PS: pausing site (0), iTSS_I: internal starting site (0)

ID: 17800−18000; Term: termination (6), NS: new start (3), PS: pausing site (3), iTSS_I: internal starting site (0)

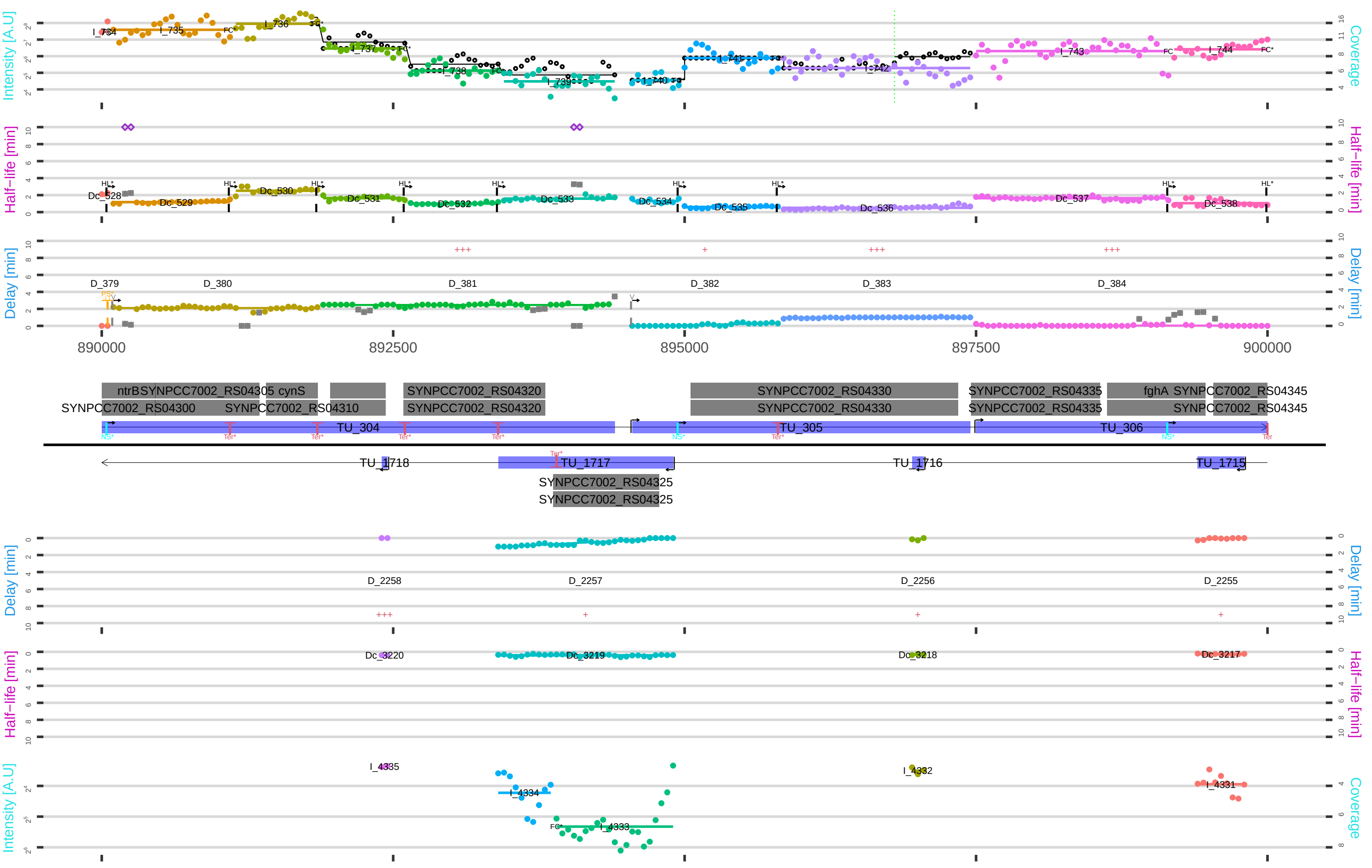

Term: termination (1), NS: new start (0), PS: pausing site (0), iTSS_I: internal starting site (0)

ID: 18000−18176; Term: termination (6), NS: new start (2), PS: pausing site (0), iTSS_I: internal starting site (0)

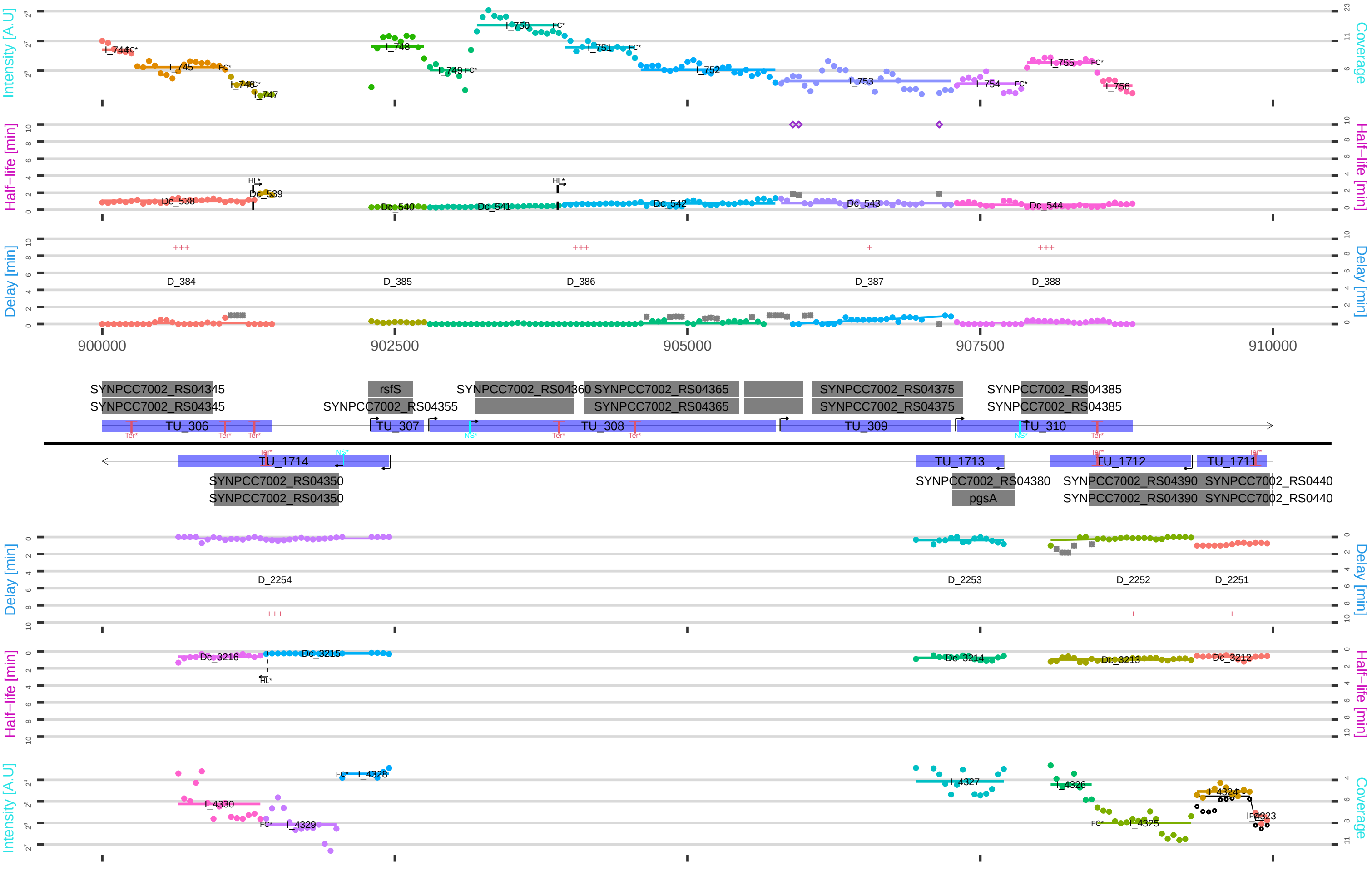

Term: termination (3), NS: new start (1), PS: pausing site (0), iTSS_I: internal starting site (0)

ID: 18212−18364; Term: termination (4), NS: new start (1), PS: pausing site (0), iTSS_I: internal starting site (0)

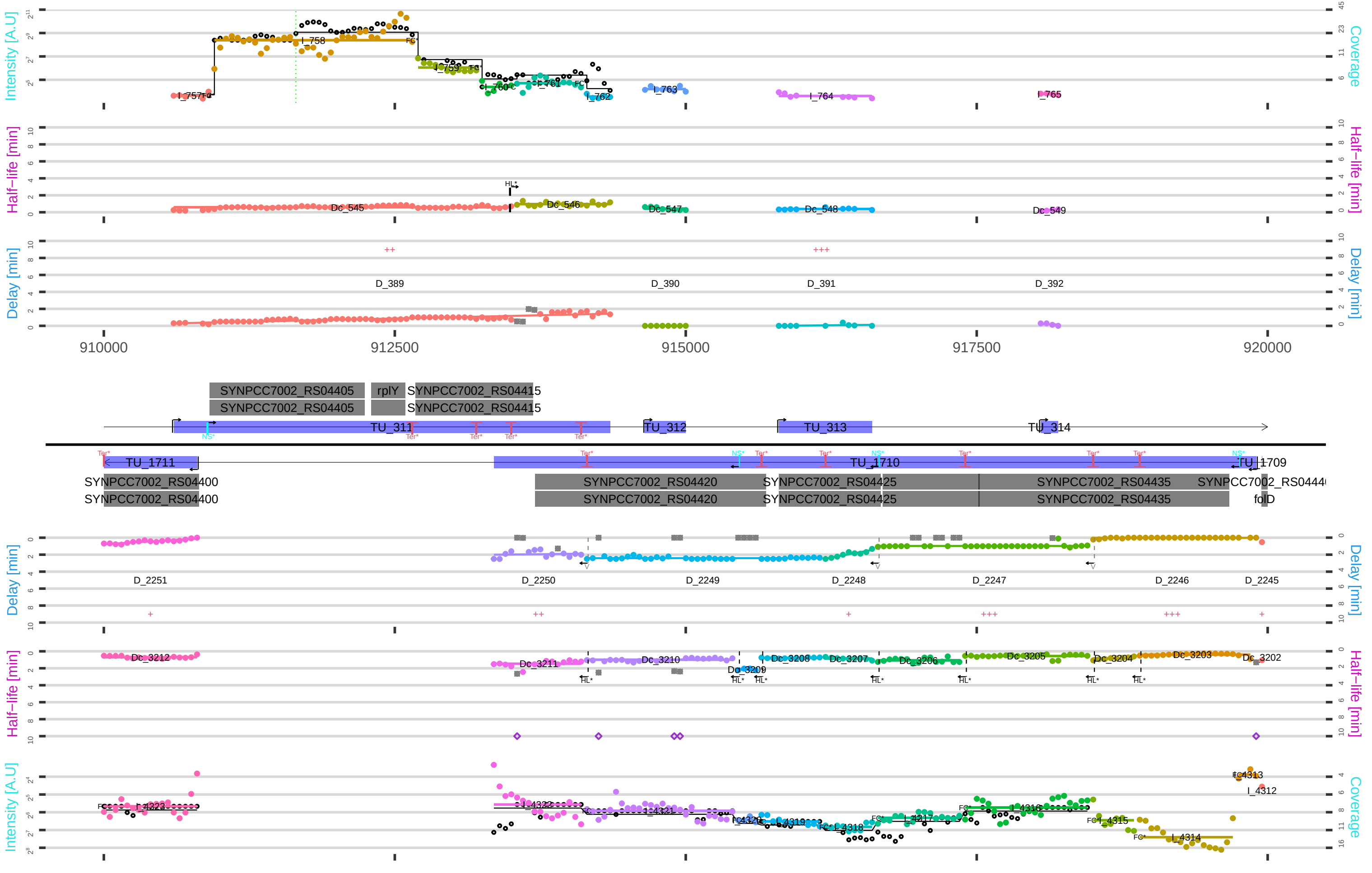

Term: termination (7), NS: new start (3), PS: pausing site (2), iTSS_I: internal starting site (2)

ID: 18529−18600; Term: termination (2), NS: new start (1), PS: pausing site (1), iTSS_I: internal starting site (0)

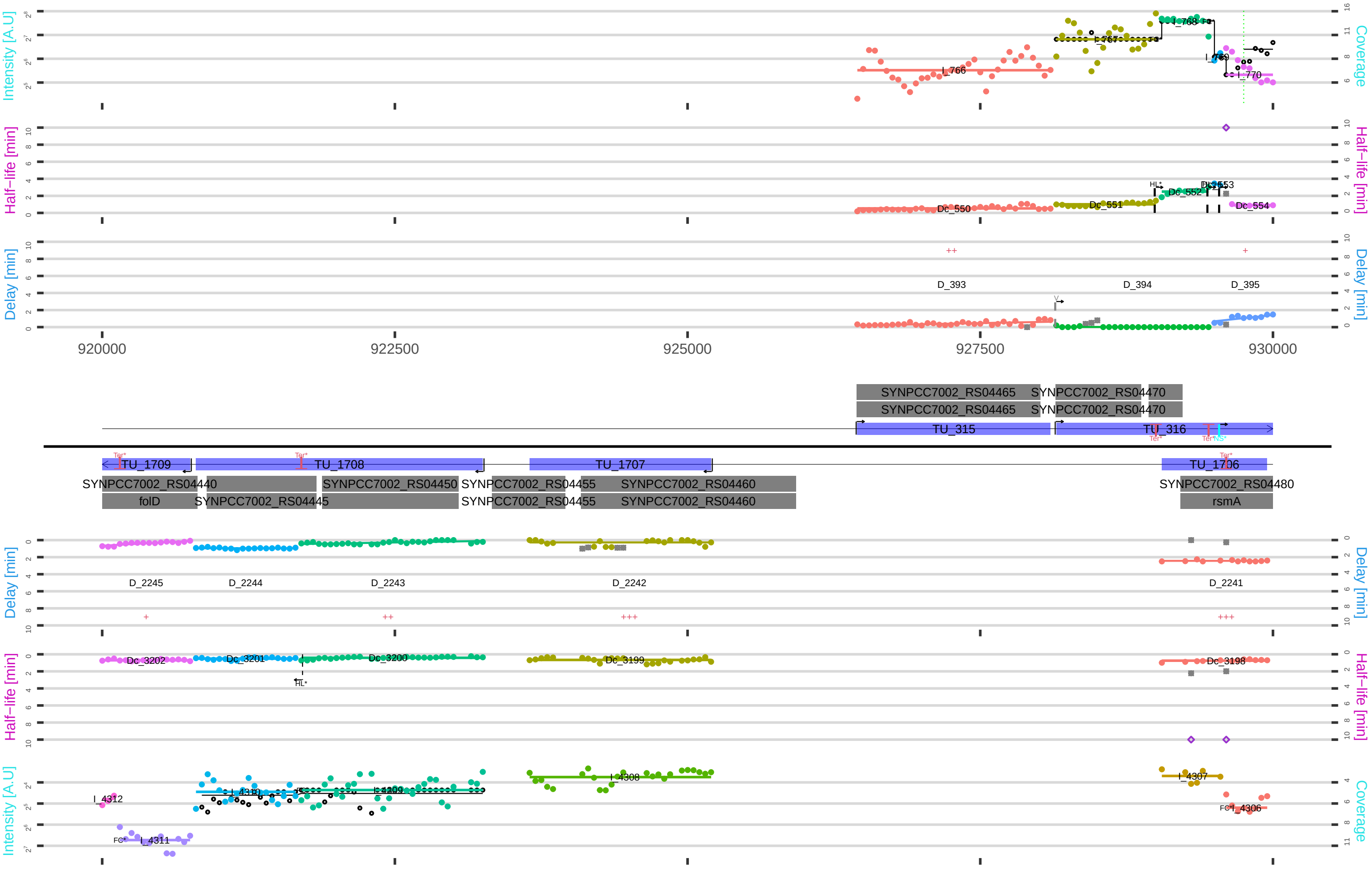

Term: termination (3), NS: new start (0), PS: pausing site (1), iTSS_I: internal starting site (0)

ID: 18600−18792; Term: termination (4), NS: new start (0), PS: pausing site (0), iTSS_I: internal starting site (0)

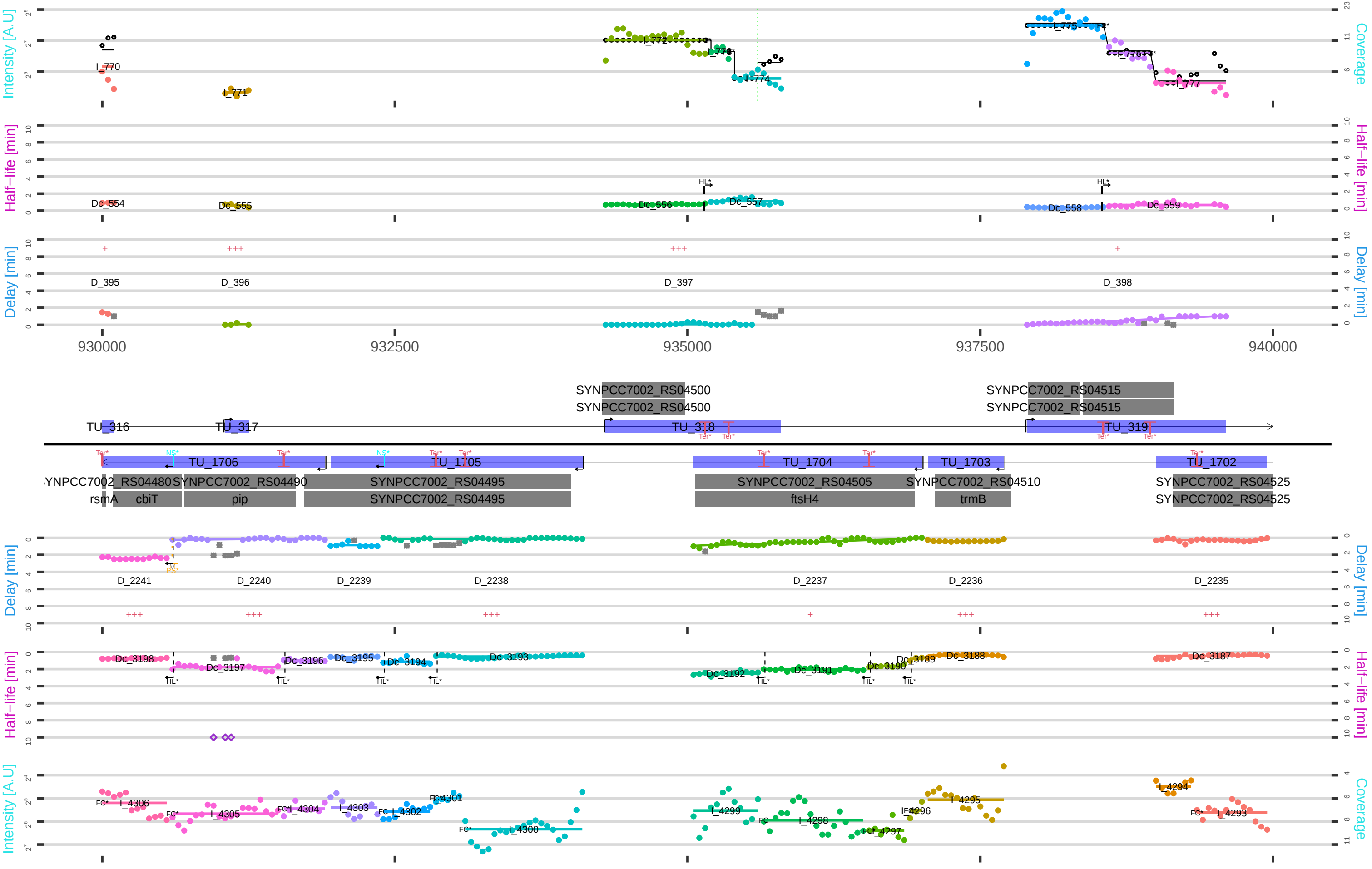

Term: termination (7), NS: new start (2), PS: pausing site (2), iTSS_I: internal starting site (0)

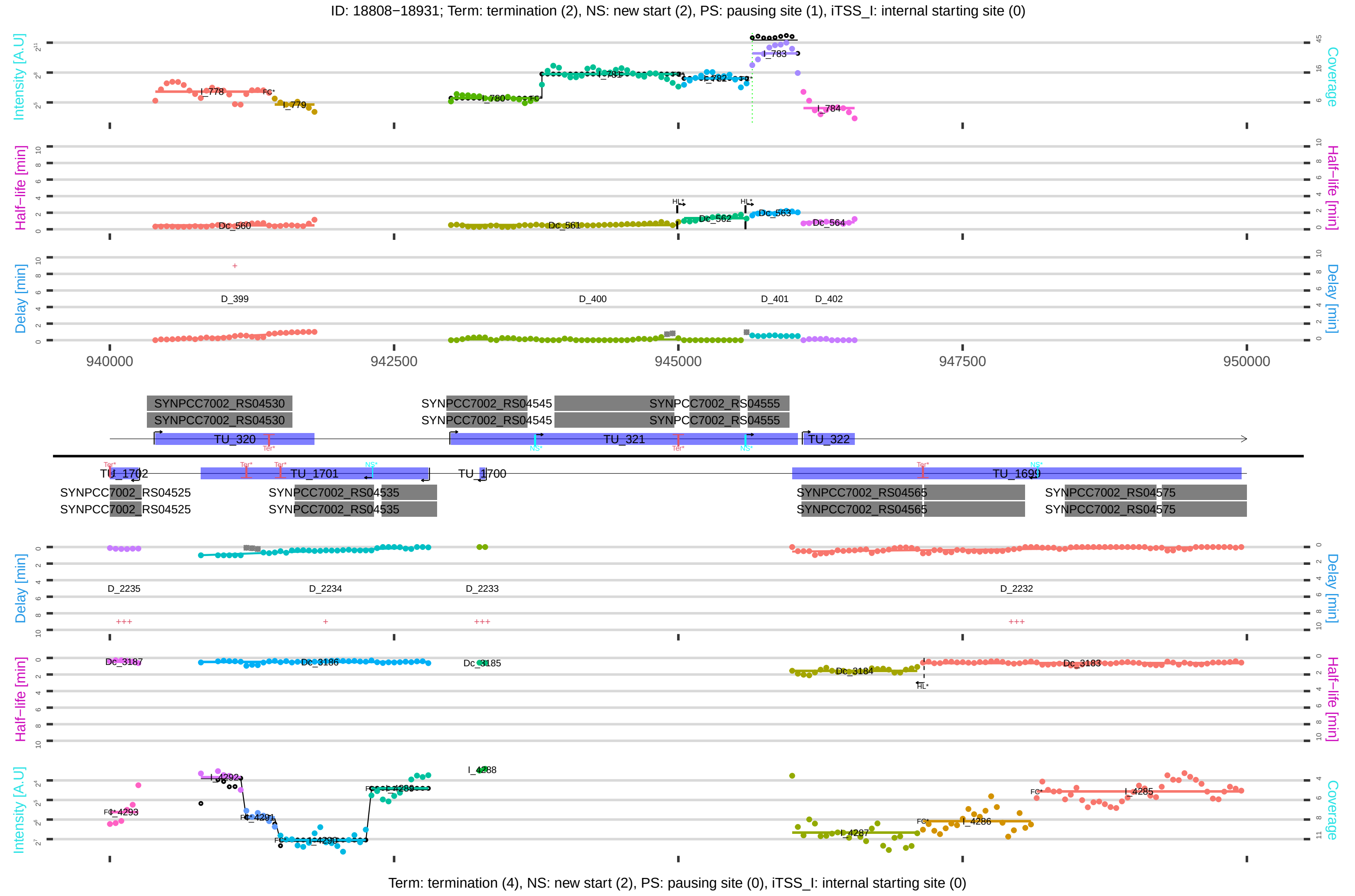

ID: 18808−18931; Term: termination (2), NS: new start (2), PS: pausing site (1), iTSS_I: internal starting site (0)
Intensity [A.U]
Coverage
Half−life [min]
Delay [min]
I_778
I_779
I_780
I_781
I_782
I_783
I_784
Dc_560
Dc_561
Dc_562
Dc_563
Dc_564
D_399
D_400
D_401
D_402
940000
942500
945000
947500
950000
SYNPCC7002_RS04530
SYNPCC7002_RS04545
SYNPCC7002_RS04555
TU_320
TU_321
TU_322
TU_1702
TU_1701
TU_1700
TU_1699
SYNPCC7002_RS04525
SYNPCC7002_RS04535
SYNPCC7002_RS04565
SYNPCC7002_RS04575
D_2235
D_2234
D_2233
D_2232
Dc_3187
Dc_3186
Dc_3185
Dc_3184
Dc_3183
I_4293
I_4292
I_4291
I_4290
I_4289
I_4288
I_4287
I_4286
I_4285
Term: termination (4), NS: new start (2), PS: pausing site (0), iTSS_I: internal starting site (0)

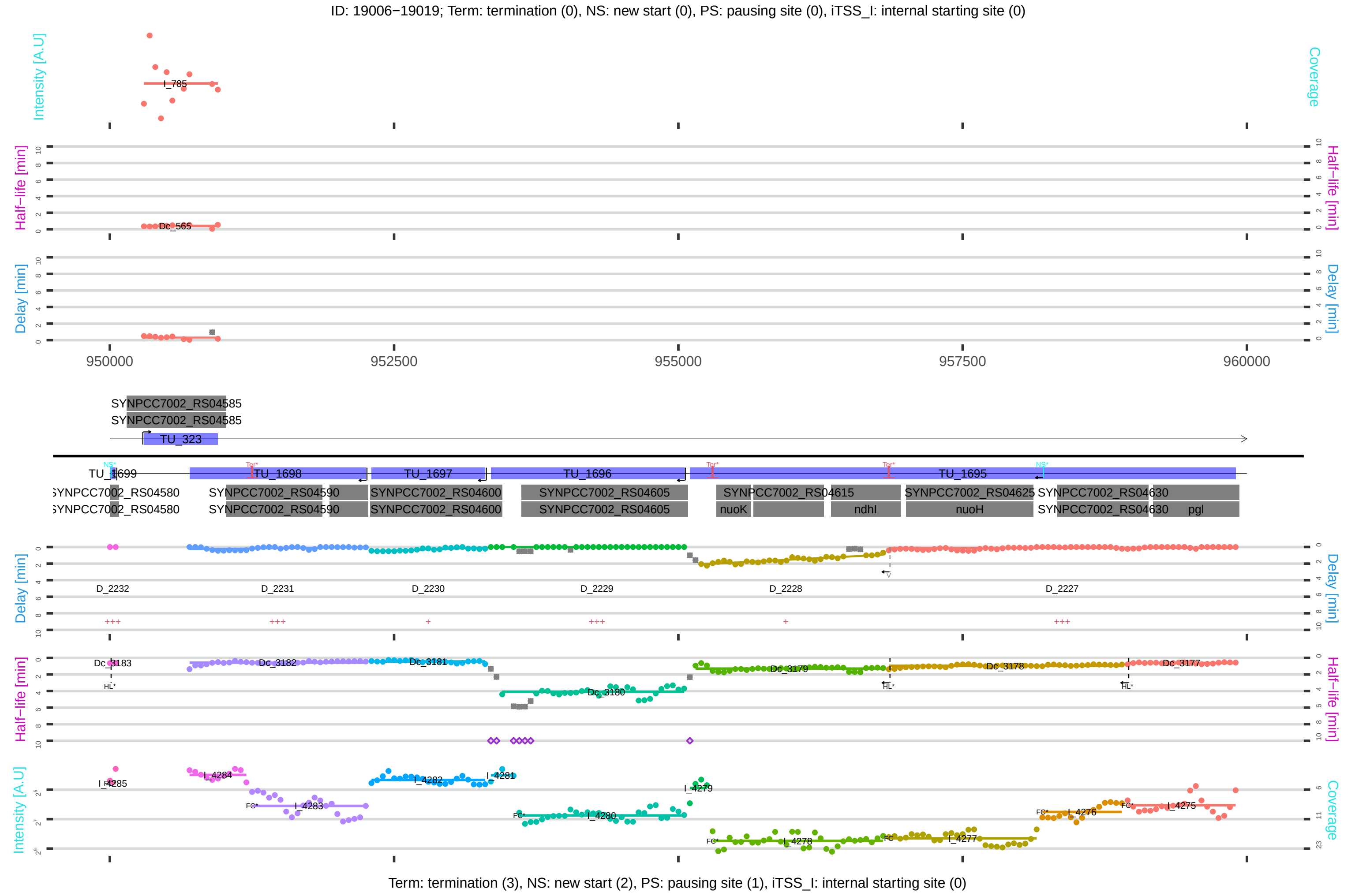
ID: 19006−19019; Term: termination (0), NS: new start (0), PS: pausing site (0), iTSS_I: internal starting site (0)
Intensity [A.U]
Coverage
I_785
Half−life [min]
Dc_565
Delay [min]
950000
952500
955000
957500
960000
SYNPCC7002_RS04585
TU_323
TU_1699
TU_1698
TU_1697
TU_1696
TU_1695
SYNPCC7002_RS04580
SYNPCC7002_RS04590
SYNPCC7002_RS04600
SYNPCC7002_RS04605
SYNPCC7002_RS04615
SYNPCC7002_RS04625
SYNPCC7002_RS04630
nuoK
ndhI
nuoH
pgl
D_2232
D_2231
D_2230
D_2229
D_2228
D_2227
Dc_3183
Dc_3182
Dc_3181
Dc_3180
Dc_3179
Dc_3178
Dc_3177
I_4285
I_4284
I_4283
I_4282
I_4281
I_4280
I_4279
I_4278
I_4277
I_4276
I_4275
Term: termination (3), NS: new start (2), PS: pausing site (1), iTSS_I: internal starting site (0)

ID: 19201−19400; Term: termination (4), NS: new start (2), PS: pausing site (0), iTSS_I: internal starting site (0)

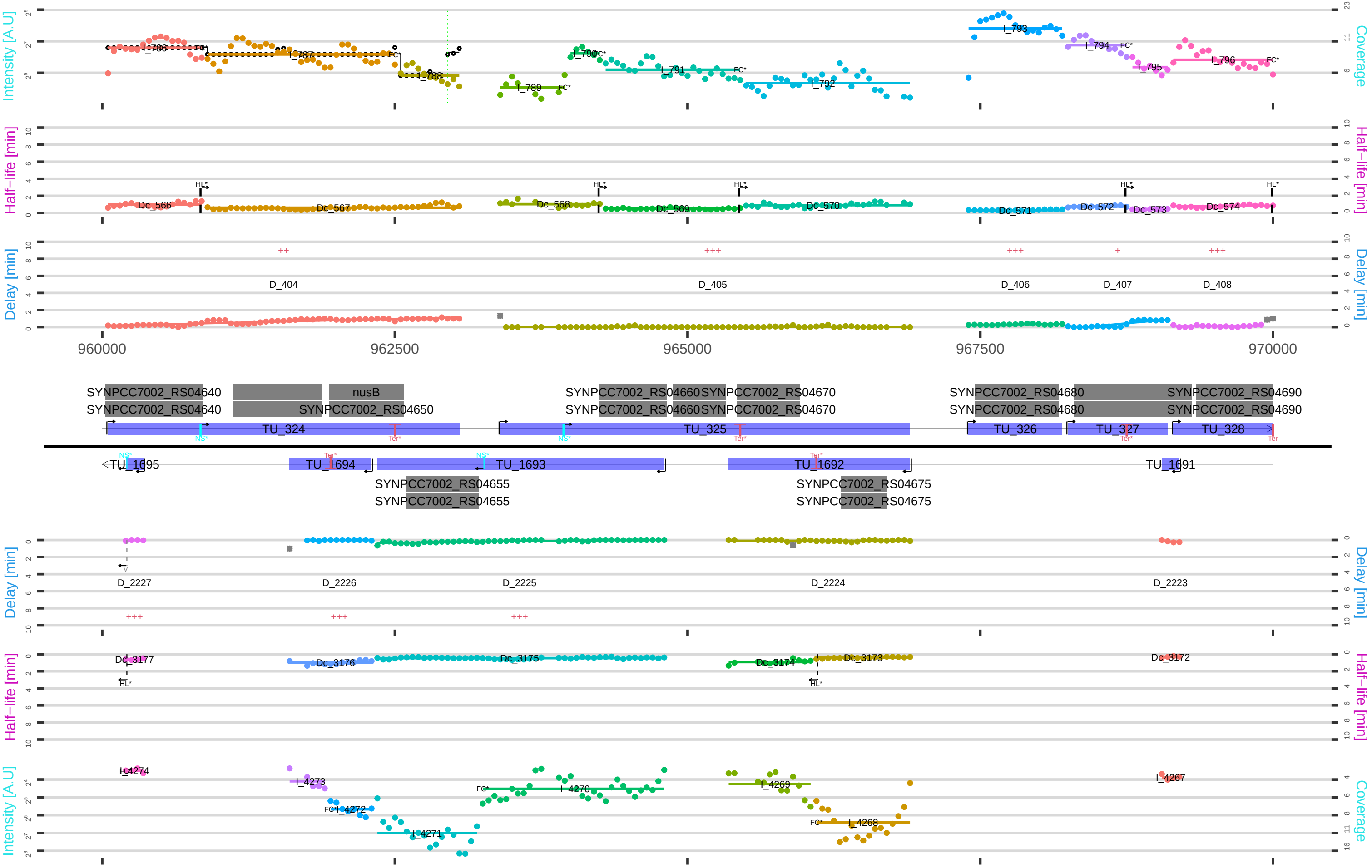

Term: termination (2), NS: new start (2), PS: pausing site (0), iTSS_I: internal starting site (0)

ID: 19400−19600; Term: termination (7), NS: new start (1), PS: pausing site (3), iTSS_I: internal starting site (0)

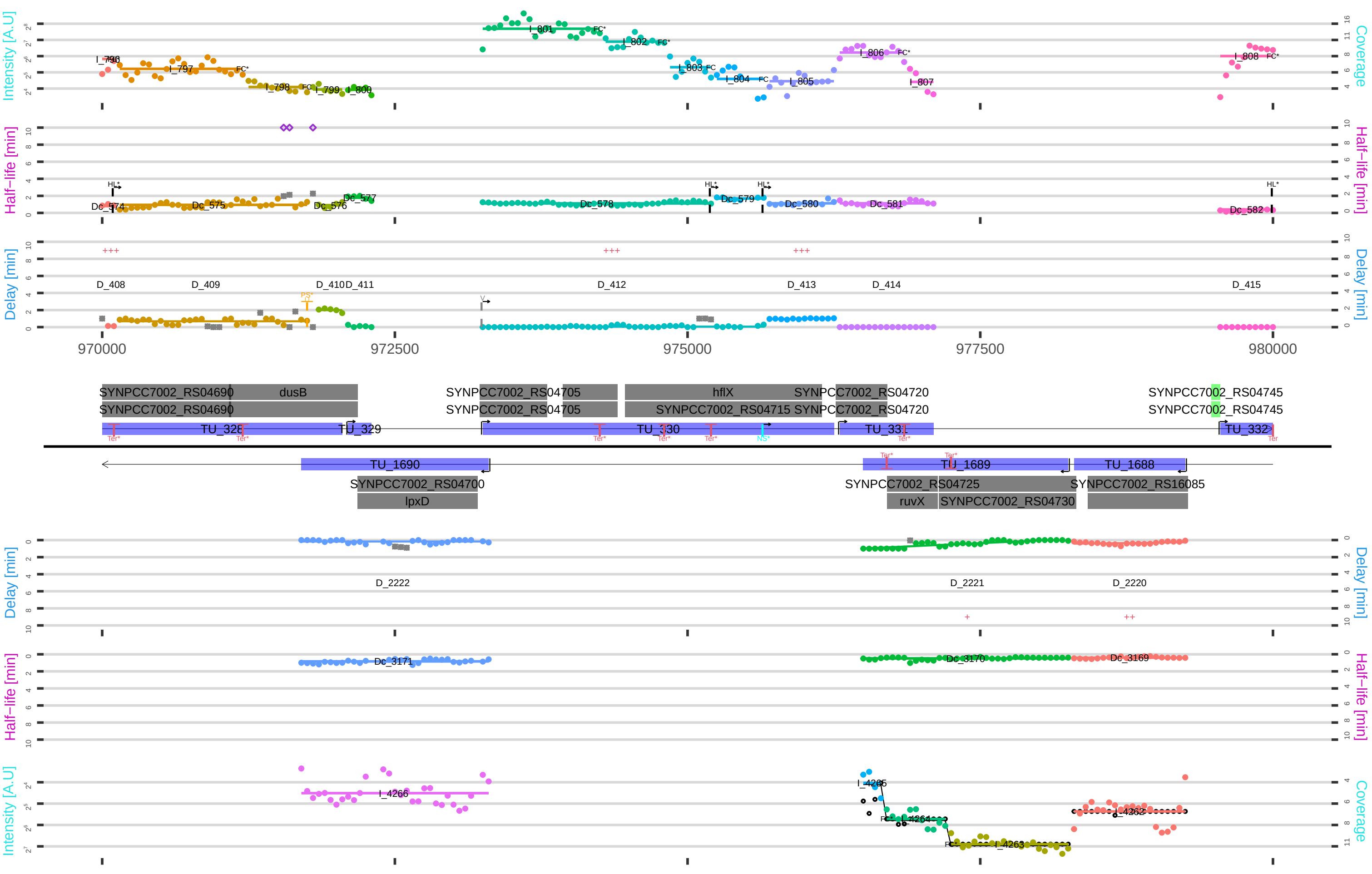

Term: termination (2), NS: new start (0), PS: pausing site (0), iTSS_I: internal starting site (0)

ID: 19600−19786; Term: termination (1), NS: new start (1), PS: pausing site (0), iTSS_I: internal starting site (0)

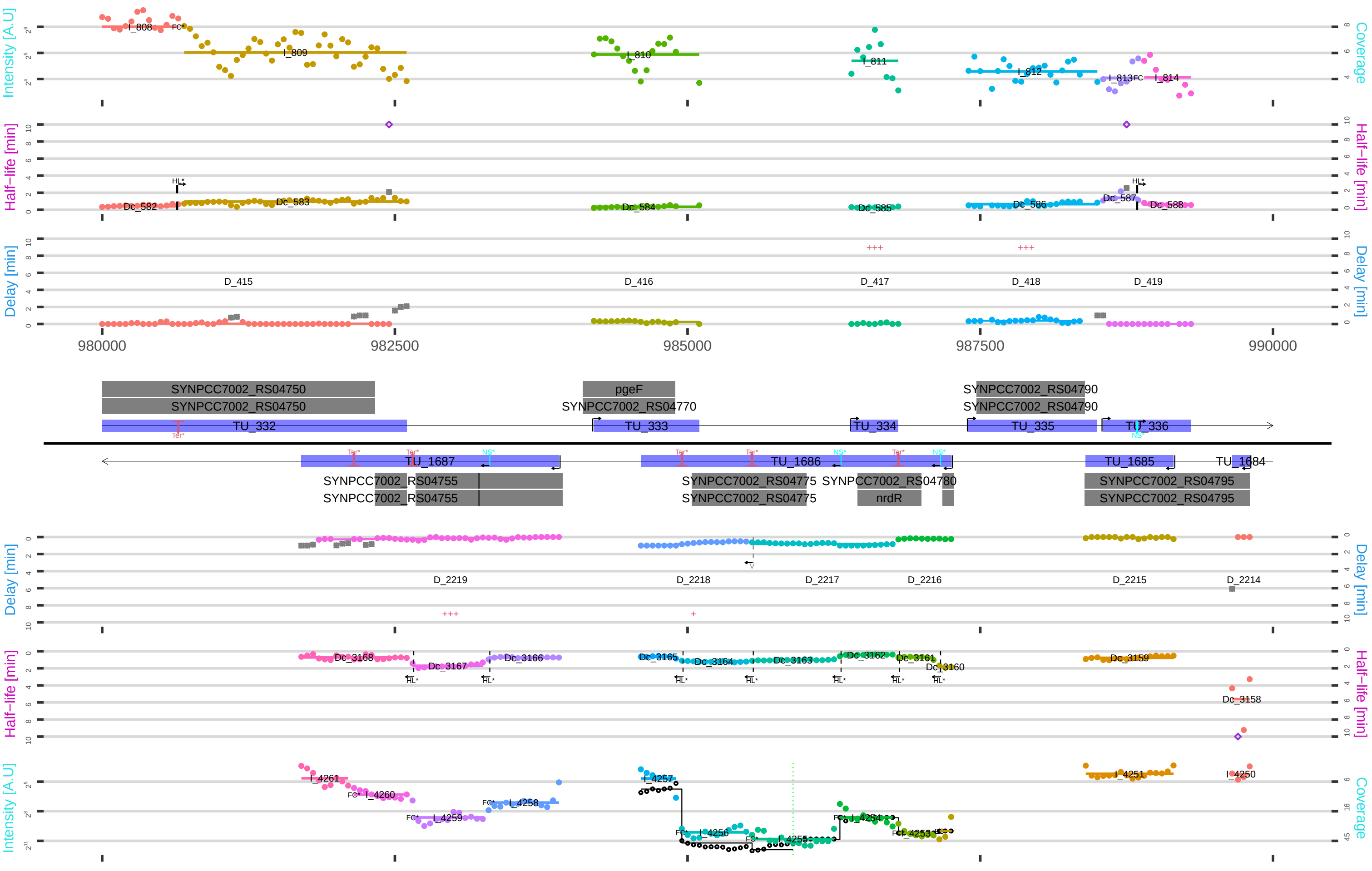

Term: termination (5), NS: new start (3), PS: pausing site (1), iTSS_I: internal starting site (1)

ID: 19802−20000; Term: termination (6), NS: new start (2), PS: pausing site (0), iTSS_I: internal starting site (0)

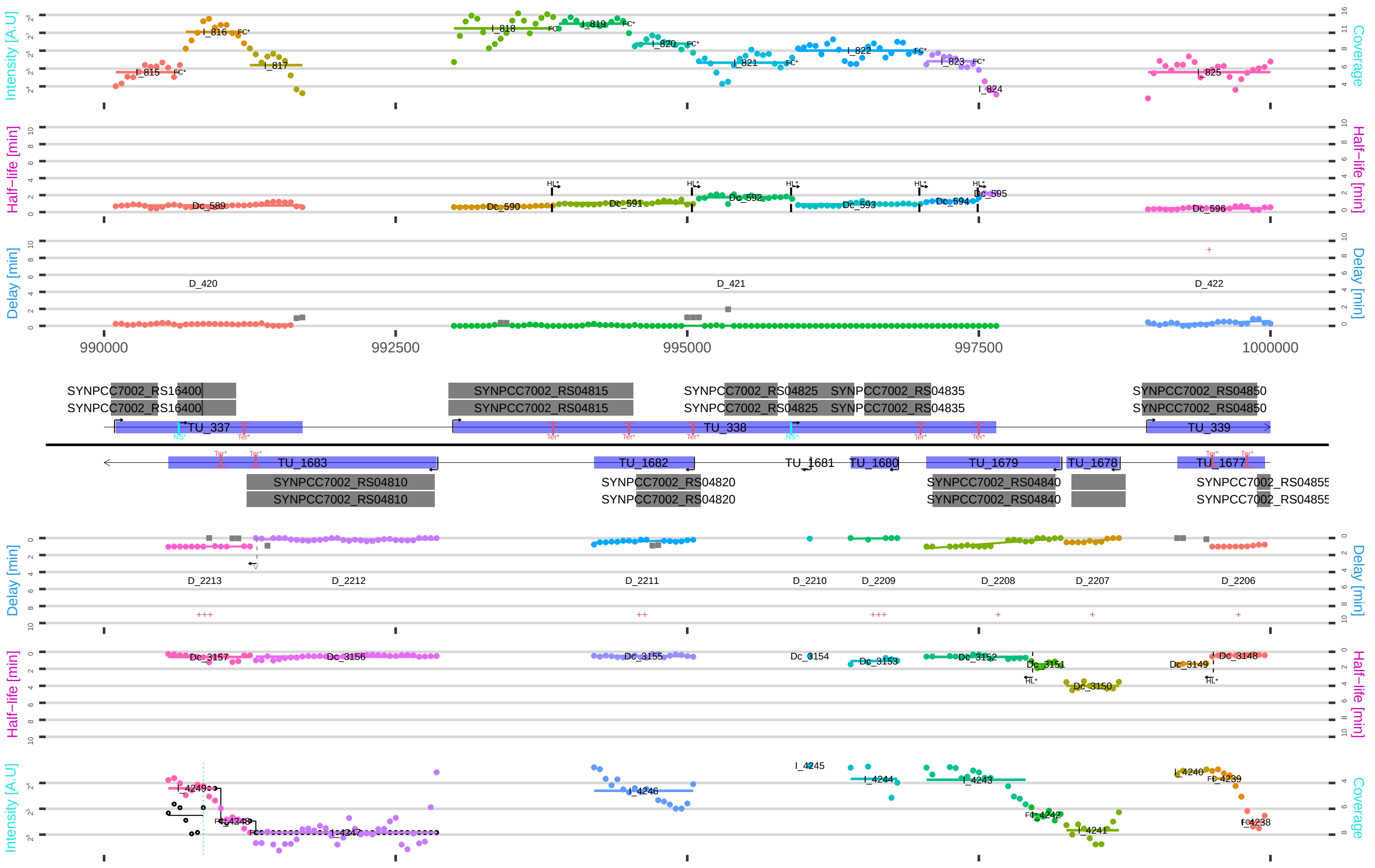

Term: termination (4), NS: new start (0), PS: pausing site (1), iTSS_I: internal starting site (0)

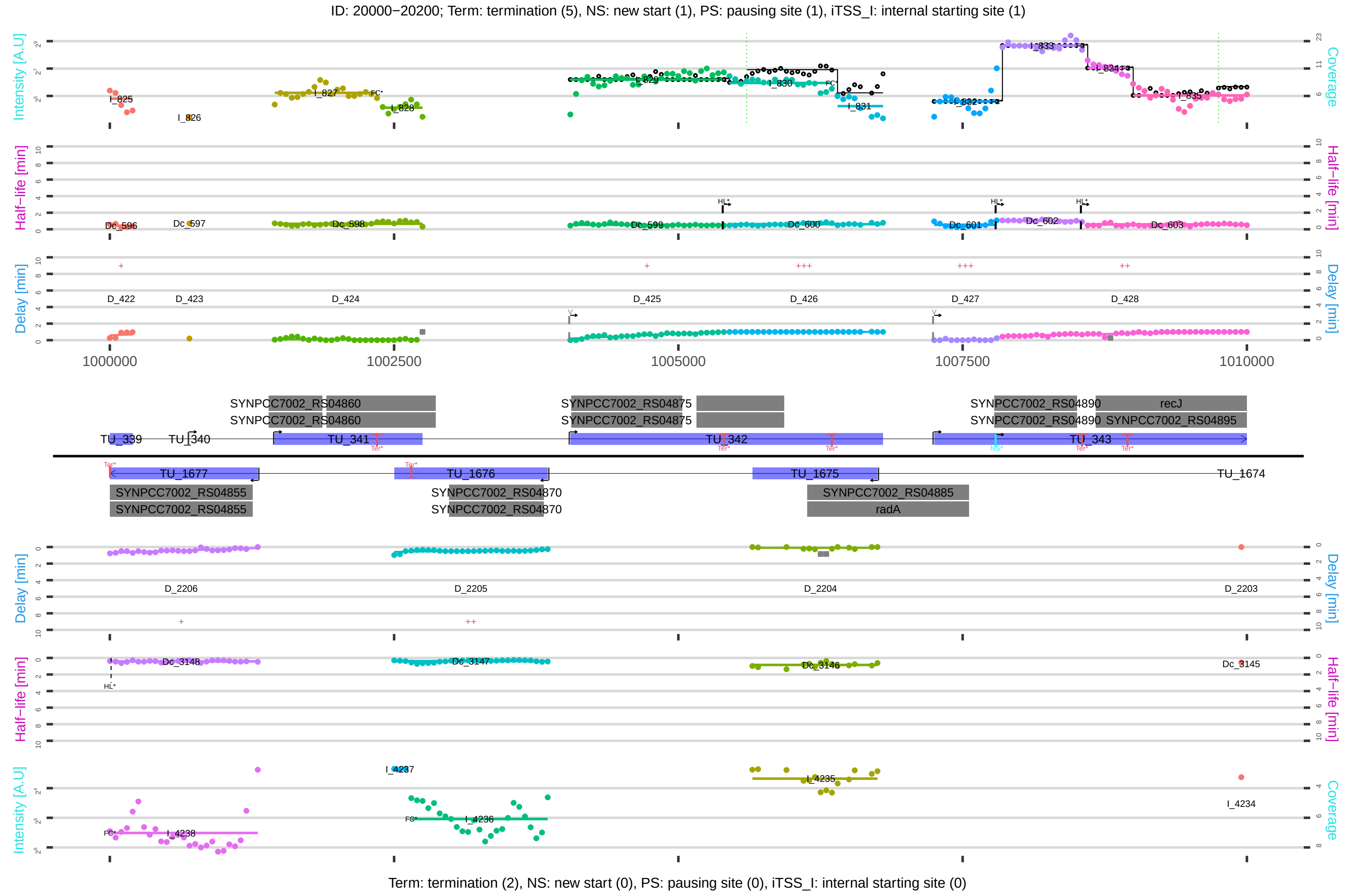

ID: 20000−20200; Term: termination (5), NS: new start (1), PS: pausing site (1), iTSS_I: internal starting site (1)
Intensity [A.U]
Coverage
Half−life [min]
Delay [min]
1000000
1002500
1005000
1007500
1010000
SYNPCC7002_RS04860
SYNPCC7002_RS04875
SYNPCC7002_RS04890
recJ
SYNPCC7002_RS04895
TU_339
TU_340
TU_341
TU_342
TU_343
TU_1677
TU_1676
TU_1675
TU_1674
SYNPCC7002_RS04855
SYNPCC7002_RS04870
SYNPCC7002_RS04885
radA
Term: termination (2), NS: new start (0), PS: pausing site (0), iTSS_I: internal starting site (0)

ID: 20200−20400; Term: termination (5), NS: new start (4), PS: pausing site (1), iTSS_I: internal starting site (0)

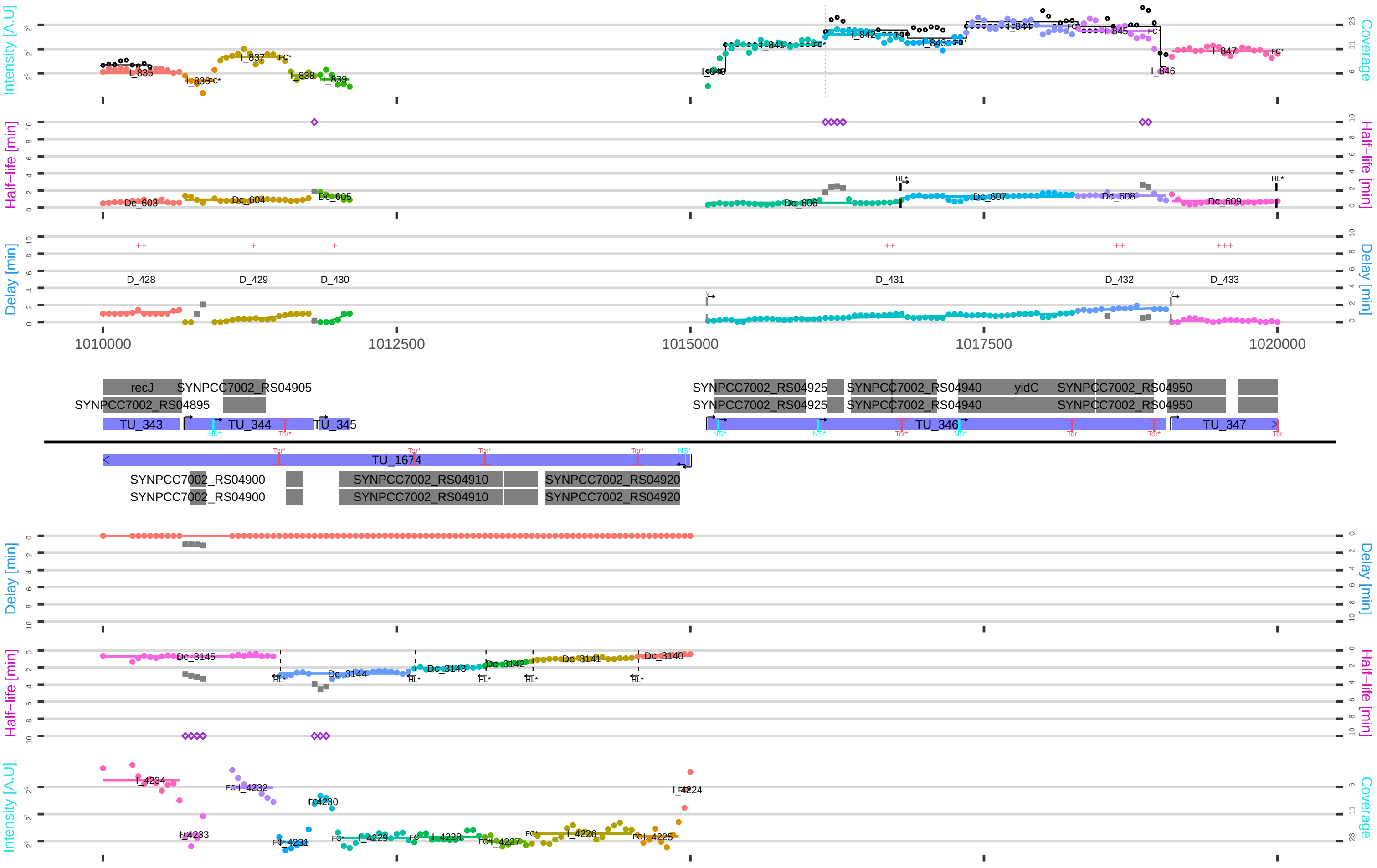

Term: termination (4), NS: new start (1), PS: pausing site (0), iTSS_I: internal starting site (0)

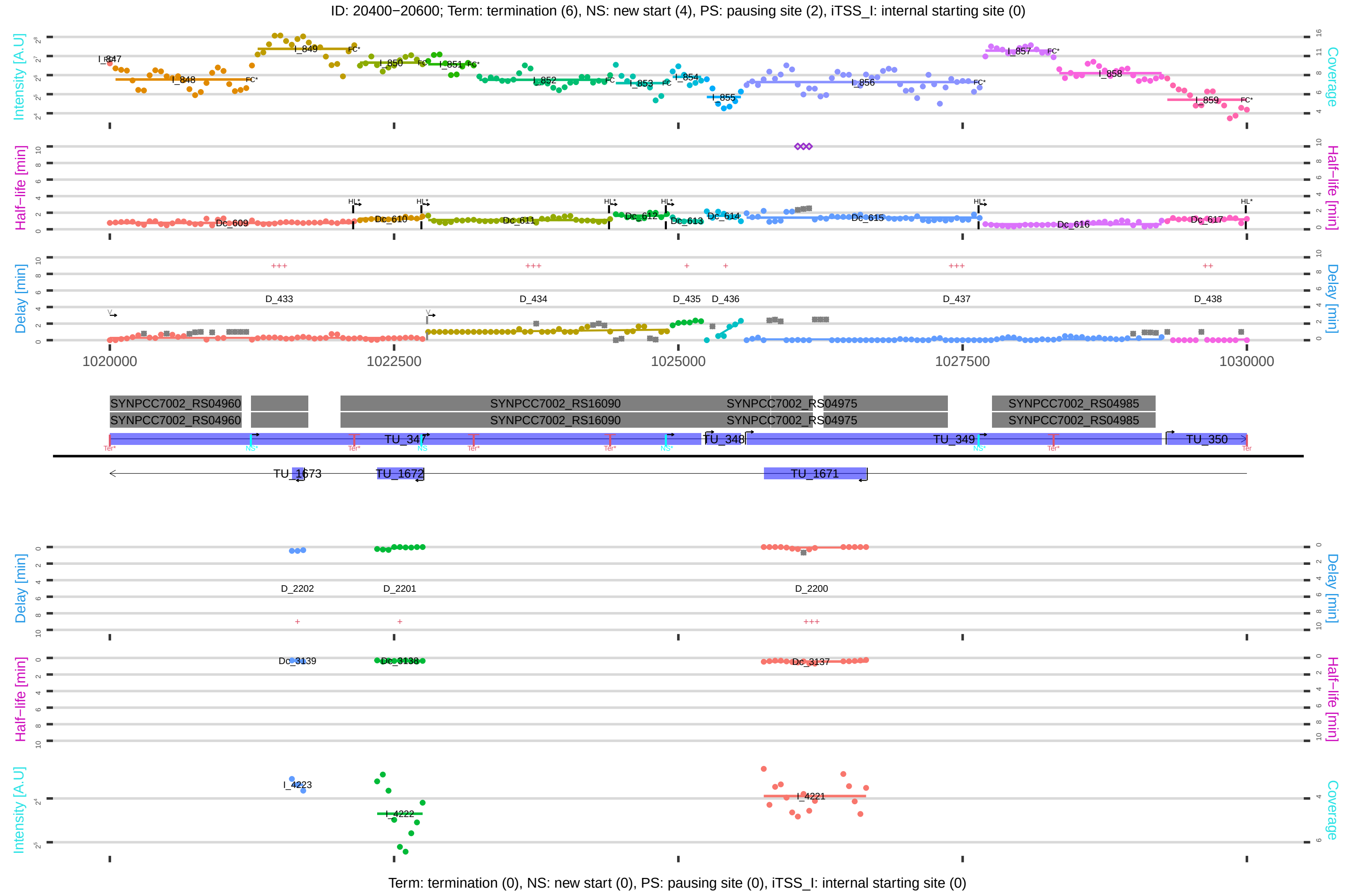

ID: 20400−20600; Term: termination (6), NS: new start (4), PS: pausing site (2), iTSS_I: internal starting site (0)
Intensity [A.U]
Coverage
Half−life [min]
Delay [min]
D_433
D_434
D_435
D_436
D_437
D_438
1020000
1022500
1025000
1027500
1030000
SYNPCC7002_RS04960
SYNPCC7002_RS16090
SYNPCC7002_RS04975
SYNPCC7002_RS04985
TU_347
TU_348
TU_349
TU_350
TU_1673
TU_1672
TU_1671
D_2202
D_2201
D_2200
Dc_3139
Dc_3138
Dc_3137
I_4223
I_4222
I_4221
Term: termination (0), NS: new start (0), PS: pausing site (0), iTSS_I: internal starting site (0)

ID: 20600−20800; Term: termination (4), NS: new start (1), PS: pausing site (1), iTSS_I: internal starting site (0)

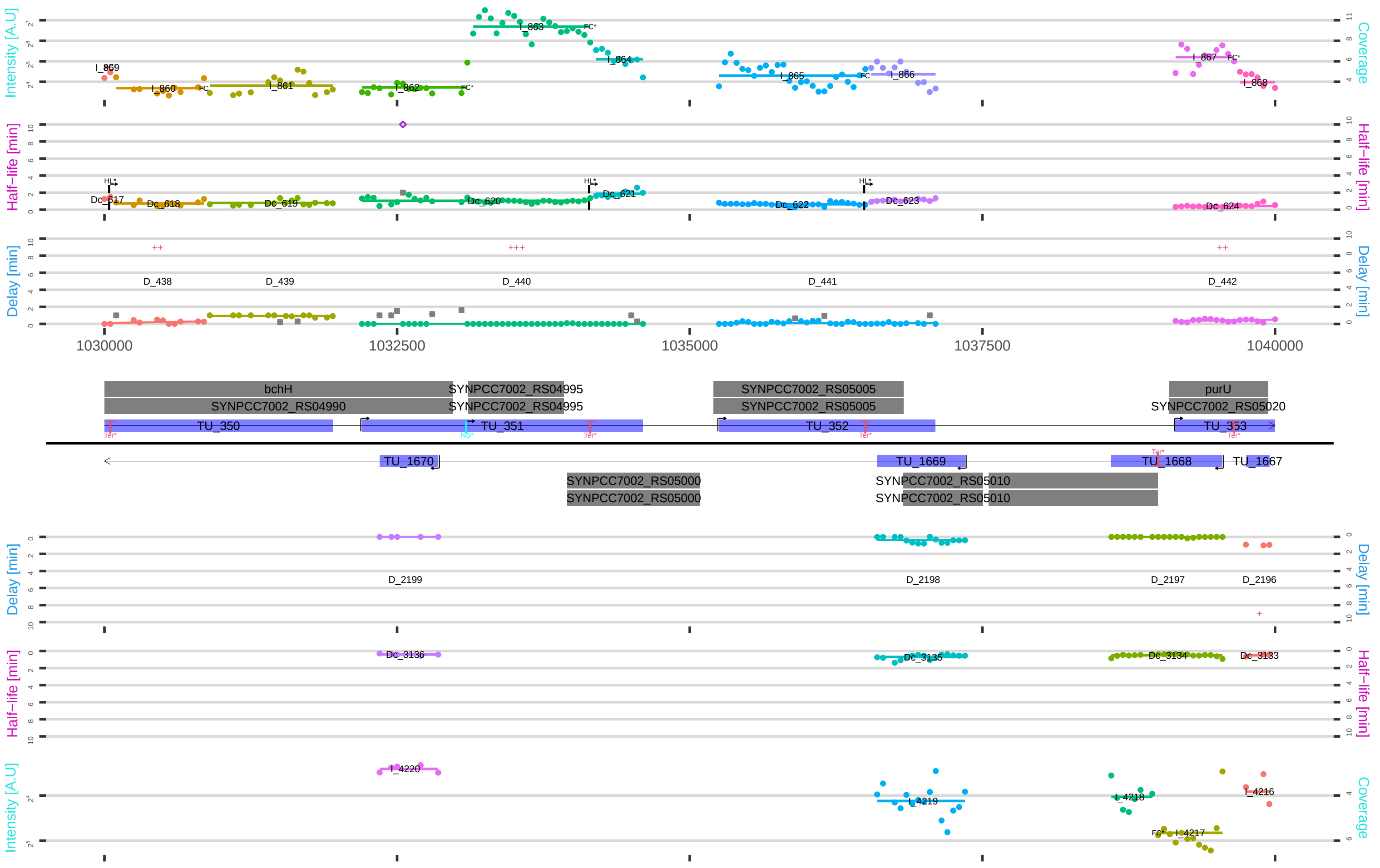

Term: termination (1), NS: new start (0), PS: pausing site (0), iTSS_I: internal starting site (0)

ID: 20800−20977; Term: termination (2), NS: new start (0), PS: pausing site (1), iTSS_I: internal starting site (1)

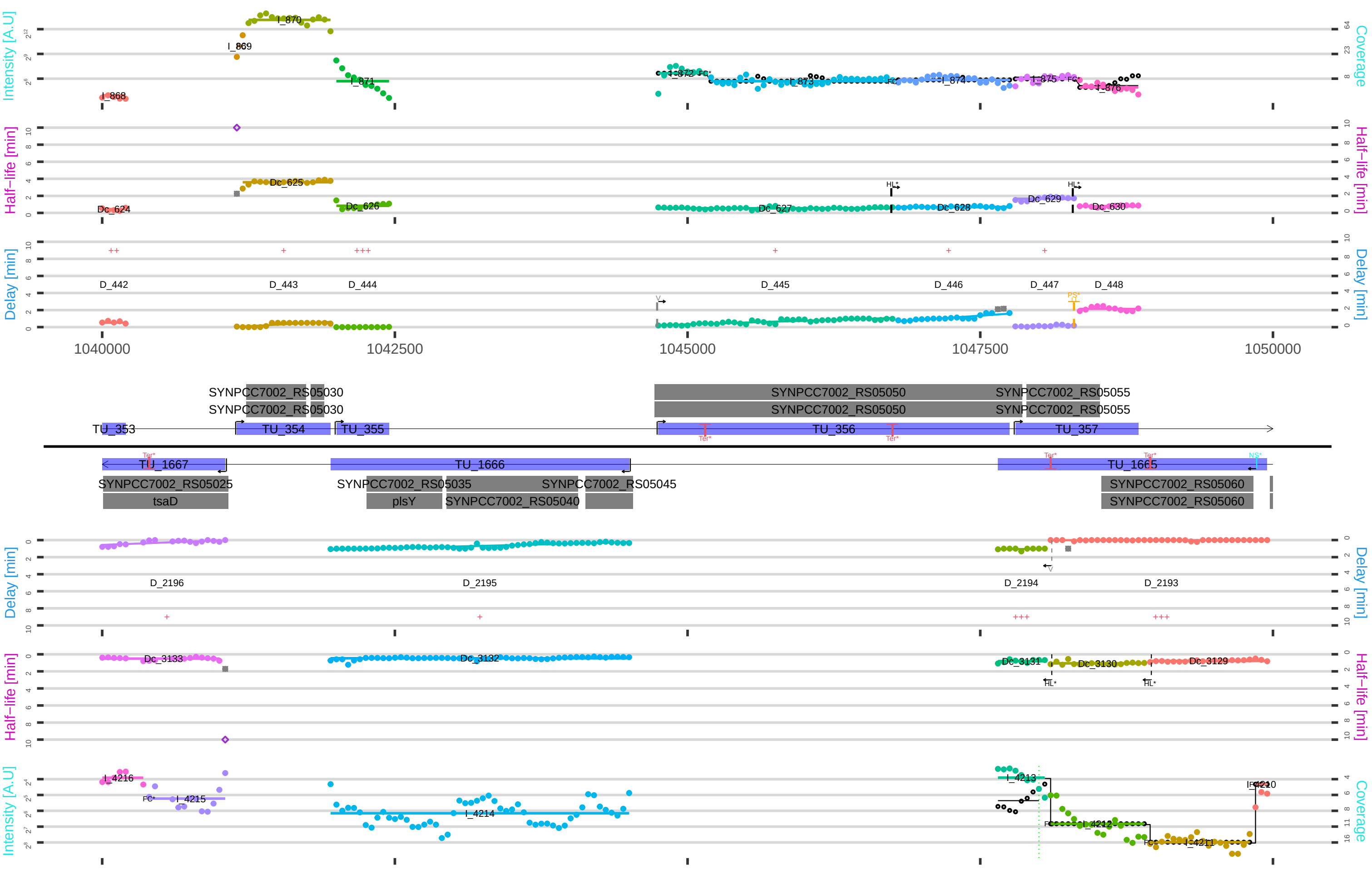

Term: termination (3), NS: new start (1), PS: pausing site (1), iTSS_I: internal starting site (0)

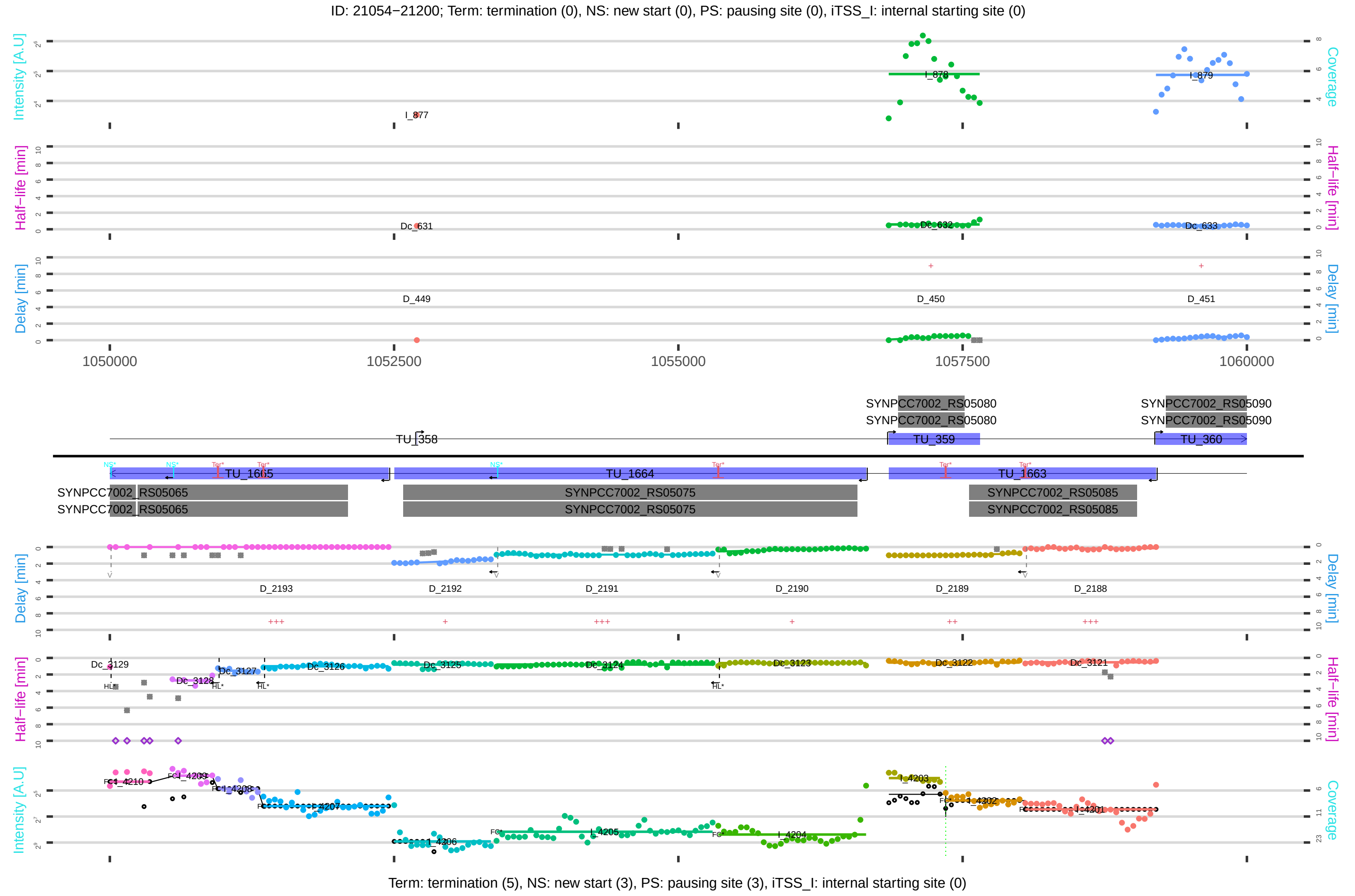
ID: 21054−21200; Term: termination (0), NS: new start (0), PS: pausing site (0), iTSS_I: internal starting site (0)
Intensity [A.U]
Coverage
Half−life [min]
Delay [min]
I_877
I_878
I_879
Dc_631
Dc_632
Dc_633
D_449
D_450
D_451
1050000
1052500
1055000
1057500
1060000
SYNPCC7002_RS05080
SYNPCC7002_RS05090
TU_358
TU_359
TU_360
TU_1665
TU_1664
TU_1663
SYNPCC7002_RS05065
SYNPCC7002_RS05075
SYNPCC7002_RS05085
D_2193
D_2192
D_2191
D_2190
D_2189
D_2188
Dc_3129
Dc_3128
Dc_3127
Dc_3126
Dc_3125
Dc_3124
Dc_3123
Dc_3122
Dc_3121
I_4210
I_4209
I_4208
I_4207
I_4206
I_4205
I_4204
I_4203
I_4202
I_4201
Term: termination (5), NS: new start (3), PS: pausing site (3), iTSS_I: internal starting site (0)

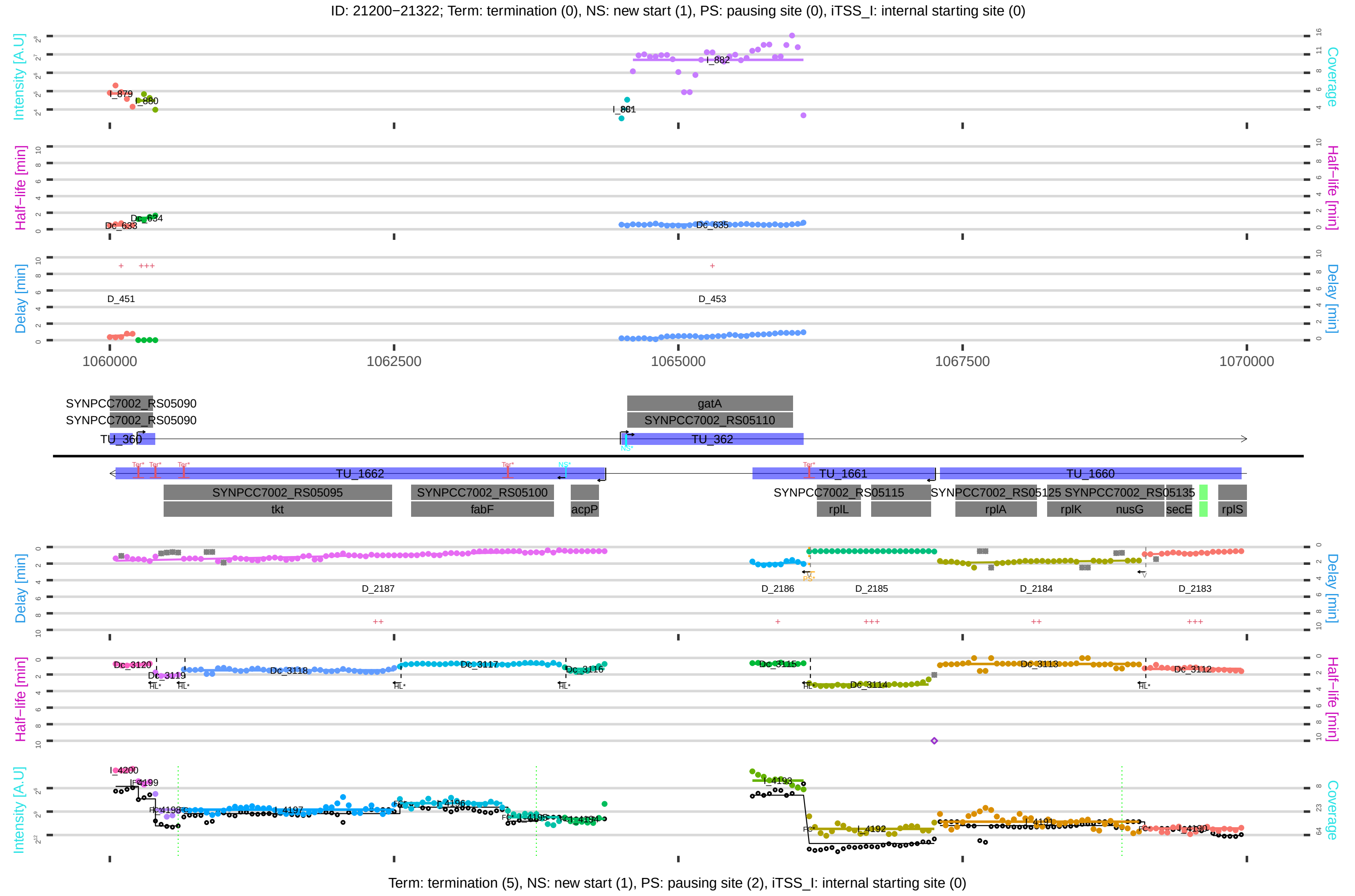
ID: 21200−21322; Term: termination (0), NS: new start (1), PS: pausing site (0), iTSS_I: internal starting site (0)
Intensity [A.U]
Coverage
Half−life [min]
Delay [min]
I_879
I_880
I_881
I_882
Dc_633
Dc_634
Dc_635
D_451
D_453
1060000
1062500
1065000
1067500
1070000
SYNPCC7002_RS05090
SYNPCC7002_RS05090
TU_360
gatA
SYNPCC7002_RS05110
TU_362
TU_1662
TU_1661
TU_1660
SYNPCC7002_RS05095
tkt
SYNPCC7002_RS05100
fabF
acpP
SYNPCC7002_RS05115
rplL
SYNPCC7002_RS05125
rplA
SYNPCC7002_RS05135
rplK
nusG
secE
rplS
D_2187
D_2186
D_2185
D_2184
D_2183
Dc_3120
Dc_3119
Dc_3118
Dc_3117
Dc_3116
Dc_3115
Dc_3114
Dc_3113
Dc_3112
I_4200
I_4199
I_4198
I_4197
I_4196
I_4195
I_4194
I_4193
I_4192
I_4191
I_4190
Term: termination (5), NS: new start (1), PS: pausing site (2), iTSS_I: internal starting site (0)

ID: 21405−21472; Term: termination (1), NS: new start (0), PS: pausing site (0), iTSS_I: internal starting site (0)

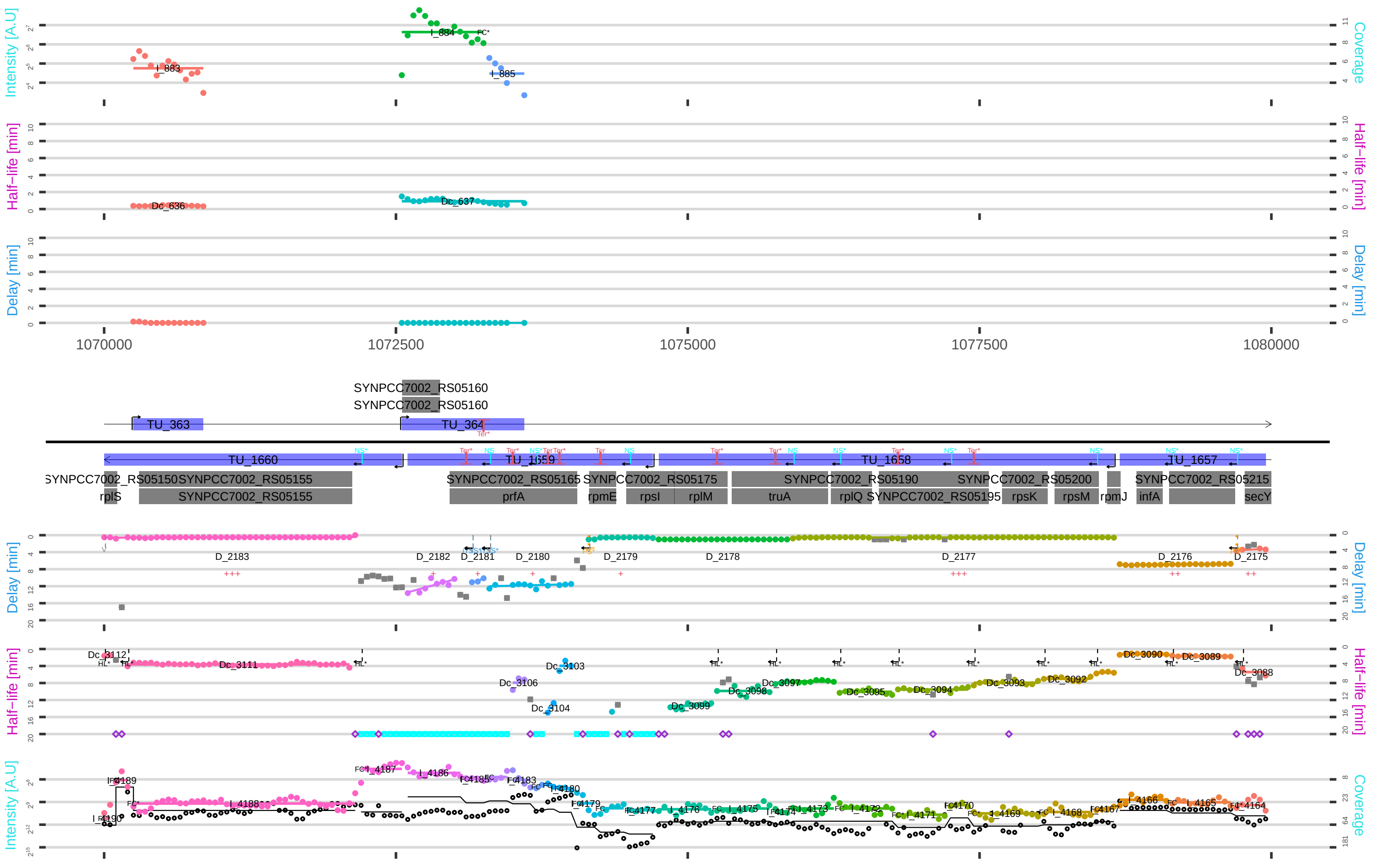

Term: termination (9), NS: new start (10), PS: pausing site (3), iTSS_I: internal starting site (2)

ID: 21619−21620; Term: termination (0), NS: new start (0), PS: pausing site (0), iTSS_I: internal starting site (0)

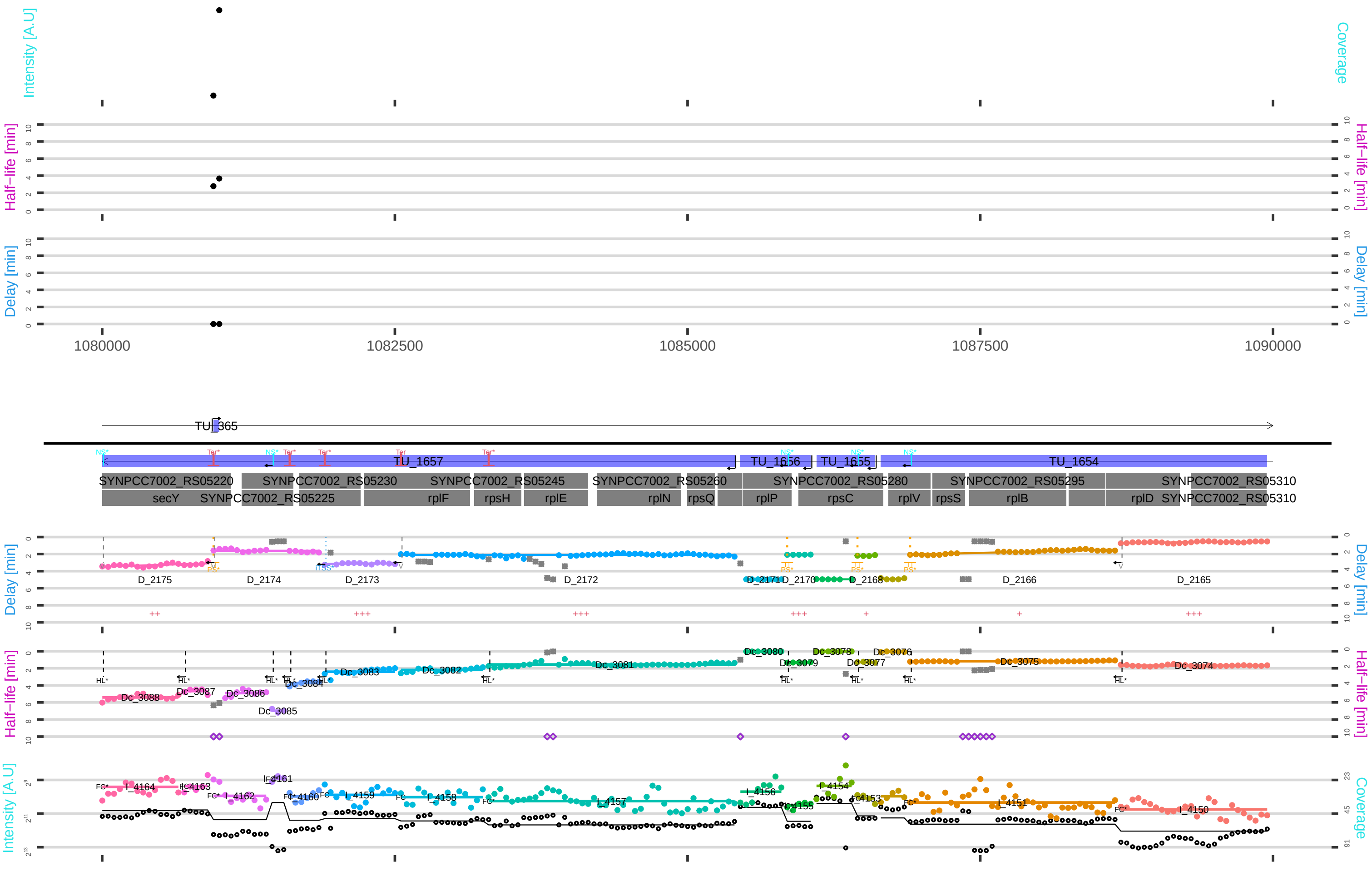

Term: termination (5), NS: new start (5), PS: pausing site (6), iTSS_I: internal starting site (1)

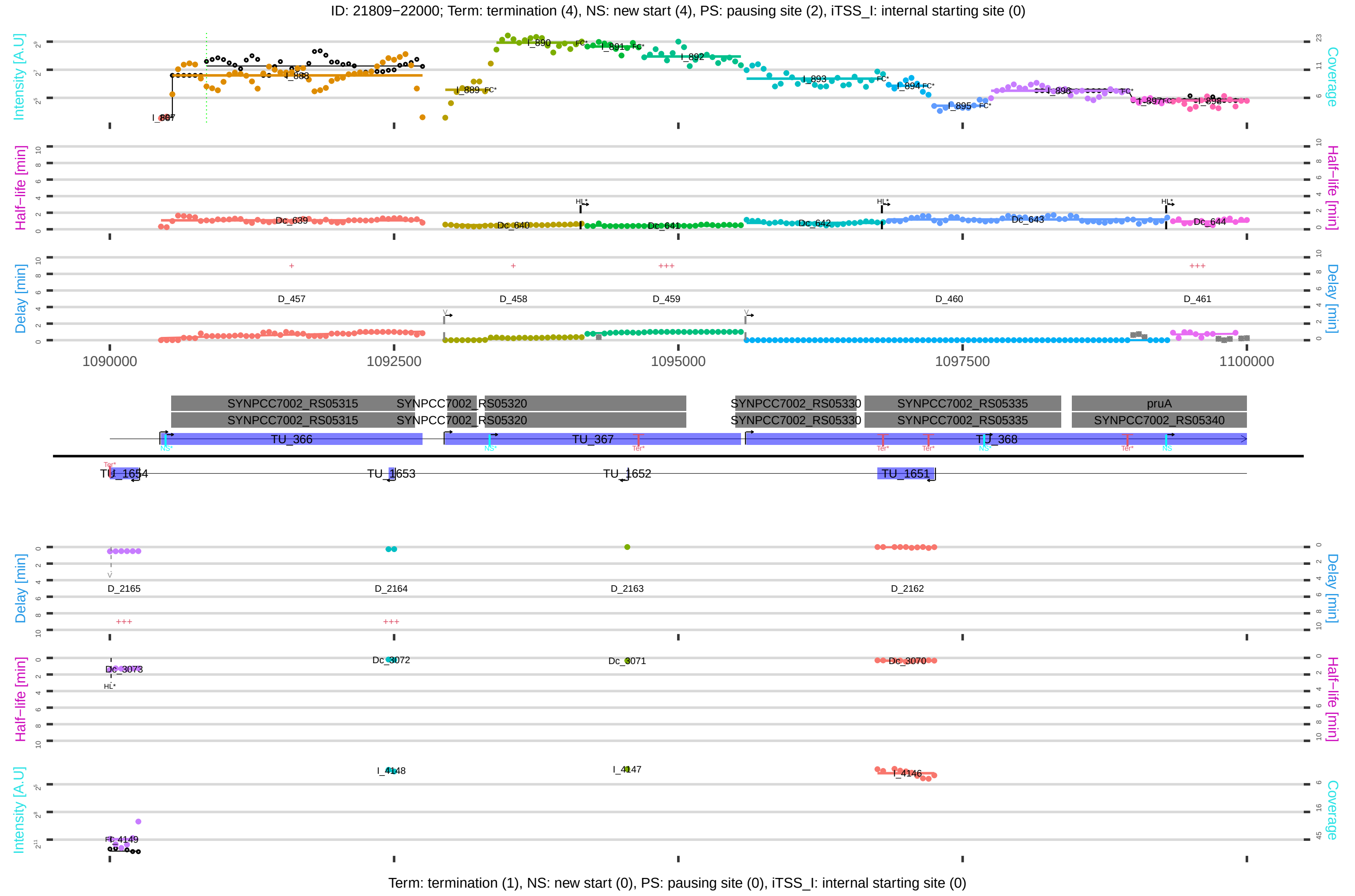

ID: 21809−22000; Term: termination (4), NS: new start (4), PS: pausing site (2), iTSS_I: internal starting site (0)
Intensity [A.U]
Coverage
Half−life [min]
Delay [min]
1090000
1092500
1095000
1097500
1100000
SYNPCC7002_RS05315
SYNPCC7002_RS05320
SYNPCC7002_RS05330
SYNPCC7002_RS05335
pruA
SYNPCC7002_RS05340
TU_366
TU_367
TU_368
TU_1654
TU_1653
TU_1652
TU_1651
D_457
D_458
D_459
D_460
D_461
D_2165
D_2164
D_2163
D_2162
Term: termination (1), NS: new start (0), PS: pausing site (0), iTSS_I: internal starting site (0)

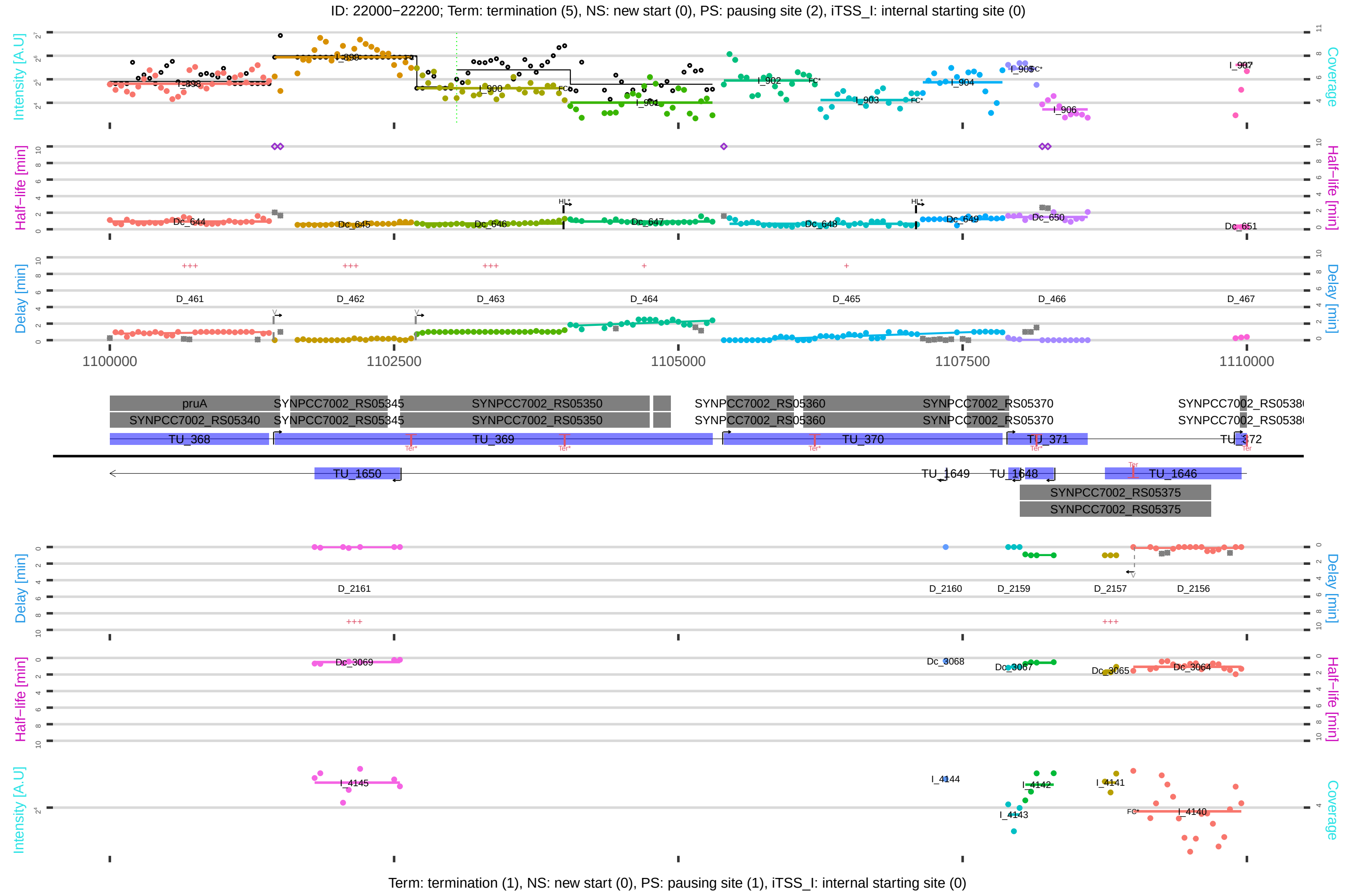
ID: 22000−22200; Term: termination (5), NS: new start (0), PS: pausing site (2), iTSS_I: internal starting site (0)
Intensity [A.U]
Coverage
Half−life [min]
Delay [min]
I_898
I_899
I_900
I_901
I_902
I_903
I_904
I_905
I_906
I_907
Dc_644
Dc_645
Dc_646
Dc_647
Dc_648
Dc_649
Dc_650
Dc_651
D_461
D_462
D_463
D_464
D_465
D_466
D_467
1100000
1102500
1105000
1107500
1110000
pruA
SYNPCC7002_RS05340
SYNPCC7002_RS05345
SYNPCC7002_RS05350
SYNPCC7002_RS05360
SYNPCC7002_RS05370
SYNPCC7002_RS05375
TU_368
TU_369
TU_370
TU_371
TU_372
TU_1650
TU_1649
TU_1648
TU_1646
D_2161
D_2160
D_2159
D_2157
D_2156
Dc_3069
Dc_3068
Dc_3067
Dc_3065
Dc_3064
I_4145
I_4144
I_4143
I_4142
I_4141
I_4140
Term: termination (1), NS: new start (0), PS: pausing site (1), iTSS_I: internal starting site (0)

ID: 22200−22390; Term: termination (4), NS: new start (1), PS: pausing site (0), iTSS_I: internal starting site (0)

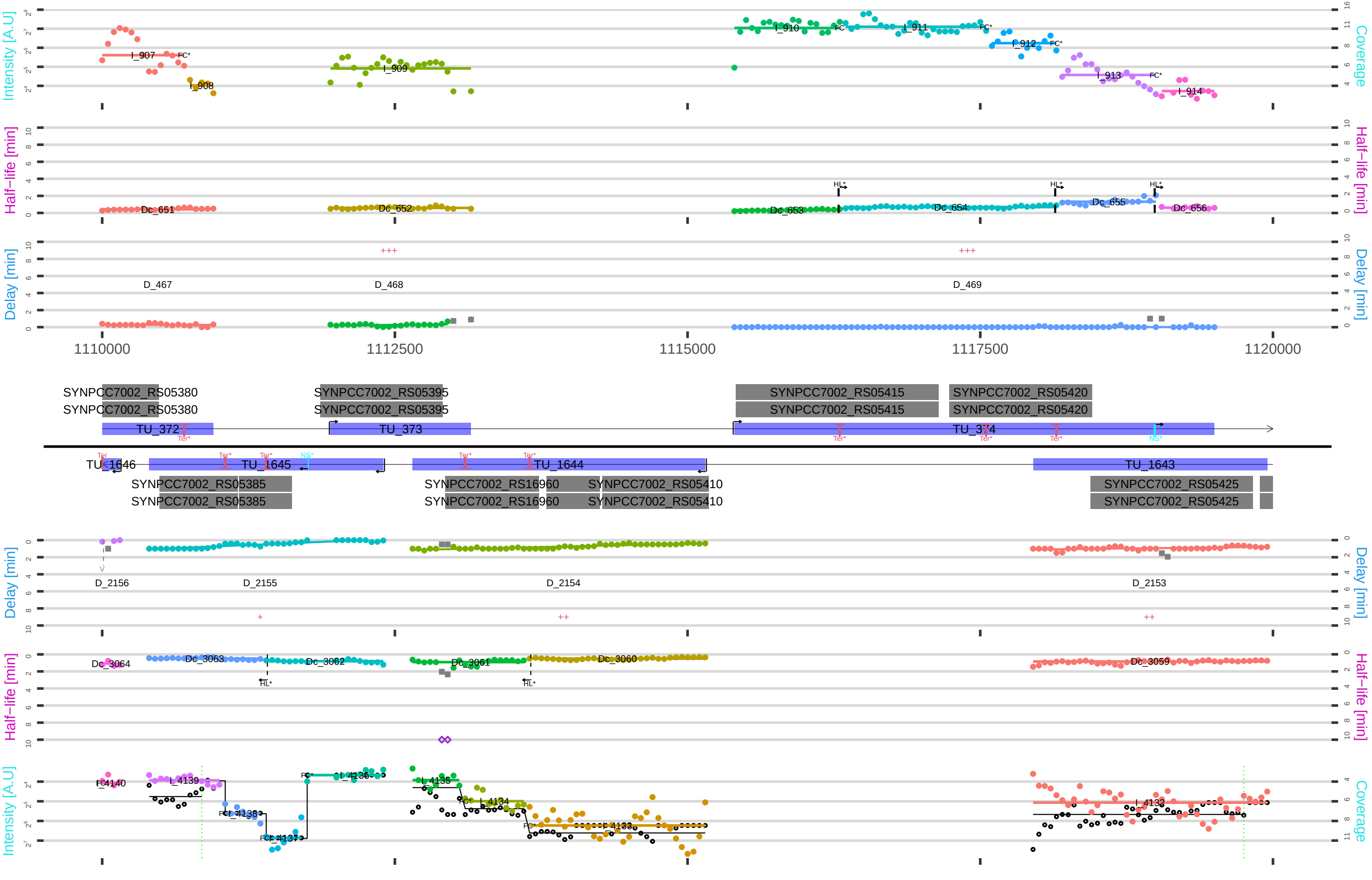

Term: termination (5), NS: new start (1), PS: pausing site (0), iTSS_I: internal starting site (0)

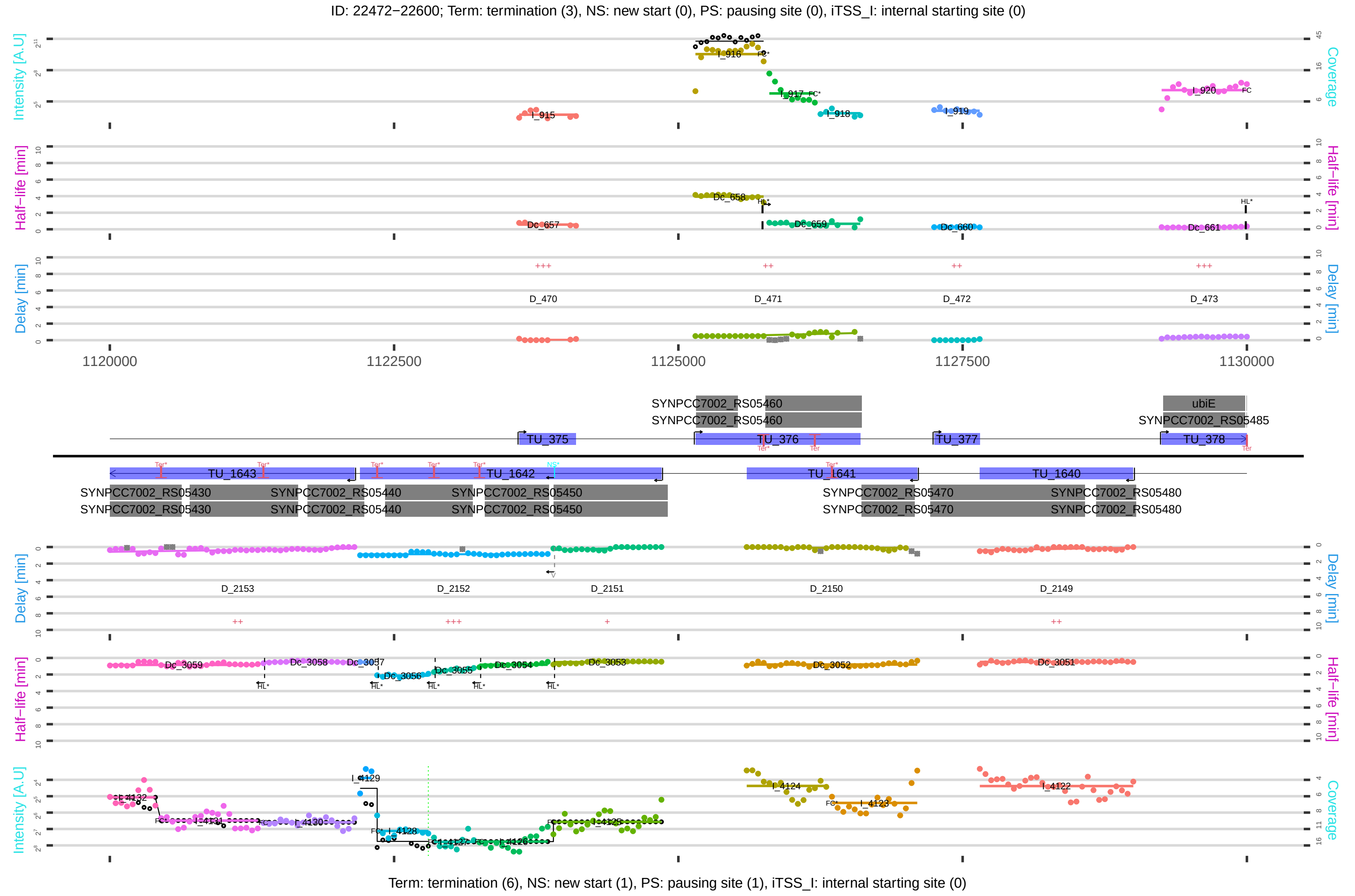
ID: 22472−22600; Term: termination (3), NS: new start (0), PS: pausing site (0), iTSS_I: internal starting site (0)
Intensity [A.U]
Coverage
Half−life [min]
Delay [min]
I_915
I_916
I_917
I_918
I_919
I_920
Dc_657
Dc_658
Dc_659
Dc_660
Dc_661
D_470
D_471
D_472
D_473
1120000
1122500
1125000
1127500
1130000
SYNPCC7002_RS05460
ubiE
SYNPCC7002_RS05485
TU_375
TU_376
TU_377
TU_378
TU_1643
TU_1642
TU_1641
TU_1640
SYNPCC7002_RS05430
SYNPCC7002_RS05440
SYNPCC7002_RS05450
SYNPCC7002_RS05470
SYNPCC7002_RS05480
D_2153
D_2152
D_2151
D_2150
D_2149
Dc_3059
Dc_3058
Dc_3057
Dc_3056
Dc_3055
Dc_3054
Dc_3053
Dc_3052
Dc_3051
I_4129
I_4132
I_4131
I_4130
I_4128
I_4127
I_4126
I_4125
I_4124
I_4123
I_4122
Term: termination (6), NS: new start (1), PS: pausing site (1), iTSS_I: internal starting site (0)

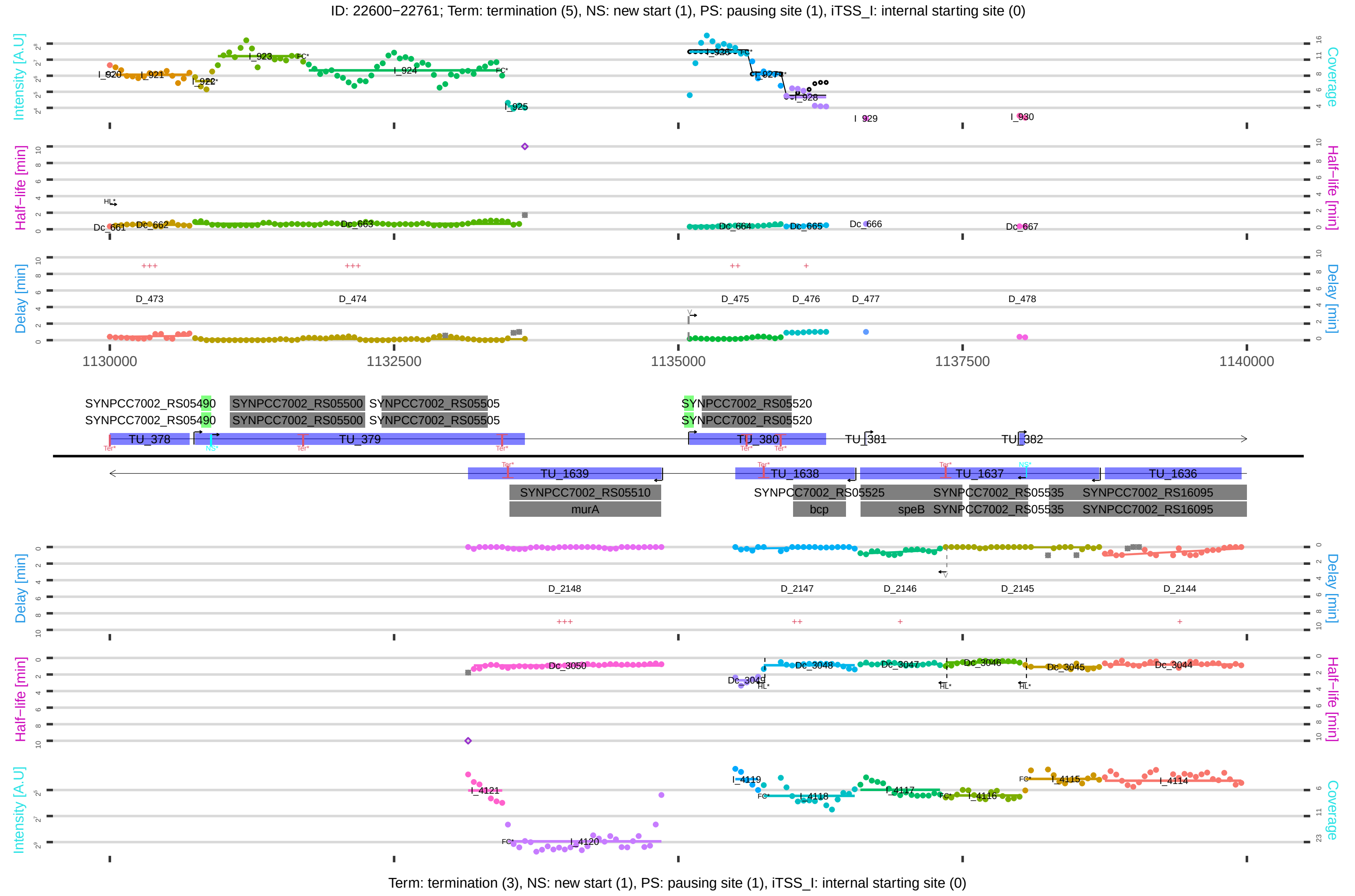
ID: 22600−22761; Term: termination (5), NS: new start (1), PS: pausing site (1), iTSS_I: internal starting site (0)
Intensity [A.U]
Coverage
Half−life [min]
Delay [min]
1130000
1132500
1135000
1137500
1140000
TU_378
TU_379
TU_380
TU_381
TU_382
TU_1639
TU_1638
TU_1637
TU_1636
SYNPCC7002_RS05490
SYNPCC7002_RS05500
SYNPCC7002_RS05505
SYNPCC7002_RS05520
SYNPCC7002_RS05510
murA
SYNPCC7002_RS05525
bcp
speB
SYNPCC7002_RS05535
SYNPCC7002_RS16095
Term: termination (3), NS: new start (1), PS: pausing site (1), iTSS_I: internal starting site (0)

ID: 22818−23000; Term: termination (5), NS: new start (1), PS: pausing site (2), iTSS_I: internal starting site (0)

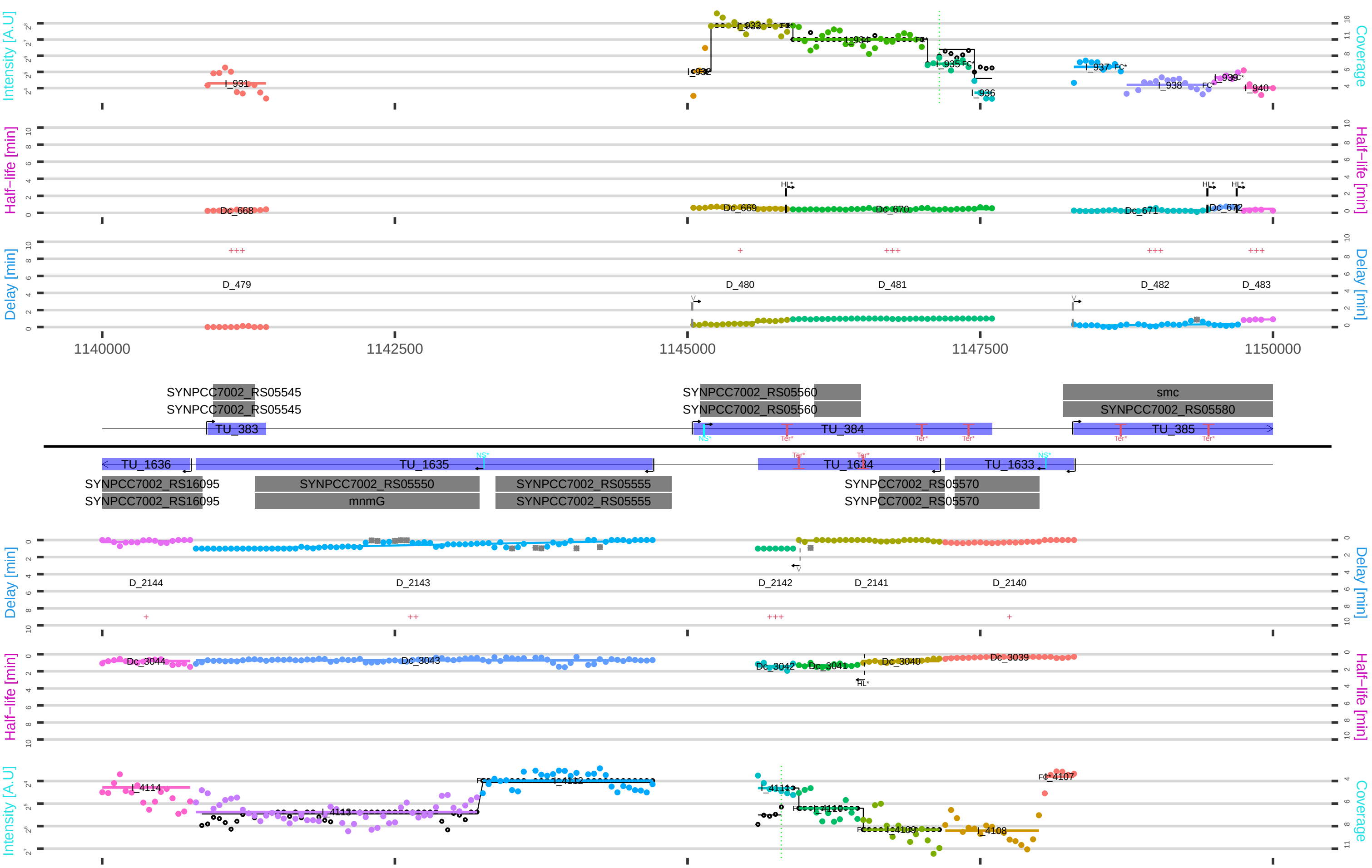

Term: termination (2), NS: new start (2), PS: pausing site (1), iTSS_I: internal starting site (0)

ID: 23000−23200; Term: termination (1), NS: new start (0), PS: pausing site (0), iTSS_I: internal starting site (0)

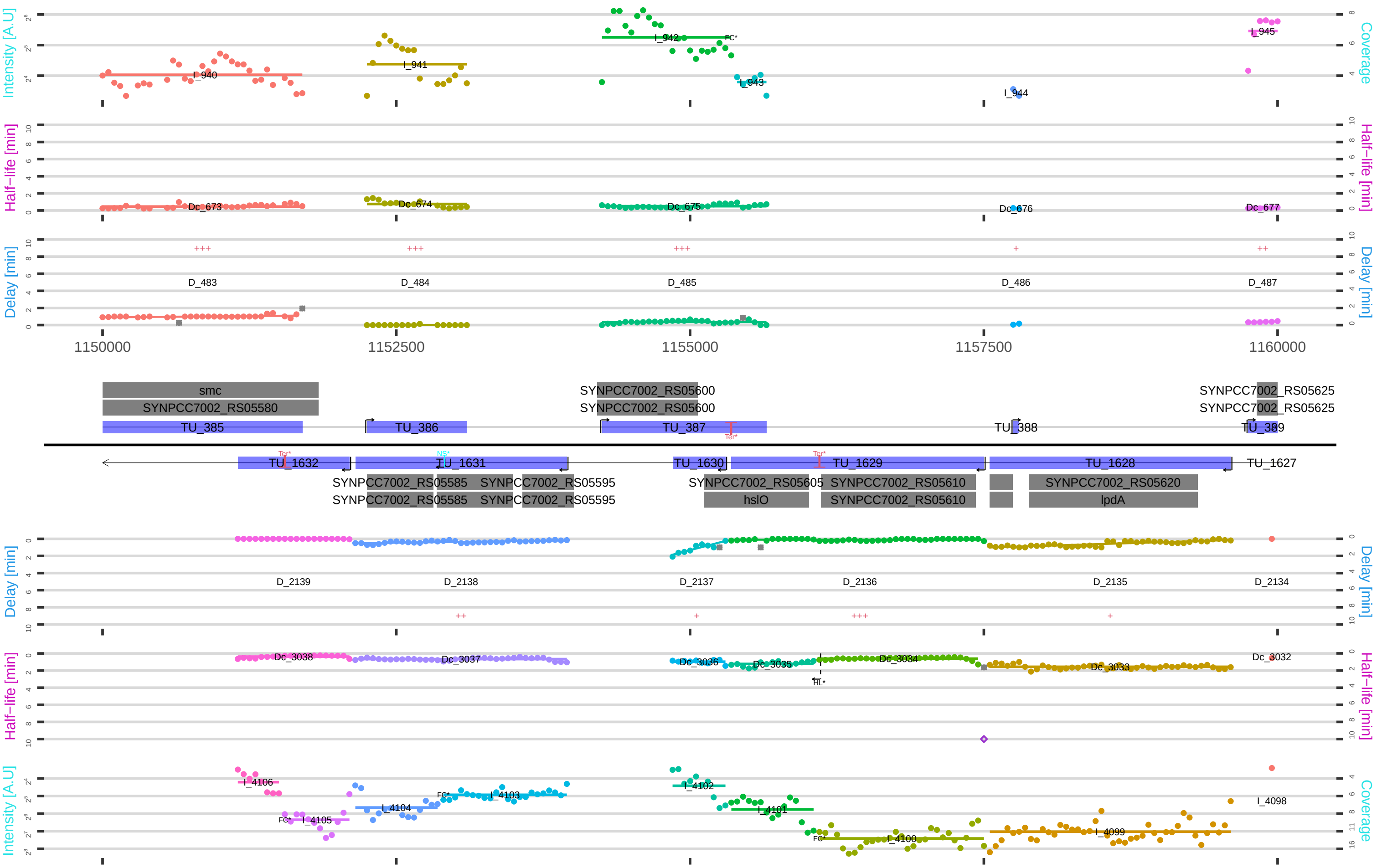

Term: termination (2), NS: new start (1), PS: pausing site (0), iTSS_I: internal starting site (0)

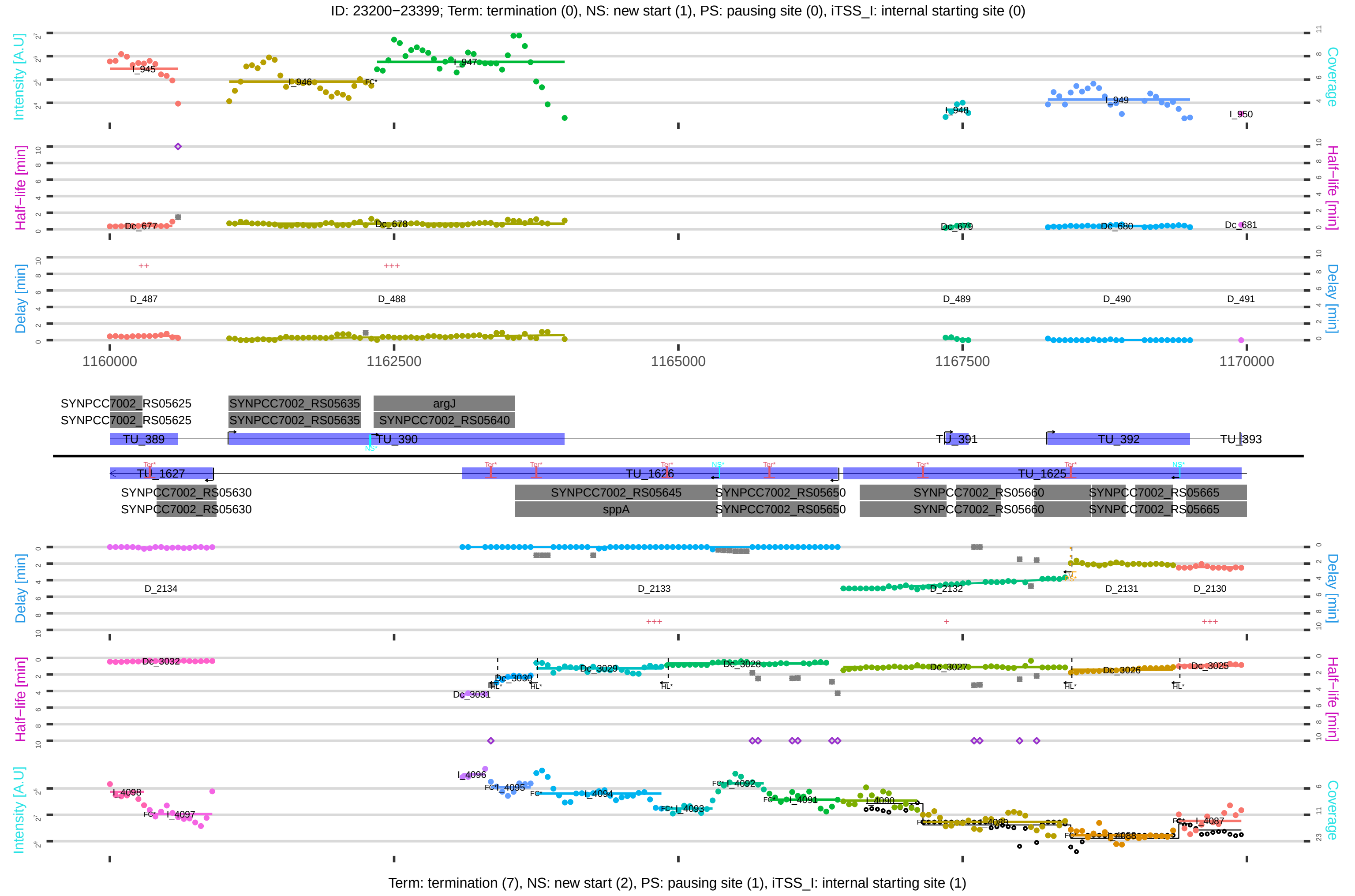

ID: 23200−23399; Term: termination (0), NS: new start (1), PS: pausing site (0), iTSS_I: internal starting site (0)
Intensity [A.U]
Coverage
Half−life [min]
Delay [min]
1160000
1162500
1165000
1167500
1170000
SYNPCC7002_RS05625
SYNPCC7002_RS05635
argJ
SYNPCC7002_RS05640
TU_389
TU_390
TU_391
TU_392
TU_393
TU_1627
TU_1626
TU_1625
SYNPCC7002_RS05630
SYNPCC7002_RS05645
sppA
SYNPCC7002_RS05650
SYNPCC7002_RS05660
SYNPCC7002_RS05665
D_2134
D_2133
D_2132
D_2131
D_2130
Term: termination (7), NS: new start (2), PS: pausing site (1), iTSS_I: internal starting site (1)

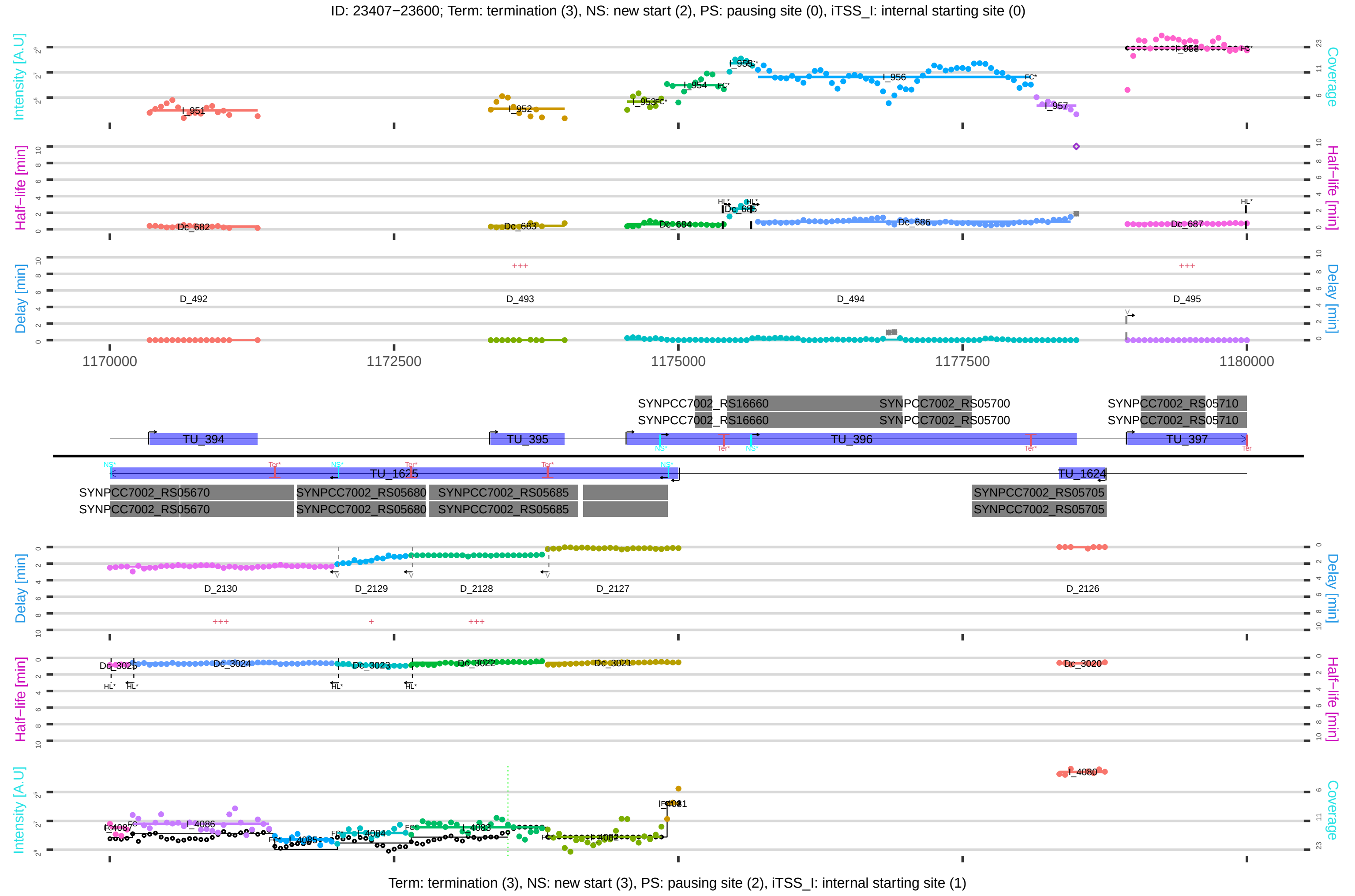

ID: 23407−23600; Term: termination (3), NS: new start (2), PS: pausing site (0), iTSS_I: internal starting site (0)
Intensity [A.U]
Coverage
Half−life [min]
Delay [min]
I_951
I_952
I_953
I_954
I_955
I_956
I_957
I_958
Dc_682
Dc_683
Dc_684
Dc_685
Dc_686
Dc_687
D_492
D_493
D_494
D_495
1170000
1172500
1175000
1177500
1180000
SYNPCC7002_RS16660
SYNPCC7002_RS05700
SYNPCC7002_RS05710
TU_394
TU_395
TU_396
TU_397
TU_1625
TU_1624
SYNPCC7002_RS05670
SYNPCC7002_RS05680
SYNPCC7002_RS05685
SYNPCC7002_RS05705
D_2130
D_2129
D_2128
D_2127
D_2126
Dc_3025
Dc_3024
Dc_3023
Dc_3022
Dc_3021
Dc_3020
I_4086
I_4085
I_4084
I_4083
I_4082
I_4081
I_4080
I_4087
Term: termination (3), NS: new start (3), PS: pausing site (2), iTSS_I: internal starting site (1)

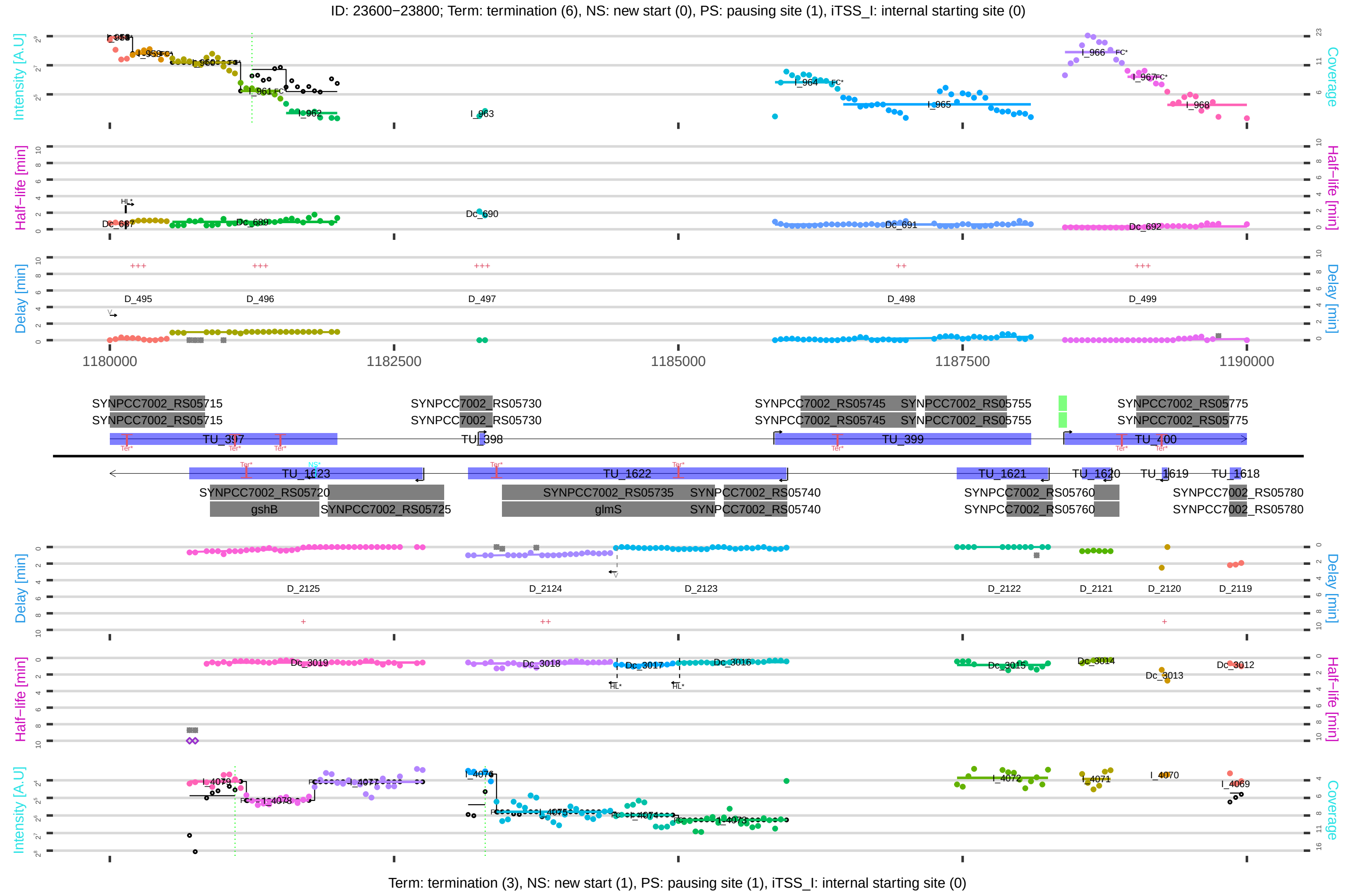
ID: 23600−23800; Term: termination (6), NS: new start (0), PS: pausing site (1), iTSS_I: internal starting site (0)
Intensity [A.U]
Coverage
Half−life [min]
Delay [min]
1180000
1182500
1185000
1187500
1190000
SYNPCC7002_RS05715
SYNPCC7002_RS05730
SYNPCC7002_RS05745
SYNPCC7002_RS05755
SYNPCC7002_RS05775
TU_397
TU_398
TU_399
TU_400
TU_1623
TU_1622
TU_1621
TU_1620
TU_1619
TU_1618
SYNPCC7002_RS05720
gshB
SYNPCC7002_RS05725
SYNPCC7002_RS05735
glmS
SYNPCC7002_RS05740
SYNPCC7002_RS05760
SYNPCC7002_RS05780
Term: termination (3), NS: new start (1), PS: pausing site (1), iTSS_I: internal starting site (0)

ID: 23800−24000; Term: termination (5), NS: new start (1), PS: pausing site (0), iTSS_I: internal starting site (0)

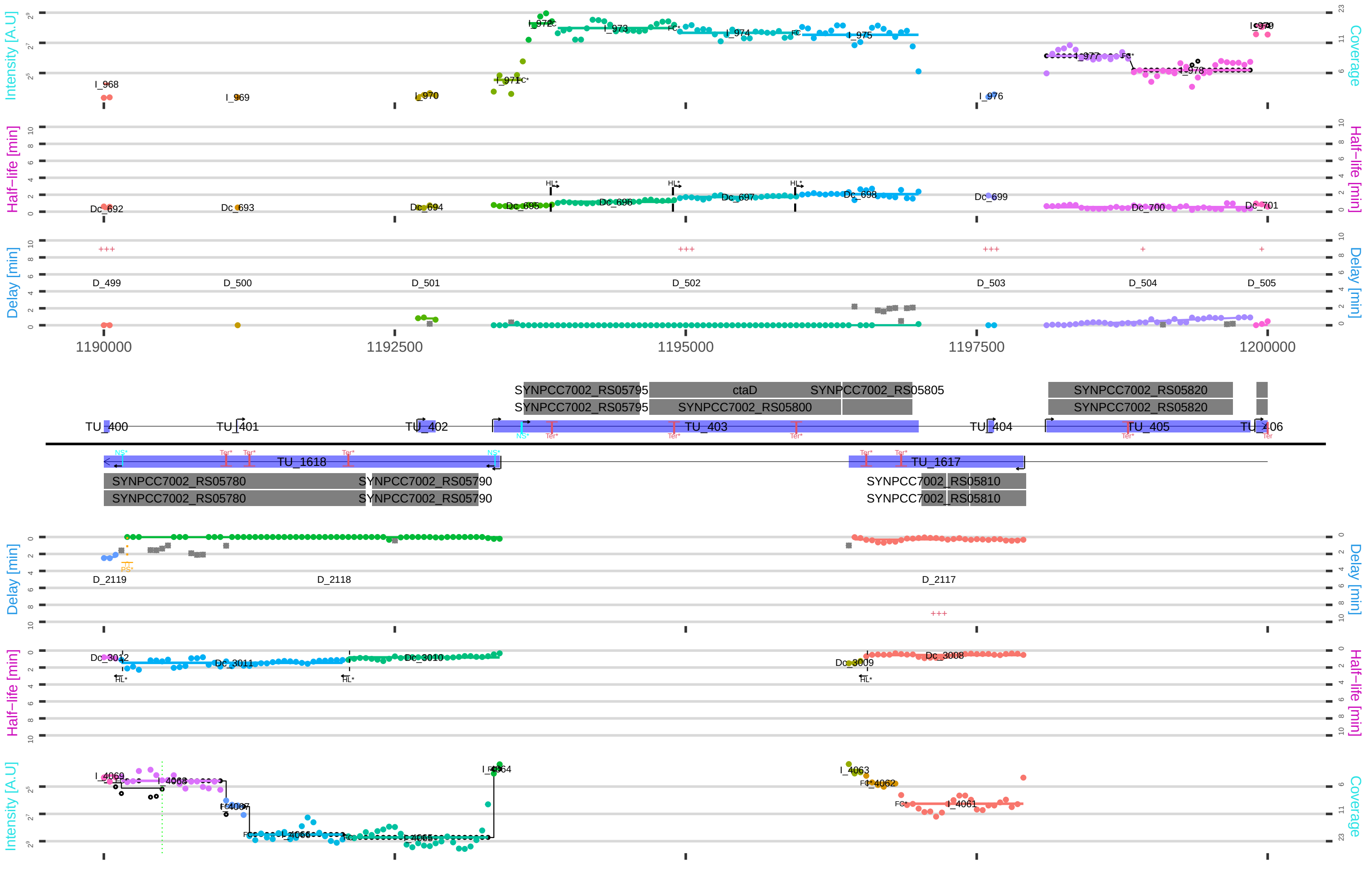

Term: termination (5), NS: new start (2), PS: pausing site (1), iTSS_I: internal starting site (0)

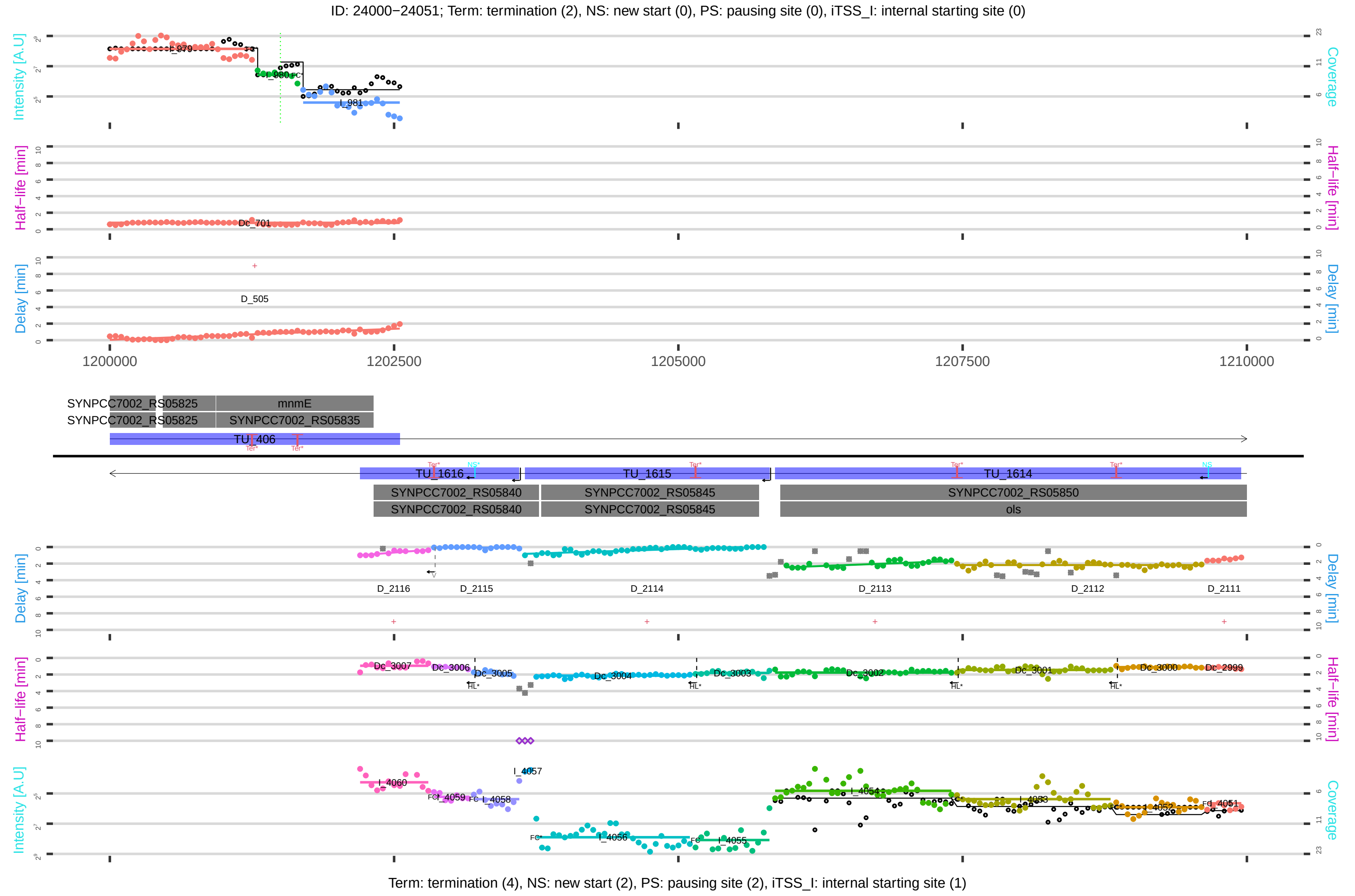
ID: 24000−24051; Term: termination (2), NS: new start (0), PS: pausing site (0), iTSS_I: internal starting site (0)
Intensity [A.U]
Coverage
Half−life [min]
Delay [min]
I_979
I_980
I_981
Dc_701
D_505
1200000
1202500
1205000
1207500
1210000
SYNPCC7002_RS05825
mnmE
SYNPCC7002_RS05835
TU_406
TU_1616
TU_1615
TU_1614
SYNPCC7002_RS05840
SYNPCC7002_RS05845
SYNPCC7002_RS05850
ols
D_2116
D_2115
D_2114
D_2113
D_2112
D_2111
Dc_3007
Dc_3006
Dc_3005
Dc_3004
Dc_3003
Dc_3002
Dc_3001
Dc_3000
Dc_2999
I_4060
I_4059
I_4058
I_4057
I_4056
I_4055
I_4054
I_4053
I_4052
I_4051
Term: termination (4), NS: new start (2), PS: pausing site (2), iTSS_I: internal starting site (1)

ID: 24276−24400; Term: termination (2), NS: new start (1), PS: pausing site (0), iTSS_I: internal starting site (0)

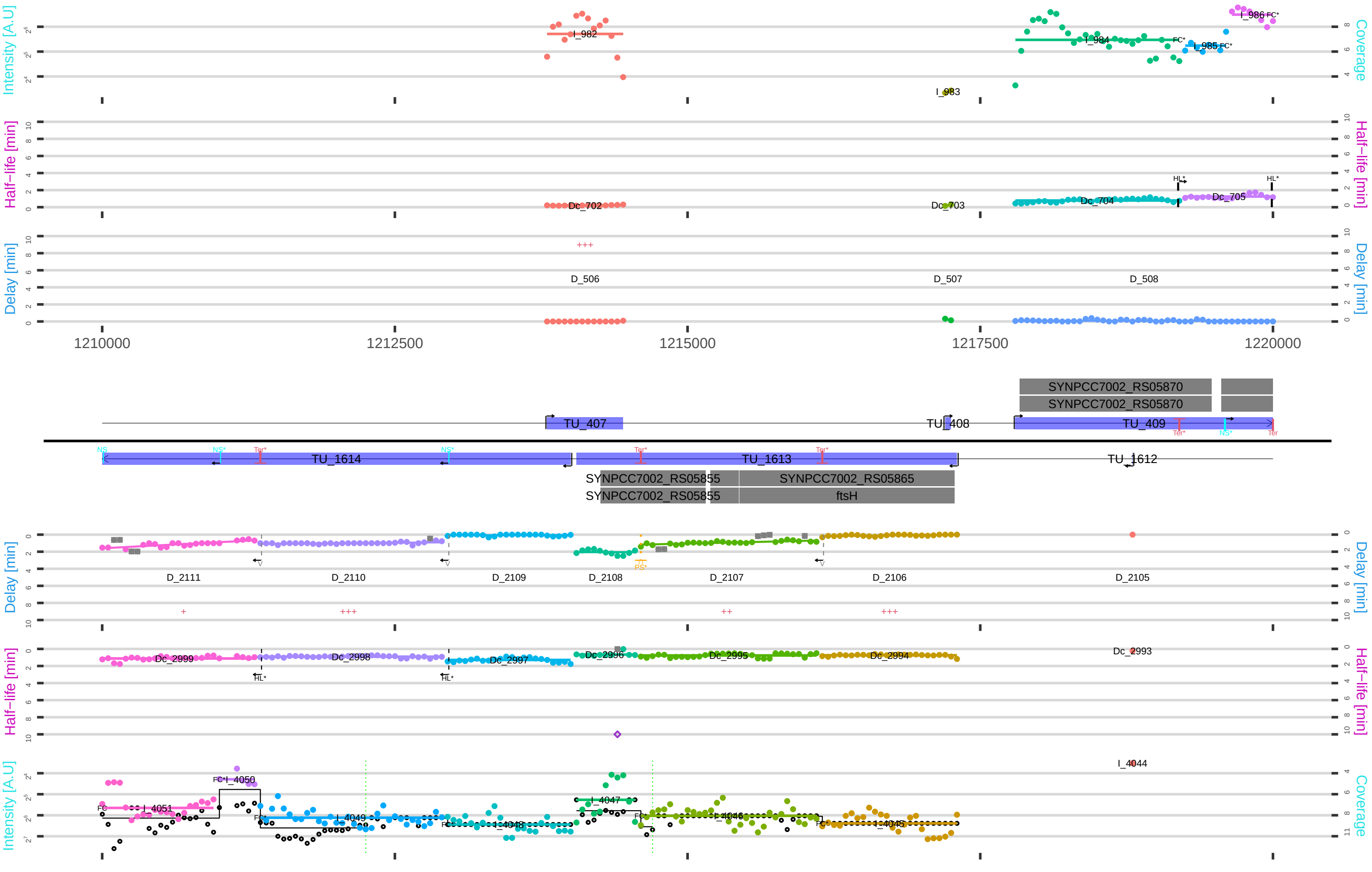

Term: termination (3), NS: new start (3), PS: pausing site (3), iTSS_I: internal starting site (1)

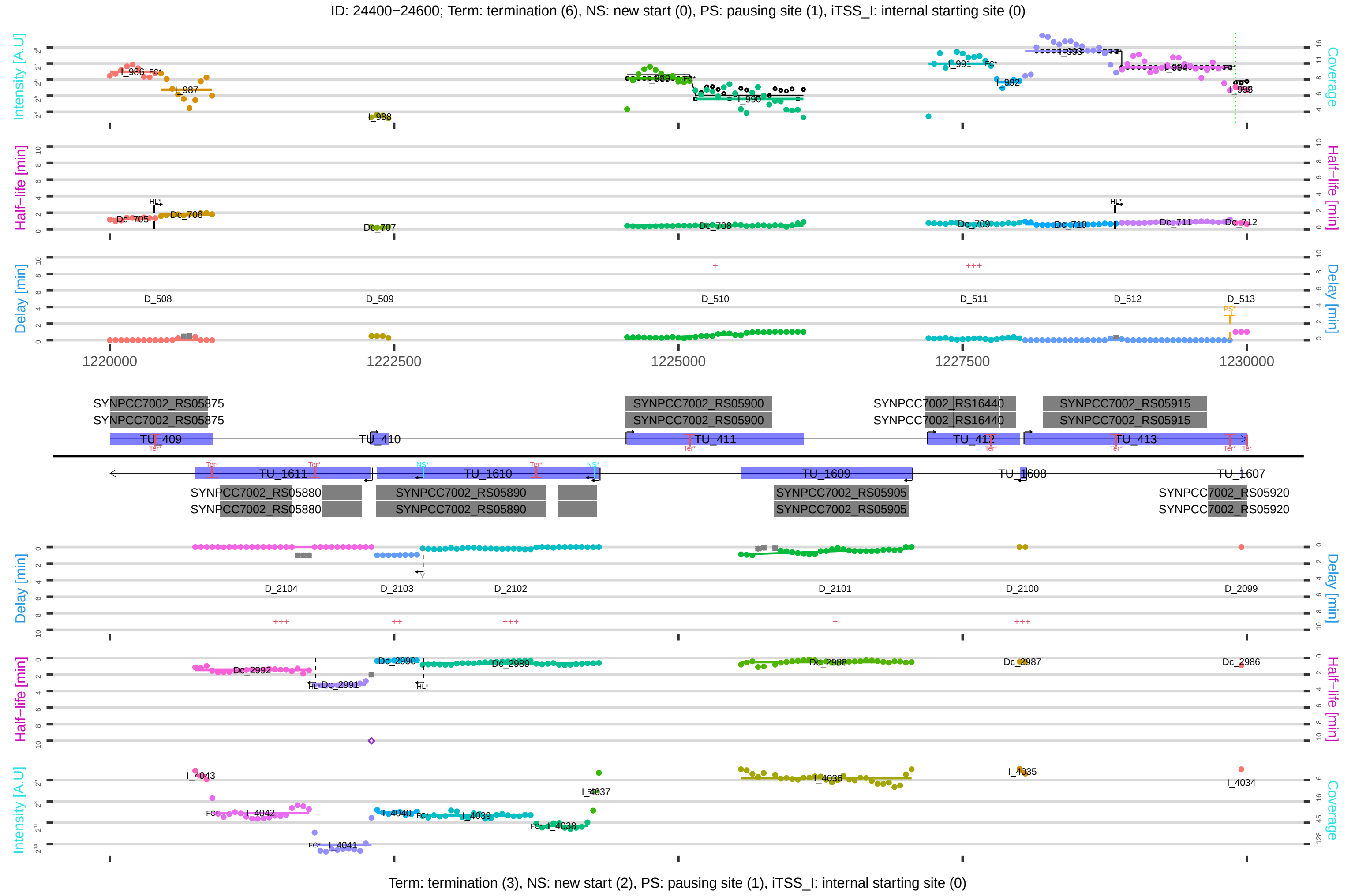

ID: 24400−24600; Term: termination (6), NS: new start (0), PS: pausing site (1), iTSS_I: internal starting site (0)
Intensity [A.U]
Coverage
Half−life [min]
Delay [min]
I_986
I_987
I_988
I_989
I_990
I_991
I_992
I_993
I_994
I_995
Dc_705
Dc_706
Dc_707
Dc_708
Dc_709
Dc_710
Dc_711
Dc_712
D_508
D_509
D_510
D_511
D_512
D_513
1220000
1222500
1225000
1227500
1230000
SYNPCC7002_RS05875
SYNPCC7002_RS05900
SYNPCC7002_RS16440
SYNPCC7002_RS05915
TU_409
TU_410
TU_411
TU_412
TU_413
TU_1611
TU_1610
TU_1609
TU_1608
TU_1607
SYNPCC7002_RS05880
SYNPCC7002_RS05890
SYNPCC7002_RS05905
SYNPCC7002_RS05920
D_2104
D_2103
D_2102
D_2101
D_2100
D_2099
Dc_2992
Dc_2991
Dc_2990
Dc_2989
Dc_2988
Dc_2987
Dc_2986
I_4043
I_4042
I_4041
I_4040
I_4039
I_4038
I_4037
I_4036
I_4035
I_4034
Term: termination (3), NS: new start (2), PS: pausing site (1), iTSS_I: internal starting site (0)

ID: 24600−24794; Term: termination (3), NS: new start (2), PS: pausing site (3), iTSS_I: internal starting site (0)

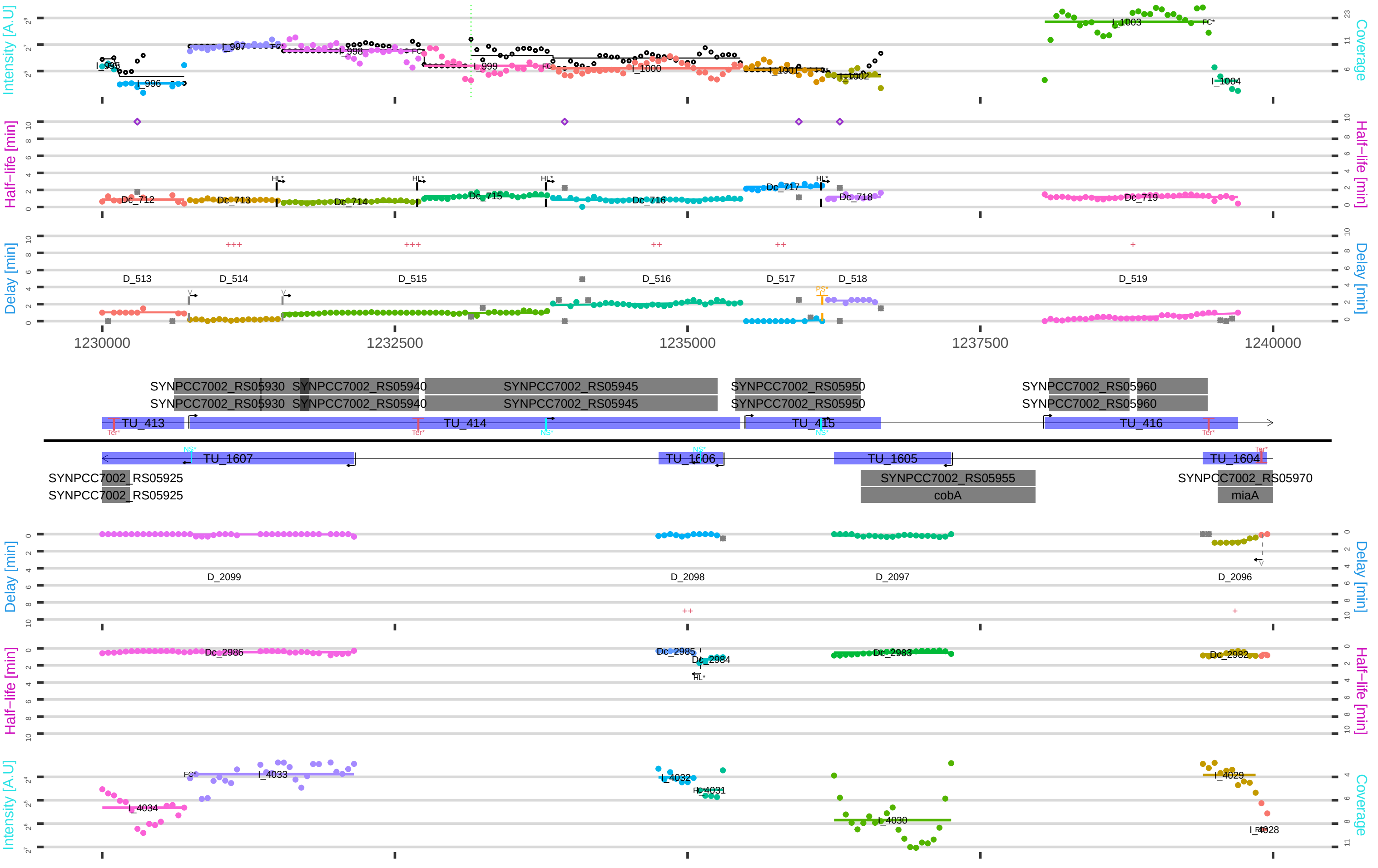

Term: termination (1), NS: new start (2), PS: pausing site (1), iTSS_I: internal starting site (0)

ID: 24831−24987; Term: termination (2), NS: new start (1), PS: pausing site (0), iTSS_I: internal starting site (0)

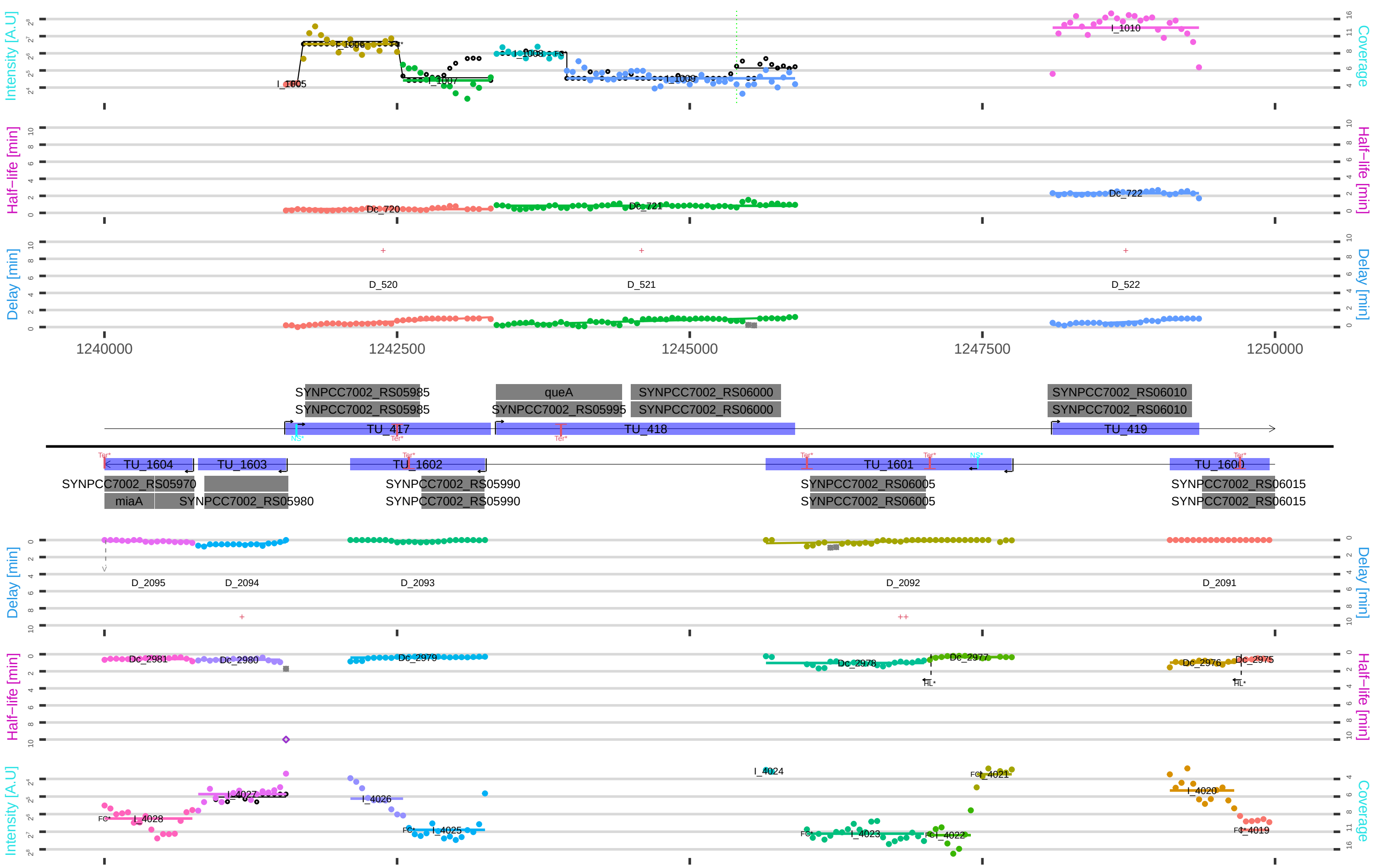

Term: termination (5), NS: new start (1), PS: pausing site (0), iTSS_I: internal starting site (0)

ID: 25007−25200; Term: termination (5), NS: new start (1), PS: pausing site (2), iTSS_I: internal starting site (0)

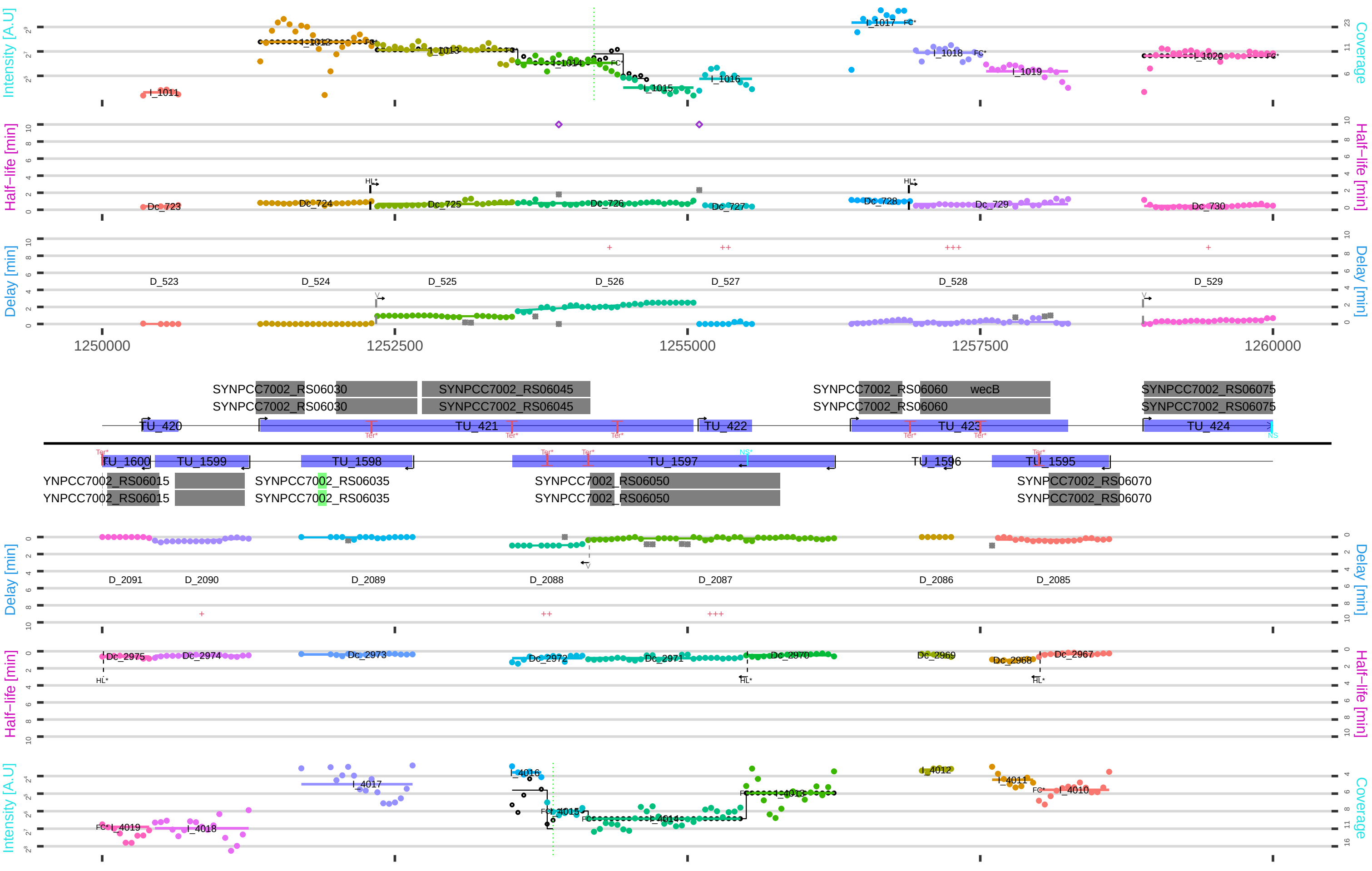

Term: termination (4), NS: new start (1), PS: pausing site (1), iTSS_I: internal starting site (0)

ID: 25200−25394; Term: termination (4), NS: new start (1), PS: pausing site (1), iTSS_I: internal starting site (0)

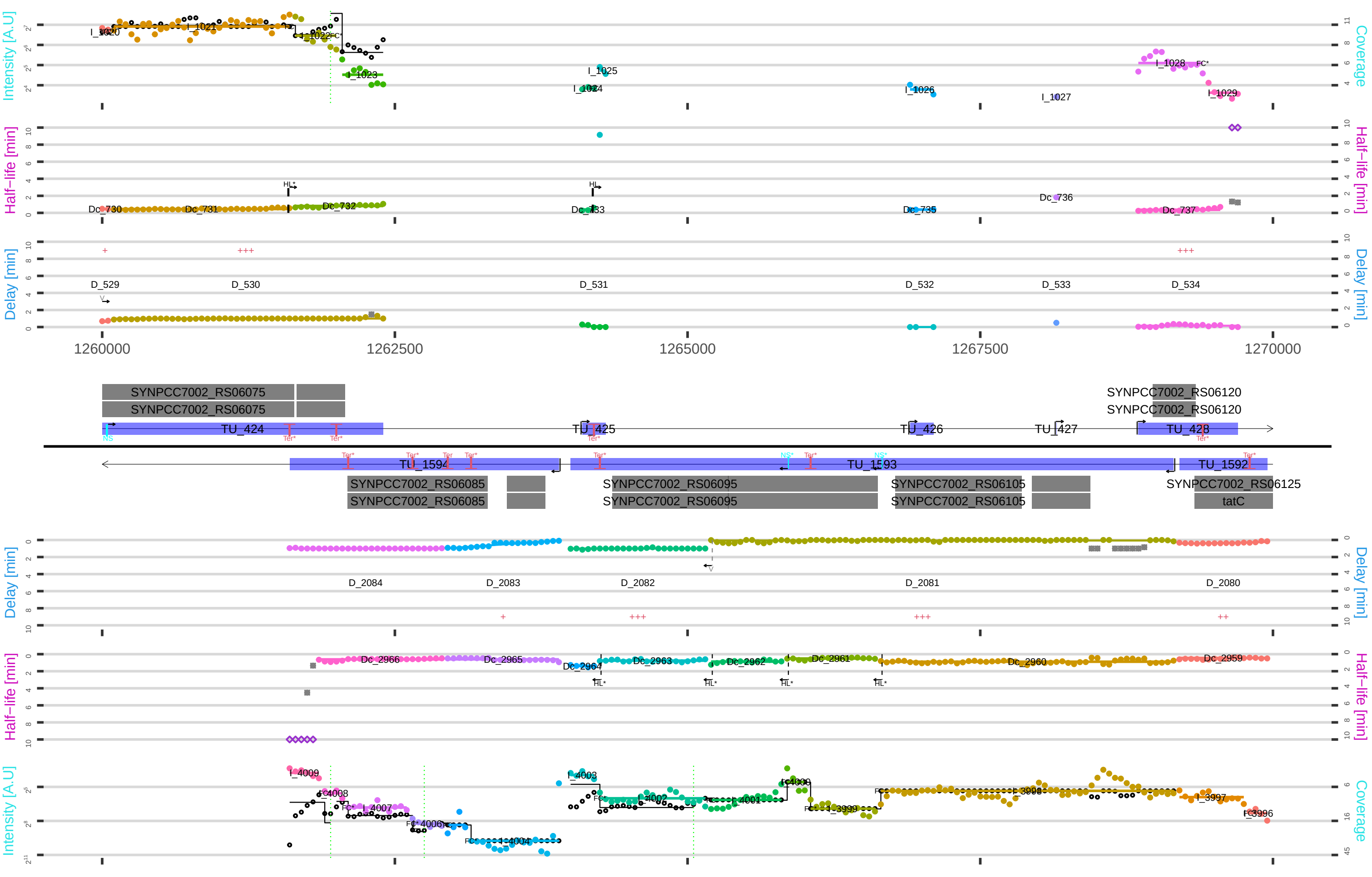

Term: termination (7), NS: new start (2), PS: pausing site (2), iTSS_I: internal starting site (0)

ID: 25414−25600; Term: termination (5), NS: new start (3), PS: pausing site (2), iTSS_I: internal starting site (0)

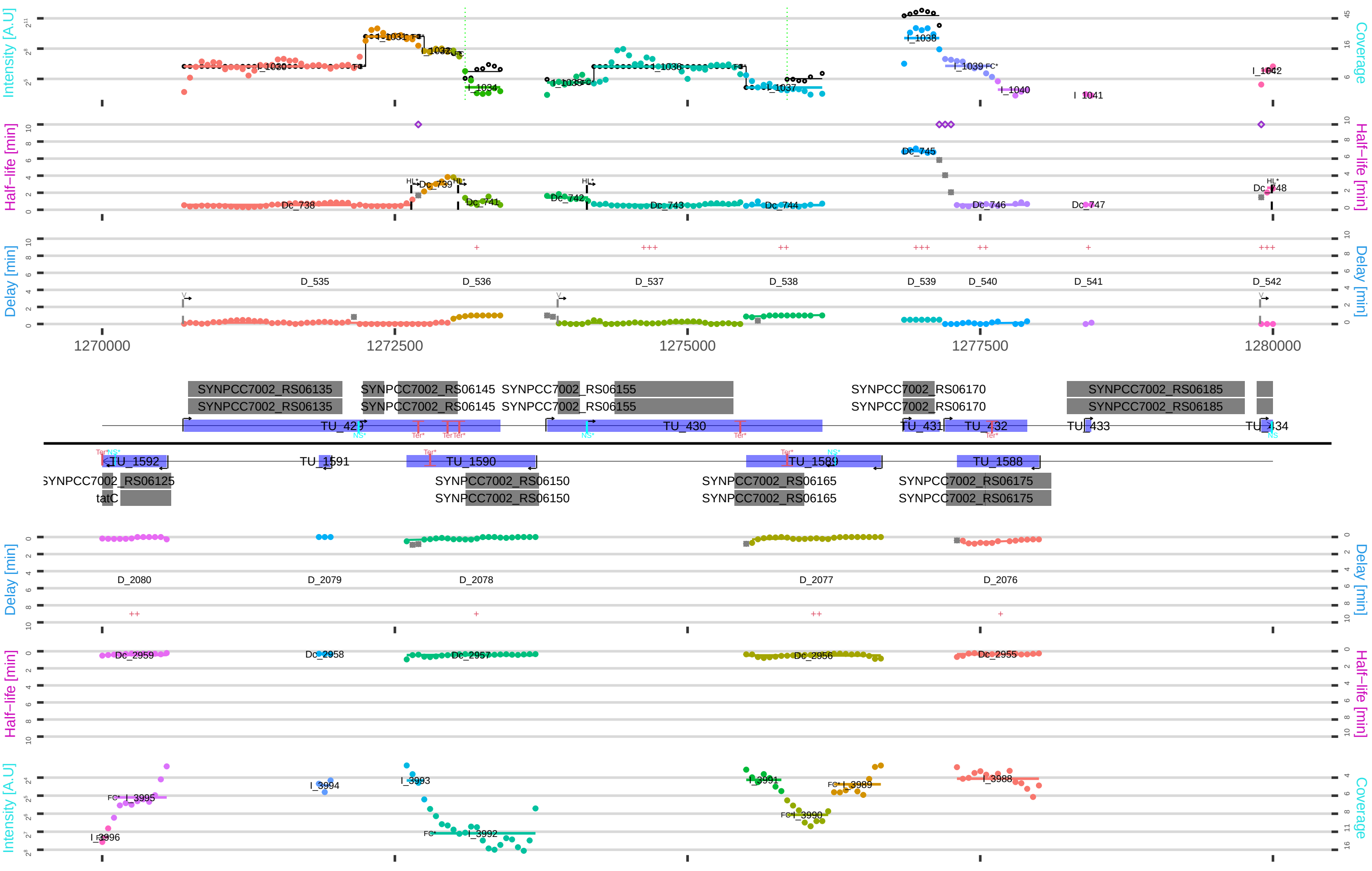

Term: termination (3), NS: new start (2), PS: pausing site (0), iTSS_I: internal starting site (0)

ID: 25600−25800; Term: termination (4), NS: new start (7), PS: pausing site (2), iTSS_I: internal starting site (0)

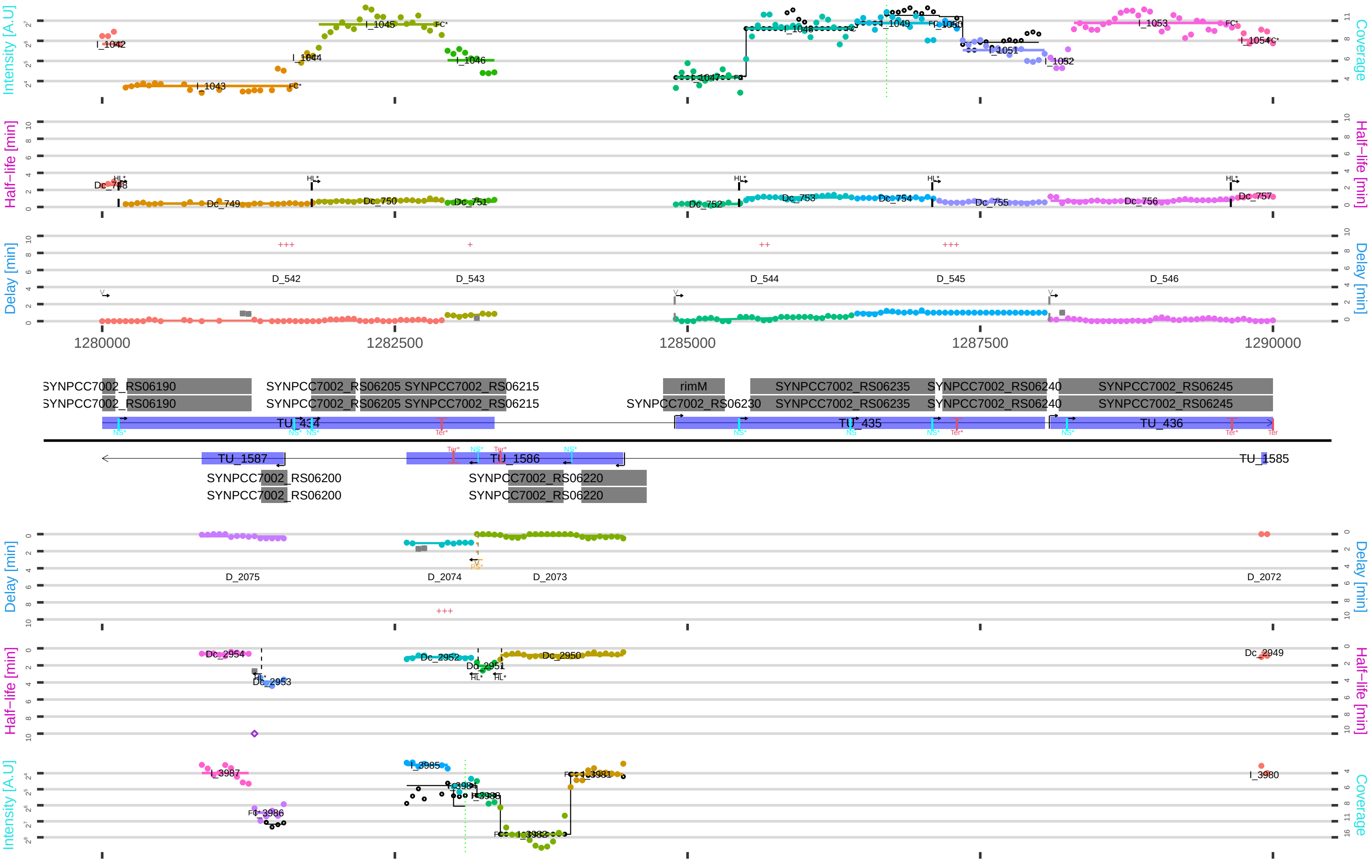

Term: termination (2), NS: new start (2), PS: pausing site (1), iTSS_I: internal starting site (0)

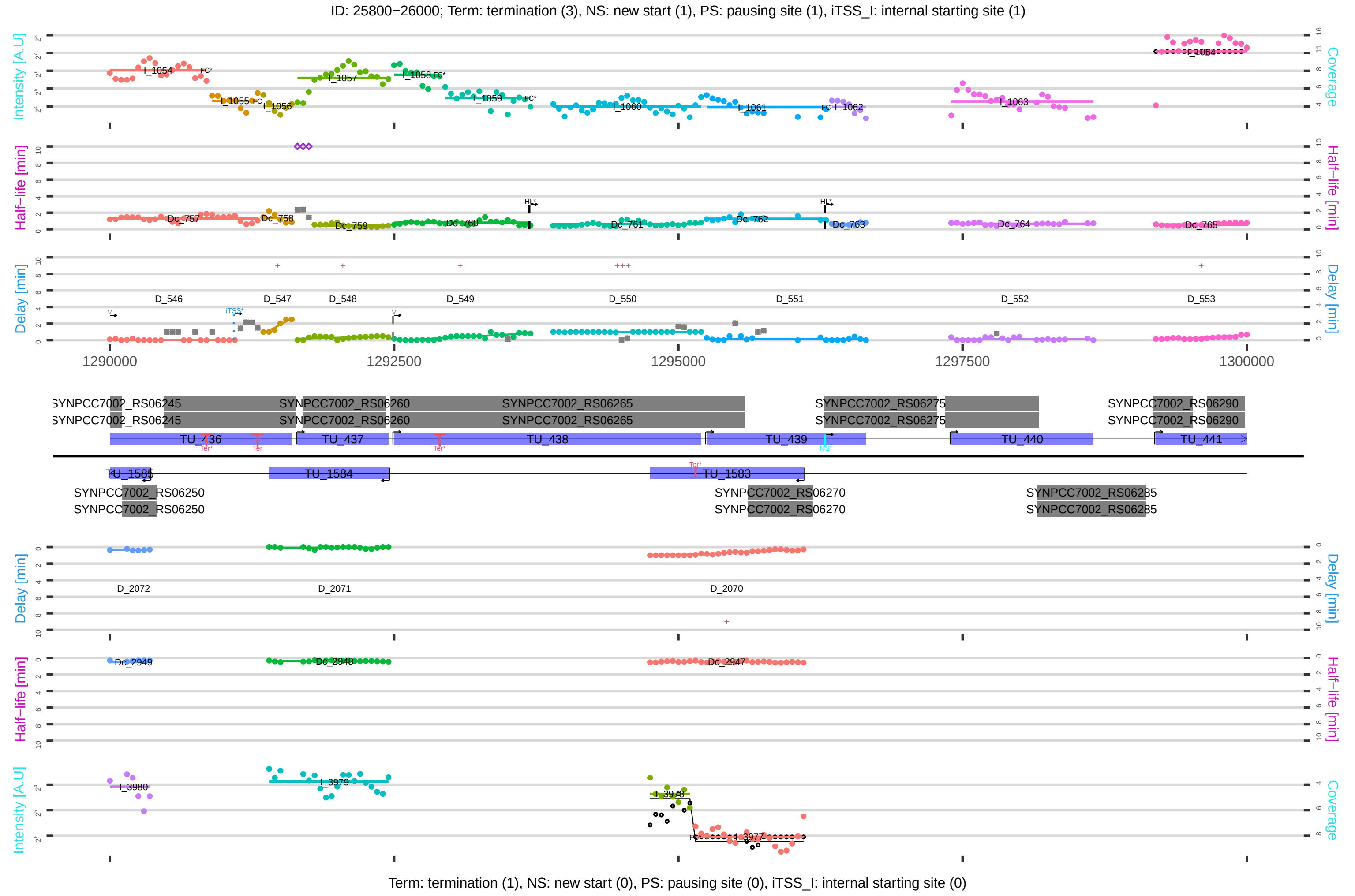
ID: 25800−26000; Term: termination (3), NS: new start (1), PS: pausing site (1), iTSS_I: internal starting site (1)
Intensity [A.U]
Coverage
Half−life [min]
Delay [min]
1290000
1292500
1295000
1297500
1300000
SYNPCC7002_RS06245
SYNPCC7002_RS06260
SYNPCC7002_RS06265
SYNPCC7002_RS06275
SYNPCC7002_RS06290
TU_436
TU_437
TU_438
TU_439
TU_440
TU_441
TU_1585
TU_1584
TU_1583
SYNPCC7002_RS06250
SYNPCC7002_RS06270
SYNPCC7002_RS06285
D_2072
D_2071
D_2070
Term: termination (1), NS: new start (0), PS: pausing site (0), iTSS_I: internal starting site (0)

ID: 26000−26194; Term: termination (3), NS: new start (2), PS: pausing site (2), iTSS_I: internal starting site (0)

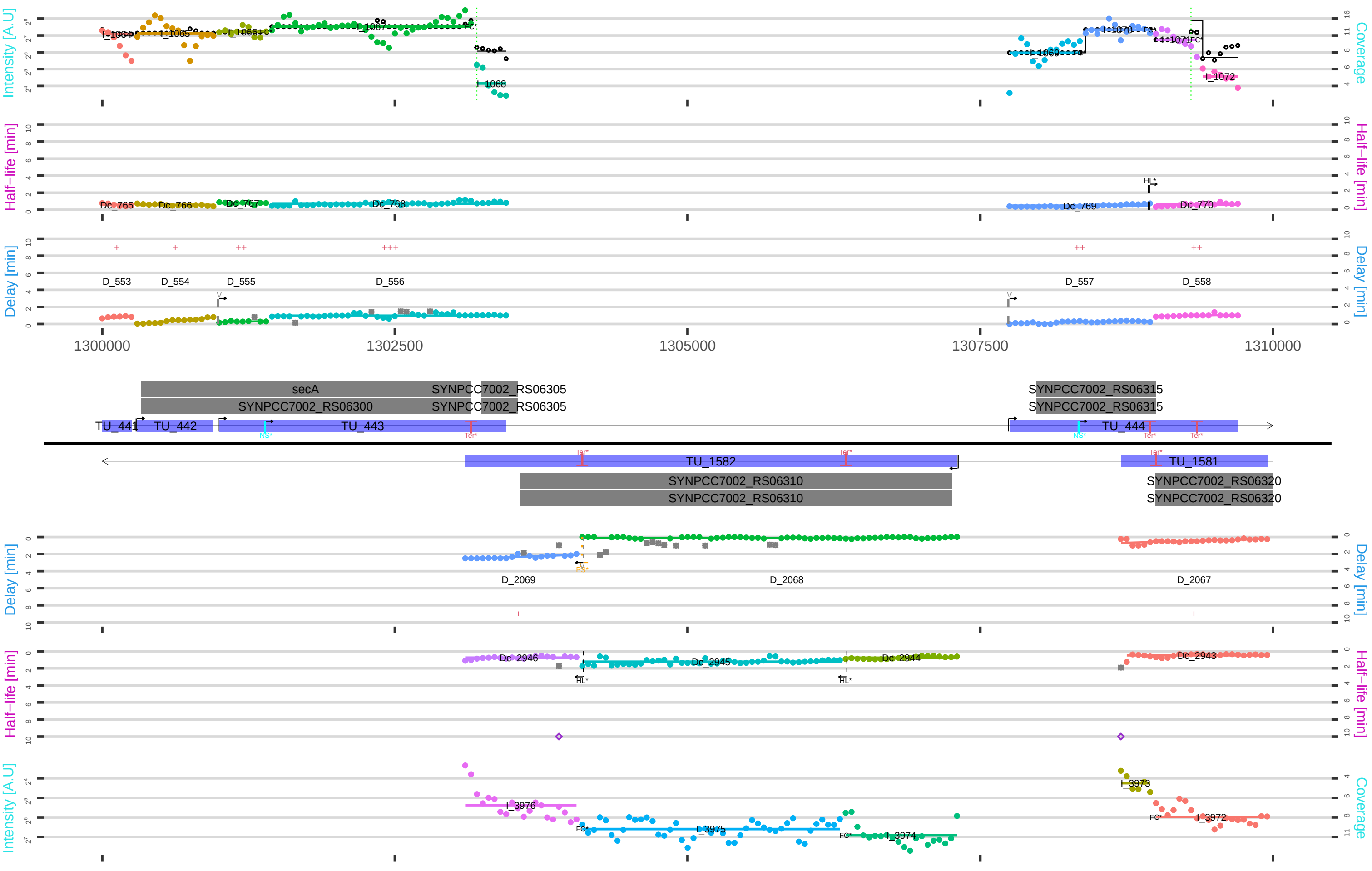

Term: termination (3), NS: new start (0), PS: pausing site (1), iTSS_I: internal starting site (0)

ID: 26207−26377; Term: termination (5), NS: new start (2), PS: pausing site (0), iTSS_I: internal starting site (0)

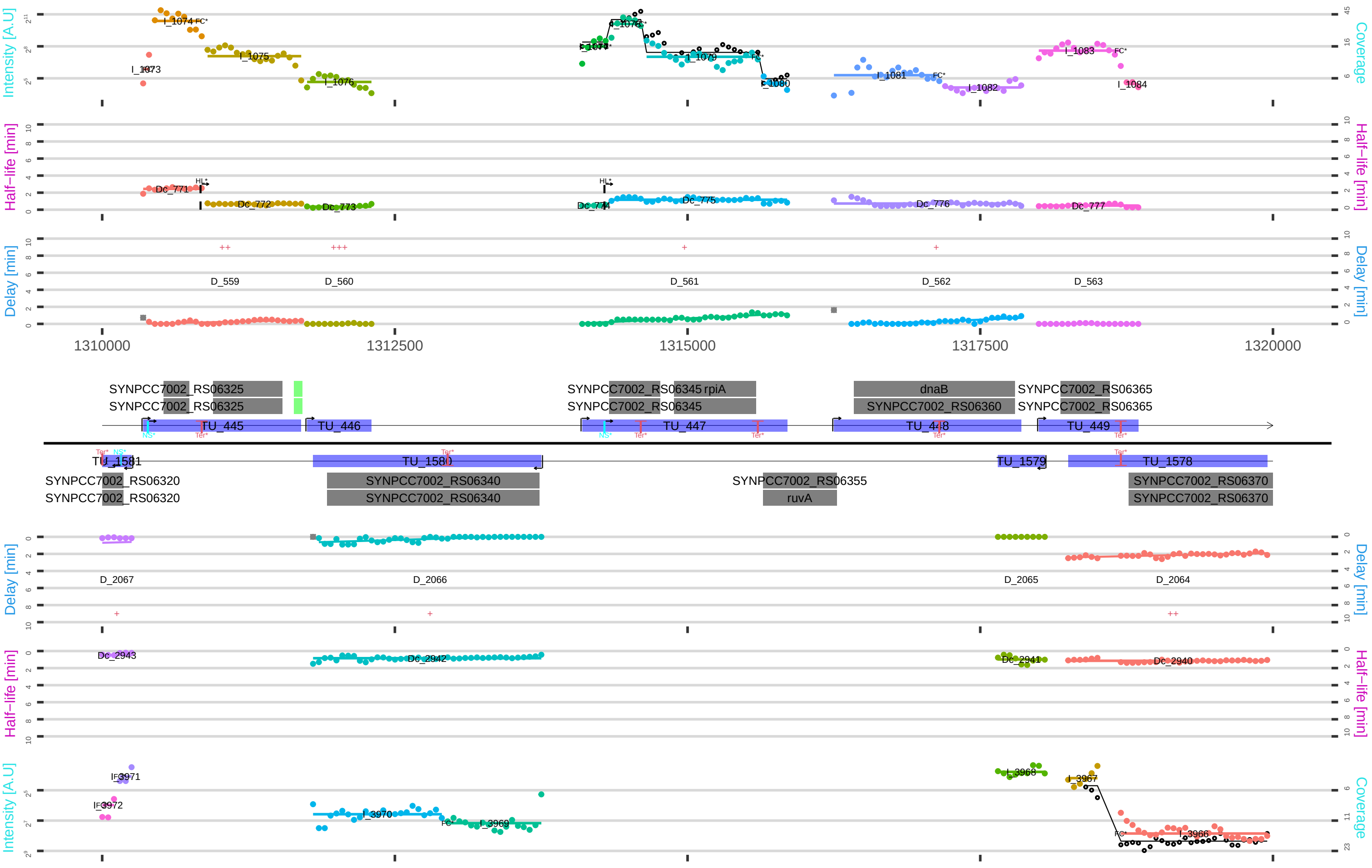

Term: termination (3), NS: new start (1), PS: pausing site (0), iTSS_I: internal starting site (0)

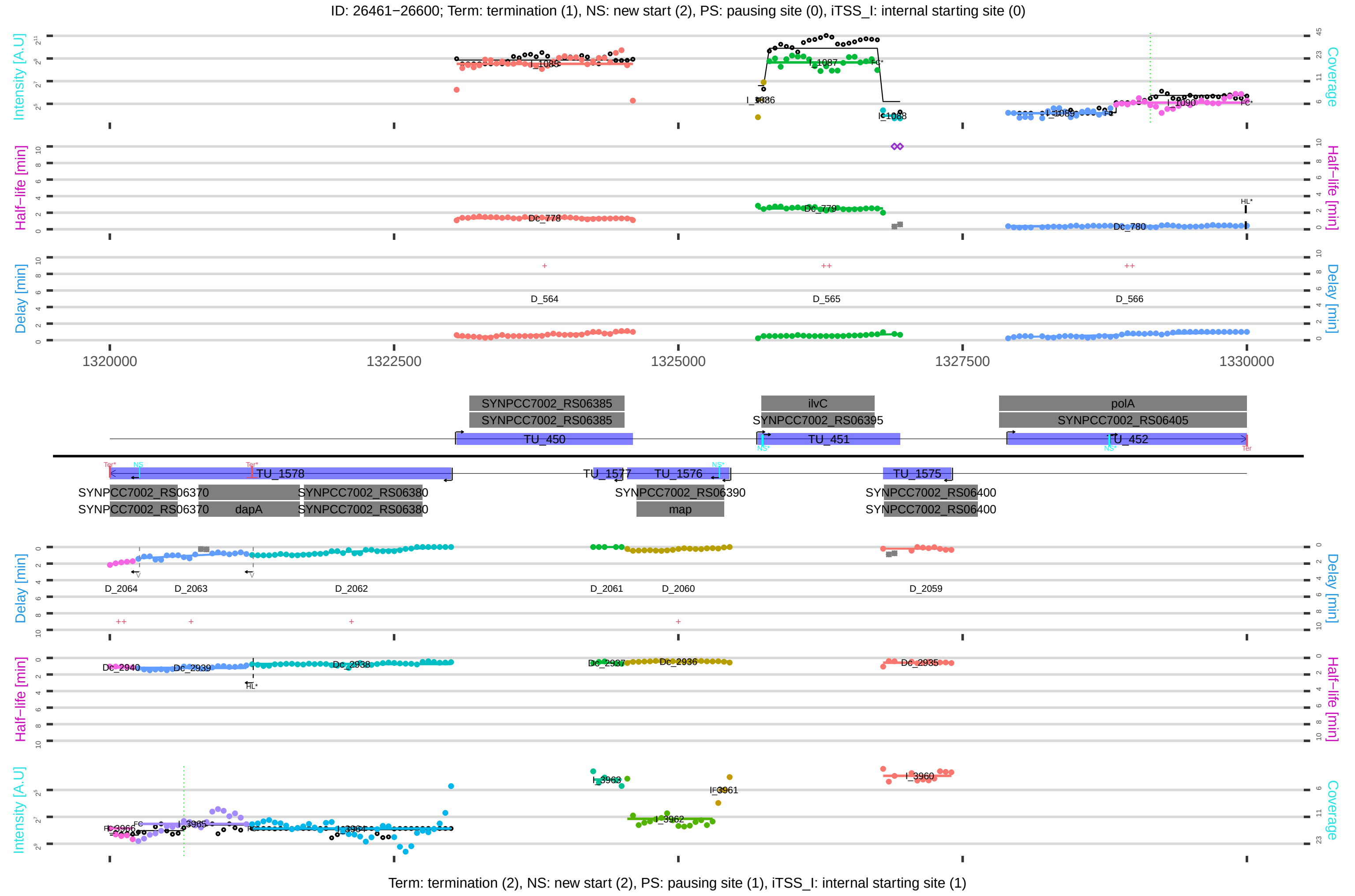

ID: 26461−26600; Term: termination (1), NS: new start (2), PS: pausing site (0), iTSS_I: internal starting site (0)
Intensity [A.U]
Coverage
Half−life [min]
Delay [min]
1320000
1322500
1325000
1327500
1330000
SYNPCC7002_RS06385
ilvC
SYNPCC7002_RS06395
polA
SYNPCC7002_RS06405
TU_450
TU_451
TU_452
TU_1578
TU_1577
TU_1576
TU_1575
SYNPCC7002_RS06370
SYNPCC7002_RS06380
dapA
SYNPCC7002_RS06390
map
SYNPCC7002_RS06400
D_564
D_565
D_566
D_2064
D_2063
D_2062
D_2061
D_2060
D_2059
Term: termination (2), NS: new start (2), PS: pausing site (1), iTSS_I: internal starting site (1)

ID: 26600−26711; Term: termination (2), NS: new start (2), PS: pausing site (0), iTSS_I: internal starting site (0)

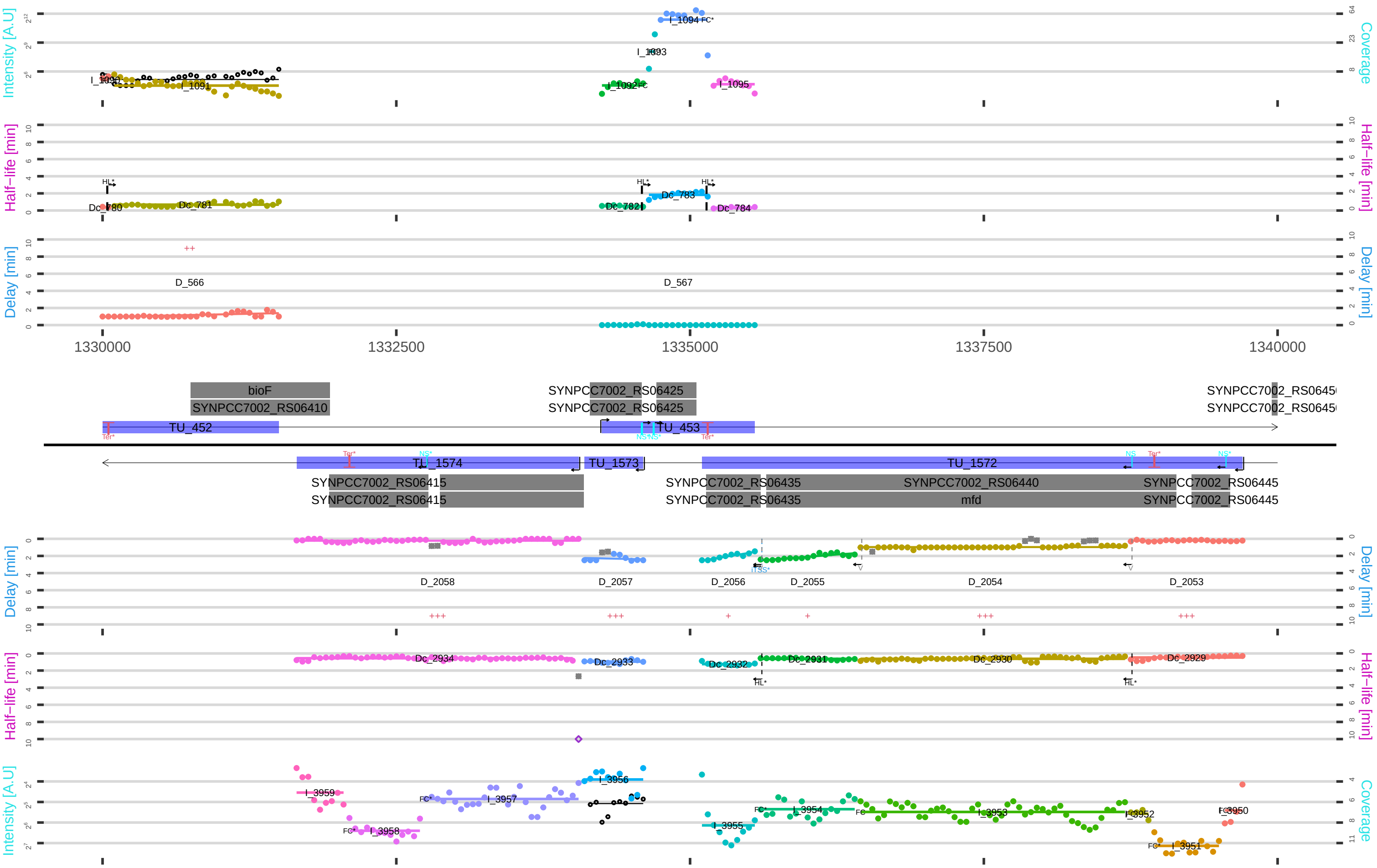

Term: termination (2), NS: new start (3), PS: pausing site (2), iTSS_I: internal starting site (1)

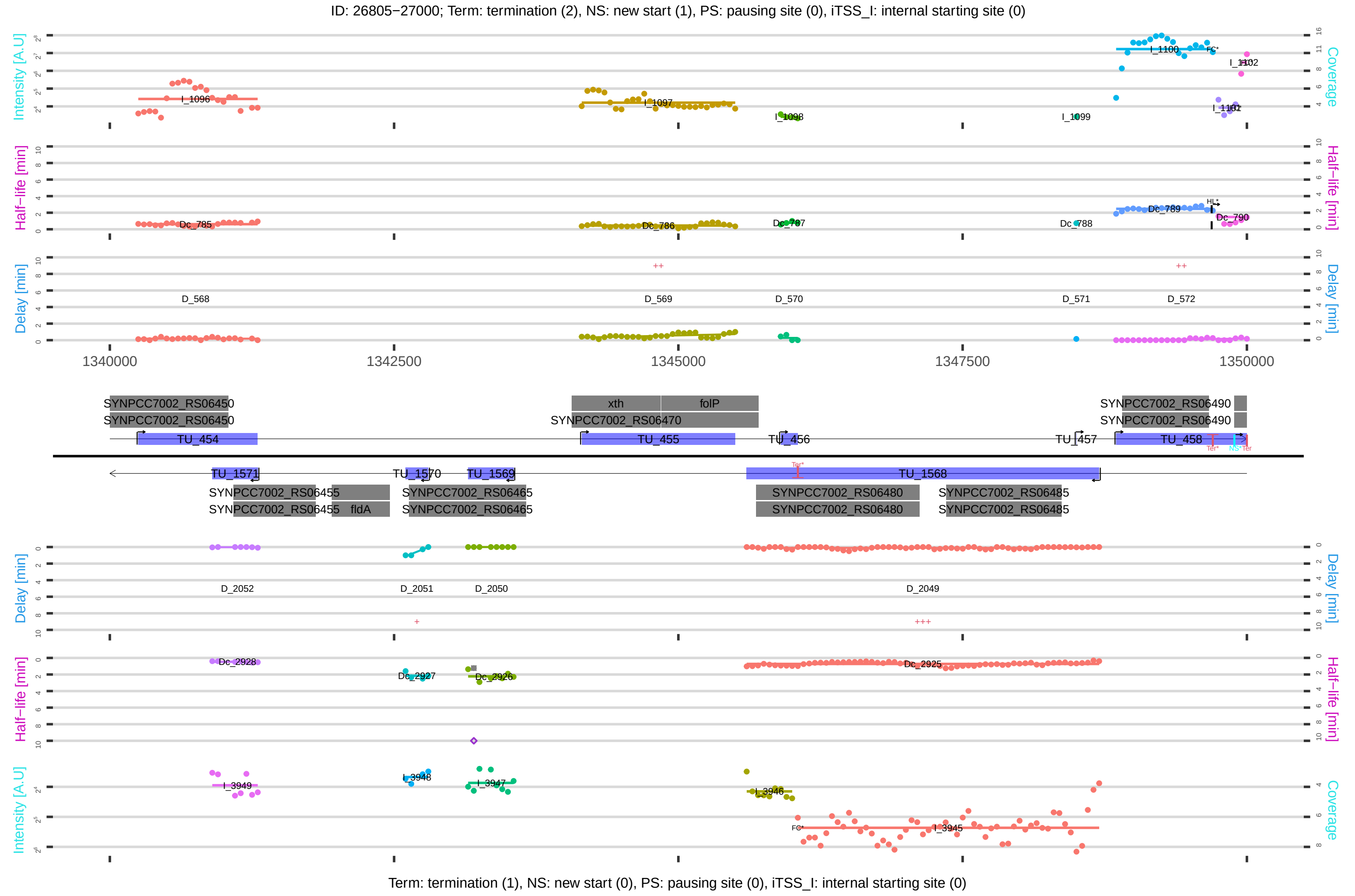
ID: 26805−27000; Term: termination (2), NS: new start (1), PS: pausing site (0), iTSS_I: internal starting site (0)
Intensity [A.U]
Coverage
I_1096
I_1097
I_1098
I_1099
I_1100
I_1101
I_1102
Half−life [min]
Dc_785
Dc_786
Dc_787
Dc_788
Dc_789
Dc_790
Delay [min]
D_568
D_569
D_570
D_571
D_572
1340000
1342500
1345000
1347500
1350000
SYNPCC7002_RS06450
xth
folP
SYNPCC7002_RS06470
SYNPCC7002_RS06490
TU_454
TU_455
TU_456
TU_457
TU_458
TU_1571
TU_1570
TU_1569
TU_1568
SYNPCC7002_RS06455
fldA
SYNPCC7002_RS06465
SYNPCC7002_RS06480
SYNPCC7002_RS06485
D_2052
D_2051
D_2050
D_2049
Dc_2928
Dc_2927
Dc_2926
Dc_2925
I_3949
I_3948
I_3947
I_3946
I_3945
Term: termination (1), NS: new start (0), PS: pausing site (0), iTSS_I: internal starting site (0)

ID: 27000−27200; Term: termination (5), NS: new start (3), PS: pausing site (1), iTSS_I: internal starting site (1)

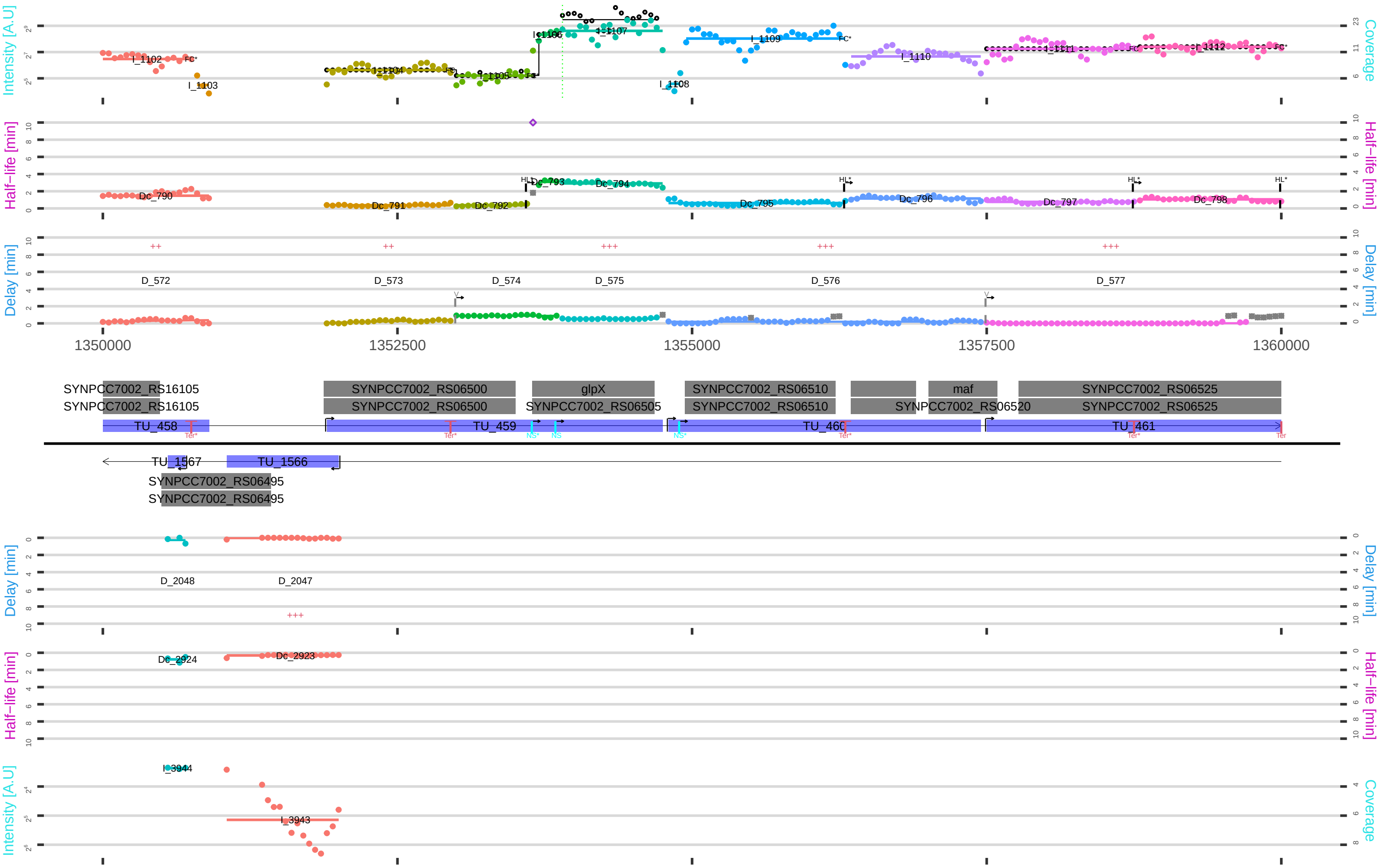

Term: termination (0), NS: new start (0), PS: pausing site (0), iTSS_I: internal starting site (0)

ID: 27200−27400; Term: termination (7), NS: new start (3), PS: pausing site (1), iTSS_I: internal starting site (0)

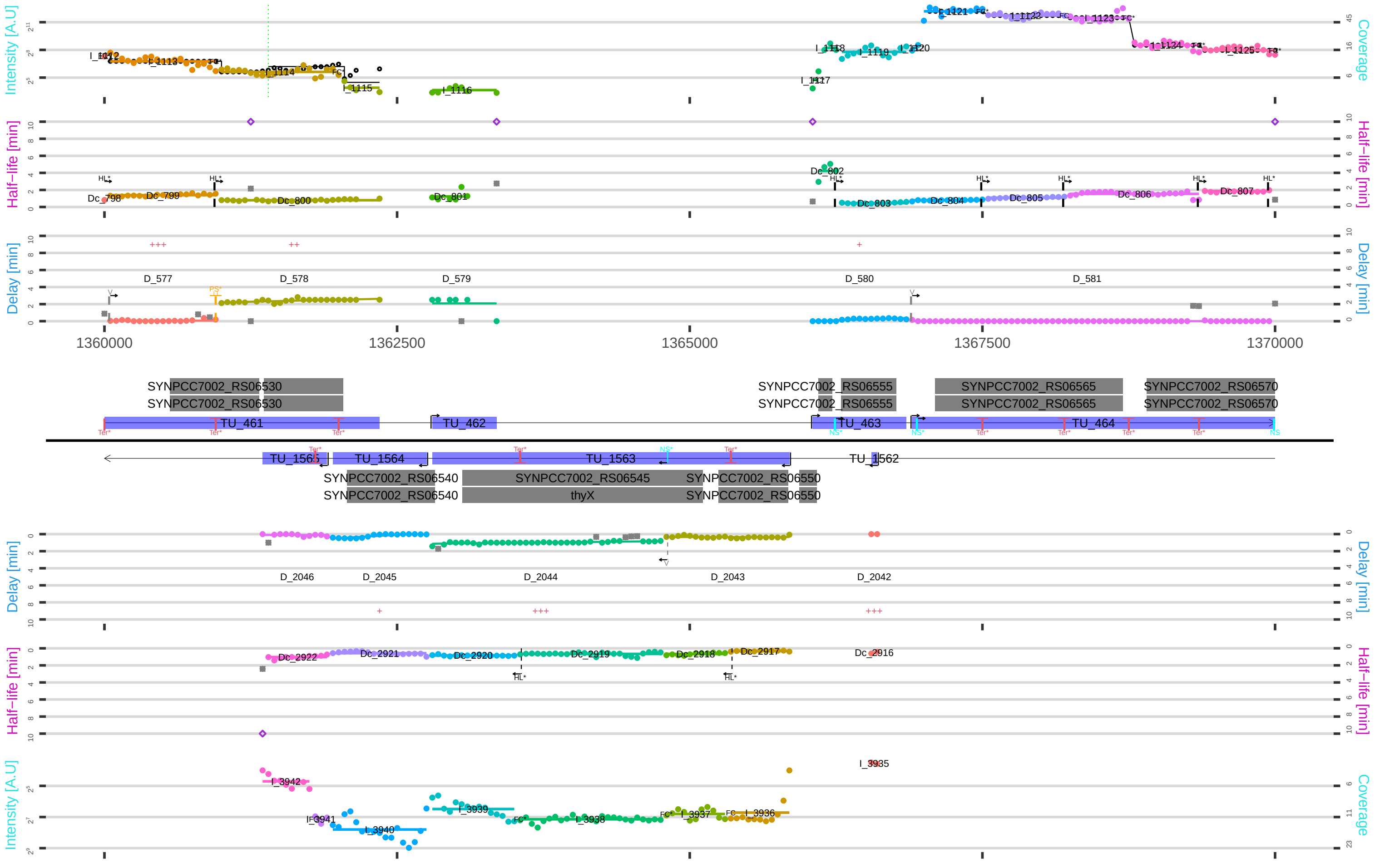

Term: termination (3), NS: new start (1), PS: pausing site (1), iTSS_I: internal starting site (0)

ID: 27400−27600; Term: termination (5), NS: new start (2), PS: pausing site (2), iTSS_I: internal starting site (0)

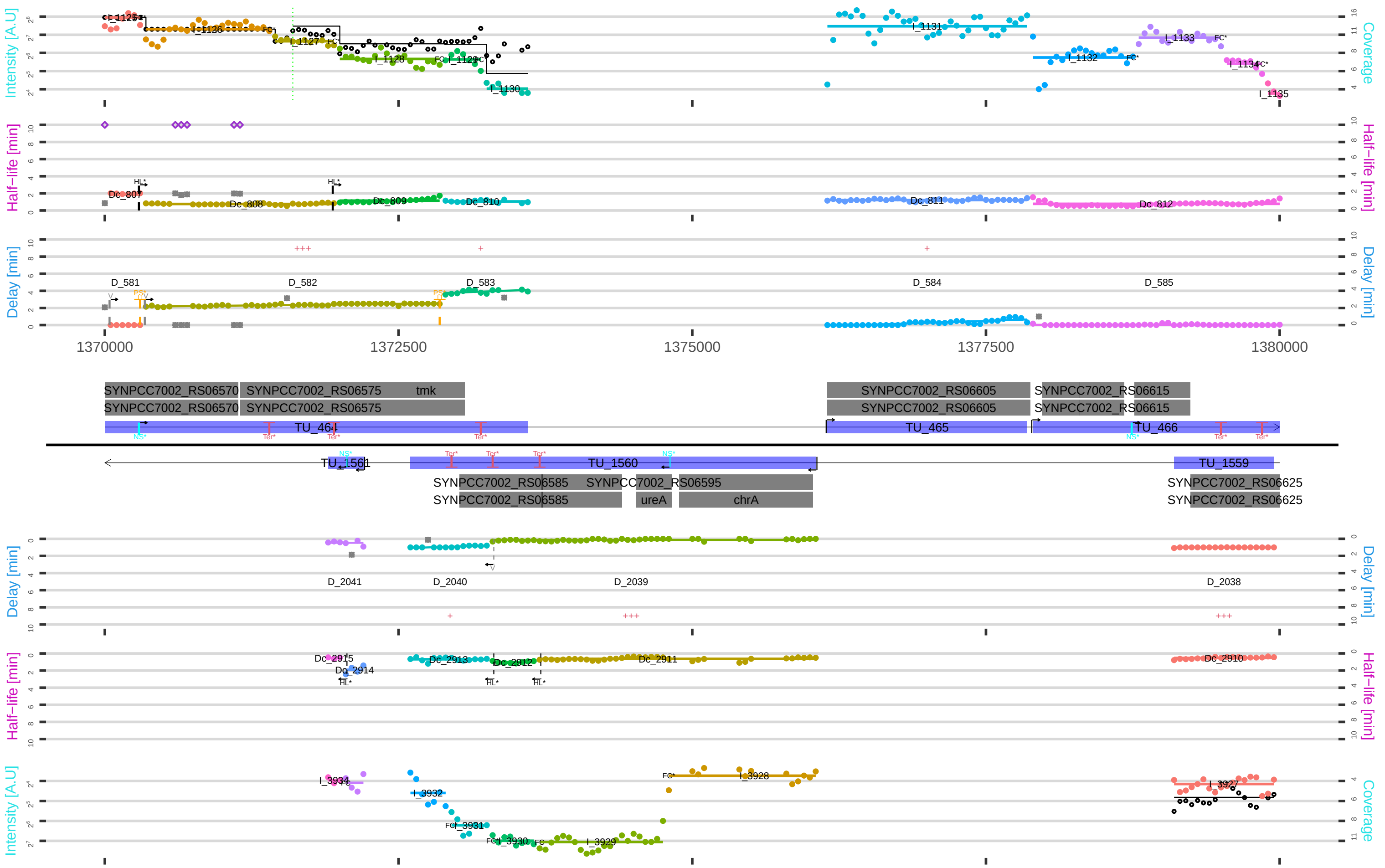

Term: termination (3), NS: new start (2), PS: pausing site (1), iTSS_I: internal starting site (0)

ID: 27600−27789; Term: termination (3), NS: new start (2), PS: pausing site (0), iTSS_I: internal starting site (0)

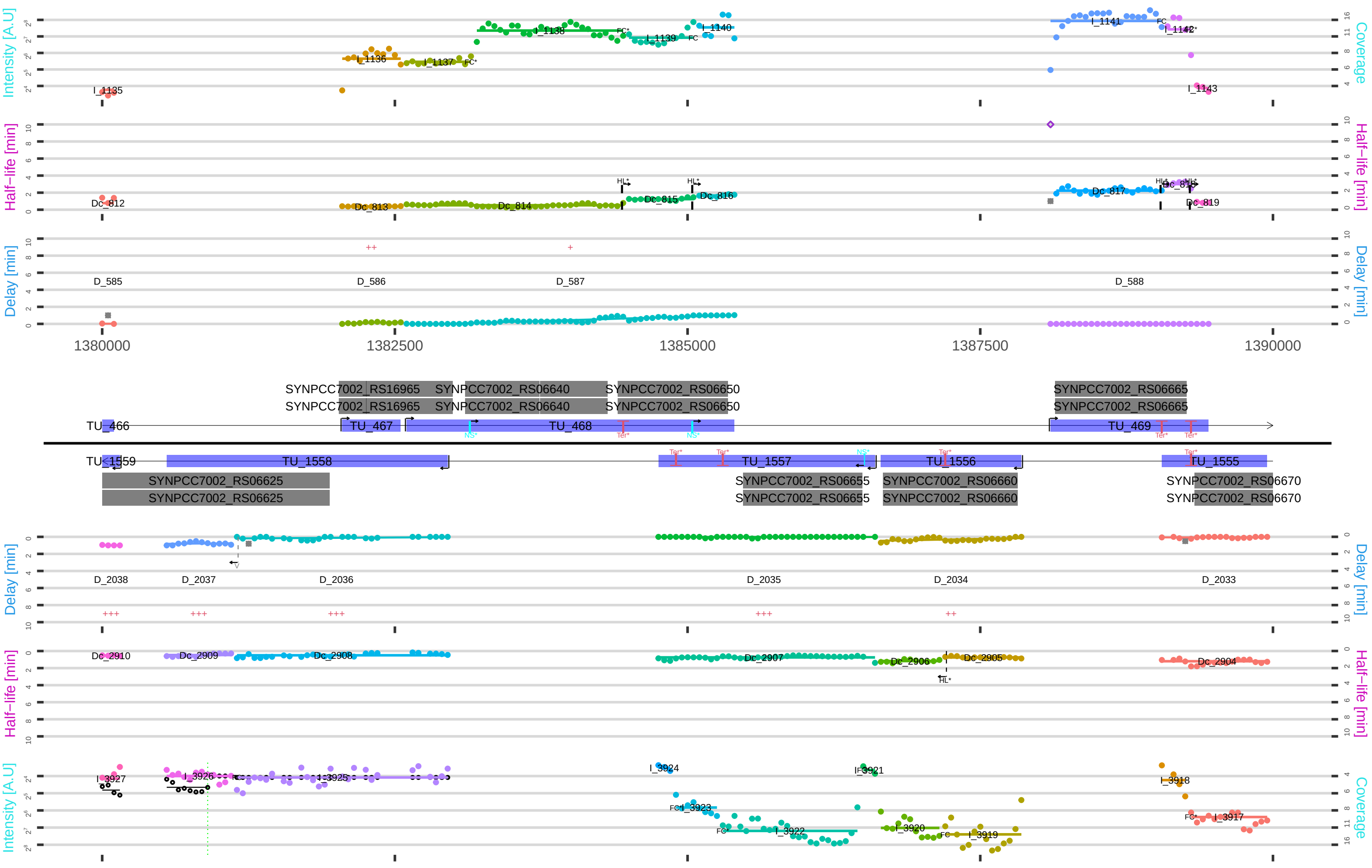

Term: termination (4), NS: new start (1), PS: pausing site (1), iTSS_I: internal starting site (0)

ID: 27809−27961; Term: termination (3), NS: new start (3), PS: pausing site (1), iTSS_I: internal starting site (0)

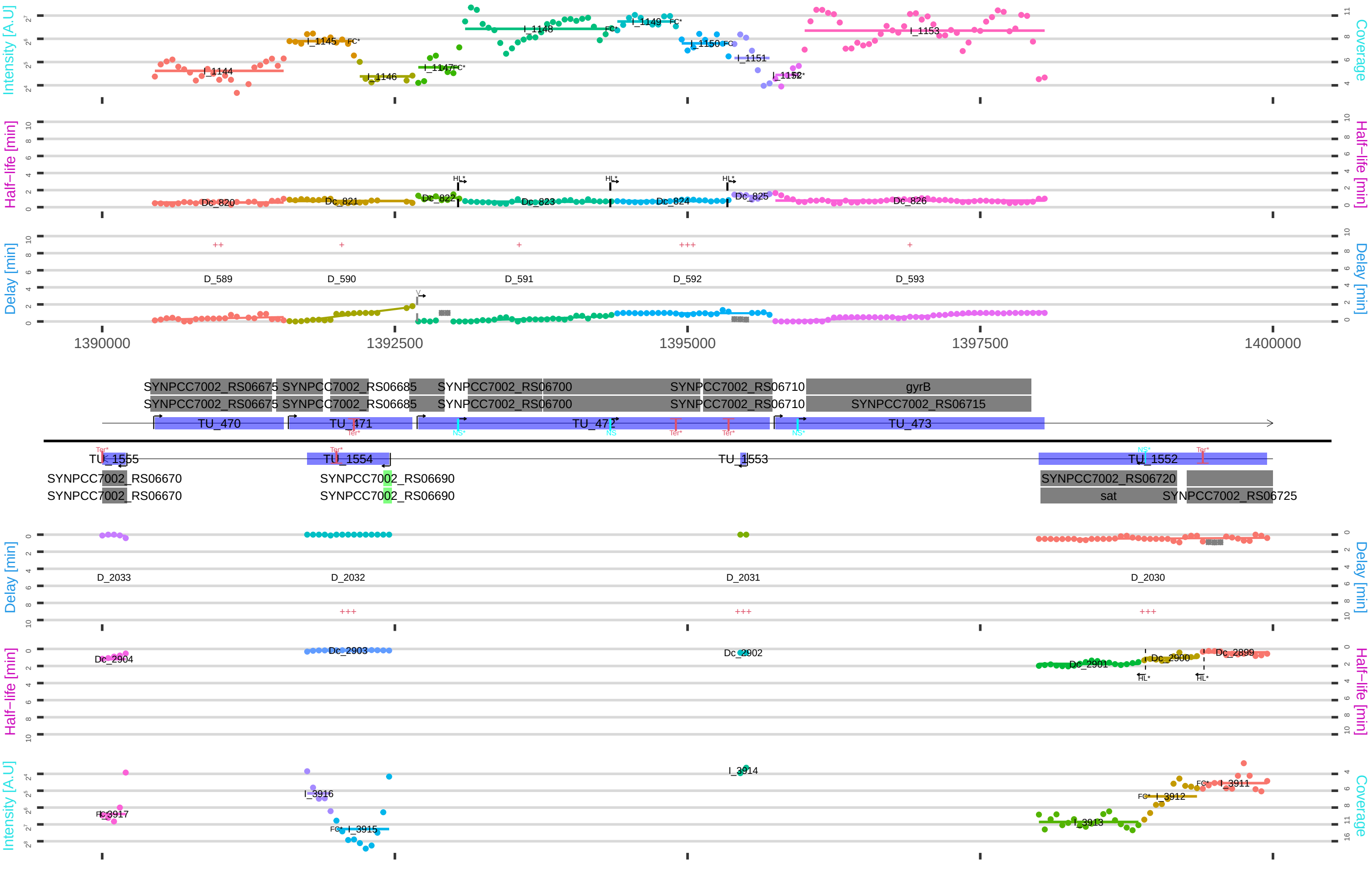

Term: termination (3), NS: new start (1), PS: pausing site (0), iTSS_I: internal starting site (0)

ID: 28057−28200; Term: termination (3), NS: new start (1), PS: pausing site (1), iTSS_I: internal starting site (0)

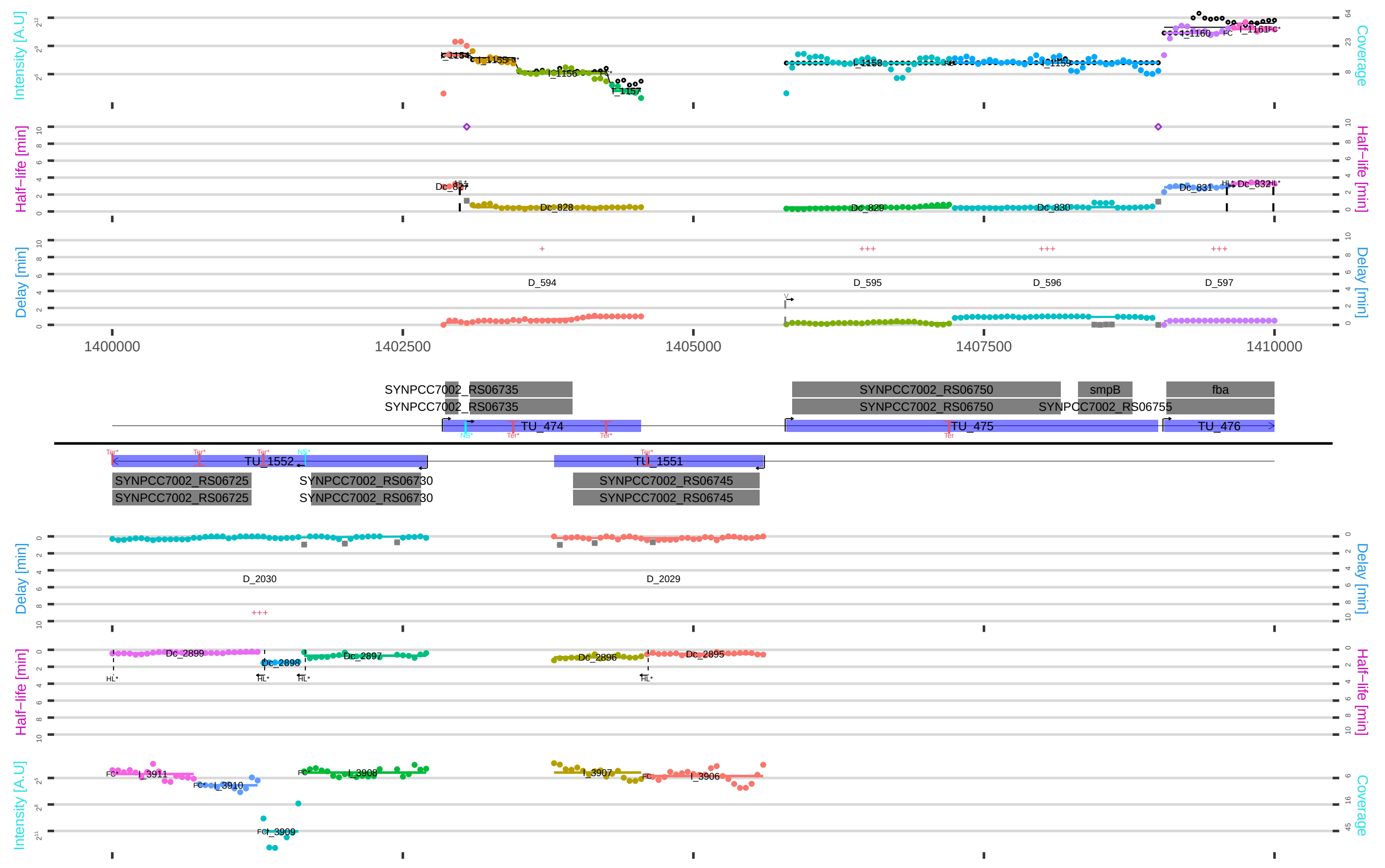

Term: termination (4), NS: new start (1), PS: pausing site (0), iTSS_I: internal starting site (0)

ID: 28200−28400; Term: termination (2), NS: new start (1), PS: pausing site (1), iTSS_I: internal starting site (0)

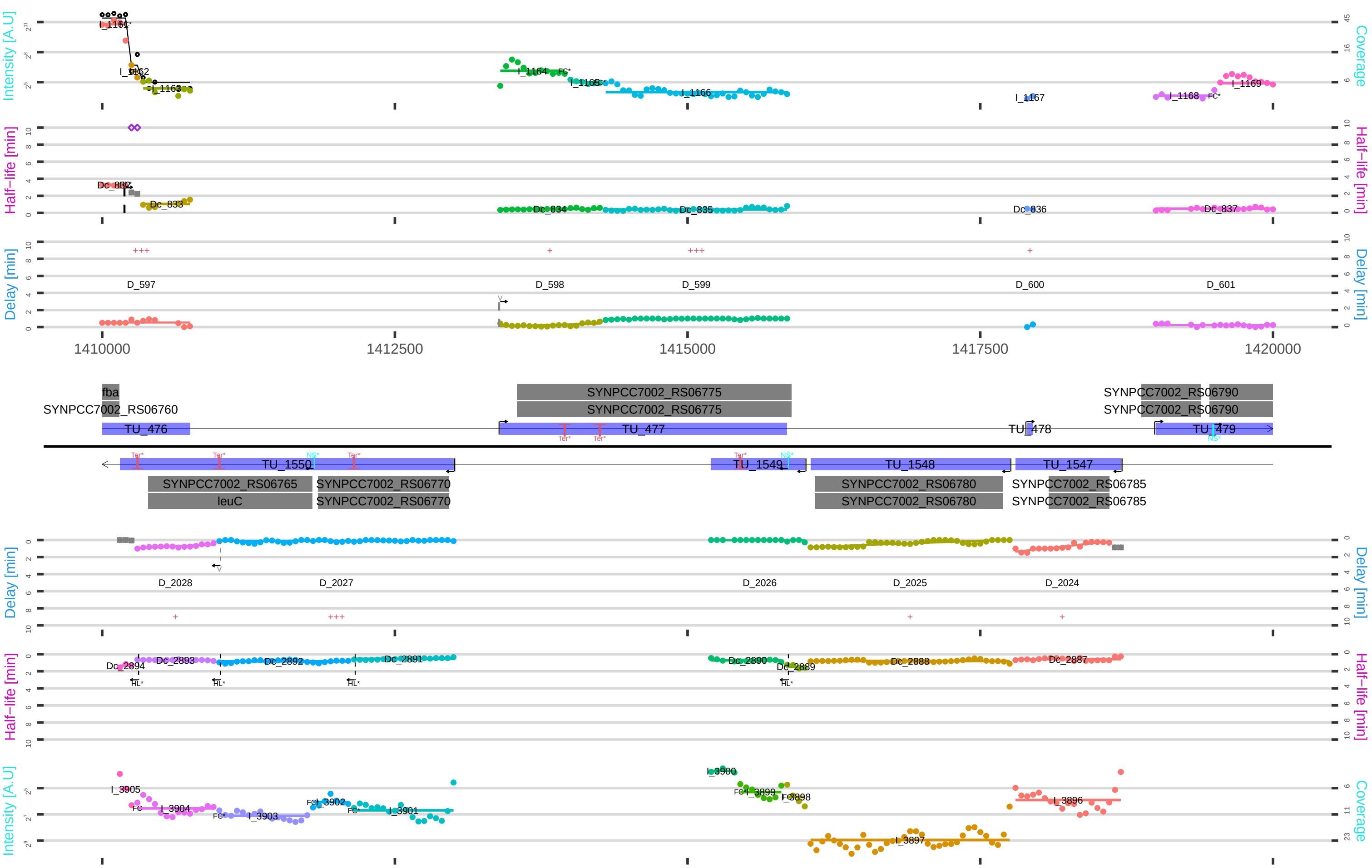

Term: termination (4), NS: new start (2), PS: pausing site (1), iTSS_I: internal starting site (0)

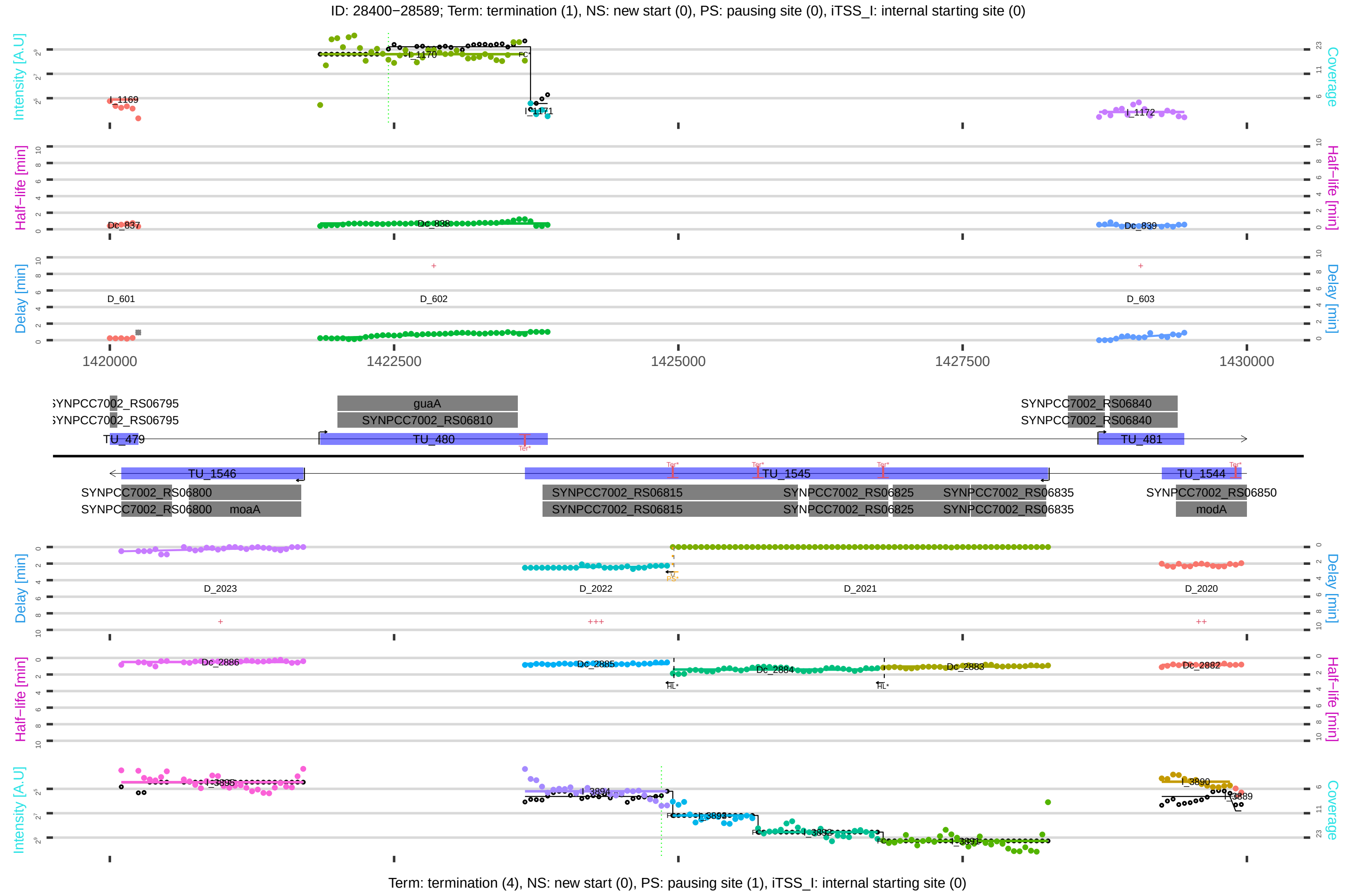

ID: 28400−28589; Term: termination (1), NS: new start (0), PS: pausing site (0), iTSS_I: internal starting site (0)
Intensity [A.U]
Coverage
Half−life [min]
Delay [min]
I_1169
I_1170
I_1171
I_1172
Dc_837
Dc_838
Dc_839
D_601
D_602
D_603
1420000
1422500
1425000
1427500
1430000
SYNPCC7002_RS06795
guaA
SYNPCC7002_RS06810
SYNPCC7002_RS06840
TU_479
TU_480
TU_481
TU_1546
TU_1545
TU_1544
SYNPCC7002_RS06800
moaA
SYNPCC7002_RS06815
SYNPCC7002_RS06825
SYNPCC7002_RS06835
SYNPCC7002_RS06850
modA
D_2023
D_2022
D_2021
D_2020
Dc_2886
Dc_2885
Dc_2884
Dc_2883
Dc_2882
I_3895
I_3894
I_3893
I_3892
I_3891
I_3890
I_3889
Term: termination (4), NS: new start (0), PS: pausing site (1), iTSS_I: internal starting site (0)

ID: 28624−28800; Term: termination (2), NS: new start (2), PS: pausing site (1), iTSS_I: internal starting site (0)

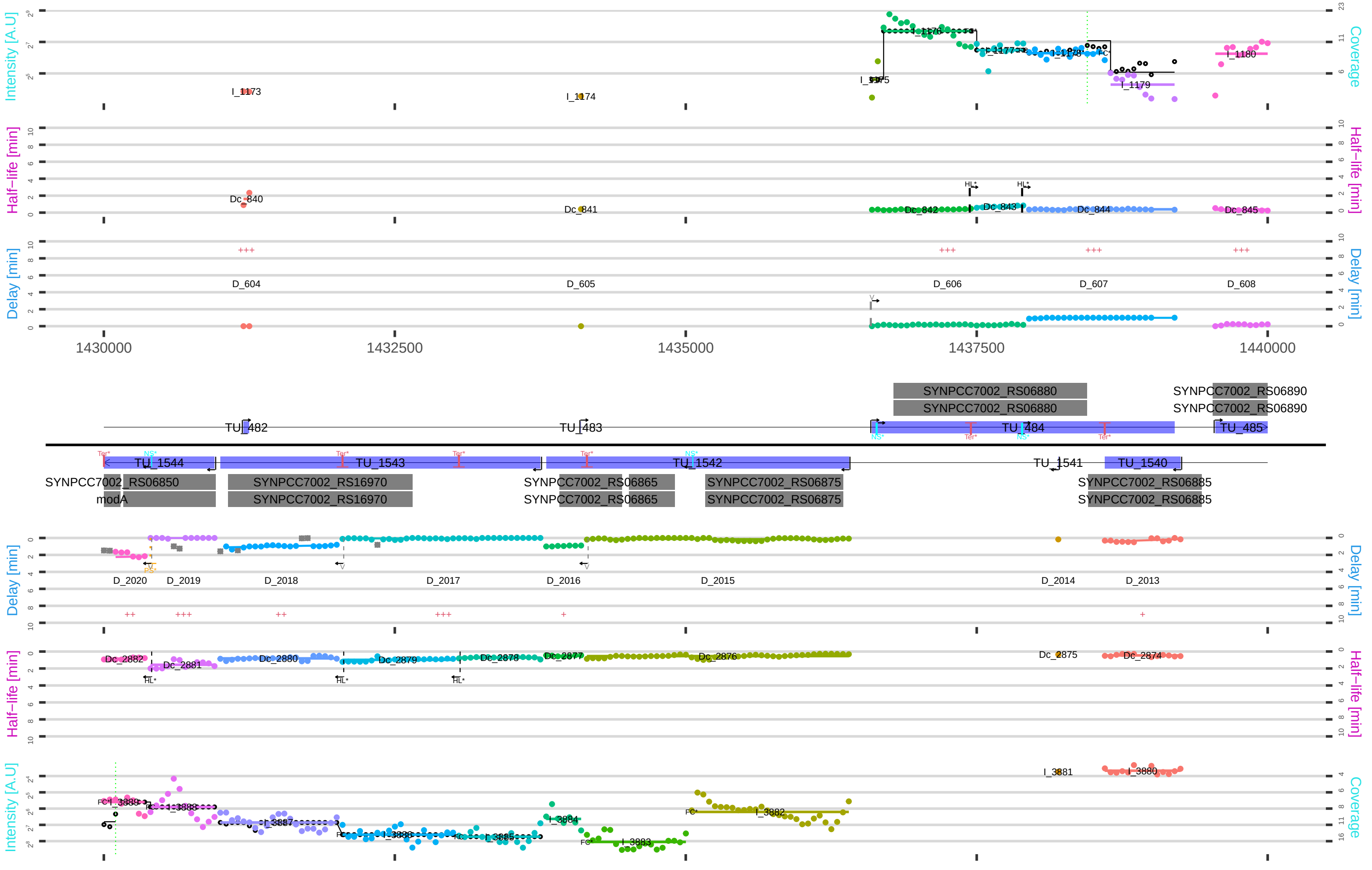

Term: termination (4), NS: new start (2), PS: pausing site (3), iTSS_I: internal starting site (0)

ID: 28800−28974; Term: termination (5), NS: new start (0), PS: pausing site (1), iTSS_I: internal starting site (0)

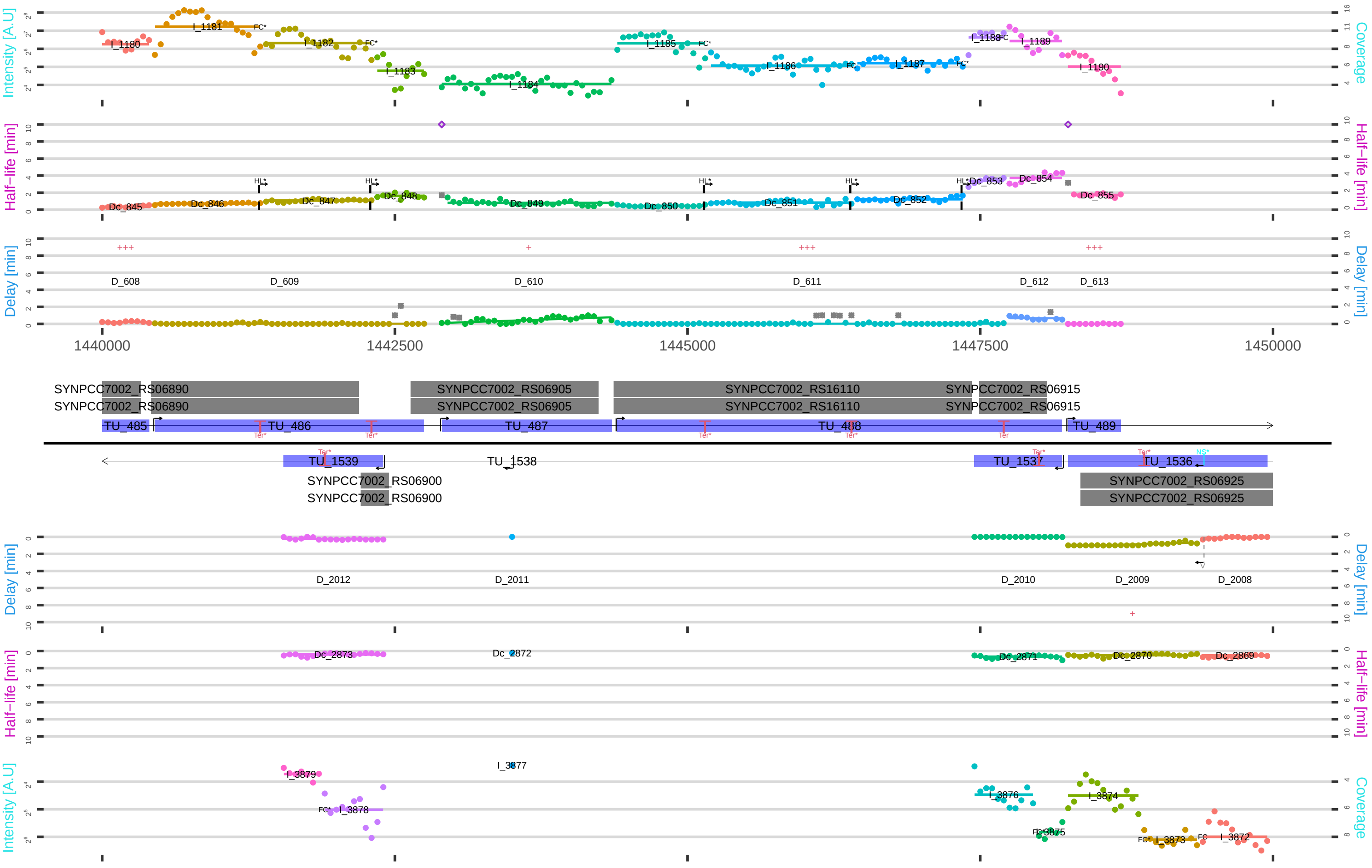

Term: termination (3), NS: new start (1), PS: pausing site (1), iTSS_I: internal starting site (0)

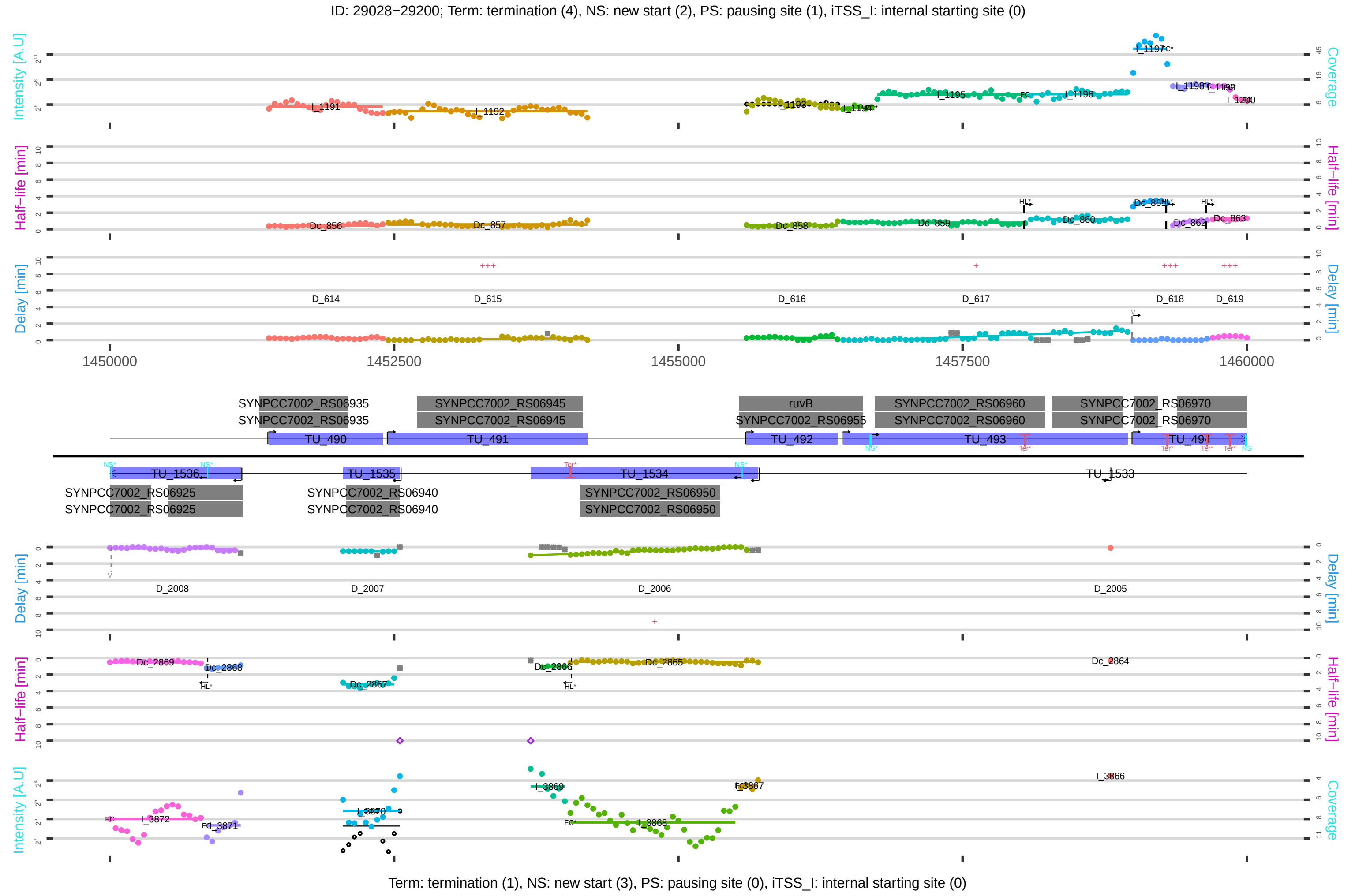
ID: 29028−29200; Term: termination (4), NS: new start (2), PS: pausing site (1), iTSS_I: internal starting site (0)
Intensity [A.U]
Coverage
Half−life [min]
Delay [min]
1450000
1452500
1455000
1457500
1460000
TU_490
TU_491
TU_492
TU_493
TU_494
TU_1536
TU_1535
TU_1534
TU_1533
SYNPCC7002_RS06935
SYNPCC7002_RS06945
ruvB
SYNPCC7002_RS06955
SYNPCC7002_RS06960
SYNPCC7002_RS06970
SYNPCC7002_RS06925
SYNPCC7002_RS06940
SYNPCC7002_RS06950
D_614
D_615
D_616
D_617
D_618
D_619
D_2008
D_2007
D_2006
D_2005
Term: termination (1), NS: new start (3), PS: pausing site (0), iTSS_I: internal starting site (0)

ID: 29200−29400; Term: termination (10), NS: new start (11), PS: pausing site (2), iTSS_I: internal starting site (0)

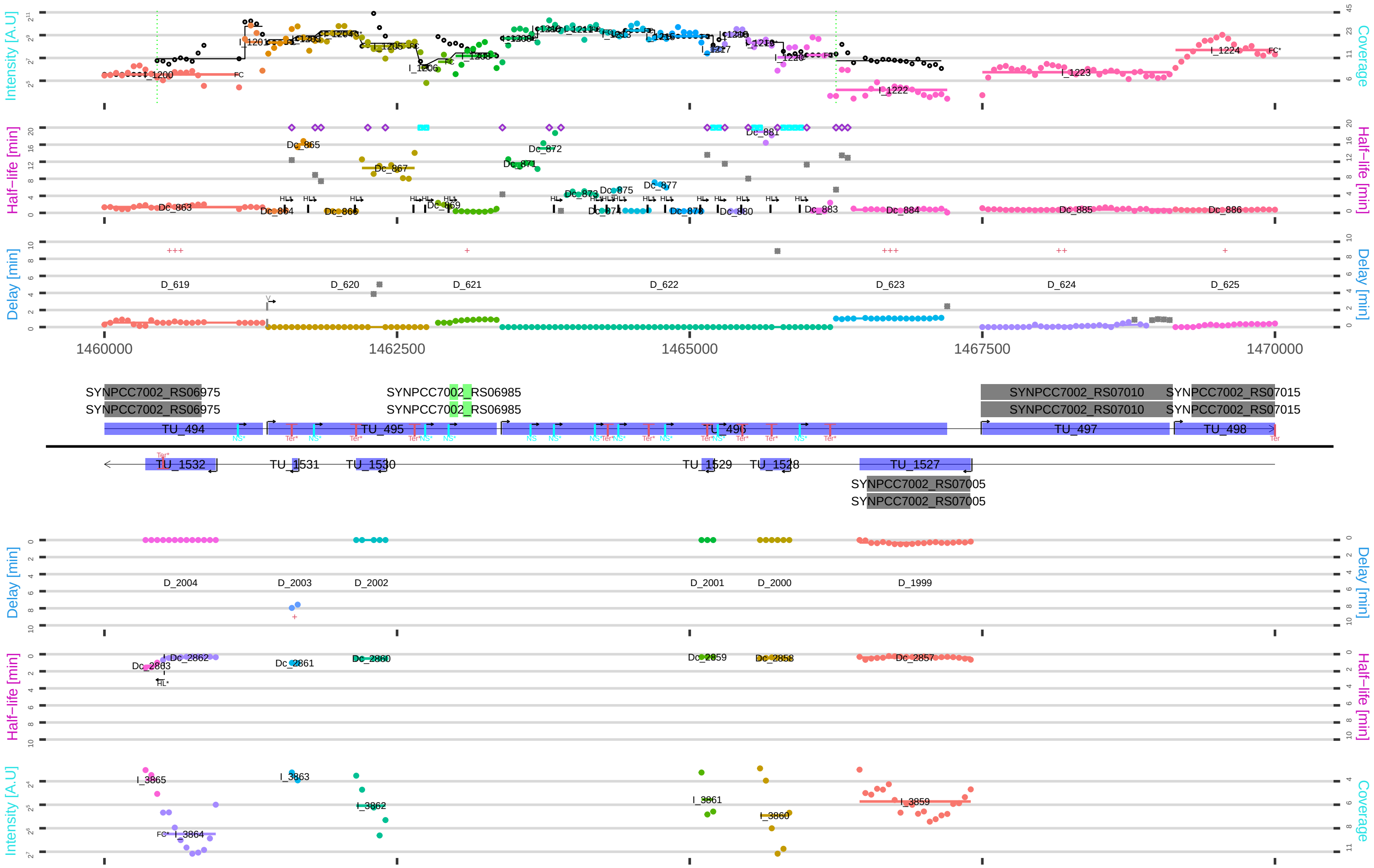

Term: termination (1), NS: new start (0), PS: pausing site (0), iTSS_I: internal starting site (0)

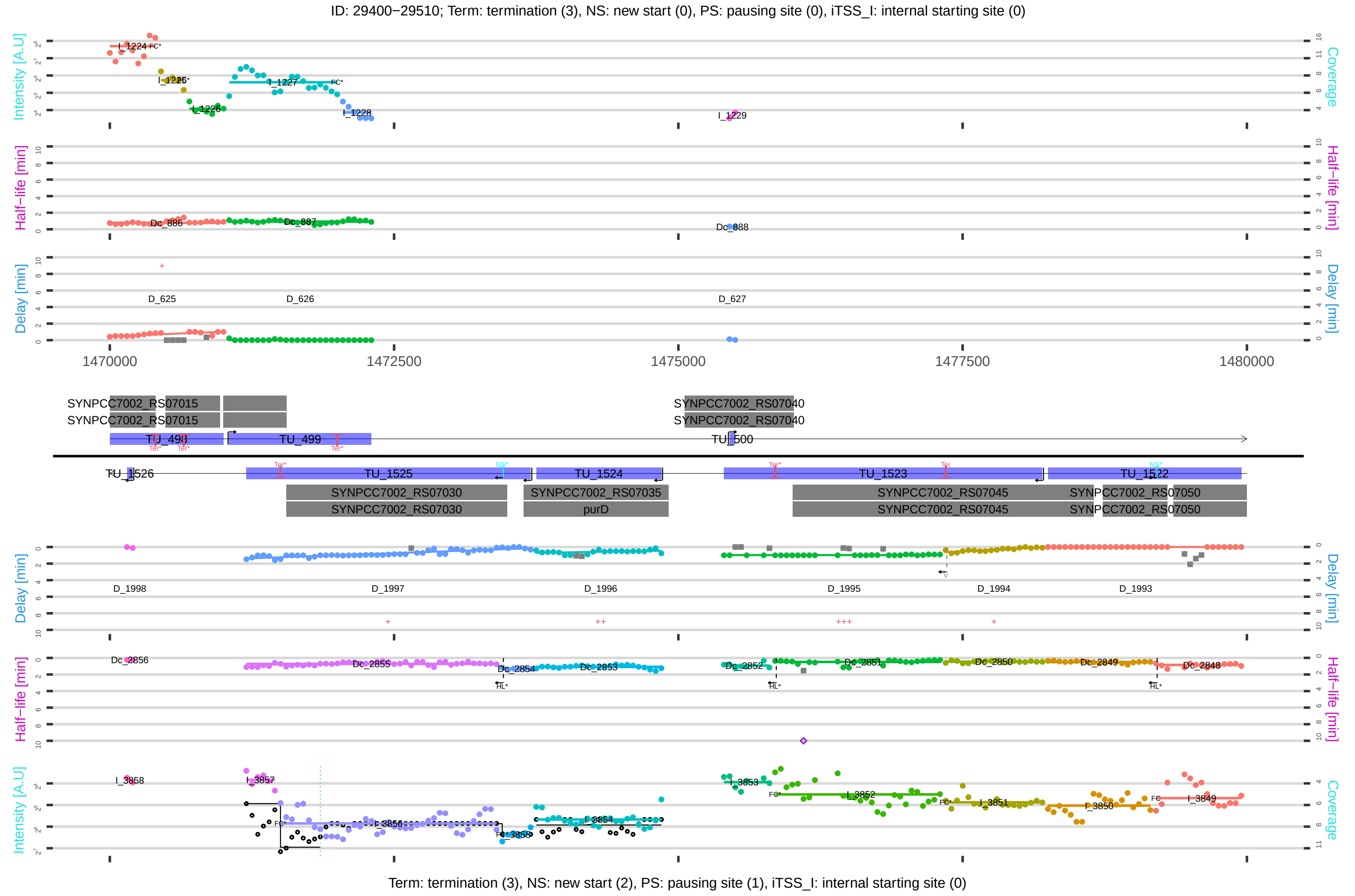

ID: 29400−29510; Term: termination (3), NS: new start (0), PS: pausing site (0), iTSS_I: internal starting site (0)
Intensity [A.U]
Coverage
Half−life [min]
Delay [min]
I_1224
I_1225
I_1226
I_1227
I_1228
I_1229
Dc_886
Dc_887
Dc_888
D_625
D_626
D_627
1470000
1472500
1475000
1477500
1480000
SYNPCC7002_RS07015
SYNPCC7002_RS07040
TU_498
TU_499
TU_500
TU_1526
TU_1525
TU_1524
TU_1523
TU_1522
SYNPCC7002_RS07030
SYNPCC7002_RS07035
purD
SYNPCC7002_RS07045
SYNPCC7002_RS07050
D_1998
D_1997
D_1996
D_1995
D_1994
D_1993
Dc_2856
Dc_2855
Dc_2854
Dc_2853
Dc_2852
Dc_2851
Dc_2850
Dc_2849
Dc_2848
I_3858
I_3857
I_3856
I_3855
I_3854
I_3853
I_3852
I_3851
I_3850
I_3849
Term: termination (3), NS: new start (2), PS: pausing site (1), iTSS_I: internal starting site (0)

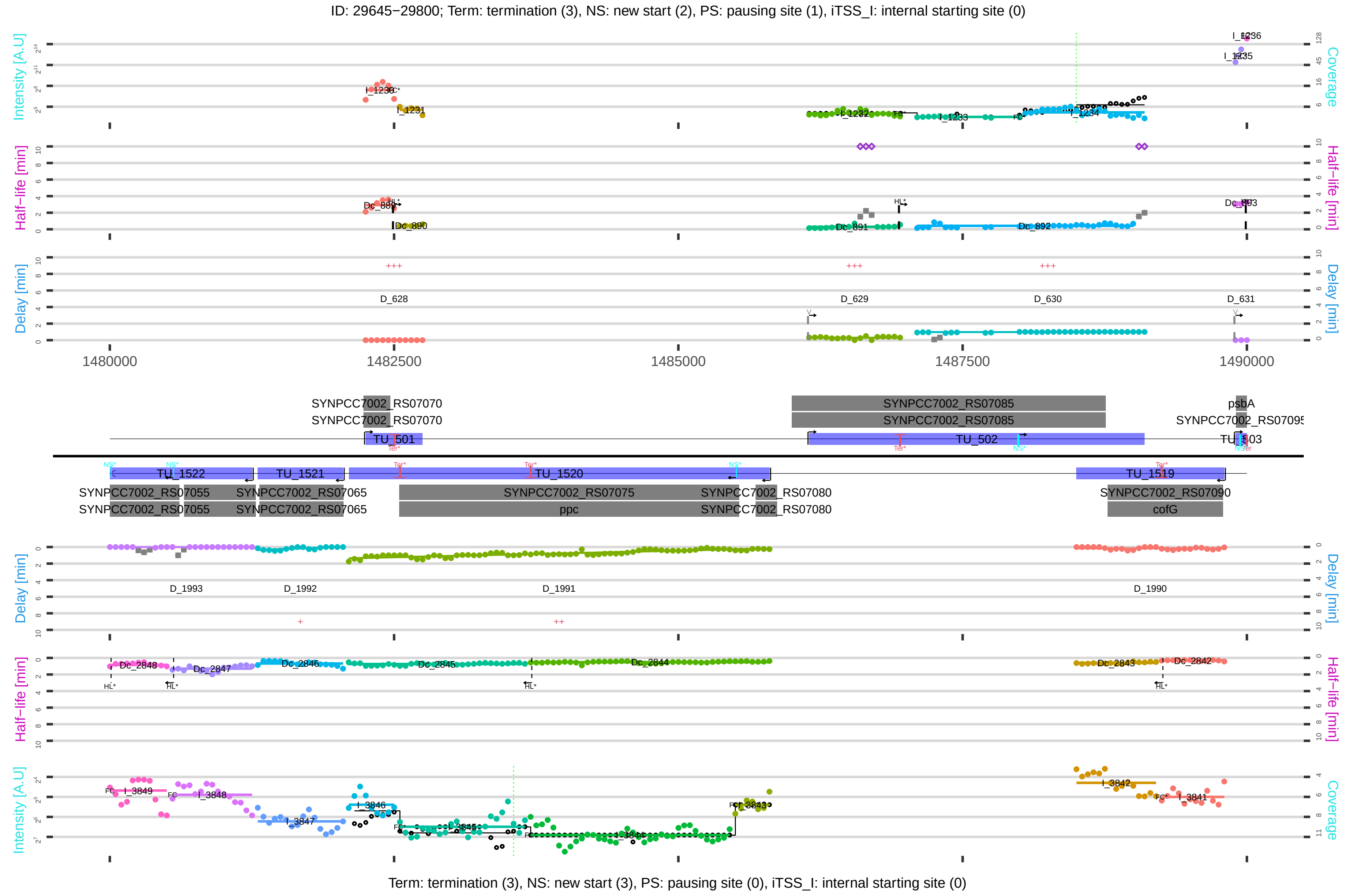

ID: 29645−29800; Term: termination (3), NS: new start (2), PS: pausing site (1), iTSS_I: internal starting site (0)
Intensity [A.U]
Coverage
Half−life [min]
Delay [min]
I_1230
I_1231
I_1232
I_1233
I_1234
I_1235
I_1236
Dc_889
Dc_890
Dc_891
Dc_892
Dc_893
D_628
D_629
D_630
D_631
1480000
1482500
1485000
1487500
1490000
SYNPCC7002_RS07070
SYNPCC7002_RS07085
psbA
SYNPCC7002_RS07095
TU_501
TU_502
TU_503
TU_1522
TU_1521
TU_1520
TU_1519
SYNPCC7002_RS07055
SYNPCC7002_RS07065
SYNPCC7002_RS07075
ppc
SYNPCC7002_RS07080
SYNPCC7002_RS07090
cofG
D_1993
D_1992
D_1991
D_1990
Dc_2848
Dc_2847
Dc_2846
Dc_2845
Dc_2844
Dc_2843
Dc_2842
I_3849
I_3848
I_3847
I_3846
I_3845
I_3844
I_3843
I_3842
I_3841
Term: termination (3), NS: new start (3), PS: pausing site (0), iTSS_I: internal starting site (0)

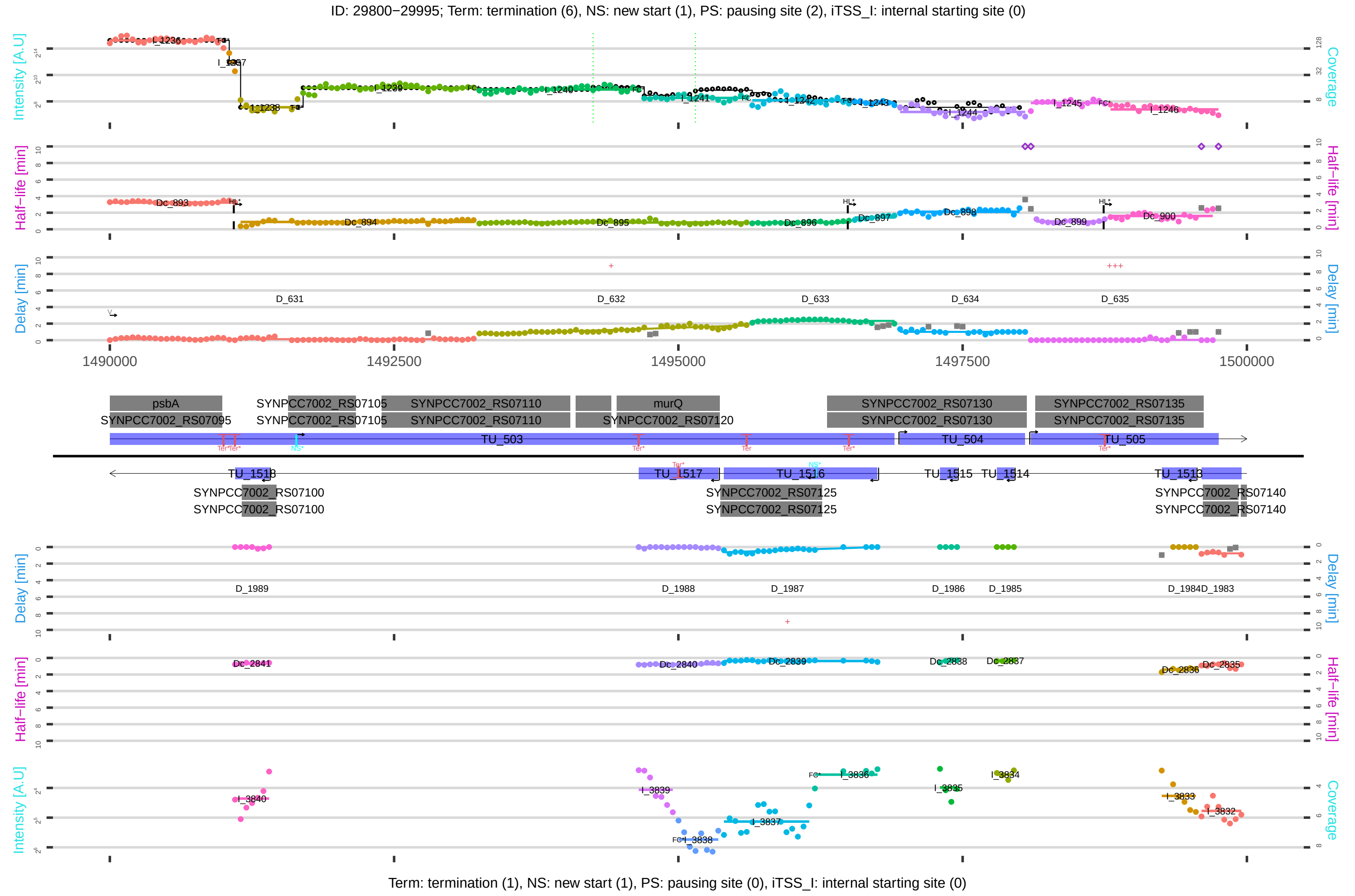

ID: 29800−29995; Term: termination (6), NS: new start (1), PS: pausing site (2), iTSS_I: internal starting site (0)
Intensity [A.U]
Coverage
Half−life [min]
Delay [min]
1490000
1492500
1495000
1497500
1500000
psbA
SYNPCC7002_RS07095
SYNPCC7002_RS07105
SYNPCC7002_RS07110
murQ
SYNPCC7002_RS07120
SYNPCC7002_RS07130
SYNPCC7002_RS07135
TU_503
TU_504
TU_505
TU_1518
TU_1517
TU_1516
TU_1515
TU_1514
TU_1513
SYNPCC7002_RS07100
SYNPCC7002_RS07125
SYNPCC7002_RS07140
Term: termination (1), NS: new start (1), PS: pausing site (0), iTSS_I: internal starting site (0)

ID: 30001−30192; Term: termination (4), NS: new start (1), PS: pausing site (1), iTSS_I: internal starting site (0)

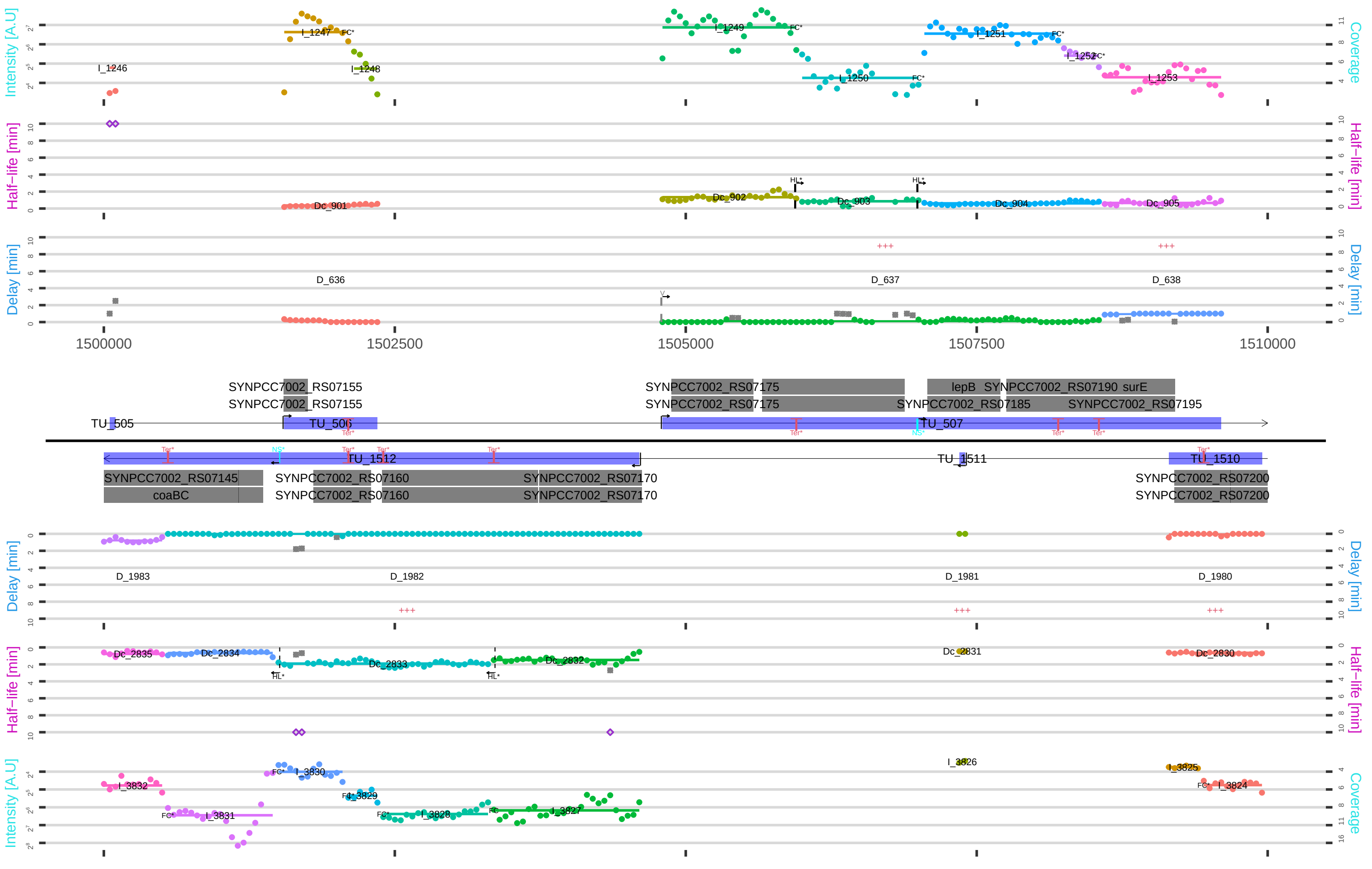

Term: termination (5), NS: new start (1), PS: pausing site (1), iTSS_I: internal starting site (0)

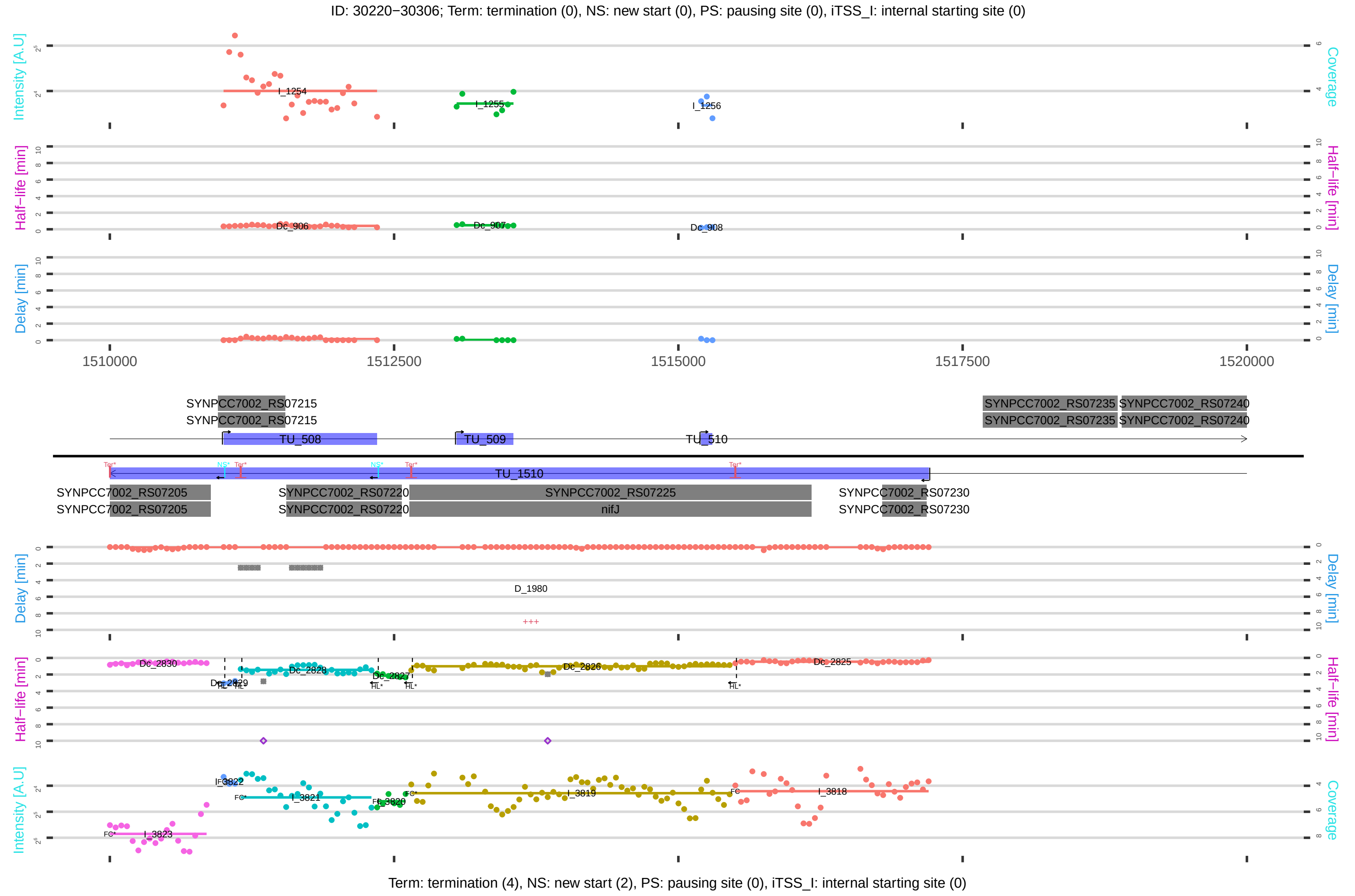

ID: 30220−30306; Term: termination (0), NS: new start (0), PS: pausing site (0), iTSS_I: internal starting site (0)
Intensity [A.U]
Coverage
Half−life [min]
Delay [min]
I_1254
I_1255
I_1256
Dc_906
Dc_907
Dc_908
1510000
1512500
1515000
1517500
1520000
SYNPCC7002_RS07215
SYNPCC7002_RS07235
SYNPCC7002_RS07240
TU_508
TU_509
TU_510
TU_1510
SYNPCC7002_RS07205
SYNPCC7002_RS07220
SYNPCC7002_RS07225
nifJ
SYNPCC7002_RS07230
D_1980
Dc_2830
Dc_2829
Dc_2828
Dc_2827
Dc_2826
Dc_2825
I_3823
I_3822
I_3821
I_3820
I_3819
I_3818
Term: termination (4), NS: new start (2), PS: pausing site (0), iTSS_I: internal starting site (0)

ID: 30474−30599; Term: termination (2), NS: new start (2), PS: pausing site (1), iTSS_I: internal starting site (0)

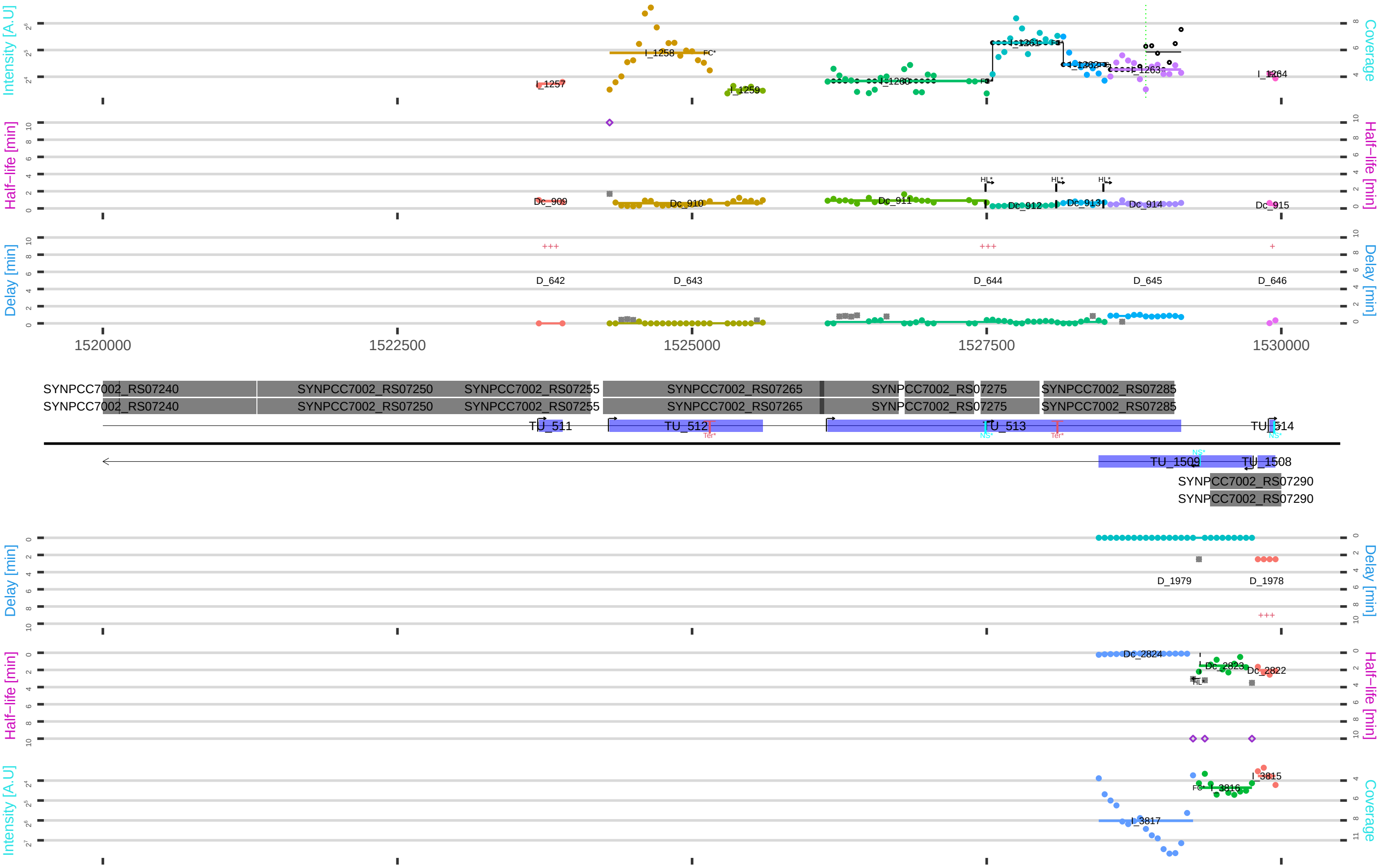

Term: termination (0), NS: new start (1), PS: pausing site (0), iTSS_I: internal starting site (0)

ID: 30603−30774; Term: termination (1), NS: new start (1), PS: pausing site (0), iTSS_I: internal starting site (0)

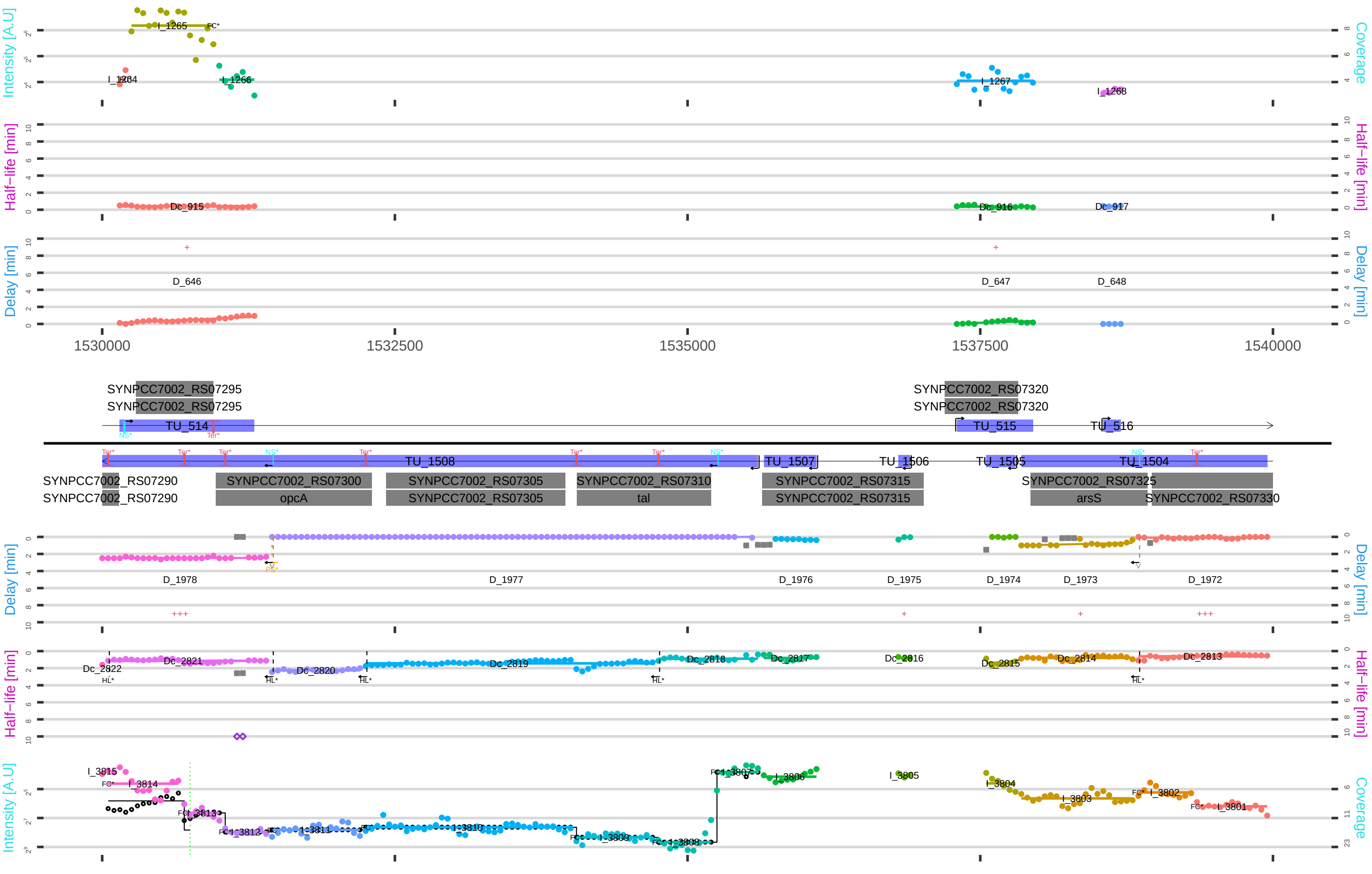

Term: termination (7), NS: new start (3), PS: pausing site (2), iTSS_I: internal starting site (0)

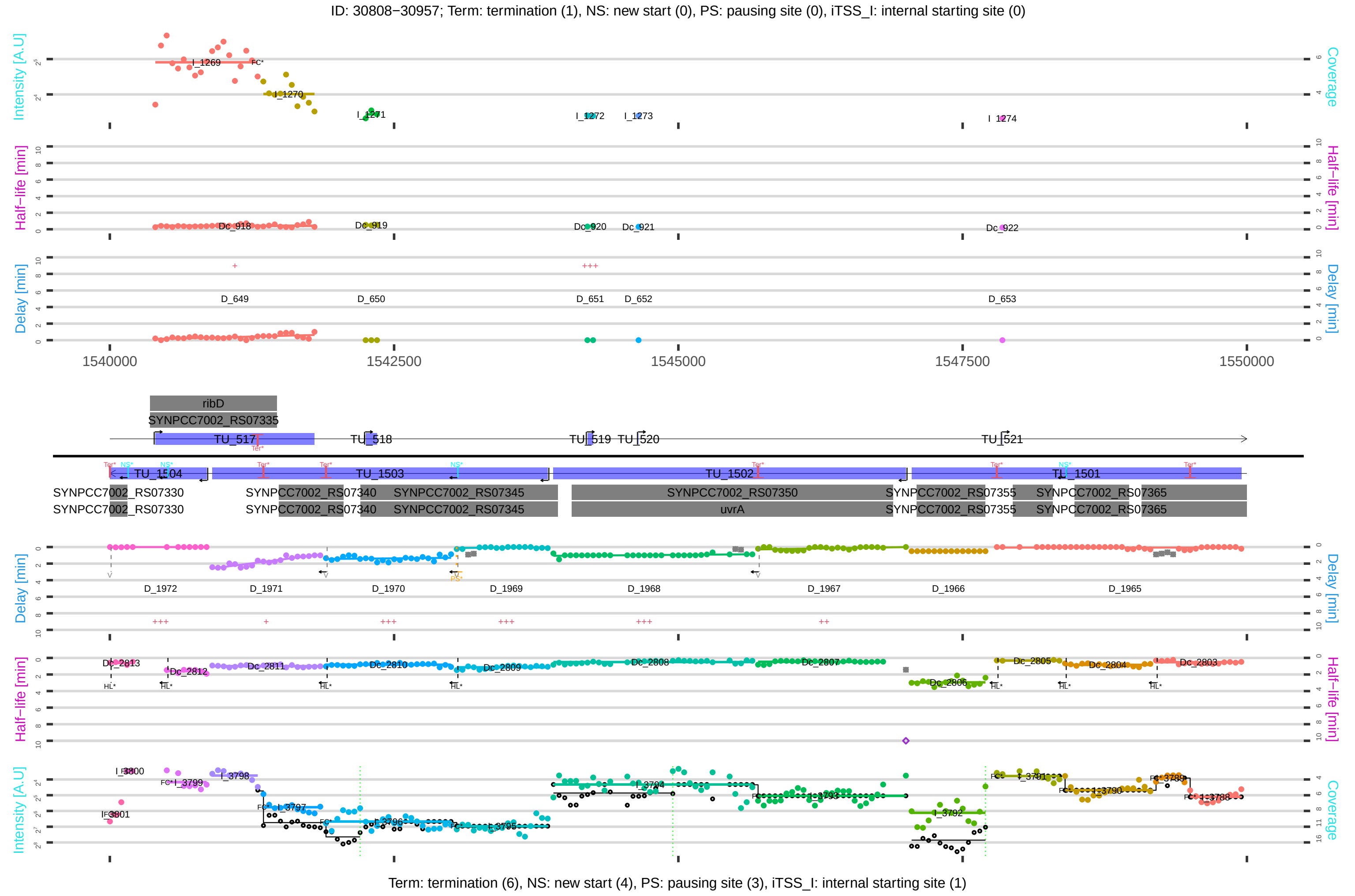

ID: 30808−30957; Term: termination (1), NS: new start (0), PS: pausing site (0), iTSS_I: internal starting site (0)
Intensity [A.U]
Coverage
Half−life [min]
Delay [min]
1540000
1542500
1545000
1547500
1550000
ribD
SYNPCC7002_RS07335
TU_517
TU_518
TU_519
TU_520
TU_521
TU_1504
TU_1503
TU_1502
TU_1501
SYNPCC7002_RS07330
SYNPCC7002_RS07340
SYNPCC7002_RS07345
SYNPCC7002_RS07350
uvrA
SYNPCC7002_RS07355
SYNPCC7002_RS07365
Term: termination (6), NS: new start (4), PS: pausing site (3), iTSS_I: internal starting site (1)

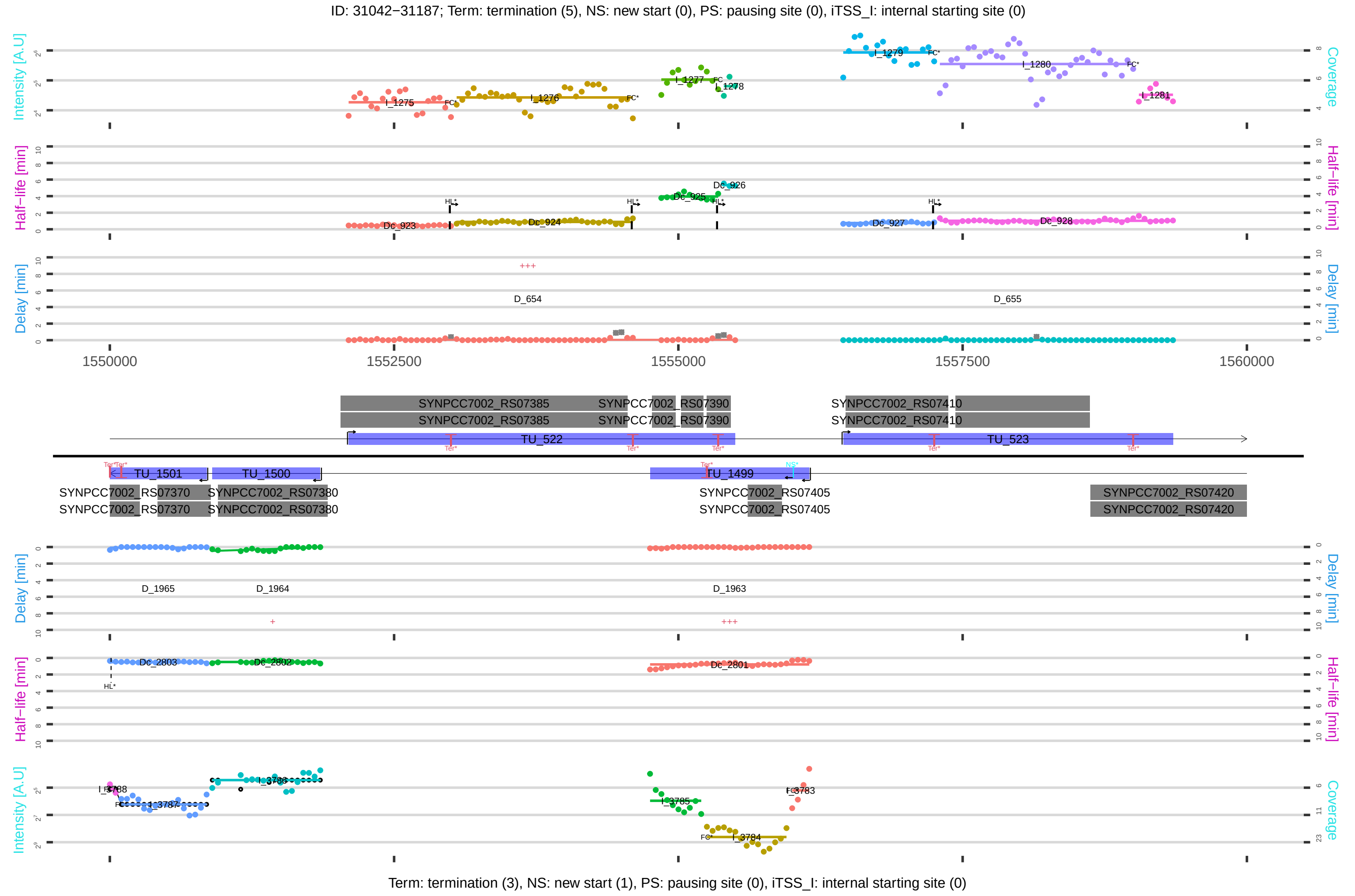
ID: 31042−31187; Term: termination (5), NS: new start (0), PS: pausing site (0), iTSS_I: internal starting site (0)
Intensity [A.U]
Coverage
Half−life [min]
Delay [min]
I_1275
I_1276
I_1277
I_1278
I_1279
I_1280
I_1281
Dc_923
Dc_924
Dc_925
Dc_926
Dc_927
Dc_928
D_654
D_655
1550000
1552500
1555000
1557500
1560000
SYNPCC7002_RS07385
SYNPCC7002_RS07390
SYNPCC7002_RS07410
TU_522
TU_523
TU_1501
TU_1500
TU_1499
SYNPCC7002_RS07370
SYNPCC7002_RS07380
SYNPCC7002_RS07405
SYNPCC7002_RS07420
D_1965
D_1964
D_1963
Dc_2803
Dc_2802
Dc_2801
I_3788
I_3787
I_3786
I_3785
I_3784
I_3783
Term: termination (3), NS: new start (1), PS: pausing site (0), iTSS_I: internal starting site (0)

ID: 31206−31400; Term: termination (1), NS: new start (1), PS: pausing site (1), iTSS_I: internal starting site (0)

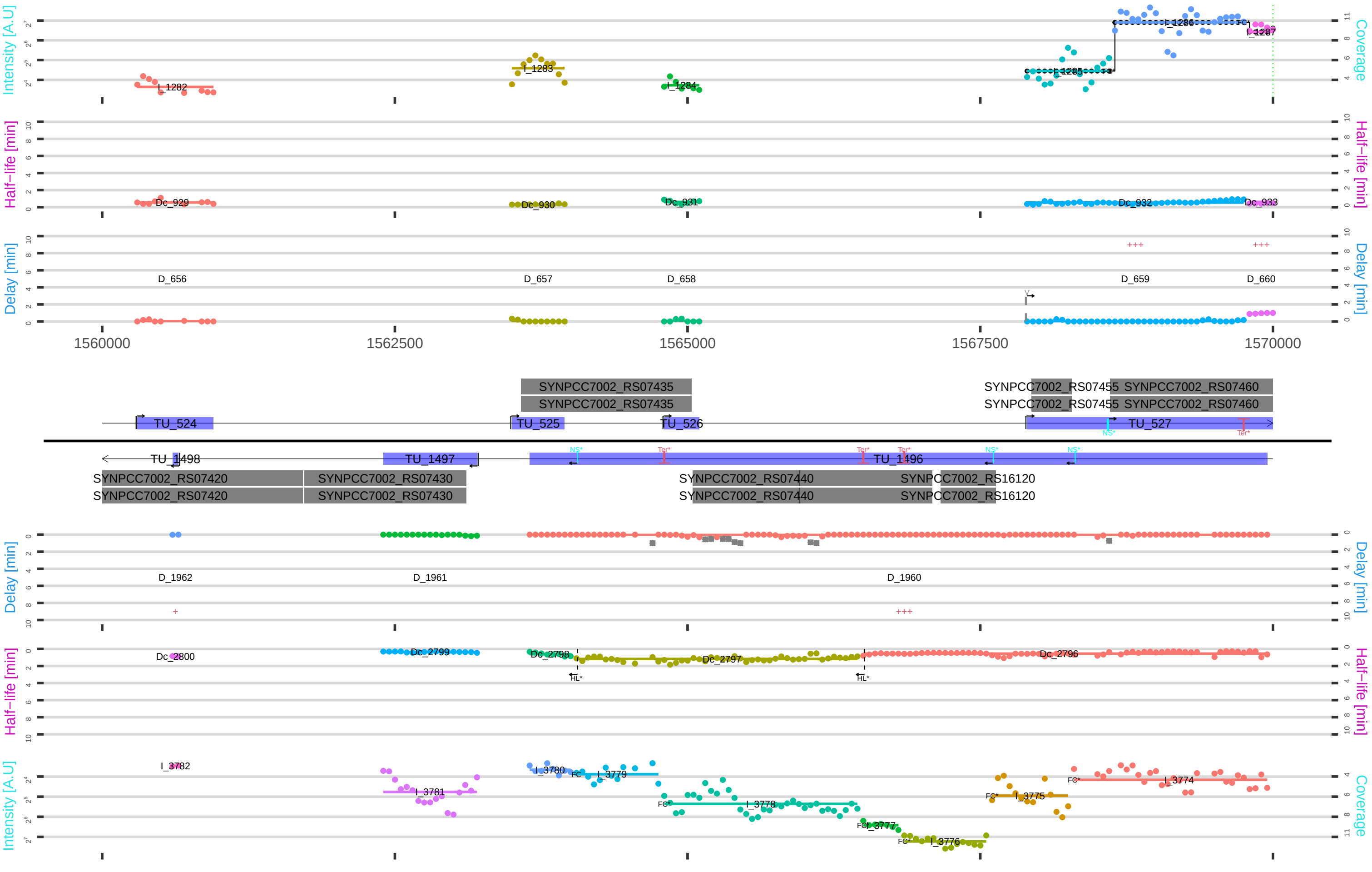

Term: termination (3), NS: new start (3), PS: pausing site (0), iTSS_I: internal starting site (0)

ID: 31400−31600; Term: termination (0), NS: new start (0), PS: pausing site (1), iTSS_I: internal starting site (0)

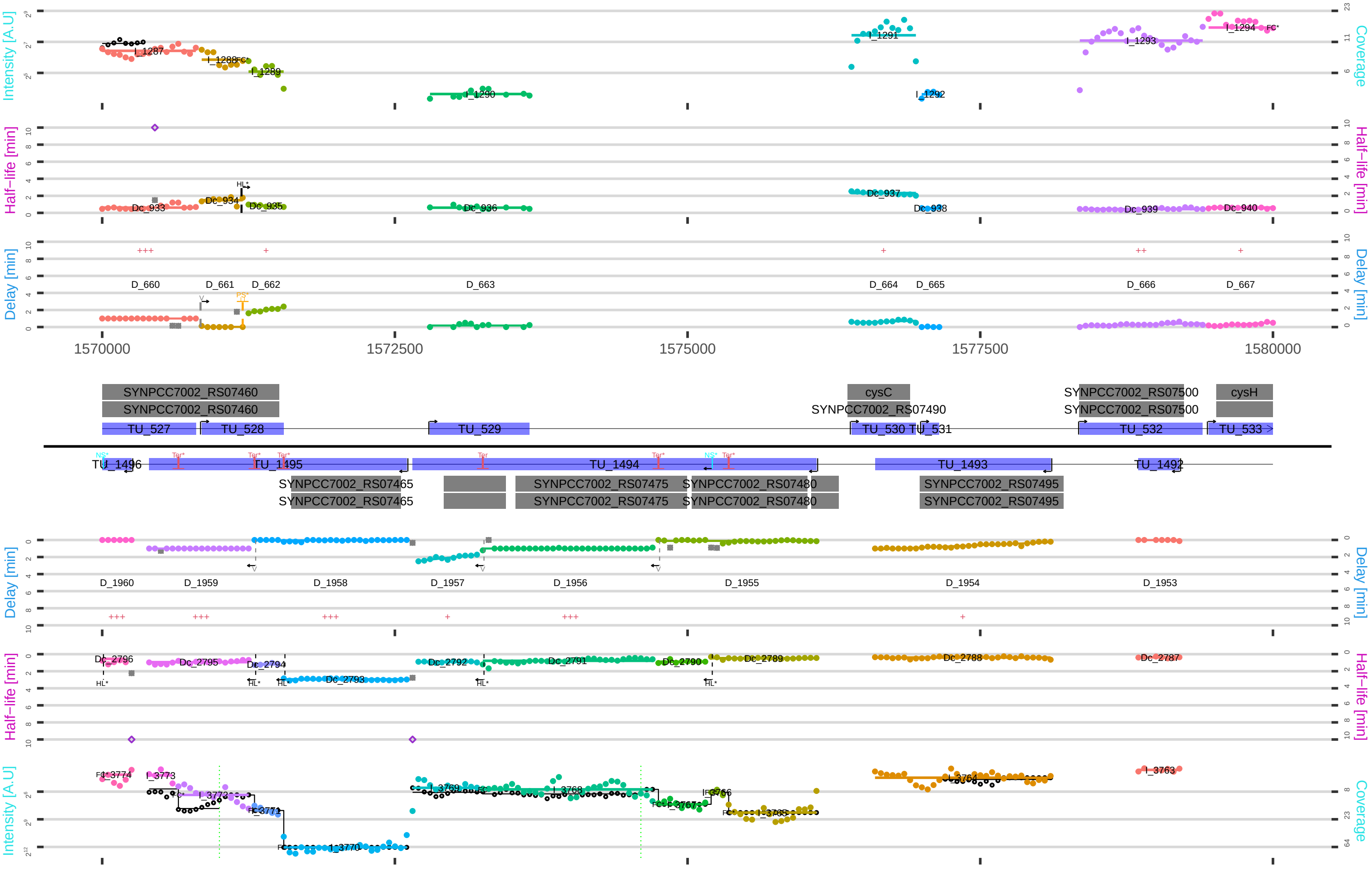

Term: termination (6), NS: new start (2), PS: pausing site (3), iTSS_I: internal starting site (0)

ID: 31600−31800; Term: termination (1), NS: new start (1), PS: pausing site (1), iTSS_I: internal starting site (0)

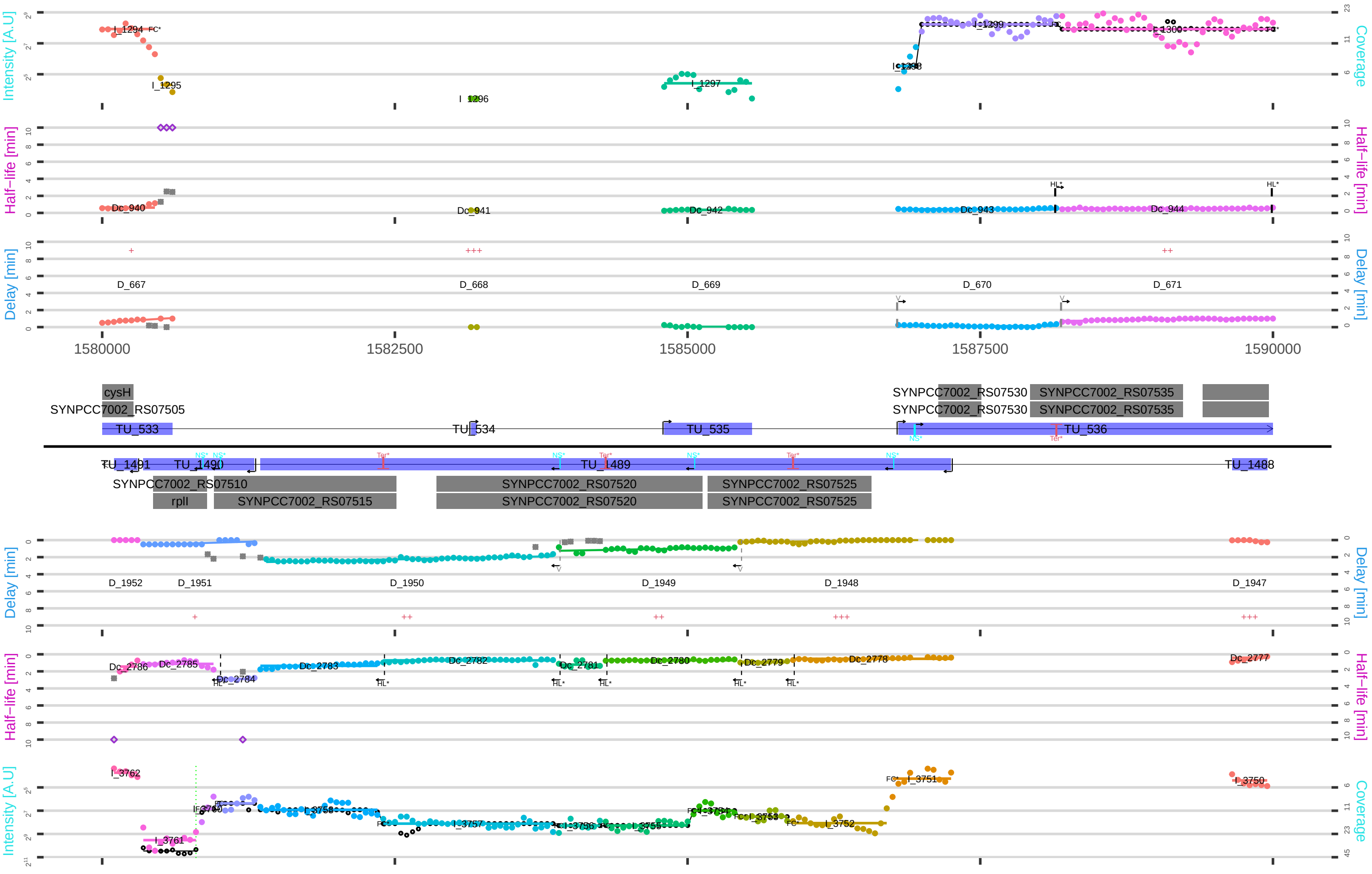

Term: termination (3), NS: new start (5), PS: pausing site (2), iTSS_I: internal starting site (0)

ID: 31800−32000; Term: termination (3), NS: new start (3), PS: pausing site (2), iTSS_I: internal starting site (3)

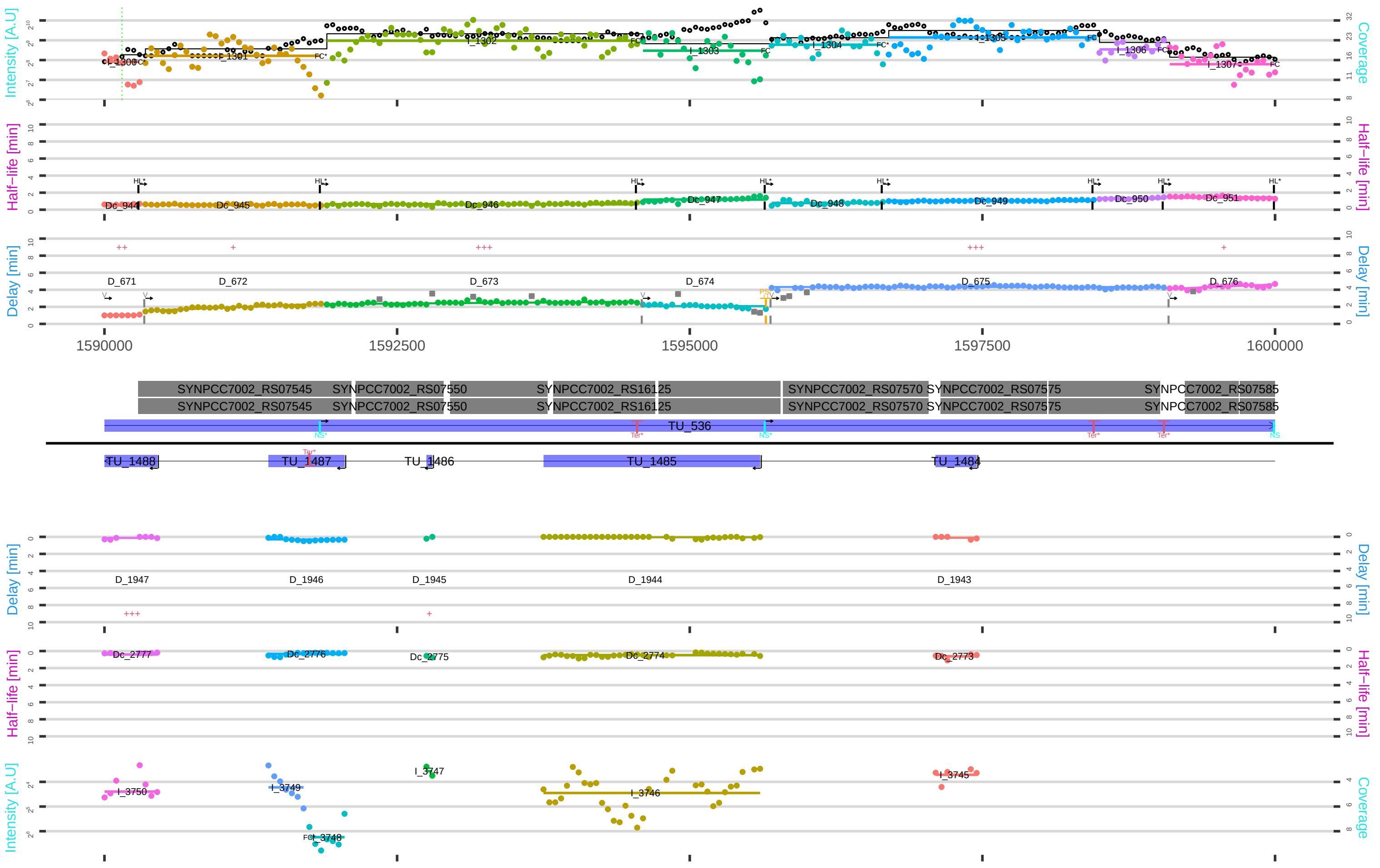

Term: termination (1), NS: new start (0), PS: pausing site (0), iTSS_I: internal starting site (0)

ID: 32000−32200; Term: termination (10), NS: new start (10), PS: pausing site (7), iTSS_I: internal starting site (3)

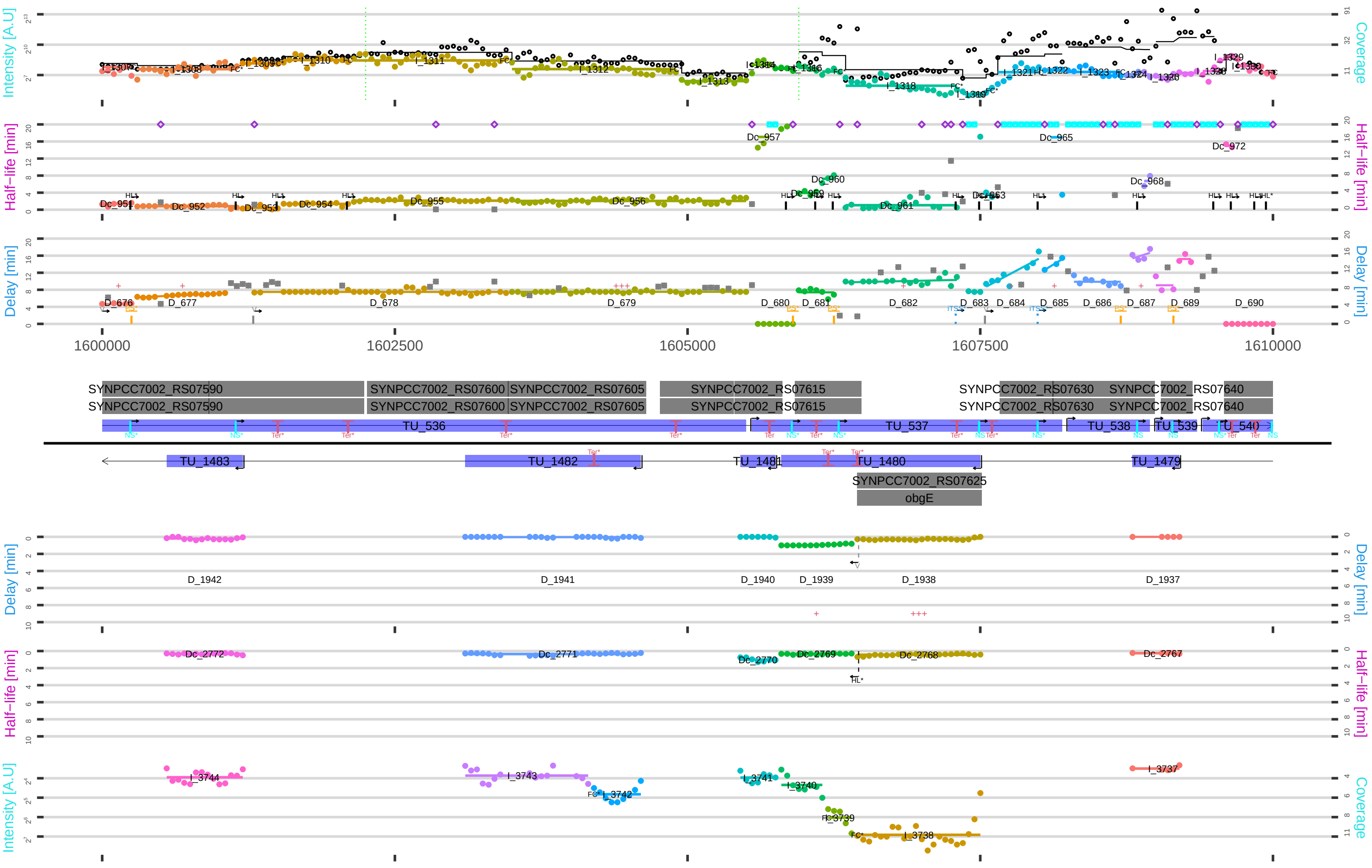

Term: termination (3), NS: new start (0), PS: pausing site (1), iTSS_I: internal starting site (0)

ID: 32200−32400; Term: termination (10), NS: new start (7), PS: pausing site (3), iTSS_I: internal starting site (7)

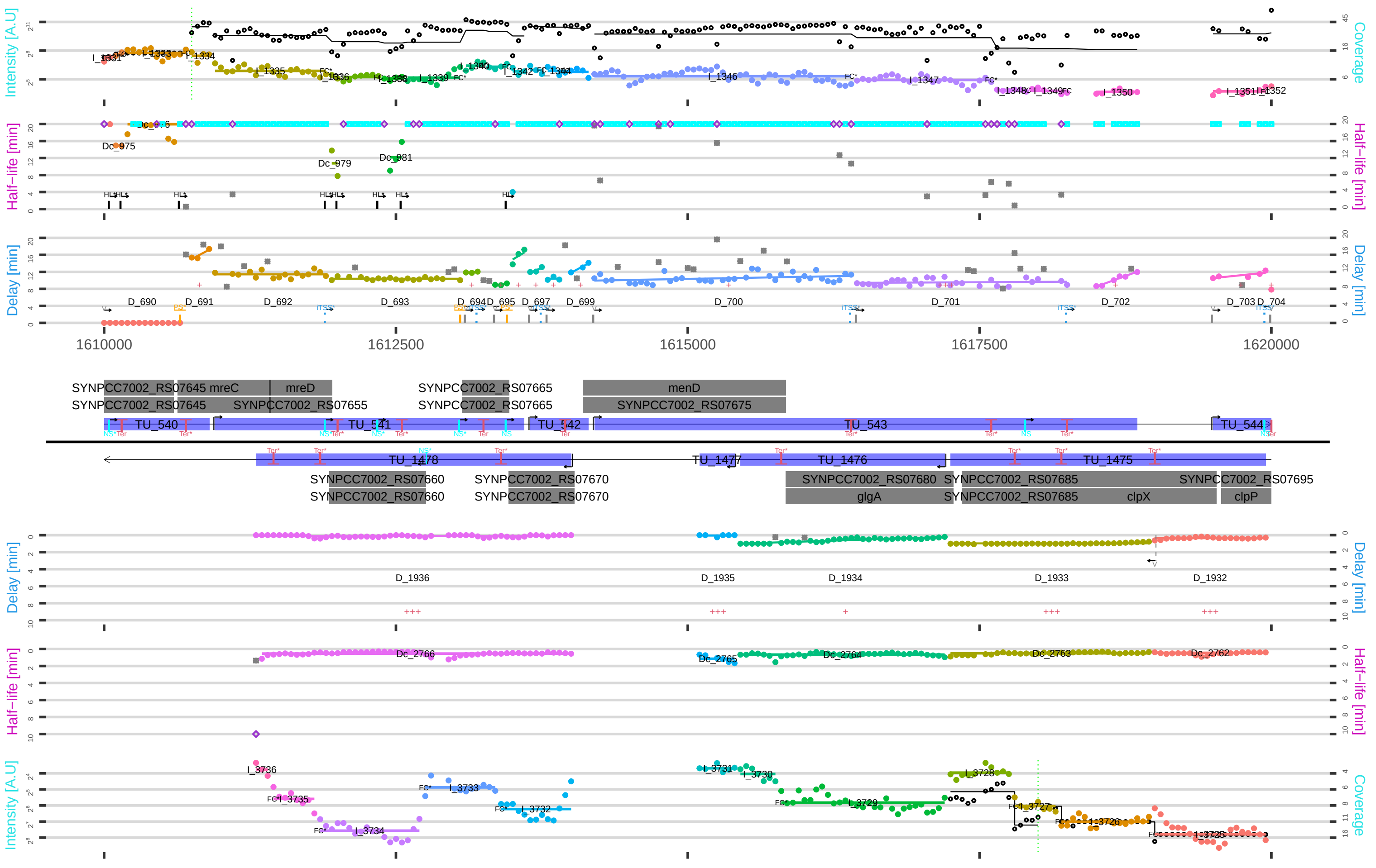

Term: termination (7), NS: new start (1), PS: pausing site (1), iTSS_I: internal starting site (0)

ID: 32400−32598; Term: termination (8), NS: new start (3), PS: pausing site (4), iTSS_I: internal starting site (6)

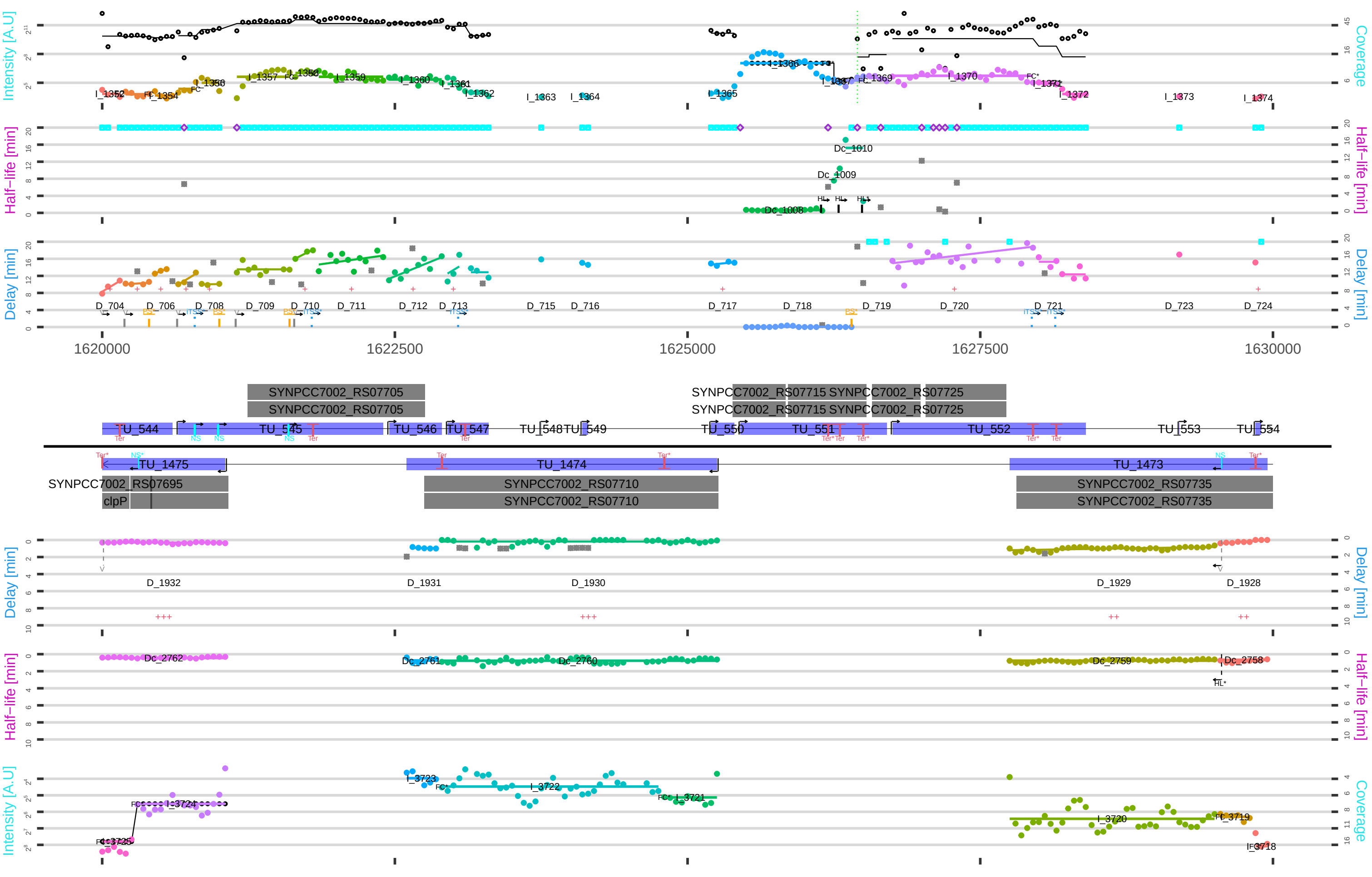

Term: termination (4), NS: new start (2), PS: pausing site (2), iTSS_I: internal starting site (0)

ID: 32606−32800; Term: termination (14), NS: new start (11), PS: pausing site (4), iTSS_I: internal starting site (2)

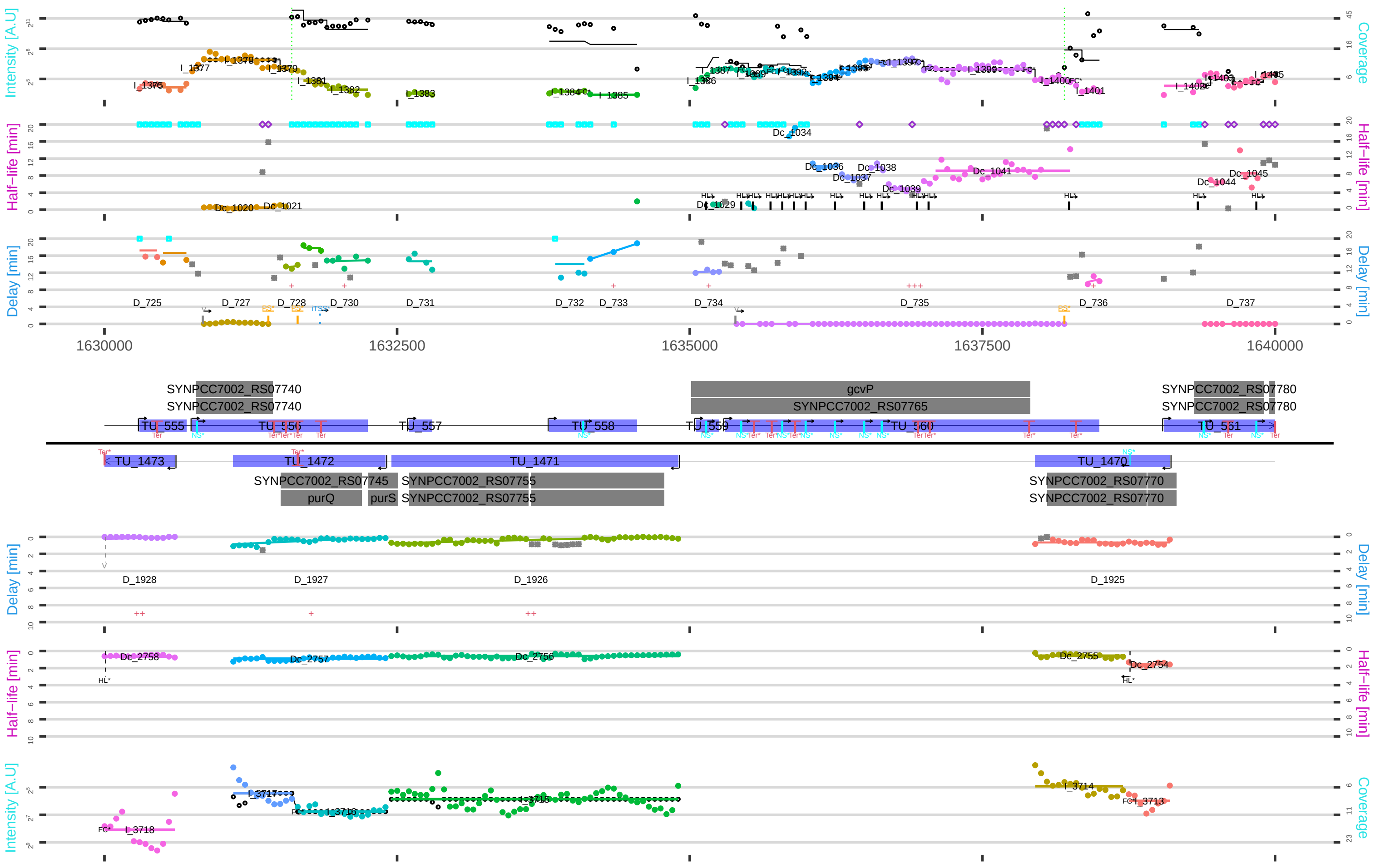

Term: termination (2), NS: new start (1), PS: pausing site (0), iTSS_I: internal starting site (0)

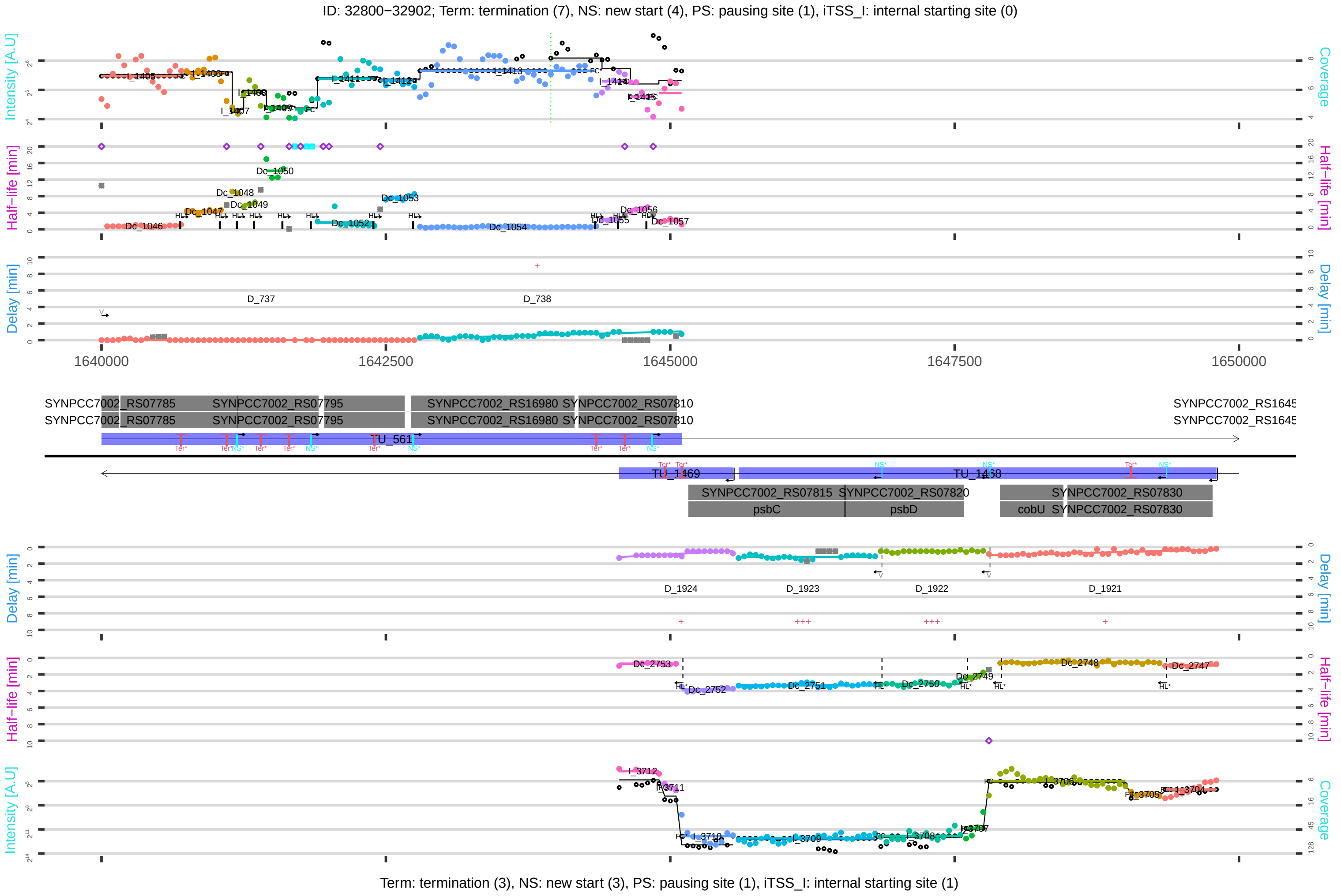
ID: 32800−32902; Term: termination (7), NS: new start (4), PS: pausing site (1), iTSS_I: internal starting site (0)
Intensity [A.U]
Coverage
Half−life [min]
Delay [min]
1640000
1642500
1645000
1647500
1650000
D_737
D_738
TU_561
TU_1469
TU_1468
SYNPCC7002_RS07785
SYNPCC7002_RS07795
SYNPCC7002_RS16980
SYNPCC7002_RS07810
SYNPCC7002_RS07815
SYNPCC7002_RS07820
SYNPCC7002_RS07830
psbC
psbD
cobU
D_1924
D_1923
D_1922
D_1921
Term: termination (3), NS: new start (3), PS: pausing site (1), iTSS_I: internal starting site (1)

ID: 33001−33143; Term: termination (5), NS: new start (4), PS: pausing site (0), iTSS_I: internal starting site (0)

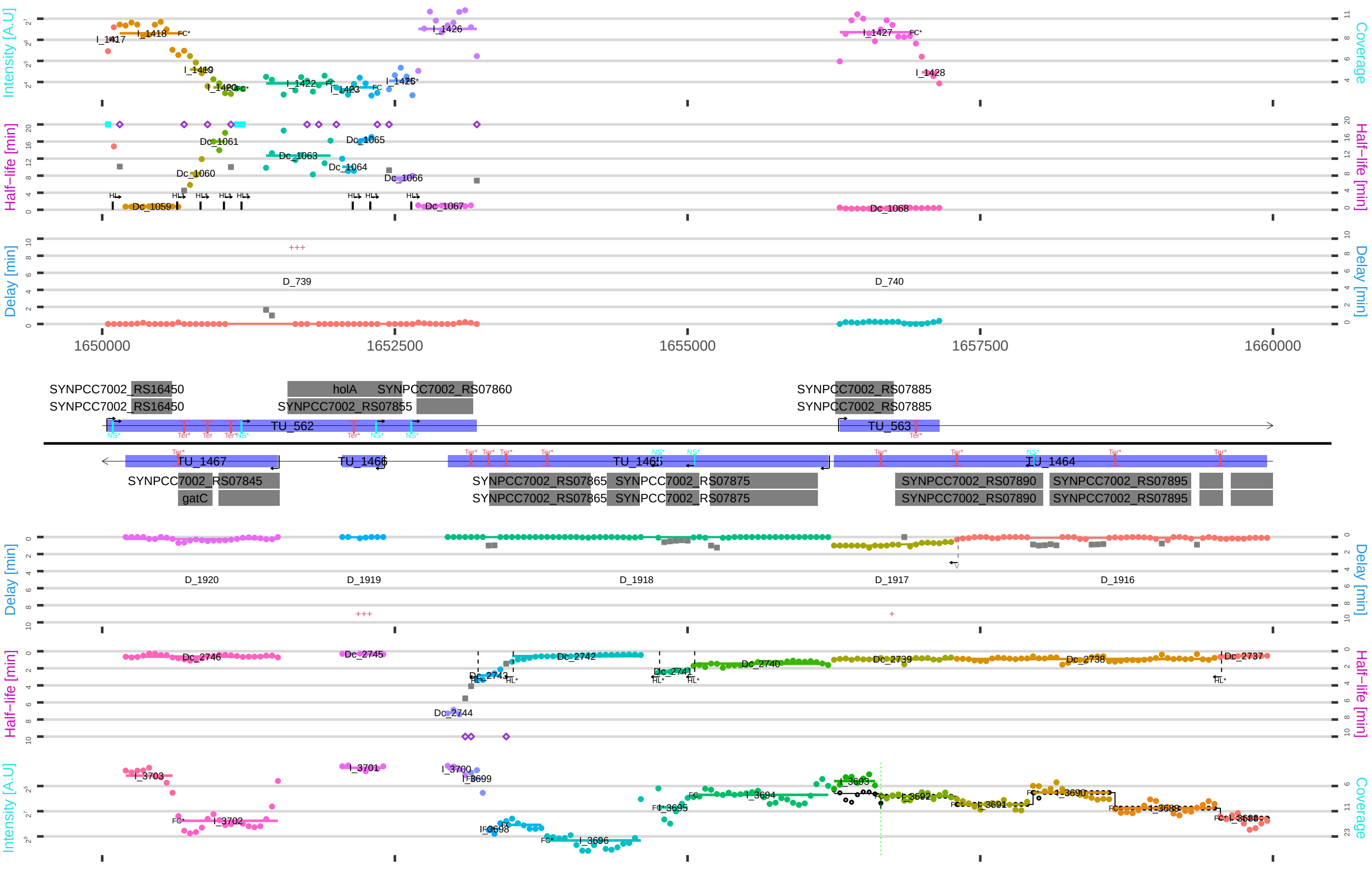

Term: termination (9), NS: new start (3), PS: pausing site (1), iTSS_I: internal starting site (0)

ID: 33209−33400; Term: termination (6), NS: new start (3), PS: pausing site (1), iTSS_I: internal starting site (0)

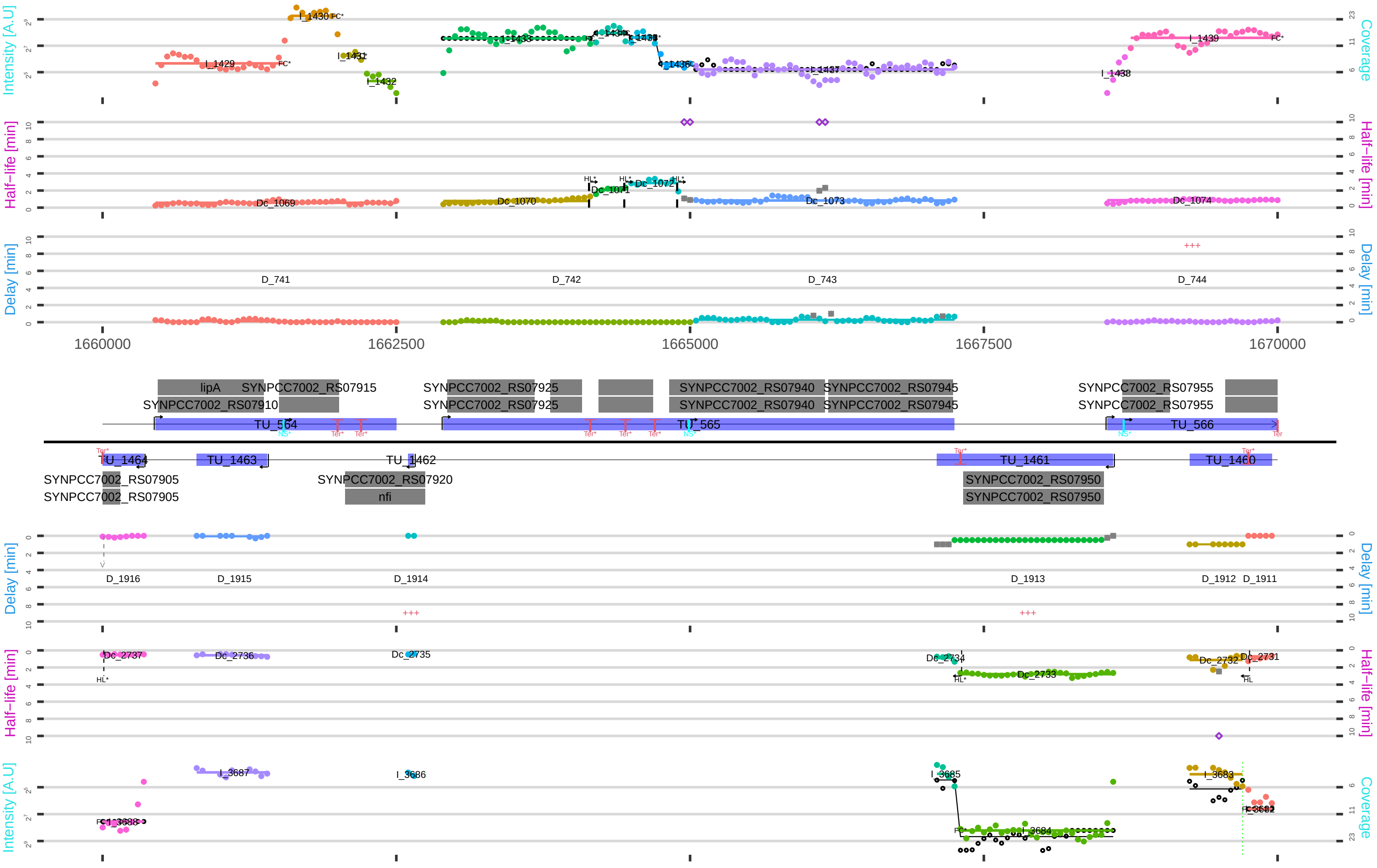

Term: termination (3), NS: new start (0), PS: pausing site (1), iTSS_I: internal starting site (0)

ID: 33400−33600; Term: termination (6), NS: new start (4), PS: pausing site (3), iTSS_I: internal starting site (0)

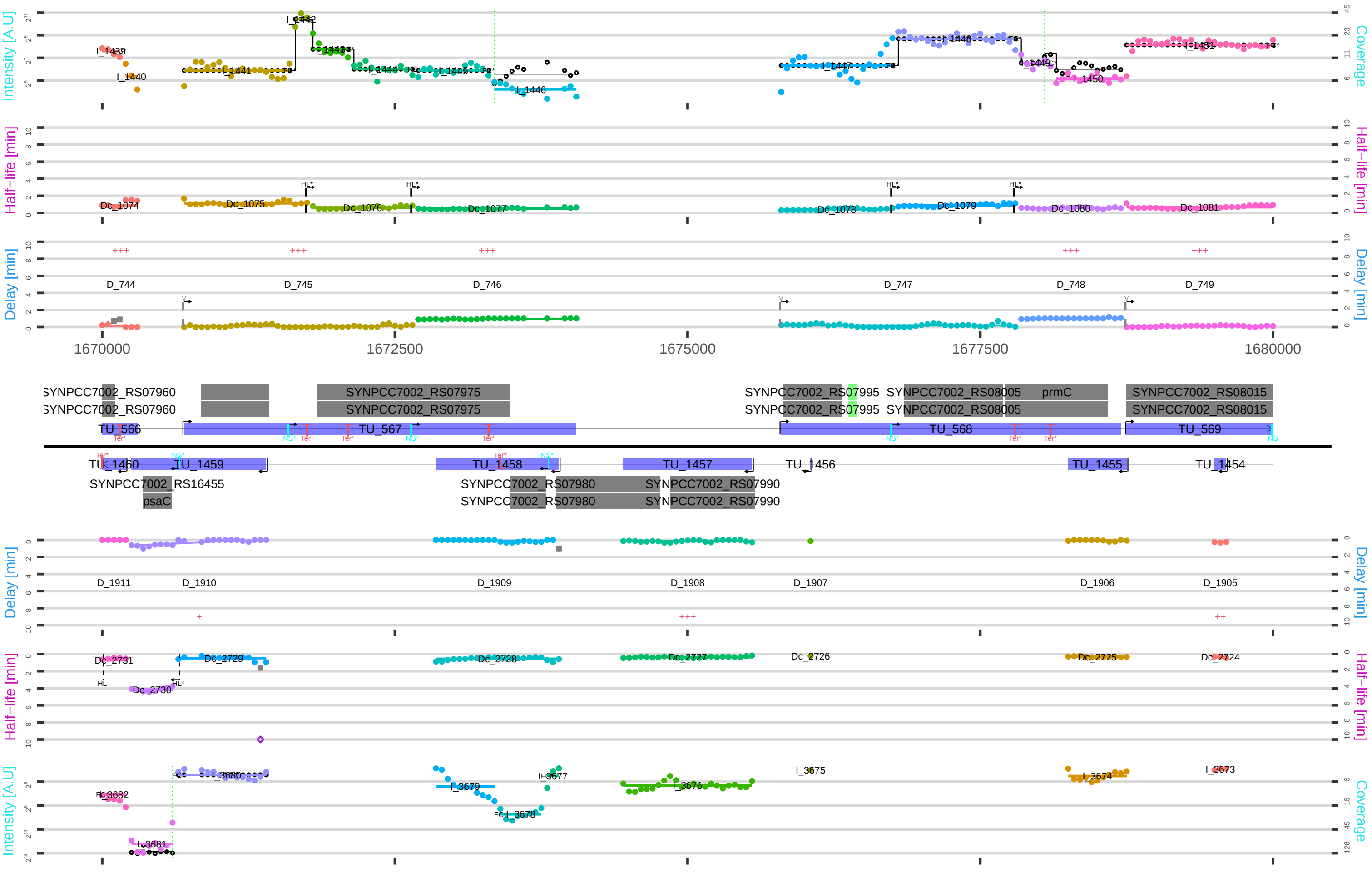

Term: termination (2), NS: new start (2), PS: pausing site (0), iTSS_I: internal starting site (0)

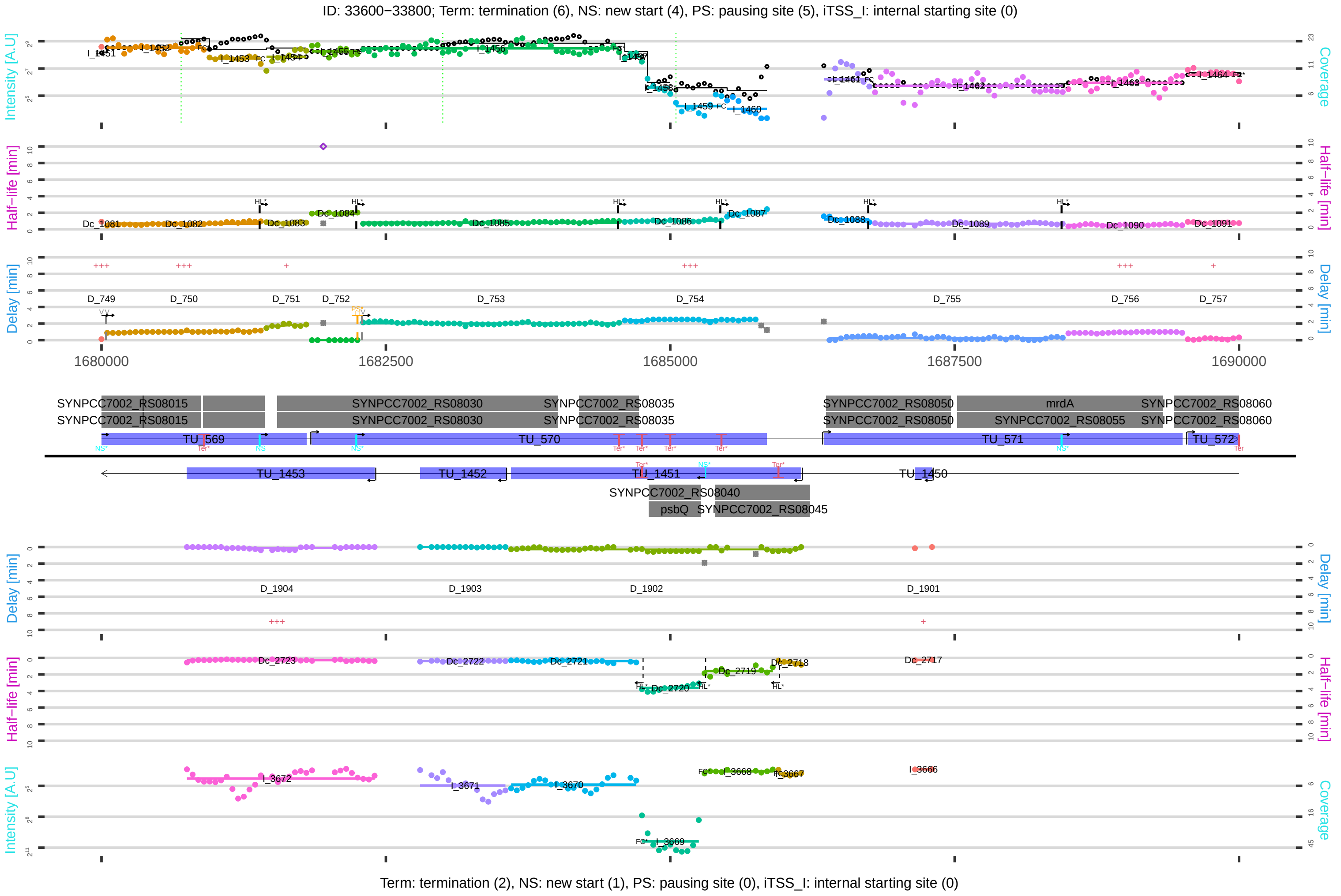

ID: 33600−33800; Term: termination (6), NS: new start (4), PS: pausing site (5), iTSS_I: internal starting site (0)
Intensity [A.U]
Coverage
Half−life [min]
Delay [min]
1680000
1682500
1685000
1687500
1690000
SYNPCC7002_RS08015
SYNPCC7002_RS08030
SYNPCC7002_RS08035
SYNPCC7002_RS08050
mrdA
SYNPCC7002_RS08055
SYNPCC7002_RS08060
TU_569
TU_570
TU_571
TU_572
TU_1453
TU_1452
TU_1451
TU_1450
SYNPCC7002_RS08040
psbQ
SYNPCC7002_RS08045
Term: termination (2), NS: new start (1), PS: pausing site (0), iTSS_I: internal starting site (0)

ID: 33800−33927; Term: termination (3), NS: new start (0), PS: pausing site (0), iTSS_I: internal starting site (0)

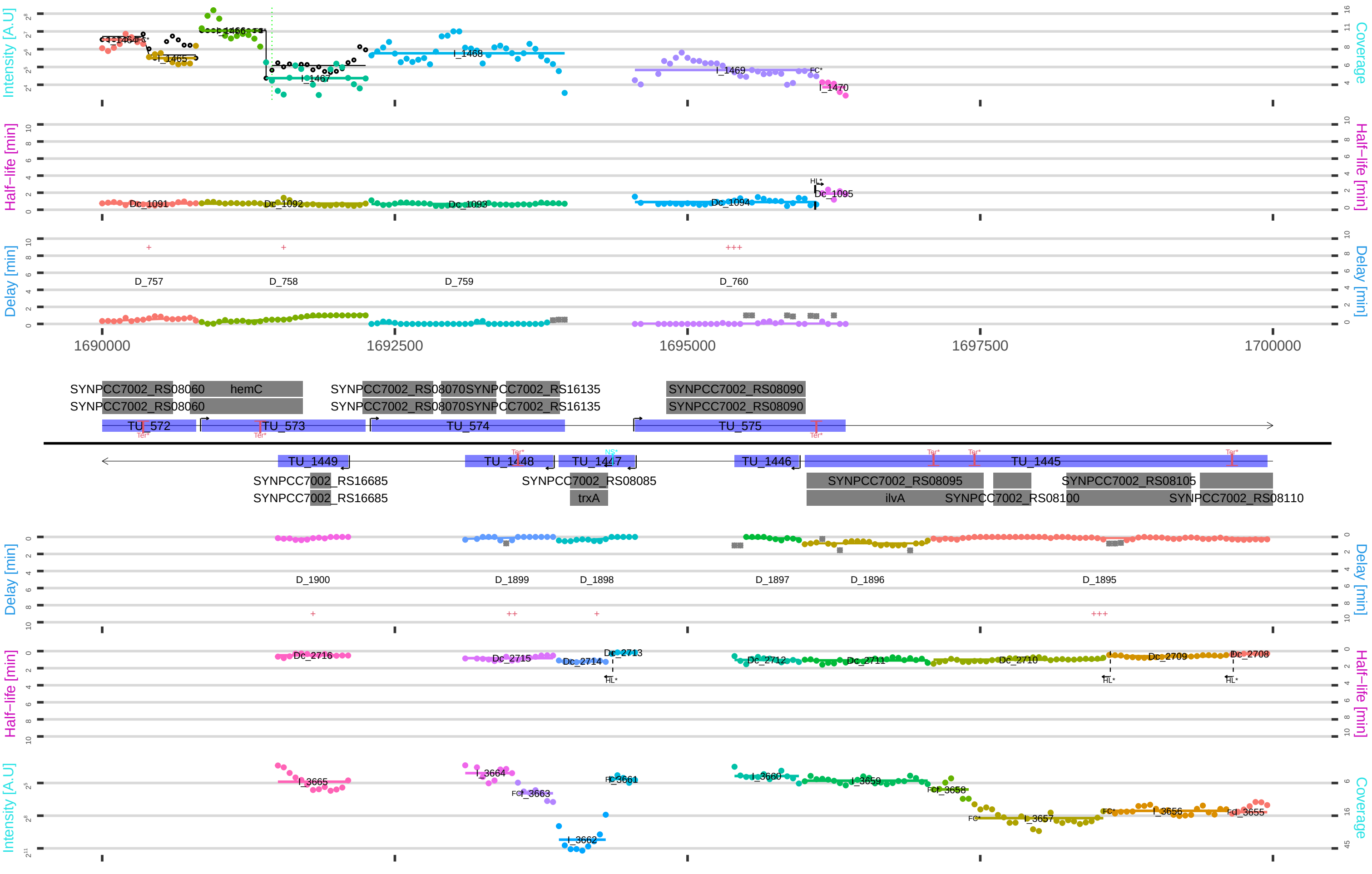

Term: termination (4), NS: new start (1), PS: pausing site (1), iTSS_I: internal starting site (0)

ID: 34055−34187; Term: termination (2), NS: new start (0), PS: pausing site (0), iTSS_I: internal starting site (0)

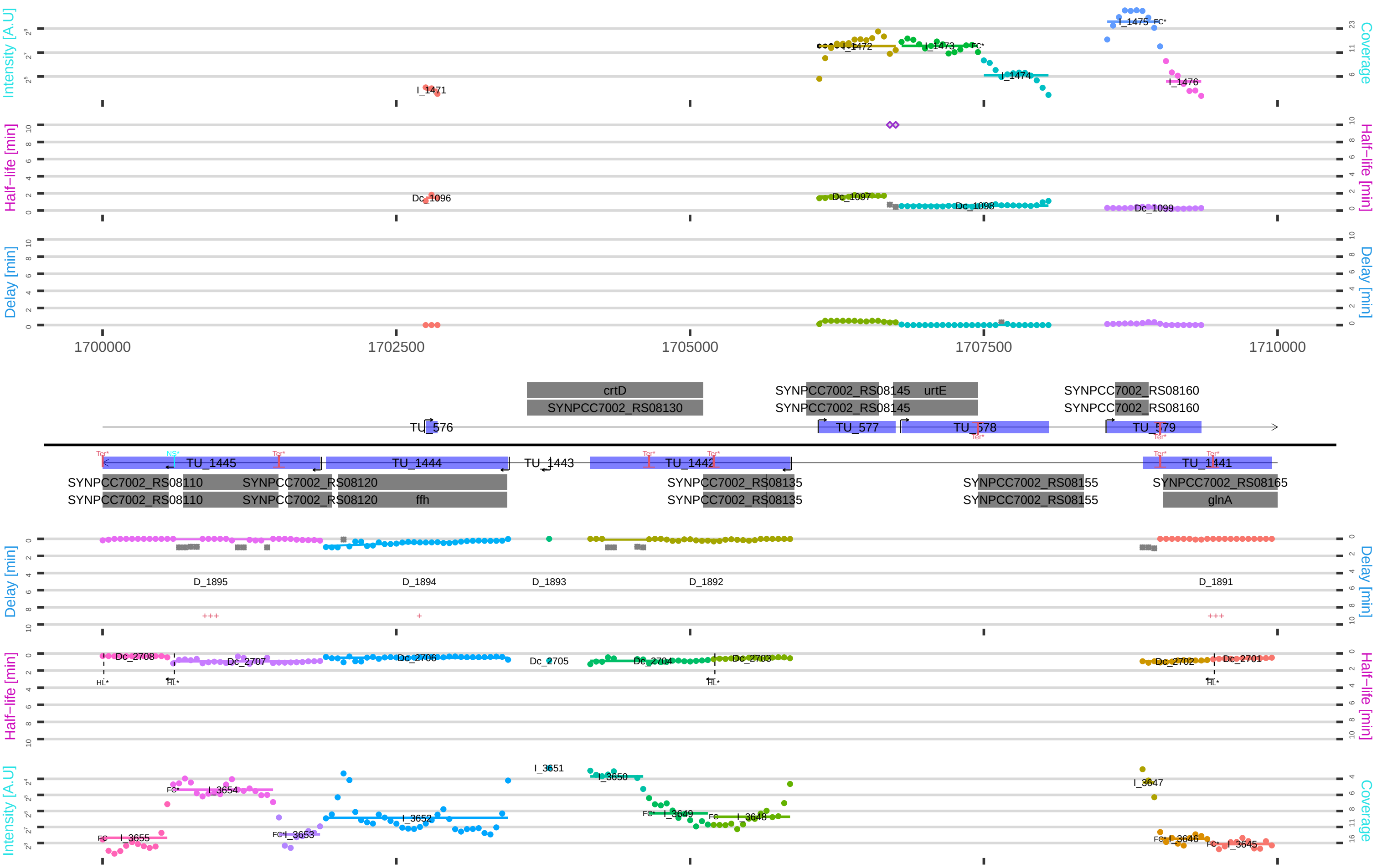

Term: termination (6), NS: new start (1), PS: pausing site (0), iTSS_I: internal starting site (0)

ID: 34216−34308; Term: termination (2), NS: new start (1), PS: pausing site (0), iTSS_I: internal starting site (0)

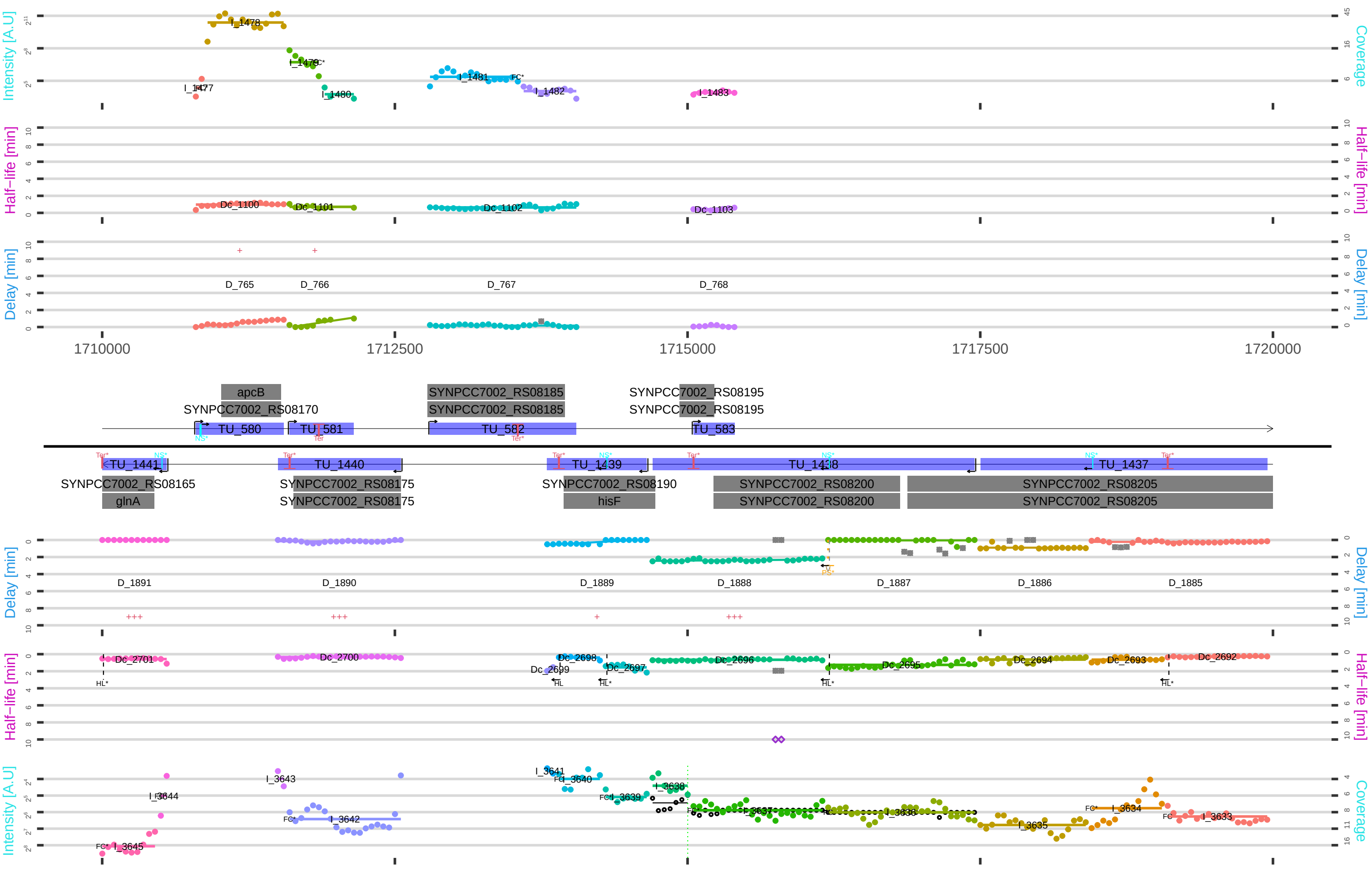

Term: termination (5), NS: new start (4), PS: pausing site (2), iTSS_I: internal starting site (0)

ID: 34404−34559; Term: termination (5), NS: new start (2), PS: pausing site (0), iTSS_I: internal starting site (0)

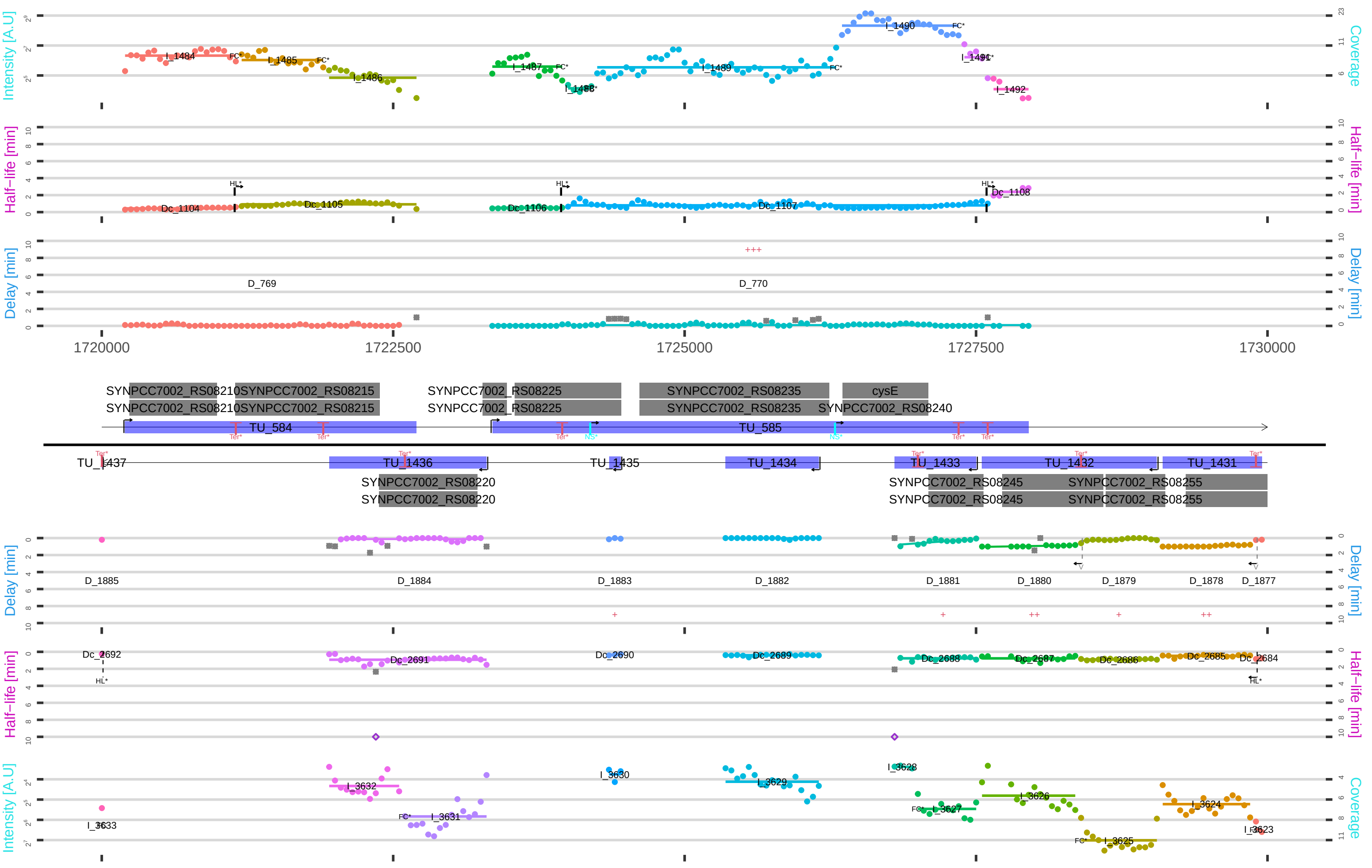

Term: termination (5), NS: new start (0), PS: pausing site (2), iTSS_I: internal starting site (0)

ID: 34631−34800; Term: termination (4), NS: new start (0), PS: pausing site (1), iTSS_I: internal starting site (0)

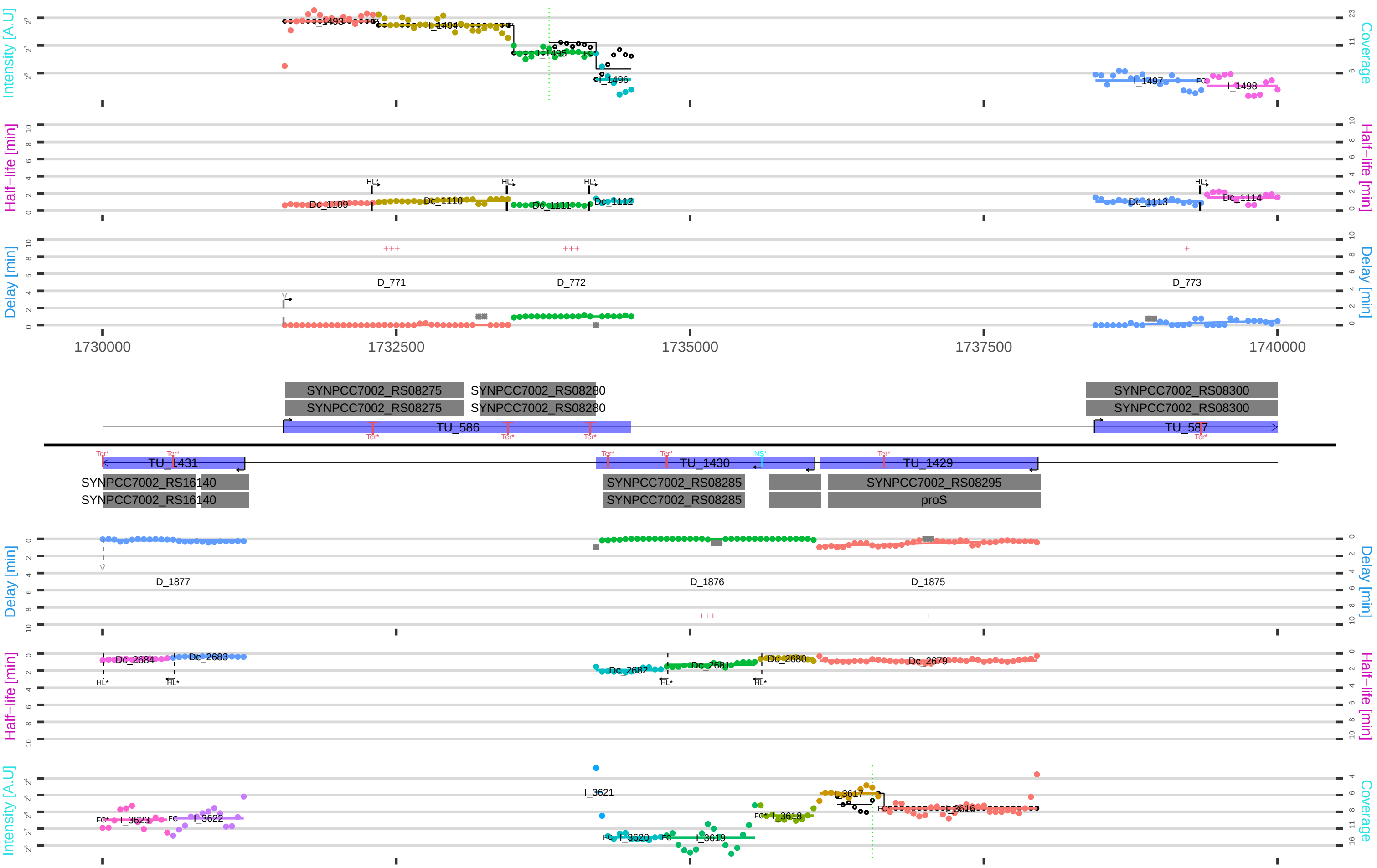

Term: termination (5), NS: new start (1), PS: pausing site (0), iTSS_I: internal starting site (0)

ID: 34800−34978; Term: termination (4), NS: new start (3), PS: pausing site (1), iTSS_I: internal starting site (0)

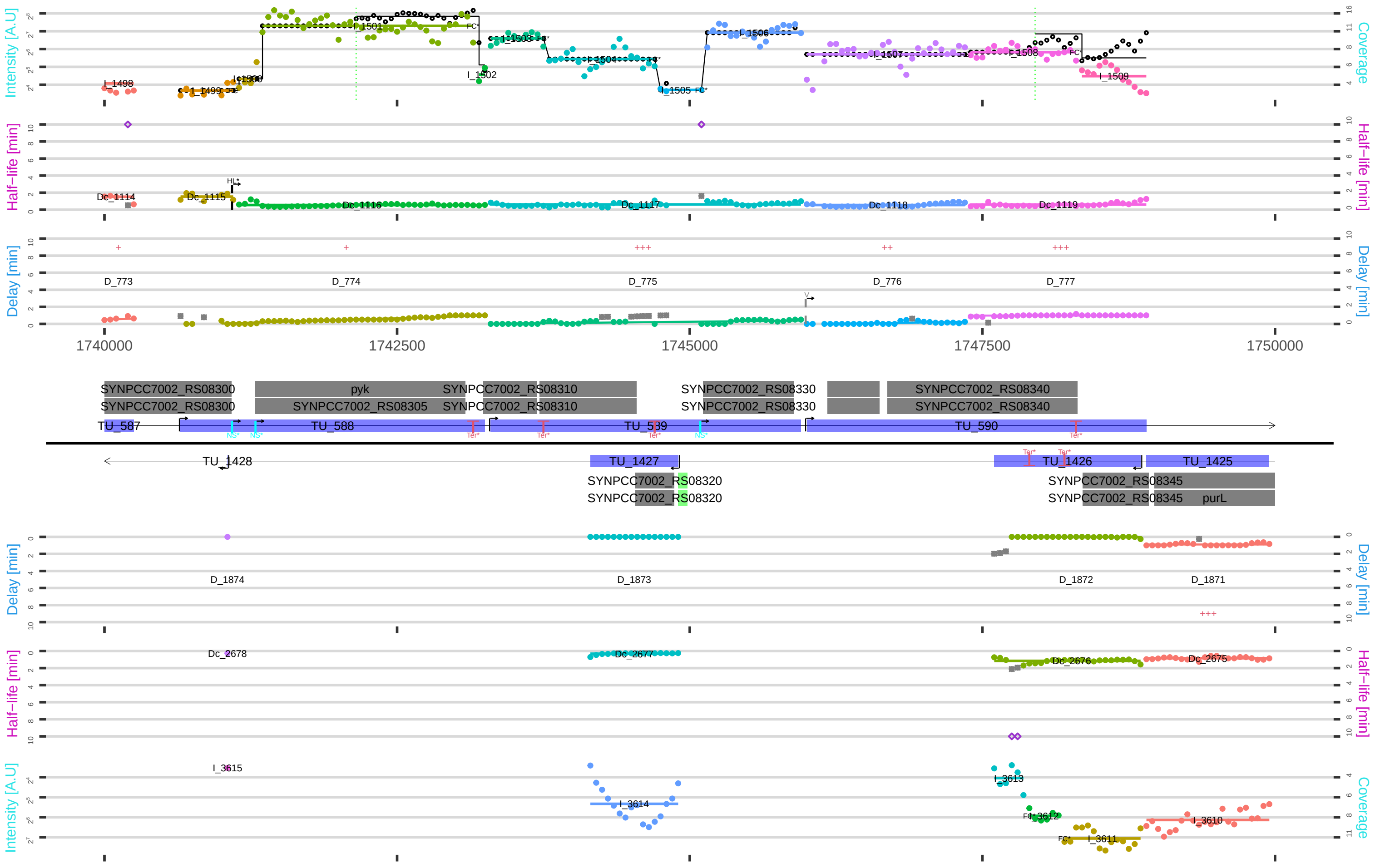

Term: termination (2), NS: new start (0), PS: pausing site (0), iTSS_I: internal starting site (0)

ID: 35038−35200; Term: termination (5), NS: new start (2), PS: pausing site (2), iTSS_I: internal starting site (0)

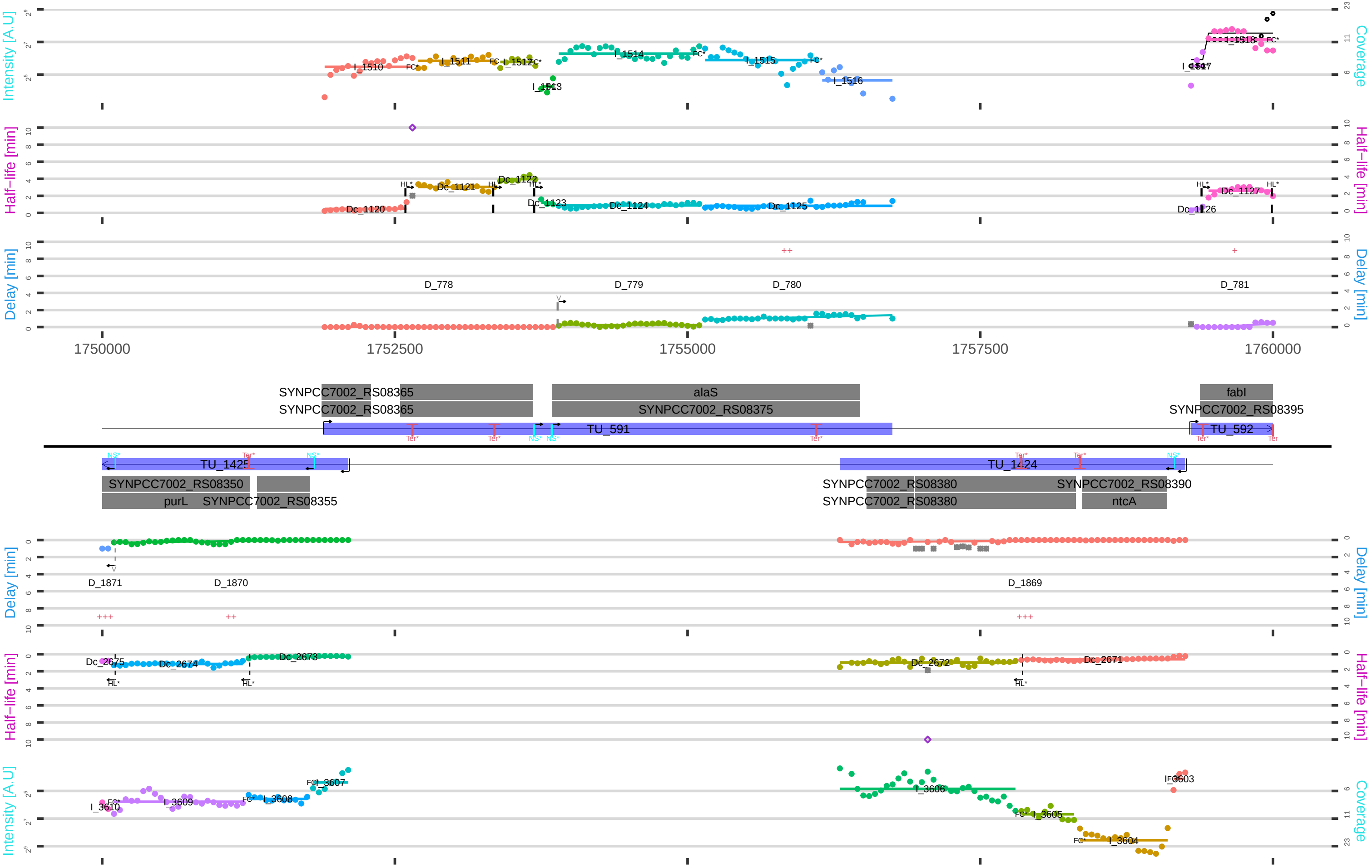

Term: termination (3), NS: new start (3), PS: pausing site (1), iTSS_I: internal starting site (0)

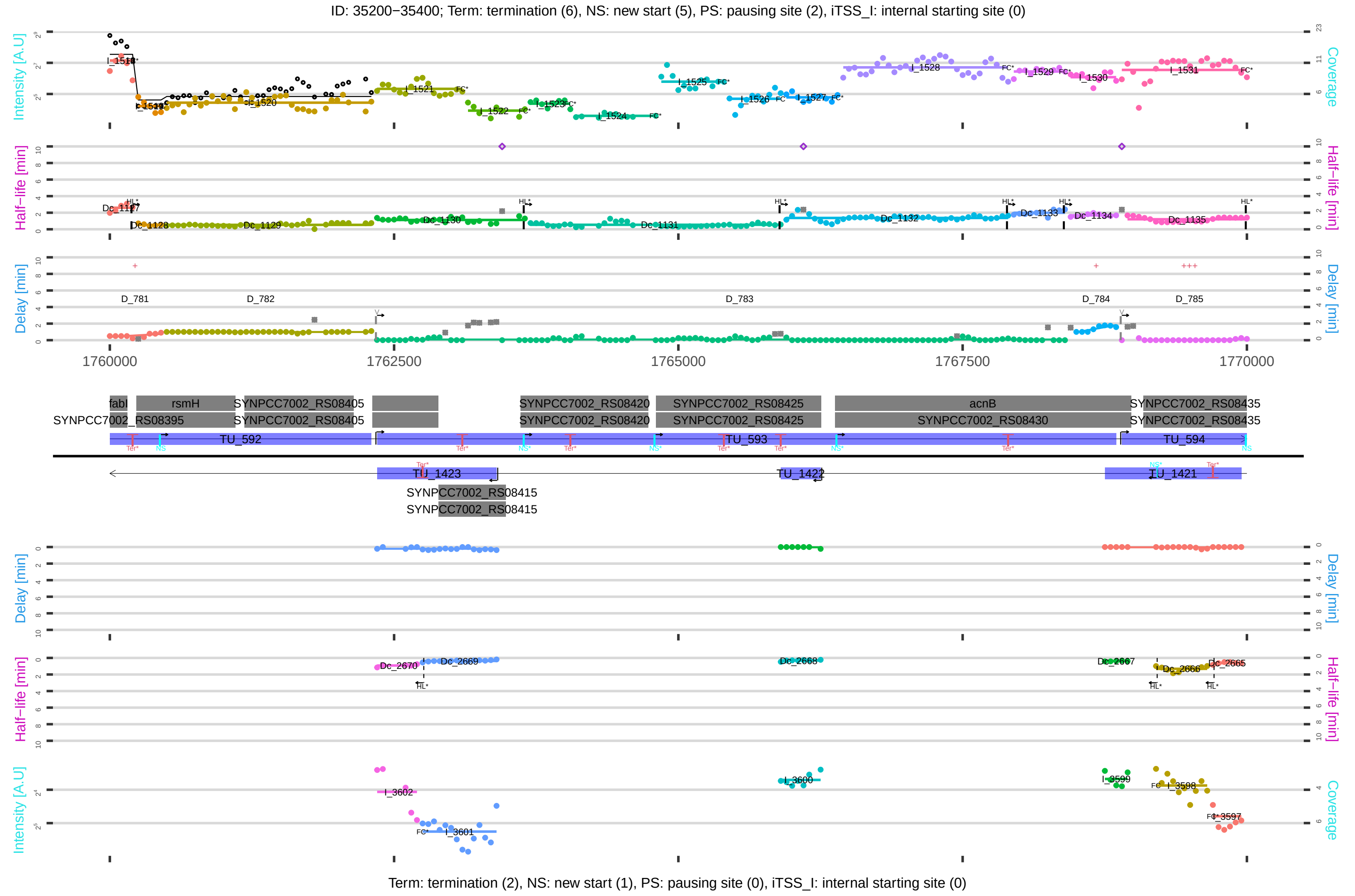

ID: 35200−35400; Term: termination (6), NS: new start (5), PS: pausing site (2), iTSS_I: internal starting site (0)
Intensity [A.U]
Coverage
Half−life [min]
Delay [min]
D_781
D_782
D_783
D_784
D_785
1760000
1762500
1765000
1767500
1770000
fabI
rsmH
SYNPCC7002_RS08395
SYNPCC7002_RS08405
SYNPCC7002_RS08420
SYNPCC7002_RS08425
acnB
SYNPCC7002_RS08430
SYNPCC7002_RS08435
TU_592
TU_593
TU_594
TU_1423
TU_1422
TU_1421
SYNPCC7002_RS08415
Term: termination (2), NS: new start (1), PS: pausing site (0), iTSS_I: internal starting site (0)

ID: 35400−35600; Term: termination (4), NS: new start (3), PS: pausing site (2), iTSS_I: internal starting site (0)

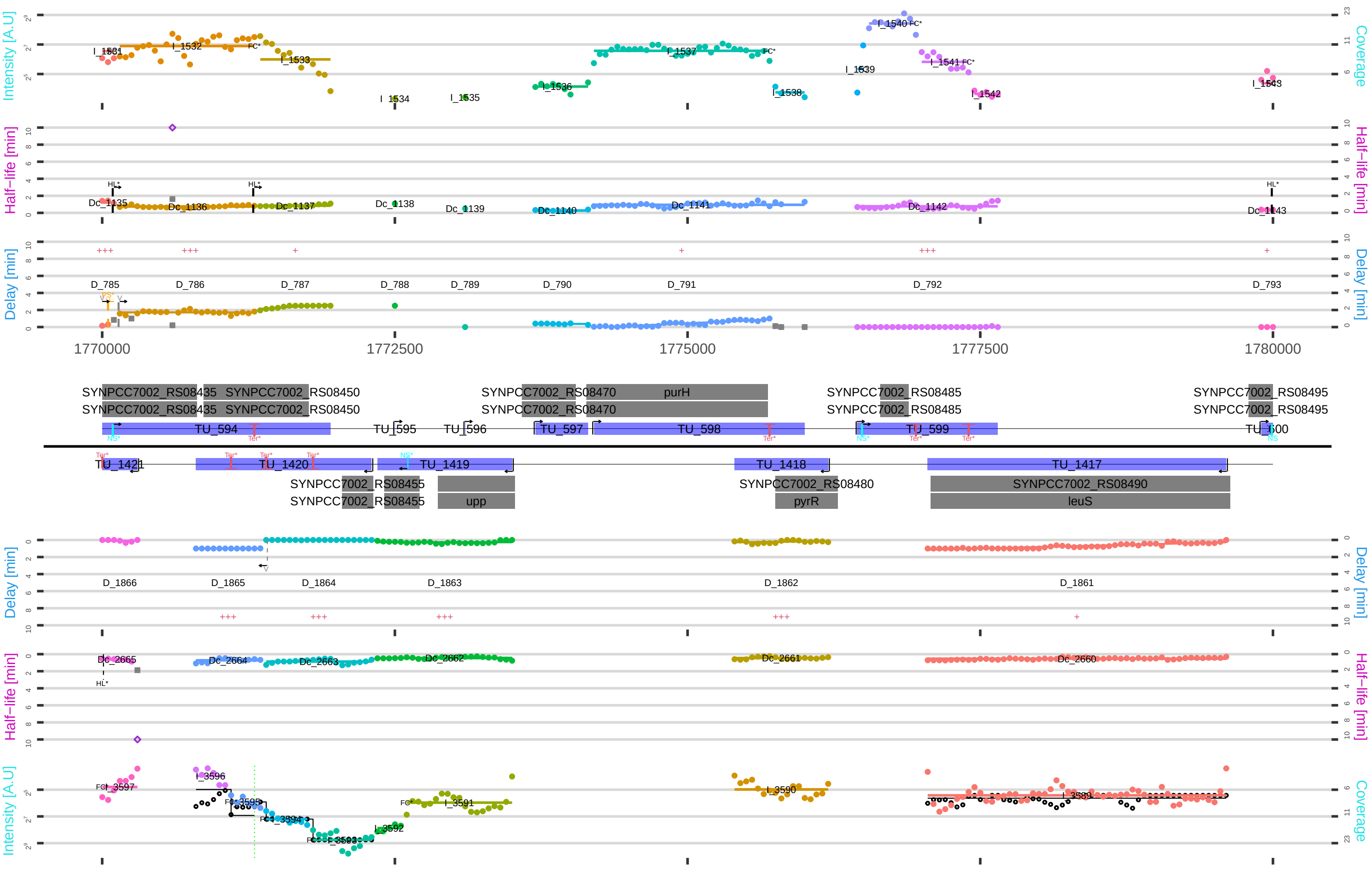

Term: termination (4), NS: new start (1), PS: pausing site (1), iTSS_I: internal starting site (0)

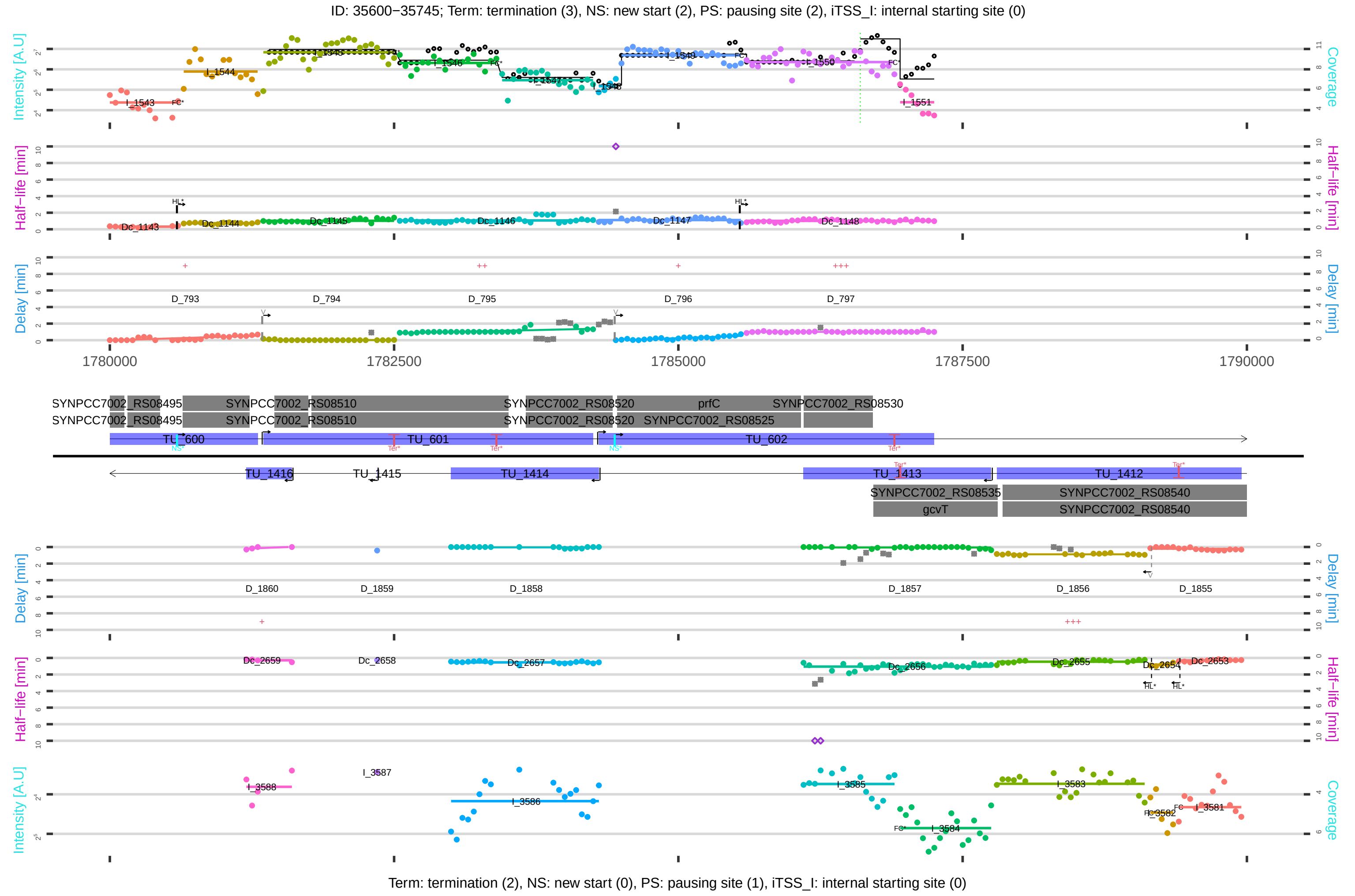

ID: 35600−35745; Term: termination (3), NS: new start (2), PS: pausing site (2), iTSS_I: internal starting site (0)
Intensity [A.U]
Coverage
Half−life [min]
Delay [min]
I_1543
I_1544
I_1545
I_1546
I_1547
I_1548
I_1549
I_1550
I_1551
Dc_1143
Dc_1144
Dc_1145
Dc_1146
Dc_1147
Dc_1148
D_793
D_794
D_795
D_796
D_797
1780000
1782500
1785000
1787500
1790000
SYNPCC7002_RS08495
SYNPCC7002_RS08510
SYNPCC7002_RS08520
prfC
SYNPCC7002_RS08525
SYNPCC7002_RS08530
TU_600
TU_601
TU_602
TU_1416
TU_1415
TU_1414
TU_1413
TU_1412
SYNPCC7002_RS08535
gcvT
SYNPCC7002_RS08540
D_1860
D_1859
D_1858
D_1857
D_1856
D_1855
Dc_2659
Dc_2658
Dc_2657
Dc_2656
Dc_2655
Dc_2654
Dc_2653
I_3588
I_3587
I_3586
I_3585
I_3584
I_3583
I_3582
I_3581
Term: termination (2), NS: new start (0), PS: pausing site (1), iTSS_I: internal starting site (0)

ID: 35806−36000; Term: termination (6), NS: new start (4), PS: pausing site (2), iTSS_I: internal starting site (0)

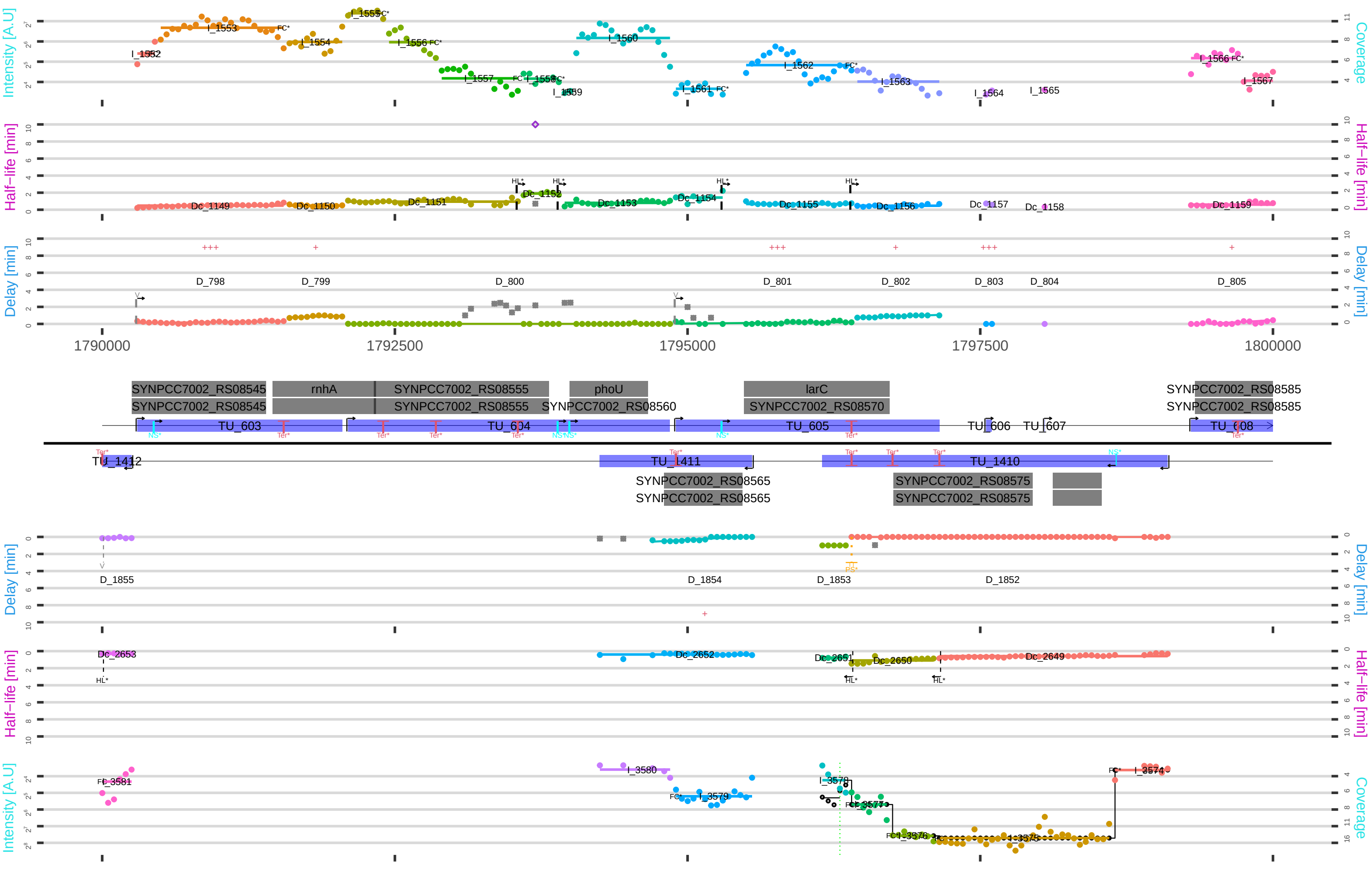

Term: termination (5), NS: new start (1), PS: pausing site (1), iTSS_I: internal starting site (0)

ID: 36000−36123; Term: termination (3), NS: new start (0), PS: pausing site (1), iTSS_I: internal starting site (0)

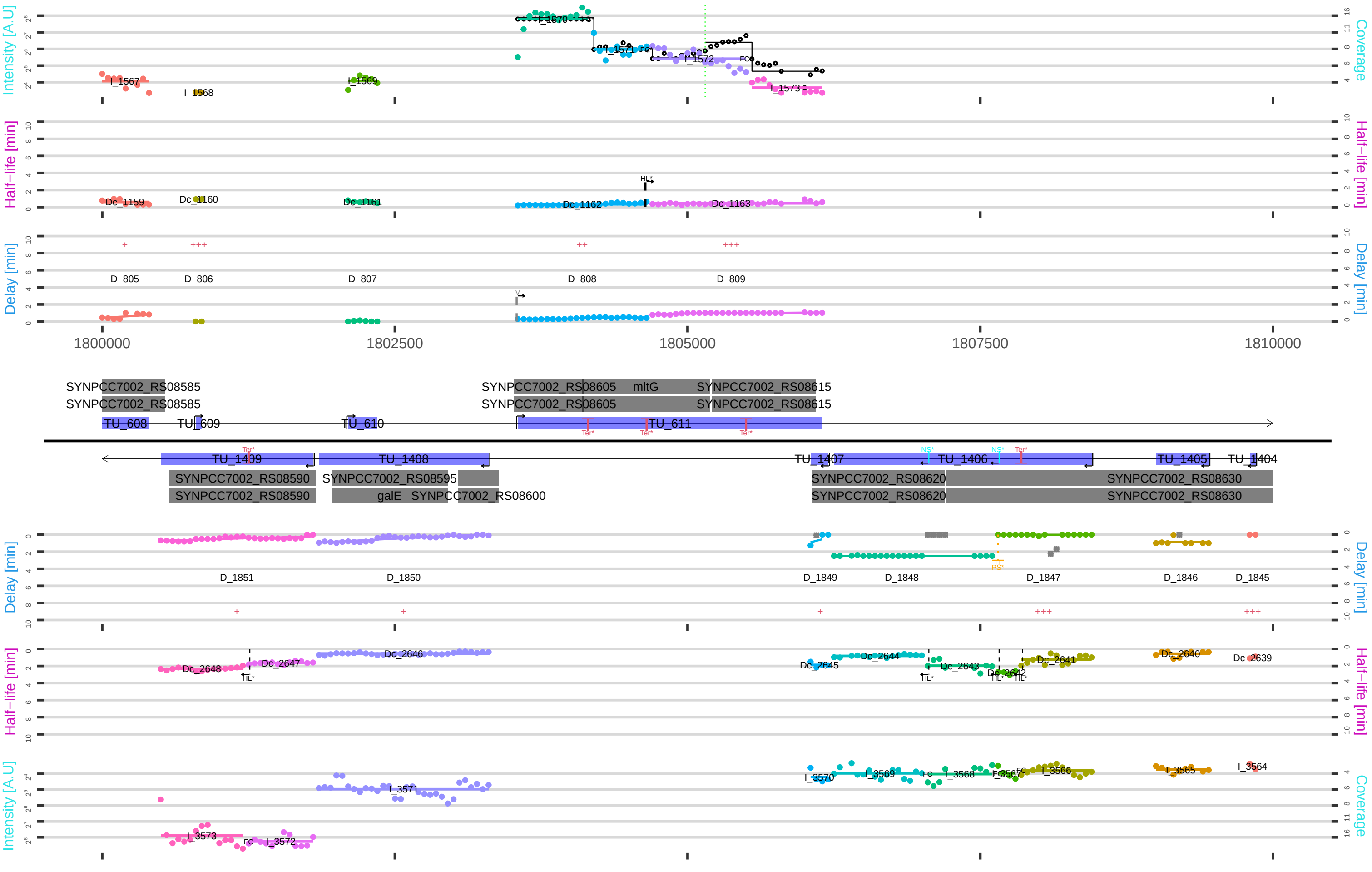

Term: termination (2), NS: new start (2), PS: pausing site (1), iTSS_I: internal starting site (0)

ID: 36235−36391; Term: termination (5), NS: new start (1), PS: pausing site (2), iTSS_I: internal starting site (0)

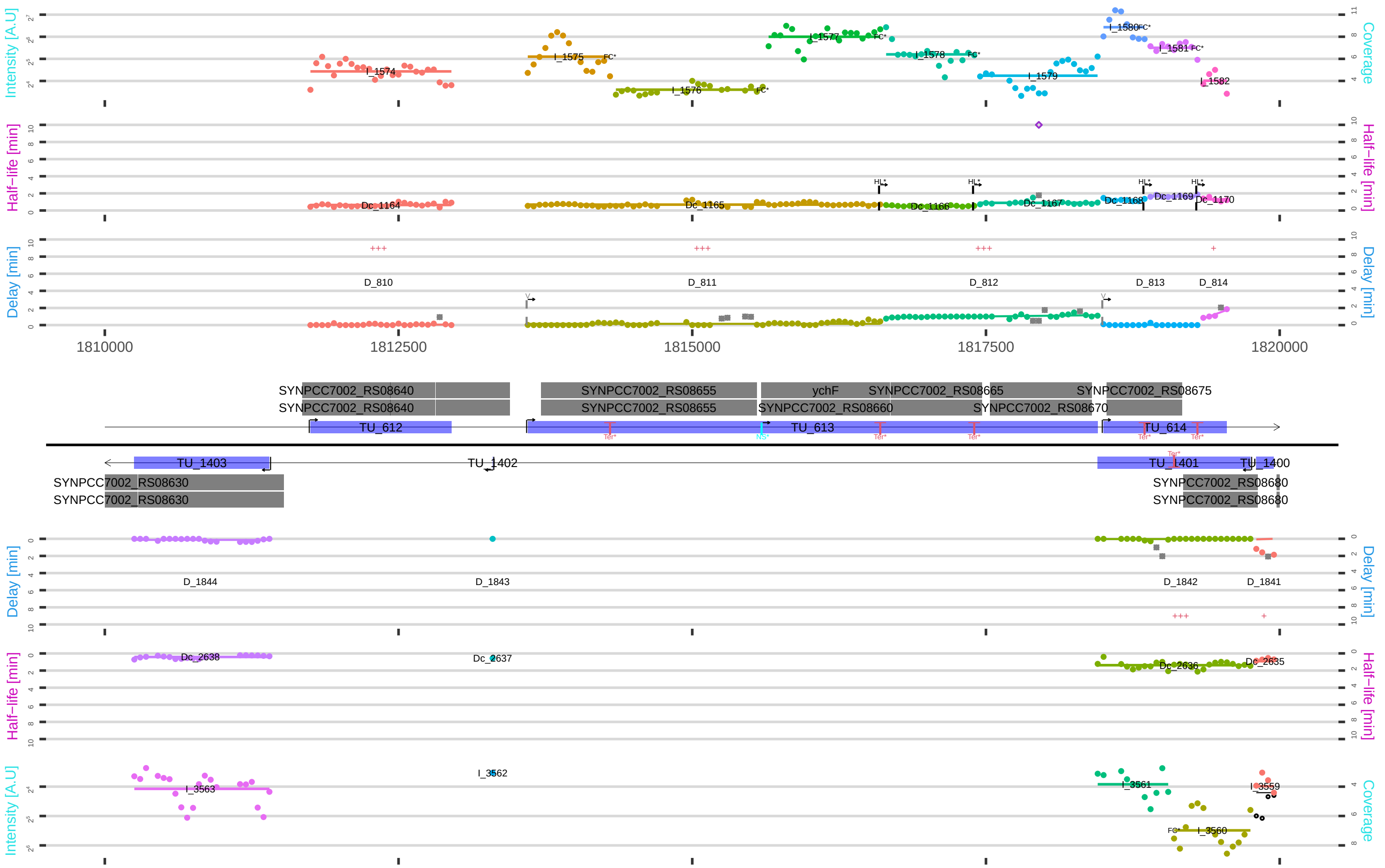

Term: termination (1), NS: new start (0), PS: pausing site (0), iTSS_I: internal starting site (0)

ID: 36401−36600; Term: termination (4), NS: new start (1), PS: pausing site (1), iTSS_I: internal starting site (0)

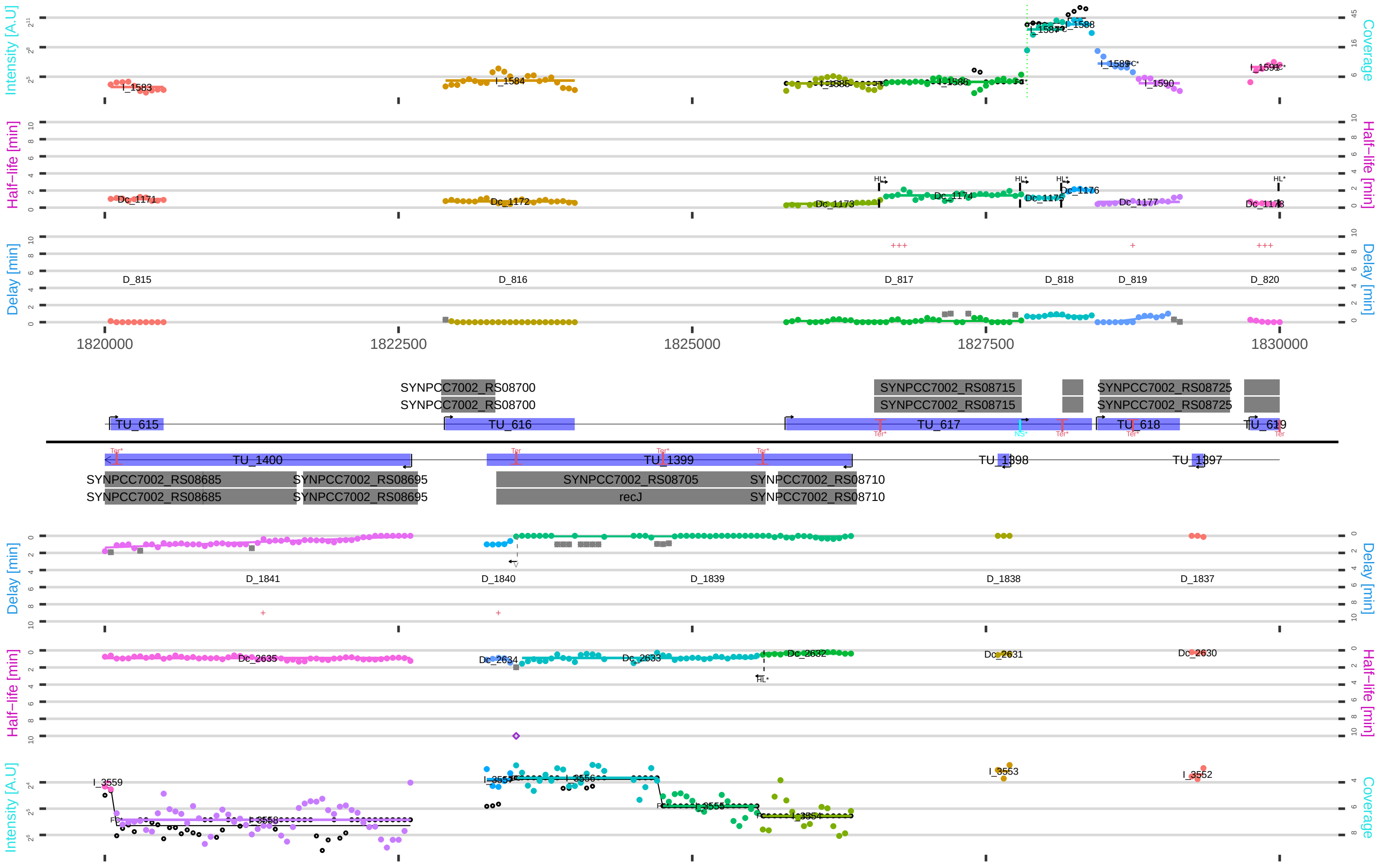

Term: termination (4), NS: new start (0), PS: pausing site (1), iTSS_I: internal starting site (0)

ID: 36600−36800; Term: termination (8), NS: new start (2), PS: pausing site (1), iTSS_I: internal starting site (0)

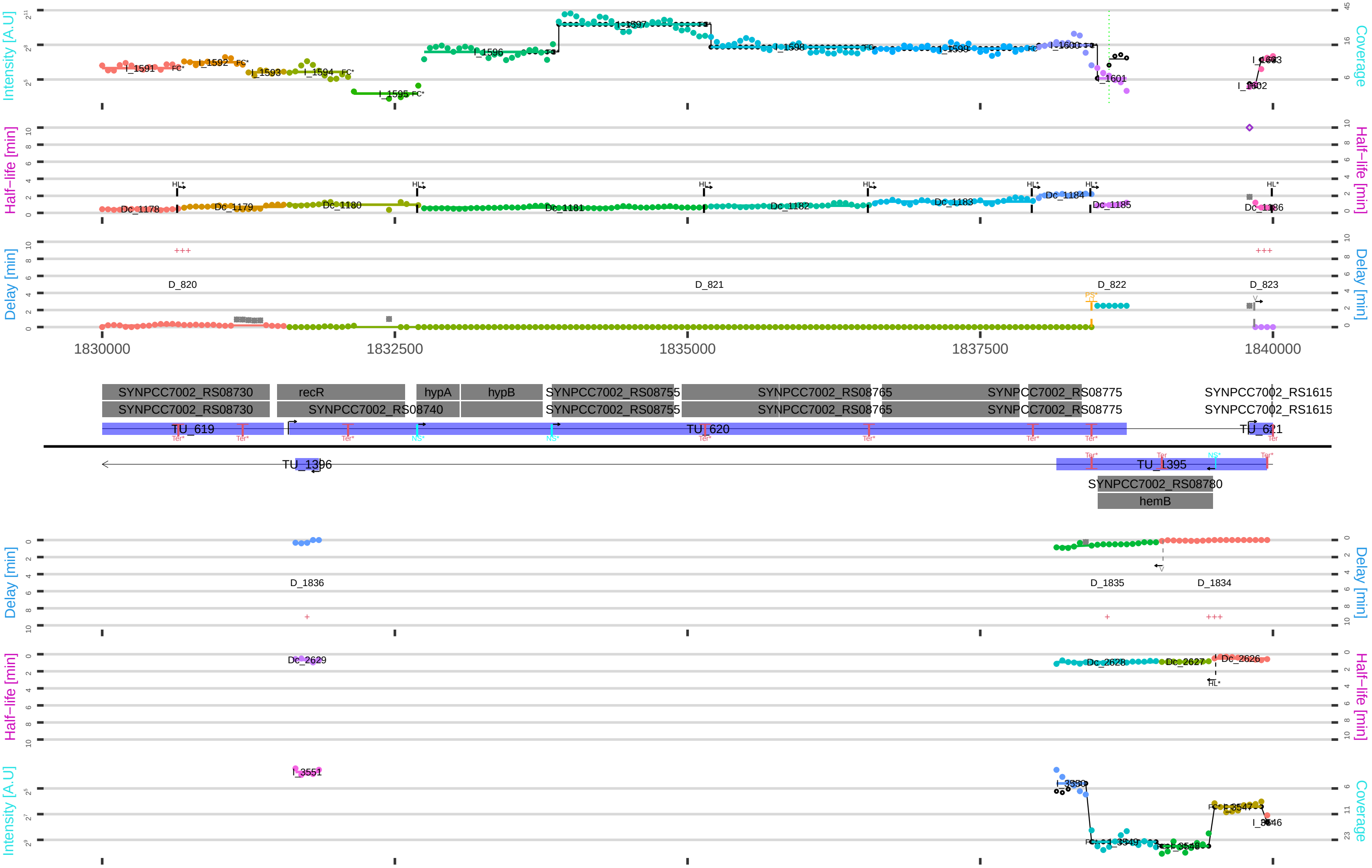

Term: termination (3), NS: new start (1), PS: pausing site (1), iTSS_I: internal starting site (0)

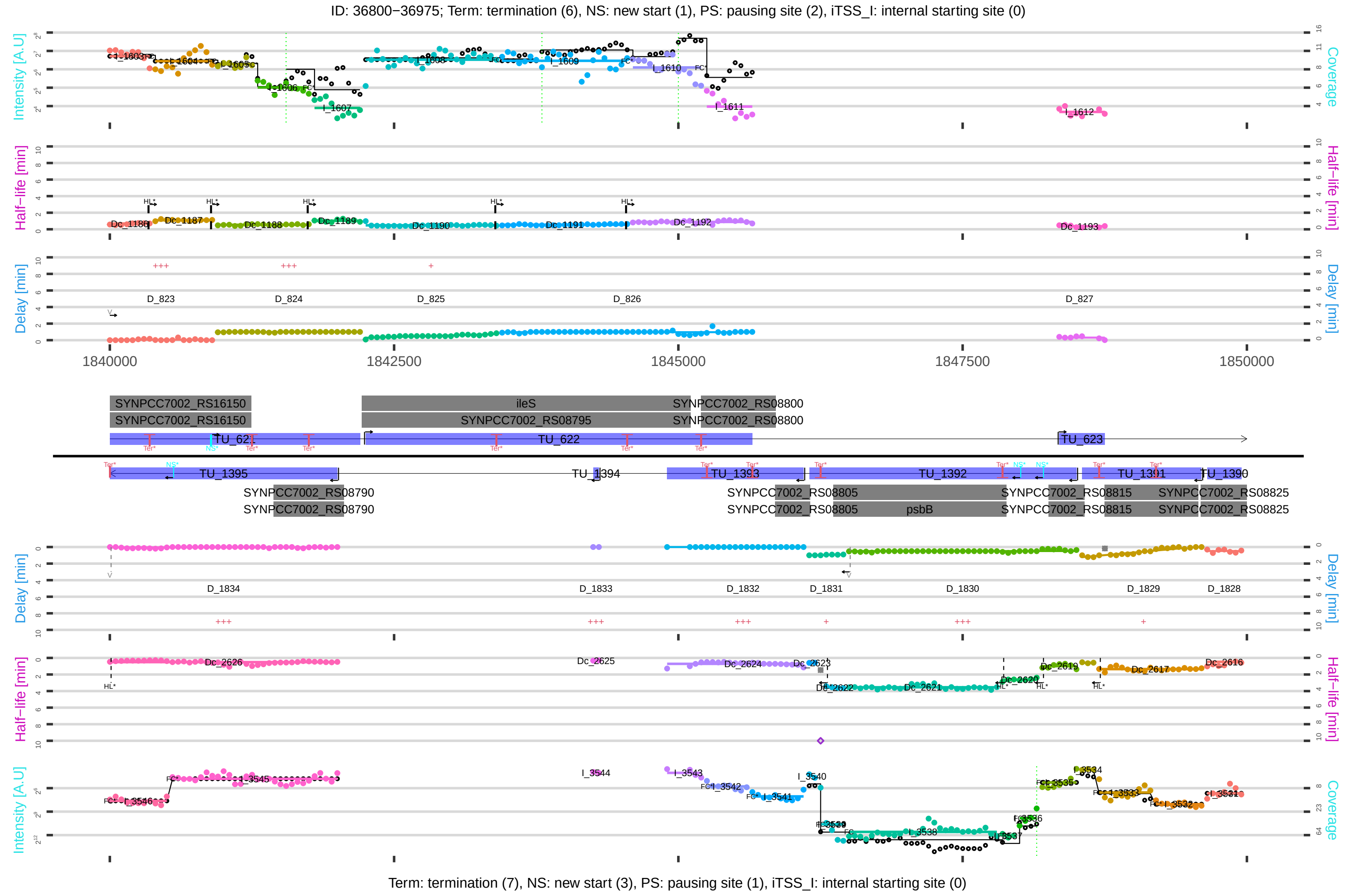
ID: 36800−36975; Term: termination (6), NS: new start (1), PS: pausing site (2), iTSS_I: internal starting site (0)
Intensity [A.U]
Coverage
Half−life [min]
Delay [min]
1840000
1842500
1845000
1847500
1850000
SYNPCC7002_RS16150
ileS
SYNPCC7002_RS08795
SYNPCC7002_RS08800
TU_621
TU_622
TU_623
TU_1395
TU_1394
TU_1393
TU_1392
TU_1391
TU_1390
SYNPCC7002_RS08790
SYNPCC7002_RS08805
psbB
SYNPCC7002_RS08815
SYNPCC7002_RS08825
Term: termination (7), NS: new start (3), PS: pausing site (1), iTSS_I: internal starting site (0)

ID: 37035−37200; Term: termination (3), NS: new start (0), PS: pausing site (1), iTSS_I: internal starting site (0)

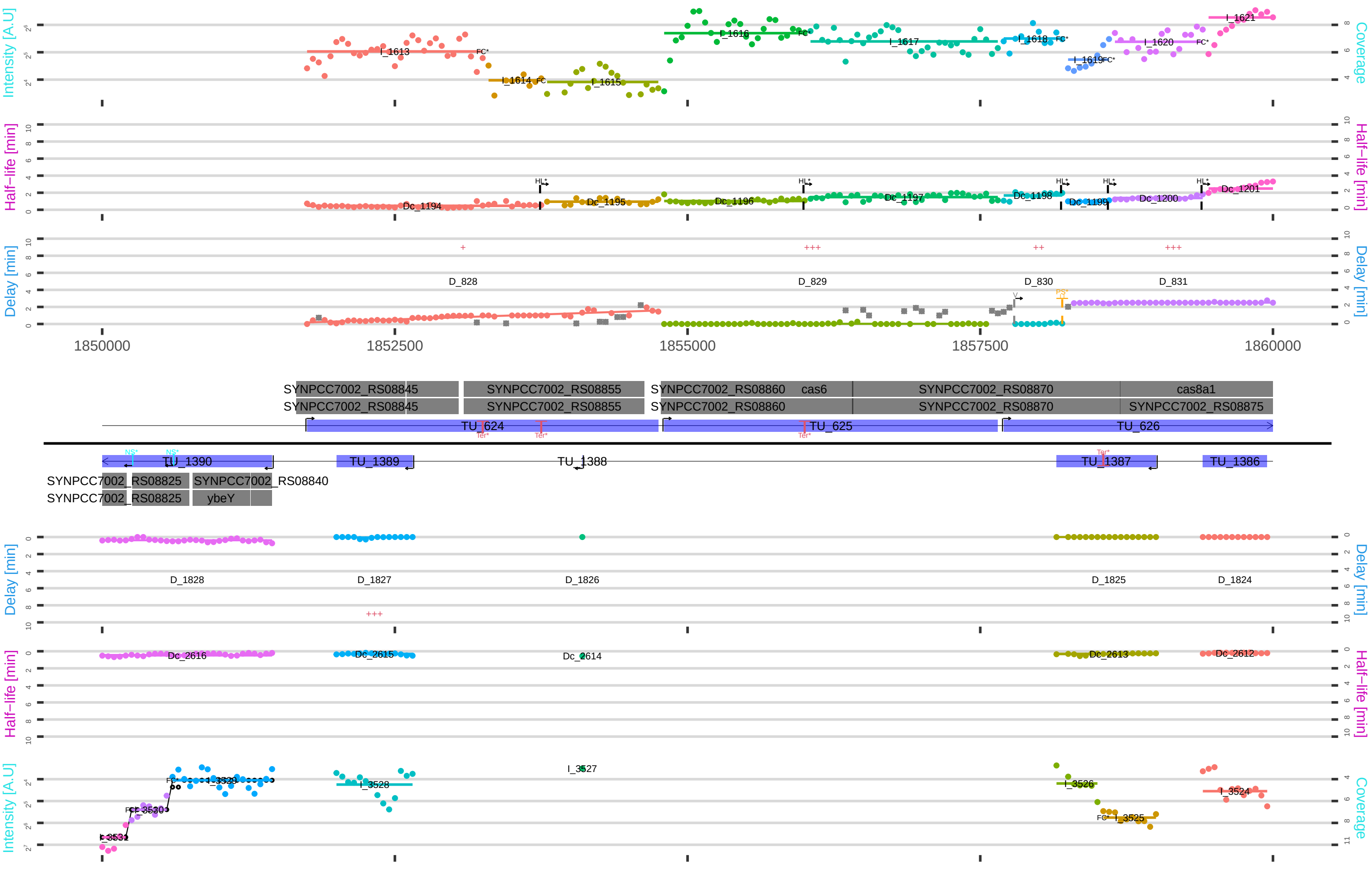

Term: termination (1), NS: new start (2), PS: pausing site (0), iTSS_I: internal starting site (0)

ID: 37200−37400; Term: termination (4), NS: new start (2), PS: pausing site (1), iTSS_I: internal starting site (0)

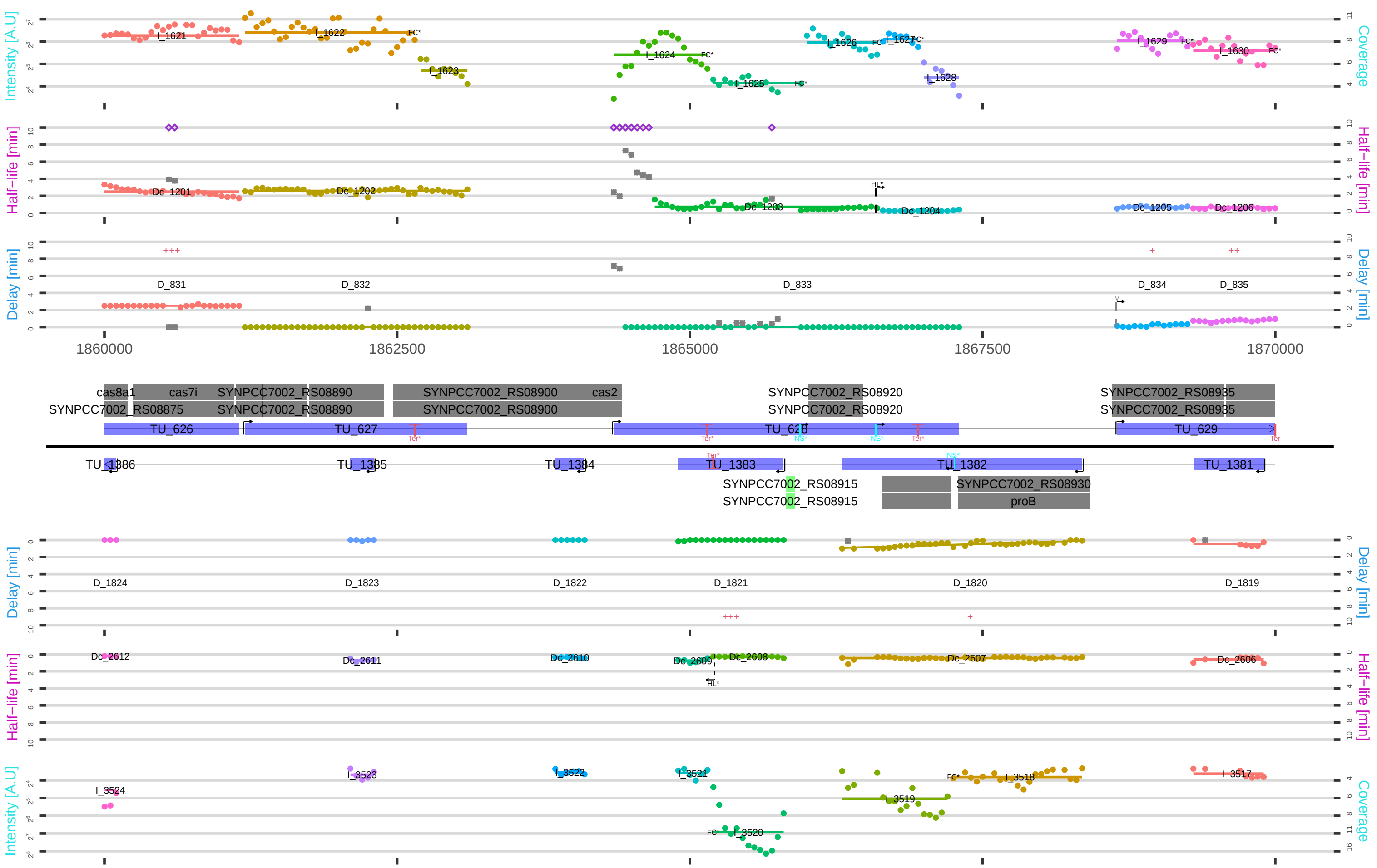

Term: termination (1), NS: new start (1), PS: pausing site (0), iTSS_I: internal starting site (0)

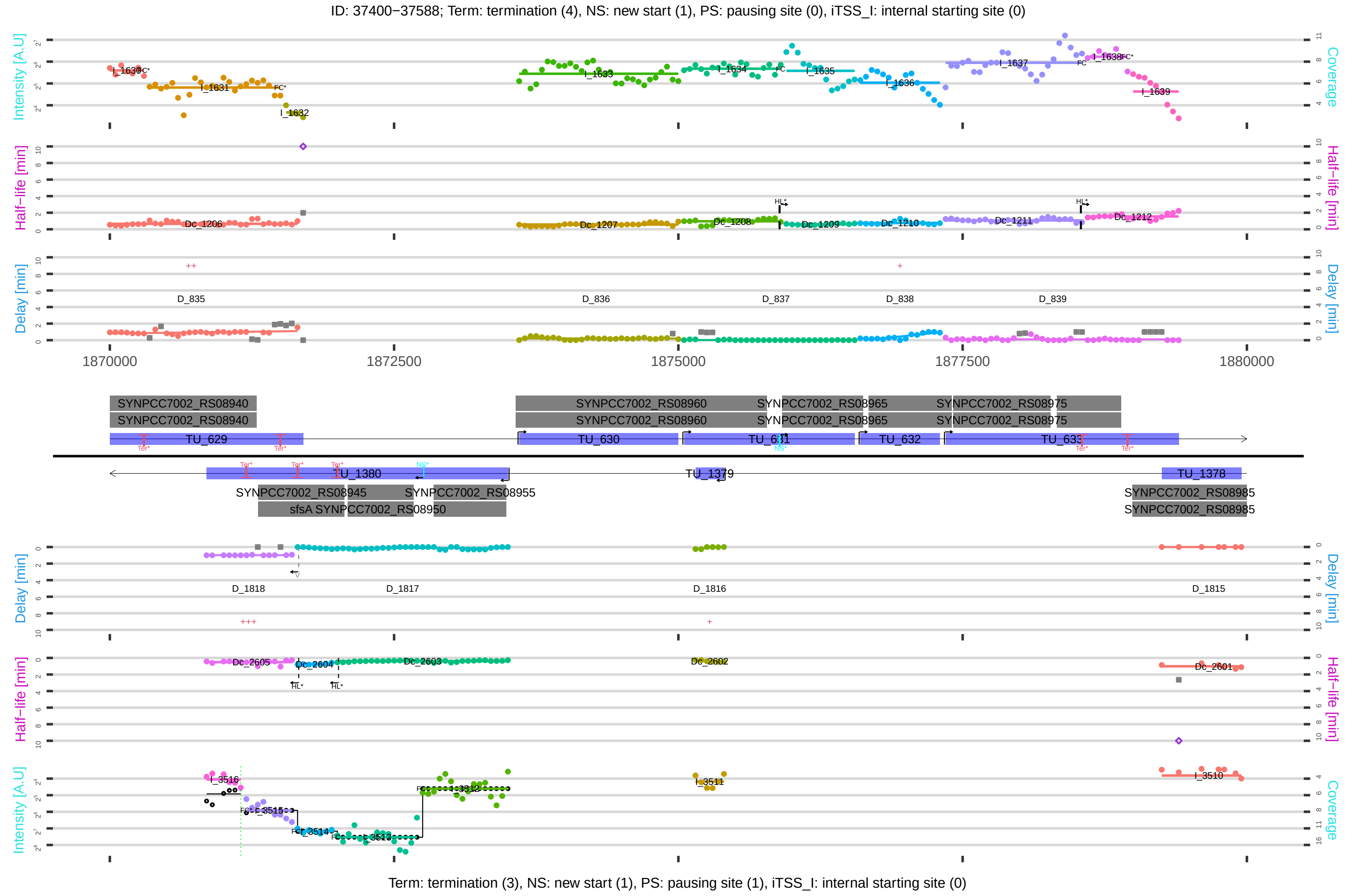
ID: 37400−37588; Term: termination (4), NS: new start (1), PS: pausing site (0), iTSS_I: internal starting site (0)
Intensity [A.U]
Coverage
Half−life [min]
Delay [min]
1870000
1872500
1875000
1877500
1880000
TU_629
TU_630
TU_631
TU_632
TU_633
TU_1380
TU_1379
TU_1378
SYNPCC7002_RS08940
SYNPCC7002_RS08960
SYNPCC7002_RS08965
SYNPCC7002_RS08975
SYNPCC7002_RS08945
SYNPCC7002_RS08955
sfsA SYNPCC7002_RS08950
SYNPCC7002_RS08985
Term: termination (3), NS: new start (1), PS: pausing site (1), iTSS_I: internal starting site (0)

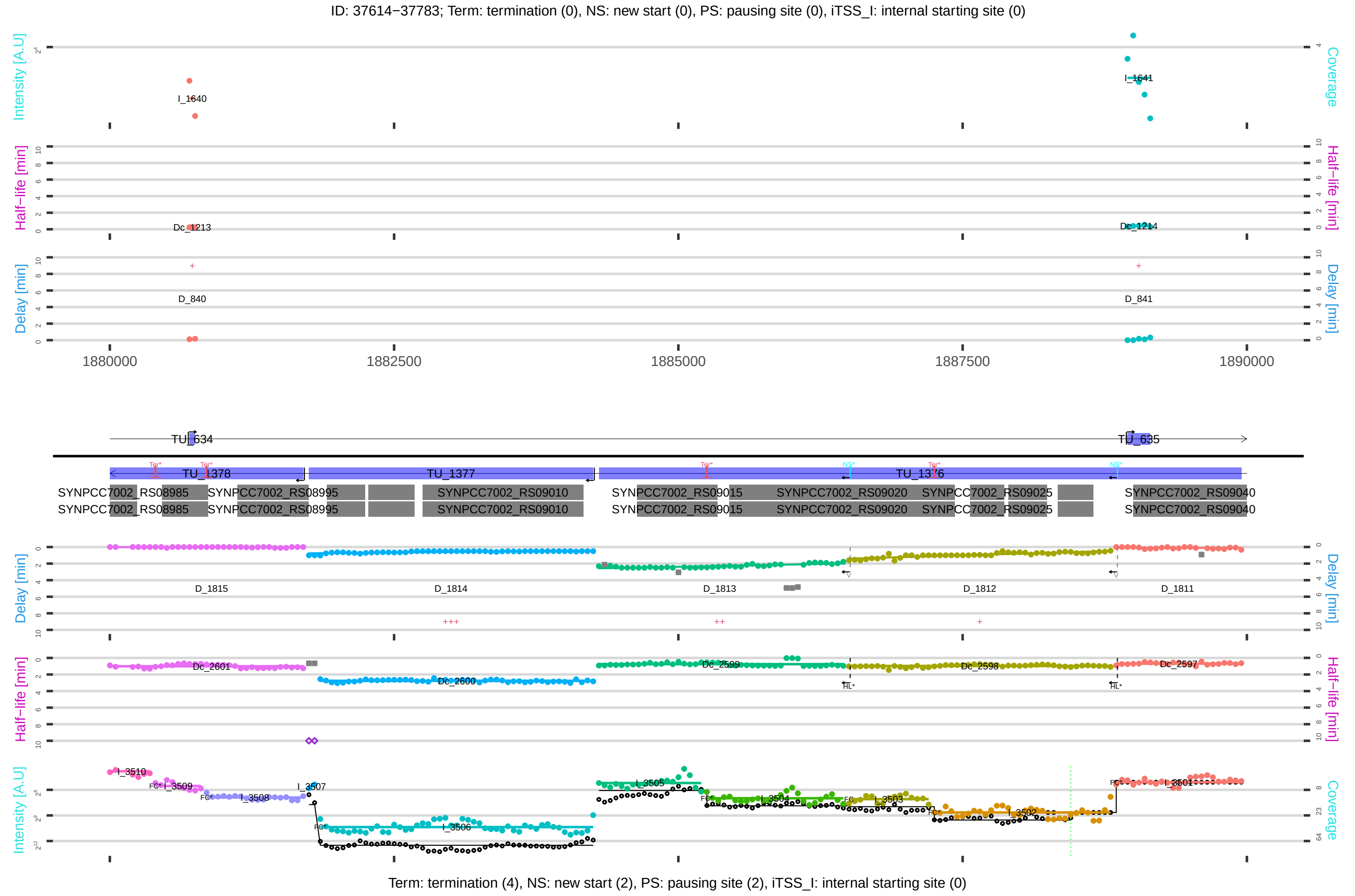
ID: 37614−37783; Term: termination (0), NS: new start (0), PS: pausing site (0), iTSS_I: internal starting site (0)
Intensity [A.U]
Coverage
Half−life [min]
Delay [min]
I_1640
I_1641
Dc_1213
Dc_1214
D_840
D_841
1880000
1882500
1885000
1887500
1890000
TU_634
TU_635
TU_1378
TU_1377
TU_1376
SYNPCC7002_RS08985
SYNPCC7002_RS08995
SYNPCC7002_RS09010
SYNPCC7002_RS09015
SYNPCC7002_RS09020
SYNPCC7002_RS09025
SYNPCC7002_RS09040
D_1815
D_1814
D_1813
D_1812
D_1811
Dc_2601
Dc_2600
Dc_2599
Dc_2598
Dc_2597
I_3510
I_3509
I_3508
I_3507
I_3506
I_3505
I_3504
I_3503
I_3502
I_3501
Term: termination (4), NS: new start (2), PS: pausing site (2), iTSS_I: internal starting site (0)

ID: 37818−37996; Term: termination (6), NS: new start (2), PS: pausing site (2), iTSS_I: internal starting site (0)

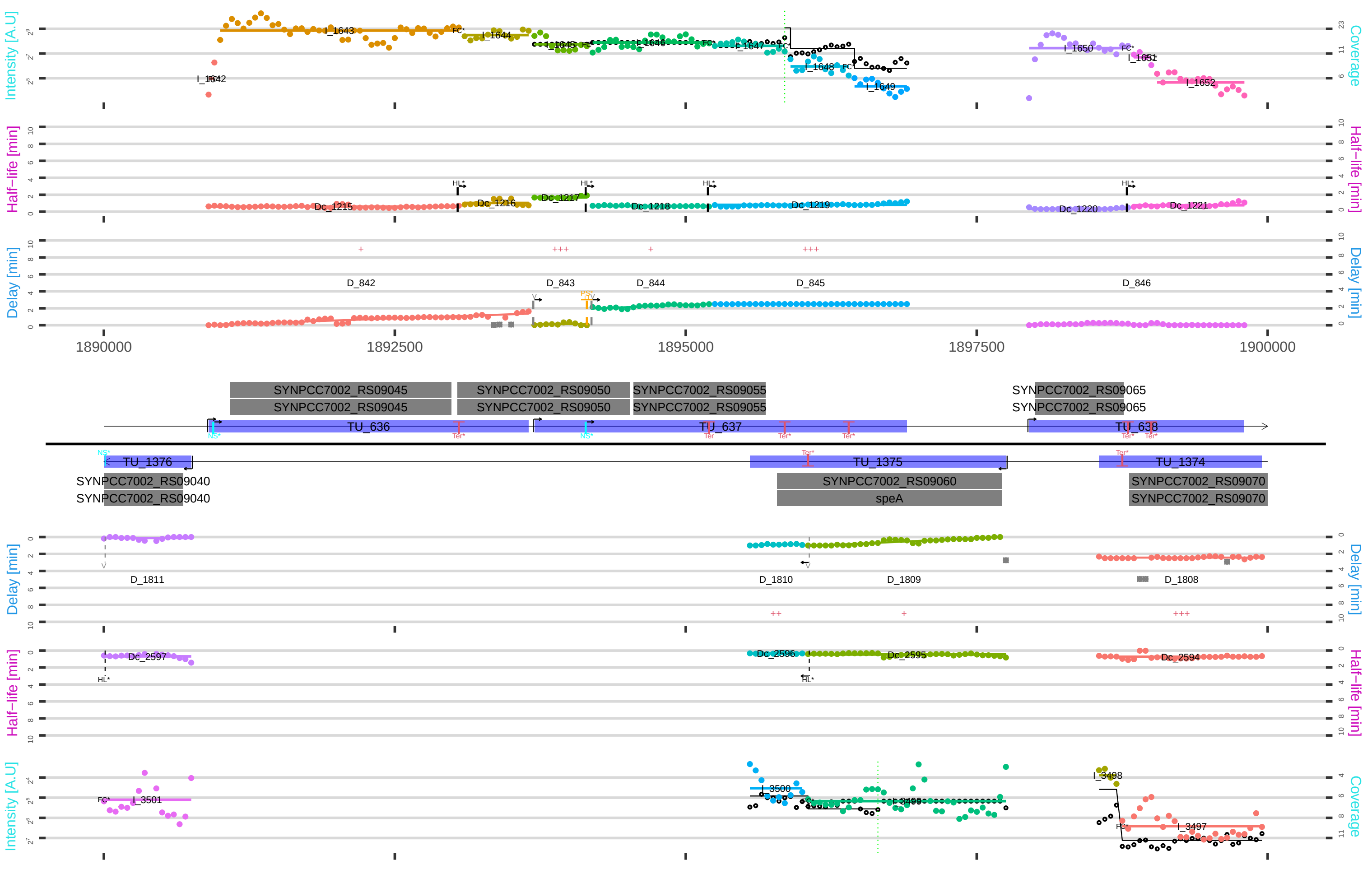

Term: termination (2), NS: new start (1), PS: pausing site (0), iTSS_I: internal starting site (1)

ID: 38078−38200; Term: termination (3), NS: new start (2), PS: pausing site (0), iTSS_I: internal starting site (1)

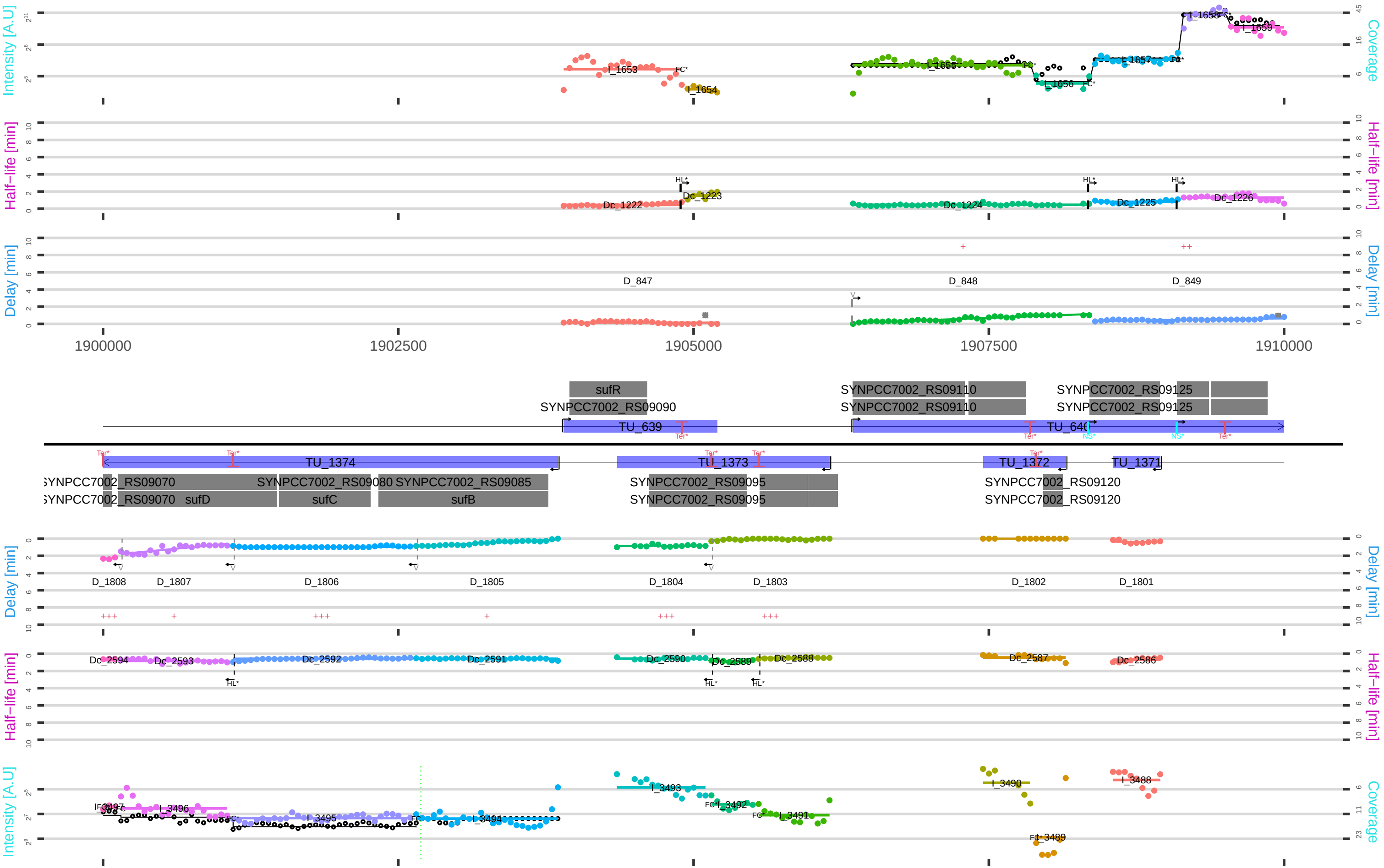

Term: termination (5), NS: new start (0), PS: pausing site (2), iTSS_I: internal starting site (2)

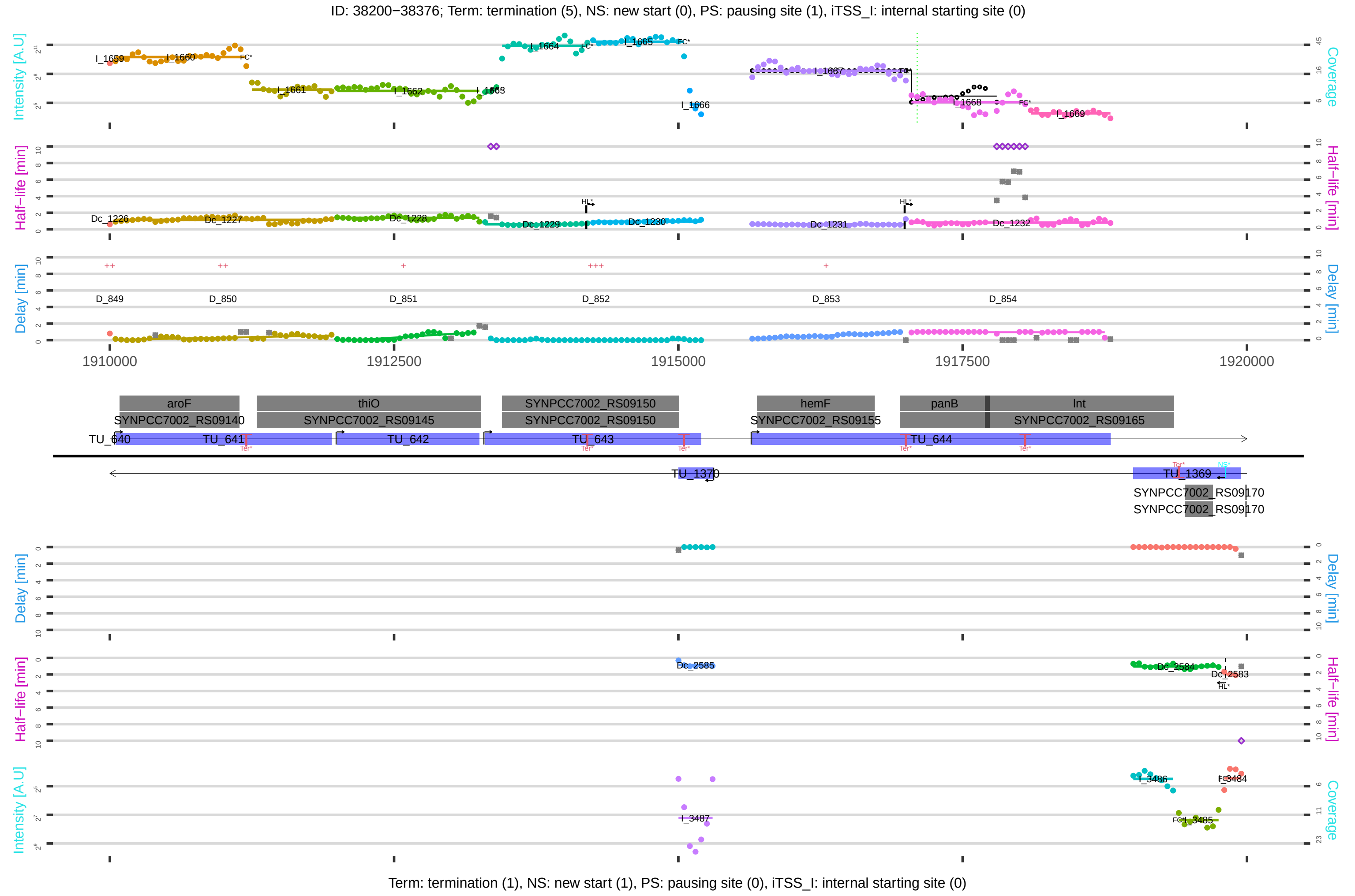

ID: 38200−38376; Term: termination (5), NS: new start (0), PS: pausing site (1), iTSS_I: internal starting site (0)
Intensity [A.U]
Coverage
Half−life [min]
Delay [min]
I_1659
I_1660
I_1661
I_1662
I_1663
I_1664
I_1665
I_1666
I_1667
I_1668
I_1669
Dc_1226
Dc_1227
Dc_1228
Dc_1229
Dc_1230
Dc_1231
Dc_1232
D_849
D_850
D_851
D_852
D_853
D_854
1910000
1912500
1915000
1917500
1920000
aroF
SYNPCC7002_RS09140
thiO
SYNPCC7002_RS09145
SYNPCC7002_RS09150
SYNPCC7002_RS09150
hemF
SYNPCC7002_RS09155
panB
Int
SYNPCC7002_RS09165
TU_640
TU_641
TU_642
TU_643
TU_644
TU_1370
TU_1369
SYNPCC7002_RS09170
SYNPCC7002_RS09170
Dc_2585
Dc_2584
Dc_2583
I_3487
I_3486
I_3485
I_3484
Term: termination (1), NS: new start (1), PS: pausing site (0), iTSS_I: internal starting site (0)

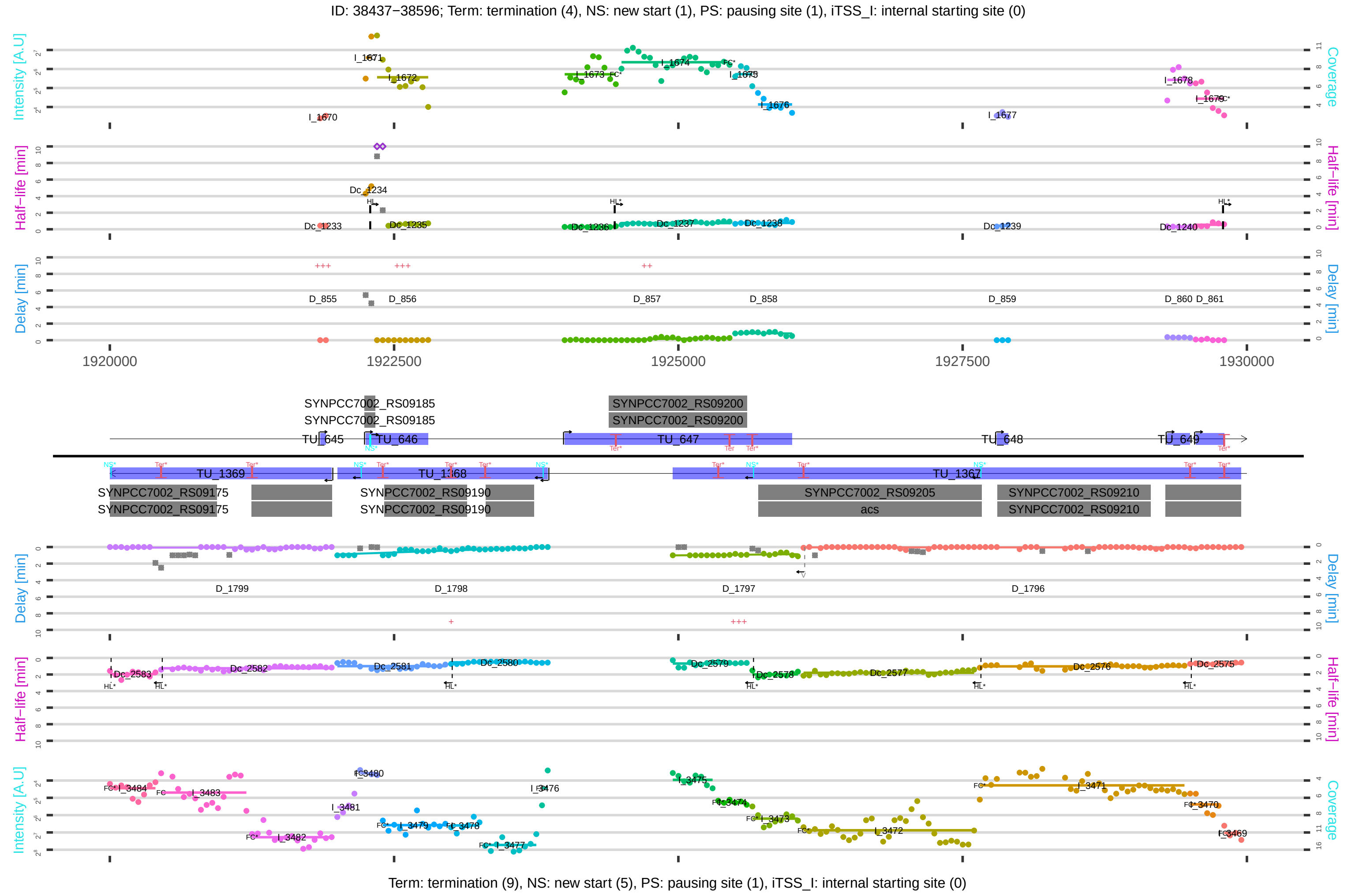

ID: 38437–38596; Term: termination (4), NS: new start (1), PS: pausing site (1), iTSS_I: internal starting site (0)
Intensity [A.U]
Coverage
Half−life [min]
Delay [min]
1920000
1922500
1925000
1927500
1930000
SYNPCC7002_RS09185
SYNPCC7002_RS09200
TU_645
TU_646
TU_647
TU_648
TU_649
TU_1369
TU_1368
TU_1367
SYNPCC7002_RS09175
SYNPCC7002_RS09190
SYNPCC7002_RS09205
acs
SYNPCC7002_RS09210
D_1799
D_1798
D_1797
D_1796
Term: termination (9), NS: new start (5), PS: pausing site (1), iTSS_I: internal starting site (0)

ID: 38602−38800; Term: termination (6), NS: new start (3), PS: pausing site (1), iTSS_I: internal starting site (0)

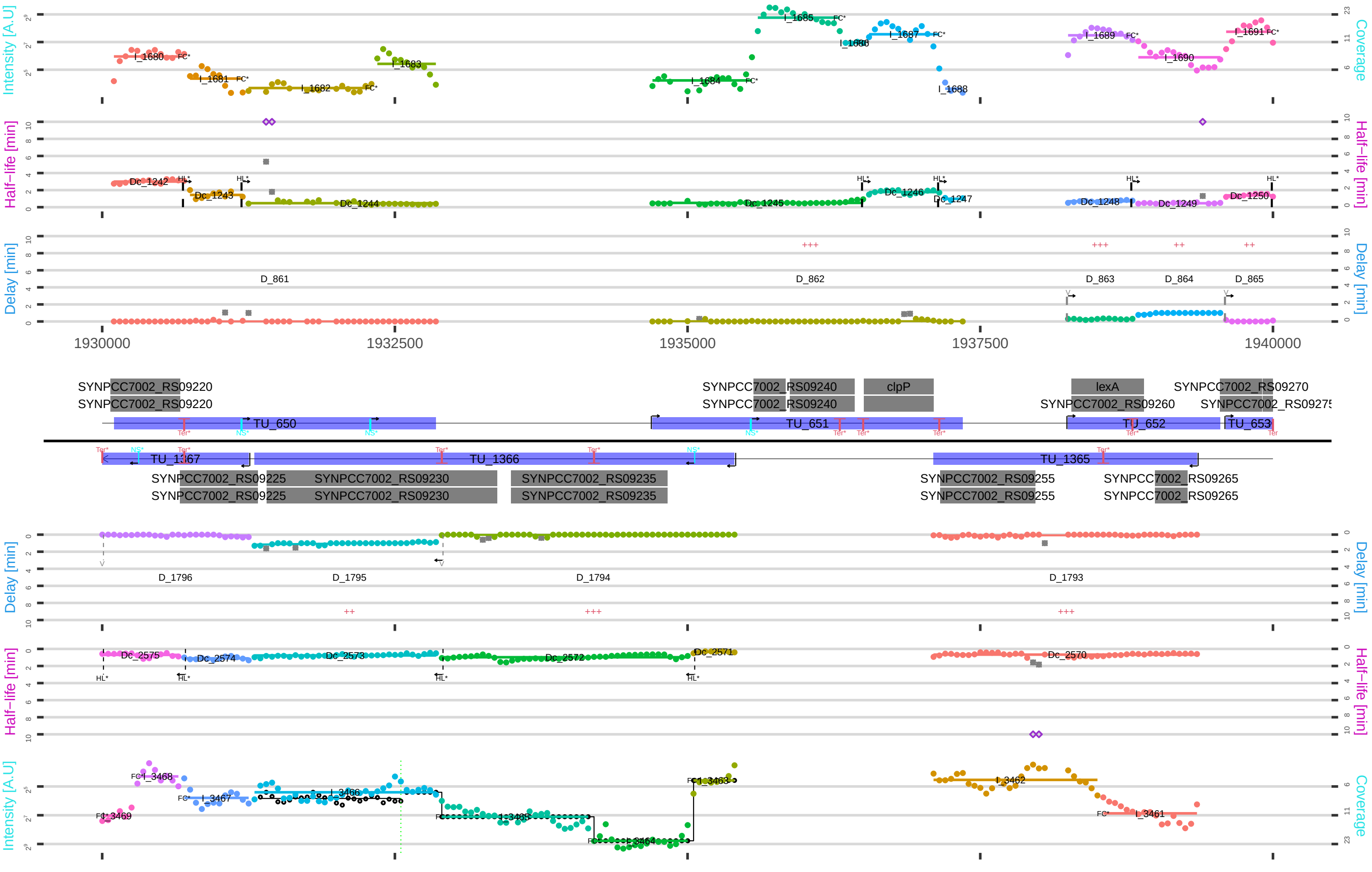

Term: termination (5), NS: new start (2), PS: pausing site (1), iTSS_I: internal starting site (0)

ID: 38800−38969; Term: termination (4), NS: new start (1), PS: pausing site (1), iTSS_I: internal starting site (0)

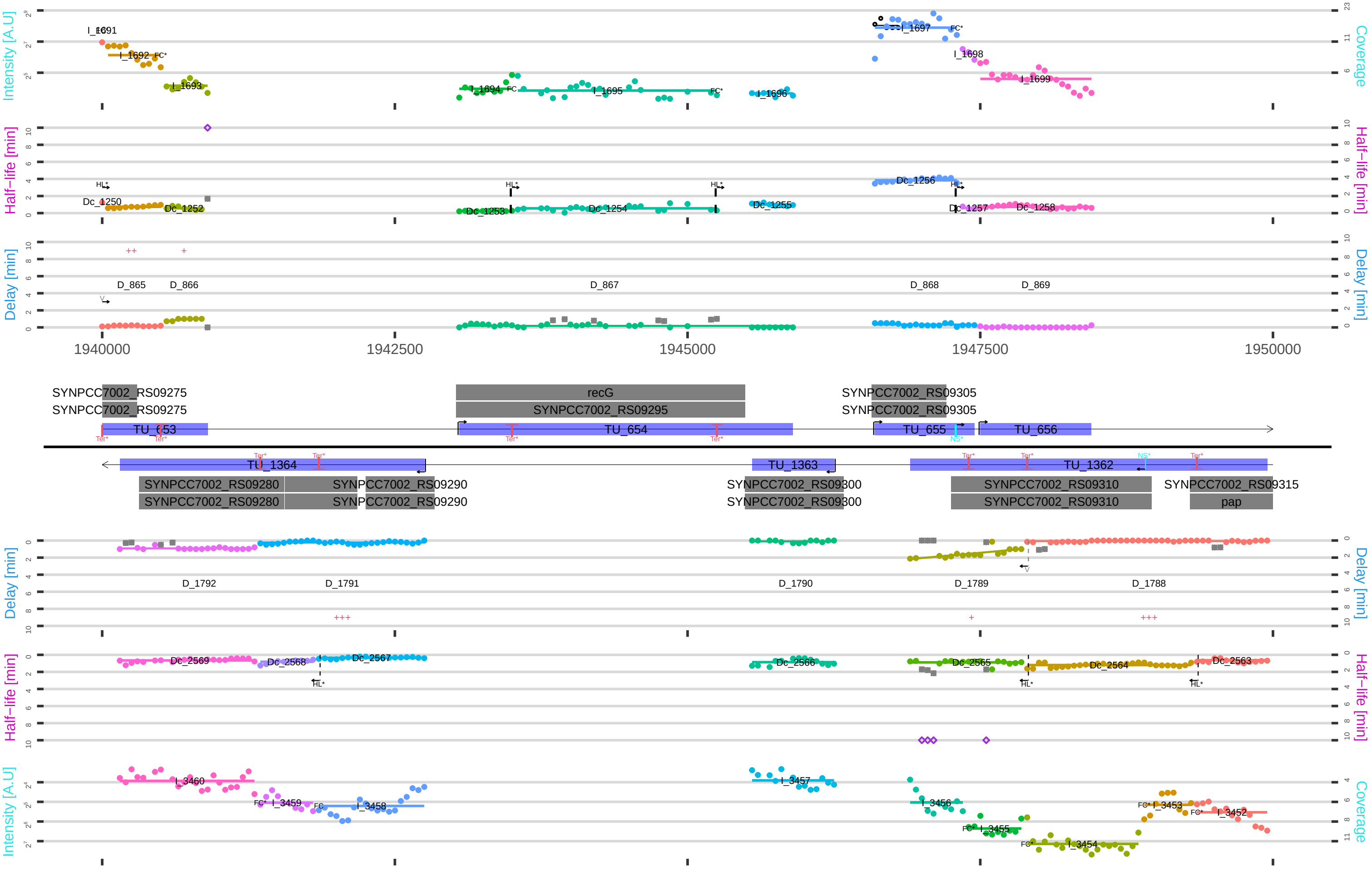

Term: termination (5), NS: new start (1), PS: pausing site (2), iTSS_I: internal starting site (0)

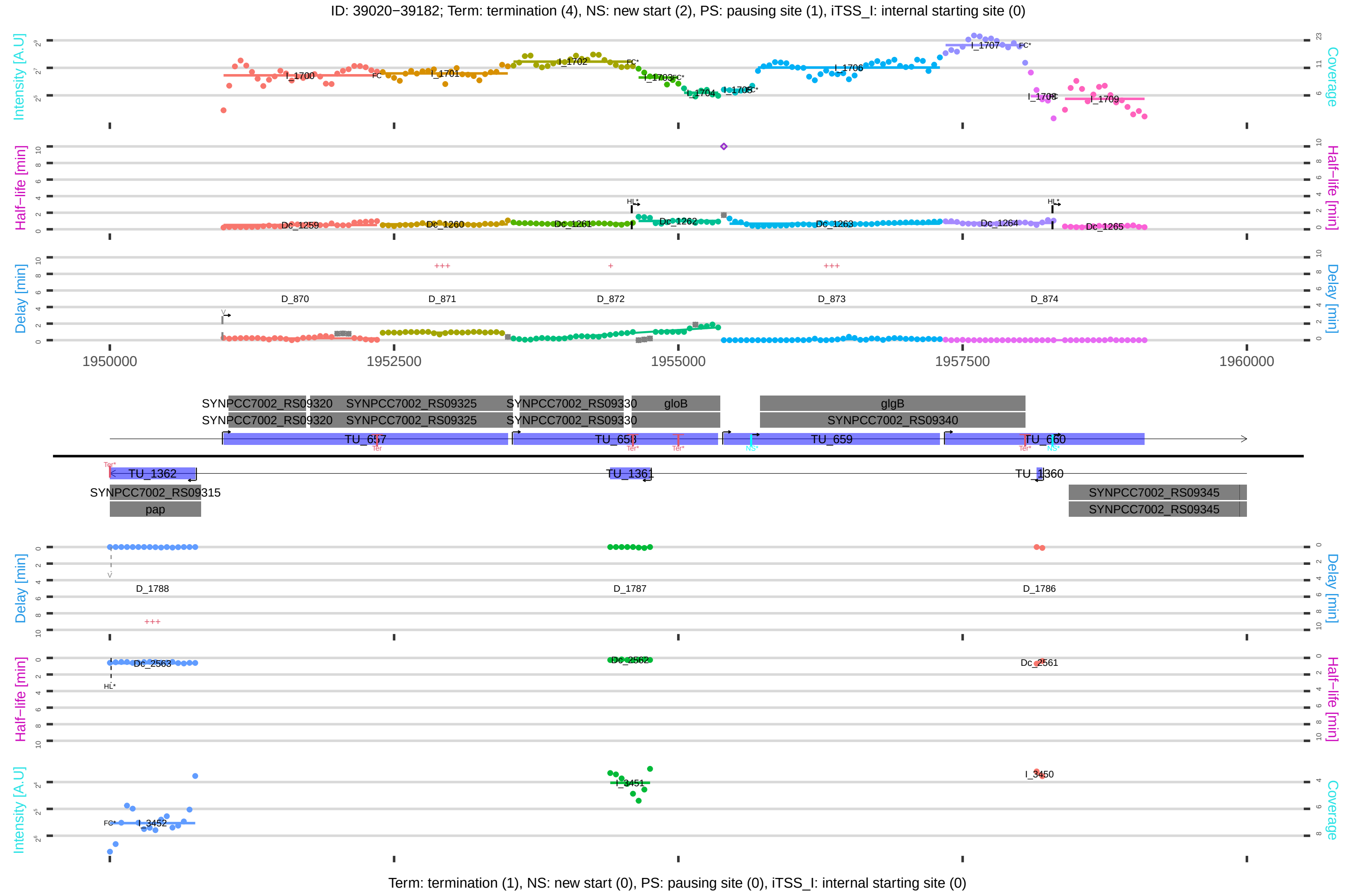
ID: 39020−39182; Term: termination (4), NS: new start (2), PS: pausing site (1), iTSS_I: internal starting site (0)
Intensity [A.U]
Coverage
Half−life [min]
Delay [min]
1950000
1952500
1955000
1957500
1960000
SYNPCC7002_RS09320
SYNPCC7002_RS09325
SYNPCC7002_RS09330
gloB
glgB
SYNPCC7002_RS09340
TU_657
TU_658
TU_659
TU_660
TU_1362
TU_1361
TU_1360
SYNPCC7002_RS09315
pap
SYNPCC7002_RS09345
D_870
D_871
D_872
D_873
D_874
D_1788
D_1787
D_1786
Dc_1259
Dc_1260
Dc_1261
Dc_1262
Dc_1263
Dc_1264
Dc_1265
Dc_2563
Dc_2562
Dc_2561
I_1700
I_1701
I_1702
I_1703
I_1704
I_1705
I_1706
I_1707
I_1708
I_1709
I_3452
I_3451
I_3450
Term: termination (1), NS: new start (0), PS: pausing site (0), iTSS_I: internal starting site (0)

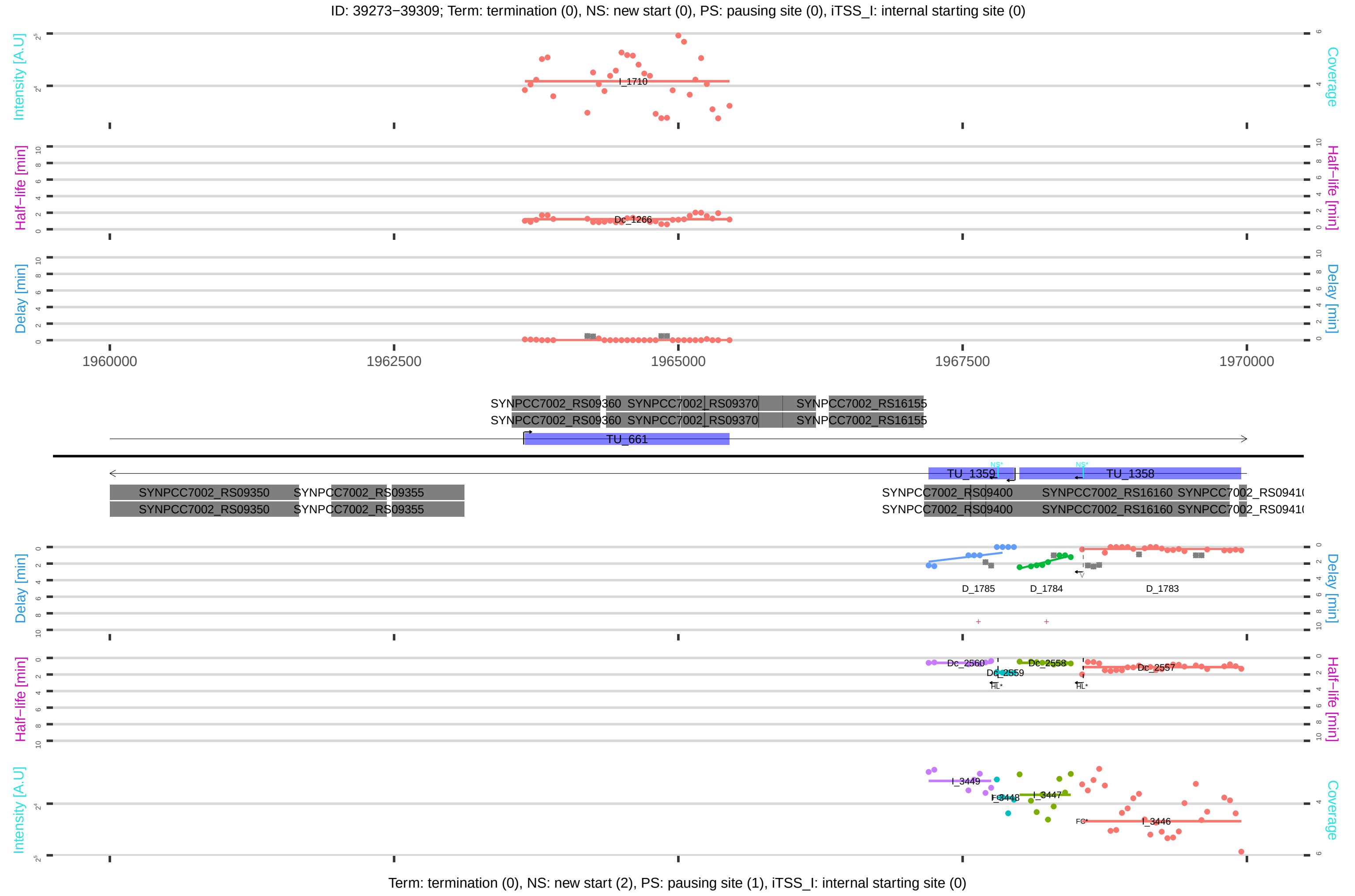

ID: 39273−39309; Term: termination (0), NS: new start (0), PS: pausing site (0), iTSS_I: internal starting site (0)
Intensity [A.U]
Coverage
I_1710
Half−life [min]
Dc_1266
Delay [min]
1960000
1962500
1965000
1967500
1970000
SYNPCC7002_RS09360
SYNPCC7002_RS09370
SYNPCC7002_RS16155
TU_661
TU_1359
TU_1358
NS*
SYNPCC7002_RS09350
SYNPCC7002_RS09355
SYNPCC7002_RS09400
SYNPCC7002_RS16160
D_1785
D_1784
D_1783
Dc_2560
Dc_2559
Dc_2558
Dc_2557
HL*
I_3449
I_3448
I_3447
I_3446
FC*
Term: termination (0), NS: new start (2), PS: pausing site (1), iTSS_I: internal starting site (0)

ID: 39412−39600; Term: termination (4), NS: new start (4), PS: pausing site (0), iTSS_I: internal starting site (0)

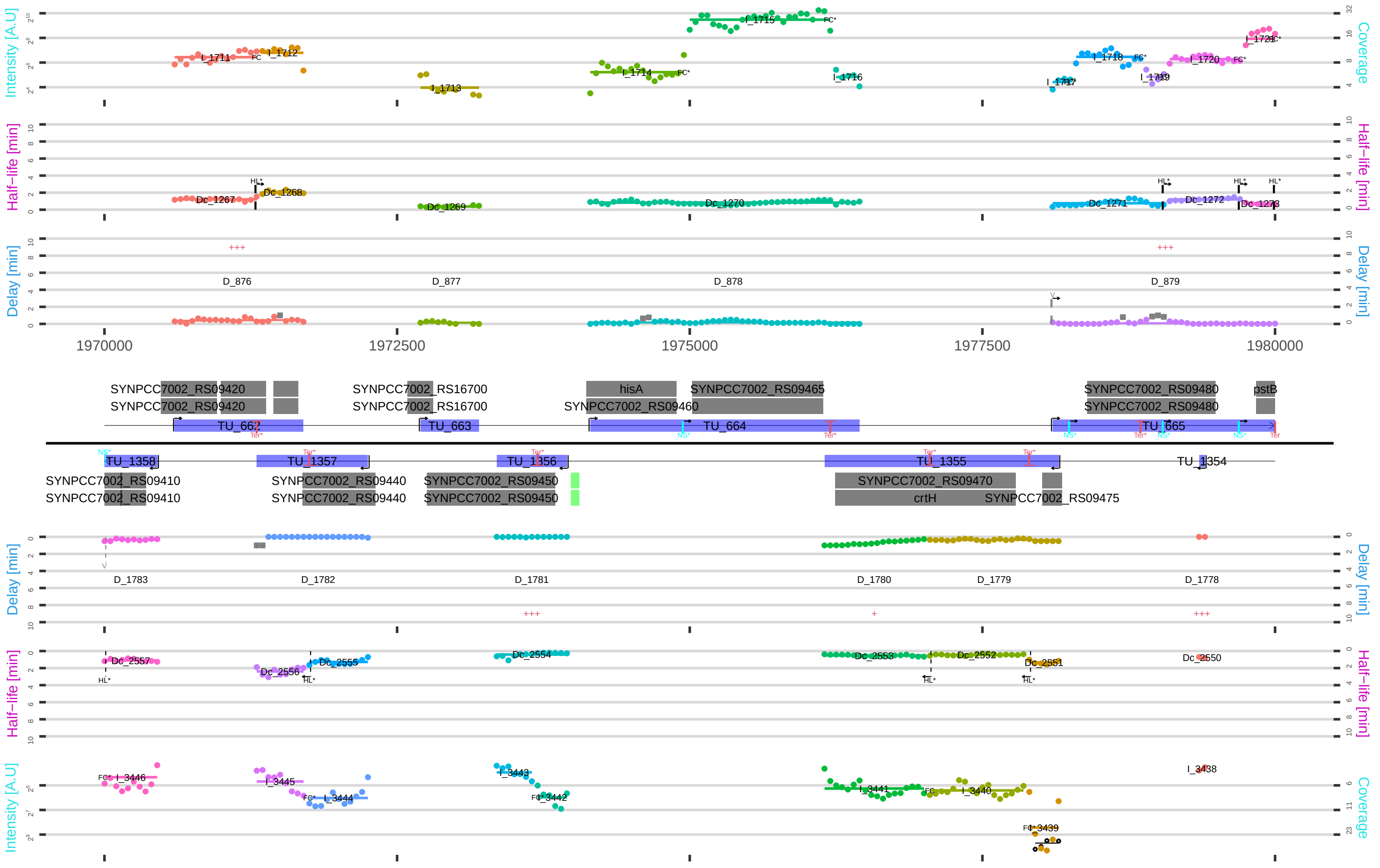

Term: termination (4), NS: new start (1), PS: pausing site (0), iTSS_I: internal starting site (1)

ID: 39600−39700; Term: termination (3), NS: new start (0), PS: pausing site (1), iTSS_I: internal starting site (0)

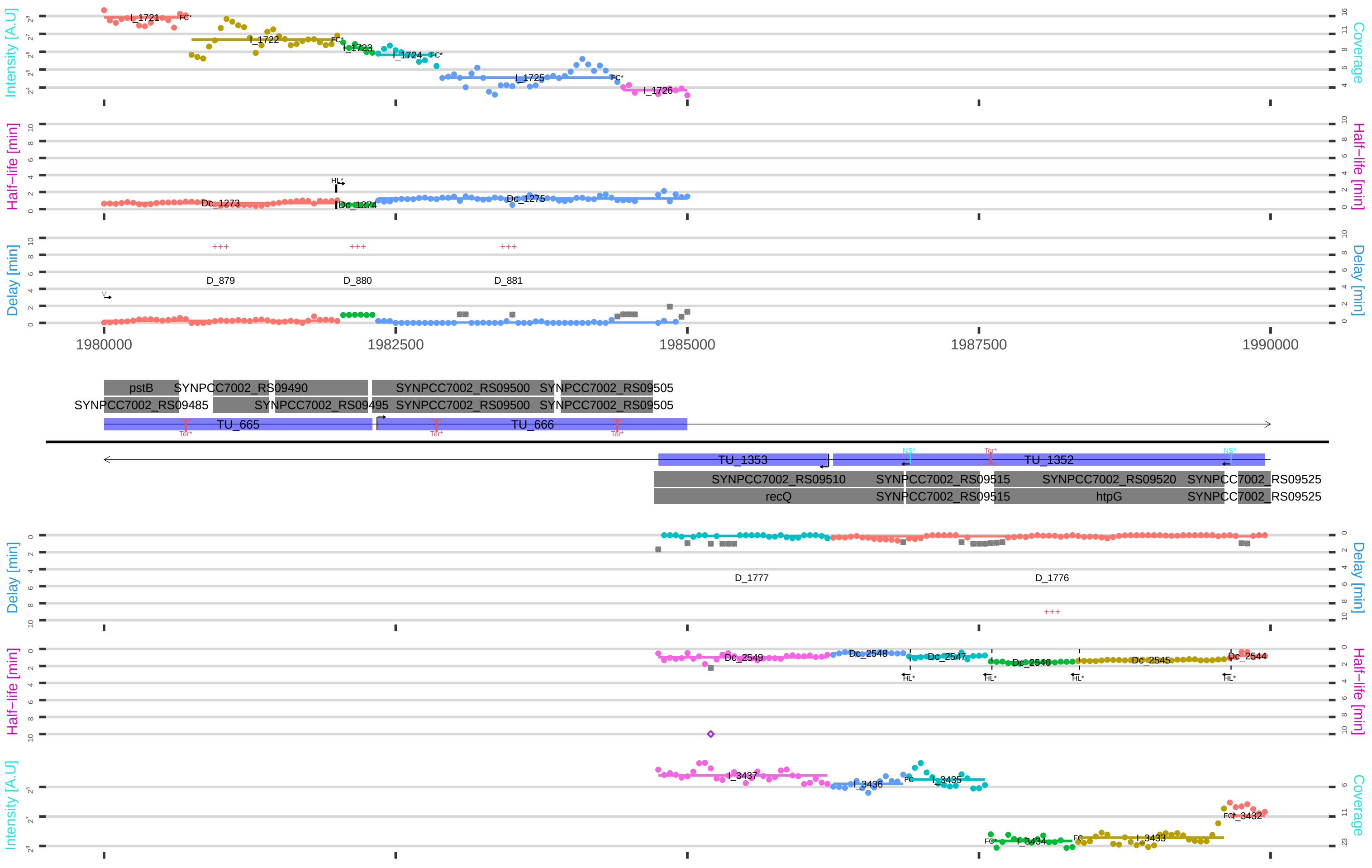

Term: termination (1), NS: new start (2), PS: pausing site (0), iTSS_I: internal starting site (0)

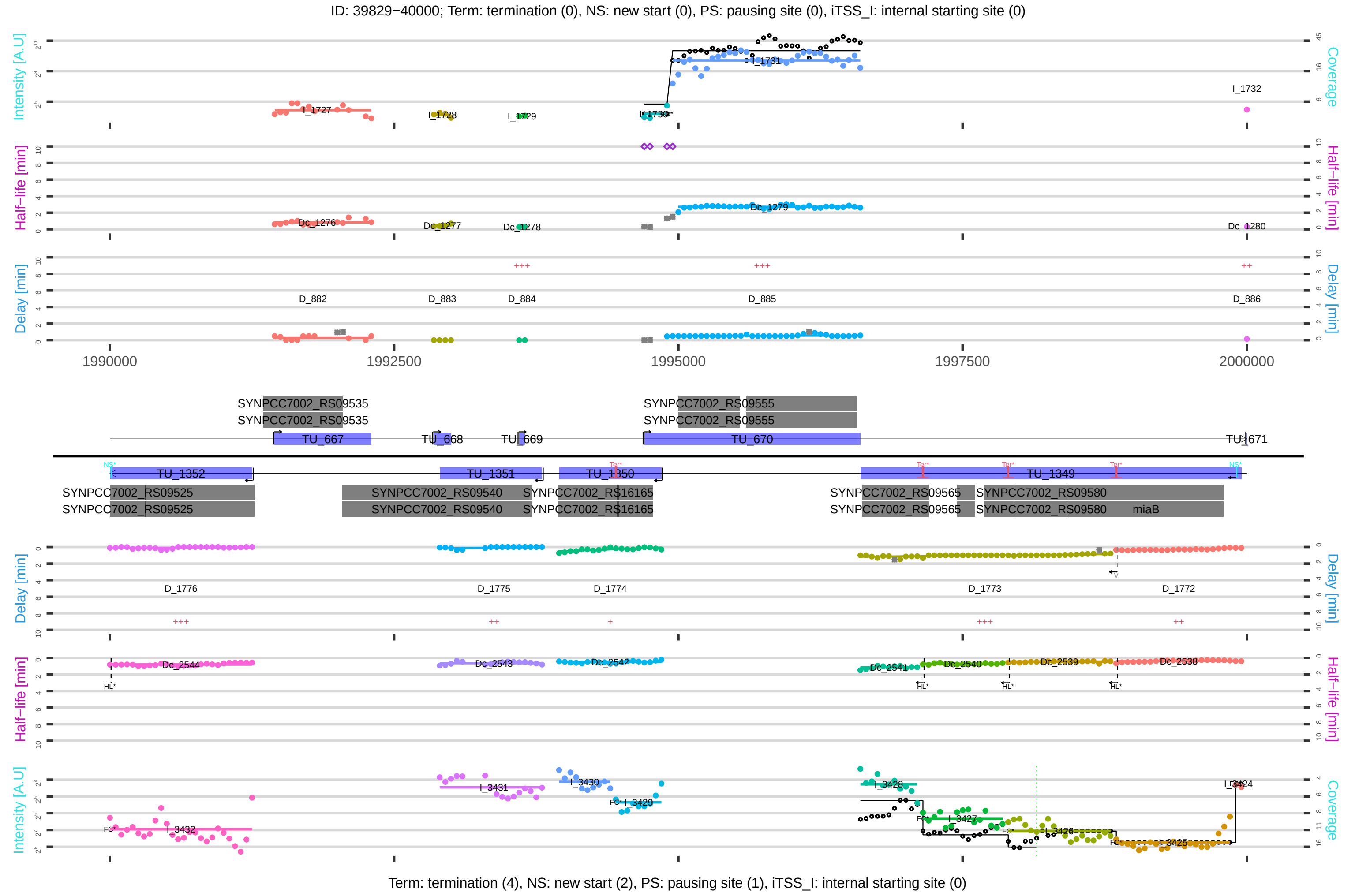

ID: 39829−40000; Term: termination (0), NS: new start (0), PS: pausing site (0), iTSS_I: internal starting site (0)
Intensity [A.U]
Coverage
Half−life [min]
Delay [min]
1990000
1992500
1995000
1997500
2000000
TU_667
TU_668
TU_669
TU_670
TU_671
TU_1352
TU_1351
TU_1350
TU_1349
SYNPCC7002_RS09535
SYNPCC7002_RS09555
SYNPCC7002_RS09525
SYNPCC7002_RS09540
SYNPCC7002_RS16165
SYNPCC7002_RS09565
SYNPCC7002_RS09580
miaB
Term: termination (4), NS: new start (2), PS: pausing site (1), iTSS_I: internal starting site (0)

ID: 40000−40200; Term: termination (6), NS: new start (2), PS: pausing site (3), iTSS_I: internal starting site (0)

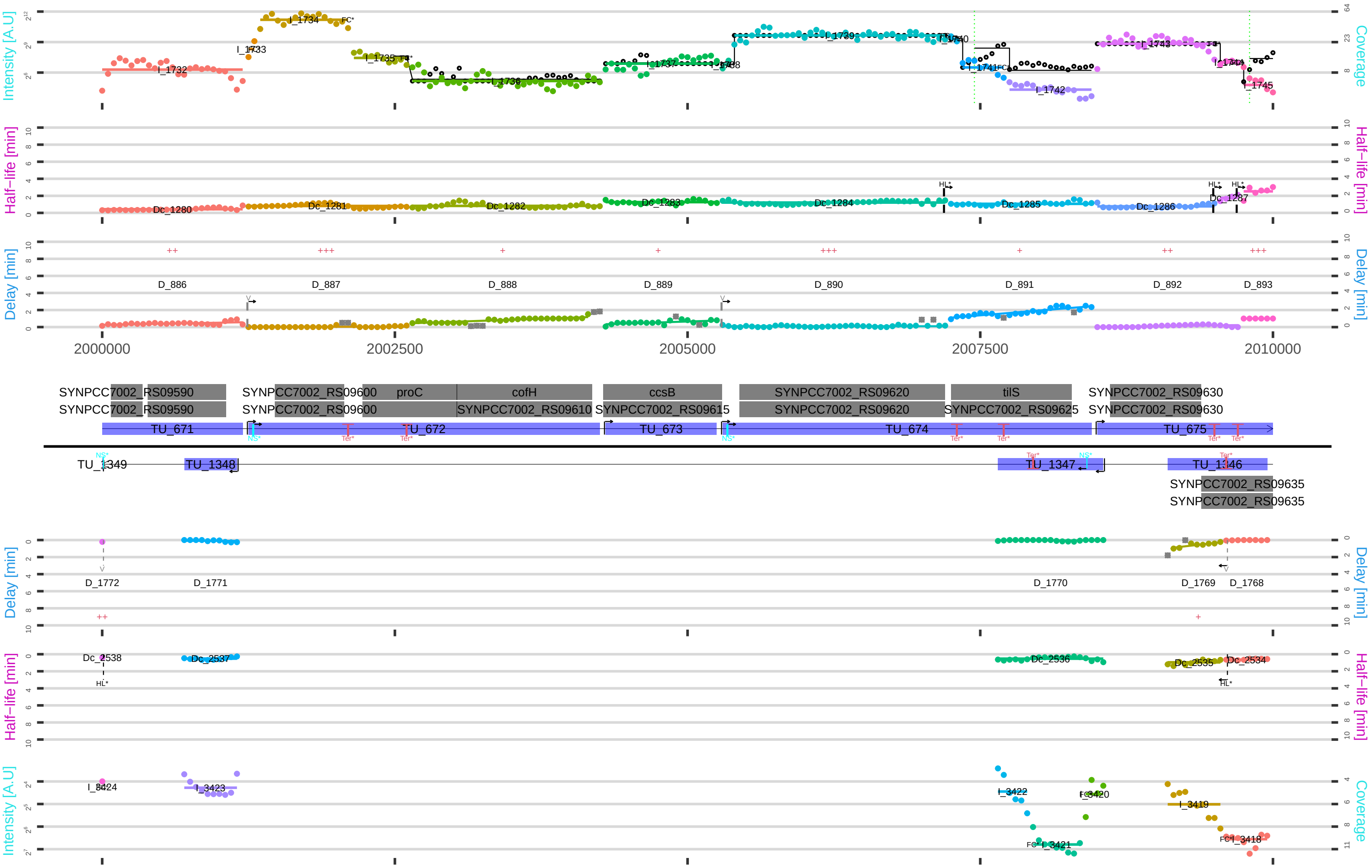

Term: termination (2), NS: new start (2), PS: pausing site (1), iTSS_I: internal starting site (0)

ID: 40200−40400; Term: termination (4), NS: new start (1), PS: pausing site (2), iTSS_I: internal starting site (0)

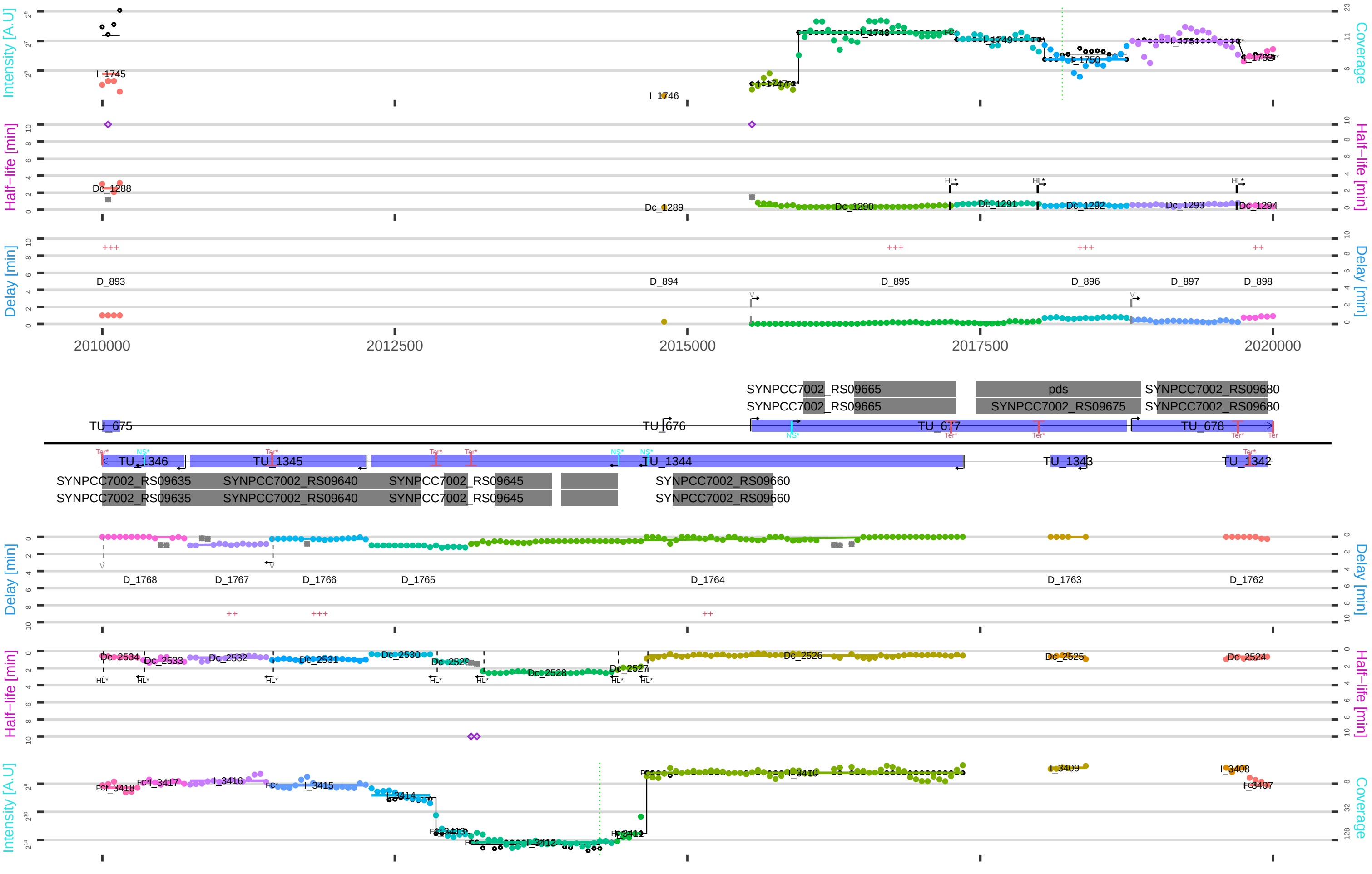

Term: termination (5), NS: new start (3), PS: pausing site (2), iTSS_I: internal starting site (0)

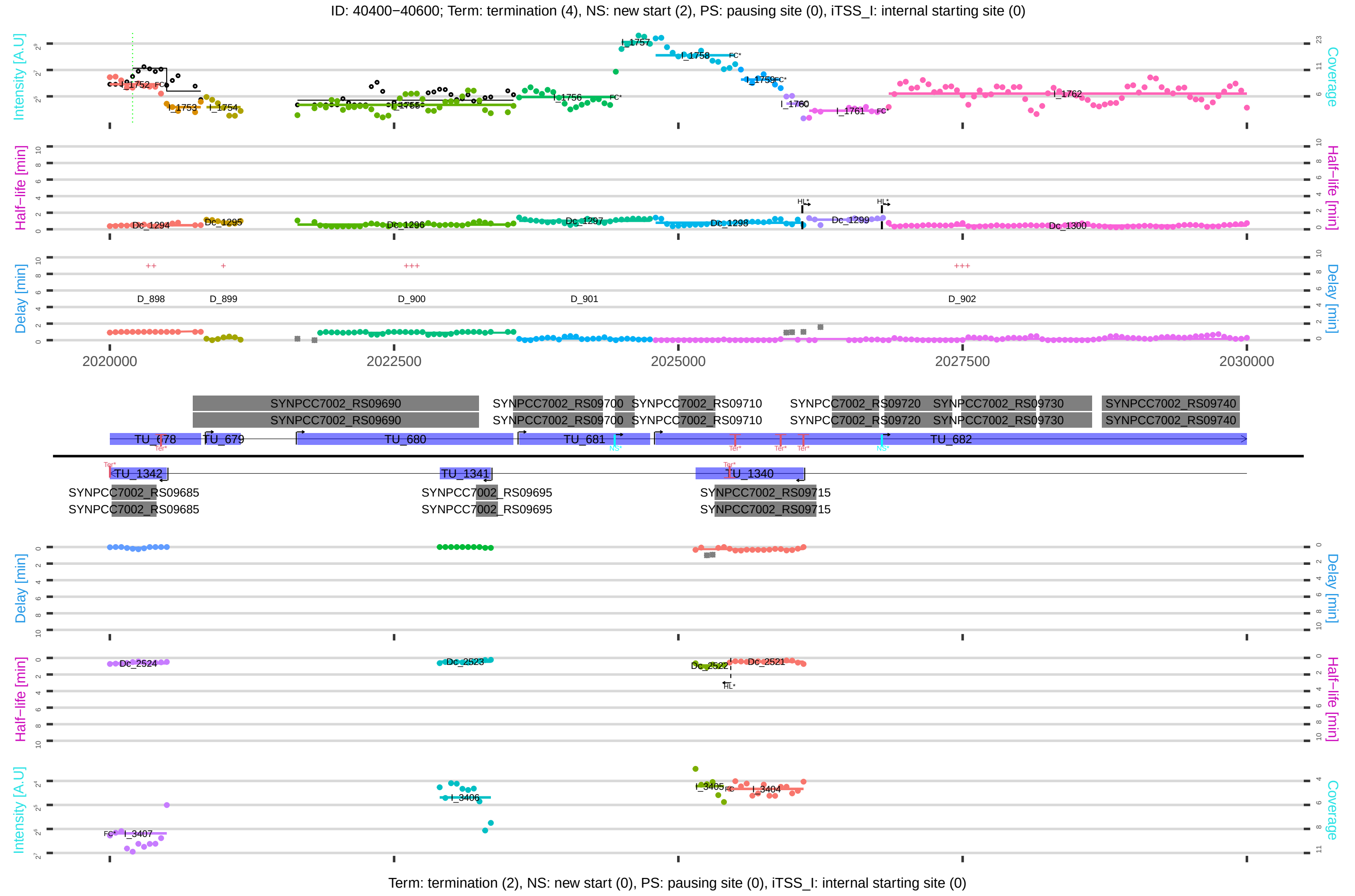
ID: 40400−40600; Term: termination (4), NS: new start (2), PS: pausing site (0), iTSS_I: internal starting site (0)
Intensity [A.U]
Coverage
Half−life [min]
Delay [min]
I_1752 FC
I_1753
I_1754
I_1755
I_1756 FC*
I_1757
I_1758 FC*
I_1759 FC*
I_1760
I_1761 FC*
I_1762
Dc_1294
Dc_1295
Dc_1296
Dc_1297
Dc_1298
Dc_1299
Dc_1300
HL*
D_898
D_899
D_900
D_901
D_902
2020000
2022500
2025000
2027500
2030000
SYNPCC7002_RS09690
SYNPCC7002_RS09700
SYNPCC7002_RS09710
SYNPCC7002_RS09720
SYNPCC7002_RS09730
SYNPCC7002_RS09740
TU_678
TU_679
TU_680
TU_681
TU_682
Ter*
NS*
TU_1342
TU_1341
TU_1340
SYNPCC7002_RS09685
SYNPCC7002_RS09695
SYNPCC7002_RS09715
Dc_2524
Dc_2523
Dc_2522
Dc_2521
I_3407 FC*
I_3406
I_3405 FC
I_3404
Term: termination (2), NS: new start (0), PS: pausing site (0), iTSS_I: internal starting site (0)

ID: 40600−40800; Term: termination (9), NS: new start (3), PS: pausing site (2), iTSS_I: internal starting site (0)

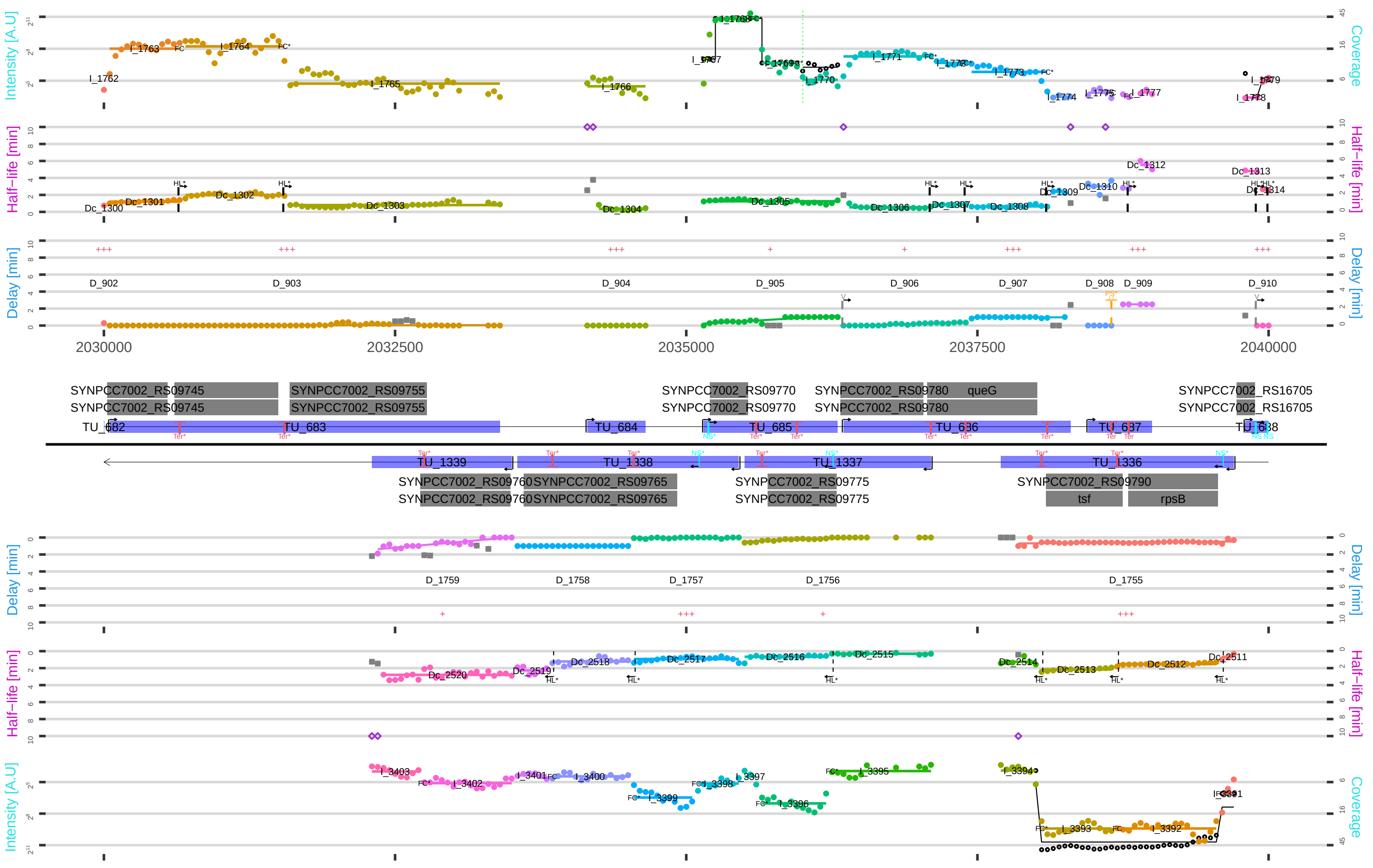

Term: termination (6), NS: new start (3), PS: pausing site (1), iTSS_I: internal starting site (0)

ID: 40800−40993; Term: termination (5), NS: new start (4), PS: pausing site (2), iTSS_I: internal starting site (0)

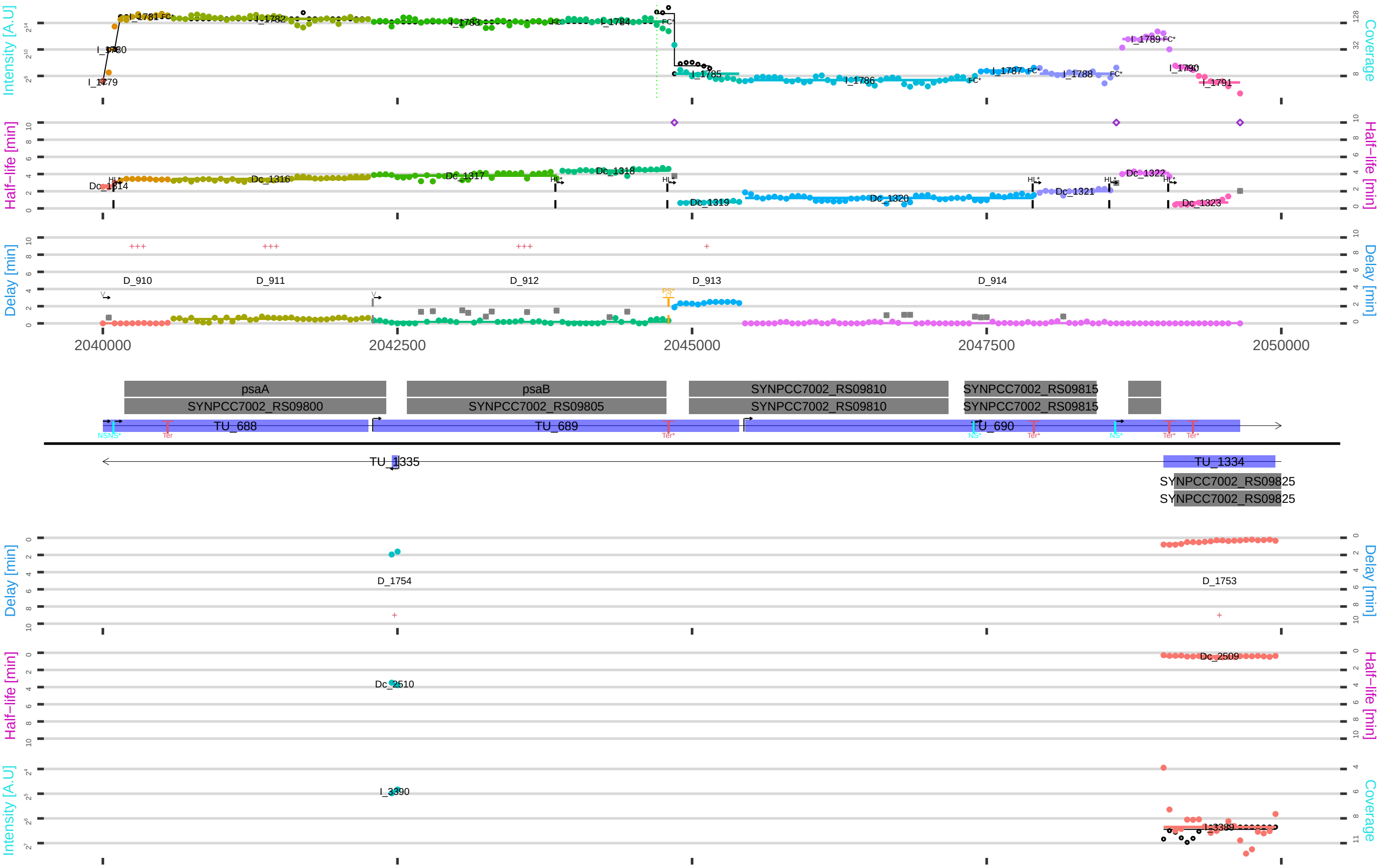

Term: termination (0), NS: new start (0), PS: pausing site (0), iTSS_I: internal starting site (0)

ID: 41008−41163; Term: termination (3), NS: new start (1), PS: pausing site (1), iTSS_I: internal starting site (0)

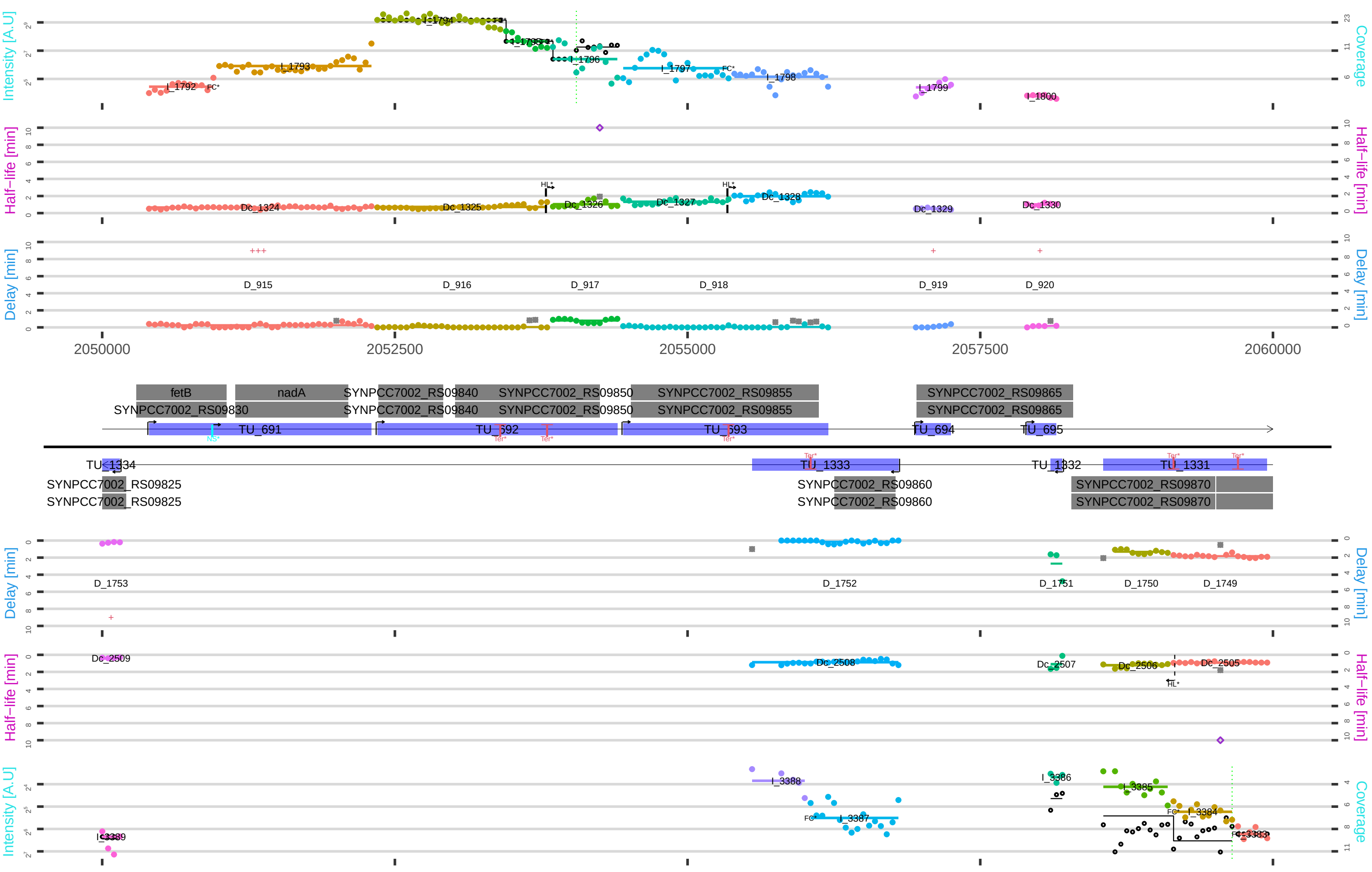

Term: termination (3), NS: new start (0), PS: pausing site (0), iTSS_I: internal starting site (1)

ID: 41206−41400; Term: termination (5), NS: new start (1), PS: pausing site (1), iTSS_I: internal starting site (0)

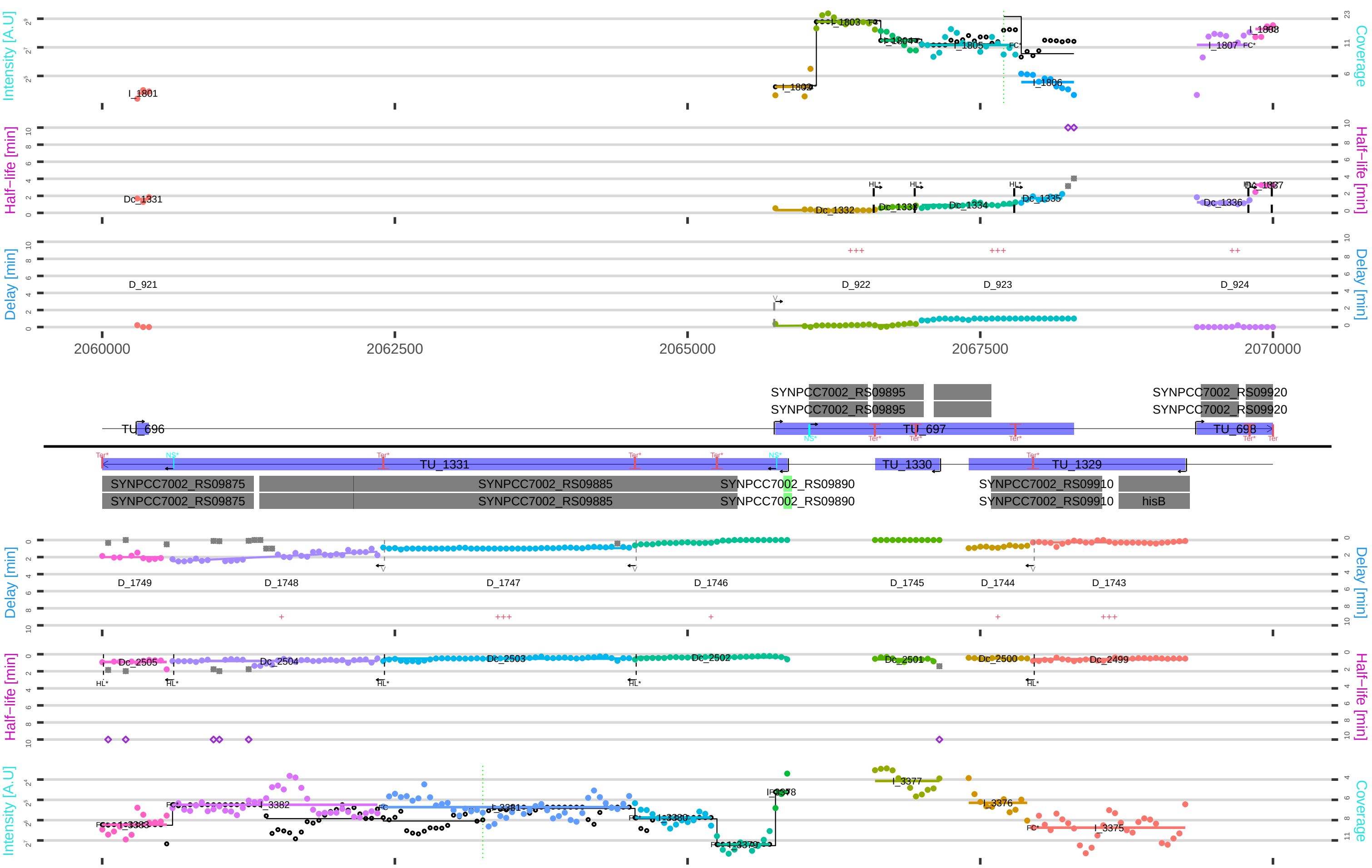

Term: termination (5), NS: new start (2), PS: pausing site (3), iTSS_I: internal starting site (1)

ID: 41400−41586; Term: termination (3), NS: new start (0), PS: pausing site (0), iTSS_I: internal starting site (0)

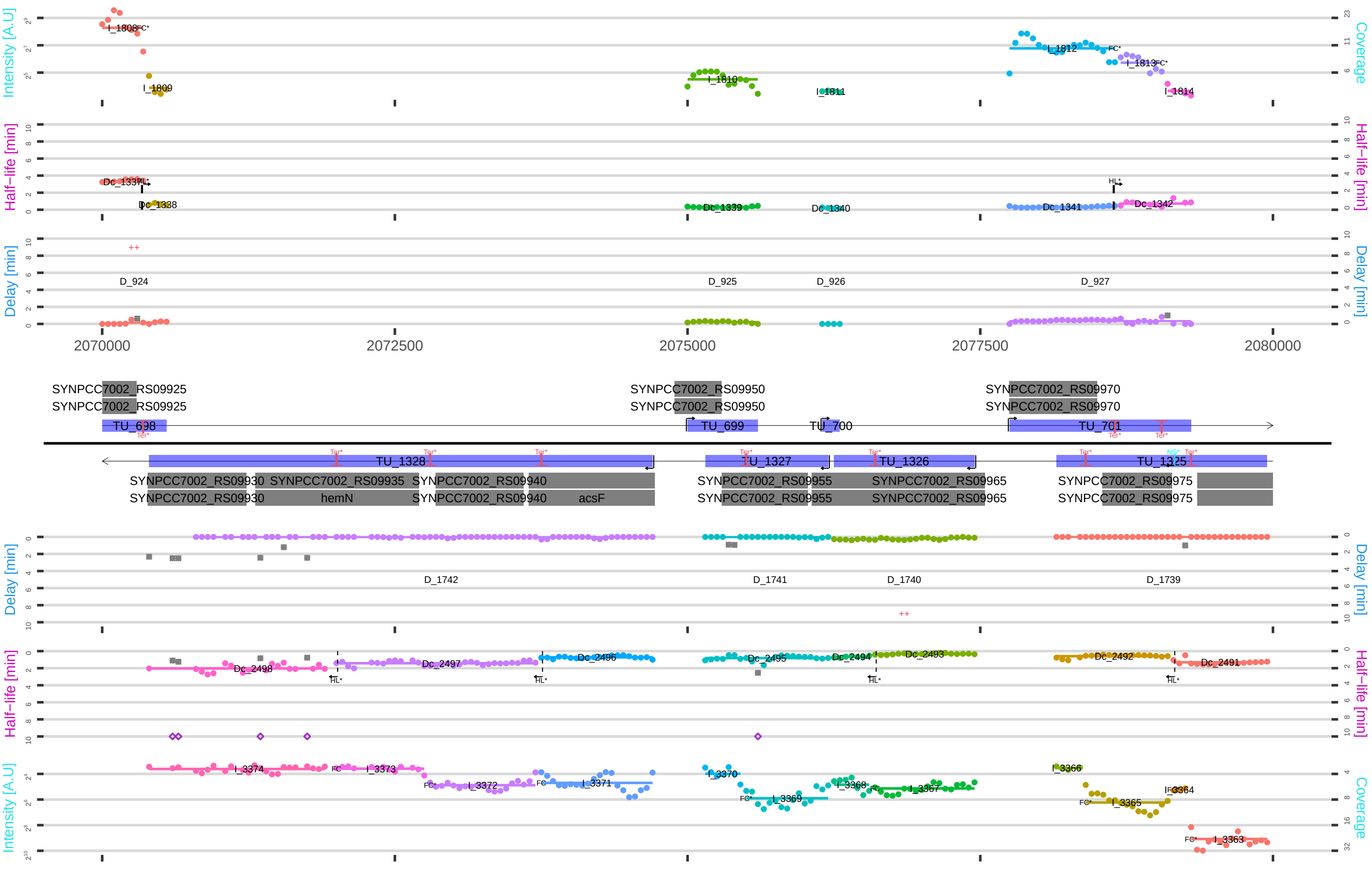

Term: termination (7), NS: new start (1), PS: pausing site (0), iTSS_I: internal starting site (0)

ID: 41622−41800; Term: termination (3), NS: new start (3), PS: pausing site (2), iTSS_I: internal starting site (0)

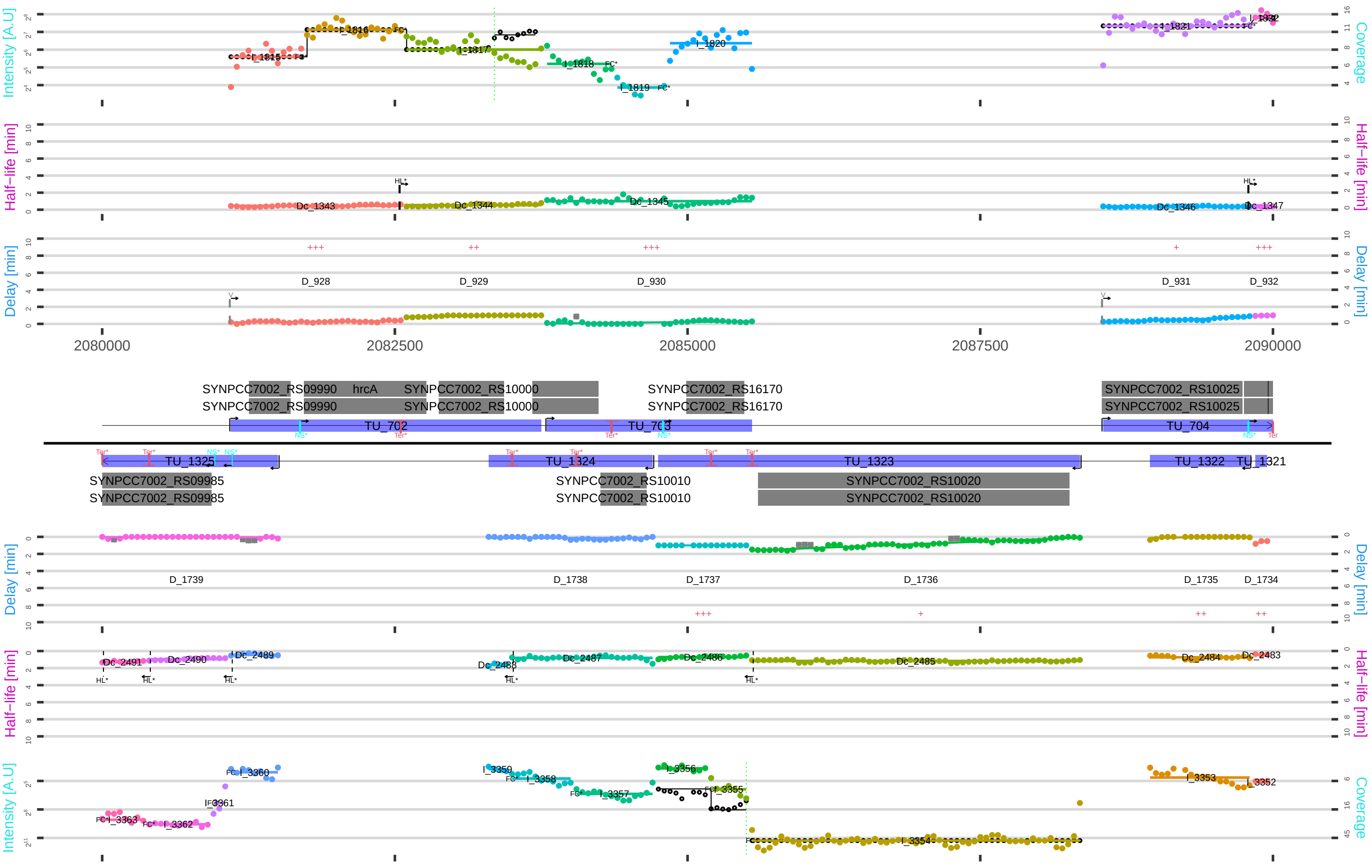

Term: termination (6), NS: new start (2), PS: pausing site (0), iTSS_I: internal starting site (1)

ID: 41800−41949; Term: termination (6), NS: new start (3), PS: pausing site (0), iTSS_I: internal starting site (0)

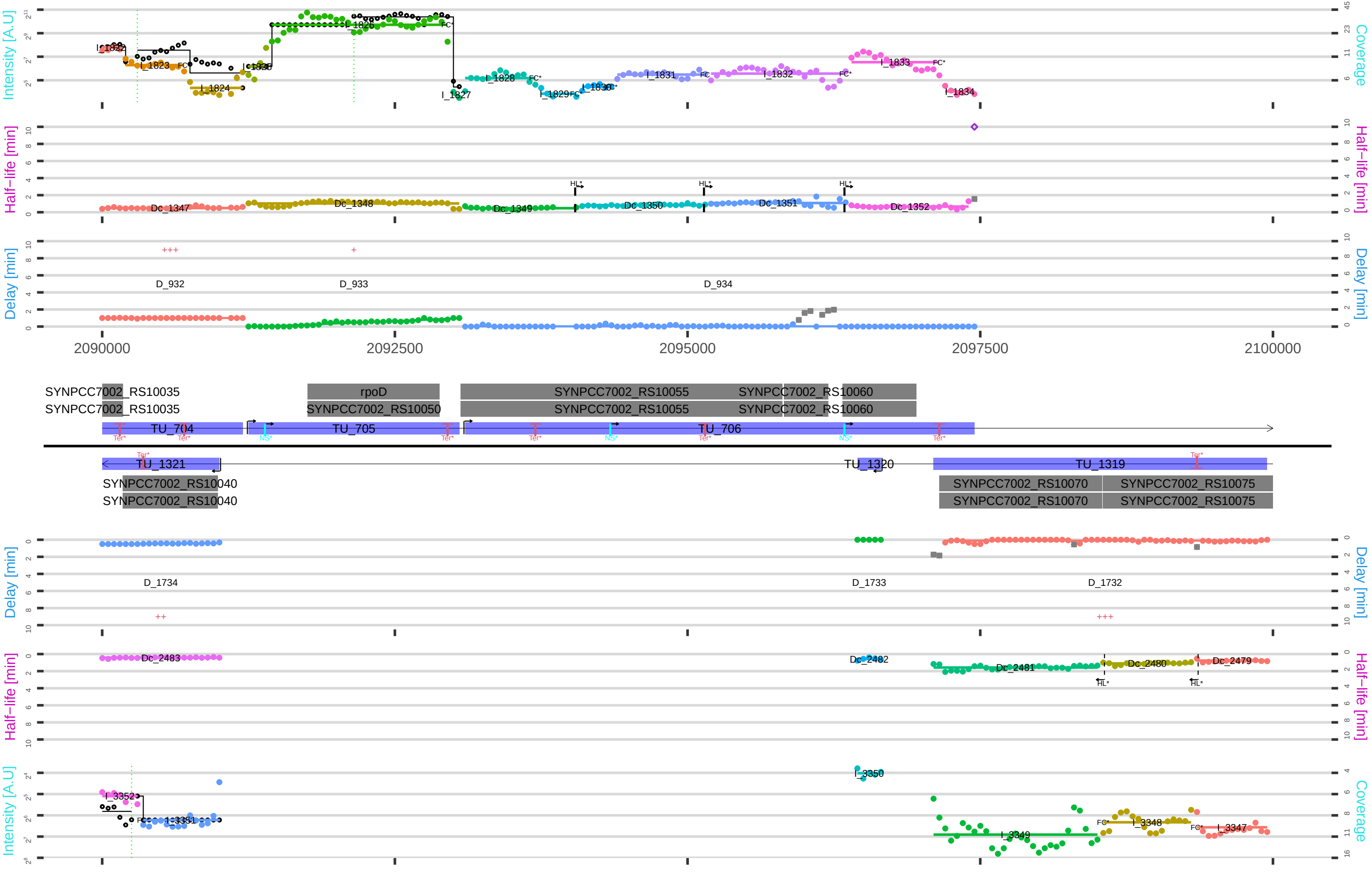

Term: termination (2), NS: new start (0), PS: pausing site (0), iTSS_I: internal starting site (0)

ID: 42045−42200; Term: termination (6), NS: new start (1), PS: pausing site (1), iTSS_I: internal starting site (0)

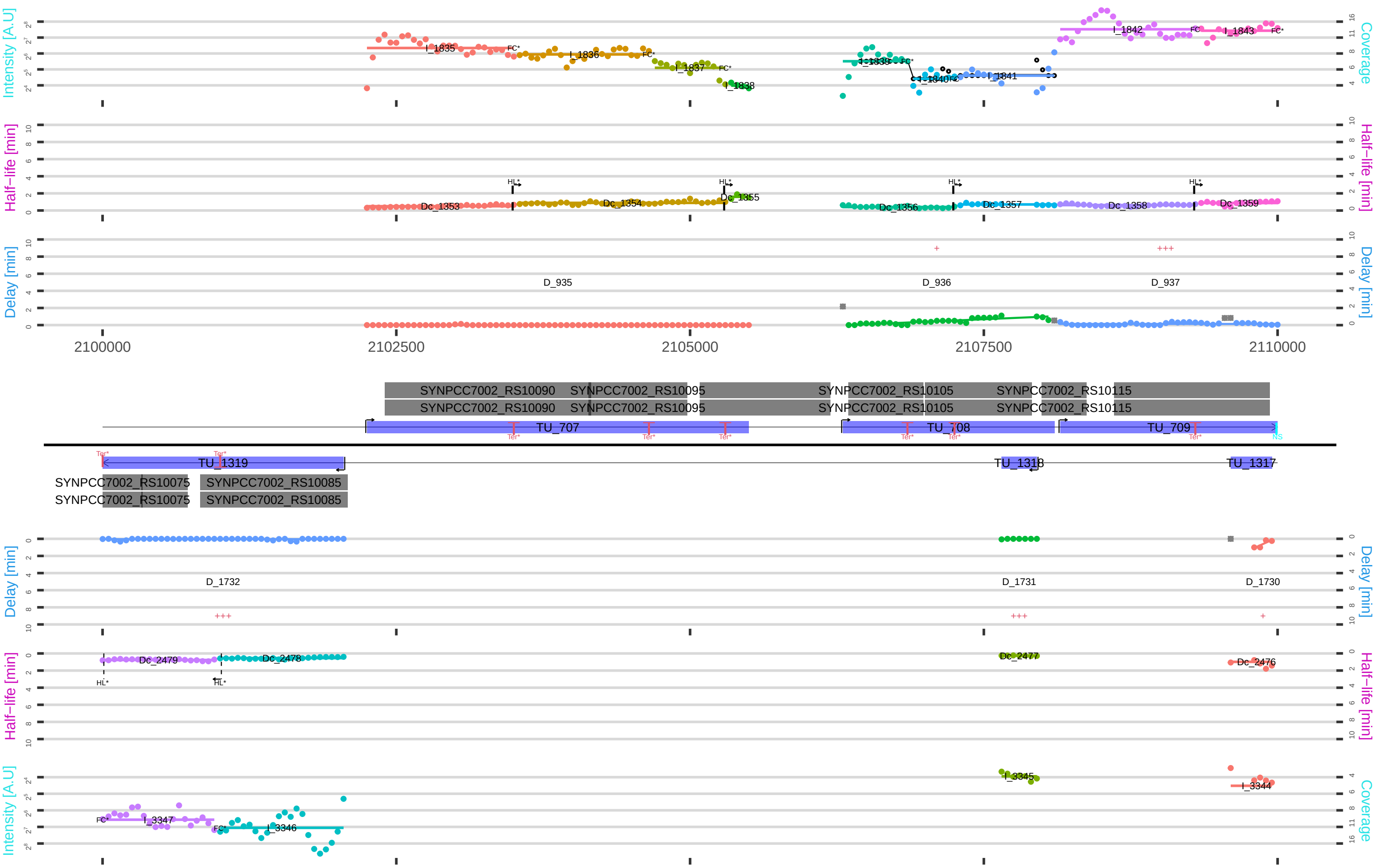

Term: termination (2), NS: new start (0), PS: pausing site (0), iTSS_I: internal starting site (0)

ID: 42200−42347; Term: termination (3), NS: new start (2), PS: pausing site (2), iTSS_I: internal starting site (0)

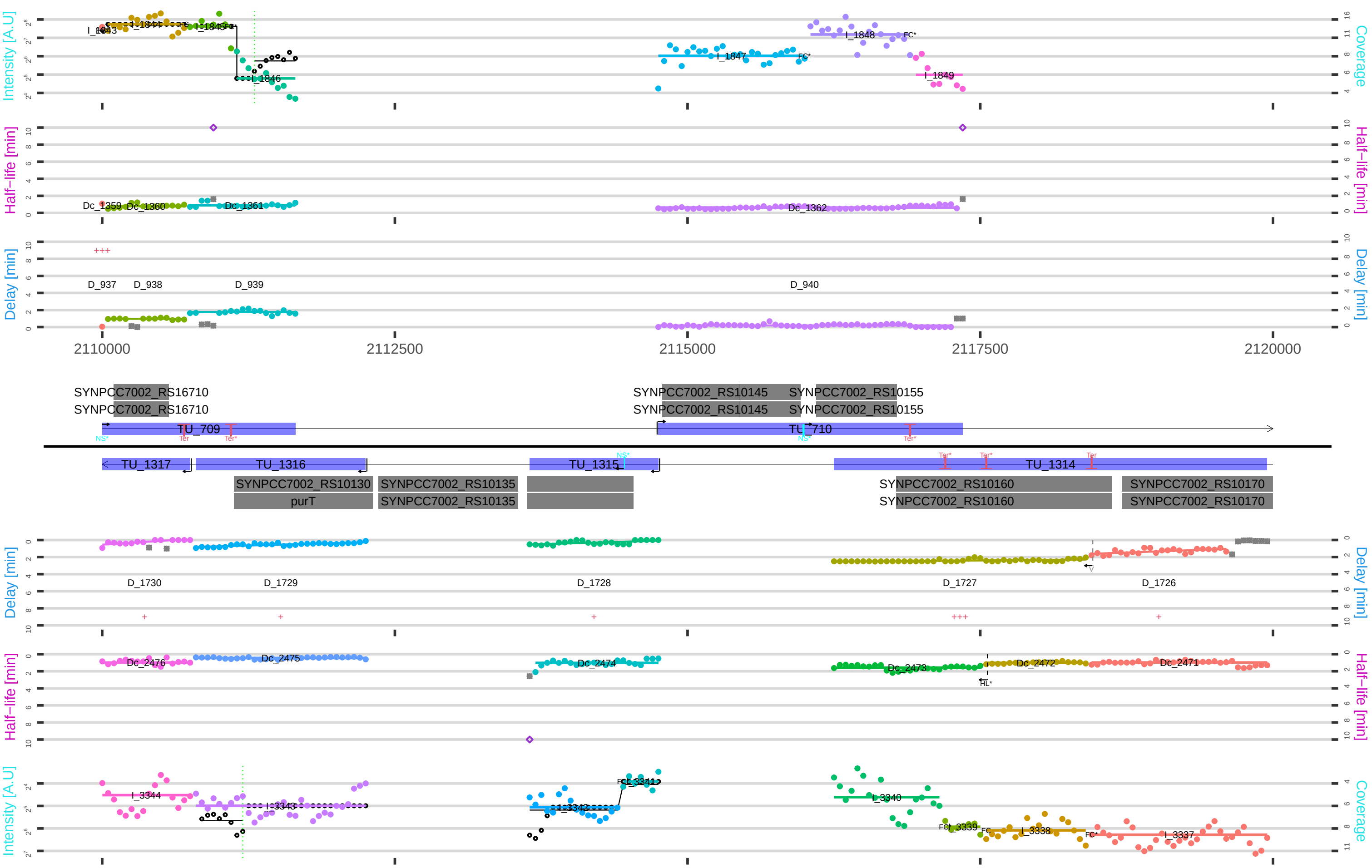

Term: termination (3), NS: new start (1), PS: pausing site (1), iTSS_I: internal starting site (0)

ID: 42443−42494; Term: termination (2), NS: new start (1), PS: pausing site (0), iTSS_I: internal starting site (0)

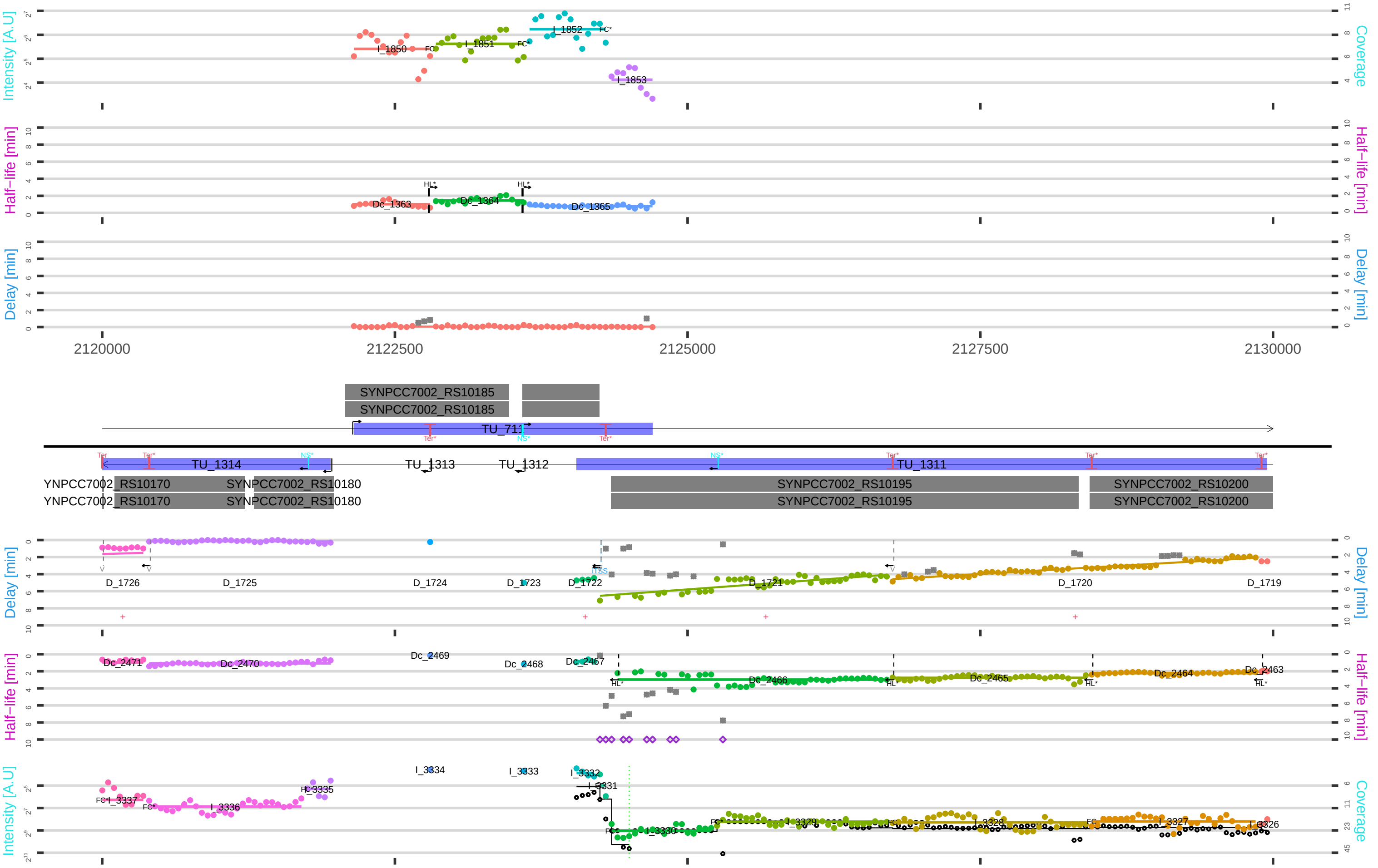

Term: termination (5), NS: new start (2), PS: pausing site (1), iTSS_I: internal starting site (3)

ID: 42707−42794; Term: termination (2), NS: new start (1), PS: pausing site (0), iTSS_I: internal starting site (0)

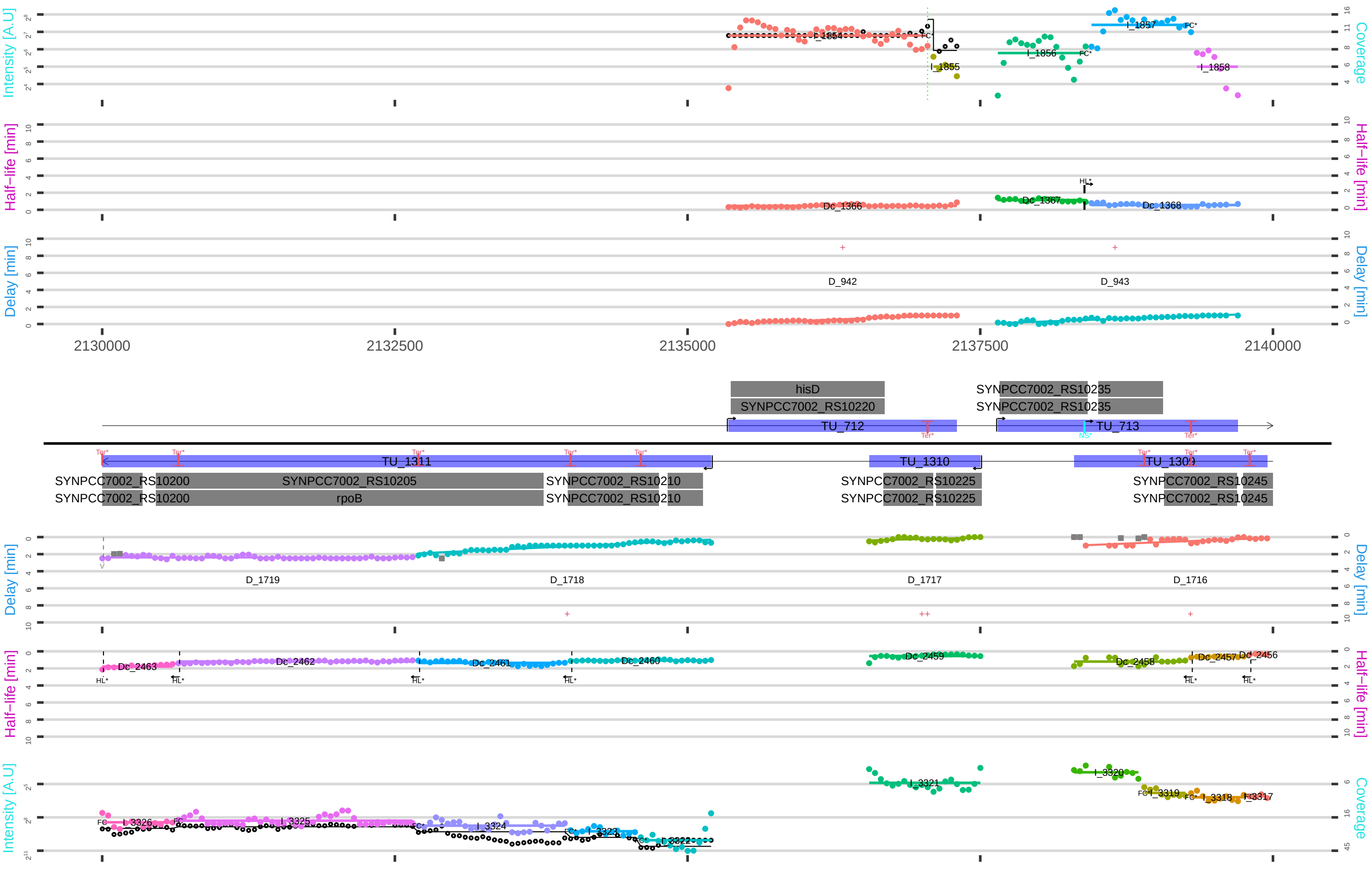

Term: termination (8), NS: new start (0), PS: pausing site (1), iTSS_I: internal starting site (0)

ID: 42809−42991; Term: termination (1), NS: new start (0), PS: pausing site (0), iTSS_I: internal starting site (0)

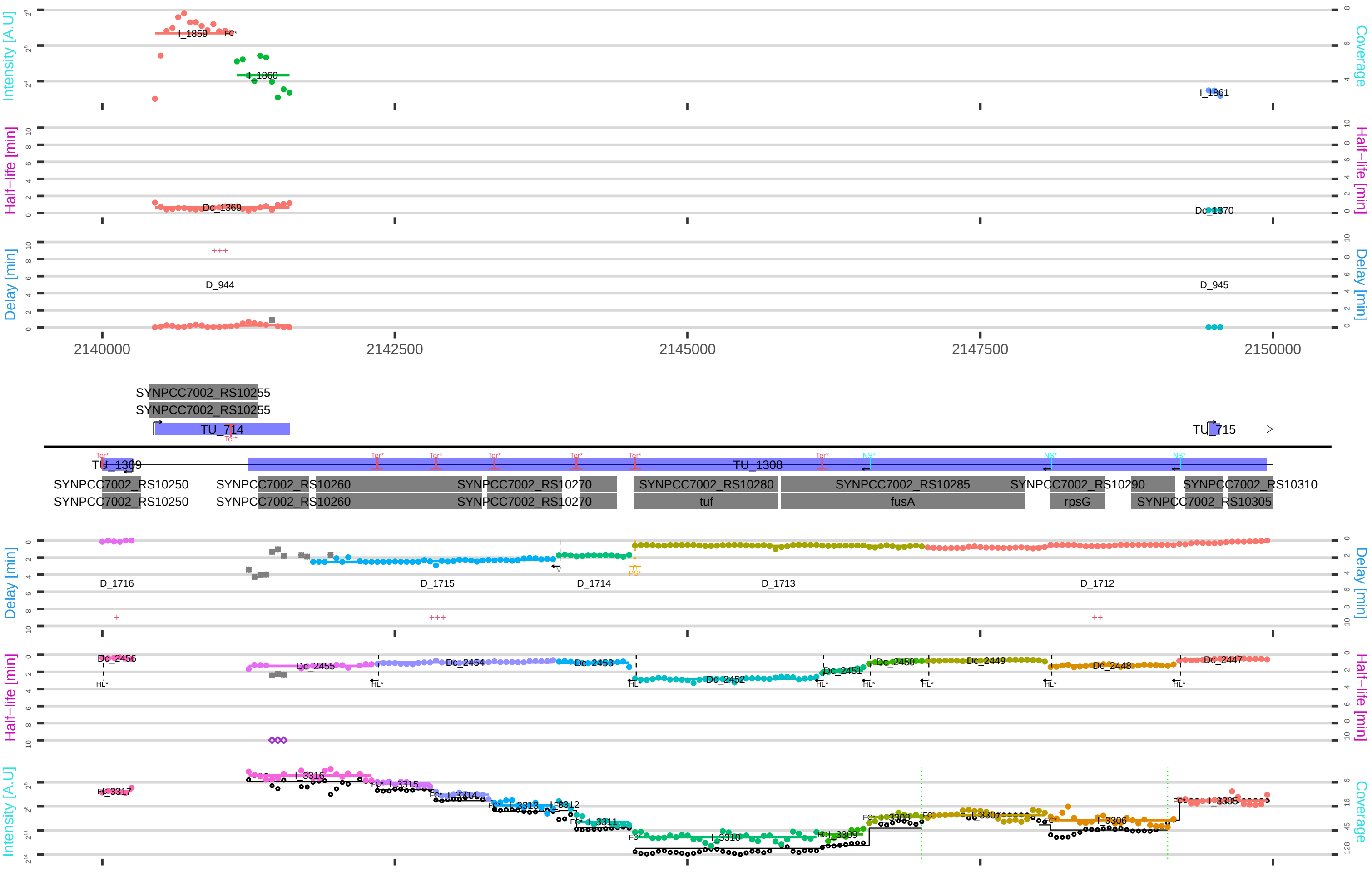

Term: termination (7), NS: new start (3), PS: pausing site (2), iTSS_I: internal starting site (1)

ID: 43020−43200; Term: termination (3), NS: new start (1), PS: pausing site (1), iTSS_I: internal starting site (0)

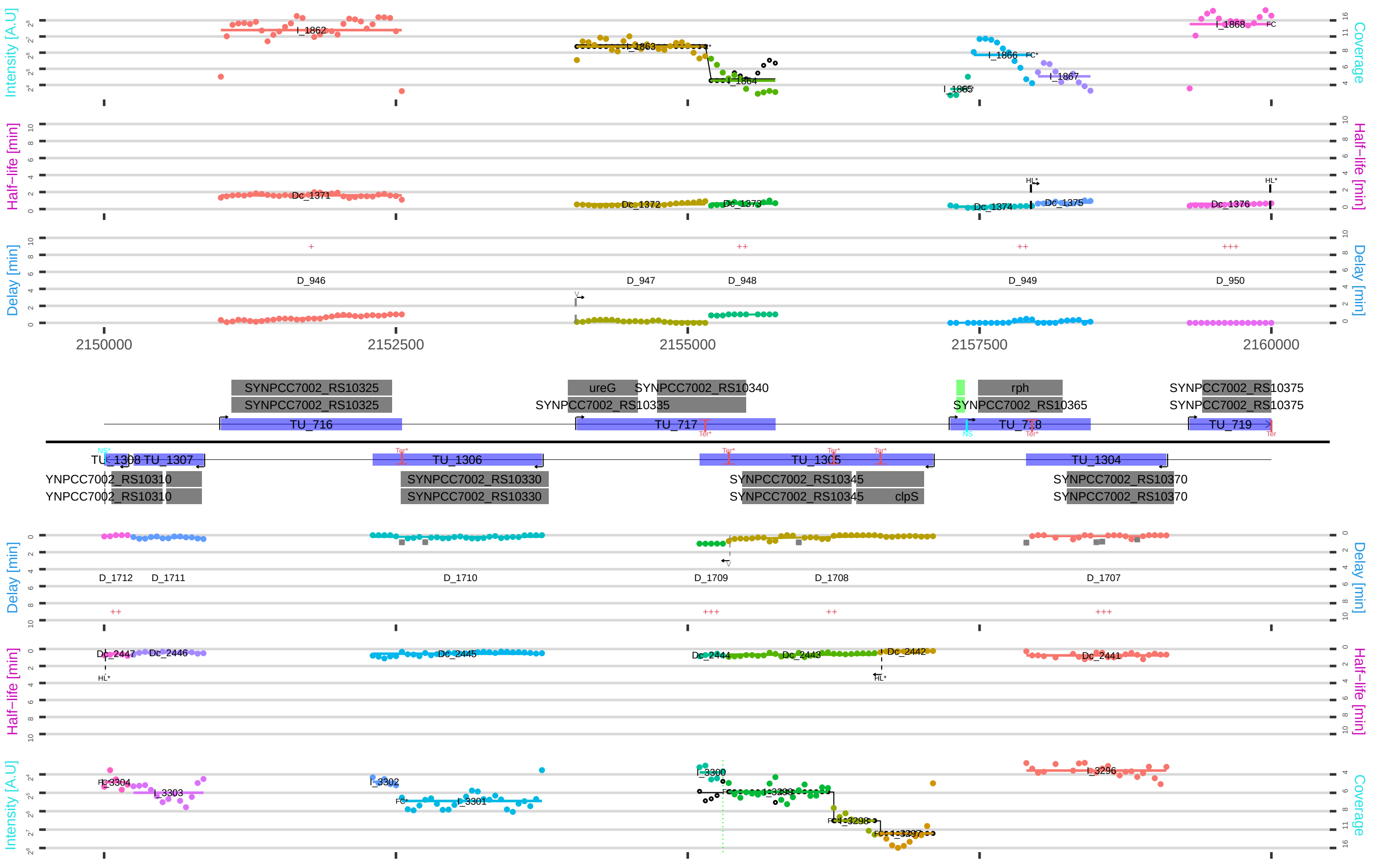

Term: termination (4), NS: new start (1), PS: pausing site (1), iTSS_I: internal starting site (0)

ID: 43200−43343; Term: termination (4), NS: new start (0), PS: pausing site (0), iTSS_I: internal starting site (0)

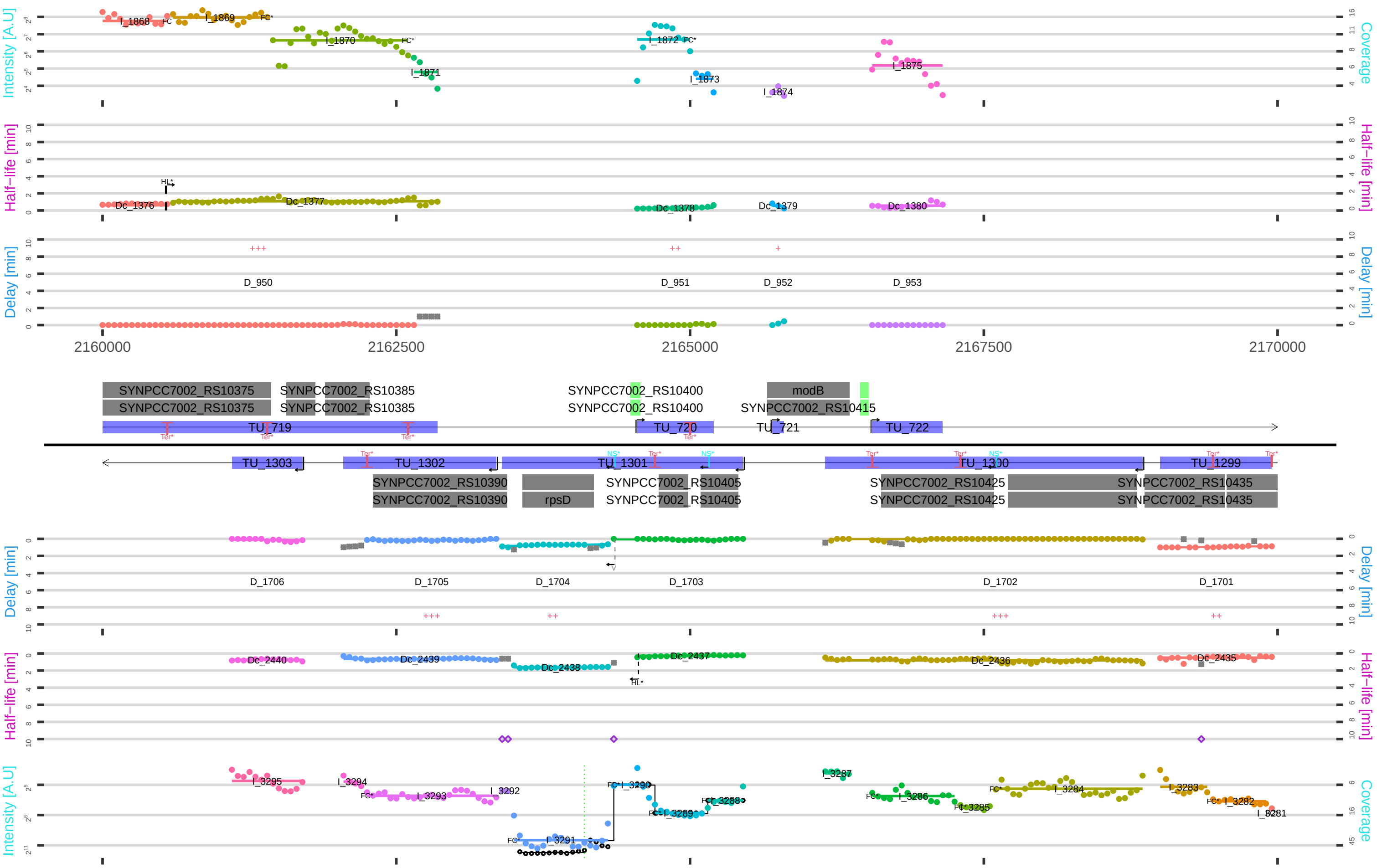

Term: termination (6), NS: new start (3), PS: pausing site (1), iTSS_I: internal starting site (0)

ID: 43432−43600; Term: termination (3), NS: new start (1), PS: pausing site (0), iTSS_I: internal starting site (0)

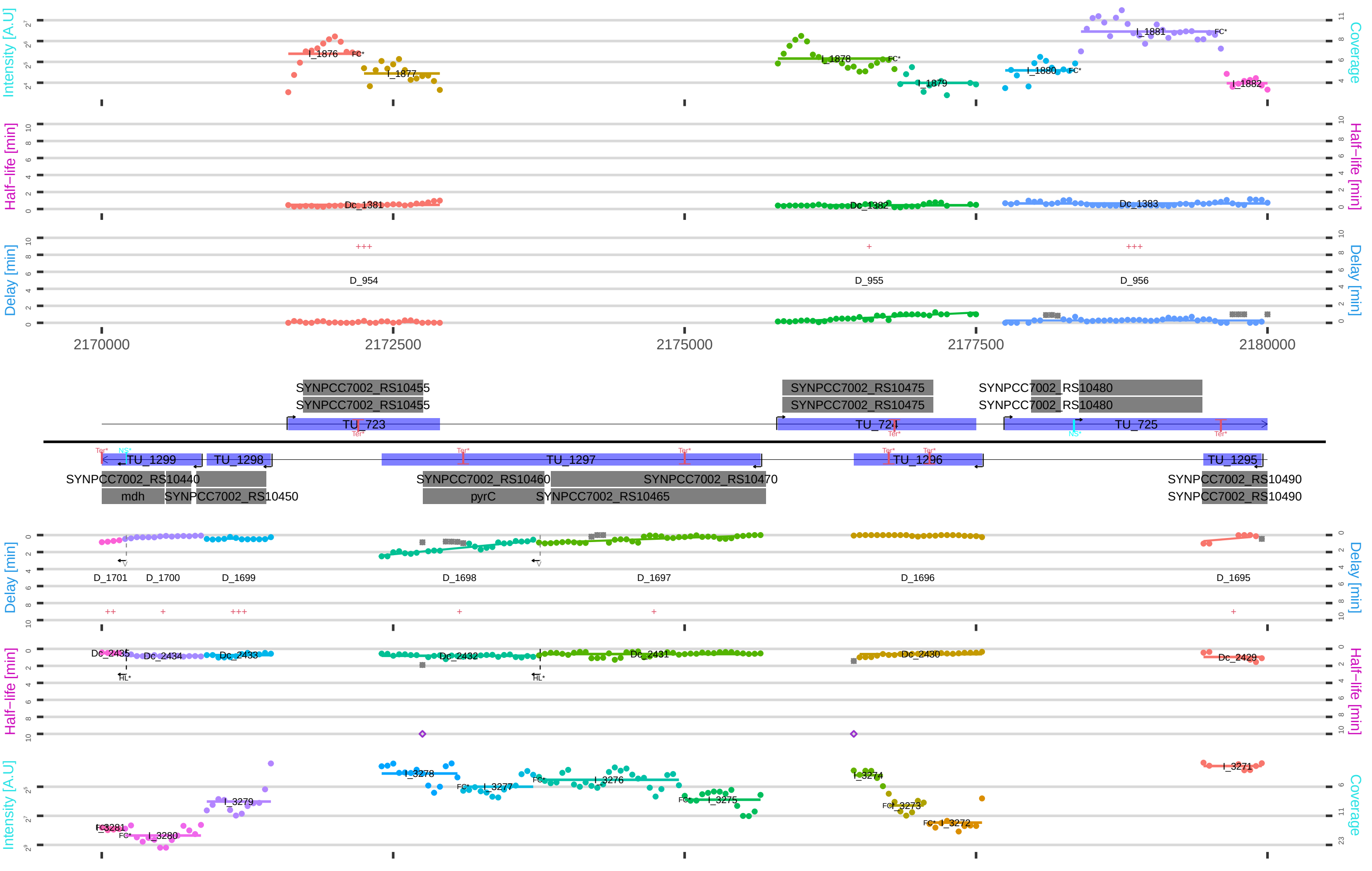

Term: termination (5), NS: new start (1), PS: pausing site (1), iTSS_I: internal starting site (1)

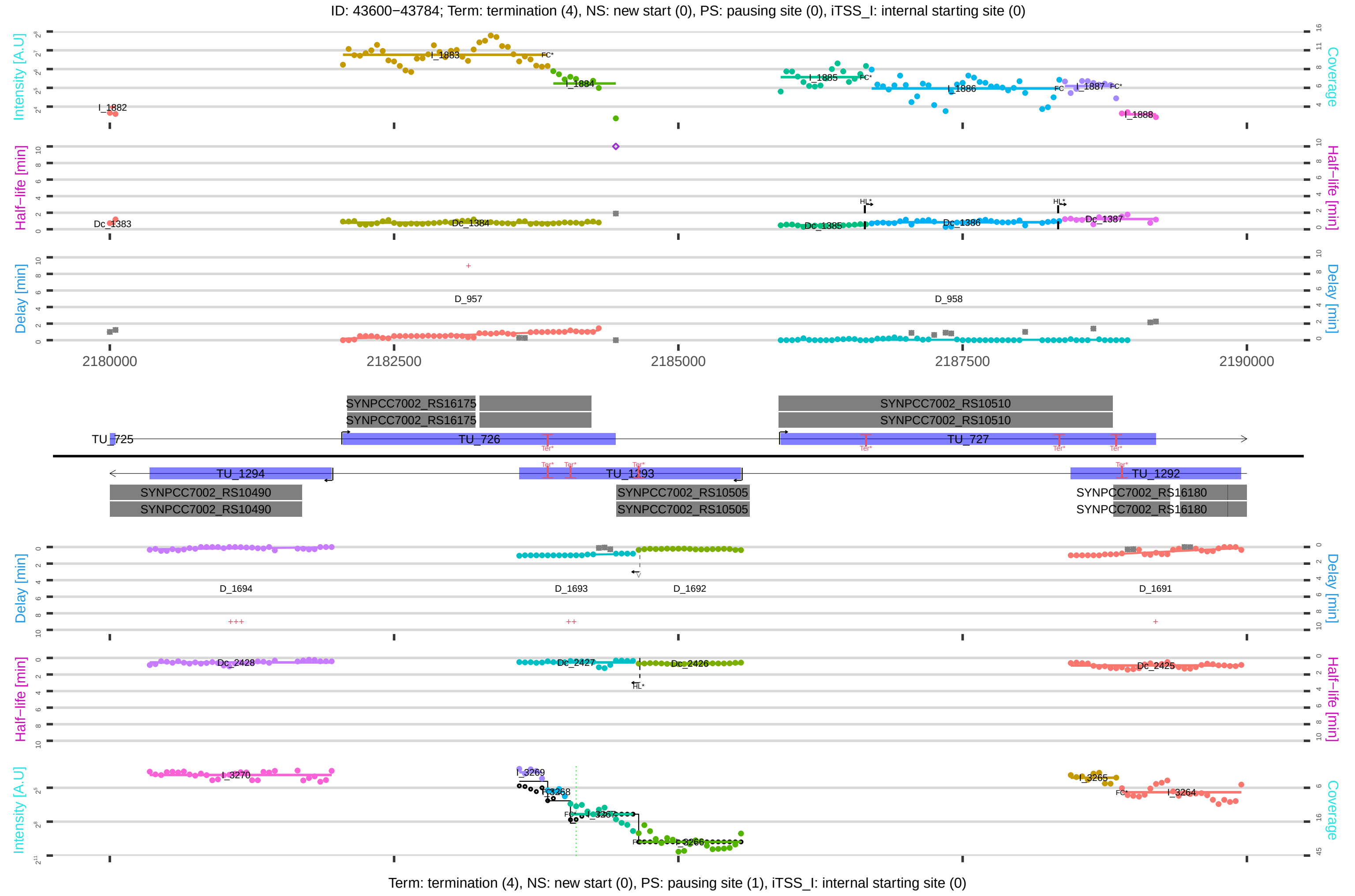
ID: 43600−43784; Term: termination (4), NS: new start (0), PS: pausing site (0), iTSS_I: internal starting site (0)
Intensity [A.U]
Coverage
Half−life [min]
Delay [min]
I_1882
I_1883
I_1884
I_1885
I_1886
I_1887
I_1888
Dc_1383
Dc_1384
Dc_1385
Dc_1386
Dc_1387
D_957
D_958
2180000
2182500
2185000
2187500
2190000
SYNPCC7002_RS16175
SYNPCC7002_RS10510
TU_725
TU_726
TU_727
TU_1294
TU_1293
TU_1292
SYNPCC7002_RS10490
SYNPCC7002_RS10505
SYNPCC7002_RS16180
D_1694
D_1693
D_1692
D_1691
Dc_2428
Dc_2427
Dc_2426
Dc_2425
I_3270
I_3269
I_3268
I_3267
I_3266
I_3265
I_3264
Term: termination (4), NS: new start (0), PS: pausing site (1), iTSS_I: internal starting site (0)

ID: 43812−44000; Term: termination (10), NS: new start (1), PS: pausing site (3), iTSS_I: internal starting site (0)

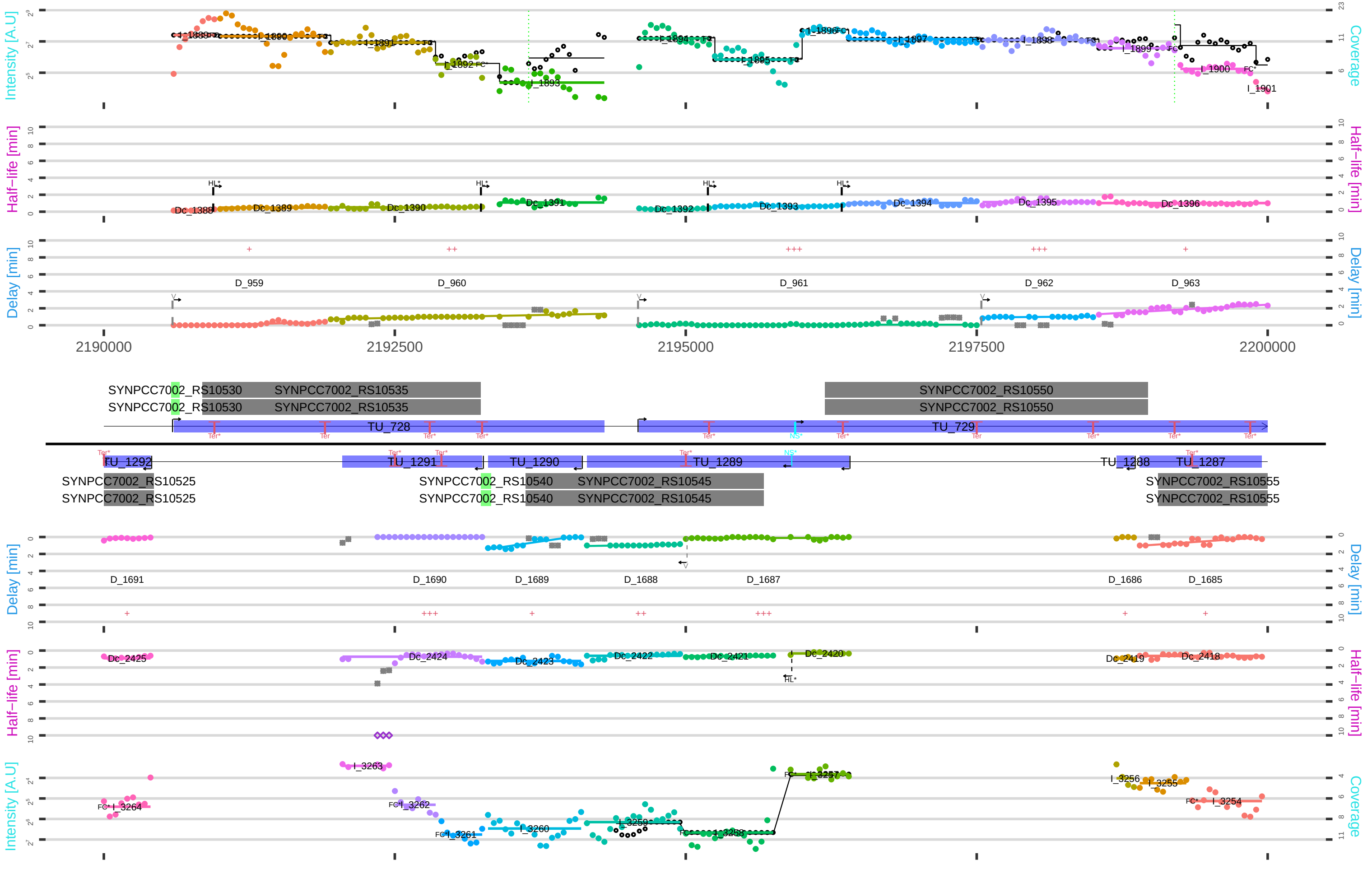

Term: termination (5), NS: new start (1), PS: pausing site (1), iTSS_I: internal starting site (0)

ID: 44000−44200; Term: termination (8), NS: new start (2), PS: pausing site (2), iTSS_I: internal starting site (0)

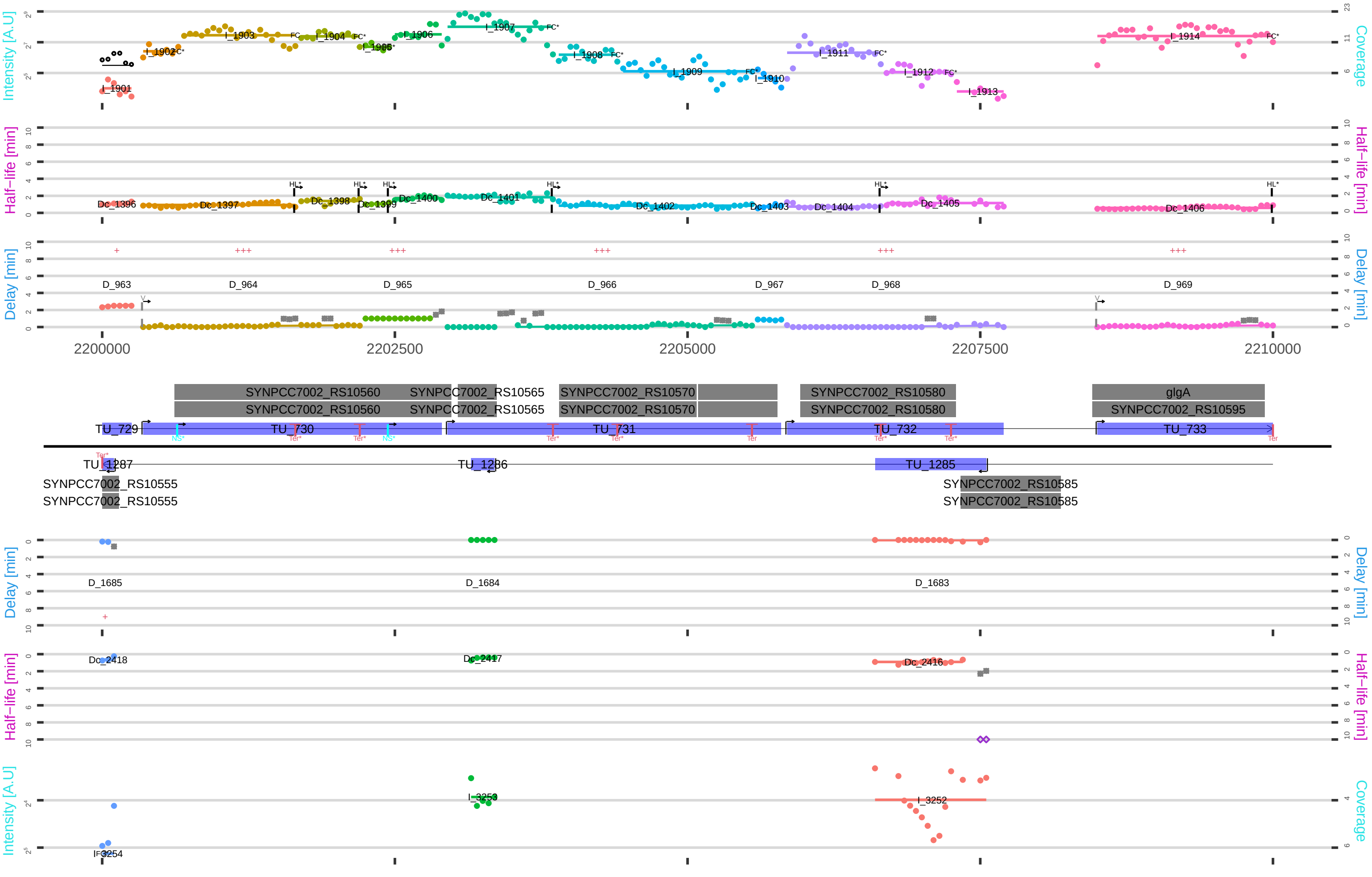

Term: termination (1), NS: new start (0), PS: pausing site (0), iTSS_I: internal starting site (0)

ID: 44200−44400; Term: termination (5), NS: new start (4), PS: pausing site (3), iTSS_I: internal starting site (0)

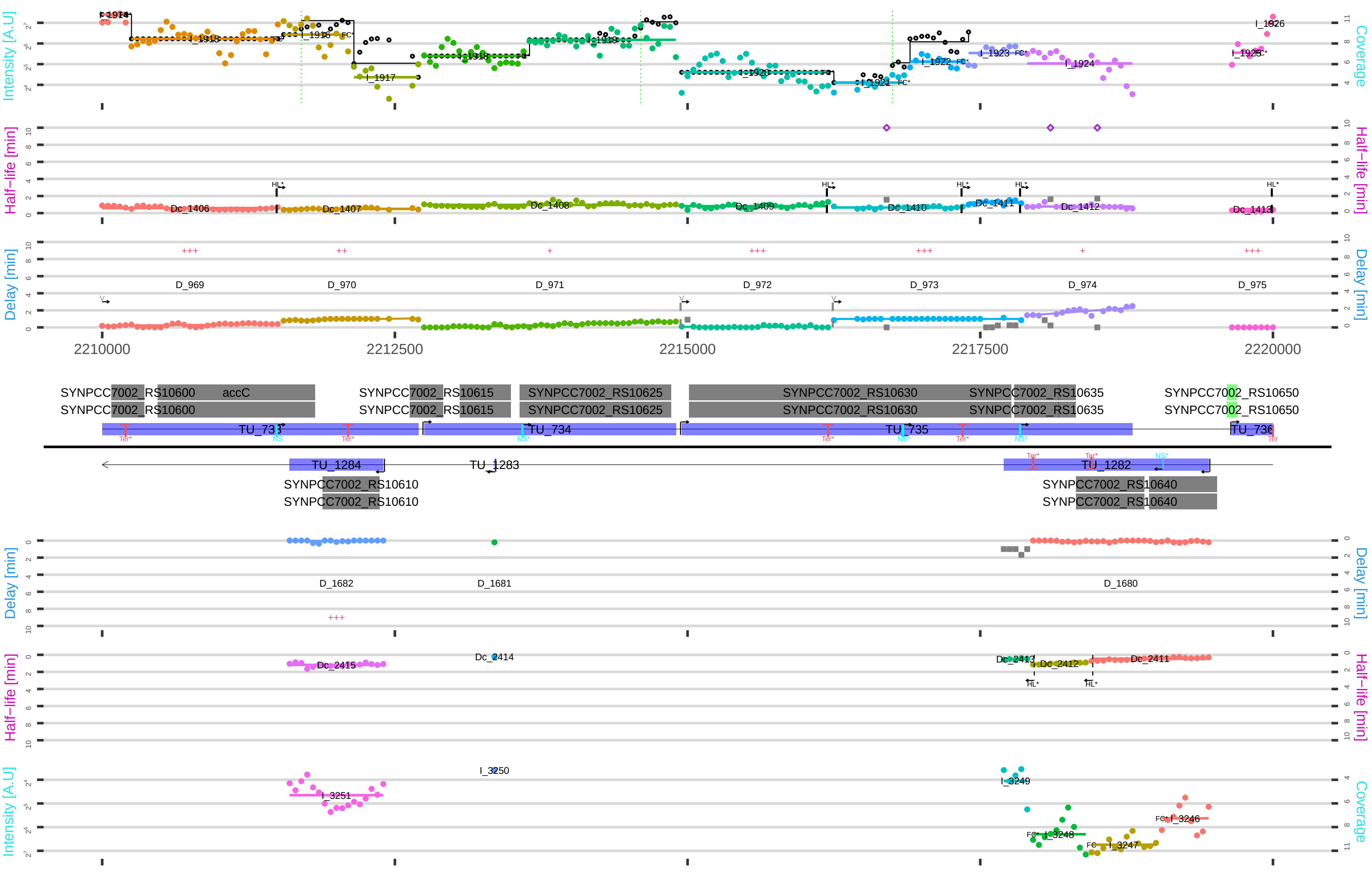

Term: termination (2), NS: new start (1), PS: pausing site (0), iTSS_I: internal starting site (0)

ID: 44400−44581; Term: termination (2), NS: new start (1), PS: pausing site (1), iTSS_I: internal starting site (0)

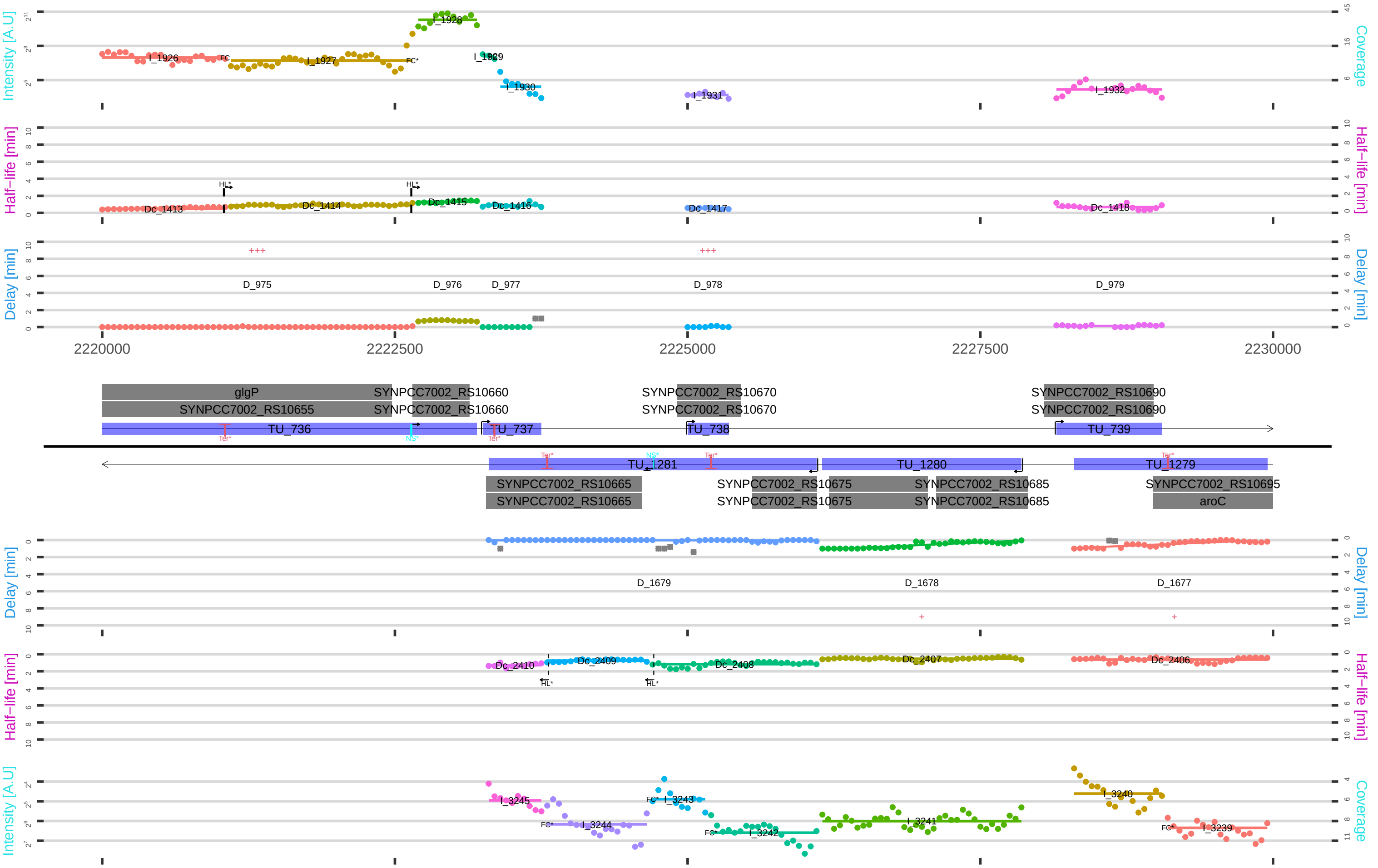

Term: termination (3), NS: new start (1), PS: pausing site (0), iTSS_I: internal starting site (0)

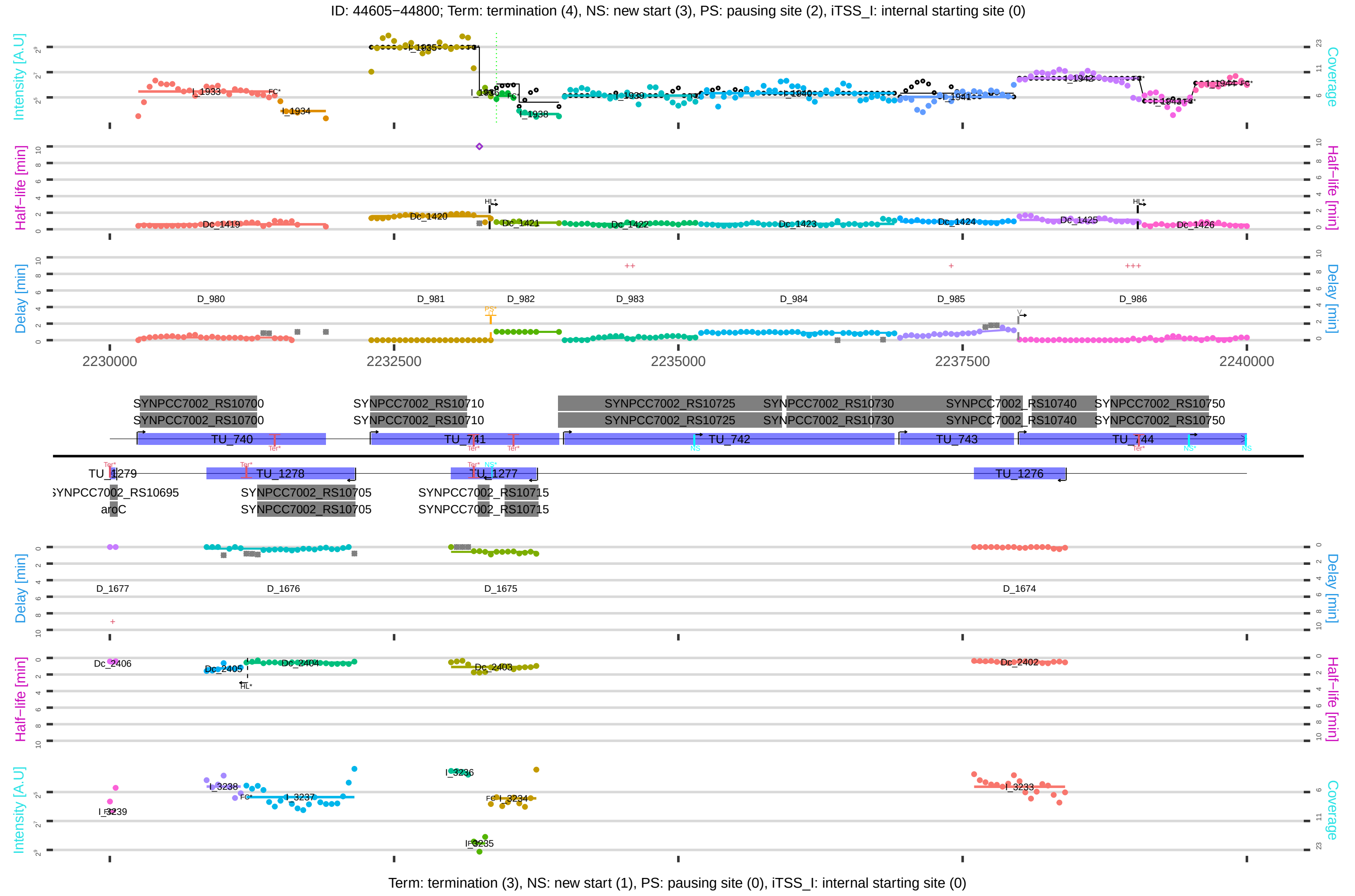
ID: 44605−44800; Term: termination (4), NS: new start (3), PS: pausing site (2), iTSS_I: internal starting site (0)
Intensity [A.U]
Coverage
Half−life [min]
Delay [min]
2230000
2232500
2235000
2237500
2240000
SYNPCC7002_RS10700
SYNPCC7002_RS10710
SYNPCC7002_RS10725
SYNPCC7002_RS10730
SYNPCC7002_RS10740
SYNPCC7002_RS10750
TU_740
TU_741
TU_742
TU_743
TU_744
TU_1279
TU_1278
TU_1277
TU_1276
SYNPCC7002_RS10705
SYNPCC7002_RS10715
aroC
Term: termination (3), NS: new start (1), PS: pausing site (0), iTSS_I: internal starting site (0)

ID: 44800−45000; Term: termination (7), NS: new start (5), PS: pausing site (1), iTSS_I: internal starting site (1)

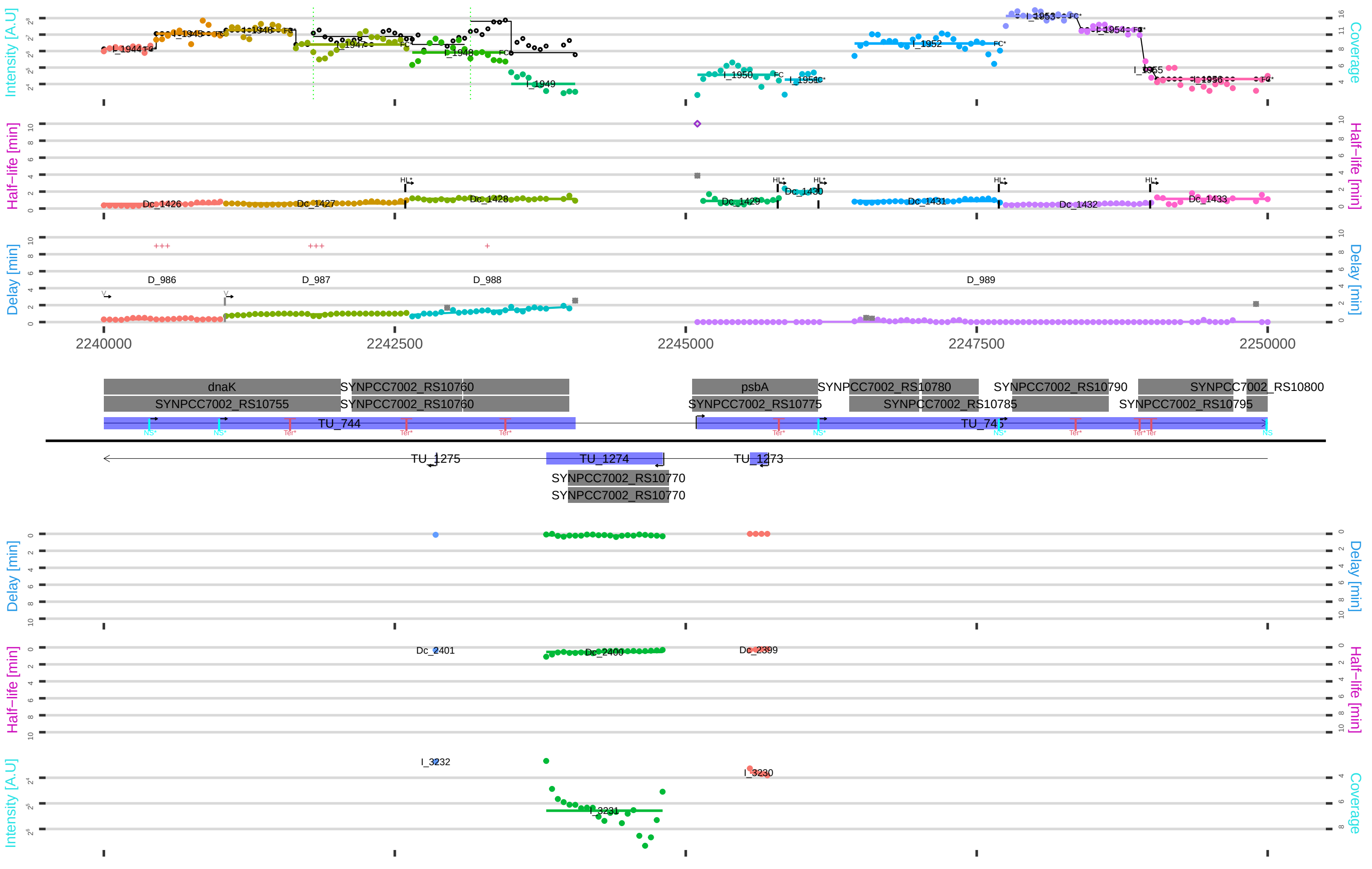

Term: termination (0), NS: new start (0), PS: pausing site (0), iTSS_I: internal starting site (0)

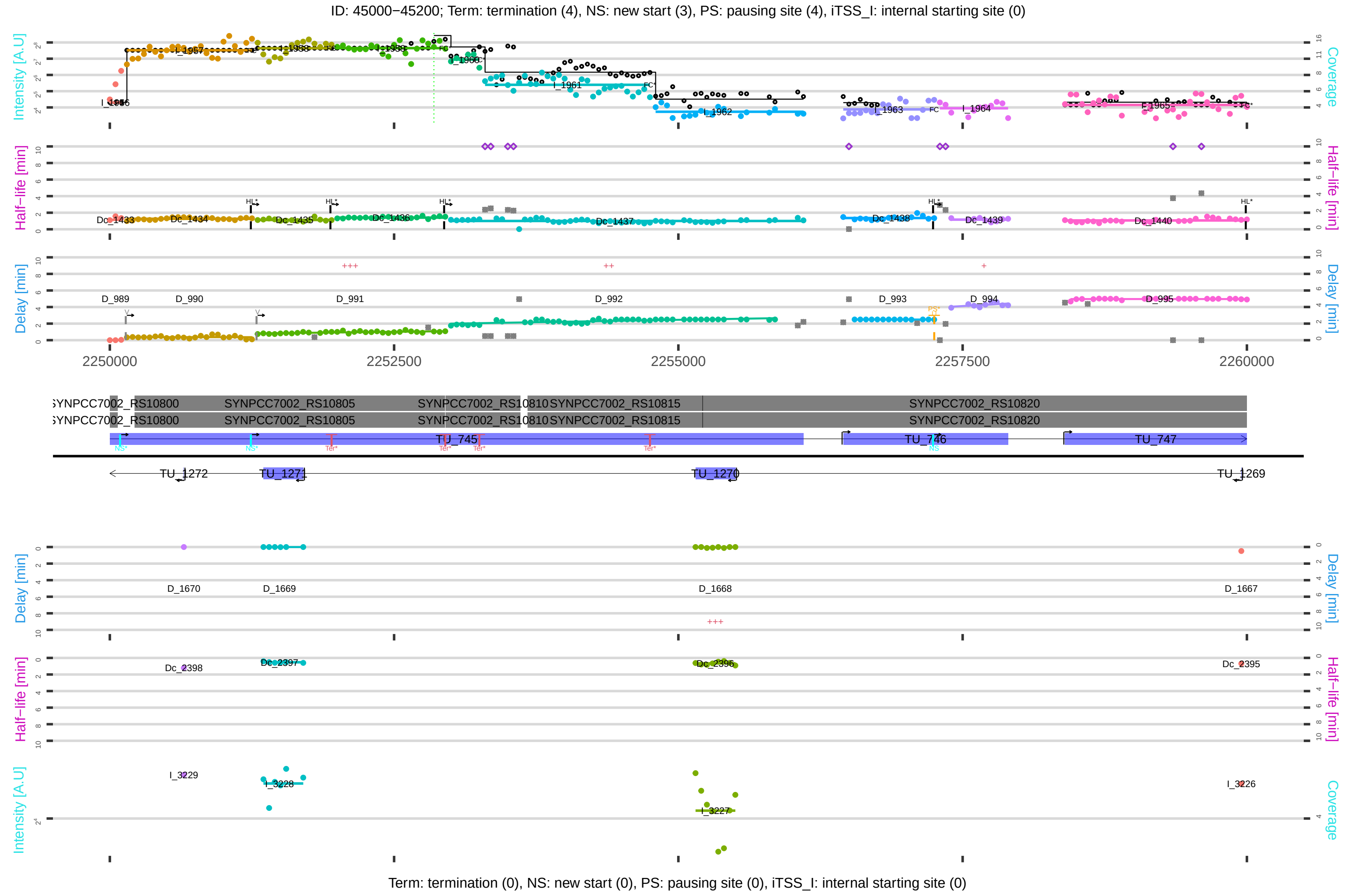

ID: 45000−45200; Term: termination (4), NS: new start (3), PS: pausing site (4), iTSS_I: internal starting site (0)
Intensity [A.U]
Coverage
Half−life [min]
Delay [min]
2250000
2252500
2255000
2257500
2260000
SYNPCC7002_RS10800
SYNPCC7002_RS10805
SYNPCC7002_RS10810
SYNPCC7002_RS10815
SYNPCC7002_RS10820
TU_745
TU_746
TU_747
TU_1272
TU_1271
TU_1270
TU_1269
D_1670
D_1669
D_1668
D_1667
Dc_2398
Dc_2397
Dc_2396
Dc_2395
I_3229
I_3228
I_3227
I_3226
Term: termination (0), NS: new start (0), PS: pausing site (0), iTSS_I: internal starting site (0)

ID: 45200−45400; Term: termination (8), NS: new start (7), PS: pausing site (3), iTSS_I: internal starting site (2)

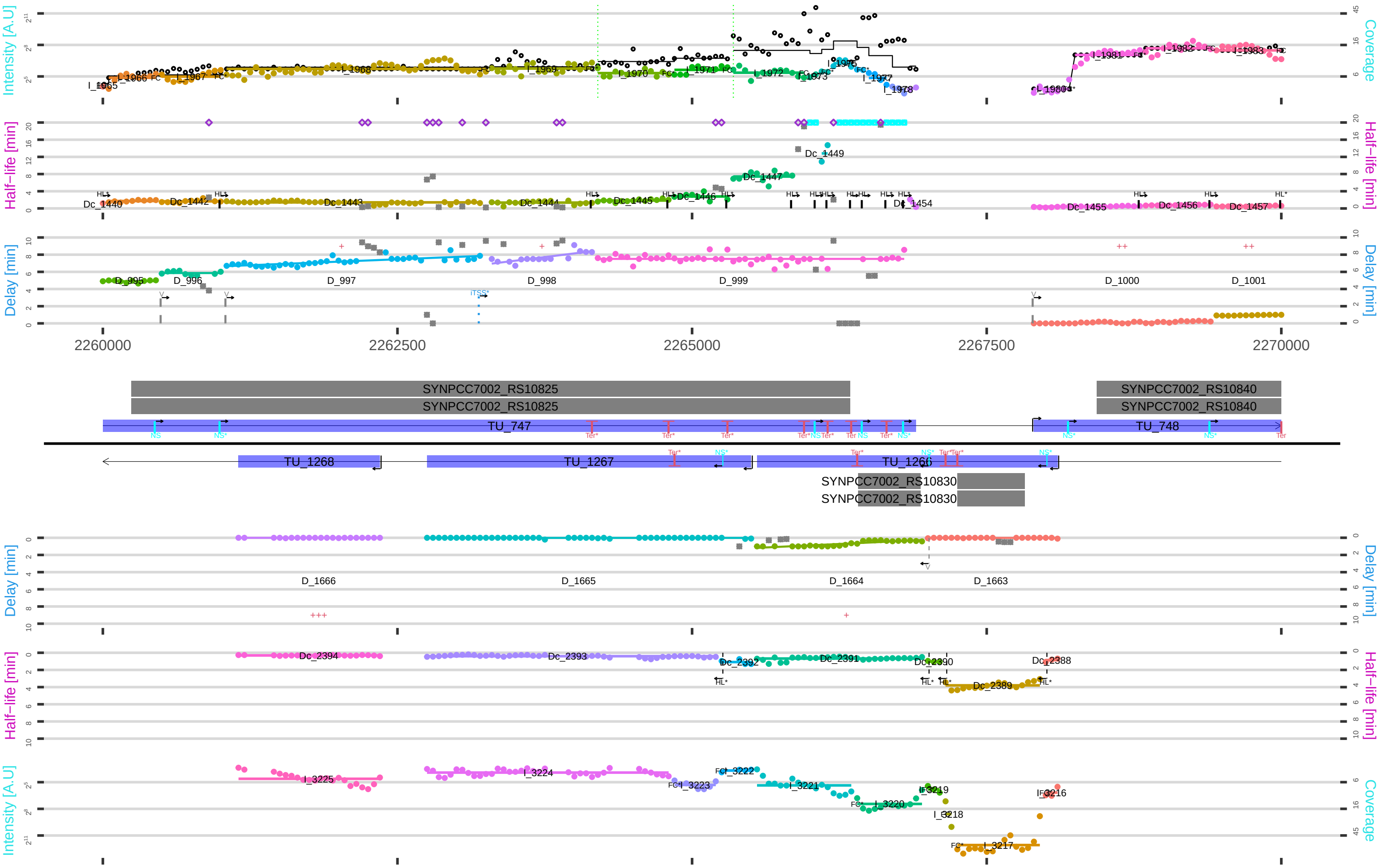

Term: termination (4), NS: new start (3), PS: pausing site (1), iTSS_I: internal starting site (0)

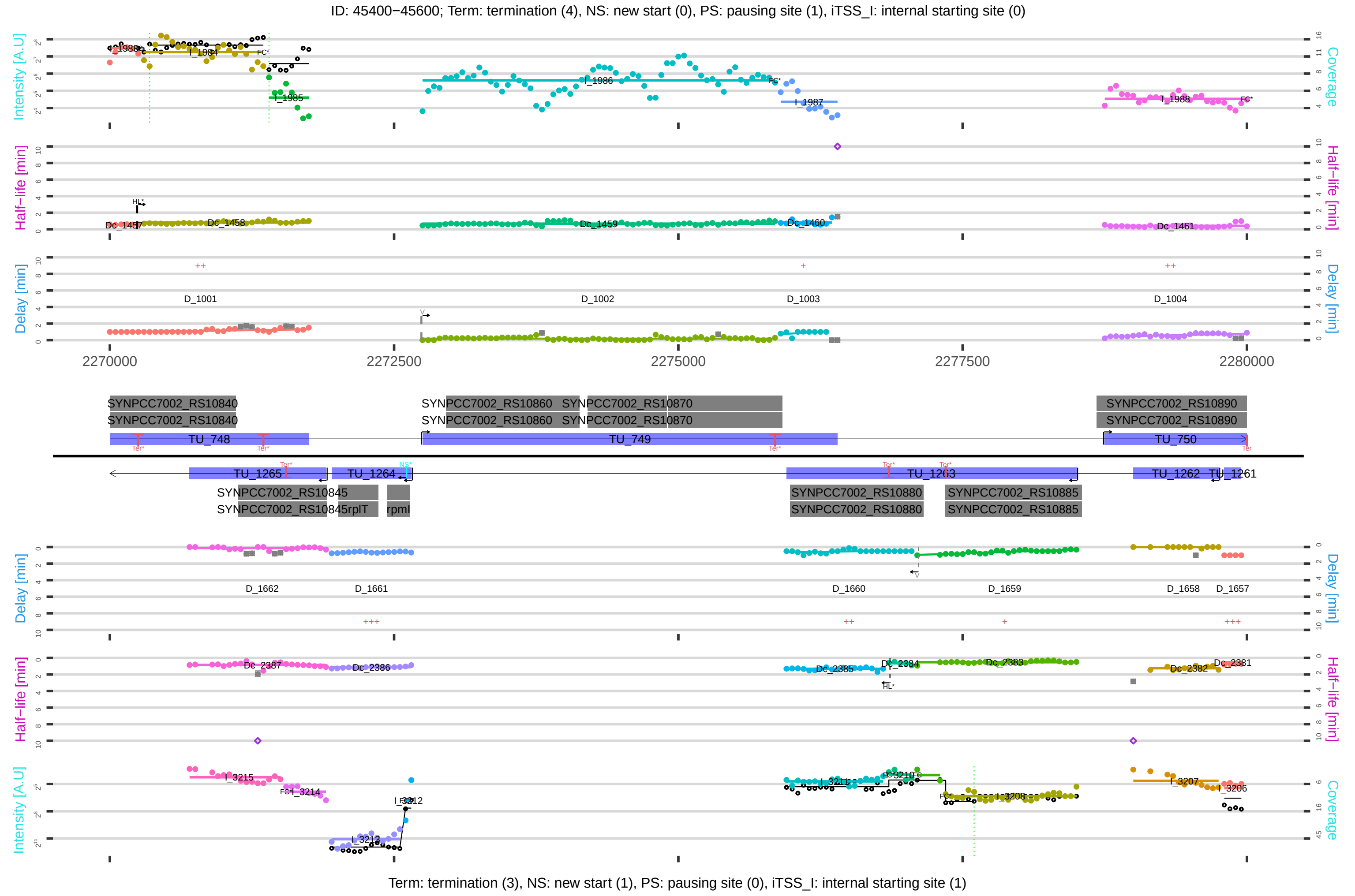

ID: 45400−45600; Term: termination (4), NS: new start (0), PS: pausing site (1), iTSS_I: internal starting site (0)
Intensity [A.U]
Coverage
Half−life [min]
Delay [min]
I_1984
I_1985
I_1986
I_1987
I_1988
Dc_1457
Dc_1458
Dc_1459
Dc_1460
Dc_1461
D_1001
D_1002
D_1003
D_1004
2270000
2272500
2275000
2277500
2280000
SYNPCC7002_RS10840
SYNPCC7002_RS10860
SYNPCC7002_RS10870
SYNPCC7002_RS10890
TU_748
TU_749
TU_750
TU_1265
TU_1264
TU_1263
TU_1262
TU_1261
SYNPCC7002_RS10845
rplT
rpmI
SYNPCC7002_RS10880
SYNPCC7002_RS10885
D_1662
D_1661
D_1660
D_1659
D_1658
D_1657
Dc_2387
Dc_2386
Dc_2385
Dc_2384
Dc_2383
Dc_2382
Dc_2381
I_3215
I_3214
I_3213
I_3212
I_3211
I_3210
I_3208
I_3207
I_3206
Term: termination (3), NS: new start (1), PS: pausing site (0), iTSS_I: internal starting site (1)

ID: 45600−45787; Term: termination (4), NS: new start (2), PS: pausing site (0), iTSS_I: internal starting site (0)

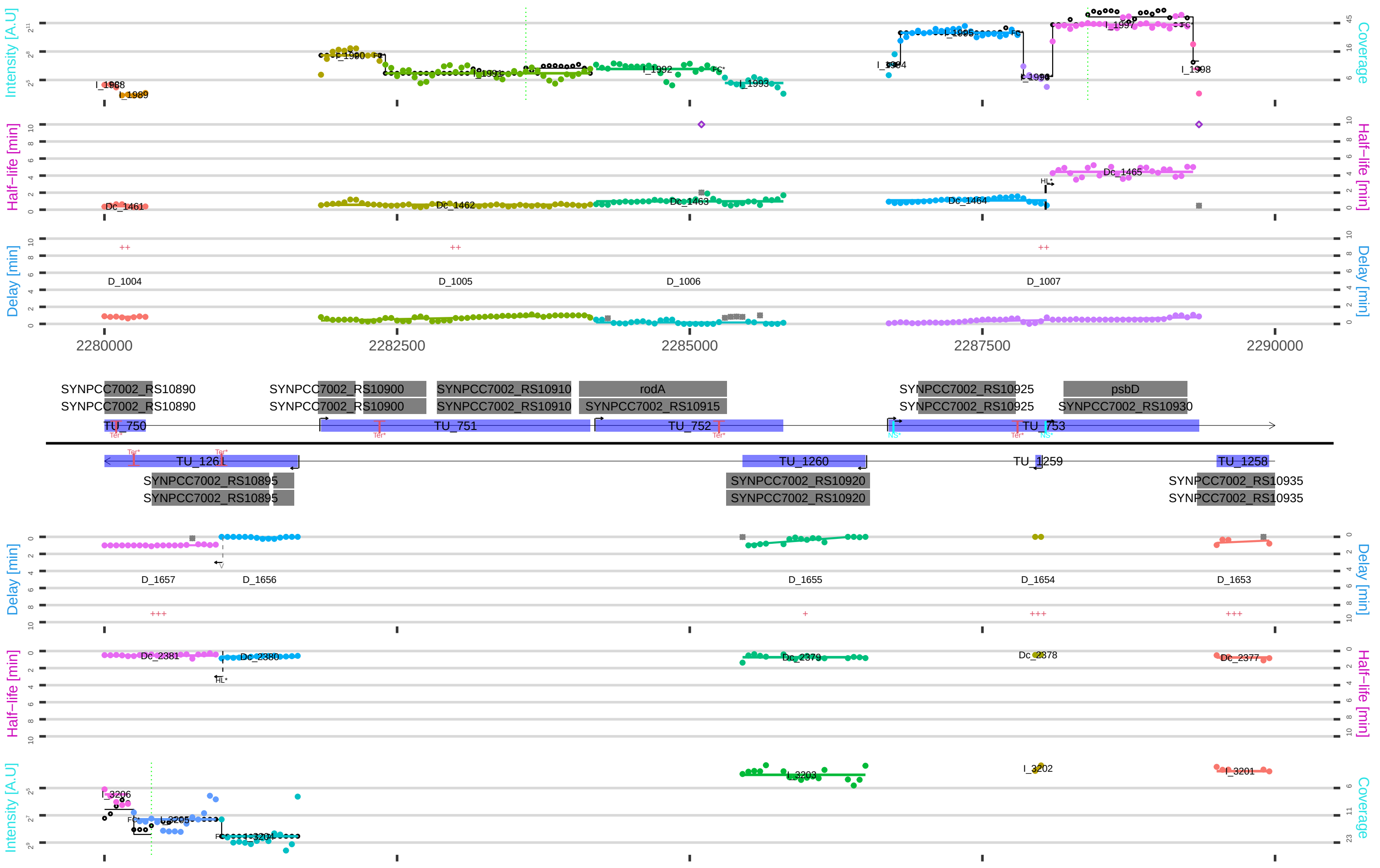

Term: termination (2), NS: new start (0), PS: pausing site (1), iTSS_I: internal starting site (0)

ID: 45814−46000; Term: termination (5), NS: new start (1), PS: pausing site (1), iTSS_I: internal starting site (0)

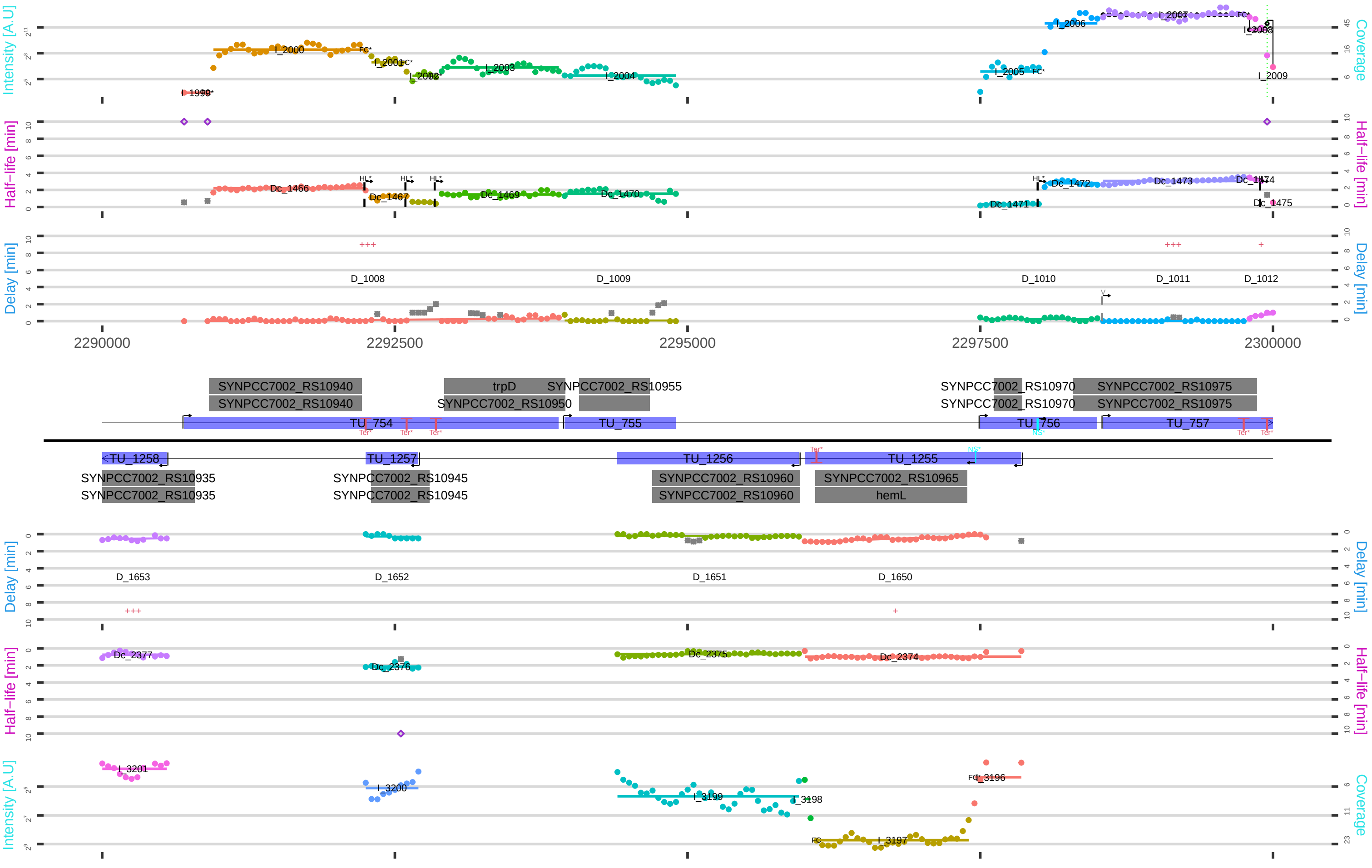

Term: termination (1), NS: new start (1), PS: pausing site (0), iTSS_I: internal starting site (0)

ID: 46000−46200; Term: termination (5), NS: new start (0), PS: pausing site (2), iTSS_I: internal starting site (0)

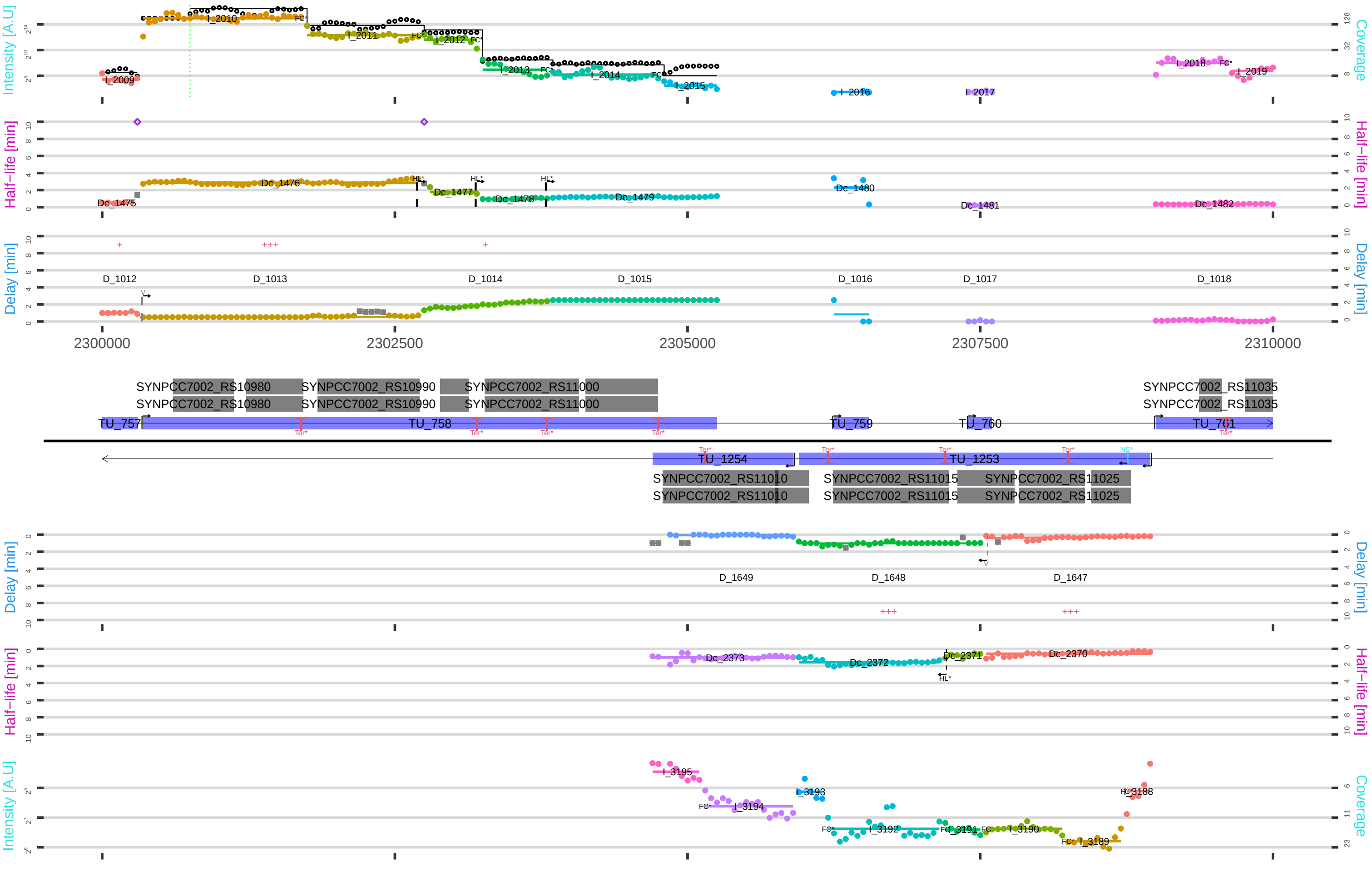

Term: termination (4), NS: new start (1), PS: pausing site (1), iTSS_I: internal starting site (0)

ID: 46200−46400; Term: termination (6), NS: new start (2), PS: pausing site (1), iTSS_I: internal starting site (0)

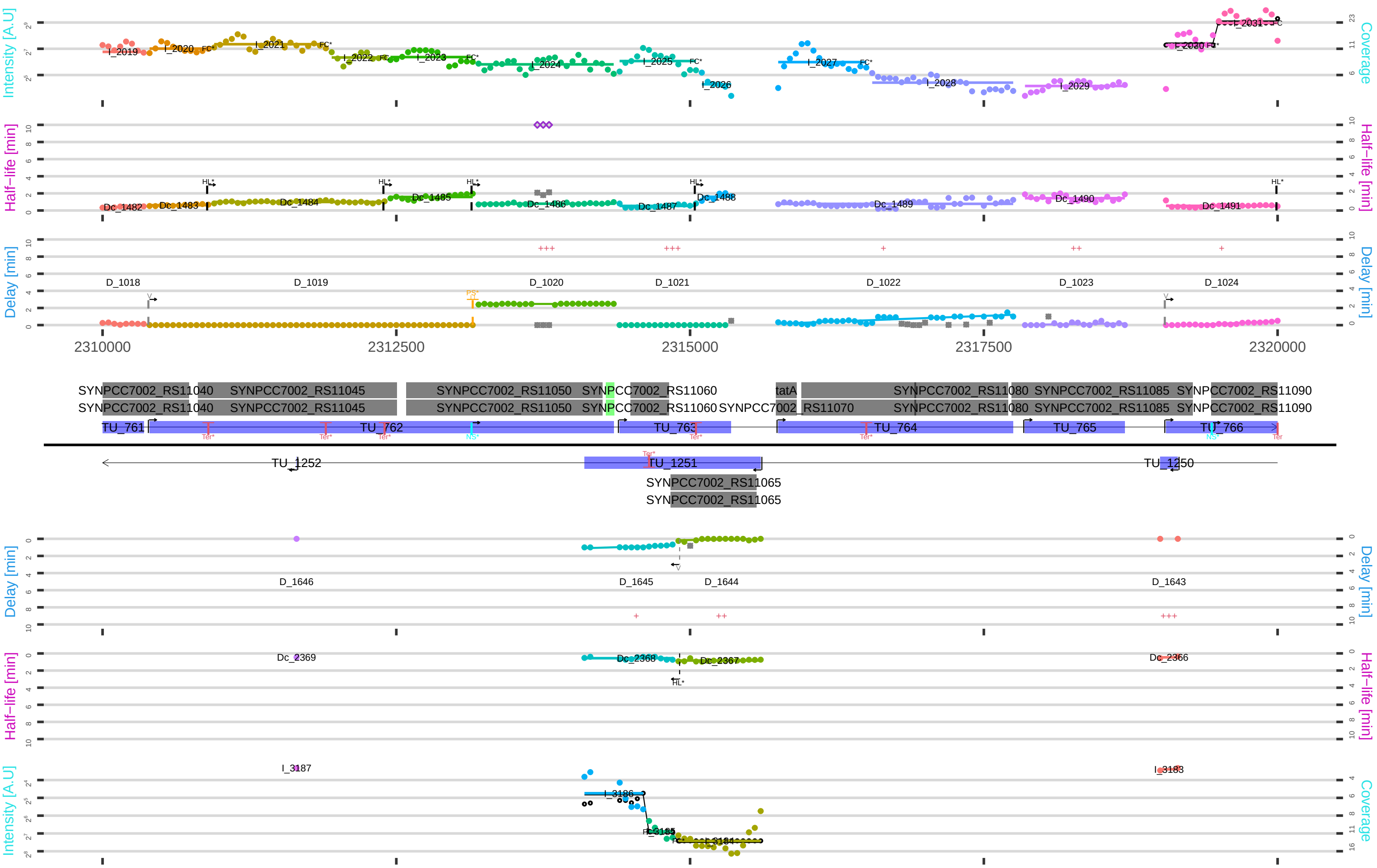

Term: termination (1), NS: new start (0), PS: pausing site (1), iTSS_I: internal starting site (0)

ID: 46400−46600; Term: termination (7), NS: new start (1), PS: pausing site (2), iTSS_I: internal starting site (0)

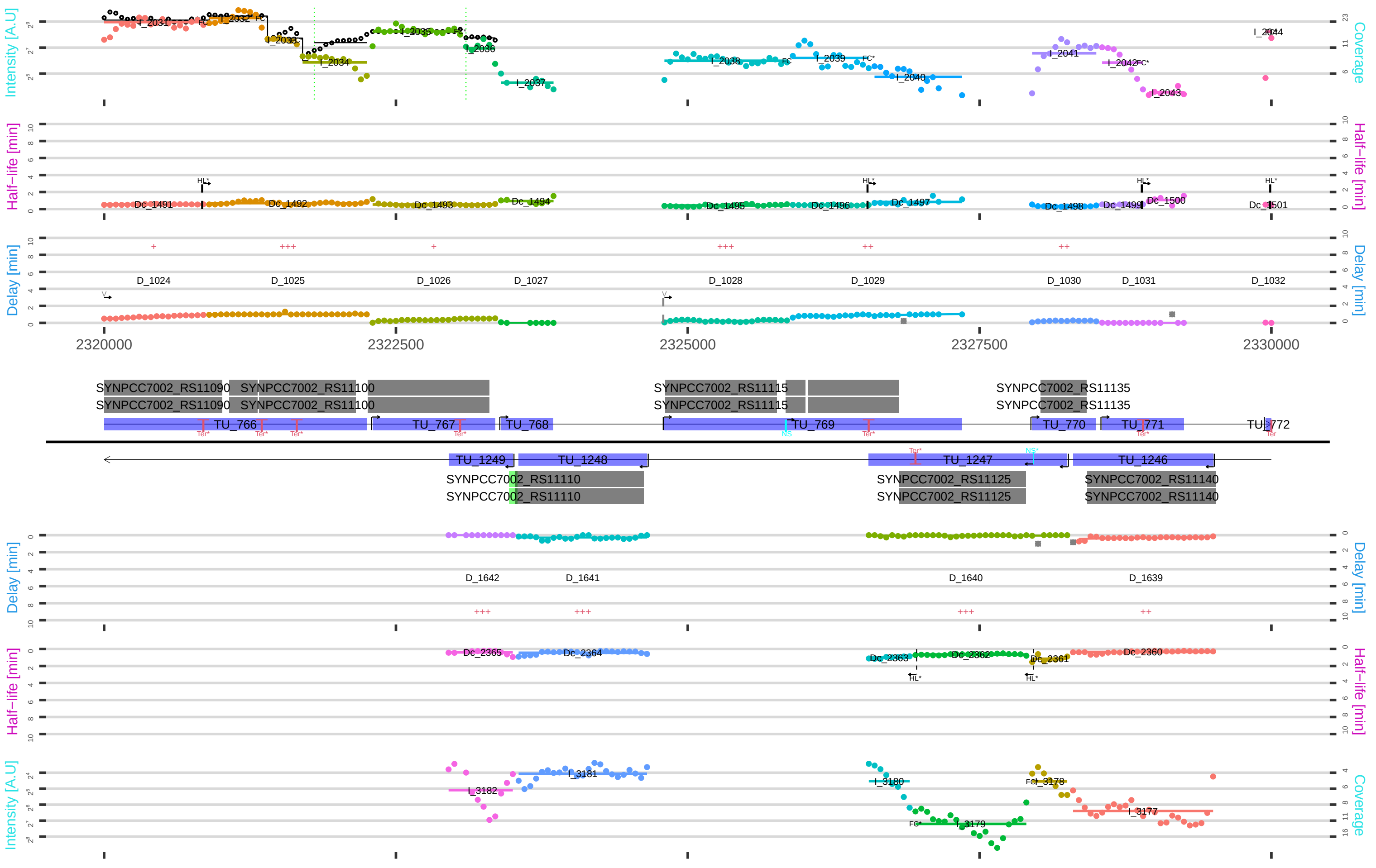

Term: termination (1), NS: new start (1), PS: pausing site (0), iTSS_I: internal starting site (0)

ID: 46600−46800; Term: termination (5), NS: new start (0), PS: pausing site (1), iTSS_I: internal starting site (0)

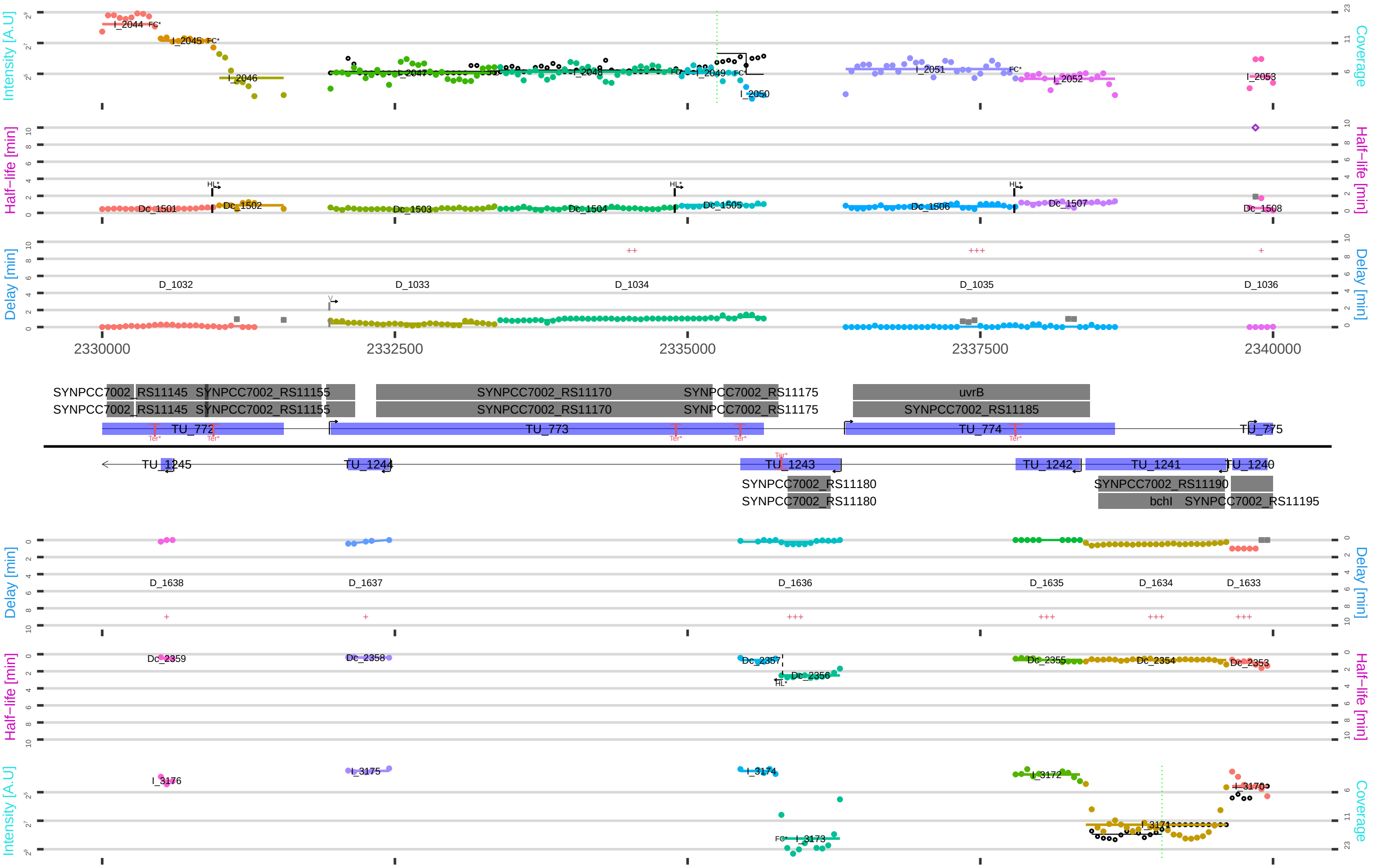

Term: termination (1), NS: new start (0), PS: pausing site (0), iTSS_I: internal starting site (0)

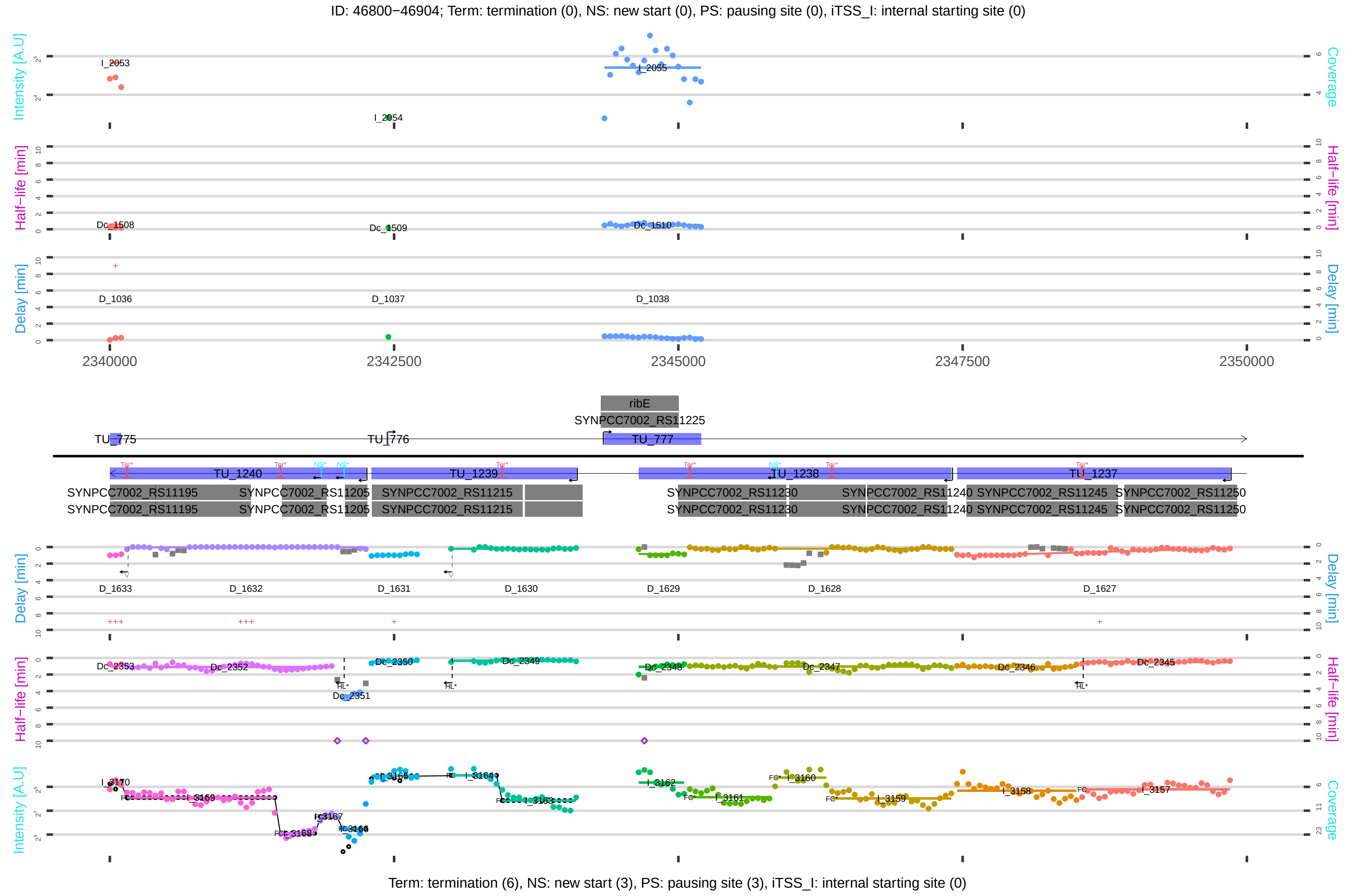

ID: 46800−46904; Term: termination (0), NS: new start (0), PS: pausing site (0), iTSS_I: internal starting site (0)
Intensity [A.U]
Coverage
Half−life [min]
Delay [min]
I_2053
I_2054
I_2055
Dc_1508
Dc_1509
Dc_1510
D_1036
D_1037
D_1038
2340000
2342500
2345000
2347500
2350000
ribE
SYNPCC7002_RS11225
TU_775
TU_776
TU_777
TU_1240
TU_1239
TU_1238
TU_1237
SYNPCC7002_RS11195
SYNPCC7002_RS11205
SYNPCC7002_RS11215
SYNPCC7002_RS11230
SYNPCC7002_RS11240
SYNPCC7002_RS11245
SYNPCC7002_RS11250
D_1633
D_1632
D_1631
D_1630
D_1629
D_1628
D_1627
Dc_2353
Dc_2352
Dc_2351
Dc_2350
Dc_2349
Dc_2348
Dc_2347
Dc_2346
Dc_2345
I_3170
I_3169
I_3168
I_3167
I_3166
I_3165
I_3164
I_3163
I_3162
I_3161
I_3160
I_3159
I_3158
I_3157
Term: termination (6), NS: new start (3), PS: pausing site (3), iTSS_I: internal starting site (0)

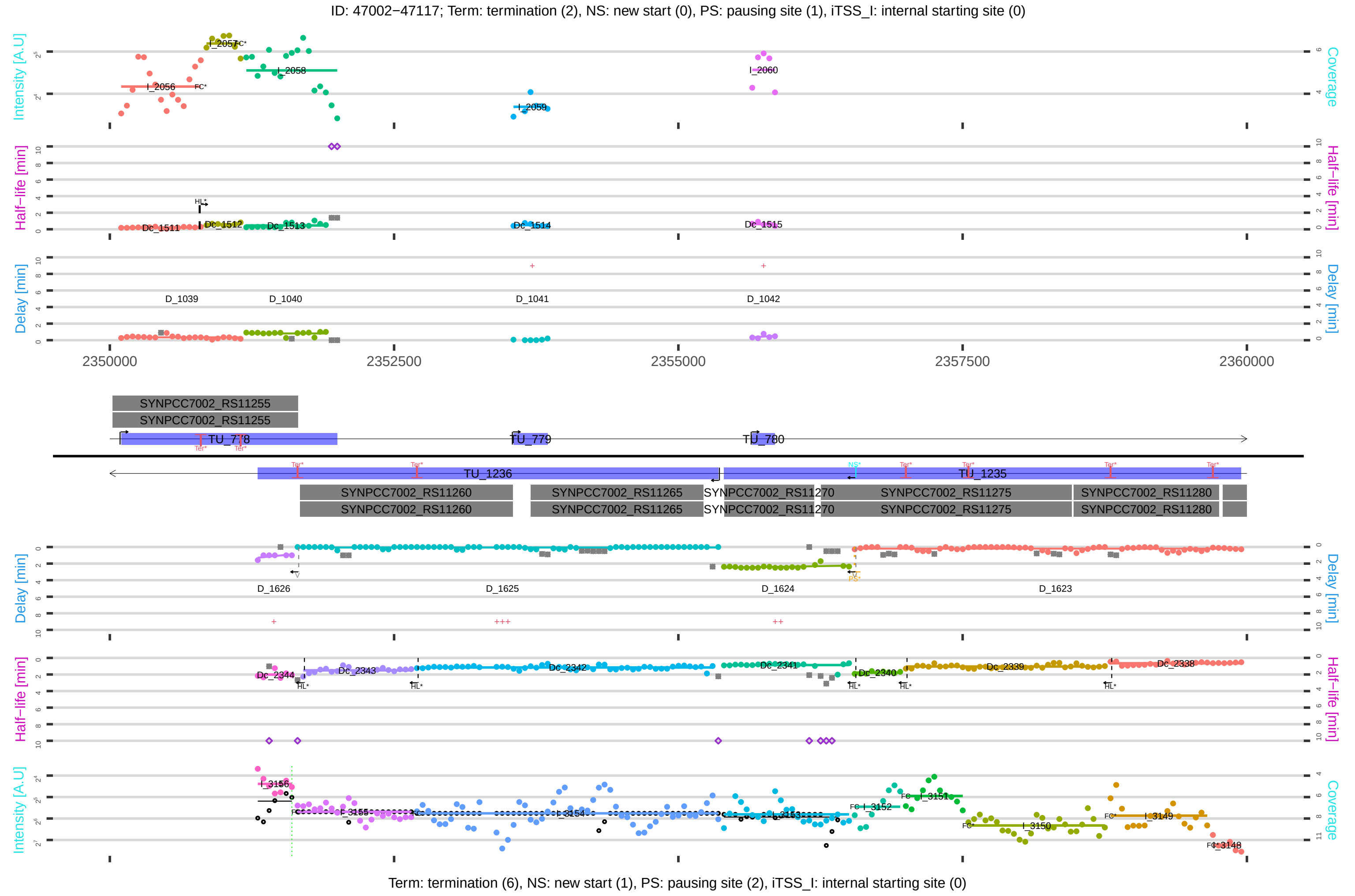

ID: 47002−47117; Term: termination (2), NS: new start (0), PS: pausing site (1), iTSS_I: internal starting site (0)
Intensity [A.U]
Coverage
Half−life [min]
Delay [min]
I_2056
I_2057
I_2058
I_2059
I_2060
Dc_1511
Dc_1512
Dc_1513
Dc_1514
Dc_1515
D_1039
D_1040
D_1041
D_1042
2350000
2352500
2355000
2357500
2360000
SYNPCC7002_RS11255
TU_778
TU_779
TU_780
TU_1236
TU_1235
SYNPCC7002_RS11260
SYNPCC7002_RS11265
SYNPCC7002_RS11270
SYNPCC7002_RS11275
SYNPCC7002_RS11280
D_1626
D_1625
D_1624
D_1623
Dc_2344
Dc_2343
Dc_2342
Dc_2341
Dc_2340
Dc_2339
Dc_2338
I_3156
I_3155
I_3154
I_3153
I_3152
I_3151
I_3150
I_3149
I_3148
Term: termination (6), NS: new start (1), PS: pausing site (2), iTSS_I: internal starting site (0)

ID: 47280−47400; Term: termination (4), NS: new start (1), PS: pausing site (2), iTSS_I: internal starting site (0)

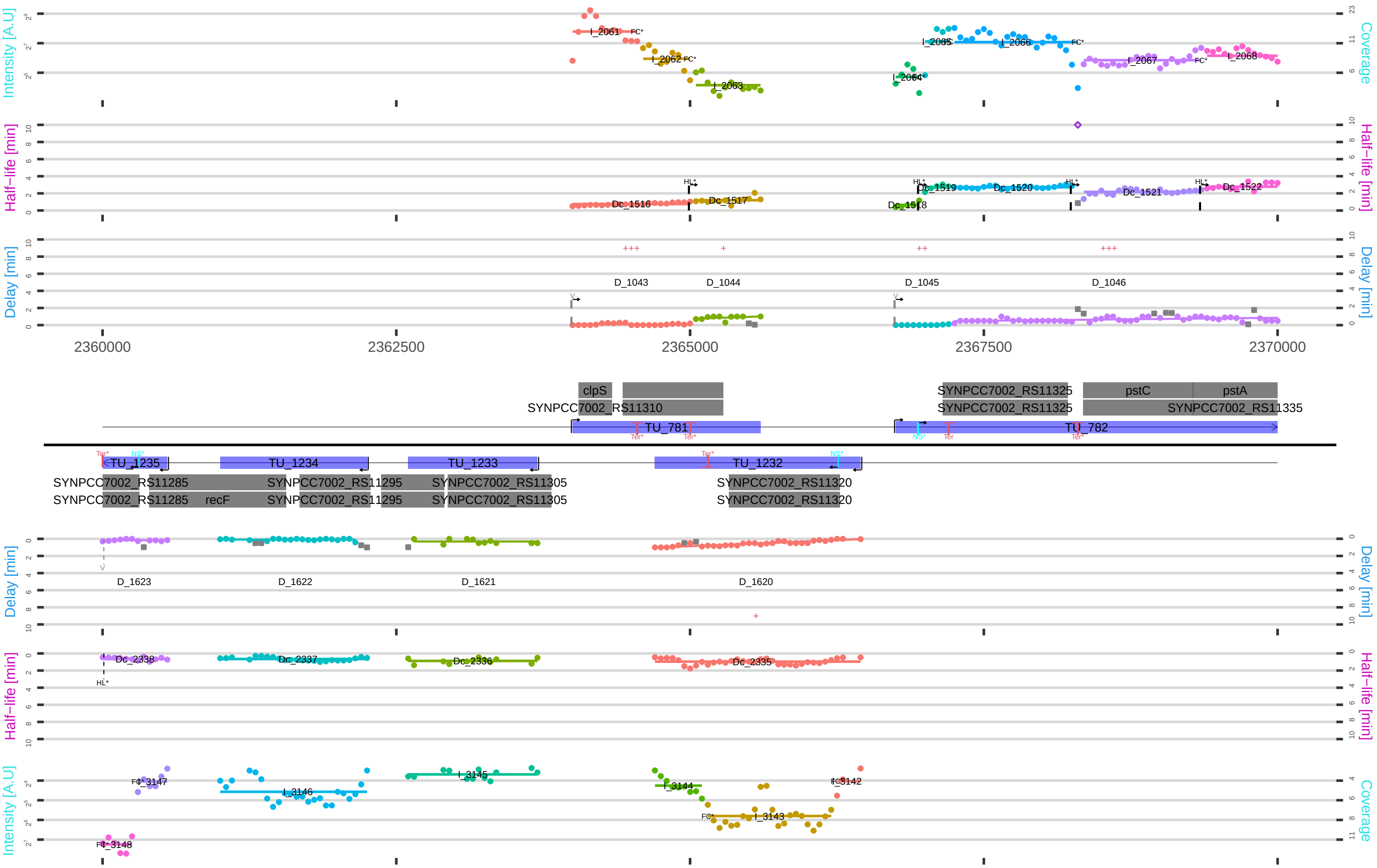

Term: termination (2), NS: new start (2), PS: pausing site (0), iTSS_I: internal starting site (0)

ID: 47400−47600; Term: termination (4), NS: new start (2), PS: pausing site (2), iTSS_I: internal starting site (0)

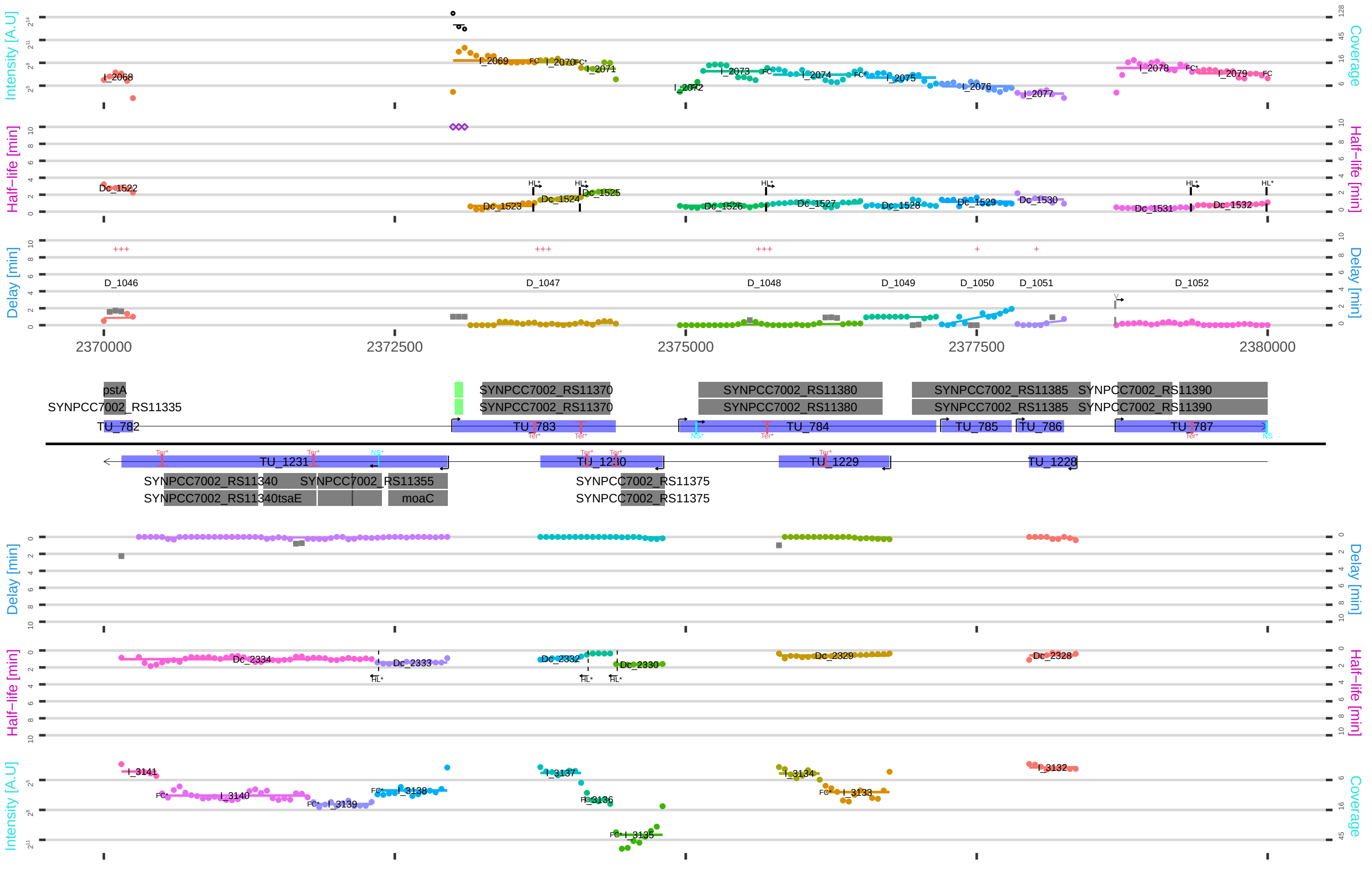

Term: termination (5), NS: new start (1), PS: pausing site (0), iTSS_I: internal starting site (0)

ID: 47600−47800; Term: termination (5), NS: new start (4), PS: pausing site (2), iTSS_I: internal starting site (0)

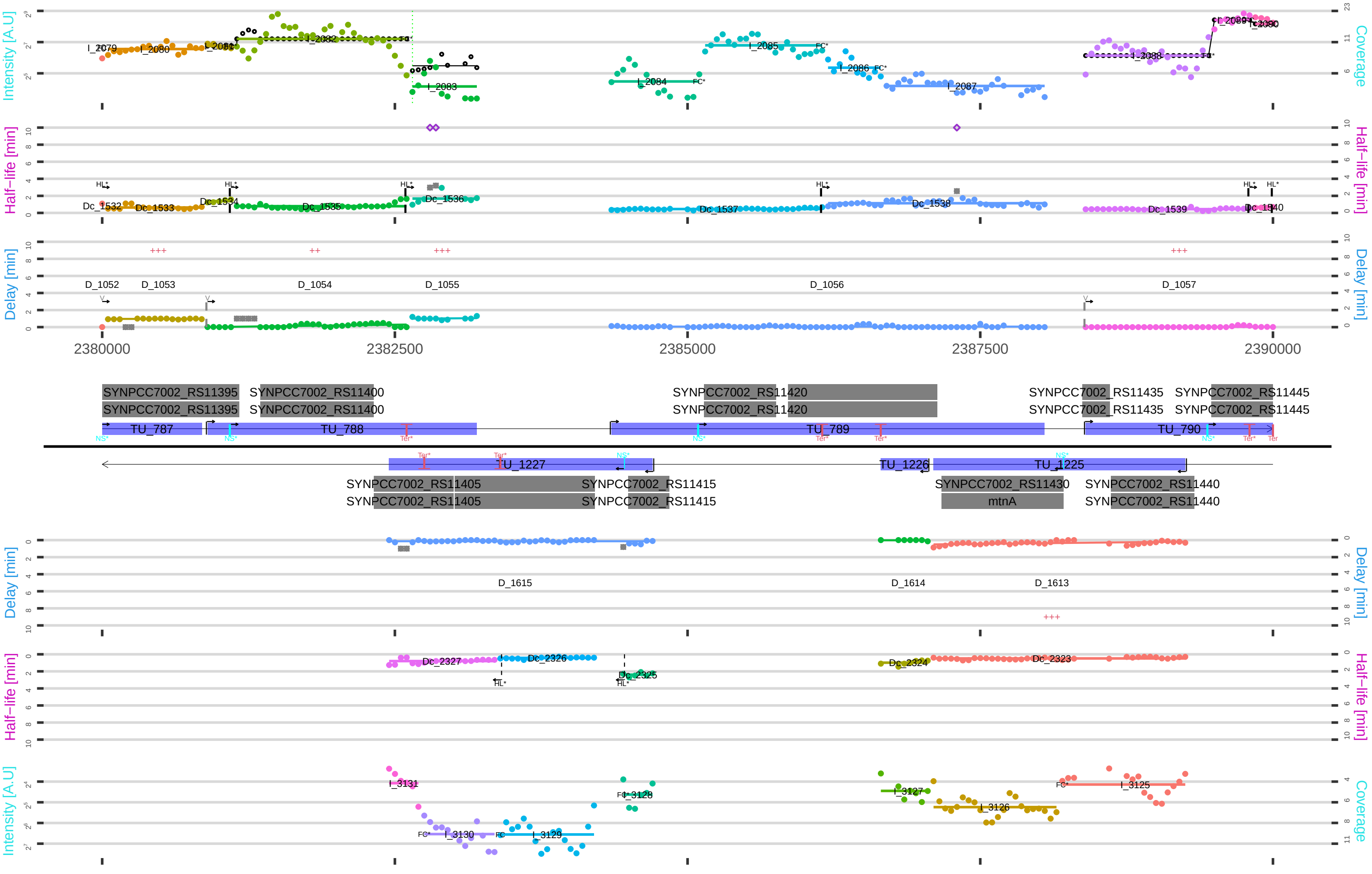

Term: termination (2), NS: new start (2), PS: pausing site (0), iTSS_I: internal starting site (0)

ID: 47800−47999; Term: termination (2), NS: new start (1), PS: pausing site (2), iTSS_I: internal starting site (0)

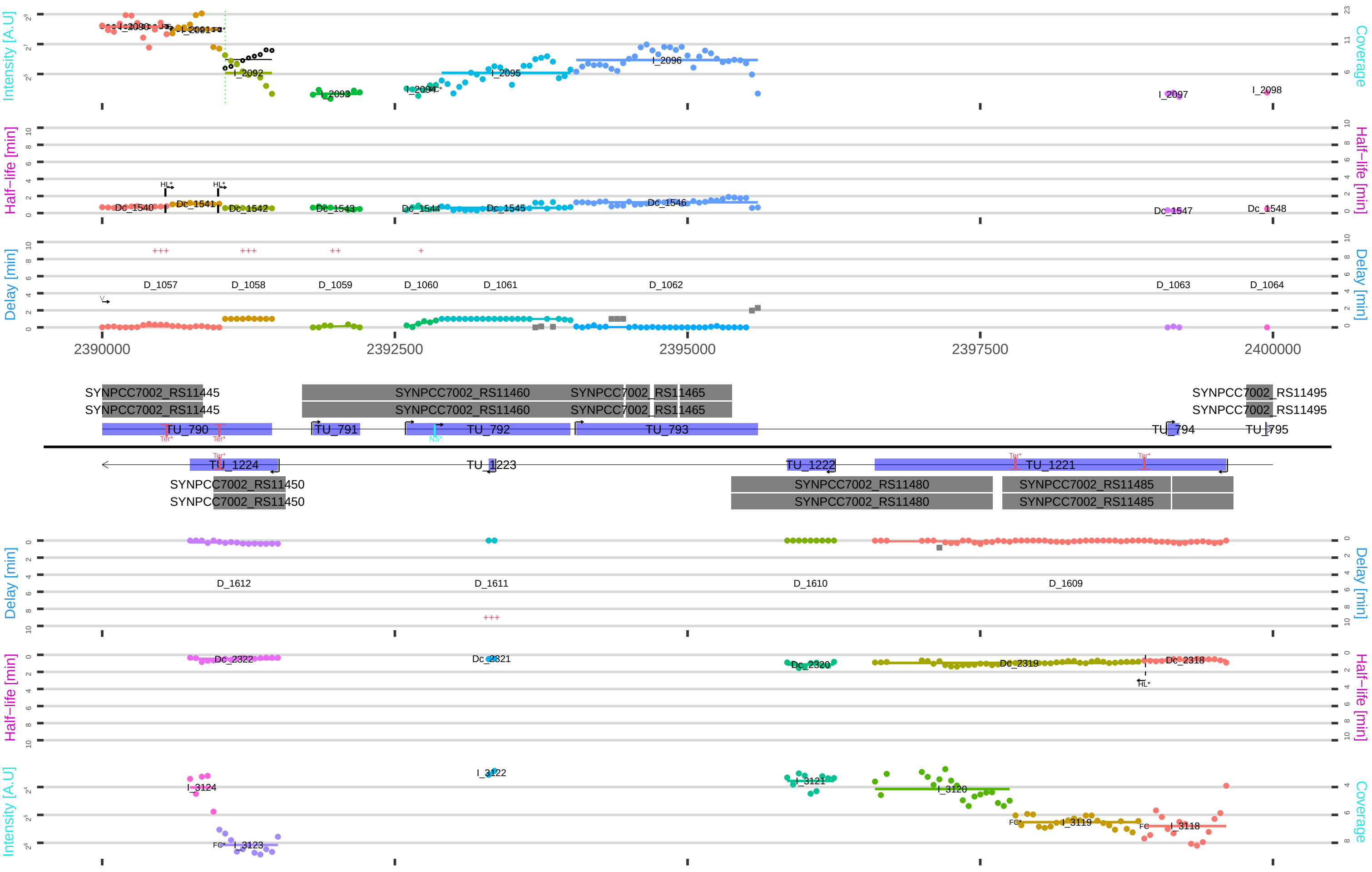

Term: termination (3), NS: new start (0), PS: pausing site (0), iTSS_I: internal starting site (0)

ID: 48004−48200; Term: termination (4), NS: new start (1), PS: pausing site (0), iTSS_I: internal starting site (0)

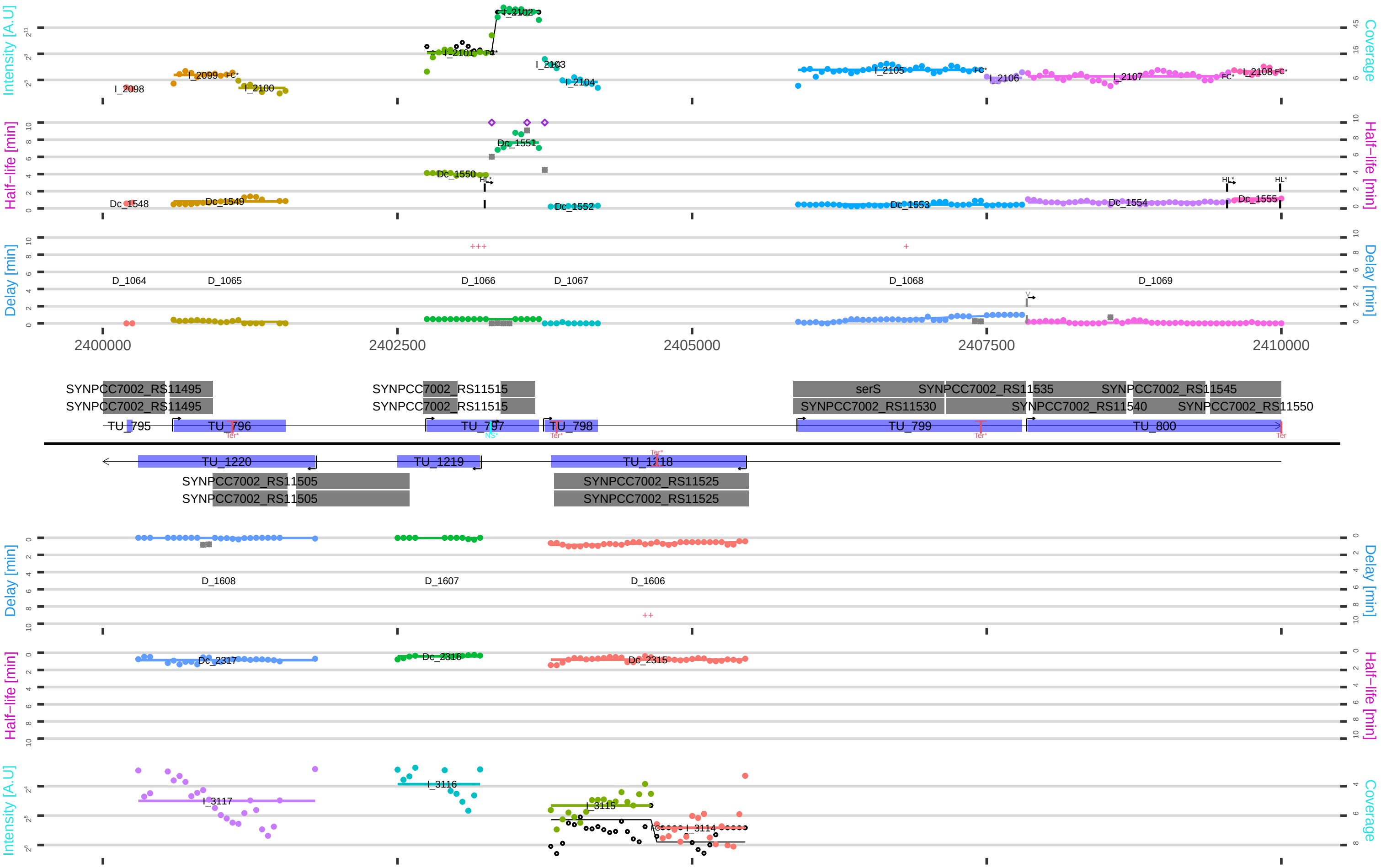

Term: termination (1), NS: new start (0), PS: pausing site (0), iTSS_I: internal starting site (0)

ID: 48200−48400; Term: termination (5), NS: new start (1), PS: pausing site (3), iTSS_I: internal starting site (0)

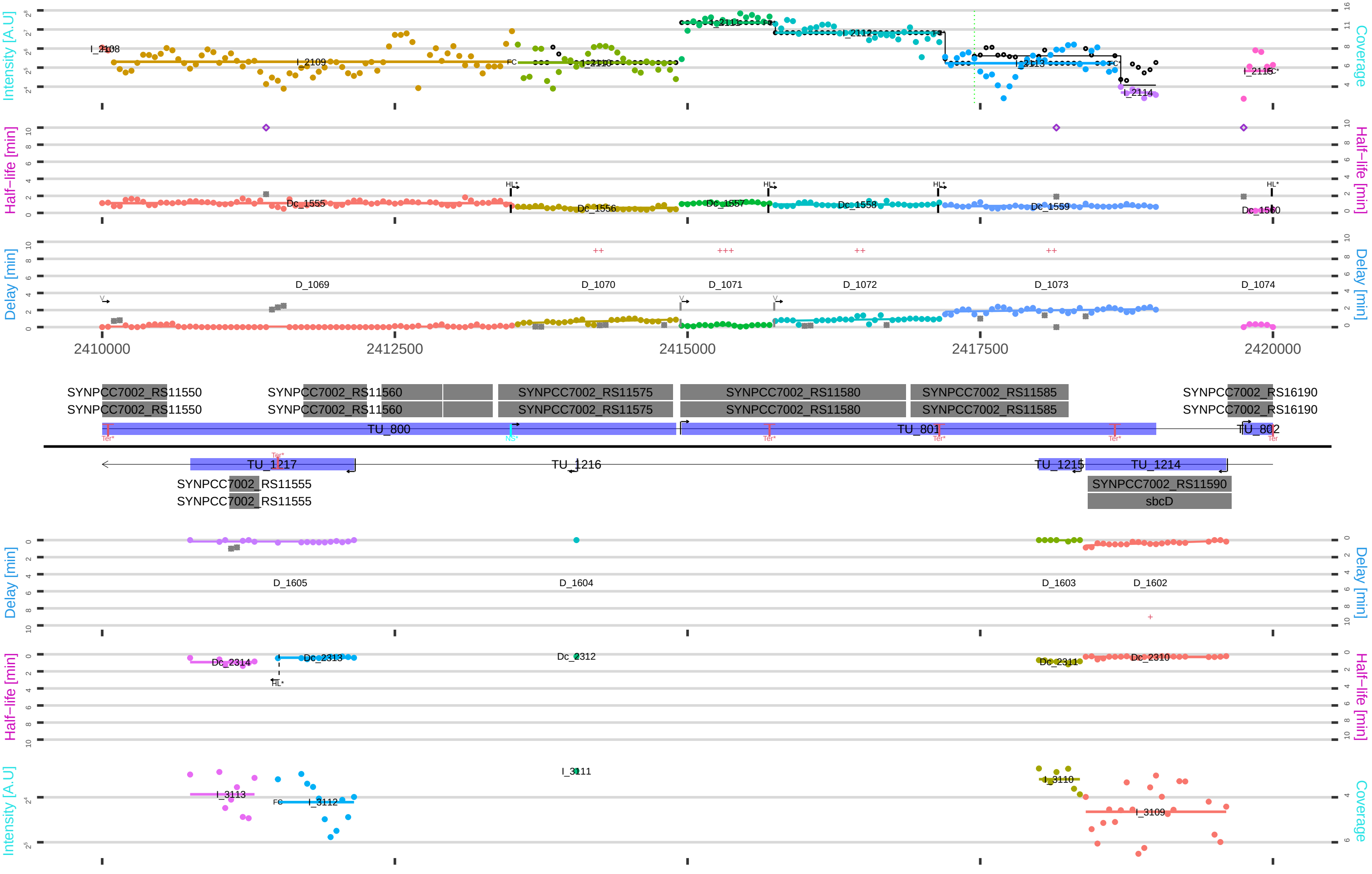

Term: termination (1), NS: new start (0), PS: pausing site (0), iTSS_I: internal starting site (0)

ID: 48400−48594; Term: termination (4), NS: new start (2), PS: pausing site (2), iTSS_I: internal starting site (0)

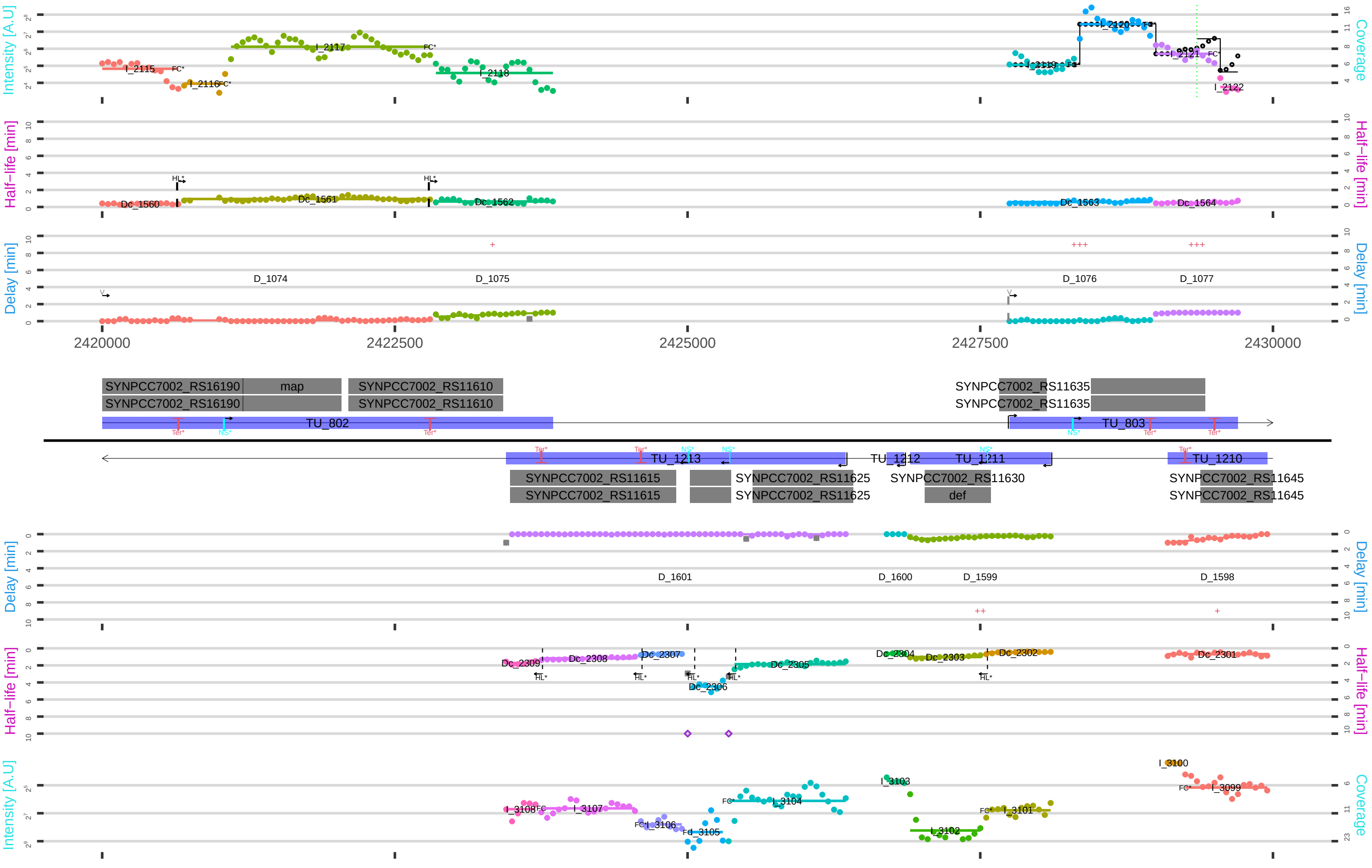

Term: termination (3), NS: new start (3), PS: pausing site (0), iTSS_I: internal starting site (0)

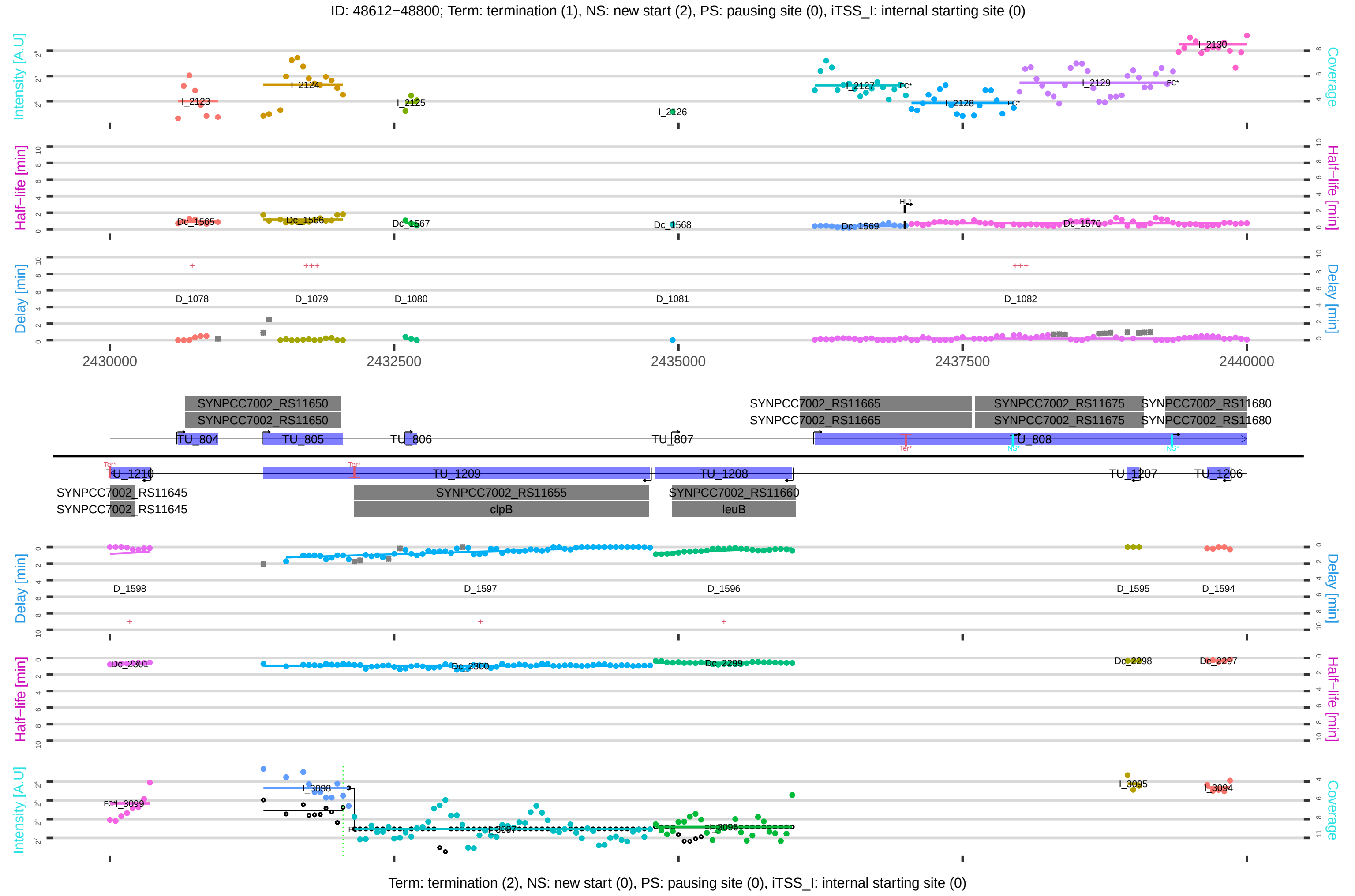

ID: 48612−48800; Term: termination (1), NS: new start (2), PS: pausing site (0), iTSS_I: internal starting site (0)
Intensity [A.U]
Coverage
Half−life [min]
Delay [min]
I_2123
I_2124
I_2125
I_2126
I_2127
I_2128
I_2129
I_2130
Dc_1565
Dc_1566
Dc_1567
Dc_1568
Dc_1569
Dc_1570
D_1078
D_1079
D_1080
D_1081
D_1082
2430000
2432500
2435000
2437500
2440000
SYNPCC7002_RS11650
SYNPCC7002_RS11665
SYNPCC7002_RS11675
SYNPCC7002_RS11680
TU_804
TU_805
TU_806
TU_807
TU_808
TU_1210
TU_1209
TU_1208
TU_1207
TU_1206
SYNPCC7002_RS11645
SYNPCC7002_RS11655
clpB
SYNPCC7002_RS11660
leuB
D_1598
D_1597
D_1596
D_1595
D_1594
Dc_2301
Dc_2300
Dc_2299
Dc_2298
Dc_2297
I_3099
I_3098
I_3097
I_3096
I_3095
I_3094
Term: termination (2), NS: new start (0), PS: pausing site (0), iTSS_I: internal starting site (0)

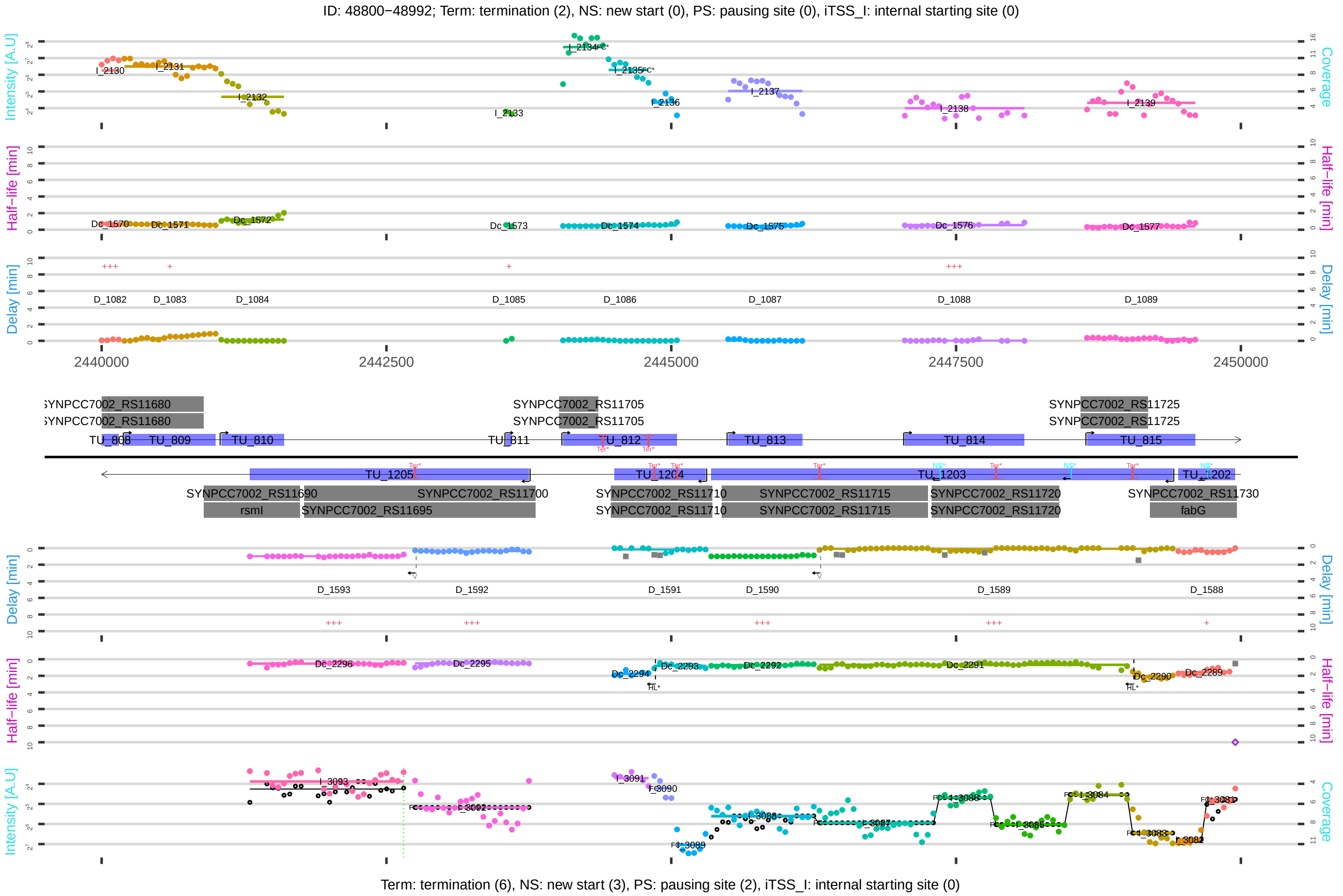

ID: 48800−48992; Term: termination (2), NS: new start (0), PS: pausing site (0), iTSS_I: internal starting site (0)
Intensity [A.U]
Coverage
Half−life [min]
Delay [min]
2440000
2442500
2445000
2447500
2450000
SYNPCC7002_RS11680
SYNPCC7002_RS11705
SYNPCC7002_RS11725
TU_808
TU_809
TU_810
TU_811
TU_812
TU_813
TU_814
TU_815
TU_1205
TU_1204
TU_1203
TU_1202
SYNPCC7002_RS11690
SYNPCC7002_RS11700
SYNPCC7002_RS11695
rsmI
SYNPCC7002_RS11710
SYNPCC7002_RS11715
SYNPCC7002_RS11720
SYNPCC7002_RS11730
fabG
Term: termination (6), NS: new start (3), PS: pausing site (2), iTSS_I: internal starting site (0)

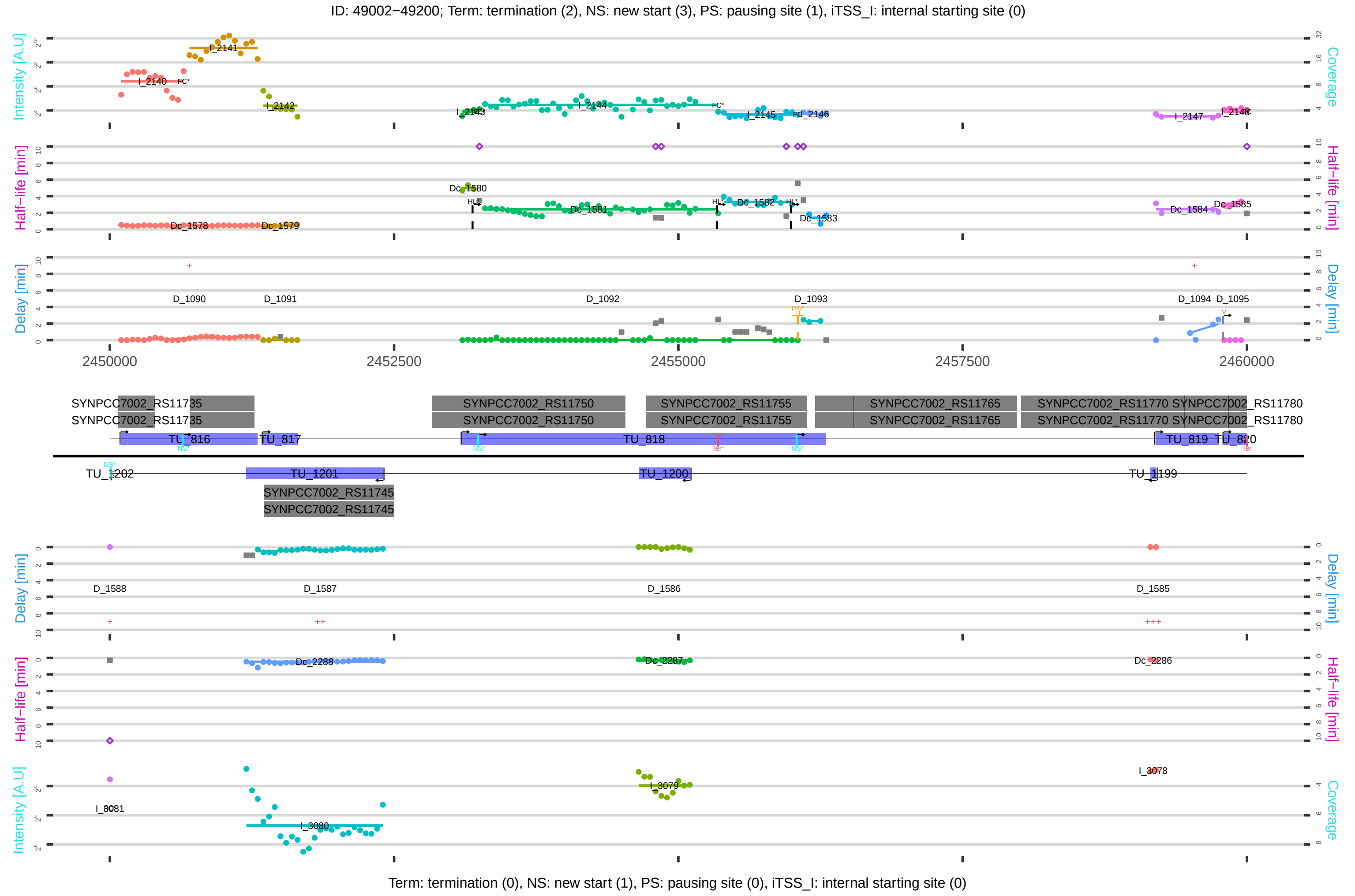
ID: 49002−49200; Term: termination (2), NS: new start (3), PS: pausing site (1), iTSS_I: internal starting site (0)
Intensity [A.U]
Coverage
Half−life [min]
Delay [min]
2450000
2452500
2455000
2457500
2460000
SYNPCC7002_RS11735
SYNPCC7002_RS11750
SYNPCC7002_RS11755
SYNPCC7002_RS11765
SYNPCC7002_RS11770
SYNPCC7002_RS11780
SYNPCC7002_RS11745
TU_816
TU_817
TU_818
TU_819
TU_820
TU_1202
TU_1201
TU_1200
TU_1199
Term: termination (0), NS: new start (1), PS: pausing site (0), iTSS_I: internal starting site (0)

ID: 49200−49400; Term: termination (3), NS: new start (0), PS: pausing site (1), iTSS_I: internal starting site (0)

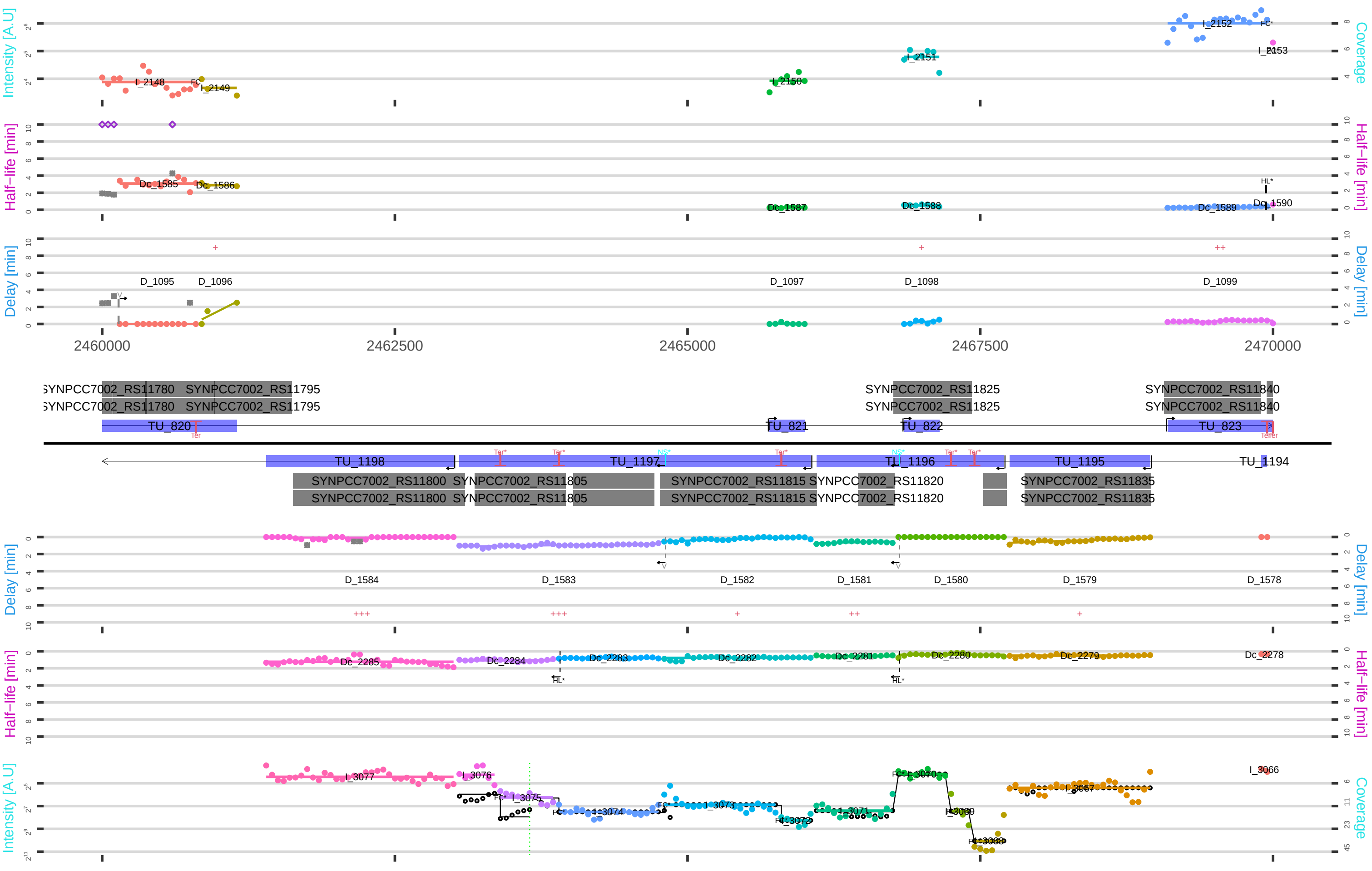

Term: termination (5), NS: new start (2), PS: pausing site (2), iTSS_I: internal starting site (0)

ID: 49400−49577; Term: termination (5), NS: new start (1), PS: pausing site (1), iTSS_I: internal starting site (1)

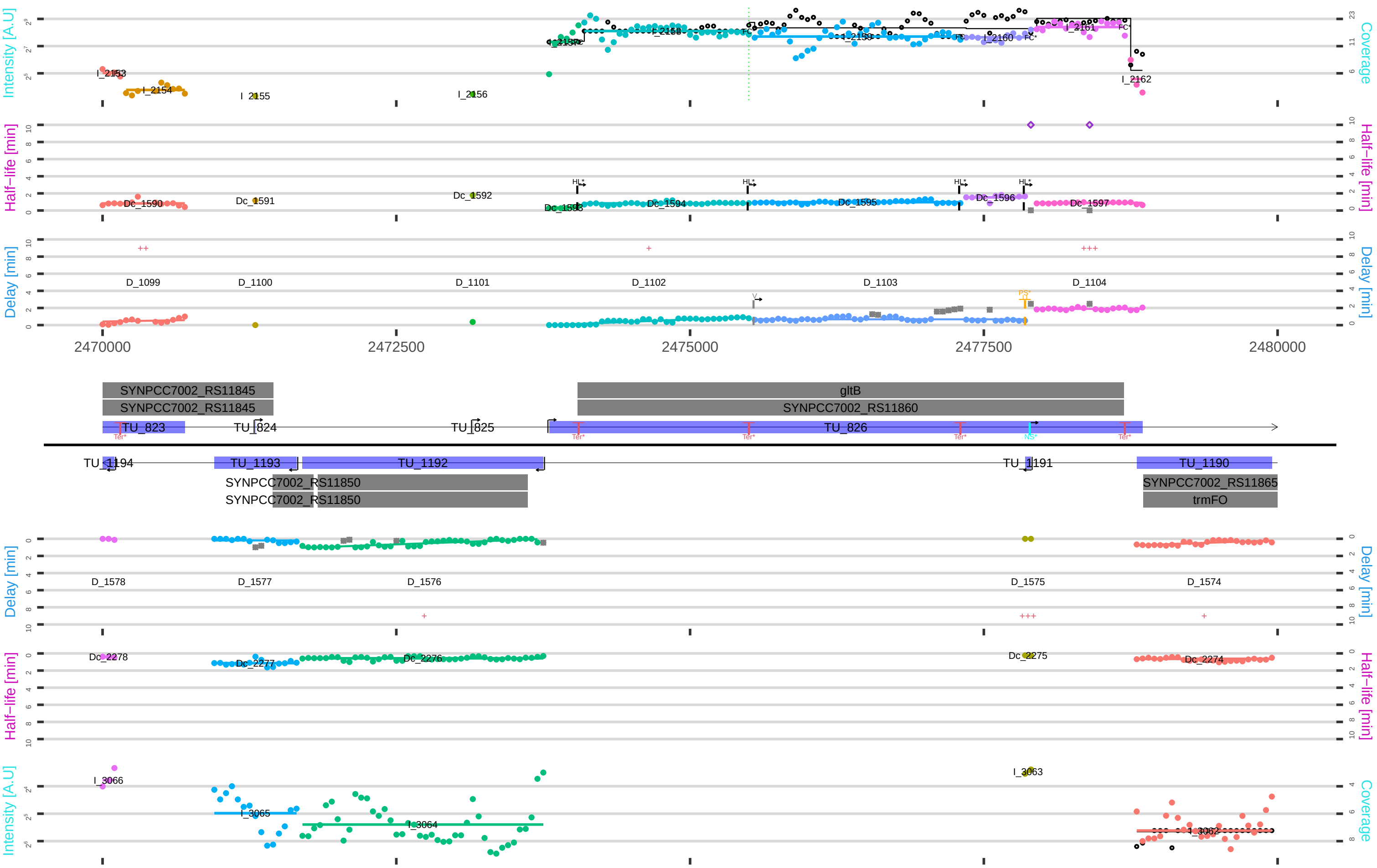

Term: termination (0), NS: new start (0), PS: pausing site (0), iTSS_I: internal starting site (0)

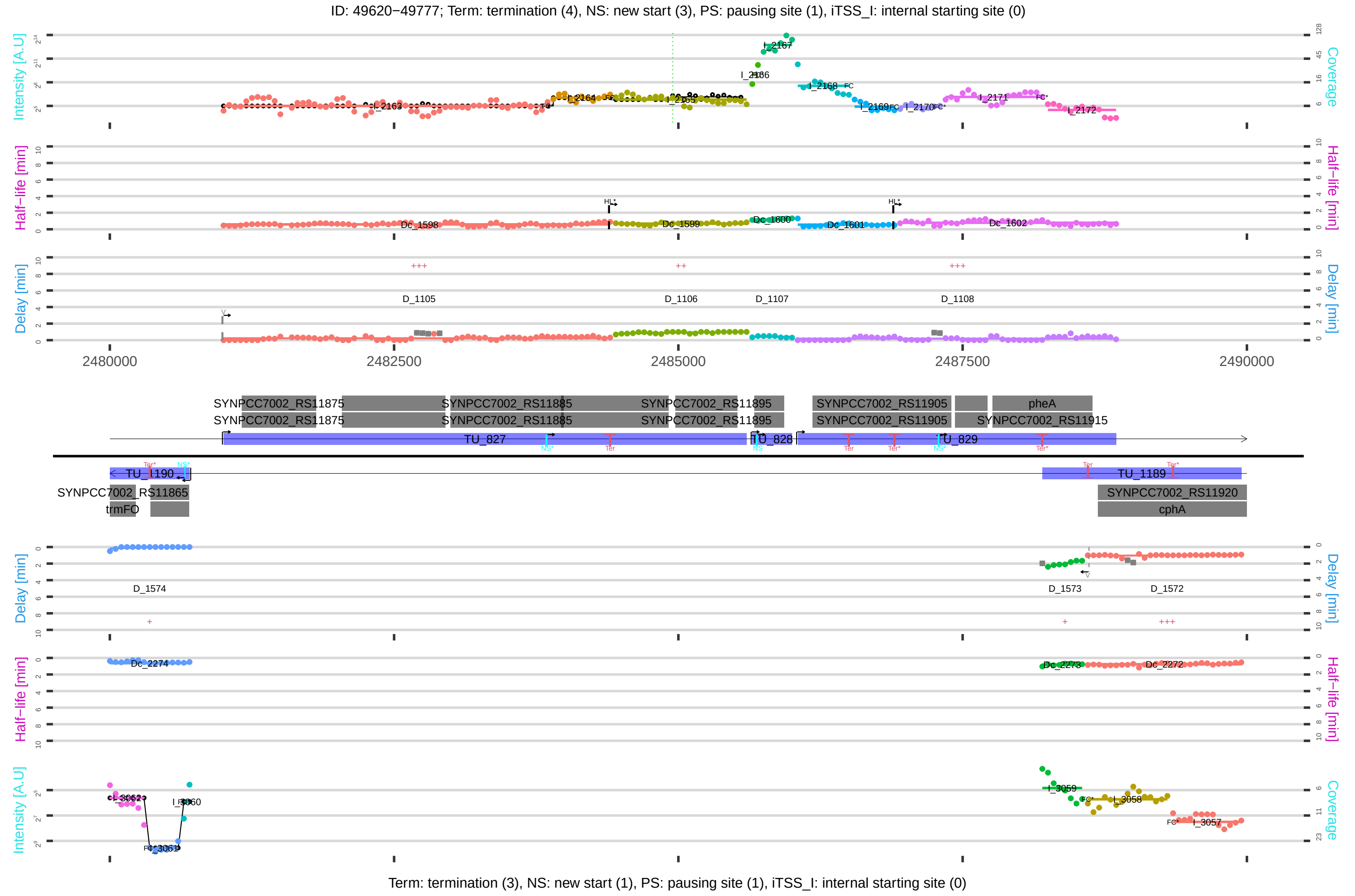
ID: 49620−49777; Term: termination (4), NS: new start (3), PS: pausing site (1), iTSS_I: internal starting site (0)
Intensity [A.U]
Coverage
Half−life [min]
Delay [min]
2480000
2482500
2485000
2487500
2490000
SYNPCC7002_RS11875
SYNPCC7002_RS11885
SYNPCC7002_RS11895
SYNPCC7002_RS11905
pheA
SYNPCC7002_RS11915
TU_827
TU_828
TU_829
TU_1190
TU_1189
SYNPCC7002_RS11865
trmFO
SYNPCC7002_RS11920
cphA
Term: termination (3), NS: new start (1), PS: pausing site (1), iTSS_I: internal starting site (0)

ID: 49852−49999; Term: termination (4), NS: new start (0), PS: pausing site (0), iTSS_I: internal starting site (1)

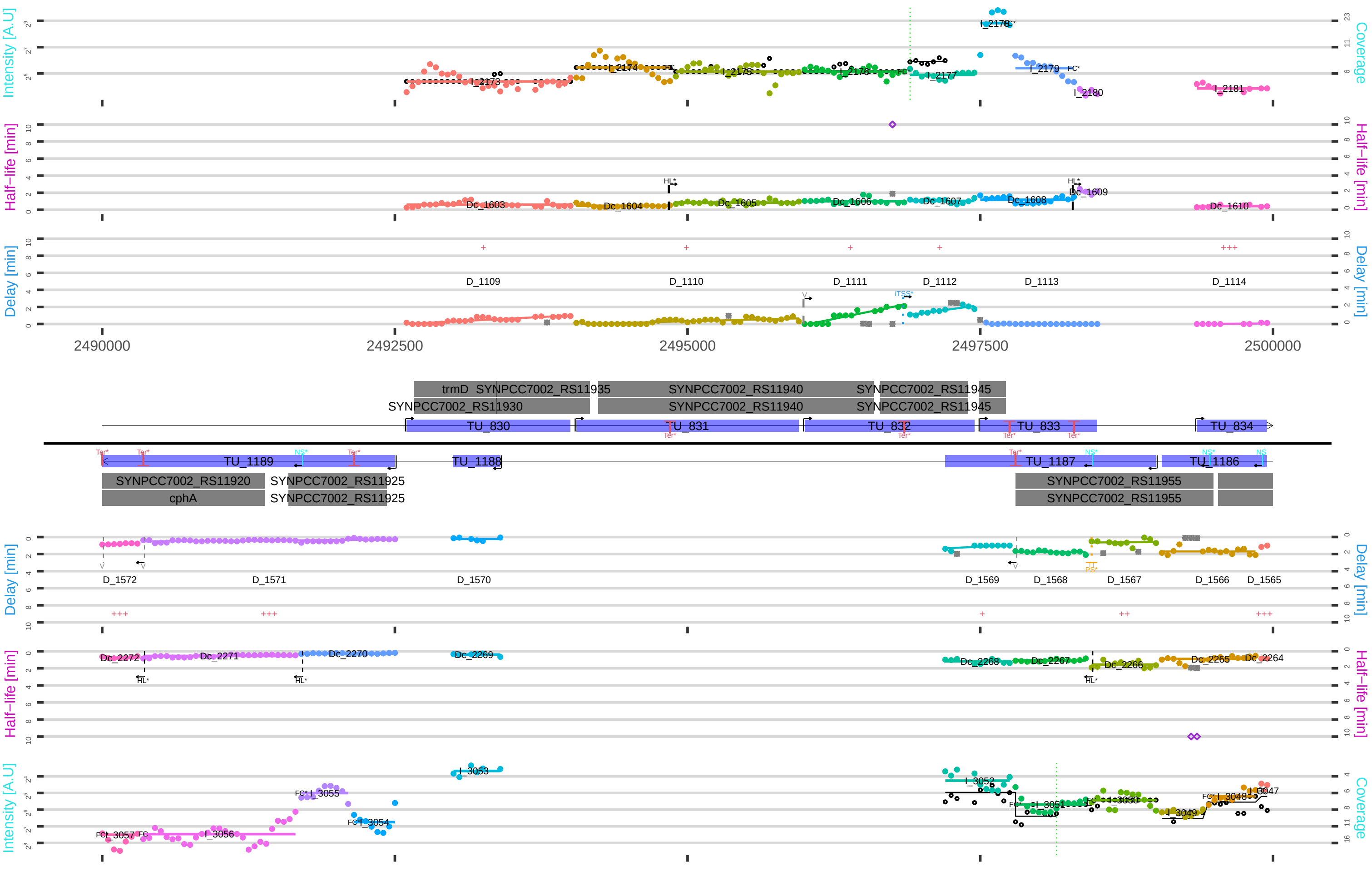

Term: termination (4), NS: new start (4), PS: pausing site (3), iTSS_I: internal starting site (1)

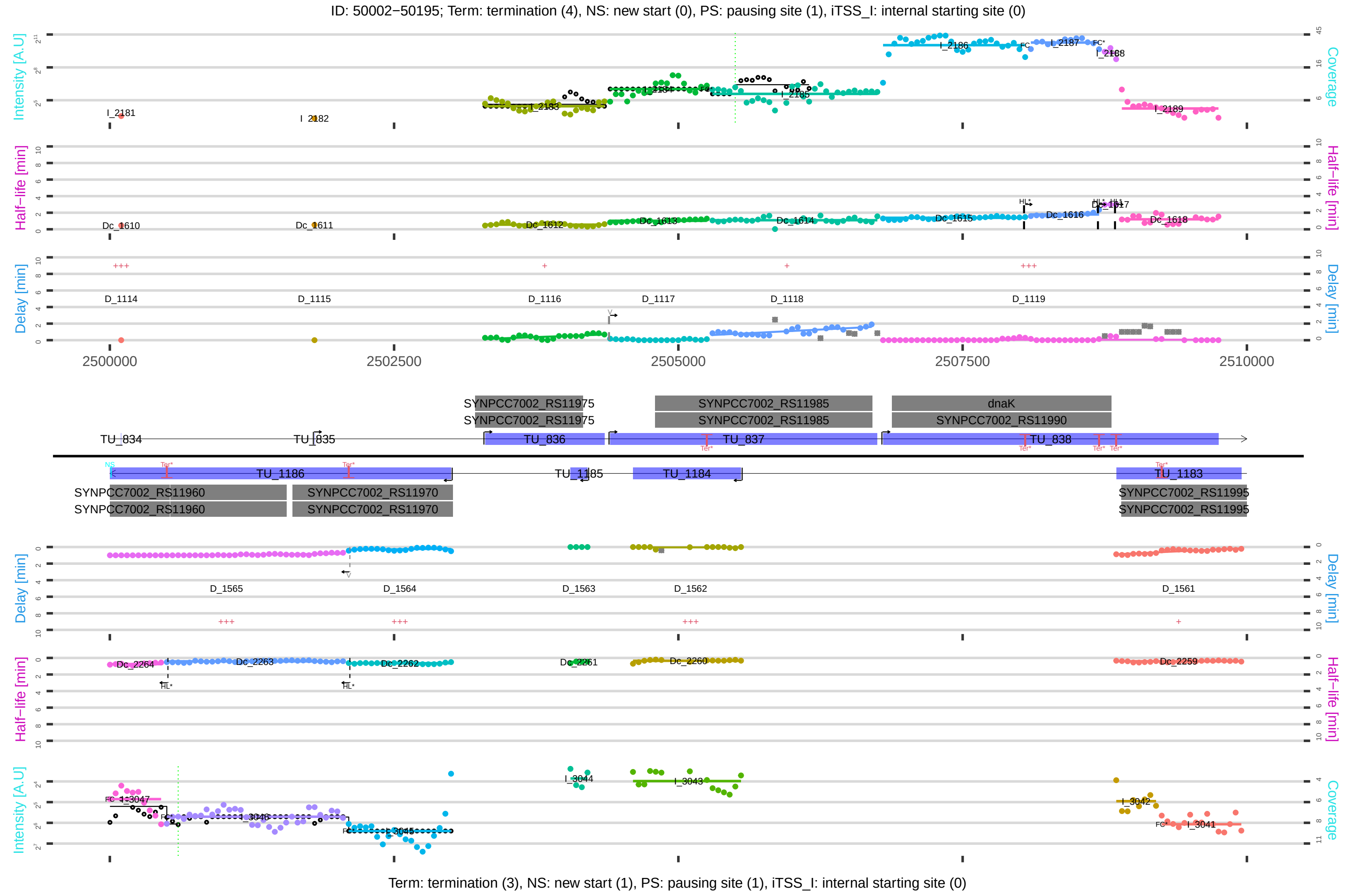

ID: 50002−50195; Term: termination (4), NS: new start (0), PS: pausing site (1), iTSS_I: internal starting site (0)
Intensity [A.U]
Coverage
Half−life [min]
Delay [min]
I_2181
I_2182
I_2183
I_2184
I_2185
I_2186
I_2187
I_2188
I_2189
Dc_1610
Dc_1611
Dc_1612
Dc_1613
Dc_1614
Dc_1615
Dc_1616
Dc_1617
Dc_1618
D_1114
D_1115
D_1116
D_1117
D_1118
D_1119
2500000
2502500
2505000
2507500
2510000
SYNPCC7002_RS11975
SYNPCC7002_RS11985
dnaK
SYNPCC7002_RS11990
TU_834
TU_835
TU_836
TU_837
TU_838
TU_1186
TU_1185
TU_1184
TU_1183
SYNPCC7002_RS11960
SYNPCC7002_RS11970
SYNPCC7002_RS11995
D_1565
D_1564
D_1563
D_1562
D_1561
Dc_2264
Dc_2263
Dc_2262
Dc_2261
Dc_2260
Dc_2259
I_3047
I_3046
I_3045
I_3044
I_3043
I_3042
I_3041
Term: termination (3), NS: new start (1), PS: pausing site (1), iTSS_I: internal starting site (0)

ID: 50208−50400; Term: termination (1), NS: new start (1), PS: pausing site (0), iTSS_I: internal starting site (0)

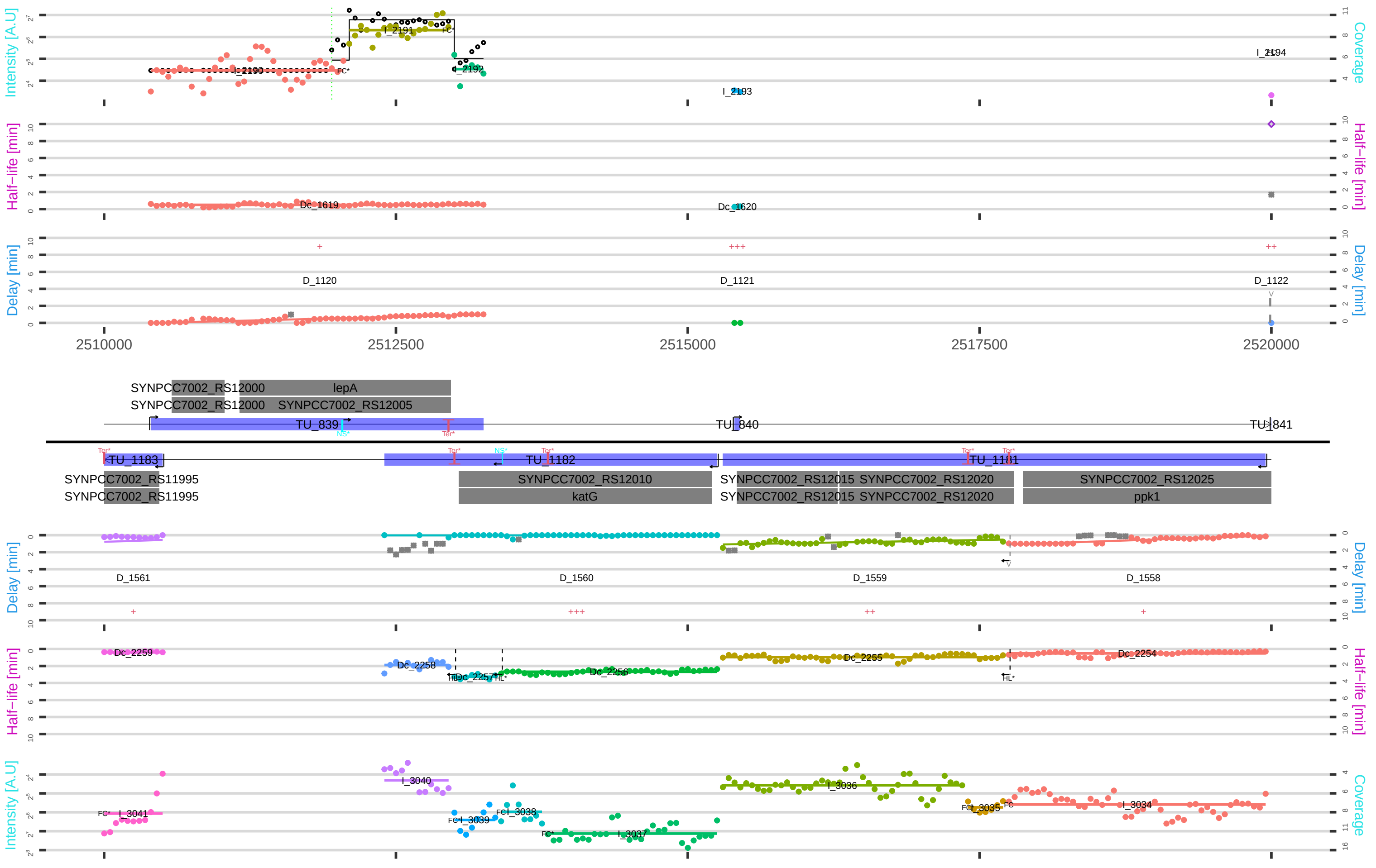

Term: termination (5), NS: new start (1), PS: pausing site (0), iTSS_I: internal starting site (1)

ID: 50400−50600; Term: termination (3), NS: new start (2), PS: pausing site (1), iTSS_I: internal starting site (0)

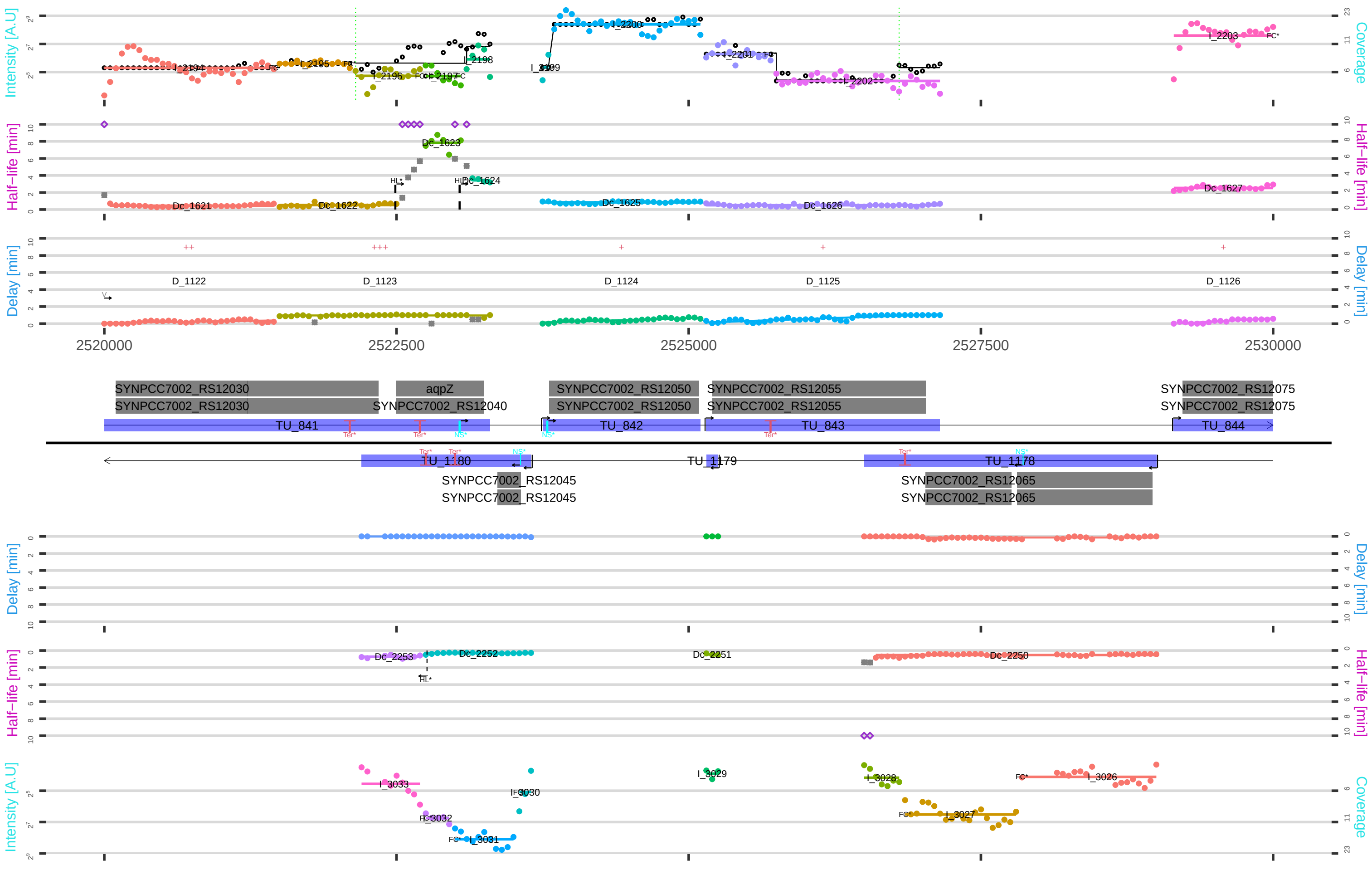

Term: termination (3), NS: new start (2), PS: pausing site (0), iTSS_I: internal starting site (0)

ID: 50600−50800; Term: termination (3), NS: new start (4), PS: pausing site (0), iTSS_I: internal starting site (0)

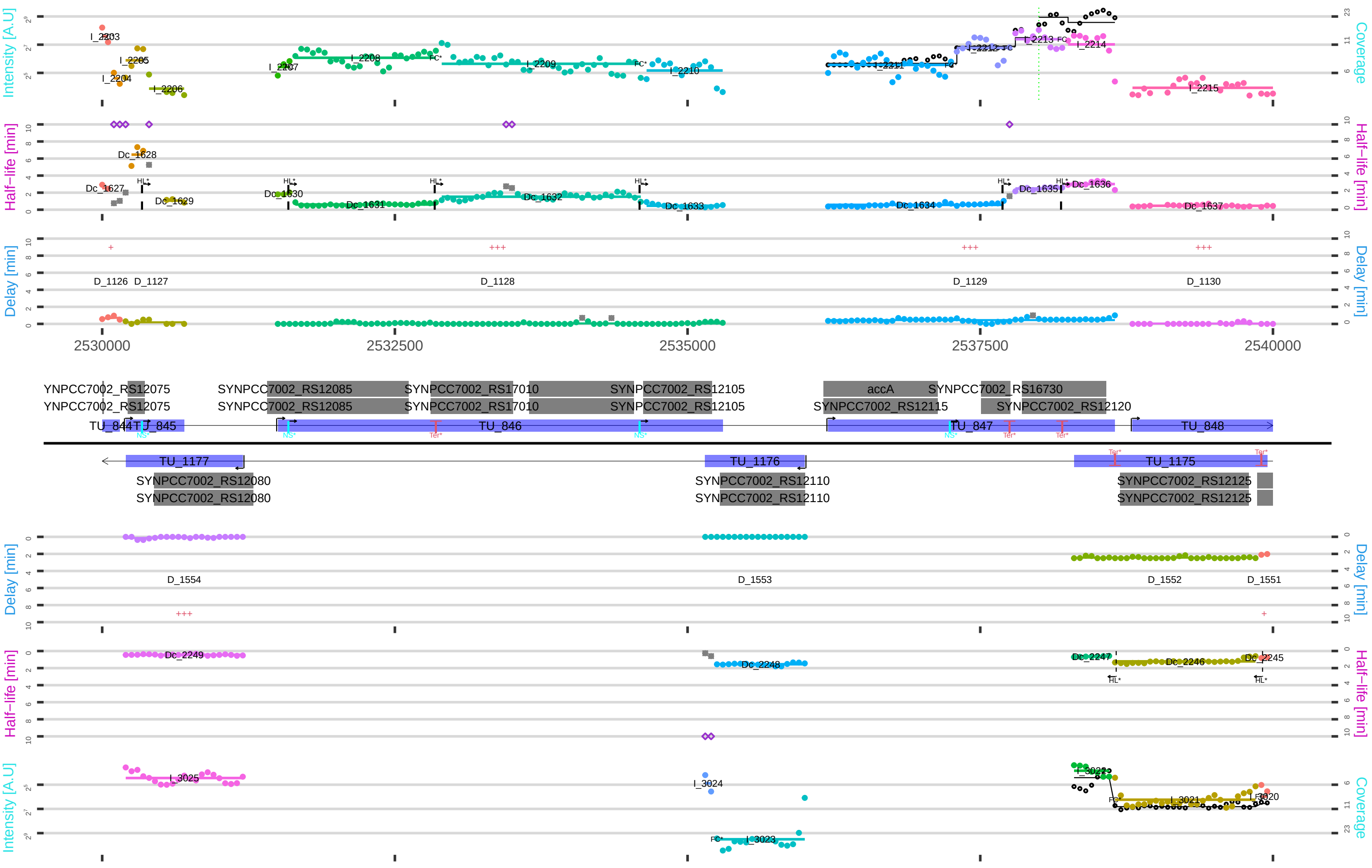

Term: termination (2), NS: new start (0), PS: pausing site (1), iTSS_I: internal starting site (0)

ID: 50800−50968; Term: termination (2), NS: new start (1), PS: pausing site (1), iTSS_I: internal starting site (0)

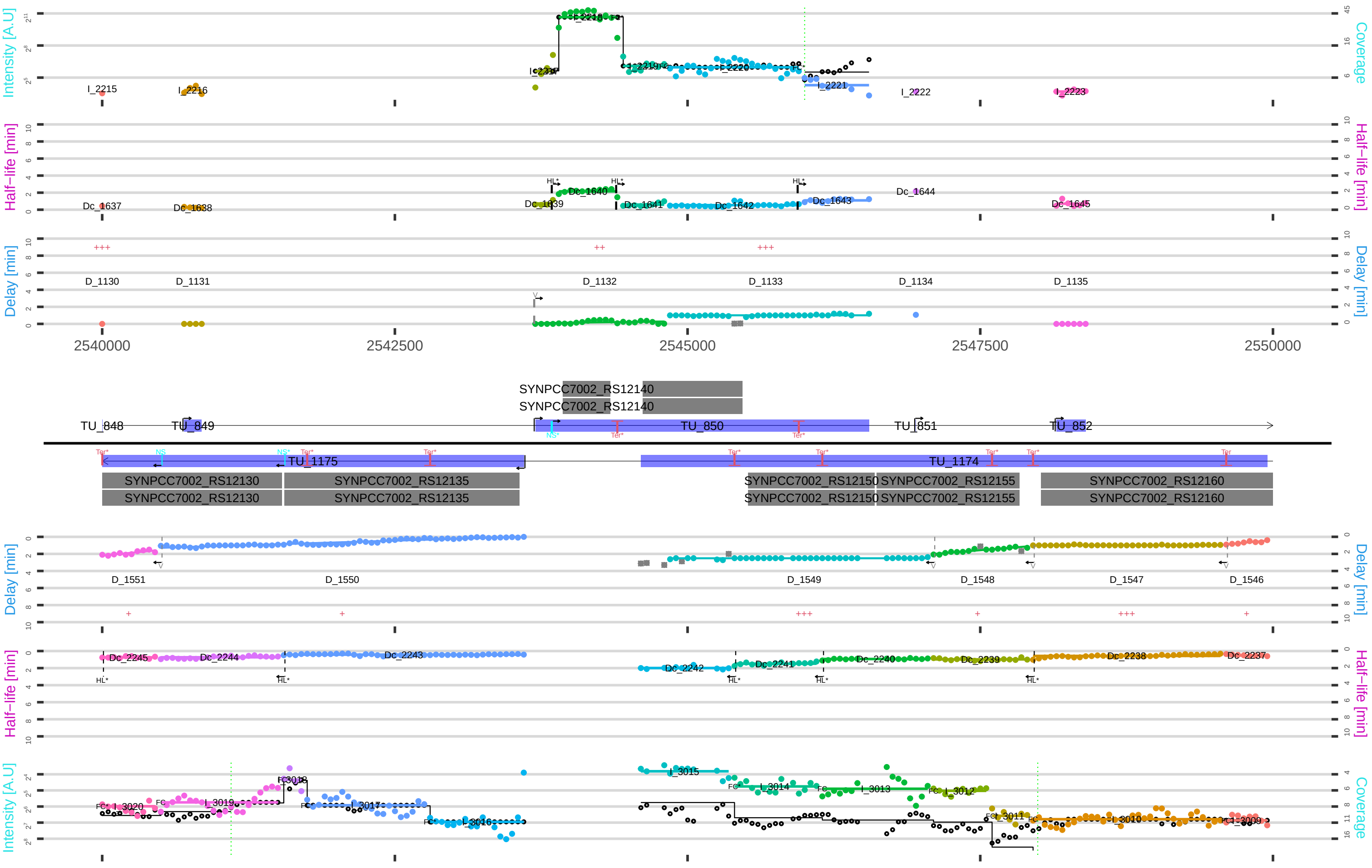

Term: termination (8), NS: new start (2), PS: pausing site (4), iTSS_I: internal starting site (0)

ID: 51026−51170; Term: termination (3), NS: new start (2), PS: pausing site (1), iTSS_I: internal starting site (0)

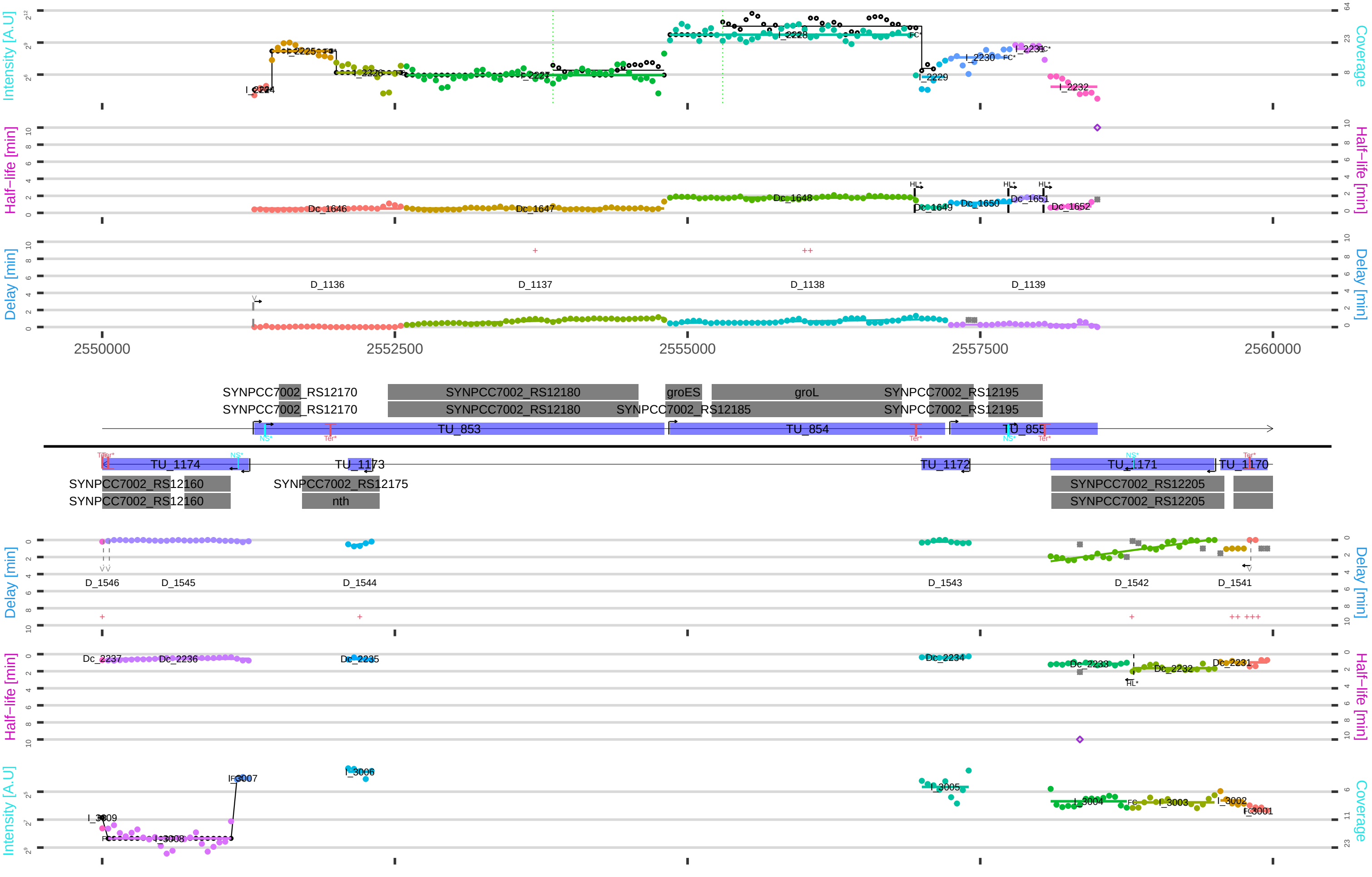

Term: termination (3), NS: new start (2), PS: pausing site (2), iTSS_I: internal starting site (0)

ID: 51229−51400; Term: termination (2), NS: new start (4), PS: pausing site (3), iTSS_I: internal starting site (0)

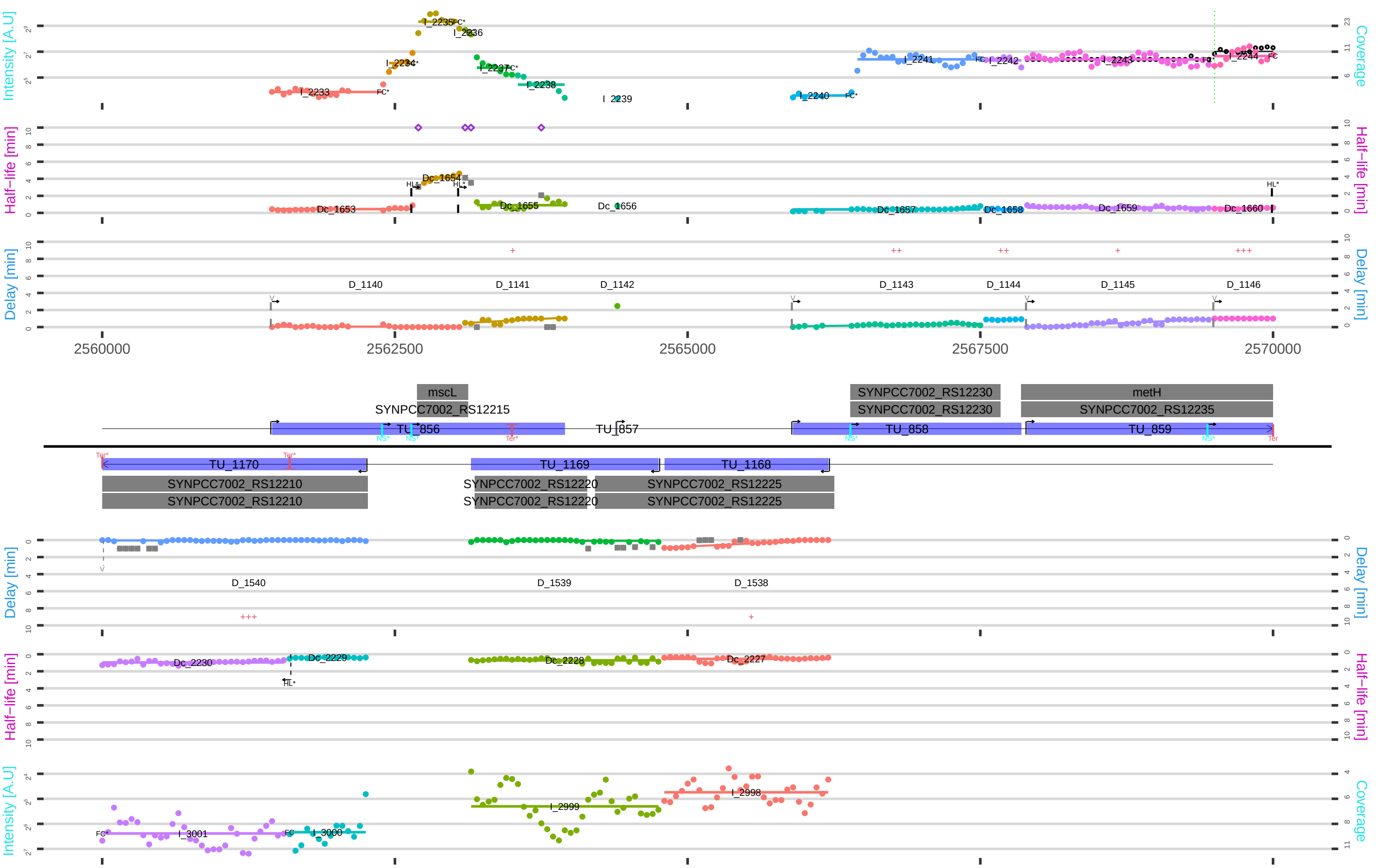

Term: termination (2), NS: new start (0), PS: pausing site (0), iTSS_I: internal starting site (0)

ID: 51400−51600; Term: termination (6), NS: new start (2), PS: pausing site (1), iTSS_I: internal starting site (1)

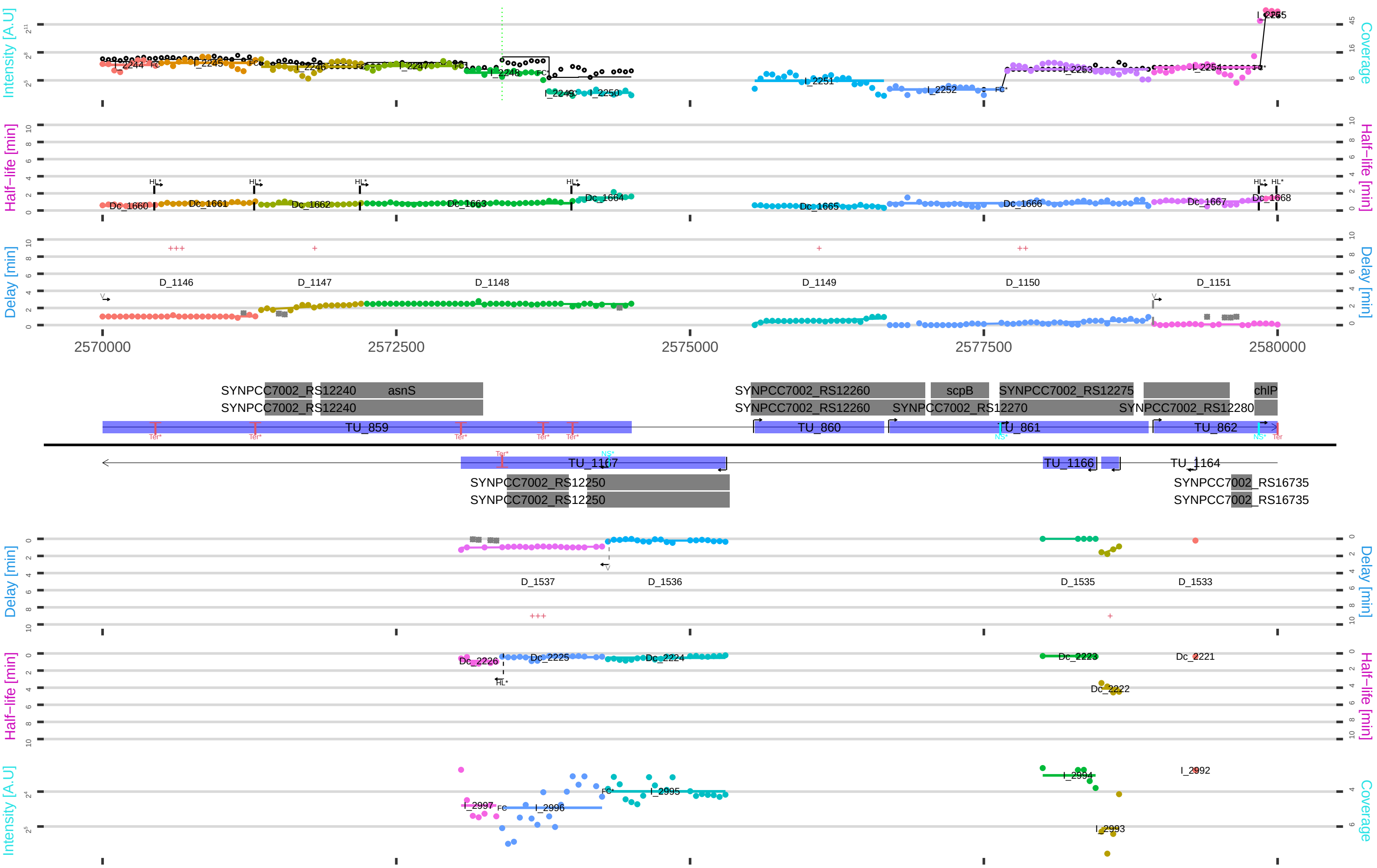

Term: termination (1), NS: new start (1), PS: pausing site (1), iTSS_I: internal starting site (0)

ID: 51600−51794; Term: termination (9), NS: new start (1), PS: pausing site (3), iTSS_I: internal starting site (0)

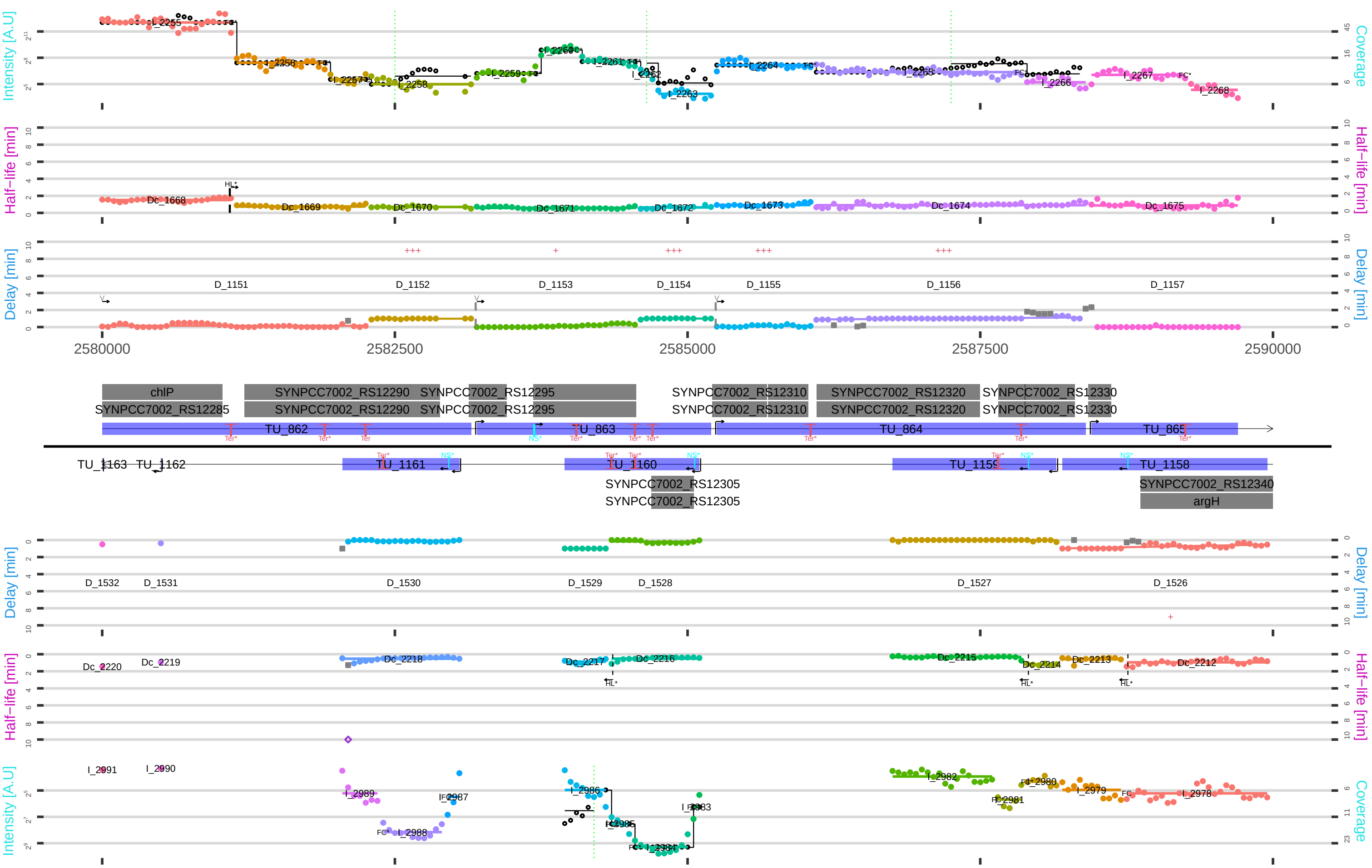

Term: termination (4), NS: new start (4), PS: pausing site (1), iTSS_I: internal starting site (0)

ID: 51811−52000; Term: termination (3), NS: new start (0), PS: pausing site (0), iTSS_I: internal starting site (0)

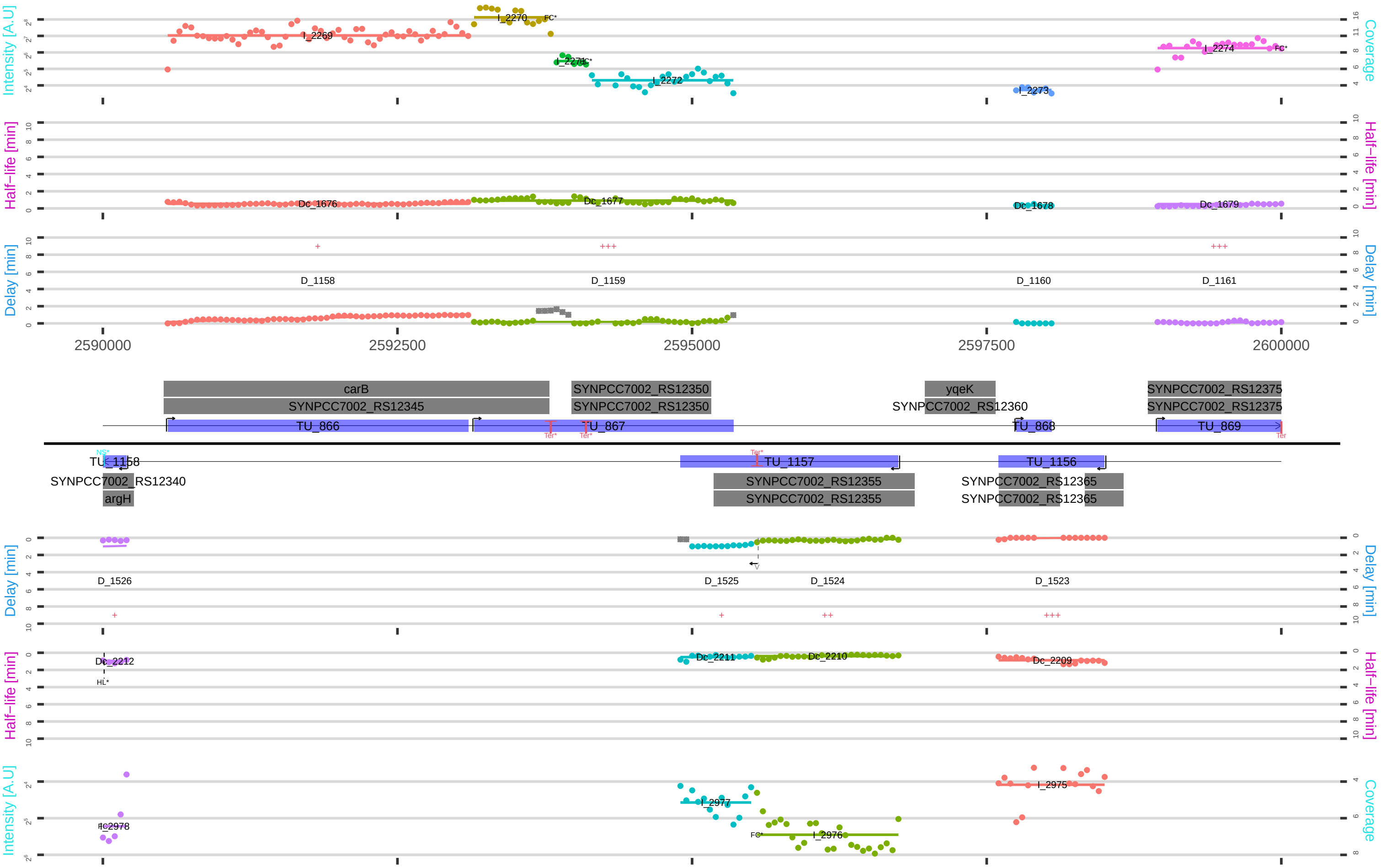

Term: termination (1), NS: new start (1), PS: pausing site (1), iTSS_I: internal starting site (0)

ID: 52000−52200; Term: termination (5), NS: new start (1), PS: pausing site (1), iTSS_I: internal starting site (0)

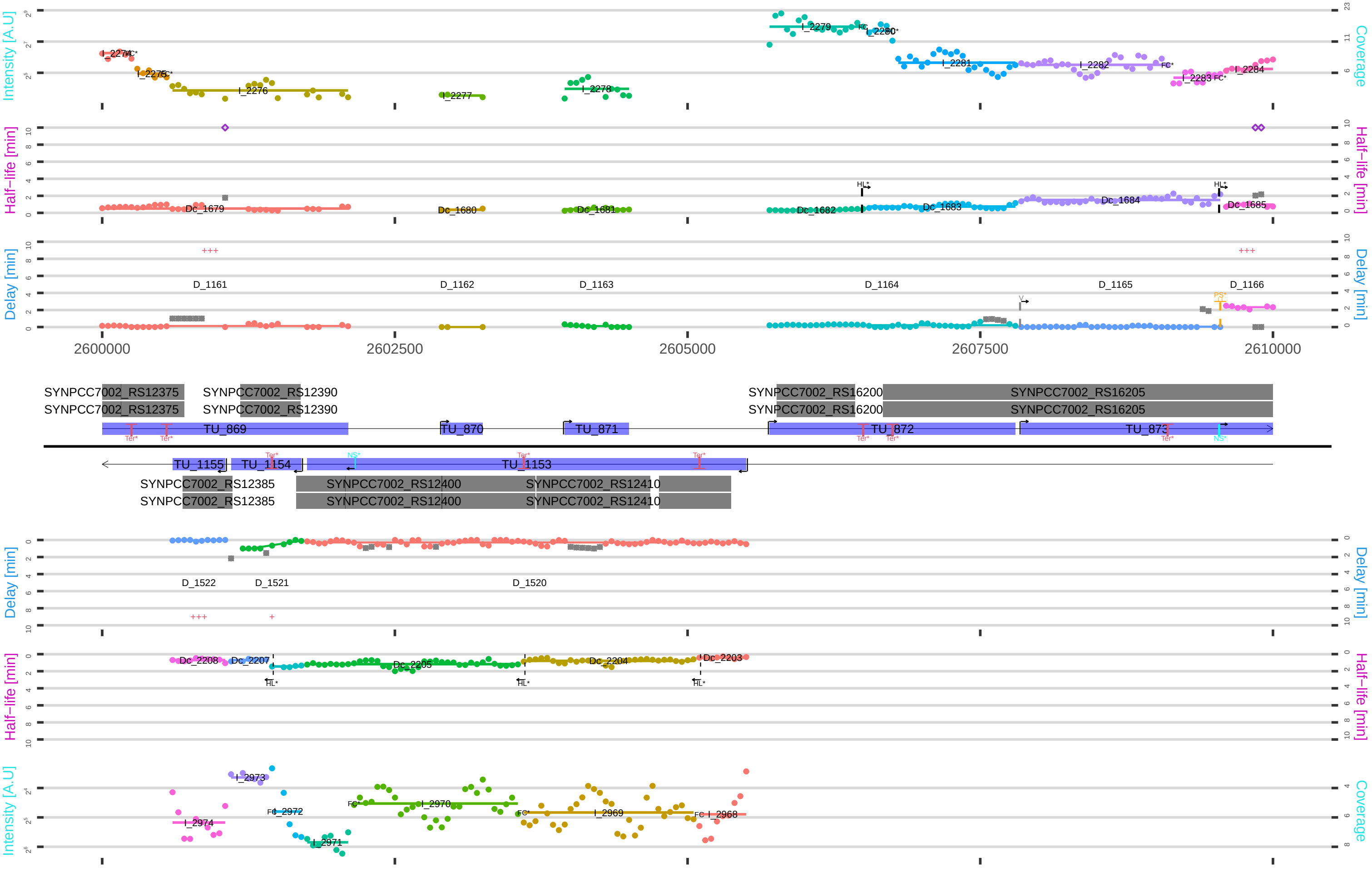

Term: termination (3), NS: new start (1), PS: pausing site (0), iTSS_I: internal starting site (0)

ID: 52200−52400; Term: termination (4), NS: new start (2), PS: pausing site (0), iTSS_I: internal starting site (0)

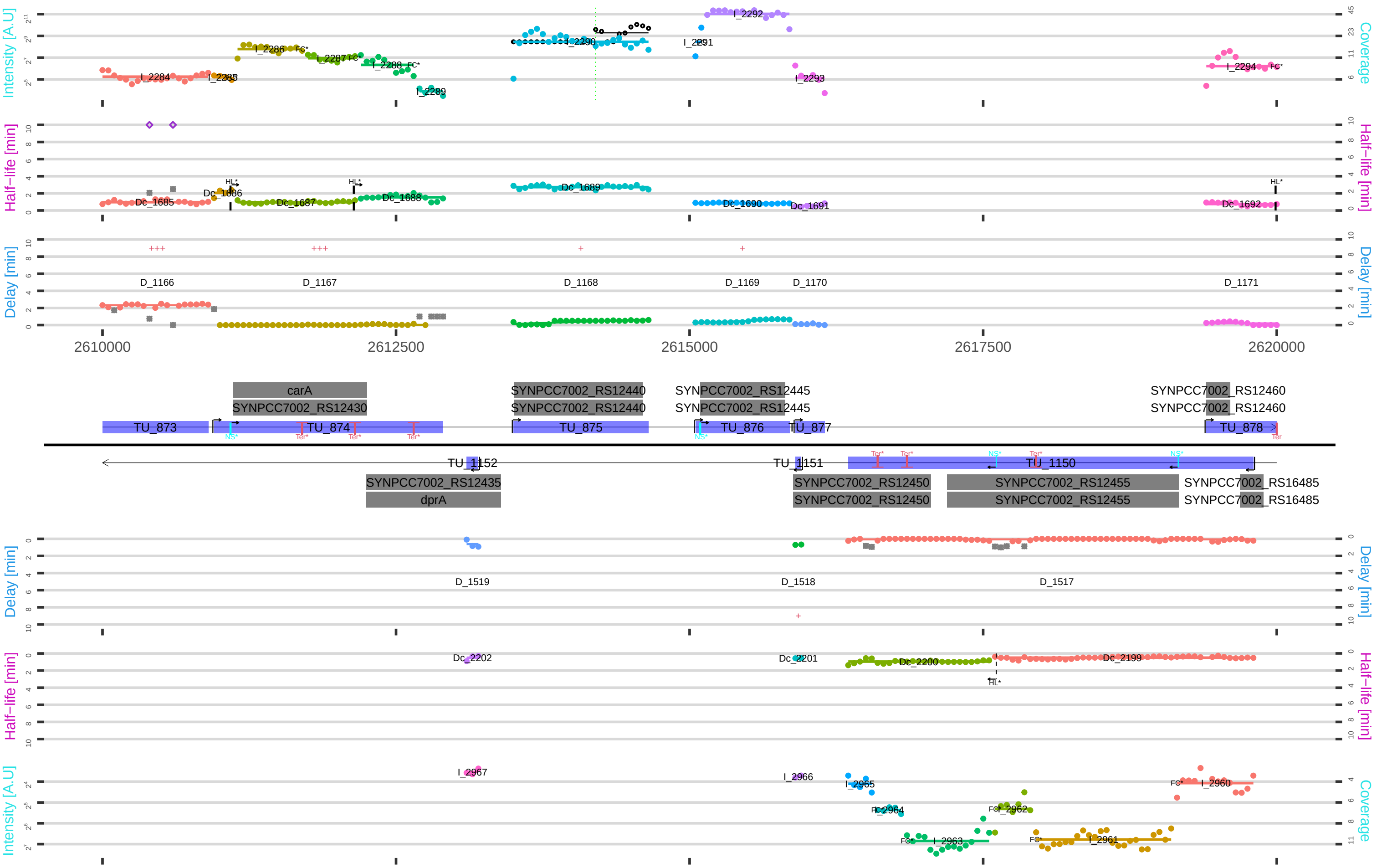

Term: termination (3), NS: new start (2), PS: pausing site (0), iTSS_I: internal starting site (0)

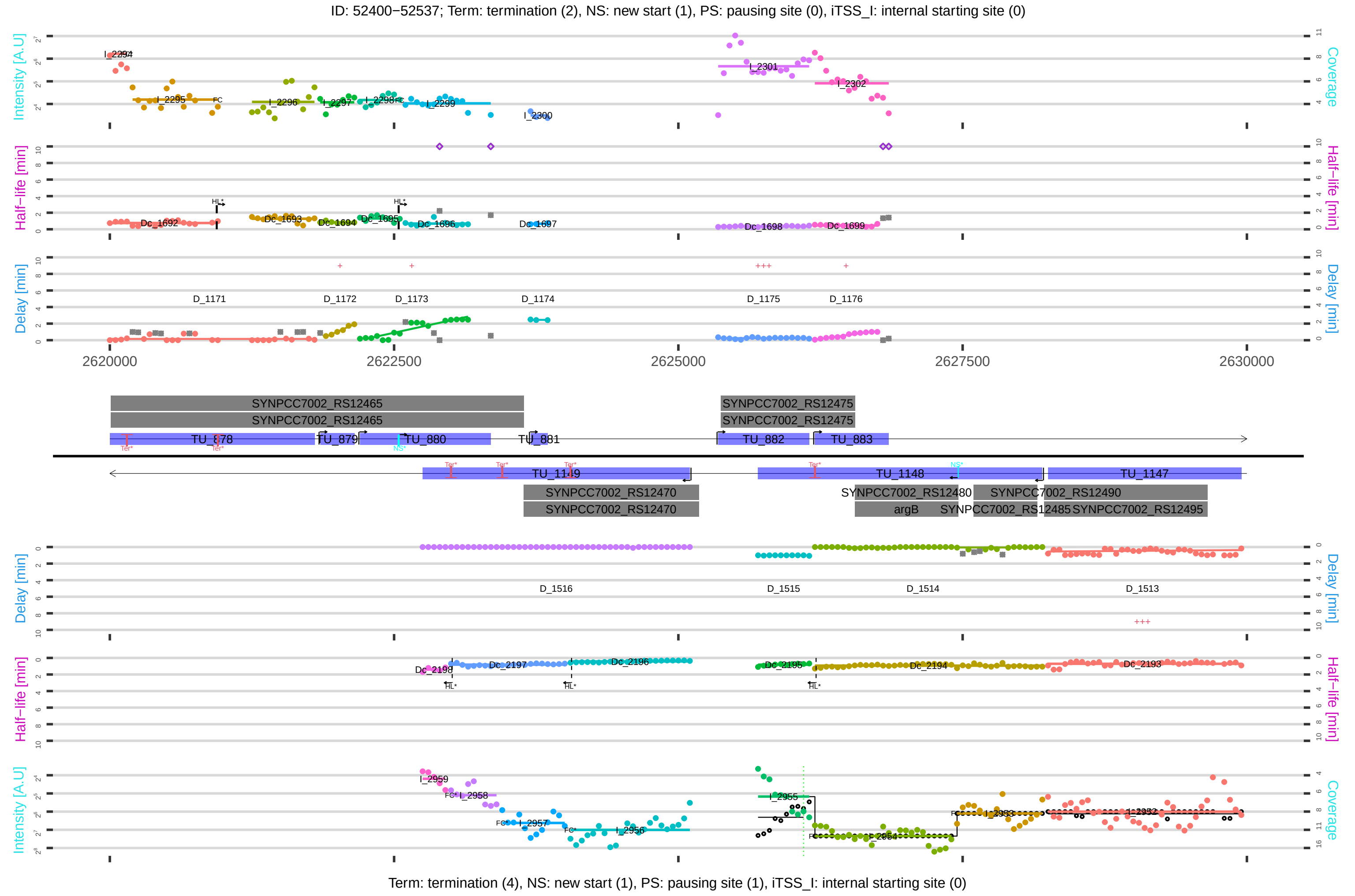
ID: 52400−52537; Term: termination (2), NS: new start (1), PS: pausing site (0), iTSS_I: internal starting site (0)
Intensity [A.U]
Coverage
Half−life [min]
Delay [min]
2620000
2622500
2625000
2627500
2630000
SYNPCC7002_RS12465
SYNPCC7002_RS12475
TU_878
TU_879
TU_880
TU_881
TU_882
TU_883
TU_1149
TU_1148
TU_1147
SYNPCC7002_RS12470
SYNPCC7002_RS12480
SYNPCC7002_RS12490
argB
SYNPCC7002_RS12485
SYNPCC7002_RS12495
D_1516
D_1515
D_1514
D_1513
Term: termination (4), NS: new start (1), PS: pausing site (1), iTSS_I: internal starting site (0)

ID: 52602−52800; Term: termination (7), NS: new start (3), PS: pausing site (2), iTSS_I: internal starting site (0)

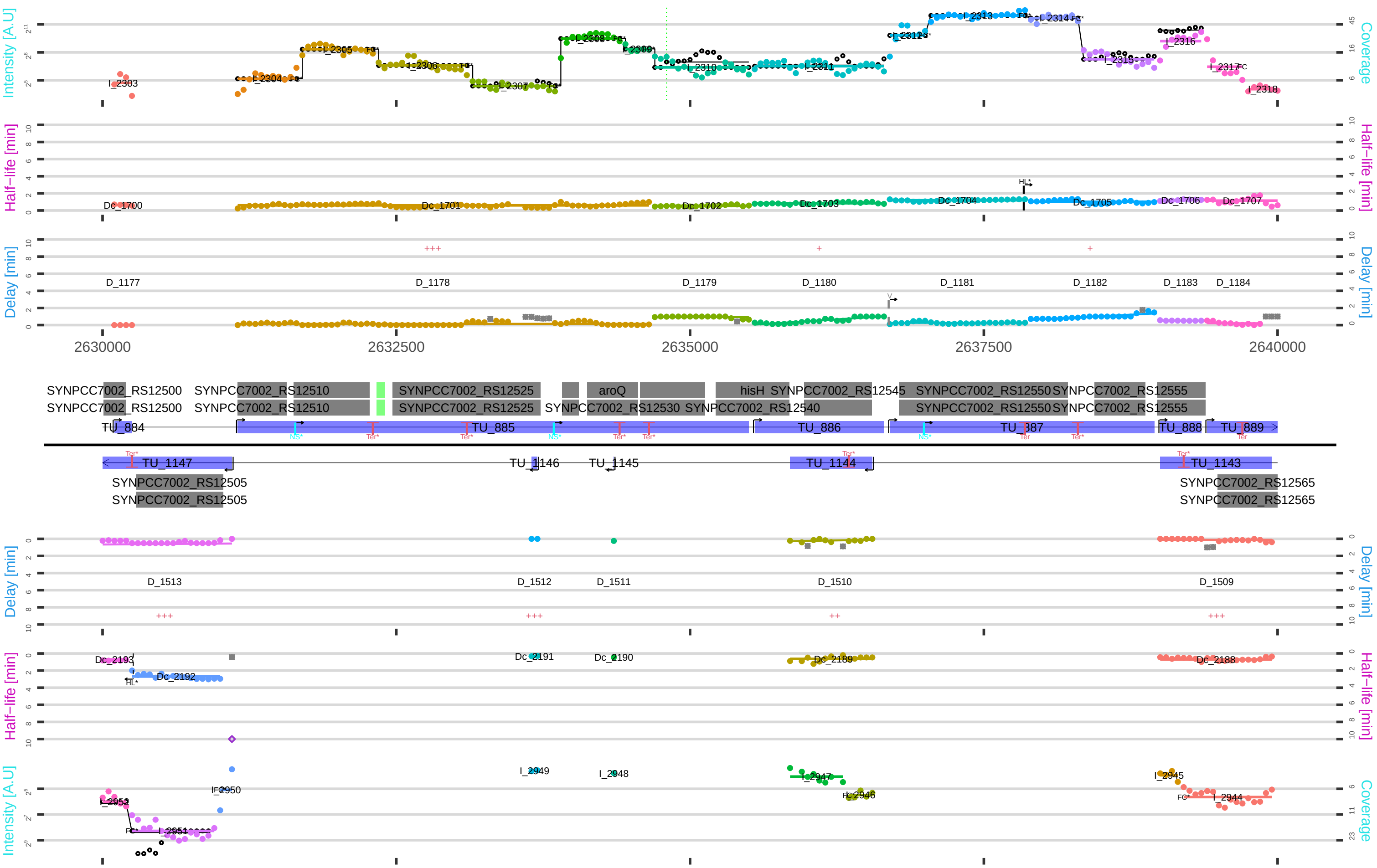

Term: termination (3), NS: new start (0), PS: pausing site (0), iTSS_I: internal starting site (0)

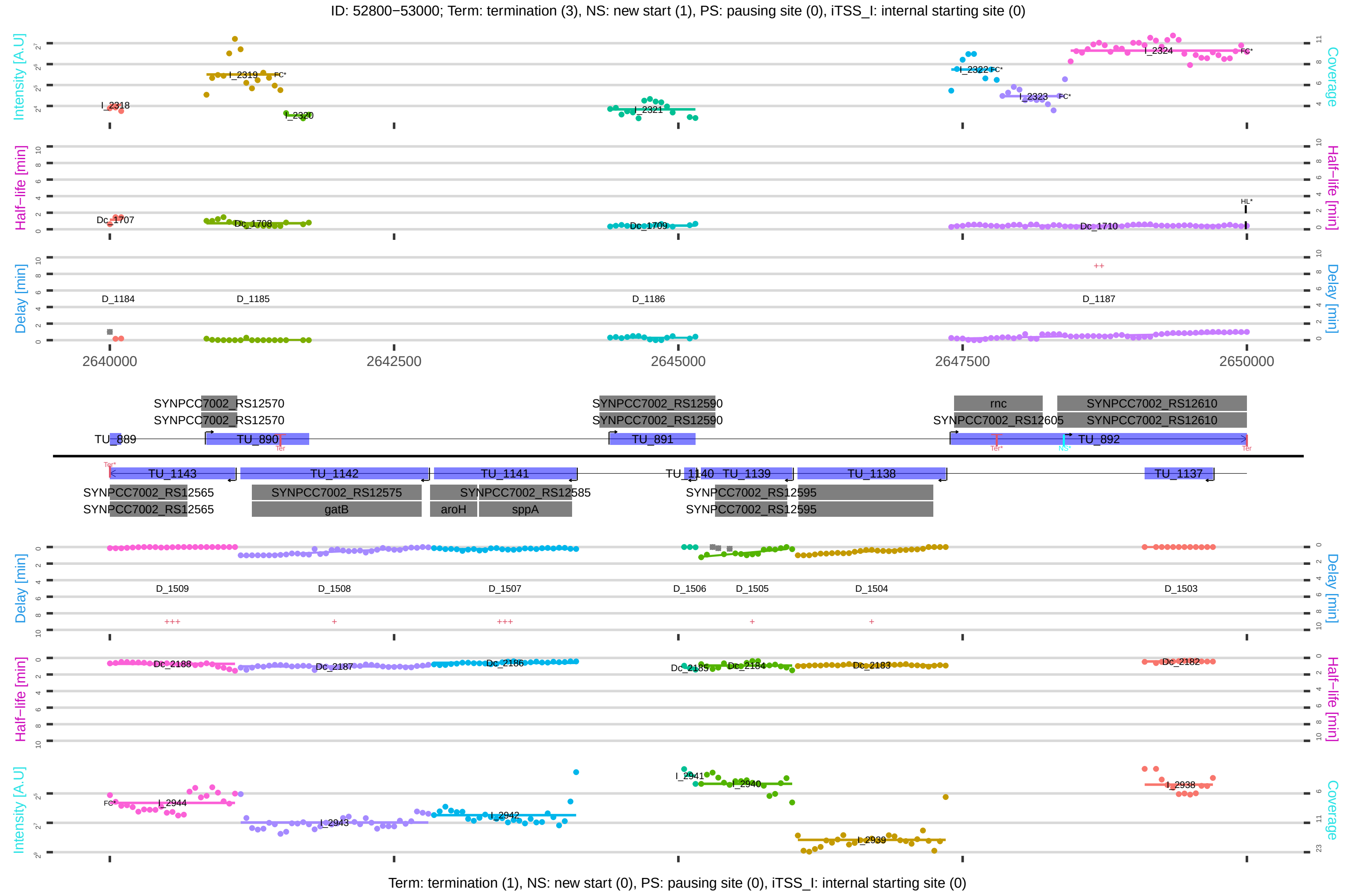
ID: 52800−53000; Term: termination (3), NS: new start (1), PS: pausing site (0), iTSS_I: internal starting site (0)
Intensity [A.U]
Coverage
Half−life [min]
Delay [min]
I_2318
I_2319
I_2320
I_2321
I_2322
I_2323
I_2324
Dc_1707
Dc_1708
Dc_1709
Dc_1710
D_1184
D_1185
D_1186
D_1187
2640000
2642500
2645000
2647500
2650000
SYNPCC7002_RS12570
SYNPCC7002_RS12590
rnc
SYNPCC7002_RS12605
SYNPCC7002_RS12610
TU_889
TU_890
TU_891
TU_892
TU_1143
TU_1142
TU_1141
TU_1140
TU_1139
TU_1138
TU_1137
SYNPCC7002_RS12565
SYNPCC7002_RS12575
gatB
aroH
SYNPCC7002_RS12585
sppA
SYNPCC7002_RS12595
D_1509
D_1508
D_1507
D_1506
D_1505
D_1504
D_1503
Dc_2188
Dc_2187
Dc_2186
Dc_2185
Dc_2184
Dc_2183
Dc_2182
I_2944
I_2943
I_2942
I_2941
I_2940
I_2939
I_2938
Term: termination (1), NS: new start (0), PS: pausing site (0), iTSS_I: internal starting site (0)

ID: 53000−53200; Term: termination (7), NS: new start (3), PS: pausing site (0), iTSS_I: internal starting site (0)

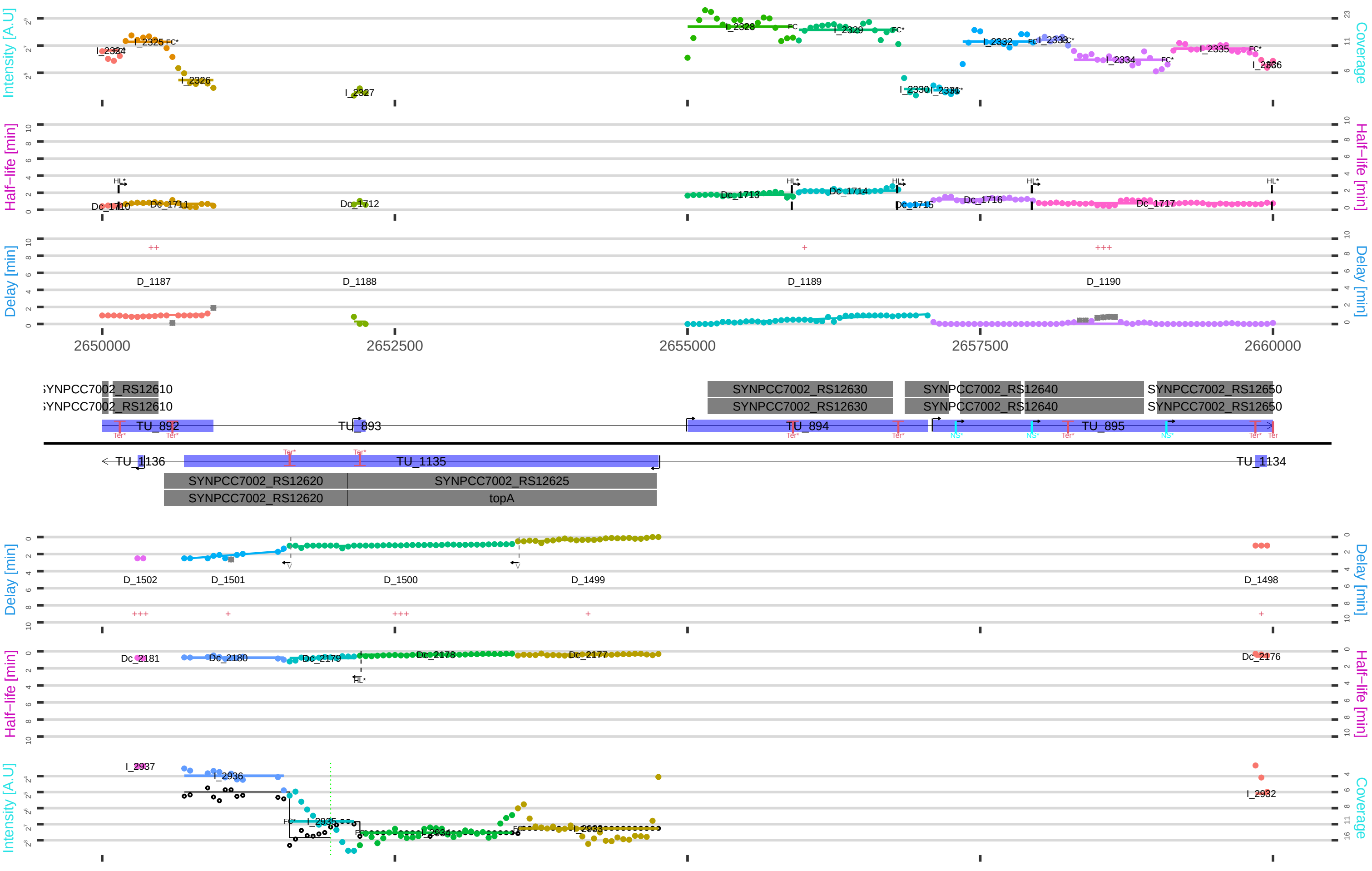

Term: termination (2), NS: new start (0), PS: pausing site (2), iTSS_I: internal starting site (0)

ID: 53200−53318; Term: termination (1), NS: new start (0), PS: pausing site (0), iTSS_I: internal starting site (0)

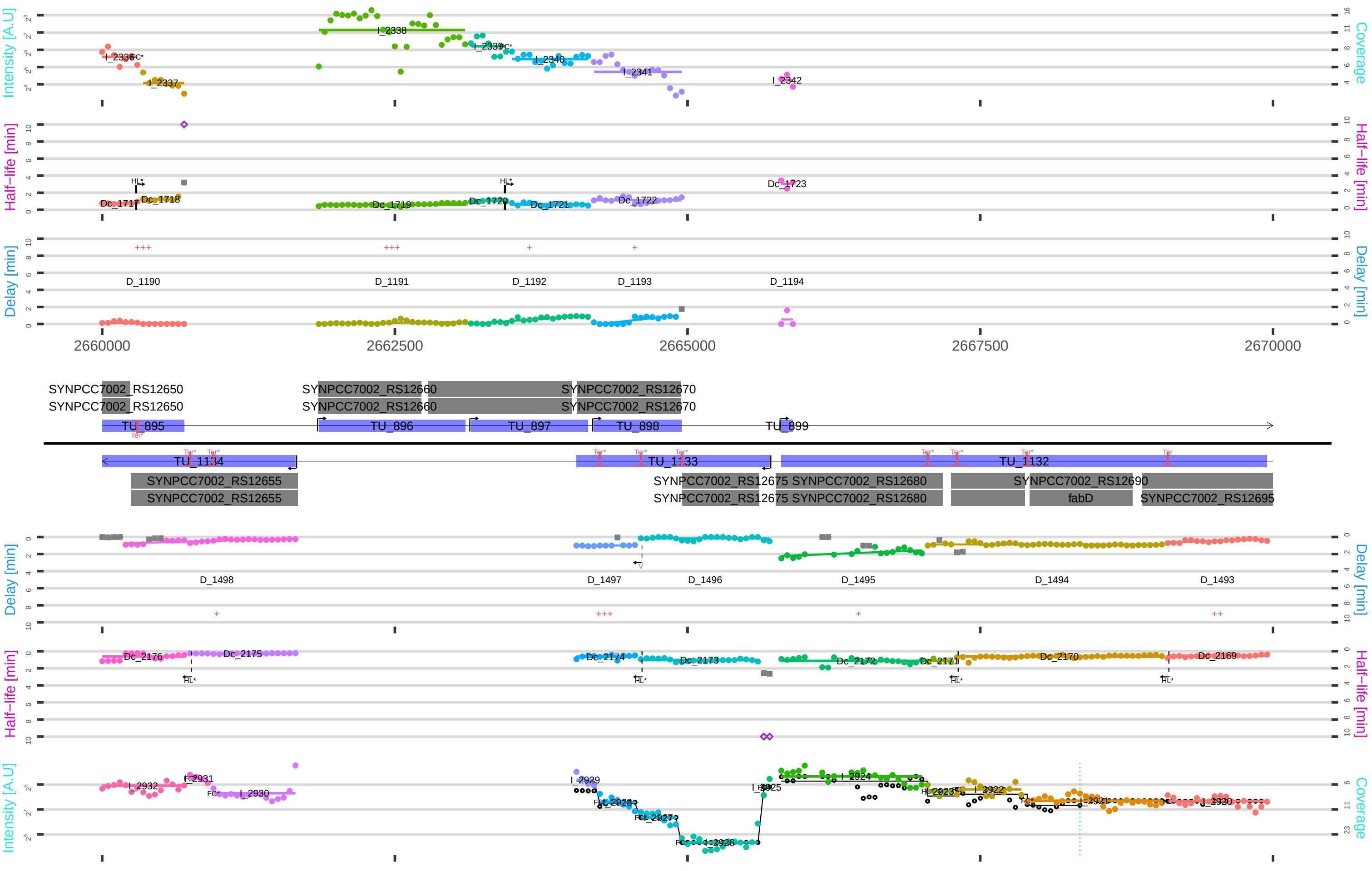

Term: termination (9), NS: new start (0), PS: pausing site (3), iTSS_I: internal starting site (0)

ID: 53409−53600; Term: termination (4), NS: new start (2), PS: pausing site (1), iTSS_I: internal starting site (0)

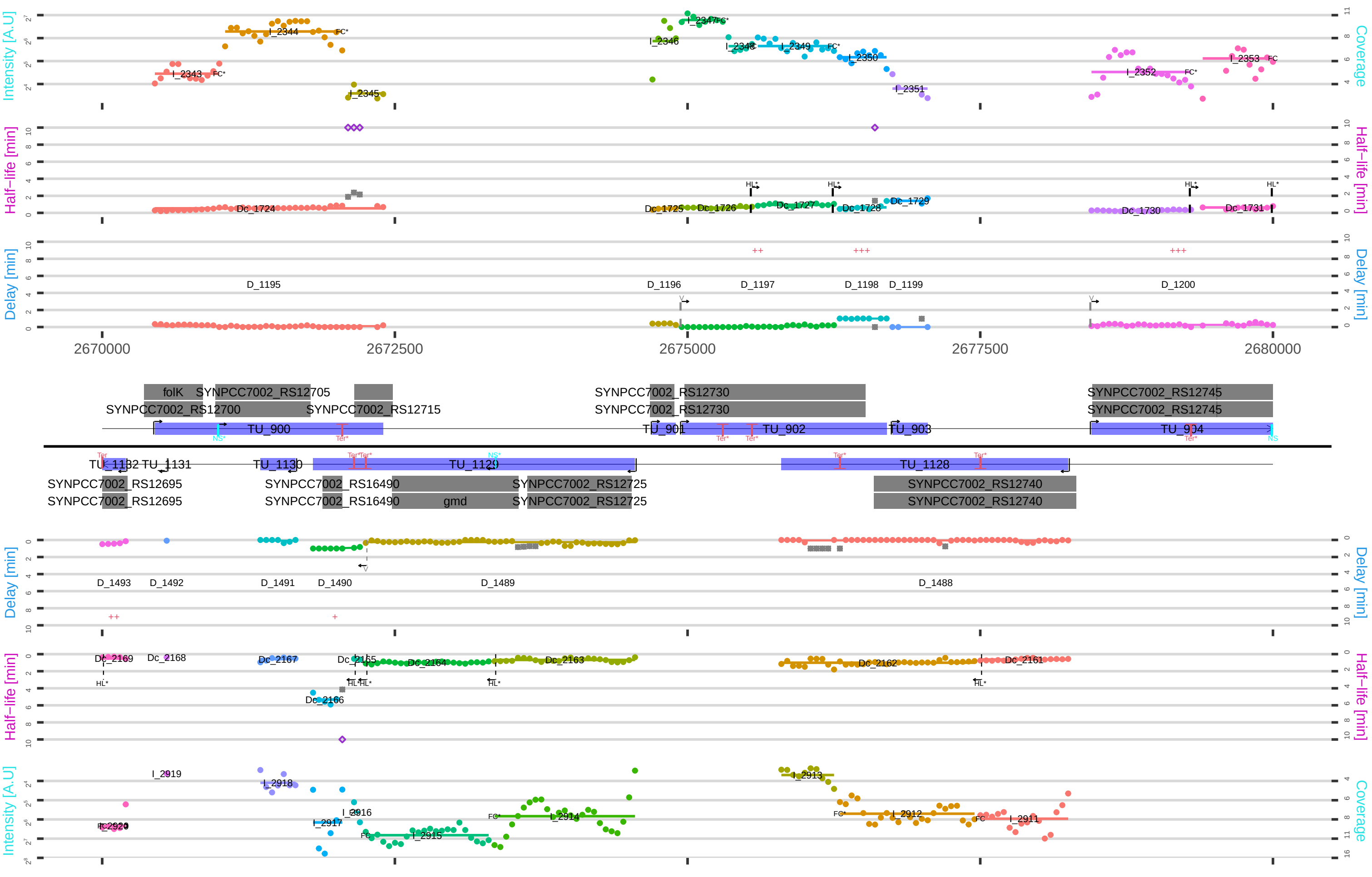

Term: termination (5), NS: new start (1), PS: pausing site (1), iTSS_I: internal starting site (0)

ID: 53600−53799; Term: termination (5), NS: new start (1), PS: pausing site (1), iTSS_I: internal starting site (0)

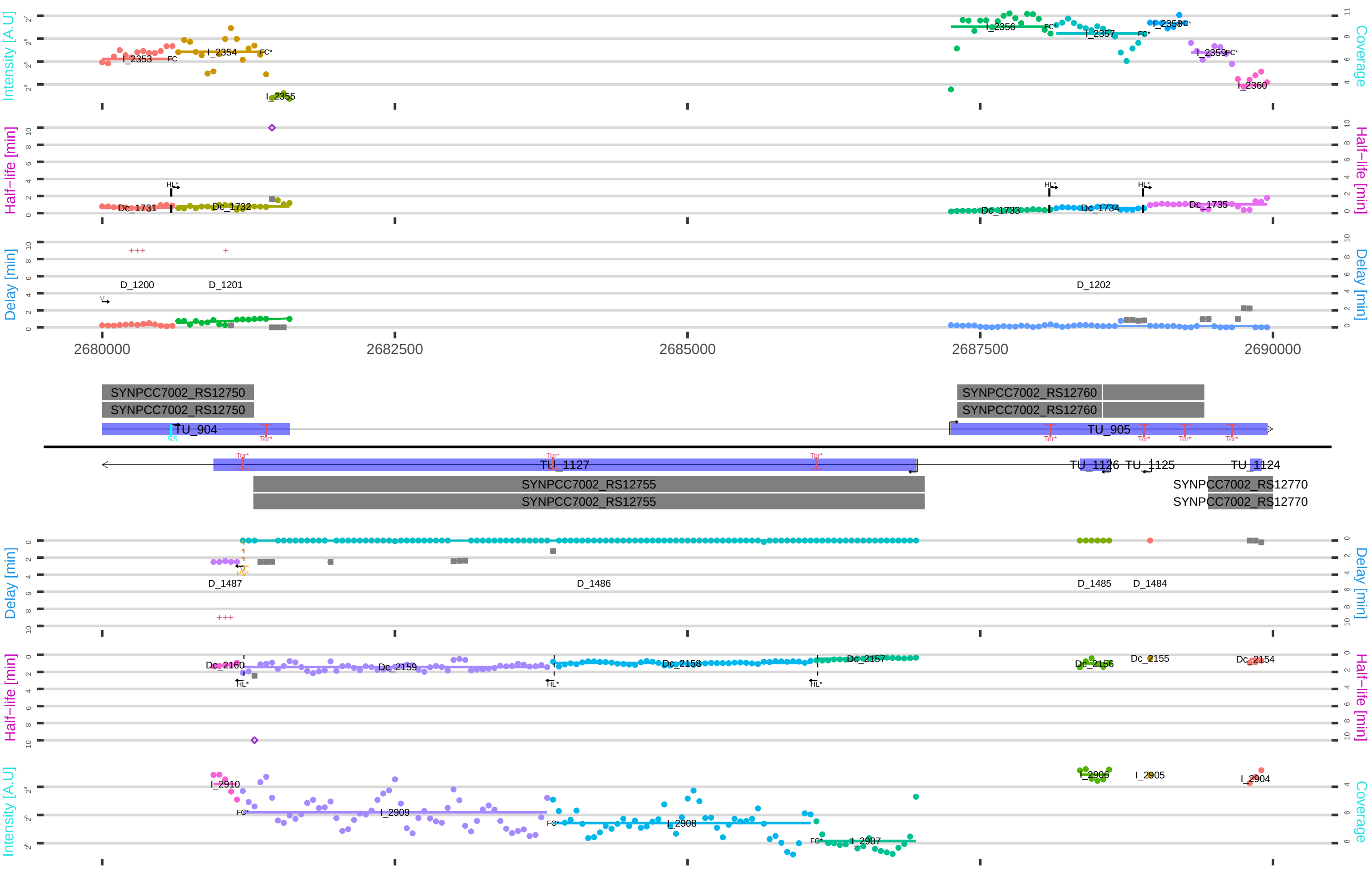

Term: termination (3), NS: new start (0), PS: pausing site (1), iTSS_I: internal starting site (0)

ID: 53805−54000; Term: termination (5), NS: new start (1), PS: pausing site (3), iTSS_I: internal starting site (0)

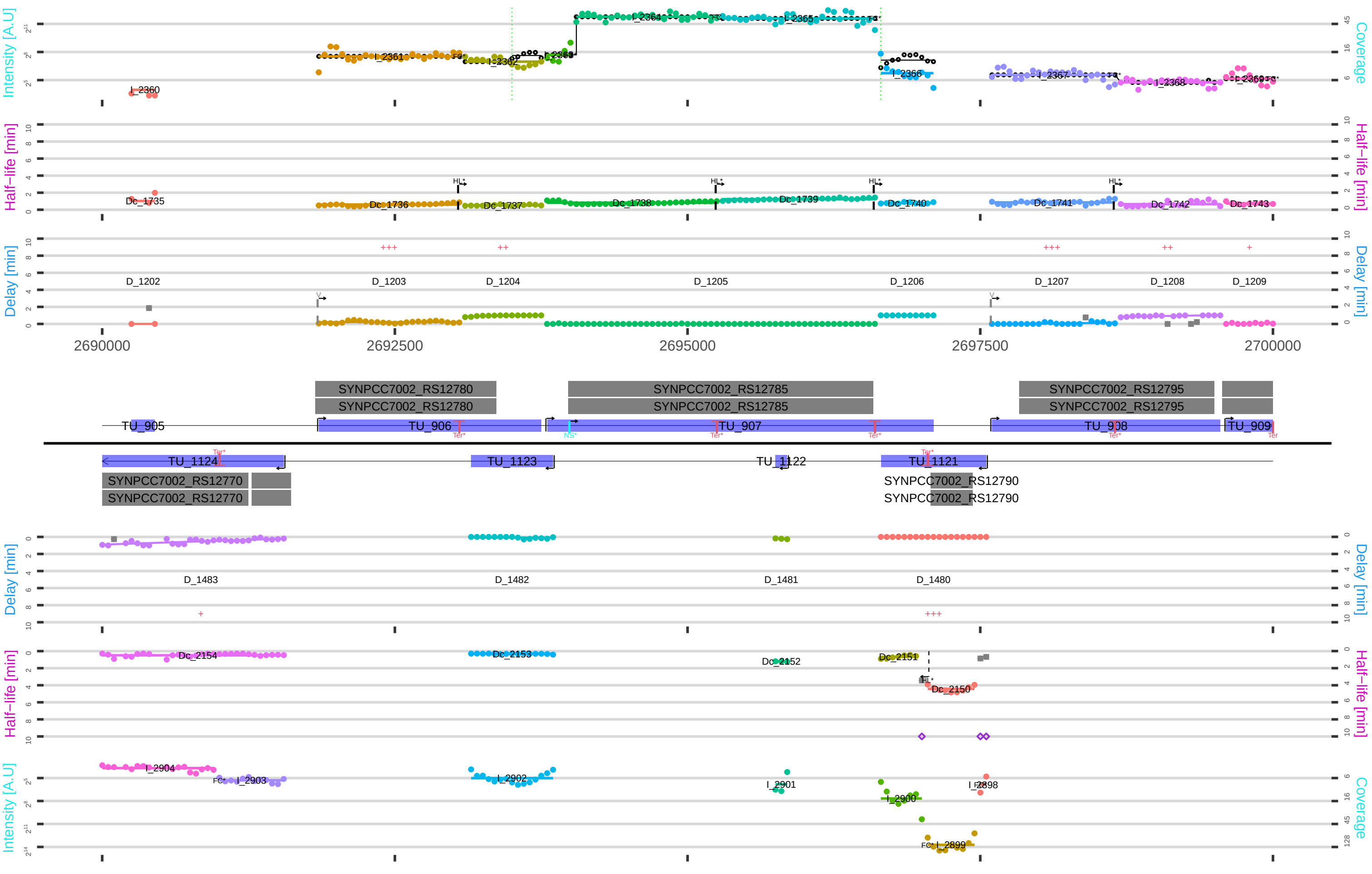

Term: termination (2), NS: new start (0), PS: pausing site (0), iTSS_I: internal starting site (0)

ID: 54000−54200; Term: termination (4), NS: new start (2), PS: pausing site (1), iTSS_I: internal starting site (0)

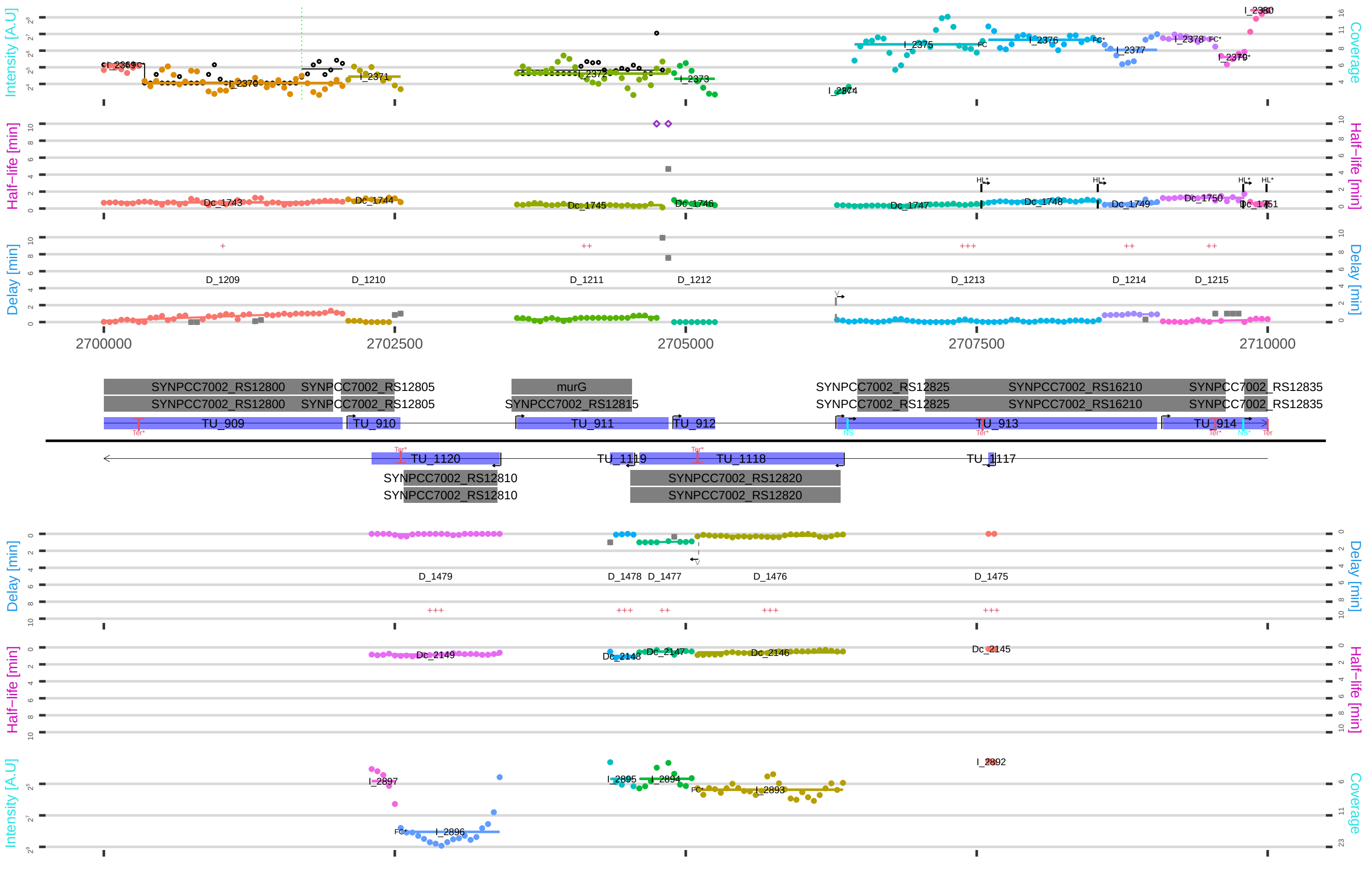

Term: termination (2), NS: new start (0), PS: pausing site (1), iTSS_I: internal starting site (0)

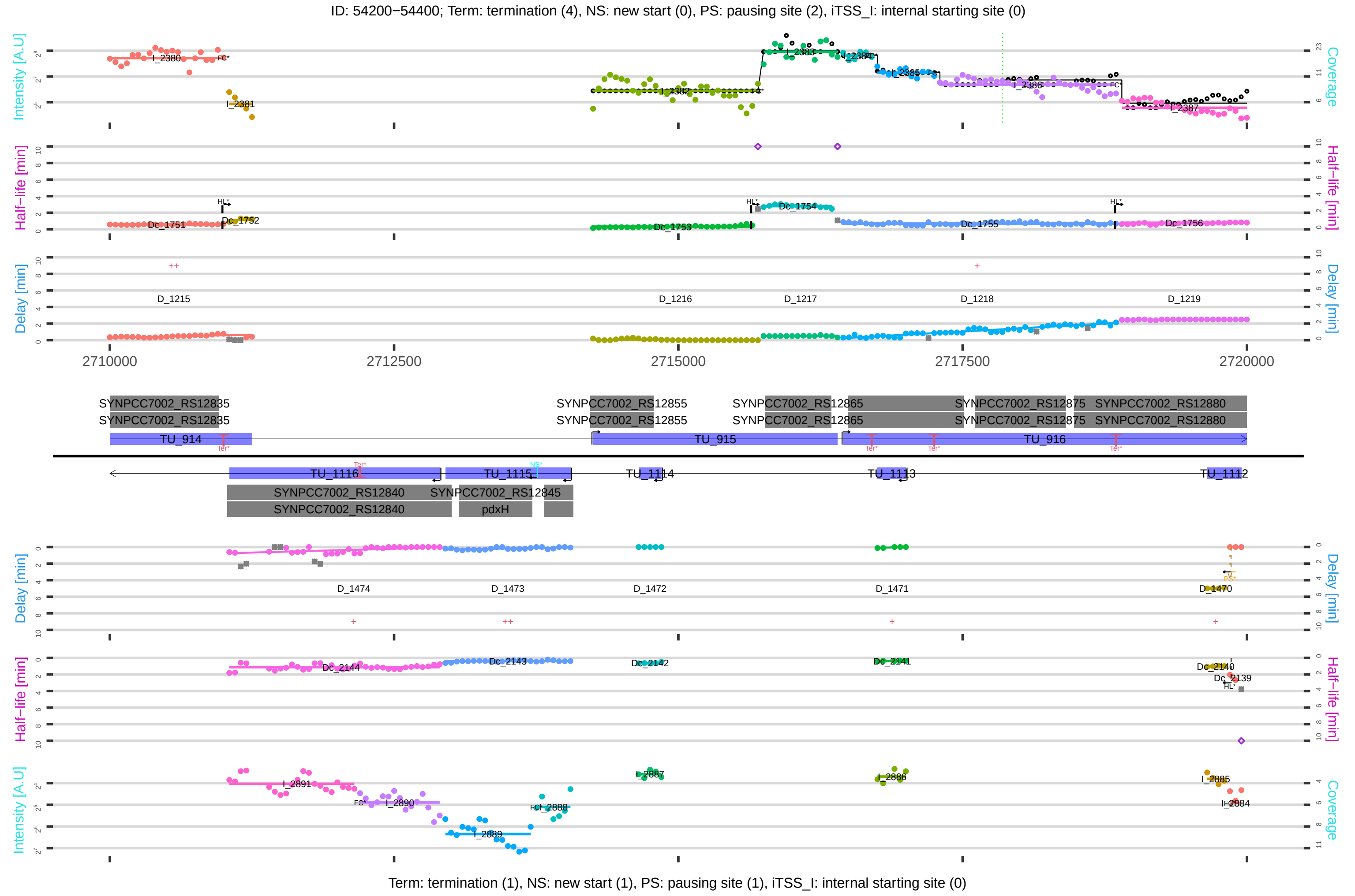
ID: 54200−54400; Term: termination (4), NS: new start (0), PS: pausing site (2), iTSS_I: internal starting site (0)
Intensity [A.U]
Coverage
Half−life [min]
Delay [min]
2710000
2712500
2715000
2717500
2720000
SYNPCC7002_RS12835
SYNPCC7002_RS12855
SYNPCC7002_RS12865
SYNPCC7002_RS12875
SYNPCC7002_RS12880
TU_914
TU_915
TU_916
TU_1116
TU_1115
TU_1114
TU_1113
TU_1112
SYNPCC7002_RS12840
SYNPCC7002_RS12845
pdxH
Term: termination (1), NS: new start (1), PS: pausing site (1), iTSS_I: internal starting site (0)

ID: 54400−54596; Term: termination (0), NS: new start (0), PS: pausing site (0), iTSS_I: internal starting site (0)

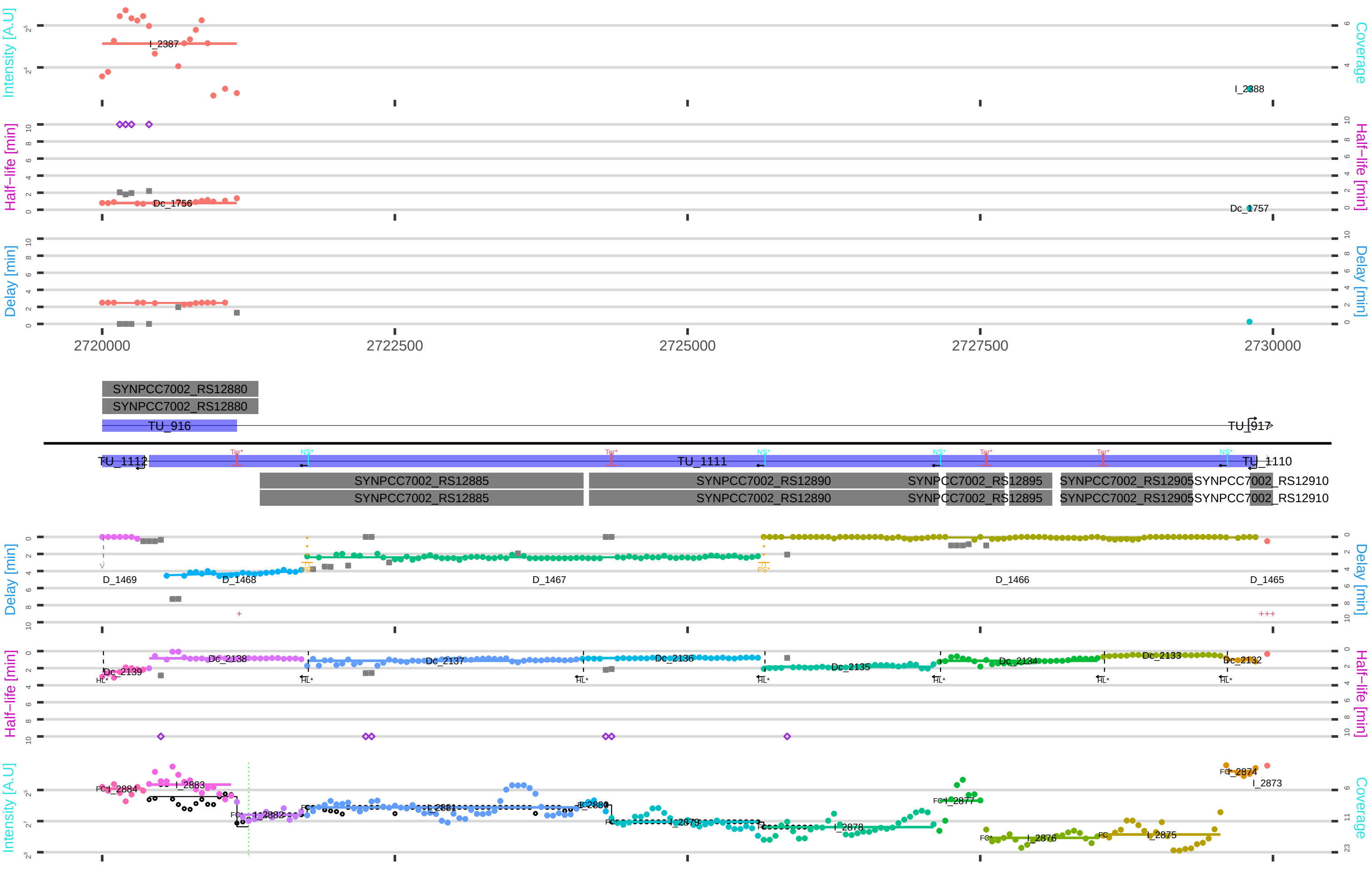

Term: termination (4), NS: new start (4), PS: pausing site (2), iTSS_I: internal starting site (0)

ID: 54731−54767; Term: termination (1), NS: new start (0), PS: pausing site (0), iTSS_I: internal starting site (0)

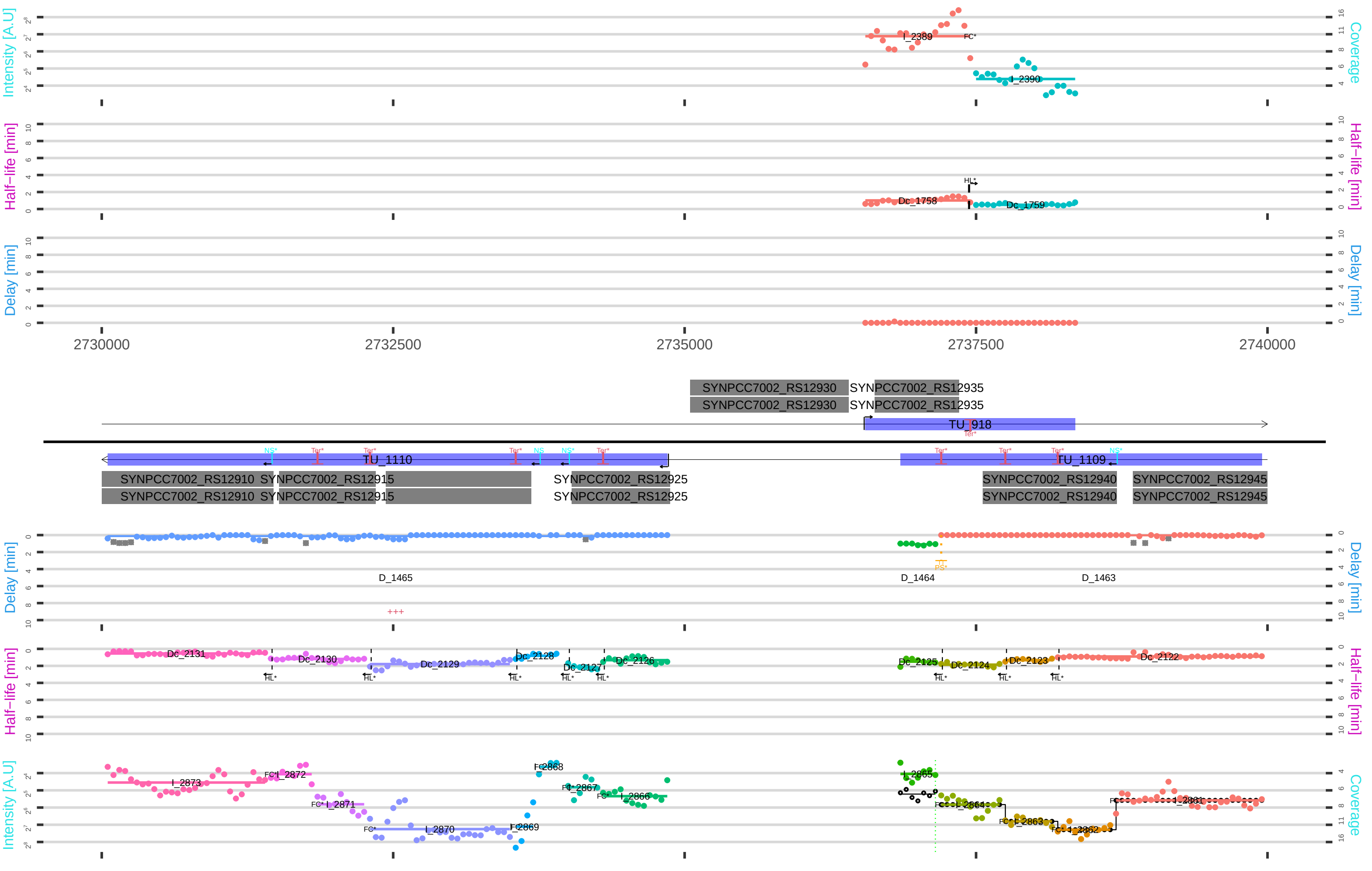

Term: termination (7), NS: new start (4), PS: pausing site (1), iTSS_I: internal starting site (0)

ID: 54842−54998; Term: termination (5), NS: new start (2), PS: pausing site (1), iTSS_I: internal starting site (0)

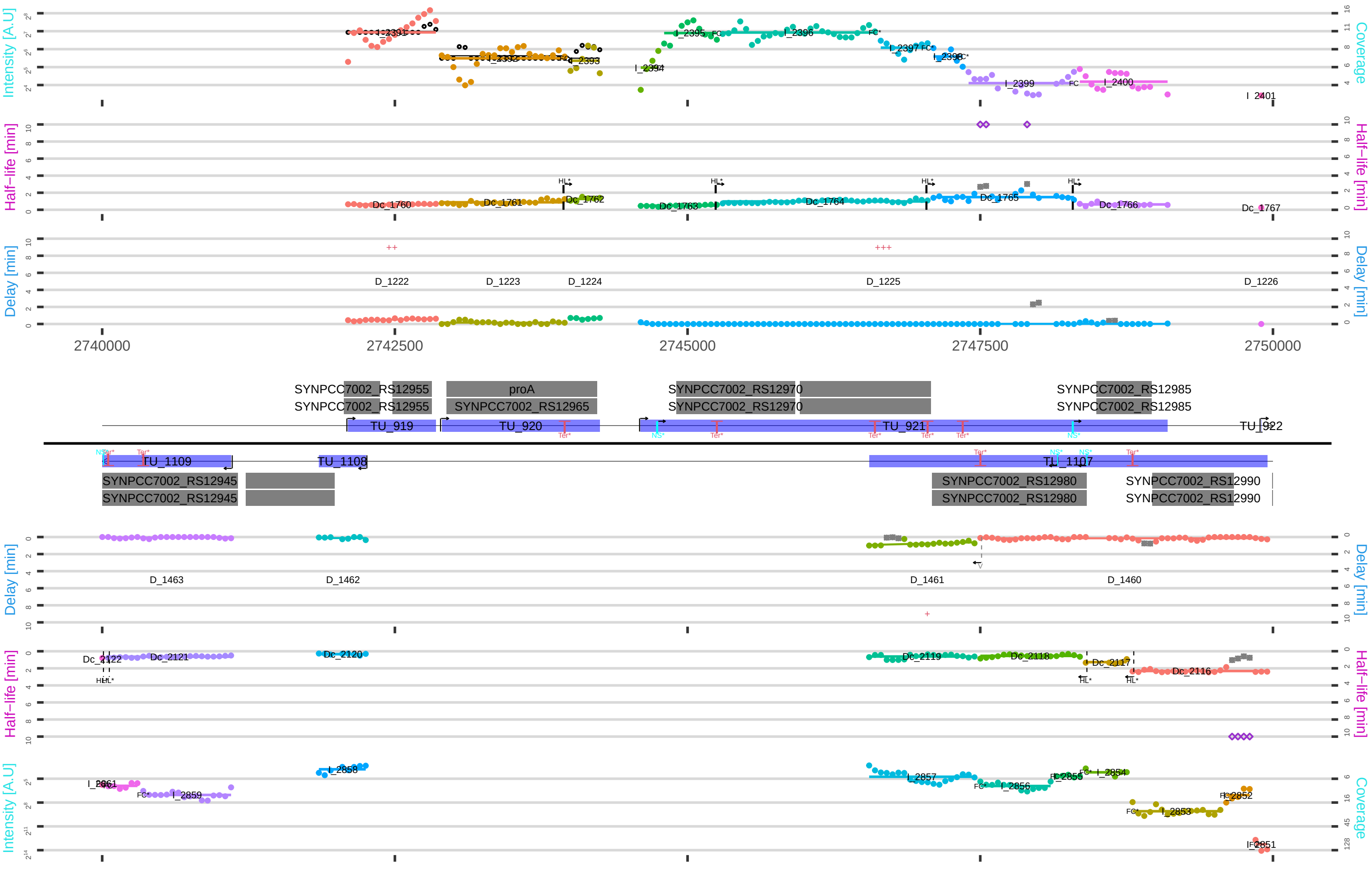

Term: termination (4), NS: new start (3), PS: pausing site (1), iTSS_I: internal starting site (0)

ID: 55011−55199; Term: termination (1), NS: new start (2), PS: pausing site (2), iTSS_I: internal starting site (0)

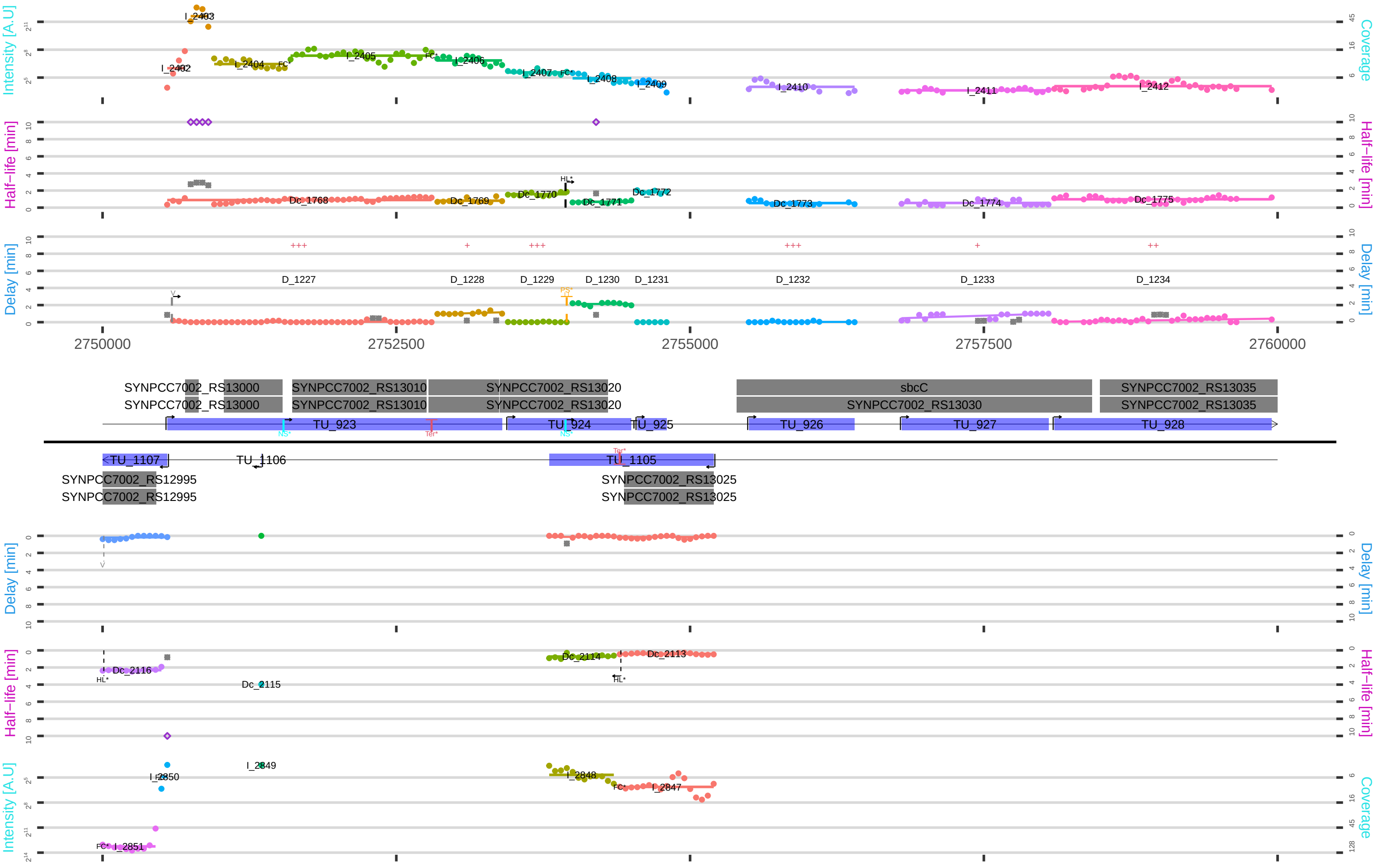

Term: termination (1), NS: new start (0), PS: pausing site (0), iTSS_I: internal starting site (0)

ID: 55201−55400; Term: termination (5), NS: new start (0), PS: pausing site (2), iTSS_I: internal starting site (0)

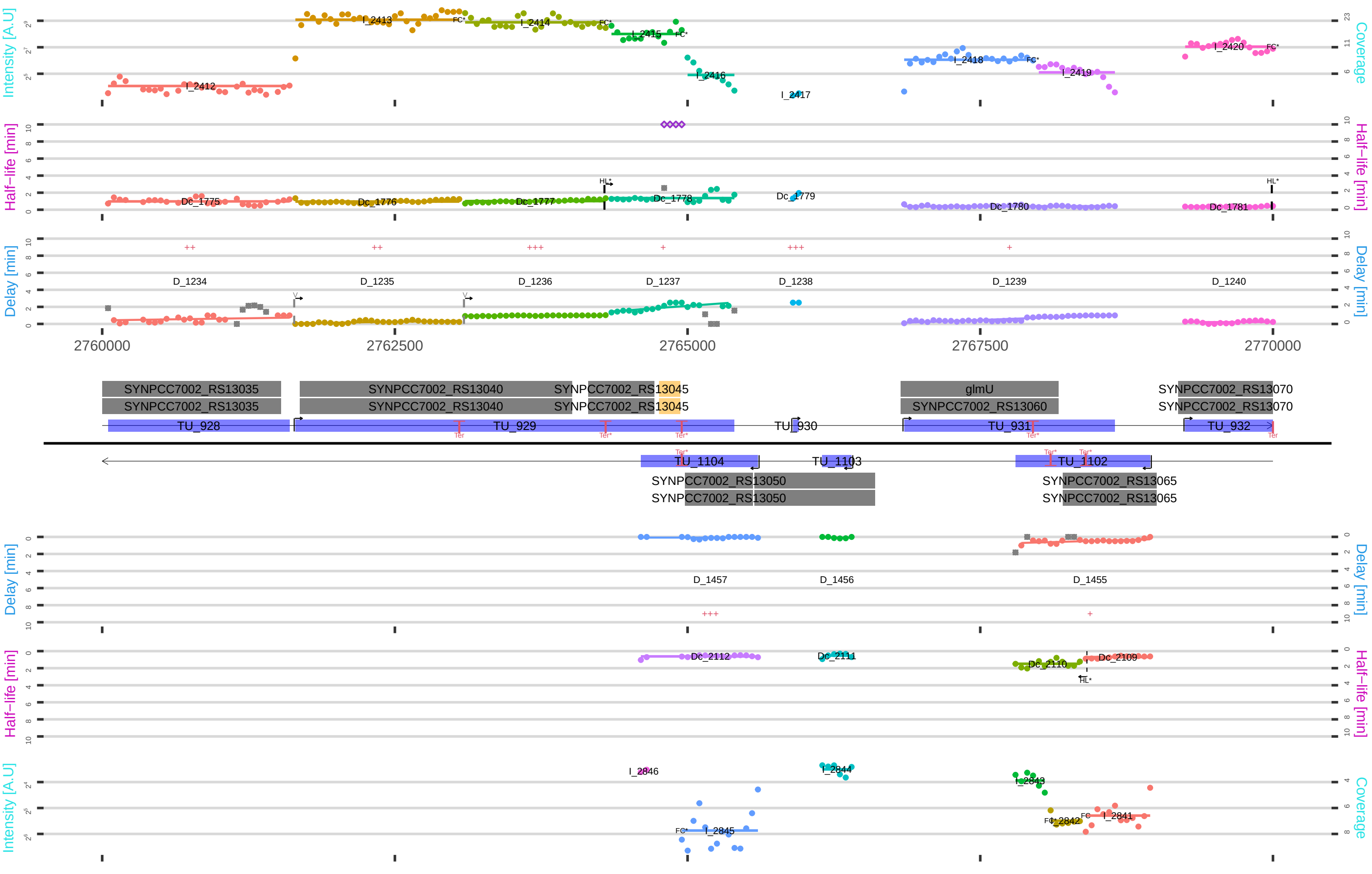

Term: termination (3), NS: new start (0), PS: pausing site (0), iTSS_I: internal starting site (0)

ID: 55400−55600; Term: termination (5), NS: new start (2), PS: pausing site (2), iTSS_I: internal starting site (0)

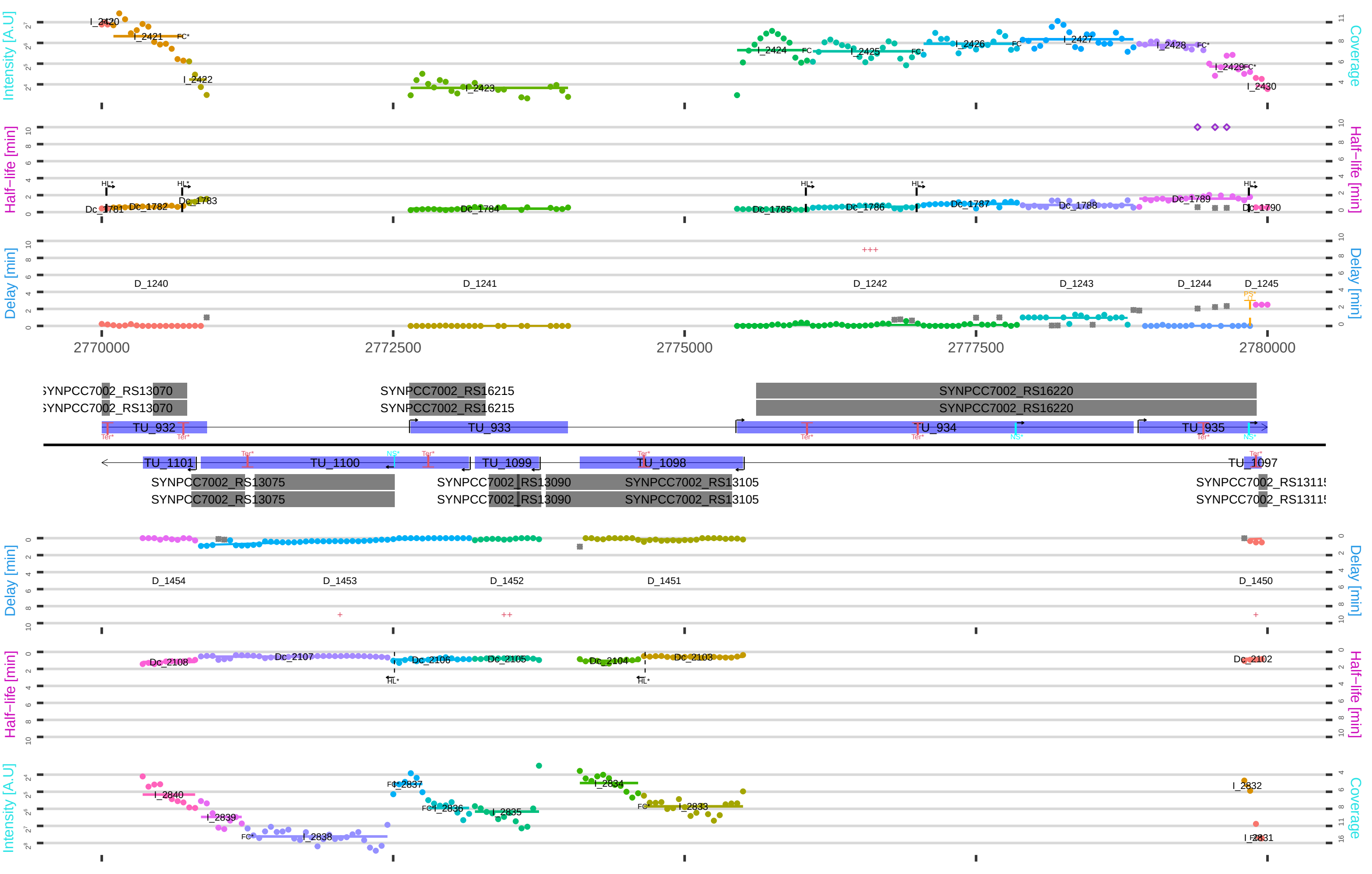

Term: termination (4), NS: new start (1), PS: pausing site (0), iTSS_I: internal starting site (0)

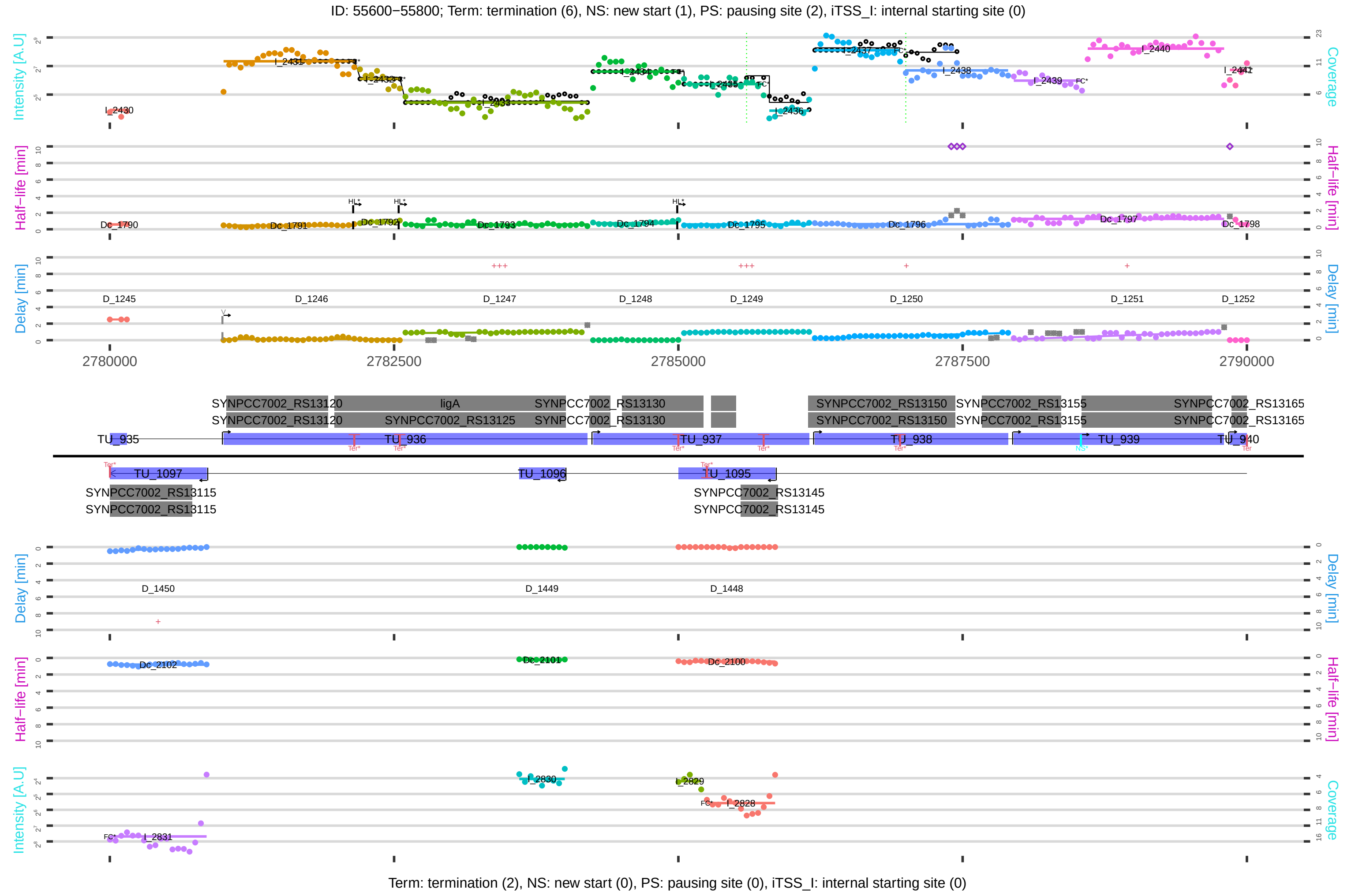

ID: 55600−55800; Term: termination (6), NS: new start (1), PS: pausing site (2), iTSS_I: internal starting site (0)
Intensity [A.U]
Coverage
Half−life [min]
Delay [min]
2780000
2782500
2785000
2787500
2790000
SYNPCC7002_RS13120
ligA
SYNPCC7002_RS13125
SYNPCC7002_RS13130
SYNPCC7002_RS13150
SYNPCC7002_RS13155
SYNPCC7002_RS13165
TU_935
TU_936
TU_937
TU_938
TU_939
TU_940
TU_1097
TU_1096
TU_1095
SYNPCC7002_RS13115
SYNPCC7002_RS13145
Term: termination (2), NS: new start (0), PS: pausing site (0), iTSS_I: internal starting site (0)

ID: 55800−56000; Term: termination (6), NS: new start (1), PS: pausing site (0), iTSS_I: internal starting site (0)

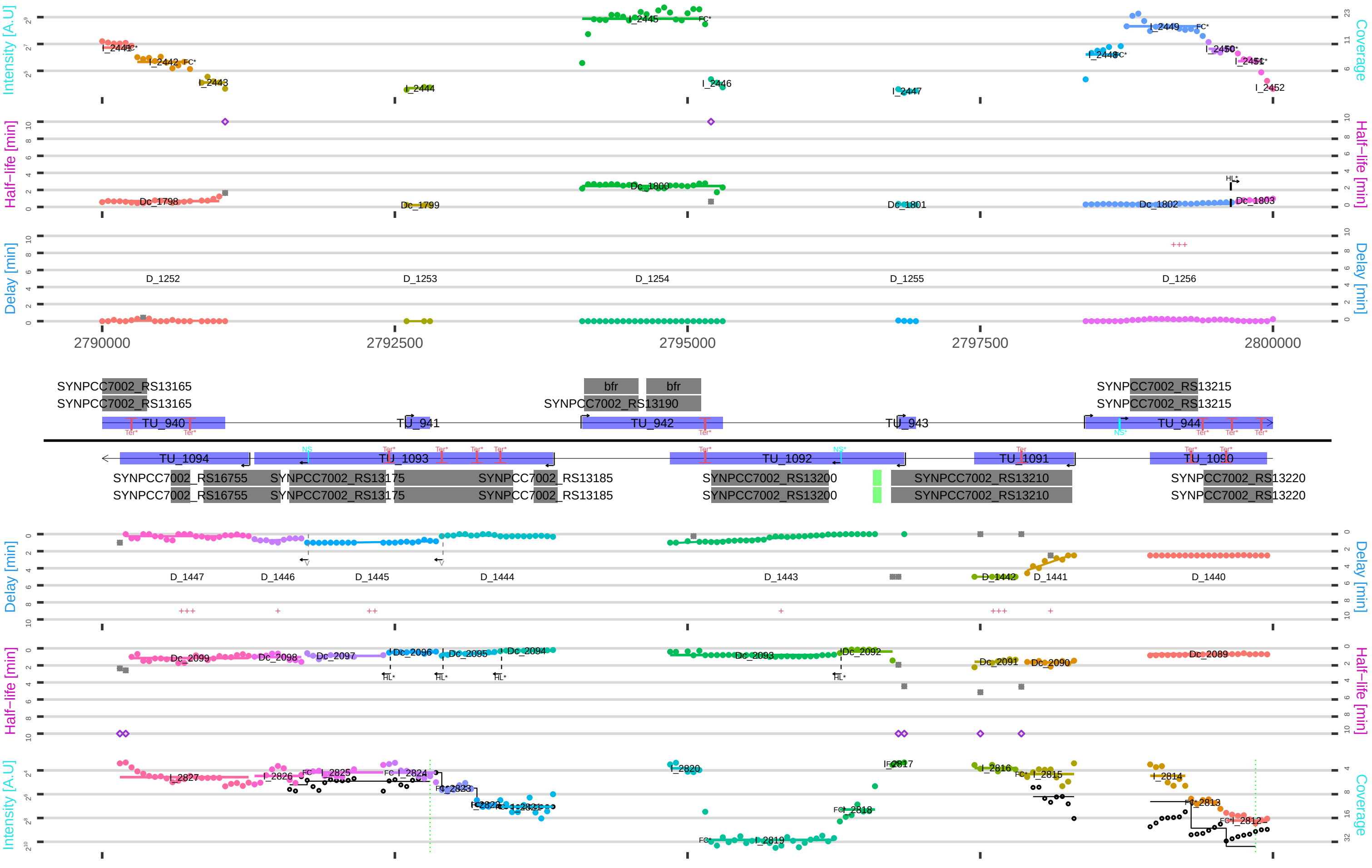

Term: termination (8), NS: new start (2), PS: pausing site (2), iTSS_I: internal starting site (1)

ID: 56000−56200; Term: termination (3), NS: new start (2), PS: pausing site (1), iTSS_I: internal starting site (0)

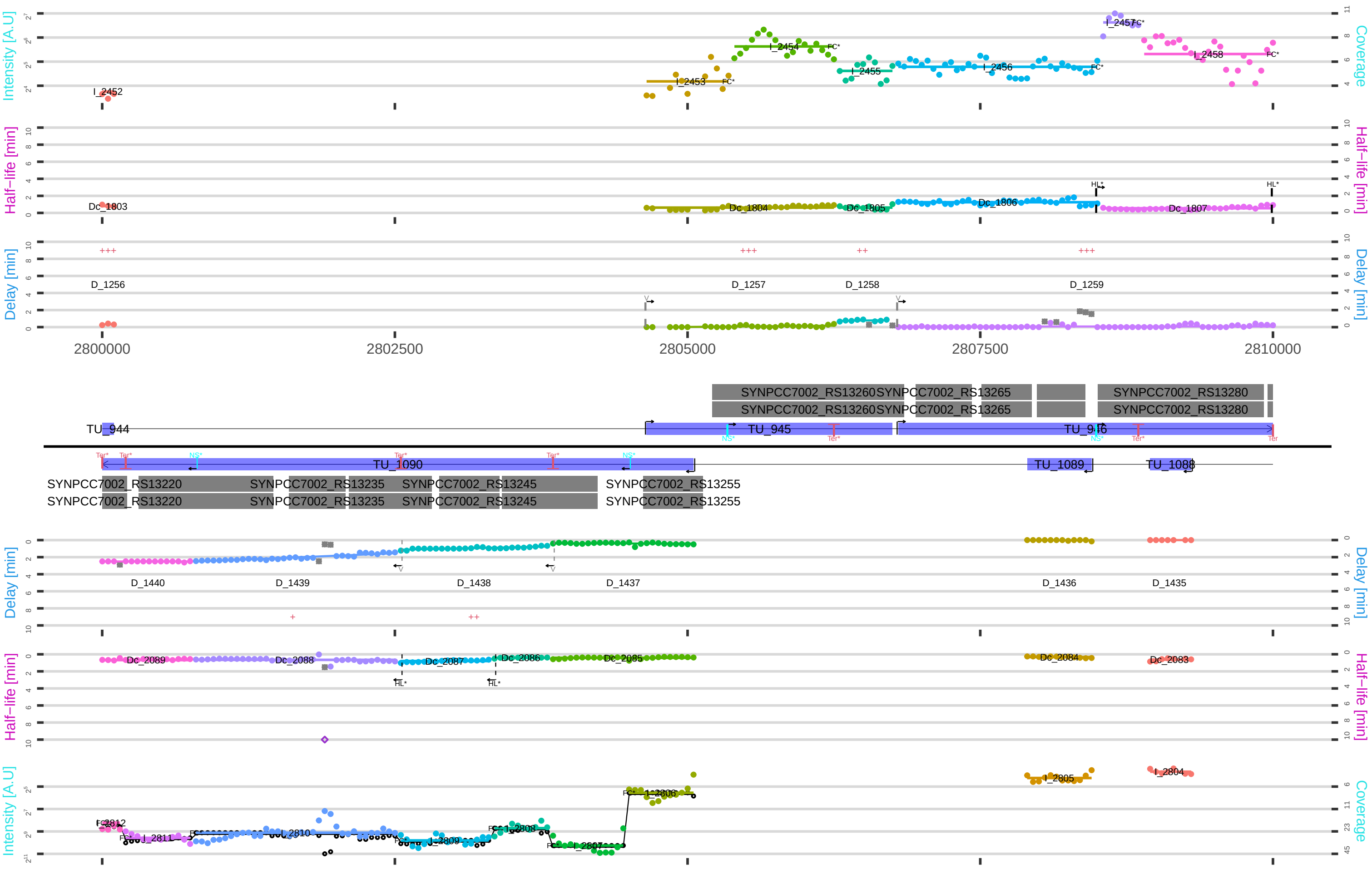

Term: termination (4), NS: new start (2), PS: pausing site (2), iTSS_I: internal starting site (1)

ID: 56200−56400; Term: termination (5), NS: new start (1), PS: pausing site (3), iTSS_I: internal starting site (0)

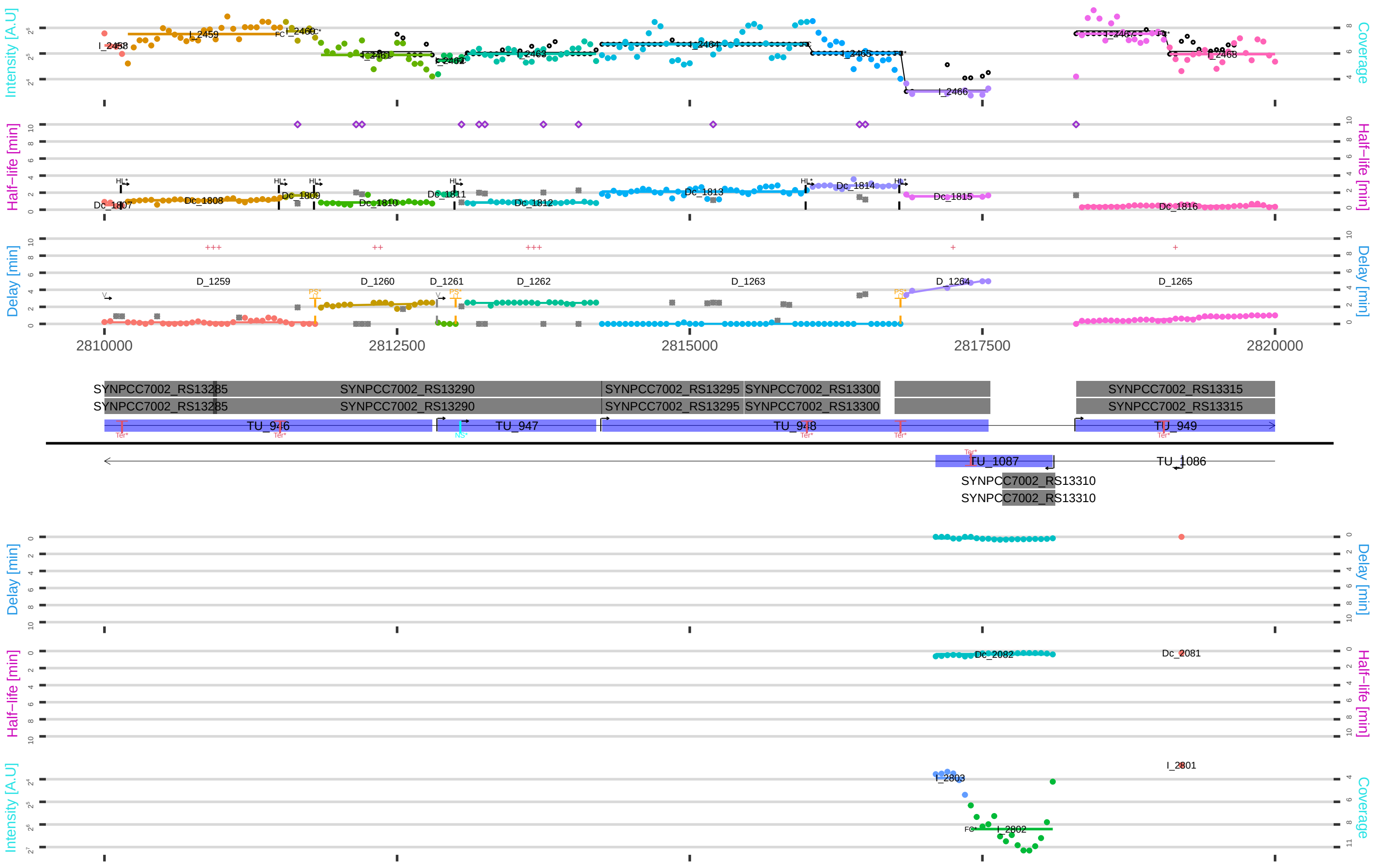

Term: termination (1), NS: new start (0), PS: pausing site (0), iTSS_I: internal starting site (0)

ID: 56400−56600; Term: termination (6), NS: new start (0), PS: pausing site (1), iTSS_I: internal starting site (0)

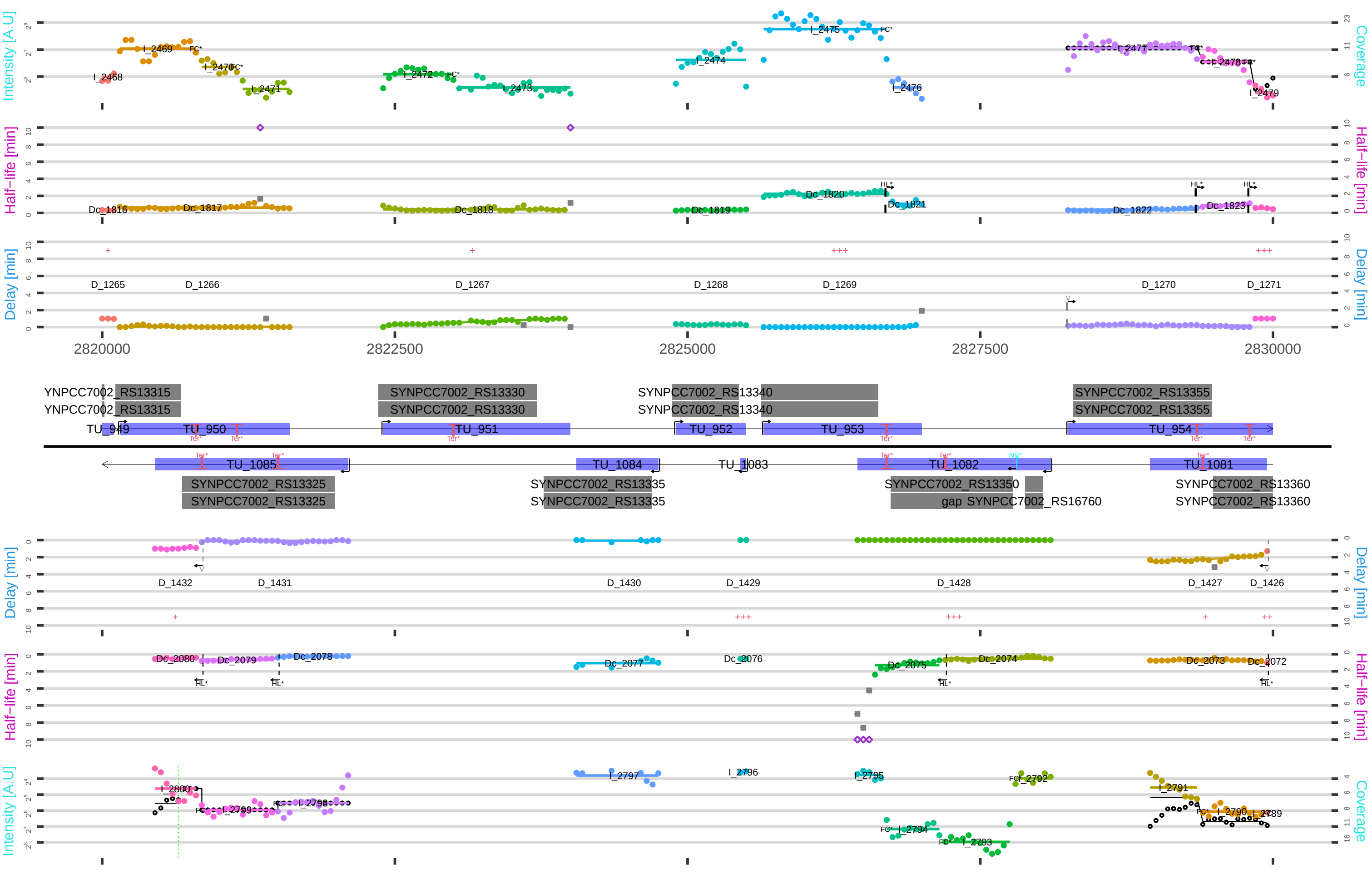

Term: termination (5), NS: new start (1), PS: pausing site (2), iTSS_I: internal starting site (0)

ID: 56600−56800; Term: termination (5), NS: new start (0), PS: pausing site (0), iTSS_I: internal starting site (0)

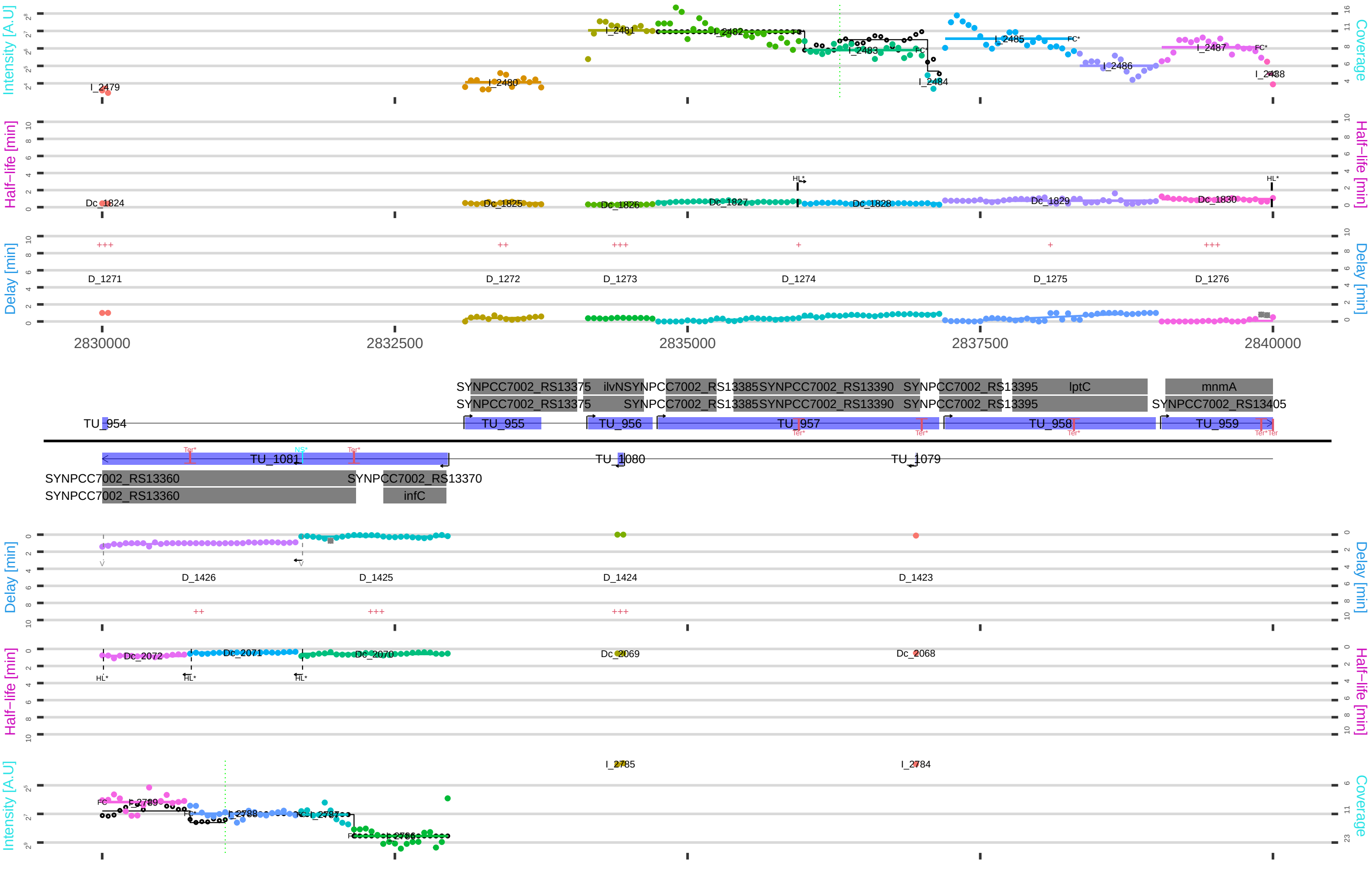

Term: termination (2), NS: new start (1), PS: pausing site (1), iTSS_I: internal starting site (0)

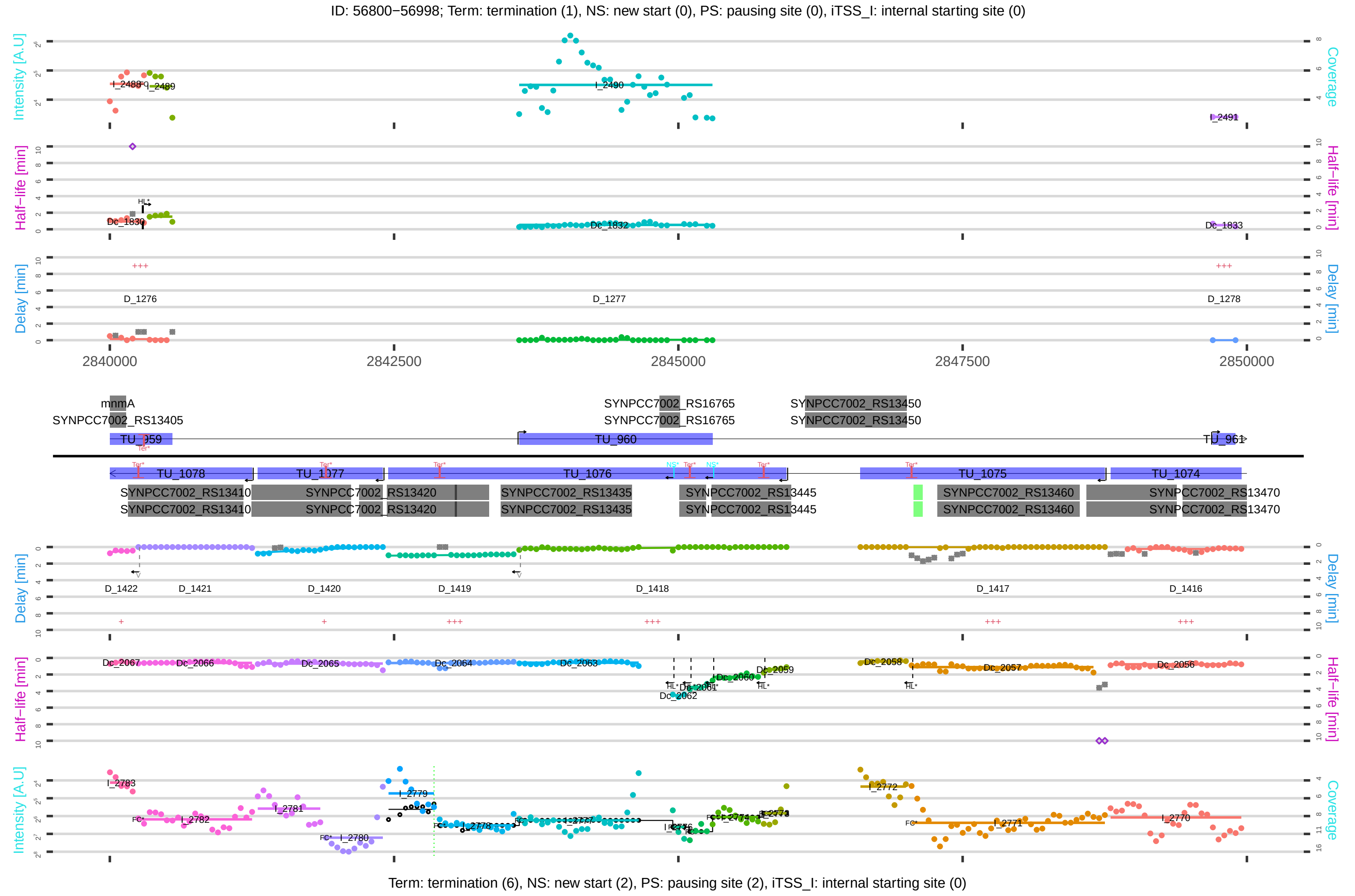

ID: 56800−56998; Term: termination (1), NS: new start (0), PS: pausing site (0), iTSS_I: internal starting site (0)
Intensity [A.U]
Coverage
Half−life [min]
Delay [min]
2840000
2842500
2845000
2847500
2850000
mnmA
SYNPCC7002_RS13405
SYNPCC7002_RS16765
SYNPCC7002_RS13450
TU_959
TU_960
TU_961
TU_1078
TU_1077
TU_1076
TU_1075
TU_1074
SYNPCC7002_RS13410
SYNPCC7002_RS13420
SYNPCC7002_RS13435
SYNPCC7002_RS13445
SYNPCC7002_RS13460
SYNPCC7002_RS13470
Term: termination (6), NS: new start (2), PS: pausing site (2), iTSS_I: internal starting site (0)

ID: 57010−57200; Term: termination (6), NS: new start (4), PS: pausing site (1), iTSS_I: internal starting site (0)

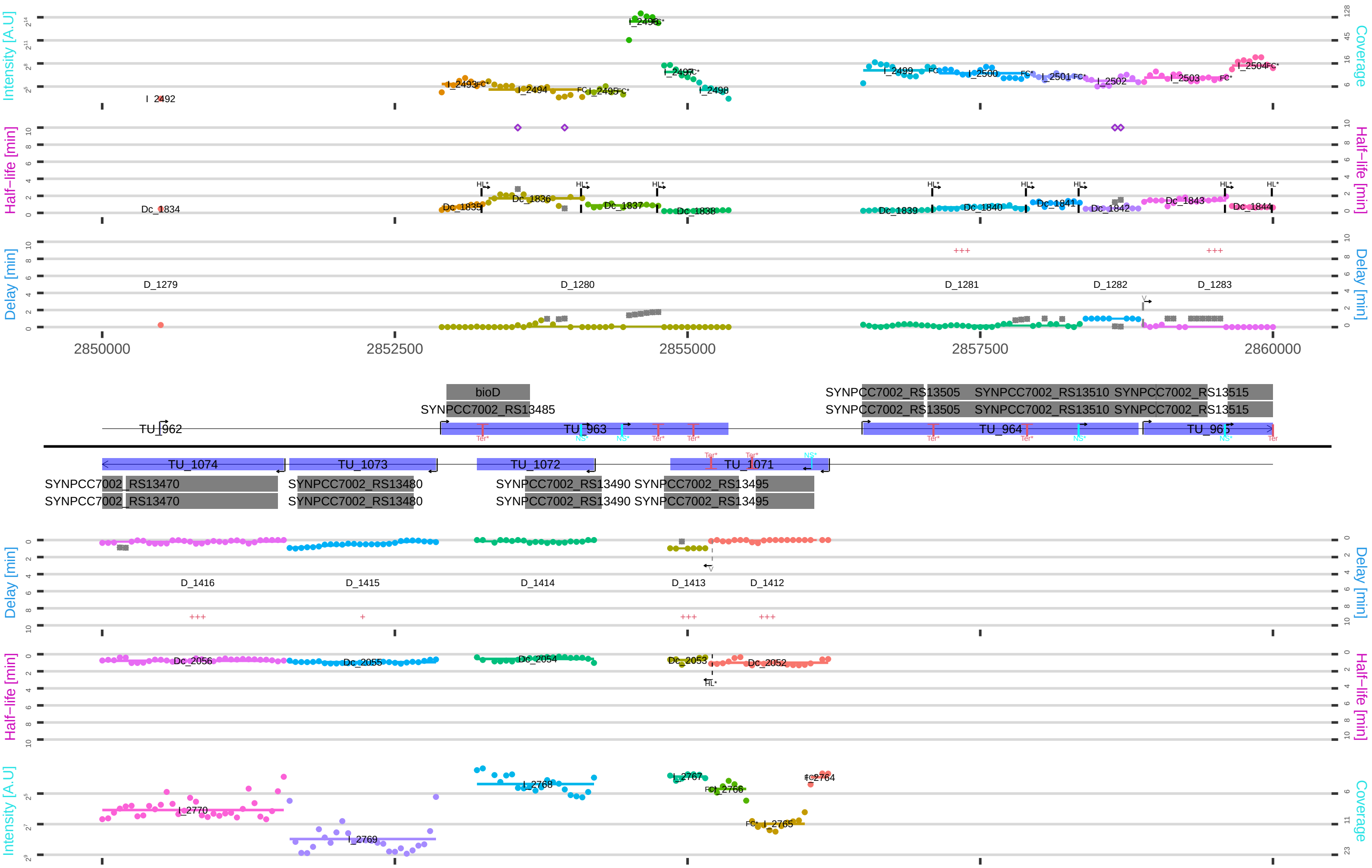

Term: termination (2), NS: new start (1), PS: pausing site (1), iTSS_I: internal starting site (0)

ID: 57200−57400; Term: termination (5), NS: new start (0), PS: pausing site (1), iTSS_I: internal starting site (0)

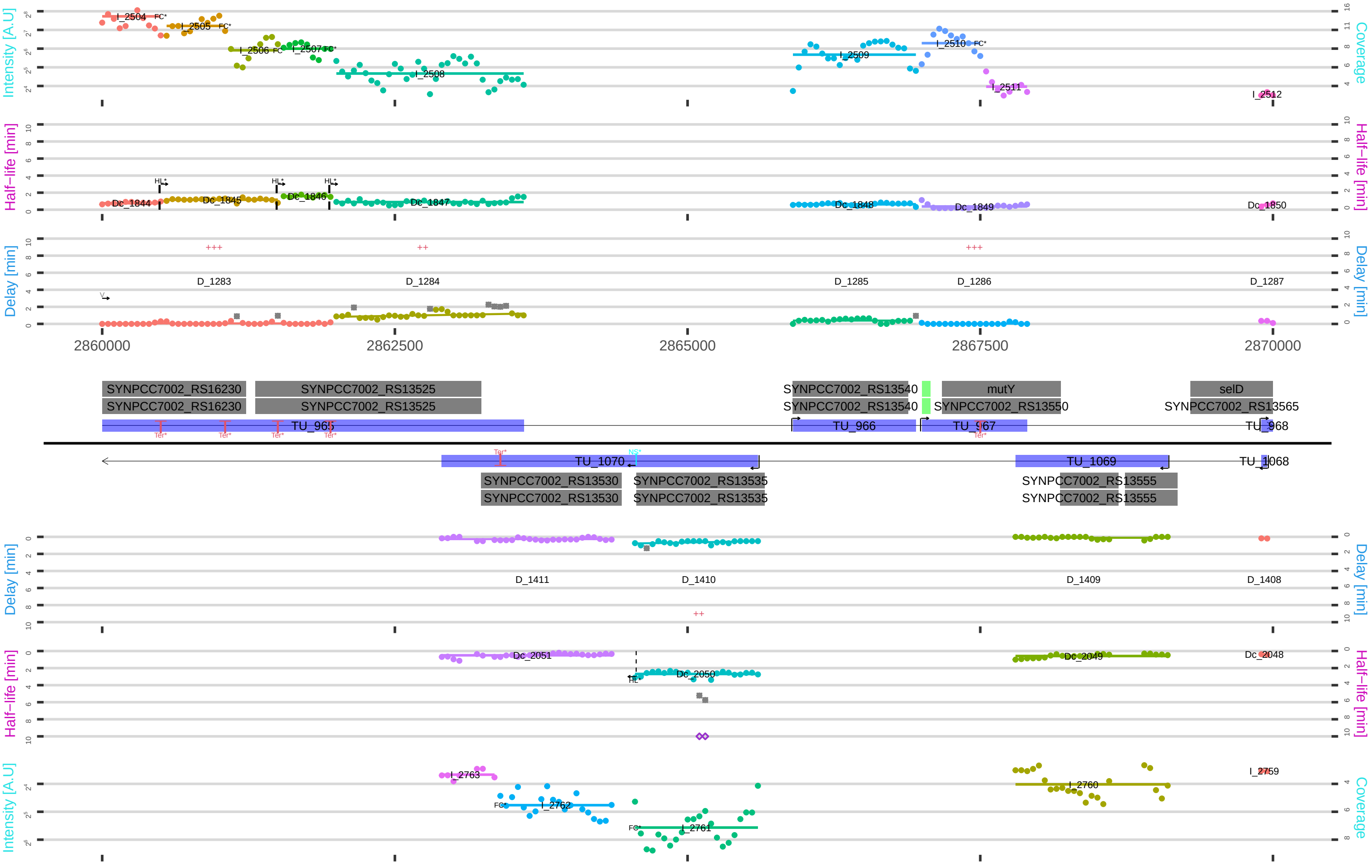

Term: termination (1), NS: new start (1), PS: pausing site (0), iTSS_I: internal starting site (1)

ID: 57400−57600; Term: termination (5), NS: new start (5), PS: pausing site (1), iTSS_I: internal starting site (0)

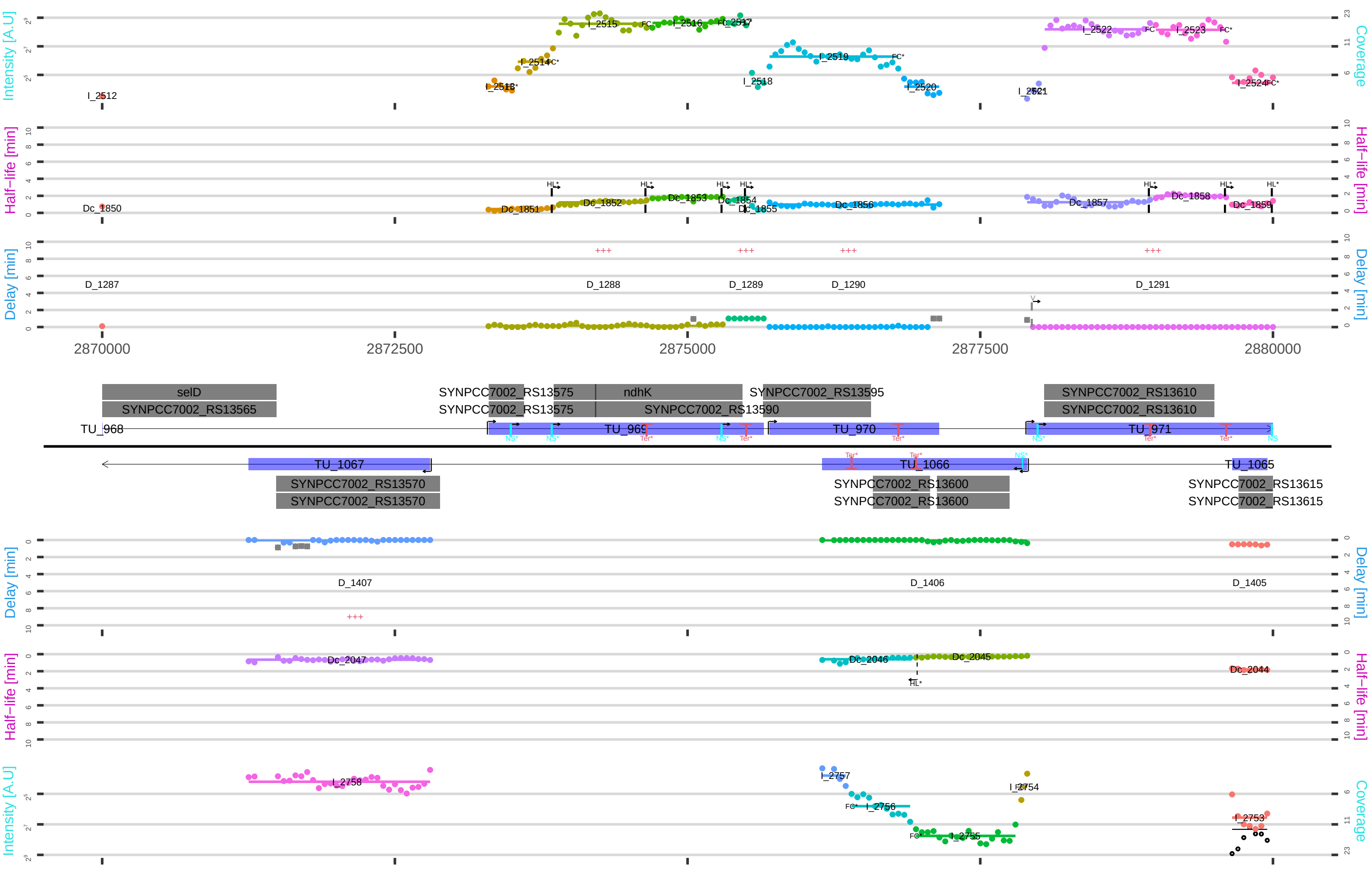

Term: termination (2), NS: new start (1), PS: pausing site (0), iTSS_I: internal starting site (0)

ID: 57600−57781; Term: termination (1), NS: new start (1), PS: pausing site (1), iTSS_I: internal starting site (0)

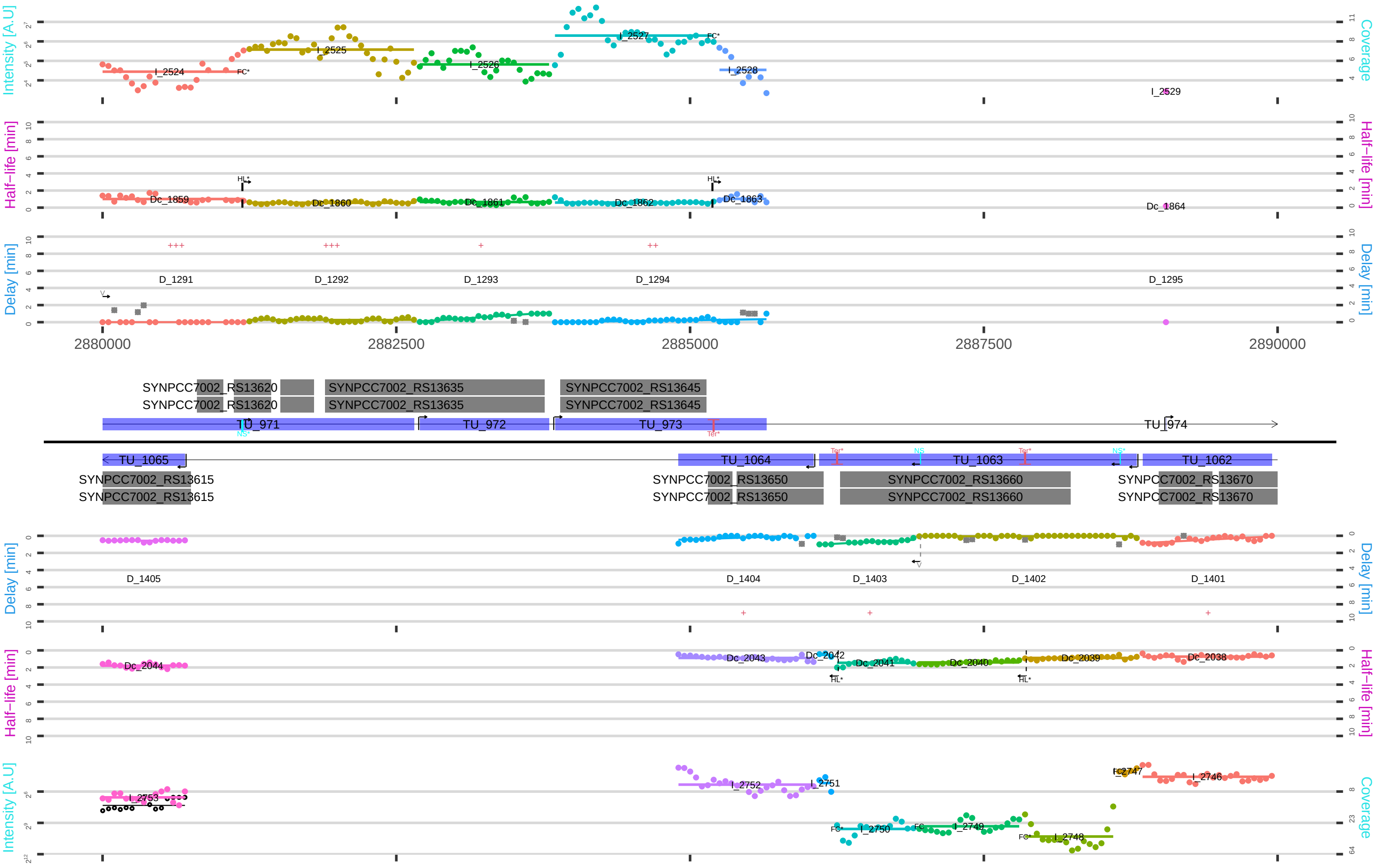

Term: termination (2), NS: new start (2), PS: pausing site (1), iTSS_I: internal starting site (0)

ID: 57817−57987; Term: termination (2), NS: new start (1), PS: pausing site (0), iTSS_I: internal starting site (0)

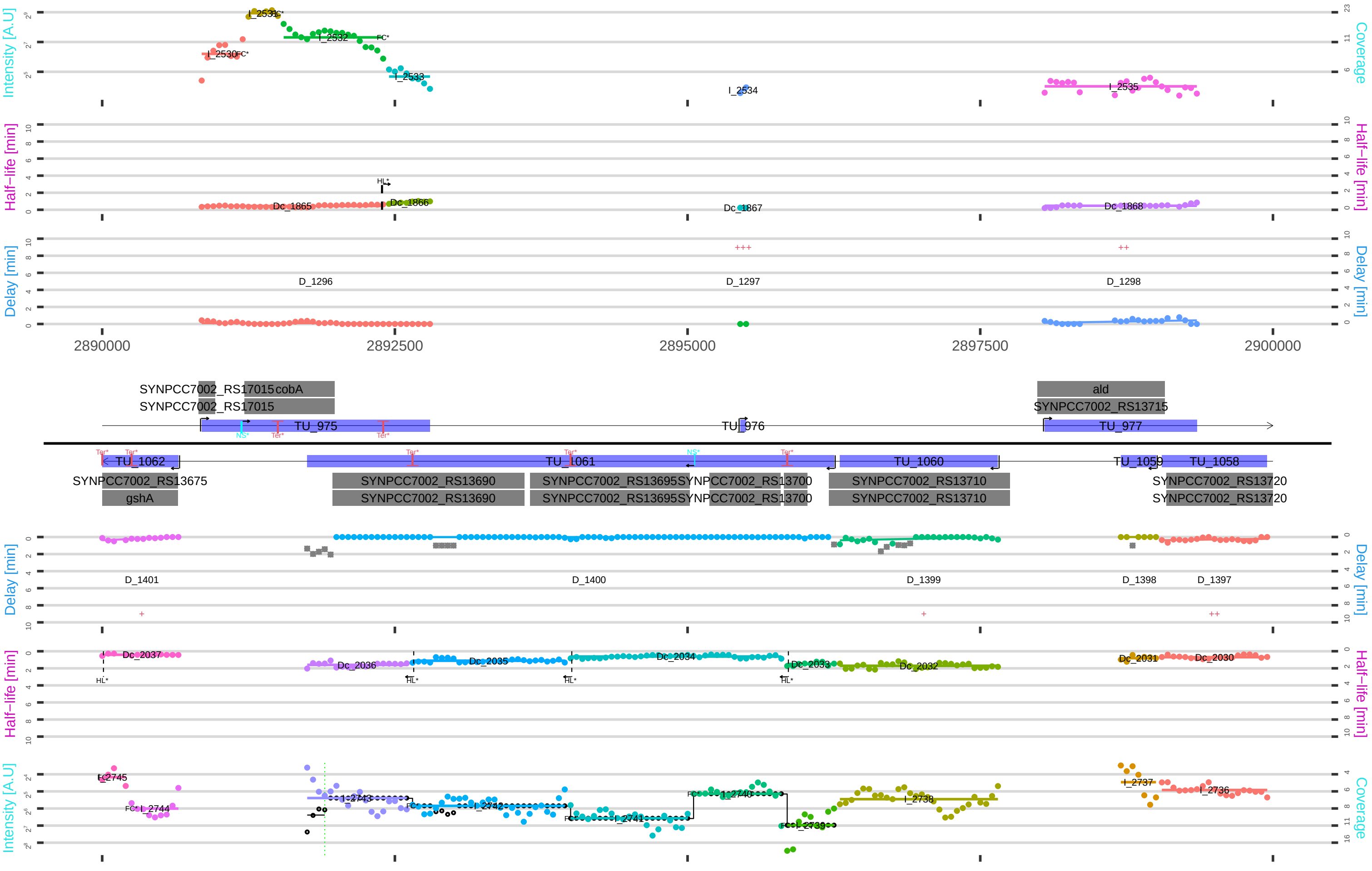

Term: termination (5), NS: new start (1), PS: pausing site (0), iTSS_I: internal starting site (0)

ID: 58023−58200; Term: termination (4), NS: new start (0), PS: pausing site (1), iTSS_I: internal starting site (0)

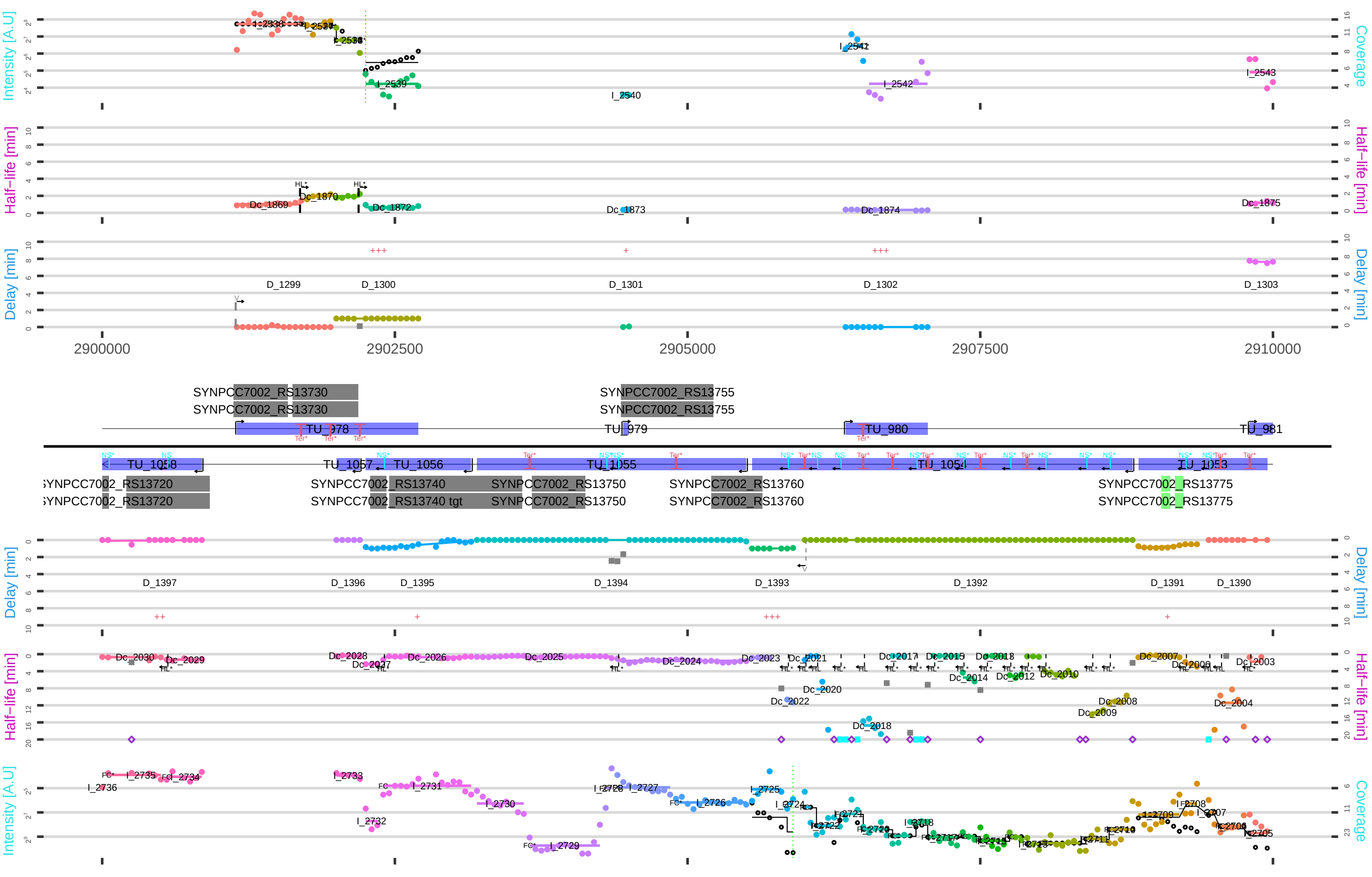

Term: termination (10), NS: new start (16), PS: pausing site (2), iTSS_I: internal starting site (0)

ID: 58200−58385; Term: termination (2), NS: new start (1), PS: pausing site (0), iTSS_I: internal starting site (0)

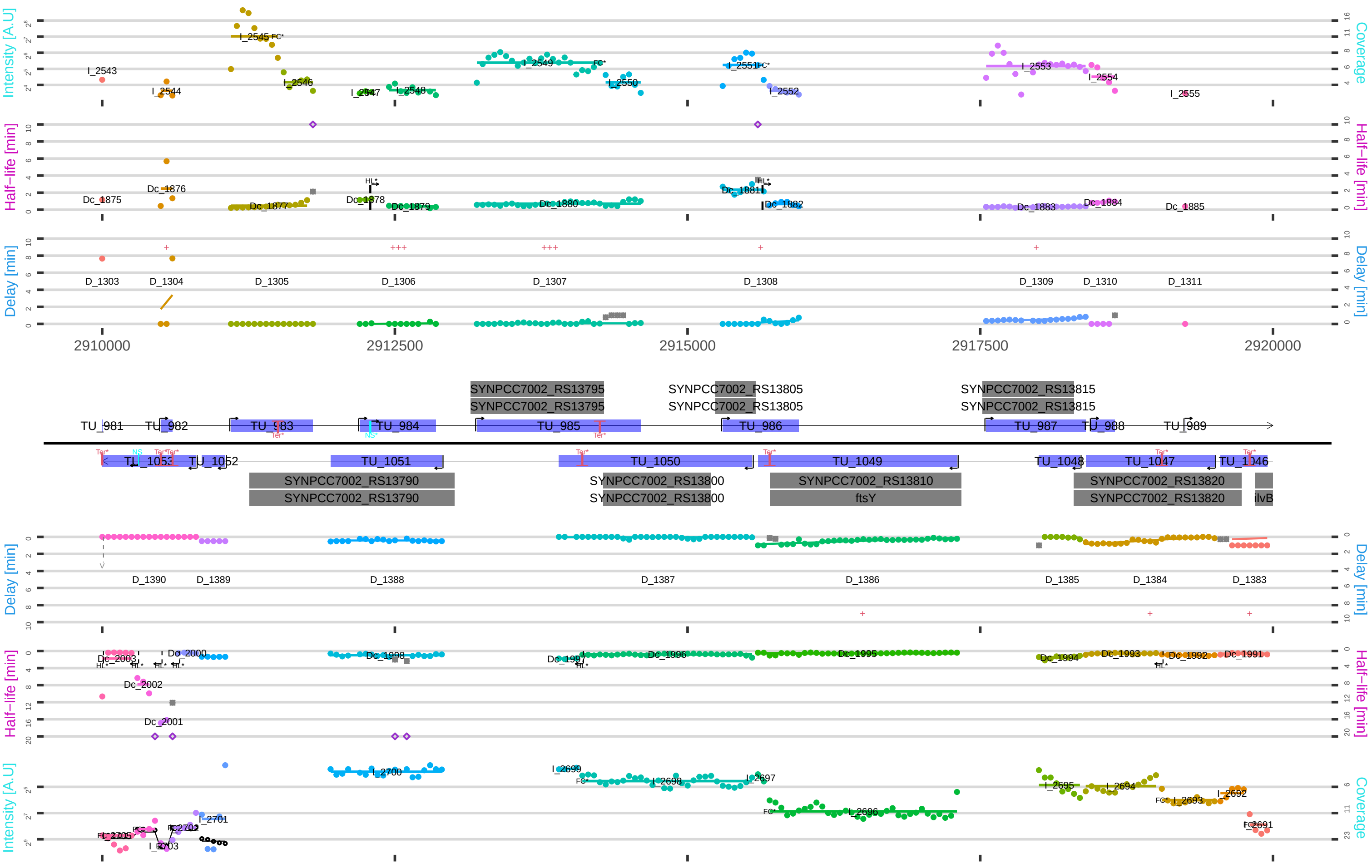

Term: termination (7), NS: new start (1), PS: pausing site (0), iTSS_I: internal starting site (0)

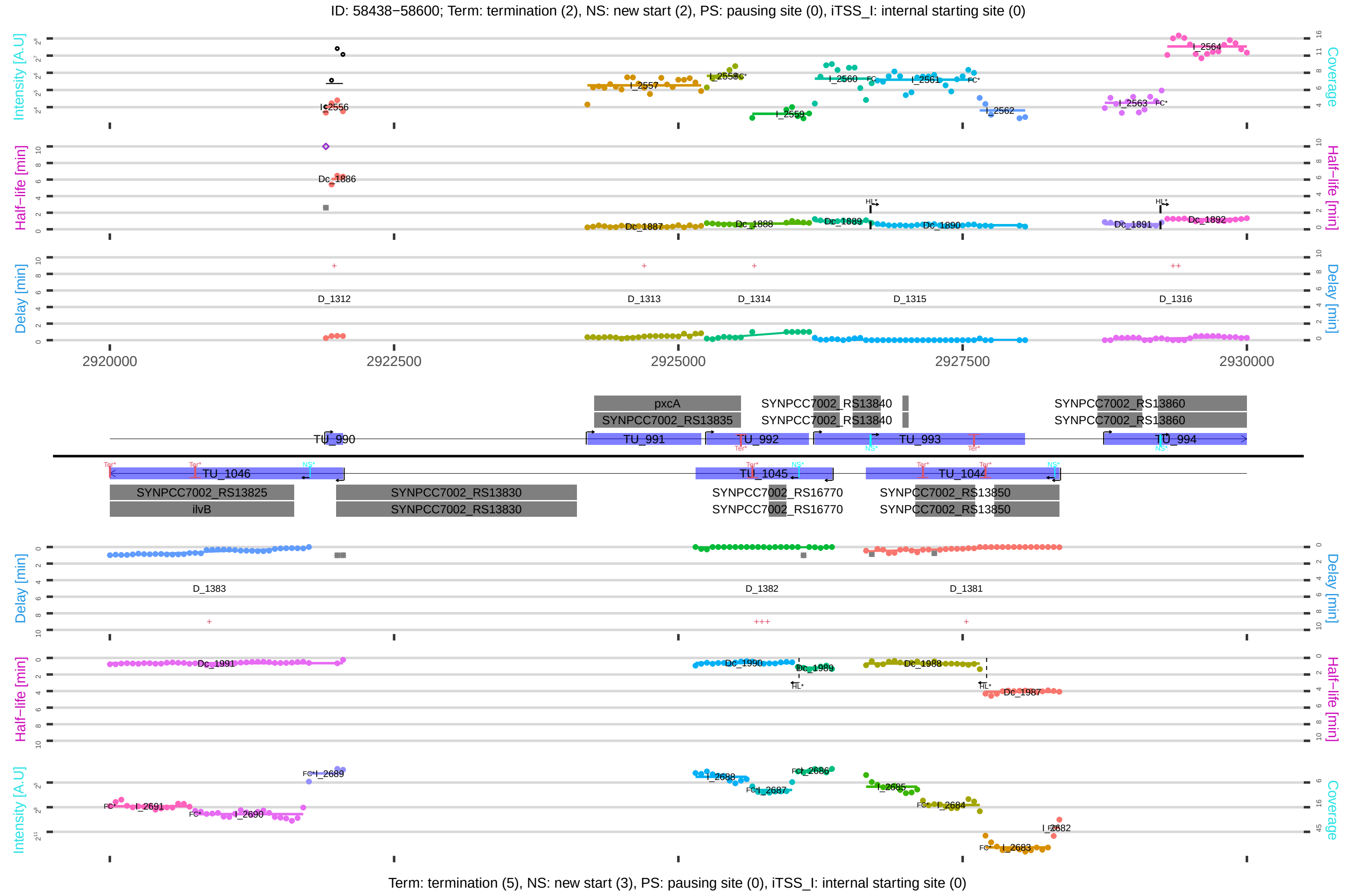
ID: 58438−58600; Term: termination (2), NS: new start (2), PS: pausing site (0), iTSS_I: internal starting site (0)
Intensity [A.U]
Coverage
Half−life [min]
Delay [min]
2920000
2922500
2925000
2927500
2930000
pxcA
SYNPCC7002_RS13835
SYNPCC7002_RS13840
SYNPCC7002_RS13860
TU_990
TU_991
TU_992
TU_993
TU_994
TU_1046
TU_1045
TU_1044
SYNPCC7002_RS13825
ilvB
SYNPCC7002_RS13830
SYNPCC7002_RS16770
SYNPCC7002_RS13850
D_1312
D_1313
D_1314
D_1315
D_1316
D_1383
D_1382
D_1381
Dc_1886
Dc_1887
Dc_1888
Dc_1889
Dc_1890
Dc_1891
Dc_1892
Dc_1991
Dc_1990
Dc_1989
Dc_1988
Dc_1987
I_2556
I_2557
I_2558
I_2559
I_2560
I_2561
I_2562
I_2563
I_2564
I_2691
I_2690
I_2689
I_2688
I_2687
I_2686
I_2685
I_2684
I_2683
I_2682
Term: termination (5), NS: new start (3), PS: pausing site (0), iTSS_I: internal starting site (0)

ID: 58600−58800; Term: termination (5), NS: new start (4), PS: pausing site (2), iTSS_I: internal starting site (0)

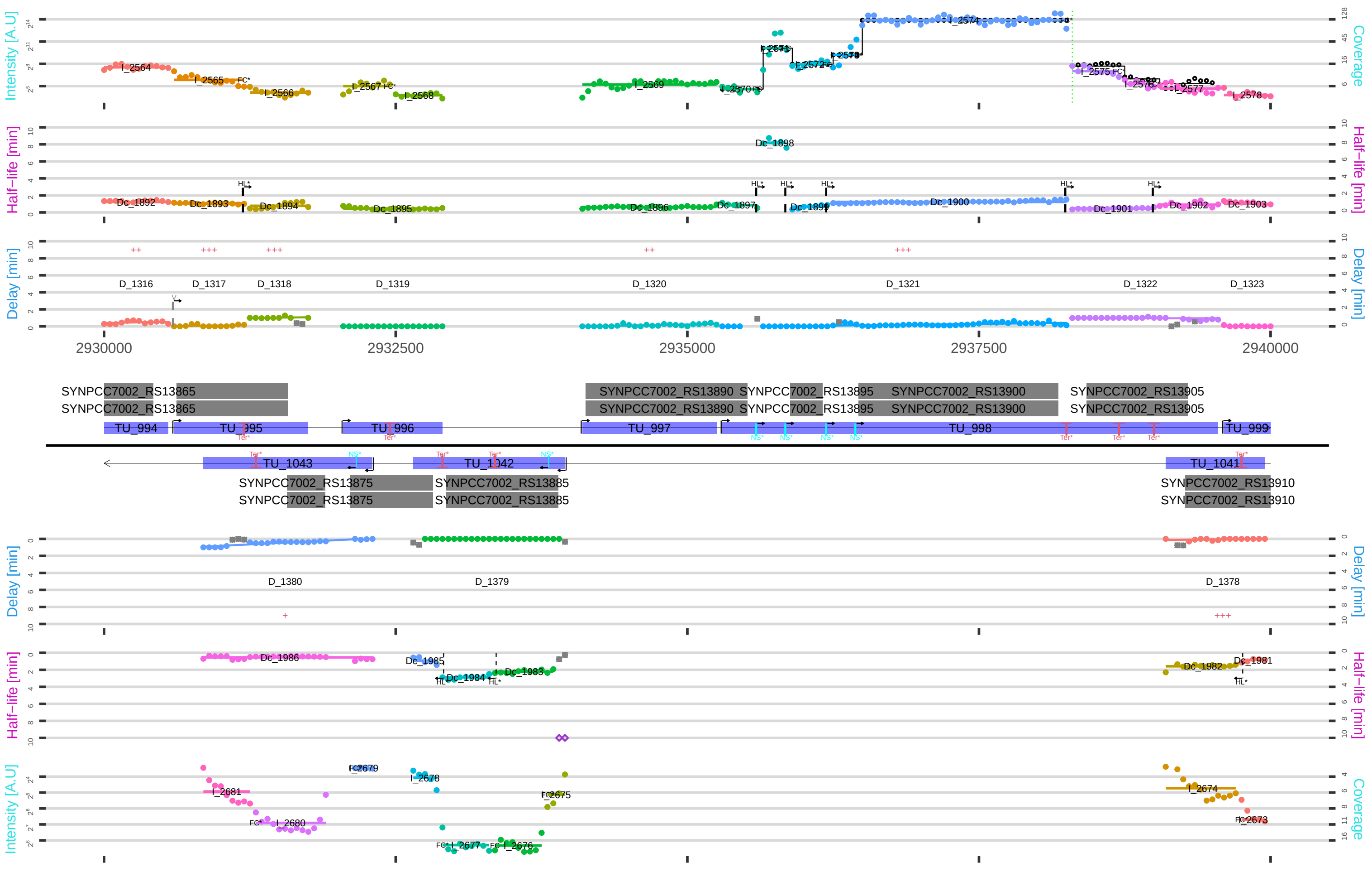

Term: termination (4), NS: new start (2), PS: pausing site (0), iTSS_I: internal starting site (0)

ID: 58800−58998; Term: termination (3), NS: new start (1), PS: pausing site (0), iTSS_I: internal starting site (0)

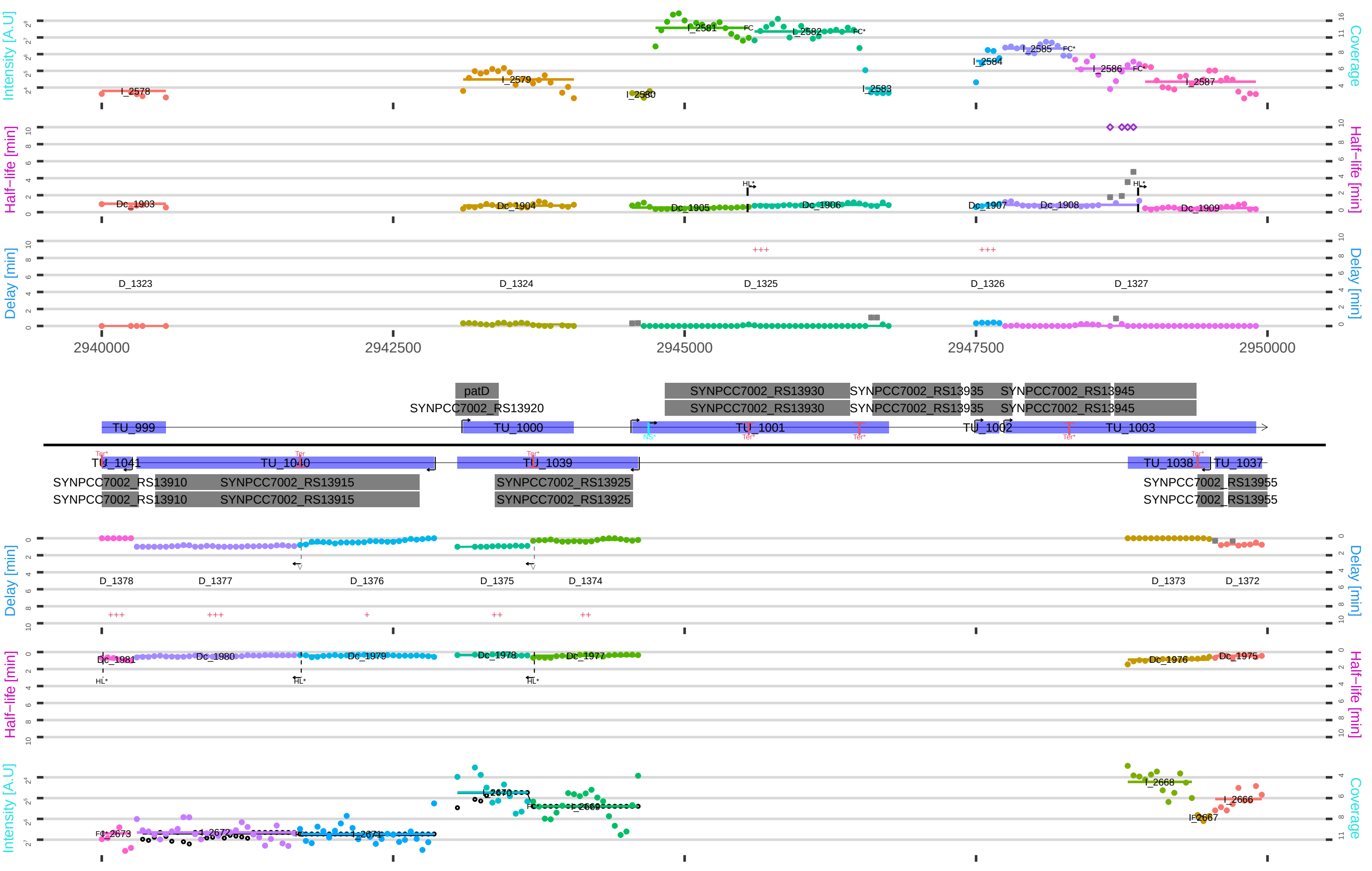

Term: termination (4), NS: new start (0), PS: pausing site (2), iTSS_I: internal starting site (0)

ID: 59005−59143; Term: termination (2), NS: new start (0), PS: pausing site (0), iTSS_I: internal starting site (0)

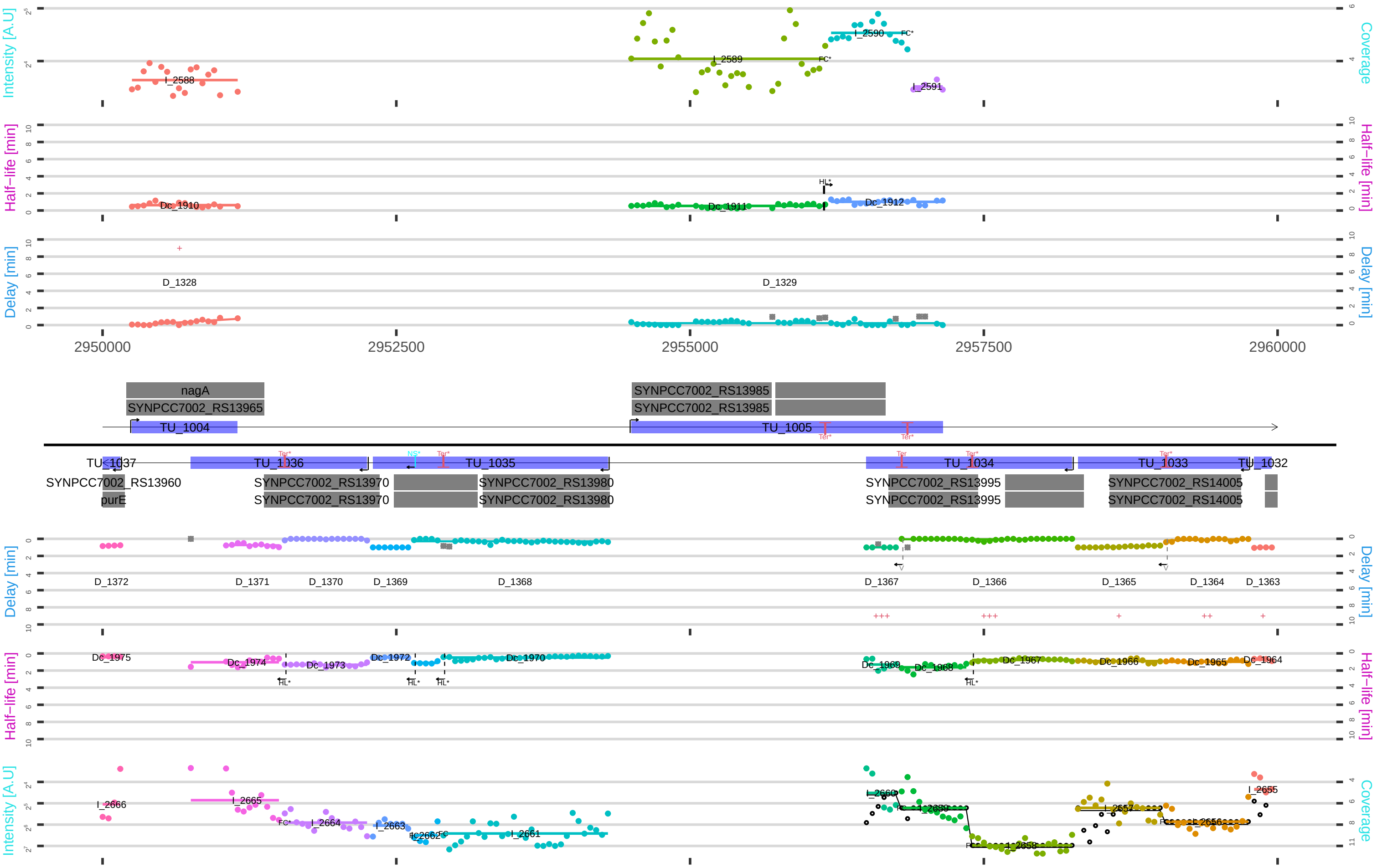

Term: termination (5), NS: new start (1), PS: pausing site (4), iTSS_I: internal starting site (0)

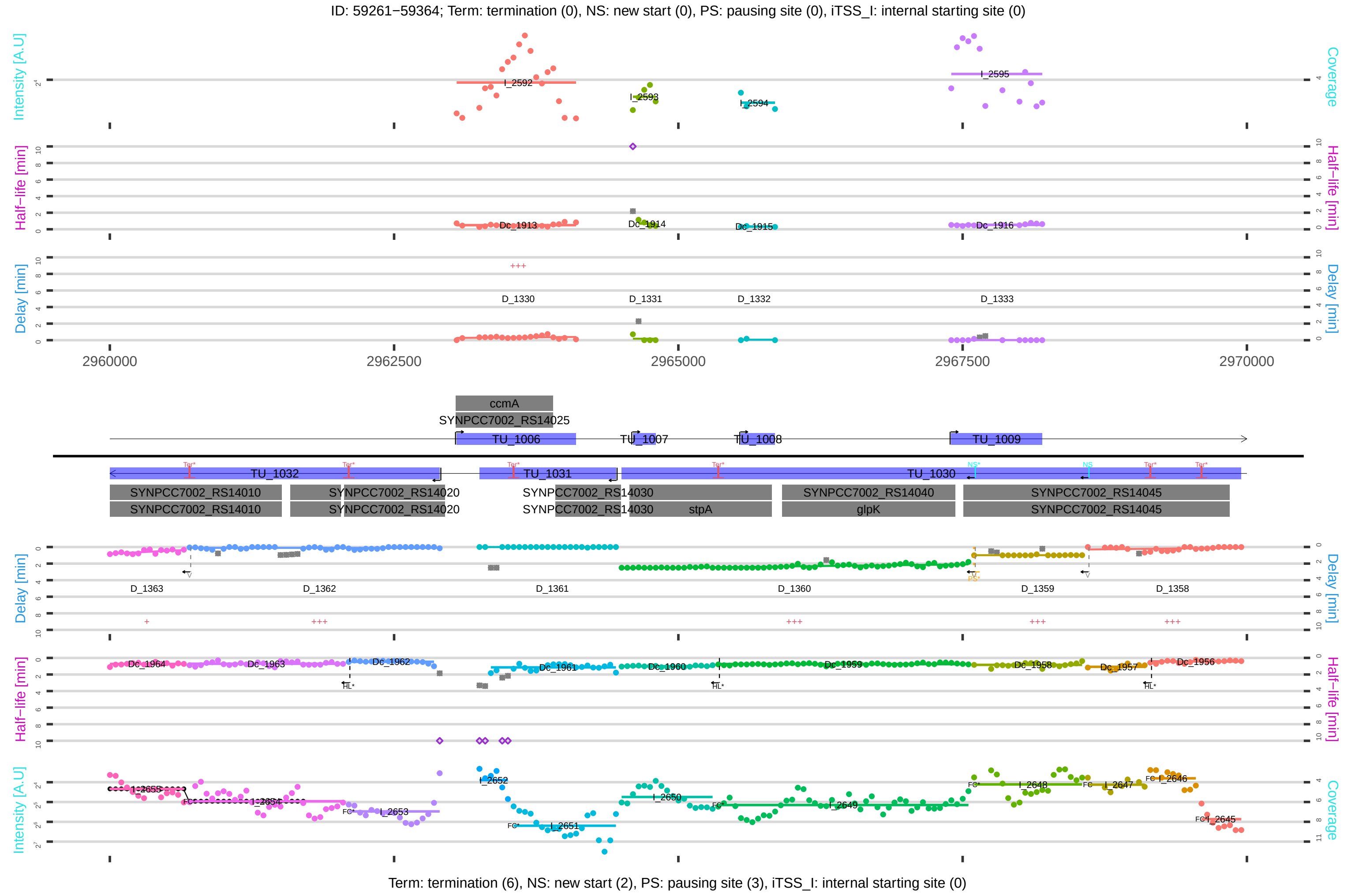
ID: 59261−59364; Term: termination (0), NS: new start (0), PS: pausing site (0), iTSS_I: internal starting site (0)
Intensity [A.U]
Coverage
I_2592
I_2593
I_2594
I_2595
Half−life [min]
Dc_1913
Dc_1914
Dc_1915
Dc_1916
Delay [min]
D_1330
D_1331
D_1332
D_1333
2960000
2962500
2965000
2967500
2970000
ccmA
SYNPCC7002_RS14025
TU_1006
TU_1007
TU_1008
TU_1009
TU_1032
TU_1031
TU_1030
SYNPCC7002_RS14010
SYNPCC7002_RS14020
SYNPCC7002_RS14030
stpA
SYNPCC7002_RS14040
glpK
SYNPCC7002_RS14045
D_1363
D_1362
D_1361
D_1360
D_1359
D_1358
Dc_1964
Dc_1963
Dc_1962
Dc_1961
Dc_1960
Dc_1959
Dc_1958
Dc_1957
Dc_1956
I_2655
I_2654
I_2653
I_2652
I_2651
I_2650
I_2649
I_2648
I_2647
I_2646
I_2645
Term: termination (6), NS: new start (2), PS: pausing site (3), iTSS_I: internal starting site (0)

ID: 59409−59600; Term: termination (4), NS: new start (3), PS: pausing site (0), iTSS_I: internal starting site (0)

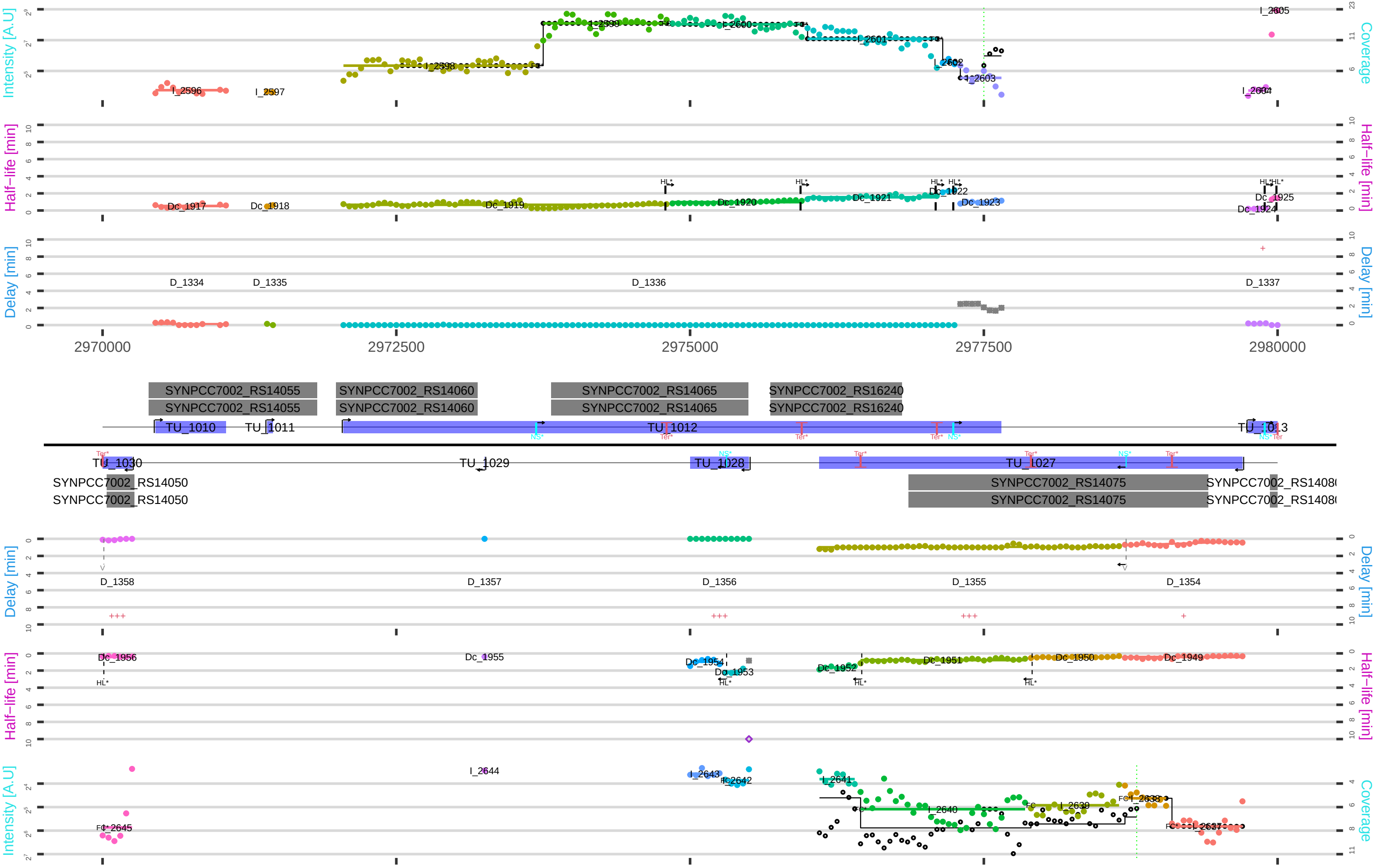

Term: termination (4), NS: new start (2), PS: pausing site (1), iTSS_I: internal starting site (0)

ID: 59600−59798; Term: termination (7), NS: new start (2), PS: pausing site (0), iTSS_I: internal starting site (1)

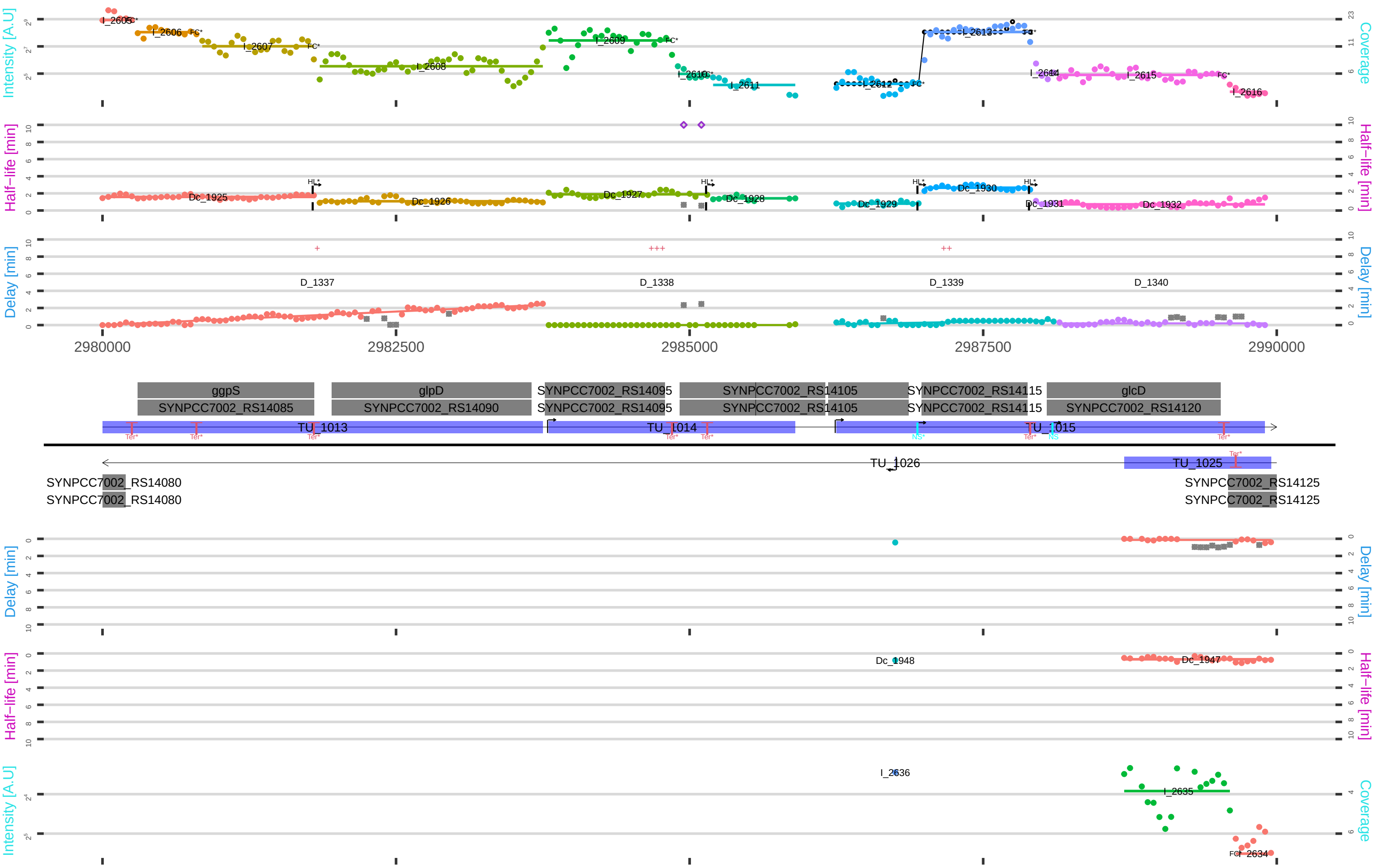

Term: termination (1), NS: new start (0), PS: pausing site (0), iTSS_I: internal starting site (0)

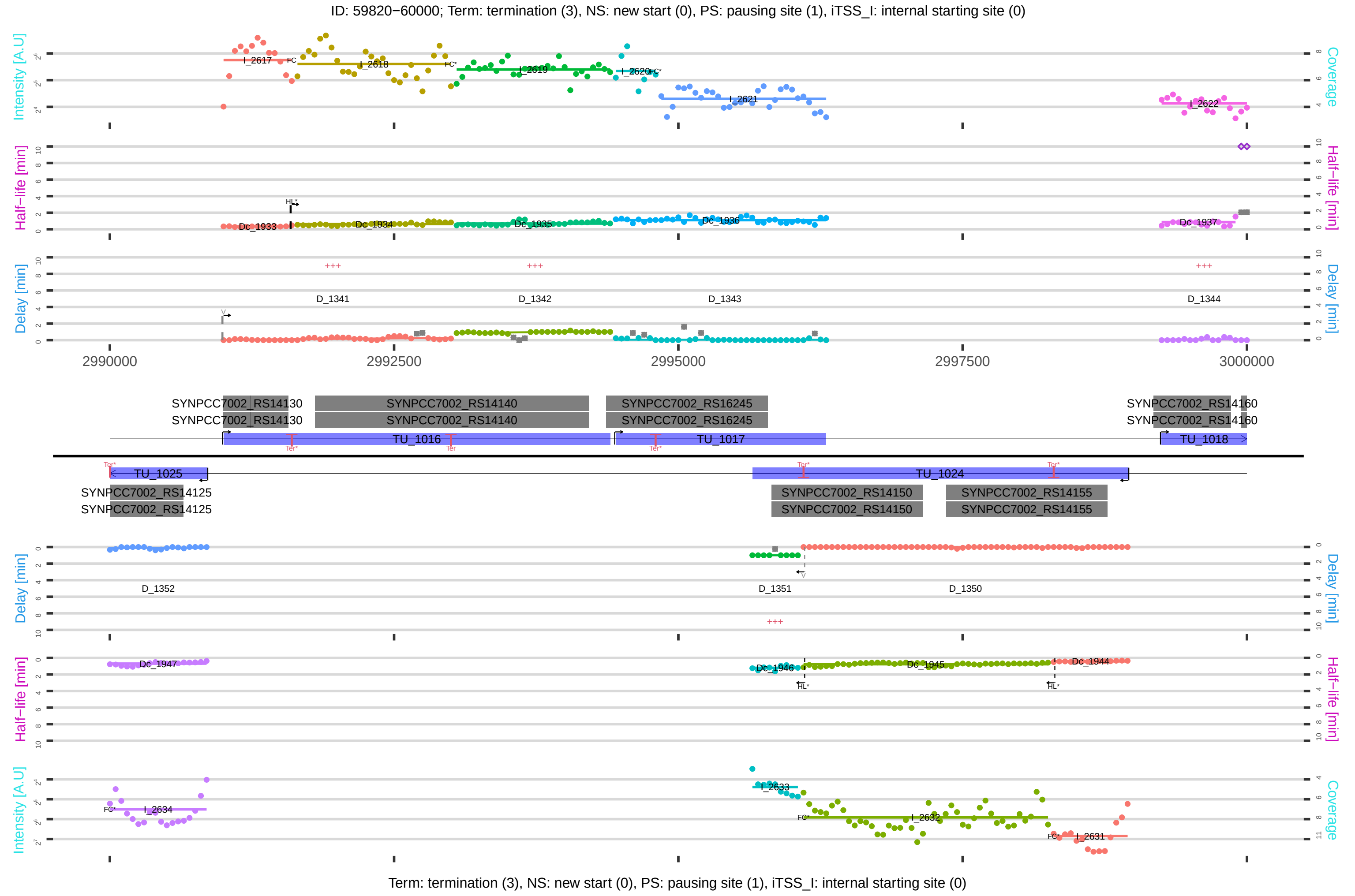

ID: 59820−60000; Term: termination (3), NS: new start (0), PS: pausing site (1), iTSS_I: internal starting site (0)
Intensity [A.U]
Coverage
Half−life [min]
Delay [min]
I_2617
I_2618
I_2619
I_2620
I_2621
I_2622
Dc_1933
Dc_1934
Dc_1935
Dc_1936
Dc_1937
D_1341
D_1342
D_1343
D_1344
2990000
2992500
2995000
2997500
3000000
SYNPCC7002_RS14130
SYNPCC7002_RS14140
SYNPCC7002_RS16245
SYNPCC7002_RS14160
TU_1016
TU_1017
TU_1018
TU_1025
TU_1024
SYNPCC7002_RS14125
SYNPCC7002_RS14150
SYNPCC7002_RS14155
D_1352
D_1351
D_1350
Dc_1947
Dc_1946
Dc_1945
Dc_1944
I_2634
I_2633
I_2632
I_2631
Term: termination (3), NS: new start (0), PS: pausing site (1), iTSS_I: internal starting site (0)

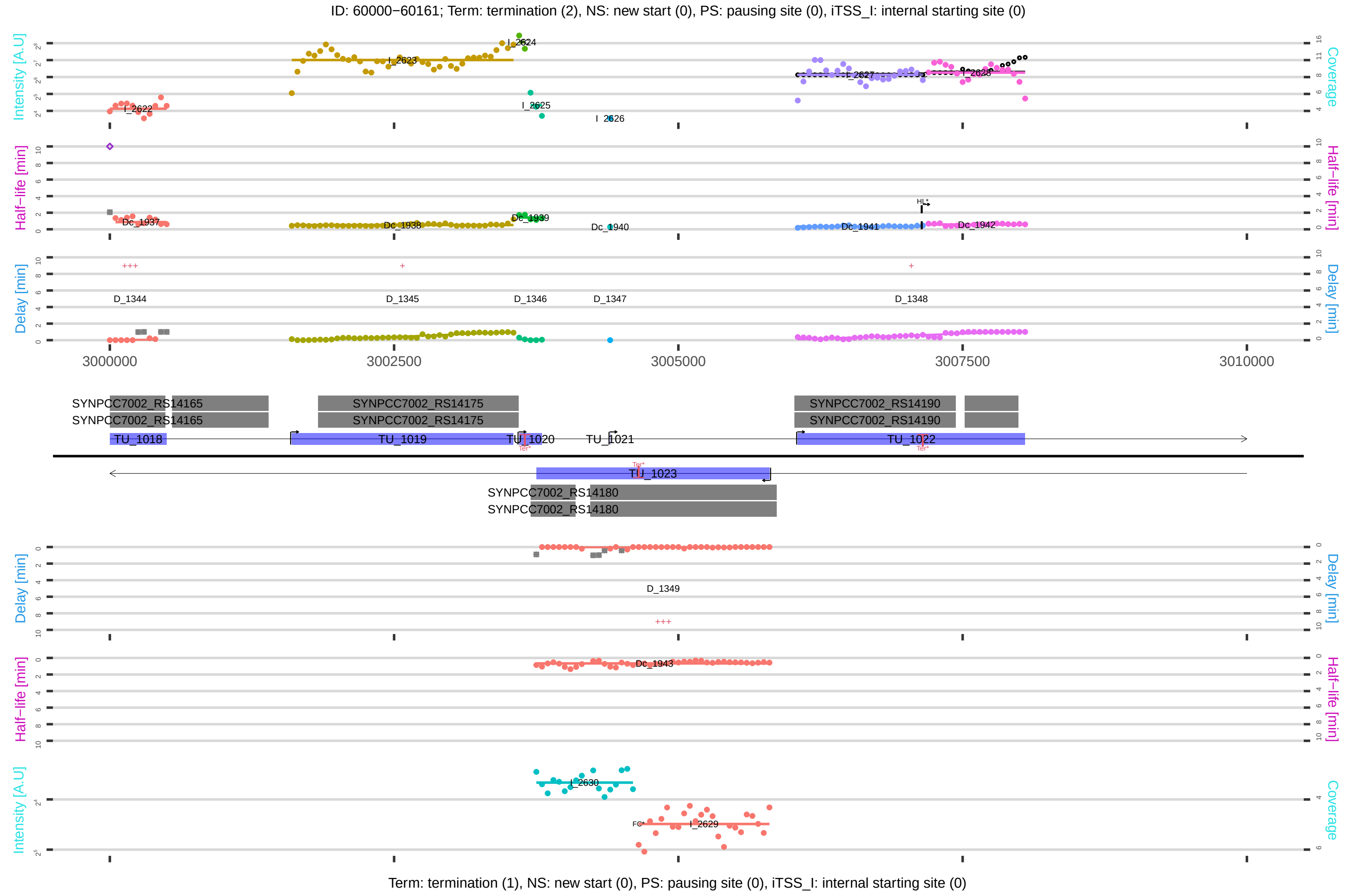

ID: 60000−60161; Term: termination (2), NS: new start (0), PS: pausing site (0), iTSS_I: internal starting site (0)
Intensity [A.U]
Coverage
Half−life [min]
Delay [min]
I_2622
I_2623
I_2624
I_2625
I_2626
I_2627
I_2628
Dc_1937
Dc_1938
Dc_1939
Dc_1940
Dc_1941
Dc_1942
HL*
D_1344
D_1345
D_1346
D_1347
D_1348
3000000
3002500
3005000
3007500
3010000
SYNPCC7002_RS14165
SYNPCC7002_RS14175
SYNPCC7002_RS14190
TU_1018
TU_1019
TU_1020
TU_1021
TU_1022
Ter*
TU_1023
SYNPCC7002_RS14180
D_1349
Dc_1943
I_2630
I_2629
FC*
Term: termination (1), NS: new start (0), PS: pausing site (0), iTSS_I: internal starting site (0)
