# Supplementary material for: Improved RNA stability estimation indicates that transcriptional interference is frequent in diverse bacteria
Source: Commun Biol. 2023 Jul 15;6:732. doi: 10.1038/s42003-023-05097-2 (PMC10349824; doi:10.1038/s42003-023-05097-2)
Supplement: Supplementary file 6 — Supplementary Data 3 [file 42003_2023_5097_MOESM6_ESM.zip › Synechocystis_PCC6803.pdf]

ID: 1−72; FC*: significant t−test of two consecutive segments;
Term: termination (3), NS: new start (0), PS: pausing site (0), iTSS_I: internal starting site (1), (*): p_value below 0.05; TI: transcription interferance.

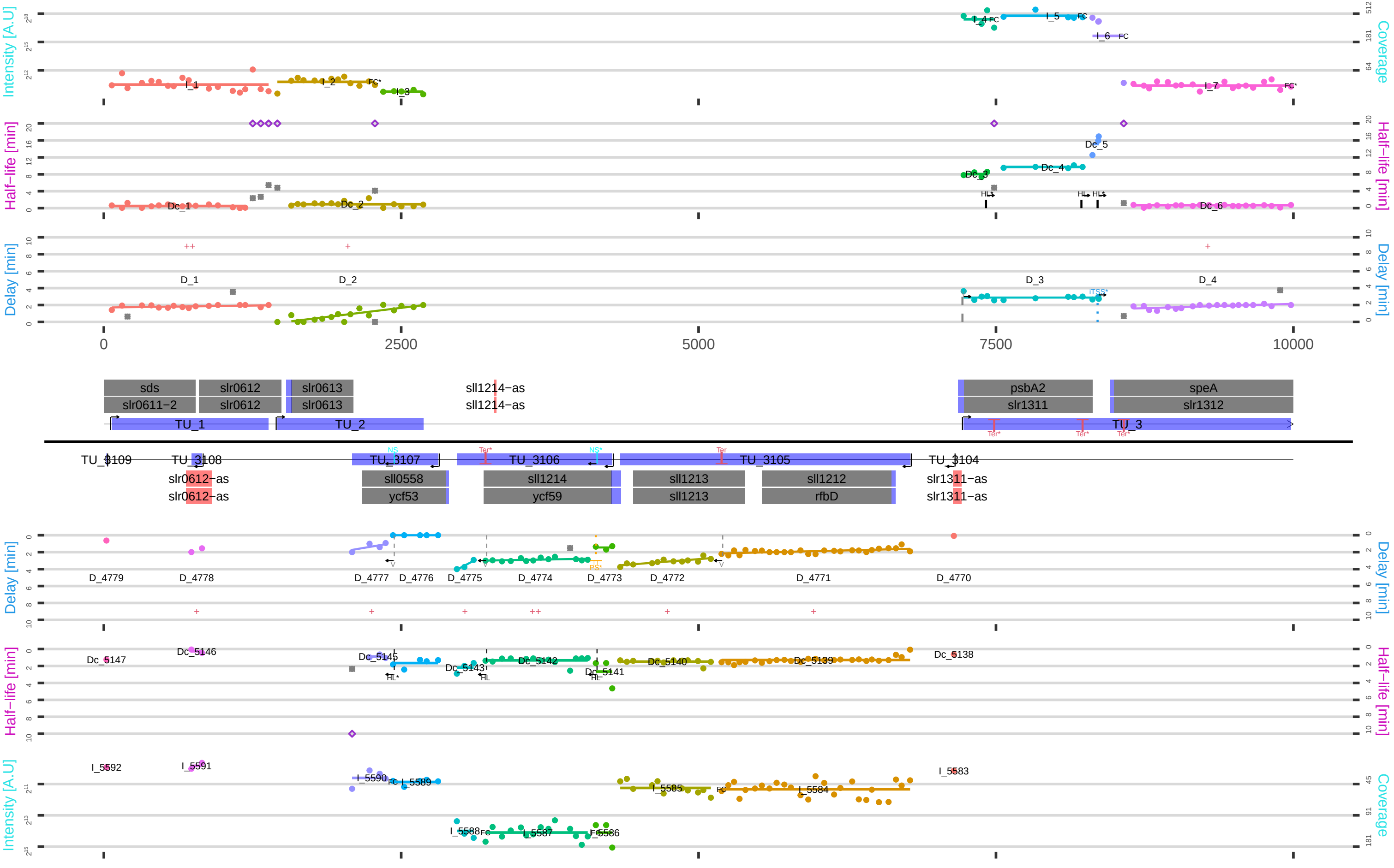

Term: termination (2), NS: new start (2), PS: pausing site (3), iTSS_I: internal starting site (1)

ID: 73−153; Term: termination (1), NS: new start (0), PS: pausing site (1), iTSS_I: internal starting site (0)

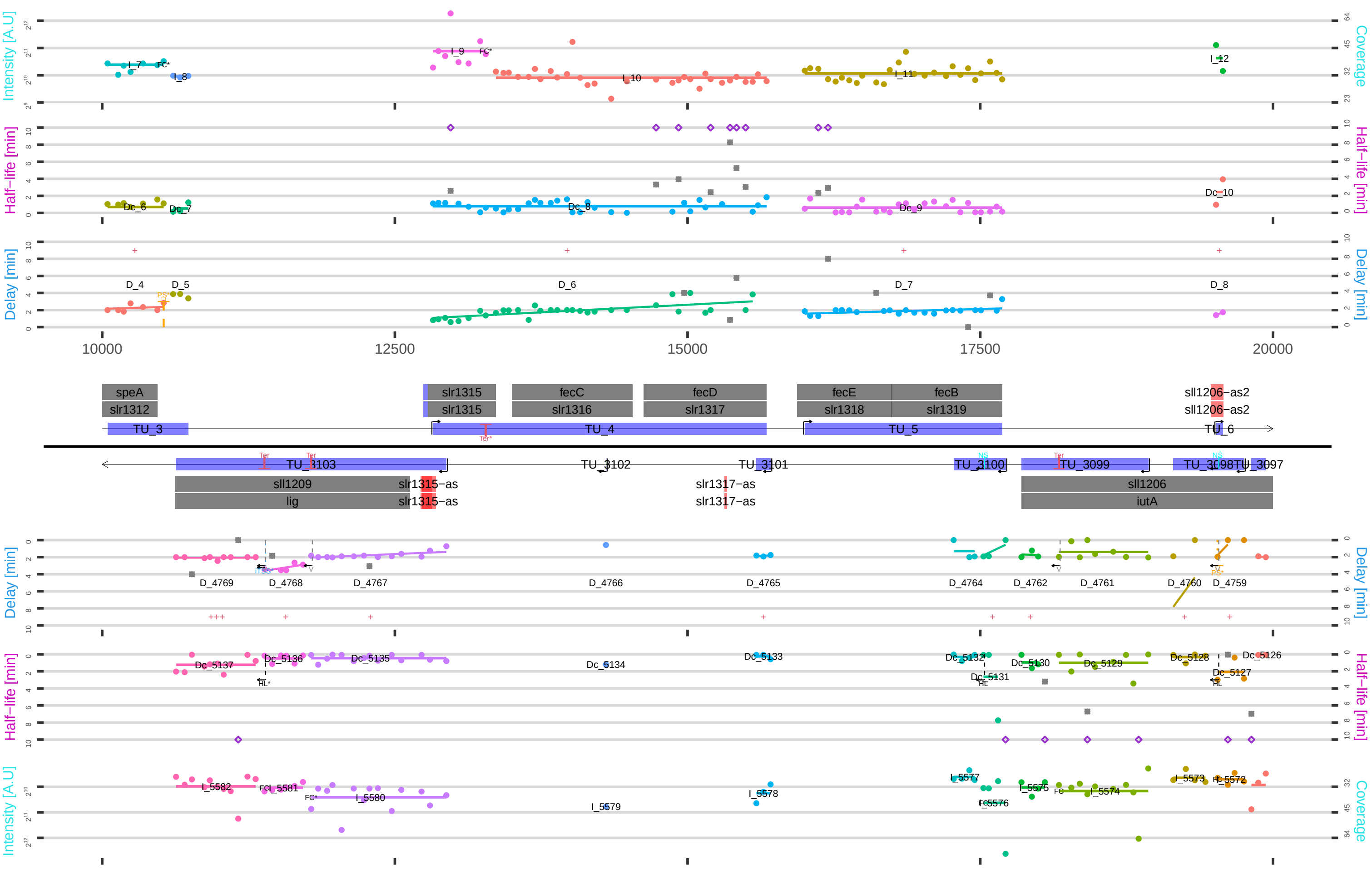

Term: termination (3), NS: new start (2), PS: pausing site (4), iTSS_I: internal starting site (1)

ID: 155−162; Term: termination (0), NS: new start (0), PS: pausing site (0), iTSS_I: internal starting site (0)

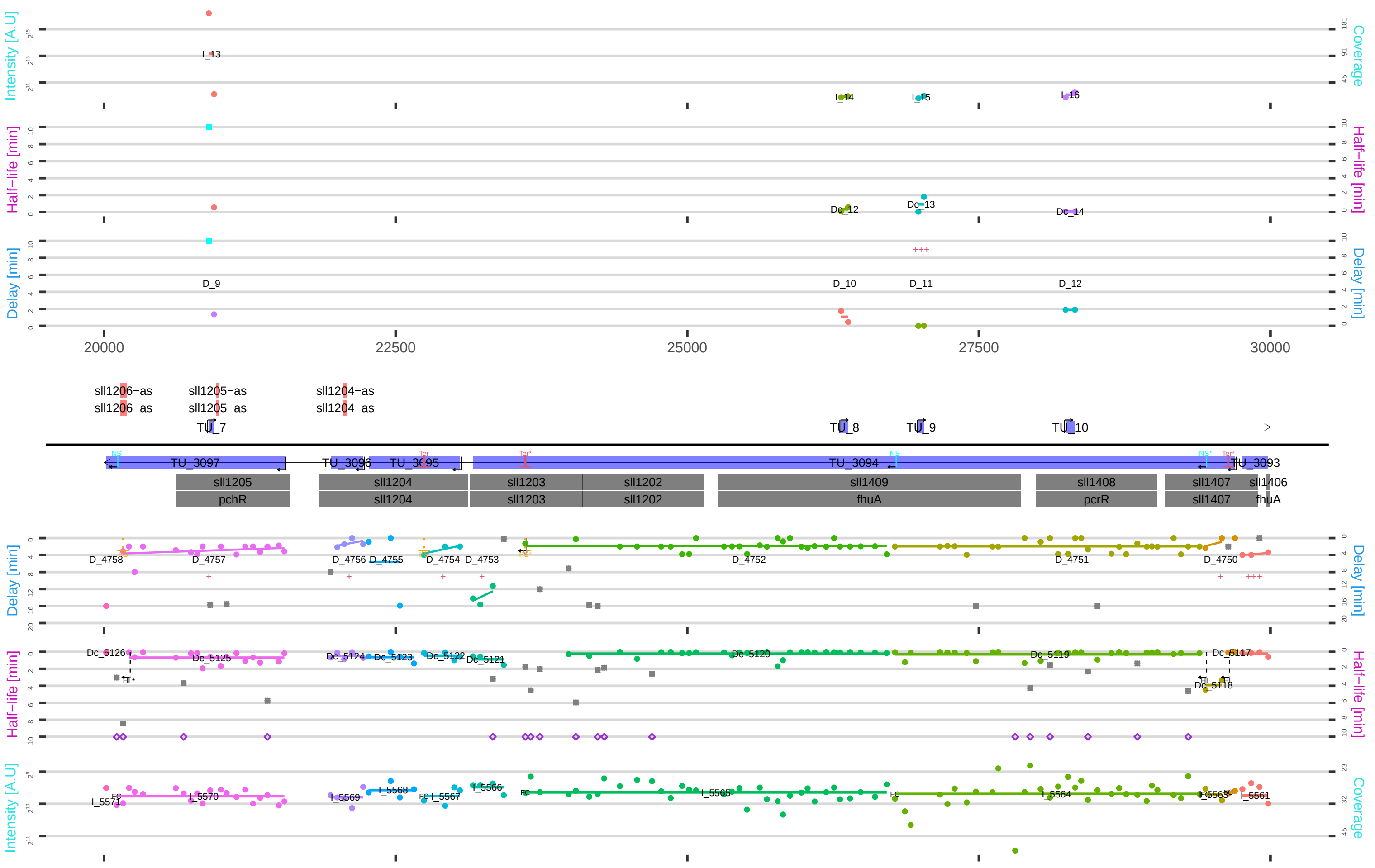

Term: termination (3), NS: new start (3), PS: pausing site (4), iTSS_I: internal starting site (1)

ID: 163−258; Term: termination (2), NS: new start (0), PS: pausing site (0), iTSS_I: internal starting site (1)

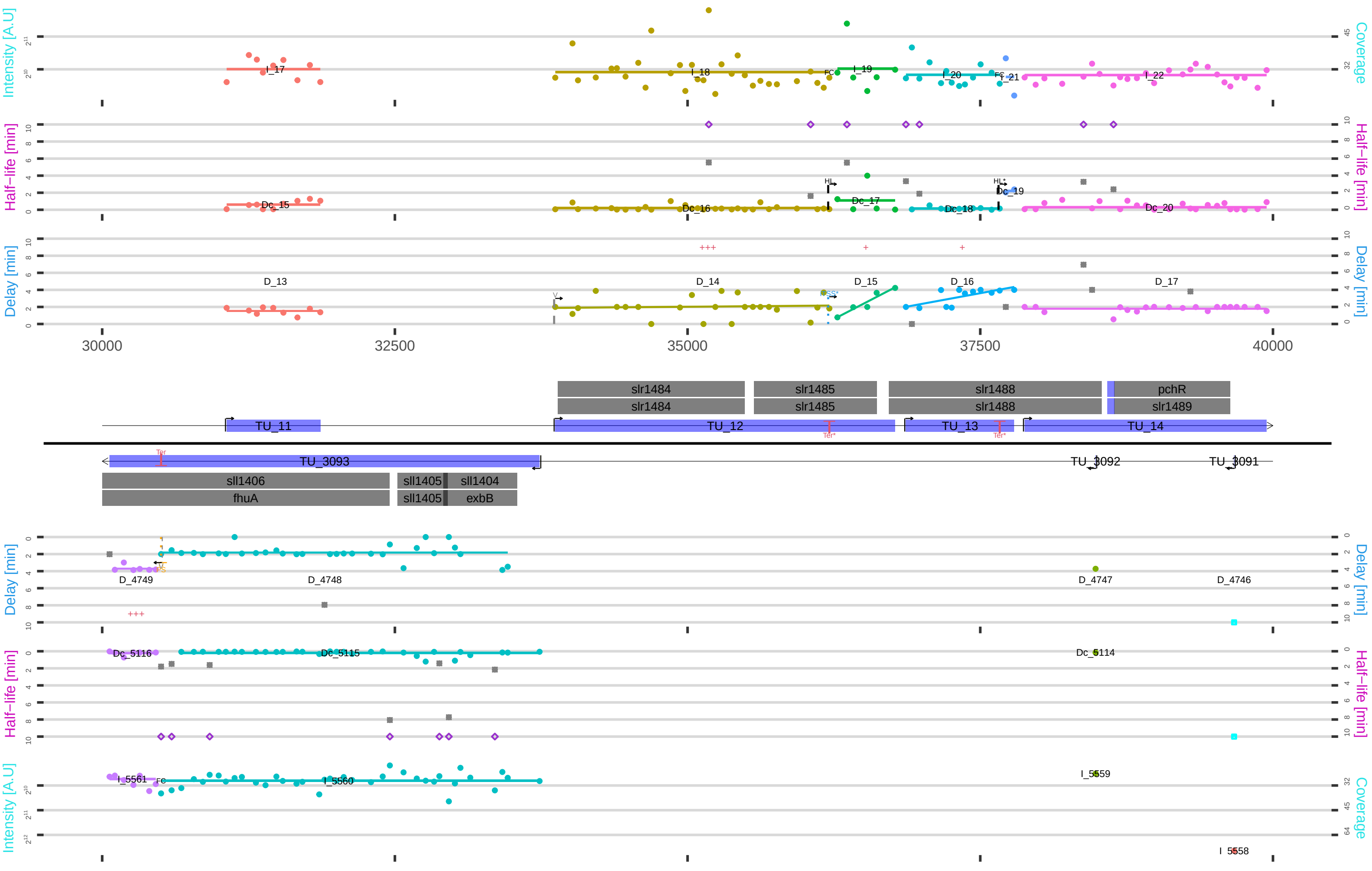

Term: termination (1), NS: new start (0), PS: pausing site (1), iTSS_I: internal starting site (0)

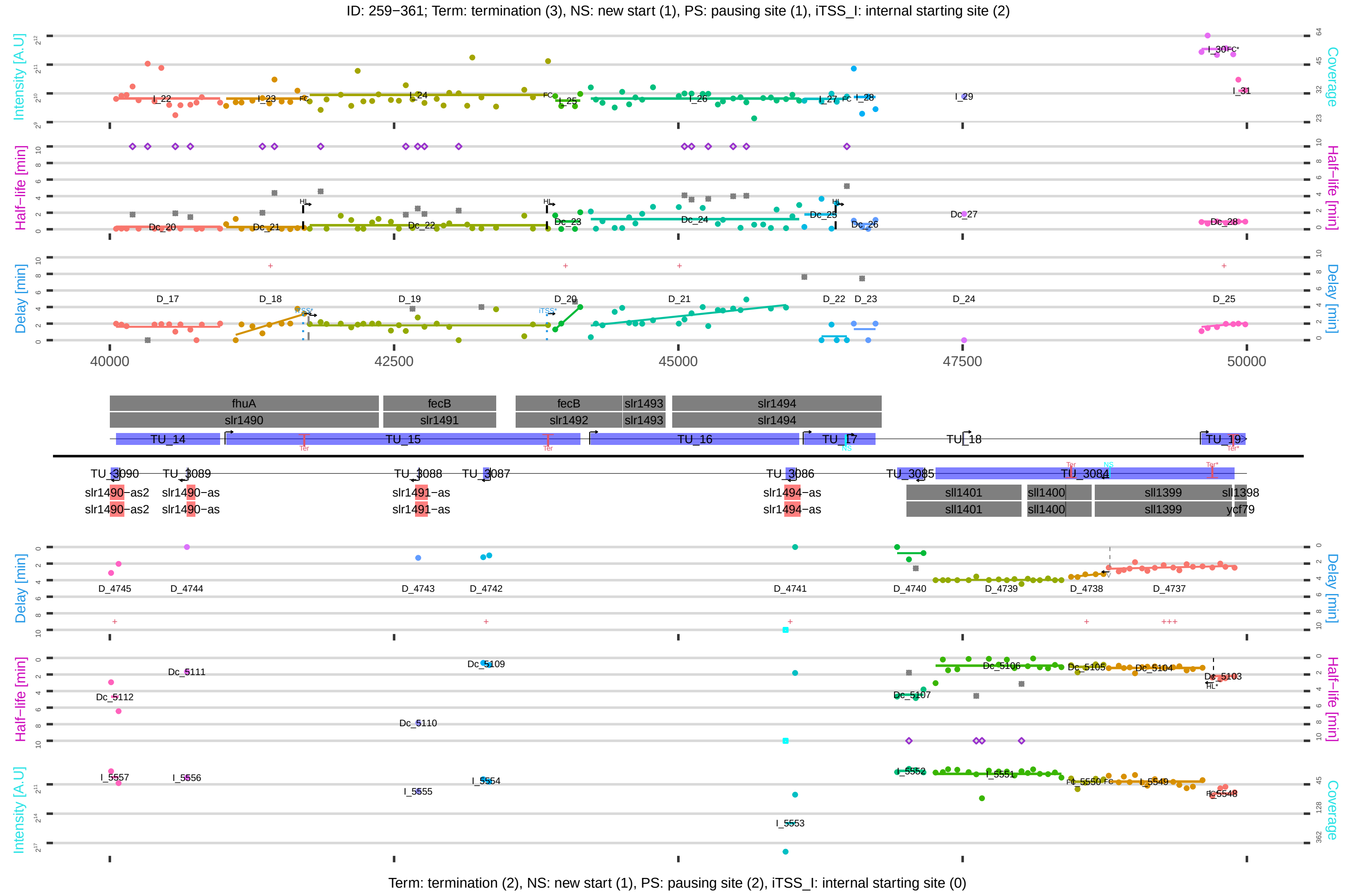

ID: 259−361; Term: termination (3), NS: new start (1), PS: pausing site (1), iTSS_I: internal starting site (2)
Intensity [A.U]
Coverage
Half−life [min]
Delay [min]
40000
42500
45000
47500
50000
fhuA
slr1490
fecB
slr1491
fecB
slr1492
slr1493
slr1493
slr1494
slr1494
TU_14
TU_15
TU_16
TU_17
TU_18
TU_19
TU_3090
TU_3089
TU_3088
TU_3087
TU_3086
TU_3085
TU_3084
slr1490−as2
slr1490−as
slr1491−as
slr1494−as
sll1401
sll1400
sll1399
sll1398
ycf79
Term: termination (2), NS: new start (1), PS: pausing site (2), iTSS_I: internal starting site (0)

ID: 362−416; Term: termination (0), NS: new start (1), PS: pausing site (0), iTSS_I: internal starting site (1)

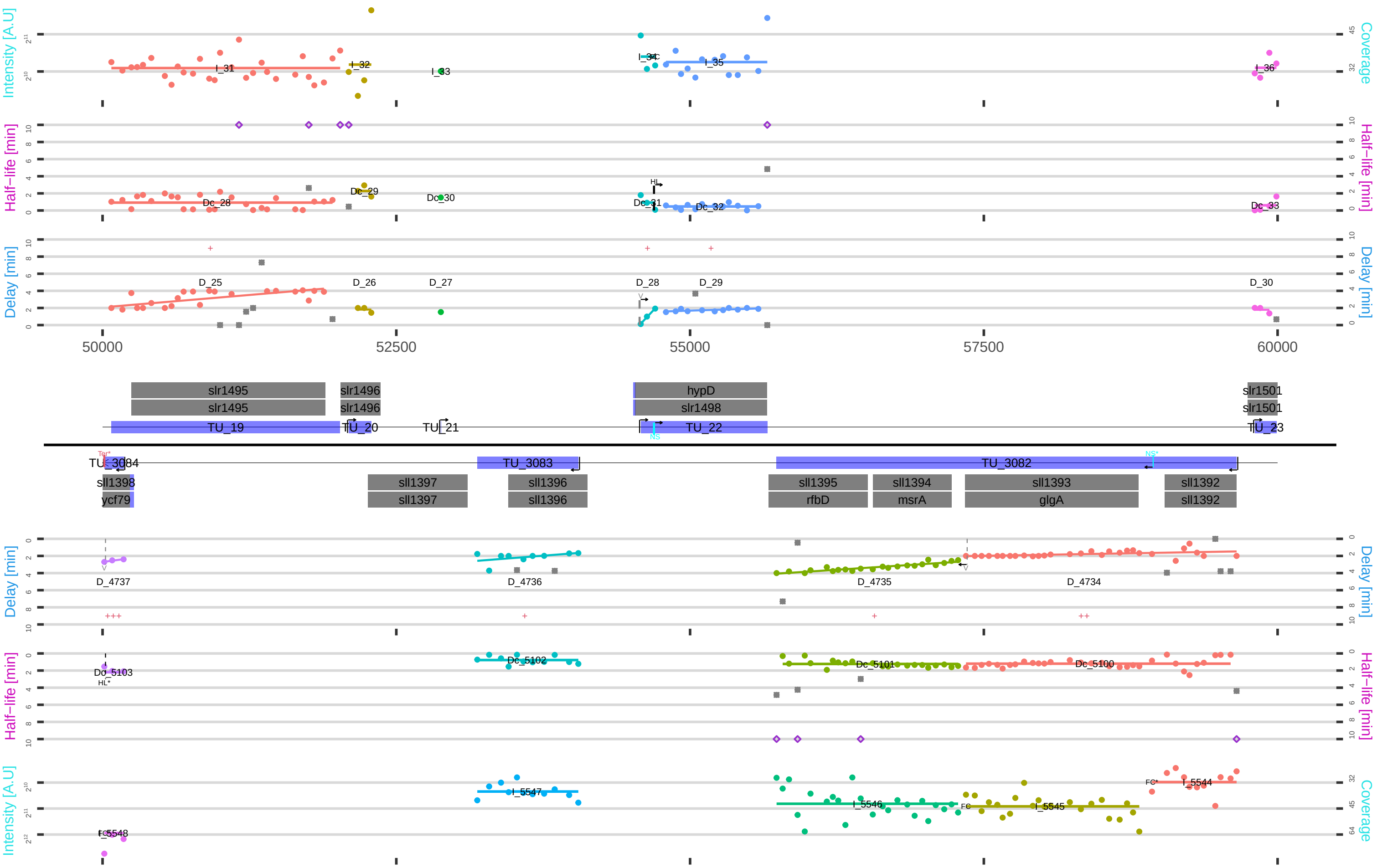

Term: termination (1), NS: new start (1), PS: pausing site (1), iTSS_I: internal starting site (0)

ID: 417−529; Term: termination (1), NS: new start (2), PS: pausing site (2), iTSS_I: internal starting site (1)

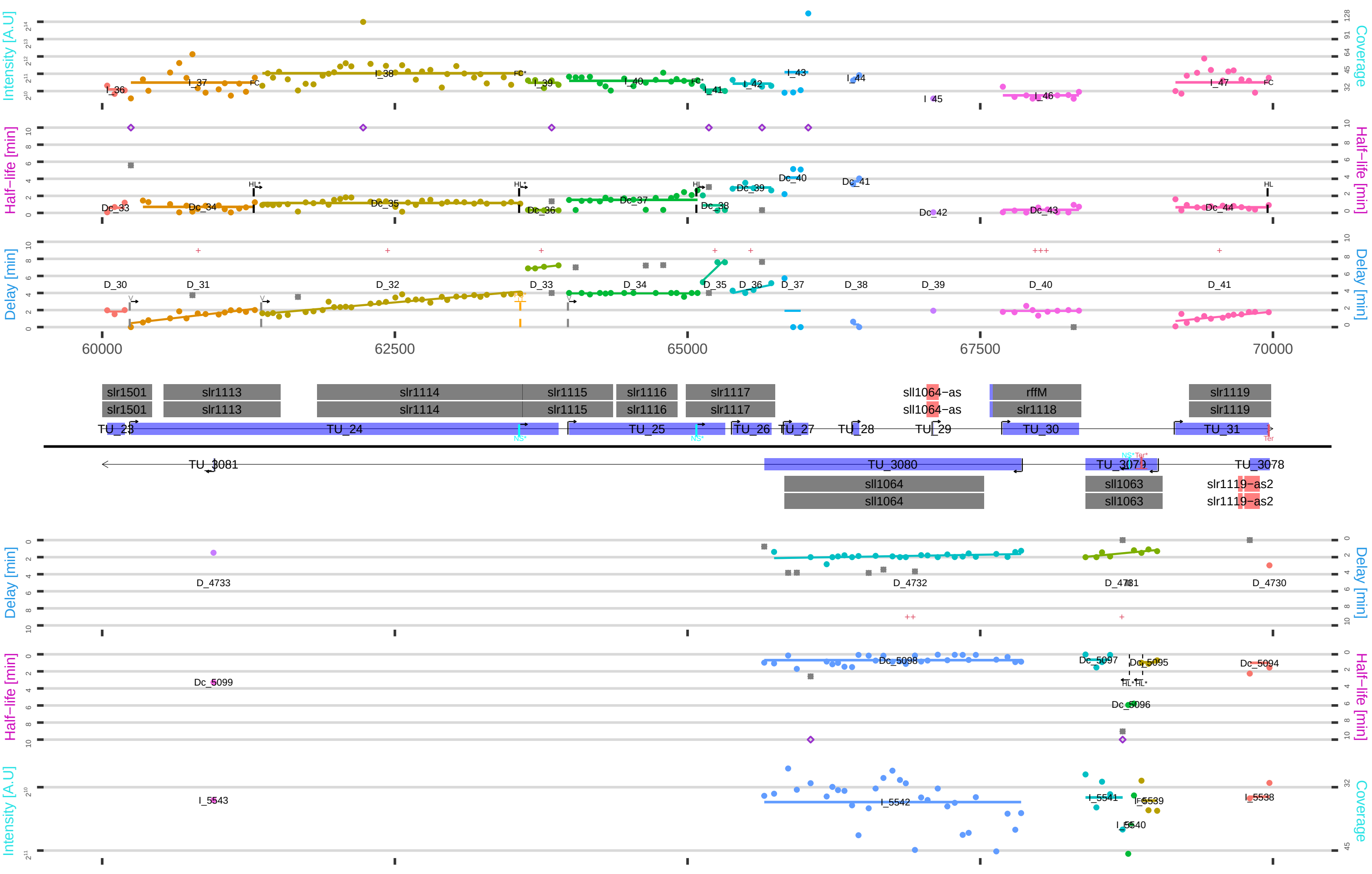

Term: termination (1), NS: new start (1), PS: pausing site (0), iTSS_I: internal starting site (0)

ID: 530−559; Term: termination (1), NS: new start (1), PS: pausing site (1), iTSS_I: internal starting site (0)

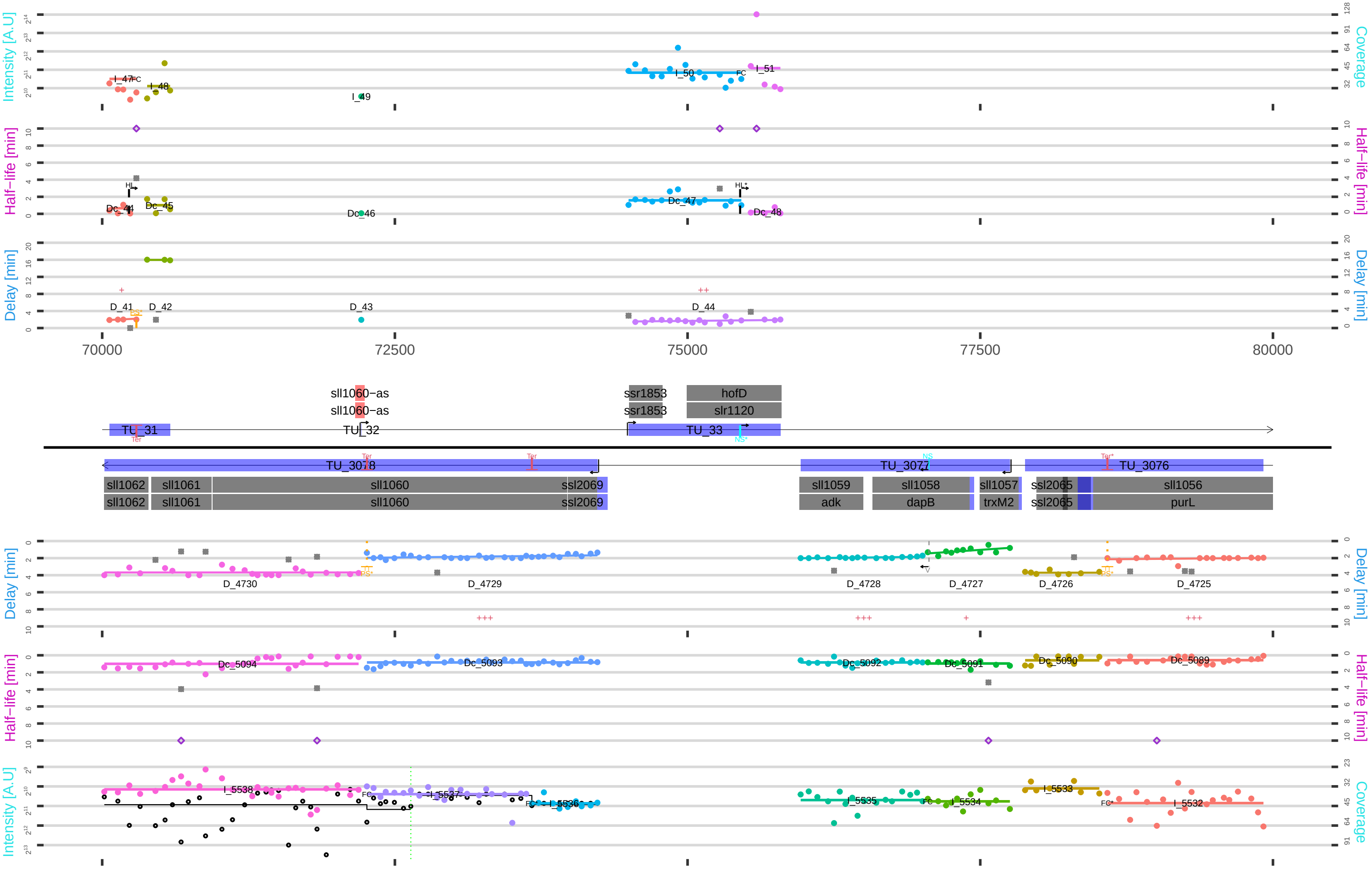

Term: termination (3), NS: new start (1), PS: pausing site (3), iTSS_I: internal starting site (0)

ID: 560−622; Term: termination (1), NS: new start (0), PS: pausing site (1), iTSS_I: internal starting site (0)

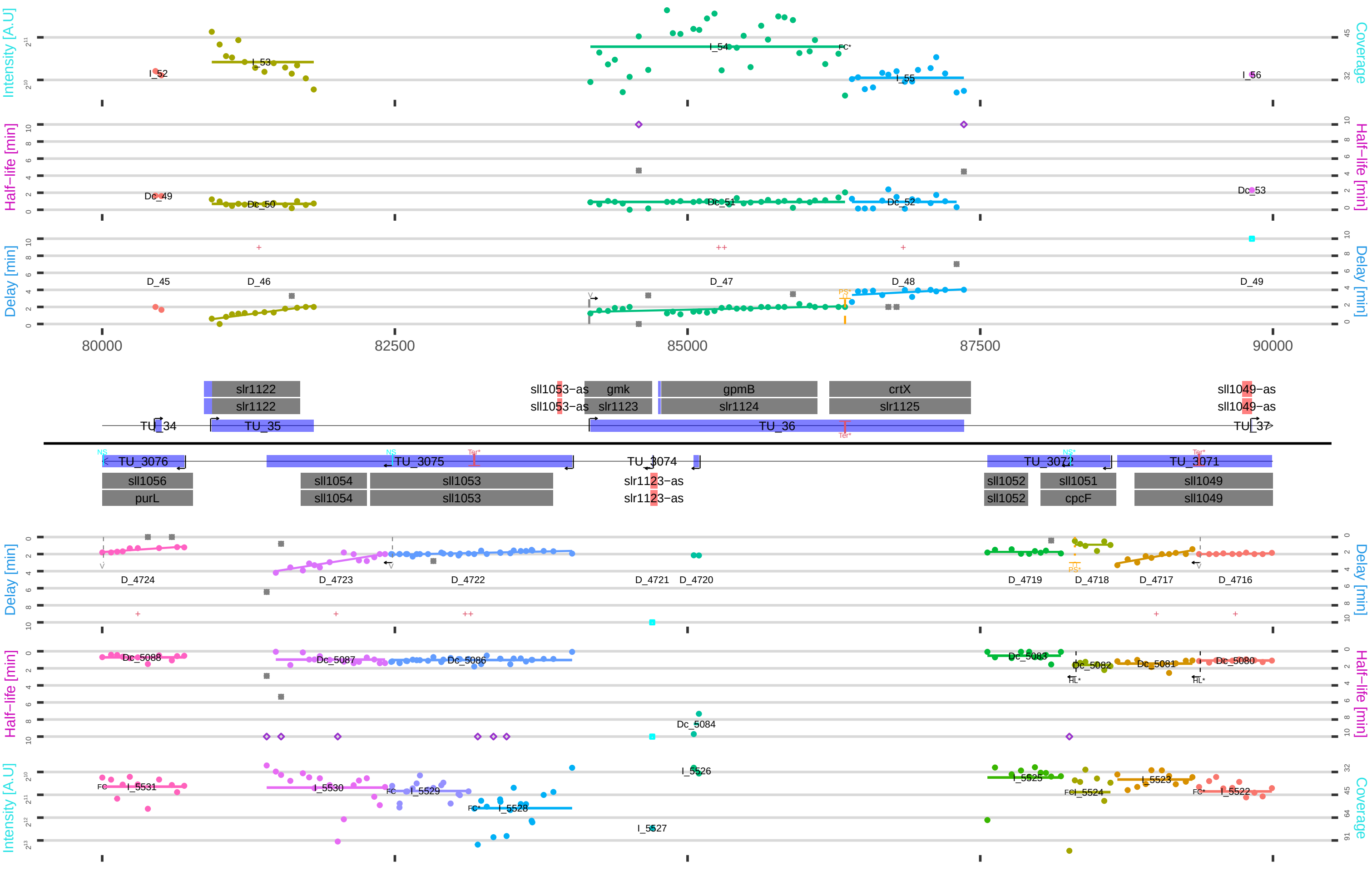

Term: termination (2), NS: new start (3), PS: pausing site (2), iTSS_I: internal starting site (2)

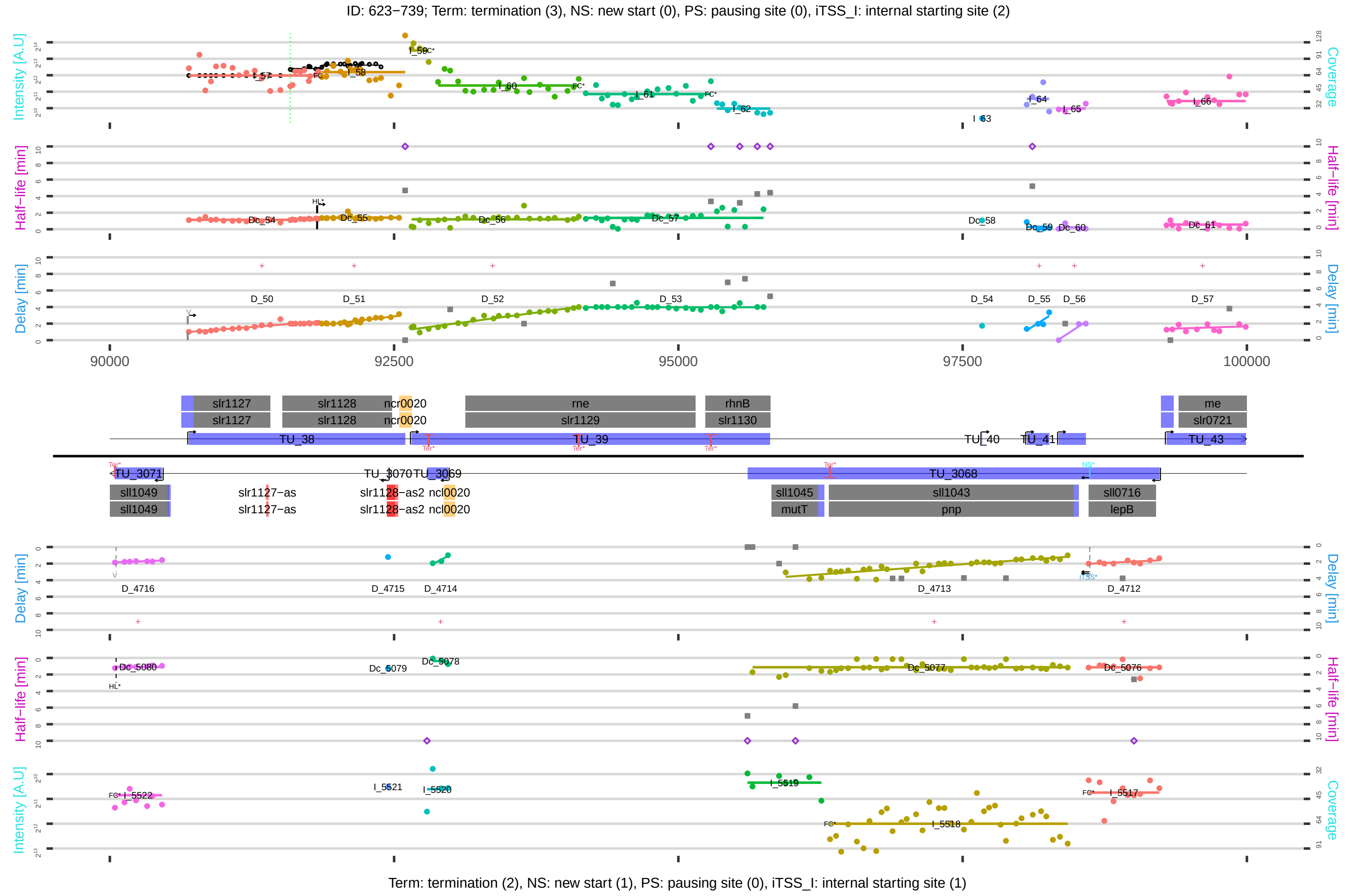
ID: 623−739; Term: termination (3), NS: new start (0), PS: pausing site (0), iTSS_I: internal starting site (2)
Intensity [A.U]
Coverage
Half−life [min]
Delay [min]
90000
92500
95000
97500
100000
slr1127
slr1128
ncr0020
rne
slr1129
rhnB
slr1130
me
slr0721
TU_38
TU_39
TU_40
TU_41
TU_43
TU_3071
TU_3070
TU_3069
TU_3068
sll1049
slr1127−as
slr1128−as2
ncl0020
sll1045
mutT
sll1043
pnp
sll0716
lepB
Term: termination (2), NS: new start (1), PS: pausing site (0), iTSS_I: internal starting site (1)

ID: 740−782; Term: termination (0), NS: new start (1), PS: pausing site (0), iTSS_I: internal starting site (0)

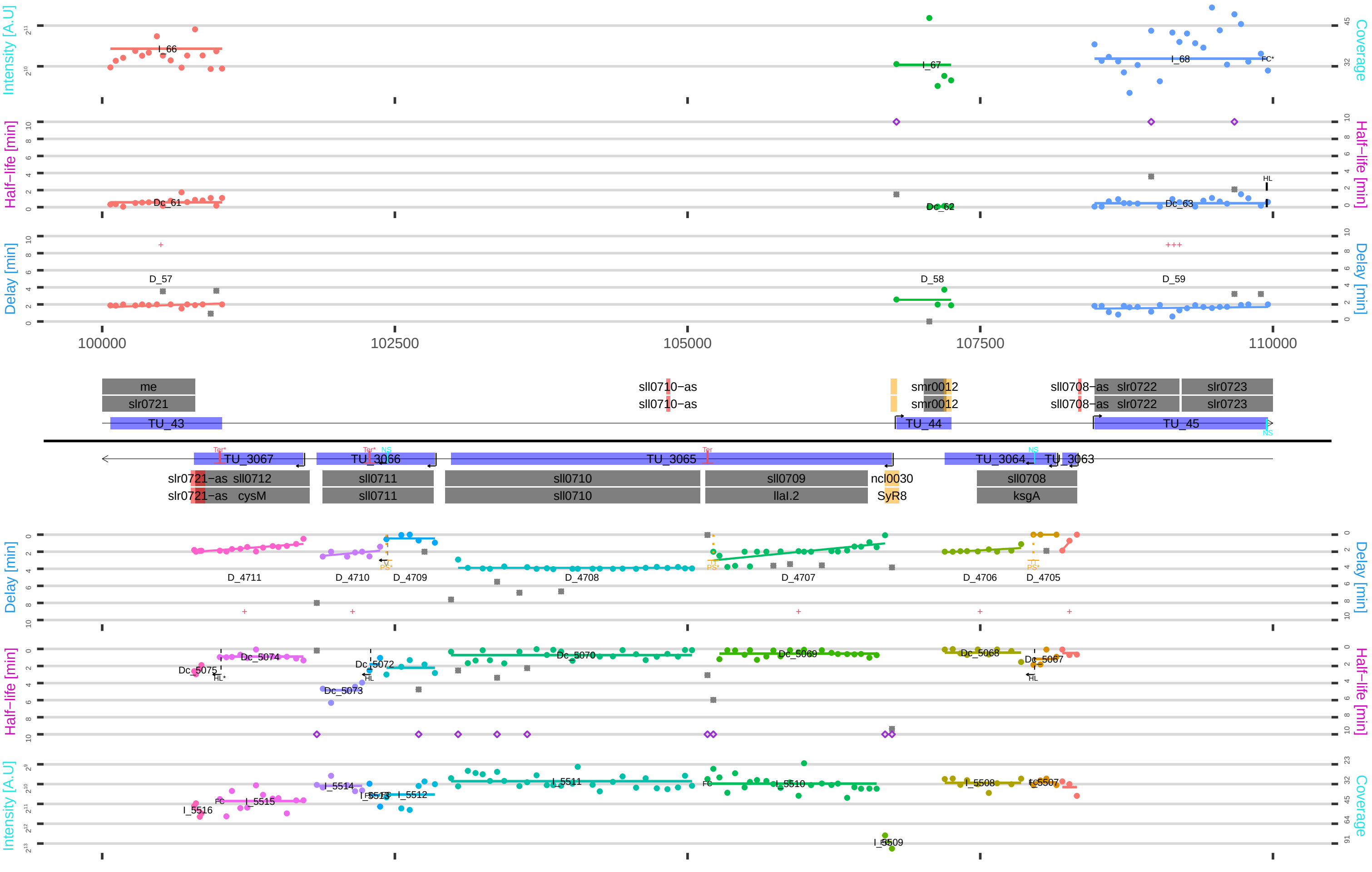

Term: termination (3), NS: new start (2), PS: pausing site (3), iTSS_I: internal starting site (0)

ID: 783−875; Term: termination (1), NS: new start (2), PS: pausing site (2), iTSS_I: internal starting site (1)

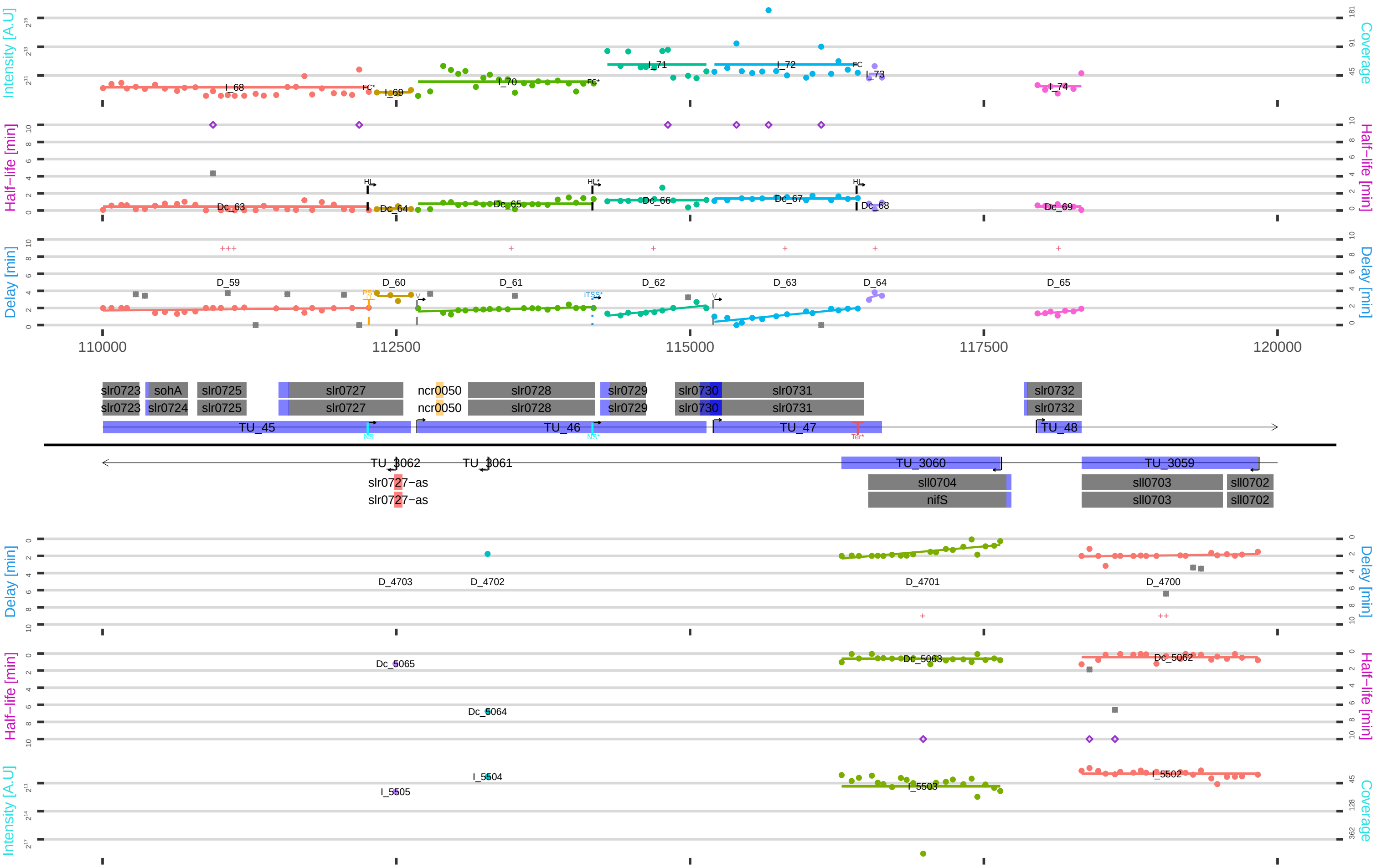

Term: termination (0), NS: new start (0), PS: pausing site (0), iTSS_I: internal starting site (0)

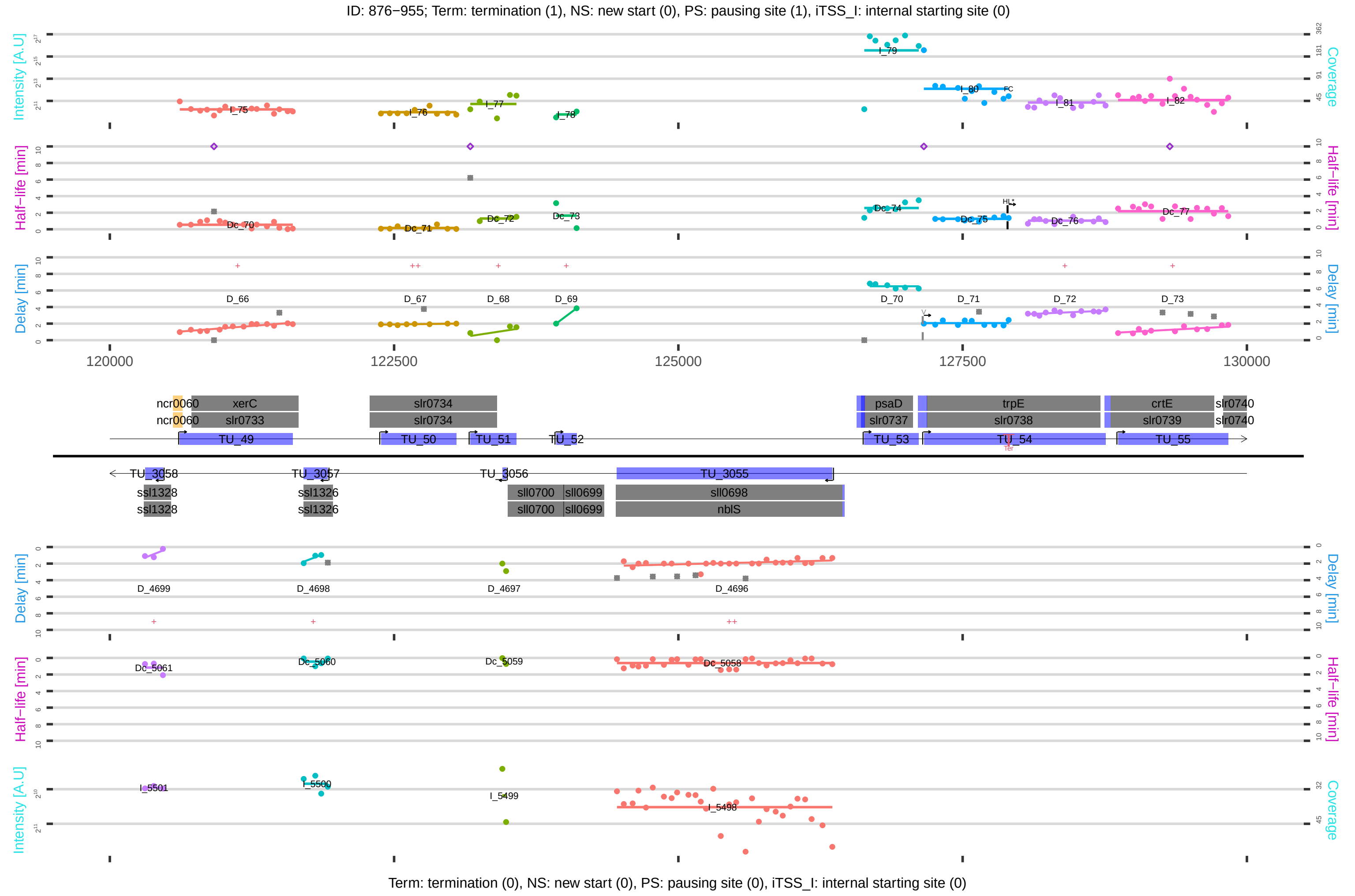

ID: 876−955; Term: termination (1), NS: new start (0), PS: pausing site (1), iTSS_I: internal starting site (0)
Intensity [A.U]
Coverage
Half−life [min]
Delay [min]
I_75
I_76
I_77
I_78
I_79
I_80
I_81
I_82
FC
Dc_70
Dc_71
Dc_72
Dc_73
Dc_74
Dc_75
Dc_76
Dc_77
HL*
D_66
D_67
D_68
D_69
D_70
D_71
D_72
D_73
120000
122500
125000
127500
130000
ncr0060
xerC
slr0733
slr0734
psaD
slr0737
trpE
slr0738
crtE
slr0739
slr0740
TU_49
TU_50
TU_51
TU_52
TU_53
TU_54
TU_55
Ter
TU_3058
TU_3057
TU_3056
TU_3055
ssl1328
ssl1326
sll0700
sll0699
sll0698
nblS
D_4699
D_4698
D_4697
D_4696
Dc_5061
Dc_5060
Dc_5059
Dc_5058
I_5501
I_5500
I_5499
I_5498
Term: termination (0), NS: new start (0), PS: pausing site (0), iTSS_I: internal starting site (0)

ID: 956−1095; Term: termination (4), NS: new start (1), PS: pausing site (2), iTSS_I: internal starting site (1)

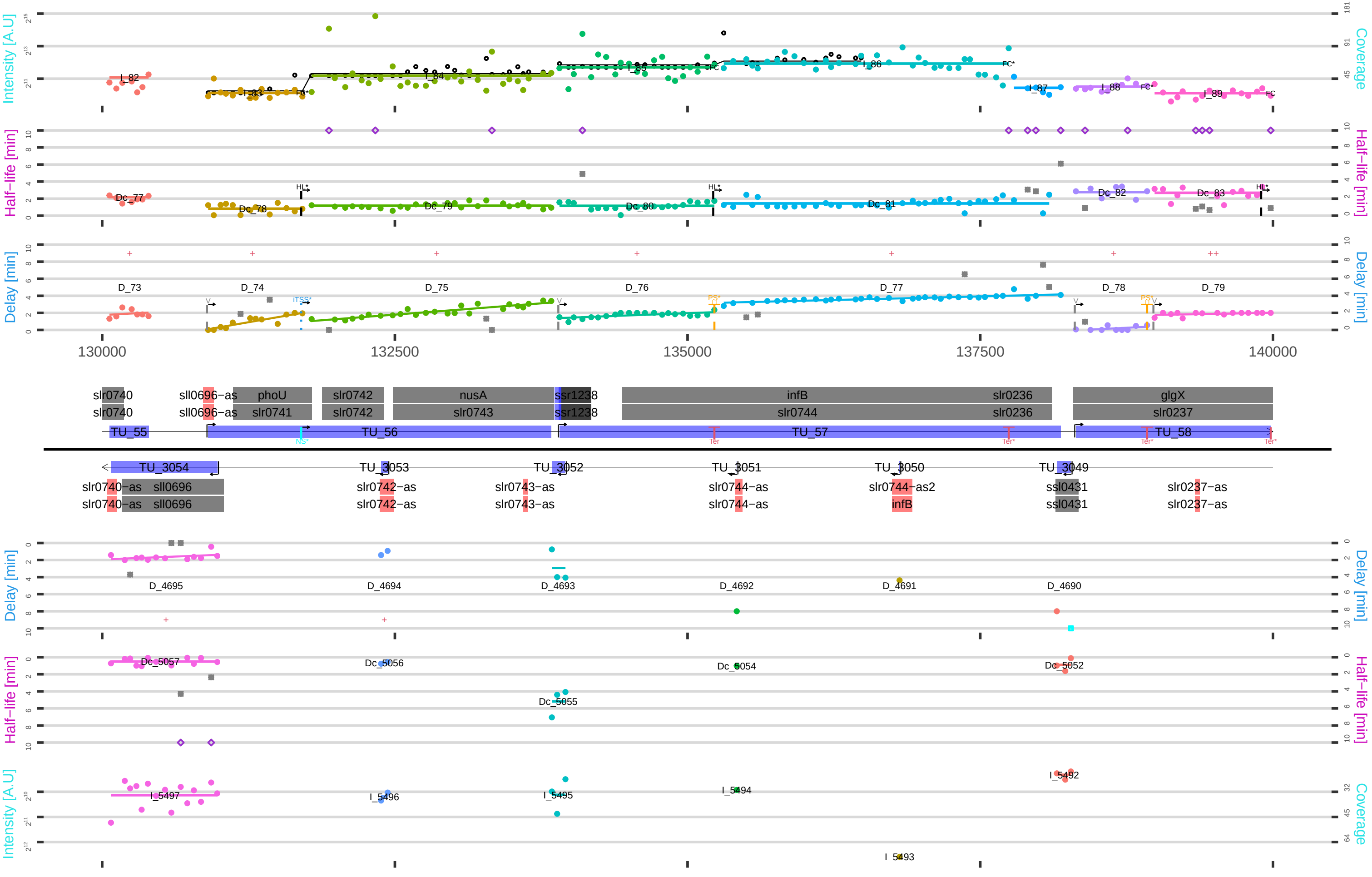

Term: termination (0), NS: new start (0), PS: pausing site (0), iTSS_I: internal starting site (0)

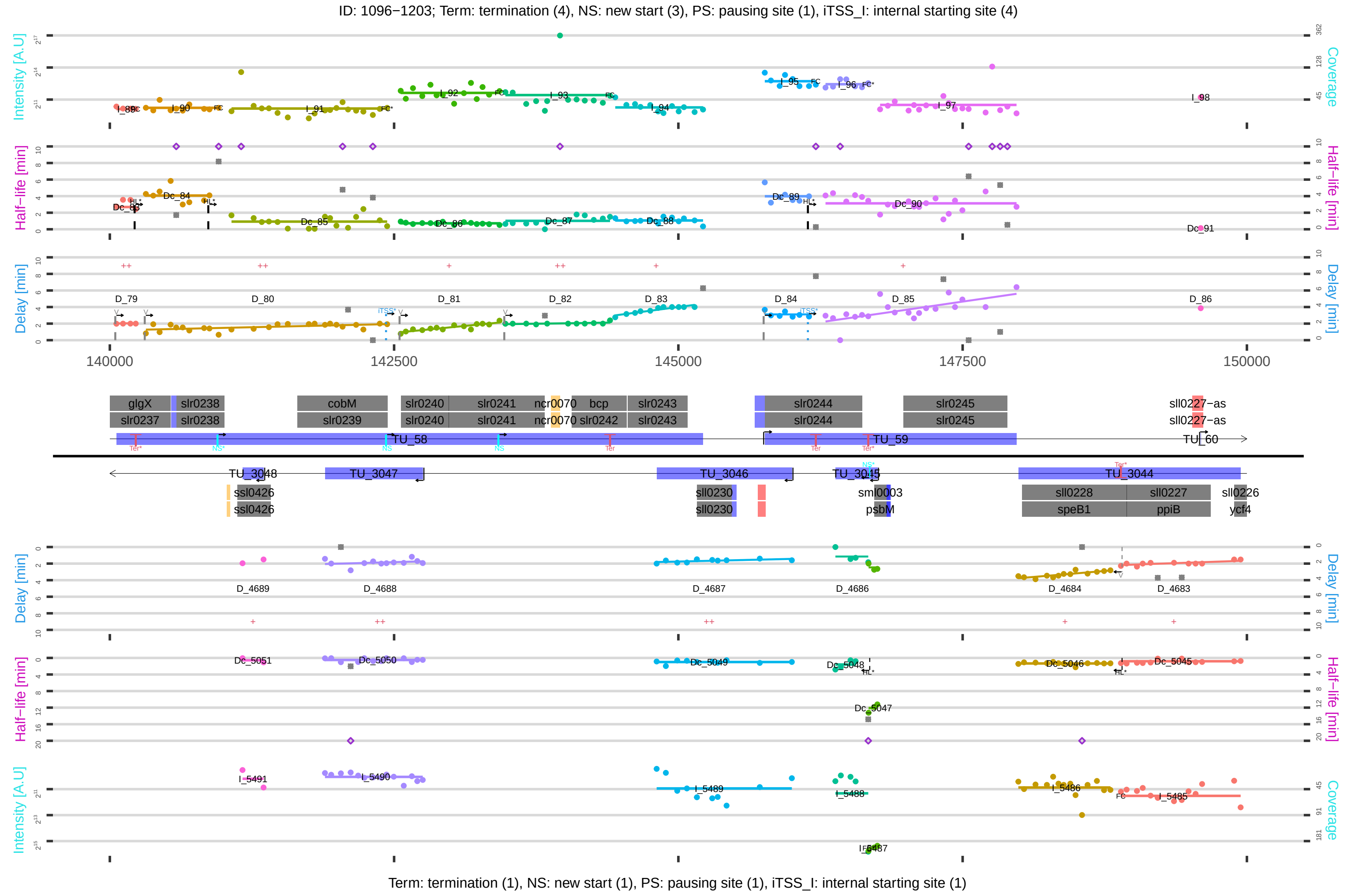
ID: 1096−1203; Term: termination (4), NS: new start (3), PS: pausing site (1), iTSS_I: internal starting site (4)
Intensity [A.U]
Coverage
Half−life [min]
Delay [min]
I_89
I_90
I_91
I_92
I_93
I_94
I_95
I_96
I_97
I_98
Dc_83
Dc_84
Dc_85
Dc_86
Dc_87
Dc_88
Dc_89
Dc_90
Dc_91
D_79
D_80
D_81
D_82
D_83
D_84
D_85
D_86
140000
142500
145000
147500
150000
glgX
slr0237
slr0238
cobM
slr0239
slr0240
slr0241
ncr0070
bcp
slr0242
slr0243
slr0244
slr0245
sll0227−as
TU_58
TU_59
TU_60
TU_3048
TU_3047
TU_3046
TU_3045
TU_3044
ssl0426
sll0230
sml0003
psbM
sll0228
speB1
sll0227
ppiB
sll0226
ycf4
D_4689
D_4688
D_4687
D_4686
D_4684
D_4683
Dc_5051
Dc_5050
Dc_5049
Dc_5048
Dc_5047
Dc_5046
Dc_5045
I_5491
I_5490
I_5489
I_5488
I_5487
I_5486
I_5485
Term: termination (1), NS: new start (1), PS: pausing site (1), iTSS_I: internal starting site (1)

ID: 1204−1259; Term: termination (1), NS: new start (1), PS: pausing site (1), iTSS_I: internal starting site (0)

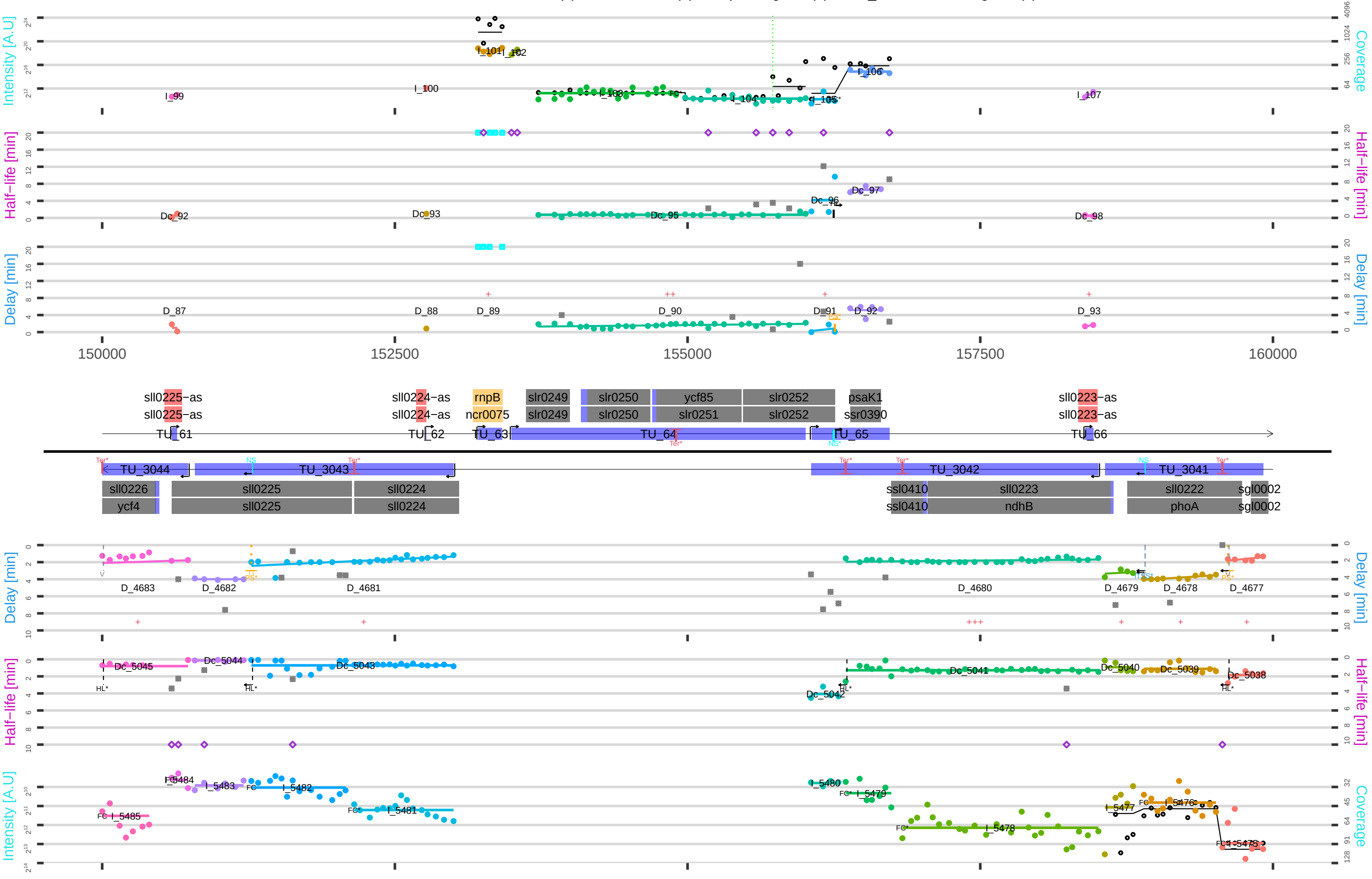

Term: termination (5), NS: new start (2), PS: pausing site (2), iTSS_I: internal starting site (1)

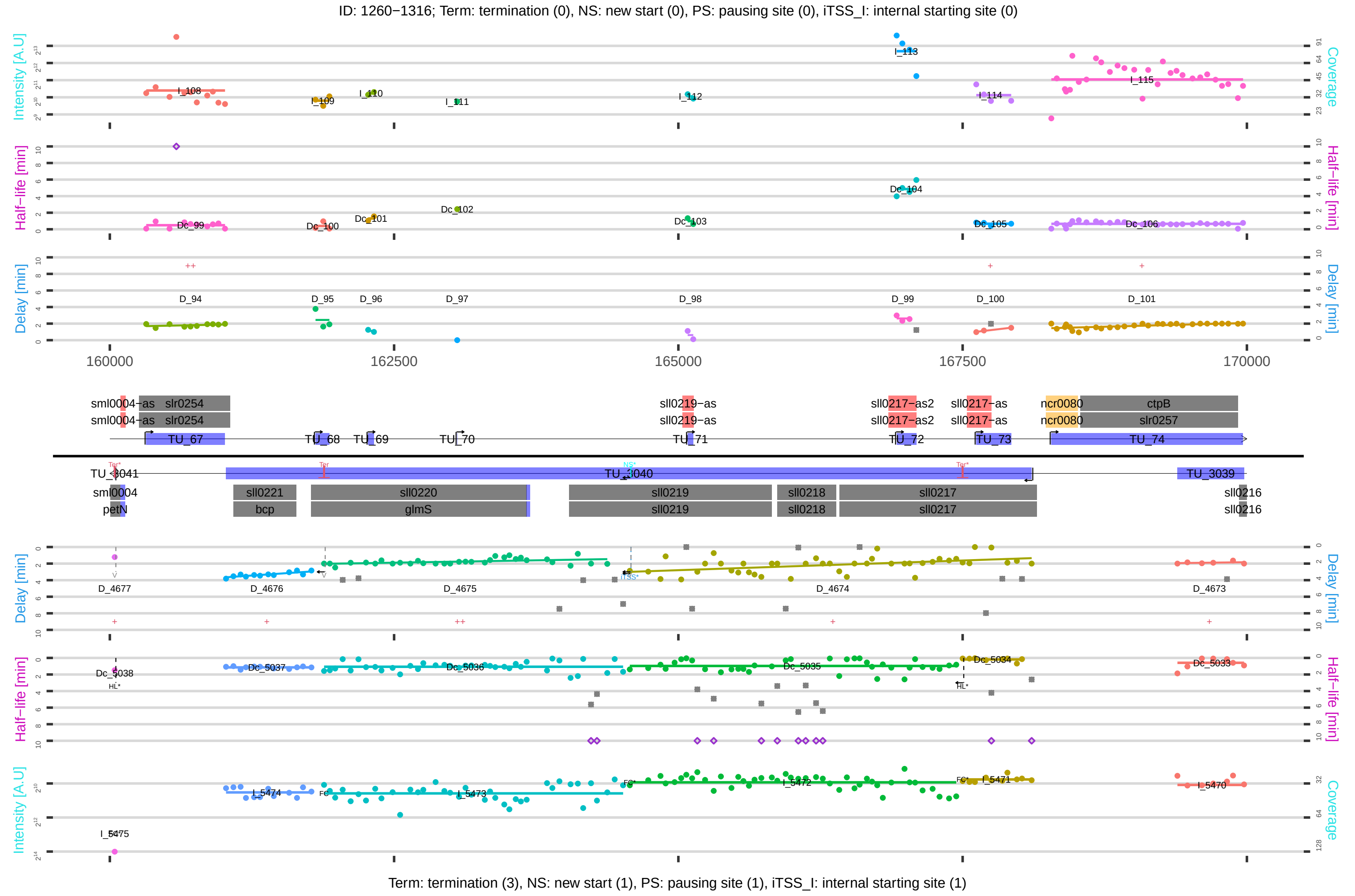
ID: 1260−1316; Term: termination (0), NS: new start (0), PS: pausing site (0), iTSS_I: internal starting site (0)
Intensity [A.U]
Coverage
Half−life [min]
Delay [min]
160000
162500
165000
167500
170000
sml0004−as slr0254
sll0219−as
sll0217−as2
sll0217−as
ncr0080
ctpB
slr0257
TU_67
TU_68
TU_69
TU_70
TU_71
TU_72
TU_73
TU_74
TU_3041
TU_3040
TU_3039
sml0004
petN
sll0221
bcp
sll0220
glmS
sll0219
sll0218
sll0217
sll0216
Term: termination (3), NS: new start (1), PS: pausing site (1), iTSS_I: internal starting site (1)

ID: 1317−1375; Term: termination (1), NS: new start (1), PS: pausing site (1), iTSS_I: internal starting site (1)

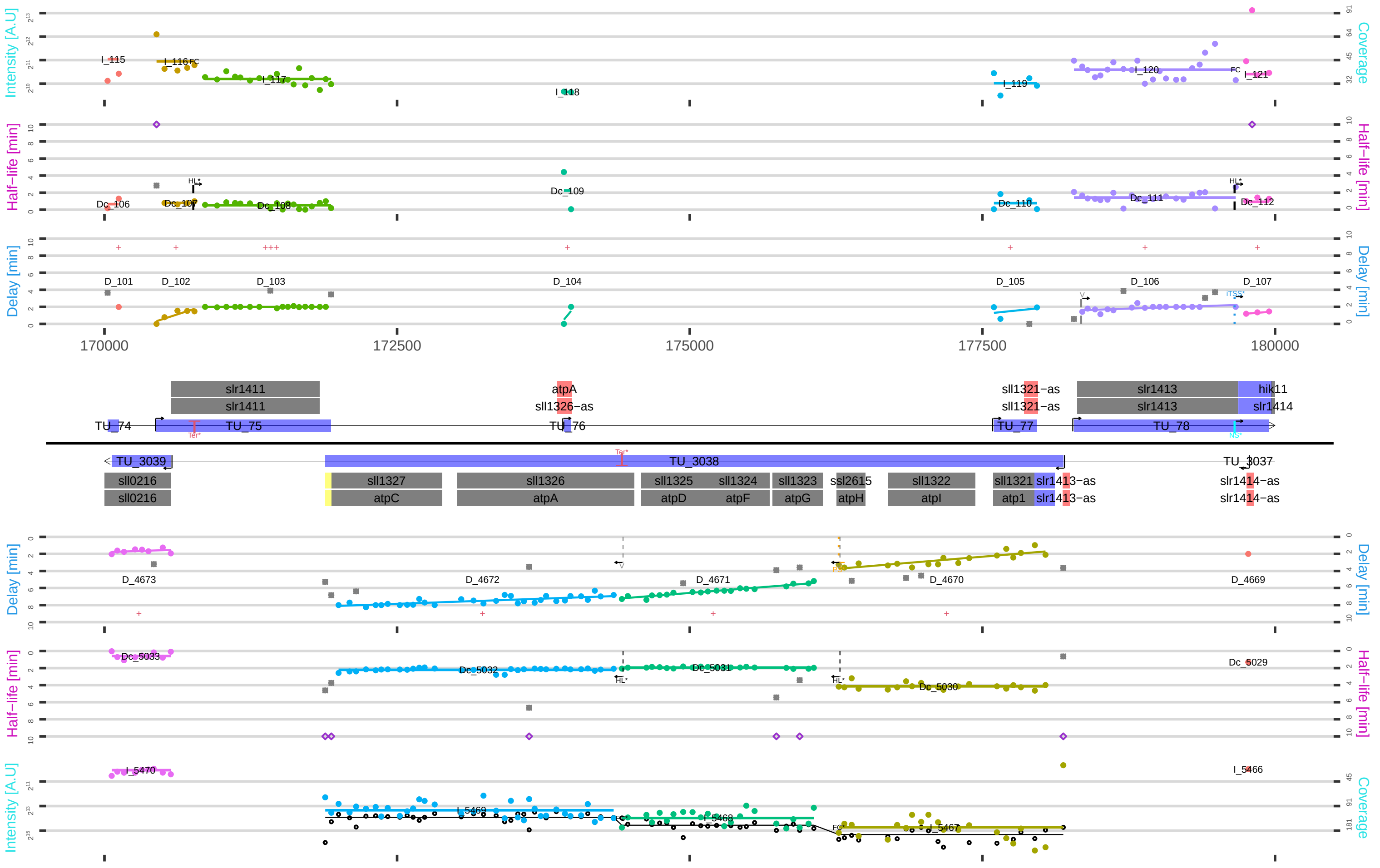

Term: termination (1), NS: new start (0), PS: pausing site (1), iTSS_I: internal starting site (1)

ID: 1376−1442; Term: termination (0), NS: new start (2), PS: pausing site (0), iTSS_I: internal starting site (0)

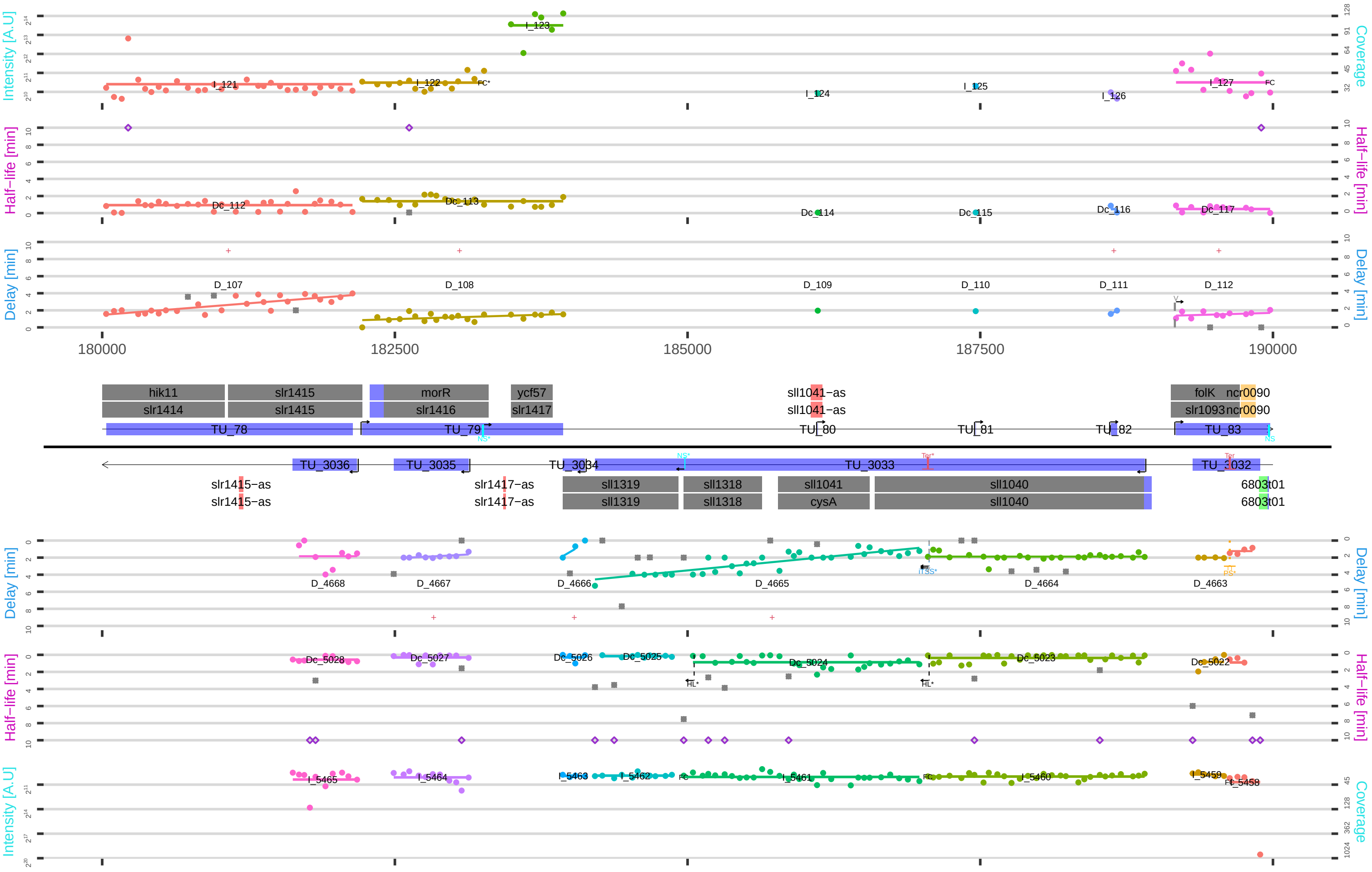

Term: termination (2), NS: new start (1), PS: pausing site (1), iTSS_I: internal starting site (1)

ID: 1443−1567; Term: termination (4), NS: new start (4), PS: pausing site (3), iTSS_I: internal starting site (5)

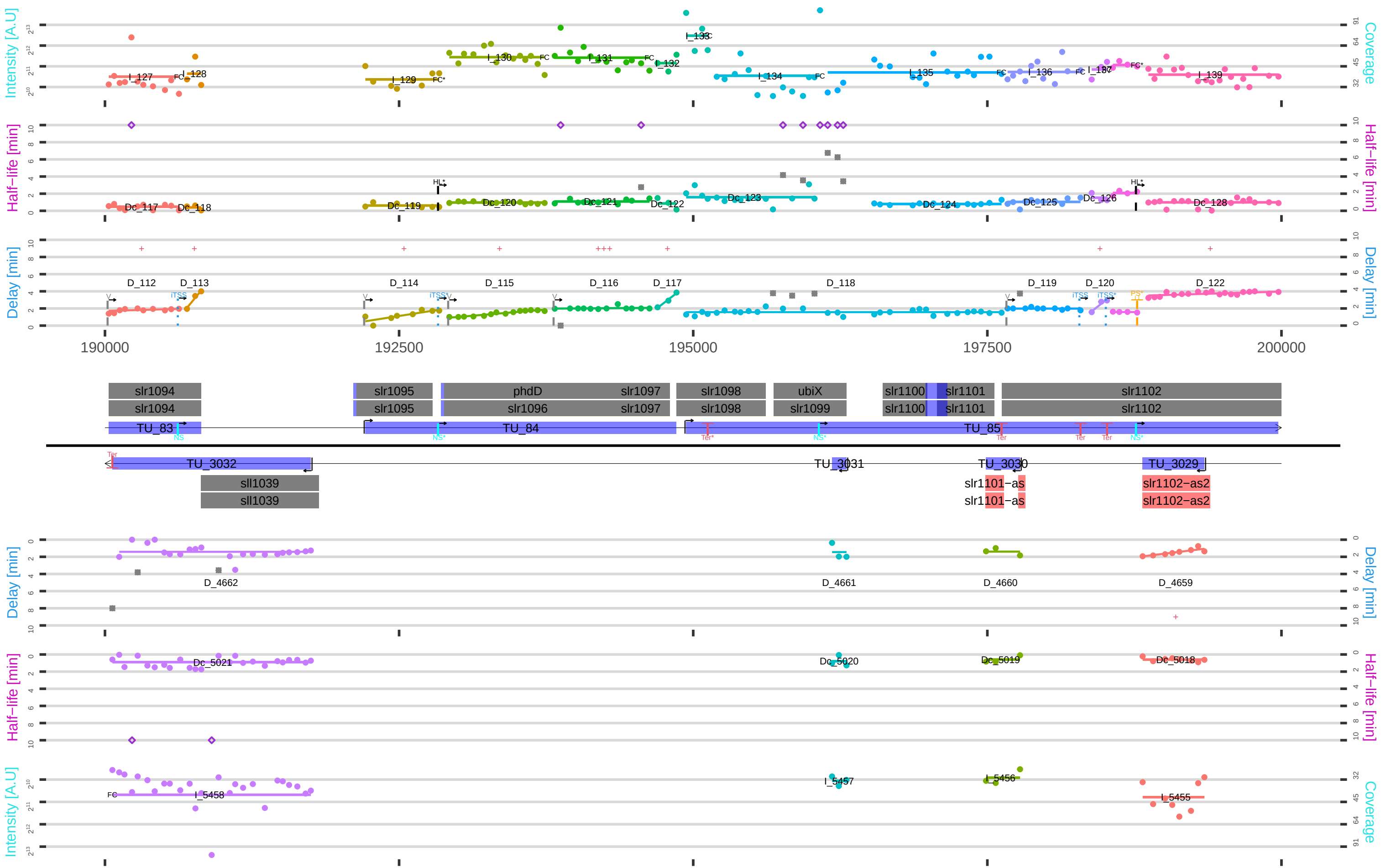

Term: termination (1), NS: new start (0), PS: pausing site (0), iTSS_I: internal starting site (0)

ID: 1568−1662; Term: termination (3), NS: new start (2), PS: pausing site (2), iTSS_I: internal starting site (2)

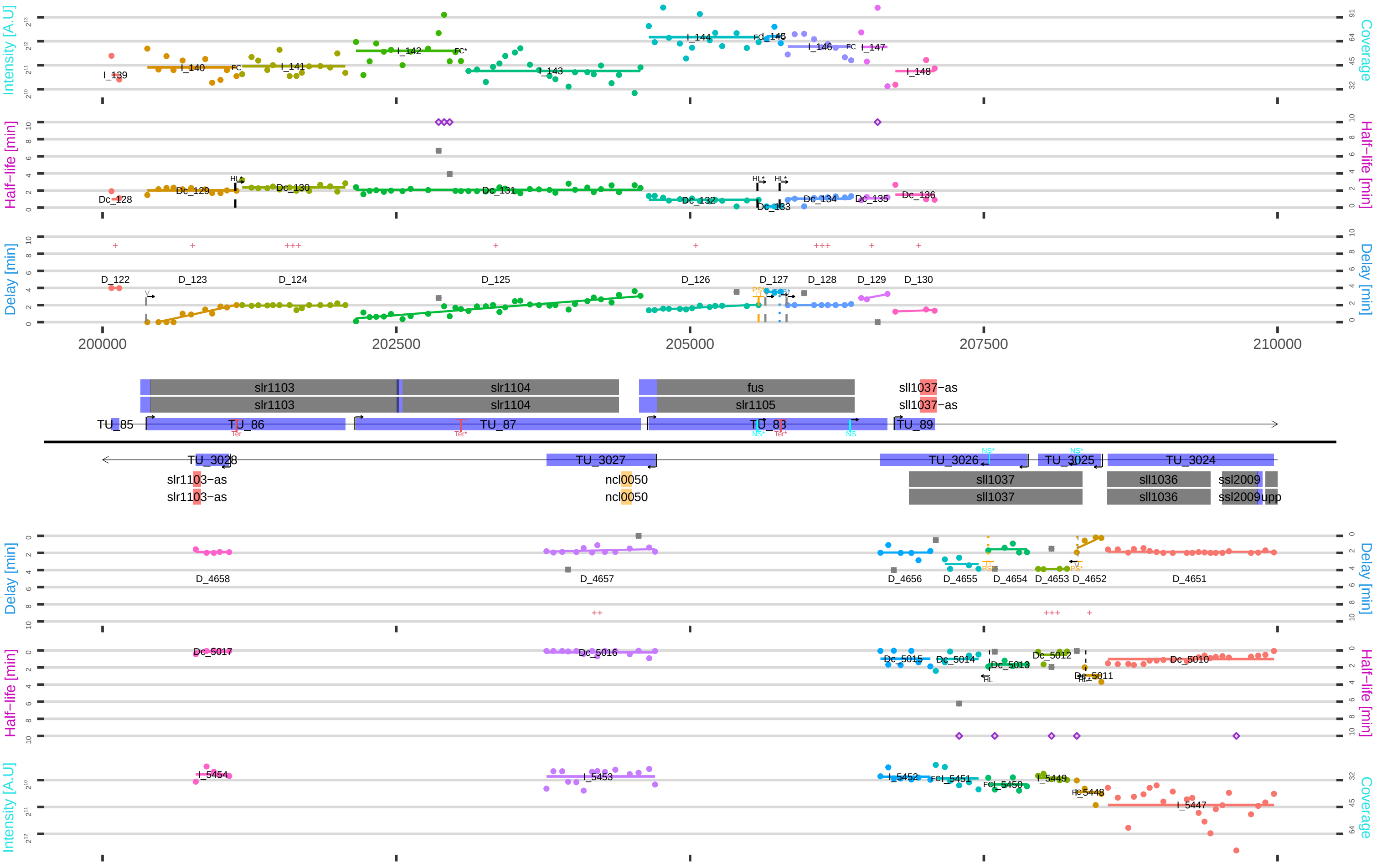

Term: termination (0), NS: new start (2), PS: pausing site (2), iTSS_I: internal starting site (1)

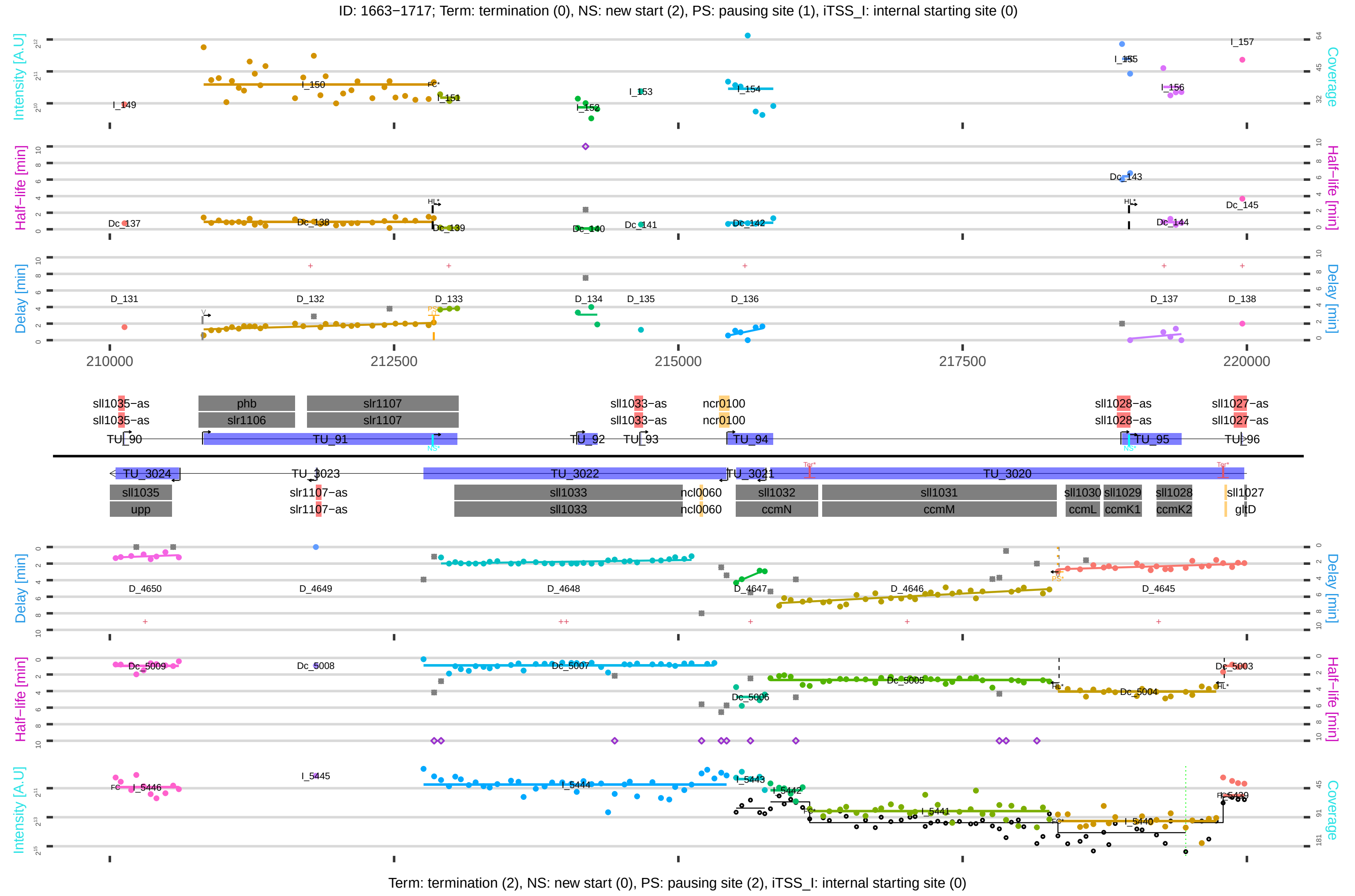
ID: 1663−1717; Term: termination (0), NS: new start (2), PS: pausing site (1), iTSS_I: internal starting site (0)
Intensity [A.U]
Coverage
Half−life [min]
Delay [min]
210000
212500
215000
217500
220000
sll1035−as
phb
slr1106
slr1107
sll1033−as
ncr0100
sll1028−as
sll1027−as
TU_90
TU_91
TU_92
TU_93
TU_94
TU_95
TU_96
TU_3024
TU_3023
TU_3022
TU_3021
TU_3020
sll1035
upp
slr1107−as
sll1033
ncl0060
sll1032
ccmN
sll1031
ccmM
sll1030
ccmL
sll1029
ccmK1
sll1028
ccmK2
sll1027
gltD
I_149
I_150
I_151
I_152
I_153
I_154
I_155
I_156
I_157
Dc_137
Dc_138
Dc_139
Dc_140
Dc_141
Dc_142
Dc_143
Dc_144
Dc_145
D_131
D_132
D_133
D_134
D_135
D_136
D_137
D_138
D_4650
D_4649
D_4648
D_4647
D_4646
D_4645
Dc_5009
Dc_5008
Dc_5007
Dc_5006
Dc_5005
Dc_5004
Dc_5003
I_5446
I_5445
I_5444
I_5443
I_5442
I_5441
I_5440
I_5439
Term: termination (2), NS: new start (0), PS: pausing site (2), iTSS_I: internal starting site (0)

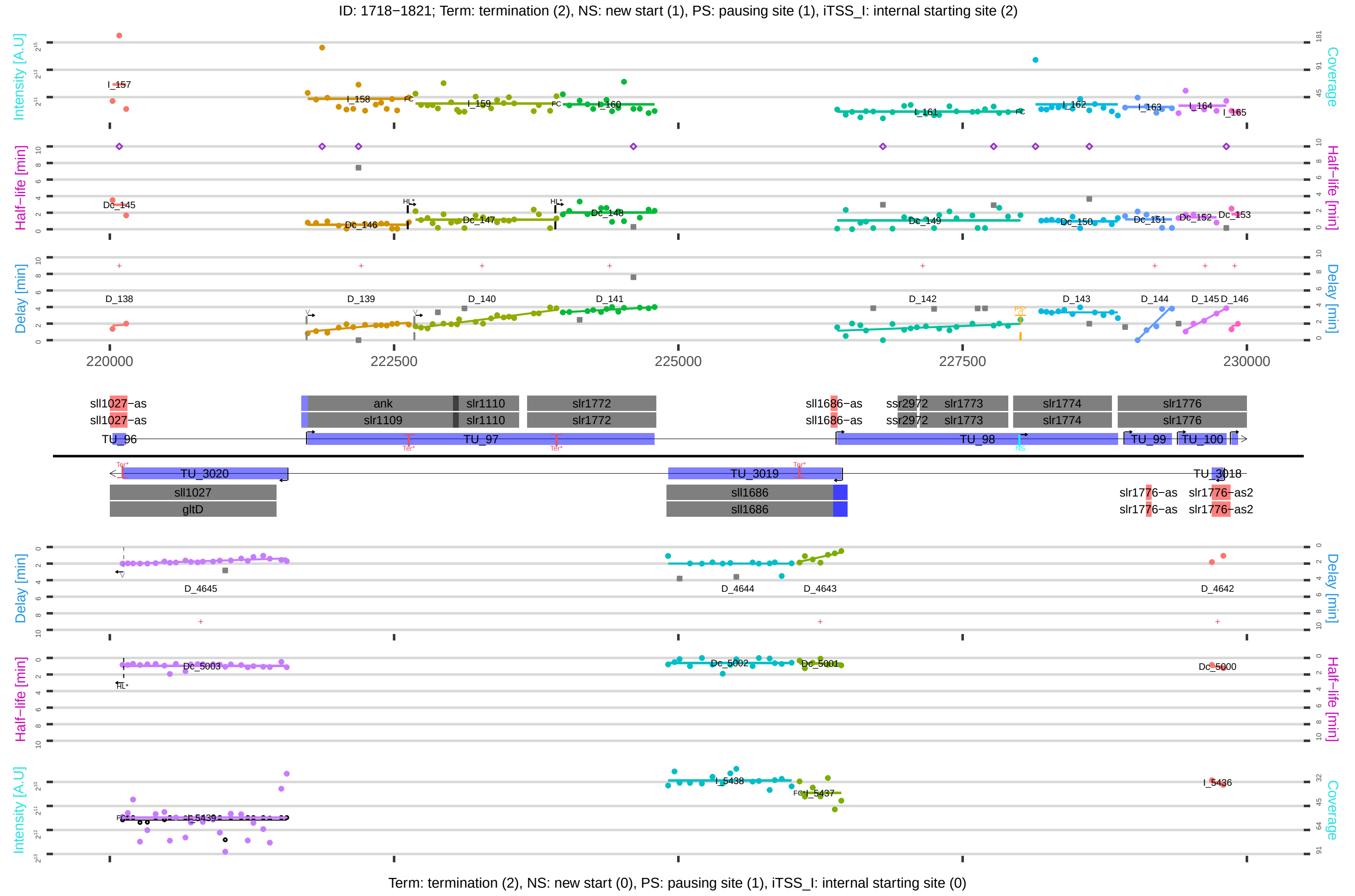

ID: 1718−1821; Term: termination (2), NS: new start (1), PS: pausing site (1), iTSS_I: internal starting site (2)
Intensity [A.U]
Coverage
Half−life [min]
Delay [min]
220000
222500
225000
227500
230000
sll1027−as
ank
slr1109
slr1110
slr1772
sll1686−as
ssr2972
slr1773
slr1774
slr1776
TU_96
TU_97
TU_98
TU_99
TU_100
TU_3020
TU_3019
TU_3018
sll1027
gltD
sll1686
slr1776−as
slr1776−as2
Term: termination (2), NS: new start (0), PS: pausing site (1), iTSS_I: internal starting site (0)

ID: 1822−1900; Term: termination (4), NS: new start (1), PS: pausing site (2), iTSS_I: internal starting site (2)

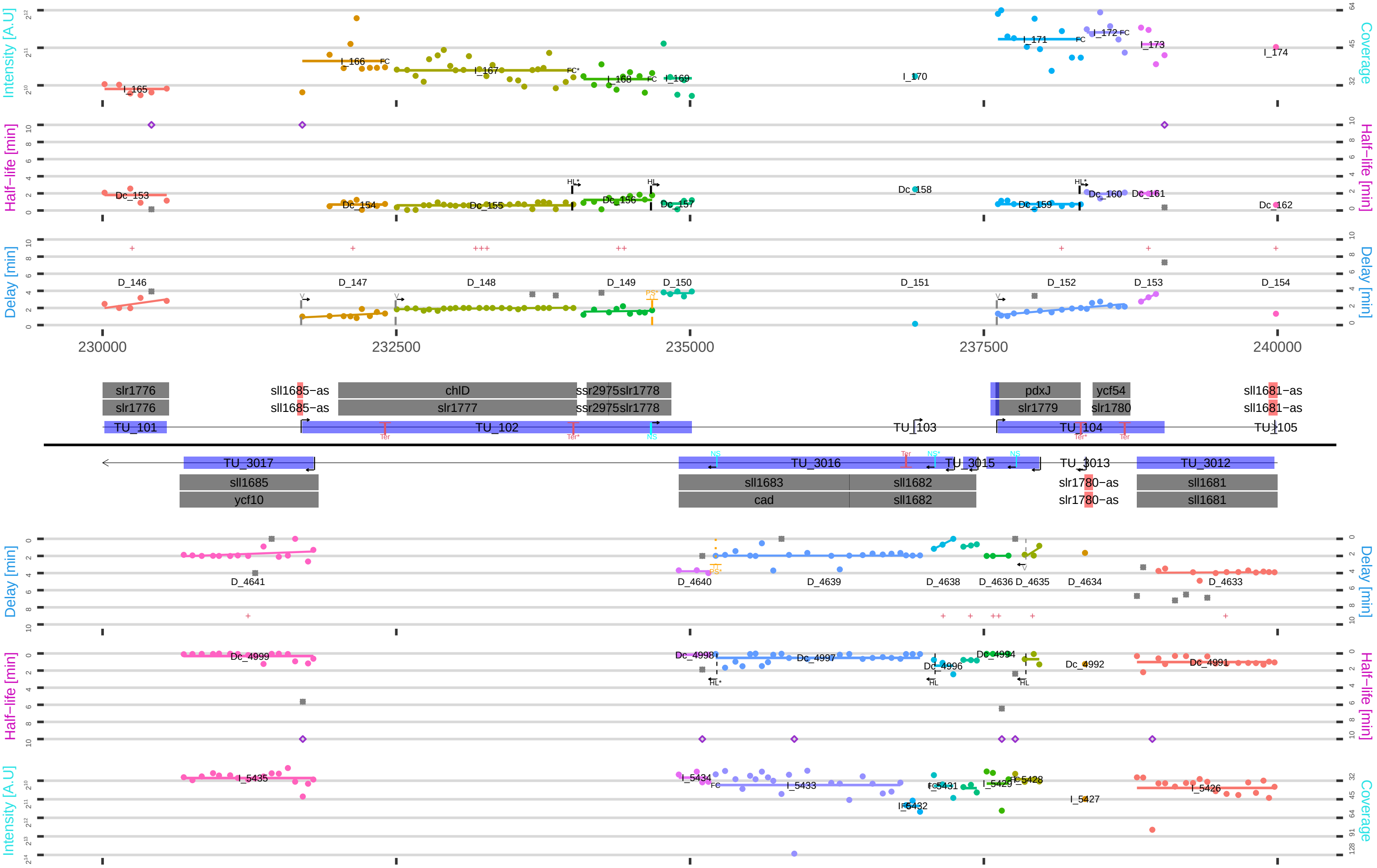

Term: termination (1), NS: new start (3), PS: pausing site (2), iTSS_I: internal starting site (1)

ID: 1901−1960; Term: termination (2), NS: new start (0), PS: pausing site (1), iTSS_I: internal starting site (0)

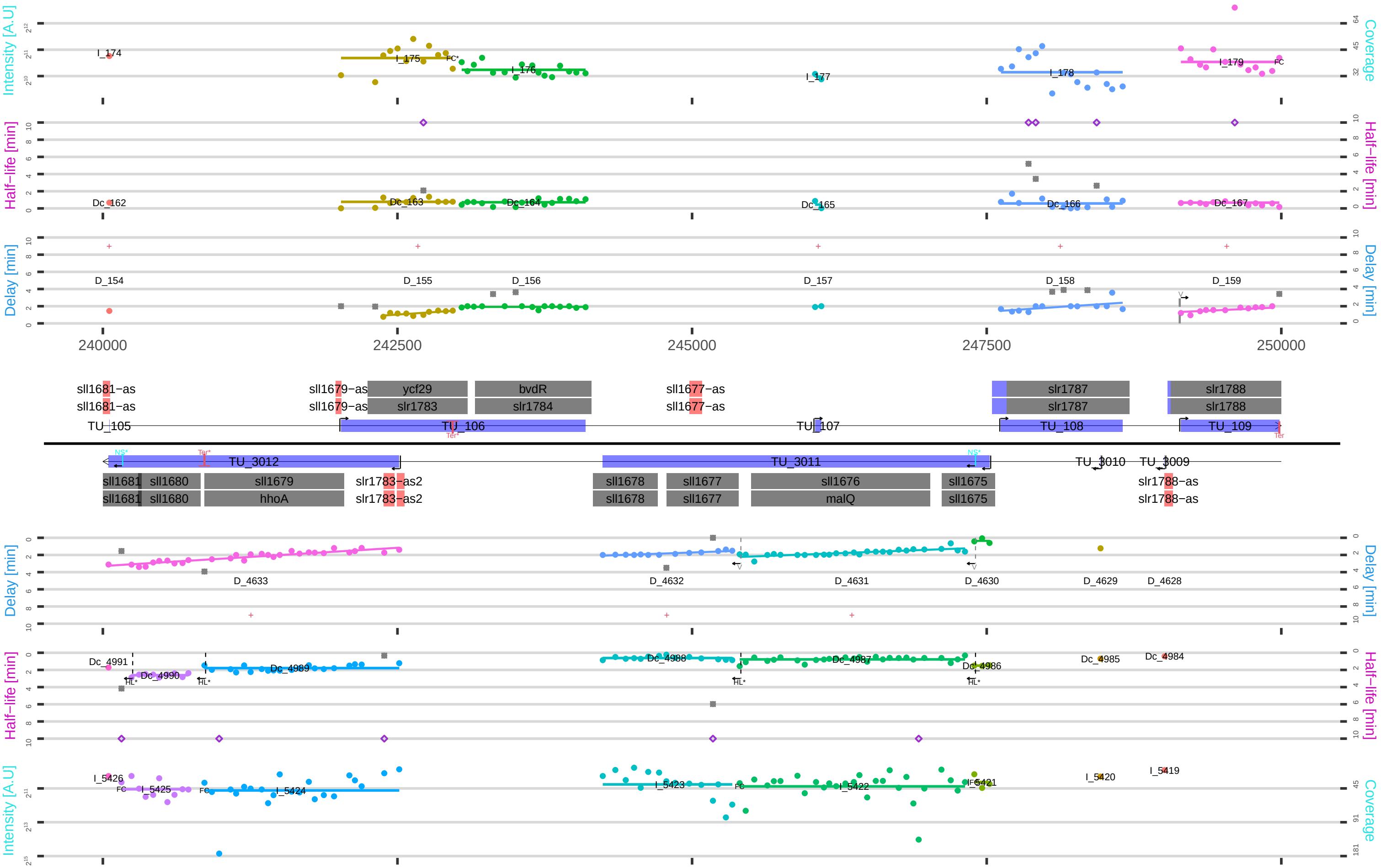

Term: termination (1), NS: new start (2), PS: pausing site (1), iTSS_I: internal starting site (1)

ID: 1961−2015; Term: termination (1), NS: new start (0), PS: pausing site (1), iTSS_I: internal starting site (0)

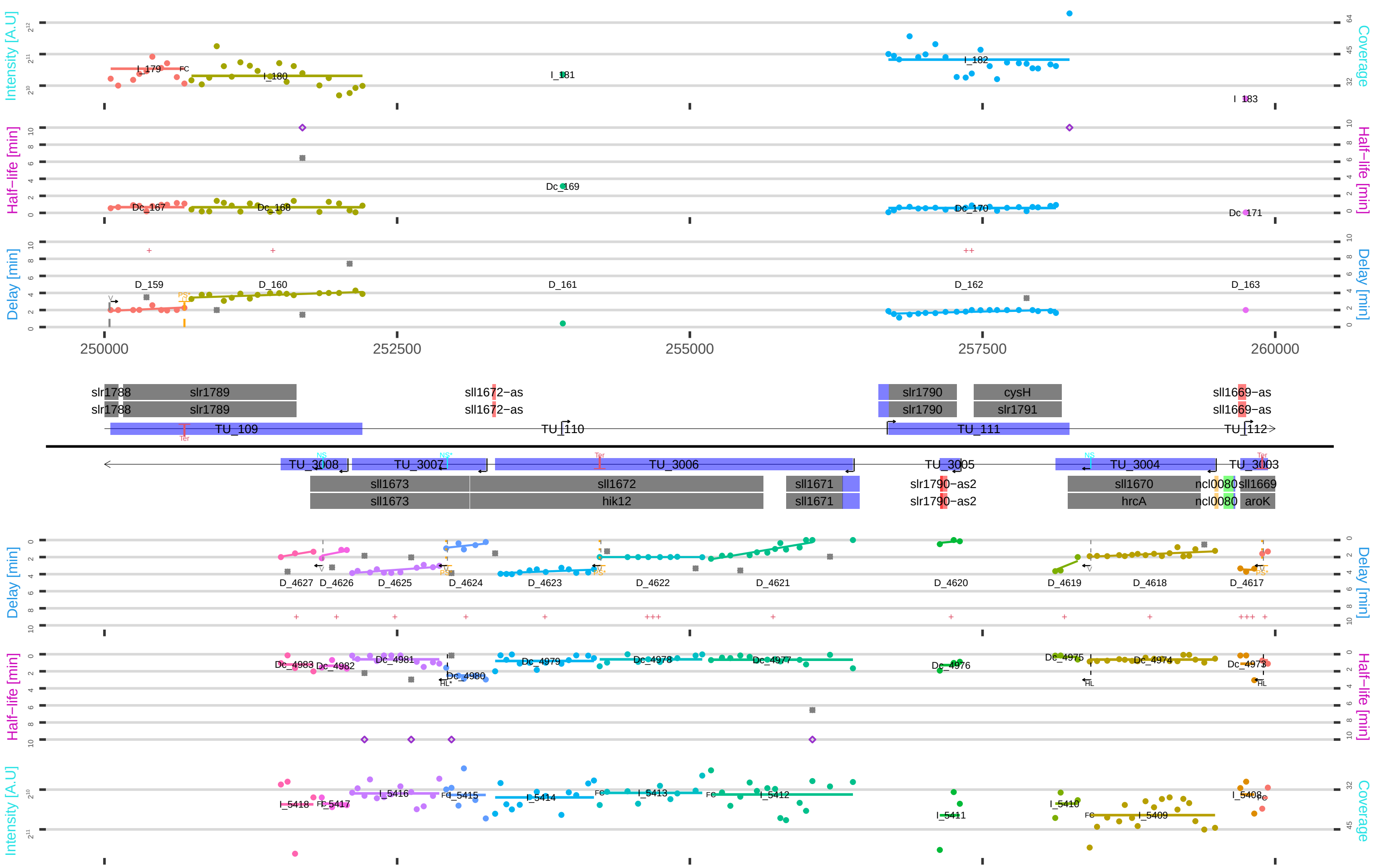

Term: termination (2), NS: new start (3), PS: pausing site (3), iTSS_I: internal starting site (3)

ID: 2016−2098; Term: termination (2), NS: new start (1), PS: pausing site (1), iTSS_I: internal starting site (1)

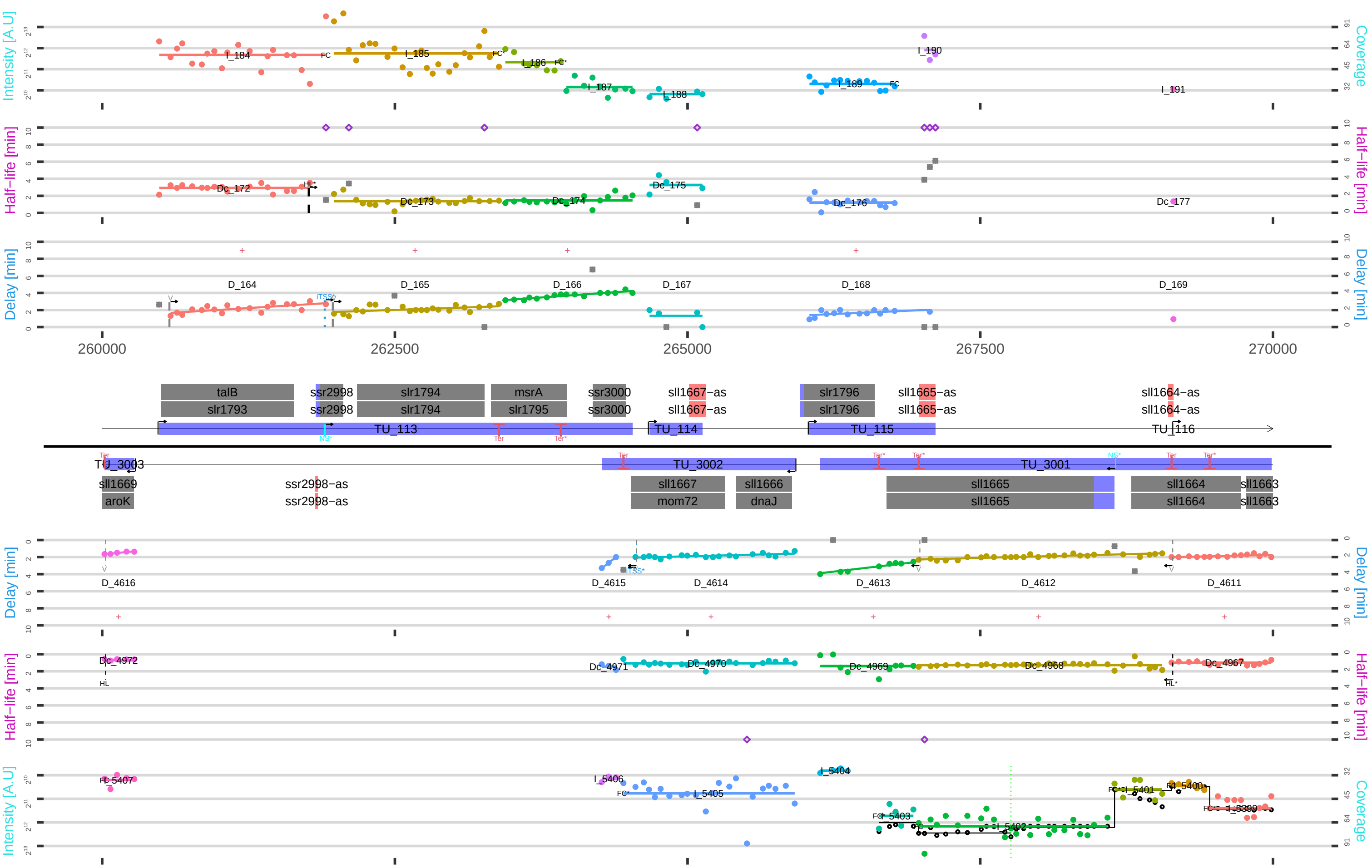

Term: termination (6), NS: new start (1), PS: pausing site (1), iTSS_I: internal starting site (2)

ID: 2099−2183; Term: termination (3), NS: new start (1), PS: pausing site (3), iTSS_I: internal starting site (1)

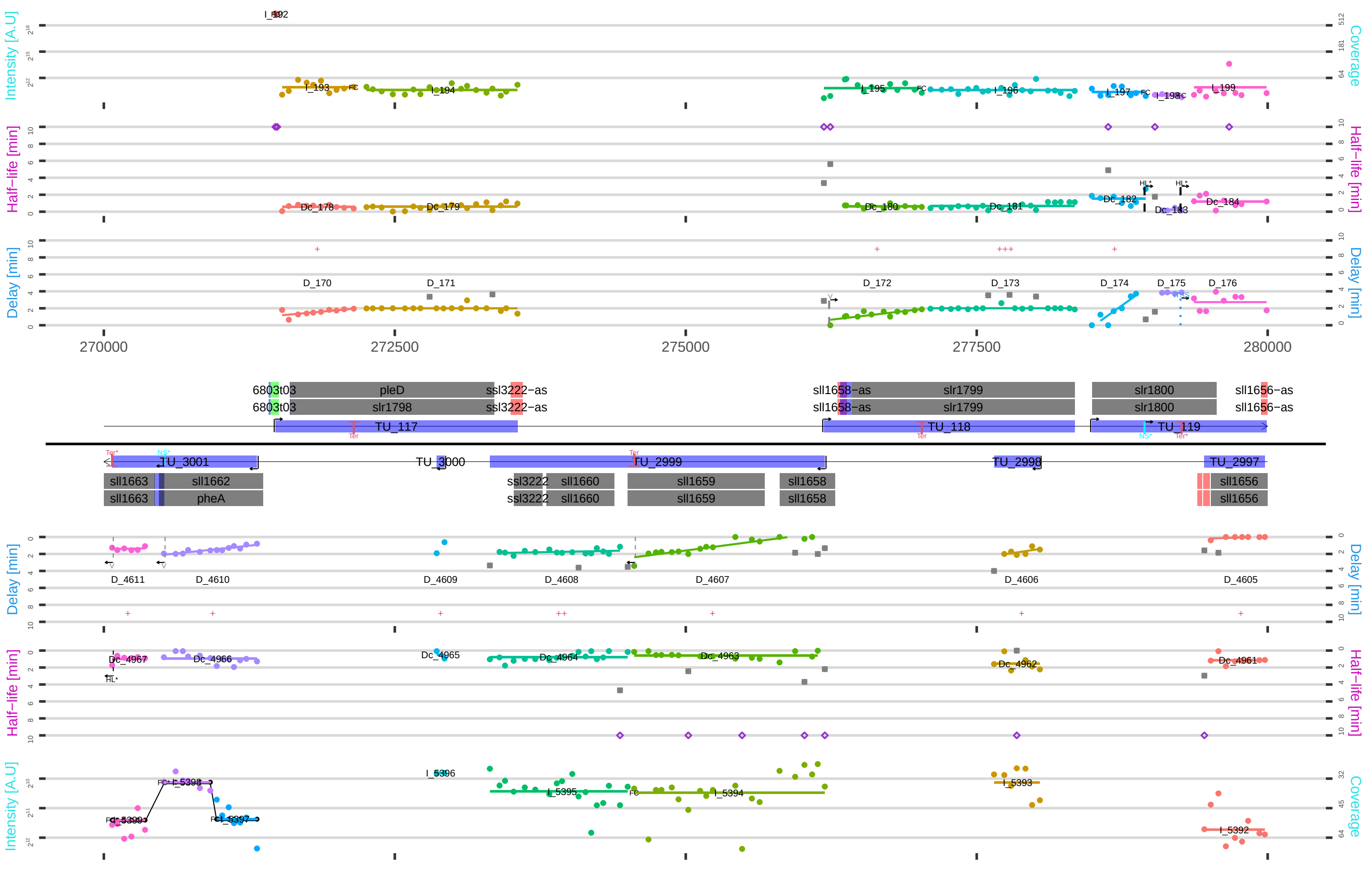

Term: termination (2), NS: new start (1), PS: pausing site (0), iTSS_I: internal starting site (2)

ID: 2184−2257; Term: termination (4), NS: new start (0), PS: pausing site (0), iTSS_I: internal starting site (1)

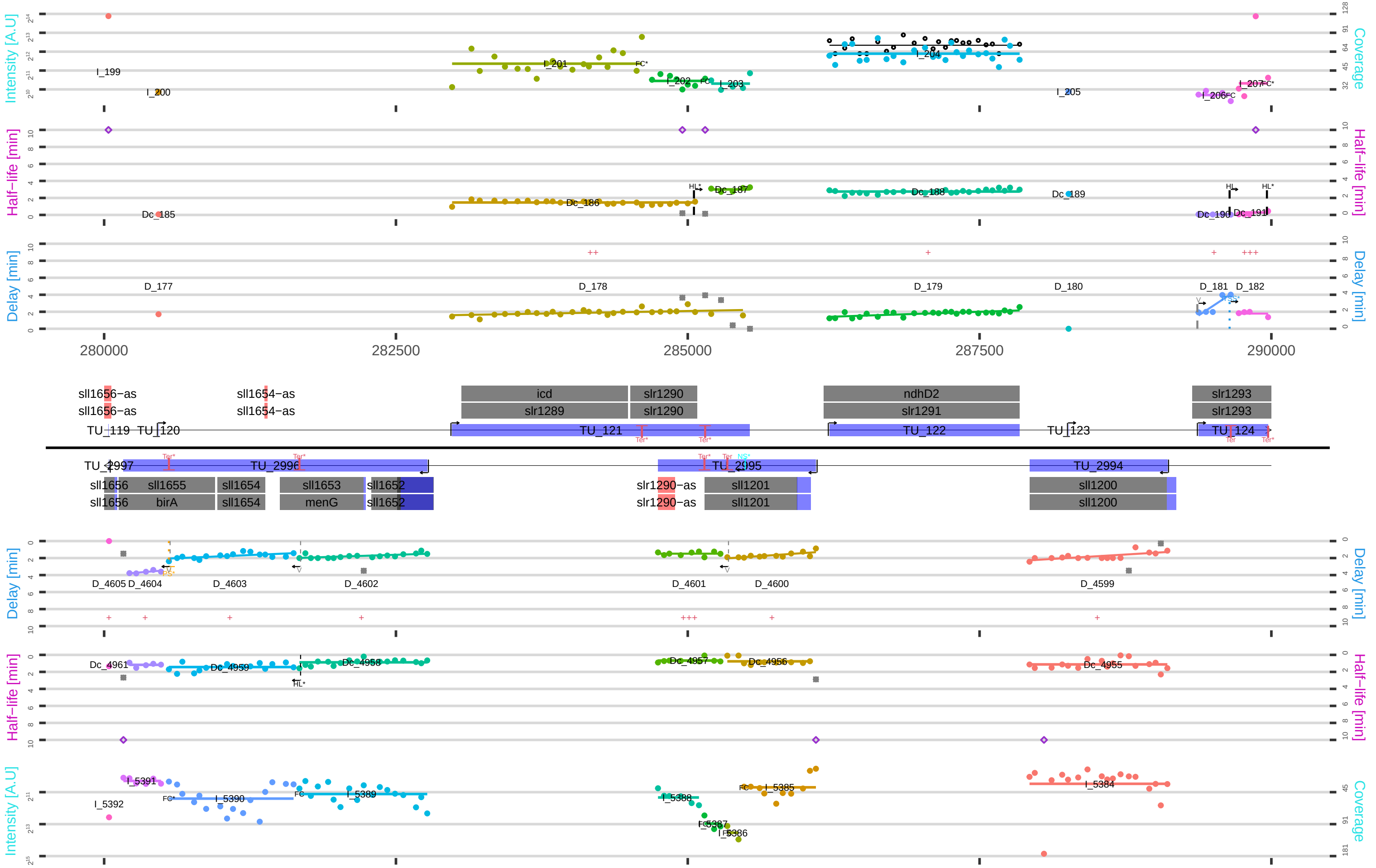

Term: termination (4), NS: new start (1), PS: pausing site (1), iTSS_I: internal starting site (2)

ID: 2258−2346; Term: termination (5), NS: new start (2), PS: pausing site (2), iTSS_I: internal starting site (0)

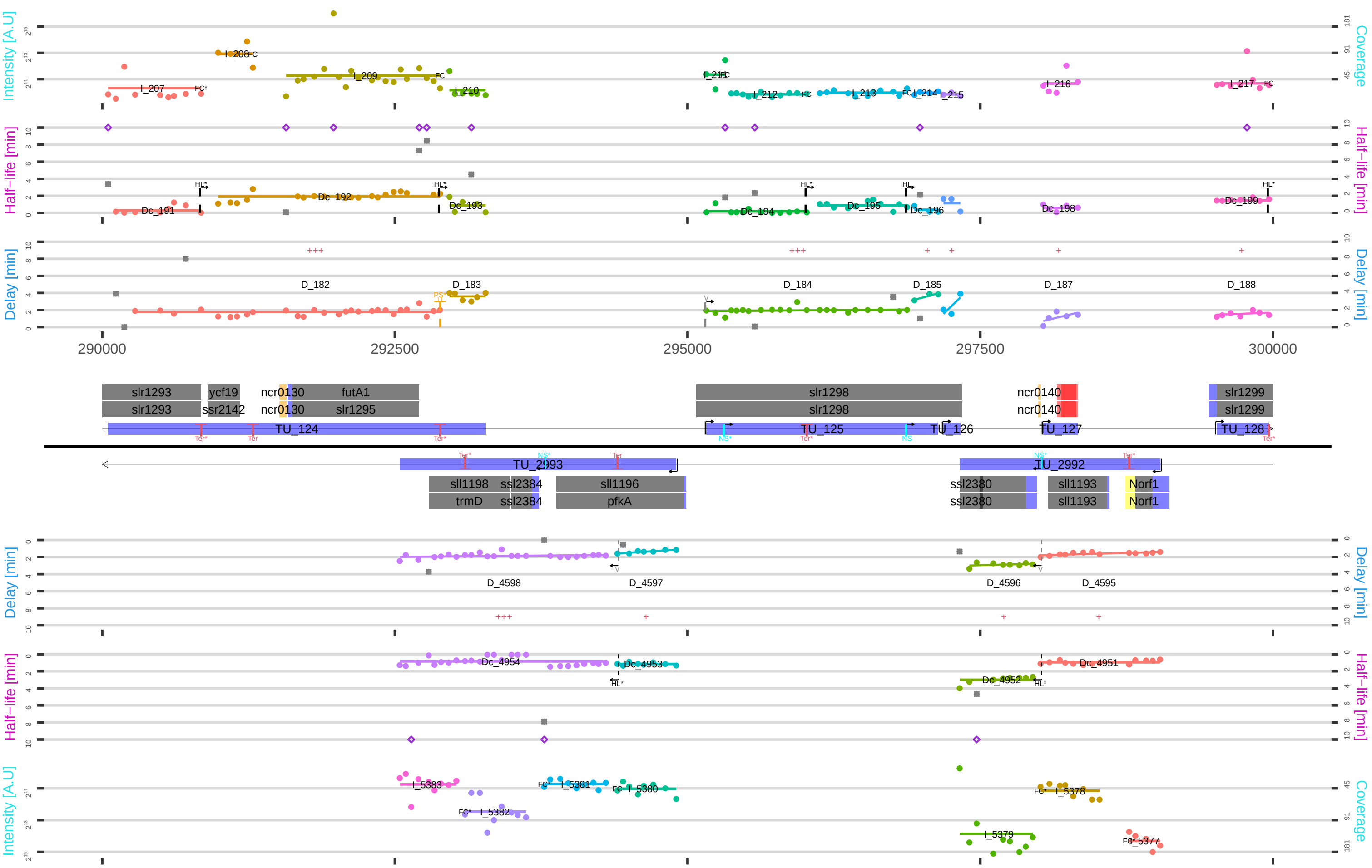

Term: termination (3), NS: new start (2), PS: pausing site (2), iTSS_I: internal starting site (0)

ID: 2347−2463; Term: termination (1), NS: new start (1), PS: pausing site (0), iTSS_I: internal starting site (1)

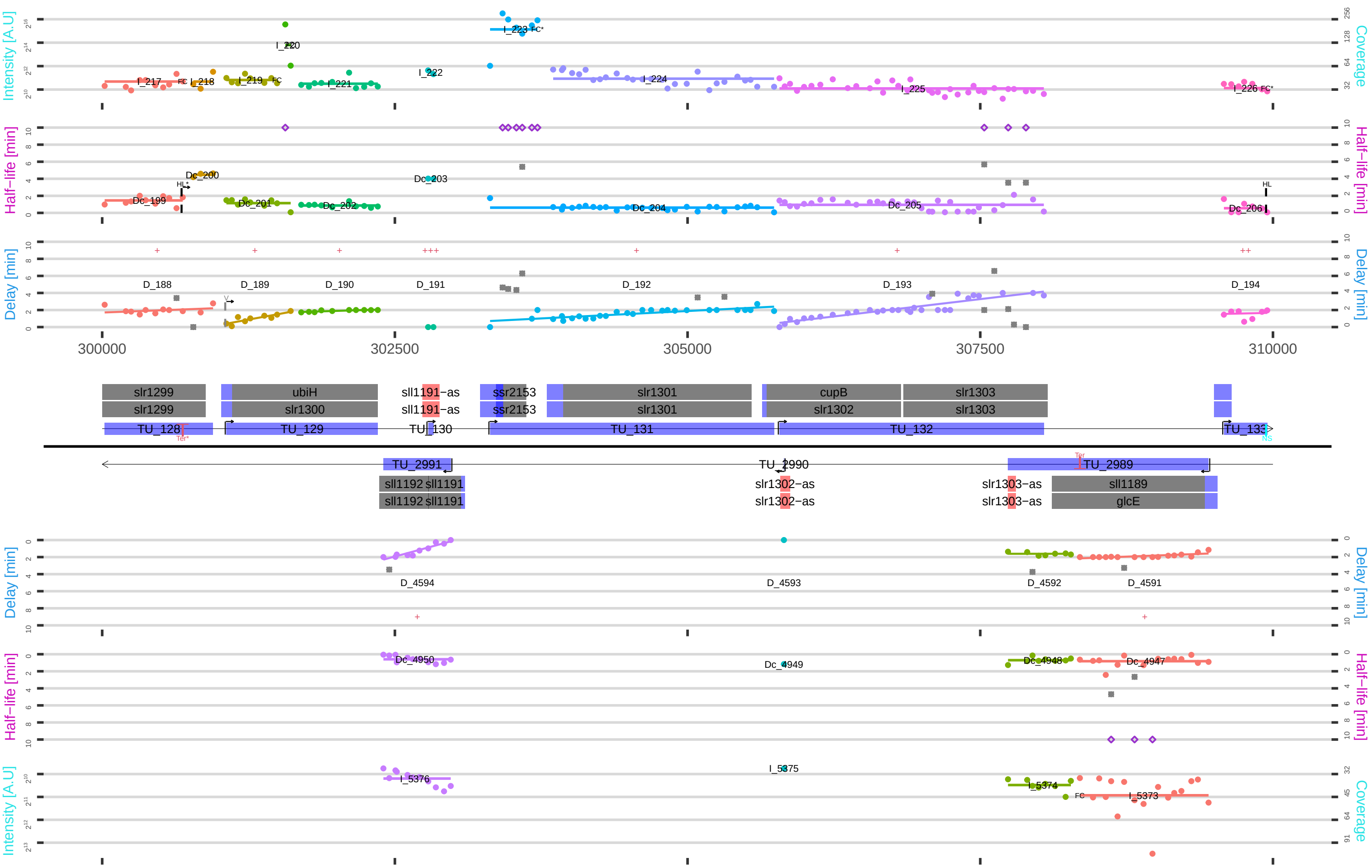

Term: termination (1), NS: new start (0), PS: pausing site (0), iTSS_I: internal starting site (1)

ID: 2464−2548; Term: termination (3), NS: new start (3), PS: pausing site (2), iTSS_I: internal starting site (0)

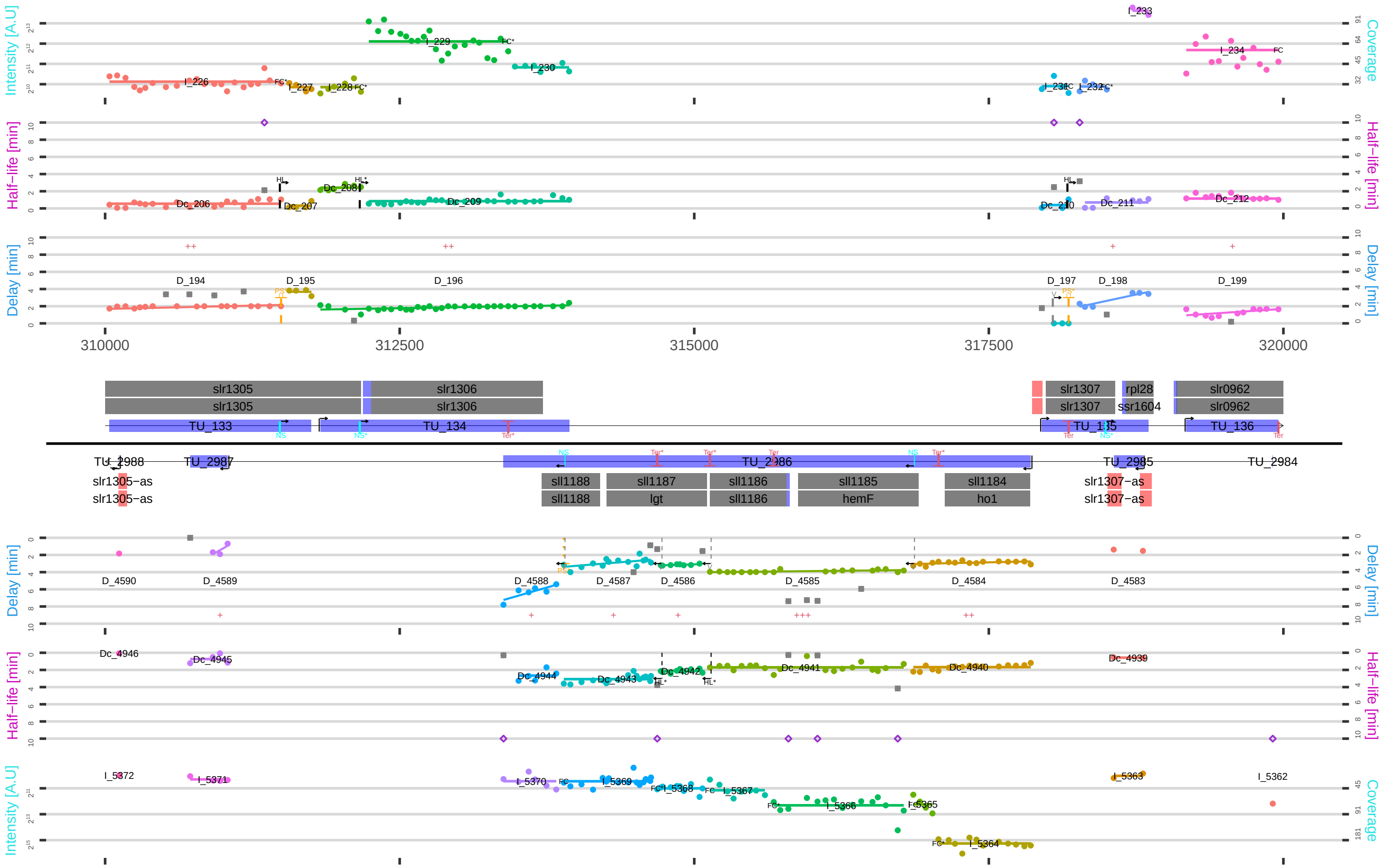

Term: termination (4), NS: new start (2), PS: pausing site (2), iTSS_I: internal starting site (2)

ID: 2549−2657; Term: termination (4), NS: new start (2), PS: pausing site (3), iTSS_I: internal starting site (3)

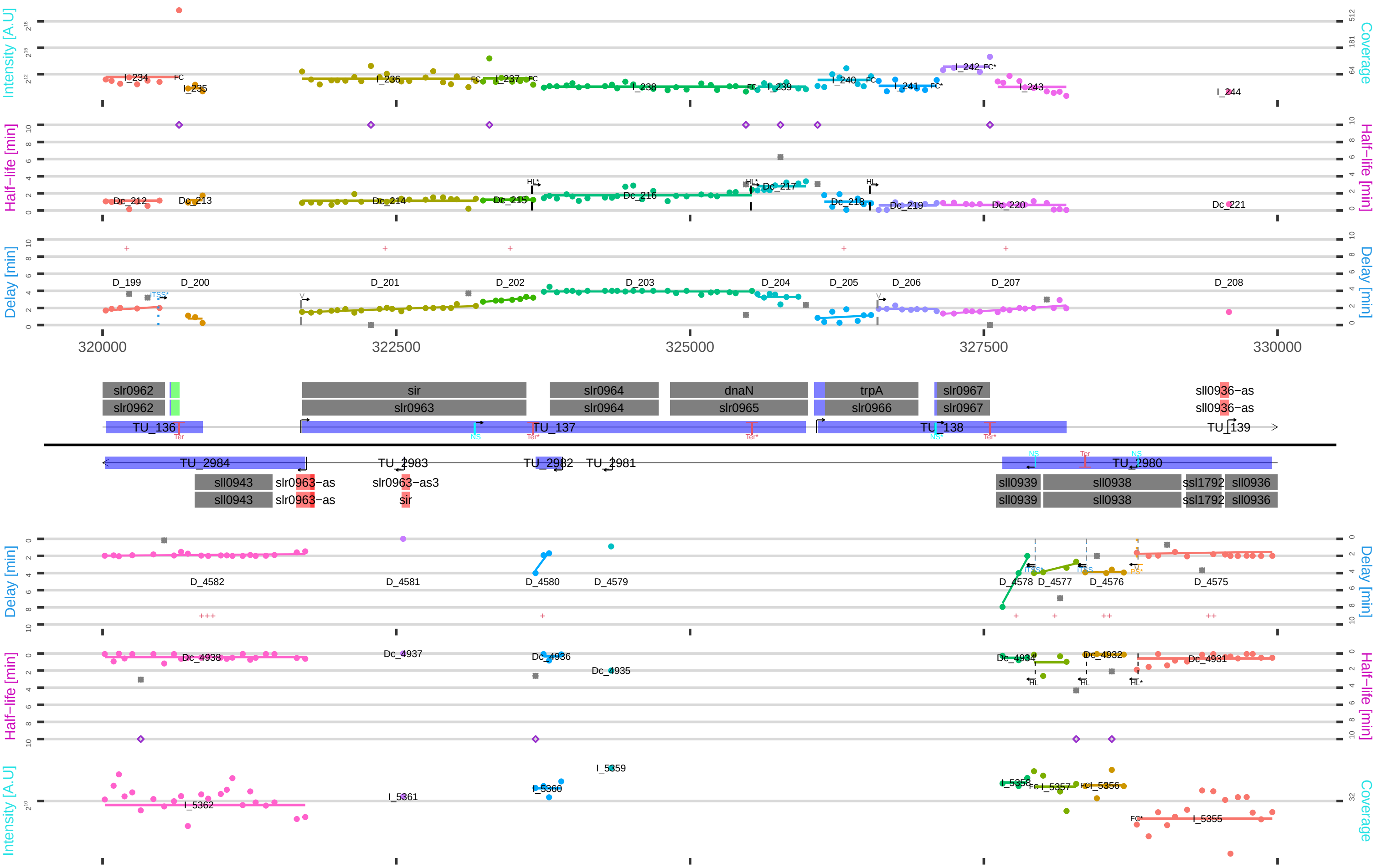

Term: termination (1), NS: new start (2), PS: pausing site (1), iTSS_I: internal starting site (2)

ID: 2658−2718; Term: termination (1), NS: new start (0), PS: pausing site (0), iTSS_I: internal starting site (1)

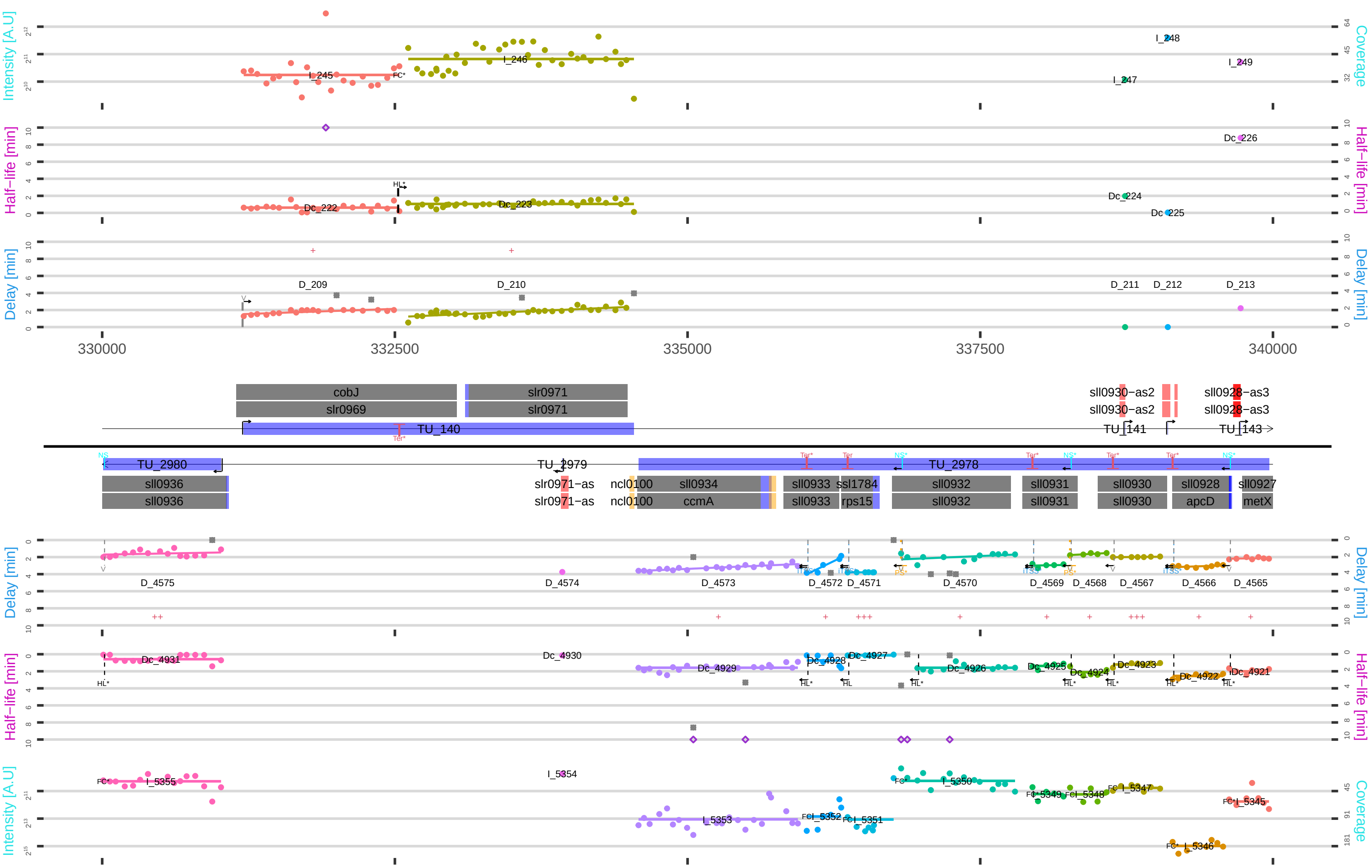

Term: termination (5), NS: new start (4), PS: pausing site (3), iTSS_I: internal starting site (5)

ID: 2719−2793; Term: termination (4), NS: new start (1), PS: pausing site (1), iTSS_I: internal starting site (1)

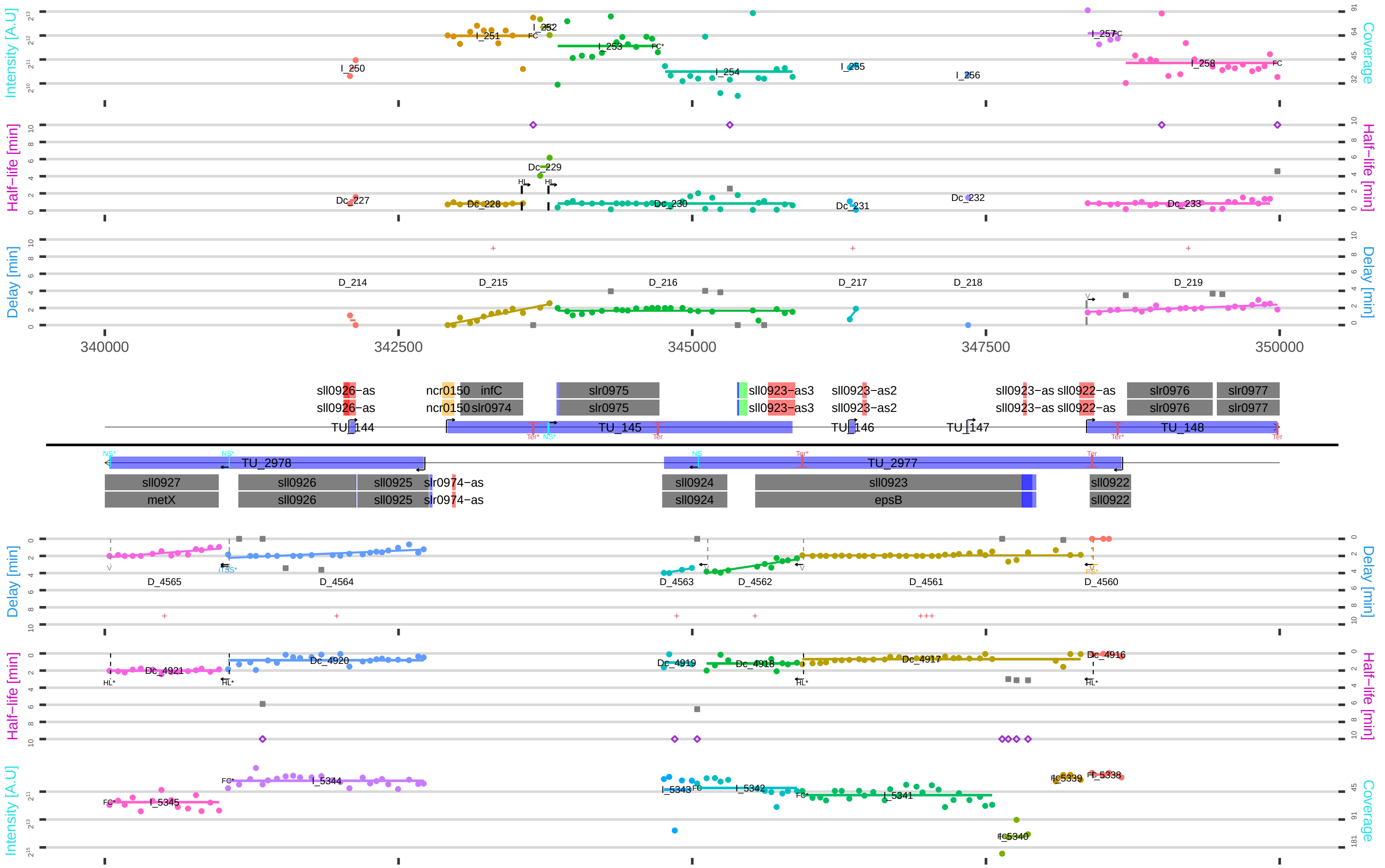

Term: termination (2), NS: new start (3), PS: pausing site (2), iTSS_I: internal starting site (2)

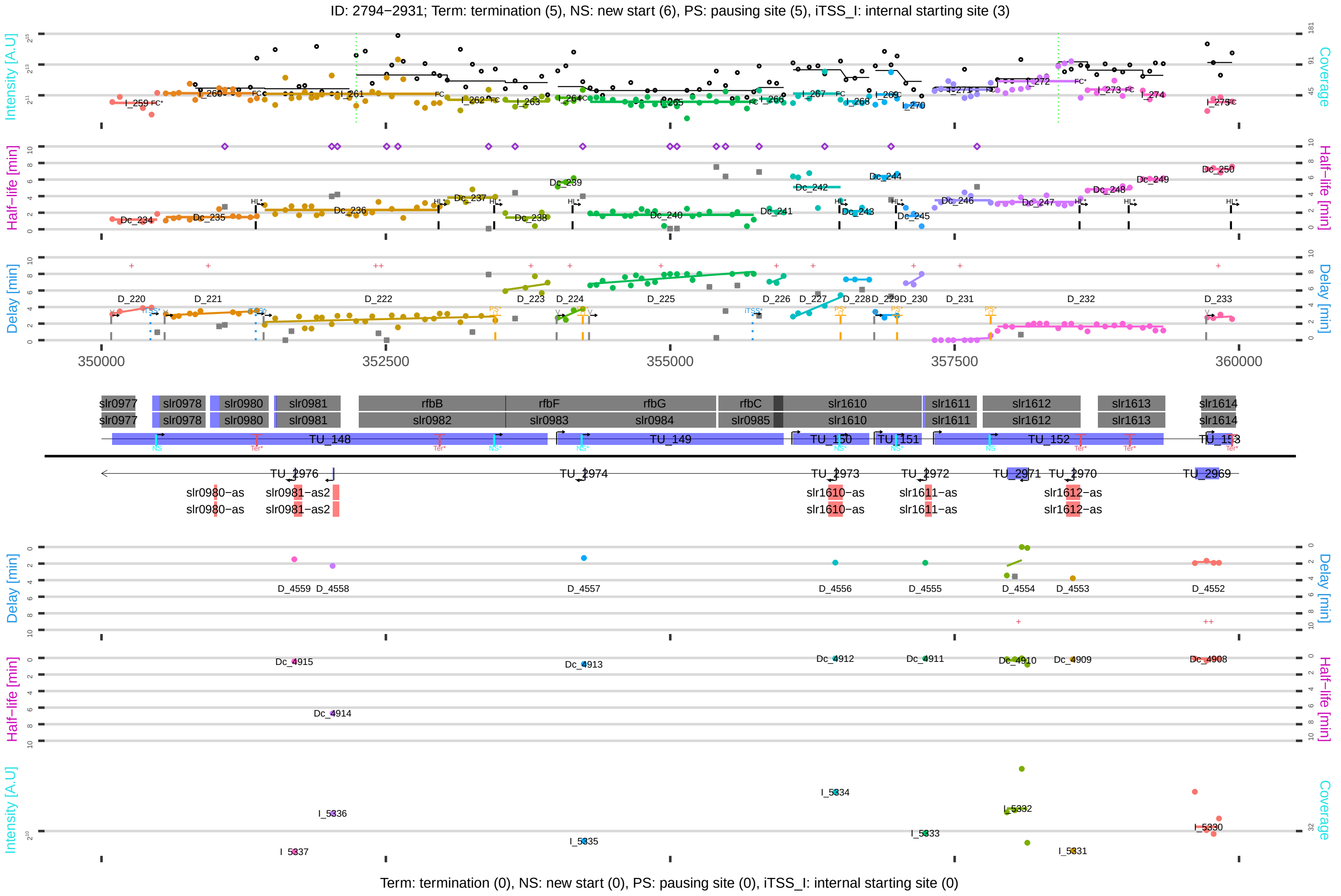

ID: 2794−2931; Term: termination (5), NS: new start (6), PS: pausing site (5), iTSS_I: internal starting site (3)
Intensity [A.U]
Coverage
Half−life [min]
Delay [min]
350000
352500
355000
357500
360000
slr0977
slr0978
slr0980
slr0981
rfbB
slr0982
rfbF
slr0983
rfbG
slr0984
rfbC
slr0985
slr1610
slr1611
slr1612
slr1613
slr1614
TU_148
TU_149
TU_150
TU_151
TU_152
TU_153
TU_2976
TU_2974
TU_2973
TU_2972
TU_2971
TU_2970
TU_2969
slr0980−as
slr0981−as2
slr1610−as
slr1611−as
slr1612−as
Term: termination (0), NS: new start (0), PS: pausing site (0), iTSS_I: internal starting site (0)

ID: 2932−3056; Term: termination (8), NS: new start (11), PS: pausing site (4), iTSS_I: internal starting site (5)

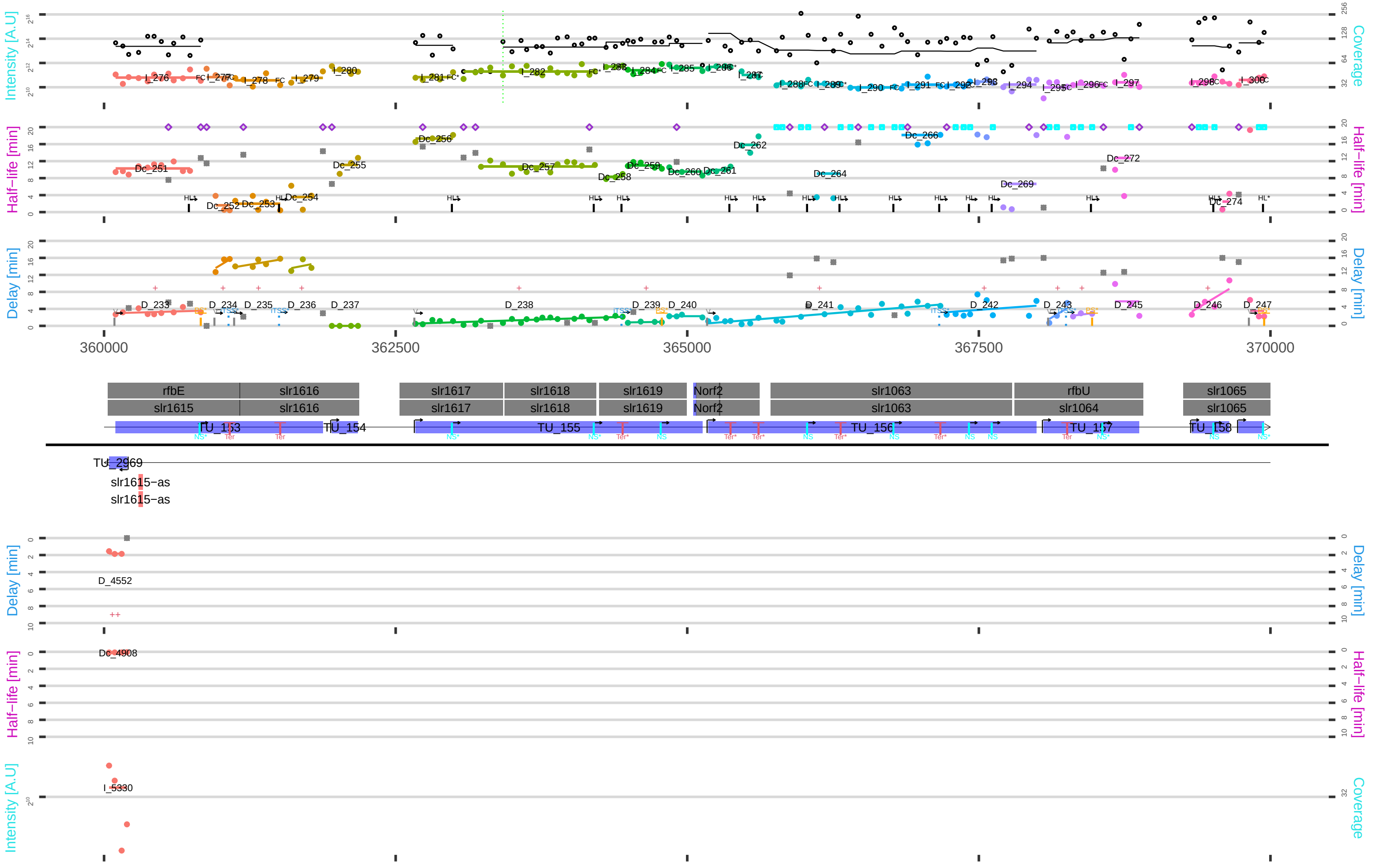

Term: termination (0), NS: new start (0), PS: pausing site (0), iTSS_I: internal starting site (0)

ID: 3057−3171; Term: termination (2), NS: new start (8), PS: pausing site (7), iTSS_I: internal starting site (3)

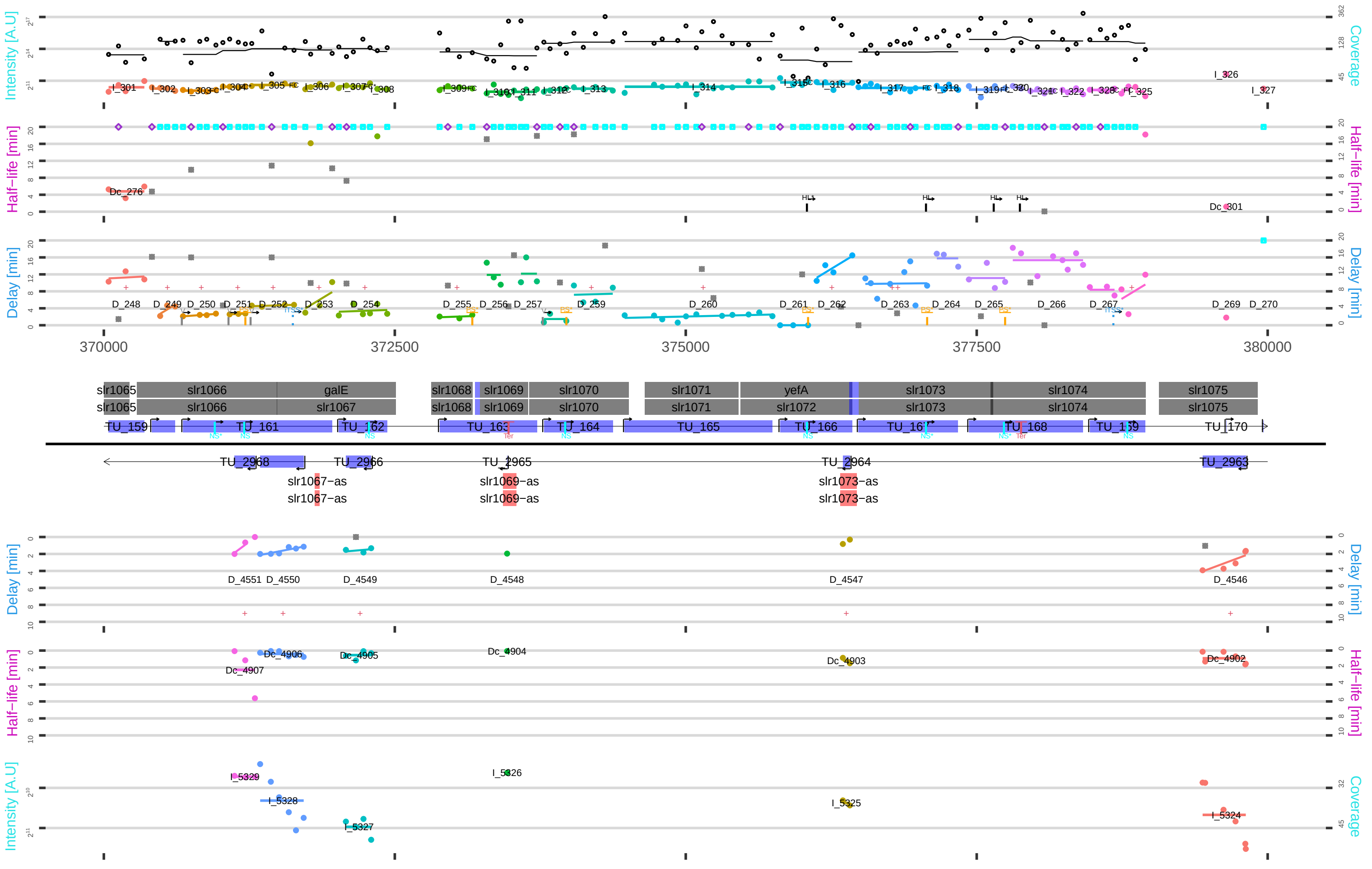

Term: termination (0), NS: new start (0), PS: pausing site (0), iTSS_I: internal starting site (0)

ID: 3172−3307; Term: termination (7), NS: new start (11), PS: pausing site (7), iTSS_I: internal starting site (1)

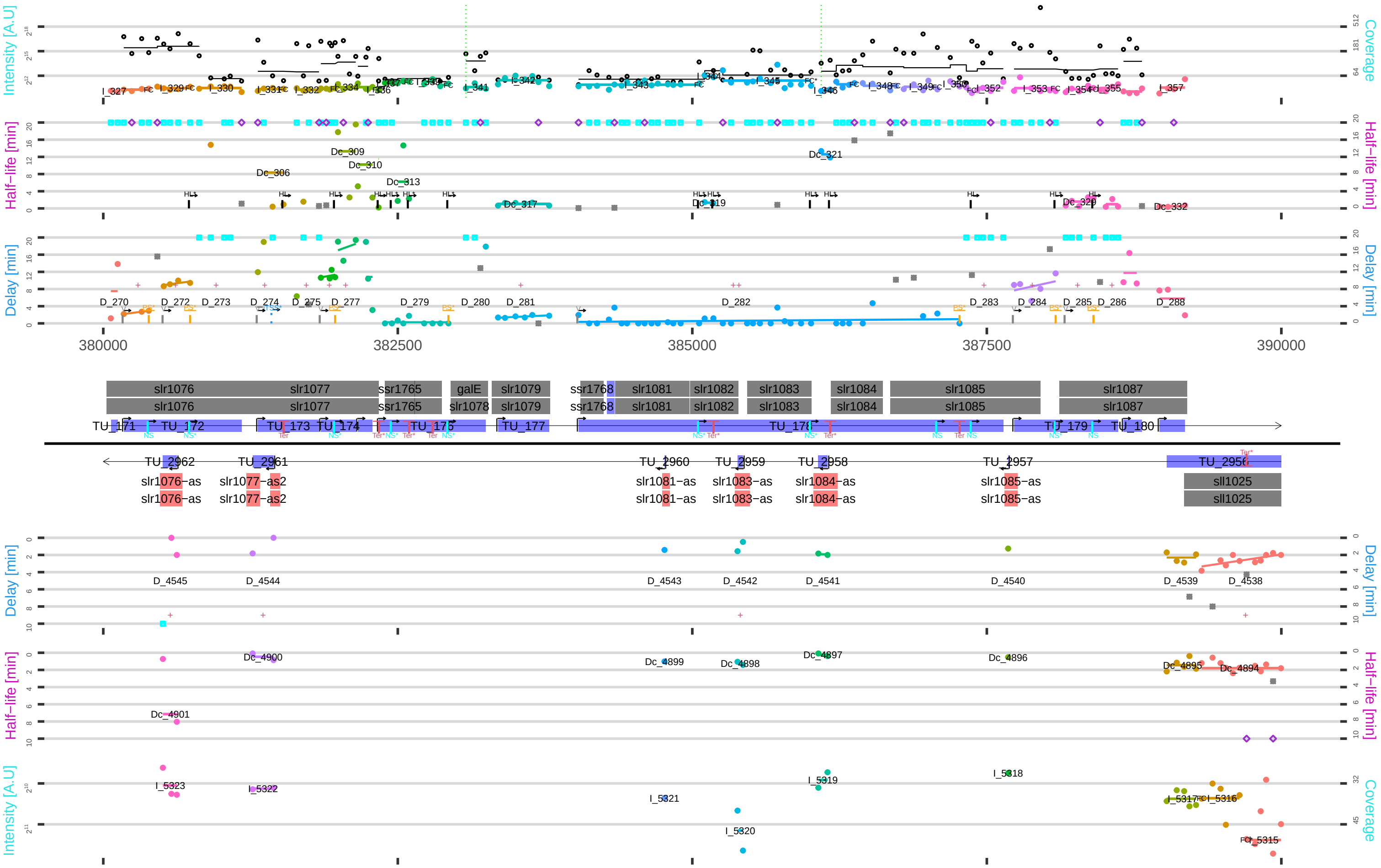

Term: termination (1), NS: new start (0), PS: pausing site (0), iTSS_I: internal starting site (1)

ID: 3308−3335; Term: termination (1), NS: new start (0), PS: pausing site (1), iTSS_I: internal starting site (0)

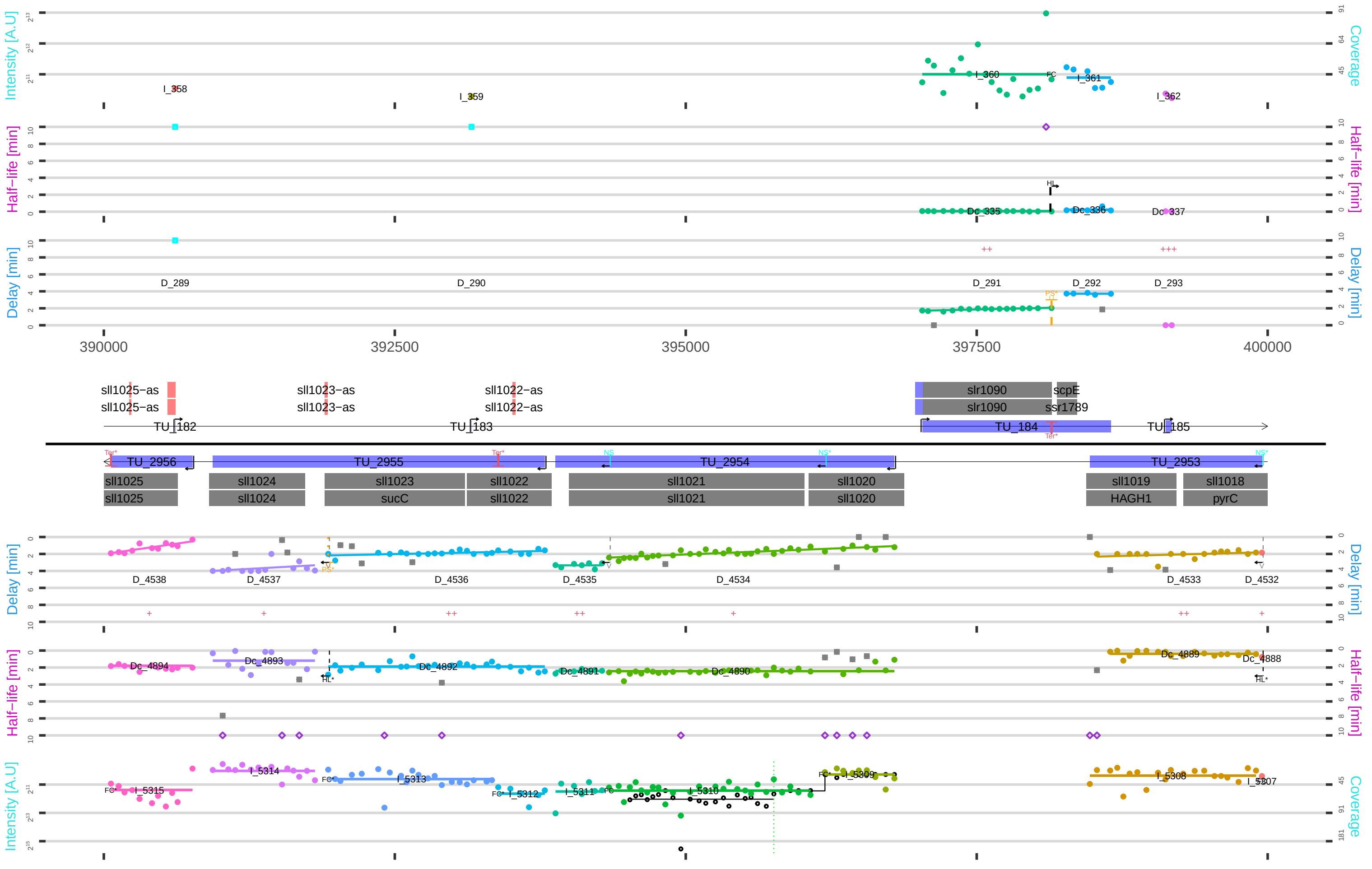

Term: termination (2), NS: new start (3), PS: pausing site (2), iTSS_I: internal starting site (1)

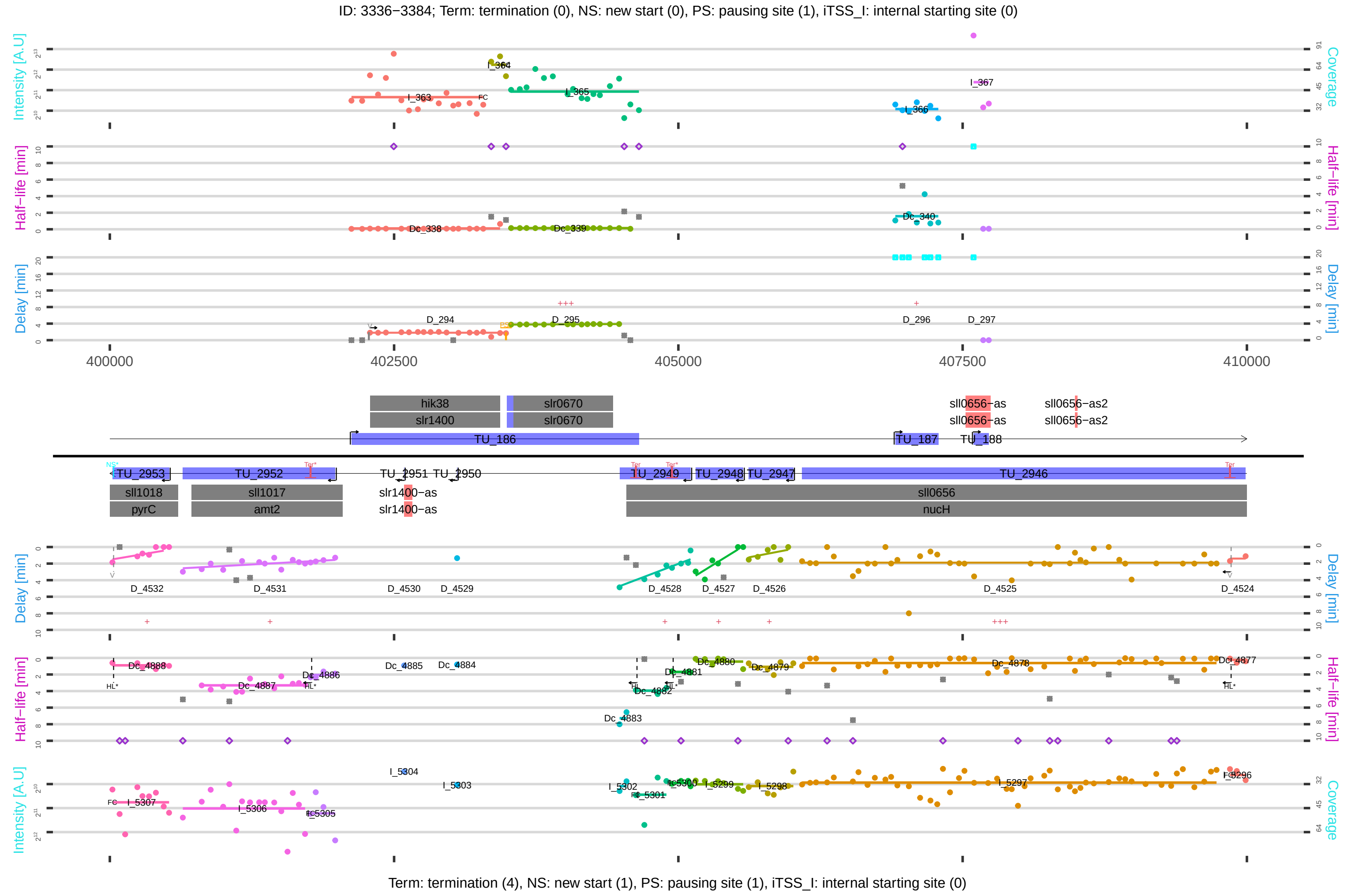

ID: 3336−3384; Term: termination (0), NS: new start (0), PS: pausing site (1), iTSS_I: internal starting site (0)
Intensity [A.U]
Coverage
Half−life [min]
Delay [min]
400000
402500
405000
407500
410000
I_363
I_364
I_365
I_366
I_367
Dc_338
Dc_339
Dc_340
D_294
D_295
D_296
D_297
hik38
slr1400
slr0670
sll0656−as
sll0656−as2
TU_186
TU_187
TU_188
TU_2953
TU_2952
TU_2951
TU_2950
TU_2949
TU_2948
TU_2947
TU_2946
sll1018
pyrC
sll1017
amt2
slr1400−as
sll0656
nucH
D_4532
D_4531
D_4530
D_4529
D_4528
D_4527
D_4526
D_4525
D_4524
Dc_4888
Dc_4887
Dc_4886
Dc_4885
Dc_4884
Dc_4883
Dc_4882
Dc_4881
Dc_4880
Dc_4879
Dc_4878
Dc_4877
I_5307
I_5306
I_5305
I_5304
I_5303
I_5302
I_5301
I_5300
I_5299
I_5298
I_5297
I_5296
Term: termination (4), NS: new start (1), PS: pausing site (1), iTSS_I: internal starting site (0)

ID: 3386−3465; Term: termination (1), NS: new start (2), PS: pausing site (2), iTSS_I: internal starting site (1)

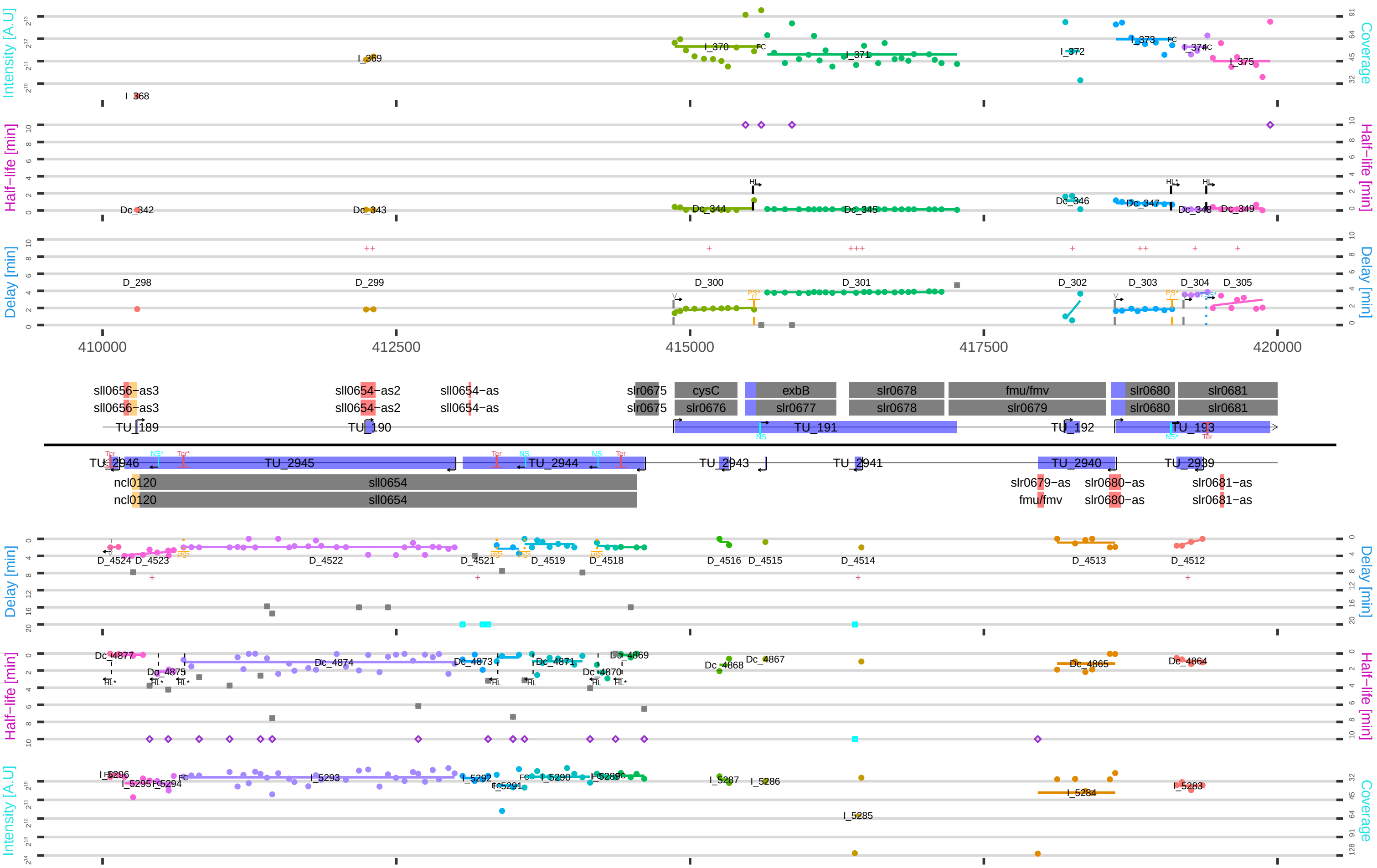

Term: termination (4), NS: new start (3), PS: pausing site (5), iTSS_I: internal starting site (0)

ID: 3466−3502; Term: termination (0), NS: new start (0), PS: pausing site (0), iTSS_I: internal starting site (0)

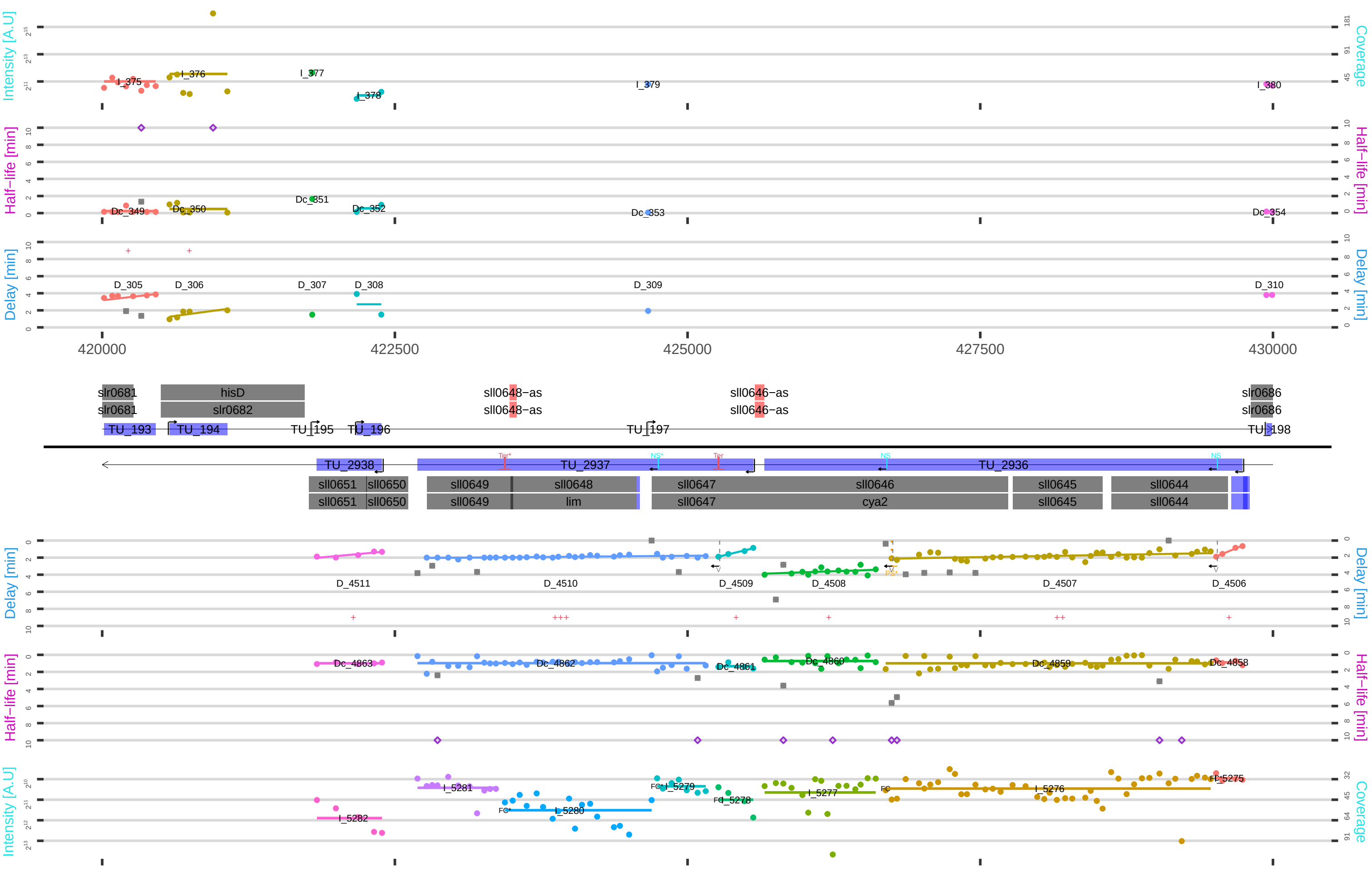

Term: termination (2), NS: new start (3), PS: pausing site (1), iTSS_I: internal starting site (2)

ID: 3503−3581; Term: termination (2), NS: new start (1), PS: pausing site (1), iTSS_I: internal starting site (1)

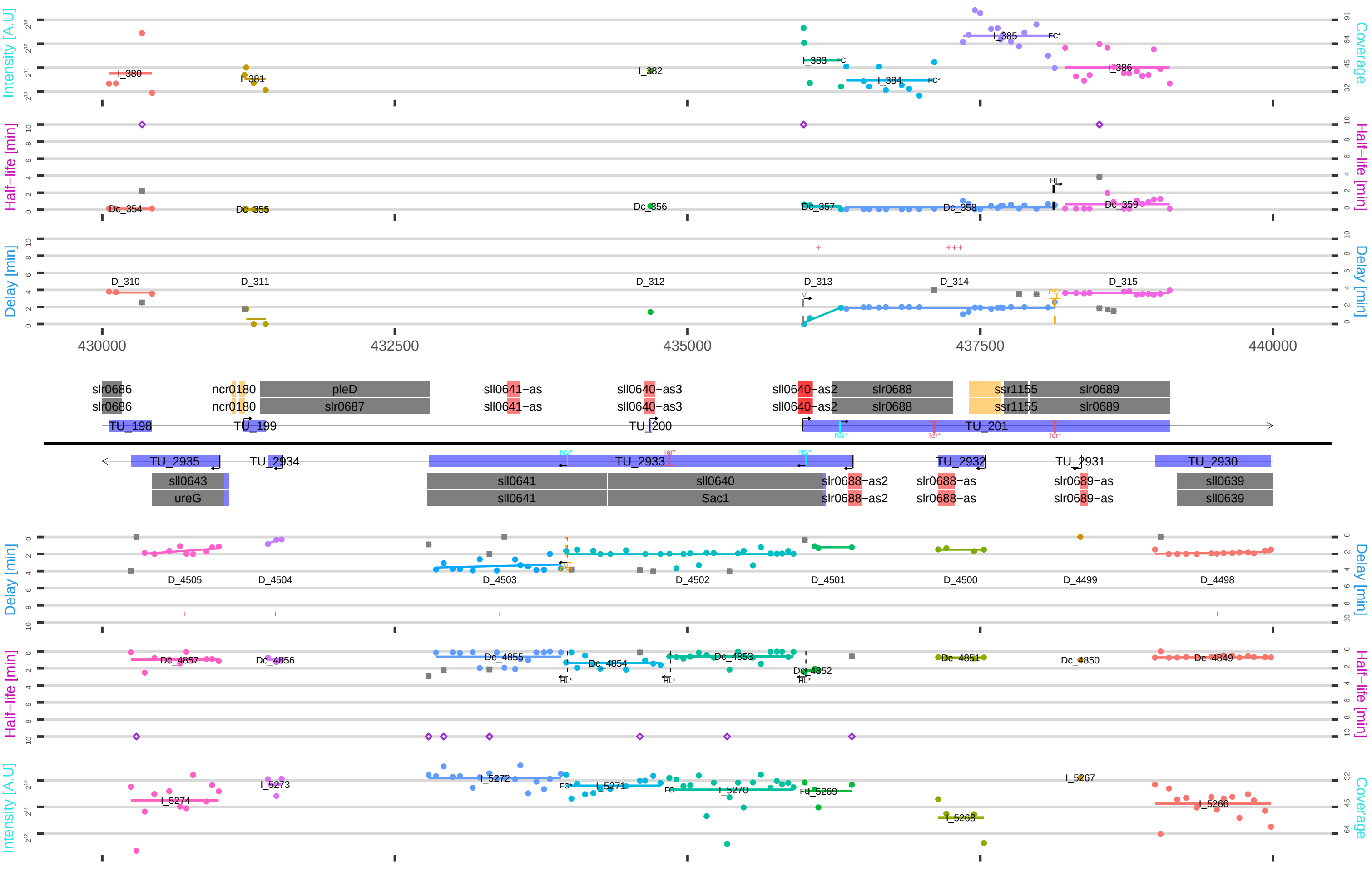

Term: termination (1), NS: new start (2), PS: pausing site (2), iTSS_I: internal starting site (0)

ID: 3582−3659; Term: termination (1), NS: new start (0), PS: pausing site (1), iTSS_I: internal starting site (0)

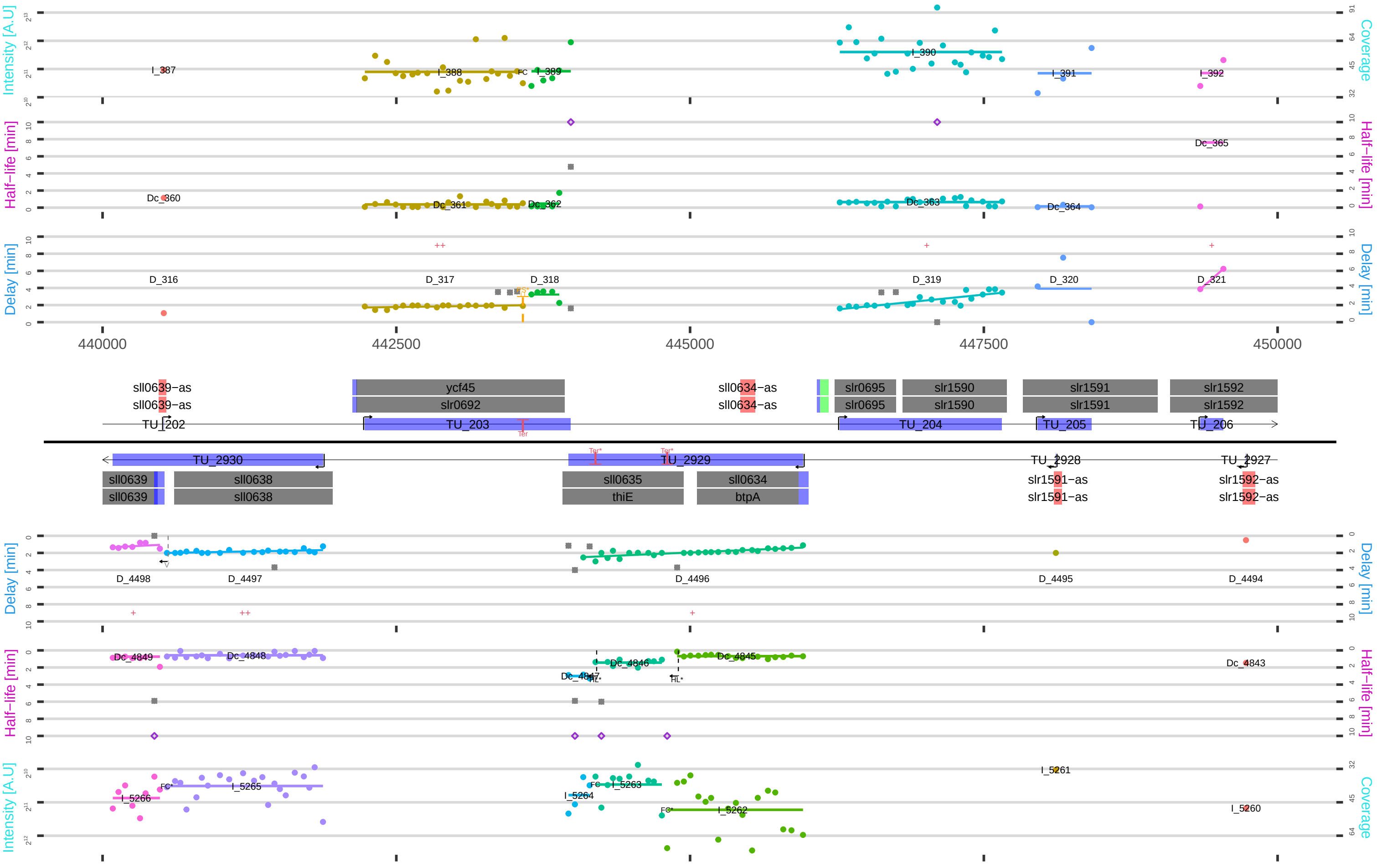

Term: termination (2), NS: new start (0), PS: pausing site (0), iTSS_I: internal starting site (1)

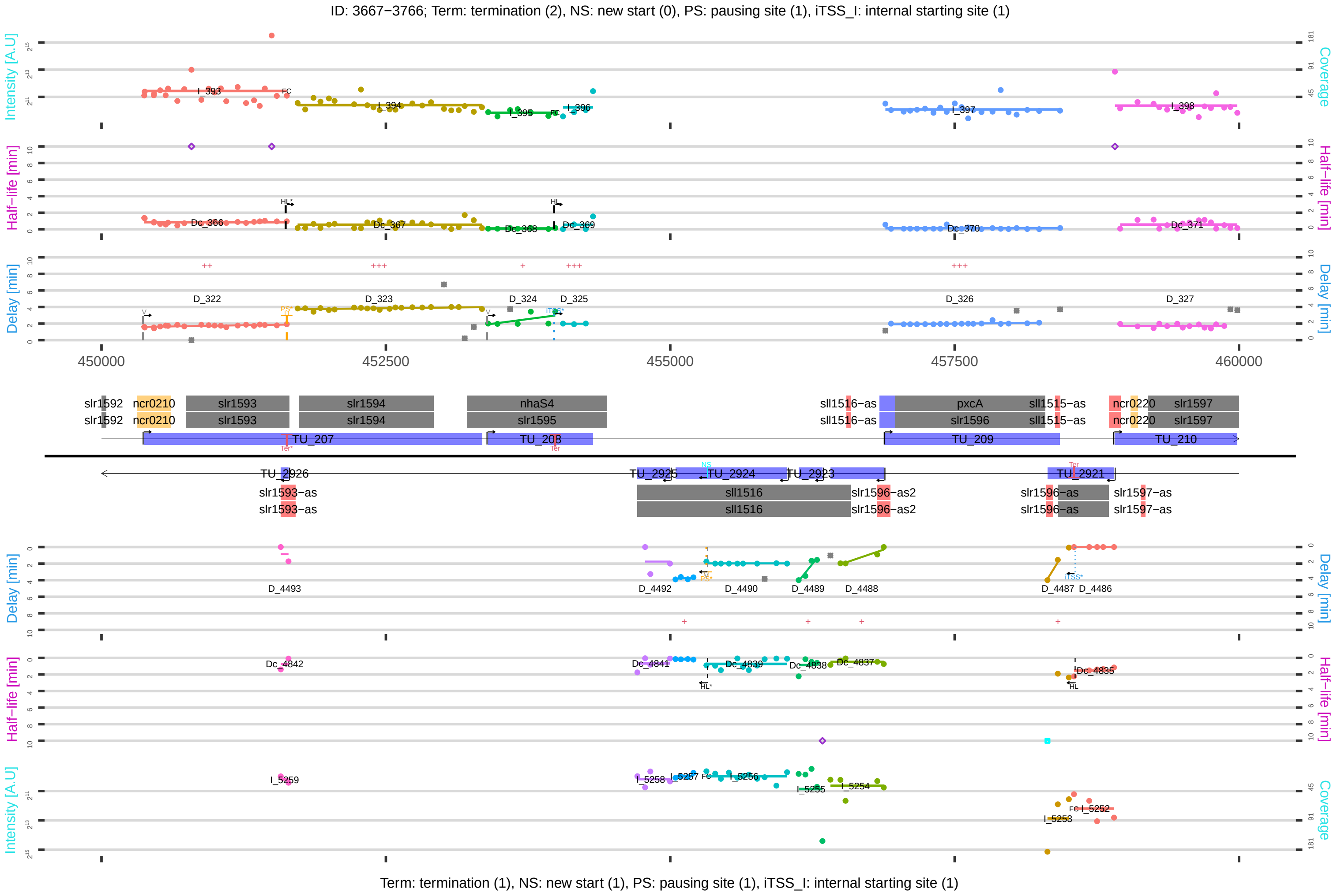

ID: 3667−3766; Term: termination (2), NS: new start (0), PS: pausing site (1), iTSS_I: internal starting site (1)
Intensity [A.U]
Coverage
Half−life [min]
Delay [min]
450000
452500
455000
457500
460000
TU_207
TU_208
TU_209
TU_210
TU_2926
TU_2925
TU_2924
TU_2923
TU_2921
slr1592
ncr0210
slr1593
slr1594
nhaS4
slr1595
sll1516−as
pxcA
slr1596
sll1515−as
ncr0220
slr1597
slr1593−as
sll1516
slr1596−as2
slr1596−as
slr1597−as
Term: termination (1), NS: new start (1), PS: pausing site (1), iTSS_I: internal starting site (1)

ID: 3767−3833; Term: termination (2), NS: new start (4), PS: pausing site (3), iTSS_I: internal starting site (2)

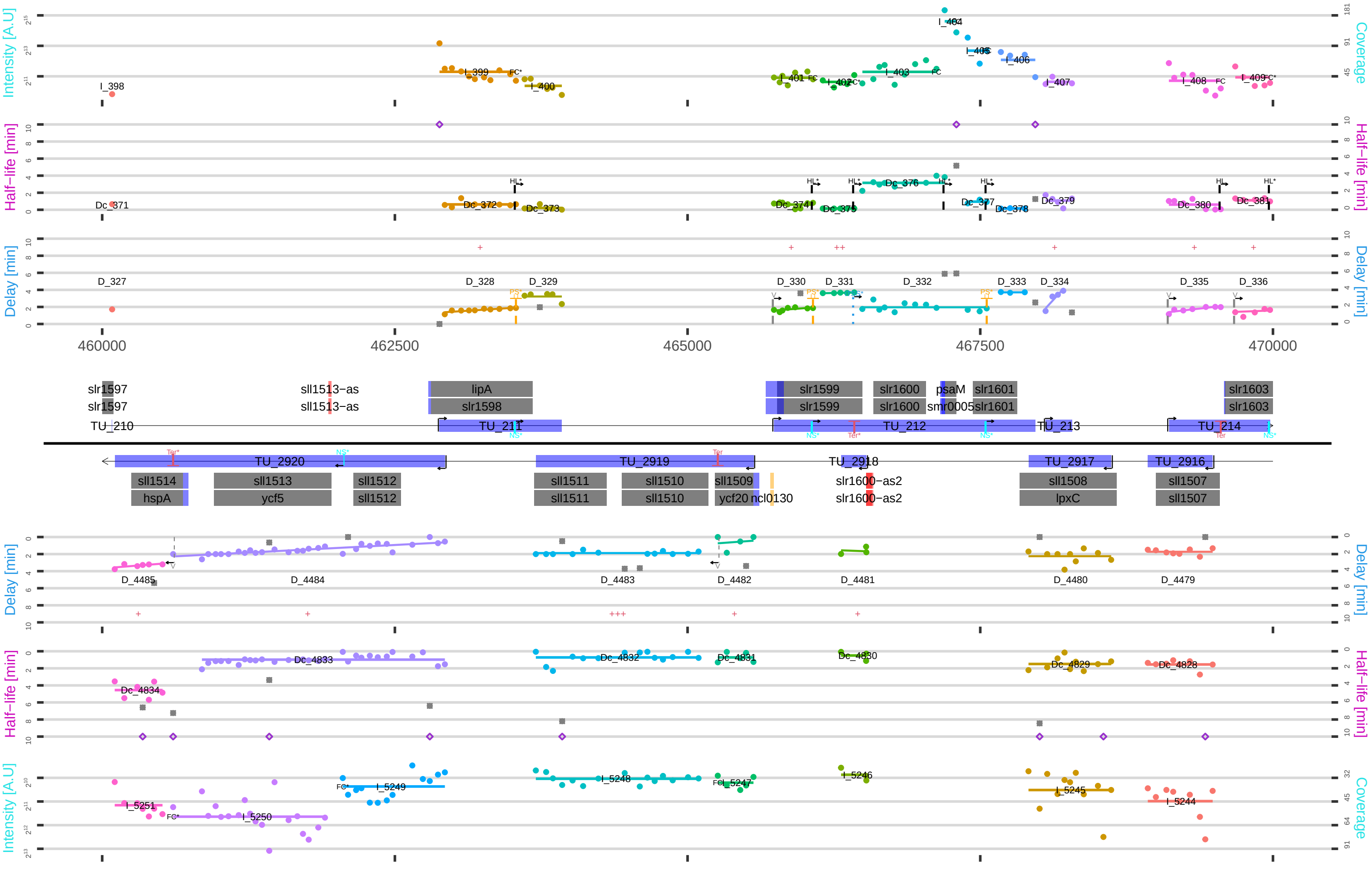

Term: termination (2), NS: new start (1), PS: pausing site (2), iTSS_I: internal starting site (0)

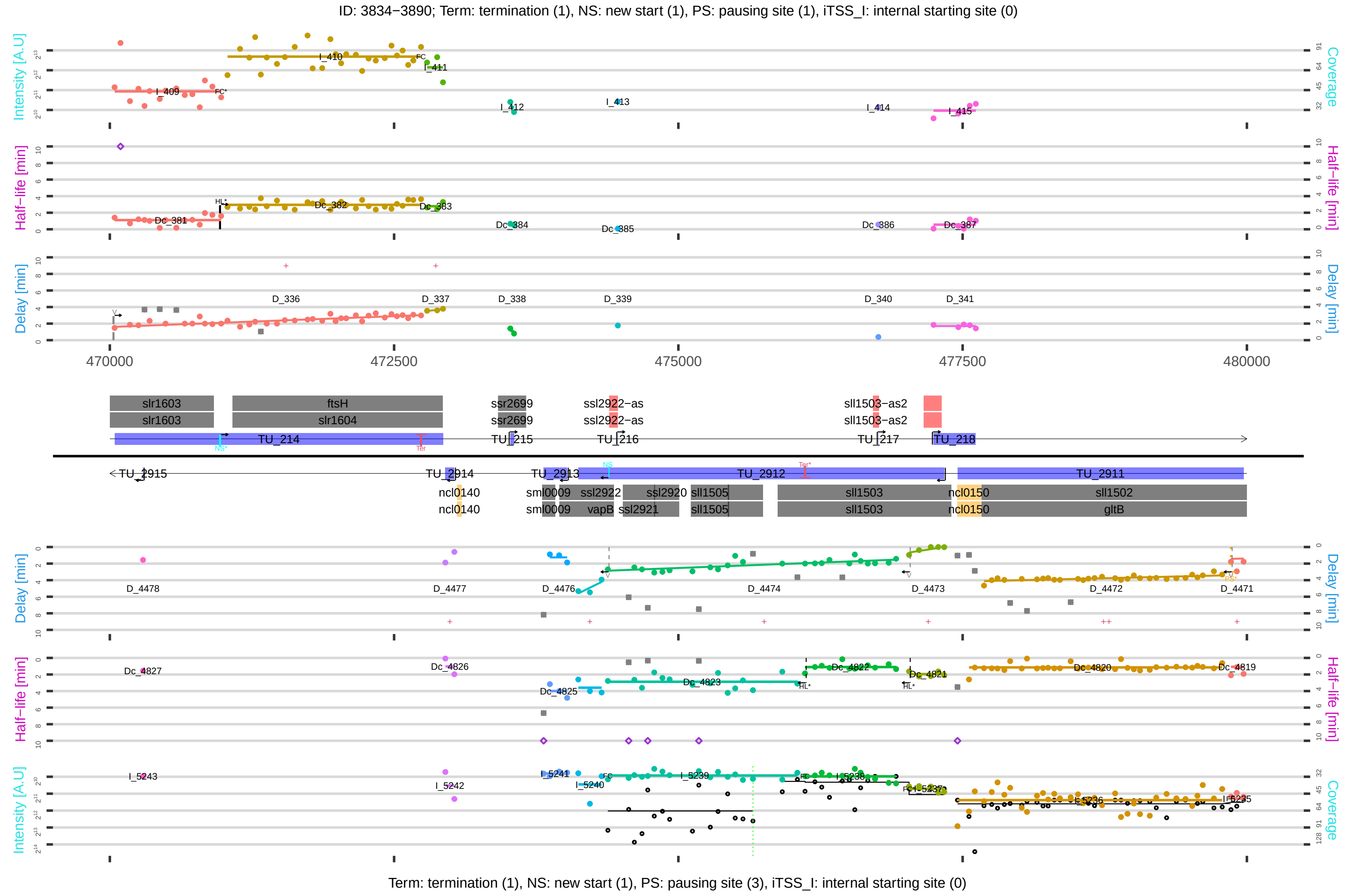

ID: 3834−3890; Term: termination (1), NS: new start (1), PS: pausing site (1), iTSS_I: internal starting site (0)
Intensity [A.U]
Coverage
Half−life [min]
Delay [min]
470000
472500
475000
477500
480000
slr1603
ftsH
slr1604
ssr2699
ssl2922−as
sll1503−as2
TU_214
TU_215
TU_216
TU_217
TU_218
TU_2915
TU_2914
TU_2913
TU_2912
TU_2911
ncl0140
sml0009
ssl2922
vapB
ssl2920
ssl2921
sll1505
sll1503
ncl0150
sll1502
gltB
Term: termination (1), NS: new start (1), PS: pausing site (3), iTSS_I: internal starting site (0)

ID: 3891−3971; Term: termination (2), NS: new start (2), PS: pausing site (2), iTSS_I: internal starting site (2)

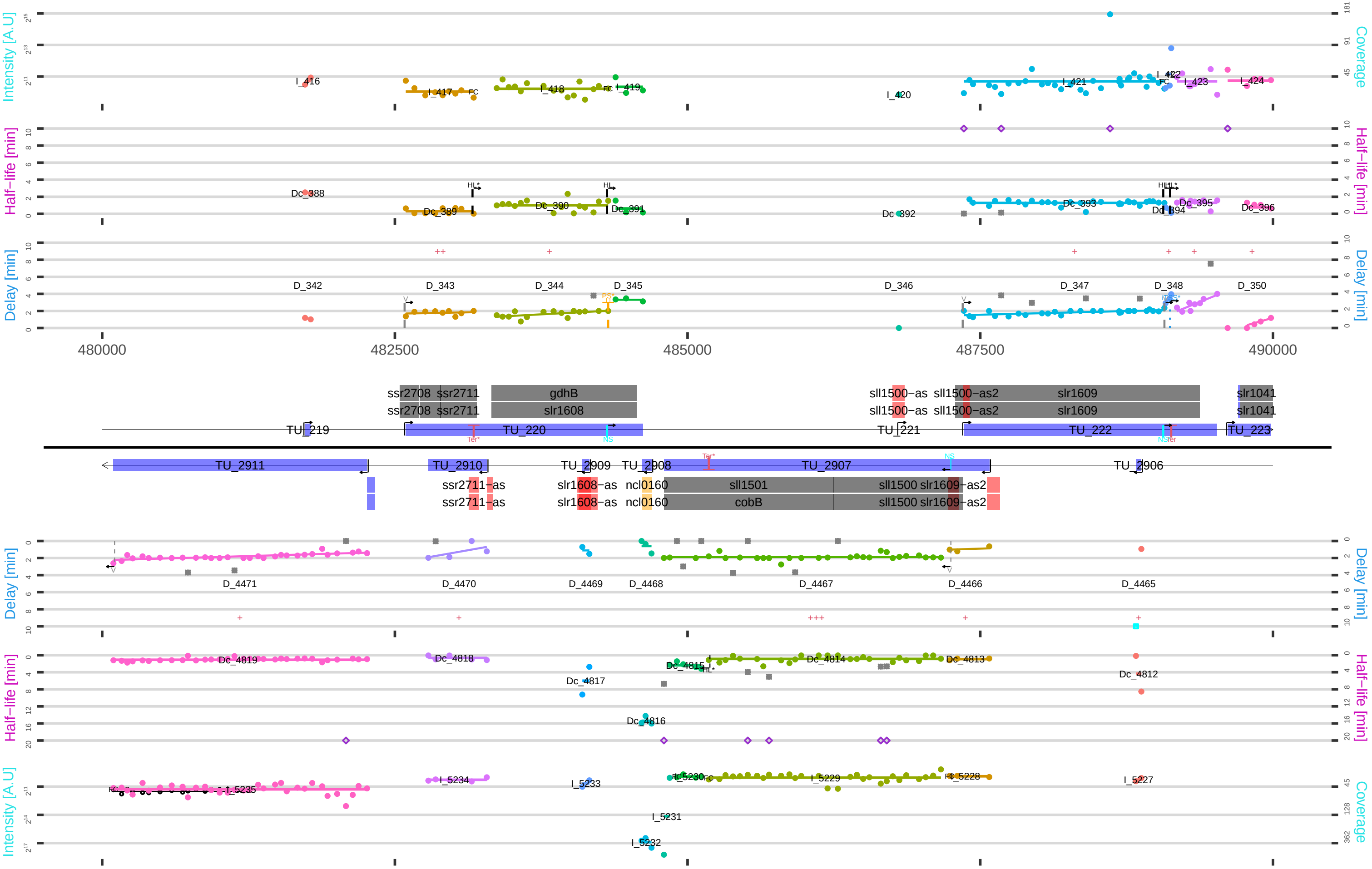

Term: termination (1), NS: new start (1), PS: pausing site (1), iTSS_I: internal starting site (0)

ID: 3972−4107; Term: termination (3), NS: new start (4), PS: pausing site (4), iTSS_I: internal starting site (3)

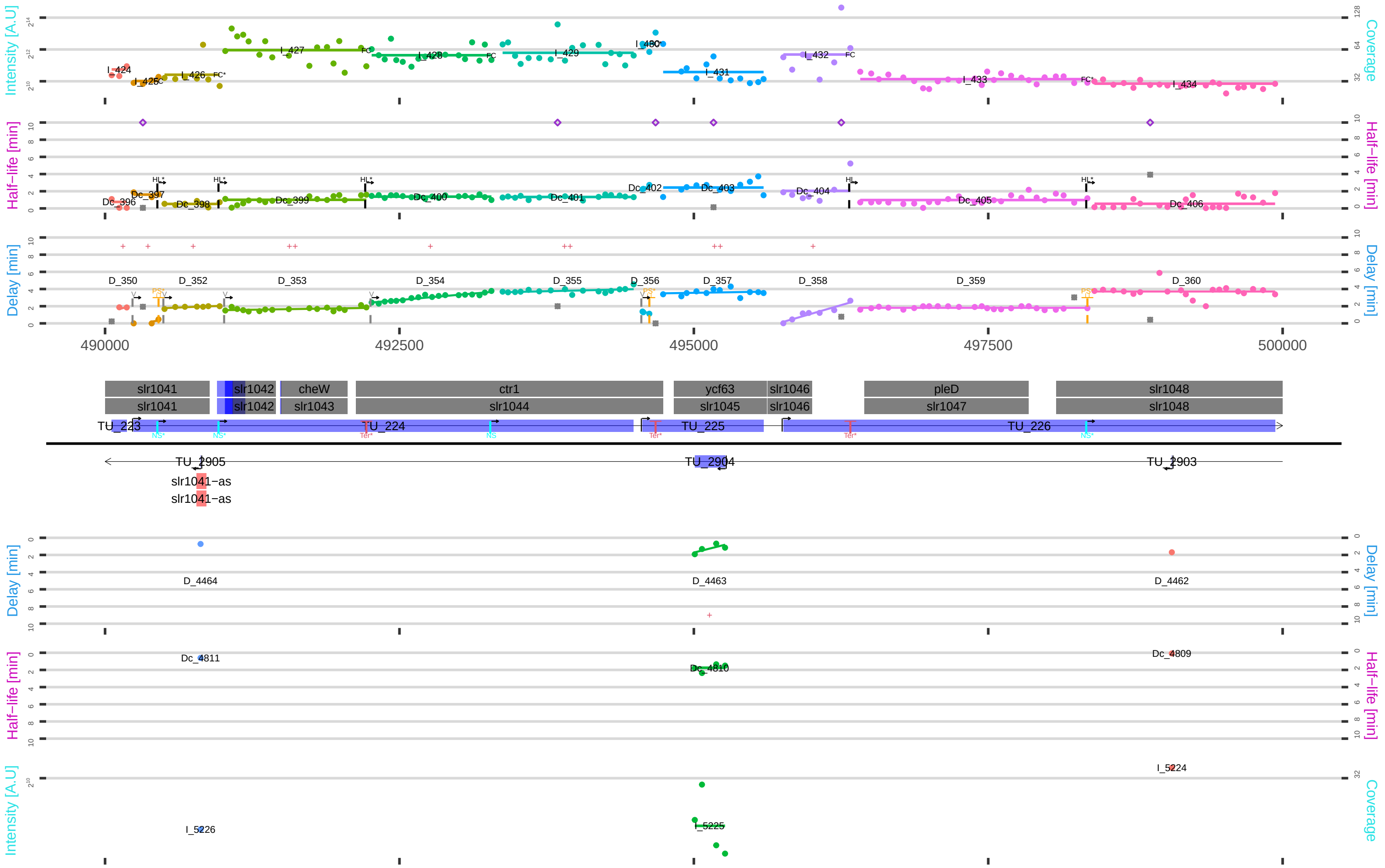

Term: termination (0), NS: new start (0), PS: pausing site (0), iTSS_I: internal starting site (0)

ID: 4108−4205; Term: termination (3), NS: new start (1), PS: pausing site (1), iTSS_I: internal starting site (1)

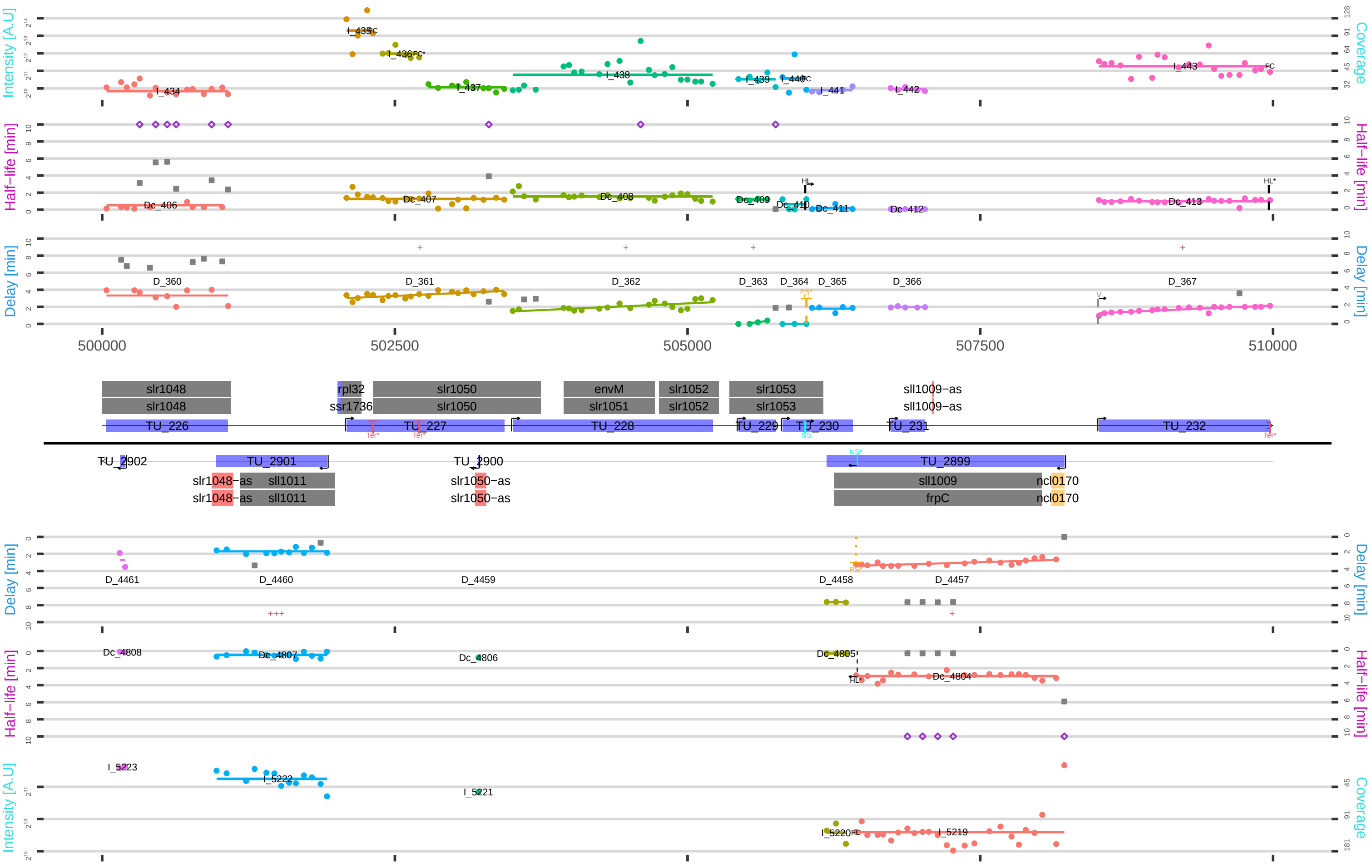

Term: termination (0), NS: new start (1), PS: pausing site (1), iTSS_I: internal starting site (0)

ID: 4206−4275; Term: termination (1), NS: new start (0), PS: pausing site (1), iTSS_I: internal starting site (0)

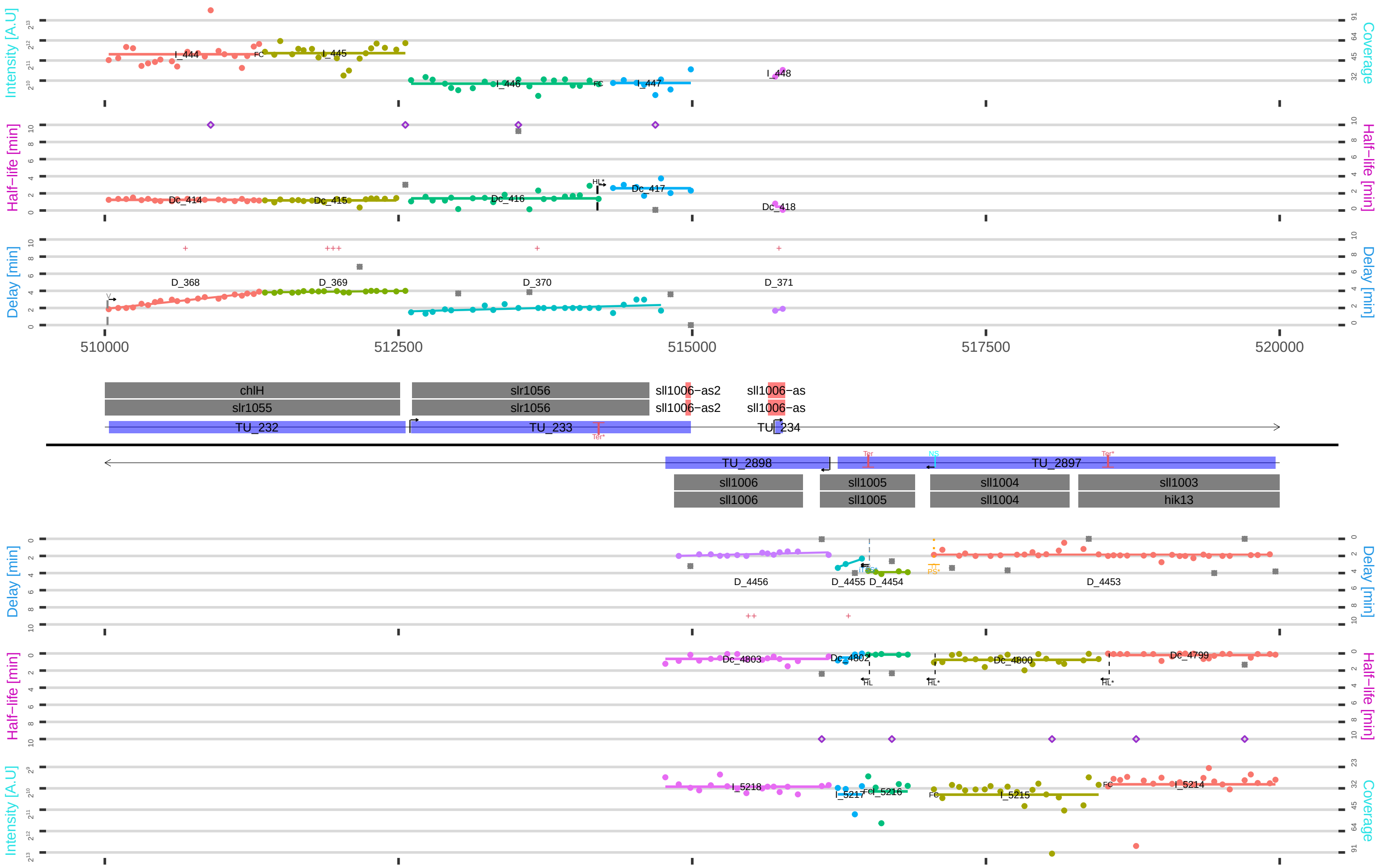

Term: termination (2), NS: new start (1), PS: pausing site (1), iTSS_I: internal starting site (1)

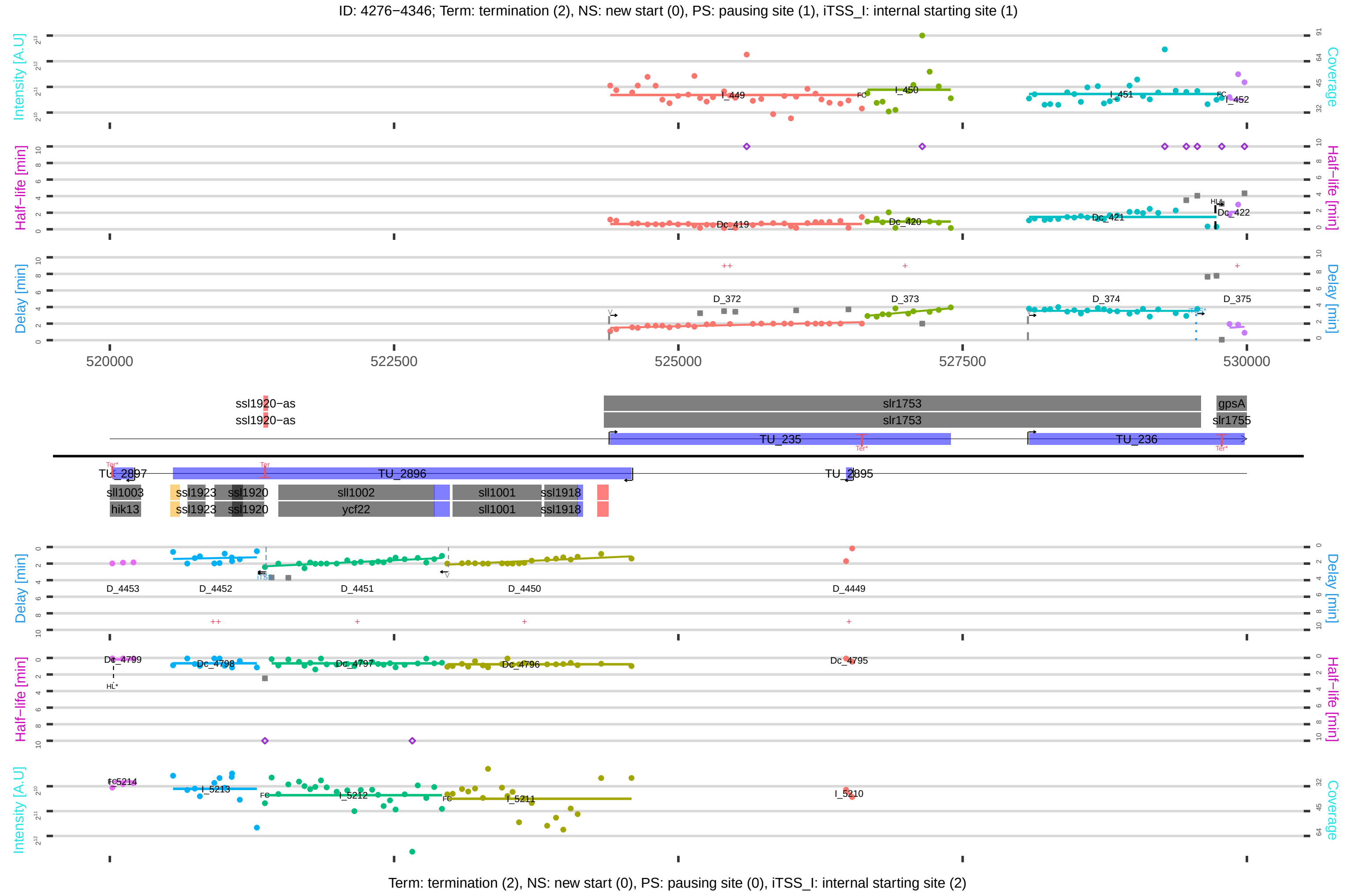
ID: 4276−4346; Term: termination (2), NS: new start (0), PS: pausing site (1), iTSS_I: internal starting site (1)
Intensity [A.U]
Coverage
Half−life [min]
Delay [min]
I_449
I_450
I_451
I_452
Dc_419
Dc_420
Dc_421
Dc_422
D_372
D_373
D_374
D_375
520000
522500
525000
527500
530000
ssl1920−as
slr1753
gpsA
slr1755
TU_235
TU_236
TU_2897
TU_2896
TU_2895
sll1003
hik13
ssl1923
ssl1920
sll1002
ycf22
sll1001
ssl1918
D_4453
D_4452
D_4451
D_4450
D_4449
Dc_4799
Dc_4798
Dc_4797
Dc_4796
Dc_4795
I_5214
I_5213
I_5212
I_5211
I_5210
Term: termination (2), NS: new start (0), PS: pausing site (0), iTSS_I: internal starting site (2)

ID: 4347−4479; Term: termination (4), NS: new start (2), PS: pausing site (1), iTSS_I: internal starting site (1)

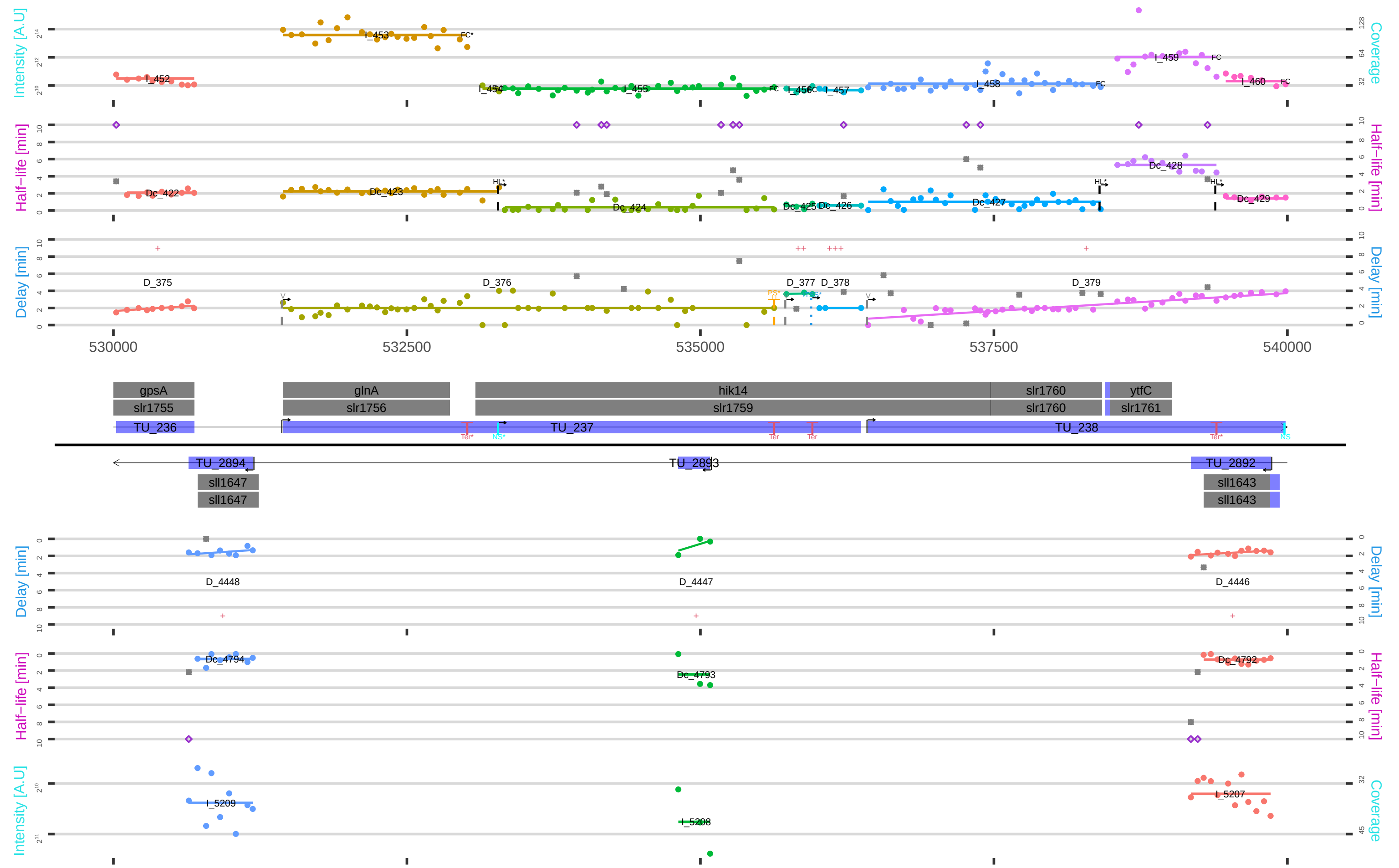

Term: termination (0), NS: new start (0), PS: pausing site (0), iTSS_I: internal starting site (0)

ID: 4480−4544; Term: termination (1), NS: new start (1), PS: pausing site (0), iTSS_I: internal starting site (1)

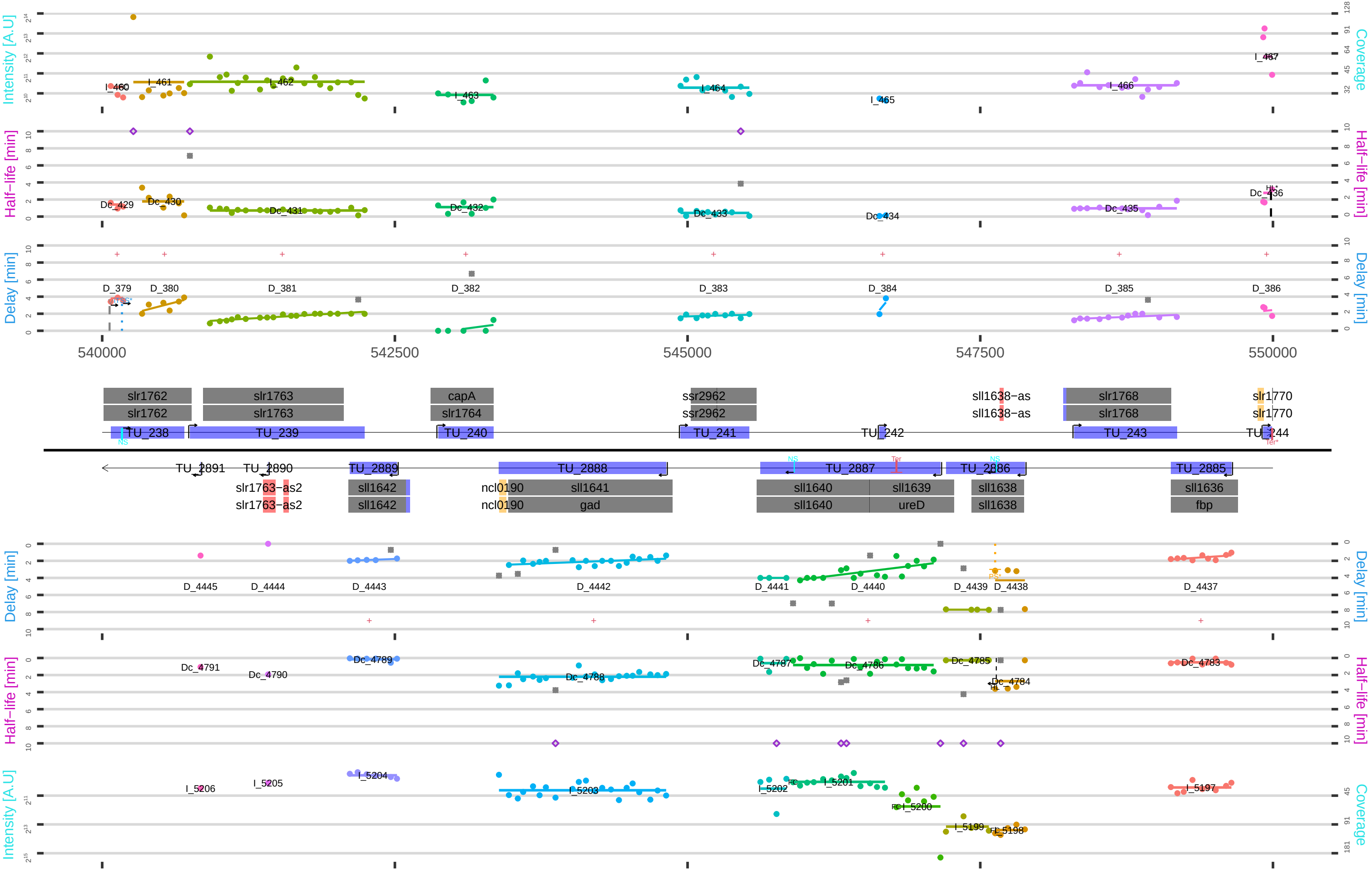

Term: termination (1), NS: new start (2), PS: pausing site (1), iTSS_I: internal starting site (1)

ID: 4545−4643; Term: termination (3), NS: new start (3), PS: pausing site (2), iTSS_I: internal starting site (1)

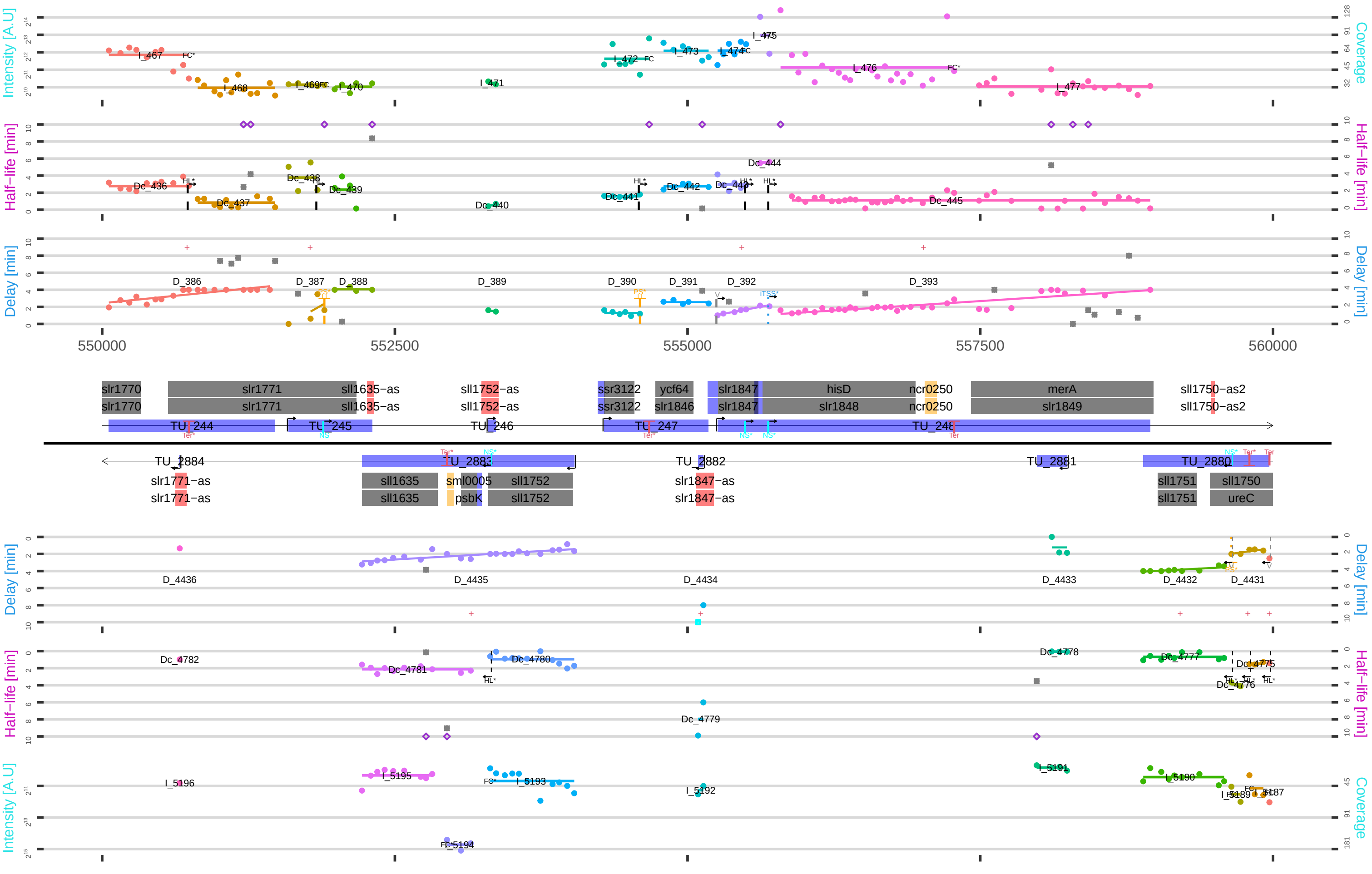

Term: termination (3), NS: new start (2), PS: pausing site (1), iTSS_I: internal starting site (1)

ID: 4644−4690; Term: termination (1), NS: new start (2), PS: pausing site (1), iTSS_I: internal starting site (0)

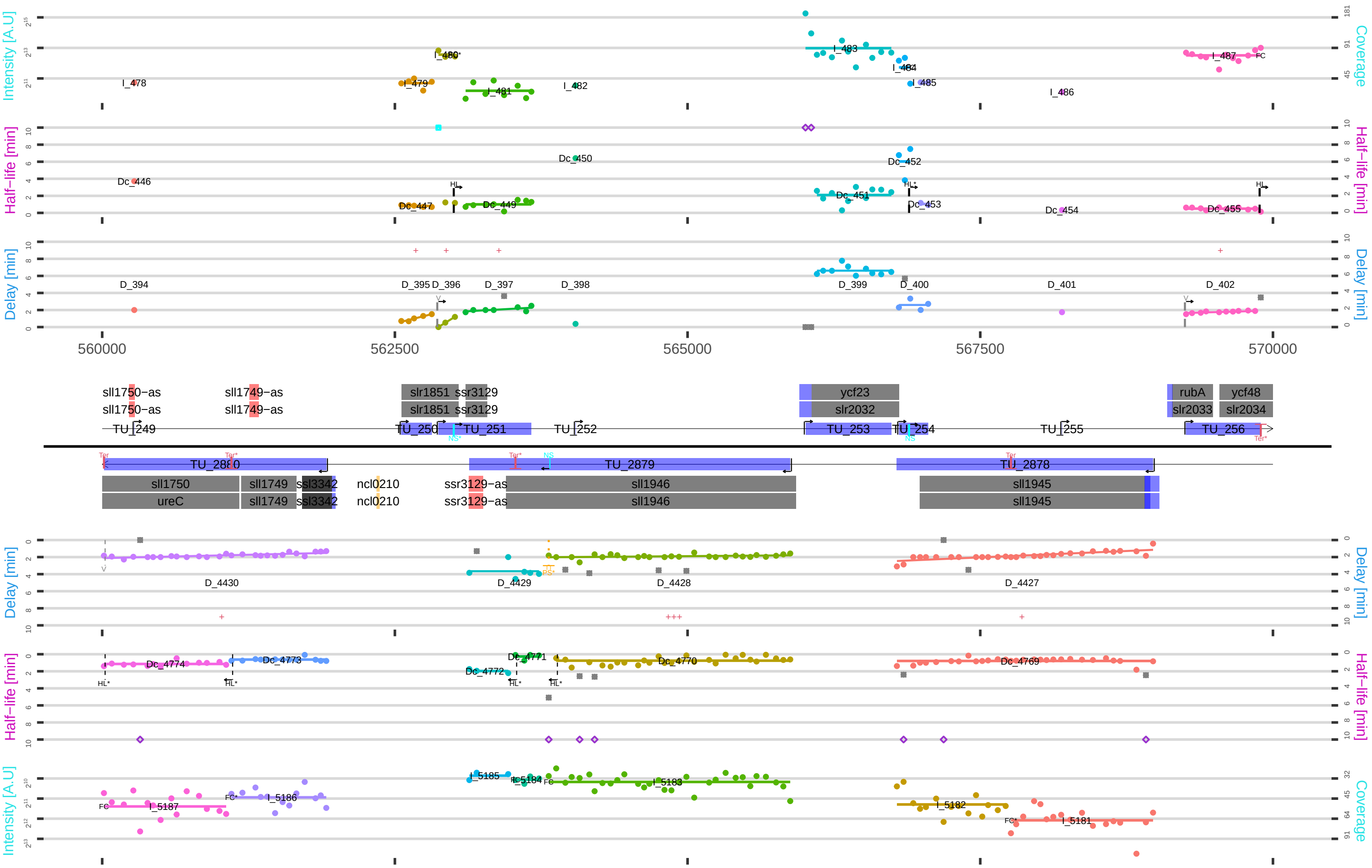

Term: termination (4), NS: new start (1), PS: pausing site (1), iTSS_I: internal starting site (0)

ID: 4691−4772; Term: termination (3), NS: new start (2), PS: pausing site (3), iTSS_I: internal starting site (2)

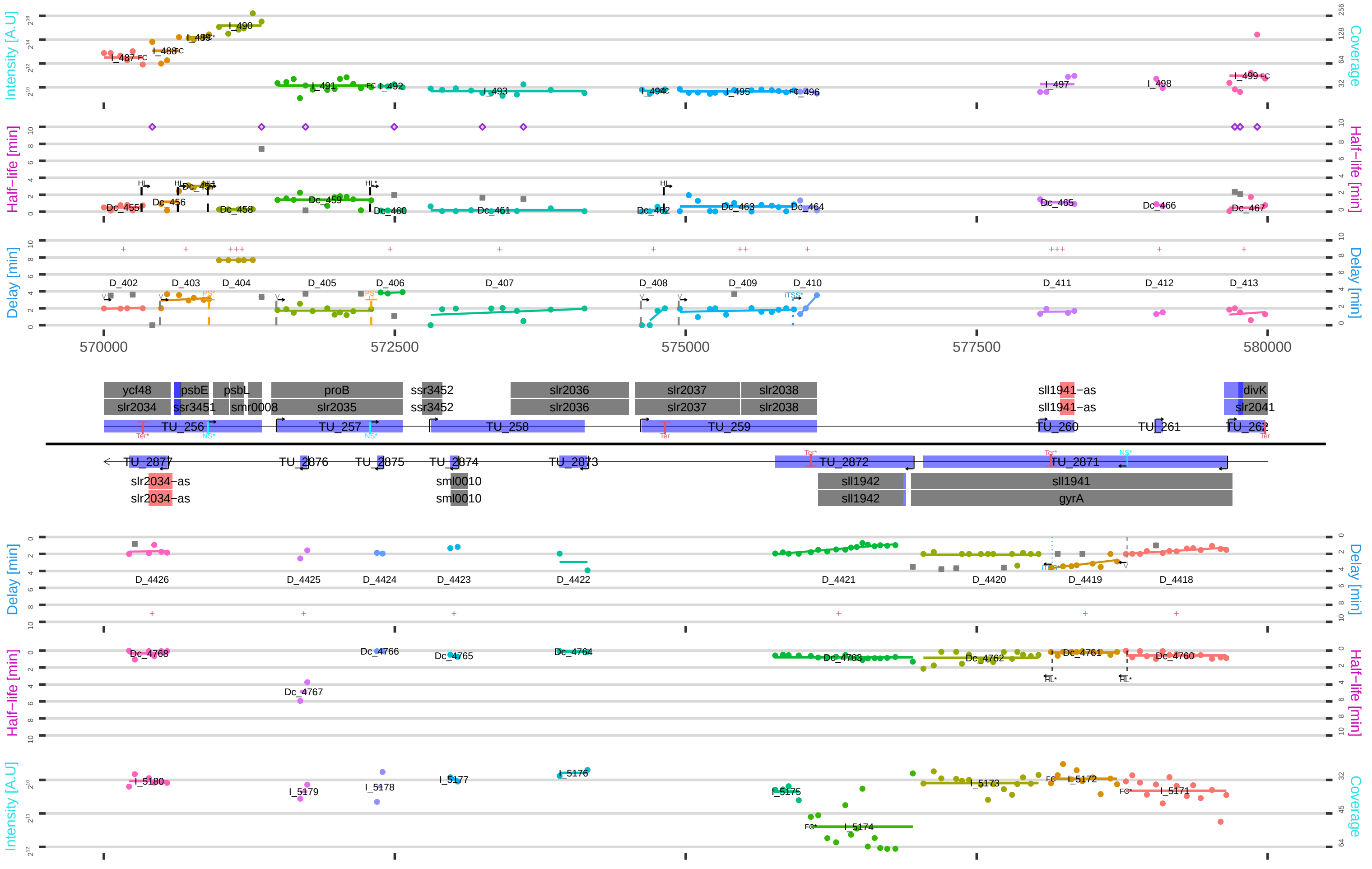

Term: termination (2), NS: new start (1), PS: pausing site (1), iTSS_I: internal starting site (1)

ID: 4773−4885; Term: termination (3), NS: new start (2), PS: pausing site (4), iTSS_I: internal starting site (0)

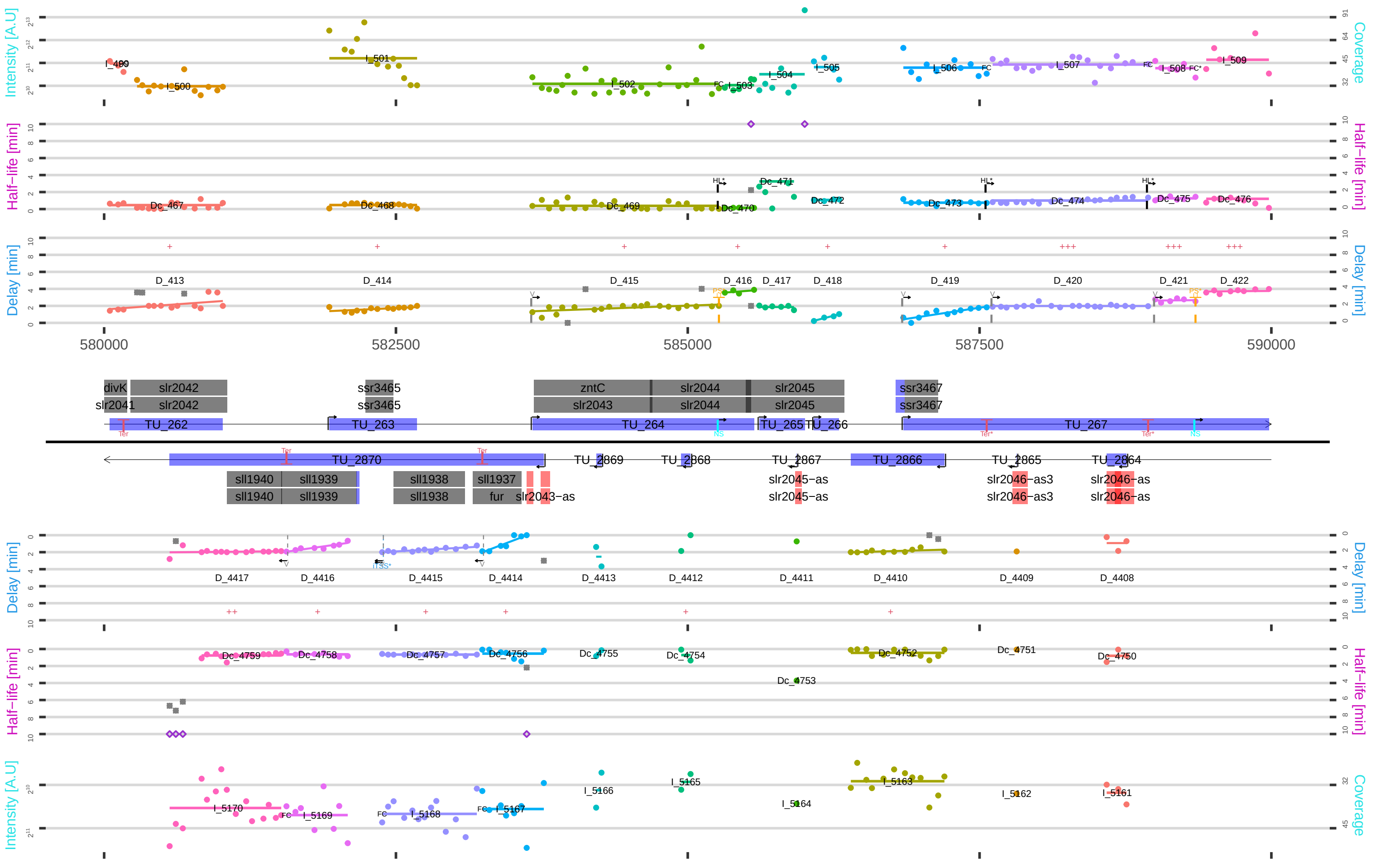

Term: termination (2), NS: new start (0), PS: pausing site (0), iTSS_I: internal starting site (3)

ID: 4886−5019; Term: termination (2), NS: new start (3), PS: pausing site (3), iTSS_I: internal starting site (0)

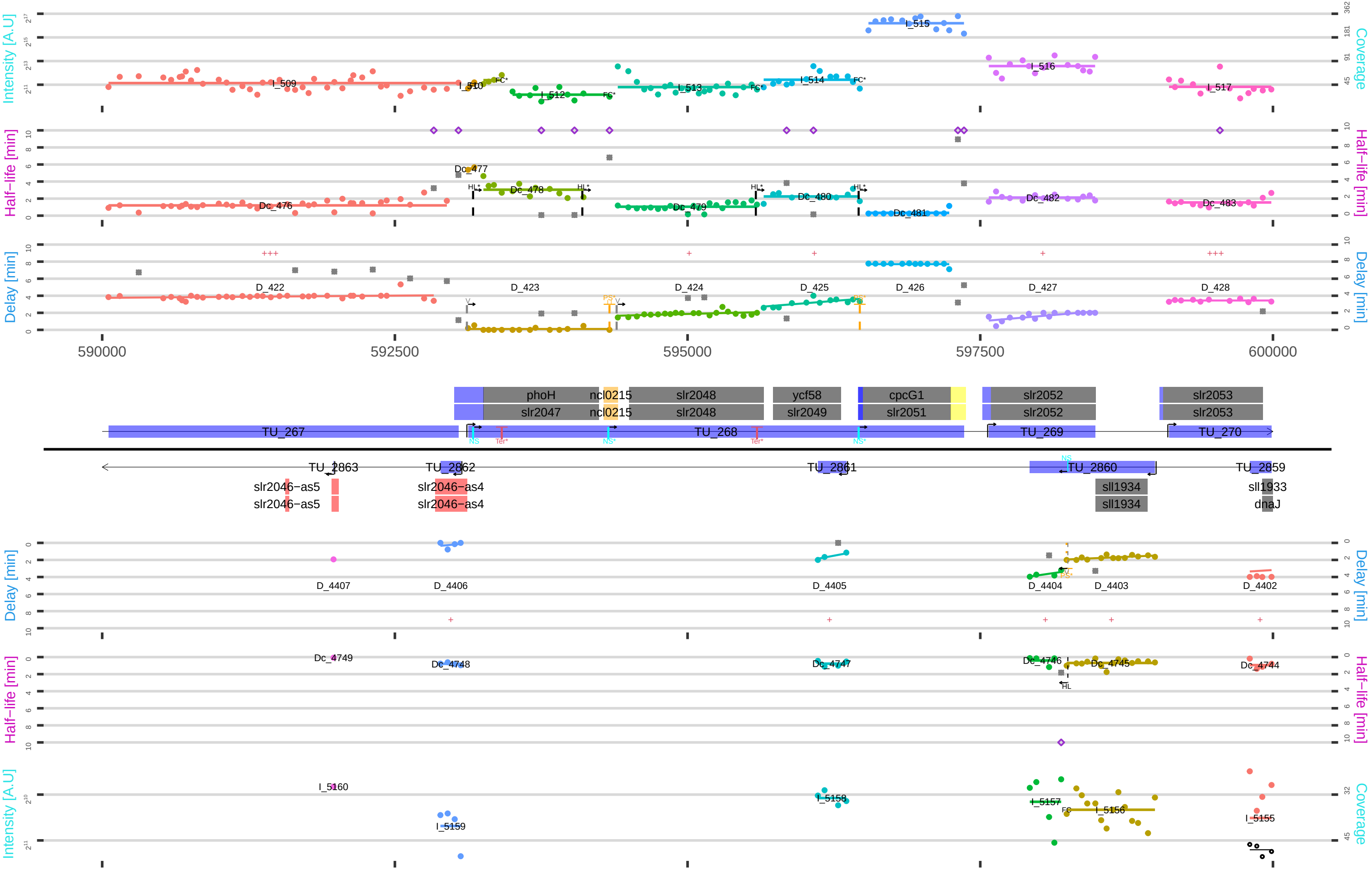

Term: termination (0), NS: new start (1), PS: pausing site (1), iTSS_I: internal starting site (0)

ID: 5020−5045; Term: termination (0), NS: new start (1), PS: pausing site (0), iTSS_I: internal starting site (0)

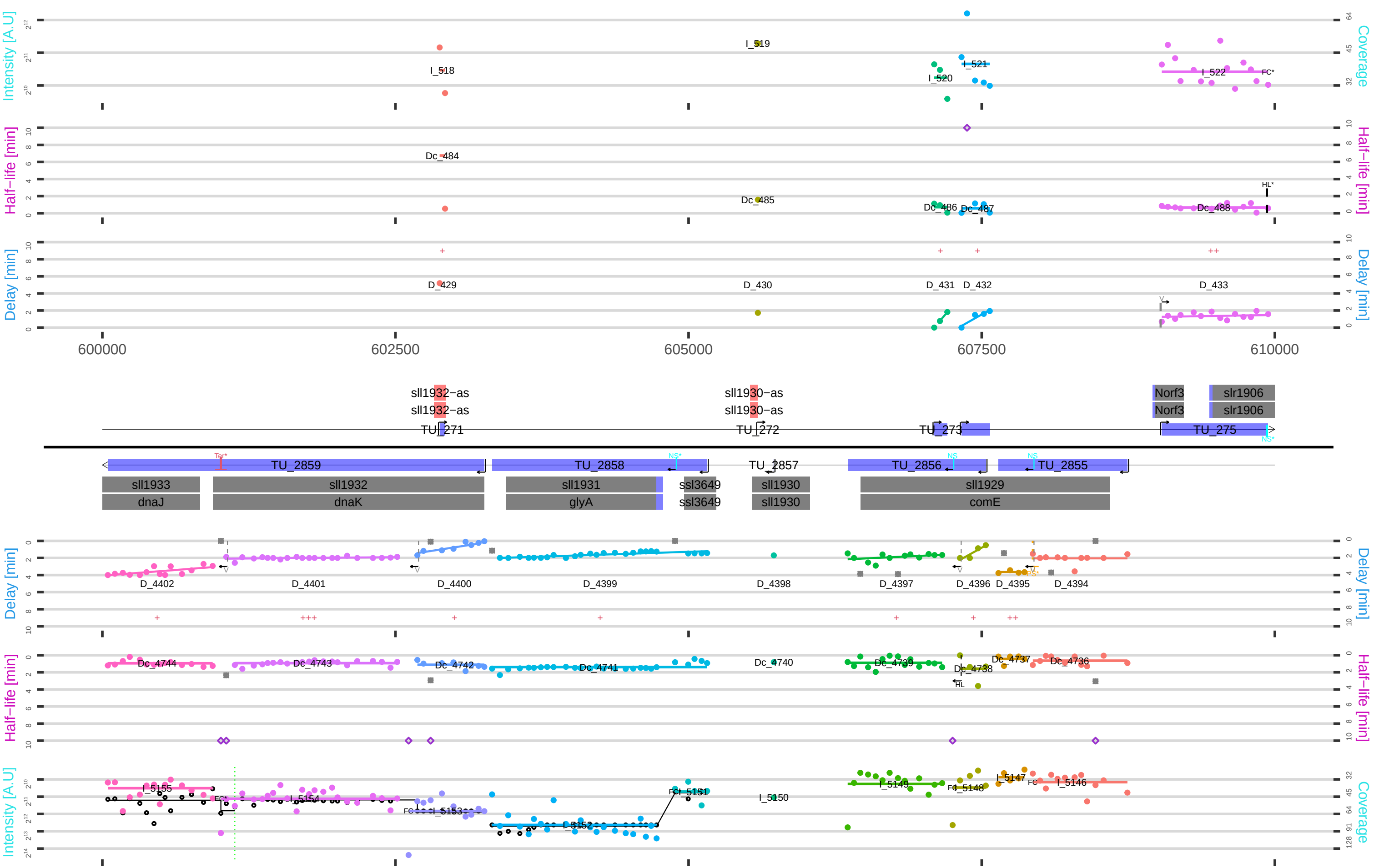

Term: termination (1), NS: new start (3), PS: pausing site (3), iTSS_I: internal starting site (1)

ID: 5046−5161; Term: termination (2), NS: new start (4), PS: pausing site (1), iTSS_I: internal starting site (3)

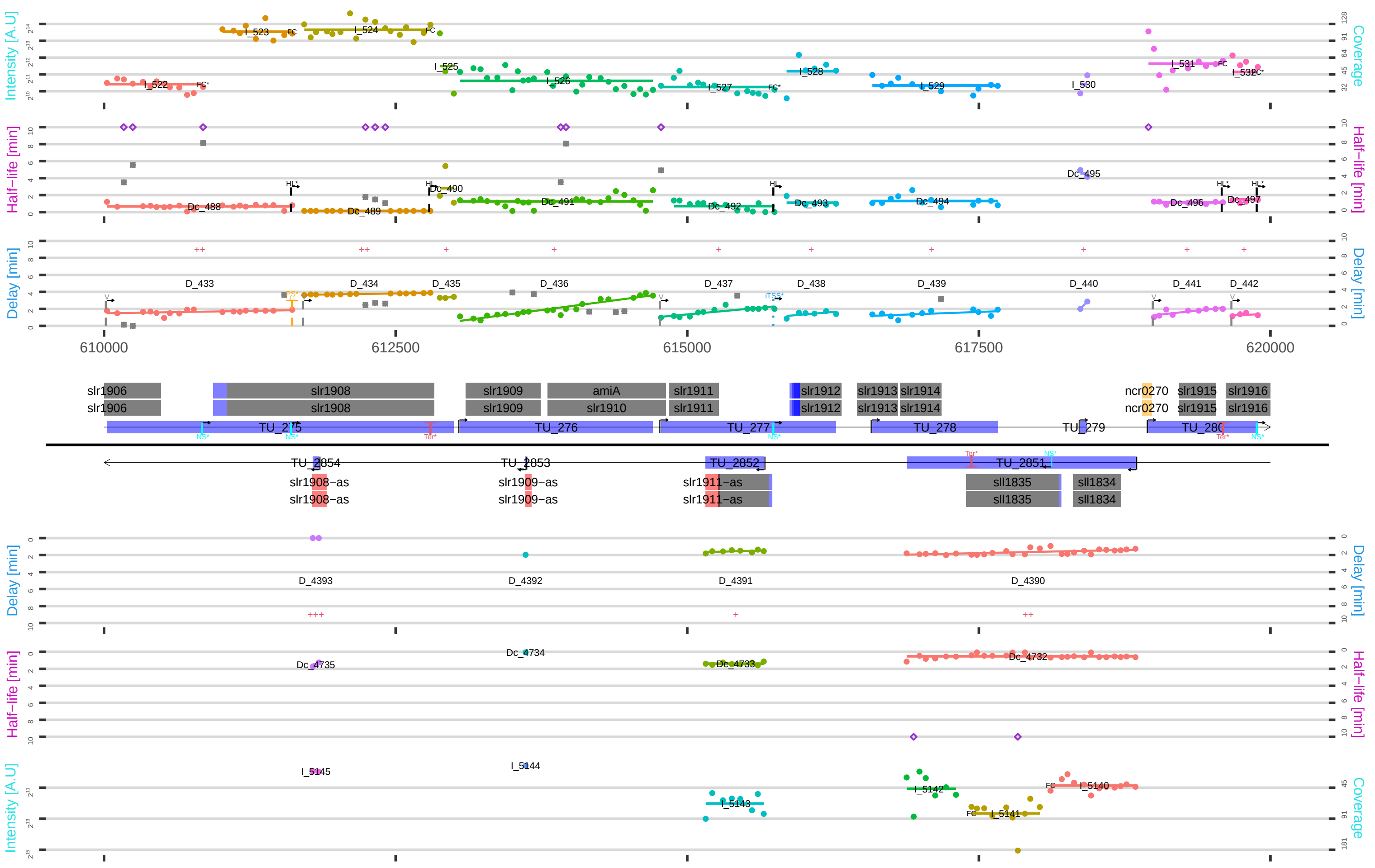

Term: termination (1), NS: new start (1), PS: pausing site (0), iTSS_I: internal starting site (0)

ID: 5164−5236; Term: termination (1), NS: new start (2), PS: pausing site (0), iTSS_I: internal starting site (4)

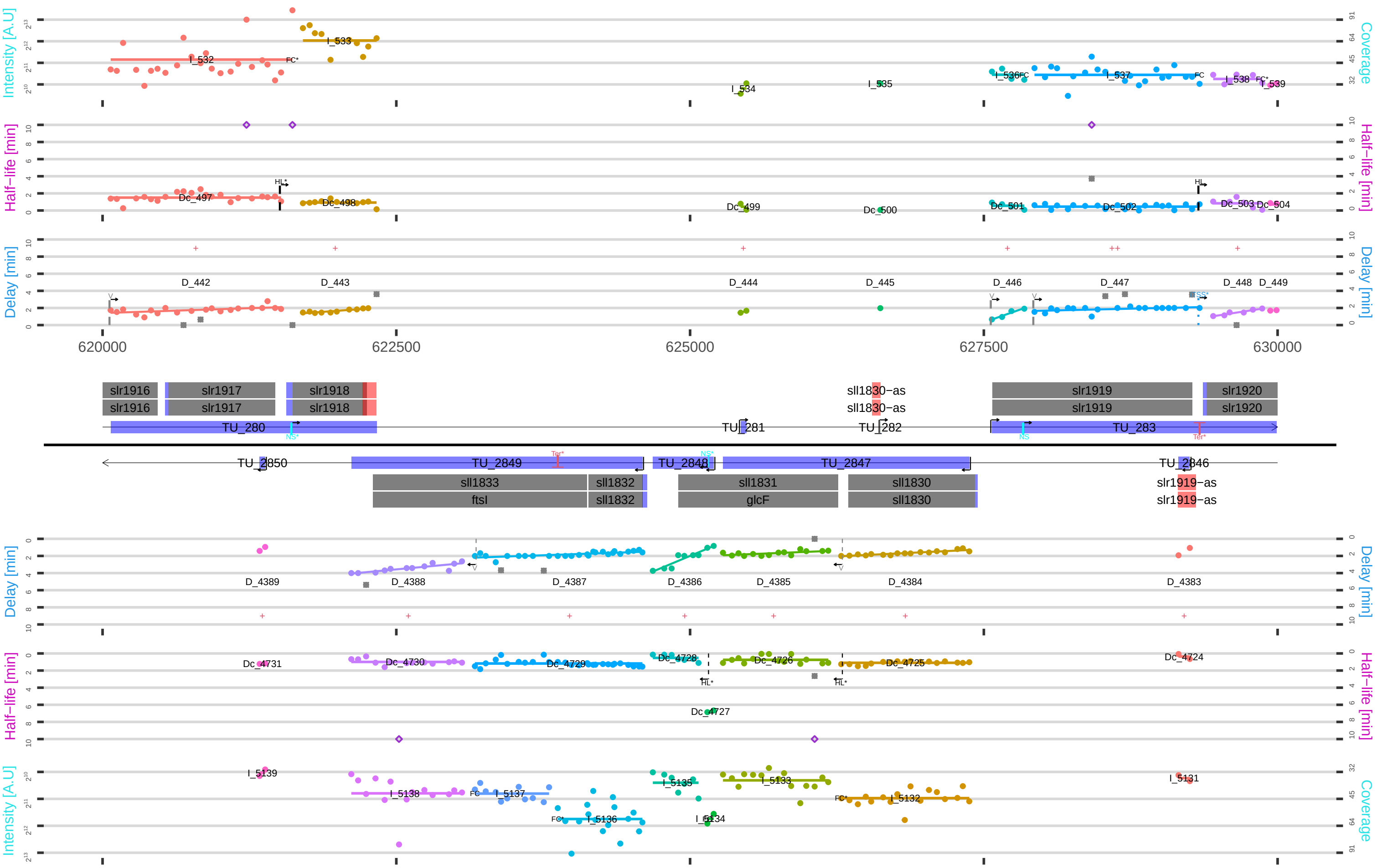

Term: termination (1), NS: new start (1), PS: pausing site (1), iTSS_I: internal starting site (1)

ID: 5237−5302; Term: termination (2), NS: new start (0), PS: pausing site (1), iTSS_I: internal starting site (0)

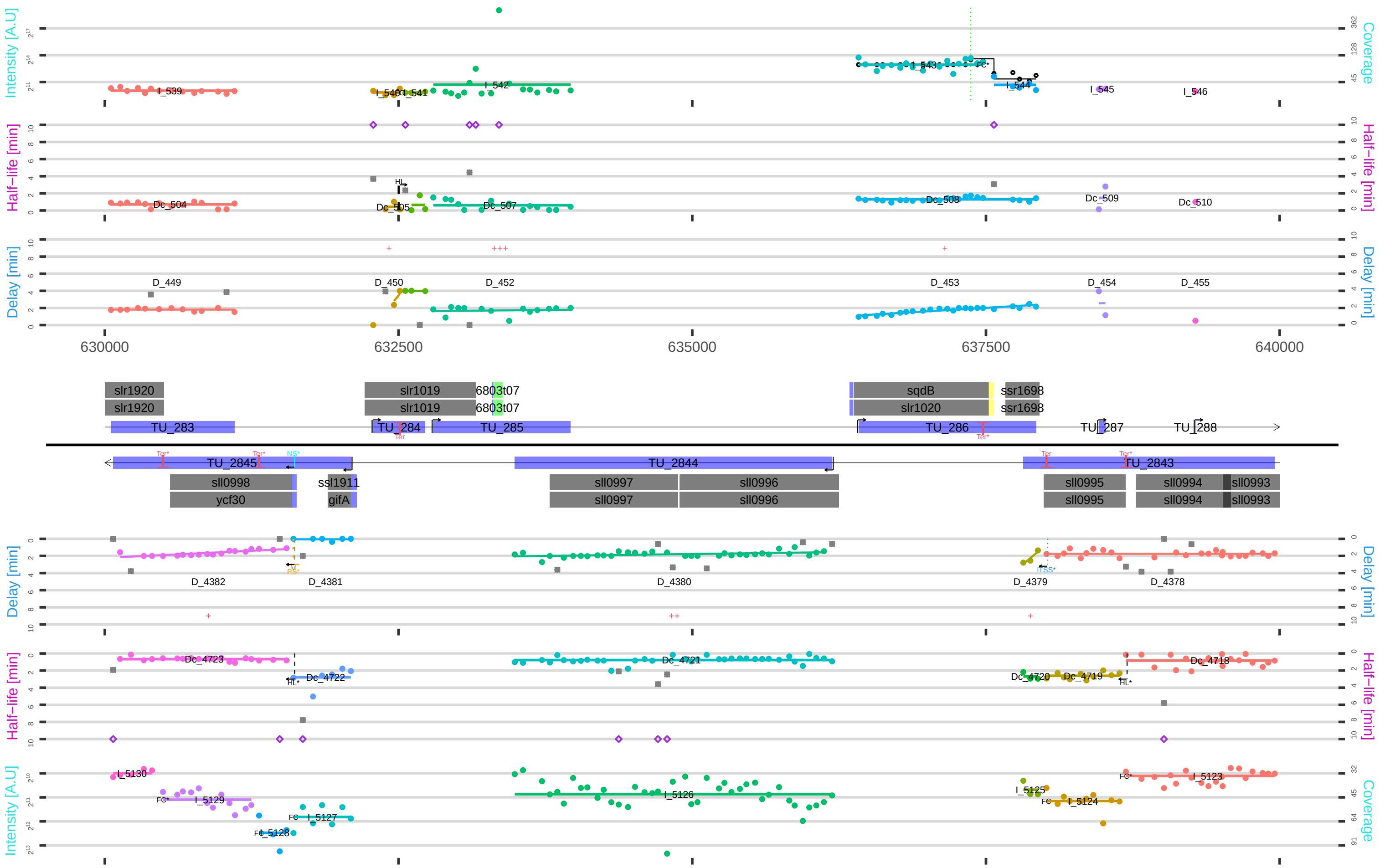

Term: termination (4), NS: new start (1), PS: pausing site (1), iTSS_I: internal starting site (1)

ID: 5303−5412; Term: termination (3), NS: new start (1), PS: pausing site (1), iTSS_I: internal starting site (2)

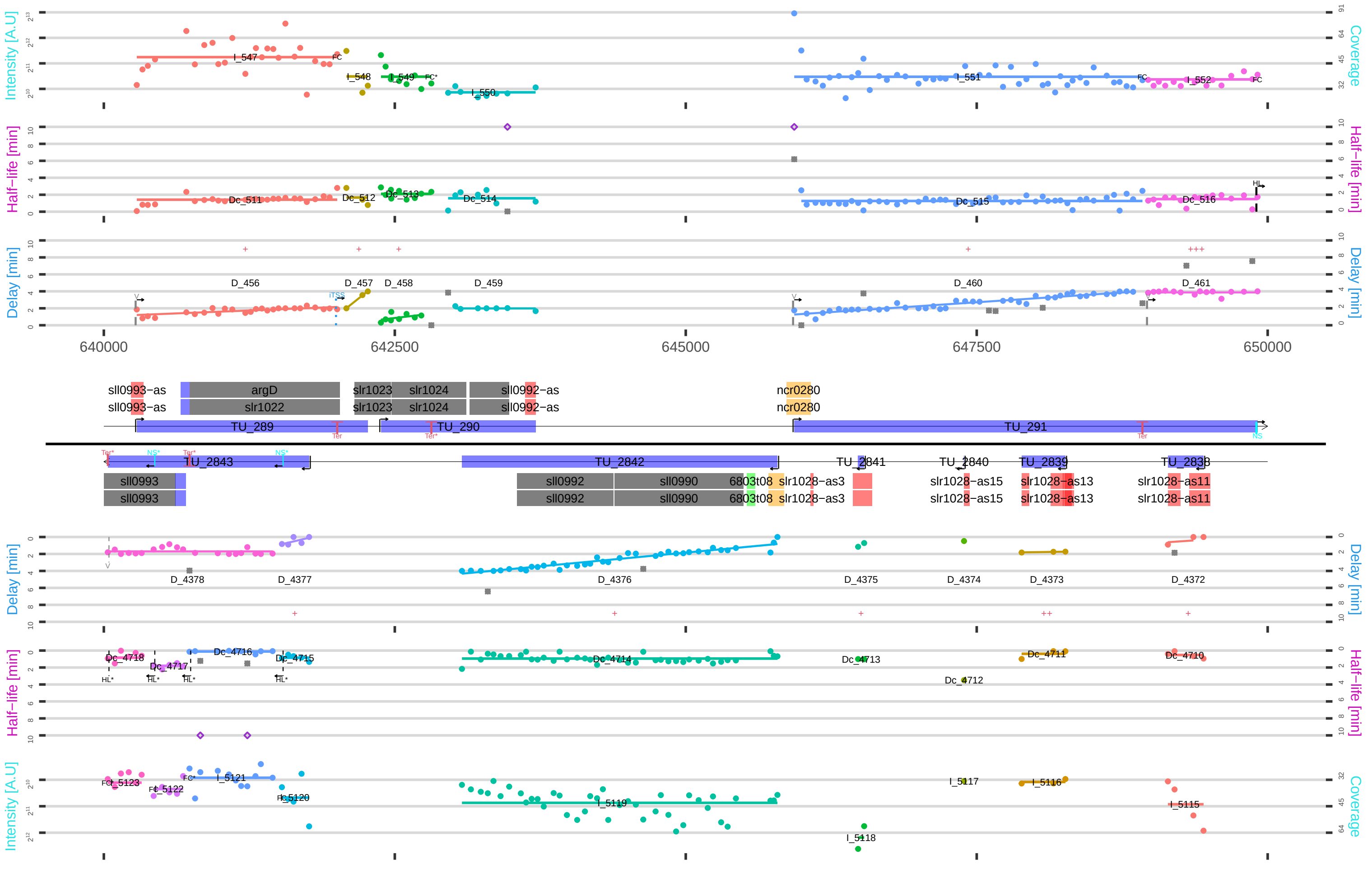

Term: termination (2), NS: new start (2), PS: pausing site (1), iTSS_I: internal starting site (0)

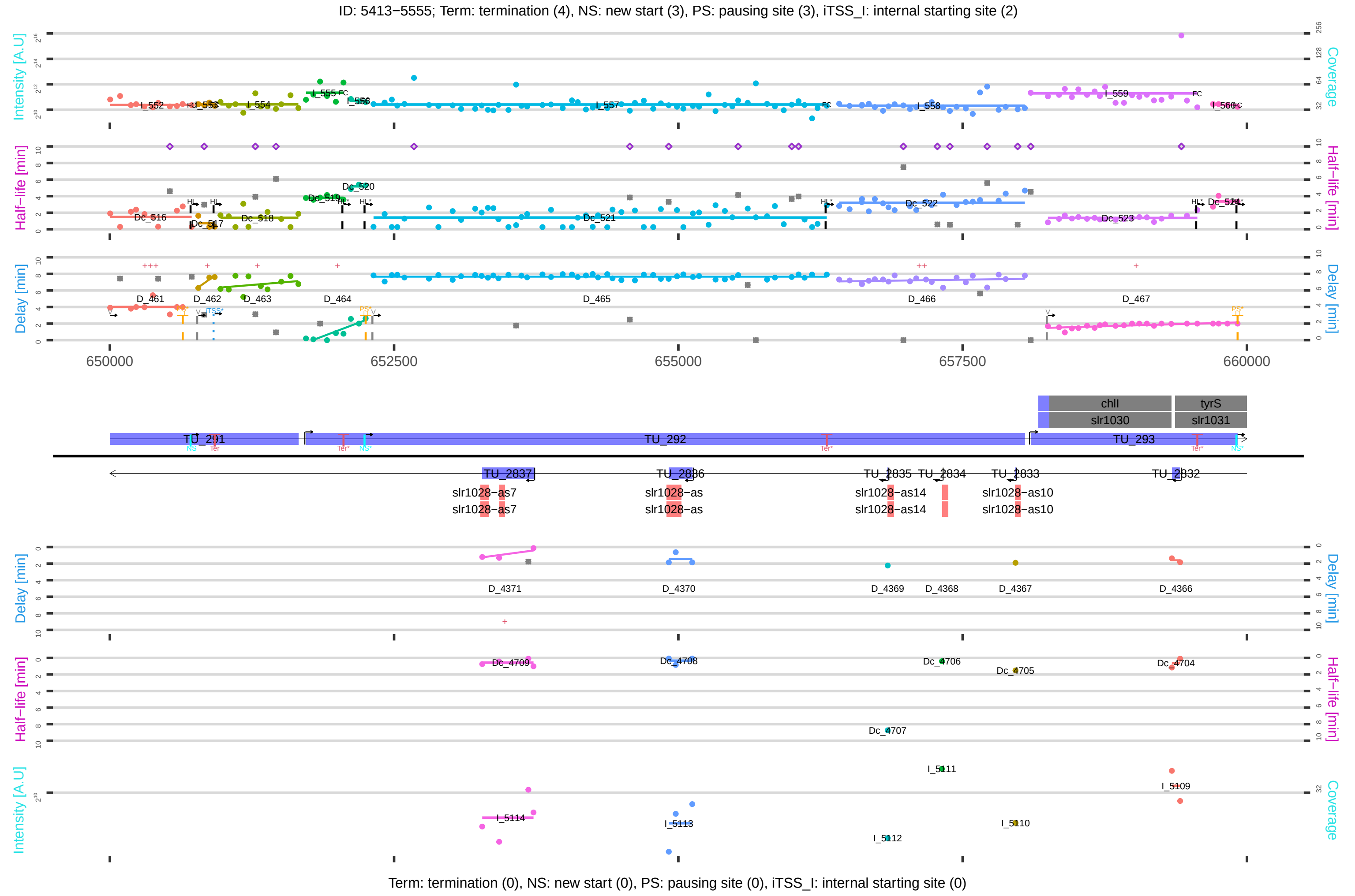
ID: 5413−5555; Term: termination (4), NS: new start (3), PS: pausing site (3), iTSS_I: internal starting site (2)
Intensity [A.U]
Coverage
Half−life [min]
Delay [min]
650000
652500
655000
657500
660000
chlI
slr1030
tyrS
slr1031
TU_291
TU_292
TU_293
TU_2837
TU_2836
TU_2835
TU_2834
TU_2833
TU_2832
slr1028−as7
slr1028−as
slr1028−as14
slr1028−as10
D_4371
D_4370
D_4369
D_4368
D_4367
D_4366
Dc_4709
Dc_4708
Dc_4707
Dc_4706
Dc_4705
Dc_4704
I_5114
I_5113
I_5112
I_5111
I_5110
I_5109
Term: termination (0), NS: new start (0), PS: pausing site (0), iTSS_I: internal starting site (0)

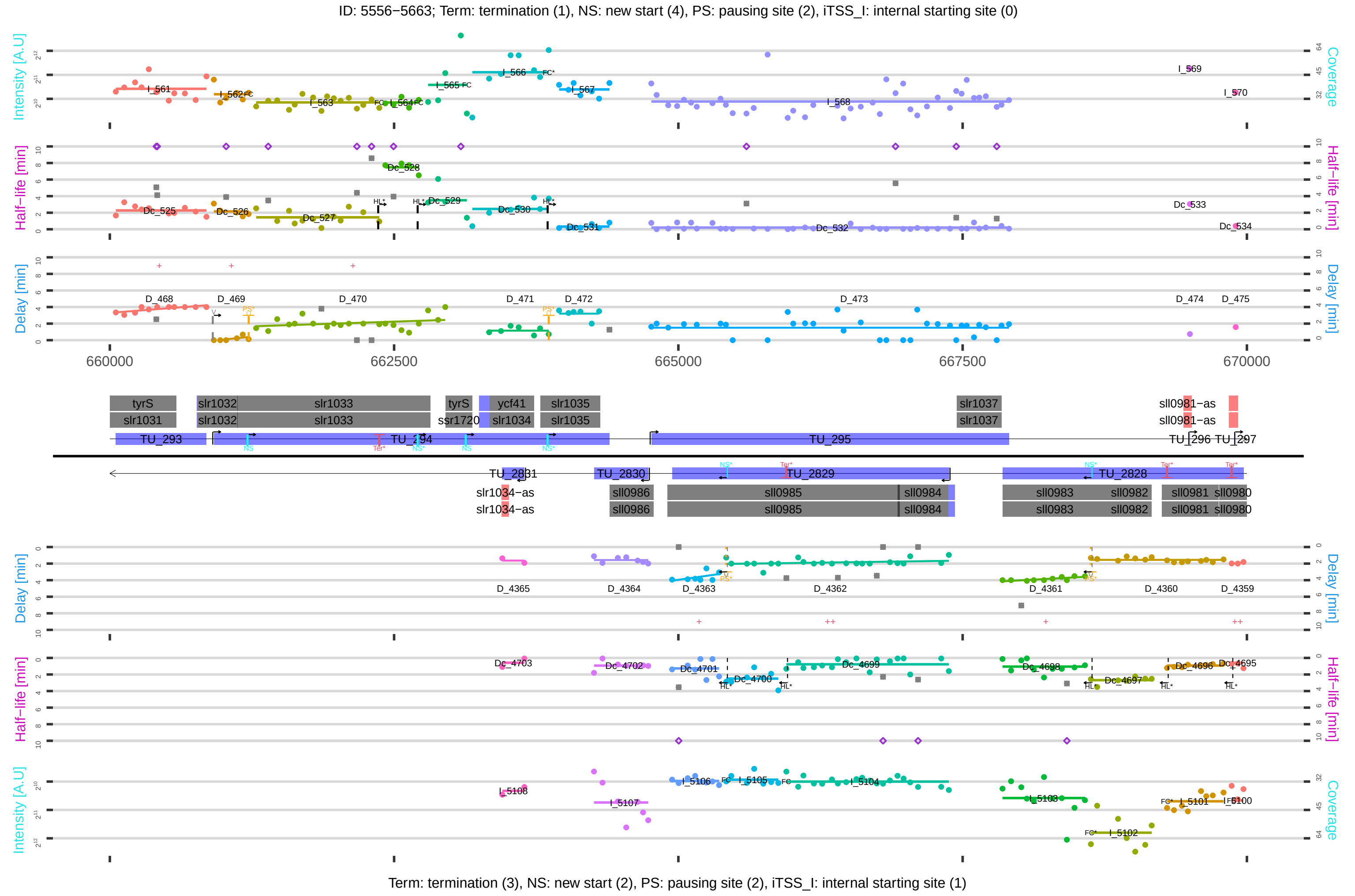
ID: 5556−5663; Term: termination (1), NS: new start (4), PS: pausing site (2), iTSS_I: internal starting site (0)
Intensity [A.U]
Coverage
Half−life [min]
Delay [min]
I_561
I_562
I_563
I_564
I_565
I_566
I_567
I_568
I_569
I_570
Dc_525
Dc_526
Dc_527
Dc_528
Dc_529
Dc_530
Dc_531
Dc_532
Dc_533
Dc_534
D_468
D_469
D_470
D_471
D_472
D_473
D_474
D_475
660000
662500
665000
667500
670000
tyrS
slr1031
slr1032
slr1033
ssr1720
ycf41
slr1034
slr1035
slr1037
sll0981−as
TU_293
TU_294
TU_295
TU_296
TU_297
TU_2831
TU_2830
TU_2829
TU_2828
slr1034−as
sll0986
sll0985
sll0984
sll0983
sll0982
sll0981
sll0980
D_4365
D_4364
D_4363
D_4362
D_4361
D_4360
D_4359
Dc_4703
Dc_4702
Dc_4701
Dc_4700
Dc_4699
Dc_4698
Dc_4697
Dc_4696
Dc_4695
I_5108
I_5107
I_5106
I_5105
I_5104
I_5103
I_5102
I_5101
I_5100
Term: termination (3), NS: new start (2), PS: pausing site (2), iTSS_I: internal starting site (1)

ID: 5664−5760; Term: termination (3), NS: new start (2), PS: pausing site (2), iTSS_I: internal starting site (0)

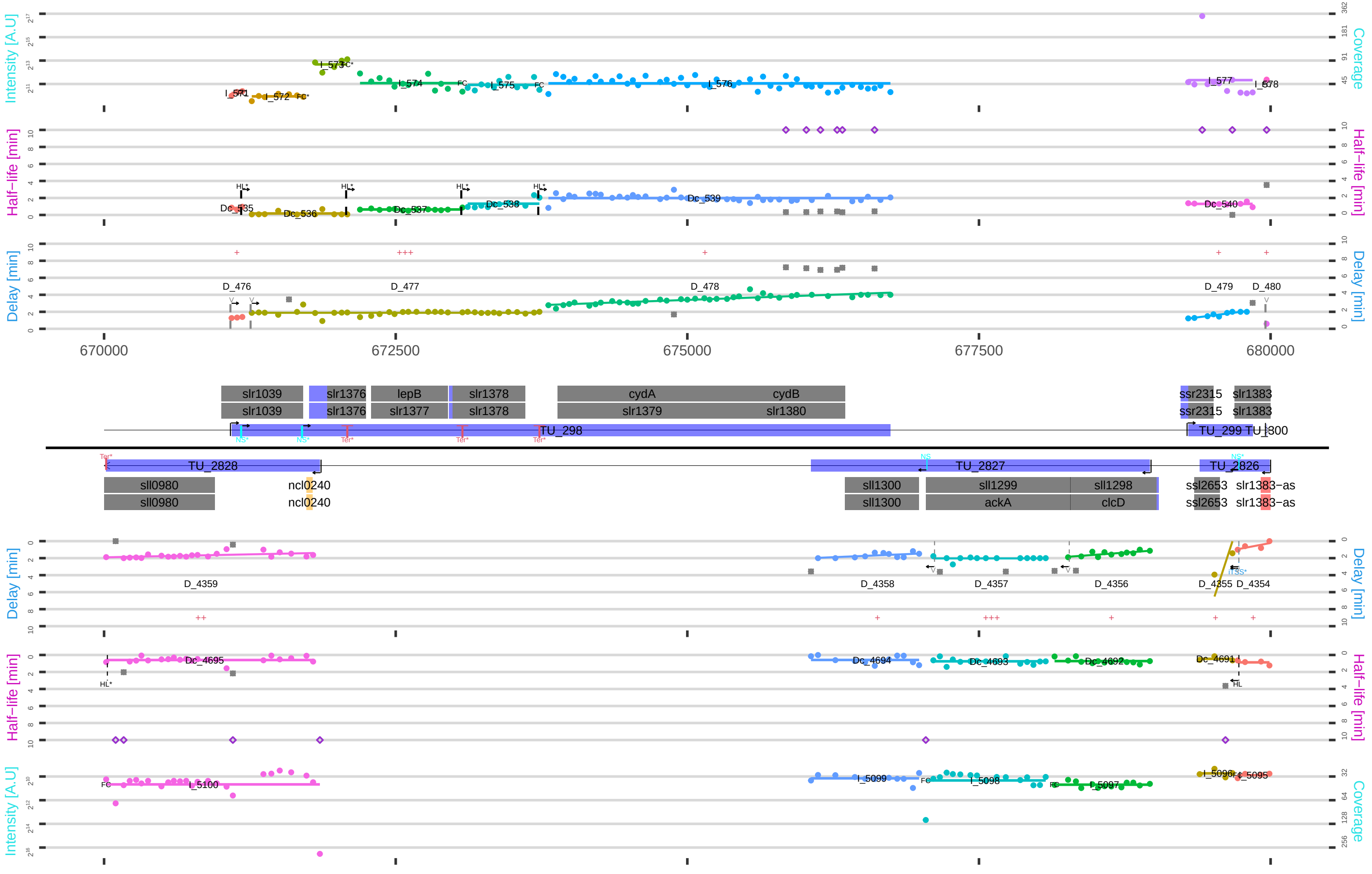

Term: termination (1), NS: new start (2), PS: pausing site (1), iTSS_I: internal starting site (2)

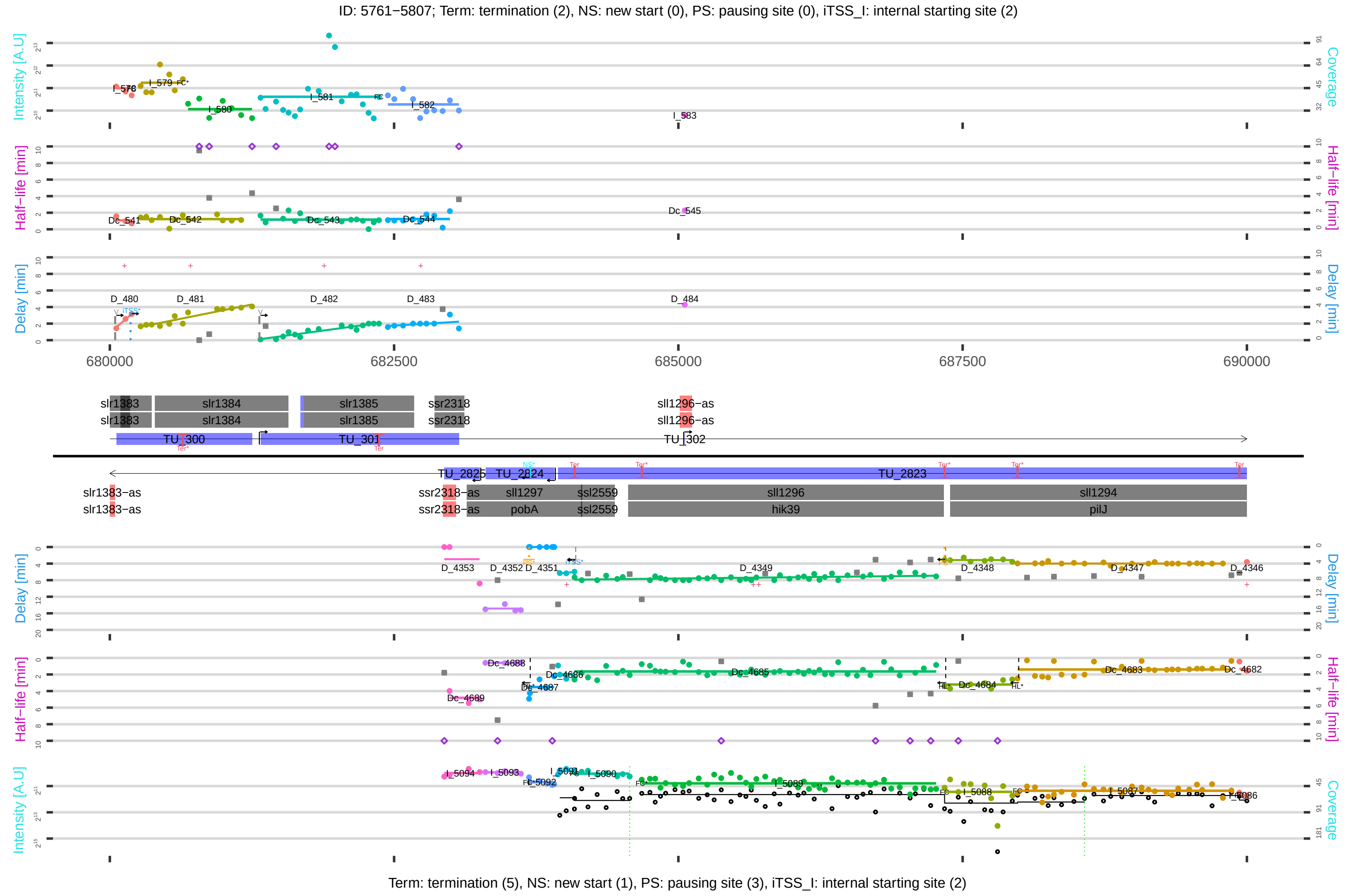

ID: 5761−5807; Term: termination (2), NS: new start (0), PS: pausing site (0), iTSS_I: internal starting site (2)
Intensity [A.U]
Coverage
Half−life [min]
Delay [min]
680000
682500
685000
687500
690000
slr1383
slr1384
slr1385
ssr2318
sll1296−as
TU_300
TU_301
TU_302
TU_2825
TU_2824
TU_2823
slr1383−as
ssr2318−as
sll1297
pobA
ssl2559
sll1296
hik39
sll1294
pilJ
Term: termination (5), NS: new start (1), PS: pausing site (3), iTSS_I: internal starting site (2)

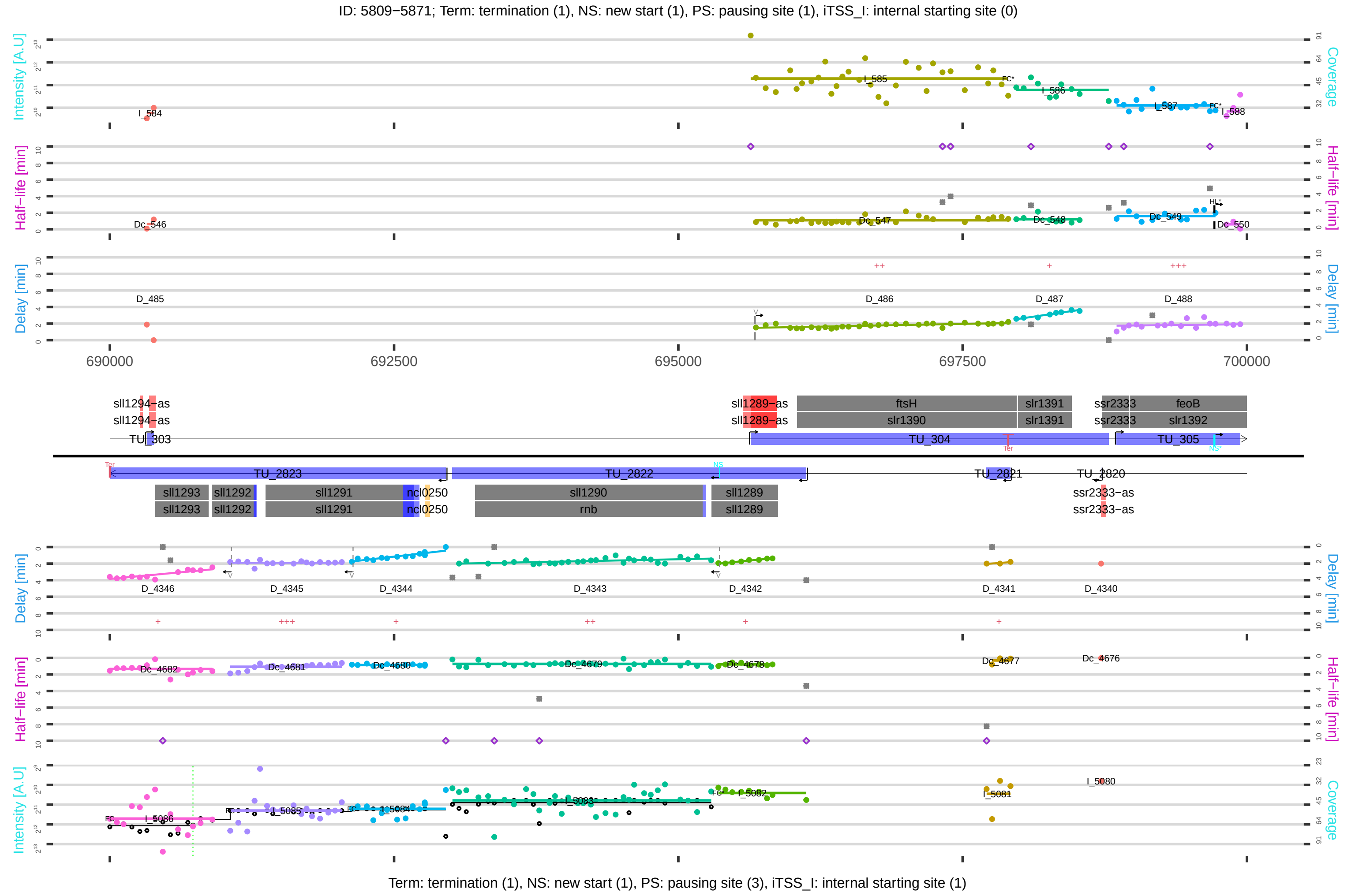
ID: 5809−5871; Term: termination (1), NS: new start (1), PS: pausing site (1), iTSS_I: internal starting site (0)
Intensity [A.U]
Coverage
Half−life [min]
Delay [min]
I_584
I_585
I_586
I_587
I_588
Dc_546
Dc_547
Dc_548
Dc_549
Dc_550
D_485
D_486
D_487
D_488
690000
692500
695000
697500
700000
sll1294−as
sll1289−as
ftsH
slr1390
slr1391
ssr2333
feoB
slr1392
TU_303
TU_304
TU_305
TU_2823
TU_2822
TU_2821
TU_2820
sll1293
sll1292
sll1291
ncl0250
sll1290
rnb
sll1289
ssr2333−as
D_4346
D_4345
D_4344
D_4343
D_4342
D_4341
D_4340
Dc_4682
Dc_4681
Dc_4680
Dc_4679
Dc_4678
Dc_4677
Dc_4676
I_5086
I_5085
I_5084
I_5083
I_5082
I_5081
I_5080
Term: termination (1), NS: new start (1), PS: pausing site (3), iTSS_I: internal starting site (1)

ID: 5872−6007; Term: termination (2), NS: new start (3), PS: pausing site (2), iTSS_I: internal starting site (2)

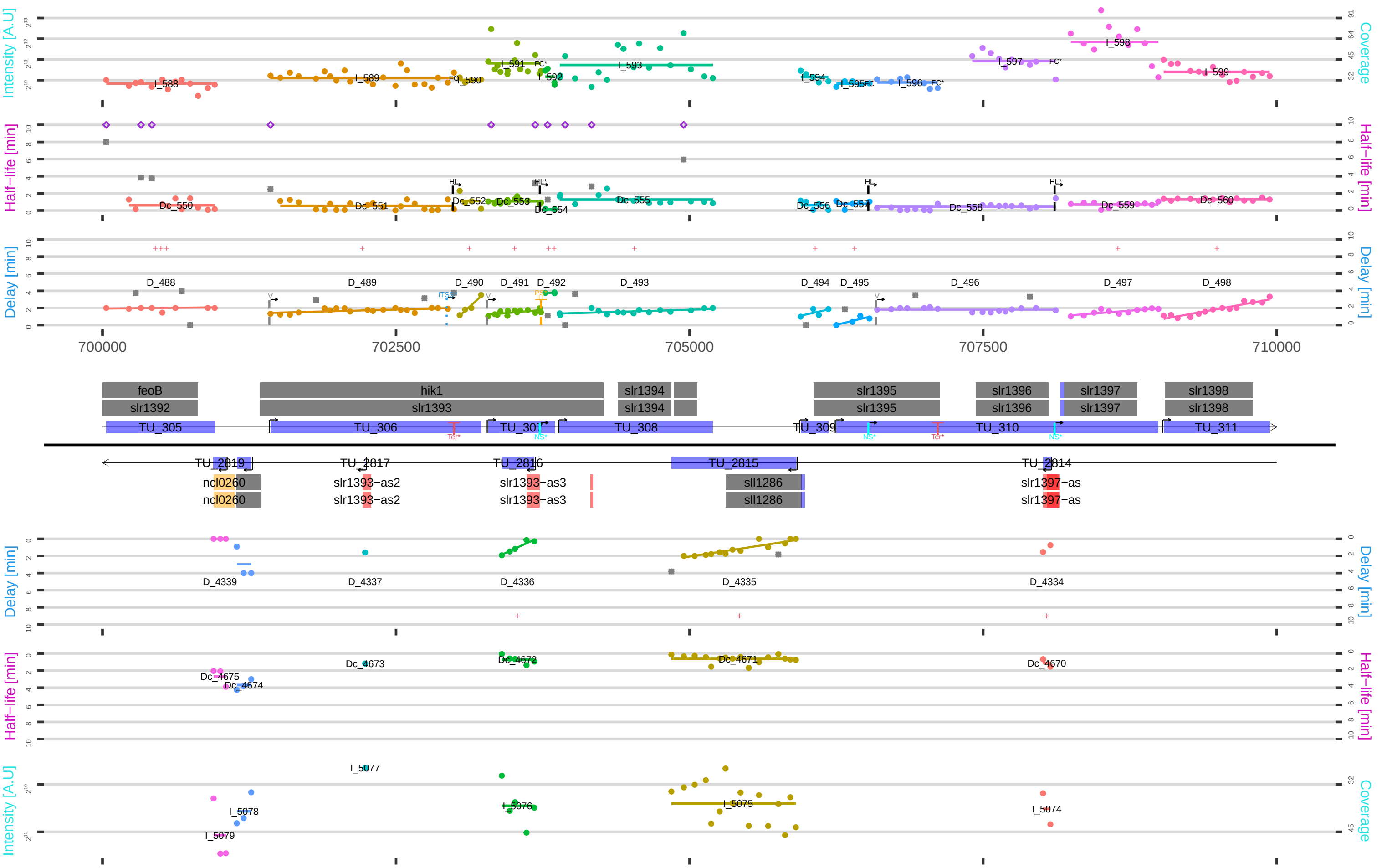

Term: termination (0), NS: new start (0), PS: pausing site (0), iTSS_I: internal starting site (0)

ID: 6009−6021; Term: termination (0), NS: new start (0), PS: pausing site (0), iTSS_I: internal starting site (0)

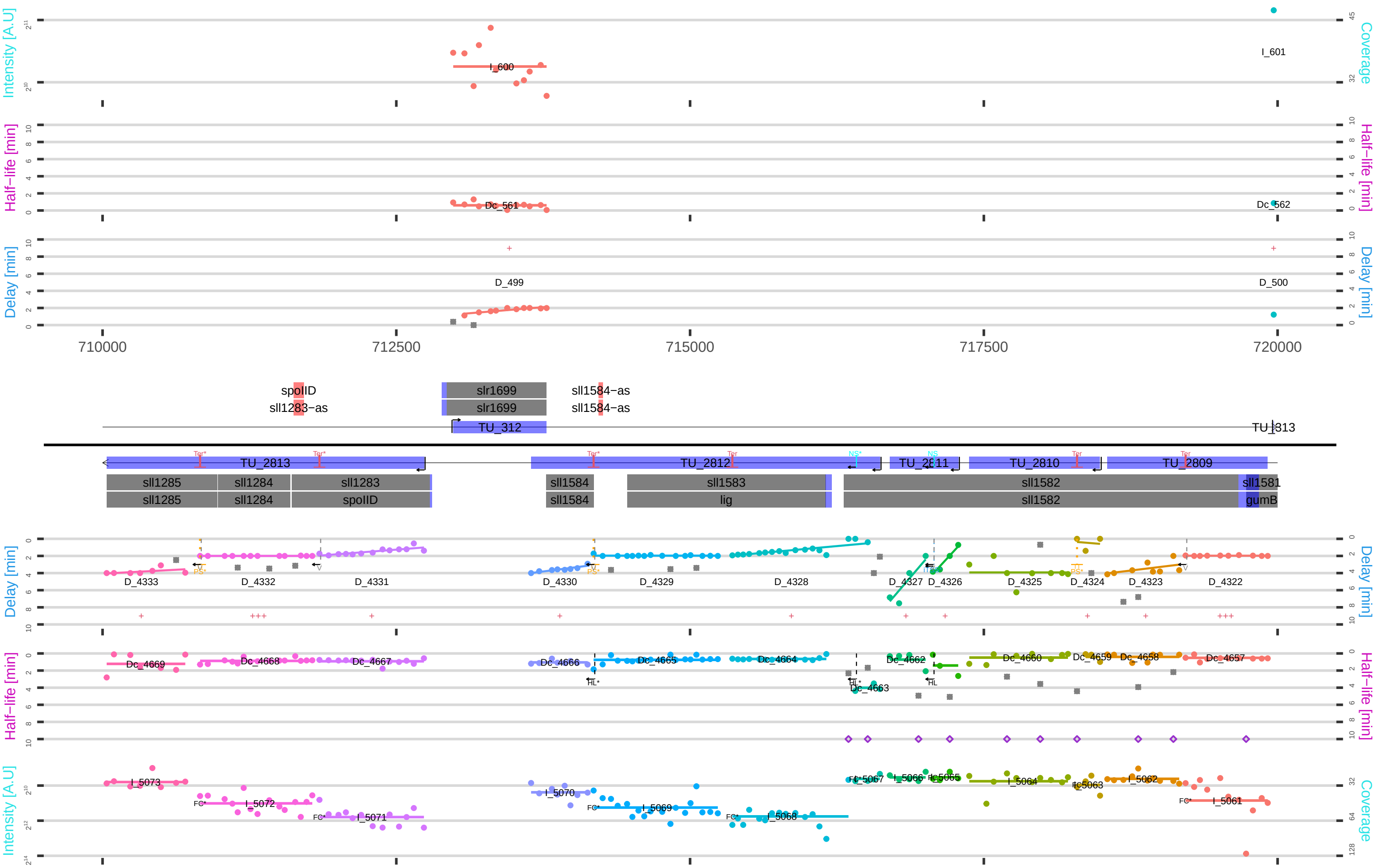

Term: termination (6), NS: new start (2), PS: pausing site (5), iTSS_I: internal starting site (2)

ID: 6022−6111; Term: termination (4), NS: new start (1), PS: pausing site (1), iTSS_I: internal starting site (3)

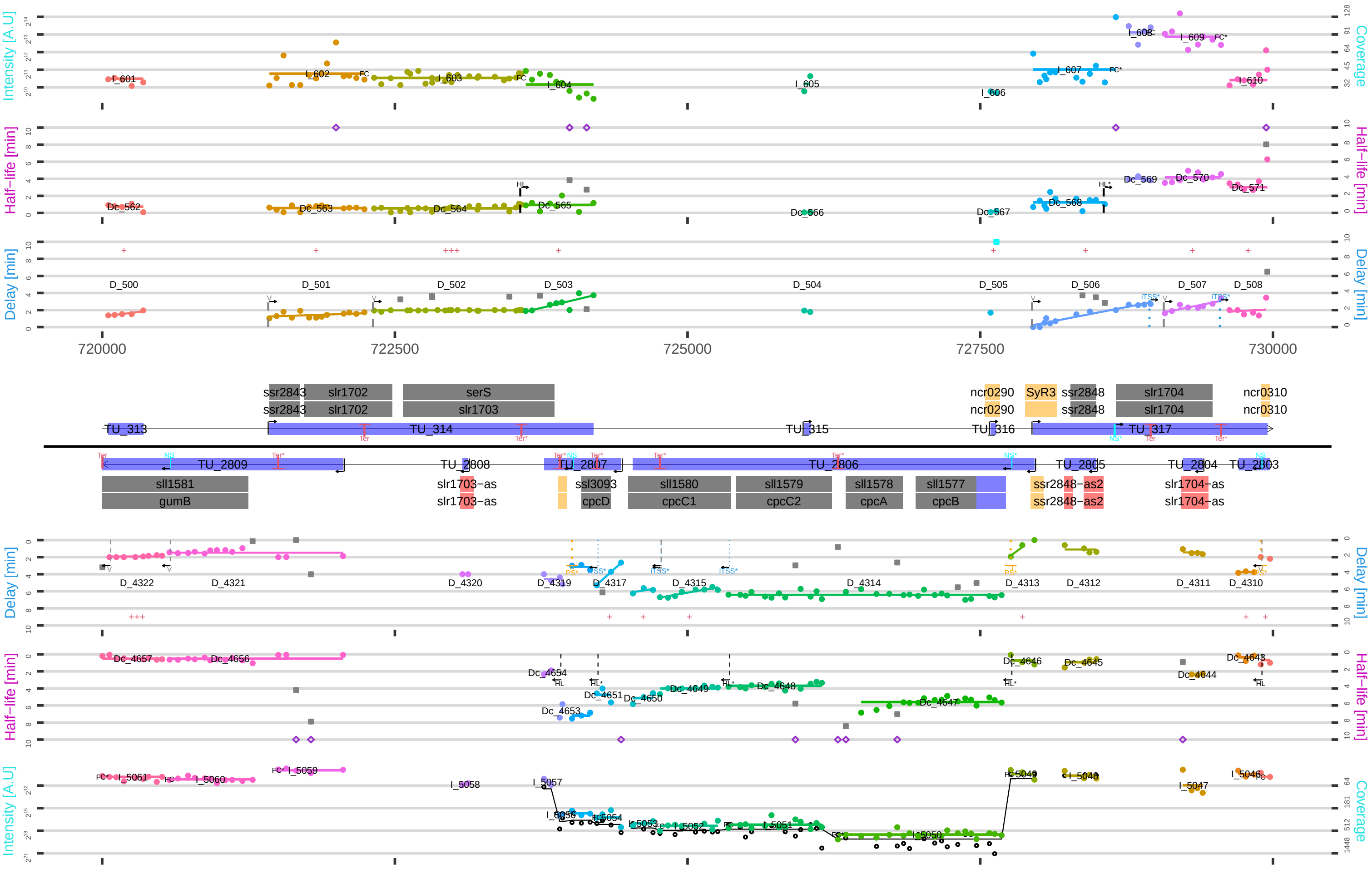

Term: termination (6), NS: new start (4), PS: pausing site (4), iTSS_I: internal starting site (3)

ID: 6112−6189; Term: termination (1), NS: new start (1), PS: pausing site (0), iTSS_I: internal starting site (0)

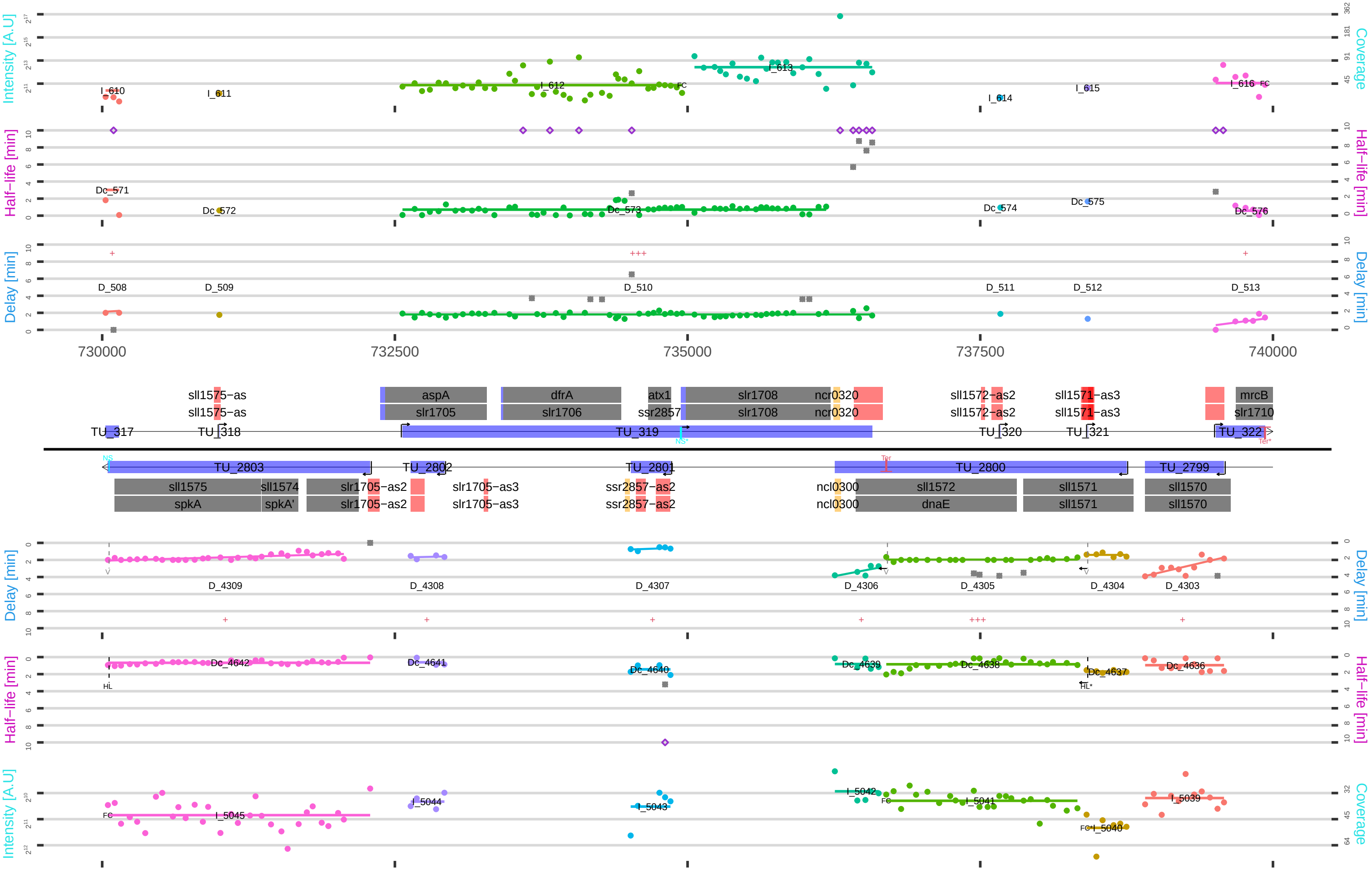

Term: termination (1), NS: new start (1), PS: pausing site (2), iTSS_I: internal starting site (0)

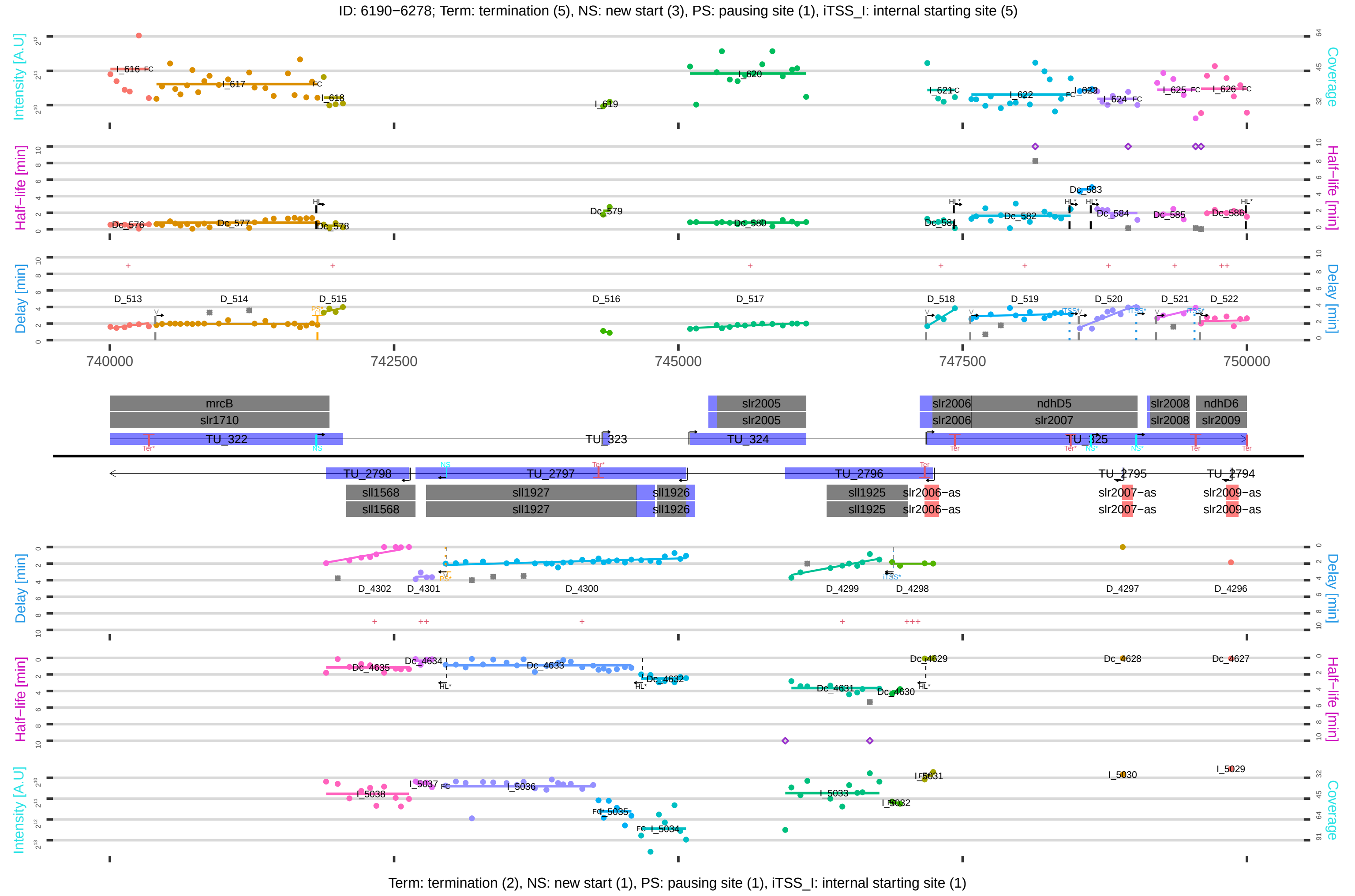

ID: 6190−6278; Term: termination (5), NS: new start (3), PS: pausing site (1), iTSS_I: internal starting site (5)
Intensity [A.U]
Coverage
Half−life [min]
Delay [min]
740000
742500
745000
747500
750000
mrcB
slr1710
TU_322
TU_323
TU_324
slr2005
slr2006
ndhD5
slr2007
slr2008
ndhD6
slr2009
TU_325
TU_2798
TU_2797
TU_2796
TU_2795
TU_2794
sll1568
sll1927
sll1926
sll1925
slr2006−as
slr2007−as
slr2009−as
Term: termination (2), NS: new start (1), PS: pausing site (1), iTSS_I: internal starting site (1)

ID: 6278−6404; Term: termination (6), NS: new start (4), PS: pausing site (4), iTSS_I: internal starting site (4)

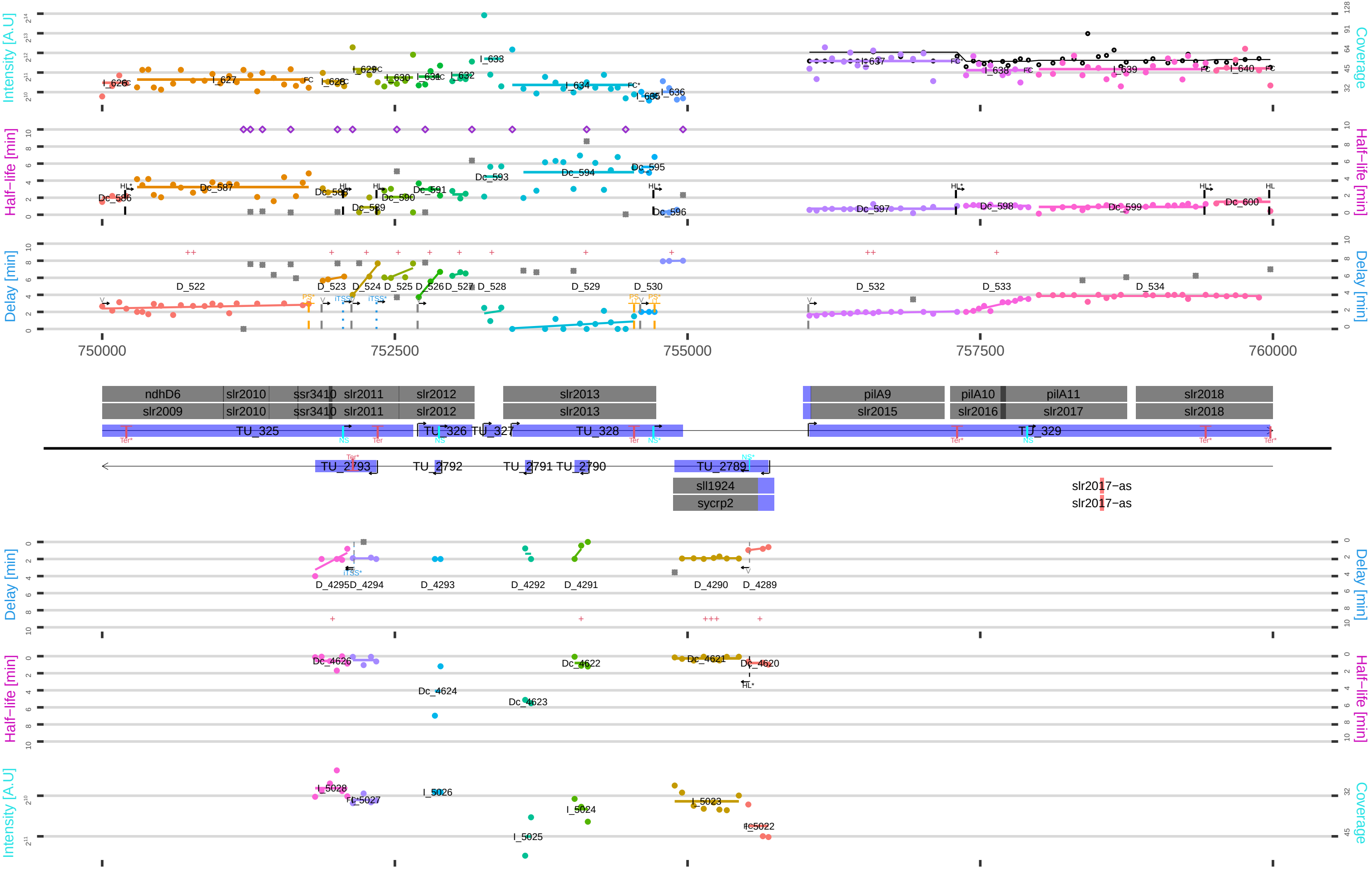

Term: termination (1), NS: new start (1), PS: pausing site (1), iTSS_I: internal starting site (1)

ID: 6405−6490; Term: termination (2), NS: new start (5), PS: pausing site (2), iTSS_I: internal starting site (5)

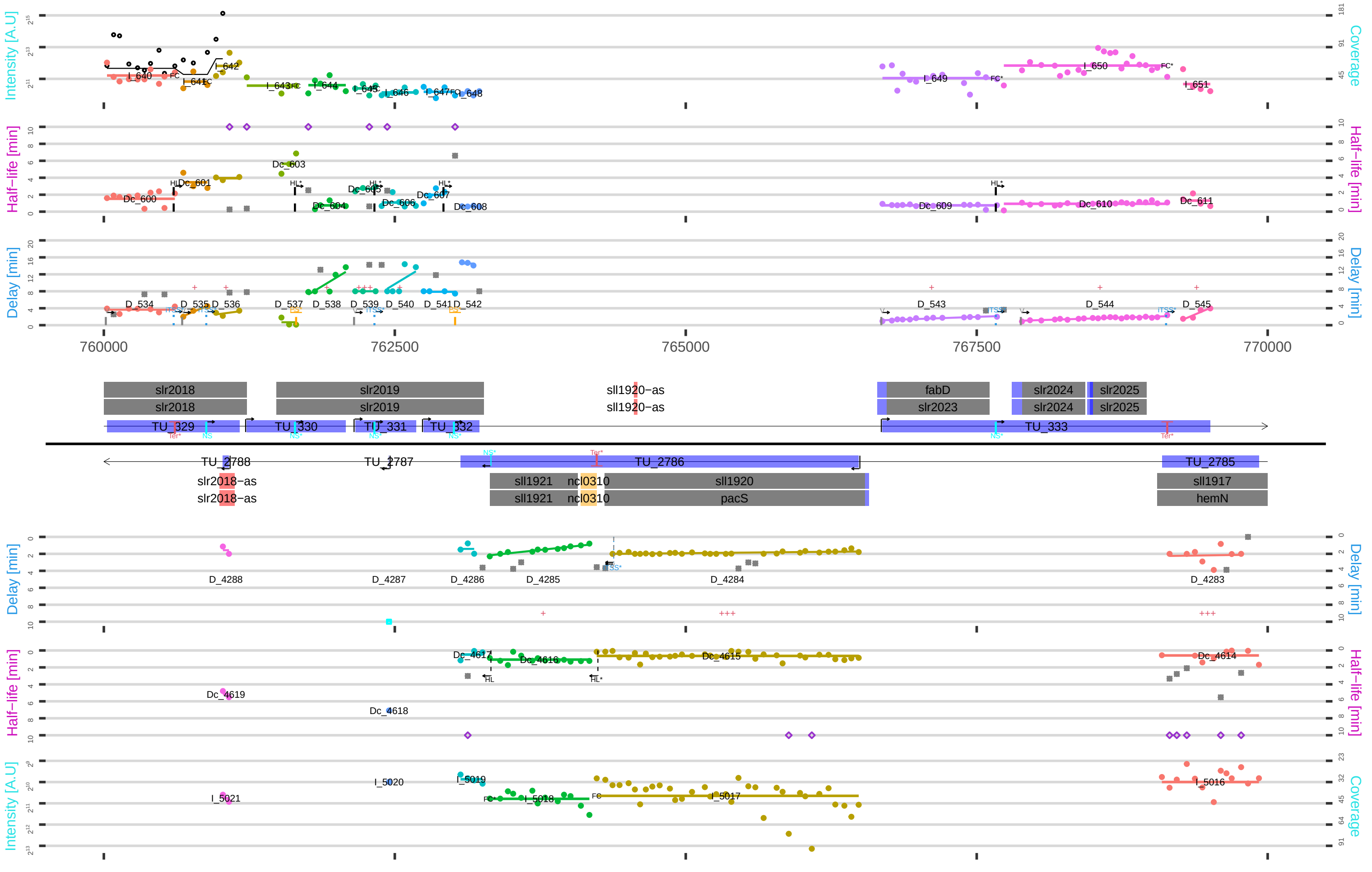

Term: termination (1), NS: new start (1), PS: pausing site (0), iTSS_I: internal starting site (2)

ID: 6491−6540; Term: termination (0), NS: new start (1), PS: pausing site (1), iTSS_I: internal starting site (0)

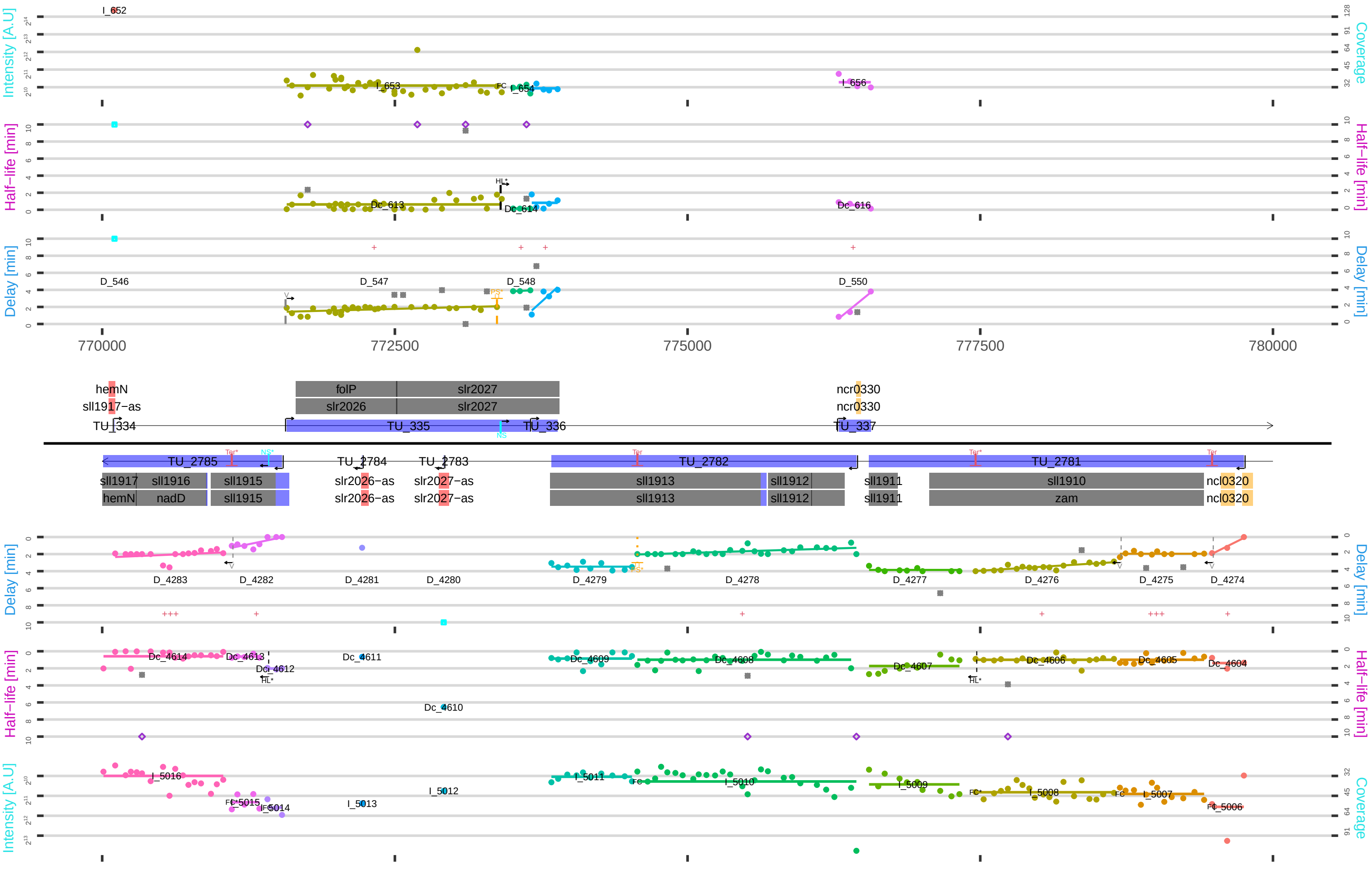

Term: termination (4), NS: new start (1), PS: pausing site (4), iTSS_I: internal starting site (1)

ID: 6541−6645; Term: termination (1), NS: new start (1), PS: pausing site (0), iTSS_I: internal starting site (1)

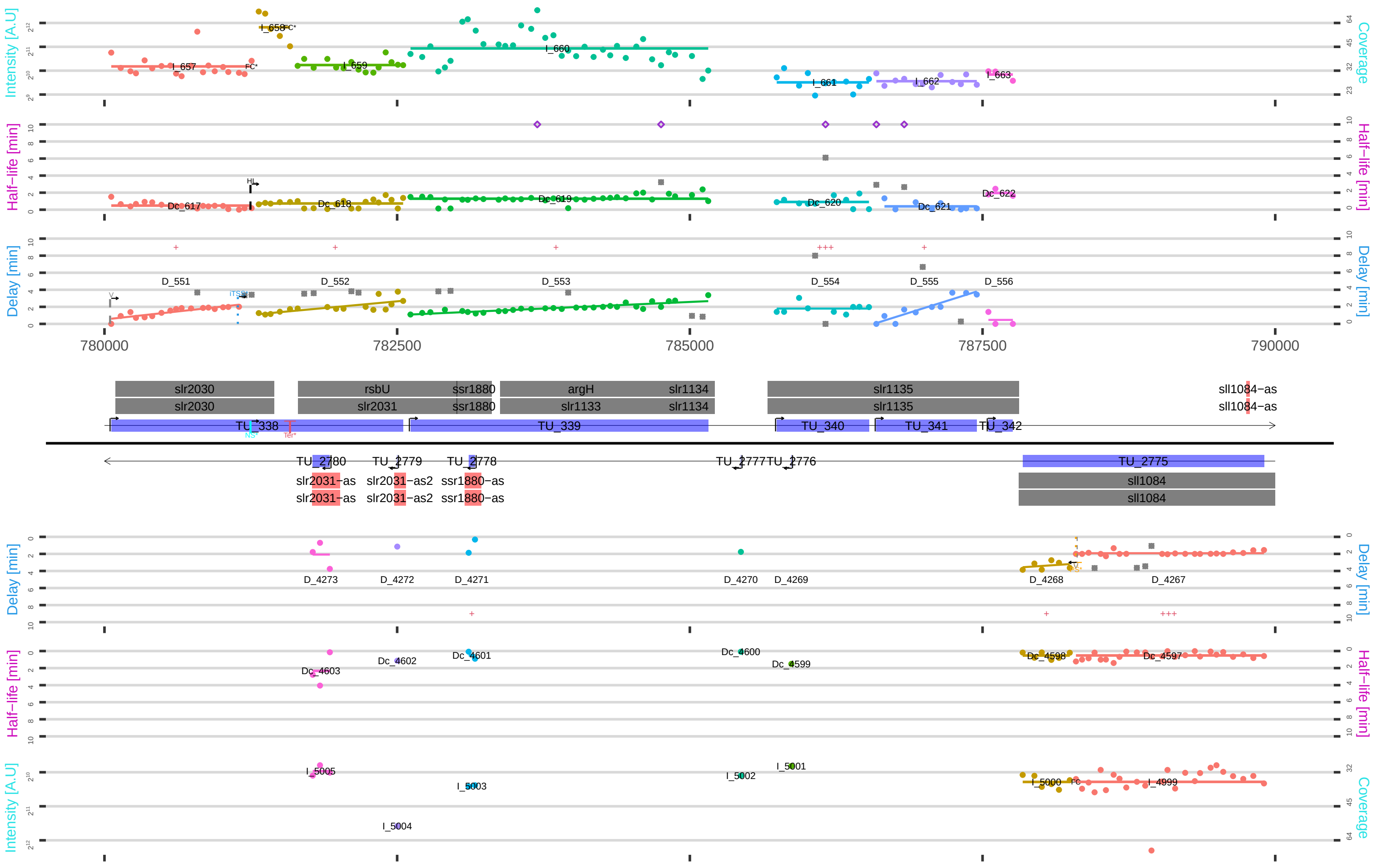

Term: termination (0), NS: new start (0), PS: pausing site (1), iTSS_I: internal starting site (0)

ID: 6646−6726; Term: termination (2), NS: new start (0), PS: pausing site (1), iTSS_I: internal starting site (0)

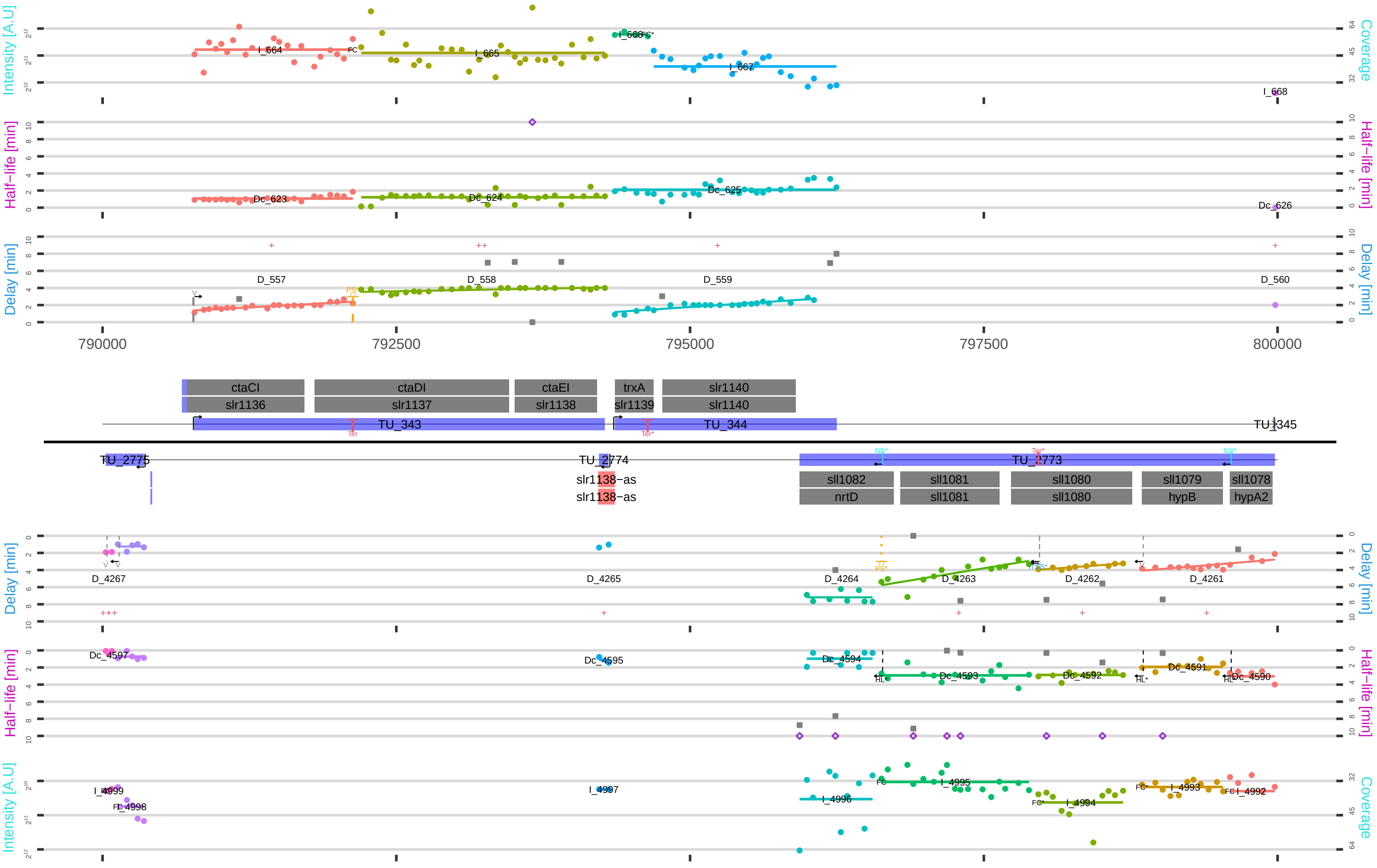

Term: termination (1), NS: new start (2), PS: pausing site (2), iTSS_I: internal starting site (2)

ID: 6727−6765; Term: termination (2), NS: new start (0), PS: pausing site (1), iTSS_I: internal starting site (0)

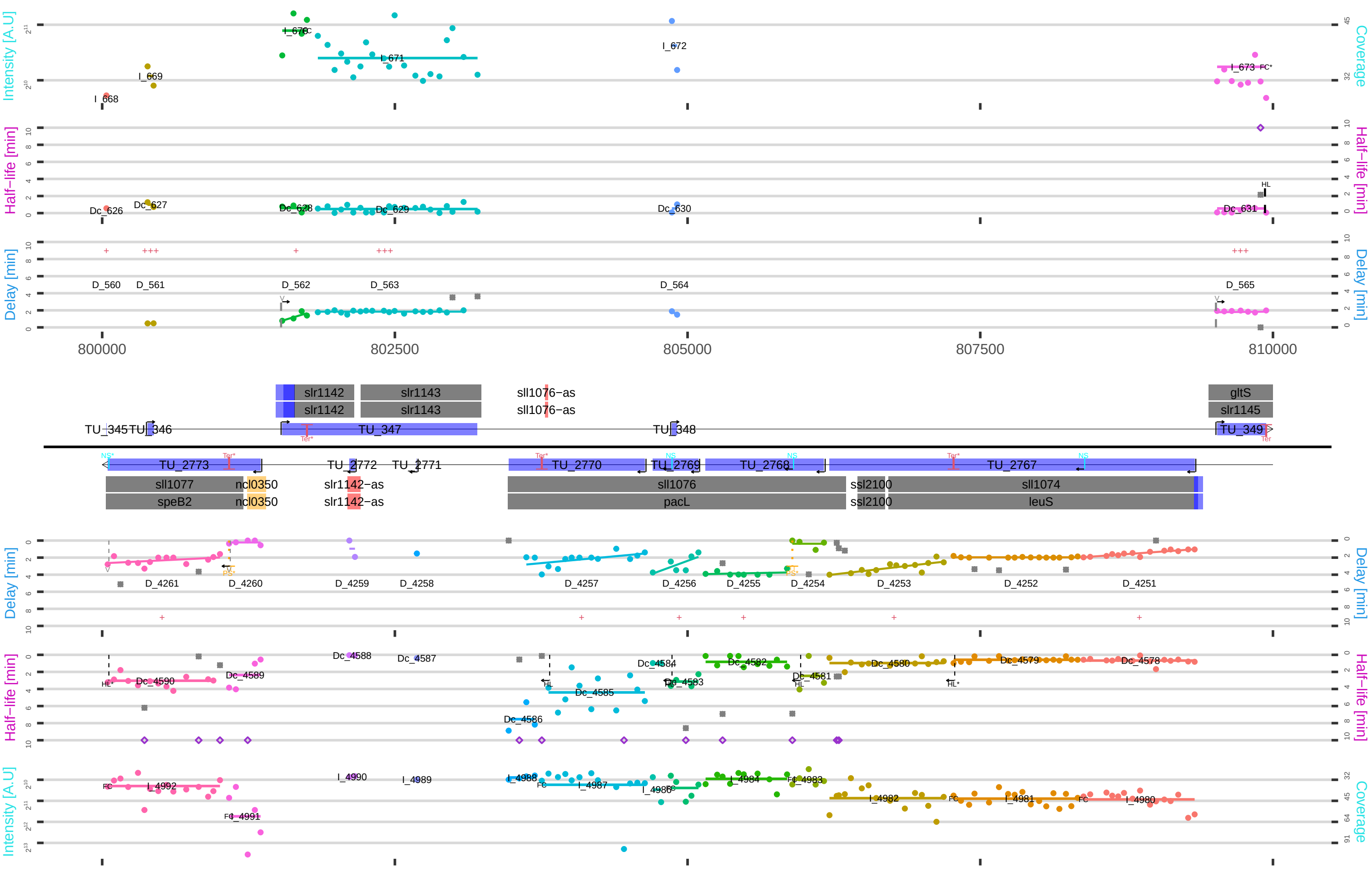

Term: termination (3), NS: new start (4), PS: pausing site (4), iTSS_I: internal starting site (0)

ID: 6766−6864; Term: termination (1), NS: new start (2), PS: pausing site (0), iTSS_I: internal starting site (4)

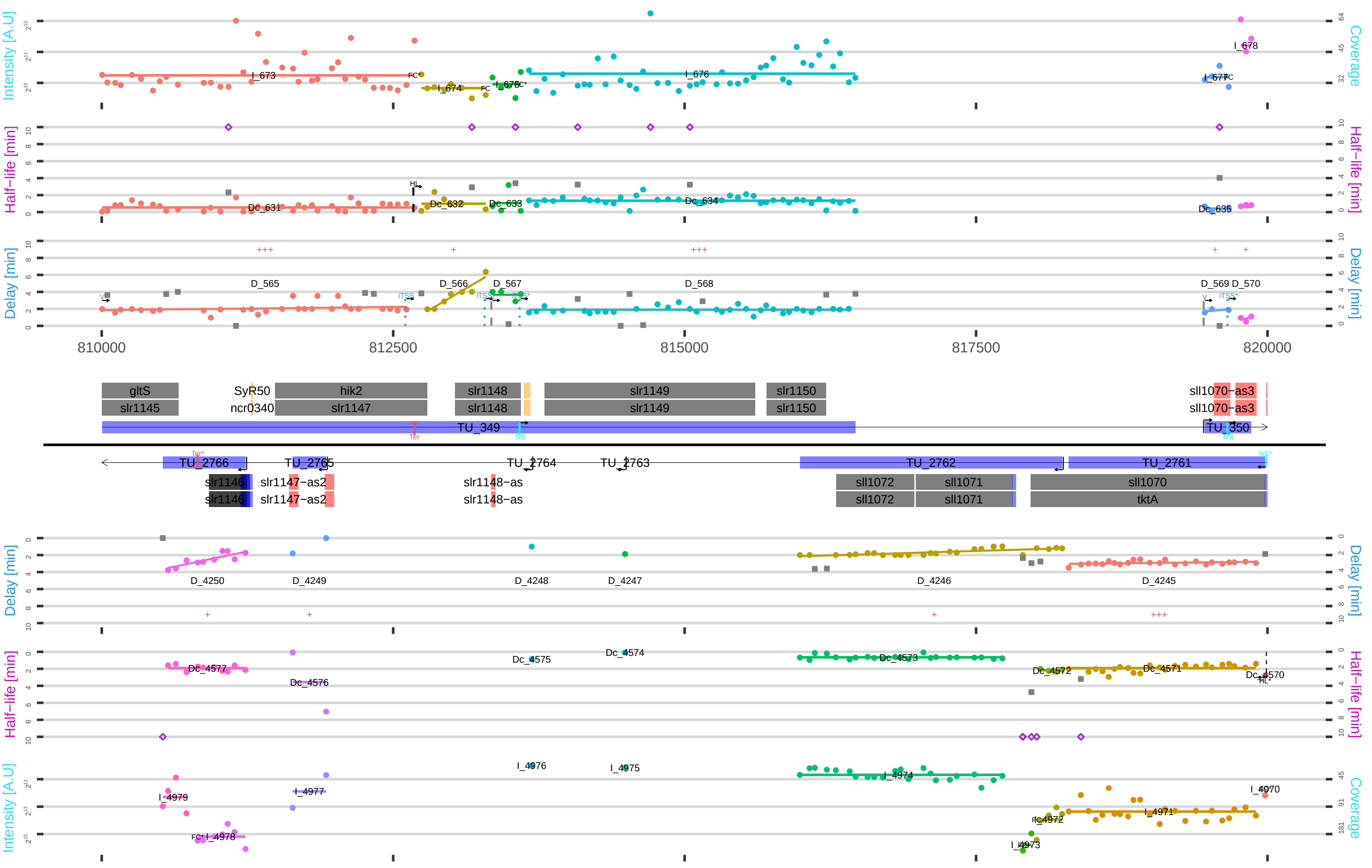

Term: termination (1), NS: new start (1), PS: pausing site (0), iTSS_I: internal starting site (0)

ID: 6866−6890; Term: termination (0), NS: new start (0), PS: pausing site (0), iTSS_I: internal starting site (0)

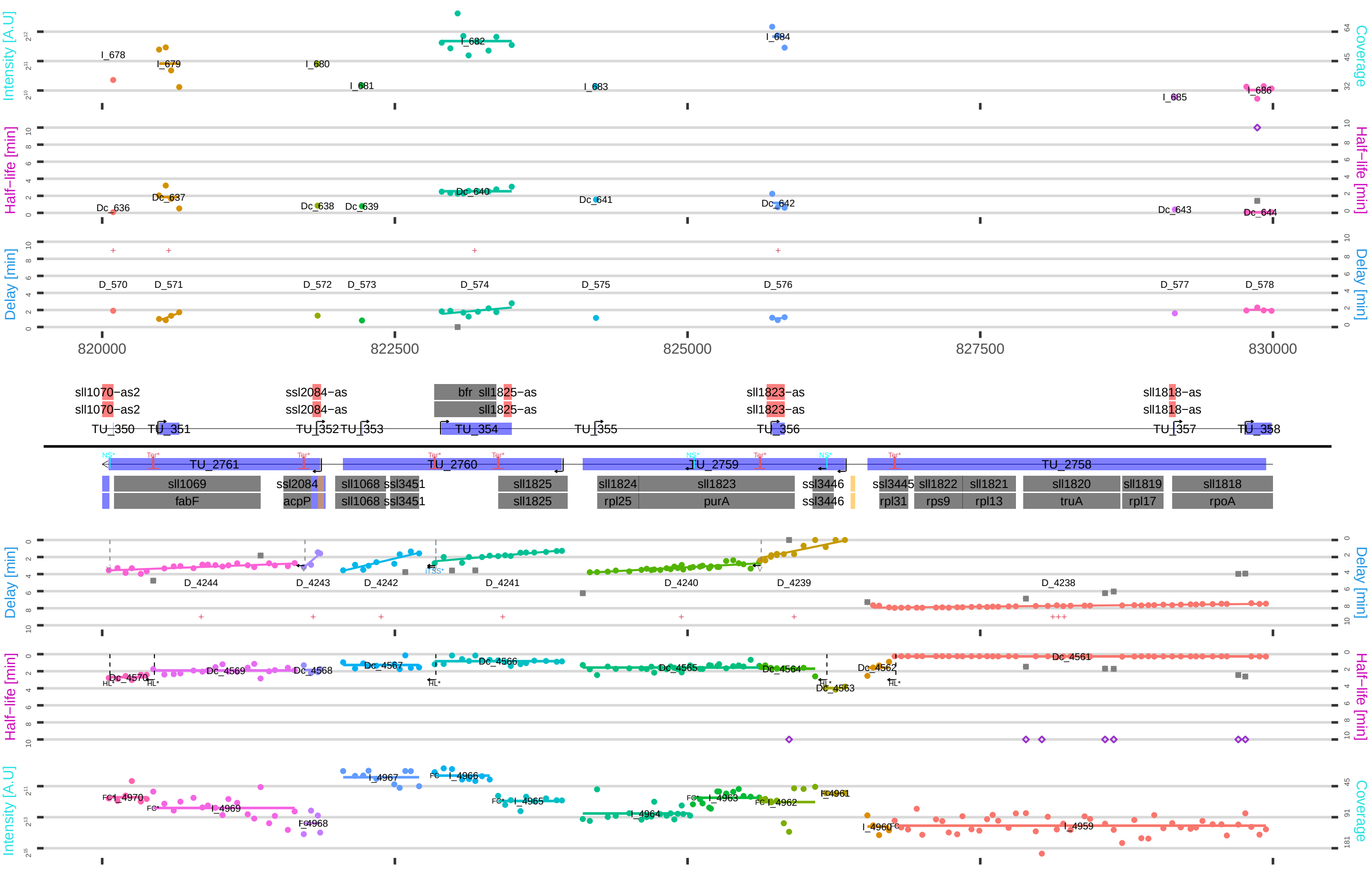

Term: termination (6), NS: new start (3), PS: pausing site (1), iTSS_I: internal starting site (3)

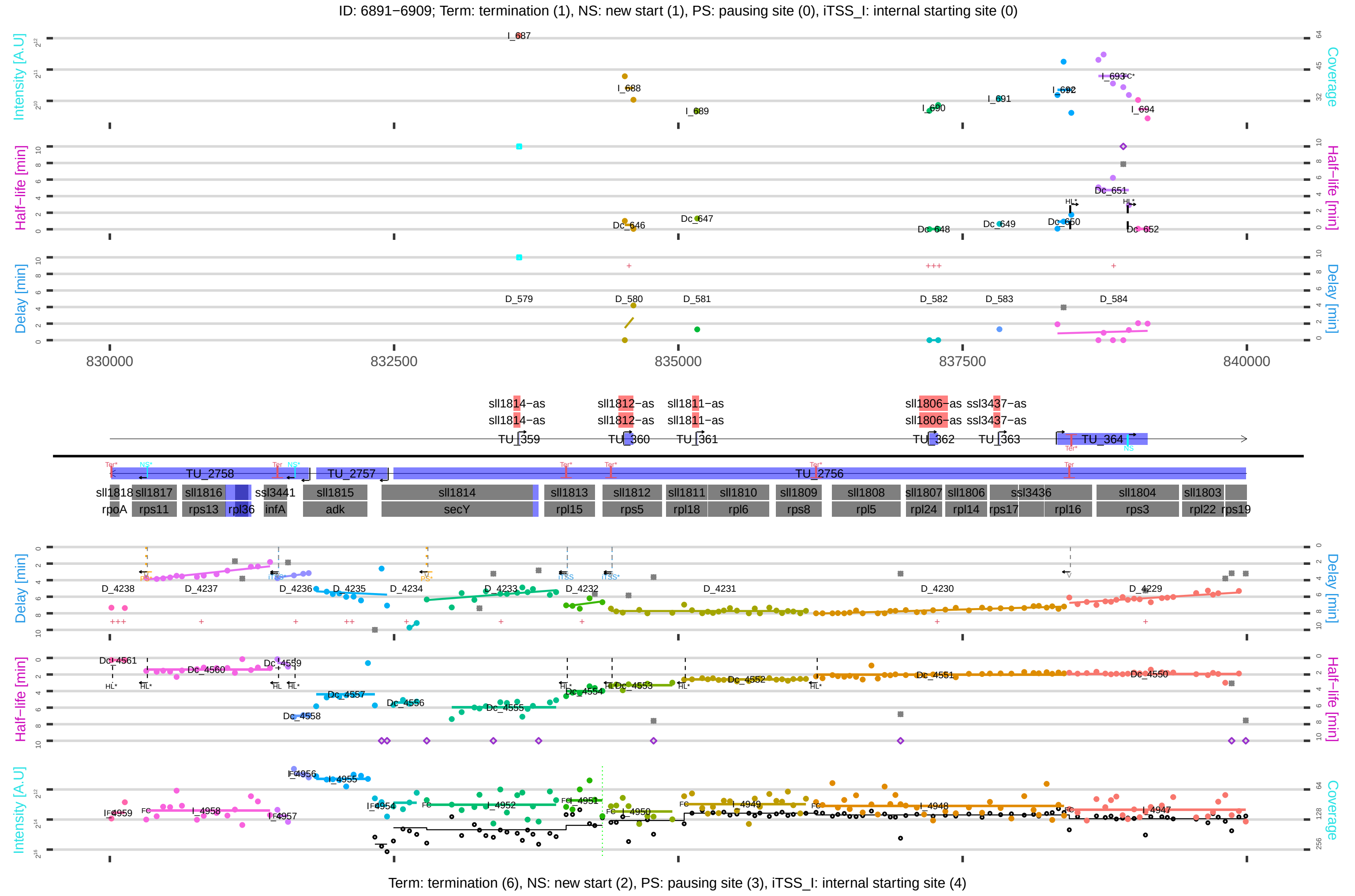
ID: 6891−6909; Term: termination (1), NS: new start (1), PS: pausing site (0), iTSS_I: internal starting site (0)
Intensity [A.U]
Coverage
Half−life [min]
Delay [min]
830000
832500
835000
837500
840000
TU_2758
TU_2757
TU_2756
sll1818
sll1817
sll1816
ssl3441
sll1815
sll1814
sll1813
sll1812
sll1811
sll1810
sll1809
sll1808
sll1807
sll1806
ssl3436
sll1804
sll1803
rpoA
rps11
rps13
rpl36
infA
adk
secY
rpl15
rps5
rpl18
rpl6
rps8
rpl5
rpl24
rpl14
rps17
rpl16
rps3
rpl22
rps19
Term: termination (6), NS: new start (2), PS: pausing site (3), iTSS_I: internal starting site (4)

ID: 6910−7001; Term: termination (4), NS: new start (2), PS: pausing site (1), iTSS_I: internal starting site (2)

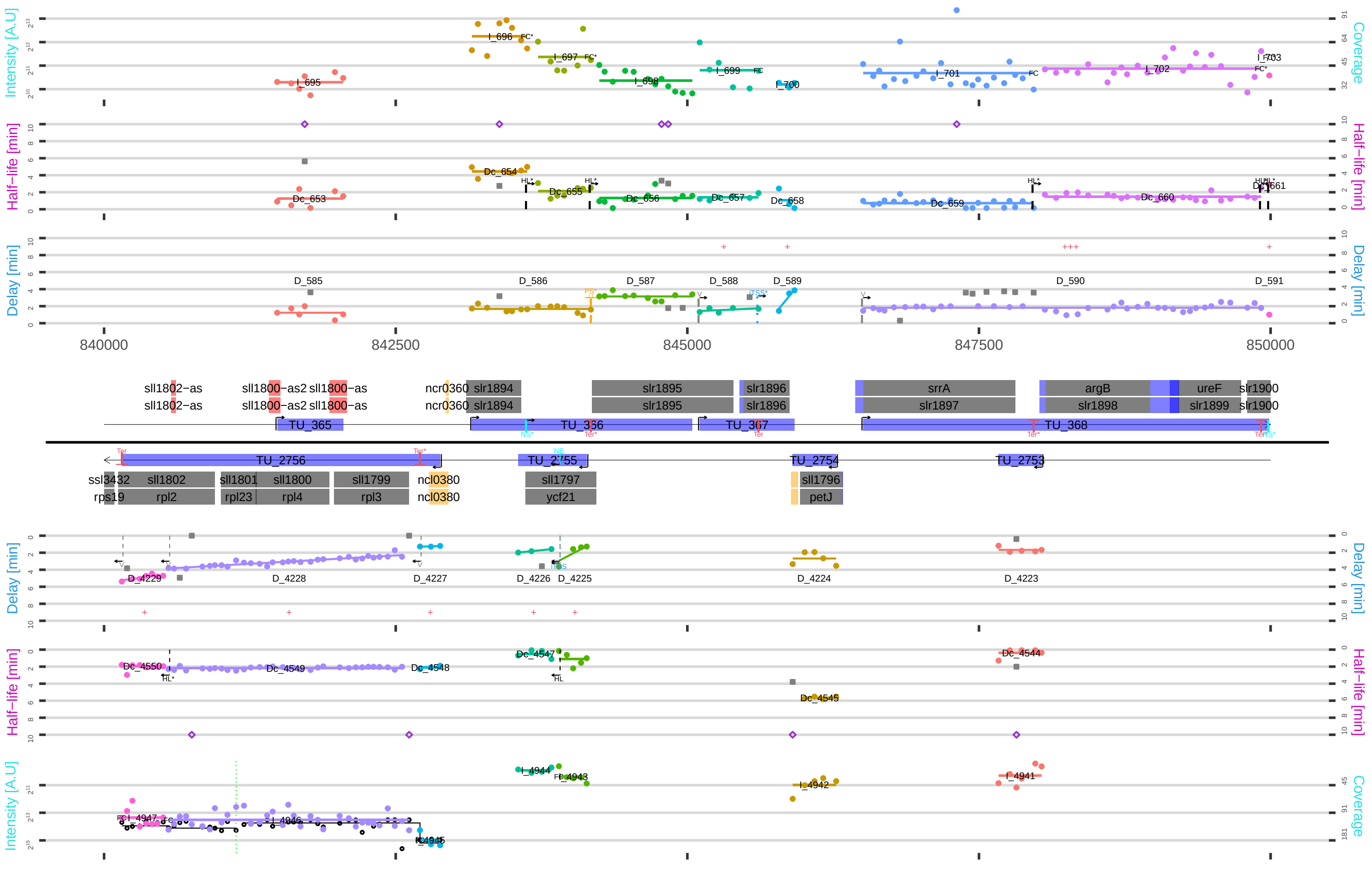

Term: termination (2), NS: new start (1), PS: pausing site (2), iTSS_I: internal starting site (1)

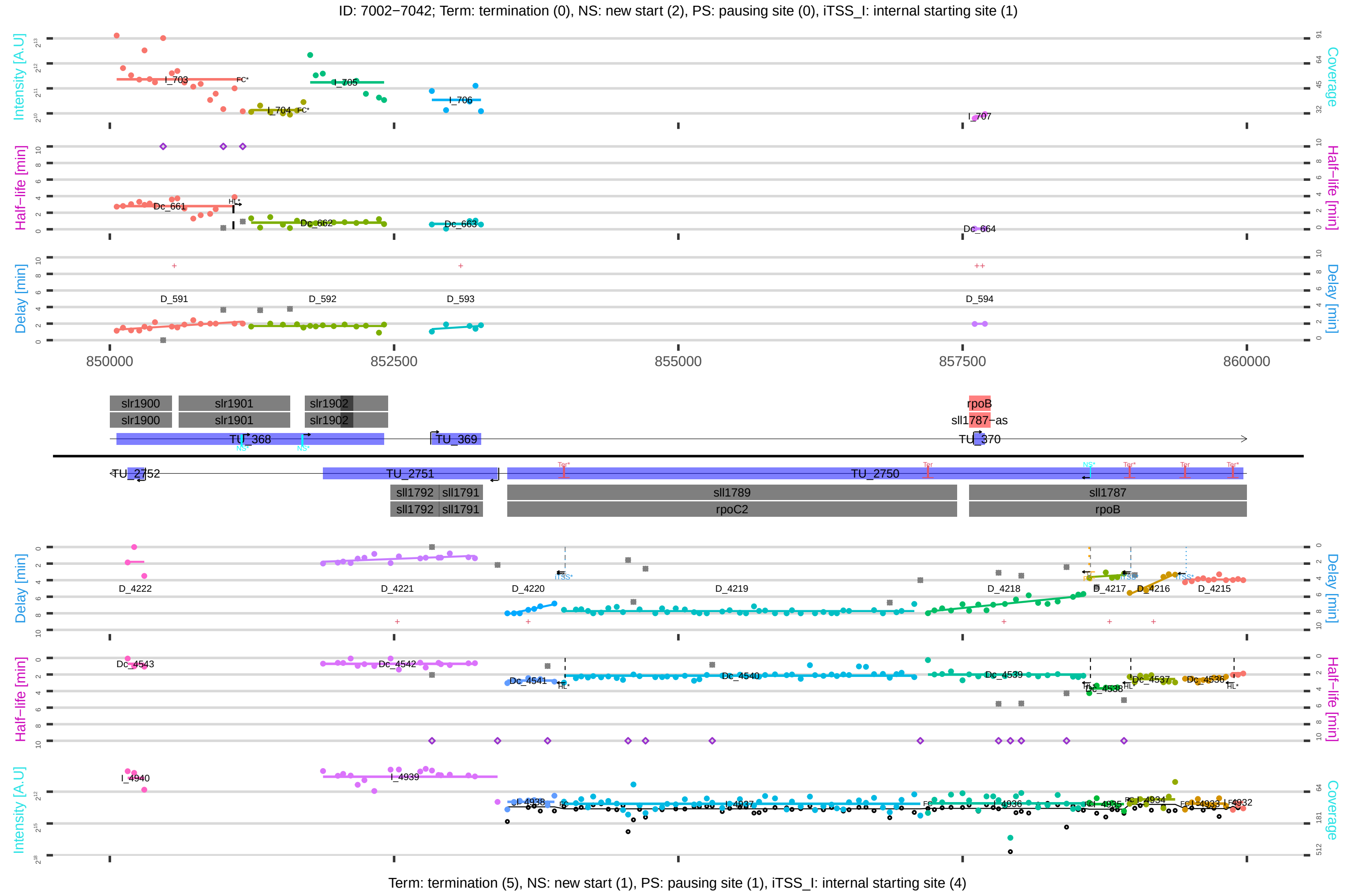
ID: 7002−7042; Term: termination (0), NS: new start (2), PS: pausing site (0), iTSS_I: internal starting site (1)
Intensity [A.U]
Coverage
Half−life [min]
Delay [min]
I_703
I_704
I_705
I_706
I_707
Dc_661
Dc_662
Dc_663
Dc_664
D_591
D_592
D_593
D_594
850000
852500
855000
857500
860000
slr1900
slr1901
slr1902
rpoB
sll1787−as
TU_368
TU_369
TU_370
TU_2752
TU_2751
TU_2750
sll1792
sll1791
sll1789
rpoC2
sll1787
D_4222
D_4221
D_4220
D_4219
D_4218
D_4217
D_4216
D_4215
Dc_4543
Dc_4542
Dc_4541
Dc_4540
Dc_4539
Dc_4538
Dc_4537
Dc_4536
I_4940
I_4939
I_4938
I_4937
I_4936
I_4935
I_4934
I_4933
I_4932
Term: termination (5), NS: new start (1), PS: pausing site (1), iTSS_I: internal starting site (4)

ID: 7043−7069; Term: termination (0), NS: new start (1), PS: pausing site (0), iTSS_I: internal starting site (0)

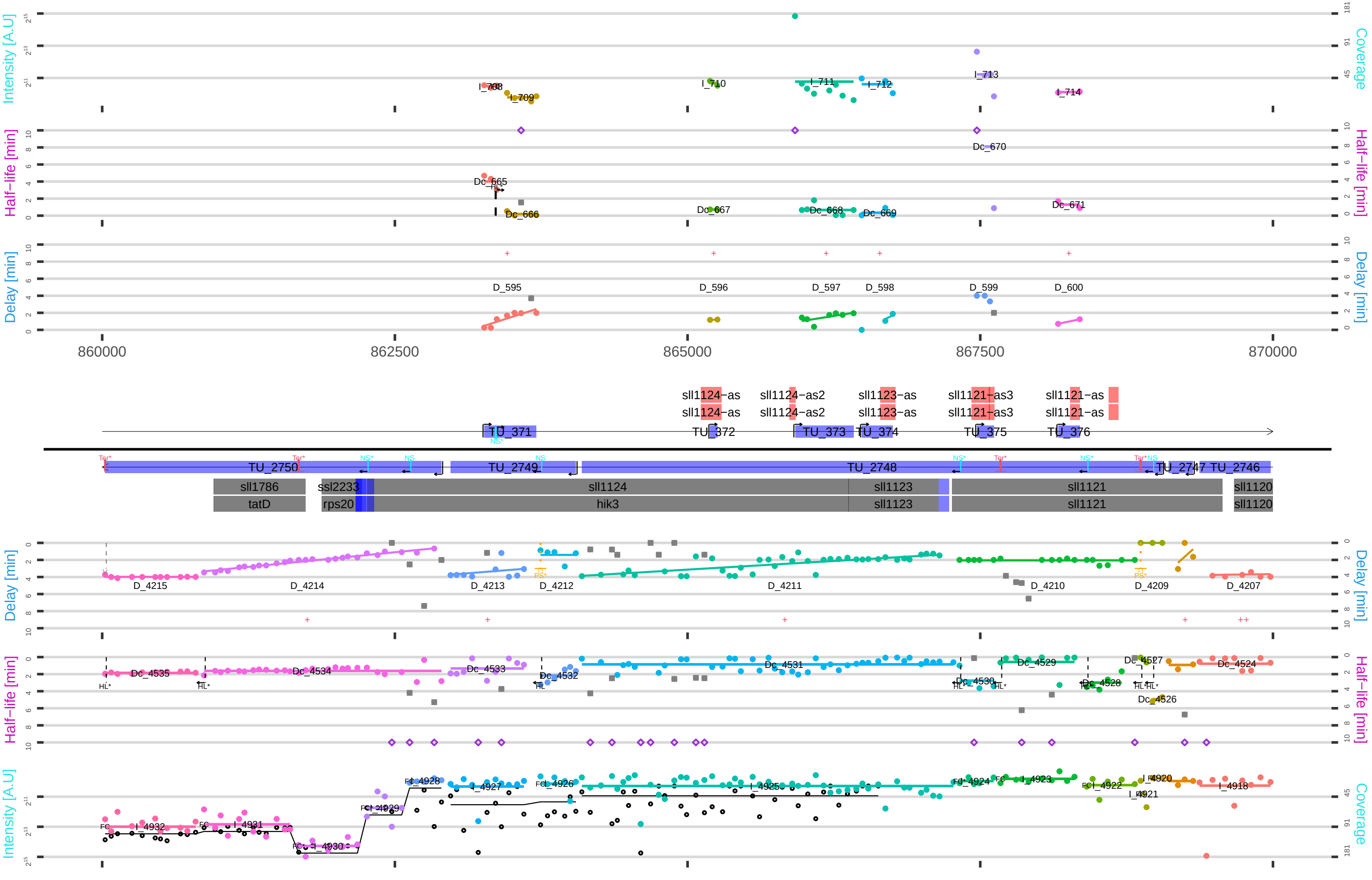

Term: termination (4), NS: new start (6), PS: pausing site (3), iTSS_I: internal starting site (1)

ID: 7071−7143; Term: termination (2), NS: new start (1), PS: pausing site (1), iTSS_I: internal starting site (0)

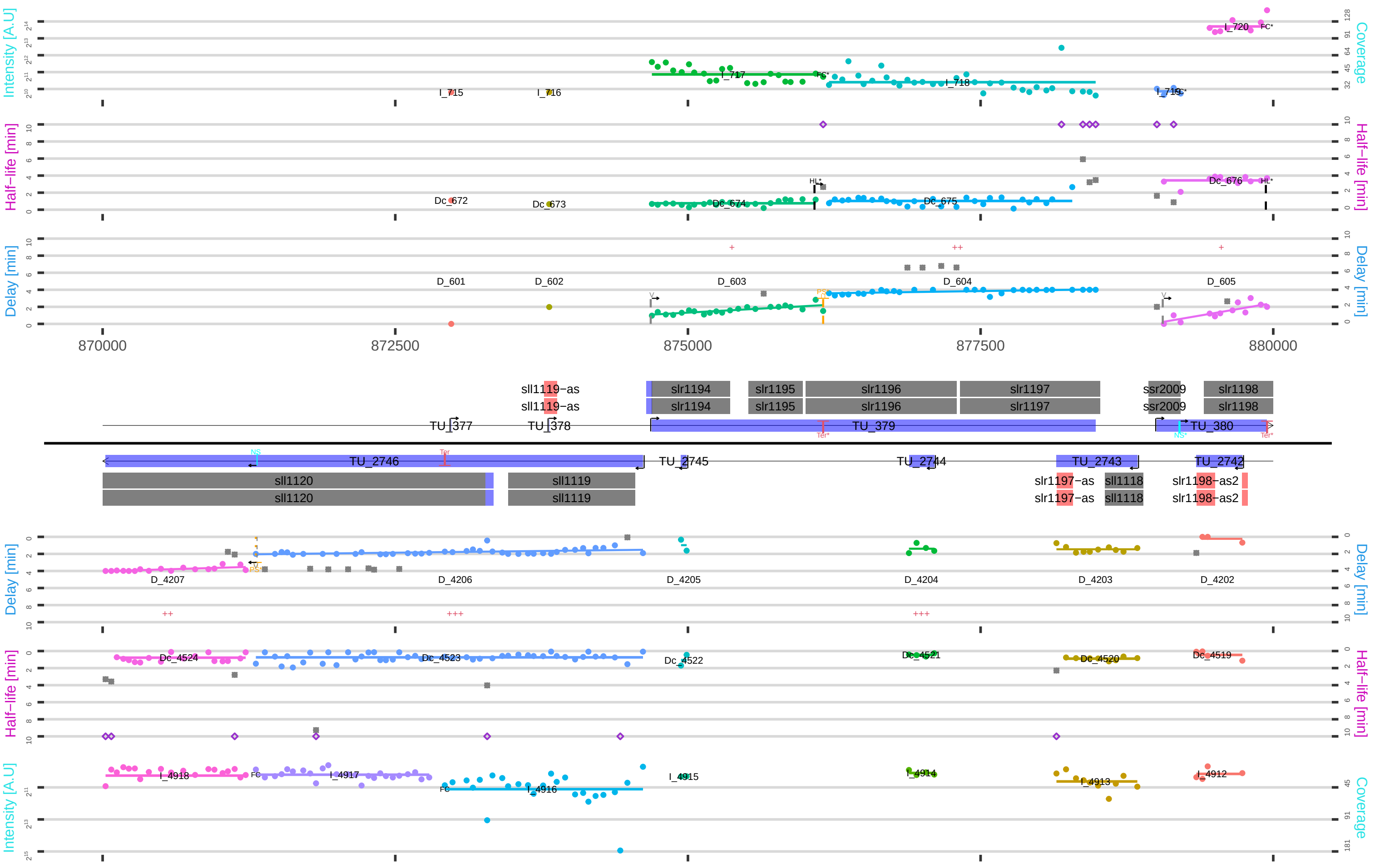

Term: termination (1), NS: new start (1), PS: pausing site (1), iTSS_I: internal starting site (0)

ID: 7144−7284; Term: termination (6), NS: new start (3), PS: pausing site (1), iTSS_I: internal starting site (4)

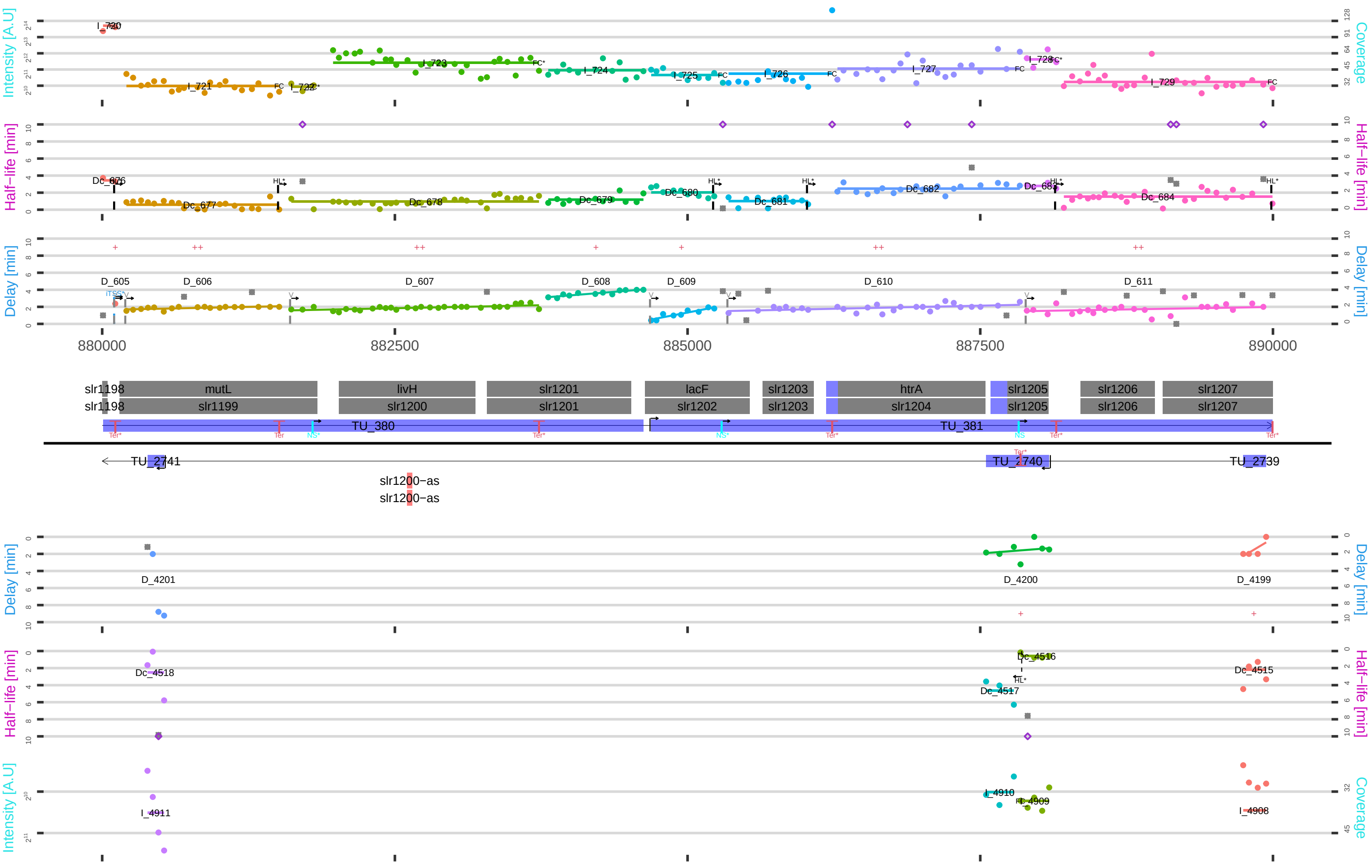

Term: termination (1), NS: new start (0), PS: pausing site (0), iTSS_I: internal starting site (0)

ID: 7285−7421; Term: termination (5), NS: new start (2), PS: pausing site (4), iTSS_I: internal starting site (1)

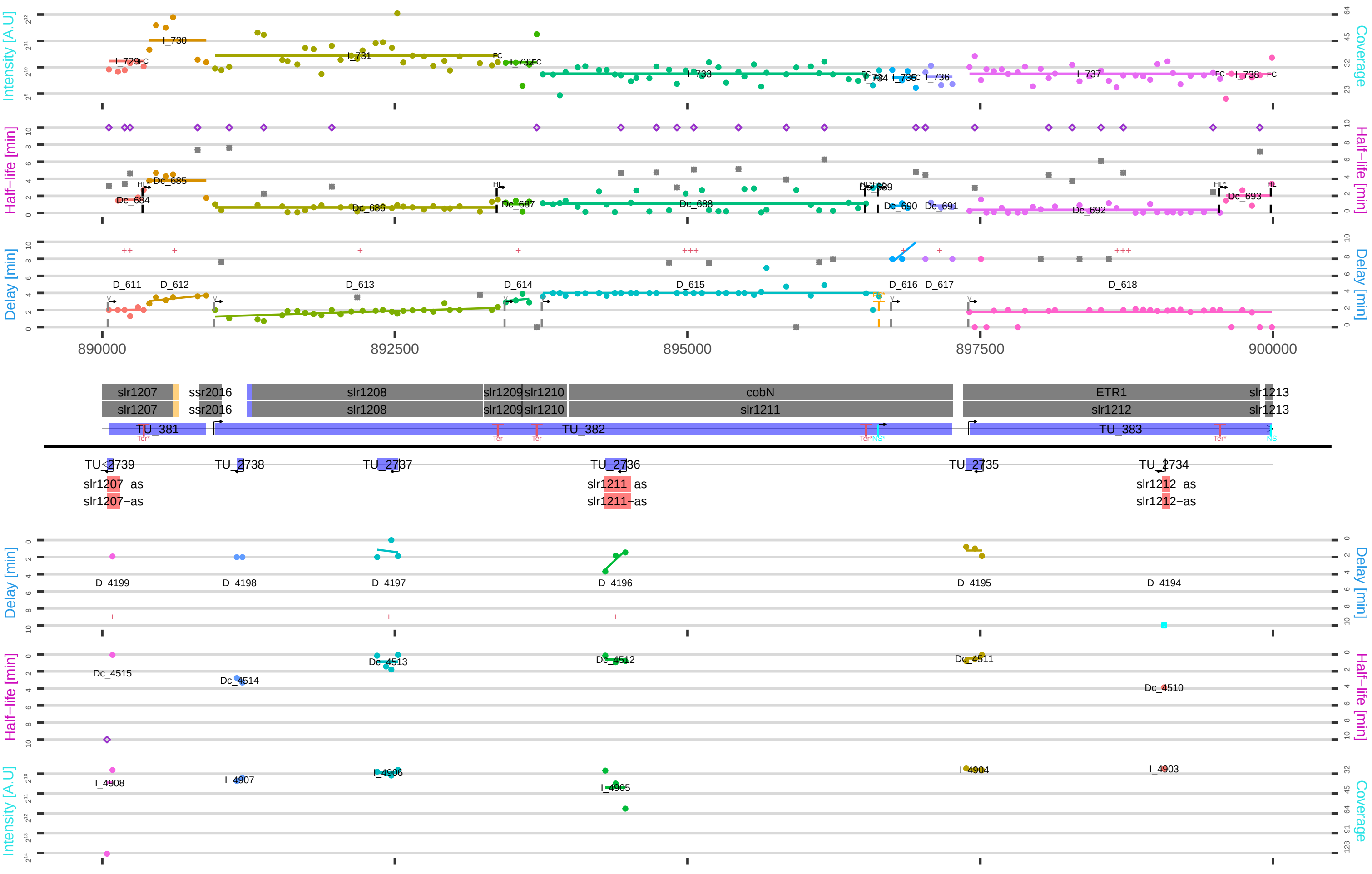

Term: termination (0), NS: new start (0), PS: pausing site (0), iTSS_I: internal starting site (0)

ID: 7422−7517; Term: termination (4), NS: new start (2), PS: pausing site (2), iTSS_I: internal starting site (2)

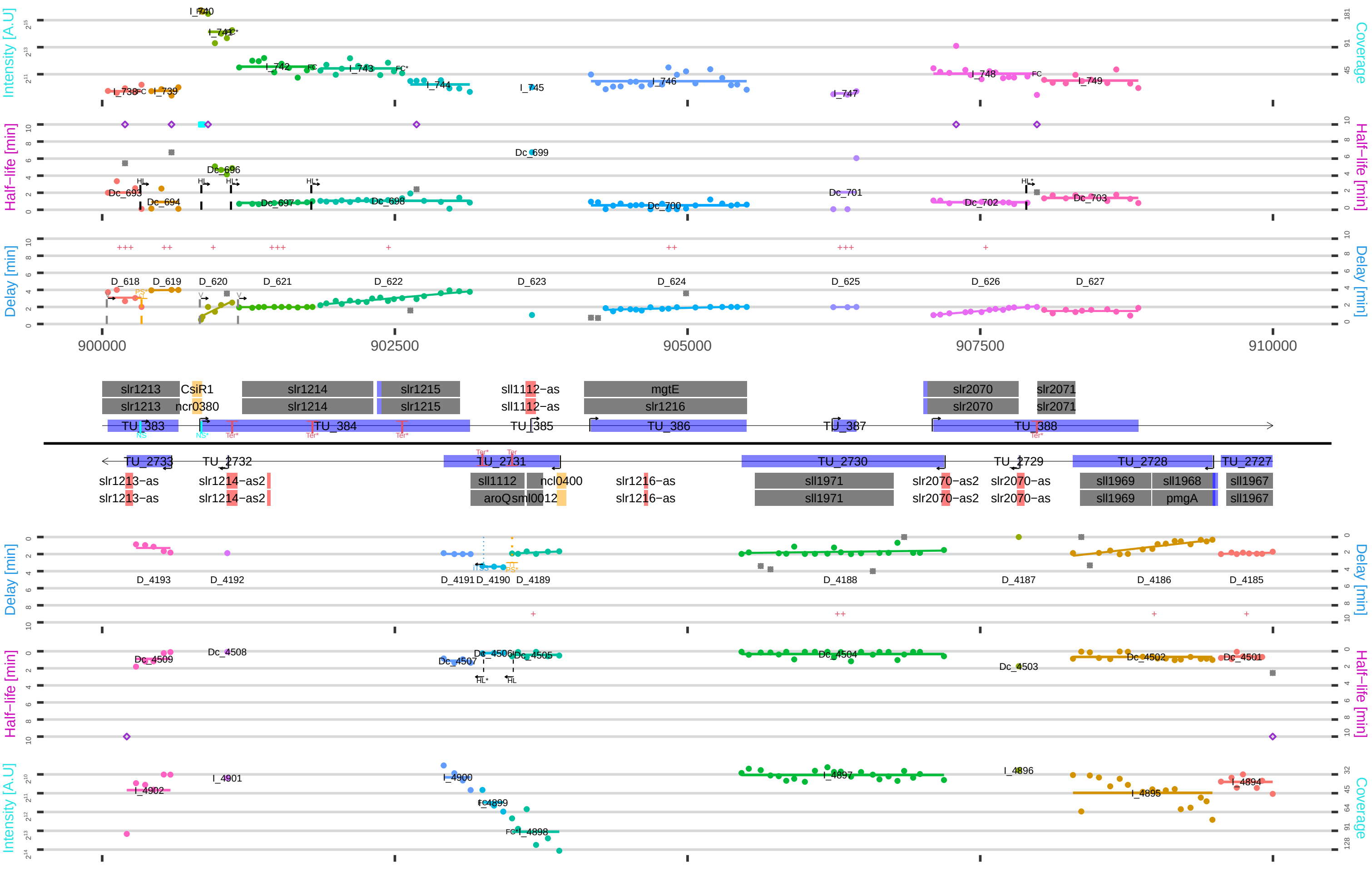

Term: termination (2), NS: new start (0), PS: pausing site (1), iTSS_I: internal starting site (1)

ID: 7518−7636; Term: termination (4), NS: new start (2), PS: pausing site (1), iTSS_I: internal starting site (4)

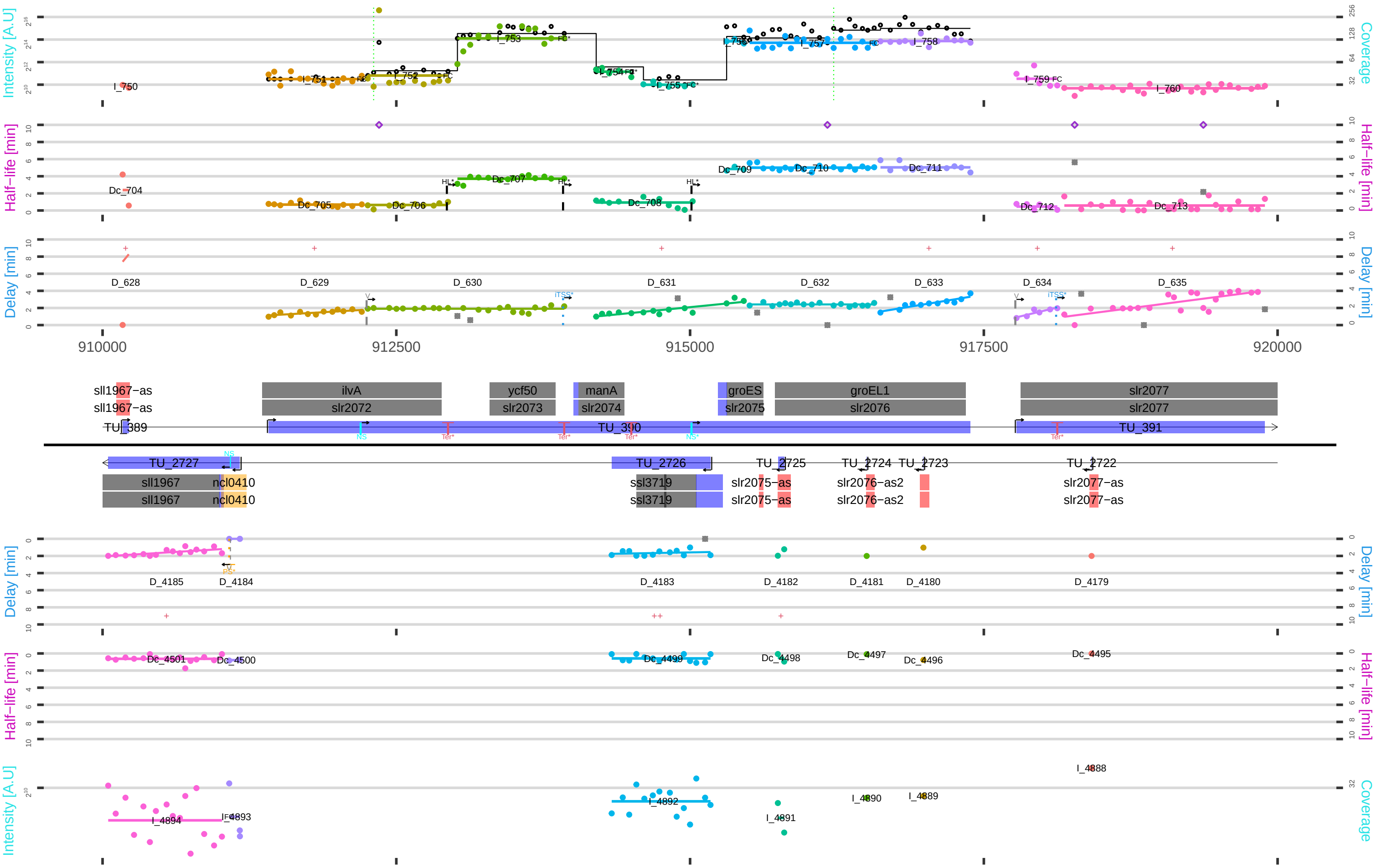

Term: termination (0), NS: new start (1), PS: pausing site (1), iTSS_I: internal starting site (0)

ID: 7637−7681; Term: termination (1), NS: new start (0), PS: pausing site (0), iTSS_I: internal starting site (0)

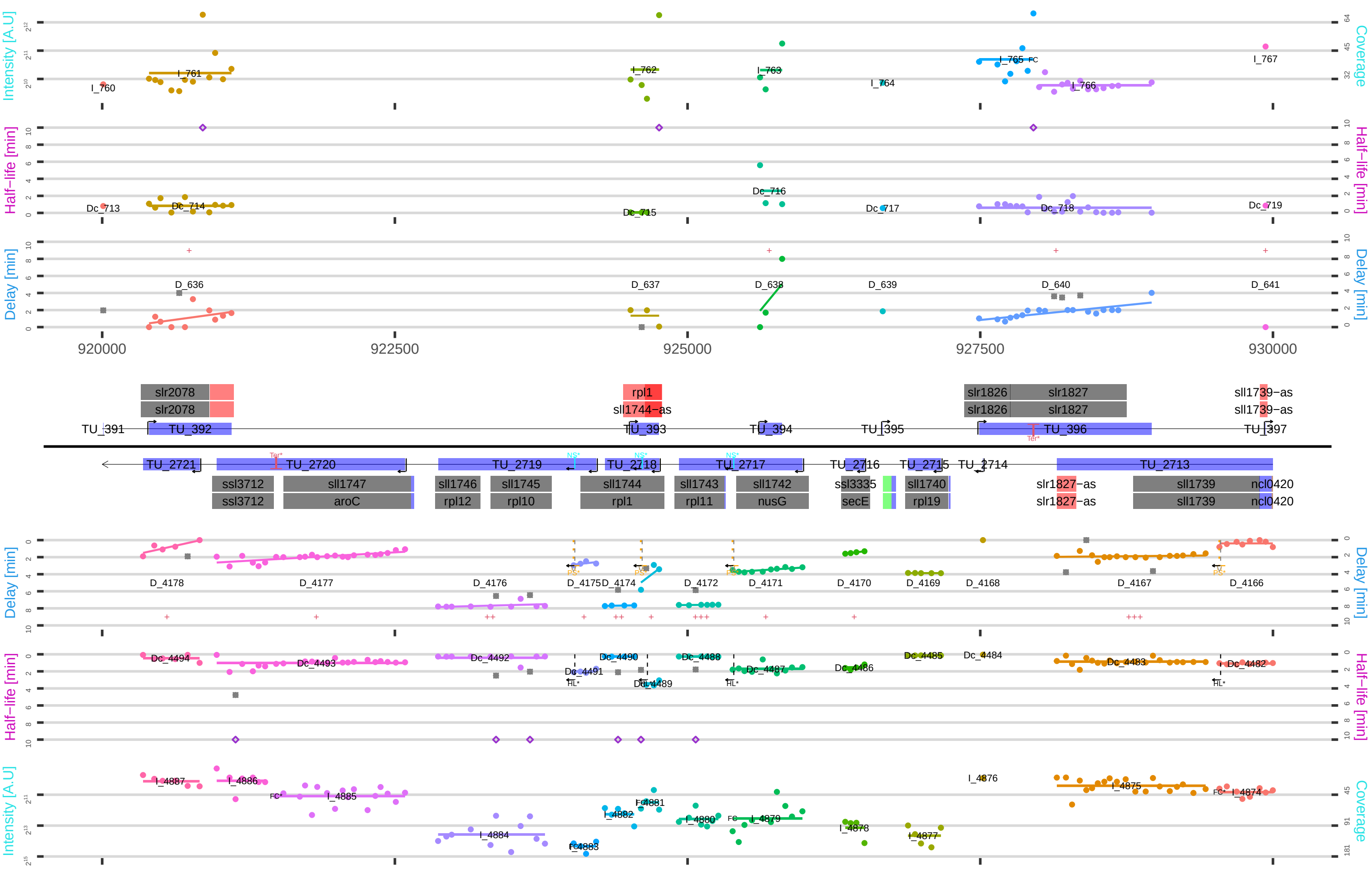

Term: termination (1), NS: new start (3), PS: pausing site (4), iTSS_I: internal starting site (0)

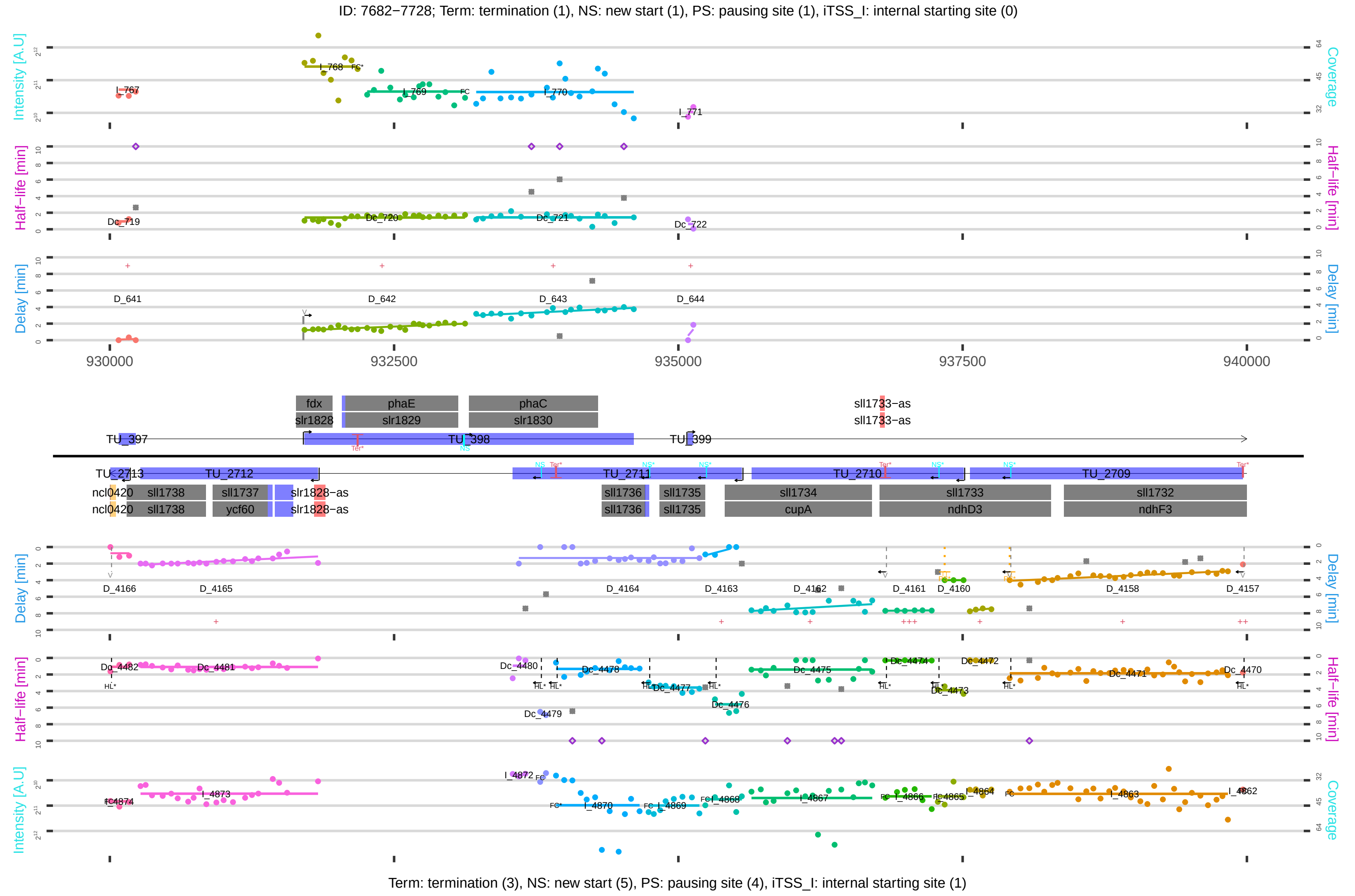
ID: 7682−7728; Term: termination (1), NS: new start (1), PS: pausing site (1), iTSS_I: internal starting site (0)
Intensity [A.U]
Coverage
Half−life [min]
Delay [min]
930000
932500
935000
937500
940000
fdx
slr1828
phaE
slr1829
phaC
slr1830
sll1733−as
TU_397
TU_398
TU_399
TU_2713
TU_2712
TU_2711
TU_2710
TU_2709
ncl0420
sll1738
sll1737
ycf60
slr1828−as
sll1736
sll1735
sll1734
cupA
sll1733
ndhD3
sll1732
ndhF3
Term: termination (3), NS: new start (5), PS: pausing site (4), iTSS_I: internal starting site (1)

ID: 7729−7830; Term: termination (5), NS: new start (3), PS: pausing site (2), iTSS_I: internal starting site (2)

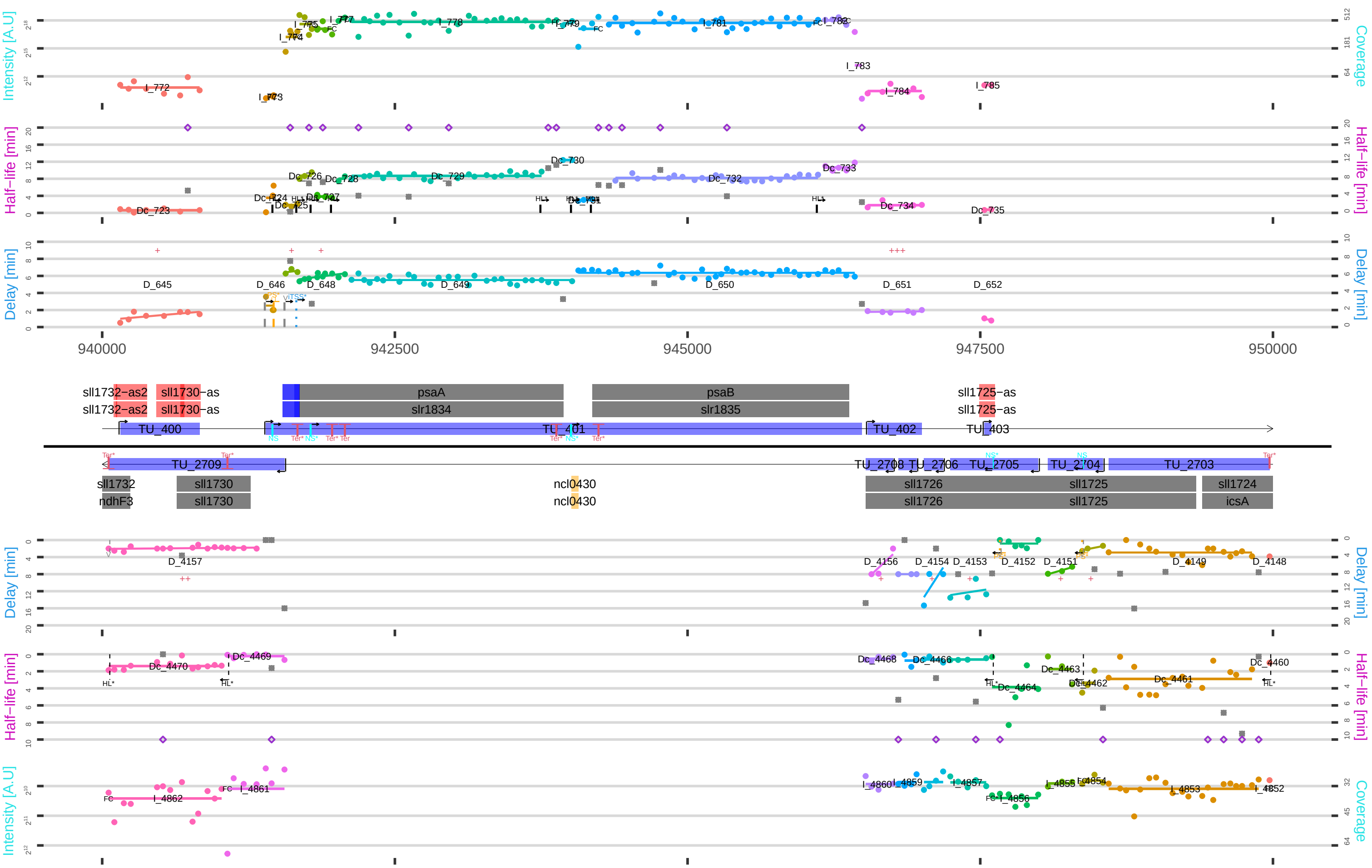

Term: termination (3), NS: new start (2), PS: pausing site (3), iTSS_I: internal starting site (0)

ID: 7831−7911; Term: termination (3), NS: new start (2), PS: pausing site (1), iTSS_I: internal starting site (1)

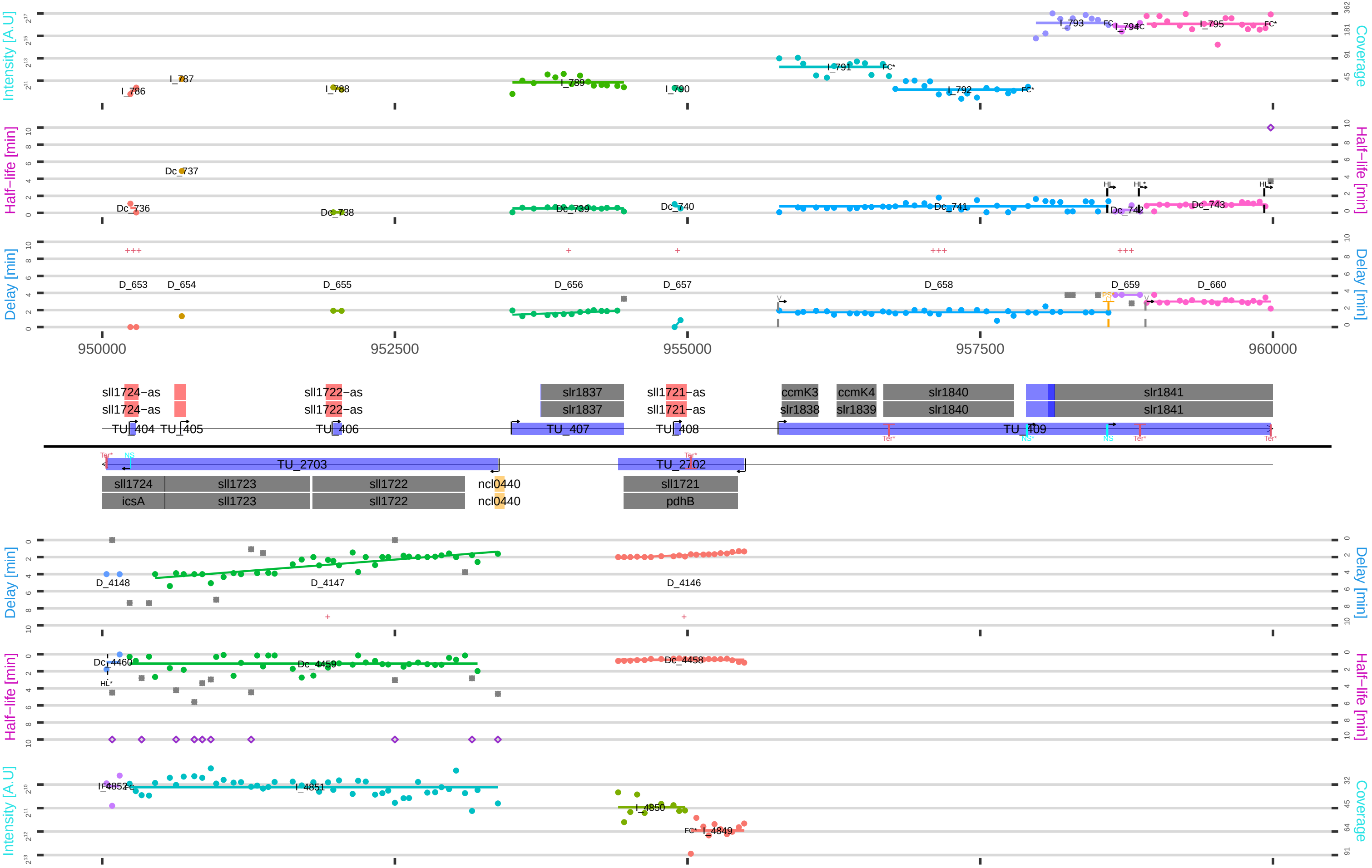

Term: termination (2), NS: new start (1), PS: pausing site (0), iTSS_I: internal starting site (0)

ID: 7912−8008; Term: termination (4), NS: new start (2), PS: pausing site (2), iTSS_I: internal starting site (3)

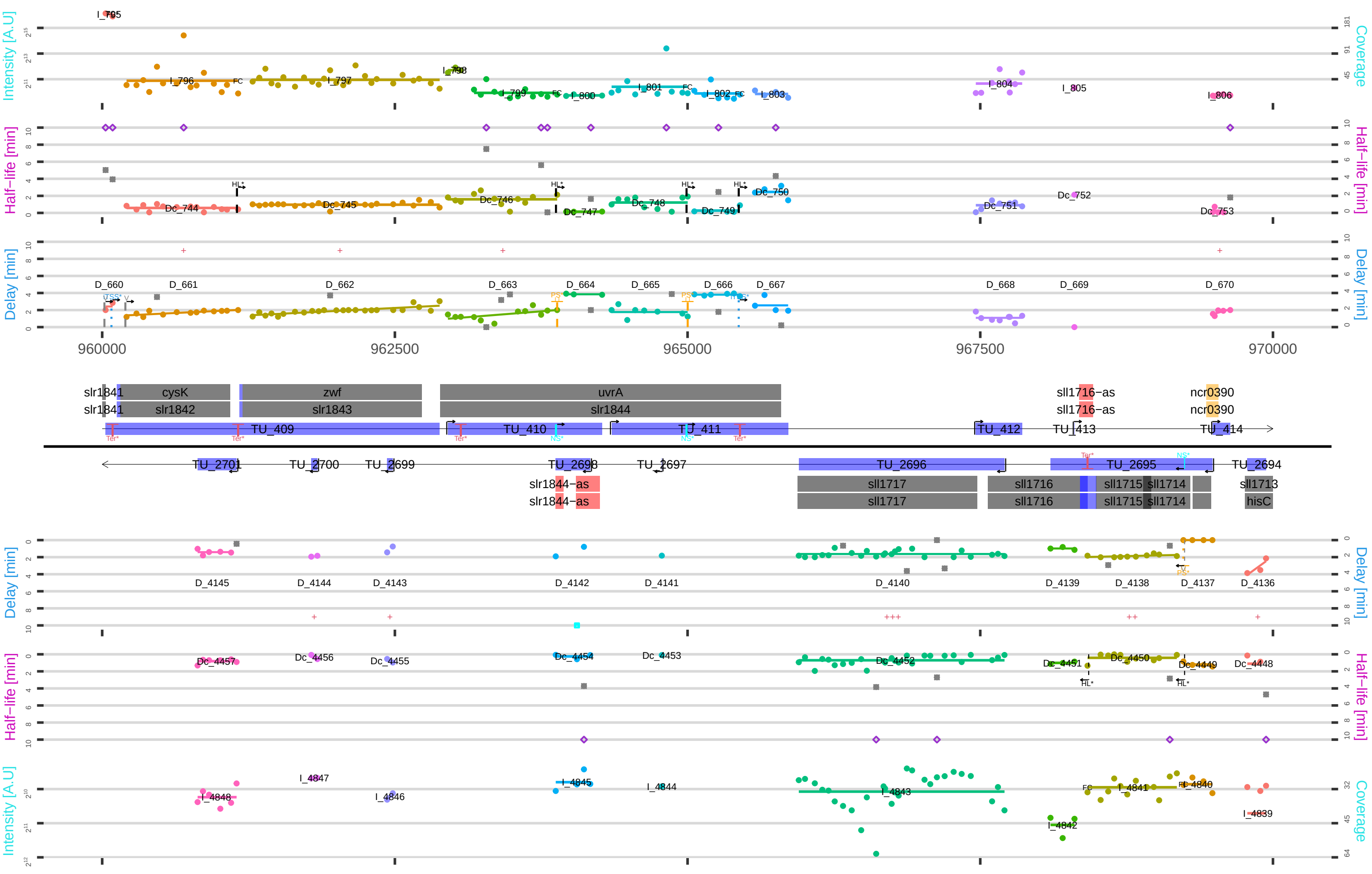

Term: termination (1), NS: new start (1), PS: pausing site (1), iTSS_I: internal starting site (1)

ID: 8010−8118; Term: termination (2), NS: new start (4), PS: pausing site (3), iTSS_I: internal starting site (2)

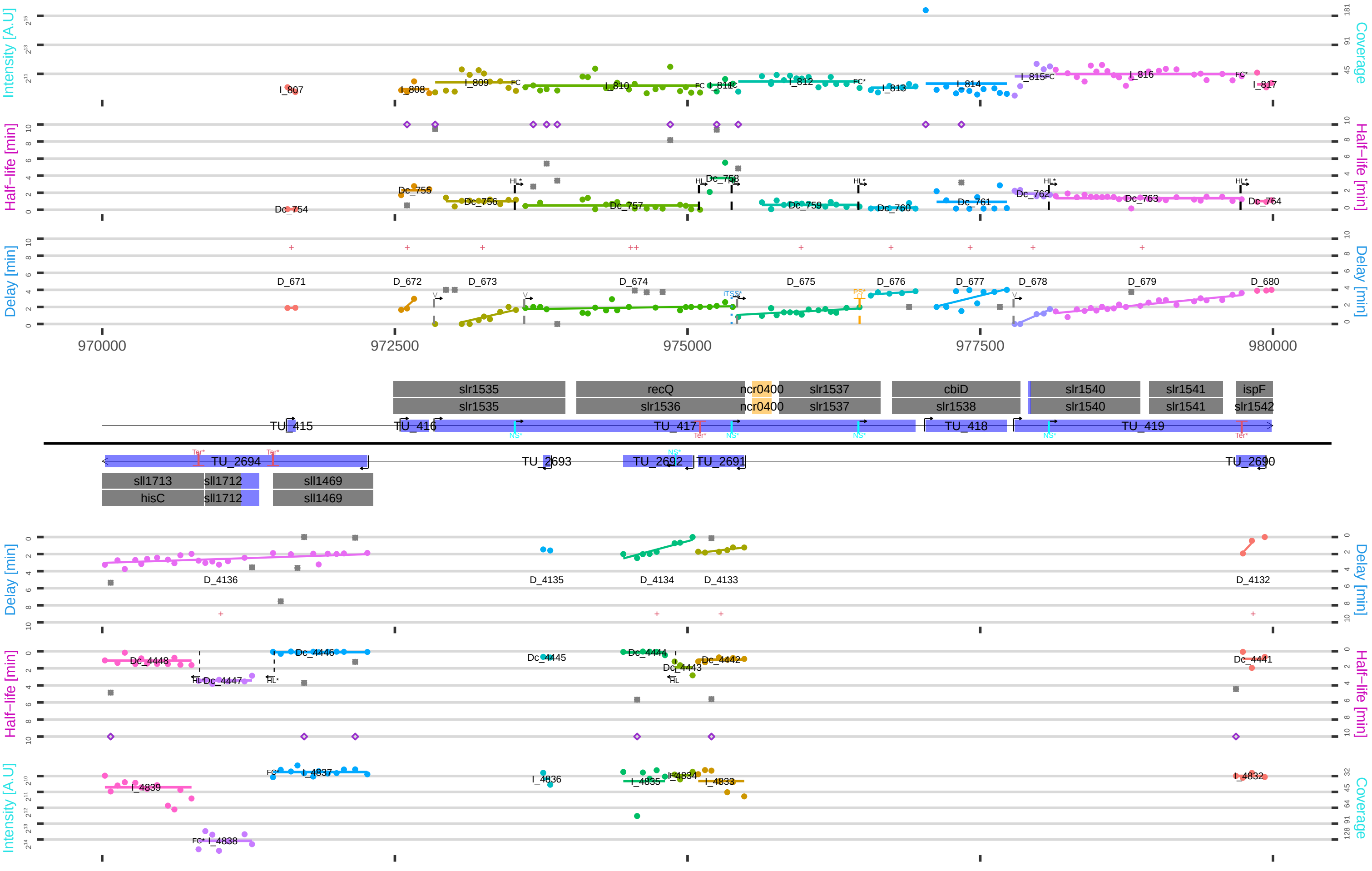

Term: termination (2), NS: new start (1), PS: pausing site (0), iTSS_I: internal starting site (0)

ID: 8119−8204; Term: termination (3), NS: new start (1), PS: pausing site (0), iTSS_I: internal starting site (1)

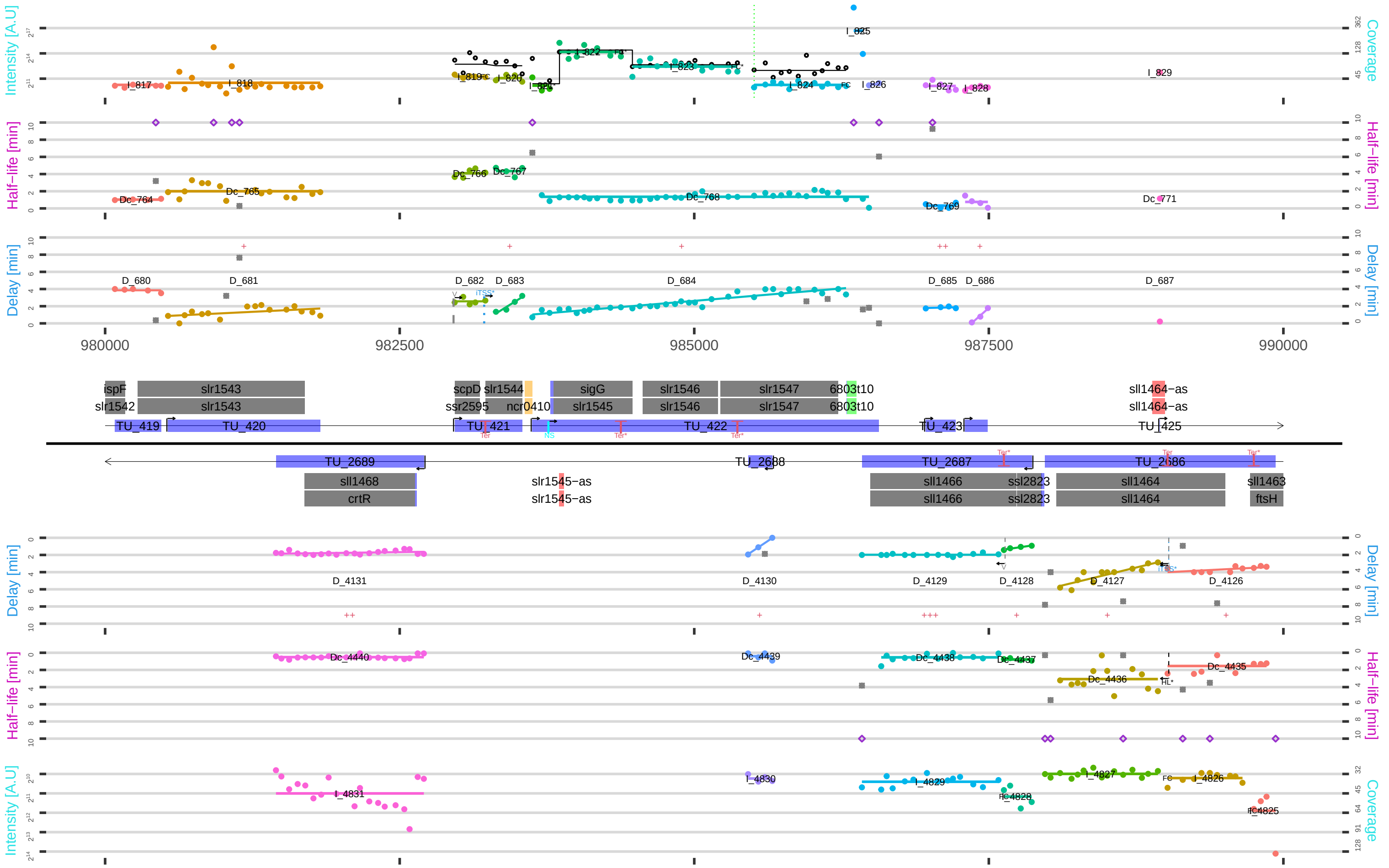

Term: termination (3), NS: new start (0), PS: pausing site (1), iTSS_I: internal starting site (1)

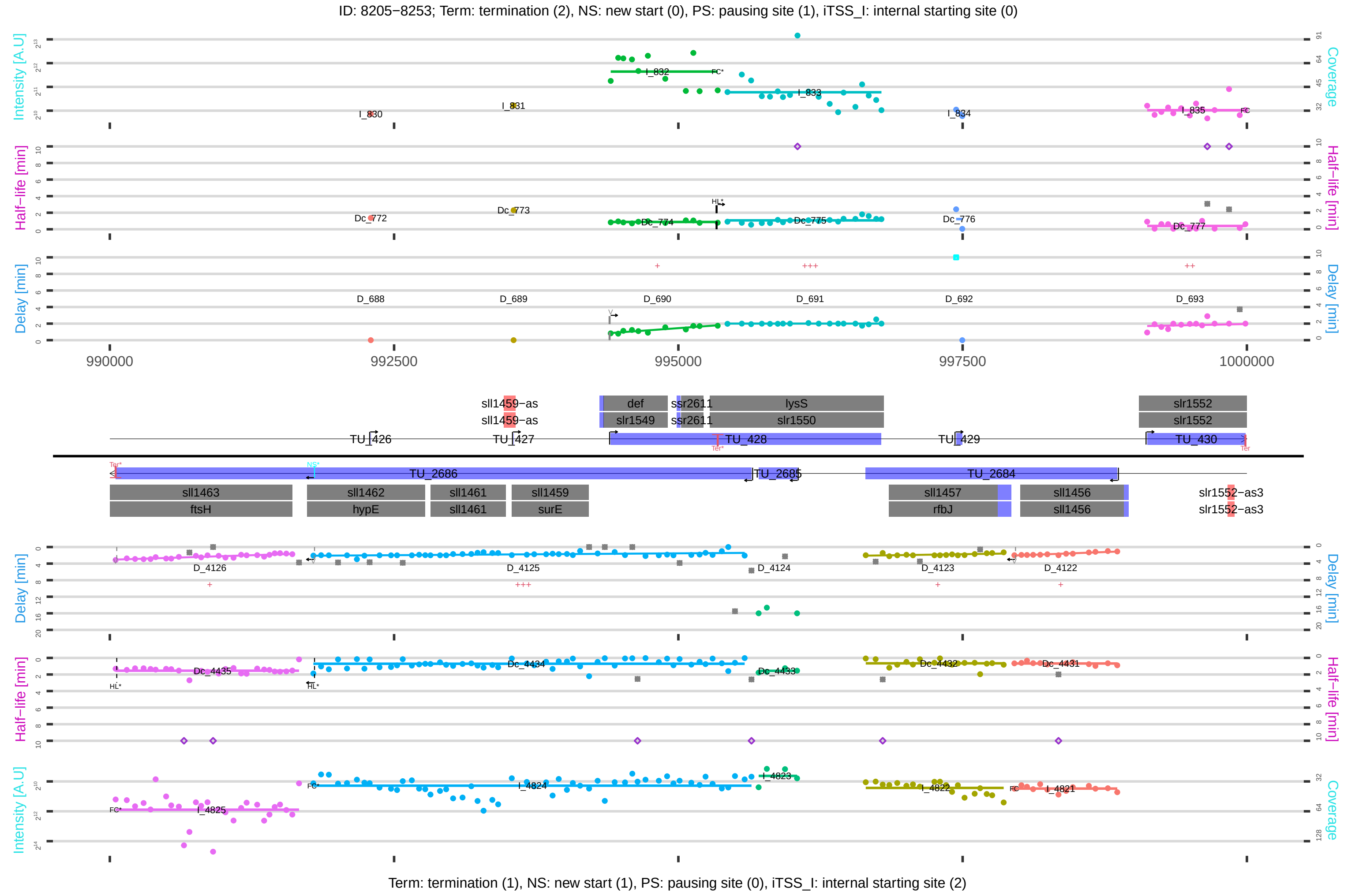
ID: 8205−8253; Term: termination (2), NS: new start (0), PS: pausing site (1), iTSS_I: internal starting site (0)
Intensity [A.U]
Coverage
Half−life [min]
Delay [min]
I_830
I_831
I_832
FC*
I_833
I_834
I_835
FC
Dc_772
Dc_773
Dc_774
HL*
Dc_775
Dc_776
Dc_777
D_688
D_689
D_690
D_691
D_692
D_693
990000
992500
995000
997500
1000000
sll1459−as
def
ssr2611
lysS
slr1549
slr1550
slr1552
TU_426
TU_427
TU_428
TU_429
TU_430
Ter*
Ter
NS*
TU_2686
TU_2685
TU_2684
sll1463
sll1462
sll1461
sll1459
sll1457
sll1456
slr1552−as3
ftsH
hypE
surE
rfbJ
D_4126
D_4125
D_4124
D_4123
D_4122
Dc_4435
Dc_4434
Dc_4433
Dc_4432
Dc_4431
I_4825
I_4824
I_4823
I_4822
I_4821
Term: termination (1), NS: new start (1), PS: pausing site (0), iTSS_I: internal starting site (2)

ID: 8254−8282; Term: termination (1), NS: new start (0), PS: pausing site (1), iTSS_I: internal starting site (0)

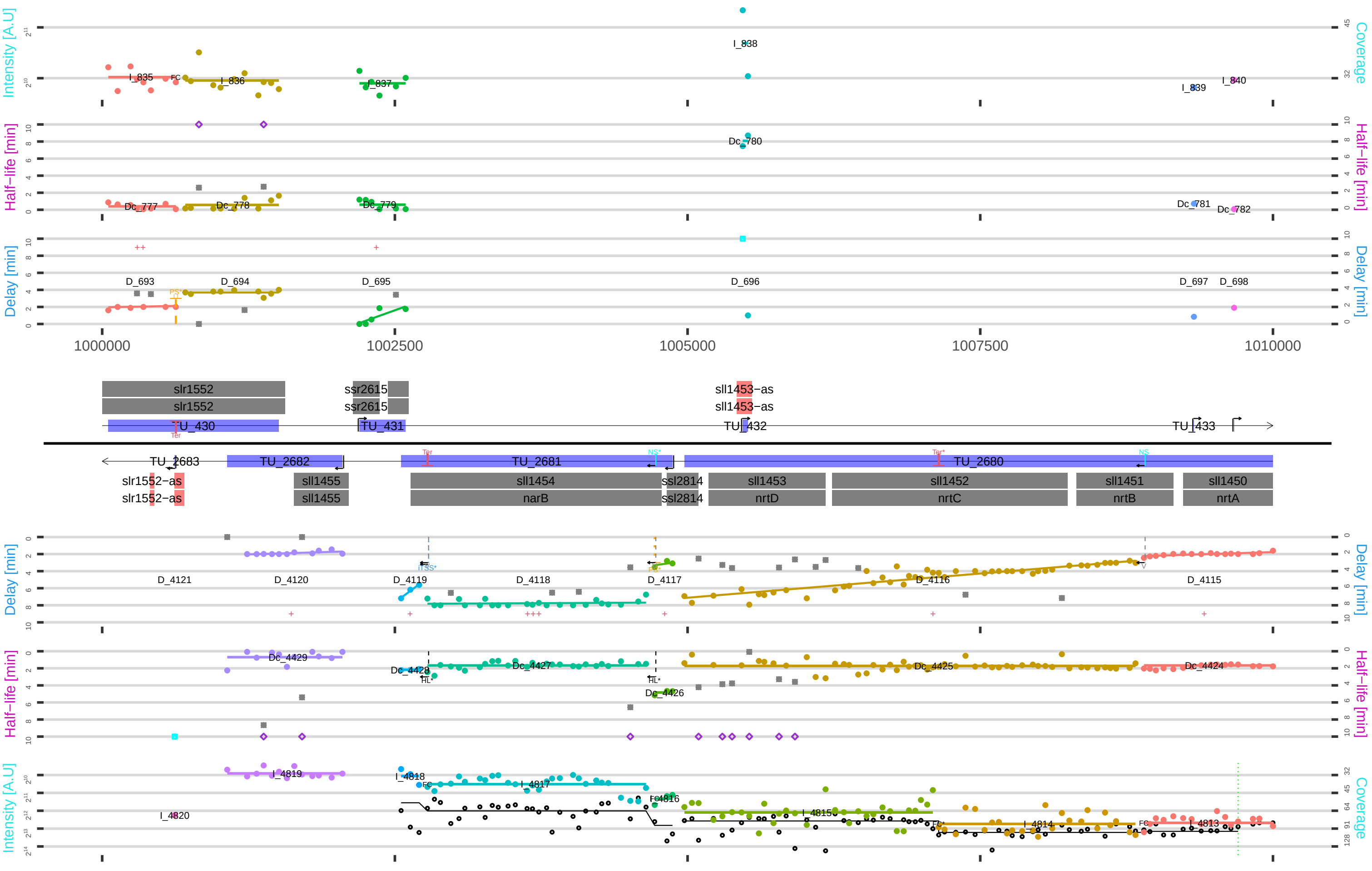

Term: termination (2), NS: new start (2), PS: pausing site (2), iTSS_I: internal starting site (1)

ID: 8283−8374; Term: termination (3), NS: new start (2), PS: pausing site (1), iTSS_I: internal starting site (4)

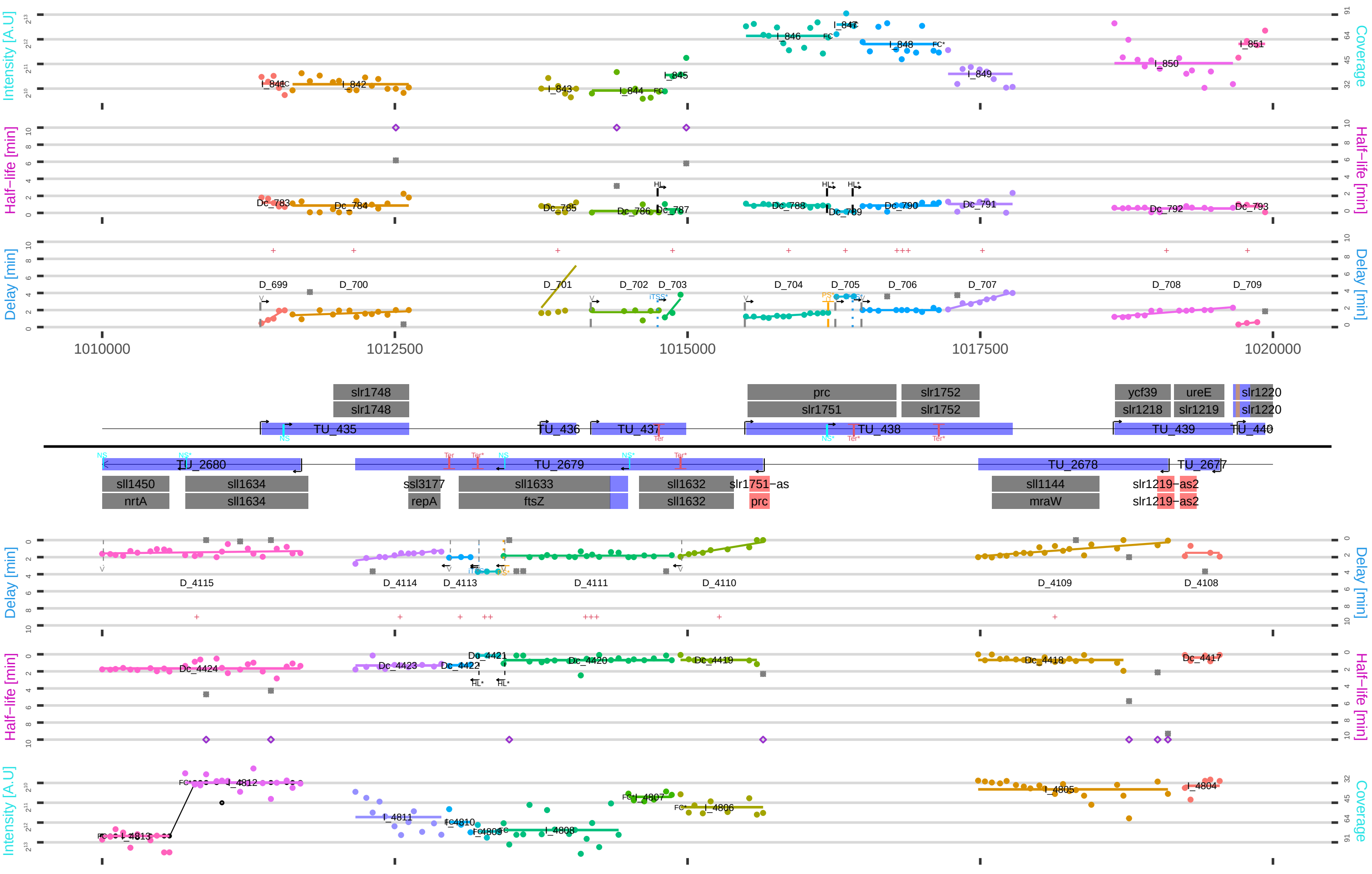

Term: termination (3), NS: new start (4), PS: pausing site (1), iTSS_I: internal starting site (3)

ID: 8375−8463; Term: termination (1), NS: new start (2), PS: pausing site (1), iTSS_I: internal starting site (2)

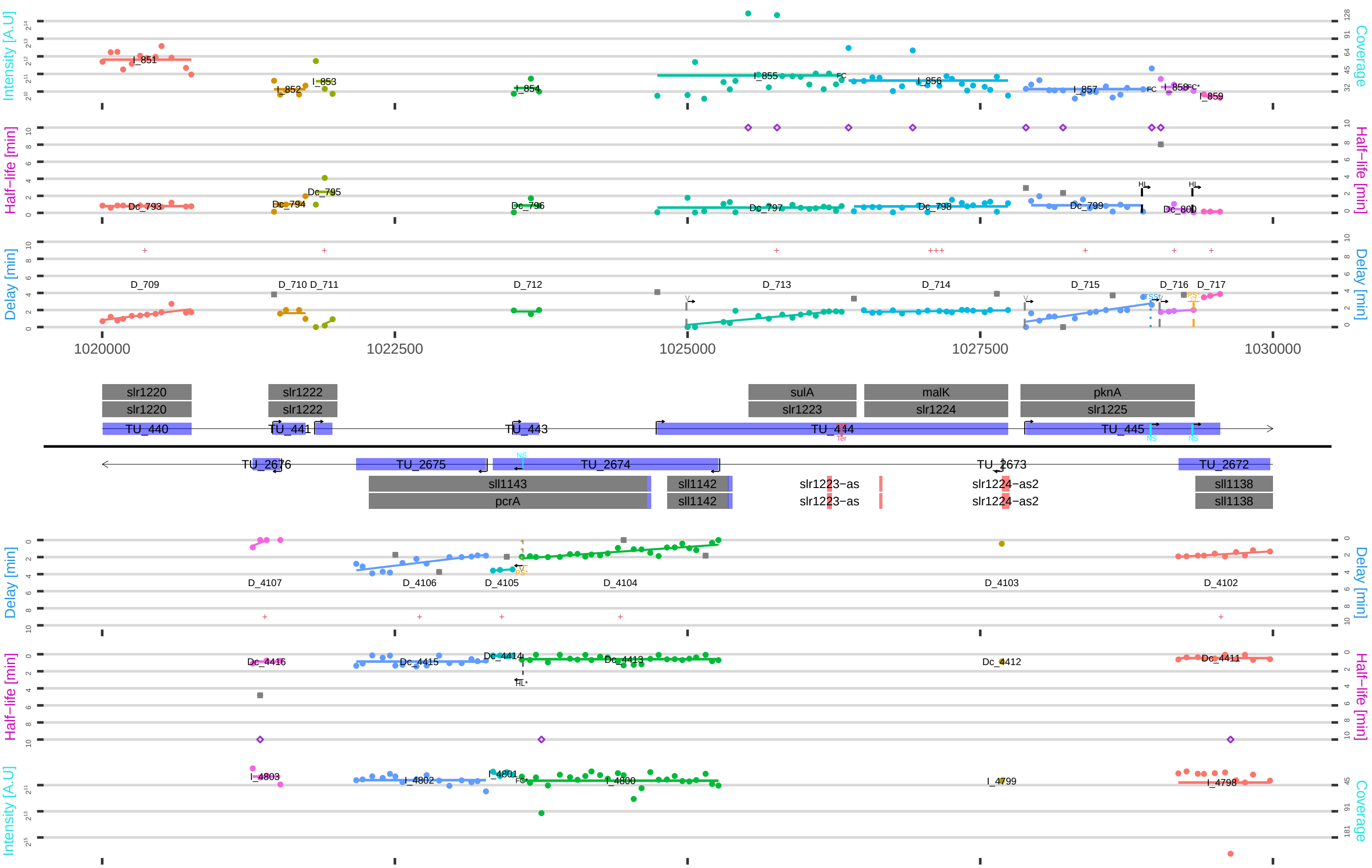

Term: termination (0), NS: new start (1), PS: pausing site (1), iTSS_I: internal starting site (0)

ID: 8464−8594; Term: termination (4), NS: new start (3), PS: pausing site (1), iTSS_I: internal starting site (1)

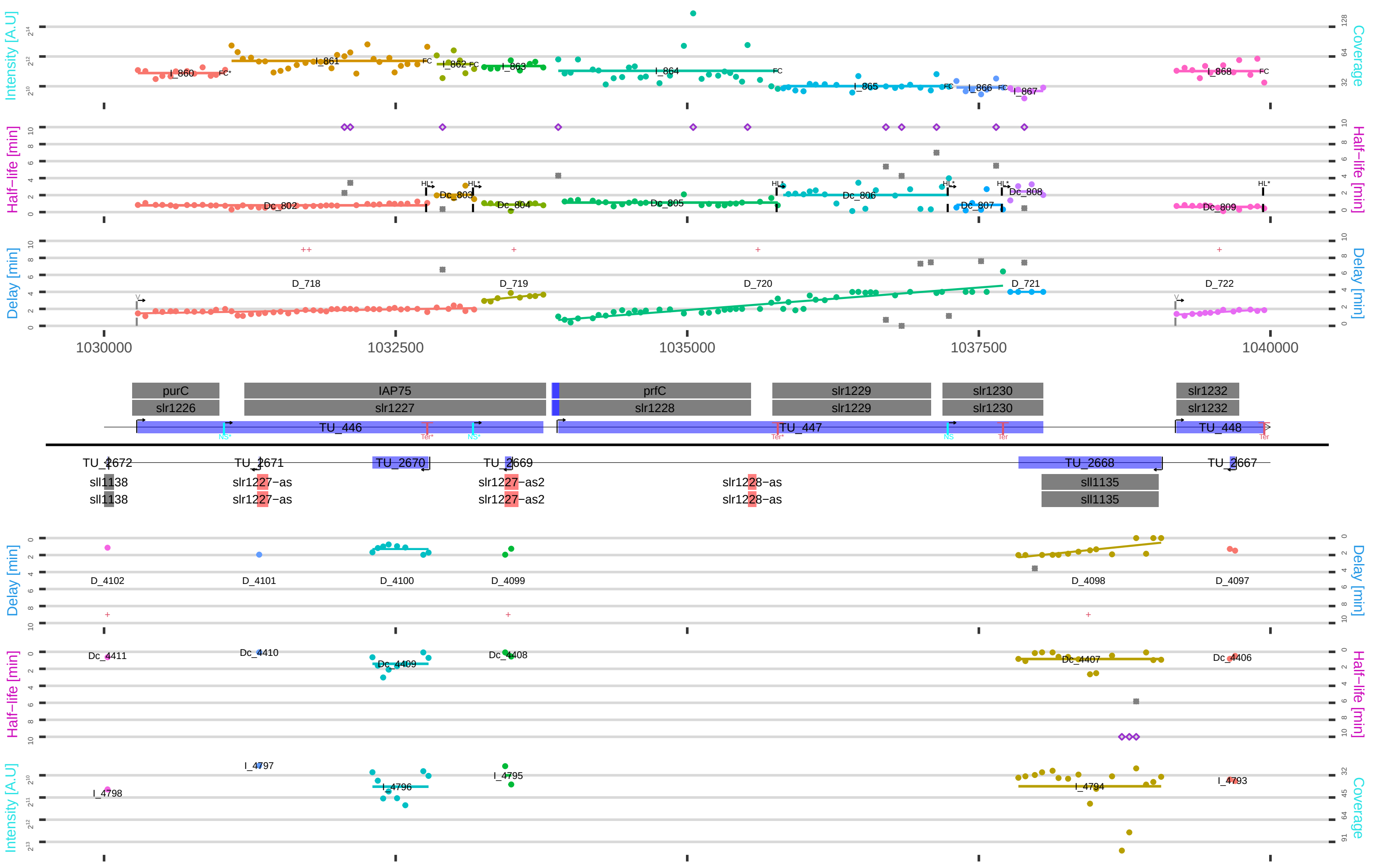

Term: termination (0), NS: new start (0), PS: pausing site (0), iTSS_I: internal starting site (0)

ID: 8595−8696; Term: termination (2), NS: new start (1), PS: pausing site (2), iTSS_I: internal starting site (1)

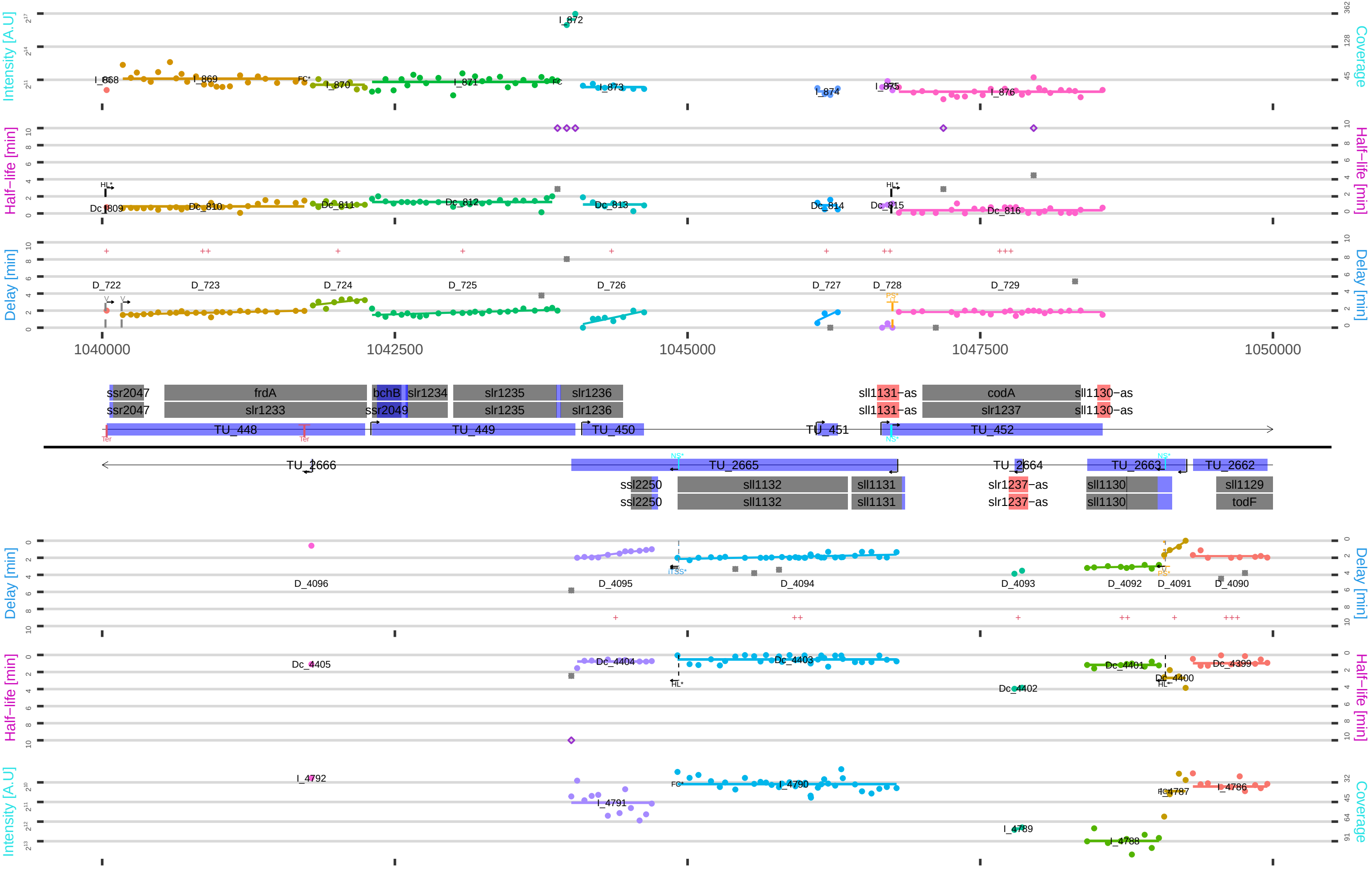

Term: termination (0), NS: new start (2), PS: pausing site (1), iTSS_I: internal starting site (1)

ID: 8698−8823; Term: termination (5), NS: new start (4), PS: pausing site (2), iTSS_I: internal starting site (3)

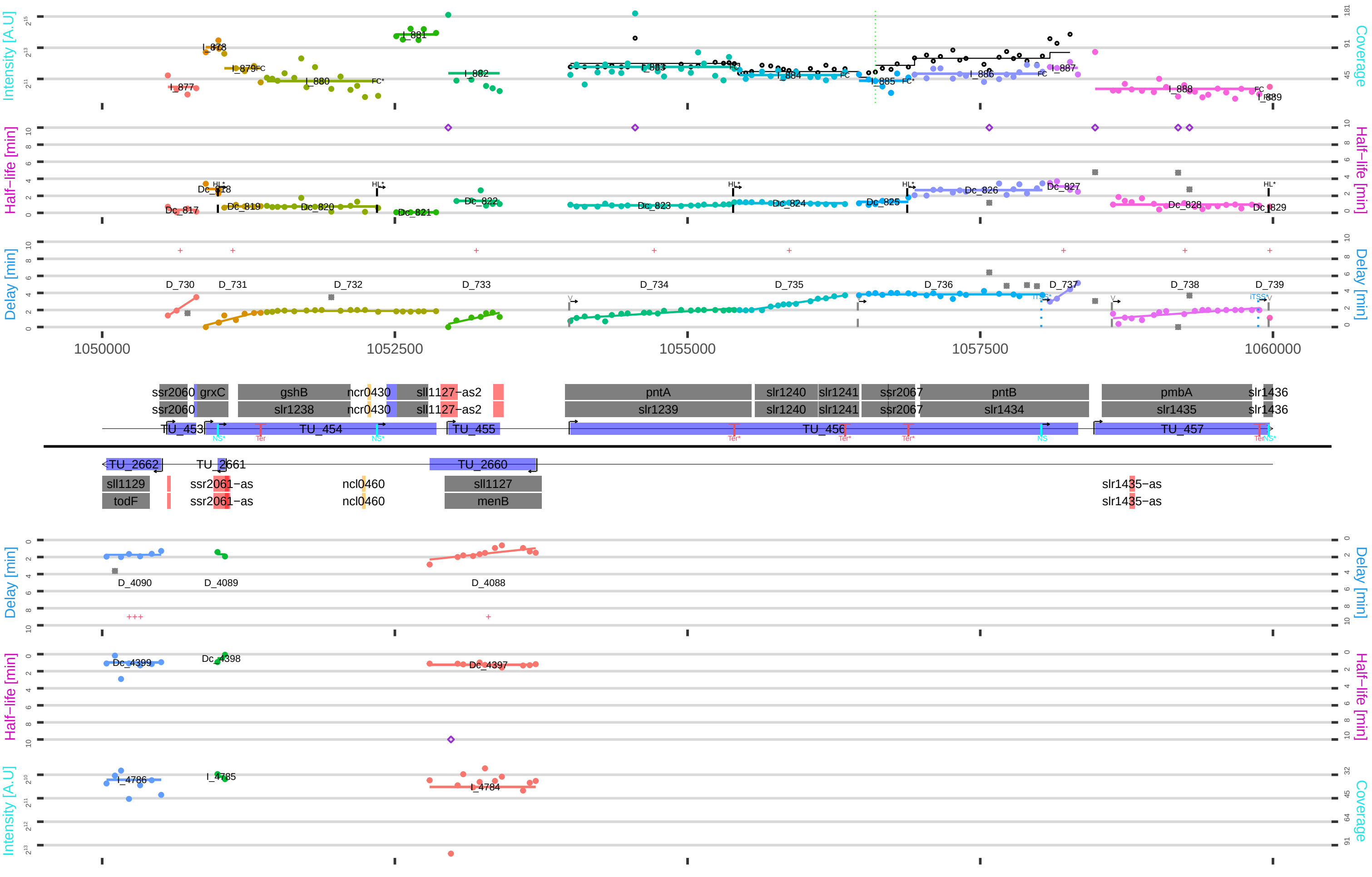

Term: termination (0), NS: new start (0), PS: pausing site (0), iTSS_I: internal starting site (0)

ID: 8824−8884; Term: termination (1), NS: new start (1), PS: pausing site (0), iTSS_I: internal starting site (2)

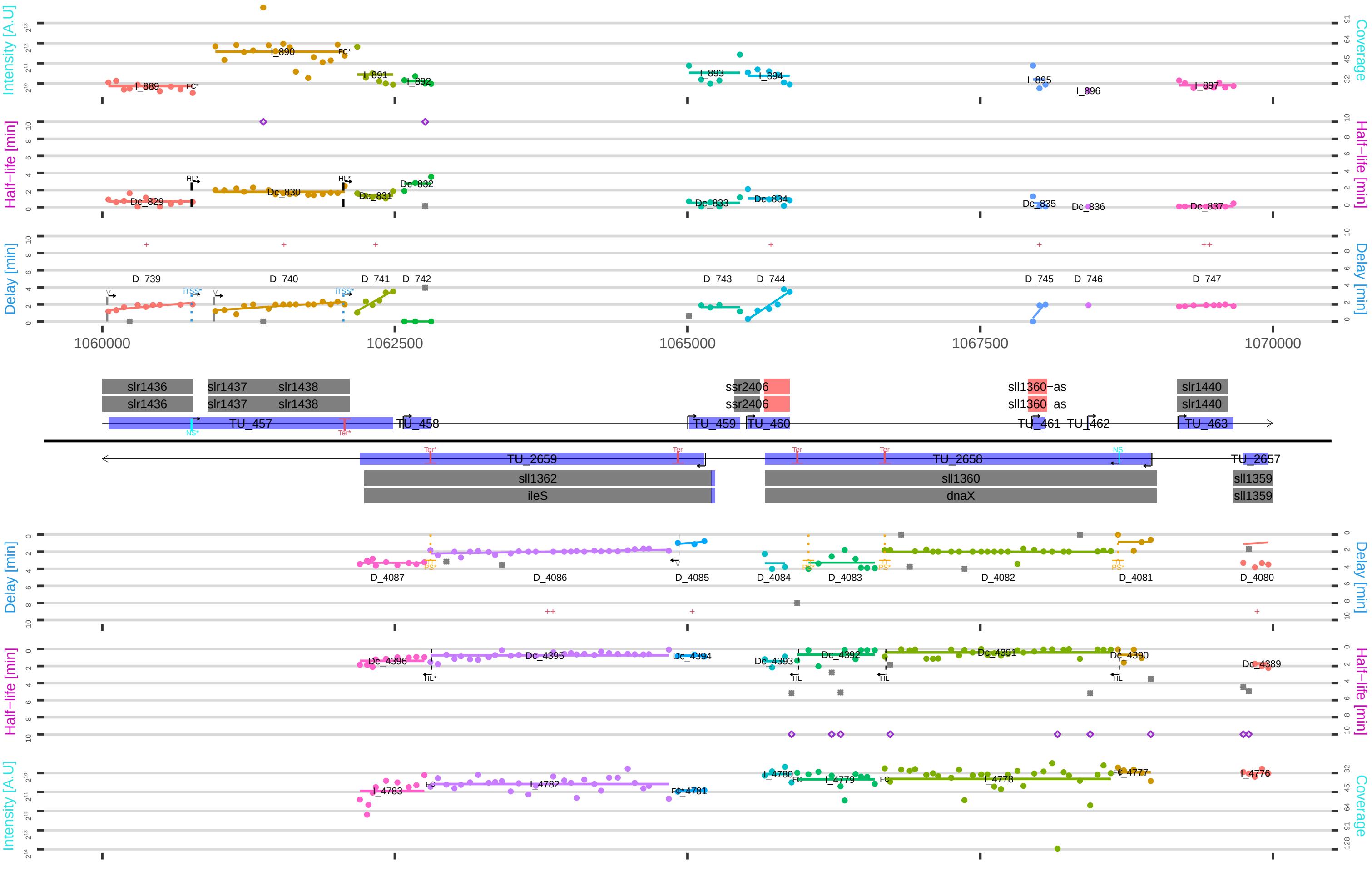

Term: termination (4), NS: new start (1), PS: pausing site (5), iTSS_I: internal starting site (0)

ID: 8885−8925; Term: termination (1), NS: new start (1), PS: pausing site (0), iTSS_I: internal starting site (0)

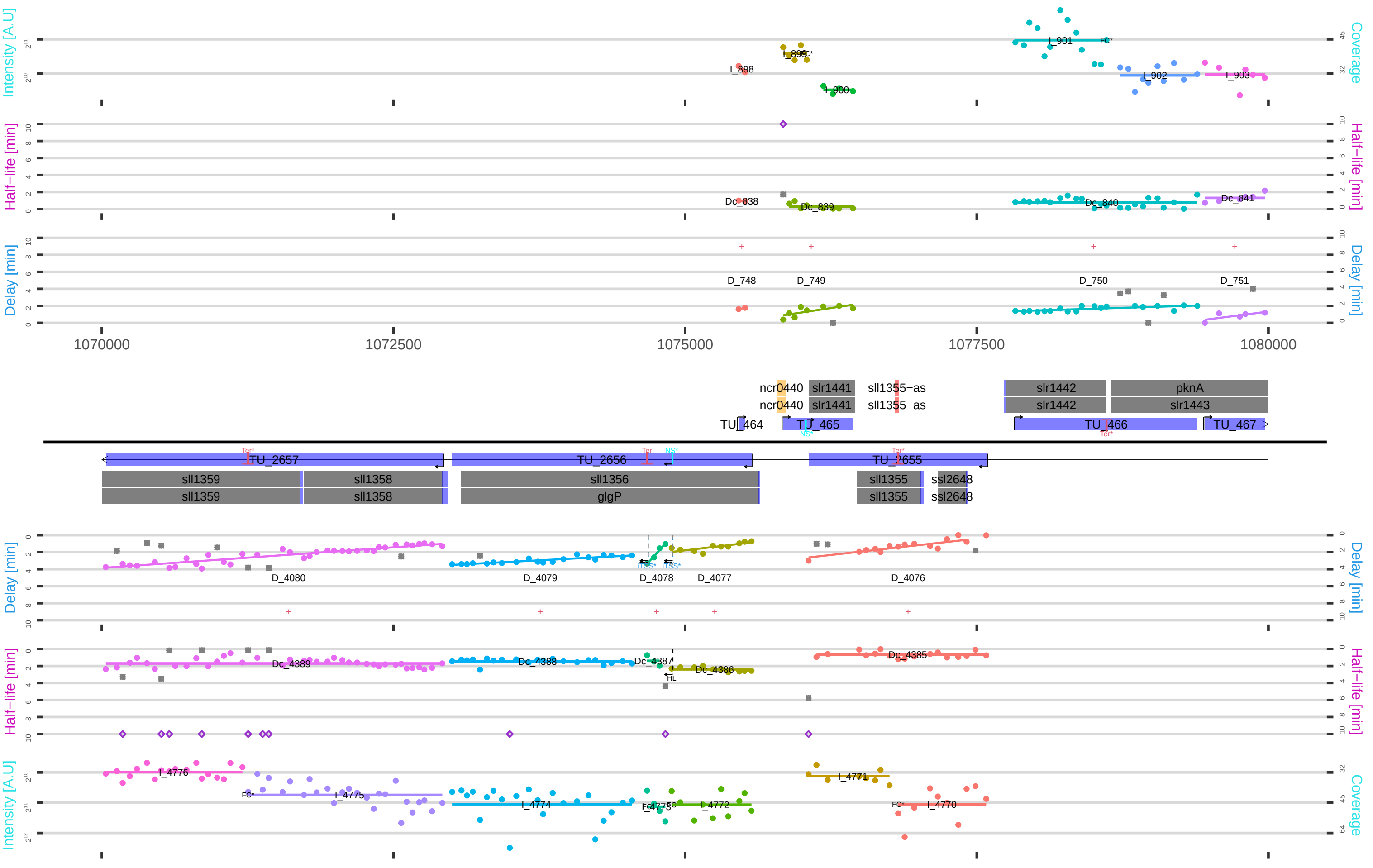

Term: termination (3), NS: new start (1), PS: pausing site (0), iTSS_I: internal starting site (2)

ID: 8926−8977; Term: termination (0), NS: new start (1), PS: pausing site (1), iTSS_I: internal starting site (0)

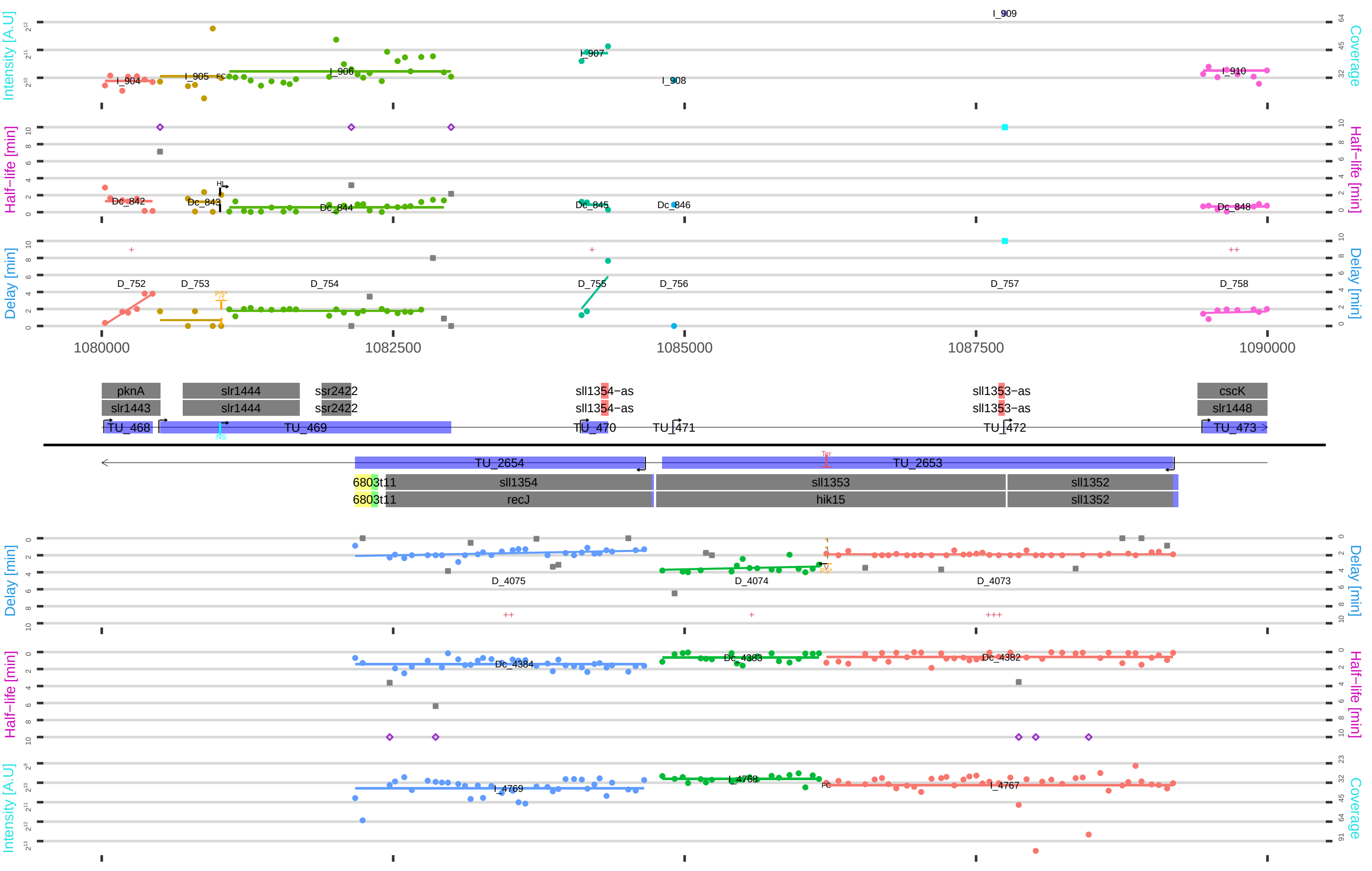

Term: termination (1), NS: new start (0), PS: pausing site (1), iTSS_I: internal starting site (0)

ID: 8978−9024; Term: termination (2), NS: new start (0), PS: pausing site (1), iTSS_I: internal starting site (2)

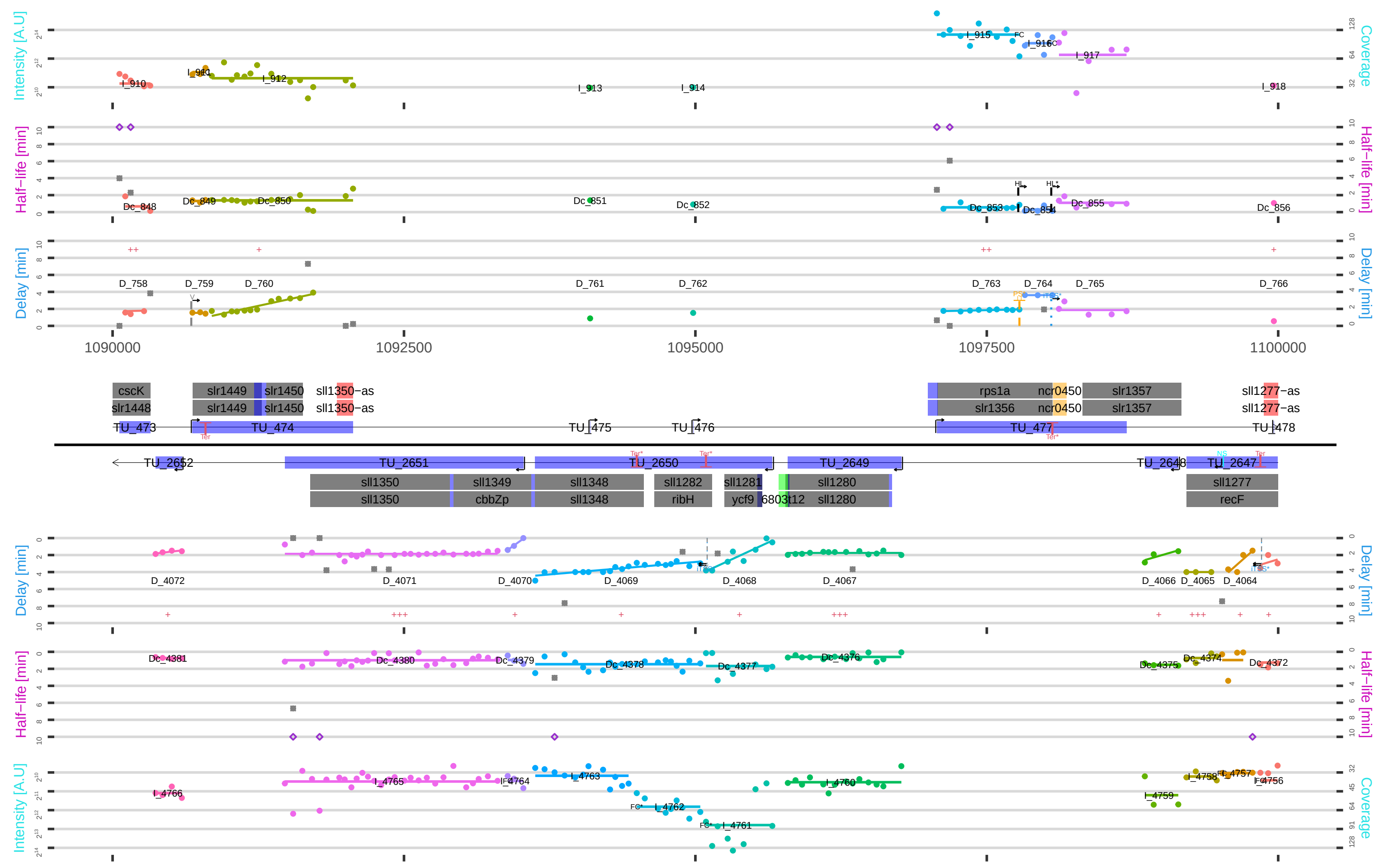

Term: termination (3), NS: new start (1), PS: pausing site (1), iTSS_I: internal starting site (3)

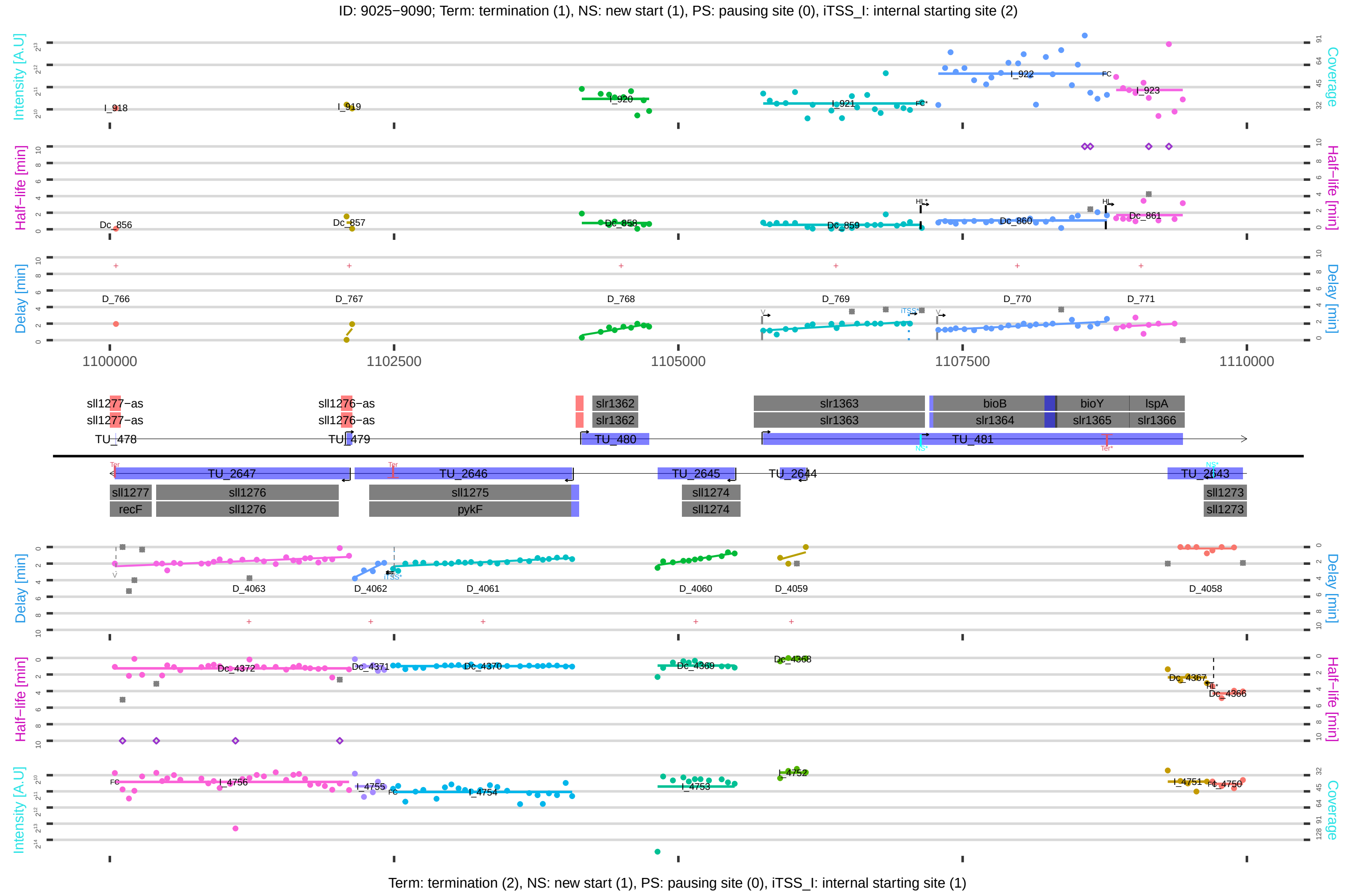
ID: 9025−9090; Term: termination (1), NS: new start (1), PS: pausing site (0), iTSS_I: internal starting site (2)
Intensity [A.U]
Coverage
Half−life [min]
Delay [min]
I_918
I_919
I_920
I_921
I_922
I_923
Dc_856
Dc_857
Dc_858
Dc_859
Dc_860
Dc_861
D_766
D_767
D_768
D_769
D_770
D_771
1100000
1102500
1105000
1107500
1110000
sll1277−as
sll1276−as
slr1362
slr1363
bioB
slr1364
bioY
slr1365
lspA
slr1366
TU_478
TU_479
TU_480
TU_481
TU_2647
TU_2646
TU_2645
TU_2644
TU_2643
sll1277
recF
sll1276
sll1275
pykF
sll1274
sll1273
D_4063
D_4062
D_4061
D_4060
D_4059
D_4058
Dc_4372
Dc_4371
Dc_4370
Dc_4369
Dc_4368
Dc_4367
Dc_4366
I_4756
I_4755
I_4754
I_4753
I_4752
I_4751
I_4750
Term: termination (2), NS: new start (1), PS: pausing site (0), iTSS_I: internal starting site (1)

ID: 9091−9140; Term: termination (2), NS: new start (1), PS: pausing site (1), iTSS_I: internal starting site (0)

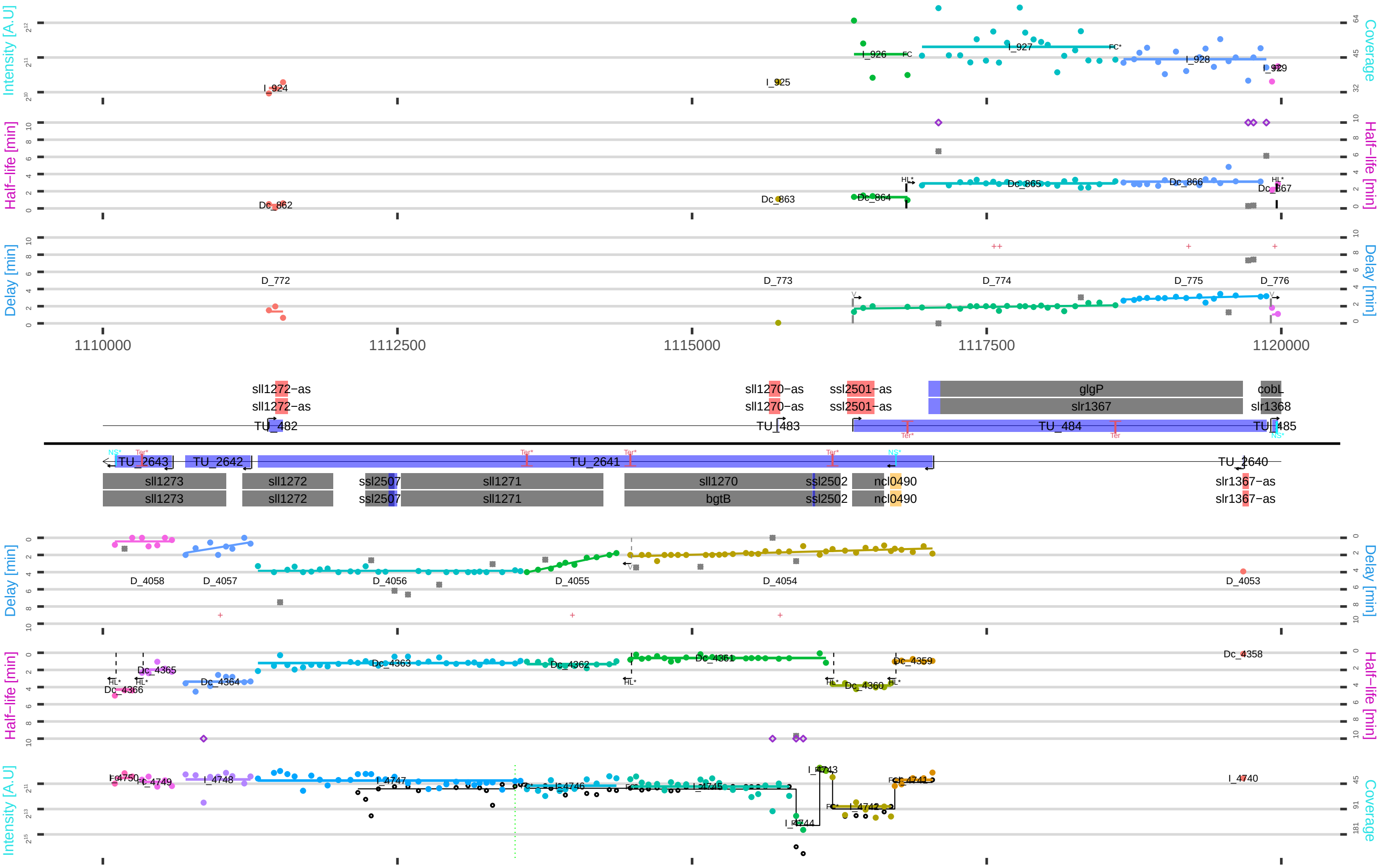

Term: termination (4), NS: new start (2), PS: pausing site (0), iTSS_I: internal starting site (2)

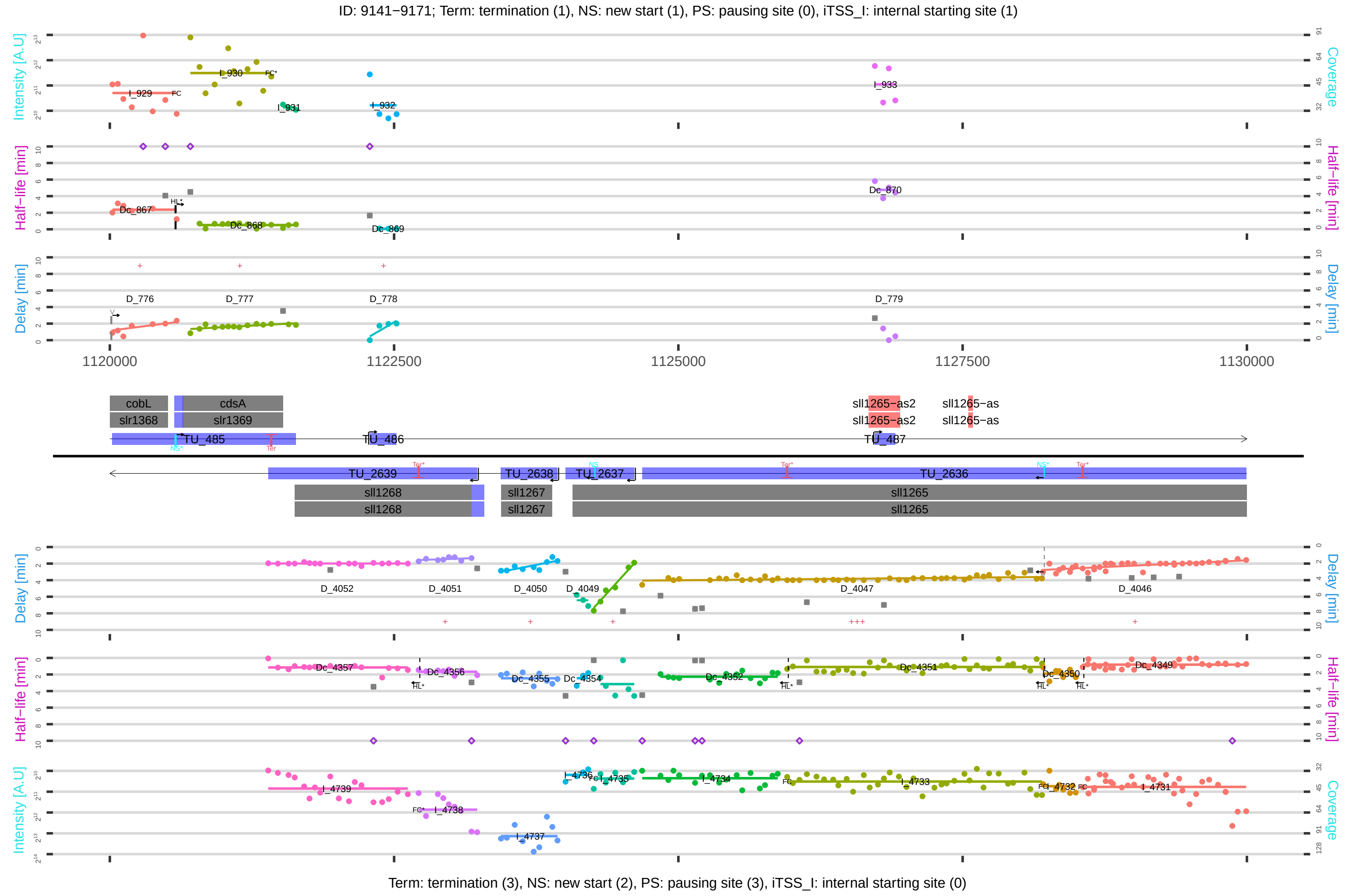

ID: 9141−9171; Term: termination (1), NS: new start (1), PS: pausing site (0), iTSS_I: internal starting site (1)
Intensity [A.U]
Coverage
Half−life [min]
Delay [min]
1120000
1122500
1125000
1127500
1130000
cobL
slr1368
cdsA
slr1369
sll1265−as2
sll1265−as
TU_485
TU_486
TU_487
TU_2639
TU_2638
TU_2637
TU_2636
sll1268
sll1267
sll1265
Term: termination (3), NS: new start (2), PS: pausing site (3), iTSS_I: internal starting site (0)

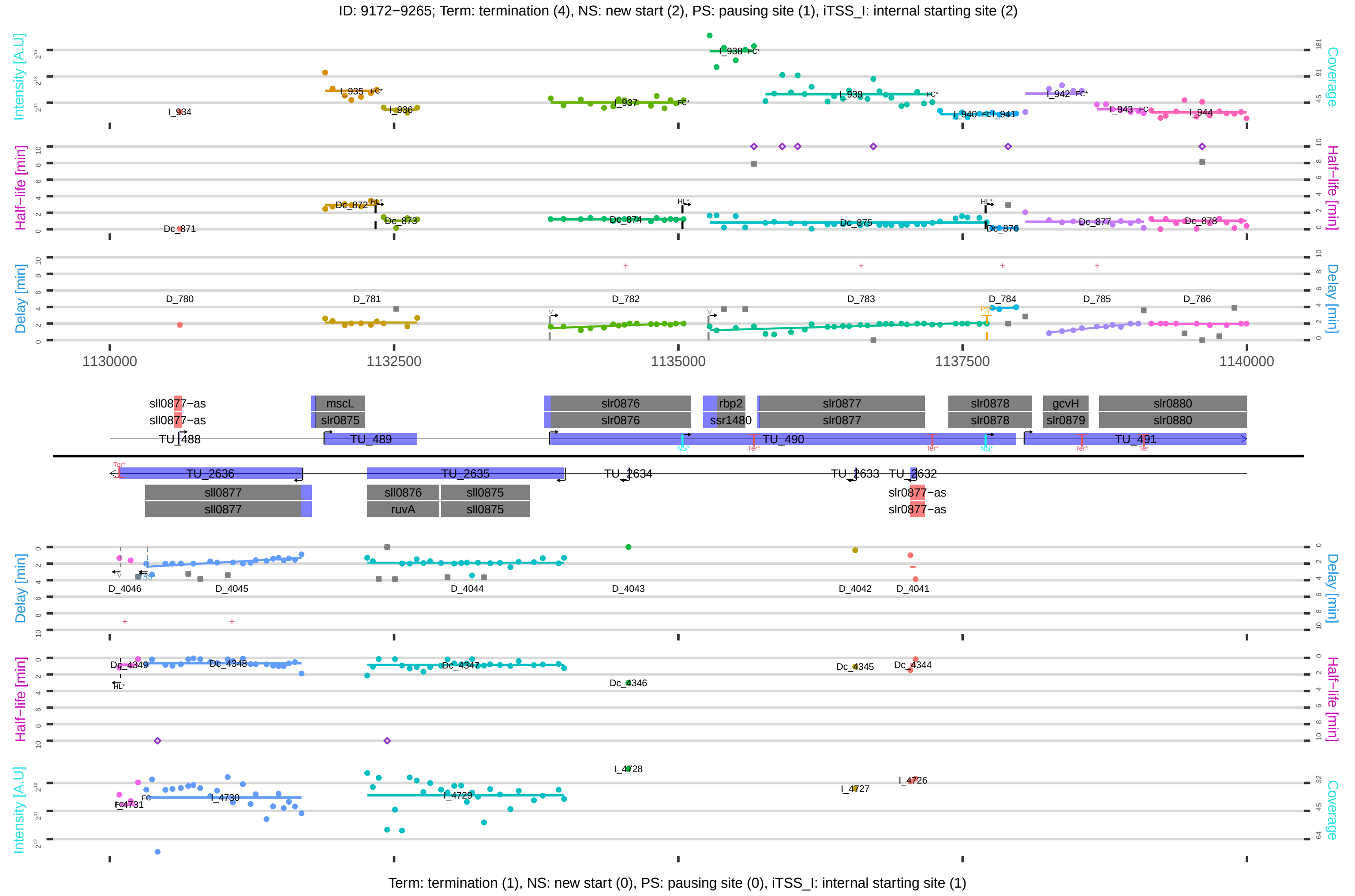
ID: 9172−9265; Term: termination (4), NS: new start (2), PS: pausing site (1), iTSS_I: internal starting site (2)
Intensity [A.U]
Coverage
Half−life [min]
Delay [min]
1130000
1132500
1135000
1137500
1140000
sll0877−as
mscL
slr0875
slr0876
rbp2
ssr1480
slr0877
slr0878
gcvH
slr0879
slr0880
TU_488
TU_489
TU_490
TU_491
TU_2636
TU_2635
TU_2634
TU_2633
TU_2632
sll0877
sll0876
ruvA
slr0877−as
Term: termination (1), NS: new start (0), PS: pausing site (0), iTSS_I: internal starting site (1)

ID: 9266−9366; Term: termination (1), NS: new start (2), PS: pausing site (1), iTSS_I: internal starting site (1)

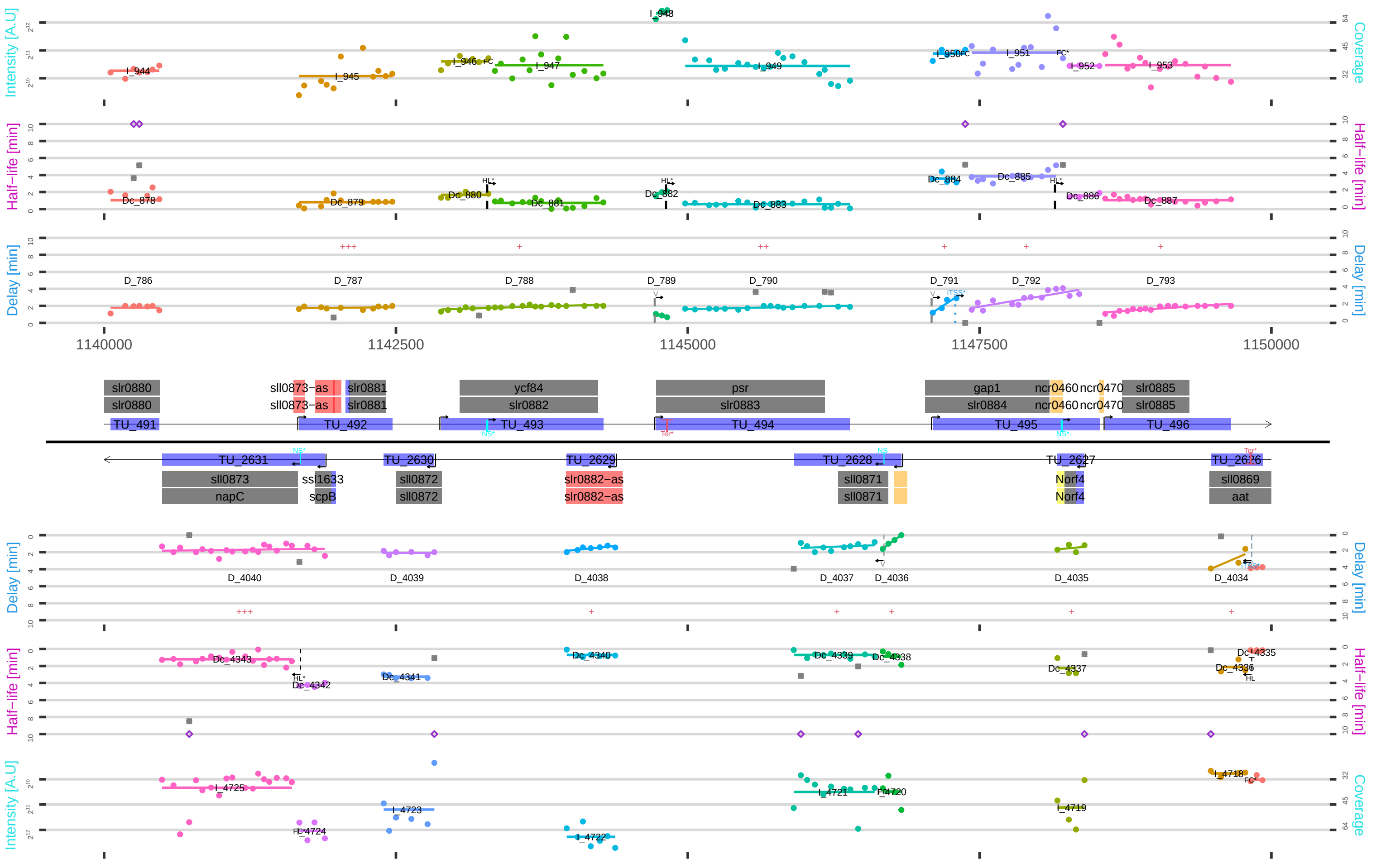

Term: termination (1), NS: new start (2), PS: pausing site (0), iTSS_I: internal starting site (2)

ID: 9367−9449; Term: termination (1), NS: new start (0), PS: pausing site (0), iTSS_I: internal starting site (1)

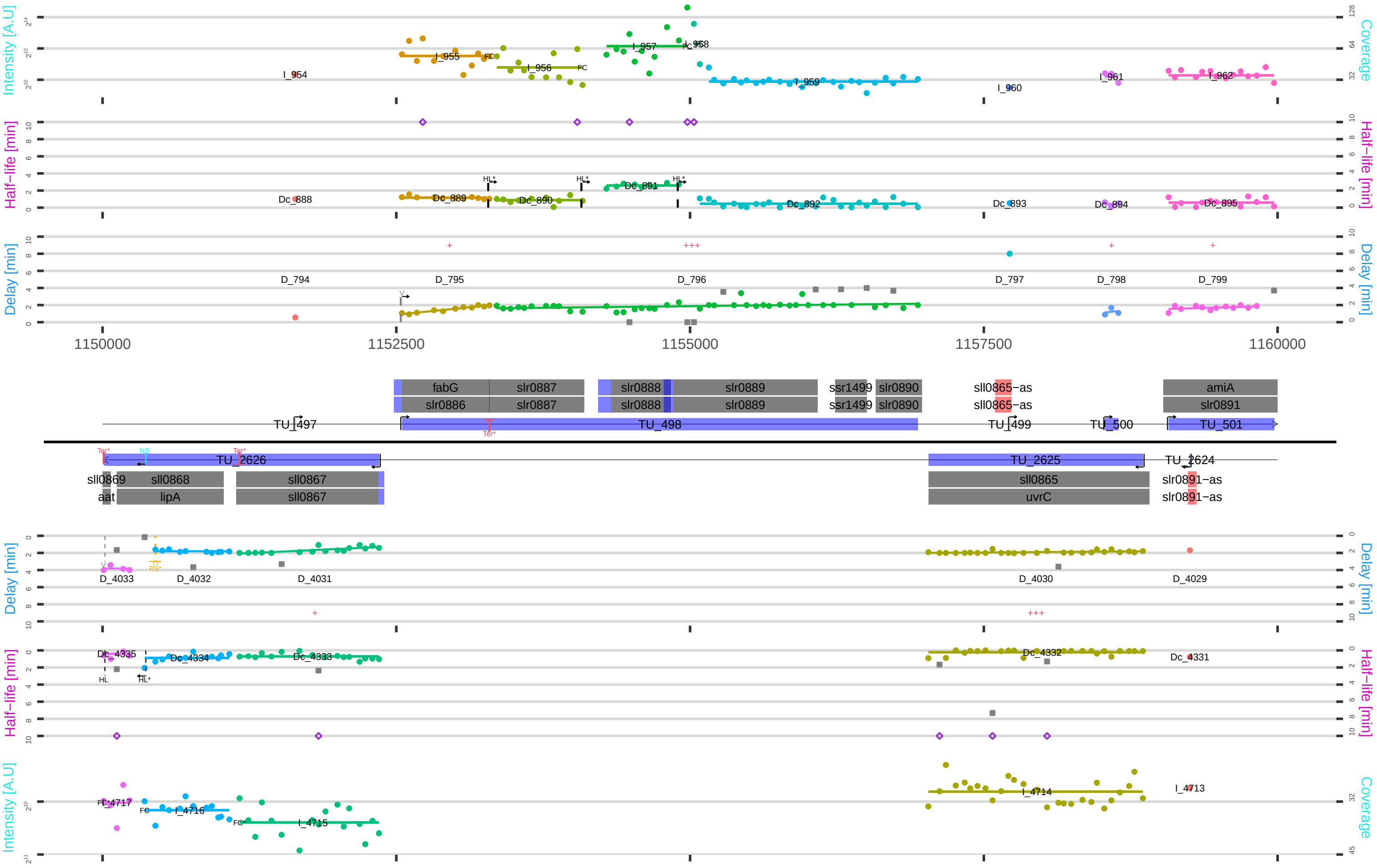

Term: termination (2), NS: new start (1), PS: pausing site (1), iTSS_I: internal starting site (1)

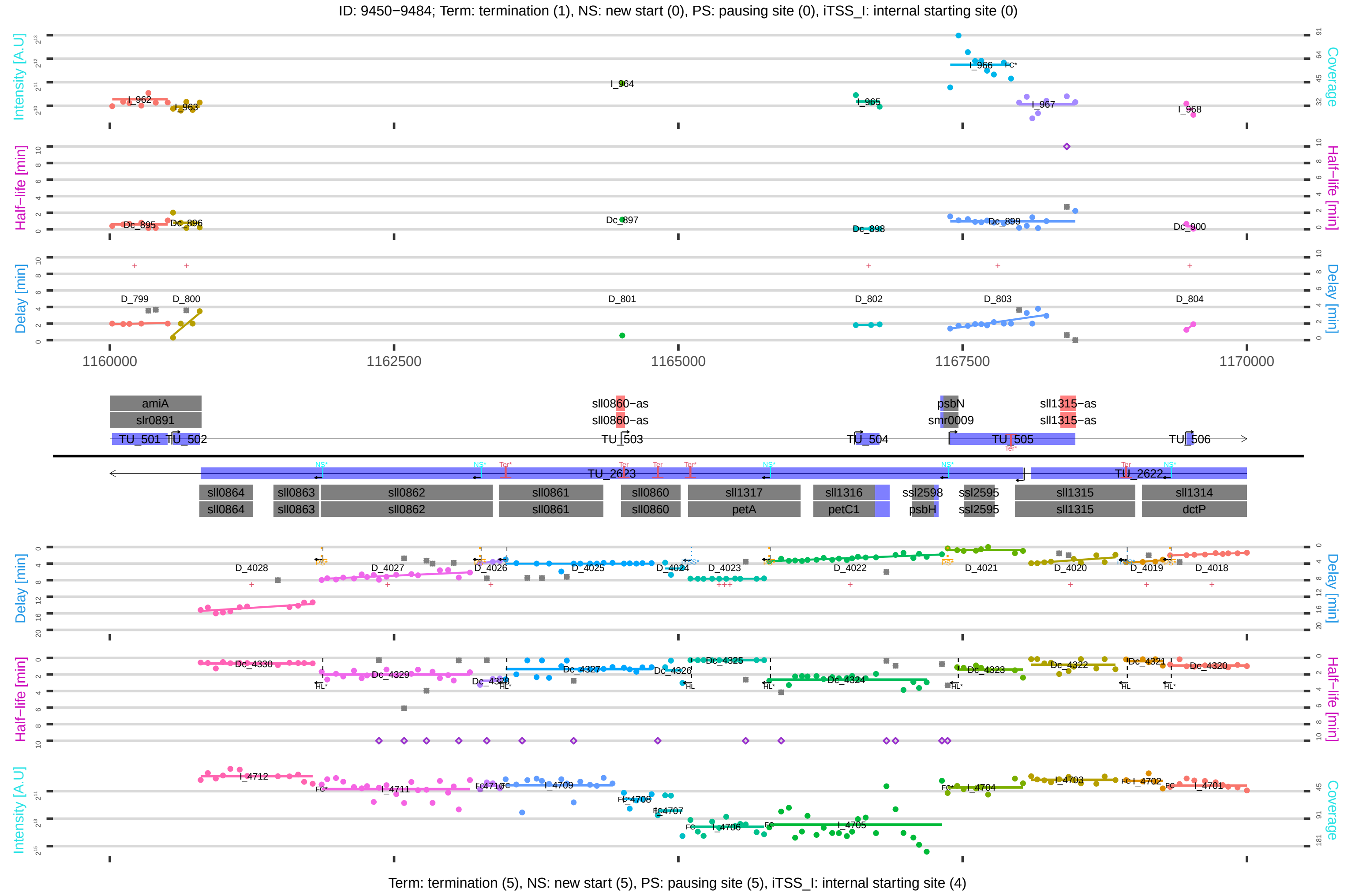

ID: 9450−9484; Term: termination (1), NS: new start (0), PS: pausing site (0), iTSS_I: internal starting site (0)
Intensity [A.U]
Coverage
Half−life [min]
Delay [min]
I_962
I_963
I_964
I_965
I_966
I_967
I_968
Dc_895
Dc_896
Dc_897
Dc_898
Dc_899
Dc_900
D_799
D_800
D_801
D_802
D_803
D_804
1160000
1162500
1165000
1167500
1170000
amiA
slr0891
sll0860−as
psbN
smr0009
sll1315−as
TU_501
TU_502
TU_503
TU_504
TU_505
TU_506
TU_2623
TU_2622
sll0864
sll0863
sll0862
sll0861
sll0860
sll1317
petA
sll1316
petC1
ssl2598
psbH
ssl2595
sll1315
sll1314
dctP
D_4028
D_4027
D_4026
D_4025
D_4024
D_4023
D_4022
D_4021
D_4020
D_4019
D_4018
Dc_4330
Dc_4329
Dc_4328
Dc_4327
Dc_4326
Dc_4325
Dc_4324
Dc_4323
Dc_4322
Dc_4321
Dc_4320
I_4712
I_4711
I_4710
I_4709
I_4708
I_4707
I_4706
I_4705
I_4704
I_4703
I_4702
I_4701
Term: termination (5), NS: new start (5), PS: pausing site (5), iTSS_I: internal starting site (4)

ID: 9485−9632; Term: termination (3), NS: new start (0), PS: pausing site (0), iTSS_I: internal starting site (2)

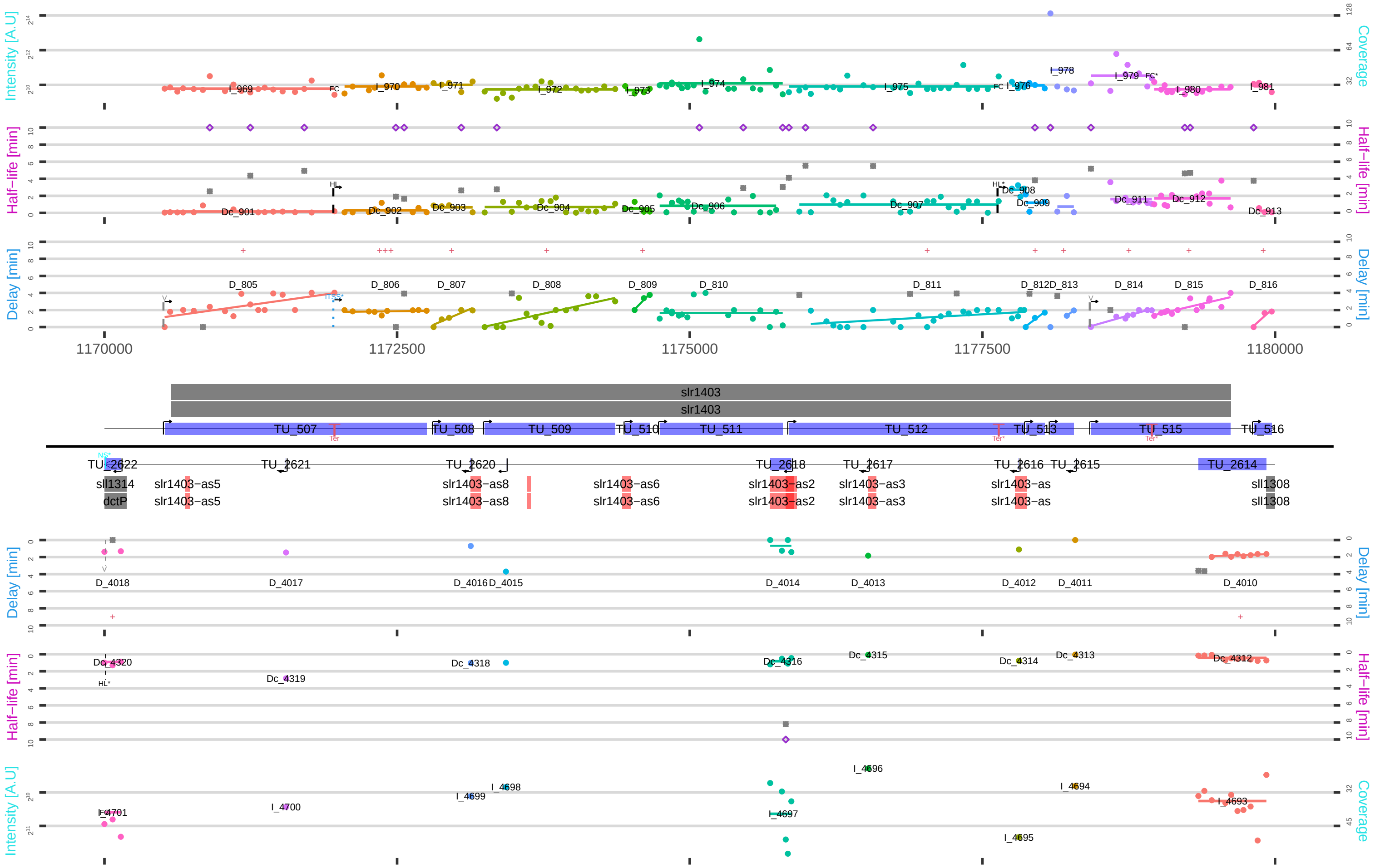

Term: termination (0), NS: new start (1), PS: pausing site (0), iTSS_I: internal starting site (0)

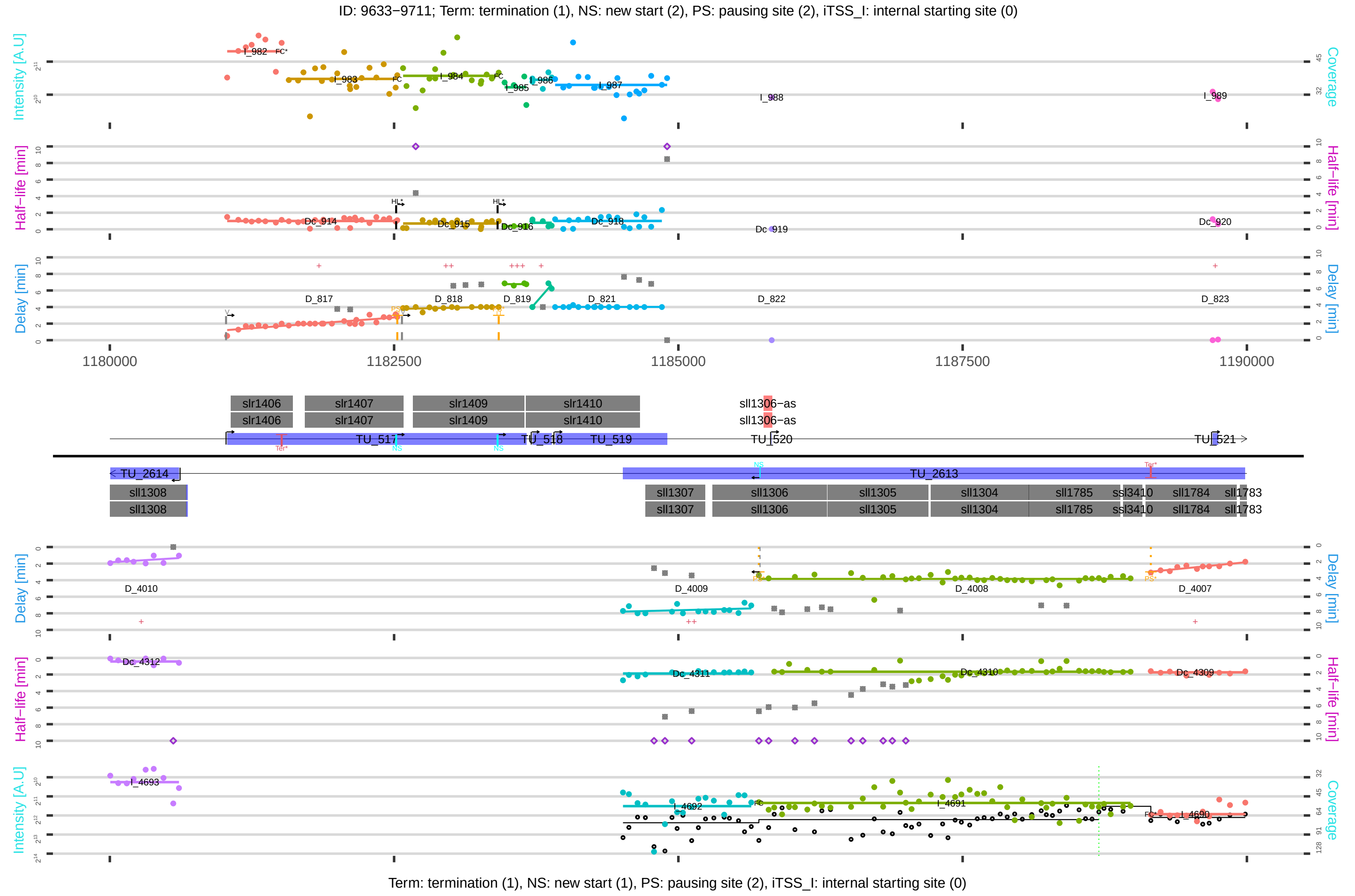

ID: 9633−9711; Term: termination (1), NS: new start (2), PS: pausing site (2), iTSS_I: internal starting site (0)
Intensity [A.U]
Coverage
Half−life [min]
Delay [min]
1180000
1182500
1185000
1187500
1190000
slr1406
slr1407
slr1409
slr1410
sll1306−as
TU_517
TU_518
TU_519
TU_520
TU_521
TU_2614
TU_2613
sll1308
sll1307
sll1306
sll1305
sll1304
sll1785
ssl3410
sll1784
sll1783
Term: termination (1), NS: new start (1), PS: pausing site (2), iTSS_I: internal starting site (0)

ID: 9712−9838; Term: termination (4), NS: new start (2), PS: pausing site (2), iTSS_I: internal starting site (3)

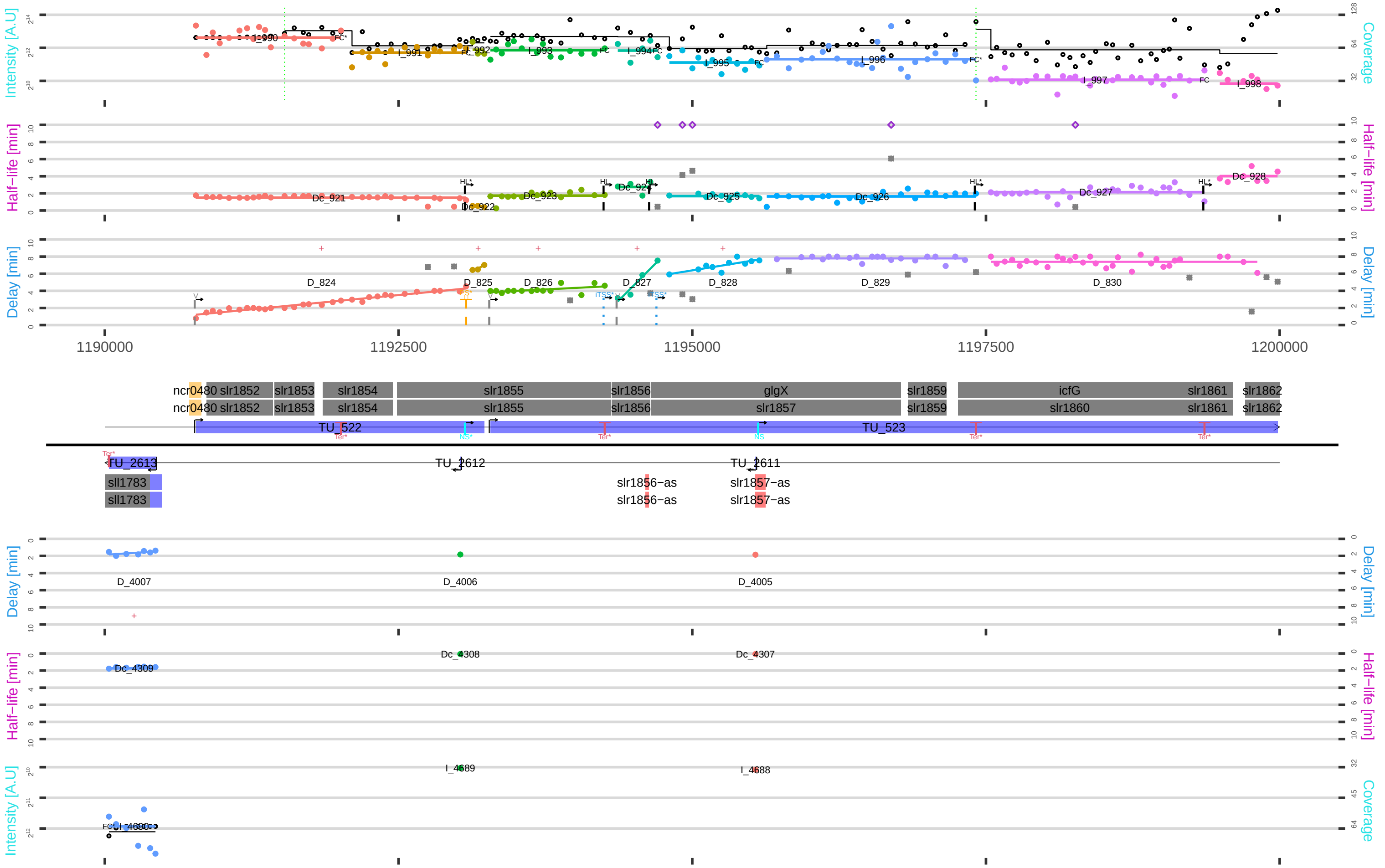

Term: termination (1), NS: new start (0), PS: pausing site (0), iTSS_I: internal starting site (0)

ID: 9839−9947; Term: termination (7), NS: new start (7), PS: pausing site (4), iTSS_I: internal starting site (2)

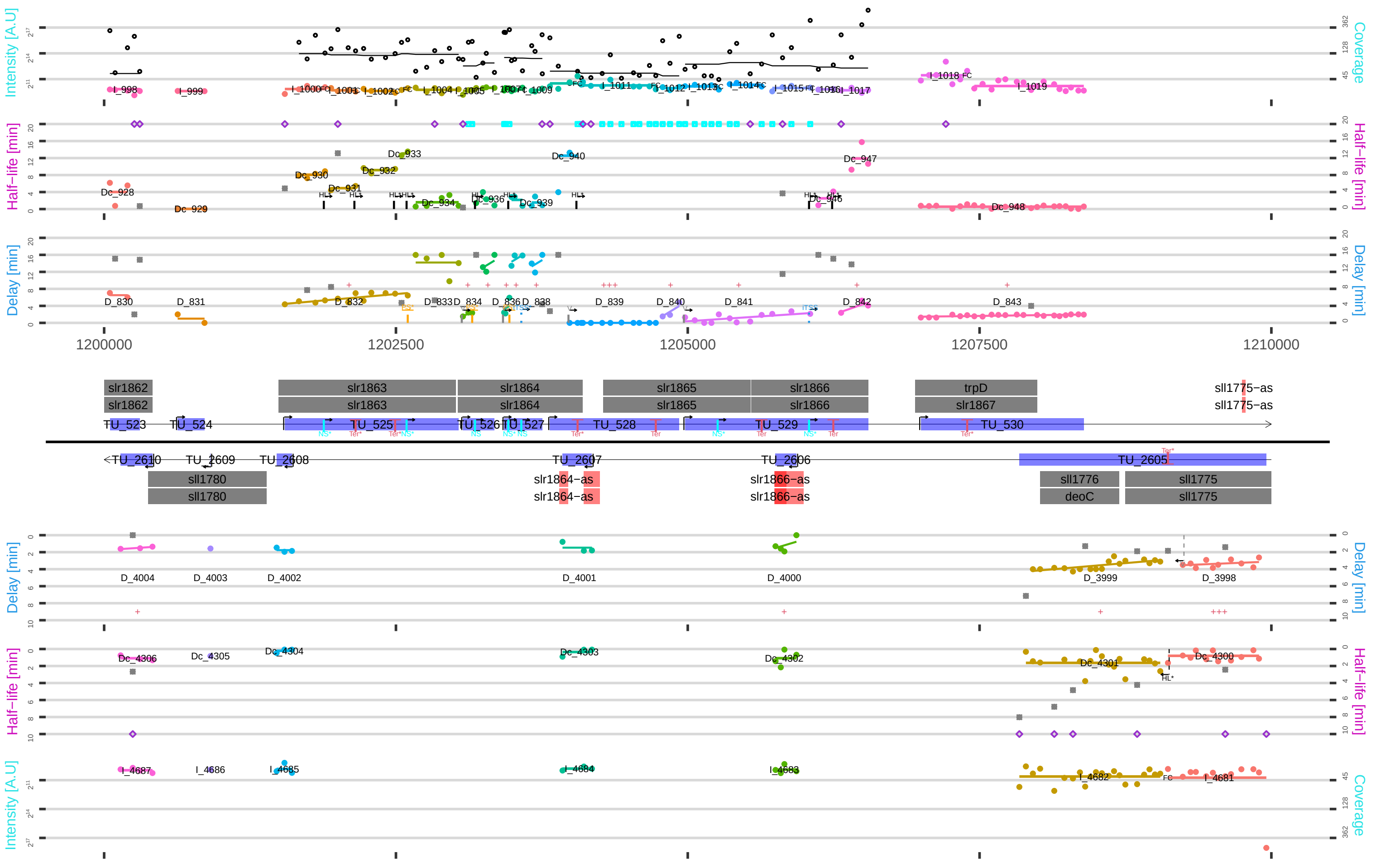

Term: termination (1), NS: new start (0), PS: pausing site (0), iTSS_I: internal starting site (1)

ID: 9948−10004; Term: termination (2), NS: new start (0), PS: pausing site (1), iTSS_I: internal starting site (1)

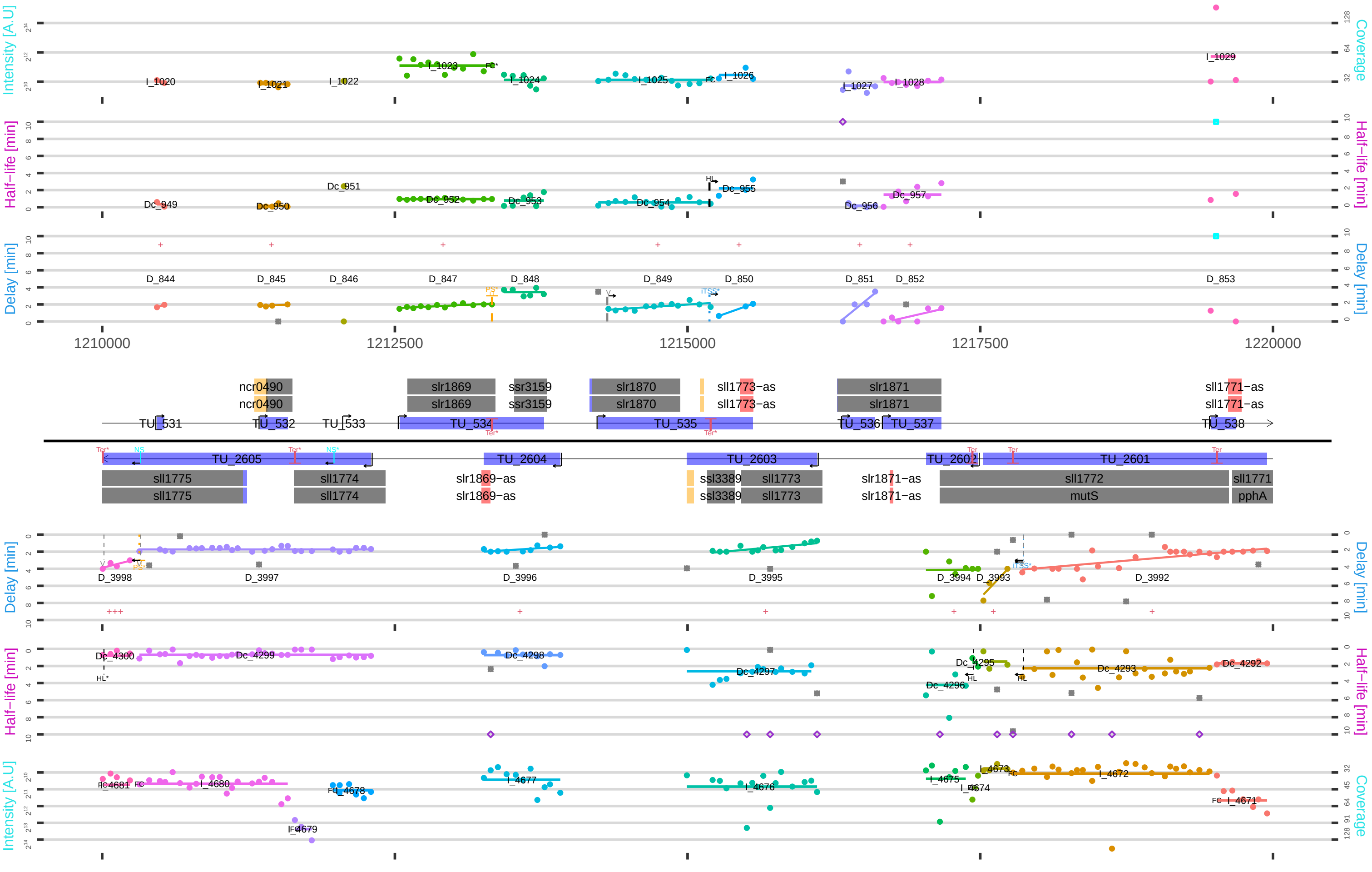

Term: termination (5), NS: new start (2), PS: pausing site (1), iTSS_I: internal starting site (1)

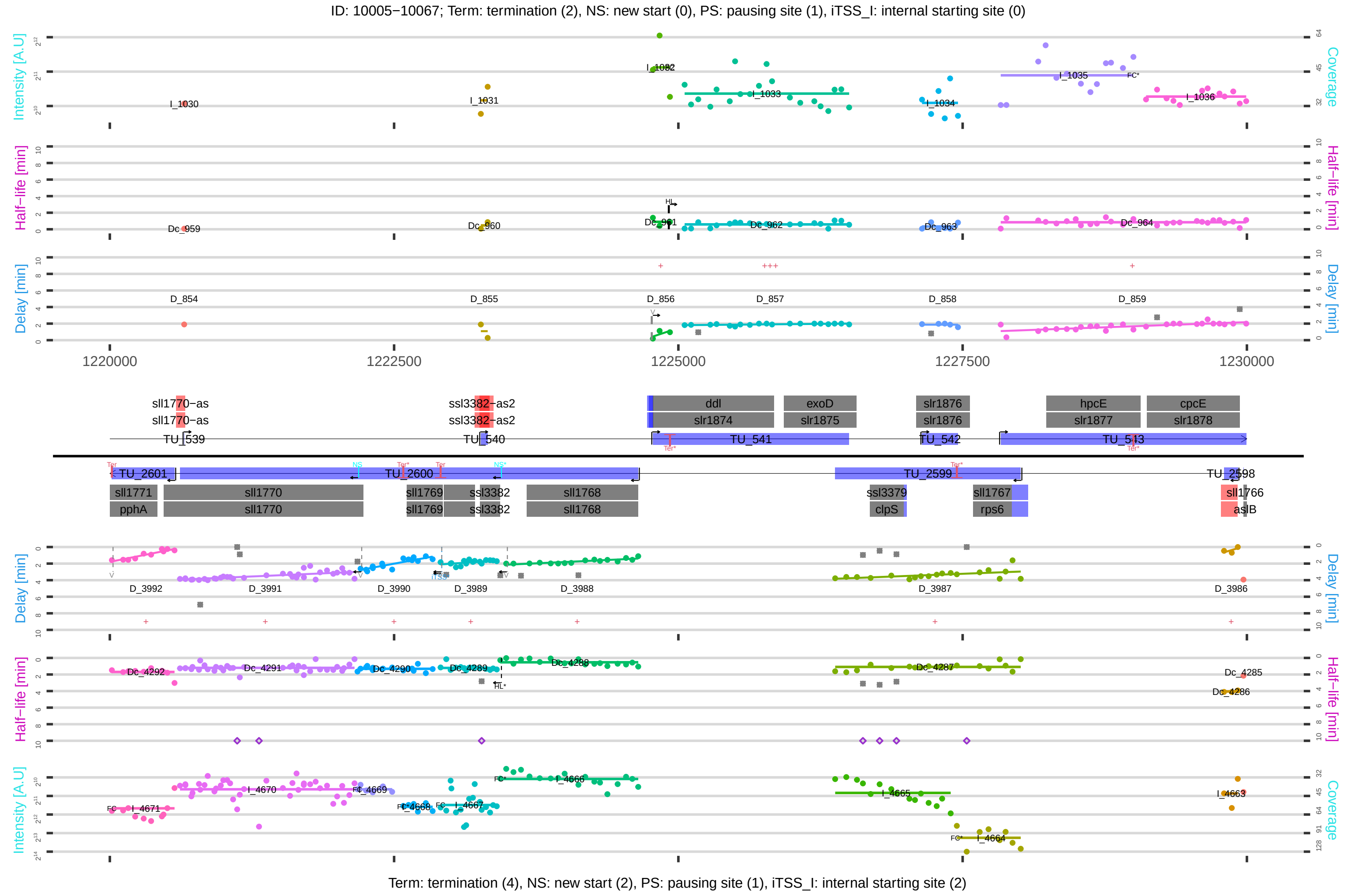
ID: 10005−10067; Term: termination (2), NS: new start (0), PS: pausing site (1), iTSS_I: internal starting site (0)
Intensity [A.U]
Coverage
Half−life [min]
Delay [min]
1220000
1222500
1225000
1227500
1230000
I_1030
I_1031
I_1032
I_1033
I_1034
I_1035
I_1036
Dc_959
Dc_960
Dc_961
Dc_962
Dc_963
Dc_964
D_854
D_855
D_856
D_857
D_858
D_859
sll1770−as
ssl3382−as2
ddl
slr1874
exoD
slr1875
slr1876
hpcE
slr1877
cpcE
slr1878
TU_539
TU_540
TU_541
TU_542
TU_543
TU_2601
TU_2600
TU_2599
TU_2598
sll1771
pphA
sll1770
sll1769
ssl3382
sll1768
ssl3379
clpS
sll1767
rps6
sll1766
aslB
D_3992
D_3991
D_3990
D_3989
D_3988
D_3987
D_3986
Dc_4292
Dc_4291
Dc_4290
Dc_4289
Dc_4288
Dc_4287
Dc_4285
Dc_4286
I_4671
I_4670
I_4669
I_4668
I_4667
I_4666
I_4665
I_4664
I_4663
Term: termination (4), NS: new start (2), PS: pausing site (1), iTSS_I: internal starting site (2)

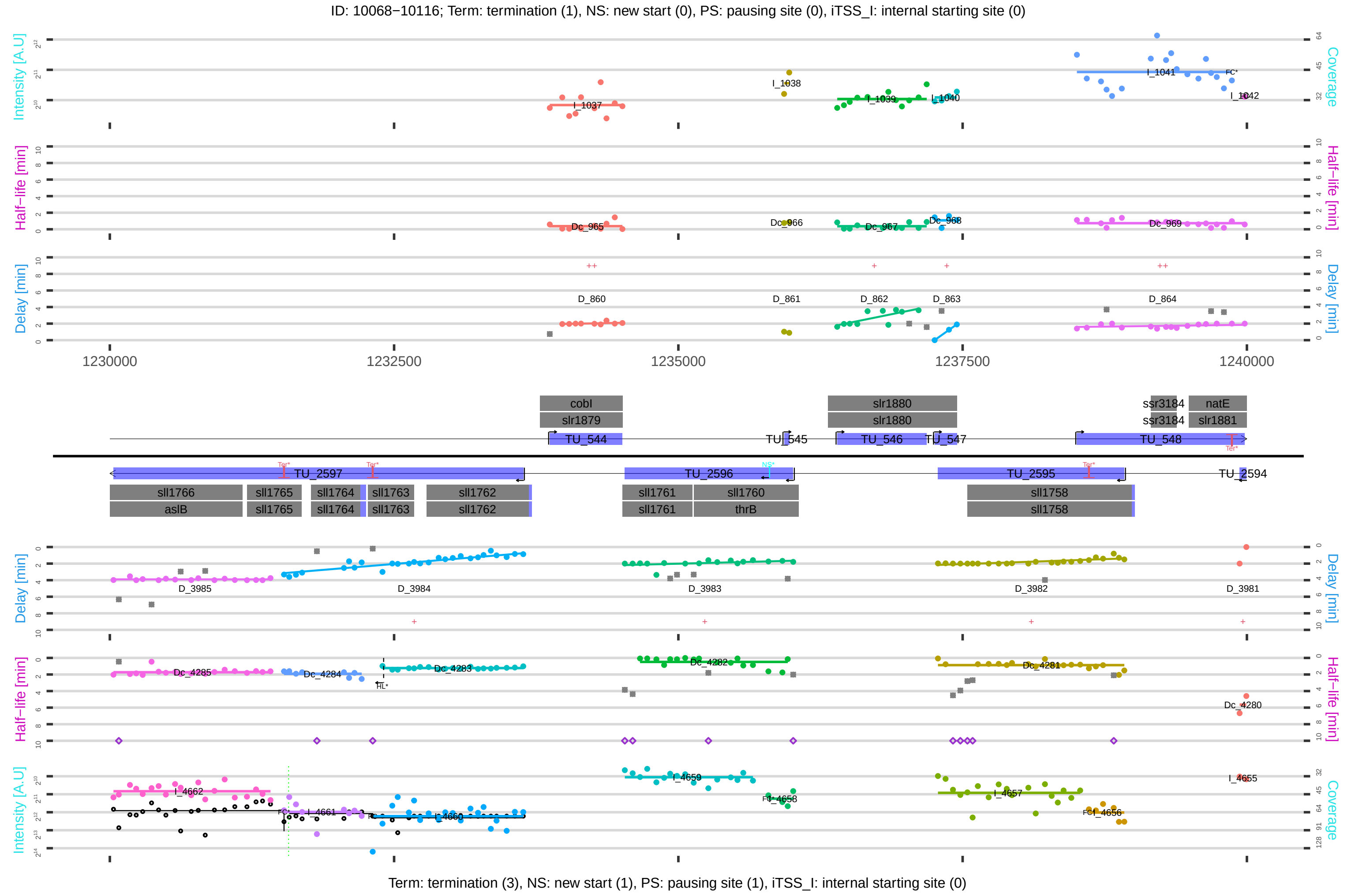

ID: 10068−10116; Term: termination (1), NS: new start (0), PS: pausing site (0), iTSS_I: internal starting site (0)
Intensity [A.U]
Coverage
Half−life [min]
Delay [min]
1230000
1232500
1235000
1237500
1240000
I_1037
I_1038
I_1039
I_1040
I_1041
I_1042
Dc_965
Dc_966
Dc_967
Dc_968
Dc_969
D_860
D_861
D_862
D_863
D_864
cobI
slr1879
slr1880
ssr3184
natE
slr1881
TU_544
TU_545
TU_546
TU_547
TU_548
TU_2597
TU_2596
TU_2595
TU_2594
sll1766
asIB
sll1765
sll1764
sll1763
sll1762
sll1761
sll1760
thrB
sll1758
D_3985
D_3984
D_3983
D_3982
D_3981
Dc_4285
Dc_4284
Dc_4283
Dc_4282
Dc_4281
Dc_4280
I_4662
I_4661
I_4660
I_4659
I_4658
I_4657
I_4656
I_4655
Term: termination (3), NS: new start (1), PS: pausing site (1), iTSS_I: internal starting site (0)

ID: 10117−10237; Term: termination (3), NS: new start (2), PS: pausing site (2), iTSS_I: internal starting site (2)

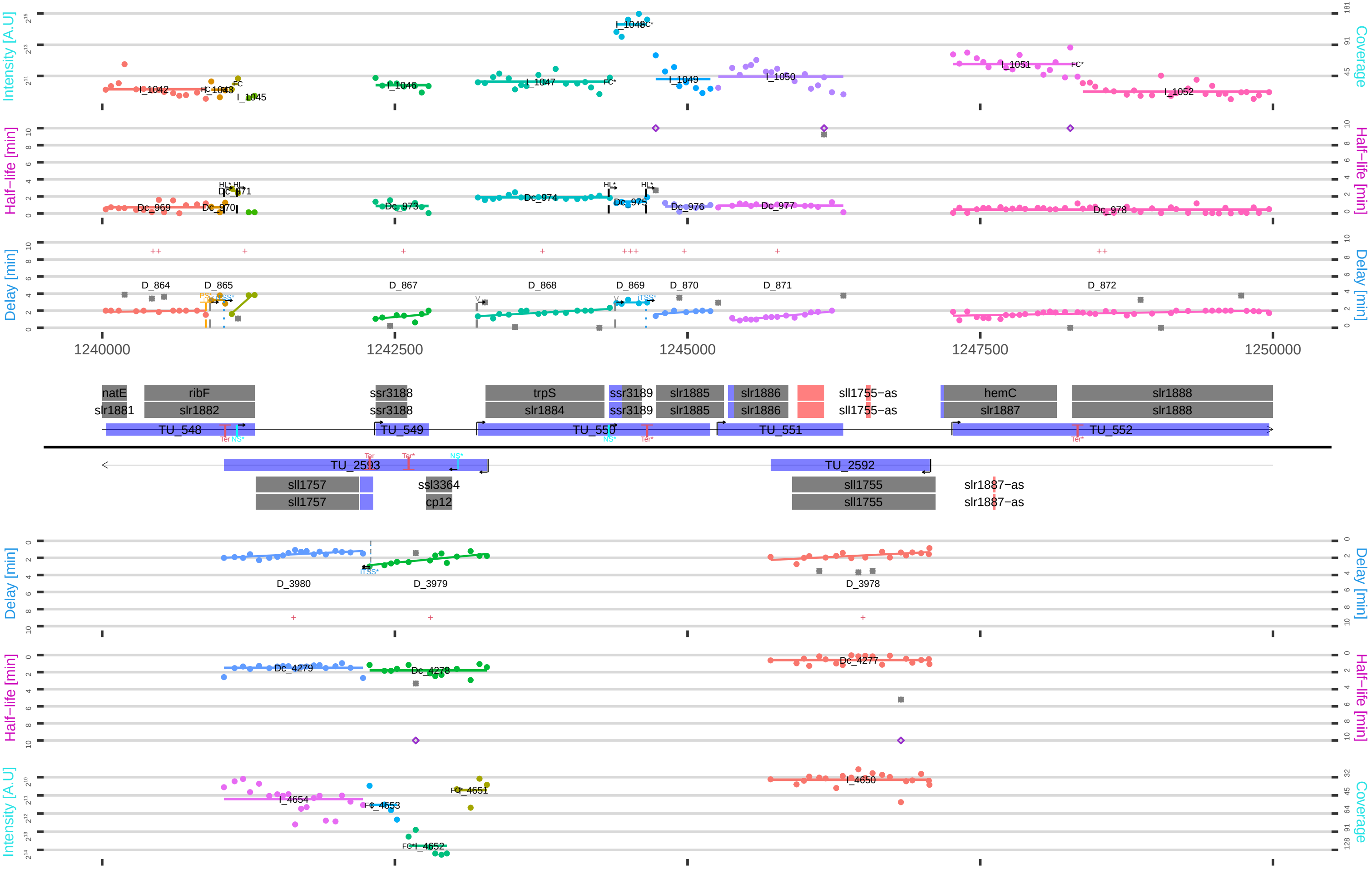

Term: termination (2), NS: new start (1), PS: pausing site (0), iTSS_I: internal starting site (1)

ID: 10238−10294; Term: termination (0), NS: new start (1), PS: pausing site (1), iTSS_I: internal starting site (0)

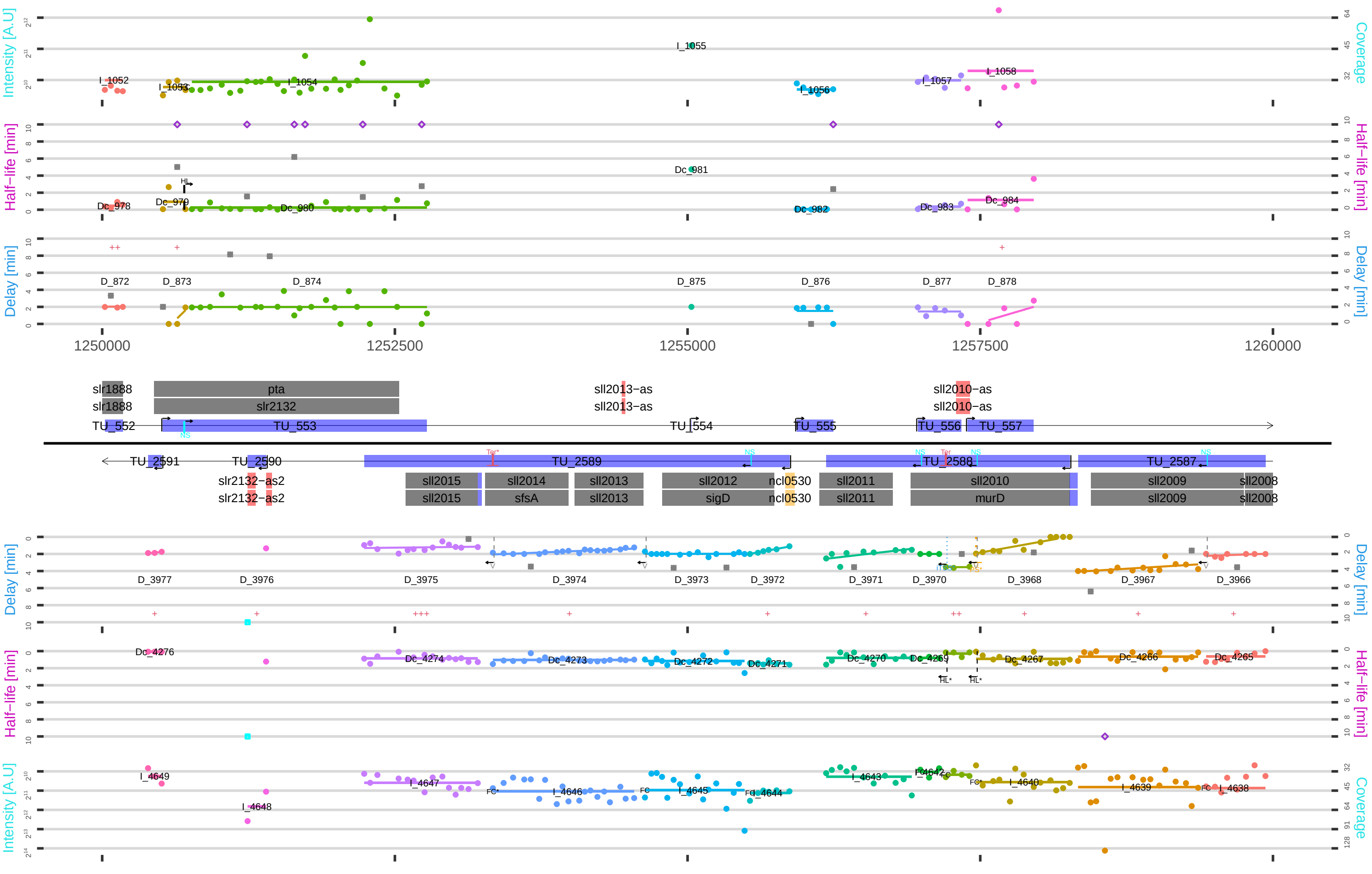

Term: termination (2), NS: new start (4), PS: pausing site (3), iTSS_I: internal starting site (4)

ID: 10295−10340; Term: termination (1), NS: new start (2), PS: pausing site (1), iTSS_I: internal starting site (1)

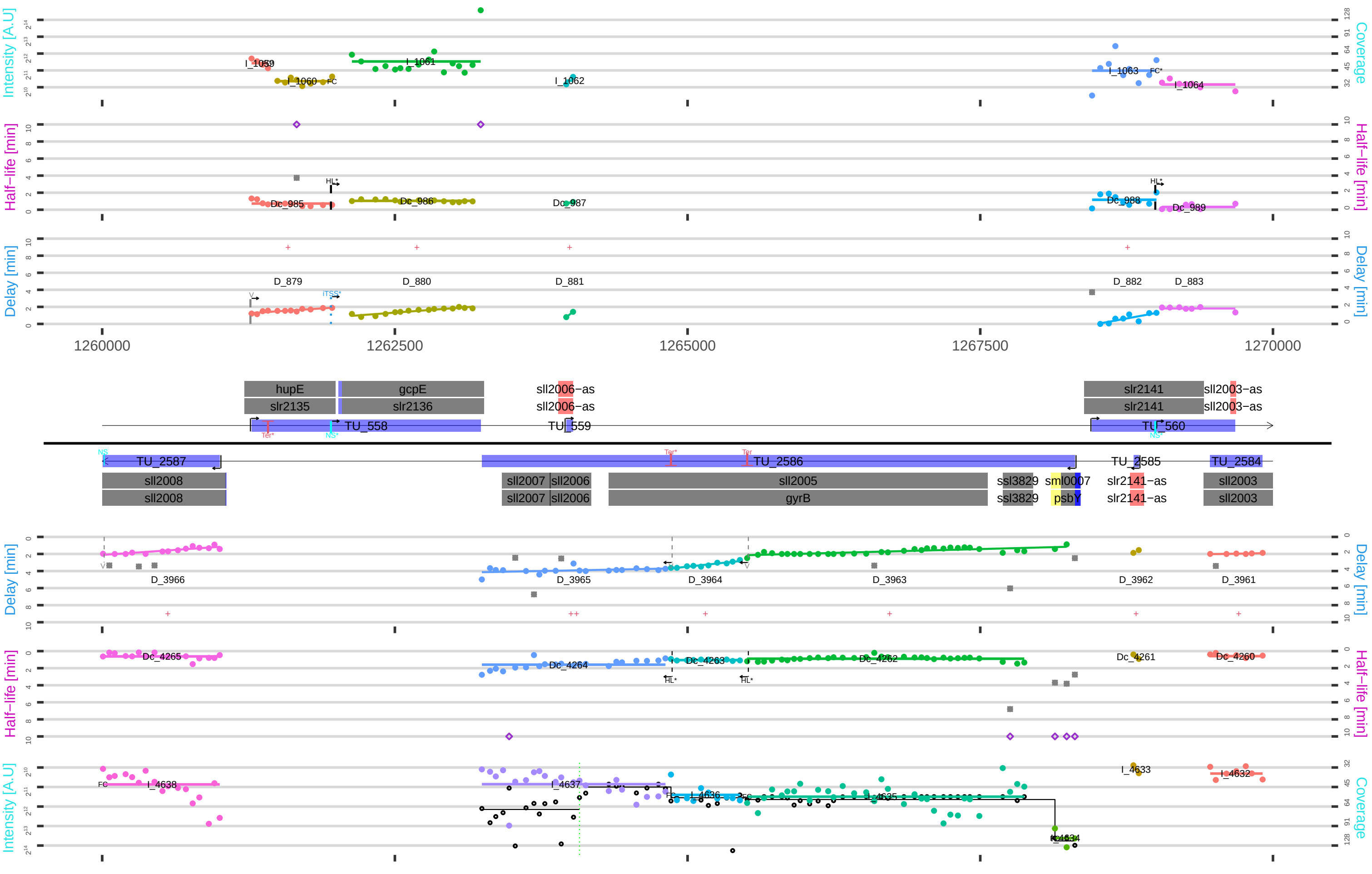

Term: termination (2), NS: new start (1), PS: pausing site (2), iTSS_I: internal starting site (0)

ID: 10341−10428; Term: termination (2), NS: new start (0), PS: pausing site (1), iTSS_I: internal starting site (1)

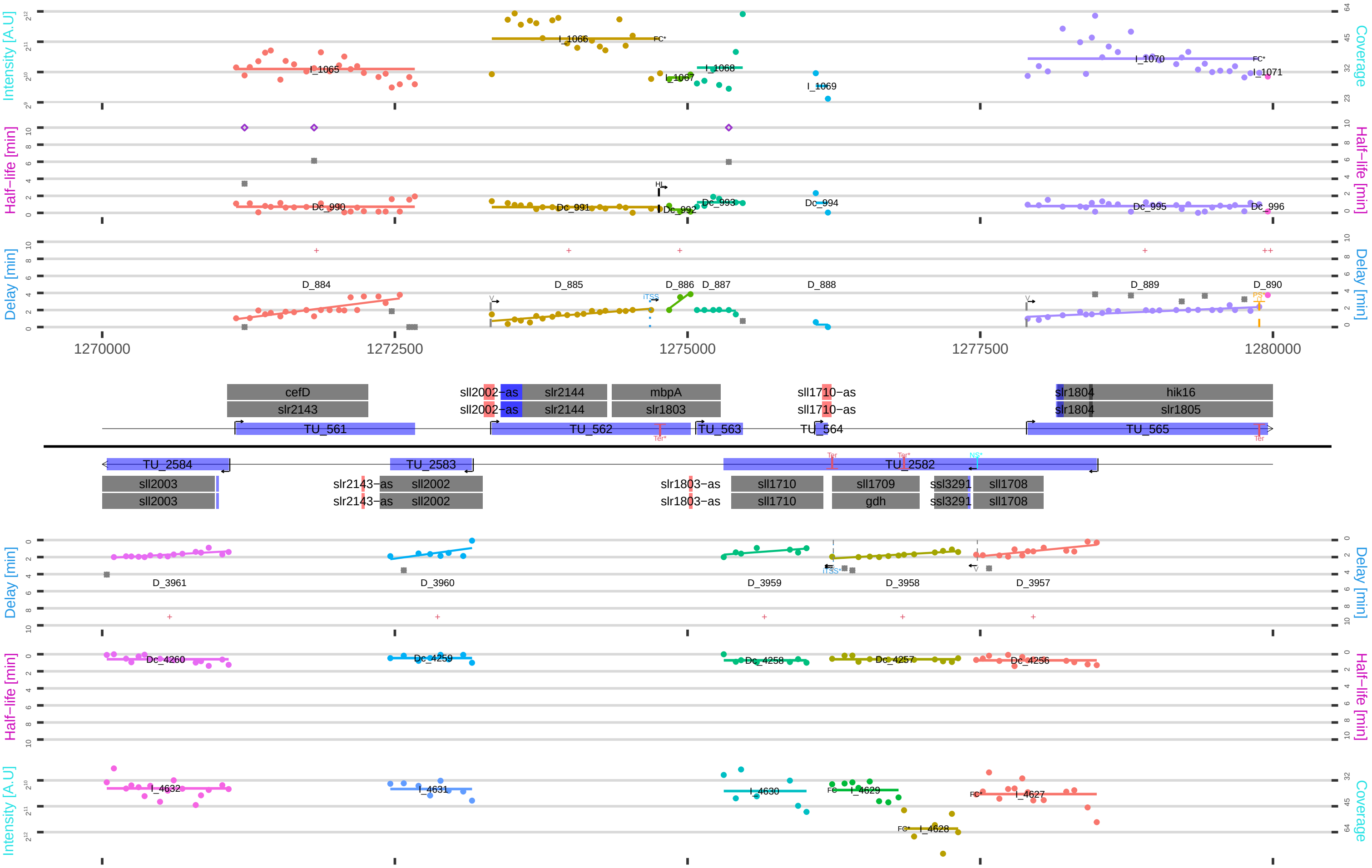

Term: termination (2), NS: new start (1), PS: pausing site (0), iTSS_I: internal starting site (2)

ID: 10429−10497; Term: termination (0), NS: new start (1), PS: pausing site (0), iTSS_I: internal starting site (1)

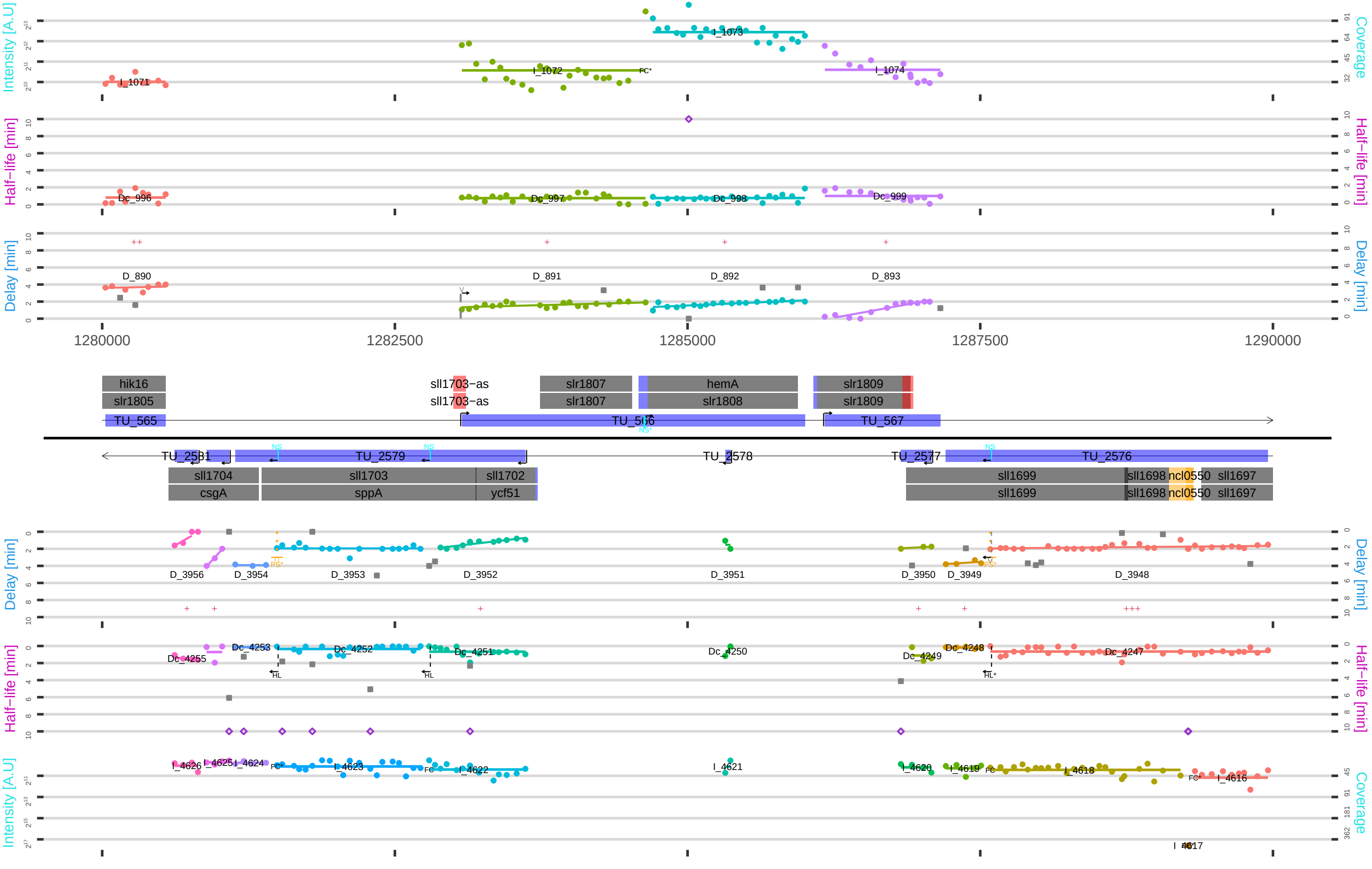

Term: termination (0), NS: new start (3), PS: pausing site (3), iTSS_I: internal starting site (0)

ID: 10498−10588; Term: termination (2), NS: new start (1), PS: pausing site (2), iTSS_I: internal starting site (1)

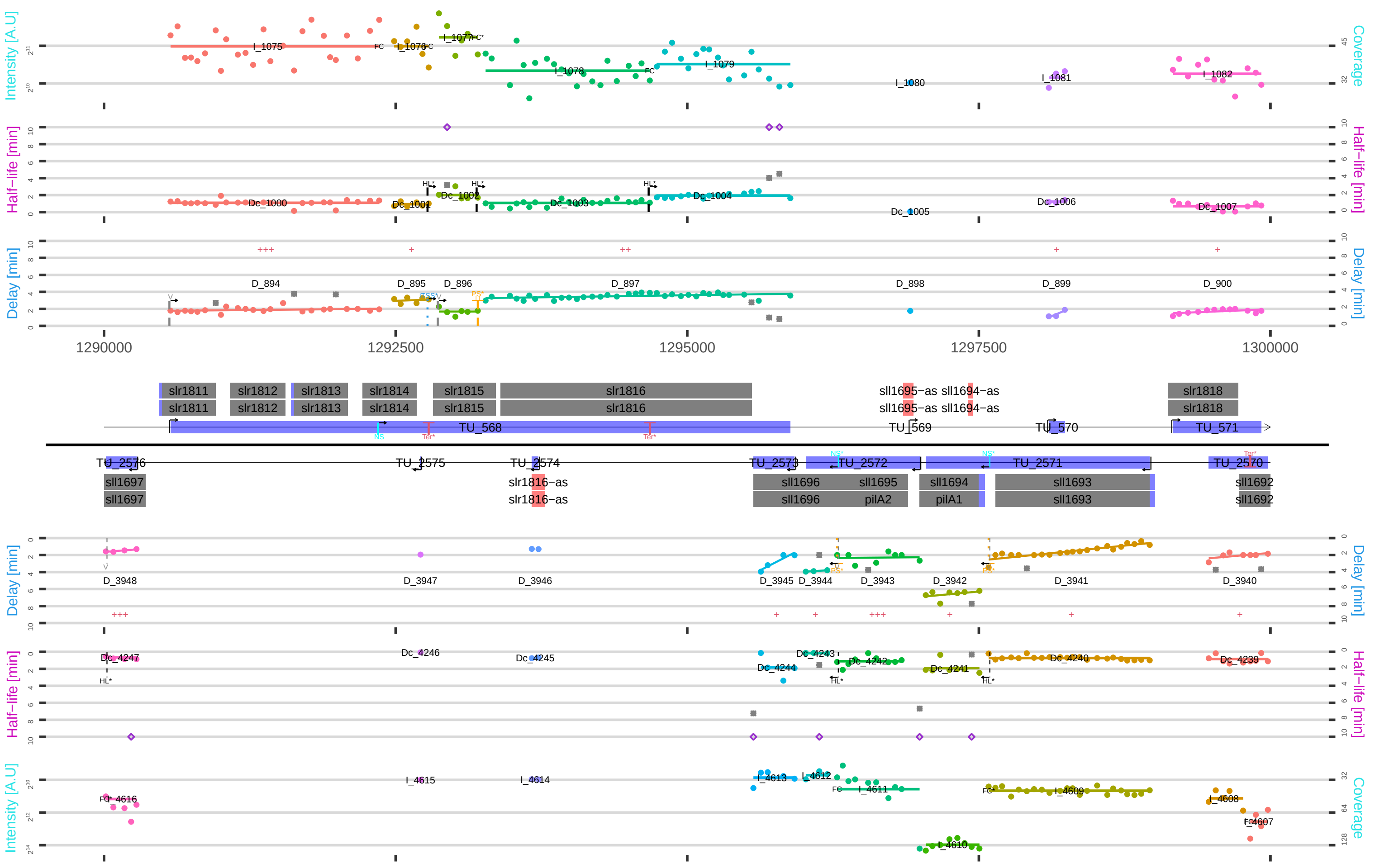

Term: termination (1), NS: new start (2), PS: pausing site (2), iTSS_I: internal starting site (0)

ID: 10589−10635; Term: termination (1), NS: new start (0), PS: pausing site (0), iTSS_I: internal starting site (0)

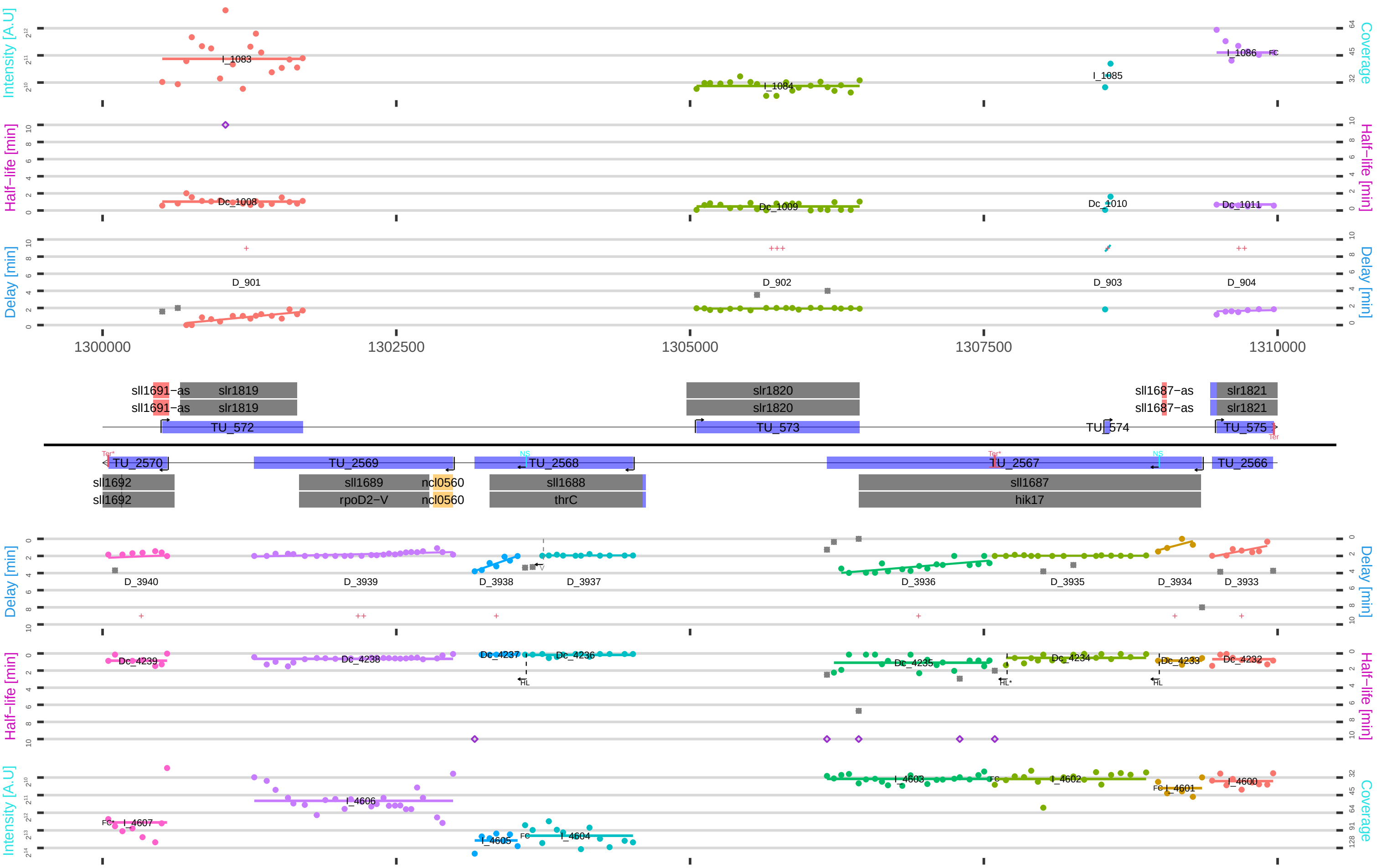

Term: termination (2), NS: new start (2), PS: pausing site (2), iTSS_I: internal starting site (1)

ID: 10636−10700; Term: termination (1), NS: new start (1), PS: pausing site (0), iTSS_I: internal starting site (1)

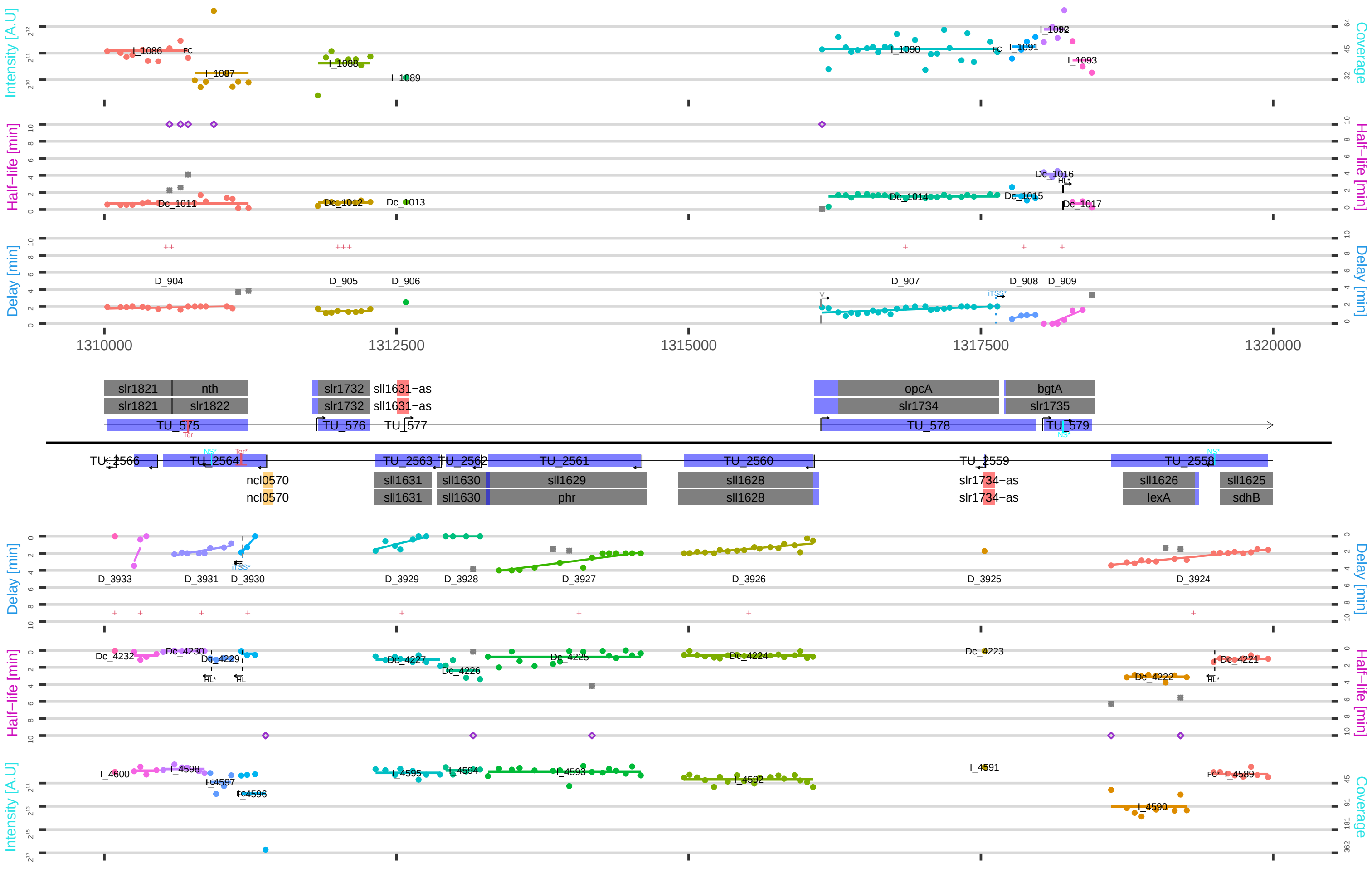

Term: termination (1), NS: new start (2), PS: pausing site (0), iTSS_I: internal starting site (1)

ID: 10701−10783; Term: termination (0), NS: new start (3), PS: pausing site (1), iTSS_I: internal starting site (1)

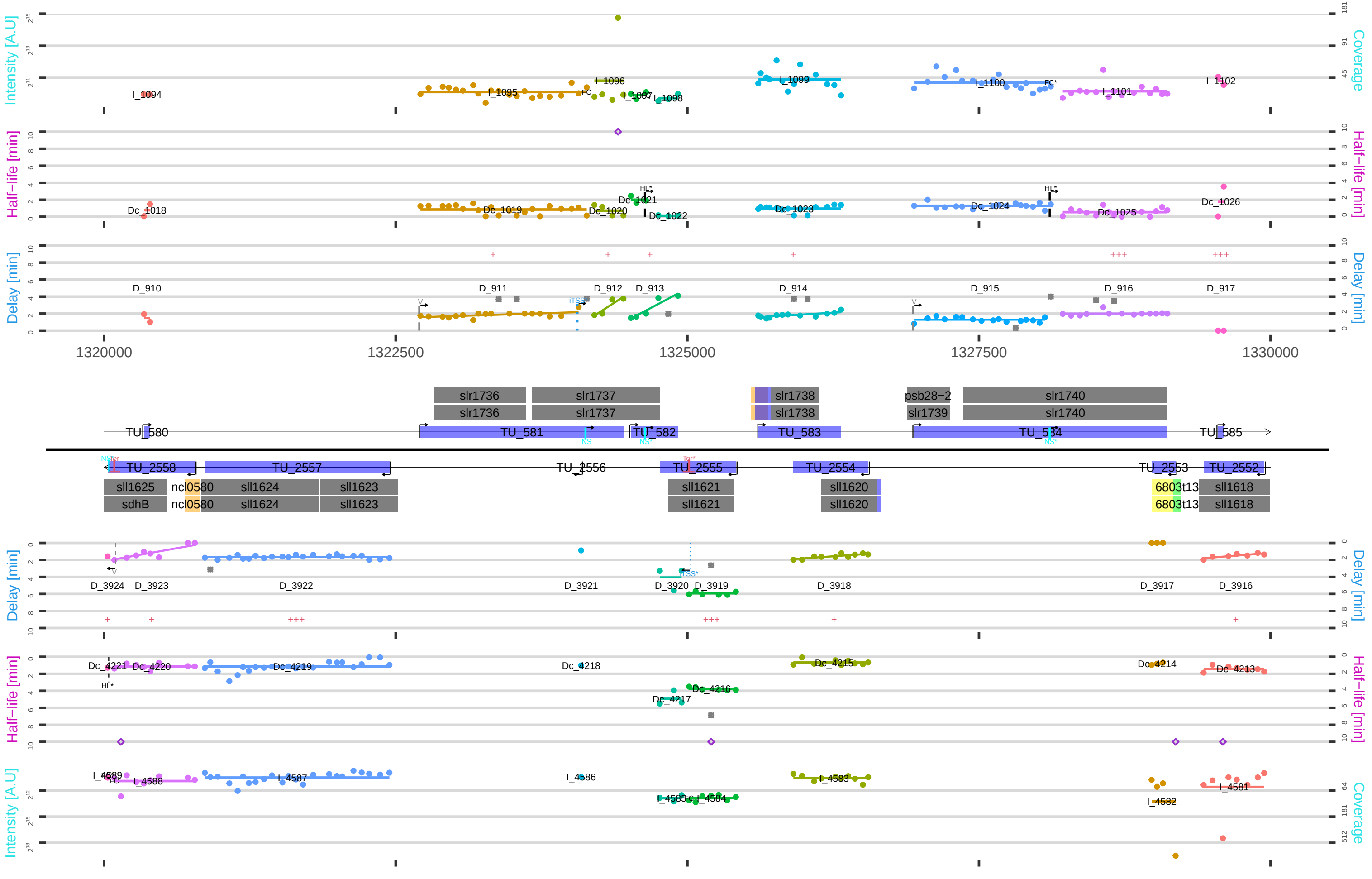

Term: termination (2), NS: new start (1), PS: pausing site (0), iTSS_I: internal starting site (2)

ID: 10784−10926; Term: termination (3), NS: new start (1), PS: pausing site (1), iTSS_I: internal starting site (3)

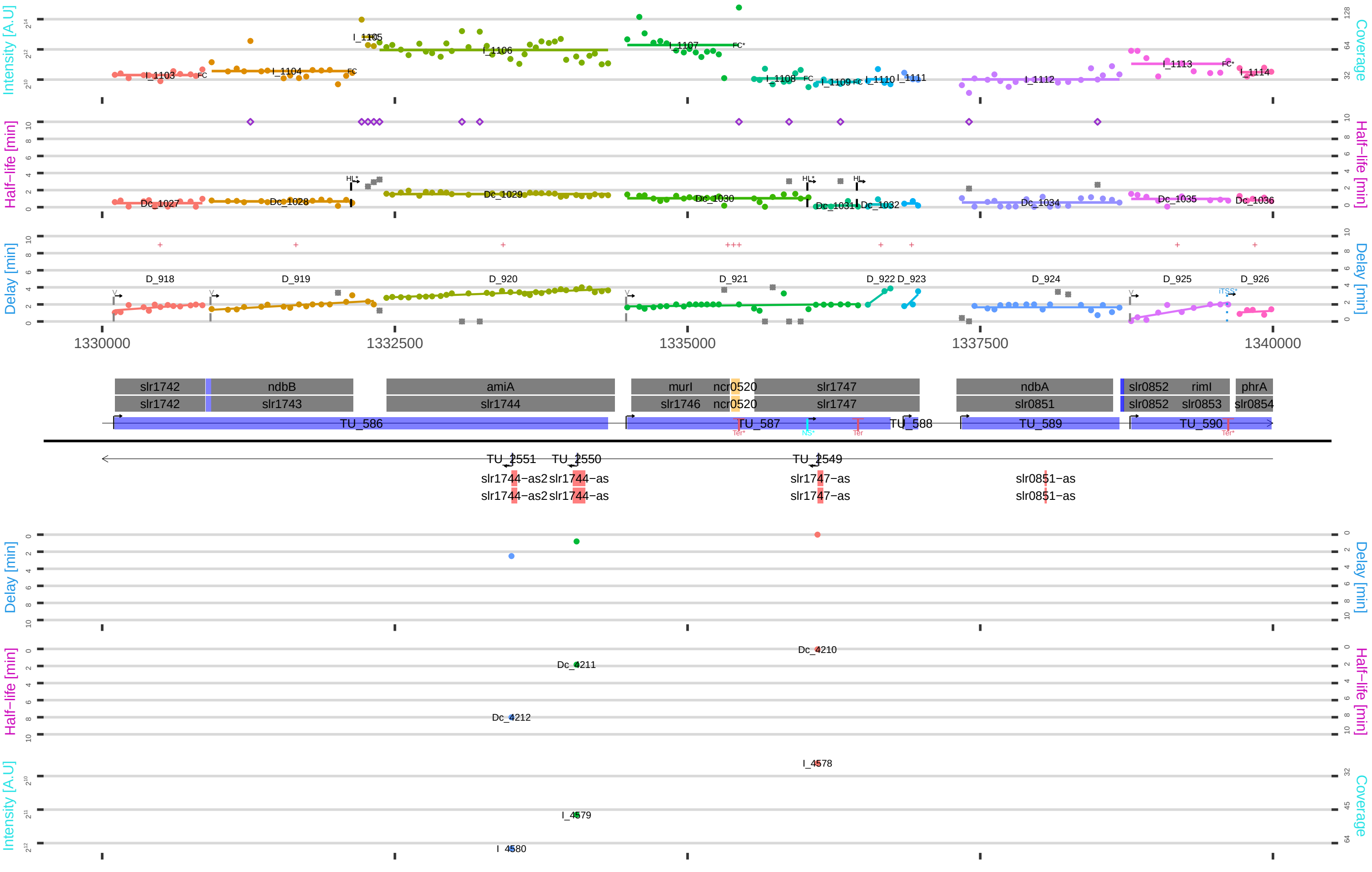

Term: termination (0), NS: new start (0), PS: pausing site (0), iTSS_I: internal starting site (0)

ID: 10927−10964; Term: termination (0), NS: new start (0), PS: pausing site (0), iTSS_I: internal starting site (0)

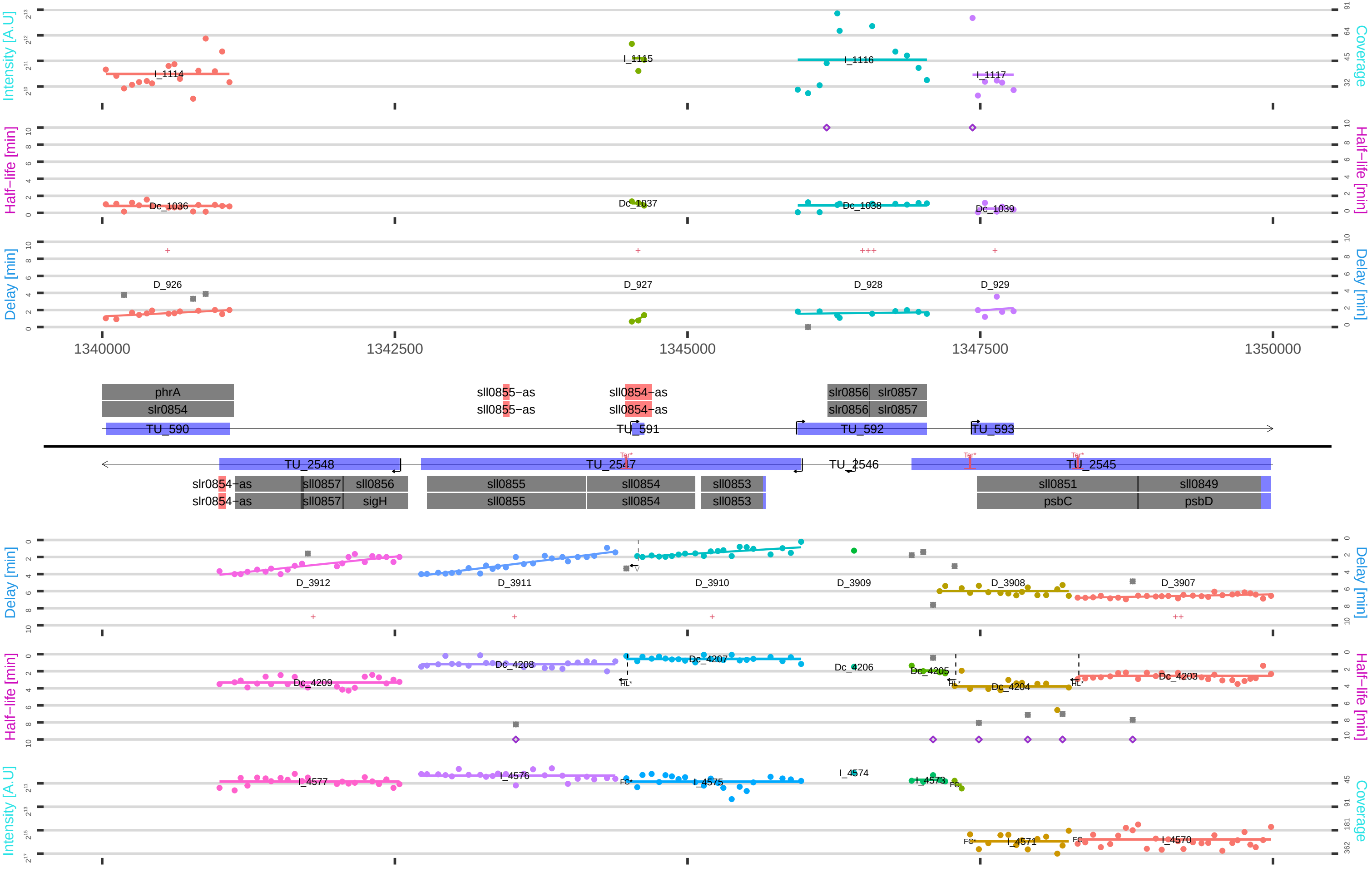

Term: termination (3), NS: new start (0), PS: pausing site (0), iTSS_I: internal starting site (2)

ID: 10965−11027; Term: termination (1), NS: new start (0), PS: pausing site (0), iTSS_I: internal starting site (1)

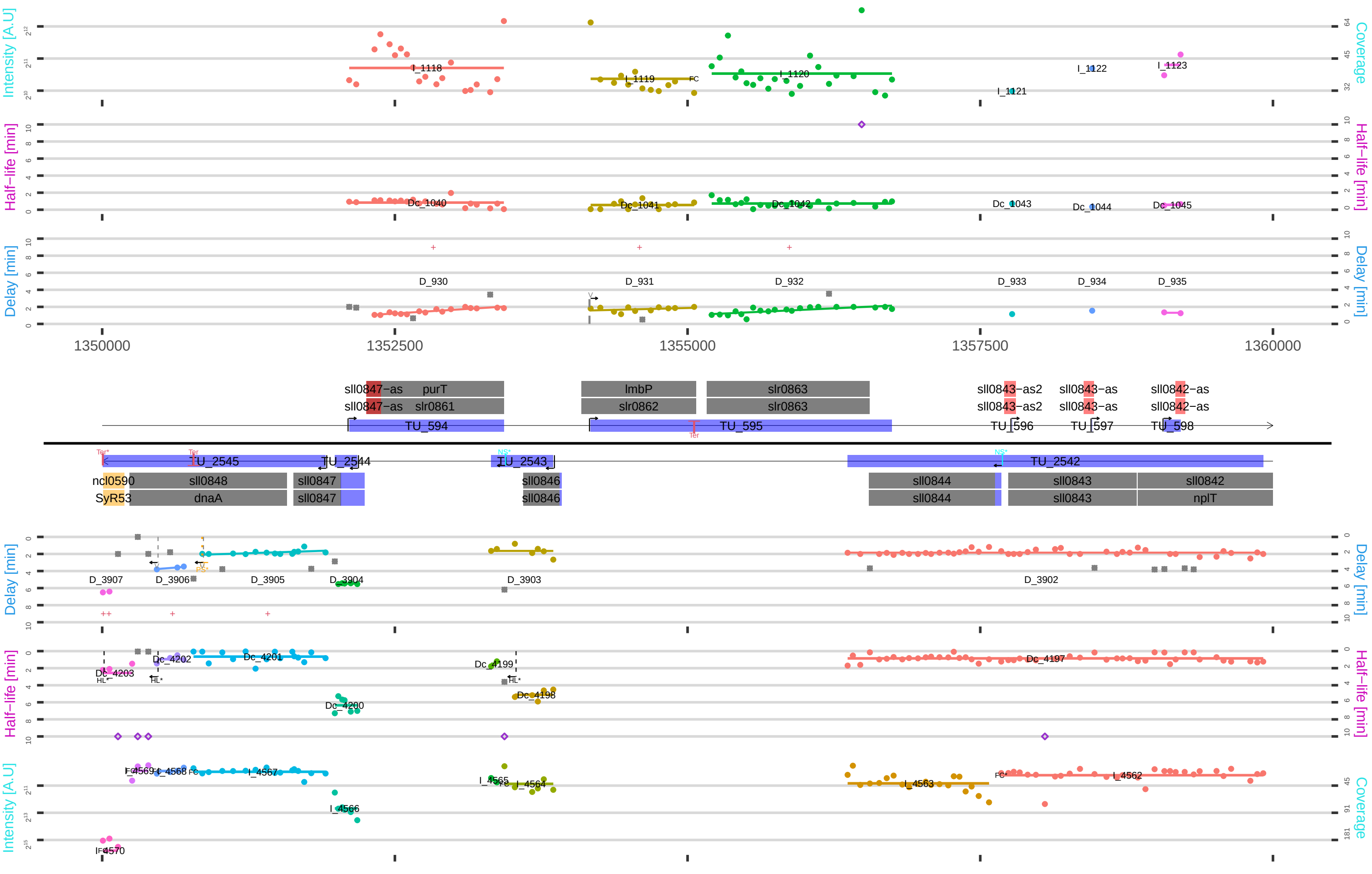

Term: termination (2), NS: new start (2), PS: pausing site (1), iTSS_I: internal starting site (0)

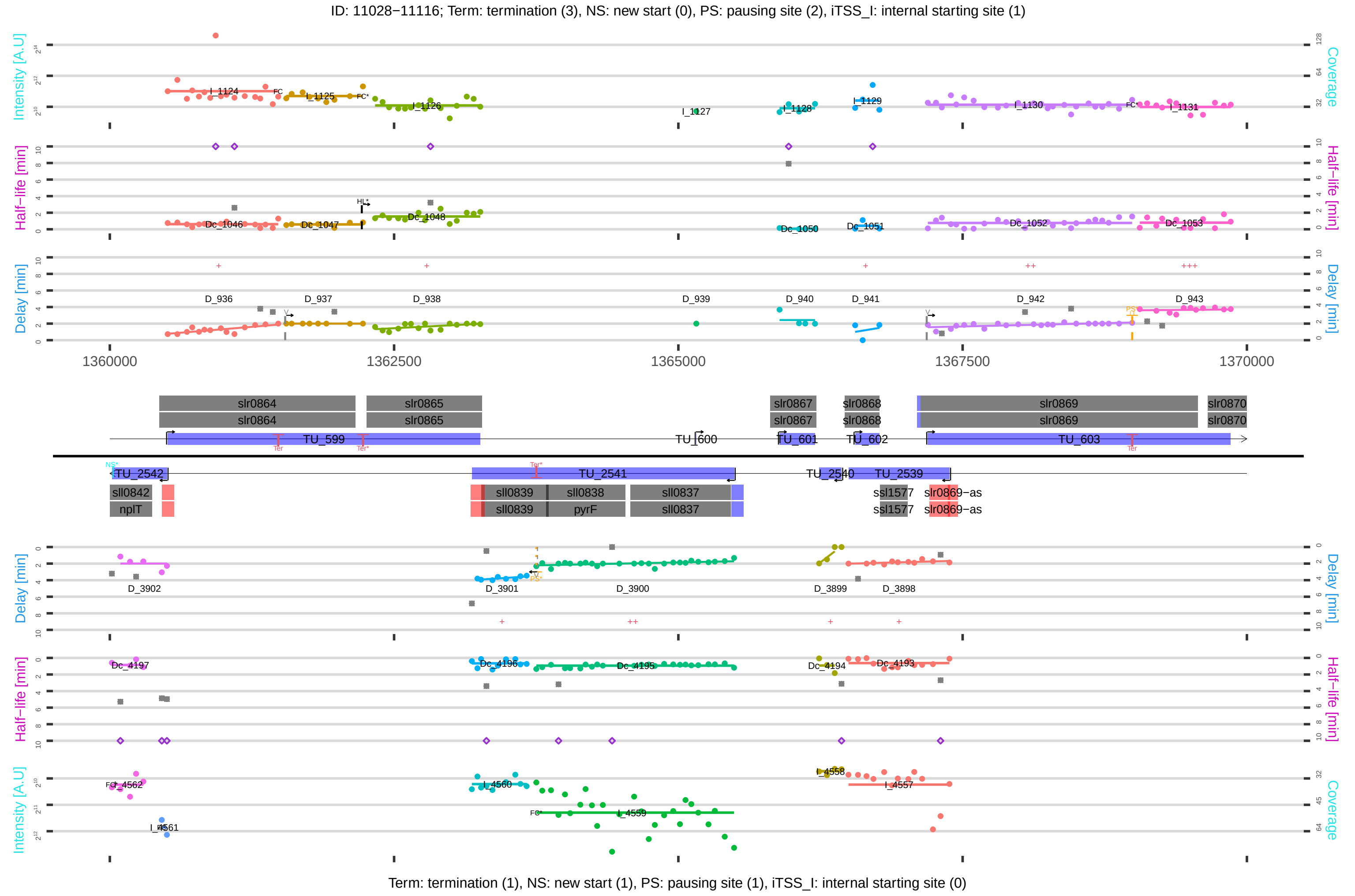

ID: 11028−11116; Term: termination (3), NS: new start (0), PS: pausing site (2), iTSS_I: internal starting site (1)
Intensity [A.U]
Coverage
Half−life [min]
Delay [min]
I_1124
I_1125
I_1126
I_1127
I_1128
I_1129
I_1130
I_1131
Dc_1046
Dc_1047
Dc_1048
Dc_1050
Dc_1051
Dc_1052
Dc_1053
D_936
D_937
D_938
D_939
D_940
D_941
D_942
D_943
1360000
1362500
1365000
1367500
1370000
slr0864
slr0865
slr0867
slr0868
slr0869
slr0870
TU_599
TU_600
TU_601
TU_602
TU_603
TU_2542
TU_2541
TU_2540
TU_2539
sll0842
nplT
sll0839
sll0838
pyrF
sll0837
ssl1577
slr0869−as
D_3902
D_3901
D_3900
D_3899
D_3898
Dc_4197
Dc_4196
Dc_4195
Dc_4194
Dc_4193
I_4562
I_4561
I_4560
I_4559
I_4558
I_4557
Term: termination (1), NS: new start (1), PS: pausing site (1), iTSS_I: internal starting site (0)

ID: 11117−11207; Term: termination (1), NS: new start (1), PS: pausing site (2), iTSS_I: internal starting site (0)

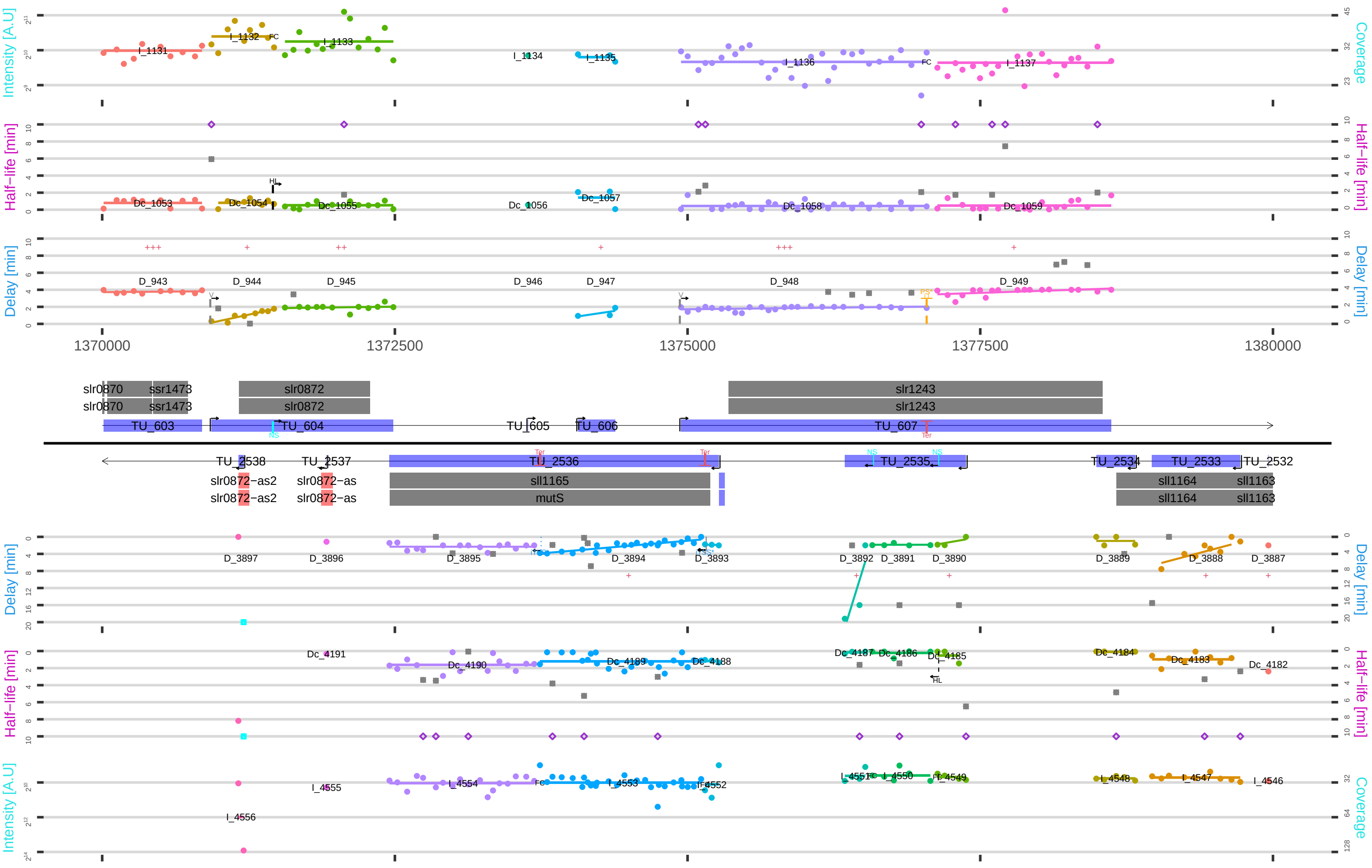

Term: termination (2), NS: new start (2), PS: pausing site (0), iTSS_I: internal starting site (4)

ID: 11208−11255; Term: termination (0), NS: new start (1), PS: pausing site (0), iTSS_I: internal starting site (0)

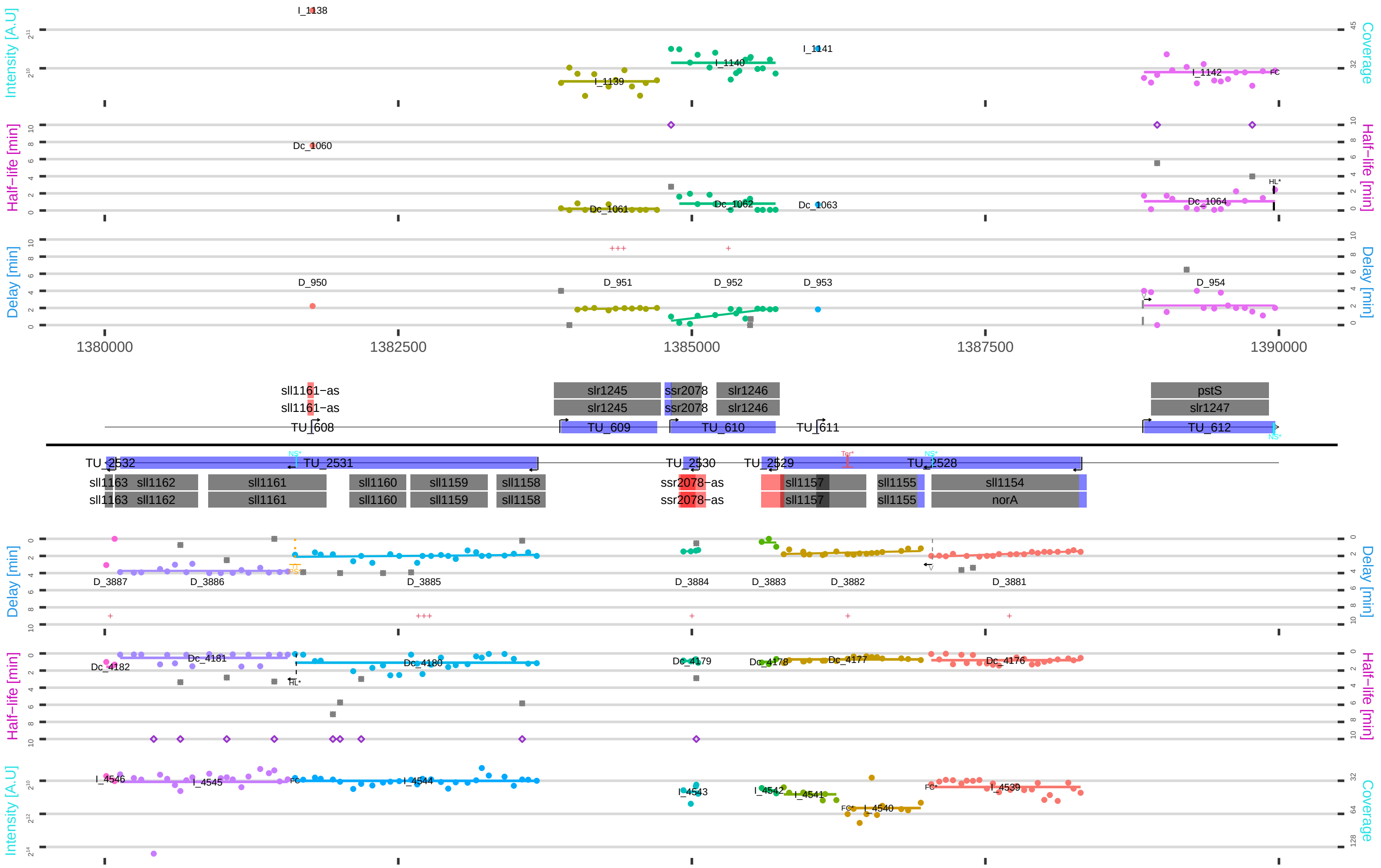

Term: termination (1), NS: new start (2), PS: pausing site (1), iTSS_I: internal starting site (1)

ID: 11258−11354; Term: termination (3), NS: new start (1), PS: pausing site (0), iTSS_I: internal starting site (2)

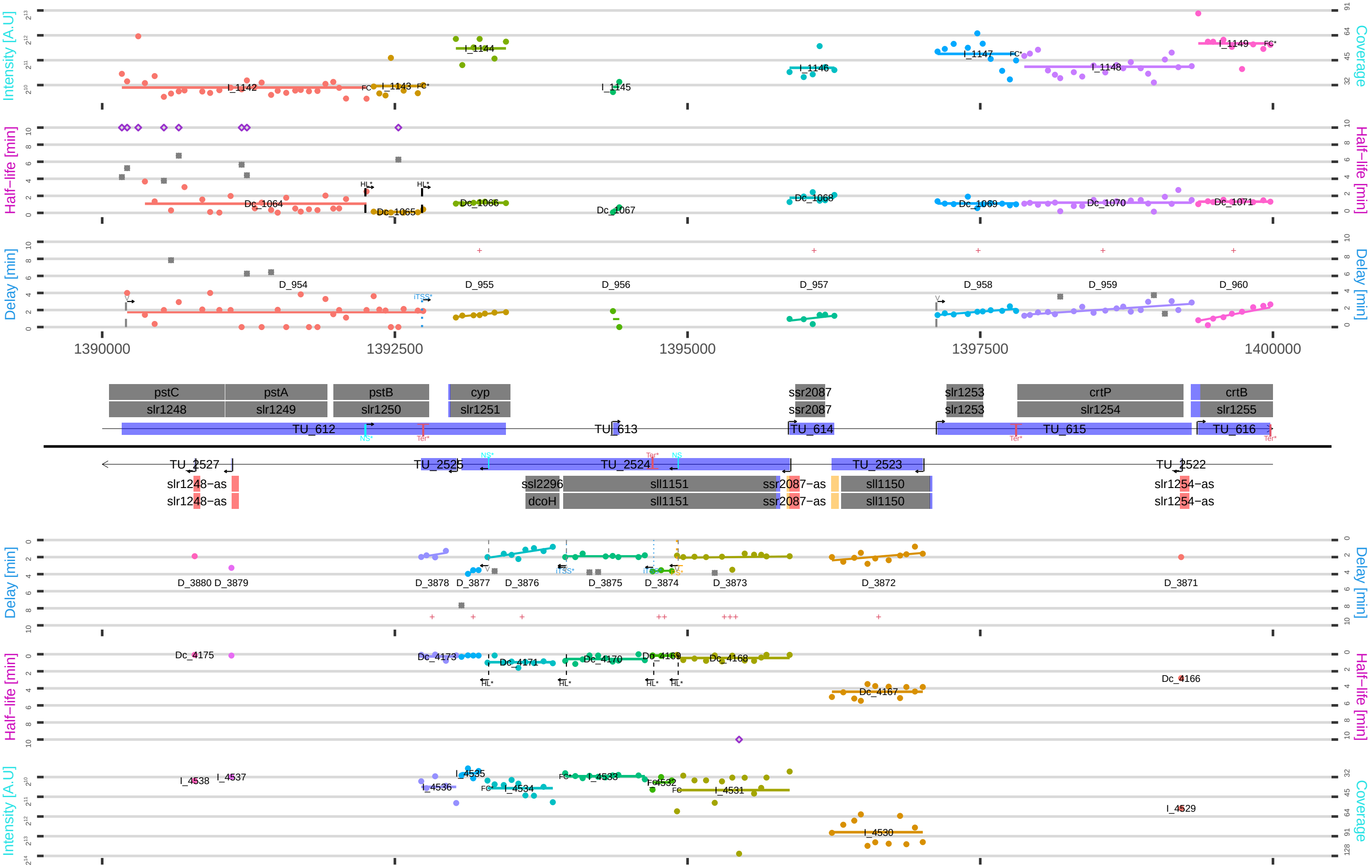

Term: termination (1), NS: new start (2), PS: pausing site (2), iTSS_I: internal starting site (2)

ID: 11355−11462; Term: termination (3), NS: new start (2), PS: pausing site (2), iTSS_I: internal starting site (2)

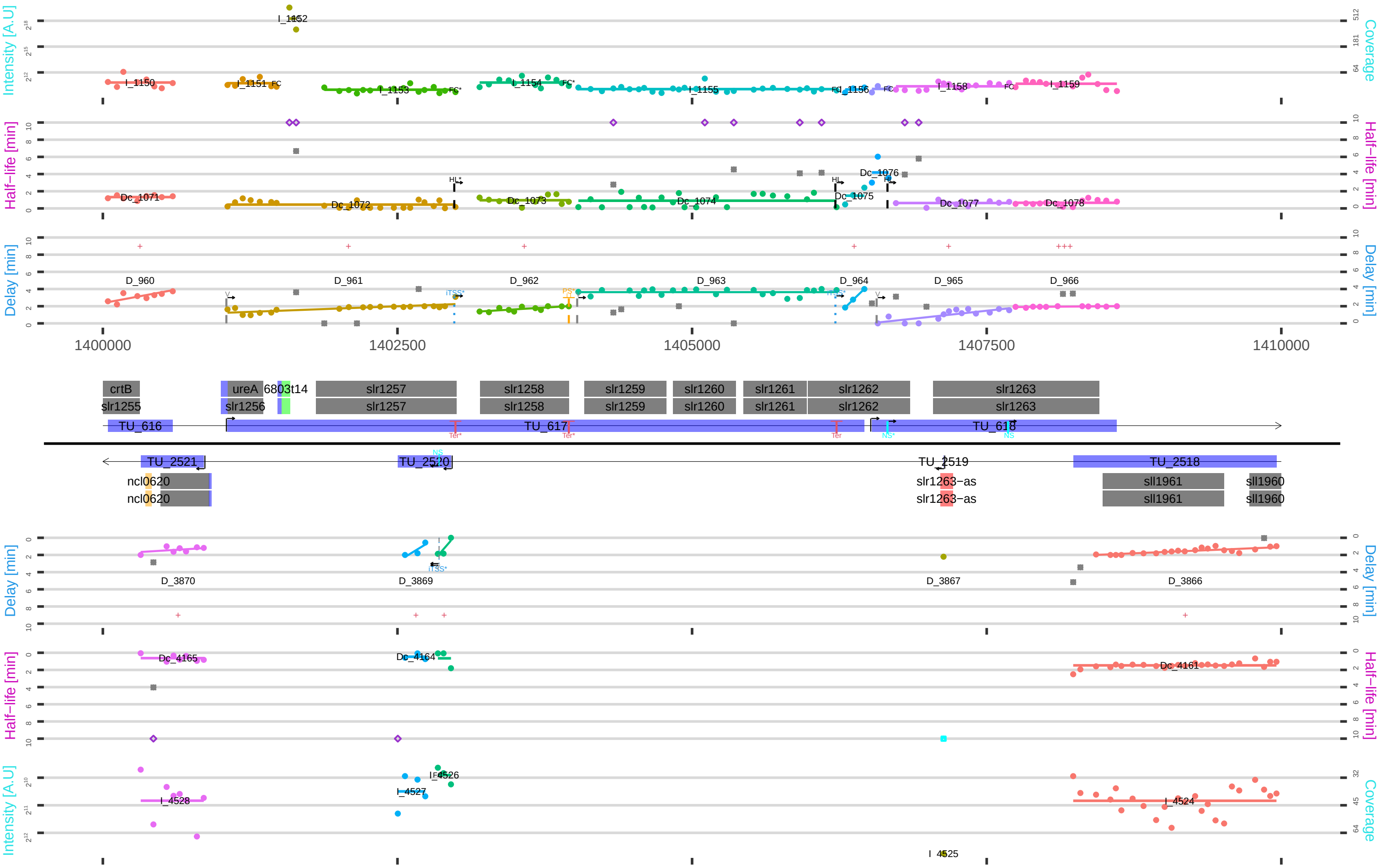

Term: termination (0), NS: new start (1), PS: pausing site (0), iTSS_I: internal starting site (1)

ID: 11463−11547; Term: termination (2), NS: new start (1), PS: pausing site (2), iTSS_I: internal starting site (1)

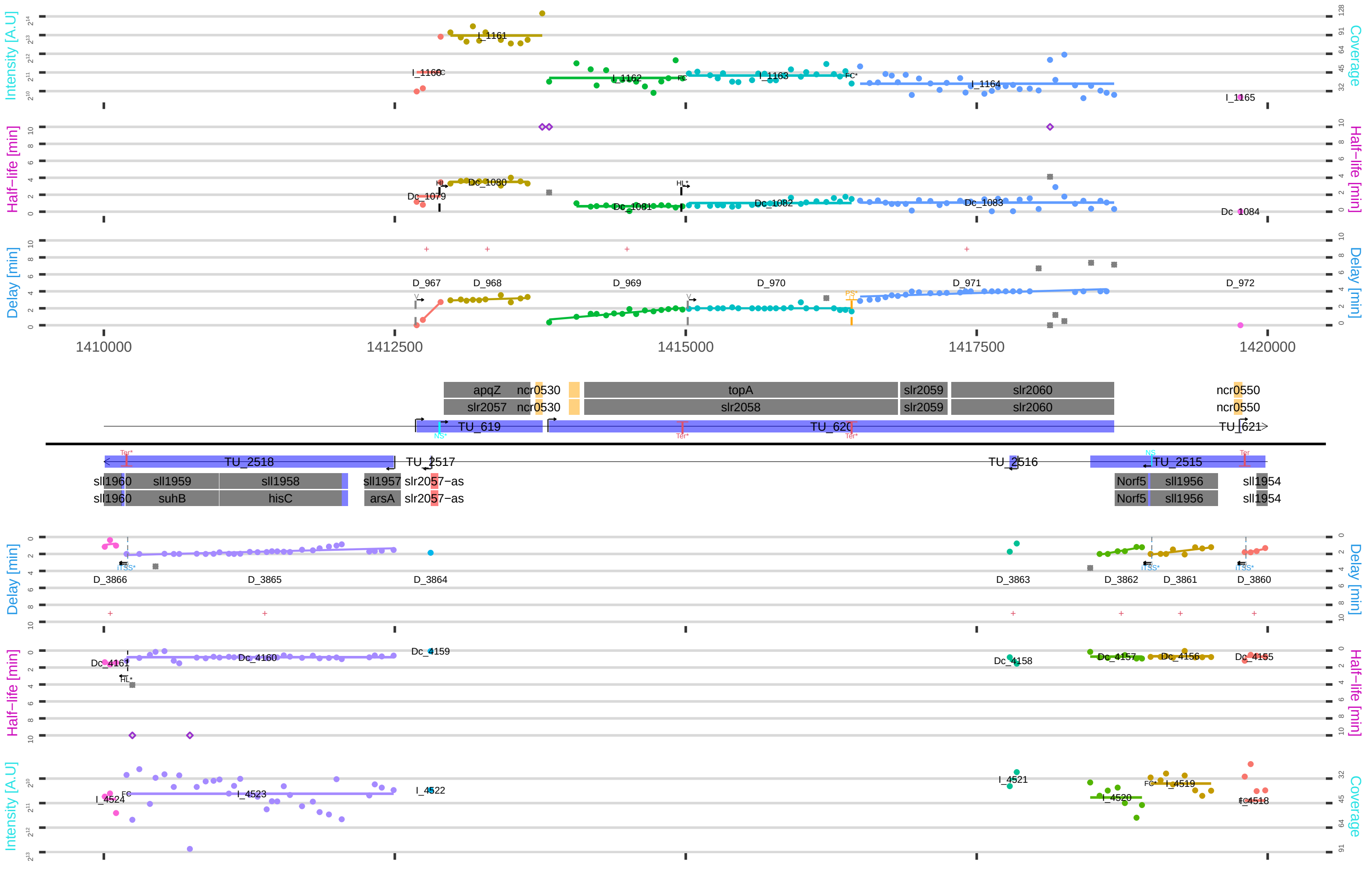

Term: termination (2), NS: new start (1), PS: pausing site (0), iTSS_I: internal starting site (3)

ID: 11548−11575; Term: termination (2), NS: new start (0), PS: pausing site (0), iTSS_I: internal starting site (1)

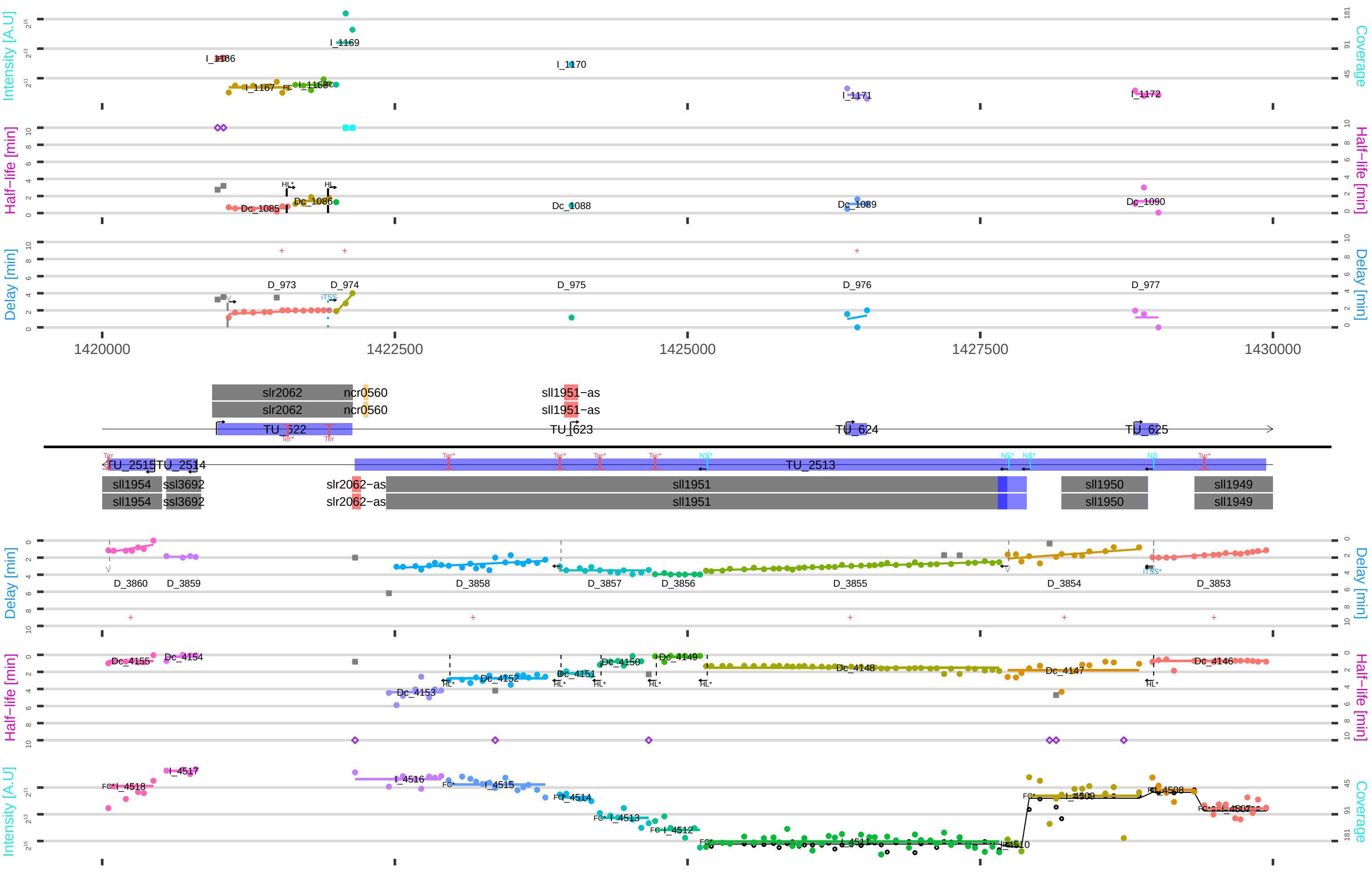

Term: termination (6), NS: new start (4), PS: pausing site (2), iTSS_I: internal starting site (3)

ID: 11576−11673; Term: termination (3), NS: new start (2), PS: pausing site (2), iTSS_I: internal starting site (2)

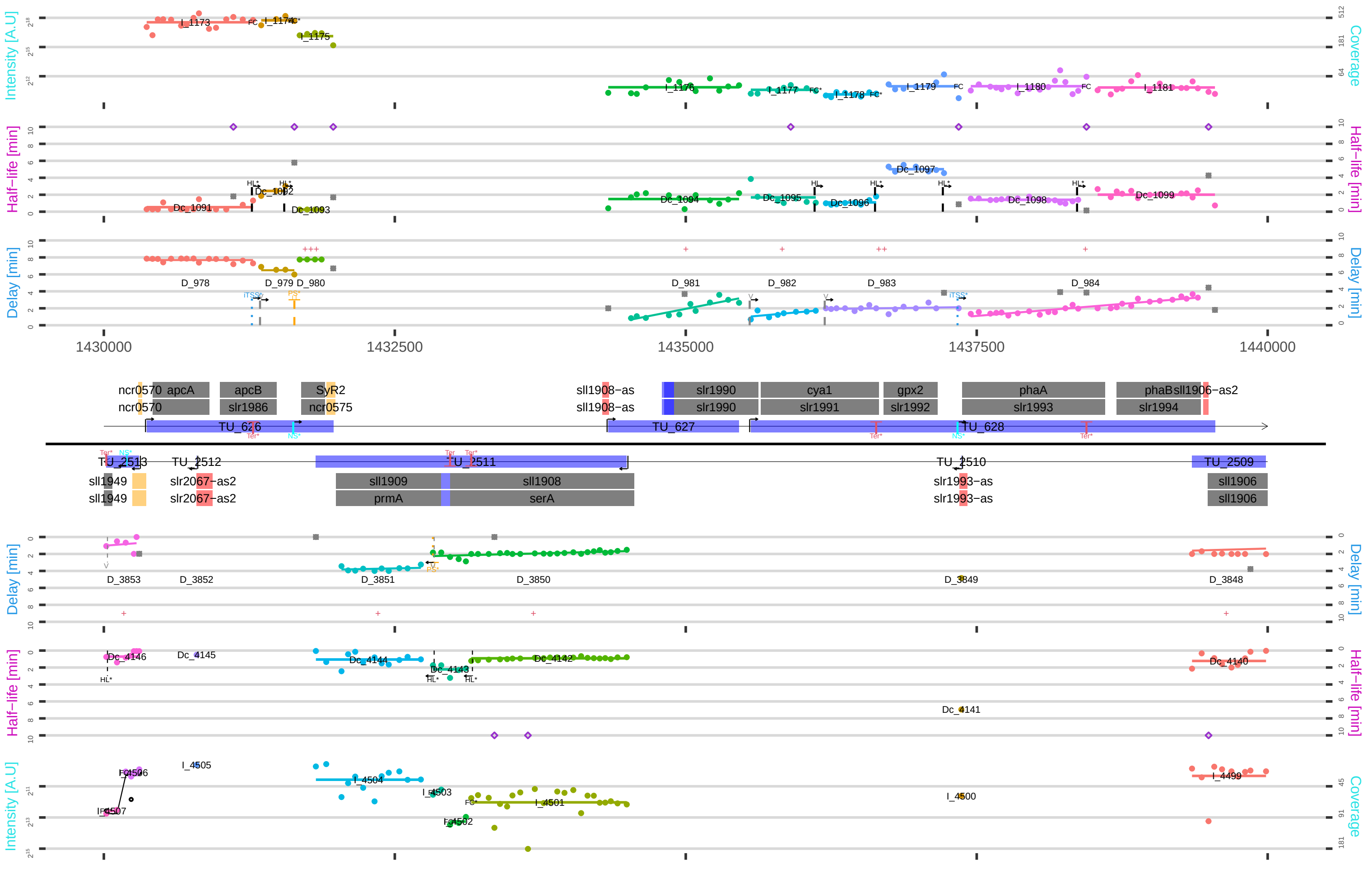

Term: termination (3), NS: new start (1), PS: pausing site (1), iTSS_I: internal starting site (0)

ID: 11674−11752; Term: termination (2), NS: new start (1), PS: pausing site (2), iTSS_I: internal starting site (1)

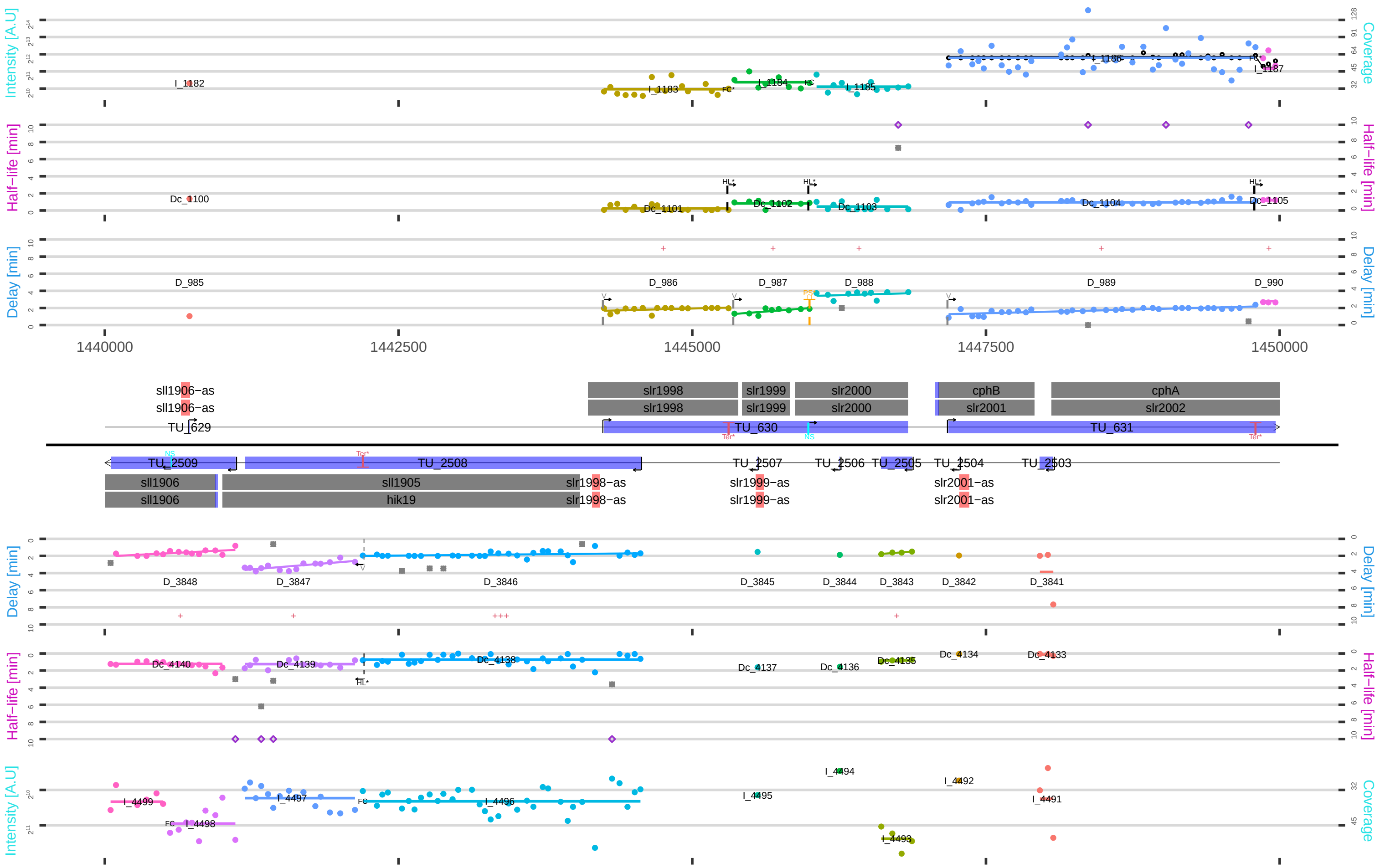

Term: termination (1), NS: new start (1), PS: pausing site (1), iTSS_I: internal starting site (0)

ID: 11753−11811; Term: termination (1), NS: new start (1), PS: pausing site (0), iTSS_I: internal starting site (2)

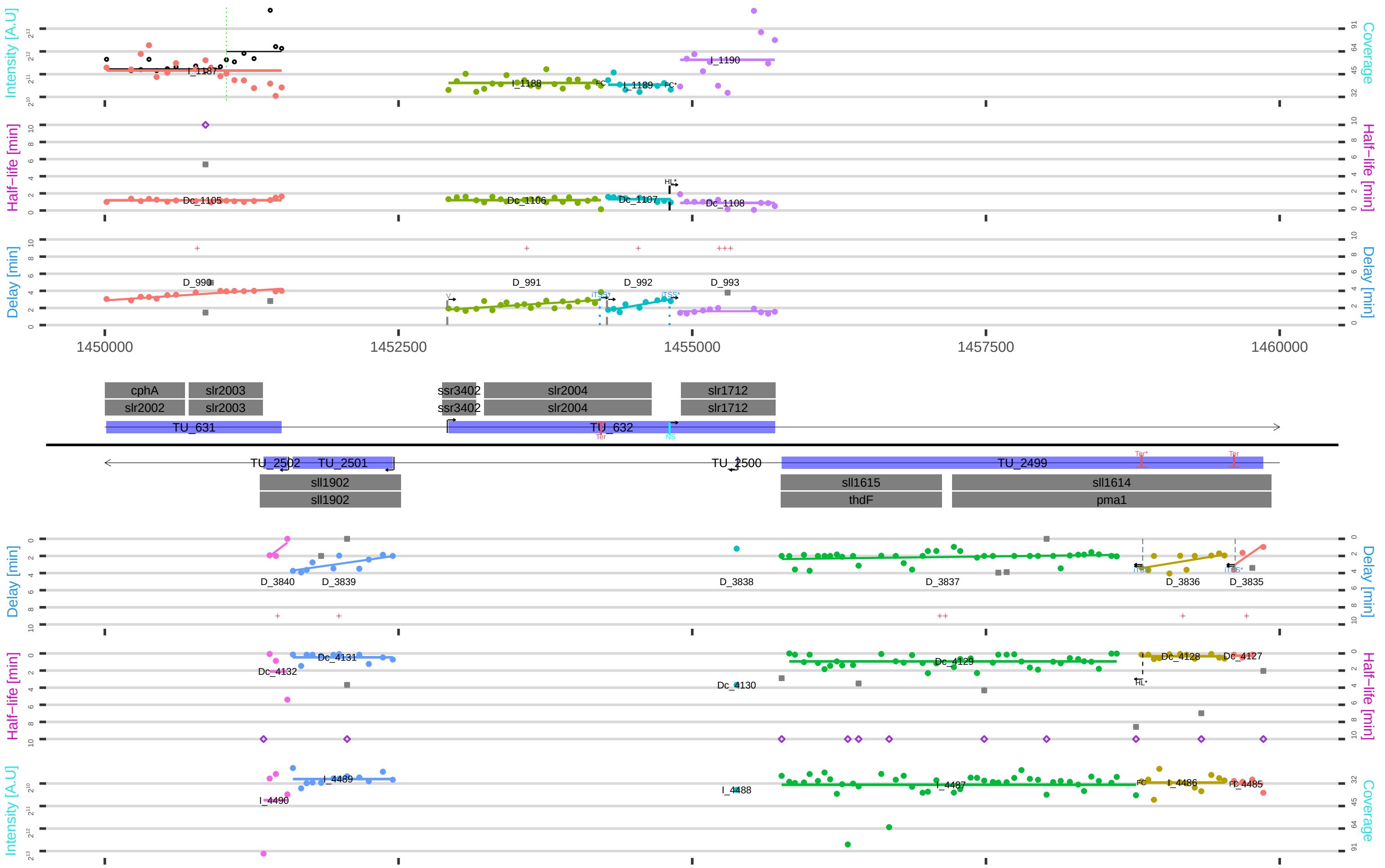

Term: termination (2), NS: new start (0), PS: pausing site (0), iTSS_I: internal starting site (2)

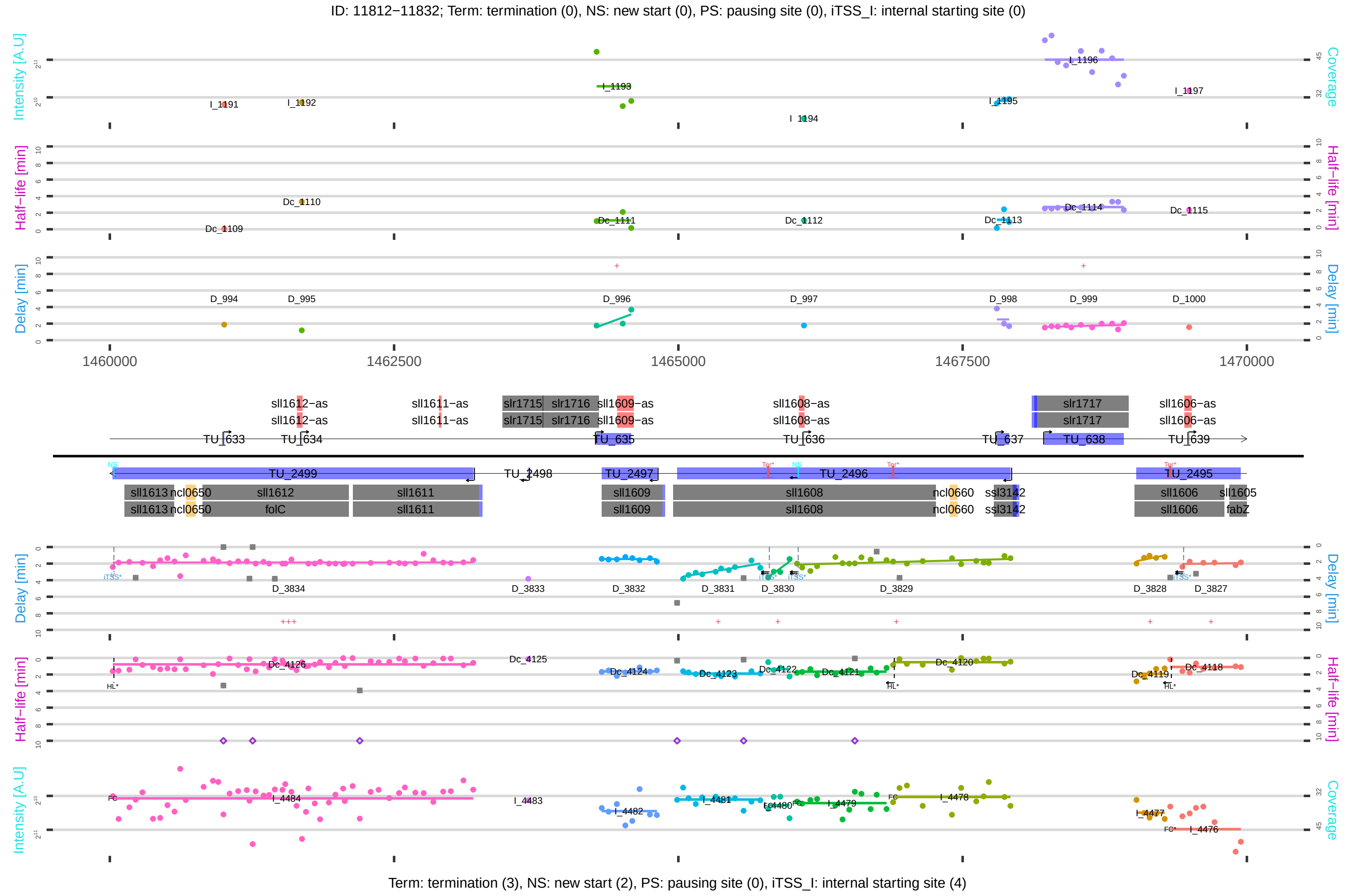

ID: 11812−11832; Term: termination (0), NS: new start (0), PS: pausing site (0), iTSS_I: internal starting site (0)
Intensity [A.U]
Coverage
Half−life [min]
Delay [min]
1460000
1462500
1465000
1467500
1470000
TU_2499
TU_2498
TU_2497
TU_2496
TU_2495
Term: termination (3), NS: new start (2), PS: pausing site (0), iTSS_I: internal starting site (4)

ID: 11833−11957; Term: termination (5), NS: new start (2), PS: pausing site (1), iTSS_I: internal starting site (2)

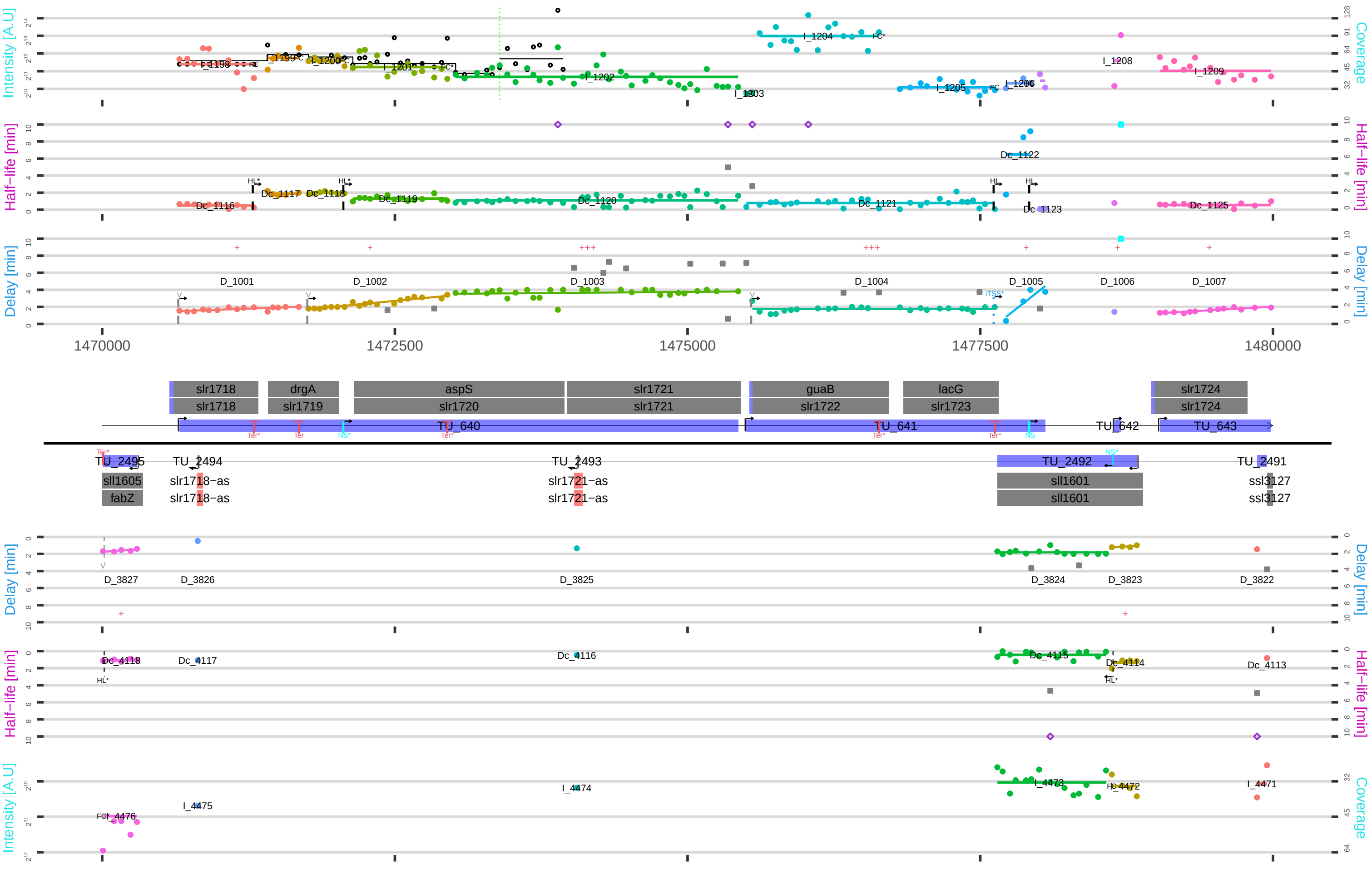

Term: termination (1), NS: new start (1), PS: pausing site (1), iTSS_I: internal starting site (0)

ID: 11958−12016; Term: termination (0), NS: new start (0), PS: pausing site (0), iTSS_I: internal starting site (0)

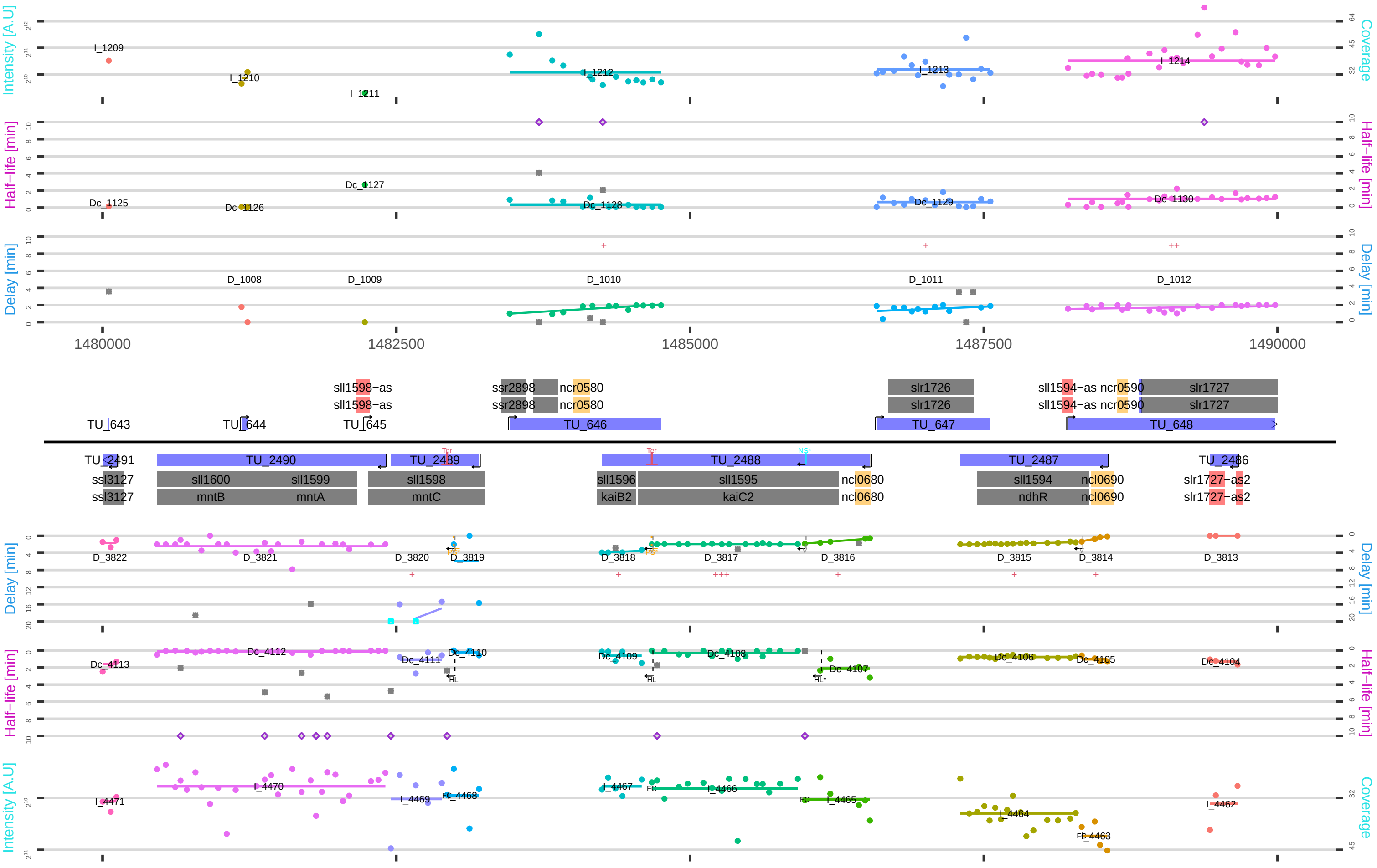

Term: termination (2), NS: new start (1), PS: pausing site (4), iTSS_I: internal starting site (0)

ID: 12017−12117; Term: termination (1), NS: new start (1), PS: pausing site (2), iTSS_I: internal starting site (1)

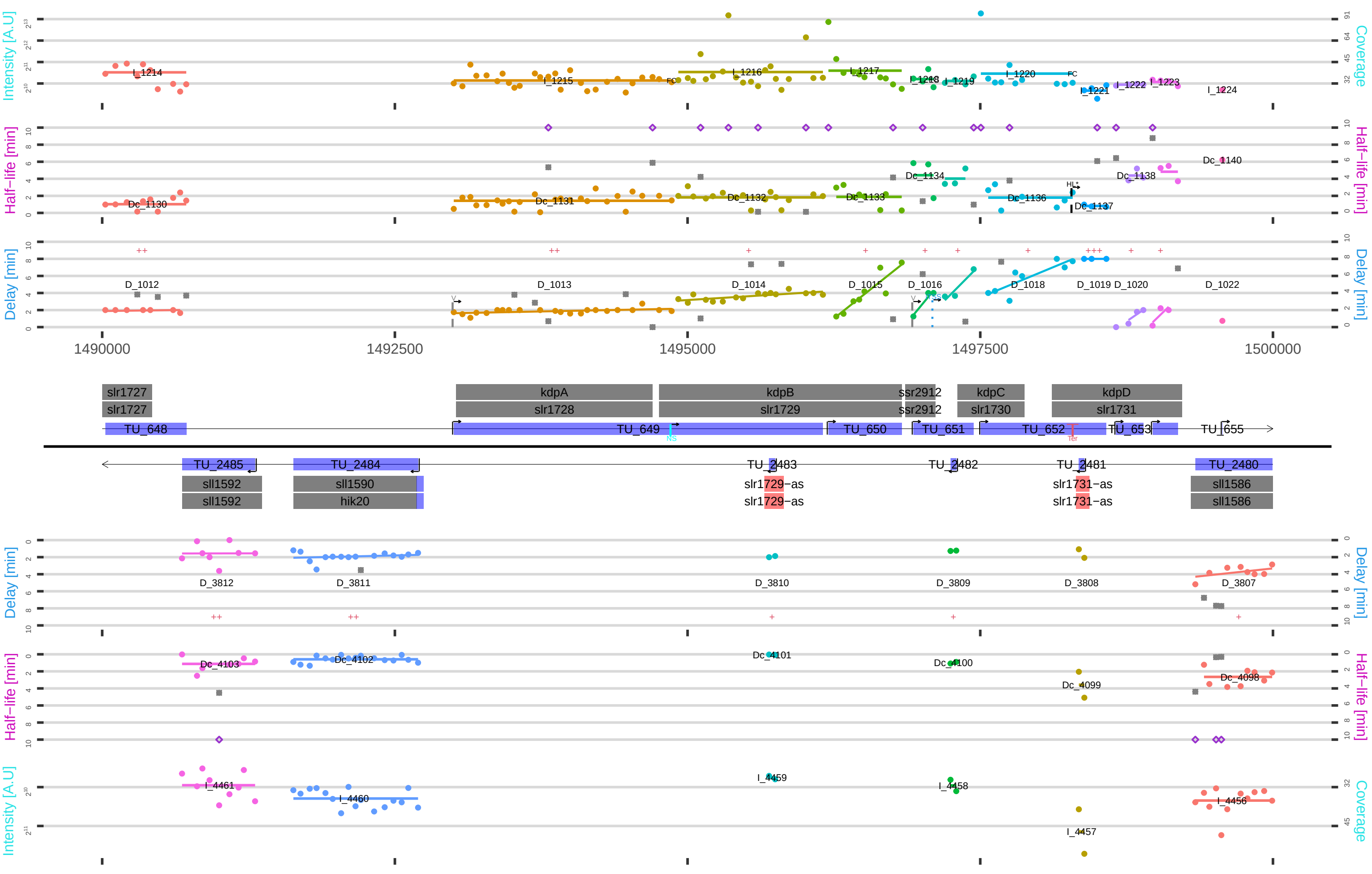

Term: termination (0), NS: new start (0), PS: pausing site (0), iTSS_I: internal starting site (0)

ID: 12118−12161; Term: termination (1), NS: new start (2), PS: pausing site (1), iTSS_I: internal starting site (1)

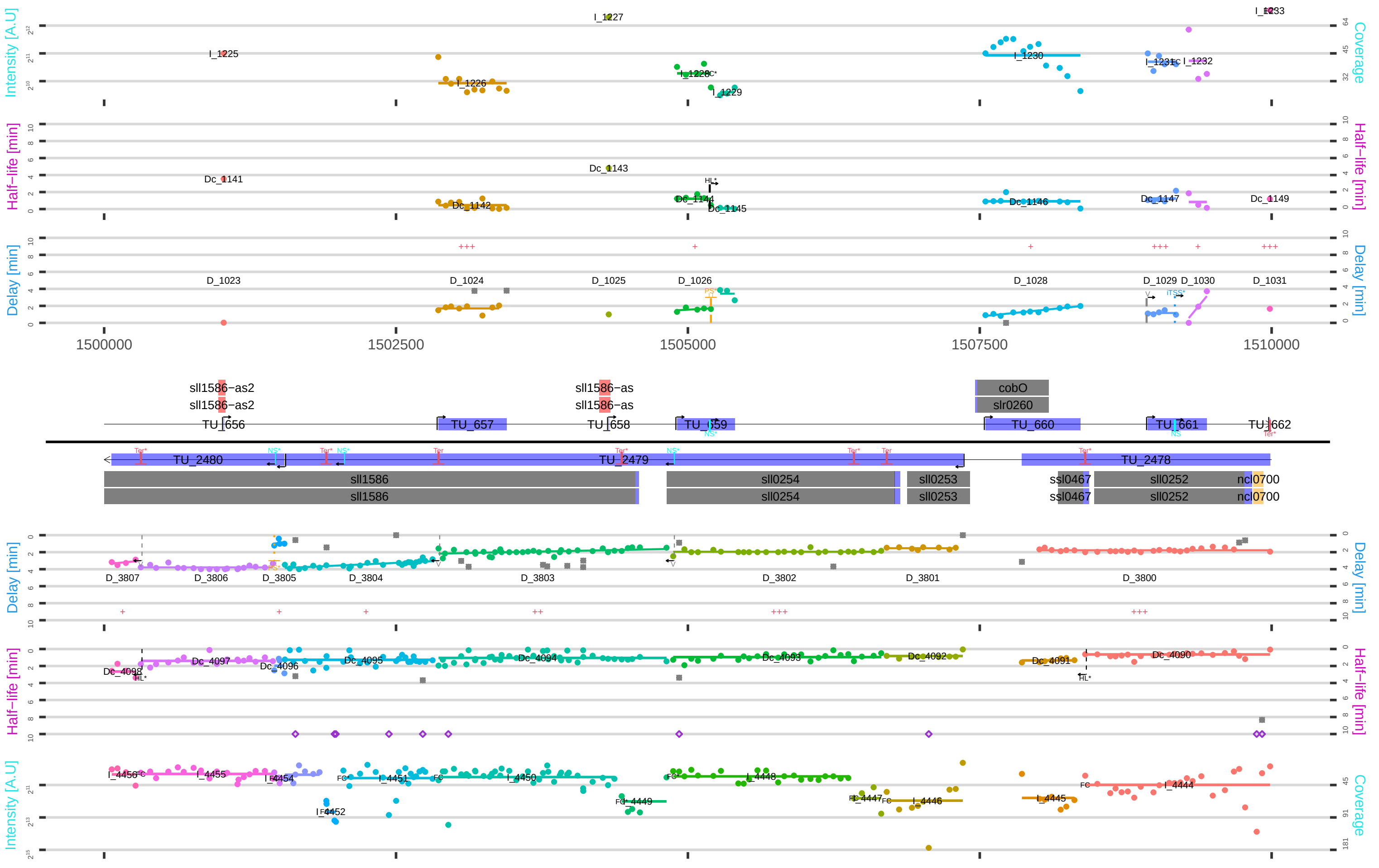

Term: termination (7), NS: new start (3), PS: pausing site (3), iTSS_I: internal starting site (2)

ID: 12162−12240; Term: termination (3), NS: new start (0), PS: pausing site (1), iTSS_I: internal starting site (1)

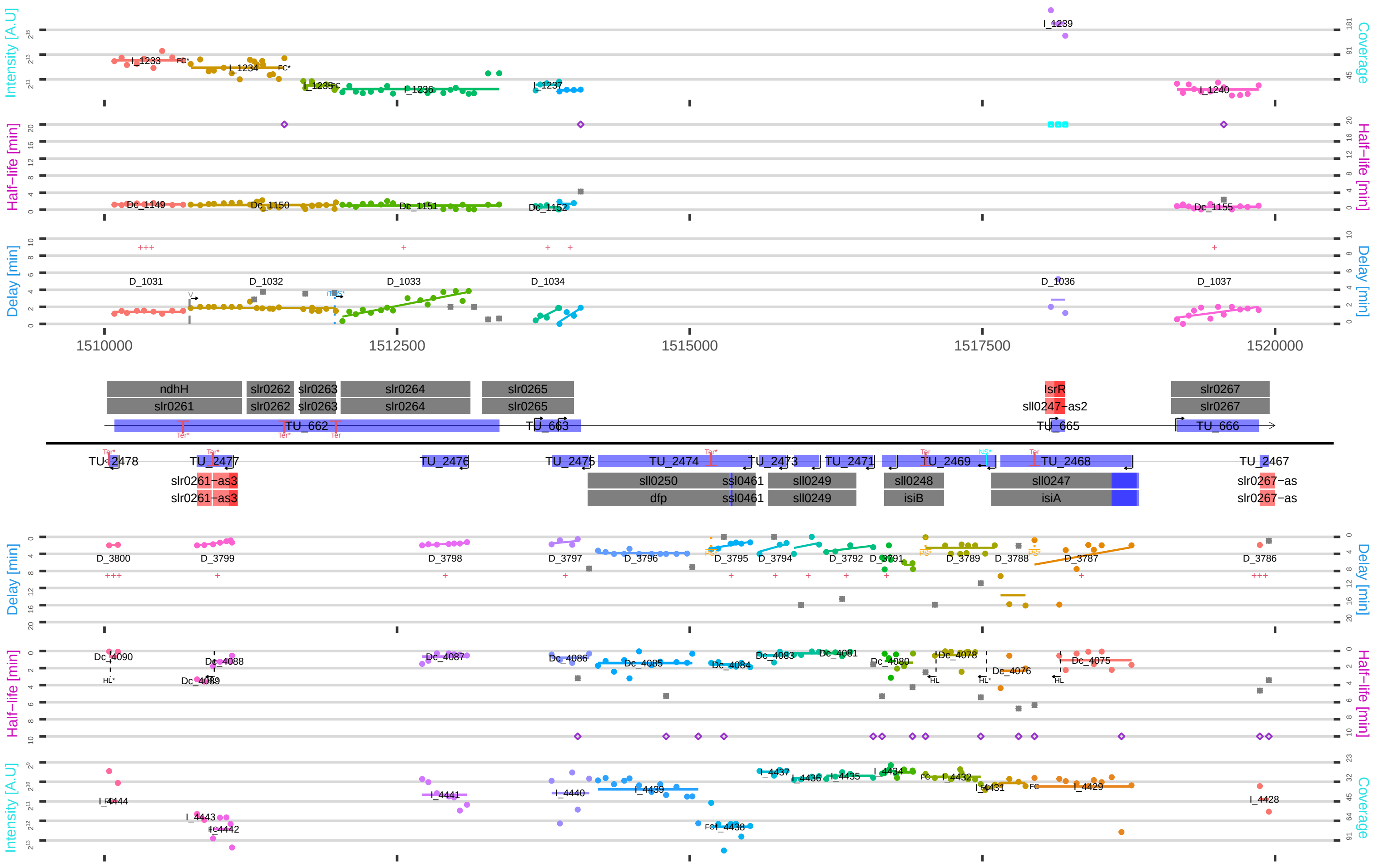

Term: termination (5), NS: new start (1), PS: pausing site (3), iTSS_I: internal starting site (0)

ID: 12241−12297; Term: termination (0), NS: new start (0), PS: pausing site (0), iTSS_I: internal starting site (0)

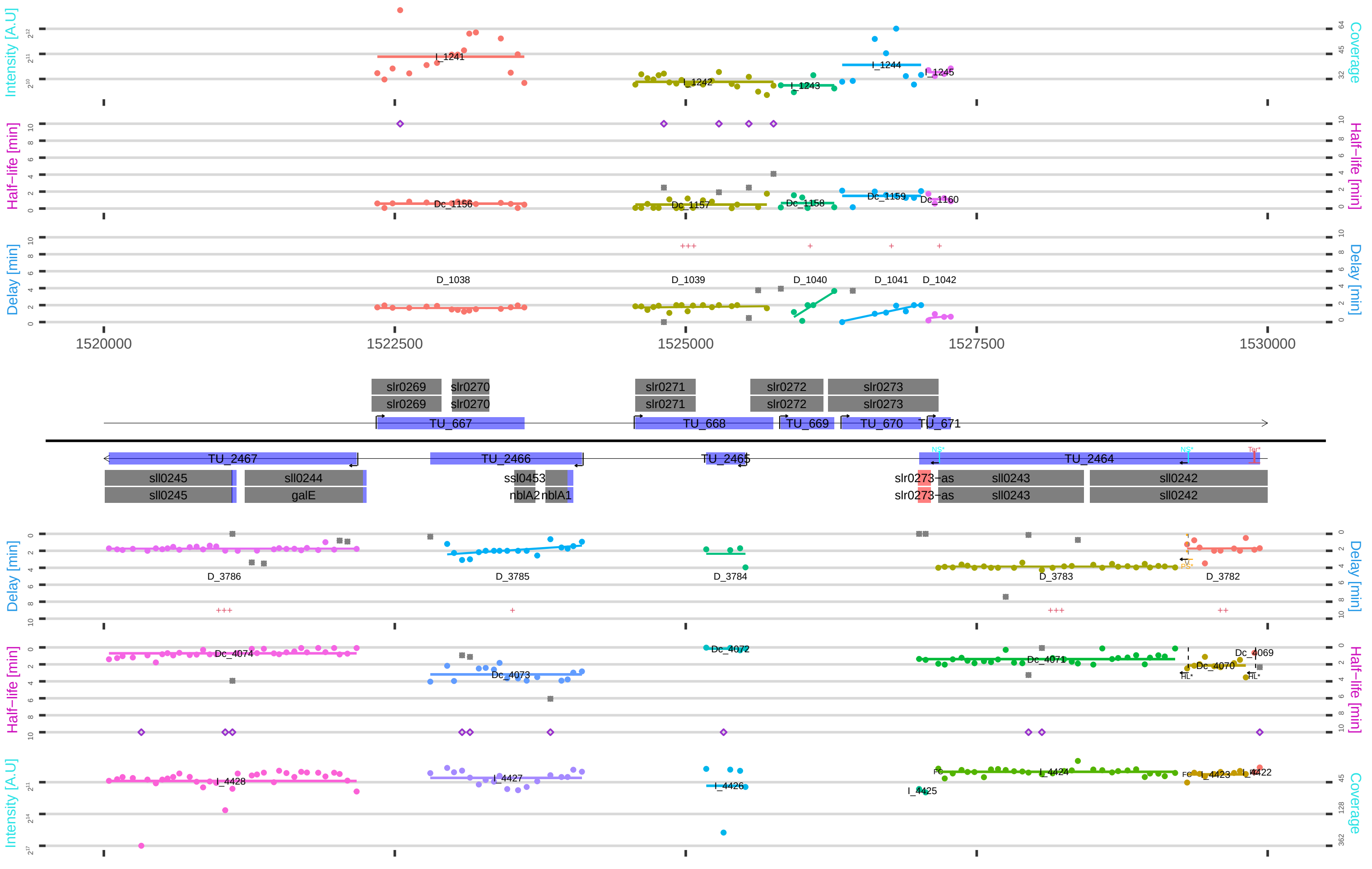

Term: termination (1), NS: new start (2), PS: pausing site (1), iTSS_I: internal starting site (0)

ID: 12298−12330; Term: termination (0), NS: new start (0), PS: pausing site (0), iTSS_I: internal starting site (0)

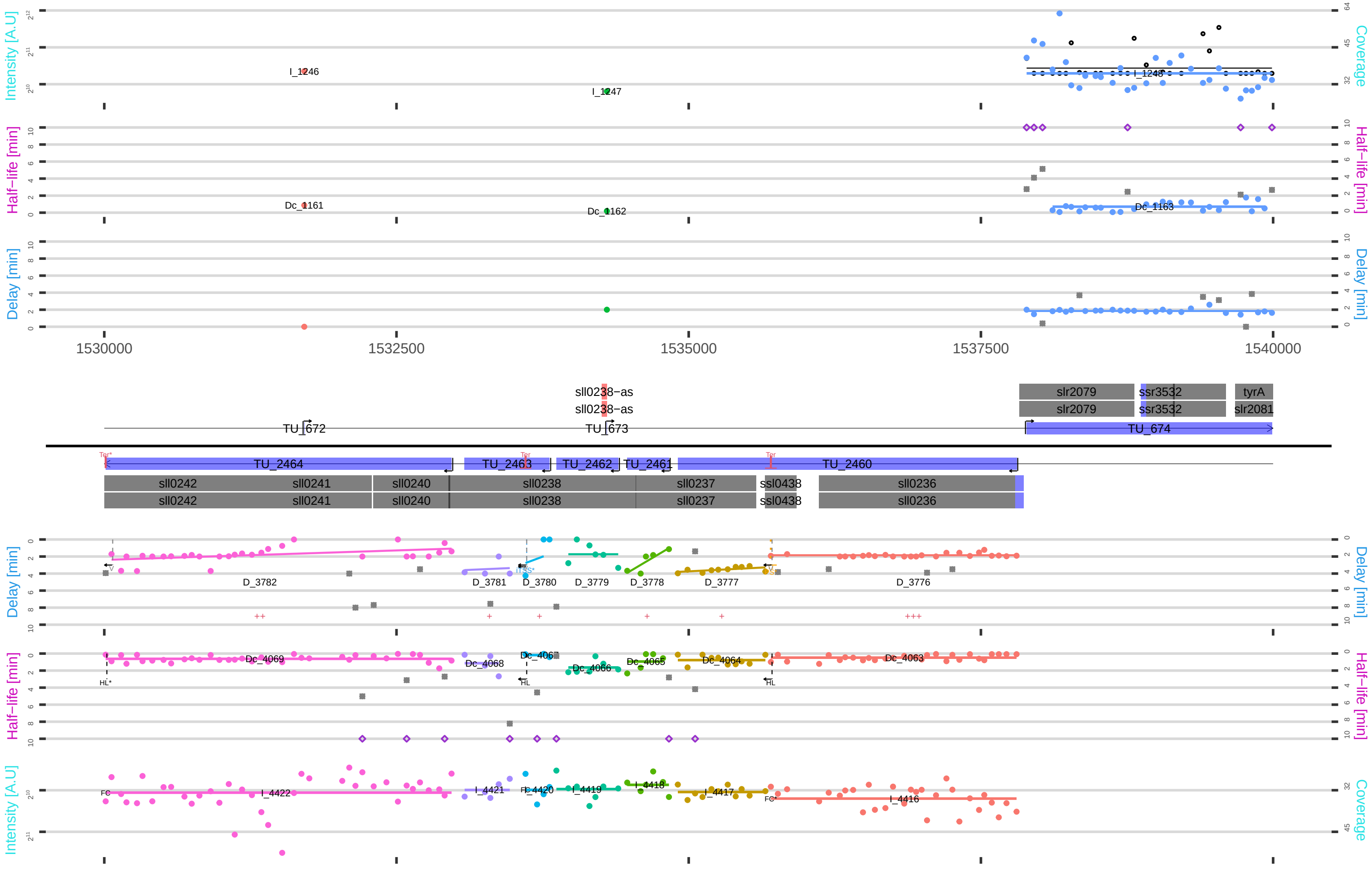

Term: termination (3), NS: new start (0), PS: pausing site (1), iTSS_I: internal starting site (1)

ID: 12331−12441; Term: termination (0), NS: new start (4), PS: pausing site (1), iTSS_I: internal starting site (2)

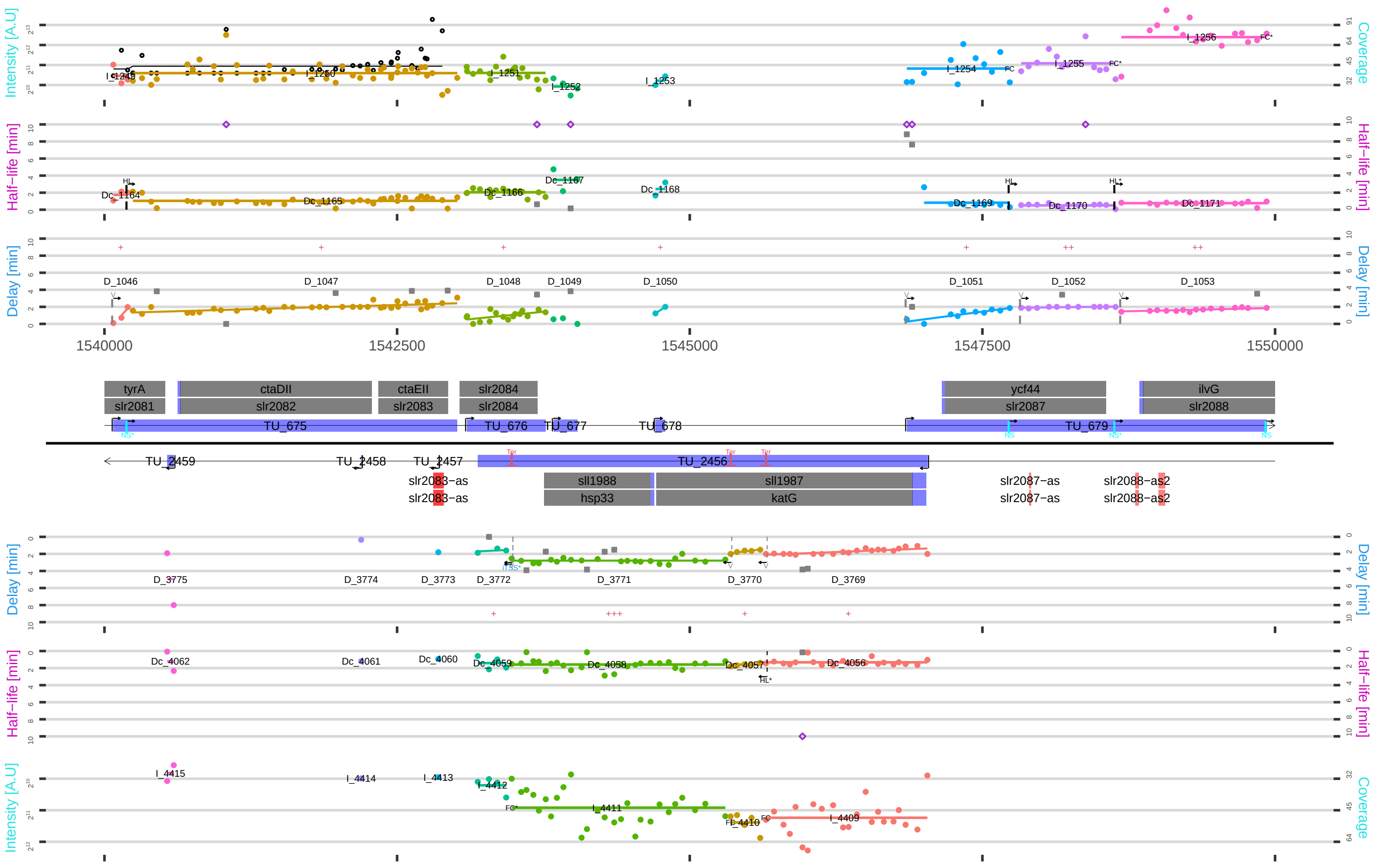

Term: termination (3), NS: new start (0), PS: pausing site (1), iTSS_I: internal starting site (2)

ID: 12442−12507; Term: termination (2), NS: new start (2), PS: pausing site (1), iTSS_I: internal starting site (2)

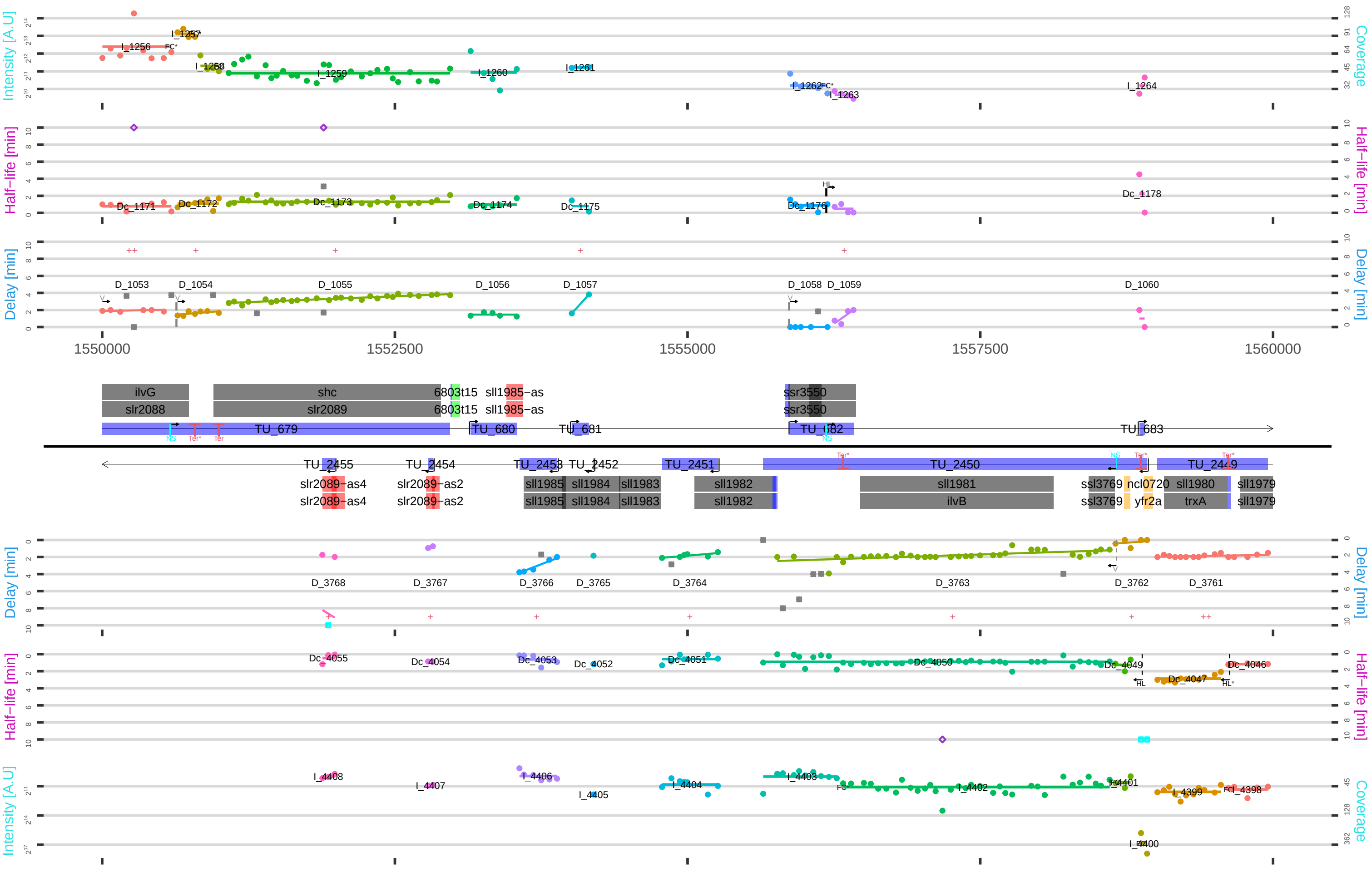

Term: termination (3), NS: new start (1), PS: pausing site (1), iTSS_I: internal starting site (0)

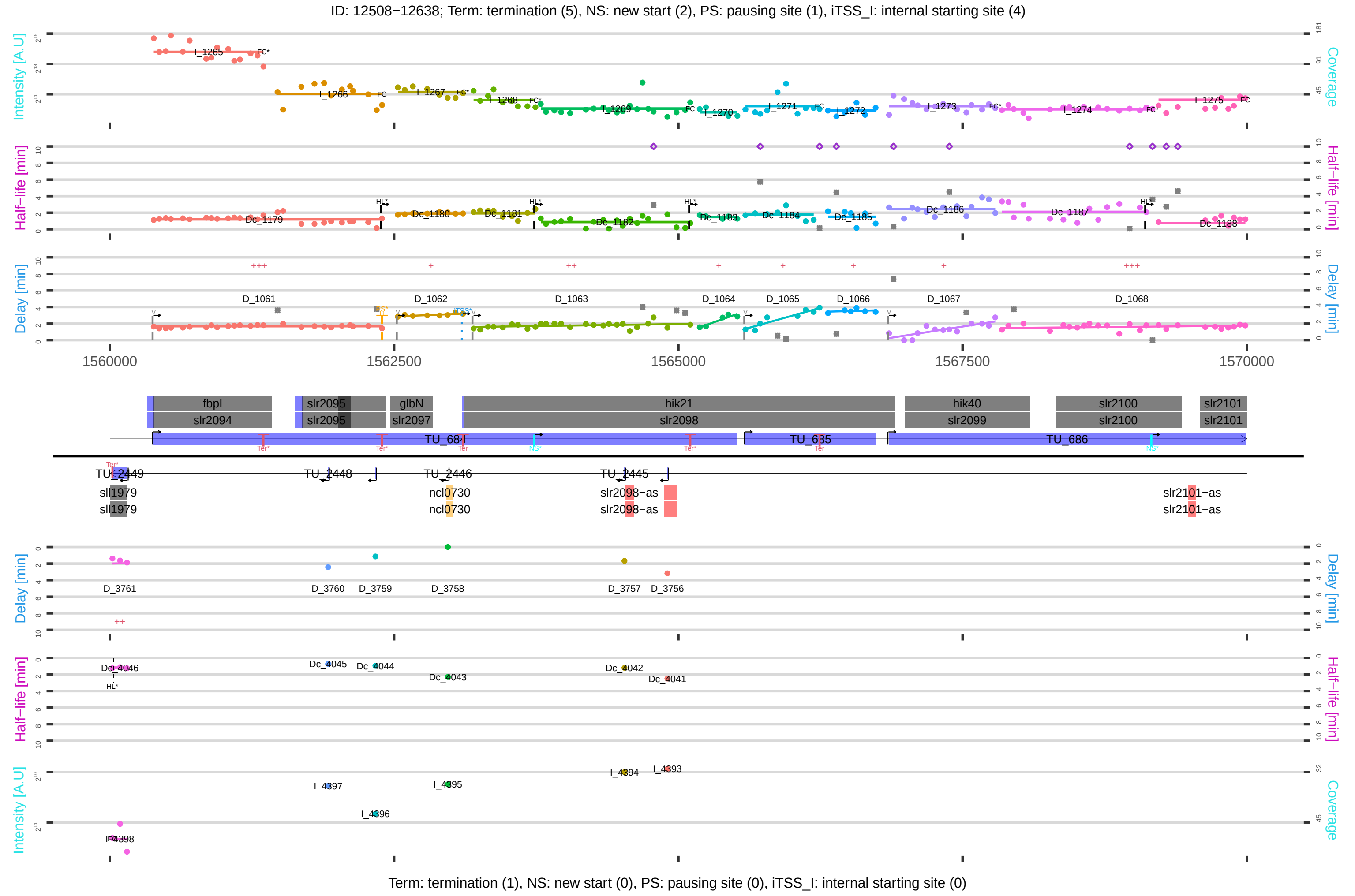
ID: 12508−12638; Term: termination (5), NS: new start (2), PS: pausing site (1), iTSS_I: internal starting site (4)
Intensity [A.U]
Coverage
Half−life [min]
Delay [min]
I_1265
I_1266
I_1267
I_1268
I_1269
I_1270
I_1271
I_1272
I_1273
I_1274
I_1275
Dc_1179
Dc_1180
Dc_1181
Dc_1182
Dc_1183
Dc_1184
Dc_1185
Dc_1186
Dc_1187
Dc_1188
D_1061
D_1062
D_1063
D_1064
D_1065
D_1066
D_1067
D_1068
1560000
1562500
1565000
1567500
1570000
fbpI
slr2094
slr2095
glbN
slr2097
hik21
slr2098
hik40
slr2099
slr2100
slr2101
TU_684
TU_685
TU_686
TU_2449
TU_2448
TU_2446
TU_2445
sll1979
ncl0730
slr2098−as
slr2101−as
D_3761
D_3760
D_3759
D_3758
D_3757
D_3756
Dc_4046
Dc_4045
Dc_4044
Dc_4043
Dc_4042
Dc_4041
I_4398
I_4397
I_4396
I_4395
I_4394
I_4393
Term: termination (1), NS: new start (0), PS: pausing site (0), iTSS_I: internal starting site (0)

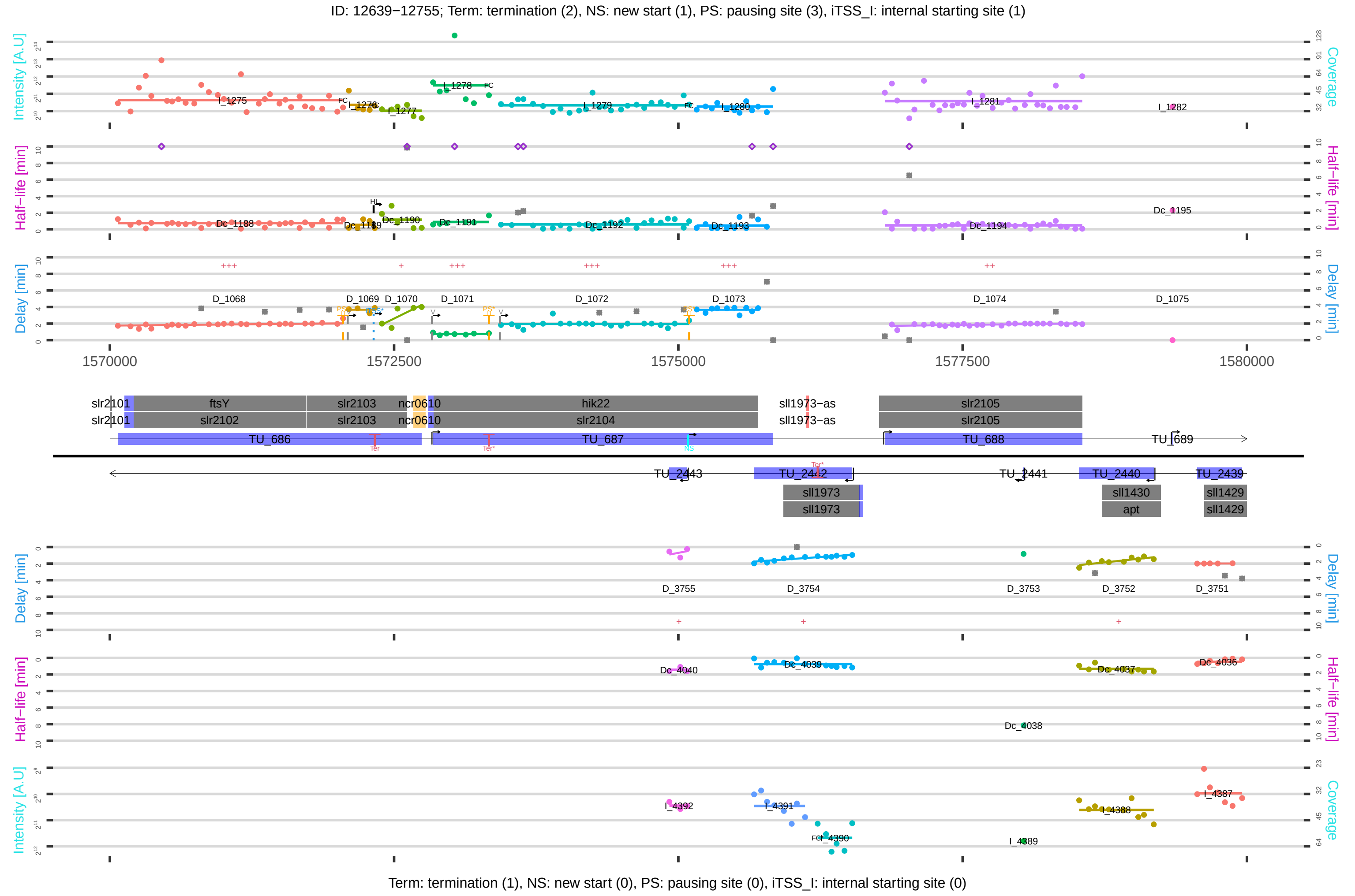
ID: 12639−12755; Term: termination (2), NS: new start (1), PS: pausing site (3), iTSS_I: internal starting site (1)
Intensity [A.U]
Coverage
Half−life [min]
Delay [min]
I_1275
I_1276
I_1277
I_1278
I_1279
I_1280
I_1281
I_1282
Dc_1188
Dc_1189
Dc_1190
Dc_1191
Dc_1192
Dc_1193
Dc_1194
Dc_1195
D_1068
D_1069
D_1070
D_1071
D_1072
D_1073
D_1074
D_1075
1570000
1572500
1575000
1577500
1580000
slr2101
ftsY
slr2102
slr2103
ncr0610
hik22
slr2104
sll1973−as
slr2105
TU_686
TU_687
TU_688
TU_689
Ter
Ter*
NS
TU_2443
TU_2442
TU_2441
TU_2440
TU_2439
sll1973
sll1430
apt
sll1429
D_3755
D_3754
D_3753
D_3752
D_3751
Dc_4040
Dc_4039
Dc_4038
Dc_4037
Dc_4036
I_4392
I_4391
I_4390
I_4389
I_4388
I_4387
Term: termination (1), NS: new start (0), PS: pausing site (0), iTSS_I: internal starting site (0)

ID: 12757−12804; Term: termination (1), NS: new start (0), PS: pausing site (0), iTSS_I: internal starting site (1)

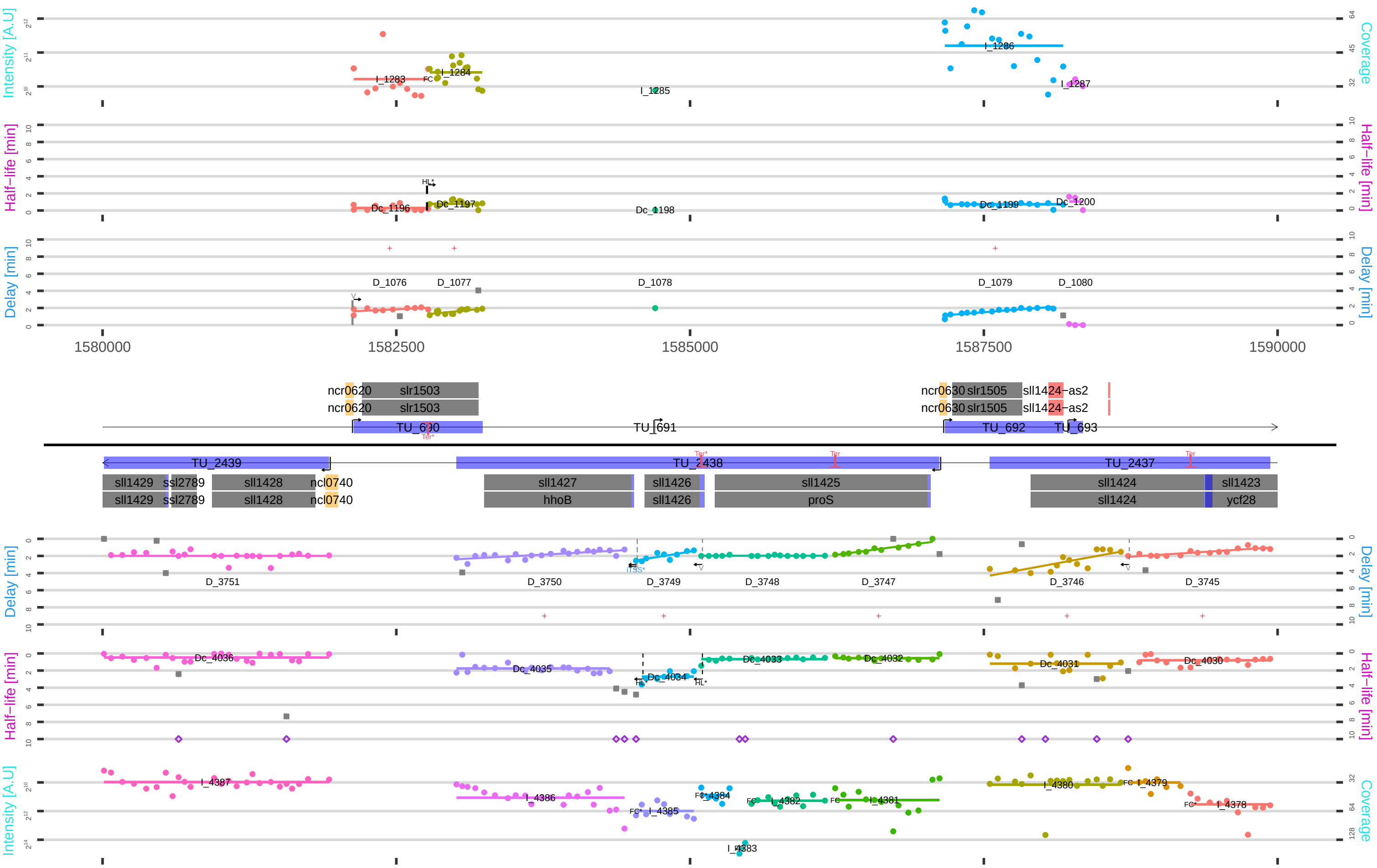

Term: termination (3), NS: new start (0), PS: pausing site (1), iTSS_I: internal starting site (3)

ID: 12805−12938; Term: termination (4), NS: new start (3), PS: pausing site (3), iTSS_I: internal starting site (3)

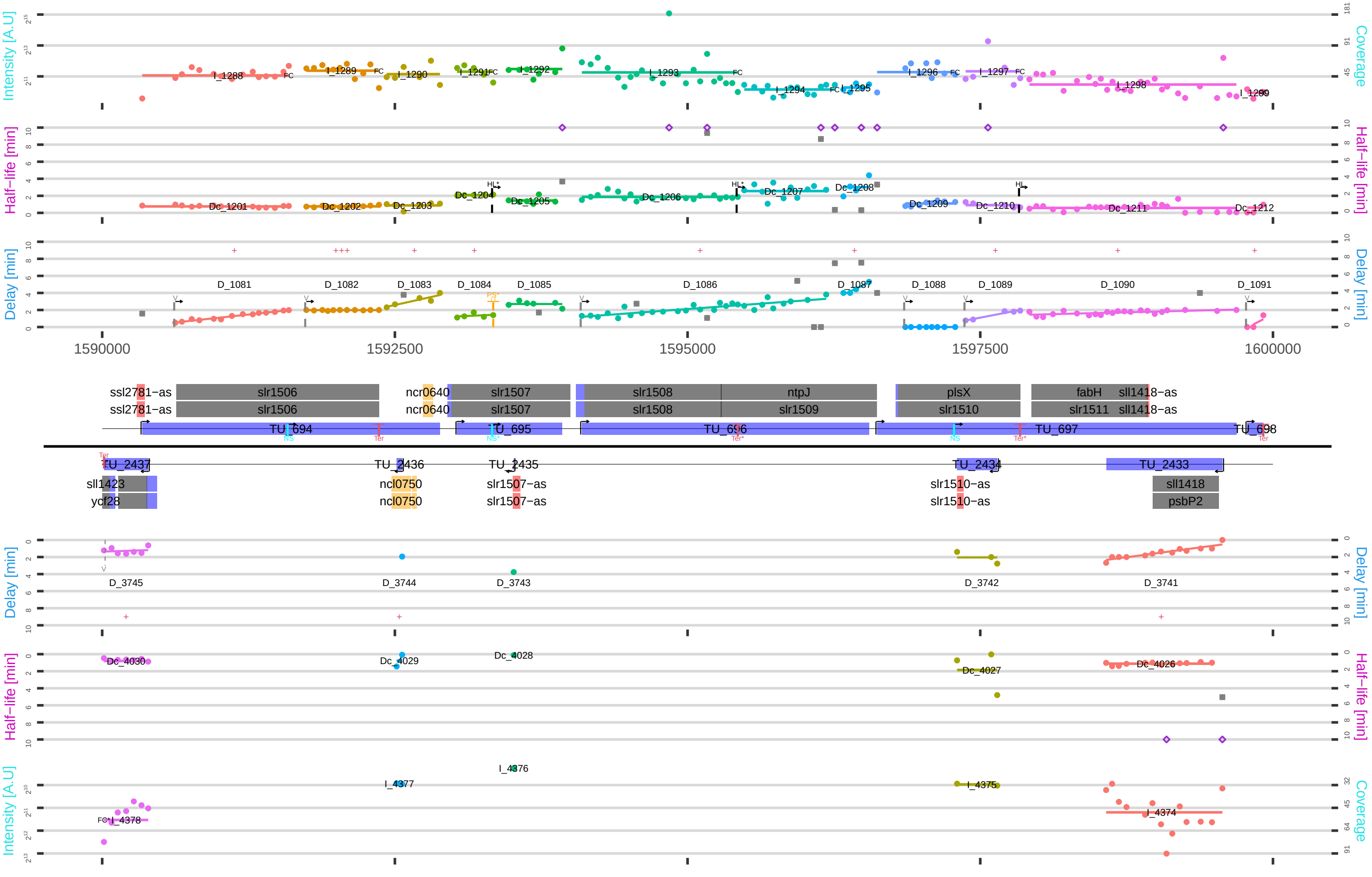

Term: termination (1), NS: new start (0), PS: pausing site (0), iTSS_I: internal starting site (0)

ID: 12939−13049; Term: termination (4), NS: new start (4), PS: pausing site (1), iTSS_I: internal starting site (2)

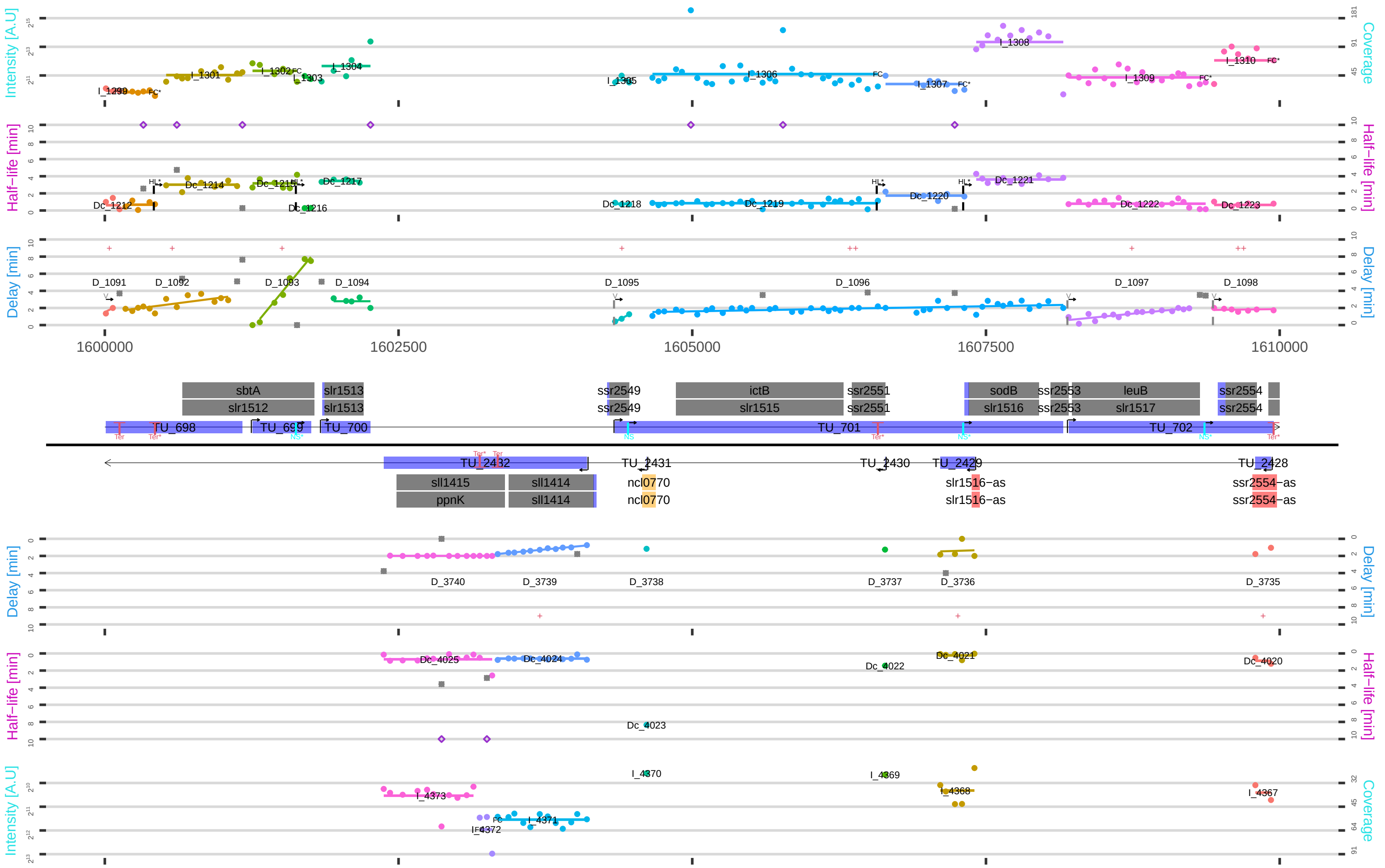

Term: termination (2), NS: new start (0), PS: pausing site (1), iTSS_I: internal starting site (0)

ID: 13050−13150; Term: termination (7), NS: new start (1), PS: pausing site (2), iTSS_I: internal starting site (3)

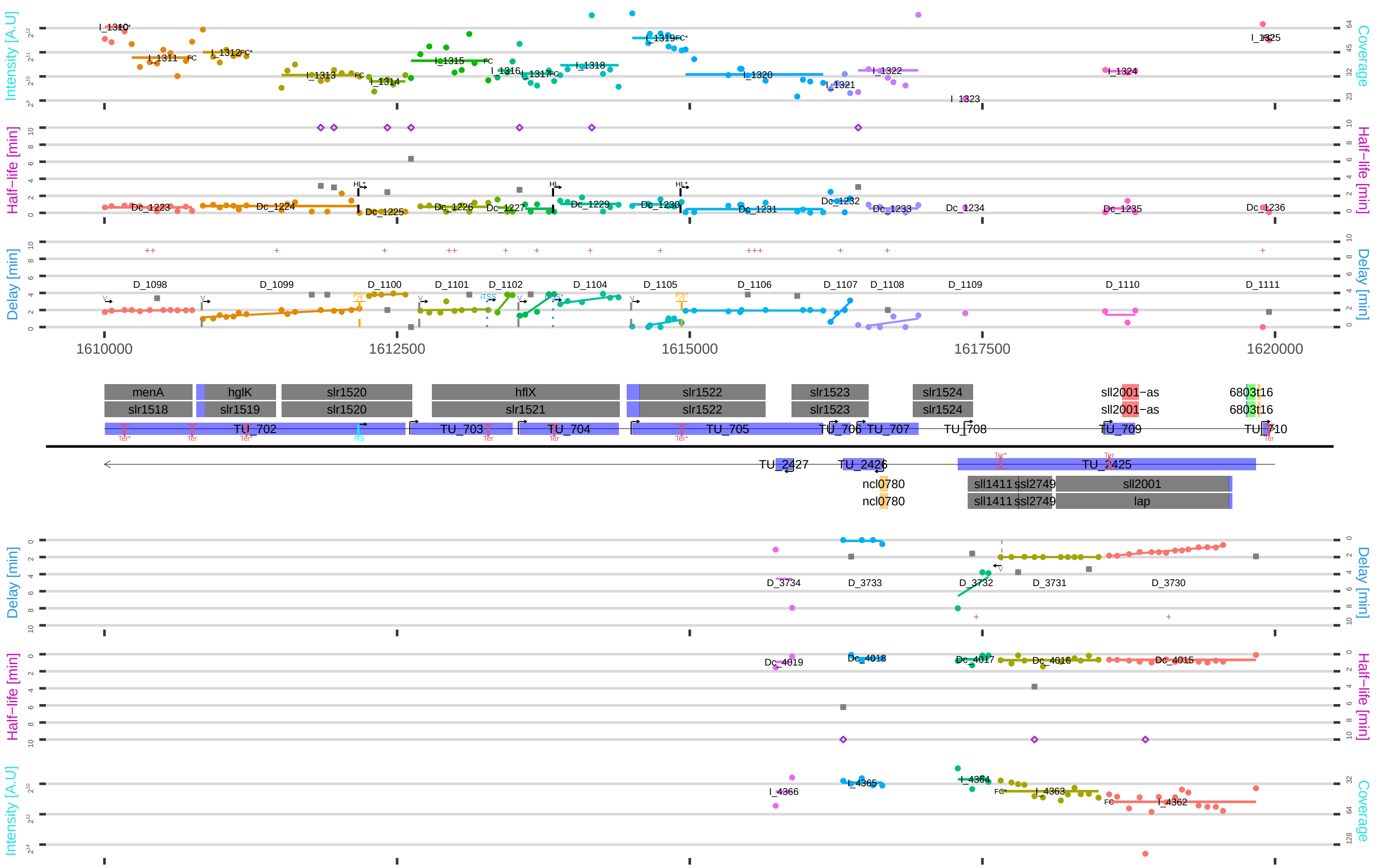

Term: termination (2), NS: new start (0), PS: pausing site (1), iTSS_I: internal starting site (1)

ID: 13151−13257; Term: termination (4), NS: new start (1), PS: pausing site (4), iTSS_I: internal starting site (1)

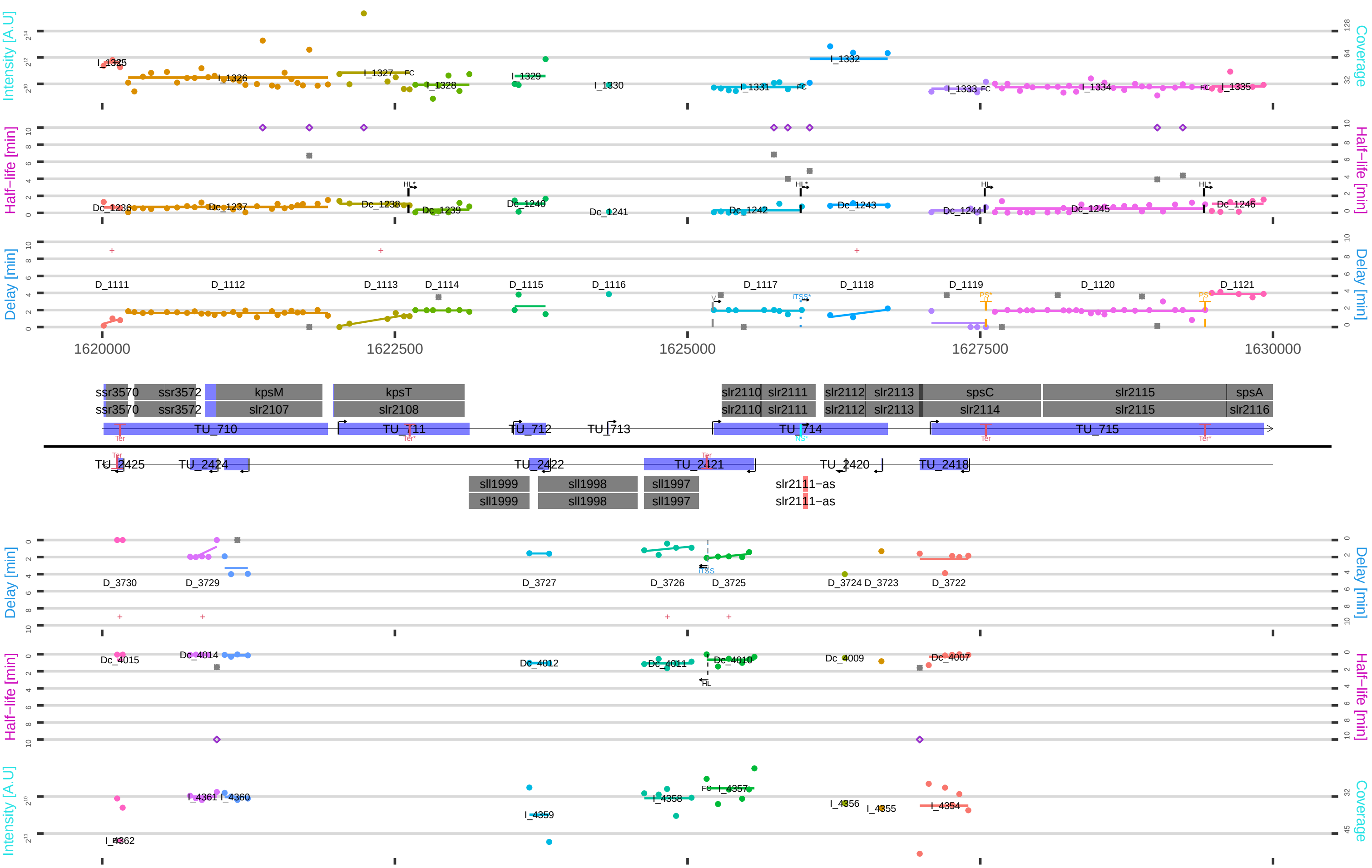

Term: termination (2), NS: new start (0), PS: pausing site (0), iTSS_I: internal starting site (1)

ID: 13258−13387; Term: termination (1), NS: new start (4), PS: pausing site (2), iTSS_I: internal starting site (1)

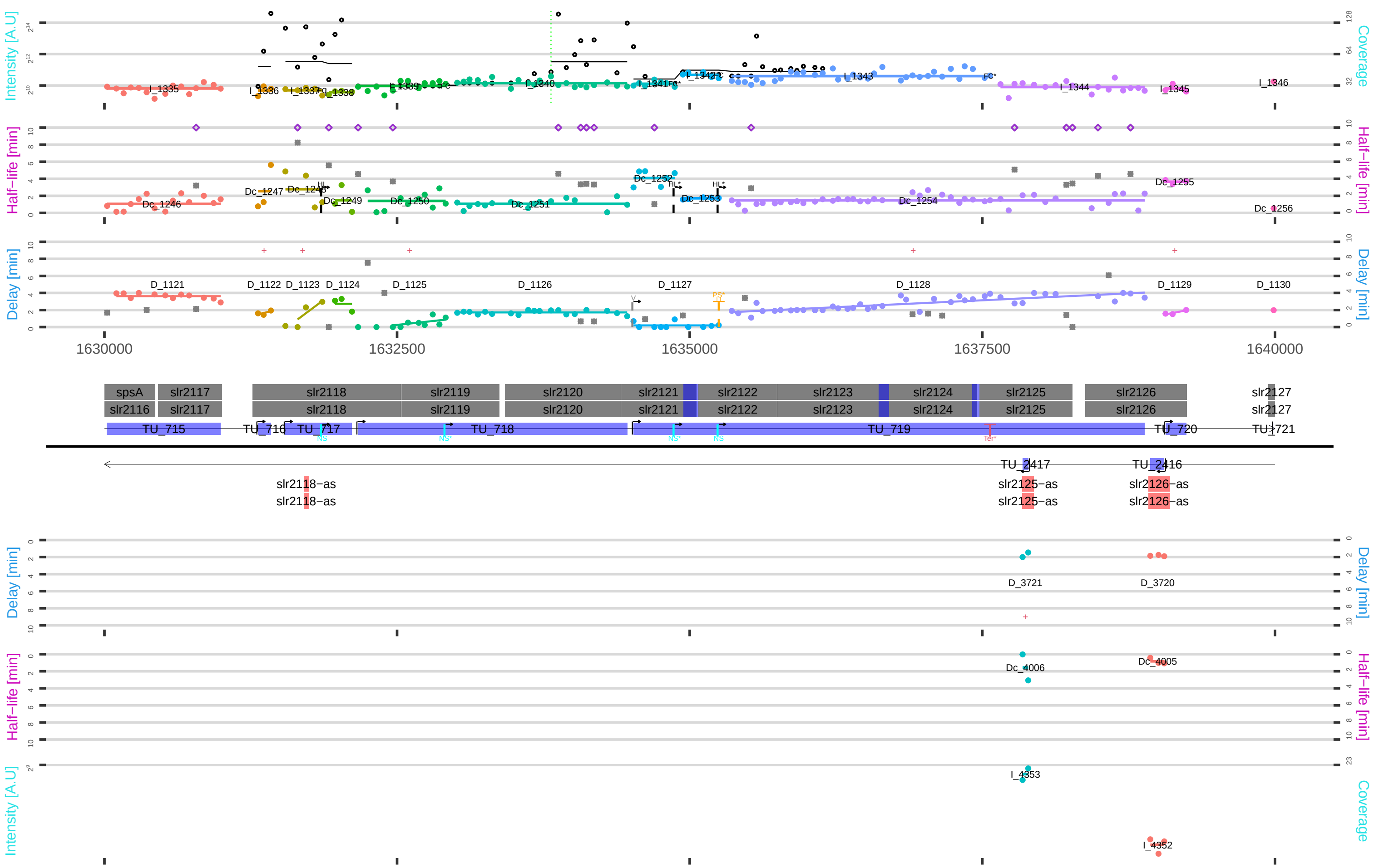

Term: termination (0), NS: new start (0), PS: pausing site (0), iTSS_I: internal starting site (0)

ID: 13388−13482; Term: termination (1), NS: new start (3), PS: pausing site (2), iTSS_I: internal starting site (2)

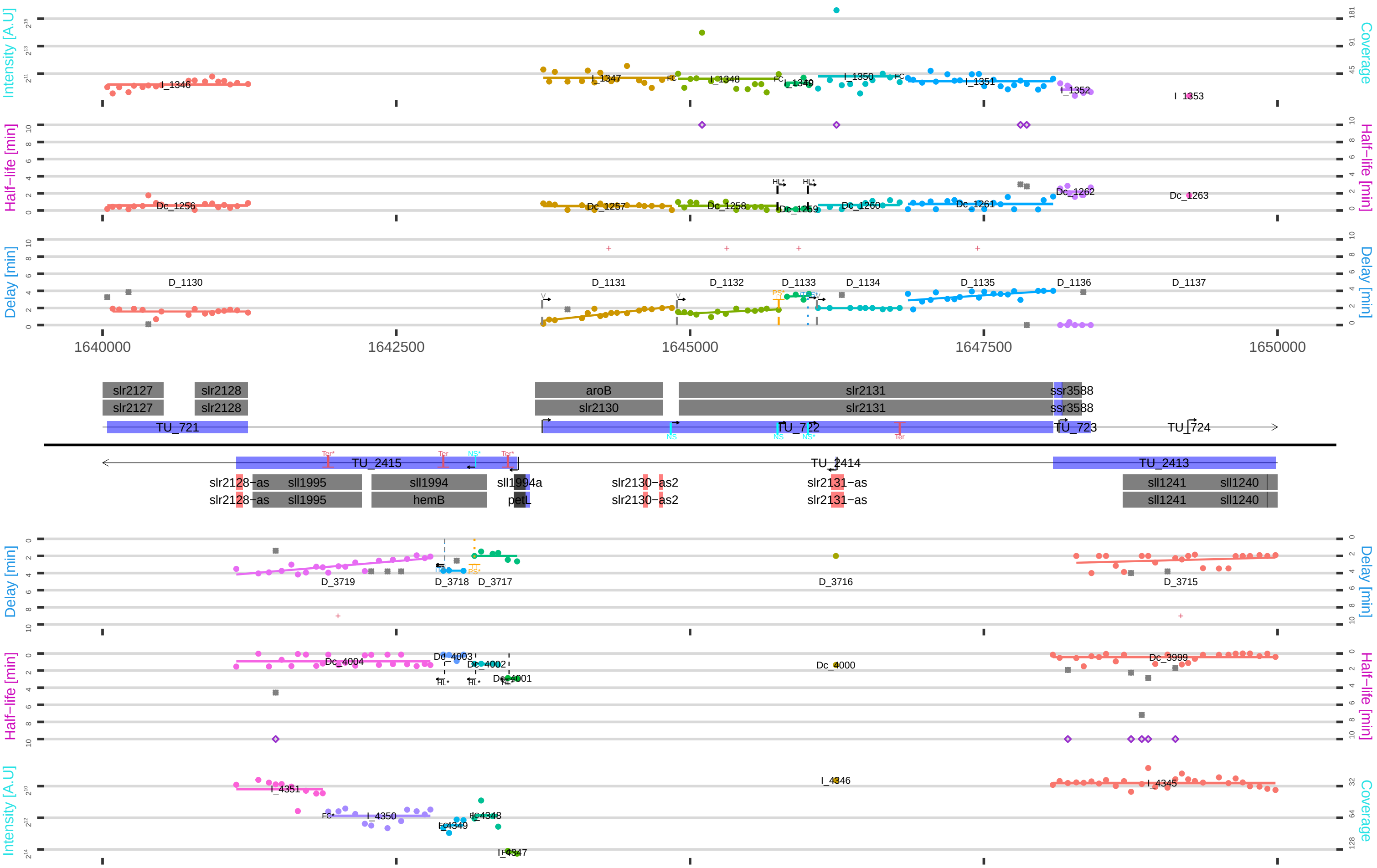

Term: termination (3), NS: new start (1), PS: pausing site (1), iTSS_I: internal starting site (1)

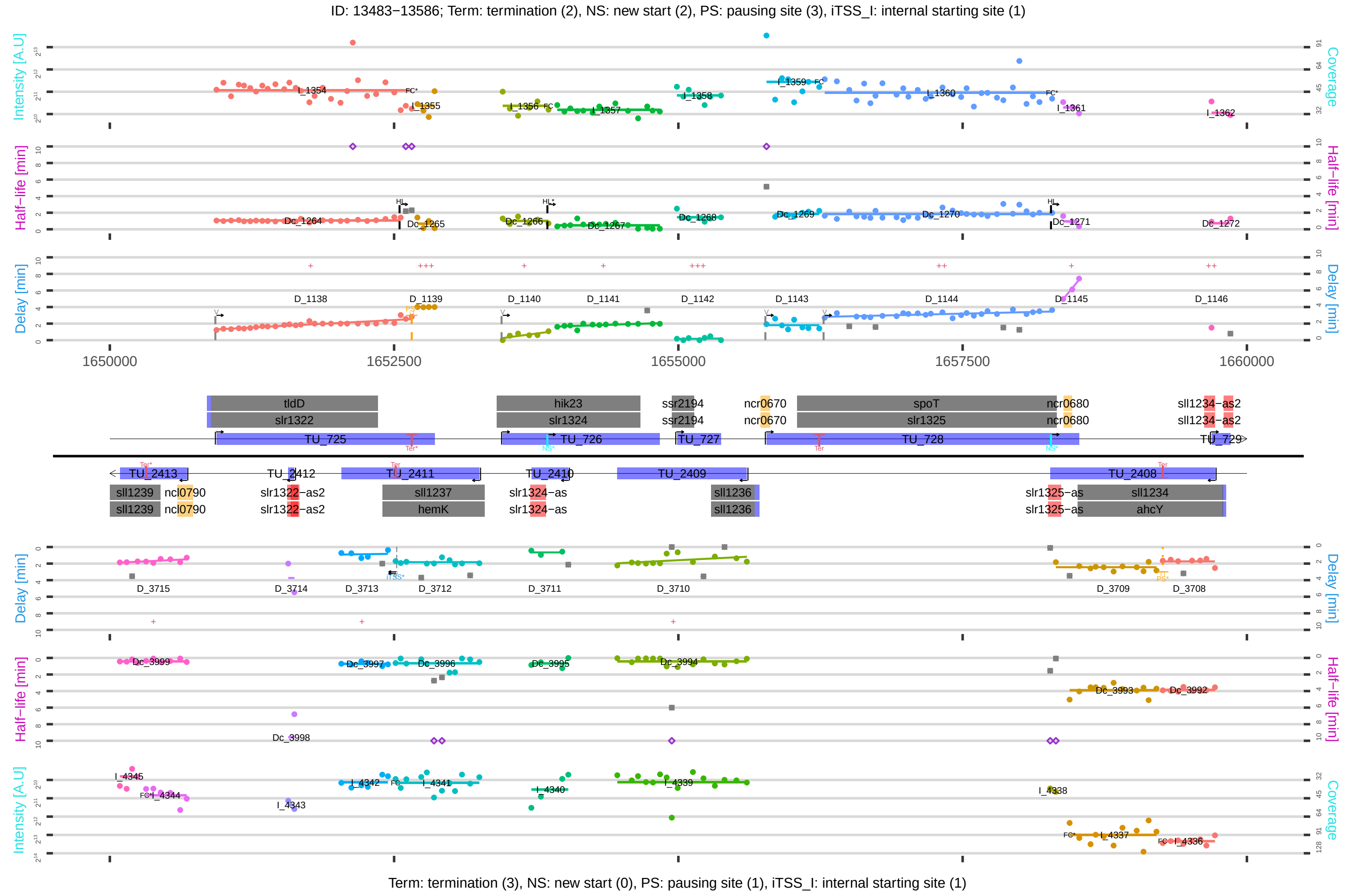
ID: 13483−13586; Term: termination (2), NS: new start (2), PS: pausing site (3), iTSS_I: internal starting site (1)
Intensity [A.U]
Coverage
Half−life [min]
Delay [min]
I_1354
I_1355
I_1356
I_1357
I_1358
I_1359
I_1360
I_1361
I_1362
Dc_1264
Dc_1265
Dc_1266
Dc_1267
Dc_1268
Dc_1269
Dc_1270
Dc_1271
Dc_1272
D_1138
D_1139
D_1140
D_1141
D_1142
D_1143
D_1144
D_1145
D_1146
1650000
1652500
1655000
1657500
1660000
tldD
slr1322
hik23
slr1324
ssr2194
ncr0670
spoT
slr1325
ncr0680
sll1234−as2
TU_725
TU_726
TU_727
TU_728
TU_729
TU_2413
TU_2412
TU_2411
TU_2410
TU_2409
TU_2408
sll1239
ncl0790
slr1322−as2
sll1237
hemK
slr1324−as
sll1236
slr1325−as
sll1234
ahcY
D_3715
D_3714
D_3713
D_3712
D_3711
D_3710
D_3709
D_3708
Dc_3999
Dc_3998
Dc_3997
Dc_3996
Dc_3995
Dc_3994
Dc_3993
Dc_3992
I_4345
I_4344
I_4343
I_4342
I_4341
I_4340
I_4339
I_4338
I_4337
I_4336
Term: termination (3), NS: new start (0), PS: pausing site (1), iTSS_I: internal starting site (1)

ID: 13587−13642; Term: termination (3), NS: new start (0), PS: pausing site (1), iTSS_I: internal starting site (0)

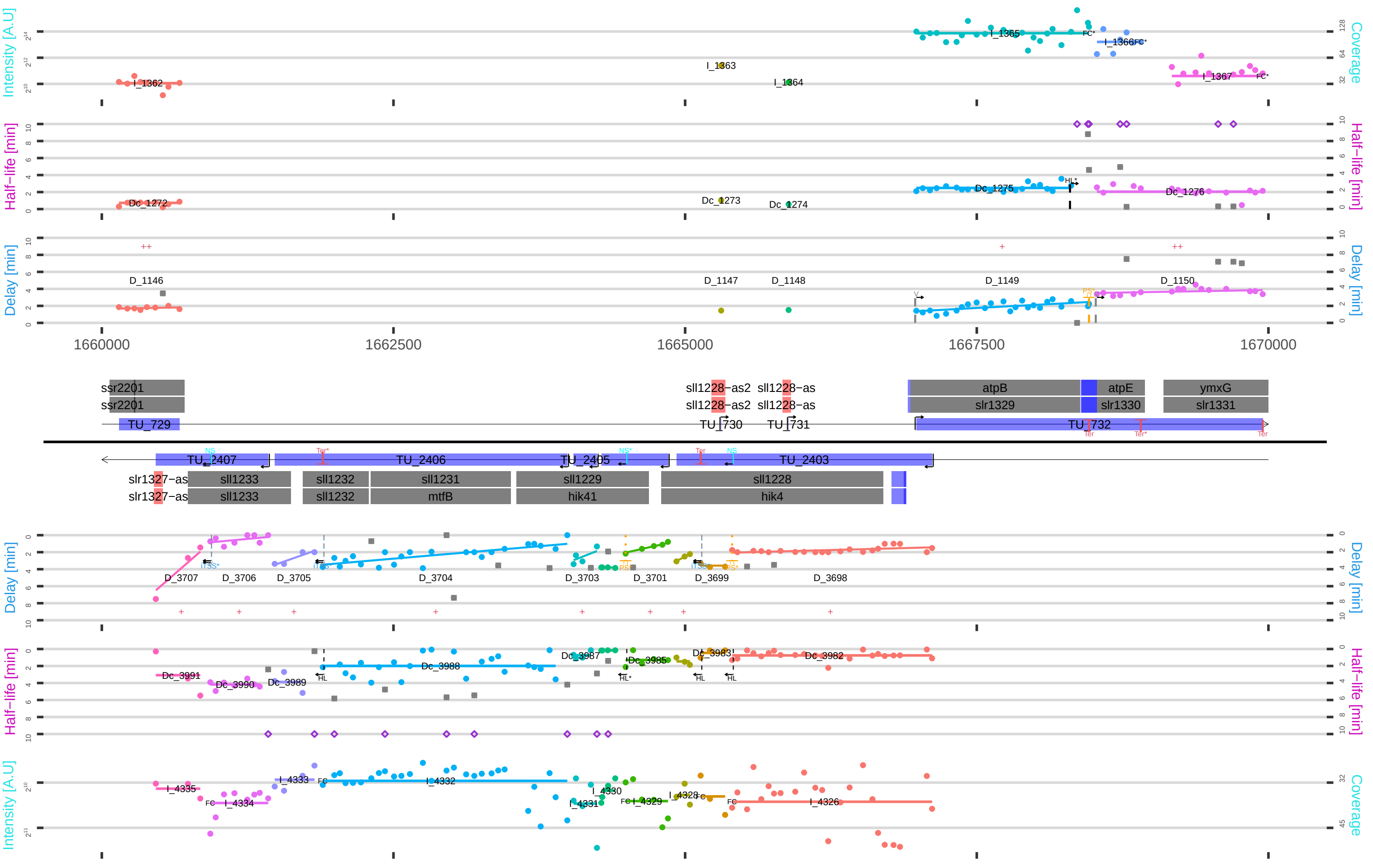

Term: termination (2), NS: new start (3), PS: pausing site (2), iTSS_I: internal starting site (3)

ID: 13643−13682; Term: termination (3), NS: new start (2), PS: pausing site (2), iTSS_I: internal starting site (1)

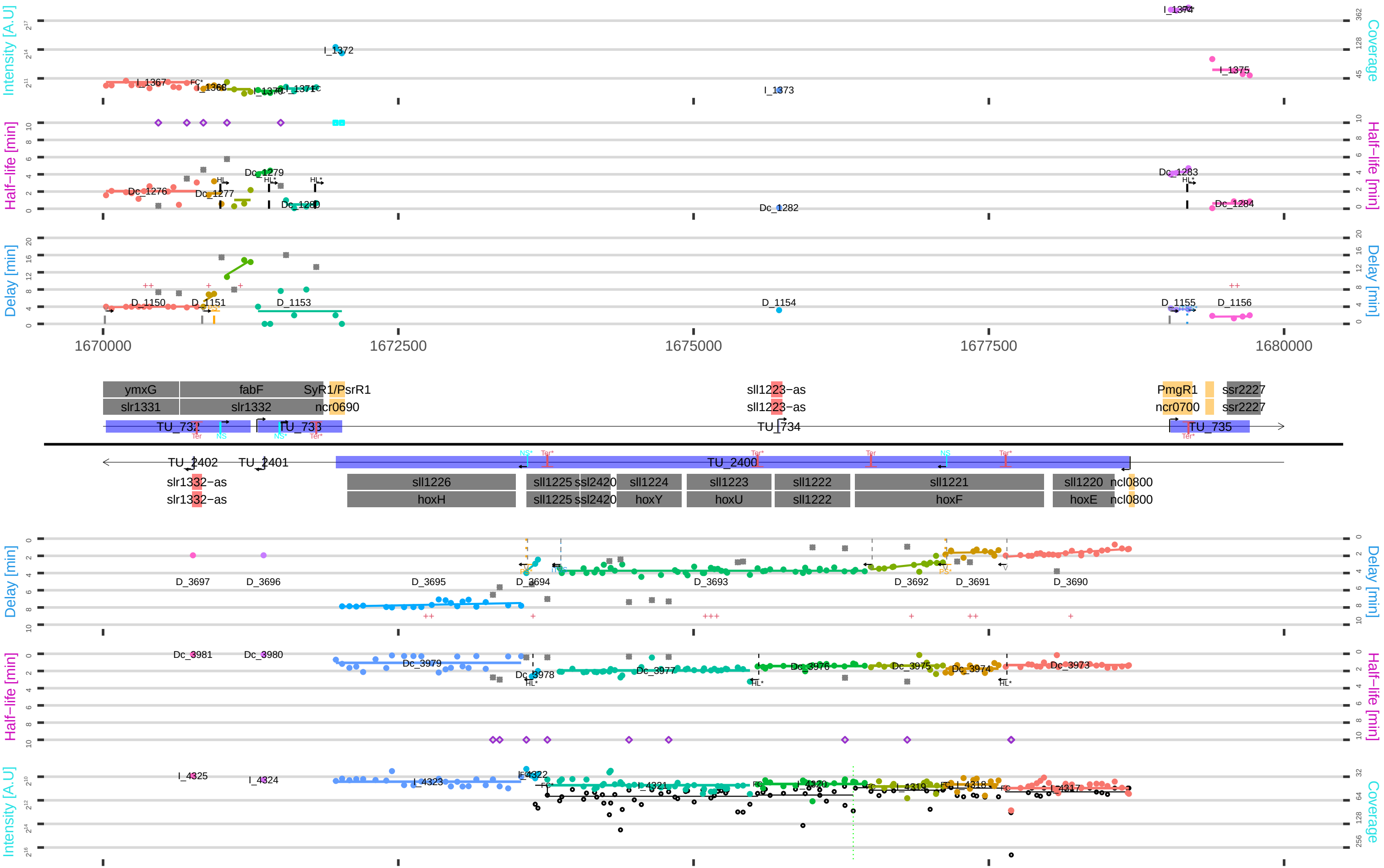

Term: termination (4), NS: new start (2), PS: pausing site (3), iTSS_I: internal starting site (2)

ID: 13684−13790; Term: termination (3), NS: new start (1), PS: pausing site (2), iTSS_I: internal starting site (0)

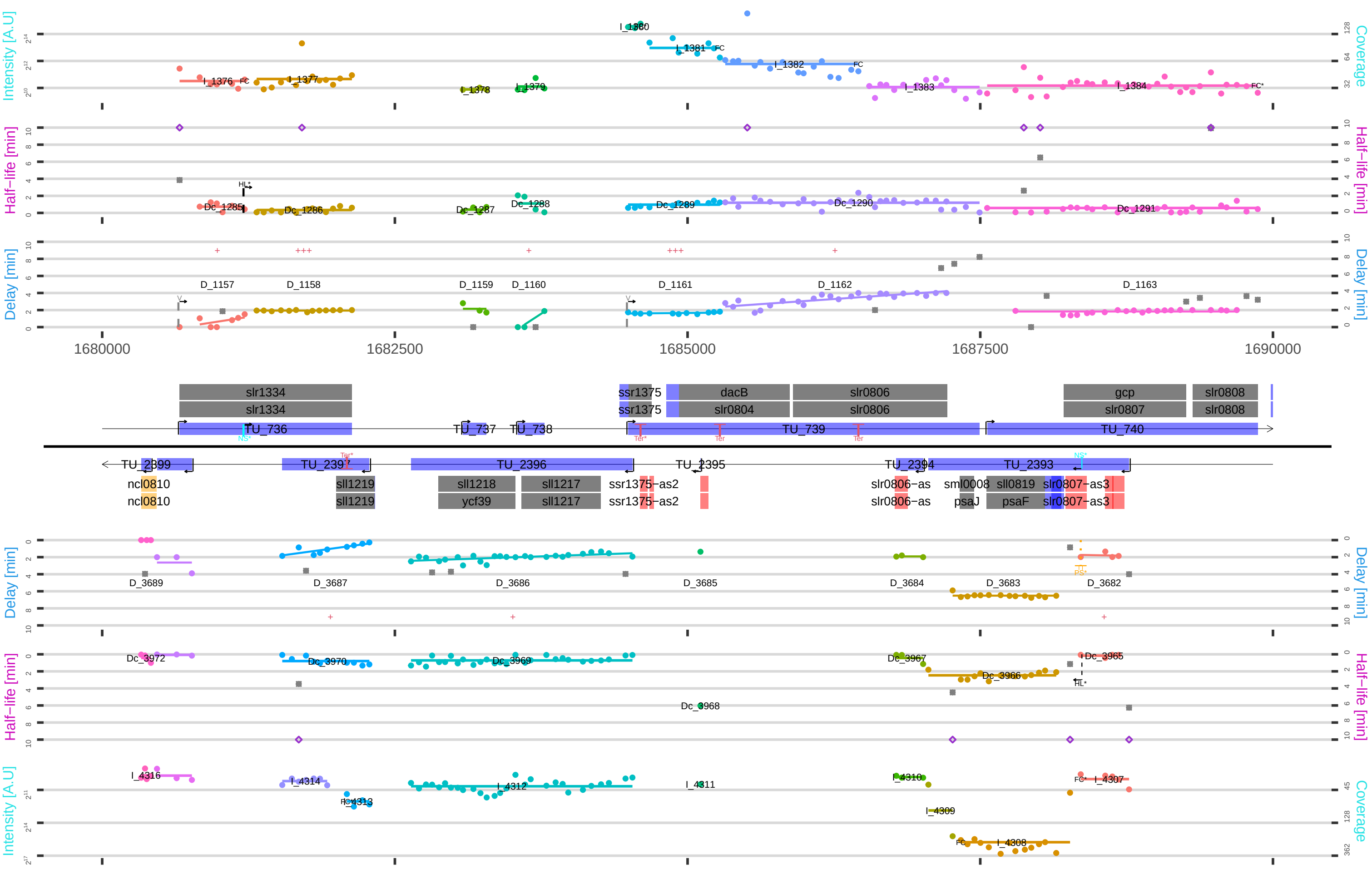

Term: termination (1), NS: new start (1), PS: pausing site (1), iTSS_I: internal starting site (0)

ID: 13791−13860; Term: termination (1), NS: new start (0), PS: pausing site (0), iTSS_I: internal starting site (0)

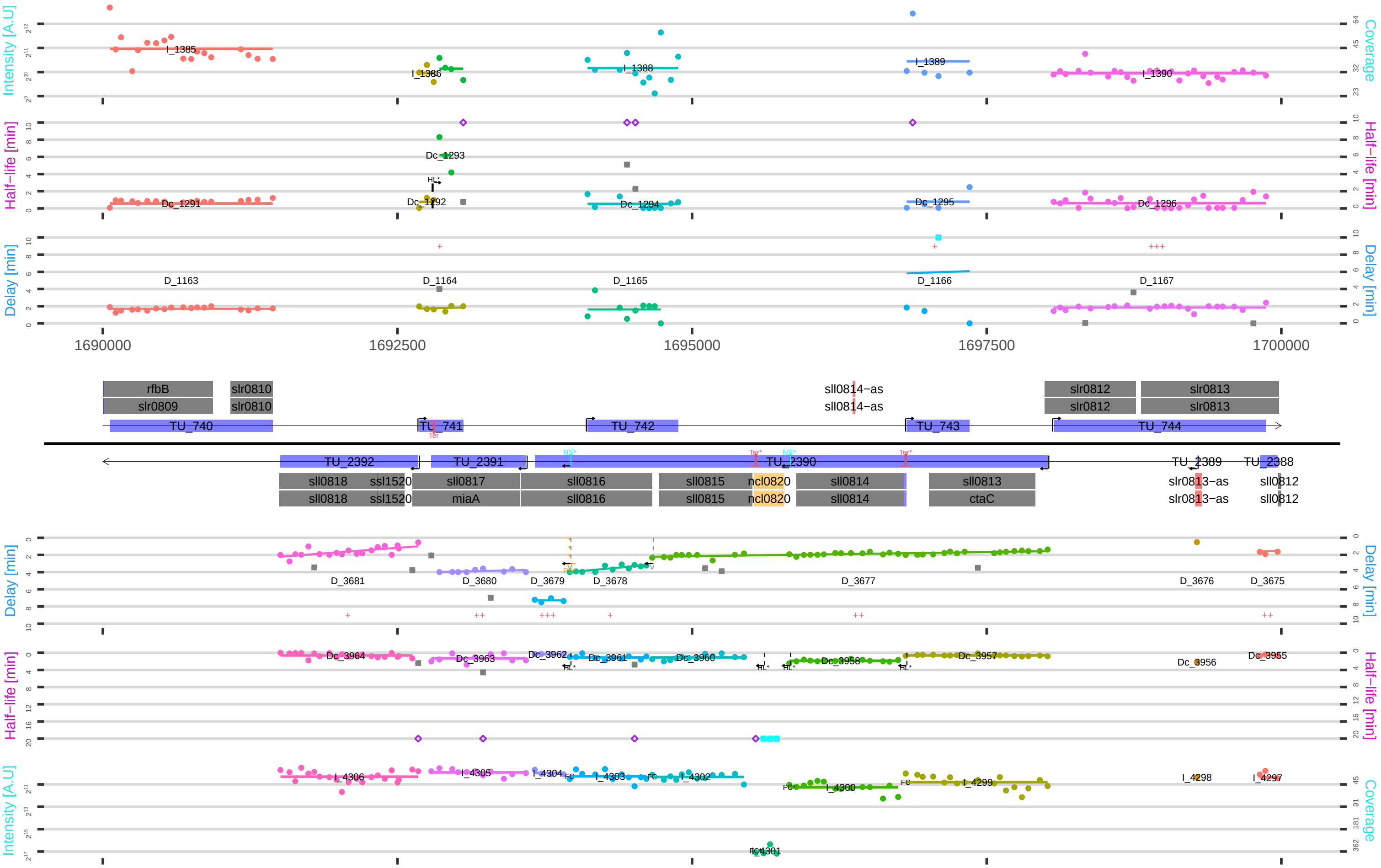

Term: termination (2), NS: new start (2), PS: pausing site (2), iTSS_I: internal starting site (0)

ID: 13862−13978; Term: termination (3), NS: new start (1), PS: pausing site (1), iTSS_I: internal starting site (1)

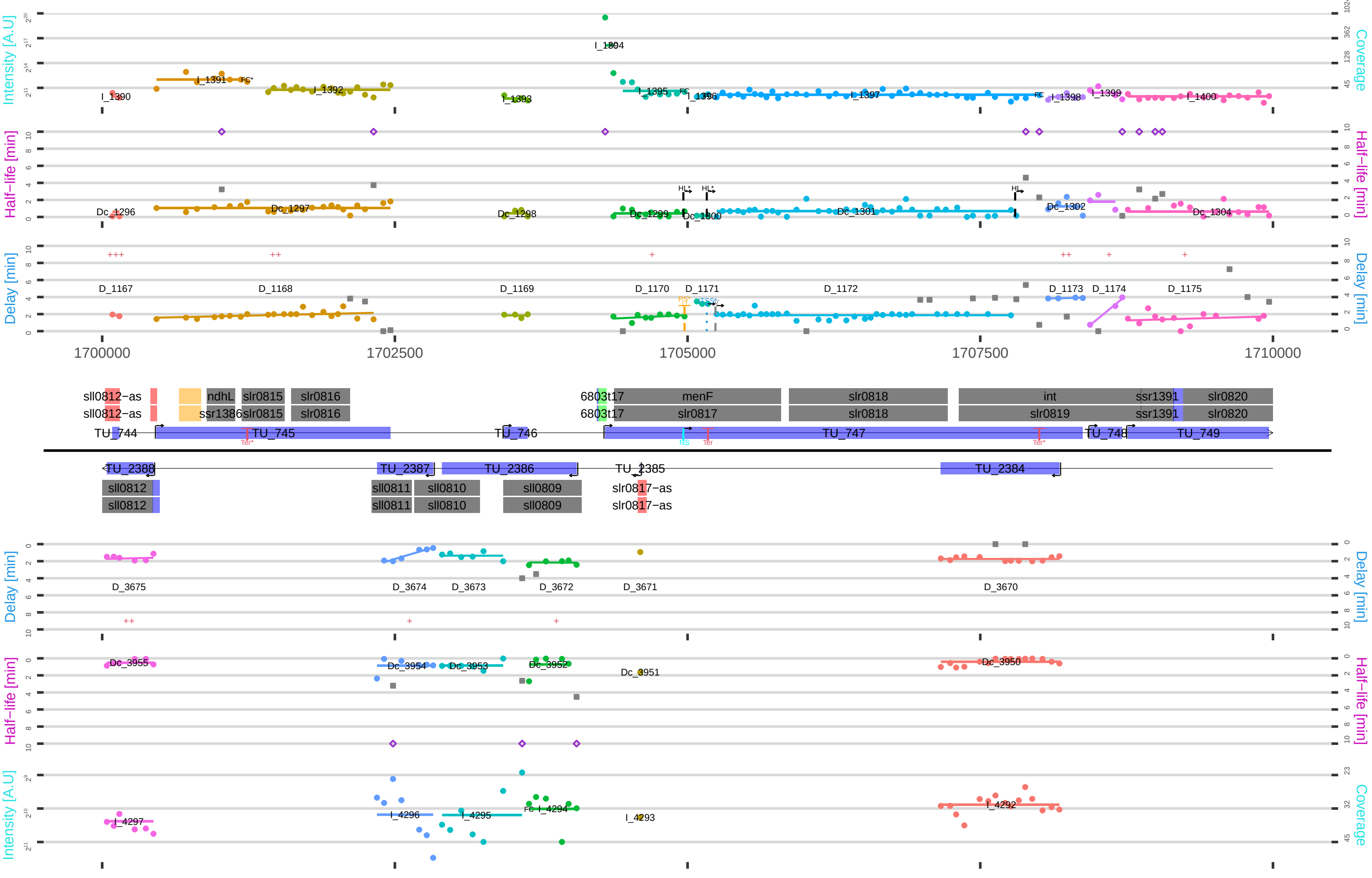

Term: termination (0), NS: new start (0), PS: pausing site (0), iTSS_I: internal starting site (1)

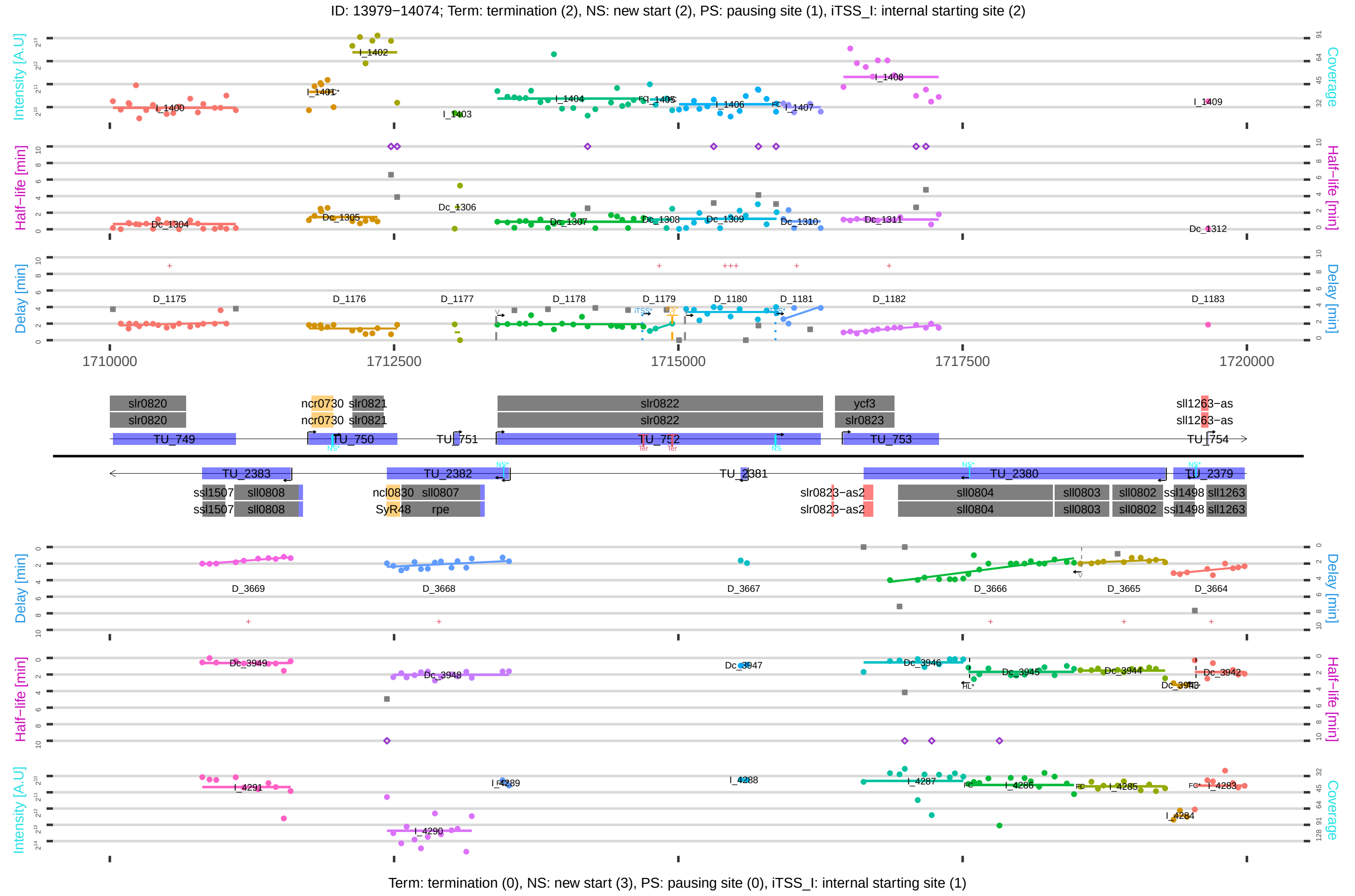

ID: 13979−14074; Term: termination (2), NS: new start (2), PS: pausing site (1), iTSS_I: internal starting site (2)
Intensity [A.U]
Coverage
Half−life [min]
Delay [min]
1710000
1712500
1715000
1717500
1720000
slr0820
ncr0730
slr0821
slr0822
ycf3
slr0823
sll1263−as
TU_749
TU_750
TU_751
TU_752
TU_753
TU_754
TU_2383
TU_2382
TU_2381
TU_2380
TU_2379
ssl1507
sll0808
ncl0830
sll0807
SyR48
rpe
slr0823−as2
sll0804
sll0803
sll0802
ssl1498
sll1263
Term: termination (0), NS: new start (3), PS: pausing site (0), iTSS_I: internal starting site (1)

ID: 14075−14126; Term: termination (1), NS: new start (0), PS: pausing site (0), iTSS_I: internal starting site (1)

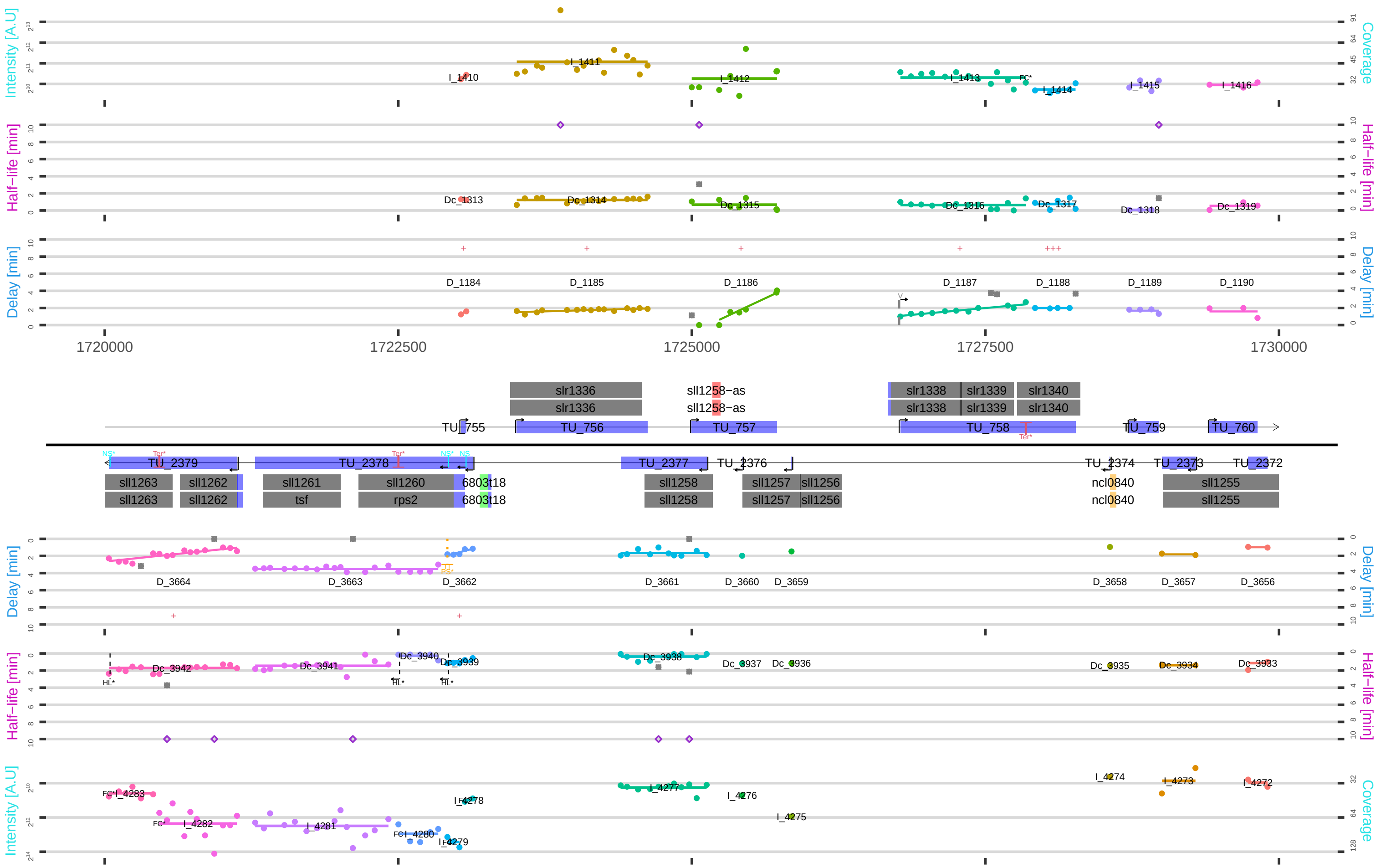

Term: termination (2), NS: new start (3), PS: pausing site (1), iTSS_I: internal starting site (0)

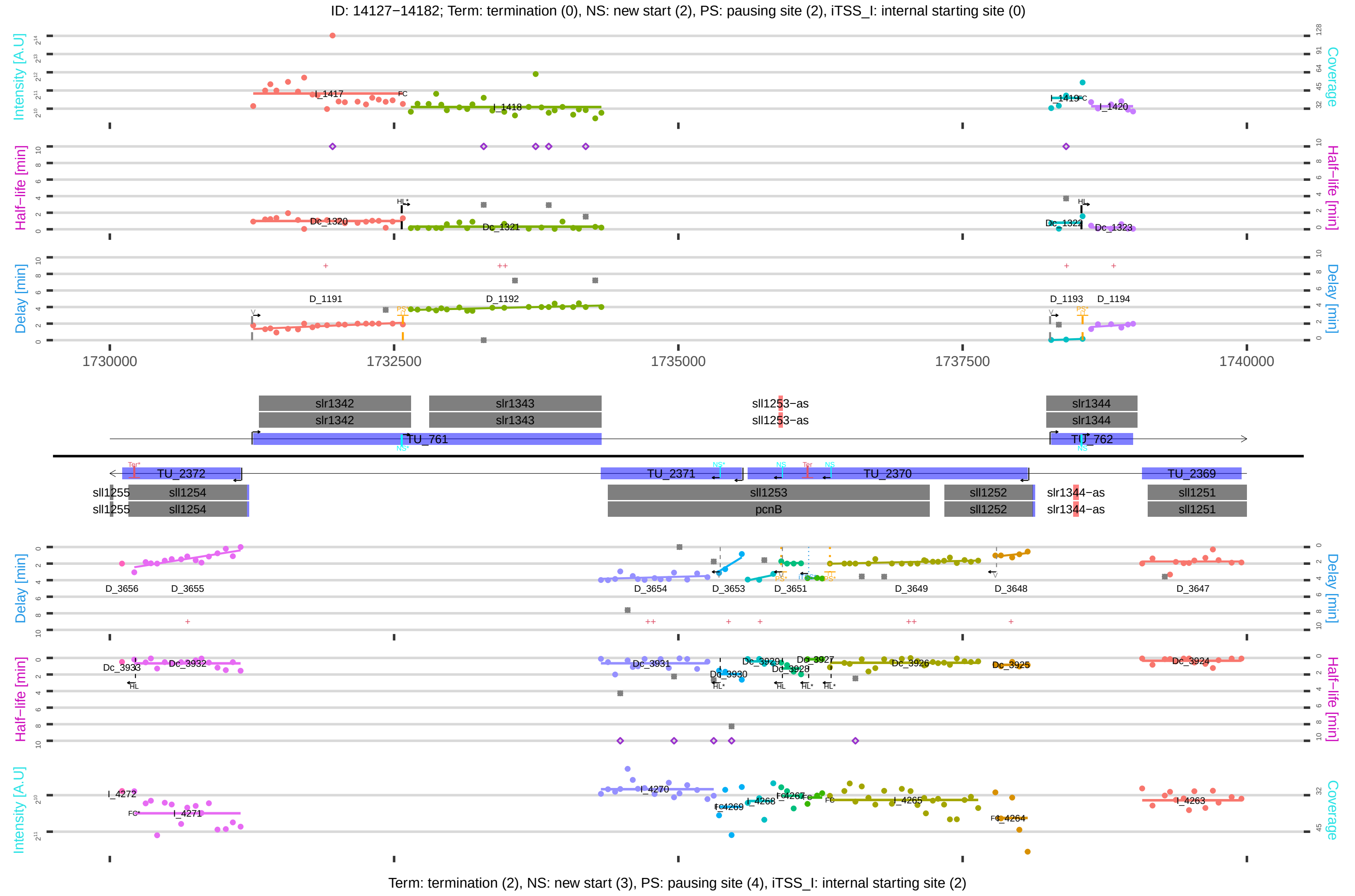
ID: 14127−14182; Term: termination (0), NS: new start (2), PS: pausing site (2), iTSS_I: internal starting site (0)
Intensity [A.U]
Coverage
Half−life [min]
Delay [min]
I_1417
I_1418
I_1419
I_1420
Dc_1320
Dc_1321
Dc_1322
Dc_1323
D_1191
D_1192
D_1193
D_1194
1730000
1732500
1735000
1737500
1740000
slr1342
slr1343
sll1253−as
slr1344
TU_761
TU_762
TU_2372
TU_2371
TU_2370
TU_2369
sll1255
sll1254
sll1253
pcnB
sll1252
slr1344−as
sll1251
D_3656
D_3655
D_3654
D_3653
D_3651
D_3649
D_3648
D_3647
Dc_3933
Dc_3932
Dc_3931
Dc_3930
Dc_3929
Dc_3928
Dc_3927
Dc_3926
Dc_3925
Dc_3924
I_4272
I_4271
I_4270
I_4269
I_4268
I_4267
I_4265
I_4264
I_4263
Term: termination (2), NS: new start (3), PS: pausing site (4), iTSS_I: internal starting site (2)

ID: 14183−14272; Term: termination (1), NS: new start (1), PS: pausing site (1), iTSS_I: internal starting site (2)

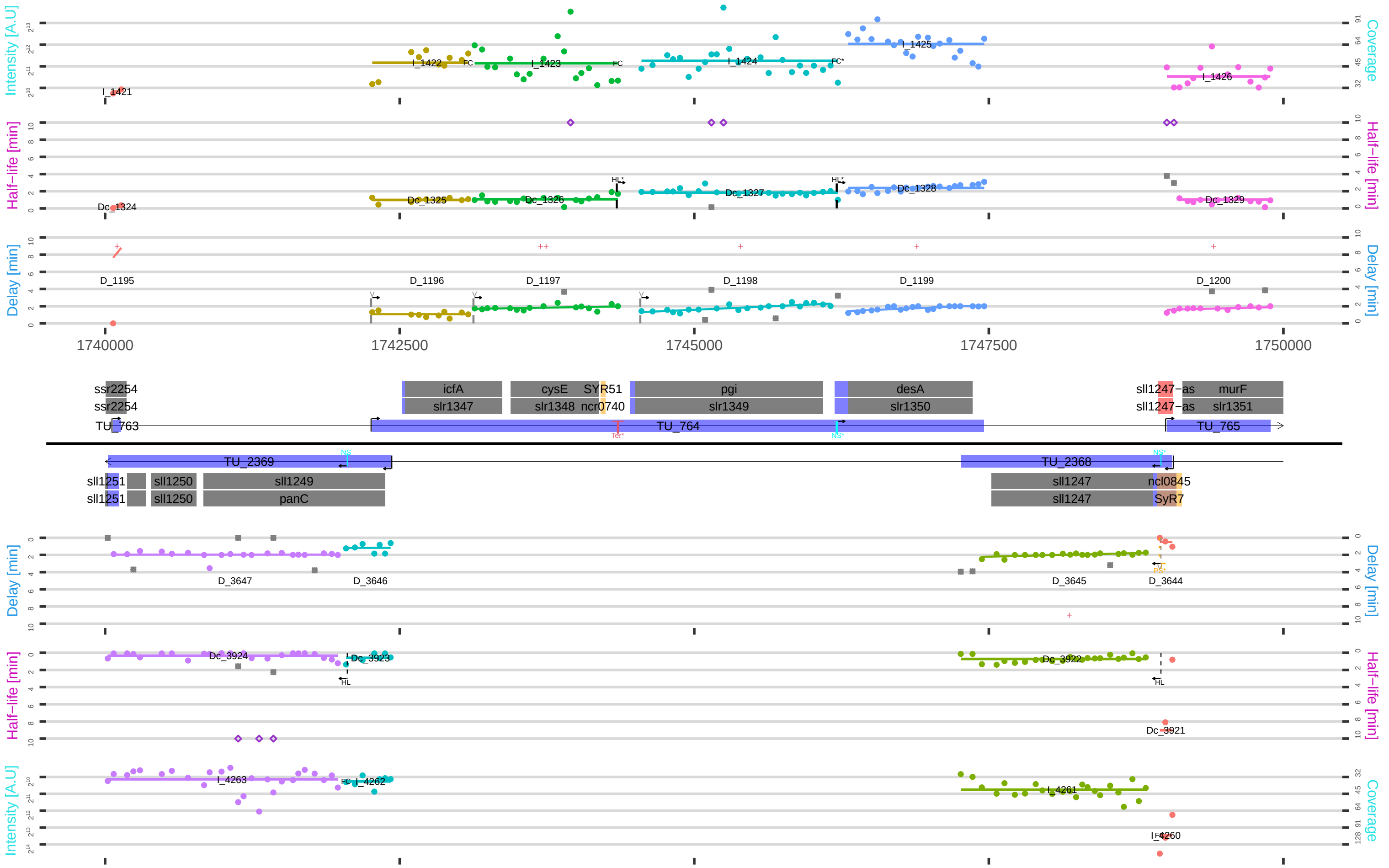

Term: termination (0), NS: new start (2), PS: pausing site (2), iTSS_I: internal starting site (0)

ID: 14273−14326; Term: termination (1), NS: new start (1), PS: pausing site (0), iTSS_I: internal starting site (1)

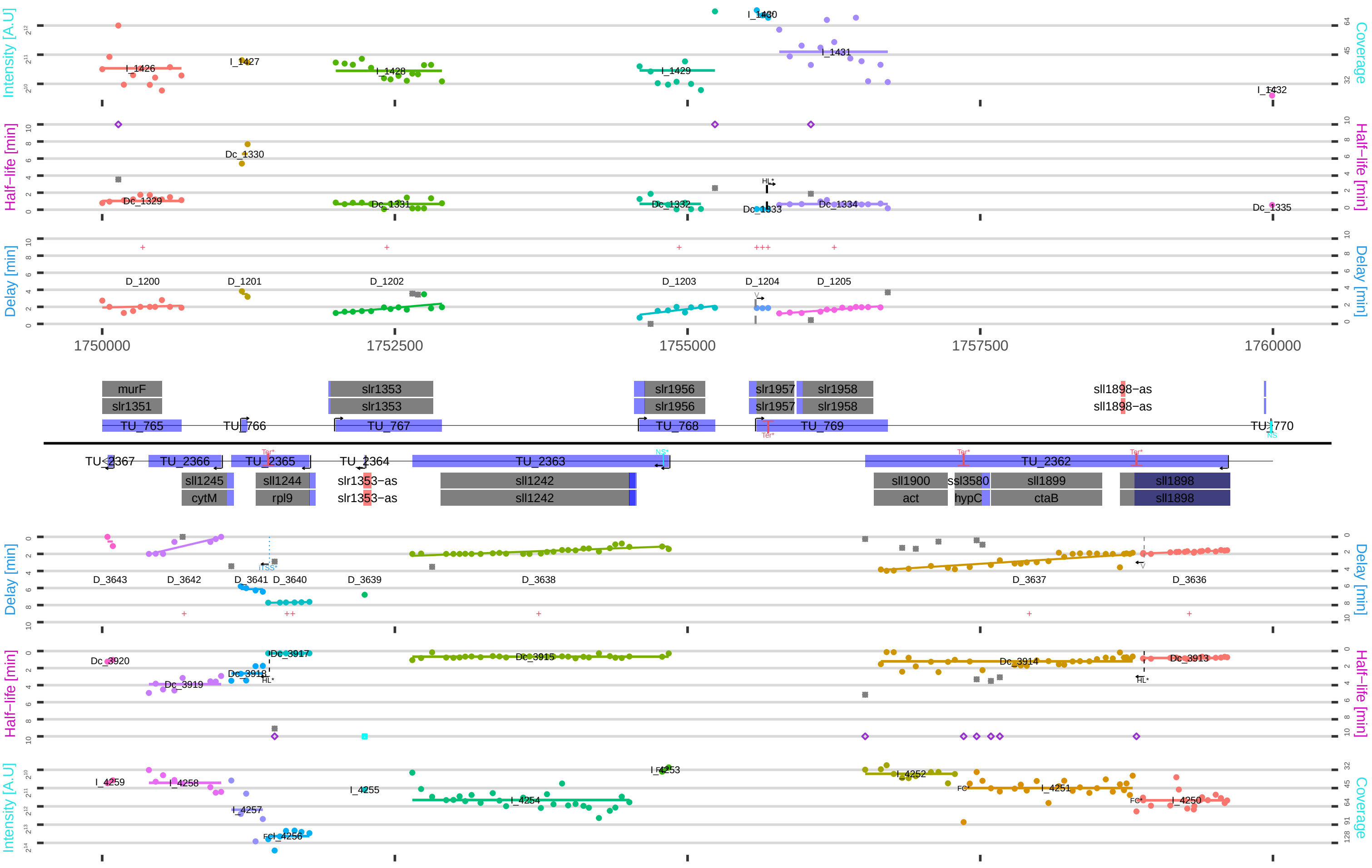

Term: termination (3), NS: new start (1), PS: pausing site (0), iTSS_I: internal starting site (2)

ID: 14327−14411; Term: termination (1), NS: new start (1), PS: pausing site (1), iTSS_I: internal starting site (1)

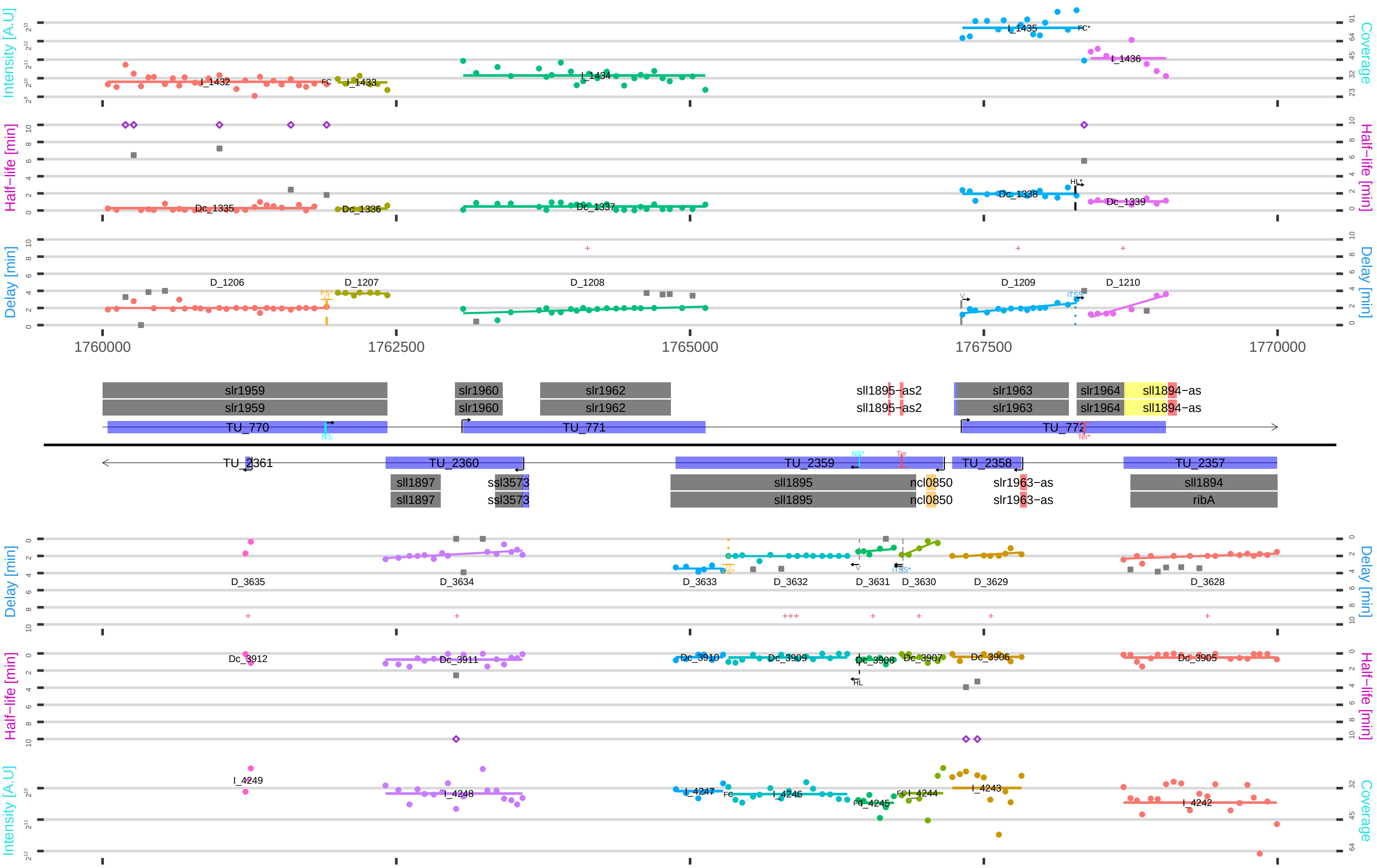

Term: termination (1), NS: new start (1), PS: pausing site (2), iTSS_I: internal starting site (1)

ID: 14413−14502; Term: termination (1), NS: new start (2), PS: pausing site (1), iTSS_I: internal starting site (2)

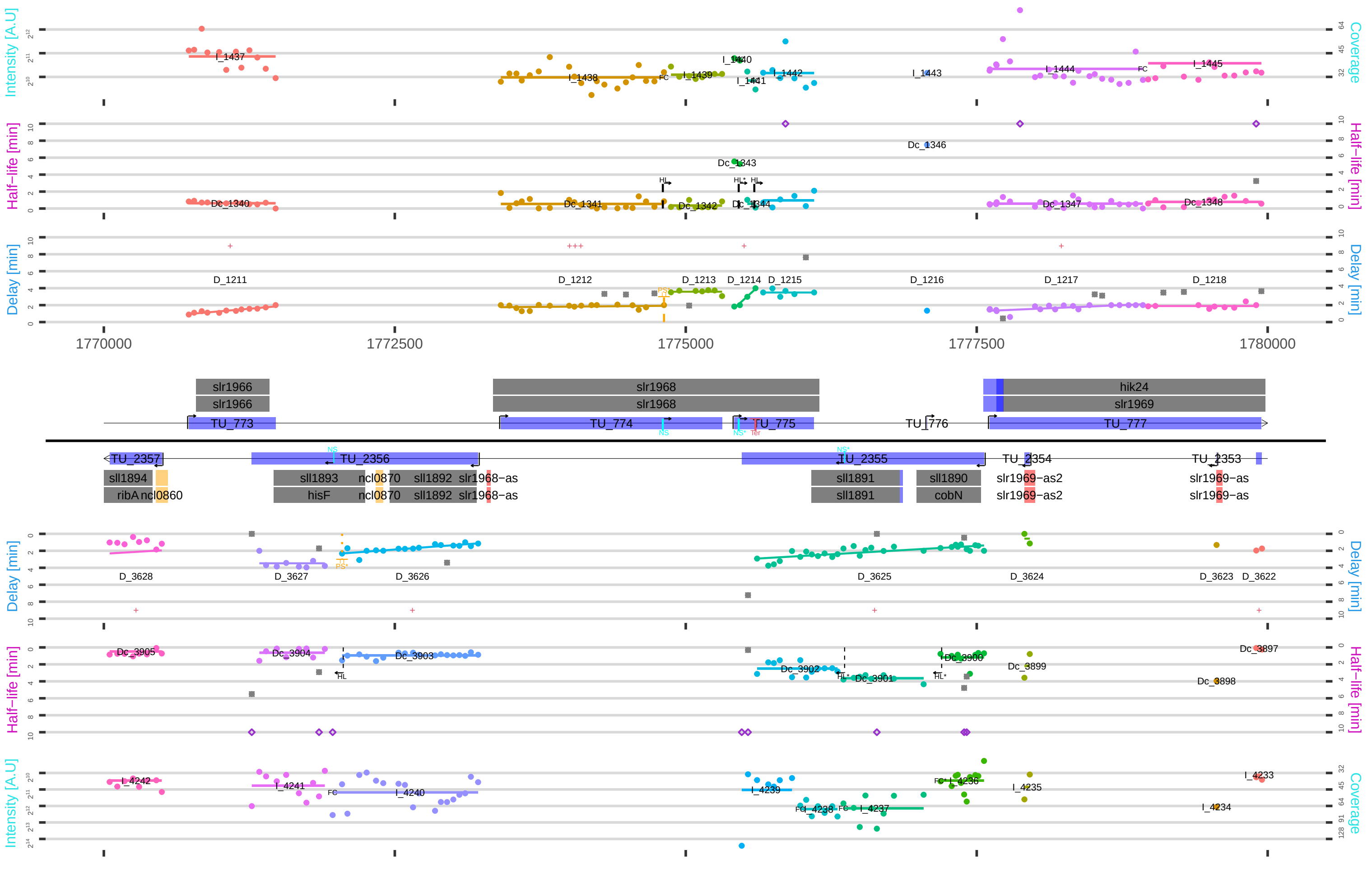

Term: termination (0), NS: new start (2), PS: pausing site (1), iTSS_I: internal starting site (0)

ID: 14503−14578; Term: termination (1), NS: new start (1), PS: pausing site (2), iTSS_I: internal starting site (0)

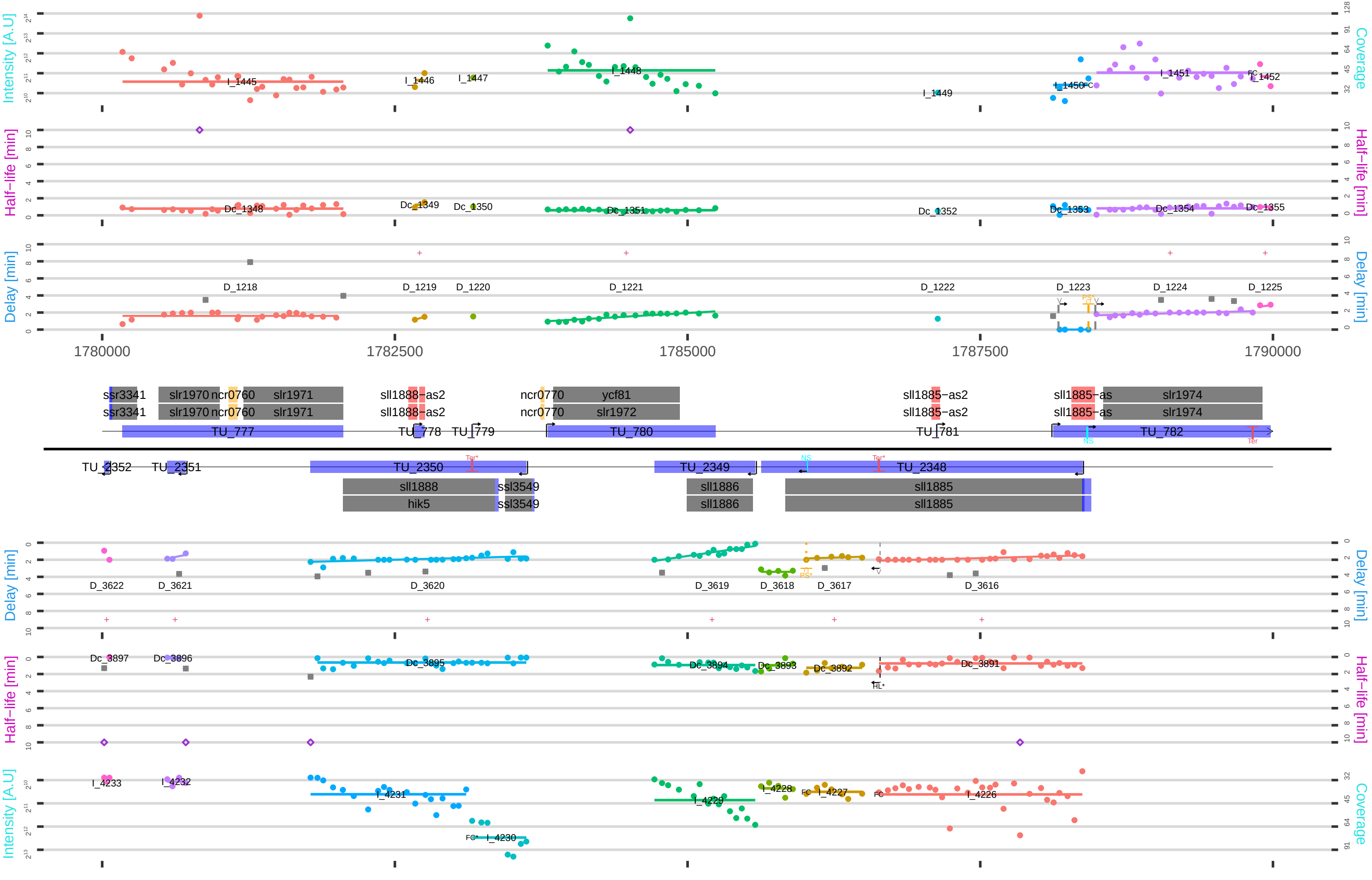

Term: termination (2), NS: new start (1), PS: pausing site (1), iTSS_I: internal starting site (1)

ID: 14579−14622; Term: termination (1), NS: new start (0), PS: pausing site (0), iTSS_I: internal starting site (0)

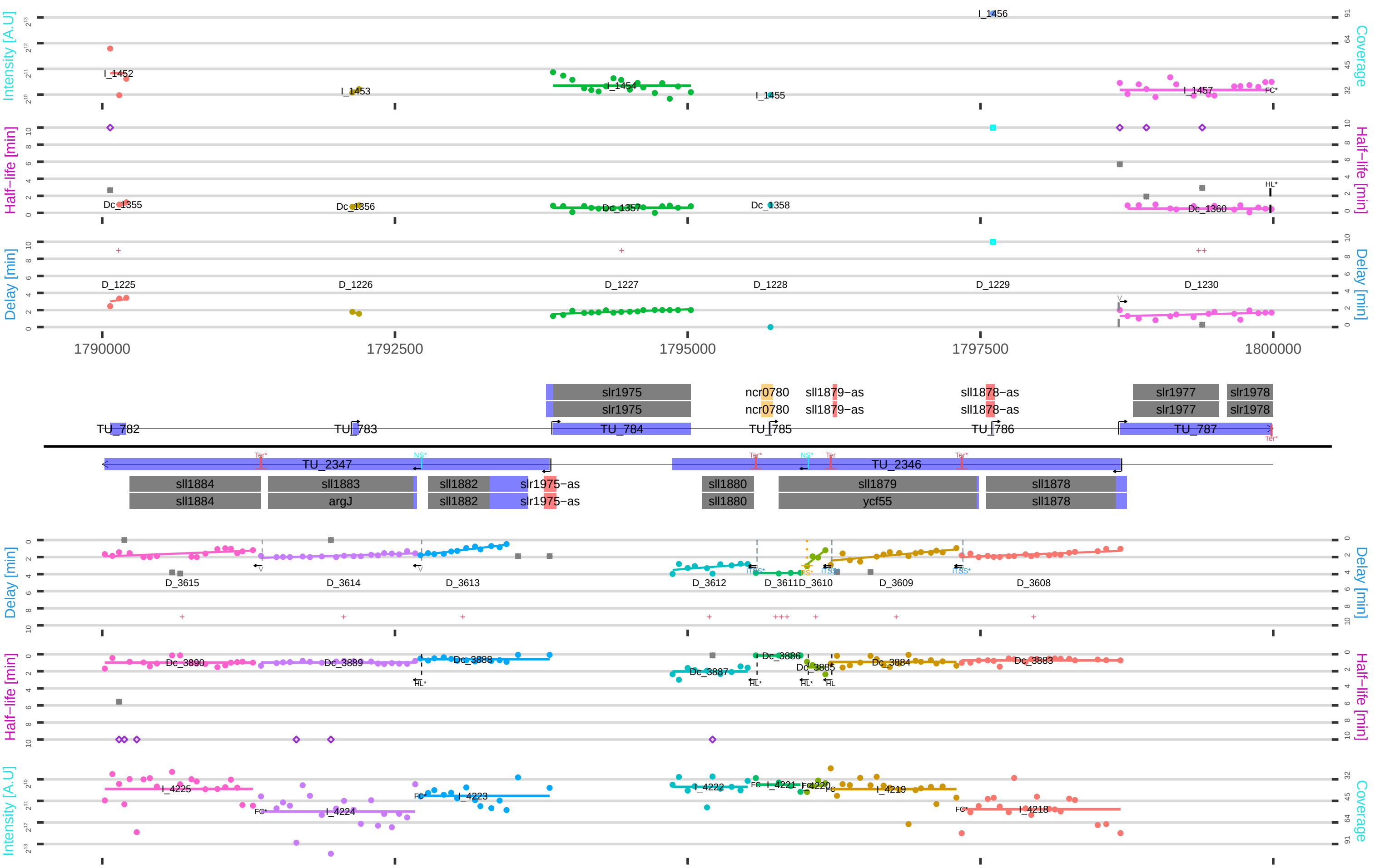

Term: termination (4), NS: new start (2), PS: pausing site (1), iTSS_I: internal starting site (5)

ID: 14623−14662; Term: termination (1), NS: new start (0), PS: pausing site (1), iTSS_I: internal starting site (0)

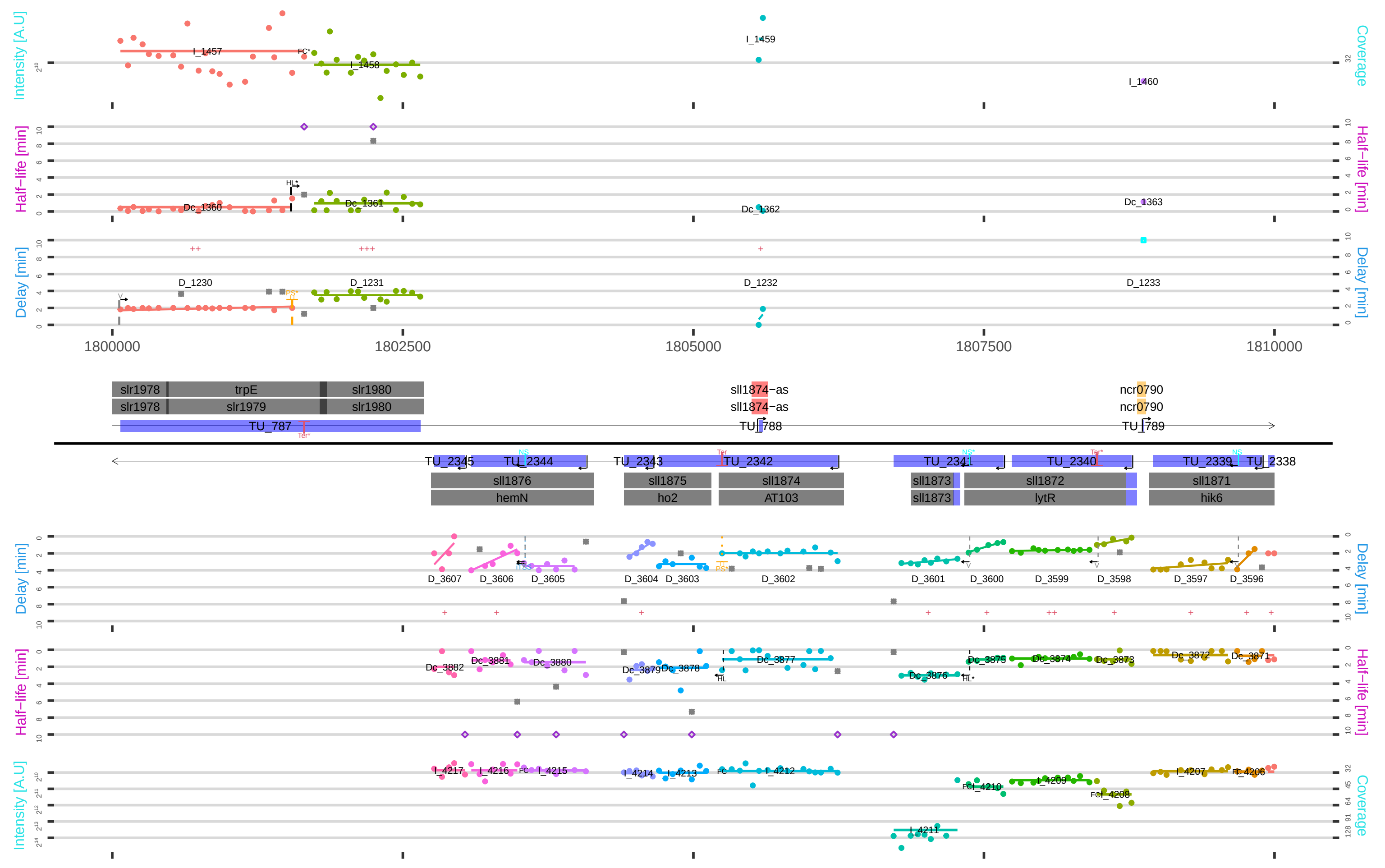

Term: termination (2), NS: new start (3), PS: pausing site (3), iTSS_I: internal starting site (2)

ID: 14663−14718; Term: termination (3), NS: new start (1), PS: pausing site (1), iTSS_I: internal starting site (1)

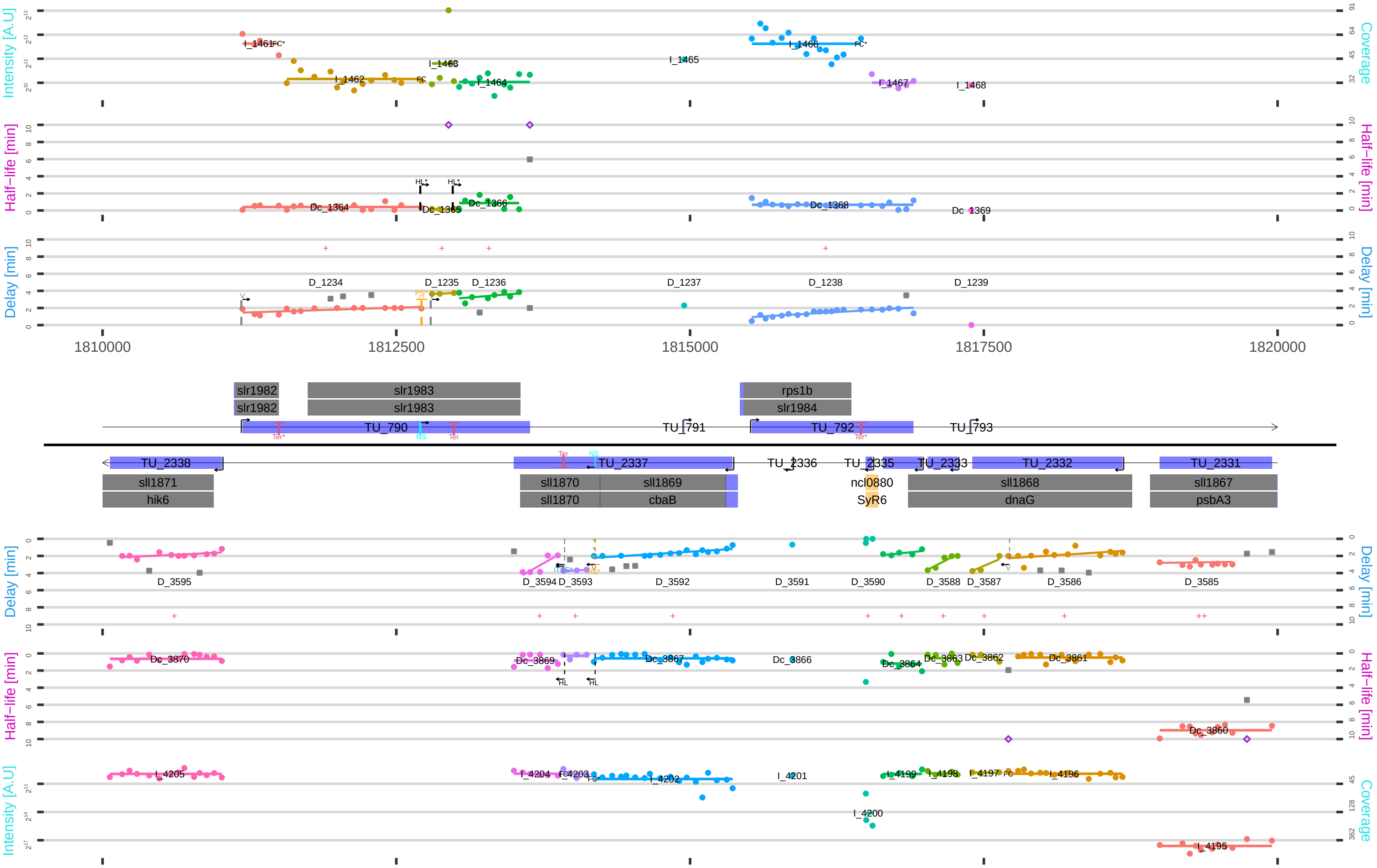

Term: termination (1), NS: new start (1), PS: pausing site (1), iTSS_I: internal starting site (2)

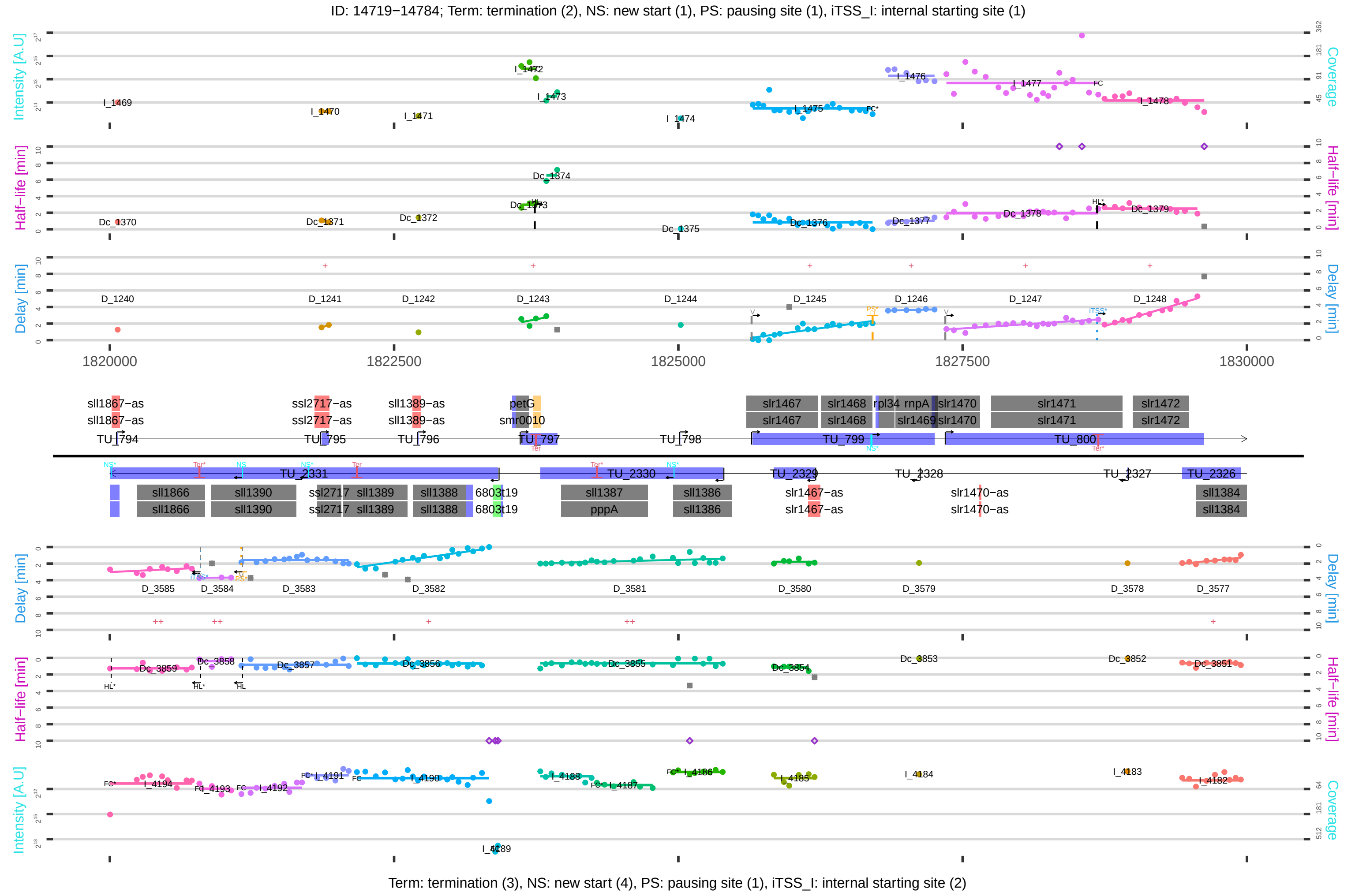
ID: 14719−14784; Term: termination (2), NS: new start (1), PS: pausing site (1), iTSS_I: internal starting site (1)
Intensity [A.U]
Coverage
Half−life [min]
Delay [min]
1820000
1822500
1825000
1827500
1830000
sll1867−as
ssl2717−as
sll1389−as
petG
smr0010
slr1467
slr1468
rpl34
rnpA
slr1469
slr1470
slr1471
slr1472
TU_794
TU_795
TU_796
TU_797
TU_798
TU_799
TU_800
TU_2331
TU_2330
TU_2329
TU_2328
TU_2327
TU_2326
sll1866
sll1390
ssl2717
sll1389
sll1388
6803t19
sll1387
pppA
sll1386
slr1467−as
slr1470−as
sll1384
Term: termination (3), NS: new start (4), PS: pausing site (1), iTSS_I: internal starting site (2)

ID: 14786−14859; Term: termination (2), NS: new start (1), PS: pausing site (1), iTSS_I: internal starting site (1)

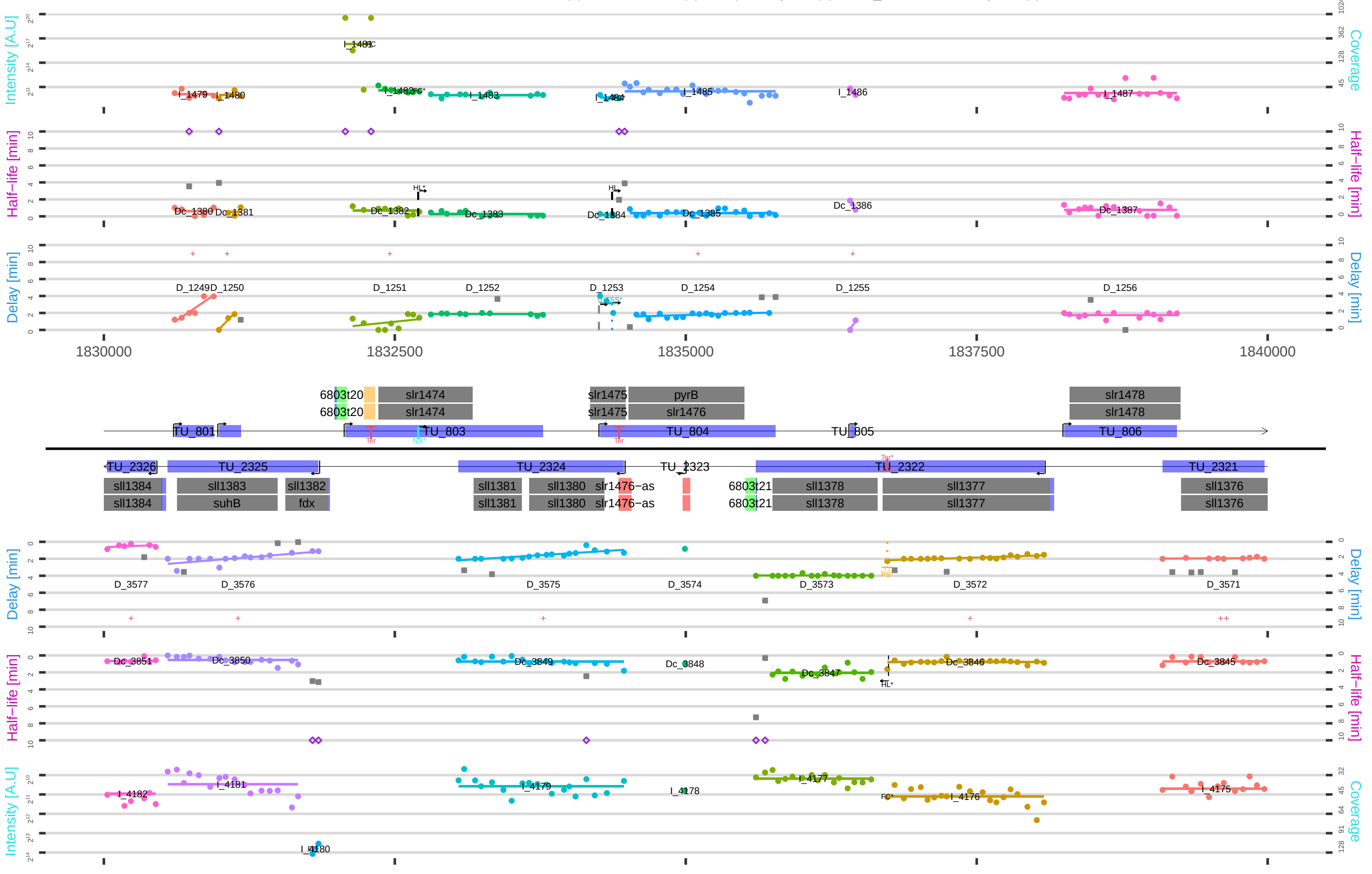

Term: termination (1), NS: new start (0), PS: pausing site (1), iTSS_I: internal starting site (0)

ID: 14860−14872; Term: termination (0), NS: new start (0), PS: pausing site (0), iTSS_I: internal starting site (0)

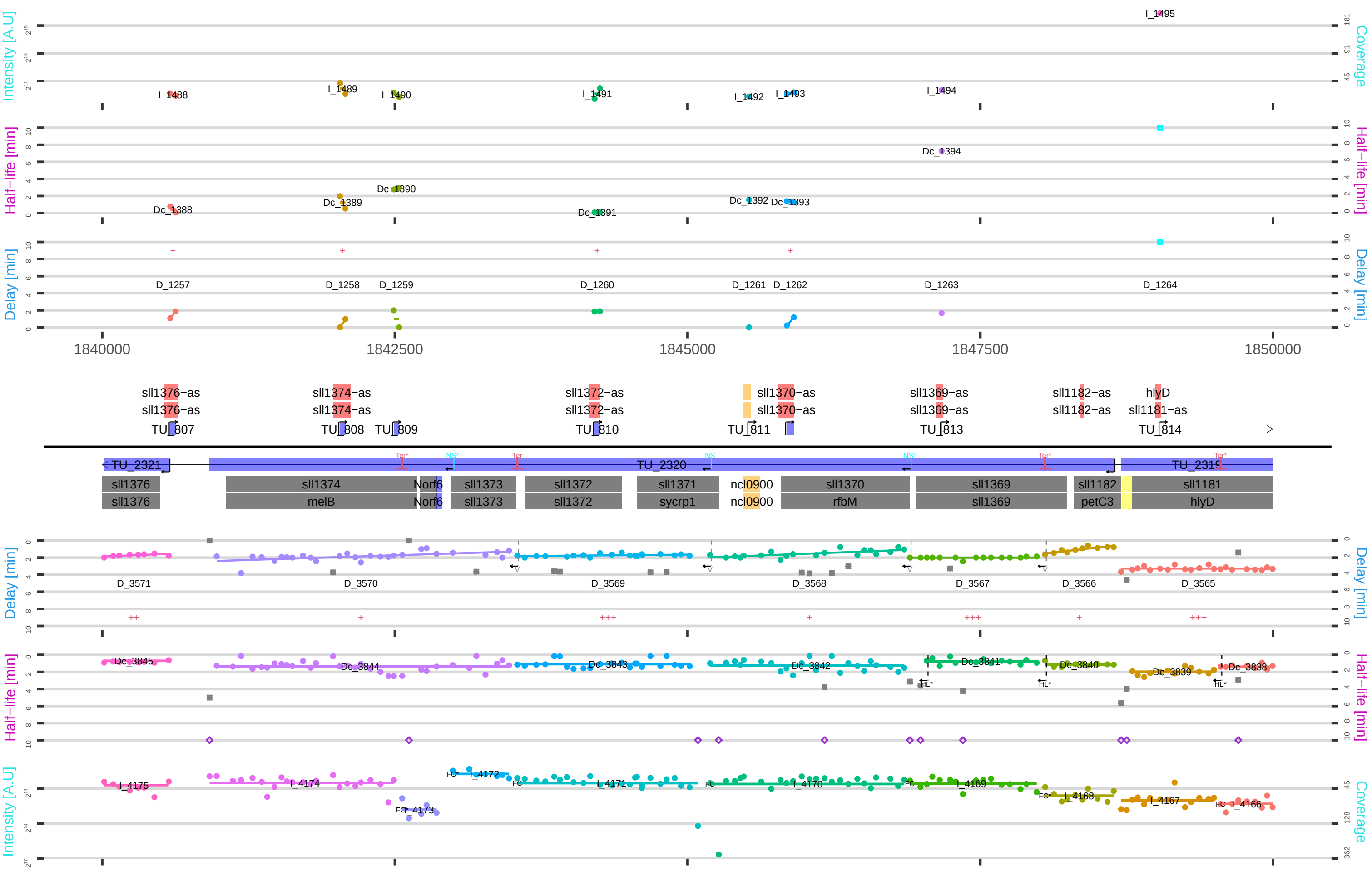

Term: termination (4), NS: new start (3), PS: pausing site (1), iTSS_I: internal starting site (3)

ID: 14873−14963; Term: termination (1), NS: new start (0), PS: pausing site (0), iTSS_I: internal starting site (0)

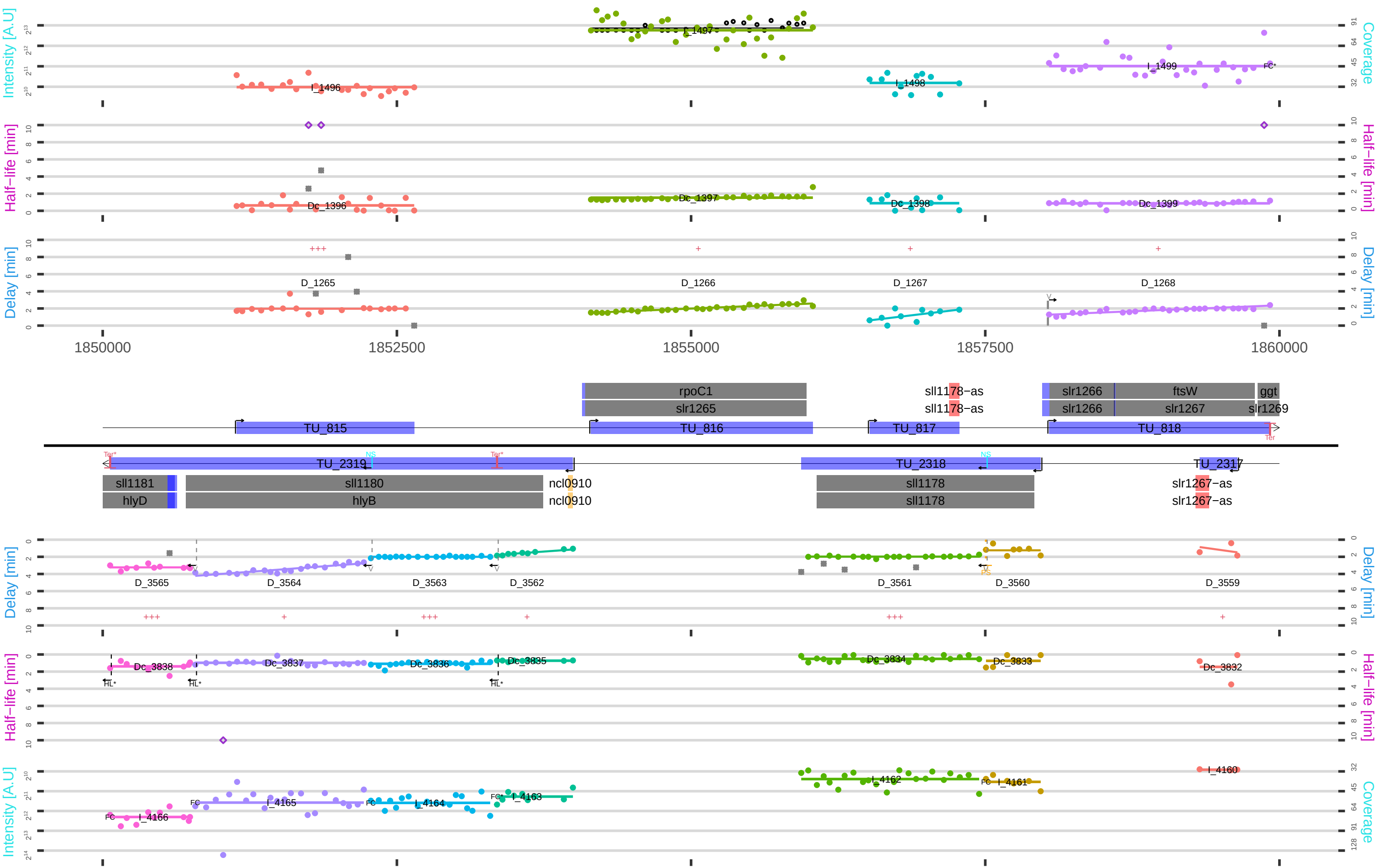

Term: termination (2), NS: new start (2), PS: pausing site (3), iTSS_I: internal starting site (1)

ID: 14965−15114; Term: termination (7), NS: new start (2), PS: pausing site (3), iTSS_I: internal starting site (5)

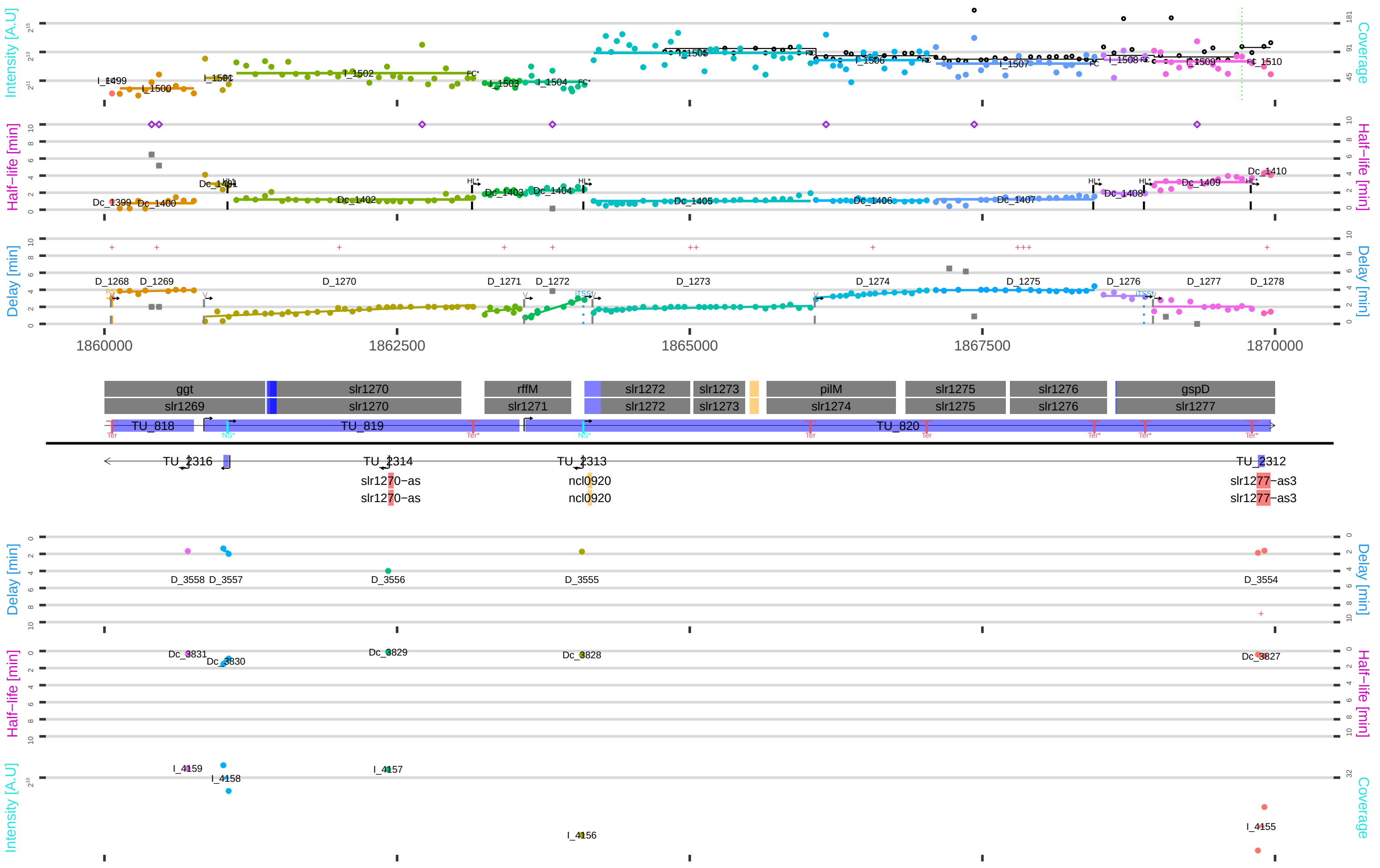

Term: termination (0), NS: new start (0), PS: pausing site (0), iTSS_I: internal starting site (0)

ID: 15115−15186; Term: termination (1), NS: new start (0), PS: pausing site (0), iTSS_I: internal starting site (1)

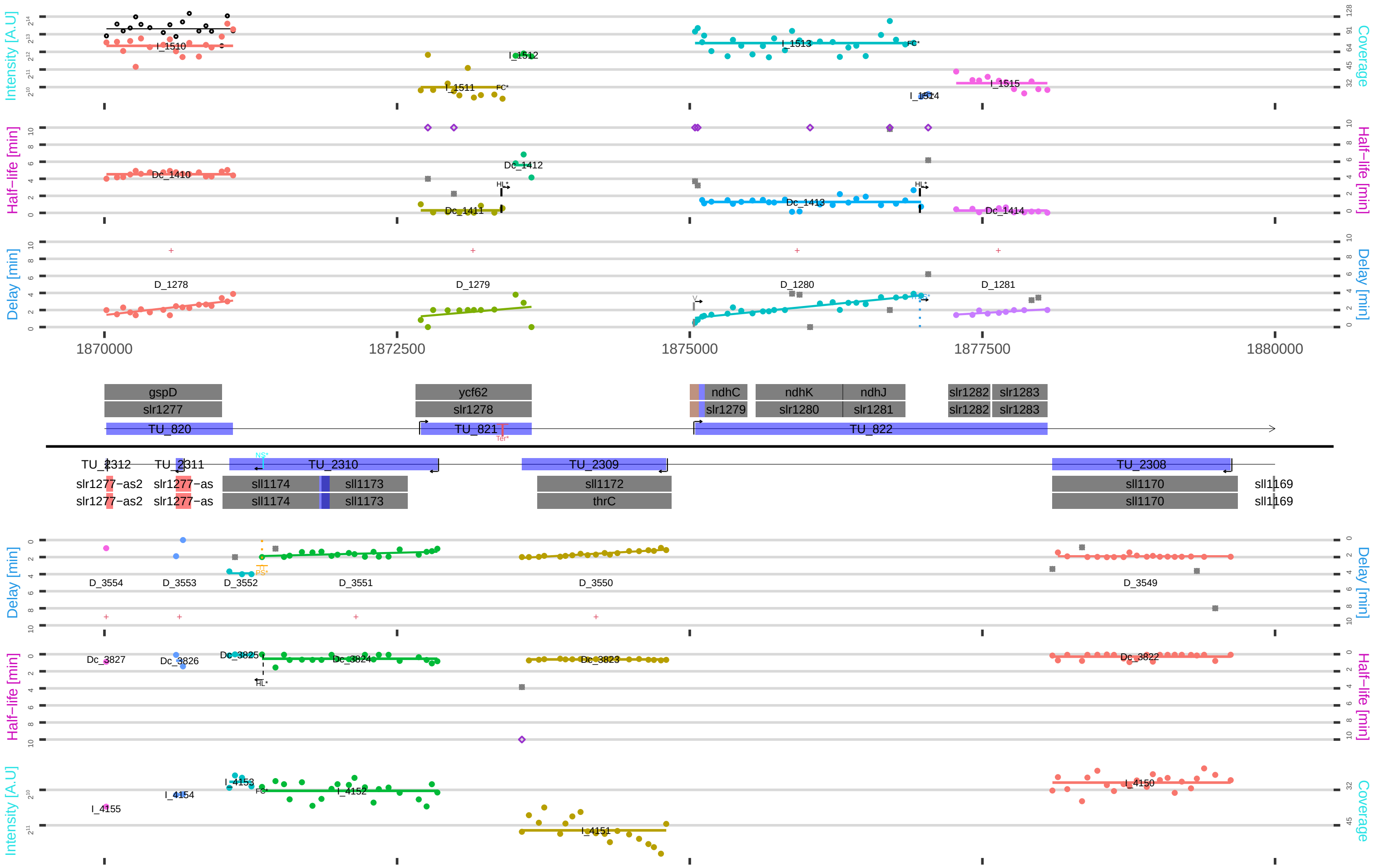

Term: termination (0), NS: new start (1), PS: pausing site (1), iTSS_I: internal starting site (0)

ID: 15187−15255; Term: termination (1), NS: new start (0), PS: pausing site (1), iTSS_I: internal starting site (1)

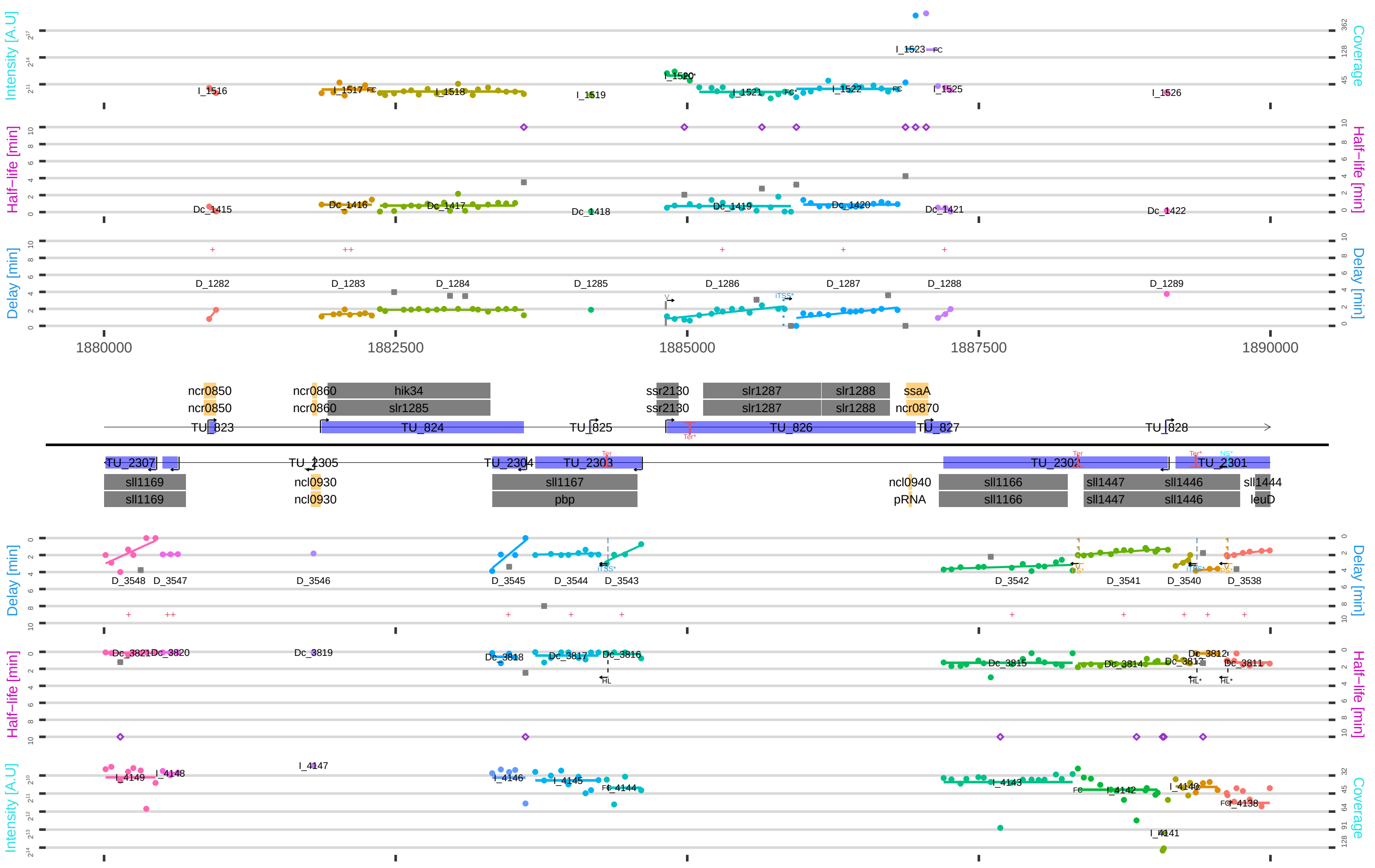

Term: termination (3), NS: new start (1), PS: pausing site (2), iTSS_I: internal starting site (2)

ID: 15256−15311; Term: termination (1), NS: new start (3), PS: pausing site (2), iTSS_I: internal starting site (2)

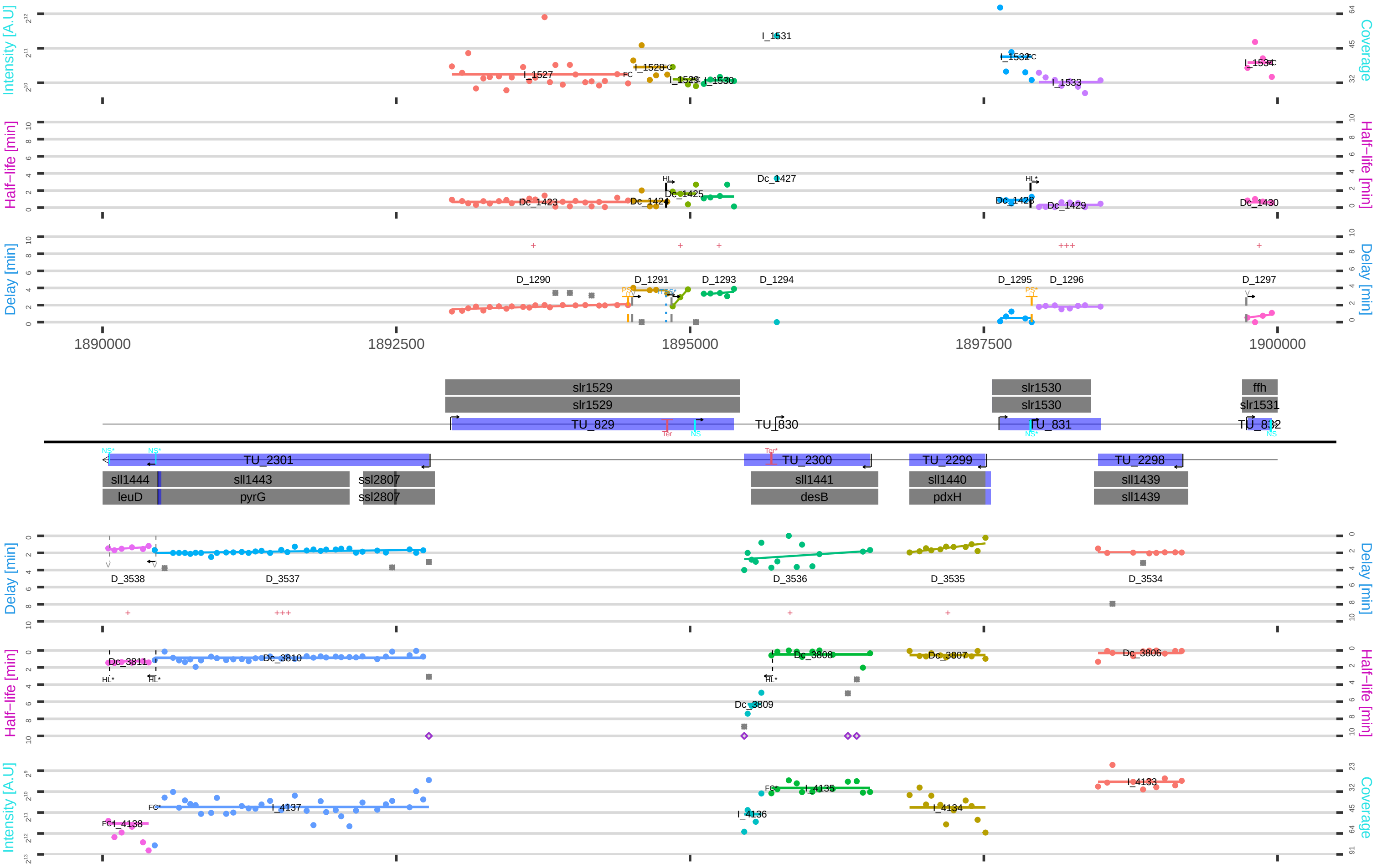

Term: termination (1), NS: new start (2), PS: pausing site (0), iTSS_I: internal starting site (1)

ID: 15312−15356; Term: termination (0), NS: new start (2), PS: pausing site (2), iTSS_I: internal starting site (0)

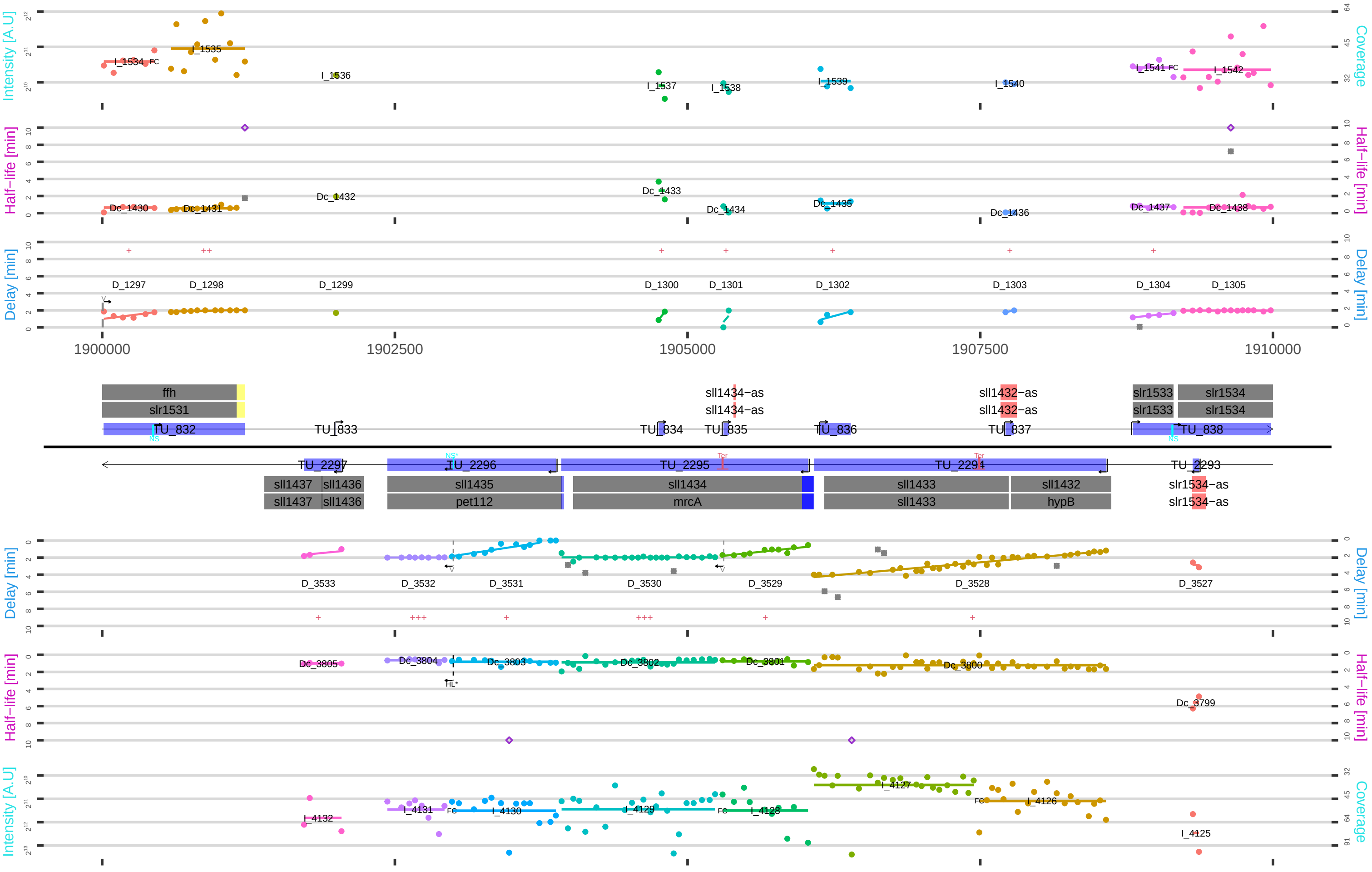

Term: termination (2), NS: new start (1), PS: pausing site (2), iTSS_I: internal starting site (0)

ID: 15357−15395; Term: termination (1), NS: new start (0), PS: pausing site (0), iTSS_I: internal starting site (1)

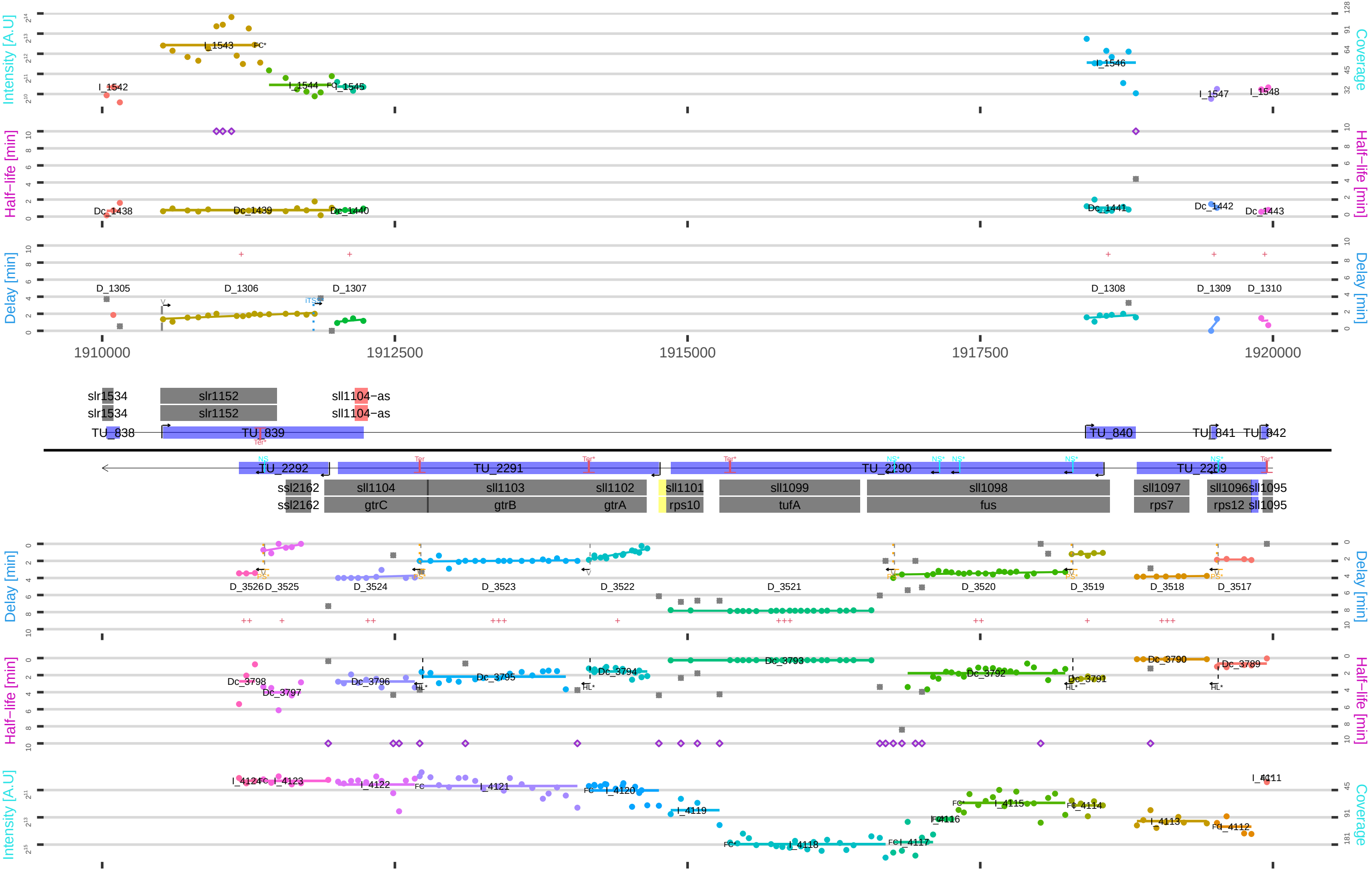

Term: termination (4), NS: new start (6), PS: pausing site (6), iTSS_I: internal starting site (0)

ID: 15396−15510; Term: termination (3), NS: new start (2), PS: pausing site (1), iTSS_I: internal starting site (2)

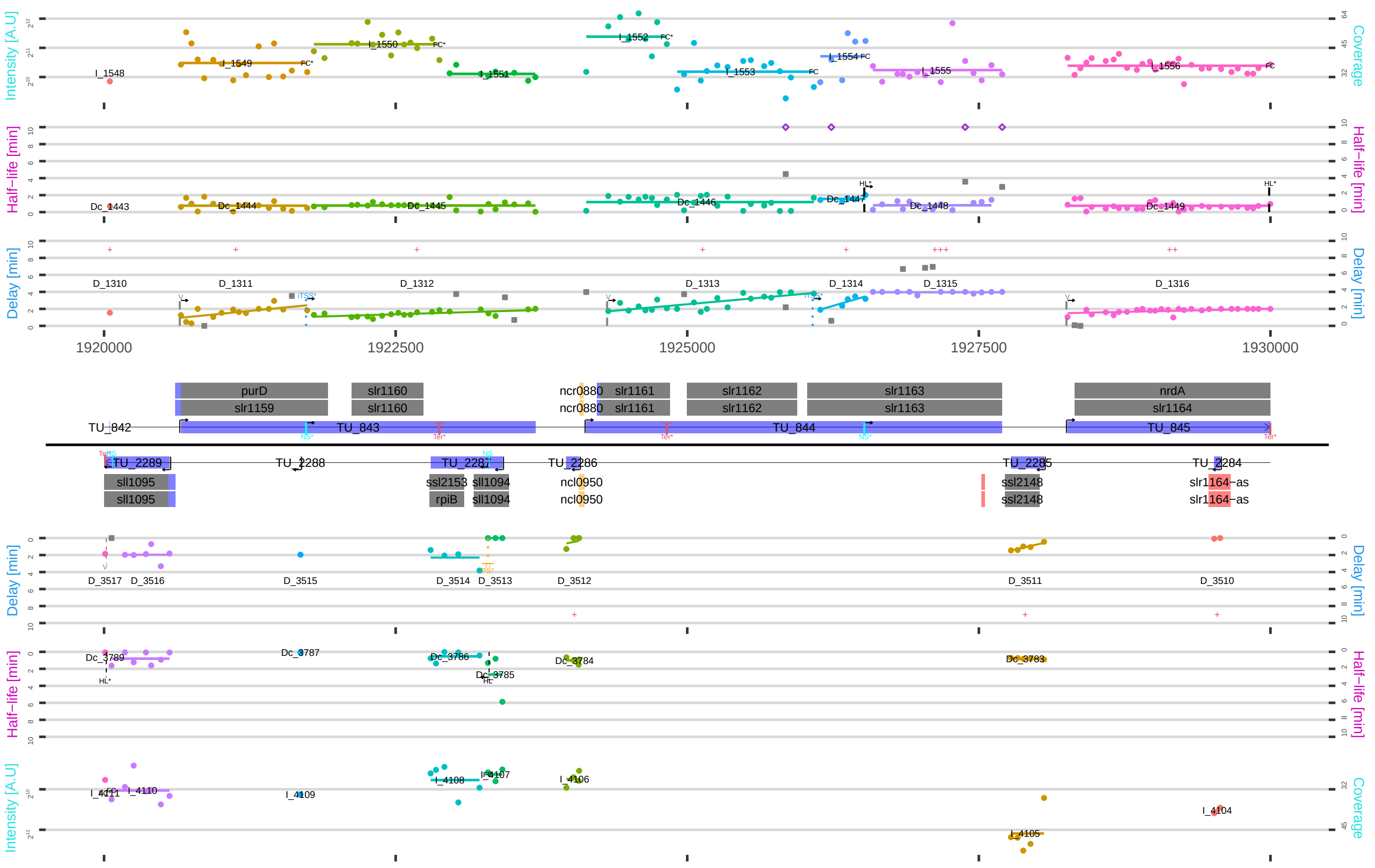

Term: termination (1), NS: new start (2), PS: pausing site (2), iTSS_I: internal starting site (0)

ID: 15511−15615; Term: termination (4), NS: new start (1), PS: pausing site (3), iTSS_I: internal starting site (1)

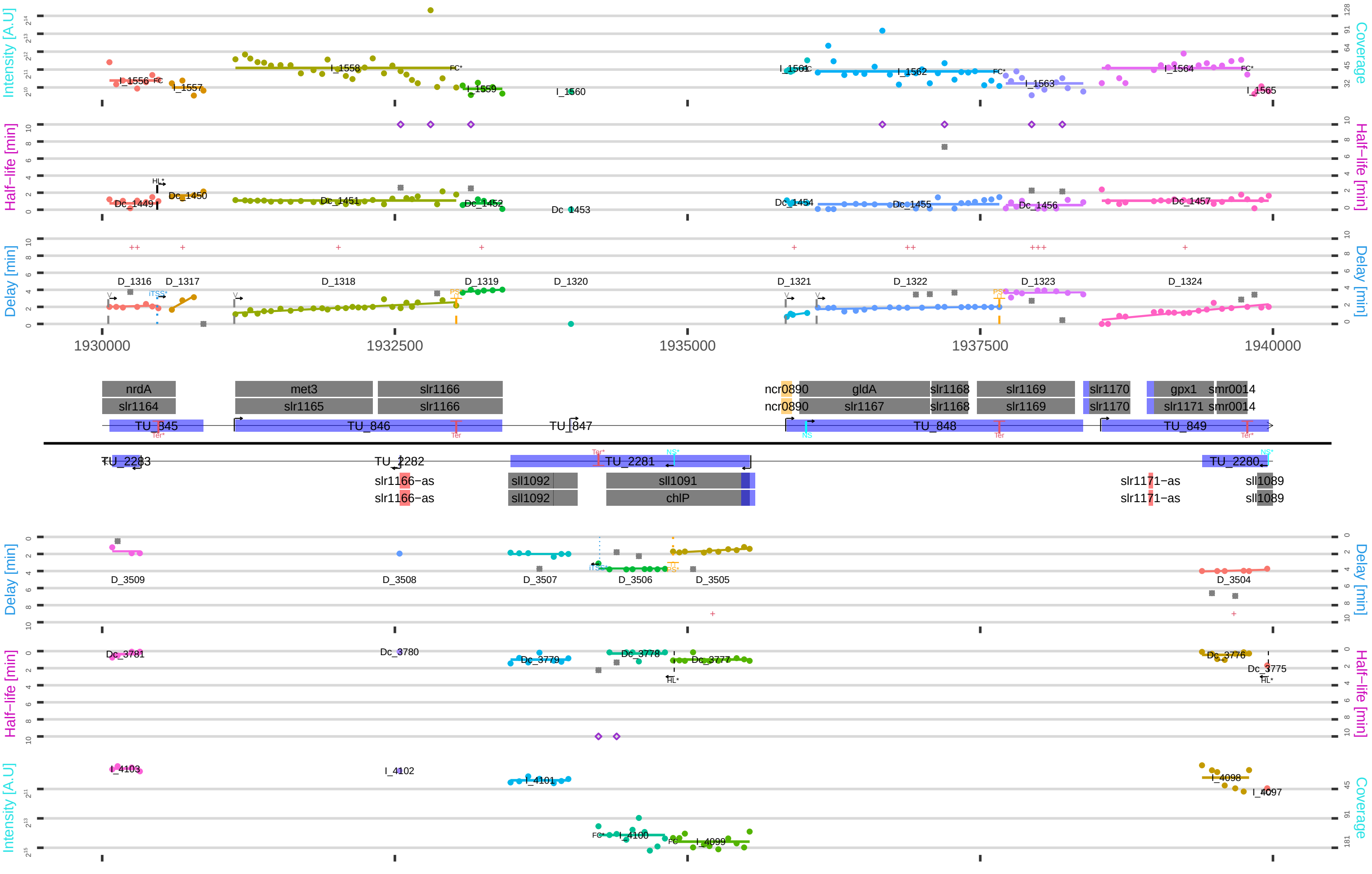

Term: termination (1), NS: new start (2), PS: pausing site (1), iTSS_I: internal starting site (1)

ID: 15616−15668; Term: termination (3), NS: new start (1), PS: pausing site (0), iTSS_I: internal starting site (2)

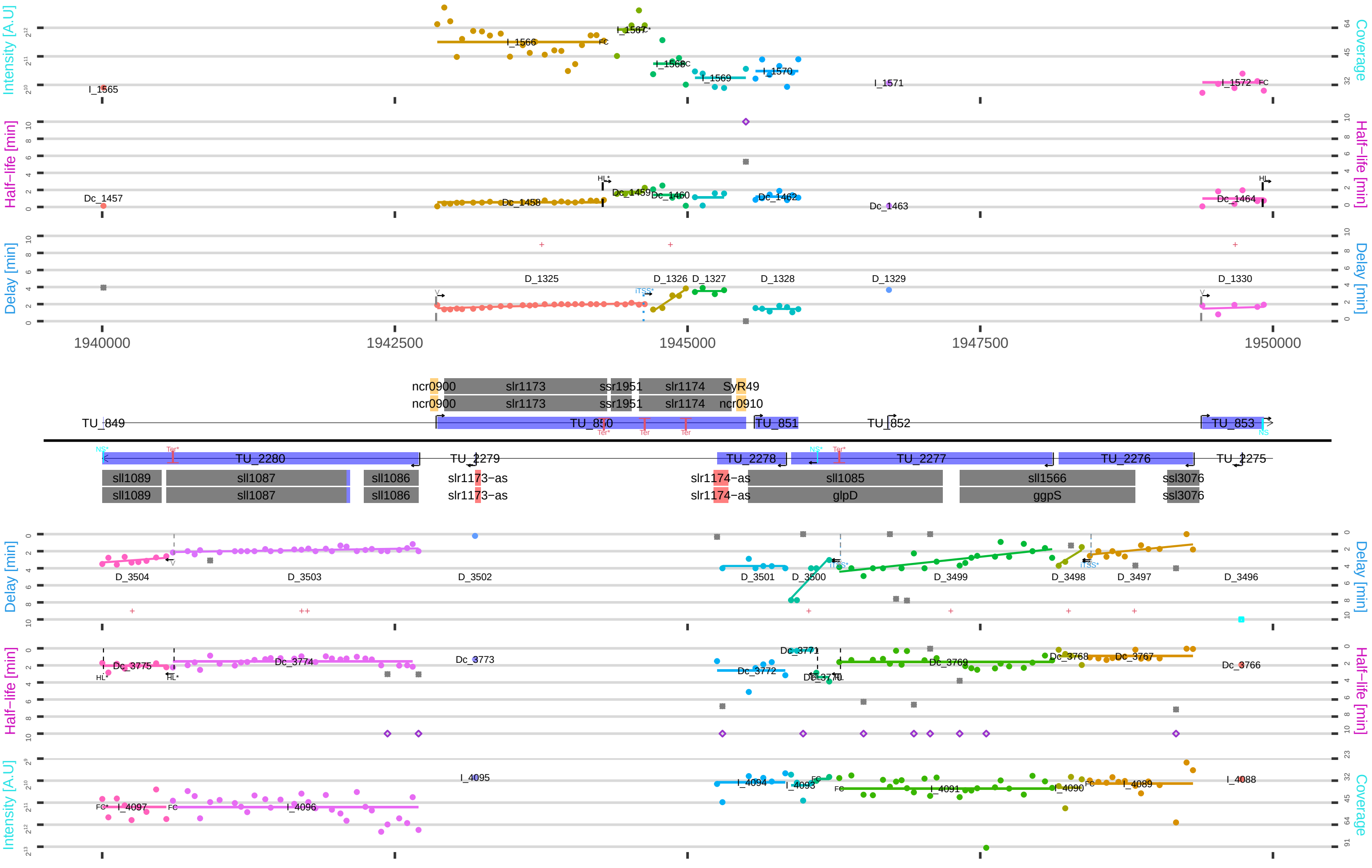

Term: termination (2), NS: new start (2), PS: pausing site (1), iTSS_I: internal starting site (2)

ID: 15669−15810; Term: termination (5), NS: new start (4), PS: pausing site (2), iTSS_I: internal starting site (3)

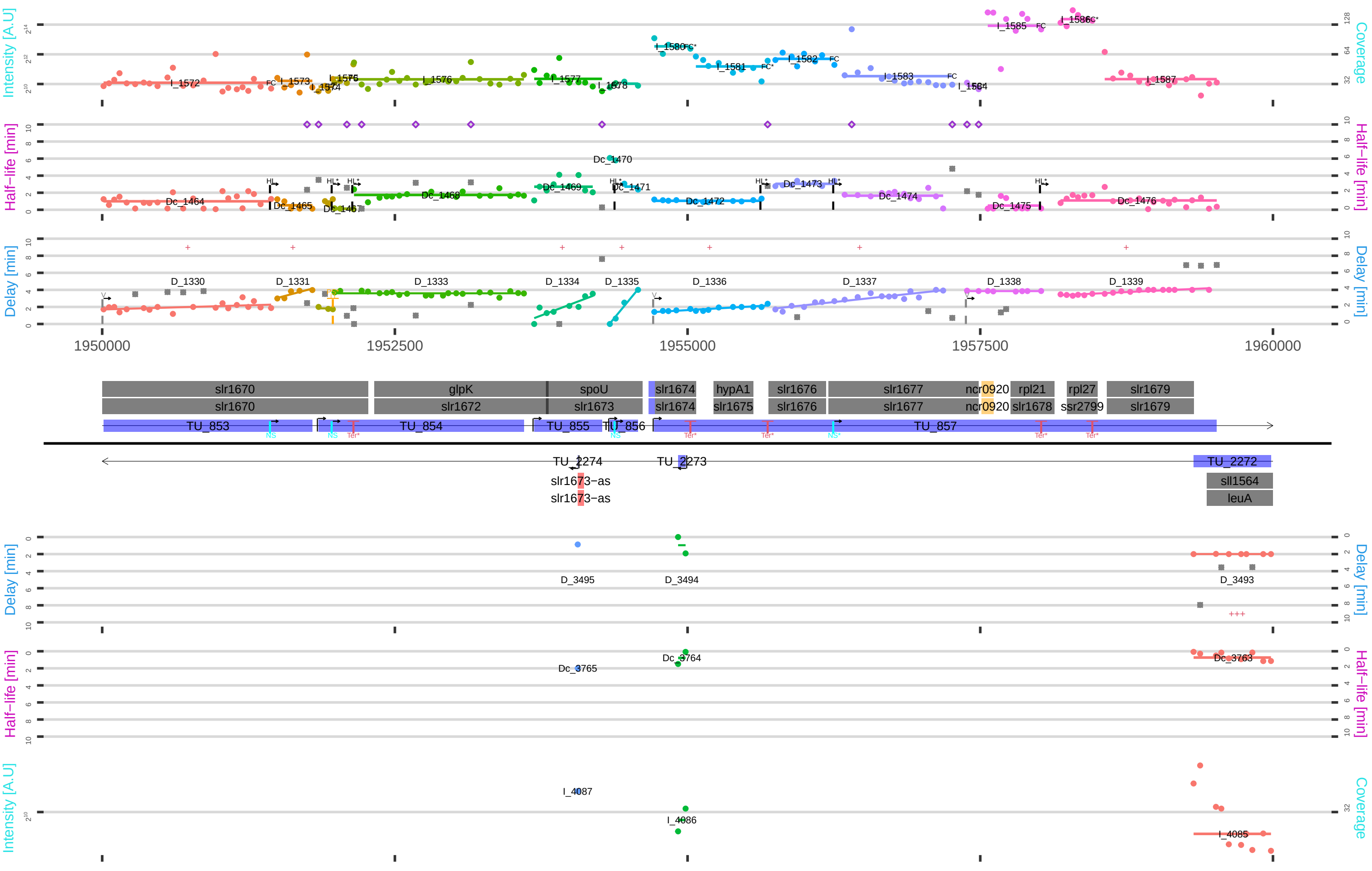

Term: termination (0), NS: new start (0), PS: pausing site (0), iTSS_I: internal starting site (0)

ID: 15811−15835; Term: termination (1), NS: new start (0), PS: pausing site (0), iTSS_I: internal starting site (1)

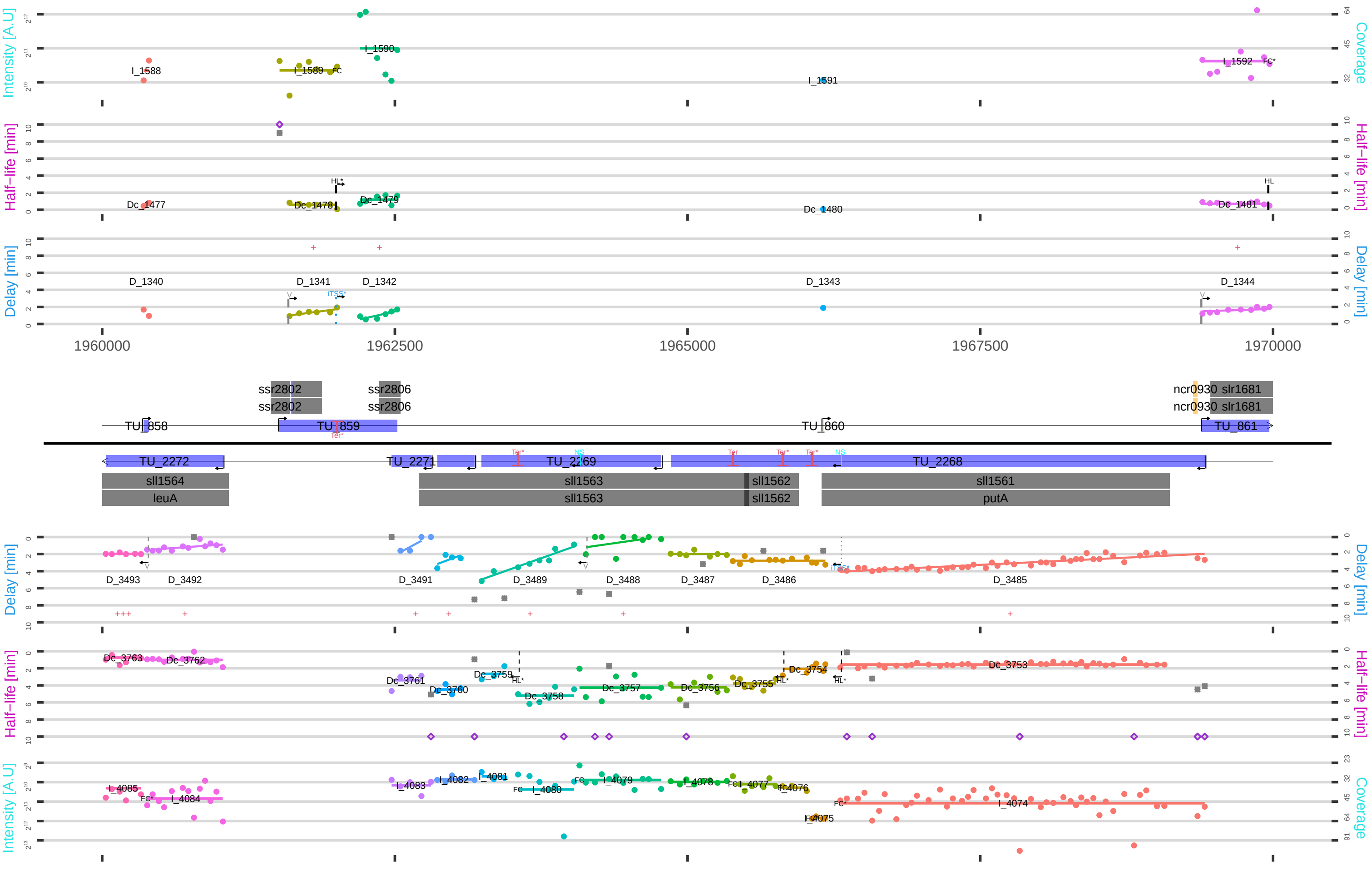

Term: termination (4), NS: new start (2), PS: pausing site (1), iTSS_I: internal starting site (3)

ID: 15836−15893; Term: termination (1), NS: new start (2), PS: pausing site (0), iTSS_I: internal starting site (2)

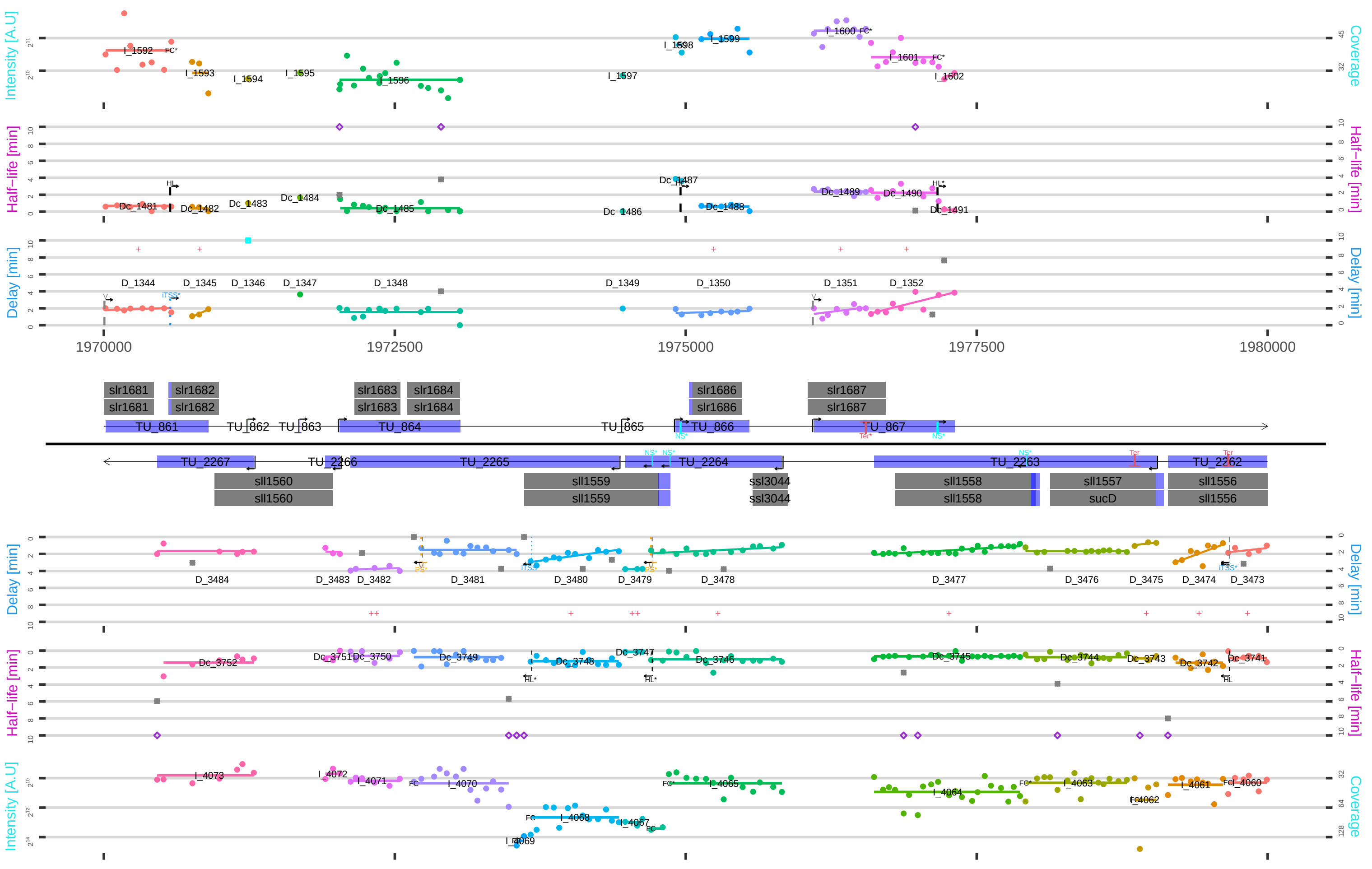

Term: termination (2), NS: new start (3), PS: pausing site (3), iTSS_I: internal starting site (3)

ID: 15894−15994; Term: termination (4), NS: new start (3), PS: pausing site (3), iTSS_I: internal starting site (5)

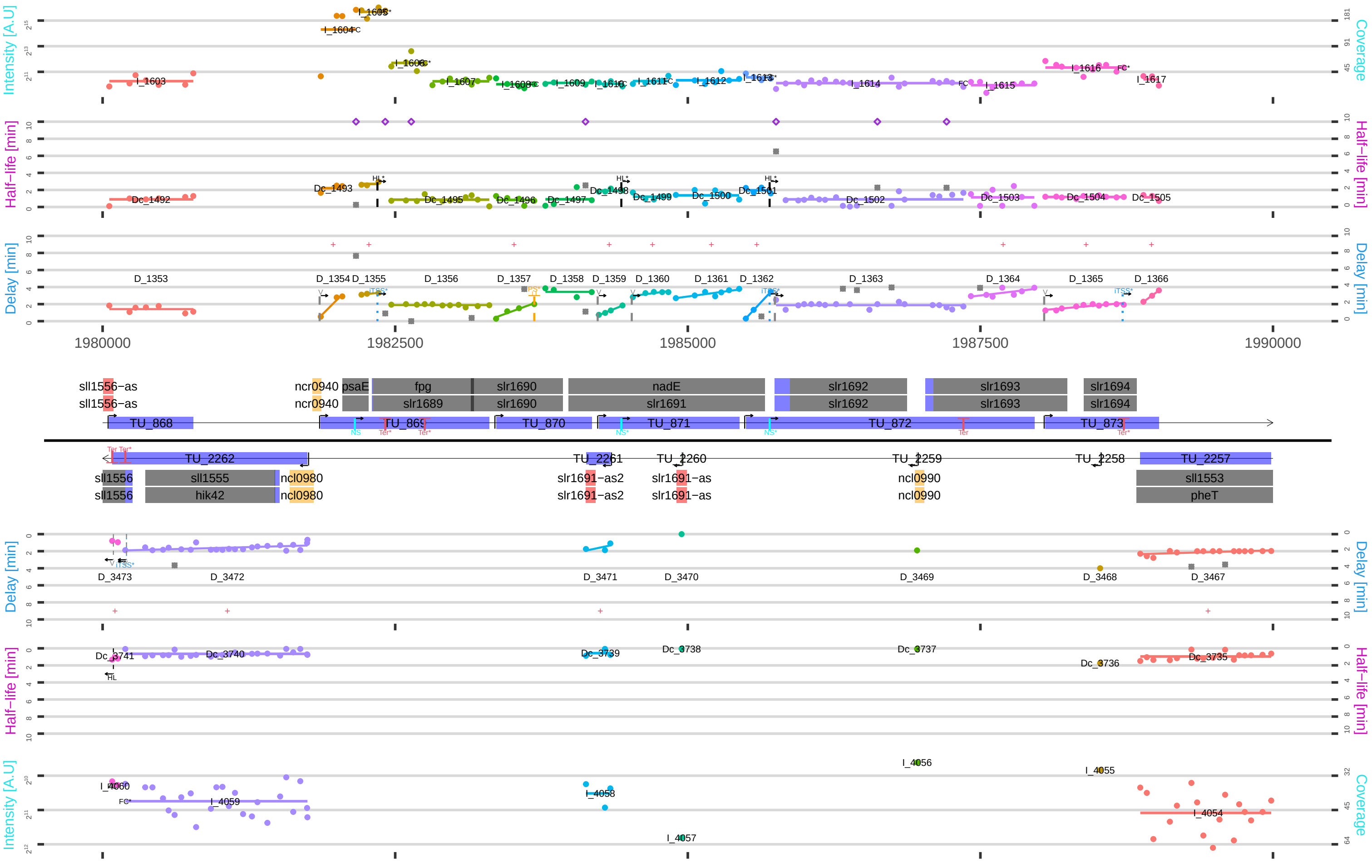

Term: termination (2), NS: new start (0), PS: pausing site (0), iTSS_I: internal starting site (1)

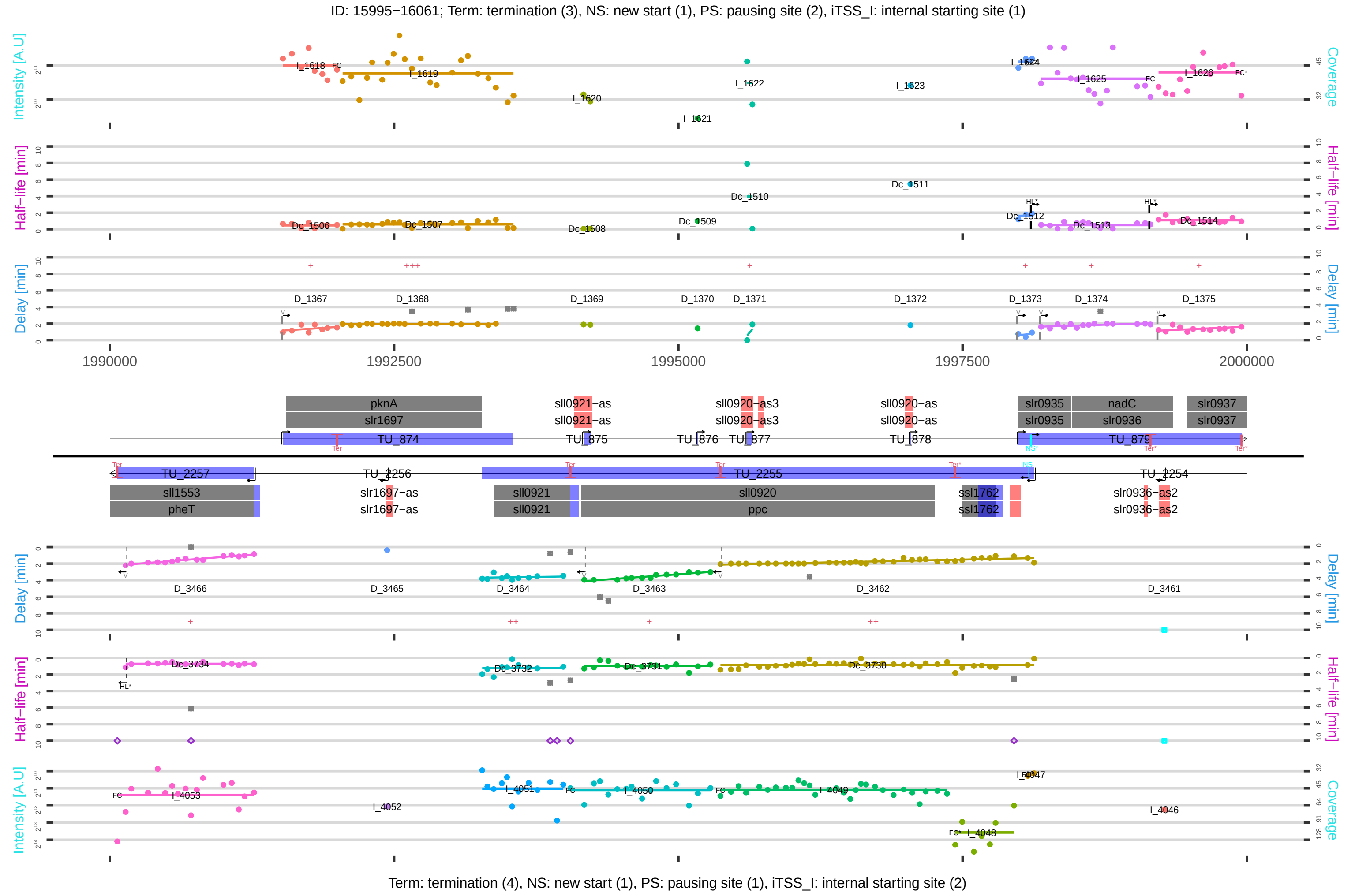

ID: 15995−16061; Term: termination (3), NS: new start (1), PS: pausing site (2), iTSS_I: internal starting site (1)
Intensity [A.U]
Coverage
Half−life [min]
Delay [min]
1990000
1992500
1995000
1997500
2000000
pknA
slr1697
sll0921−as
sll0920−as3
sll0920−as
slr0935
nadC
slr0936
slr0937
TU_874
TU_875
TU_876
TU_877
TU_878
TU_879
TU_2257
TU_2256
TU_2255
TU_2254
sll1553
pheT
slr1697−as
sll0921
sll0920
ppc
ssl1762
slr0936−as2
Term: termination (4), NS: new start (1), PS: pausing site (1), iTSS_I: internal starting site (2)

ID: 16062−16157; Term: termination (2), NS: new start (2), PS: pausing site (3), iTSS_I: internal starting site (1)

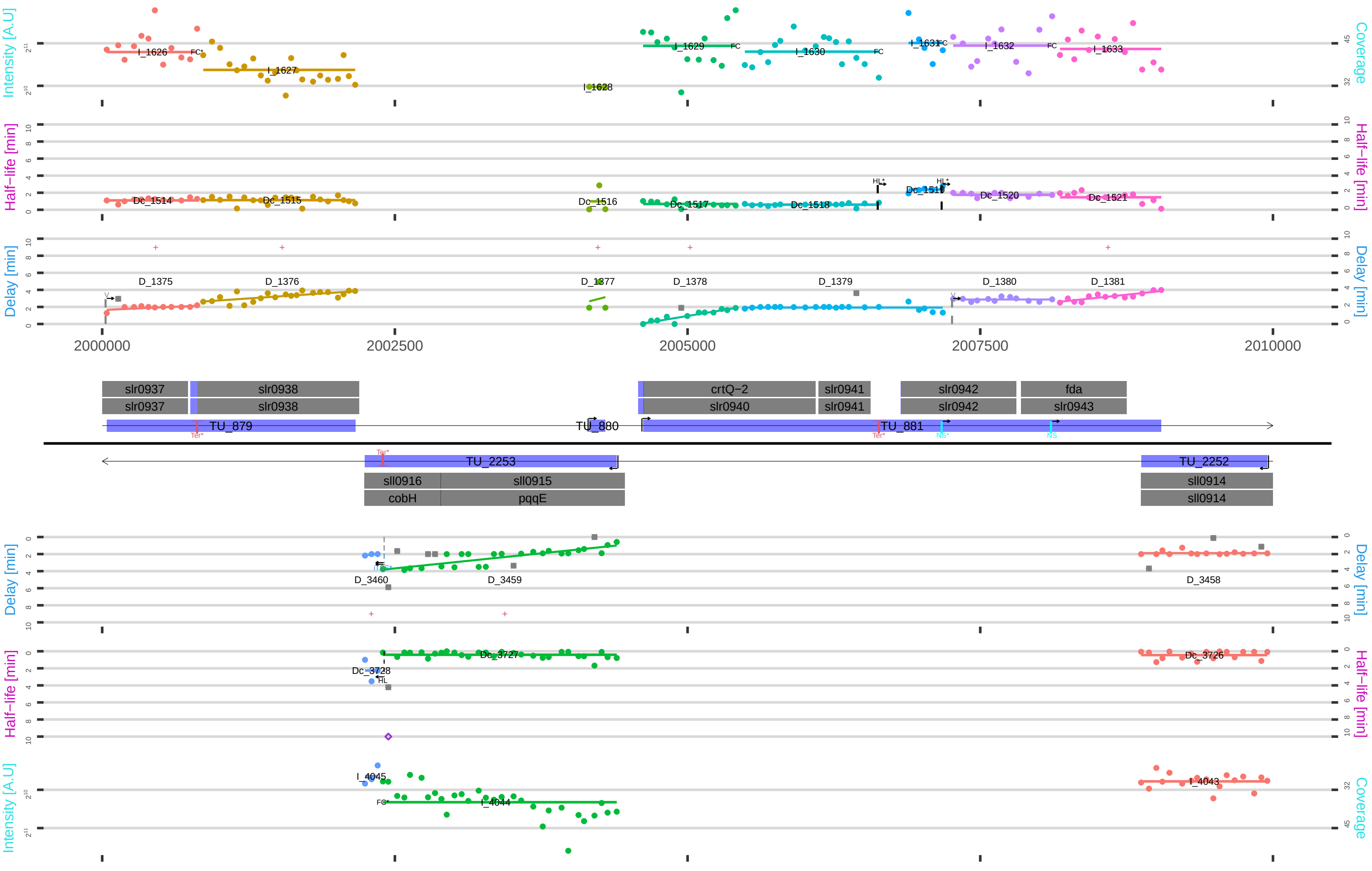

Term: termination (1), NS: new start (0), PS: pausing site (0), iTSS_I: internal starting site (1)

ID: 16158−16246; Term: termination (2), NS: new start (1), PS: pausing site (0), iTSS_I: internal starting site (1)

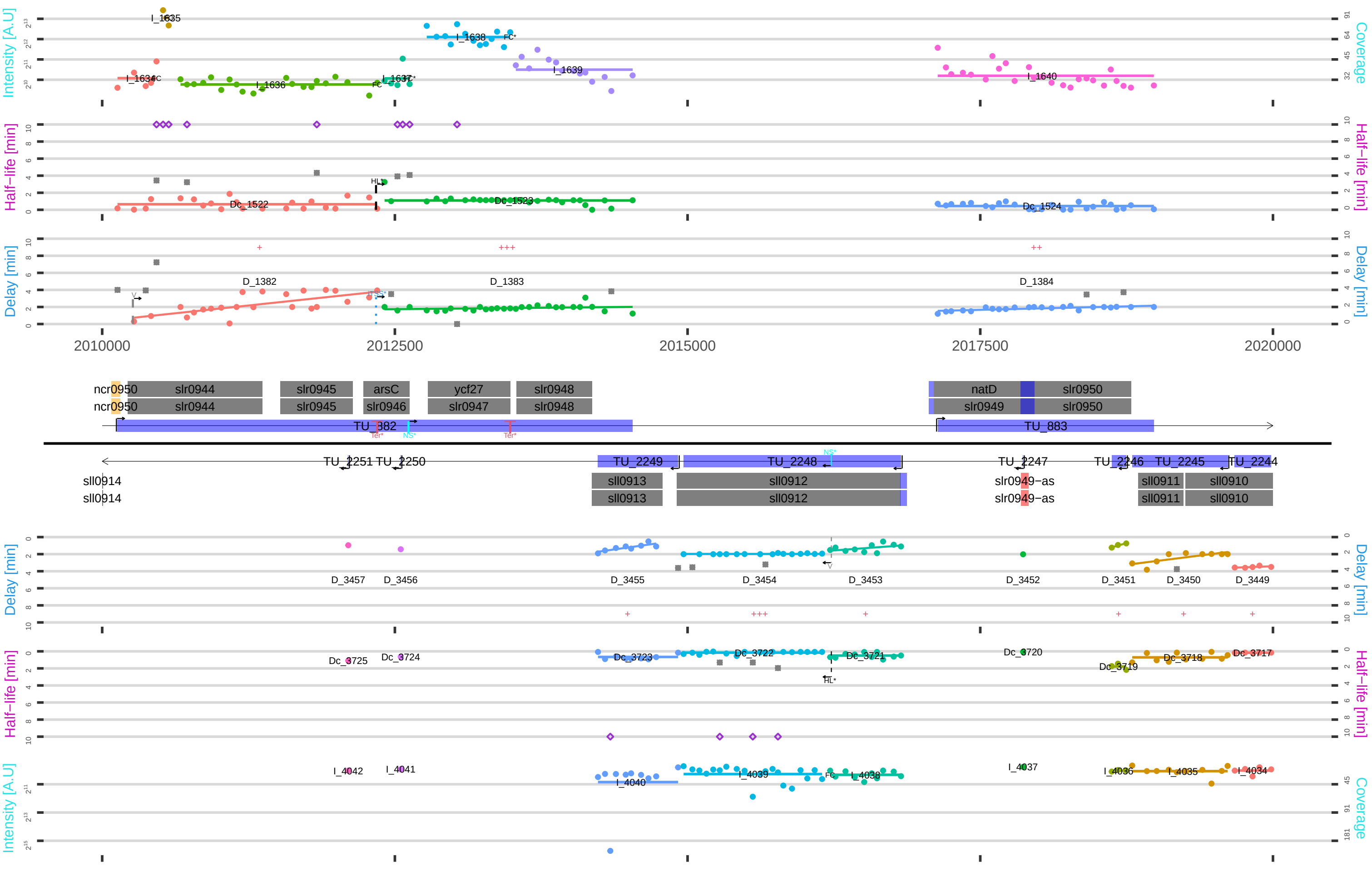

Term: termination (0), NS: new start (1), PS: pausing site (1), iTSS_I: internal starting site (0)

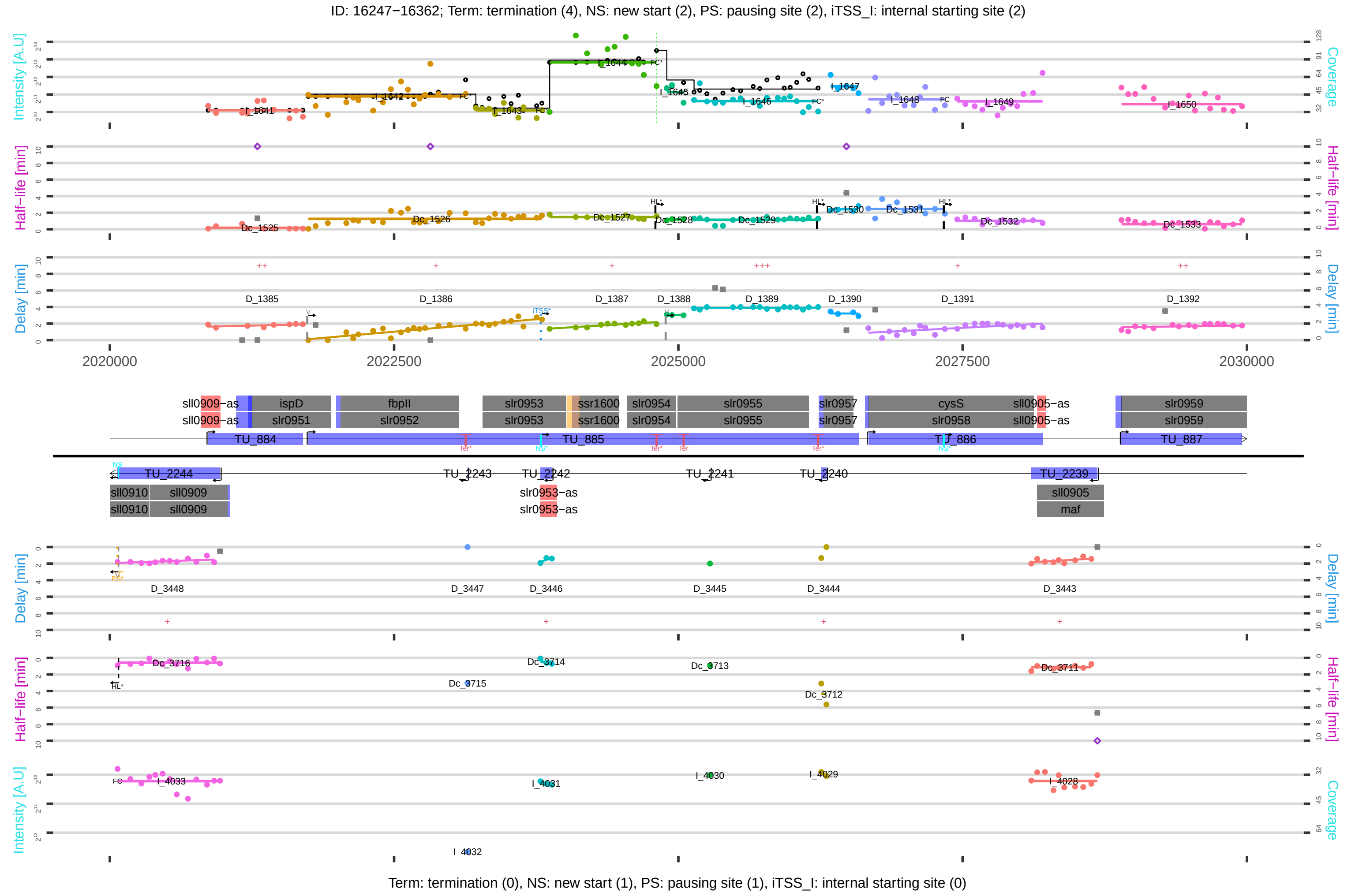
ID: 16247−16362; Term: termination (4), NS: new start (2), PS: pausing site (2), iTSS_I: internal starting site (2)
Intensity [A.U]
Coverage
Half−life [min]
Delay [min]
2020000
2022500
2025000
2027500
2030000
sll0909−as
ispD
fbpII
slr0953
ssr1600
slr0954
slr0955
slr0957
cysS
sll0905−as
slr0959
slr0951
slr0952
slr0958
TU_884
TU_885
TU_886
TU_887
TU_2244
TU_2243
TU_2242
TU_2241
TU_2240
TU_2239
sll0910
sll0909
slr0953−as
sll0905
maf
Term: termination (0), NS: new start (1), PS: pausing site (1), iTSS_I: internal starting site (0)

ID: 16363−16442; Term: termination (1), NS: new start (2), PS: pausing site (1), iTSS_I: internal starting site (1)

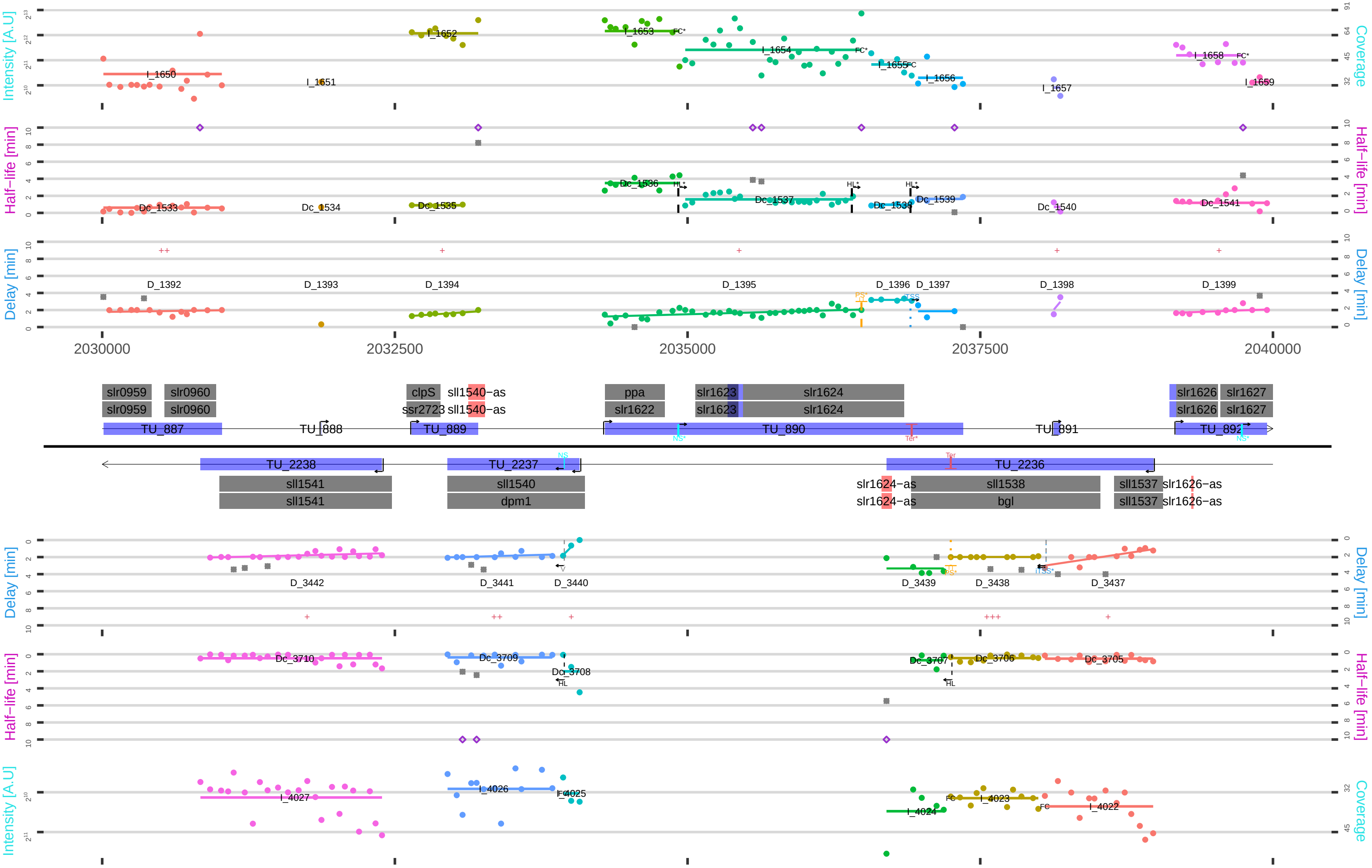

Term: termination (1), NS: new start (1), PS: pausing site (1), iTSS_I: internal starting site (2)

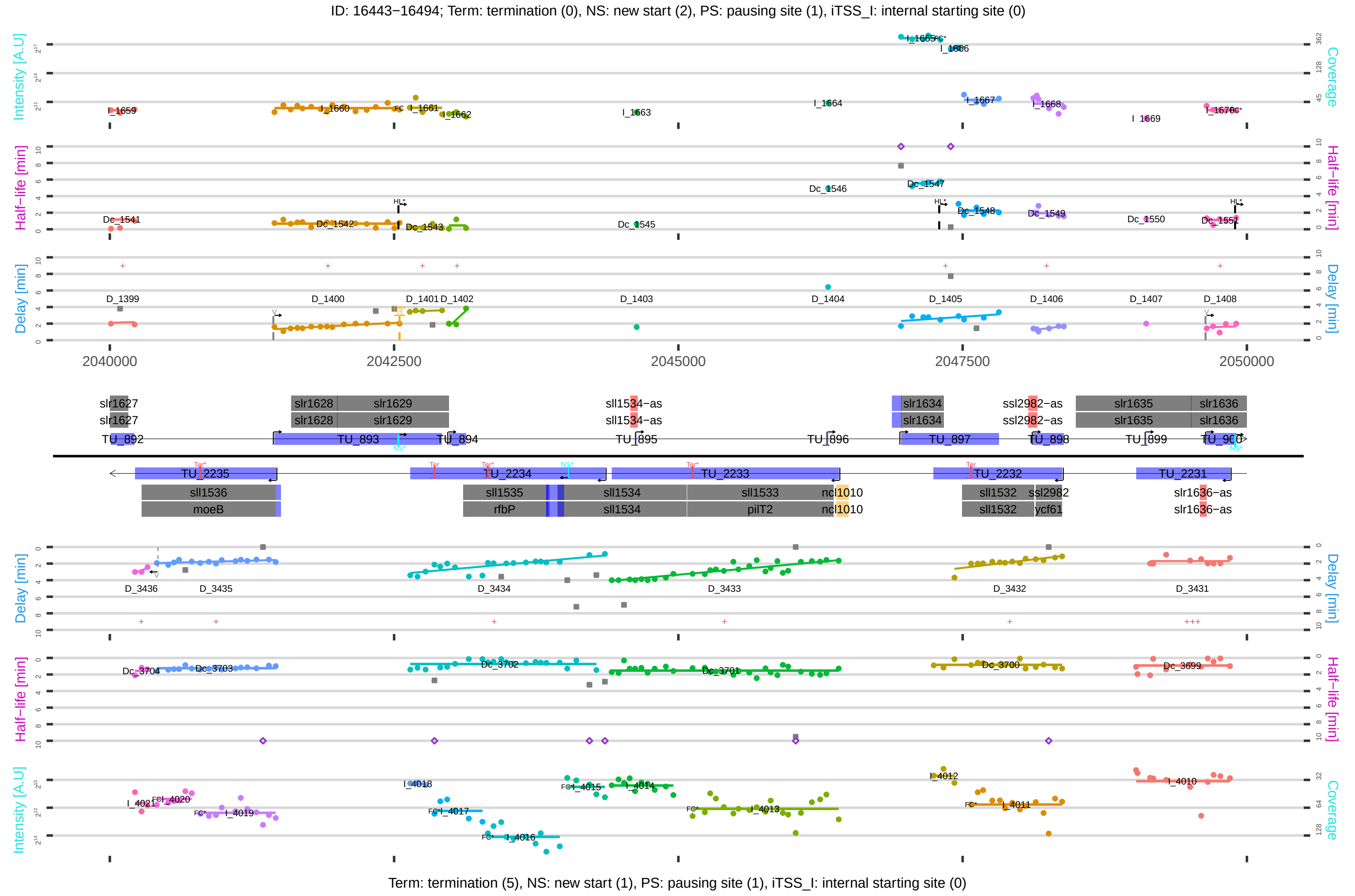
ID: 16443−16494; Term: termination (0), NS: new start (2), PS: pausing site (1), iTSS_I: internal starting site (0)
Intensity [A.U]
Coverage
Half−life [min]
Delay [min]
2040000
2042500
2045000
2047500
2050000
slr1627
slr1628
slr1629
sll1534−as
slr1634
ssl2982−as
slr1635
slr1636
TU_892
TU_893
TU_894
TU_895
TU_896
TU_897
TU_898
TU_899
TU_900
TU_2235
TU_2234
TU_2233
TU_2232
TU_2231
sll1536
moeB
sll1535
rfbP
sll1534
sll1533
pilT2
ncl1010
sll1532
ssl2982
ycf61
slr1636−as
Term: termination (5), NS: new start (1), PS: pausing site (1), iTSS_I: internal starting site (0)

ID: 16497−16522; Term: termination (2), NS: new start (1), PS: pausing site (0), iTSS_I: internal starting site (1)

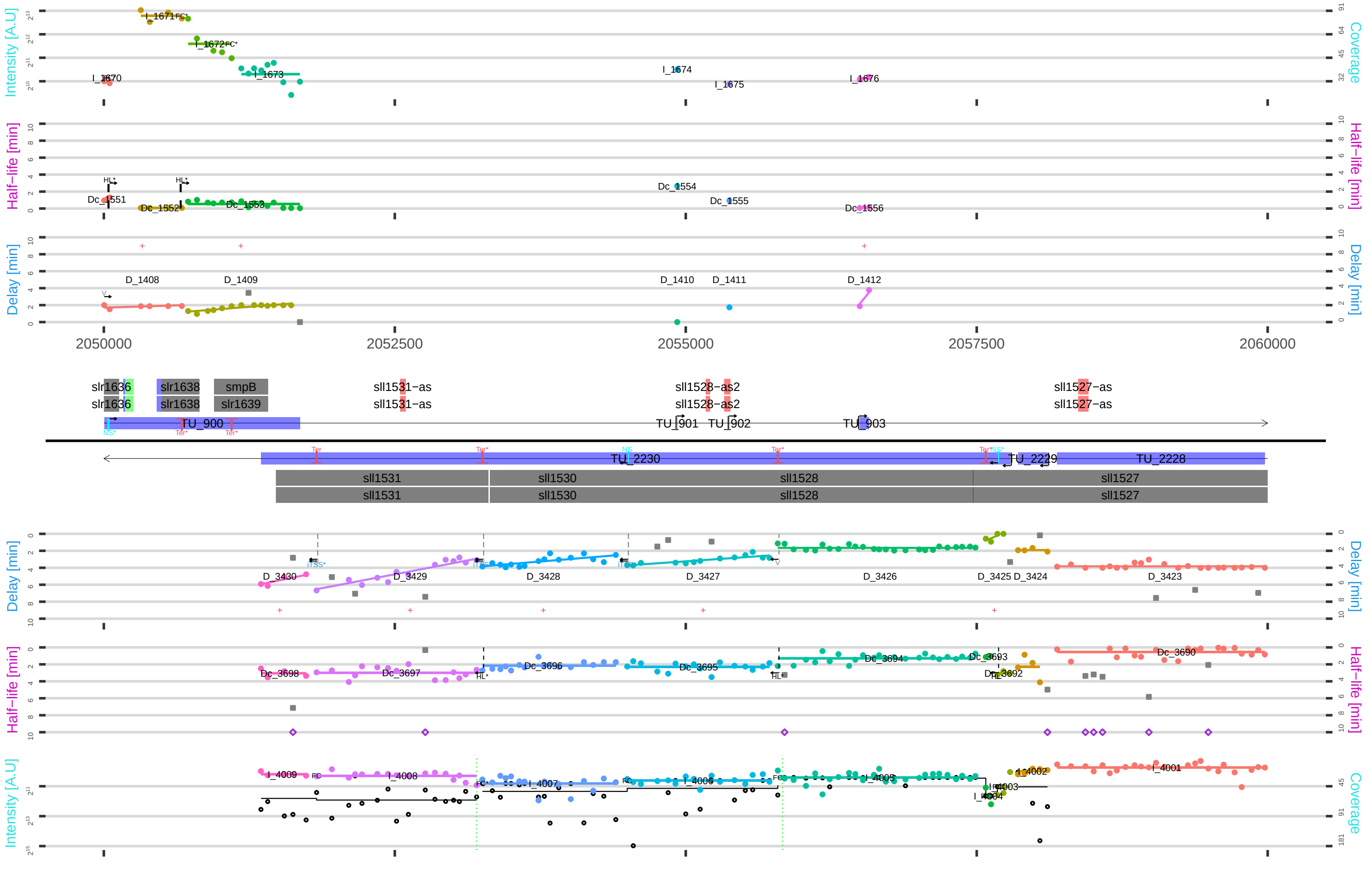

Term: termination (4), NS: new start (2), PS: pausing site (2), iTSS_I: internal starting site (3)

ID: 16523−16589; Term: termination (0), NS: new start (1), PS: pausing site (0), iTSS_I: internal starting site (0)

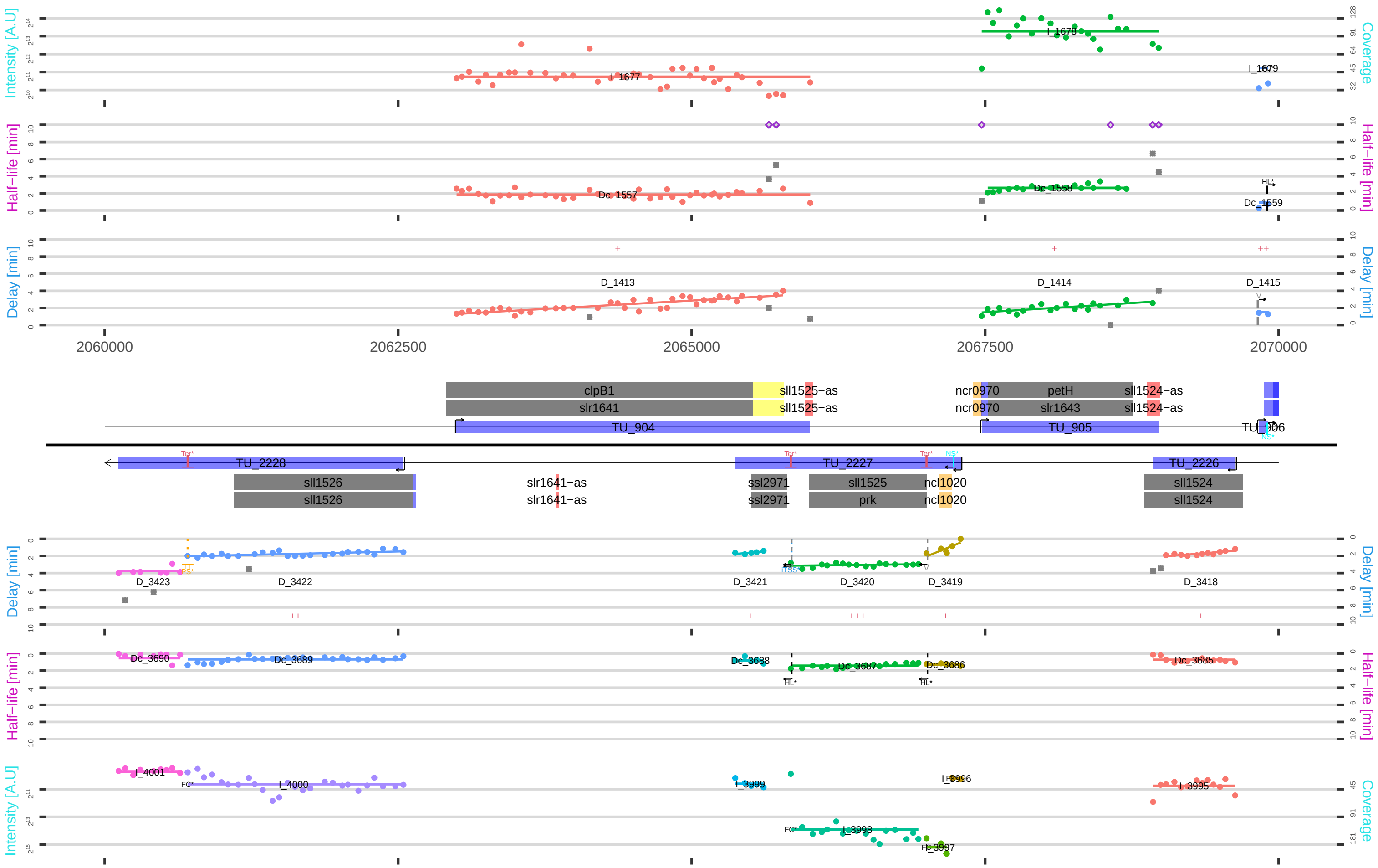

Term: termination (3), NS: new start (1), PS: pausing site (2), iTSS_I: internal starting site (1)

ID: 16590−16677; Term: termination (2), NS: new start (3), PS: pausing site (1), iTSS_I: internal starting site (1)

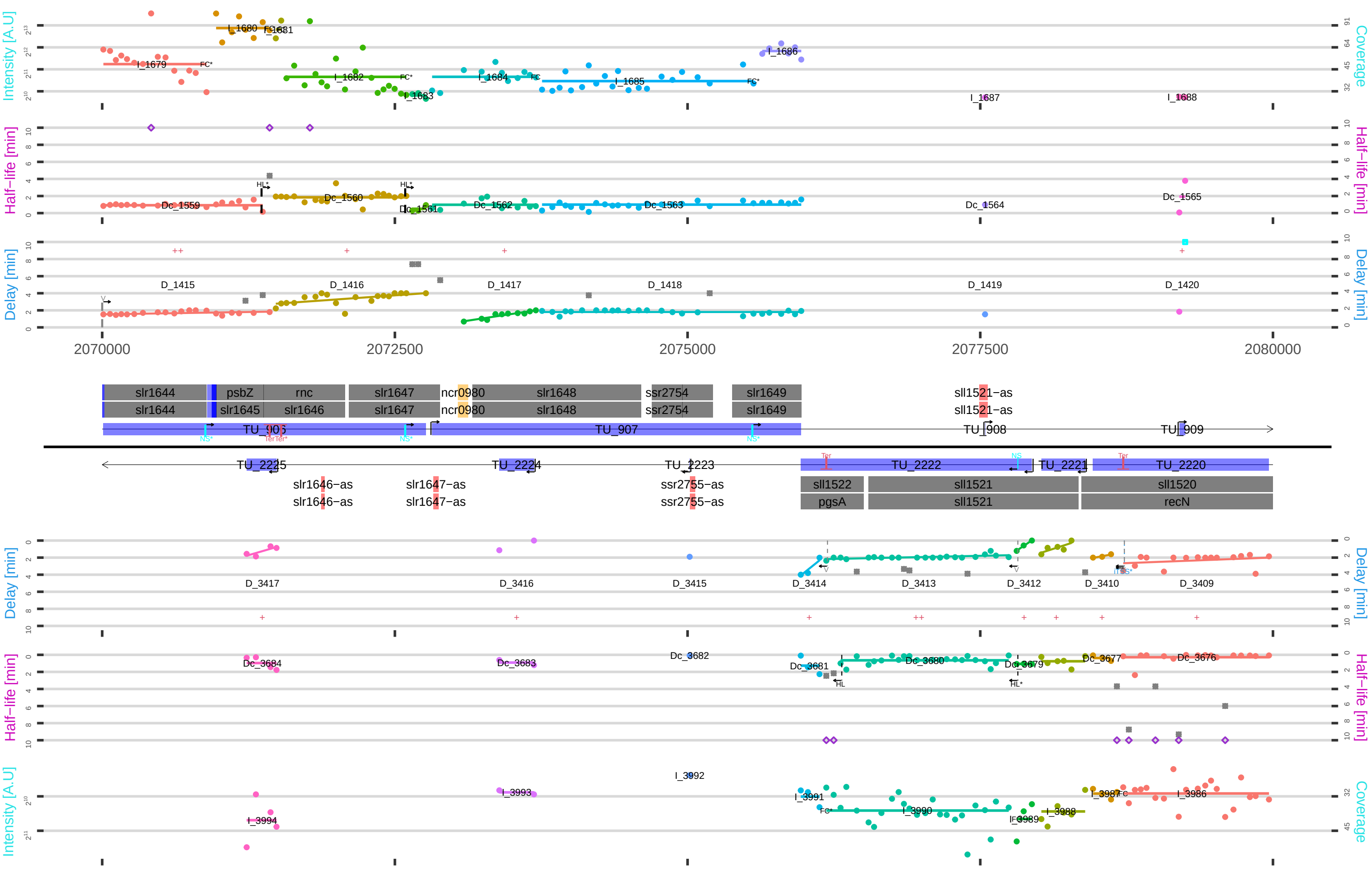

Term: termination (2), NS: new start (1), PS: pausing site (1), iTSS_I: internal starting site (2)

ID: 16678−16779; Term: termination (1), NS: new start (2), PS: pausing site (2), iTSS_I: internal starting site (1)

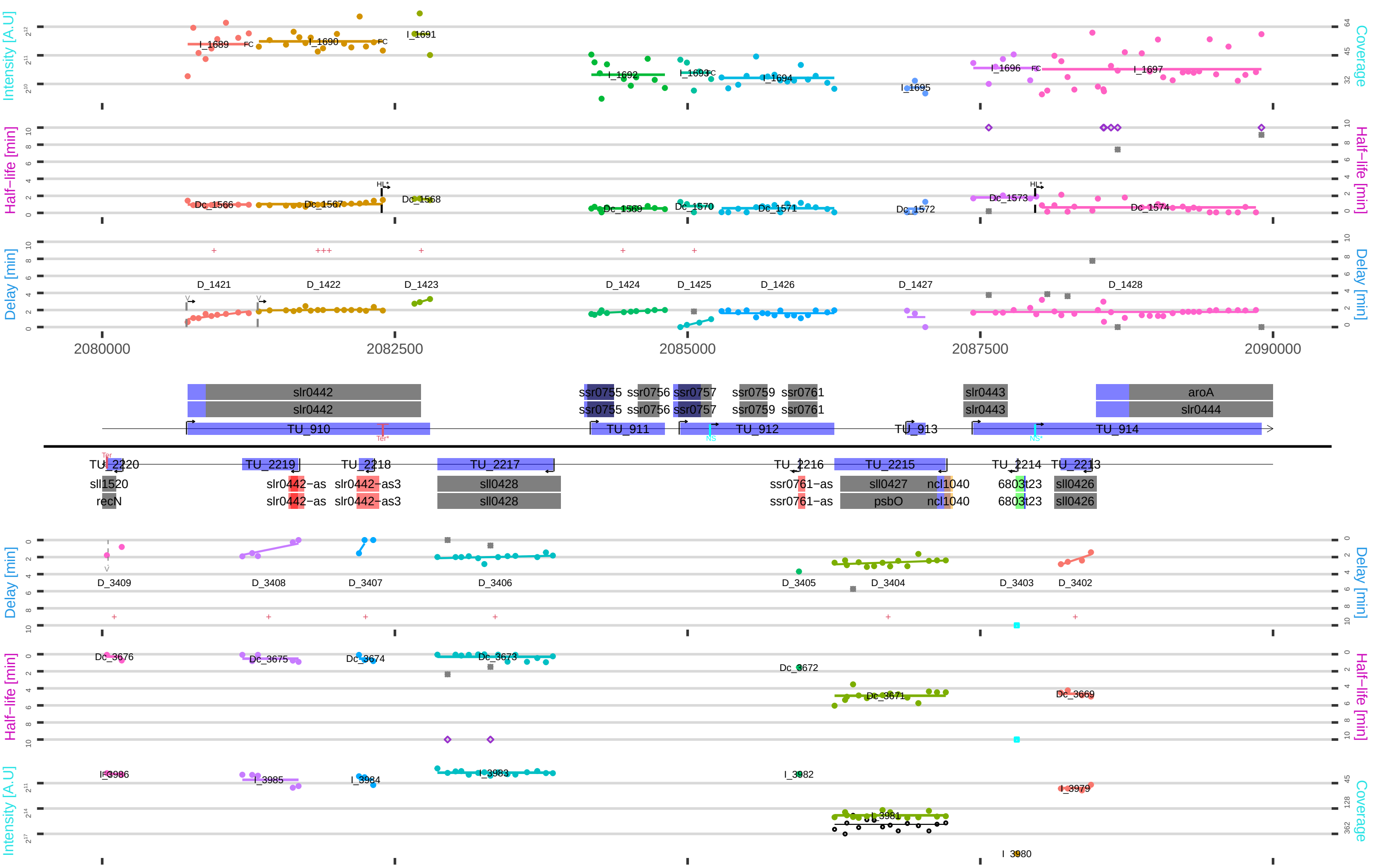

Term: termination (1), NS: new start (0), PS: pausing site (0), iTSS_I: internal starting site (0)

ID: 16781−16842; Term: termination (1), NS: new start (4), PS: pausing site (1), iTSS_I: internal starting site (0)

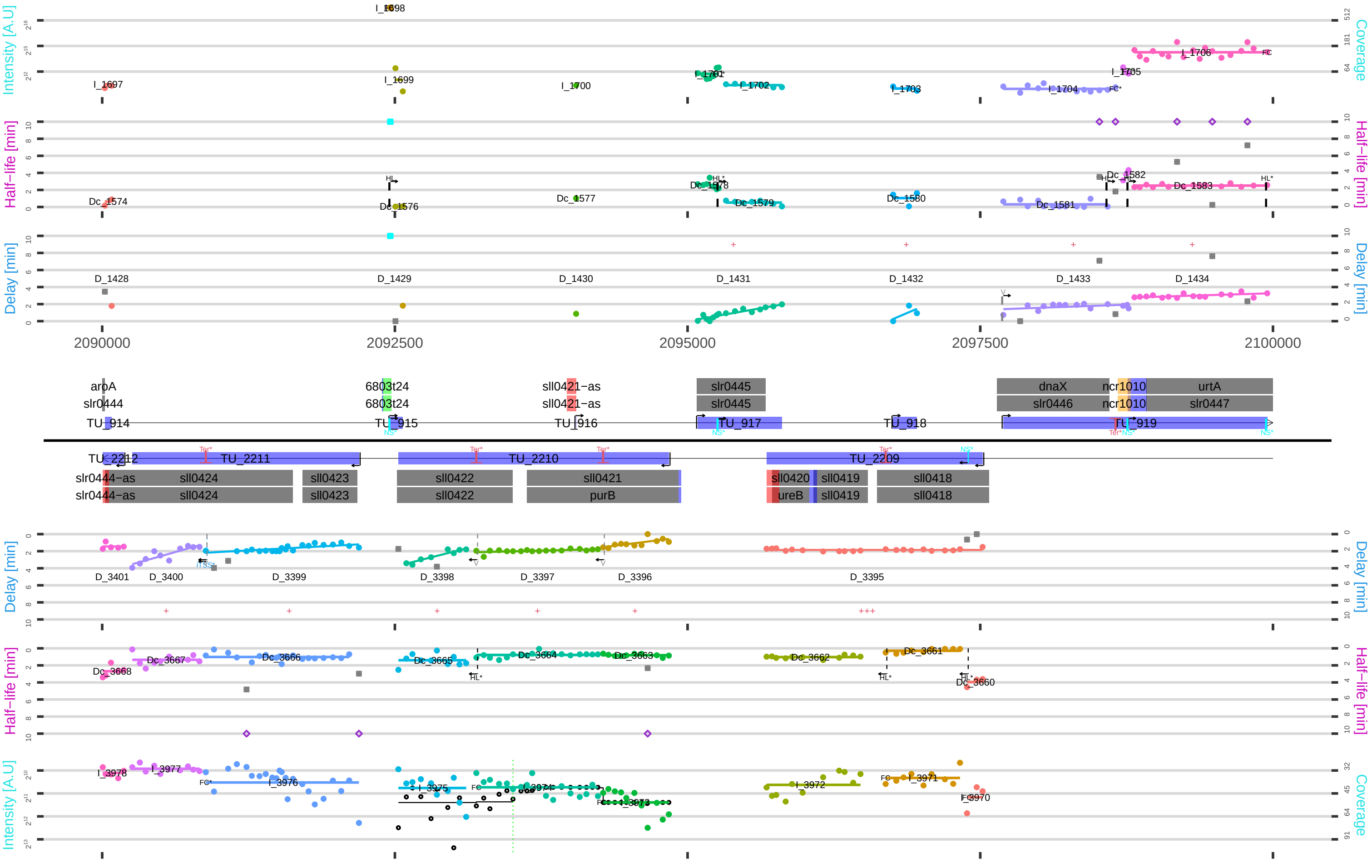

Term: termination (4), NS: new start (1), PS: pausing site (1), iTSS_I: internal starting site (2)

ID: 16843−16904; Term: termination (0), NS: new start (1), PS: pausing site (1), iTSS_I: internal starting site (0)

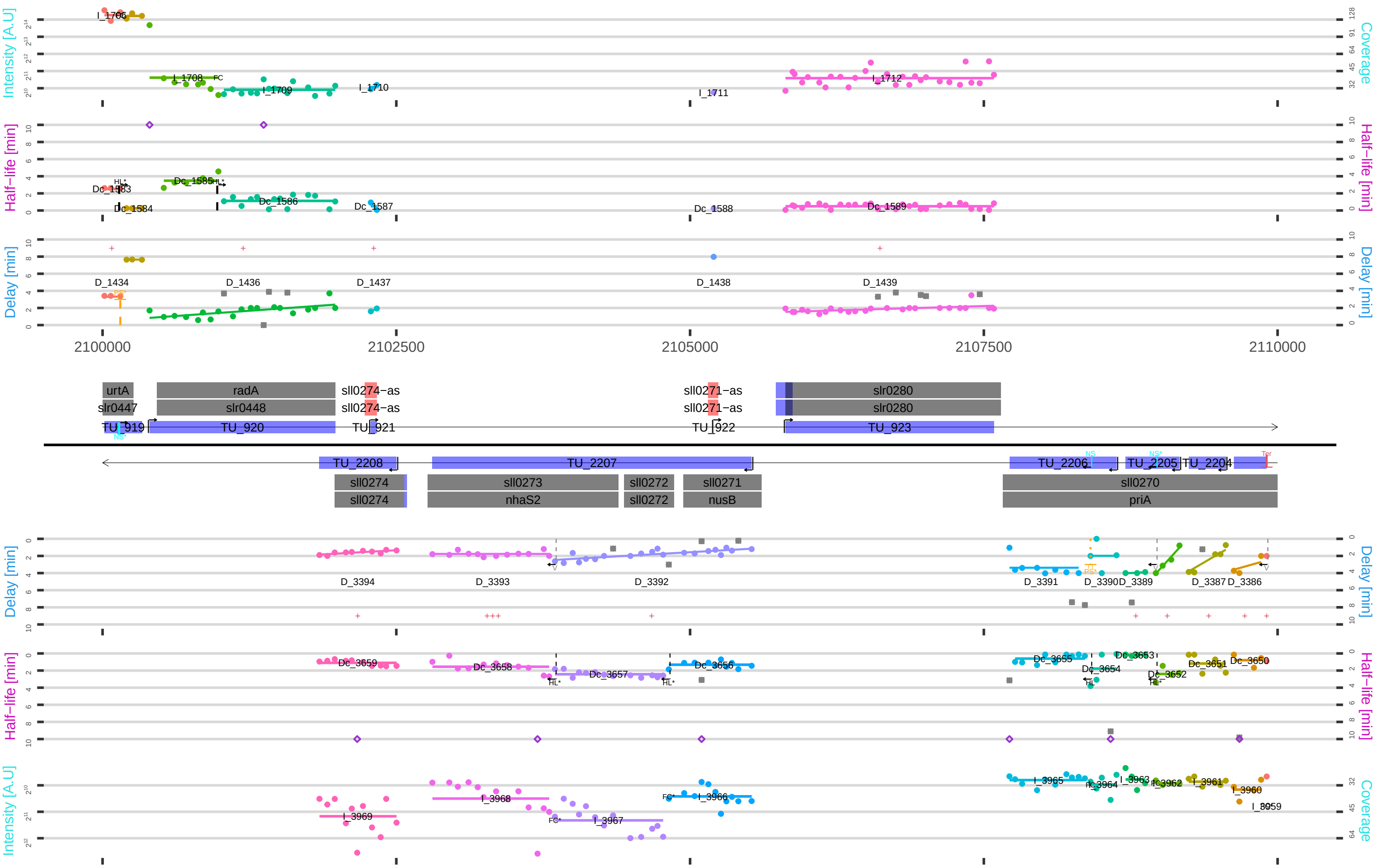

Term: termination (1), NS: new start (2), PS: pausing site (1), iTSS_I: internal starting site (3)

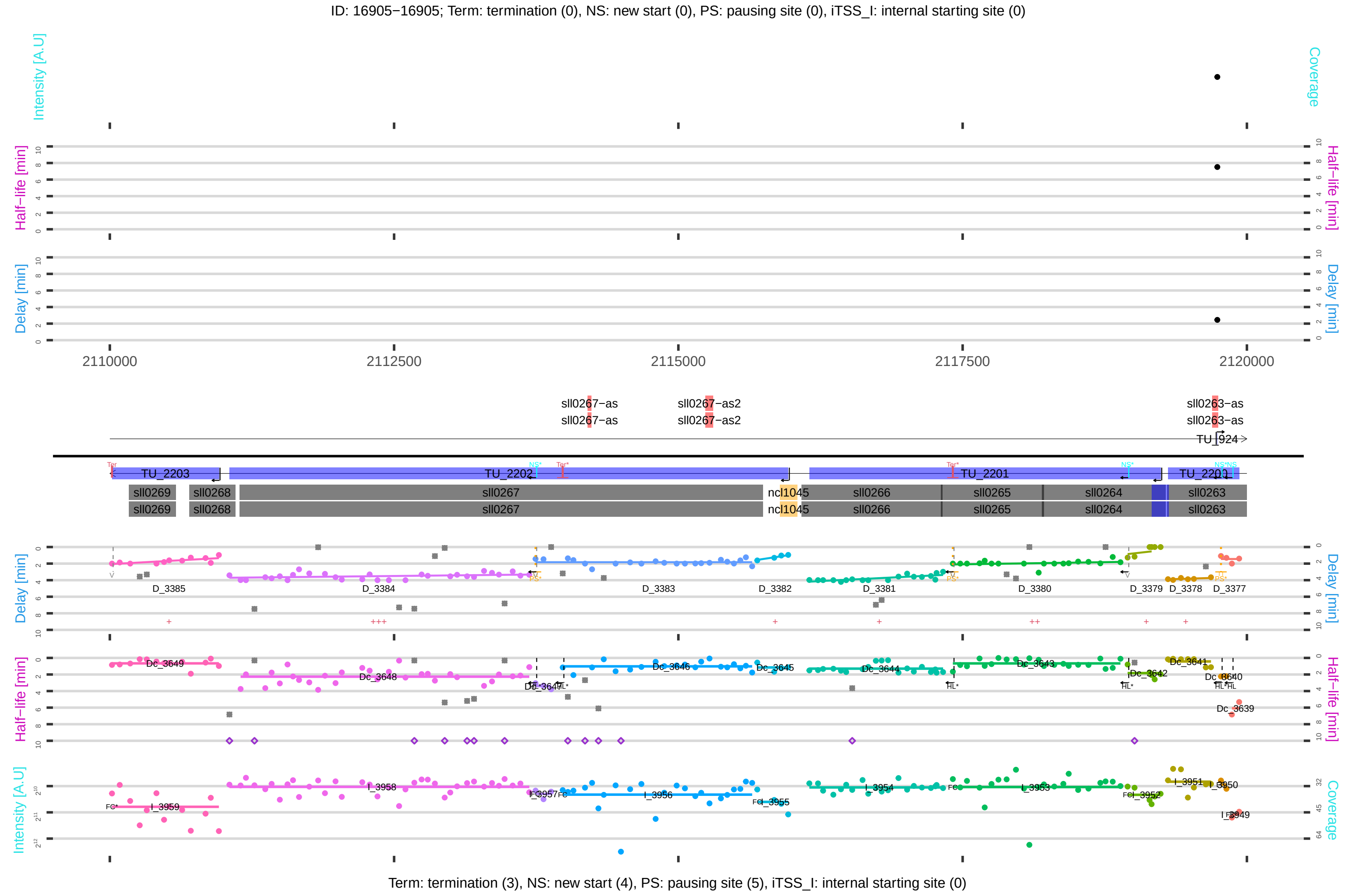
ID: 16905−16905; Term: termination (0), NS: new start (0), PS: pausing site (0), iTSS_I: internal starting site (0)
Intensity [A.U]
Coverage
Half−life [min]
Delay [min]
2110000
2112500
2115000
2117500
2120000
sll0267−as
sll0267−as2
sll0263−as
TU_924
TU_2203
TU_2202
TU_2201
TU_2200
sll0269
sll0268
sll0267
ncl1045
sll0266
sll0265
sll0264
sll0263
D_3385
D_3384
D_3383
D_3382
D_3381
D_3380
D_3379
D_3378
D_3377
Dc_3649
Dc_3648
Dc_3647
Dc_3646
Dc_3645
Dc_3644
Dc_3643
Dc_3642
Dc_3641
Dc_3640
Dc_3639
I_3959
I_3958
I_3957
I_3956
I_3955
I_3954
I_3953
I_3952
I_3951
I_3950
I_3949
Term: termination (3), NS: new start (4), PS: pausing site (5), iTSS_I: internal starting site (0)

ID: 16906−16971; Term: termination (0), NS: new start (2), PS: pausing site (2), iTSS_I: internal starting site (0)

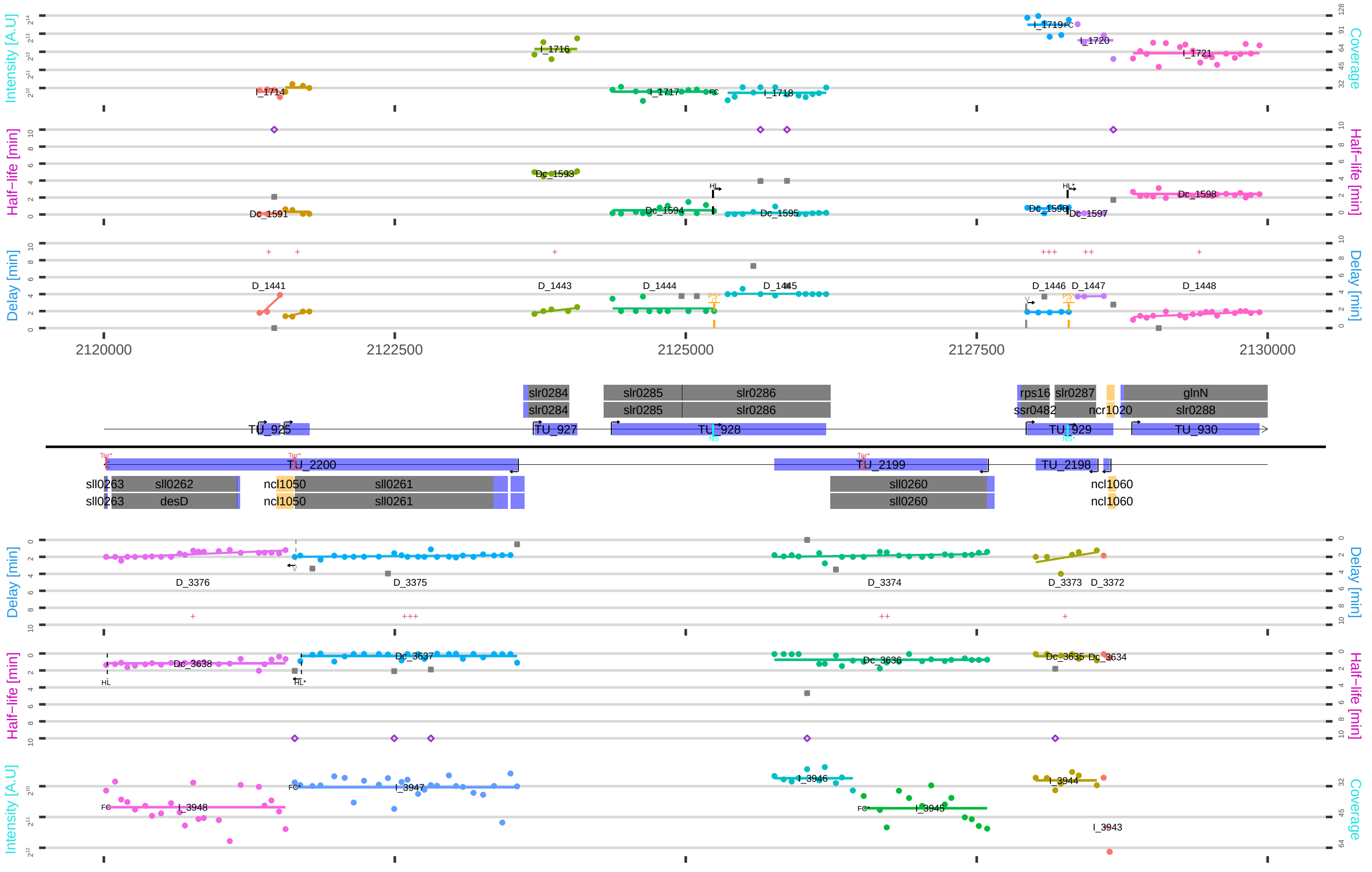

Term: termination (3), NS: new start (0), PS: pausing site (0), iTSS_I: internal starting site (2)

ID: 16972−17108; Term: termination (1), NS: new start (3), PS: pausing site (3), iTSS_I: internal starting site (1)

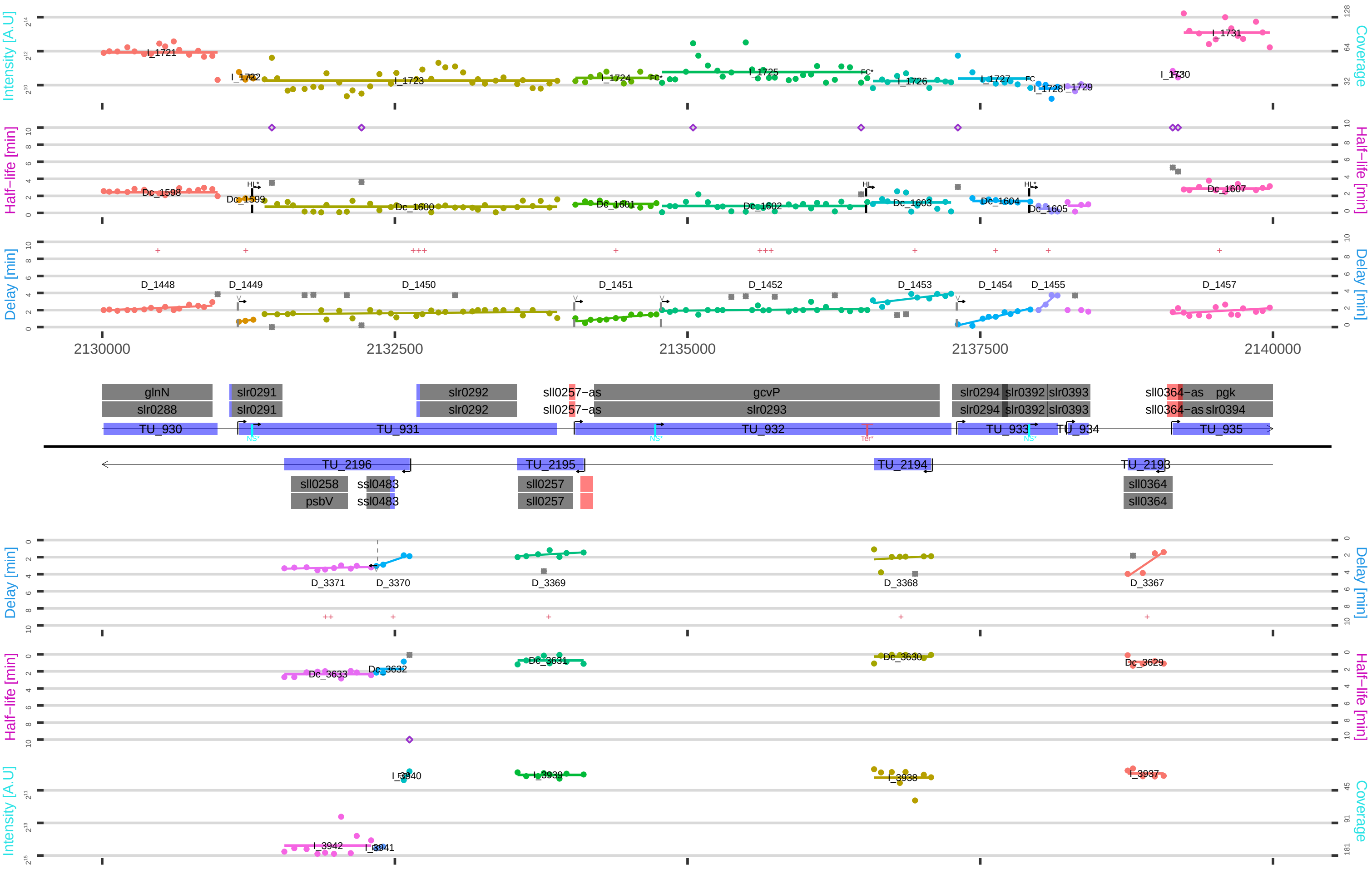

Term: termination (0), NS: new start (0), PS: pausing site (1), iTSS_I: internal starting site (0)

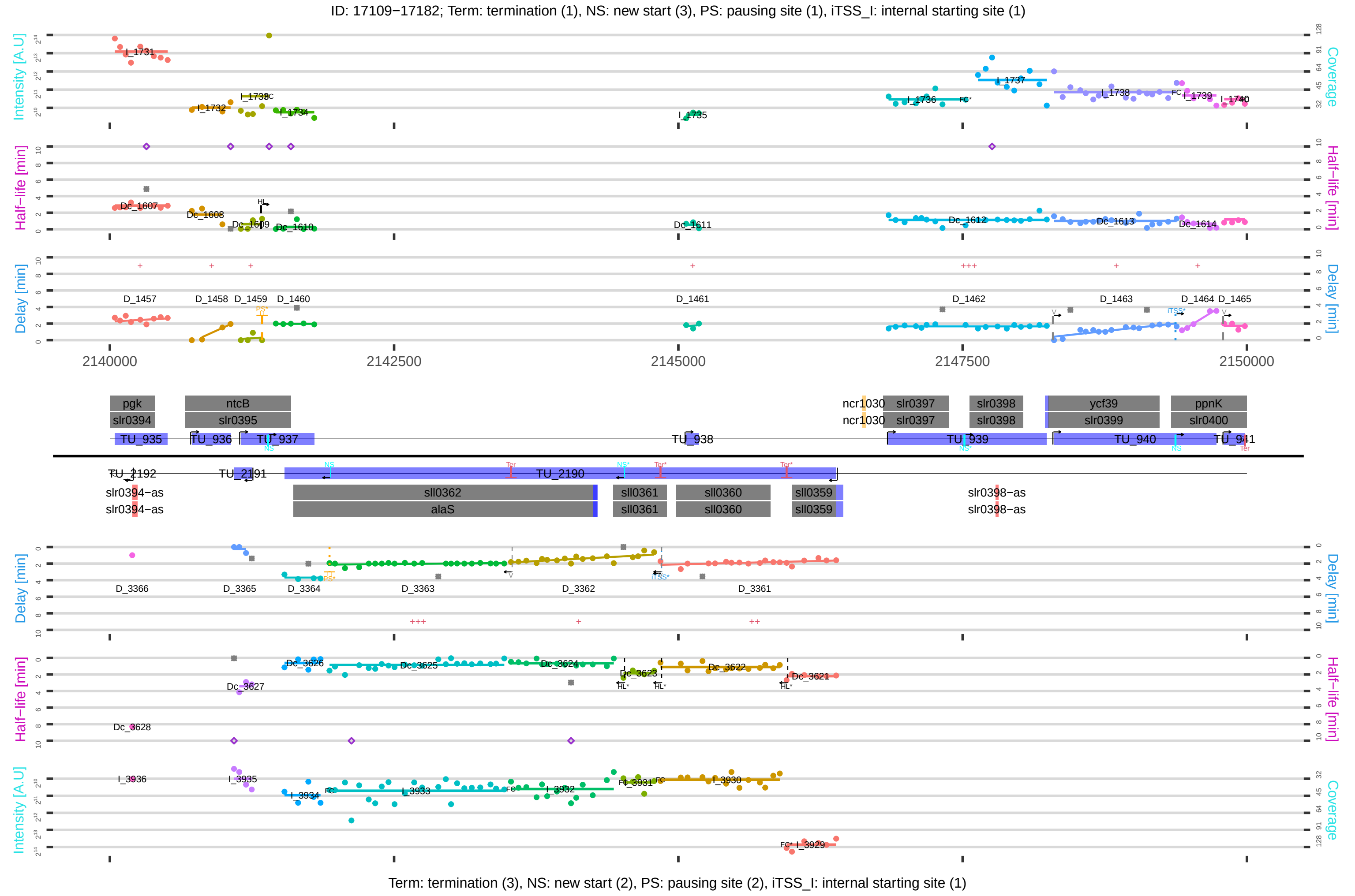
ID: 17109−17182; Term: termination (1), NS: new start (3), PS: pausing site (1), iTSS_I: internal starting site (1)
Intensity [A.U]
Coverage
Half−life [min]
Delay [min]
2140000
2142500
2145000
2147500
2150000
pgk
slr0394
ntcB
slr0395
ncr1030
slr0397
slr0398
ycf39
slr0399
ppnK
slr0400
TU_935
TU_936
TU_937
TU_938
TU_939
TU_940
TU_941
TU_2192
TU_2191
TU_2190
slr0394−as
sll0362
alaS
sll0361
sll0360
sll0359
slr0398−as
Term: termination (3), NS: new start (2), PS: pausing site (2), iTSS_I: internal starting site (1)

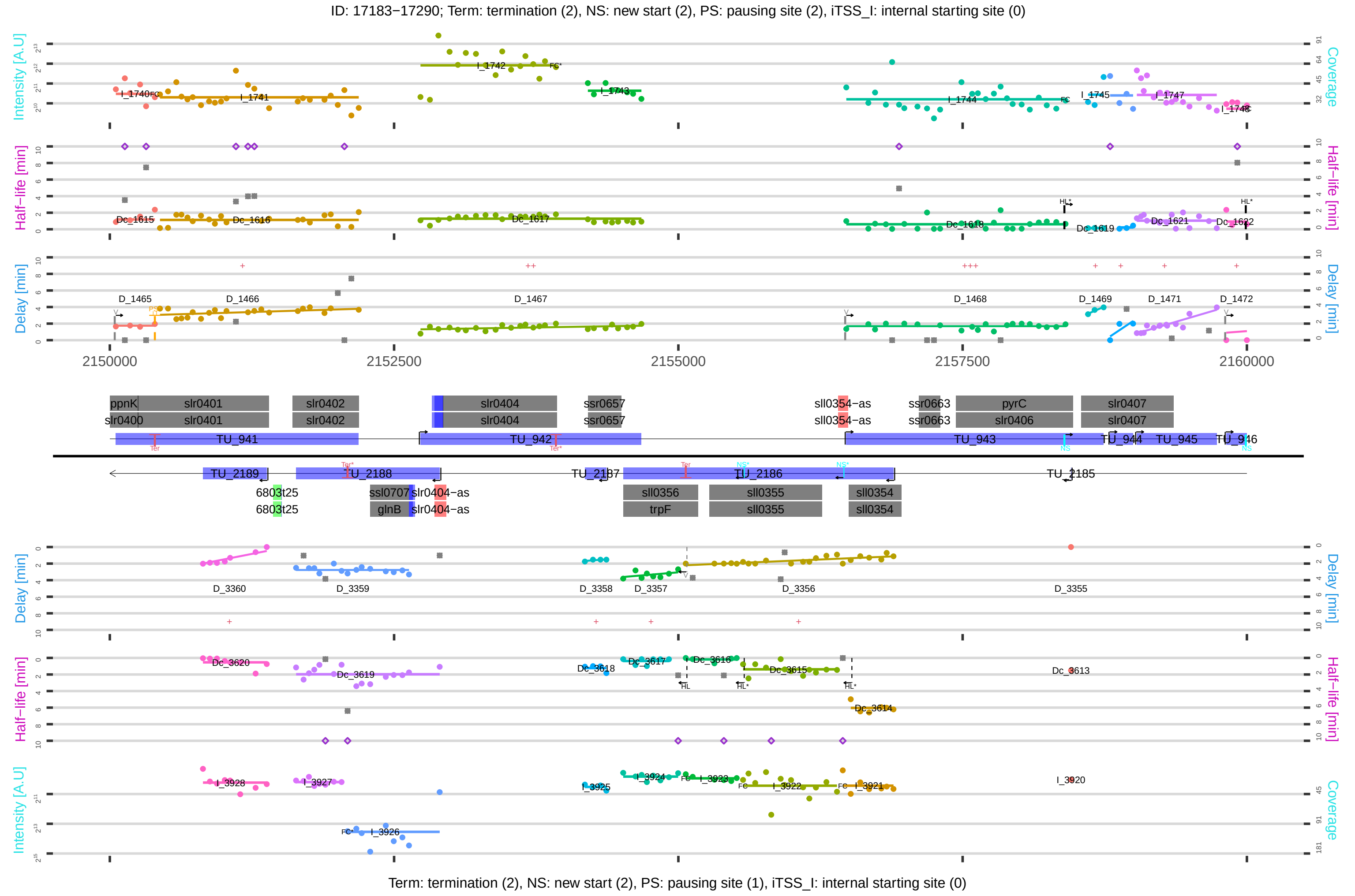
ID: 17183−17290; Term: termination (2), NS: new start (2), PS: pausing site (2), iTSS_I: internal starting site (0)
Intensity [A.U]
Coverage
Half−life [min]
Delay [min]
2150000
2152500
2155000
2157500
2160000
ppnK
slr0400
slr0401
slr0402
slr0404
ssr0657
sll0354−as
ssr0663
pyrC
slr0406
slr0407
TU_941
TU_942
TU_943
TU_944
TU_945
TU_946
TU_2189
TU_2188
TU_2187
TU_2186
TU_2185
6803t25
ssl0707
glnB
slr0404−as
sll0356
trpF
sll0355
sll0354
Term: termination (2), NS: new start (2), PS: pausing site (1), iTSS_I: internal starting site (0)

ID: 17290−17438; Term: termination (5), NS: new start (3), PS: pausing site (4), iTSS_I: internal starting site (2)

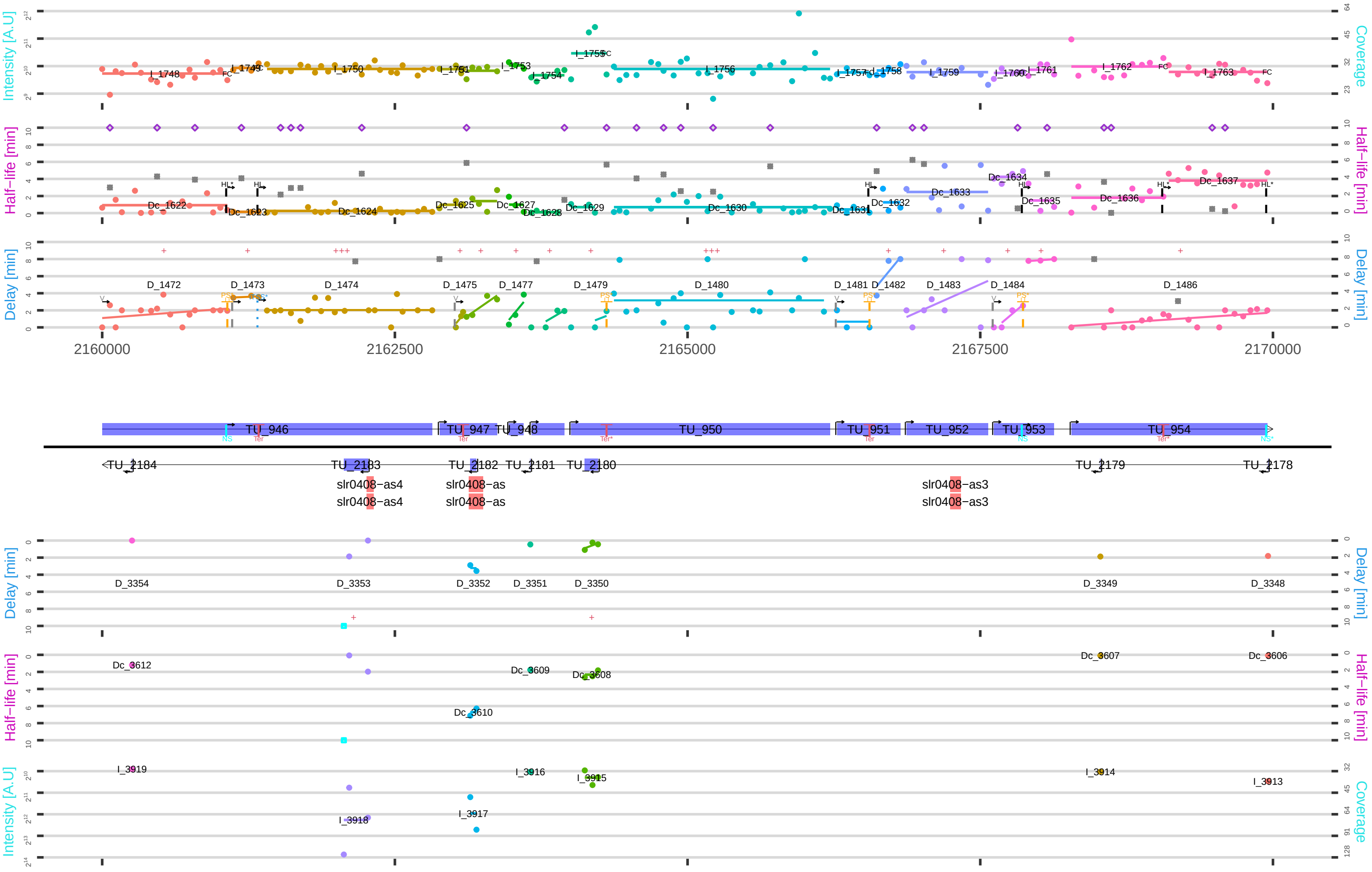

Term: termination (0), NS: new start (0), PS: pausing site (0), iTSS_I: internal starting site (0)

ID: 17439−17600; Term: termination (2), NS: new start (6), PS: pausing site (3), iTSS_I: internal starting site (1)

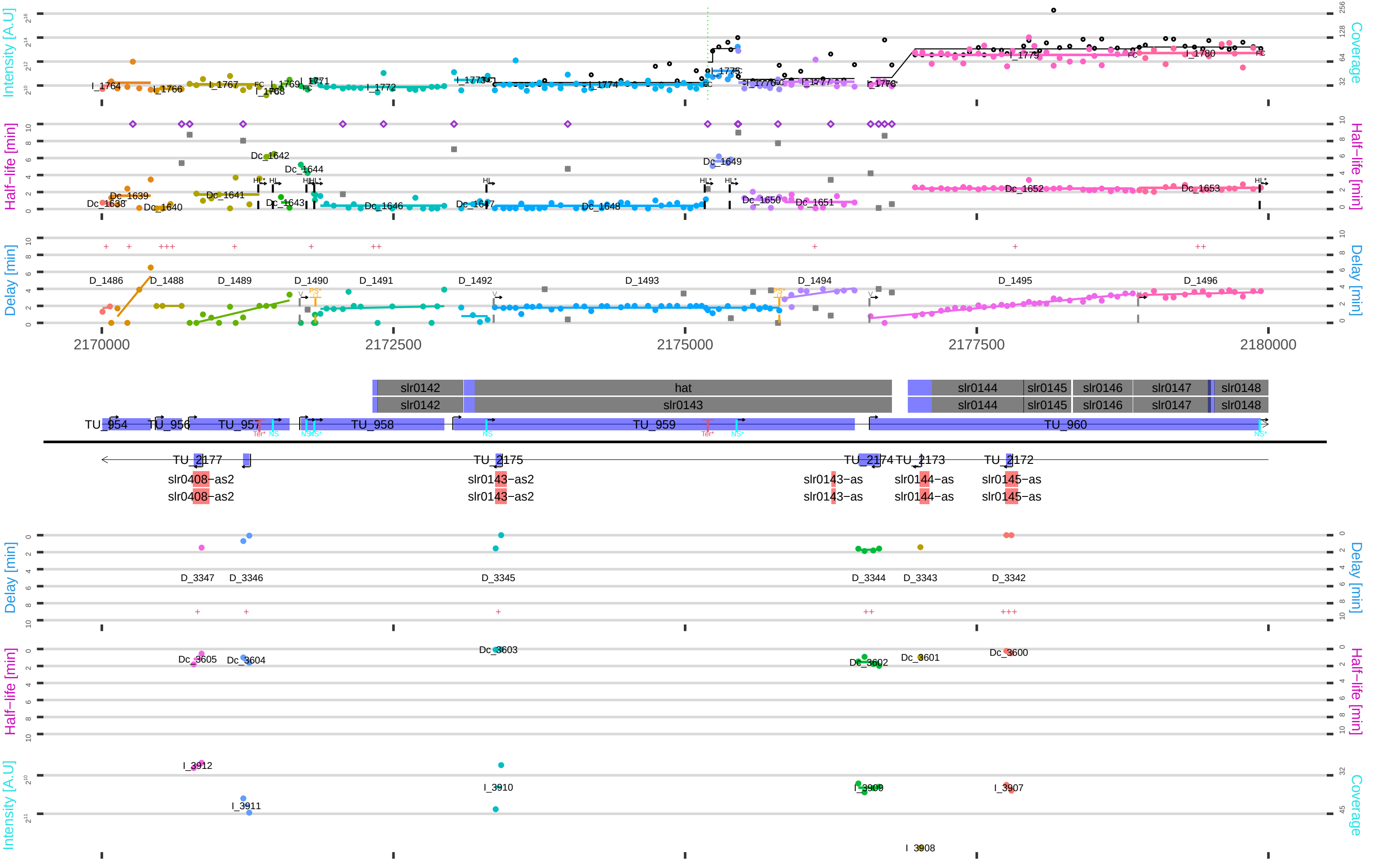

Term: termination (0), NS: new start (0), PS: pausing site (0), iTSS_I: internal starting site (0)

ID: 17601−17661; Term: termination (1), NS: new start (2), PS: pausing site (2), iTSS_I: internal starting site (1)

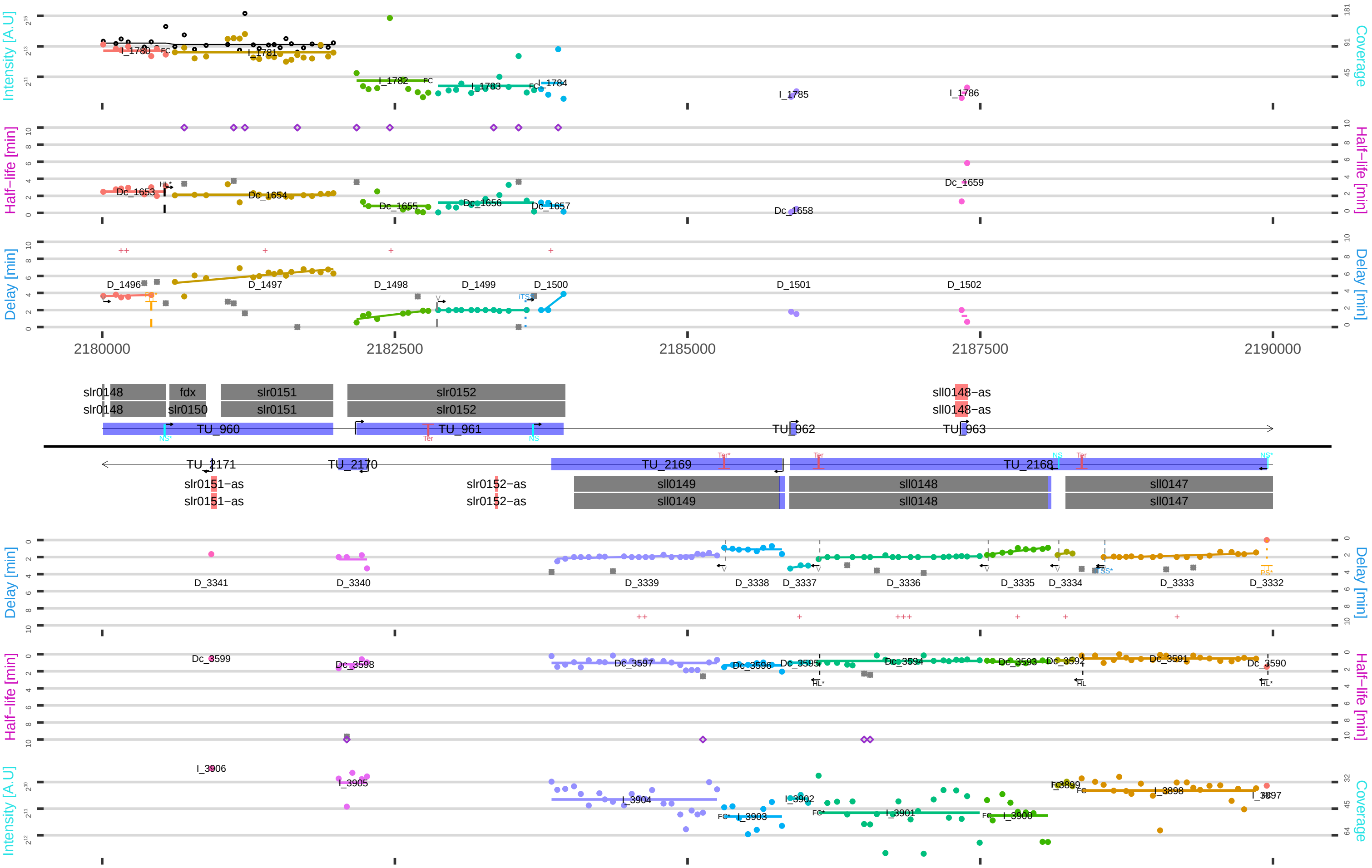

Term: termination (3), NS: new start (2), PS: pausing site (4), iTSS_I: internal starting site (2)

ID: 17662−17709; Term: termination (2), NS: new start (1), PS: pausing site (2), iTSS_I: internal starting site (1)

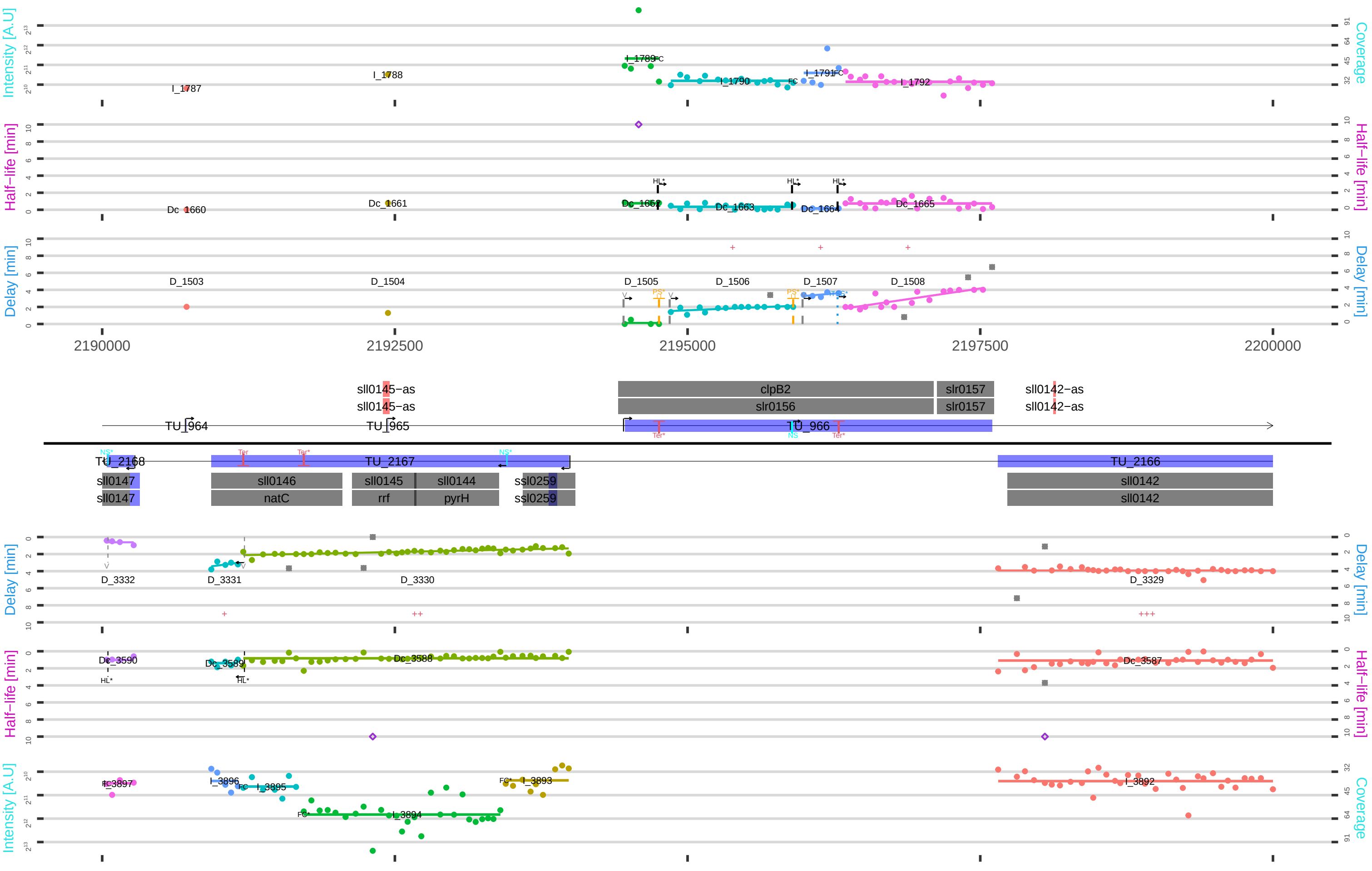

Term: termination (2), NS: new start (2), PS: pausing site (1), iTSS_I: internal starting site (0)

ID: 17710−17775; Term: termination (1), NS: new start (0), PS: pausing site (0), iTSS_I: internal starting site (1)

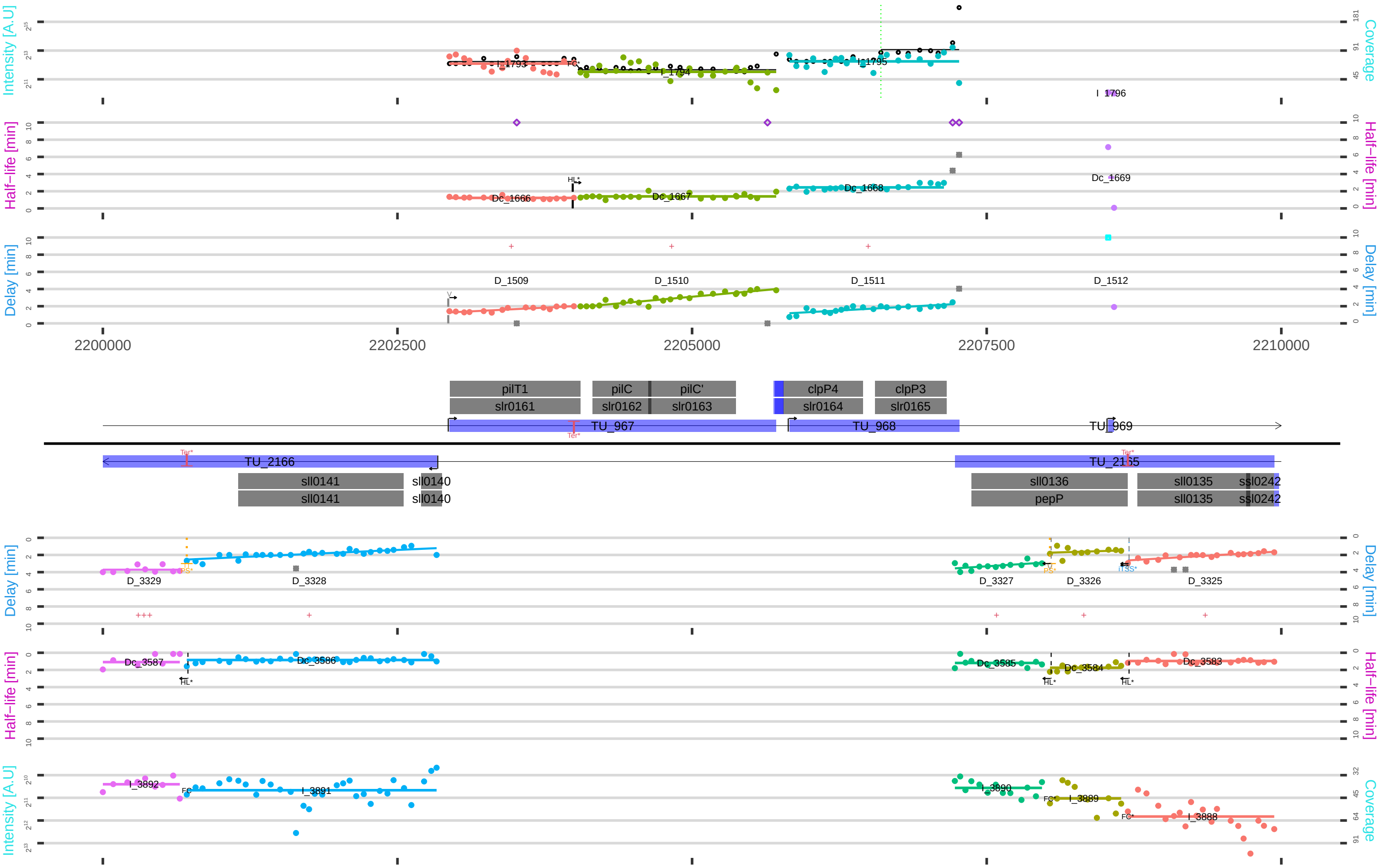

Term: termination (2), NS: new start (0), PS: pausing site (2), iTSS_I: internal starting site (1)

ID: 17776−17859; Term: termination (5), NS: new start (1), PS: pausing site (2), iTSS_I: internal starting site (1)

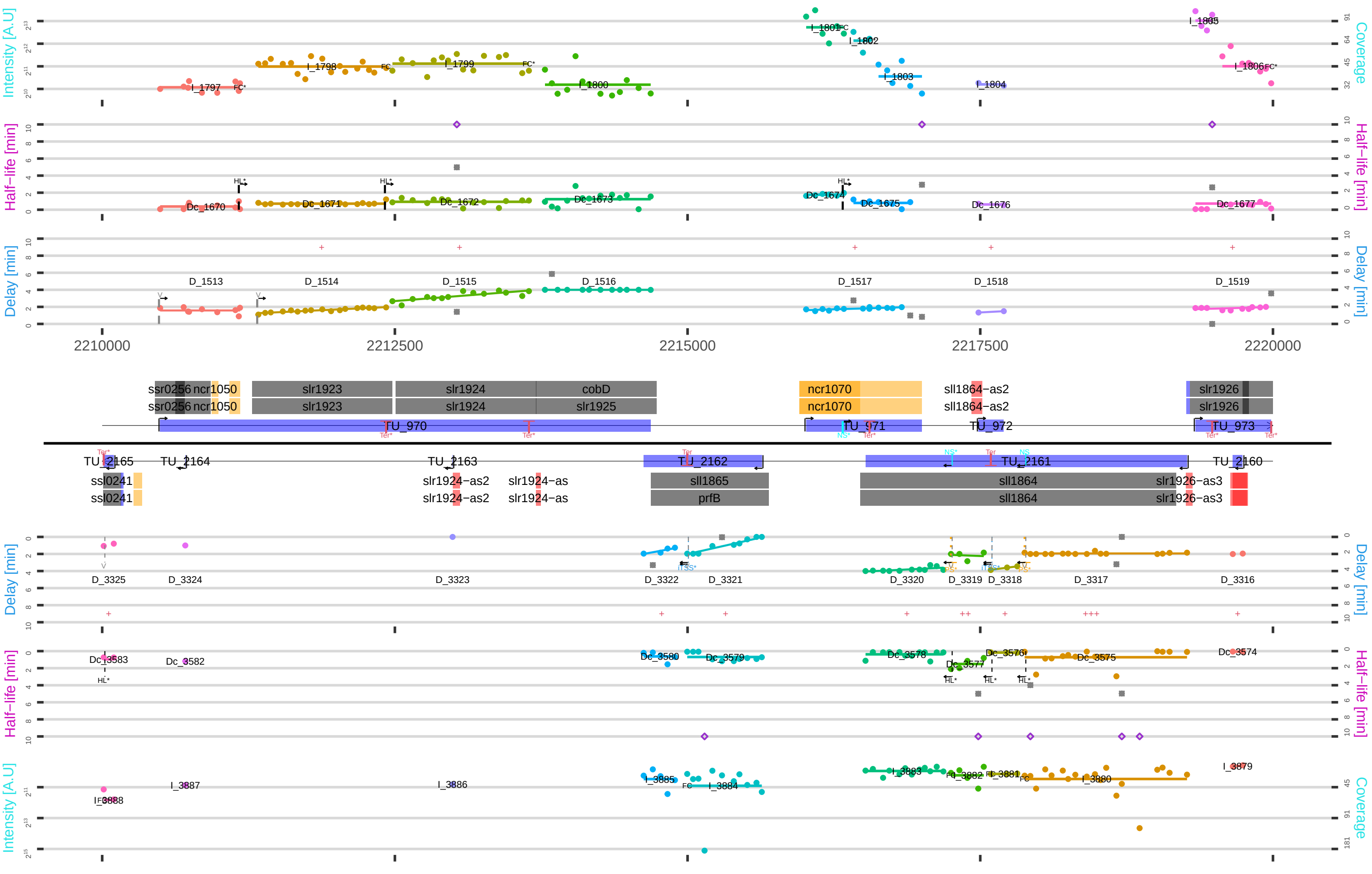

Term: termination (3), NS: new start (2), PS: pausing site (2), iTSS_I: internal starting site (2)

ID: 17860−17942; Term: termination (2), NS: new start (1), PS: pausing site (0), iTSS_I: internal starting site (1)

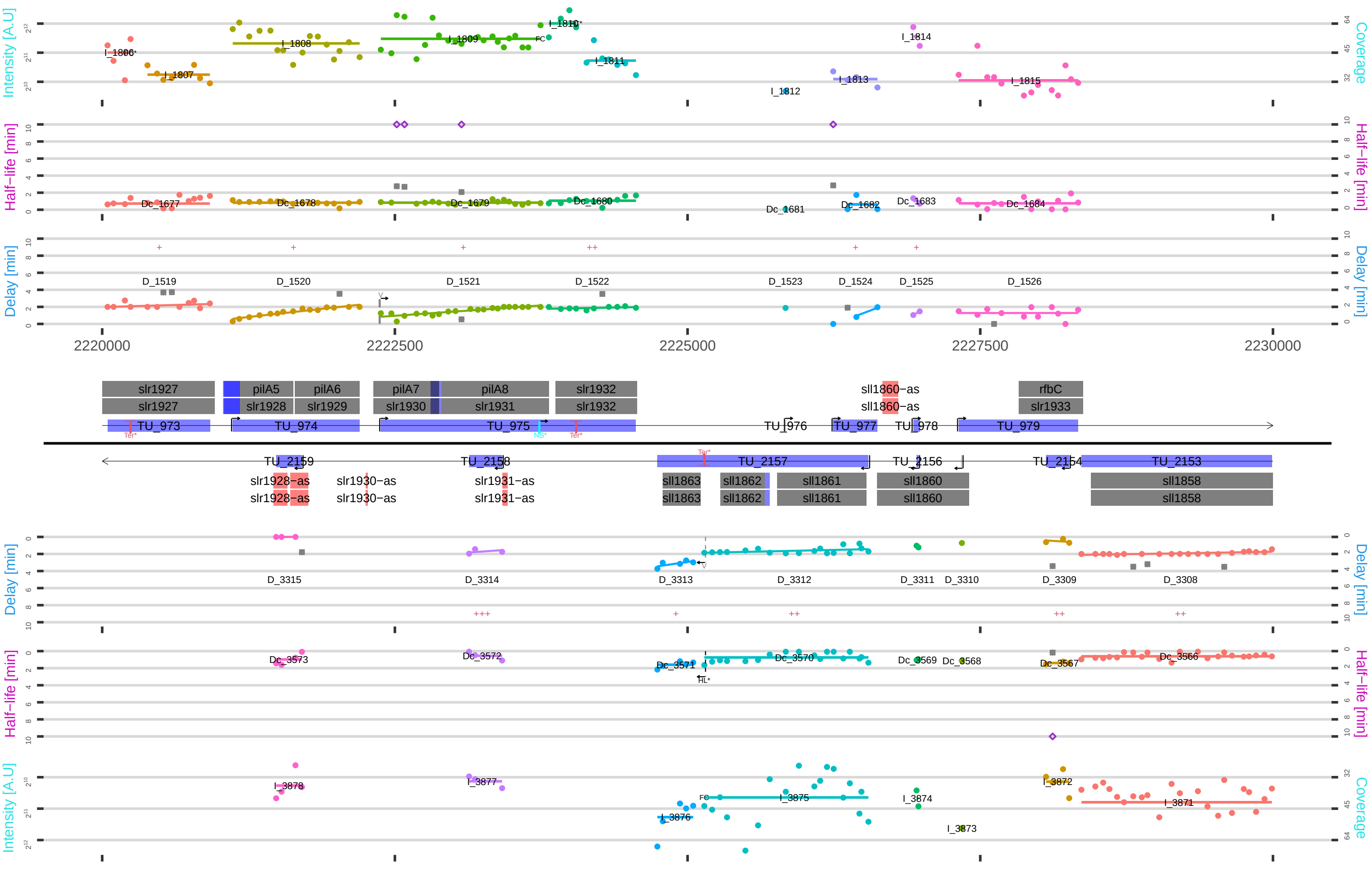

Term: termination (1), NS: new start (0), PS: pausing site (1), iTSS_I: internal starting site (0)

ID: 17943−18030; Term: termination (1), NS: new start (2), PS: pausing site (1), iTSS_I: internal starting site (1)

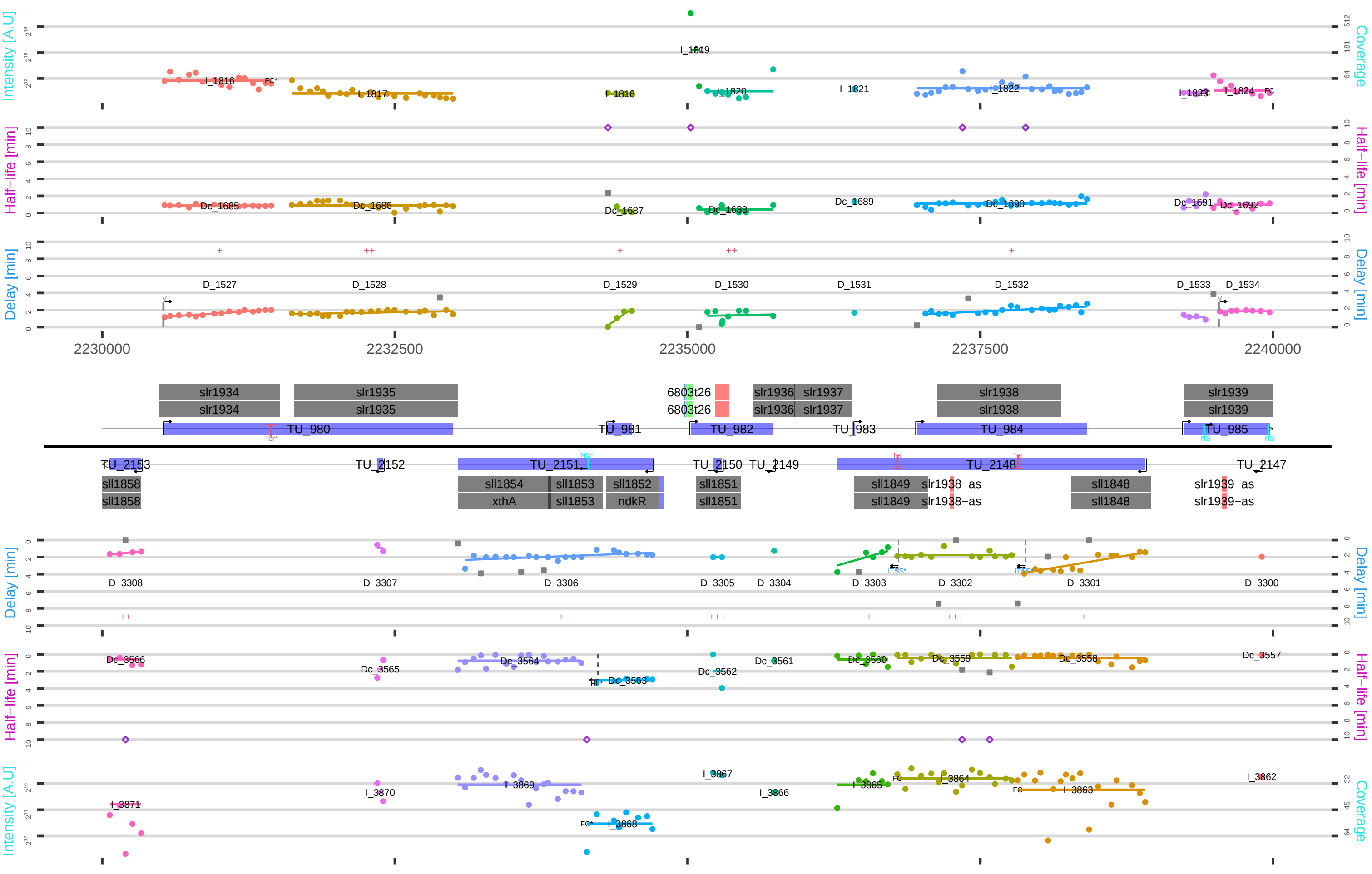

Term: termination (2), NS: new start (1), PS: pausing site (0), iTSS_I: internal starting site (2)

ID: 18031−18166; Term: termination (3), NS: new start (1), PS: pausing site (1), iTSS_I: internal starting site (1)

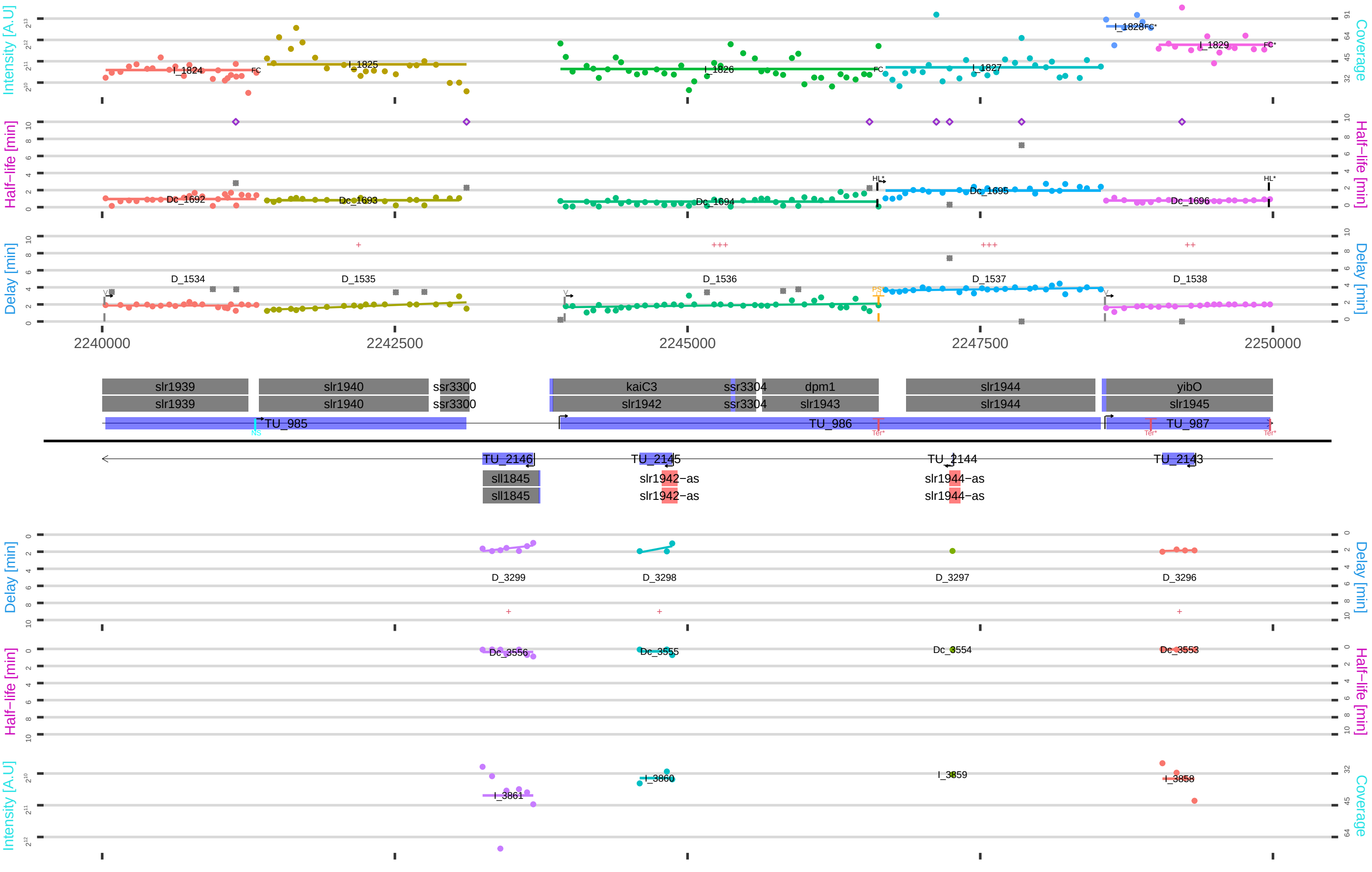

Term: termination (0), NS: new start (0), PS: pausing site (0), iTSS_I: internal starting site (0)

ID: 18167−18288; Term: termination (5), NS: new start (1), PS: pausing site (2), iTSS_I: internal starting site (2)

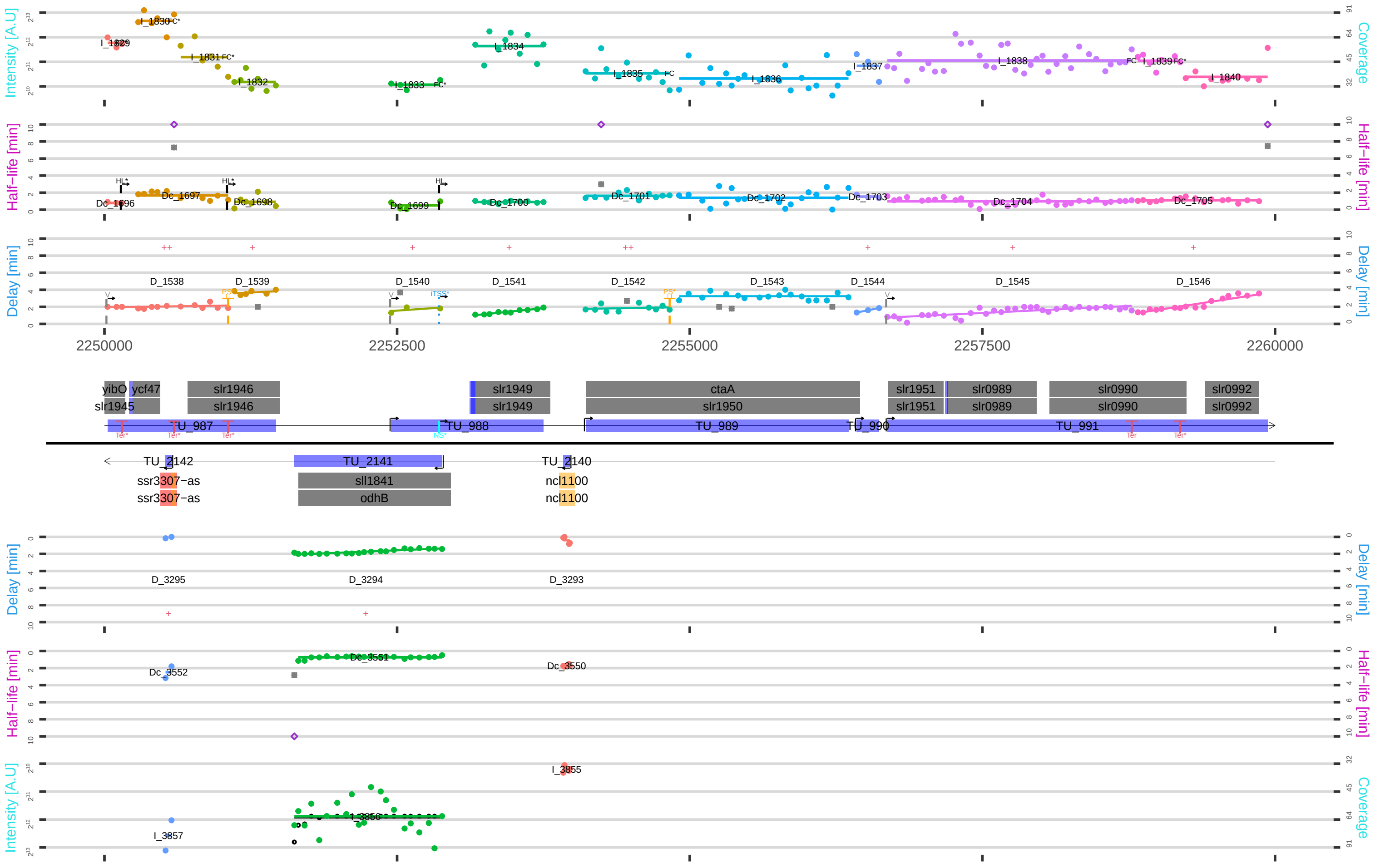

Term: termination (0), NS: new start (0), PS: pausing site (0), iTSS_I: internal starting site (0)

ID: 18289−18370; Term: termination (2), NS: new start (1), PS: pausing site (1), iTSS_I: internal starting site (2)

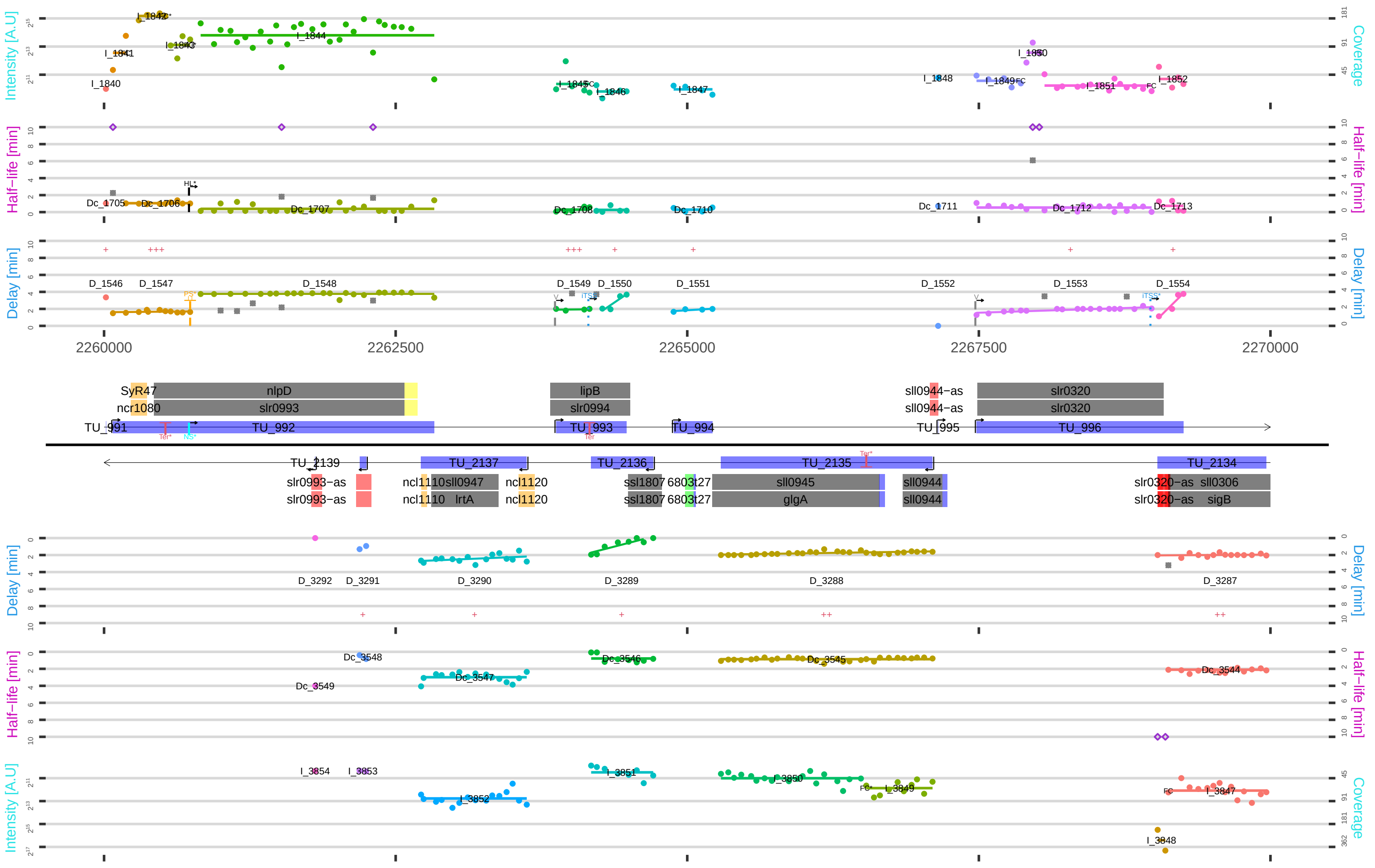

Term: termination (1), NS: new start (0), PS: pausing site (0), iTSS_I: internal starting site (0)

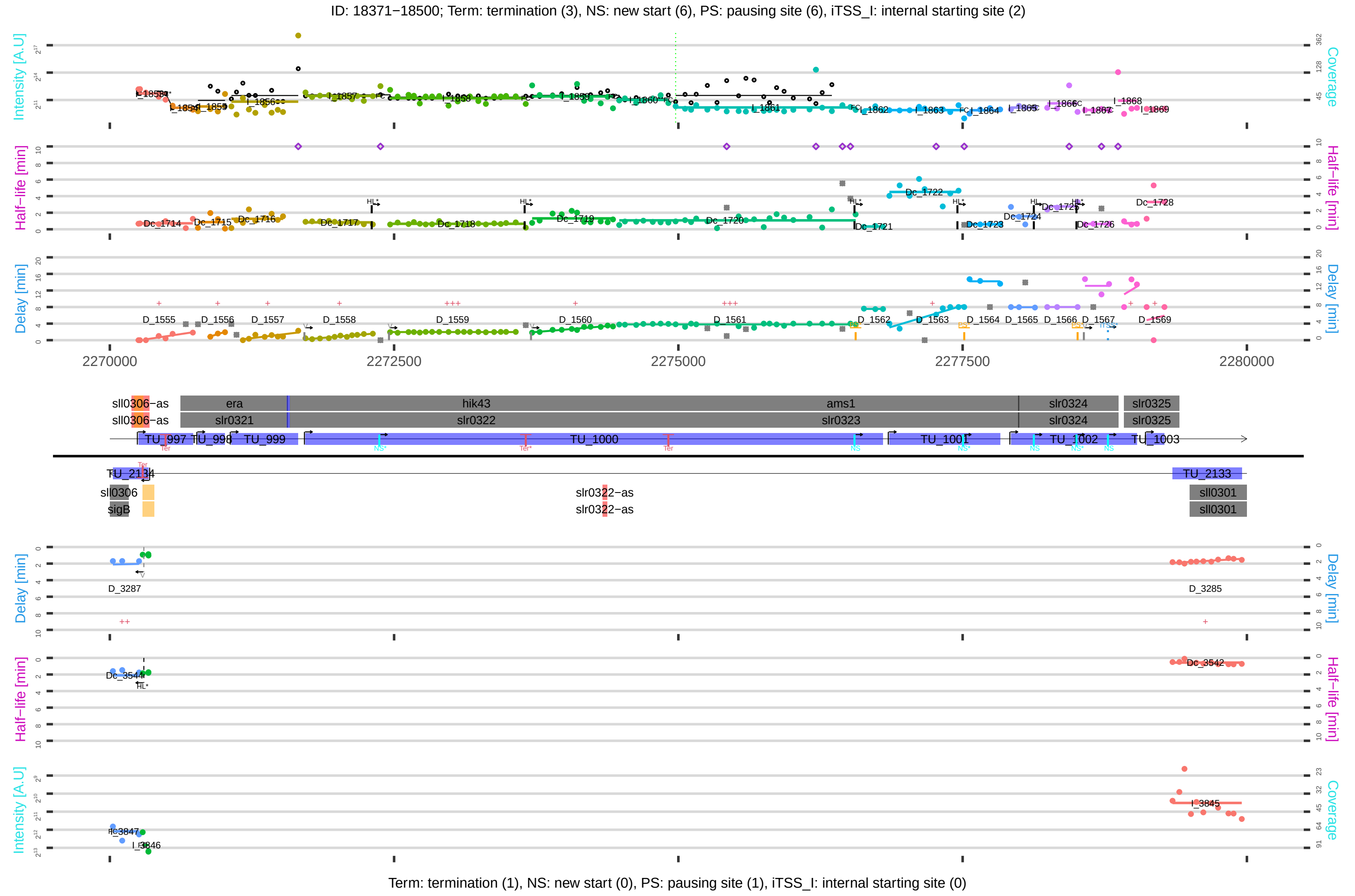

ID: 18371−18500; Term: termination (3), NS: new start (6), PS: pausing site (6), iTSS_I: internal starting site (2)
Intensity [A.U]
Coverage
Half−life [min]
Delay [min]
2270000
2272500
2275000
2277500
2280000
sll0306−as
era
hik43
ams1
slr0324
slr0325
slr0321
slr0322
slr0323
TU_997
TU_998
TU_999
TU_1000
TU_1001
TU_1002
TU_1003
TU_2134
TU_2133
sll0306
sigB
slr0322−as
sll0301
Term: termination (1), NS: new start (0), PS: pausing site (1), iTSS_I: internal starting site (0)

ID: 18502−18597; Term: termination (2), NS: new start (0), PS: pausing site (1), iTSS_I: internal starting site (1)

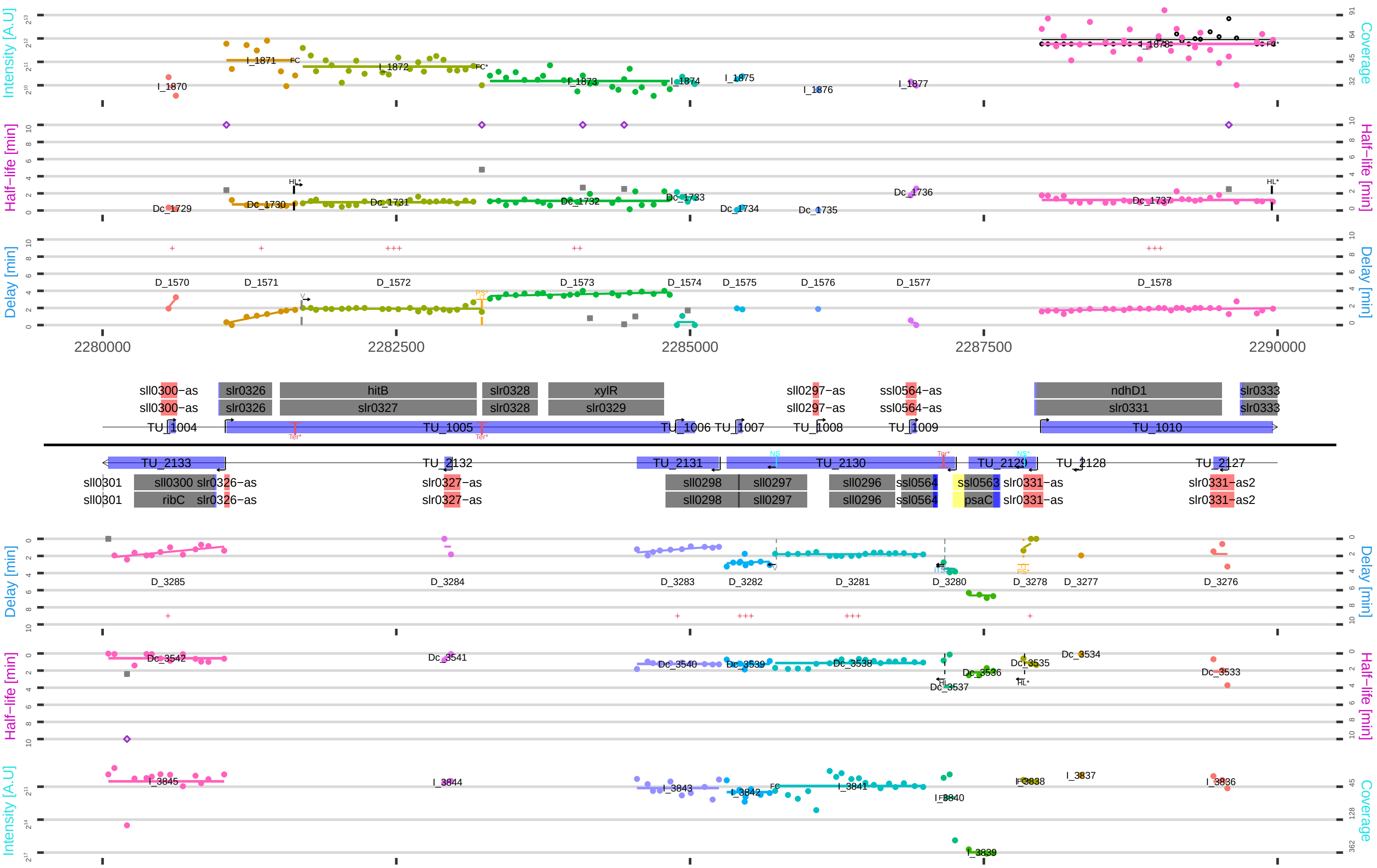

Term: termination (1), NS: new start (2), PS: pausing site (2), iTSS_I: internal starting site (1)

ID: 18598−18710; Term: termination (3), NS: new start (1), PS: pausing site (2), iTSS_I: internal starting site (1)

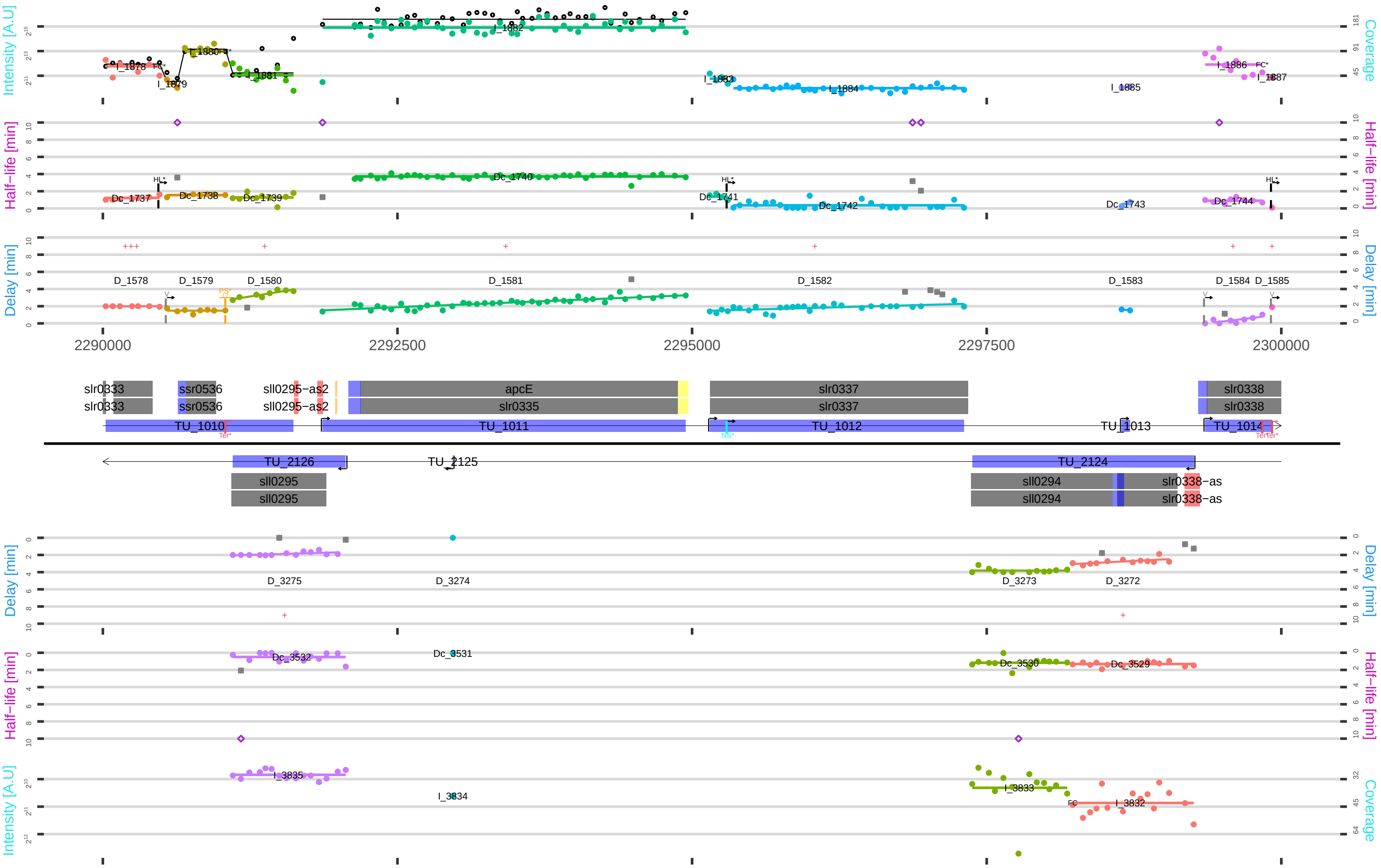

Term: termination (0), NS: new start (0), PS: pausing site (1), iTSS_I: internal starting site (0)

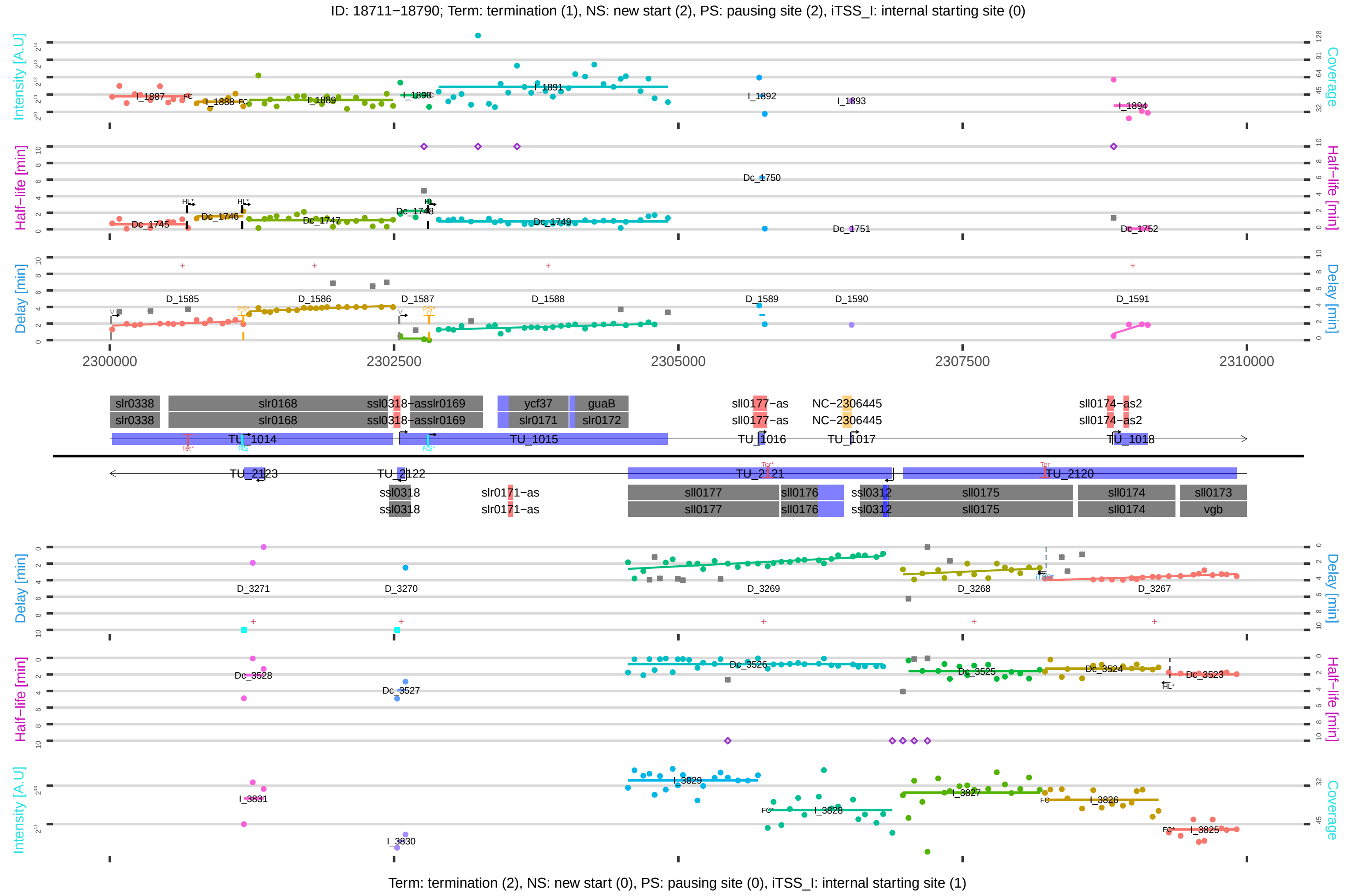
ID: 18711−18790; Term: termination (1), NS: new start (2), PS: pausing site (2), iTSS_I: internal starting site (0)
Intensity [A.U]
Coverage
Half−life [min]
Delay [min]
2300000
2302500
2305000
2307500
2310000
I_1887
I_1888
I_1889
I_1890
I_1891
I_1892
I_1893
I_1894
Dc_1745
Dc_1746
Dc_1747
Dc_1748
Dc_1749
Dc_1750
Dc_1751
Dc_1752
D_1585
D_1586
D_1587
D_1588
D_1589
D_1590
D_1591
slr0338
slr0168
ssl0318−asslr0169
ycf37
guaB
slr0171
slr0172
sll0177−as
NC−2306445
sll0174−as2
TU_1014
TU_1015
TU_1016
TU_1017
TU_1018
TU_2123
TU_2122
TU_2121
TU_2120
ssl0318
slr0171−as
sll0177
sll0176
ssl0312
sll0175
sll0174
sll0173
vgb
D_3271
D_3270
D_3269
D_3268
D_3267
Dc_3528
Dc_3527
Dc_3526
Dc_3525
Dc_3524
Dc_3523
I_3831
I_3830
I_3829
I_3828
I_3827
I_3826
I_3825
Term: termination (2), NS: new start (0), PS: pausing site (0), iTSS_I: internal starting site (1)

ID: 18791−18804; Term: termination (1), NS: new start (0), PS: pausing site (0), iTSS_I: internal starting site (0)

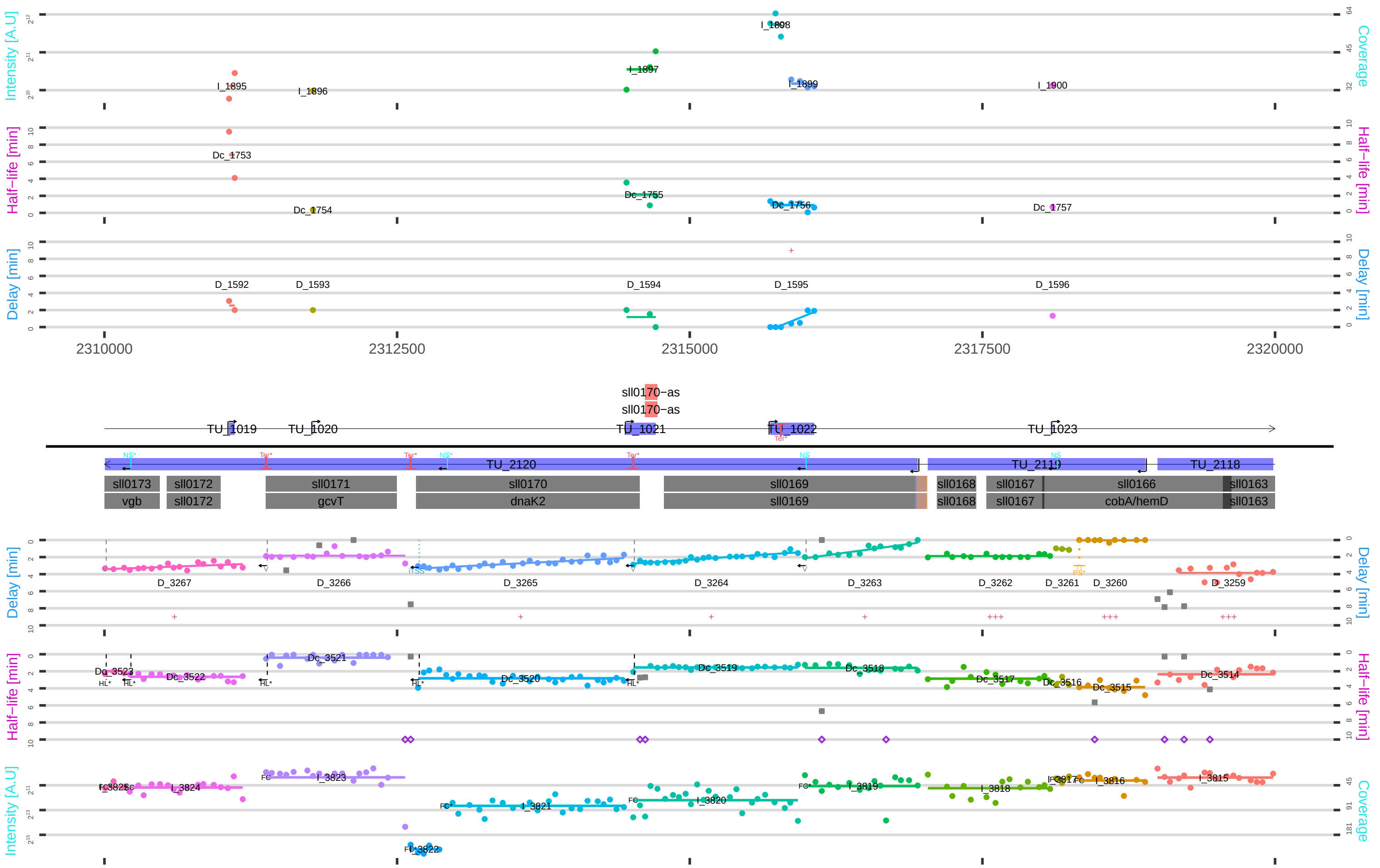

Term: termination (3), NS: new start (4), PS: pausing site (3), iTSS_I: internal starting site (3)

ID: 18805−18845; Term: termination (1), NS: new start (0), PS: pausing site (0), iTSS_I: internal starting site (1)

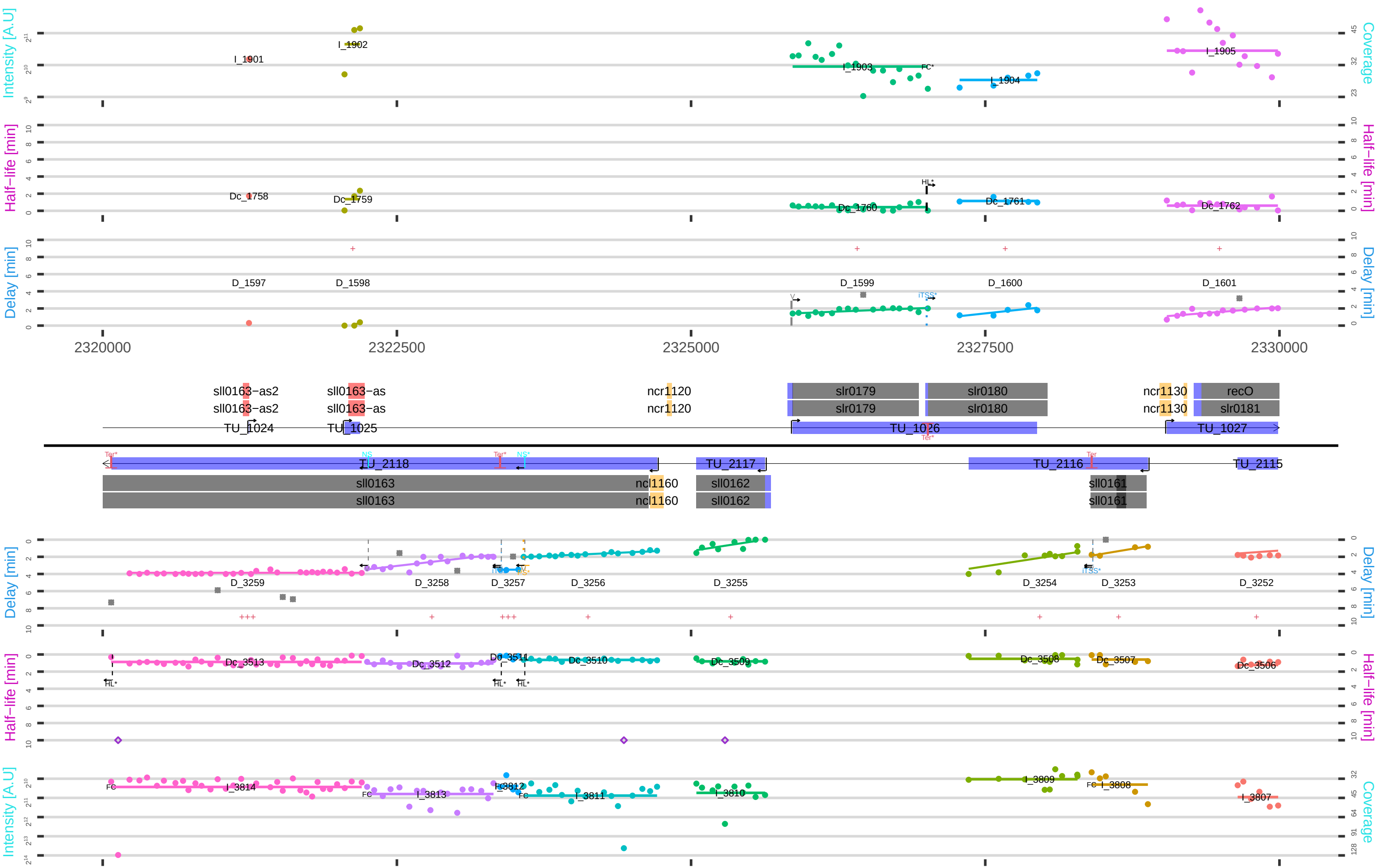

Term: termination (3), NS: new start (2), PS: pausing site (2), iTSS_I: internal starting site (2)

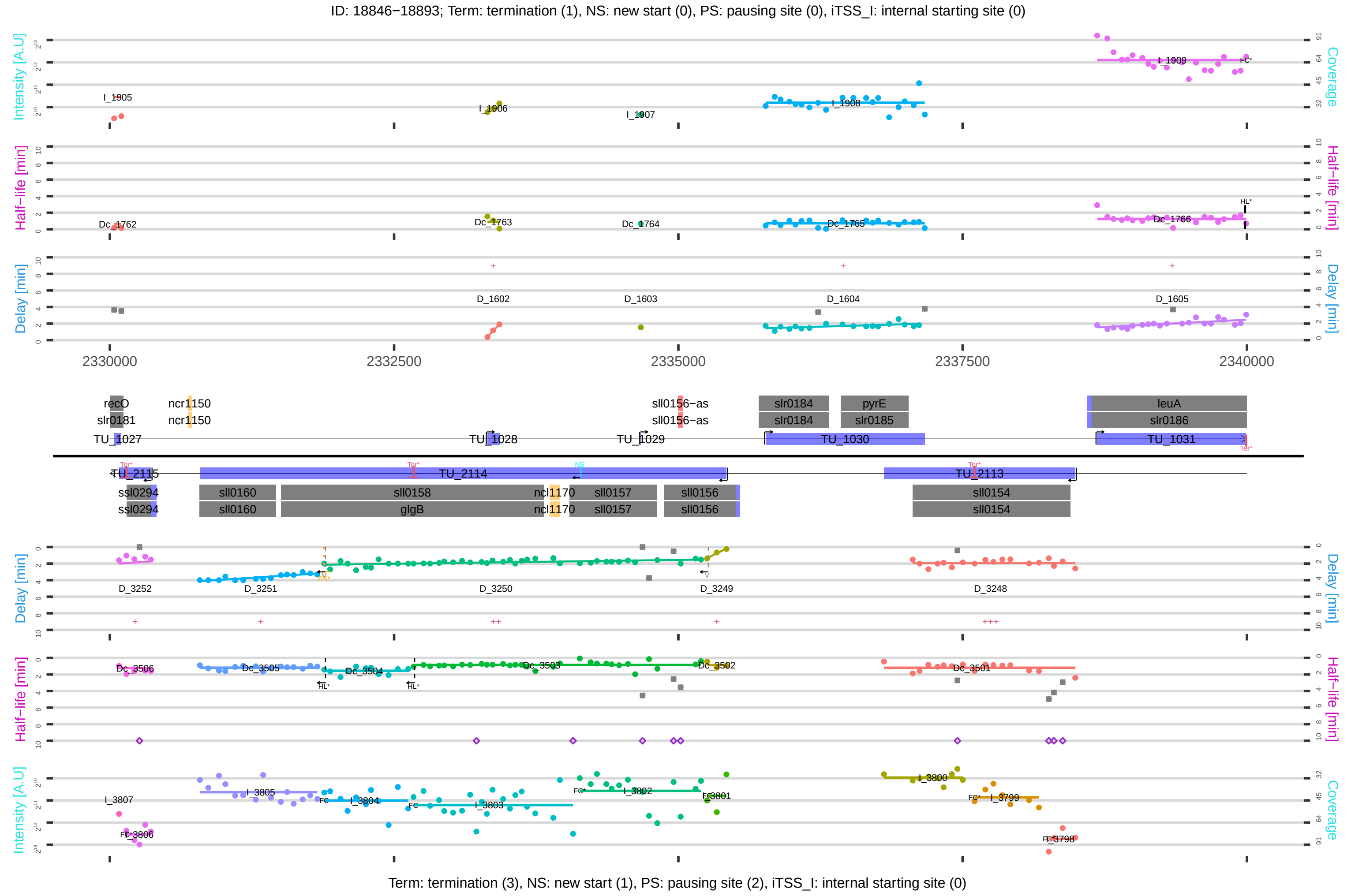

ID: 18846−18893; Term: termination (1), NS: new start (0), PS: pausing site (0), iTSS_I: internal starting site (0)
Intensity [A.U]
Coverage
Half−life [min]
Delay [min]
I_1905
I_1906
I_1907
I_1908
I_1909
Dc_1762
Dc_1763
Dc_1764
Dc_1765
Dc_1766
D_1602
D_1603
D_1604
D_1605
2330000
2332500
2335000
2337500
2340000
recO
slr0181
ncr1150
sll0156−as
slr0184
pyrE
slr0185
leuA
slr0186
TU_1027
TU_1028
TU_1029
TU_1030
TU_1031
TU_2115
TU_2114
TU_2113
ssl0294
sll0160
sll0158
glgB
ncl1170
sll0157
sll0156
sll0154
D_3252
D_3251
D_3250
D_3249
D_3248
Dc_3506
Dc_3505
Dc_3504
Dc_3503
Dc_3502
Dc_3501
I_3807
I_3806
I_3805
I_3804
I_3803
I_3802
I_3801
I_3800
I_3799
I_3798
Term: termination (3), NS: new start (1), PS: pausing site (2), iTSS_I: internal starting site (0)

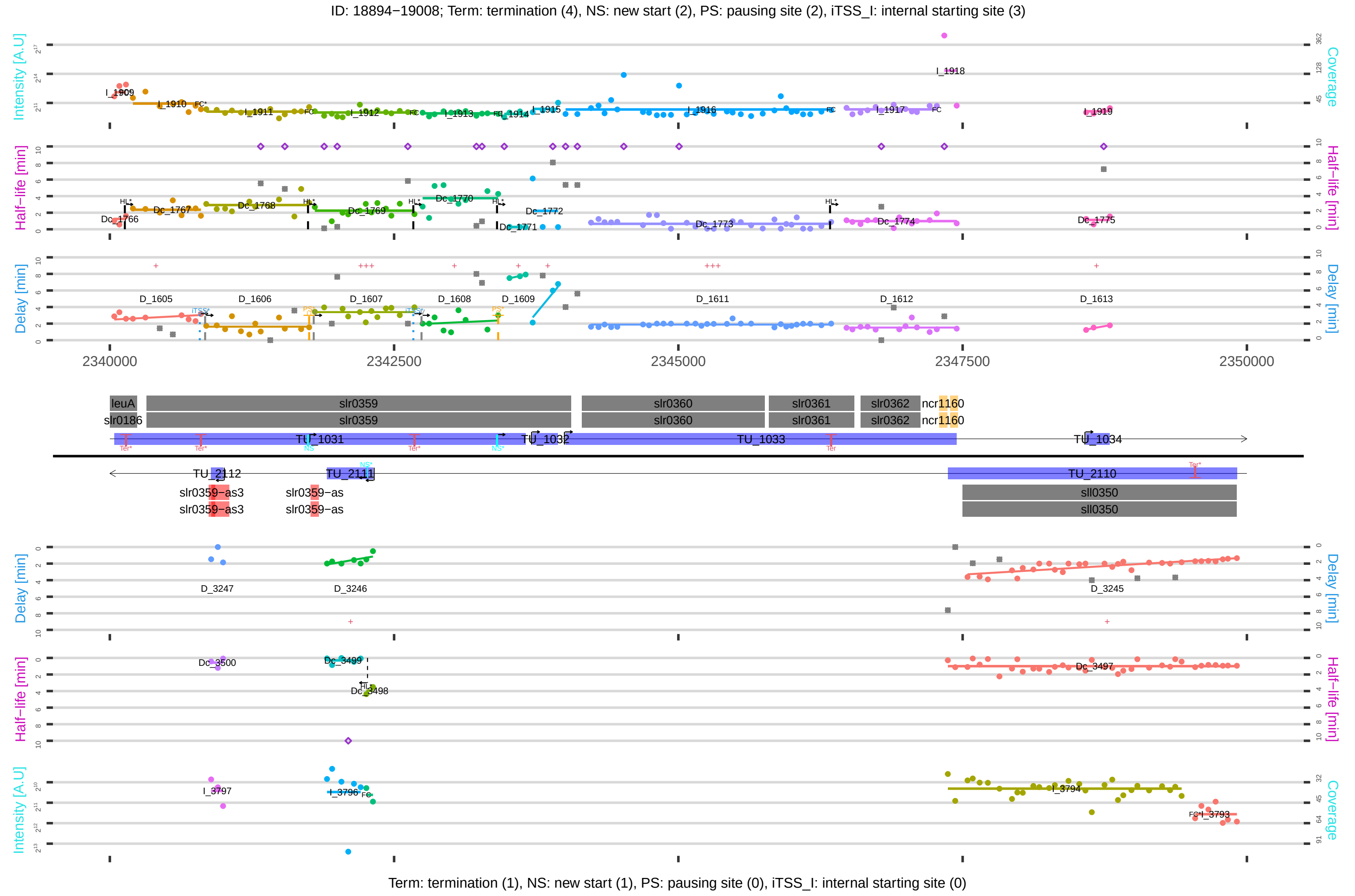
ID: 18894−19008; Term: termination (4), NS: new start (2), PS: pausing site (2), iTSS_I: internal starting site (3)
Intensity [A.U]
Coverage
Half−life [min]
Delay [min]
2340000
2342500
2345000
2347500
2350000
leuA
slr0186
slr0359
slr0360
slr0361
slr0362
ncr1160
TU_1031
TU_1032
TU_1033
TU_1034
TU_2112
TU_2111
TU_2110
slr0359−as3
slr0359−as
sll0350
Term: termination (1), NS: new start (1), PS: pausing site (0), iTSS_I: internal starting site (0)

ID: 19009−19124; Term: termination (1), NS: new start (3), PS: pausing site (2), iTSS_I: internal starting site (0)

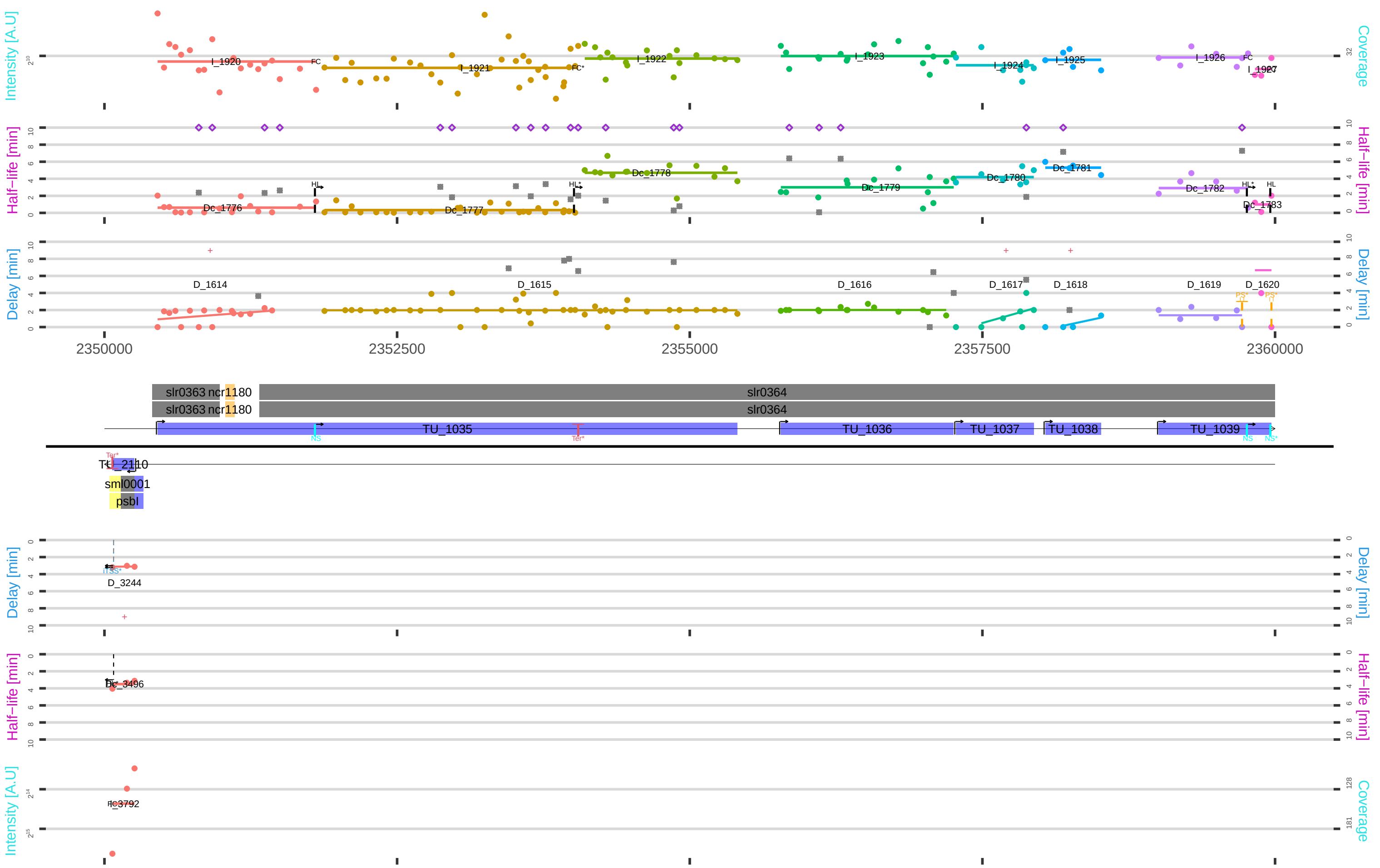

Term: termination (1), NS: new start (0), PS: pausing site (0), iTSS_I: internal starting site (1)

ID: 19125−19278; Term: termination (3), NS: new start (3), PS: pausing site (2), iTSS_I: internal starting site (2)

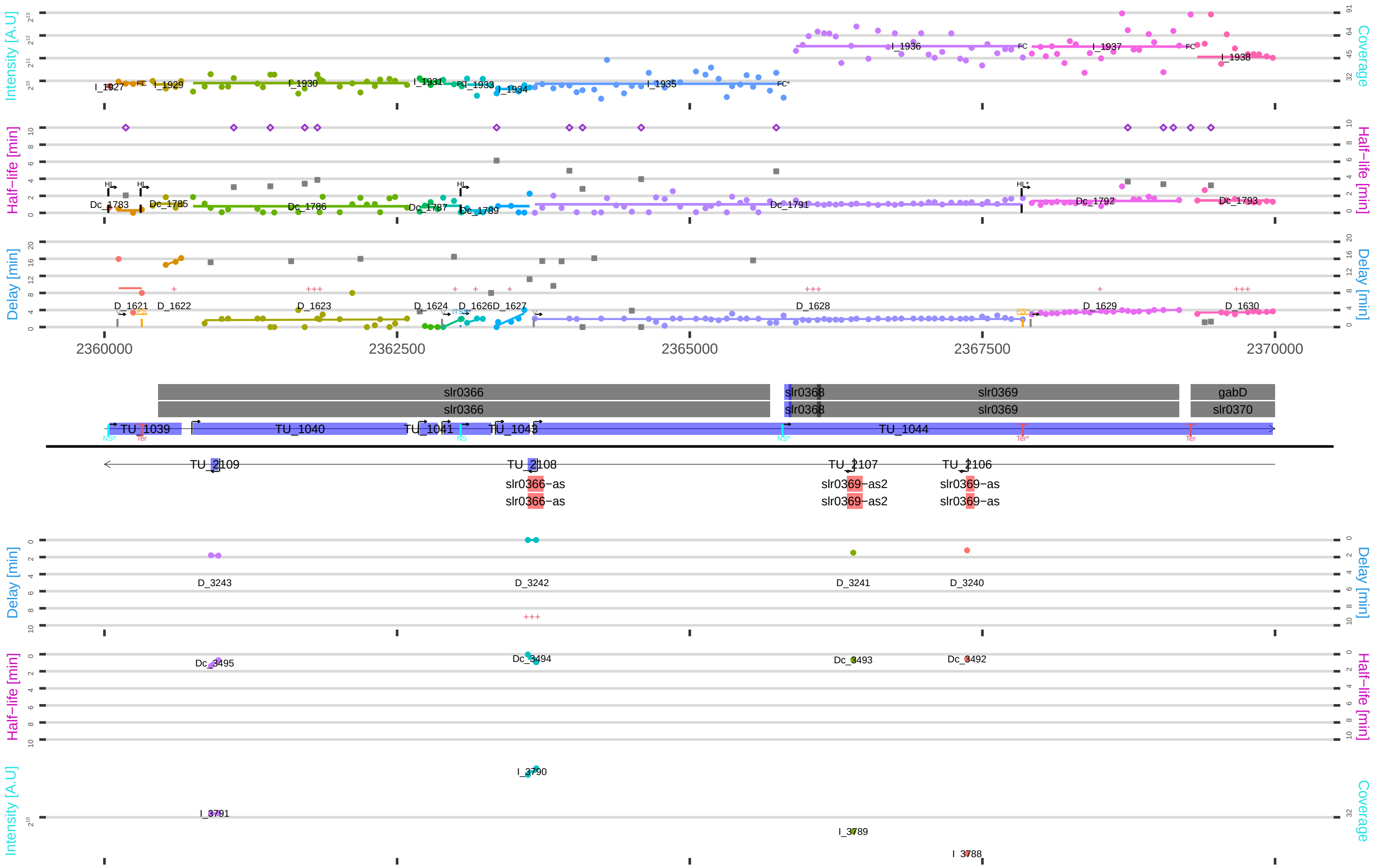

Term: termination (0), NS: new start (0), PS: pausing site (0), iTSS_I: internal starting site (0)

ID: 19279−19367; Term: termination (2), NS: new start (2), PS: pausing site (1), iTSS_I: internal starting site (1)

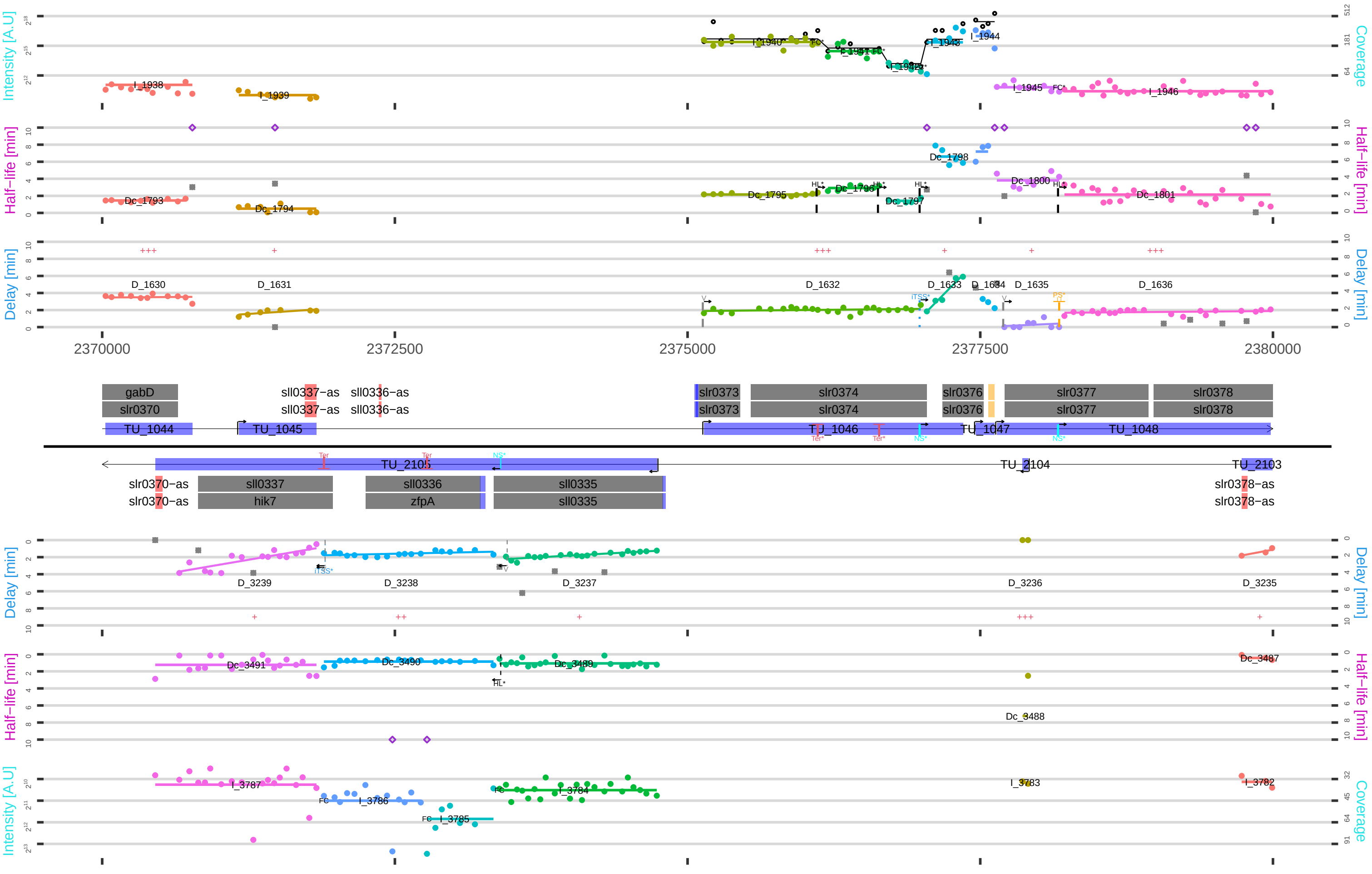

Term: termination (2), NS: new start (1), PS: pausing site (0), iTSS_I: internal starting site (2)

ID: 19368−19460; Term: termination (1), NS: new start (1), PS: pausing site (0), iTSS_I: internal starting site (2)

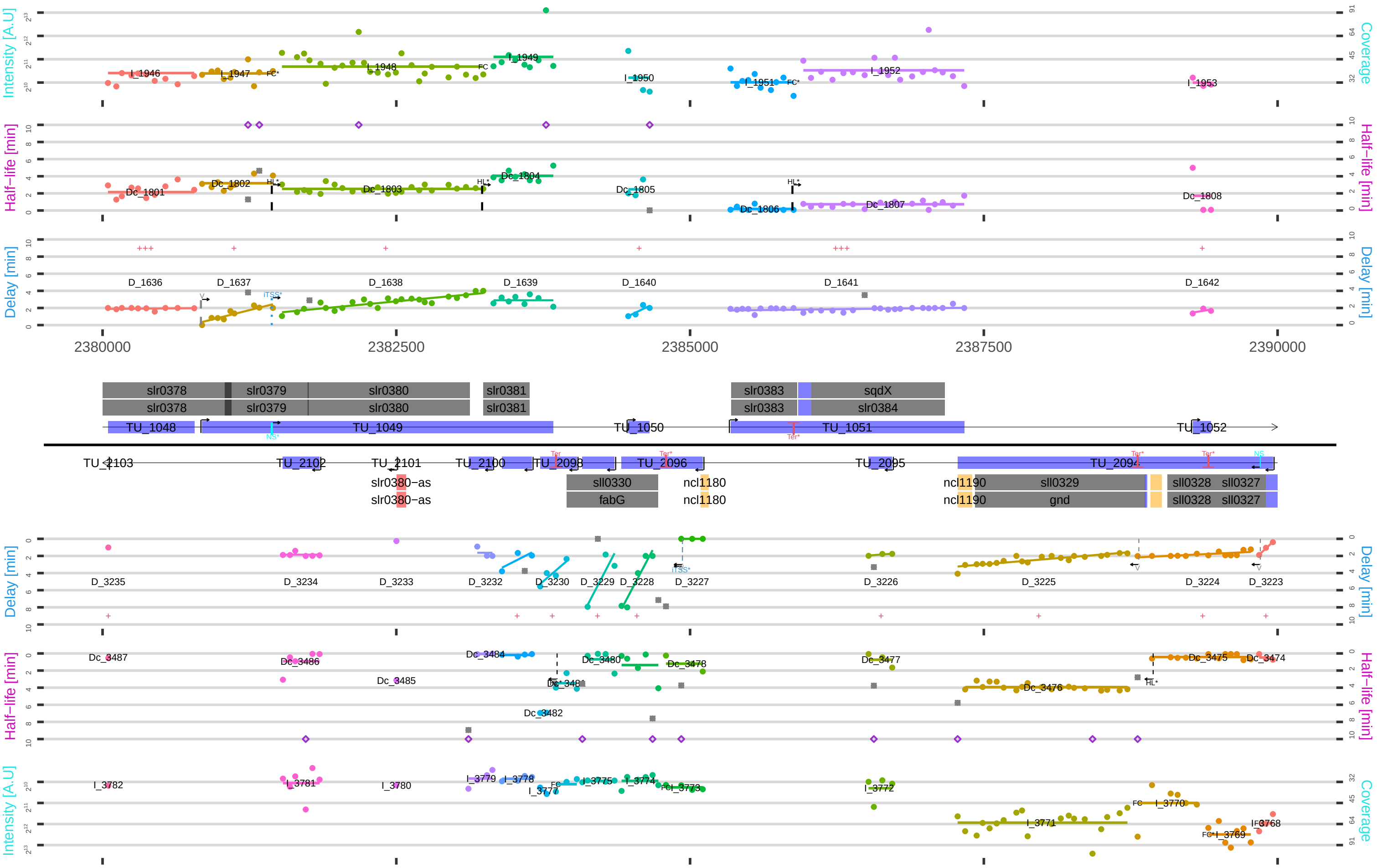

Term: termination (4), NS: new start (1), PS: pausing site (0), iTSS_I: internal starting site (3)

ID: 19462−19567; Term: termination (2), NS: new start (2), PS: pausing site (2), iTSS_I: internal starting site (3)

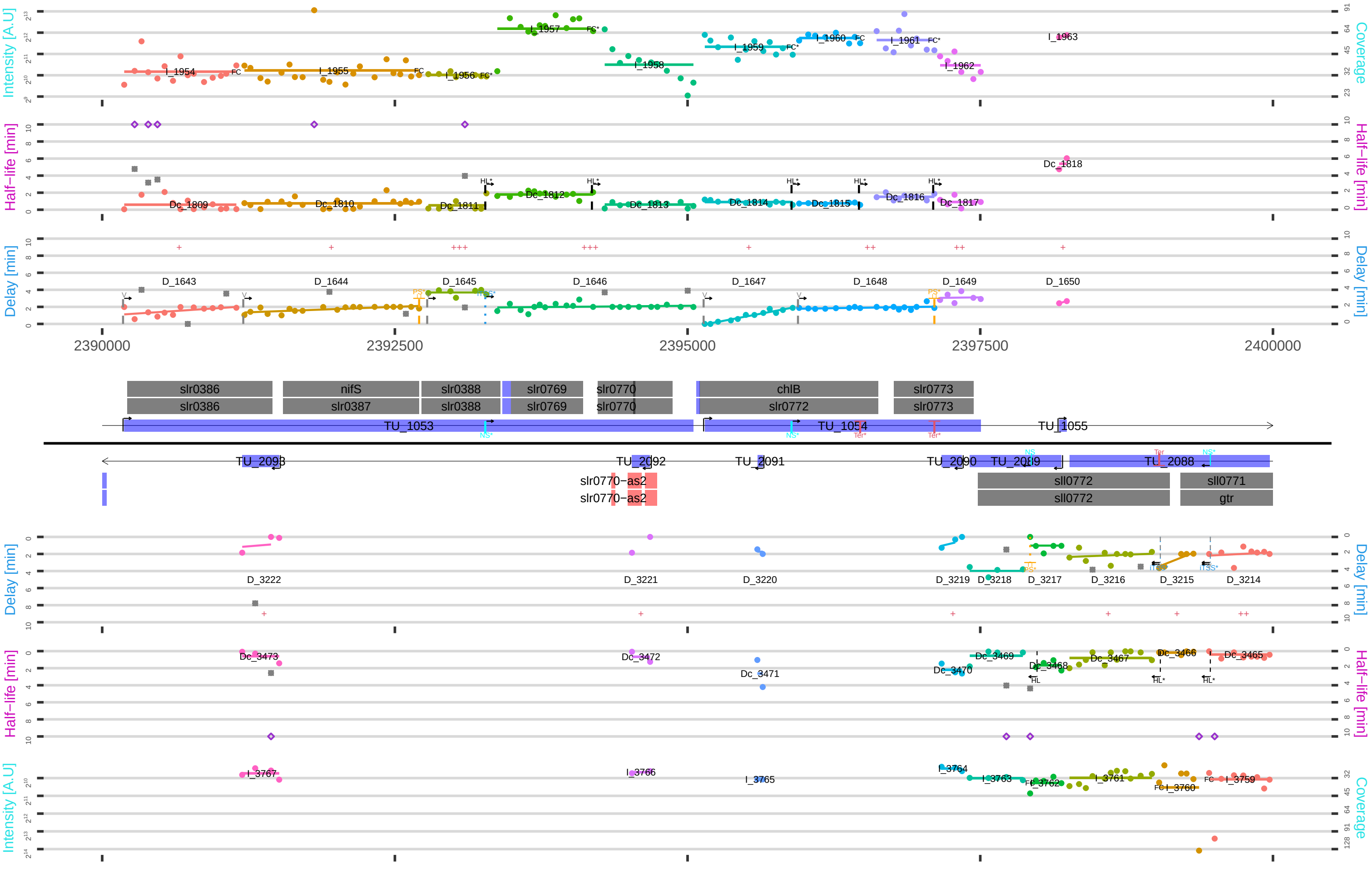

Term: termination (1), NS: new start (2), PS: pausing site (1), iTSS_I: internal starting site (2)

ID: 19568−19646; Term: termination (3), NS: new start (0), PS: pausing site (1), iTSS_I: internal starting site (1)

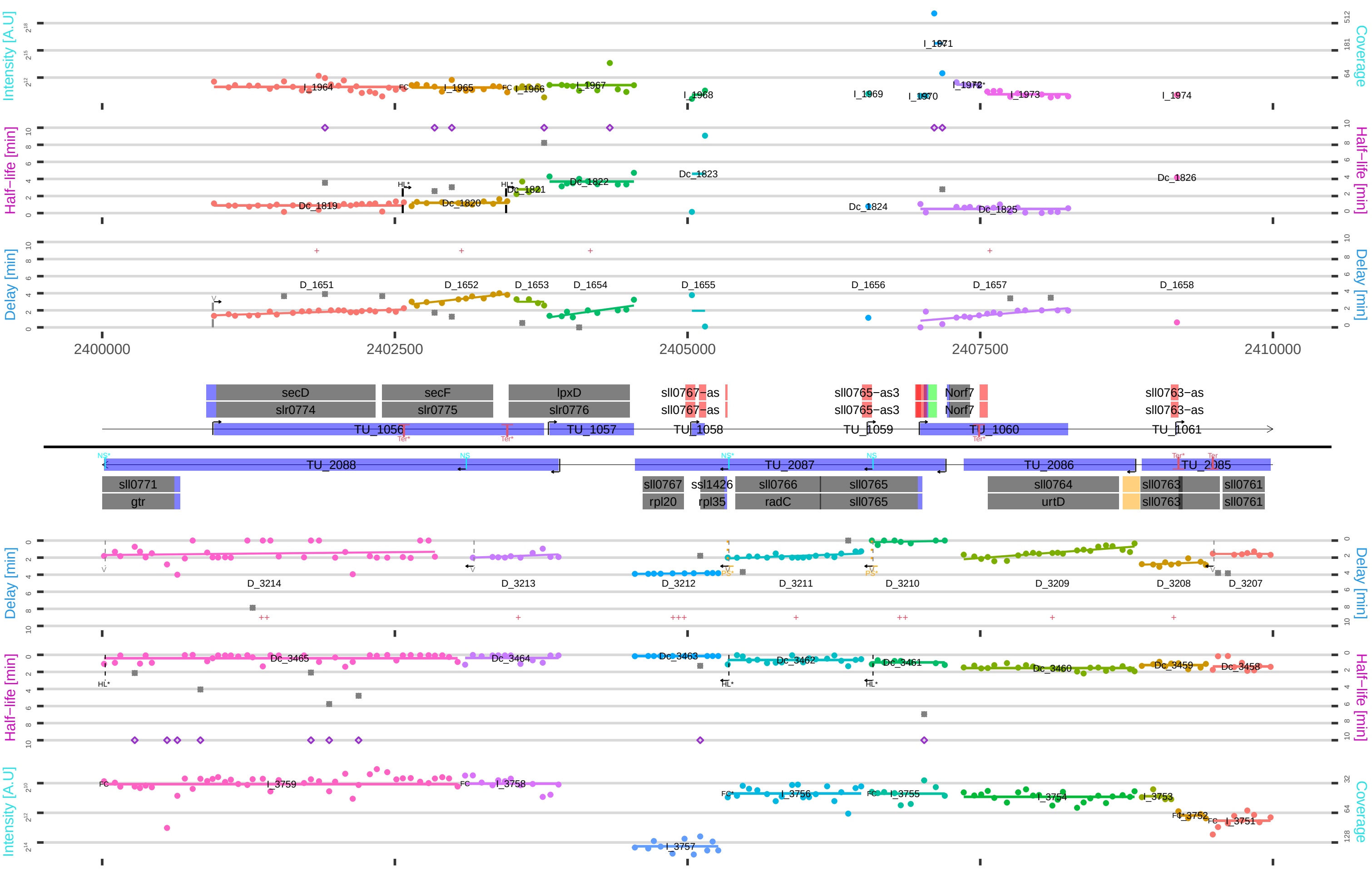

Term: termination (2), NS: new start (4), PS: pausing site (3), iTSS_I: internal starting site (0)

ID: 19647−19689; Term: termination (2), NS: new start (2), PS: pausing site (0), iTSS_I: internal starting site (0)

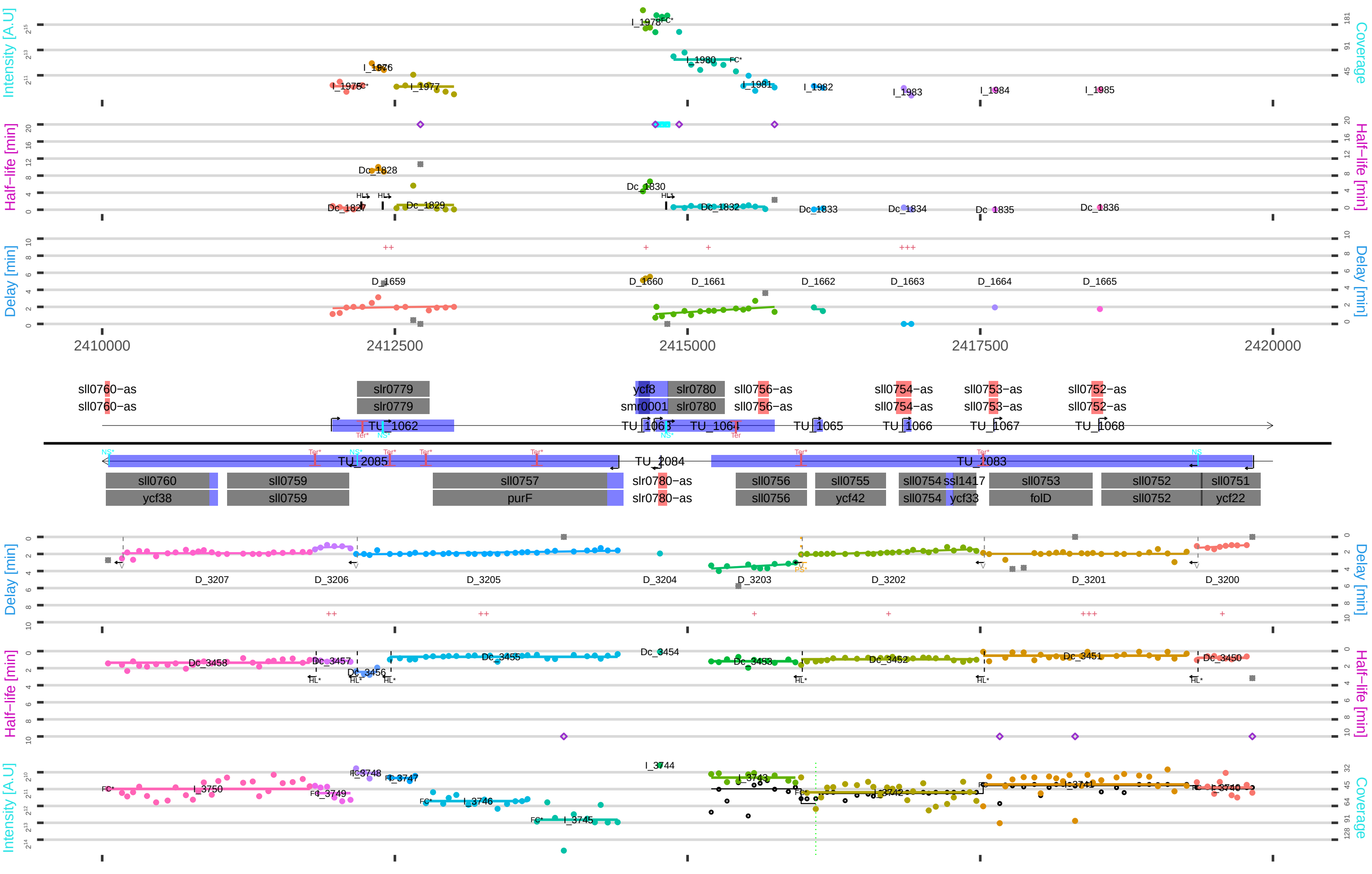

Term: termination (6), NS: new start (3), PS: pausing site (3), iTSS_I: internal starting site (2)

ID: 19690−19794; Term: termination (3), NS: new start (4), PS: pausing site (2), iTSS_I: internal starting site (3)

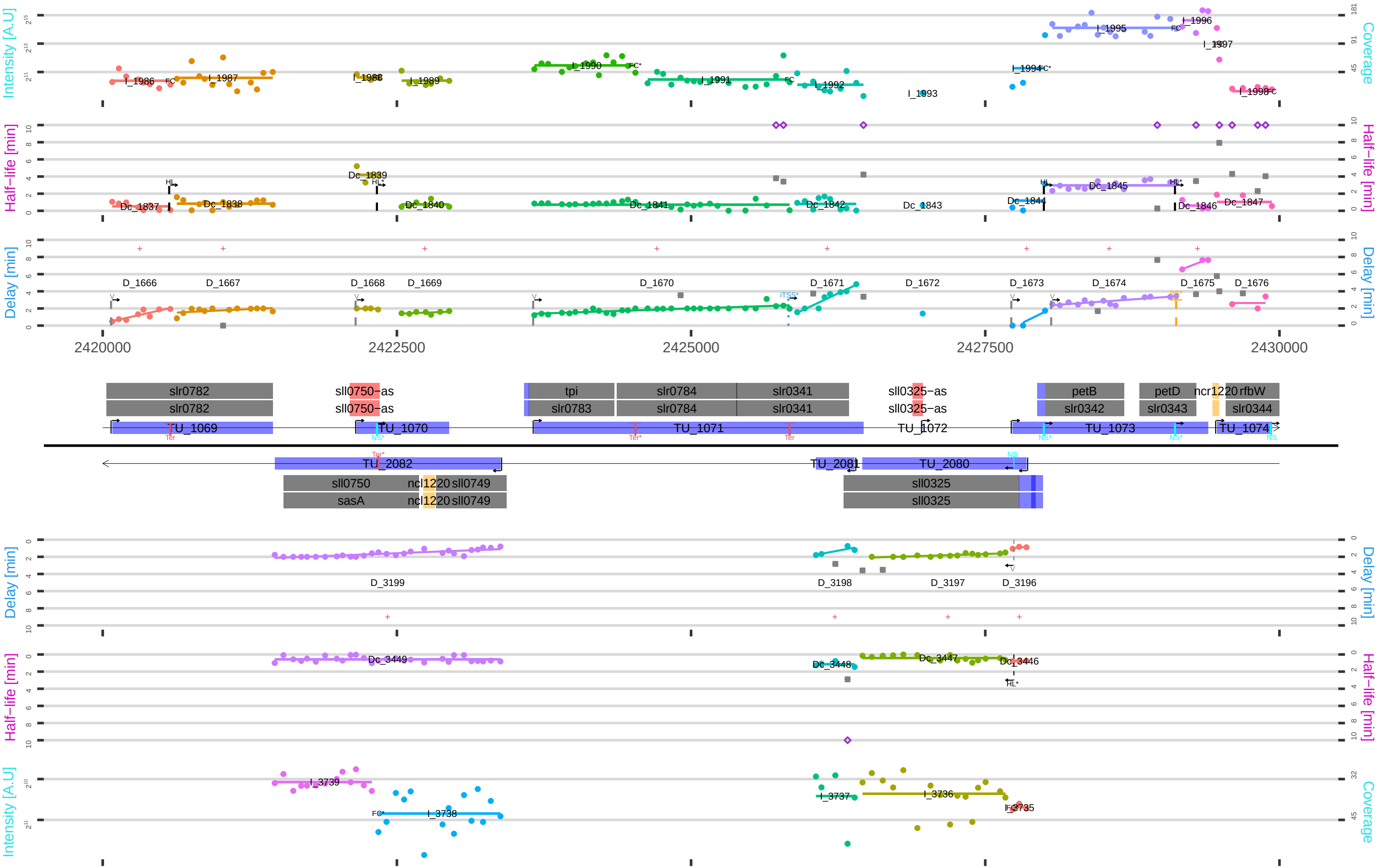

Term: termination (1), NS: new start (1), PS: pausing site (1), iTSS_I: internal starting site (0)

ID: 19795−19856; Term: termination (1), NS: new start (1), PS: pausing site (0), iTSS_I: internal starting site (0)

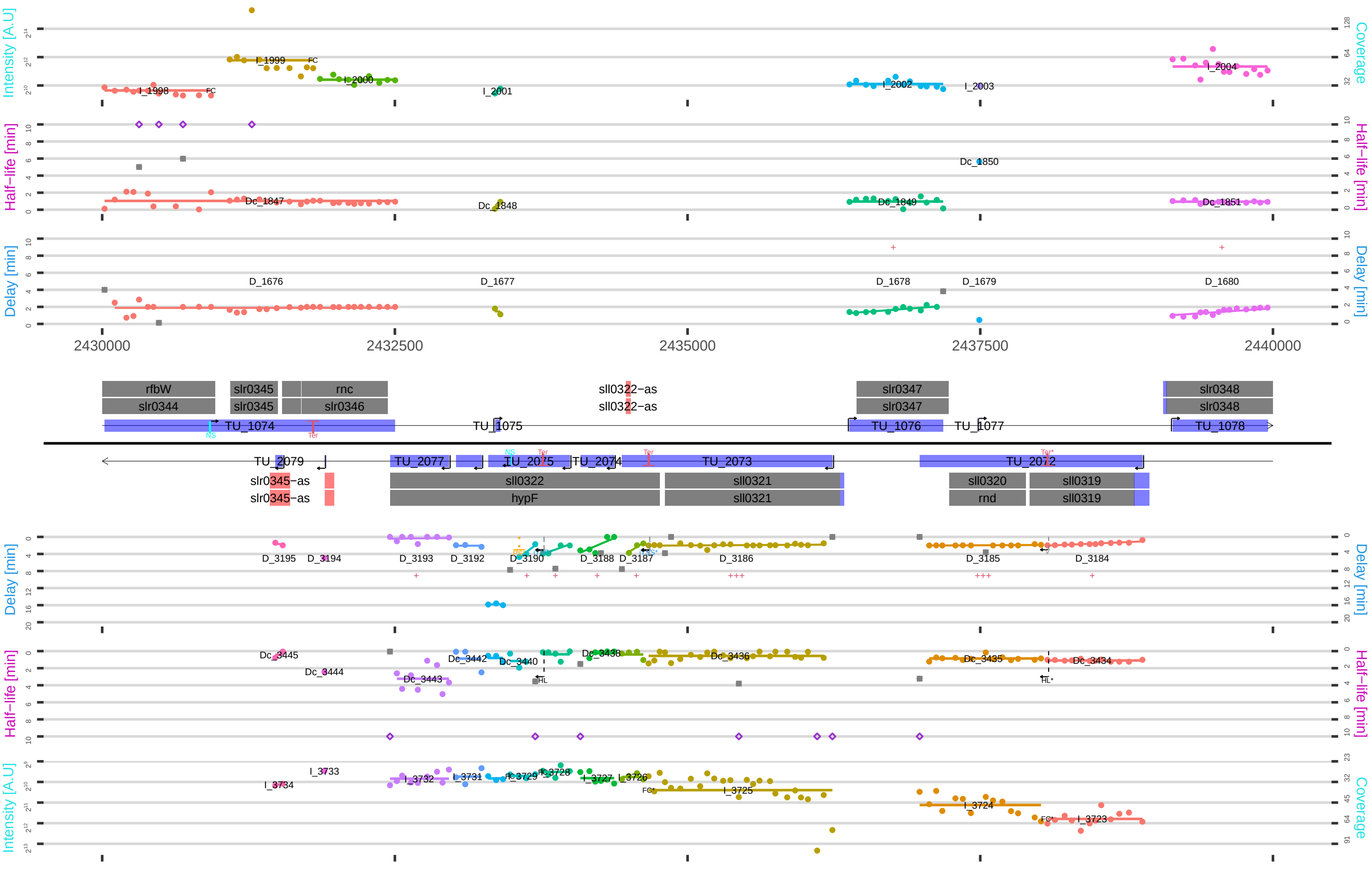

Term: termination (3), NS: new start (1), PS: pausing site (1), iTSS_I: internal starting site (3)

ID: 19857−19910; Term: termination (1), NS: new start (1), PS: pausing site (1), iTSS_I: internal starting site (1)

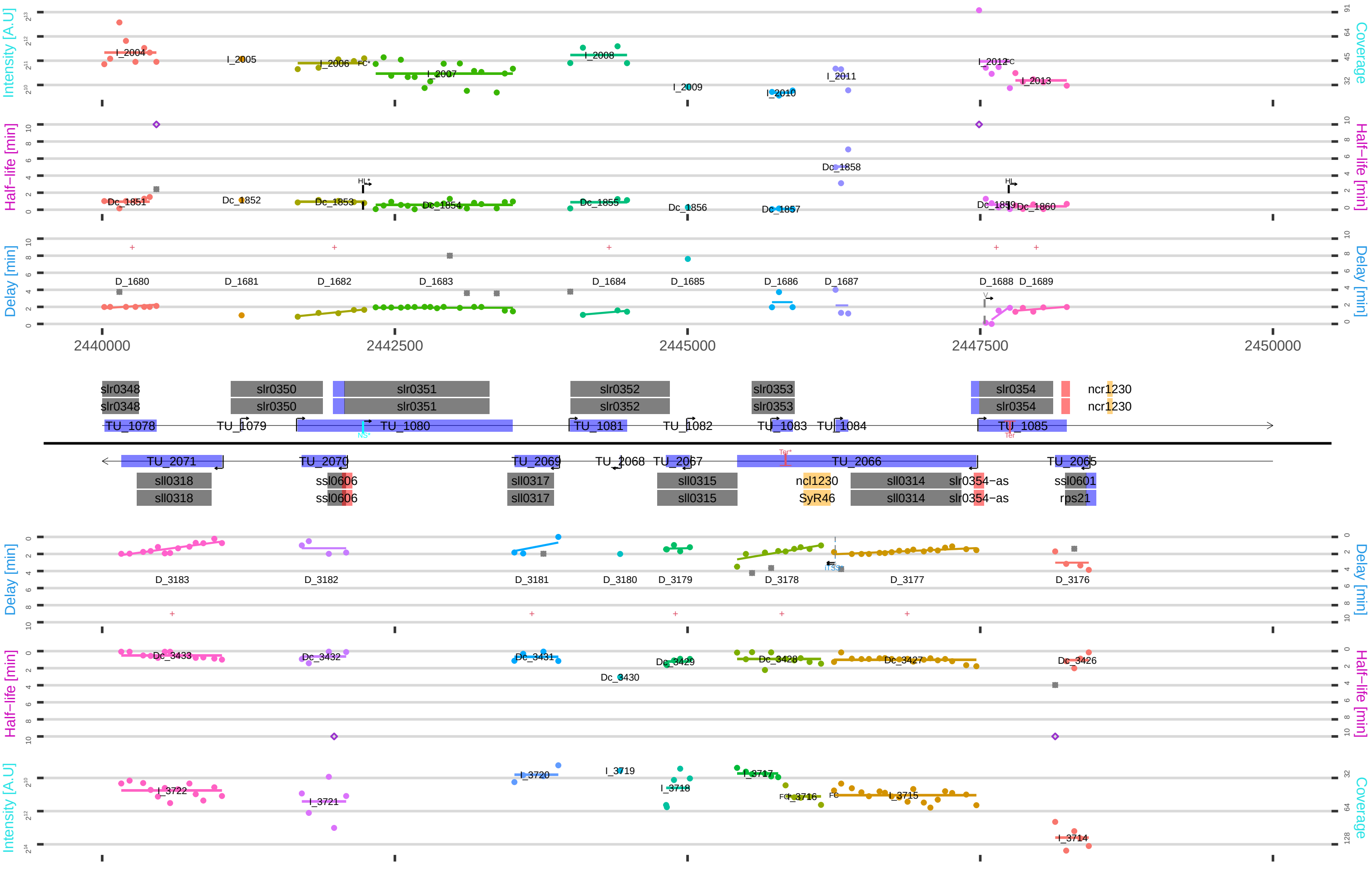

Term: termination (1), NS: new start (0), PS: pausing site (0), iTSS_I: internal starting site (1)

ID: 19912−19983; Term: termination (1), NS: new start (2), PS: pausing site (1), iTSS_I: internal starting site (2)

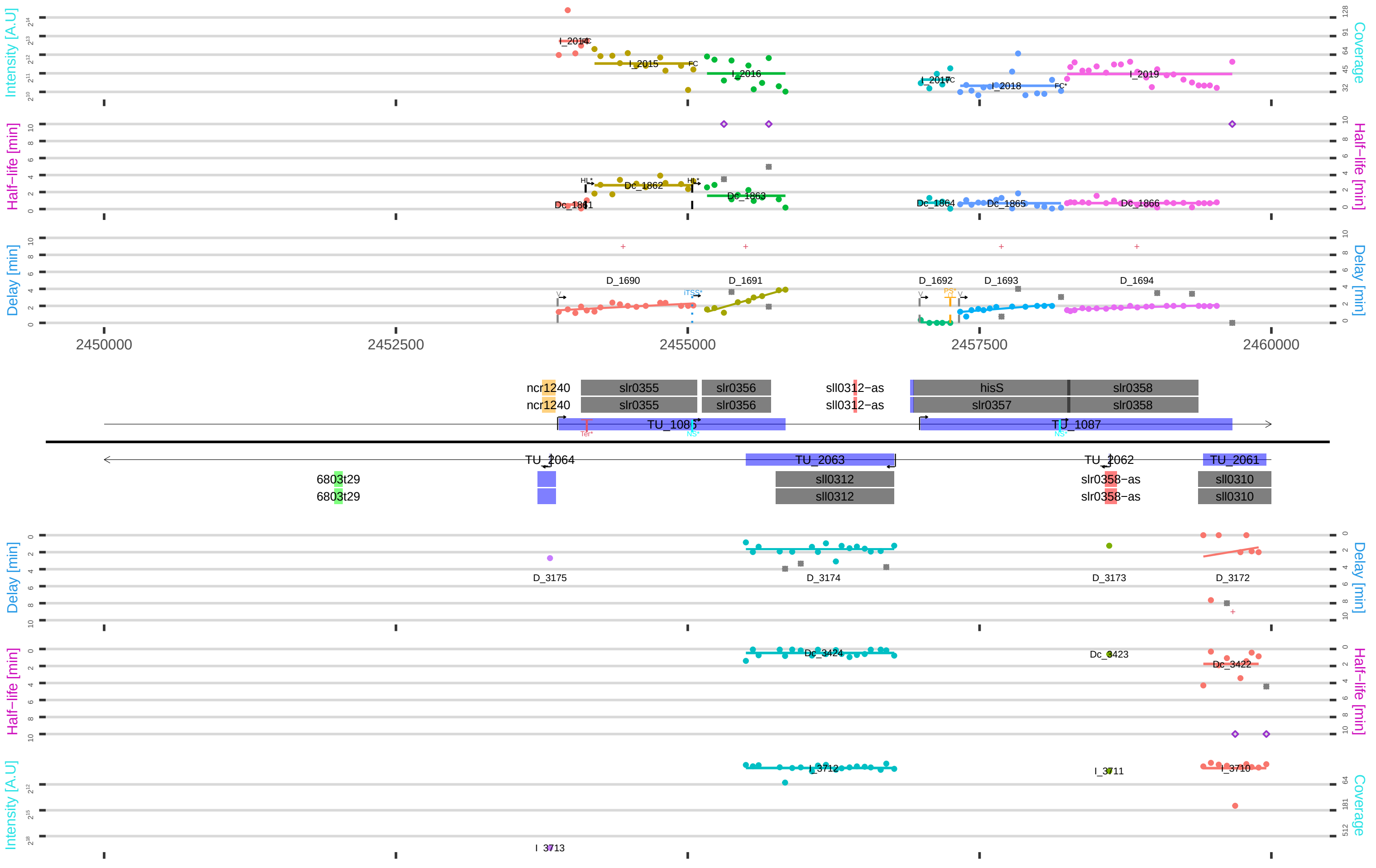

Term: termination (0), NS: new start (0), PS: pausing site (0), iTSS_I: internal starting site (0)

ID: 19984−20003; Term: termination (0), NS: new start (0), PS: pausing site (0), iTSS_I: internal starting site (0)

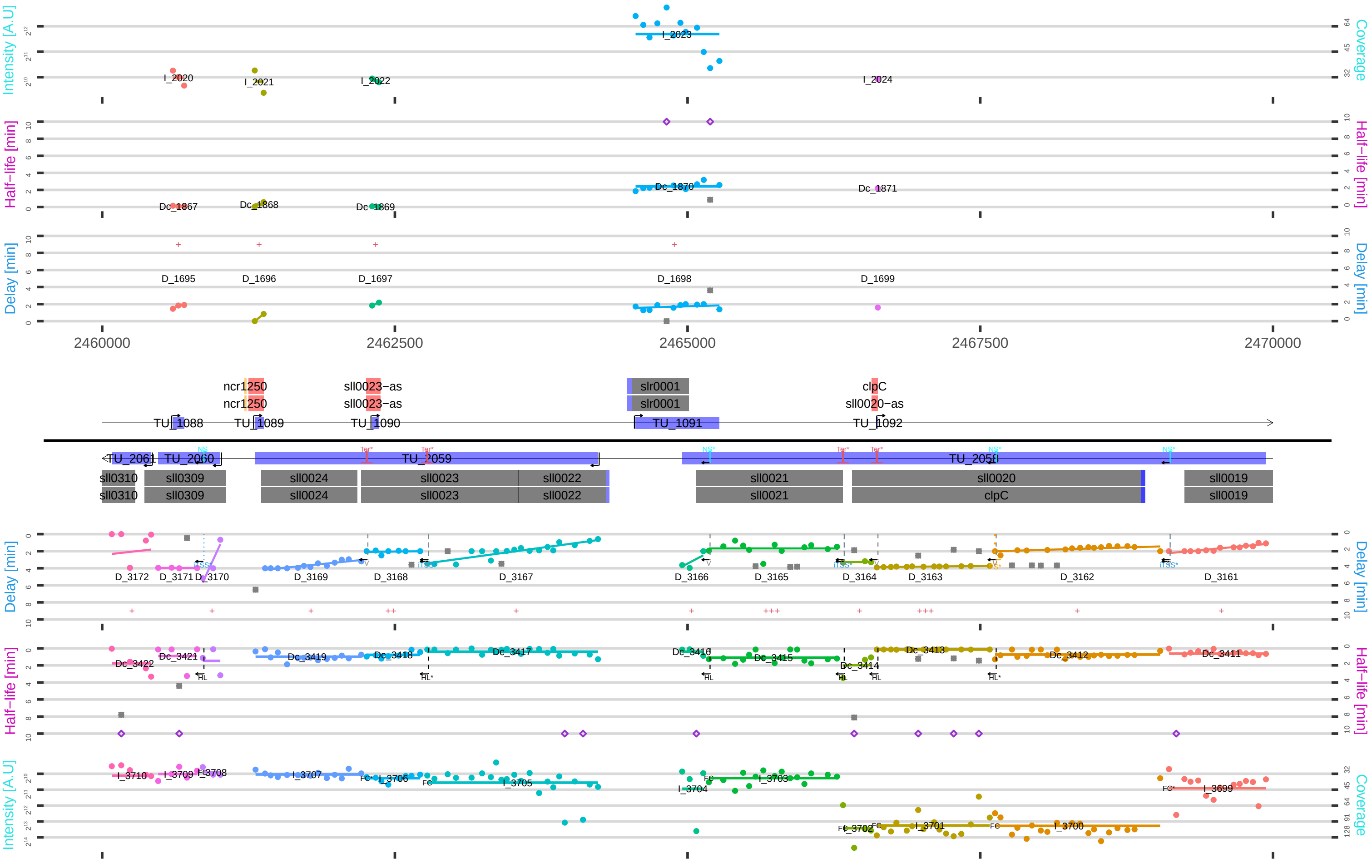

Term: termination (4), NS: new start (4), PS: pausing site (3), iTSS_I: internal starting site (5)

ID: 20004−20081; Term: termination (1), NS: new start (2), PS: pausing site (1), iTSS_I: internal starting site (1)

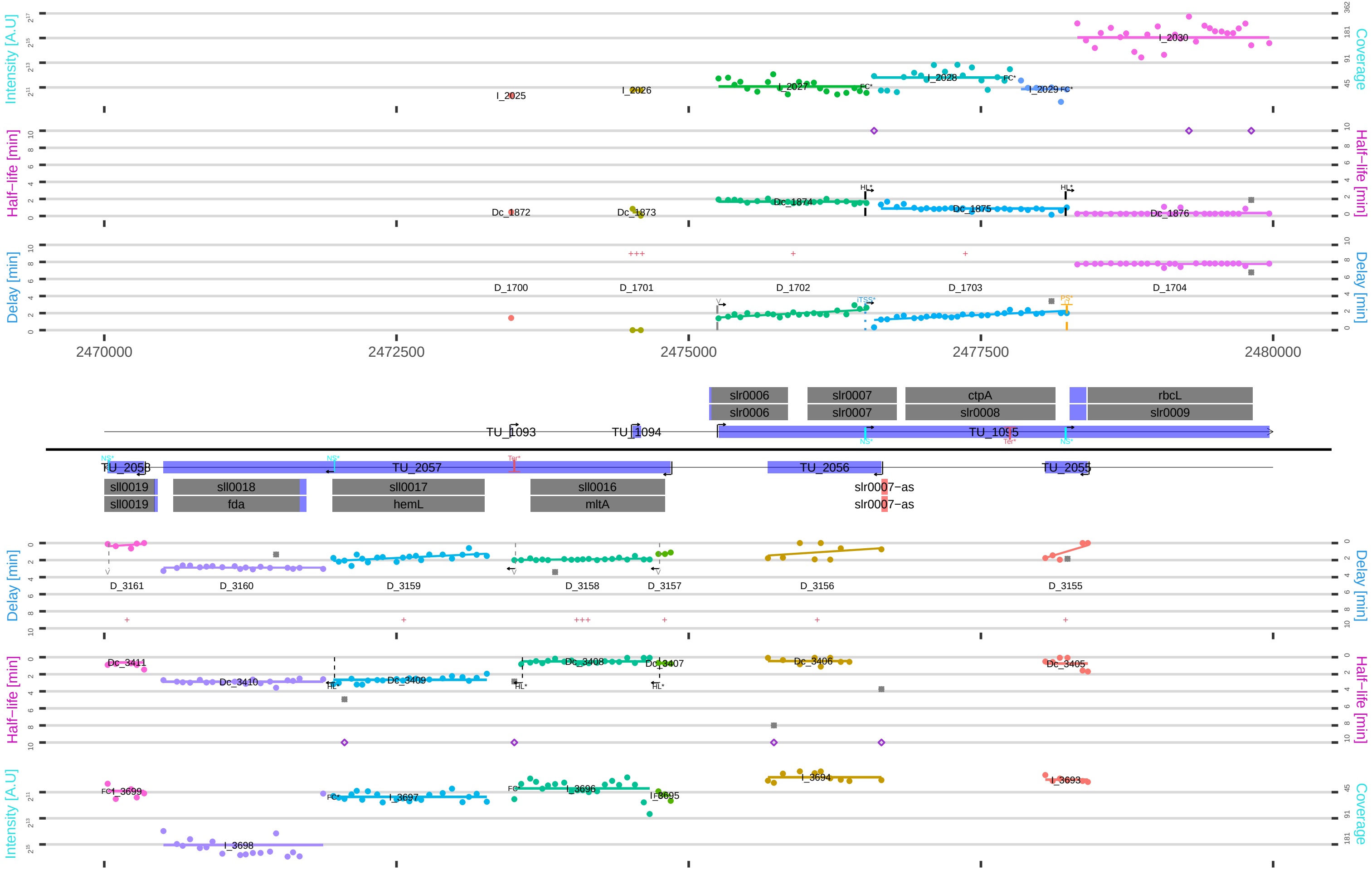

Term: termination (1), NS: new start (2), PS: pausing site (2), iTSS_I: internal starting site (1)

ID: 20082−20193; Term: termination (4), NS: new start (2), PS: pausing site (1), iTSS_I: internal starting site (1)

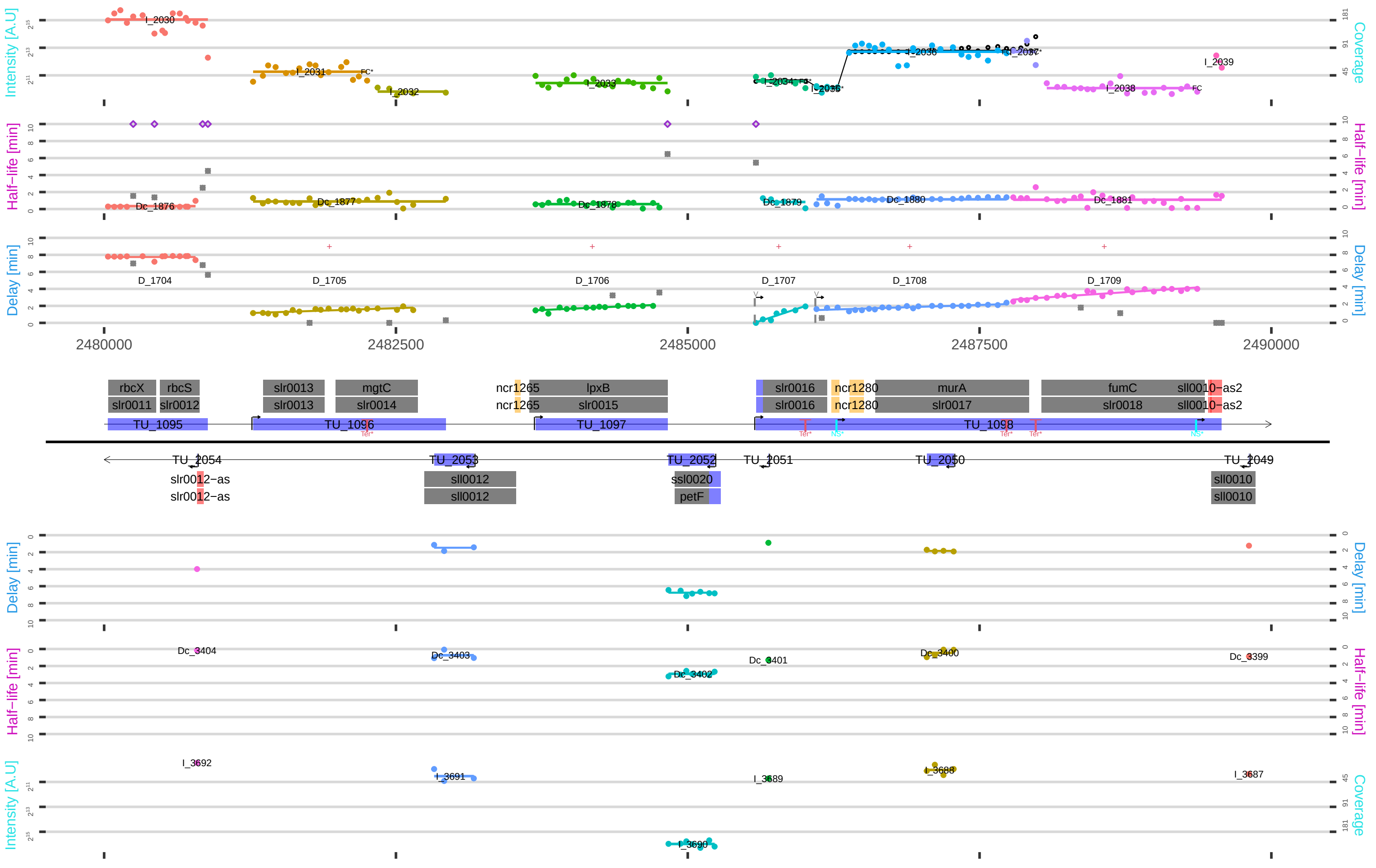

Term: termination (0), NS: new start (0), PS: pausing site (0), iTSS_I: internal starting site (0)

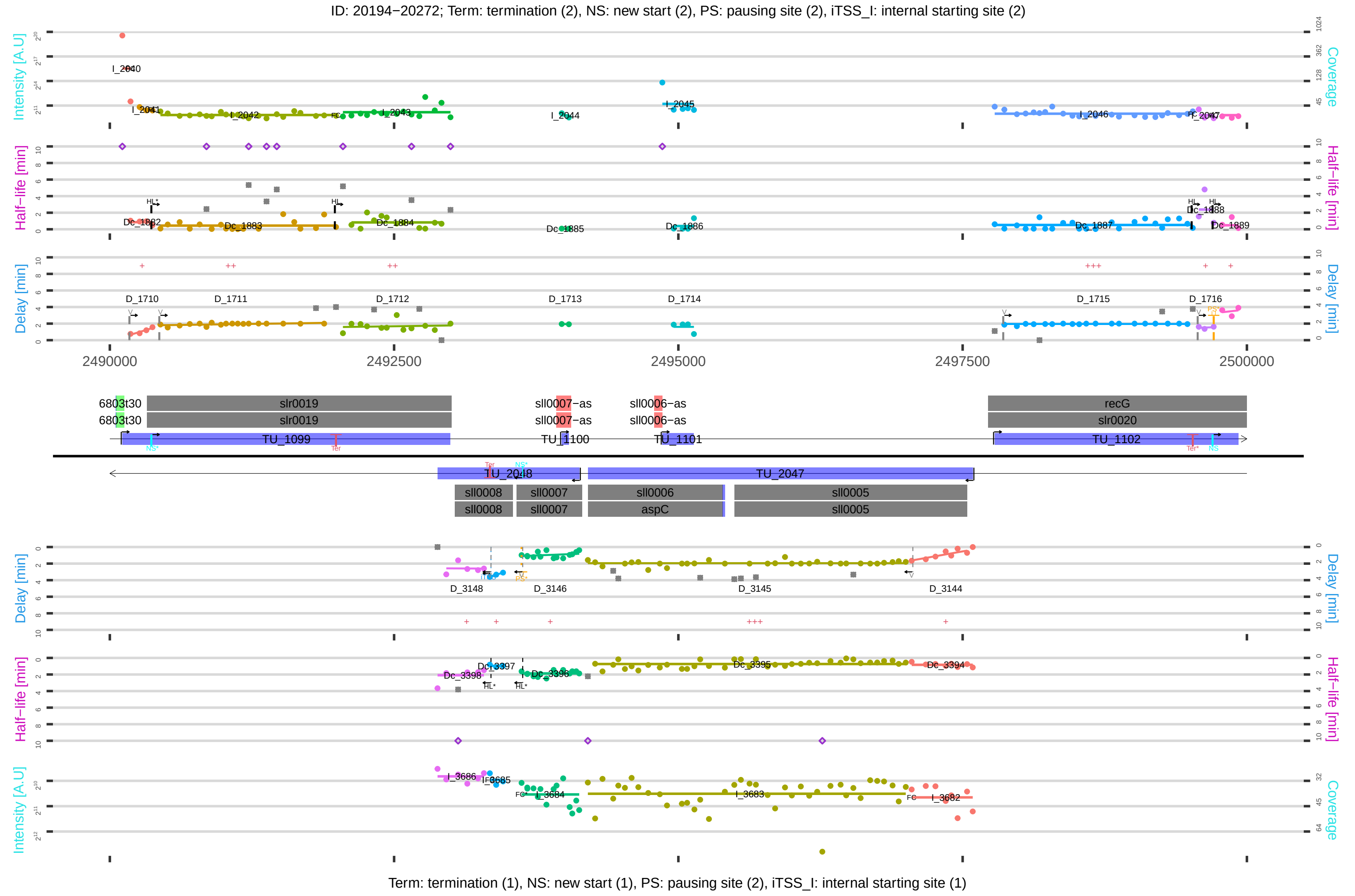
ID: 20194−20272; Term: termination (2), NS: new start (2), PS: pausing site (2), iTSS_I: internal starting site (2)
Intensity [A.U]
Coverage
Half−life [min]
Delay [min]
2490000
2492500
2495000
2497500
2500000
6803t30
slr0019
sll0007−as
sll0006−as
recG
slr0020
TU_1099
TU_1100
TU_1101
TU_1102
TU_2048
TU_2047
sll0008
sll0007
sll0006
aspC
sll0005
Term: termination (1), NS: new start (1), PS: pausing site (2), iTSS_I: internal starting site (1)

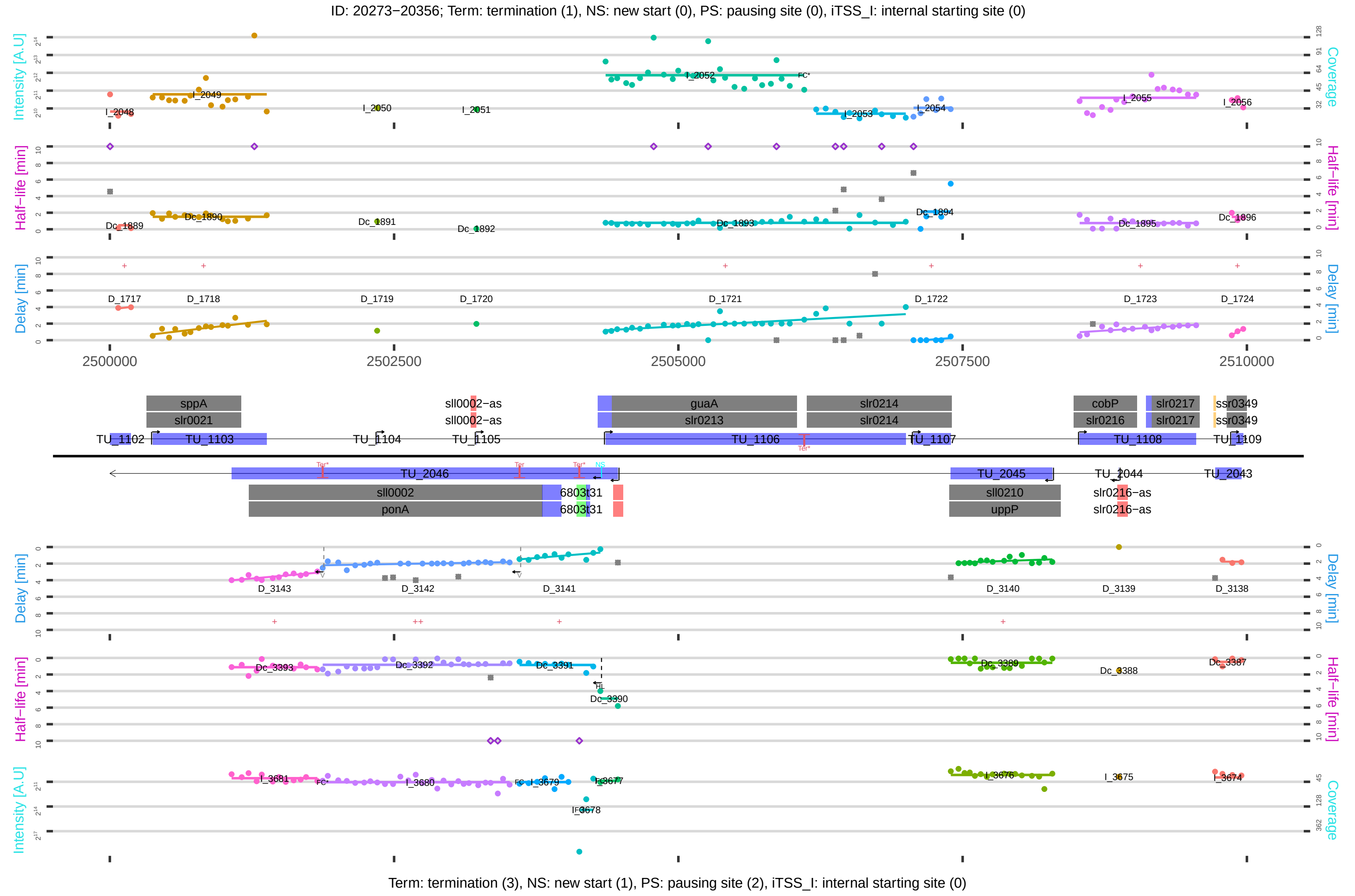

ID: 20273−20356; Term: termination (1), NS: new start (0), PS: pausing site (0), iTSS_I: internal starting site (0)
Intensity [A.U]
Coverage
Half−life [min]
Delay [min]
2500000
2502500
2505000
2507500
2510000
sppA
slr0021
sll0002−as
guaA
slr0213
slr0214
cobP
slr0216
slr0217
ssr0349
TU_1102
TU_1103
TU_1104
TU_1105
TU_1106
TU_1107
TU_1108
TU_1109
TU_2046
TU_2045
TU_2044
TU_2043
sll0002
ponA
6803t31
sll0210
uppP
slr0216−as
Term: termination (3), NS: new start (1), PS: pausing site (2), iTSS_I: internal starting site (0)

ID: 20357−20437; Term: termination (2), NS: new start (2), PS: pausing site (2), iTSS_I: internal starting site (1)

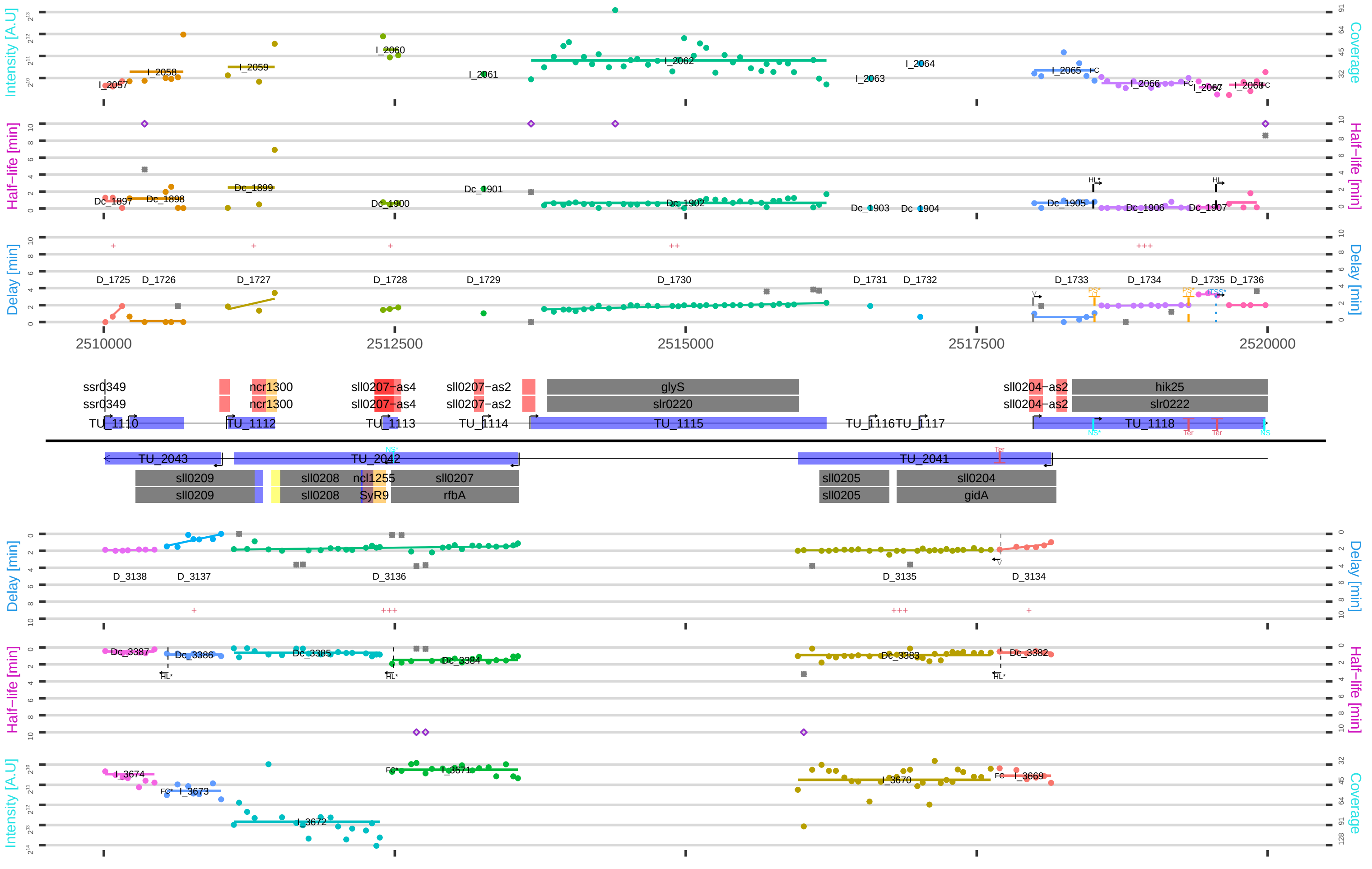

Term: termination (1), NS: new start (1), PS: pausing site (2), iTSS_I: internal starting site (0)

ID: 20438−20515; Term: termination (2), NS: new start (1), PS: pausing site (1), iTSS_I: internal starting site (0)

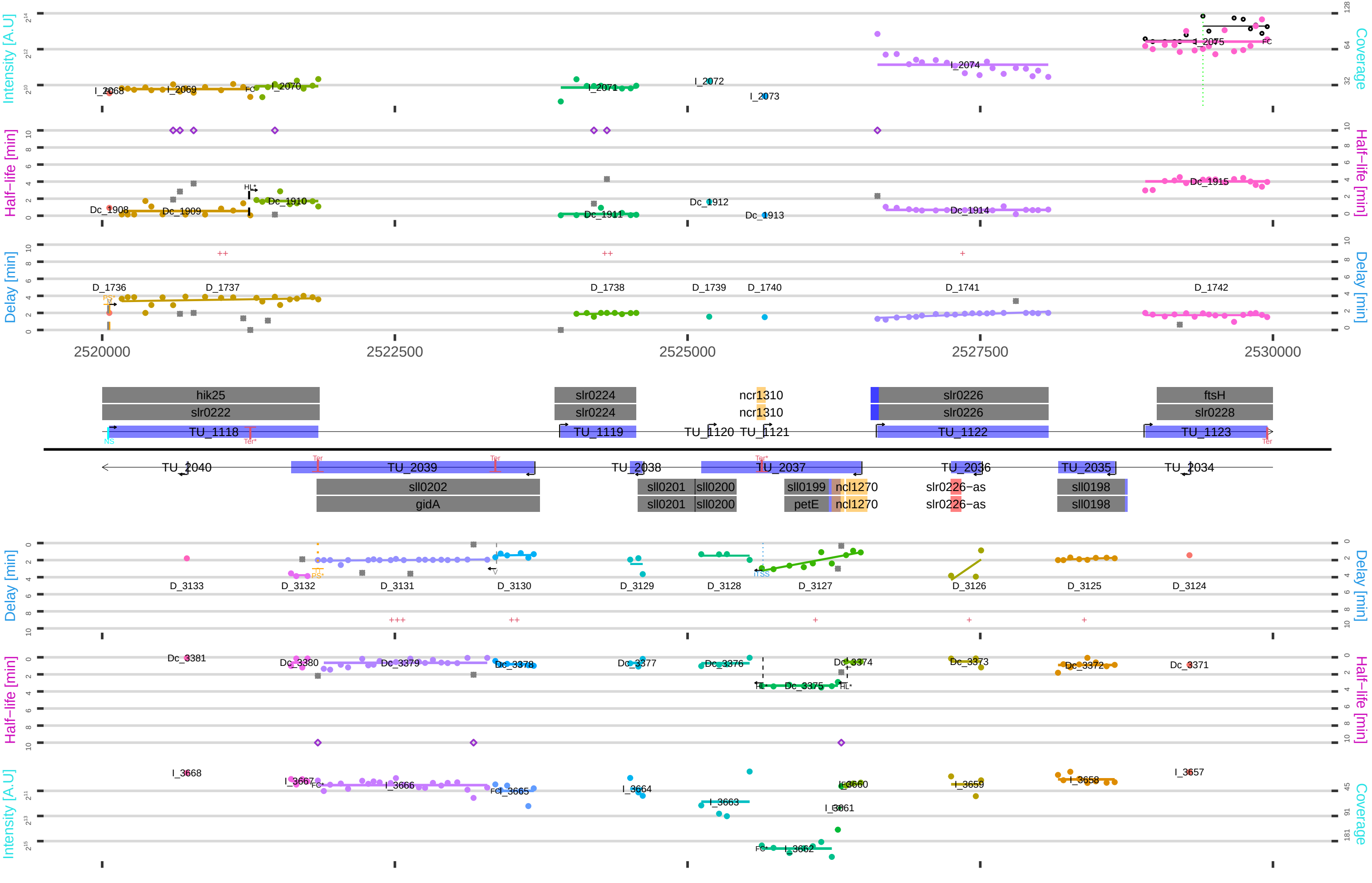

Term: termination (3), NS: new start (0), PS: pausing site (2), iTSS_I: internal starting site (1)

ID: 20516−20575; Term: termination (1), NS: new start (0), PS: pausing site (1), iTSS_I: internal starting site (0)

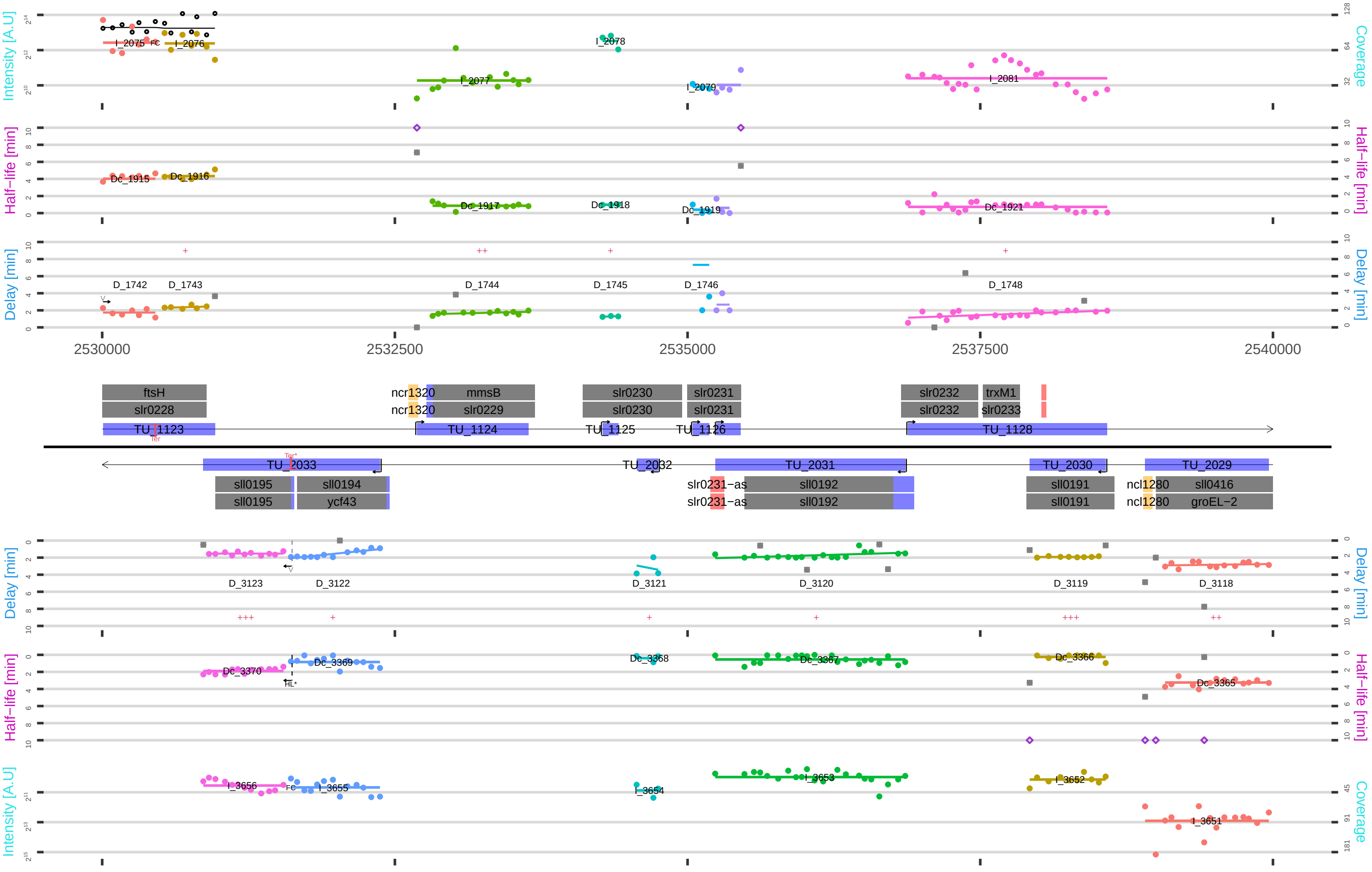

Term: termination (1), NS: new start (0), PS: pausing site (0), iTSS_I: internal starting site (1)

ID: 20576−20611; Term: termination (1), NS: new start (0), PS: pausing site (0), iTSS_I: internal starting site (0)

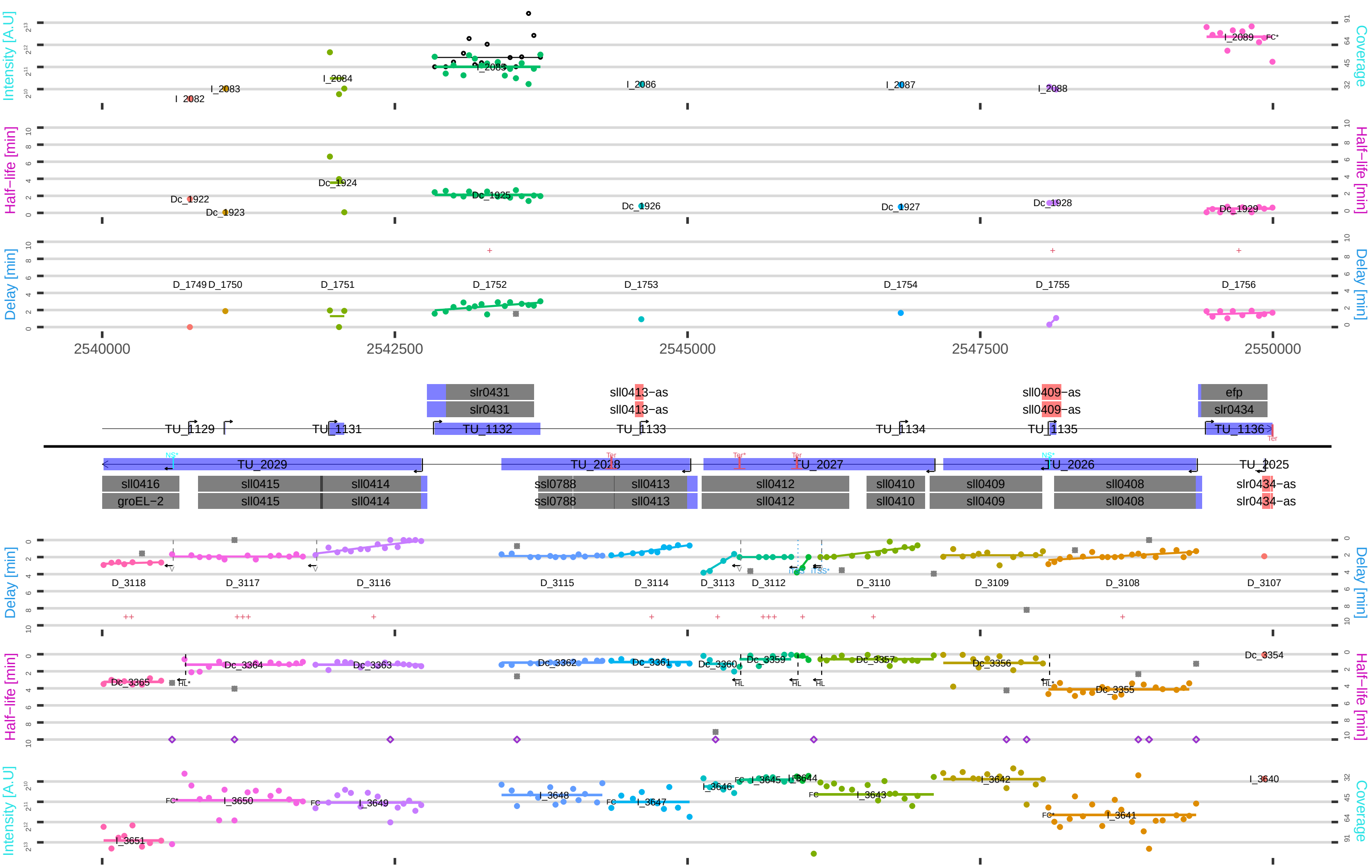

Term: termination (3), NS: new start (2), PS: pausing site (3), iTSS_I: internal starting site (4)

ID: 20612−20685; Term: termination (1), NS: new start (0), PS: pausing site (0), iTSS_I: internal starting site (0)

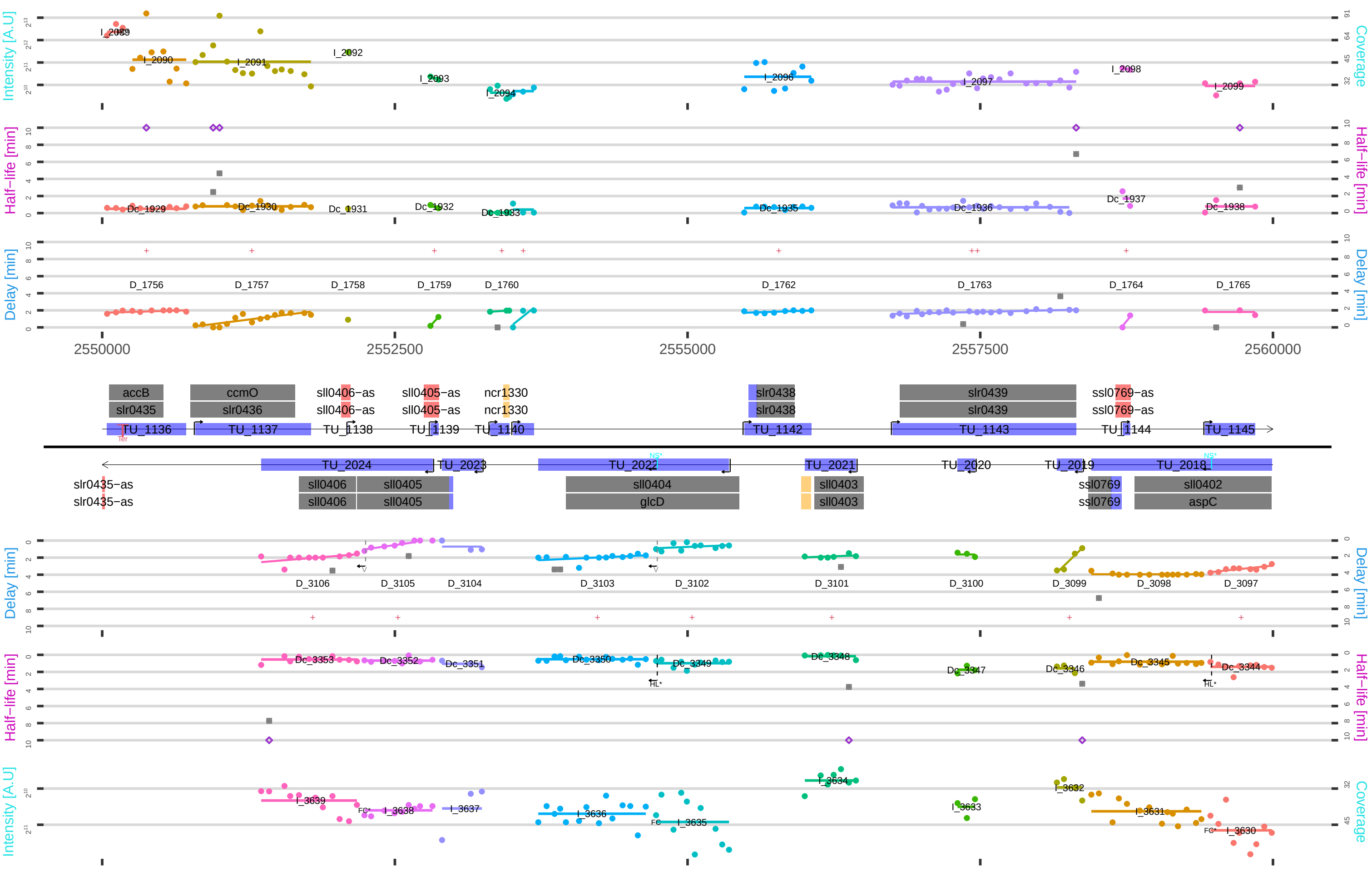

Term: termination (0), NS: new start (2), PS: pausing site (3), iTSS_I: internal starting site (0)

ID: 20686−20765; Term: termination (1), NS: new start (2), PS: pausing site (1), iTSS_I: internal starting site (2)

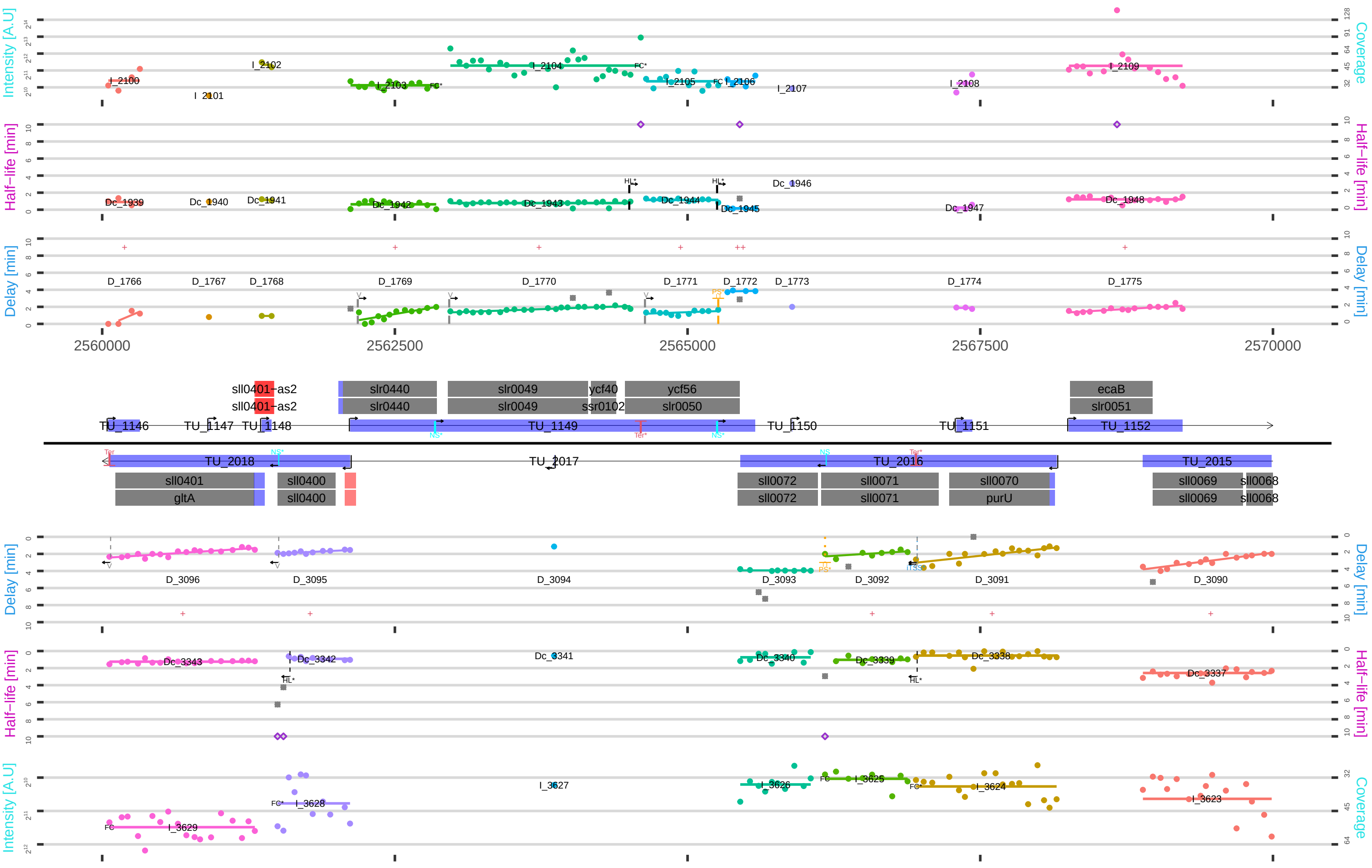

Term: termination (2), NS: new start (2), PS: pausing site (2), iTSS_I: internal starting site (2)

ID: 20766−20829; Term: termination (1), NS: new start (1), PS: pausing site (0), iTSS_I: internal starting site (1)

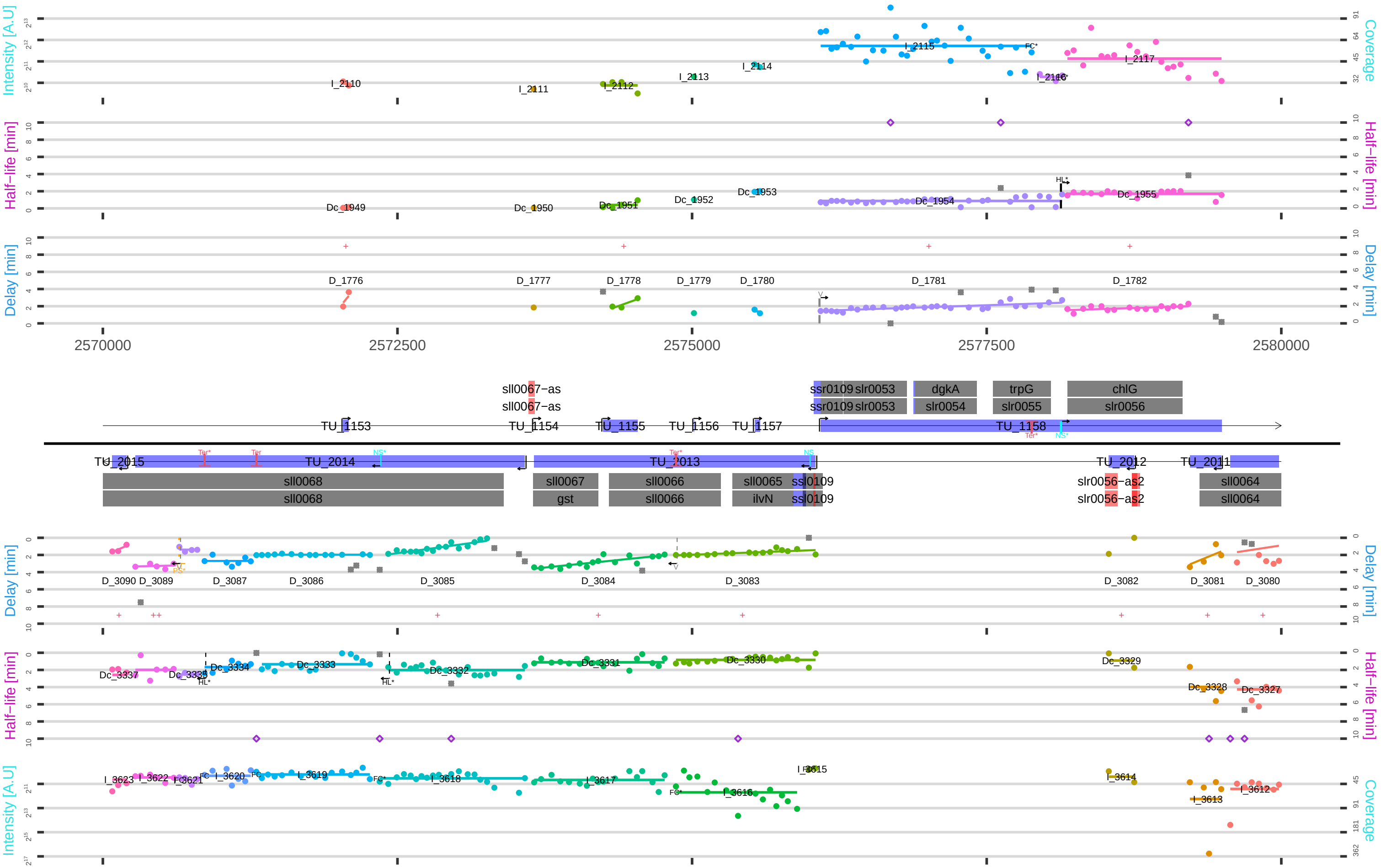

Term: termination (3), NS: new start (2), PS: pausing site (3), iTSS_I: internal starting site (2)

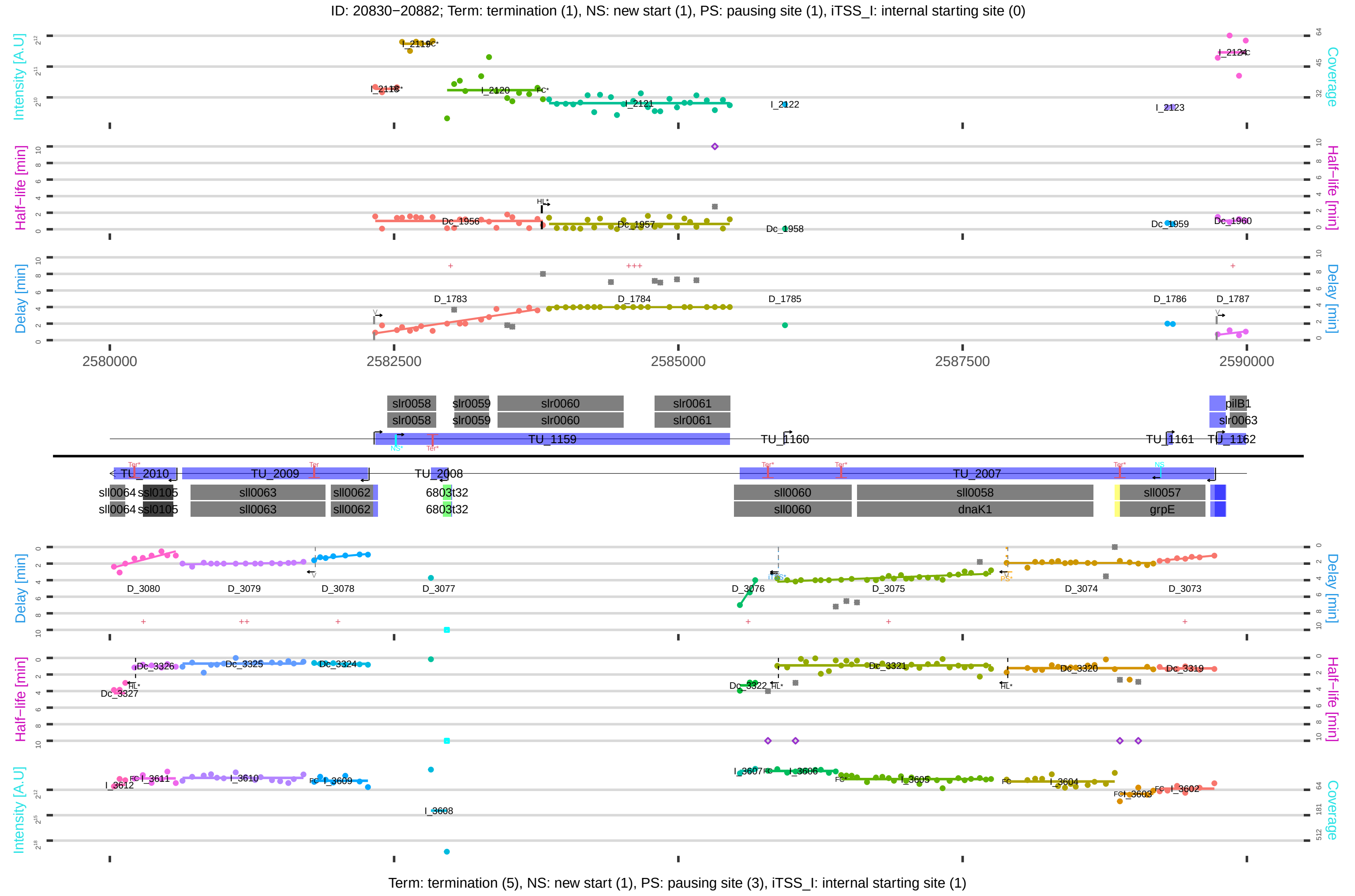

ID: 20830−20882; Term: termination (1), NS: new start (1), PS: pausing site (1), iTSS_I: internal starting site (0)
Intensity [A.U]
Coverage
Half−life [min]
Delay [min]
2580000
2582500
2585000
2587500
2590000
slr0058
slr0059
slr0060
slr0061
pilB1
slr0063
TU_1159
TU_1160
TU_1161
TU_1162
TU_2010
TU_2009
TU_2008
TU_2007
sll0064
sll0105
sll0063
sll0062
6803t32
sll0060
sll0058
dnaK1
sll0057
grpE
Term: termination (5), NS: new start (1), PS: pausing site (3), iTSS_I: internal starting site (1)

ID: 20883−20988; Term: termination (1), NS: new start (1), PS: pausing site (1), iTSS_I: internal starting site (2)

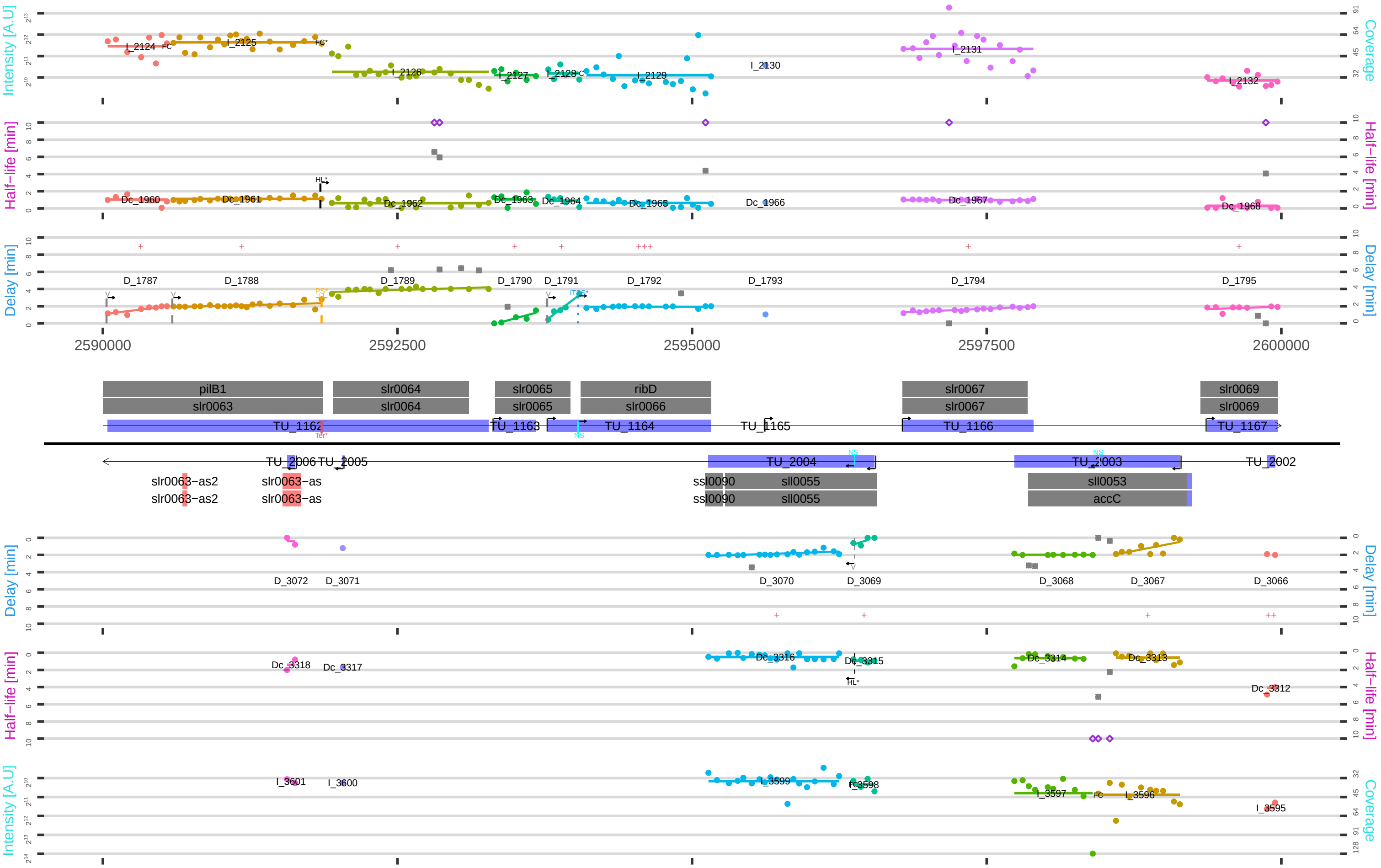

Term: termination (0), NS: new start (2), PS: pausing site (2), iTSS_I: internal starting site (0)

ID: 20989−21089; Term: termination (2), NS: new start (2), PS: pausing site (2), iTSS_I: internal starting site (2)

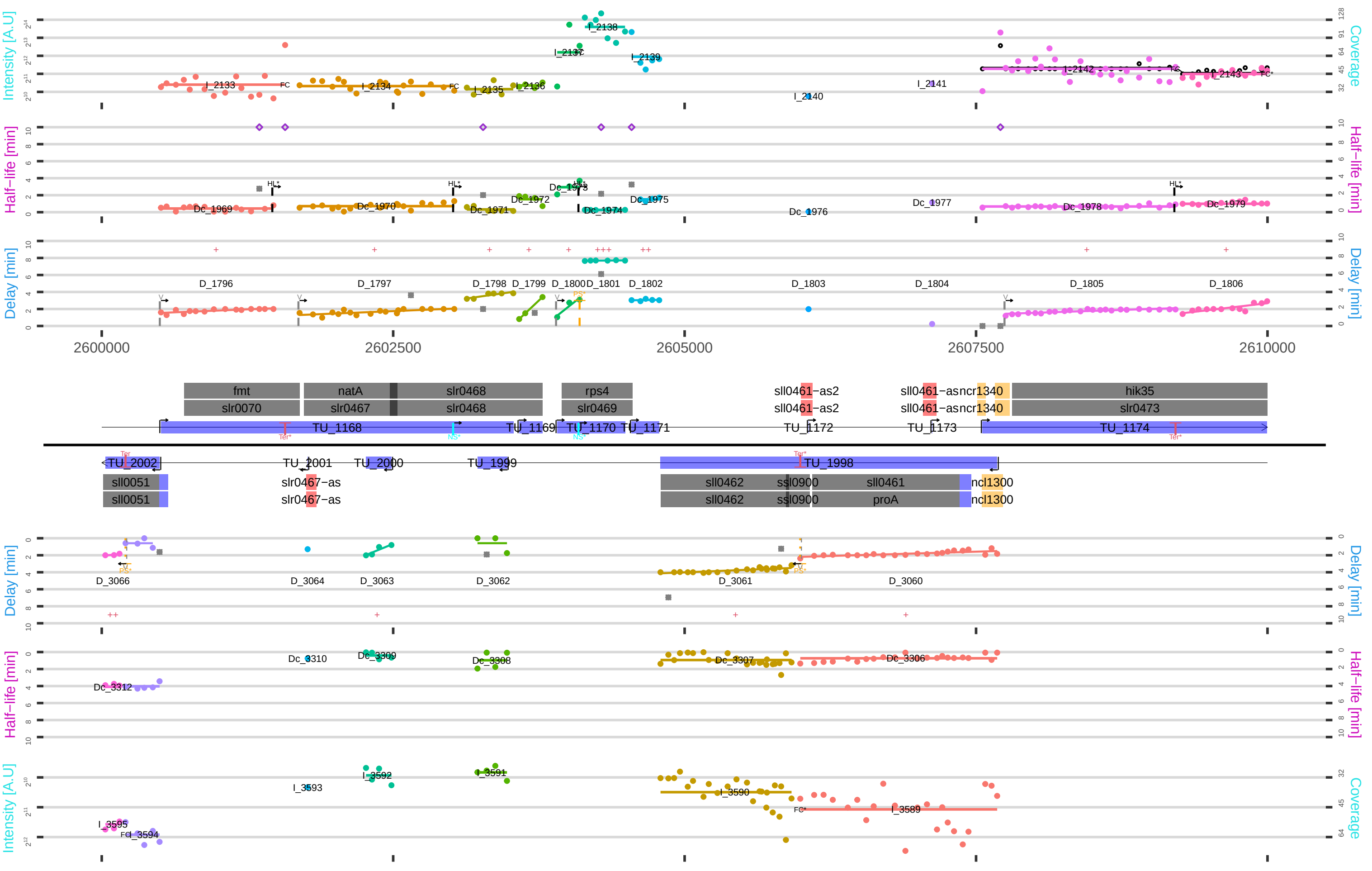

Term: termination (2), NS: new start (0), PS: pausing site (2), iTSS_I: internal starting site (0)

ID: 21090−21232; Term: termination (2), NS: new start (2), PS: pausing site (1), iTSS_I: internal starting site (0)

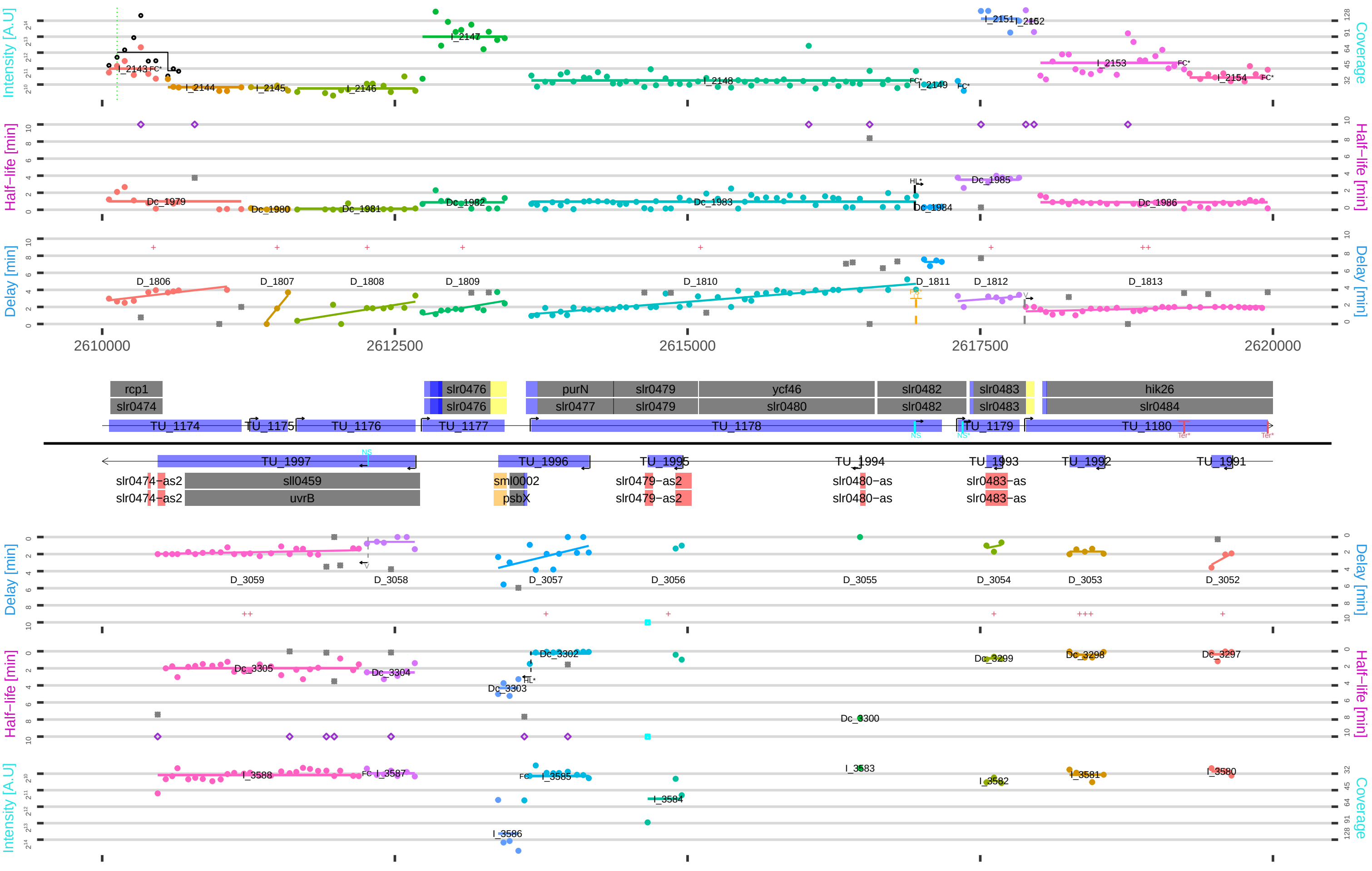

Term: termination (0), NS: new start (1), PS: pausing site (1), iTSS_I: internal starting site (0)

ID: 21233−21332; Term: termination (2), NS: new start (2), PS: pausing site (2), iTSS_I: internal starting site (1)

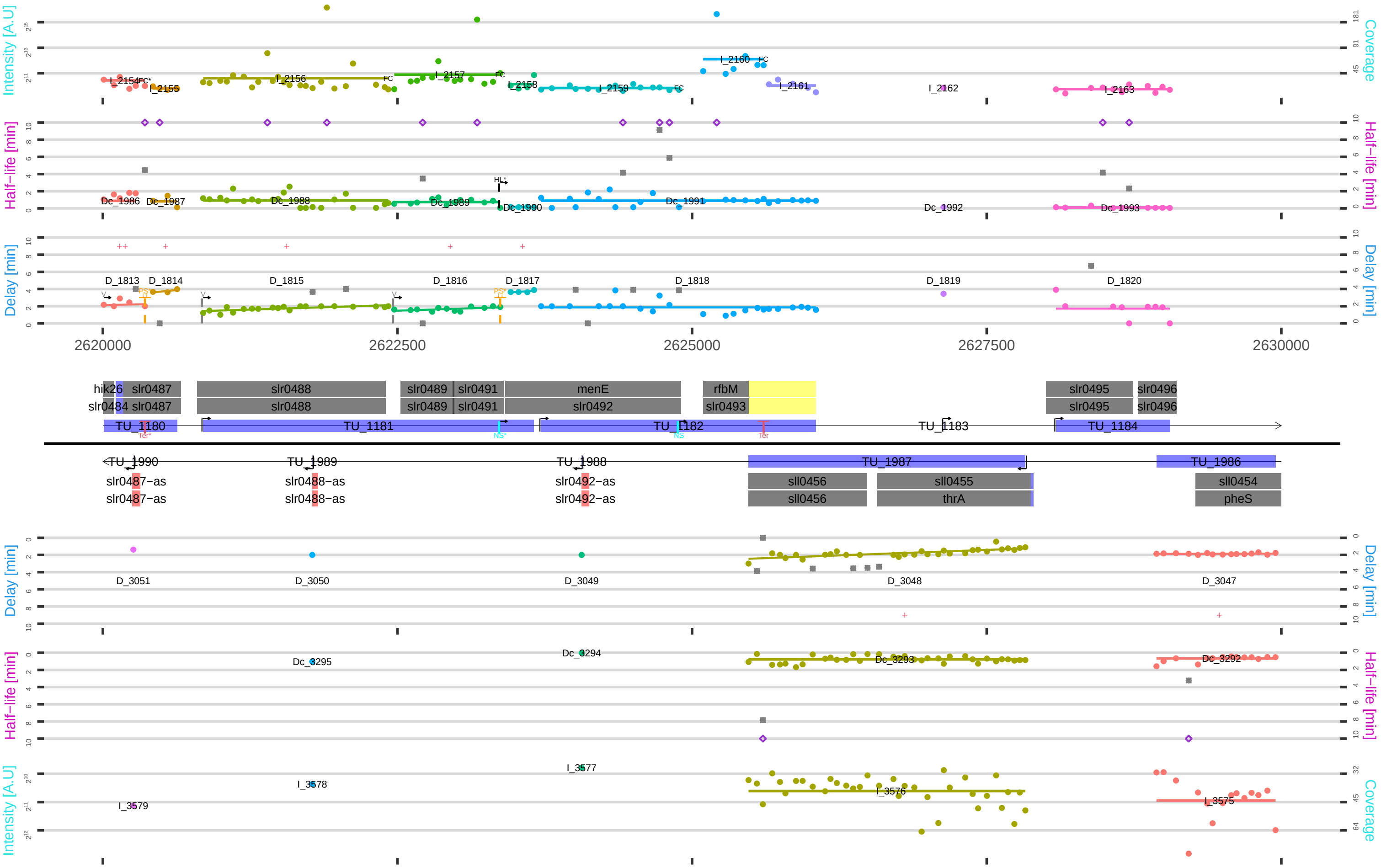

Term: termination (0), NS: new start (0), PS: pausing site (0), iTSS_I: internal starting site (0)

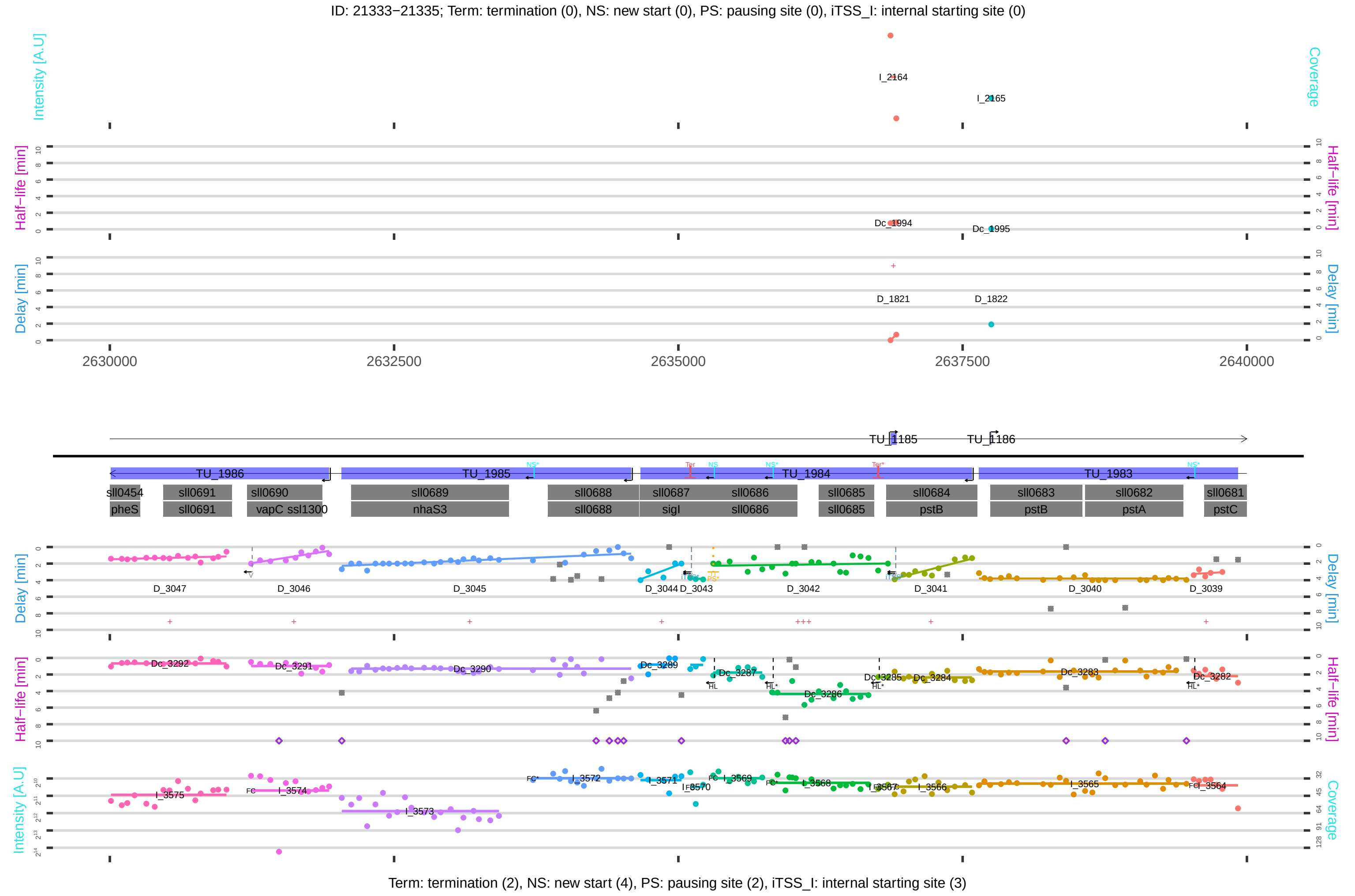

ID: 21333−21335; Term: termination (0), NS: new start (0), PS: pausing site (0), iTSS_I: internal starting site (0)
Intensity [A.U]
Coverage
Half−life [min]
Delay [min]
I_2164
I_2165
Dc_1994
Dc_1995
D_1821
D_1822
2630000
2632500
2635000
2637500
2640000
TU_1185
TU_1186
TU_1986
TU_1985
TU_1984
TU_1983
sll0454
pheS
sll0691
sll0690
vapC ssl1300
sll0689
nhaS3
sll0688
sll0687
sigI
sll0686
sll0685
sll0684
pstB
sll0683
sll0682
pstA
sll0681
pstC
D_3047
D_3046
D_3045
D_3044
D_3043
D_3042
D_3041
D_3040
D_3039
Dc_3292
Dc_3291
Dc_3290
Dc_3289
Dc_3287
Dc_3286
Dc_3285
Dc_3284
Dc_3283
Dc_3282
I_3575
I_3574
I_3573
I_3572
I_3571
I_3570
I_3569
I_3568
I_3567
I_3566
I_3565
I_3564
Term: termination (2), NS: new start (4), PS: pausing site (2), iTSS_I: internal starting site (3)

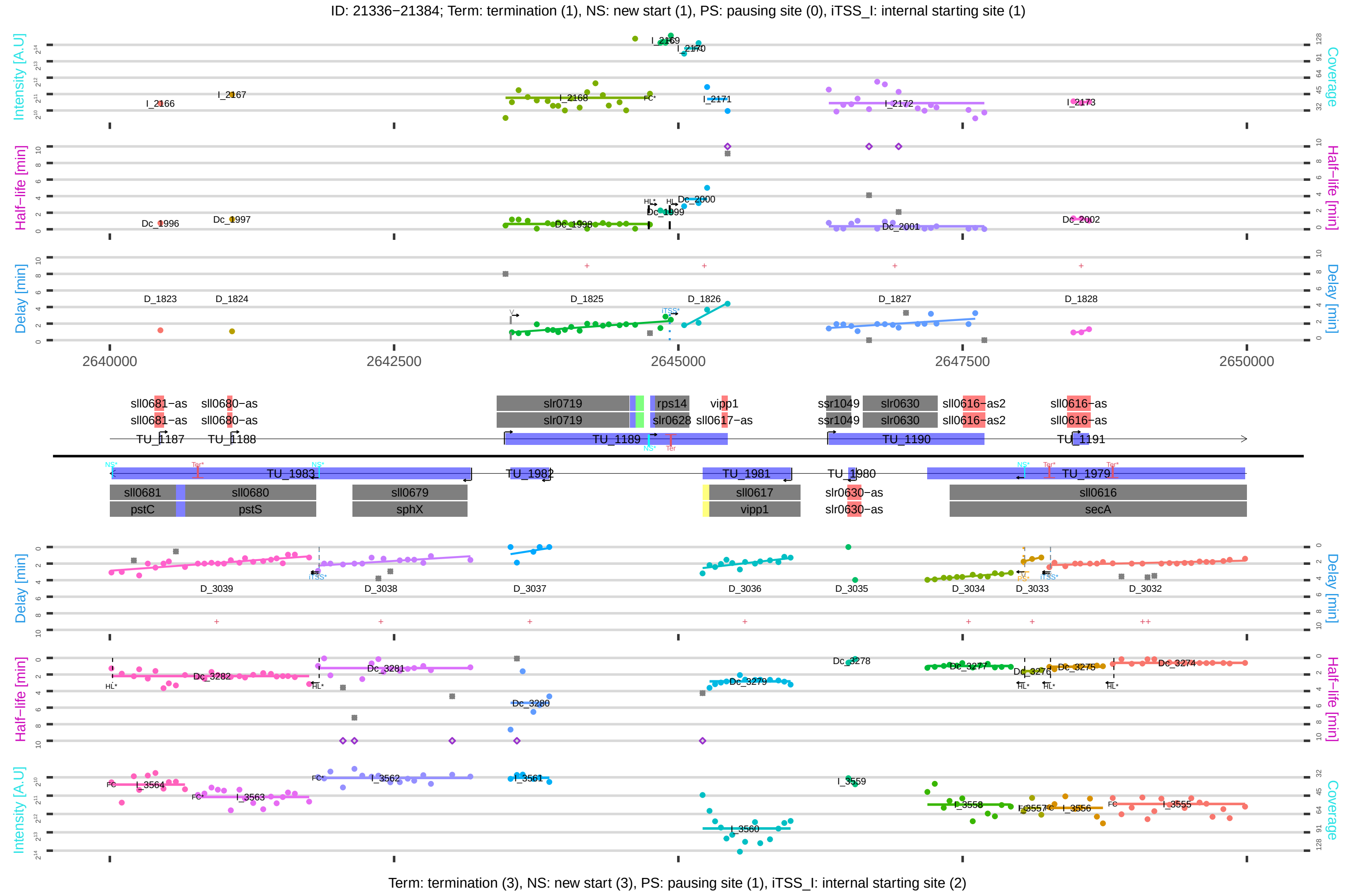

ID: 21336−21384; Term: termination (1), NS: new start (1), PS: pausing site (0), iTSS_I: internal starting site (1)
Intensity [A.U]
Coverage
Half−life [min]
Delay [min]
I_2166
I_2167
I_2168
I_2169
I_2170
I_2171
I_2172
I_2173
Dc_1996
Dc_1997
Dc_1998
Dc_1999
Dc_2000
Dc_2001
Dc_2002
D_1823
D_1824
D_1825
D_1826
D_1827
D_1828
2640000
2642500
2645000
2647500
2650000
sll0681−as
sll0680−as
slr0719
rps14
slr0628
vipp1
sll0617−as
ssr1049
slr0630
sll0616−as2
sll0616−as
TU_1187
TU_1188
TU_1189
TU_1190
TU_1191
TU_1983
TU_1982
TU_1981
TU_1980
TU_1979
sll0681
pstC
sll0680
pstS
sll0679
sphX
sll0617
vipp1
slr0630−as
sll0616
secA
D_3039
D_3038
D_3037
D_3036
D_3035
D_3034
D_3033
D_3032
Dc_3282
Dc_3281
Dc_3280
Dc_3279
Dc_3278
Dc_3277
Dc_3276
Dc_3275
Dc_3274
I_3564
I_3563
I_3562
I_3561
I_3560
I_3559
I_3558
I_3557
I_3556
I_3555
Term: termination (3), NS: new start (3), PS: pausing site (1), iTSS_I: internal starting site (2)

ID: 21385−21489; Term: termination (3), NS: new start (1), PS: pausing site (1), iTSS_I: internal starting site (1)

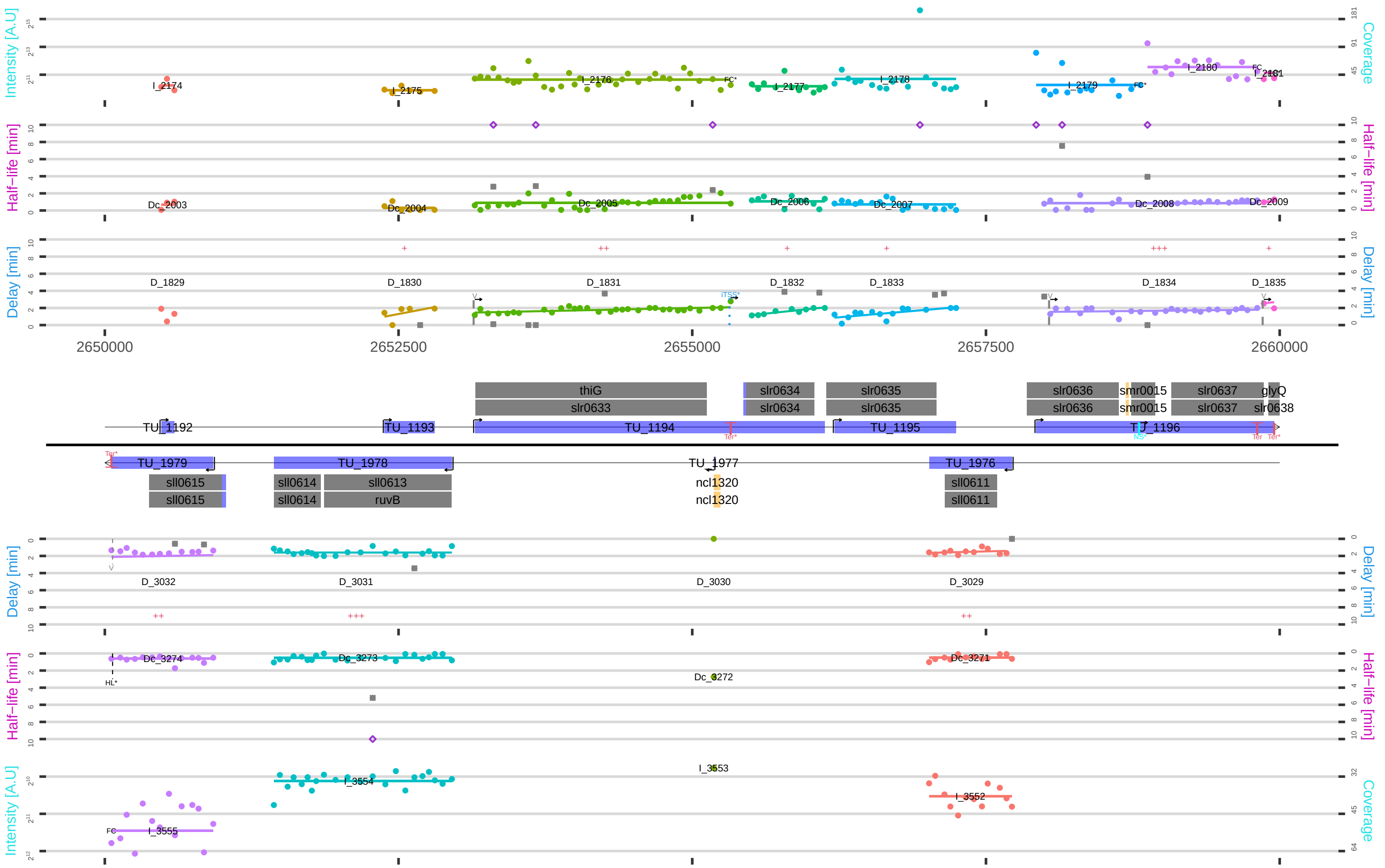

Term: termination (1), NS: new start (0), PS: pausing site (0), iTSS_I: internal starting site (0)

ID: 21490−21597; Term: termination (3), NS: new start (0), PS: pausing site (3), iTSS_I: internal starting site (0)

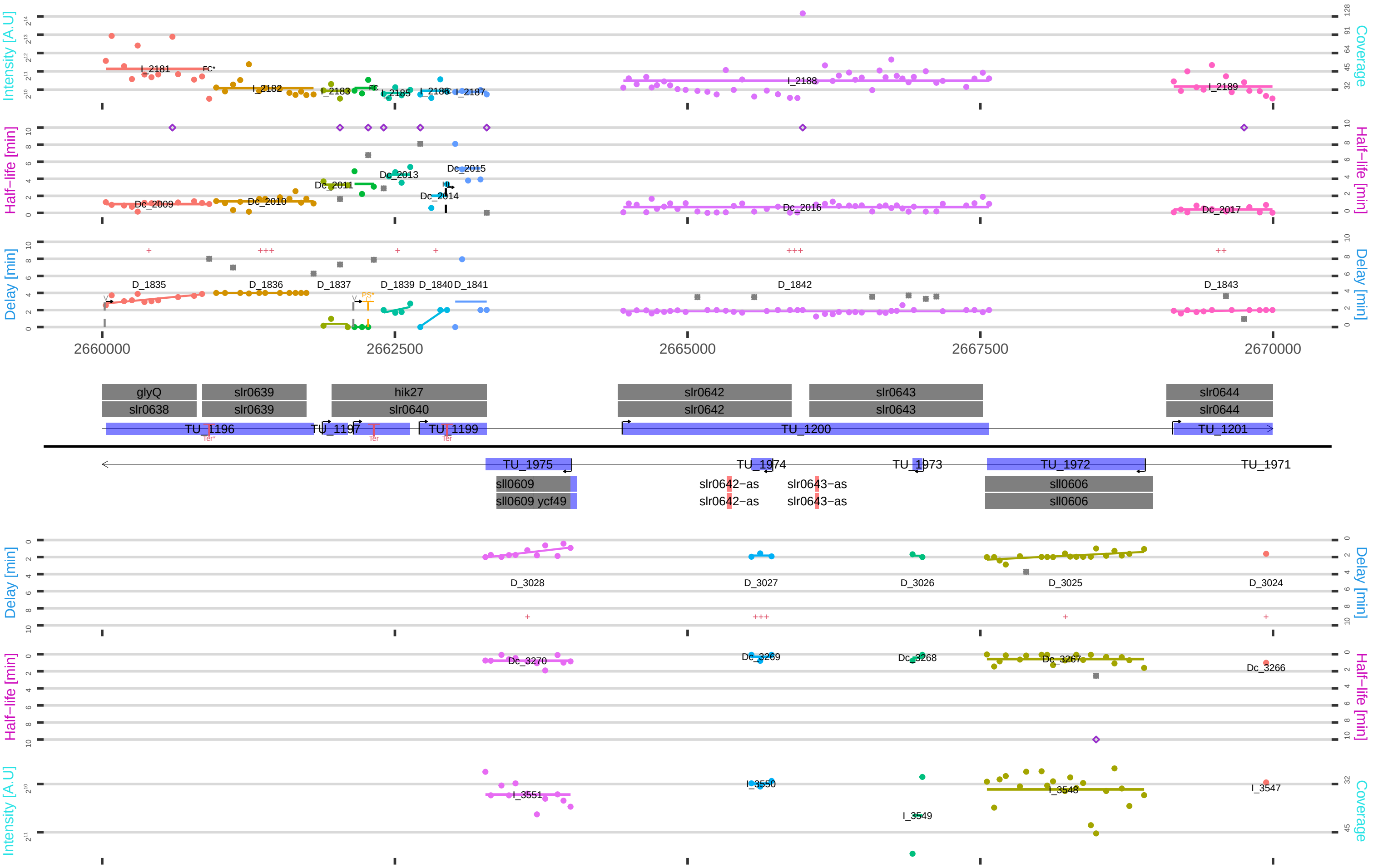

Term: termination (0), NS: new start (0), PS: pausing site (0), iTSS_I: internal starting site (0)

ID: 21598−21670; Term: termination (1), NS: new start (1), PS: pausing site (1), iTSS_I: internal starting site (1)

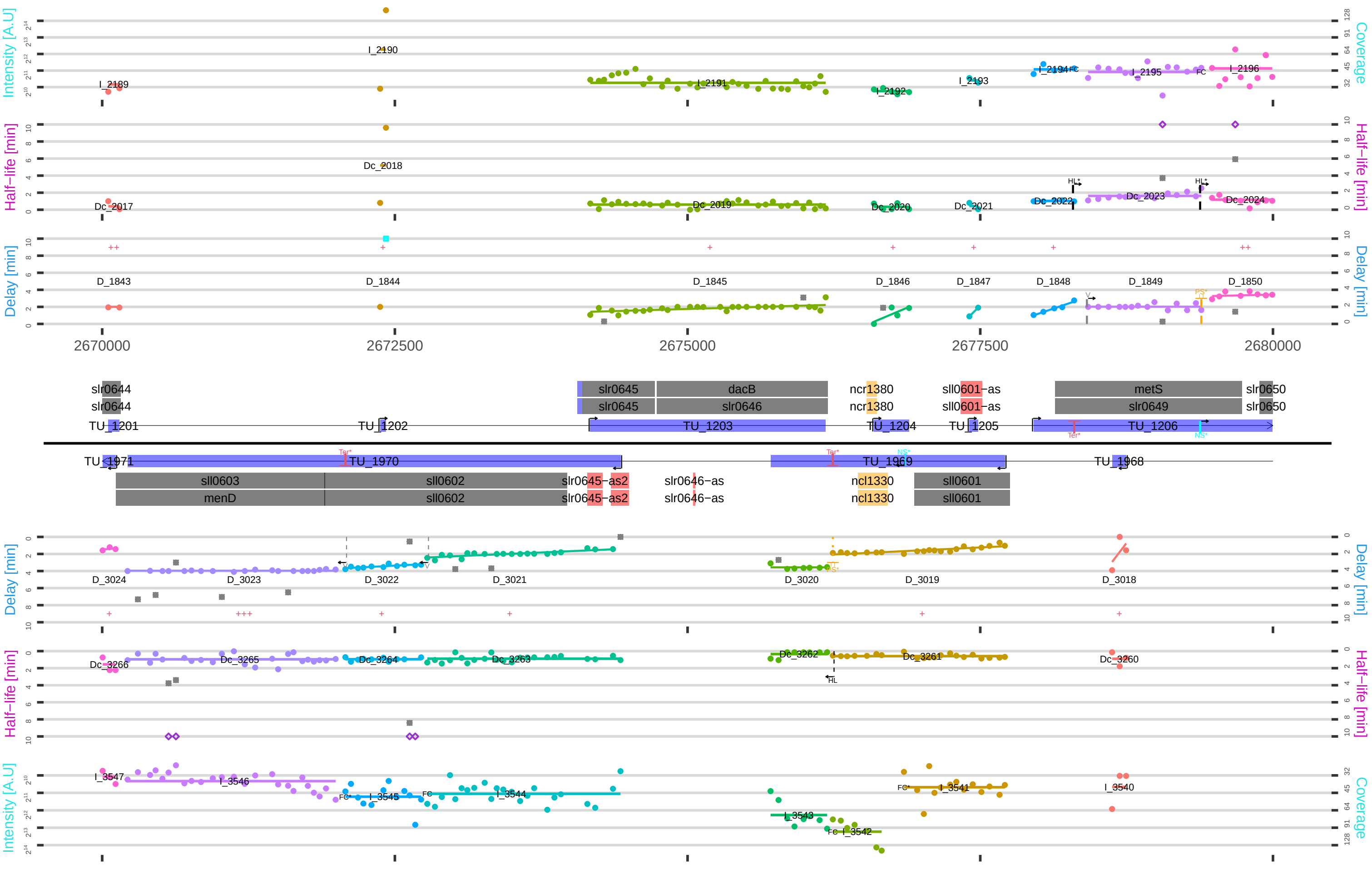

Term: termination (2), NS: new start (1), PS: pausing site (3), iTSS_I: internal starting site (0)

ID: 21671−21755; Term: termination (4), NS: new start (0), PS: pausing site (0), iTSS_I: internal starting site (1)

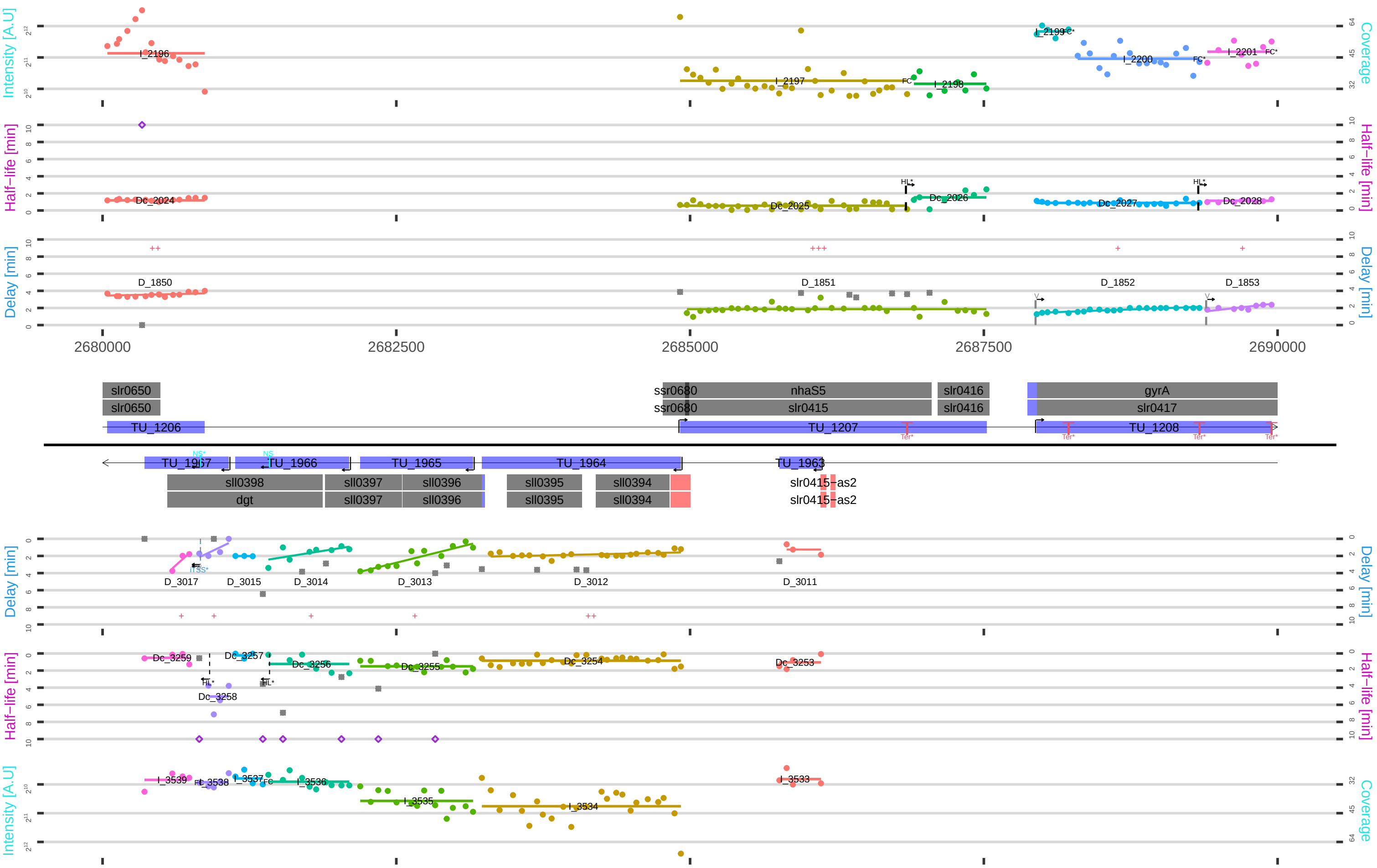

Term: termination (0), NS: new start (2), PS: pausing site (0), iTSS_I: internal starting site (2)

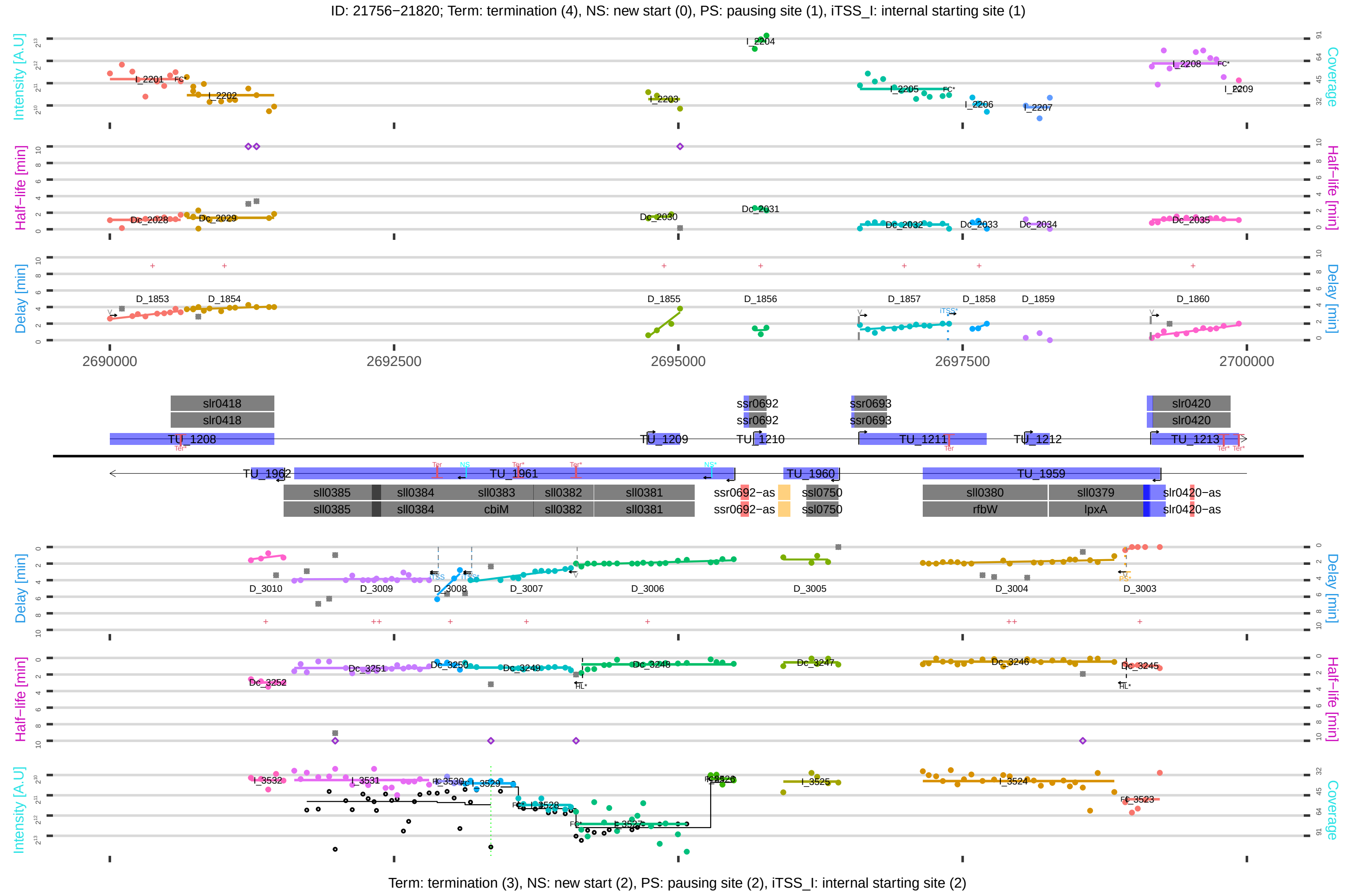

ID: 21756−21820; Term: termination (4), NS: new start (0), PS: pausing site (1), iTSS_I: internal starting site (1)
Intensity [A.U]
Coverage
Half−life [min]
Delay [min]
2690000
2692500
2695000
2697500
2700000
slr0418
ssr0692
ssr0693
slr0420
TU_1208
TU_1209
TU_1210
TU_1211
TU_1212
TU_1213
TU_1962
TU_1961
TU_1960
TU_1959
sll0385
sll0384
sll0383
cbiM
sll0382
sll0381
ssr0692−as
ssl0750
sll0380
rfbW
sll0379
lpxA
slr0420−as
Term: termination (3), NS: new start (2), PS: pausing site (2), iTSS_I: internal starting site (2)

ID: 21821−21872; Term: termination (1), NS: new start (0), PS: pausing site (0), iTSS_I: internal starting site (1)

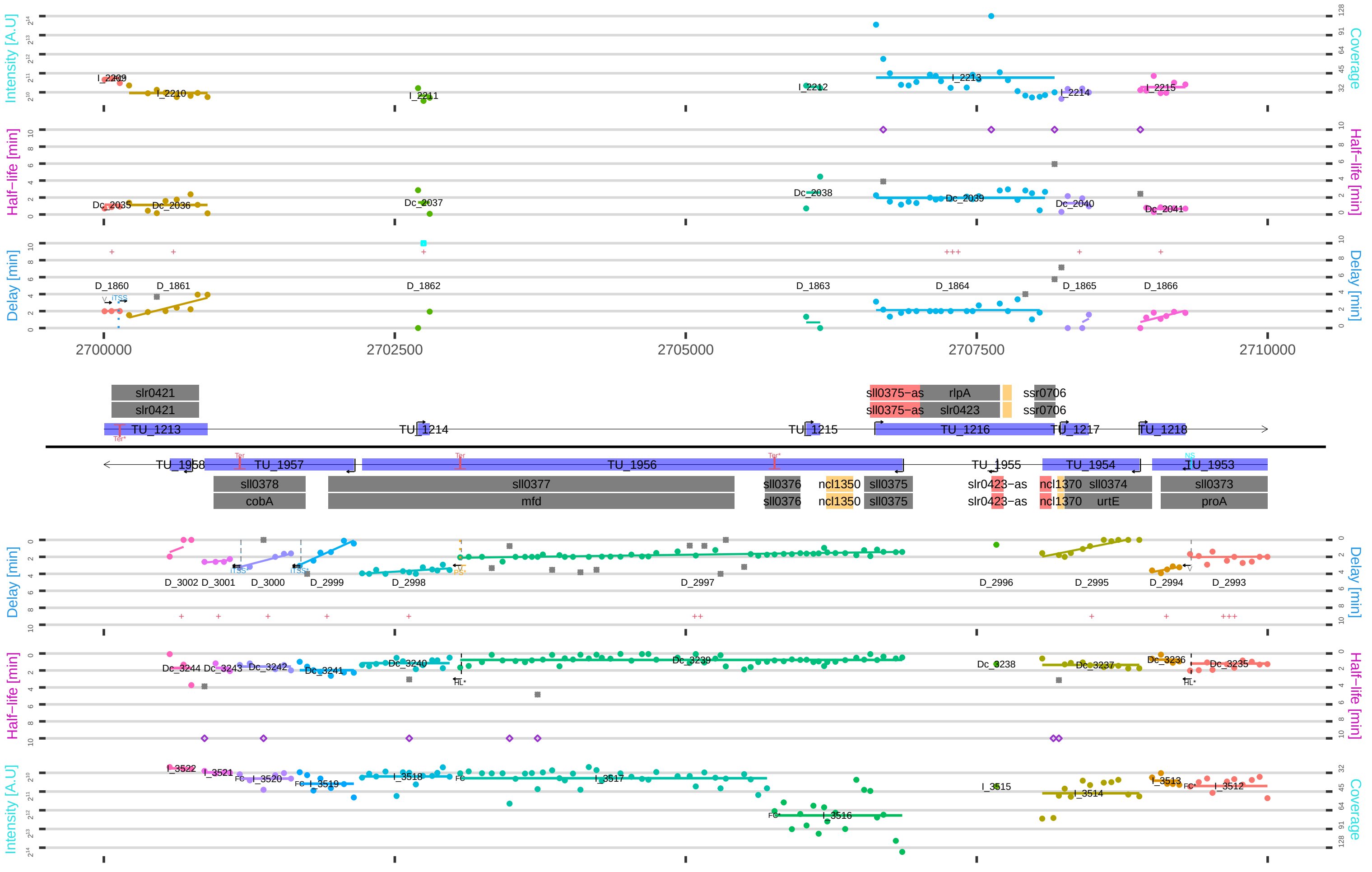

Term: termination (3), NS: new start (1), PS: pausing site (2), iTSS_I: internal starting site (2)

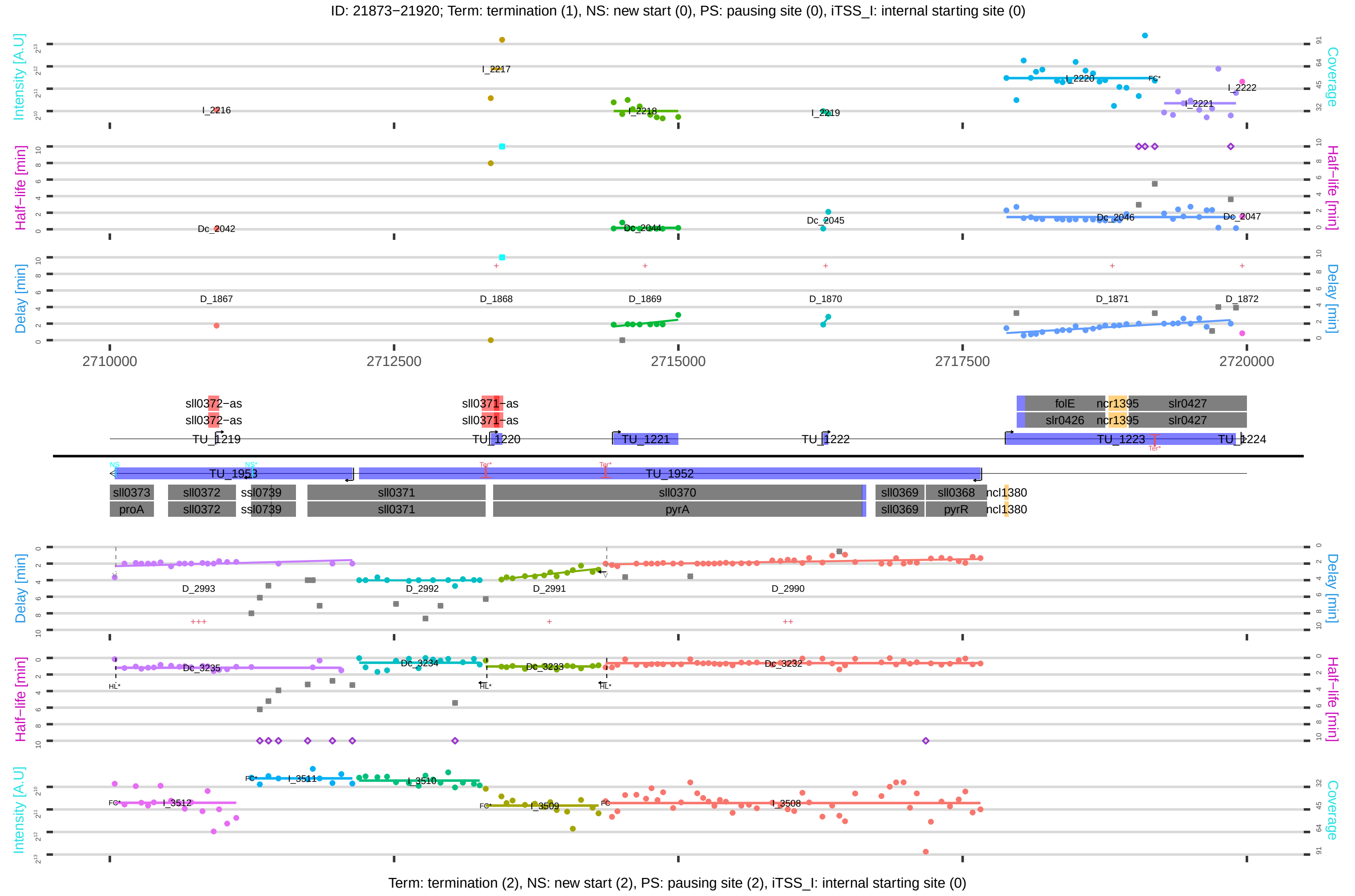

ID: 21873−21920; Term: termination (1), NS: new start (0), PS: pausing site (0), iTSS_I: internal starting site (0)
Intensity [A.U]
Coverage
Half−life [min]
Delay [min]
I_2216
I_2217
I_2218
I_2219
I_2220
I_2221
I_2222
Dc_2042
Dc_2044
Dc_2045
Dc_2046
Dc_2047
D_1867
D_1868
D_1869
D_1870
D_1871
D_1872
2710000
2712500
2715000
2717500
2720000
sll0372−as
sll0371−as
folE
ncr1395
slr0427
slr0426
TU_1219
TU_1220
TU_1221
TU_1222
TU_1223
TU_1224
TU_1953
TU_1952
sll0373
proA
sll0372
ssl0739
sll0371
sll0370
pyrA
sll0369
sll0368
pyrR
ncl1380
D_2993
D_2992
D_2991
D_2990
Dc_3235
Dc_3234
Dc_3233
Dc_3232
I_3512
I_3511
I_3510
I_3509
I_3508
Term: termination (2), NS: new start (2), PS: pausing site (2), iTSS_I: internal starting site (0)

ID: 21921−22016; Term: termination (2), NS: new start (2), PS: pausing site (1), iTSS_I: internal starting site (0)

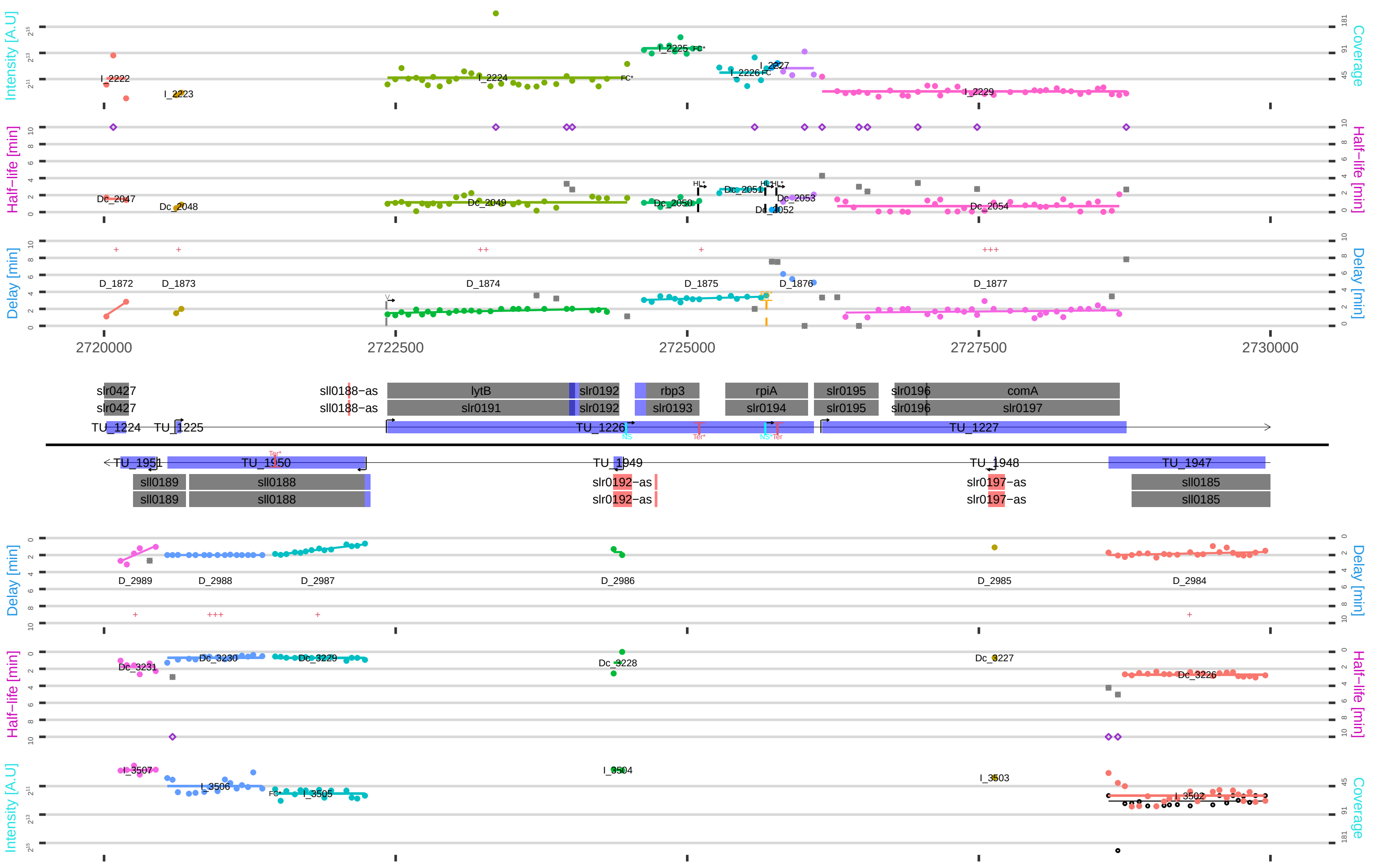

Term: termination (1), NS: new start (0), PS: pausing site (0), iTSS_I: internal starting site (1)

ID: 22017−22052; Term: termination (0), NS: new start (0), PS: pausing site (0), iTSS_I: internal starting site (0)

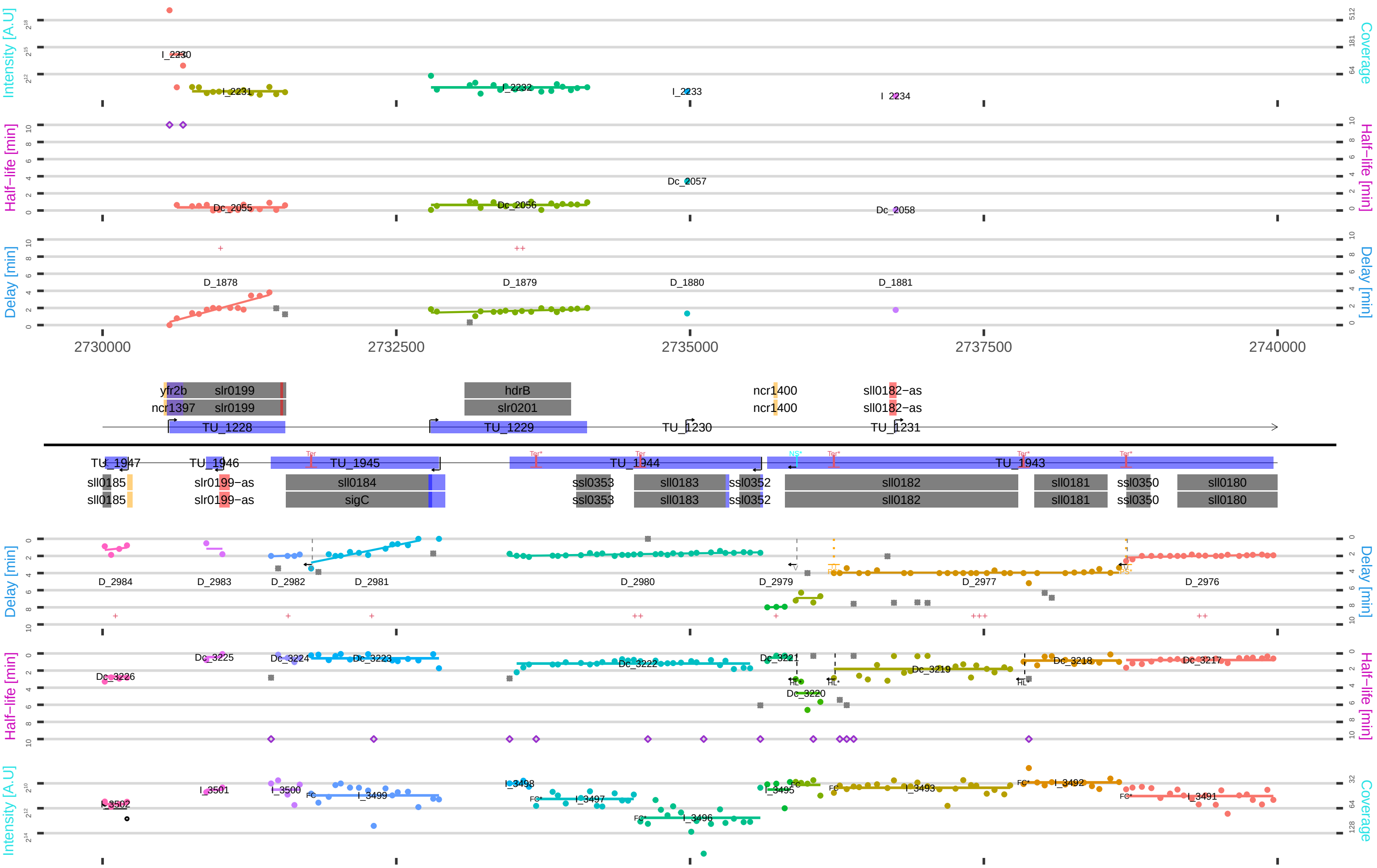

Term: termination (6), NS: new start (1), PS: pausing site (3), iTSS_I: internal starting site (1)

ID: 22053−22170; Term: termination (2), NS: new start (2), PS: pausing site (1), iTSS_I: internal starting site (3)

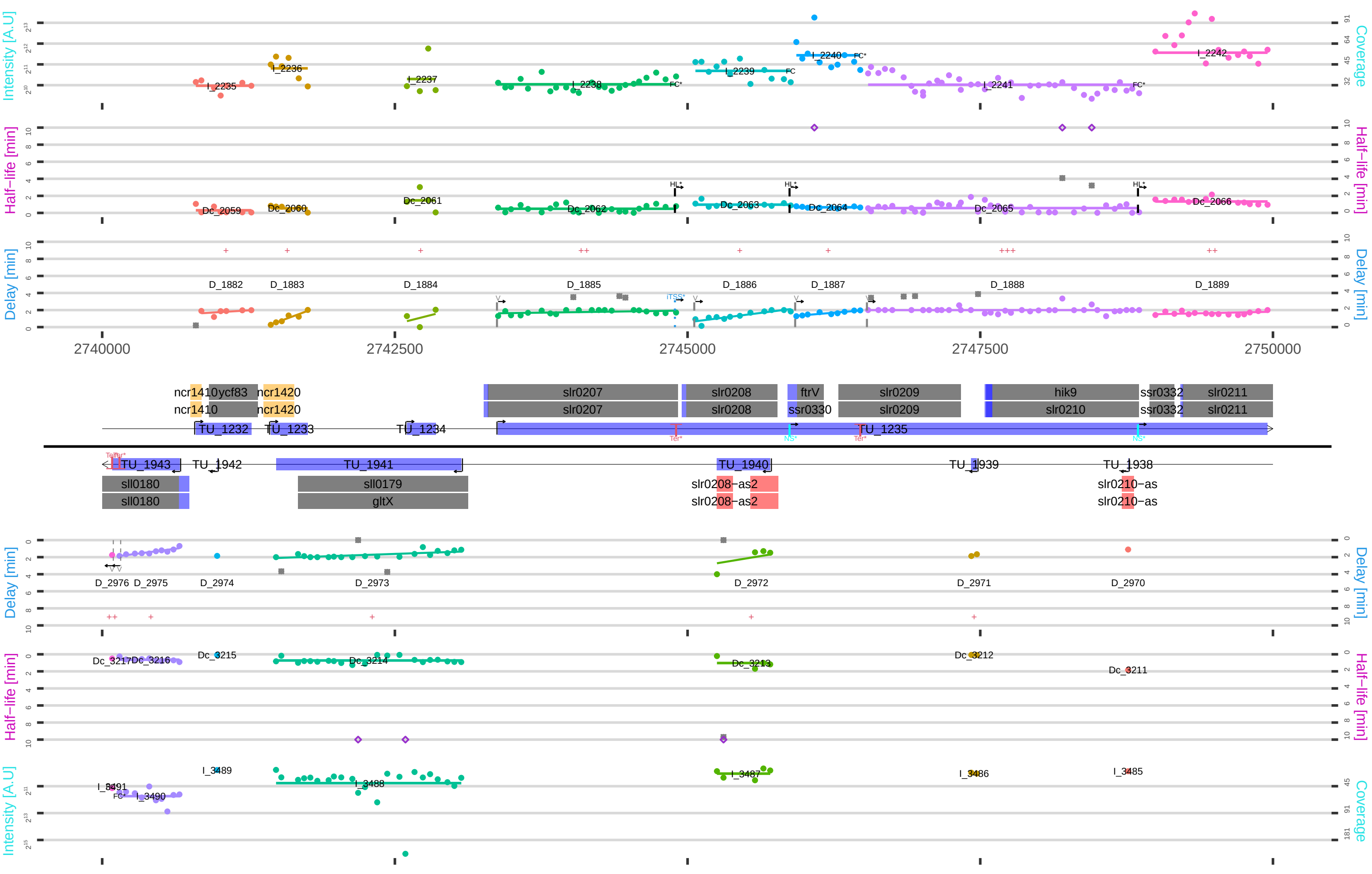

Term: termination (2), NS: new start (0), PS: pausing site (0), iTSS_I: internal starting site (1)

ID: 22171−22243; Term: termination (2), NS: new start (1), PS: pausing site (2), iTSS_I: internal starting site (1)

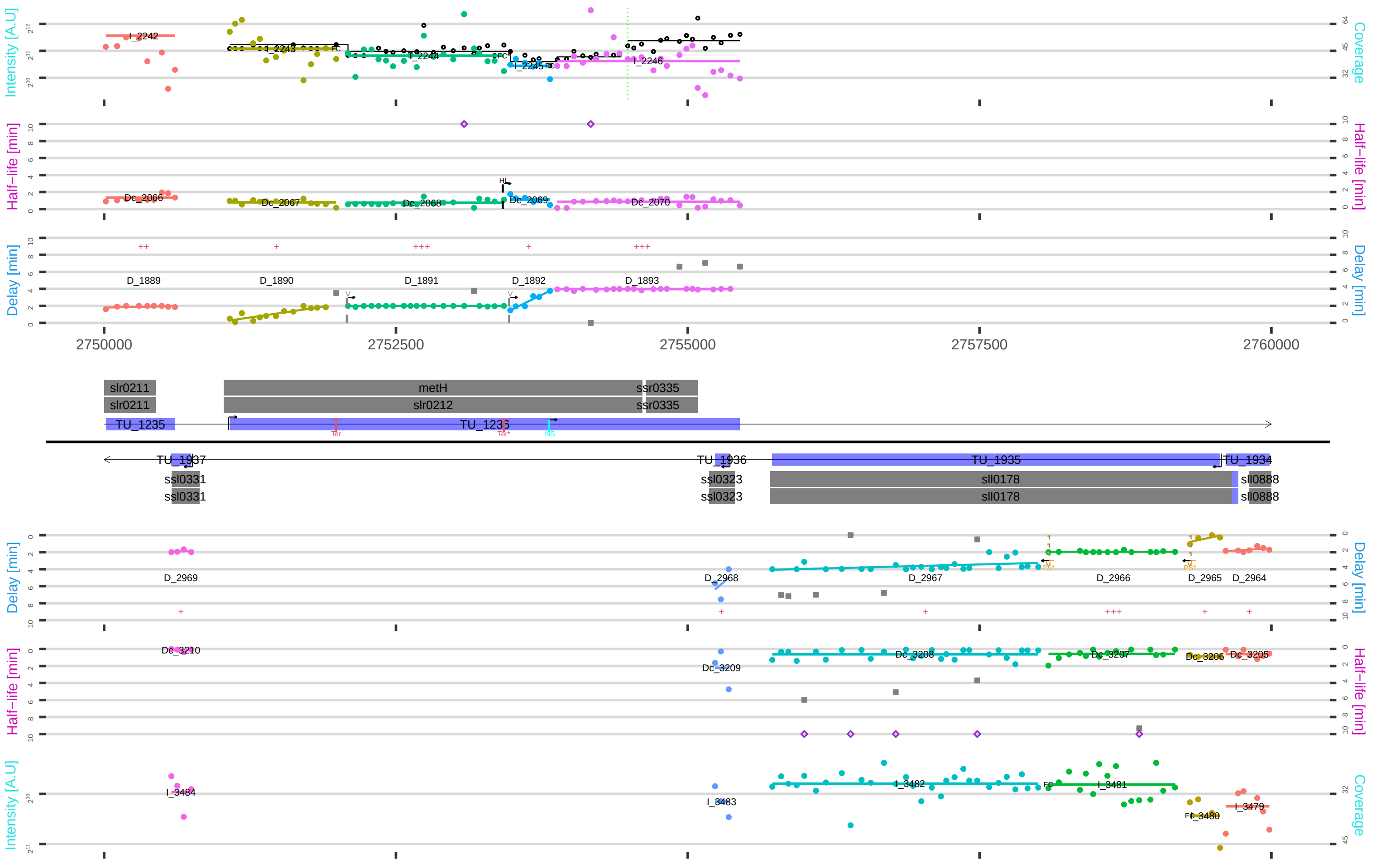

Term: termination (0), NS: new start (0), PS: pausing site (2), iTSS_I: internal starting site (0)

ID: 22244−22337; Term: termination (1), NS: new start (4), PS: pausing site (3), iTSS_I: internal starting site (1)

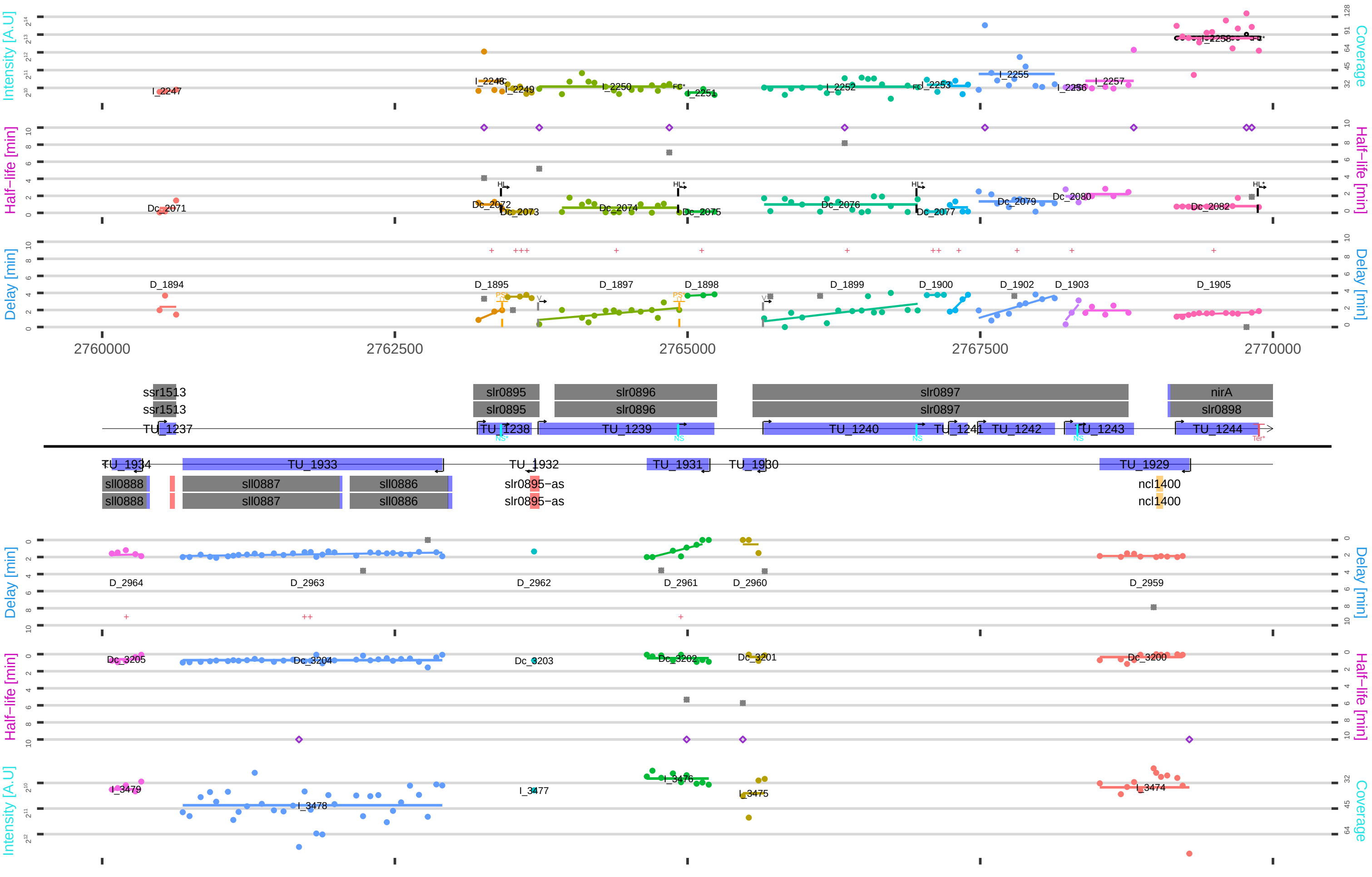

Term: termination (0), NS: new start (0), PS: pausing site (0), iTSS_I: internal starting site (0)

ID: 22338−22473; Term: termination (5), NS: new start (3), PS: pausing site (2), iTSS_I: internal starting site (3)

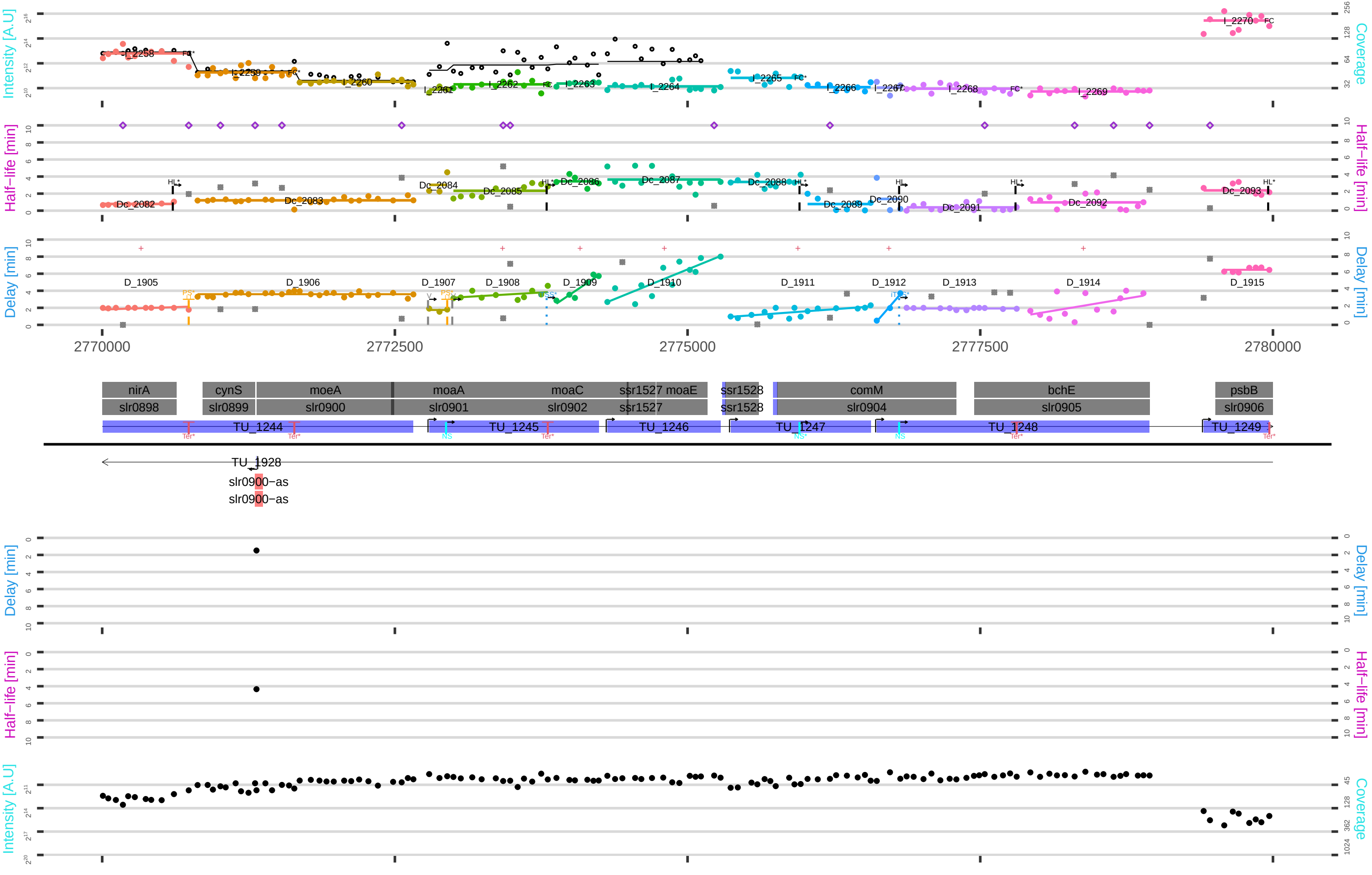

Term: termination (0), NS: new start (0), PS: pausing site (0), iTSS_I: internal starting site (0)

ID: 22474−22610; Term: termination (3), NS: new start (2), PS: pausing site (3), iTSS_I: internal starting site (2)

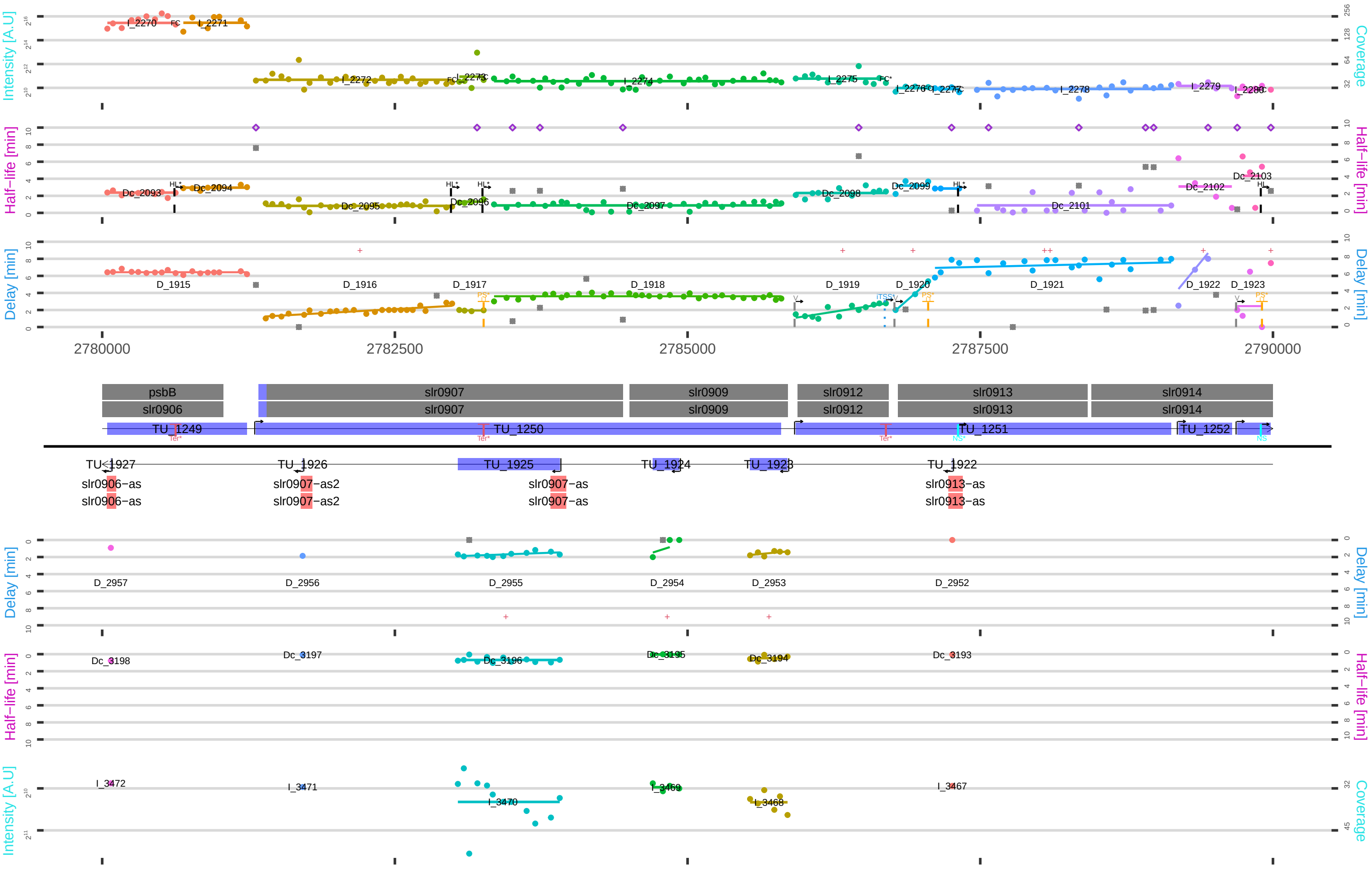

Term: termination (0), NS: new start (0), PS: pausing site (0), iTSS_I: internal starting site (0)

ID: 22611−22711; Term: termination (2), NS: new start (5), PS: pausing site (2), iTSS_I: internal starting site (3)

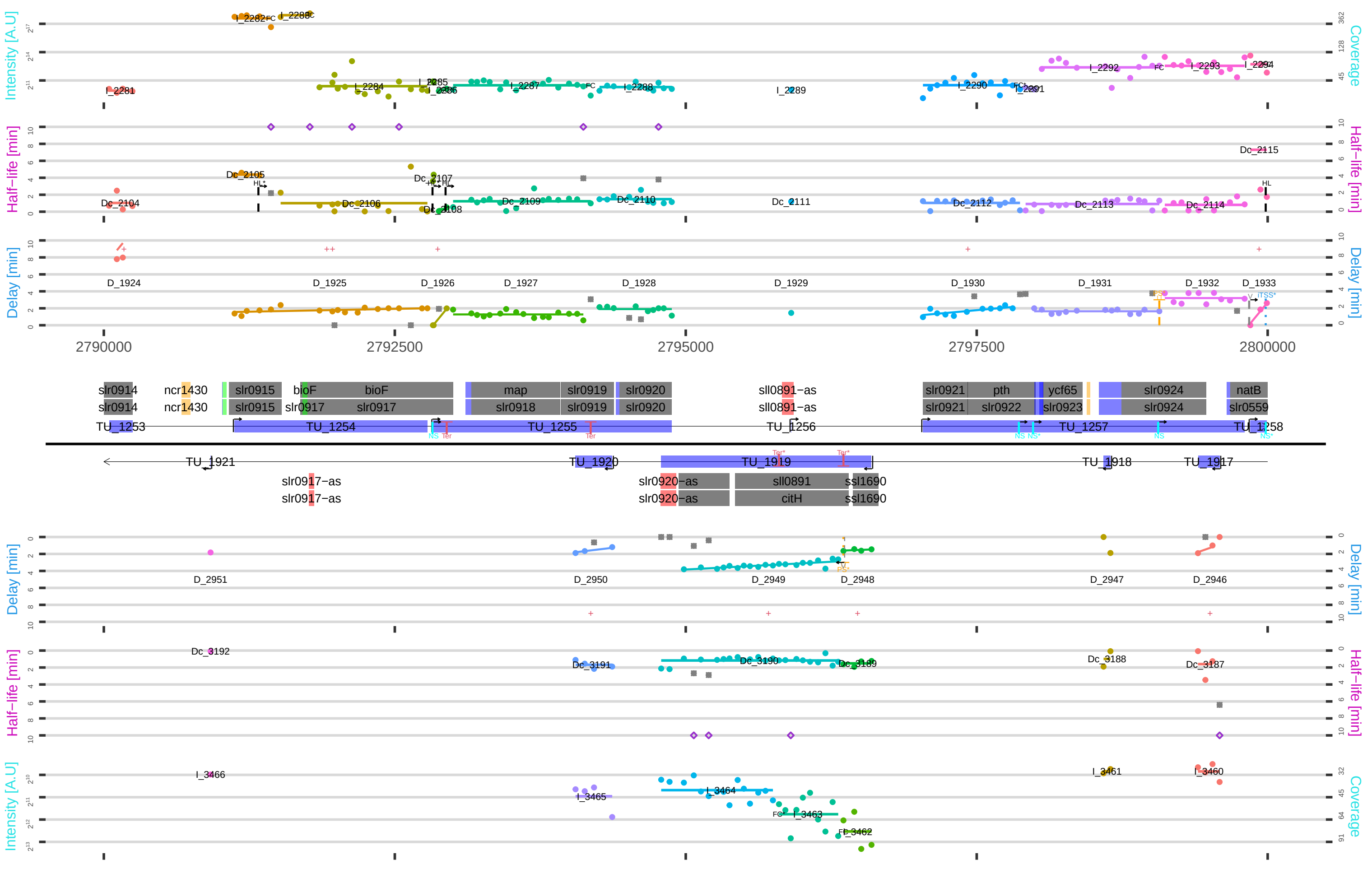

Term: termination (2), NS: new start (0), PS: pausing site (1), iTSS_I: internal starting site (0)

ID: 22712−22749; Term: termination (2), NS: new start (0), PS: pausing site (0), iTSS_I: internal starting site (0)

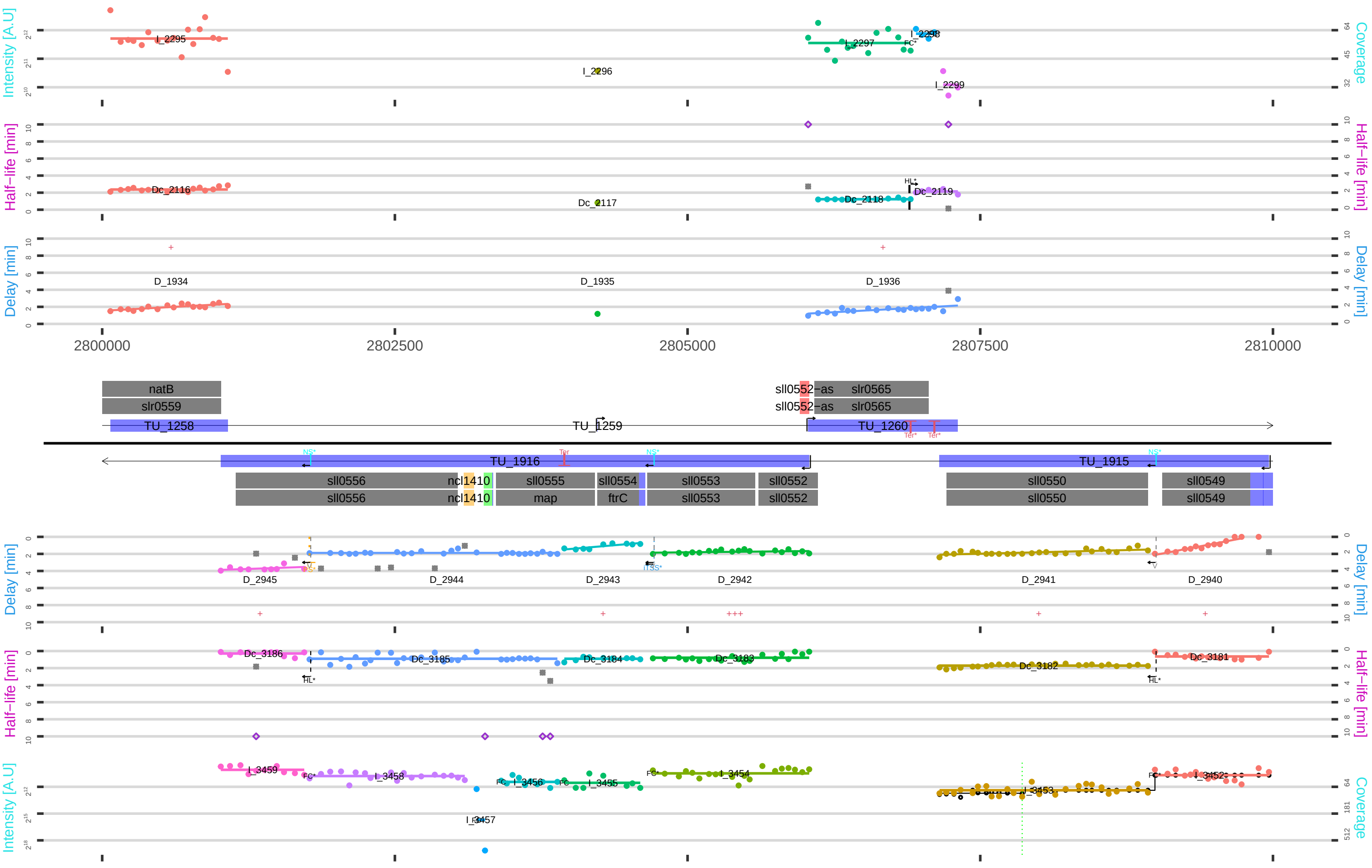

Term: termination (1), NS: new start (3), PS: pausing site (2), iTSS_I: internal starting site (2)

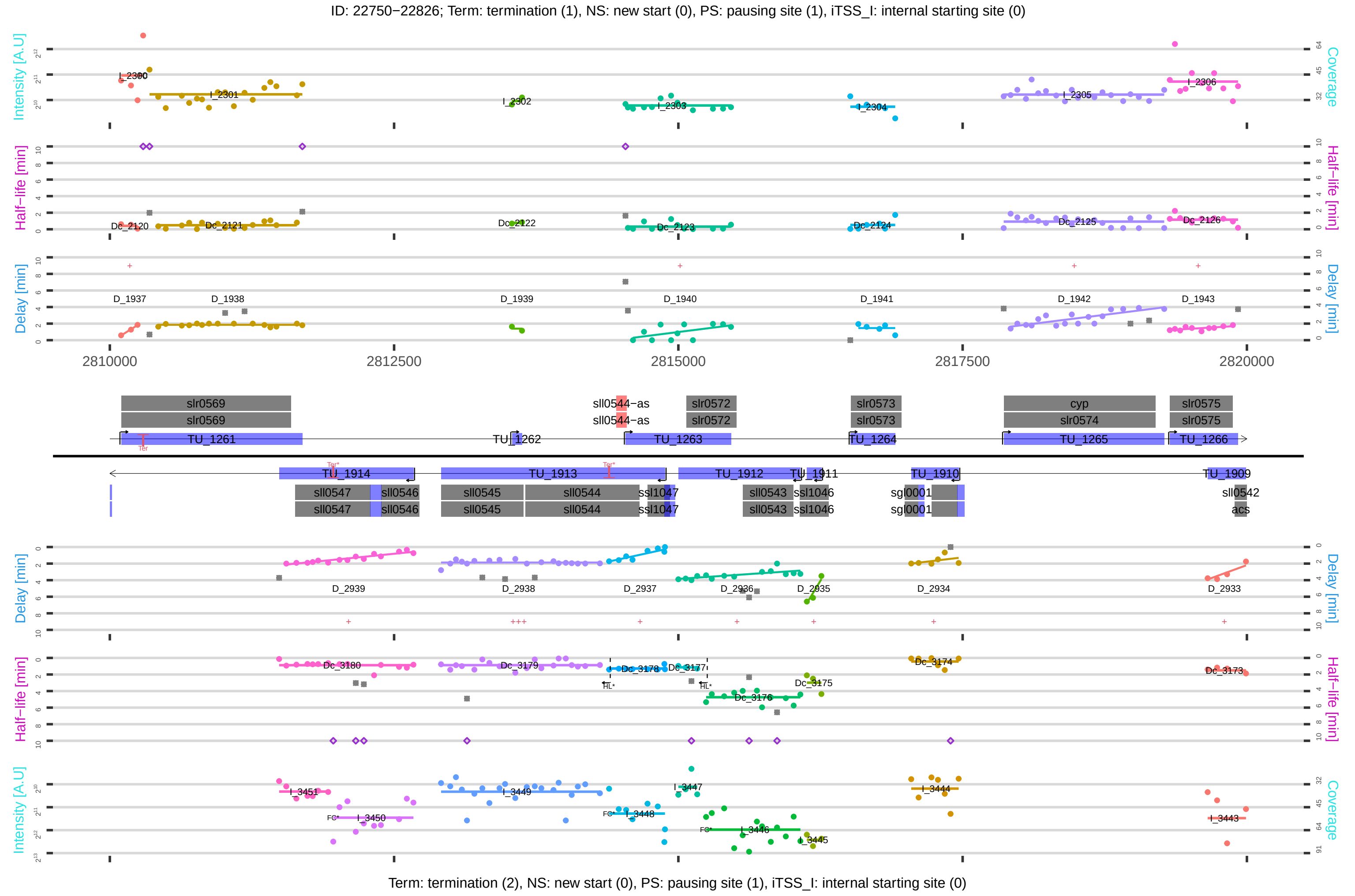

ID: 22750−22826; Term: termination (1), NS: new start (0), PS: pausing site (1), iTSS_I: internal starting site (0)
Intensity [A.U]
Coverage
Half−life [min]
Delay [min]
2810000
2812500
2815000
2817500
2820000
slr0569
sll0544−as
slr0572
slr0573
cyp
slr0574
slr0575
TU_1261
TU_1262
TU_1263
TU_1264
TU_1265
TU_1266
TU_1914
TU_1913
TU_1912
TU_1911
TU_1910
TU_1909
sll0547
sll0546
sll0545
sll0544
ssl1047
sll0543
ssl1046
sgl0001
sll0542
acs
Term: termination (2), NS: new start (0), PS: pausing site (1), iTSS_I: internal starting site (0)

ID: 22827−22898; Term: termination (1), NS: new start (2), PS: pausing site (1), iTSS_I: internal starting site (1)

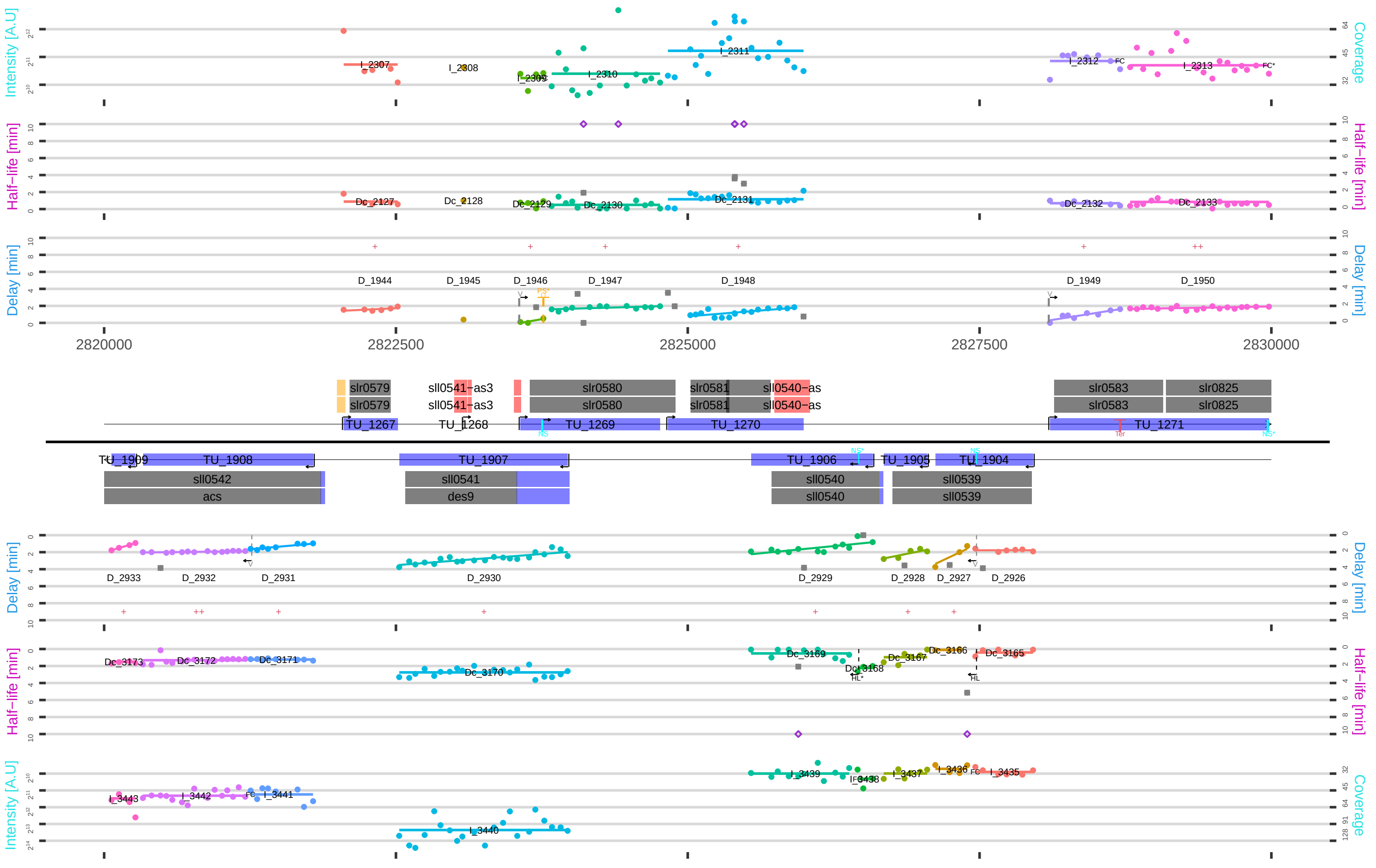

Term: termination (0), NS: new start (2), PS: pausing site (1), iTSS_I: internal starting site (1)

ID: 22899−22990; Term: termination (3), NS: new start (5), PS: pausing site (3), iTSS_I: internal starting site (3)

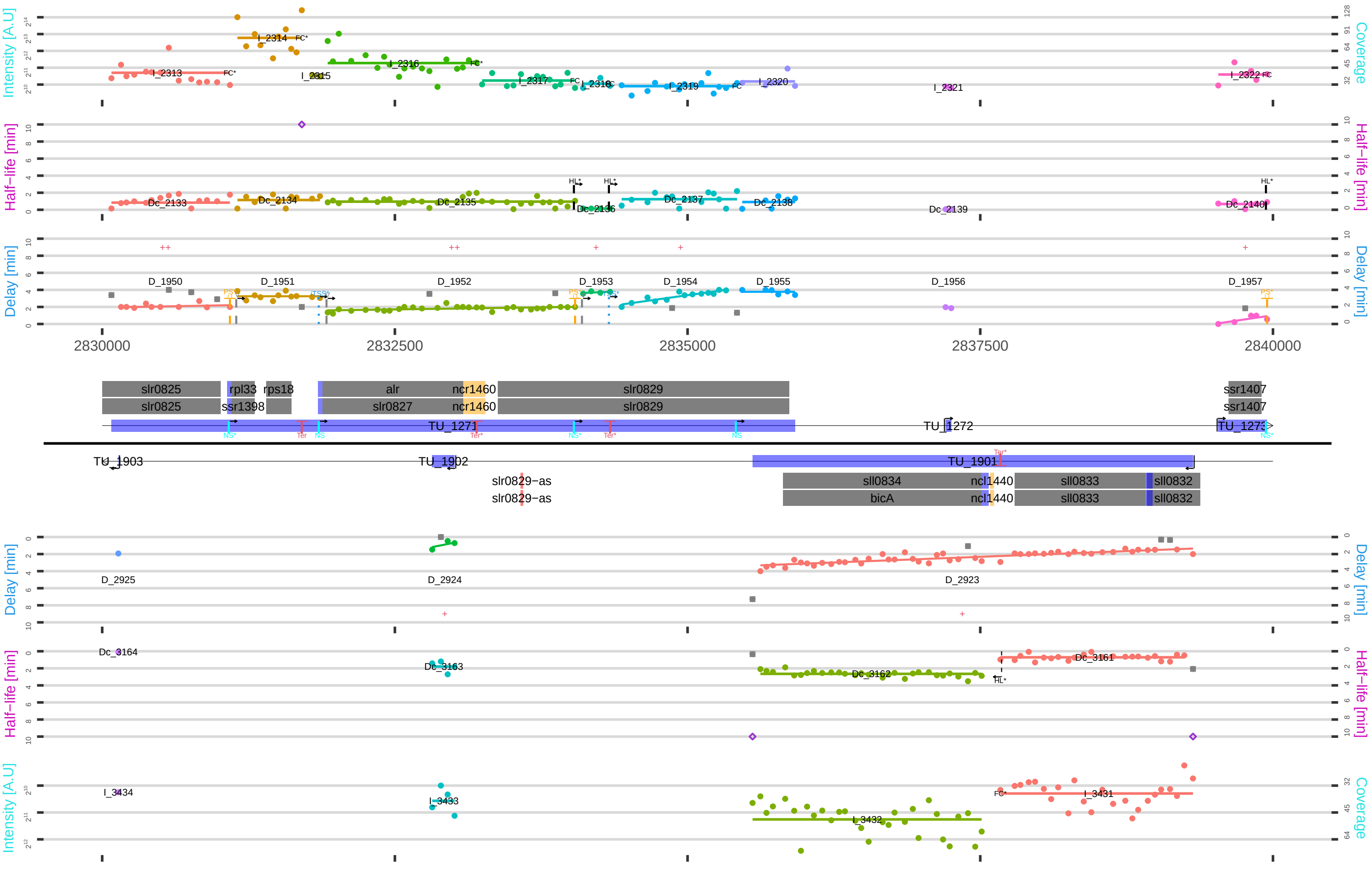

Term: termination (1), NS: new start (0), PS: pausing site (0), iTSS_I: internal starting site (0)

ID: 22992−23091; Term: termination (2), NS: new start (0), PS: pausing site (1), iTSS_I: internal starting site (1)

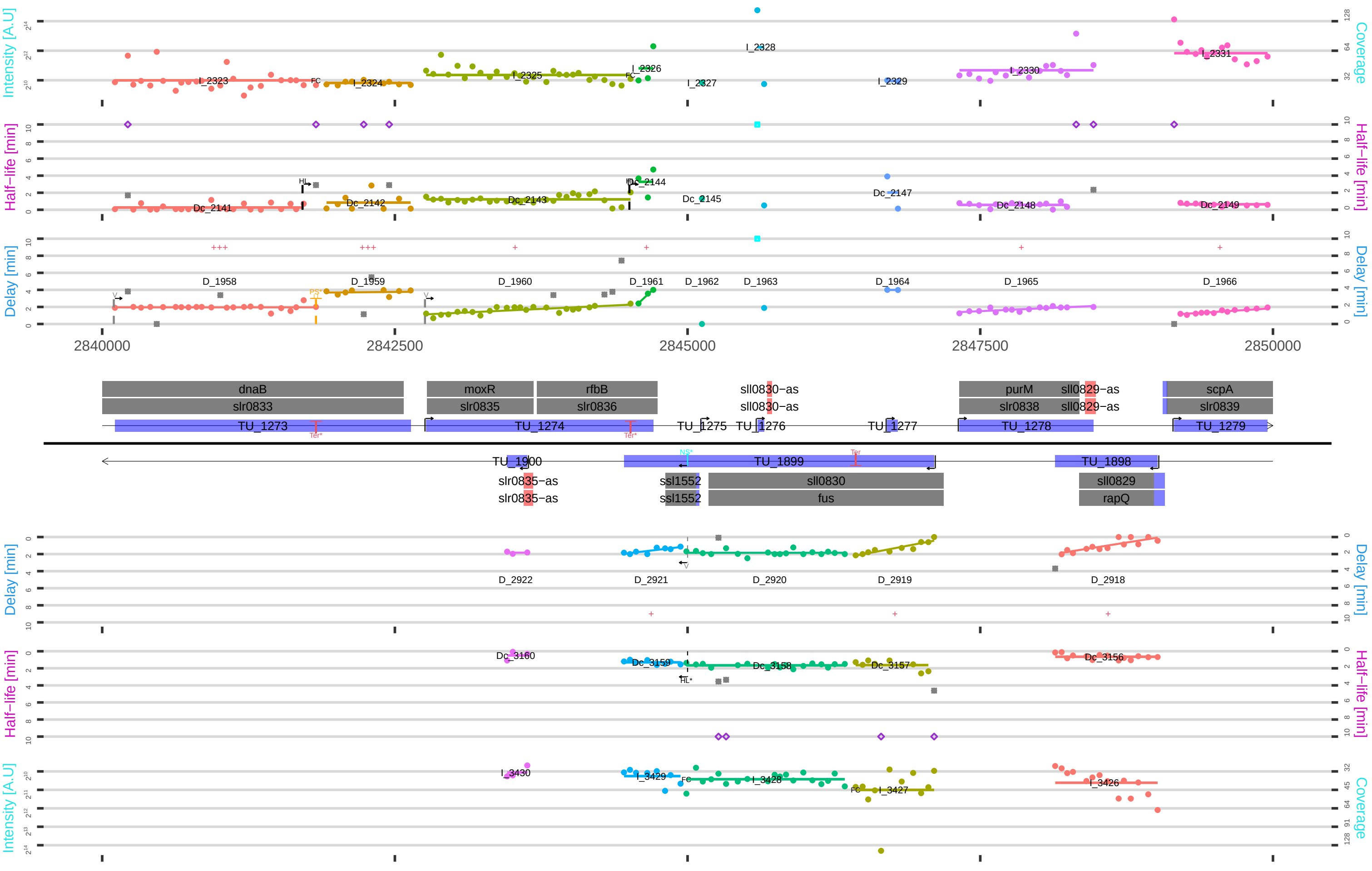

Term: termination (1), NS: new start (1), PS: pausing site (0), iTSS_I: internal starting site (2)

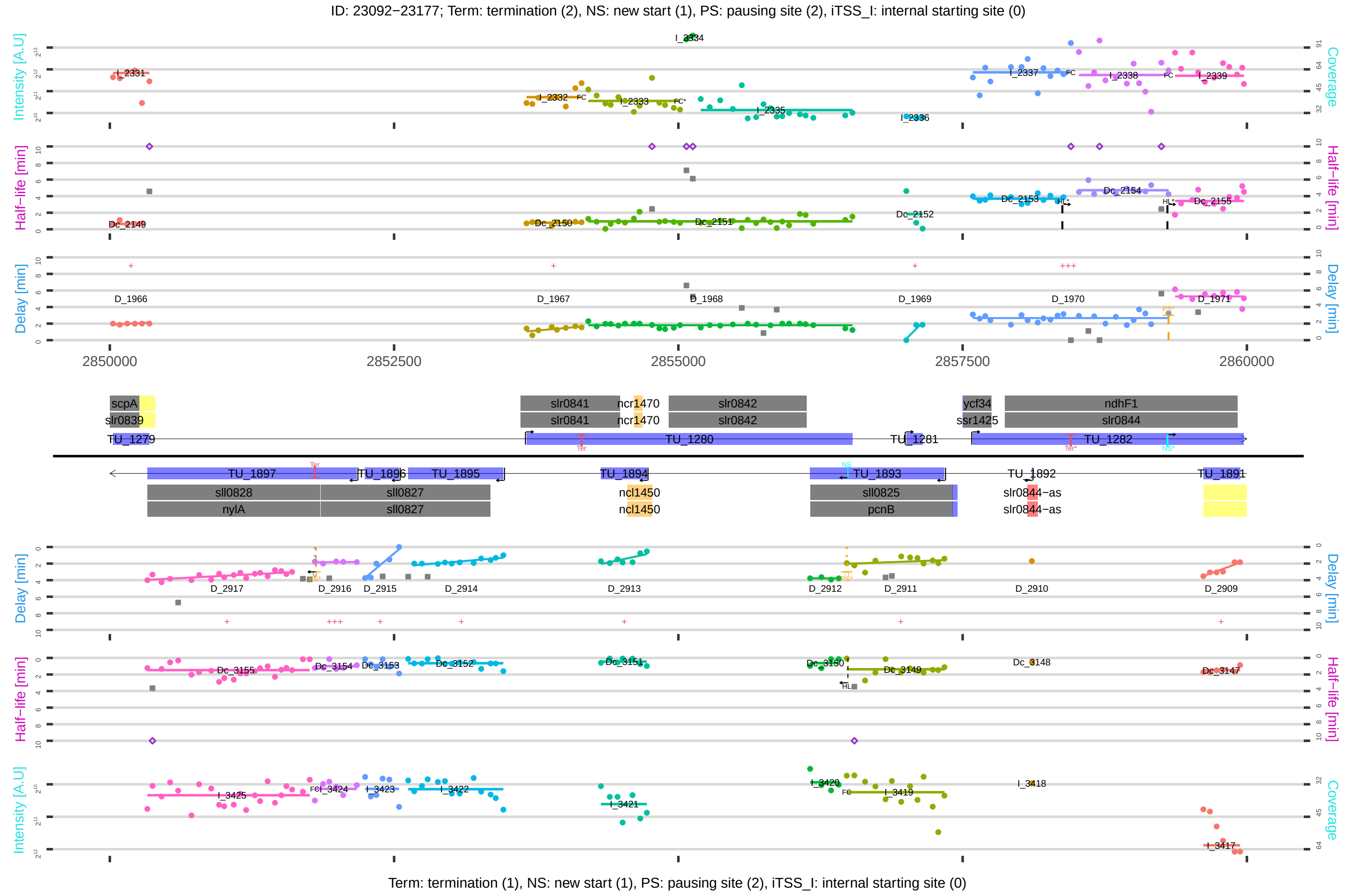

ID: 23092−23177; Term: termination (2), NS: new start (1), PS: pausing site (2), iTSS_I: internal starting site (0)
Intensity [A.U]
Coverage
Half−life [min]
Delay [min]
2850000
2852500
2855000
2857500
2860000
scpA
slr0839
slr0841
ncr1470
slr0842
ycf34
ssr1425
ndhF1
slr0844
TU_1279
TU_1280
TU_1281
TU_1282
TU_1897
TU_1896
TU_1895
TU_1894
TU_1893
TU_1892
TU_1891
sll0828
nylA
sll0827
ncl1450
sll0825
pcnB
slr0844−as
Term: termination (1), NS: new start (1), PS: pausing site (2), iTSS_I: internal starting site (0)

ID: 23178−23248; Term: termination (0), NS: new start (1), PS: pausing site (0), iTSS_I: internal starting site (0)

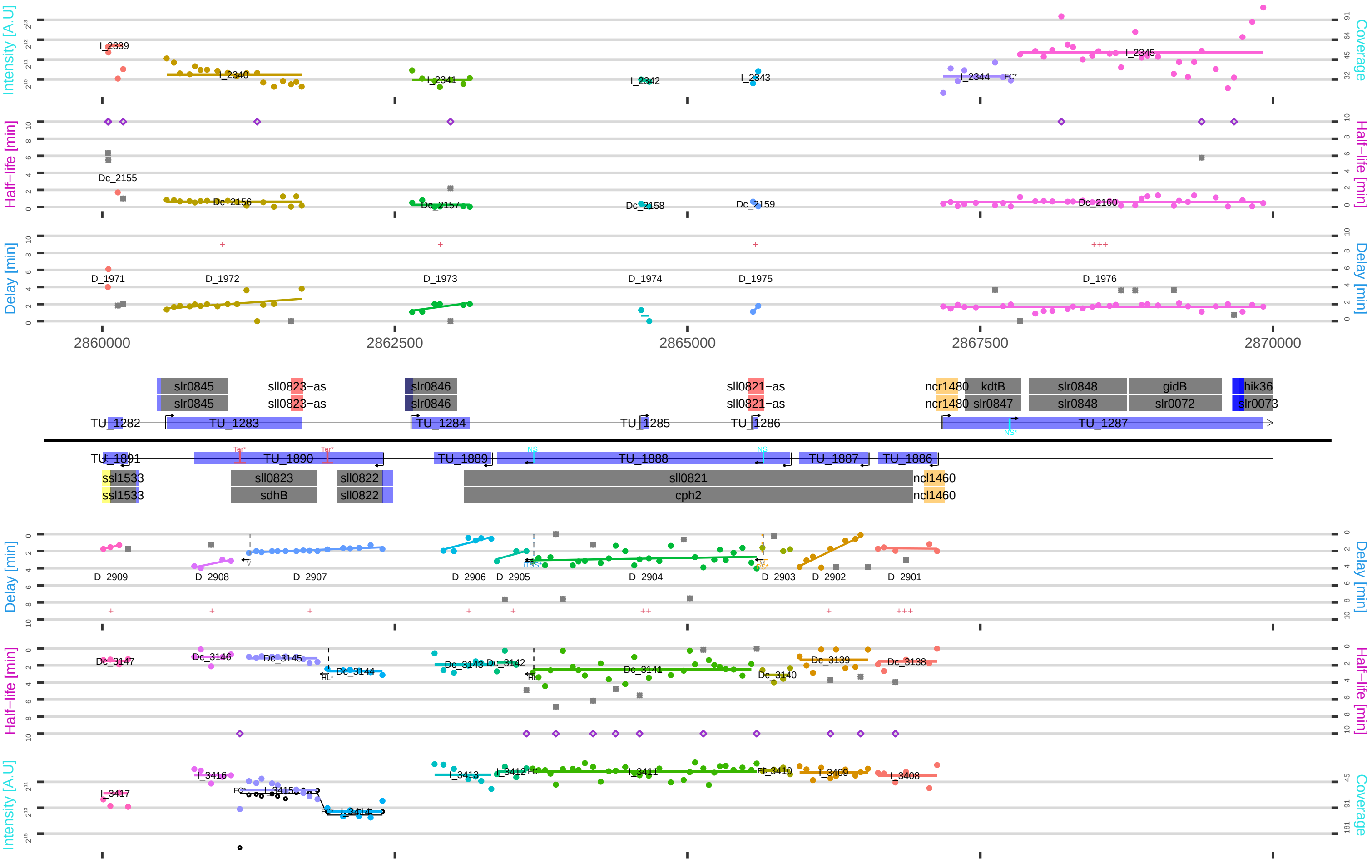

Term: termination (2), NS: new start (2), PS: pausing site (2), iTSS_I: internal starting site (1)

ID: 23249−23342; Term: termination (2), NS: new start (0), PS: pausing site (2), iTSS_I: internal starting site (0)

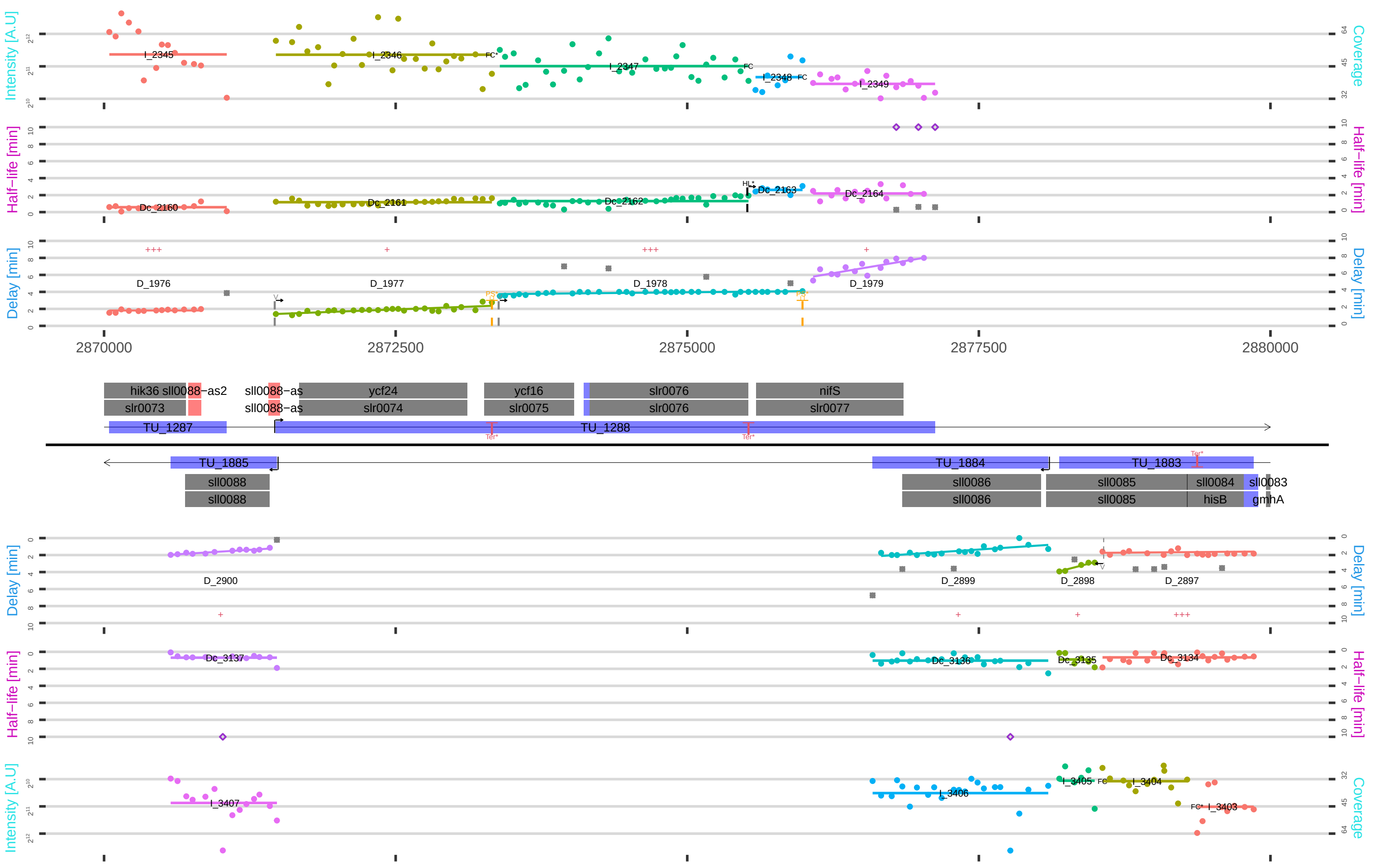

Term: termination (1), NS: new start (0), PS: pausing site (1), iTSS_I: internal starting site (0)

ID: 23343−23454; Term: termination (3), NS: new start (1), PS: pausing site (3), iTSS_I: internal starting site (1)

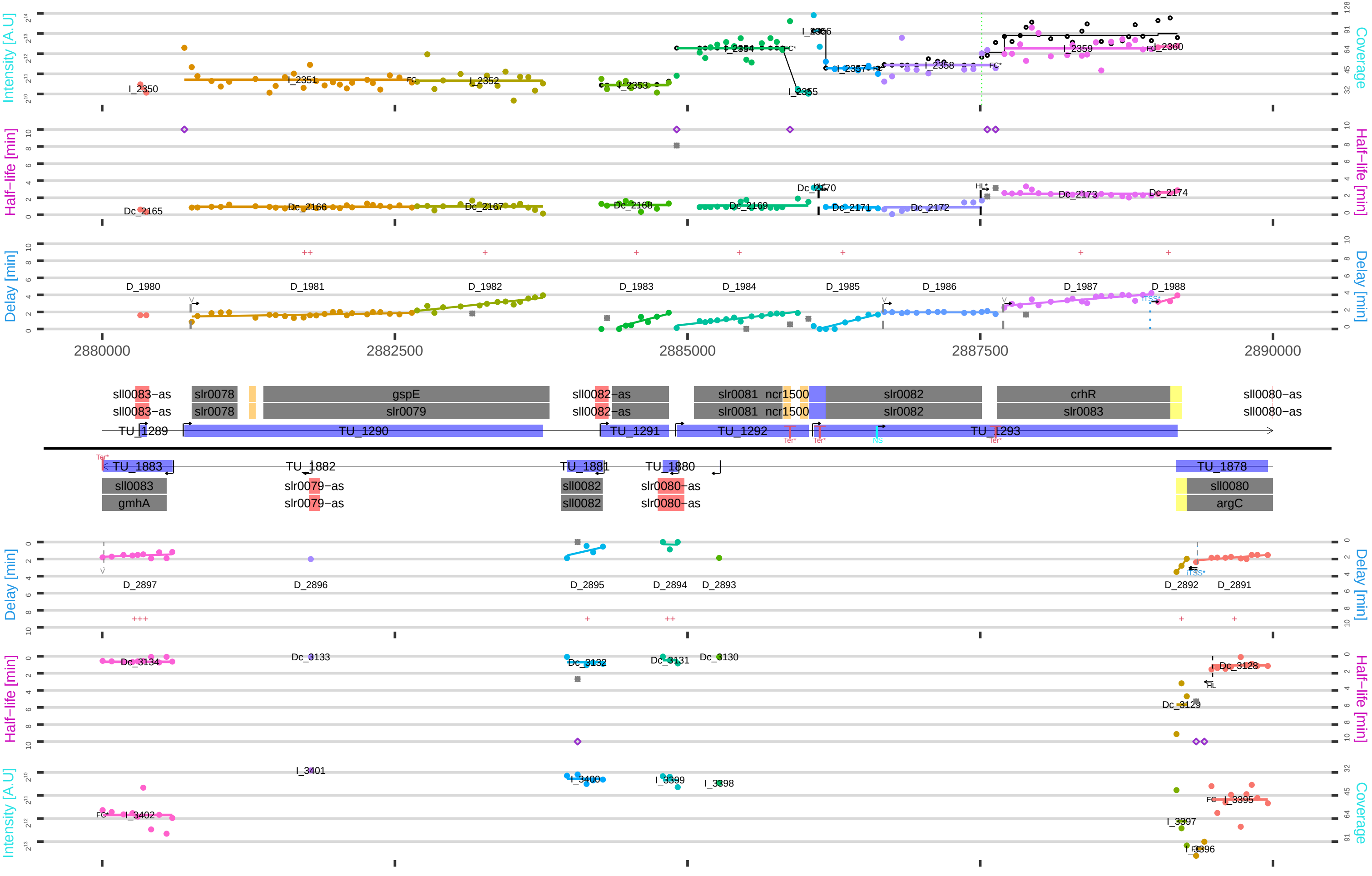

Term: termination (1), NS: new start (0), PS: pausing site (0), iTSS_I: internal starting site (1)

ID: 23455−23565; Term: termination (3), NS: new start (2), PS: pausing site (1), iTSS_I: internal starting site (2)

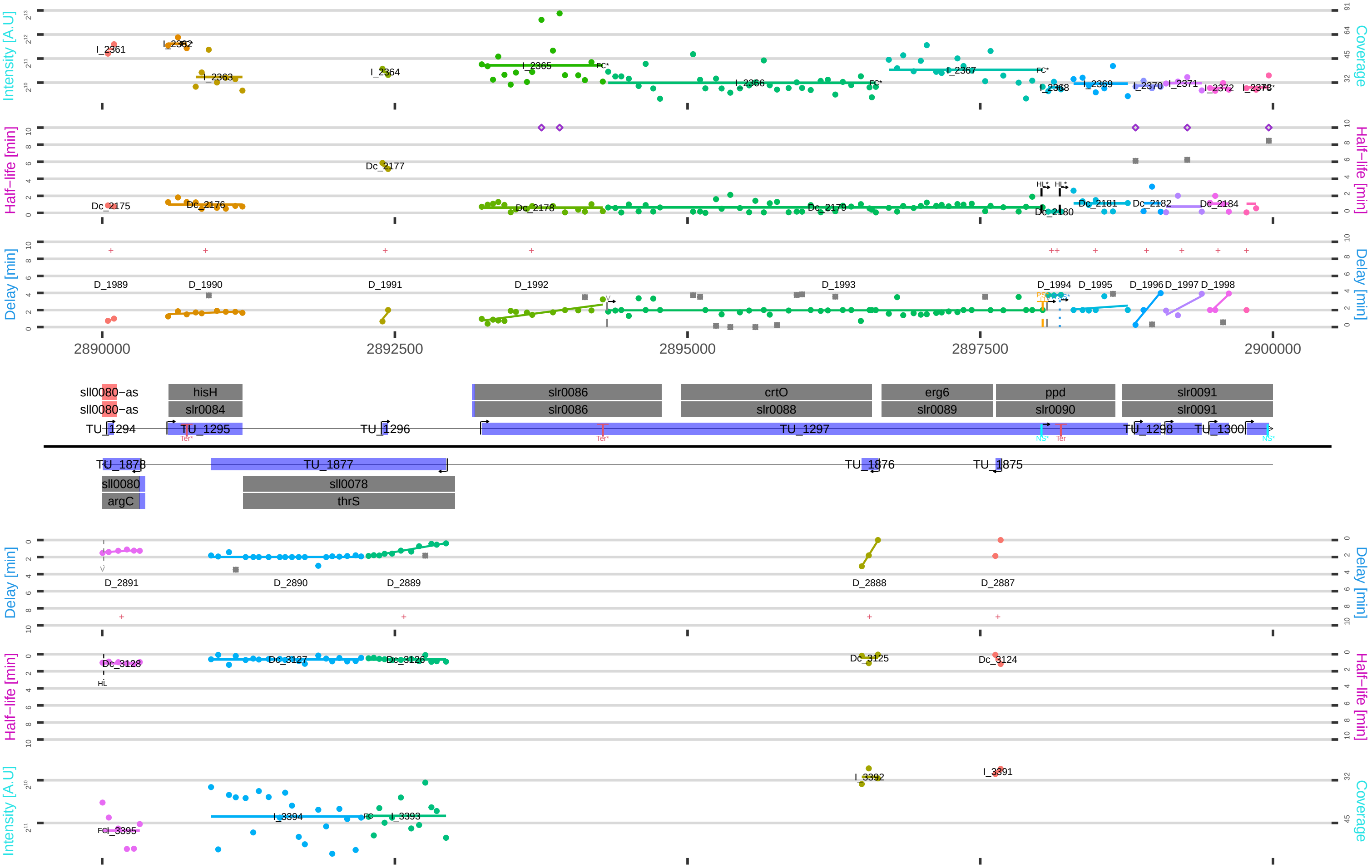

Term: termination (0), NS: new start (0), PS: pausing site (1), iTSS_I: internal starting site (0)

ID: 23566−23652; Term: termination (1), NS: new start (2), PS: pausing site (0), iTSS_I: internal starting site (2)

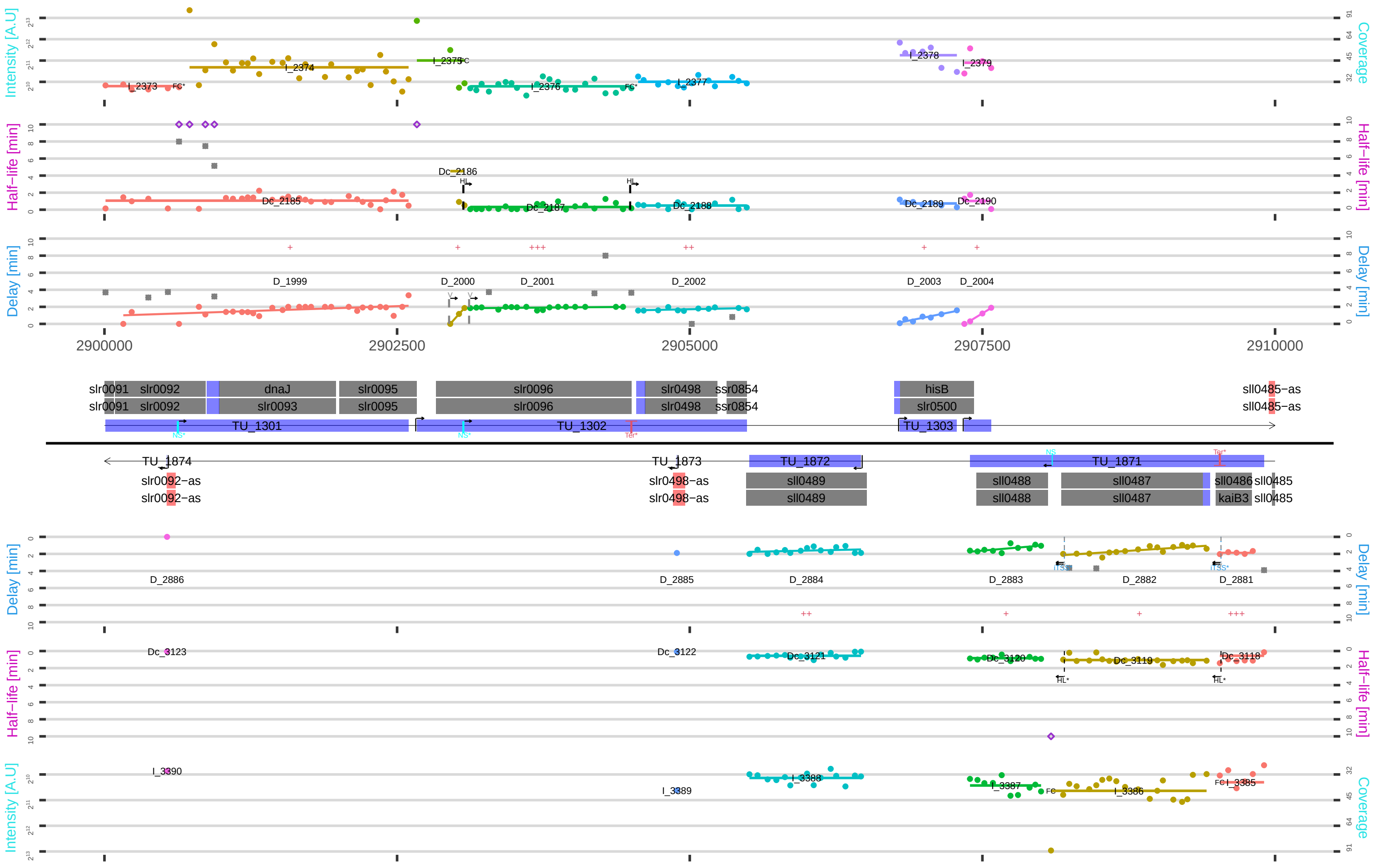

Term: termination (1), NS: new start (1), PS: pausing site (0), iTSS_I: internal starting site (2)

ID: 23654−23733; Term: termination (3), NS: new start (2), PS: pausing site (0), iTSS_I: internal starting site (2)

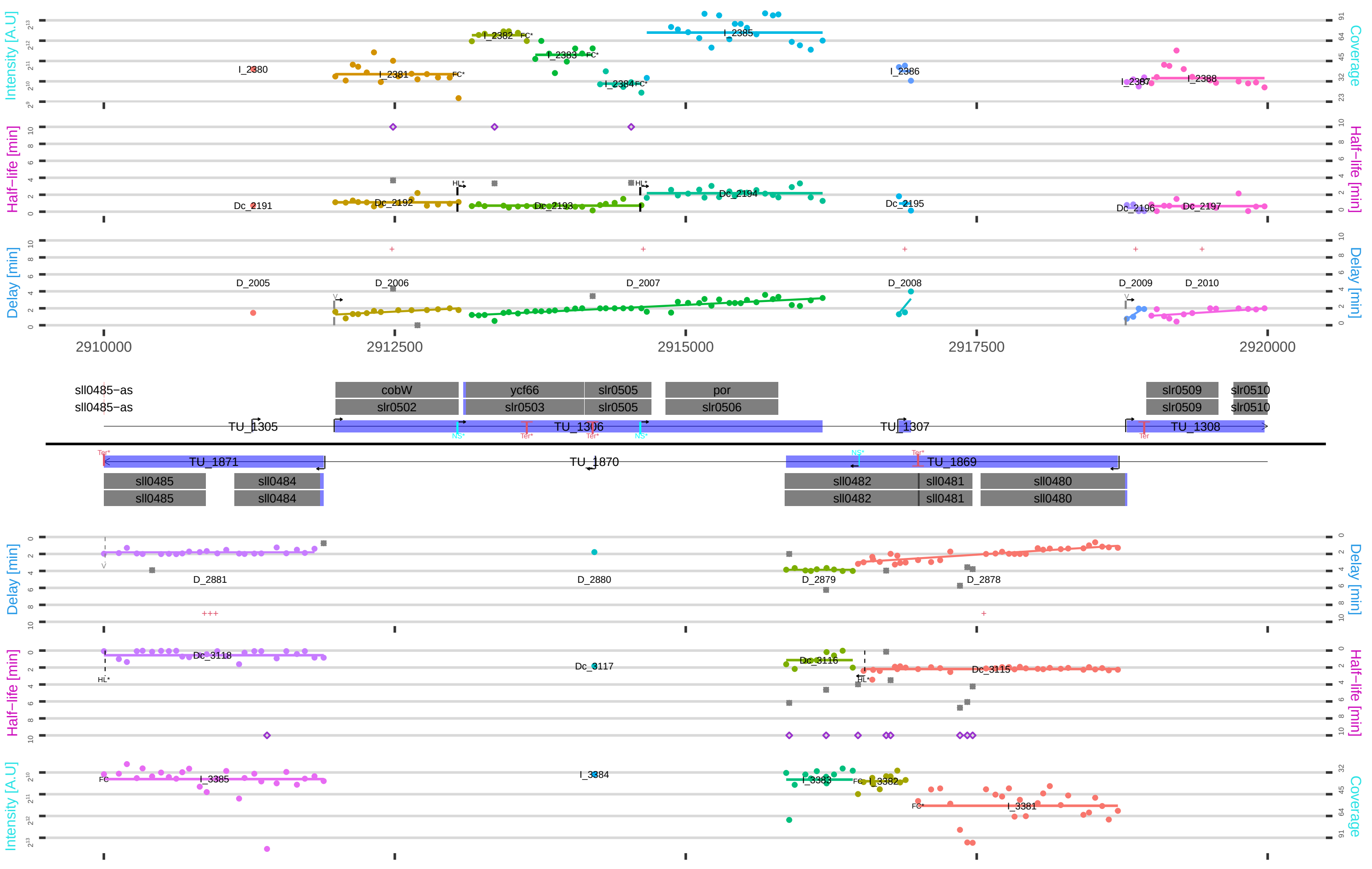

Term: termination (2), NS: new start (1), PS: pausing site (1), iTSS_I: internal starting site (0)

ID: 23736−23815; Term: termination (0), NS: new start (2), PS: pausing site (2), iTSS_I: internal starting site (0)

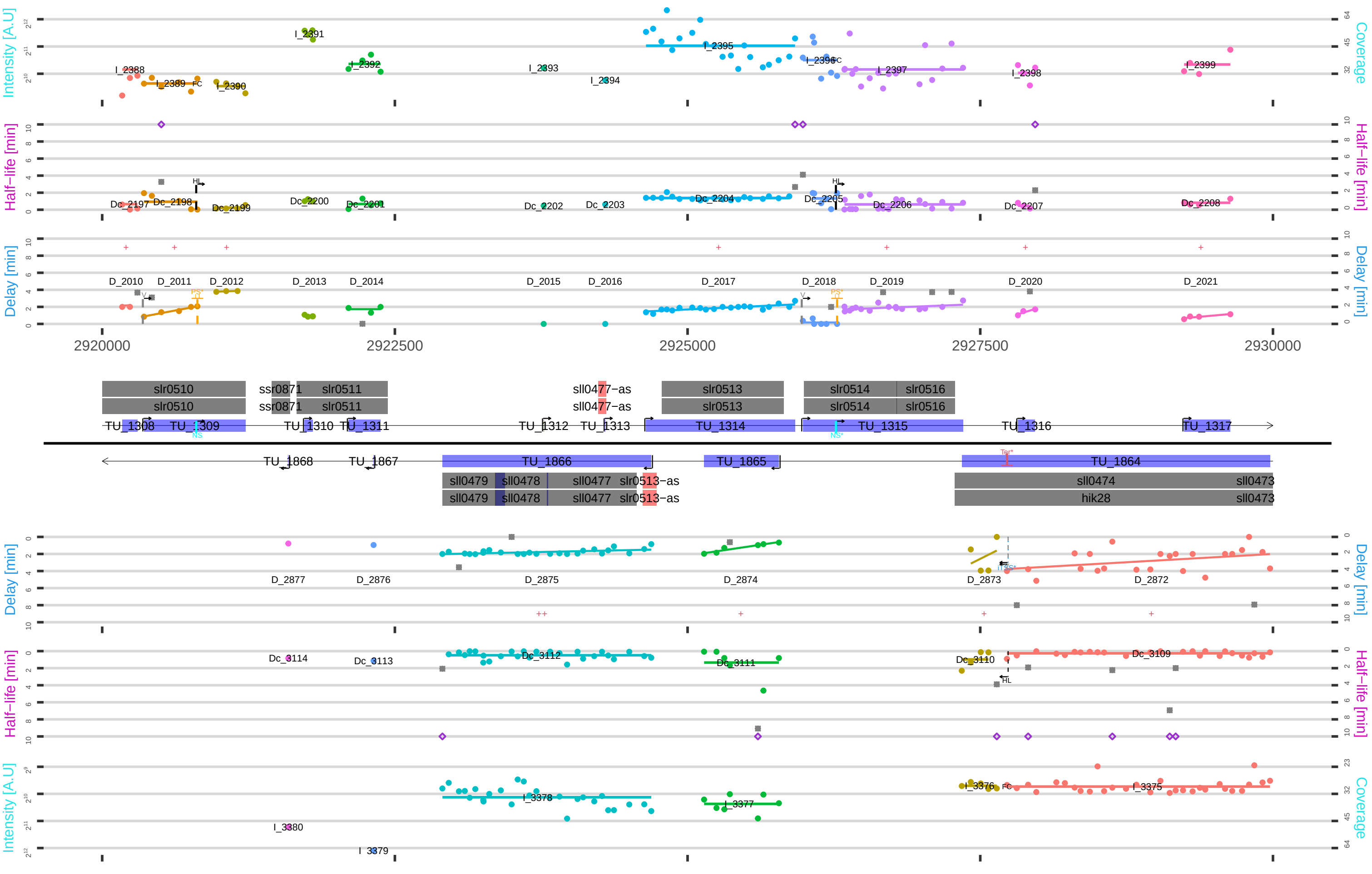

Term: termination (1), NS: new start (0), PS: pausing site (0), iTSS_I: internal starting site (1)

ID: 23816–23880; Term: termination (2), NS: new start (3), PS: pausing site (2), iTSS_I: internal starting site (3)

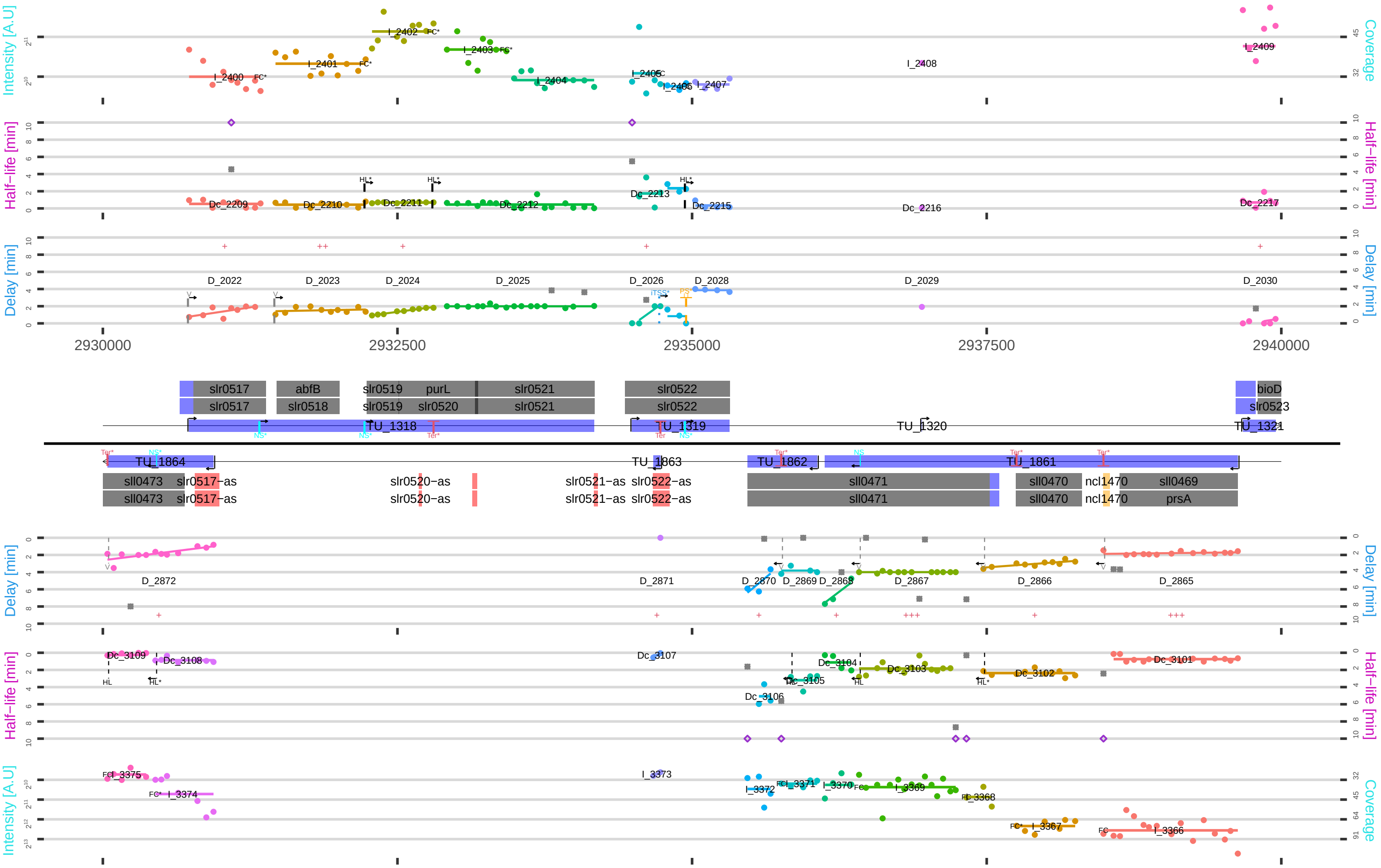

Term: termination (4), NS: new start (2), PS: pausing site (2), iTSS_I: internal starting site (2)

ID: 23881−23945; Term: termination (2), NS: new start (0), PS: pausing site (1), iTSS_I: internal starting site (0)

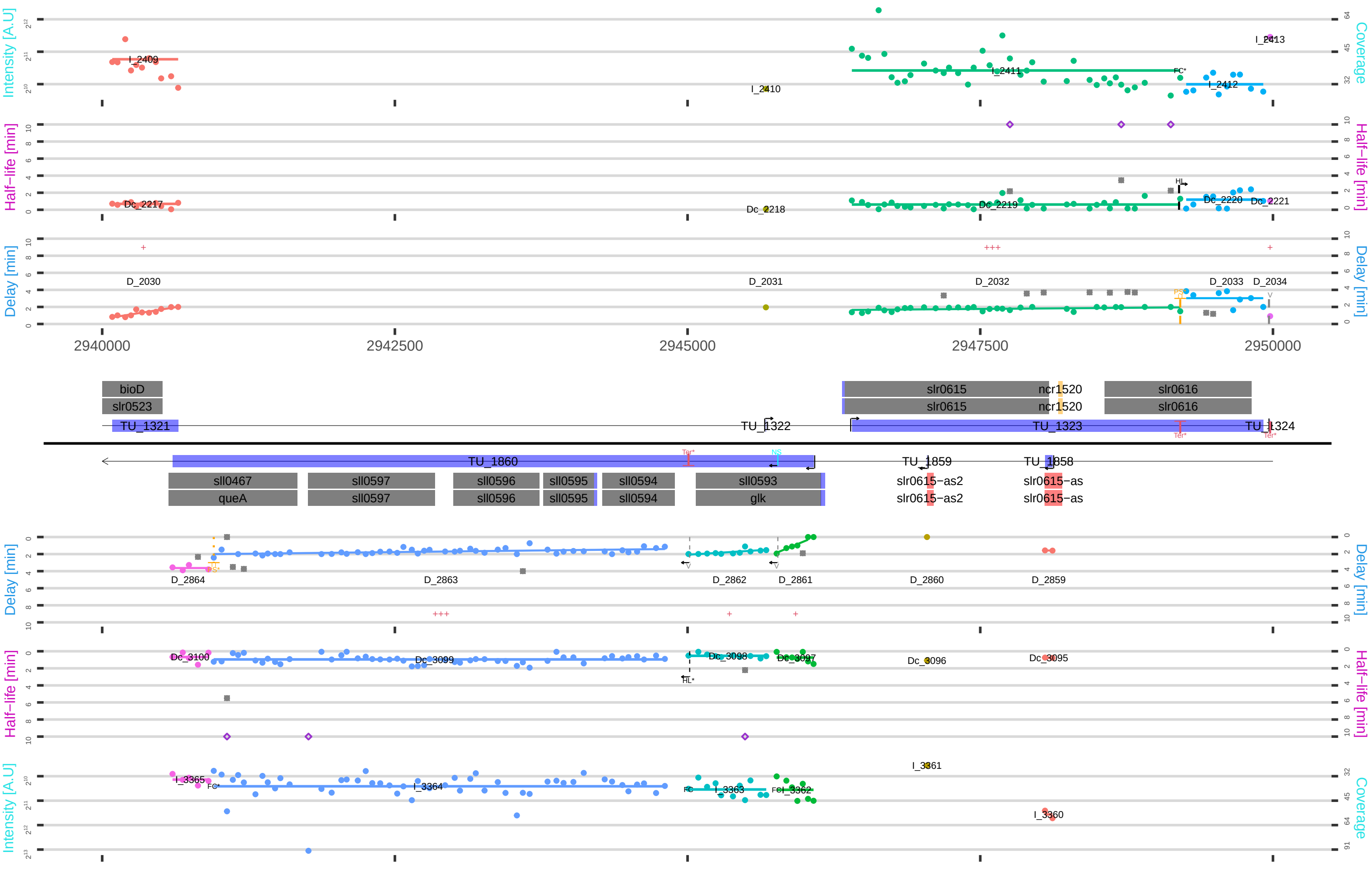

Term: termination (1), NS: new start (1), PS: pausing site (1), iTSS_I: internal starting site (2)

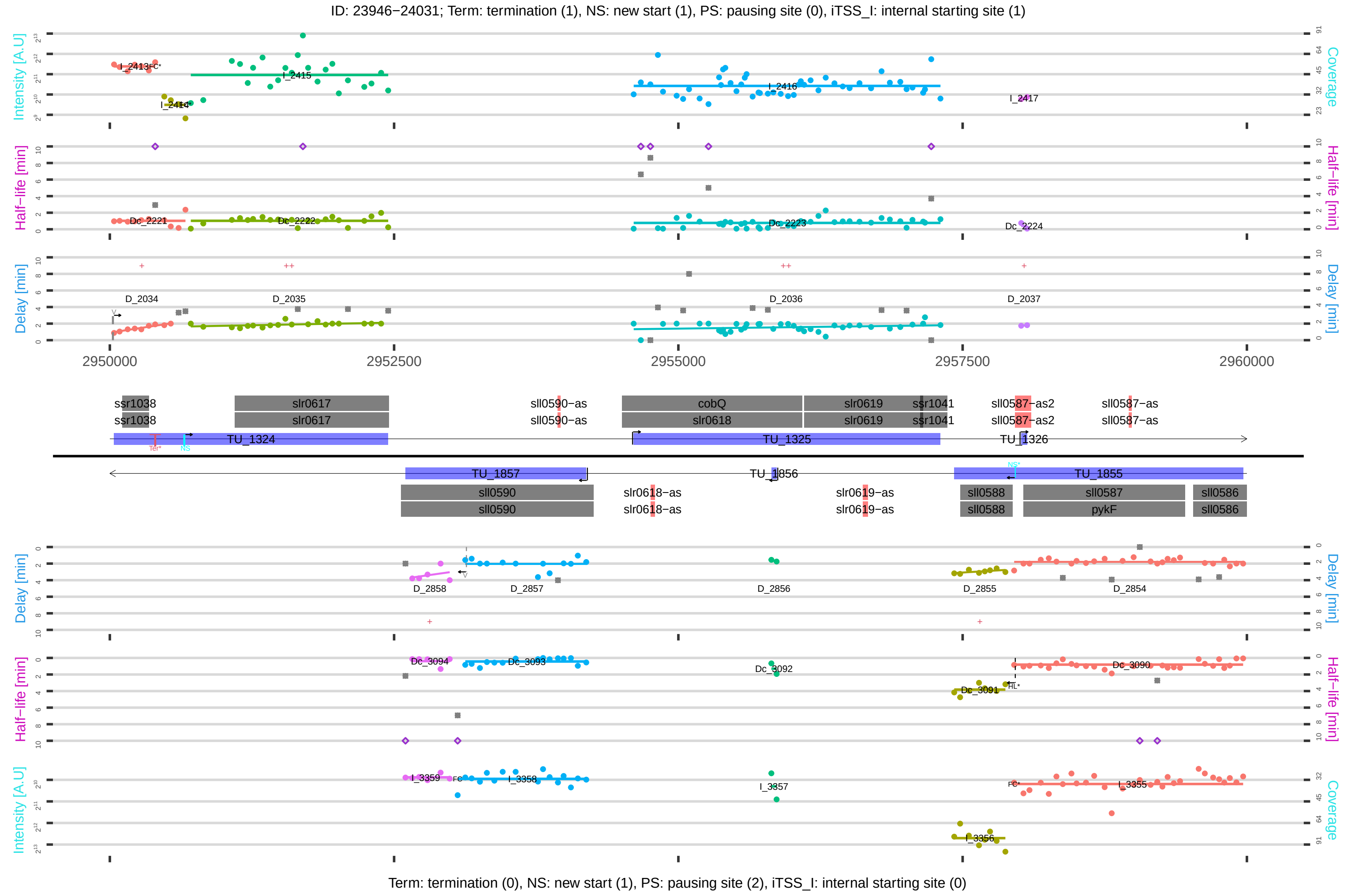
ID: 23946−24031; Term: termination (1), NS: new start (1), PS: pausing site (0), iTSS_I: internal starting site (1)
Intensity [A.U]
Coverage
Half−life [min]
Delay [min]
I_2413
I_2414
I_2415
I_2416
I_2417
Dc_2221
Dc_2222
Dc_2223
Dc_2224
D_2034
D_2035
D_2036
D_2037
2950000
2952500
2955000
2957500
2960000
ssr1038
slr0617
sll0590−as
cobQ
slr0618
slr0619
ssr1041
sll0587−as2
sll0587−as
TU_1324
TU_1325
TU_1326
TU_1857
TU_1856
TU_1855
sll0590
slr0618−as
slr0619−as
sll0588
sll0587
pykF
sll0586
D_2858
D_2857
D_2856
D_2855
D_2854
Dc_3094
Dc_3093
Dc_3092
Dc_3091
Dc_3090
I_3359
I_3358
I_3357
I_3356
I_3355
Term: termination (0), NS: new start (1), PS: pausing site (2), iTSS_I: internal starting site (0)

ID: 24032−24116; Term: termination (1), NS: new start (2), PS: pausing site (2), iTSS_I: internal starting site (0)

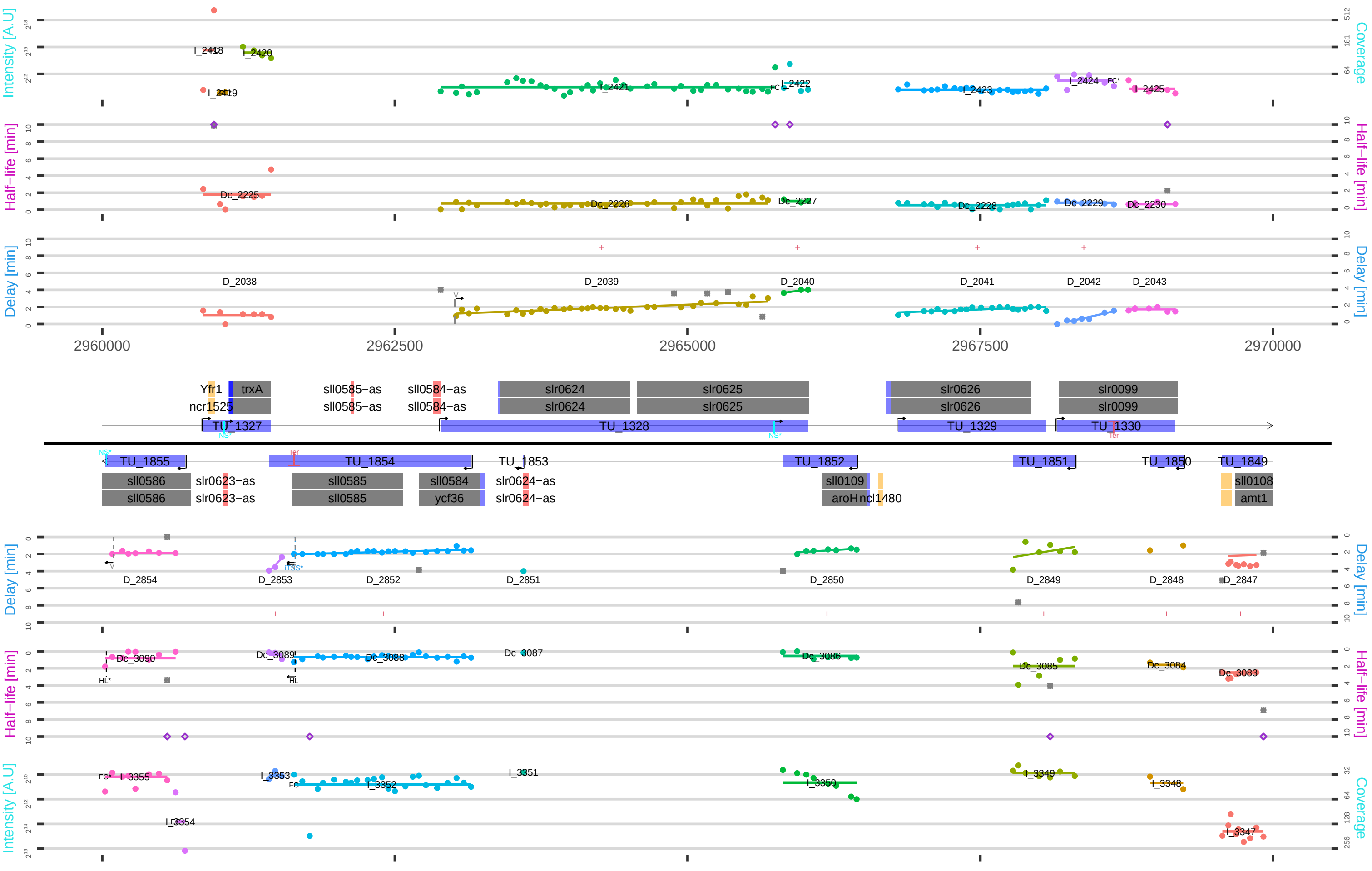

Term: termination (1), NS: new start (1), PS: pausing site (0), iTSS_I: internal starting site (1)

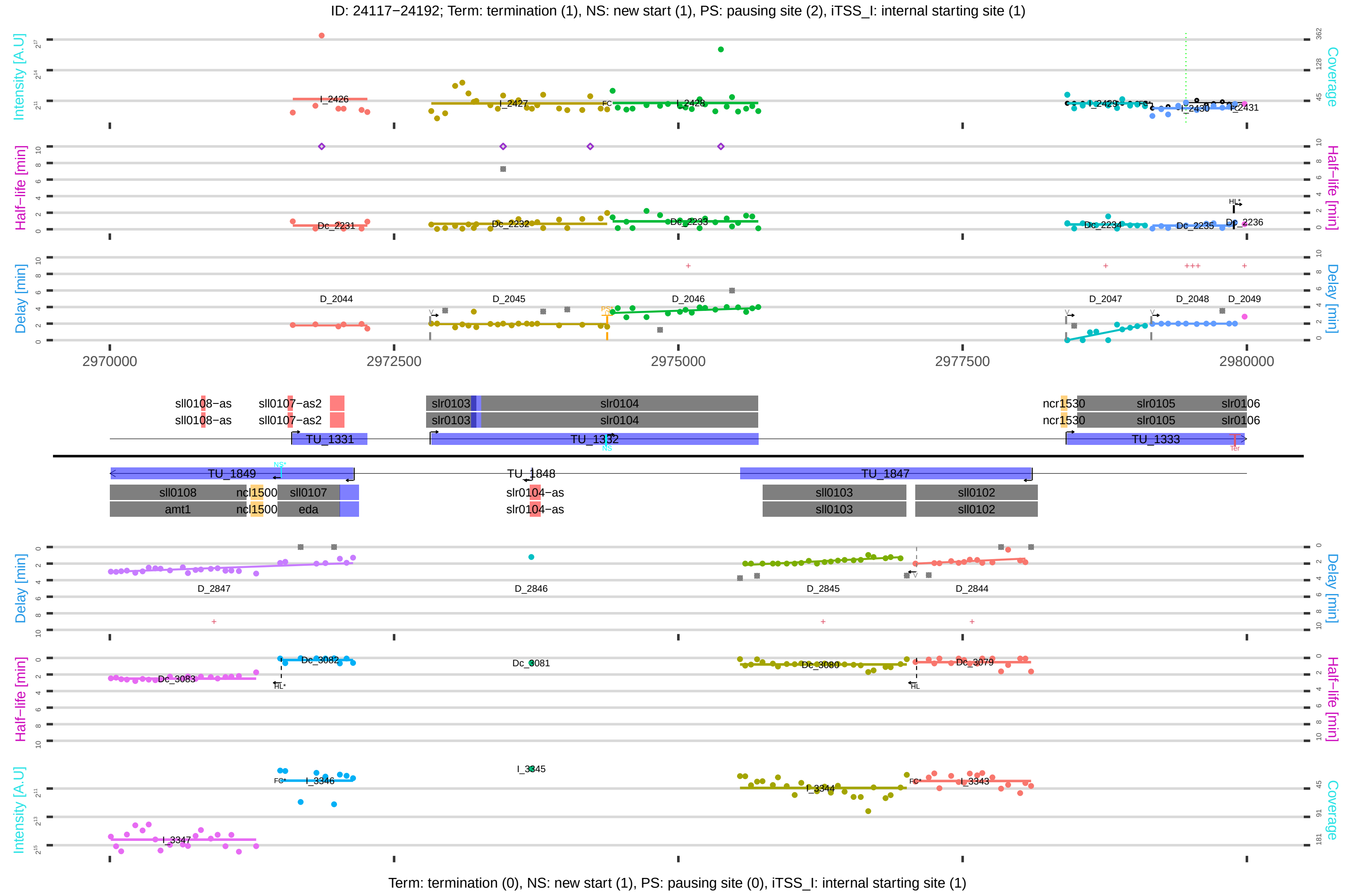

ID: 24117−24192; Term: termination (1), NS: new start (1), PS: pausing site (2), iTSS_I: internal starting site (1)
Intensity [A.U]
Coverage
Half−life [min]
Delay [min]
I_2426
I_2427
I_2428
I_2429
I_2430
I_2431
Dc_2231
Dc_2232
Dc_2233
Dc_2234
Dc_2235
Dc_2236
D_2044
D_2045
D_2046
D_2047
D_2048
D_2049
2970000
2972500
2975000
2977500
2980000
sll0108−as
sll0107−as2
slr0103
slr0104
ncr1530
slr0105
slr0106
TU_1331
TU_1332
TU_1333
TU_1849
TU_1848
TU_1847
sll0108
amt1
ncl1500
sll0107
eda
slr0104−as
sll0103
sll0102
D_2847
D_2846
D_2845
D_2844
Dc_3083
Dc_3082
Dc_3081
Dc_3080
Dc_3079
I_3347
I_3346
I_3345
I_3344
I_3343
Term: termination (0), NS: new start (1), PS: pausing site (0), iTSS_I: internal starting site (1)

ID: 24193−24243; Term: termination (0), NS: new start (0), PS: pausing site (0), iTSS_I: internal starting site (0)

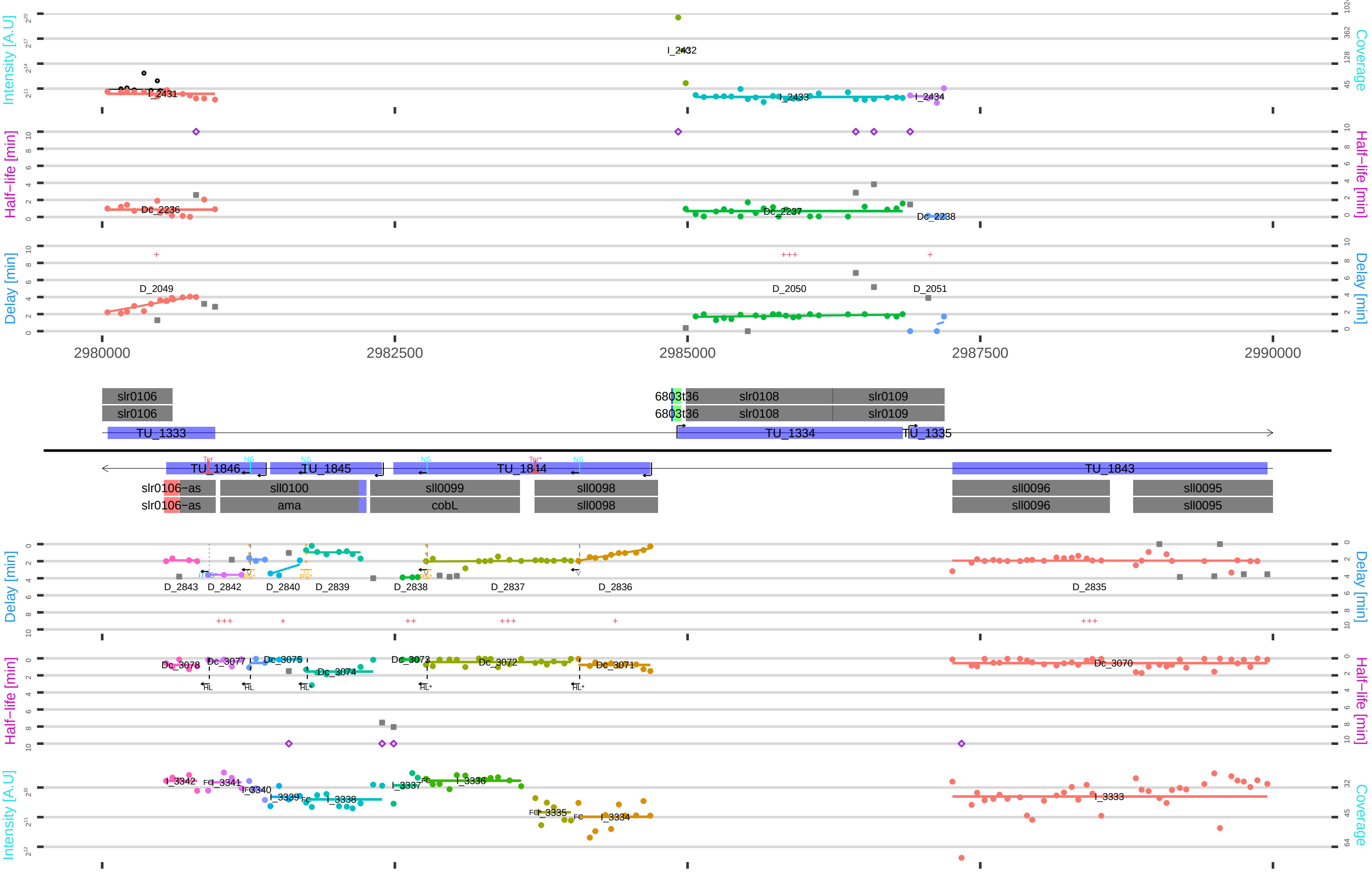

Term: termination (2), NS: new start (4), PS: pausing site (3), iTSS_I: internal starting site (2)

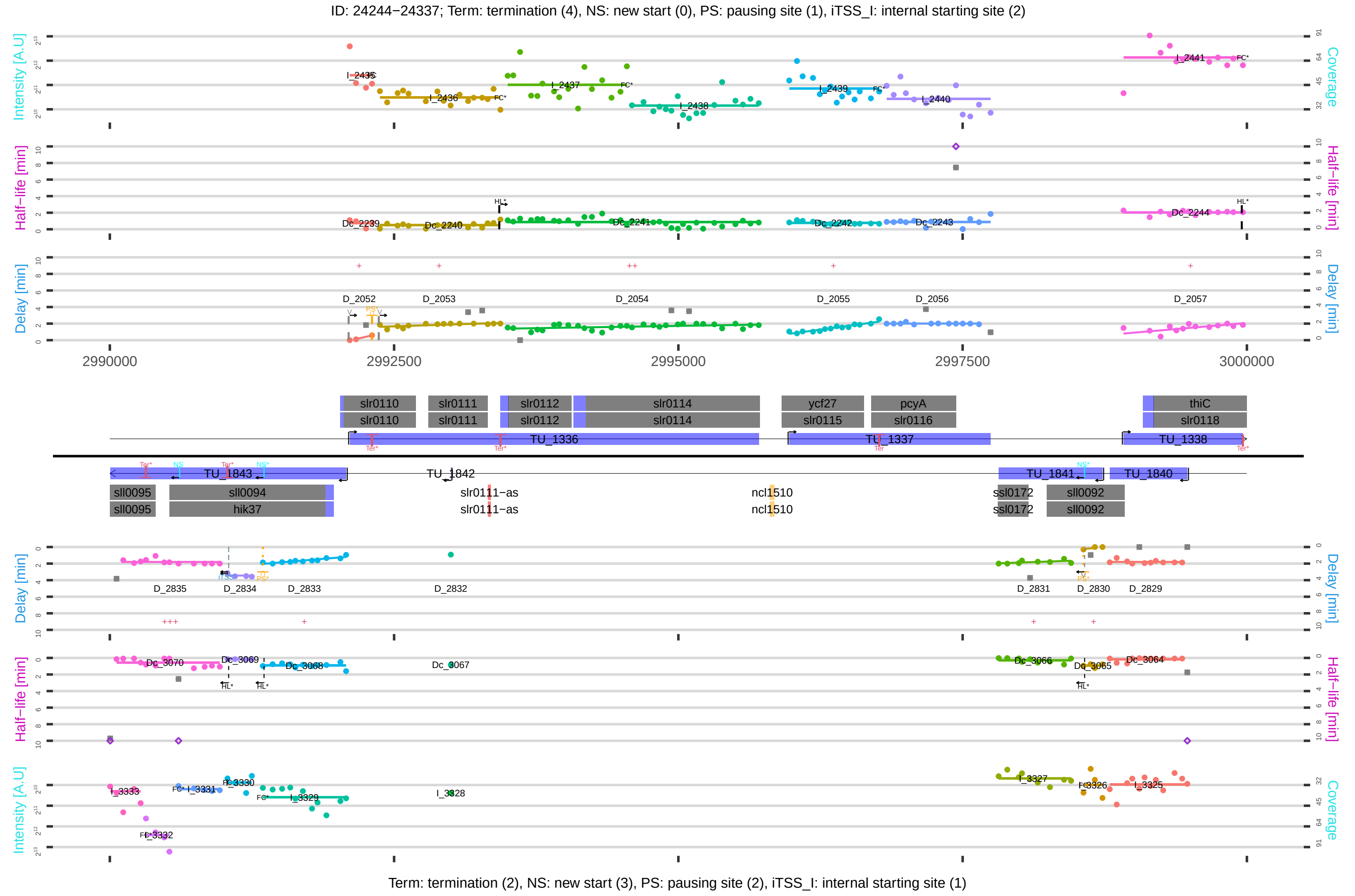
ID: 24244−24337; Term: termination (4), NS: new start (0), PS: pausing site (1), iTSS_I: internal starting site (2)
Intensity [A.U]
Coverage
Half−life [min]
Delay [min]
I_2435
I_2436
I_2437
I_2438
I_2439
I_2440
I_2441
Dc_2239
Dc_2240
Dc_2241
Dc_2242
Dc_2243
Dc_2244
D_2052
D_2053
D_2054
D_2055
D_2056
D_2057
2990000
2992500
2995000
2997500
3000000
slr0110
slr0111
slr0112
slr0114
ycf27
pcyA
thiC
slr0115
slr0116
slr0118
TU_1336
TU_1337
TU_1338
TU_1843
TU_1842
TU_1841
TU_1840
sll0095
sll0094
hik37
slr0111−as
ncl1510
ssl0172
sll0092
D_2835
D_2834
D_2833
D_2832
D_2831
D_2830
D_2829
Dc_3070
Dc_3069
Dc_3068
Dc_3067
Dc_3066
Dc_3065
Dc_3064
I_3333
I_3332
I_3331
I_3330
I_3329
I_3328
I_3327
I_3326
I_3325
Term: termination (2), NS: new start (3), PS: pausing site (2), iTSS_I: internal starting site (1)

ID: 24338−24416; Term: termination (3), NS: new start (1), PS: pausing site (1), iTSS_I: internal starting site (1)

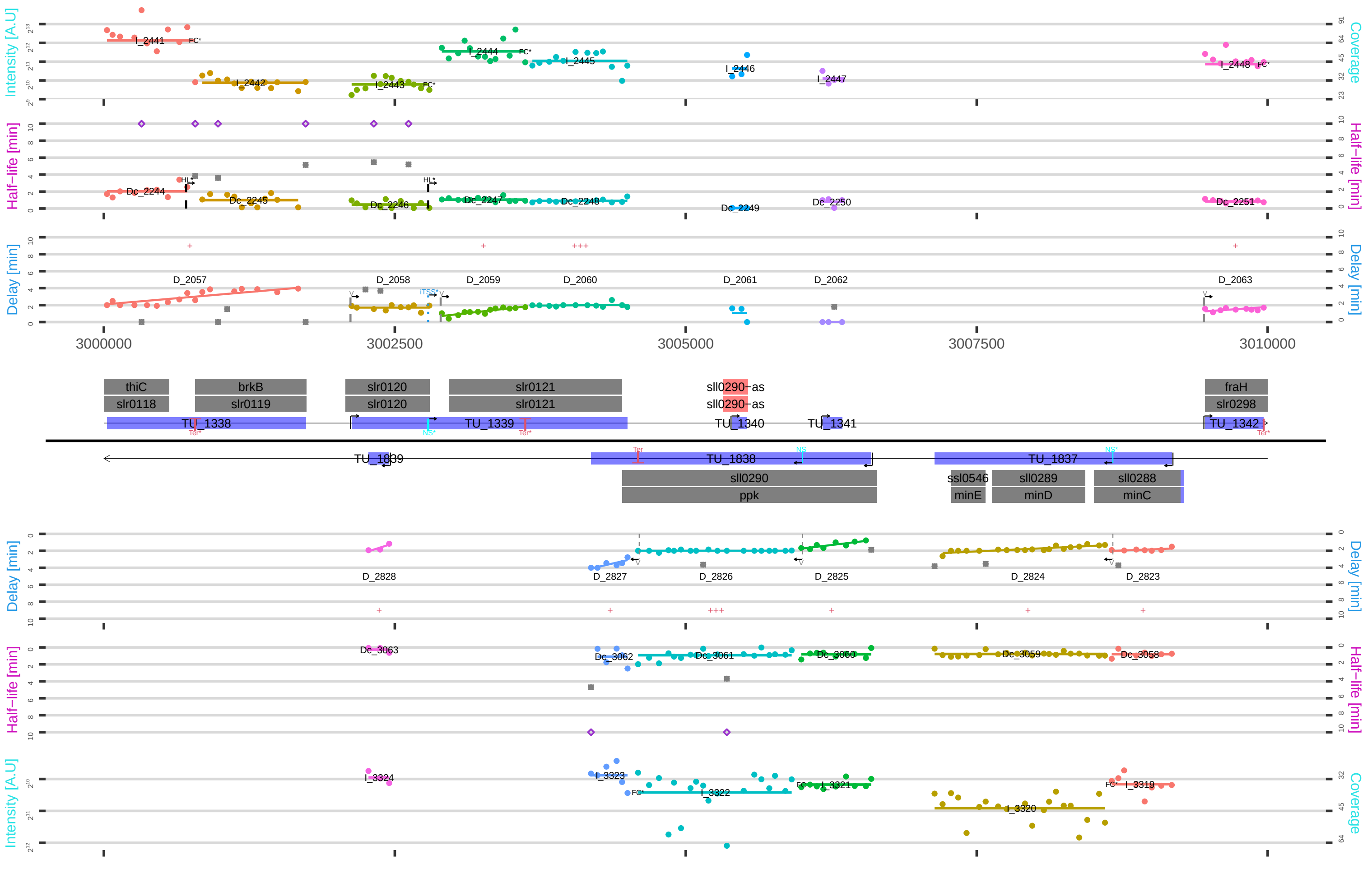

Term: termination (1), NS: new start (2), PS: pausing site (2), iTSS_I: internal starting site (1)

ID: 24417−24553; Term: termination (3), NS: new start (5), PS: pausing site (2), iTSS_I: internal starting site (4)

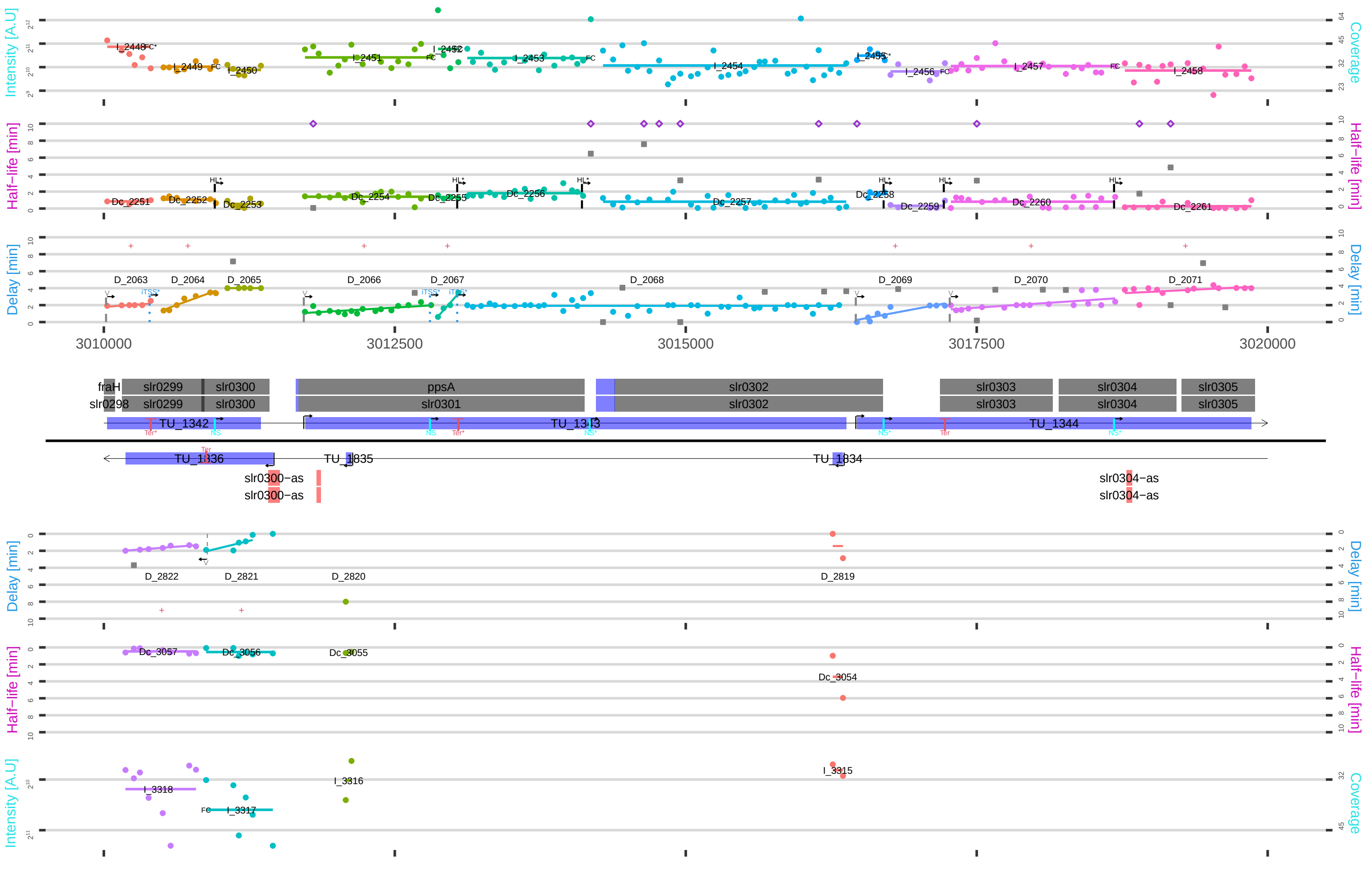

Term: termination (1), NS: new start (0), PS: pausing site (0), iTSS_I: internal starting site (1)

ID: 24554−24601; Term: termination (1), NS: new start (0), PS: pausing site (0), iTSS_I: internal starting site (1)

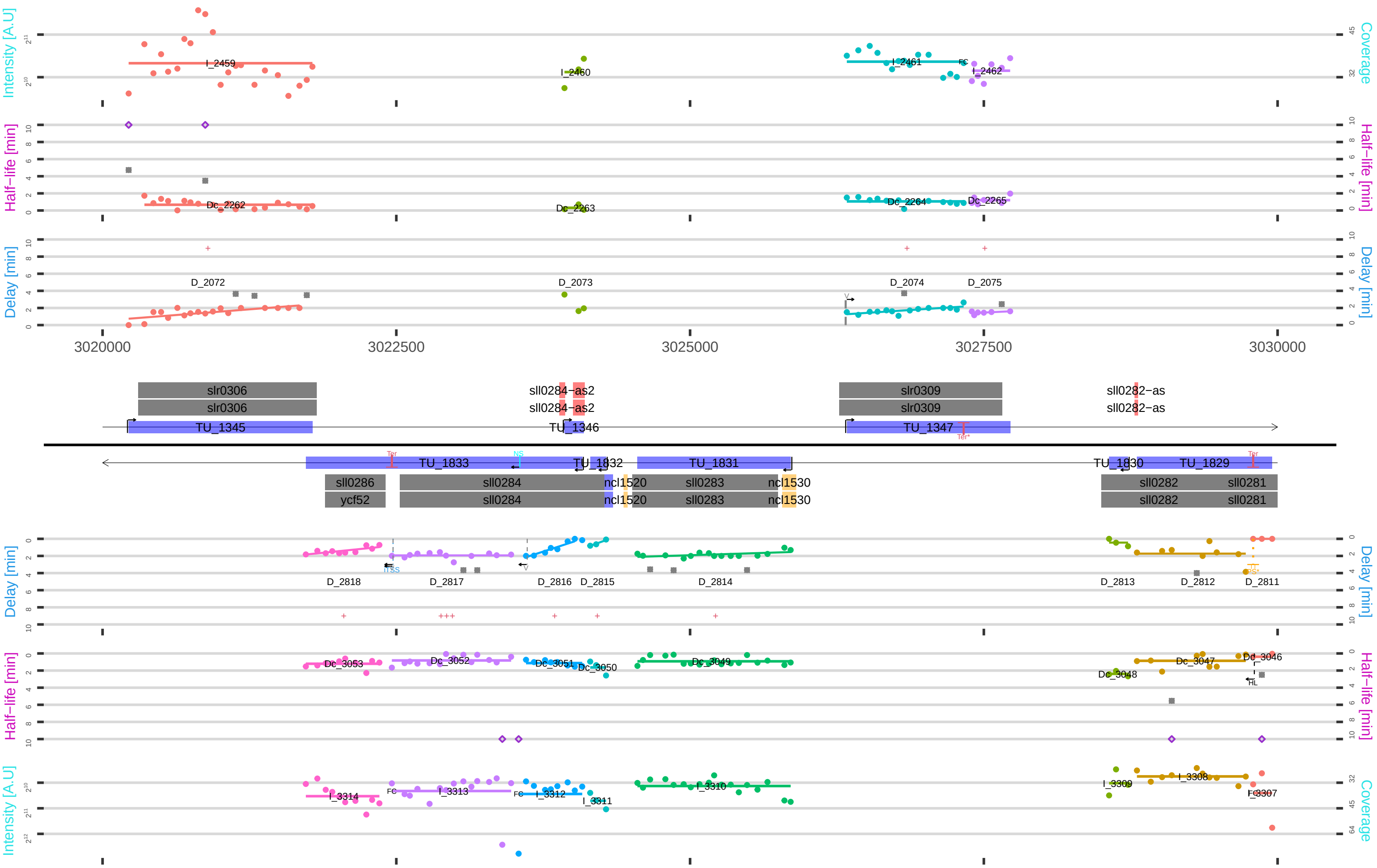

Term: termination (2), NS: new start (1), PS: pausing site (1), iTSS_I: internal starting site (2)

ID: 24602−24692; Term: termination (1), NS: new start (3), PS: pausing site (3), iTSS_I: internal starting site (1)

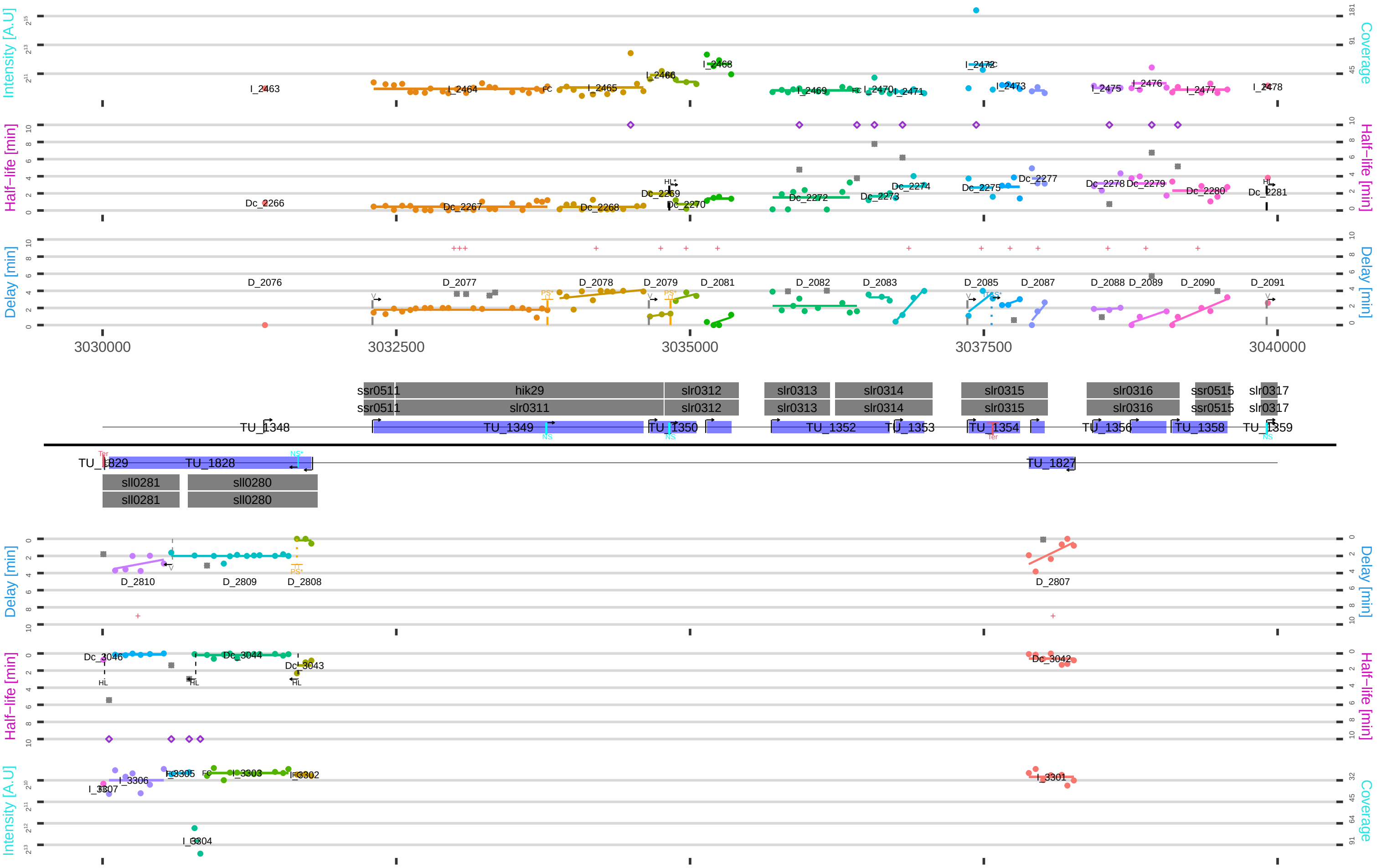

Term: termination (1), NS: new start (1), PS: pausing site (2), iTSS_I: internal starting site (0)

ID: 24693−24798; Term: termination (2), NS: new start (2), PS: pausing site (2), iTSS_I: internal starting site (2)

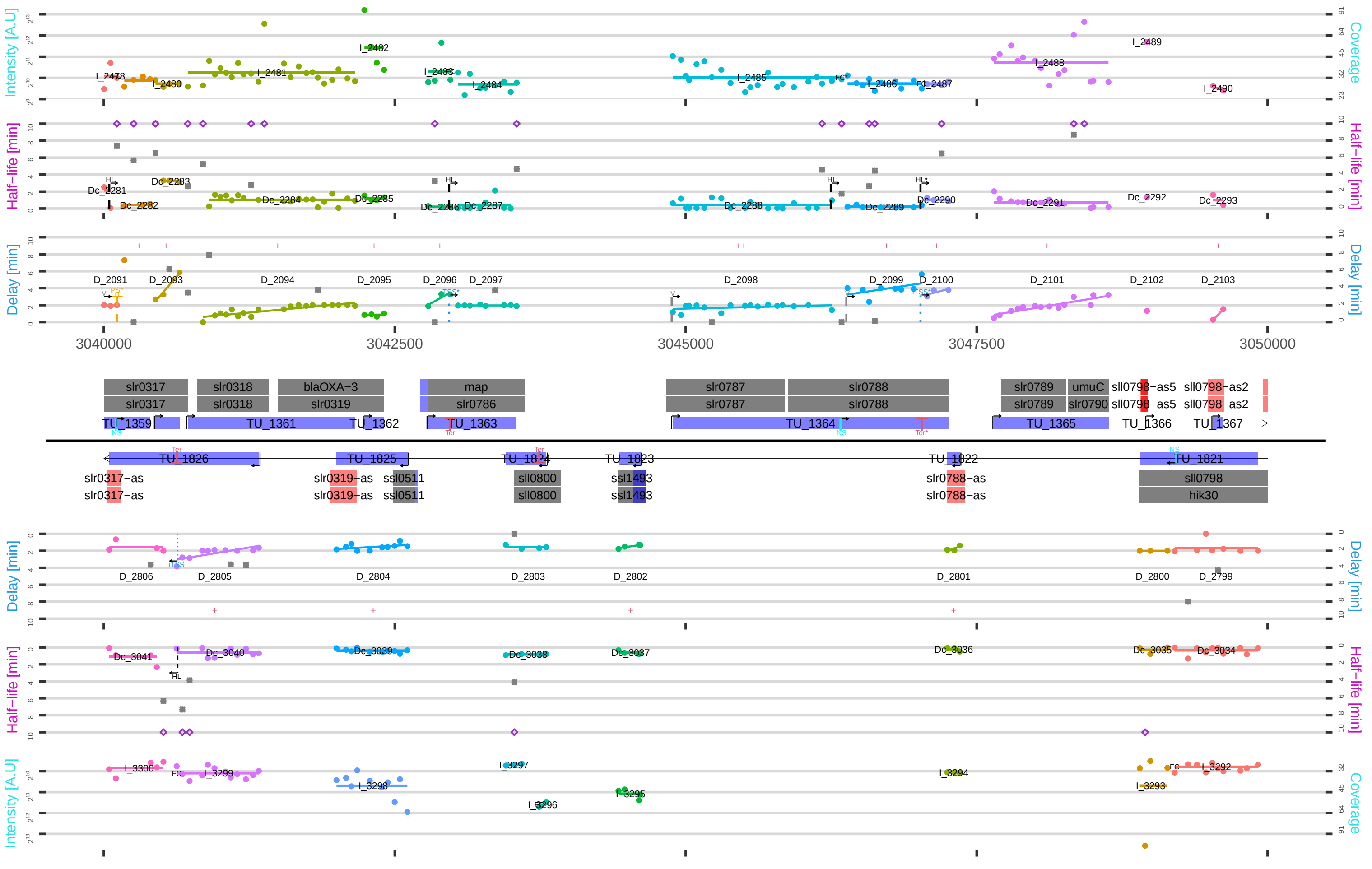

Term: termination (2), NS: new start (1), PS: pausing site (1), iTSS_I: internal starting site (1)

ID: 24799−24918; Term: termination (2), NS: new start (2), PS: pausing site (3), iTSS_I: internal starting site (0)

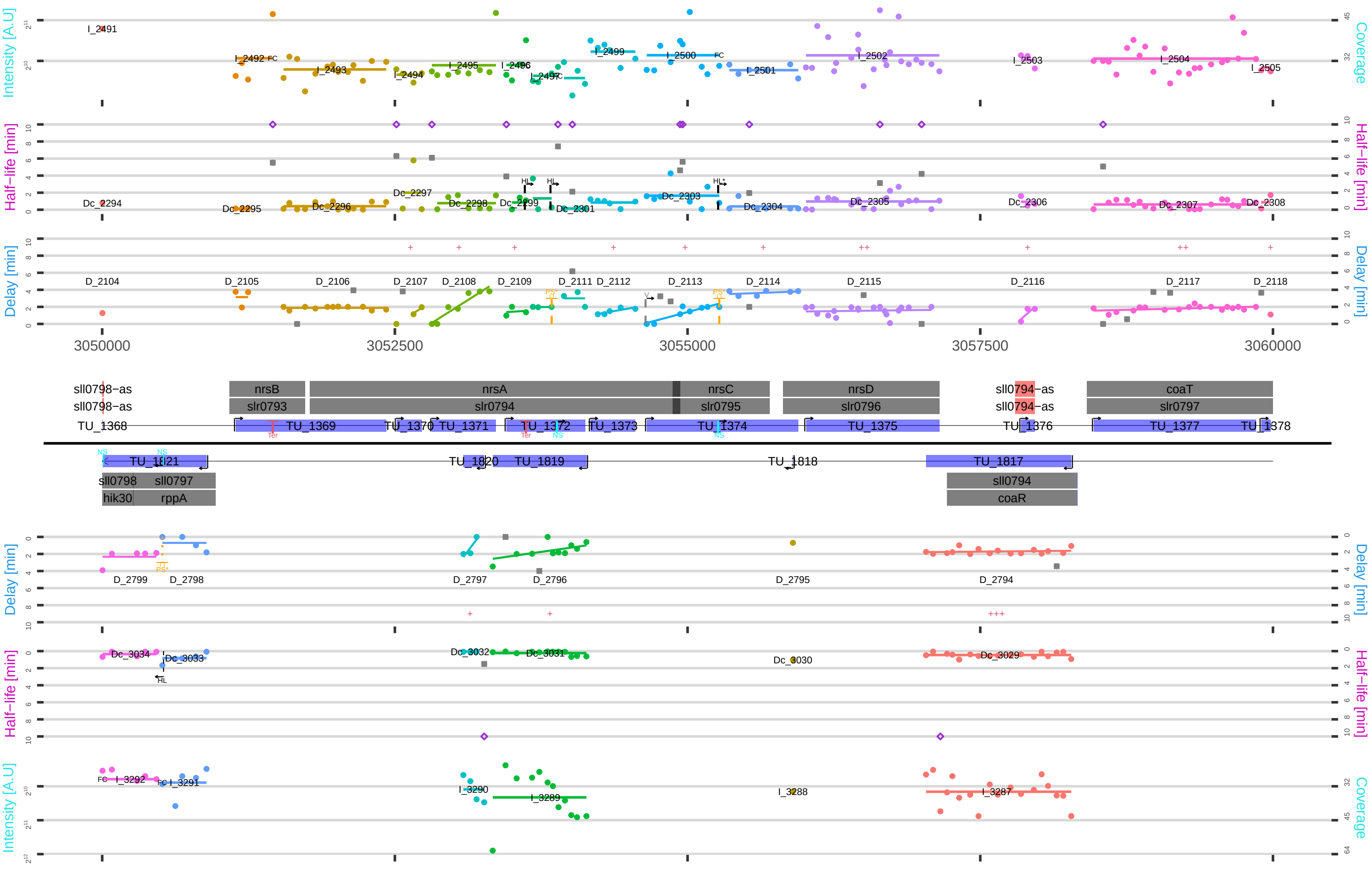

Term: termination (0), NS: new start (2), PS: pausing site (1), iTSS_I: internal starting site (0)

ID: 24919−24972; Term: termination (0), NS: new start (0), PS: pausing site (0), iTSS_I: internal starting site (0)

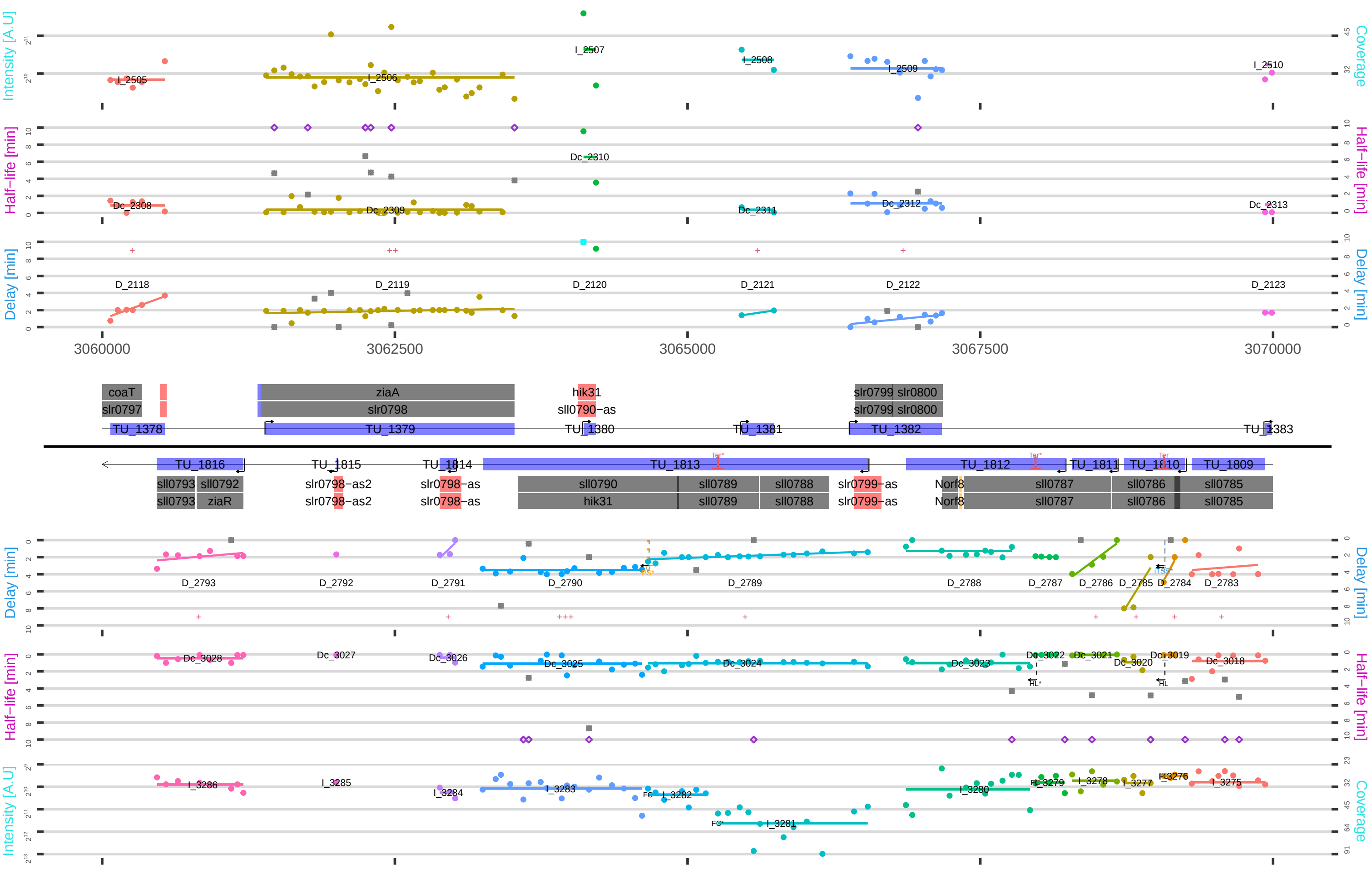

Term: termination (3), NS: new start (0), PS: pausing site (1), iTSS_I: internal starting site (2)

ID: 24973−24999; Term: termination (0), NS: new start (0), PS: pausing site (0), iTSS_I: internal starting site (0)

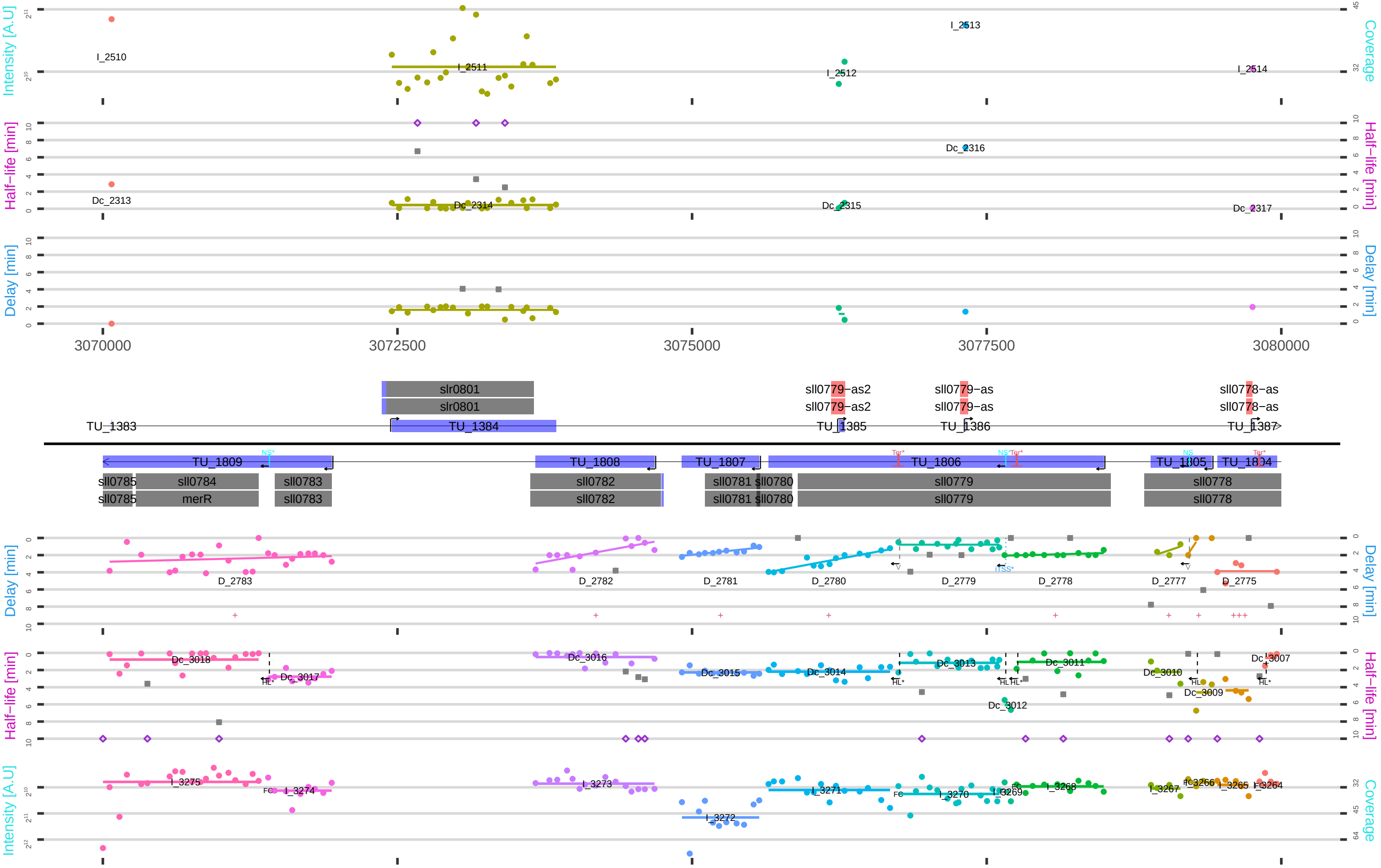

Term: termination (3), NS: new start (3), PS: pausing site (1), iTSS_I: internal starting site (2)

ID: 25000−25076; Term: termination (2), NS: new start (4), PS: pausing site (3), iTSS_I: internal starting site (2)

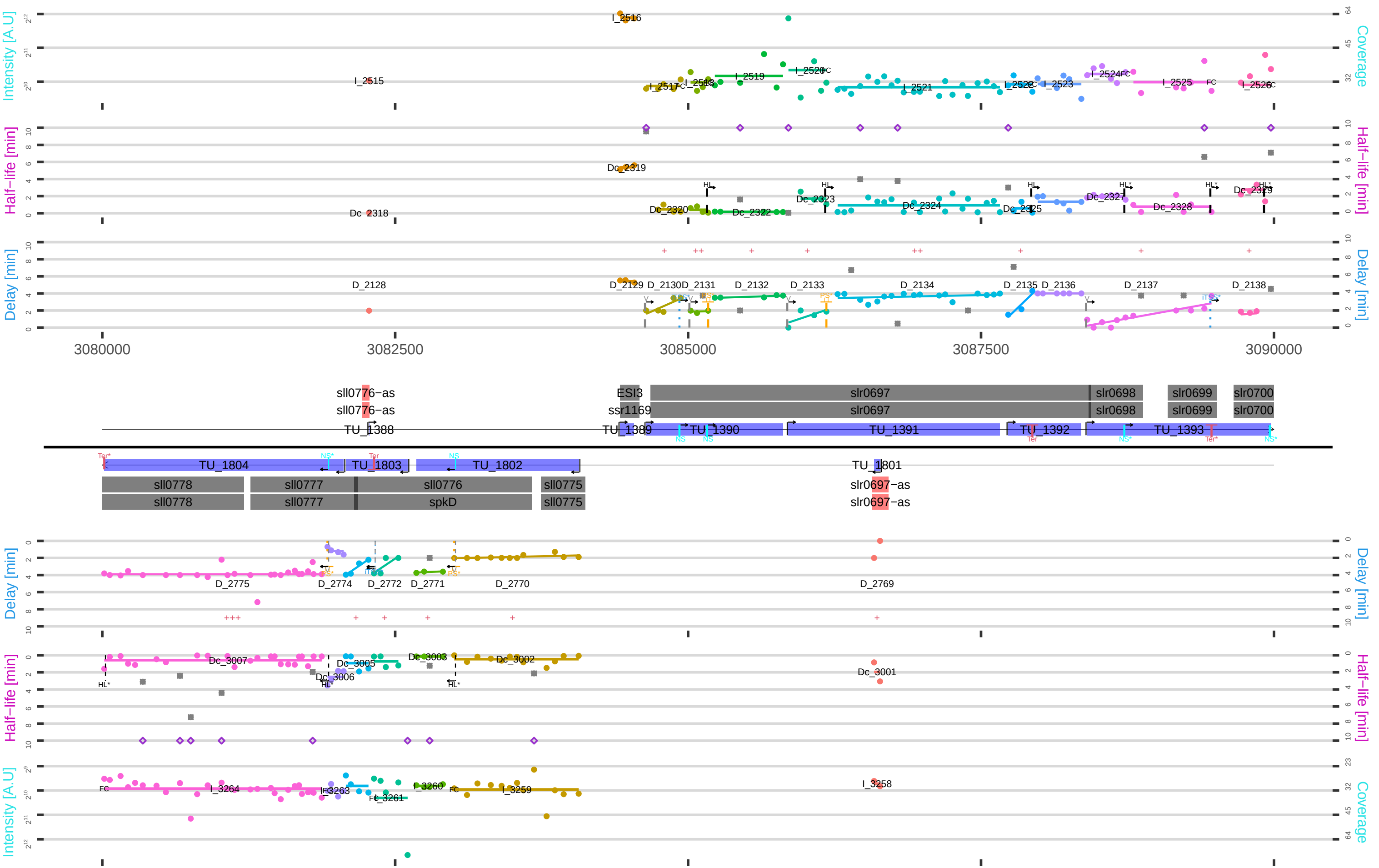

Term: termination (2), NS: new start (2), PS: pausing site (2), iTSS_I: internal starting site (1)

ID: 25077−25127; Term: termination (1), NS: new start (1), PS: pausing site (0), iTSS_I: internal starting site (0)

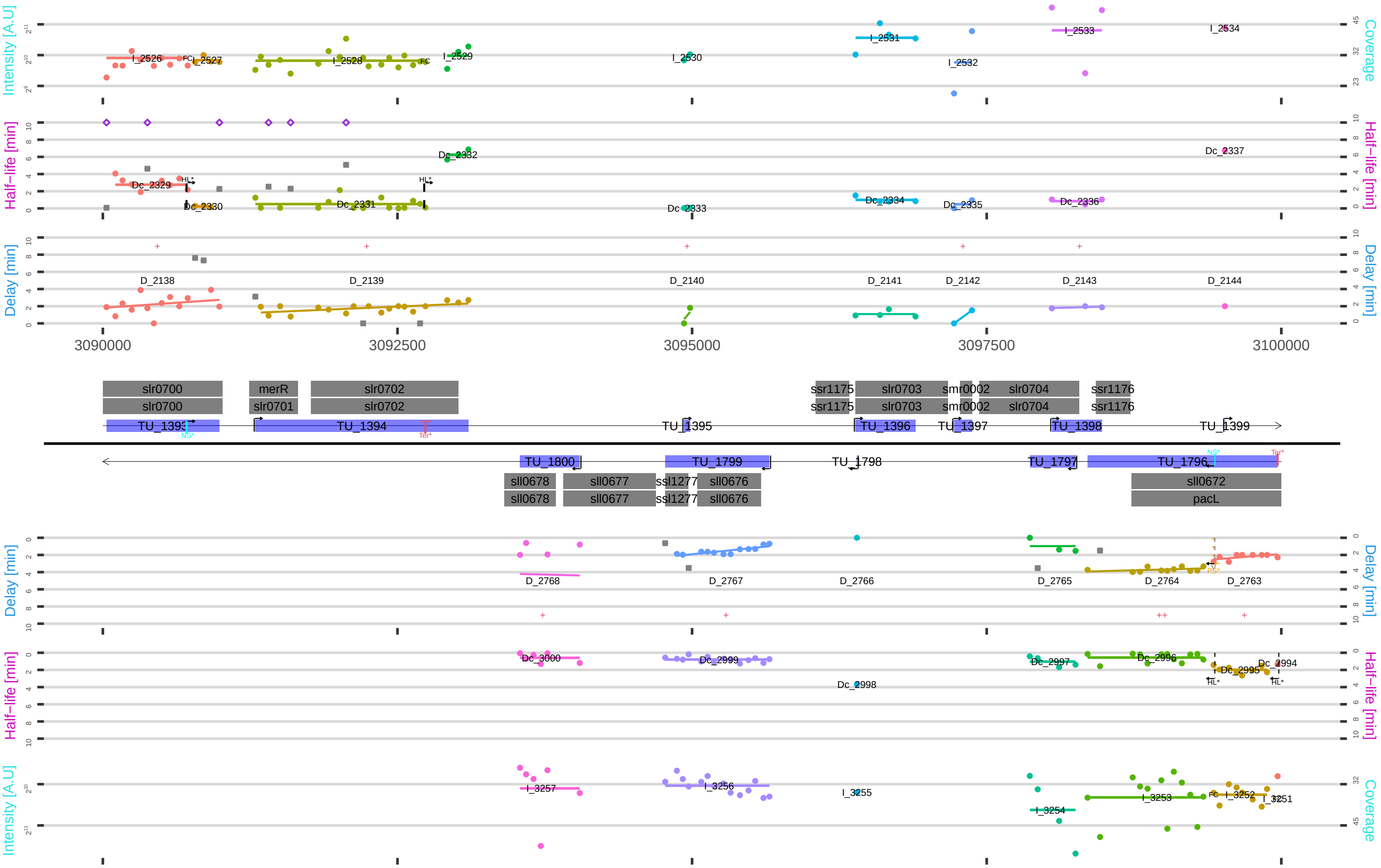

Term: termination (1), NS: new start (1), PS: pausing site (1), iTSS_I: internal starting site (0)

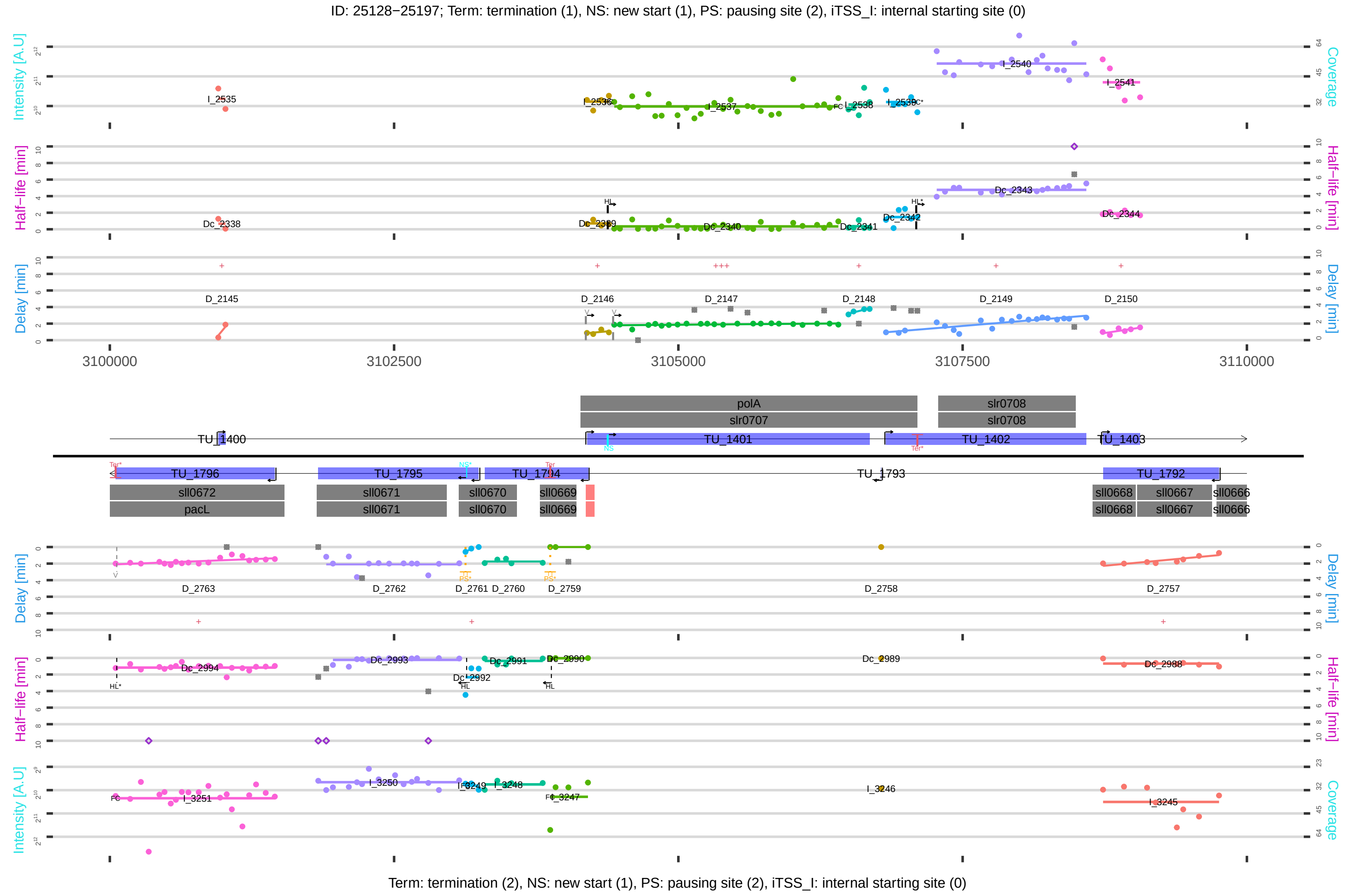
ID: 25128−25197; Term: termination (1), NS: new start (1), PS: pausing site (2), iTSS_I: internal starting site (0)
Intensity [A.U]
Coverage
Half−life [min]
Delay [min]
I_2535
I_2536
I_2537
I_2538
I_2539
I_2540
I_2541
Dc_2338
Dc_2339
Dc_2340
Dc_2341
Dc_2342
Dc_2343
Dc_2344
D_2145
D_2146
D_2147
D_2148
D_2149
D_2150
3100000
3102500
3105000
3107500
3110000
polA
slr0707
slr0708
TU_1400
TU_1401
TU_1402
TU_1403
TU_1796
TU_1795
TU_1794
TU_1793
TU_1792
sll0672
pacL
sll0671
sll0670
sll0669
sll0668
sll0667
sll0666
D_2763
D_2762
D_2761
D_2760
D_2759
D_2758
D_2757
Dc_2994
Dc_2993
Dc_2992
Dc_2991
Dc_2990
Dc_2989
Dc_2988
I_3251
I_3250
I_3249
I_3248
I_3247
I_3246
I_3245
Term: termination (2), NS: new start (1), PS: pausing site (2), iTSS_I: internal starting site (0)

ID: 25198−25273; Term: termination (1), NS: new start (0), PS: pausing site (1), iTSS_I: internal starting site (1)

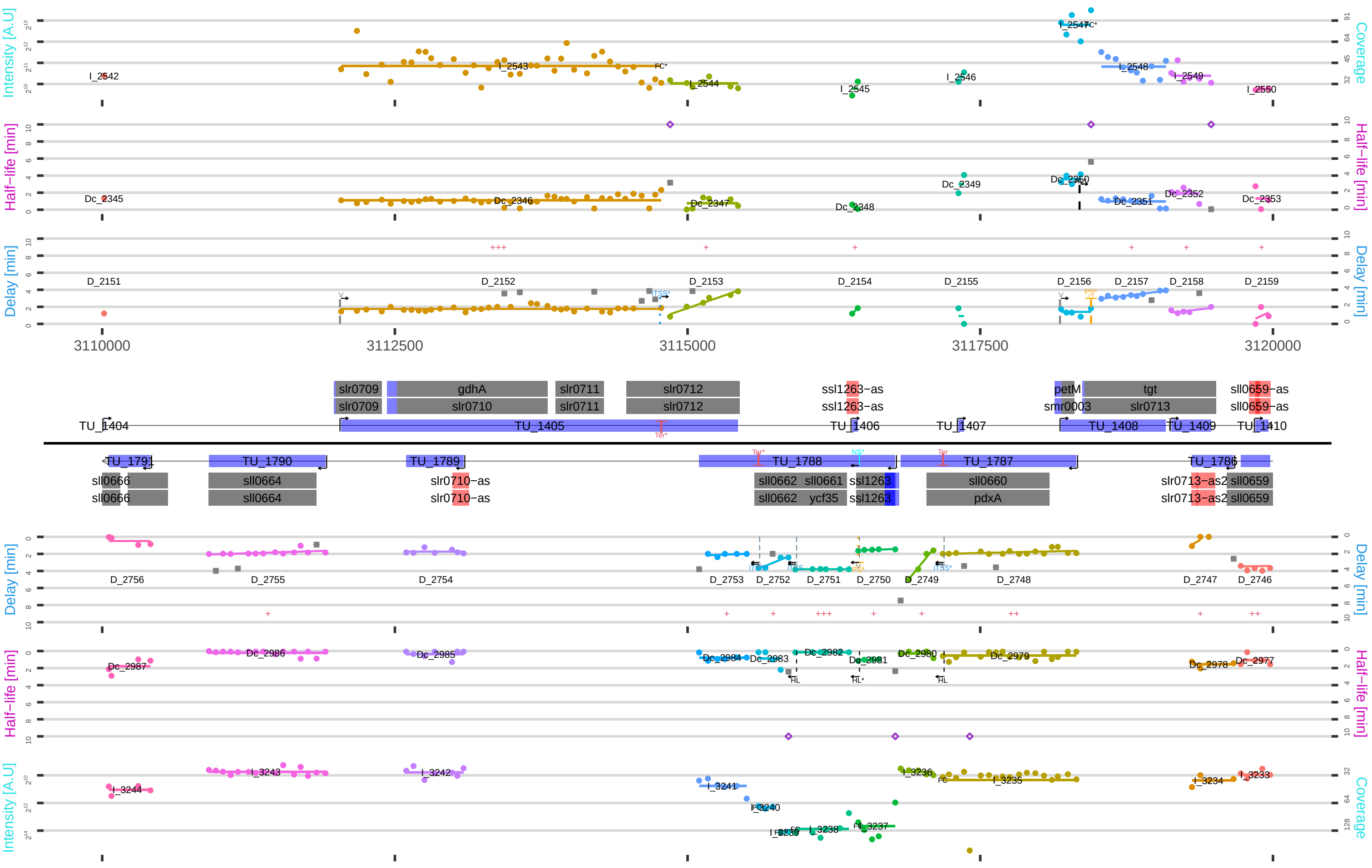

Term: termination (2), NS: new start (1), PS: pausing site (1), iTSS_I: internal starting site (3)

ID: 25274−25307; Term: termination (0), NS: new start (0), PS: pausing site (0), iTSS_I: internal starting site (0)

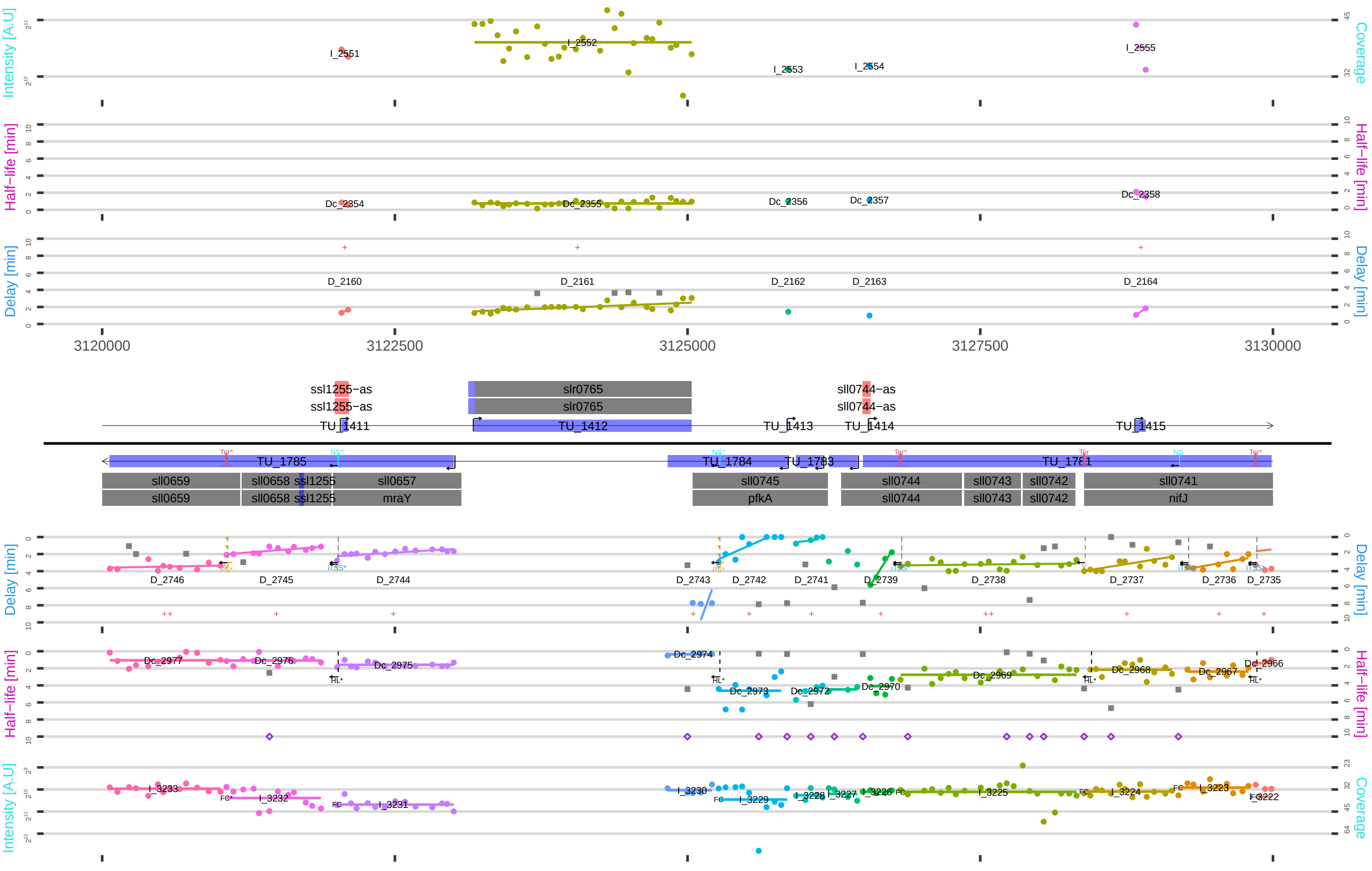

Term: termination (4), NS: new start (3), PS: pausing site (2), iTSS_I: internal starting site (5)

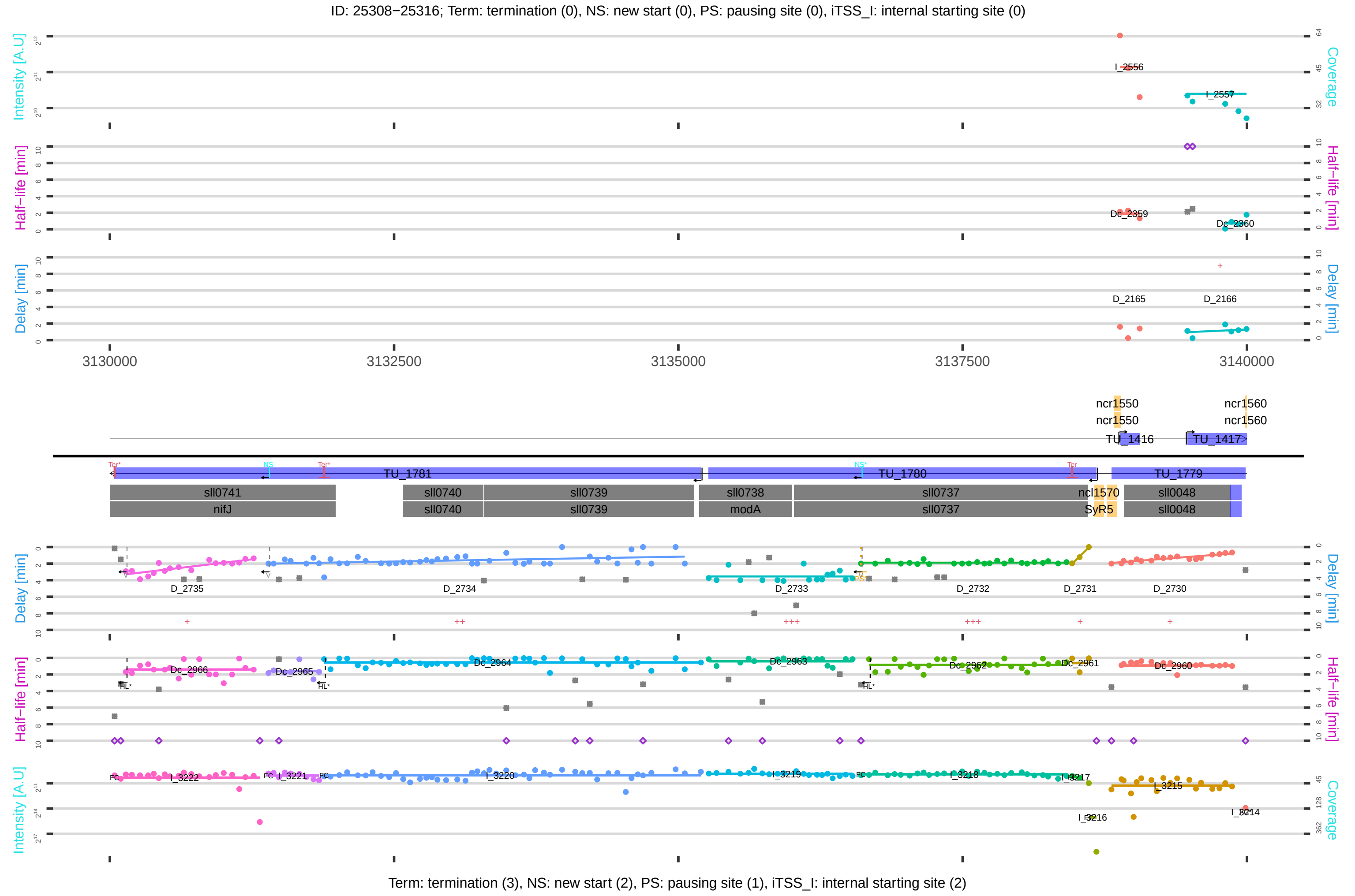

ID: 25308−25316; Term: termination (0), NS: new start (0), PS: pausing site (0), iTSS_I: internal starting site (0)
Intensity [A.U]
Coverage
Half−life [min]
Delay [min]
I_2556
I_2557
Dc_2359
Dc_2360
D_2165
D_2166
3130000
3132500
3135000
3137500
3140000
ncr1550
ncr1560
TU_1416
TU_1417
TU_1781
TU_1780
TU_1779
sll0741
nifJ
sll0740
sll0739
sll0738
modA
sll0737
ncl1570
SyR5
sll0048
D_2735
D_2734
D_2733
D_2732
D_2731
D_2730
Dc_2966
Dc_2965
Dc_2964
Dc_2963
Dc_2962
Dc_2961
Dc_2960
I_3222
I_3221
I_3220
I_3219
I_3218
I_3217
I_3216
I_3215
I_3214
Term: termination (3), NS: new start (2), PS: pausing site (1), iTSS_I: internal starting site (2)

ID: 25317−25351; Term: termination (0), NS: new start (1), PS: pausing site (0), iTSS_I: internal starting site (1)

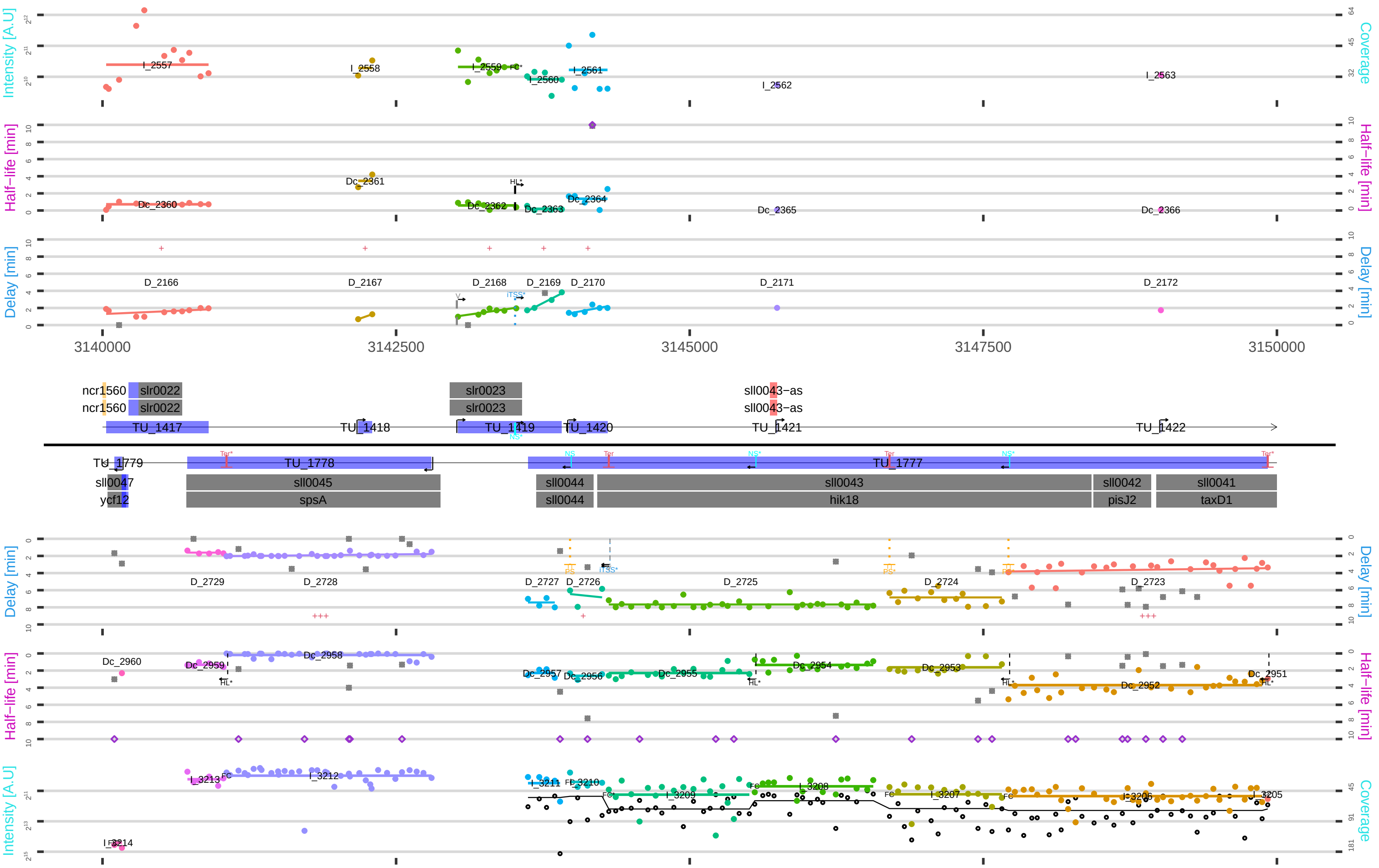

Term: termination (4), NS: new start (3), PS: pausing site (3), iTSS_I: internal starting site (2)

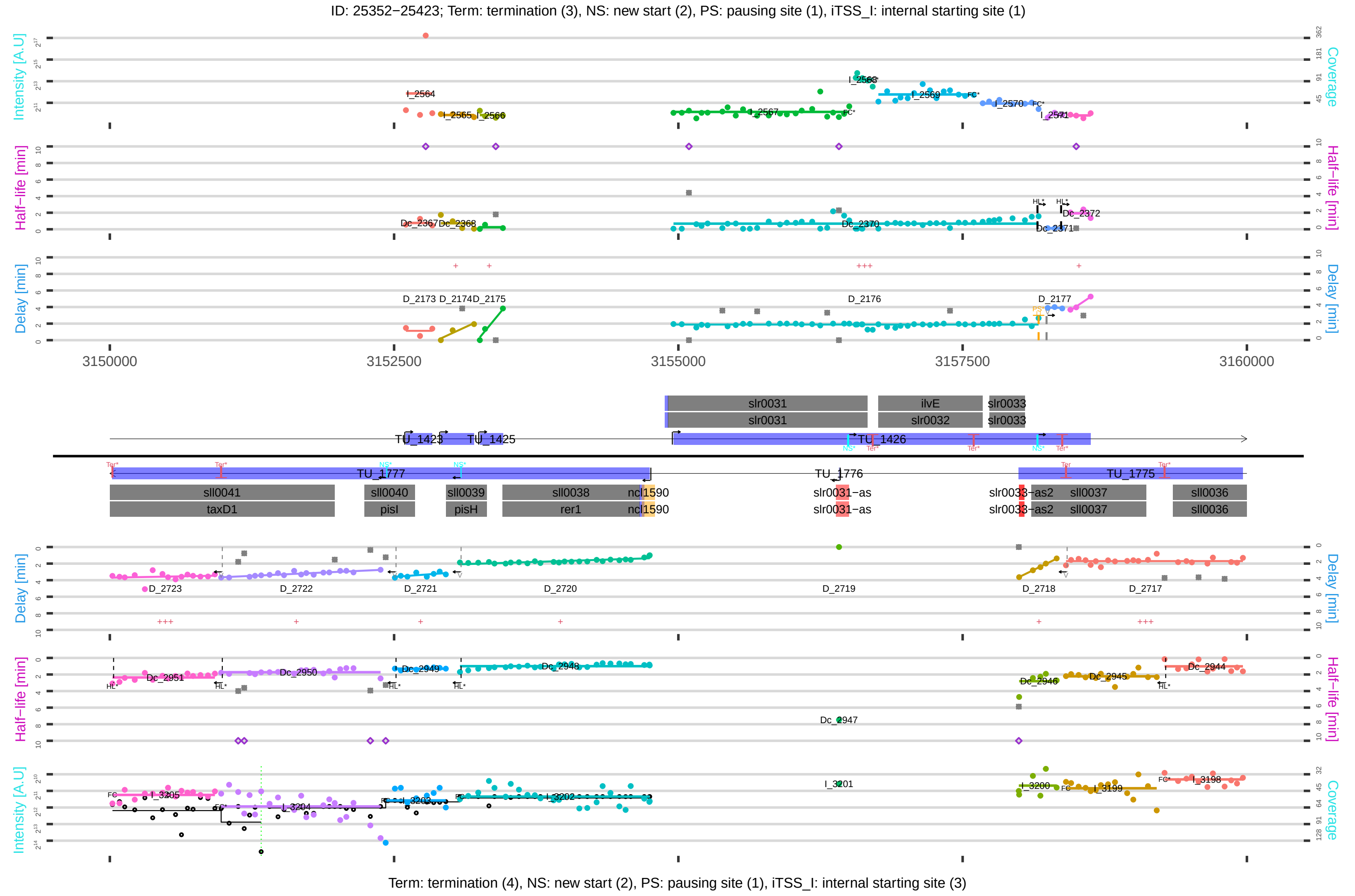
ID: 25352−25423; Term: termination (3), NS: new start (2), PS: pausing site (1), iTSS_I: internal starting site (1)
Intensity [A.U]
Coverage
Half−life [min]
Delay [min]
3150000
3152500
3155000
3157500
3160000
slr0031
ilvE
slr0033
slr0032
TU_1423
TU_1425
TU_1426
TU_1777
TU_1776
TU_1775
sll0041
taxD1
sll0040
pisI
sll0039
pisH
sll0038
rer1
ncl1590
slr0031−as
slr0033−as2
sll0037
sll0036
Term: termination (4), NS: new start (2), PS: pausing site (1), iTSS_I: internal starting site (3)

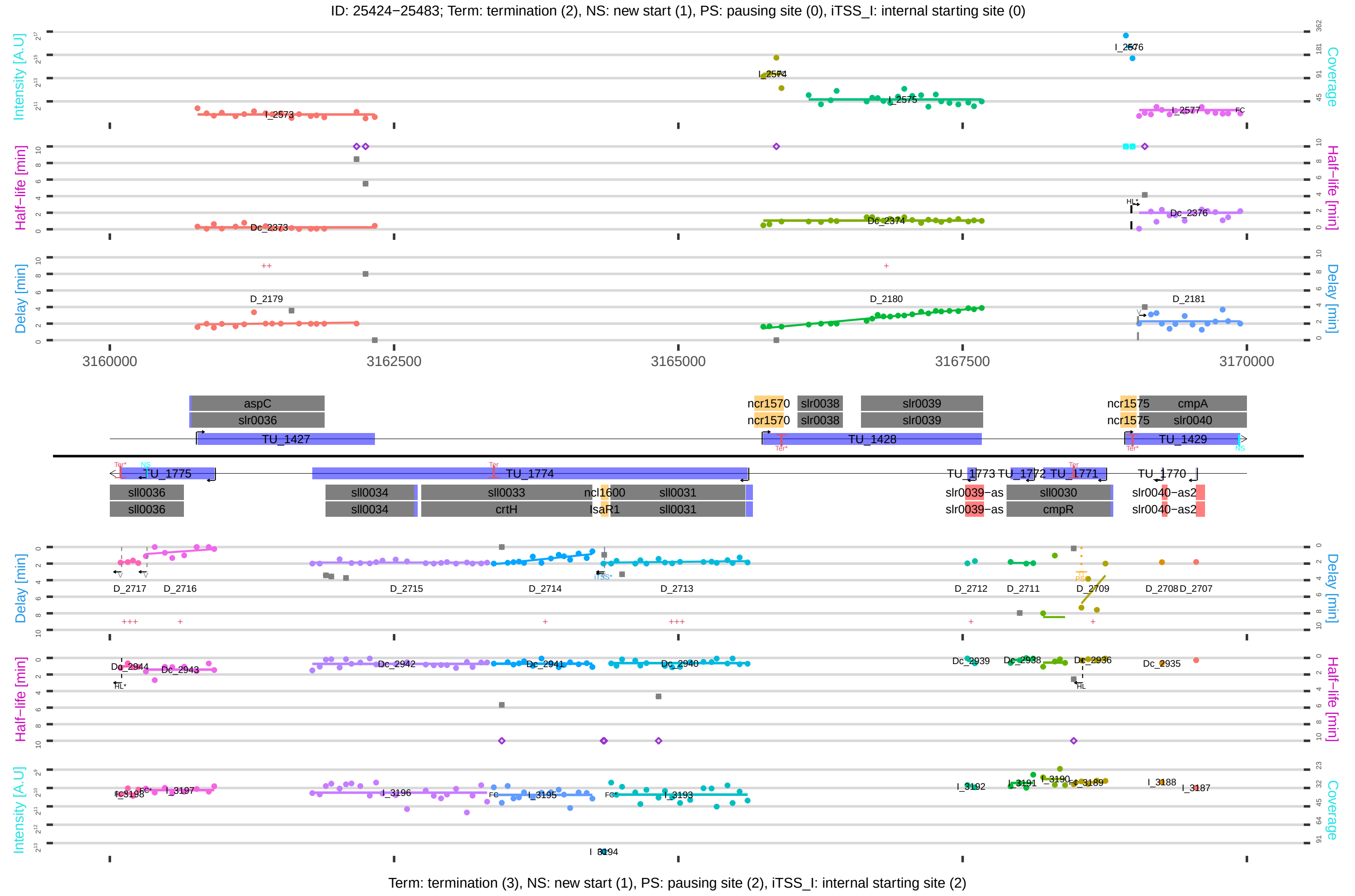

ID: 25424−25483; Term: termination (2), NS: new start (1), PS: pausing site (0), iTSS_I: internal starting site (0)
Intensity [A.U]
Coverage
Half−life [min]
Delay [min]
I_2573
I_2574
I_2575
I_2576
I_2577
FC
Dc_2373
Dc_2374
Dc_2376
HL*
D_2179
D_2180
D_2181
3160000
3162500
3165000
3167500
3170000
aspC
slr0036
ncr1570
slr0038
slr0039
ncr1575
cmpA
slr0040
TU_1427
TU_1428
TU_1429
Ter*
NS
TU_1775
TU_1774
Ter
TU_1773
TU_1772
TU_1771
TU_1770
sll0036
sll0034
sll0033
crtH
ncl1600
IsaR1
sll0031
slr0039−as
sll0030
cmpR
slr0040−as2
D_2717
D_2716
D_2715
D_2714
D_2713
D_2712
D_2711
D_2709
D_2708
D_2707
iTSS*
PS
Dc_2944
Dc_2943
Dc_2942
Dc_2941
Dc_2940
Dc_2939
Dc_2938
Dc_2936
Dc_2935
HL
I_3198
I_3197
I_3196
I_3195
I_3194
I_3193
I_3192
I_3191
I_3190
I_3189
I_3188
I_3187
FC*
Term: termination (3), NS: new start (1), PS: pausing site (2), iTSS_I: internal starting site (2)

ID: 25484−25586; Term: termination (1), NS: new start (5), PS: pausing site (2), iTSS_I: internal starting site (4)

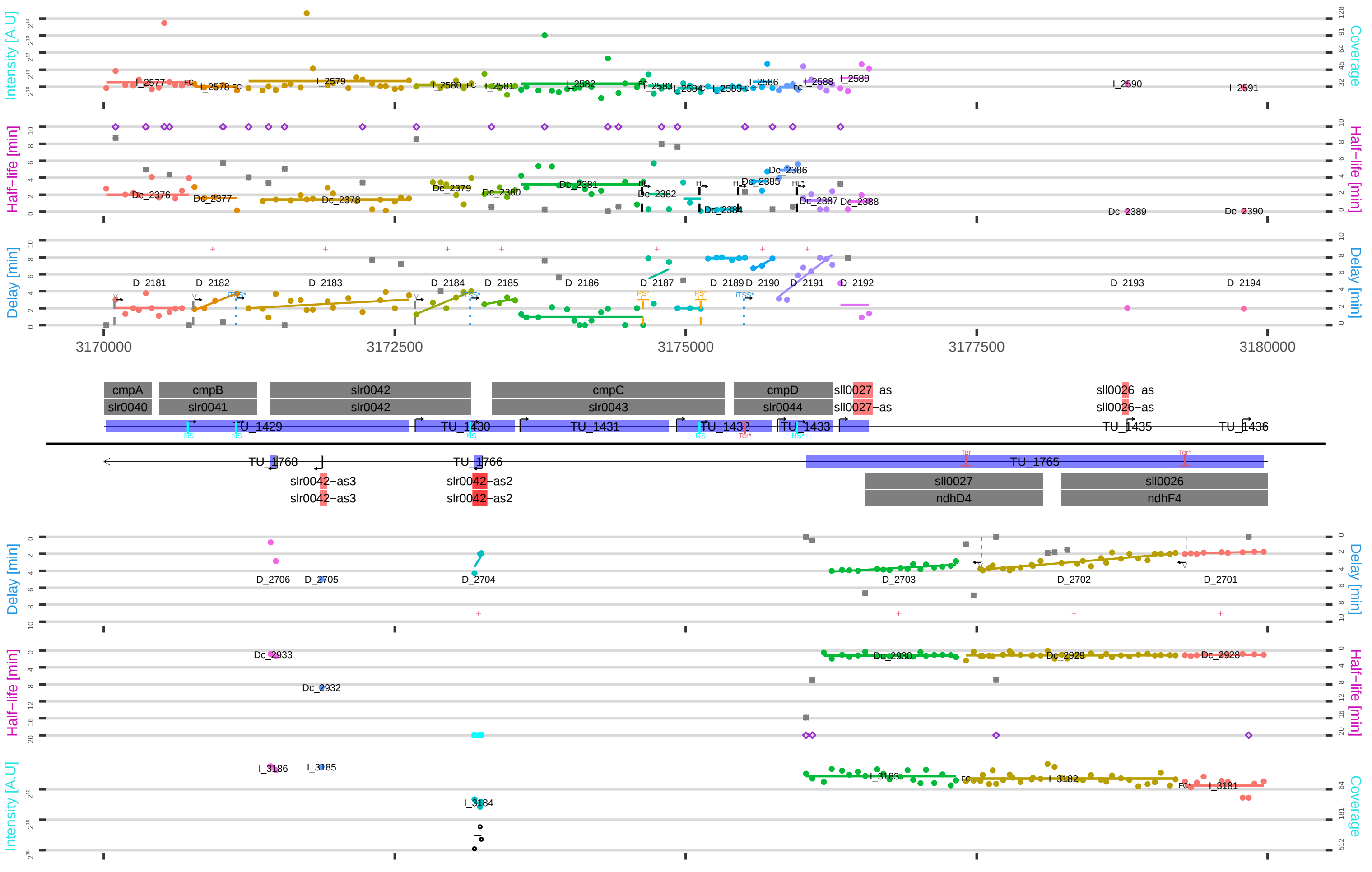

Term: termination (2), NS: new start (0), PS: pausing site (0), iTSS_I: internal starting site (2)

ID: 25587−25669; Term: termination (2), NS: new start (0), PS: pausing site (0), iTSS_I: internal starting site (0)

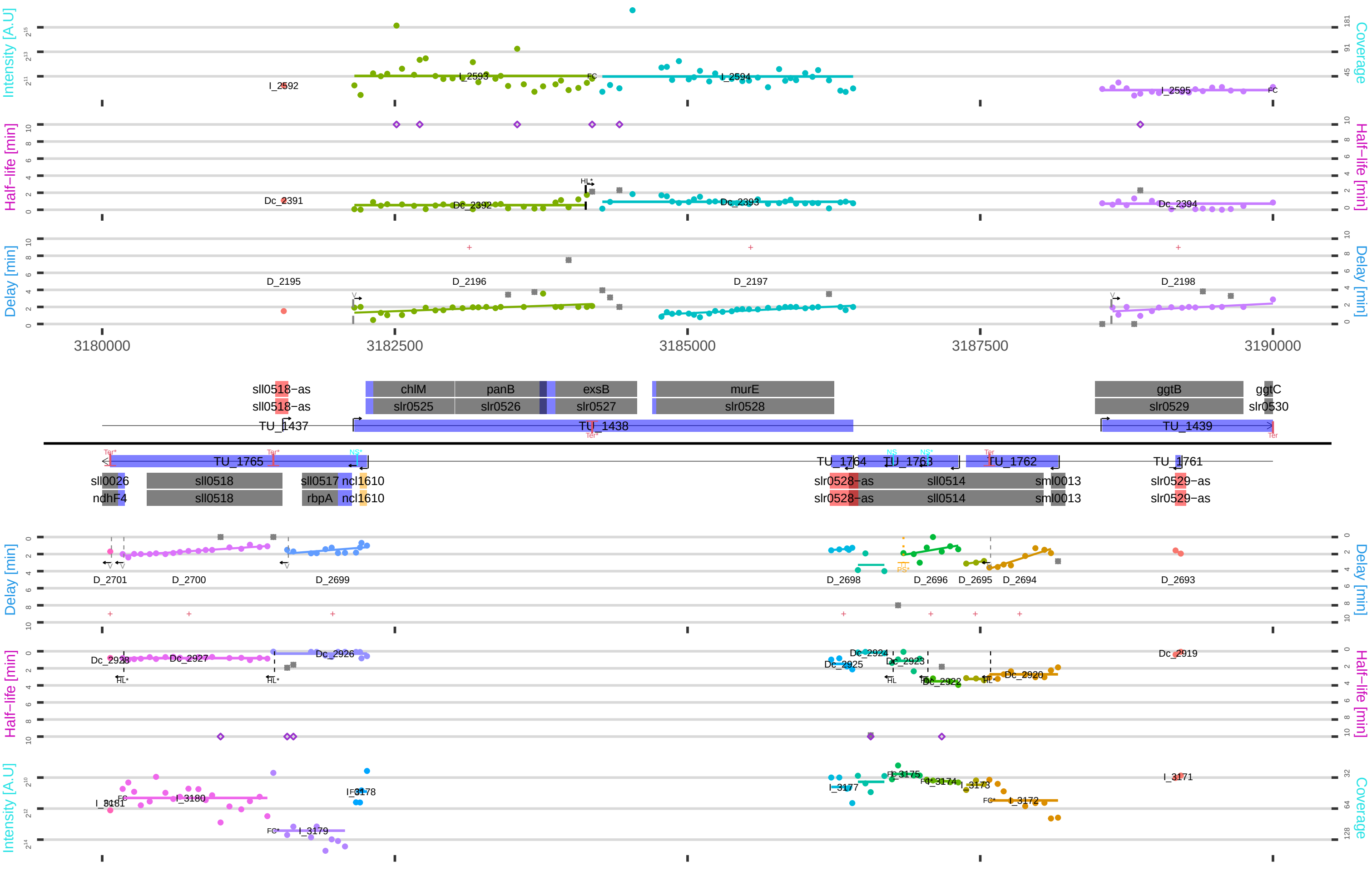

Term: termination (3), NS: new start (3), PS: pausing site (1), iTSS_I: internal starting site (3)

ID: 25669−25812; Term: termination (4), NS: new start (4), PS: pausing site (6), iTSS_I: internal starting site (1)

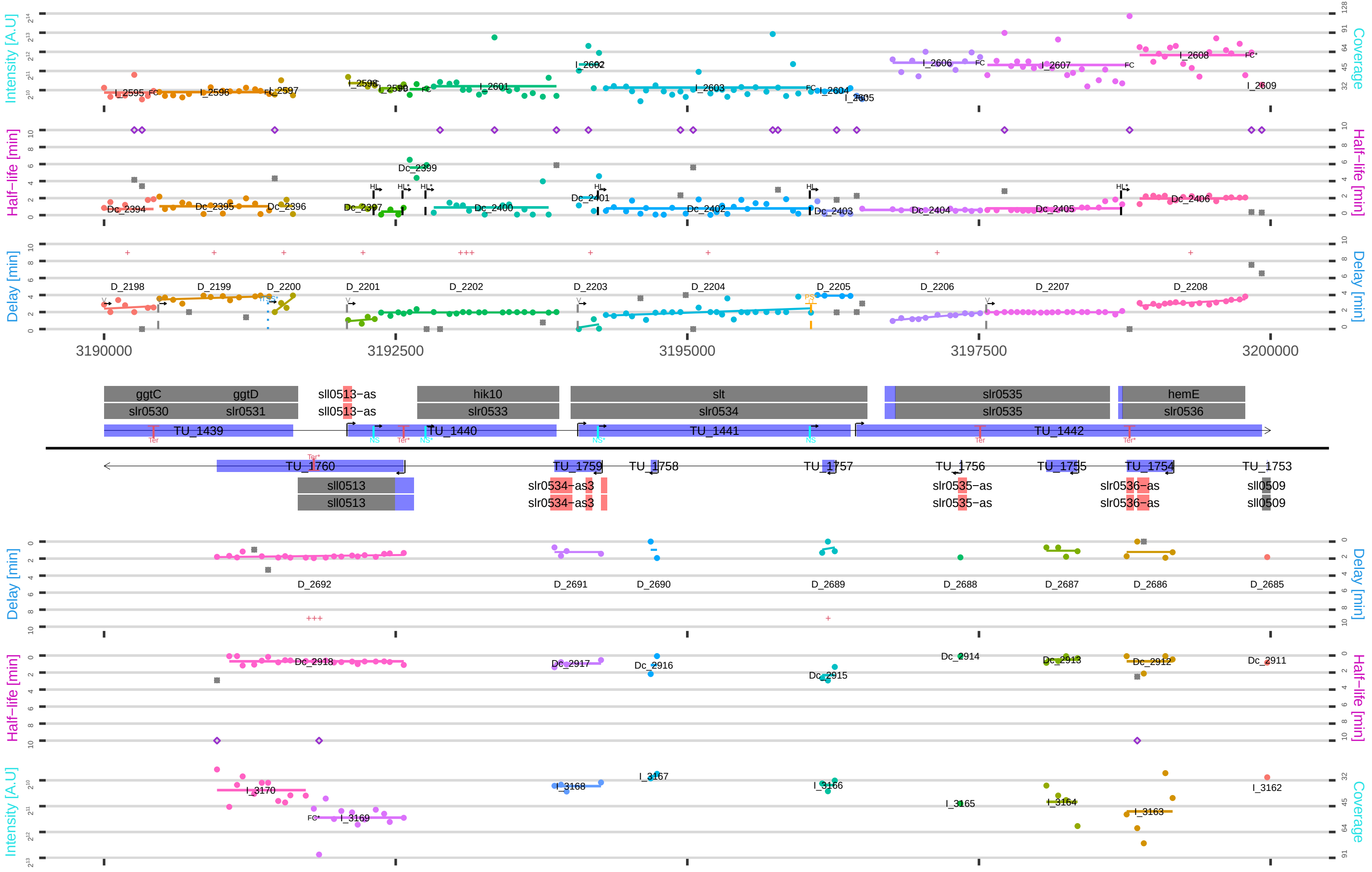

Term: termination (1), NS: new start (0), PS: pausing site (0), iTSS_I: internal starting site (0)

ID: 25813−25836; Term: termination (1), NS: new start (0), PS: pausing site (0), iTSS_I: internal starting site (0)

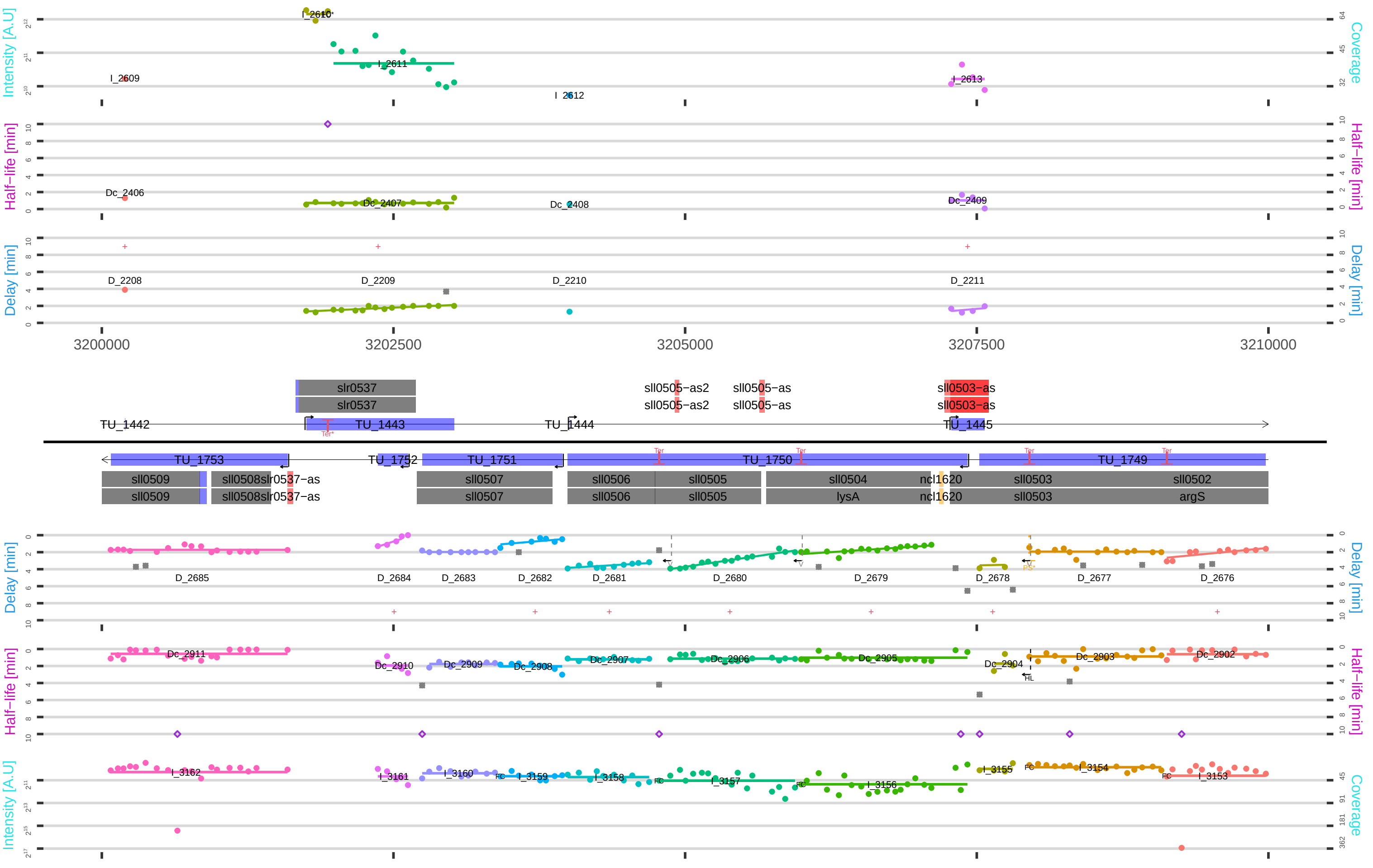

Term: termination (4), NS: new start (0), PS: pausing site (2), iTSS_I: internal starting site (3)

ID: 25837−25900; Term: termination (2), NS: new start (1), PS: pausing site (1), iTSS_I: internal starting site (2)

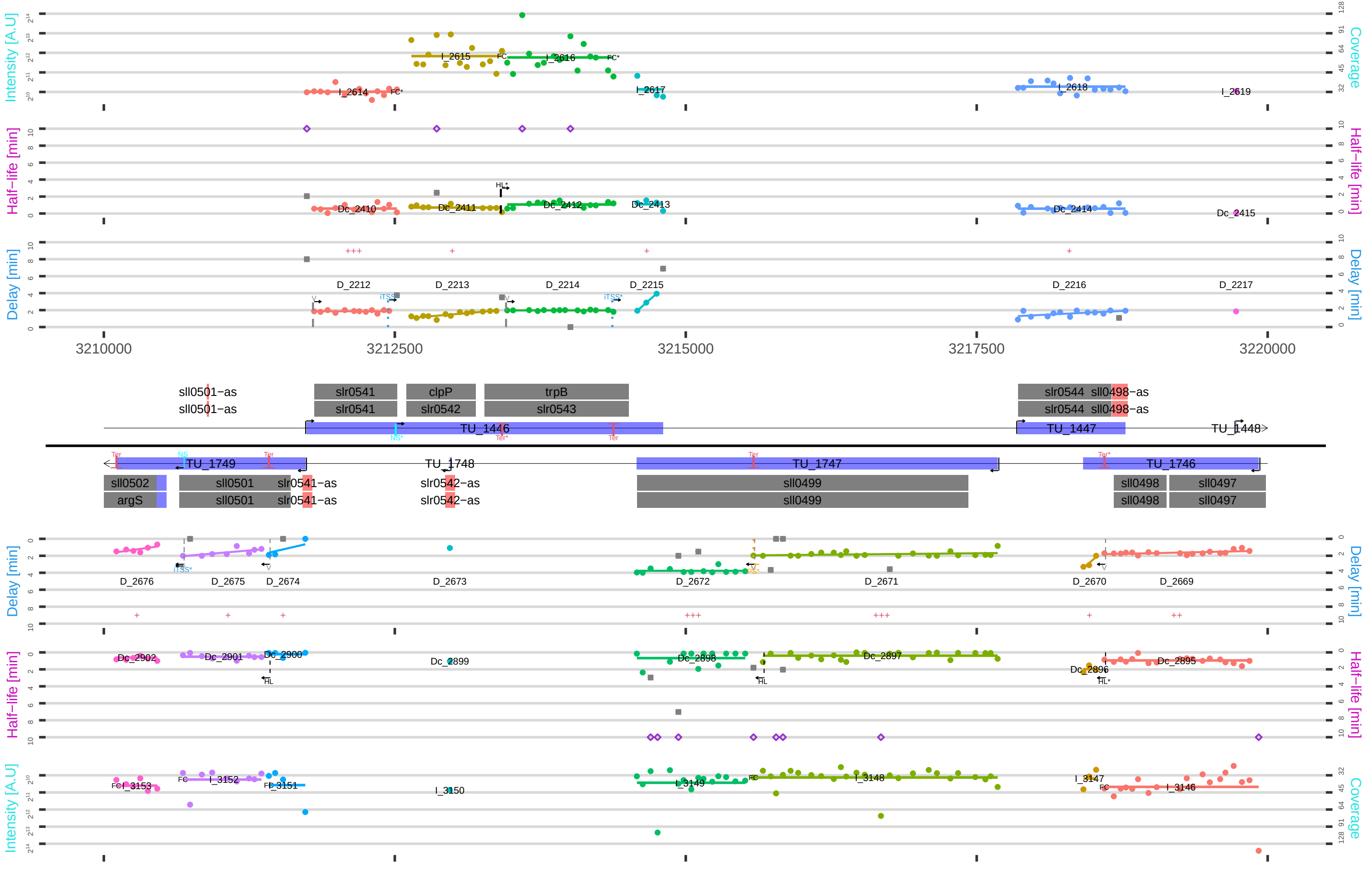

Term: termination (4), NS: new start (1), PS: pausing site (1), iTSS_I: internal starting site (3)

ID: 25901−25952; Term: termination (2), NS: new start (1), PS: pausing site (2), iTSS_I: internal starting site (0)

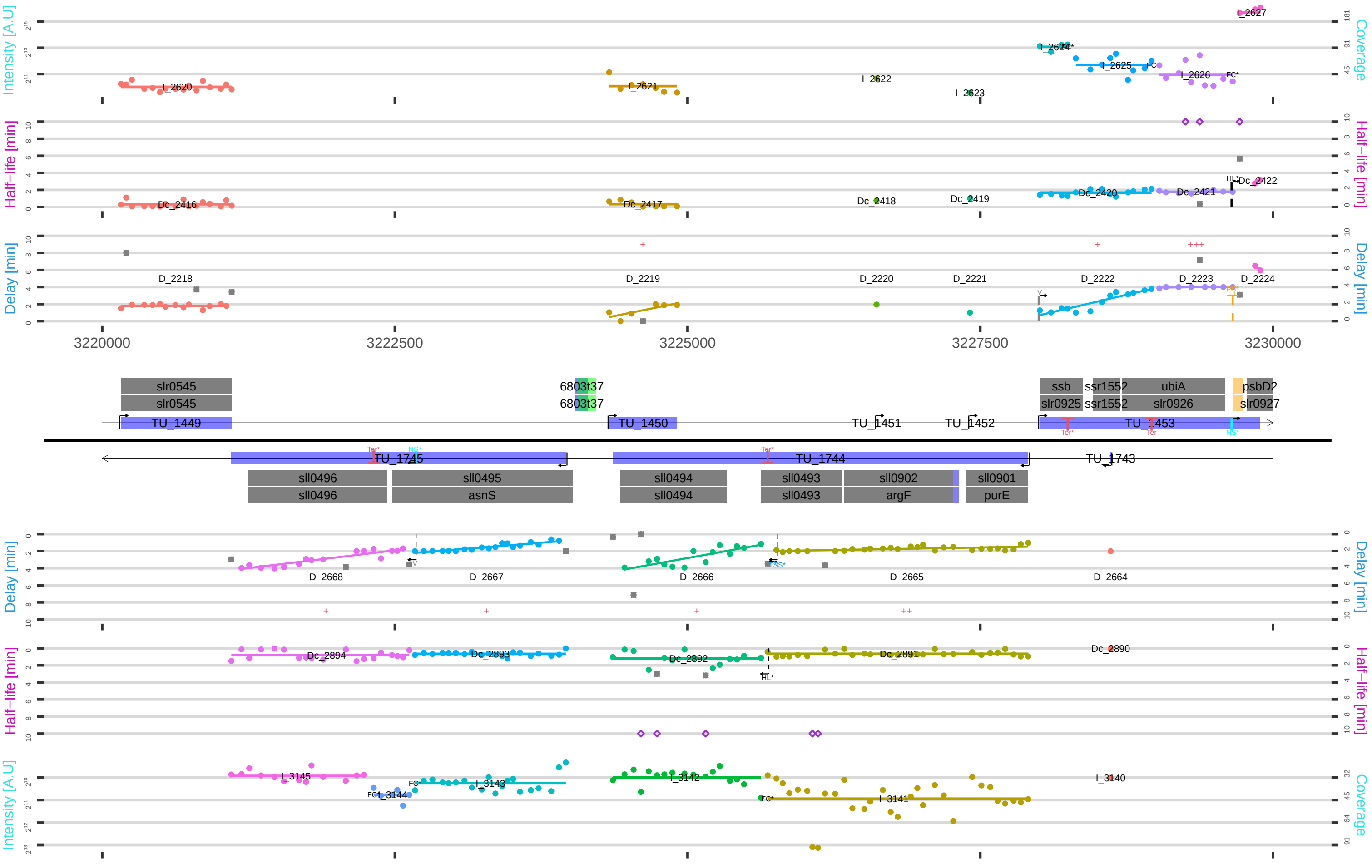

Term: termination (2), NS: new start (1), PS: pausing site (0), iTSS_I: internal starting site (2)

ID: 25953−26009; Term: termination (2), NS: new start (0), PS: pausing site (1), iTSS_I: internal starting site (1)

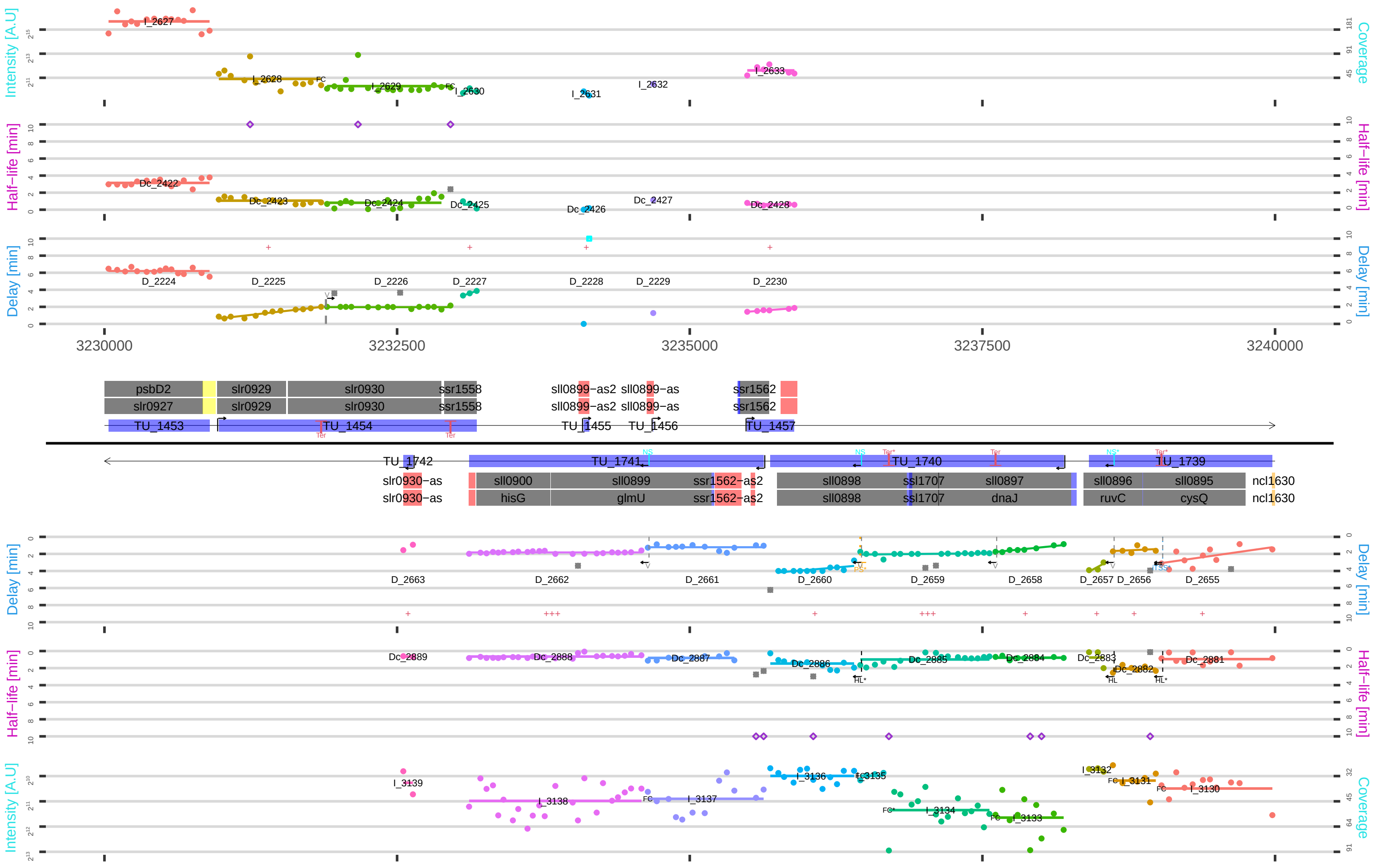

Term: termination (3), NS: new start (3), PS: pausing site (4), iTSS_I: internal starting site (1)

ID: 26010−26059; Term: termination (1), NS: new start (0), PS: pausing site (1), iTSS_I: internal starting site (0)

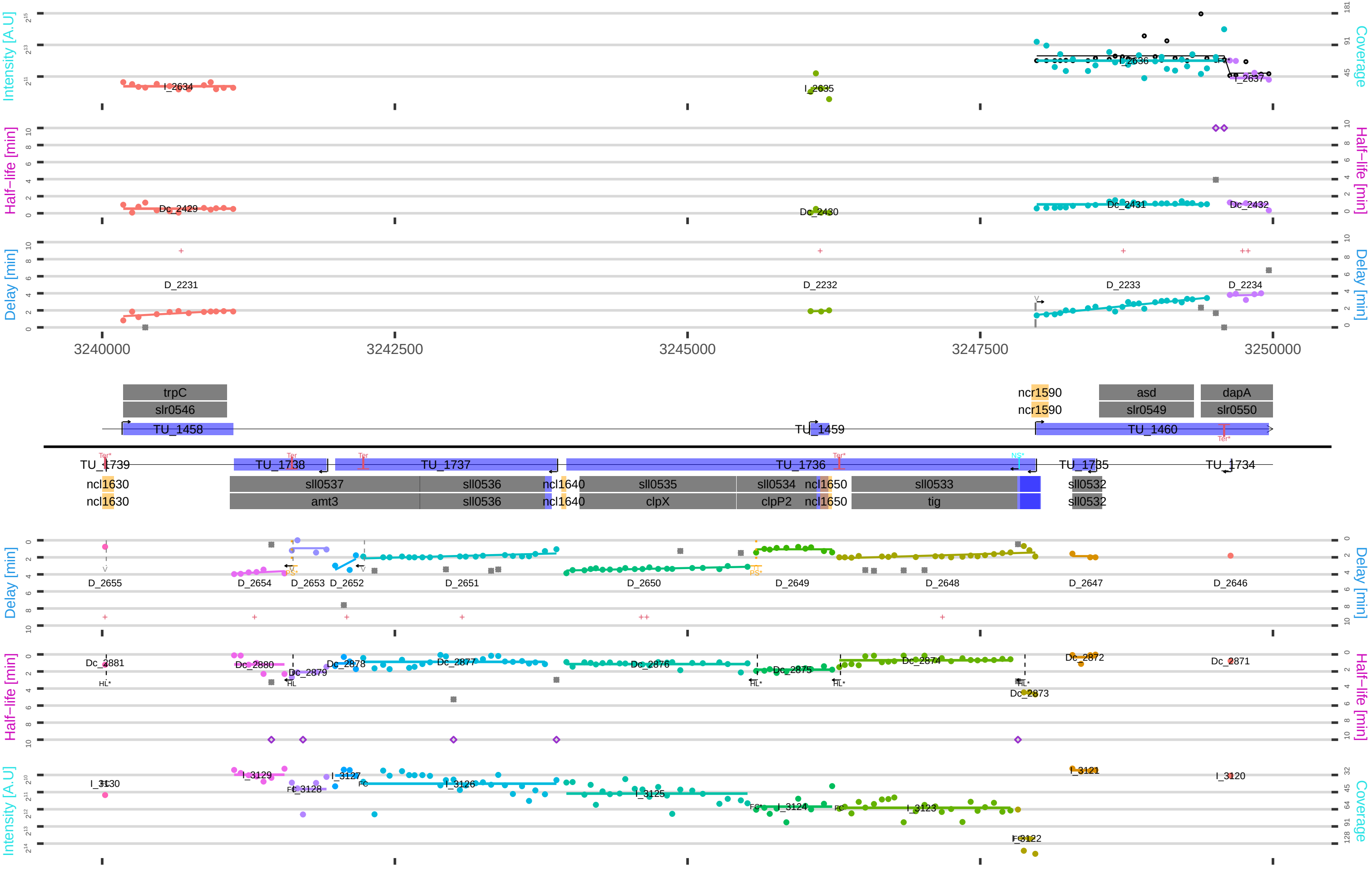

Term: termination (4), NS: new start (1), PS: pausing site (2), iTSS_I: internal starting site (2)

ID: 26060−26169; Term: termination (4), NS: new start (1), PS: pausing site (3), iTSS_I: internal starting site (1)

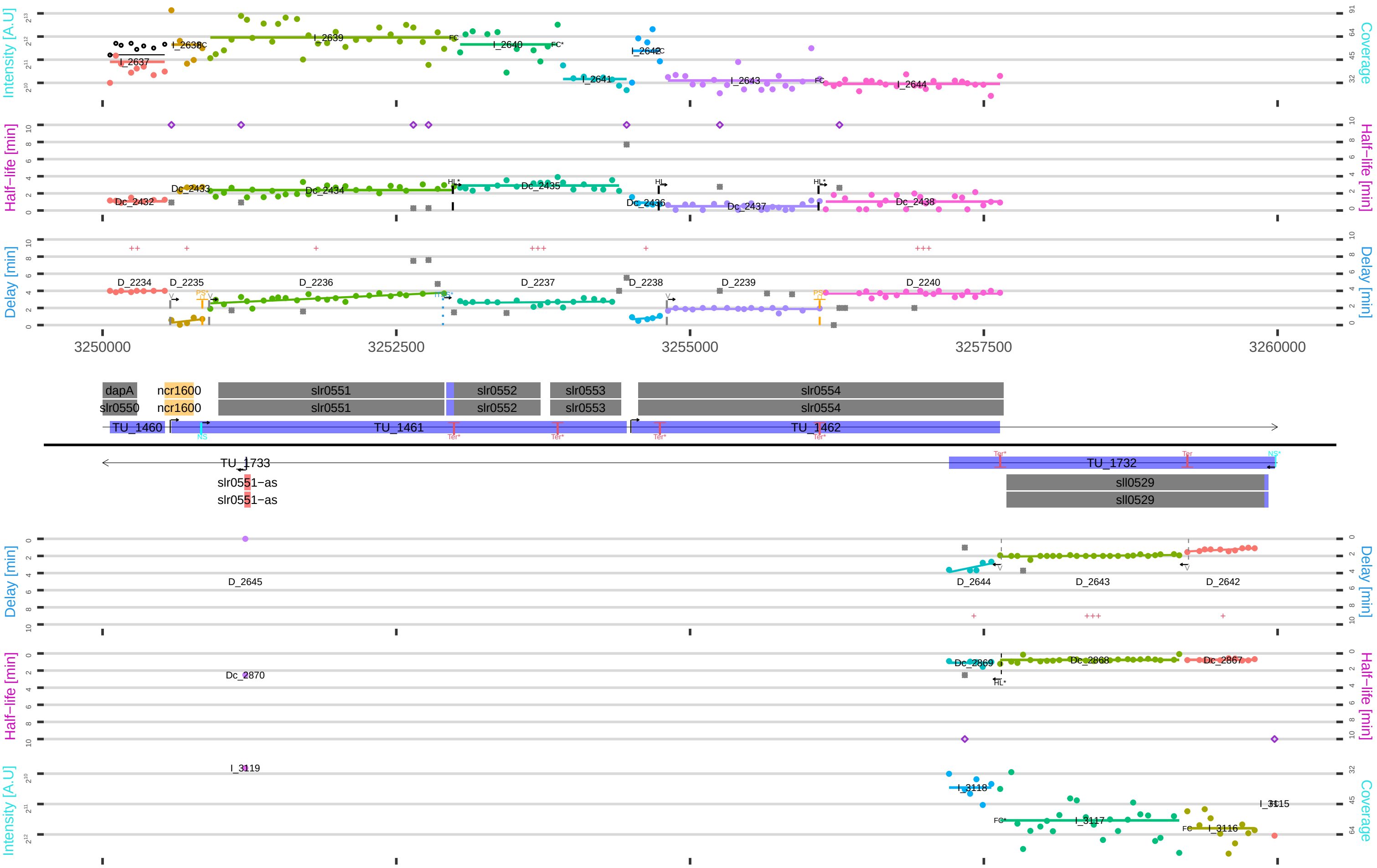

Term: termination (2), NS: new start (1), PS: pausing site (2), iTSS_I: internal starting site (0)

ID: 26170−26238; Term: termination (2), NS: new start (0), PS: pausing site (2), iTSS_I: internal starting site (0)

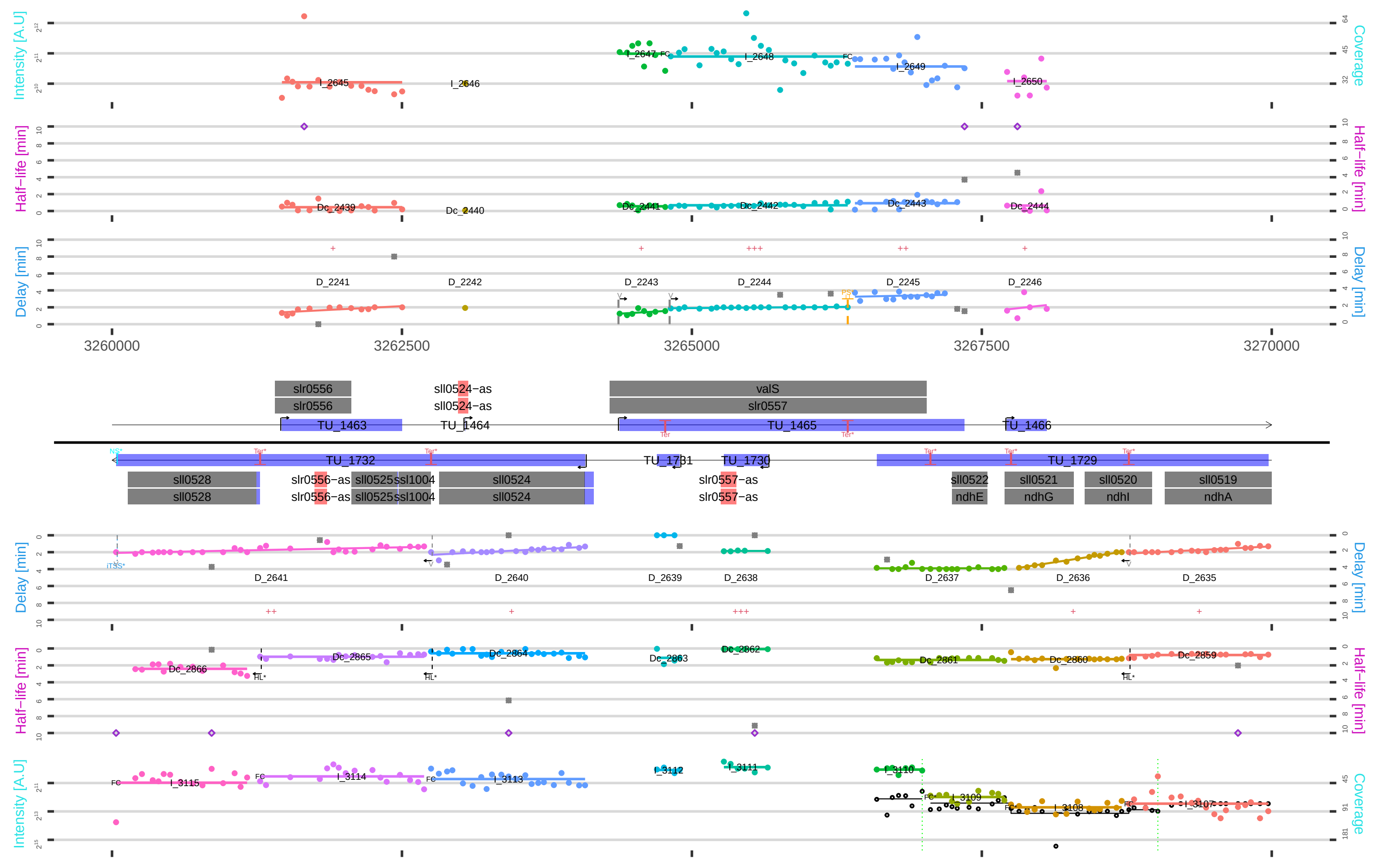

Term: termination (5), NS: new start (1), PS: pausing site (1), iTSS_I: internal starting site (3)

ID: 26239−26318; Term: termination (3), NS: new start (0), PS: pausing site (0), iTSS_I: internal starting site (1)

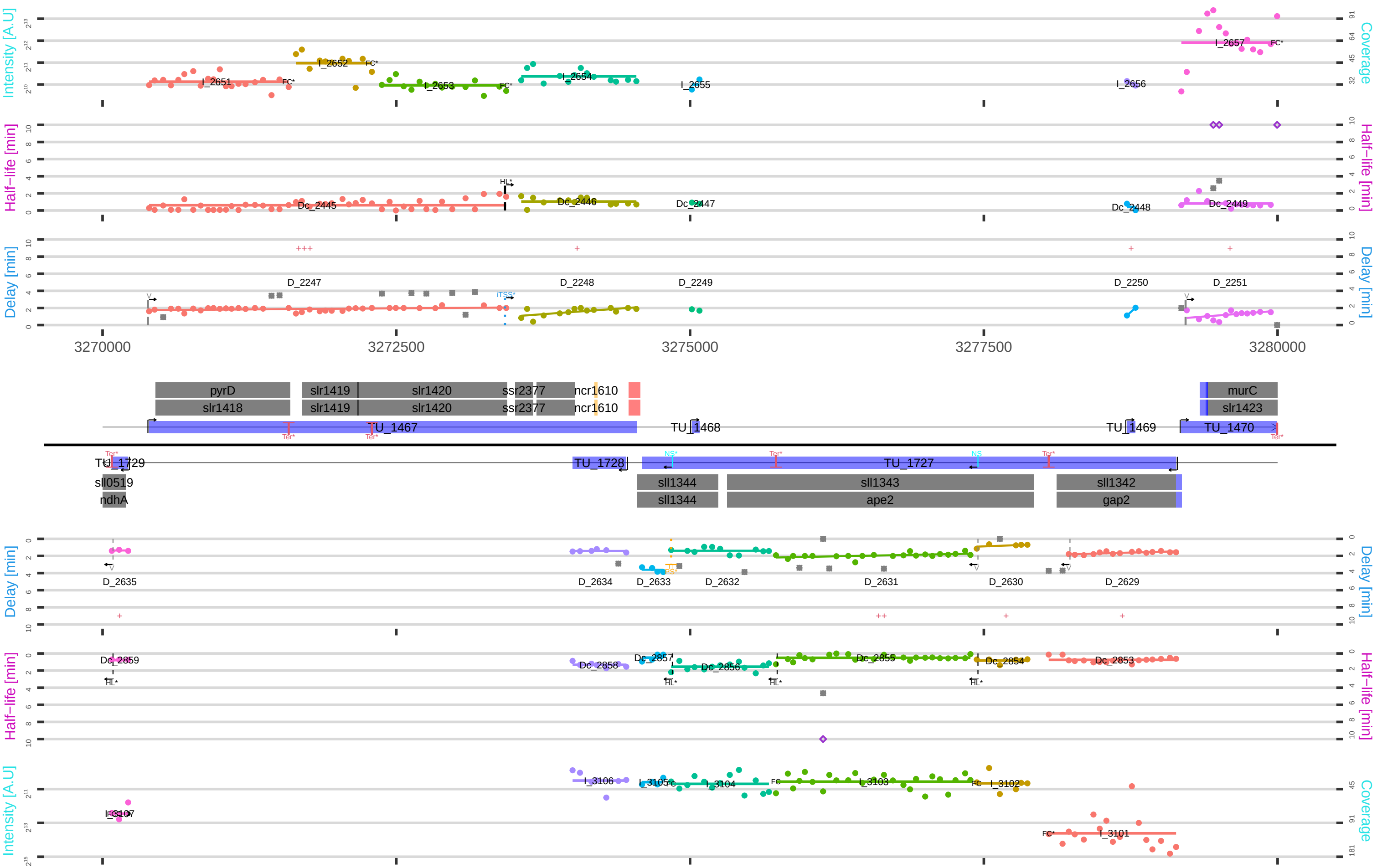

Term: termination (3), NS: new start (2), PS: pausing site (2), iTSS_I: internal starting site (1)

ID: 26319−26412; Term: termination (3), NS: new start (0), PS: pausing site (4), iTSS_I: internal starting site (0)

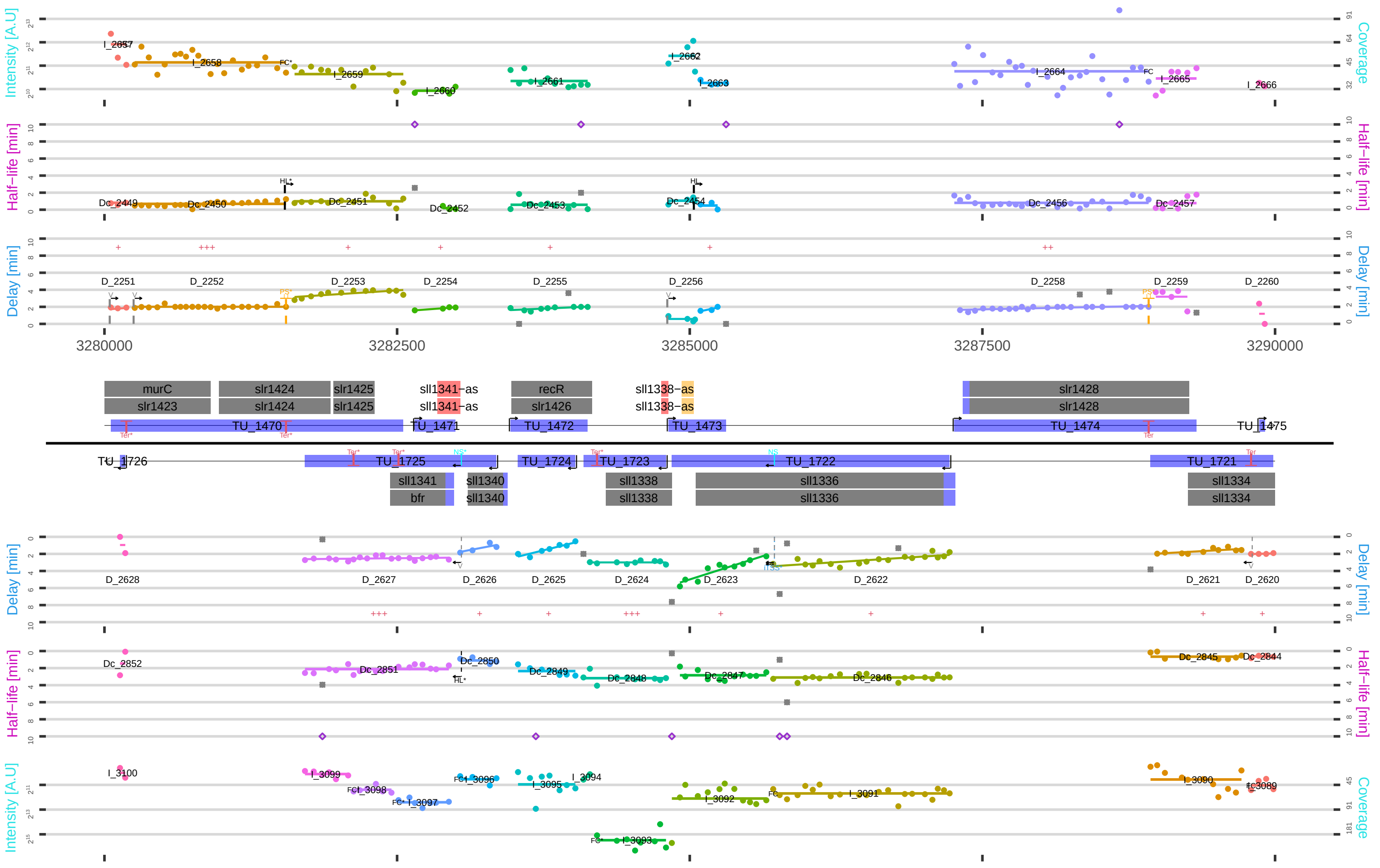

Term: termination (4), NS: new start (2), PS: pausing site (1), iTSS_I: internal starting site (2)

ID: 26413−26492; Term: termination (2), NS: new start (0), PS: pausing site (0), iTSS_I: internal starting site (0)

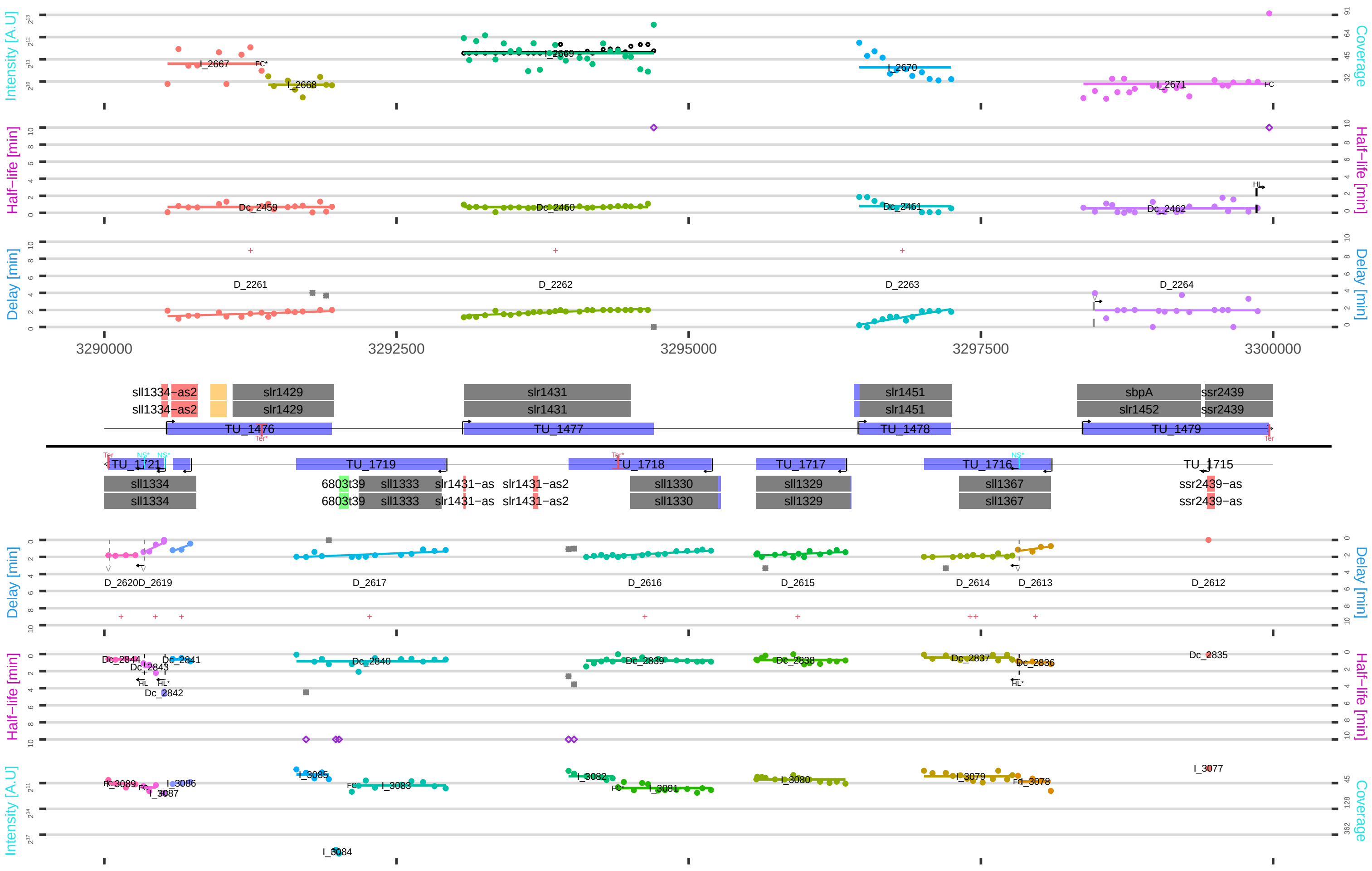

Term: termination (2), NS: new start (3), PS: pausing site (2), iTSS_I: internal starting site (0)

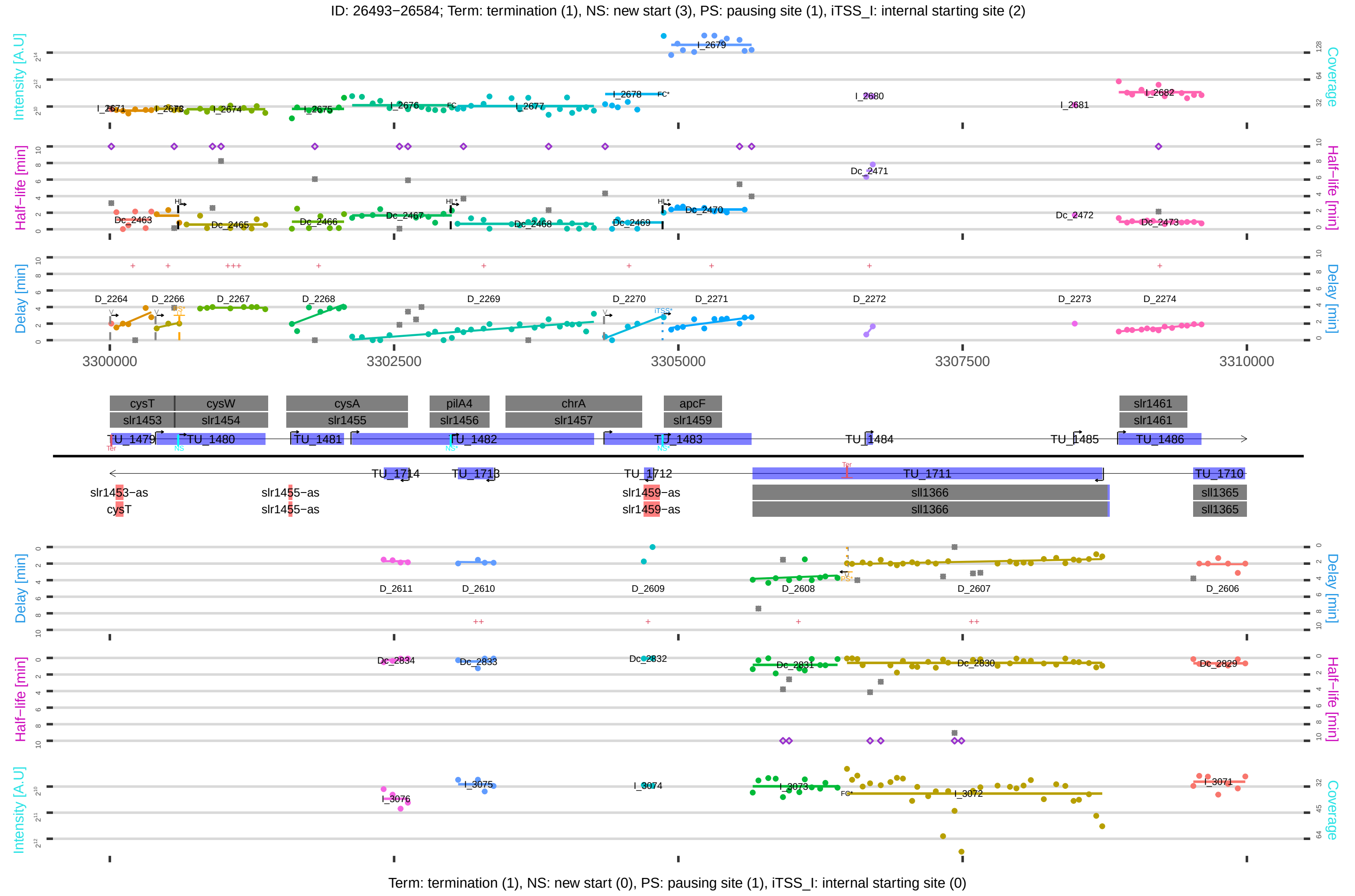
ID: 26493−26584; Term: termination (1), NS: new start (3), PS: pausing site (1), iTSS_I: internal starting site (2)
Intensity [A.U]
Coverage
Half−life [min]
Delay [min]
3300000
3302500
3305000
3307500
3310000
cysT
slr1453
cysW
slr1454
cysA
slr1455
pilA4
slr1456
chrA
slr1457
apcF
slr1459
slr1461
TU_1479
TU_1480
TU_1481
TU_1482
TU_1483
TU_1484
TU_1485
TU_1486
TU_1714
TU_1713
TU_1712
TU_1711
TU_1710
slr1453−as
cysT
slr1455−as
slr1459−as
sll1366
sll1365
Term: termination (1), NS: new start (0), PS: pausing site (1), iTSS_I: internal starting site (0)

ID: 26585−26676; Term: termination (0), NS: new start (1), PS: pausing site (1), iTSS_I: internal starting site (0)

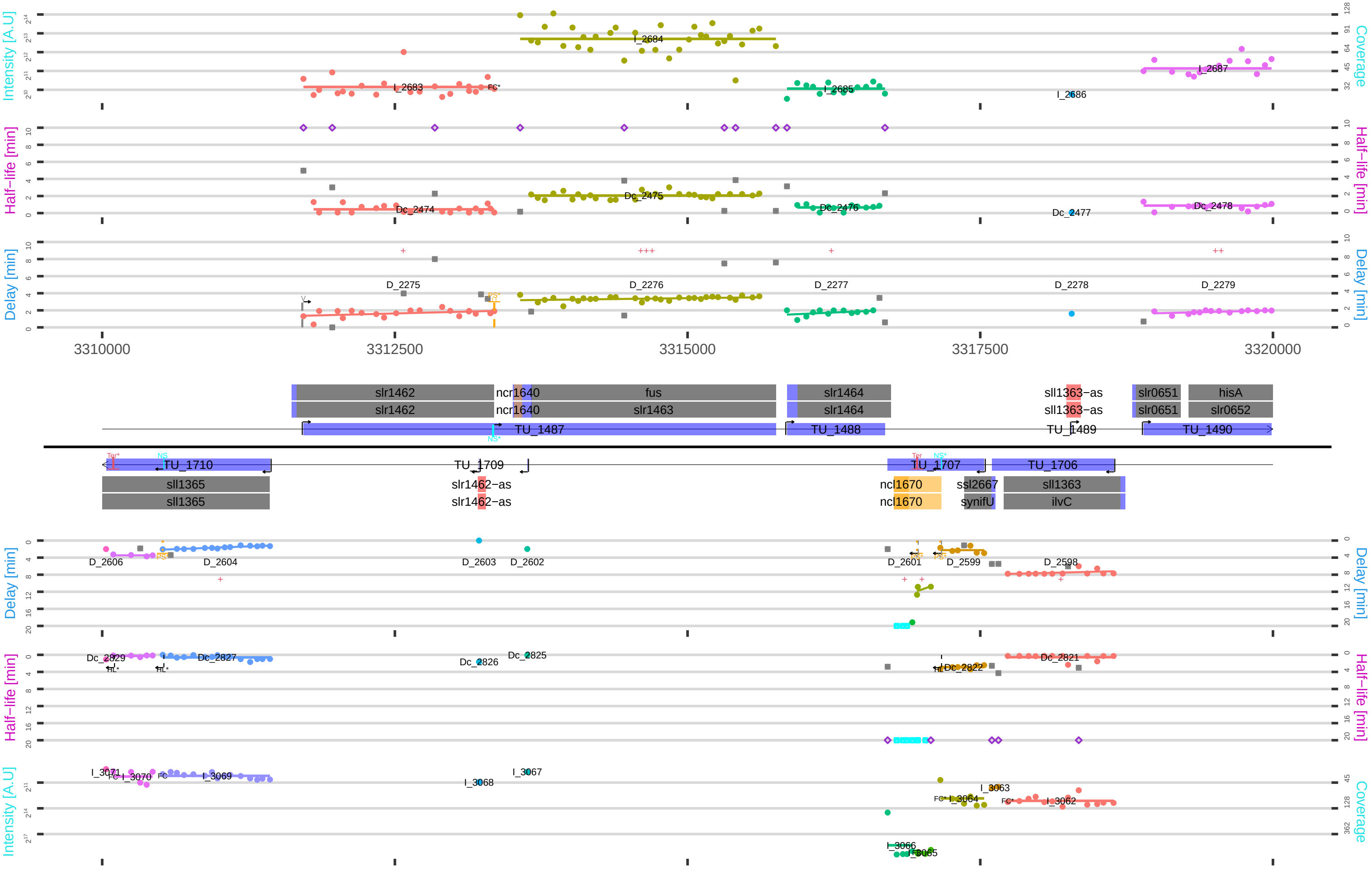

Term: termination (2), NS: new start (2), PS: pausing site (3), iTSS_I: internal starting site (1)

ID: 26677−26707; Term: termination (0), NS: new start (0), PS: pausing site (0), iTSS_I: internal starting site (0)

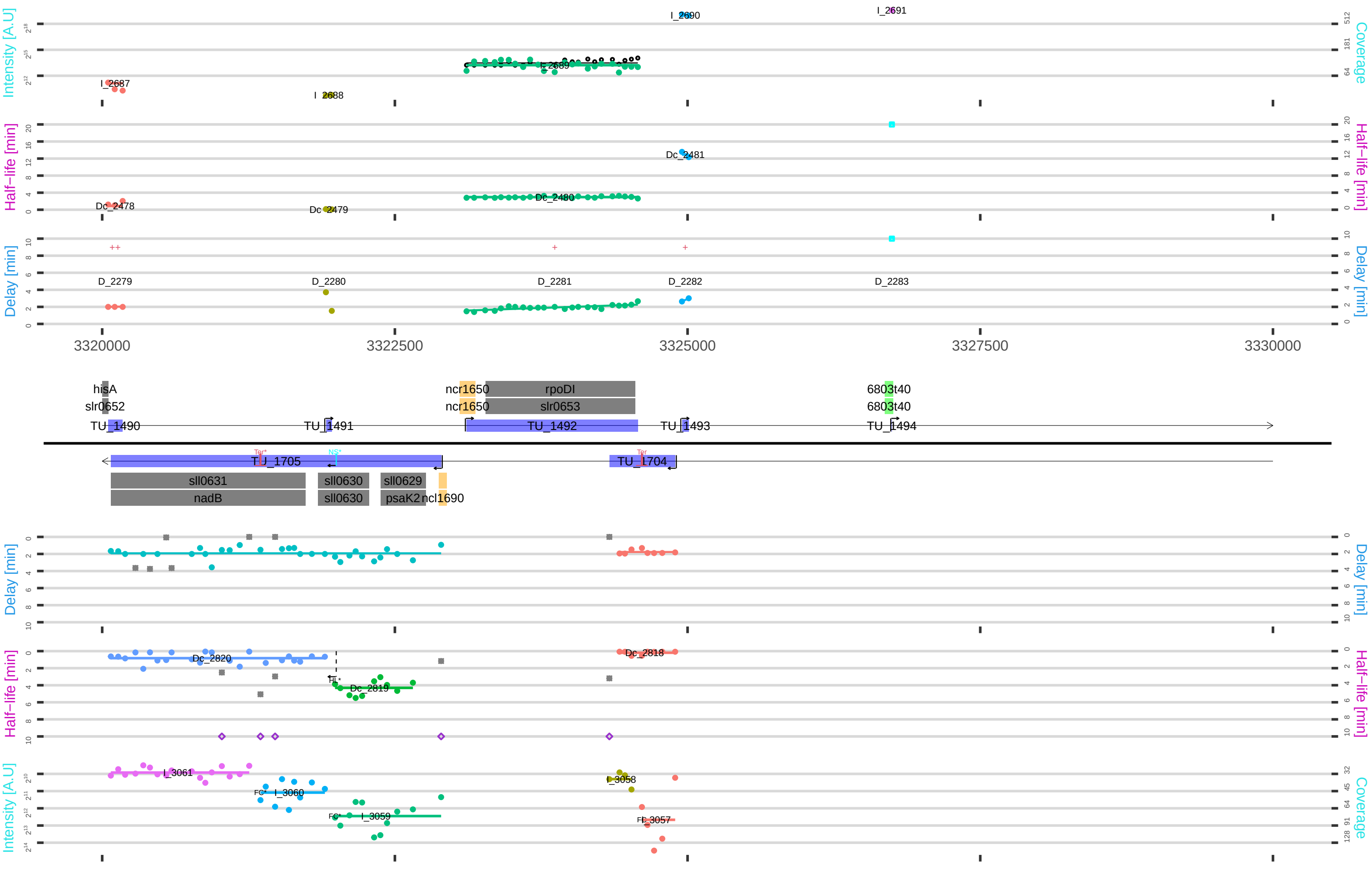

Term: termination (2), NS: new start (1), PS: pausing site (0), iTSS_I: internal starting site (0)

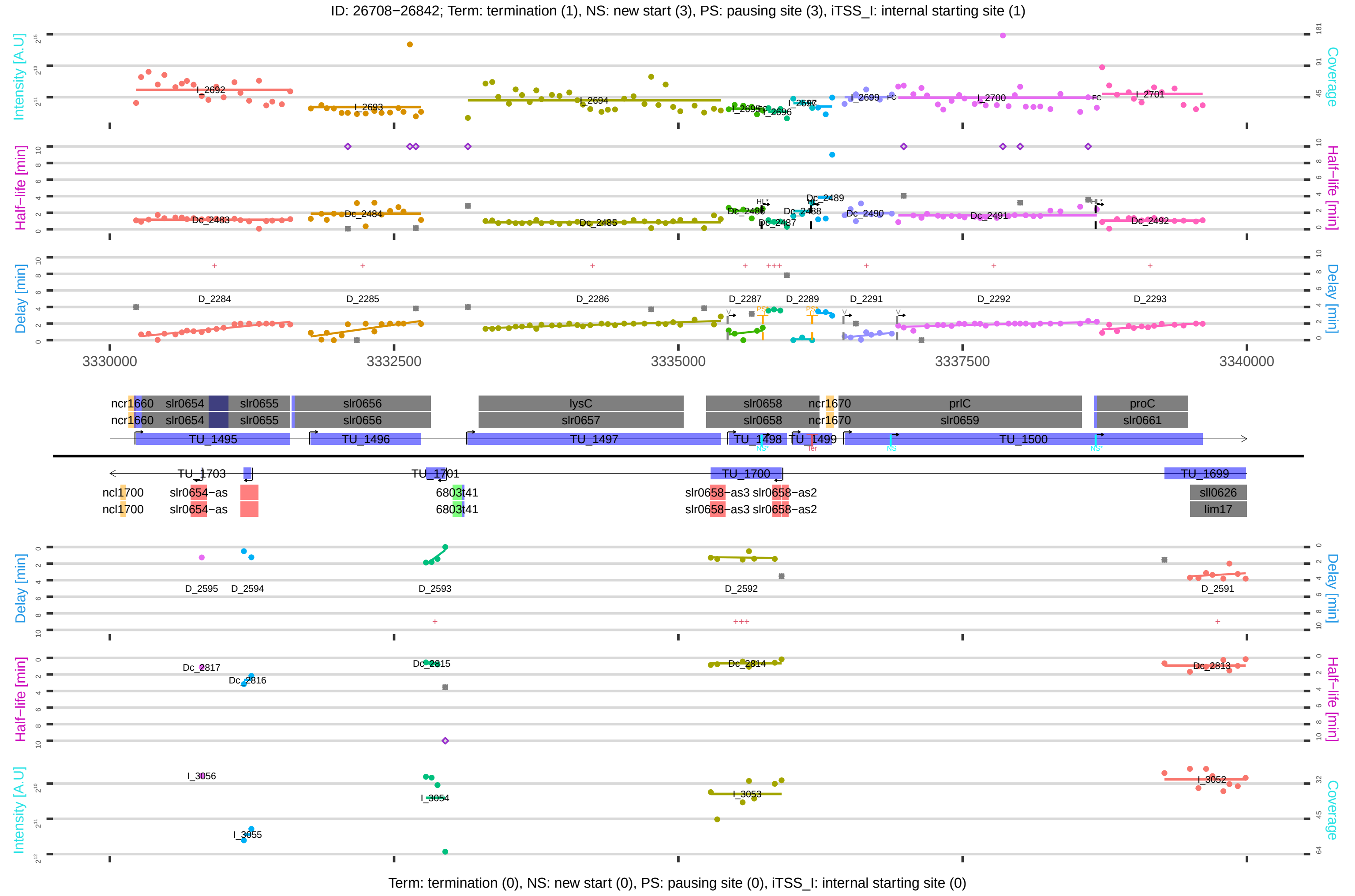
ID: 26708−26842; Term: termination (1), NS: new start (3), PS: pausing site (3), iTSS_I: internal starting site (1)
Intensity [A.U]
Coverage
Half−life [min]
Delay [min]
3330000
3332500
3335000
3337500
3340000
ncr1660
slr0654
slr0655
slr0656
lysC
slr0657
slr0658
ncr1670
prlC
slr0659
proC
slr0661
TU_1495
TU_1496
TU_1497
TU_1498
TU_1499
TU_1500
TU_1703
TU_1701
TU_1700
TU_1699
ncl1700
slr0654−as
6803t41
slr0658−as3
slr0658−as2
sll0626
lim17
Term: termination (0), NS: new start (0), PS: pausing site (0), iTSS_I: internal starting site (0)

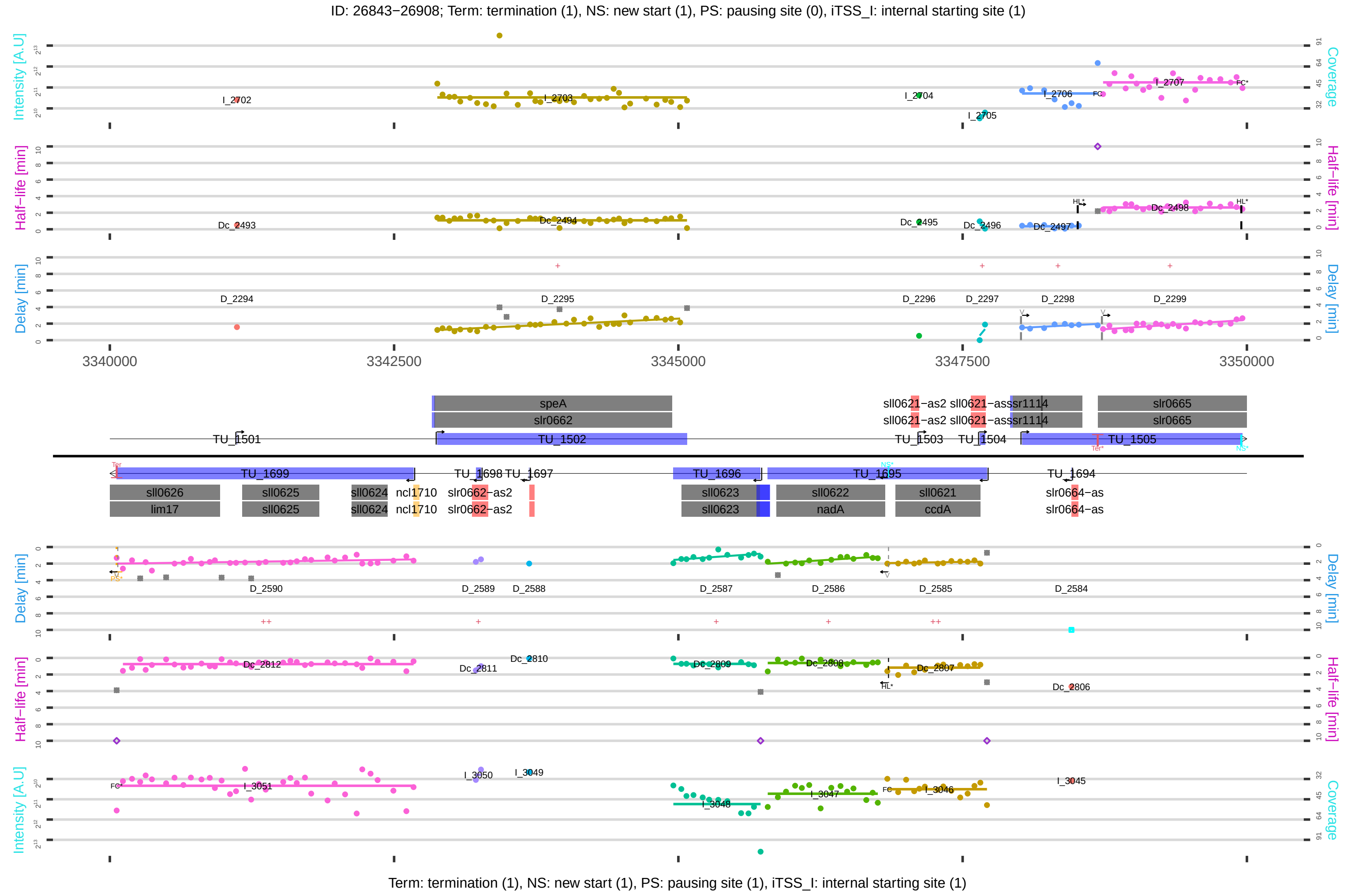

ID: 26843−26908; Term: termination (1), NS: new start (1), PS: pausing site (0), iTSS_I: internal starting site (1)
Intensity [A.U]
Coverage
Half−life [min]
Delay [min]
I_2702
I_2703
I_2704
I_2705
I_2706
I_2707
Dc_2493
Dc_2494
Dc_2495
Dc_2496
Dc_2497
Dc_2498
D_2294
D_2295
D_2296
D_2297
D_2298
D_2299
3340000
3342500
3345000
3347500
3350000
speA
slr0662
sll0621−as2
sll0621−as
ssr1114
slr0665
TU_1501
TU_1502
TU_1503
TU_1504
TU_1505
TU_1699
TU_1698
TU_1697
TU_1696
TU_1695
TU_1694
sll0626
lim17
sll0625
sll0624
ncl1710
slr0662−as2
sll0623
sll0622
nadA
sll0621
ccdA
slr0664−as
D_2590
D_2589
D_2588
D_2587
D_2586
D_2585
D_2584
Dc_2812
Dc_2811
Dc_2810
Dc_2809
Dc_2808
Dc_2807
Dc_2806
I_3051
I_3050
I_3049
I_3048
I_3047
I_3046
I_3045
Term: termination (1), NS: new start (1), PS: pausing site (1), iTSS_I: internal starting site (1)

ID: 26909−26985; Term: termination (0), NS: new start (1), PS: pausing site (0), iTSS_I: internal starting site (1)

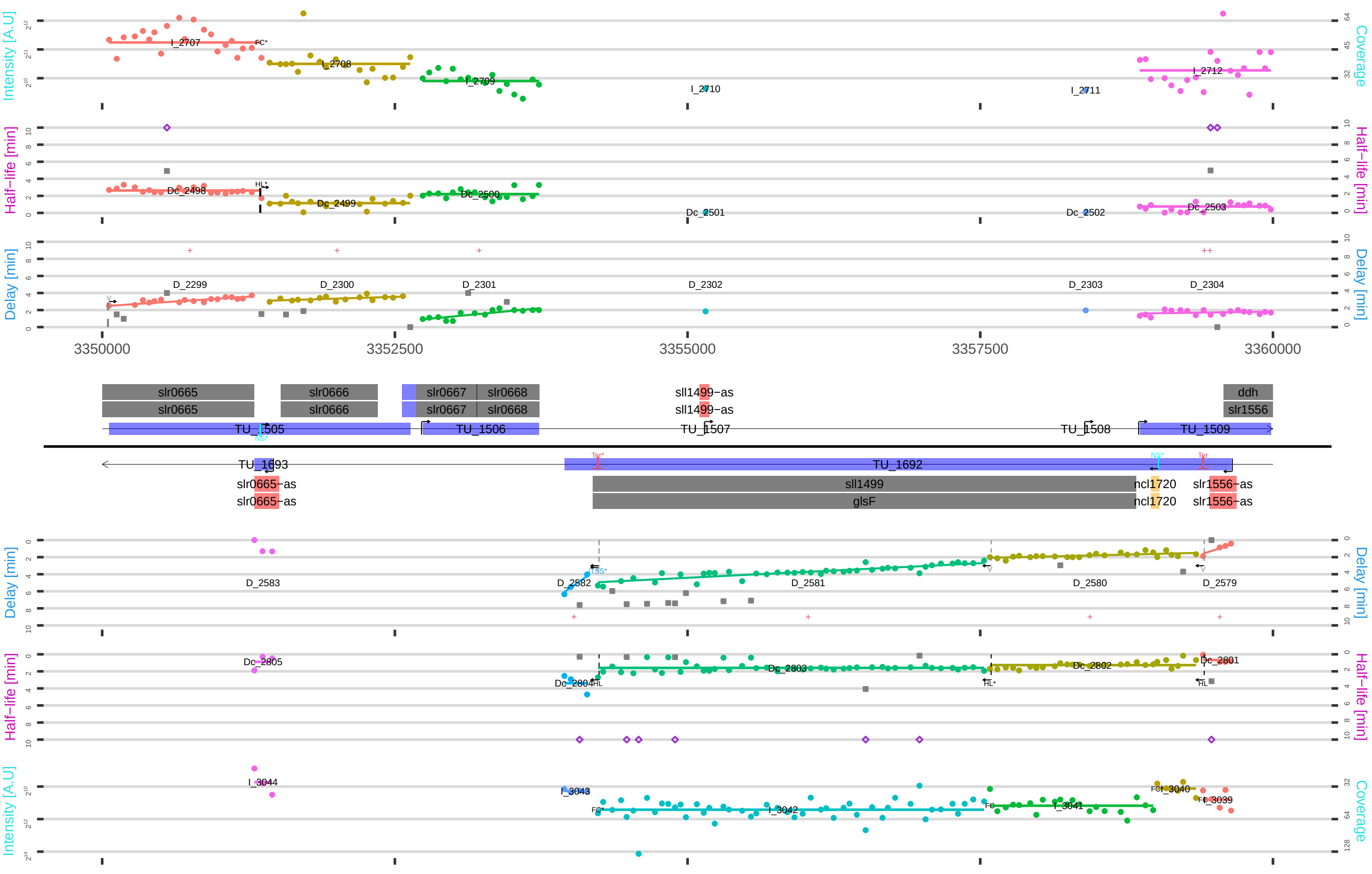

Term: termination (2), NS: new start (1), PS: pausing site (1), iTSS_I: internal starting site (2)

ID: 26986−27060; Term: termination (2), NS: new start (1), PS: pausing site (2), iTSS_I: internal starting site (0)

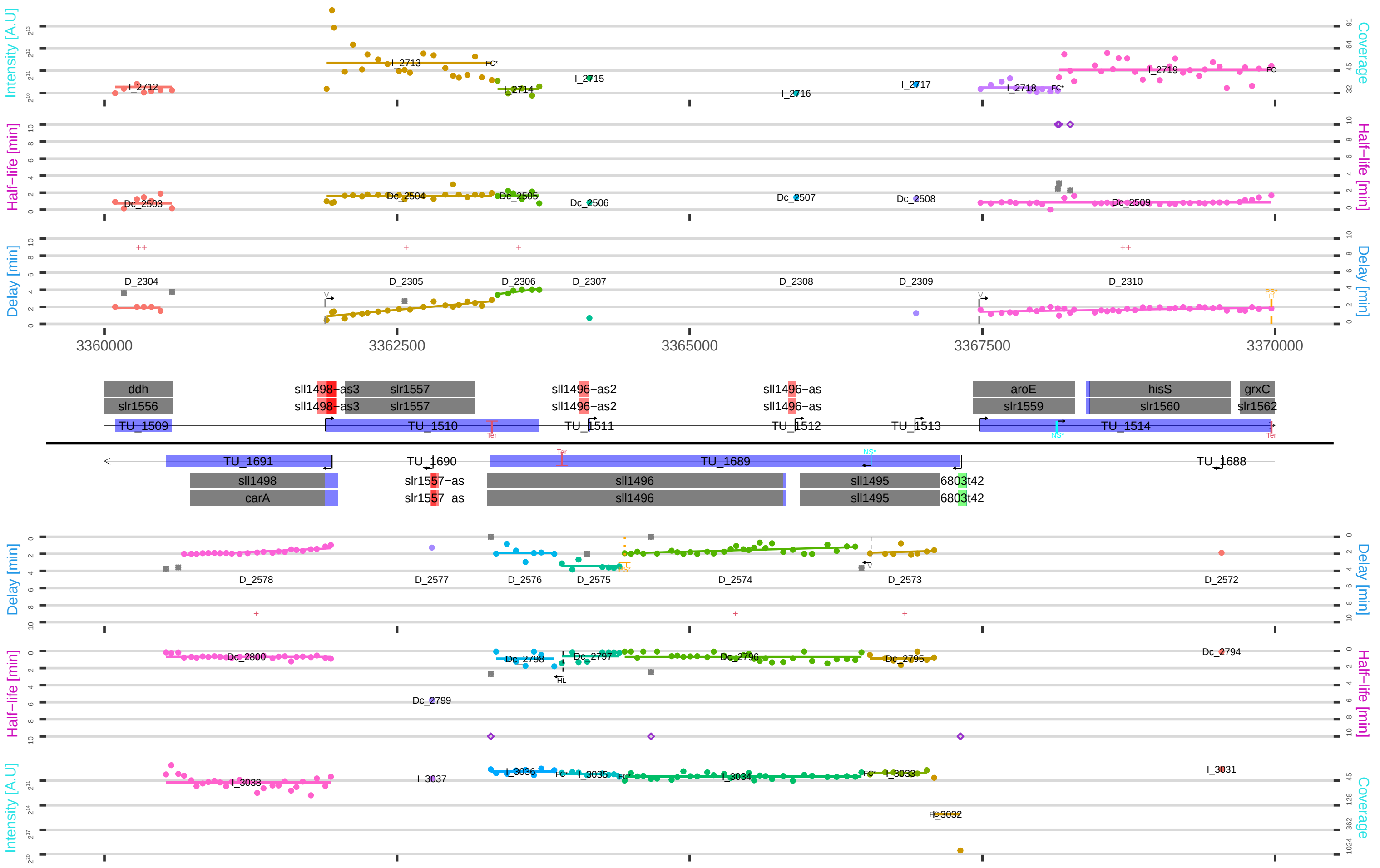

Term: termination (1), NS: new start (1), PS: pausing site (1), iTSS_I: internal starting site (2)

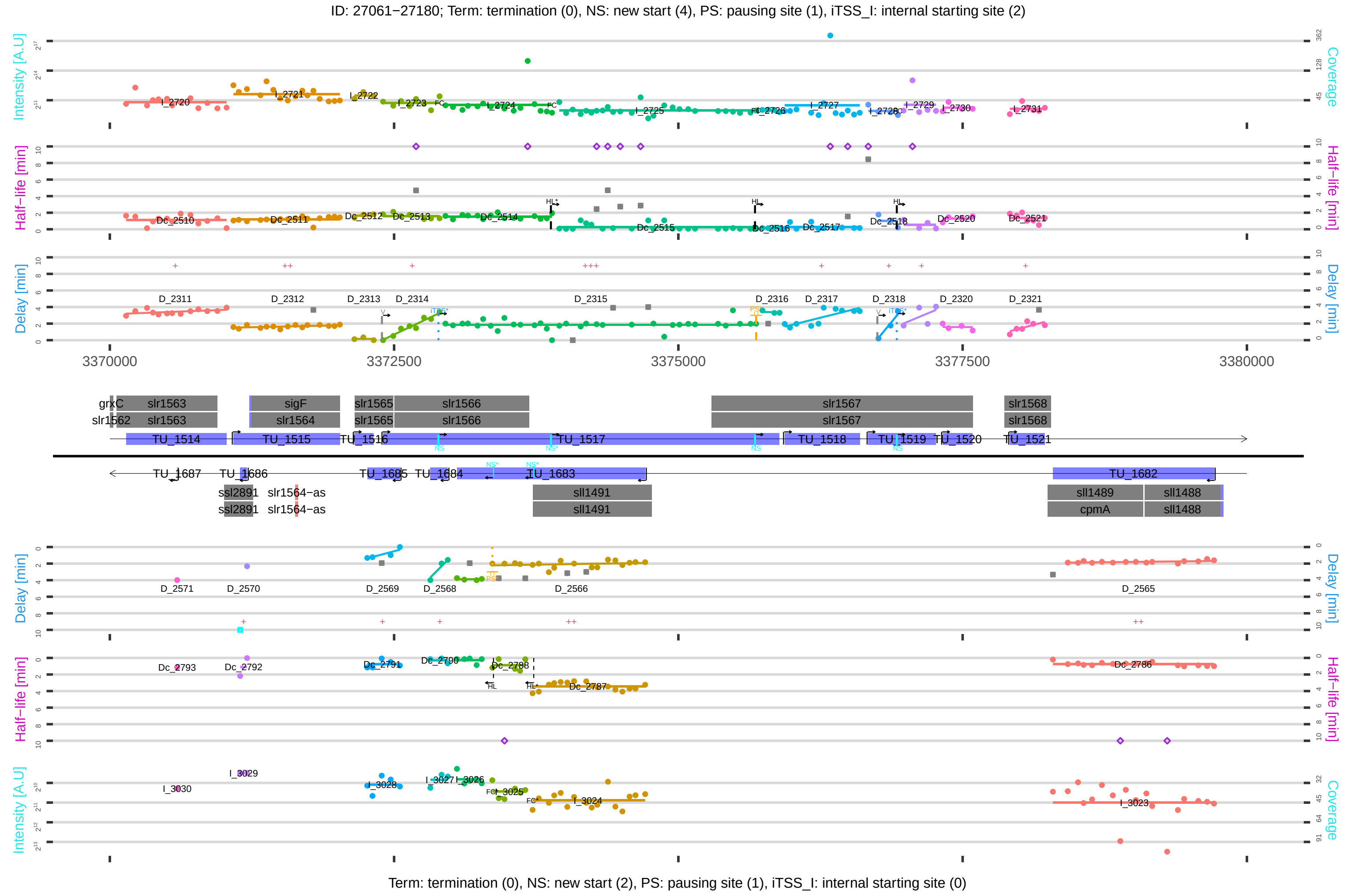
ID: 27061−27180; Term: termination (0), NS: new start (4), PS: pausing site (1), iTSS_I: internal starting site (2)
Intensity [A.U]
Coverage
Half−life [min]
Delay [min]
3370000
3372500
3375000
3377500
3380000
grxC
slr1562
slr1563
sigF
slr1564
slr1565
slr1566
slr1567
slr1568
TU_1514
TU_1515
TU_1516
TU_1517
TU_1518
TU_1519
TU_1520
TU_1521
TU_1687
TU_1686
TU_1685
TU_1684
TU_1683
TU_1682
ssl2891
slr1564−as
sll1491
sll1489
sll1488
cpmA
Term: termination (0), NS: new start (2), PS: pausing site (1), iTSS_I: internal starting site (0)

ID: 27181−27299; Term: termination (3), NS: new start (2), PS: pausing site (2), iTSS_I: internal starting site (2)

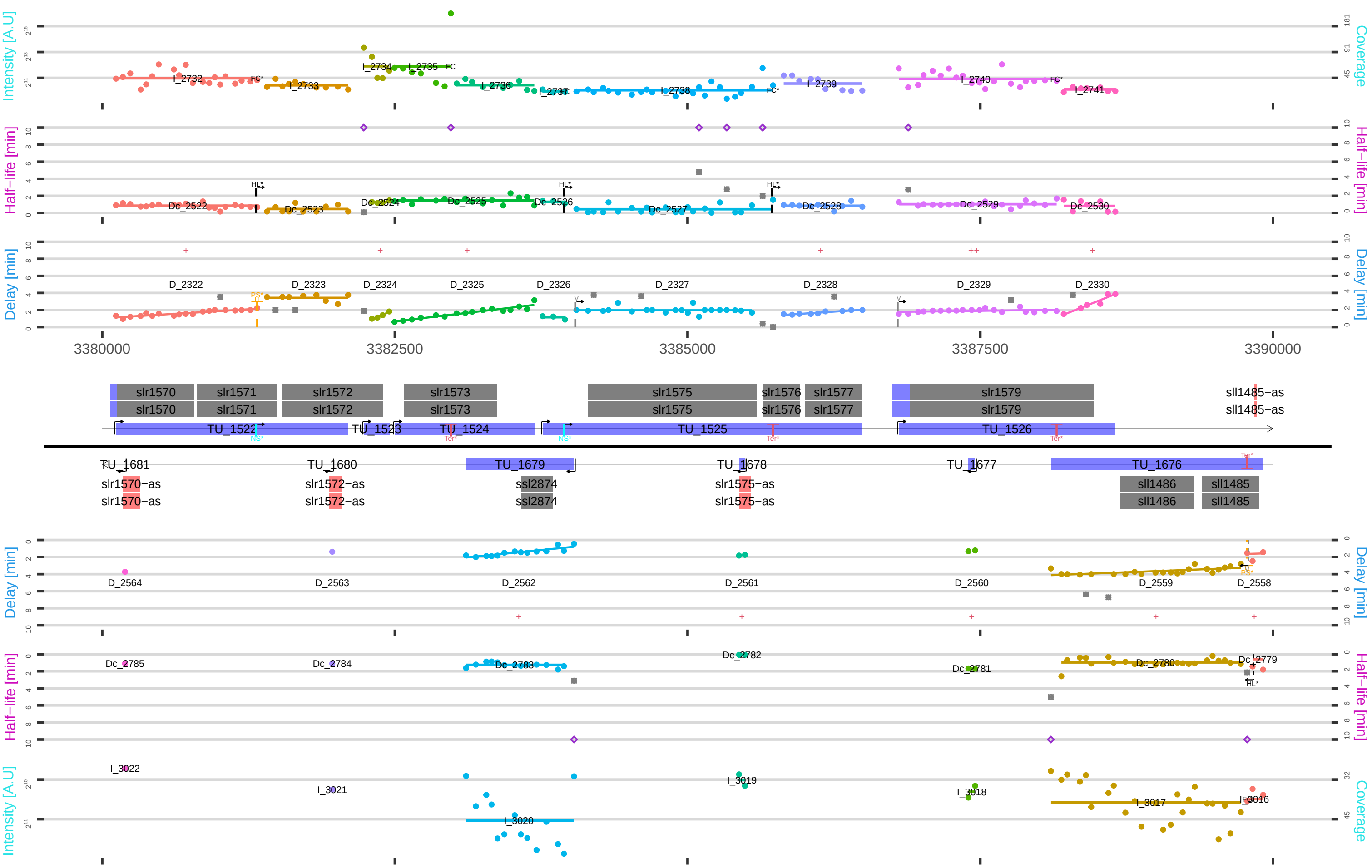

Term: termination (1), NS: new start (0), PS: pausing site (1), iTSS_I: internal starting site (0)

ID: 27300−27327; Term: termination (1), NS: new start (2), PS: pausing site (1), iTSS_I: internal starting site (0)

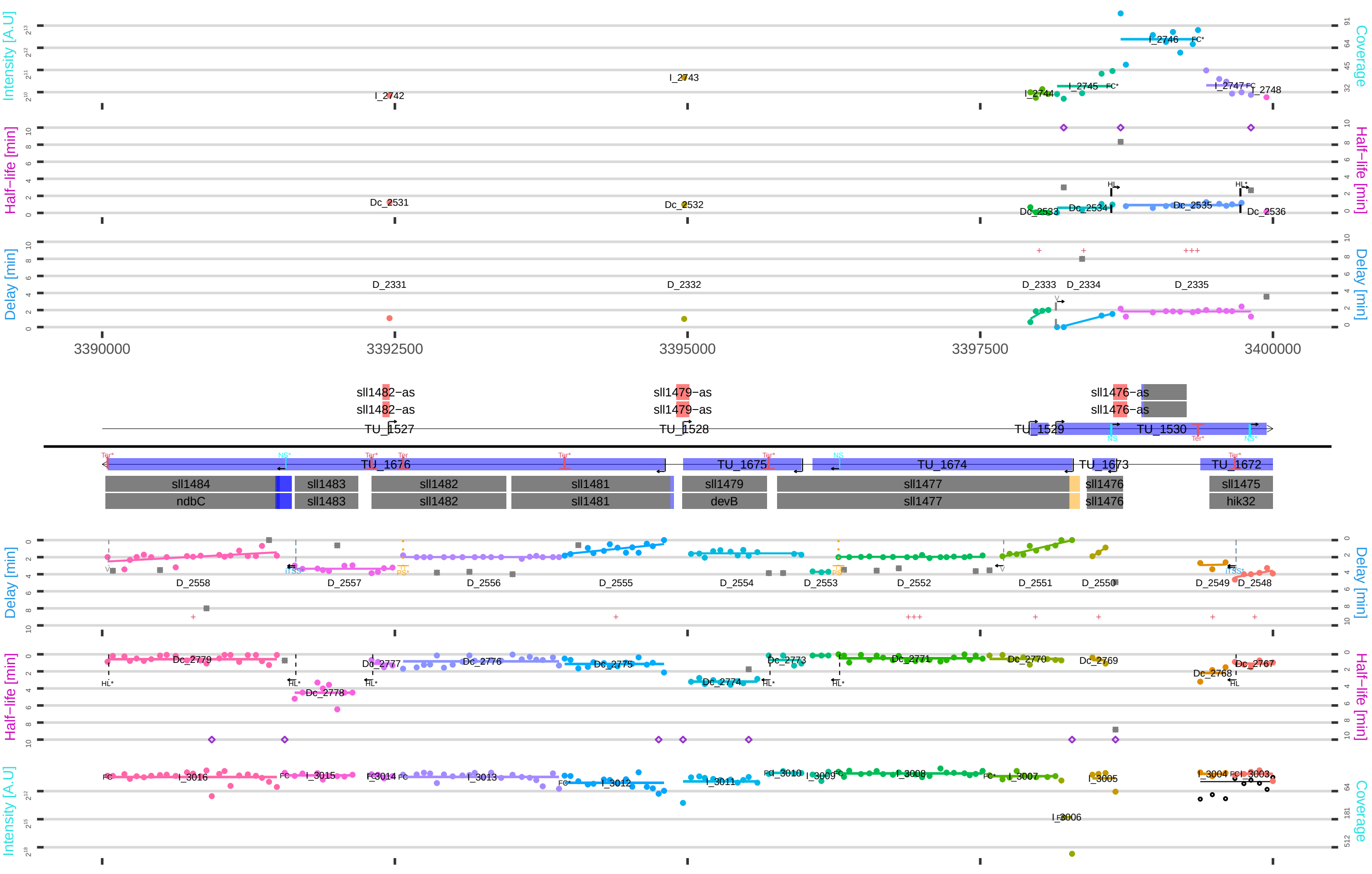

Term: termination (6), NS: new start (2), PS: pausing site (3), iTSS_I: internal starting site (3)

ID: 27328−27383; Term: termination (2), NS: new start (1), PS: pausing site (1), iTSS_I: internal starting site (0)

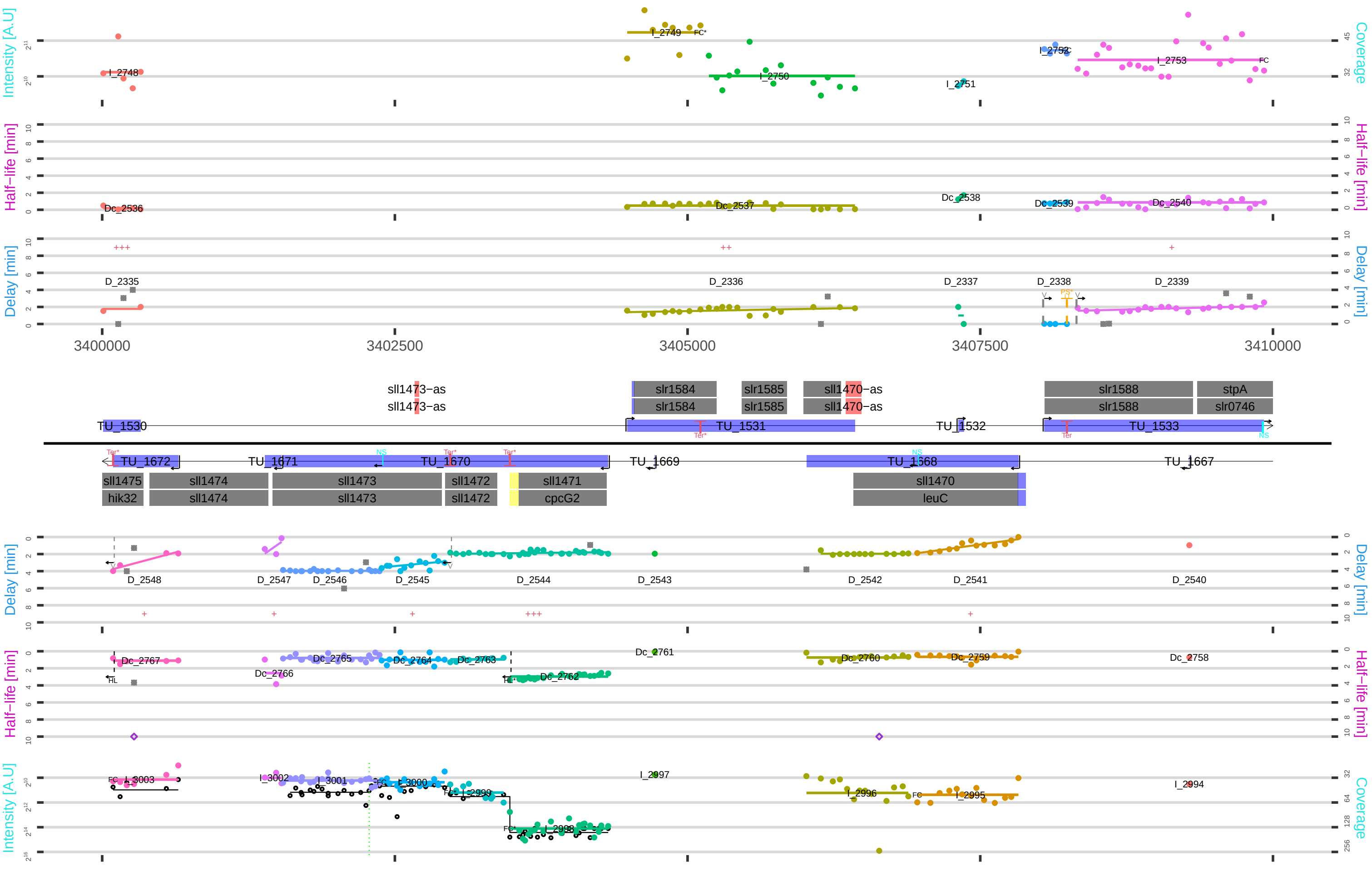

Term: termination (3), NS: new start (2), PS: pausing site (3), iTSS_I: internal starting site (0)

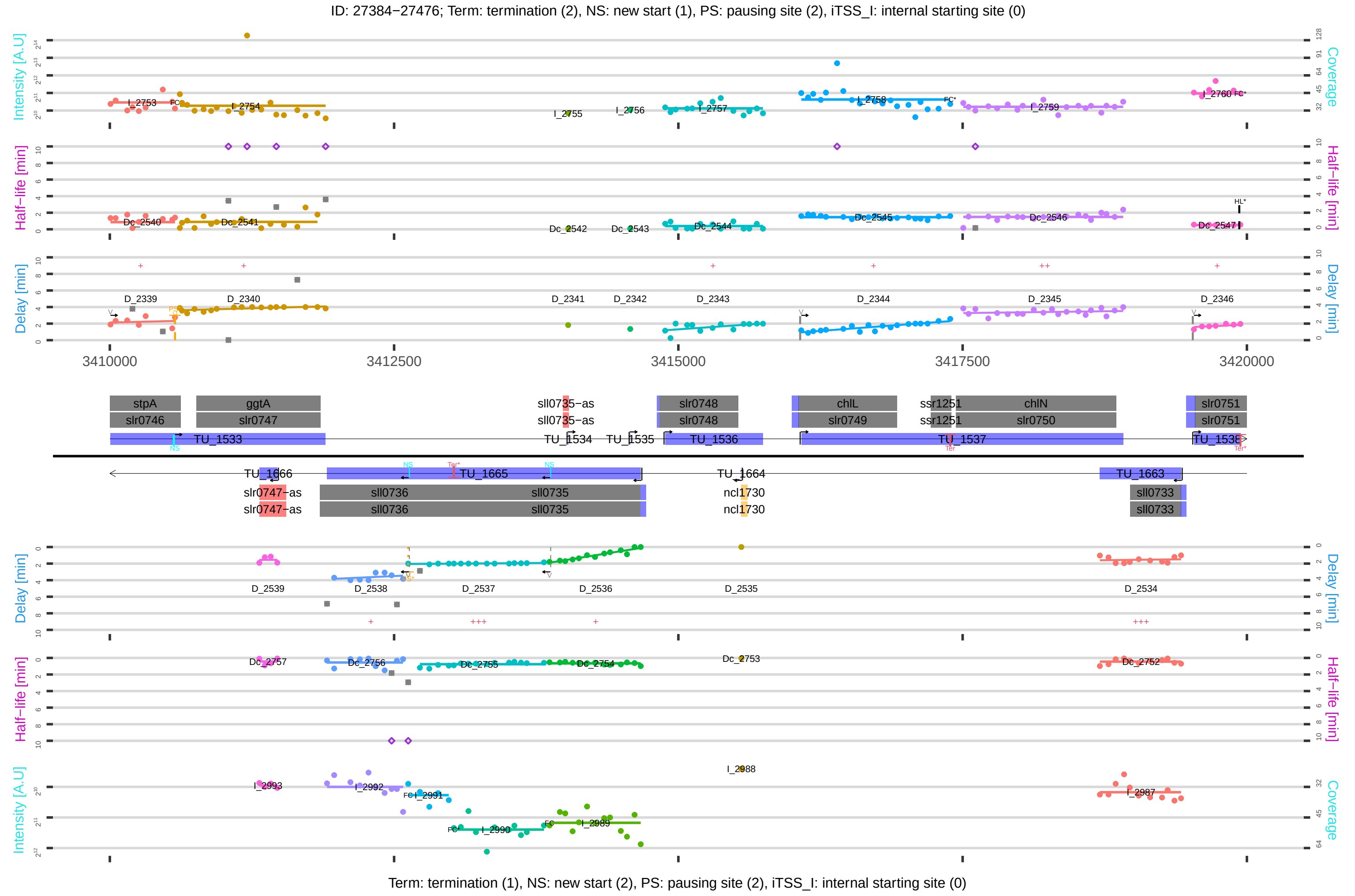
ID: 27384−27476; Term: termination (2), NS: new start (1), PS: pausing site (2), iTSS_I: internal starting site (0)
Intensity [A.U]
Coverage
Half−life [min]
Delay [min]
3410000
3412500
3415000
3417500
3420000
stpA
slr0746
ggtA
slr0747
sll0735−as
slr0748
chlL
slr0749
ssr1251
chlN
slr0750
slr0751
TU_1533
TU_1534
TU_1535
TU_1536
TU_1537
TU_1538
TU_1666
TU_1665
TU_1664
TU_1663
slr0747−as
sll0736
sll0735
ncl1730
sll0733
Term: termination (1), NS: new start (2), PS: pausing site (2), iTSS_I: internal starting site (0)

ID: 27477−27561; Term: termination (4), NS: new start (2), PS: pausing site (0), iTSS_I: internal starting site (3)

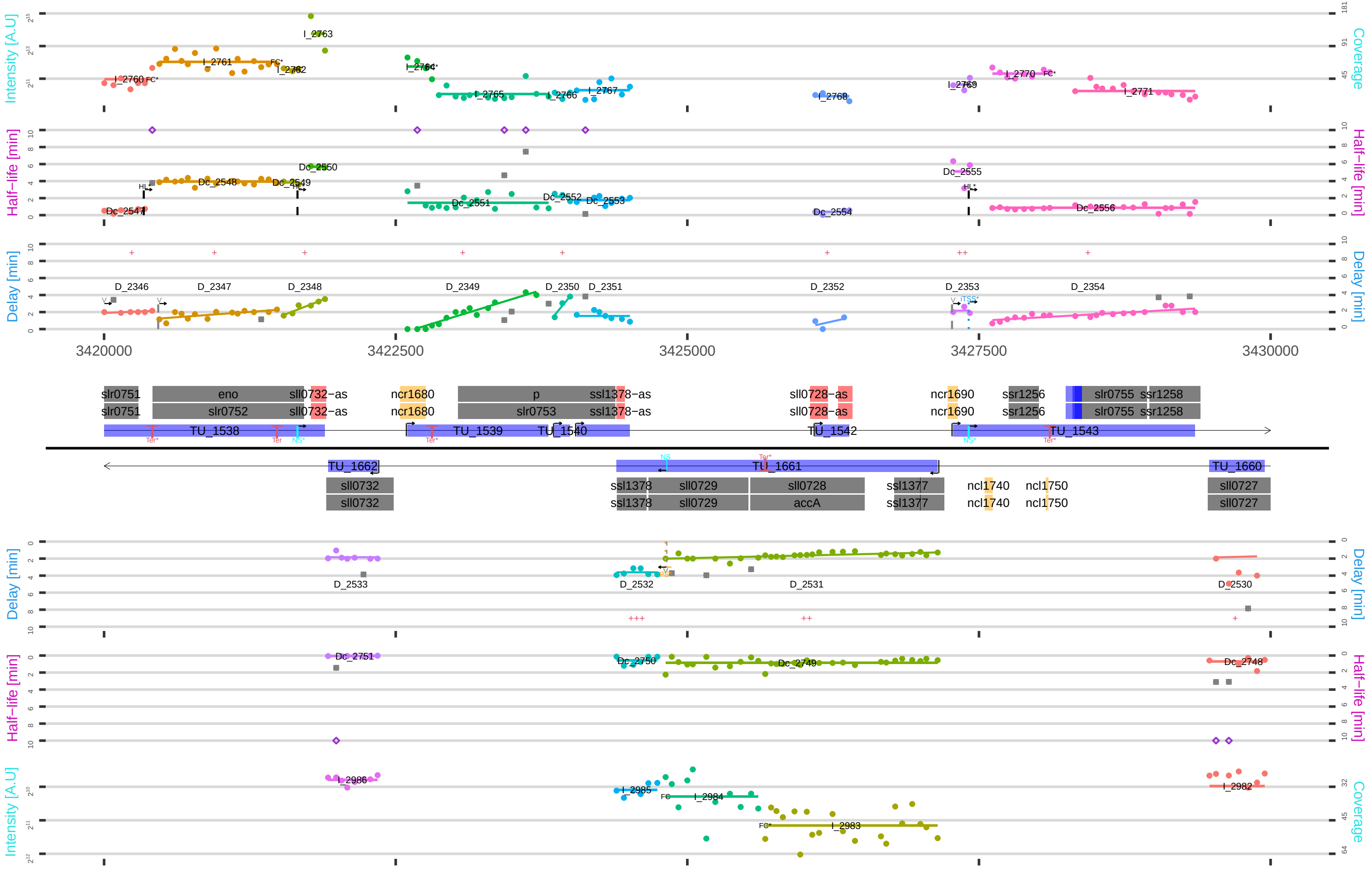

Term: termination (1), NS: new start (1), PS: pausing site (1), iTSS_I: internal starting site (0)

ID: 27562−27607; Term: termination (1), NS: new start (0), PS: pausing site (0), iTSS_I: internal starting site (1)

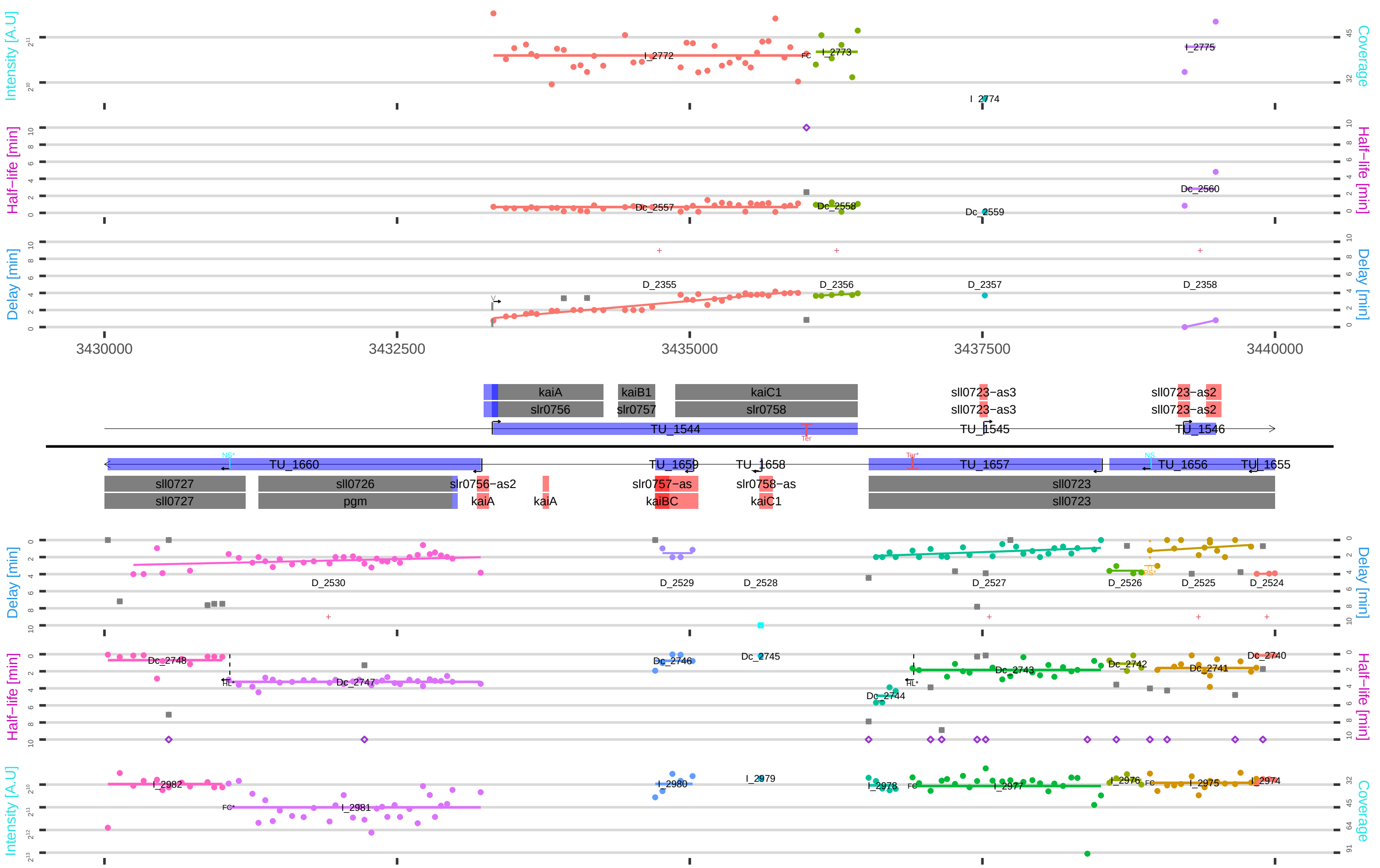

Term: termination (1), NS: new start (2), PS: pausing site (1), iTSS_I: internal starting site (0)

ID: 27609−27630; Term: termination (0), NS: new start (0), PS: pausing site (0), iTSS_I: internal starting site (0)

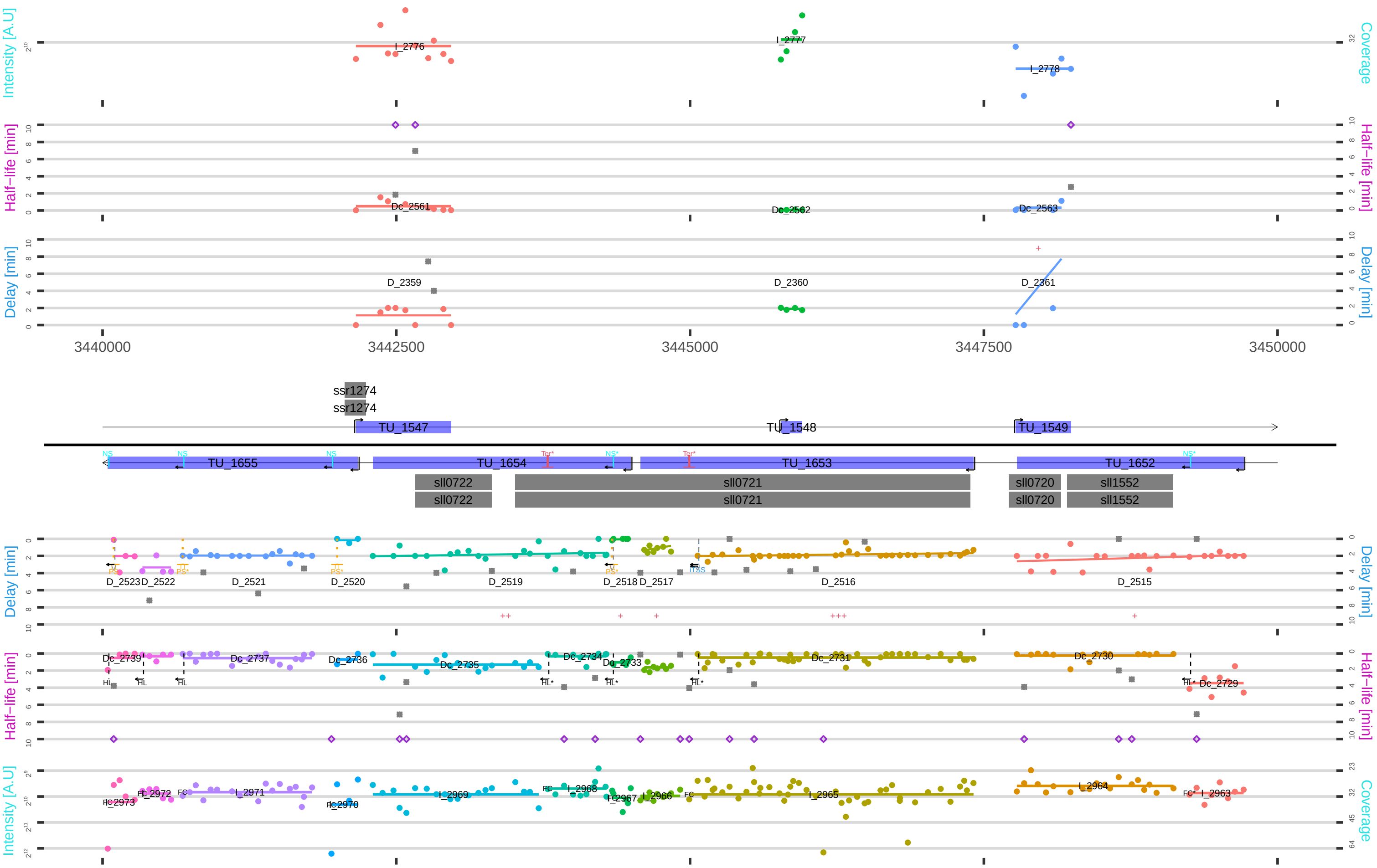

Term: termination (2), NS: new start (5), PS: pausing site (4), iTSS_I: internal starting site (2)

ID: 27631−27734; Term: termination (3), NS: new start (0), PS: pausing site (1), iTSS_I: internal starting site (2)

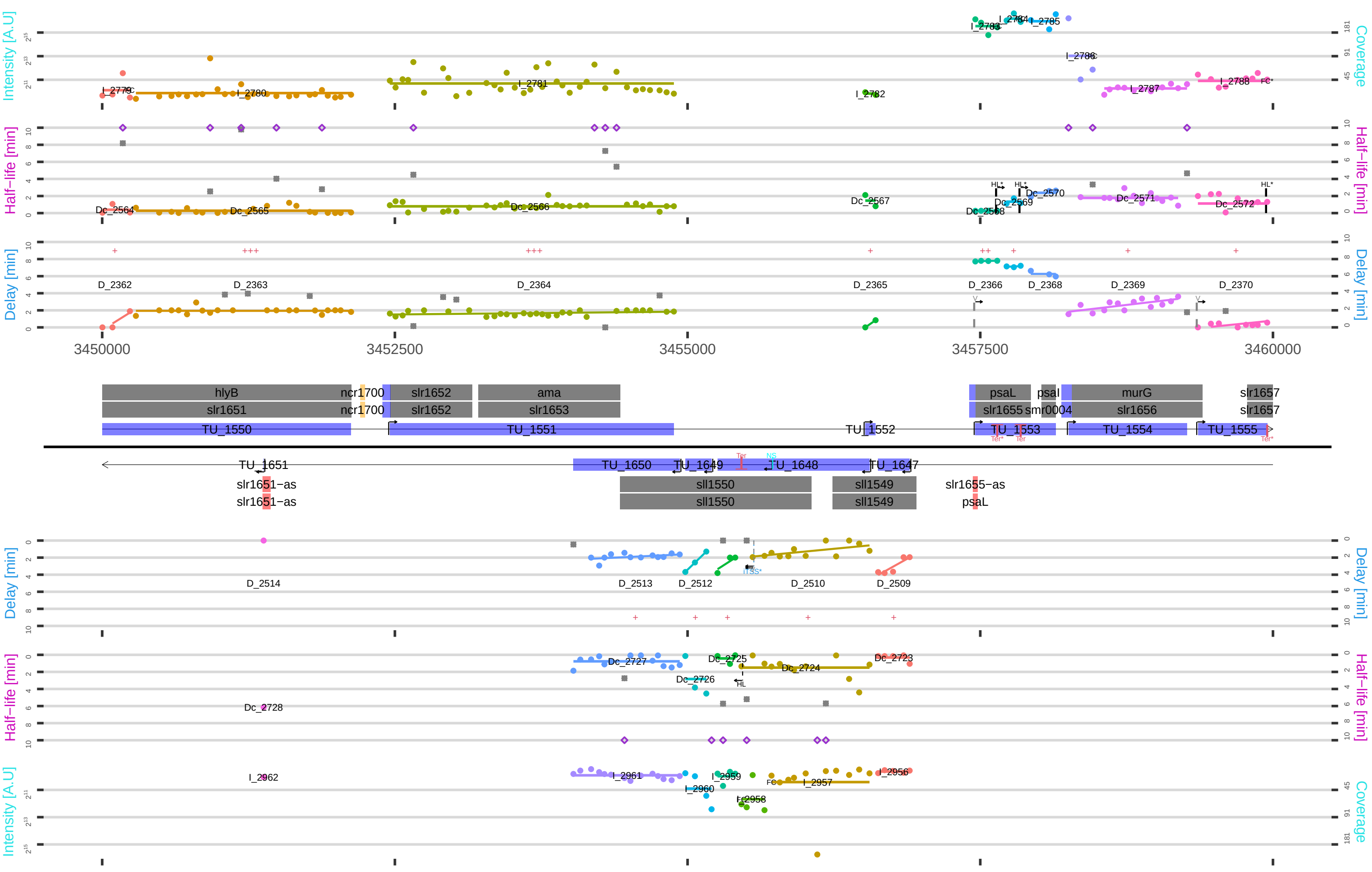

Term: termination (1), NS: new start (1), PS: pausing site (0), iTSS_I: internal starting site (1)

ID: 27735−27823; Term: termination (1), NS: new start (1), PS: pausing site (1), iTSS_I: internal starting site (1)

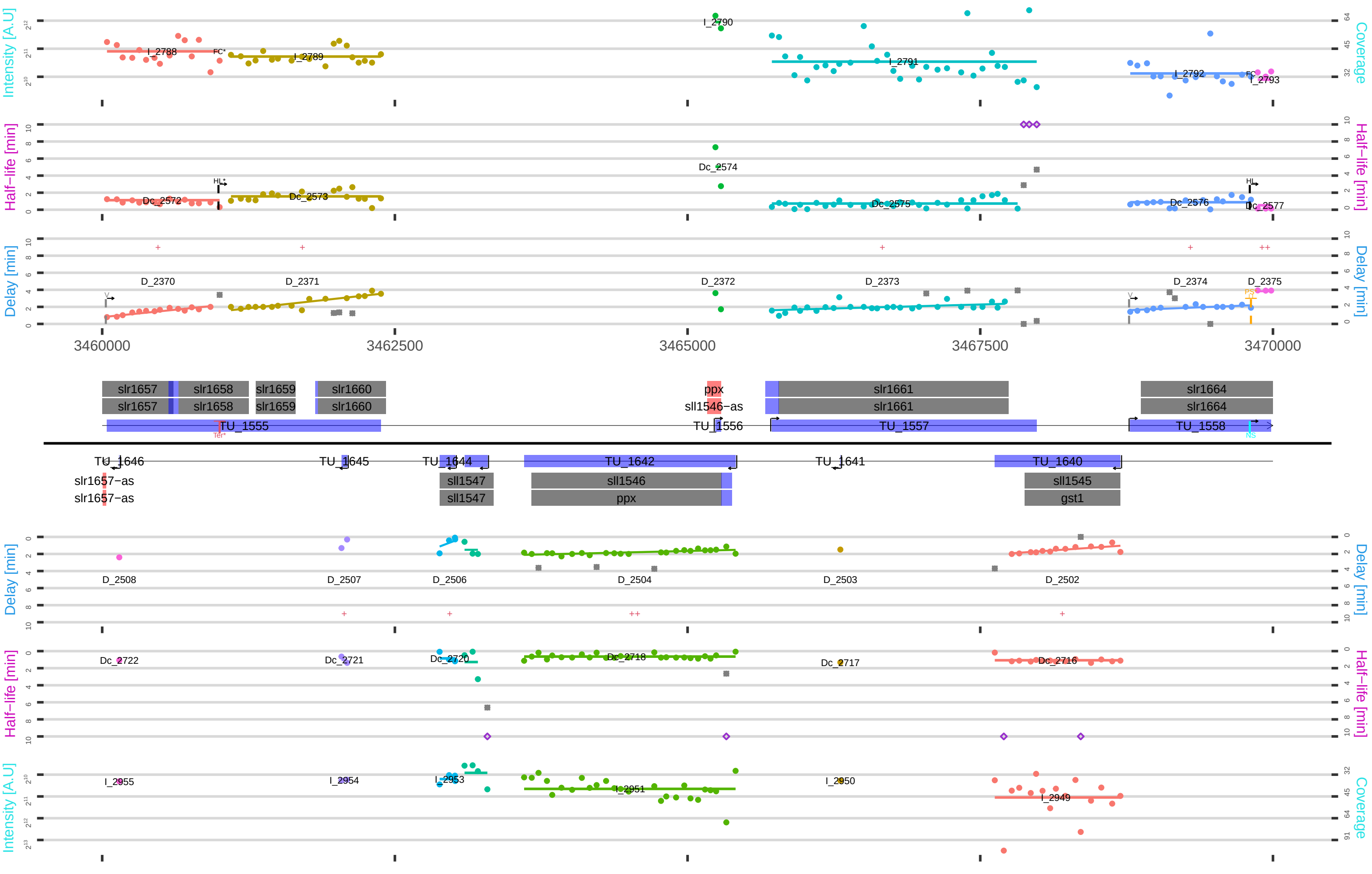

Term: termination (0), NS: new start (0), PS: pausing site (0), iTSS_I: internal starting site (0)

ID: 27824−27930; Term: termination (3), NS: new start (1), PS: pausing site (1), iTSS_I: internal starting site (2)

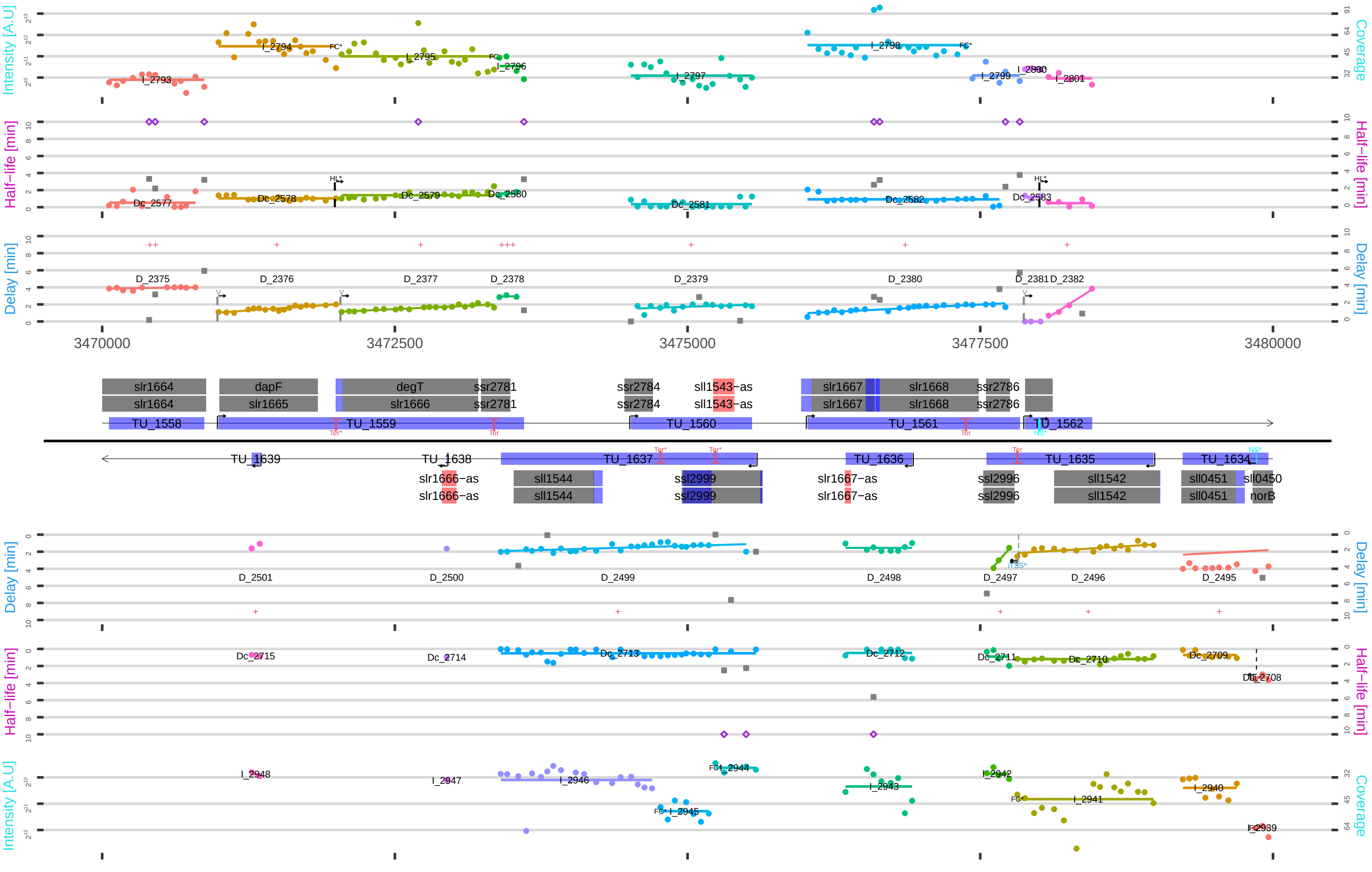

Term: termination (3), NS: new start (1), PS: pausing site (0), iTSS_I: internal starting site (1)

ID: 27931−27940; Term: termination (0), NS: new start (0), PS: pausing site (0), iTSS_I: internal starting site (0)

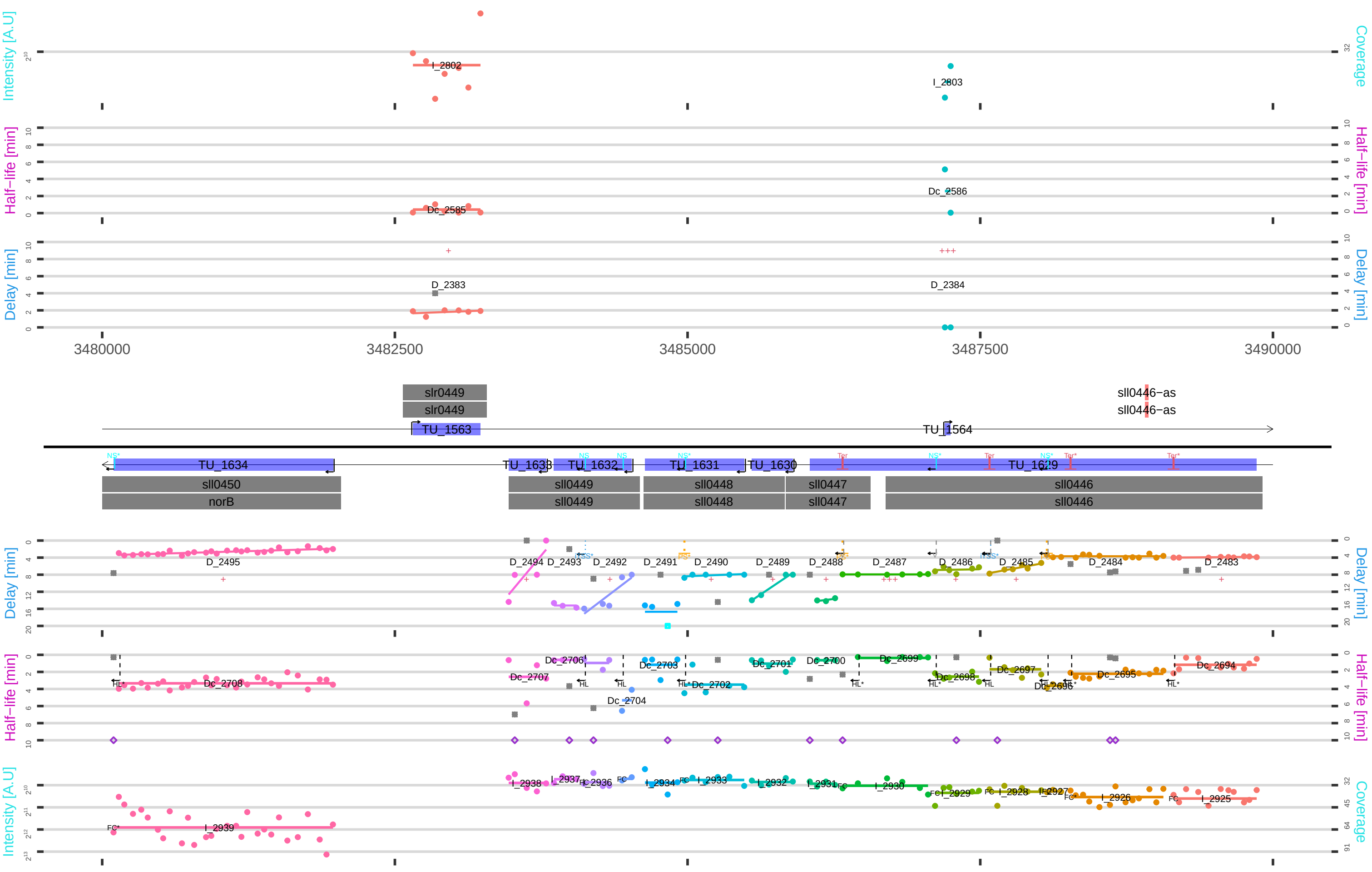

Term: termination (4), NS: new start (6), PS: pausing site (4), iTSS_I: internal starting site (3)

ID: 27941−28008; Term: termination (0), NS: new start (2), PS: pausing site (3), iTSS_I: internal starting site (0)

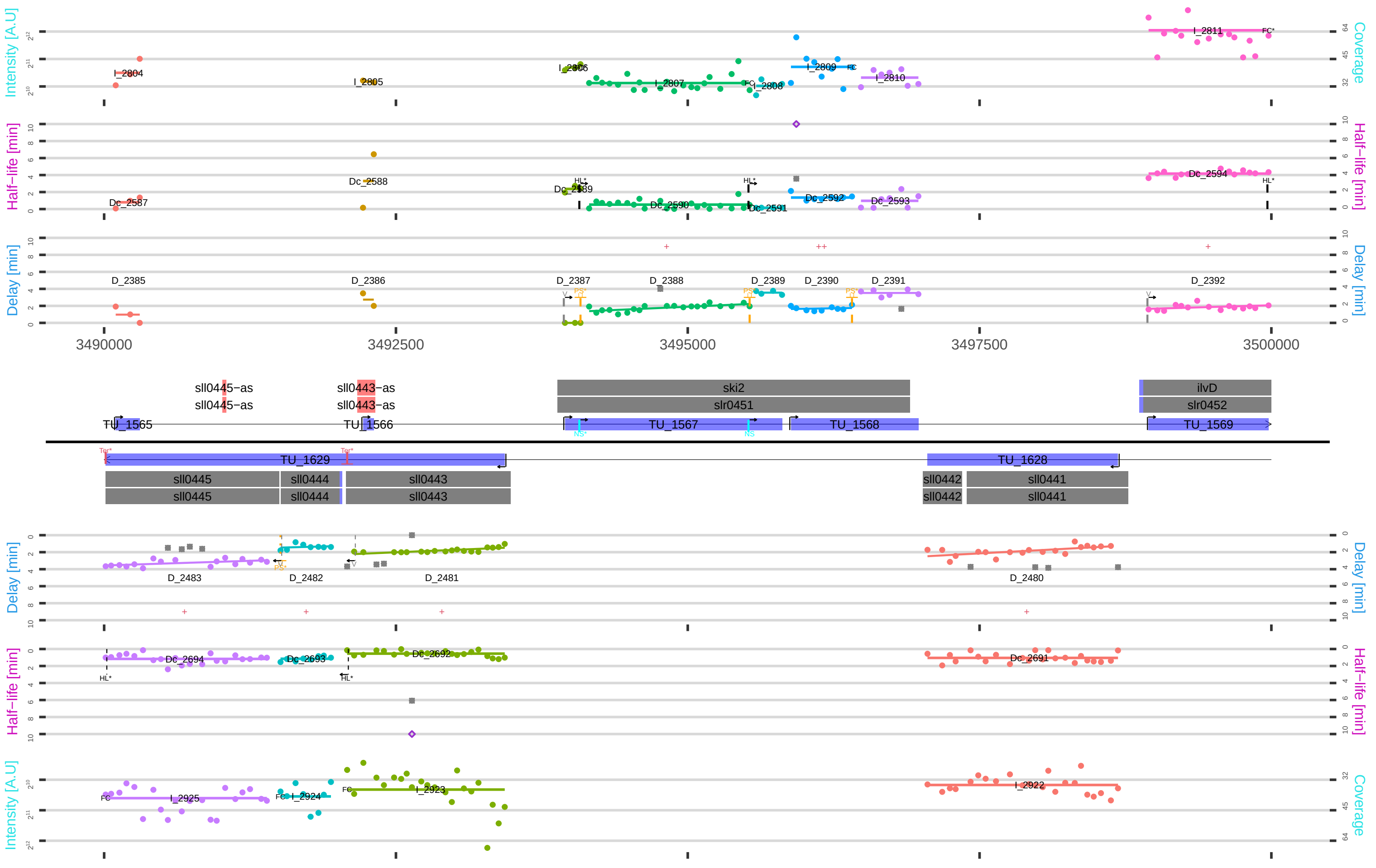

Term: termination (2), NS: new start (0), PS: pausing site (1), iTSS_I: internal starting site (1)

ID: 28009−28138; Term: termination (2), NS: new start (3), PS: pausing site (1), iTSS_I: internal starting site (1)

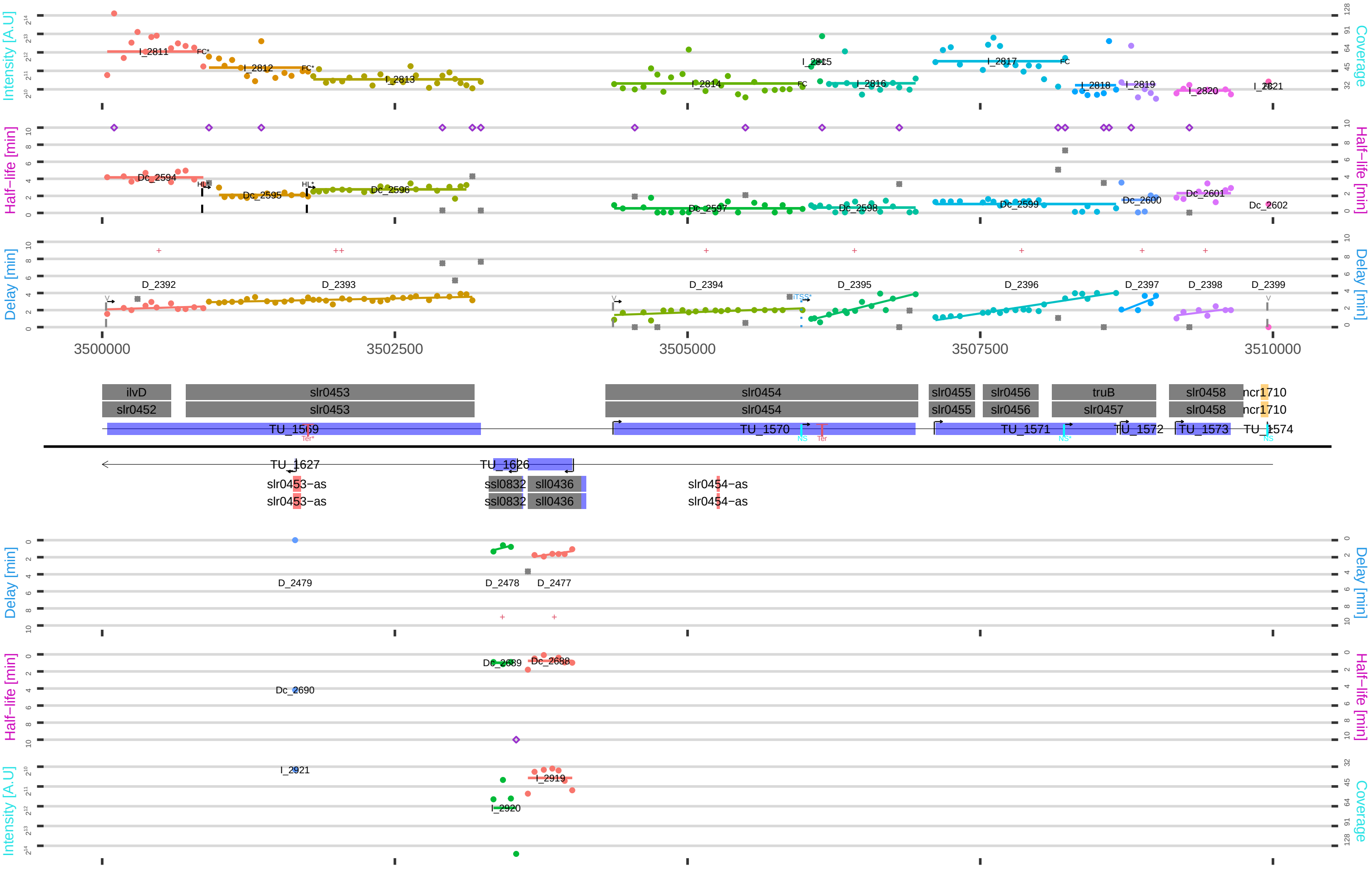

Term: termination (0), NS: new start (0), PS: pausing site (0), iTSS_I: internal starting site (0)

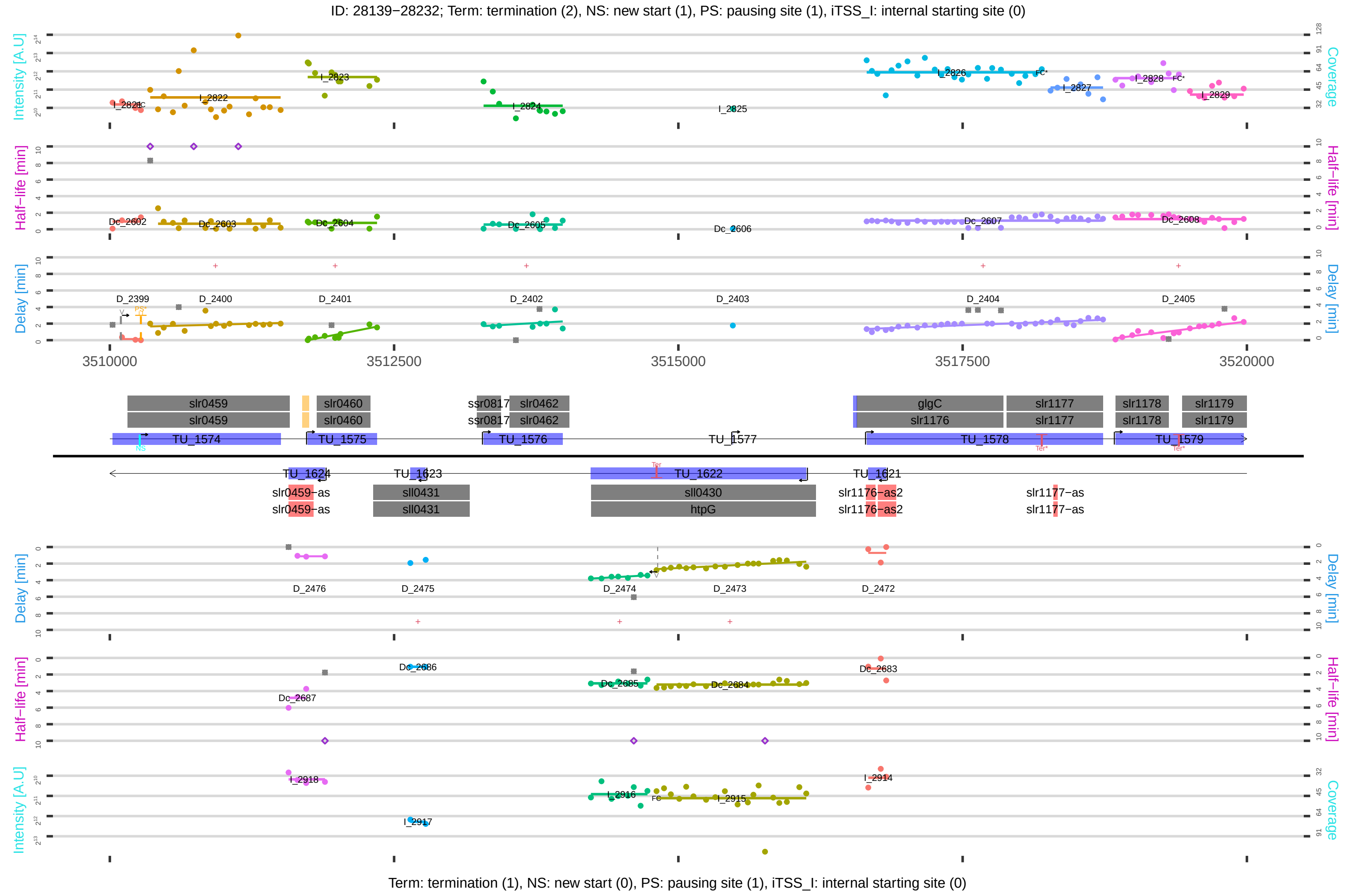

ID: 28139−28232; Term: termination (2), NS: new start (1), PS: pausing site (1), iTSS_I: internal starting site (0)
Intensity [A.U]
Coverage
Half−life [min]
Delay [min]
3510000
3512500
3515000
3517500
3520000
slr0459
slr0460
ssr0817
slr0462
glgC
slr1176
slr1177
slr1178
slr1179
TU_1574
TU_1575
TU_1576
TU_1577
TU_1578
TU_1579
TU_1624
TU_1623
TU_1622
TU_1621
slr0459−as
sll0431
sll0430
htpG
slr1176−as2
slr1177−as
Term: termination (1), NS: new start (0), PS: pausing site (1), iTSS_I: internal starting site (0)

ID: 28233−28327; Term: termination (3), NS: new start (4), PS: pausing site (0), iTSS_I: internal starting site (0)

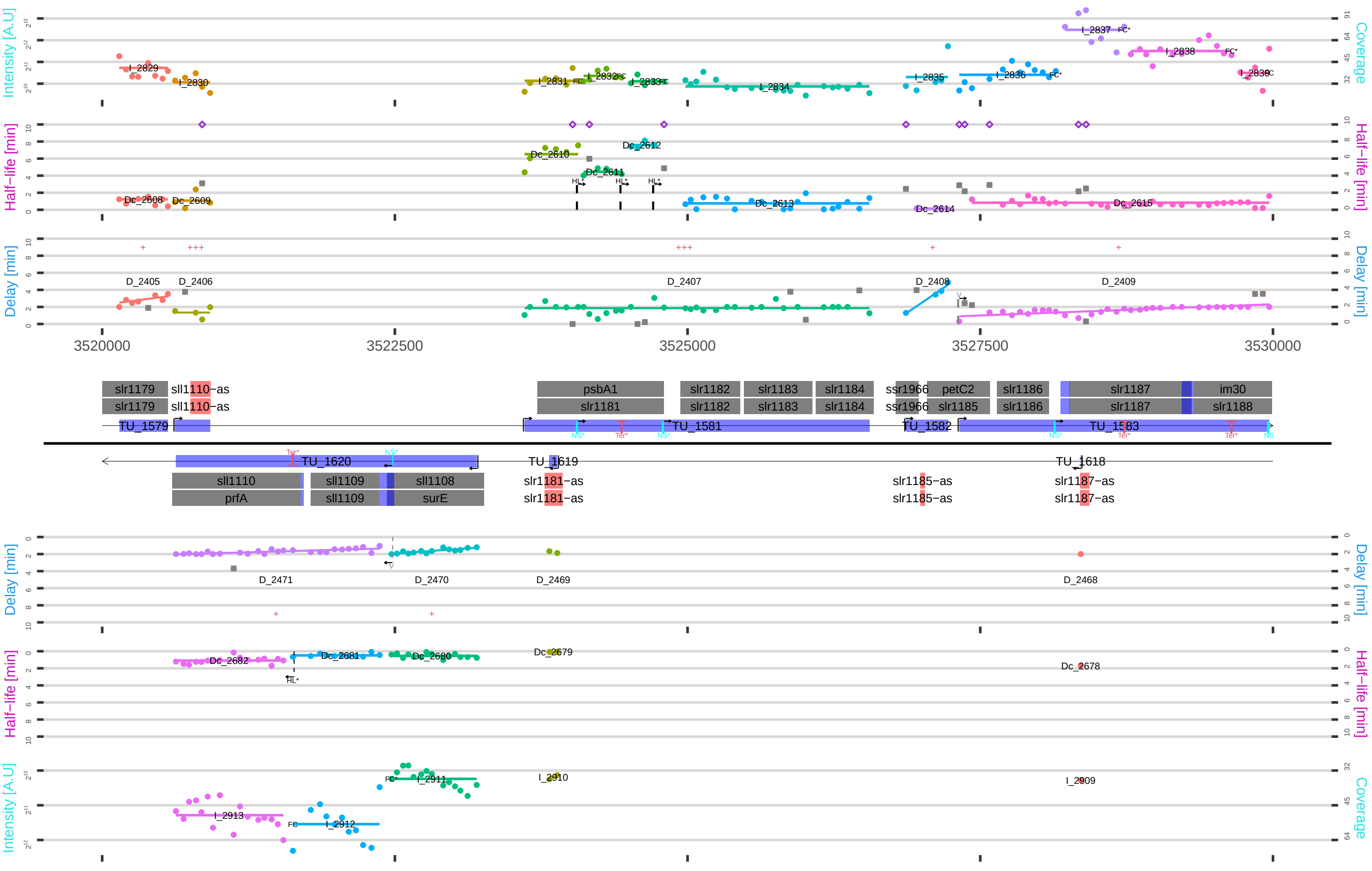

Term: termination (1), NS: new start (1), PS: pausing site (0), iTSS_I: internal starting site (1)

ID: 28328−28431; Term: termination (3), NS: new start (3), PS: pausing site (3), iTSS_I: internal starting site (1)

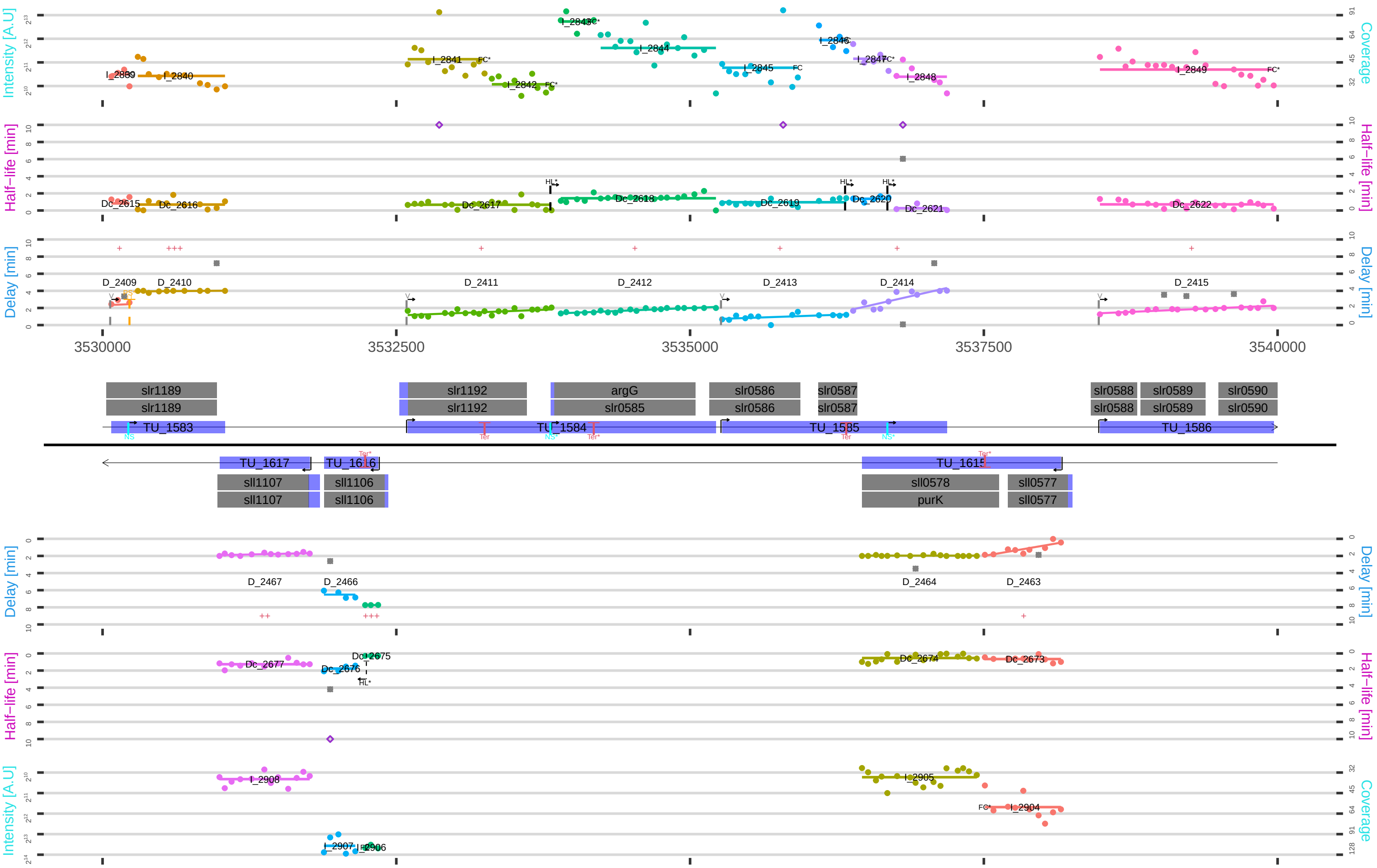

Term: termination (2), NS: new start (0), PS: pausing site (0), iTSS_I: internal starting site (2)

ID: 28432−28493; Term: termination (2), NS: new start (0), PS: pausing site (1), iTSS_I: internal starting site (1)

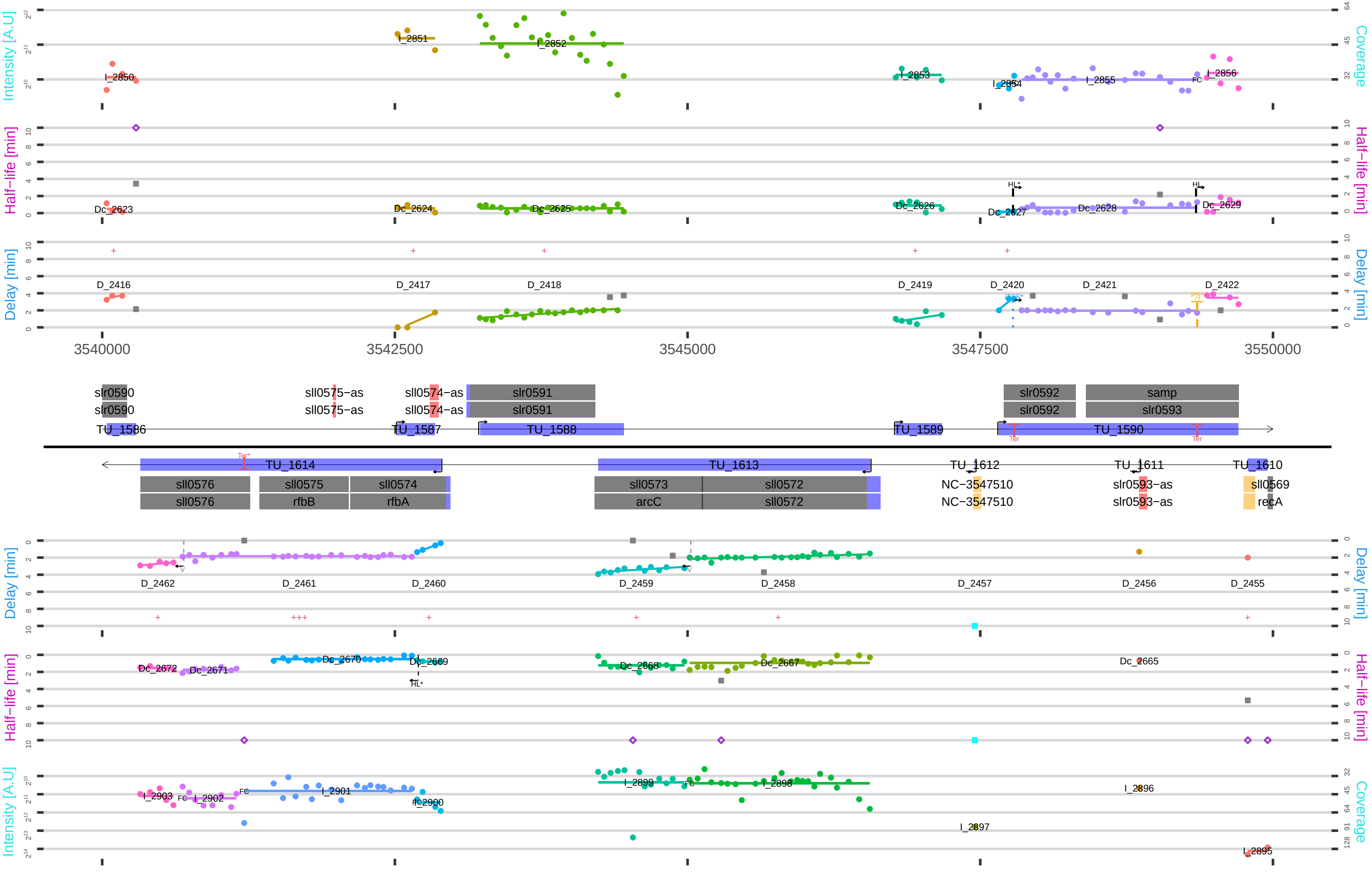

Term: termination (1), NS: new start (0), PS: pausing site (3), iTSS_I: internal starting site (0)

ID: 28494−28603; Term: termination (3), NS: new start (1), PS: pausing site (1), iTSS_I: internal starting site (3)

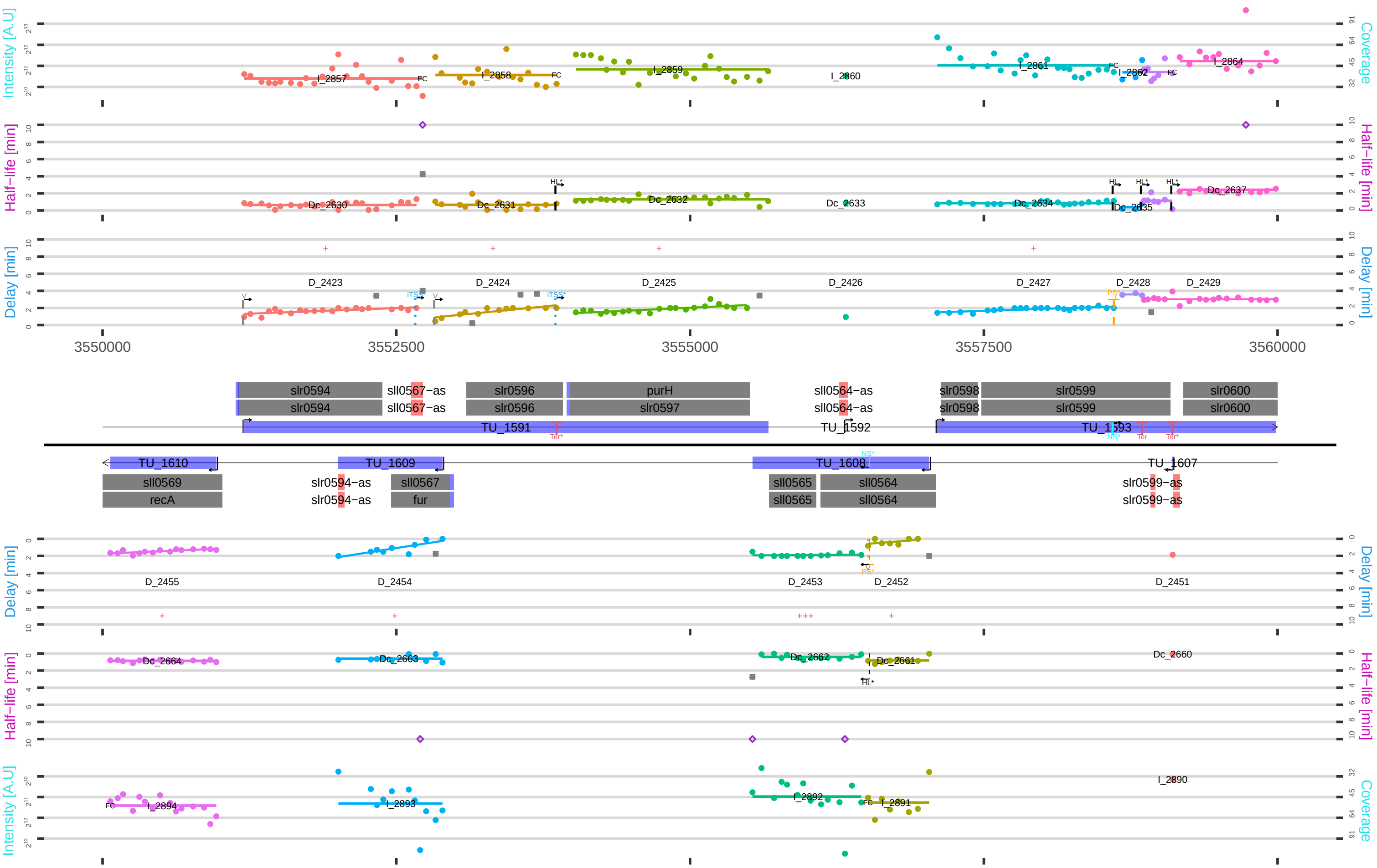

Term: termination (0), NS: new start (1), PS: pausing site (1), iTSS_I: internal starting site (0)

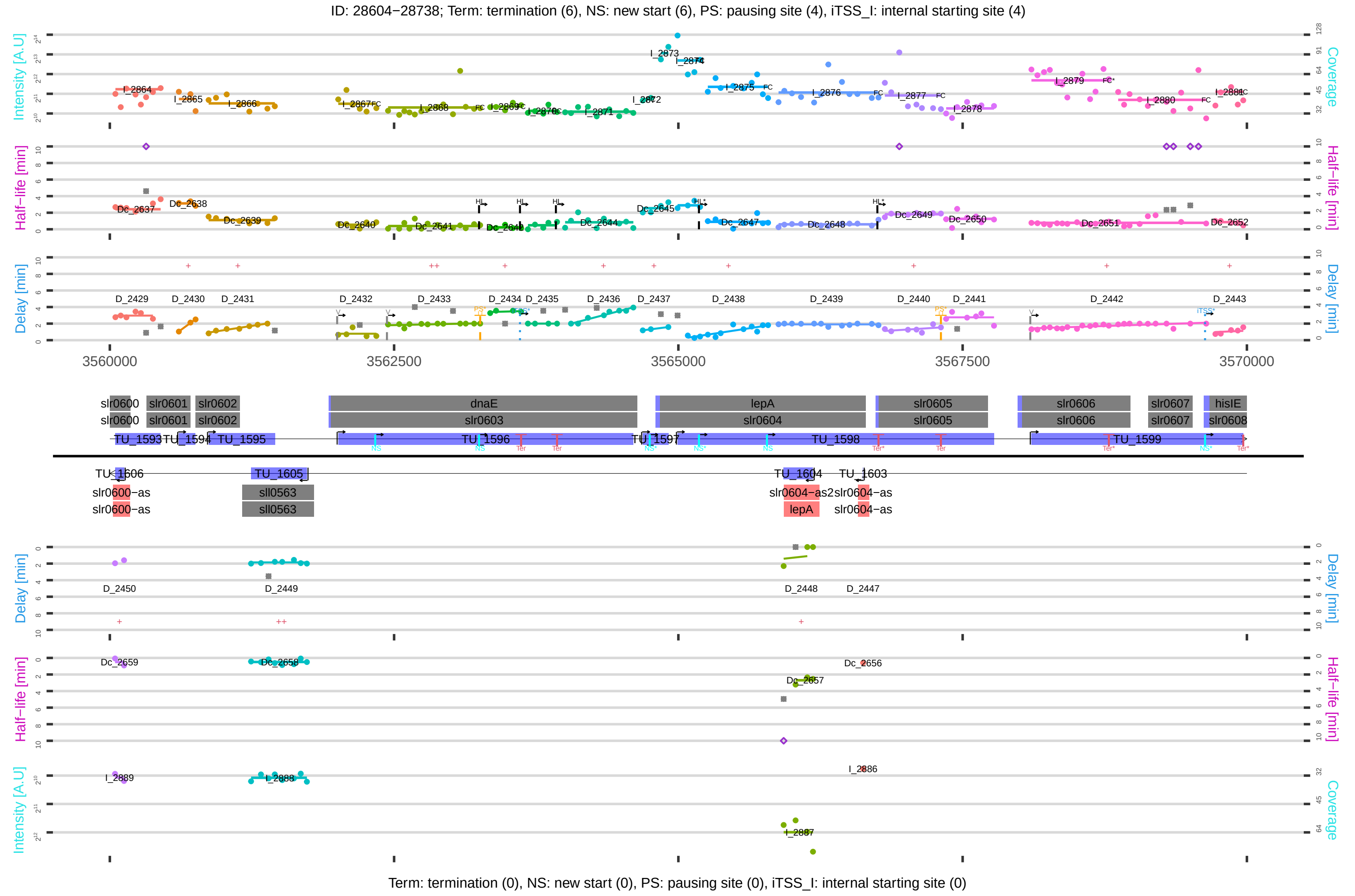

ID: 28604−28738; Term: termination (6), NS: new start (6), PS: pausing site (4), iTSS_I: internal starting site (4)
Intensity [A.U]
Coverage
Half−life [min]
Delay [min]
3560000
3562500
3565000
3567500
3570000
slr0600
slr0601
slr0602
dnaE
slr0603
lepA
slr0604
slr0605
slr0606
slr0607
hisIE
slr0608
TU_1593
TU_1594
TU_1595
TU_1596
TU_1597
TU_1598
TU_1599
TU_1606
TU_1605
TU_1604
TU_1603
slr0600−as
sll0563
slr0604−as2
slr0604−as
lepA
Term: termination (0), NS: new start (0), PS: pausing site (0), iTSS_I: internal starting site (0)

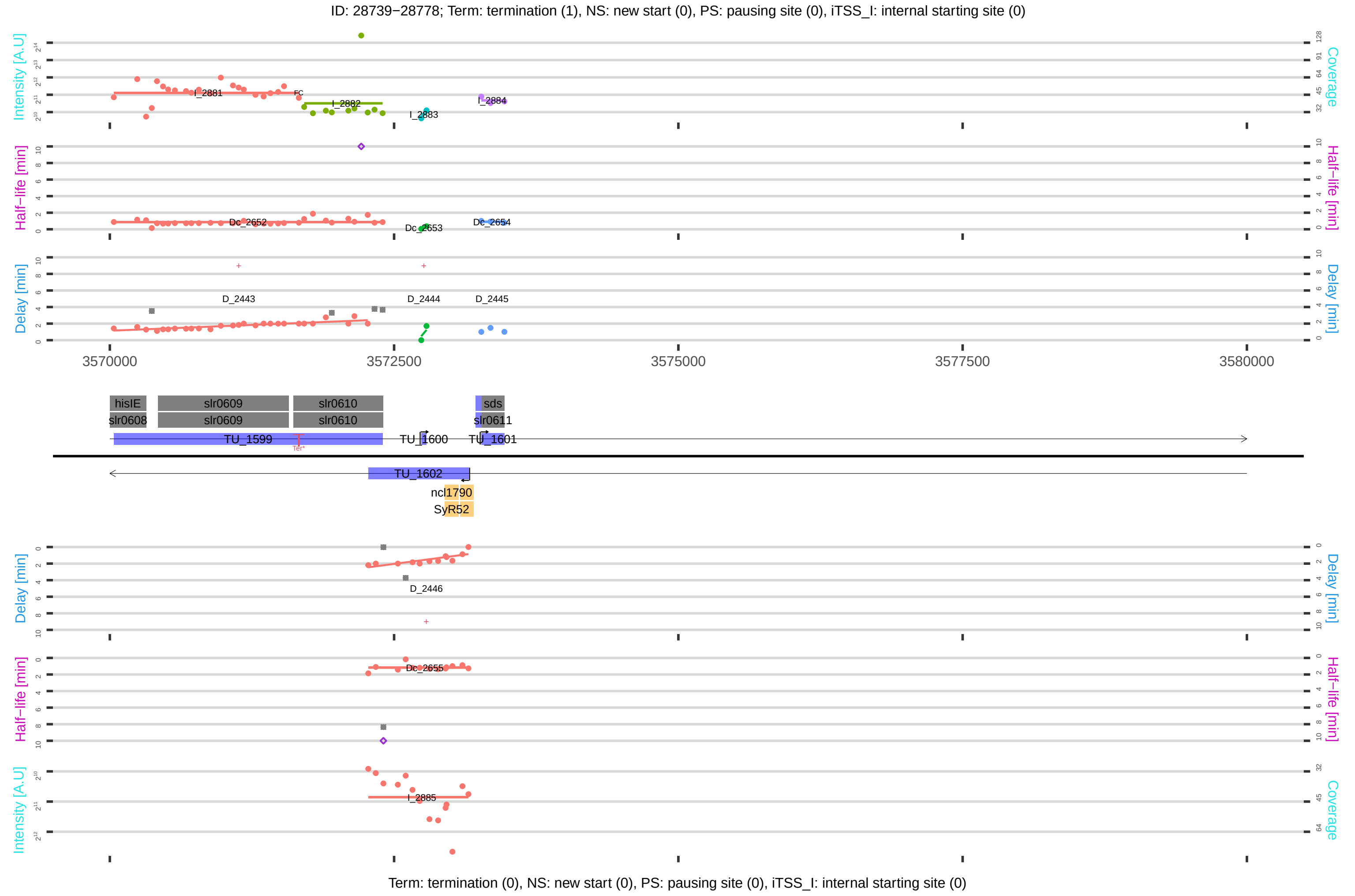
ID: 28739−28778; Term: termination (1), NS: new start (0), PS: pausing site (0), iTSS_I: internal starting site (0)
Intensity [A.U]
Coverage
I_2881
FC
I_2882
I_2883
I_2884
Half−life [min]
Dc_2652
Dc_2653
Dc_2654
Delay [min]
D_2443
D_2444
D_2445
3570000
3572500
3575000
3577500
3580000
hisIE
slr0608
slr0609
slr0610
sds
slr0611
TU_1599
Ter
TU_1600
TU_1601
TU_1602
ncl1790
SyR52
D_2446
Dc_2655
I_2885
Term: termination (0), NS: new start (0), PS: pausing site (0), iTSS_I: internal starting site (0)
