# Supplementary material for: Improved RNA stability estimation indicates that transcriptional interference is frequent in diverse bacteria
Source: Commun Biol. 2023 Jul 15;6:732. doi: 10.1038/s42003-023-05097-2 (PMC10349824; doi:10.1038/s42003-023-05097-2)
Supplement: Supplementary file 9 — Reporting Summary [file 42003_2023_5097_MOESM9_ESM.pdf]

nature portfolio

Corresponding author(s): Jens Georg

Last updated by author(s): Jun 16, 2023

Reporting Summary

Nature Portfolio wishes to improve the reproducibility of the work that we publish. This form provides structure for consistency and transparency in reporting. For further information on Nature Portfolio policies, see our Editorial Policies and the Editorial Policy Checklist.

Statistics

For all statistical analyses, confirm that the following items are present in the figure legend, table legend, main text, or Methods section.

n/a	Confirmed	
☐	☒	The exact sample size (*n*) for each experimental group/condition, given as a discrete number and unit of measurement
☐	☒	A statement on whether measurements were taken from distinct samples or whether the same sample was measured repeatedly
☐	☒	The statistical test(s) used AND whether they are one- or two-sided<br>*Only common tests should be described solely by name; describe more complex techniques in the Methods section.*
☐	☒	A description of all covariates tested
☐	☒	A description of any assumptions or corrections, such as tests of normality and adjustment for multiple comparisons
☐	☒	A full description of the statistical parameters including central tendency (e.g. means) or other basic estimates (e.g. regression coefficient) AND variation (e.g. standard deviation) or associated estimates of uncertainty (e.g. confidence intervals)
☐	☒	For null hypothesis testing, the test statistic (e.g. *F*, *t*, *r*) with confidence intervals, effect sizes, degrees of freedom and *P* value noted<br>*Give P values as exact values whenever suitable.*
☒	☐	For Bayesian analysis, information on the choice of priors and Markov chain Monte Carlo settings
☐	☒	For hierarchical and complex designs, identification of the appropriate level for tests and full reporting of outcomes
☐	☒	Estimates of effect sizes (e.g. Cohen's *d*, Pearson's *r*), indicating how they were calculated

Our web collection on statistics for biologists contains articles on many of the points above.

Software and code

Policy information about availability of computer code

Data collection	Publicly available data was downloaded from the NCBI Gene Expression Omnibus (GEO) database or the European Nucleotide Archive (ENA) database. The PCC6803 samples were hybridized to an Agilent 8 x 60K array, which was scanned with an Agilent Technologies Scanner G2505B. features were extracted with Agilent Technologies' Feature Extraction software version 10.5.1.1 (protocol GE1_105_Dec08).
Data analysis	For the RNA-Seq data the reads were aligned with BWA alignment tool version 0.7.17 to the genomes CP009273.1, NC_015663.1, NC_000913.2 and NC_010475.1. Bedtools genomecov version 2.30.0 was used to count the sequencing depth prior to the analysis with the rifi R-package version 1.2.2 from Bioconductor version 3.16. The microarray data were normalized with limma prior to the analysis with the rifi R-package version 1.2.2 from Bioconductor version 3.16.

For manuscripts utilizing custom algorithms or software that are central to the research but not yet described in published literature, software must be made available to editors and reviewers. We strongly encourage code deposition in a community repository (e.g. GitHub). See the Nature Portfolio guidelines for submitting code & software for further information.

Data

Policy information about availability of data

All manuscripts must include a data availability statement. This statement should provide the following information, where applicable:

- Accession codes, unique identifiers, or web links for publicly available datasets
- A description of any restrictions on data availability
- For clinical datasets or third party data, please ensure that the statement adheres to our policy

The Synechocystis PCC 6803 microarray data from this study have been deposited with the GEO accession number GSE209879. The data sources and respective references for the other organisms are: Escherichia coli K12 BW25113 and Klebsiella aerogenes KCTC 2190 (Dar & Sorek, 20183, ENA project ID PRJEB21982). E. coli K12 MG1655 (Moffit et al., 201618, GEO: GSE75818). Synechococcus sp. strain PCC 7002 (Gordon et al., 202019, GEO: GSE109174). The 'rifi' R objects containing the source data for all analyses and figures based on experimental data are available in Supplementary Dataset 5.

Research involving human participants, their data, or biological material

Policy information about studies with human participants or human data. See also policy information about sex, gender (identity/presentation), and sexual orientation and race, ethnicity and racism.

Reporting on sex and gender	This information is not relevant for this study which deals with bacterial organisms and has not been collected
Reporting on race, ethnicity, or other socially relevant groupings	This information is not relevant for this study which deals with bacterial organisms and has not been collected
Population characteristics	This information is not relevant for this study which deals with bacterial organisms and has not been collected
Recruitment	This information is not relevant for the study
Ethics oversight	This information is not relevant for the study

Note that full information on the approval of the study protocol must also be provided in the manuscript.

Field-specific reporting

Please select the one below that is the best fit for your research. If you are not sure, read the appropriate sections before making your selection.

☒ Life sciences ☐ Behavioural & social sciences ☐ Ecological, evolutionary & environmental sciences

For a reference copy of the document with all sections, see nature.com/documents/nr-reporting-summary-flat.pdf

Life sciences study design

All studies must disclose on these points even when the disclosure is negative.

Sample size	No sample size calculation was performed. All analyses are based on the available replicates in the previously published data (1 to 3). For the microarray data we used 3 biological replicates.
Data exclusions	For the RNA-Seq data bins with an average normalized read coverage below 10 were not used for the analysis. For the microarray experiment probes with the highest expression at the last time point or the lowest expression at the first time point were excluded.
Replication	All available replicates were used together to fit the models. Replicate variation is reflected in the fit statistics.
Randomization	Randomization is not relevant for the study, we did not allocate the data to different groups and did not compare different groups.
Blinding	Blinding is not relevant for the study, we did not allocate the data to different groups and did not compare different groups.

Reporting for specific materials, systems and methods

We require information from authors about some types of materials, experimental systems and methods used in many studies. Here, indicate whether each material, system or method listed is relevant to your study. If you are not sure if a list item applies to your research, read the appropriate section before selecting a response.

Materials & experimental systems

n/a	Involved in the study
☒	☐ Antibodies
☒	☐ Eukaryotic cell lines
☒	☐ Palaeontology and archaeology
☒	☐ Animals and other organisms
☒	☐ Clinical data
☒	☐ Dual use research of concern
☒	☐ Plants

Methods

n/a	Involved in the study
☒	☐ ChIP-seq
☒	☐ Flow cytometry
☒	☐ MRI-based neuroimaging
